# Supplementary material for: TPGS1 regulates central spindle microtubule glutamylation and remodeling during telophase and abscission
Source: EMBO Rep. 2026 Mar 23;27(8):1944–63. doi: 10.1038/s44319-026-00742-3 (PMC13121839; doi:10.1038/s44319-026-00742-3)
Supplement: Supplementary file 1 — Appendix [file 44319_2026_742_MOESM1_ESM.pdf]

Appendix Figures for "TPGS1 Regulates Central Spindle Microtubule Glutamylation and Remodeling During Telophase and Abscission"

Table of Contents **Page**

Table of Contents	Page
Appendix Figure S1	2
Appendix Figure S2	3
Appendix Figure S3	4
Appendix Figure S4	5
Appendix Figure S5	6
Appendix Figure S6	7
Appendix Figure S7	8
Appendix Figure S8	9
Appendix Figure S9	10
Appendix Figure S10	11

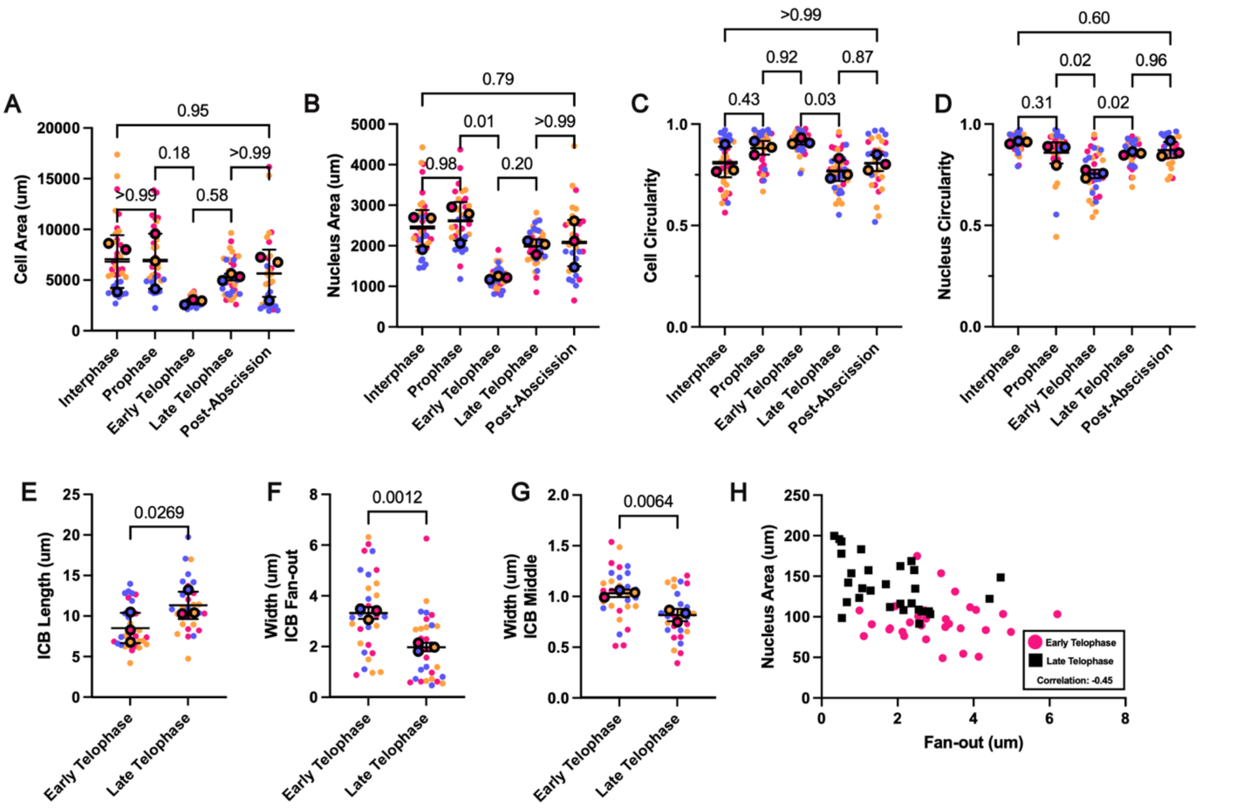

Appendix Figure S1.

(A-H) Quantification of images of fixed wild-type HeLa cells, co-stained with and anti-α-tubulin and anti-acetylated α-tubulin antibodies. Each experiment included biological replicates (n=3), with 10 cells measured per replicate, color coded. One-way ANOVA was done on (A-D), t-test on (E-G), and linear regression analysis on (H).

(A-D) Cell and nucleus area and circularity were measured by selecting α-tubulin signal (cells; A,C) and Hoechst signal (nuclei; B and D) to measure total area and circularity.

(E-G) Telophase cell ICB dimensions used for ratios in figure 1 were measured between each pair of cells, each treated as one technical replicate per one pair of cells, with 10 cells per biological replicate (n=3).

(H) Nucleus area plotted relative to paired fan-out value, indicating a negative correlation between the two. Nucleus area was a primary indicator of telophase stage used in this paper. Early telophase cells are shown in magenta and late telophase cells in black.

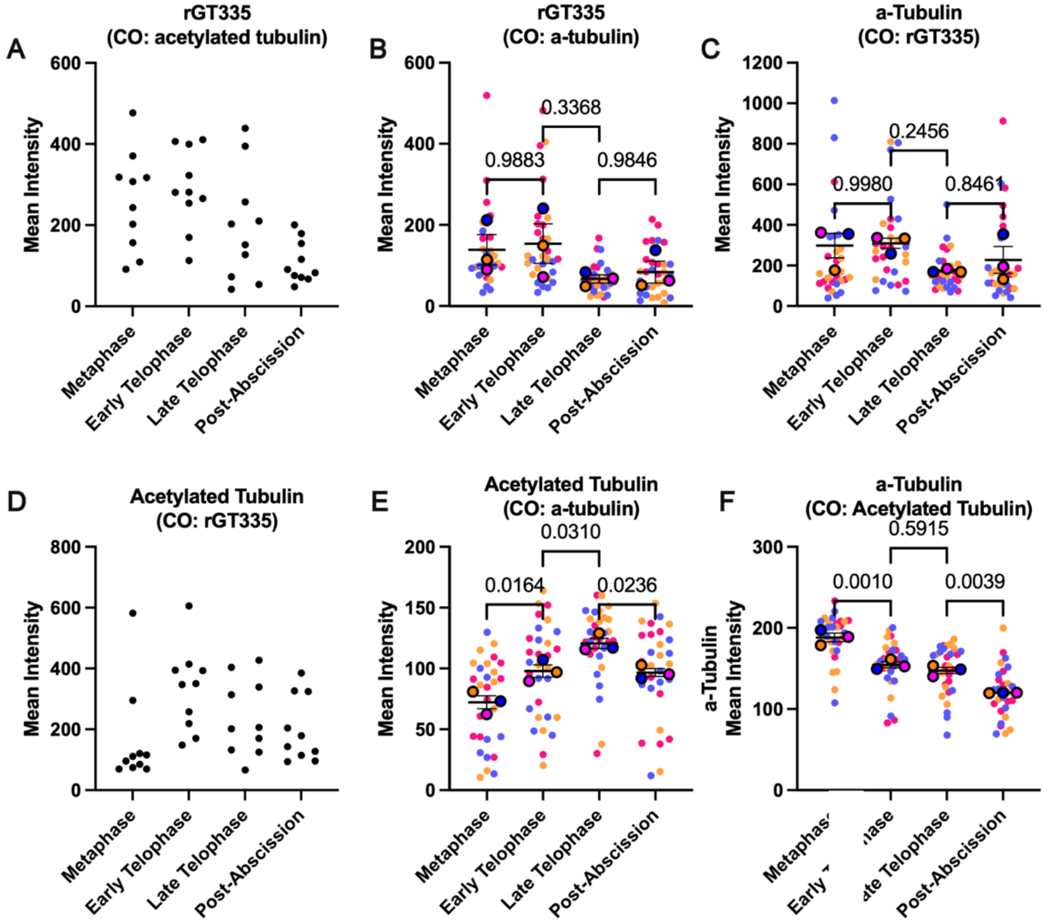

Appendix Figure S2.

(A;D) Mean fluorecence intensity values of microtubule structures per channel of cells co-stained with rGT335 (A) and acetylated α-tubulin antibodies (D). Data shows one biological replicate, with 10 cells per condition.

(B-C; E-F) Mean fluorescence intensity values of microtubule structures used for ratios shown in figure 2, given for each antibody pair: anti-GT335 (B-C) and anti-acetylated α-tubulin (E-F). Each antibody was co-stained with anti- α-tubulin antibodies (C,F). Statistics were calculated with one-way ANOVA on means from color-coded biological replicates (n=3), each including 10 cells.

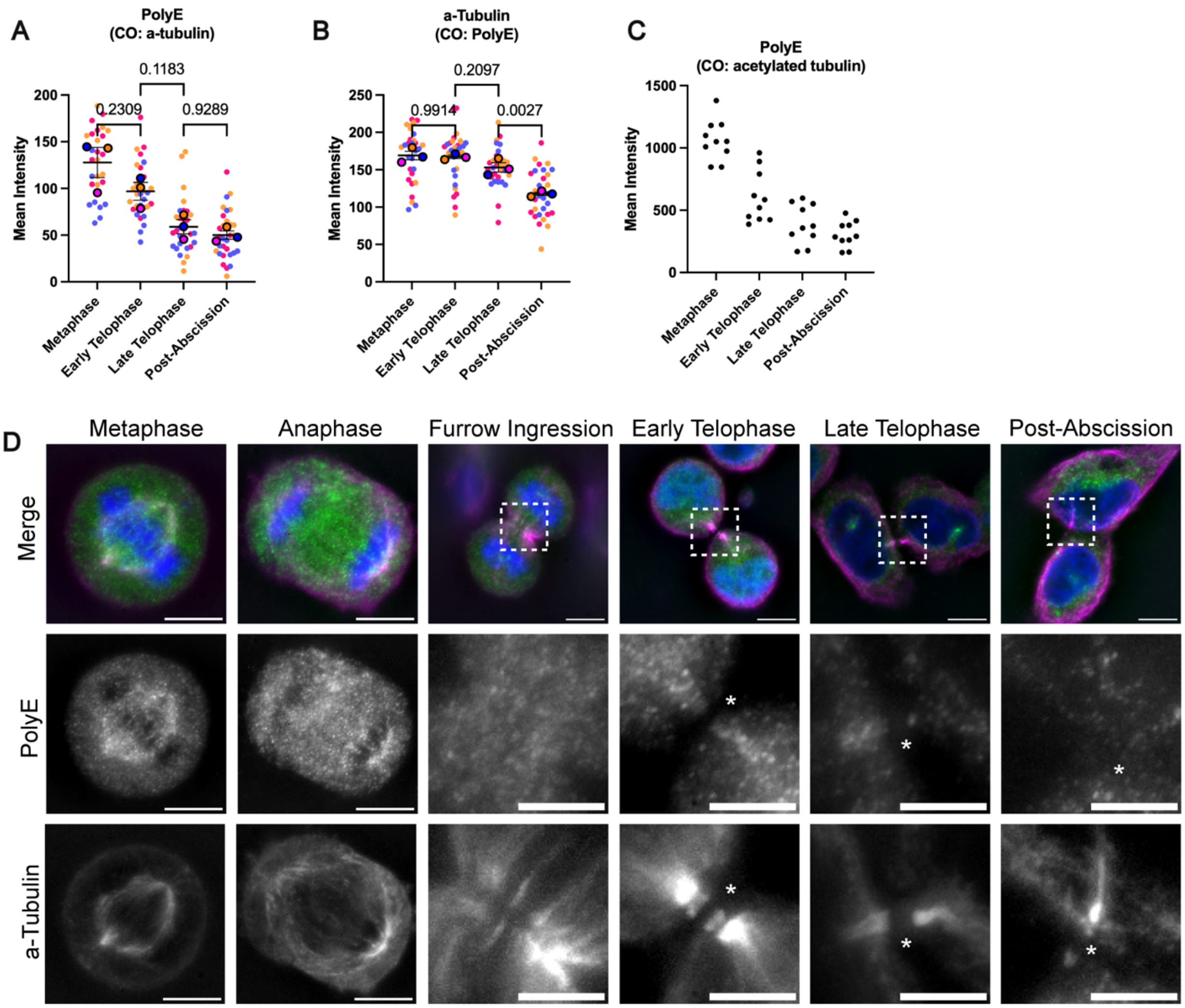

Appendix Figure S3.

(A-B) Mean fluorescence intensity values of microtubule structures used for ratios shown in Fig. 2A, given for each antibody: PolyE (A) and a-tubulin (B). Statistics were calculated with one-way ANOVA on means from color-coded biological replicates ($n=3$), each including 10 cells. One-way ANOVA was calculated on the means of each set of replicates along with a Dunnett post-test. Error bars represent SEM of biological replicates.

(E) Mean fluorecence intensity values of microtubule structures for PolyE alone of cells co-stained with PolyE and acetylated α-tubulin antibodies. Data shows one biological replicate with 10 cells per condition.

(D) Representative images of cells in different mitotic stages co-stained with PolyE (green) and anti-α-tubulin (magenta) antibodies. Full image scale bars are 10mm, zoomed-in images are 5 mm. Asterisk marks the MB, boxes represent the zoomed in area.

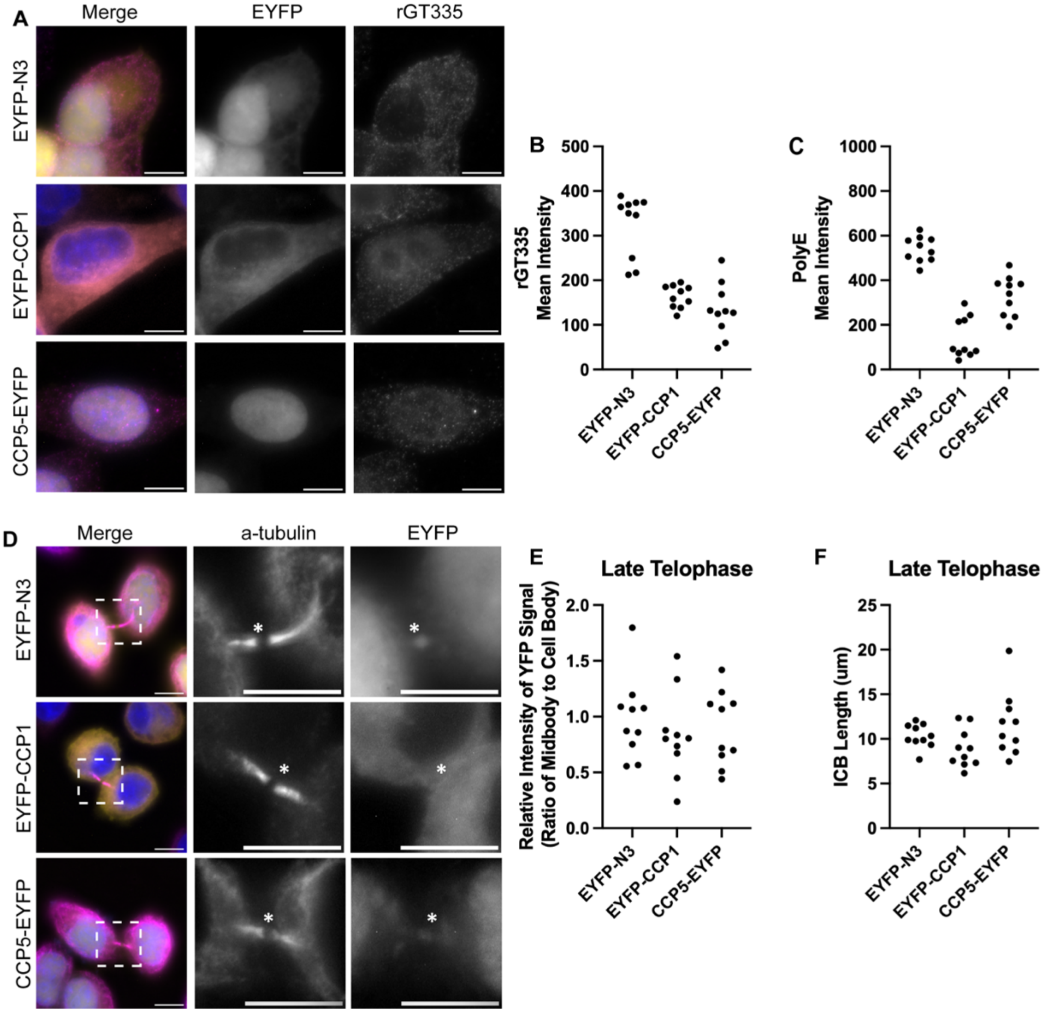

Appendix Figure S4.

(A-C) Representative images and quantification of cells transfected with EYFP-N3, EYFP-CCP1, and CCP5-EYFP plasmids (yellow), fixed and co-stained with rGT335 (magenta) antibodies (A). Quantification of rGT335 (B) and PolyE (C) signal were done on 10 cells in one biological replicate, measured for the cell body (with the nucleus subtracted). Image scale bars are 10mm.

(D) Images of telophase cells transfected with EYFP-N3, EYFP-CCP1, and CCP5-EYFP plasmids (yellow), fixed and co-stained with anti- α-tubulin (magenta) antibodies. Image scale bars are 10mm. Asterisk marks the MB.

(E) Ratio of EYFP fluorescence mean intensity of the MB divided by that of the whole cell per image, of one biological replicate containing 10 cells in each condition.

(F) ICB length measured between telophase cells in each condition of one biological replicate containing 10 cells in each condition.

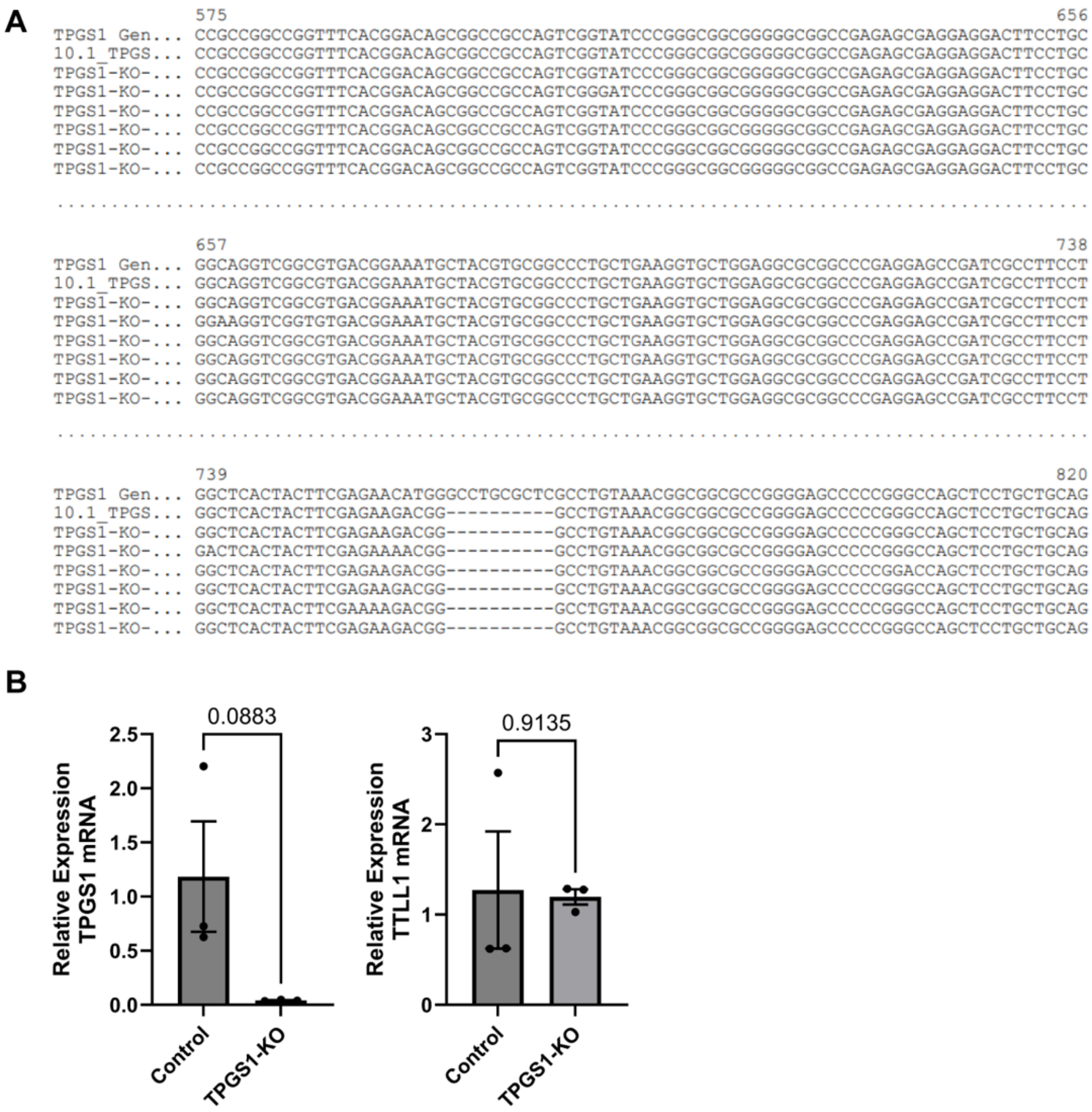

Appendix Figure S5.

(A) Sequencing results aligned to TPGS1 sequence for clones of TPGS1-KO cells. Cells exhibit a 10bp deletion in all alleles resulting in frame shift and premature stop codon.

(B) RT-qPCR results on biological replicates (n=3) of control and TPGS1-KO cells for TPGS1 and TTLL1 mRNA, normalized to GAPDH. Error bars represent SEM. Statistics were done with students t-test between the control and TPGS1-KO biological replicates.

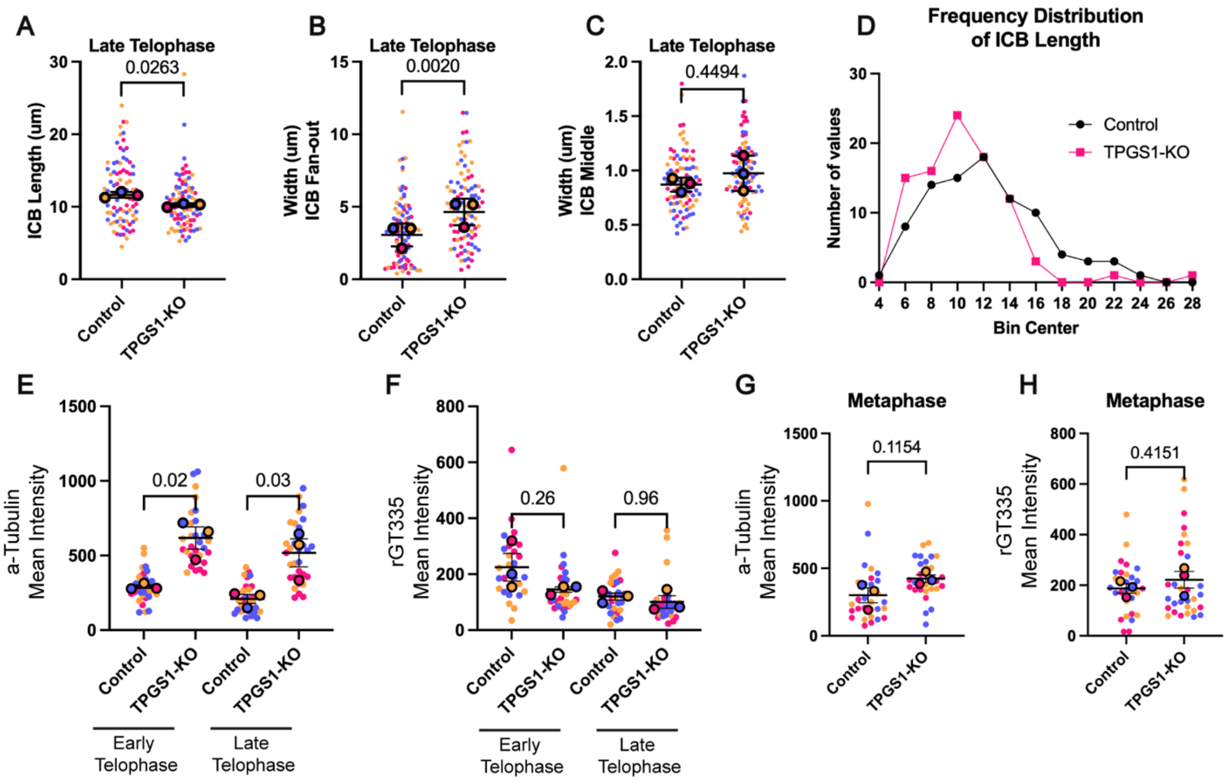

Appendix Figure S6.

(A-D) Quantification of images of fixed wild-type HeLa cells, co-stained with and anti-α-tubulin and anti-acetylated α-tubulin antibodies. Each experiment included biological re plicates (n=3), with 30 cells measured per replicate, color coded. All statistics are done on means. T-test was calculated for (A-C), Late telophase cell ICB dimensions of control and TPGS1-KO cells used for ratios in figure 7 were measured between each pair of cells, each treated as one technical replicate per one pair of cells.

(E-H) Quantification of individual channels for experiments from Figure 7A-E for early and late telophase (E-F) and metaphase (G-H). Mean fluorescence intensity values of microtubule structures are given for each antibody pair: anti-GT335 (F,H) and anti-α-tubulin (E,G). Statistics were calculated with one-way ANOVA (E-F) or student's t-test (G-H) on means from color-coded biological replicates (n=3), each including 10 cells.

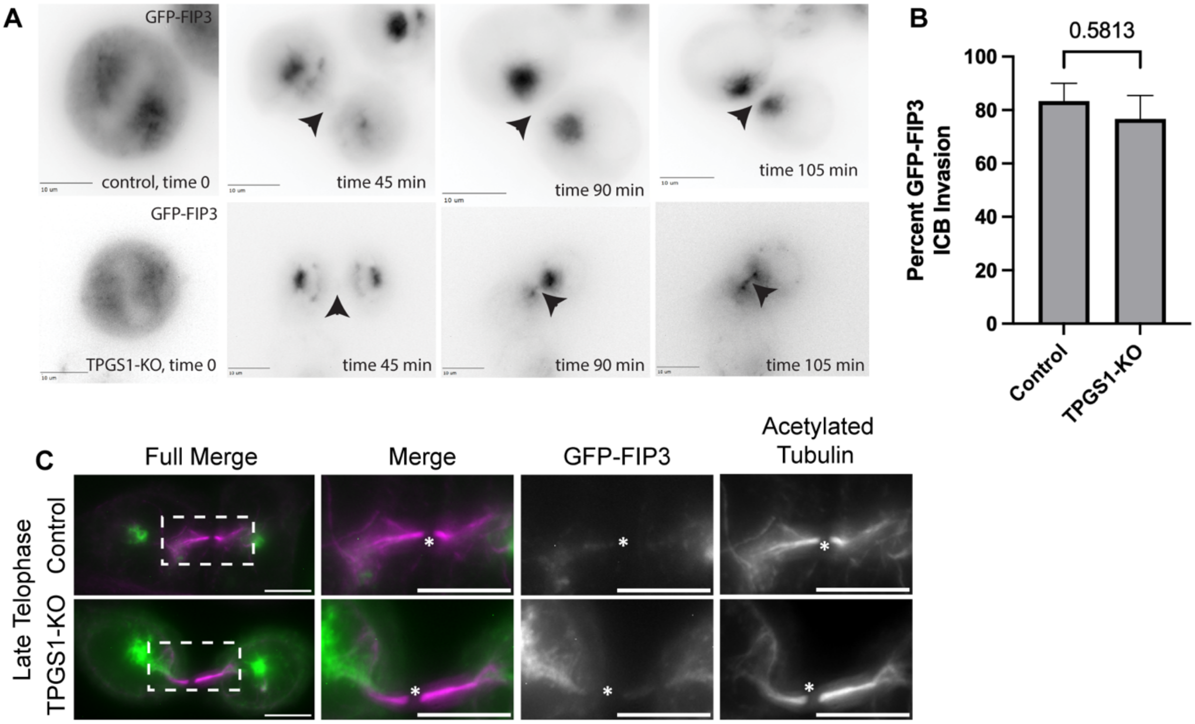

Appendix Figure S7.

(A) Time-lapse imaging of control and TPGS1-KO HeLa cells transfected with GFP-FIP3. Scale bars are 10mm, arrows point to the MB.

(B) Quantification of images from (B) of late telophase cells, measuring the percent of c ells in each biological replicate (n=3) that showed GFP-FIP3 signal in the ICB. T-t est was done on the percentages of replicates.

(C) Control and TPGS1-KO cells were transfected with GFP-FIP3 (green), fixed, and stained with anti-acetylated-α-tubulin (red) antibodies. Asterisks mark the MB. Scale bars are 10mm. Boxes mark the zoomed in area.

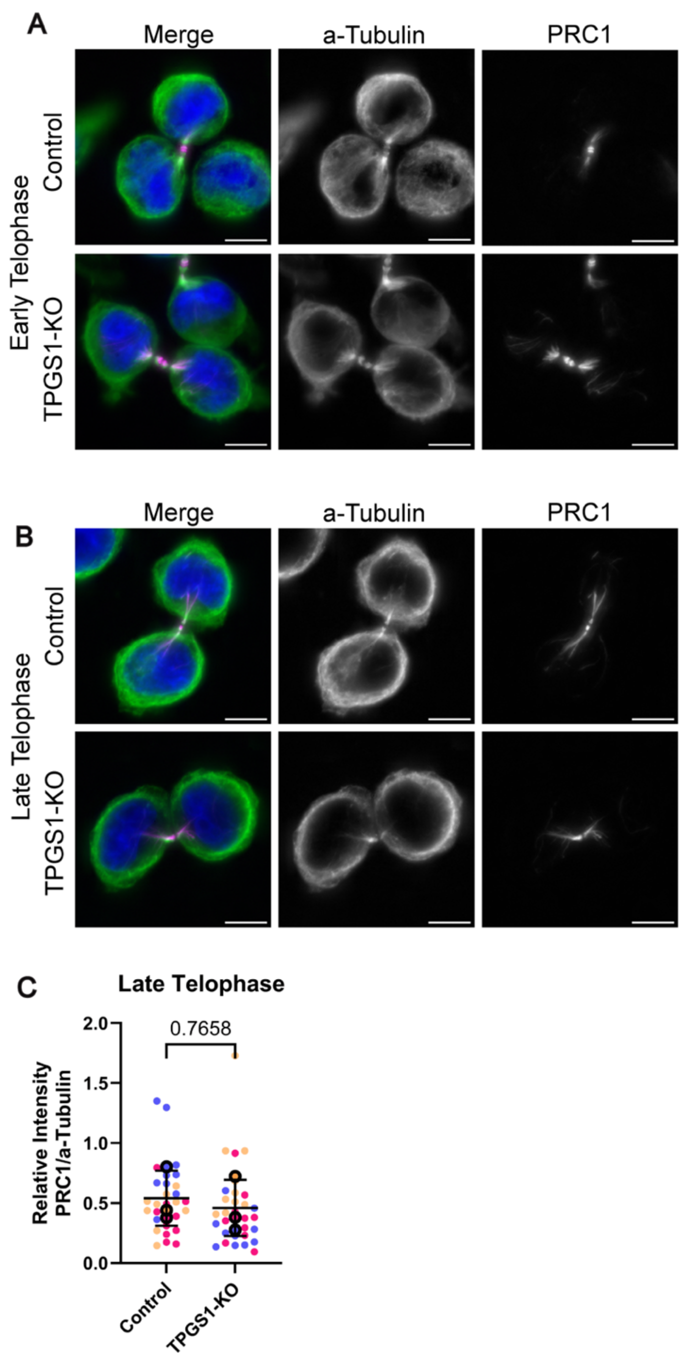

Appendix Figure S8.

(A-B) Control and TPGS1-KO cells were fixed and stained with an anti-PRC1 (green) antibody and a-tubulin (magenta) antibody for early (A) and late (B) staged telophase cells. Scale bars are 10mm.

(C) Quantification of ICB antibody intensity between control and TPGS1-KO cells from (B). Biological replicate means (n=3) are color coded and statistics were done with t-test of the means, with each mean representing 10 cells.

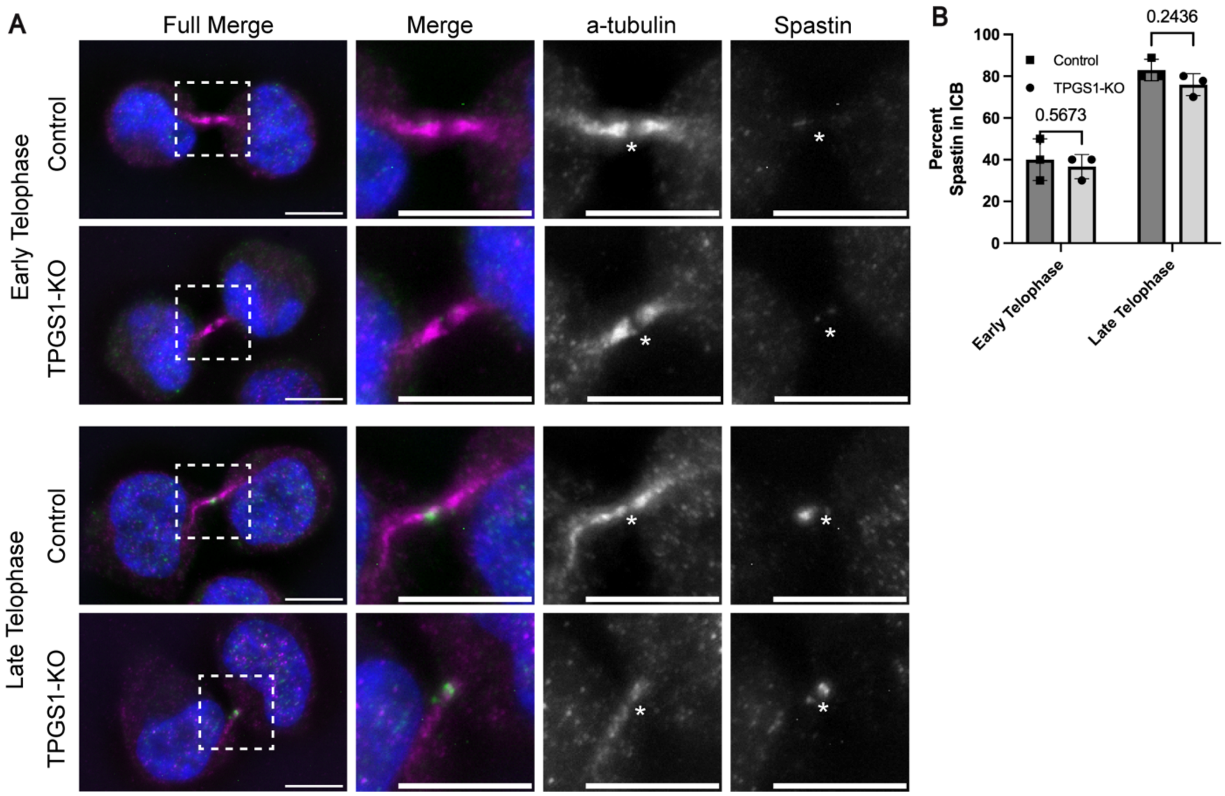

Appendix Figure S9.

(A) Control and TPGS1-KO cells were fixed and stained with anti-acetylated α-tubulin (magenta) and anti-spastin (green) antibodies. Asterisks mark the MB. Scale bars are 10mm. Boxes mark the zoomed in area.

(B) Quantification of images from (A) in early and late telophase, measuring the perce nt of cells in each biological replicate (n=3) that showed spastin localized near the MB in intact ICBs in images of cells. T-test was done on the percentages of replicates be tween each stage of telophase.

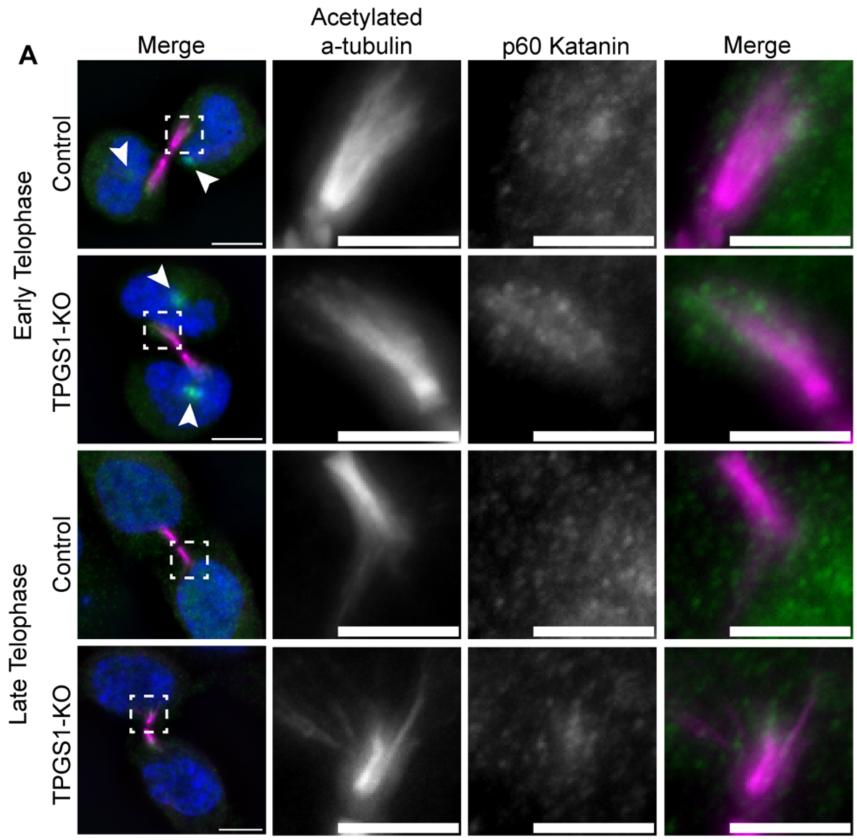

Appendix Figure S10.

(A) Control and TPGS1-KO cells were fixed and stained with anti-acetylated α-tubulin (magenta) and anti-p60 katanin (green) antibodies. Arrows point to centrosome populations in early telophase. Scale bars are 10mm. Boxes mark the zoomed in area.
