# Supplementary figures and images for: TPGS1 regulates central spindle microtubule glutamylation and remodeling during telophase and abscission (part 2 of 36)
Source: EMBO Rep. 2026 Mar 23;27(8):1944–63. doi: 10.1038/s44319-026-00742-3 (PMC13121839; doi:10.1038/s44319-026-00742-3)

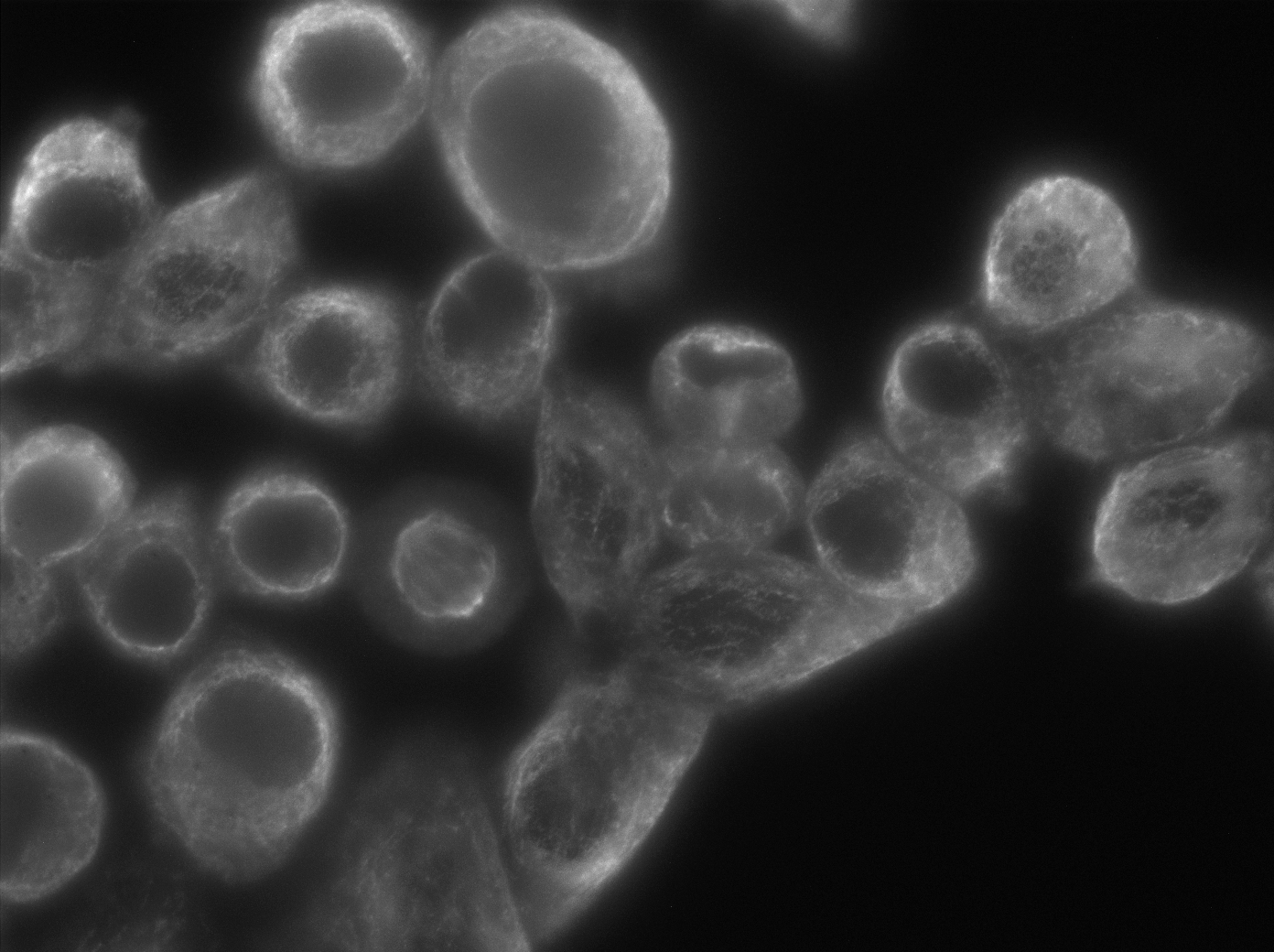

Supplement: Supplementary file 3 — Source data Fig. 1 [file 44319_2026_742_MOESM3_ESM.zip › Figure 1/Fig 1bcd WT Hela acetylated a tubulin atubulin/actub-atub 8-14-24 R3 M1 ET1.Project Maximum Z_XY1724701790_Z0_T0_C1.tif]

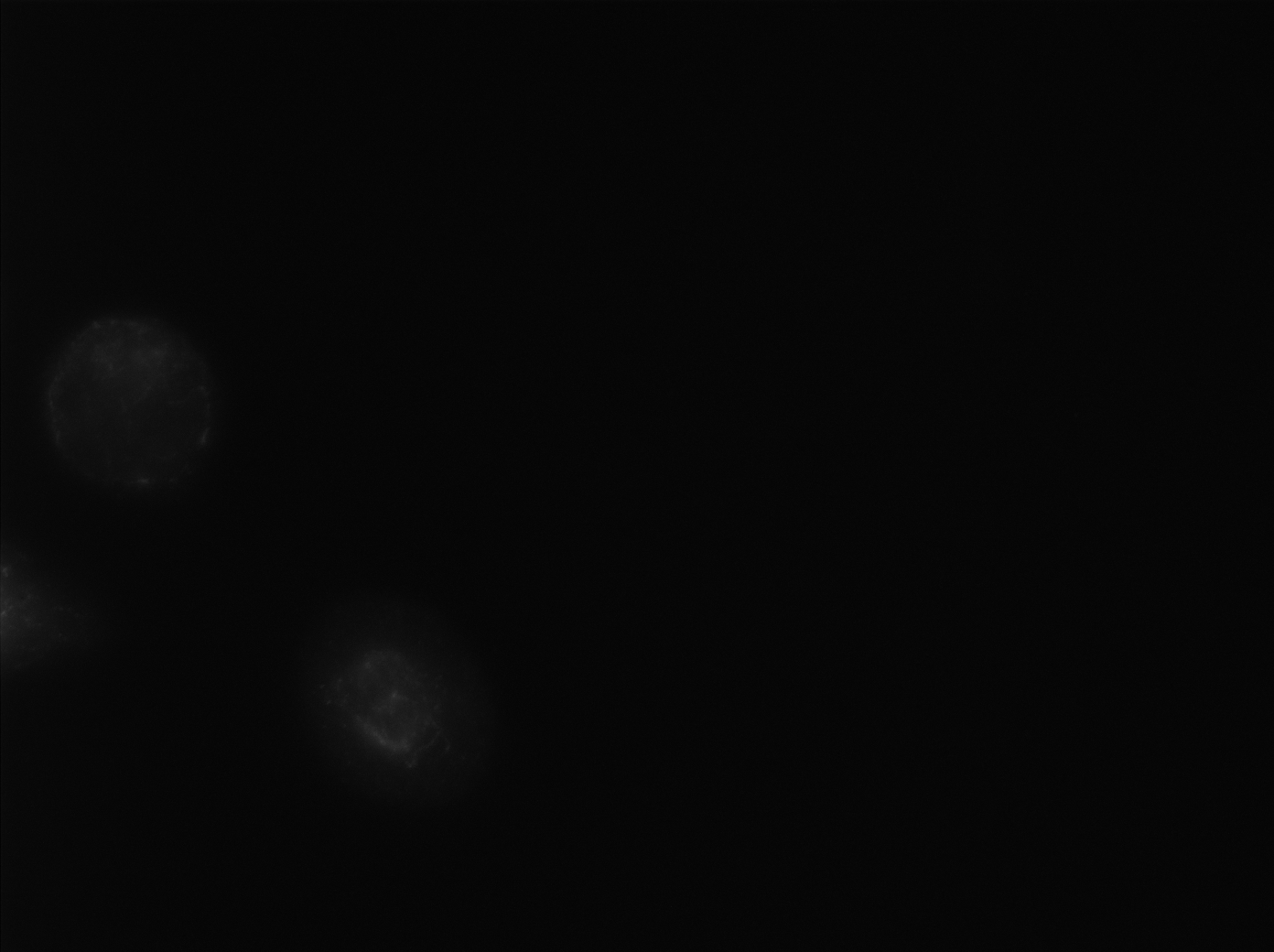

Supplement: Supplementary file 3 — Source data Fig. 1 [file 44319_2026_742_MOESM3_ESM.zip › Figure 1/Fig 1bcd WT Hela acetylated a tubulin atubulin/actub-atub 8-14-24 R2 M1.Project Maximum Z_XY1724690706_Z0_T0_C2.tif]

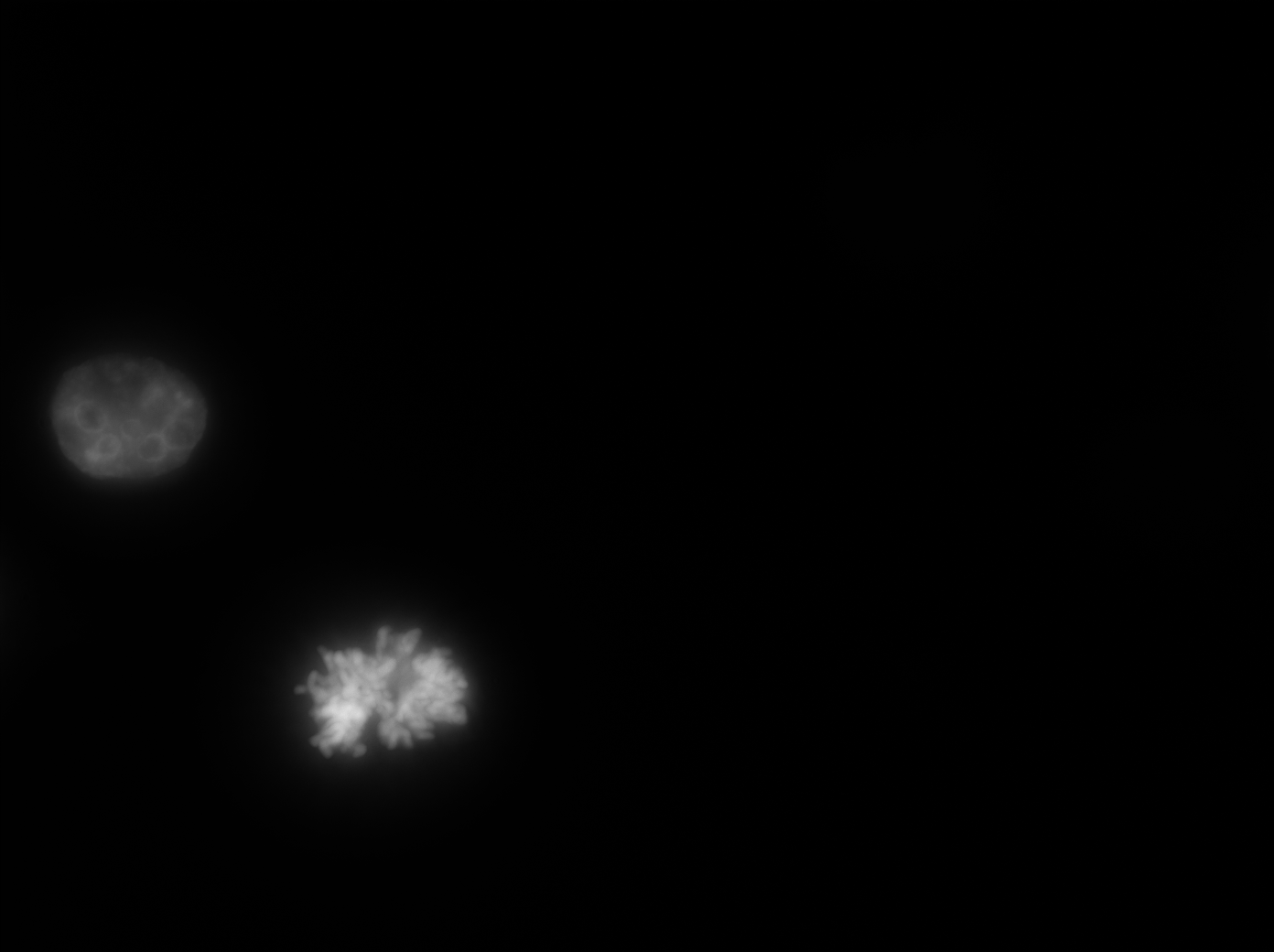

Supplement: Supplementary file 3 — Source data Fig. 1 [file 44319_2026_742_MOESM3_ESM.zip › Figure 1/Fig 1bcd WT Hela acetylated a tubulin atubulin/actub-atub 8-14-24 R2 M1.Project Maximum Z_XY1724690706_Z0_T0_C0.tif]

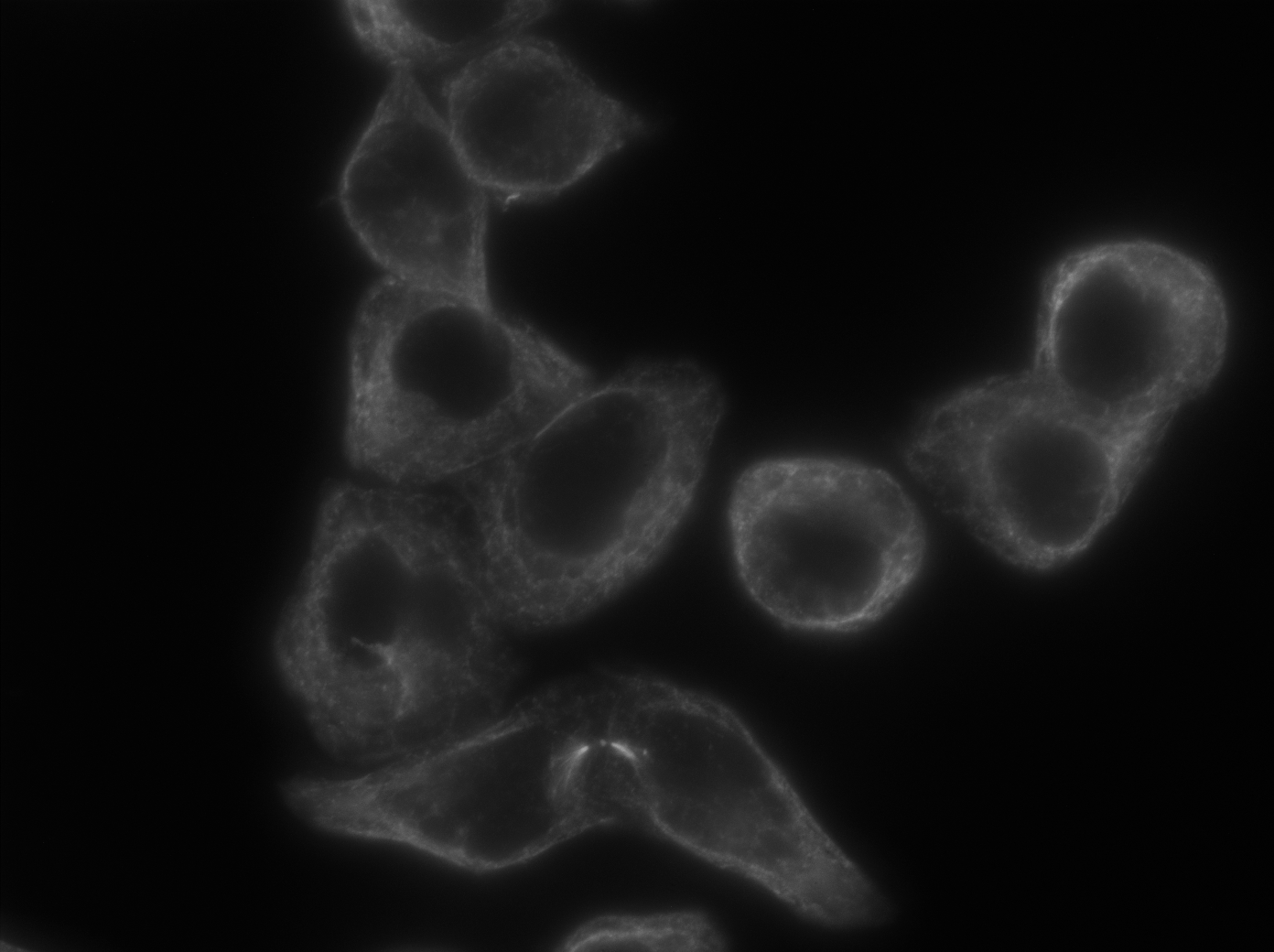

Supplement: Supplementary file 3 — Source data Fig. 1 [file 44319_2026_742_MOESM3_ESM.zip › Figure 1/Fig 1bcd WT Hela acetylated a tubulin atubulin/actub-atub 8-14-24 R1 LT6PA7.Project Maximum Z_XY1724364381_Z0_T0_C1.tif]

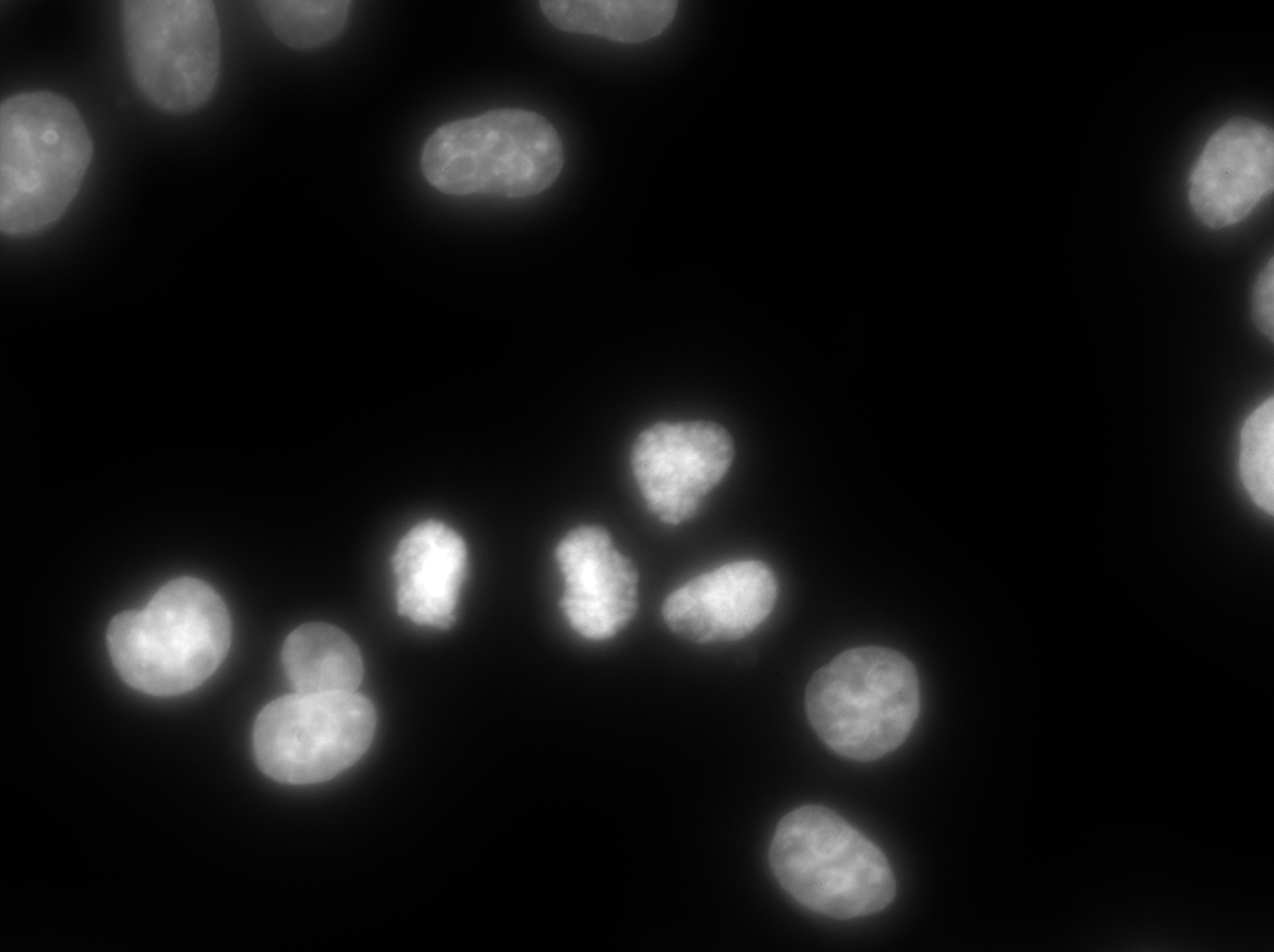

Supplement: Supplementary file 3 — Source data Fig. 1 [file 44319_2026_742_MOESM3_ESM.zip › Figure 1/Fig 1bcd WT Hela acetylated a tubulin atubulin/actub-atub 8-14-24 R3 ET9ET10.Project Maximum Z_XY1724704359_Z0_T0_C0.tif]

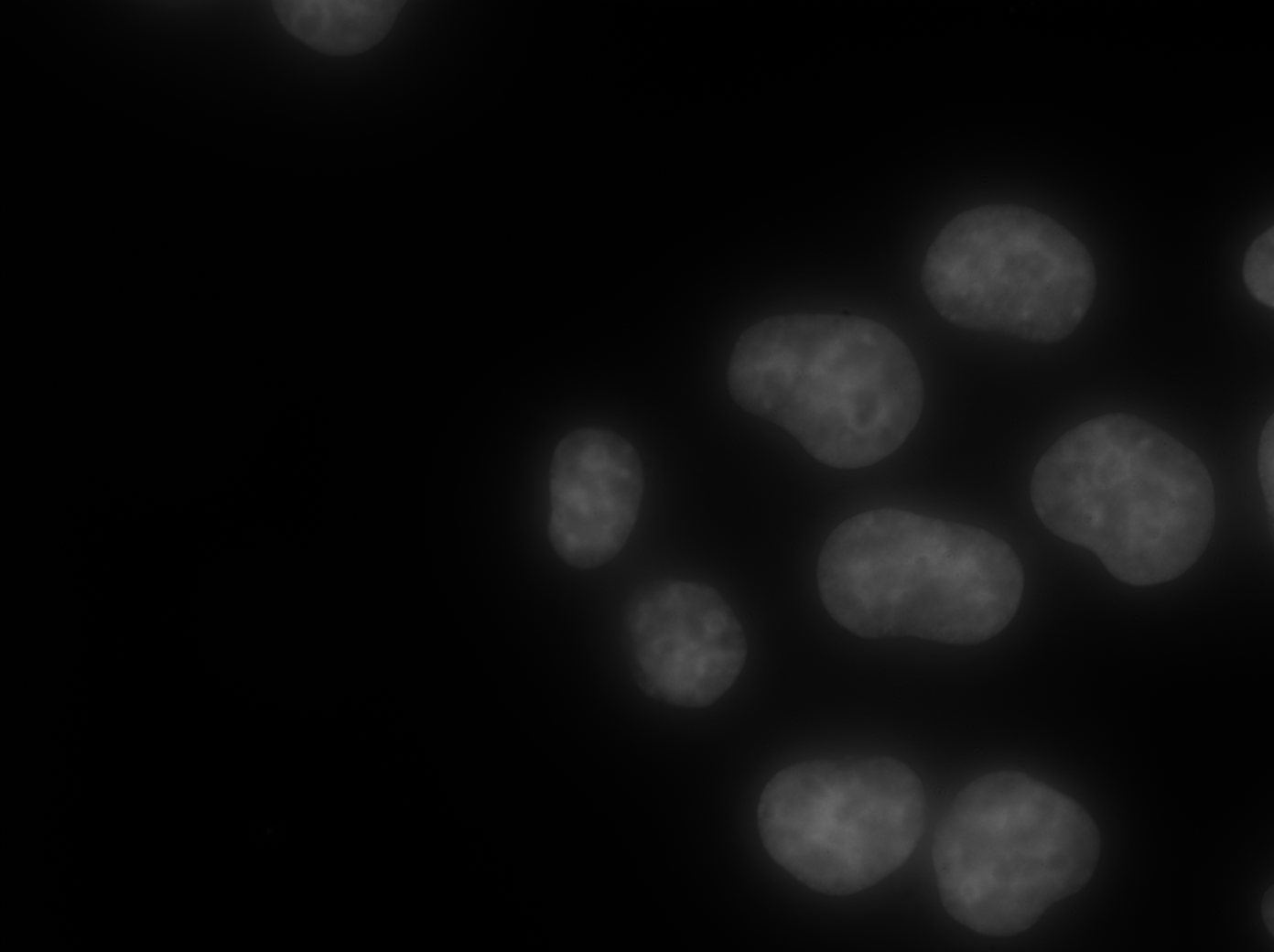

Supplement: Supplementary file 3 — Source data Fig. 1 [file 44319_2026_742_MOESM3_ESM.zip › Figure 1/Fig 1bcd WT Hela acetylated a tubulin atubulin/actub-atub 8-14-24 R2 PA9.Project Maximum Z_XY1724695303_Z0_T0_C0.tif]

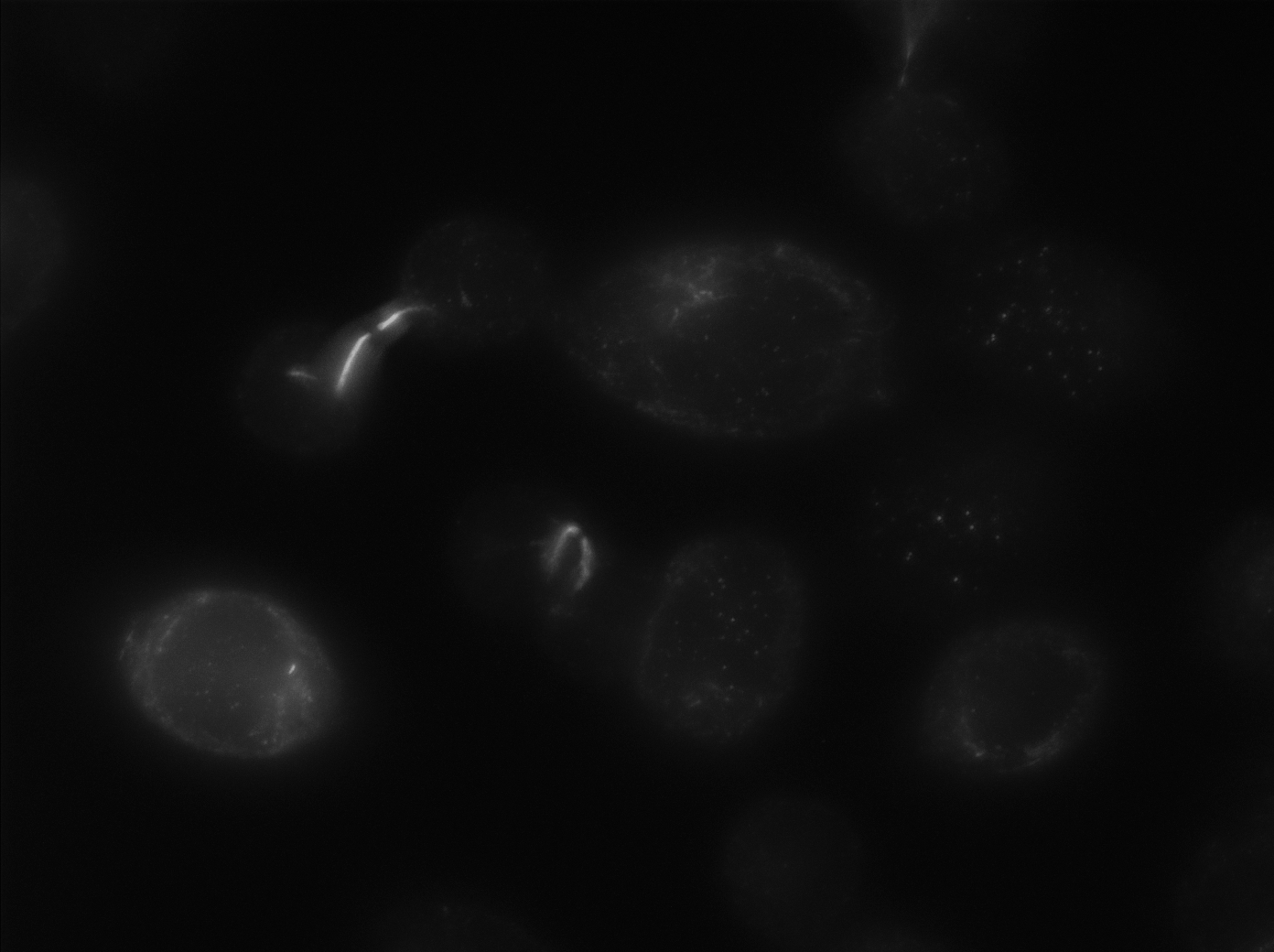

Supplement: Supplementary file 3 — Source data Fig. 1 [file 44319_2026_742_MOESM3_ESM.zip › Figure 1/Fig 1bcd WT Hela acetylated a tubulin atubulin/actub-atub 8-14-24 R3 ET4ET5.Project Maximum Z_XY1724703355_Z0_T0_C2.tif]

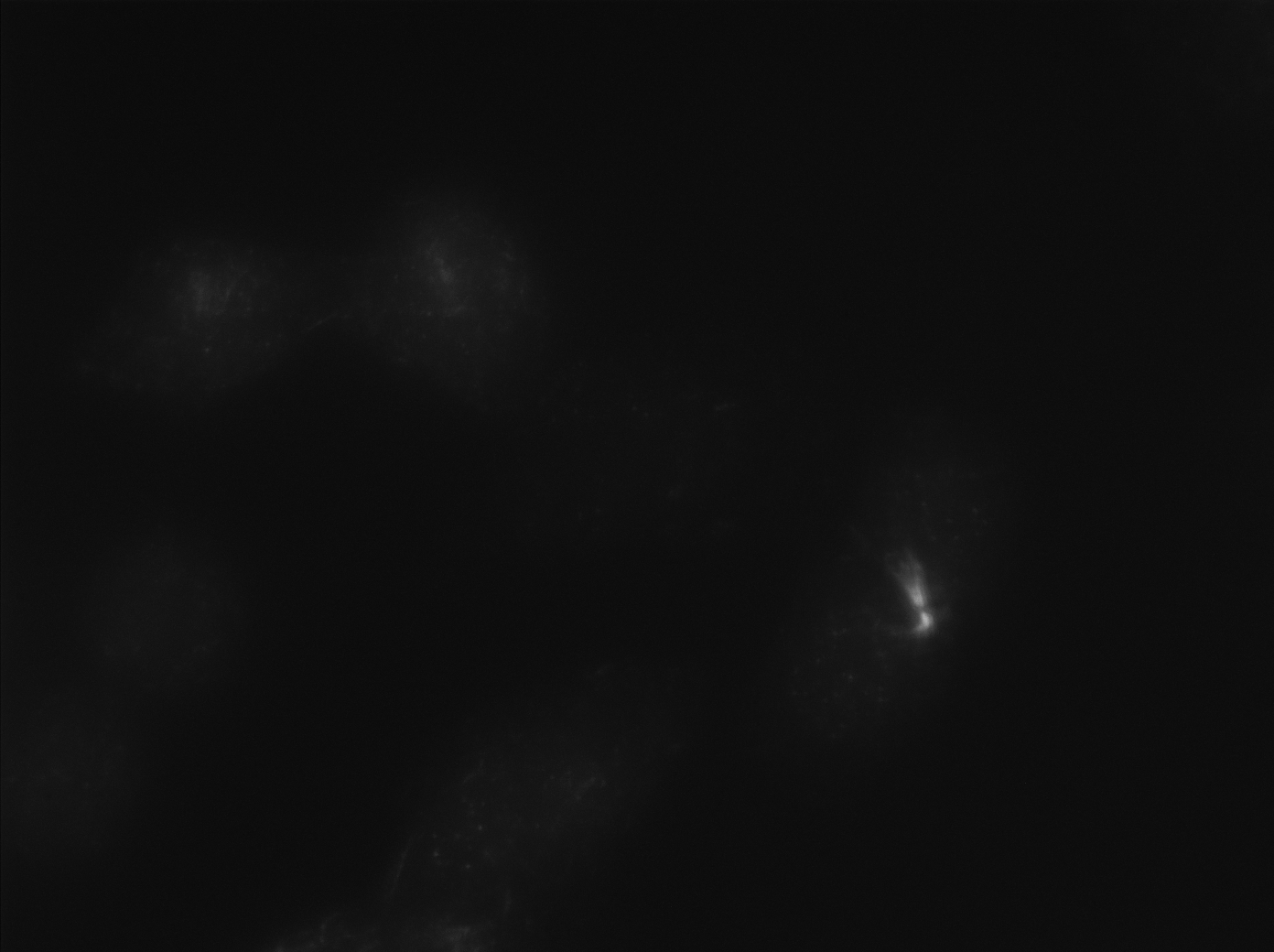

Supplement: Supplementary file 3 — Source data Fig. 1 [file 44319_2026_742_MOESM3_ESM.zip › Figure 1/Fig 1bcd WT Hela acetylated a tubulin atubulin/actub-atub 8-14-24 R1 LT7.Project Maximum Z_XY1724364950_Z0_T0_C2.tif]

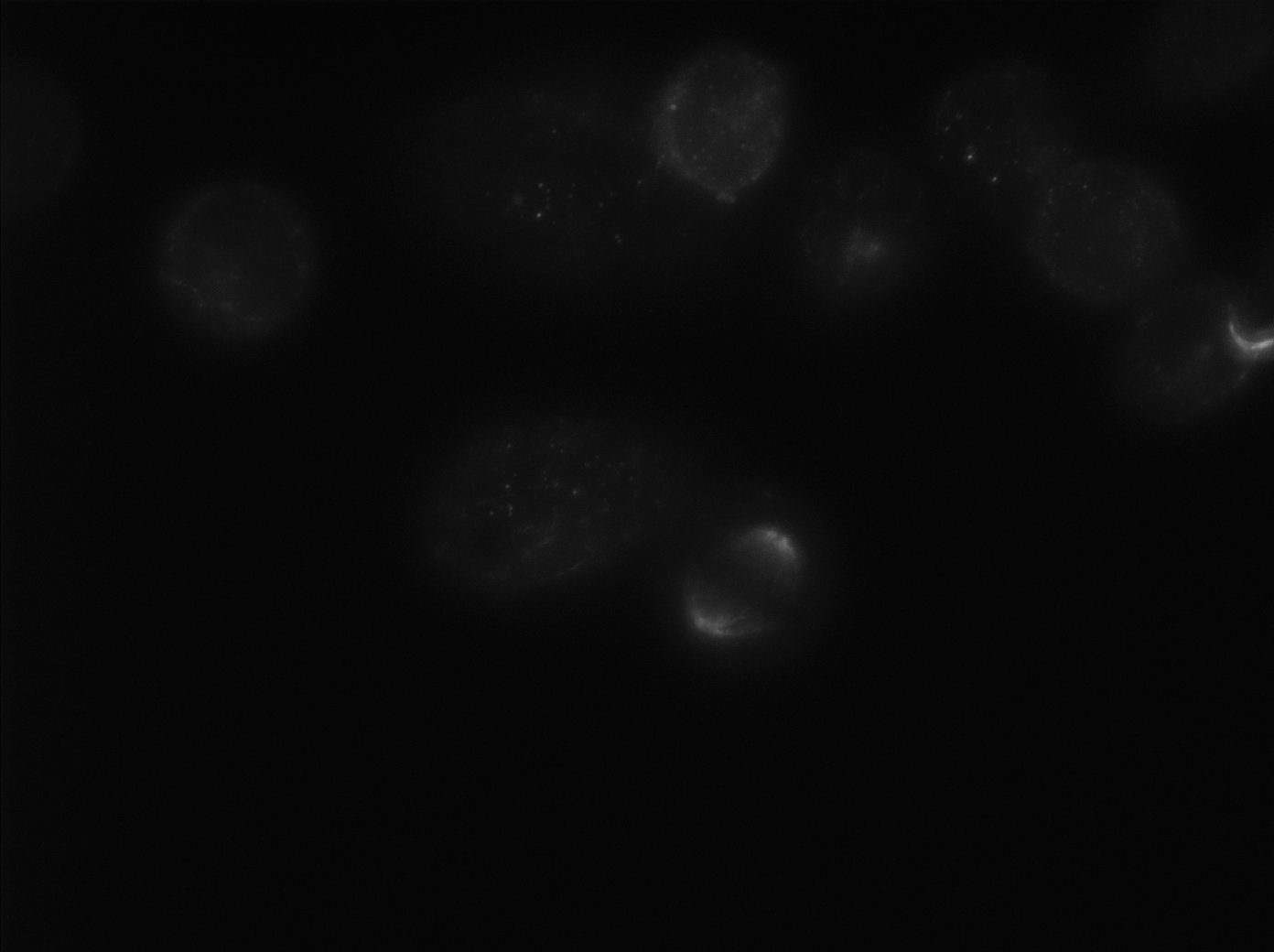

Supplement: Supplementary file 3 — Source data Fig. 1 [file 44319_2026_742_MOESM3_ESM.zip › Figure 1/Fig 1bcd WT Hela acetylated a tubulin atubulin/actub-atub 8-14-24 R3 M9.Project Maximum Z_XY1724703770_Z0_T0_C2.tif]

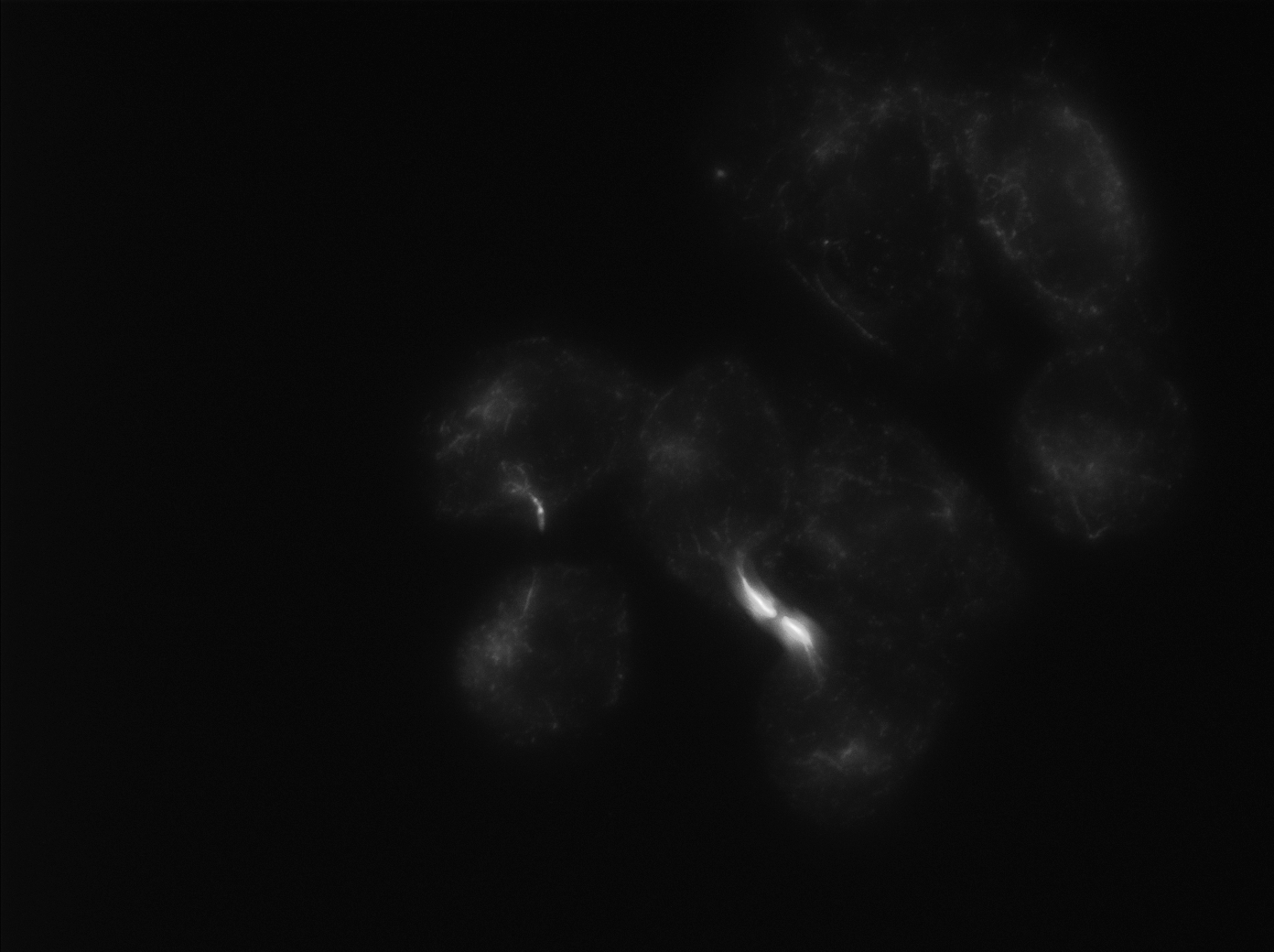

Supplement: Supplementary file 3 — Source data Fig. 1 [file 44319_2026_742_MOESM3_ESM.zip › Figure 1/Fig 1bcd WT Hela acetylated a tubulin atubulin/actub-atub 8-14-24 R1 LT4PA5.Project Maximum Z_XY1724363364_Z0_T0_C2.tif]

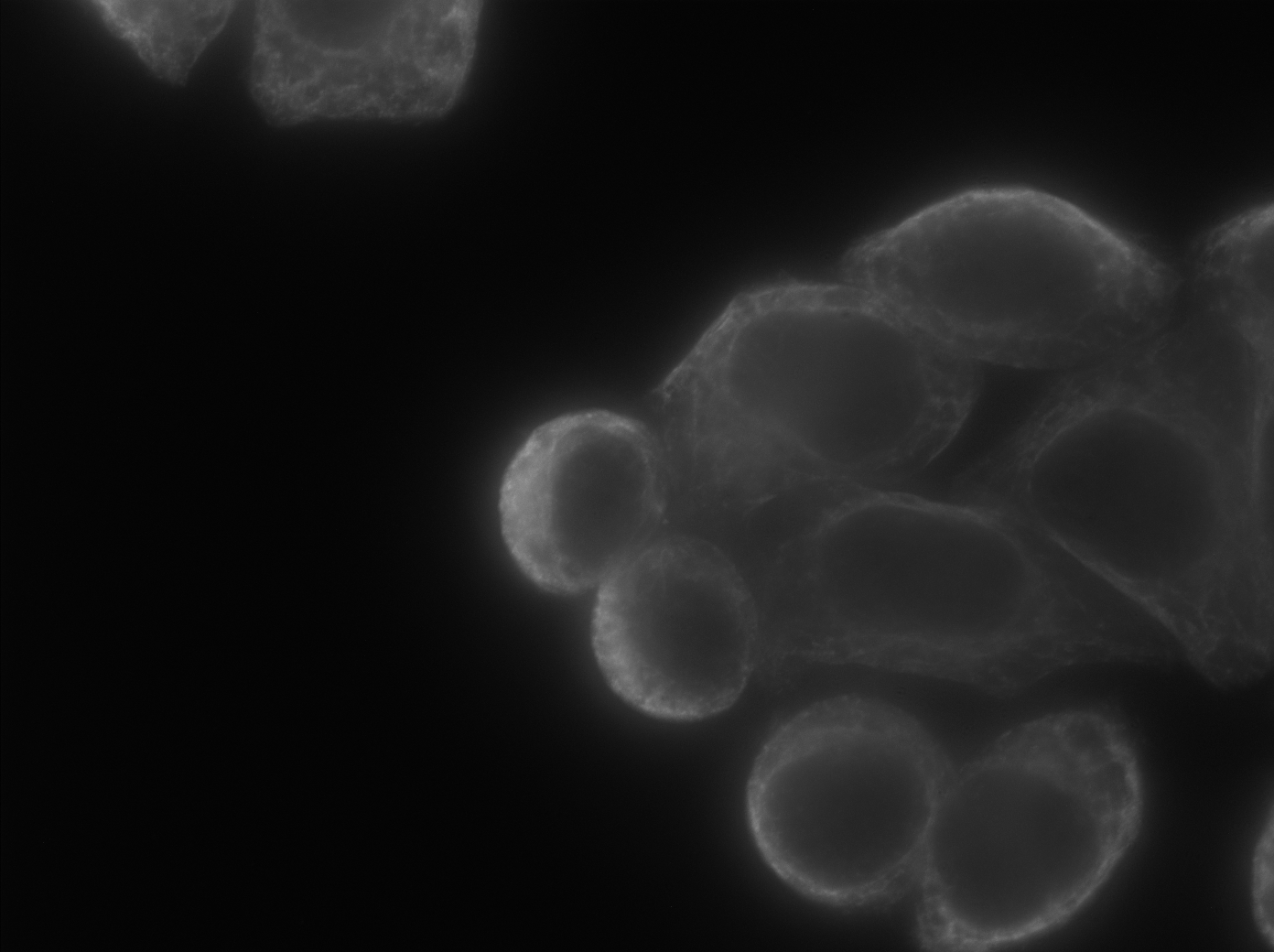

Supplement: Supplementary file 3 — Source data Fig. 1 [file 44319_2026_742_MOESM3_ESM.zip › Figure 1/Fig 1bcd WT Hela acetylated a tubulin atubulin/actub-atub 8-14-24 R2 PA9.Project Maximum Z_XY1724695303_Z0_T0_C1.tif]

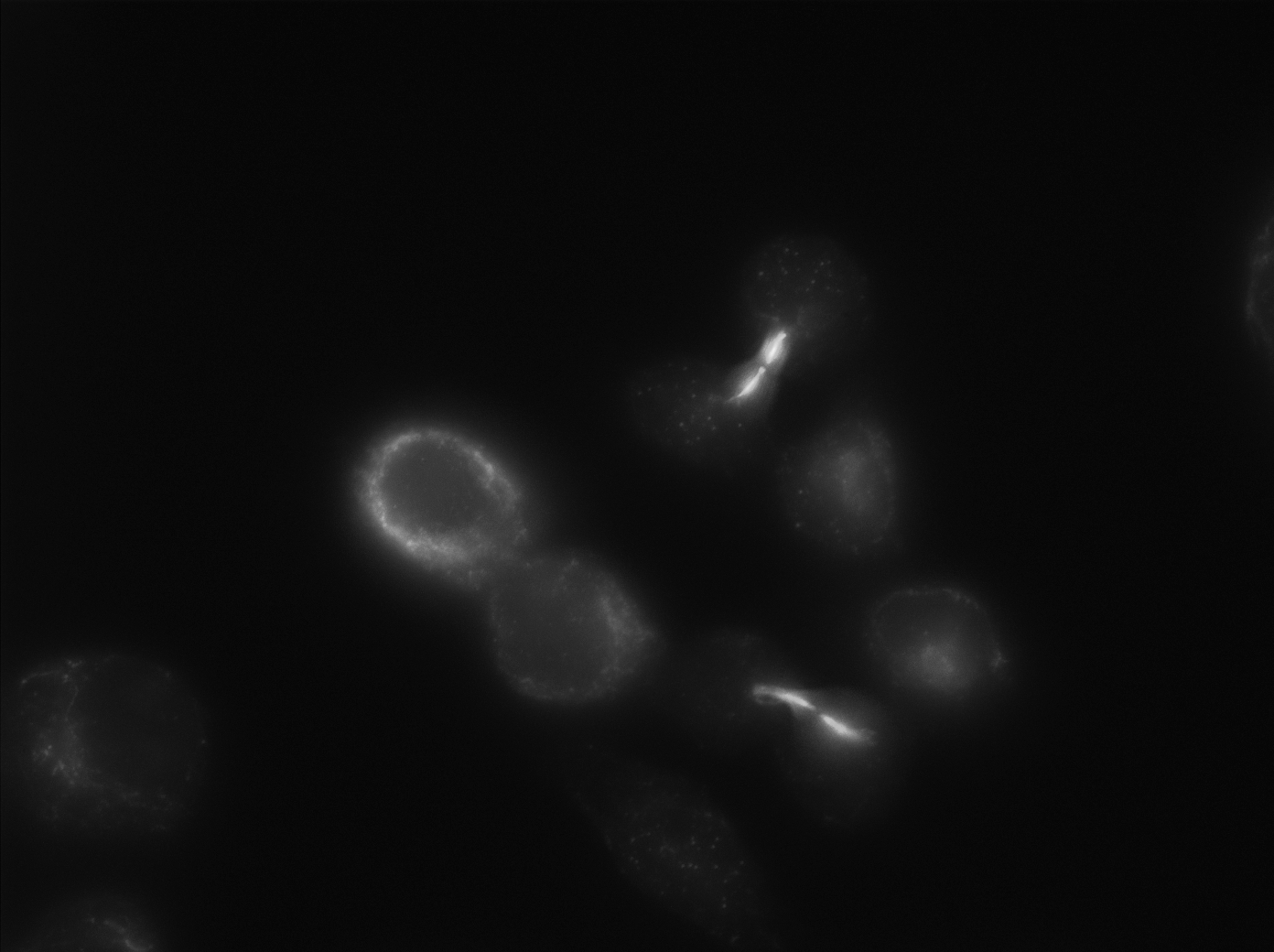

Supplement: Supplementary file 3 — Source data Fig. 1 [file 44319_2026_742_MOESM3_ESM.zip › Figure 1/Fig 1bcd WT Hela acetylated a tubulin atubulin/actub-atub 8-14-24 R1 ET5ET6.Project Maximum Z_XY1724363821_Z0_T0_C2.tif]

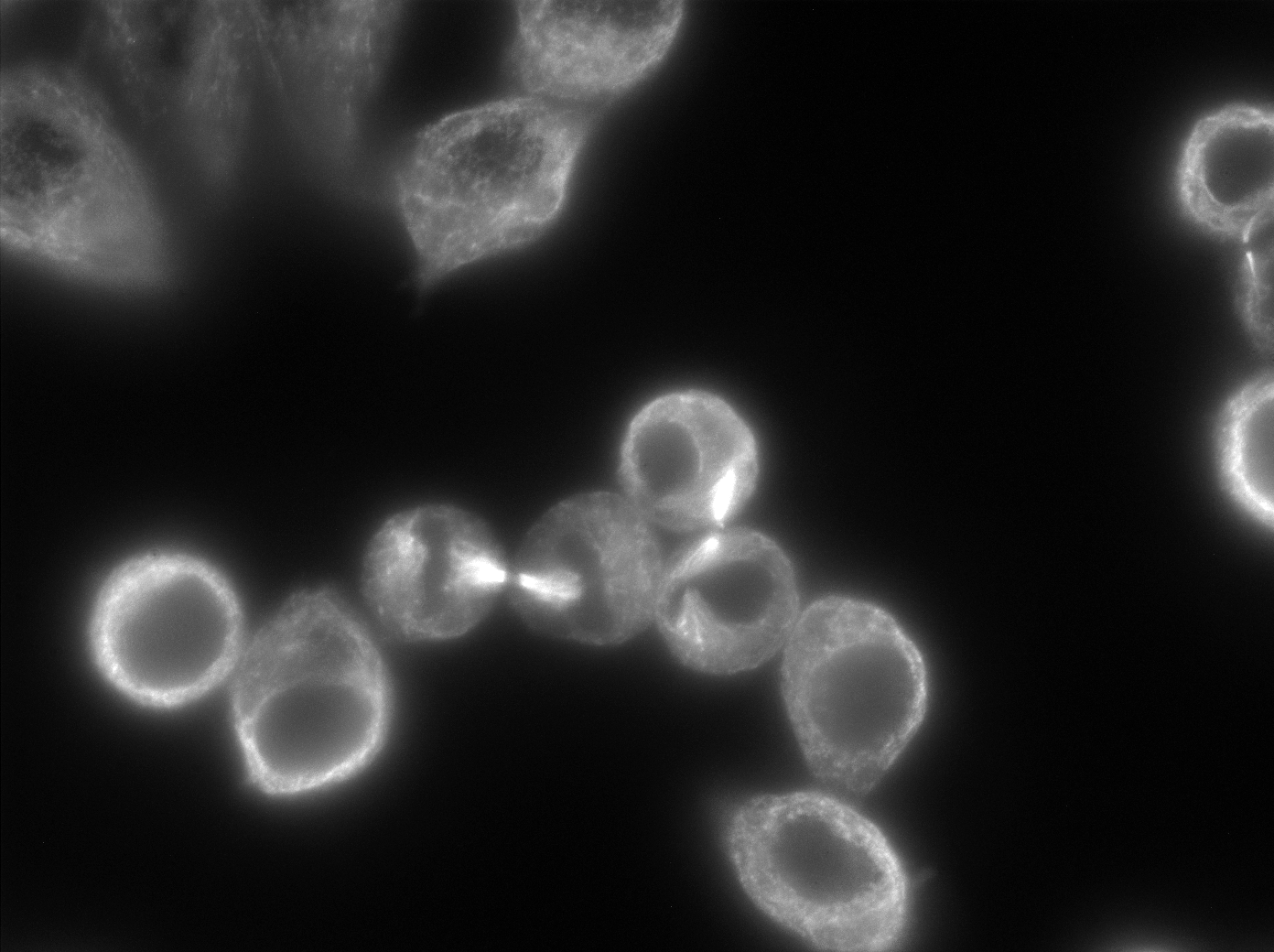

Supplement: Supplementary file 3 — Source data Fig. 1 [file 44319_2026_742_MOESM3_ESM.zip › Figure 1/Fig 1bcd WT Hela acetylated a tubulin atubulin/actub-atub 8-14-24 R3 ET9ET10.Project Maximum Z_XY1724704359_Z0_T0_C1.tif]

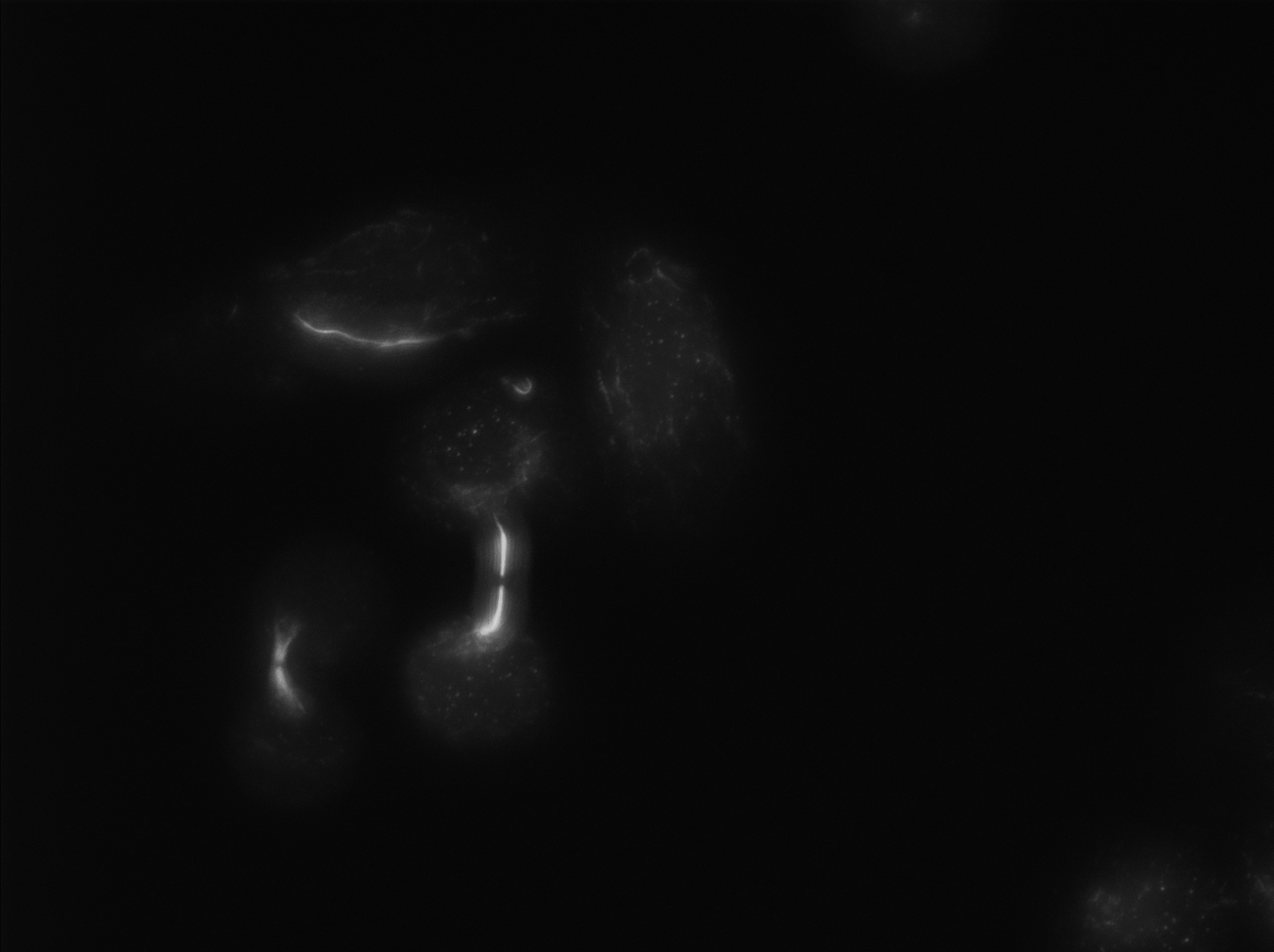

Supplement: Supplementary file 3 — Source data Fig. 1 [file 44319_2026_742_MOESM3_ESM.zip › Figure 1/Fig 1bcd WT Hela acetylated a tubulin atubulin/actub-atub 8-14-24 R2 ET5 LT4.Project Maximum Z_XY1724690247_Z0_T0_C2.tif]

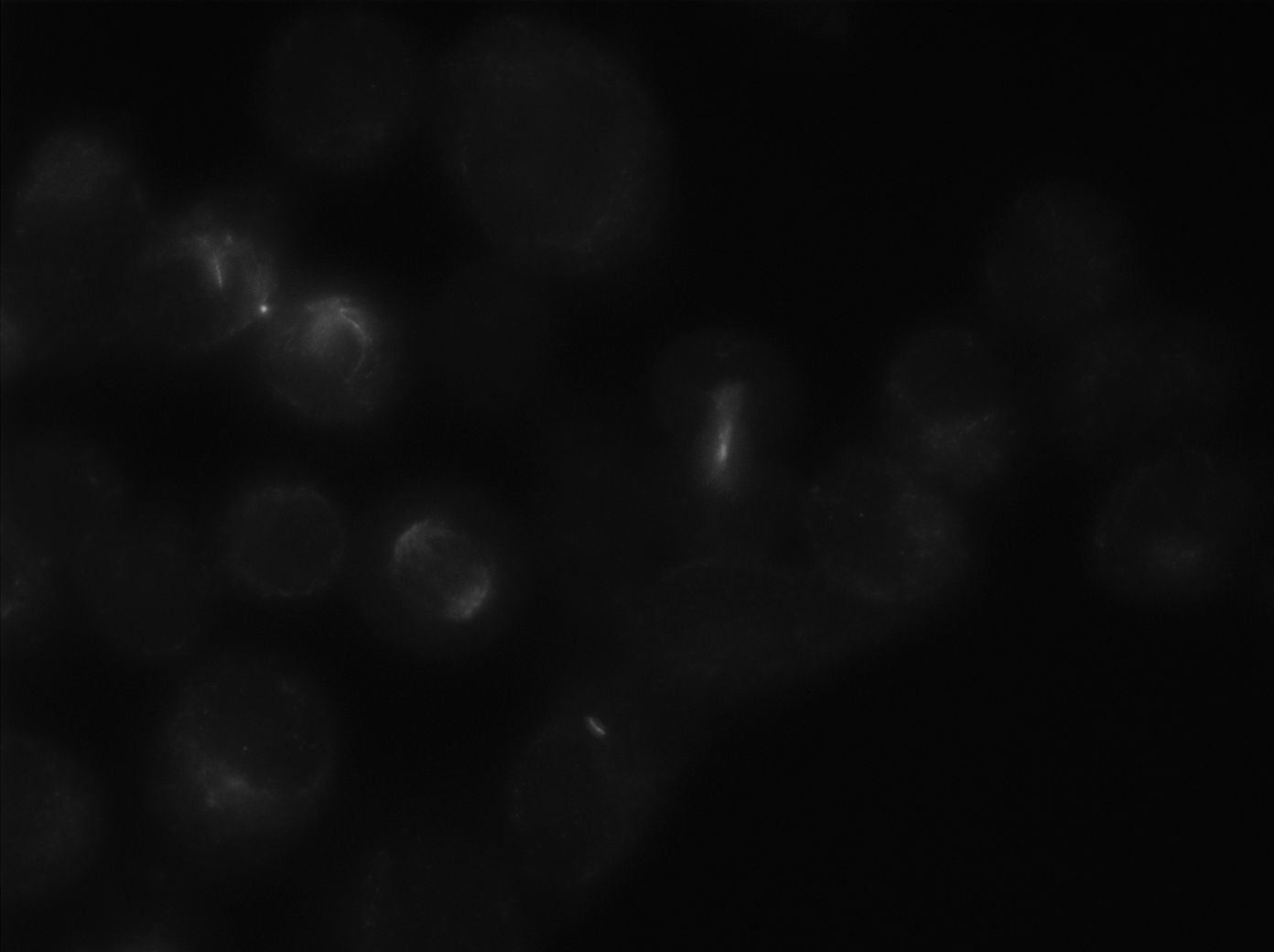

Supplement: Supplementary file 3 — Source data Fig. 1 [file 44319_2026_742_MOESM3_ESM.zip › Figure 1/Fig 1bcd WT Hela acetylated a tubulin atubulin/actub-atub 8-14-24 R3 M1 ET1.Project Maximum Z_XY1724701790_Z0_T0_C2.tif]

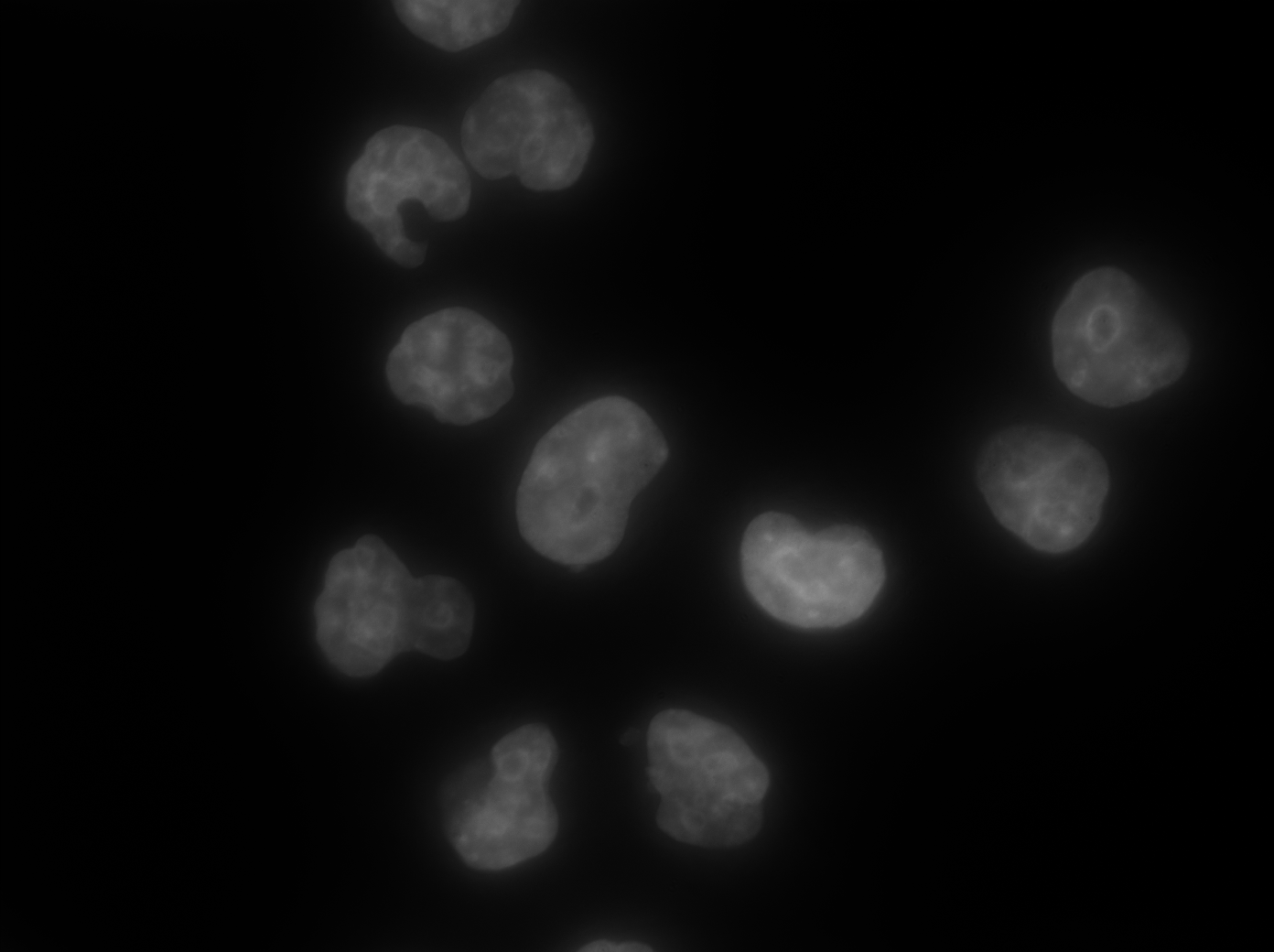

Supplement: Supplementary file 3 — Source data Fig. 1 [file 44319_2026_742_MOESM3_ESM.zip › Figure 1/Fig 1bcd WT Hela acetylated a tubulin atubulin/actub-atub 8-14-24 R1 LT6PA7.Project Maximum Z_XY1724364381_Z0_T0_C0.tif]

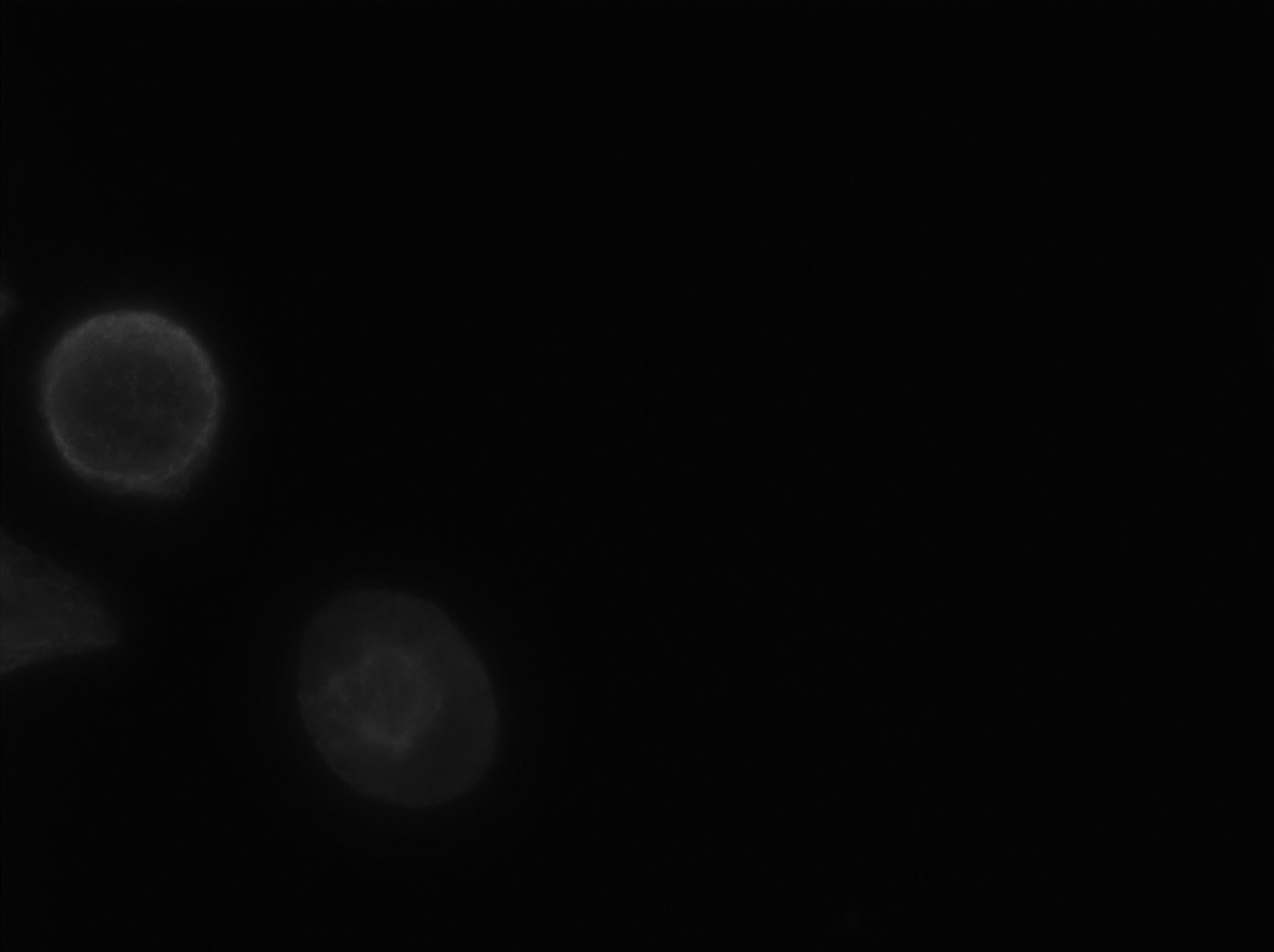

Supplement: Supplementary file 3 — Source data Fig. 1 [file 44319_2026_742_MOESM3_ESM.zip › Figure 1/Fig 1bcd WT Hela acetylated a tubulin atubulin/actub-atub 8-14-24 R2 M1.Project Maximum Z_XY1724690706_Z0_T0_C1.tif]

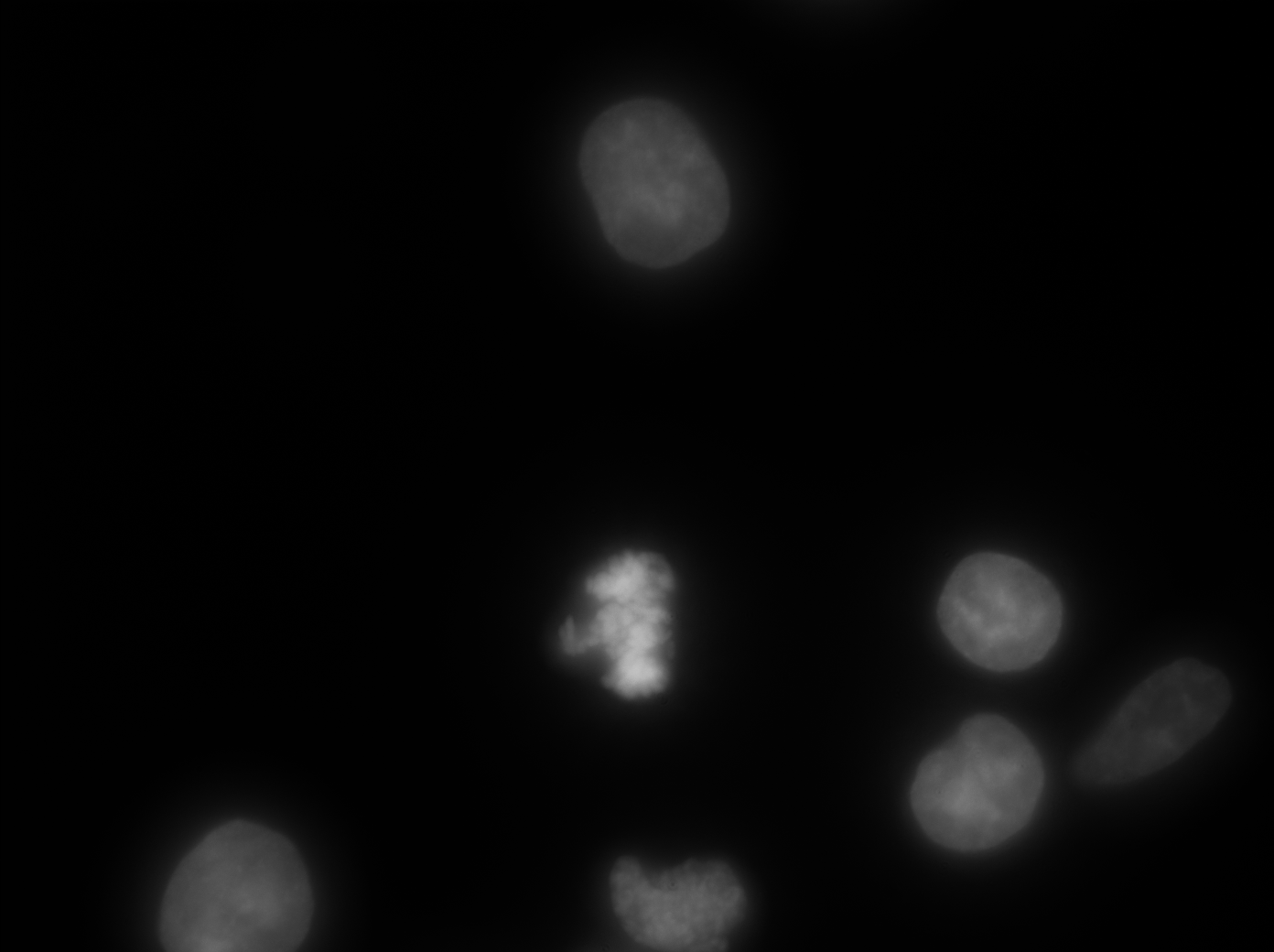

Supplement: Supplementary file 3 — Source data Fig. 1 [file 44319_2026_742_MOESM3_ESM.zip › Figure 1/Fig 1bcd WT Hela acetylated a tubulin atubulin/actub-atub 8-14-24 R1 M10.Project Maximum Z_XY1724367185_Z0_T0_C0.tif]

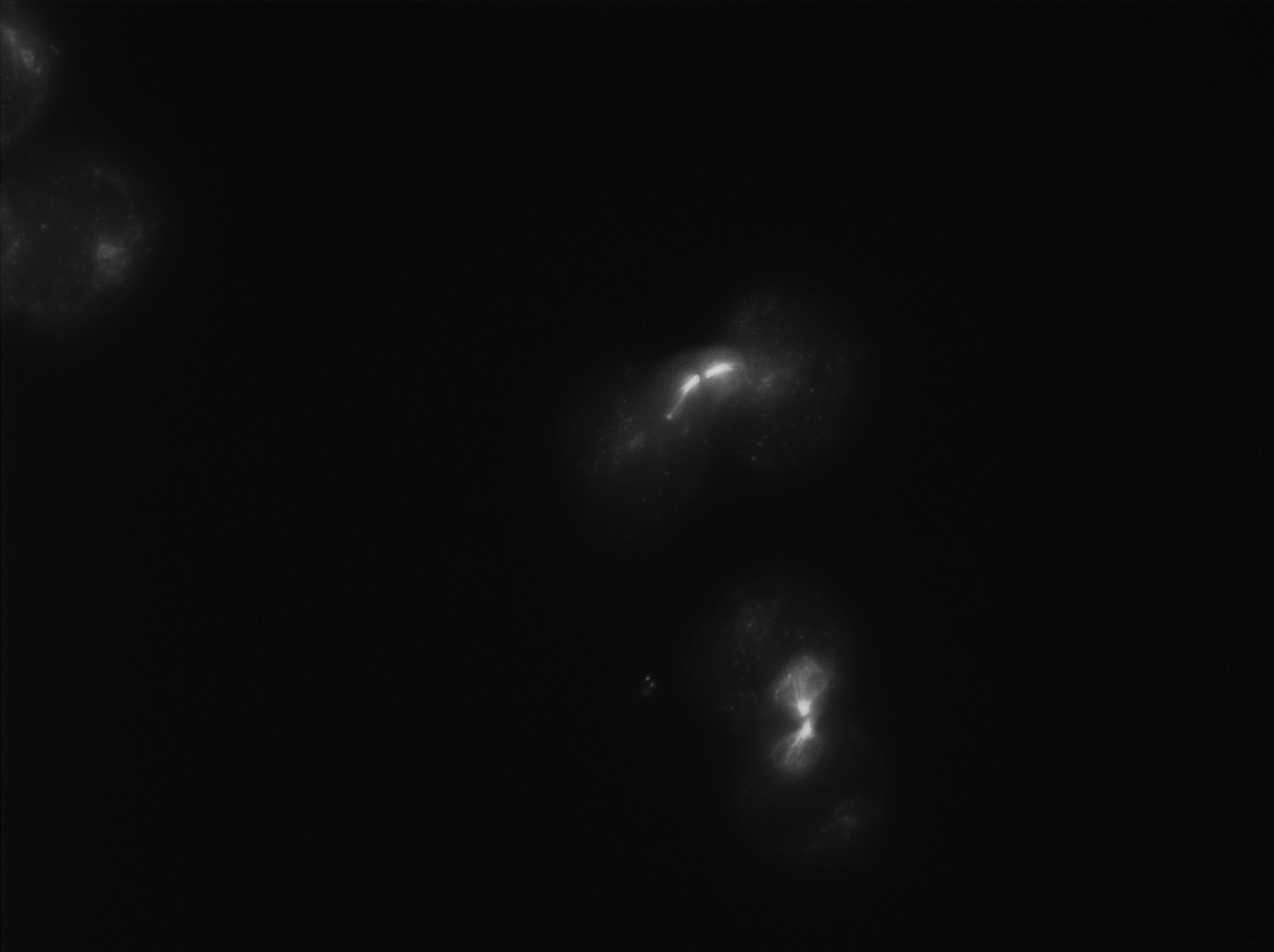

Supplement: Supplementary file 3 — Source data Fig. 1 [file 44319_2026_742_MOESM3_ESM.zip › Figure 1/Fig 1bcd WT Hela acetylated a tubulin atubulin/actub-atub 8-14-24 R1 ET9 LT8.Project Maximum Z_XY1724365615_Z0_T0_C2.tif]

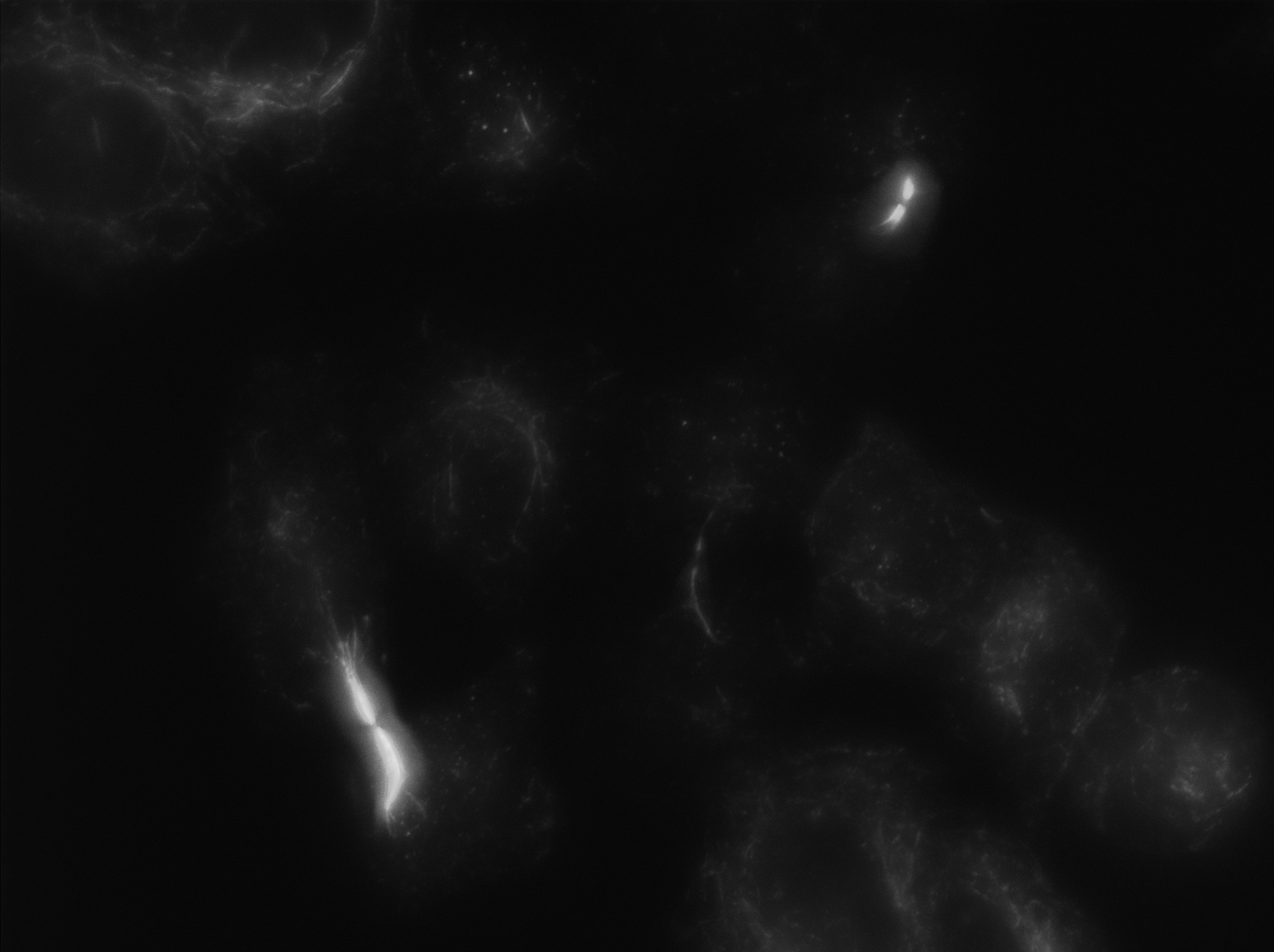

Supplement: Supplementary file 3 — Source data Fig. 1 [file 44319_2026_742_MOESM3_ESM.zip › Figure 1/Fig 1bcd WT Hela acetylated a tubulin atubulin/actub-atub 8-14-24 R2 PA6 LT11LT12.Project Maximum Z_XY1724694664_Z0_T0_C2.tif]

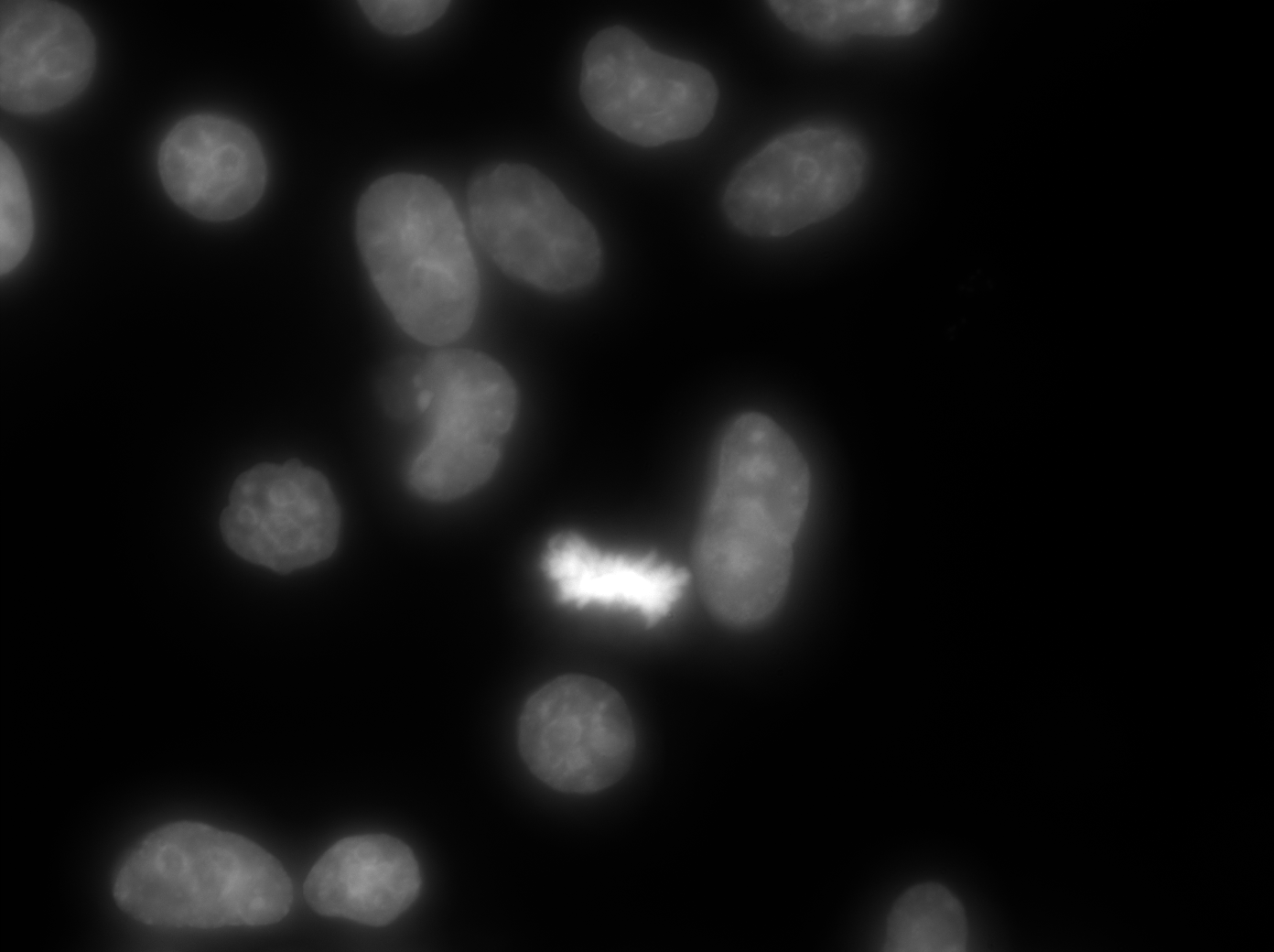

Supplement: Supplementary file 3 — Source data Fig. 1 [file 44319_2026_742_MOESM3_ESM.zip › Figure 1/Fig 1bcd WT Hela acetylated a tubulin atubulin/actub-atub 8-14-24 R3 M7.Project Maximum Z_XY1724703577_Z0_T0_C0.tif]

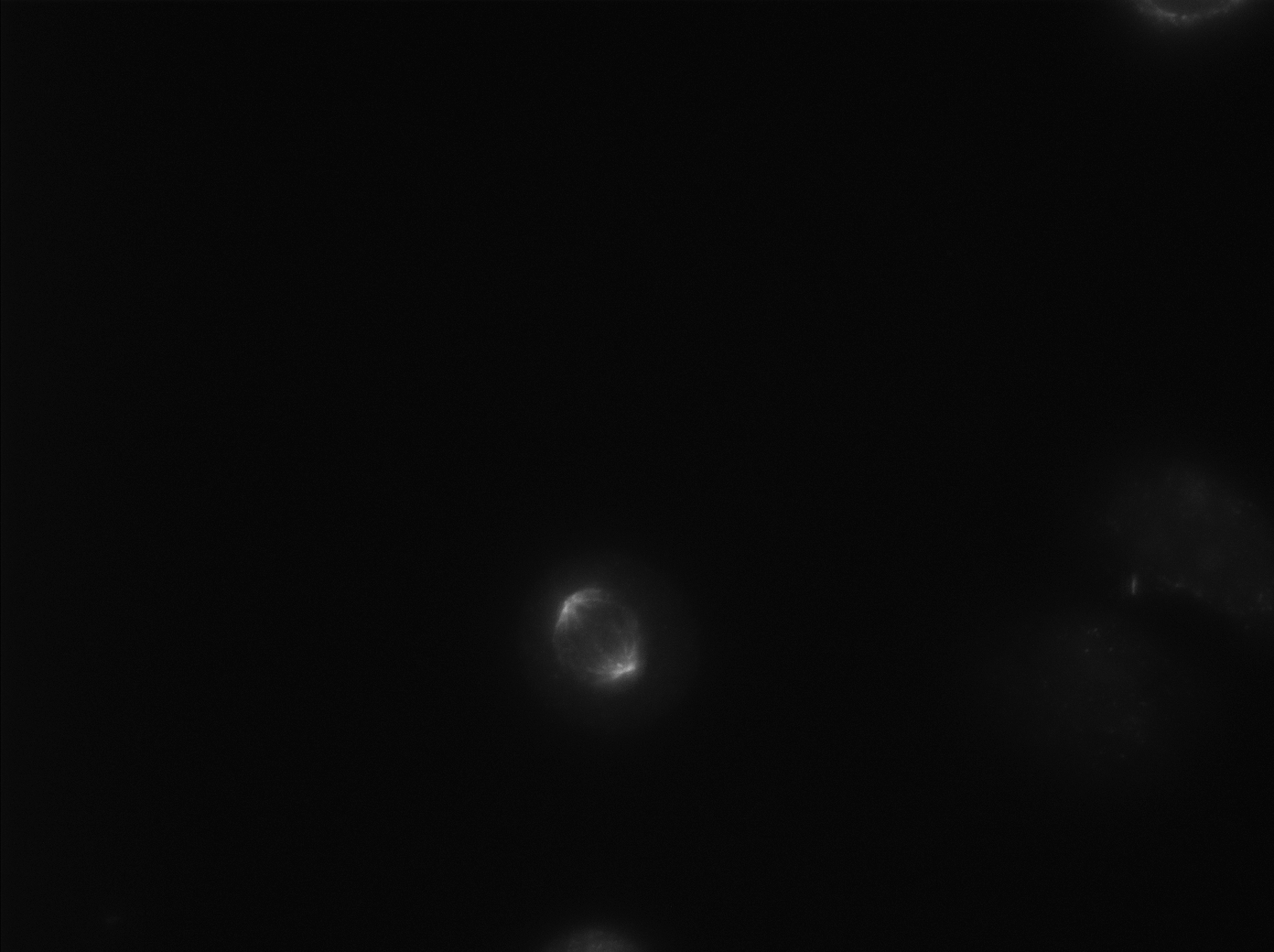

Supplement: Supplementary file 3 — Source data Fig. 1 [file 44319_2026_742_MOESM3_ESM.zip › Figure 1/Fig 1bcd WT Hela acetylated a tubulin atubulin/actub-atub 8-14-24 R1 M2.Project Maximum Z_XY1724363694_Z0_T0_C2.tif]

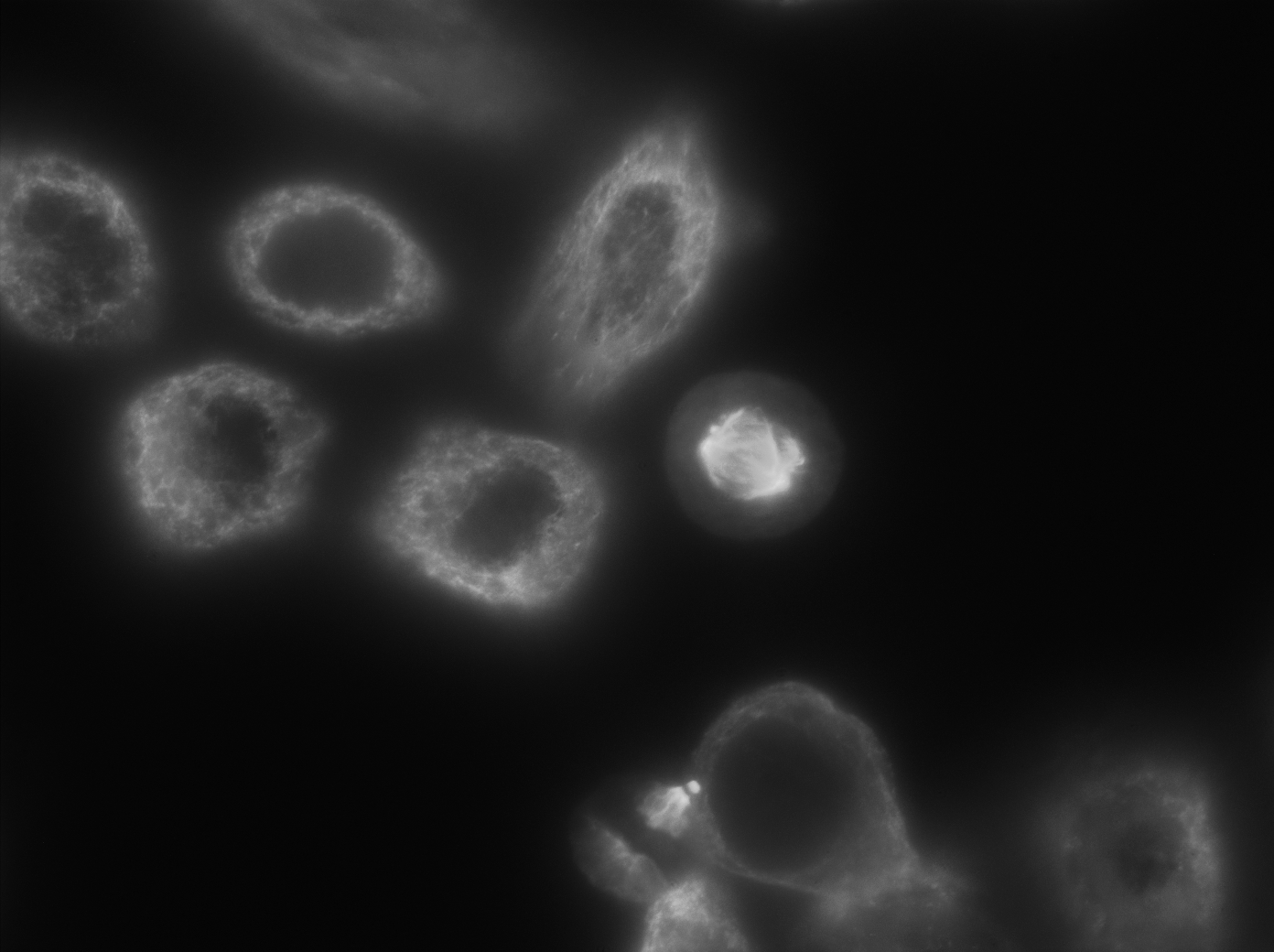

Supplement: Supplementary file 3 — Source data Fig. 1 [file 44319_2026_742_MOESM3_ESM.zip › Figure 1/Fig 1bcd WT Hela acetylated a tubulin atubulin/actub-atub 8-14-24 R1 M8.Project Maximum Z_XY1724366252_Z0_T0_C1.tif]

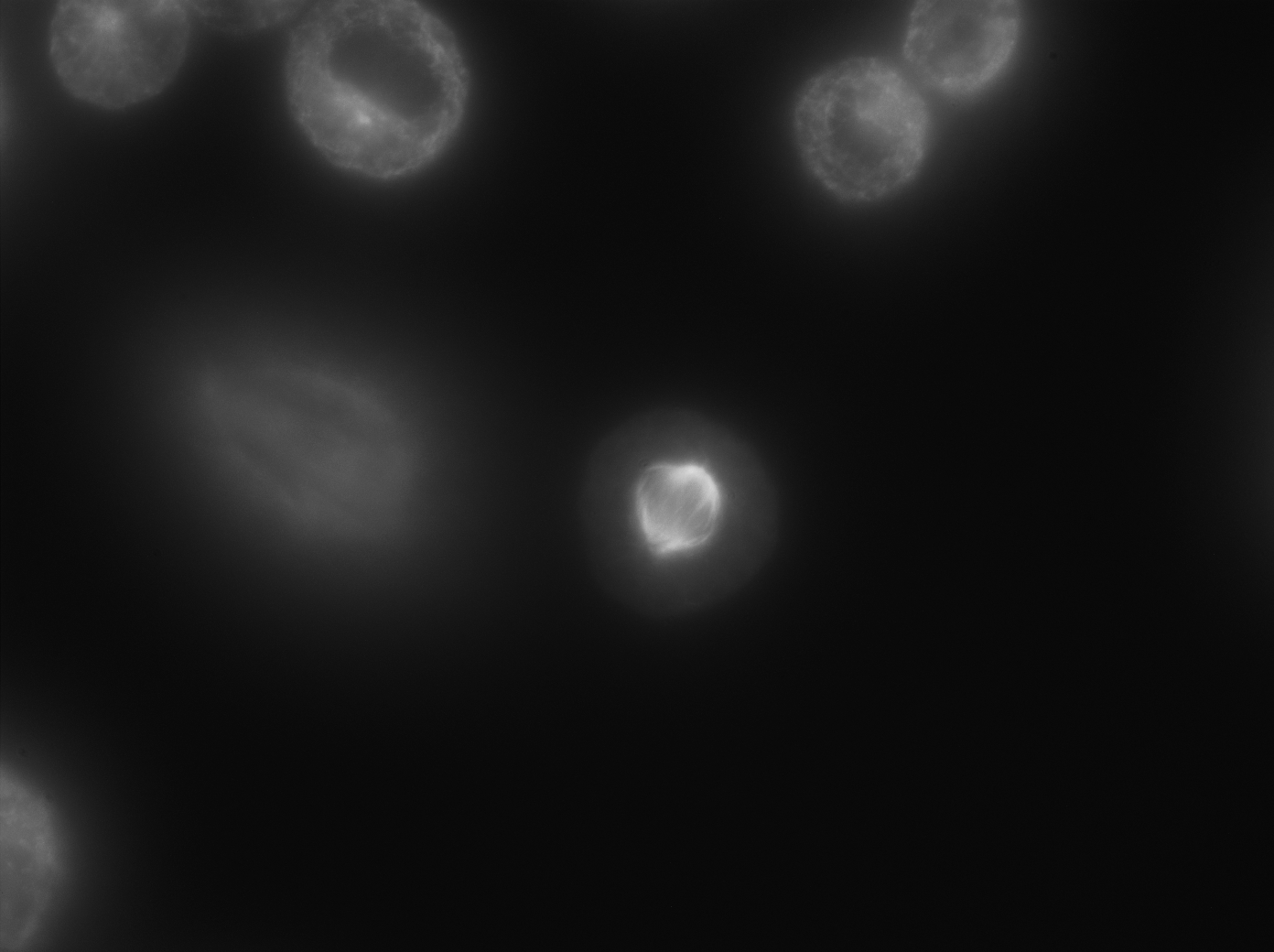

Supplement: Supplementary file 3 — Source data Fig. 1 [file 44319_2026_742_MOESM3_ESM.zip › Figure 1/Fig 1bcd WT Hela acetylated a tubulin atubulin/actub-atub 8-14-24 R2 M5.Project Maximum Z_XY1724693767_Z0_T0_C1.tif]

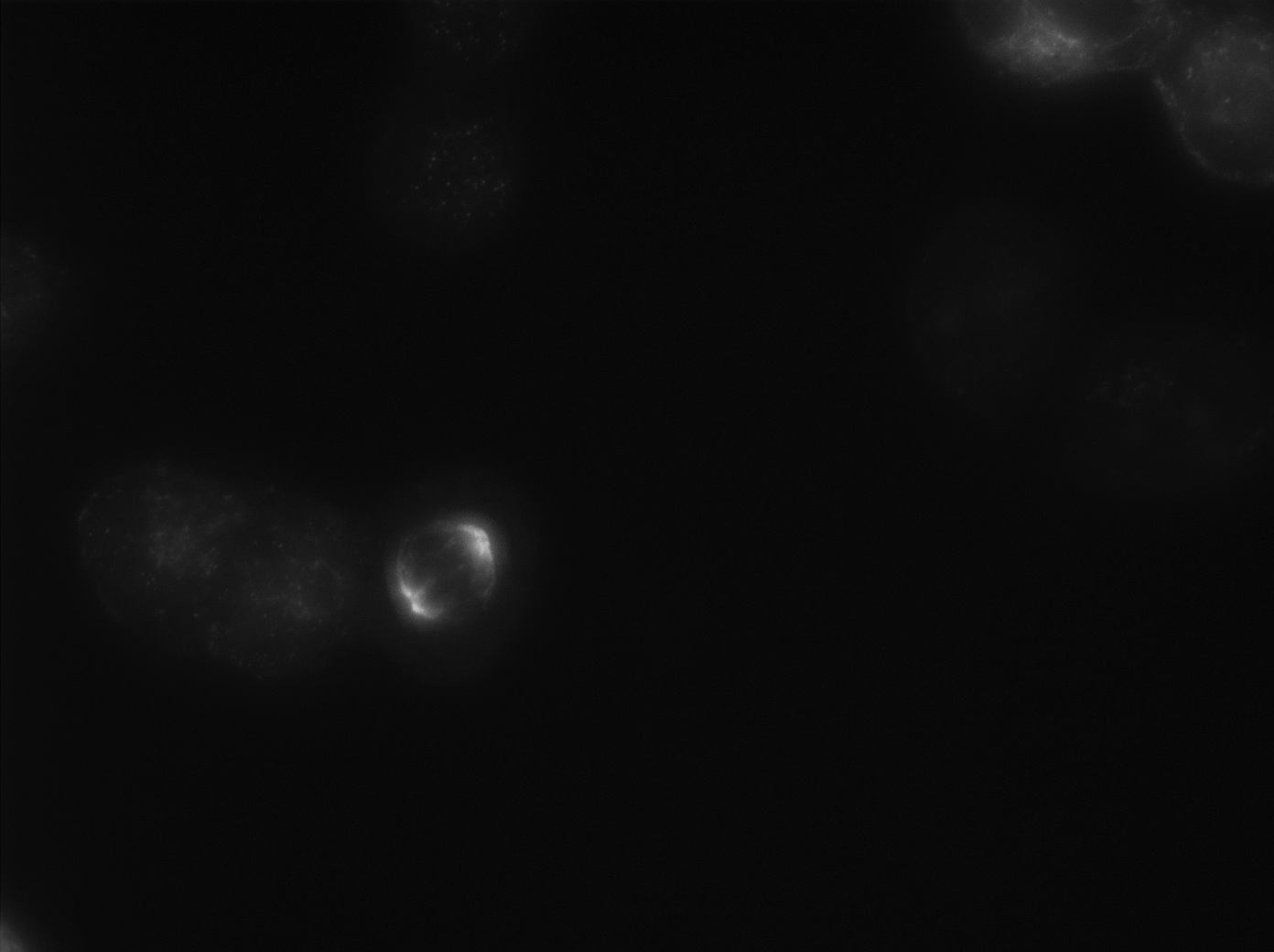

Supplement: Supplementary file 3 — Source data Fig. 1 [file 44319_2026_742_MOESM3_ESM.zip › Figure 1/Fig 1bcd WT Hela acetylated a tubulin atubulin/actub-atub 8-14-24 R1 M3.Project Maximum Z_XY1724364163_Z0_T0_C2.tif]

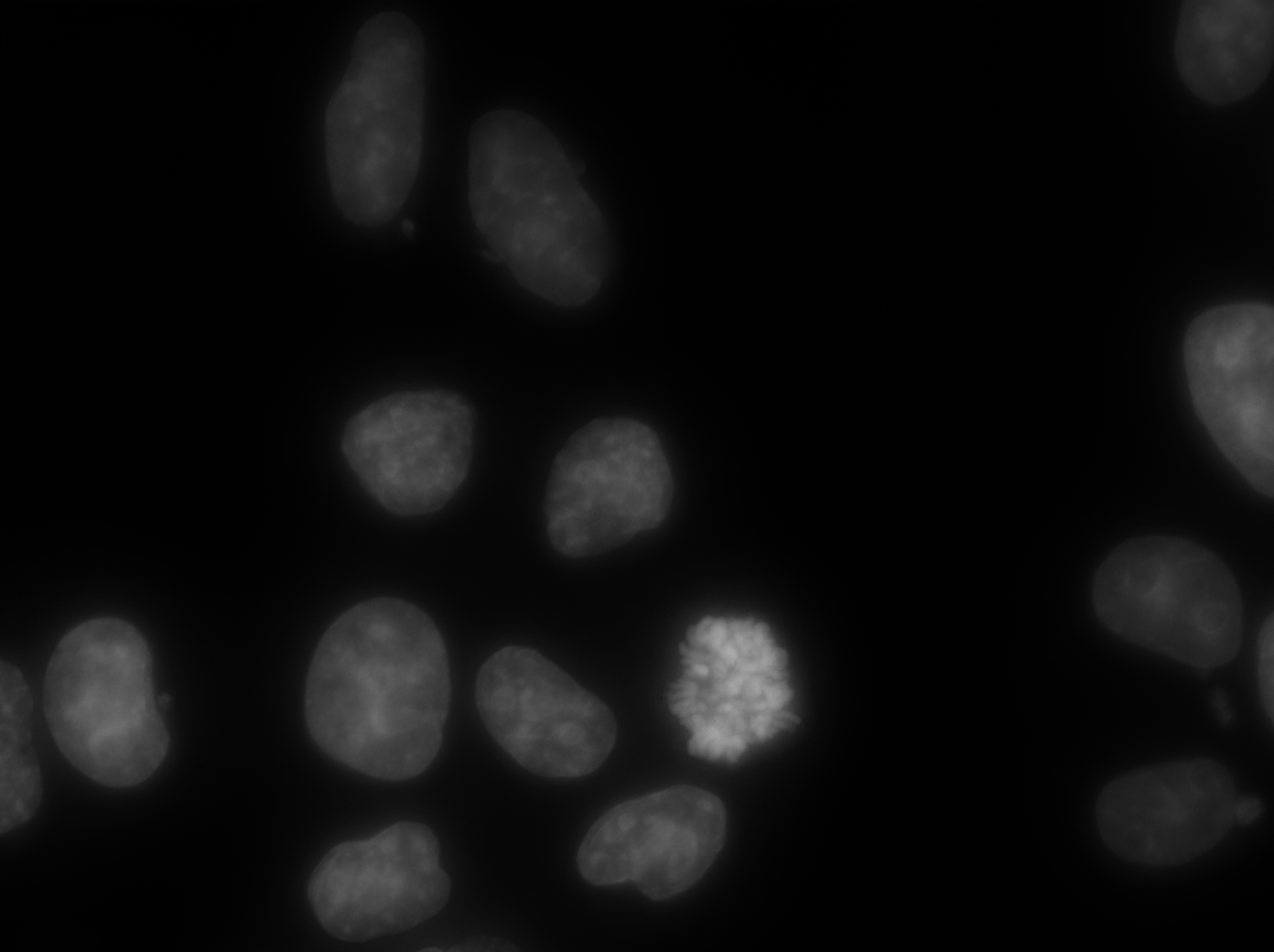

Supplement: Supplementary file 3 — Source data Fig. 1 [file 44319_2026_742_MOESM3_ESM.zip › Figure 1/Fig 1bcd WT Hela acetylated a tubulin atubulin/actub-atub 8-14-24 R2 M10.Project Maximum Z_XY1724695478_Z0_T0_C0.tif]

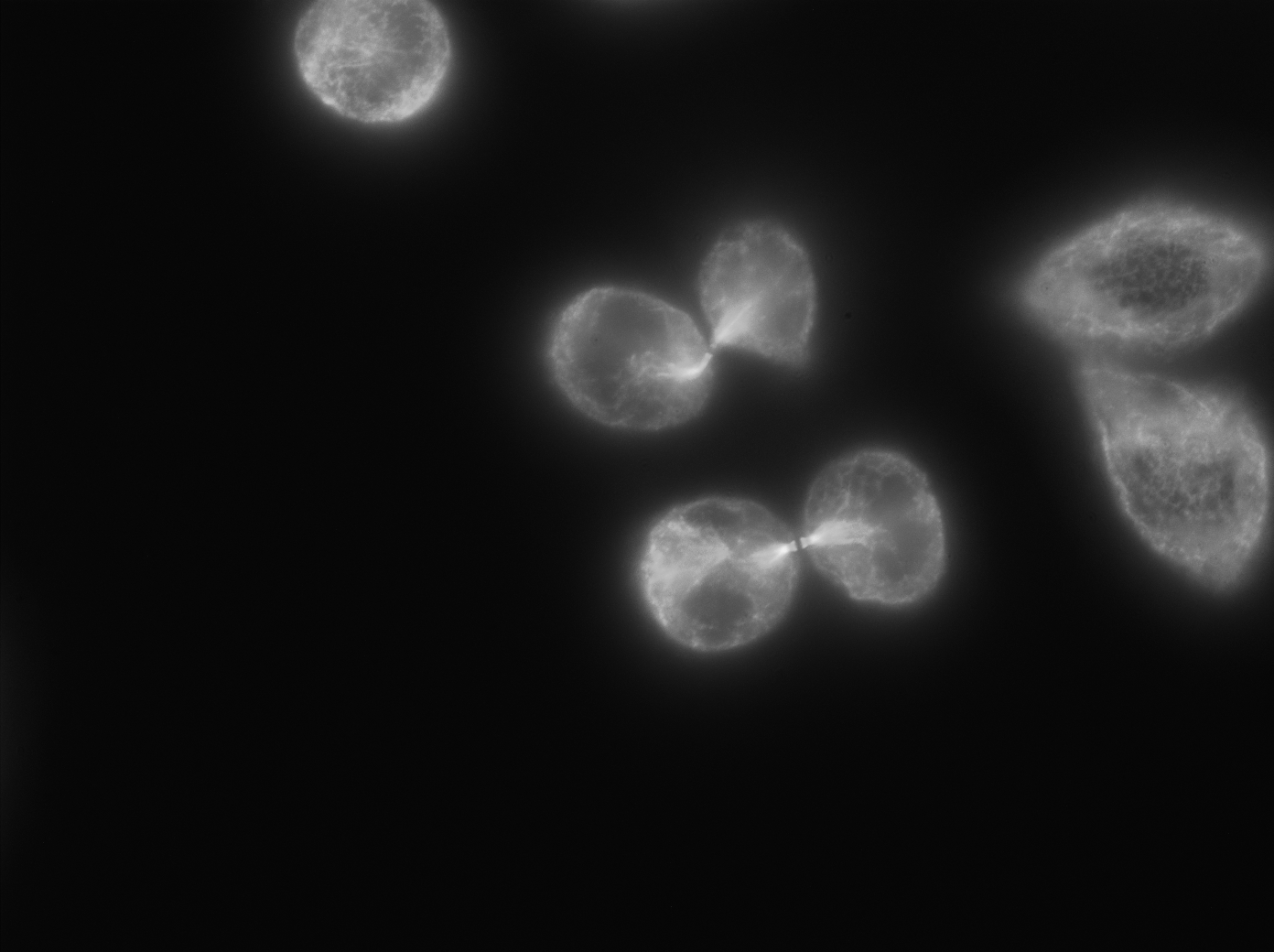

Supplement: Supplementary file 3 — Source data Fig. 1 [file 44319_2026_742_MOESM3_ESM.zip › Figure 1/Fig 1bcd WT Hela acetylated a tubulin atubulin/actub-atub 8-14-24 R1 ET10ET11.Project Maximum Z_XY1724366427_Z0_T0_C1.tif]

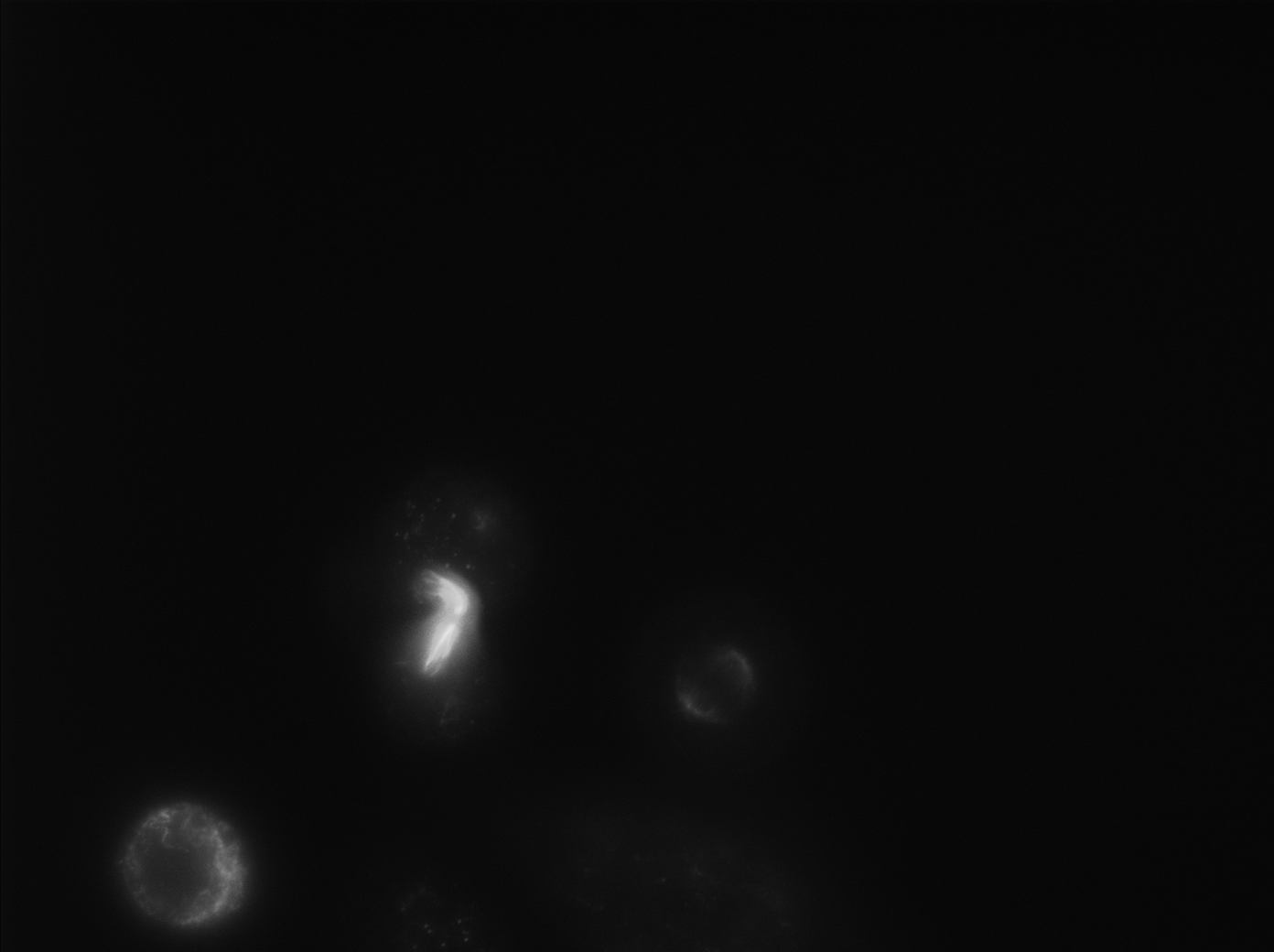

Supplement: Supplementary file 3 — Source data Fig. 1 [file 44319_2026_742_MOESM3_ESM.zip › Figure 1/Fig 1bcd WT Hela acetylated a tubulin atubulin/actub-atub 8-14-24 R2 ET9 M8.Project Maximum Z_XY1724694377_Z0_T0_C2.tif]

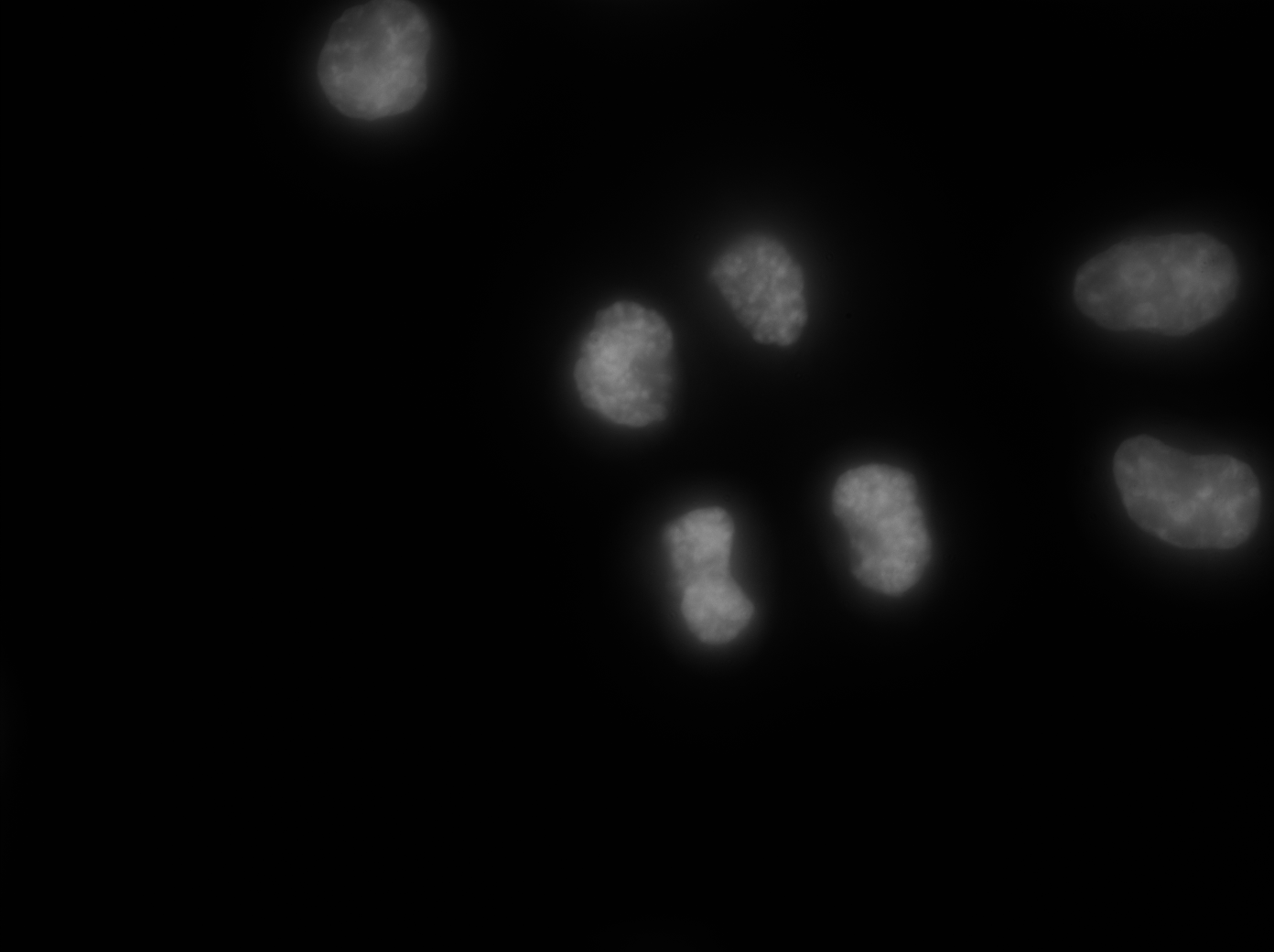

Supplement: Supplementary file 3 — Source data Fig. 1 [file 44319_2026_742_MOESM3_ESM.zip › Figure 1/Fig 1bcd WT Hela acetylated a tubulin atubulin/actub-atub 8-14-24 R1 ET10ET11.Project Maximum Z_XY1724366427_Z0_T0_C0.tif]

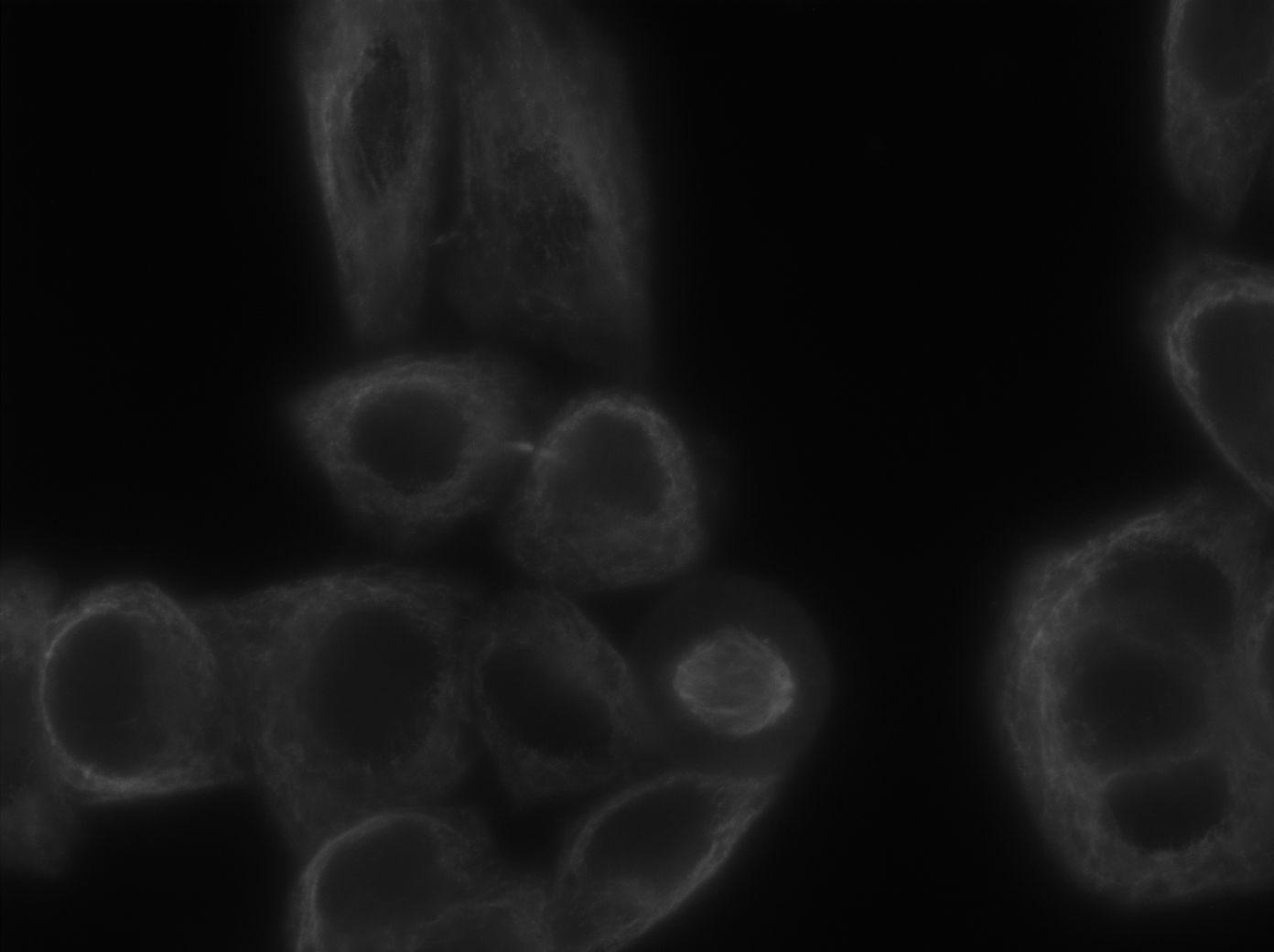

Supplement: Supplementary file 3 — Source data Fig. 1 [file 44319_2026_742_MOESM3_ESM.zip › Figure 1/Fig 1bcd WT Hela acetylated a tubulin atubulin/actub-atub 8-14-24 R2 M10.Project Maximum Z_XY1724695478_Z0_T0_C1.tif]

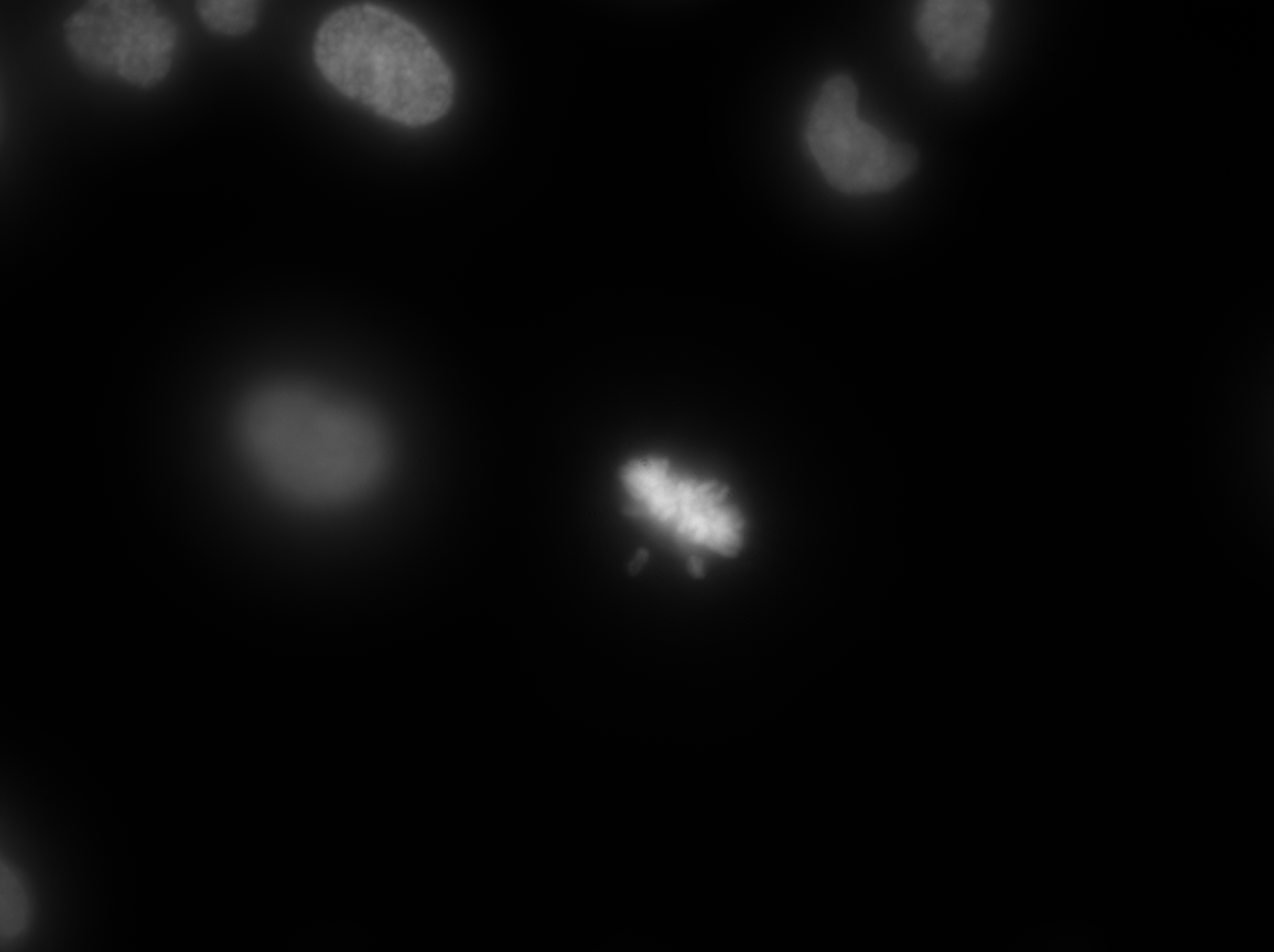

Supplement: Supplementary file 3 — Source data Fig. 1 [file 44319_2026_742_MOESM3_ESM.zip › Figure 1/Fig 1bcd WT Hela acetylated a tubulin atubulin/actub-atub 8-14-24 R2 M5.Project Maximum Z_XY1724693767_Z0_T0_C0.tif]

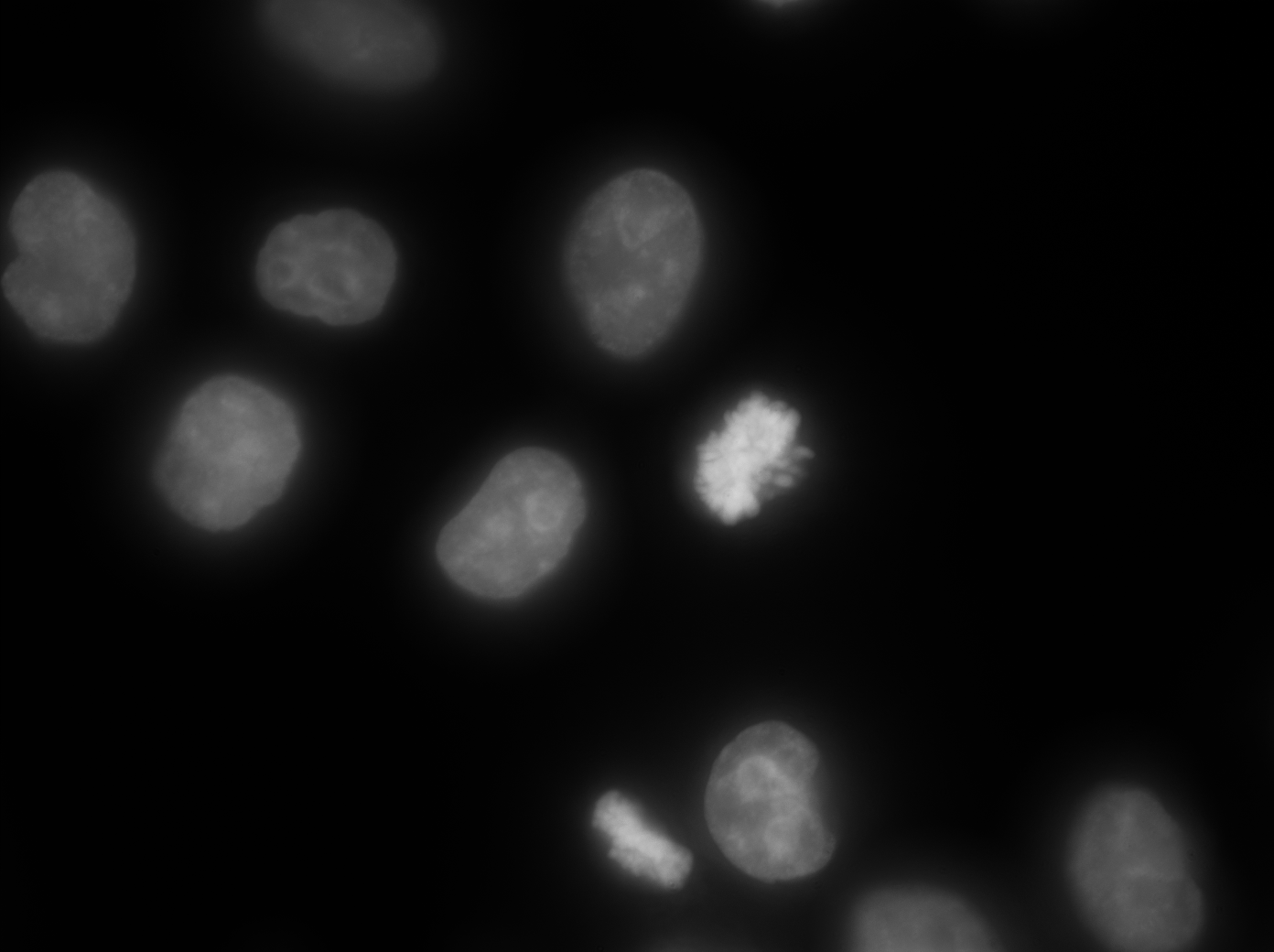

Supplement: Supplementary file 3 — Source data Fig. 1 [file 44319_2026_742_MOESM3_ESM.zip › Figure 1/Fig 1bcd WT Hela acetylated a tubulin atubulin/actub-atub 8-14-24 R1 M8.Project Maximum Z_XY1724366252_Z0_T0_C0.tif]

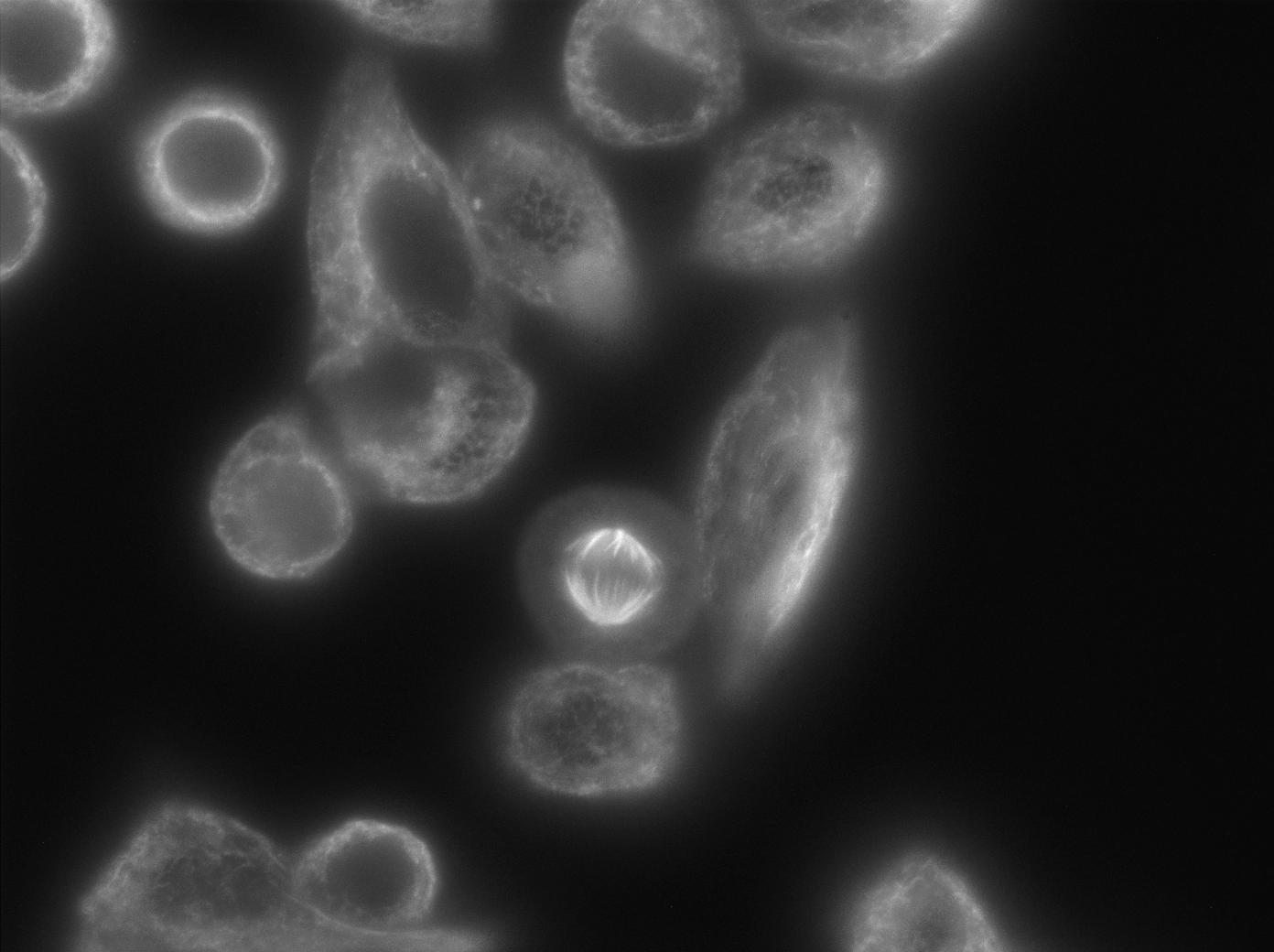

Supplement: Supplementary file 3 — Source data Fig. 1 [file 44319_2026_742_MOESM3_ESM.zip › Figure 1/Fig 1bcd WT Hela acetylated a tubulin atubulin/actub-atub 8-14-24 R3 M7.Project Maximum Z_XY1724703577_Z0_T0_C1.tif]

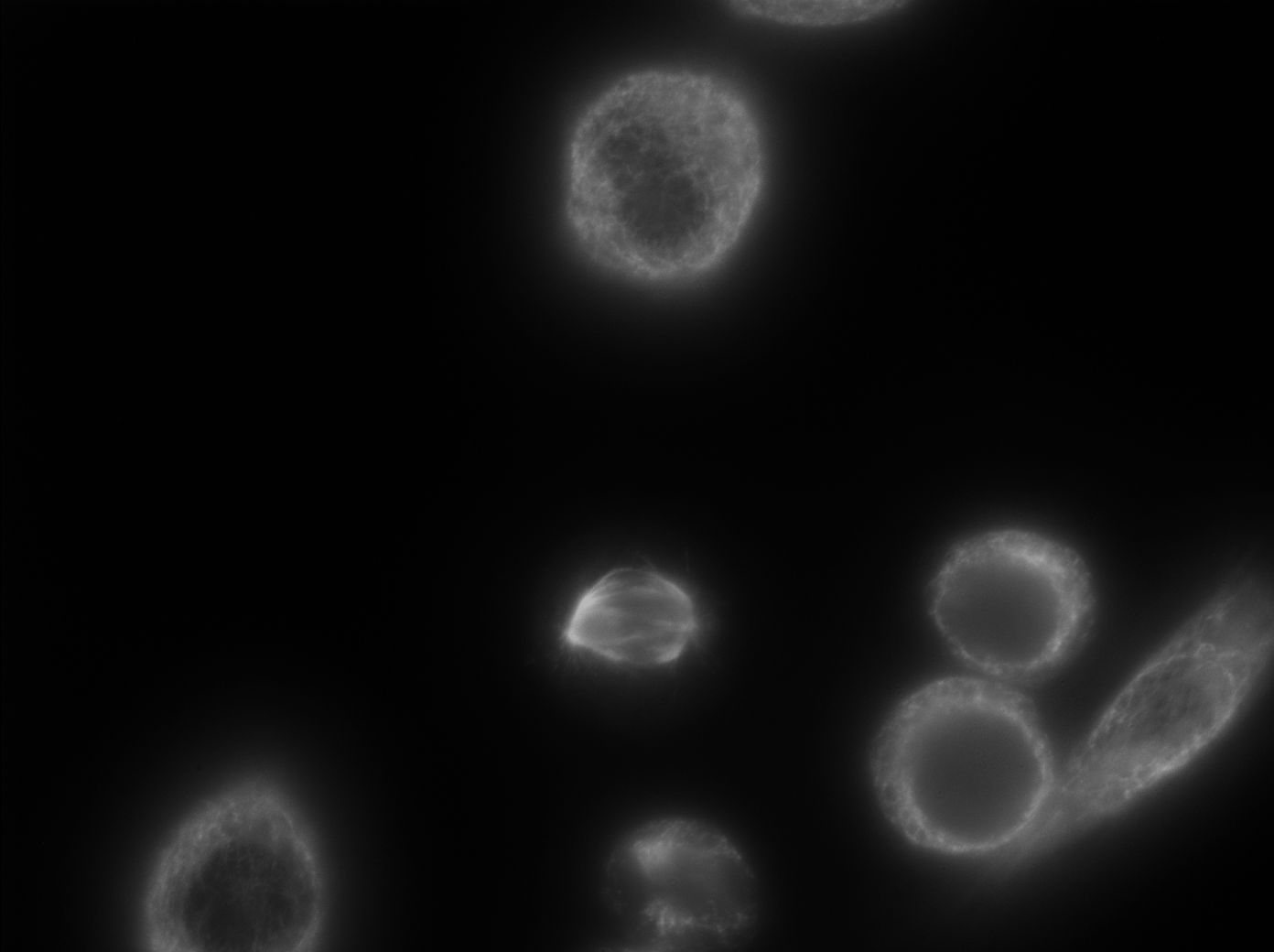

Supplement: Supplementary file 3 — Source data Fig. 1 [file 44319_2026_742_MOESM3_ESM.zip › Figure 1/Fig 1bcd WT Hela acetylated a tubulin atubulin/actub-atub 8-14-24 R1 M10.Project Maximum Z_XY1724367185_Z0_T0_C1.tif]

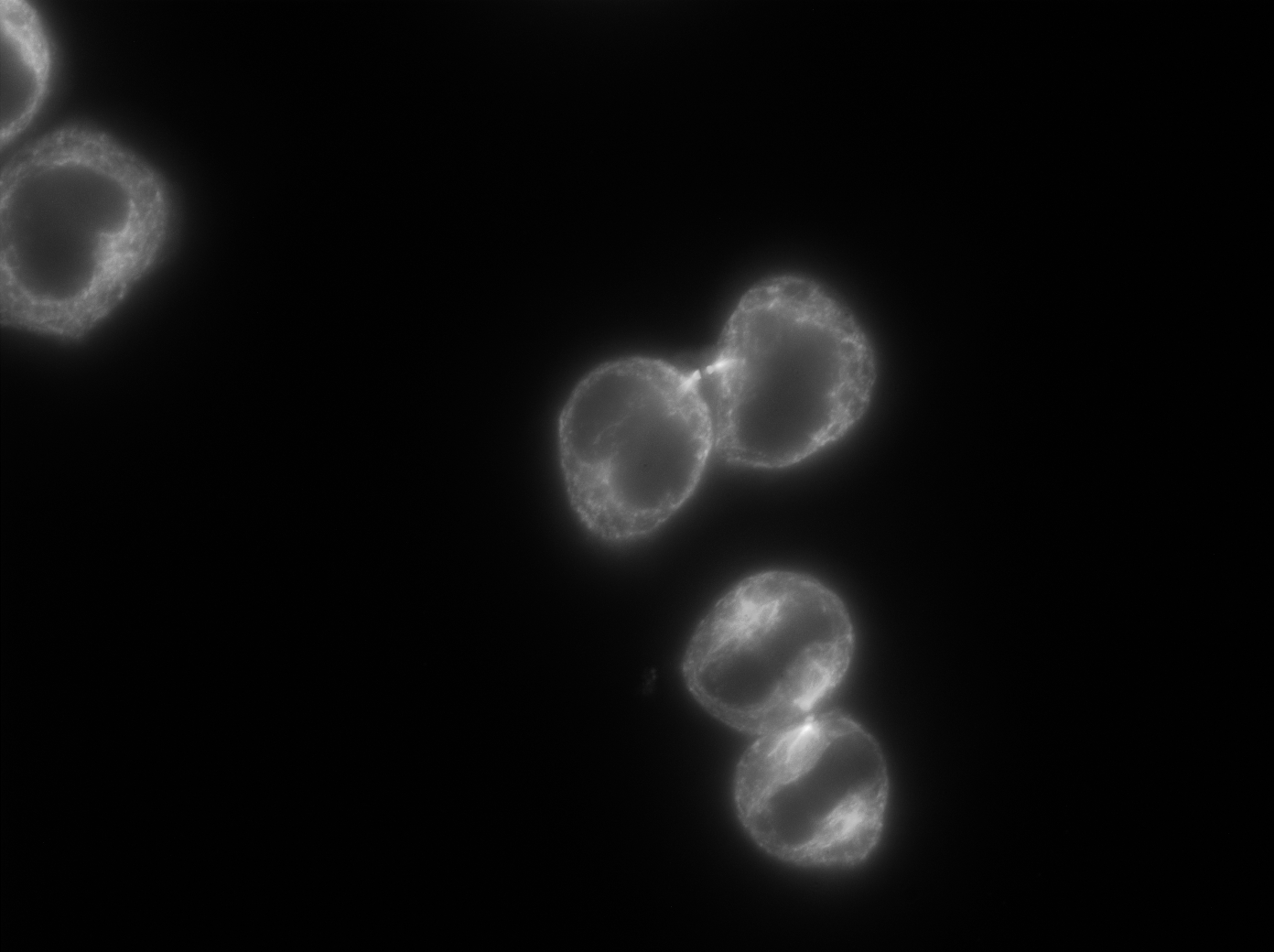

Supplement: Supplementary file 3 — Source data Fig. 1 [file 44319_2026_742_MOESM3_ESM.zip › Figure 1/Fig 1bcd WT Hela acetylated a tubulin atubulin/actub-atub 8-14-24 R1 ET9 LT8.Project Maximum Z_XY1724365615_Z0_T0_C1.tif]

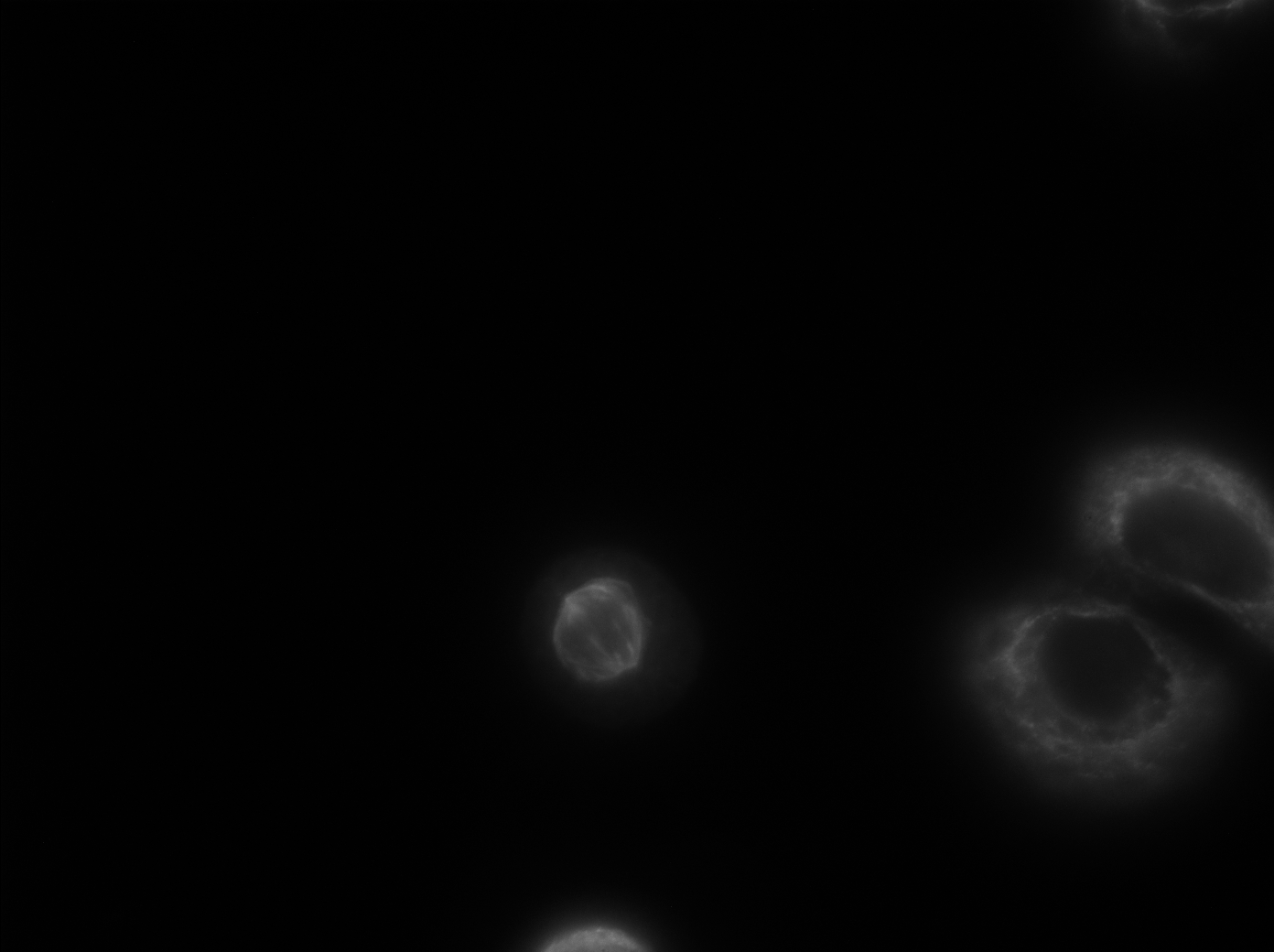

Supplement: Supplementary file 3 — Source data Fig. 1 [file 44319_2026_742_MOESM3_ESM.zip › Figure 1/Fig 1bcd WT Hela acetylated a tubulin atubulin/actub-atub 8-14-24 R1 M2.Project Maximum Z_XY1724363694_Z0_T0_C1.tif]

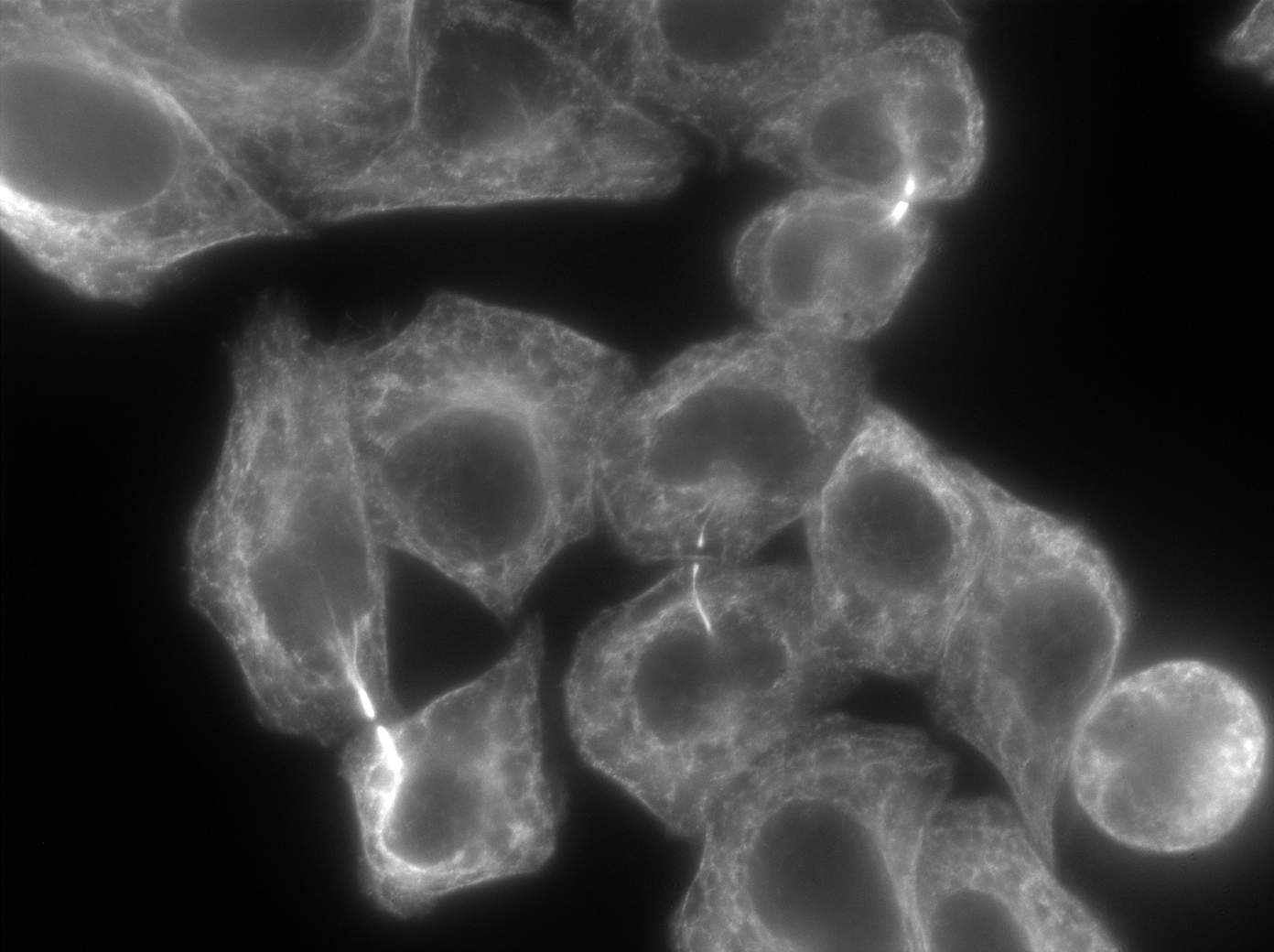

Supplement: Supplementary file 3 — Source data Fig. 1 [file 44319_2026_742_MOESM3_ESM.zip › Figure 1/Fig 1bcd WT Hela acetylated a tubulin atubulin/actub-atub 8-14-24 R2 PA6 LT11LT12.Project Maximum Z_XY1724694664_Z0_T0_C1.tif]

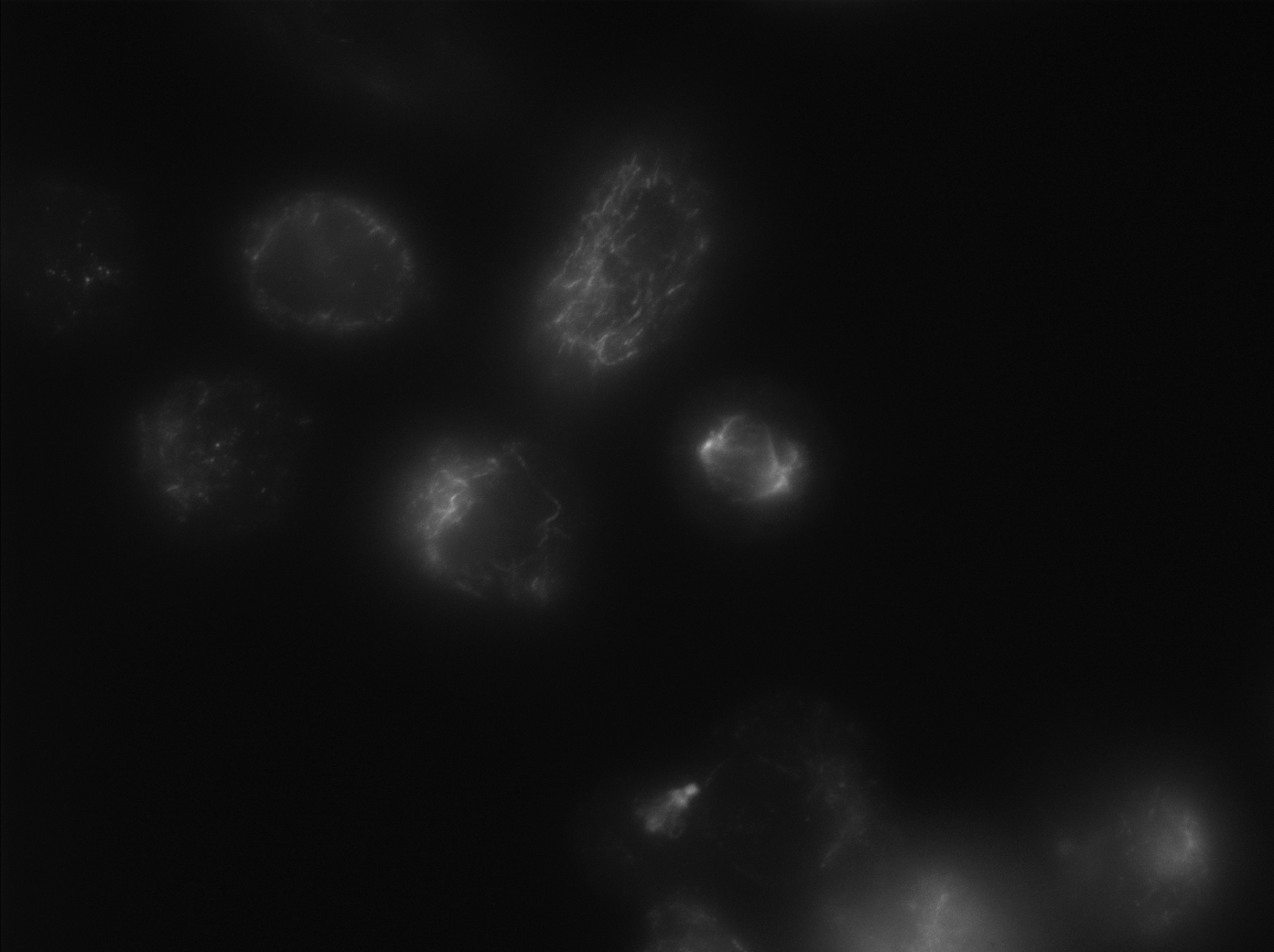

Supplement: Supplementary file 3 — Source data Fig. 1 [file 44319_2026_742_MOESM3_ESM.zip › Figure 1/Fig 1bcd WT Hela acetylated a tubulin atubulin/actub-atub 8-14-24 R1 M8.Project Maximum Z_XY1724366252_Z0_T0_C2.tif]

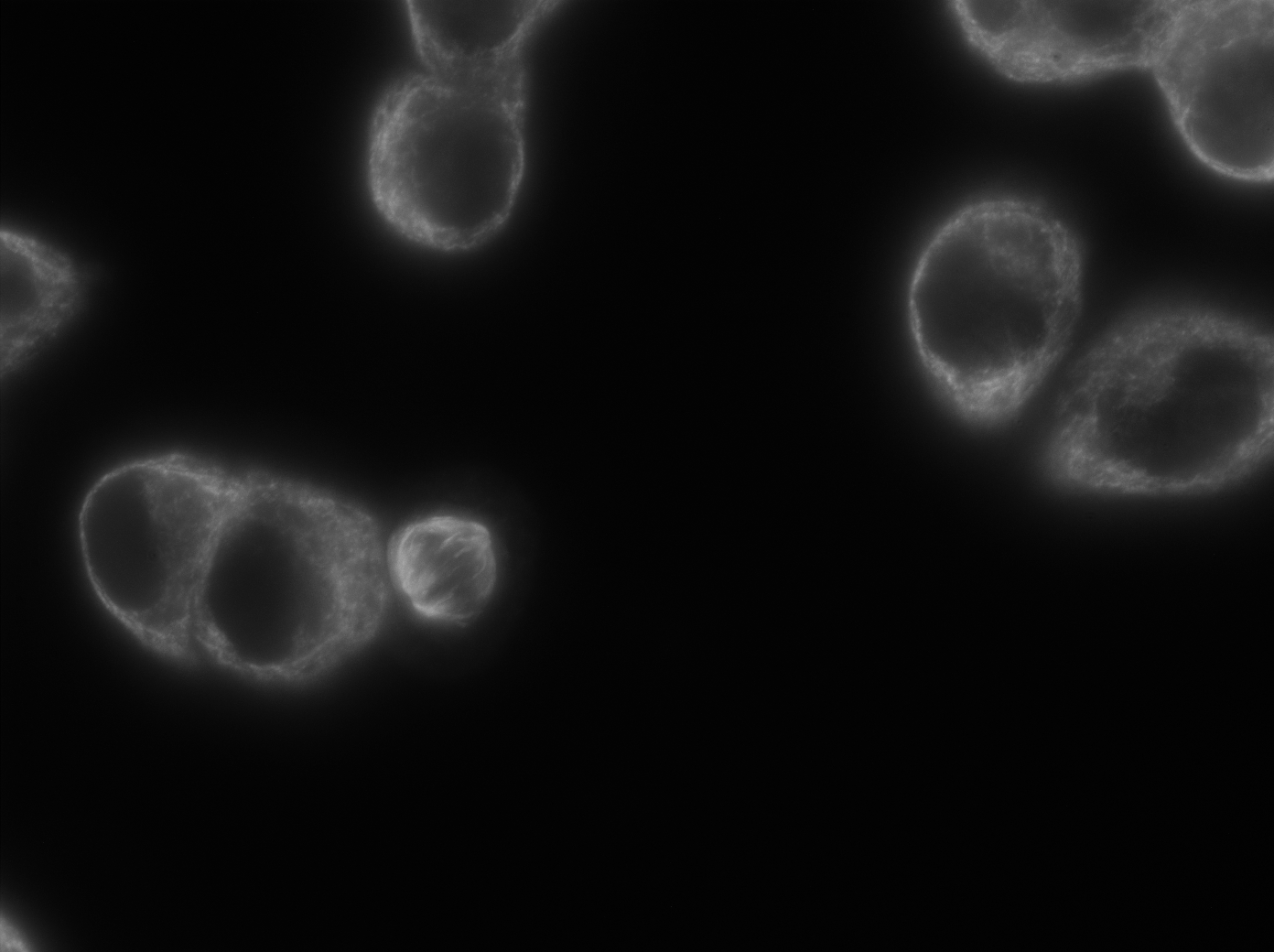

Supplement: Supplementary file 3 — Source data Fig. 1 [file 44319_2026_742_MOESM3_ESM.zip › Figure 1/Fig 1bcd WT Hela acetylated a tubulin atubulin/actub-atub 8-14-24 R1 M3.Project Maximum Z_XY1724364163_Z0_T0_C1.tif]

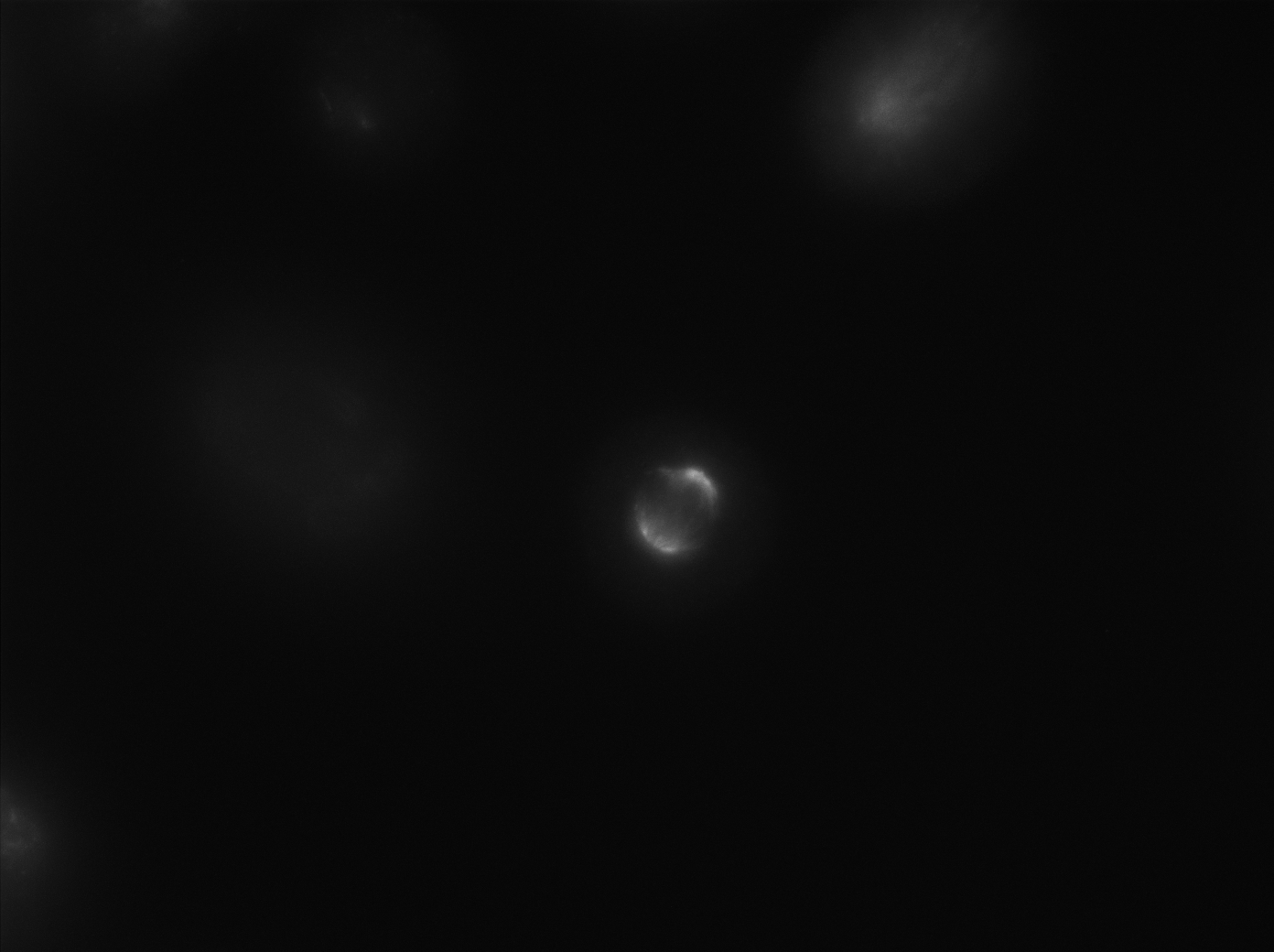

Supplement: Supplementary file 3 — Source data Fig. 1 [file 44319_2026_742_MOESM3_ESM.zip › Figure 1/Fig 1bcd WT Hela acetylated a tubulin atubulin/actub-atub 8-14-24 R2 M5.Project Maximum Z_XY1724693767_Z0_T0_C2.tif]

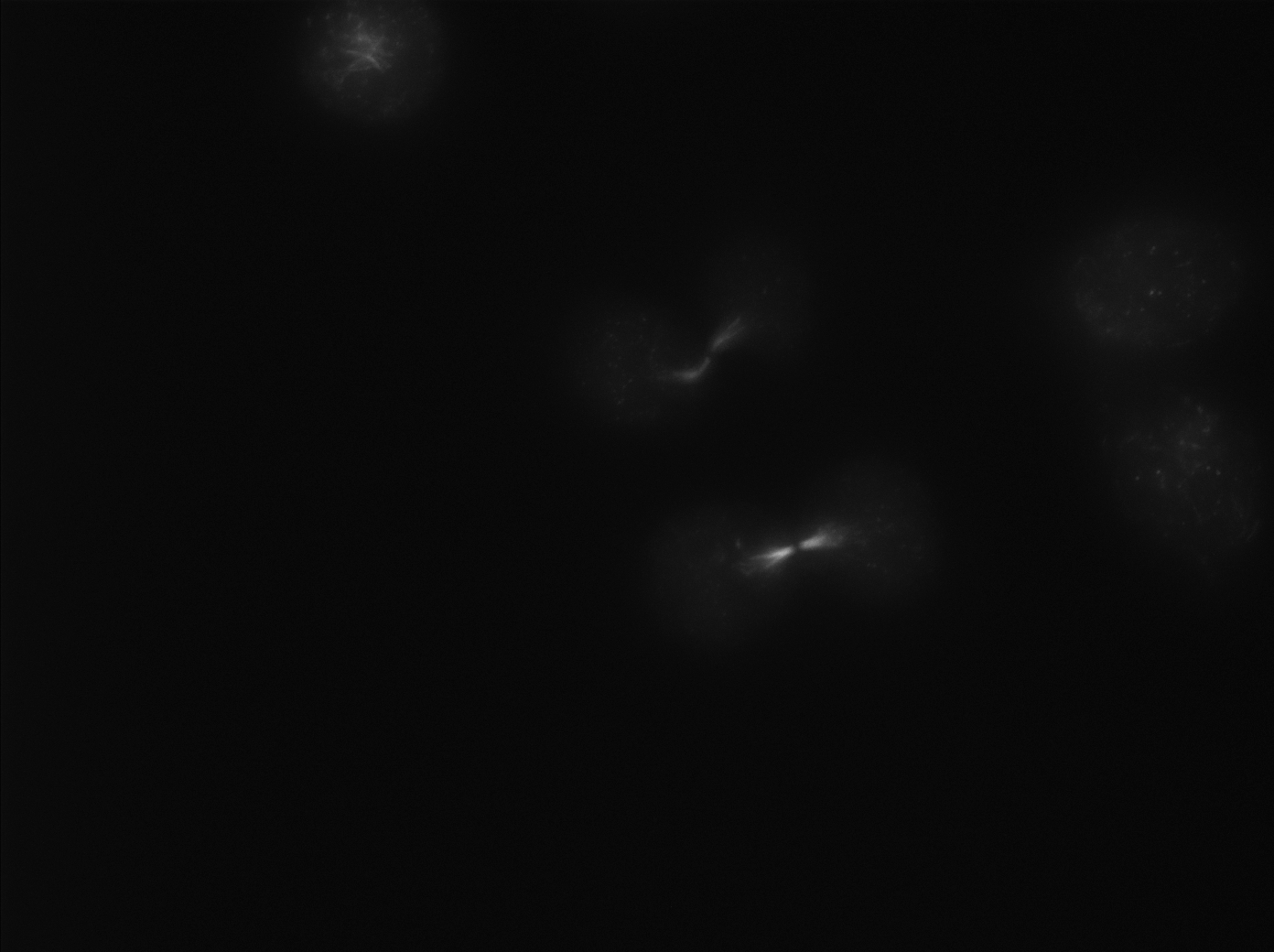

Supplement: Supplementary file 3 — Source data Fig. 1 [file 44319_2026_742_MOESM3_ESM.zip › Figure 1/Fig 1bcd WT Hela acetylated a tubulin atubulin/actub-atub 8-14-24 R1 ET10ET11.Project Maximum Z_XY1724366427_Z0_T0_C2.tif]

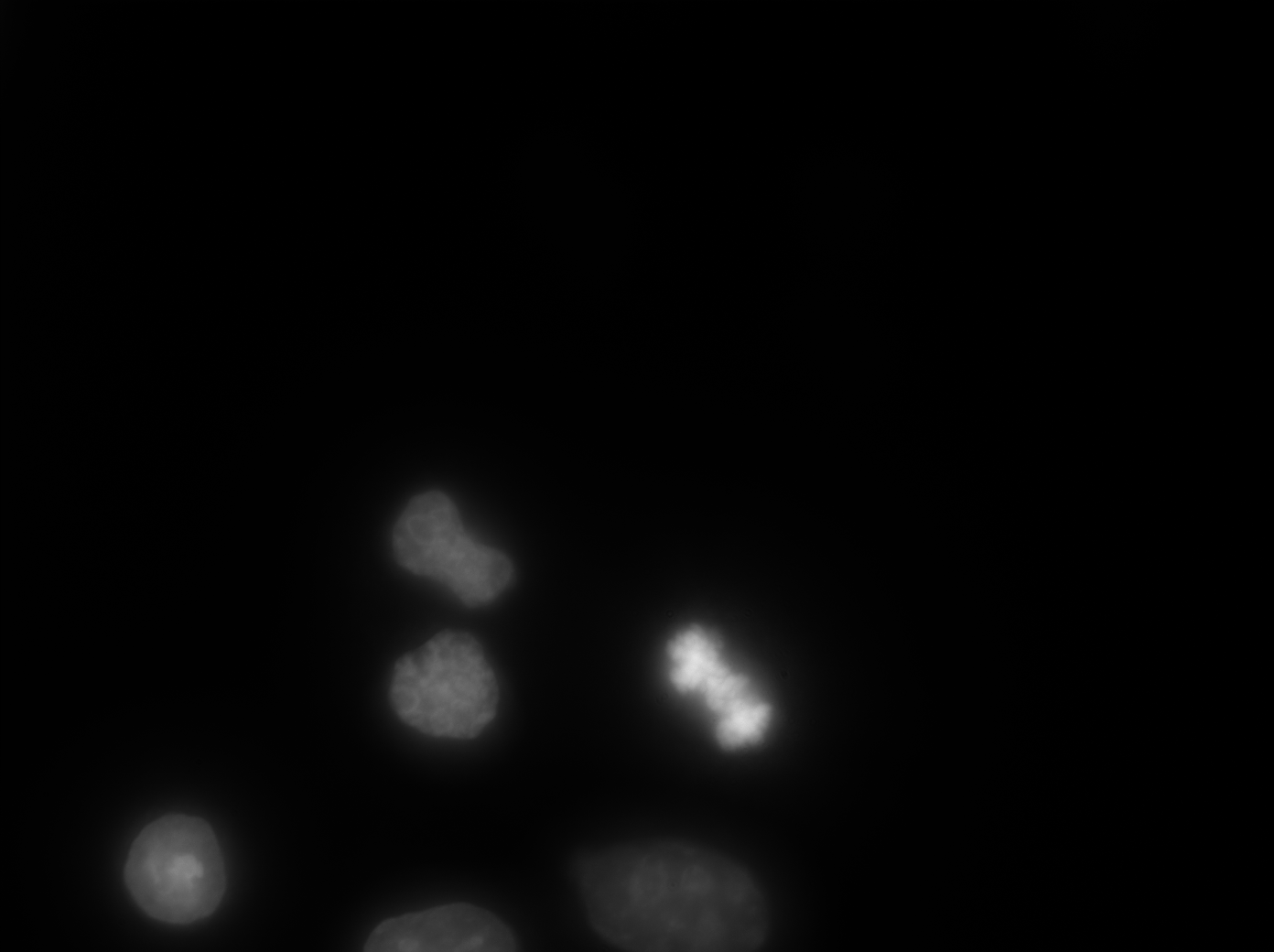

Supplement: Supplementary file 3 — Source data Fig. 1 [file 44319_2026_742_MOESM3_ESM.zip › Figure 1/Fig 1bcd WT Hela acetylated a tubulin atubulin/actub-atub 8-14-24 R2 ET9 M8.Project Maximum Z_XY1724694377_Z0_T0_C0.tif]

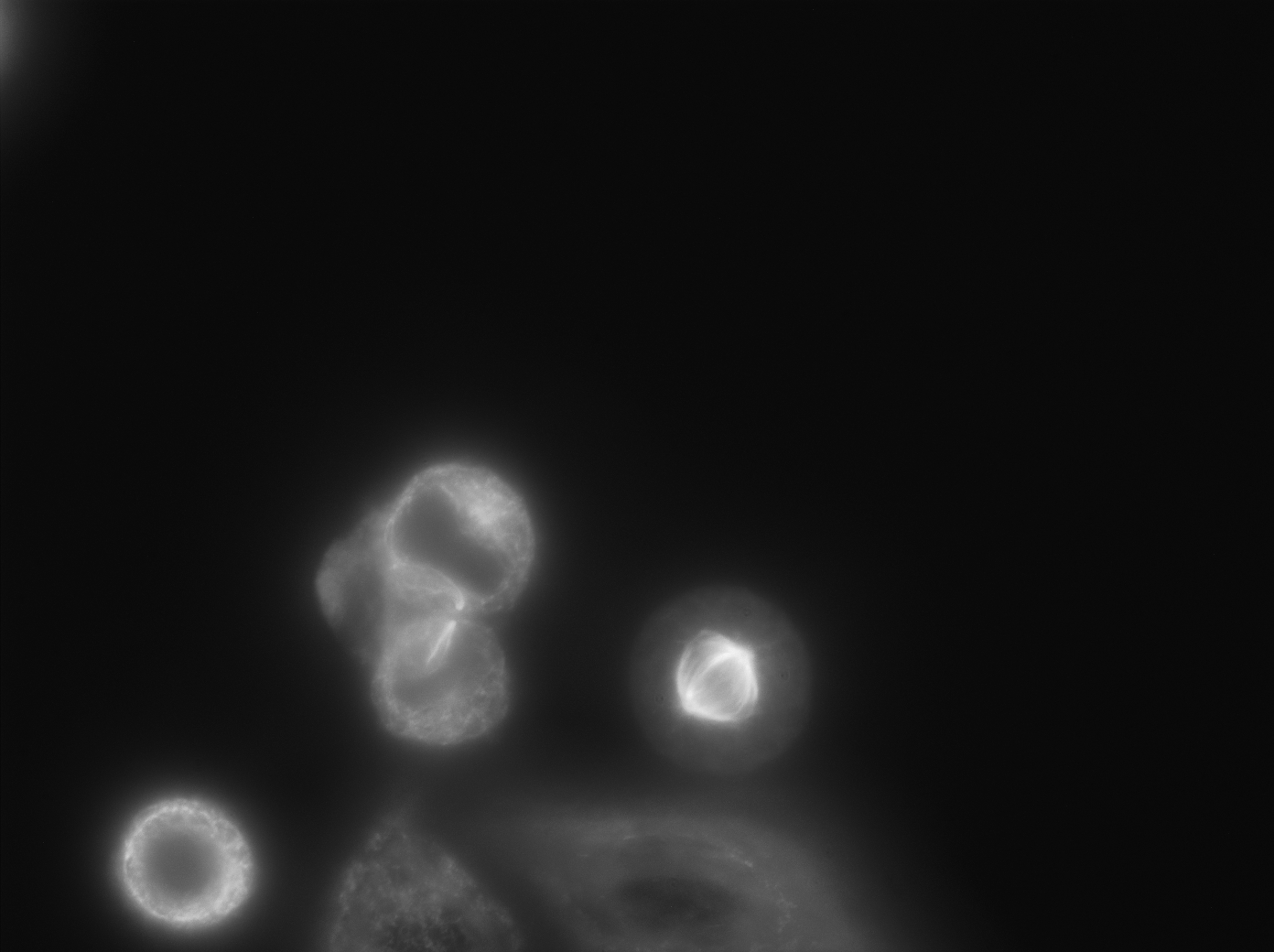

Supplement: Supplementary file 3 — Source data Fig. 1 [file 44319_2026_742_MOESM3_ESM.zip › Figure 1/Fig 1bcd WT Hela acetylated a tubulin atubulin/actub-atub 8-14-24 R2 ET9 M8.Project Maximum Z_XY1724694377_Z0_T0_C1.tif]

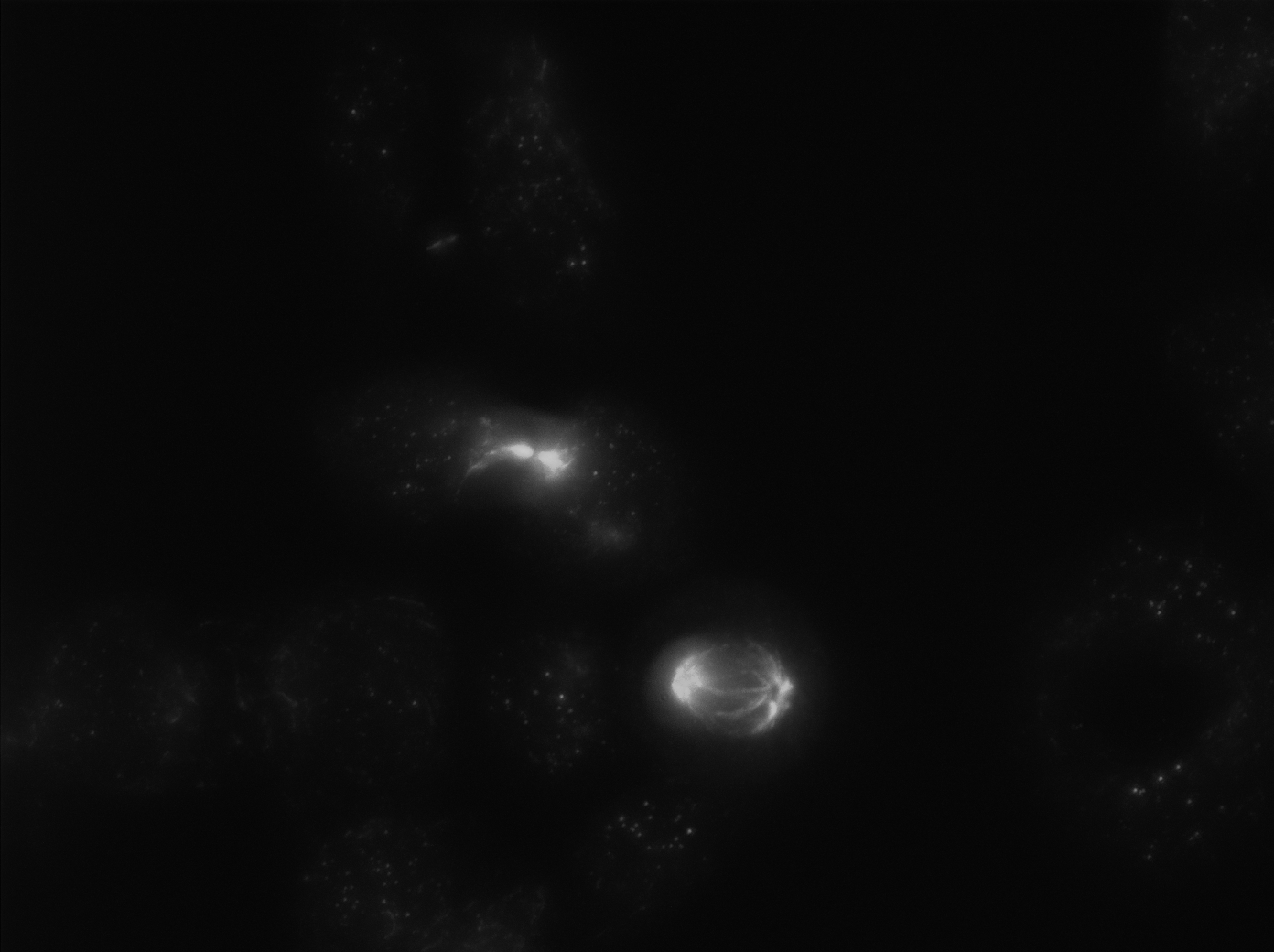

Supplement: Supplementary file 3 — Source data Fig. 1 [file 44319_2026_742_MOESM3_ESM.zip › Figure 1/Fig 1bcd WT Hela acetylated a tubulin atubulin/actub-atub 8-14-24 R2 M10.Project Maximum Z_XY1724695478_Z0_T0_C2.tif]

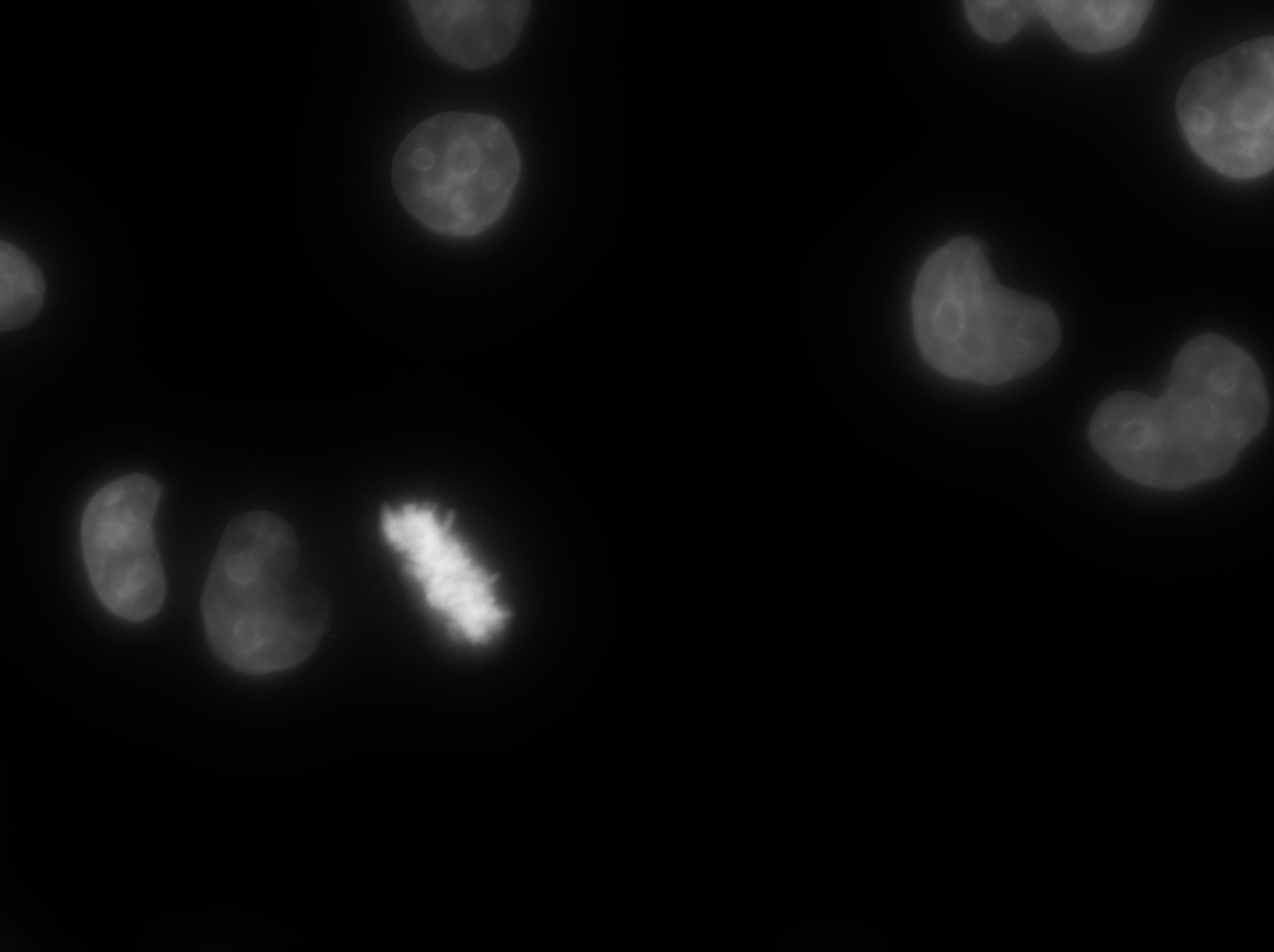

Supplement: Supplementary file 3 — Source data Fig. 1 [file 44319_2026_742_MOESM3_ESM.zip › Figure 1/Fig 1bcd WT Hela acetylated a tubulin atubulin/actub-atub 8-14-24 R1 M3.Project Maximum Z_XY1724364163_Z0_T0_C0.tif]

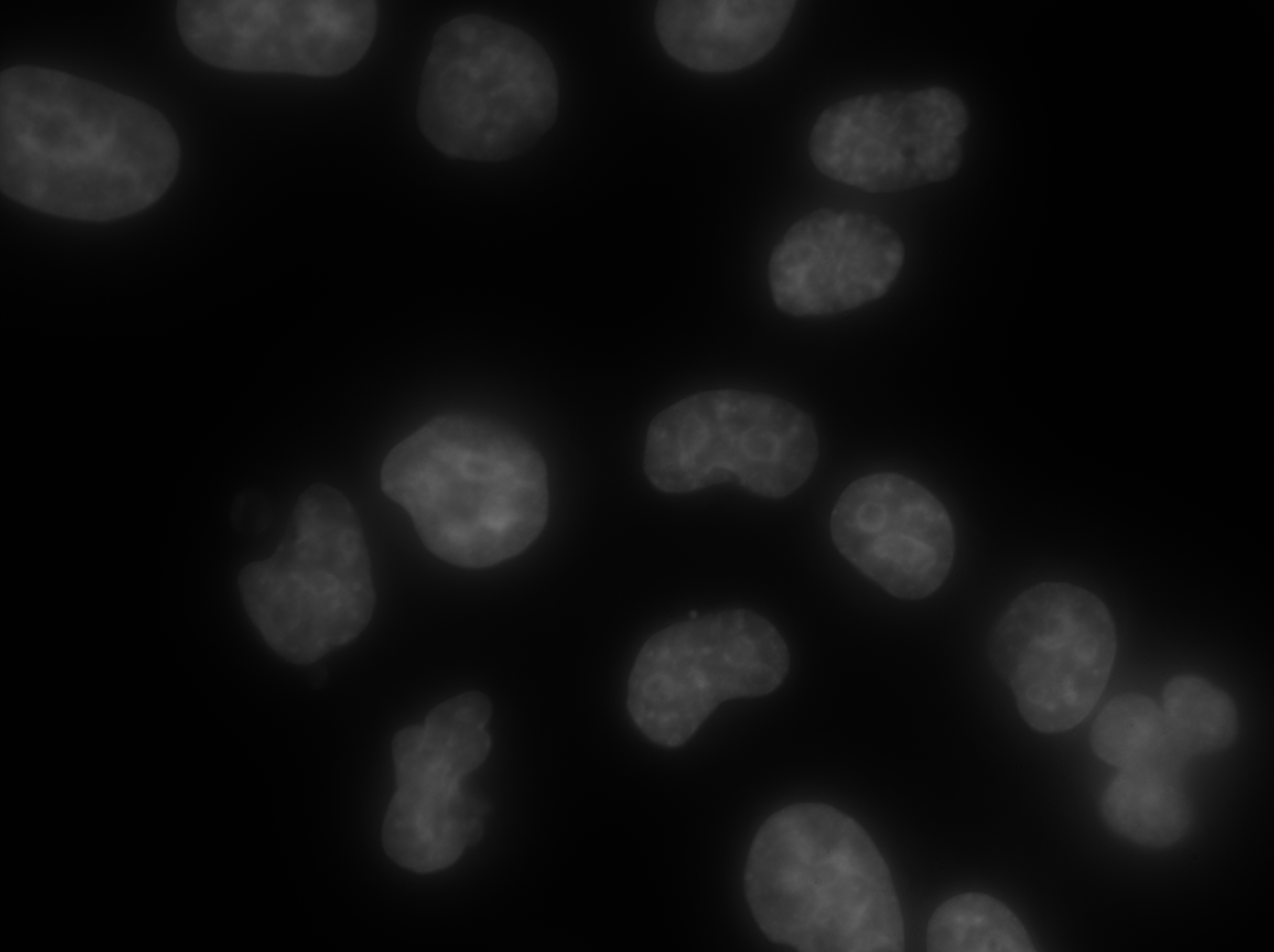

Supplement: Supplementary file 3 — Source data Fig. 1 [file 44319_2026_742_MOESM3_ESM.zip › Figure 1/Fig 1bcd WT Hela acetylated a tubulin atubulin/actub-atub 8-14-24 R2 PA6 LT11LT12.Project Maximum Z_XY1724694664_Z0_T0_C0.tif]

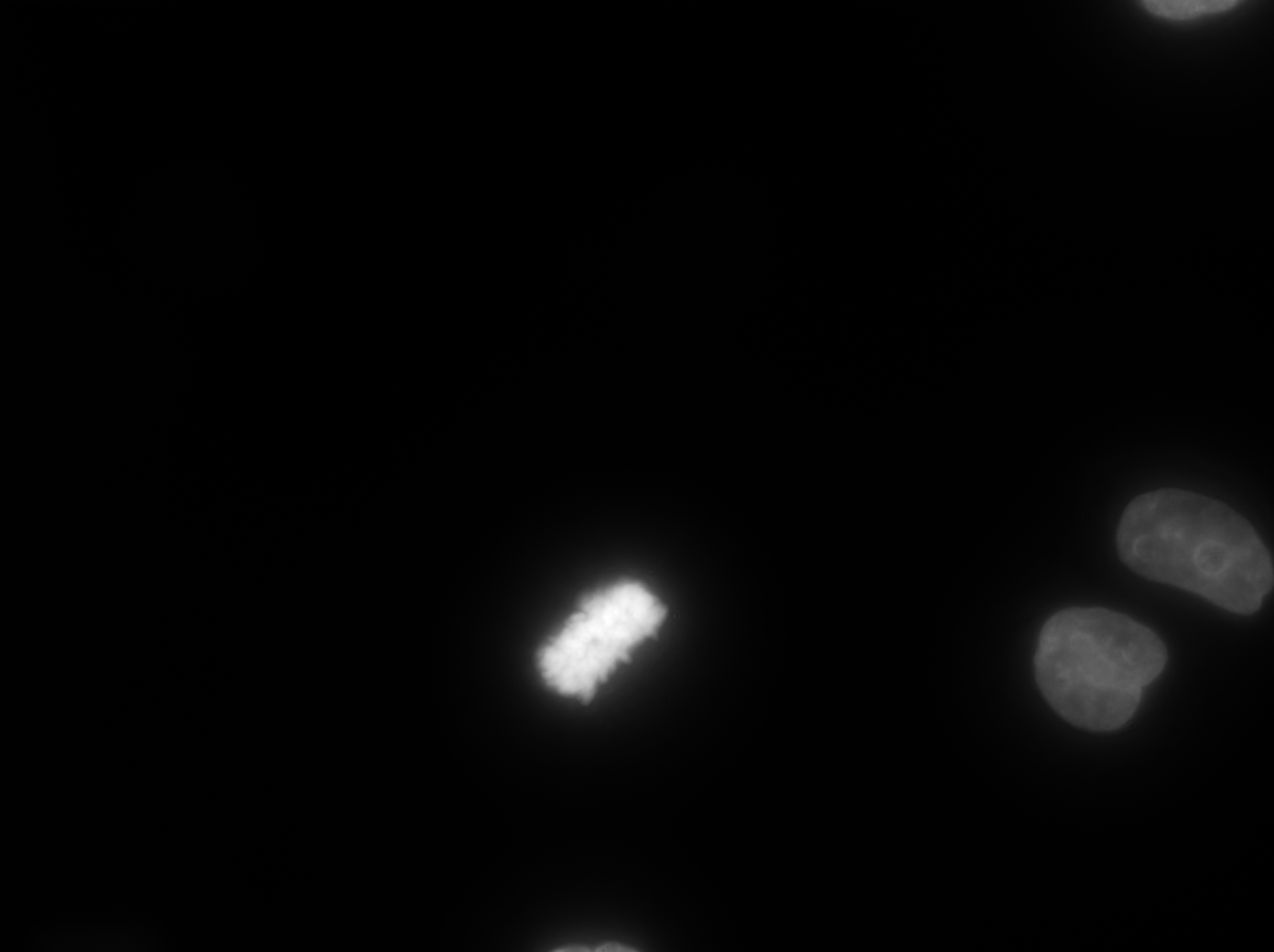

Supplement: Supplementary file 3 — Source data Fig. 1 [file 44319_2026_742_MOESM3_ESM.zip › Figure 1/Fig 1bcd WT Hela acetylated a tubulin atubulin/actub-atub 8-14-24 R1 M2.Project Maximum Z_XY1724363694_Z0_T0_C0.tif]

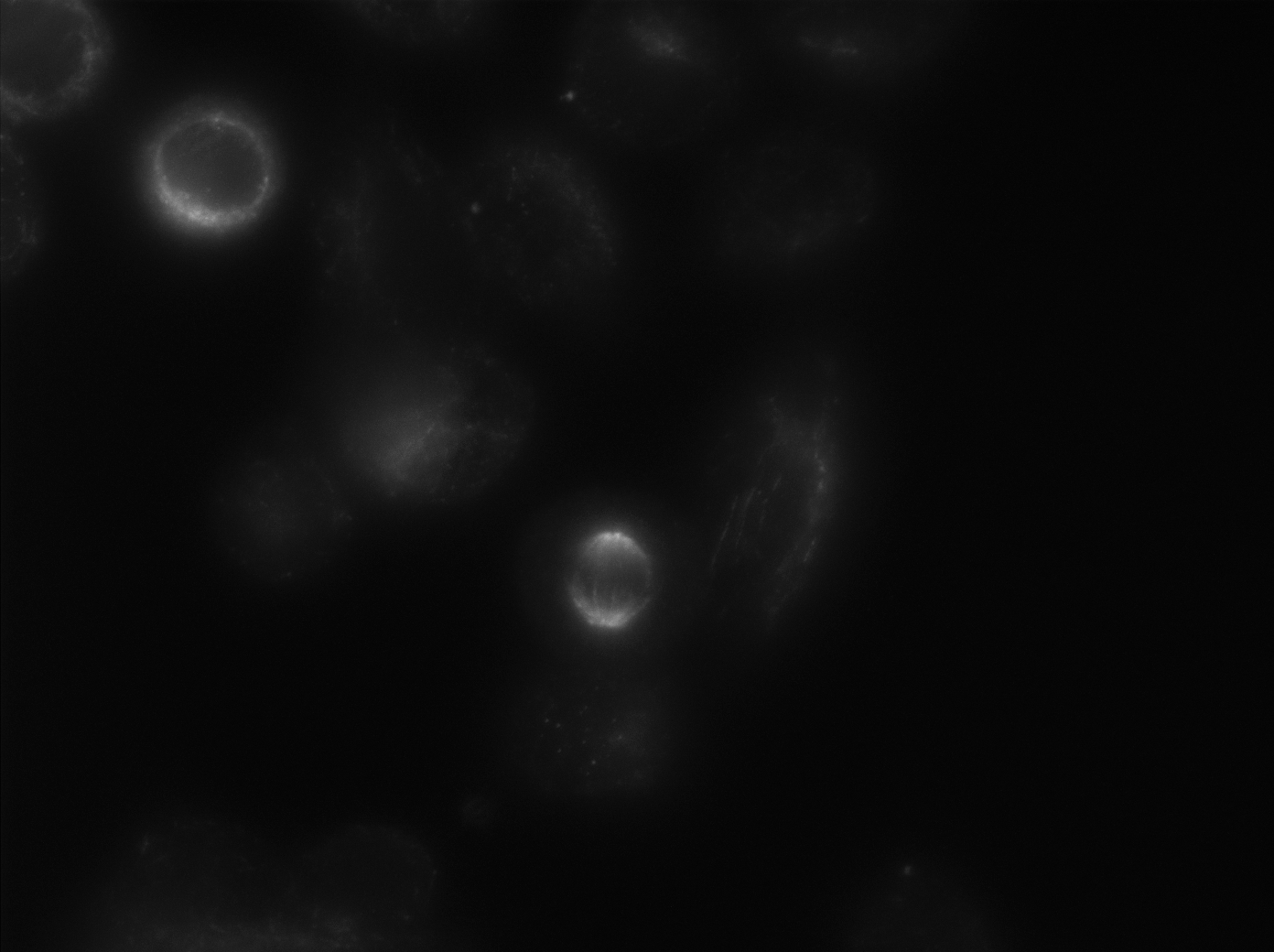

Supplement: Supplementary file 3 — Source data Fig. 1 [file 44319_2026_742_MOESM3_ESM.zip › Figure 1/Fig 1bcd WT Hela acetylated a tubulin atubulin/actub-atub 8-14-24 R3 M7.Project Maximum Z_XY1724703577_Z0_T0_C2.tif]

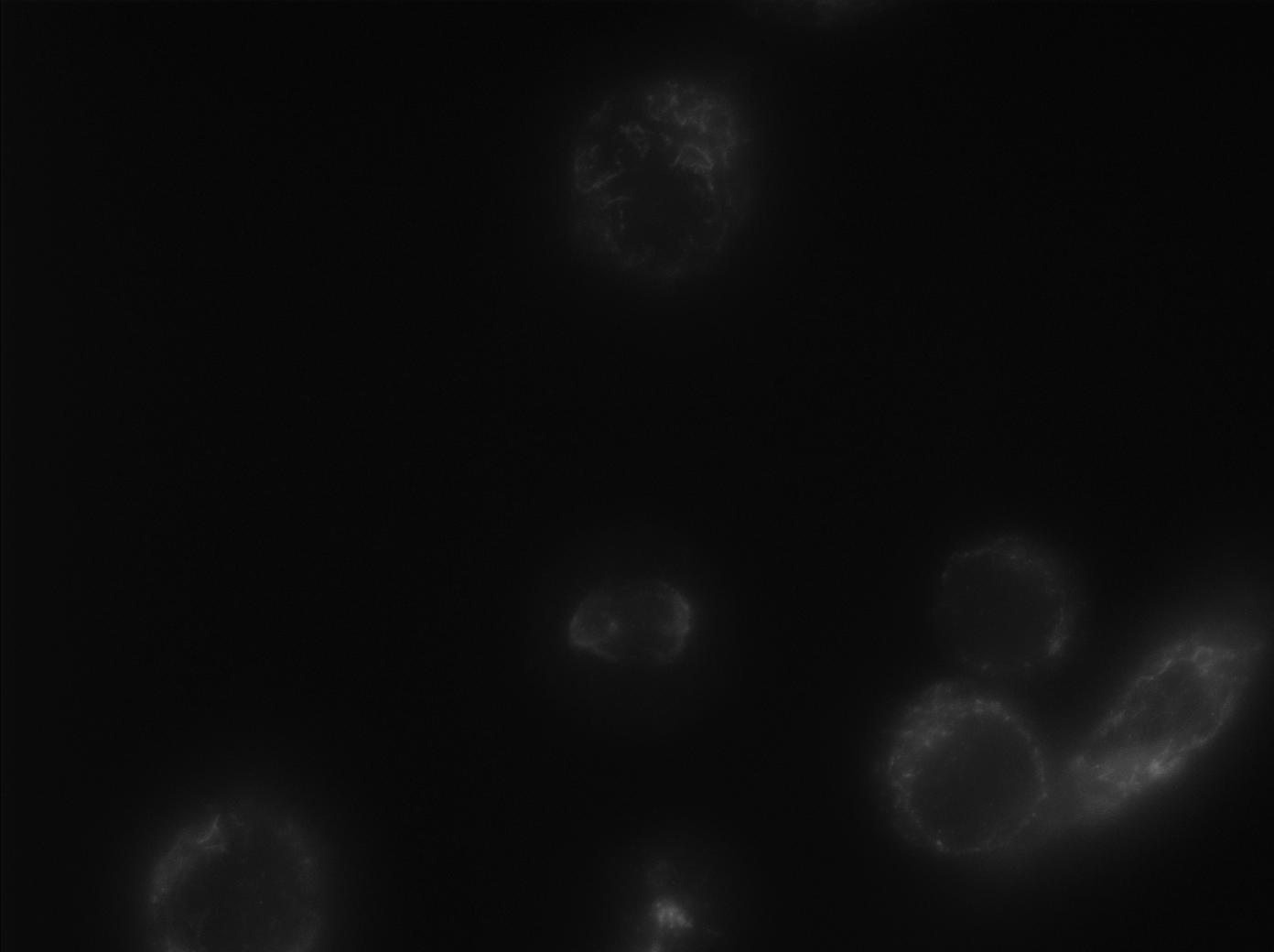

Supplement: Supplementary file 3 — Source data Fig. 1 [file 44319_2026_742_MOESM3_ESM.zip › Figure 1/Fig 1bcd WT Hela acetylated a tubulin atubulin/actub-atub 8-14-24 R1 M10.Project Maximum Z_XY1724367185_Z0_T0_C2.tif]

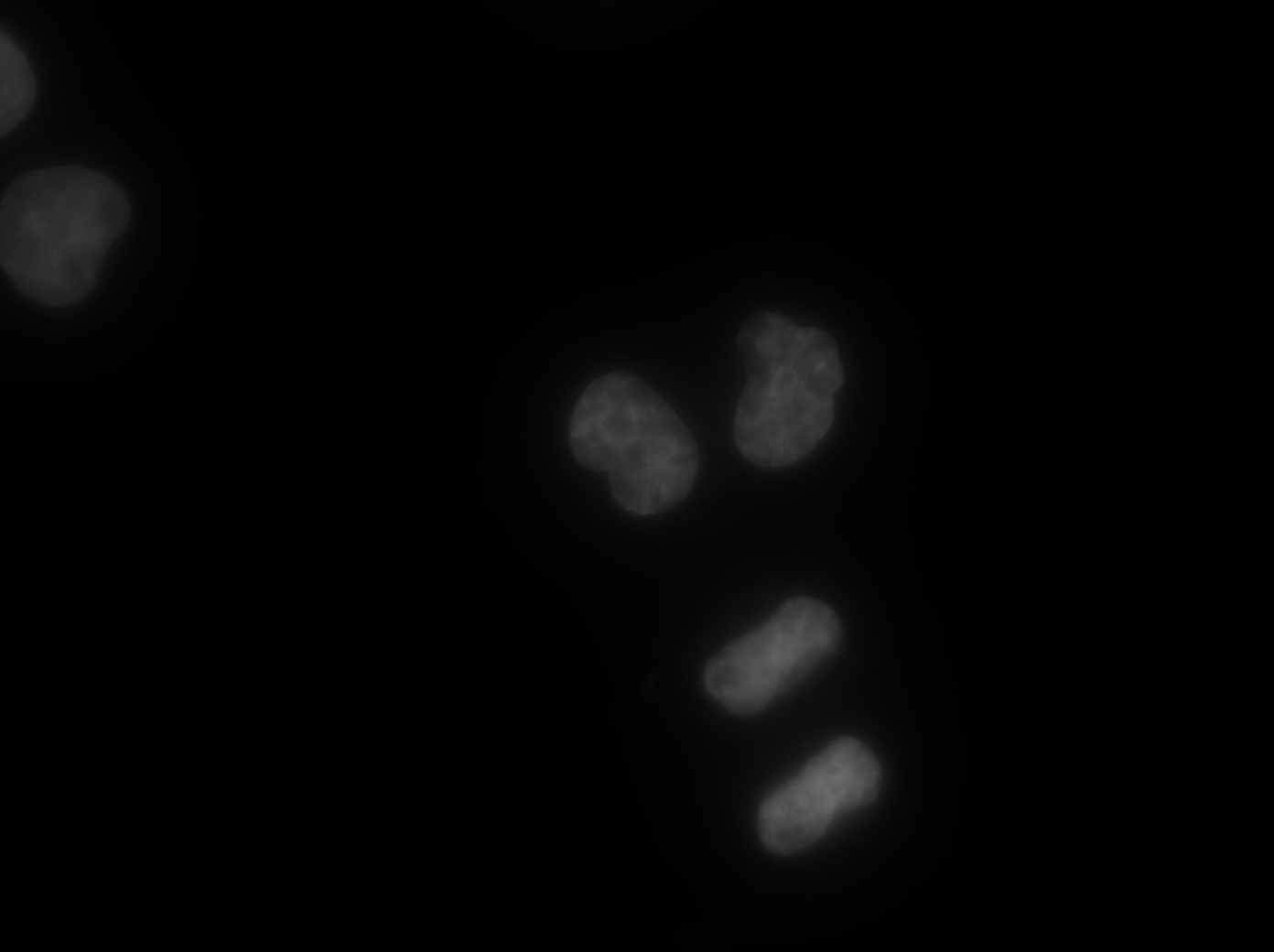

Supplement: Supplementary file 3 — Source data Fig. 1 [file 44319_2026_742_MOESM3_ESM.zip › Figure 1/Fig 1bcd WT Hela acetylated a tubulin atubulin/actub-atub 8-14-24 R1 ET9 LT8.Project Maximum Z_XY1724365615_Z0_T0_C0.tif]

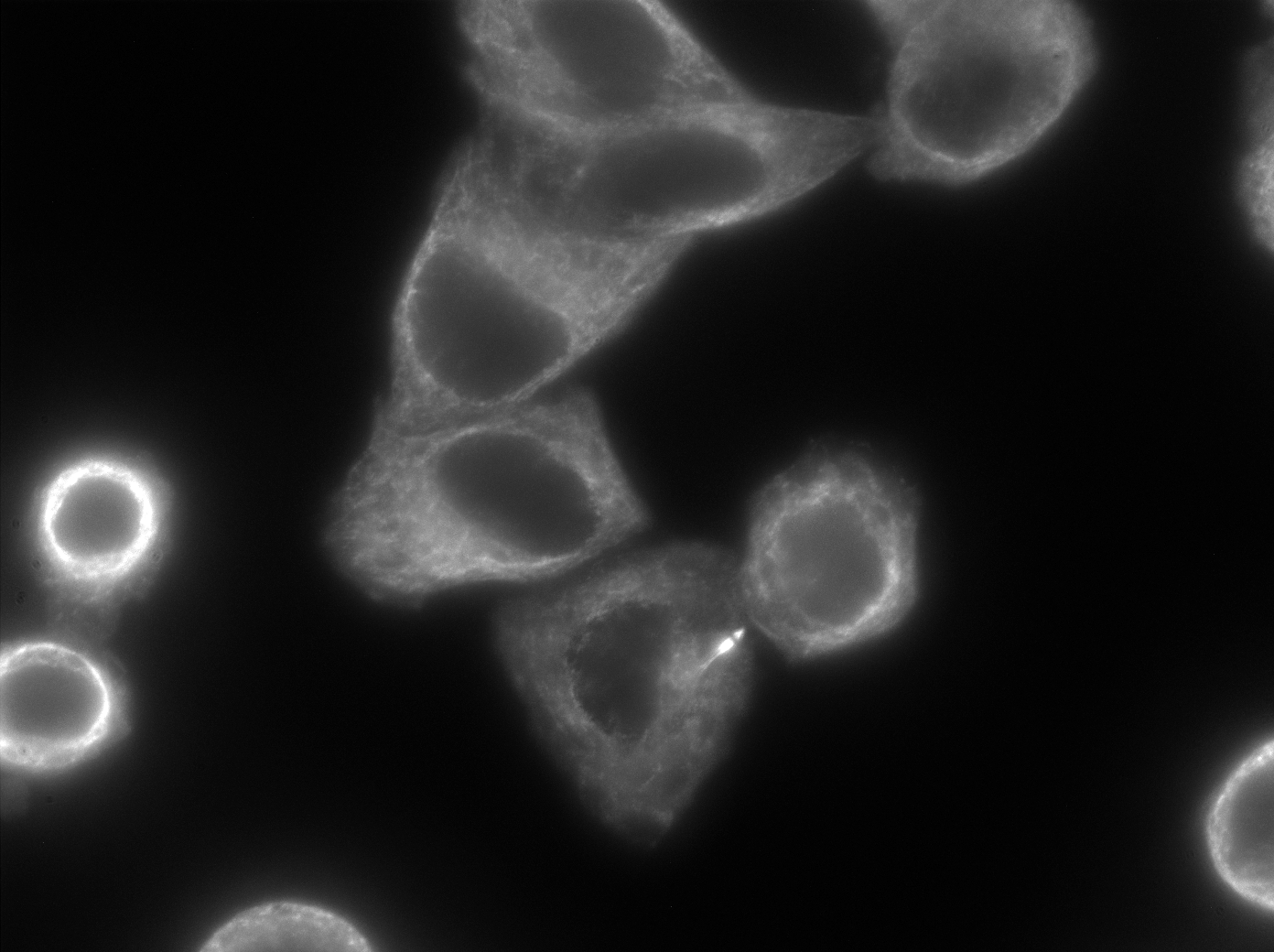

Supplement: Supplementary file 3 — Source data Fig. 1 [file 44319_2026_742_MOESM3_ESM.zip › Figure 1/Fig 1bcd WT Hela acetylated a tubulin atubulin/actub-atub 8-14-24 R3 PA1.Project Maximum Z_XY1724703264_Z0_T0_C1.tif]

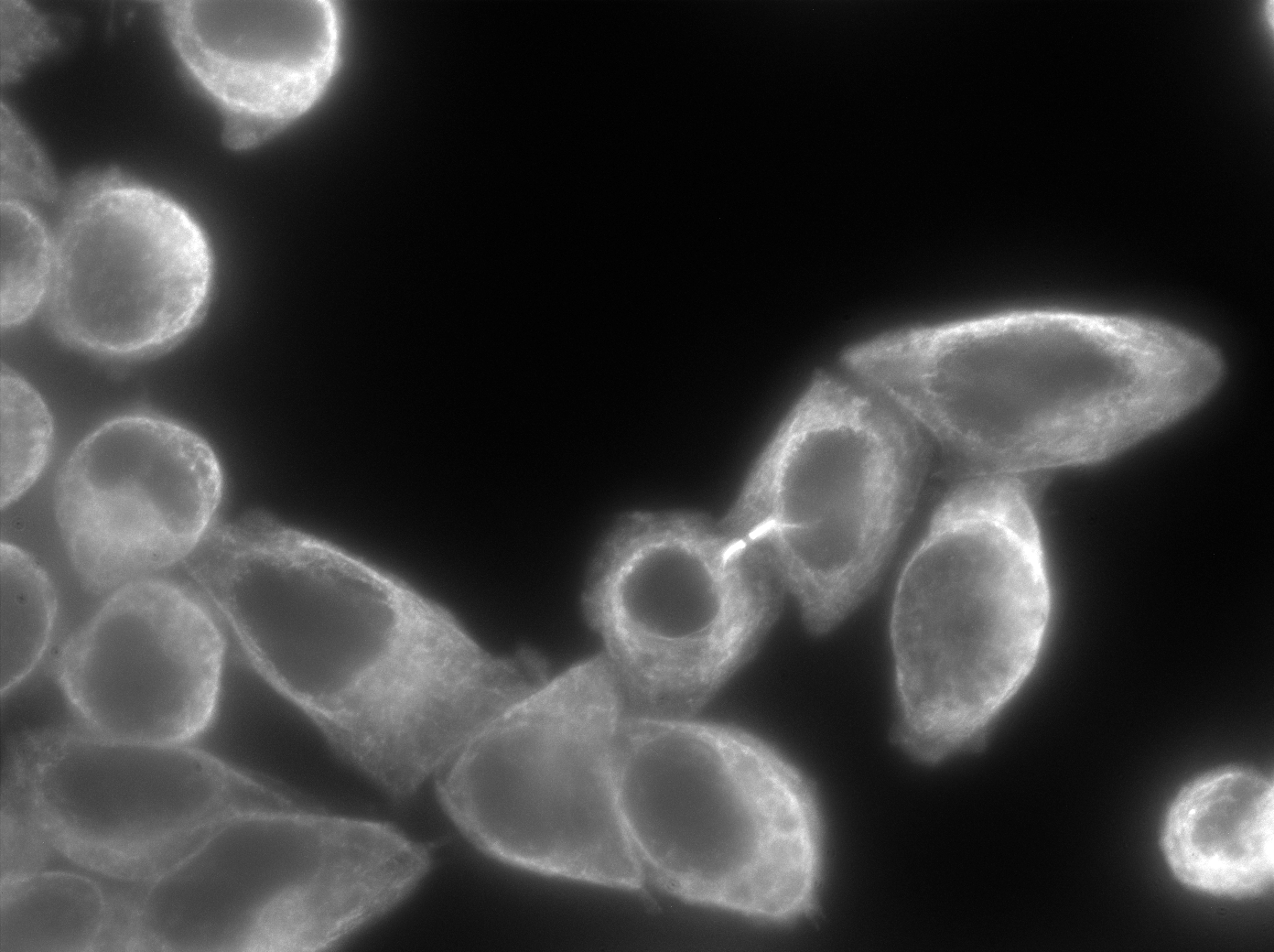

Supplement: Supplementary file 3 — Source data Fig. 1 [file 44319_2026_742_MOESM3_ESM.zip › Figure 1/Fig 1bcd WT Hela acetylated a tubulin atubulin/actub-atub 8-14-24 R3 LT10.Project Maximum Z_XY1724717708_Z0_T0_C1.tif]

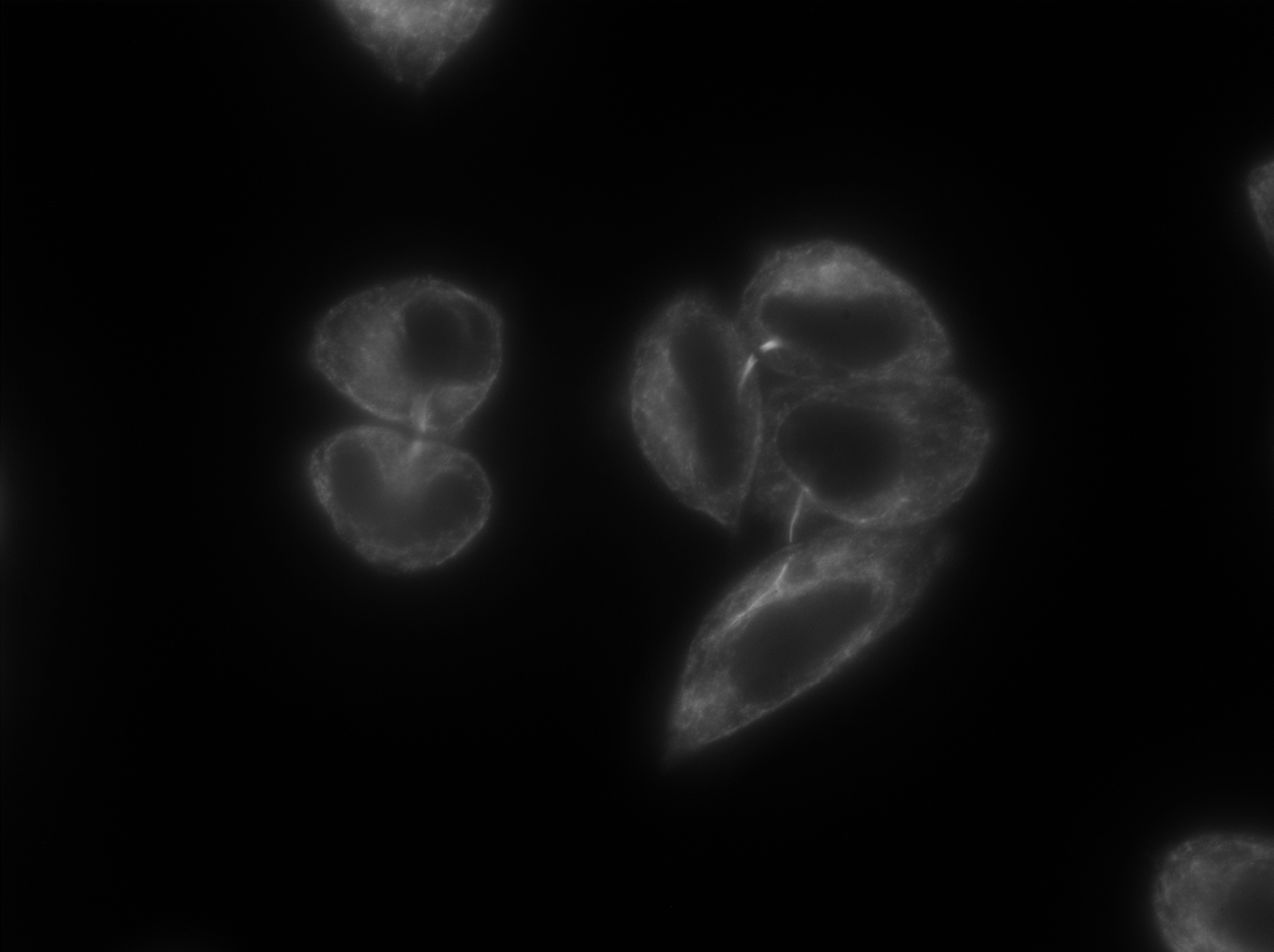

Supplement: Supplementary file 3 — Source data Fig. 1 [file 44319_2026_742_MOESM3_ESM.zip › Figure 1/Fig 1bcd WT Hela acetylated a tubulin atubulin/actub-atub 8-14-24 R1 LT9LT10LT11.Project Maximum Z_XY1724365990_Z0_T0_C1.tif]

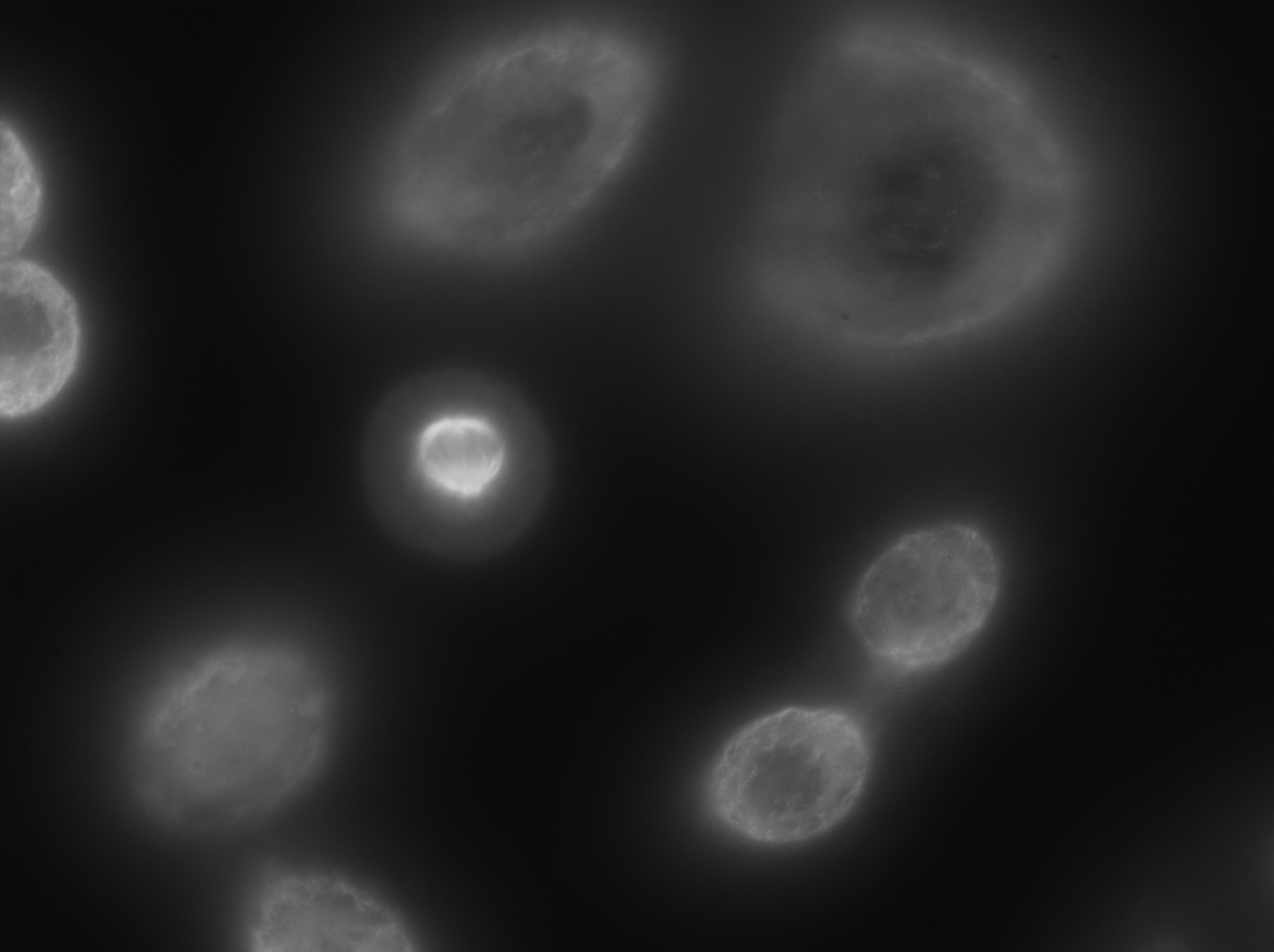

Supplement: Supplementary file 3 — Source data Fig. 1 [file 44319_2026_742_MOESM3_ESM.zip › Figure 1/Fig 1bcd WT Hela acetylated a tubulin atubulin/actub-atub 8-14-24 R2 M6.Project Maximum Z_XY1724693909_Z0_T0_C1.tif]

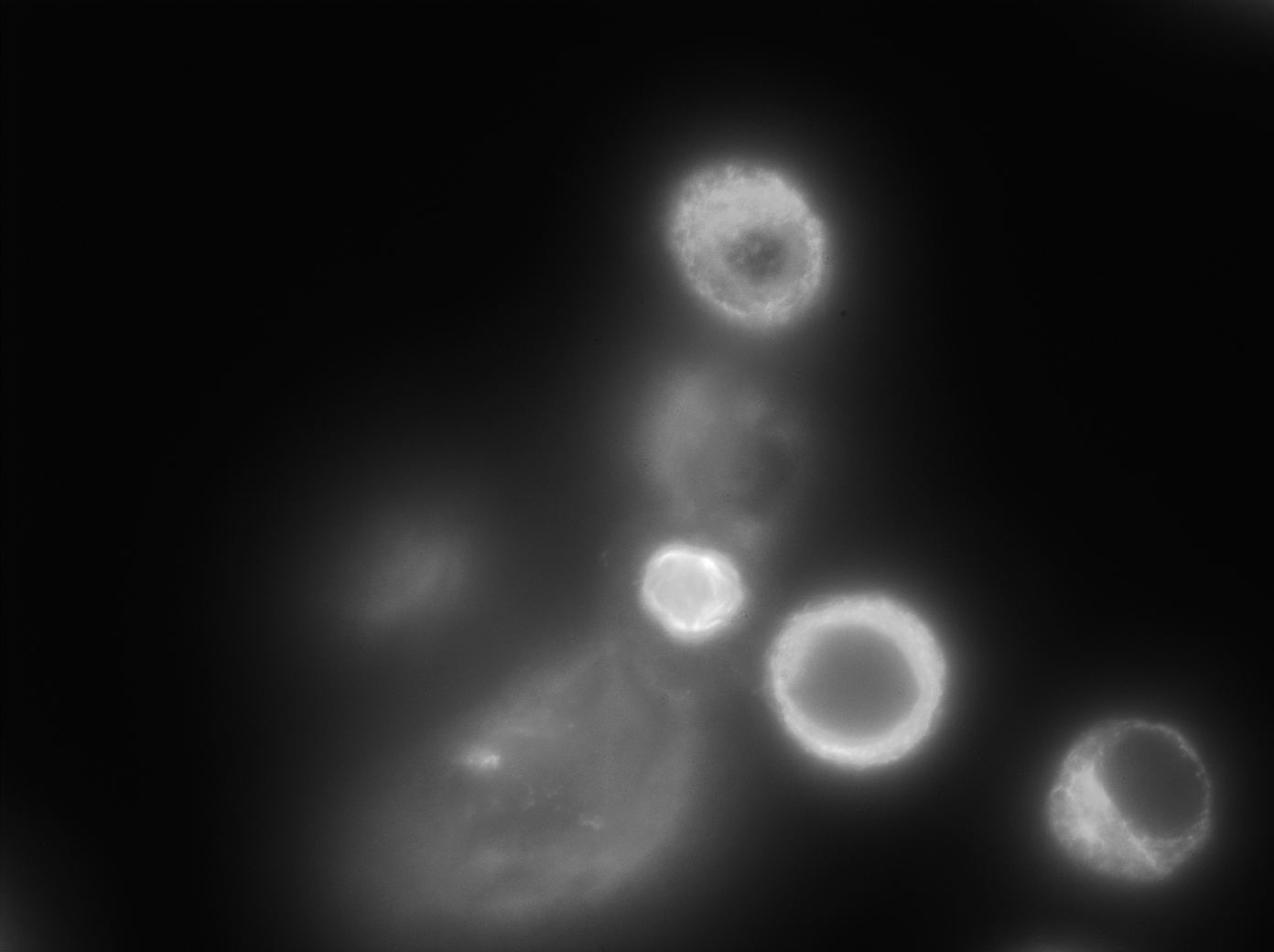

Supplement: Supplementary file 3 — Source data Fig. 1 [file 44319_2026_742_MOESM3_ESM.zip › Figure 1/Fig 1bcd WT Hela acetylated a tubulin atubulin/actub-atub 8-14-24 R1 M9.Project Maximum Z_XY1724366967_Z0_T0_C1.tif]

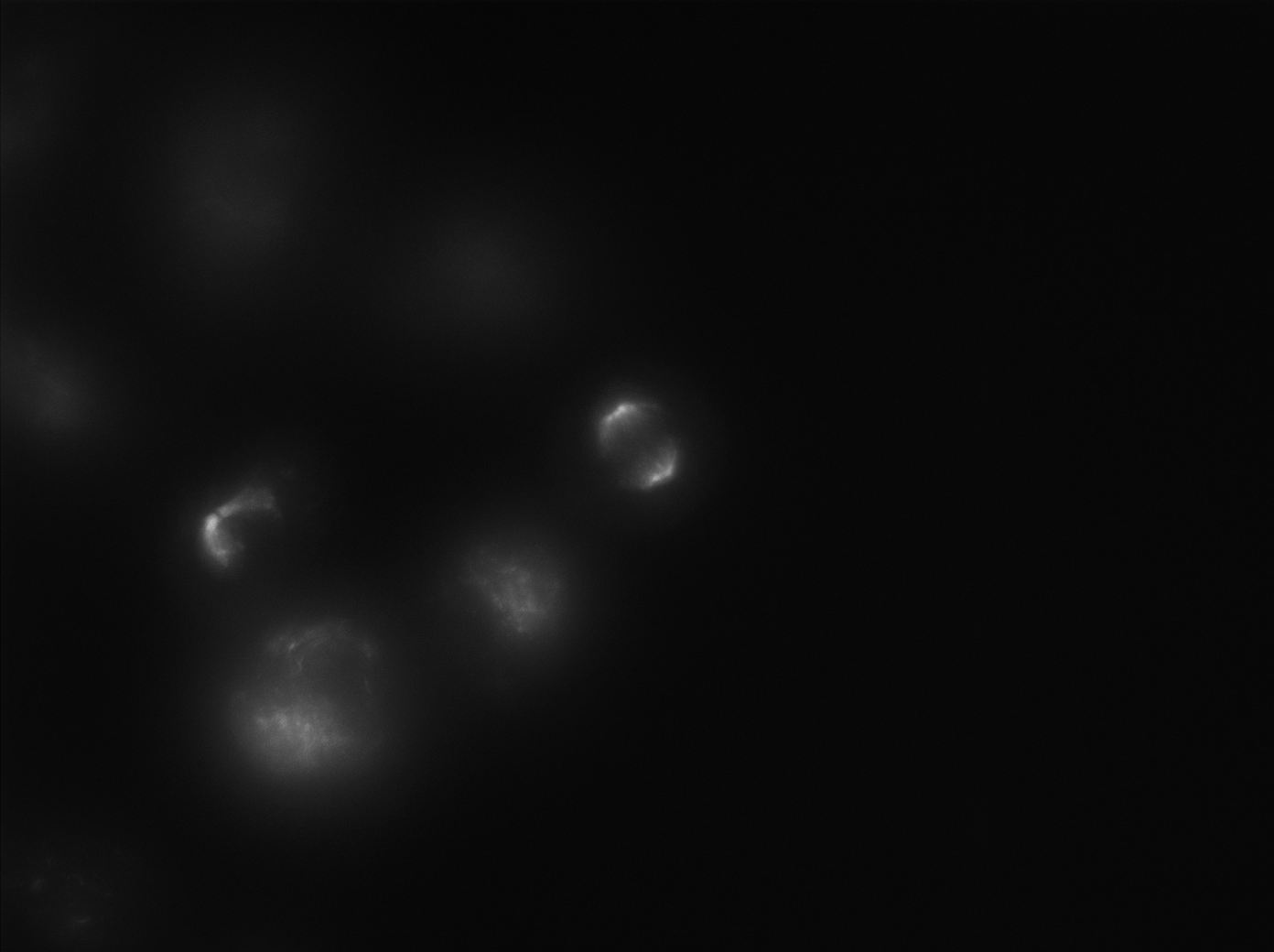

Supplement: Supplementary file 3 — Source data Fig. 1 [file 44319_2026_742_MOESM3_ESM.zip › Figure 1/Fig 1bcd WT Hela acetylated a tubulin atubulin/actub-atub 8-14-24 R2 M4.Project Maximum Z_XY1724693629_Z0_T0_C2.tif]

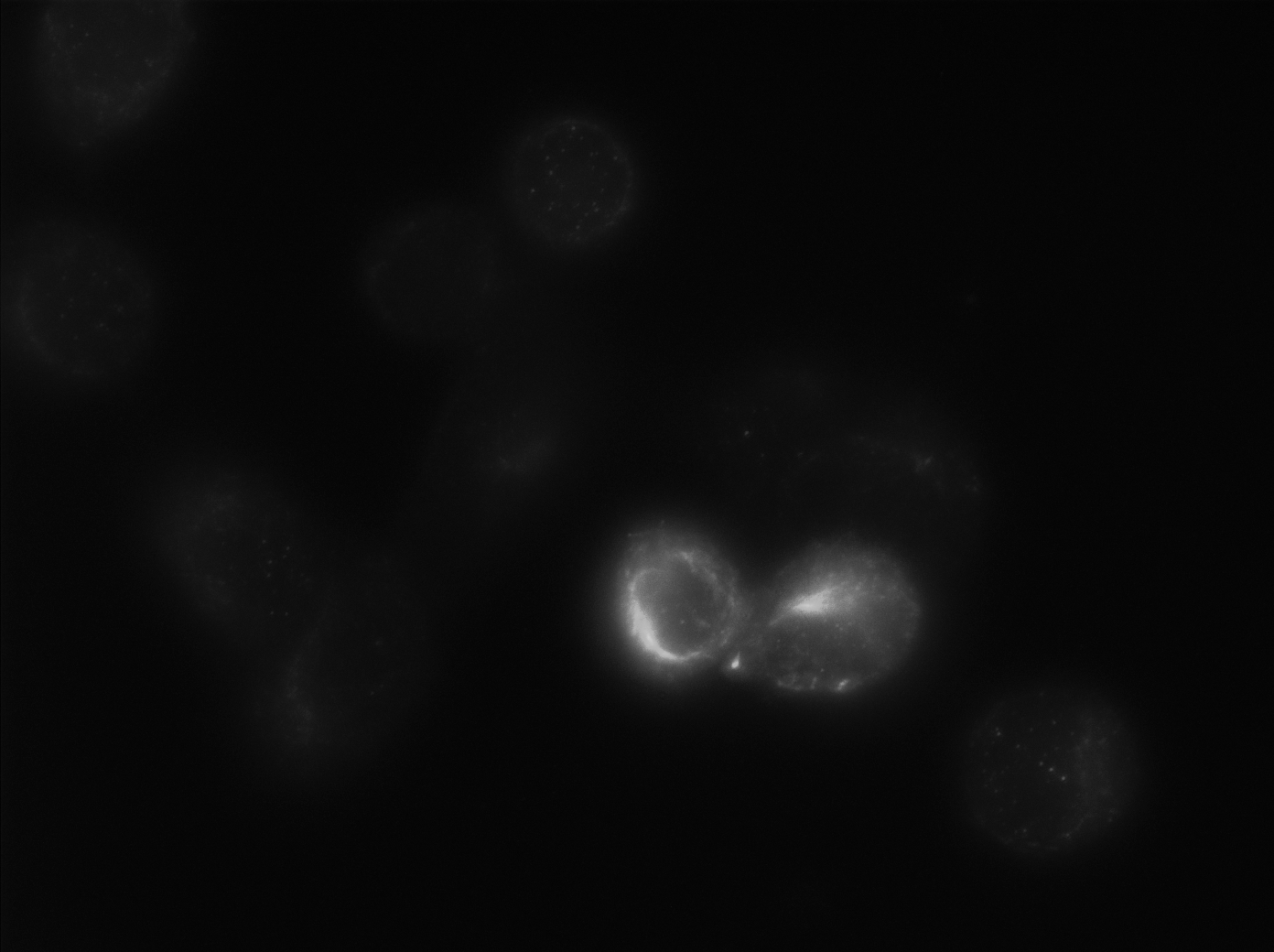

Supplement: Supplementary file 3 — Source data Fig. 1 [file 44319_2026_742_MOESM3_ESM.zip › Figure 1/Fig 1bcd WT Hela acetylated a tubulin atubulin/actub-atub 8-14-24 R3 PA4.Project Maximum Z_XY1724704169_Z0_T0_C2.tif]

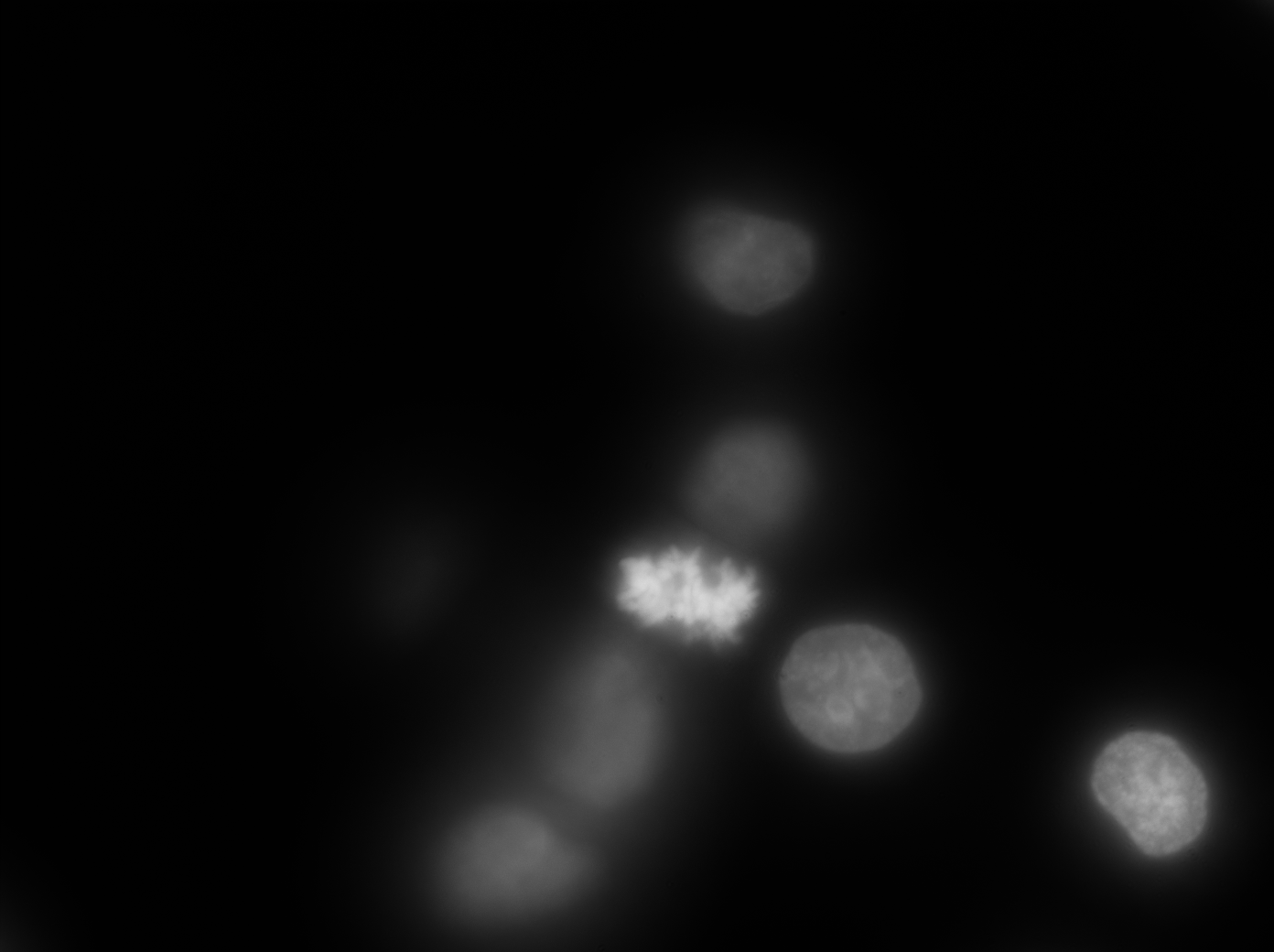

Supplement: Supplementary file 3 — Source data Fig. 1 [file 44319_2026_742_MOESM3_ESM.zip › Figure 1/Fig 1bcd WT Hela acetylated a tubulin atubulin/actub-atub 8-14-24 R1 M9.Project Maximum Z_XY1724366967_Z0_T0_C0.tif]

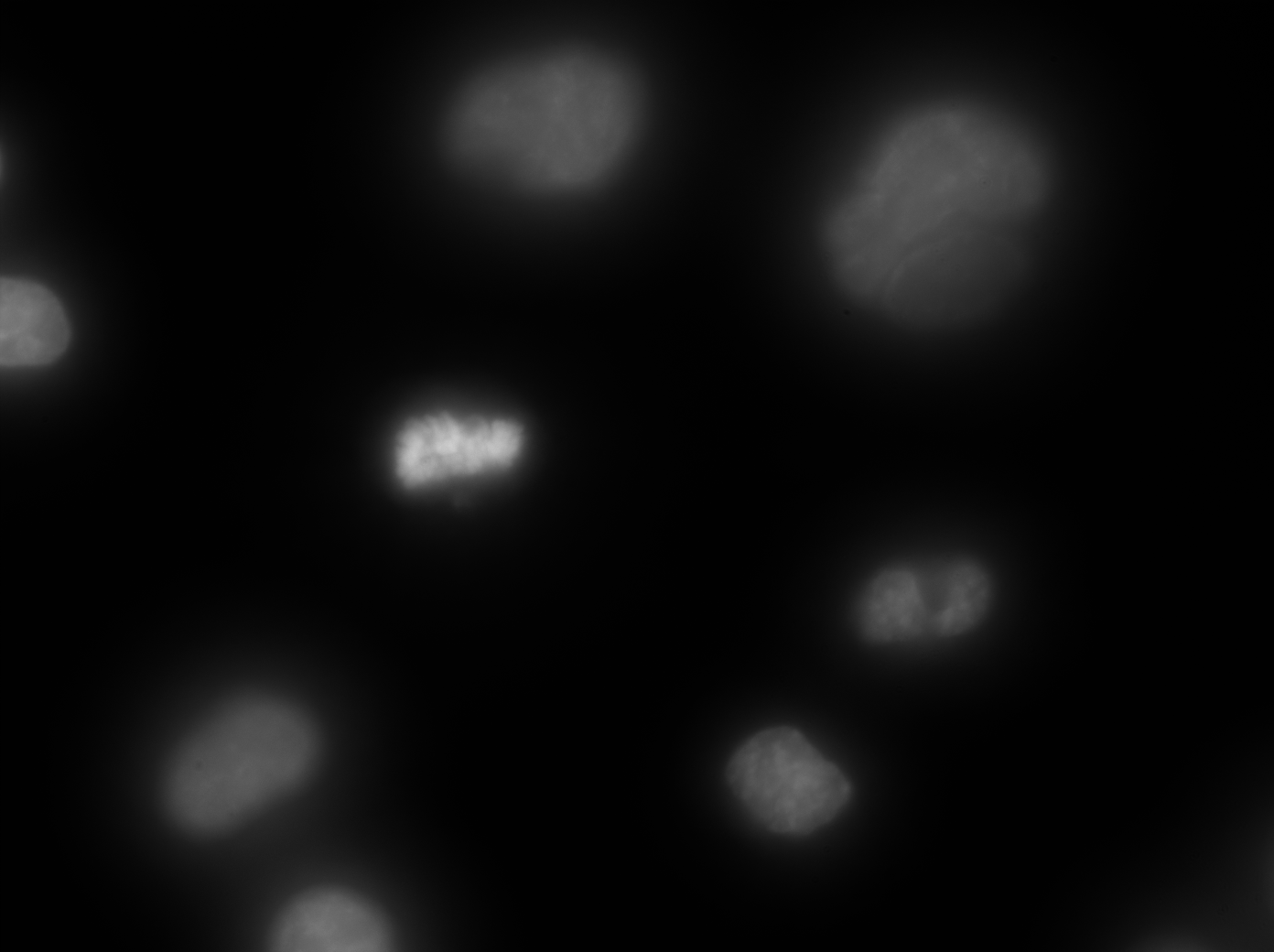

Supplement: Supplementary file 3 — Source data Fig. 1 [file 44319_2026_742_MOESM3_ESM.zip › Figure 1/Fig 1bcd WT Hela acetylated a tubulin atubulin/actub-atub 8-14-24 R2 M6.Project Maximum Z_XY1724693909_Z0_T0_C0.tif]

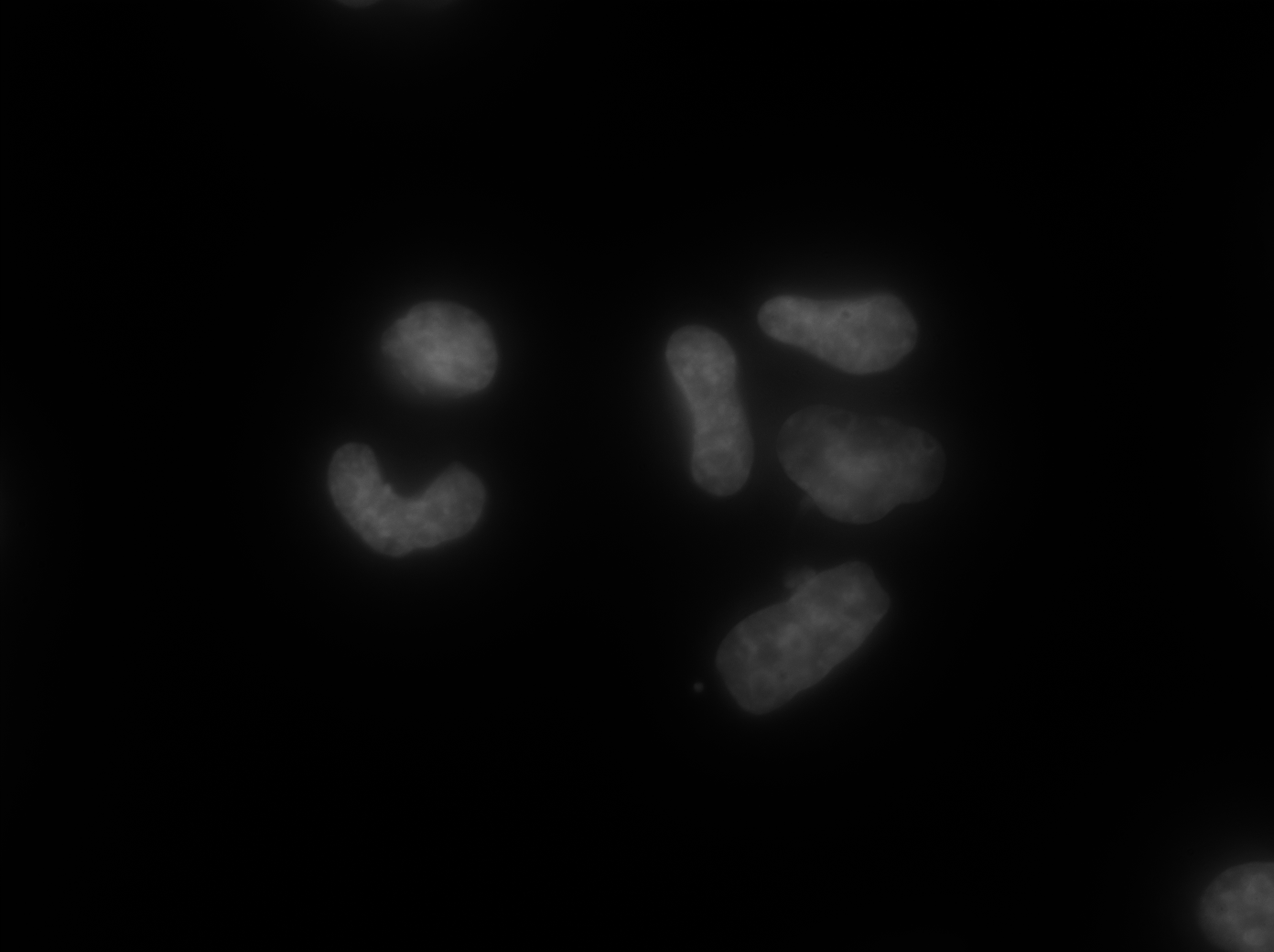

Supplement: Supplementary file 3 — Source data Fig. 1 [file 44319_2026_742_MOESM3_ESM.zip › Figure 1/Fig 1bcd WT Hela acetylated a tubulin atubulin/actub-atub 8-14-24 R1 LT9LT10LT11.Project Maximum Z_XY1724365990_Z0_T0_C0.tif]

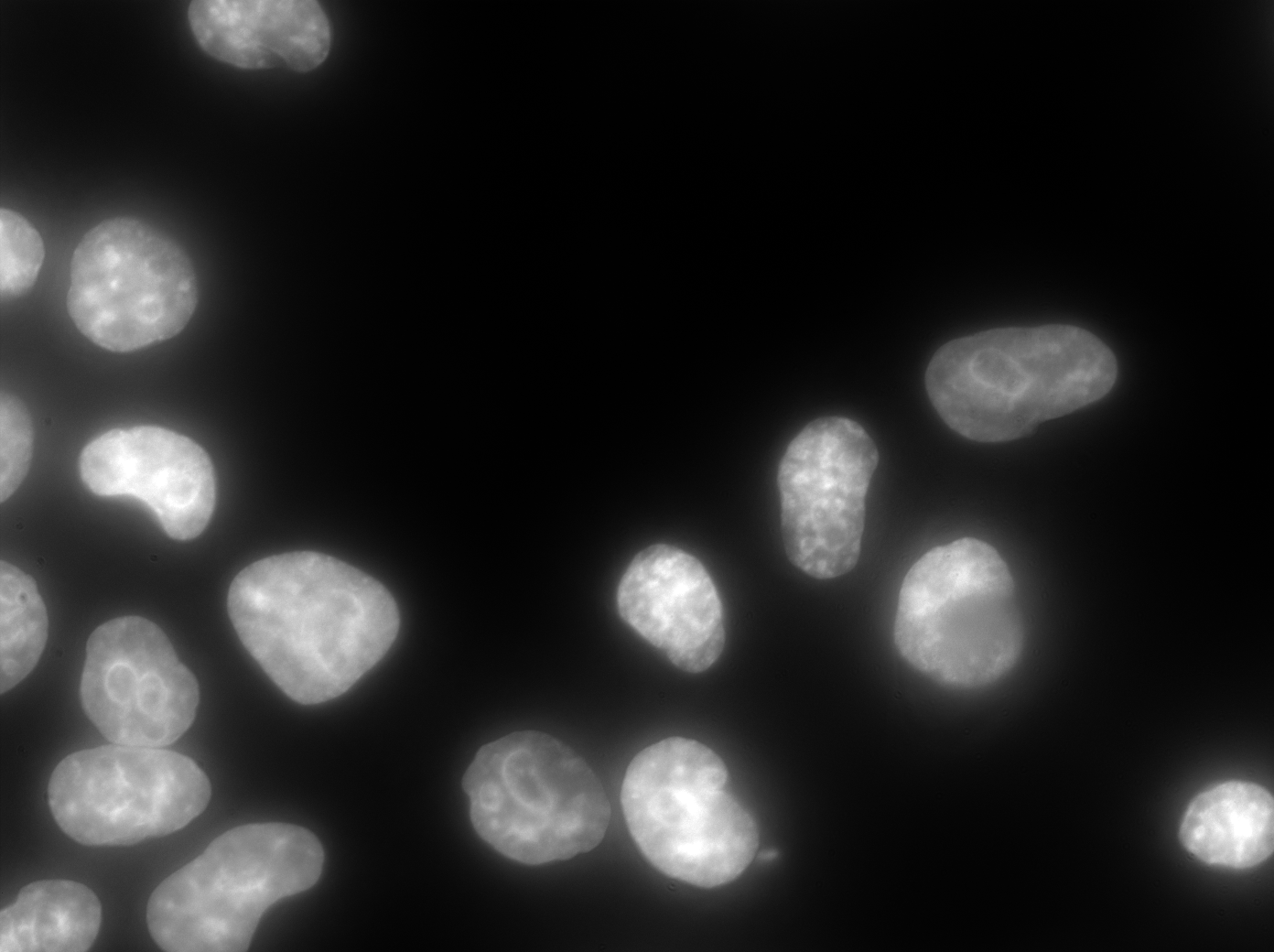

Supplement: Supplementary file 3 — Source data Fig. 1 [file 44319_2026_742_MOESM3_ESM.zip › Figure 1/Fig 1bcd WT Hela acetylated a tubulin atubulin/actub-atub 8-14-24 R3 LT10.Project Maximum Z_XY1724717708_Z0_T0_C0.tif]

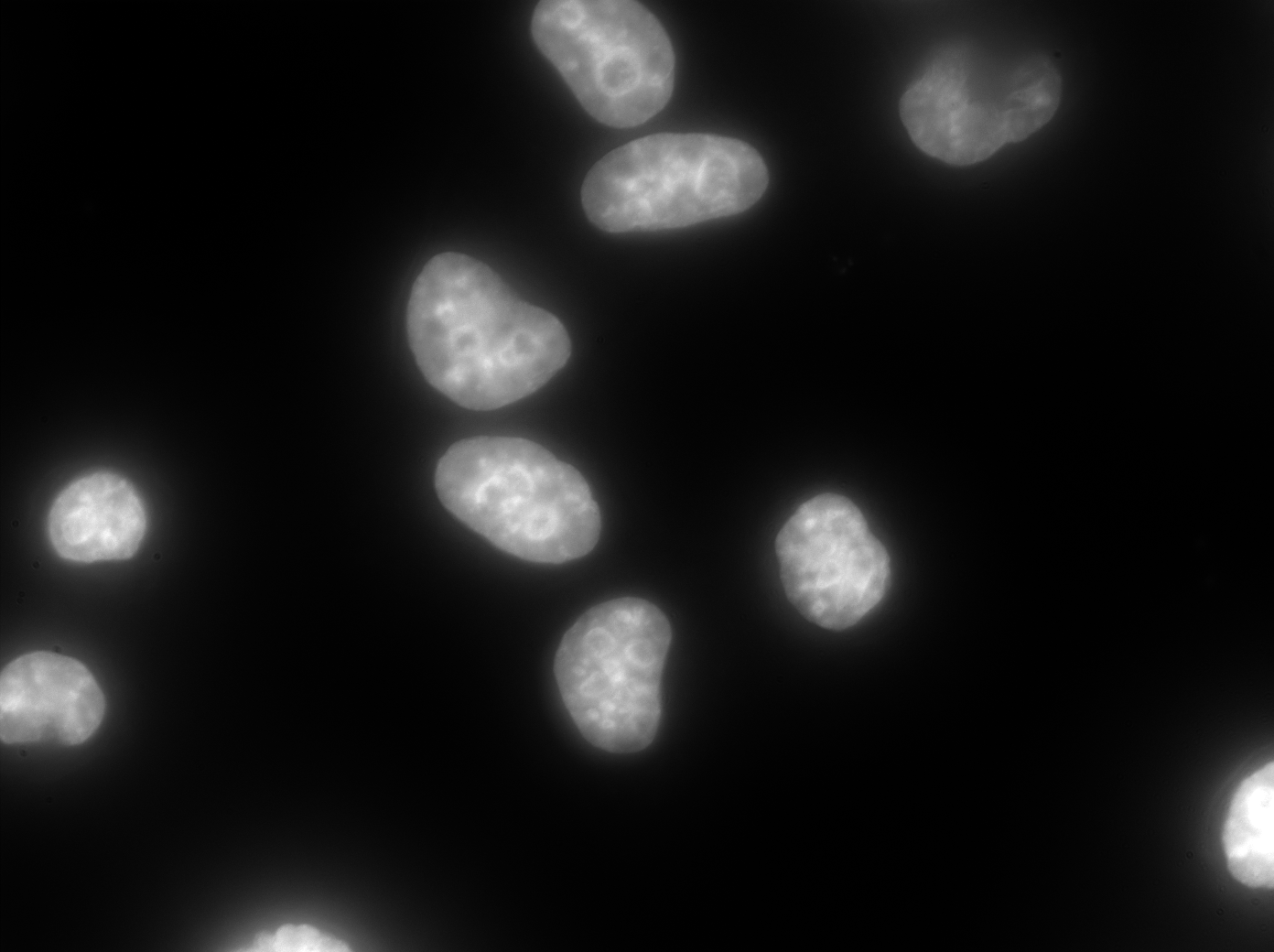

Supplement: Supplementary file 3 — Source data Fig. 1 [file 44319_2026_742_MOESM3_ESM.zip › Figure 1/Fig 1bcd WT Hela acetylated a tubulin atubulin/actub-atub 8-14-24 R3 PA1.Project Maximum Z_XY1724703264_Z0_T0_C0.tif]

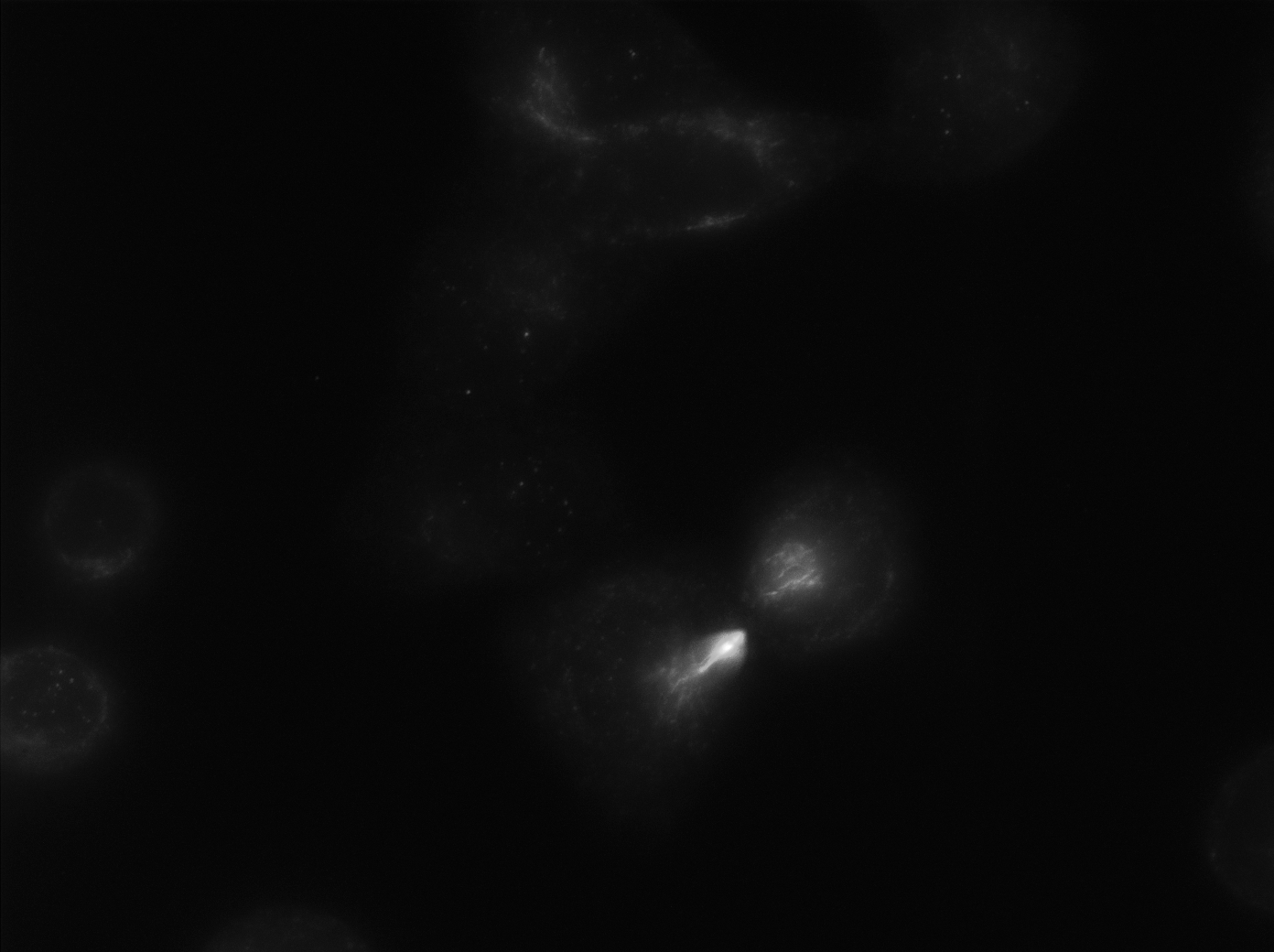

Supplement: Supplementary file 3 — Source data Fig. 1 [file 44319_2026_742_MOESM3_ESM.zip › Figure 1/Fig 1bcd WT Hela acetylated a tubulin atubulin/actub-atub 8-14-24 R3 PA1.Project Maximum Z_XY1724703264_Z0_T0_C2.tif]

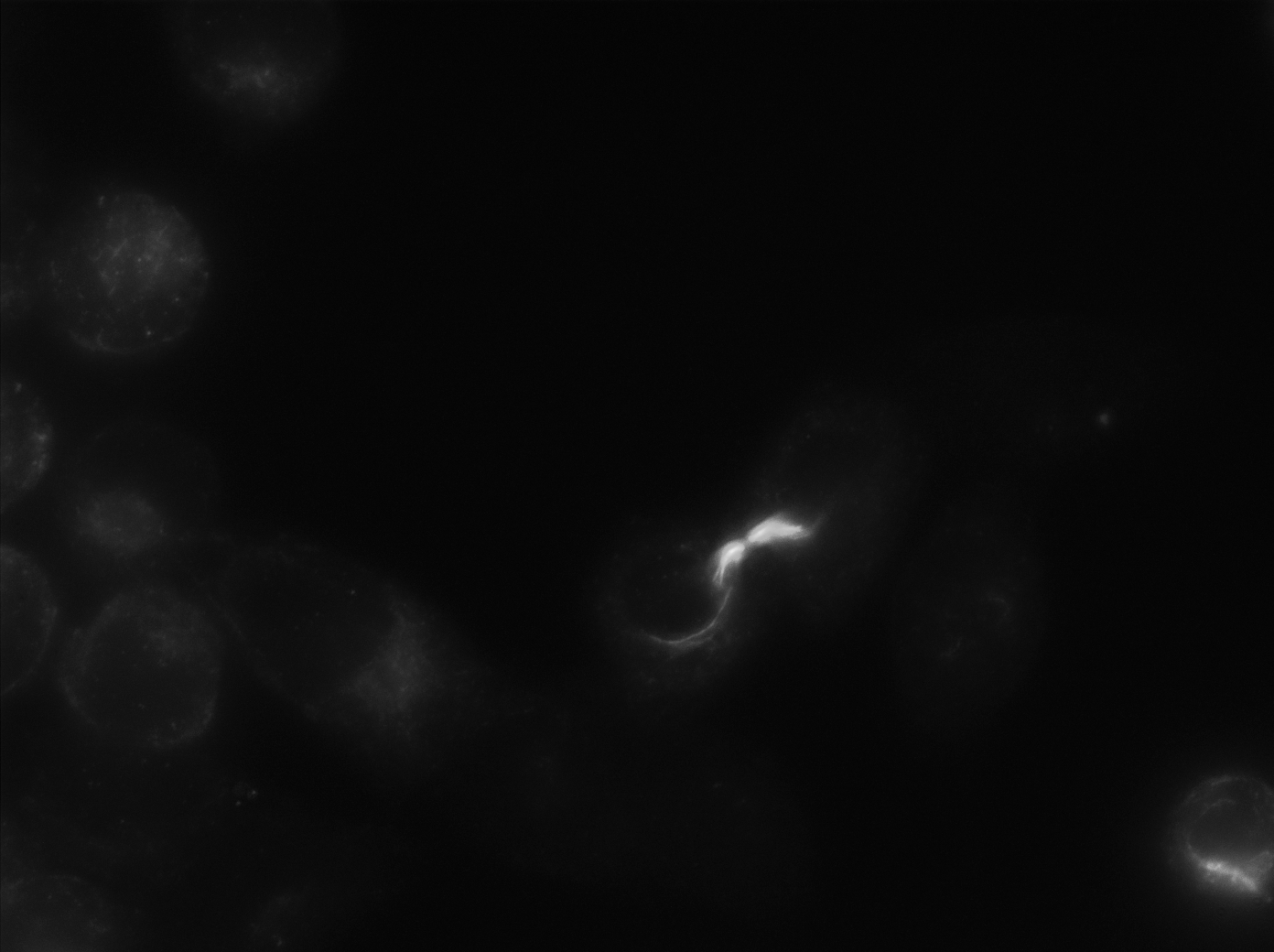

Supplement: Supplementary file 3 — Source data Fig. 1 [file 44319_2026_742_MOESM3_ESM.zip › Figure 1/Fig 1bcd WT Hela acetylated a tubulin atubulin/actub-atub 8-14-24 R3 LT10.Project Maximum Z_XY1724717708_Z0_T0_C2.tif]

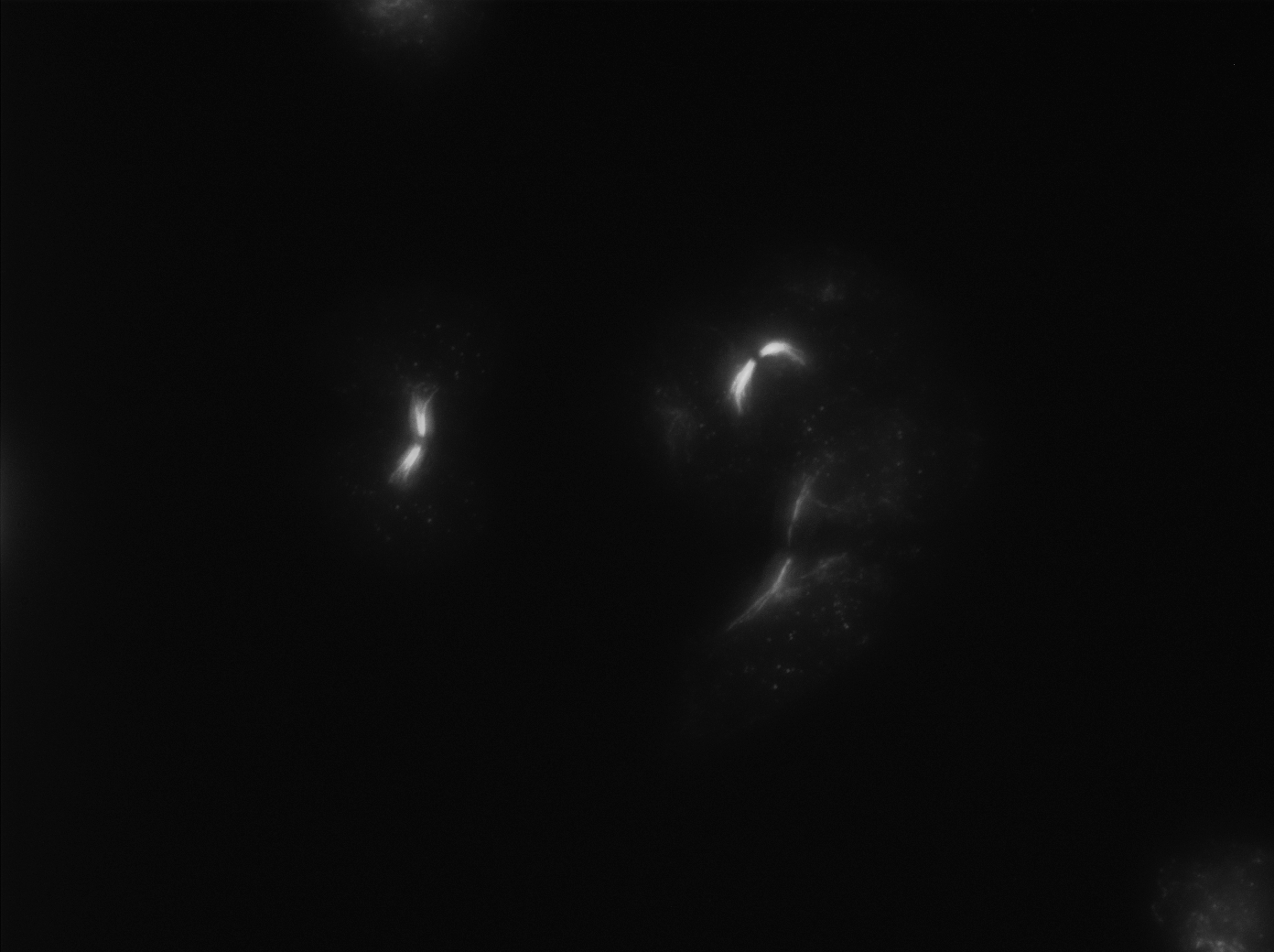

Supplement: Supplementary file 3 — Source data Fig. 1 [file 44319_2026_742_MOESM3_ESM.zip › Figure 1/Fig 1bcd WT Hela acetylated a tubulin atubulin/actub-atub 8-14-24 R1 LT9LT10LT11.Project Maximum Z_XY1724365990_Z0_T0_C2.tif]

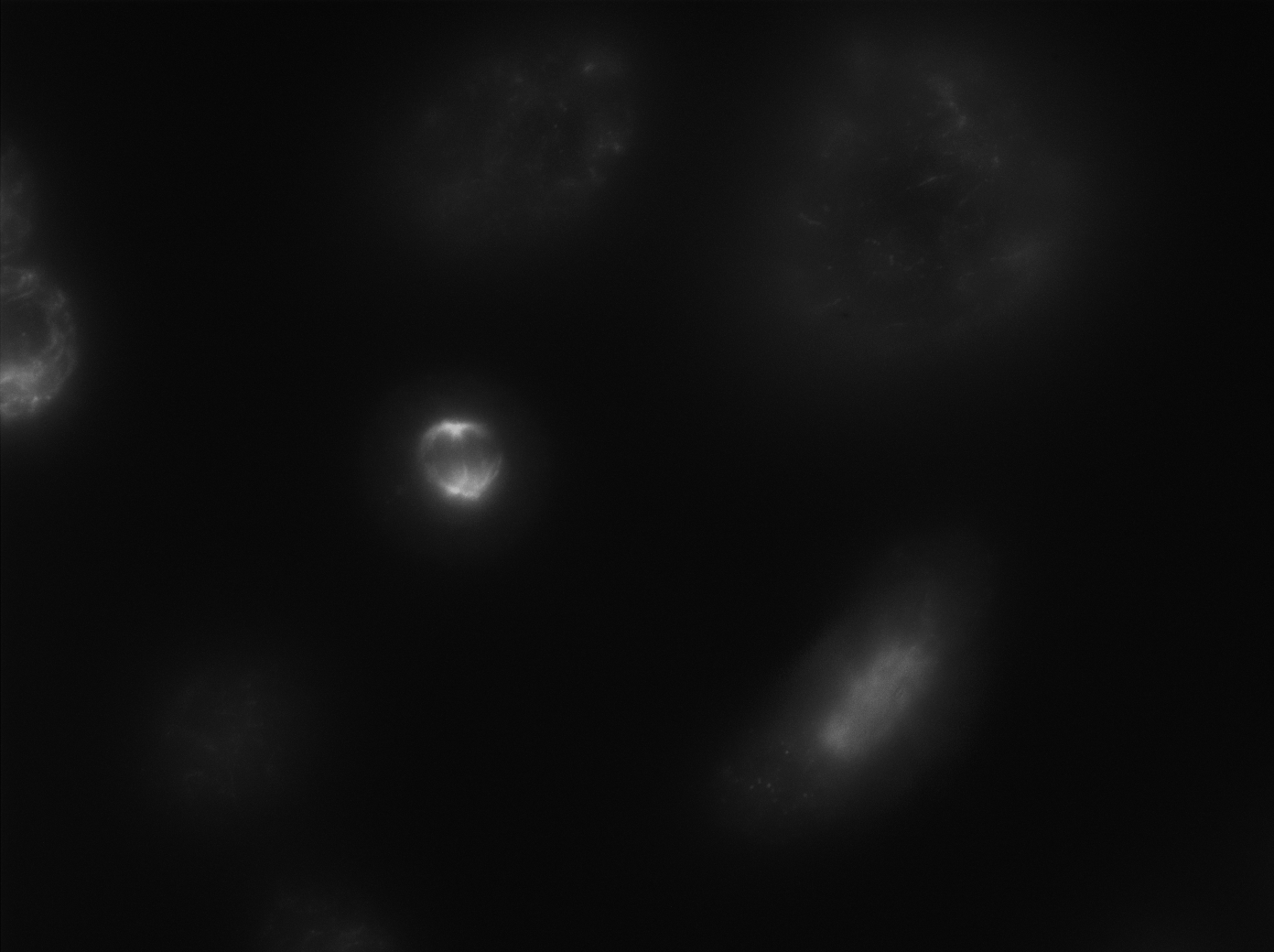

Supplement: Supplementary file 3 — Source data Fig. 1 [file 44319_2026_742_MOESM3_ESM.zip › Figure 1/Fig 1bcd WT Hela acetylated a tubulin atubulin/actub-atub 8-14-24 R2 M6.Project Maximum Z_XY1724693909_Z0_T0_C2.tif]

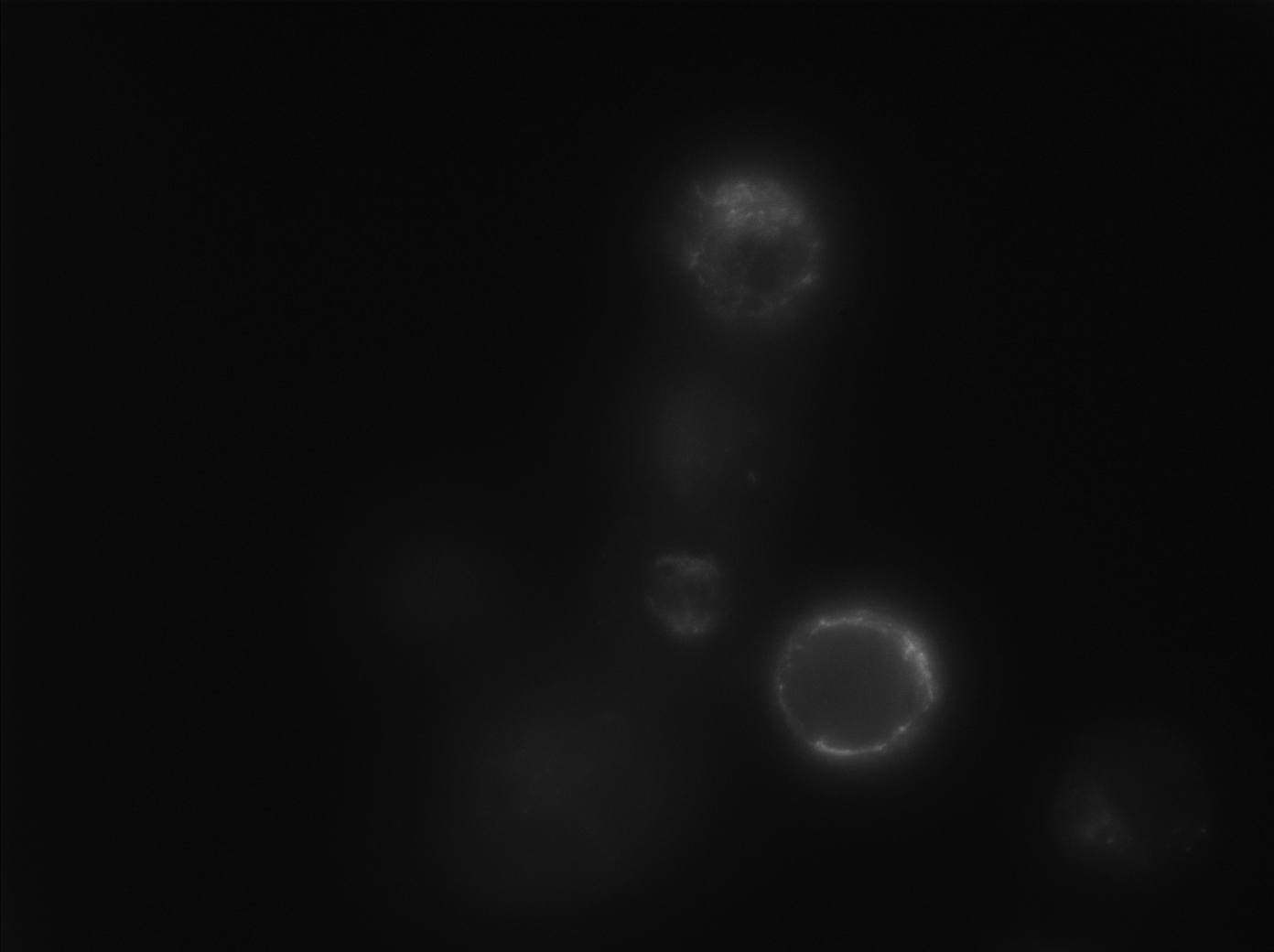

Supplement: Supplementary file 3 — Source data Fig. 1 [file 44319_2026_742_MOESM3_ESM.zip › Figure 1/Fig 1bcd WT Hela acetylated a tubulin atubulin/actub-atub 8-14-24 R1 M9.Project Maximum Z_XY1724366967_Z0_T0_C2.tif]

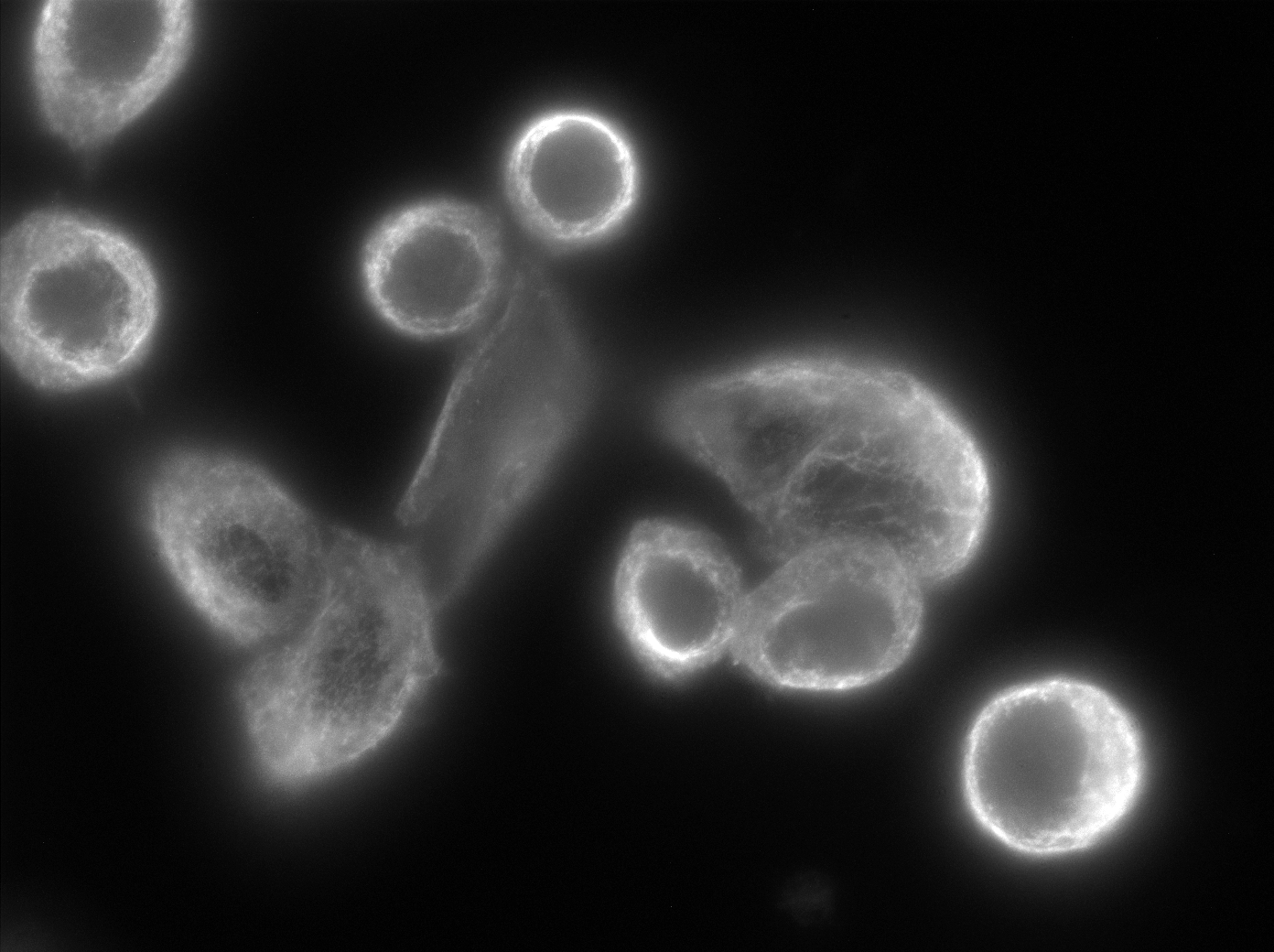

Supplement: Supplementary file 3 — Source data Fig. 1 [file 44319_2026_742_MOESM3_ESM.zip › Figure 1/Fig 1bcd WT Hela acetylated a tubulin atubulin/actub-atub 8-14-24 R3 PA4.Project Maximum Z_XY1724704169_Z0_T0_C1.tif]

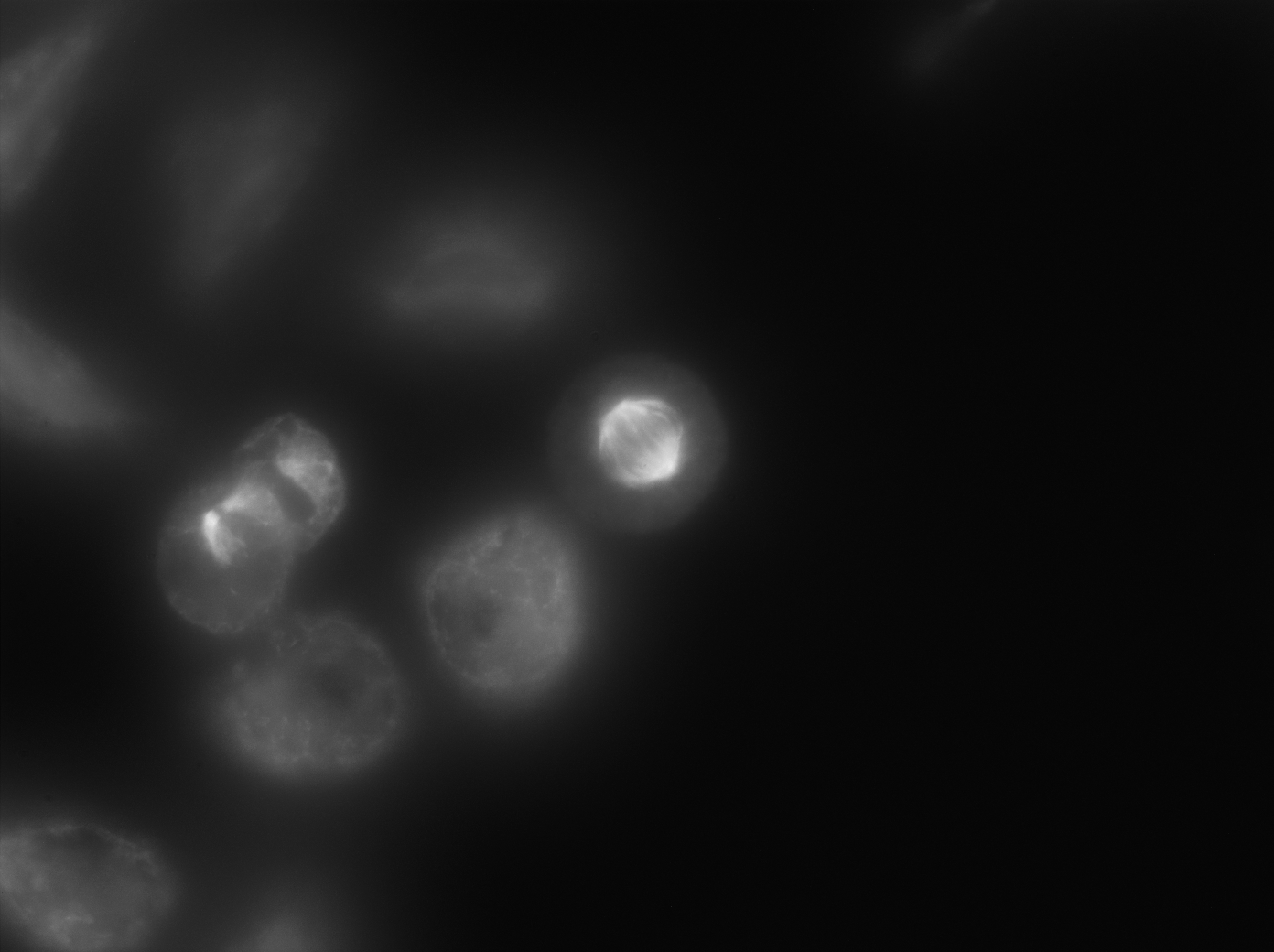

Supplement: Supplementary file 3 — Source data Fig. 1 [file 44319_2026_742_MOESM3_ESM.zip › Figure 1/Fig 1bcd WT Hela acetylated a tubulin atubulin/actub-atub 8-14-24 R2 M4.Project Maximum Z_XY1724693629_Z0_T0_C1.tif]

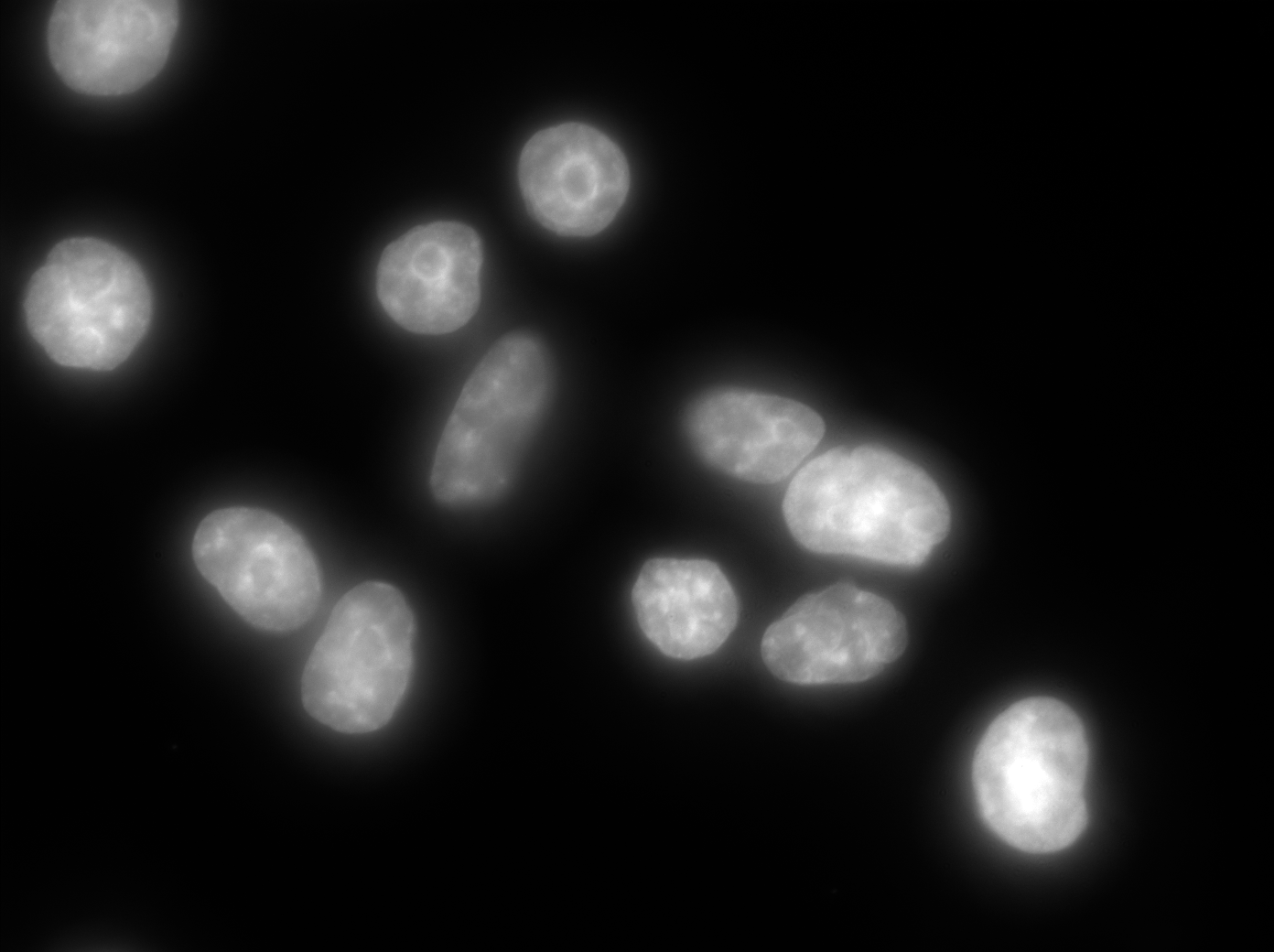

Supplement: Supplementary file 3 — Source data Fig. 1 [file 44319_2026_742_MOESM3_ESM.zip › Figure 1/Fig 1bcd WT Hela acetylated a tubulin atubulin/actub-atub 8-14-24 R3 PA4.Project Maximum Z_XY1724704169_Z0_T0_C0.tif]

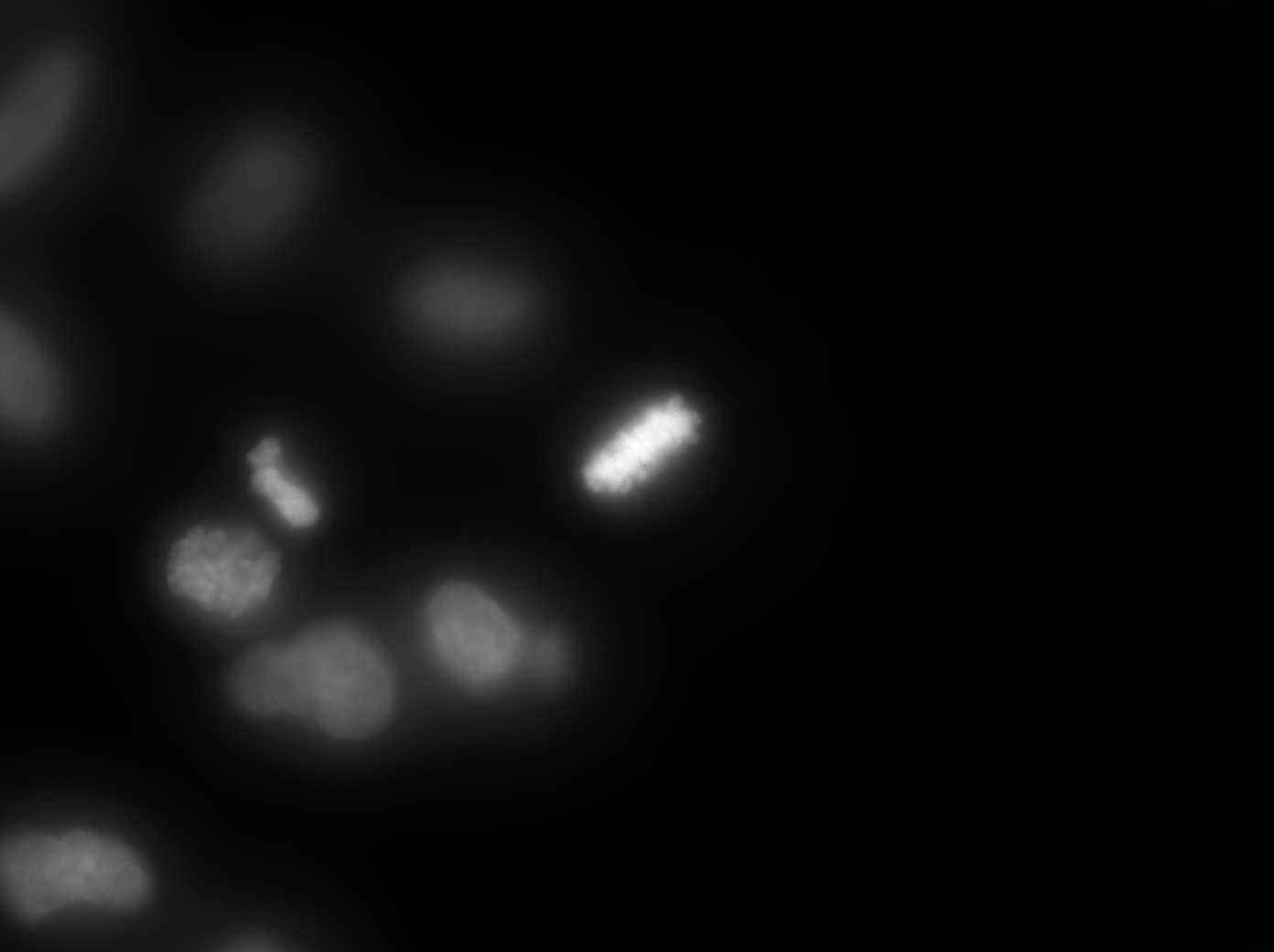

Supplement: Supplementary file 3 — Source data Fig. 1 [file 44319_2026_742_MOESM3_ESM.zip › Figure 1/Fig 1bcd WT Hela acetylated a tubulin atubulin/actub-atub 8-14-24 R2 M4.Project Maximum Z_XY1724693629_Z0_T0_C0.tif]

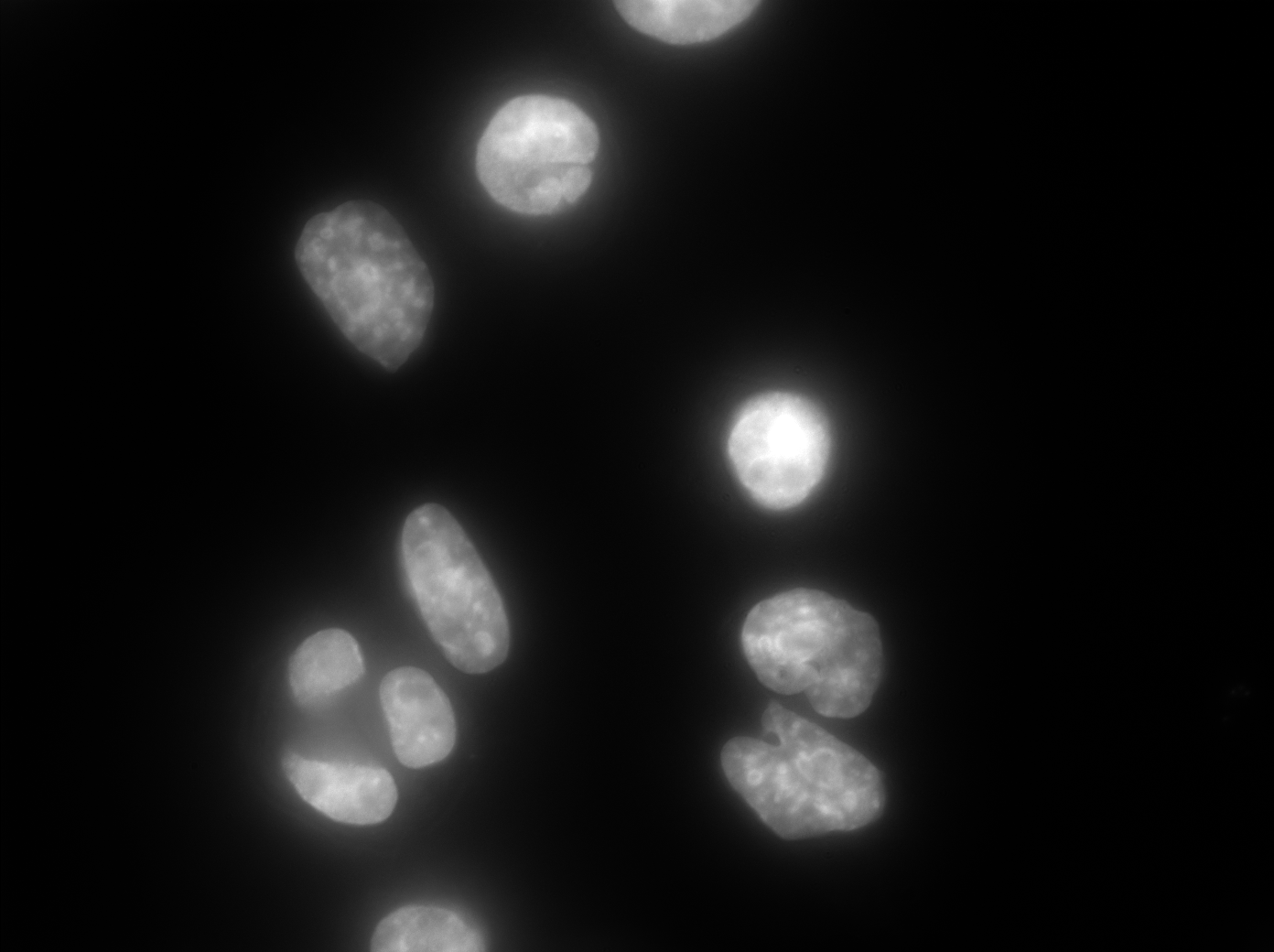

Supplement: Supplementary file 3 — Source data Fig. 1 [file 44319_2026_742_MOESM3_ESM.zip › Figure 1/Fig 1bcd WT Hela acetylated a tubulin atubulin/actub-atub 8-14-24 R3 LT3LT4.Project Maximum Z_XY1724703126_Z0_T0_C0.tif]

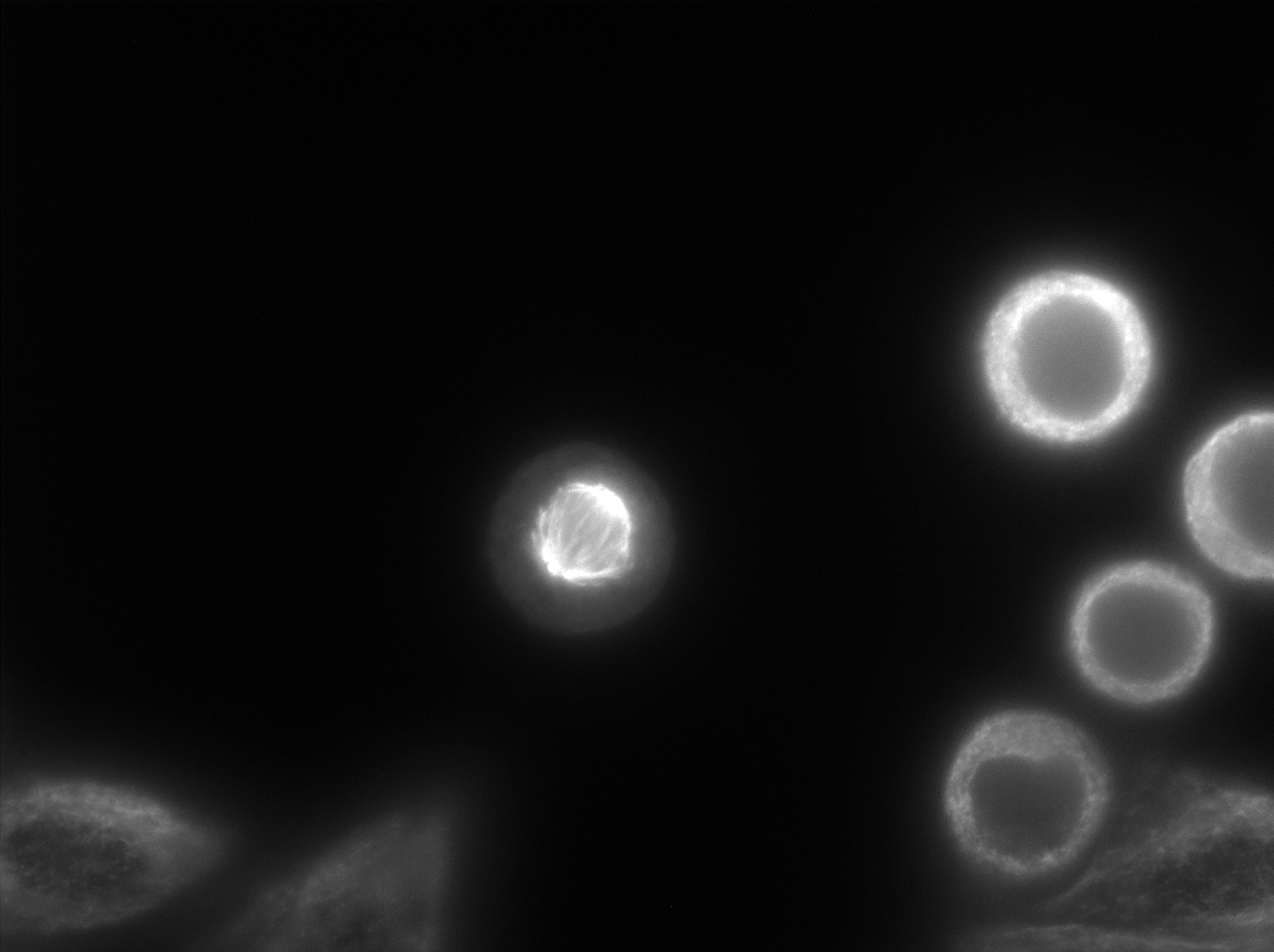

Supplement: Supplementary file 3 — Source data Fig. 1 [file 44319_2026_742_MOESM3_ESM.zip › Figure 1/Fig 1bcd WT Hela acetylated a tubulin atubulin/actub-atub 8-14-24 R3 M8.Project Maximum Z_XY1724703664_Z0_T0_C1.tif]

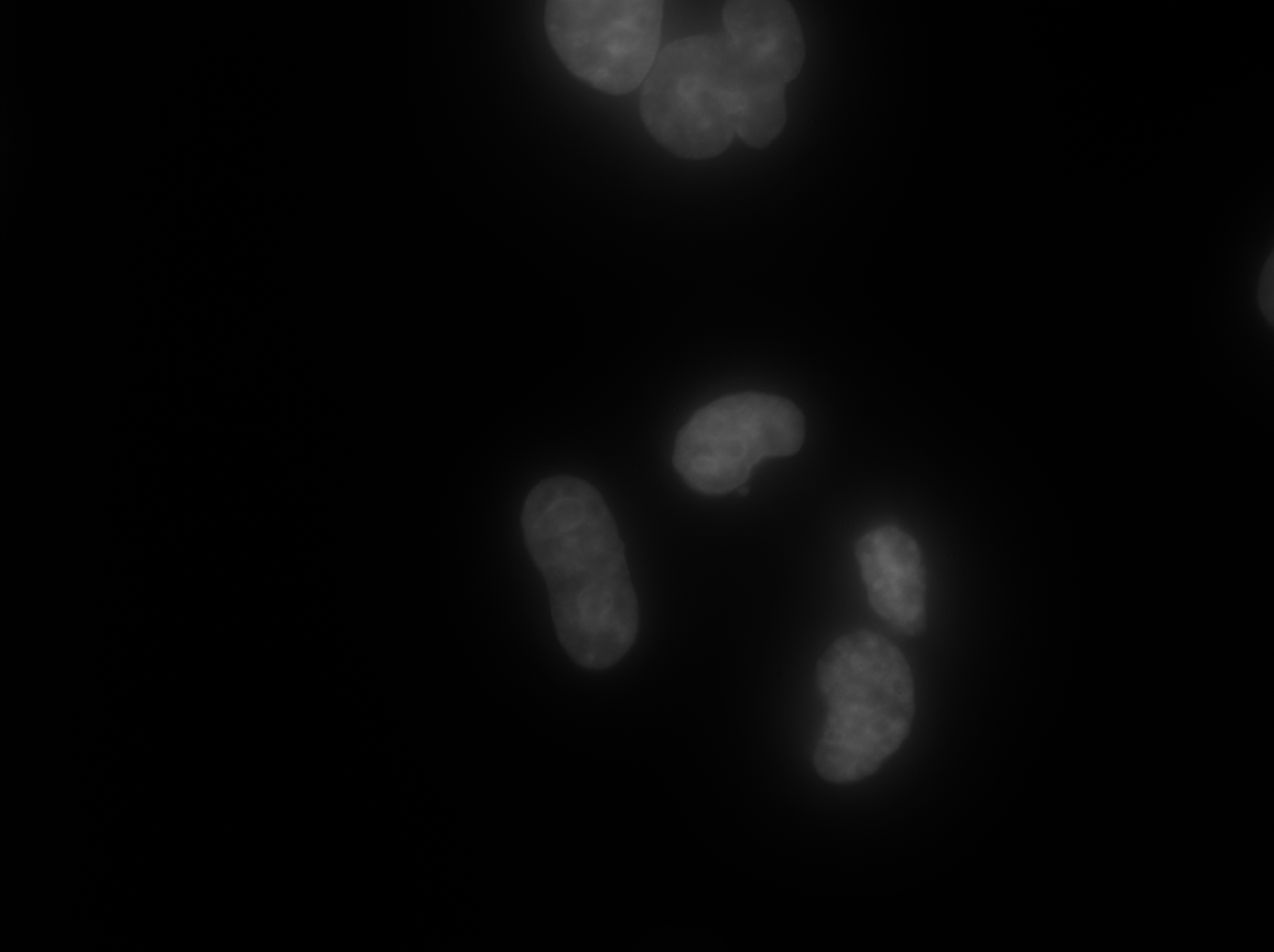

Supplement: Supplementary file 3 — Source data Fig. 1 [file 44319_2026_742_MOESM3_ESM.zip › Figure 1/Fig 1bcd WT Hela acetylated a tubulin atubulin/actub-atub 8-14-24 R2 PA7.Project Maximum Z_XY1724694856_Z0_T0_C0.tif]

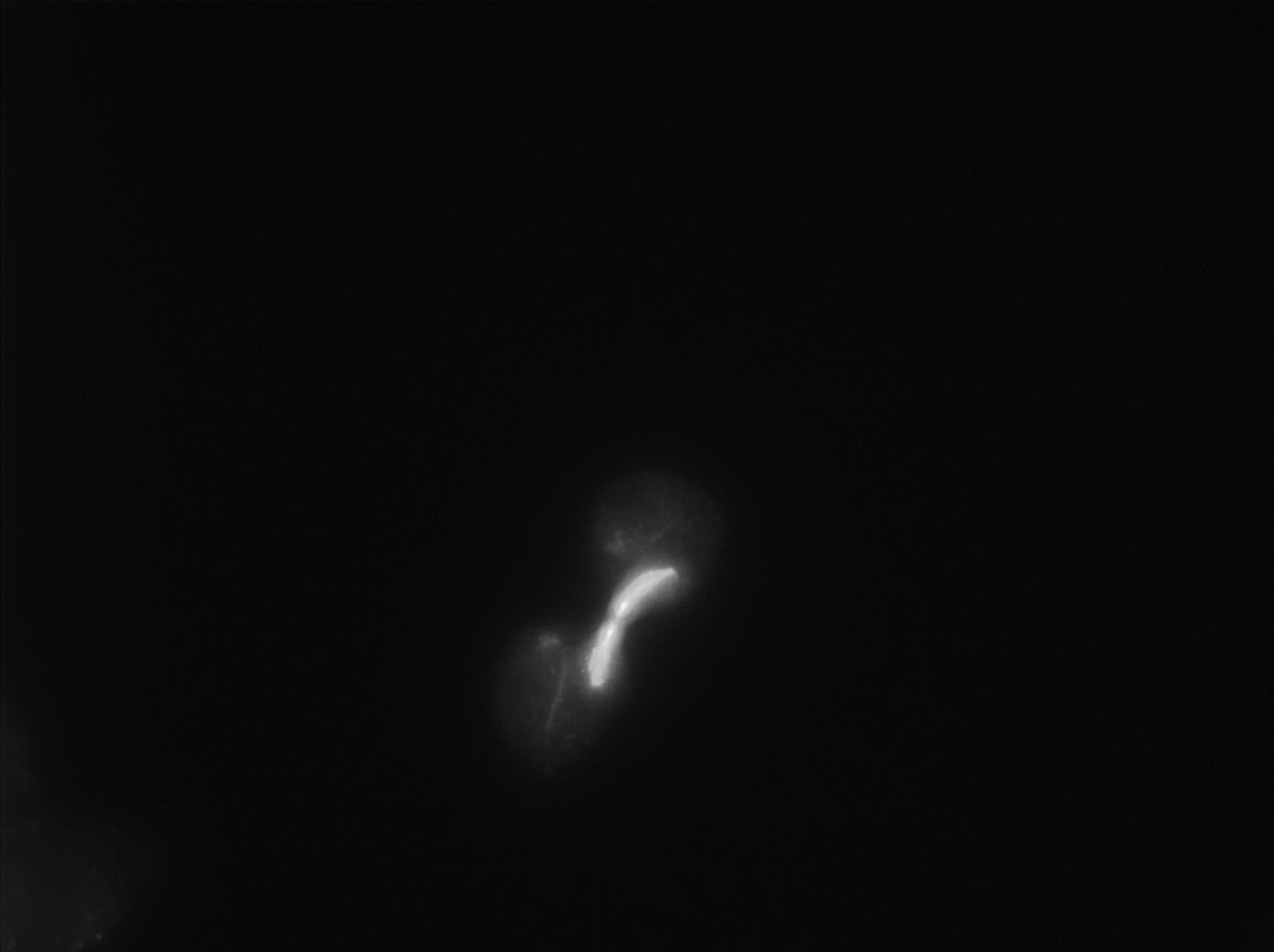

Supplement: Supplementary file 3 — Source data Fig. 1 [file 44319_2026_742_MOESM3_ESM.zip › Figure 1/Fig 1bcd WT Hela acetylated a tubulin atubulin/actub-atub 8-14-24 R1 ET2.Project Maximum Z_XY1724363021_Z0_T0_C2.tif]

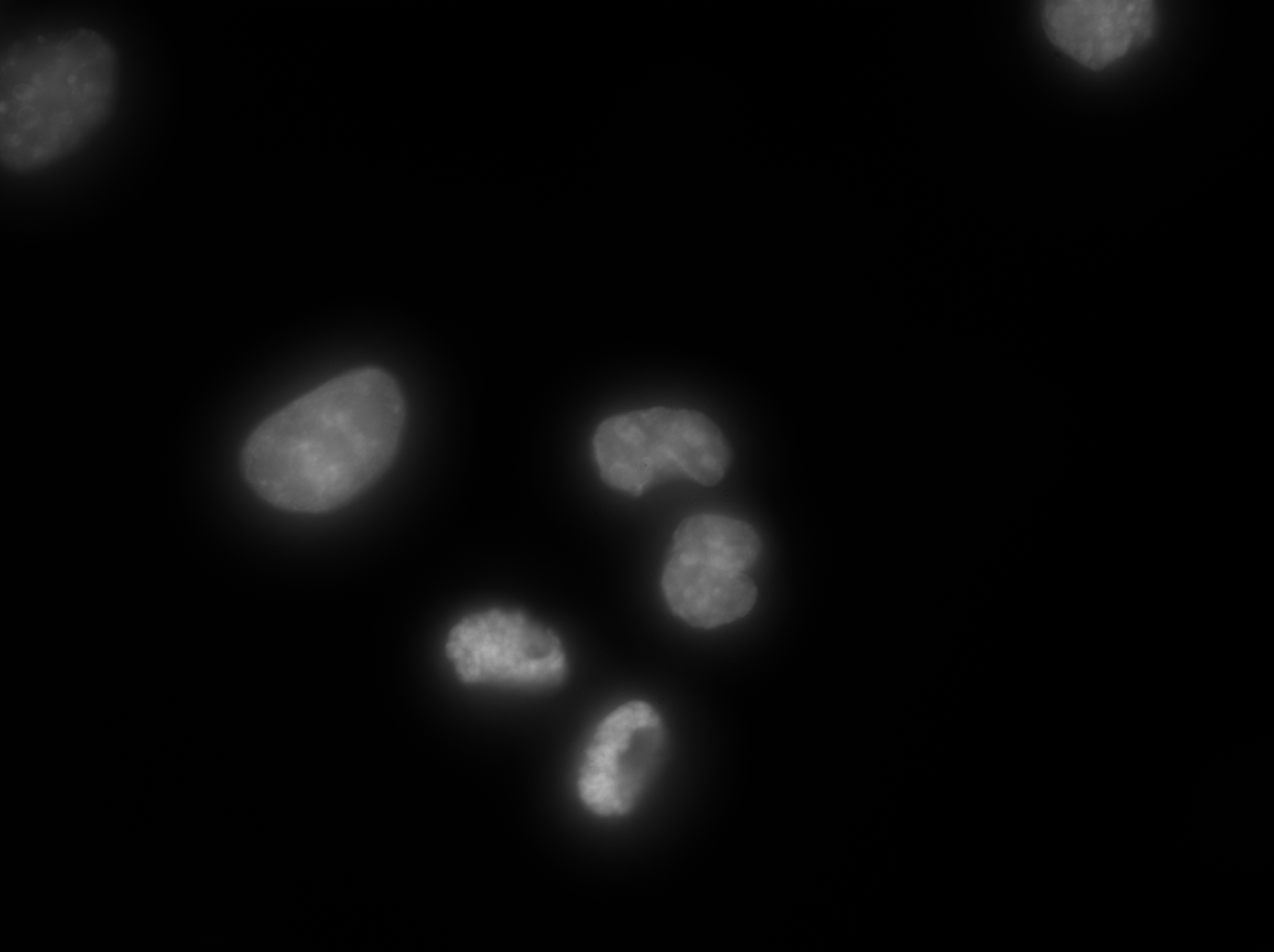

Supplement: Supplementary file 3 — Source data Fig. 1 [file 44319_2026_742_MOESM3_ESM.zip › Figure 1/Fig 1bcd WT Hela acetylated a tubulin atubulin/actub-atub 8-14-24 R2 ET6 PA4.Project Maximum Z_XY1724690530_Z0_T0_C0.tif]

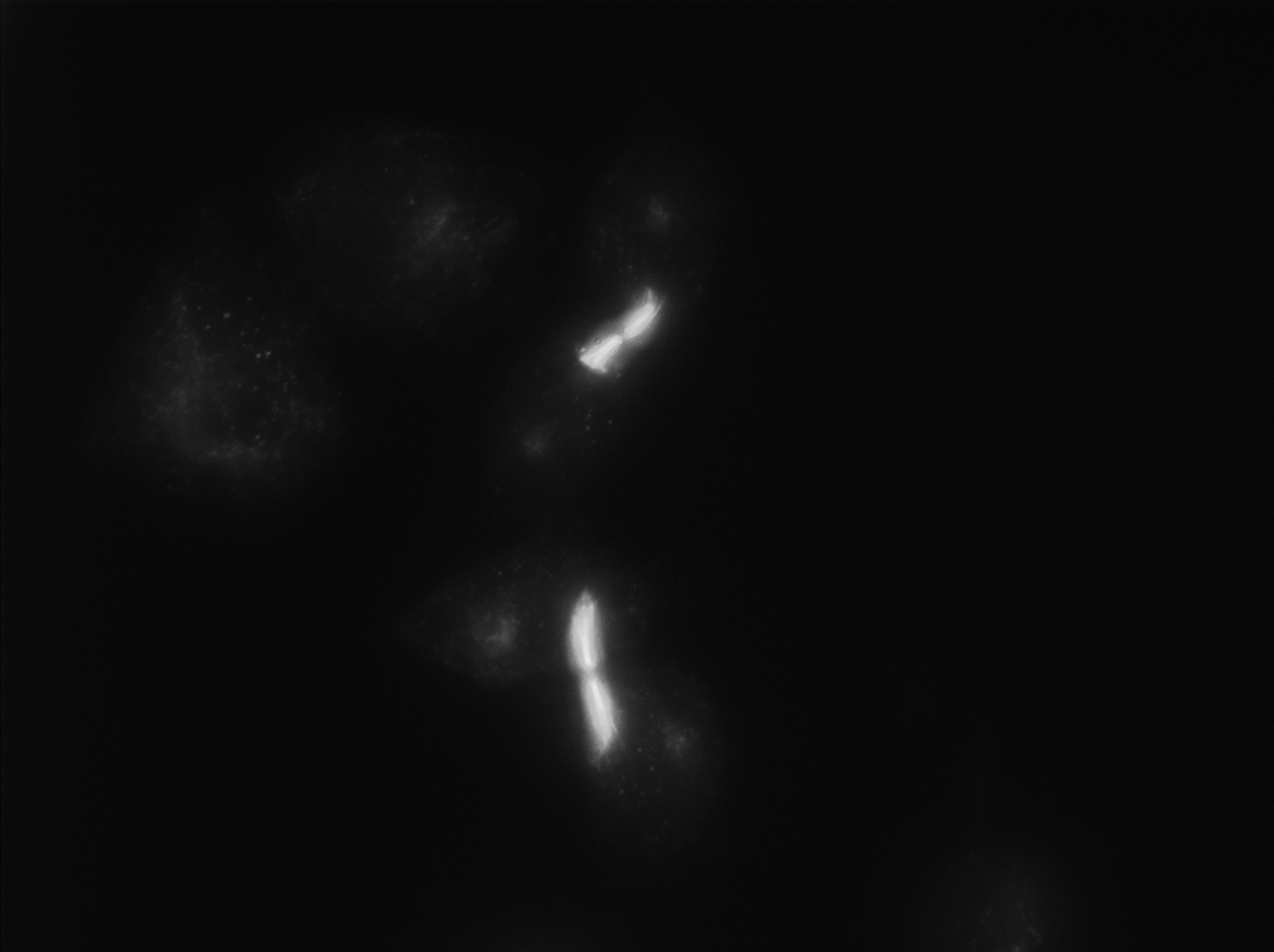

Supplement: Supplementary file 3 — Source data Fig. 1 [file 44319_2026_742_MOESM3_ESM.zip › Figure 1/Fig 1bcd WT Hela acetylated a tubulin atubulin/actub-atub 8-14-24 R2 LT7LT8.Project Maximum Z_XY1724693990_Z0_T0_C2.tif]

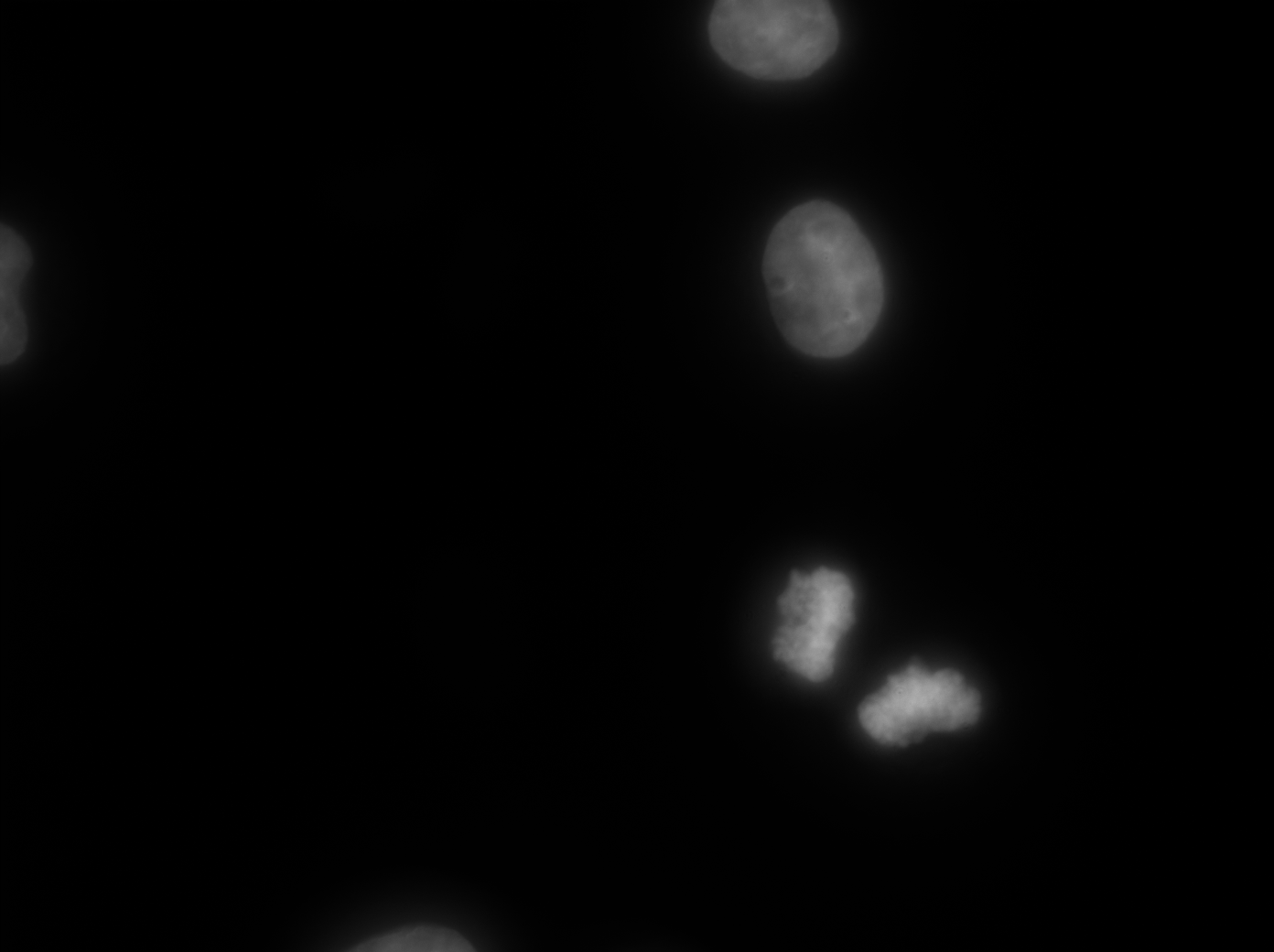

Supplement: Supplementary file 3 — Source data Fig. 1 [file 44319_2026_742_MOESM3_ESM.zip › Figure 1/Fig 1bcd WT Hela acetylated a tubulin atubulin/actub-atub 8-14-24 R2 ET4.Project Maximum Z_XY1724690168_Z0_T0_C0.tif]

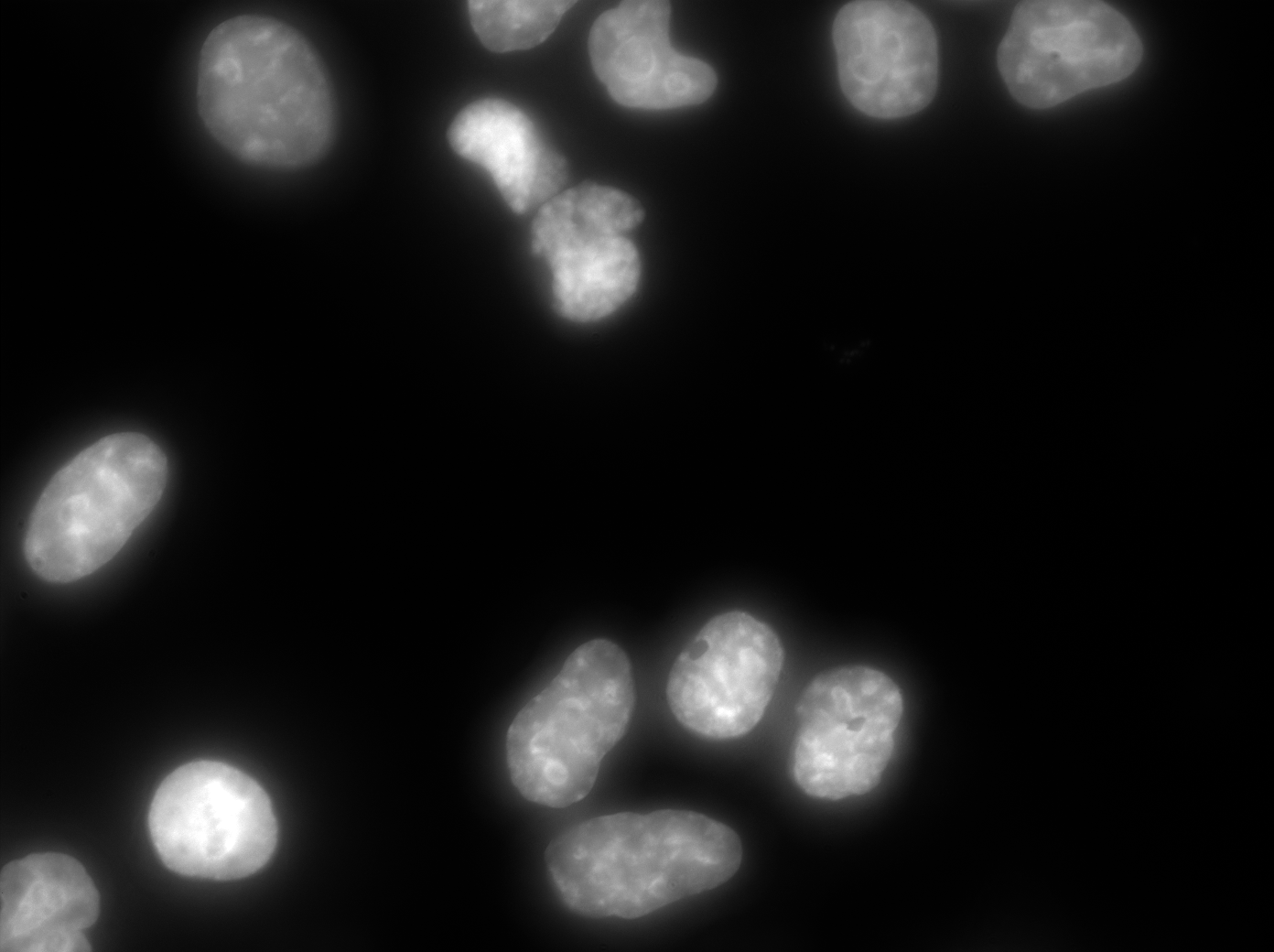

Supplement: Supplementary file 3 — Source data Fig. 1 [file 44319_2026_742_MOESM3_ESM.zip › Figure 1/Fig 1bcd WT Hela acetylated a tubulin atubulin/actub-atub 8-14-24 R3 ET6 PA2.Project Maximum Z_XY1724703496_Z0_T0_C0.tif]

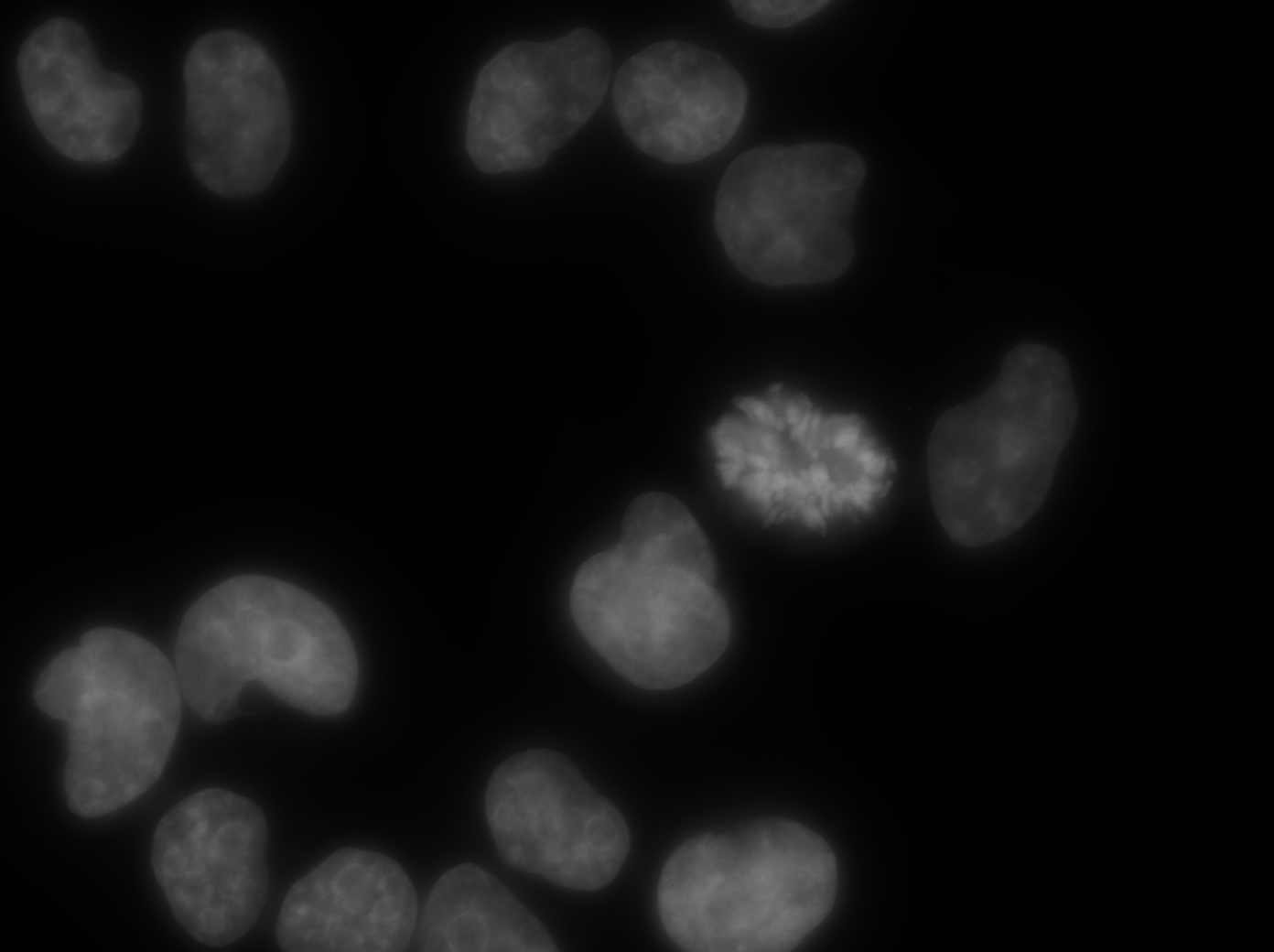

Supplement: Supplementary file 3 — Source data Fig. 1 [file 44319_2026_742_MOESM3_ESM.zip › Figure 1/Fig 1bcd WT Hela acetylated a tubulin atubulin/actub-atub 8-14-24 R2 M3.Project Maximum Z_XY1724693151_Z0_T0_C0.tif]

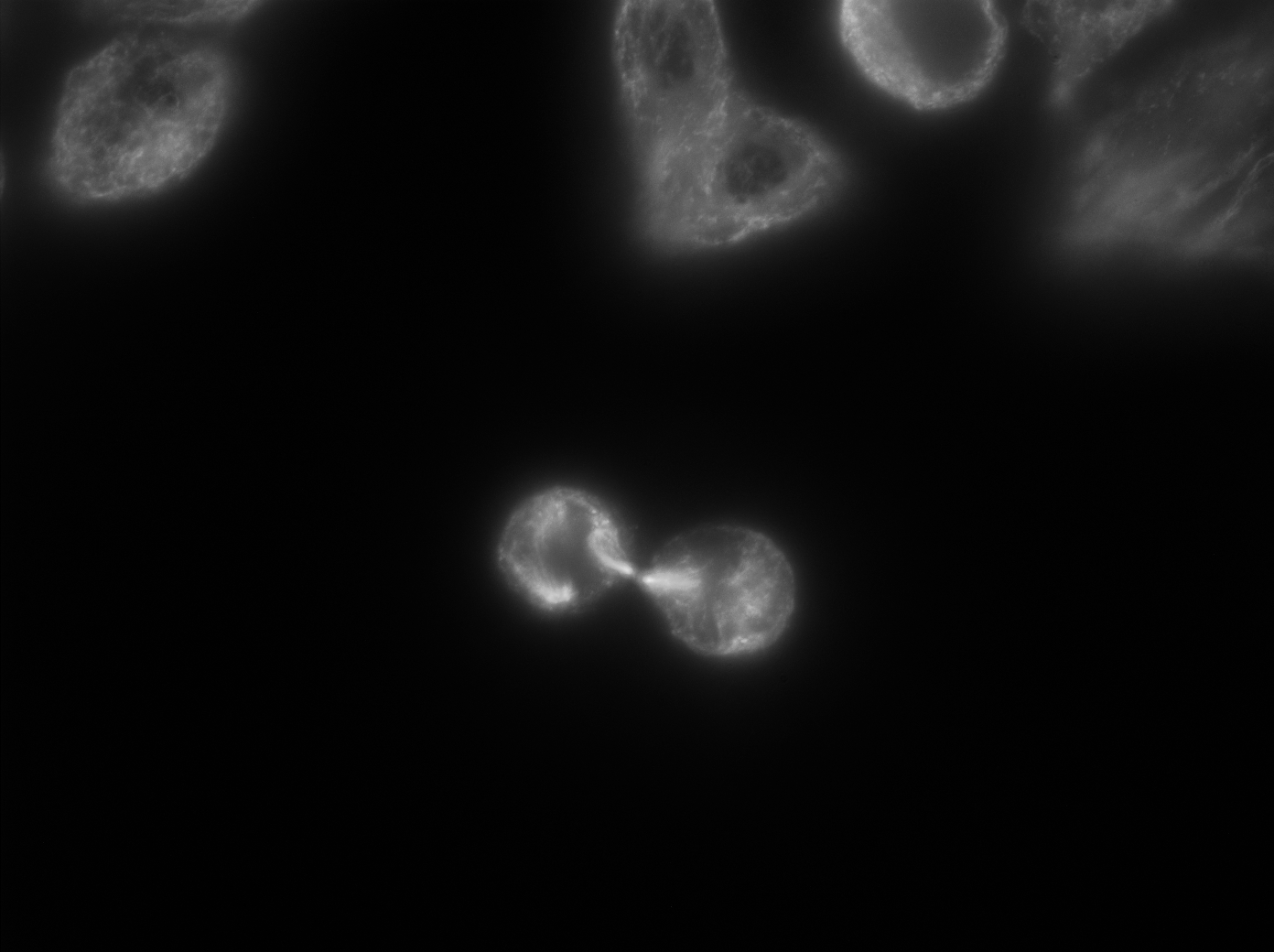

Supplement: Supplementary file 3 — Source data Fig. 1 [file 44319_2026_742_MOESM3_ESM.zip › Figure 1/Fig 1bcd WT Hela acetylated a tubulin atubulin/actub-atub 8-14-24 R1 ET1.Project Maximum Z_XY1724362932_Z0_T0_C1.tif]

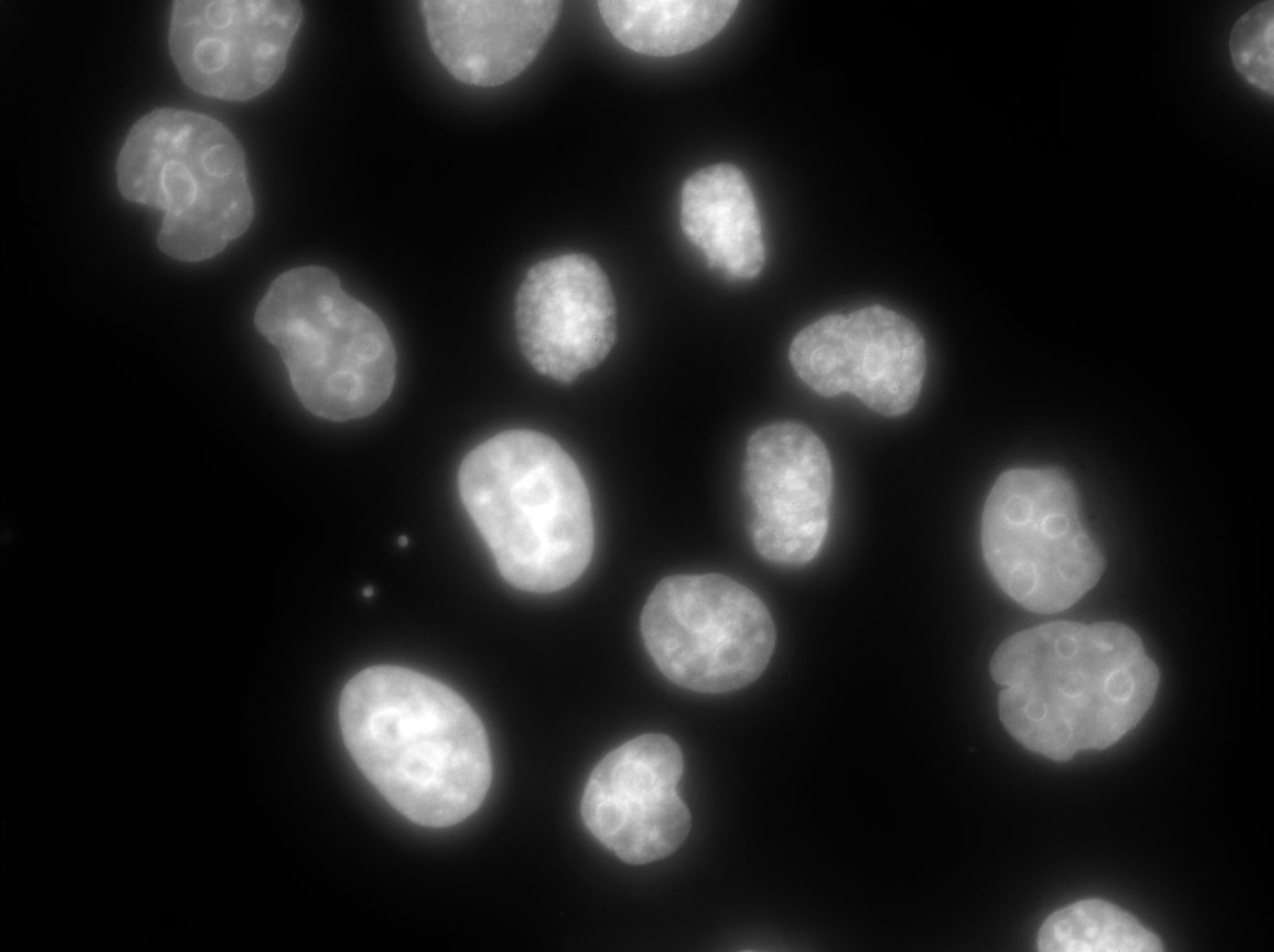

Supplement: Supplementary file 3 — Source data Fig. 1 [file 44319_2026_742_MOESM3_ESM.zip › Figure 1/Fig 1bcd WT Hela acetylated a tubulin atubulin/actub-atub 8-14-24 R3 LT6LT7.Project Maximum Z_XY1724716888_Z0_T0_C0.tif]

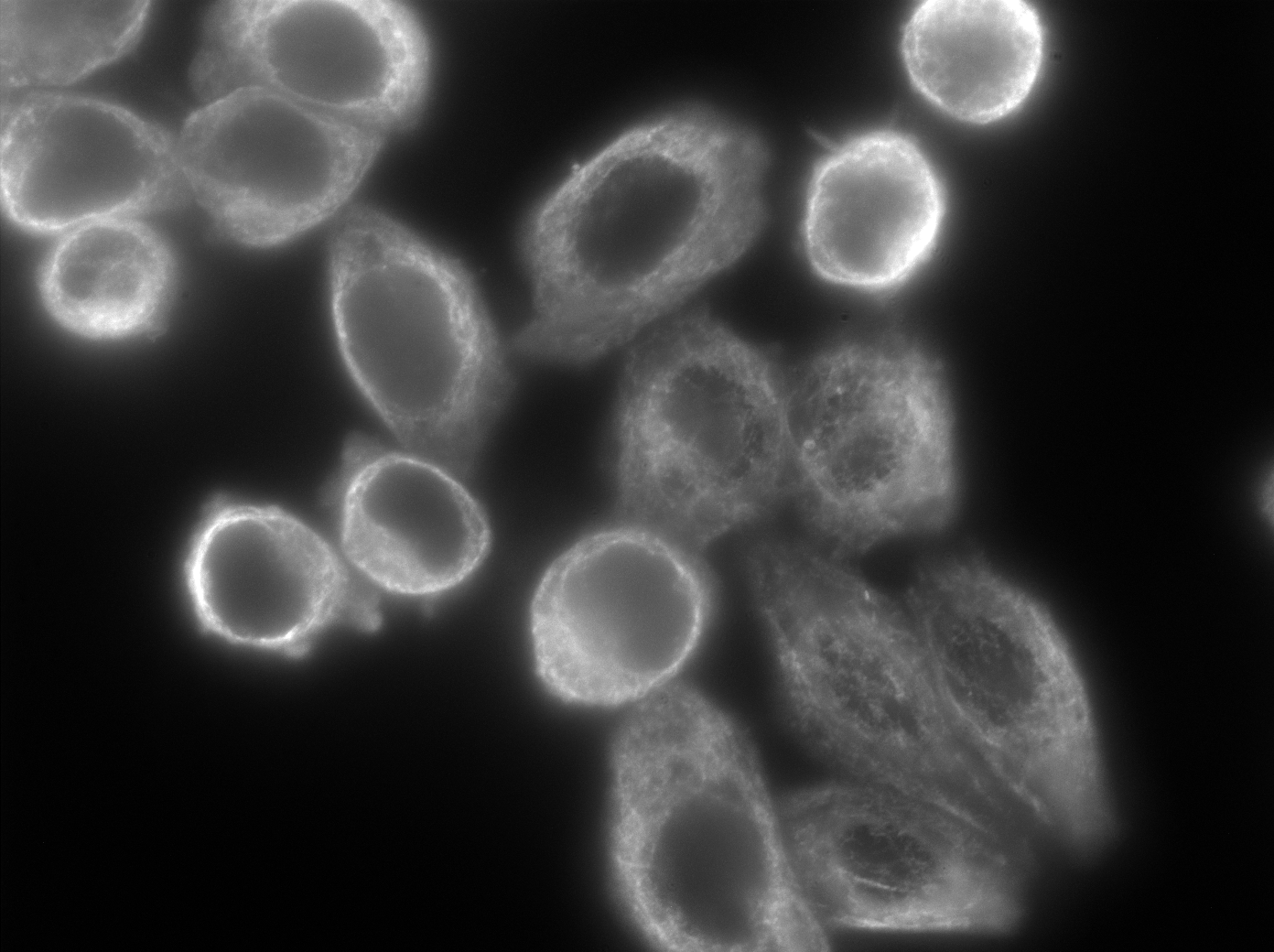

Supplement: Supplementary file 3 — Source data Fig. 1 [file 44319_2026_742_MOESM3_ESM.zip › Figure 1/Fig 1bcd WT Hela acetylated a tubulin atubulin/actub-atub 8-14-24 R3 PA8.Project Maximum Z_XY1724717079_Z0_T0_C1.tif]

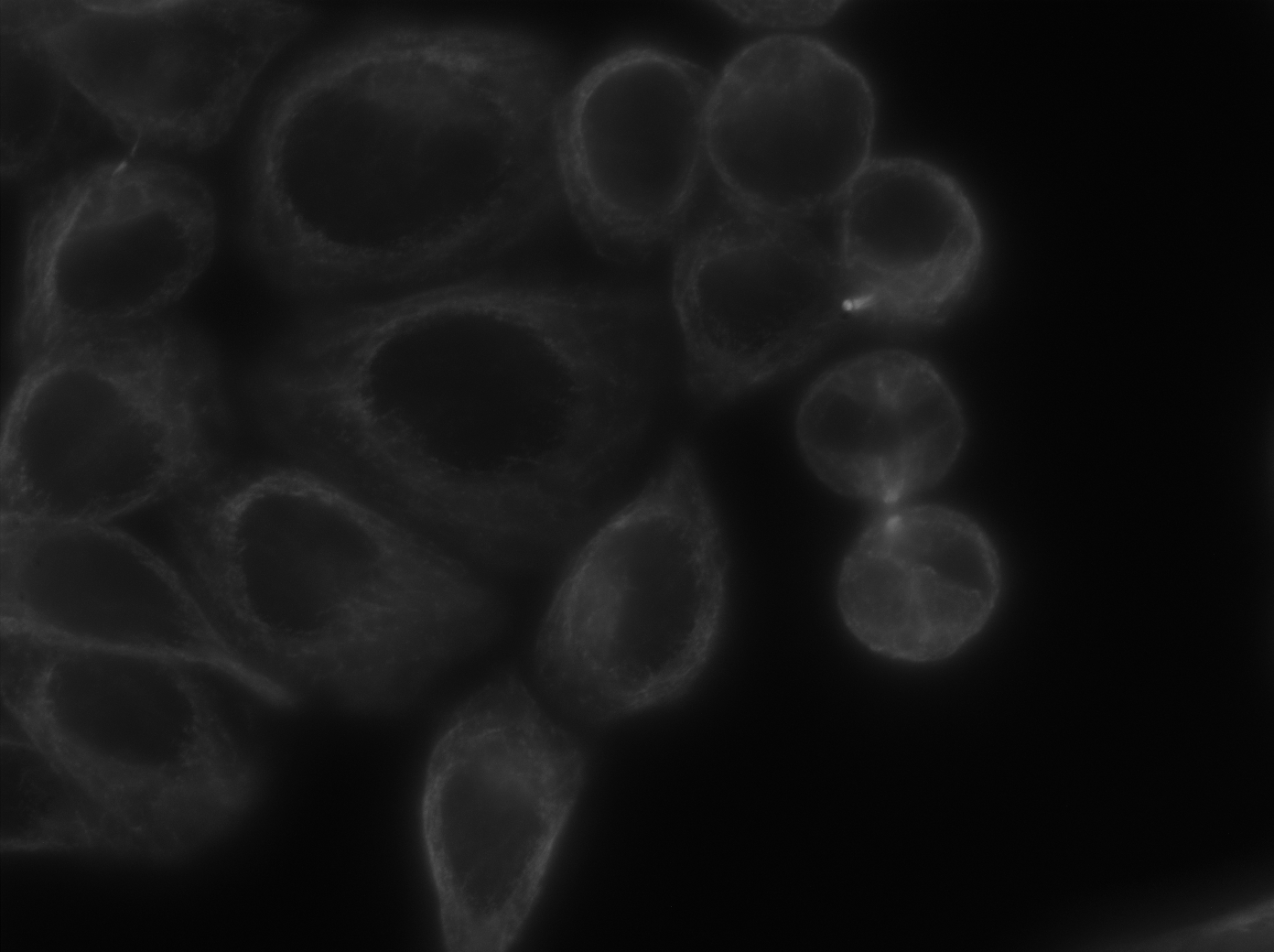

Supplement: Supplementary file 3 — Source data Fig. 1 [file 44319_2026_742_MOESM3_ESM.zip › Figure 1/Fig 1bcd WT Hela acetylated a tubulin atubulin/actub-atub 8-14-24 R2 ET10 PA10.Project Maximum Z_XY1724695557_Z0_T0_C1.tif]

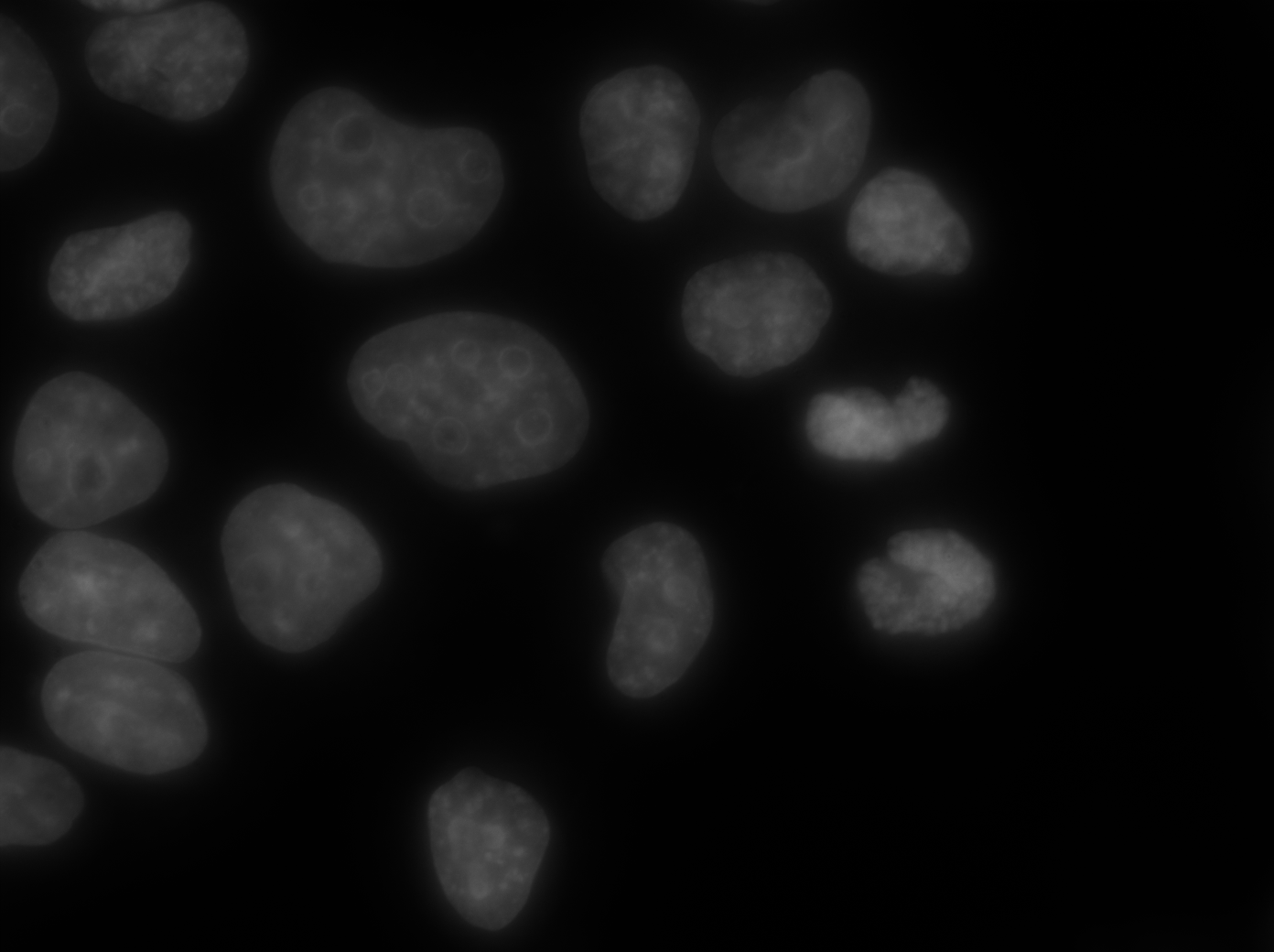

Supplement: Supplementary file 3 — Source data Fig. 1 [file 44319_2026_742_MOESM3_ESM.zip › Figure 1/Fig 1bcd WT Hela acetylated a tubulin atubulin/actub-atub 8-14-24 R2 ET10 PA10.Project Maximum Z_XY1724695557_Z0_T0_C0.tif]

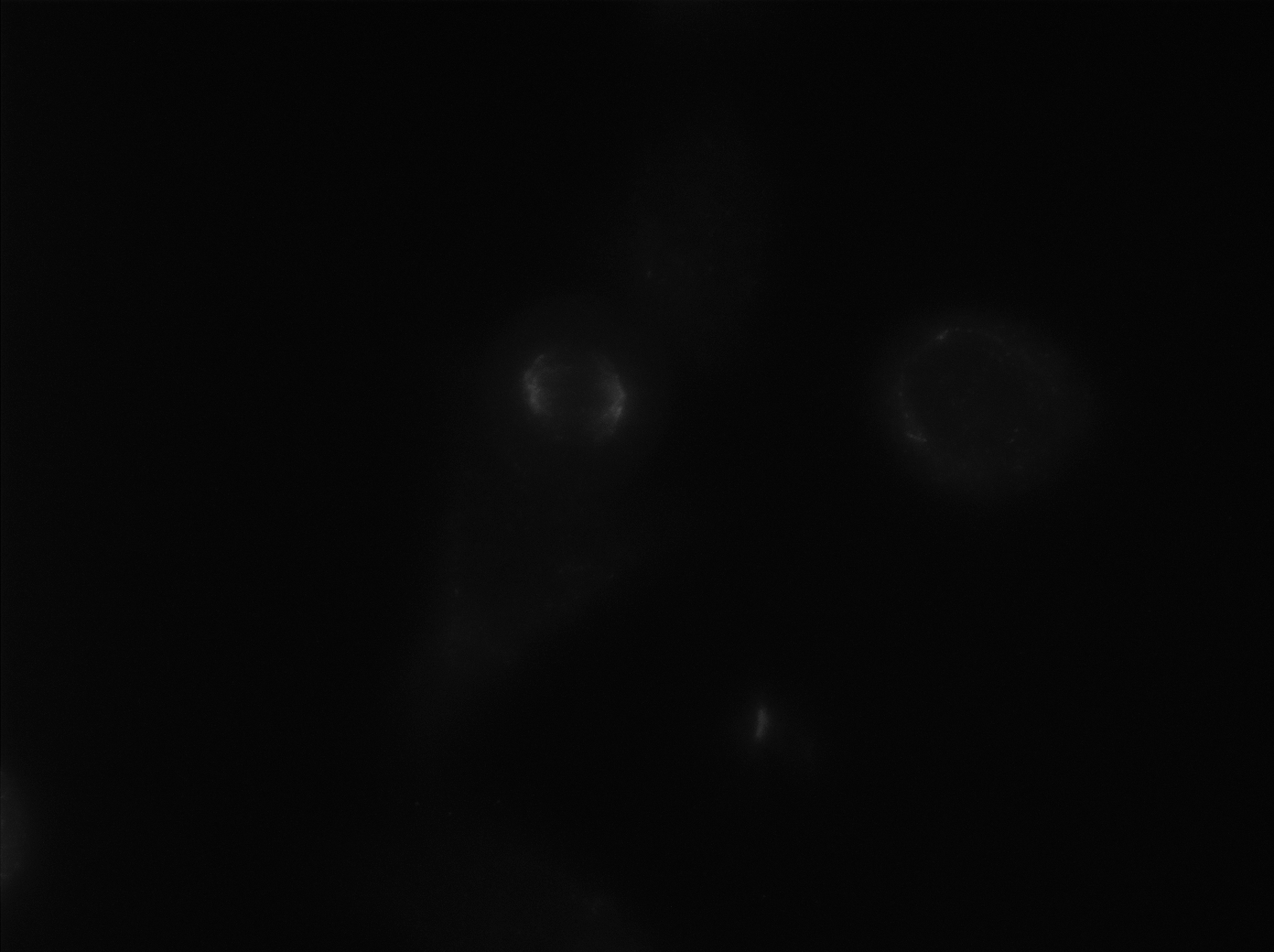

Supplement: Supplementary file 3 — Source data Fig. 1 [file 44319_2026_742_MOESM3_ESM.zip › Figure 1/Fig 1bcd WT Hela acetylated a tubulin atubulin/actub-atub 8-14-24 R3 M10.Project Maximum Z_XY1724703982_Z0_T0_C2.tif]

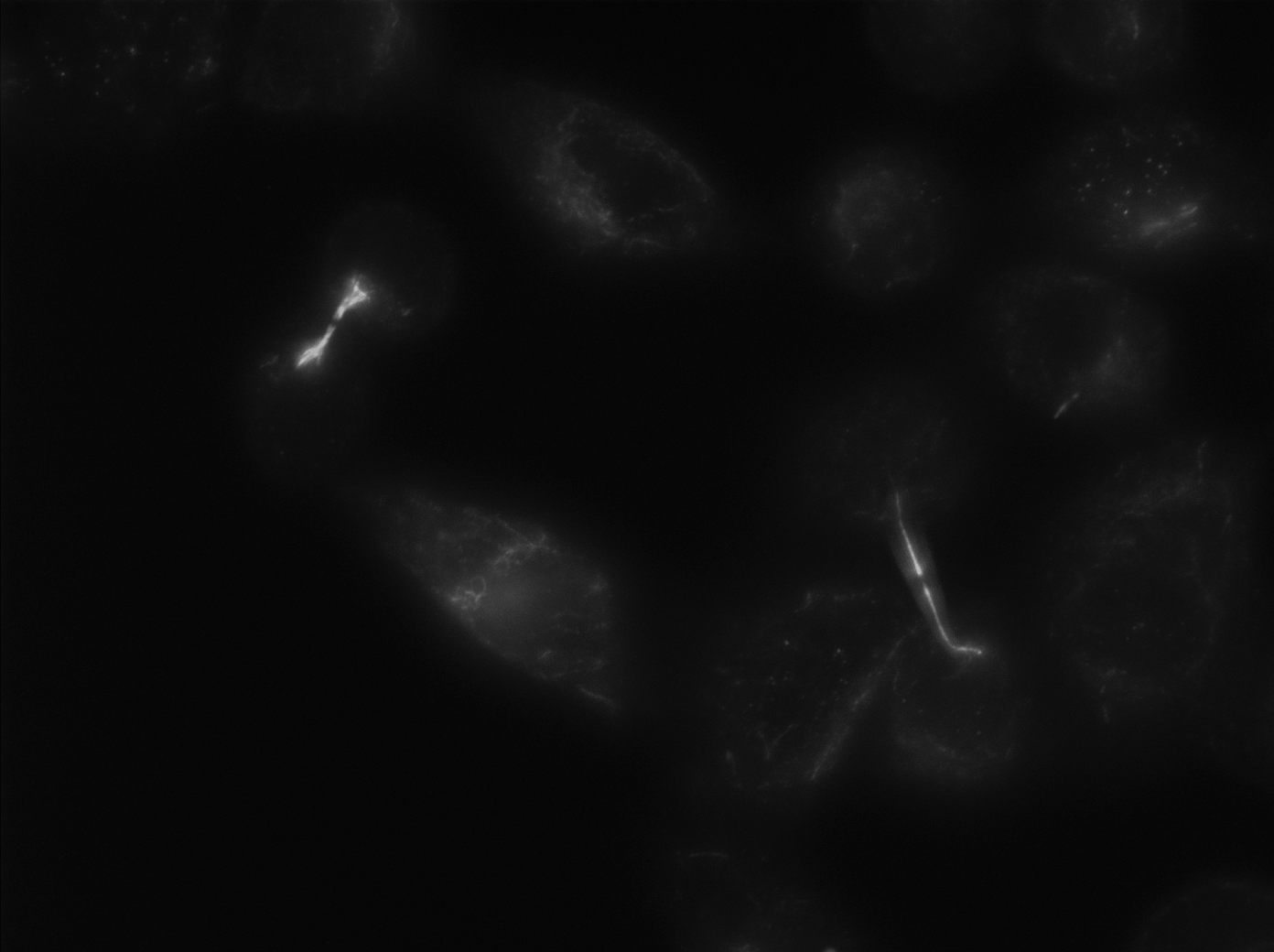

Supplement: Supplementary file 3 — Source data Fig. 1 [file 44319_2026_742_MOESM3_ESM.zip › Figure 1/Fig 1bcd WT Hela acetylated a tubulin atubulin/actub-atub 8-14-24 R3 LT8LT9.Project Maximum Z_XY1724717570_Z0_T0_C2.tif]

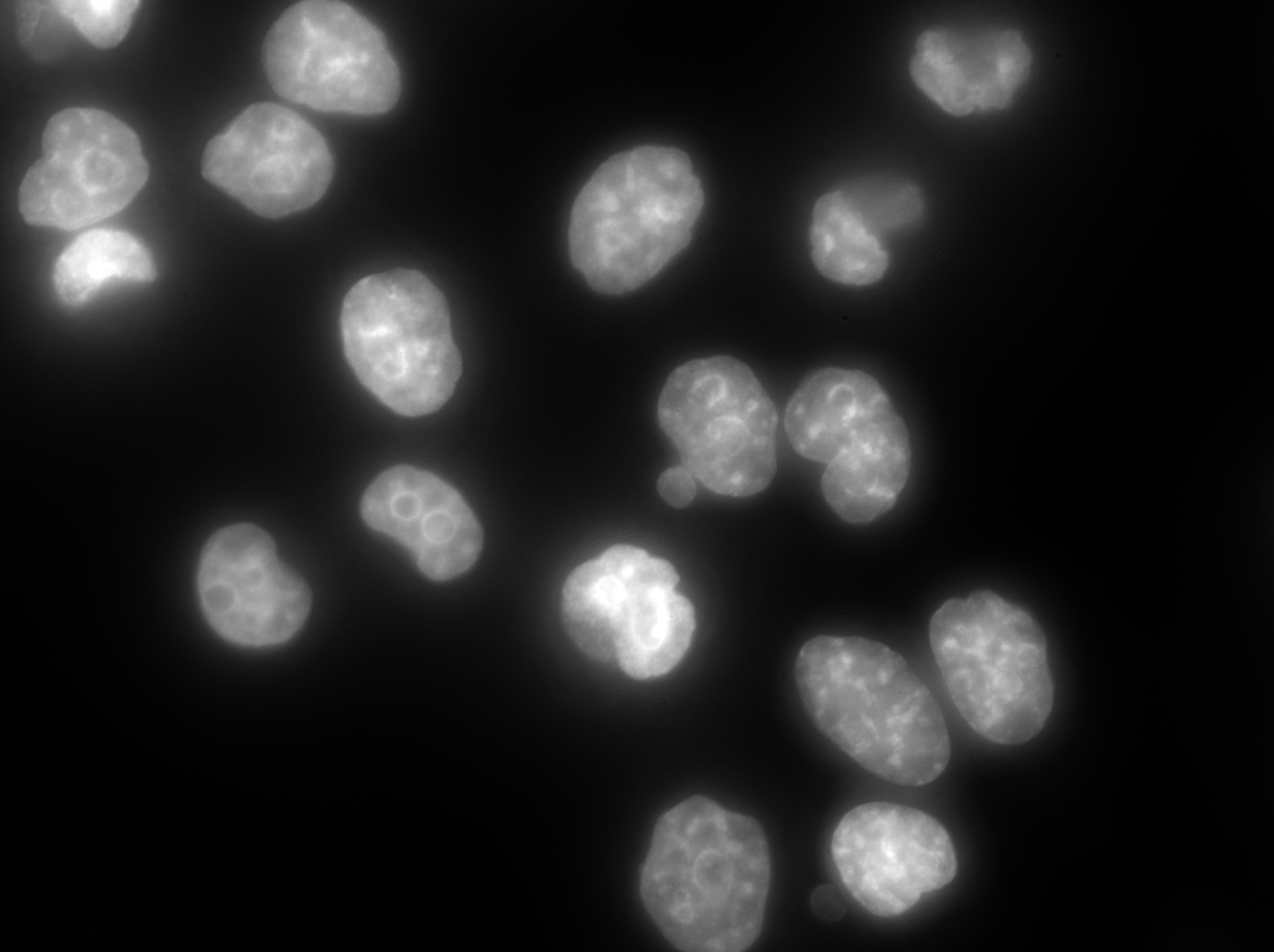

Supplement: Supplementary file 3 — Source data Fig. 1 [file 44319_2026_742_MOESM3_ESM.zip › Figure 1/Fig 1bcd WT Hela acetylated a tubulin atubulin/actub-atub 8-14-24 R3 PA8.Project Maximum Z_XY1724717079_Z0_T0_C0.tif]

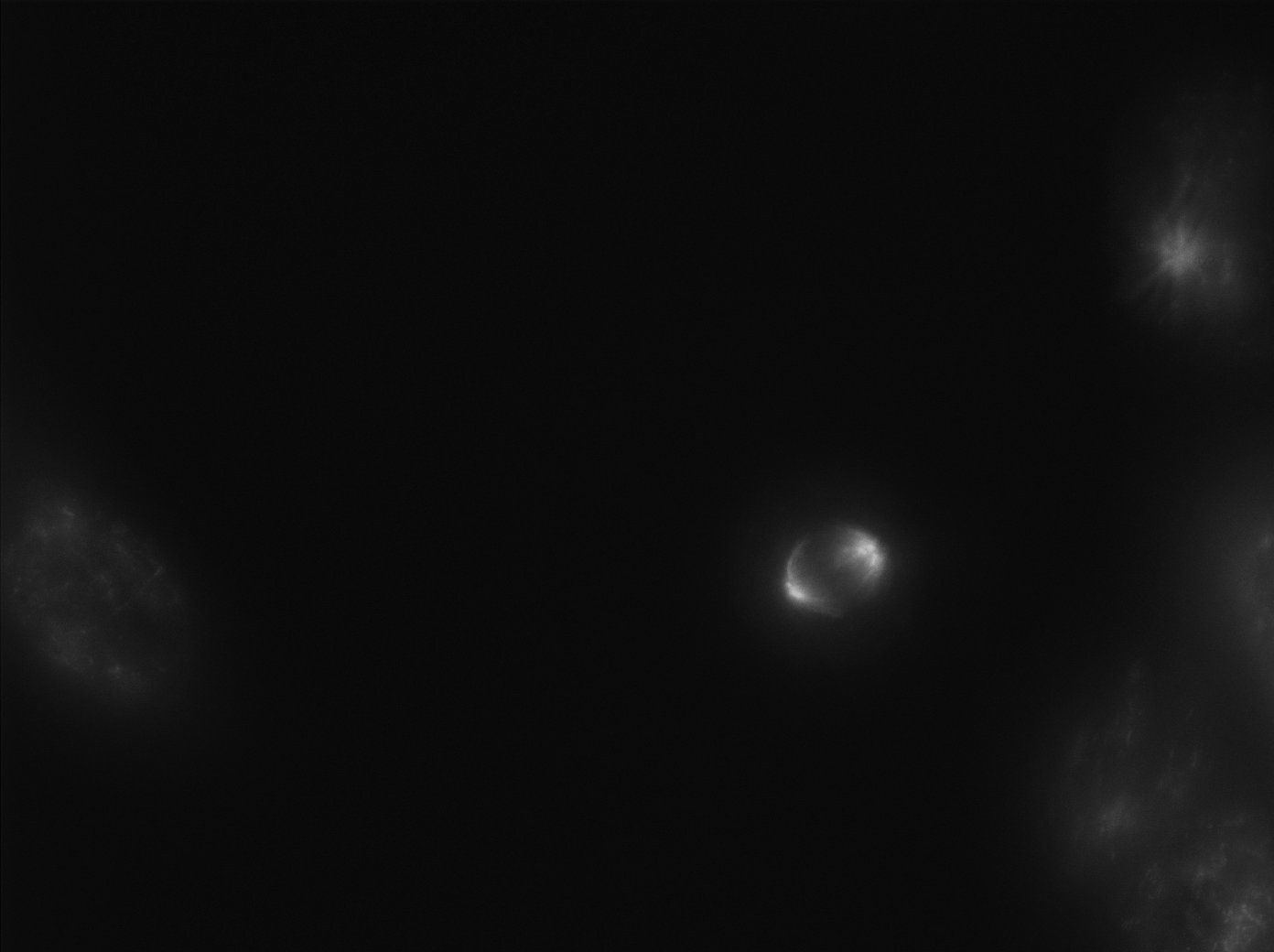

Supplement: Supplementary file 3 — Source data Fig. 1 [file 44319_2026_742_MOESM3_ESM.zip › Figure 1/Fig 1bcd WT Hela acetylated a tubulin atubulin/actub-atub 8-14-24 R1 M7.Project Maximum Z_XY1724365267_Z0_T0_C2.tif]

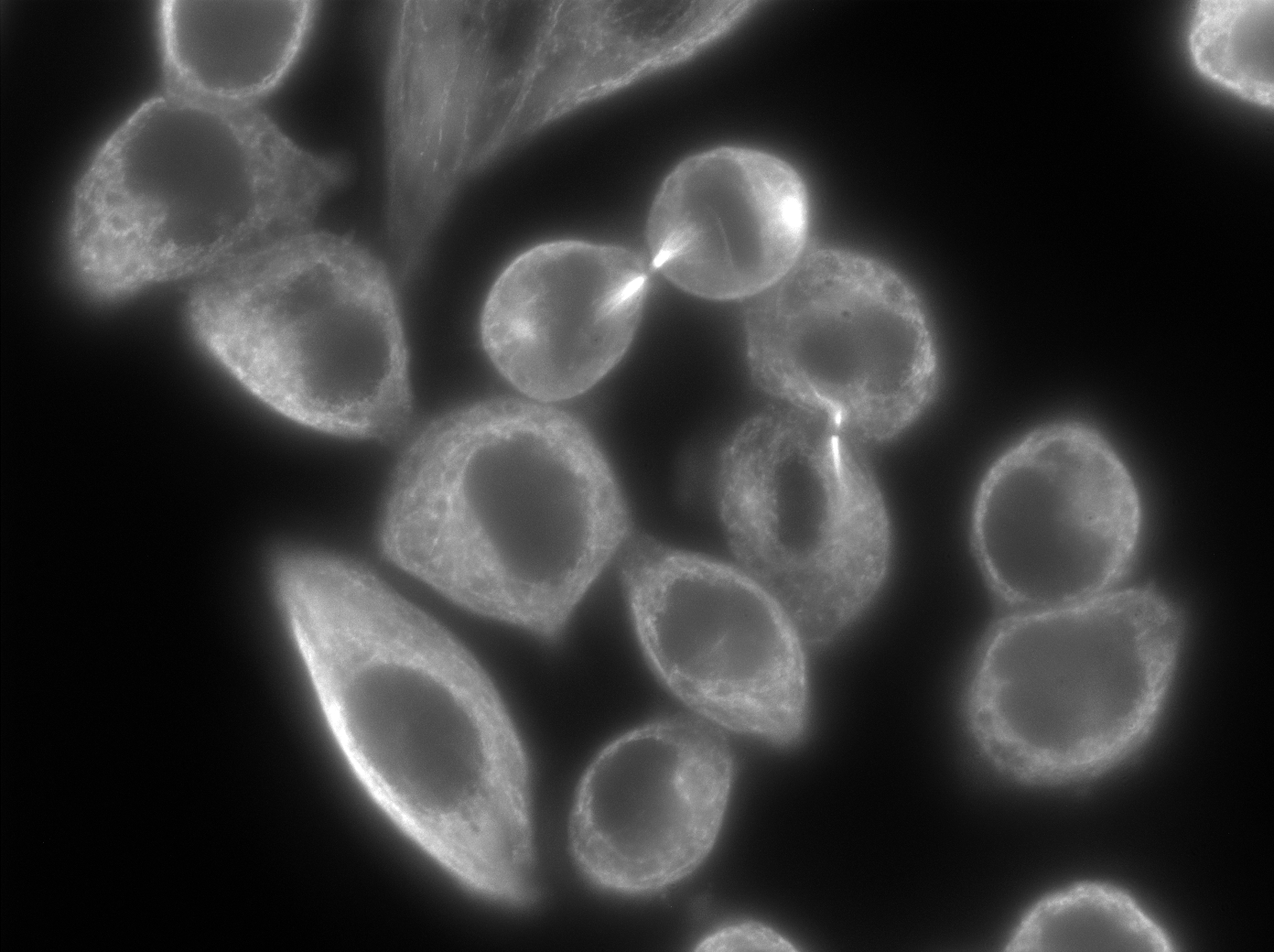

Supplement: Supplementary file 3 — Source data Fig. 1 [file 44319_2026_742_MOESM3_ESM.zip › Figure 1/Fig 1bcd WT Hela acetylated a tubulin atubulin/actub-atub 8-14-24 R3 LT6LT7.Project Maximum Z_XY1724716888_Z0_T0_C1.tif]

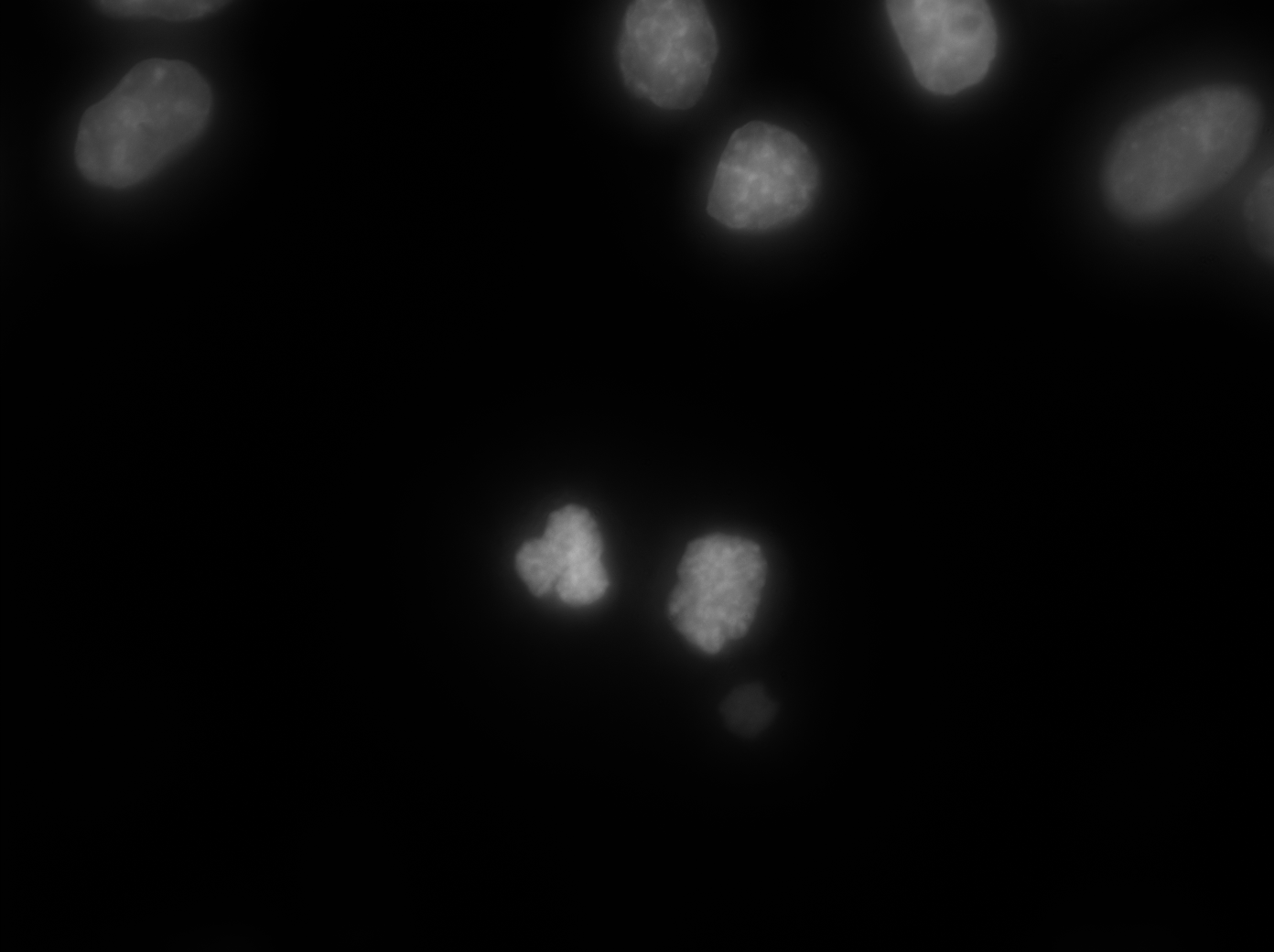

Supplement: Supplementary file 3 — Source data Fig. 1 [file 44319_2026_742_MOESM3_ESM.zip › Figure 1/Fig 1bcd WT Hela acetylated a tubulin atubulin/actub-atub 8-14-24 R1 ET1.Project Maximum Z_XY1724362932_Z0_T0_C0.tif]

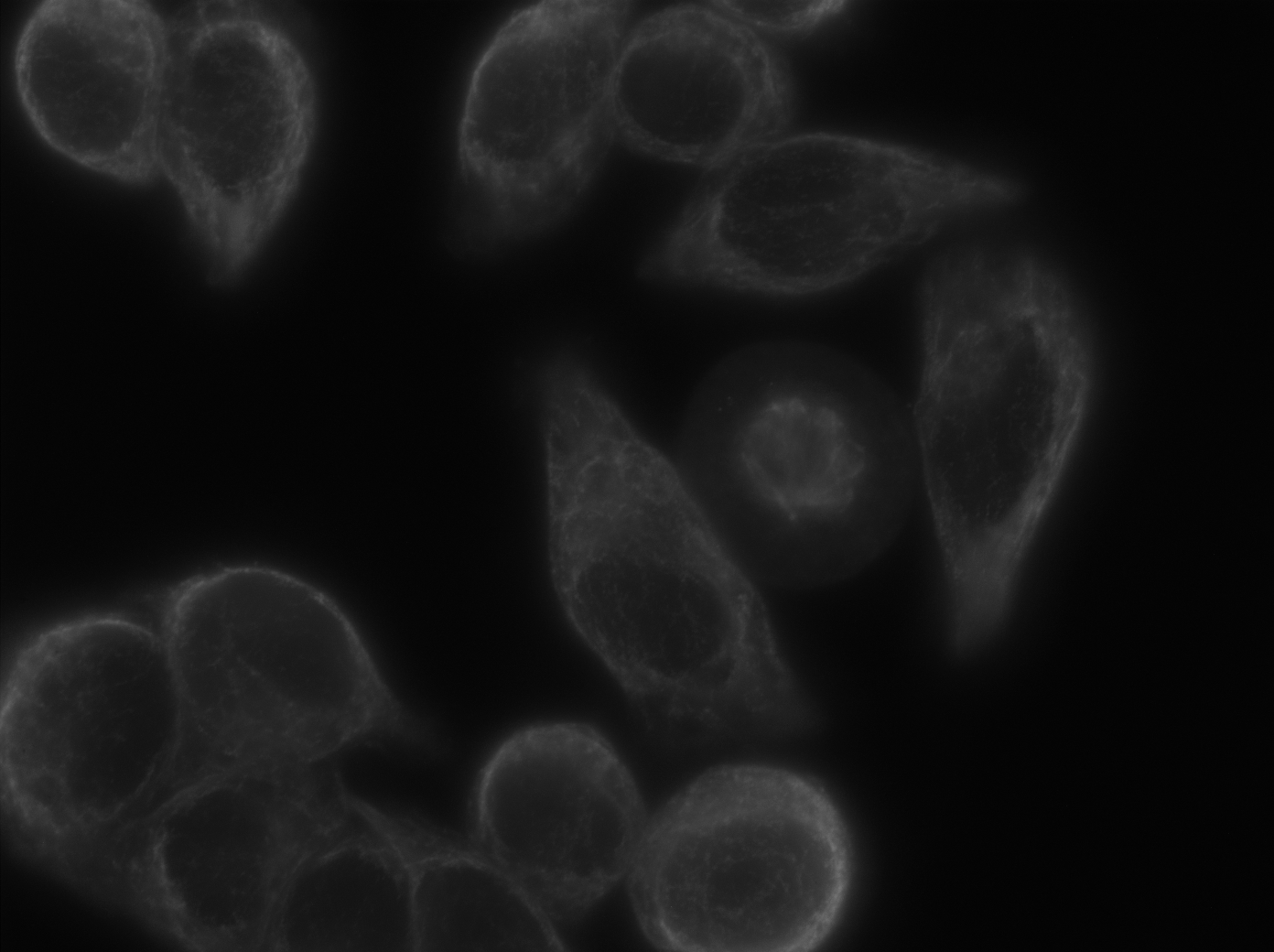

Supplement: Supplementary file 3 — Source data Fig. 1 [file 44319_2026_742_MOESM3_ESM.zip › Figure 1/Fig 1bcd WT Hela acetylated a tubulin atubulin/actub-atub 8-14-24 R2 M3.Project Maximum Z_XY1724693151_Z0_T0_C1.tif]

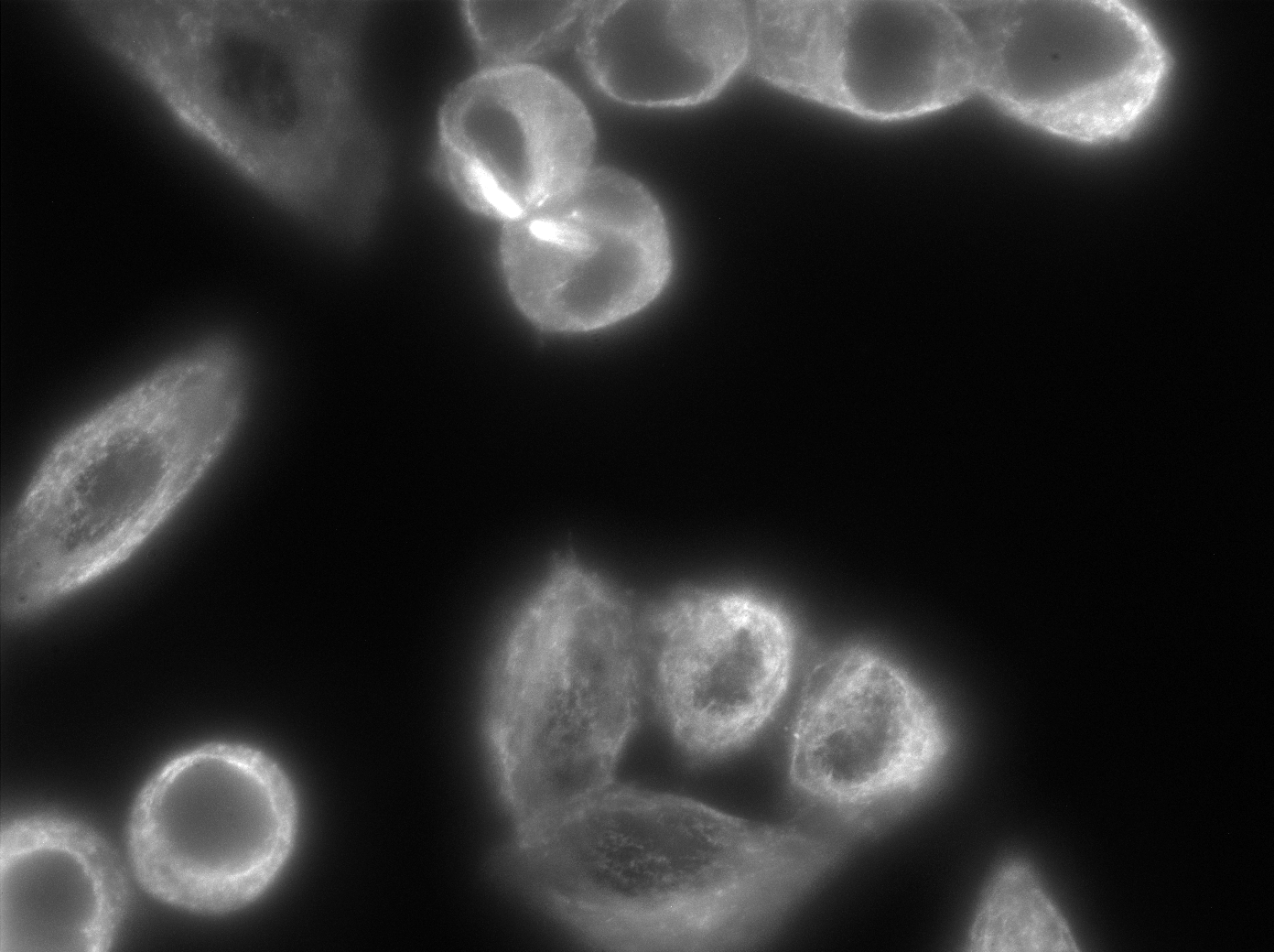

Supplement: Supplementary file 3 — Source data Fig. 1 [file 44319_2026_742_MOESM3_ESM.zip › Figure 1/Fig 1bcd WT Hela acetylated a tubulin atubulin/actub-atub 8-14-24 R3 ET6 PA2.Project Maximum Z_XY1724703496_Z0_T0_C1.tif]

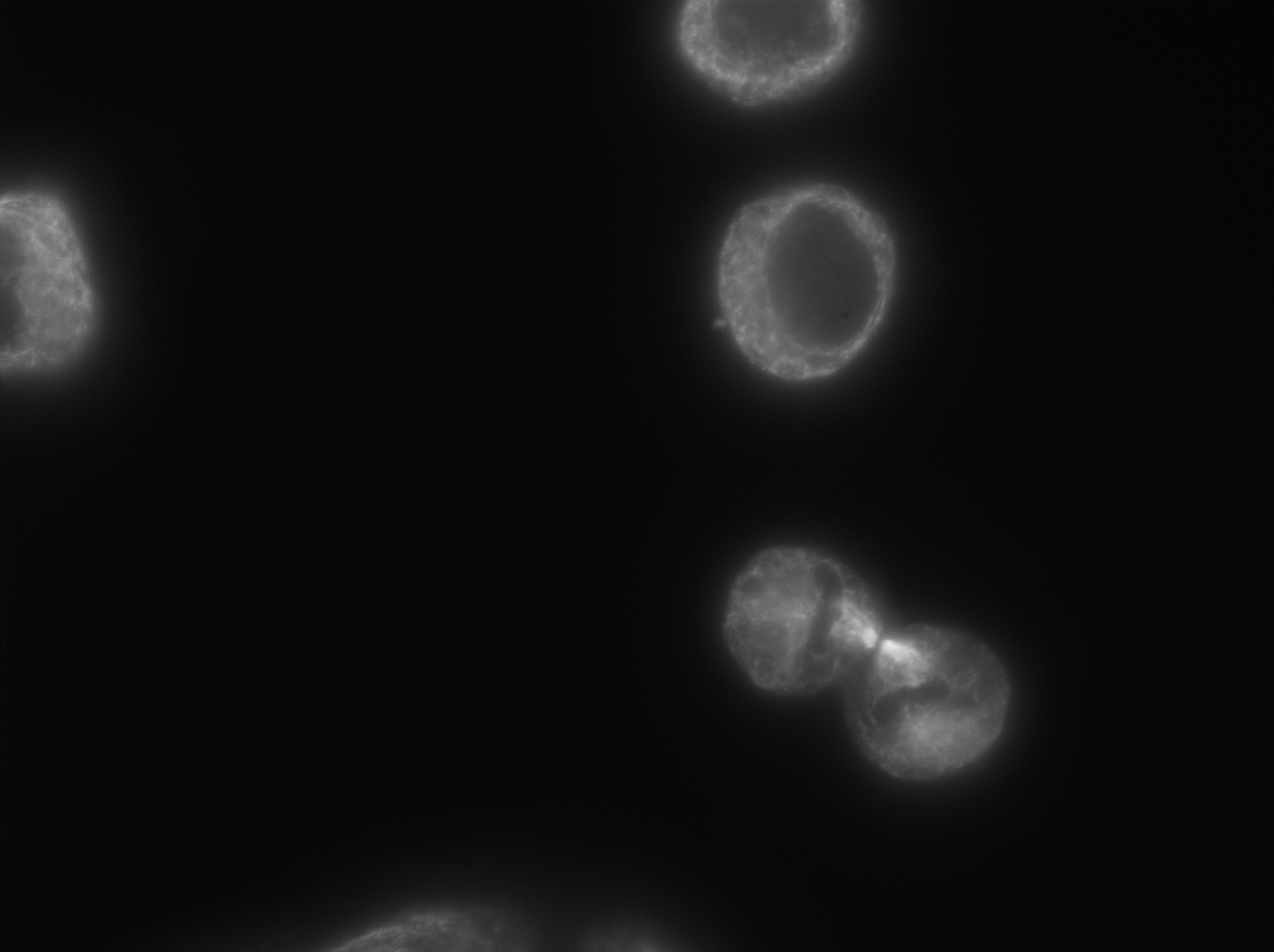

Supplement: Supplementary file 3 — Source data Fig. 1 [file 44319_2026_742_MOESM3_ESM.zip › Figure 1/Fig 1bcd WT Hela acetylated a tubulin atubulin/actub-atub 8-14-24 R2 ET4.Project Maximum Z_XY1724690168_Z0_T0_C1.tif]

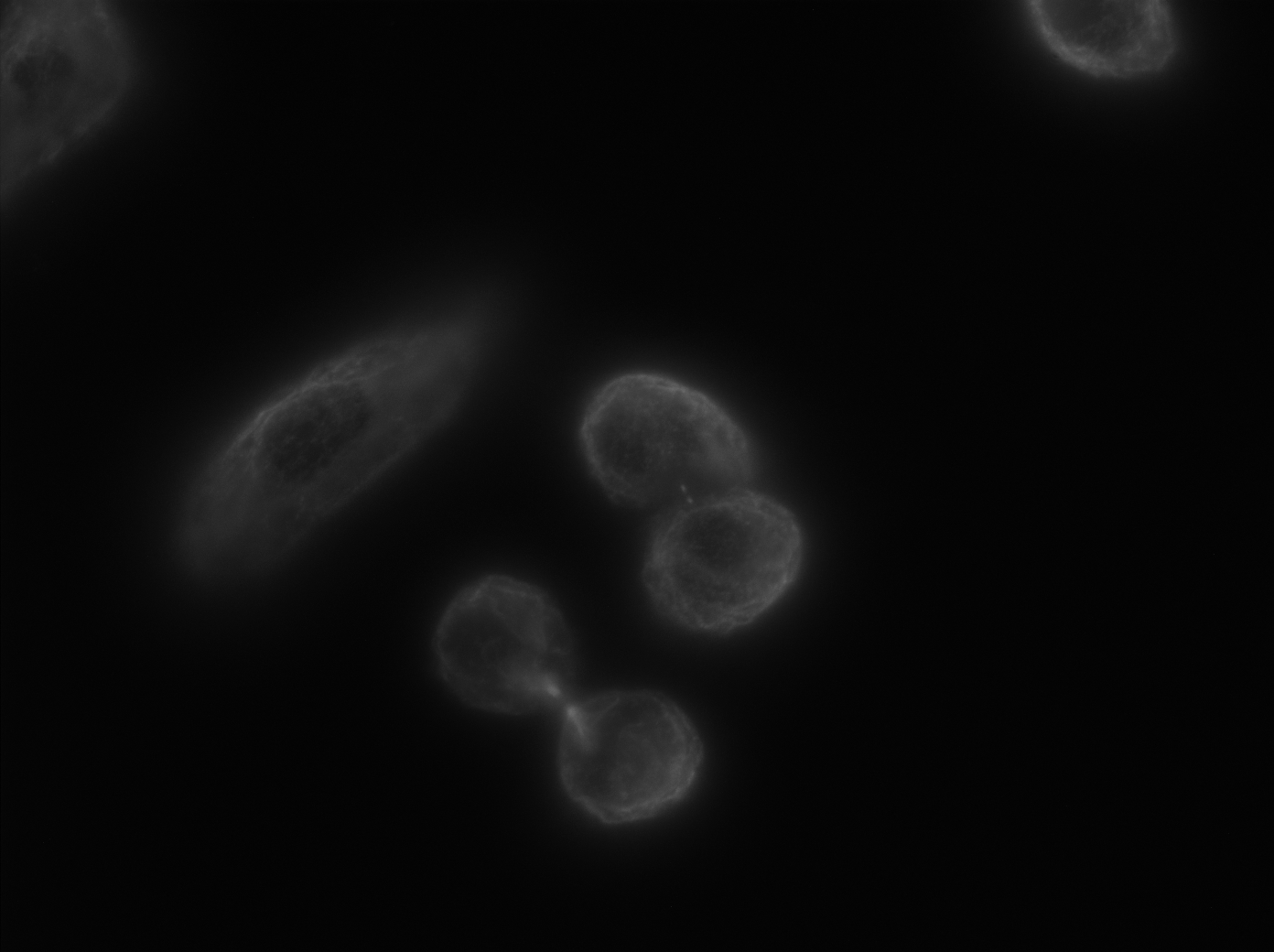

Supplement: Supplementary file 3 — Source data Fig. 1 [file 44319_2026_742_MOESM3_ESM.zip › Figure 1/Fig 1bcd WT Hela acetylated a tubulin atubulin/actub-atub 8-14-24 R2 ET6 PA4.Project Maximum Z_XY1724690530_Z0_T0_C1.tif]

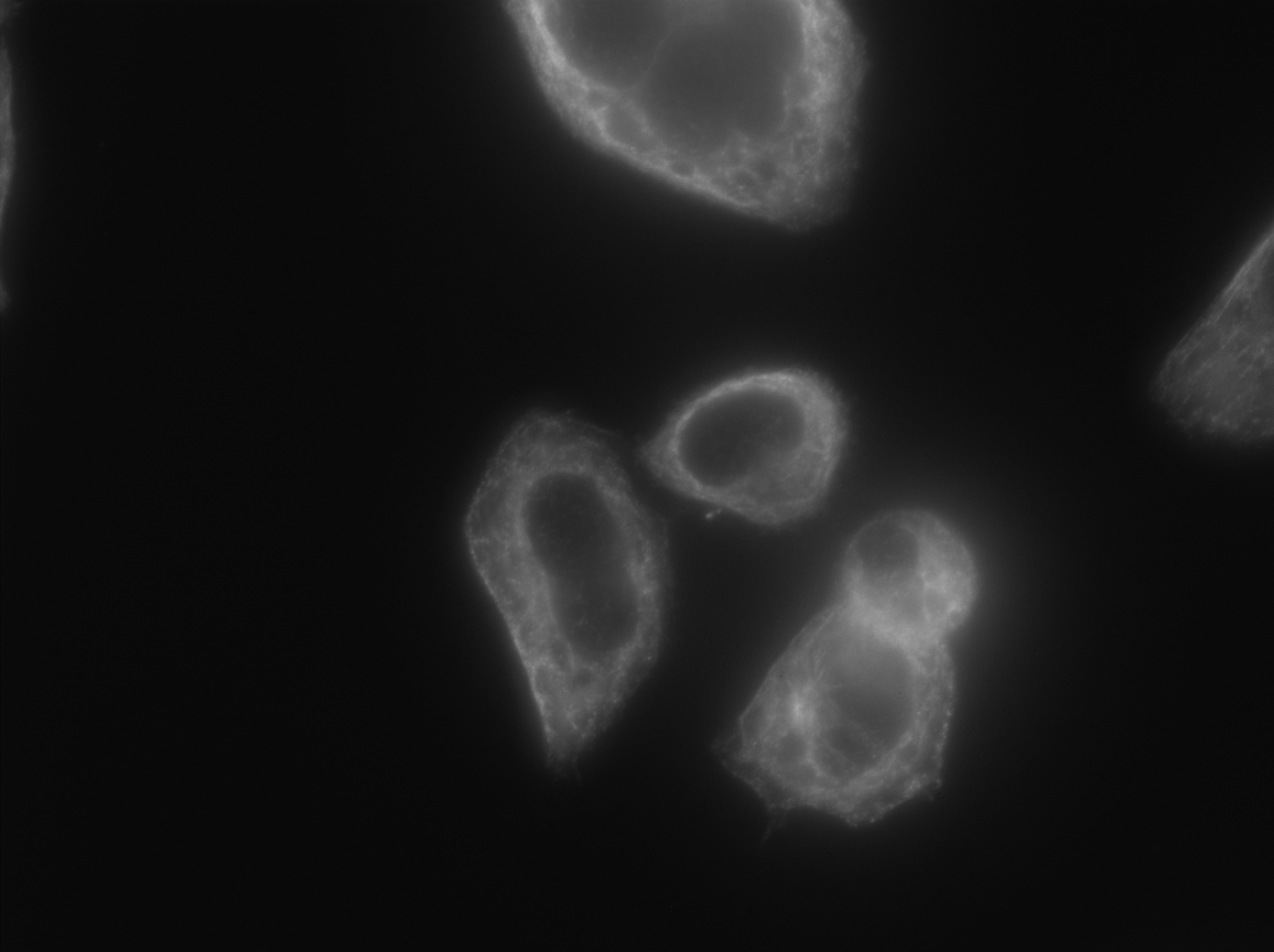

Supplement: Supplementary file 3 — Source data Fig. 1 [file 44319_2026_742_MOESM3_ESM.zip › Figure 1/Fig 1bcd WT Hela acetylated a tubulin atubulin/actub-atub 8-14-24 R2 PA7.Project Maximum Z_XY1724694856_Z0_T0_C1.tif]

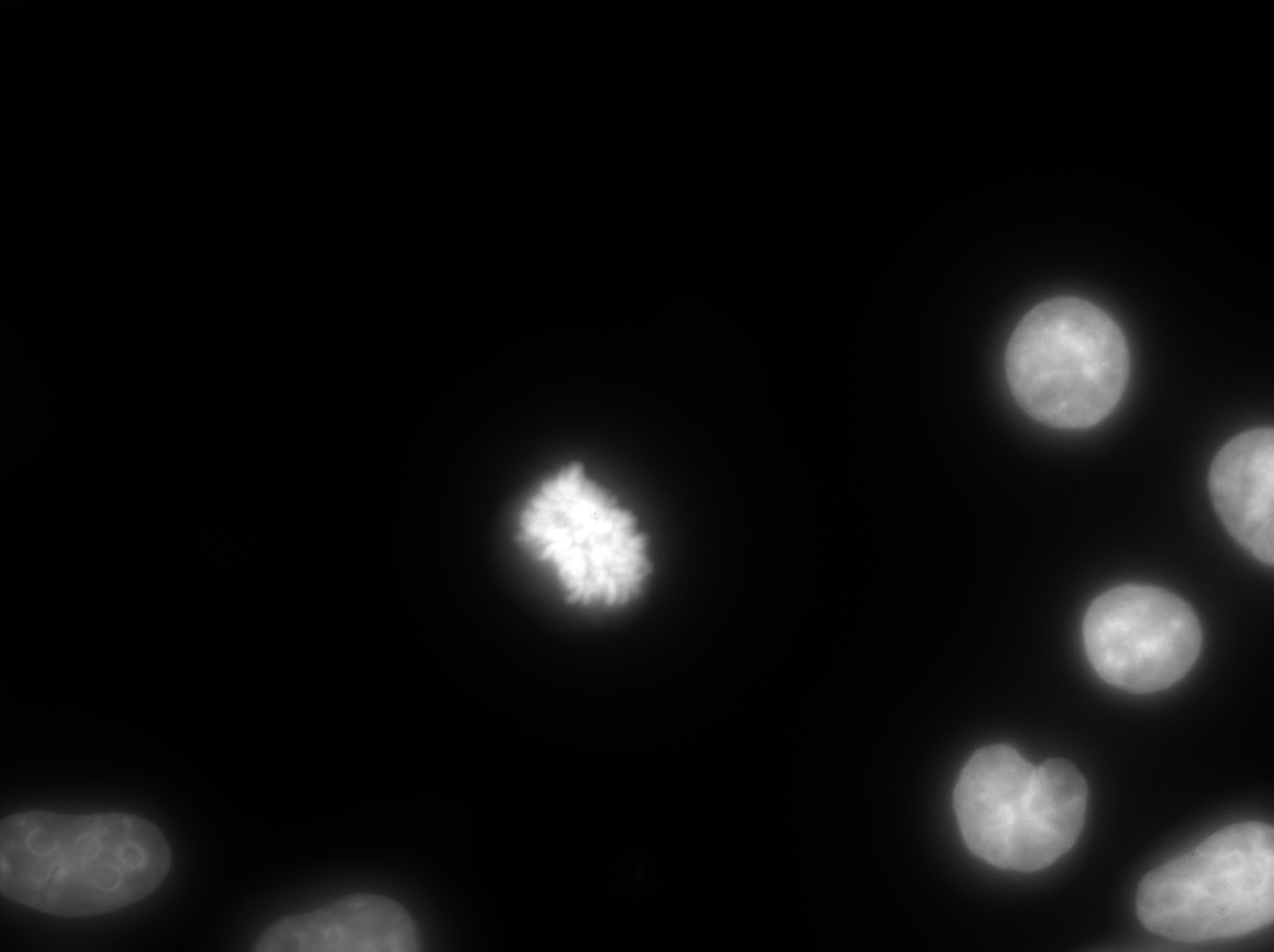

Supplement: Supplementary file 3 — Source data Fig. 1 [file 44319_2026_742_MOESM3_ESM.zip › Figure 1/Fig 1bcd WT Hela acetylated a tubulin atubulin/actub-atub 8-14-24 R3 M8.Project Maximum Z_XY1724703664_Z0_T0_C0.tif]

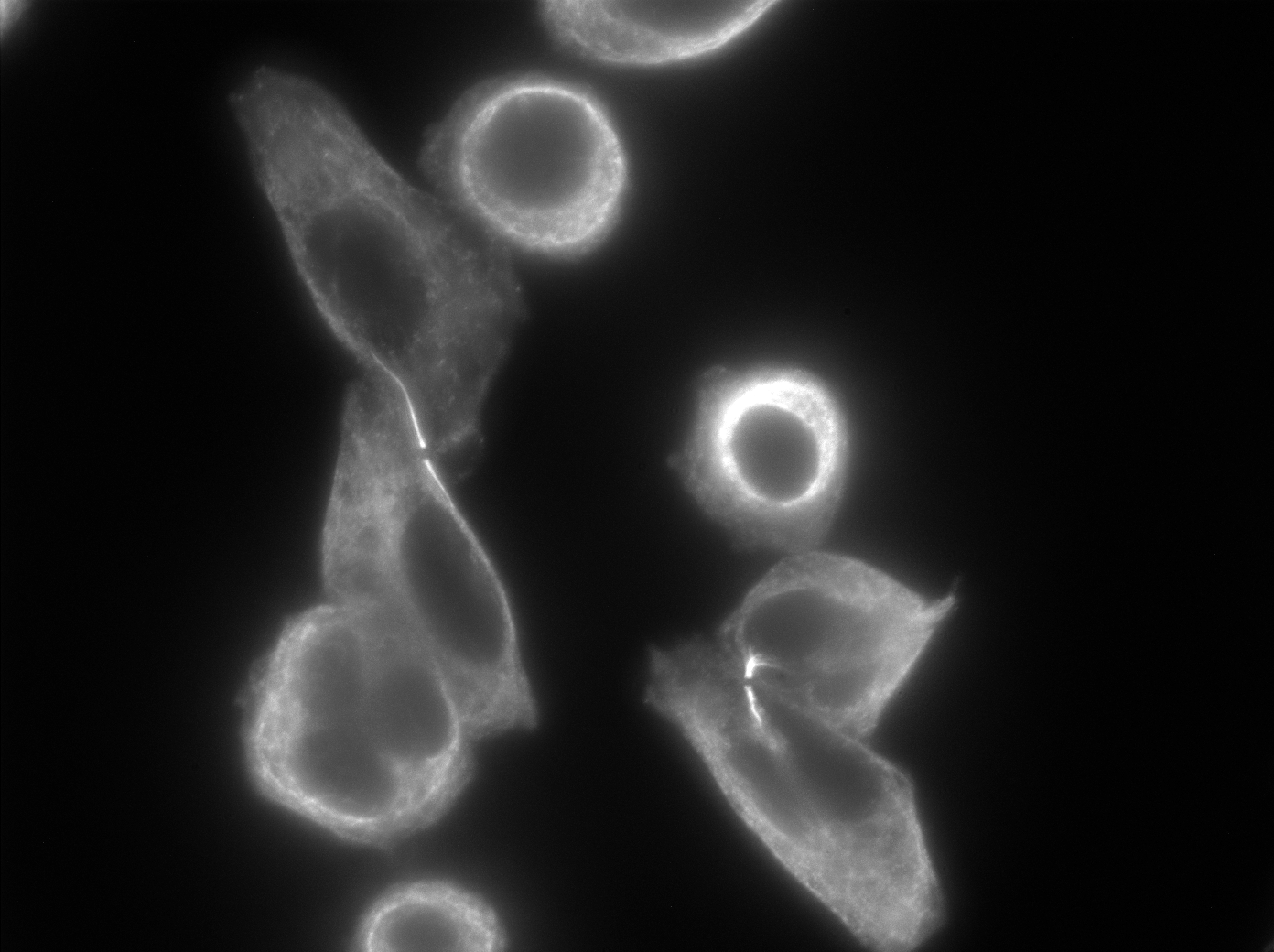

Supplement: Supplementary file 3 — Source data Fig. 1 [file 44319_2026_742_MOESM3_ESM.zip › Figure 1/Fig 1bcd WT Hela acetylated a tubulin atubulin/actub-atub 8-14-24 R3 LT3LT4.Project Maximum Z_XY1724703126_Z0_T0_C1.tif]

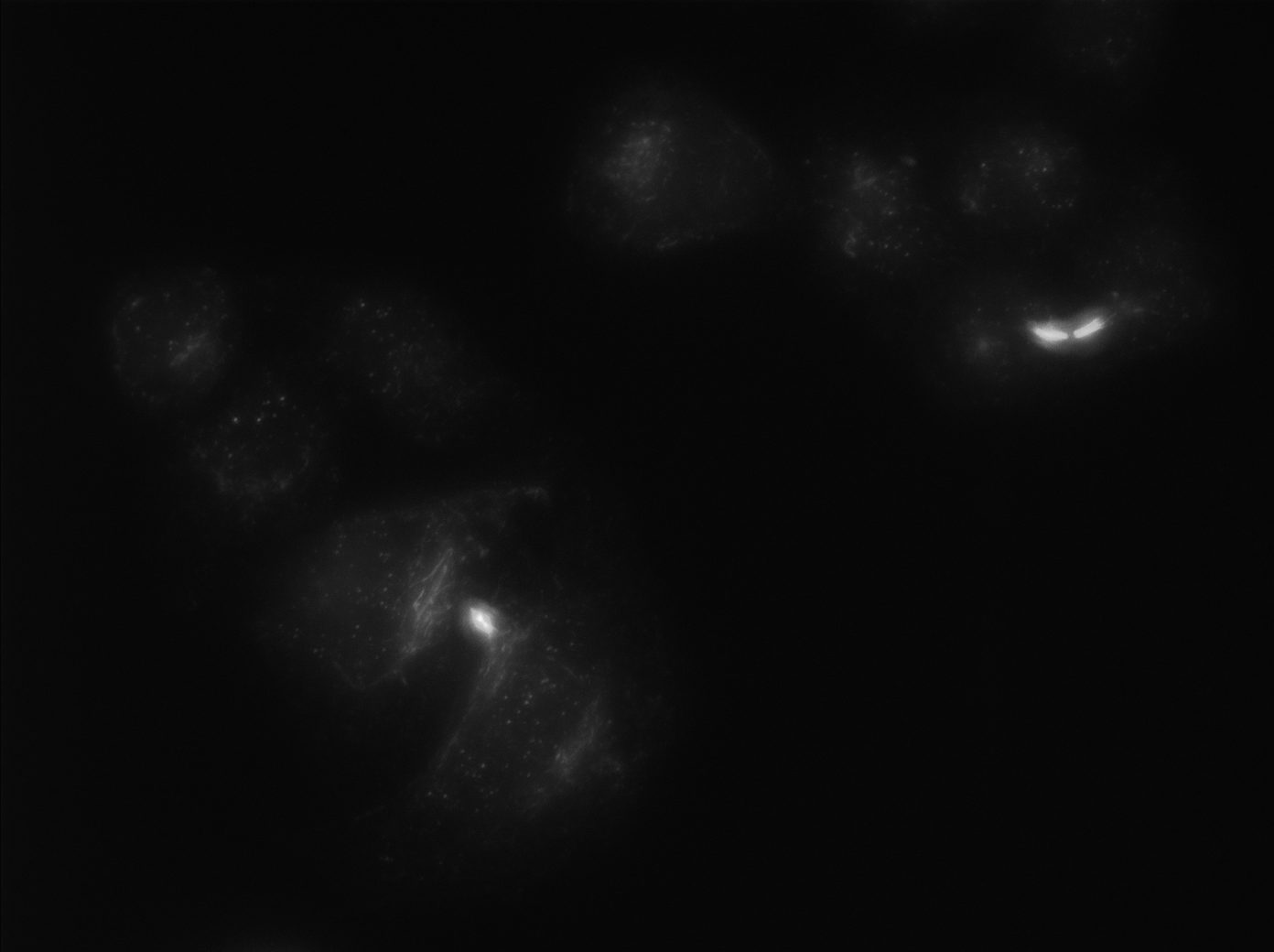

Supplement: Supplementary file 3 — Source data Fig. 1 [file 44319_2026_742_MOESM3_ESM.zip › Figure 1/Fig 1bcd WT Hela acetylated a tubulin atubulin/actub-atub 8-14-24 R2 LT3 PA1.Project Maximum Z_XY1724689811_Z0_T0_C2.tif]

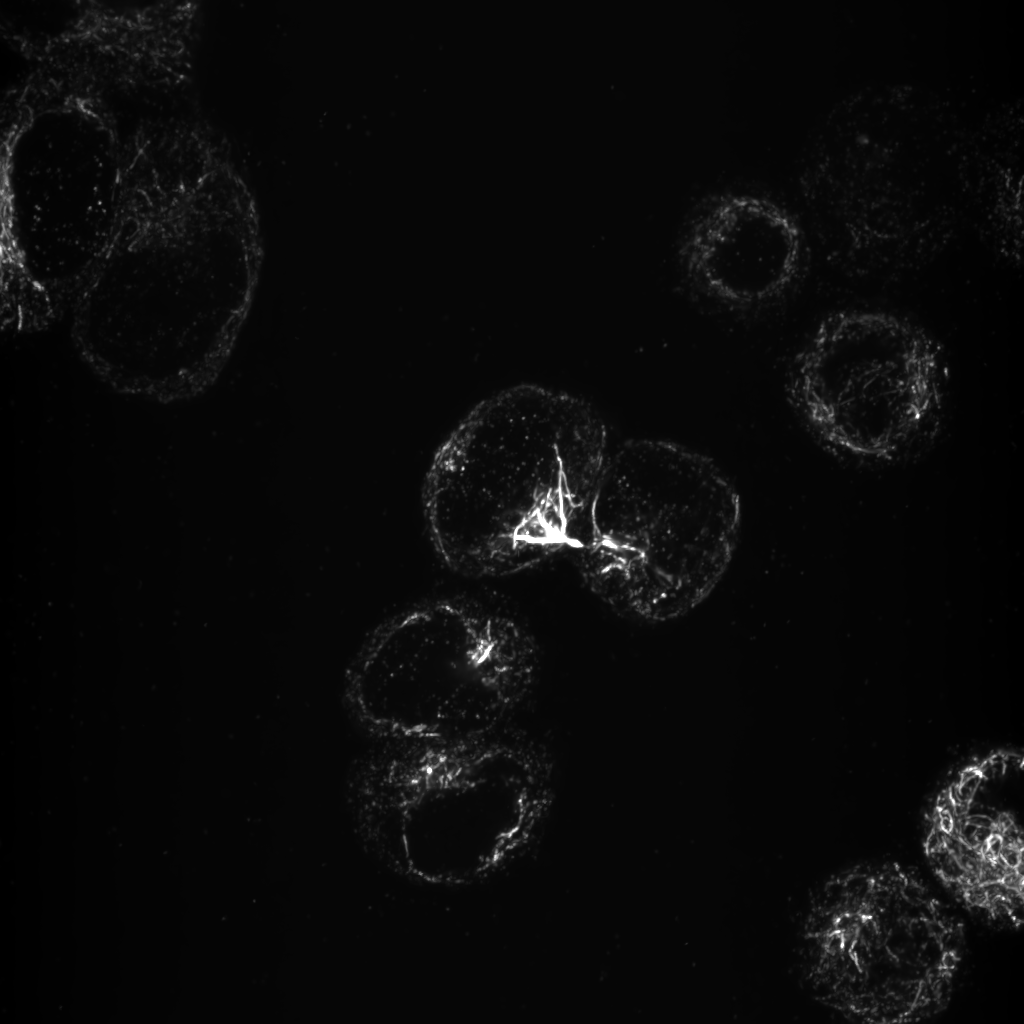

Supplement: Supplementary file 4 — Source data Fig. 2 part 1 [file 44319_2026_742_MOESM4_ESM.zip › Figure 2 Part 1/Fig 2a rgt335 acetylated tubulin control confocal/GTacPA1002-MaxIP_594.tif]
