# Supplementary figures and images for: TPGS1 regulates central spindle microtubule glutamylation and remodeling during telophase and abscission (part 1 of 36)
Source: EMBO Rep. 2026 Mar 23;27(8):1944–63. doi: 10.1038/s44319-026-00742-3 (PMC13121839; doi:10.1038/s44319-026-00742-3)

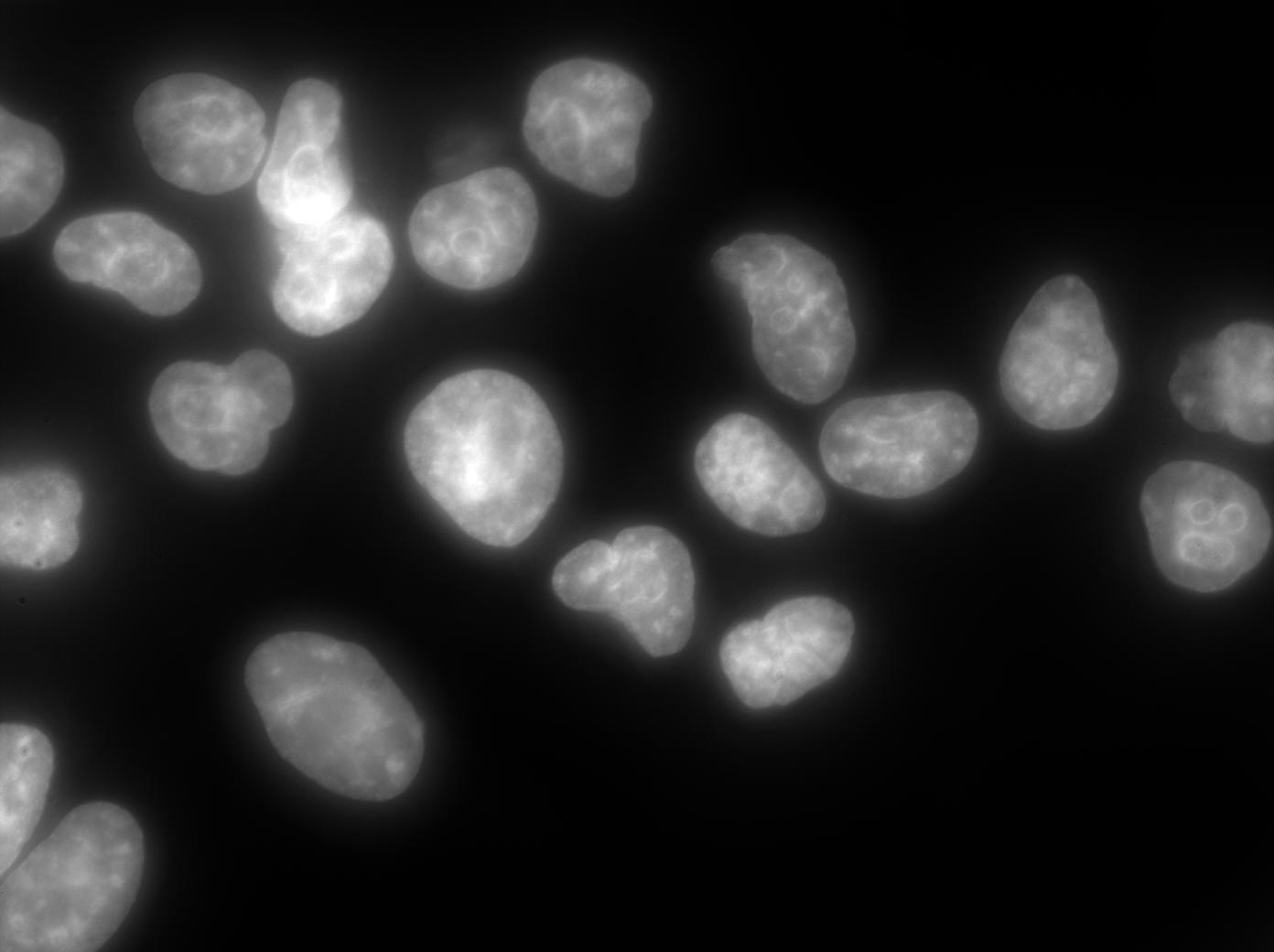

Supplement: Supplementary file 3 — Source data Fig. 1 [file 44319_2026_742_MOESM3_ESM.zip › Figure 1/Fig 1bcd WT Hela acetylated a tubulin atubulin/actub-atub 8-14-24 R3 PA6.Project Maximum Z_XY1724716567_Z0_T0_C0.tif]

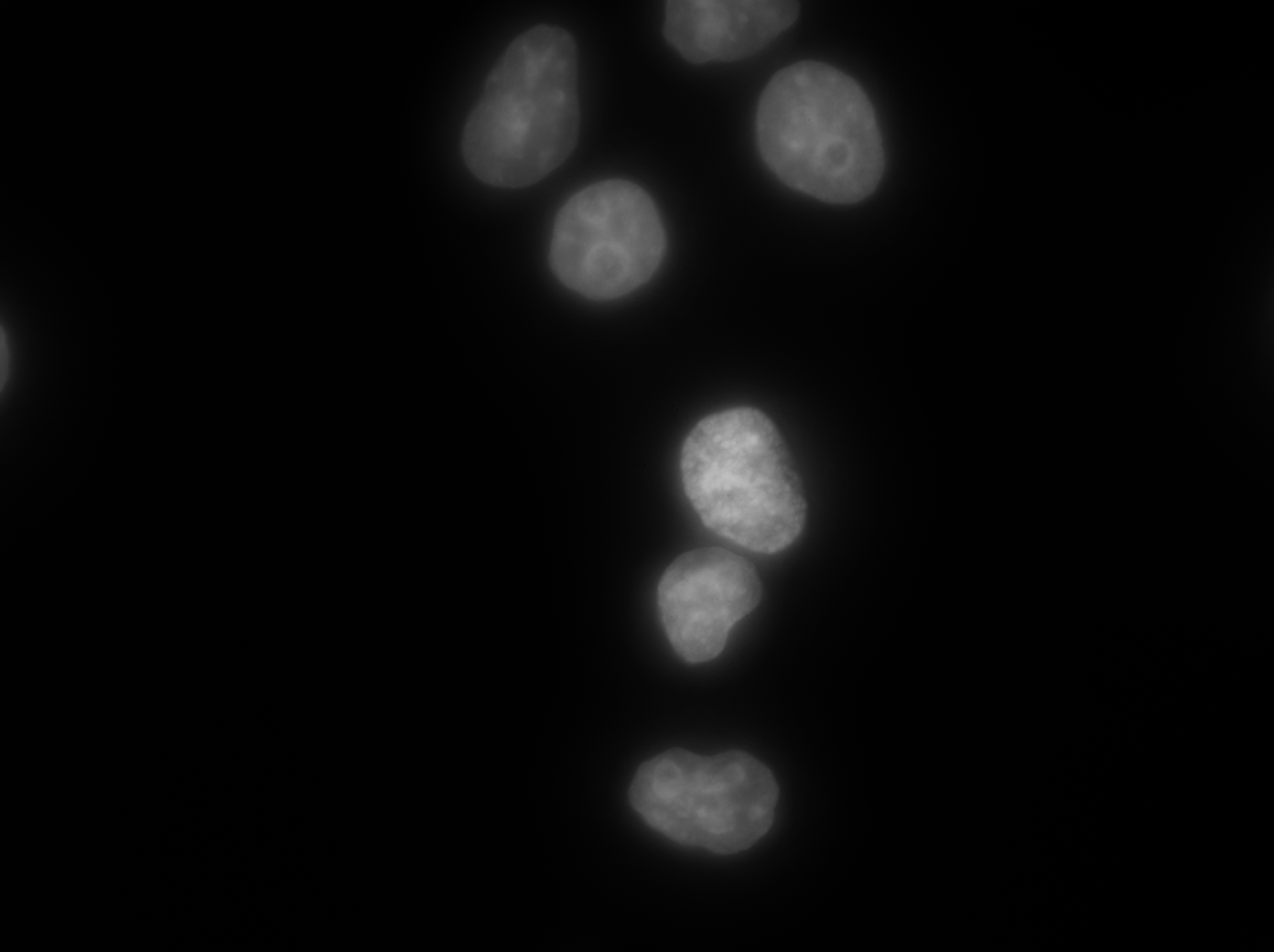

Supplement: Supplementary file 3 — Source data Fig. 1 [file 44319_2026_742_MOESM3_ESM.zip › Figure 1/Fig 1bcd WT Hela acetylated a tubulin atubulin/actub-atub 8-14-24 R1 PA2.Project Maximum Z_XY1724362613_Z0_T0_C0.tif]

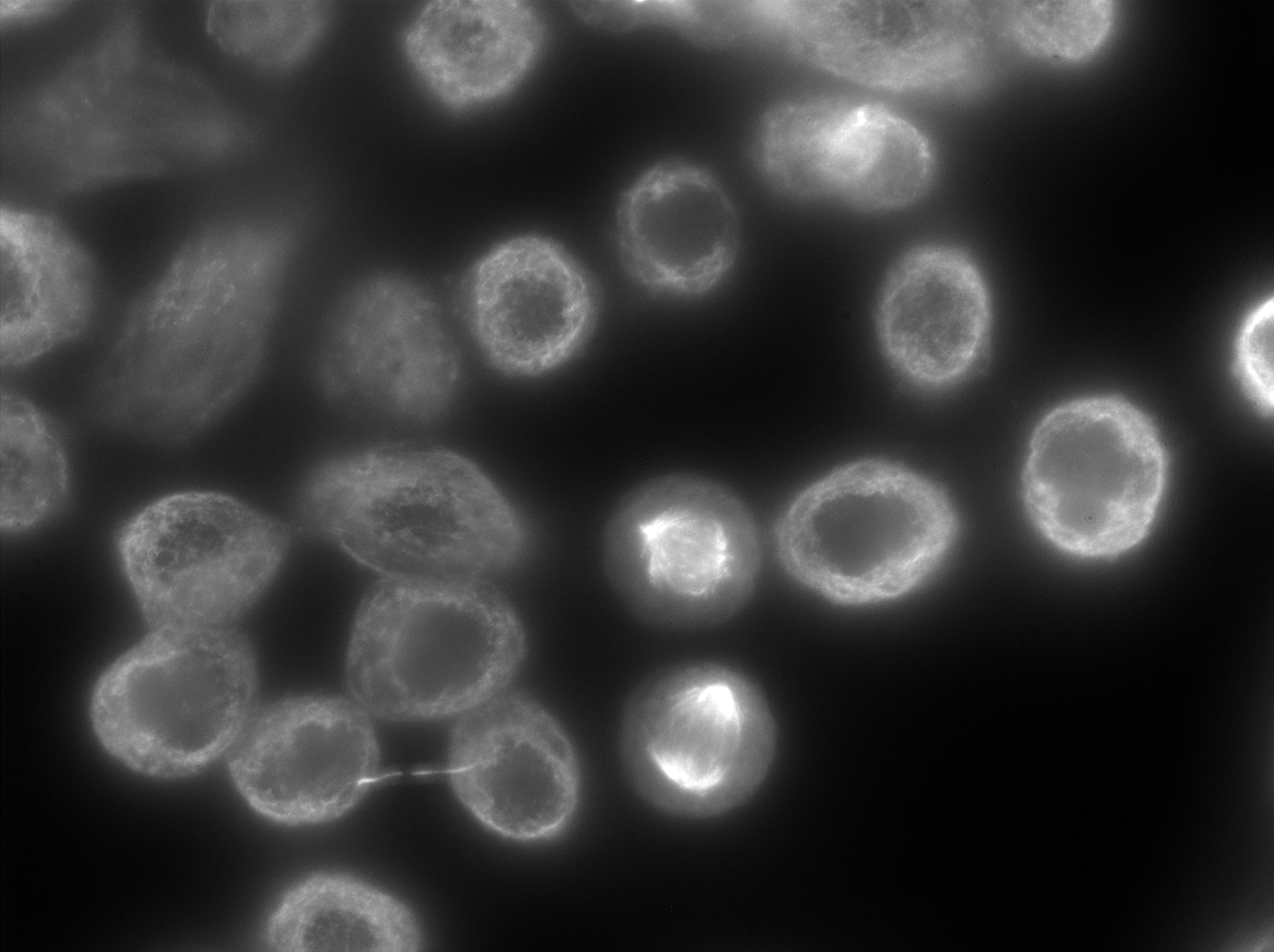

Supplement: Supplementary file 3 — Source data Fig. 1 [file 44319_2026_742_MOESM3_ESM.zip › Figure 1/Fig 1bcd WT Hela acetylated a tubulin atubulin/actub-atub 8-14-24 R3 M2M3 LT1.Project Maximum Z_XY1724701933_Z0_T0_C1.tif]

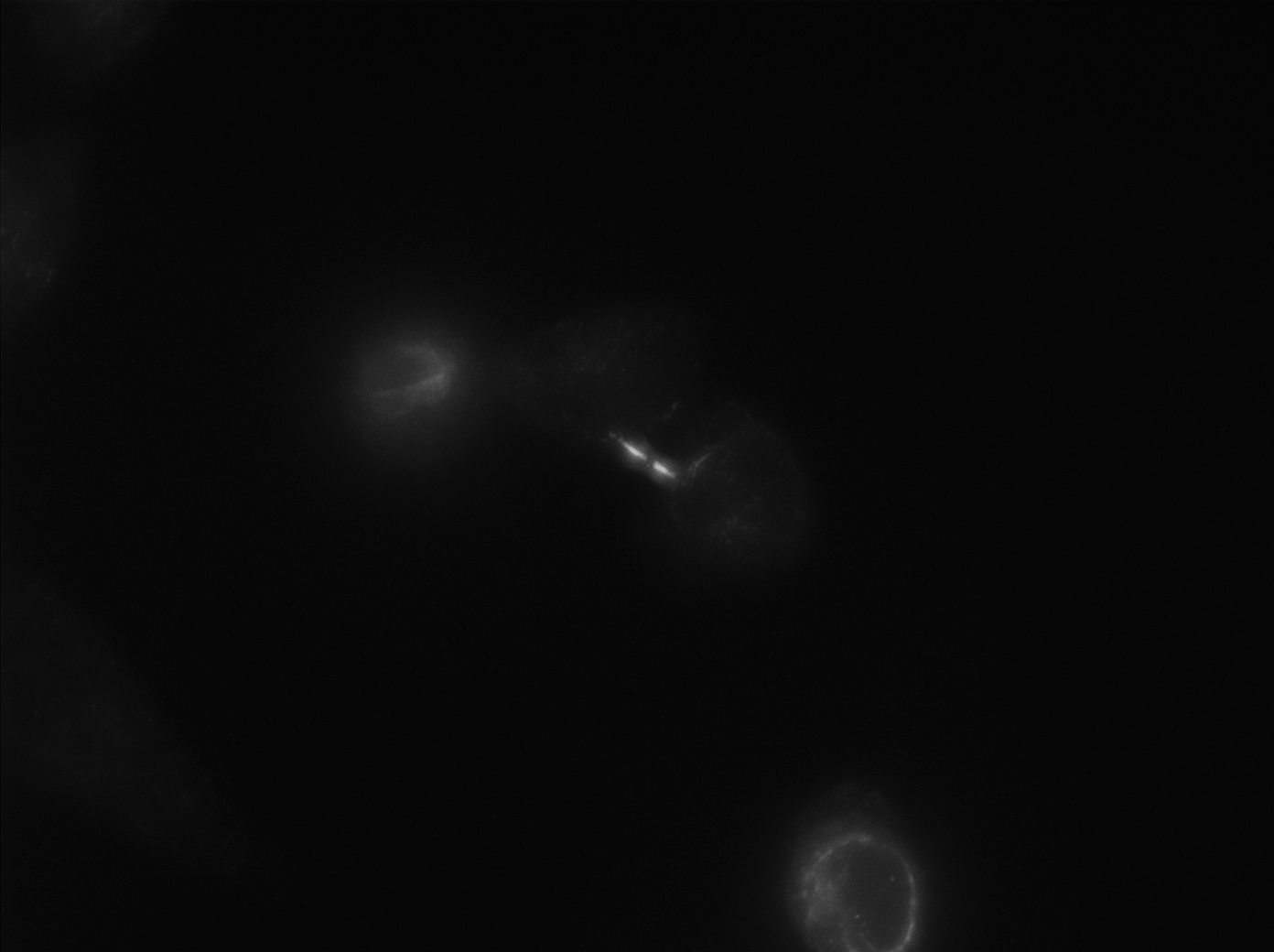

Supplement: Supplementary file 3 — Source data Fig. 1 [file 44319_2026_742_MOESM3_ESM.zip › Figure 1/Fig 1bcd WT Hela acetylated a tubulin atubulin/actub-atub 8-14-24 R3 LT2.Project Maximum Z_XY1724702329_Z0_T0_C2.tif]

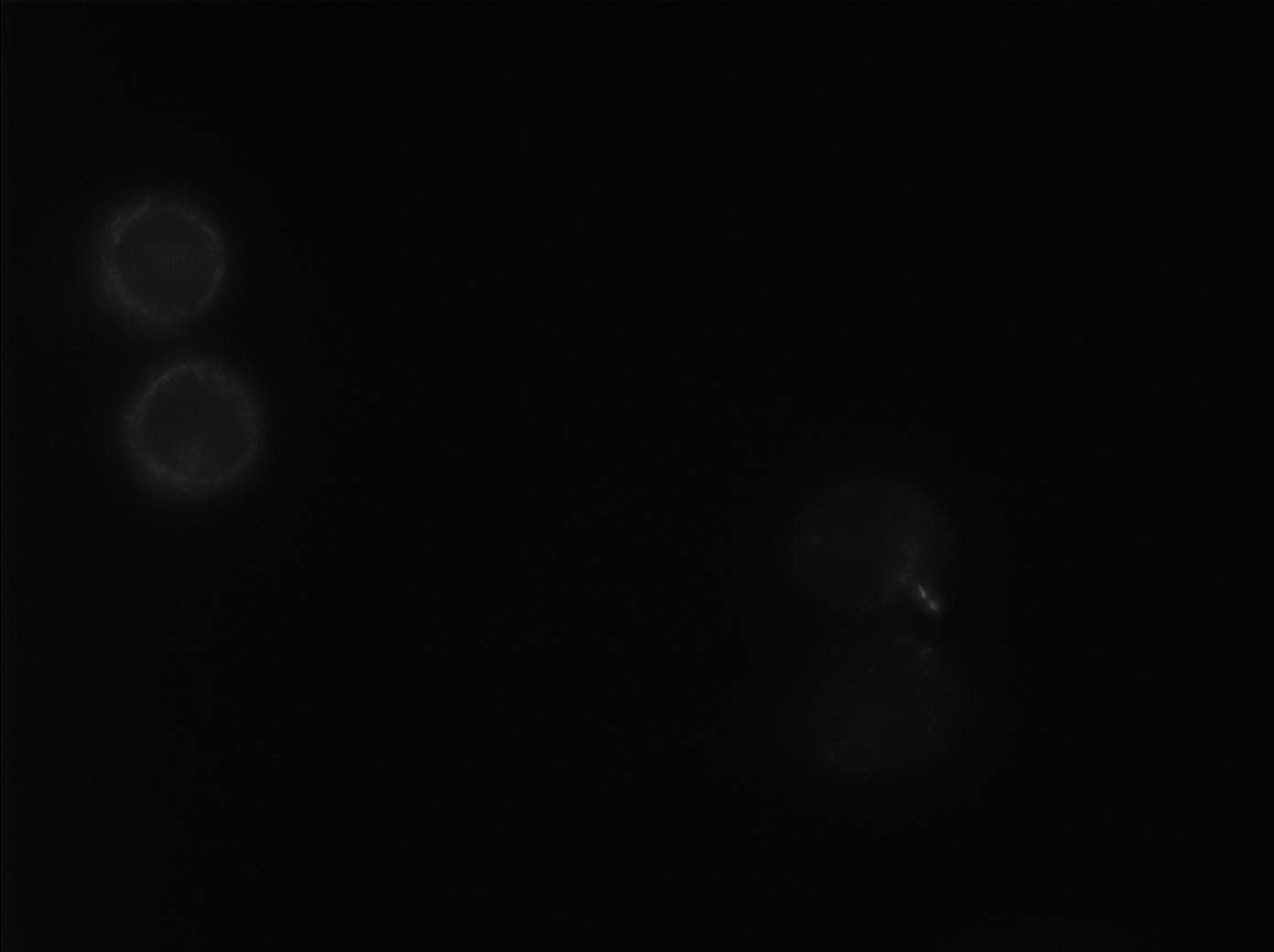

Supplement: Supplementary file 3 — Source data Fig. 1 [file 44319_2026_742_MOESM3_ESM.zip › Figure 1/Fig 1bcd WT Hela acetylated a tubulin atubulin/actub-atub 8-14-24 R3 PA5.Project Maximum Z_XY1724704270_Z0_T0_C2.tif]

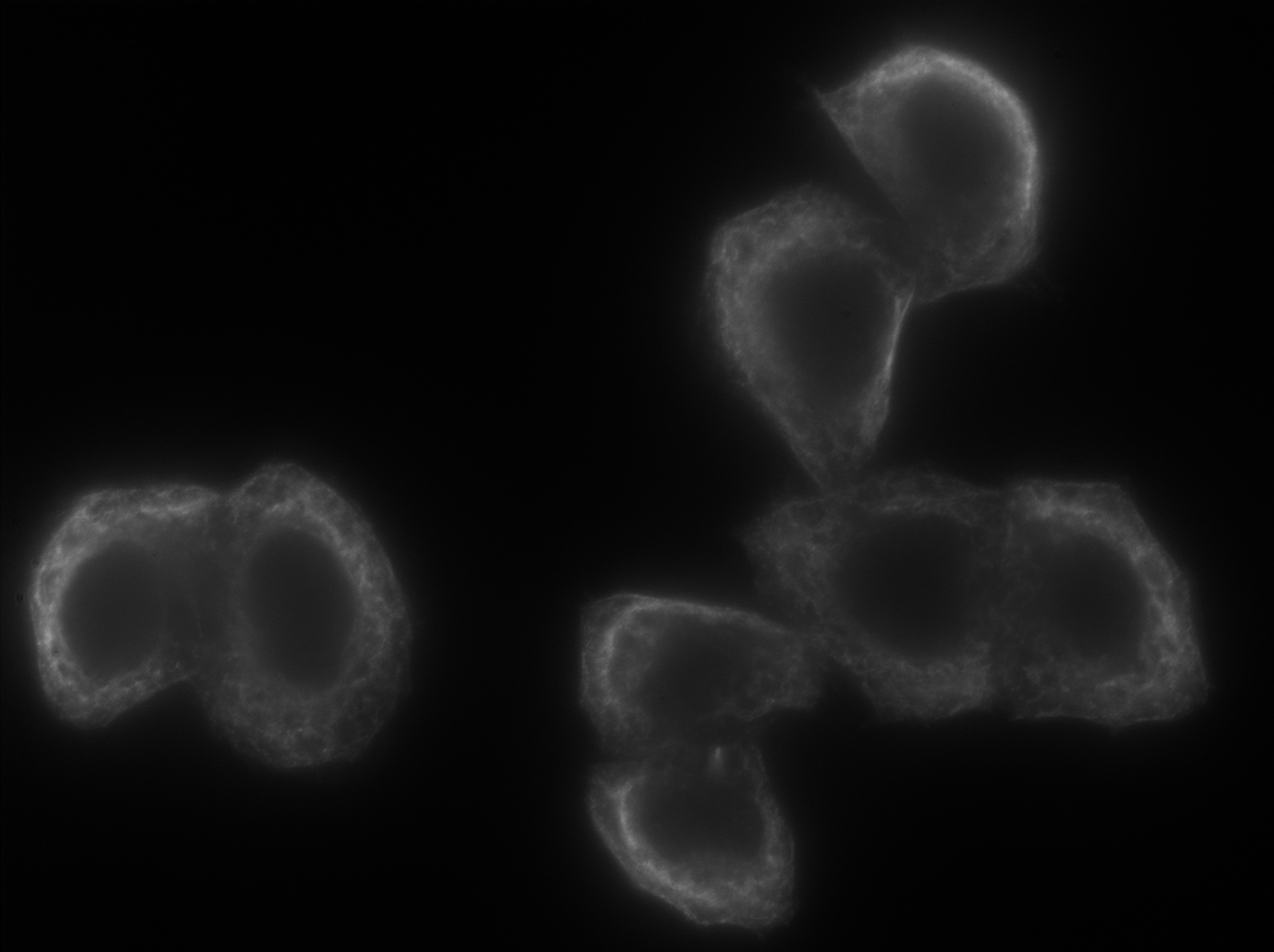

Supplement: Supplementary file 3 — Source data Fig. 1 [file 44319_2026_742_MOESM3_ESM.zip › Figure 1/Fig 1bcd WT Hela acetylated a tubulin atubulin/actub-atub 8-14-24 R1 PA6.Project Maximum Z_XY1724364026_Z0_T0_C1.tif]

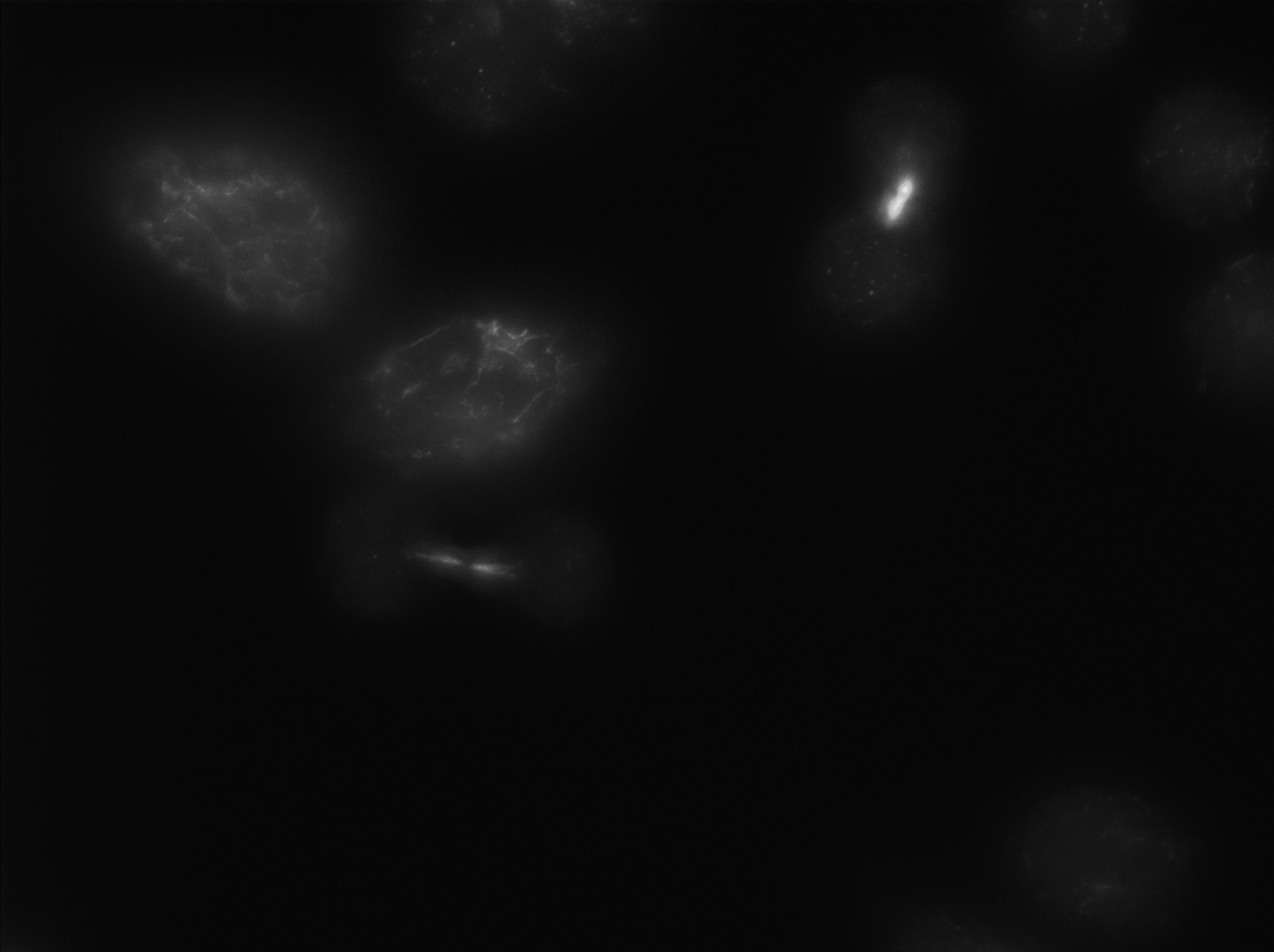

Supplement: Supplementary file 3 — Source data Fig. 1 [file 44319_2026_742_MOESM3_ESM.zip › Figure 1/Fig 1bcd WT Hela acetylated a tubulin atubulin/actub-atub 8-14-24 R2 ET2 LT1.Project Maximum Z_XY1724689540_Z0_T0_C2.tif]

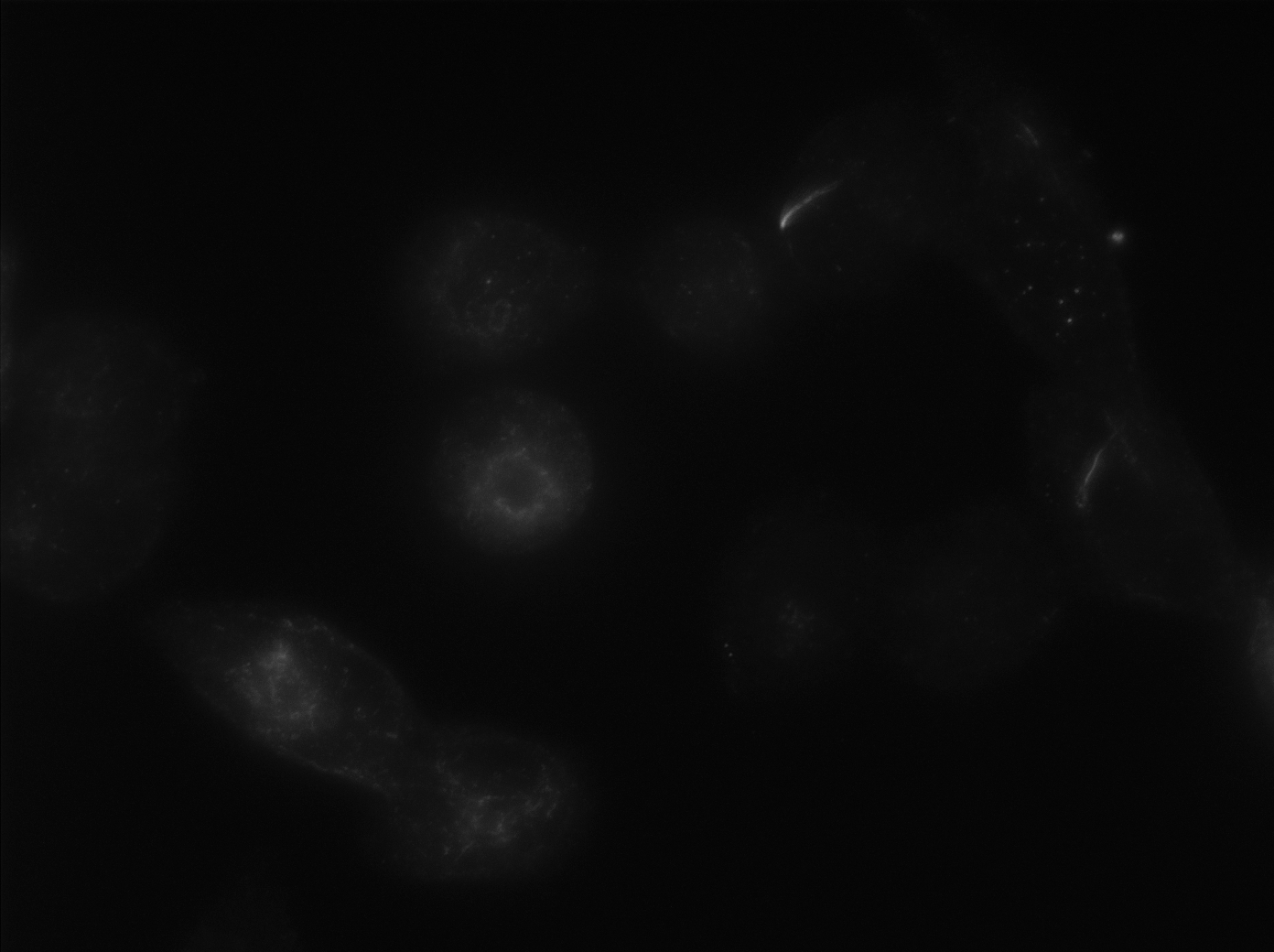

Supplement: Supplementary file 3 — Source data Fig. 1 [file 44319_2026_742_MOESM3_ESM.zip › Figure 1/Fig 1bcd WT Hela acetylated a tubulin atubulin/actub-atub 8-14-24 R3 PA7.Project Maximum Z_XY1724716637_Z0_T0_C2.tif]

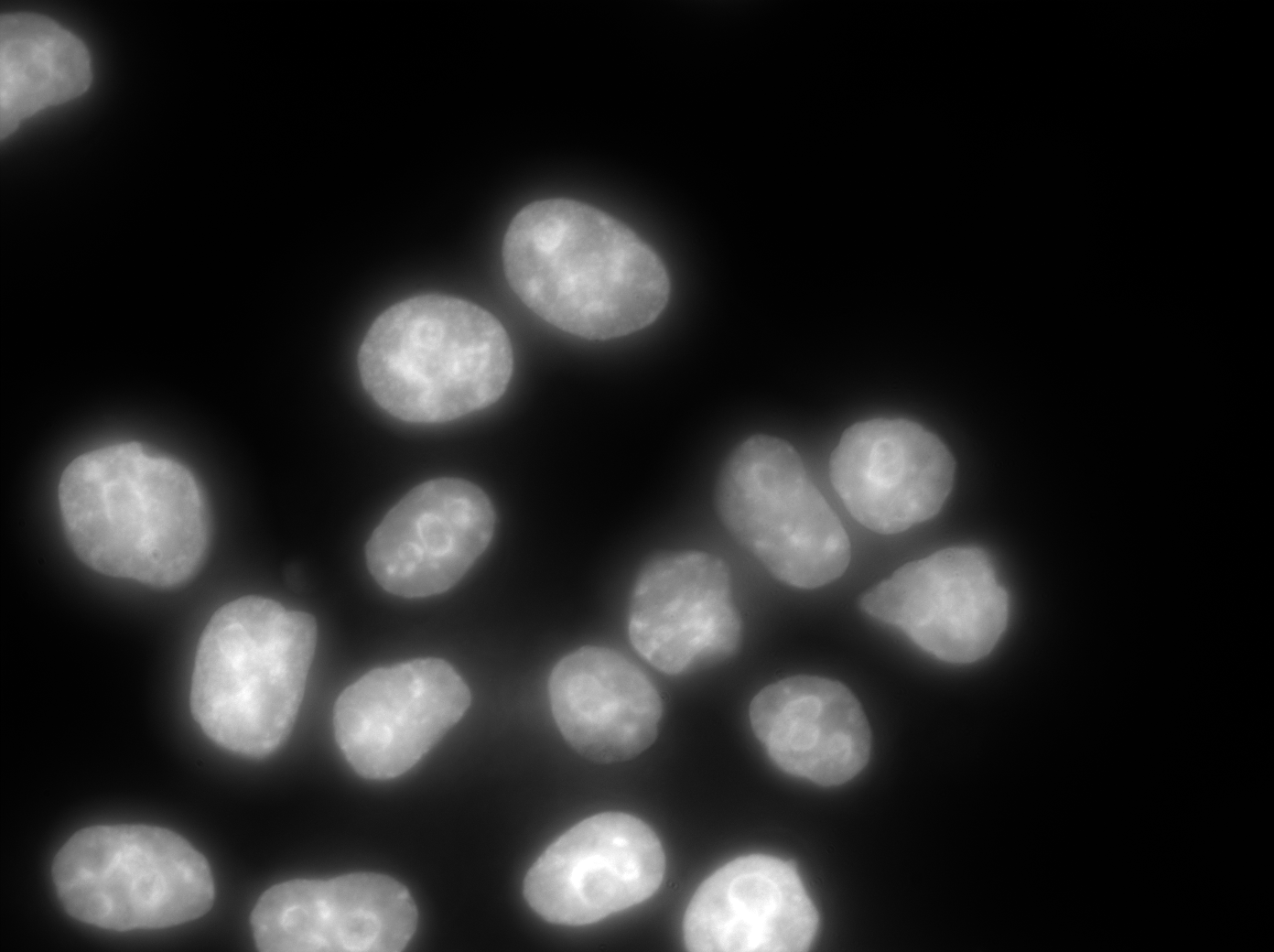

Supplement: Supplementary file 3 — Source data Fig. 1 [file 44319_2026_742_MOESM3_ESM.zip › Figure 1/Fig 1bcd WT Hela acetylated a tubulin atubulin/actub-atub 8-14-24 R3 PA9.Project Maximum Z_XY1724717294_Z0_T0_C0.tif]

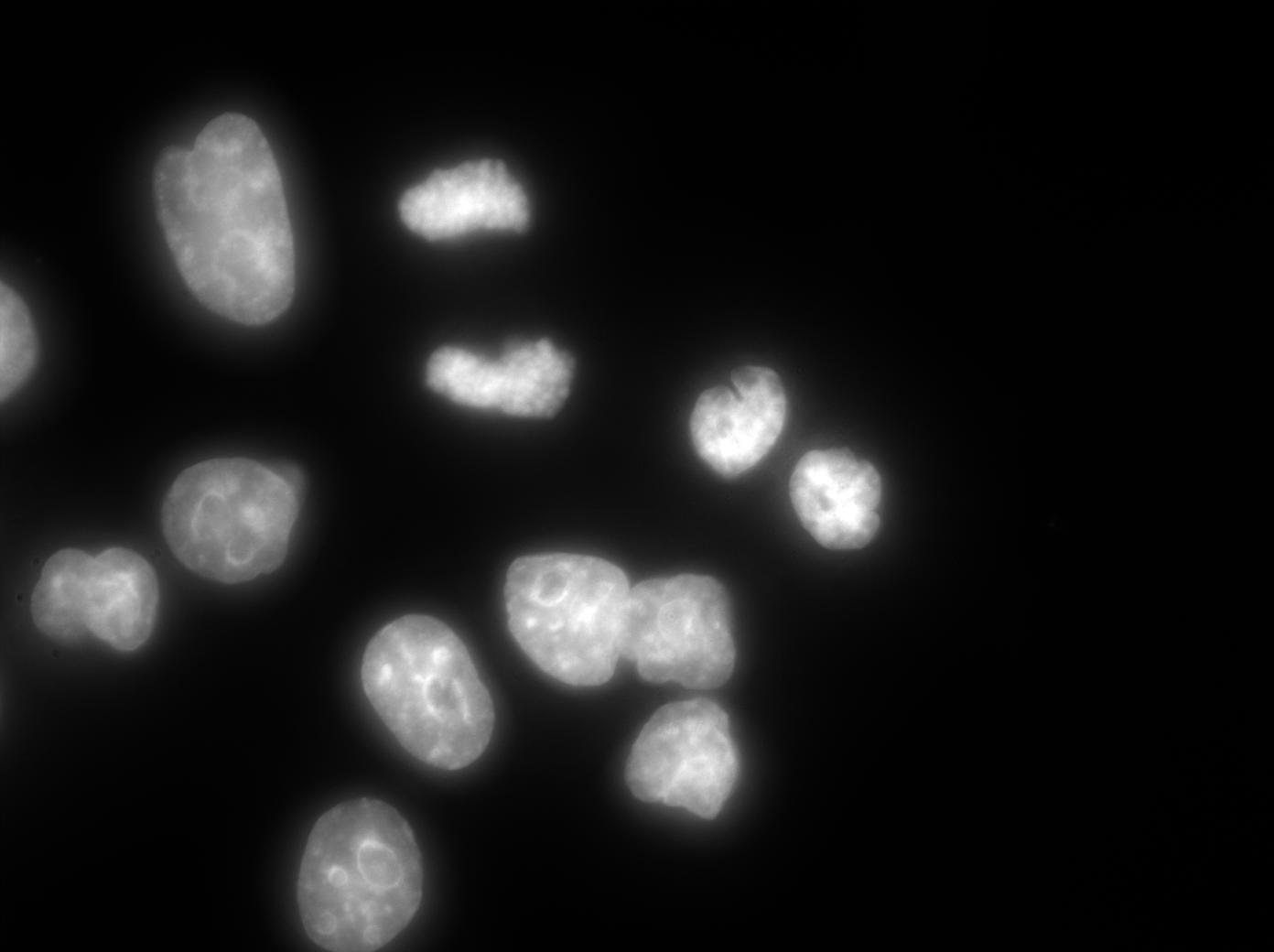

Supplement: Supplementary file 3 — Source data Fig. 1 [file 44319_2026_742_MOESM3_ESM.zip › Figure 1/Fig 1bcd WT Hela acetylated a tubulin atubulin/actub-atub 8-14-24 R3 ET7ET8 PA3.Project Maximum Z_XY1724703913_Z0_T0_C0.tif]

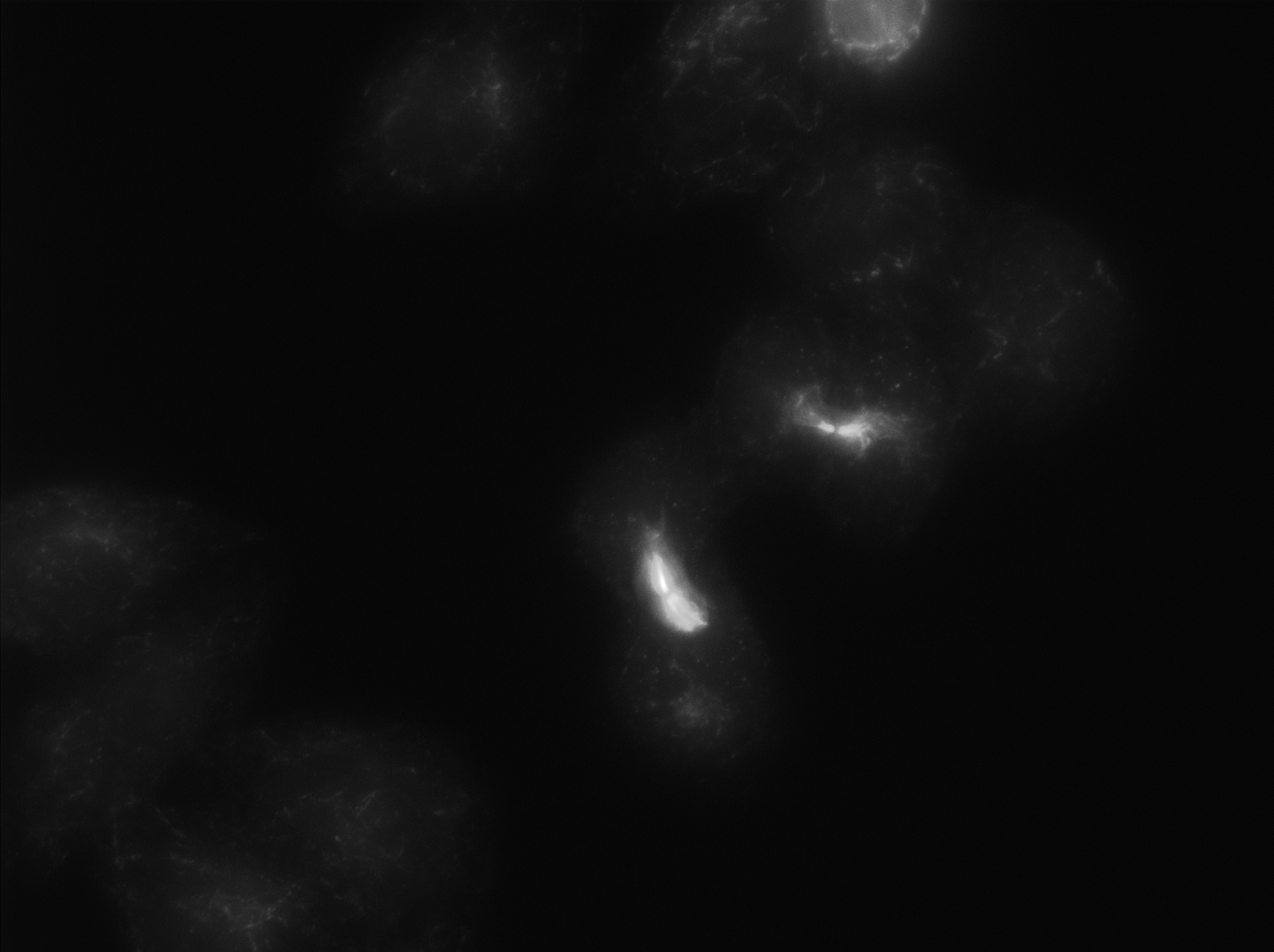

Supplement: Supplementary file 3 — Source data Fig. 1 [file 44319_2026_742_MOESM3_ESM.zip › Figure 1/Fig 1bcd WT Hela acetylated a tubulin atubulin/actub-atub 8-14-24 R2 ET3 LT2.Project Maximum Z_XY1724689652_Z0_T0_C2.tif]

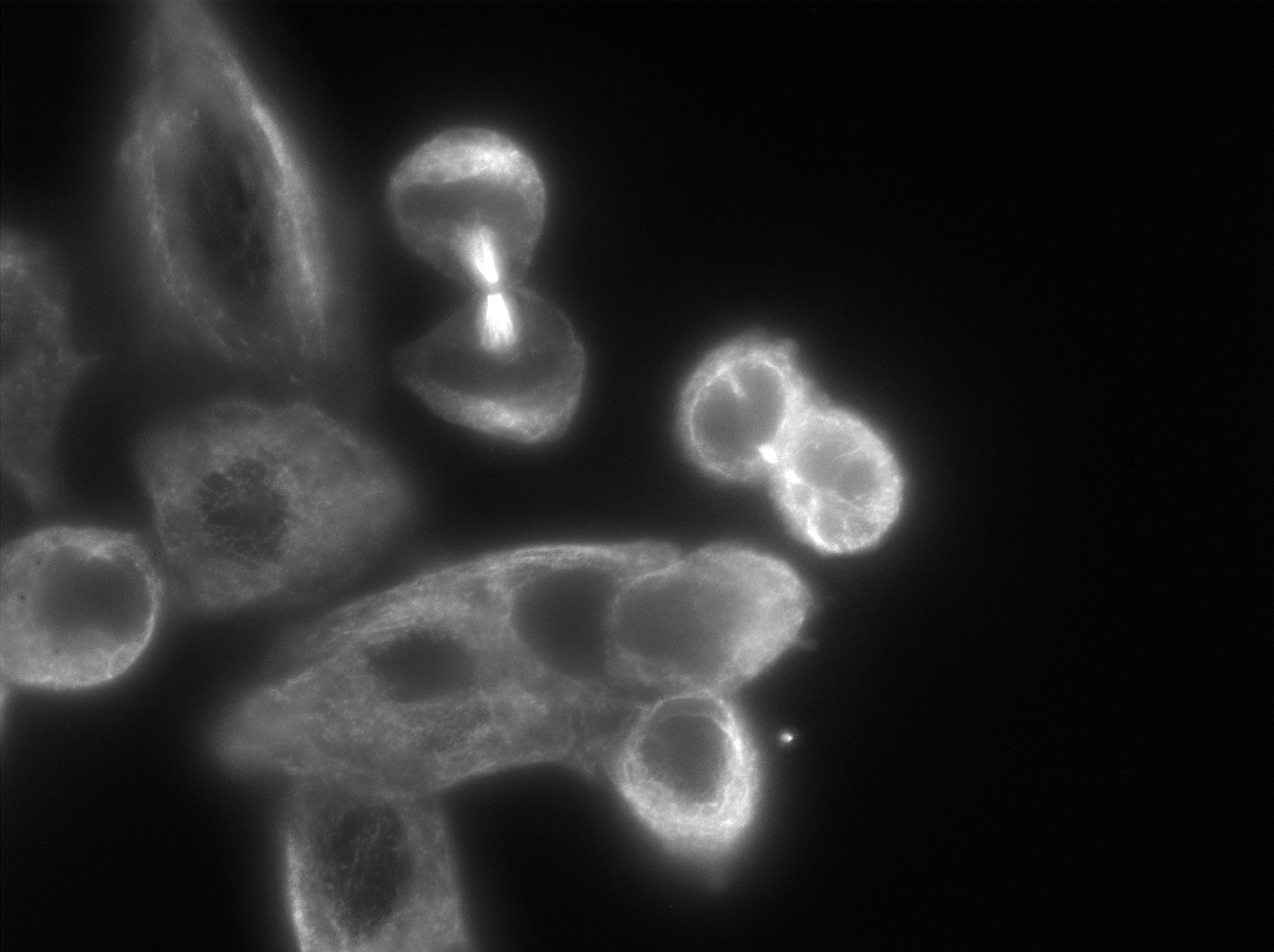

Supplement: Supplementary file 3 — Source data Fig. 1 [file 44319_2026_742_MOESM3_ESM.zip › Figure 1/Fig 1bcd WT Hela acetylated a tubulin atubulin/actub-atub 8-14-24 R3 ET7ET8 PA3.Project Maximum Z_XY1724703913_Z0_T0_C1.tif]

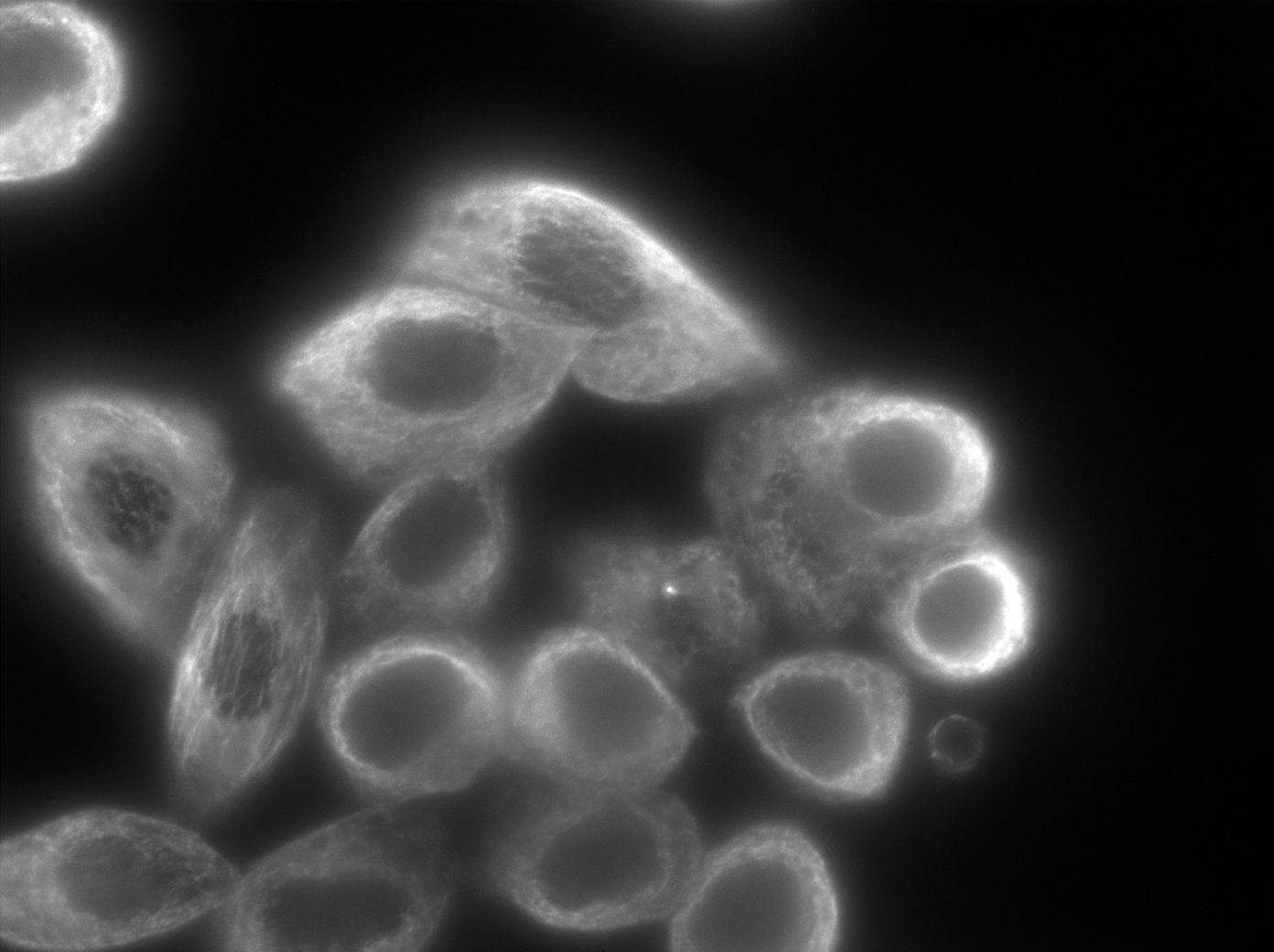

Supplement: Supplementary file 3 — Source data Fig. 1 [file 44319_2026_742_MOESM3_ESM.zip › Figure 1/Fig 1bcd WT Hela acetylated a tubulin atubulin/actub-atub 8-14-24 R3 PA9.Project Maximum Z_XY1724717294_Z0_T0_C1.tif]

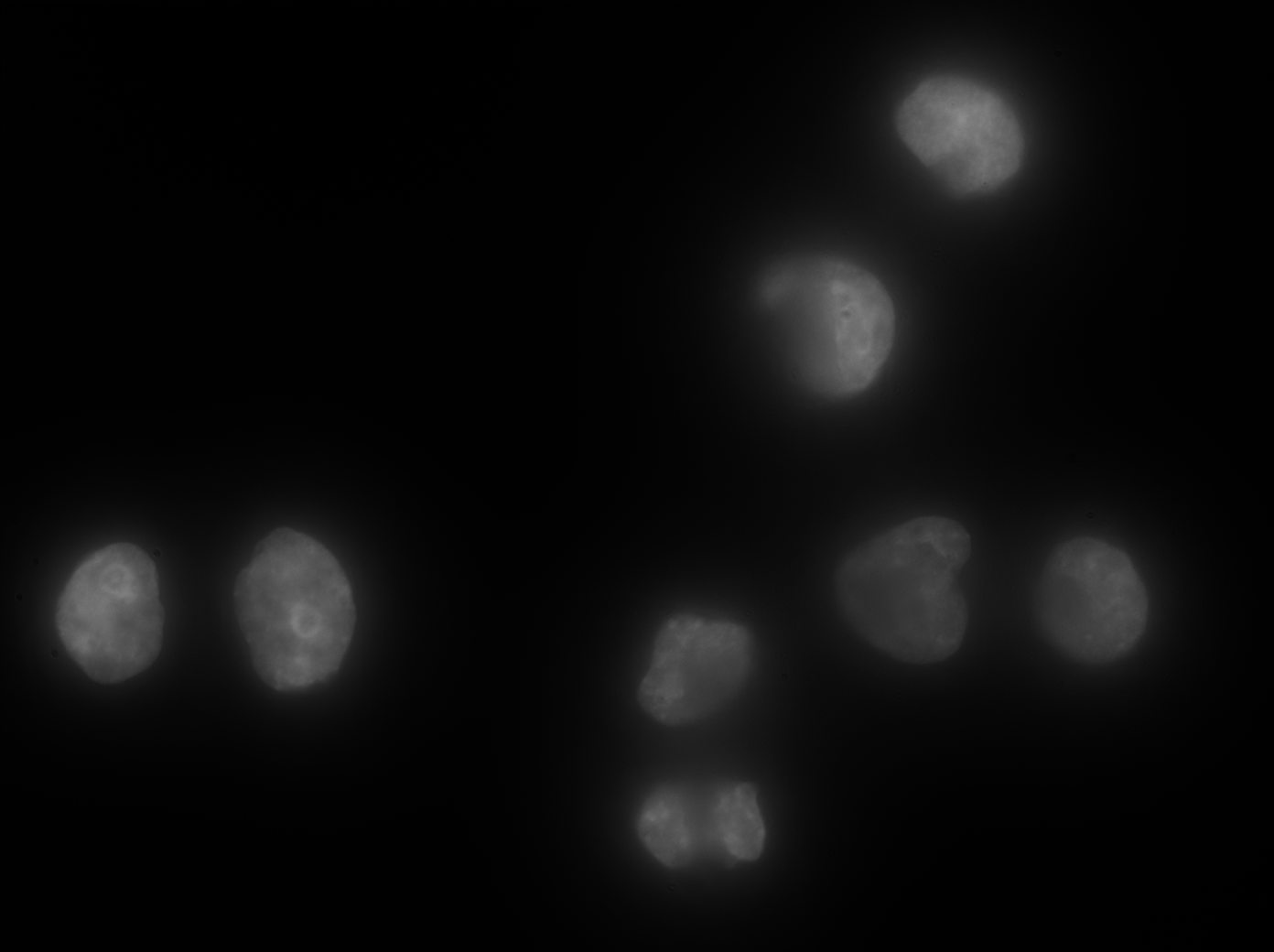

Supplement: Supplementary file 3 — Source data Fig. 1 [file 44319_2026_742_MOESM3_ESM.zip › Figure 1/Fig 1bcd WT Hela acetylated a tubulin atubulin/actub-atub 8-14-24 R1 PA6.Project Maximum Z_XY1724364026_Z0_T0_C0.tif]

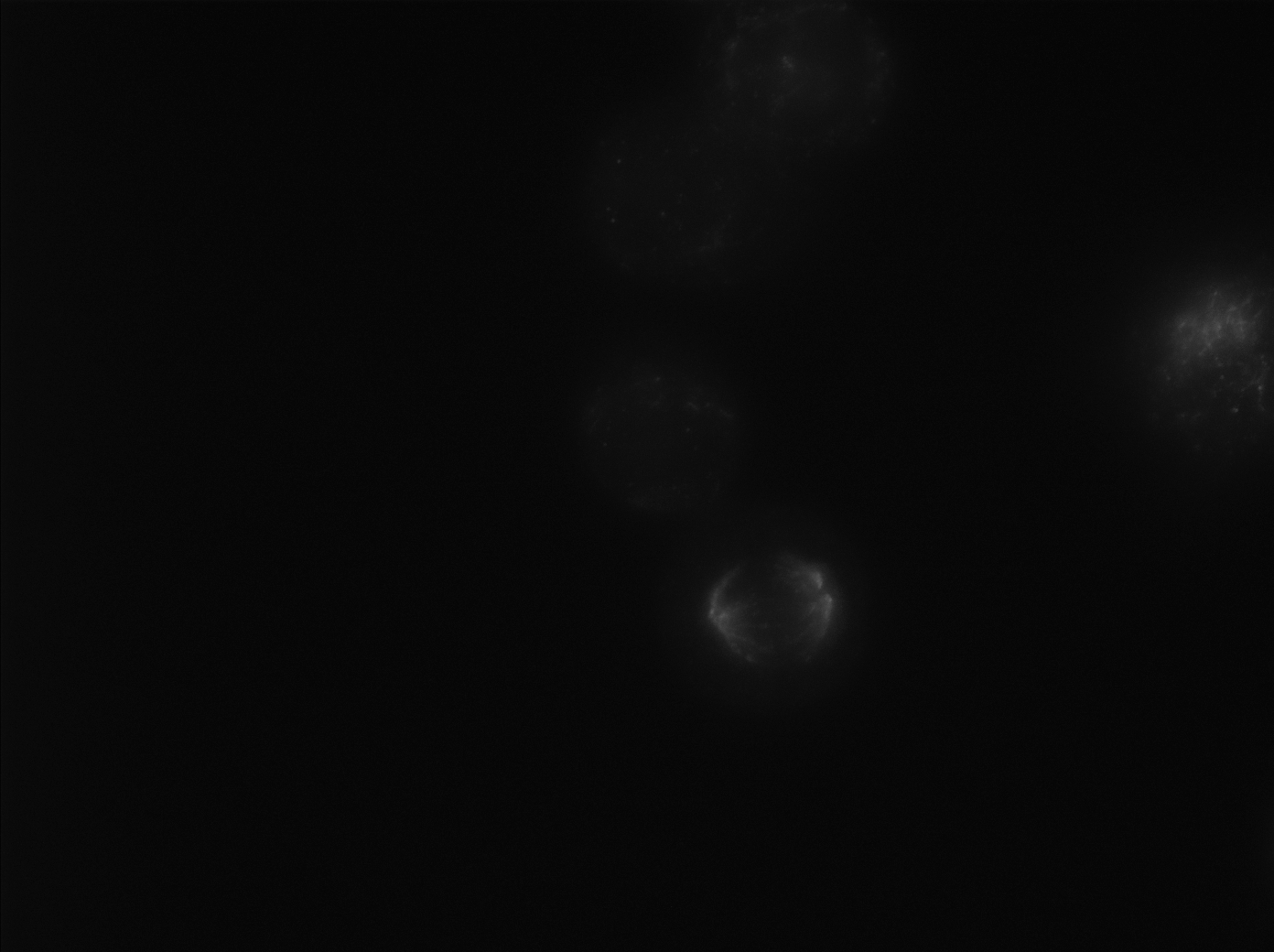

Supplement: Supplementary file 3 — Source data Fig. 1 [file 44319_2026_742_MOESM3_ESM.zip › Figure 1/Fig 1bcd WT Hela acetylated a tubulin atubulin/actub-atub 8-14-24 R1 M1.Project Maximum Z_XY1724363237_Z0_T0_C2.tif]

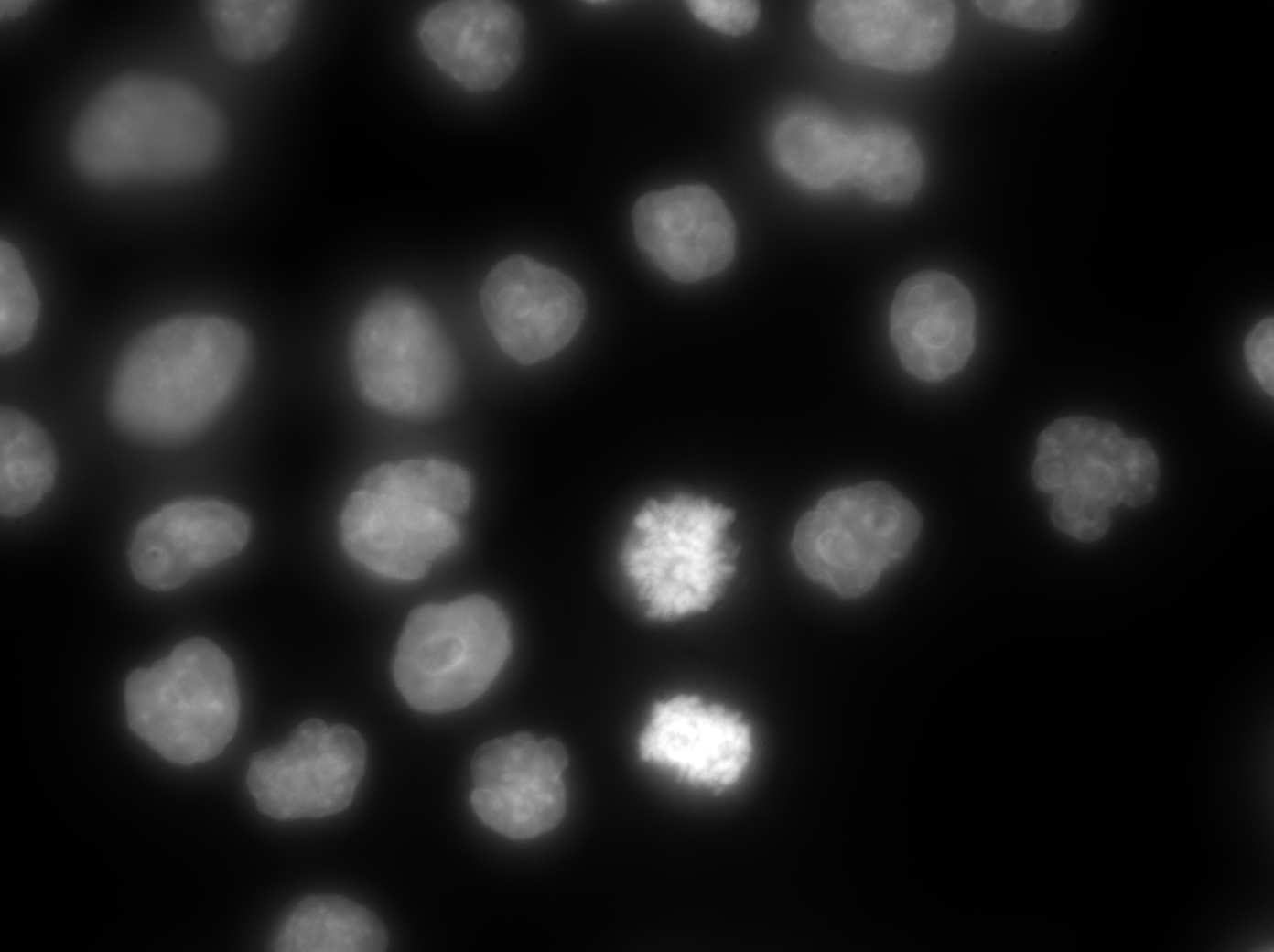

Supplement: Supplementary file 3 — Source data Fig. 1 [file 44319_2026_742_MOESM3_ESM.zip › Figure 1/Fig 1bcd WT Hela acetylated a tubulin atubulin/actub-atub 8-14-24 R3 M2M3 LT1.Project Maximum Z_XY1724701933_Z0_T0_C0.tif]

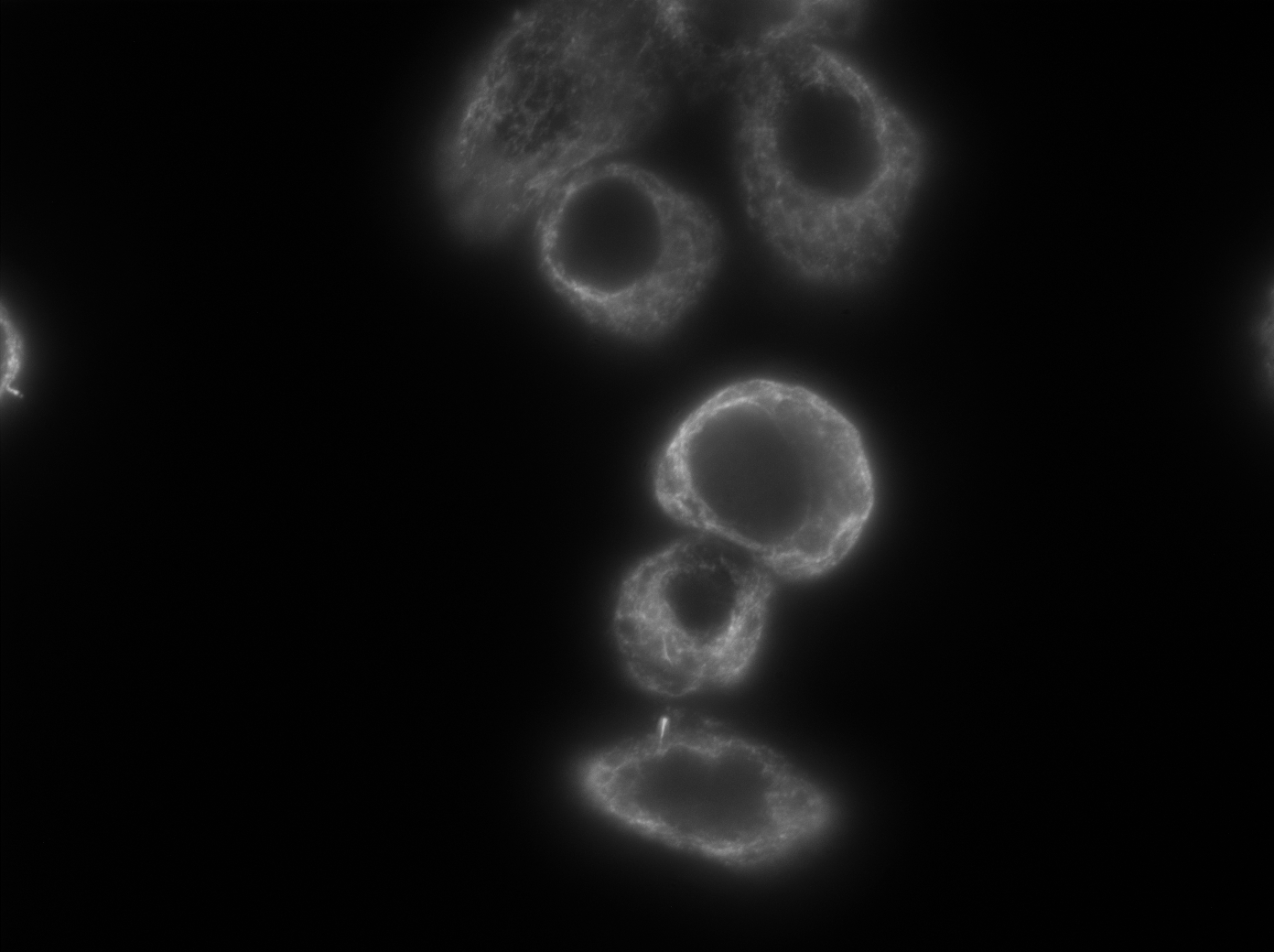

Supplement: Supplementary file 3 — Source data Fig. 1 [file 44319_2026_742_MOESM3_ESM.zip › Figure 1/Fig 1bcd WT Hela acetylated a tubulin atubulin/actub-atub 8-14-24 R1 PA2.Project Maximum Z_XY1724362613_Z0_T0_C1.tif]

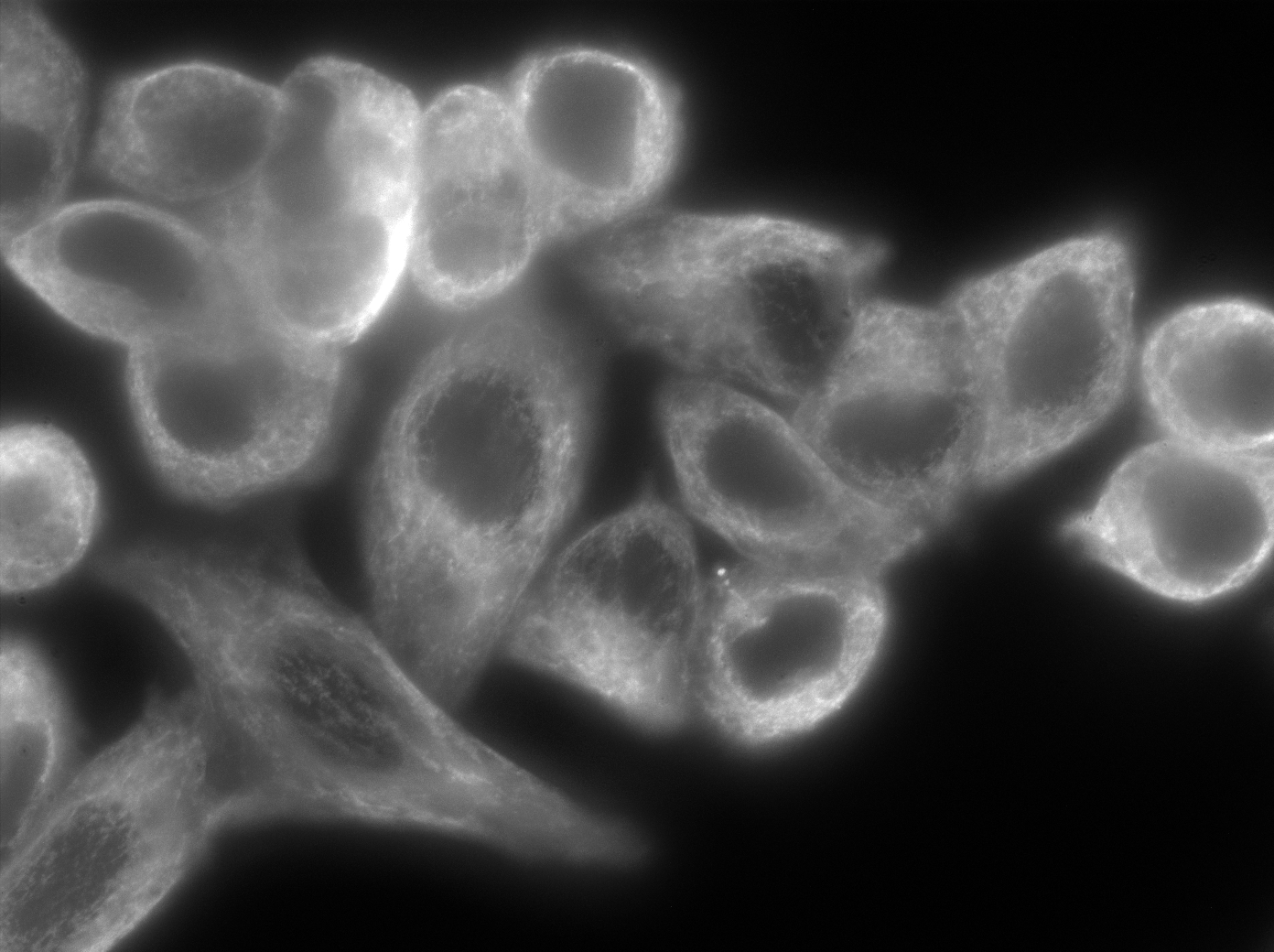

Supplement: Supplementary file 3 — Source data Fig. 1 [file 44319_2026_742_MOESM3_ESM.zip › Figure 1/Fig 1bcd WT Hela acetylated a tubulin atubulin/actub-atub 8-14-24 R3 PA6.Project Maximum Z_XY1724716567_Z0_T0_C1.tif]

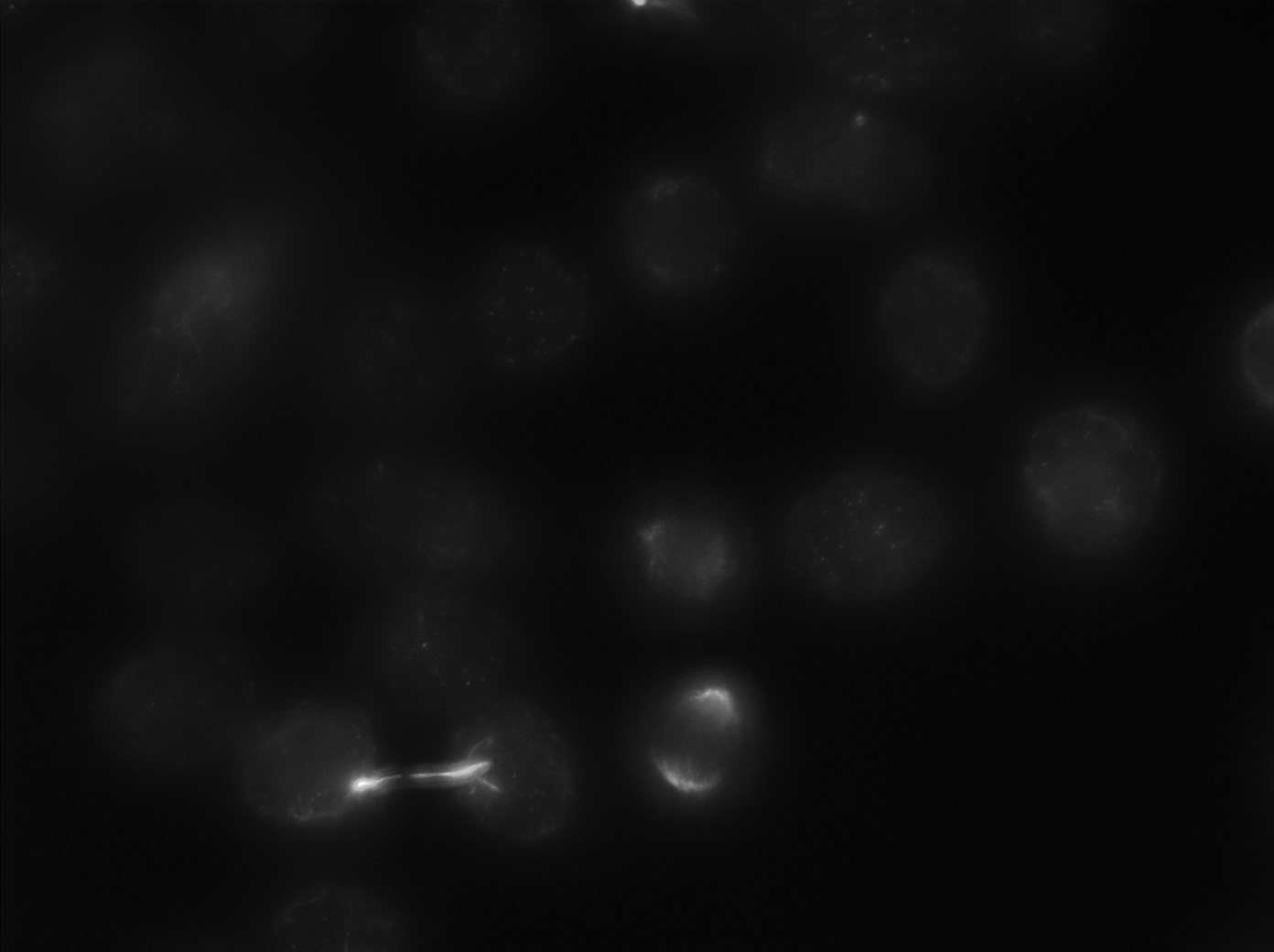

Supplement: Supplementary file 3 — Source data Fig. 1 [file 44319_2026_742_MOESM3_ESM.zip › Figure 1/Fig 1bcd WT Hela acetylated a tubulin atubulin/actub-atub 8-14-24 R3 M2M3 LT1.Project Maximum Z_XY1724701933_Z0_T0_C2.tif]

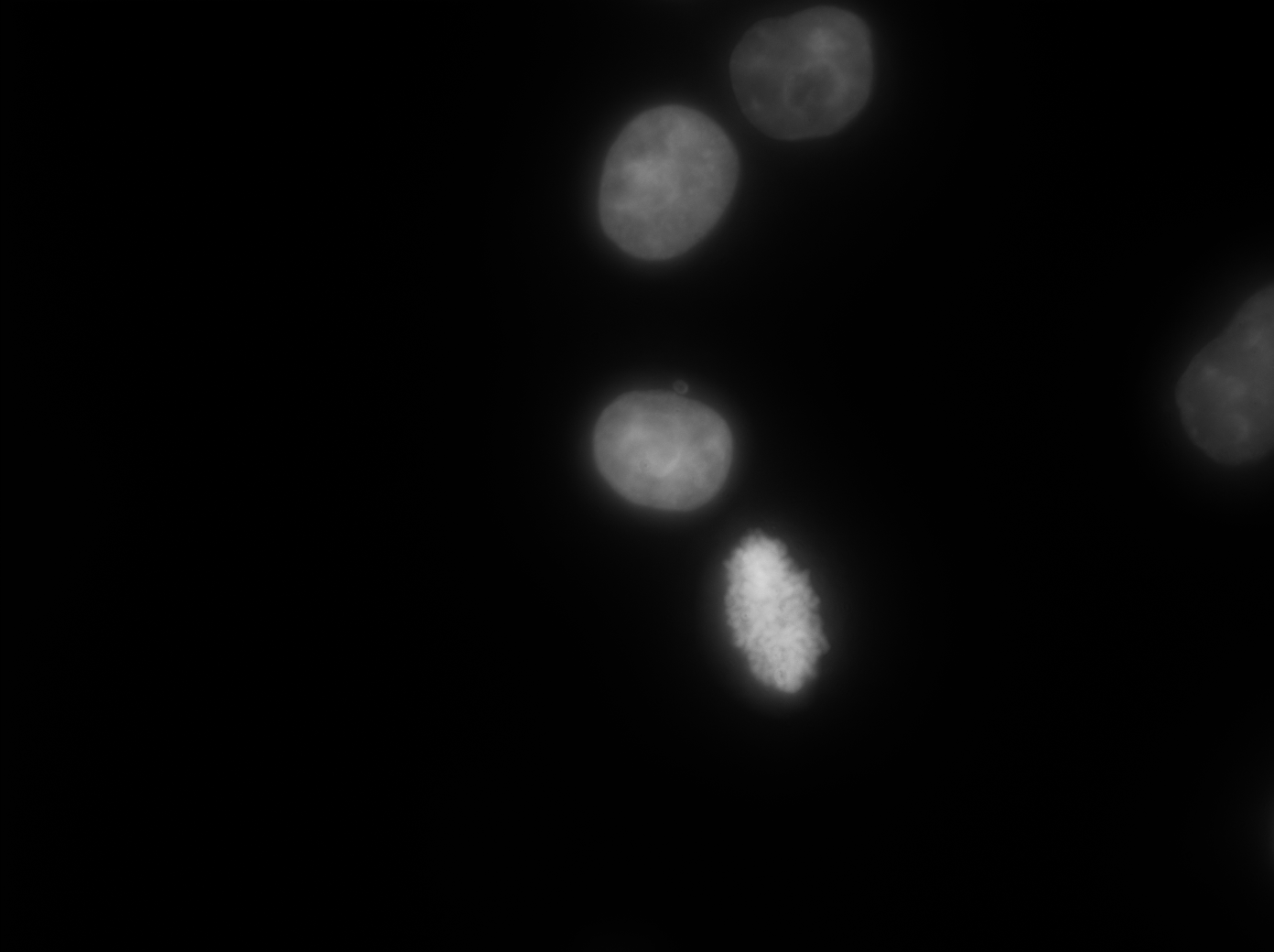

Supplement: Supplementary file 3 — Source data Fig. 1 [file 44319_2026_742_MOESM3_ESM.zip › Figure 1/Fig 1bcd WT Hela acetylated a tubulin atubulin/actub-atub 8-14-24 R1 M1.Project Maximum Z_XY1724363237_Z0_T0_C0.tif]

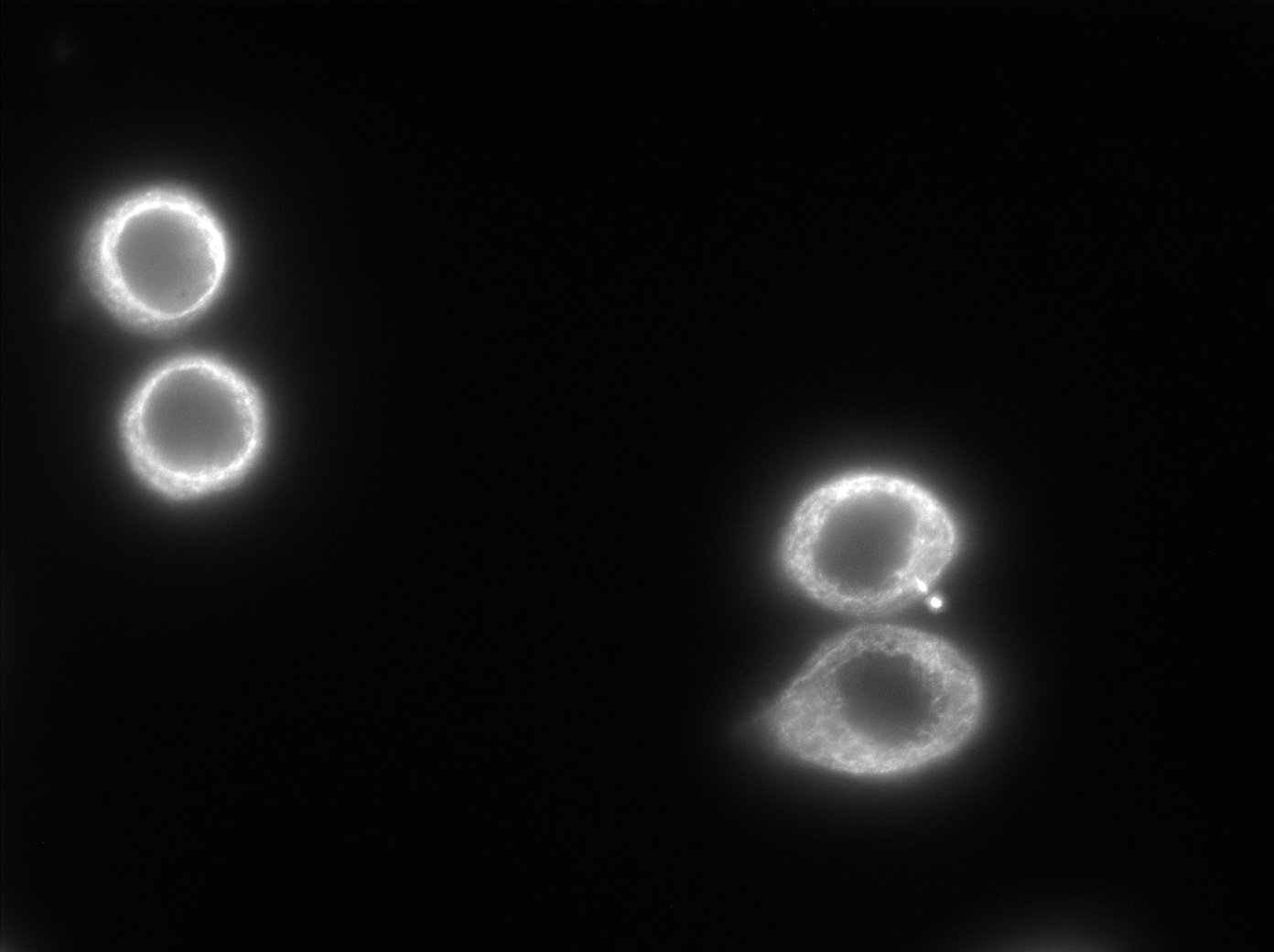

Supplement: Supplementary file 3 — Source data Fig. 1 [file 44319_2026_742_MOESM3_ESM.zip › Figure 1/Fig 1bcd WT Hela acetylated a tubulin atubulin/actub-atub 8-14-24 R3 PA5.Project Maximum Z_XY1724704270_Z0_T0_C1.tif]

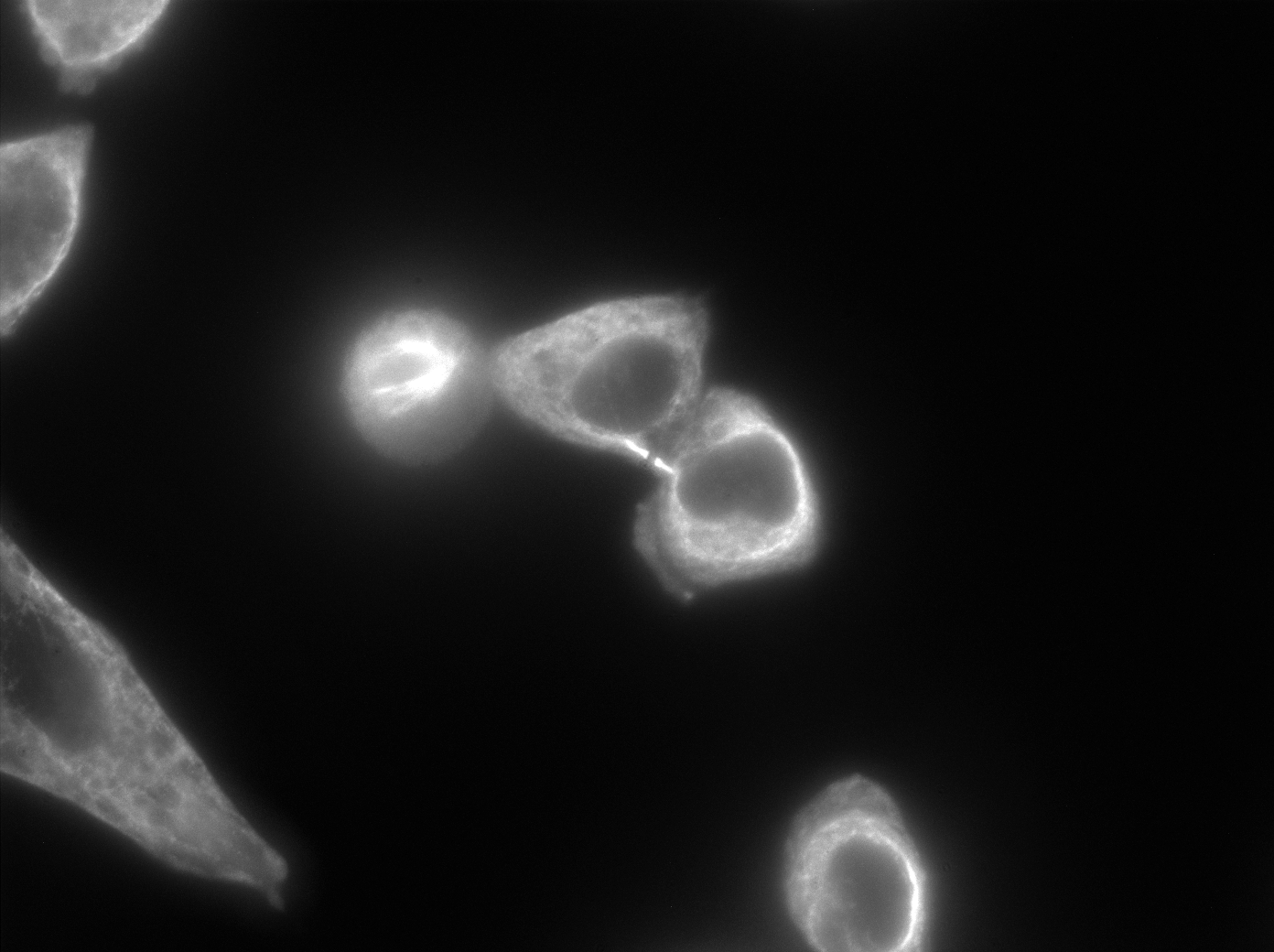

Supplement: Supplementary file 3 — Source data Fig. 1 [file 44319_2026_742_MOESM3_ESM.zip › Figure 1/Fig 1bcd WT Hela acetylated a tubulin atubulin/actub-atub 8-14-24 R3 LT2.Project Maximum Z_XY1724702329_Z0_T0_C1.tif]

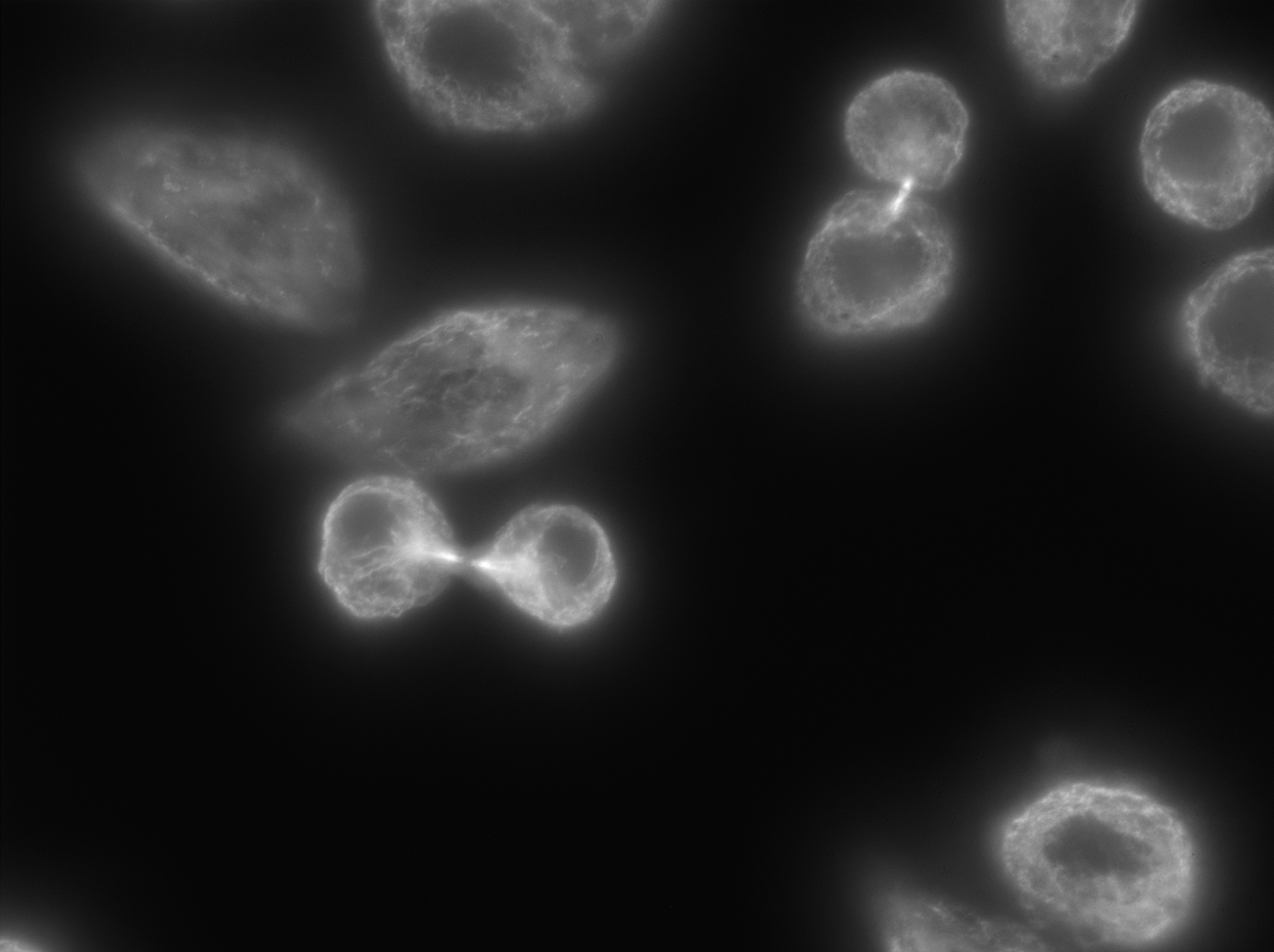

Supplement: Supplementary file 3 — Source data Fig. 1 [file 44319_2026_742_MOESM3_ESM.zip › Figure 1/Fig 1bcd WT Hela acetylated a tubulin atubulin/actub-atub 8-14-24 R2 ET2 LT1.Project Maximum Z_XY1724689540_Z0_T0_C1.tif]

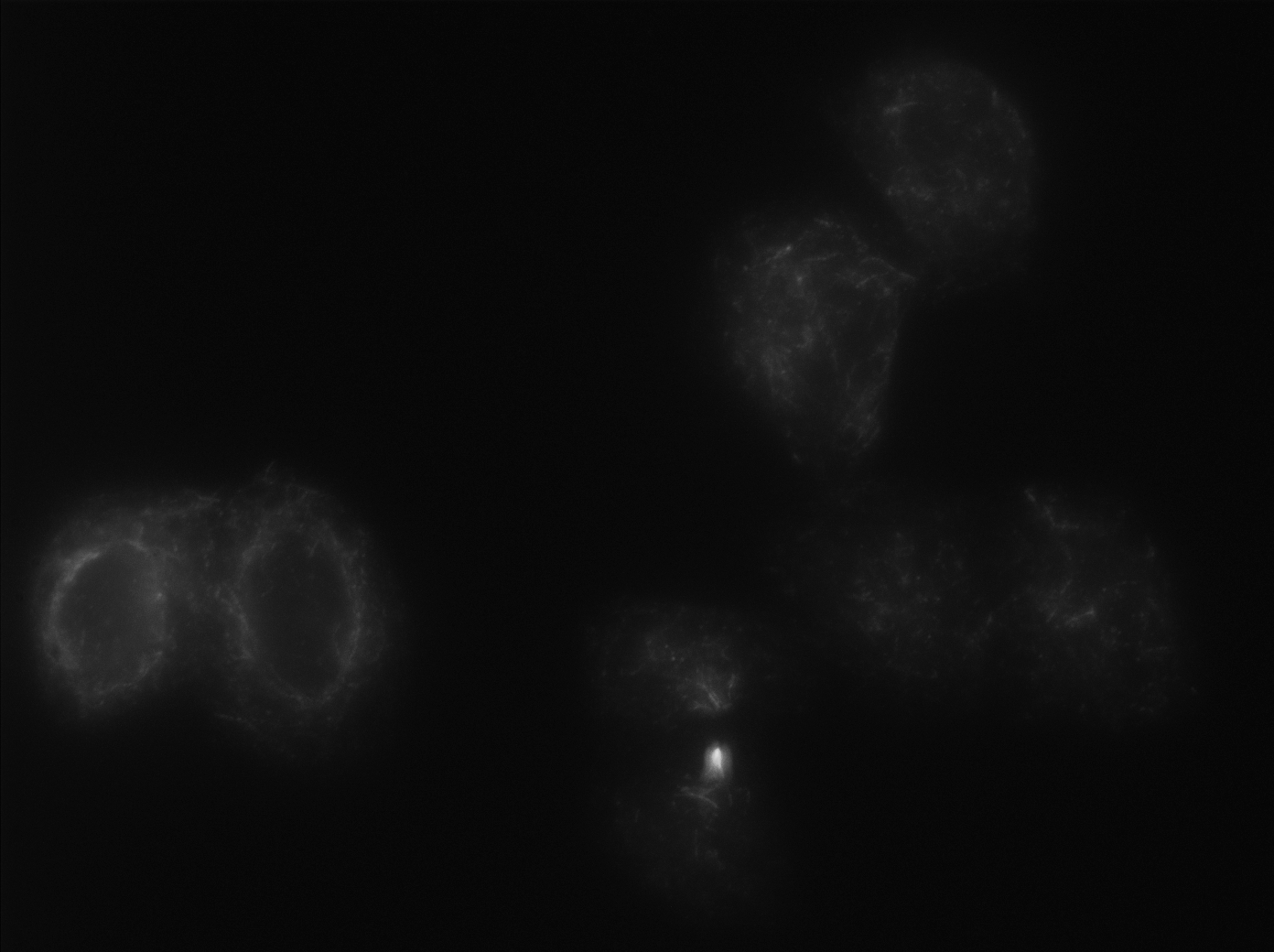

Supplement: Supplementary file 3 — Source data Fig. 1 [file 44319_2026_742_MOESM3_ESM.zip › Figure 1/Fig 1bcd WT Hela acetylated a tubulin atubulin/actub-atub 8-14-24 R1 PA6.Project Maximum Z_XY1724364026_Z0_T0_C2.tif]

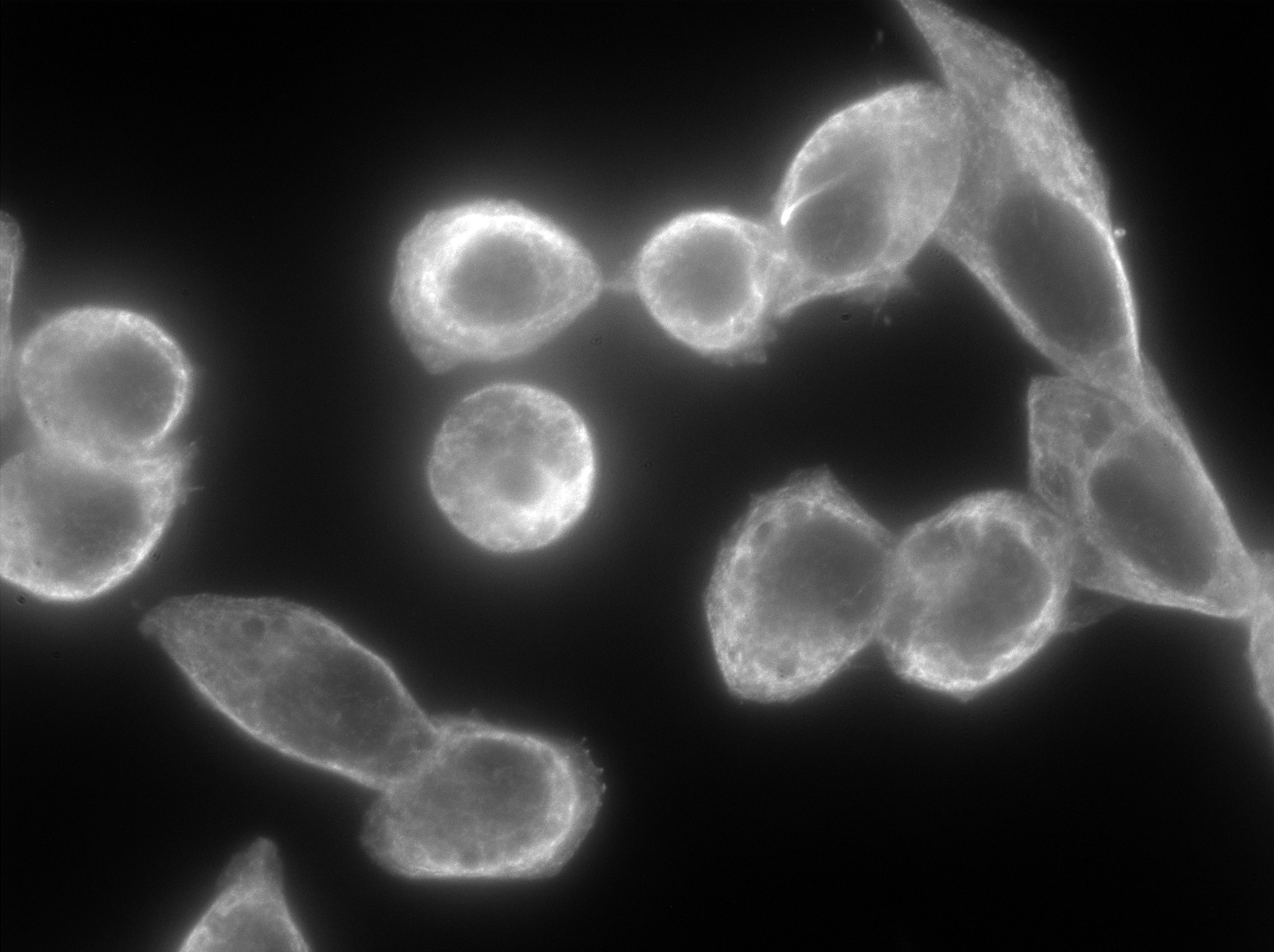

Supplement: Supplementary file 3 — Source data Fig. 1 [file 44319_2026_742_MOESM3_ESM.zip › Figure 1/Fig 1bcd WT Hela acetylated a tubulin atubulin/actub-atub 8-14-24 R3 PA7.Project Maximum Z_XY1724716637_Z0_T0_C1.tif]

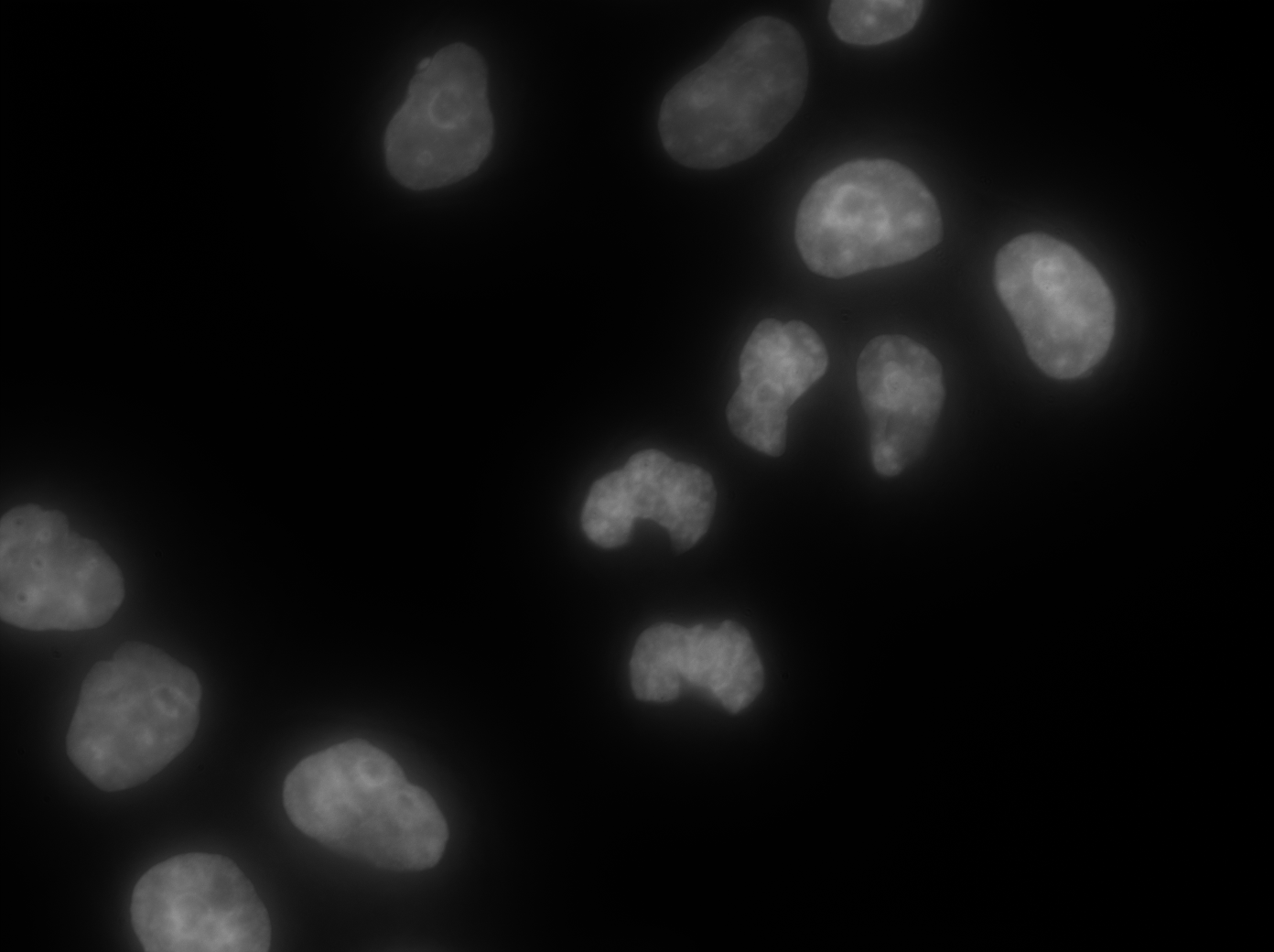

Supplement: Supplementary file 3 — Source data Fig. 1 [file 44319_2026_742_MOESM3_ESM.zip › Figure 1/Fig 1bcd WT Hela acetylated a tubulin atubulin/actub-atub 8-14-24 R2 ET3 LT2.Project Maximum Z_XY1724689652_Z0_T0_C0.tif]

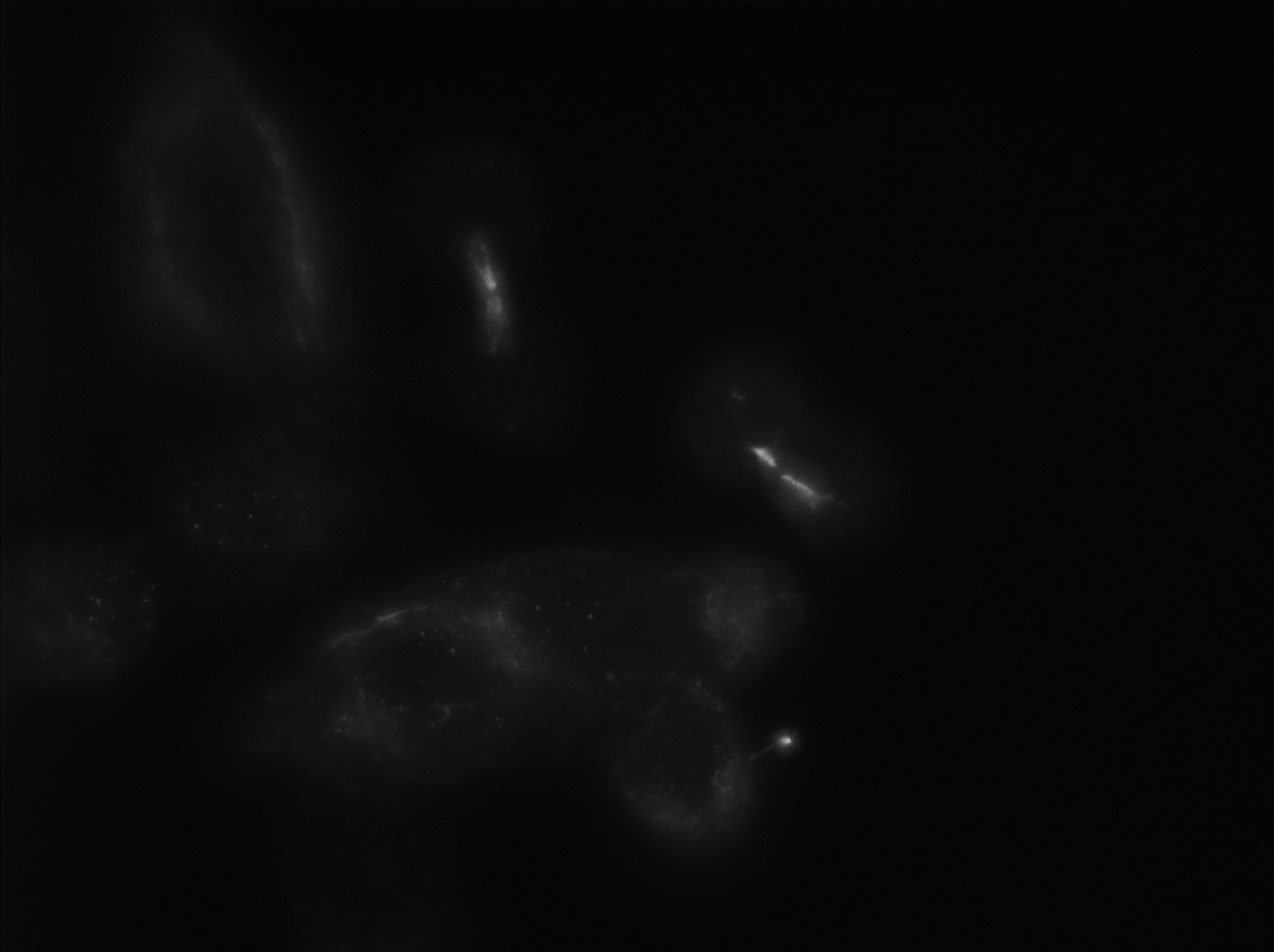

Supplement: Supplementary file 3 — Source data Fig. 1 [file 44319_2026_742_MOESM3_ESM.zip › Figure 1/Fig 1bcd WT Hela acetylated a tubulin atubulin/actub-atub 8-14-24 R3 ET7ET8 PA3.Project Maximum Z_XY1724703913_Z0_T0_C2.tif]

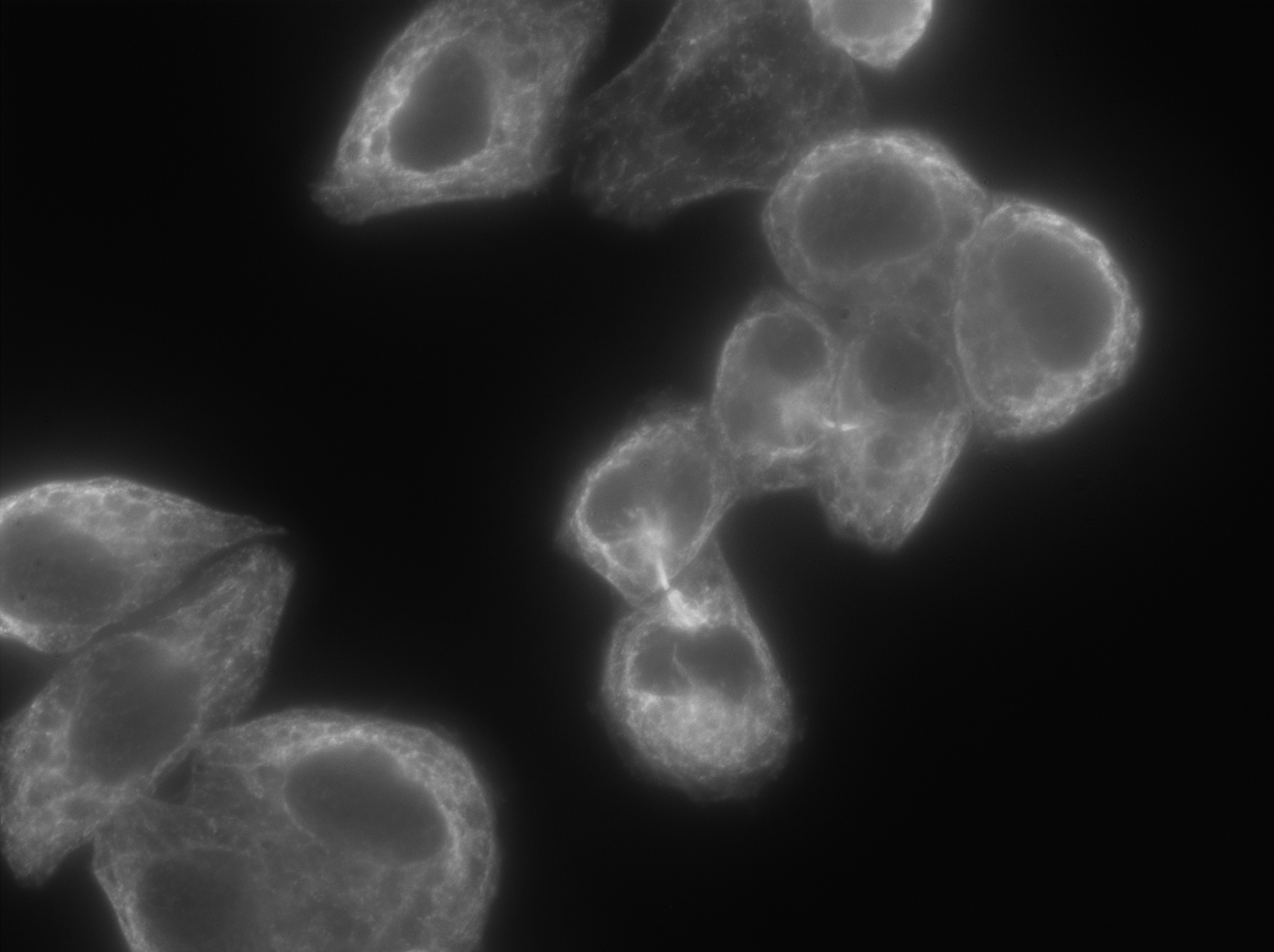

Supplement: Supplementary file 3 — Source data Fig. 1 [file 44319_2026_742_MOESM3_ESM.zip › Figure 1/Fig 1bcd WT Hela acetylated a tubulin atubulin/actub-atub 8-14-24 R2 ET3 LT2.Project Maximum Z_XY1724689652_Z0_T0_C1.tif]

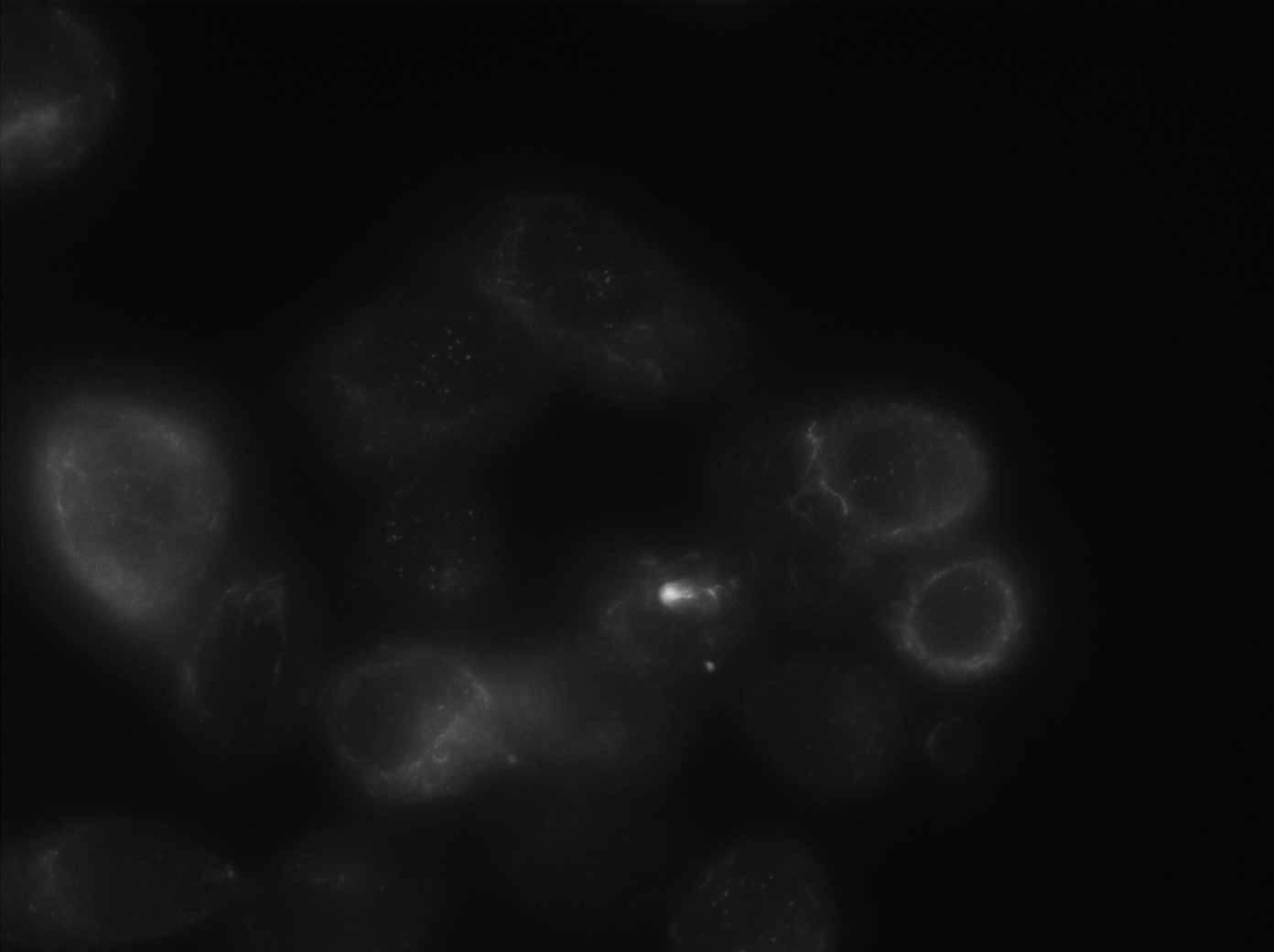

Supplement: Supplementary file 3 — Source data Fig. 1 [file 44319_2026_742_MOESM3_ESM.zip › Figure 1/Fig 1bcd WT Hela acetylated a tubulin atubulin/actub-atub 8-14-24 R3 PA9.Project Maximum Z_XY1724717294_Z0_T0_C2.tif]

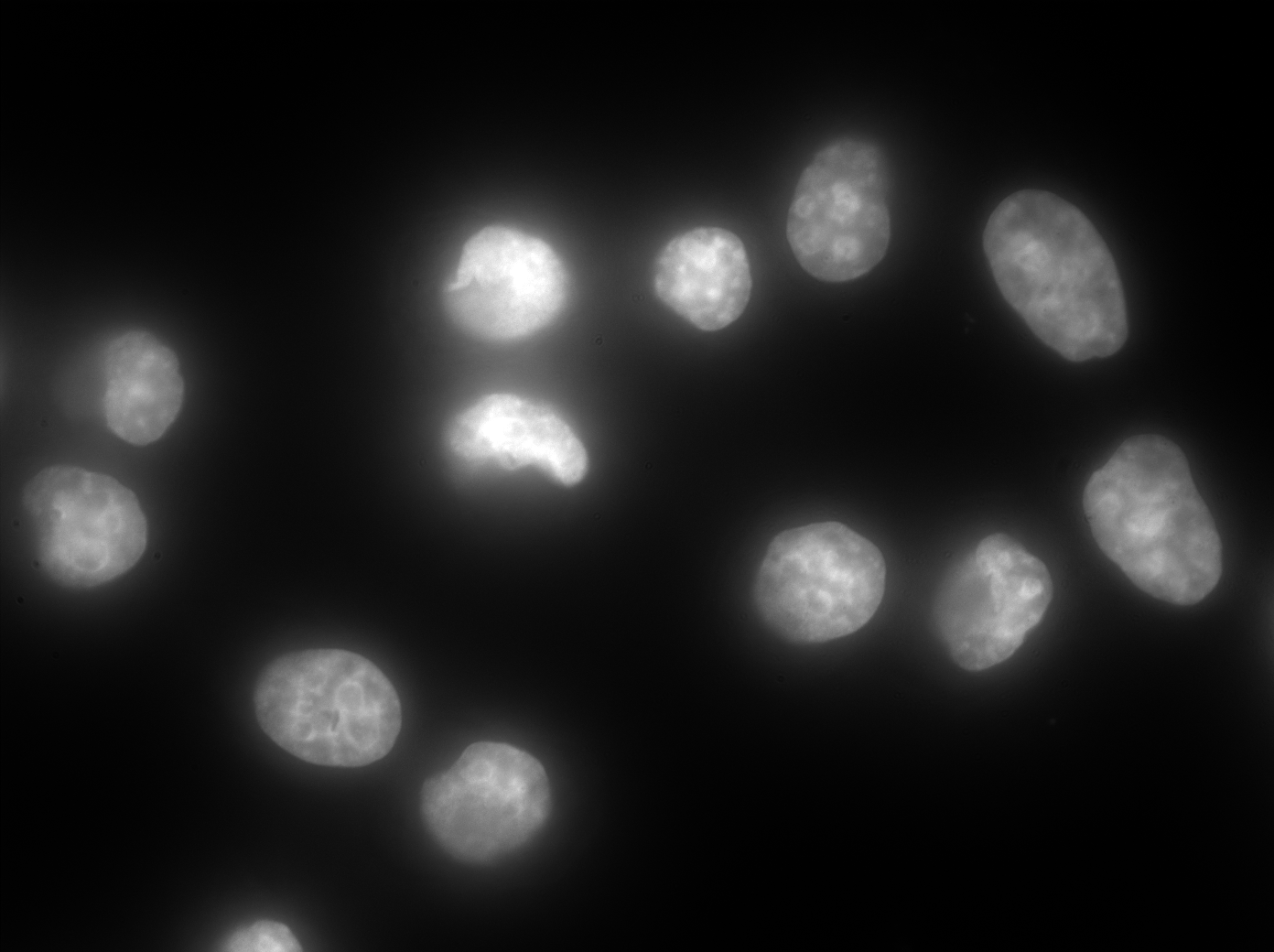

Supplement: Supplementary file 3 — Source data Fig. 1 [file 44319_2026_742_MOESM3_ESM.zip › Figure 1/Fig 1bcd WT Hela acetylated a tubulin atubulin/actub-atub 8-14-24 R3 PA7.Project Maximum Z_XY1724716637_Z0_T0_C0.tif]

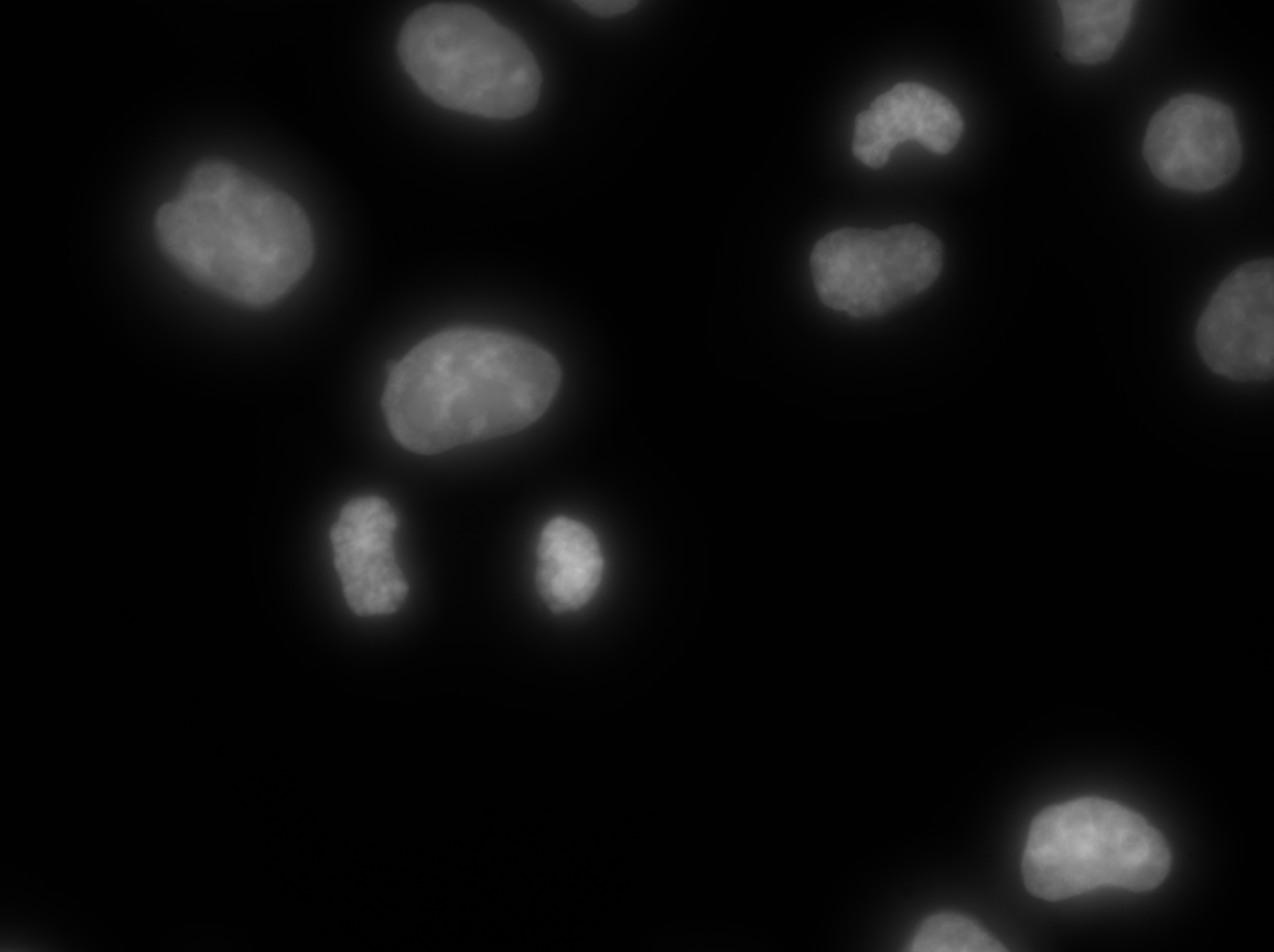

Supplement: Supplementary file 3 — Source data Fig. 1 [file 44319_2026_742_MOESM3_ESM.zip › Figure 1/Fig 1bcd WT Hela acetylated a tubulin atubulin/actub-atub 8-14-24 R2 ET2 LT1.Project Maximum Z_XY1724689540_Z0_T0_C0.tif]

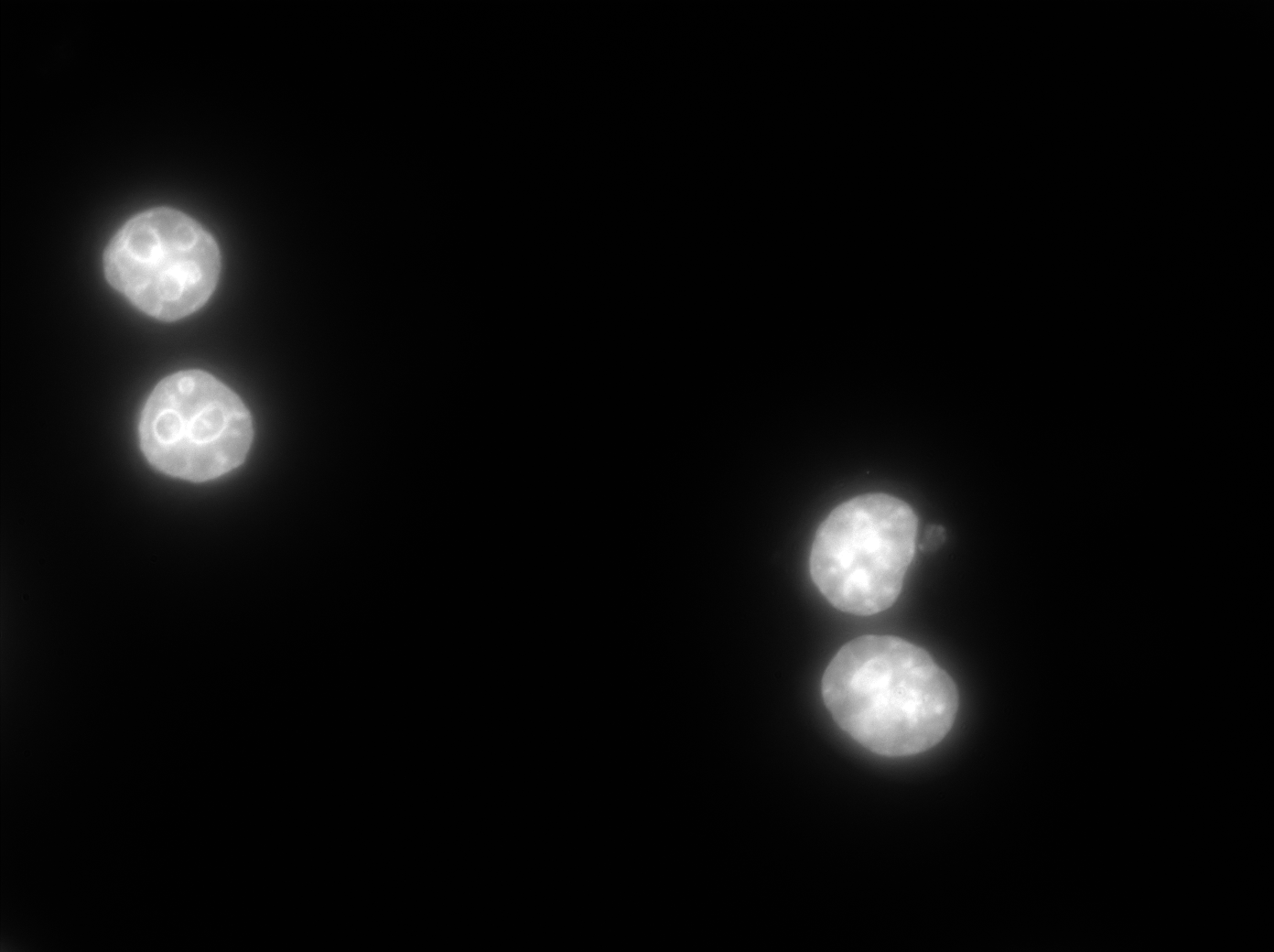

Supplement: Supplementary file 3 — Source data Fig. 1 [file 44319_2026_742_MOESM3_ESM.zip › Figure 1/Fig 1bcd WT Hela acetylated a tubulin atubulin/actub-atub 8-14-24 R3 PA5.Project Maximum Z_XY1724704270_Z0_T0_C0.tif]

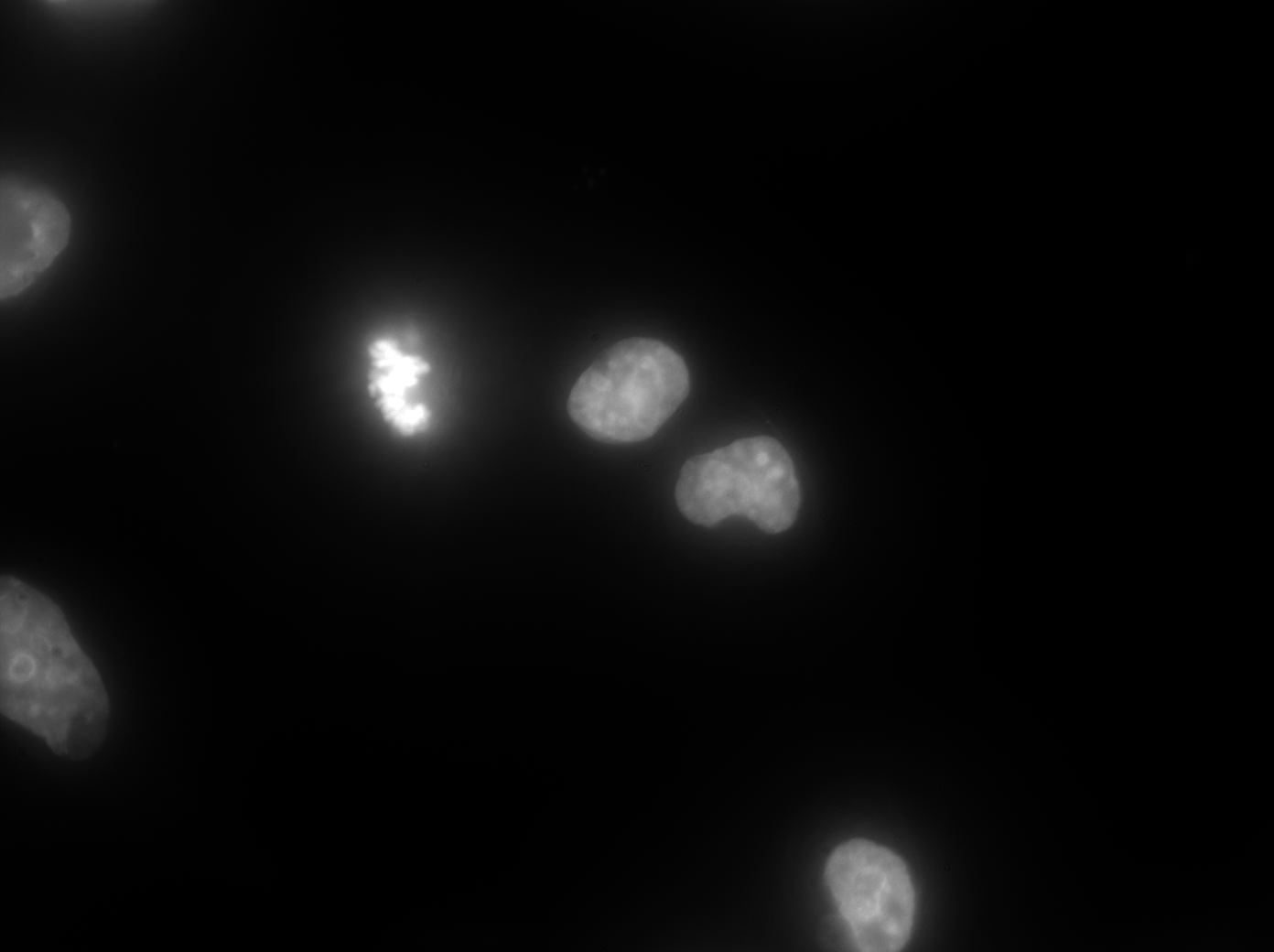

Supplement: Supplementary file 3 — Source data Fig. 1 [file 44319_2026_742_MOESM3_ESM.zip › Figure 1/Fig 1bcd WT Hela acetylated a tubulin atubulin/actub-atub 8-14-24 R3 LT2.Project Maximum Z_XY1724702329_Z0_T0_C0.tif]

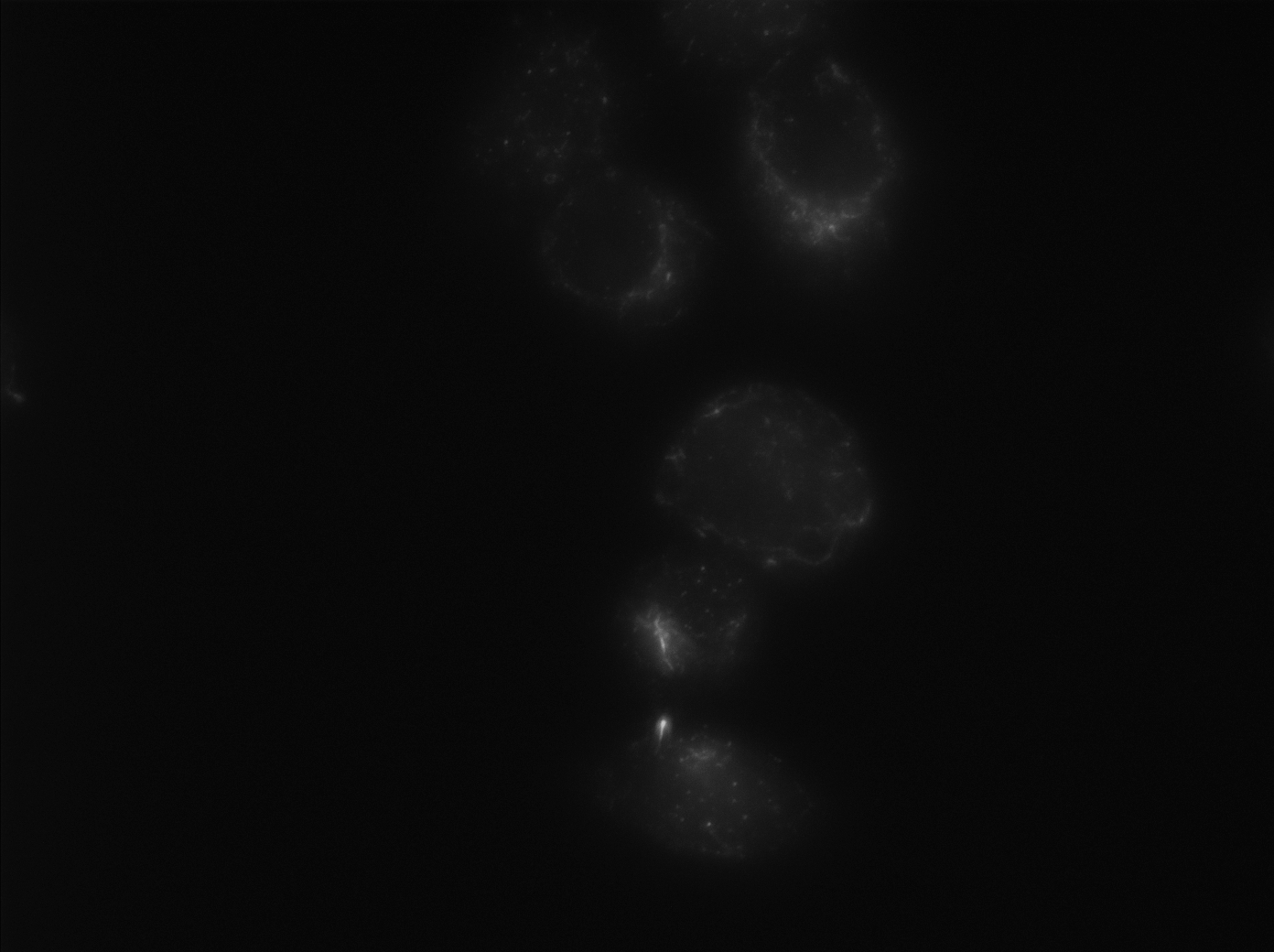

Supplement: Supplementary file 3 — Source data Fig. 1 [file 44319_2026_742_MOESM3_ESM.zip › Figure 1/Fig 1bcd WT Hela acetylated a tubulin atubulin/actub-atub 8-14-24 R1 PA2.Project Maximum Z_XY1724362613_Z0_T0_C2.tif]

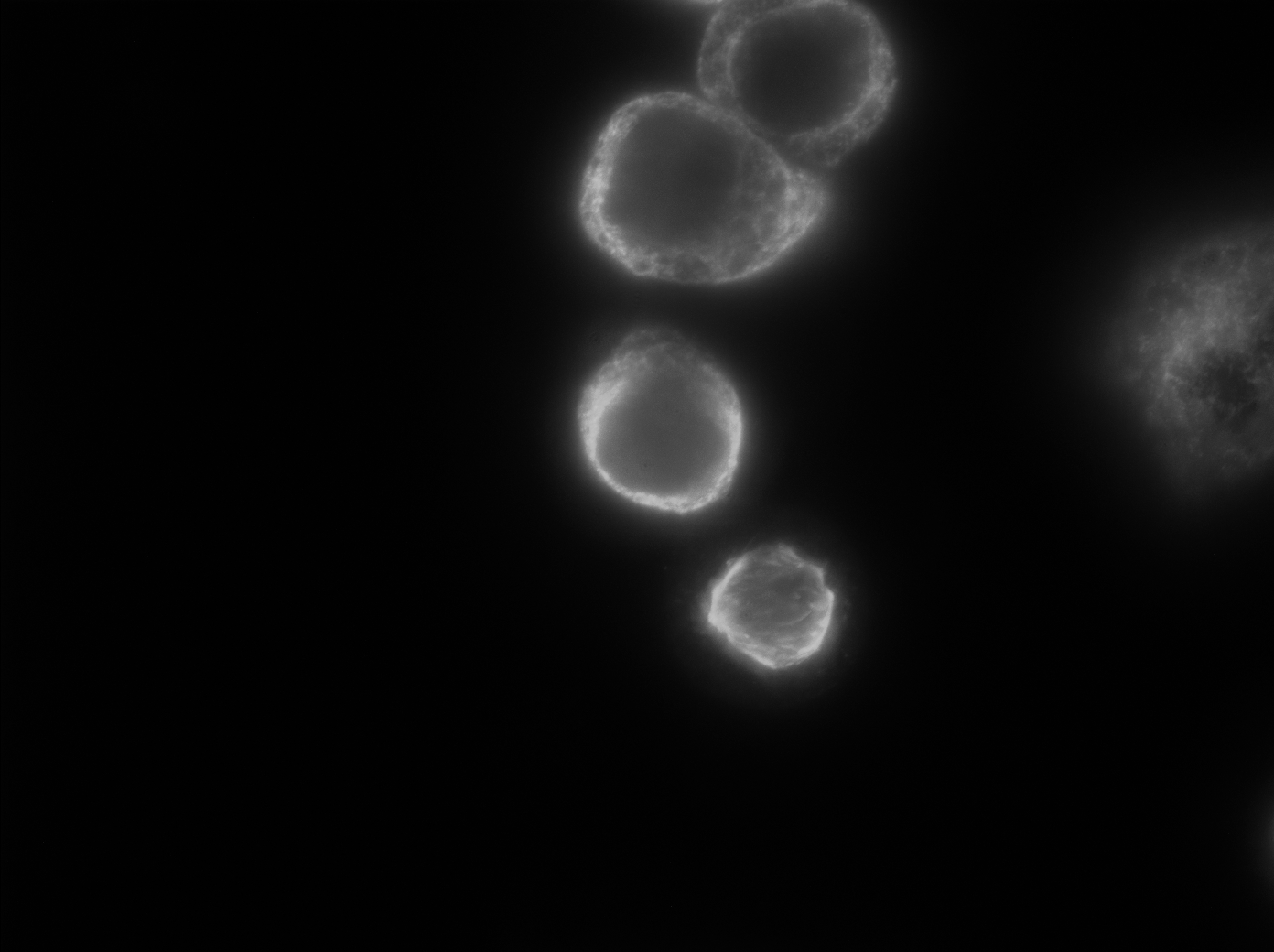

Supplement: Supplementary file 3 — Source data Fig. 1 [file 44319_2026_742_MOESM3_ESM.zip › Figure 1/Fig 1bcd WT Hela acetylated a tubulin atubulin/actub-atub 8-14-24 R1 M1.Project Maximum Z_XY1724363237_Z0_T0_C1.tif]

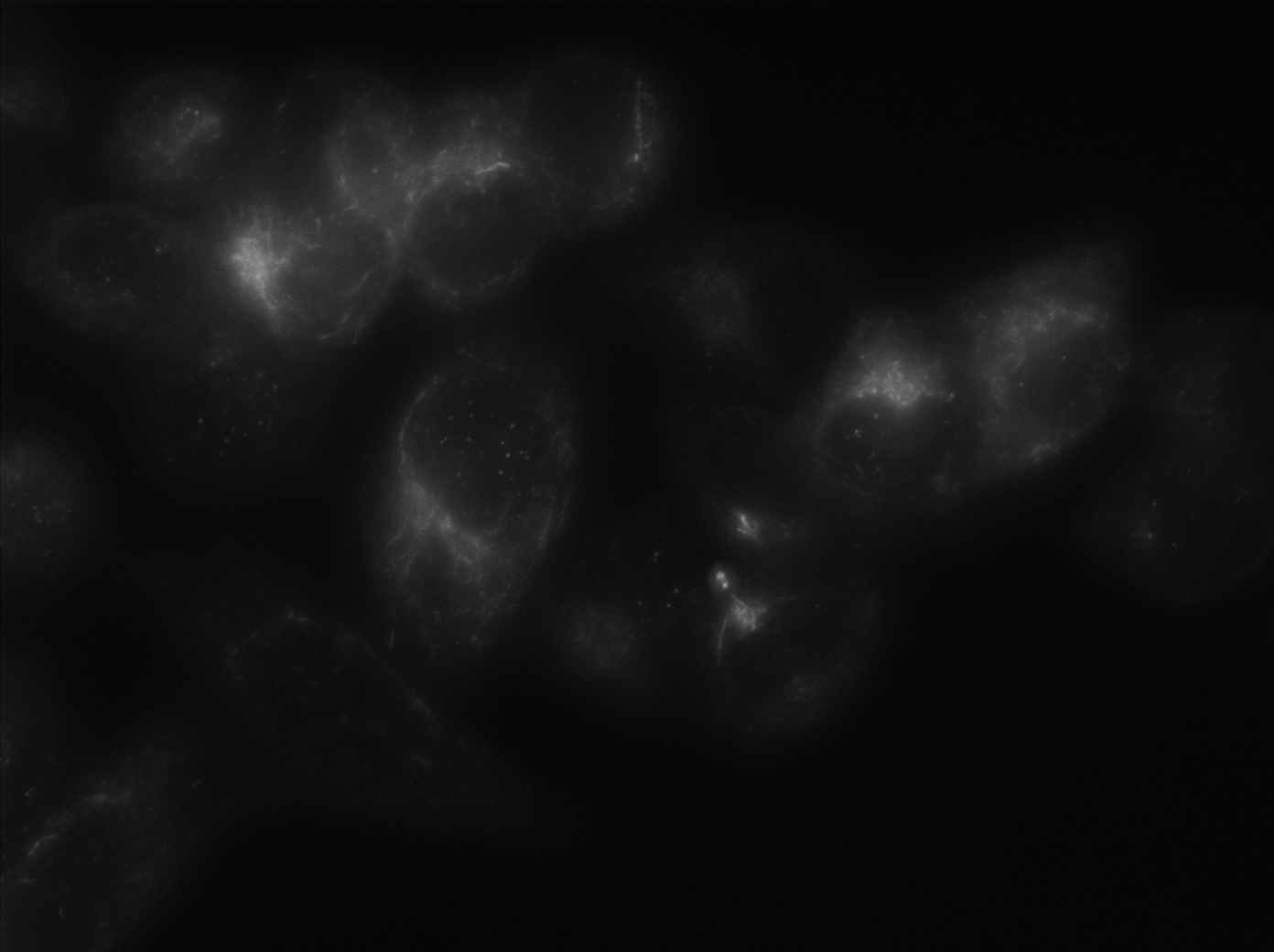

Supplement: Supplementary file 3 — Source data Fig. 1 [file 44319_2026_742_MOESM3_ESM.zip › Figure 1/Fig 1bcd WT Hela acetylated a tubulin atubulin/actub-atub 8-14-24 R3 PA6.Project Maximum Z_XY1724716567_Z0_T0_C2.tif]

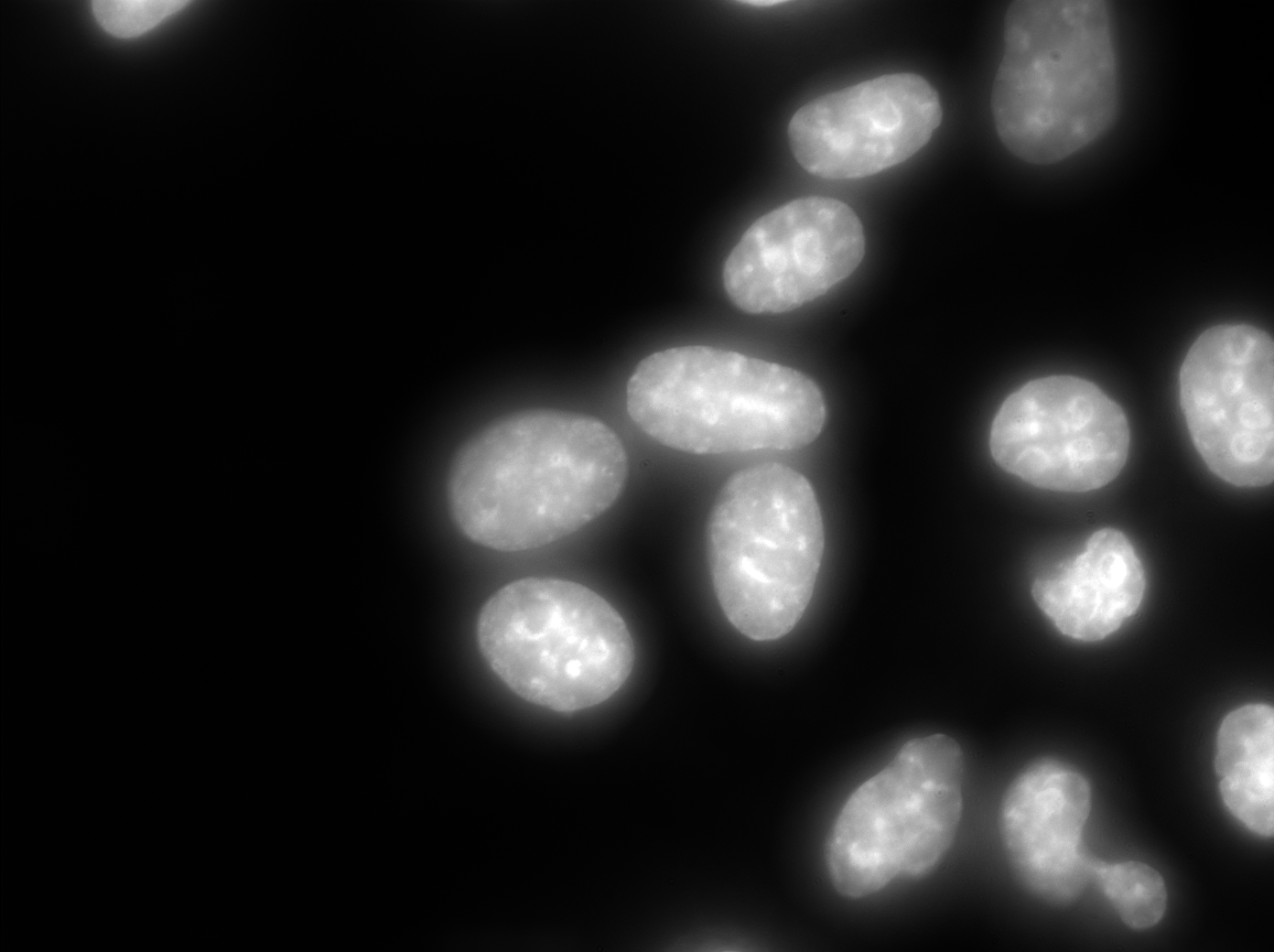

Supplement: Supplementary file 3 — Source data Fig. 1 [file 44319_2026_742_MOESM3_ESM.zip › Figure 1/Fig 1bcd WT Hela acetylated a tubulin atubulin/actub-atub 8-14-24 R3 PA10.Project Maximum Z_XY1724717416_Z0_T0_C0.tif]

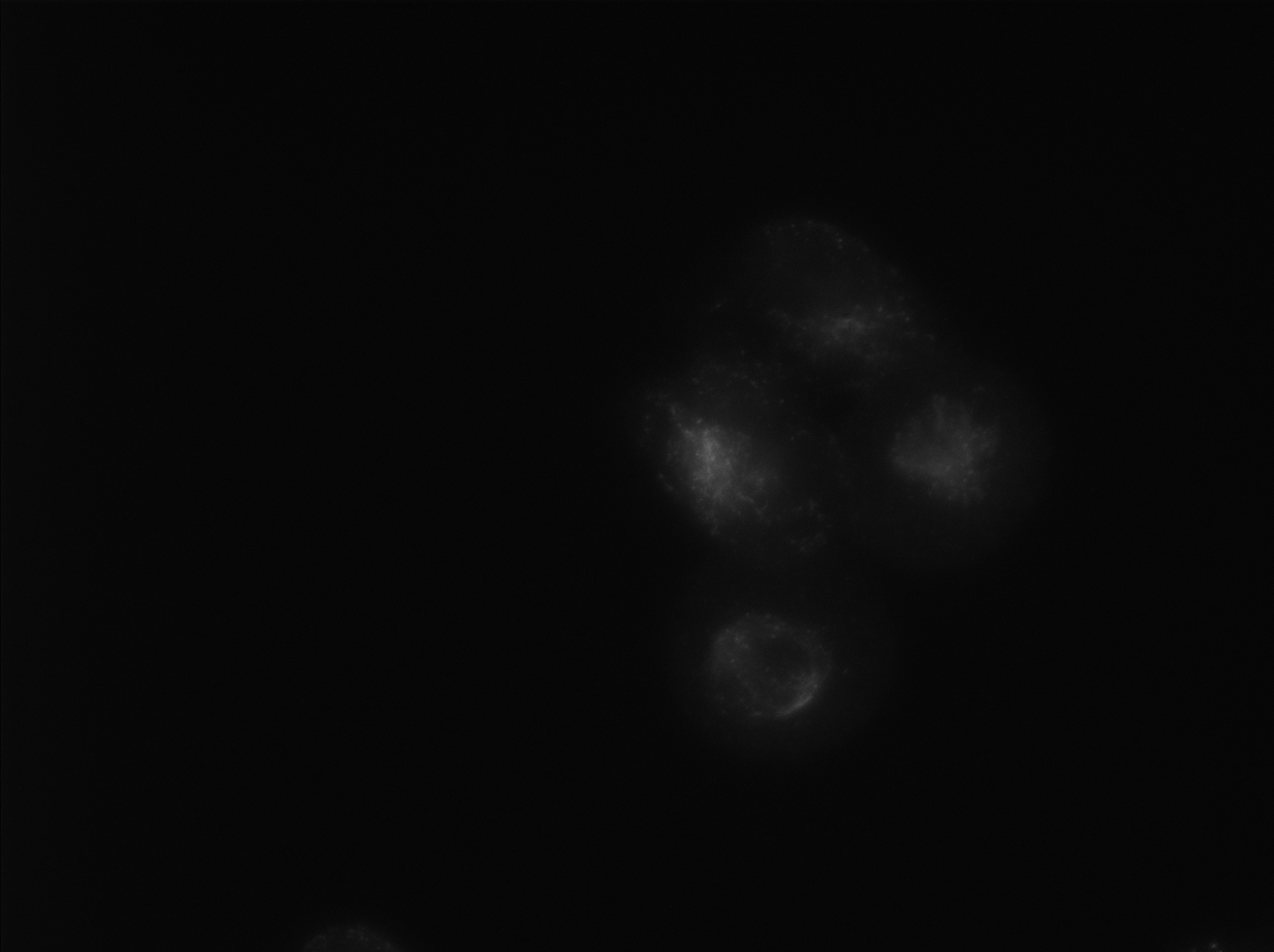

Supplement: Supplementary file 3 — Source data Fig. 1 [file 44319_2026_742_MOESM3_ESM.zip › Figure 1/Fig 1bcd WT Hela acetylated a tubulin atubulin/actub-atub 8-14-24 R2 M2.Project Maximum Z_XY1724690941_Z0_T0_C2.tif]

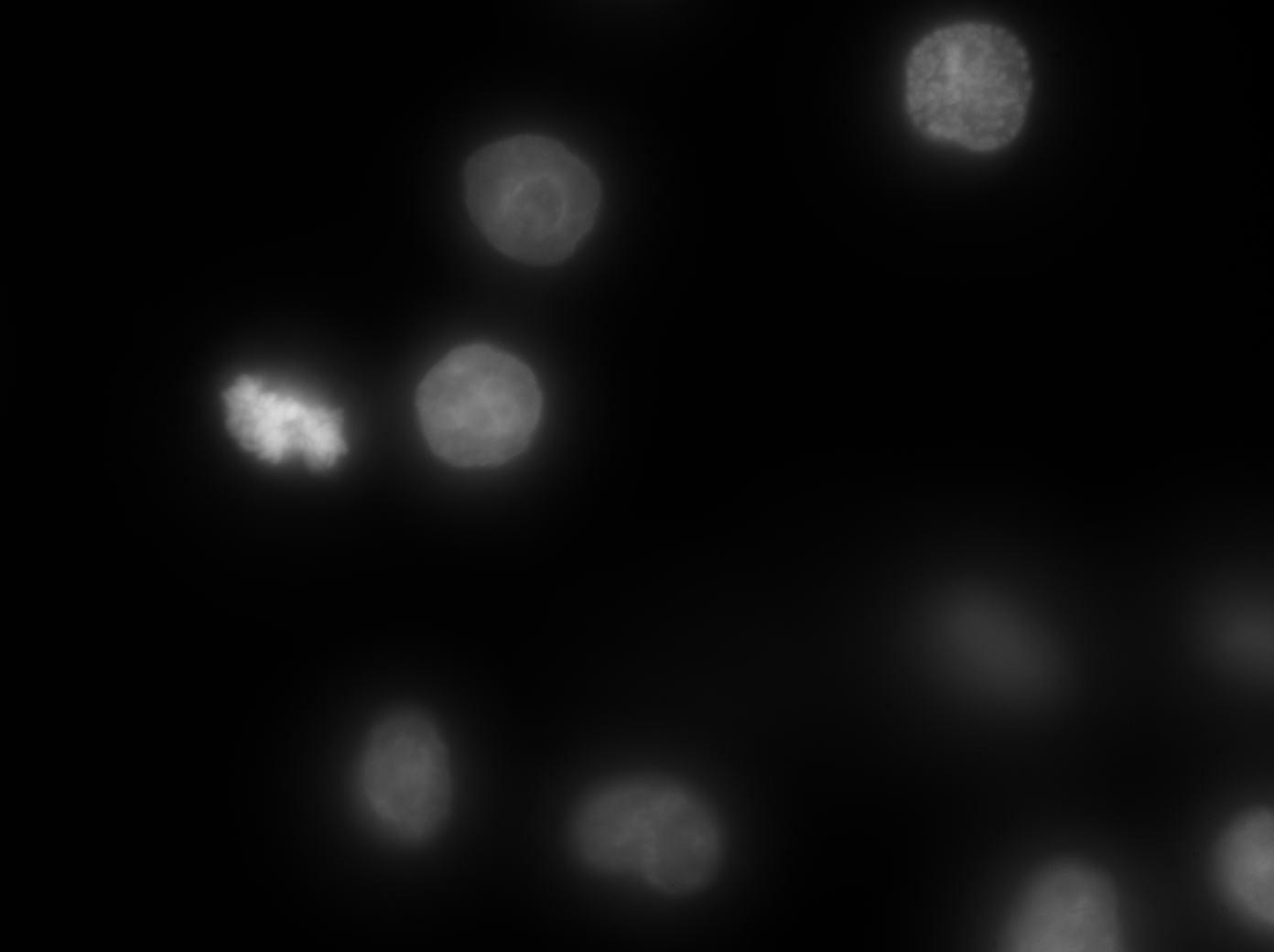

Supplement: Supplementary file 3 — Source data Fig. 1 [file 44319_2026_742_MOESM3_ESM.zip › Figure 1/Fig 1bcd WT Hela acetylated a tubulin atubulin/actub-atub 8-14-24 R2 M7.Project Maximum Z_XY1724694094_Z0_T0_C0.tif]

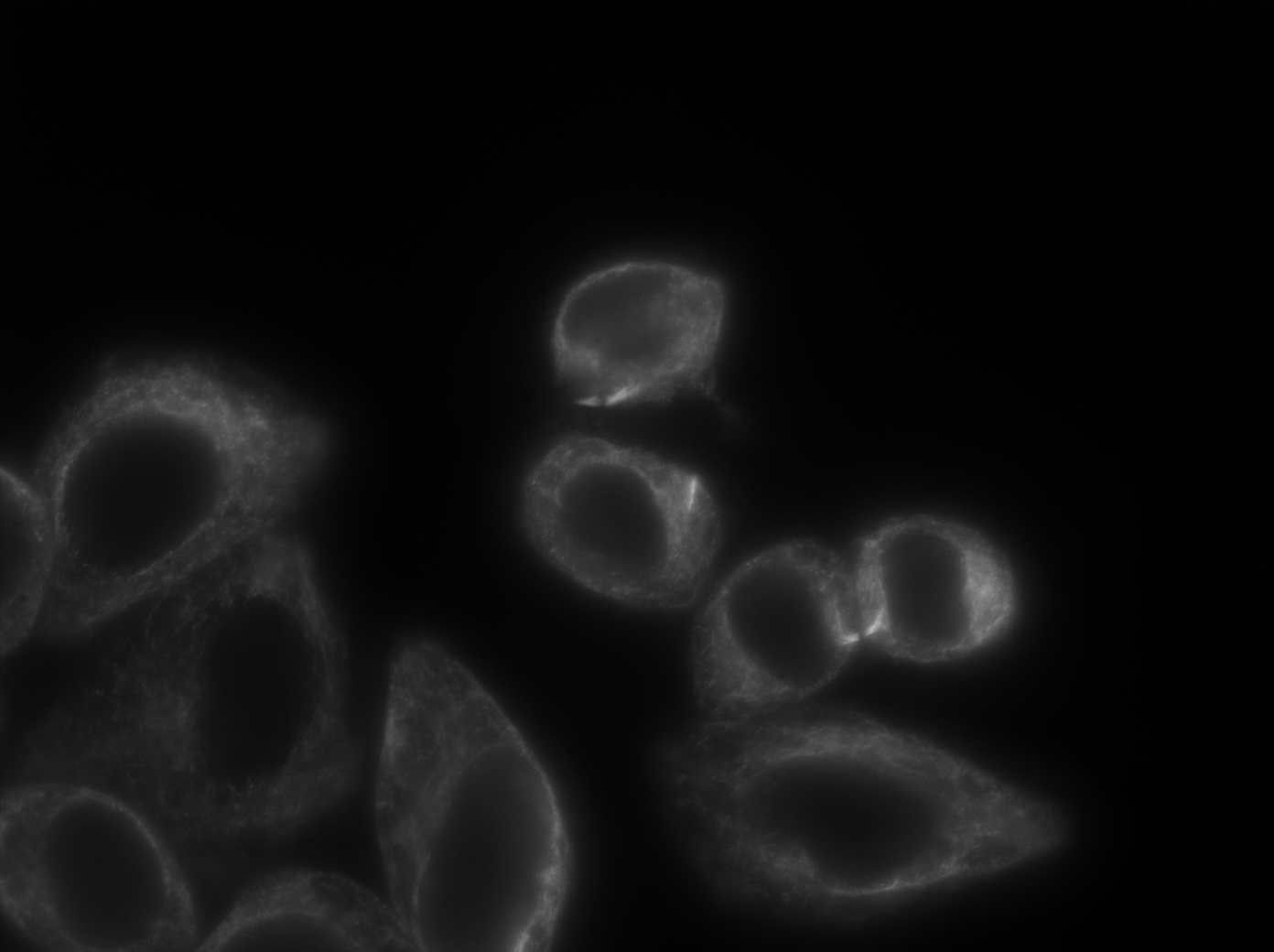

Supplement: Supplementary file 3 — Source data Fig. 1 [file 44319_2026_742_MOESM3_ESM.zip › Figure 1/Fig 1bcd WT Hela acetylated a tubulin atubulin/actub-atub 8-14-24 R1 PA8.Project Maximum Z_XY1724366086_Z0_T0_C1.tif]

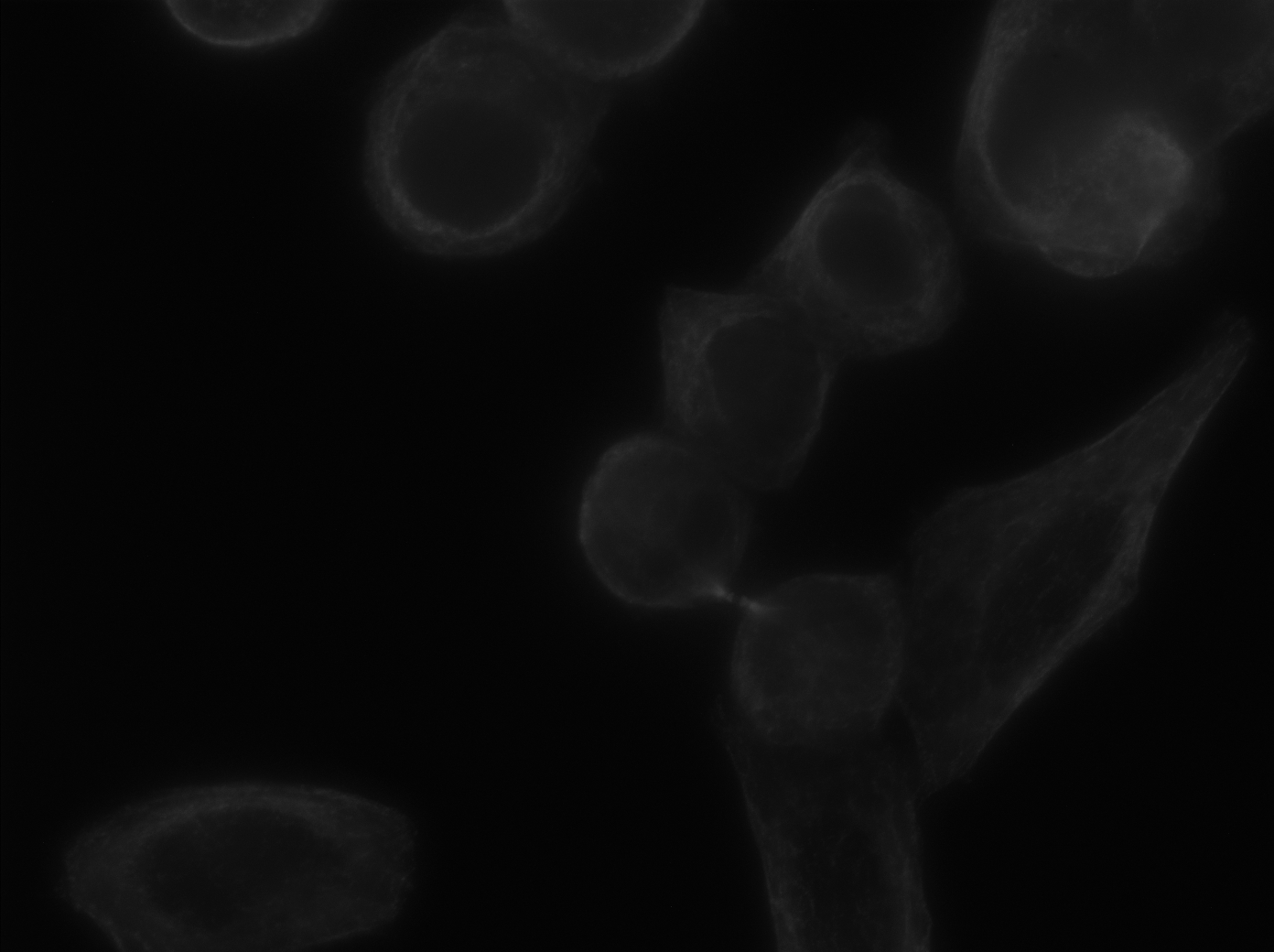

Supplement: Supplementary file 3 — Source data Fig. 1 [file 44319_2026_742_MOESM3_ESM.zip › Figure 1/Fig 1bcd WT Hela acetylated a tubulin atubulin/actub-atub 8-14-24 R2 LT5.Project Maximum Z_XY1724690793_Z0_T0_C1.tif]

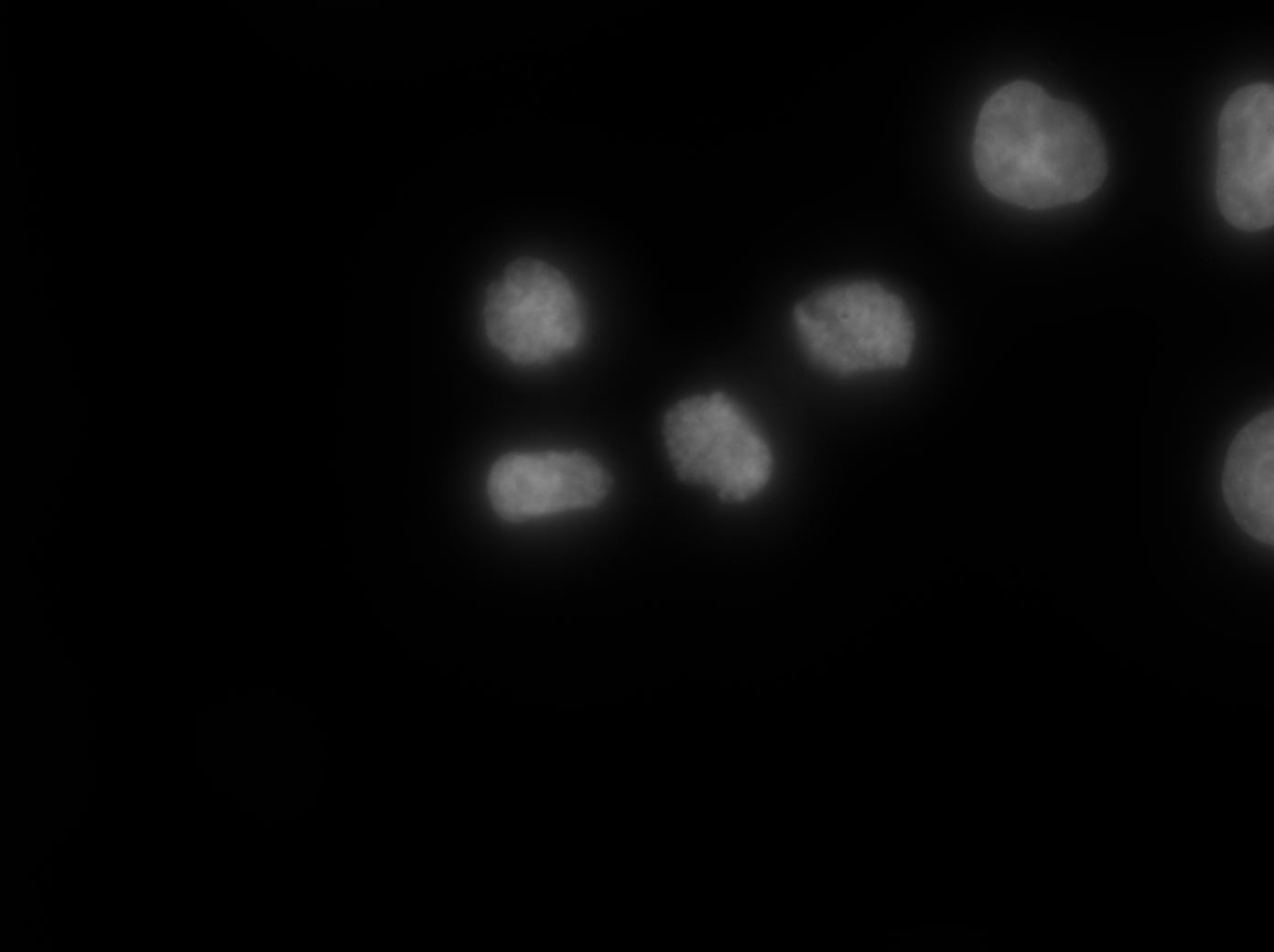

Supplement: Supplementary file 3 — Source data Fig. 1 [file 44319_2026_742_MOESM3_ESM.zip › Figure 1/Fig 1bcd WT Hela acetylated a tubulin atubulin/actub-atub 8-14-24 R1 ET3ET4.Project Maximum Z_XY1724363161_Z0_T0_C0.tif]

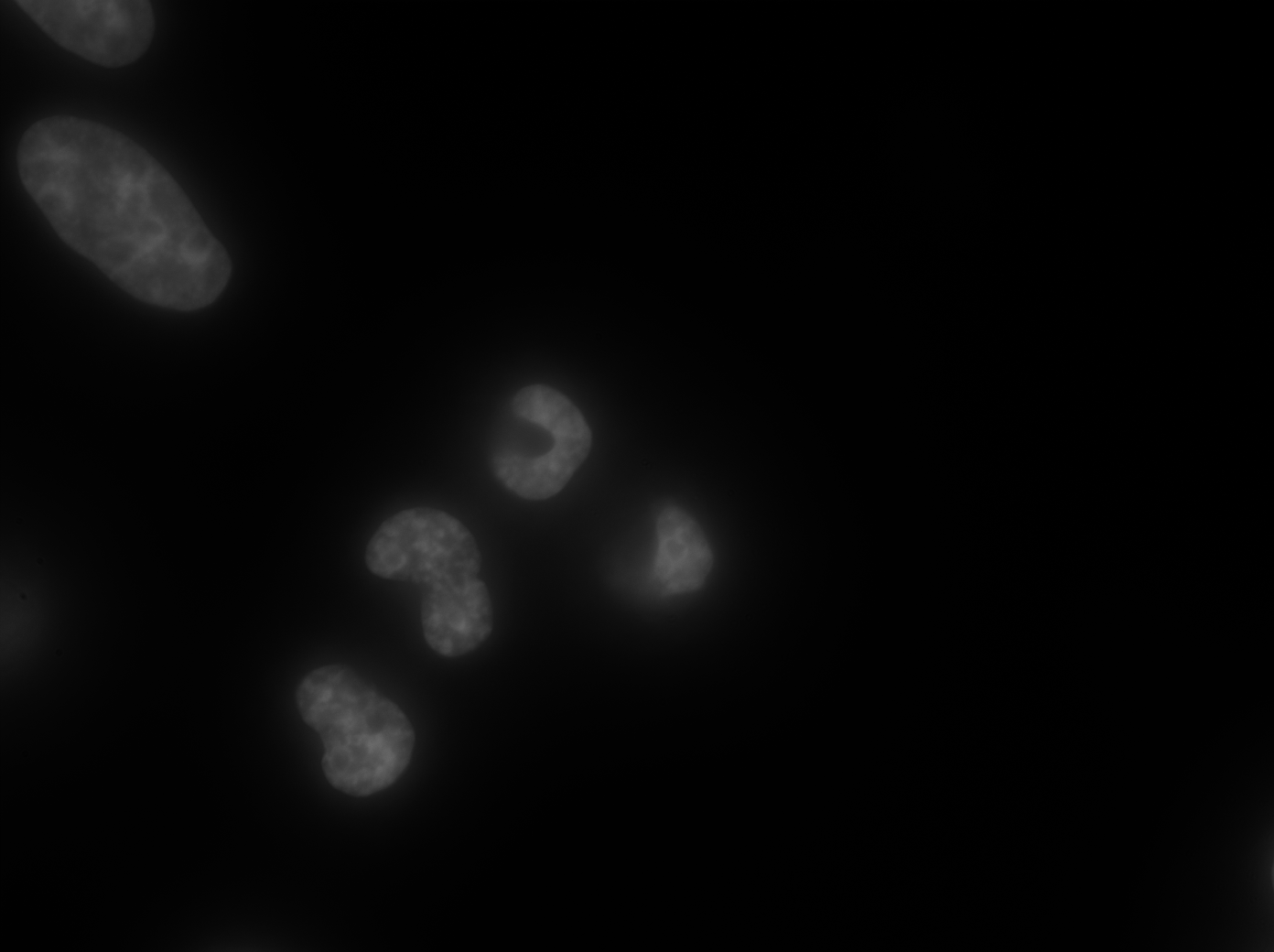

Supplement: Supplementary file 3 — Source data Fig. 1 [file 44319_2026_742_MOESM3_ESM.zip › Figure 1/Fig 1bcd WT Hela acetylated a tubulin atubulin/actub-atub 8-14-24 R2 LT9LT10.Project Maximum Z_XY1724694226_Z0_T0_C0.tif]

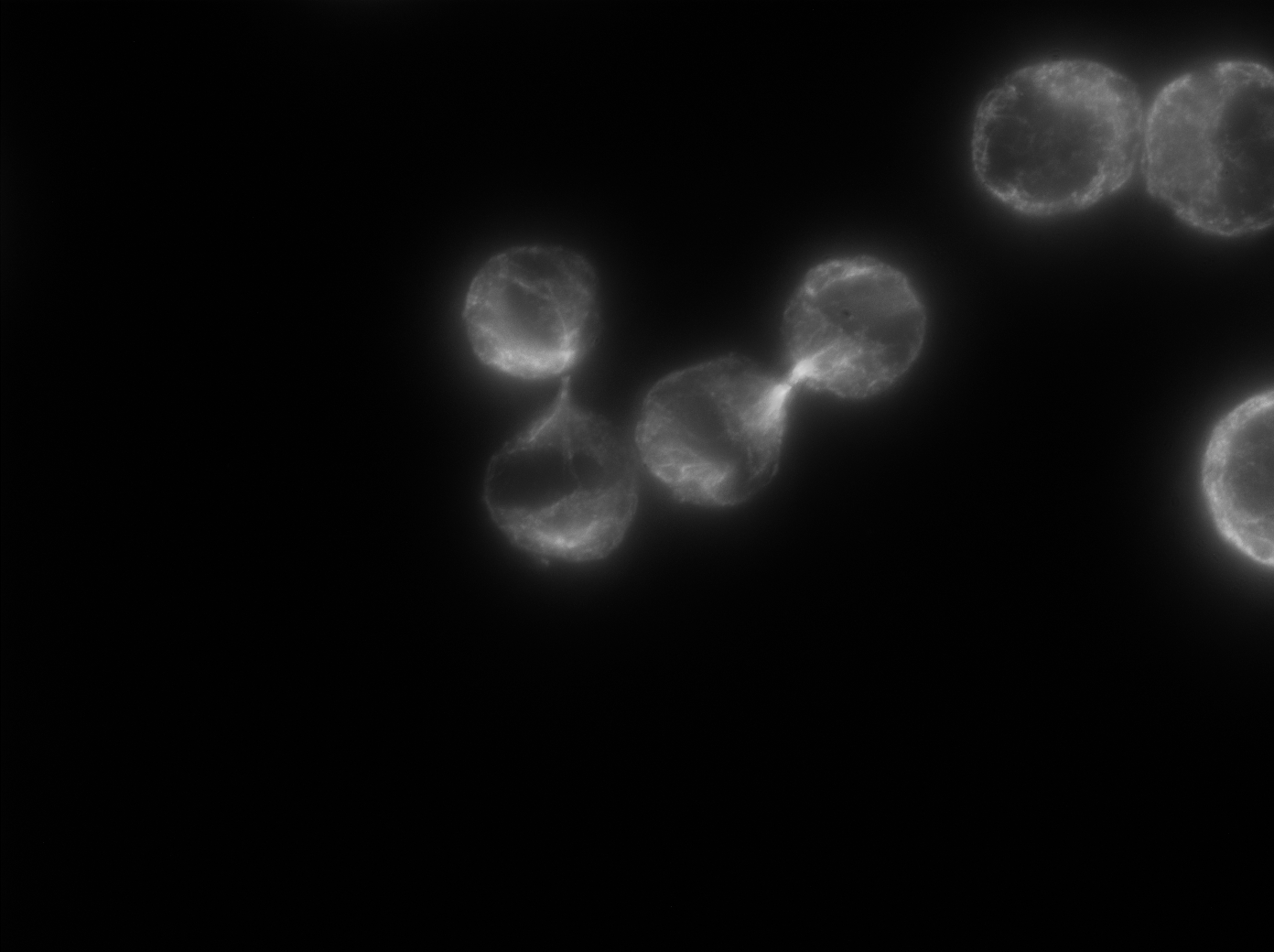

Supplement: Supplementary file 3 — Source data Fig. 1 [file 44319_2026_742_MOESM3_ESM.zip › Figure 1/Fig 1bcd WT Hela acetylated a tubulin atubulin/actub-atub 8-14-24 R1 ET3ET4.Project Maximum Z_XY1724363161_Z0_T0_C1.tif]

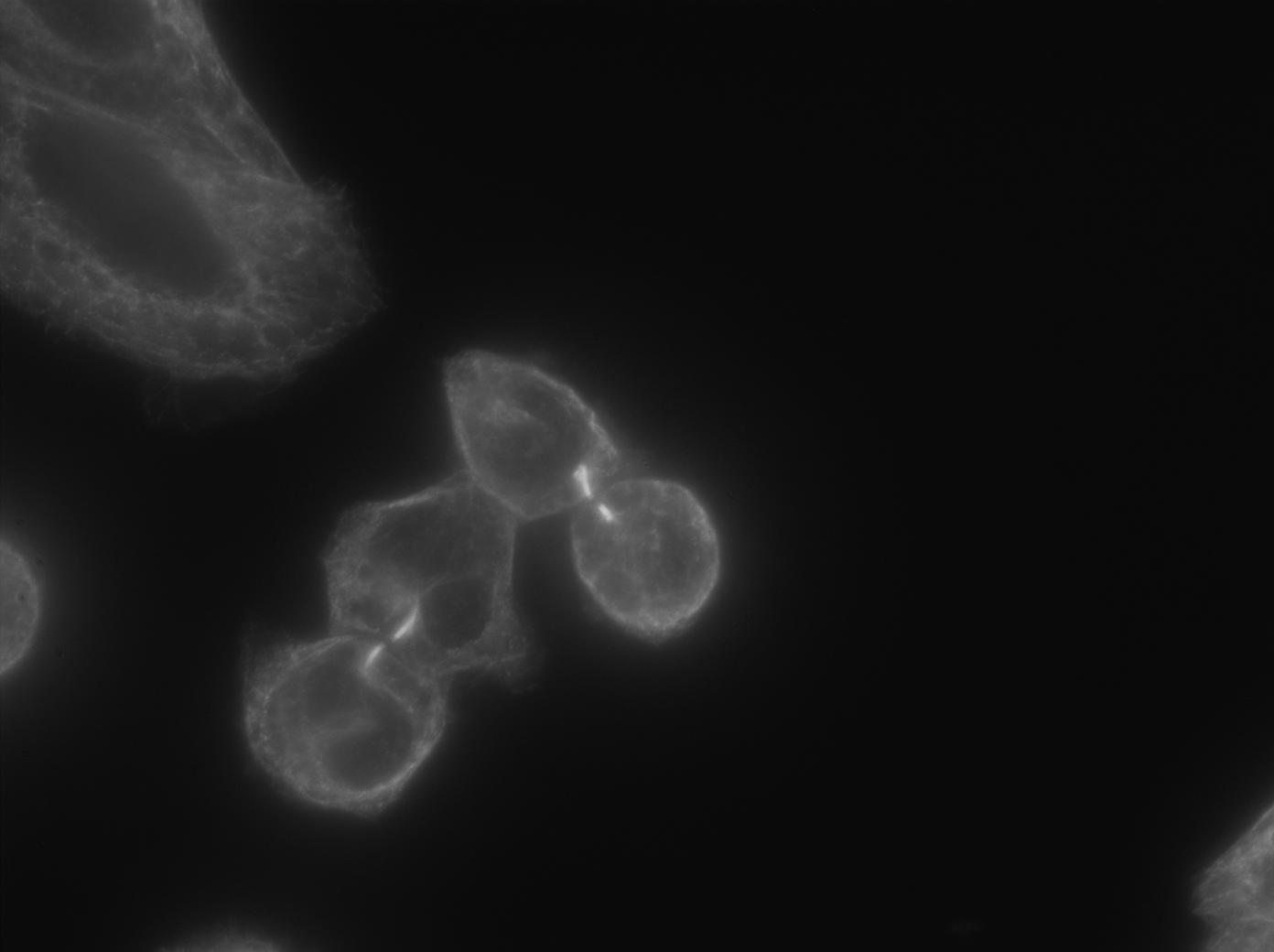

Supplement: Supplementary file 3 — Source data Fig. 1 [file 44319_2026_742_MOESM3_ESM.zip › Figure 1/Fig 1bcd WT Hela acetylated a tubulin atubulin/actub-atub 8-14-24 R2 LT9LT10.Project Maximum Z_XY1724694226_Z0_T0_C1.tif]

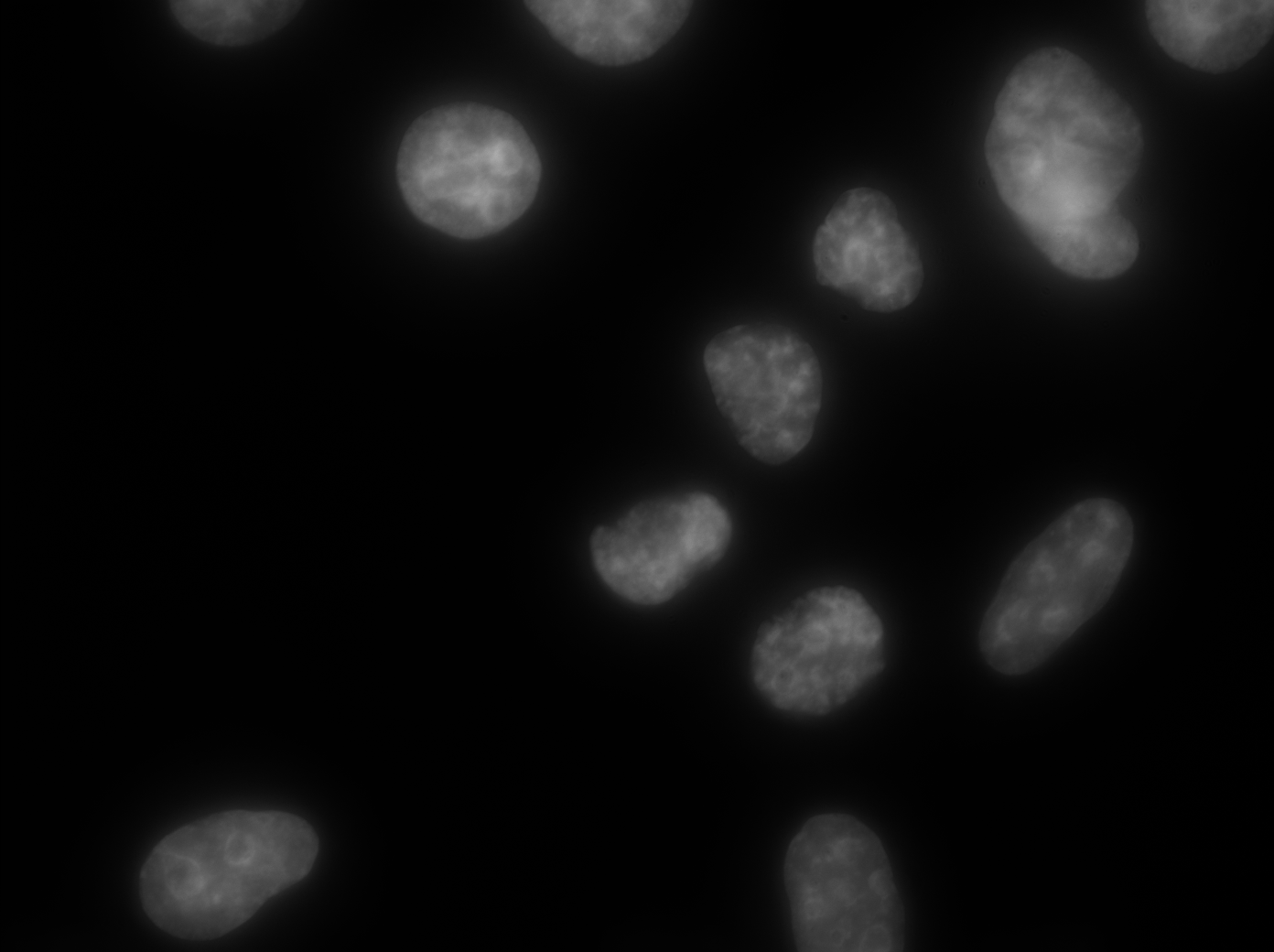

Supplement: Supplementary file 3 — Source data Fig. 1 [file 44319_2026_742_MOESM3_ESM.zip › Figure 1/Fig 1bcd WT Hela acetylated a tubulin atubulin/actub-atub 8-14-24 R2 LT5.Project Maximum Z_XY1724690793_Z0_T0_C0.tif]

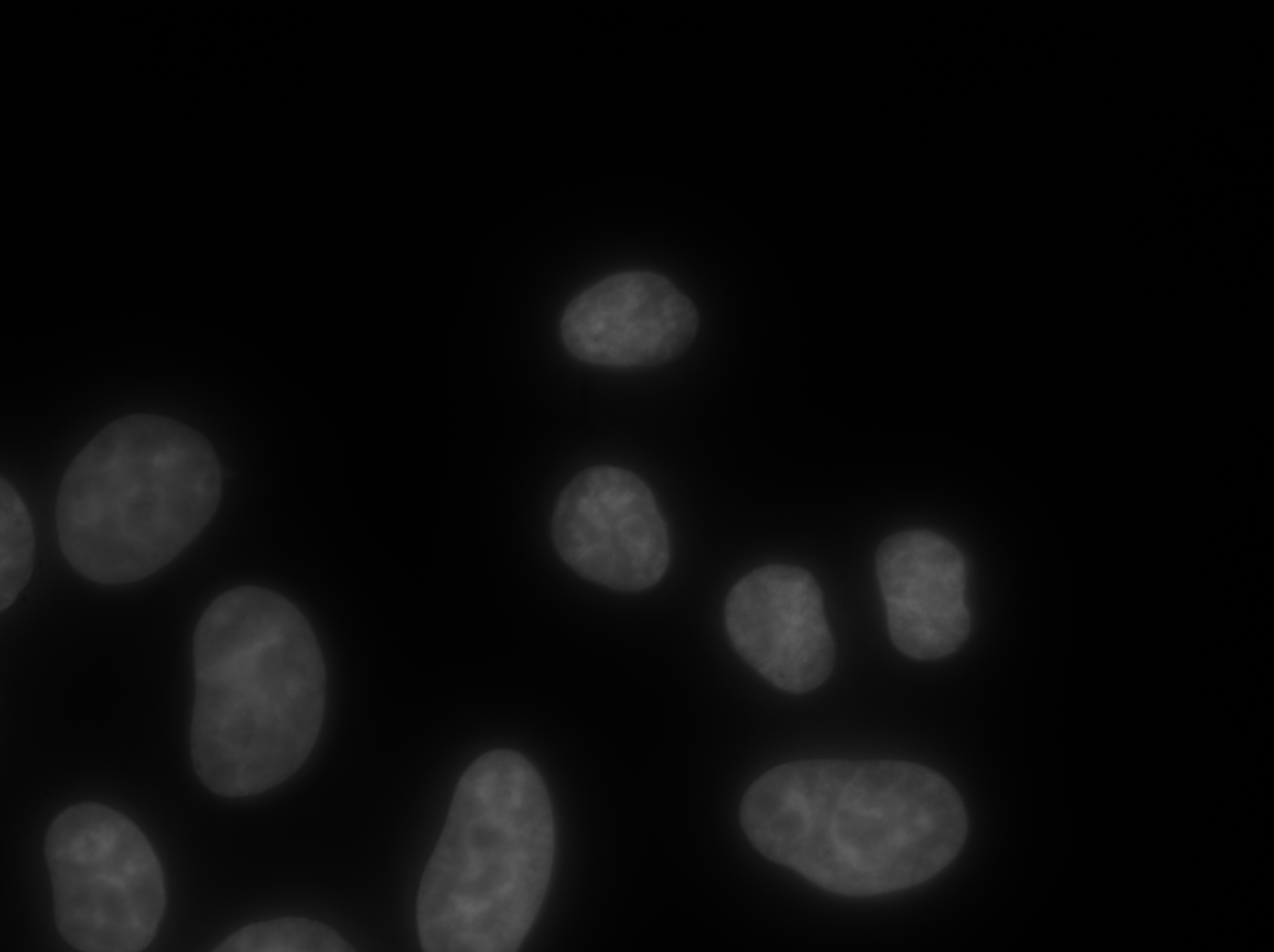

Supplement: Supplementary file 3 — Source data Fig. 1 [file 44319_2026_742_MOESM3_ESM.zip › Figure 1/Fig 1bcd WT Hela acetylated a tubulin atubulin/actub-atub 8-14-24 R1 PA8.Project Maximum Z_XY1724366086_Z0_T0_C0.tif]

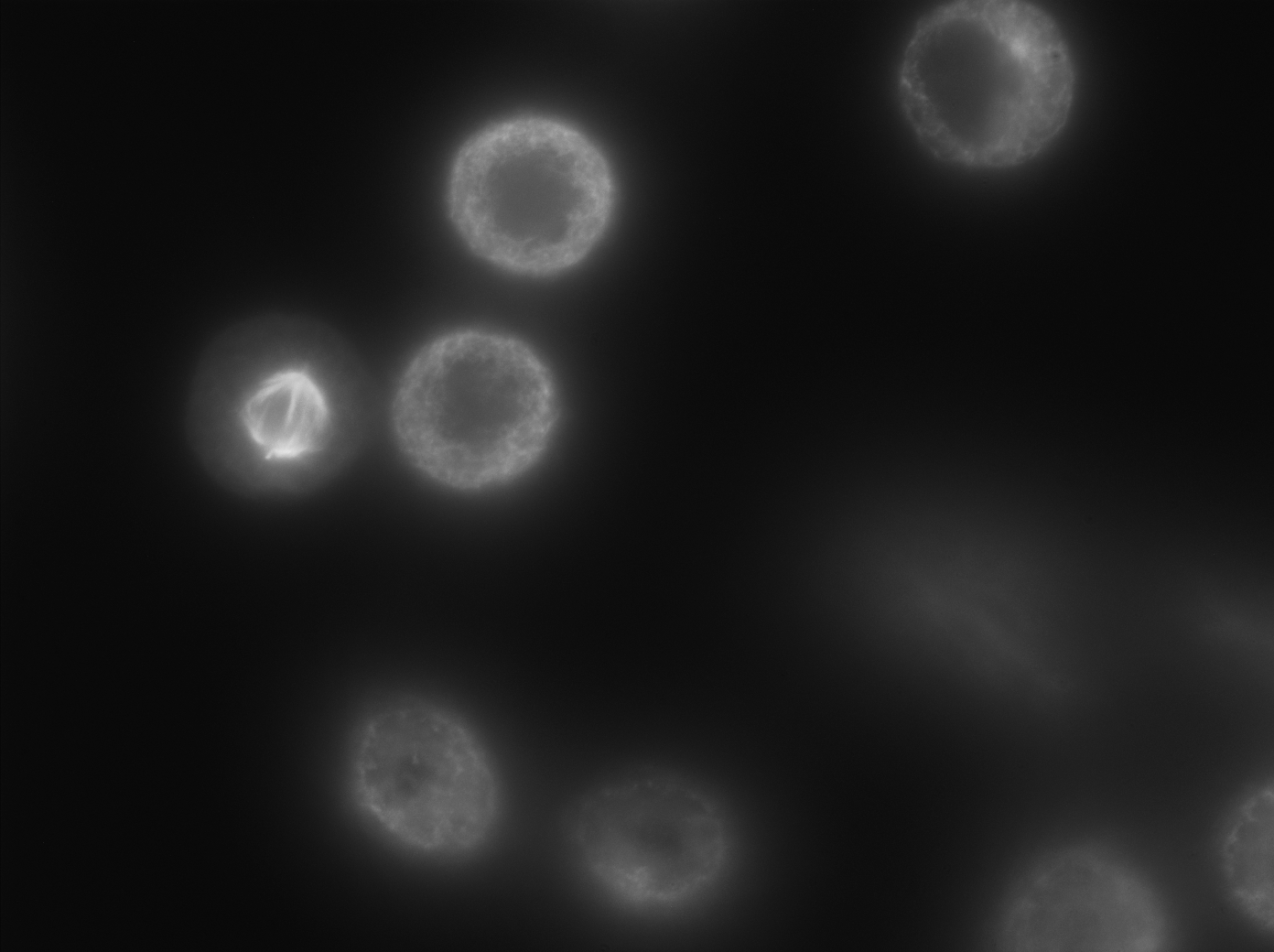

Supplement: Supplementary file 3 — Source data Fig. 1 [file 44319_2026_742_MOESM3_ESM.zip › Figure 1/Fig 1bcd WT Hela acetylated a tubulin atubulin/actub-atub 8-14-24 R2 M7.Project Maximum Z_XY1724694094_Z0_T0_C1.tif]

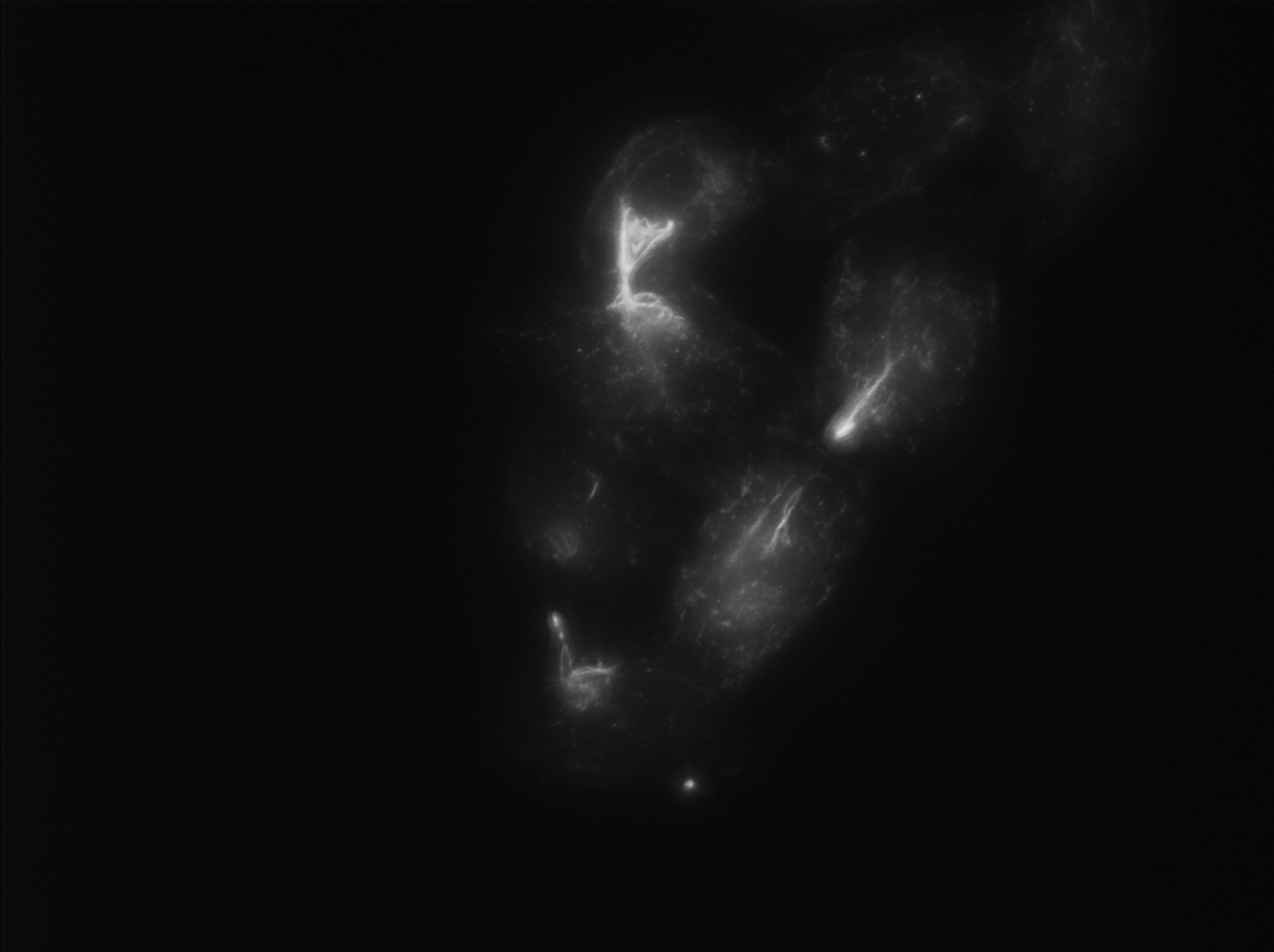

Supplement: Supplementary file 3 — Source data Fig. 1 [file 44319_2026_742_MOESM3_ESM.zip › Figure 1/Fig 1bcd WT Hela acetylated a tubulin atubulin/actub-atub 8-14-24 R1 LT1 PA3PA4.Project Maximum Z_XY1724362702_Z0_T0_C2.tif]

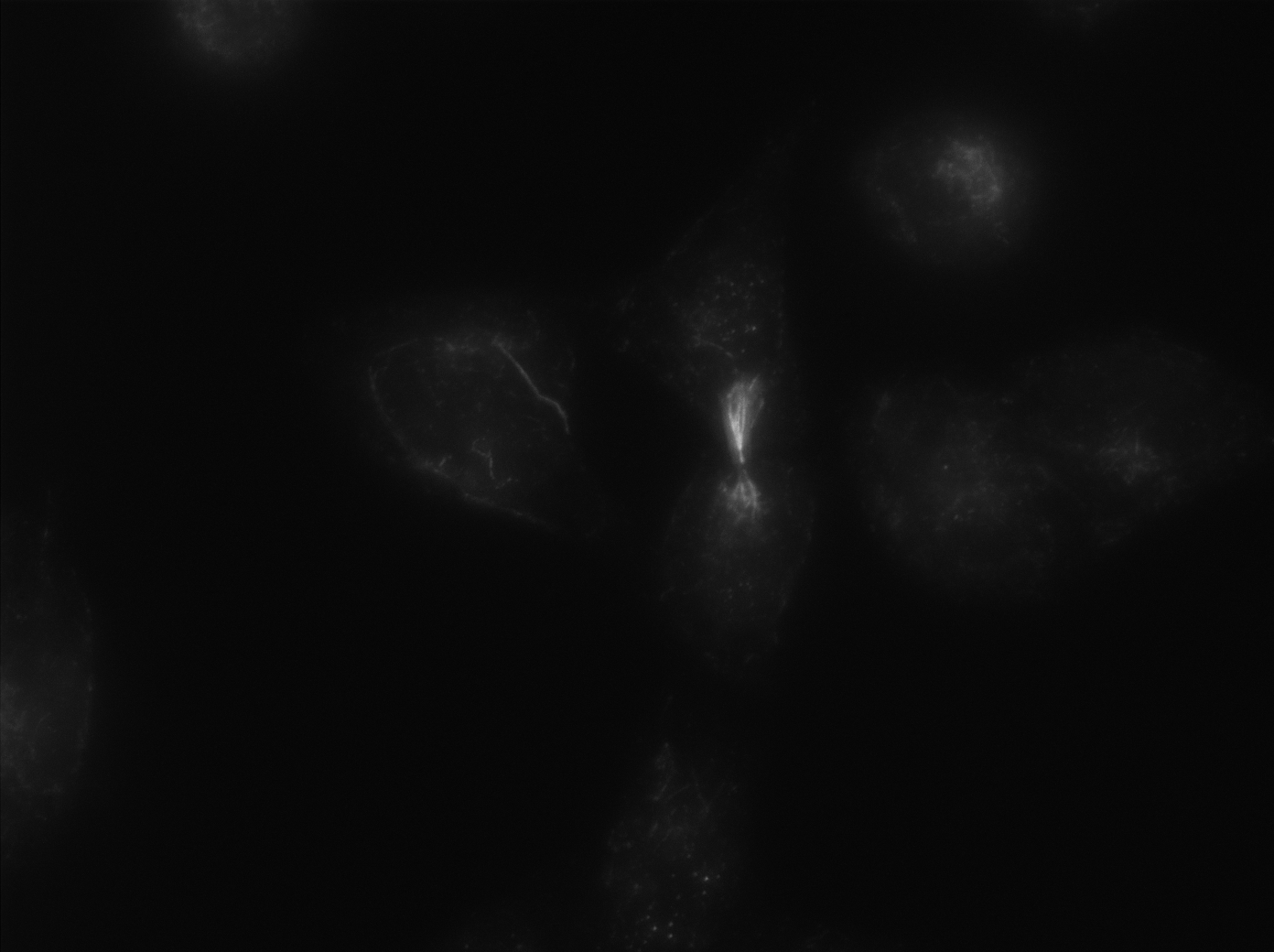

Supplement: Supplementary file 3 — Source data Fig. 1 [file 44319_2026_742_MOESM3_ESM.zip › Figure 1/Fig 1bcd WT Hela acetylated a tubulin atubulin/actub-atub 8-14-24 R2 LT6.Project Maximum Z_XY1724693513_Z0_T0_C2.tif]

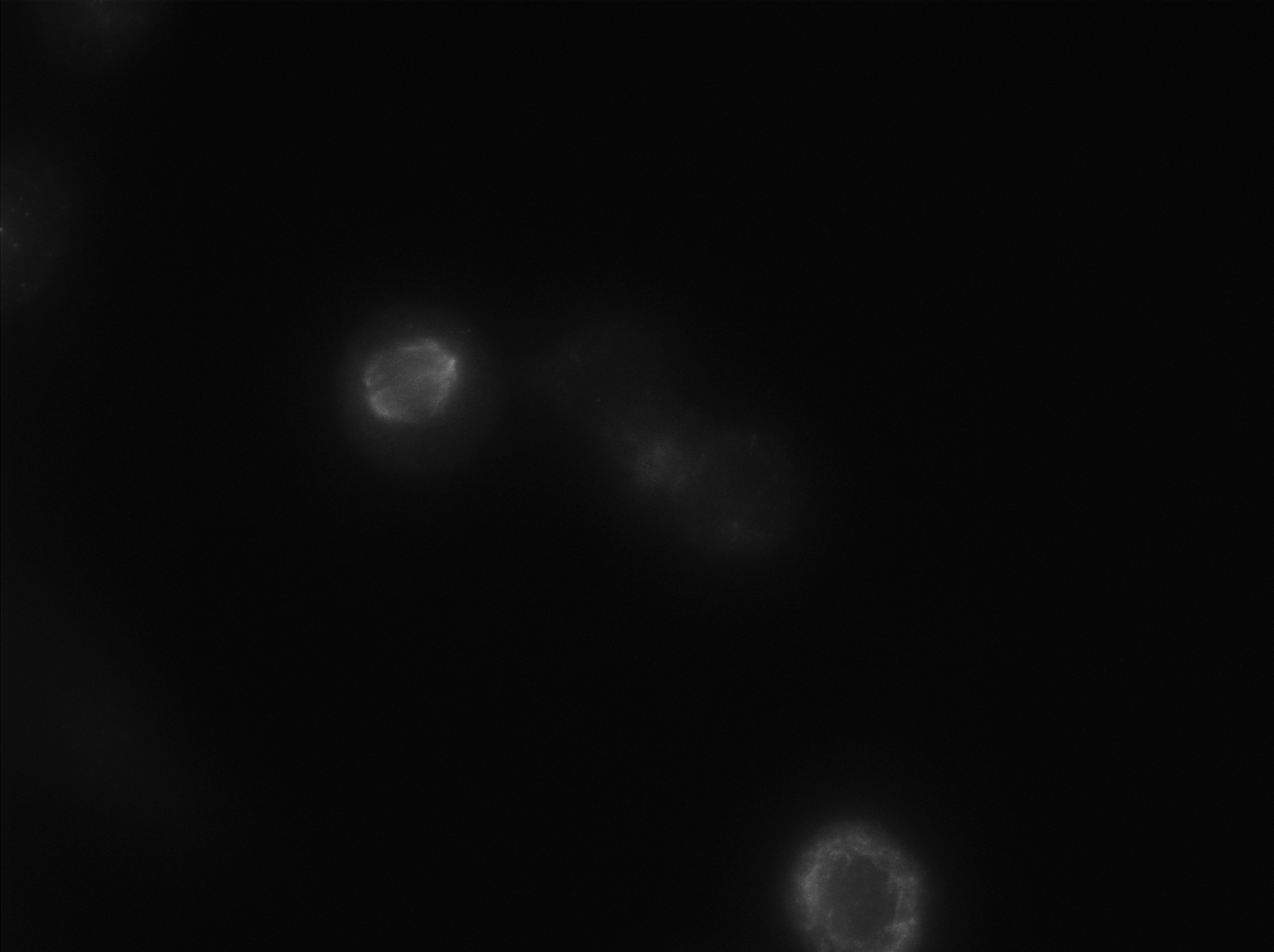

Supplement: Supplementary file 3 — Source data Fig. 1 [file 44319_2026_742_MOESM3_ESM.zip › Figure 1/Fig 1bcd WT Hela acetylated a tubulin atubulin/actub-atub 8-14-24 R3 M5.Project Maximum Z_XY1724702253_Z0_T0_C2.tif]

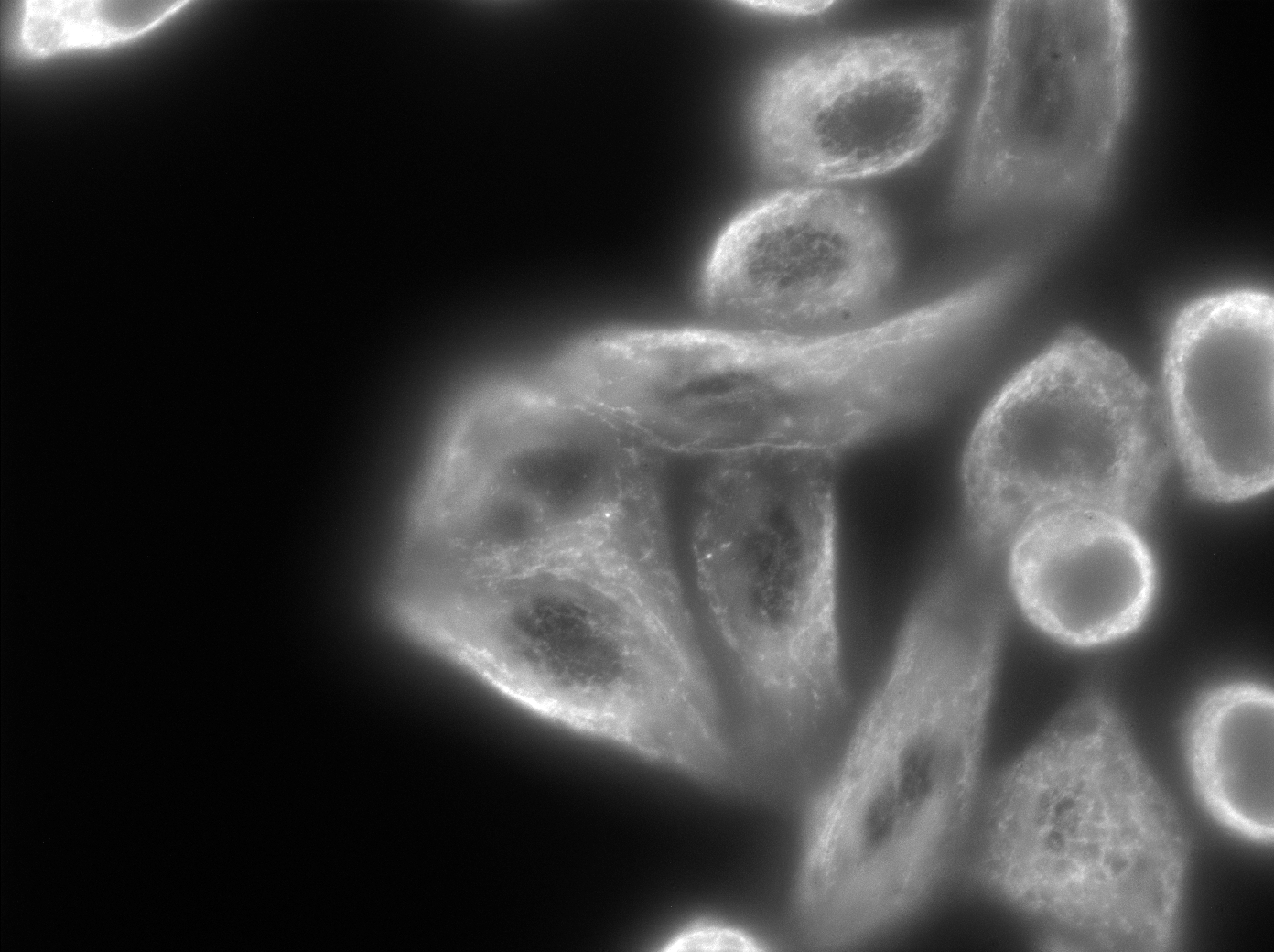

Supplement: Supplementary file 3 — Source data Fig. 1 [file 44319_2026_742_MOESM3_ESM.zip › Figure 1/Fig 1bcd WT Hela acetylated a tubulin atubulin/actub-atub 8-14-24 R3 PA10.Project Maximum Z_XY1724717416_Z0_T0_C1.tif]

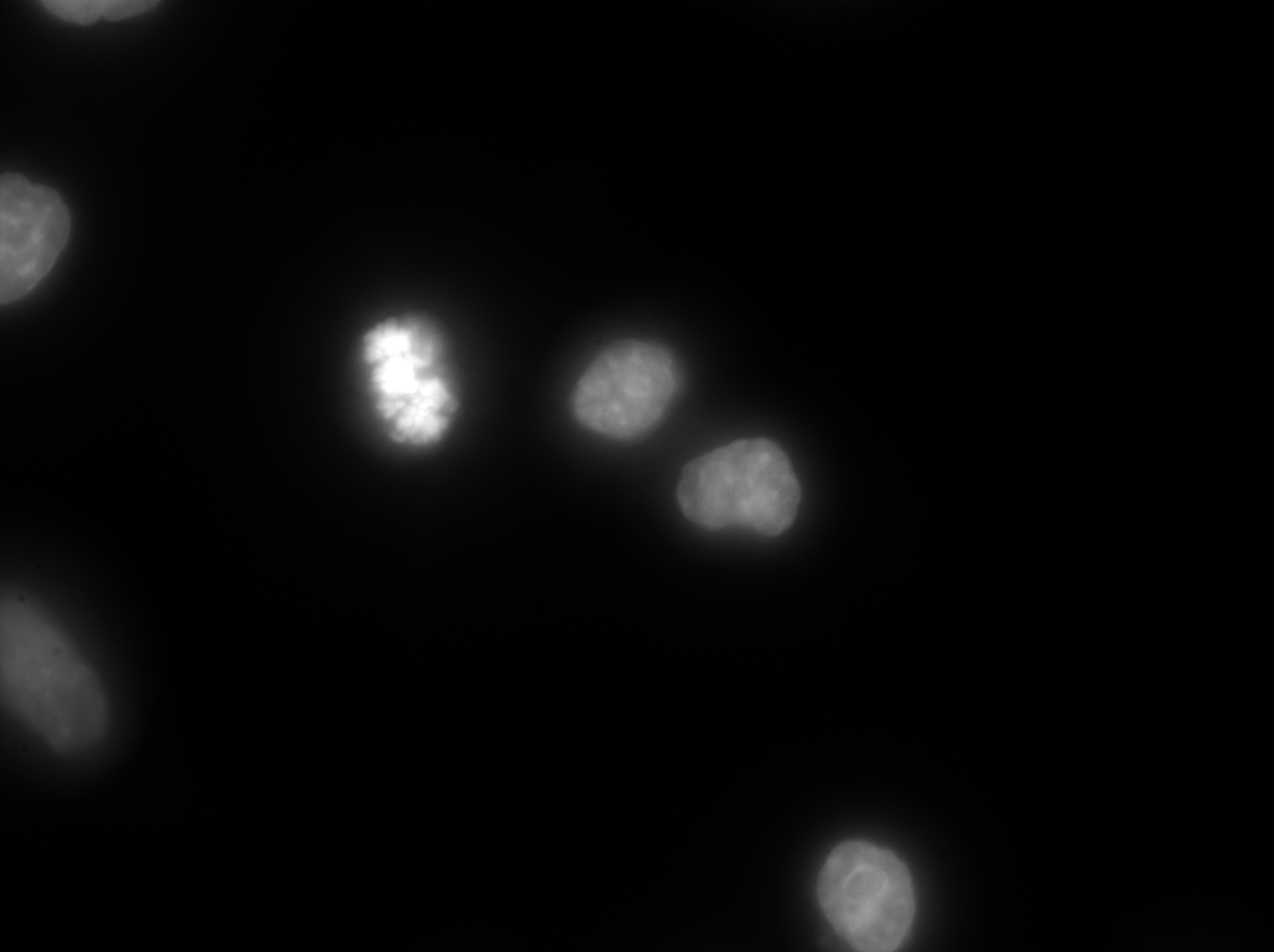

Supplement: Supplementary file 3 — Source data Fig. 1 [file 44319_2026_742_MOESM3_ESM.zip › Figure 1/Fig 1bcd WT Hela acetylated a tubulin atubulin/actub-atub 8-14-24 R3 M5.Project Maximum Z_XY1724702253_Z0_T0_C0.tif]

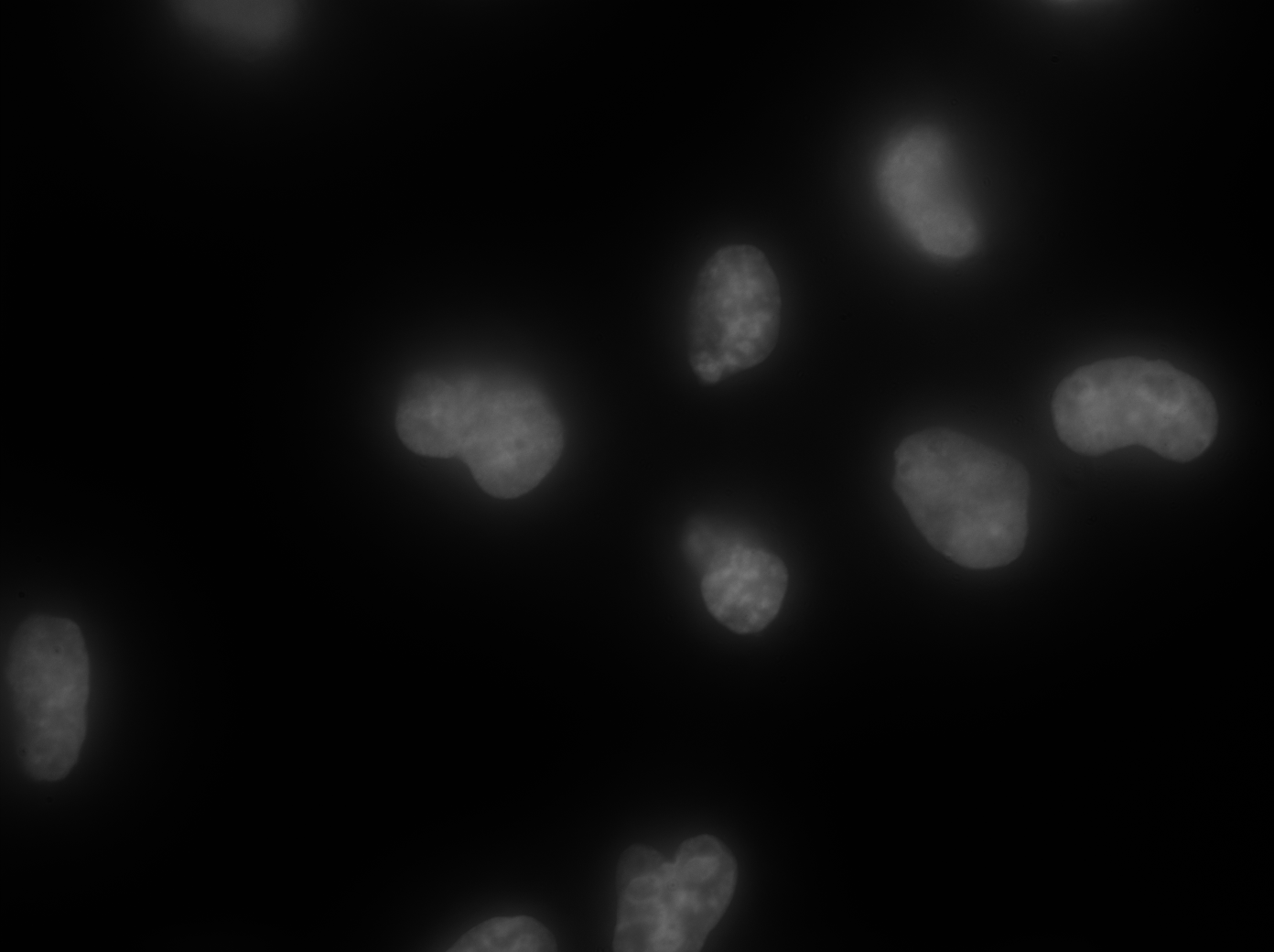

Supplement: Supplementary file 3 — Source data Fig. 1 [file 44319_2026_742_MOESM3_ESM.zip › Figure 1/Fig 1bcd WT Hela acetylated a tubulin atubulin/actub-atub 8-14-24 R2 LT6.Project Maximum Z_XY1724693513_Z0_T0_C0.tif]

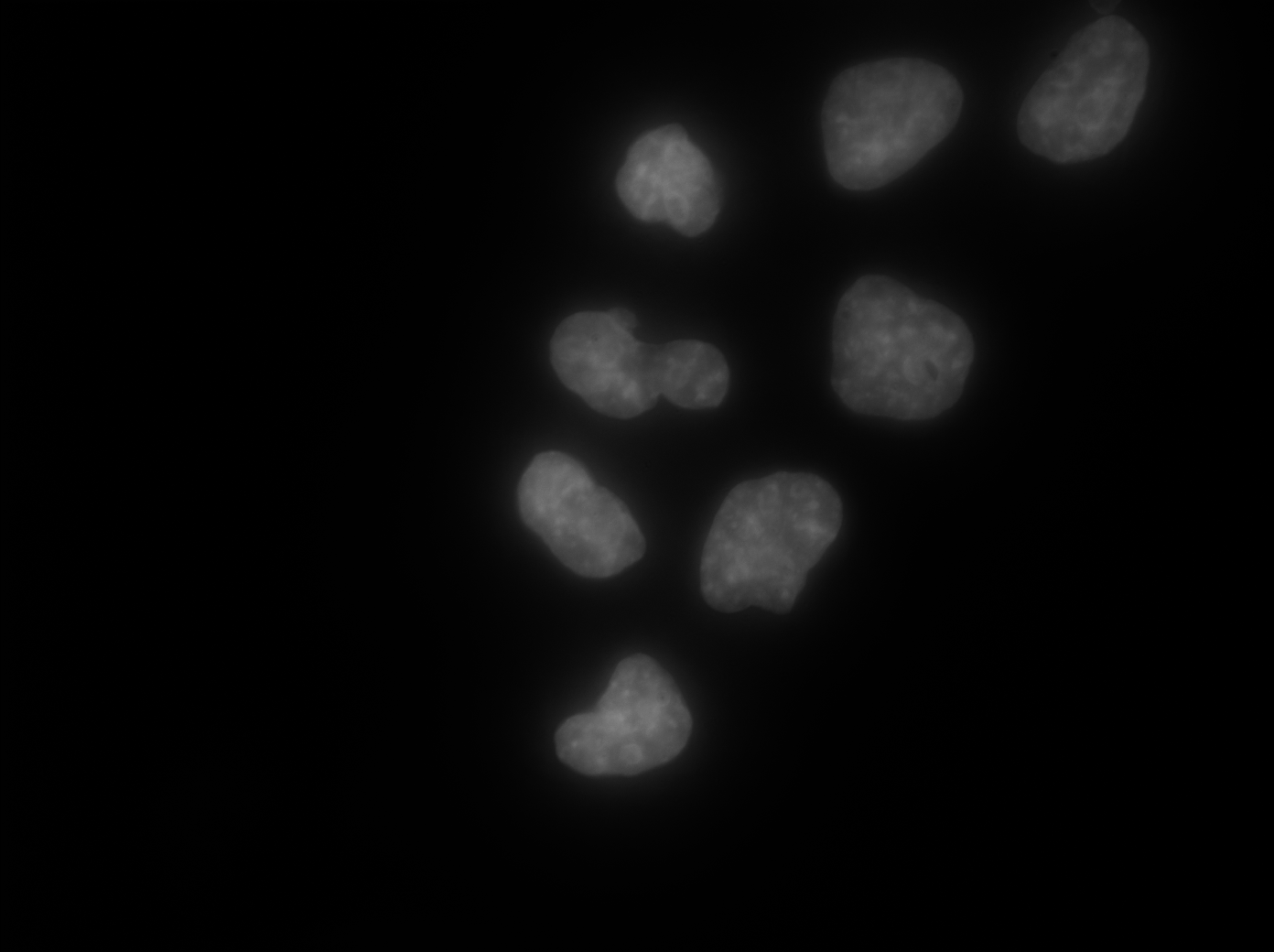

Supplement: Supplementary file 3 — Source data Fig. 1 [file 44319_2026_742_MOESM3_ESM.zip › Figure 1/Fig 1bcd WT Hela acetylated a tubulin atubulin/actub-atub 8-14-24 R1 LT1 PA3PA4.Project Maximum Z_XY1724362702_Z0_T0_C0.tif]

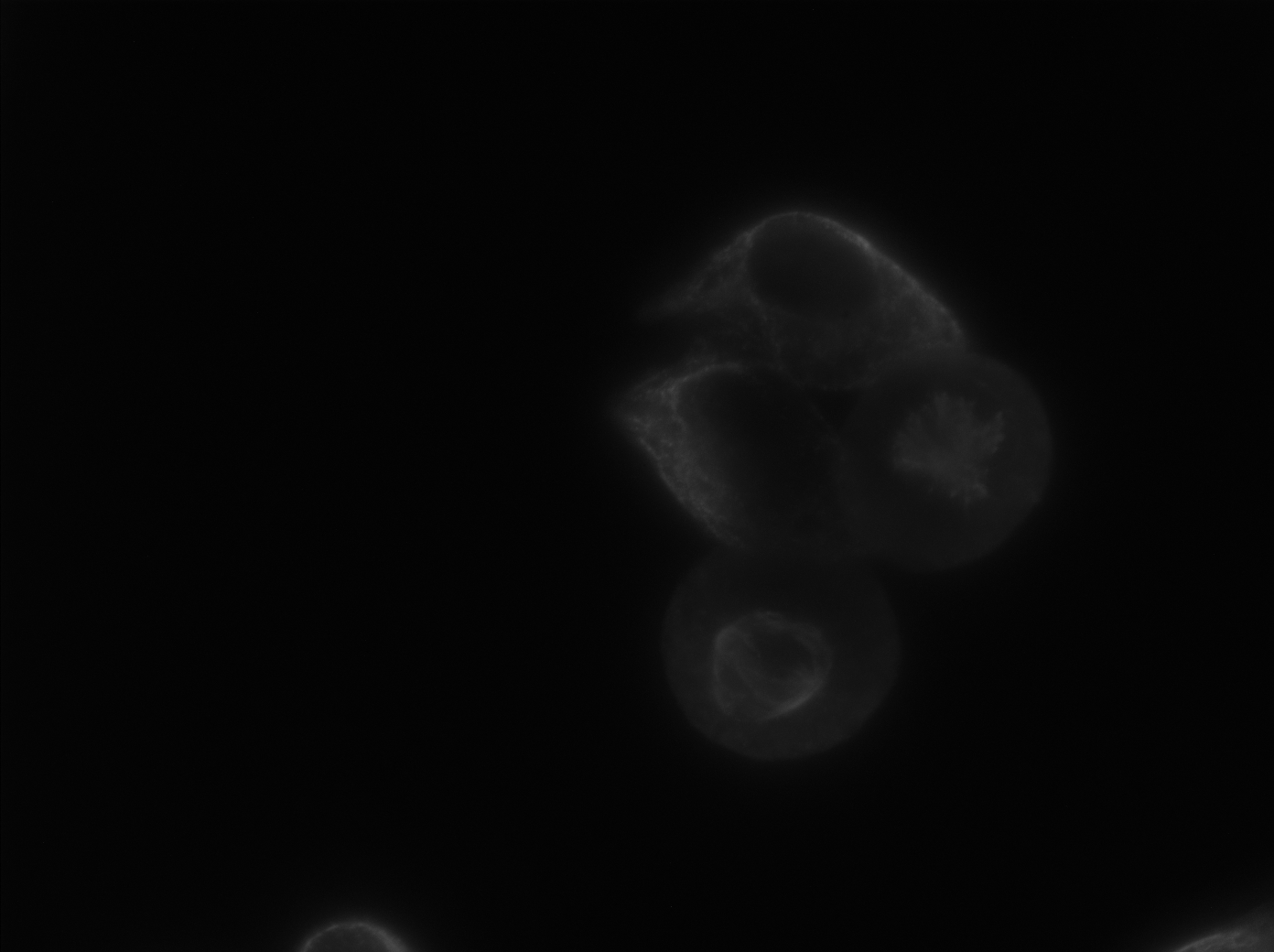

Supplement: Supplementary file 3 — Source data Fig. 1 [file 44319_2026_742_MOESM3_ESM.zip › Figure 1/Fig 1bcd WT Hela acetylated a tubulin atubulin/actub-atub 8-14-24 R2 M2.Project Maximum Z_XY1724690941_Z0_T0_C1.tif]

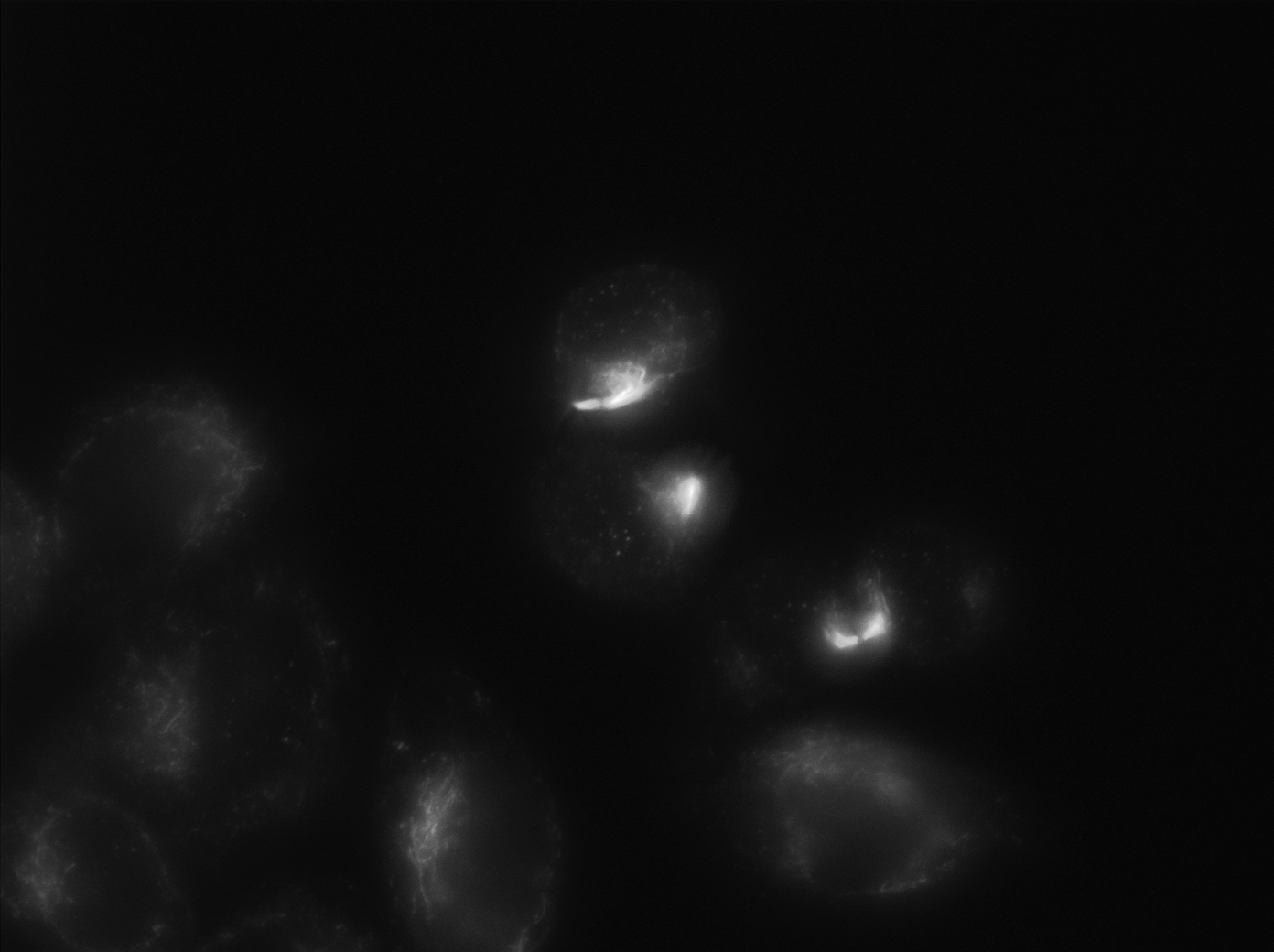

Supplement: Supplementary file 3 — Source data Fig. 1 [file 44319_2026_742_MOESM3_ESM.zip › Figure 1/Fig 1bcd WT Hela acetylated a tubulin atubulin/actub-atub 8-14-24 R1 PA8.Project Maximum Z_XY1724366086_Z0_T0_C2.tif]

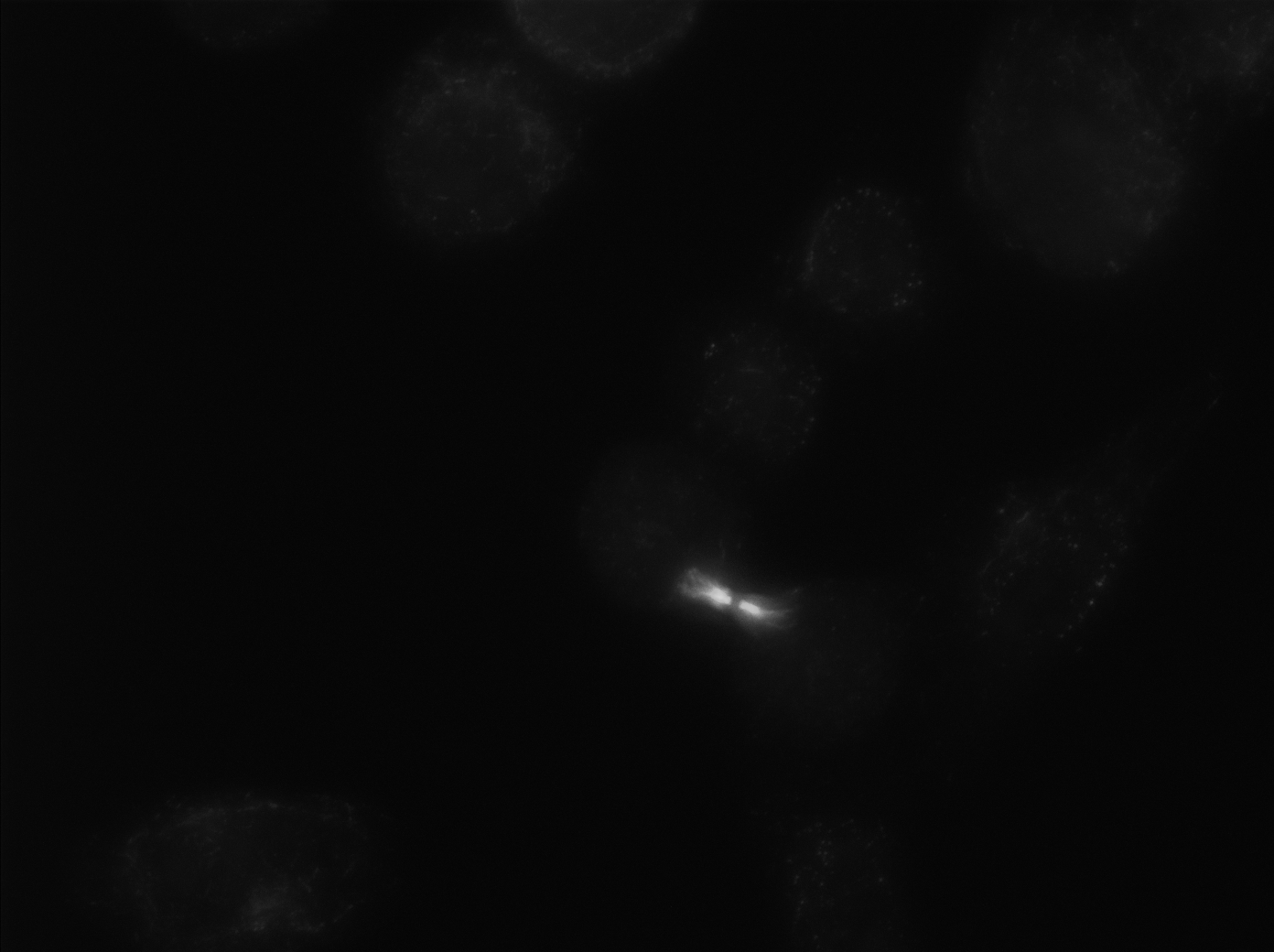

Supplement: Supplementary file 3 — Source data Fig. 1 [file 44319_2026_742_MOESM3_ESM.zip › Figure 1/Fig 1bcd WT Hela acetylated a tubulin atubulin/actub-atub 8-14-24 R2 LT5.Project Maximum Z_XY1724690793_Z0_T0_C2.tif]

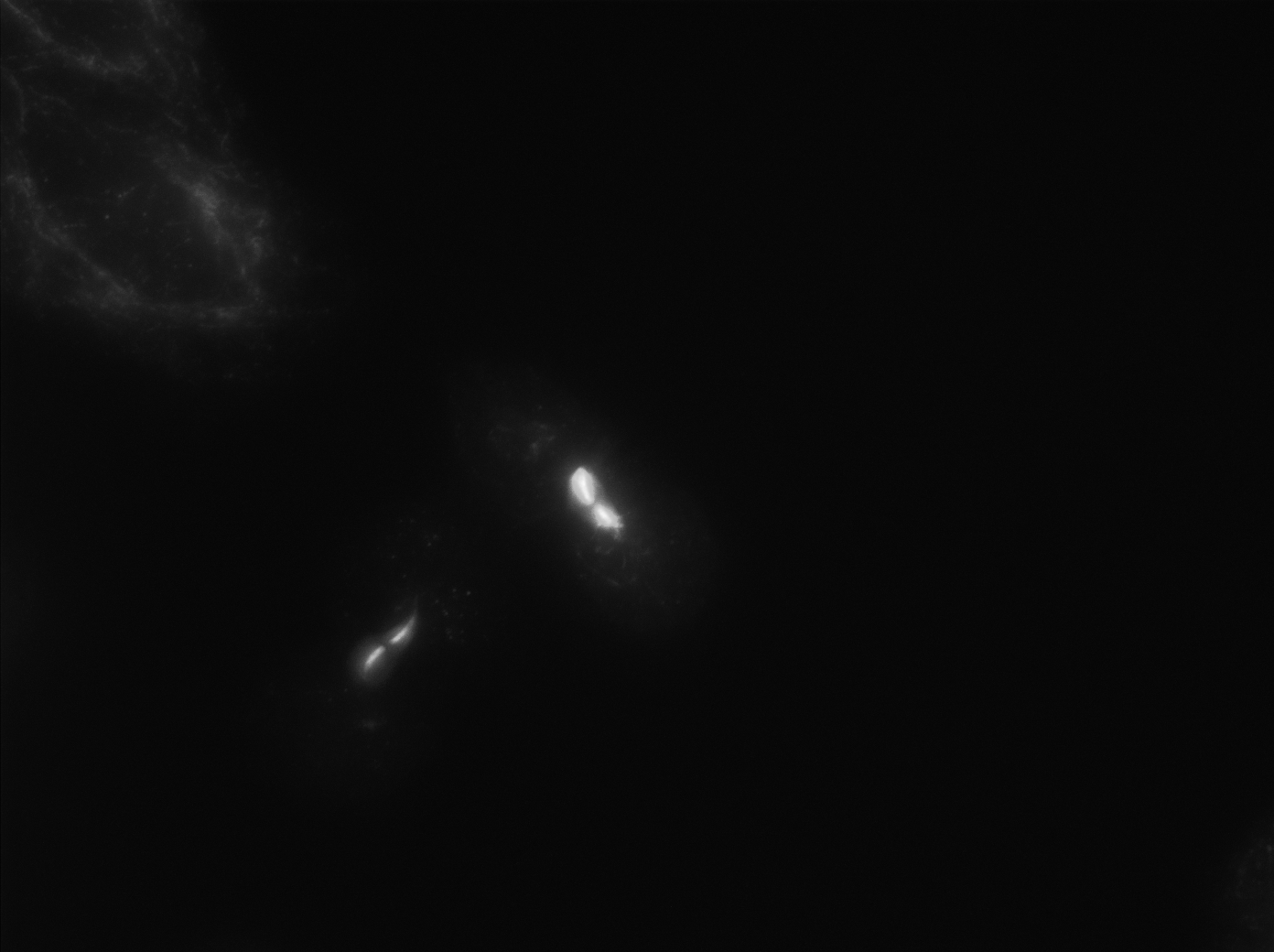

Supplement: Supplementary file 3 — Source data Fig. 1 [file 44319_2026_742_MOESM3_ESM.zip › Figure 1/Fig 1bcd WT Hela acetylated a tubulin atubulin/actub-atub 8-14-24 R2 LT9LT10.Project Maximum Z_XY1724694226_Z0_T0_C2.tif]

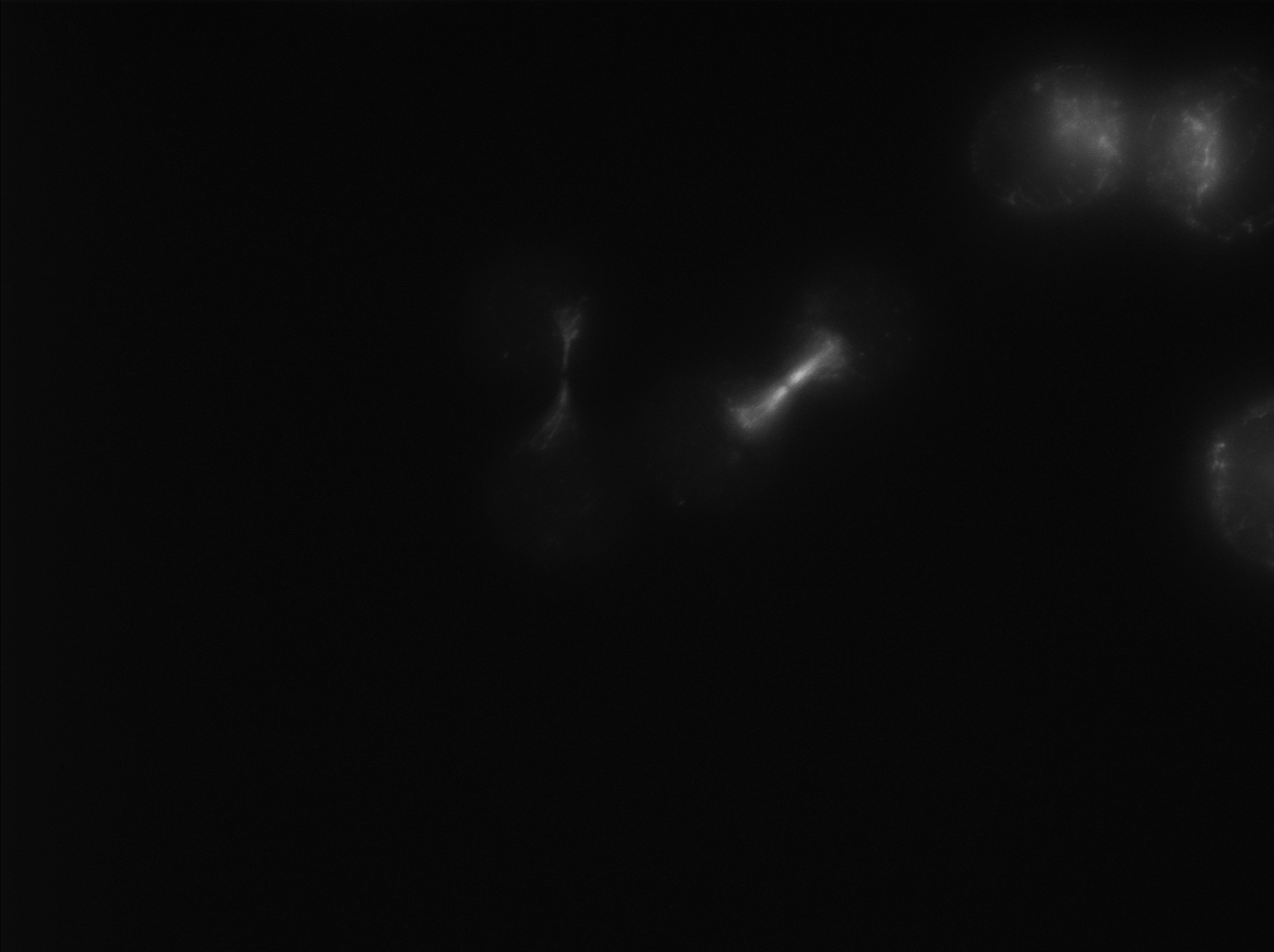

Supplement: Supplementary file 3 — Source data Fig. 1 [file 44319_2026_742_MOESM3_ESM.zip › Figure 1/Fig 1bcd WT Hela acetylated a tubulin atubulin/actub-atub 8-14-24 R1 ET3ET4.Project Maximum Z_XY1724363161_Z0_T0_C2.tif]

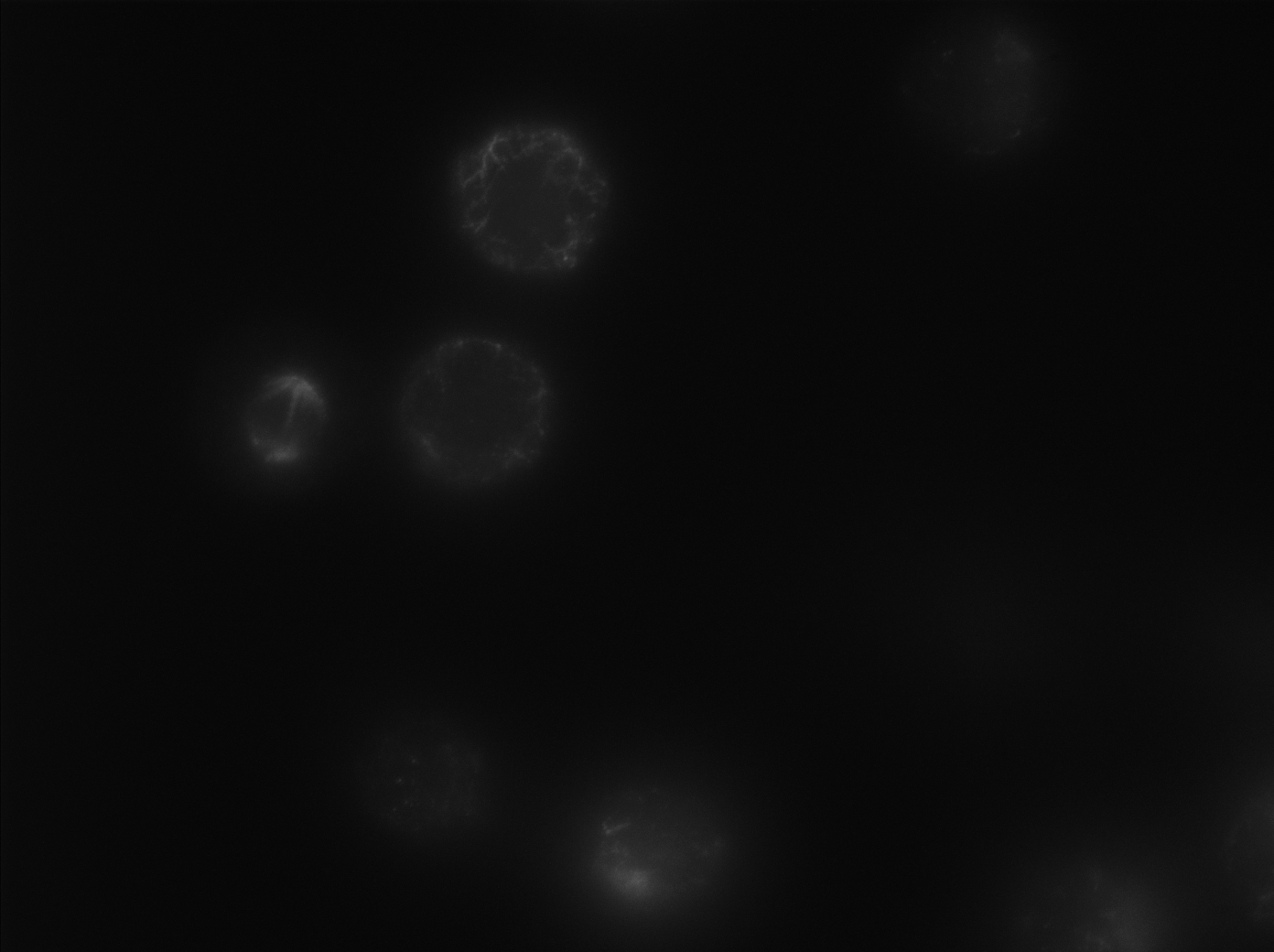

Supplement: Supplementary file 3 — Source data Fig. 1 [file 44319_2026_742_MOESM3_ESM.zip › Figure 1/Fig 1bcd WT Hela acetylated a tubulin atubulin/actub-atub 8-14-24 R2 M7.Project Maximum Z_XY1724694094_Z0_T0_C2.tif]

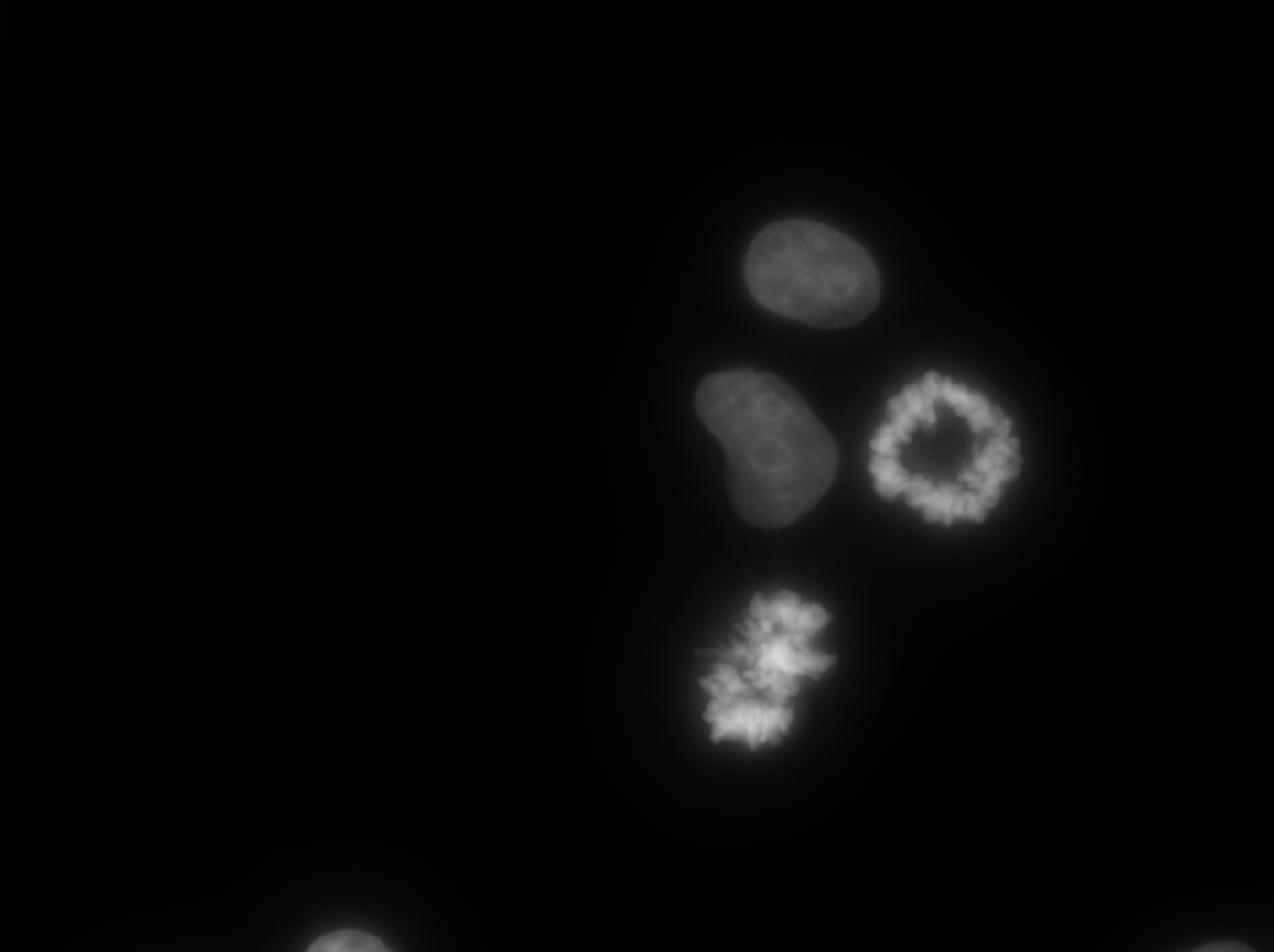

Supplement: Supplementary file 3 — Source data Fig. 1 [file 44319_2026_742_MOESM3_ESM.zip › Figure 1/Fig 1bcd WT Hela acetylated a tubulin atubulin/actub-atub 8-14-24 R2 M2.Project Maximum Z_XY1724690941_Z0_T0_C0.tif]

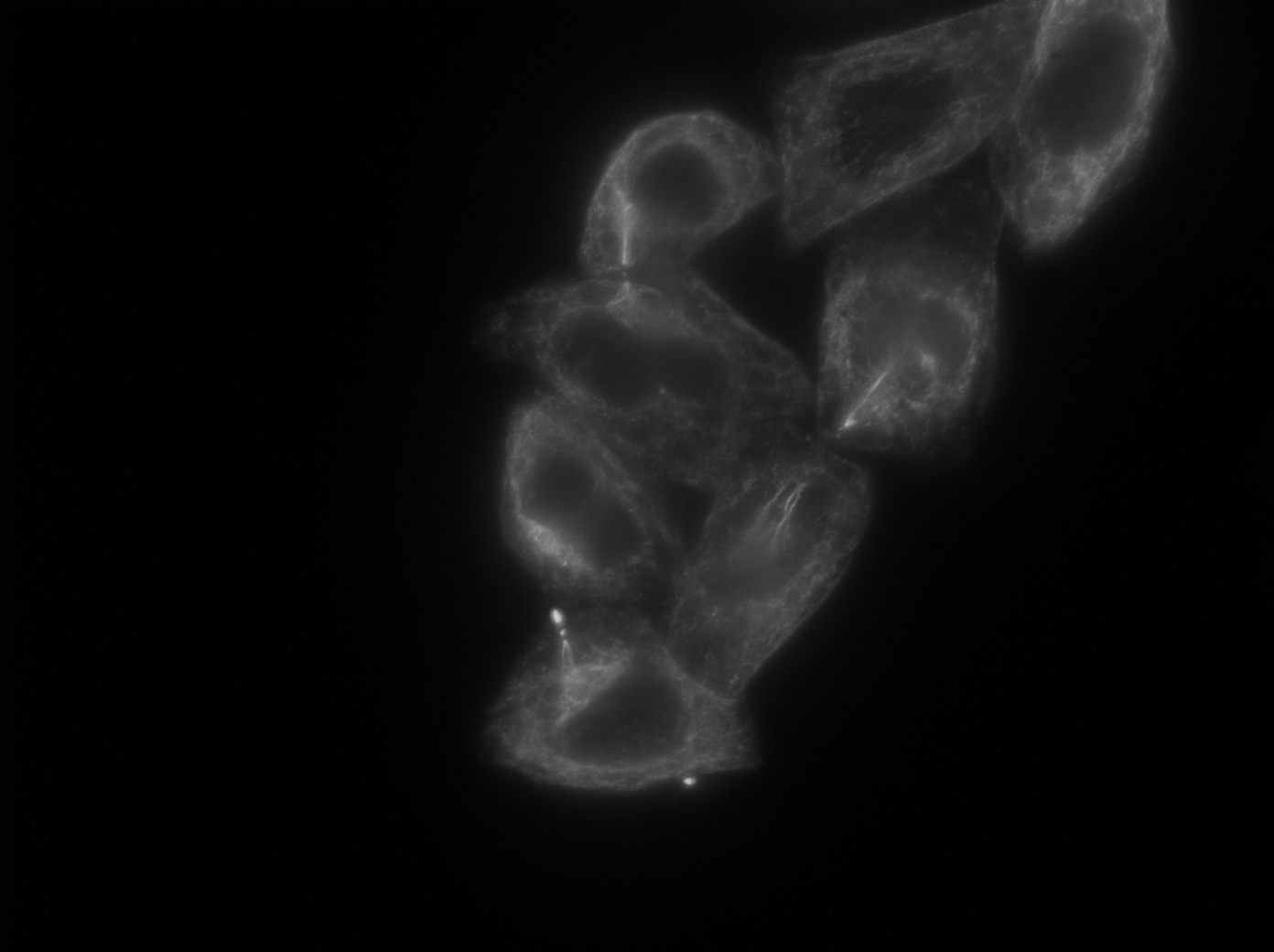

Supplement: Supplementary file 3 — Source data Fig. 1 [file 44319_2026_742_MOESM3_ESM.zip › Figure 1/Fig 1bcd WT Hela acetylated a tubulin atubulin/actub-atub 8-14-24 R1 LT1 PA3PA4.Project Maximum Z_XY1724362702_Z0_T0_C1.tif]

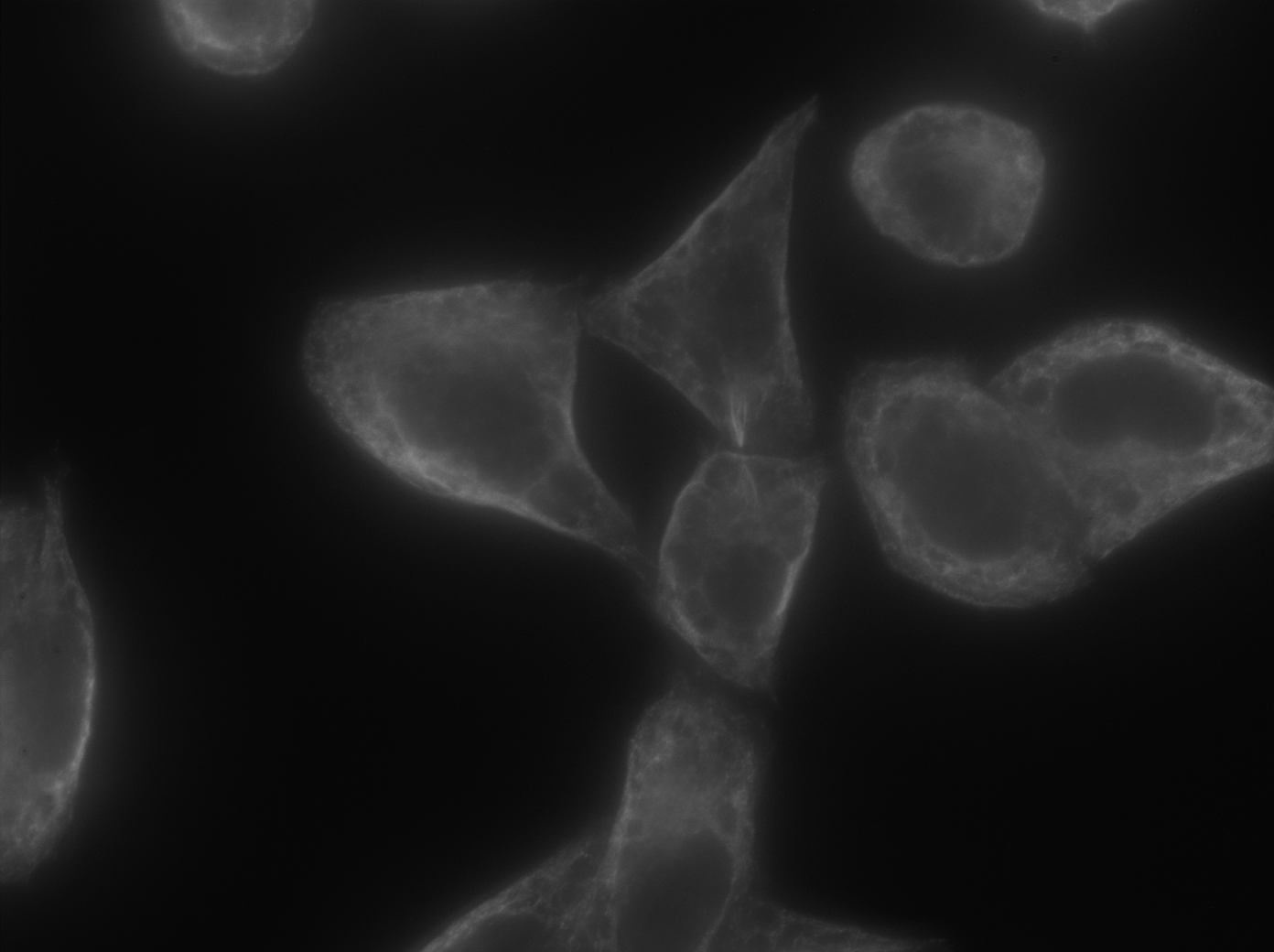

Supplement: Supplementary file 3 — Source data Fig. 1 [file 44319_2026_742_MOESM3_ESM.zip › Figure 1/Fig 1bcd WT Hela acetylated a tubulin atubulin/actub-atub 8-14-24 R2 LT6.Project Maximum Z_XY1724693513_Z0_T0_C1.tif]

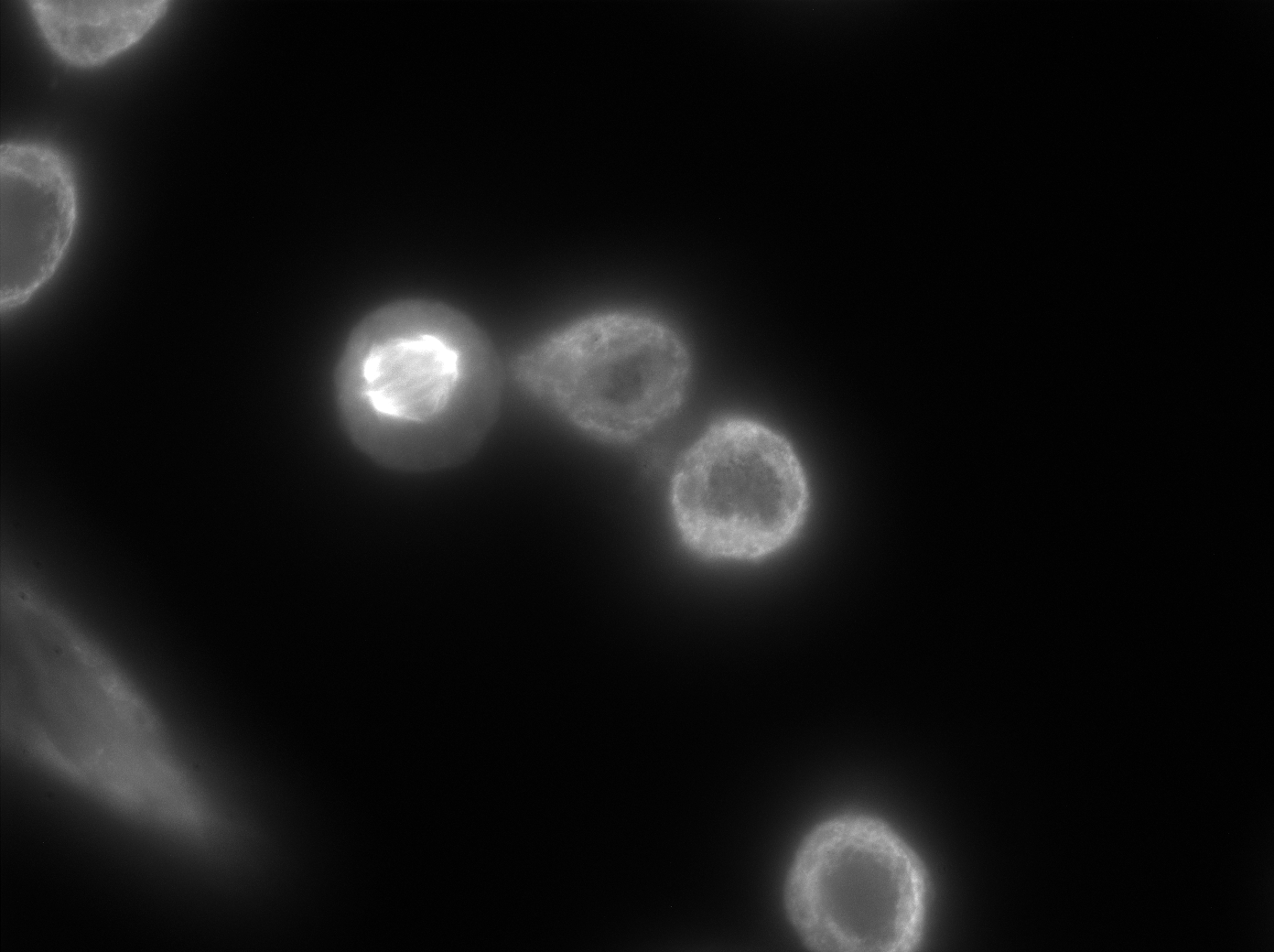

Supplement: Supplementary file 3 — Source data Fig. 1 [file 44319_2026_742_MOESM3_ESM.zip › Figure 1/Fig 1bcd WT Hela acetylated a tubulin atubulin/actub-atub 8-14-24 R3 M5.Project Maximum Z_XY1724702253_Z0_T0_C1.tif]

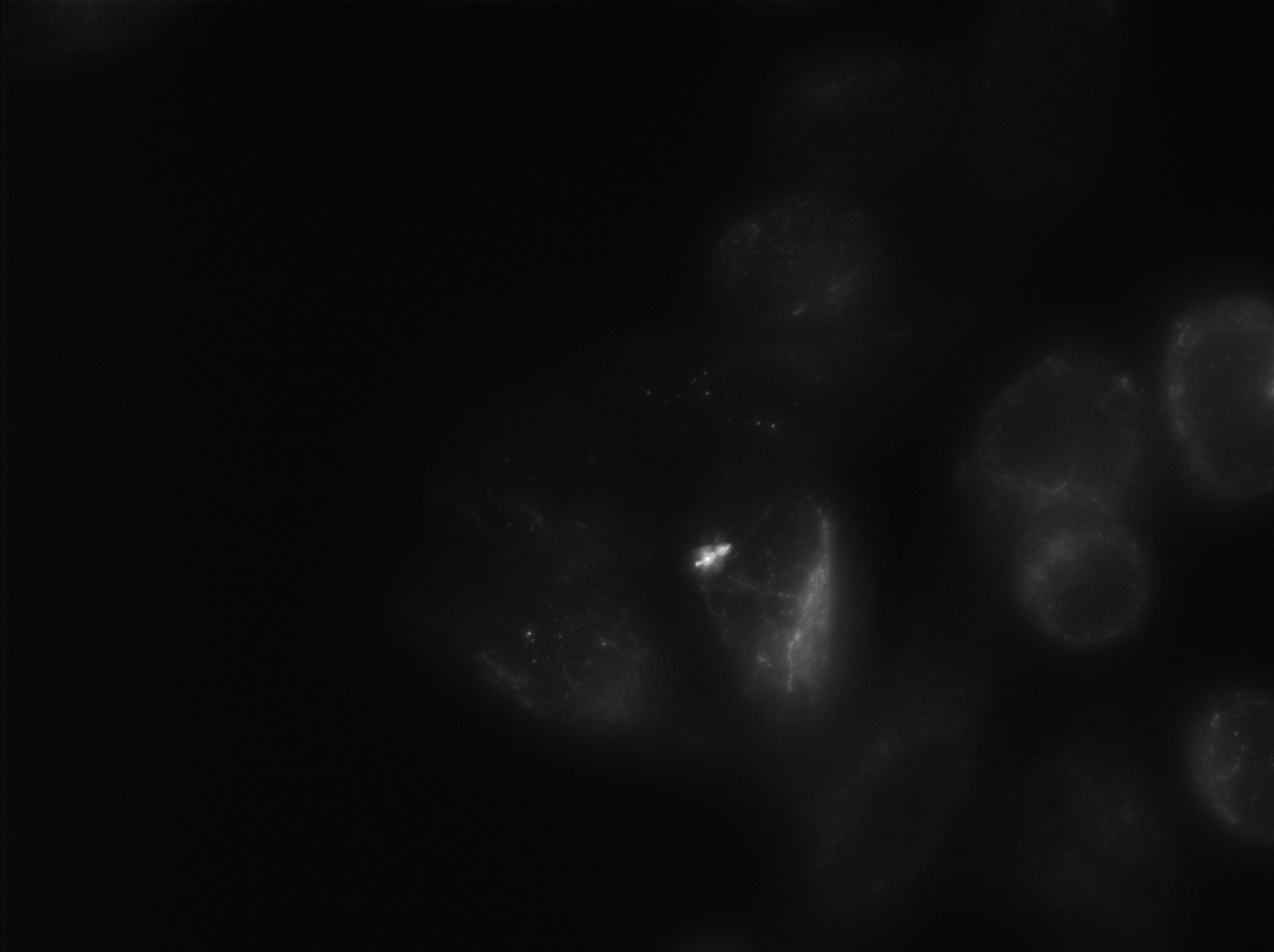

Supplement: Supplementary file 3 — Source data Fig. 1 [file 44319_2026_742_MOESM3_ESM.zip › Figure 1/Fig 1bcd WT Hela acetylated a tubulin atubulin/actub-atub 8-14-24 R3 PA10.Project Maximum Z_XY1724717416_Z0_T0_C2.tif]

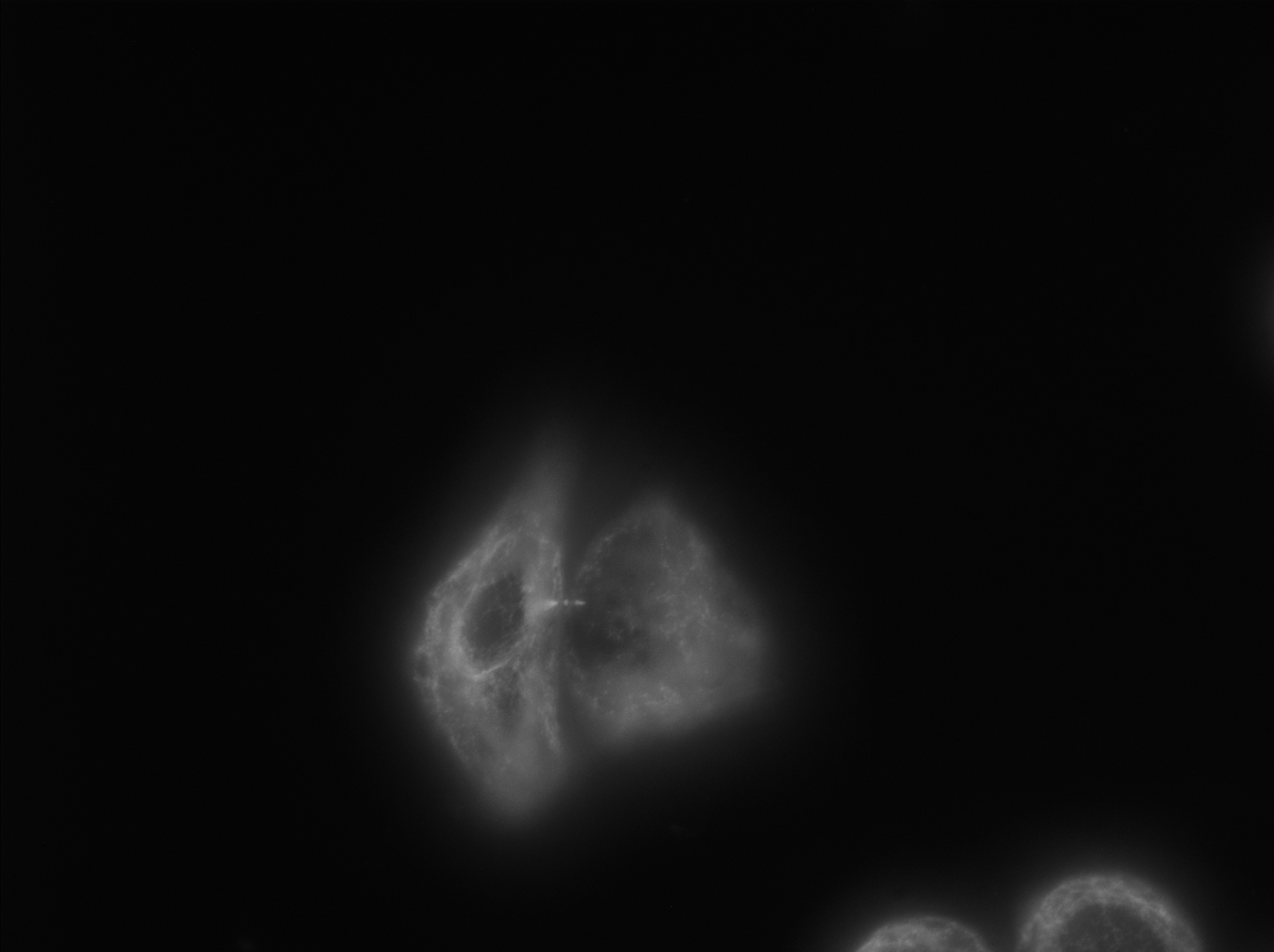

Supplement: Supplementary file 3 — Source data Fig. 1 [file 44319_2026_742_MOESM3_ESM.zip › Figure 1/Fig 1bcd WT Hela acetylated a tubulin atubulin/actub-atub 8-14-24 R2 PA8.Project Maximum Z_XY1724695165_Z0_T0_C1.tif]

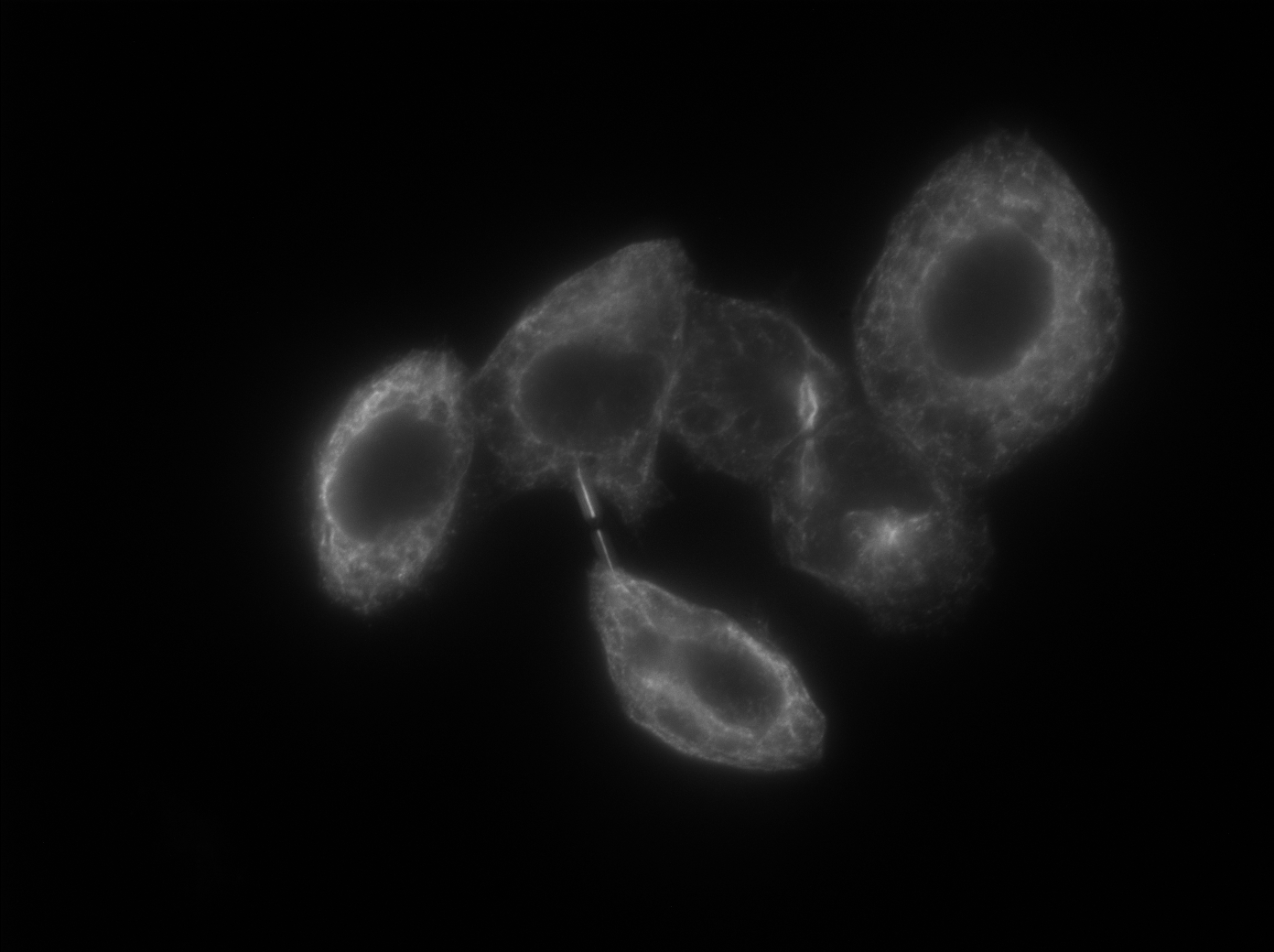

Supplement: Supplementary file 3 — Source data Fig. 1 [file 44319_2026_742_MOESM3_ESM.zip › Figure 1/Fig 1bcd WT Hela acetylated a tubulin atubulin/actub-atub 8-14-24 R1 LT2LT3.Project Maximum Z_XY1724362822_Z0_T0_C1.tif]

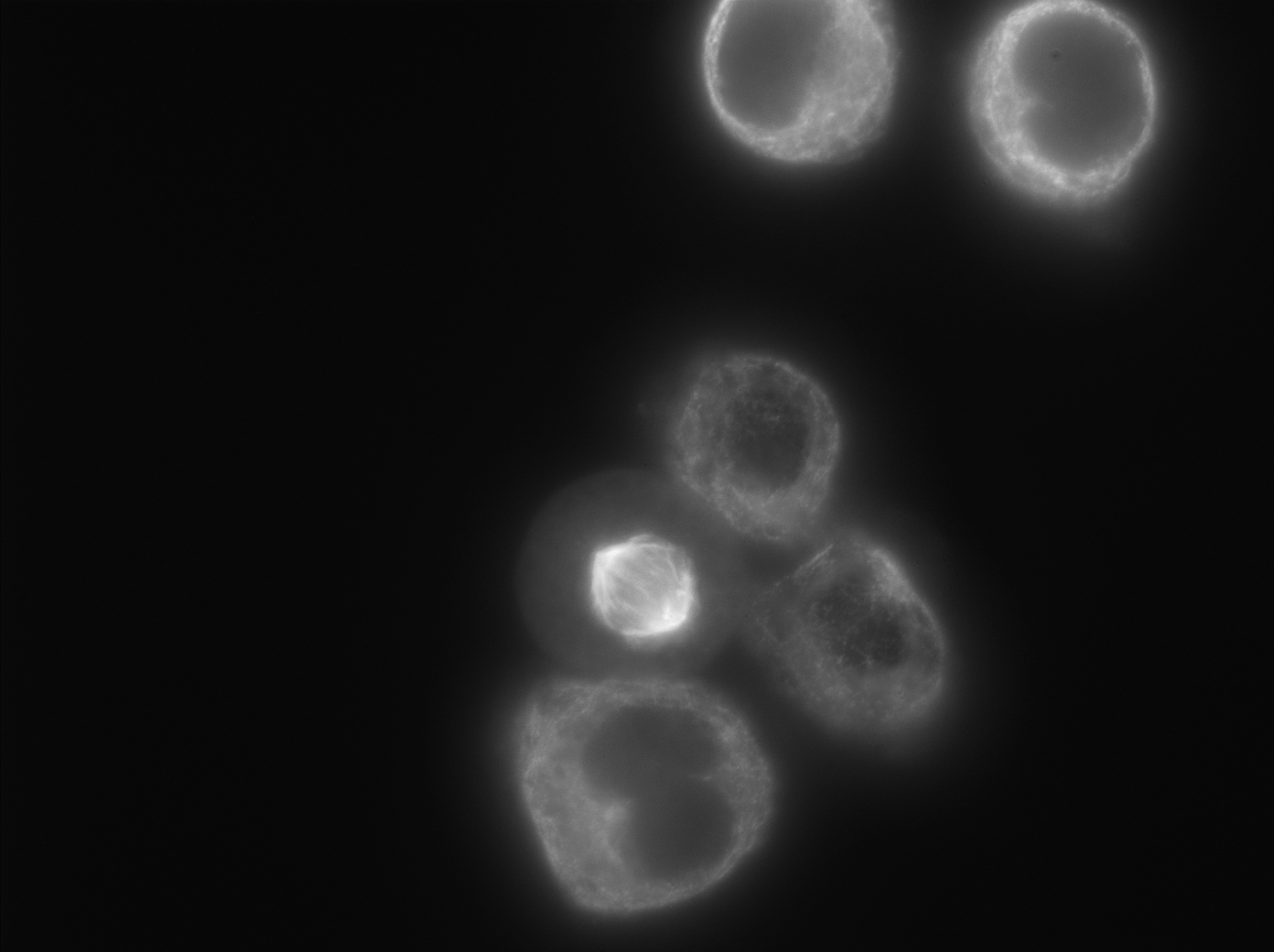

Supplement: Supplementary file 3 — Source data Fig. 1 [file 44319_2026_742_MOESM3_ESM.zip › Figure 1/Fig 1bcd WT Hela acetylated a tubulin atubulin/actub-atub 8-14-24 R2 M9.Project Maximum Z_XY1724694990_Z0_T0_C1.tif]

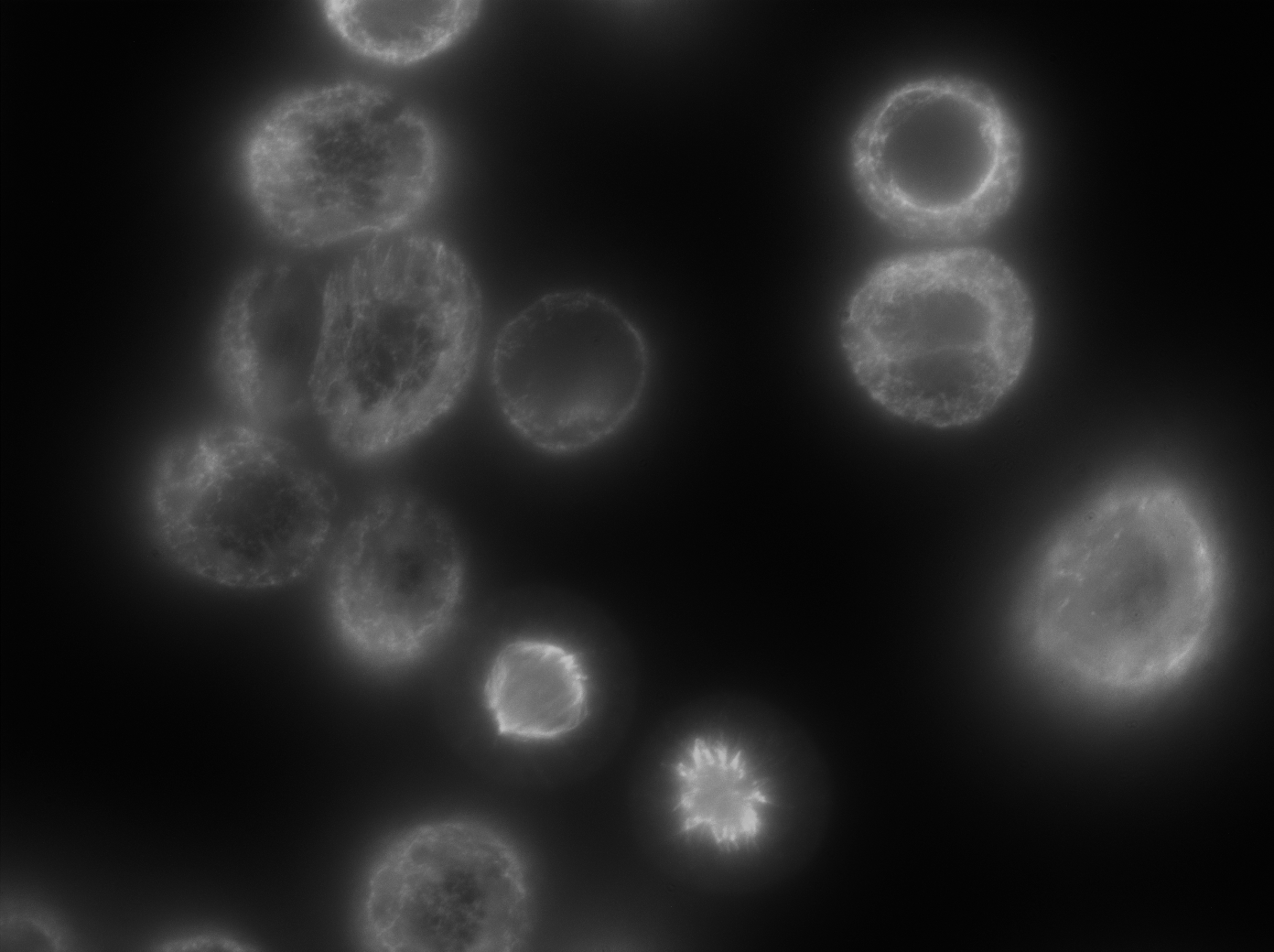

Supplement: Supplementary file 3 — Source data Fig. 1 [file 44319_2026_742_MOESM3_ESM.zip › Figure 1/Fig 1bcd WT Hela acetylated a tubulin atubulin/actub-atub 8-14-24 R1 M4.Project Maximum Z_XY1724364701_Z0_T0_C1.tif]

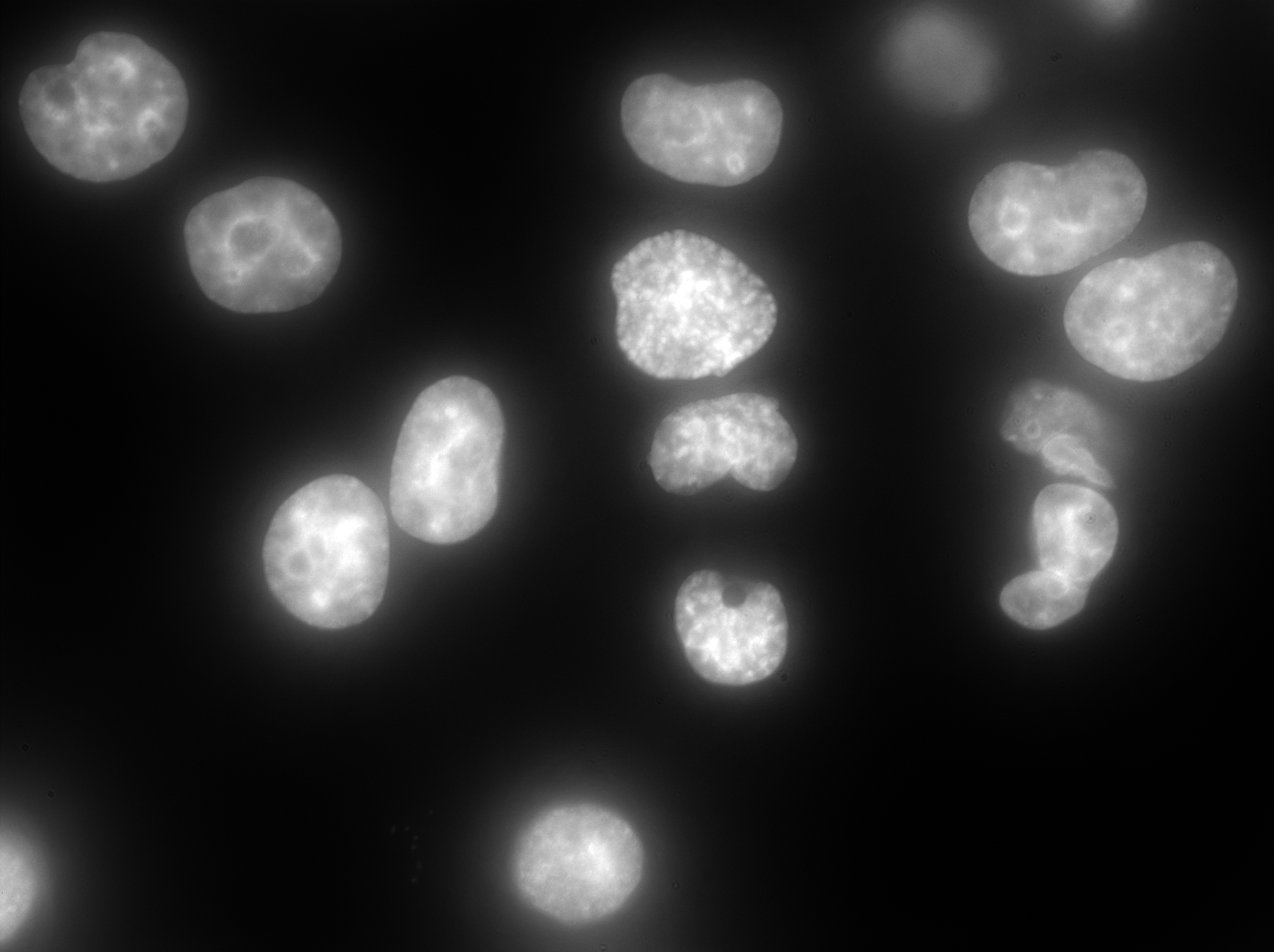

Supplement: Supplementary file 3 — Source data Fig. 1 [file 44319_2026_742_MOESM3_ESM.zip › Figure 1/Fig 1bcd WT Hela acetylated a tubulin atubulin/actub-atub 8-14-24 R3 LT5.Project Maximum Z_XY1724716758_Z0_T0_C0.tif]

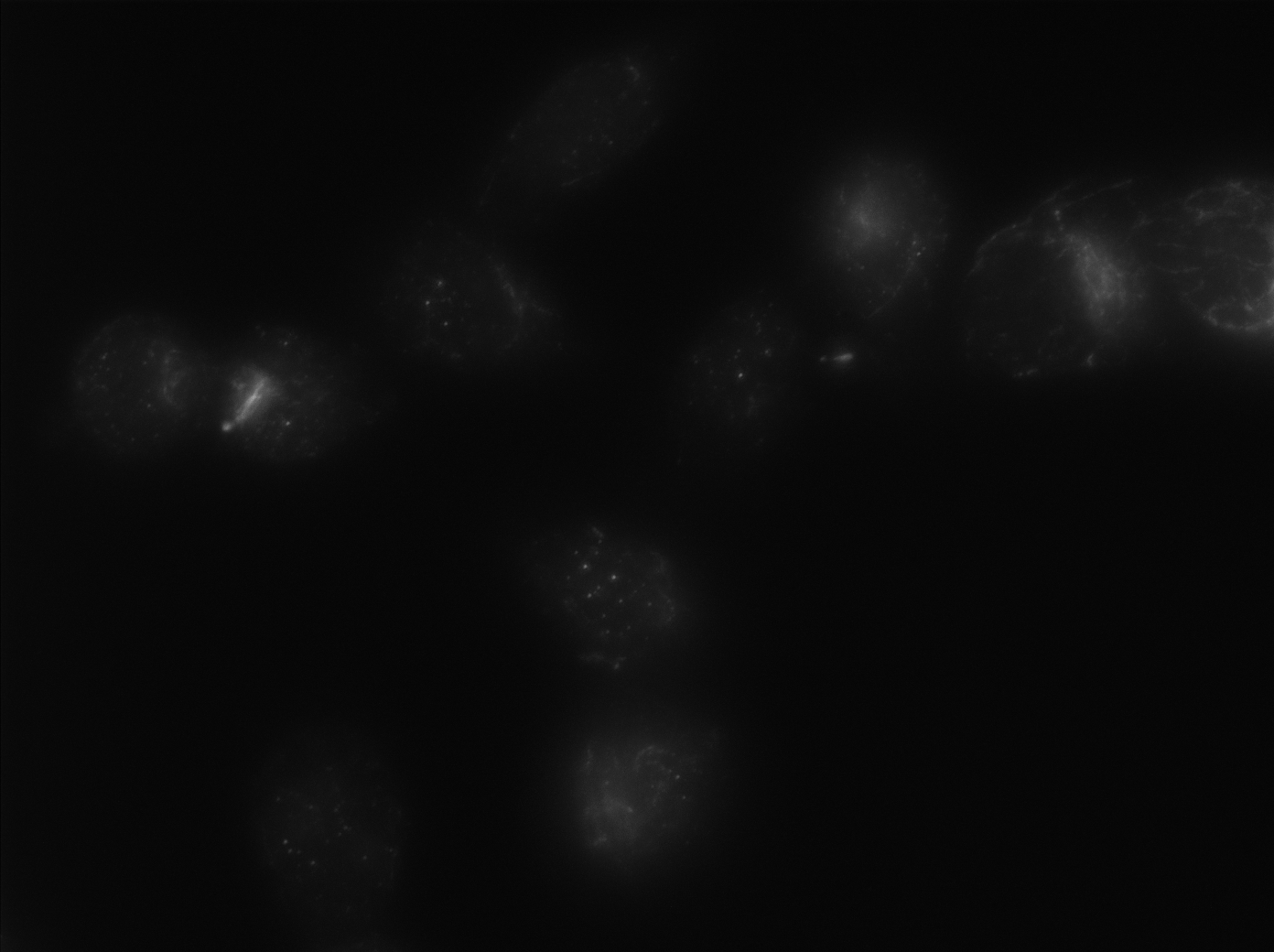

Supplement: Supplementary file 3 — Source data Fig. 1 [file 44319_2026_742_MOESM3_ESM.zip › Figure 1/Fig 1bcd WT Hela acetylated a tubulin atubulin/actub-atub 8-14-24 R2 PA2PA3.Project Maximum Z_XY1724690051_Z0_T0_C2.tif]

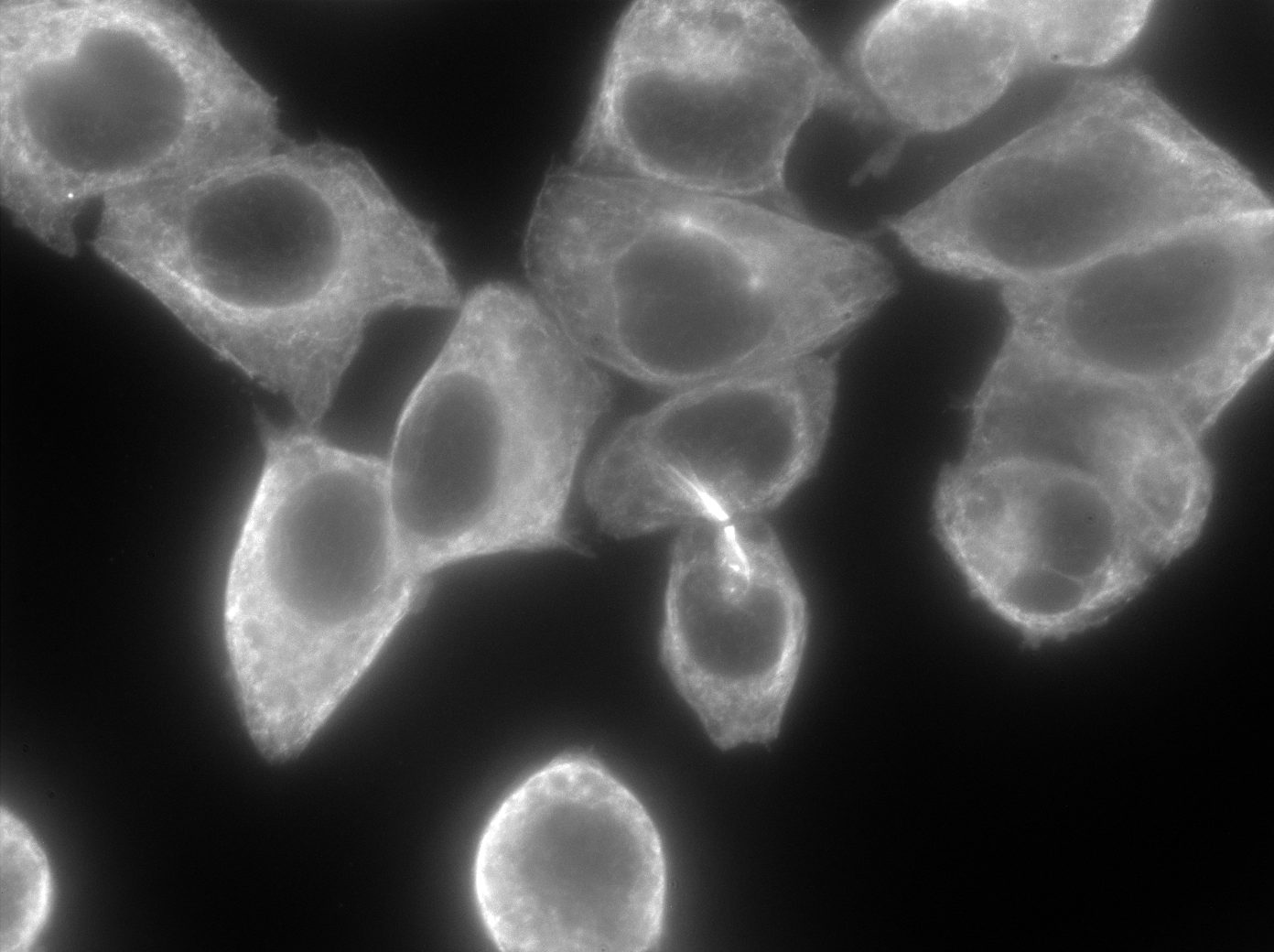

Supplement: Supplementary file 3 — Source data Fig. 1 [file 44319_2026_742_MOESM3_ESM.zip › Figure 1/Fig 1bcd WT Hela acetylated a tubulin atubulin/actub-atub 8-14-24 R3 LT5.Project Maximum Z_XY1724716758_Z0_T0_C1.tif]

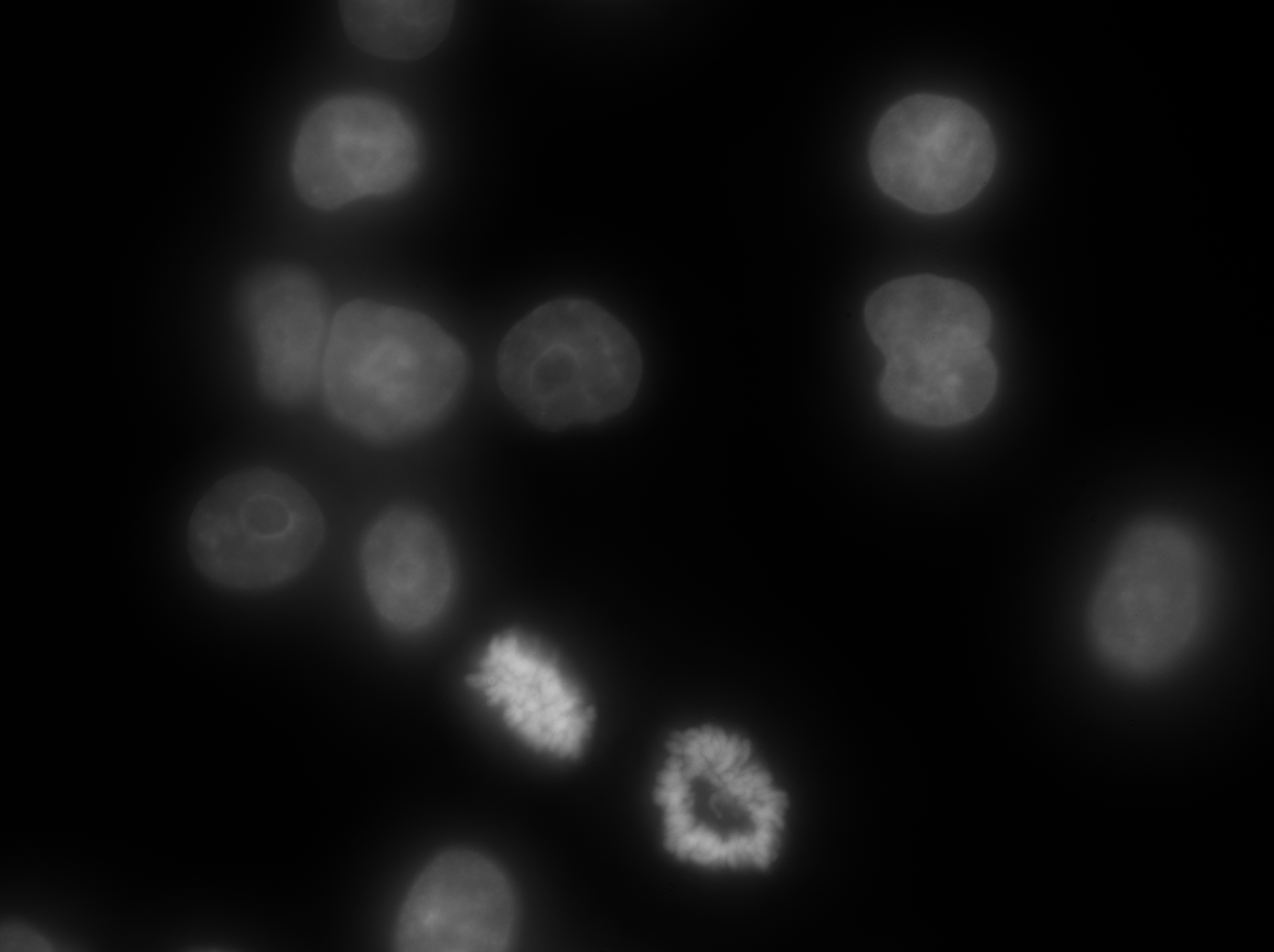

Supplement: Supplementary file 3 — Source data Fig. 1 [file 44319_2026_742_MOESM3_ESM.zip › Figure 1/Fig 1bcd WT Hela acetylated a tubulin atubulin/actub-atub 8-14-24 R1 M4.Project Maximum Z_XY1724364701_Z0_T0_C0.tif]

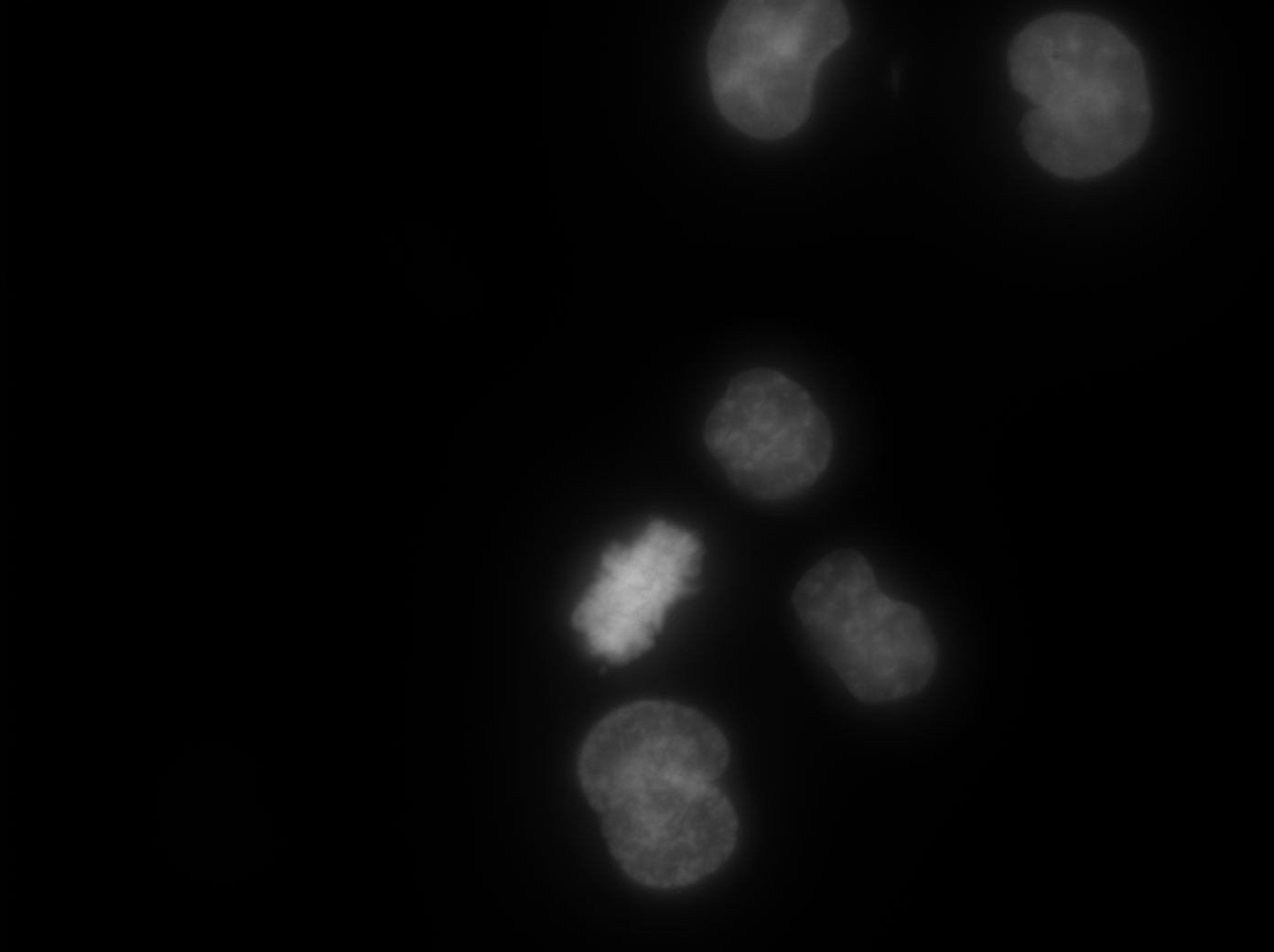

Supplement: Supplementary file 3 — Source data Fig. 1 [file 44319_2026_742_MOESM3_ESM.zip › Figure 1/Fig 1bcd WT Hela acetylated a tubulin atubulin/actub-atub 8-14-24 R2 M9.Project Maximum Z_XY1724694990_Z0_T0_C0.tif]

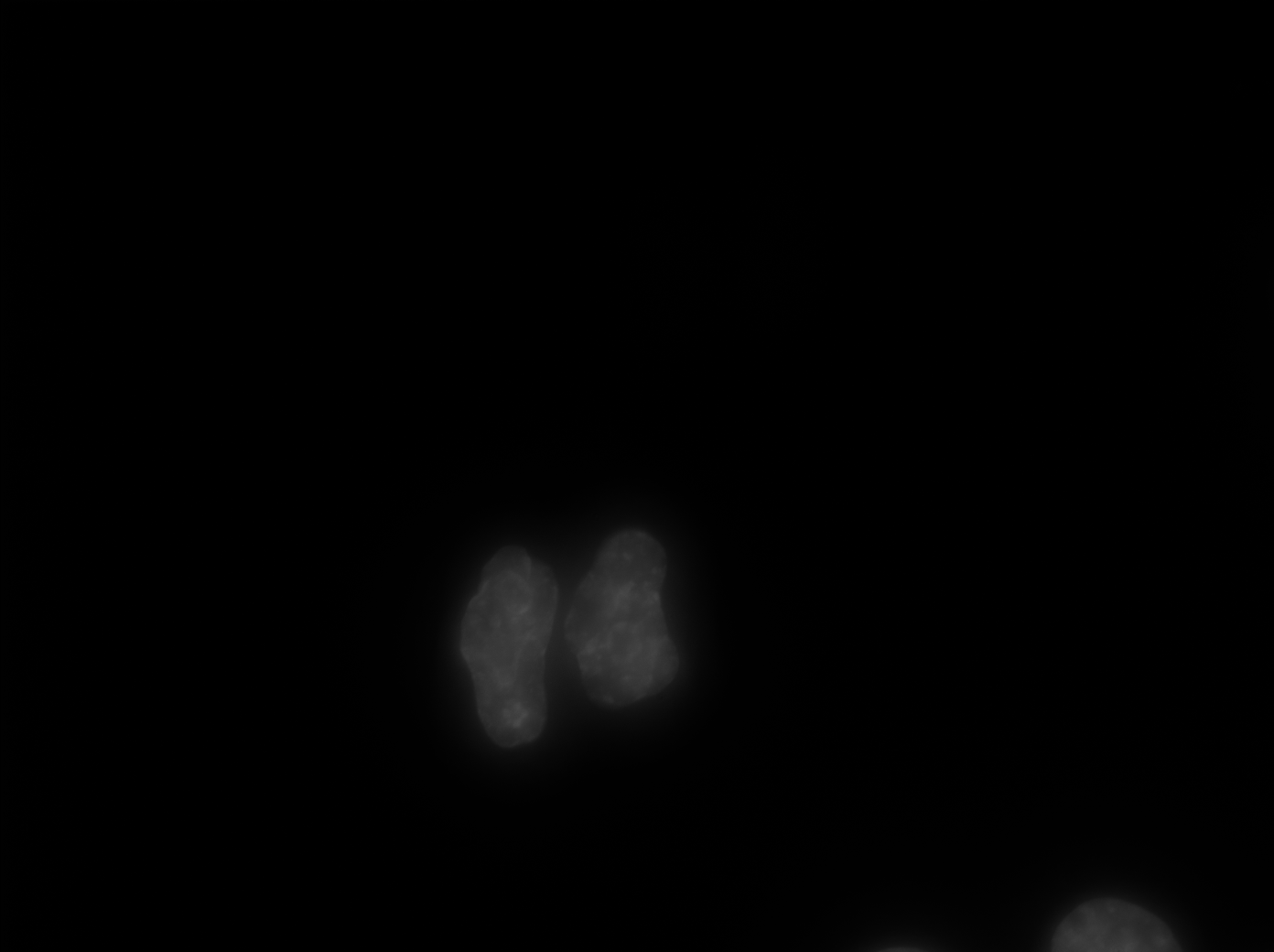

Supplement: Supplementary file 3 — Source data Fig. 1 [file 44319_2026_742_MOESM3_ESM.zip › Figure 1/Fig 1bcd WT Hela acetylated a tubulin atubulin/actub-atub 8-14-24 R2 PA8.Project Maximum Z_XY1724695165_Z0_T0_C0.tif]

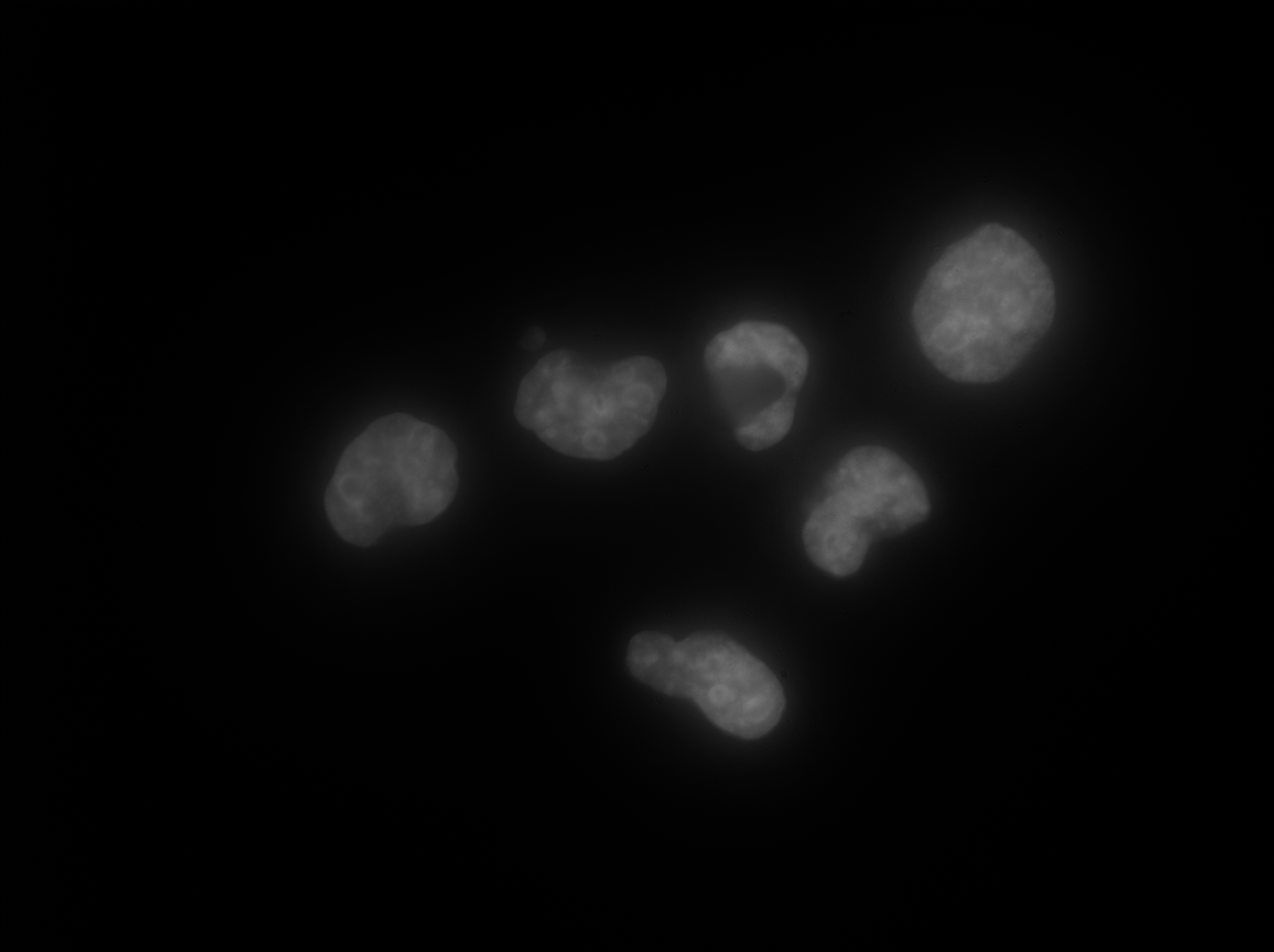

Supplement: Supplementary file 3 — Source data Fig. 1 [file 44319_2026_742_MOESM3_ESM.zip › Figure 1/Fig 1bcd WT Hela acetylated a tubulin atubulin/actub-atub 8-14-24 R1 LT2LT3.Project Maximum Z_XY1724362822_Z0_T0_C0.tif]

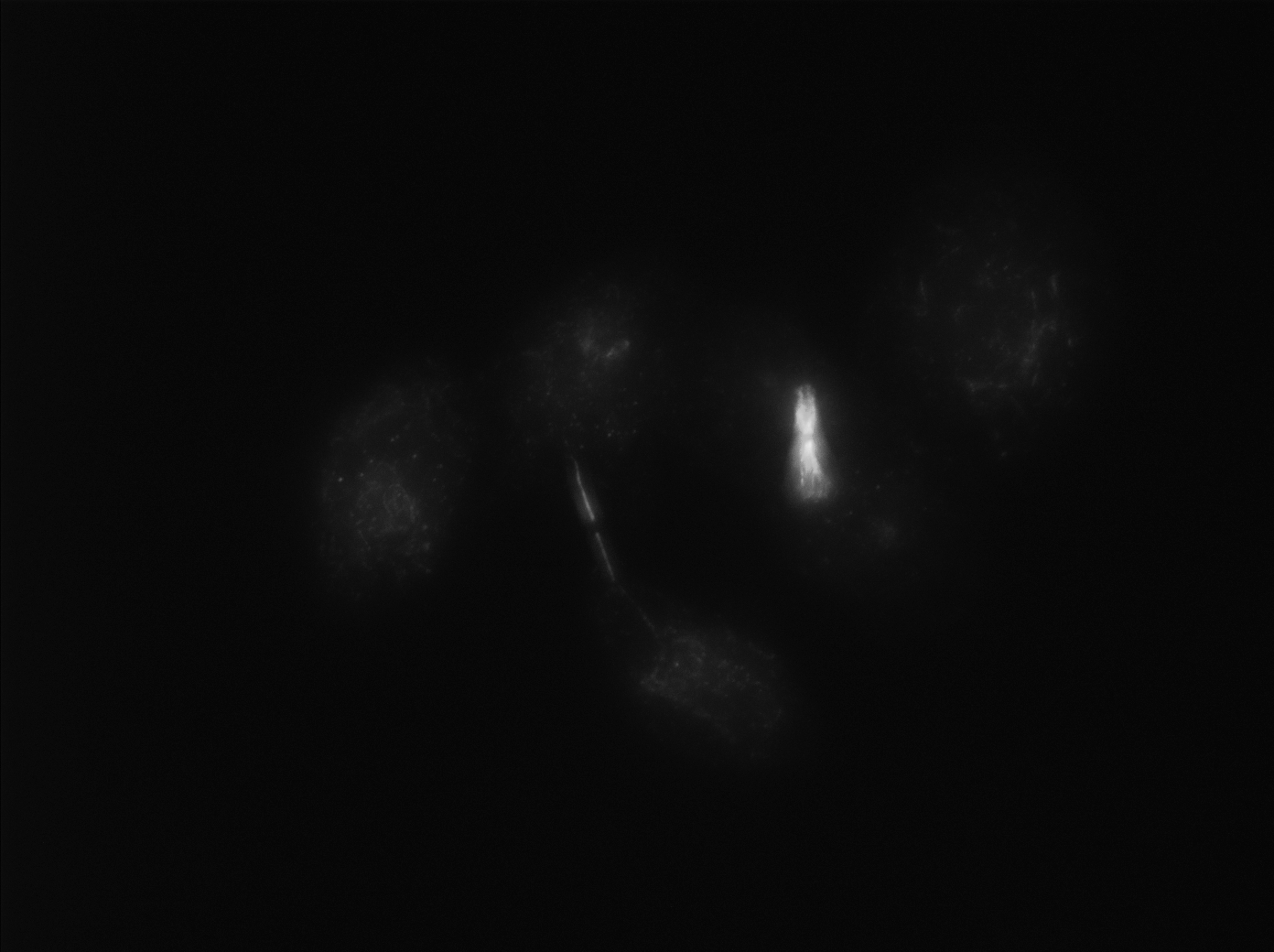

Supplement: Supplementary file 3 — Source data Fig. 1 [file 44319_2026_742_MOESM3_ESM.zip › Figure 1/Fig 1bcd WT Hela acetylated a tubulin atubulin/actub-atub 8-14-24 R1 LT2LT3.Project Maximum Z_XY1724362822_Z0_T0_C2.tif]

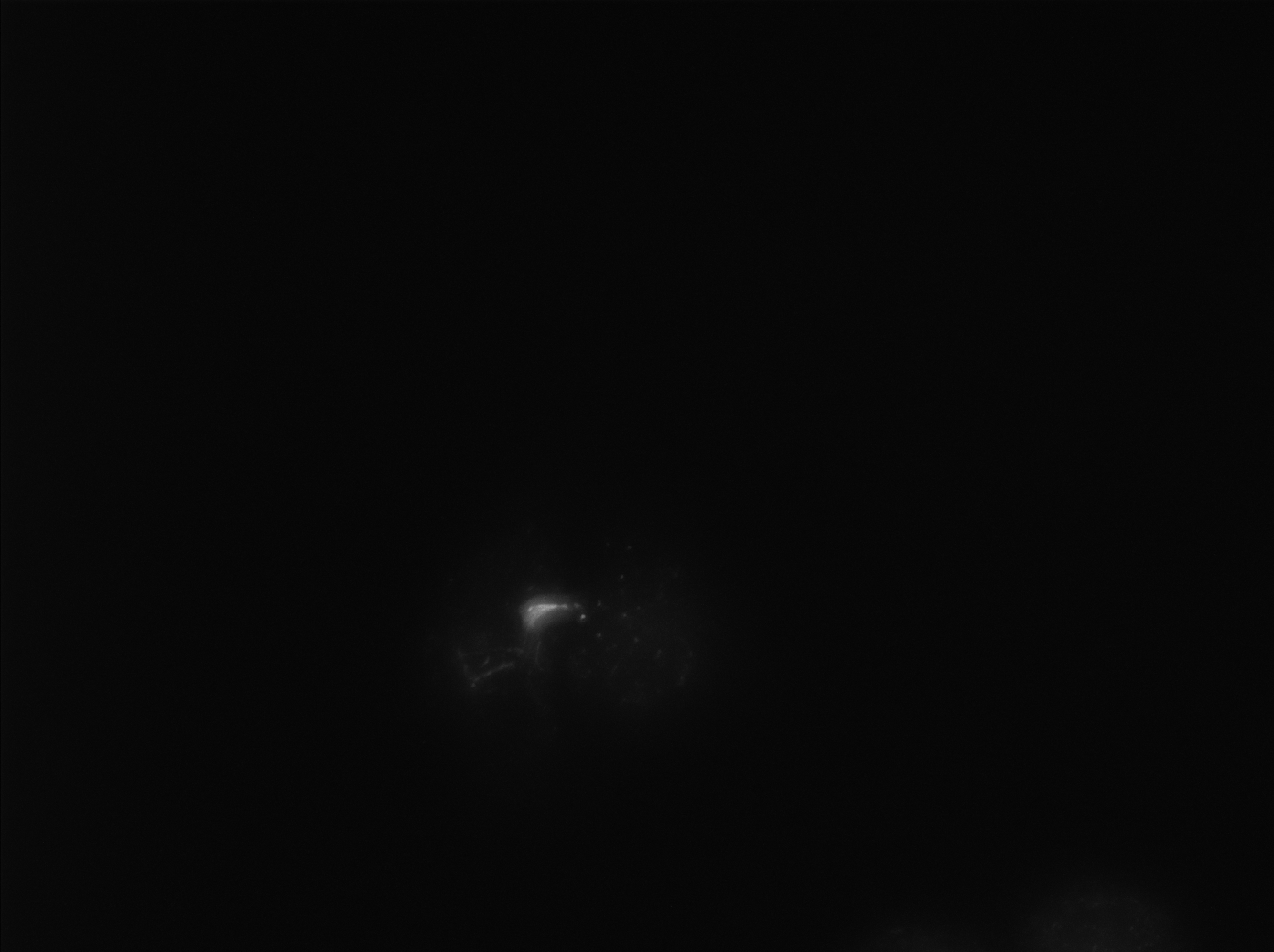

Supplement: Supplementary file 3 — Source data Fig. 1 [file 44319_2026_742_MOESM3_ESM.zip › Figure 1/Fig 1bcd WT Hela acetylated a tubulin atubulin/actub-atub 8-14-24 R2 PA8.Project Maximum Z_XY1724695165_Z0_T0_C2.tif]

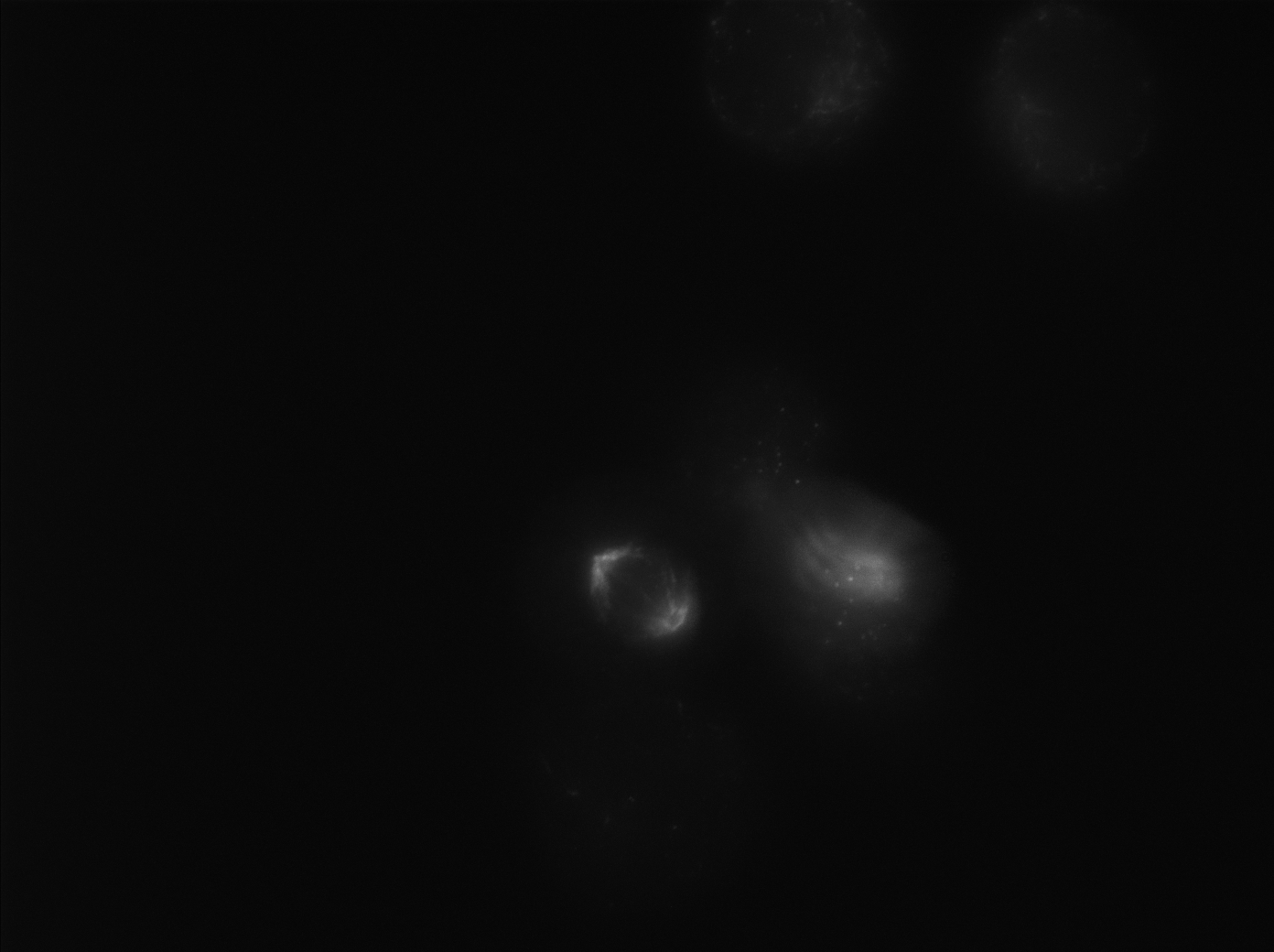

Supplement: Supplementary file 3 — Source data Fig. 1 [file 44319_2026_742_MOESM3_ESM.zip › Figure 1/Fig 1bcd WT Hela acetylated a tubulin atubulin/actub-atub 8-14-24 R2 M9.Project Maximum Z_XY1724694990_Z0_T0_C2.tif]

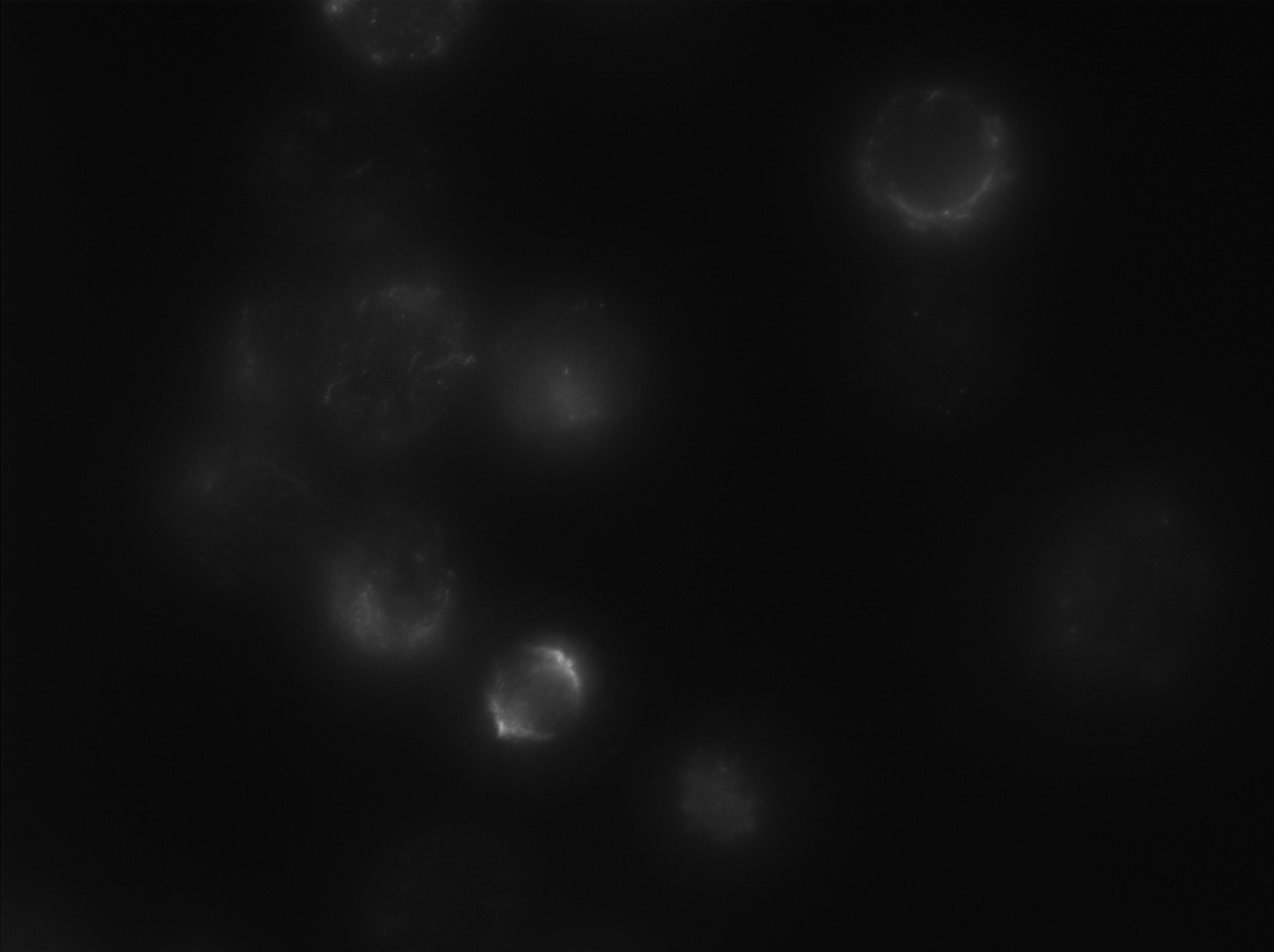

Supplement: Supplementary file 3 — Source data Fig. 1 [file 44319_2026_742_MOESM3_ESM.zip › Figure 1/Fig 1bcd WT Hela acetylated a tubulin atubulin/actub-atub 8-14-24 R1 M4.Project Maximum Z_XY1724364701_Z0_T0_C2.tif]

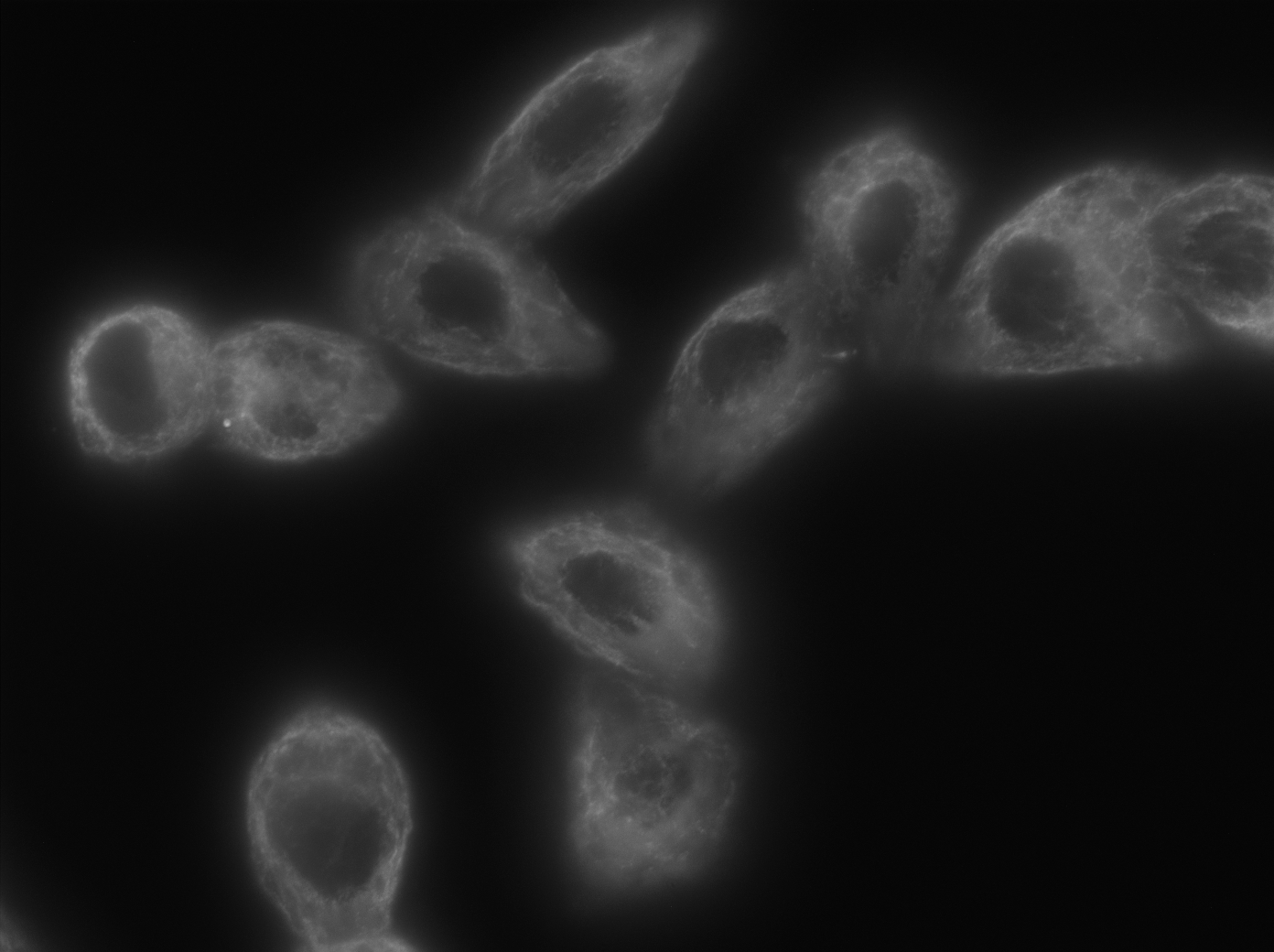

Supplement: Supplementary file 3 — Source data Fig. 1 [file 44319_2026_742_MOESM3_ESM.zip › Figure 1/Fig 1bcd WT Hela acetylated a tubulin atubulin/actub-atub 8-14-24 R2 PA2PA3.Project Maximum Z_XY1724690051_Z0_T0_C1.tif]

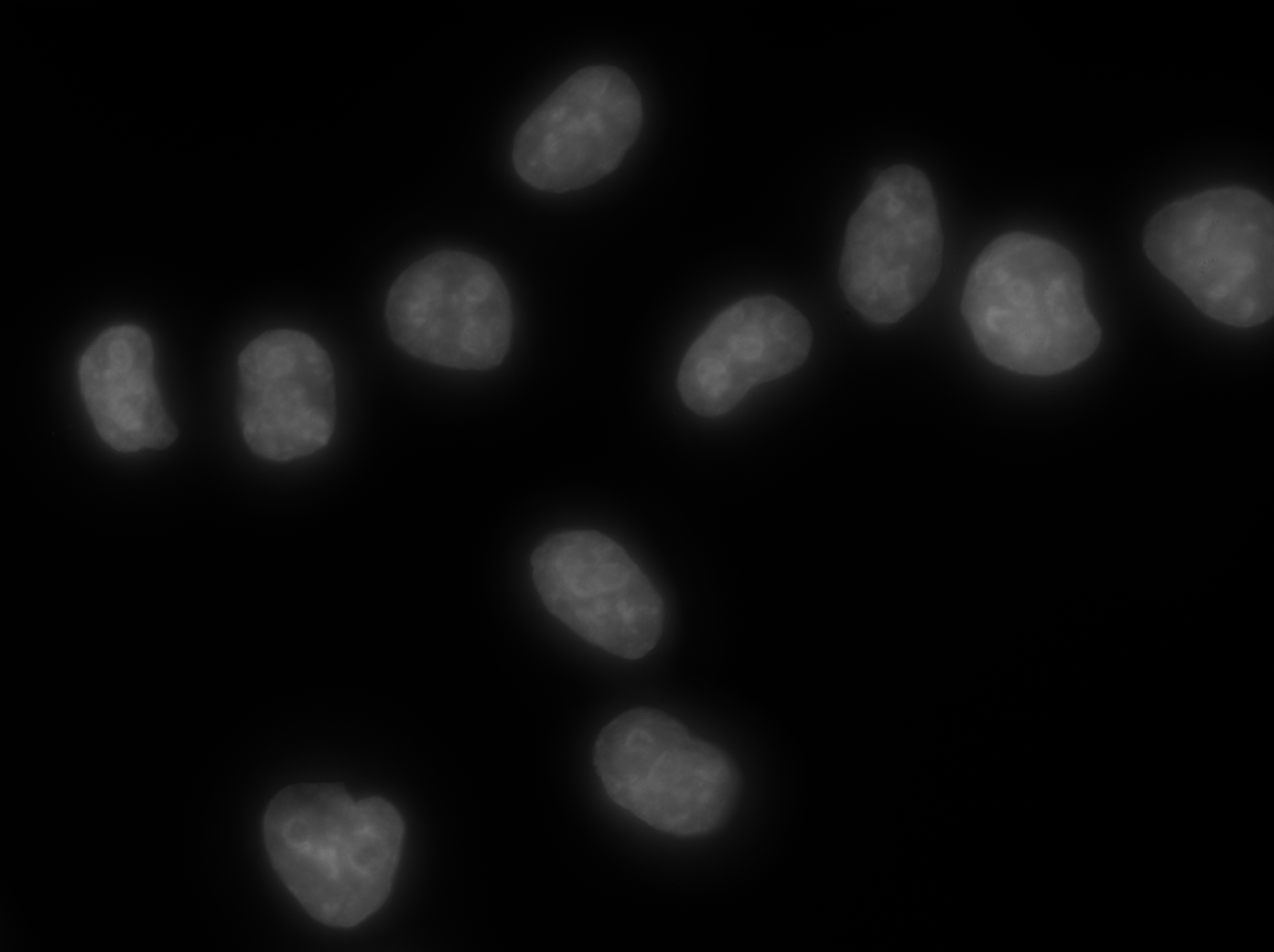

Supplement: Supplementary file 3 — Source data Fig. 1 [file 44319_2026_742_MOESM3_ESM.zip › Figure 1/Fig 1bcd WT Hela acetylated a tubulin atubulin/actub-atub 8-14-24 R2 PA2PA3.Project Maximum Z_XY1724690051_Z0_T0_C0.tif]

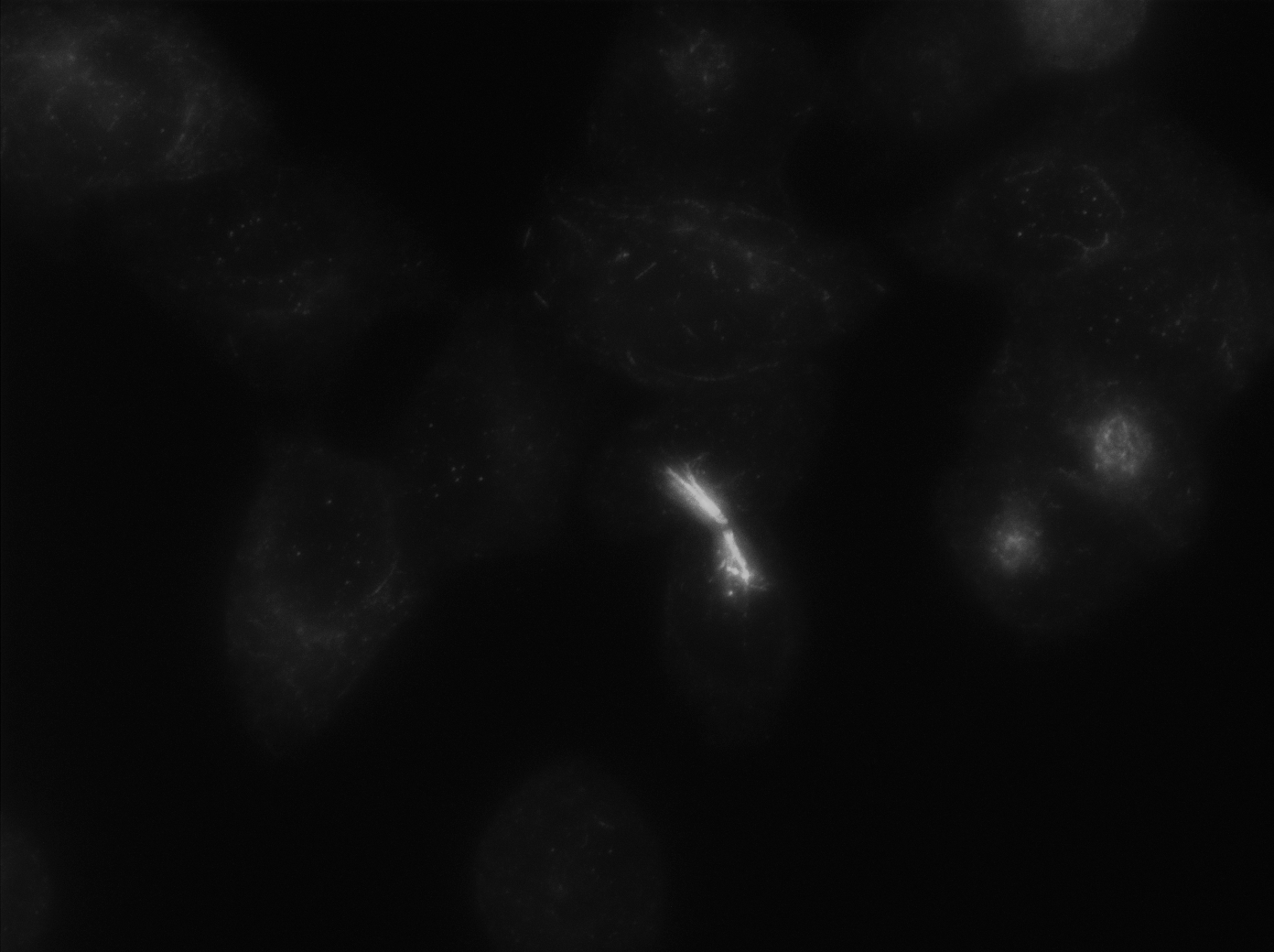

Supplement: Supplementary file 3 — Source data Fig. 1 [file 44319_2026_742_MOESM3_ESM.zip › Figure 1/Fig 1bcd WT Hela acetylated a tubulin atubulin/actub-atub 8-14-24 R3 LT5.Project Maximum Z_XY1724716758_Z0_T0_C2.tif]

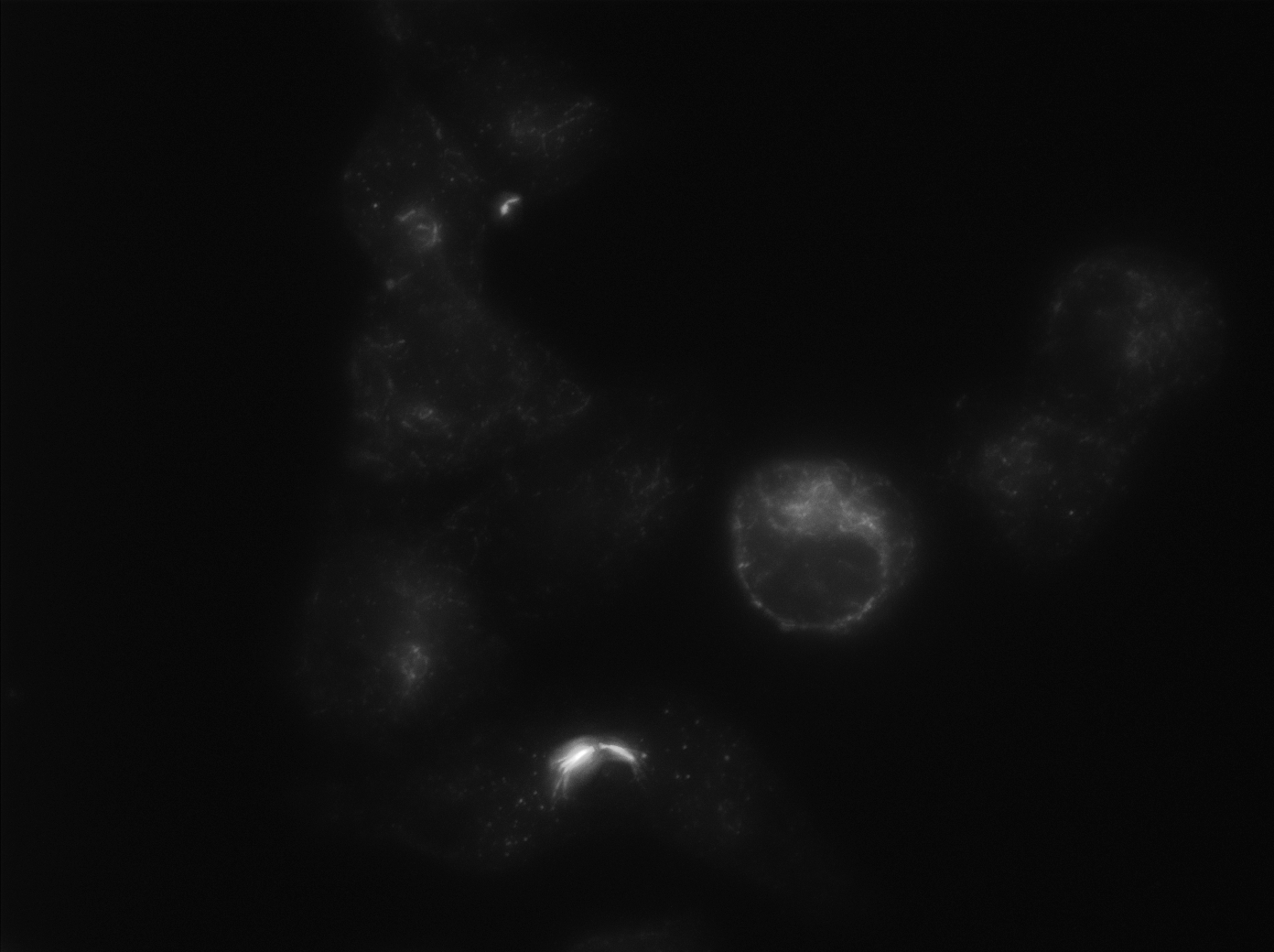

Supplement: Supplementary file 3 — Source data Fig. 1 [file 44319_2026_742_MOESM3_ESM.zip › Figure 1/Fig 1bcd WT Hela acetylated a tubulin atubulin/actub-atub 8-14-24 R1 LT6PA7.Project Maximum Z_XY1724364381_Z0_T0_C2.tif]

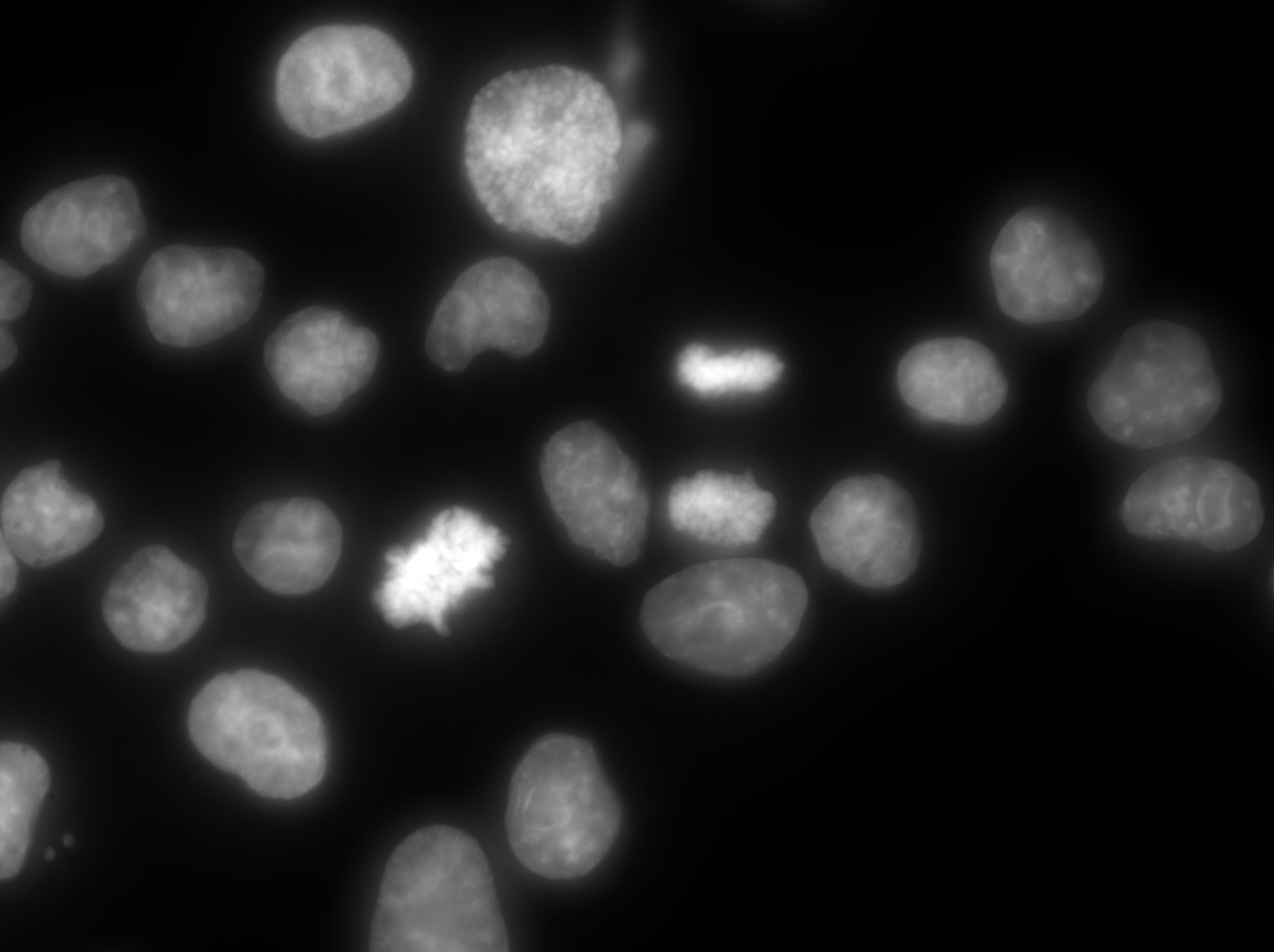

Supplement: Supplementary file 3 — Source data Fig. 1 [file 44319_2026_742_MOESM3_ESM.zip › Figure 1/Fig 1bcd WT Hela acetylated a tubulin atubulin/actub-atub 8-14-24 R3 M1 ET1.Project Maximum Z_XY1724701790_Z0_T0_C0.tif]

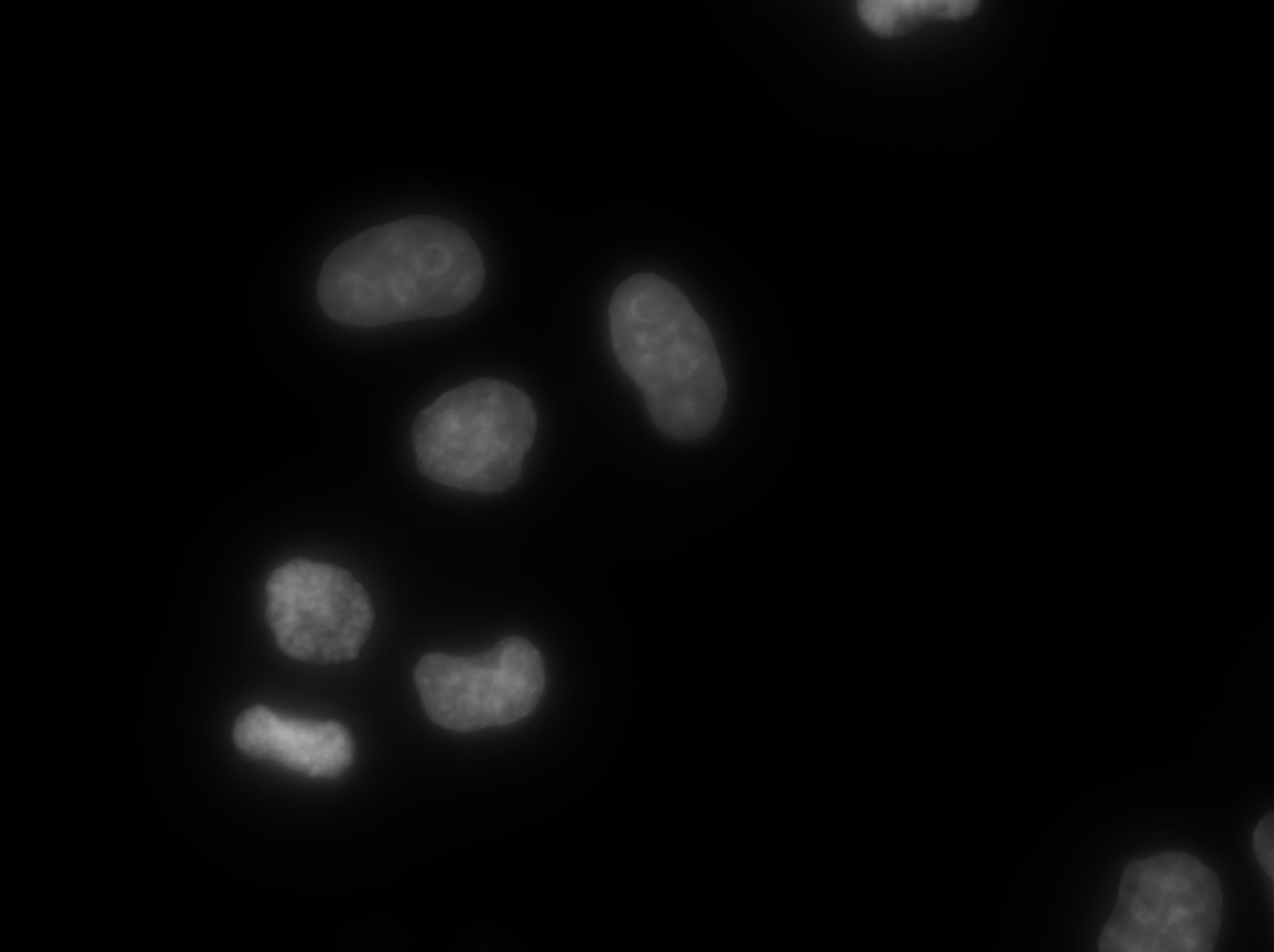

Supplement: Supplementary file 3 — Source data Fig. 1 [file 44319_2026_742_MOESM3_ESM.zip › Figure 1/Fig 1bcd WT Hela acetylated a tubulin atubulin/actub-atub 8-14-24 R2 ET5 LT4.Project Maximum Z_XY1724690247_Z0_T0_C0.tif]

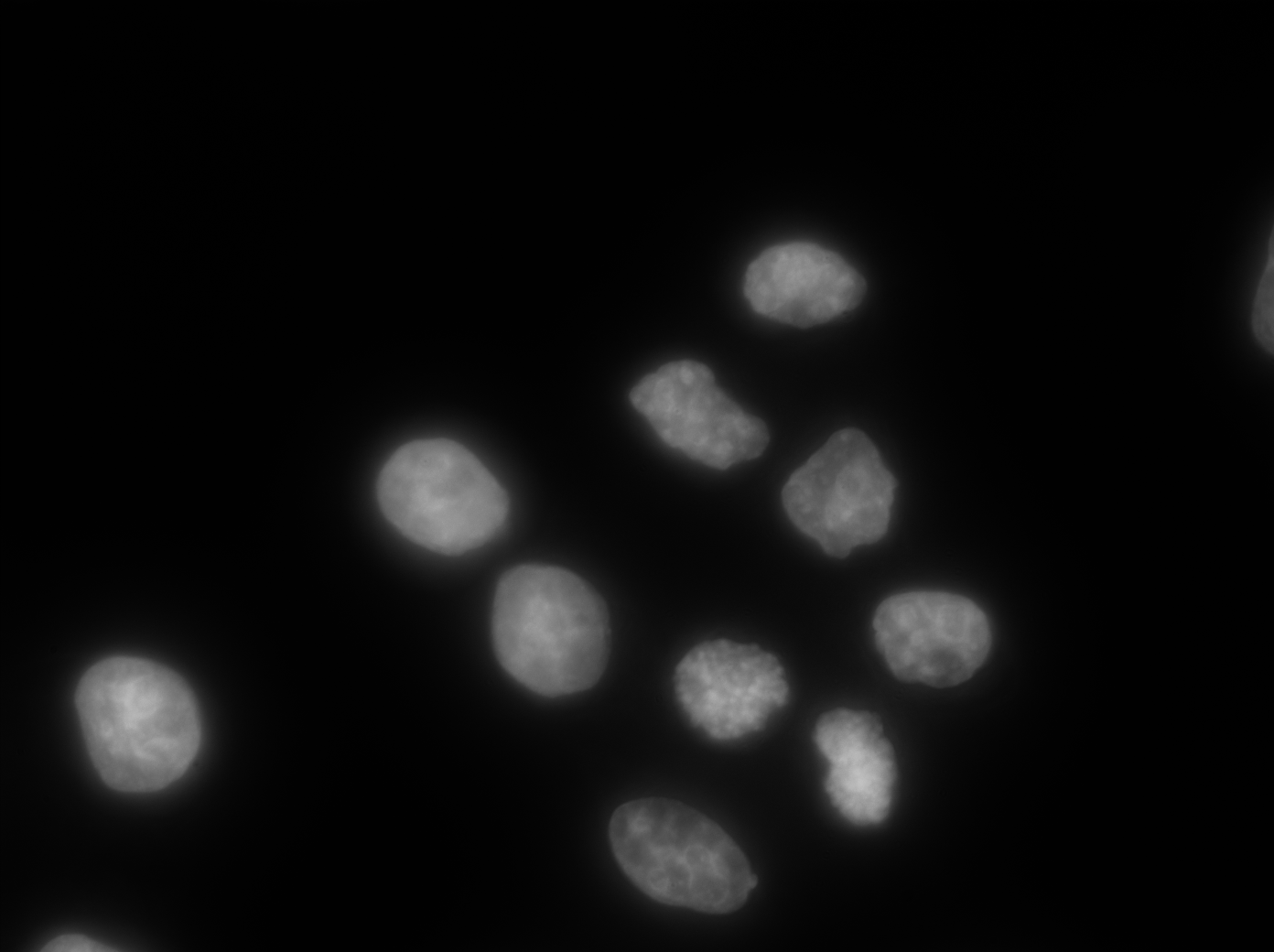

Supplement: Supplementary file 3 — Source data Fig. 1 [file 44319_2026_742_MOESM3_ESM.zip › Figure 1/Fig 1bcd WT Hela acetylated a tubulin atubulin/actub-atub 8-14-24 R1 ET5ET6.Project Maximum Z_XY1724363821_Z0_T0_C0.tif]

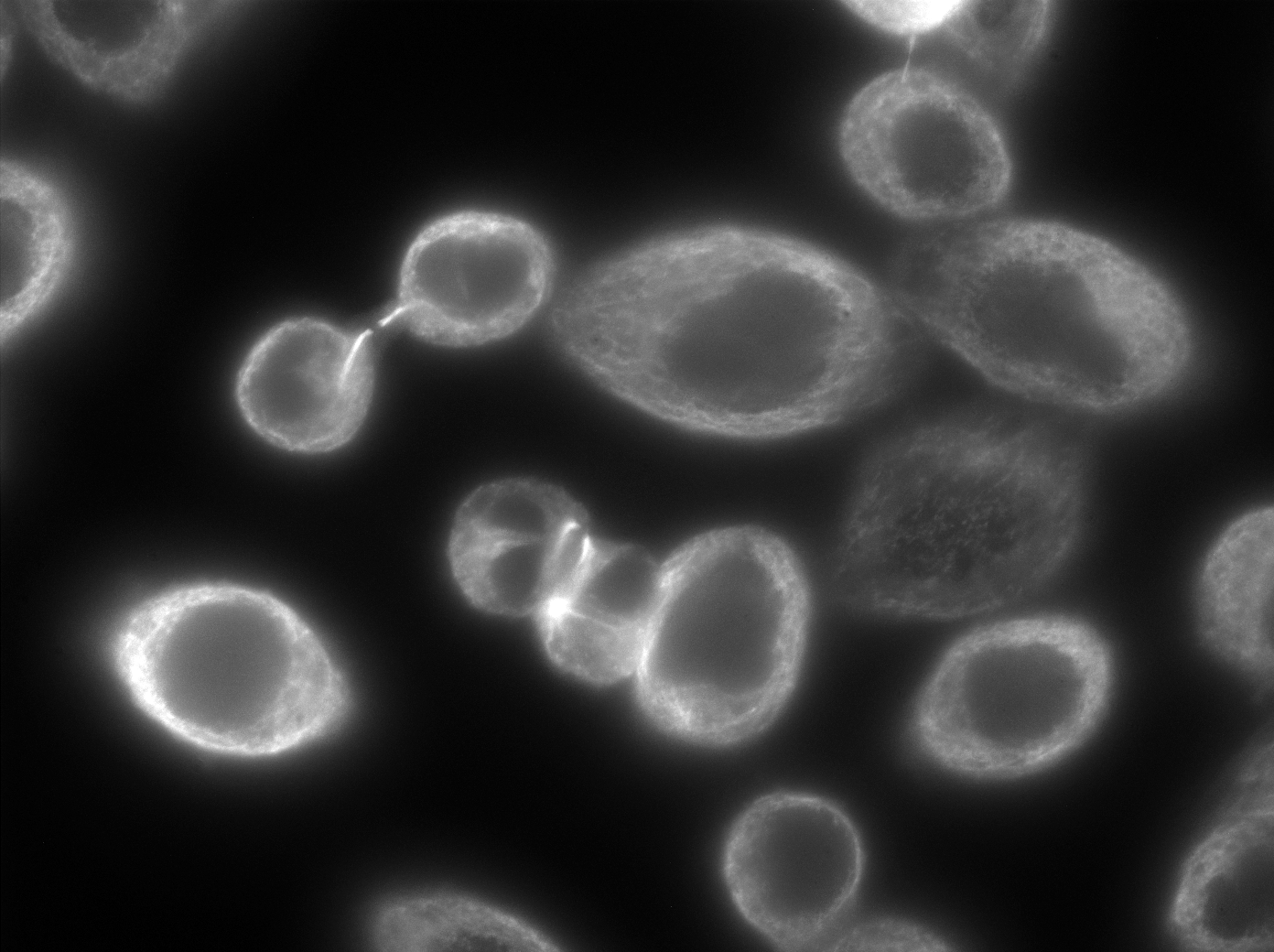

Supplement: Supplementary file 3 — Source data Fig. 1 [file 44319_2026_742_MOESM3_ESM.zip › Figure 1/Fig 1bcd WT Hela acetylated a tubulin atubulin/actub-atub 8-14-24 R3 ET4ET5.Project Maximum Z_XY1724703355_Z0_T0_C1.tif]

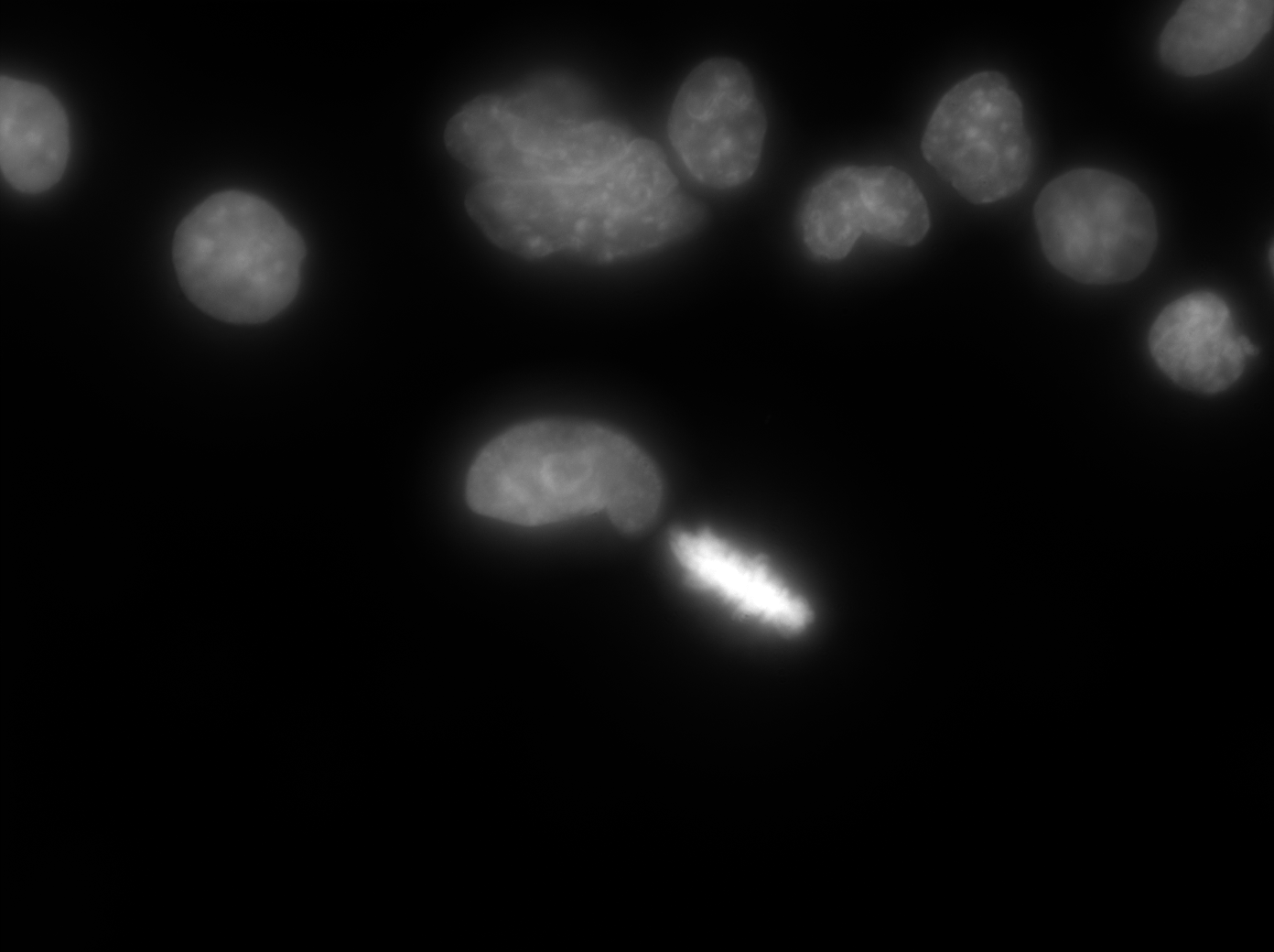

Supplement: Supplementary file 3 — Source data Fig. 1 [file 44319_2026_742_MOESM3_ESM.zip › Figure 1/Fig 1bcd WT Hela acetylated a tubulin atubulin/actub-atub 8-14-24 R3 M9.Project Maximum Z_XY1724703770_Z0_T0_C0.tif]

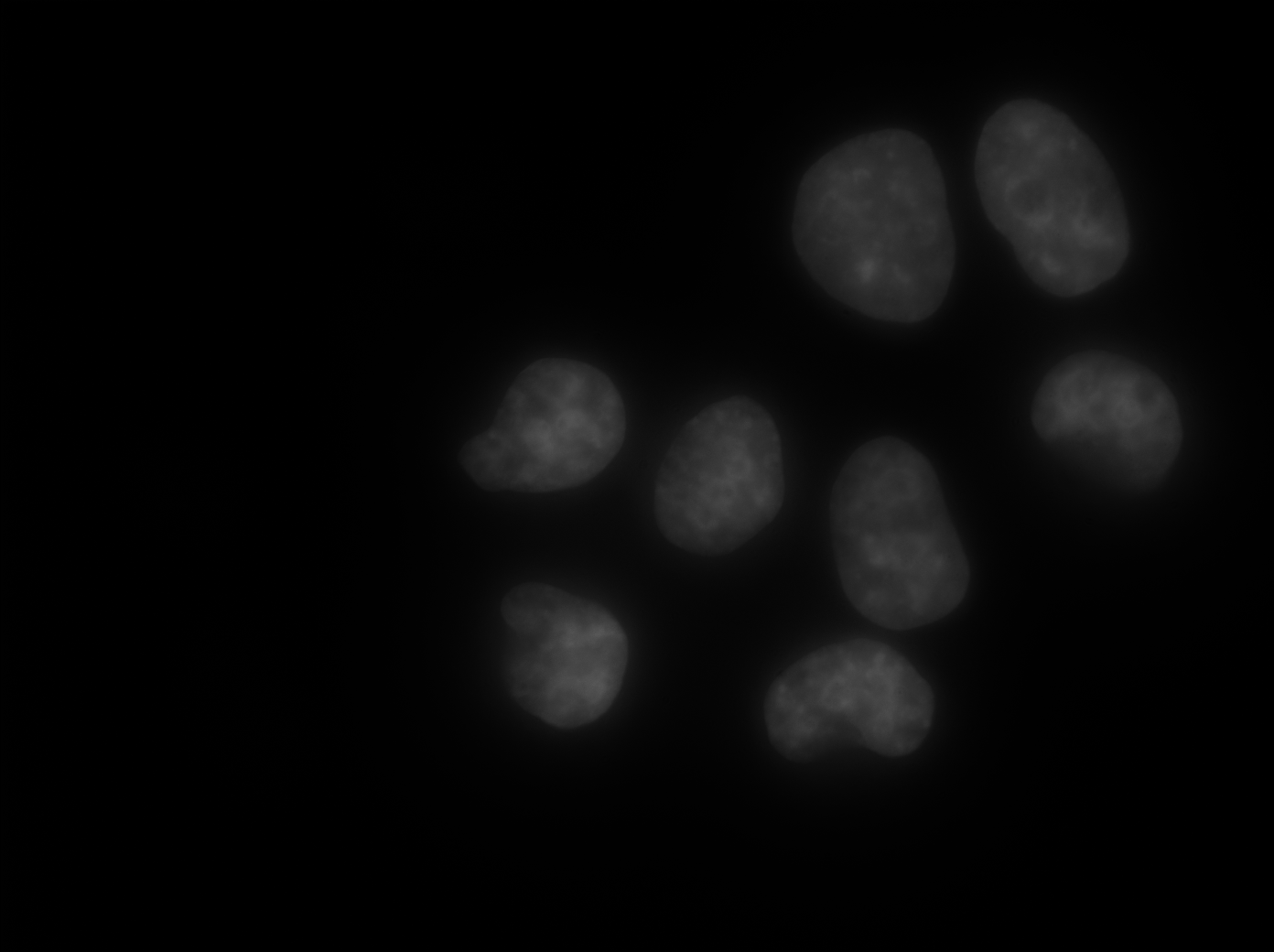

Supplement: Supplementary file 3 — Source data Fig. 1 [file 44319_2026_742_MOESM3_ESM.zip › Figure 1/Fig 1bcd WT Hela acetylated a tubulin atubulin/actub-atub 8-14-24 R1 LT4PA5.Project Maximum Z_XY1724363364_Z0_T0_C0.tif]

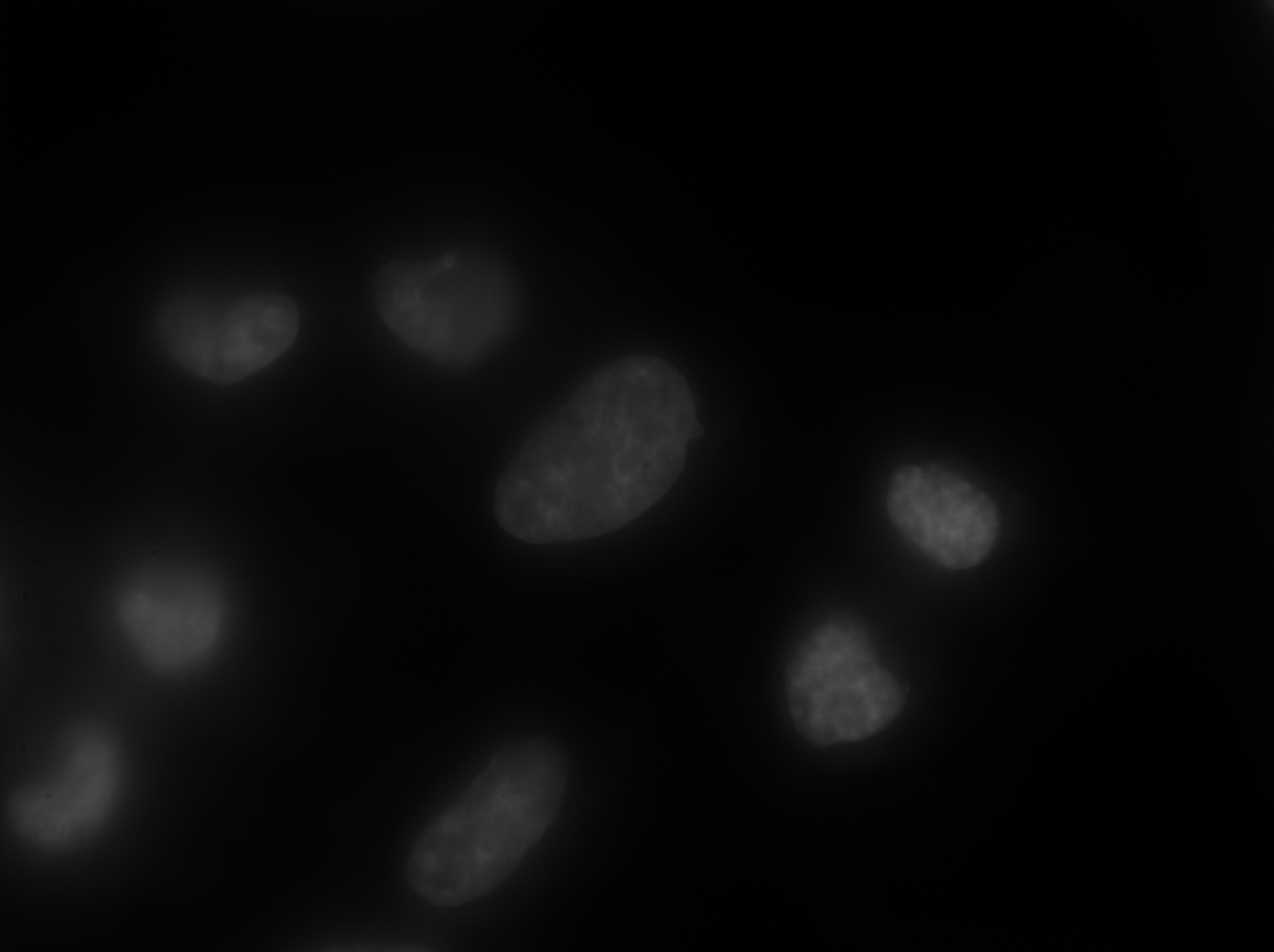

Supplement: Supplementary file 3 — Source data Fig. 1 [file 44319_2026_742_MOESM3_ESM.zip › Figure 1/Fig 1bcd WT Hela acetylated a tubulin atubulin/actub-atub 8-14-24 R1 LT7.Project Maximum Z_XY1724364950_Z0_T0_C0.tif]

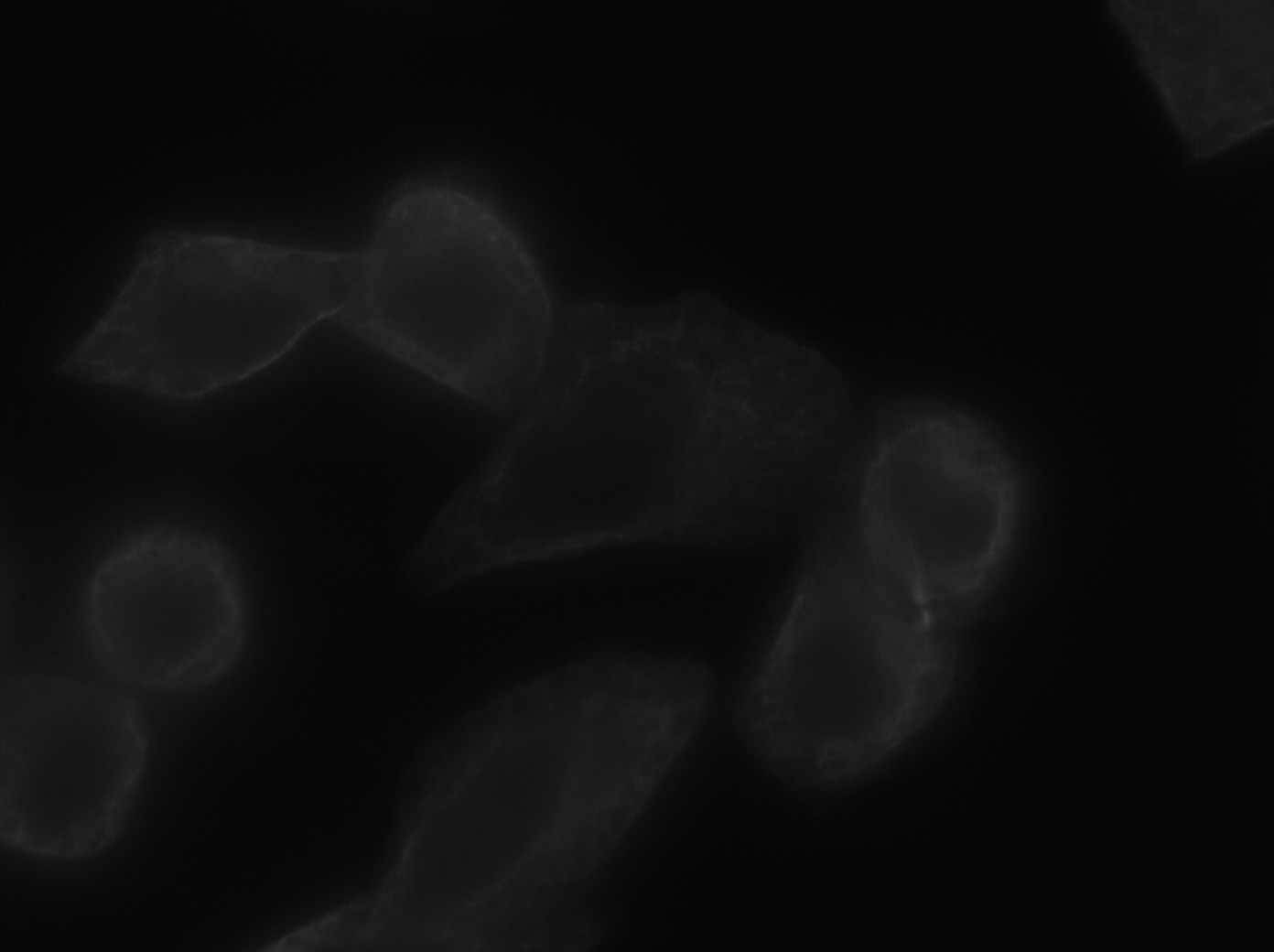

Supplement: Supplementary file 3 — Source data Fig. 1 [file 44319_2026_742_MOESM3_ESM.zip › Figure 1/Fig 1bcd WT Hela acetylated a tubulin atubulin/actub-atub 8-14-24 R1 LT7.Project Maximum Z_XY1724364950_Z0_T0_C1.tif]

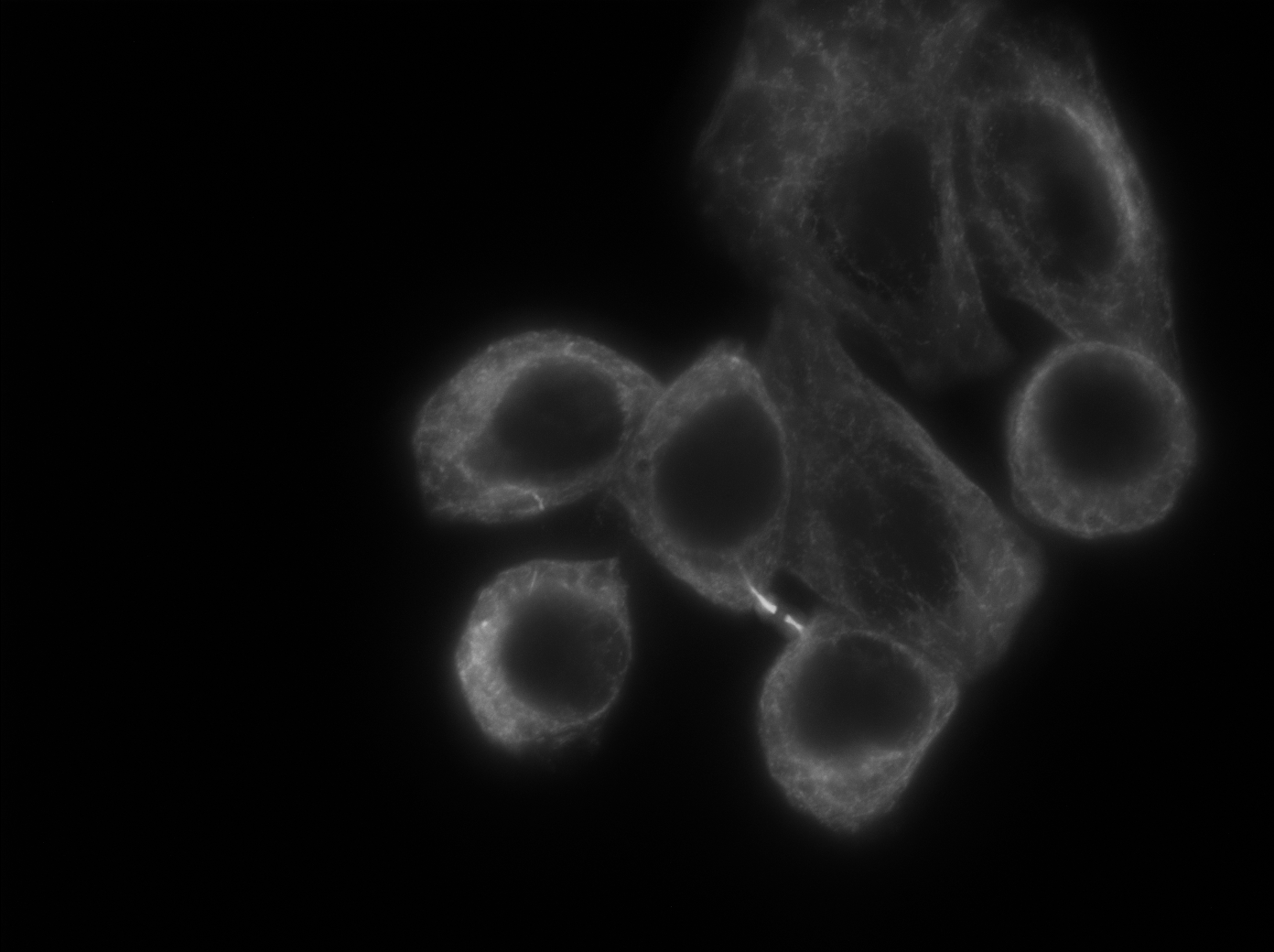

Supplement: Supplementary file 3 — Source data Fig. 1 [file 44319_2026_742_MOESM3_ESM.zip › Figure 1/Fig 1bcd WT Hela acetylated a tubulin atubulin/actub-atub 8-14-24 R1 LT4PA5.Project Maximum Z_XY1724363364_Z0_T0_C1.tif]

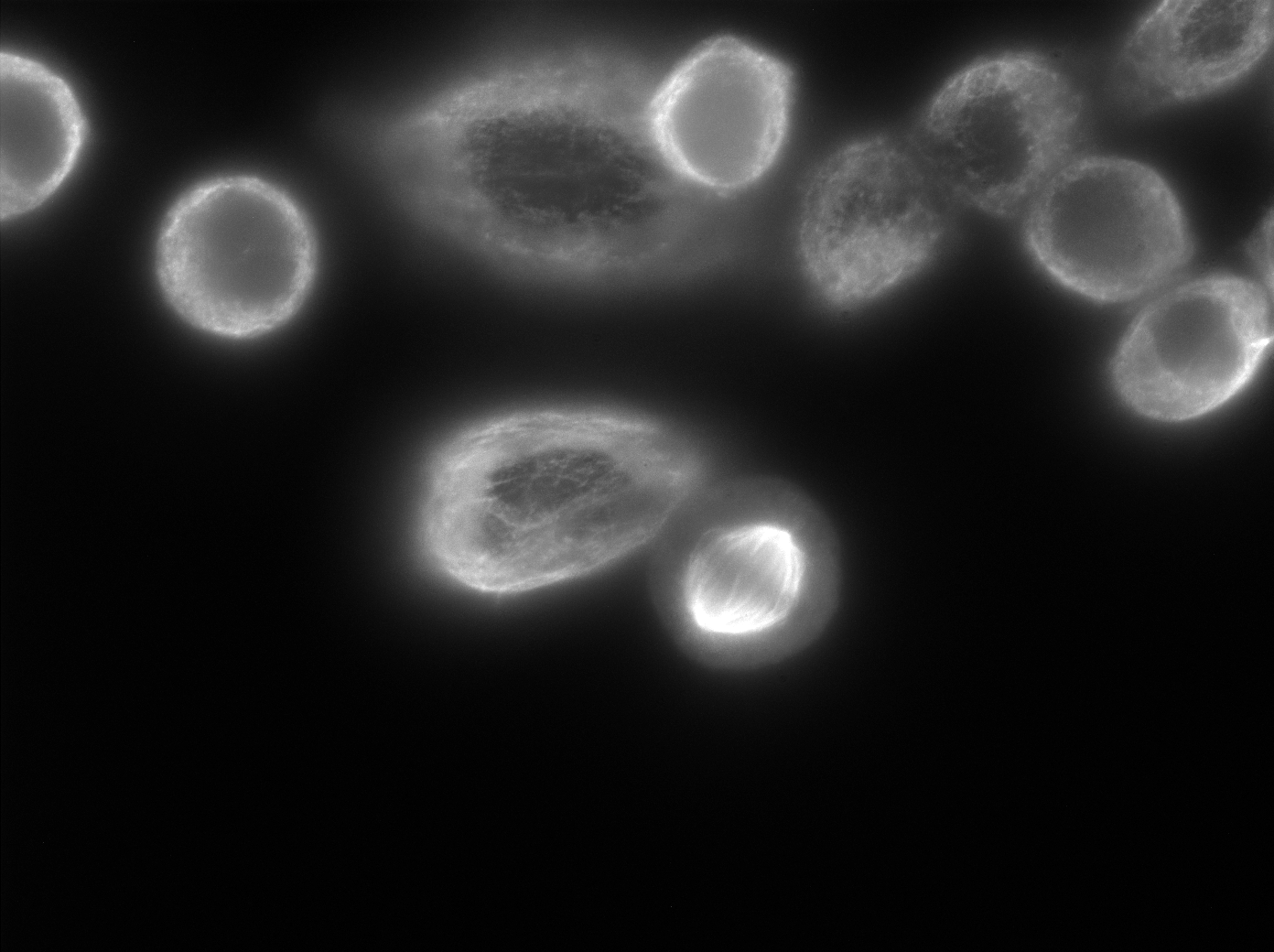

Supplement: Supplementary file 3 — Source data Fig. 1 [file 44319_2026_742_MOESM3_ESM.zip › Figure 1/Fig 1bcd WT Hela acetylated a tubulin atubulin/actub-atub 8-14-24 R3 M9.Project Maximum Z_XY1724703770_Z0_T0_C1.tif]

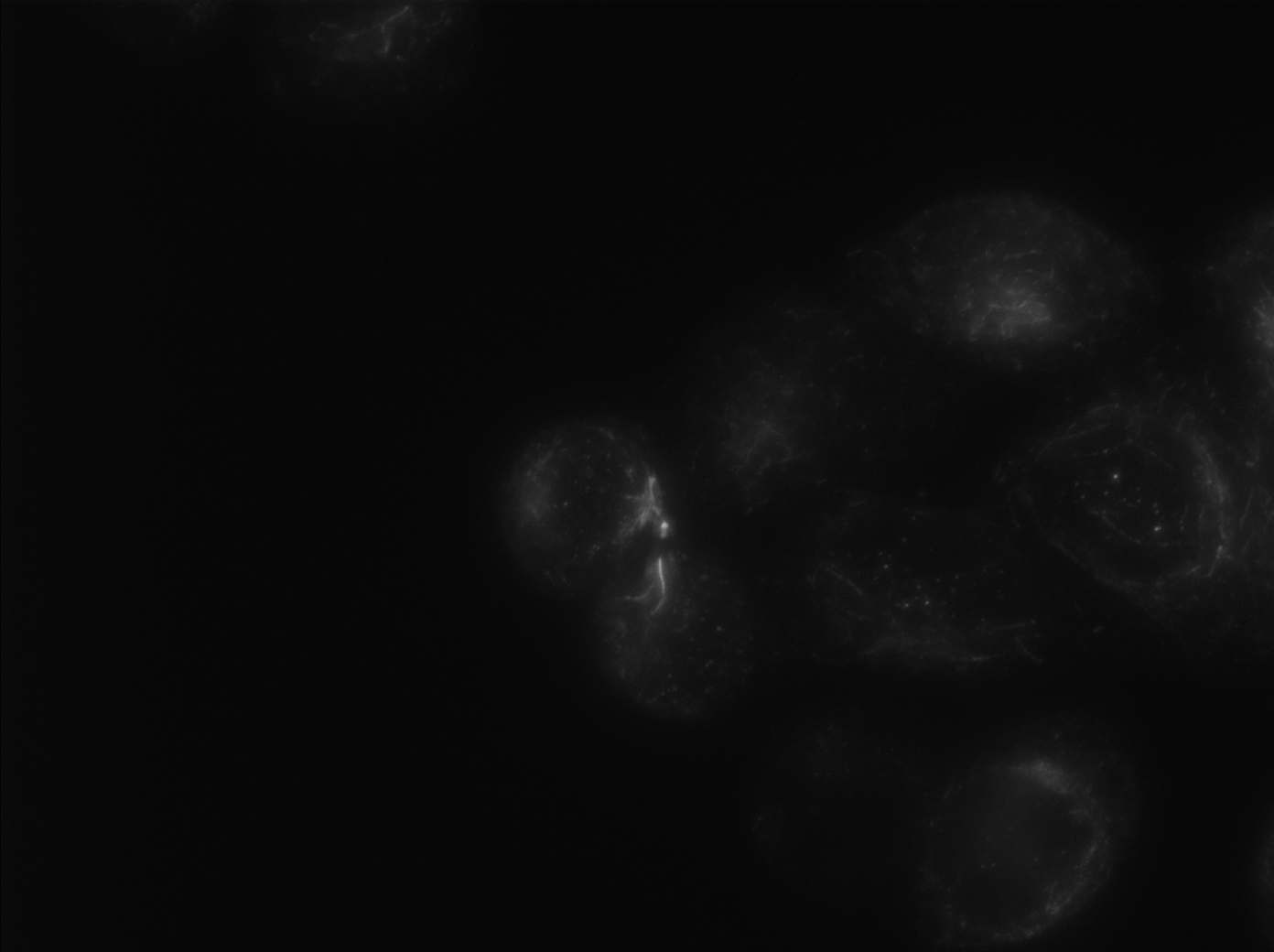

Supplement: Supplementary file 3 — Source data Fig. 1 [file 44319_2026_742_MOESM3_ESM.zip › Figure 1/Fig 1bcd WT Hela acetylated a tubulin atubulin/actub-atub 8-14-24 R2 PA9.Project Maximum Z_XY1724695303_Z0_T0_C2.tif]

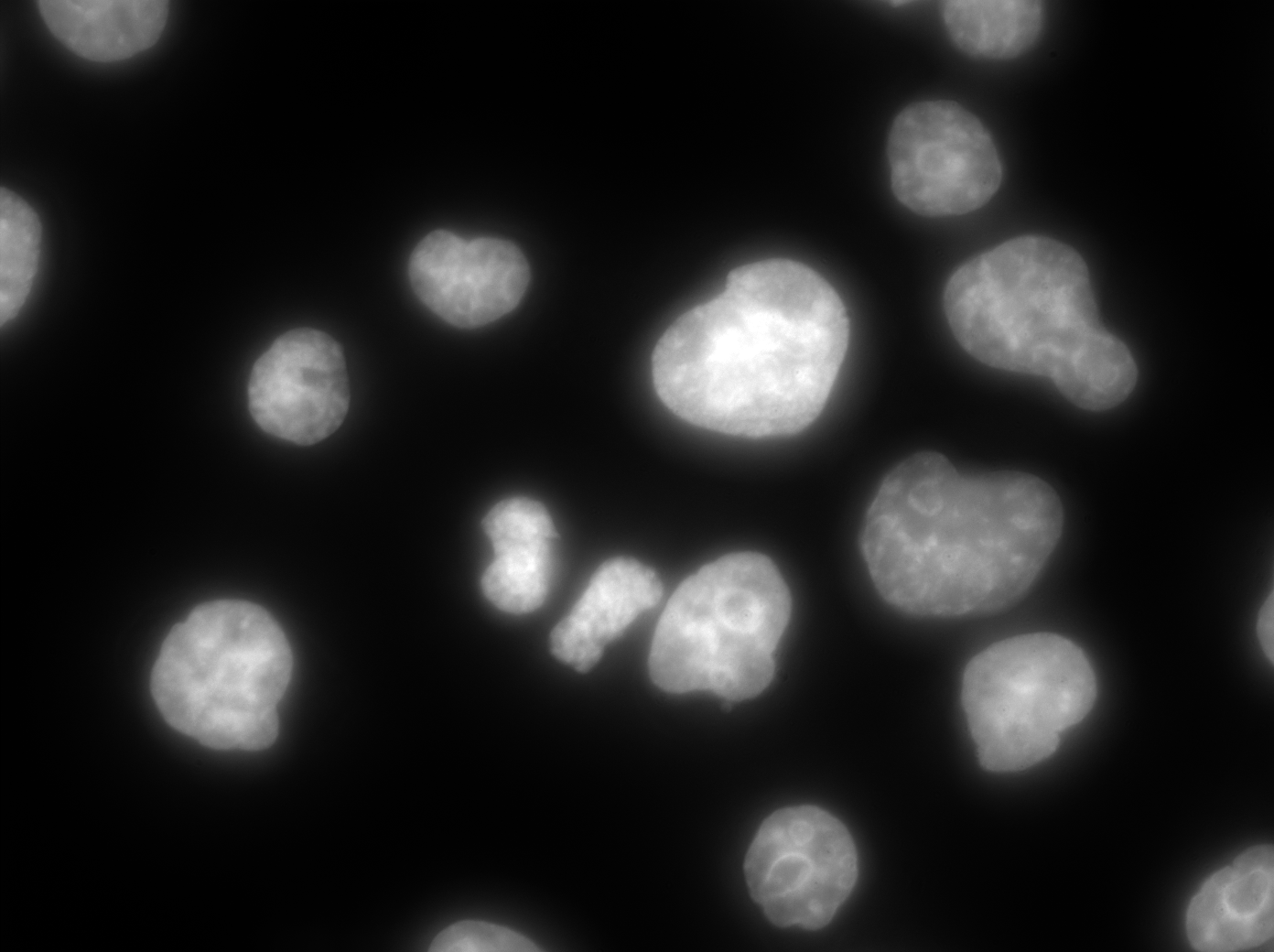

Supplement: Supplementary file 3 — Source data Fig. 1 [file 44319_2026_742_MOESM3_ESM.zip › Figure 1/Fig 1bcd WT Hela acetylated a tubulin atubulin/actub-atub 8-14-24 R3 ET4ET5.Project Maximum Z_XY1724703355_Z0_T0_C0.tif]

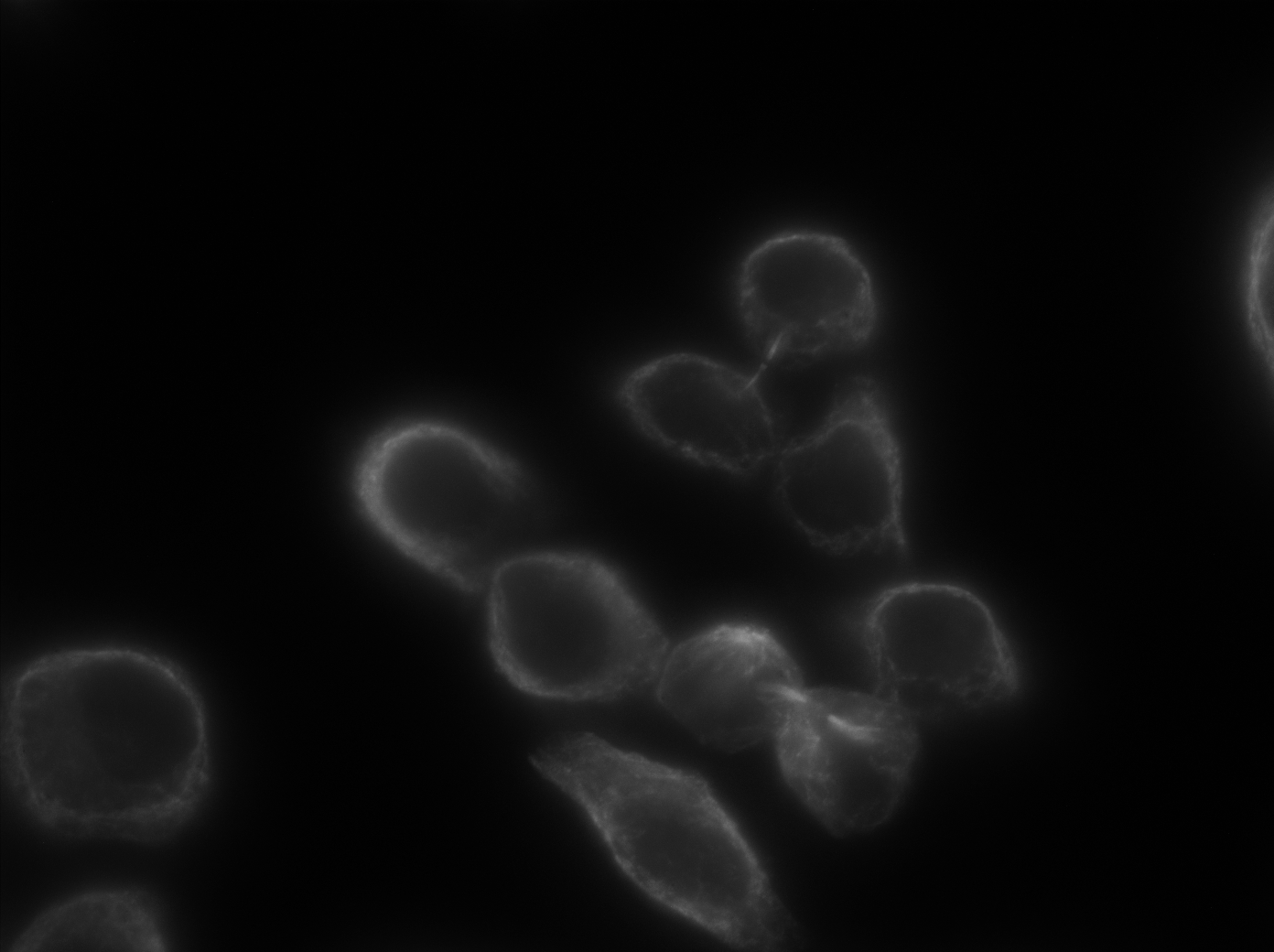

Supplement: Supplementary file 3 — Source data Fig. 1 [file 44319_2026_742_MOESM3_ESM.zip › Figure 1/Fig 1bcd WT Hela acetylated a tubulin atubulin/actub-atub 8-14-24 R1 ET5ET6.Project Maximum Z_XY1724363821_Z0_T0_C1.tif]

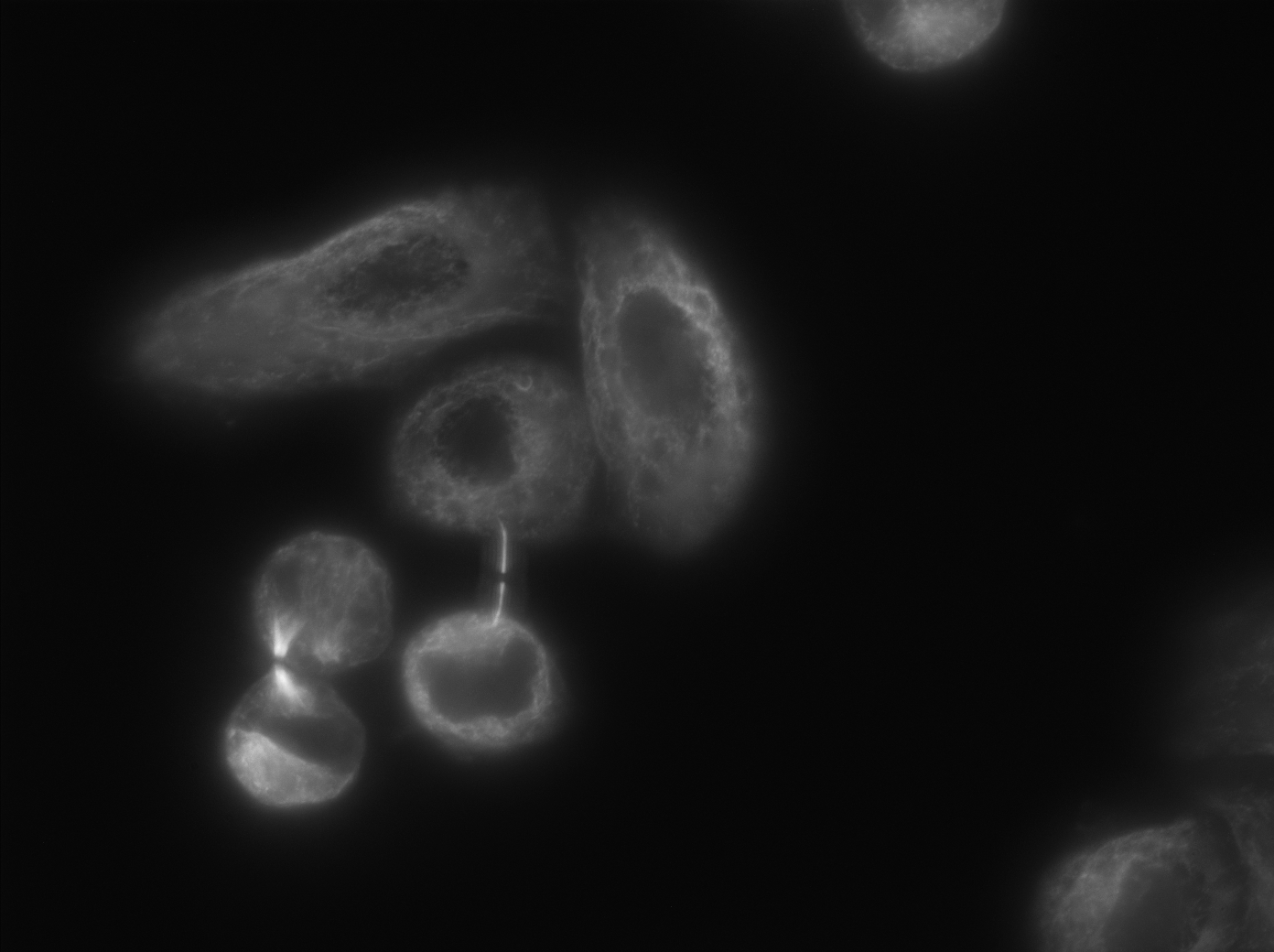

Supplement: Supplementary file 3 — Source data Fig. 1 [file 44319_2026_742_MOESM3_ESM.zip › Figure 1/Fig 1bcd WT Hela acetylated a tubulin atubulin/actub-atub 8-14-24 R2 ET5 LT4.Project Maximum Z_XY1724690247_Z0_T0_C1.tif]

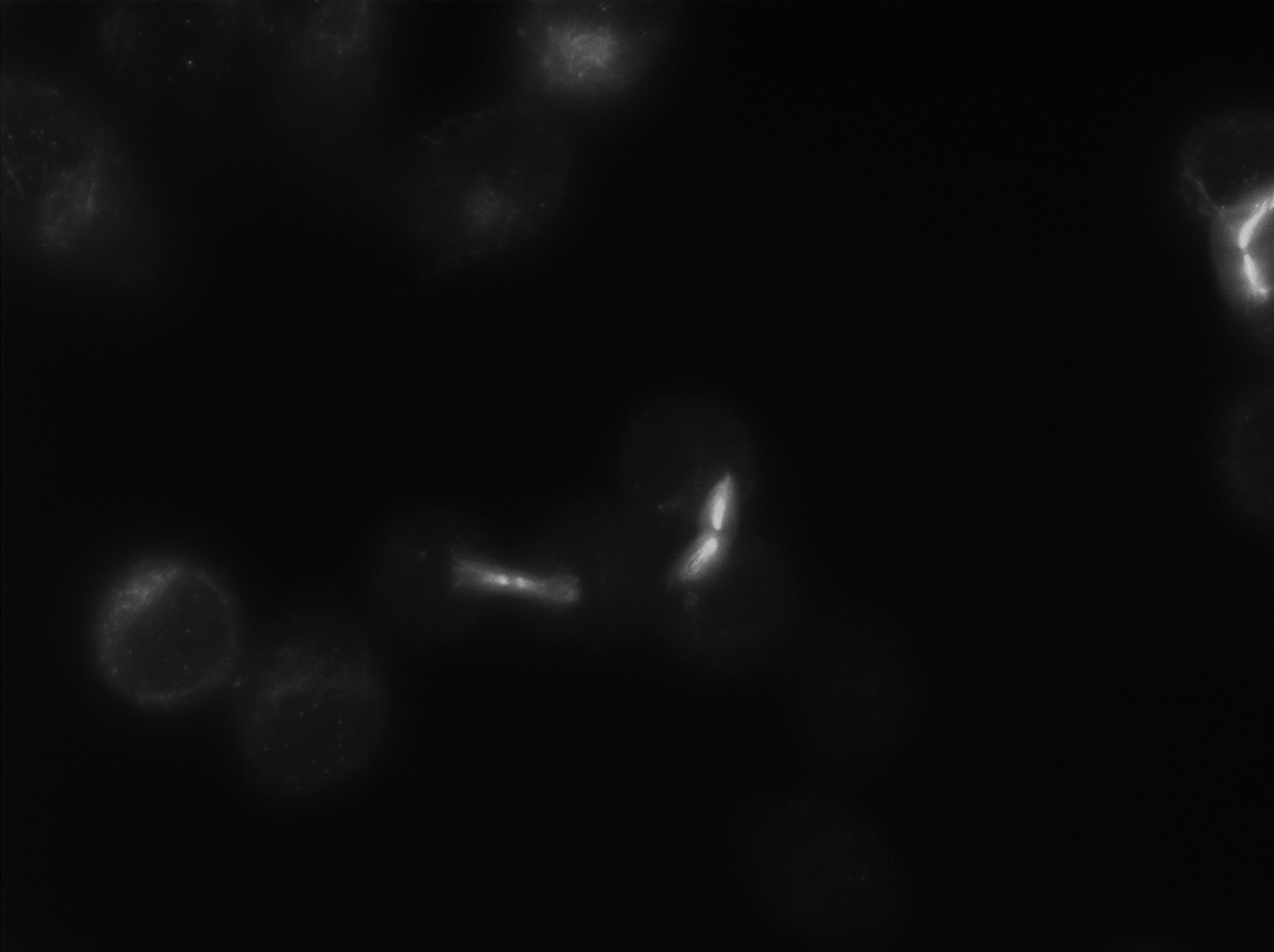

Supplement: Supplementary file 3 — Source data Fig. 1 [file 44319_2026_742_MOESM3_ESM.zip › Figure 1/Fig 1bcd WT Hela acetylated a tubulin atubulin/actub-atub 8-14-24 R3 ET9ET10.Project Maximum Z_XY1724704359_Z0_T0_C2.tif]
