# Supplementary figures and images for: TPGS1 regulates central spindle microtubule glutamylation and remodeling during telophase and abscission (part 4 of 36)
Source: EMBO Rep. 2026 Mar 23;27(8):1944–63. doi: 10.1038/s44319-026-00742-3 (PMC13121839; doi:10.1038/s44319-026-00742-3)

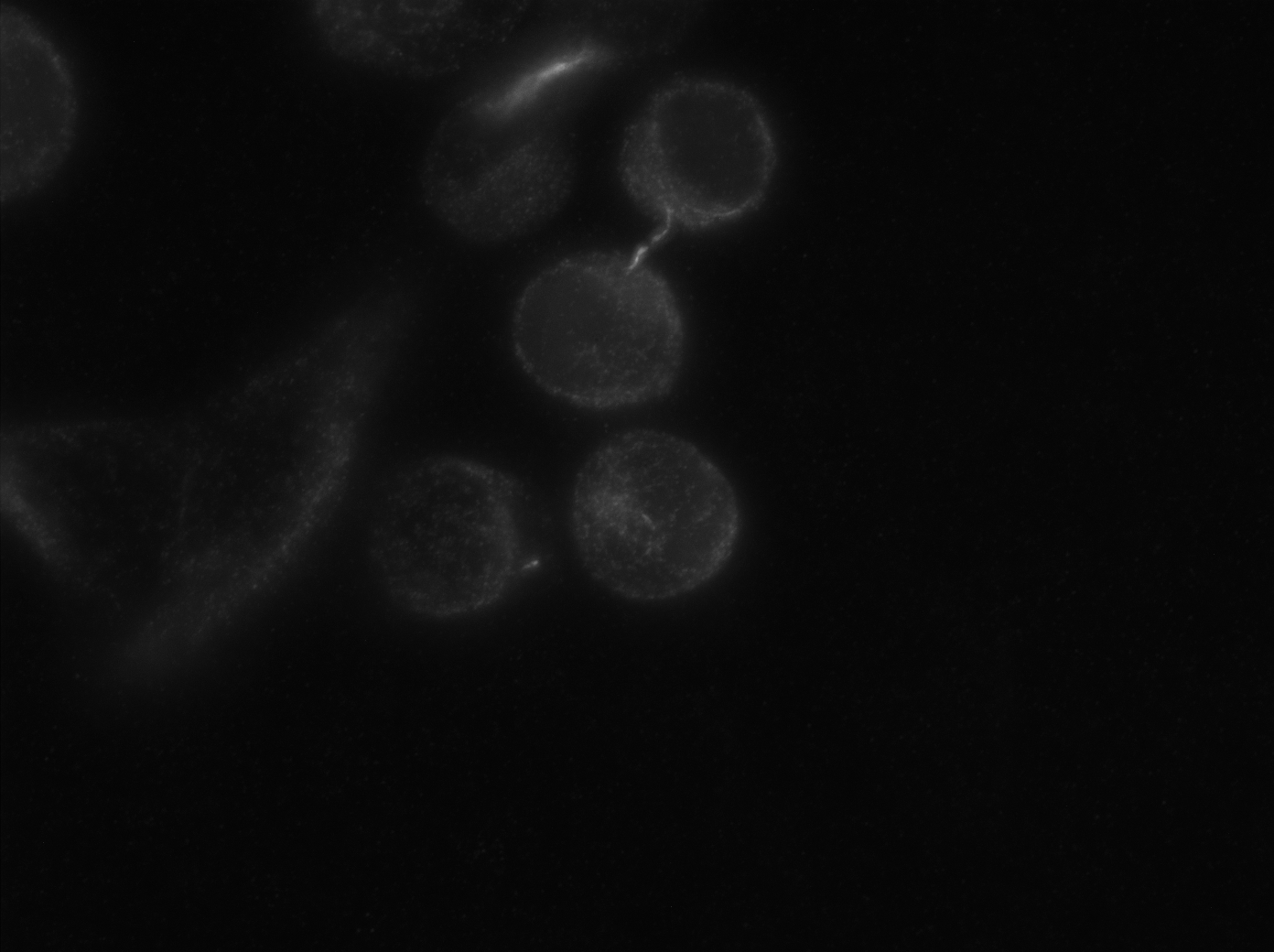

Supplement: Supplementary file 4 — Source data Fig. 2 part 1 [file 44319_2026_742_MOESM4_ESM.zip › Figure 2 Part 1/Fig 2c Cas9 Hela rGT335 atubulin/Cas9 GT335recomb atub 3-24-25 R2 LT6 PA4.Project Maximum Z_XY1743441273_Z0_T0_C2.tif]

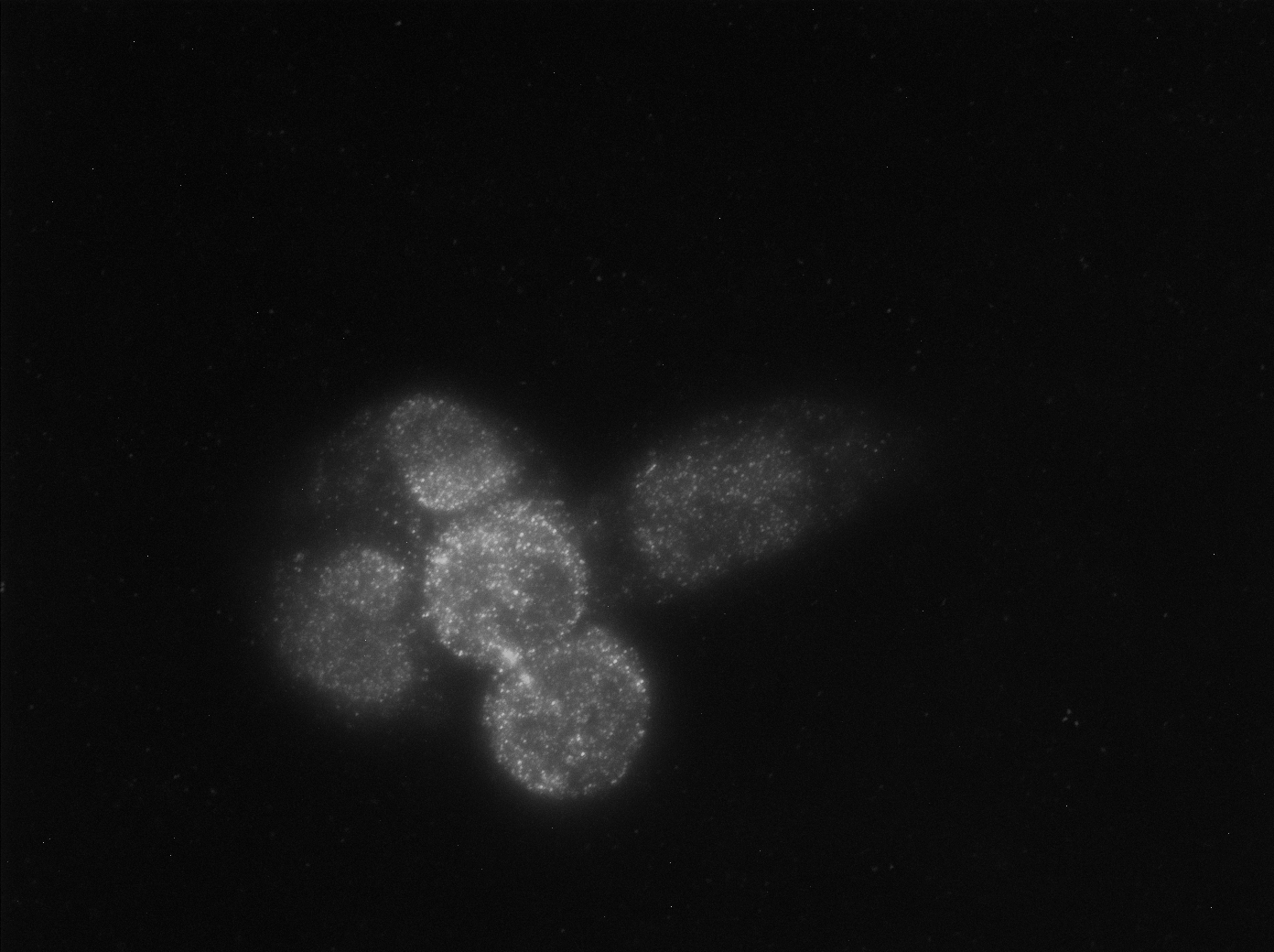

Supplement: Supplementary file 4 — Source data Fig. 2 part 1 [file 44319_2026_742_MOESM4_ESM.zip › Figure 2 Part 1/Fig 2c Cas9 Hela rGT335 atubulin/Cas9 GT335recomb atub 3-24-25 R3 ET3.Project Maximum Z_XY1743452941_Z0_T0_C1.tif]

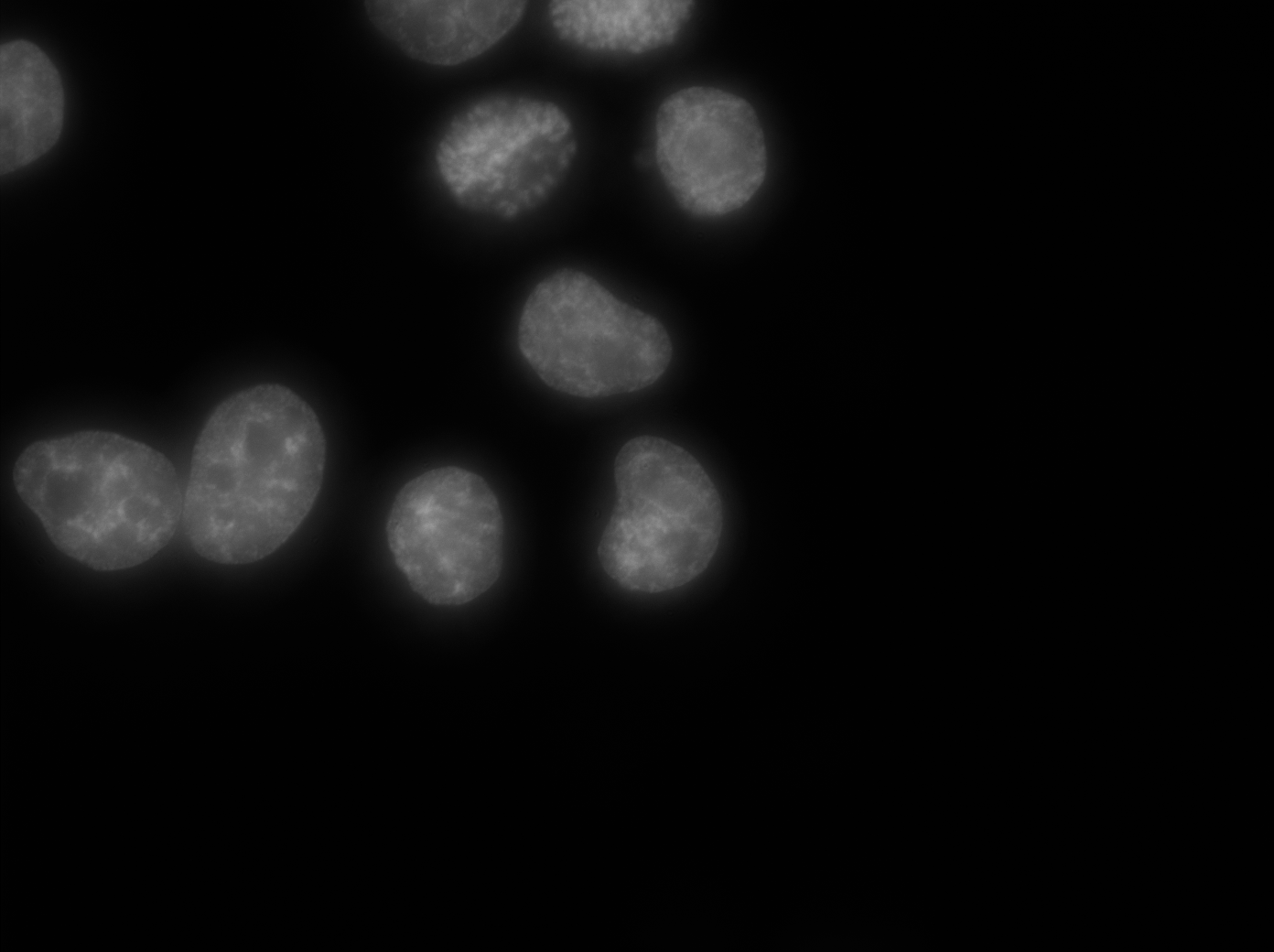

Supplement: Supplementary file 4 — Source data Fig. 2 part 1 [file 44319_2026_742_MOESM4_ESM.zip › Figure 2 Part 1/Fig 2c Cas9 Hela rGT335 atubulin/Cas9 GT335recomb atub 3-24-25 R2 LT6 PA4.Project Maximum Z_XY1743441273_Z0_T0_C0.tif]

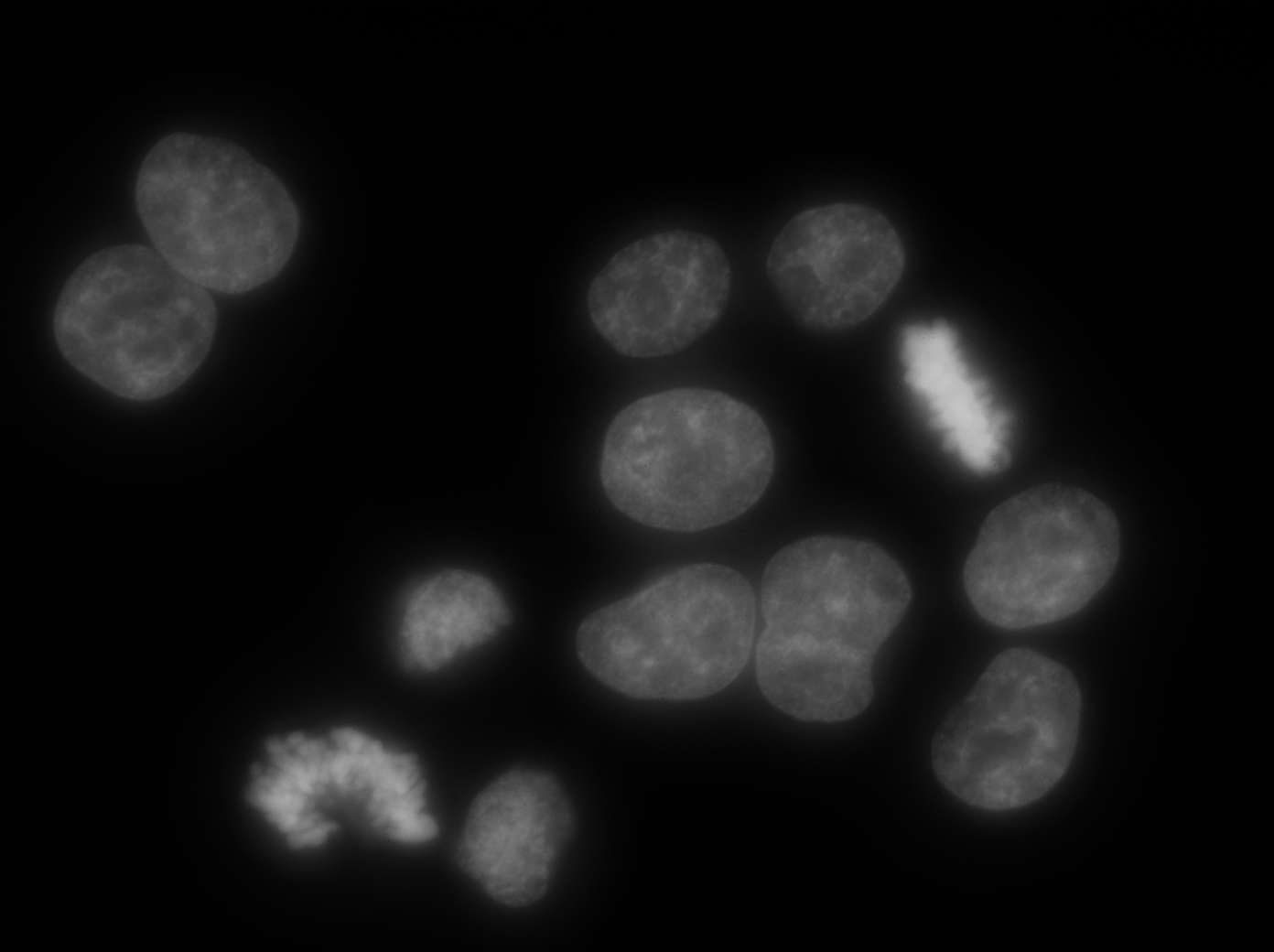

Supplement: Supplementary file 4 — Source data Fig. 2 part 1 [file 44319_2026_742_MOESM4_ESM.zip › Figure 2 Part 1/Fig 2c Cas9 Hela rGT335 atubulin/Cas9 GT335recomb atub 3-24-25 R1 PA1 M1.Project Maximum Z_XY1742835552_Z0_T0_C0.tif]

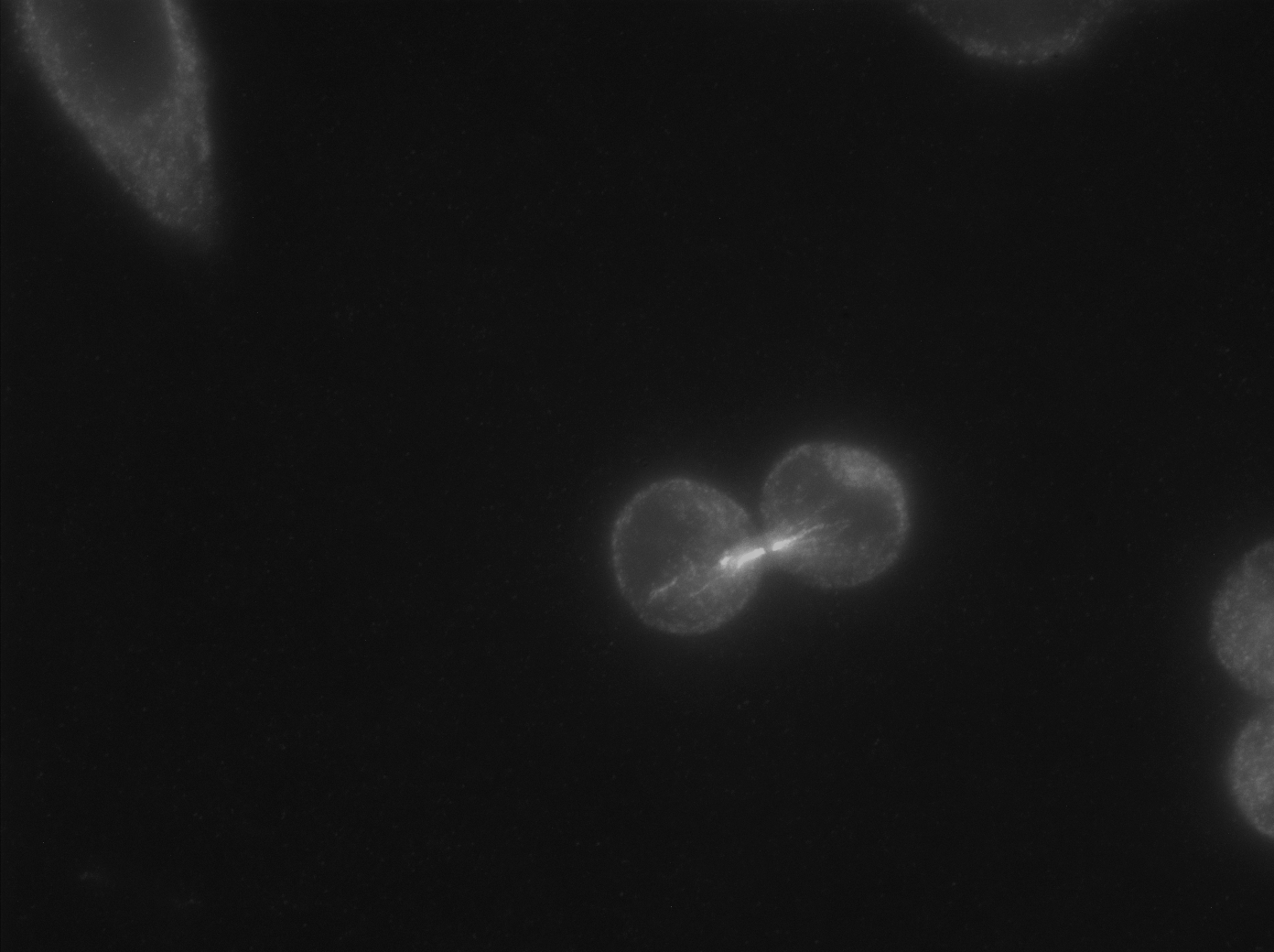

Supplement: Supplementary file 4 — Source data Fig. 2 part 1 [file 44319_2026_742_MOESM4_ESM.zip › Figure 2 Part 1/Fig 2c Cas9 Hela rGT335 atubulin/Cas9 GT335recomb atub 3-24-25 R1 ET3.Project Maximum Z_XY1743101375_Z0_T0_C2.tif]

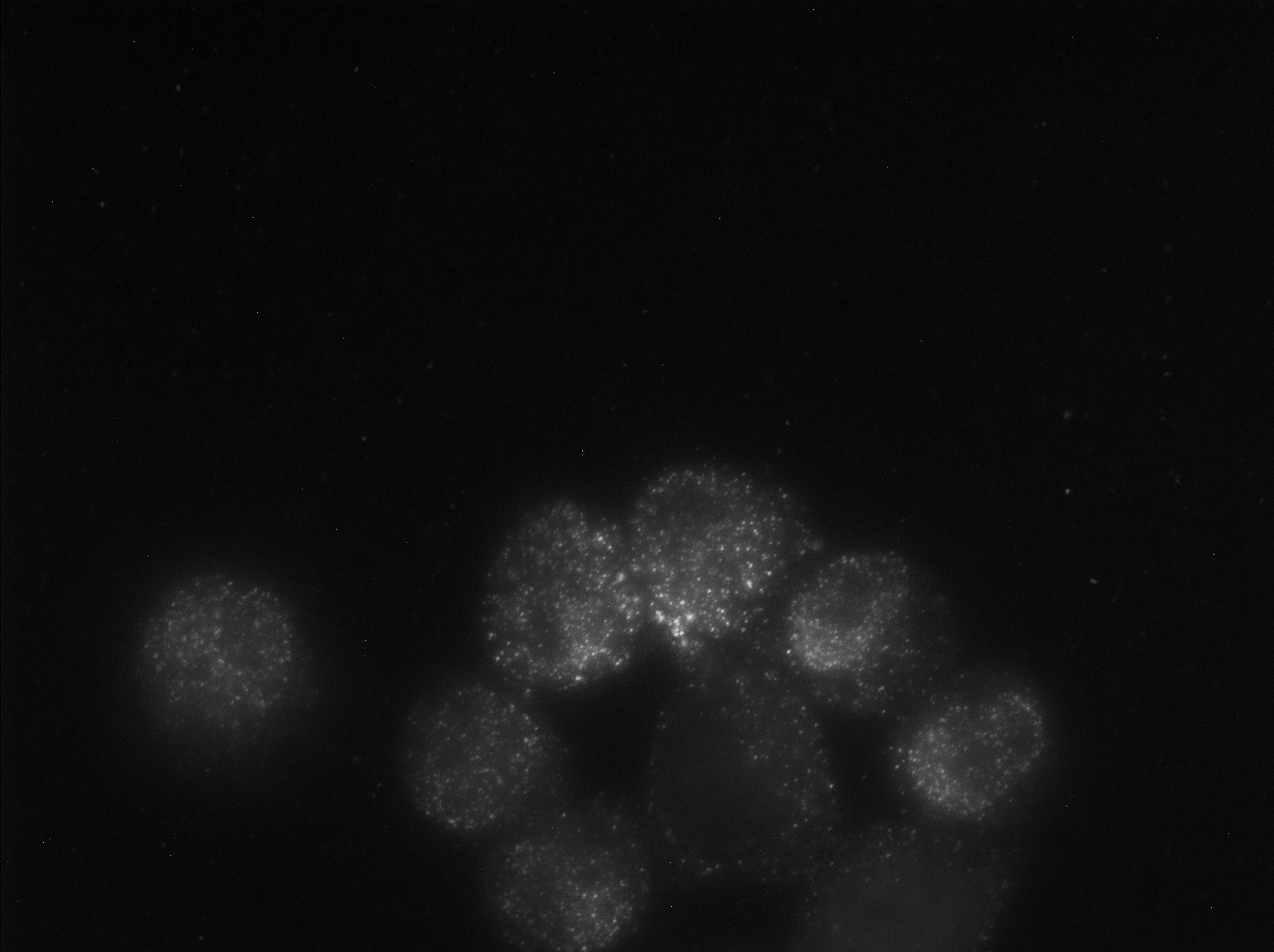

Supplement: Supplementary file 4 — Source data Fig. 2 part 1 [file 44319_2026_742_MOESM4_ESM.zip › Figure 2 Part 1/Fig 2c Cas9 Hela rGT335 atubulin/Cas9 GT335recomb atub 3-24-25 R3 LT3.Project Maximum Z_XY1743451368_Z0_T0_C1.tif]

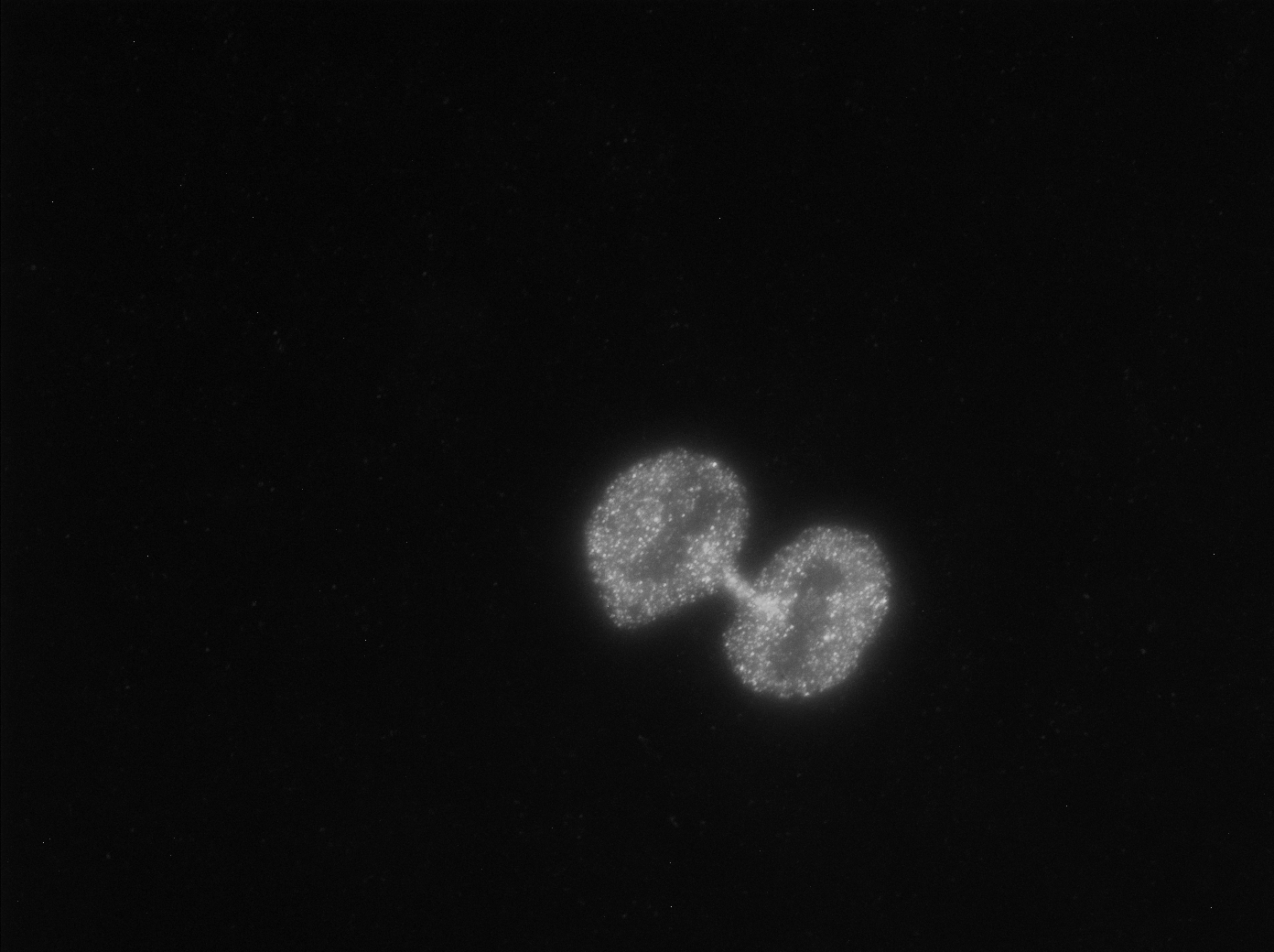

Supplement: Supplementary file 4 — Source data Fig. 2 part 1 [file 44319_2026_742_MOESM4_ESM.zip › Figure 2 Part 1/Fig 2c Cas9 Hela rGT335 atubulin/Cas9 GT335recomb atub 3-24-25 R2 ET1.Project Maximum Z_XY1743439798_Z0_T0_C1.tif]

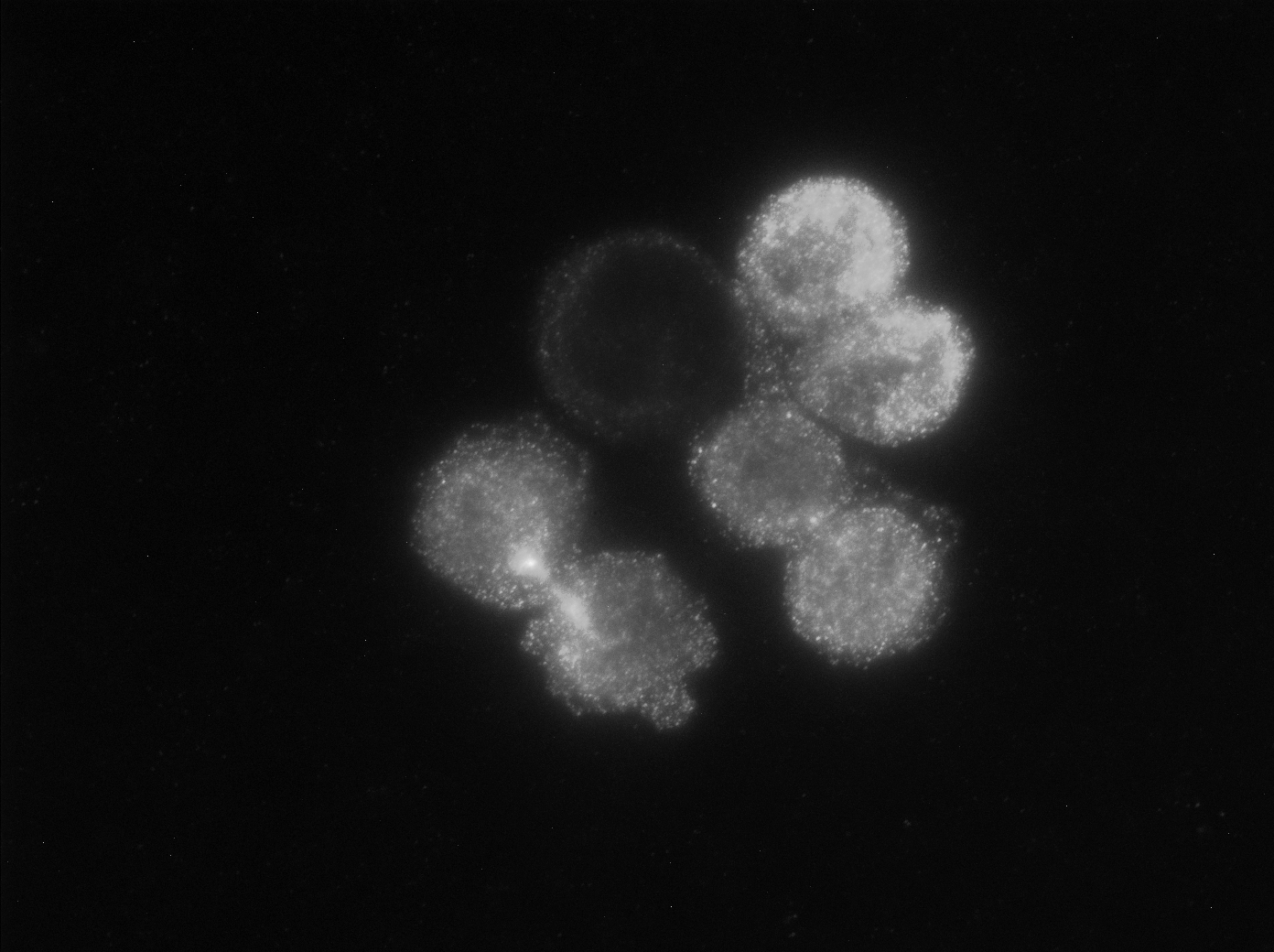

Supplement: Supplementary file 4 — Source data Fig. 2 part 1 [file 44319_2026_742_MOESM4_ESM.zip › Figure 2 Part 1/Fig 2c Cas9 Hela rGT335 atubulin/Cas9 GT335recomb atub 3-24-25 R2 A6.Project Maximum Z_XY1743446093_Z0_T0_C1.tif]

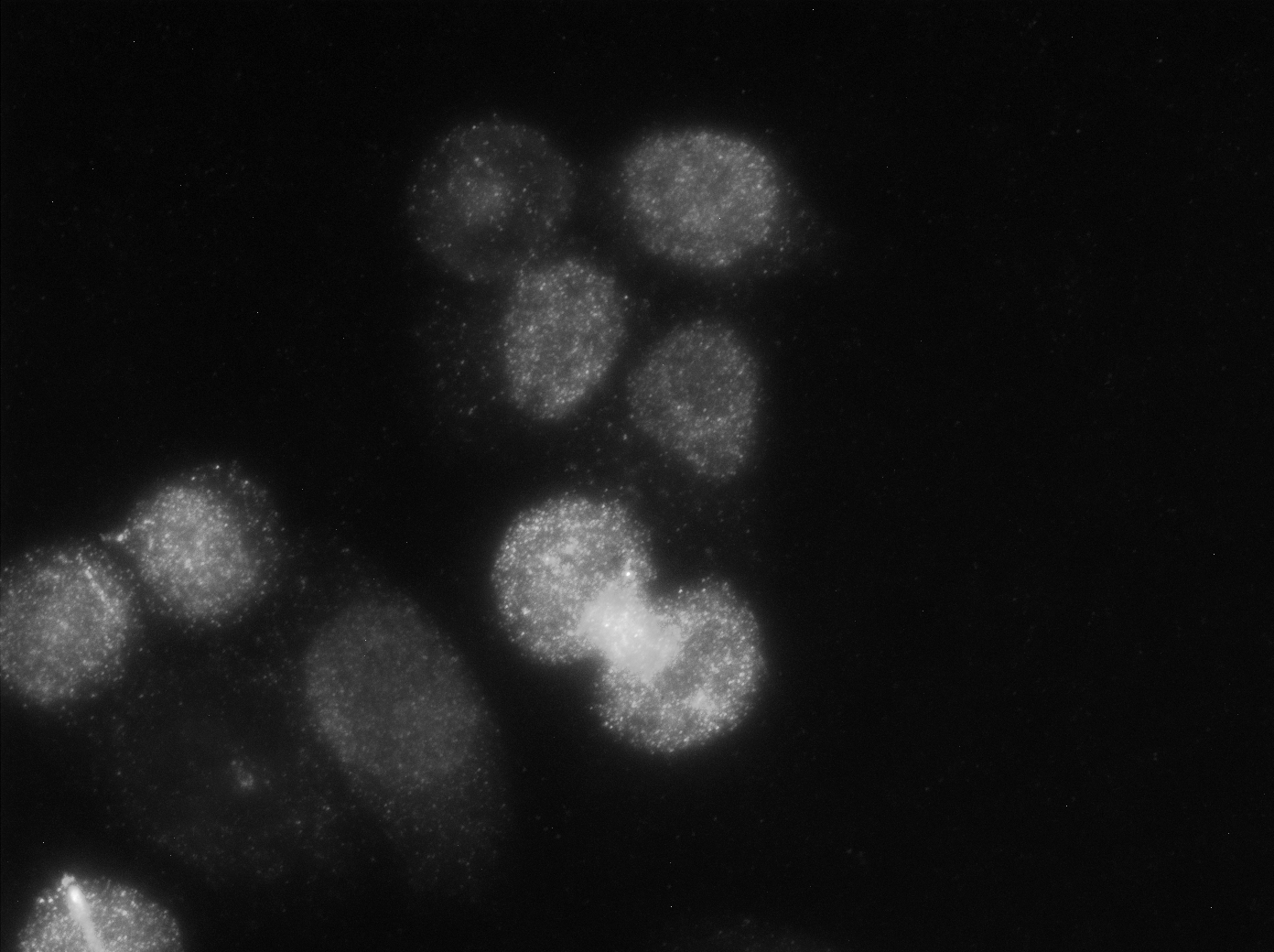

Supplement: Supplementary file 4 — Source data Fig. 2 part 1 [file 44319_2026_742_MOESM4_ESM.zip › Figure 2 Part 1/Fig 2c Cas9 Hela rGT335 atubulin/Cas9 GT335recomb atub 3-24-25 R2 preET2.Project Maximum Z_XY1743447696_Z0_T0_C1.tif]

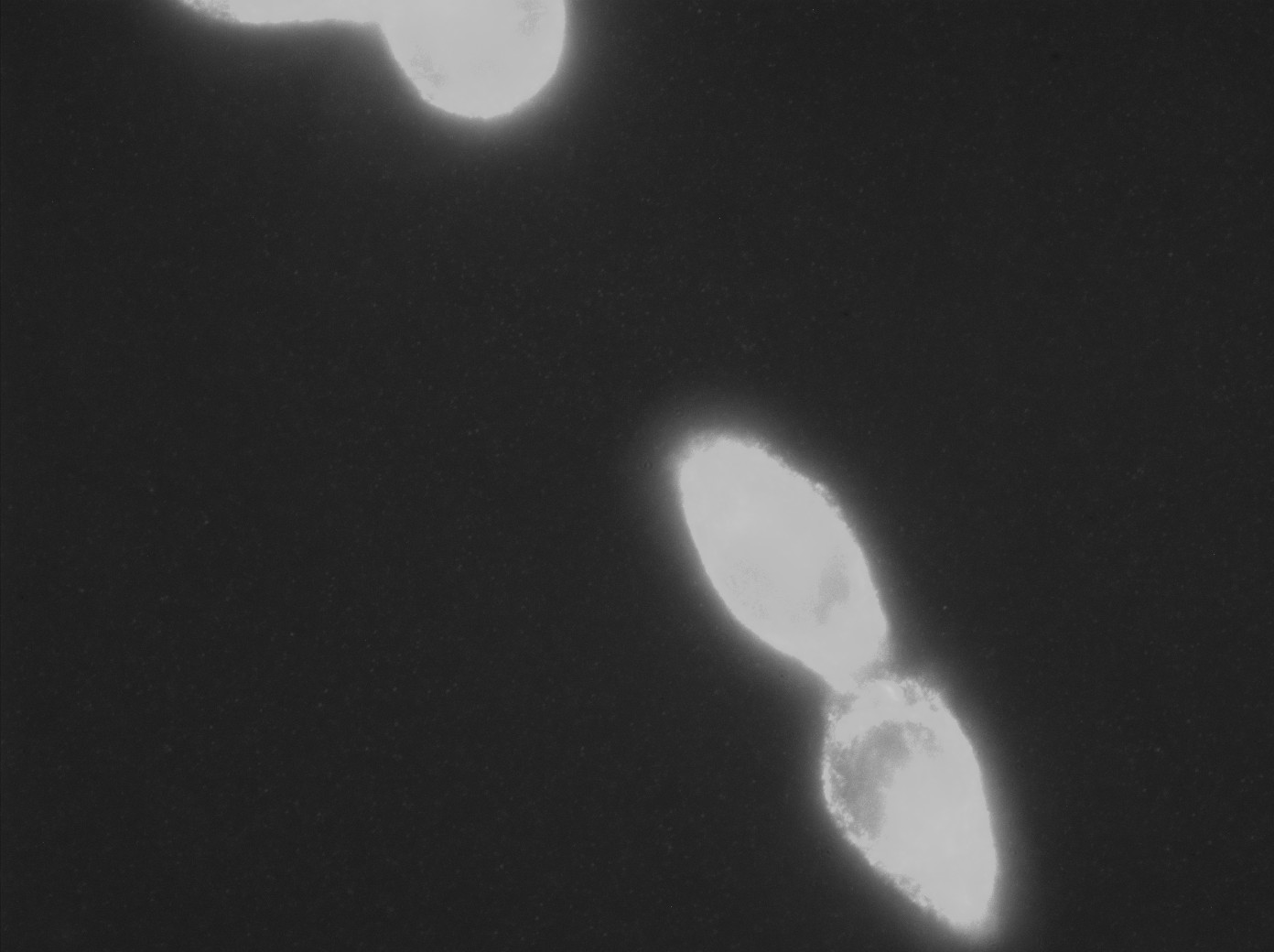

Supplement: Supplementary file 4 — Source data Fig. 2 part 1 [file 44319_2026_742_MOESM4_ESM.zip › Figure 2 Part 1/Fig 2c Cas9 Hela rGT335 atubulin/Cas9 GT335recomb atub 3-24-25 R1 PA9.Project Maximum Z_XY1743103585_Z0_T0_C2.tif]

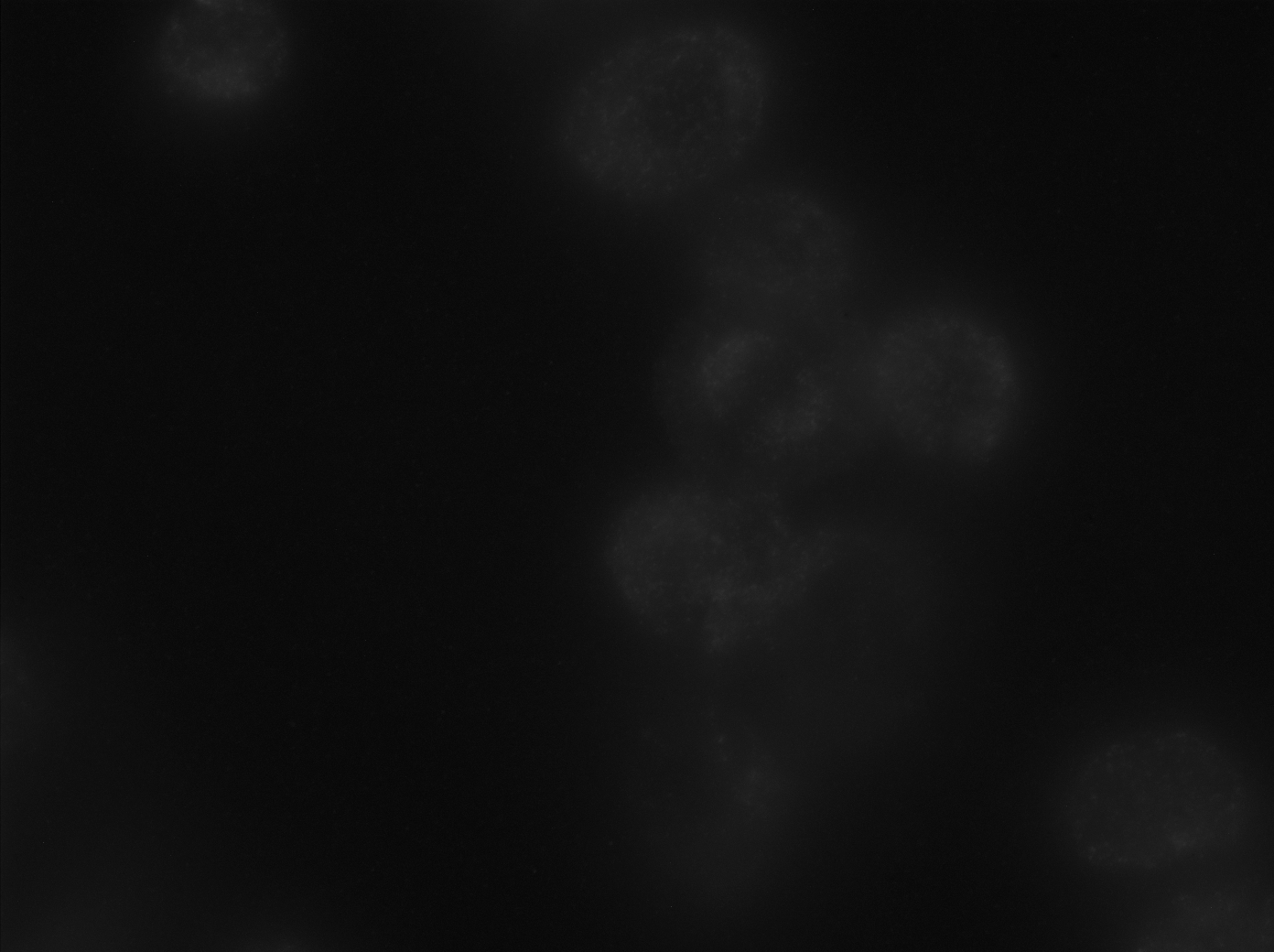

Supplement: Supplementary file 4 — Source data Fig. 2 part 1 [file 44319_2026_742_MOESM4_ESM.zip › Figure 2 Part 1/Fig 2c Cas9 Hela rGT335 atubulin/Cas9 GT335recomb atub 3-24-25 R3 M9.Project Maximum Z_XY1743454469_Z0_T0_C2.tif]

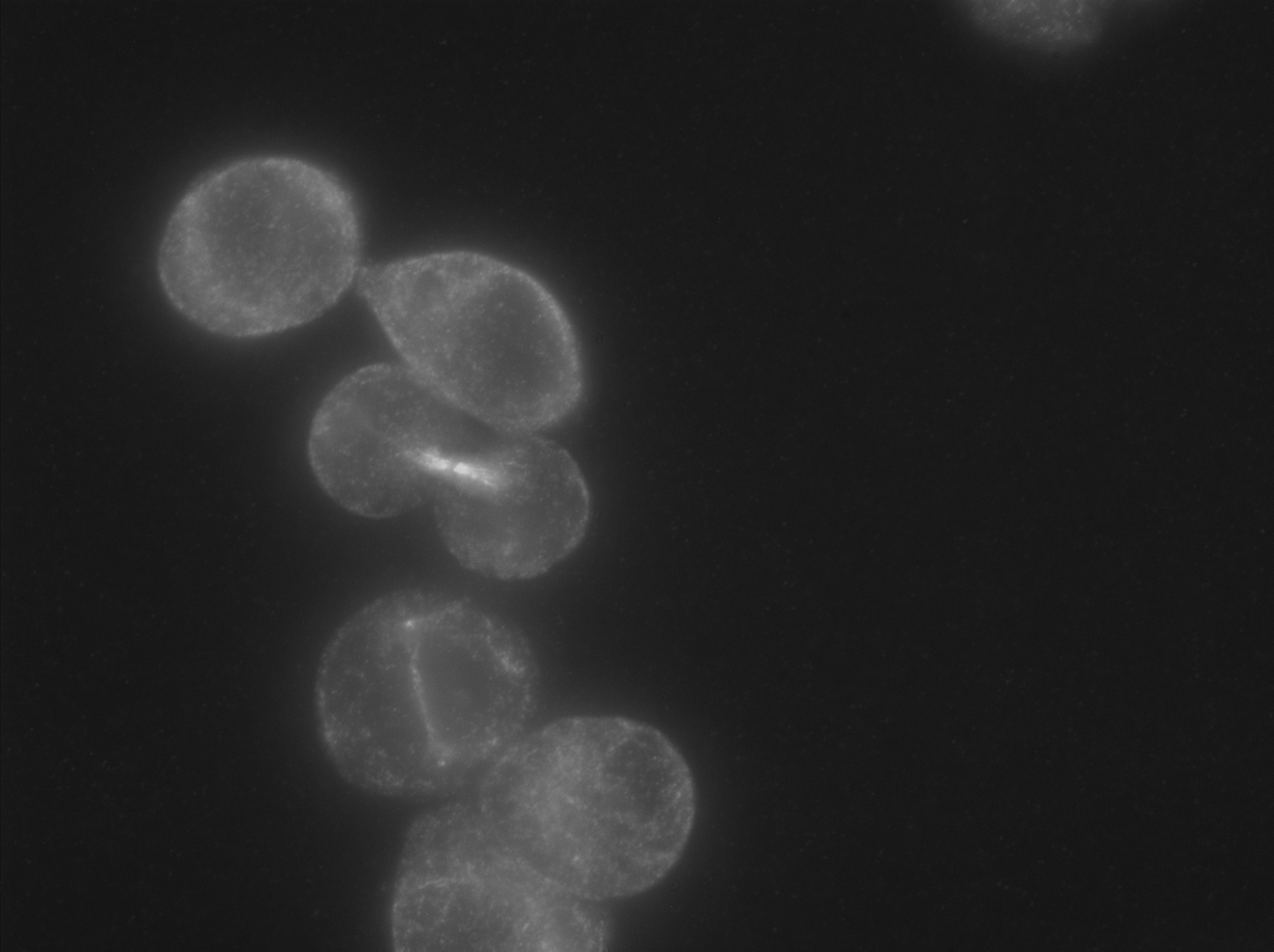

Supplement: Supplementary file 4 — Source data Fig. 2 part 1 [file 44319_2026_742_MOESM4_ESM.zip › Figure 2 Part 1/Fig 2c Cas9 Hela rGT335 atubulin/Cas9 GT335recomb atub 3-24-25 R3 ET8.Project Maximum Z_XY1743455361_Z0_T0_C2.tif]

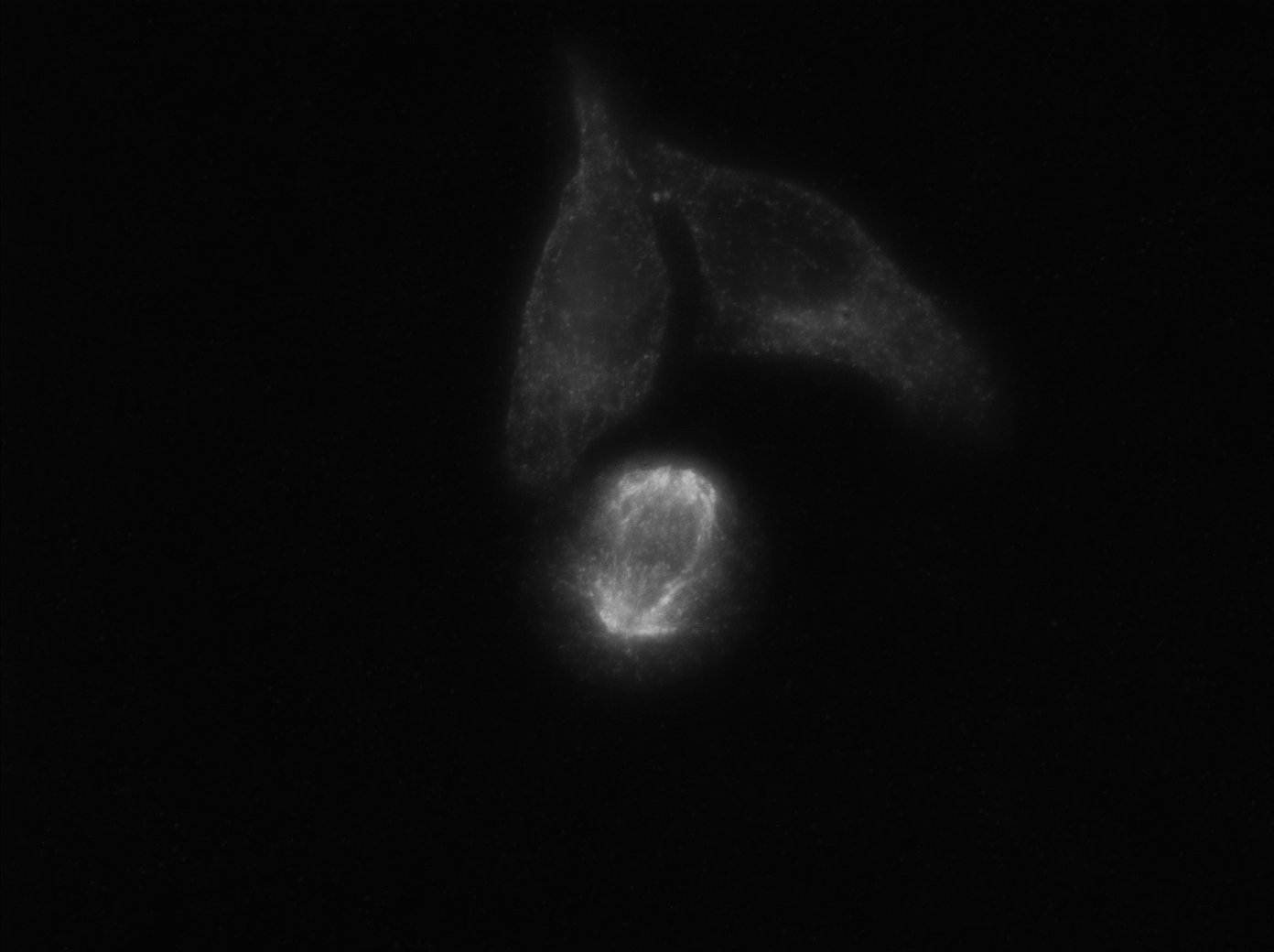

Supplement: Supplementary file 4 — Source data Fig. 2 part 1 [file 44319_2026_742_MOESM4_ESM.zip › Figure 2 Part 1/Fig 2c Cas9 Hela rGT335 atubulin/Cas9 GT335recomb atub 3-24-25 R2 A3.Project Maximum Z_XY1743442497_Z0_T0_C2.tif]

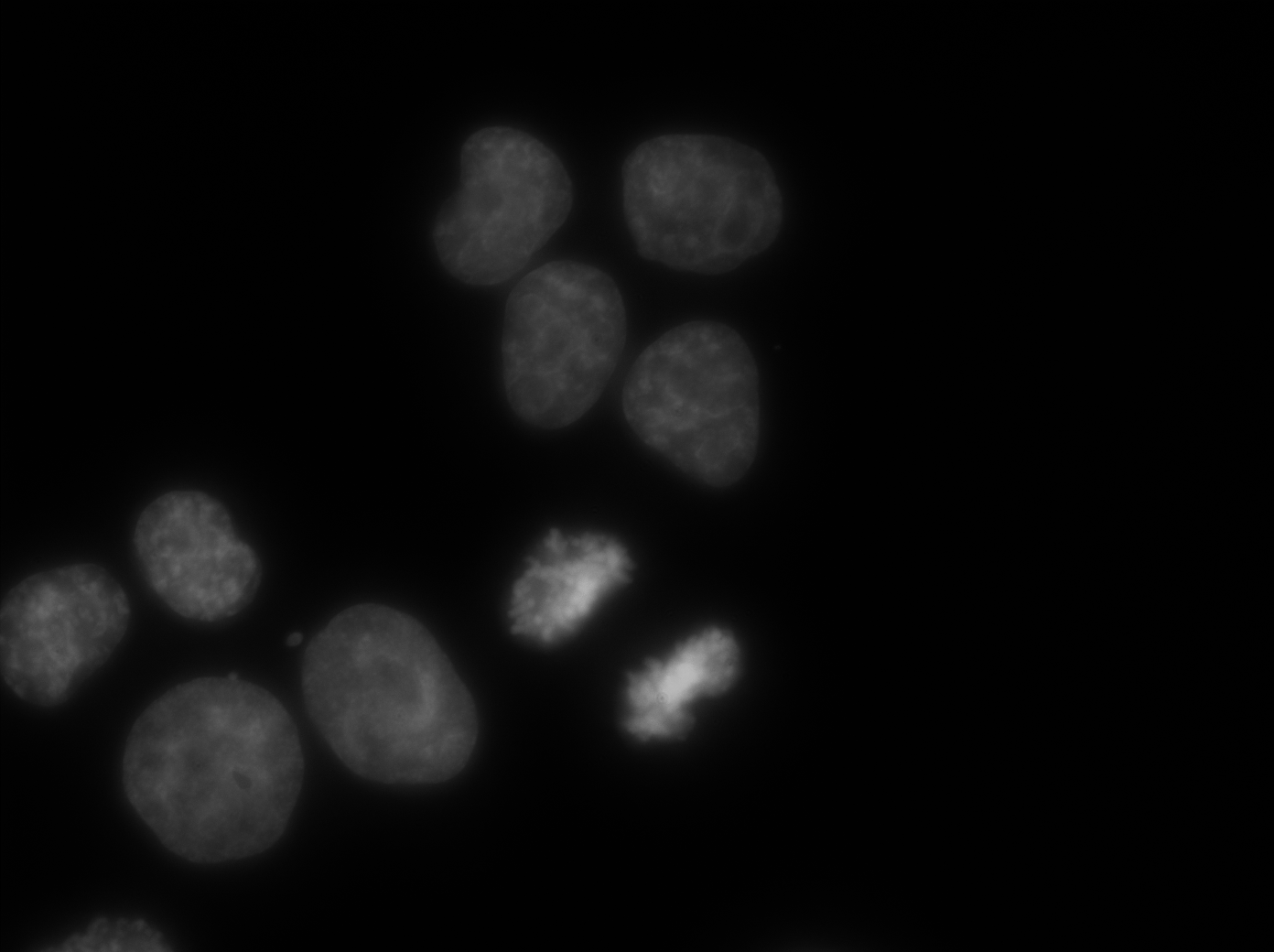

Supplement: Supplementary file 4 — Source data Fig. 2 part 1 [file 44319_2026_742_MOESM4_ESM.zip › Figure 2 Part 1/Fig 2c Cas9 Hela rGT335 atubulin/Cas9 GT335recomb atub 3-24-25 R2 preET2.Project Maximum Z_XY1743447696_Z0_T0_C0.tif]

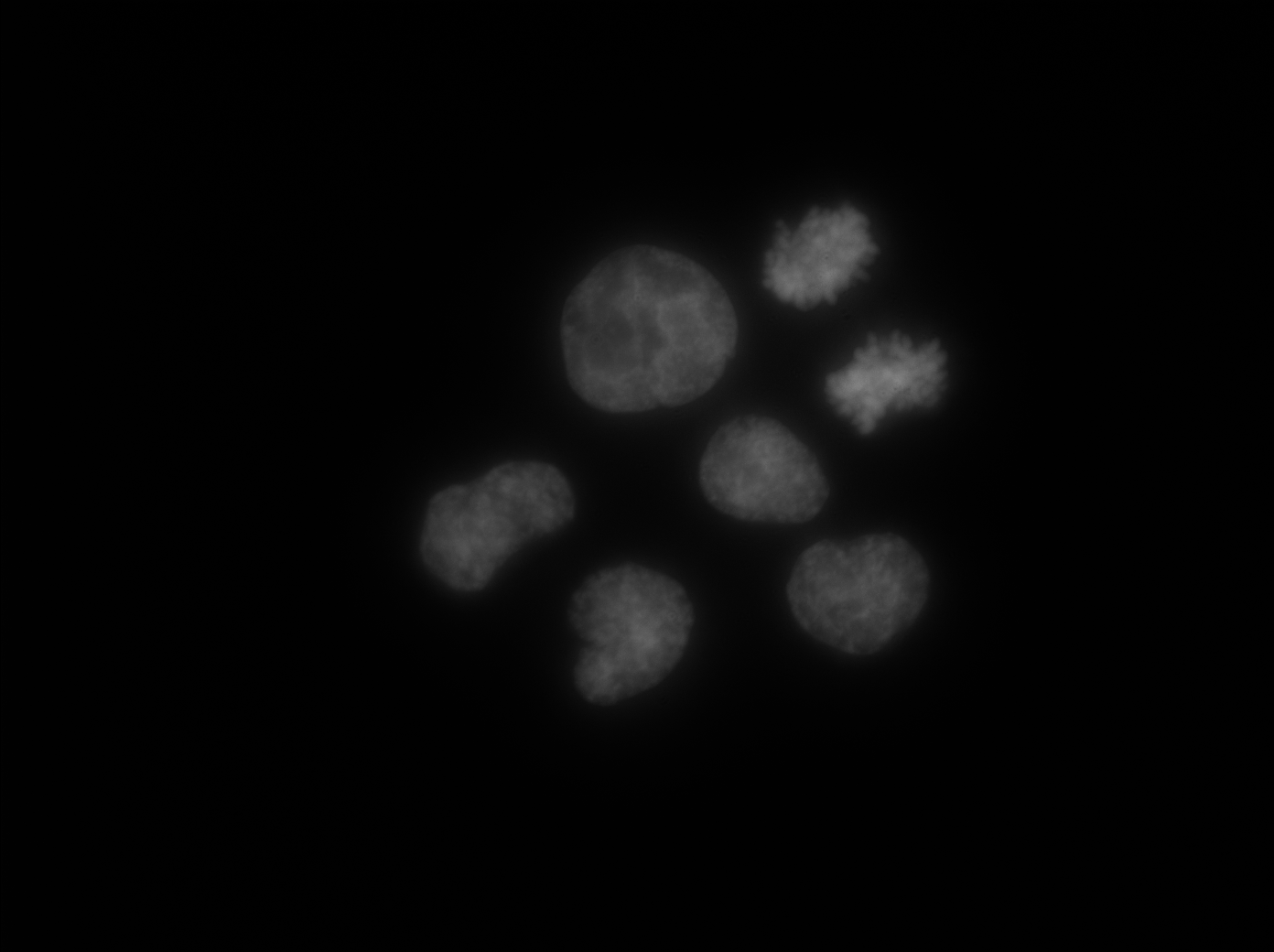

Supplement: Supplementary file 4 — Source data Fig. 2 part 1 [file 44319_2026_742_MOESM4_ESM.zip › Figure 2 Part 1/Fig 2c Cas9 Hela rGT335 atubulin/Cas9 GT335recomb atub 3-24-25 R2 A6.Project Maximum Z_XY1743446093_Z0_T0_C0.tif]

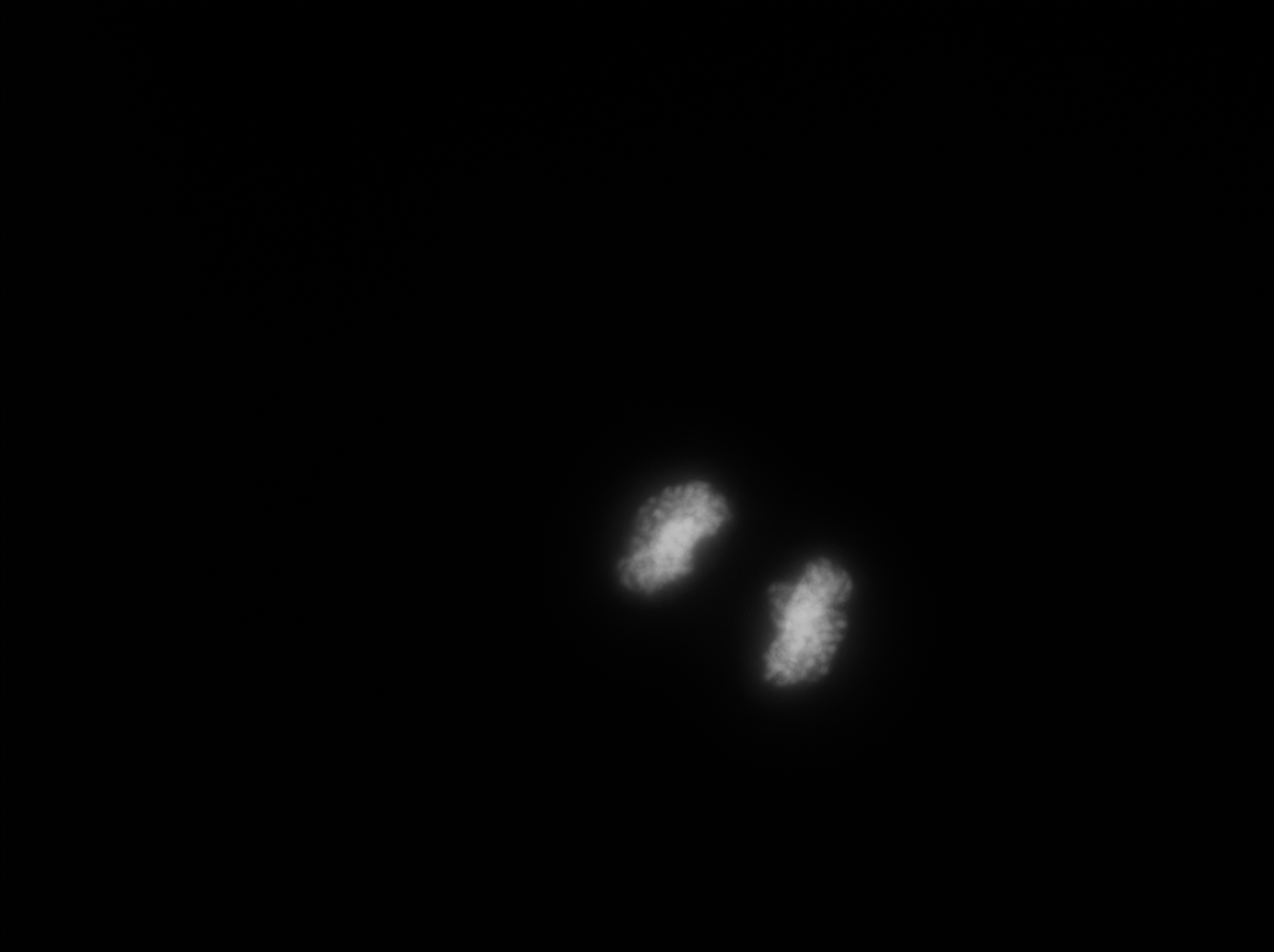

Supplement: Supplementary file 4 — Source data Fig. 2 part 1 [file 44319_2026_742_MOESM4_ESM.zip › Figure 2 Part 1/Fig 2c Cas9 Hela rGT335 atubulin/Cas9 GT335recomb atub 3-24-25 R2 ET1.Project Maximum Z_XY1743439798_Z0_T0_C0.tif]

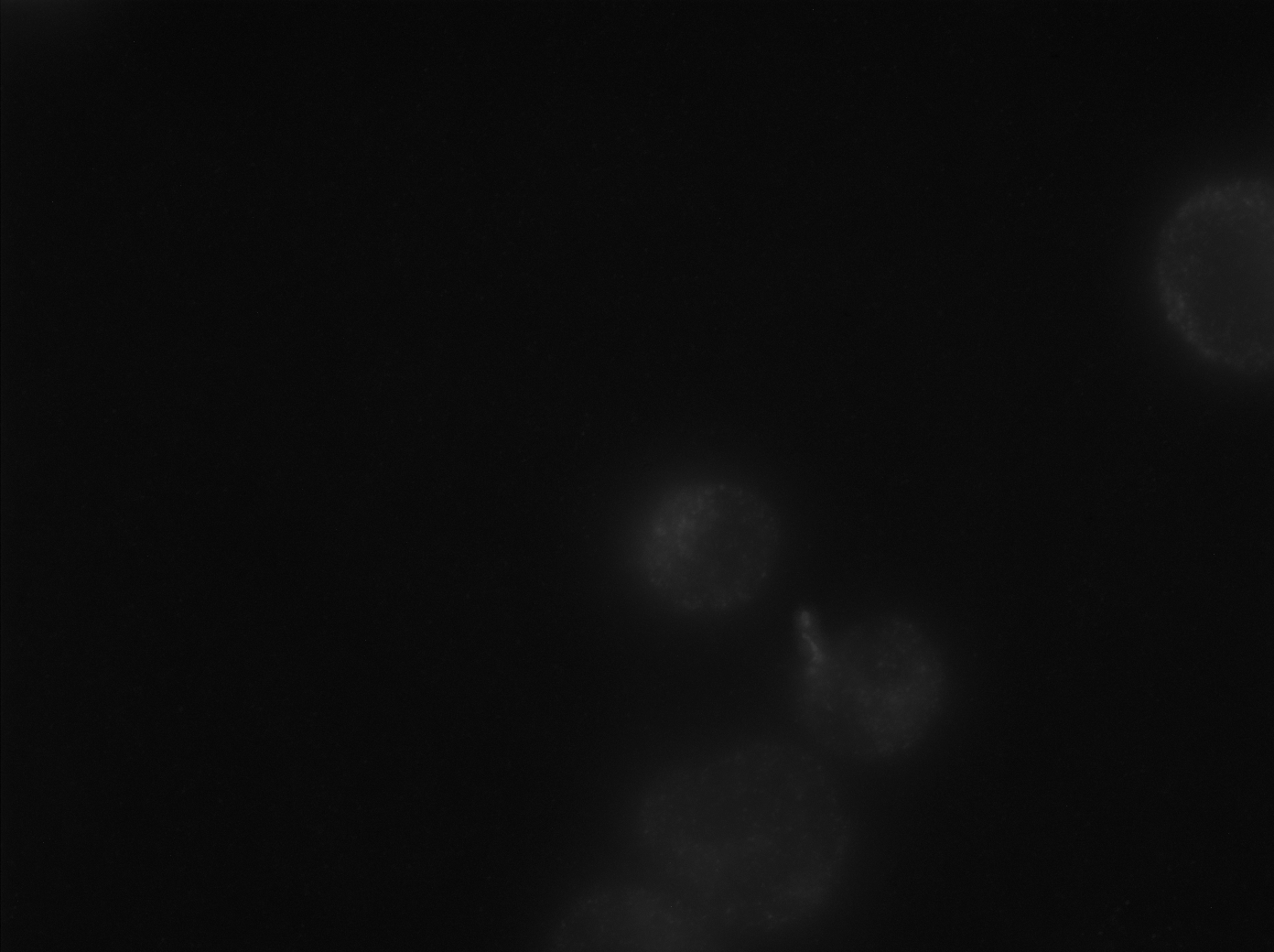

Supplement: Supplementary file 4 — Source data Fig. 2 part 1 [file 44319_2026_742_MOESM4_ESM.zip › Figure 2 Part 1/Fig 2c Cas9 Hela rGT335 atubulin/Cas9 GT335recomb atub 3-24-25 R3 PA10.Project Maximum Z_XY1743454681_Z0_T0_C2.tif]

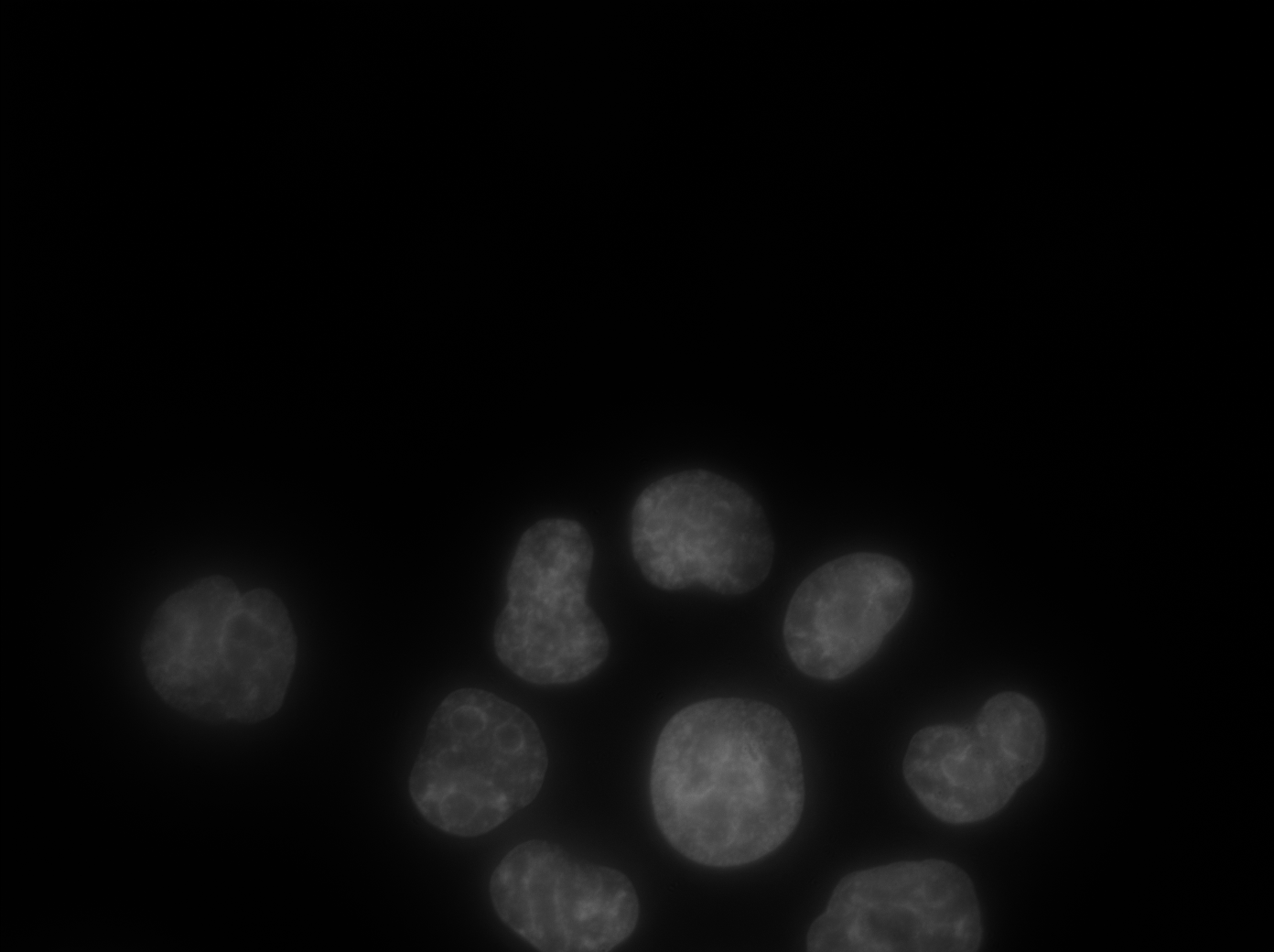

Supplement: Supplementary file 4 — Source data Fig. 2 part 1 [file 44319_2026_742_MOESM4_ESM.zip › Figure 2 Part 1/Fig 2c Cas9 Hela rGT335 atubulin/Cas9 GT335recomb atub 3-24-25 R3 LT3.Project Maximum Z_XY1743451368_Z0_T0_C0.tif]

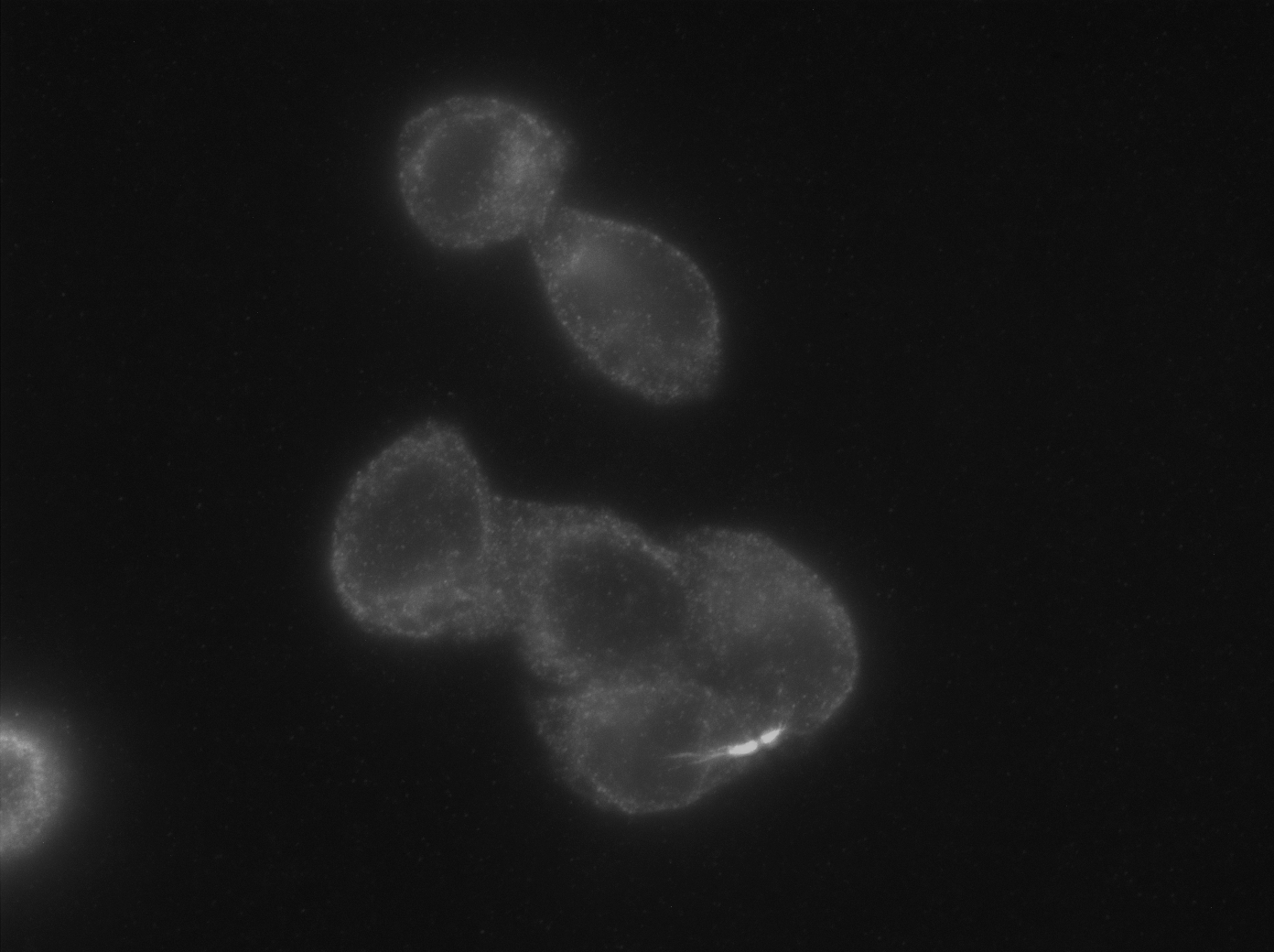

Supplement: Supplementary file 4 — Source data Fig. 2 part 1 [file 44319_2026_742_MOESM4_ESM.zip › Figure 2 Part 1/Fig 2c Cas9 Hela rGT335 atubulin/Cas9 GT335recomb atub 3-24-25 R1 LT9.Project Maximum Z_XY1743101906_Z0_T0_C2.tif]

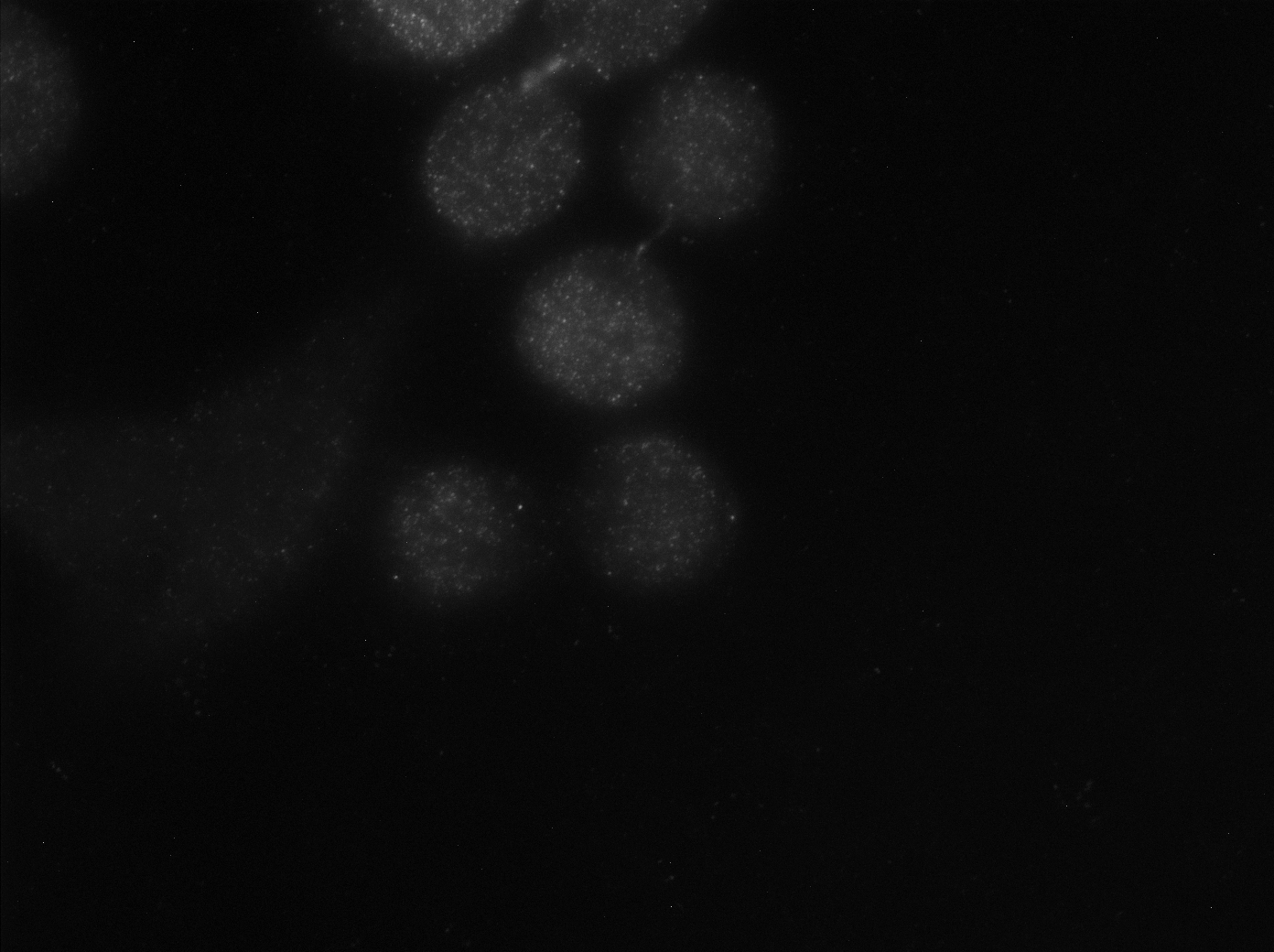

Supplement: Supplementary file 4 — Source data Fig. 2 part 1 [file 44319_2026_742_MOESM4_ESM.zip › Figure 2 Part 1/Fig 2c Cas9 Hela rGT335 atubulin/Cas9 GT335recomb atub 3-24-25 R2 LT6 PA4.Project Maximum Z_XY1743441273_Z0_T0_C1.tif]

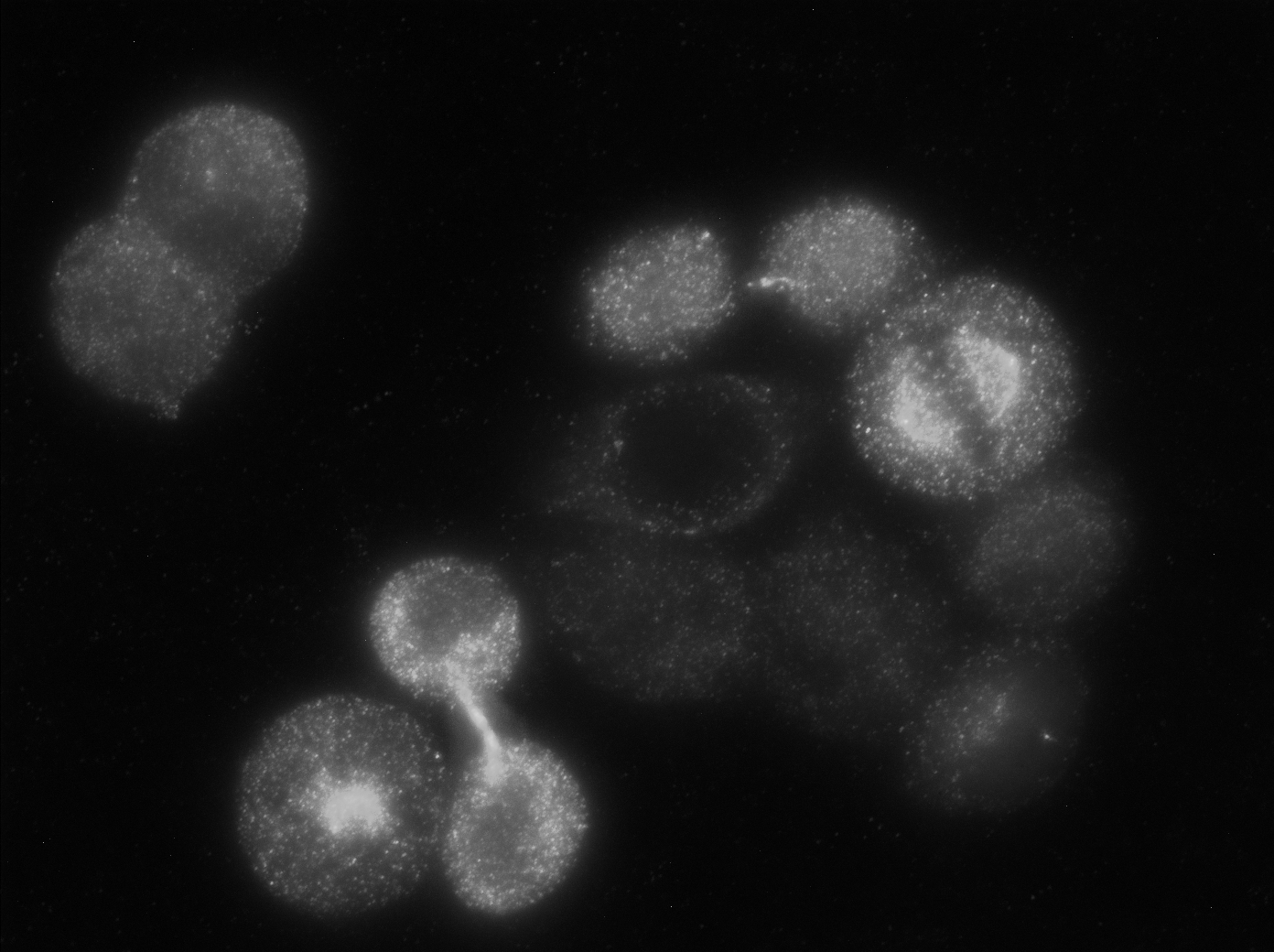

Supplement: Supplementary file 4 — Source data Fig. 2 part 1 [file 44319_2026_742_MOESM4_ESM.zip › Figure 2 Part 1/Fig 2c Cas9 Hela rGT335 atubulin/Cas9 GT335recomb atub 3-24-25 R1 PA1 M1.Project Maximum Z_XY1742835552_Z0_T0_C1.tif]

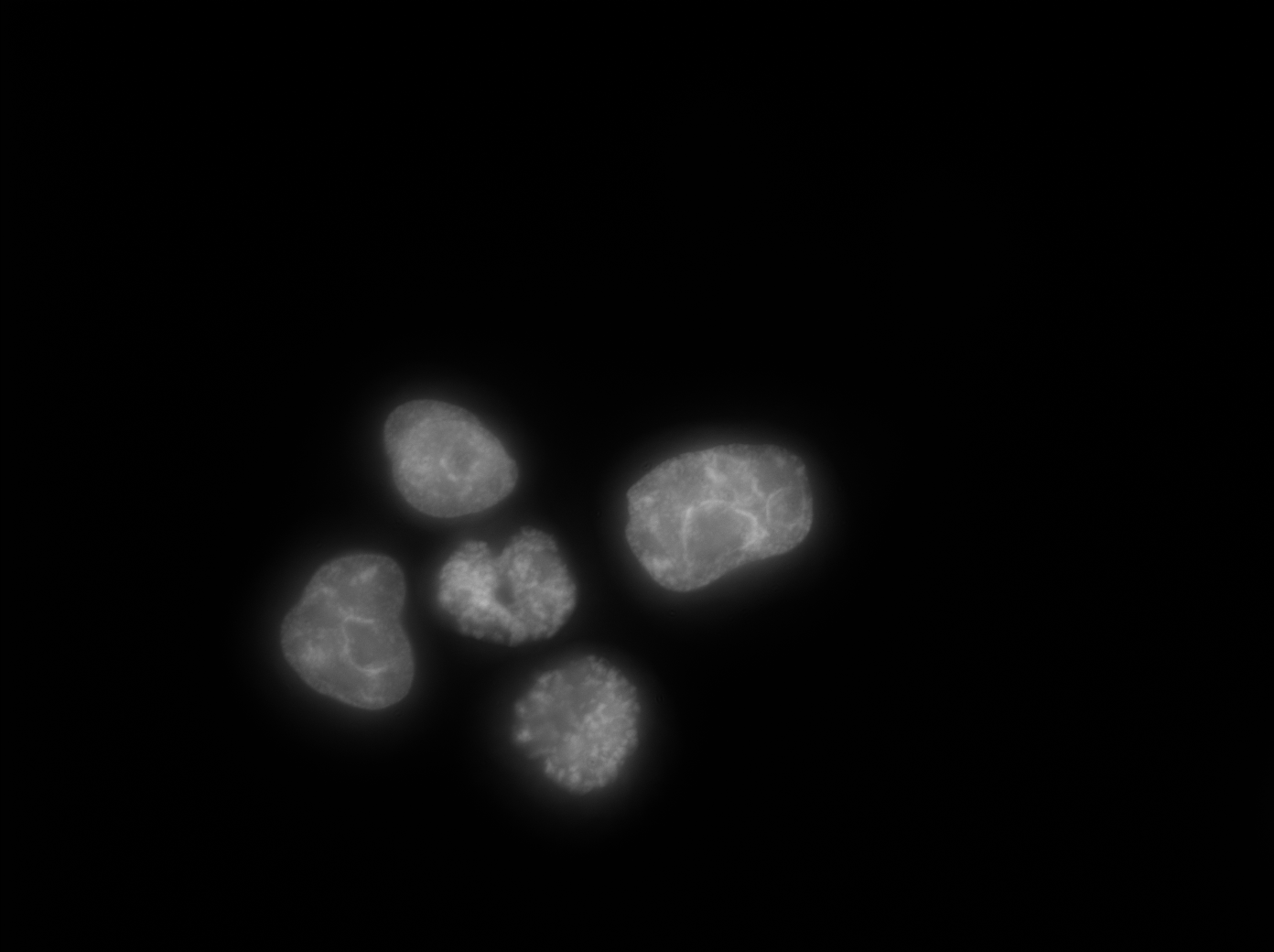

Supplement: Supplementary file 4 — Source data Fig. 2 part 1 [file 44319_2026_742_MOESM4_ESM.zip › Figure 2 Part 1/Fig 2c Cas9 Hela rGT335 atubulin/Cas9 GT335recomb atub 3-24-25 R3 ET3.Project Maximum Z_XY1743452941_Z0_T0_C0.tif]

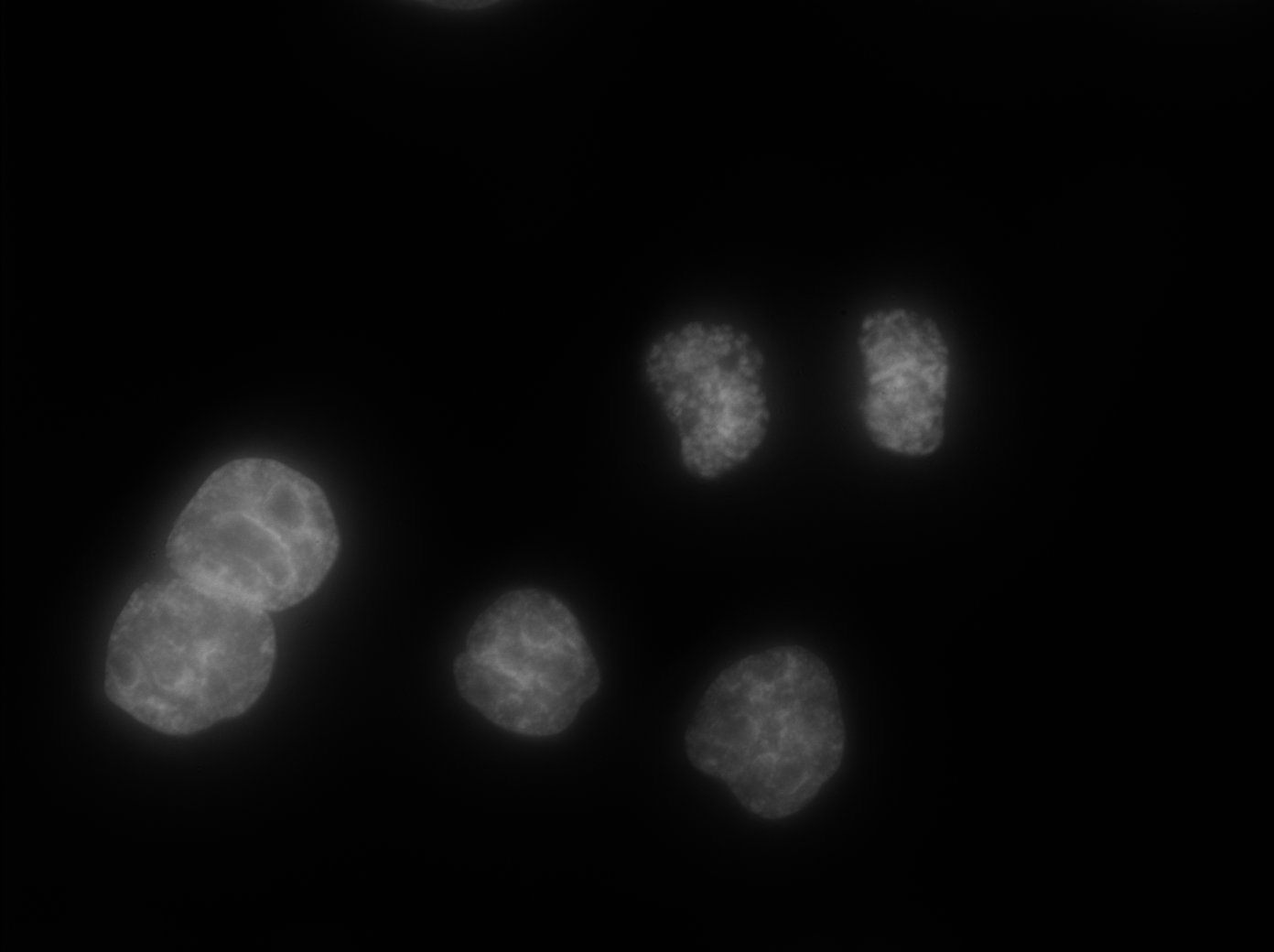

Supplement: Supplementary file 4 — Source data Fig. 2 part 1 [file 44319_2026_742_MOESM4_ESM.zip › Figure 2 Part 1/Fig 2c Cas9 Hela rGT335 atubulin/Cas9 GT335recomb atub 3-24-25 R2 ET2 LT4.Project Maximum Z_XY1743439952_Z0_T0_C0.tif]

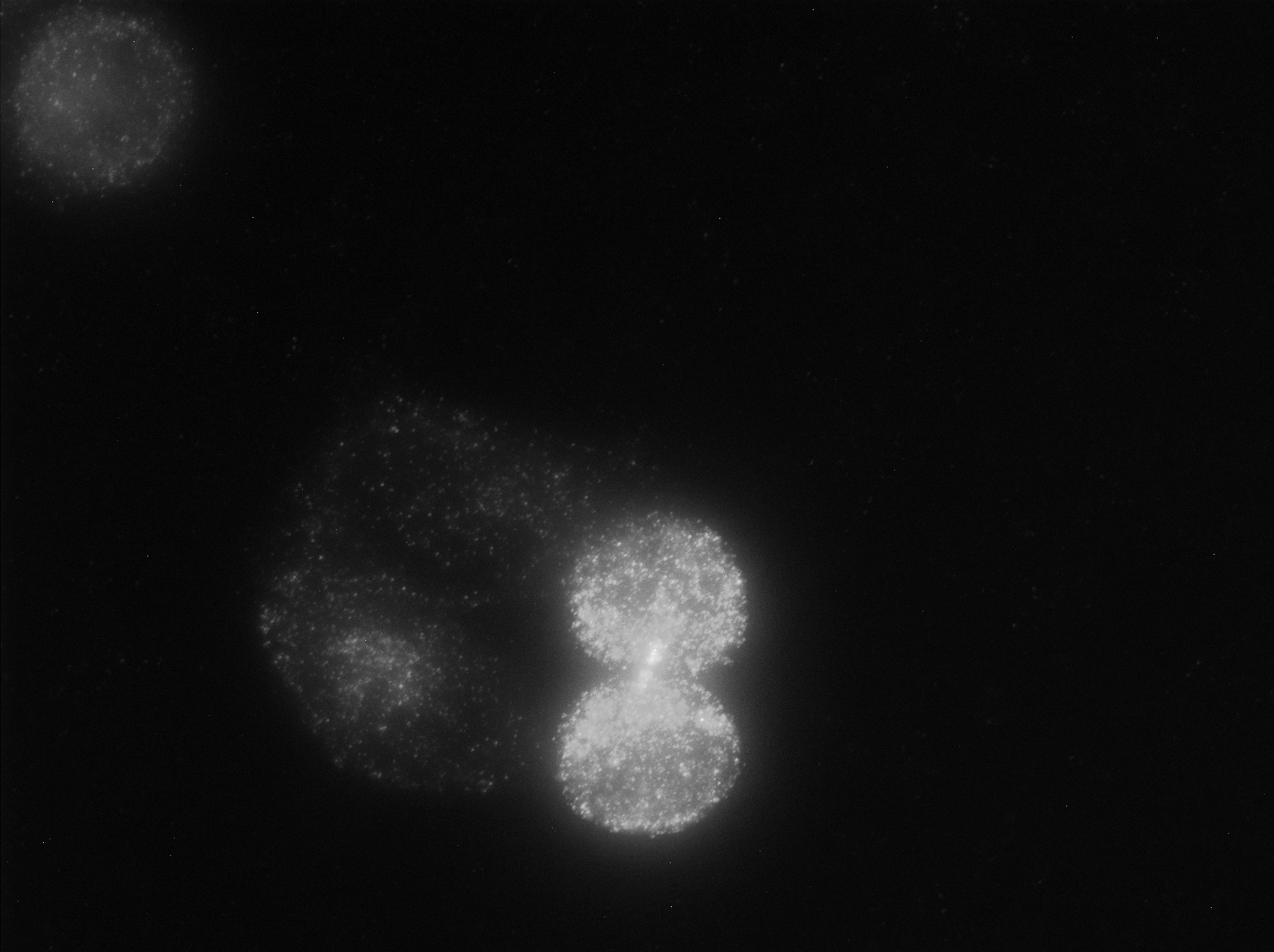

Supplement: Supplementary file 4 — Source data Fig. 2 part 1 [file 44319_2026_742_MOESM4_ESM.zip › Figure 2 Part 1/Fig 2c Cas9 Hela rGT335 atubulin/Cas9 GT335recomb atub 3-24-25 R2 ET6.Project Maximum Z_XY1743444007_Z0_T0_C1.tif]

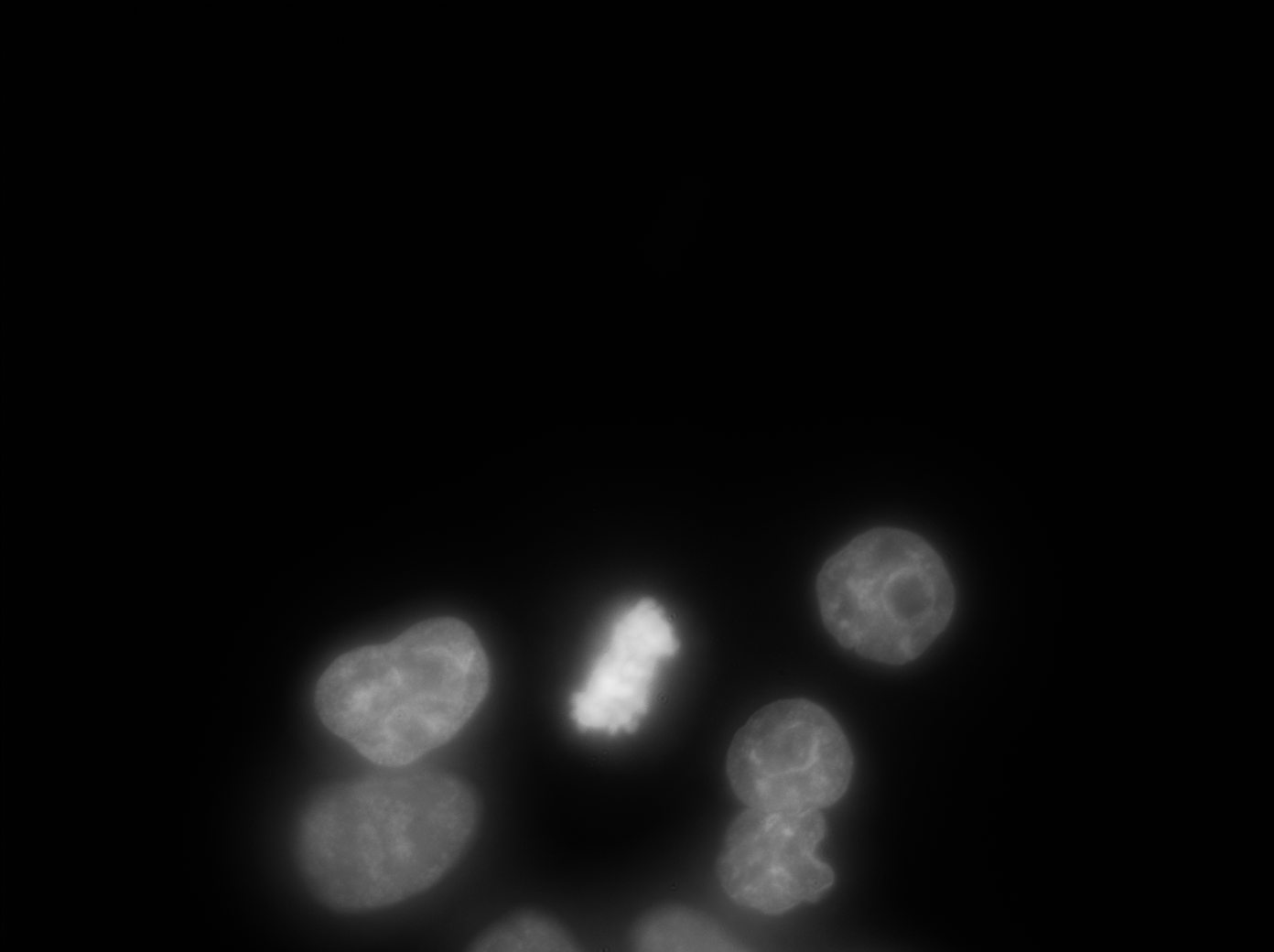

Supplement: Supplementary file 4 — Source data Fig. 2 part 1 [file 44319_2026_742_MOESM4_ESM.zip › Figure 2 Part 1/Fig 2c Cas9 Hela rGT335 atubulin/Cas9 GT335recomb atub 3-24-25 R1 M4.Project Maximum Z_XY1743104120_Z0_T0_C0.tif]

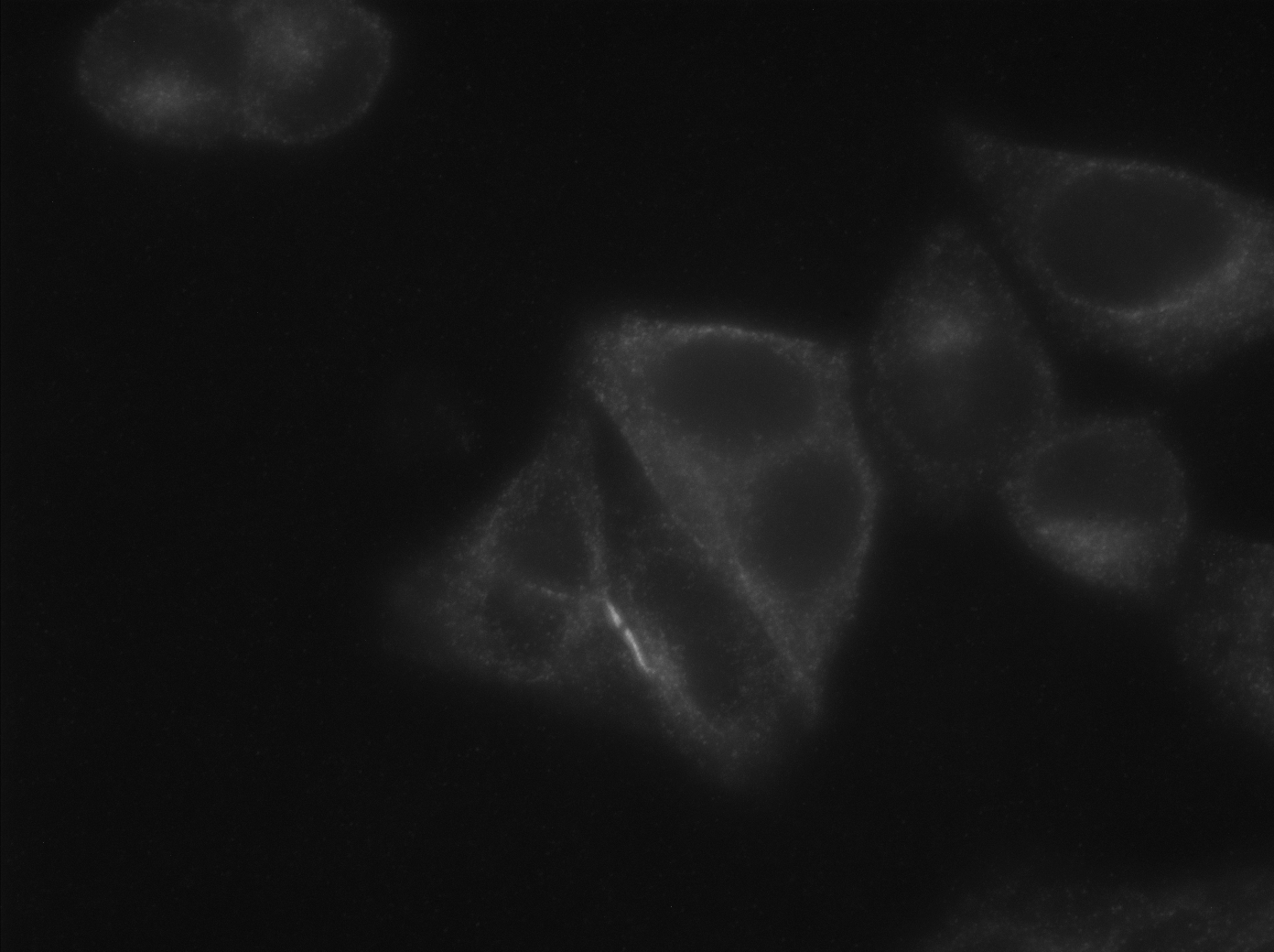

Supplement: Supplementary file 4 — Source data Fig. 2 part 1 [file 44319_2026_742_MOESM4_ESM.zip › Figure 2 Part 1/Fig 2c Cas9 Hela rGT335 atubulin/Cas9 GT335recomb atub 3-24-25 R1 LT2.Project Maximum Z_XY1743100727_Z0_T0_C2.tif]

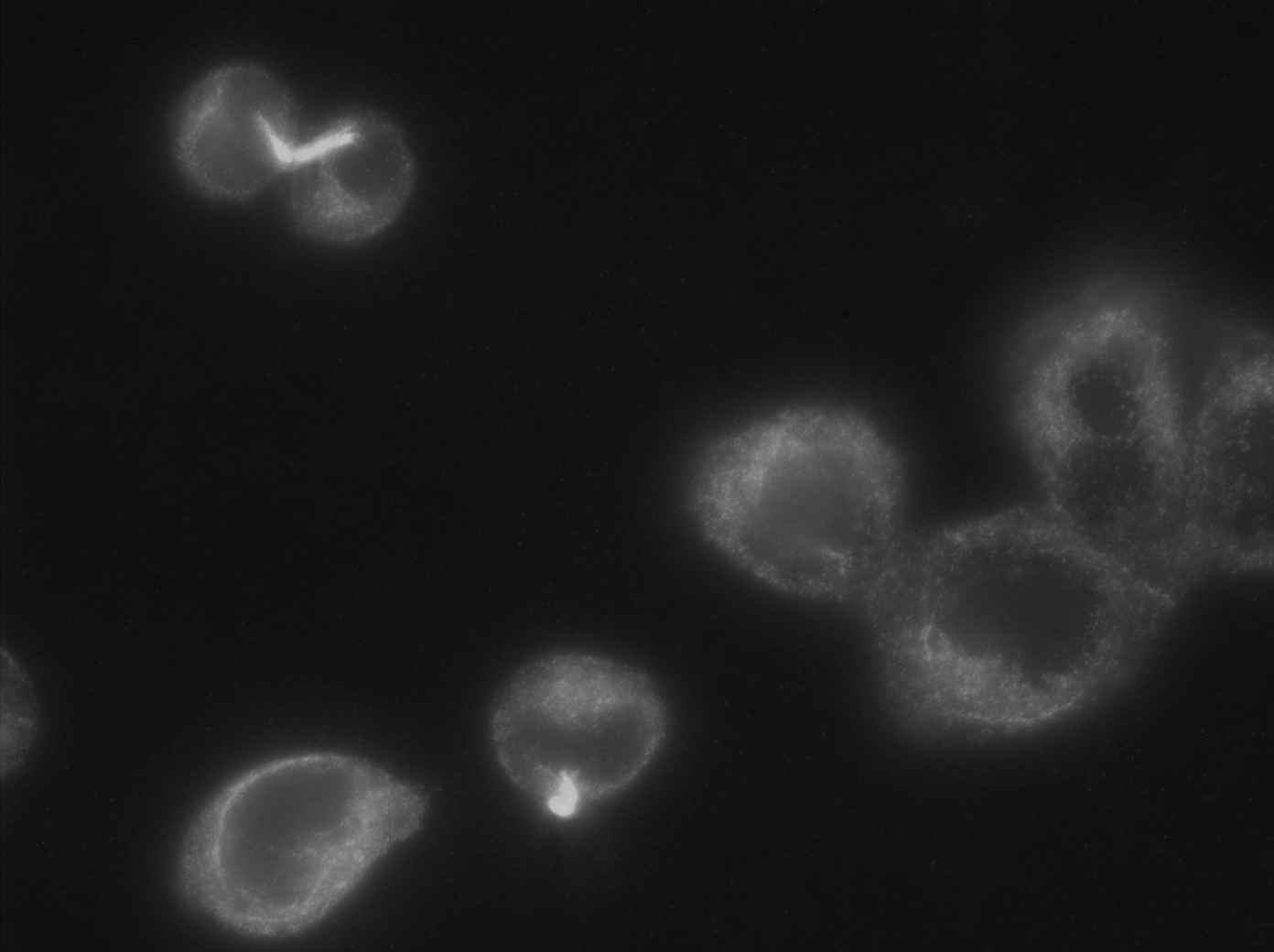

Supplement: Supplementary file 4 — Source data Fig. 2 part 1 [file 44319_2026_742_MOESM4_ESM.zip › Figure 2 Part 1/Fig 2c Cas9 Hela rGT335 atubulin/Cas9 GT335recomb atub 3-24-25 R1 ET4 PA6.Project Maximum Z_XY1743101663_Z0_T0_C2.tif]

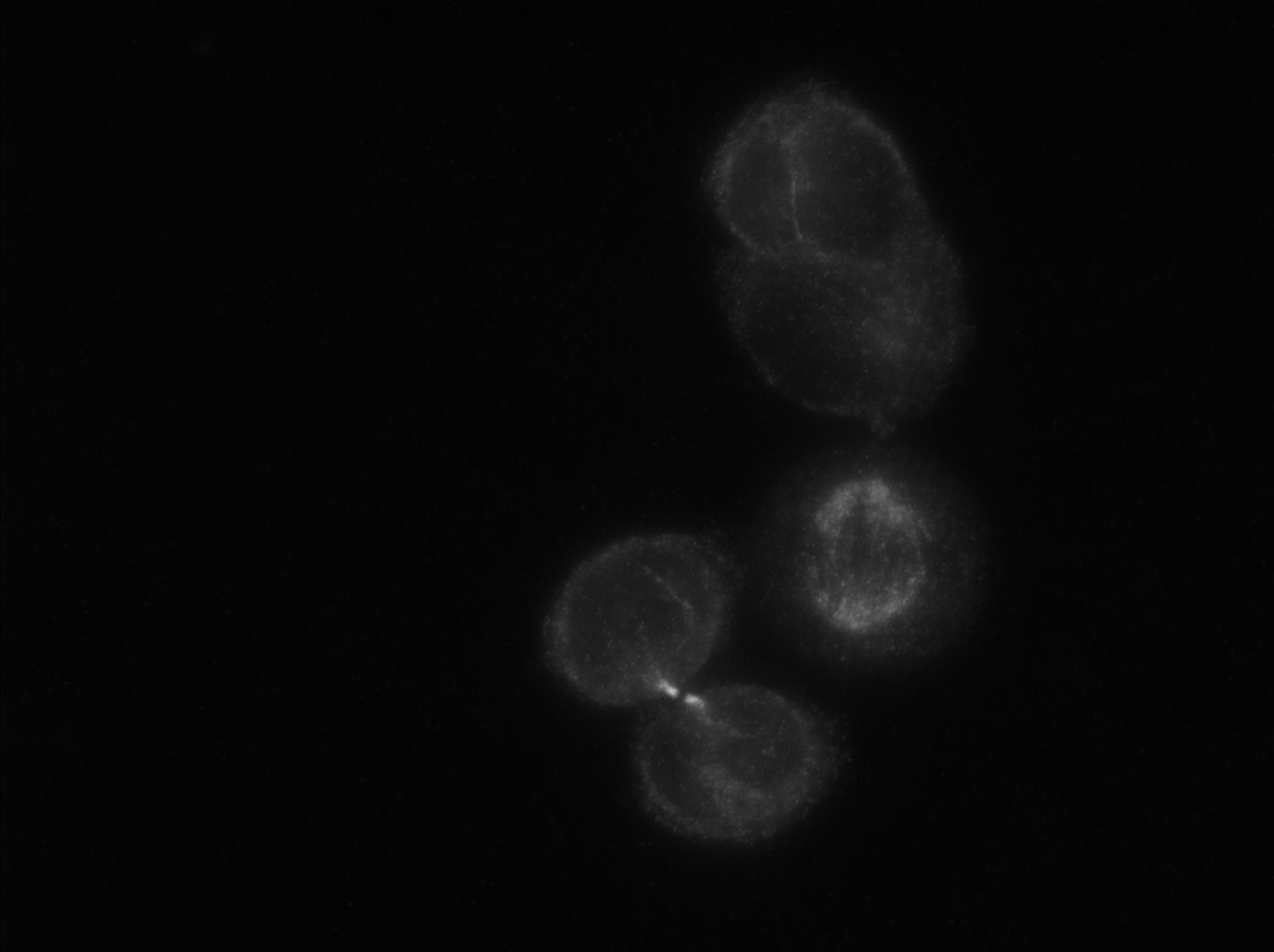

Supplement: Supplementary file 4 — Source data Fig. 2 part 1 [file 44319_2026_742_MOESM4_ESM.zip › Figure 2 Part 1/Fig 2c Cas9 Hela rGT335 atubulin/Cas9 GT335recomb atub 3-24-25 R2 M3.Project Maximum Z_XY1743444217_Z0_T0_C2.tif]

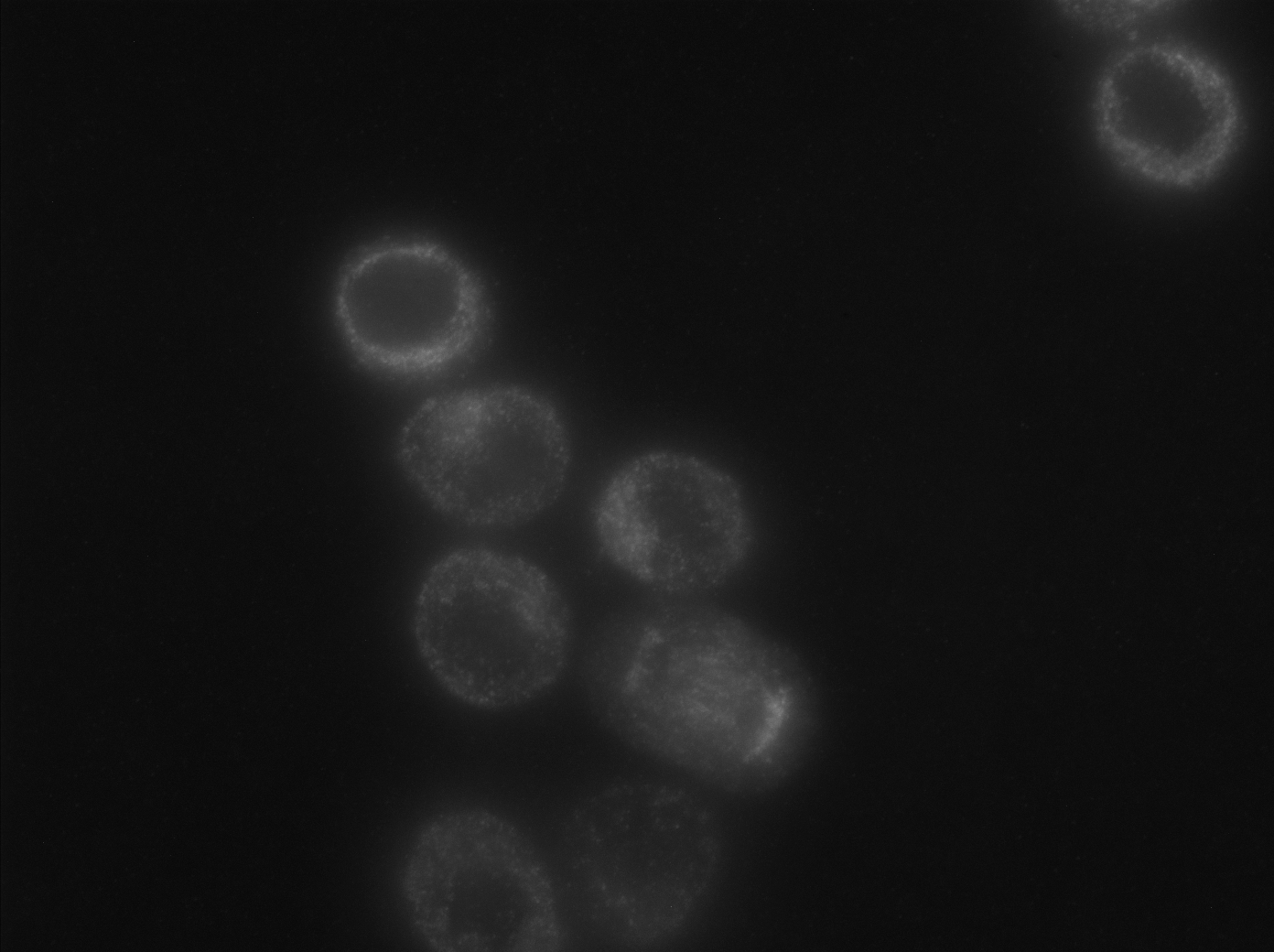

Supplement: Supplementary file 4 — Source data Fig. 2 part 1 [file 44319_2026_742_MOESM4_ESM.zip › Figure 2 Part 1/Fig 2c Cas9 Hela rGT335 atubulin/Cas9 GT335recomb atub 3-24-25 R1 A1.Project Maximum Z_XY1743104807_Z0_T0_C2.tif]

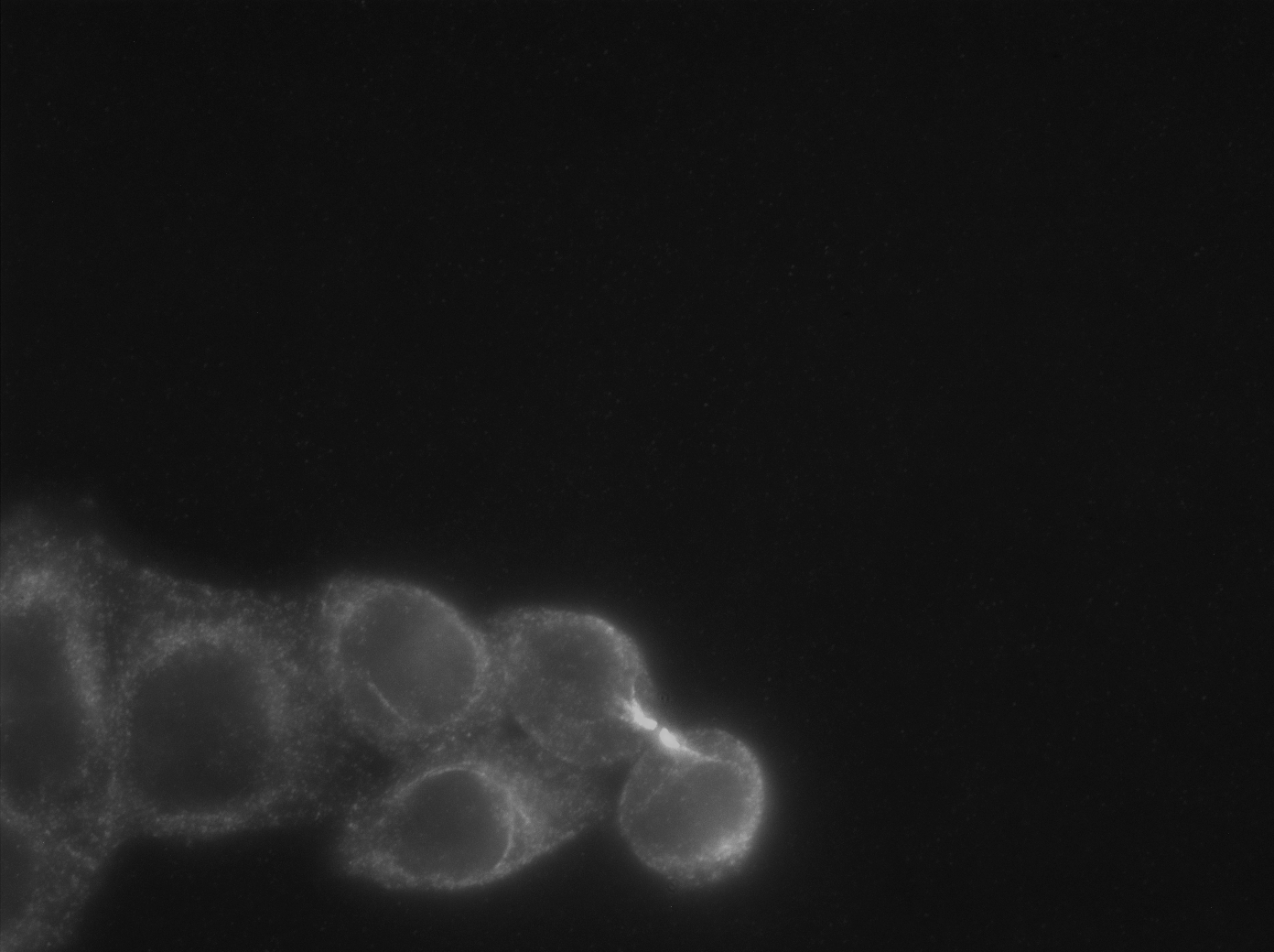

Supplement: Supplementary file 4 — Source data Fig. 2 part 1 [file 44319_2026_742_MOESM4_ESM.zip › Figure 2 Part 1/Fig 2c Cas9 Hela rGT335 atubulin/Cas9 GT335recomb atub 3-24-25 R1 LT7.Project Maximum Z_XY1743101575_Z0_T0_C2.tif]

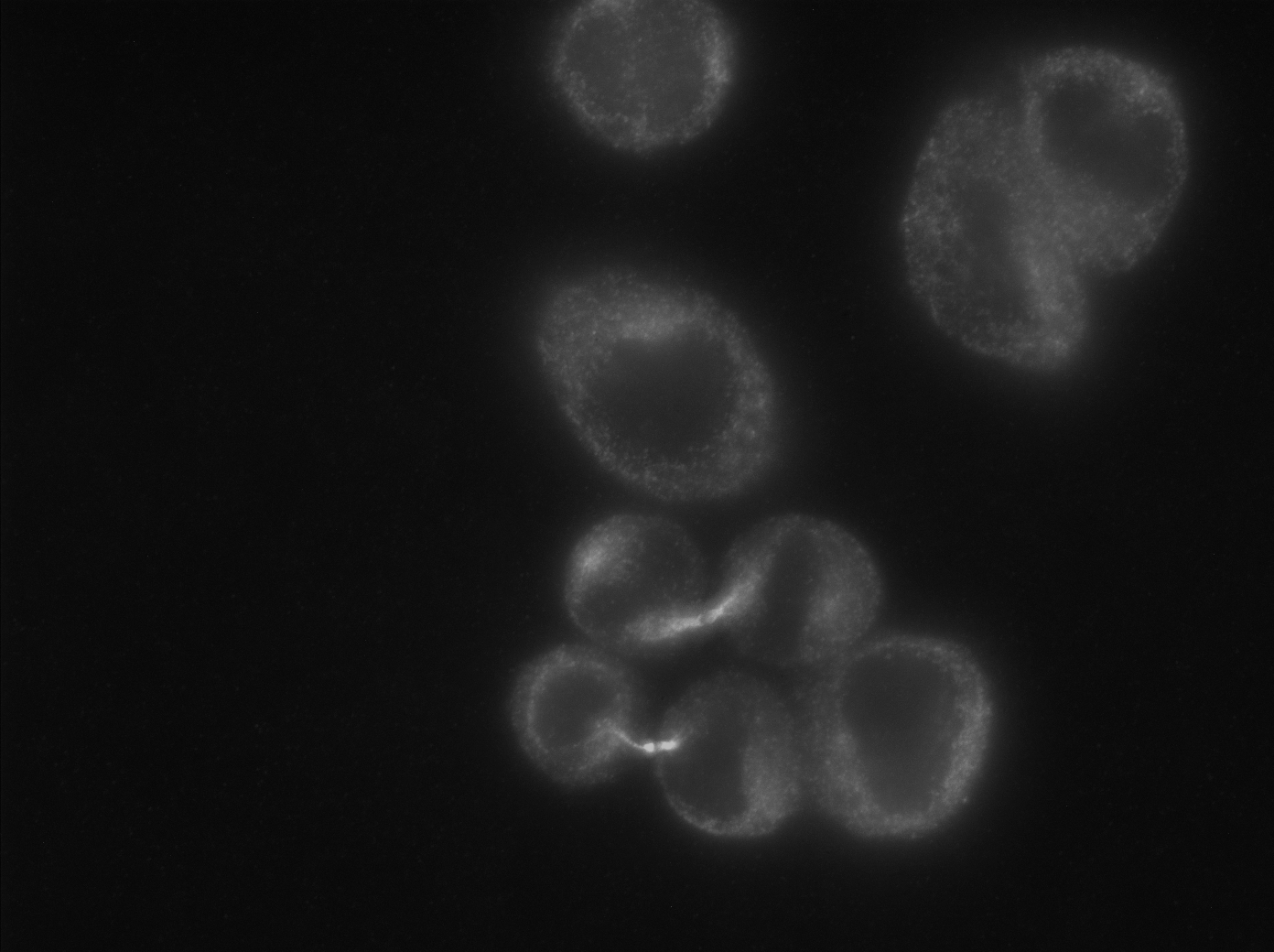

Supplement: Supplementary file 4 — Source data Fig. 2 part 1 [file 44319_2026_742_MOESM4_ESM.zip › Figure 2 Part 1/Fig 2c Cas9 Hela rGT335 atubulin/Cas9 GT335recomb atub 3-24-25 R1 PA3.Project Maximum Z_XY1742836284_Z0_T0_C2.tif]

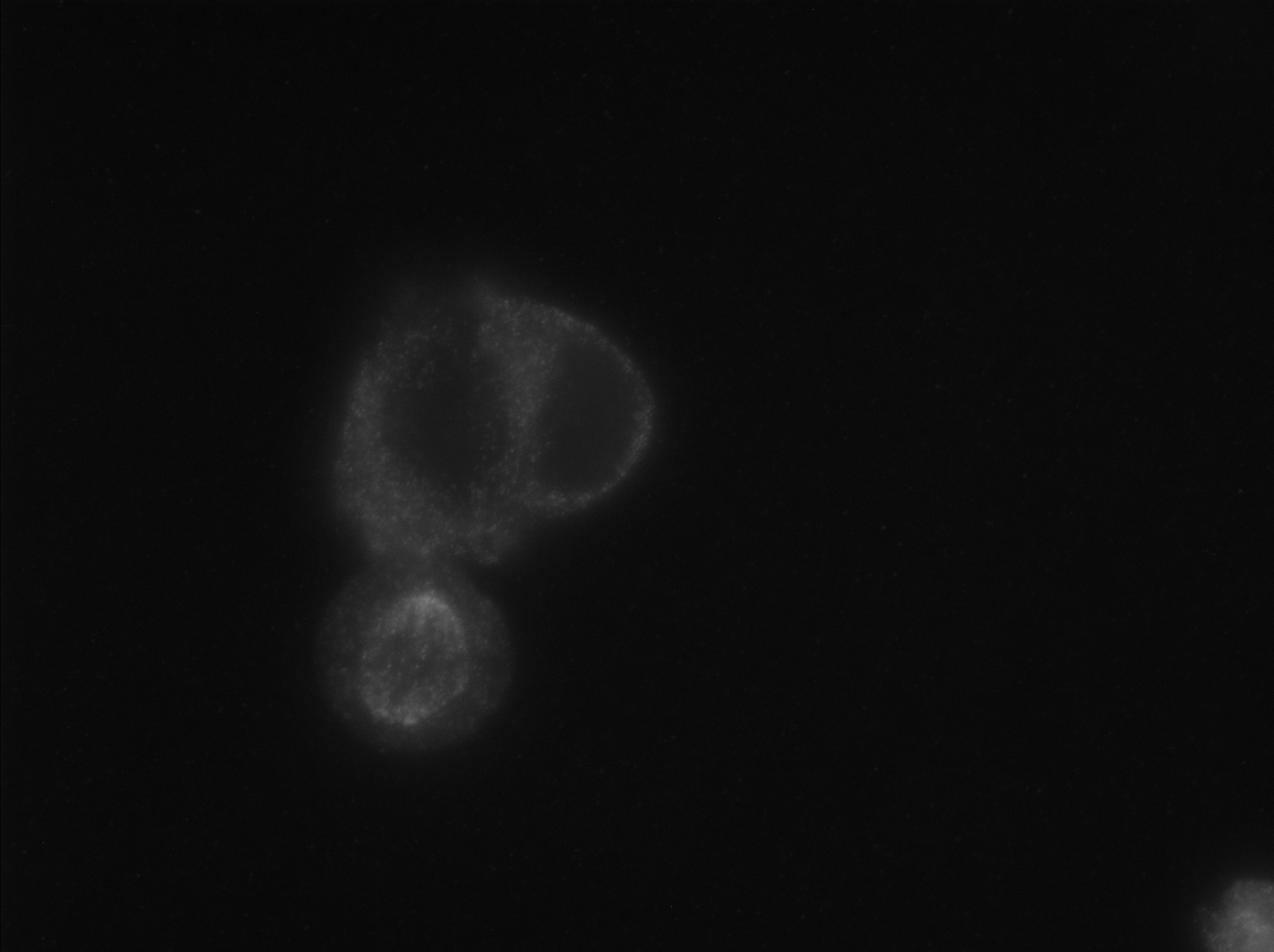

Supplement: Supplementary file 4 — Source data Fig. 2 part 1 [file 44319_2026_742_MOESM4_ESM.zip › Figure 2 Part 1/Fig 2c Cas9 Hela rGT335 atubulin/Cas9 GT335recomb atub 3-24-25 R1 M8.Project Maximum Z_XY1743105633_Z0_T0_C2.tif]

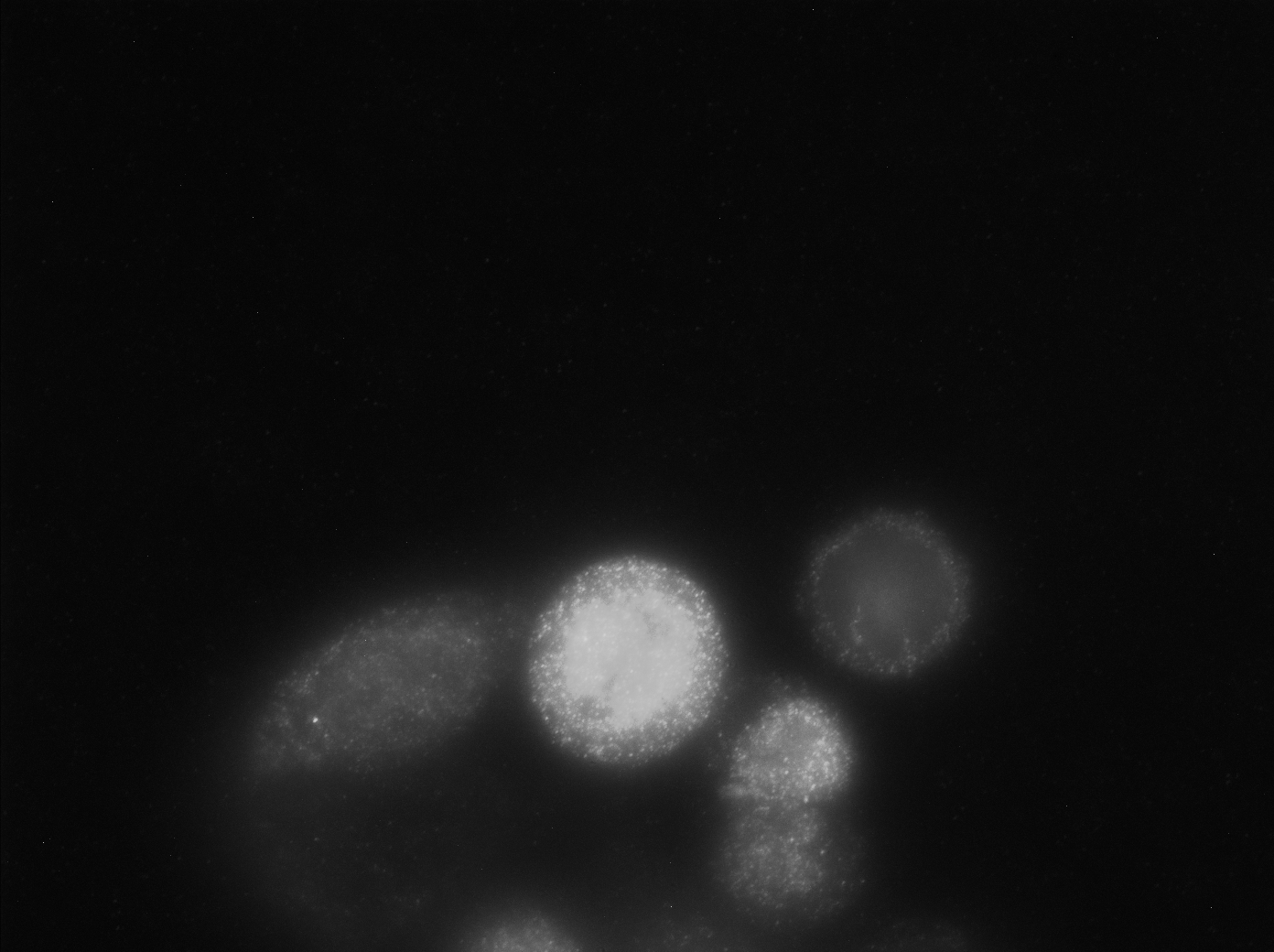

Supplement: Supplementary file 4 — Source data Fig. 2 part 1 [file 44319_2026_742_MOESM4_ESM.zip › Figure 2 Part 1/Fig 2c Cas9 Hela rGT335 atubulin/Cas9 GT335recomb atub 3-24-25 R1 M4.Project Maximum Z_XY1743104120_Z0_T0_C1.tif]

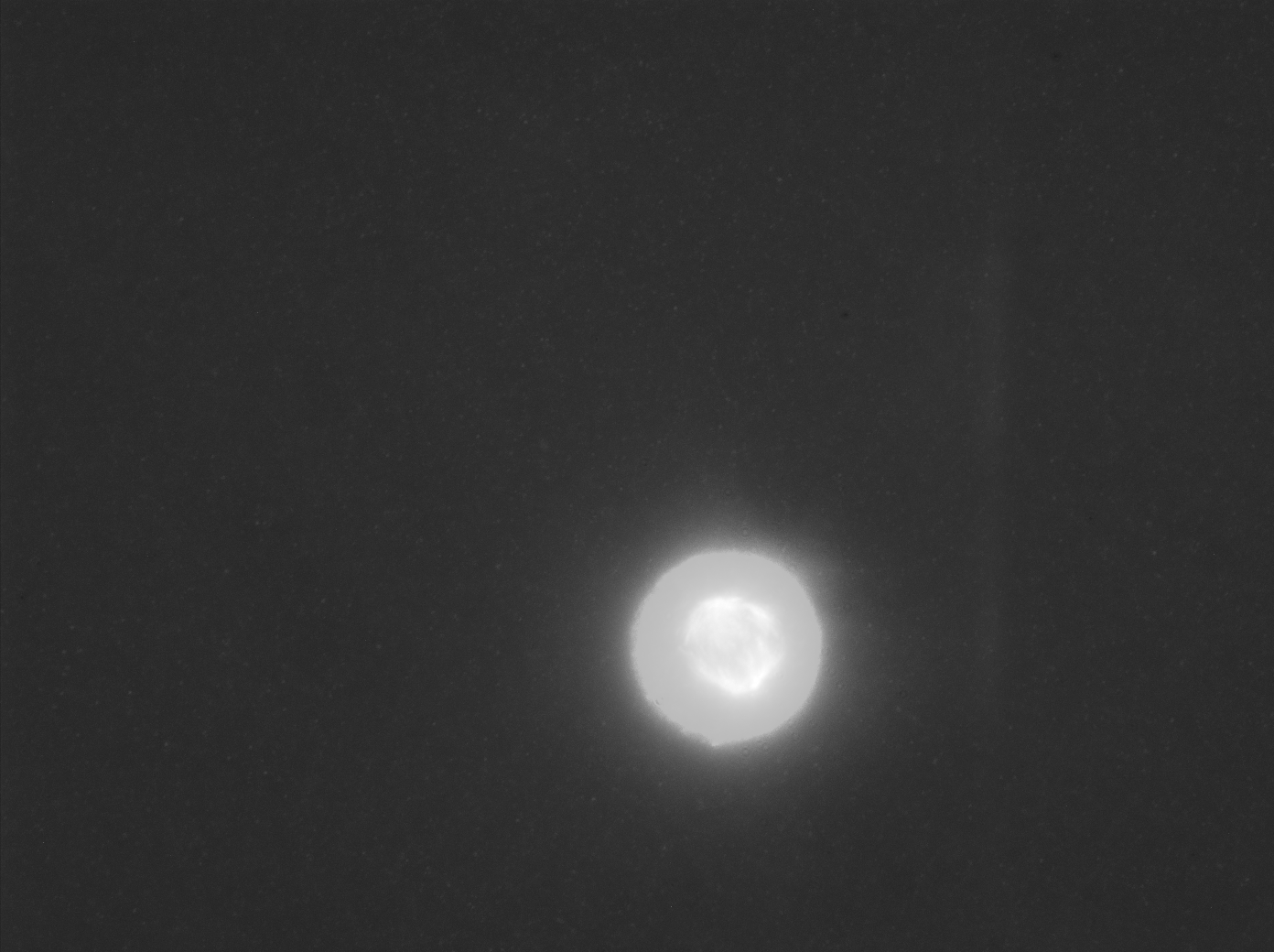

Supplement: Supplementary file 4 — Source data Fig. 2 part 1 [file 44319_2026_742_MOESM4_ESM.zip › Figure 2 Part 1/Fig 2c Cas9 Hela rGT335 atubulin/Cas9 GT335recomb atub 3-24-25 R1 M3.Project Maximum Z_XY1743103847_Z0_T0_C2.tif]

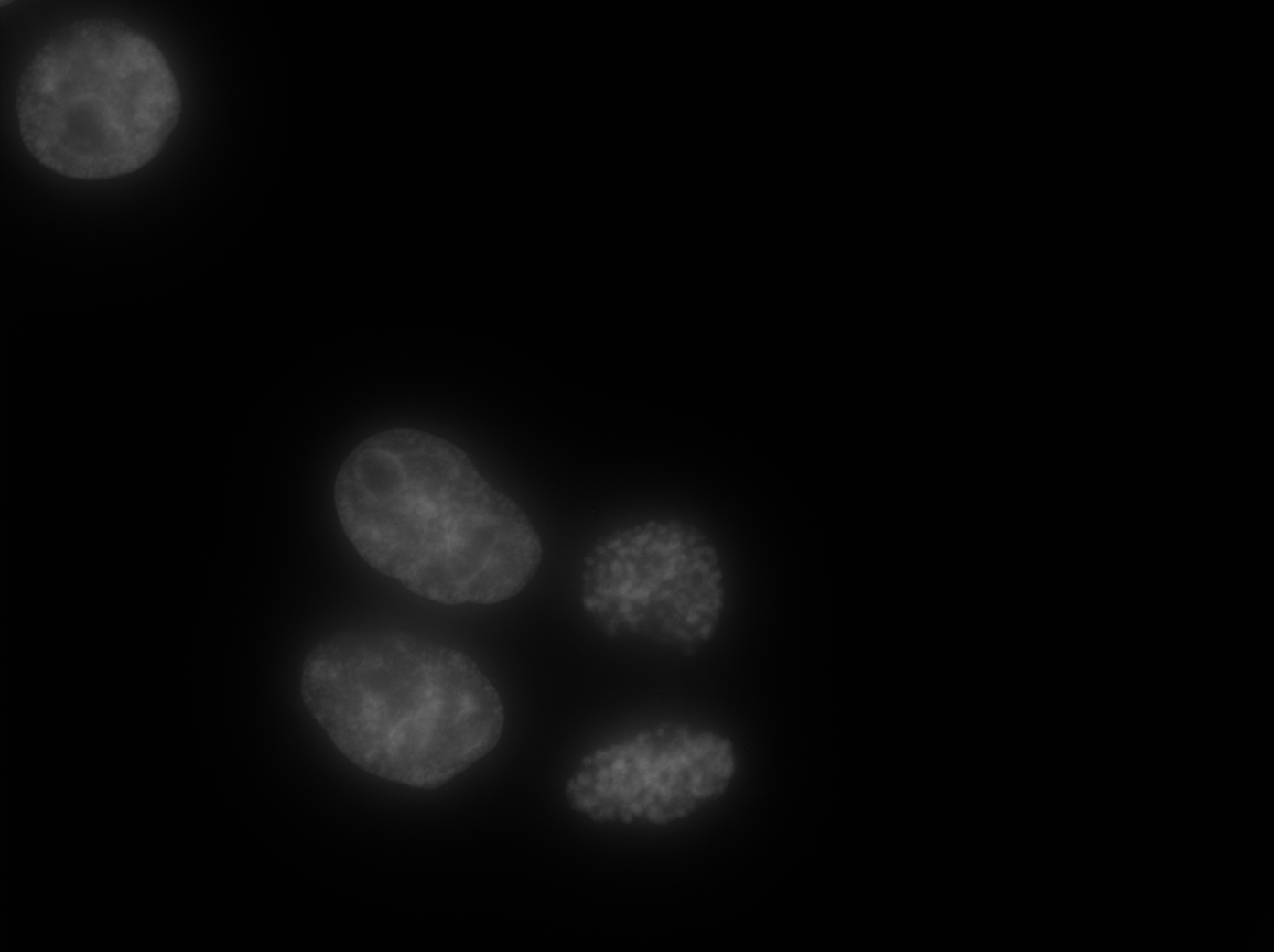

Supplement: Supplementary file 4 — Source data Fig. 2 part 1 [file 44319_2026_742_MOESM4_ESM.zip › Figure 2 Part 1/Fig 2c Cas9 Hela rGT335 atubulin/Cas9 GT335recomb atub 3-24-25 R2 ET6.Project Maximum Z_XY1743444007_Z0_T0_C0.tif]

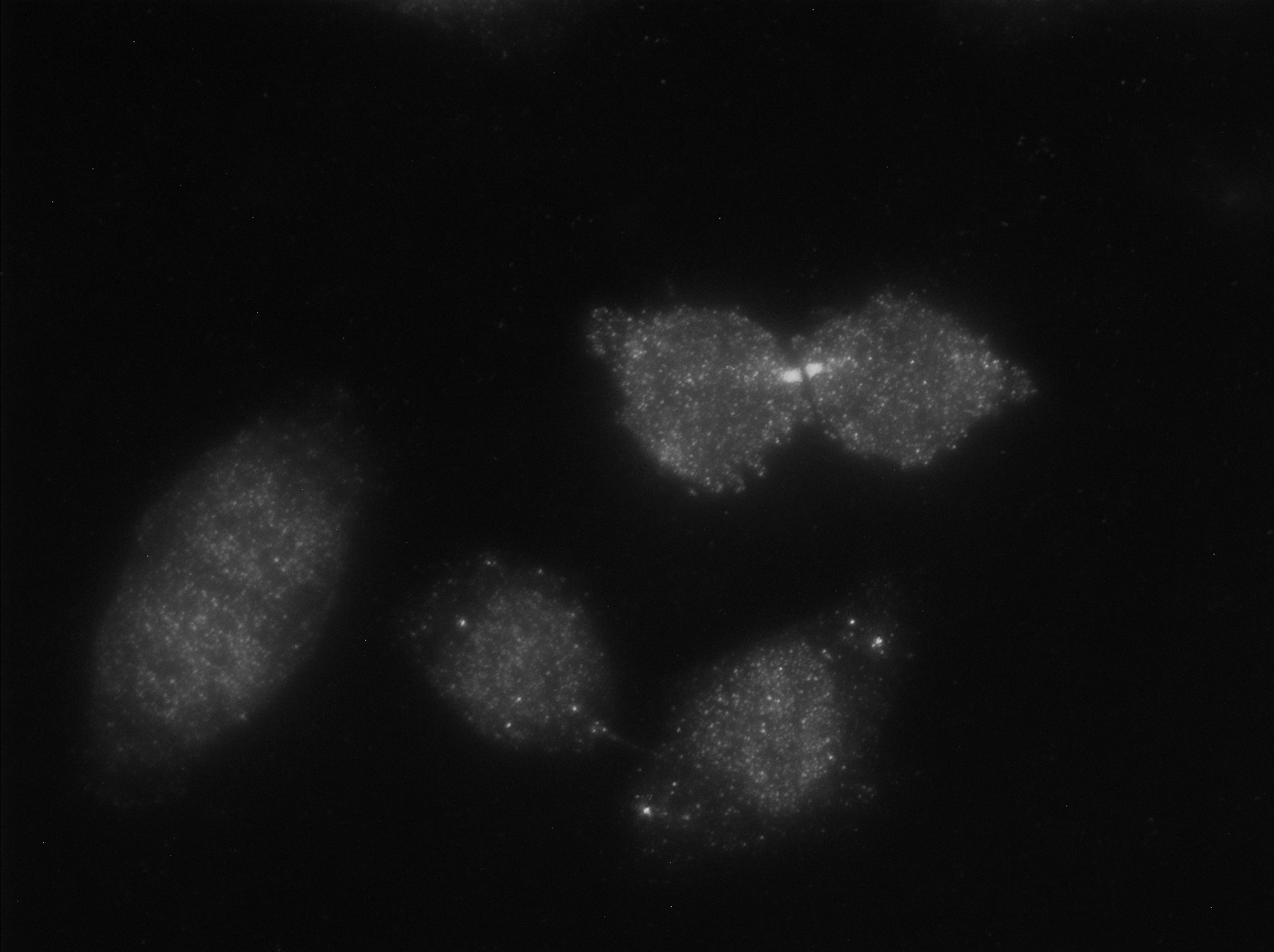

Supplement: Supplementary file 4 — Source data Fig. 2 part 1 [file 44319_2026_742_MOESM4_ESM.zip › Figure 2 Part 1/Fig 2c Cas9 Hela rGT335 atubulin/Cas9 GT335recomb atub 3-24-25 R2 ET2 LT4.Project Maximum Z_XY1743439952_Z0_T0_C1.tif]

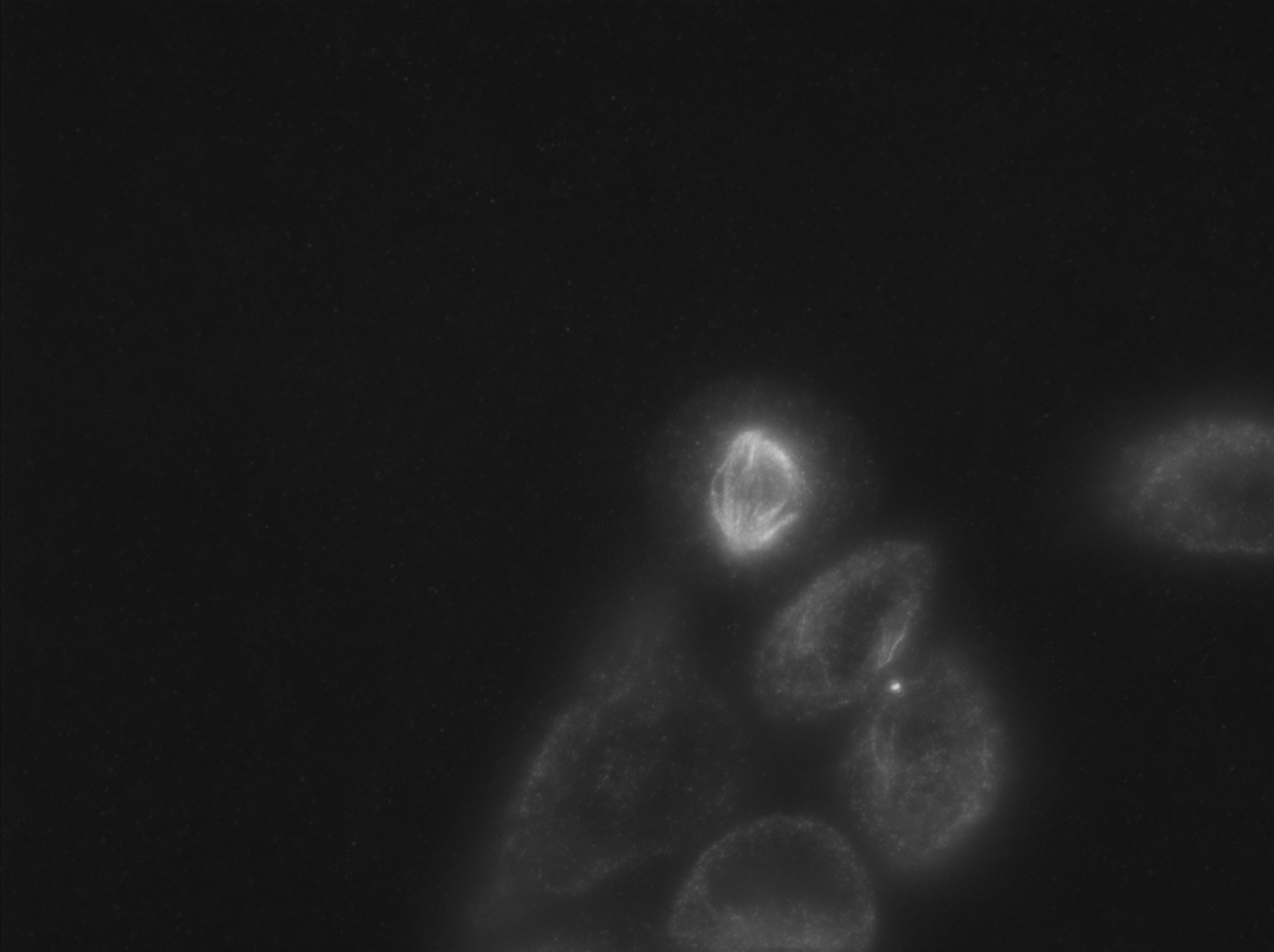

Supplement: Supplementary file 4 — Source data Fig. 2 part 1 [file 44319_2026_742_MOESM4_ESM.zip › Figure 2 Part 1/Fig 2c Cas9 Hela rGT335 atubulin/Cas9 GT335recomb atub 3-24-25 R3 M5.Project Maximum Z_XY1743453240_Z0_T0_C2.tif]

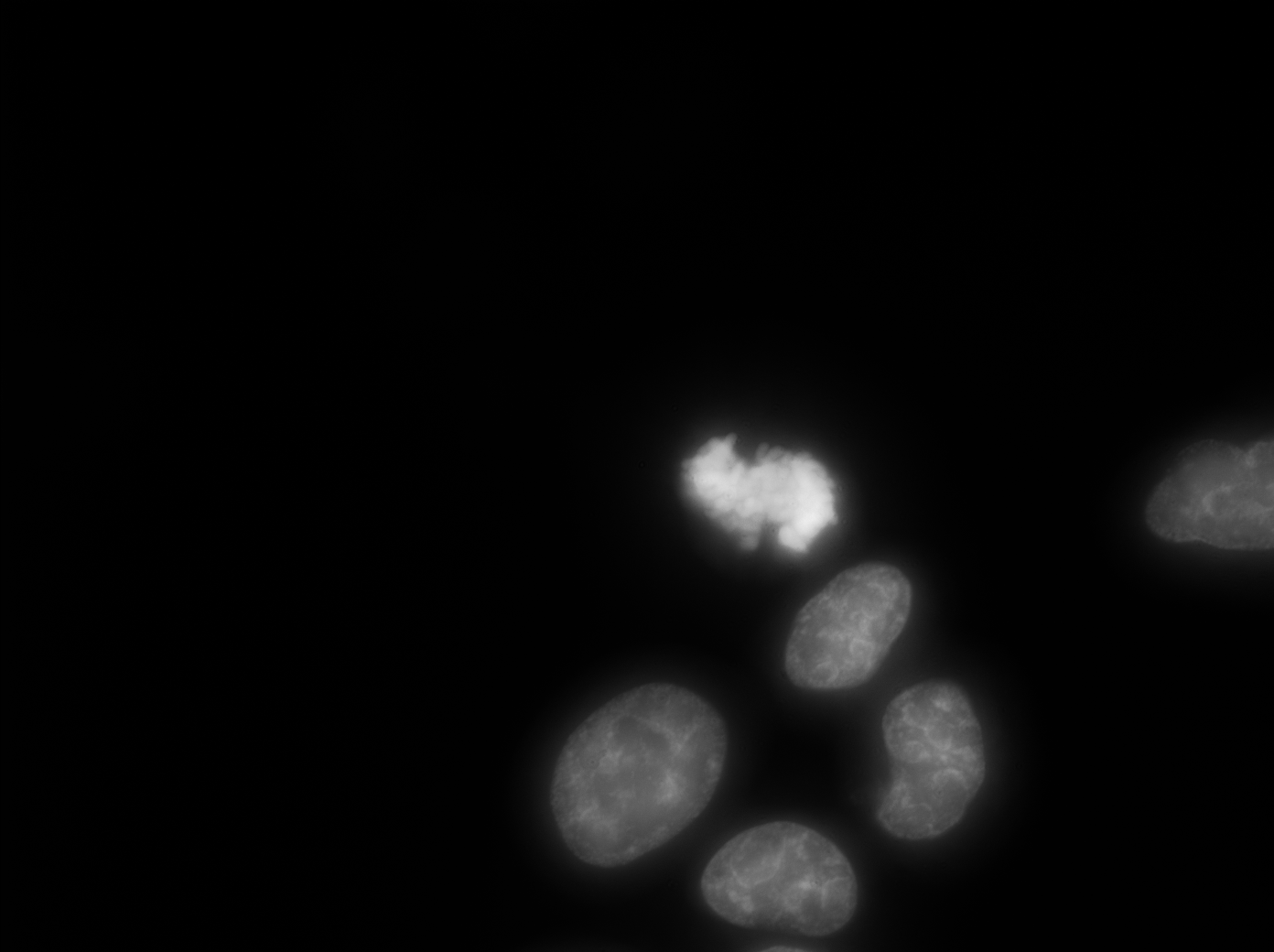

Supplement: Supplementary file 4 — Source data Fig. 2 part 1 [file 44319_2026_742_MOESM4_ESM.zip › Figure 2 Part 1/Fig 2c Cas9 Hela rGT335 atubulin/Cas9 GT335recomb atub 3-24-25 R3 M5.Project Maximum Z_XY1743453240_Z0_T0_C0.tif]

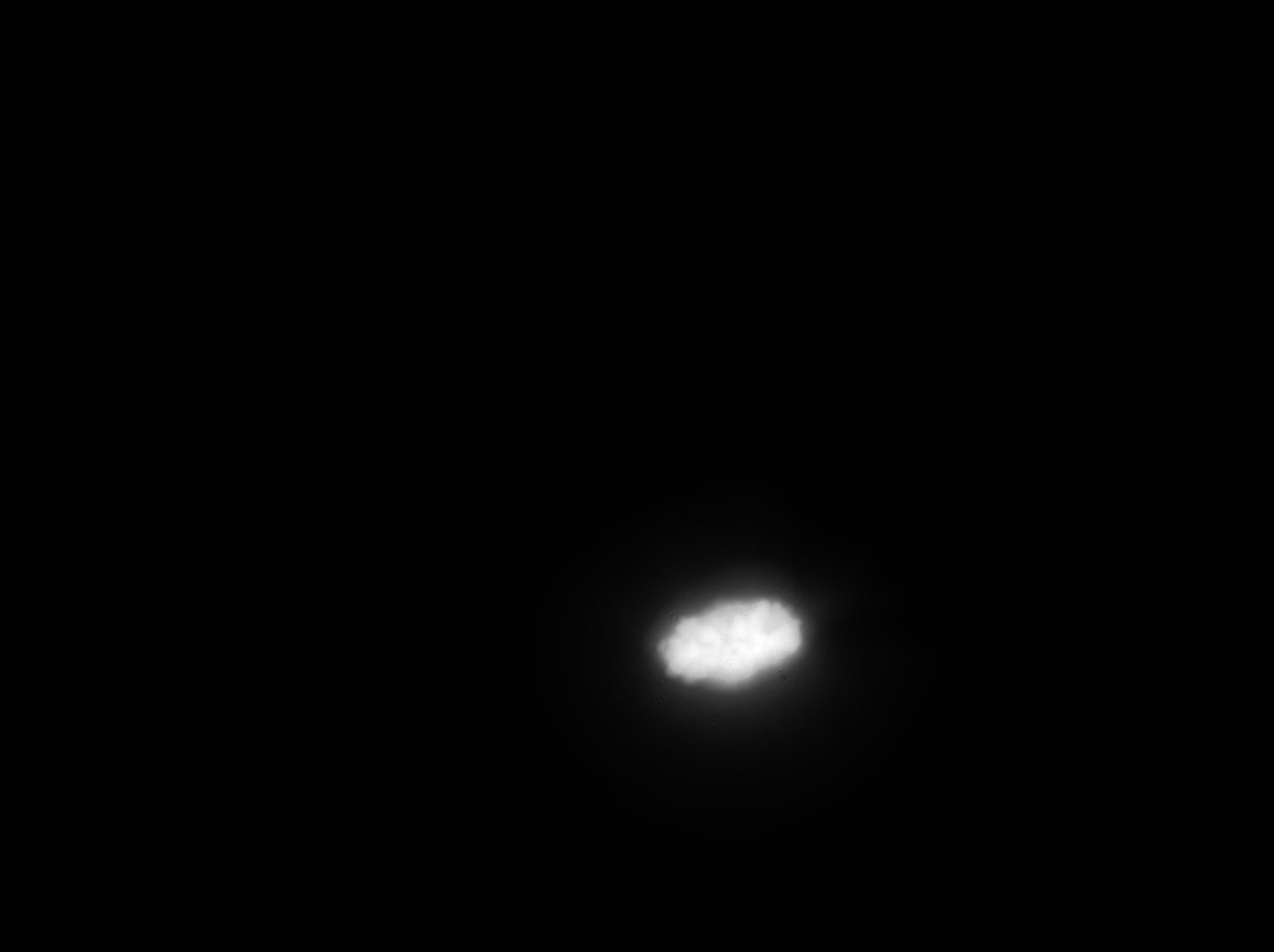

Supplement: Supplementary file 4 — Source data Fig. 2 part 1 [file 44319_2026_742_MOESM4_ESM.zip › Figure 2 Part 1/Fig 2c Cas9 Hela rGT335 atubulin/Cas9 GT335recomb atub 3-24-25 R1 M3.Project Maximum Z_XY1743103847_Z0_T0_C0.tif]

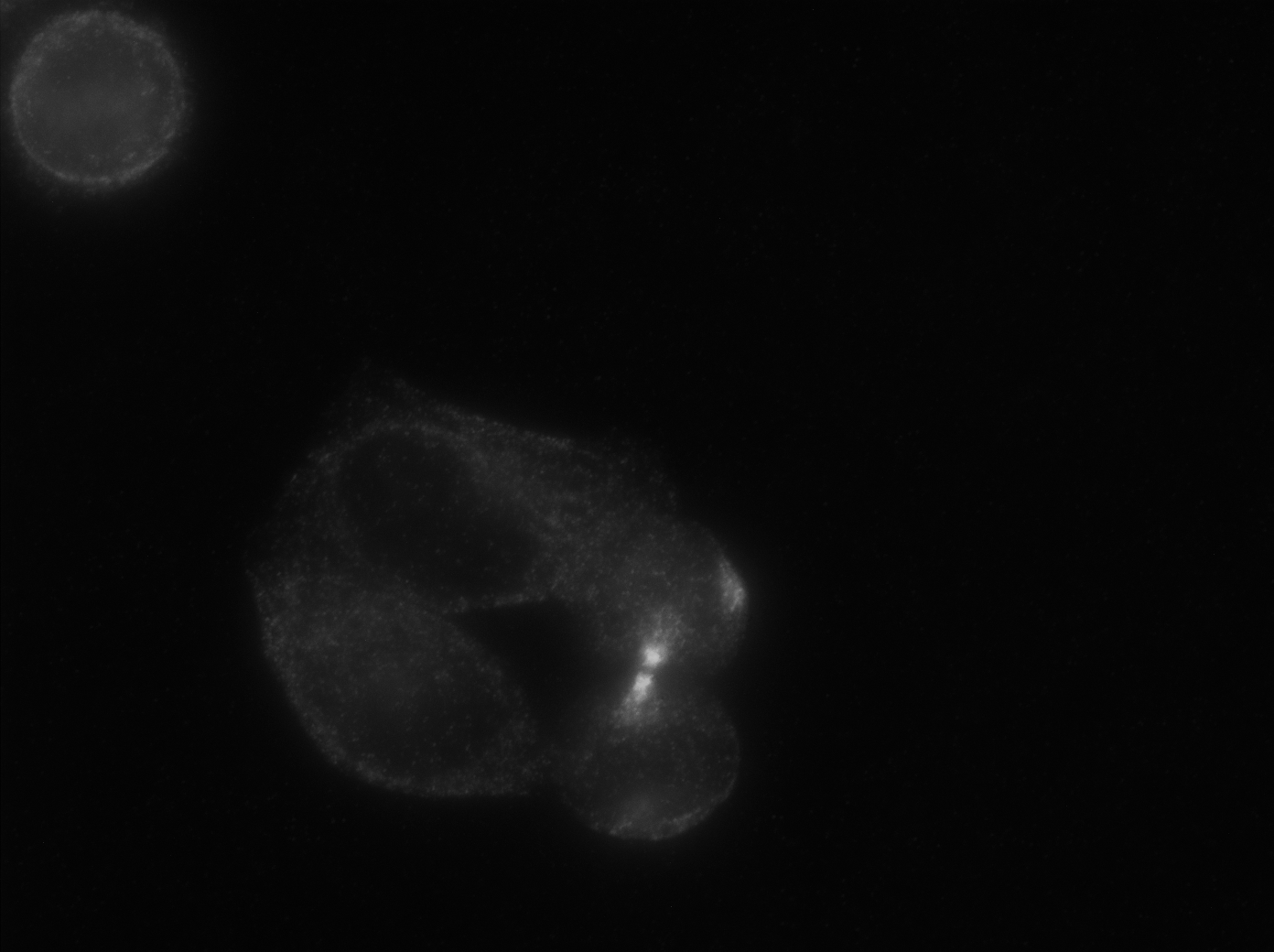

Supplement: Supplementary file 4 — Source data Fig. 2 part 1 [file 44319_2026_742_MOESM4_ESM.zip › Figure 2 Part 1/Fig 2c Cas9 Hela rGT335 atubulin/Cas9 GT335recomb atub 3-24-25 R2 ET6.Project Maximum Z_XY1743444007_Z0_T0_C2.tif]

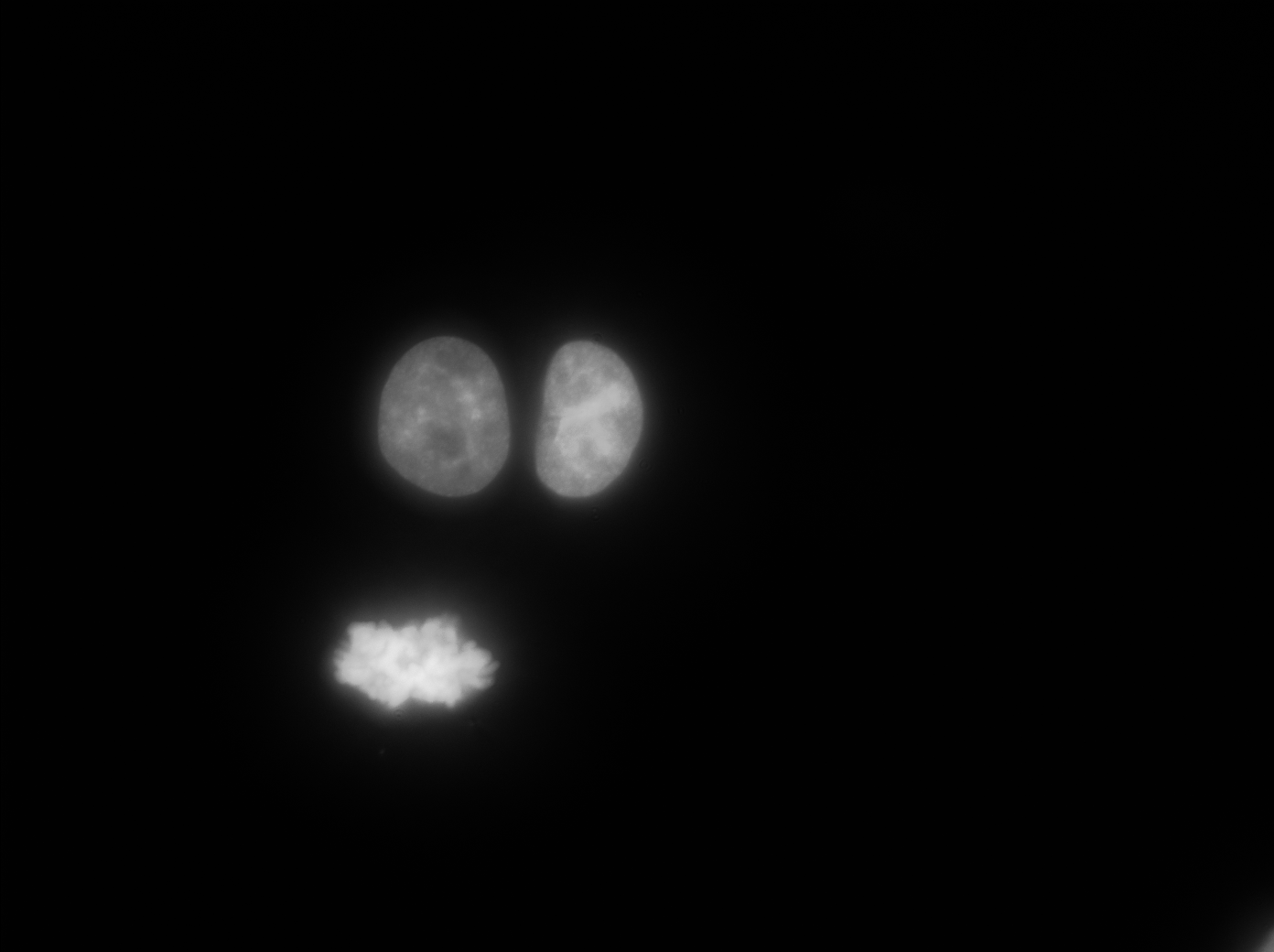

Supplement: Supplementary file 4 — Source data Fig. 2 part 1 [file 44319_2026_742_MOESM4_ESM.zip › Figure 2 Part 1/Fig 2c Cas9 Hela rGT335 atubulin/Cas9 GT335recomb atub 3-24-25 R1 M8.Project Maximum Z_XY1743105633_Z0_T0_C0.tif]

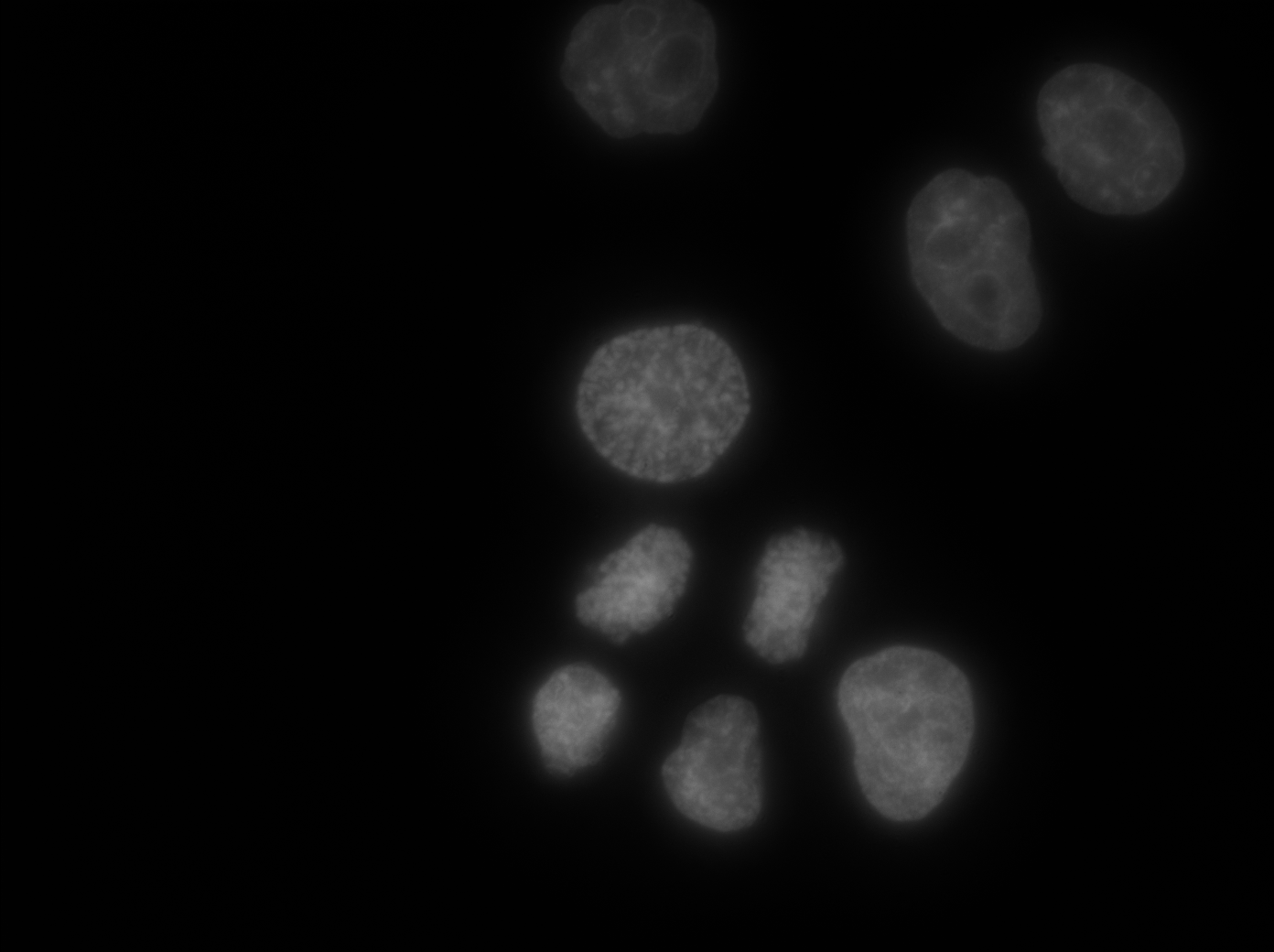

Supplement: Supplementary file 4 — Source data Fig. 2 part 1 [file 44319_2026_742_MOESM4_ESM.zip › Figure 2 Part 1/Fig 2c Cas9 Hela rGT335 atubulin/Cas9 GT335recomb atub 3-24-25 R1 PA3.Project Maximum Z_XY1742836284_Z0_T0_C0.tif]

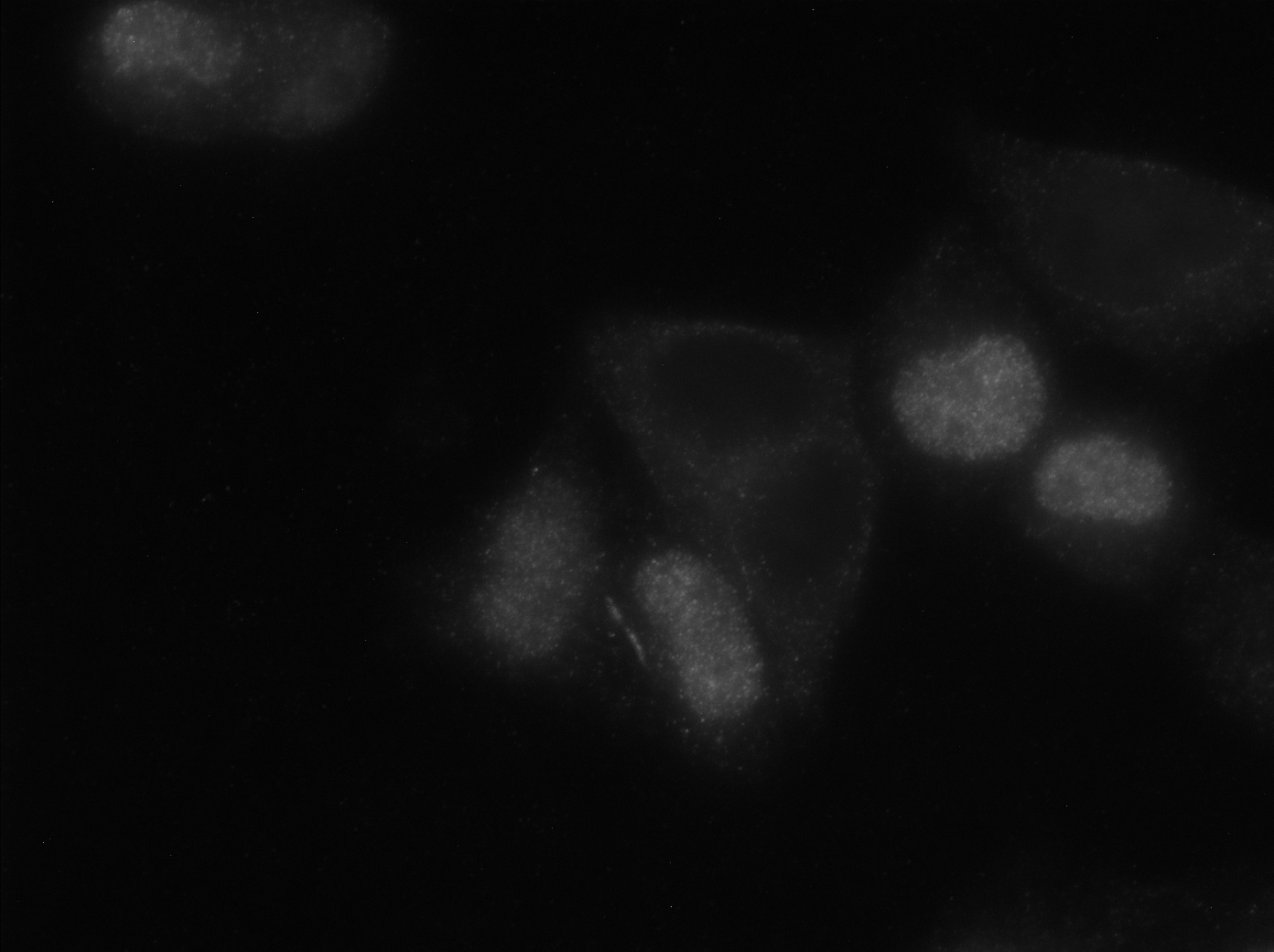

Supplement: Supplementary file 4 — Source data Fig. 2 part 1 [file 44319_2026_742_MOESM4_ESM.zip › Figure 2 Part 1/Fig 2c Cas9 Hela rGT335 atubulin/Cas9 GT335recomb atub 3-24-25 R1 LT2.Project Maximum Z_XY1743100727_Z0_T0_C1.tif]

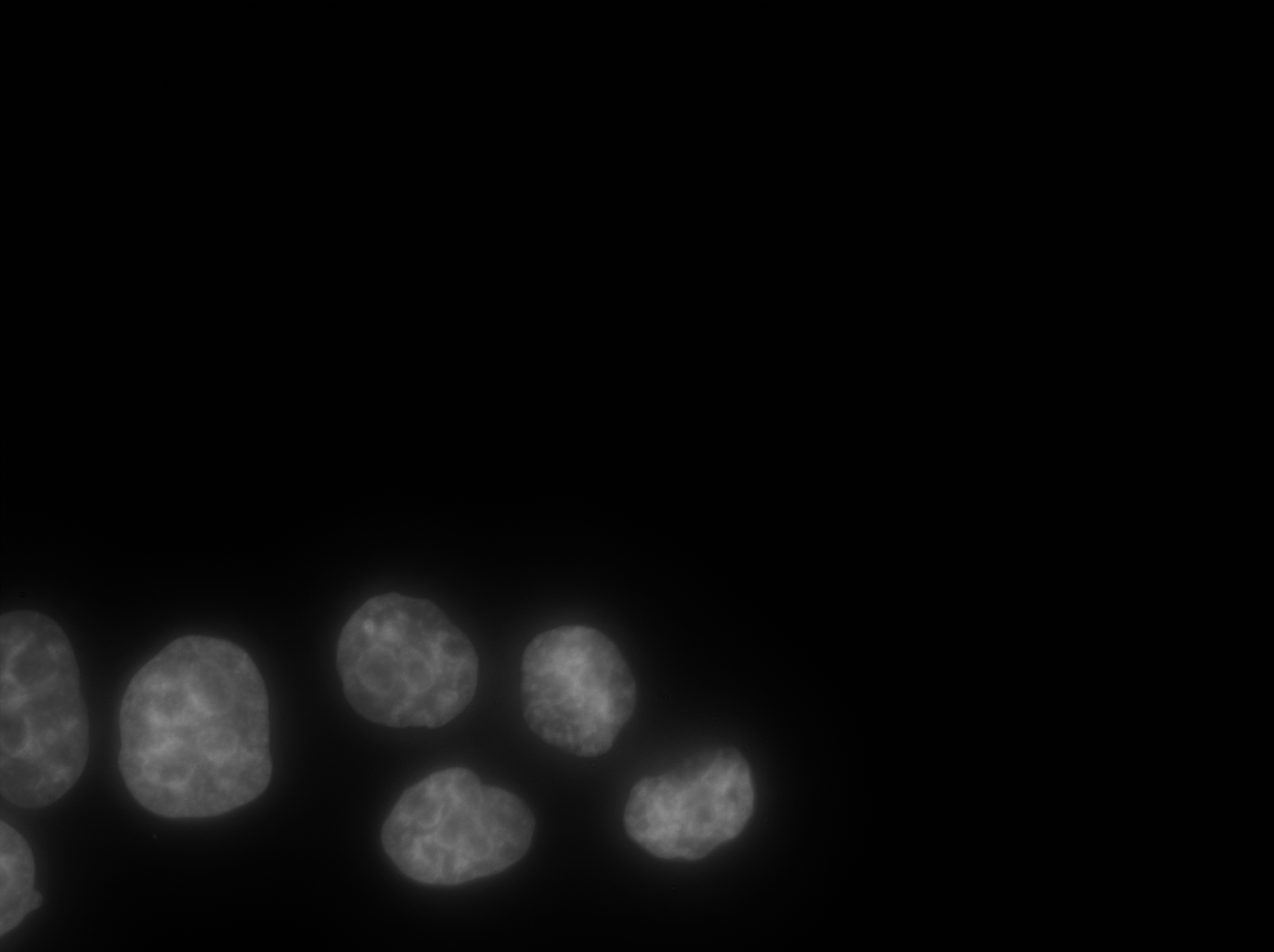

Supplement: Supplementary file 4 — Source data Fig. 2 part 1 [file 44319_2026_742_MOESM4_ESM.zip › Figure 2 Part 1/Fig 2c Cas9 Hela rGT335 atubulin/Cas9 GT335recomb atub 3-24-25 R1 LT7.Project Maximum Z_XY1743101575_Z0_T0_C0.tif]

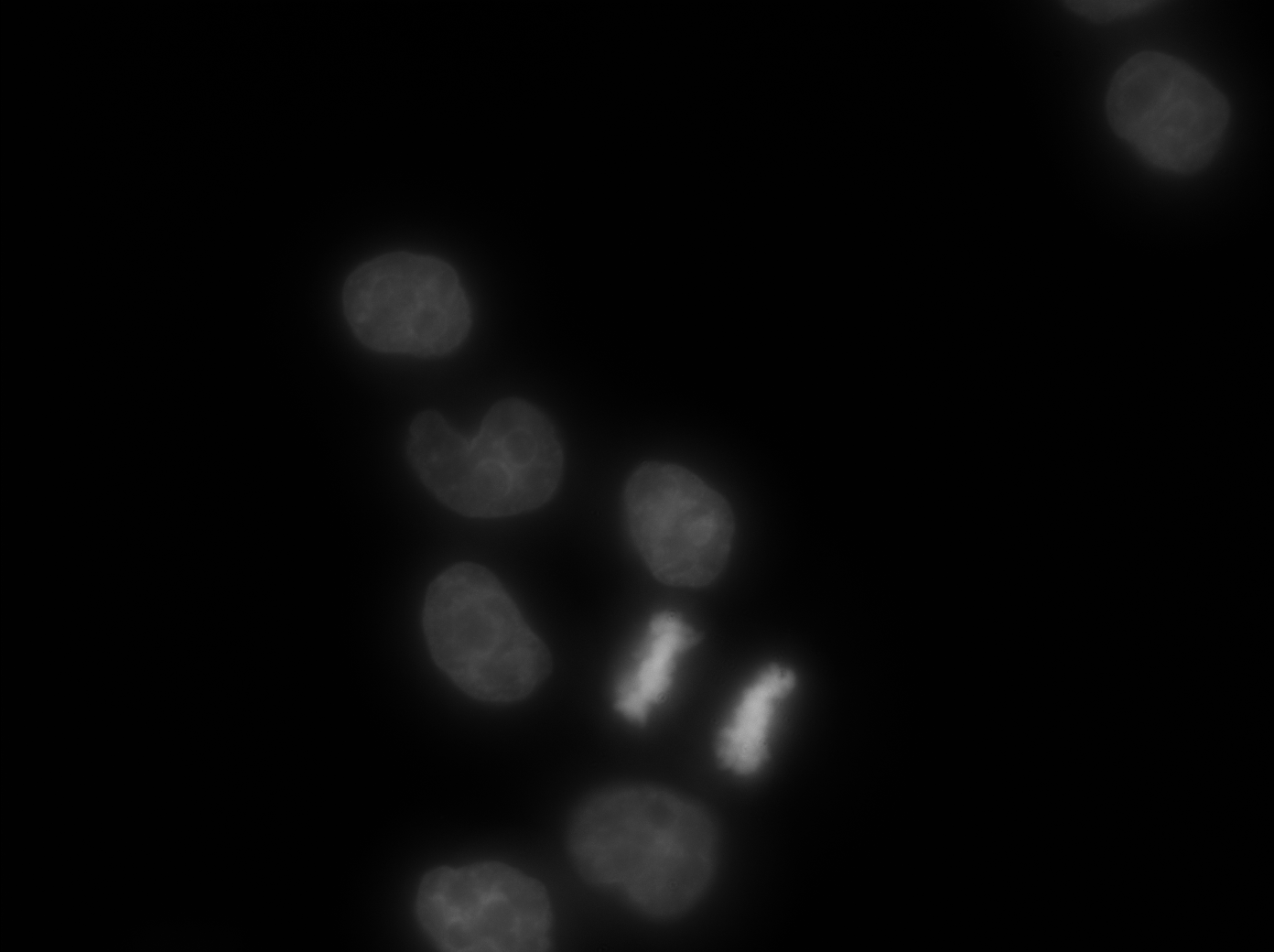

Supplement: Supplementary file 4 — Source data Fig. 2 part 1 [file 44319_2026_742_MOESM4_ESM.zip › Figure 2 Part 1/Fig 2c Cas9 Hela rGT335 atubulin/Cas9 GT335recomb atub 3-24-25 R1 A1.Project Maximum Z_XY1743104807_Z0_T0_C0.tif]

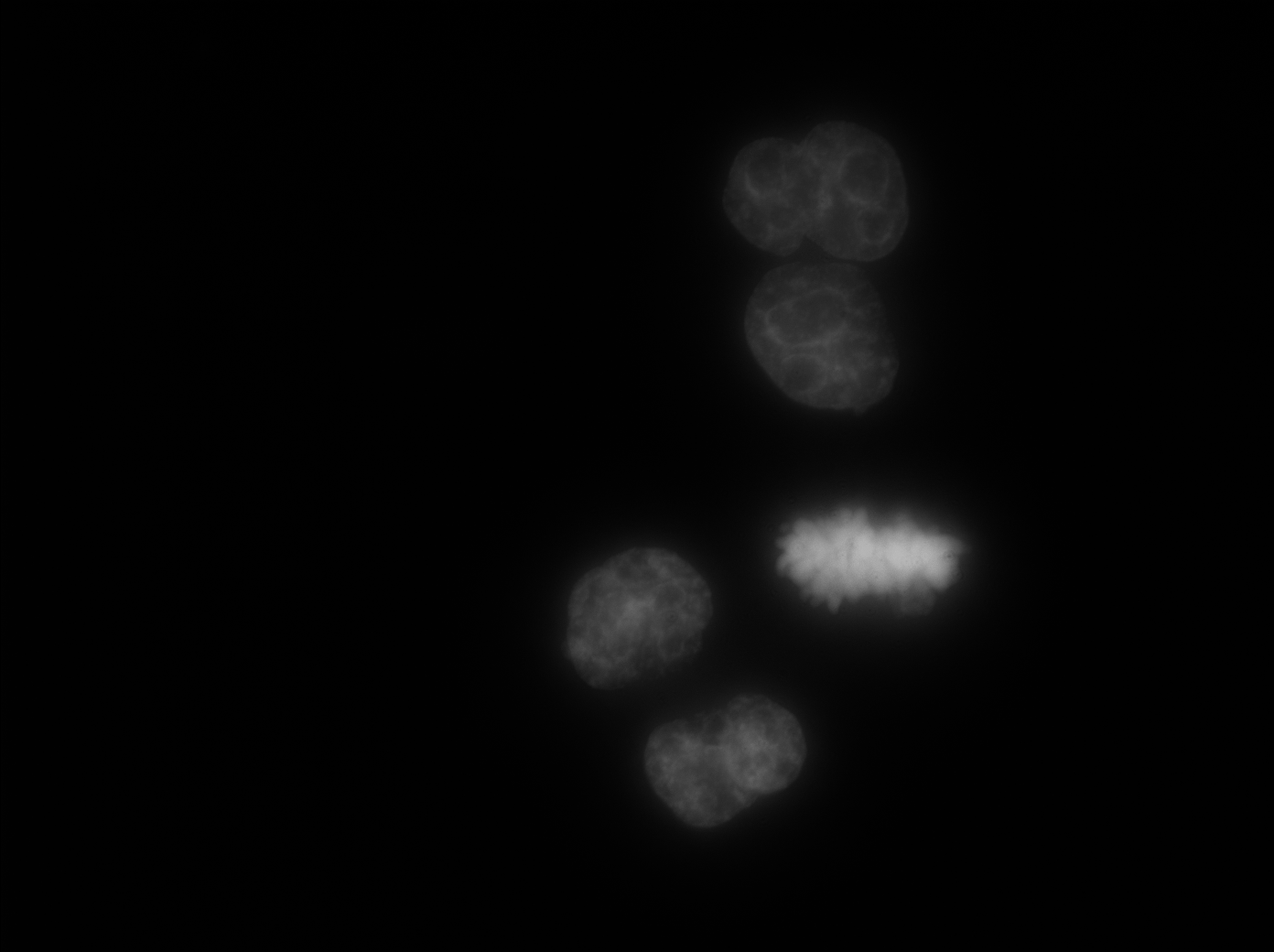

Supplement: Supplementary file 4 — Source data Fig. 2 part 1 [file 44319_2026_742_MOESM4_ESM.zip › Figure 2 Part 1/Fig 2c Cas9 Hela rGT335 atubulin/Cas9 GT335recomb atub 3-24-25 R2 M3.Project Maximum Z_XY1743444217_Z0_T0_C0.tif]

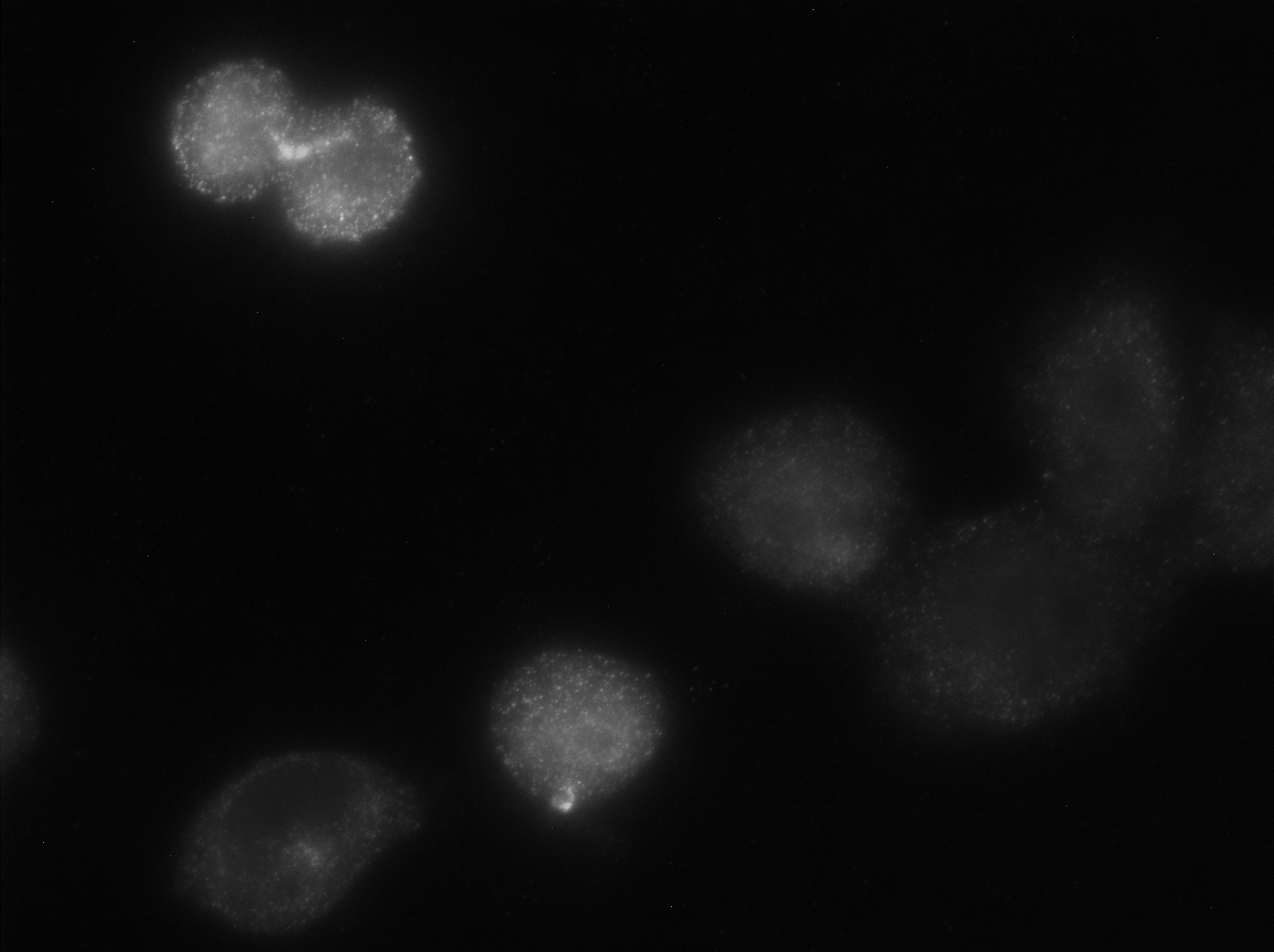

Supplement: Supplementary file 4 — Source data Fig. 2 part 1 [file 44319_2026_742_MOESM4_ESM.zip › Figure 2 Part 1/Fig 2c Cas9 Hela rGT335 atubulin/Cas9 GT335recomb atub 3-24-25 R1 ET4 PA6.Project Maximum Z_XY1743101663_Z0_T0_C1.tif]

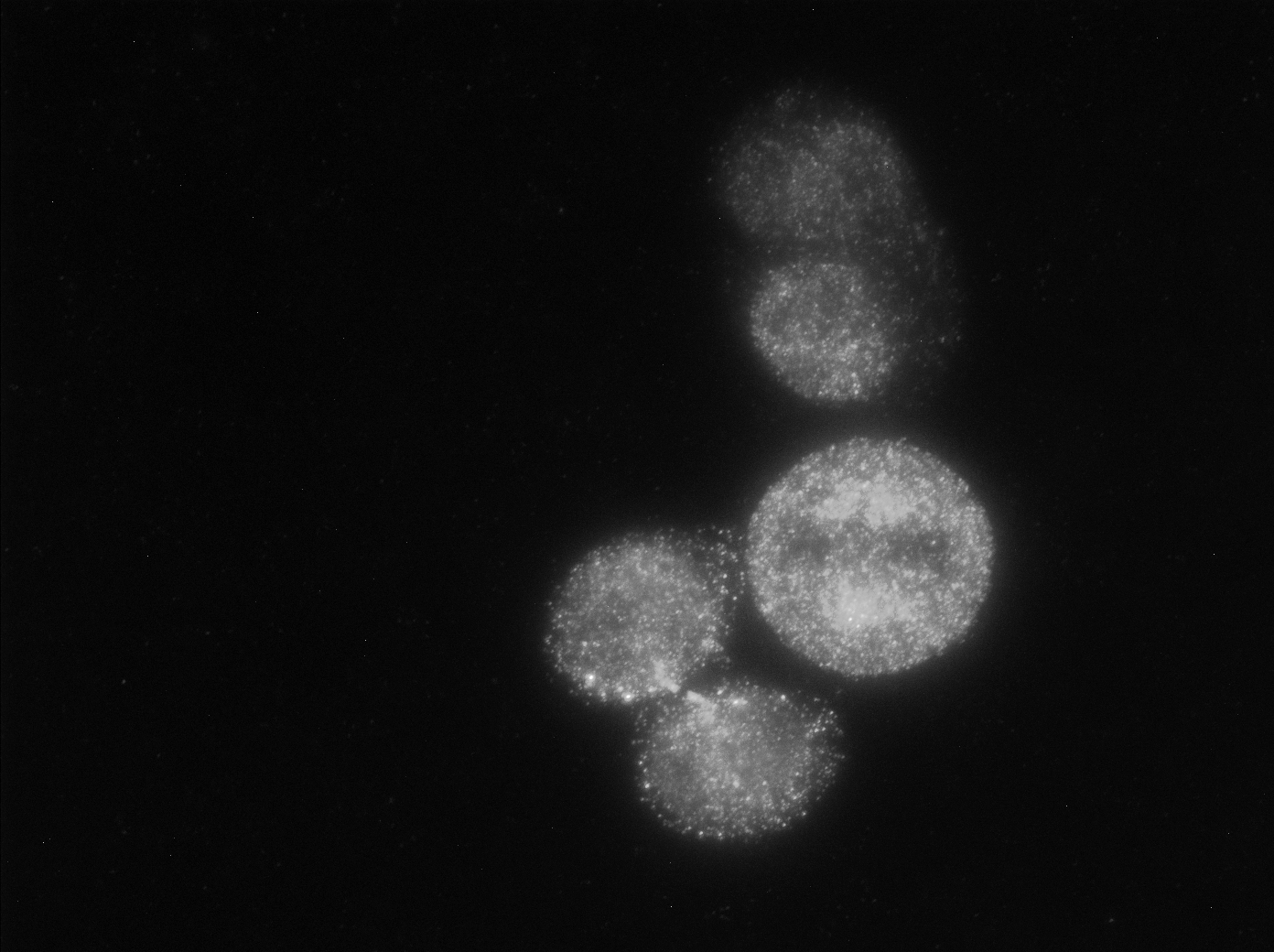

Supplement: Supplementary file 4 — Source data Fig. 2 part 1 [file 44319_2026_742_MOESM4_ESM.zip › Figure 2 Part 1/Fig 2c Cas9 Hela rGT335 atubulin/Cas9 GT335recomb atub 3-24-25 R2 M3.Project Maximum Z_XY1743444217_Z0_T0_C1.tif]

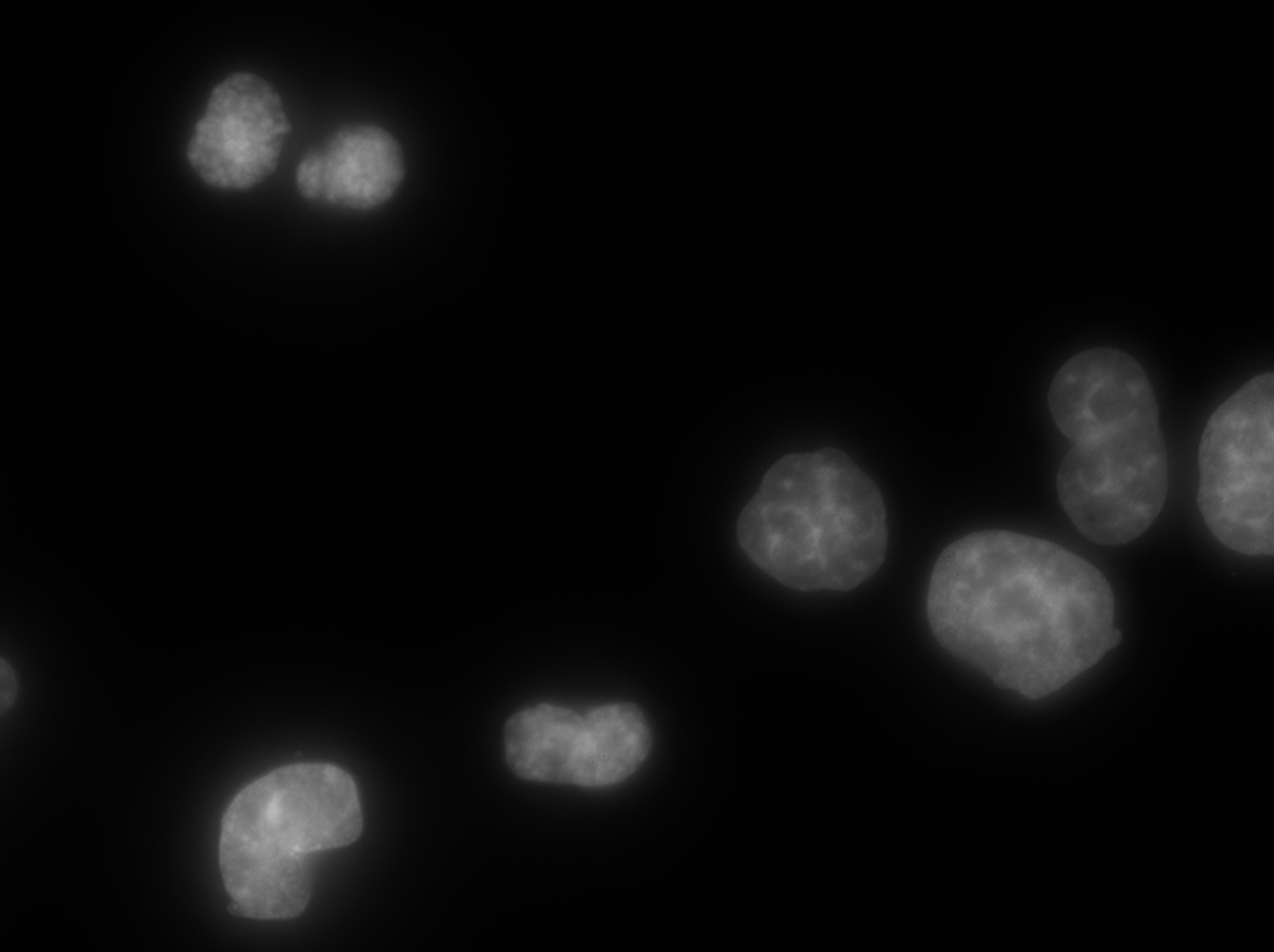

Supplement: Supplementary file 4 — Source data Fig. 2 part 1 [file 44319_2026_742_MOESM4_ESM.zip › Figure 2 Part 1/Fig 2c Cas9 Hela rGT335 atubulin/Cas9 GT335recomb atub 3-24-25 R1 ET4 PA6.Project Maximum Z_XY1743101663_Z0_T0_C0.tif]

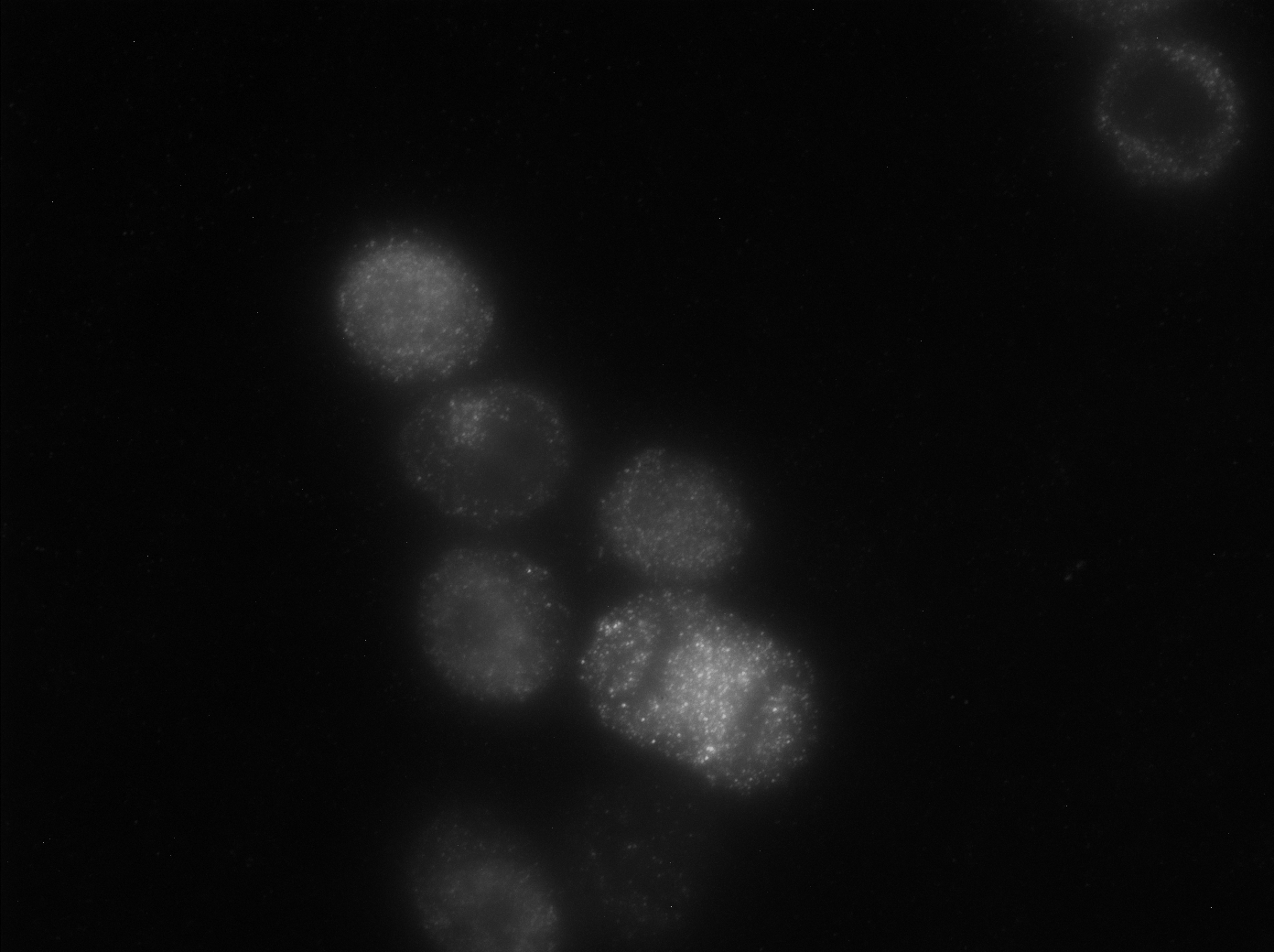

Supplement: Supplementary file 4 — Source data Fig. 2 part 1 [file 44319_2026_742_MOESM4_ESM.zip › Figure 2 Part 1/Fig 2c Cas9 Hela rGT335 atubulin/Cas9 GT335recomb atub 3-24-25 R1 A1.Project Maximum Z_XY1743104807_Z0_T0_C1.tif]

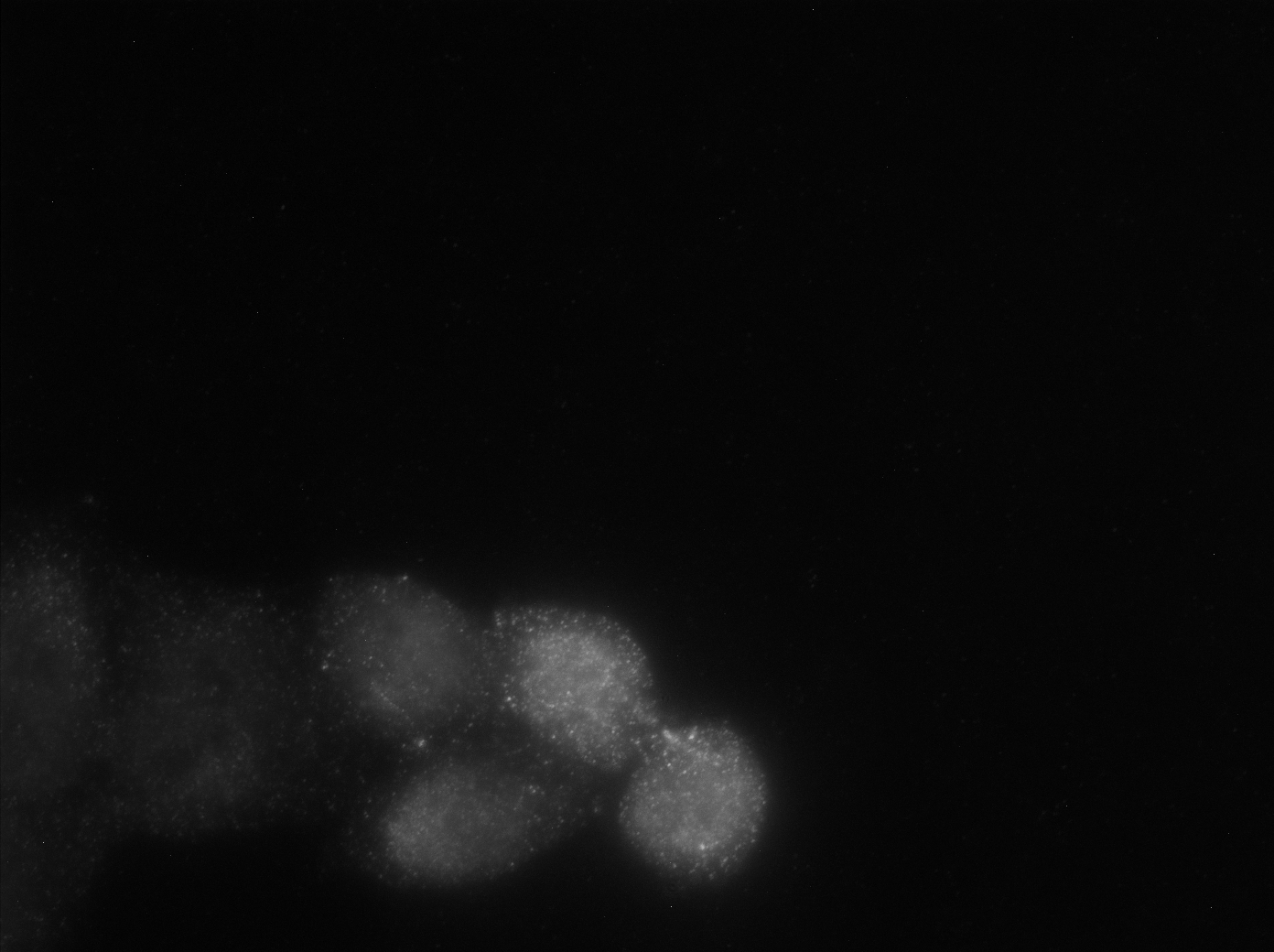

Supplement: Supplementary file 4 — Source data Fig. 2 part 1 [file 44319_2026_742_MOESM4_ESM.zip › Figure 2 Part 1/Fig 2c Cas9 Hela rGT335 atubulin/Cas9 GT335recomb atub 3-24-25 R1 LT7.Project Maximum Z_XY1743101575_Z0_T0_C1.tif]

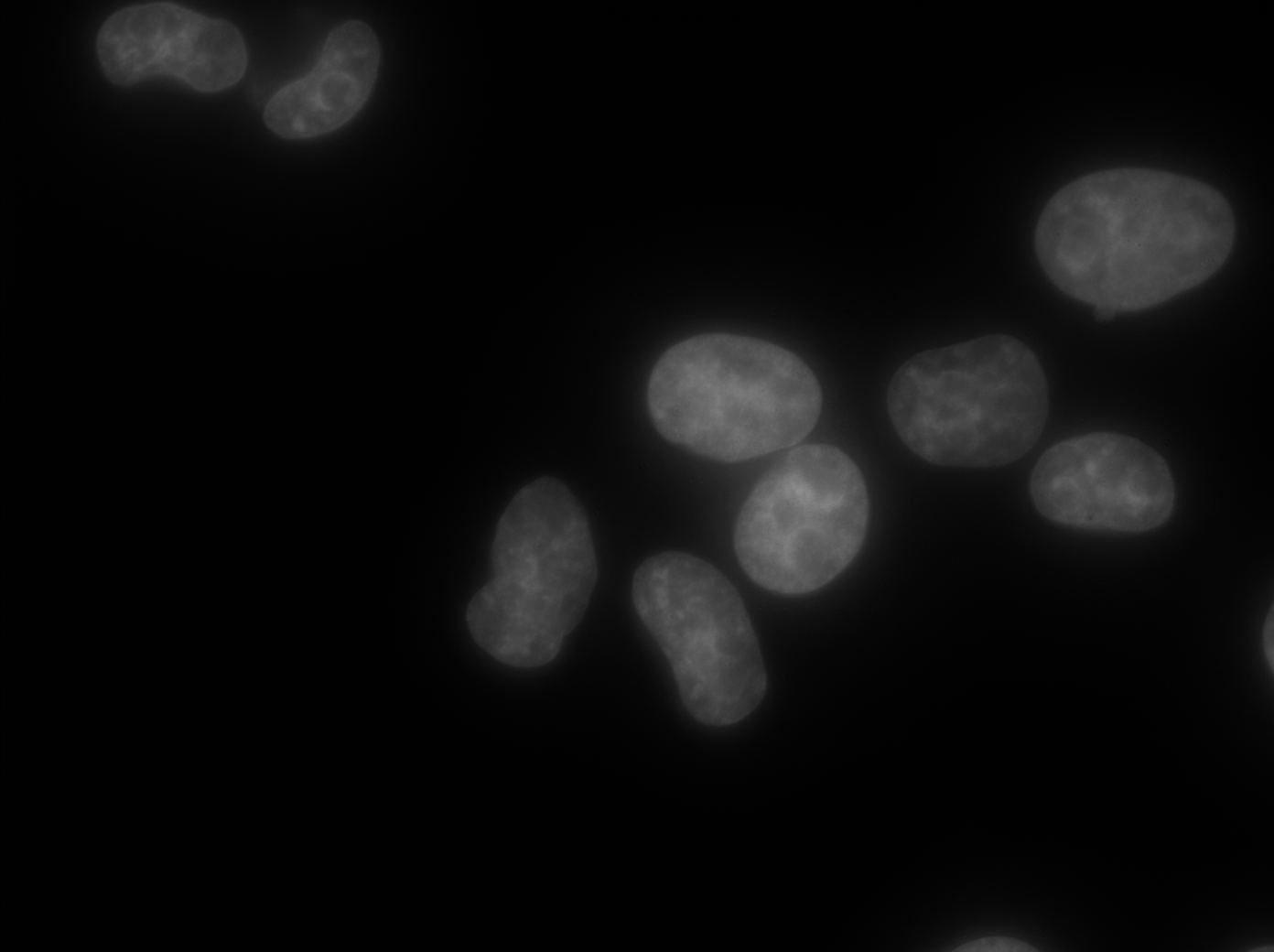

Supplement: Supplementary file 4 — Source data Fig. 2 part 1 [file 44319_2026_742_MOESM4_ESM.zip › Figure 2 Part 1/Fig 2c Cas9 Hela rGT335 atubulin/Cas9 GT335recomb atub 3-24-25 R1 LT2.Project Maximum Z_XY1743100727_Z0_T0_C0.tif]

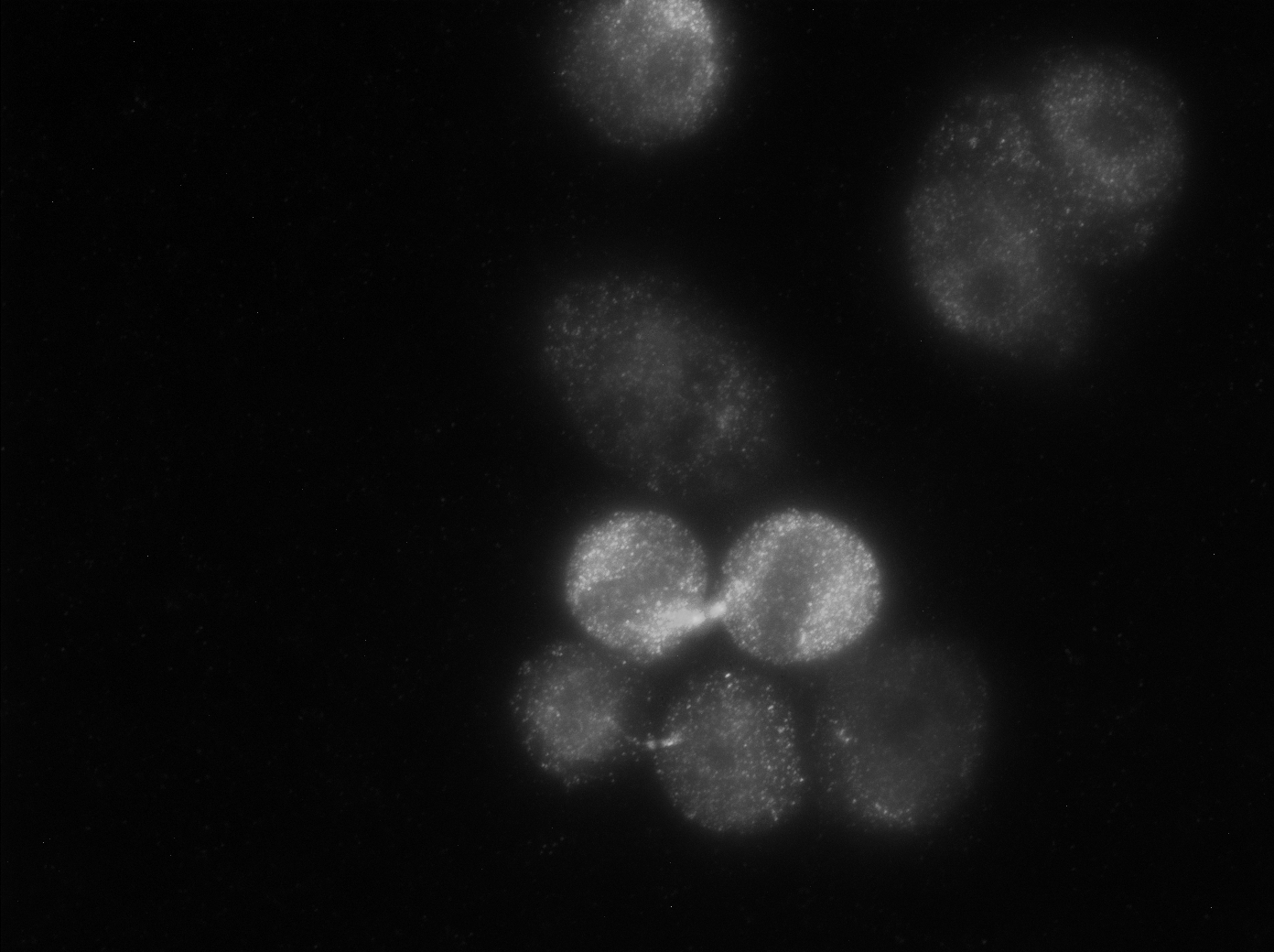

Supplement: Supplementary file 4 — Source data Fig. 2 part 1 [file 44319_2026_742_MOESM4_ESM.zip › Figure 2 Part 1/Fig 2c Cas9 Hela rGT335 atubulin/Cas9 GT335recomb atub 3-24-25 R1 PA3.Project Maximum Z_XY1742836284_Z0_T0_C1.tif]

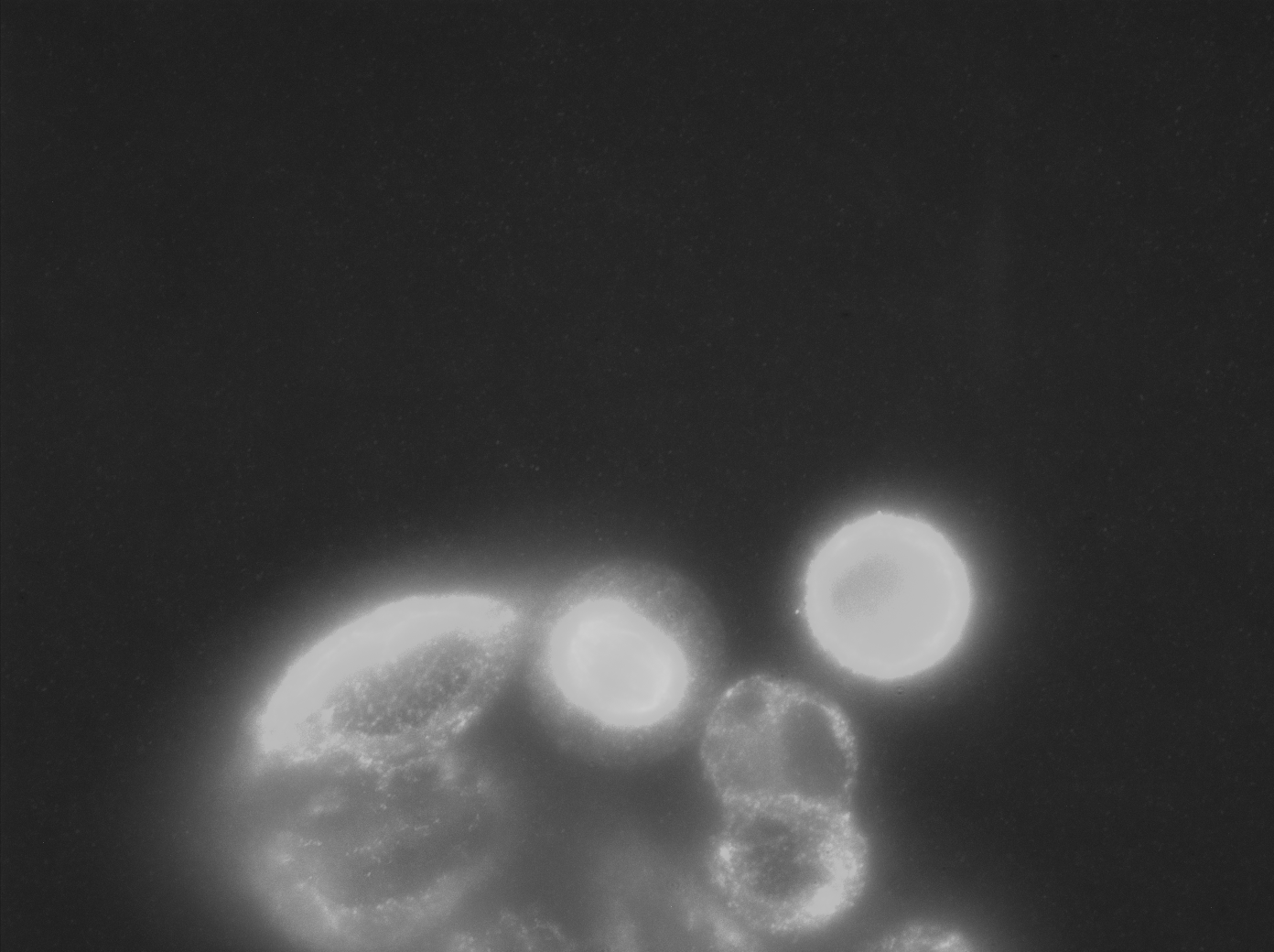

Supplement: Supplementary file 4 — Source data Fig. 2 part 1 [file 44319_2026_742_MOESM4_ESM.zip › Figure 2 Part 1/Fig 2c Cas9 Hela rGT335 atubulin/Cas9 GT335recomb atub 3-24-25 R1 M4.Project Maximum Z_XY1743104120_Z0_T0_C2.tif]

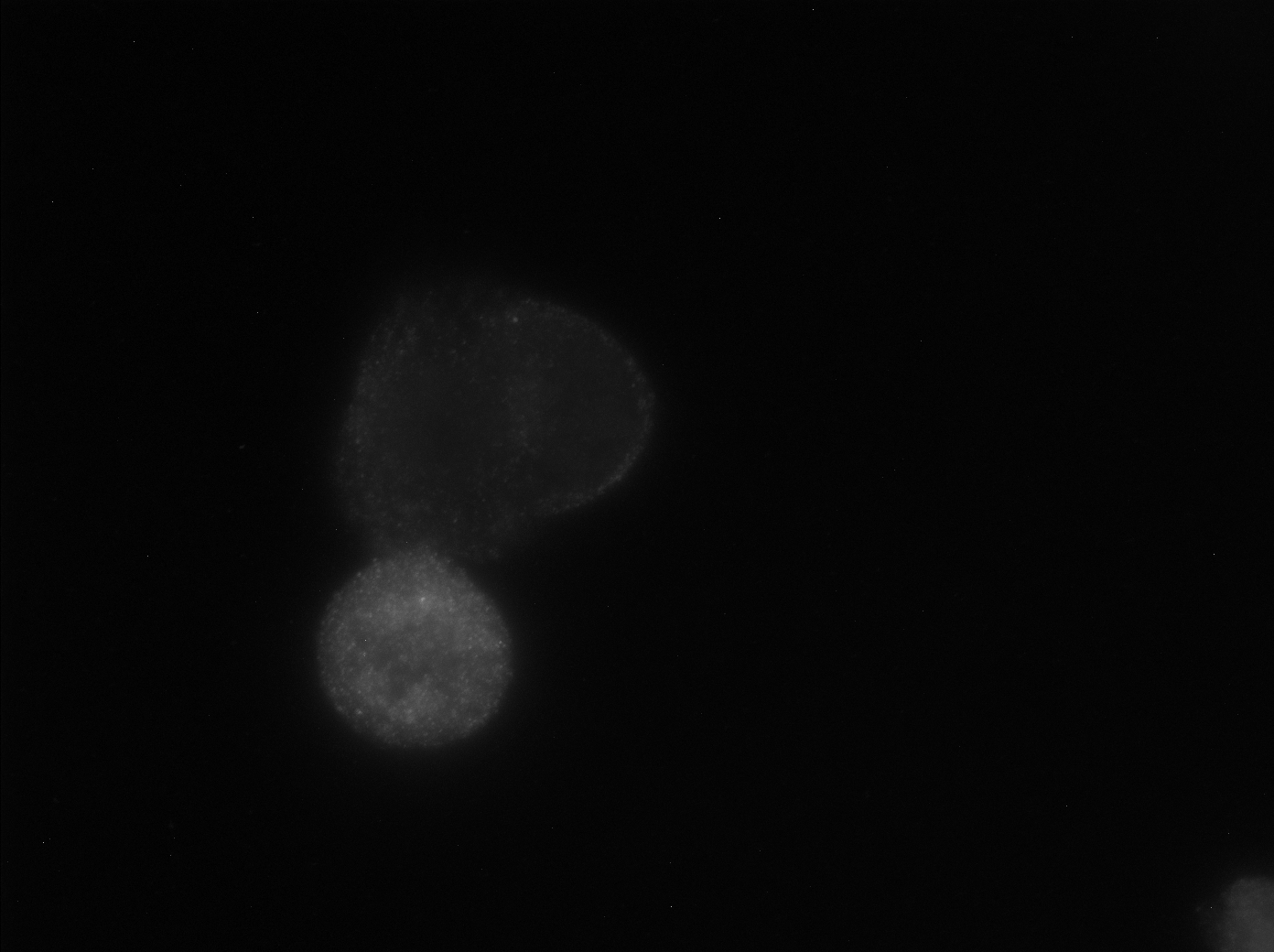

Supplement: Supplementary file 4 — Source data Fig. 2 part 1 [file 44319_2026_742_MOESM4_ESM.zip › Figure 2 Part 1/Fig 2c Cas9 Hela rGT335 atubulin/Cas9 GT335recomb atub 3-24-25 R1 M8.Project Maximum Z_XY1743105633_Z0_T0_C1.tif]

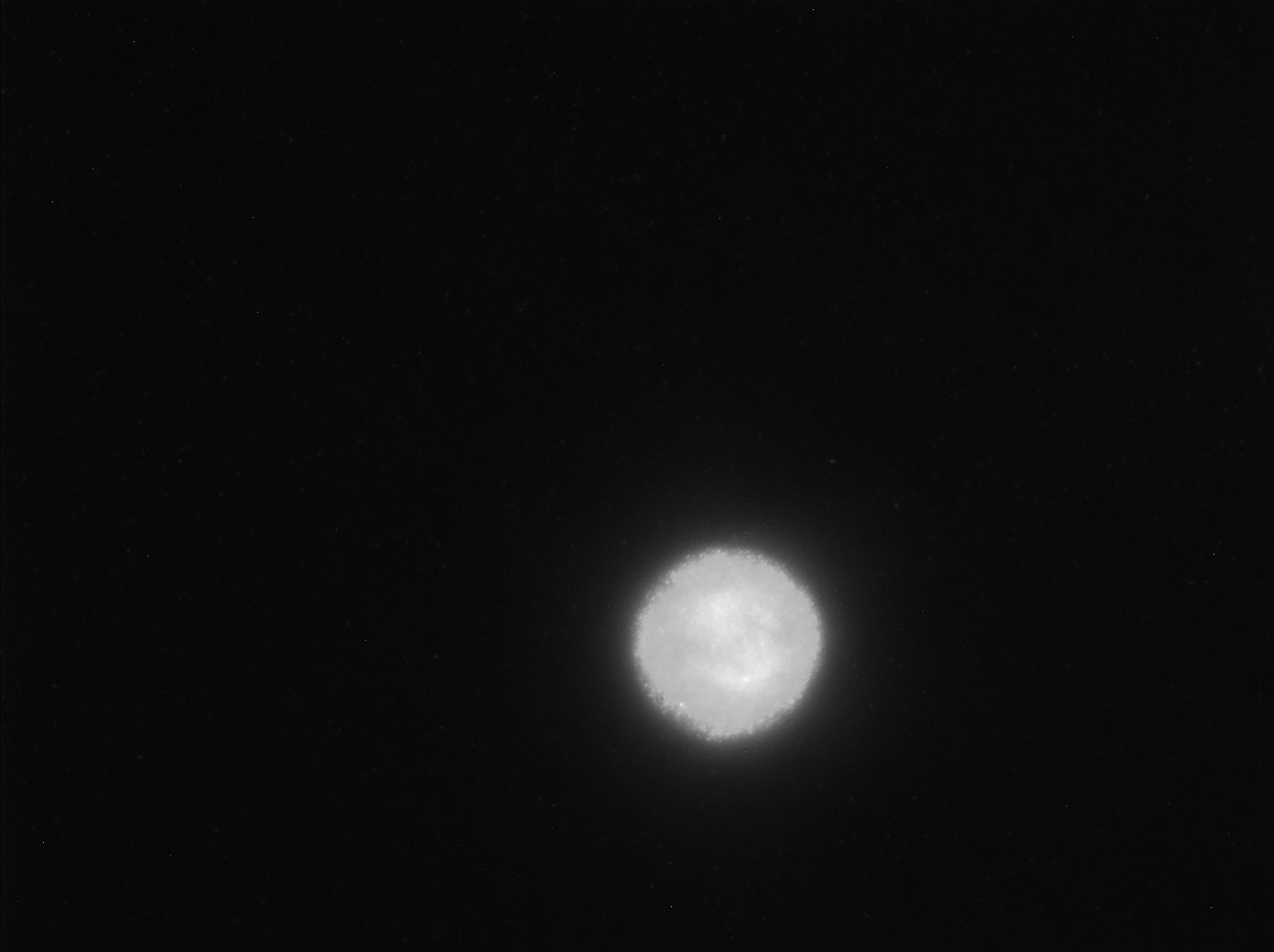

Supplement: Supplementary file 4 — Source data Fig. 2 part 1 [file 44319_2026_742_MOESM4_ESM.zip › Figure 2 Part 1/Fig 2c Cas9 Hela rGT335 atubulin/Cas9 GT335recomb atub 3-24-25 R1 M3.Project Maximum Z_XY1743103847_Z0_T0_C1.tif]

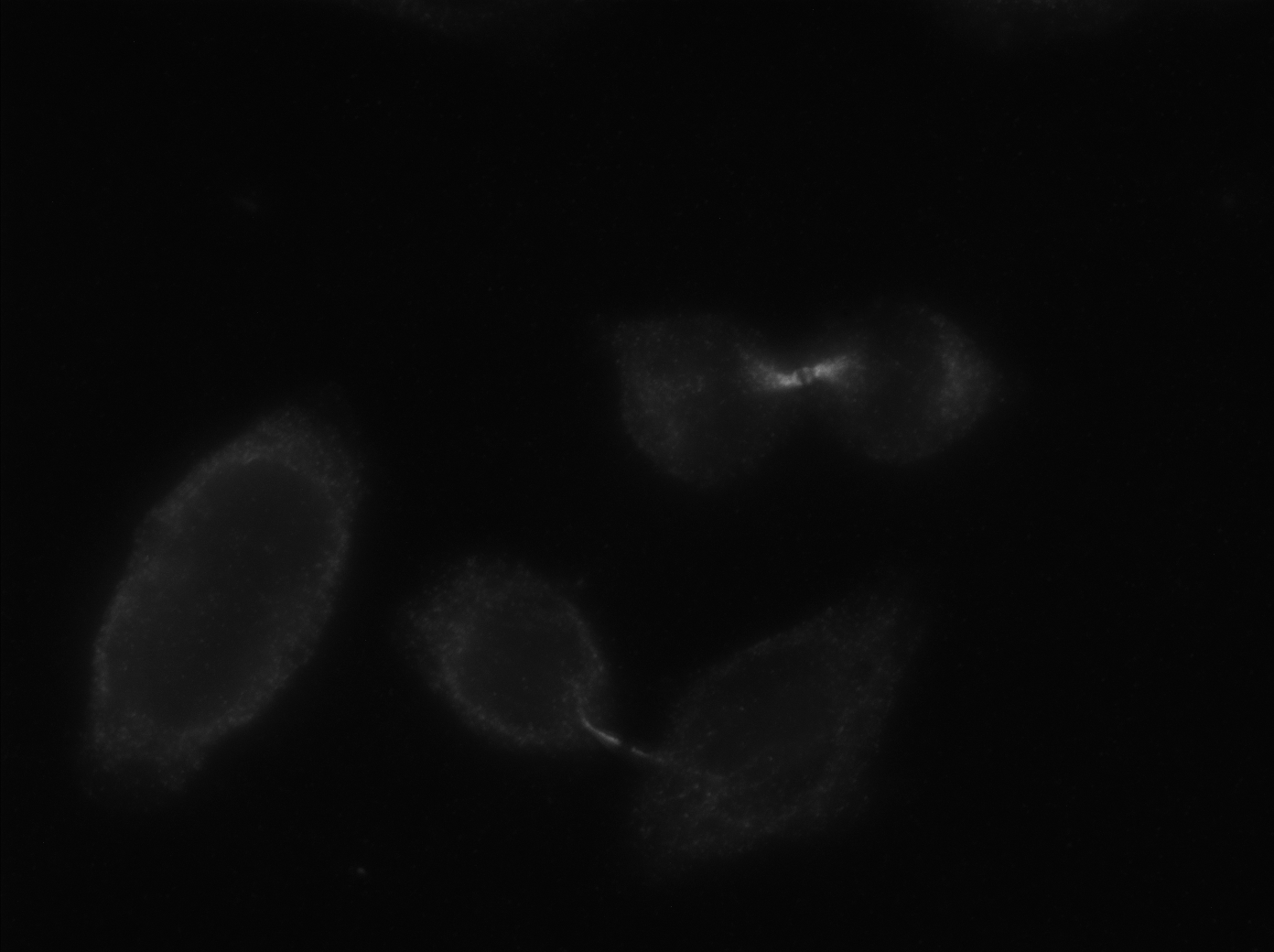

Supplement: Supplementary file 4 — Source data Fig. 2 part 1 [file 44319_2026_742_MOESM4_ESM.zip › Figure 2 Part 1/Fig 2c Cas9 Hela rGT335 atubulin/Cas9 GT335recomb atub 3-24-25 R2 ET2 LT4.Project Maximum Z_XY1743439952_Z0_T0_C2.tif]

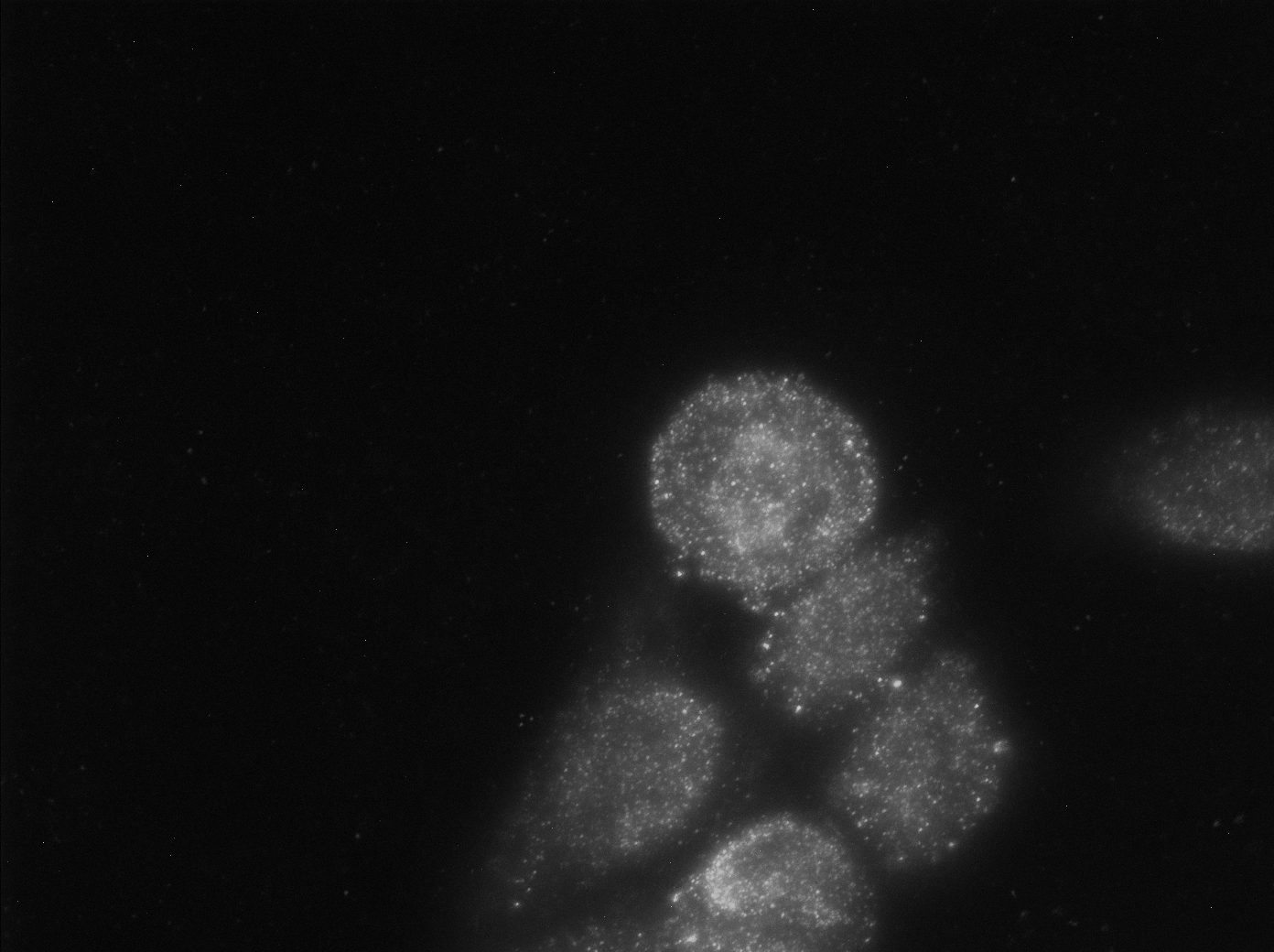

Supplement: Supplementary file 4 — Source data Fig. 2 part 1 [file 44319_2026_742_MOESM4_ESM.zip › Figure 2 Part 1/Fig 2c Cas9 Hela rGT335 atubulin/Cas9 GT335recomb atub 3-24-25 R3 M5.Project Maximum Z_XY1743453240_Z0_T0_C1.tif]

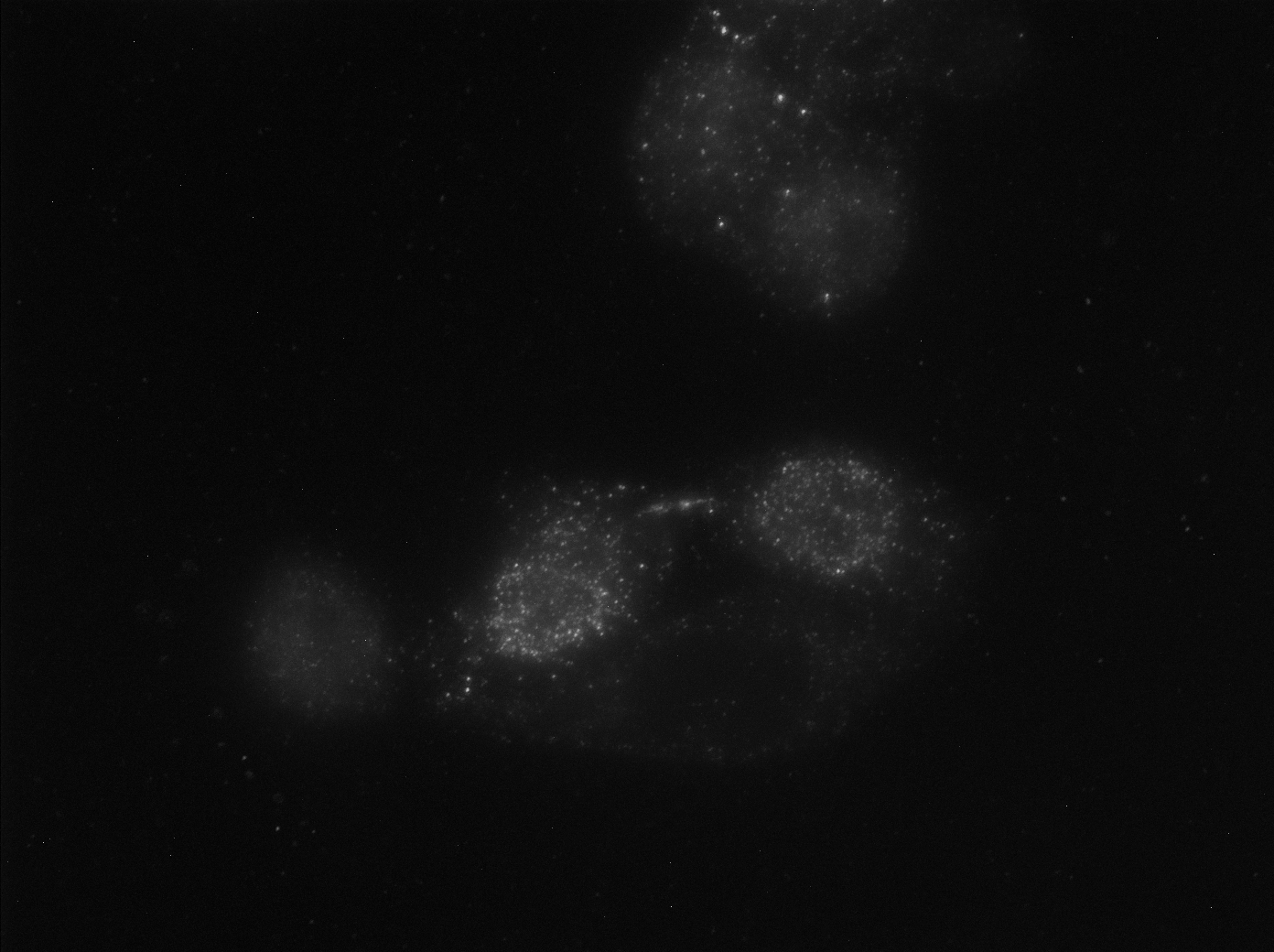

Supplement: Supplementary file 4 — Source data Fig. 2 part 1 [file 44319_2026_742_MOESM4_ESM.zip › Figure 2 Part 1/Fig 2c Cas9 Hela rGT335 atubulin/Cas9 GT335recomb atub 3-24-25 R3 LT2.Project Maximum Z_XY1743451289_Z0_T0_C1.tif]

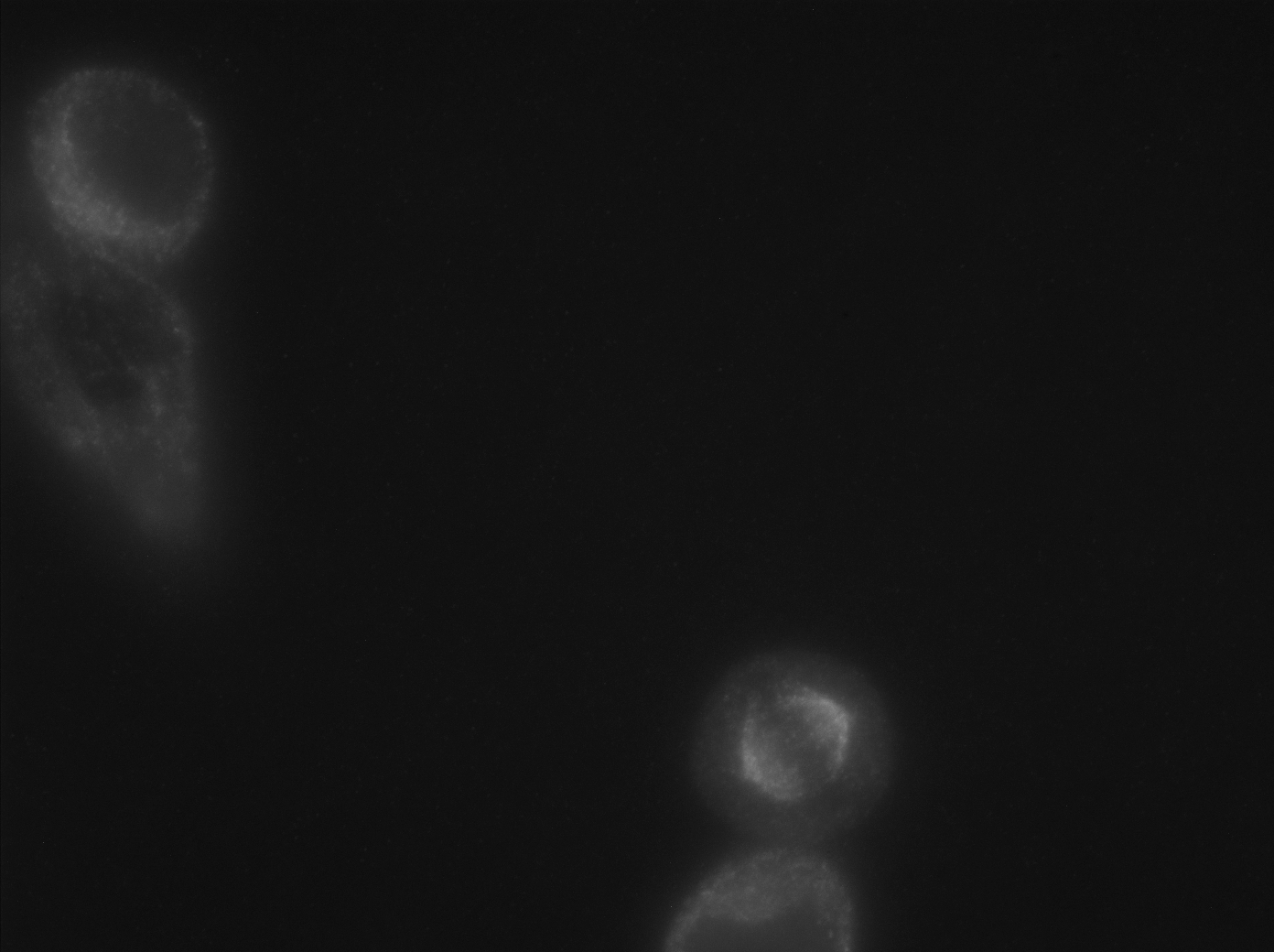

Supplement: Supplementary file 4 — Source data Fig. 2 part 1 [file 44319_2026_742_MOESM4_ESM.zip › Figure 2 Part 1/Fig 2c Cas9 Hela rGT335 atubulin/Cas9 GT335recomb atub 3-24-25 R1 M7.Project Maximum Z_XY1743105485_Z0_T0_C2.tif]

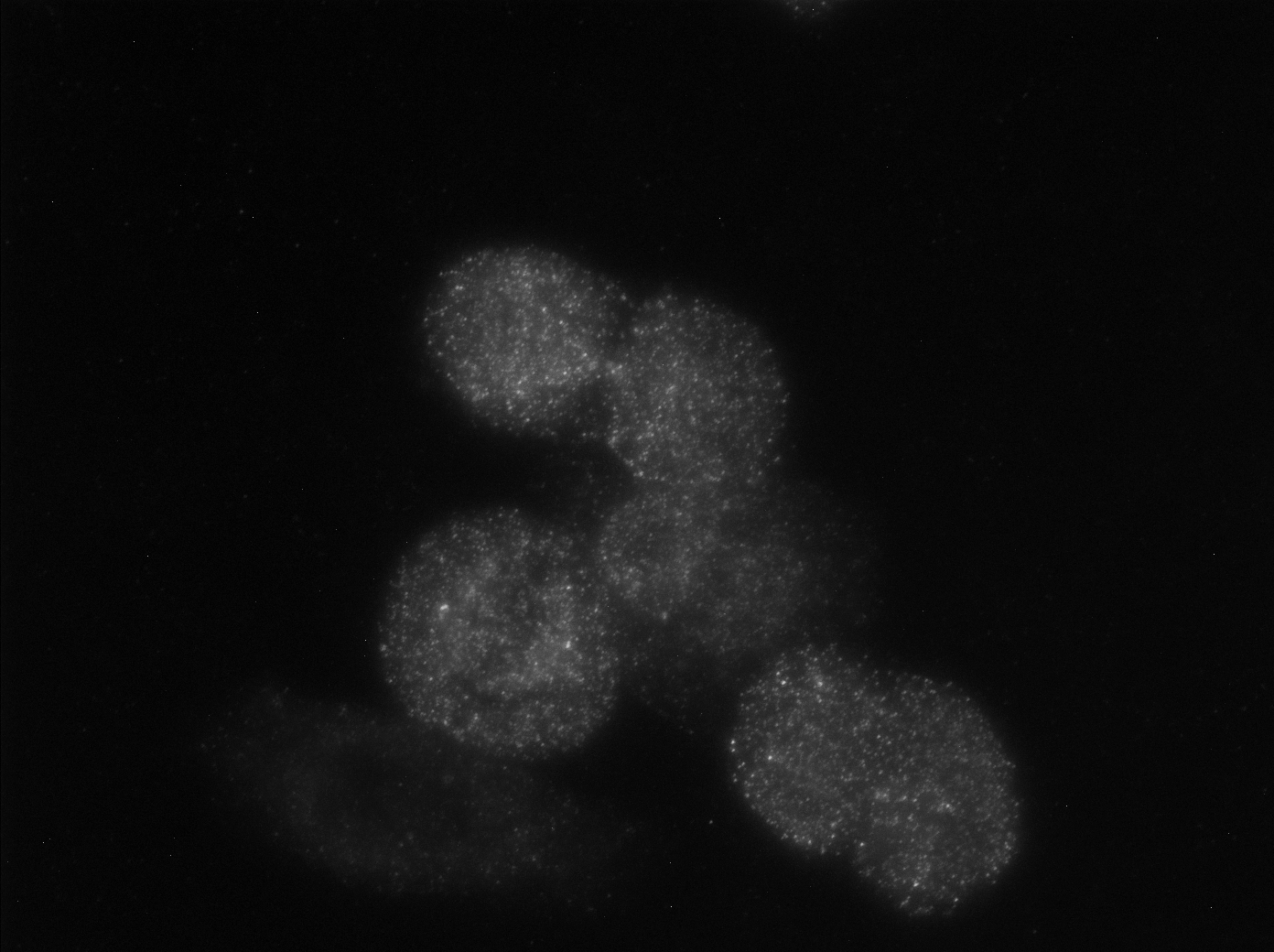

Supplement: Supplementary file 4 — Source data Fig. 2 part 1 [file 44319_2026_742_MOESM4_ESM.zip › Figure 2 Part 1/Fig 2c Cas9 Hela rGT335 atubulin/Cas9 GT335recomb atub 3-24-25 R2 M7.Project Maximum Z_XY1743446847_Z0_T0_C1.tif]

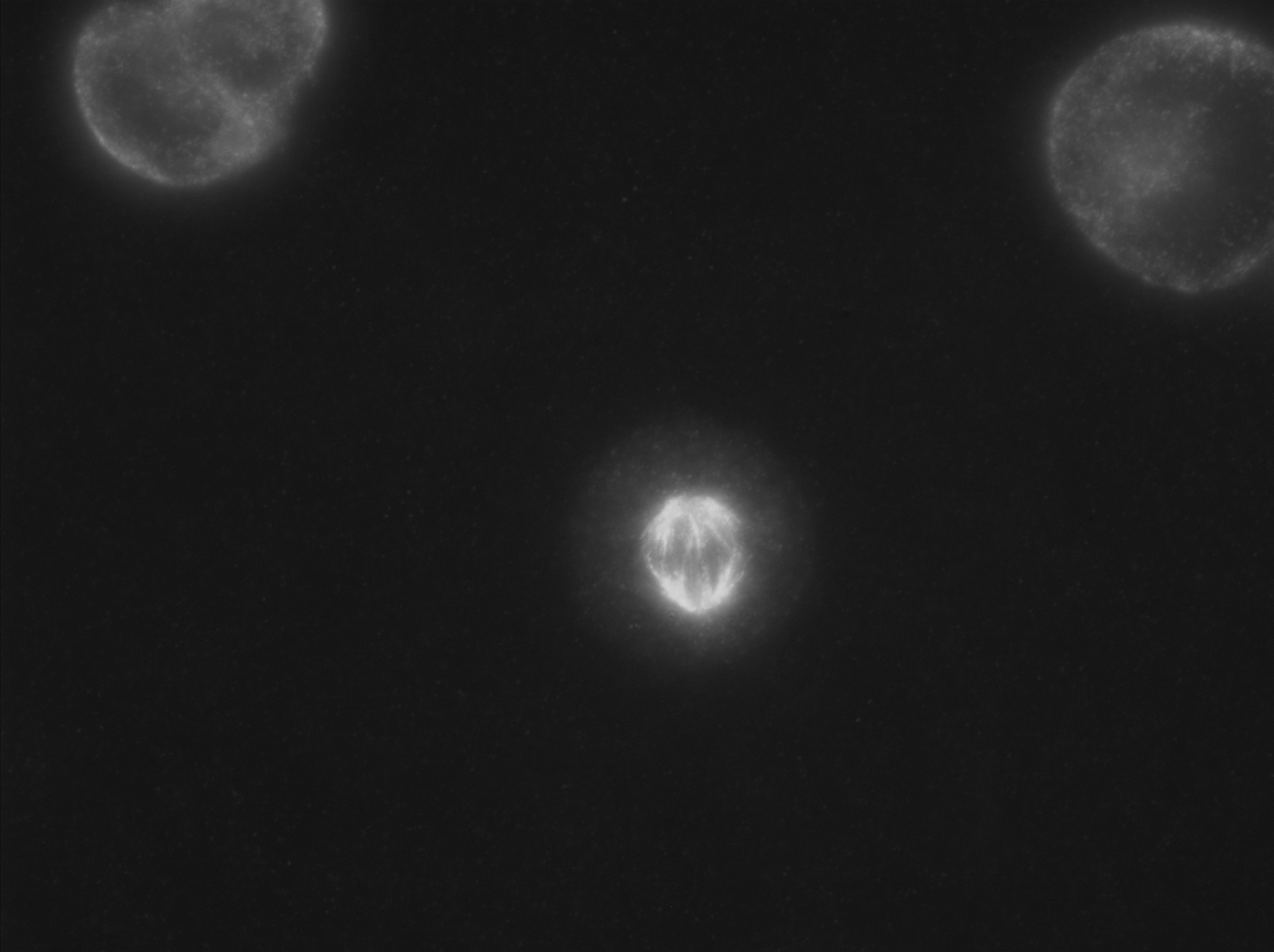

Supplement: Supplementary file 4 — Source data Fig. 2 part 1 [file 44319_2026_742_MOESM4_ESM.zip › Figure 2 Part 1/Fig 2c Cas9 Hela rGT335 atubulin/Cas9 GT335recomb atub 3-24-25 R3 M6.Project Maximum Z_XY1743453627_Z0_T0_C2.tif]

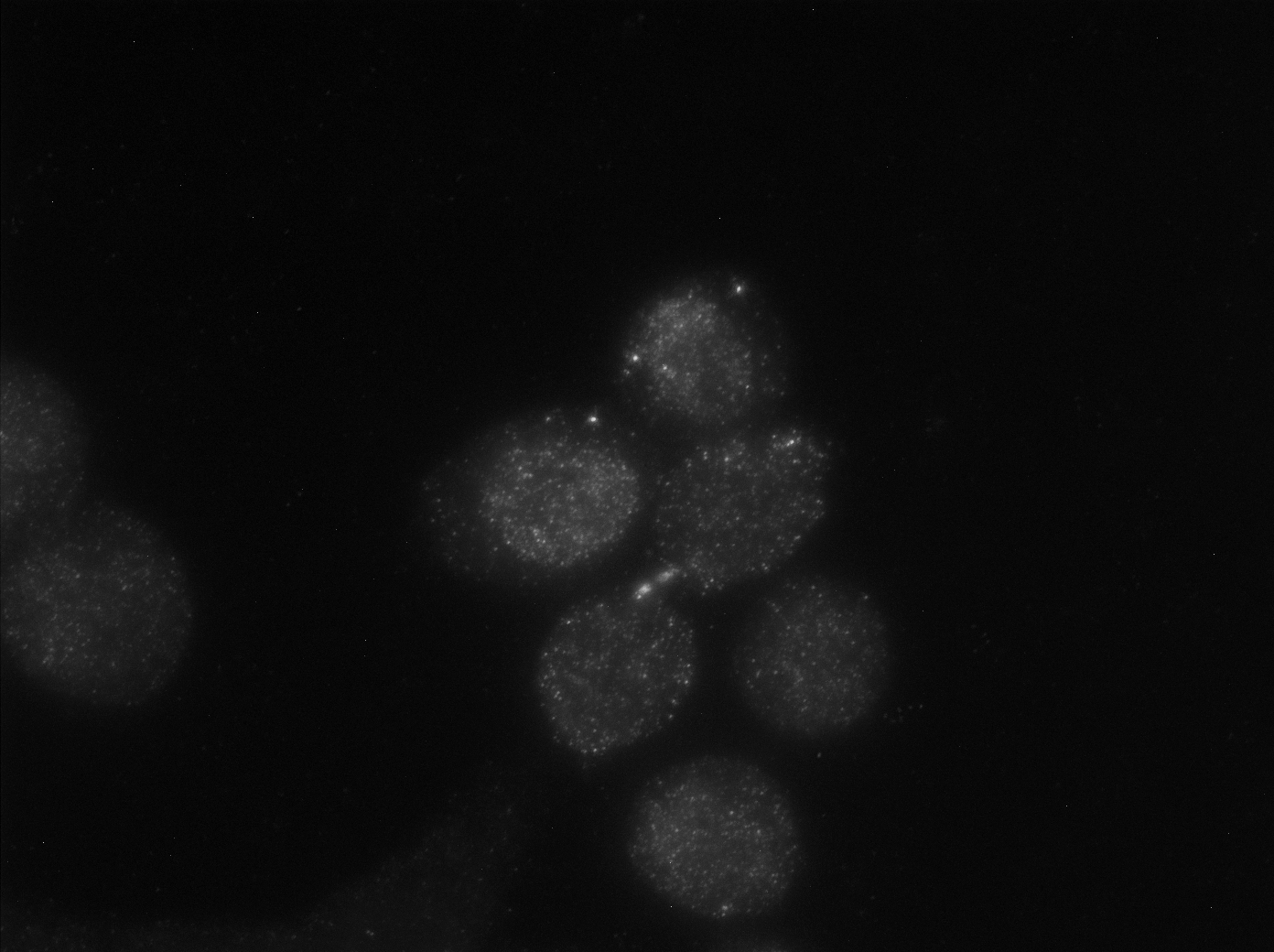

Supplement: Supplementary file 4 — Source data Fig. 2 part 1 [file 44319_2026_742_MOESM4_ESM.zip › Figure 2 Part 1/Fig 2c Cas9 Hela rGT335 atubulin/Cas9 GT335recomb atub 3-24-25 R2 ET4 PA3.Project Maximum Z_XY1743441190_Z0_T0_C1.tif]

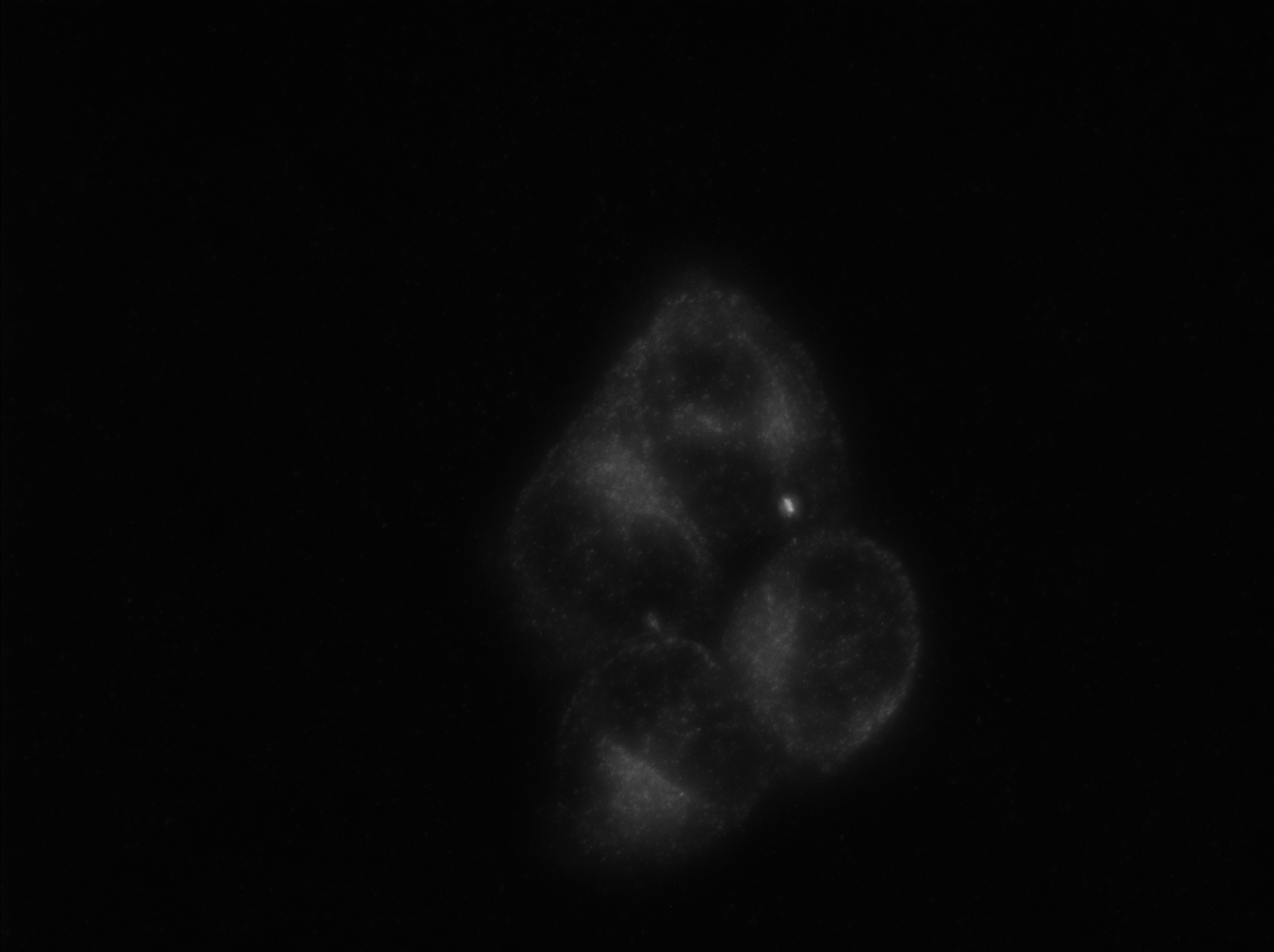

Supplement: Supplementary file 4 — Source data Fig. 2 part 1 [file 44319_2026_742_MOESM4_ESM.zip › Figure 2 Part 1/Fig 2c Cas9 Hela rGT335 atubulin/Cas9 GT335recomb atub 3-24-25 R2 PA9PA10.Project Maximum Z_XY1743446596_Z0_T0_C2.tif]

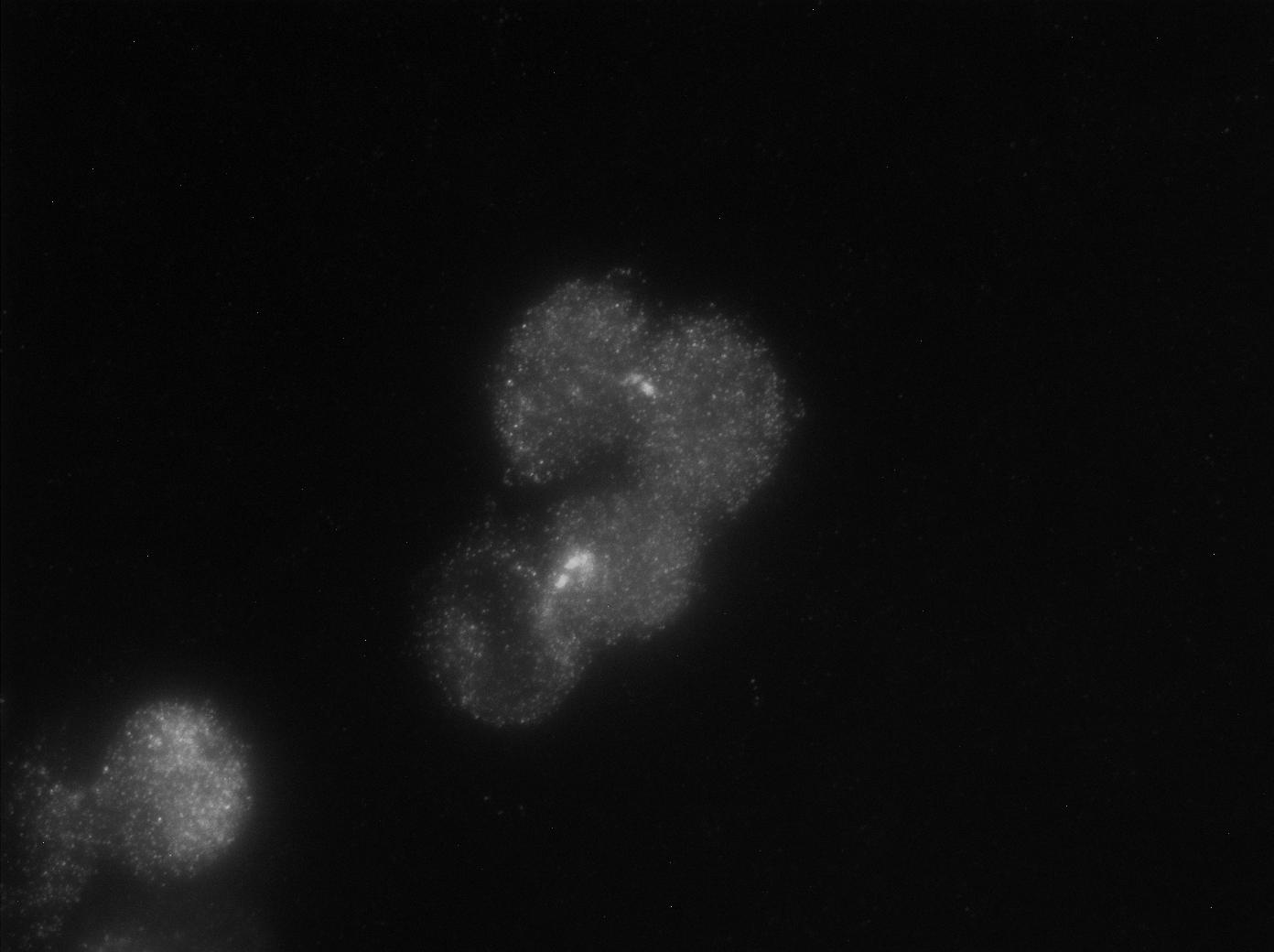

Supplement: Supplementary file 4 — Source data Fig. 2 part 1 [file 44319_2026_742_MOESM4_ESM.zip › Figure 2 Part 1/Fig 2c Cas9 Hela rGT335 atubulin/Cas9 GT335recomb atub 3-24-25 R2 ET8ET9.Project Maximum Z_XY1743444778_Z0_T0_C1.tif]

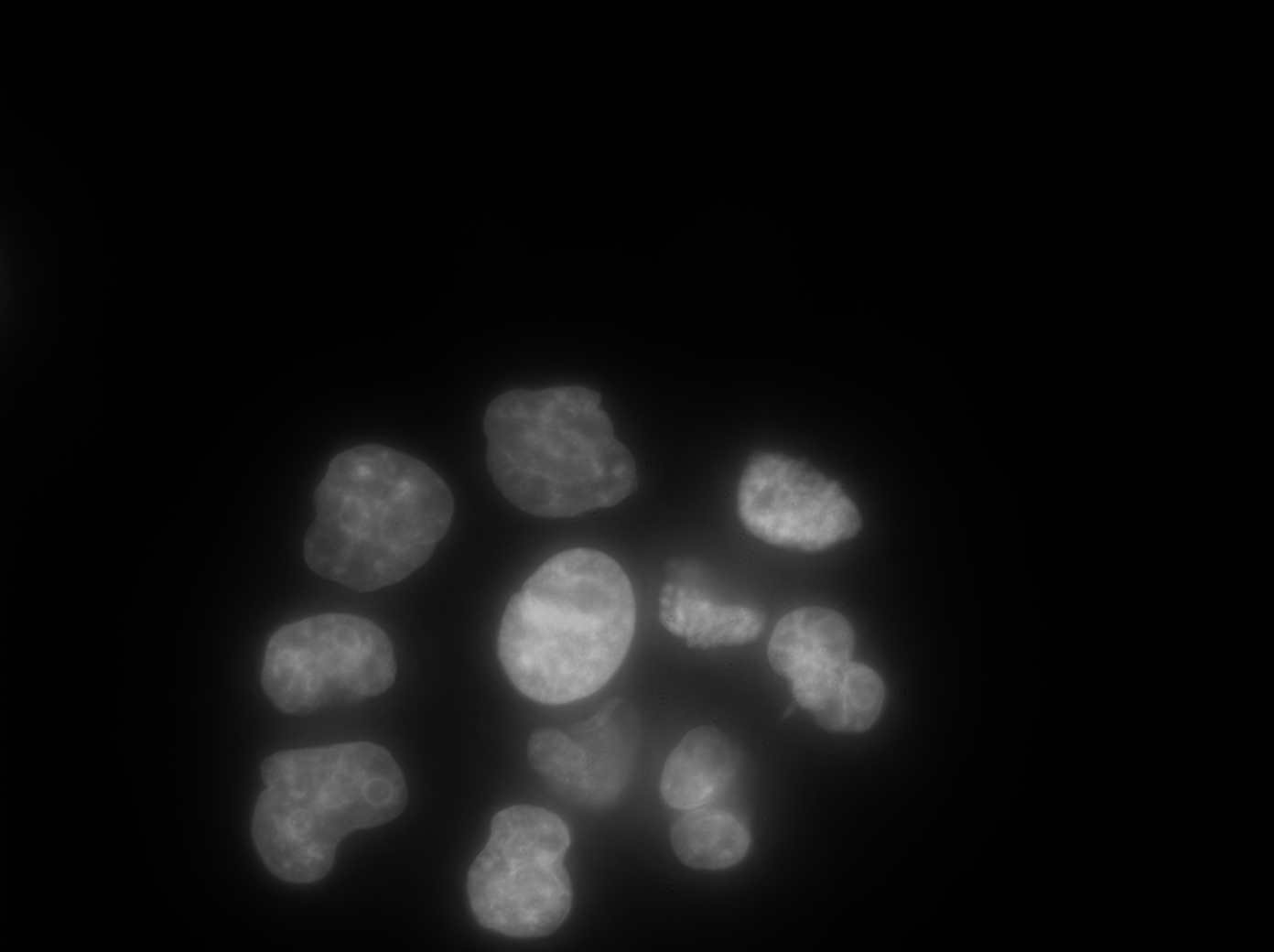

Supplement: Supplementary file 4 — Source data Fig. 2 part 1 [file 44319_2026_742_MOESM4_ESM.zip › Figure 2 Part 1/Fig 2c Cas9 Hela rGT335 atubulin/Cas9 GT335recomb atub 3-24-25 R1 ET6.Project Maximum Z_XY1743102611_Z0_T0_C0.tif]

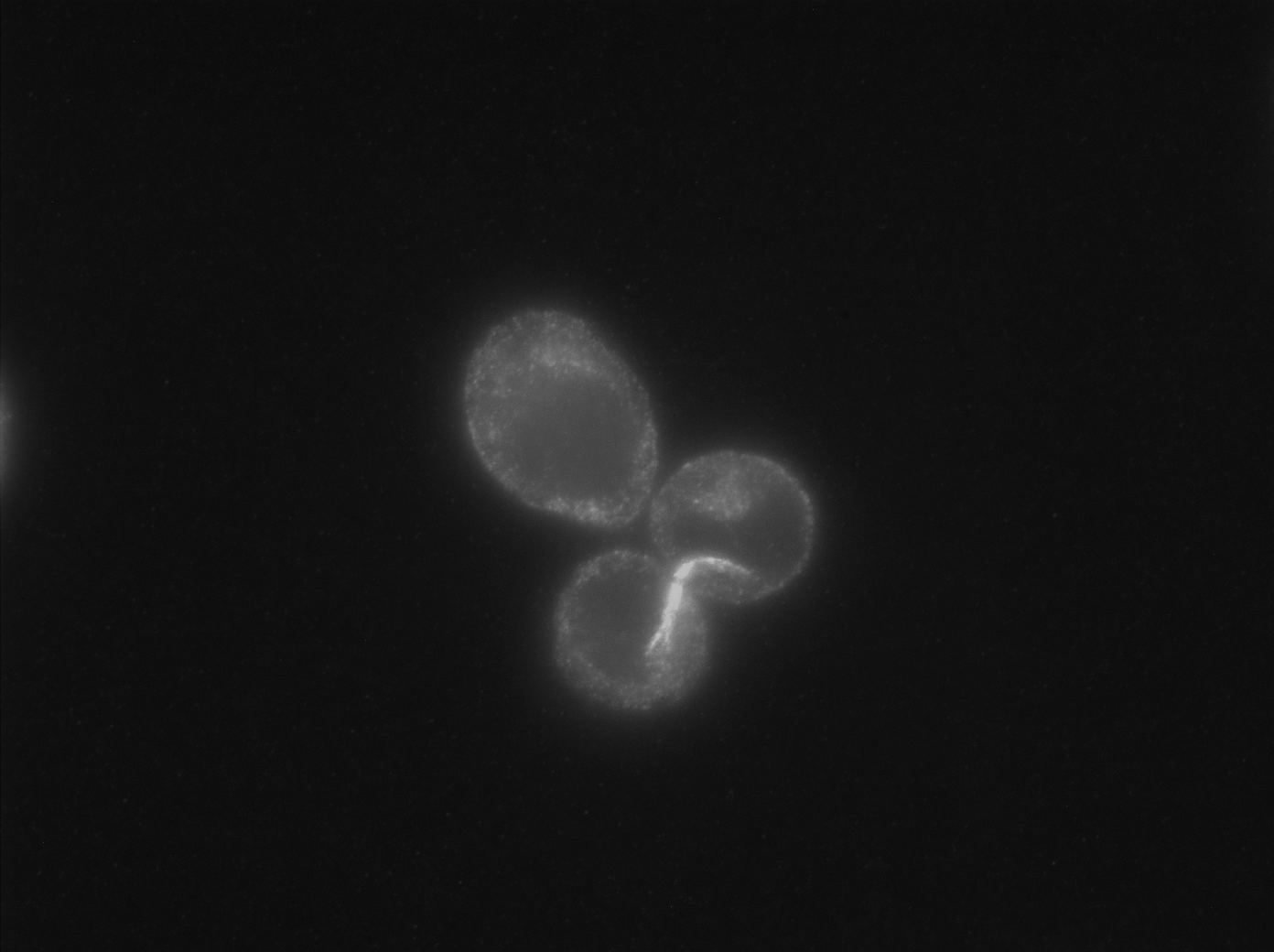

Supplement: Supplementary file 4 — Source data Fig. 2 part 1 [file 44319_2026_742_MOESM4_ESM.zip › Figure 2 Part 1/Fig 2c Cas9 Hela rGT335 atubulin/Cas9 GT335recomb atub 3-24-25 R1 ET5.Project Maximum Z_XY1743102526_Z0_T0_C2.tif]

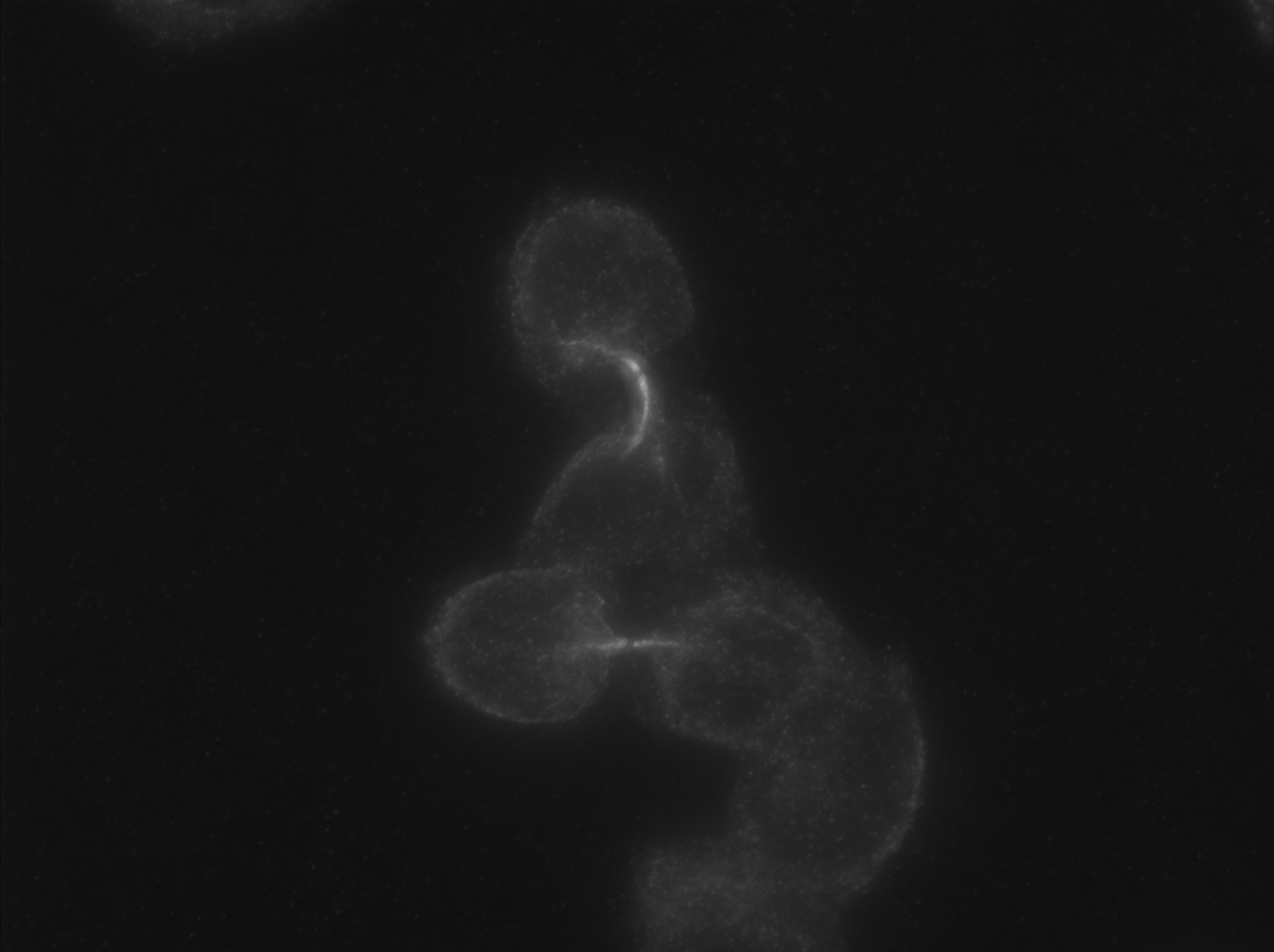

Supplement: Supplementary file 4 — Source data Fig. 2 part 1 [file 44319_2026_742_MOESM4_ESM.zip › Figure 2 Part 1/Fig 2c Cas9 Hela rGT335 atubulin/Cas9 GT335recomb atub 3-24-25 R3 LT7LT8.Project Maximum Z_XY1743452825_Z0_T0_C2.tif]

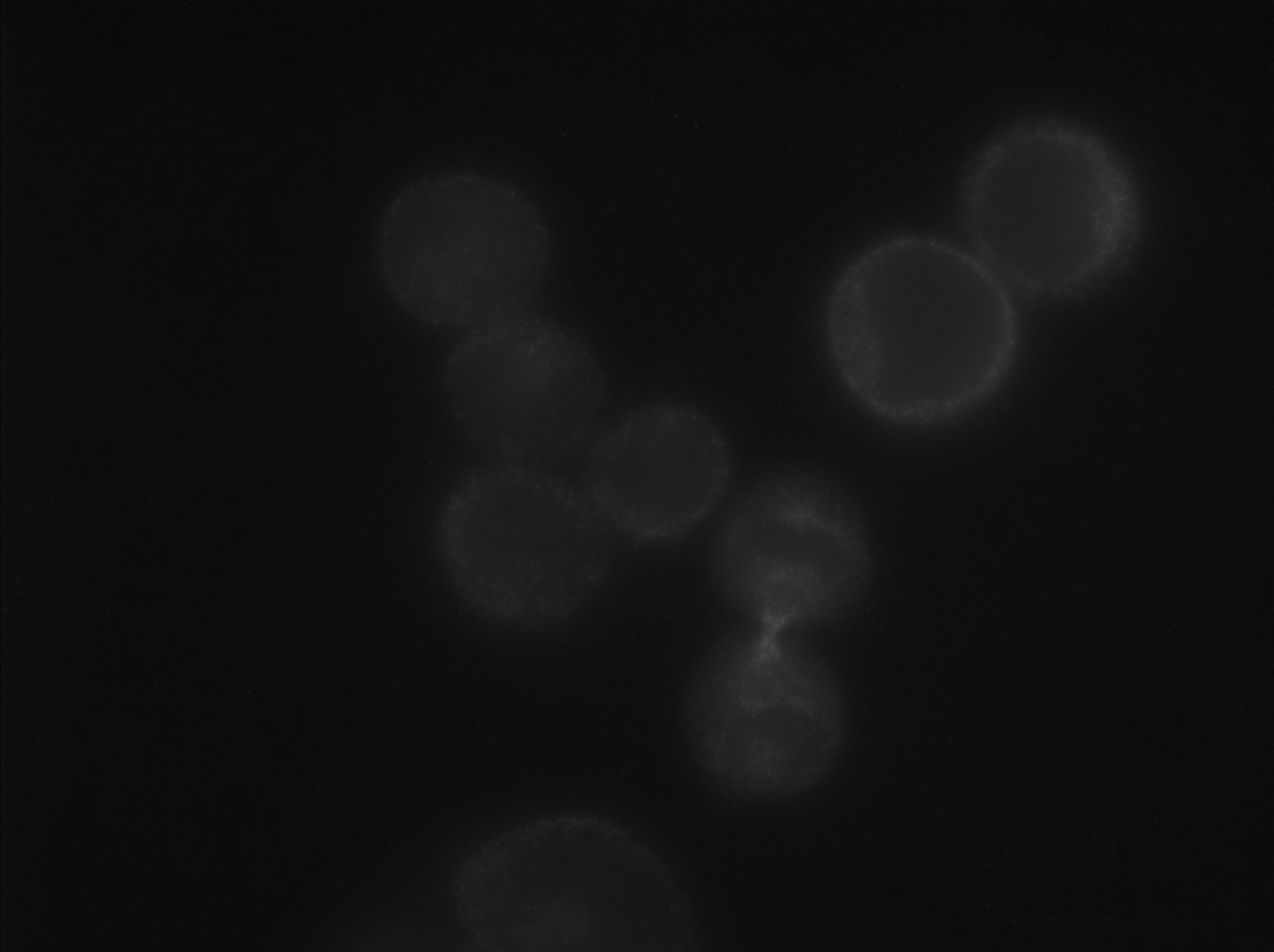

Supplement: Supplementary file 4 — Source data Fig. 2 part 1 [file 44319_2026_742_MOESM4_ESM.zip › Figure 2 Part 1/Fig 2c Cas9 Hela rGT335 atubulin/Cas9 GT335recomb atub 3-24-25 R3 ET5.Project Maximum Z_XY1743454833_Z0_T0_C2.tif]

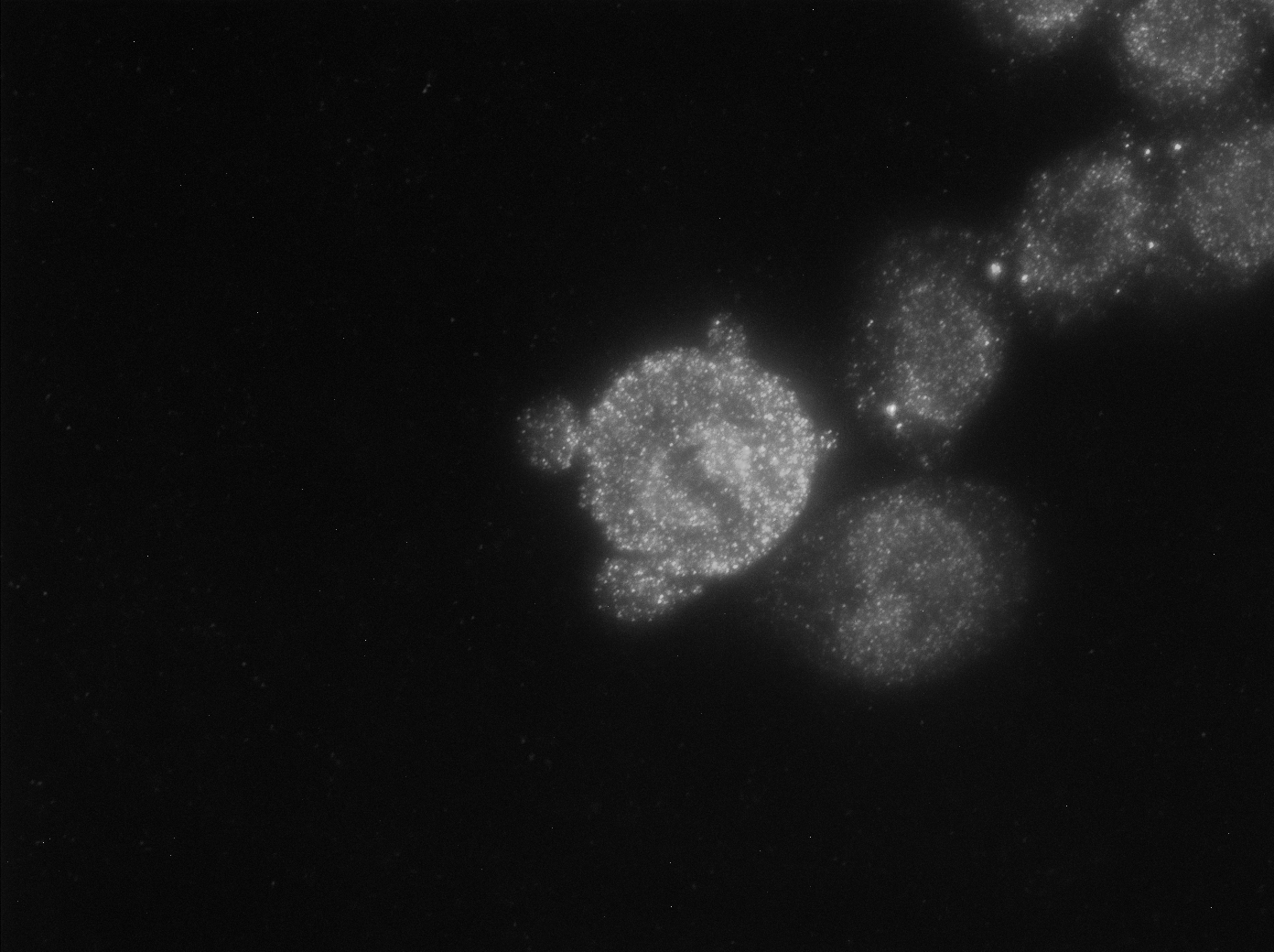

Supplement: Supplementary file 4 — Source data Fig. 2 part 1 [file 44319_2026_742_MOESM4_ESM.zip › Figure 2 Part 1/Fig 2c Cas9 Hela rGT335 atubulin/Cas9 GT335recomb atub 3-24-25 R3 M4 PA5.Project Maximum Z_XY1743453144_Z0_T0_C1.tif]

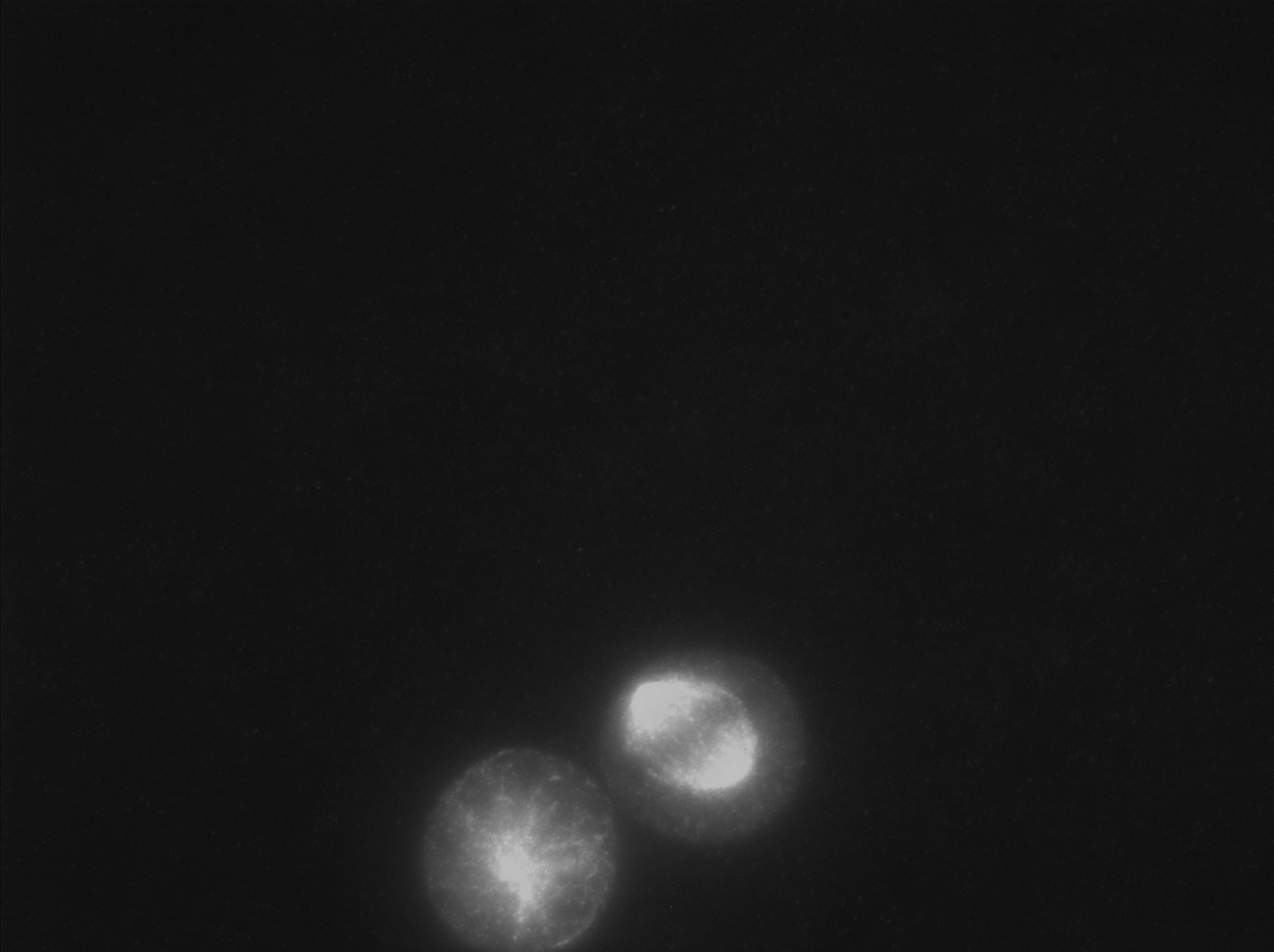

Supplement: Supplementary file 4 — Source data Fig. 2 part 1 [file 44319_2026_742_MOESM4_ESM.zip › Figure 2 Part 1/Fig 2c Cas9 Hela rGT335 atubulin/Cas9 GT335recomb atub 3-24-25 R1 M5.Project Maximum Z_XY1743104305_Z0_T0_C2.tif]

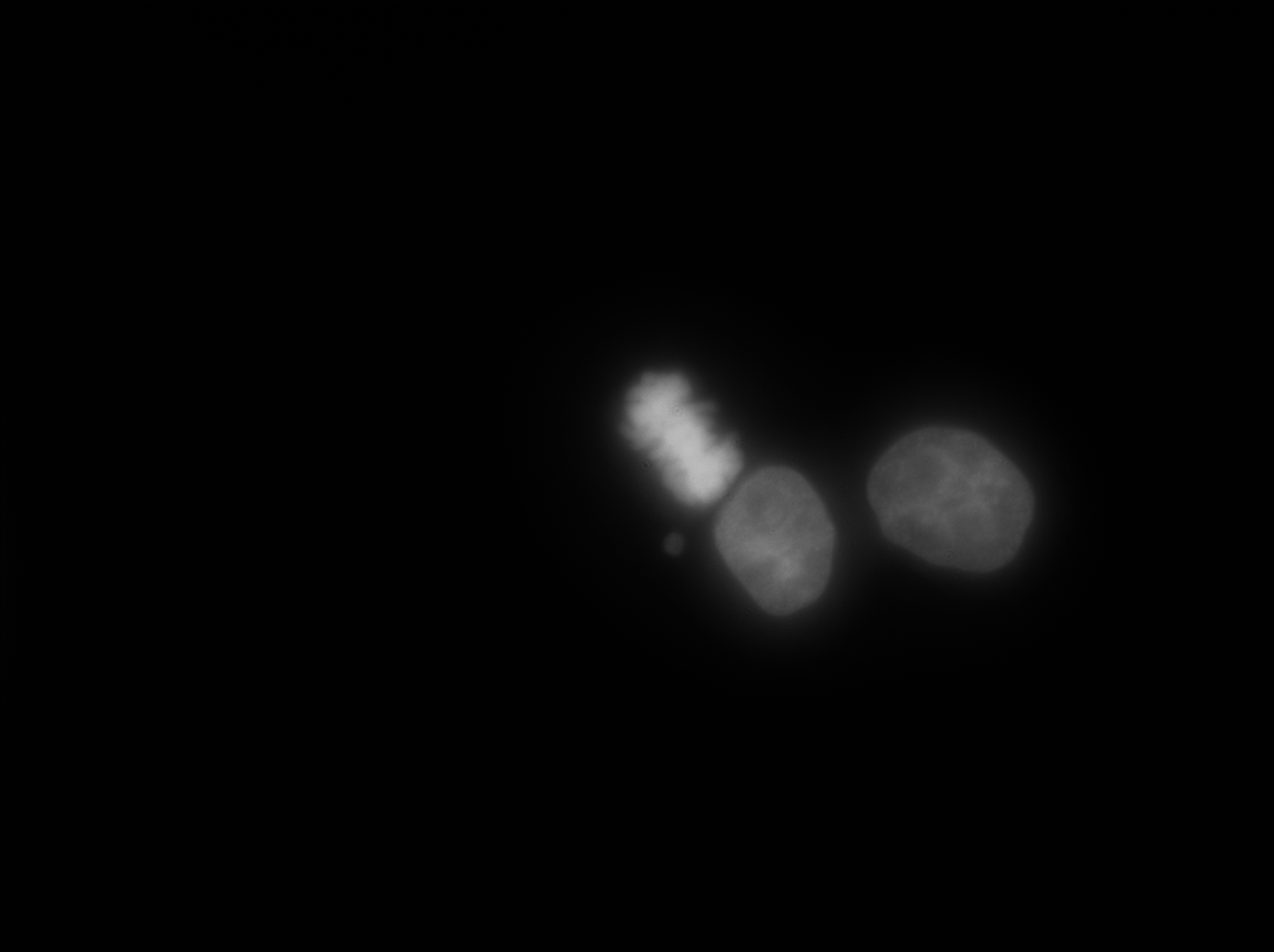

Supplement: Supplementary file 4 — Source data Fig. 2 part 1 [file 44319_2026_742_MOESM4_ESM.zip › Figure 2 Part 1/Fig 2c Cas9 Hela rGT335 atubulin/Cas9 GT335recomb atub 3-24-25 R1 M6.Project Maximum Z_XY1743104509_Z0_T0_C0.tif]

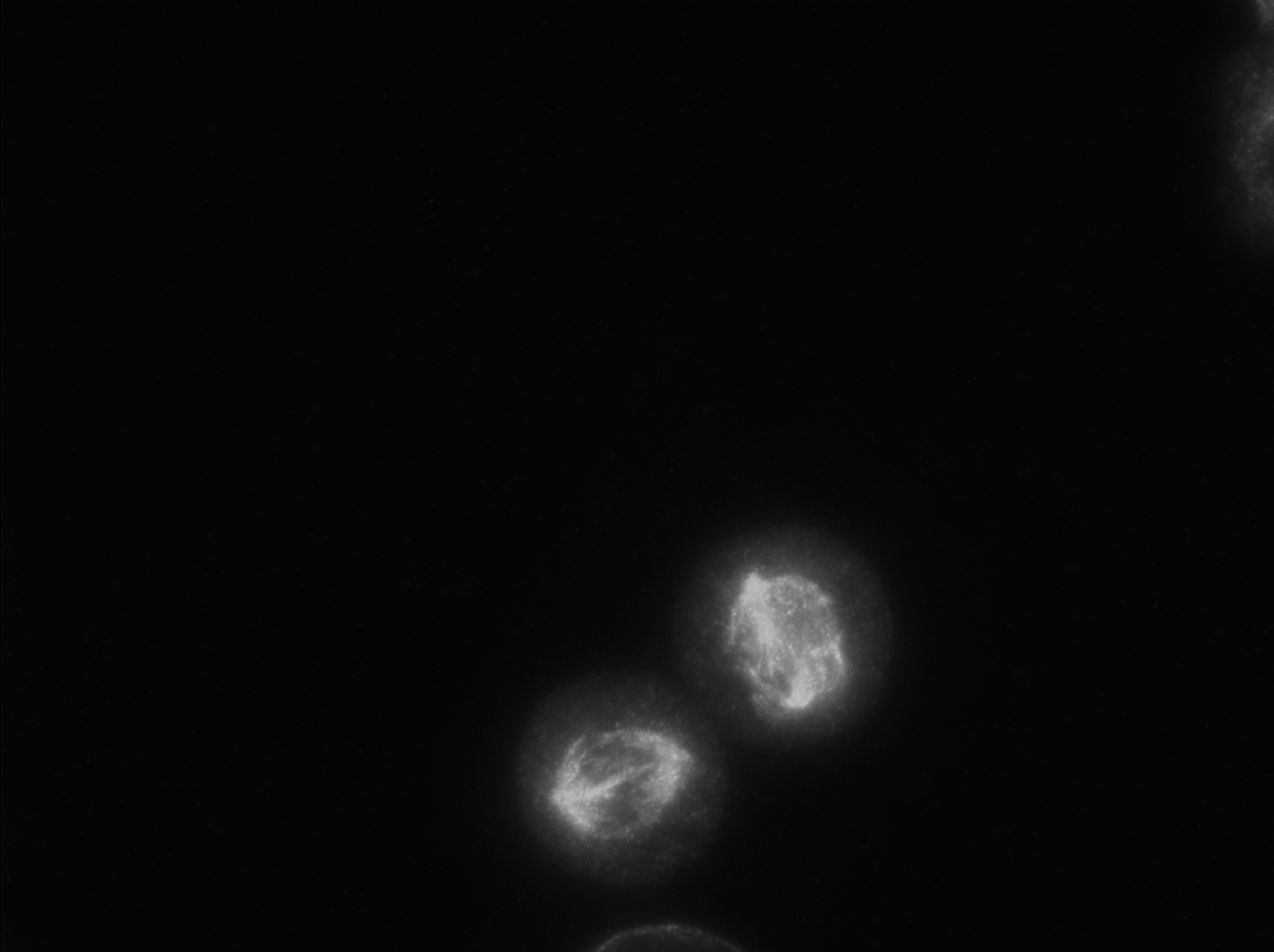

Supplement: Supplementary file 4 — Source data Fig. 2 part 1 [file 44319_2026_742_MOESM4_ESM.zip › Figure 2 Part 1/Fig 2c Cas9 Hela rGT335 atubulin/Cas9 GT335recomb atub 3-24-25 R2 M5M6.Project Maximum Z_XY1743446381_Z0_T0_C2.tif]

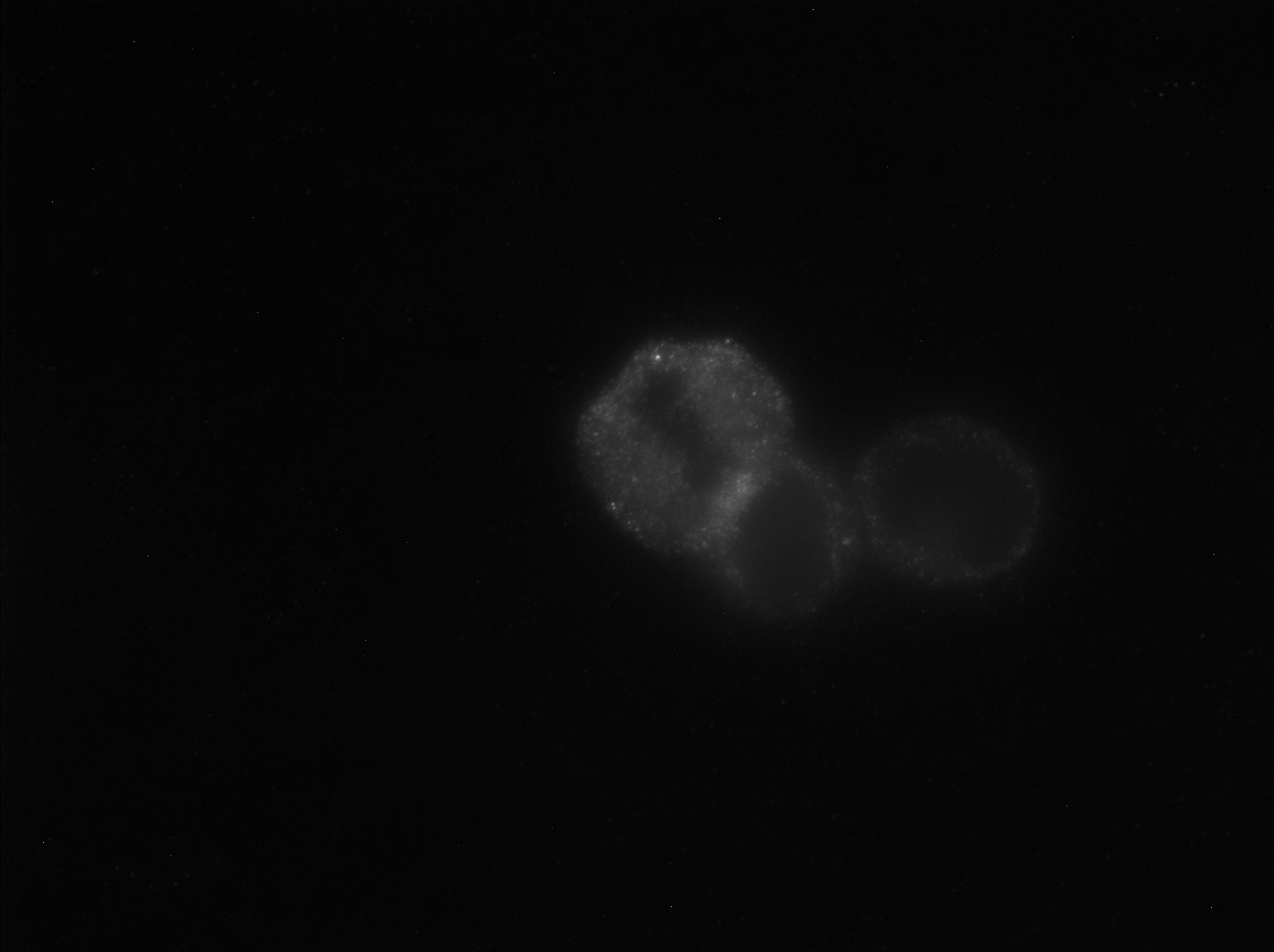

Supplement: Supplementary file 4 — Source data Fig. 2 part 1 [file 44319_2026_742_MOESM4_ESM.zip › Figure 2 Part 1/Fig 2c Cas9 Hela rGT335 atubulin/Cas9 GT335recomb atub 3-24-25 R1 M6.Project Maximum Z_XY1743104509_Z0_T0_C1.tif]

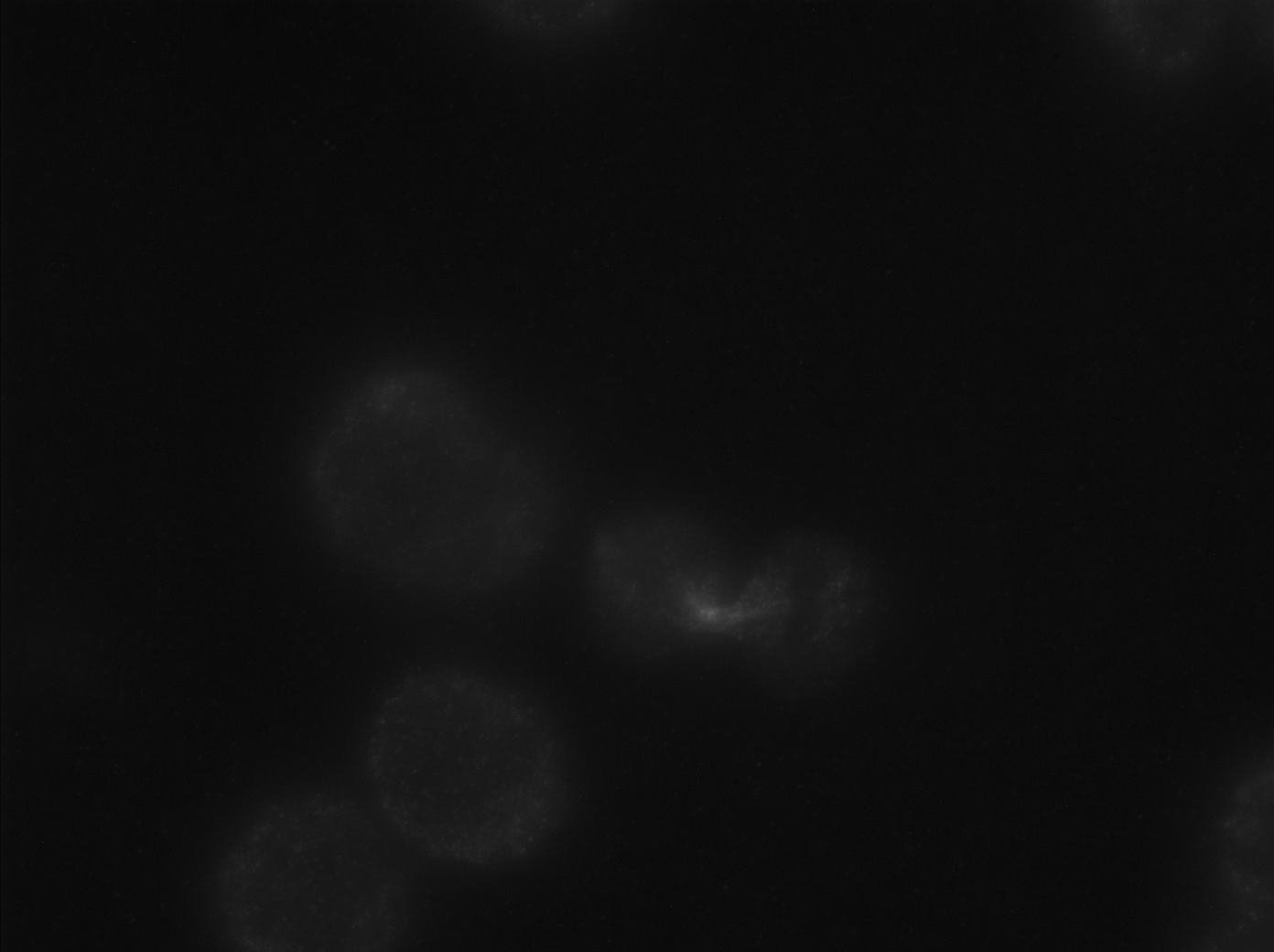

Supplement: Supplementary file 4 — Source data Fig. 2 part 1 [file 44319_2026_742_MOESM4_ESM.zip › Figure 2 Part 1/Fig 2c Cas9 Hela rGT335 atubulin/Cas9 GT335recomb atub 3-24-25 R3 ET2.Project Maximum Z_XY1743451921_Z0_T0_C2.tif]

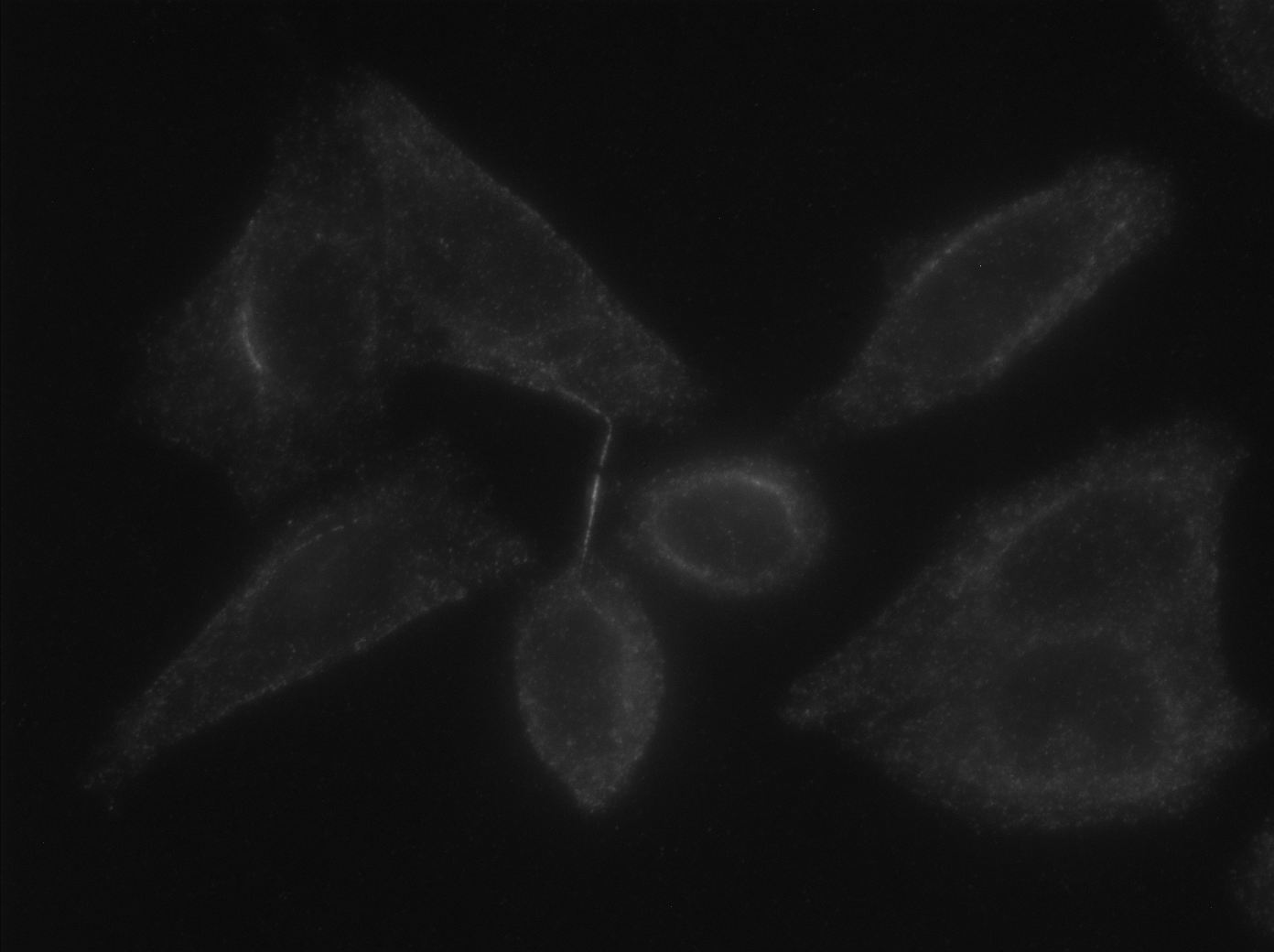

Supplement: Supplementary file 4 — Source data Fig. 2 part 1 [file 44319_2026_742_MOESM4_ESM.zip › Figure 2 Part 1/Fig 2c Cas9 Hela rGT335 atubulin/Cas9 GT335recomb atub 3-24-25 R1 LT3.Project Maximum Z_XY1743100808_Z0_T0_C2.tif]

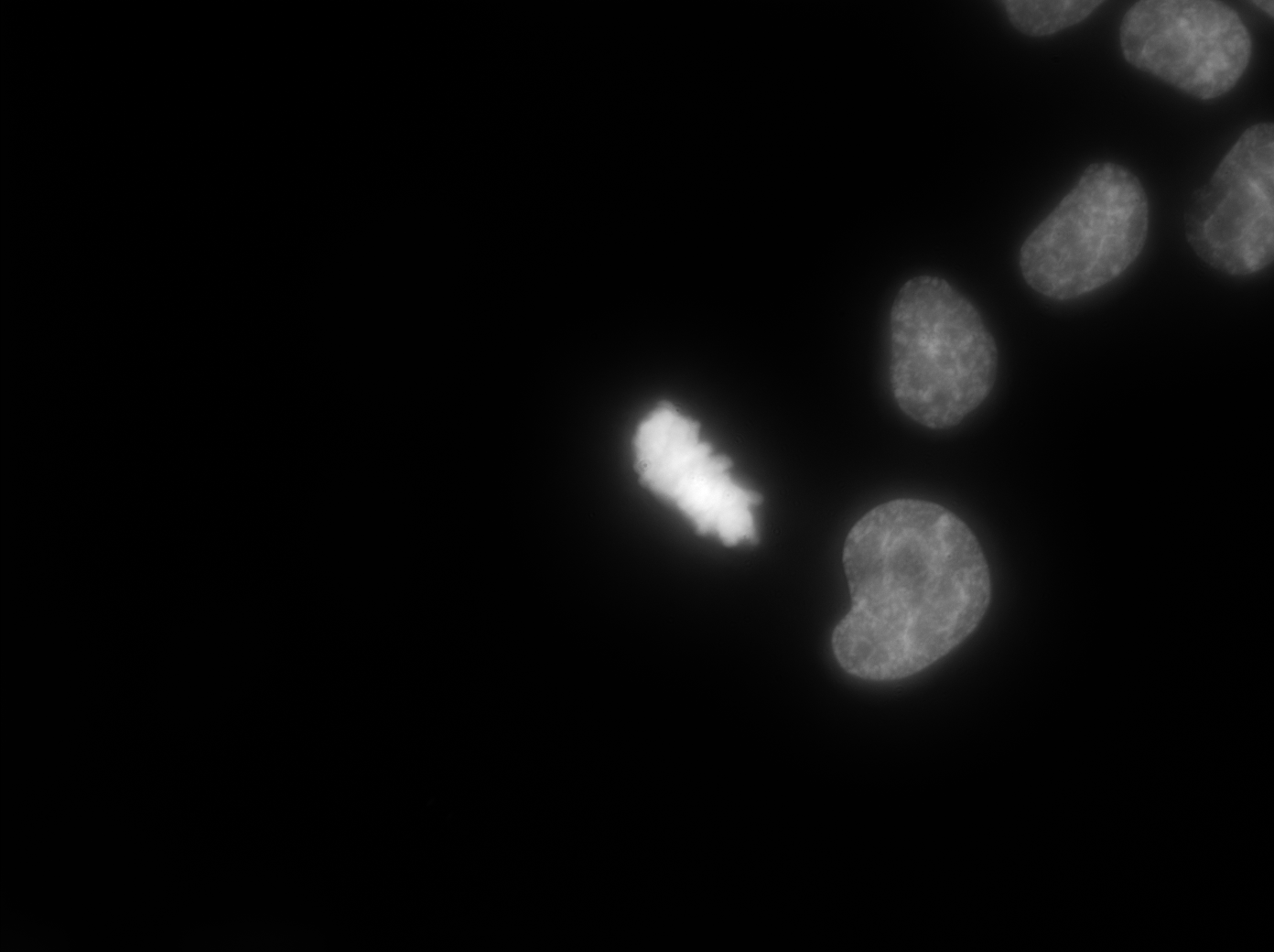

Supplement: Supplementary file 4 — Source data Fig. 2 part 1 [file 44319_2026_742_MOESM4_ESM.zip › Figure 2 Part 1/Fig 2c Cas9 Hela rGT335 atubulin/Cas9 GT335recomb atub 3-24-25 R3 M4 PA5.Project Maximum Z_XY1743453144_Z0_T0_C0.tif]

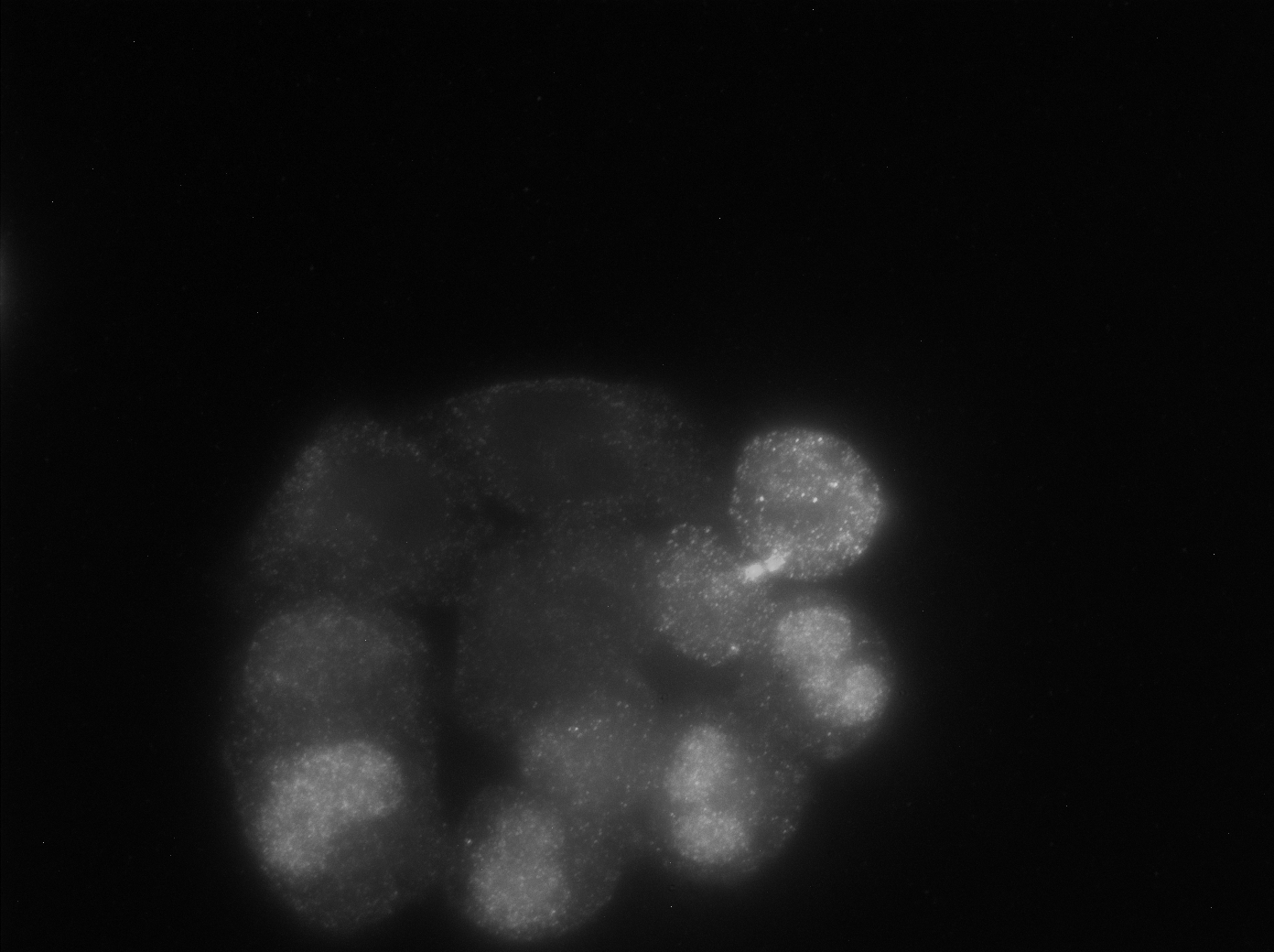

Supplement: Supplementary file 4 — Source data Fig. 2 part 1 [file 44319_2026_742_MOESM4_ESM.zip › Figure 2 Part 1/Fig 2c Cas9 Hela rGT335 atubulin/Cas9 GT335recomb atub 3-24-25 R1 ET6.Project Maximum Z_XY1743102611_Z0_T0_C1.tif]

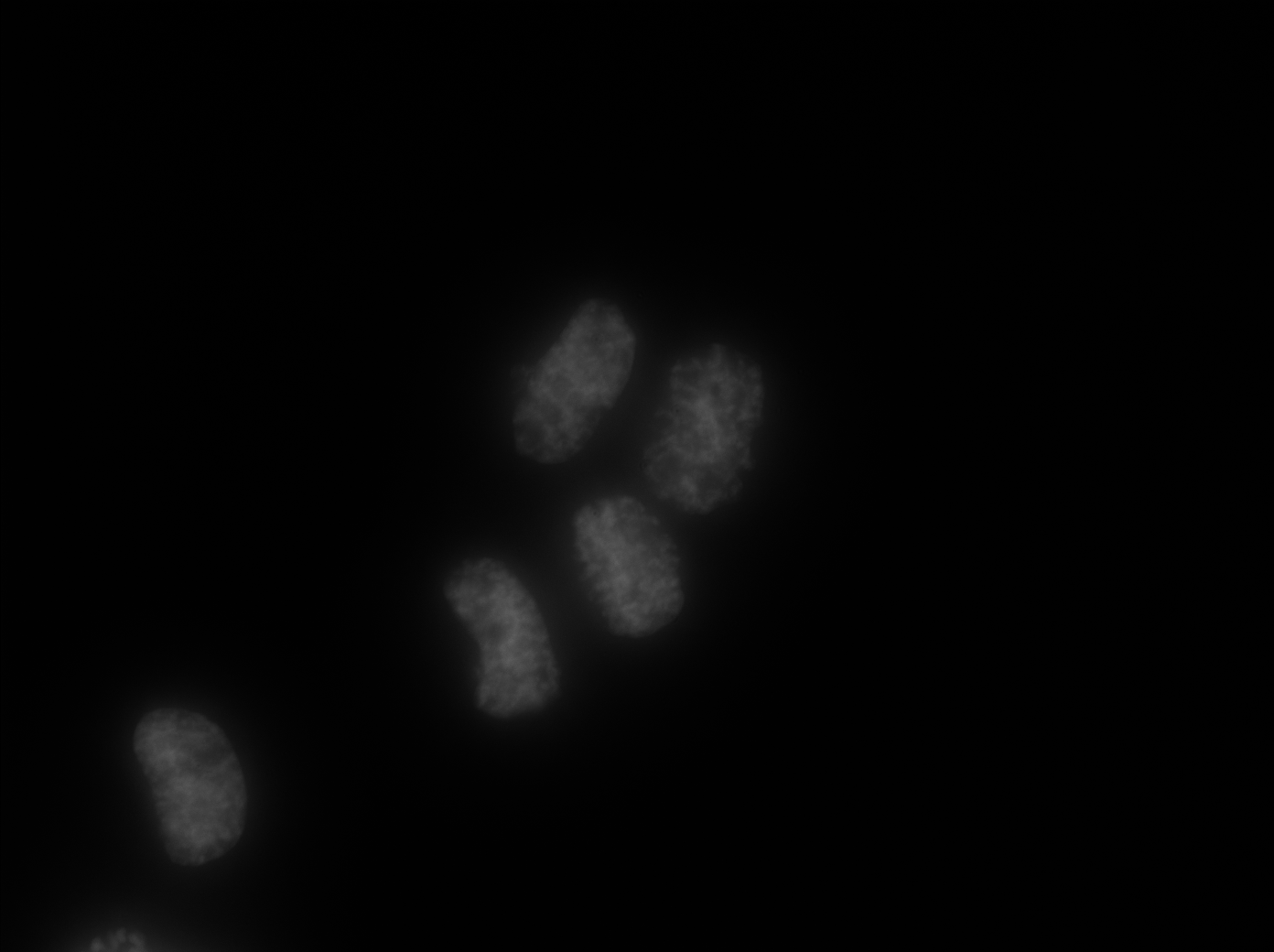

Supplement: Supplementary file 4 — Source data Fig. 2 part 1 [file 44319_2026_742_MOESM4_ESM.zip › Figure 2 Part 1/Fig 2c Cas9 Hela rGT335 atubulin/Cas9 GT335recomb atub 3-24-25 R2 ET8ET9.Project Maximum Z_XY1743444778_Z0_T0_C0.tif]

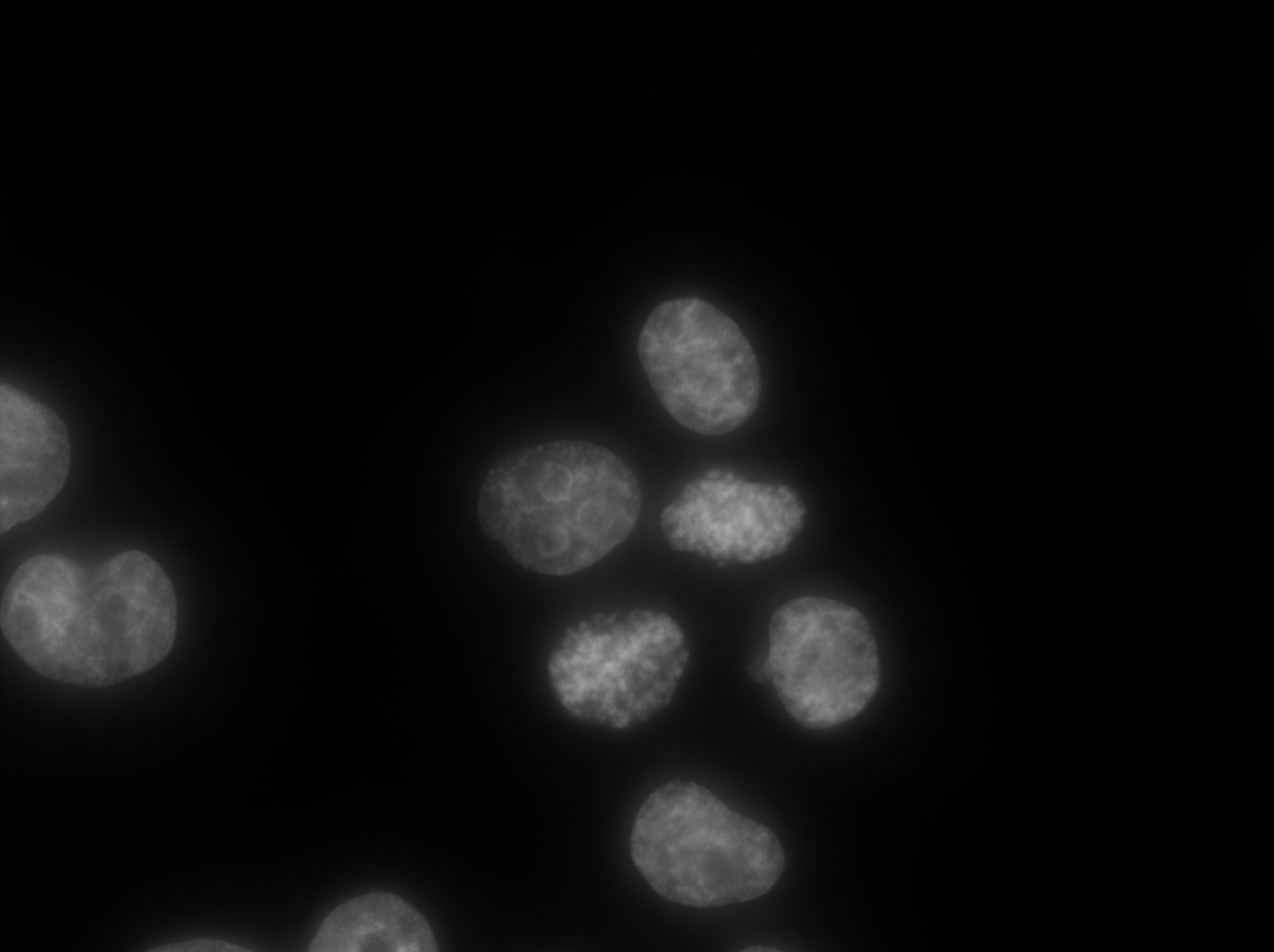

Supplement: Supplementary file 4 — Source data Fig. 2 part 1 [file 44319_2026_742_MOESM4_ESM.zip › Figure 2 Part 1/Fig 2c Cas9 Hela rGT335 atubulin/Cas9 GT335recomb atub 3-24-25 R2 ET4 PA3.Project Maximum Z_XY1743441190_Z0_T0_C0.tif]

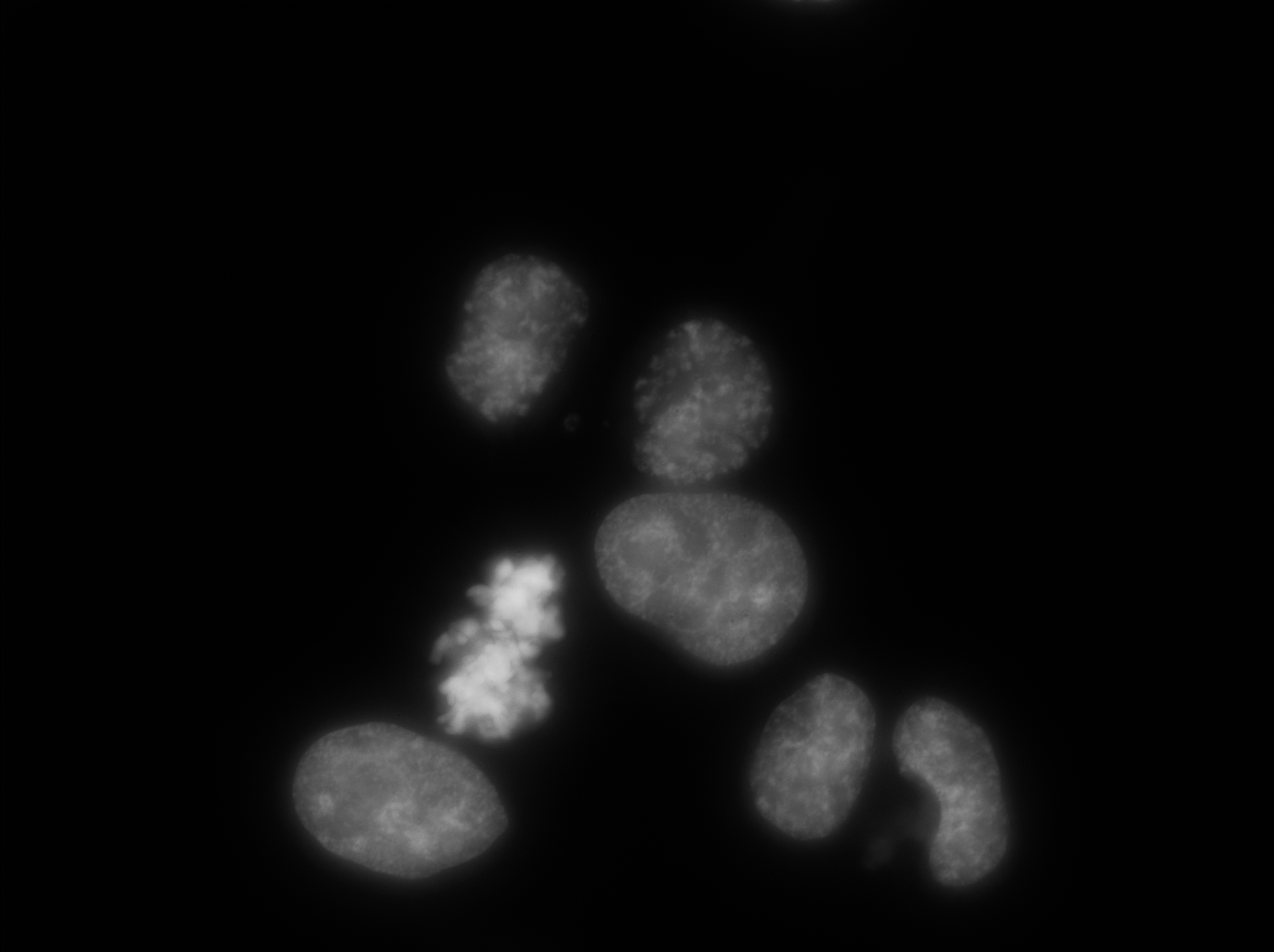

Supplement: Supplementary file 4 — Source data Fig. 2 part 1 [file 44319_2026_742_MOESM4_ESM.zip › Figure 2 Part 1/Fig 2c Cas9 Hela rGT335 atubulin/Cas9 GT335recomb atub 3-24-25 R2 M7.Project Maximum Z_XY1743446847_Z0_T0_C0.tif]

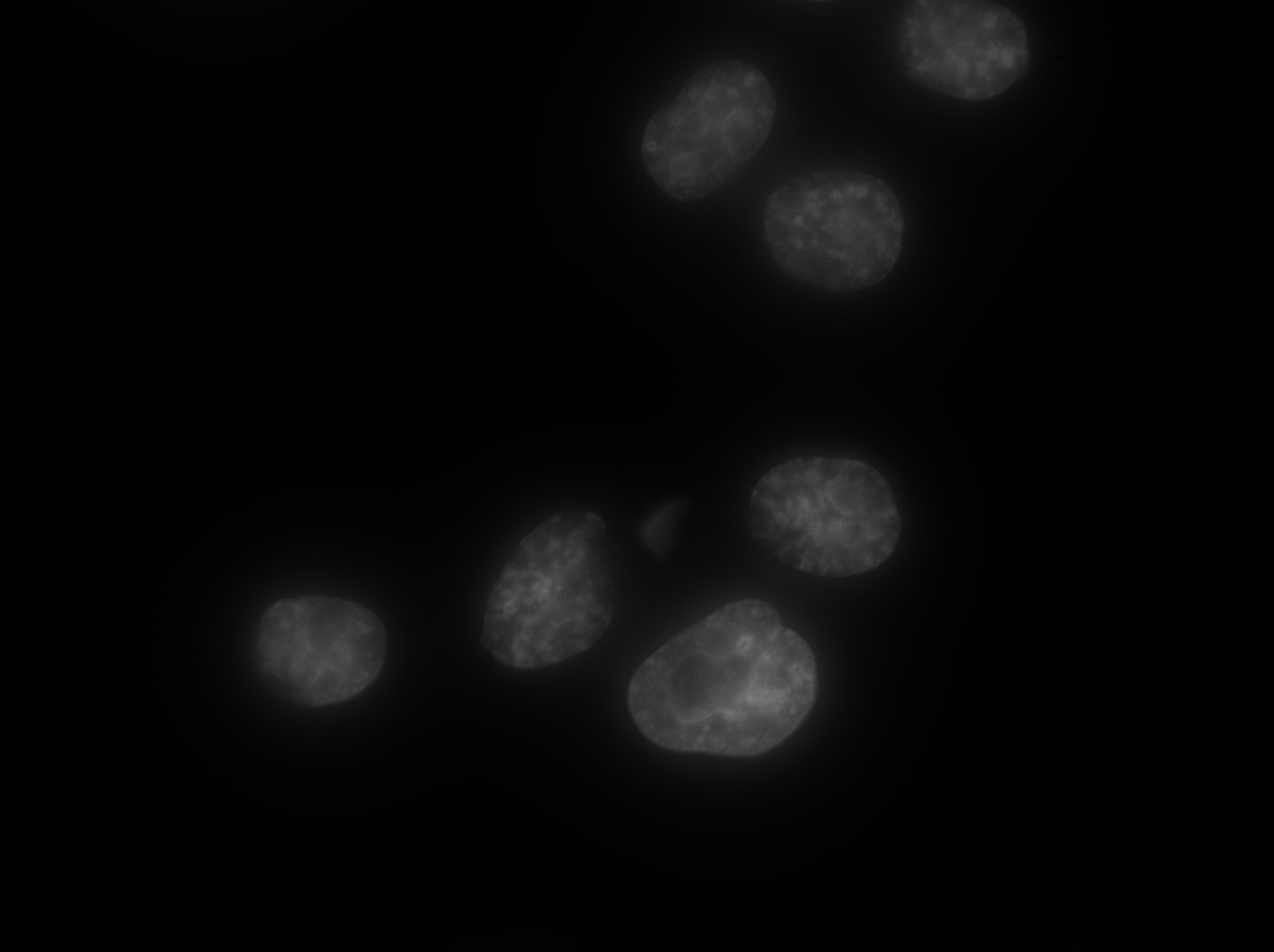

Supplement: Supplementary file 4 — Source data Fig. 2 part 1 [file 44319_2026_742_MOESM4_ESM.zip › Figure 2 Part 1/Fig 2c Cas9 Hela rGT335 atubulin/Cas9 GT335recomb atub 3-24-25 R3 LT2.Project Maximum Z_XY1743451289_Z0_T0_C0.tif]

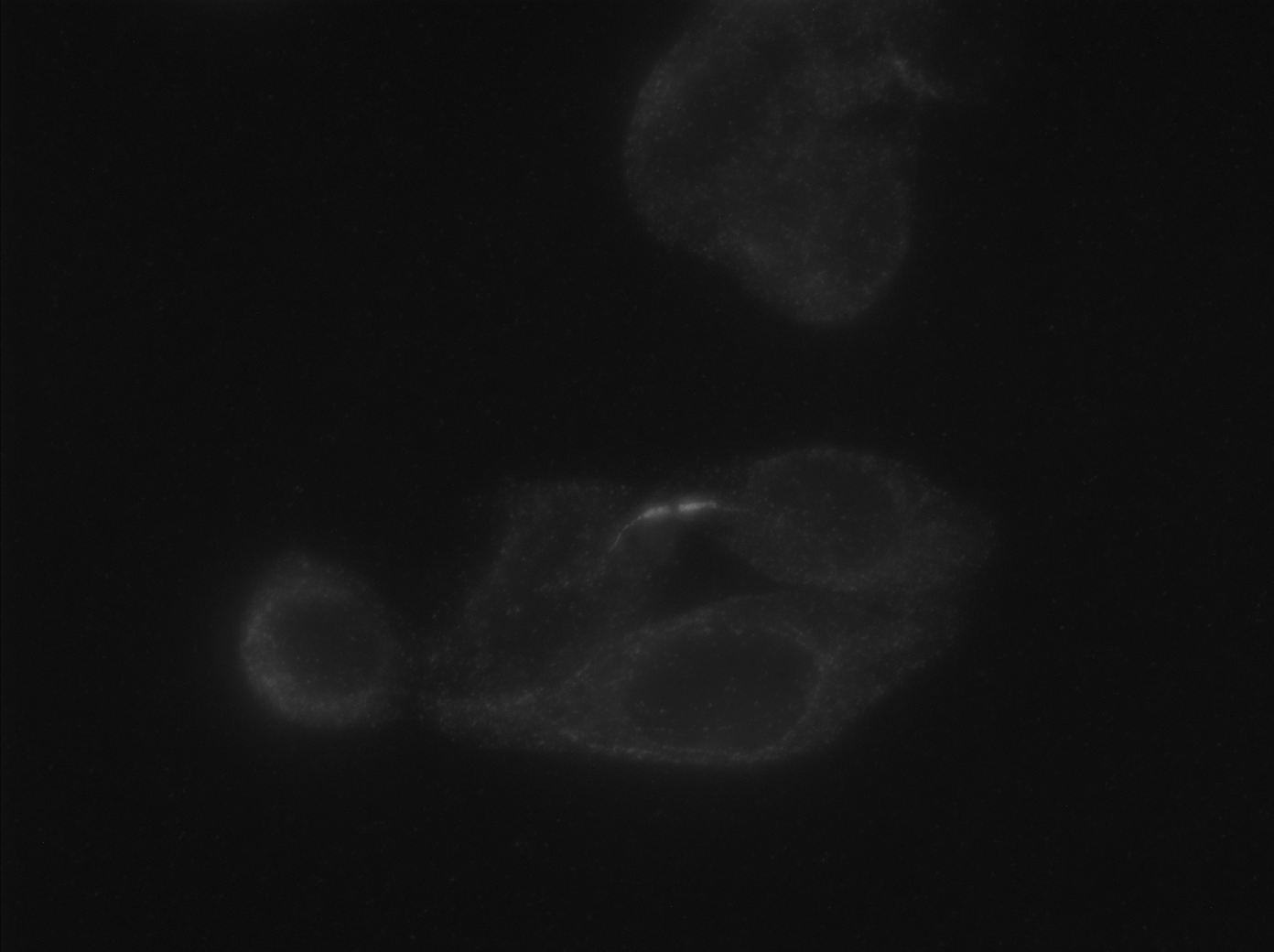

Supplement: Supplementary file 4 — Source data Fig. 2 part 1 [file 44319_2026_742_MOESM4_ESM.zip › Figure 2 Part 1/Fig 2c Cas9 Hela rGT335 atubulin/Cas9 GT335recomb atub 3-24-25 R3 LT2.Project Maximum Z_XY1743451289_Z0_T0_C2.tif]

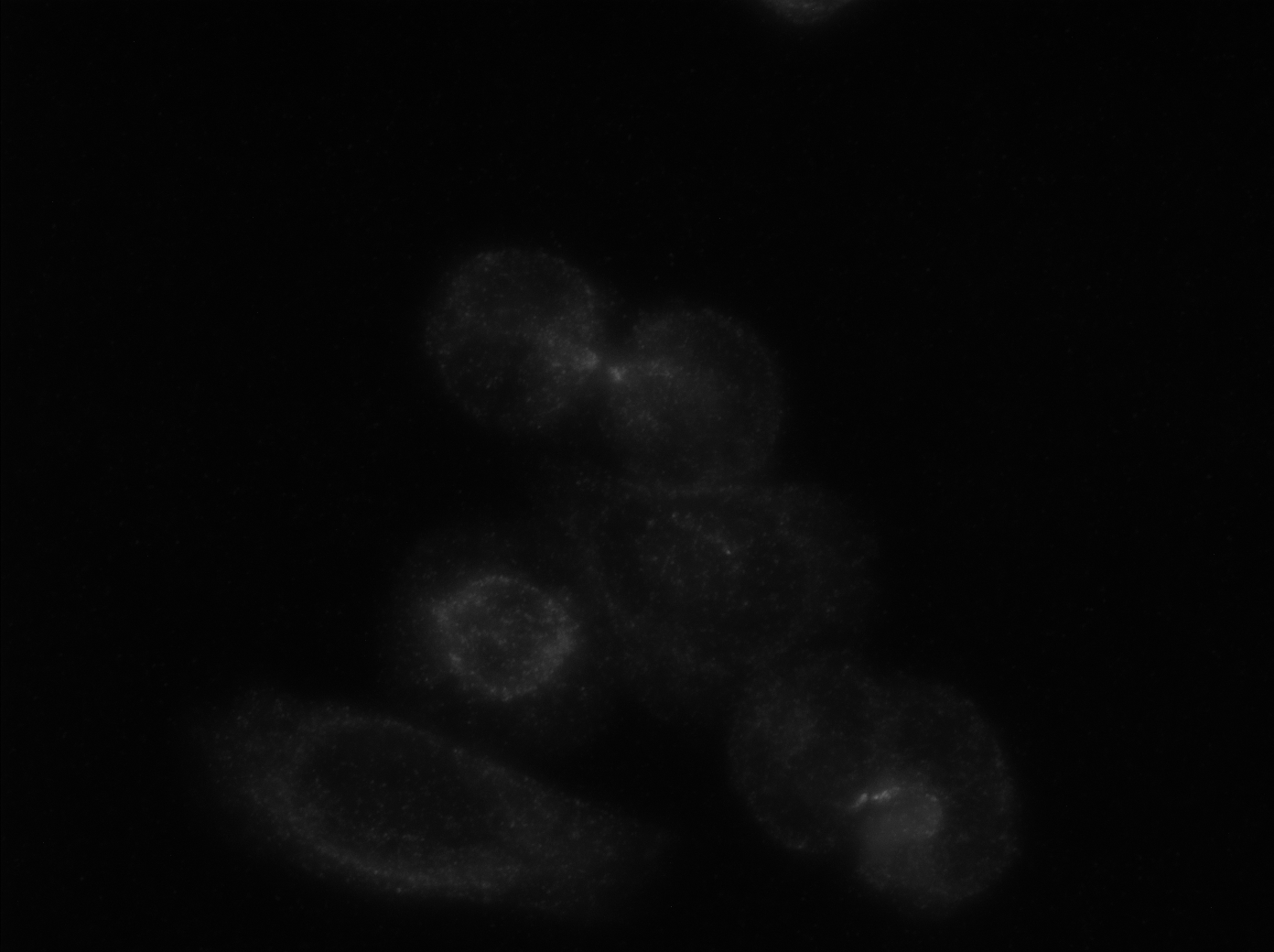

Supplement: Supplementary file 4 — Source data Fig. 2 part 1 [file 44319_2026_742_MOESM4_ESM.zip › Figure 2 Part 1/Fig 2c Cas9 Hela rGT335 atubulin/Cas9 GT335recomb atub 3-24-25 R2 M7.Project Maximum Z_XY1743446847_Z0_T0_C2.tif]

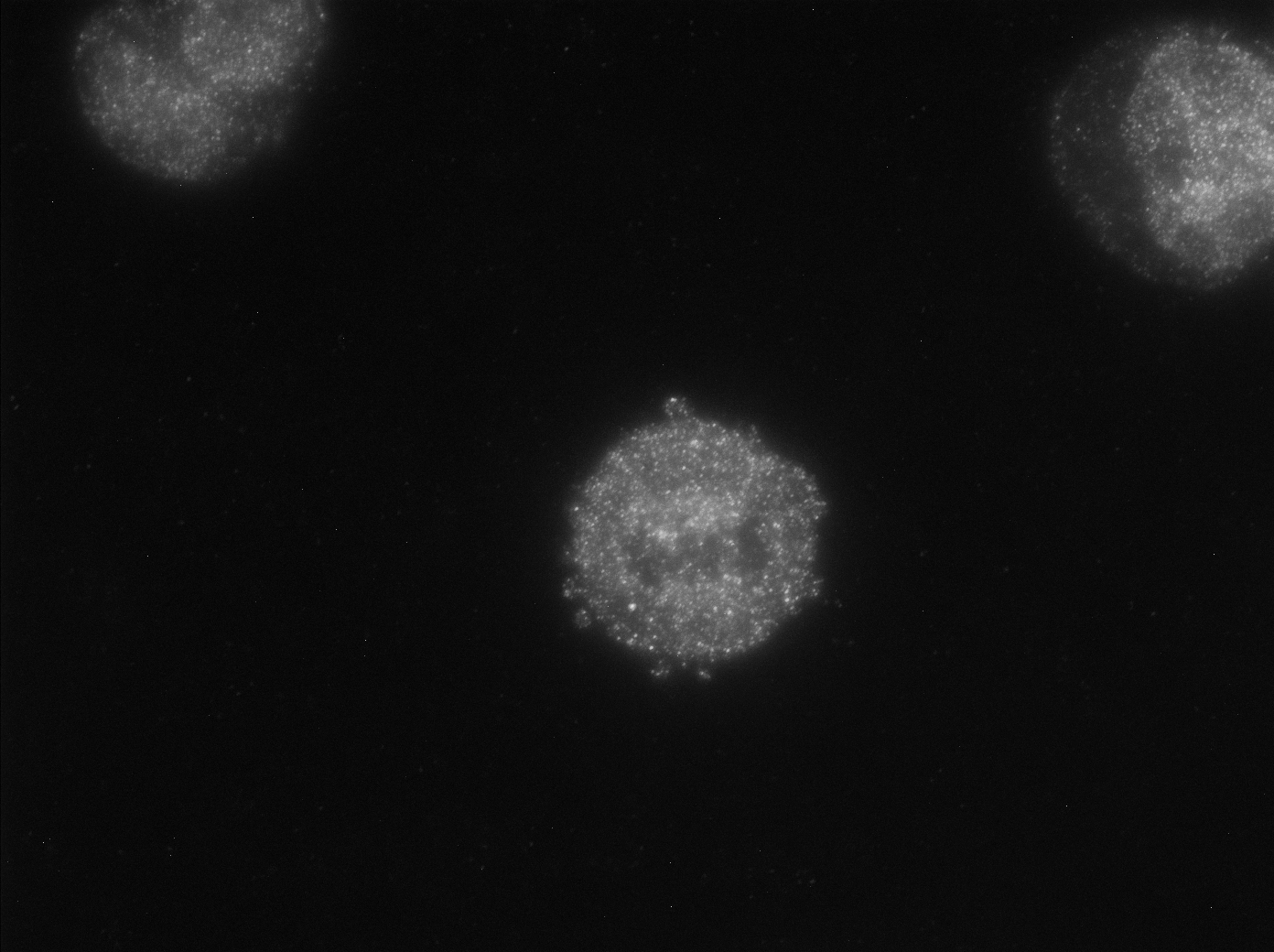

Supplement: Supplementary file 4 — Source data Fig. 2 part 1 [file 44319_2026_742_MOESM4_ESM.zip › Figure 2 Part 1/Fig 2c Cas9 Hela rGT335 atubulin/Cas9 GT335recomb atub 3-24-25 R3 M6.Project Maximum Z_XY1743453627_Z0_T0_C1.tif]

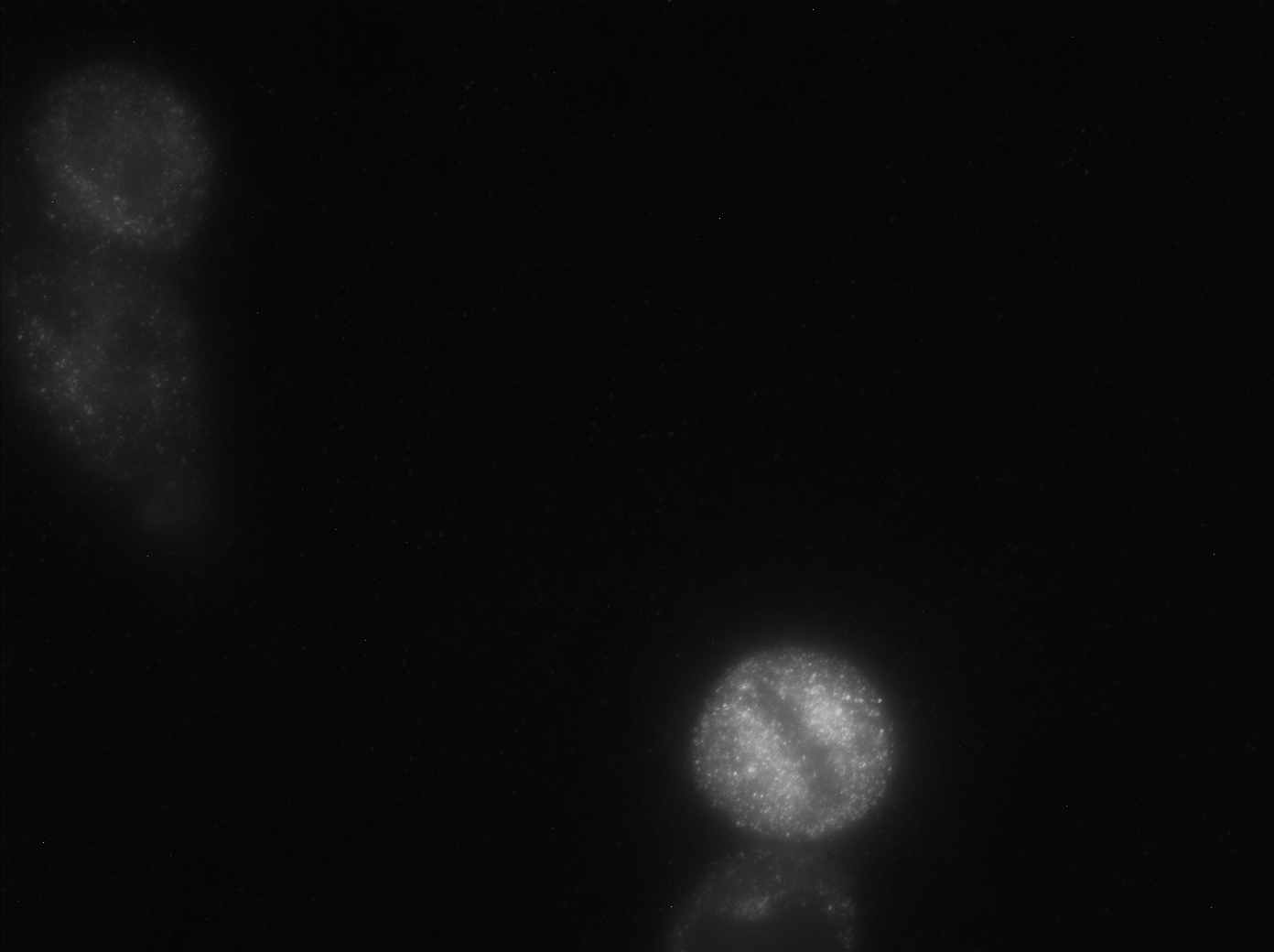

Supplement: Supplementary file 4 — Source data Fig. 2 part 1 [file 44319_2026_742_MOESM4_ESM.zip › Figure 2 Part 1/Fig 2c Cas9 Hela rGT335 atubulin/Cas9 GT335recomb atub 3-24-25 R1 M7.Project Maximum Z_XY1743105485_Z0_T0_C1.tif]

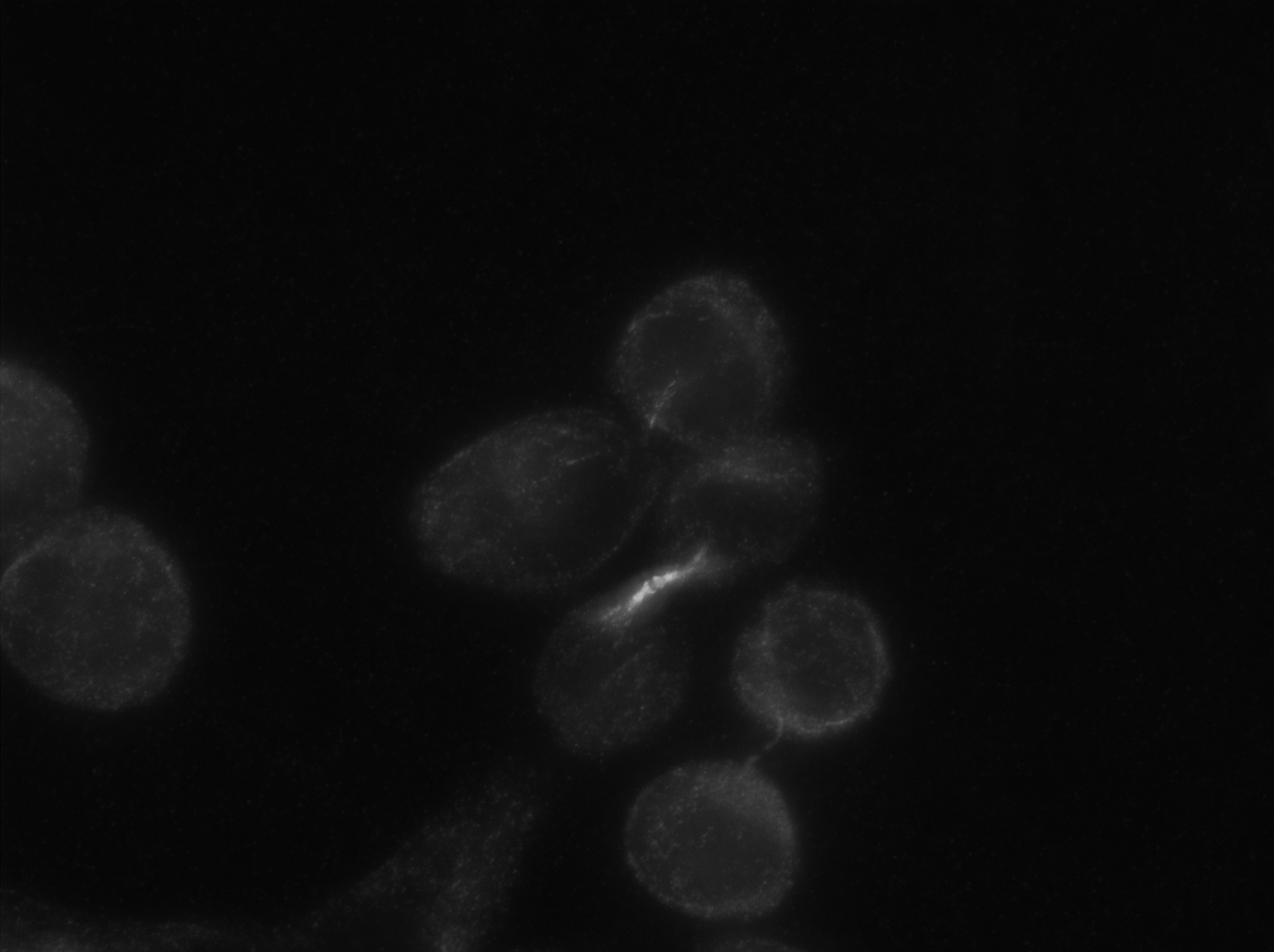

Supplement: Supplementary file 4 — Source data Fig. 2 part 1 [file 44319_2026_742_MOESM4_ESM.zip › Figure 2 Part 1/Fig 2c Cas9 Hela rGT335 atubulin/Cas9 GT335recomb atub 3-24-25 R2 ET4 PA3.Project Maximum Z_XY1743441190_Z0_T0_C2.tif]

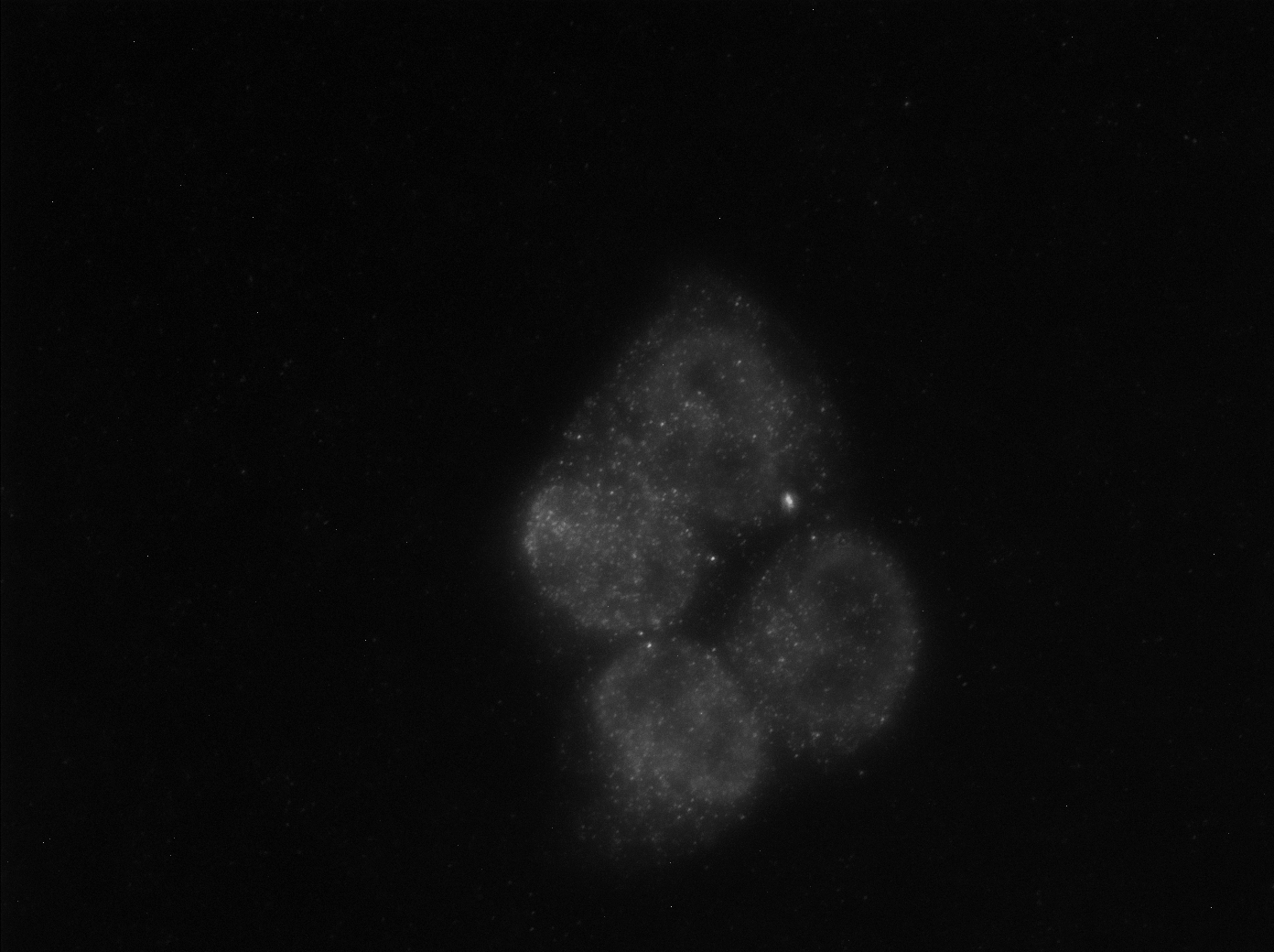

Supplement: Supplementary file 4 — Source data Fig. 2 part 1 [file 44319_2026_742_MOESM4_ESM.zip › Figure 2 Part 1/Fig 2c Cas9 Hela rGT335 atubulin/Cas9 GT335recomb atub 3-24-25 R2 PA9PA10.Project Maximum Z_XY1743446596_Z0_T0_C1.tif]

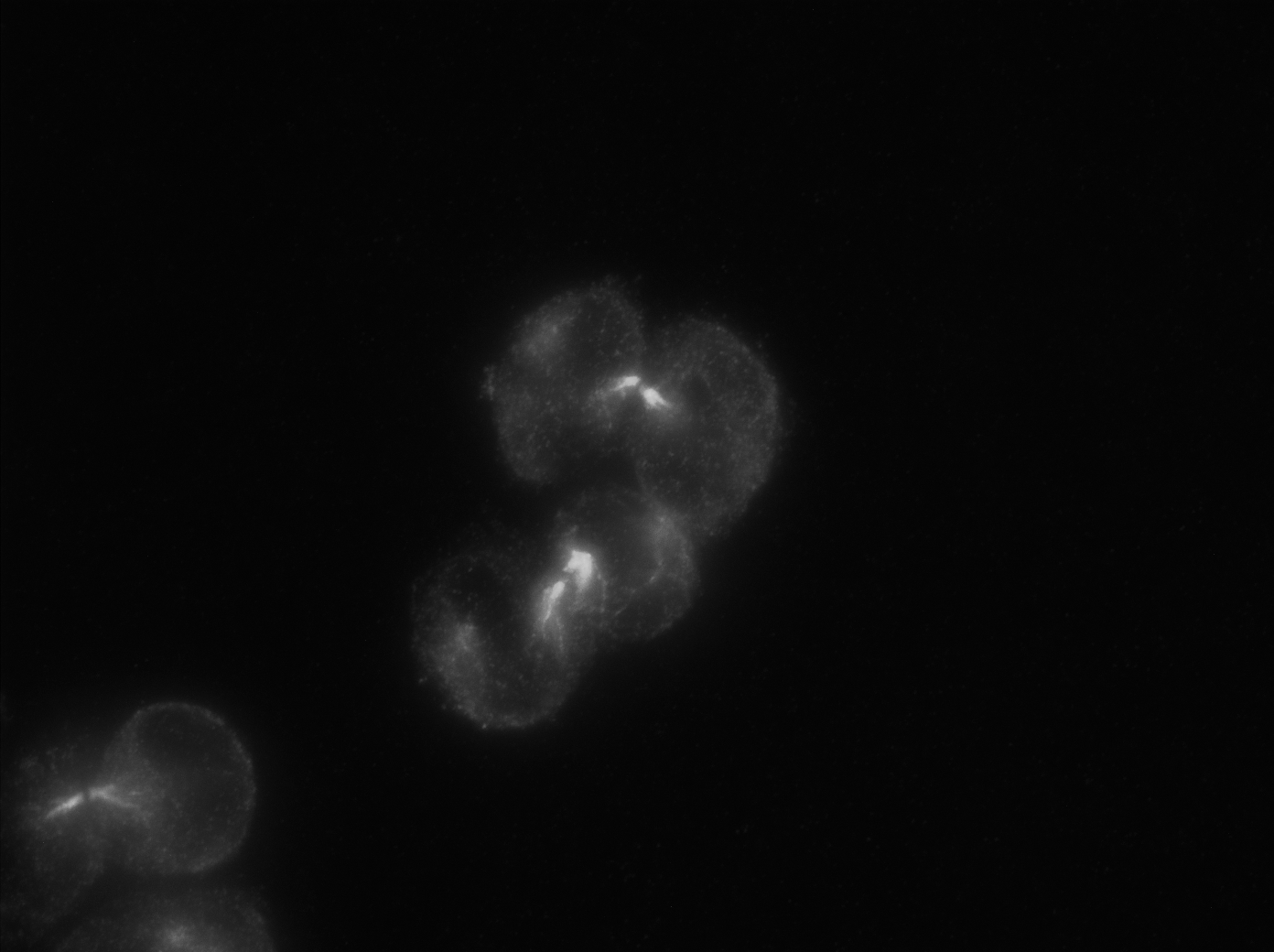

Supplement: Supplementary file 4 — Source data Fig. 2 part 1 [file 44319_2026_742_MOESM4_ESM.zip › Figure 2 Part 1/Fig 2c Cas9 Hela rGT335 atubulin/Cas9 GT335recomb atub 3-24-25 R2 ET8ET9.Project Maximum Z_XY1743444778_Z0_T0_C2.tif]

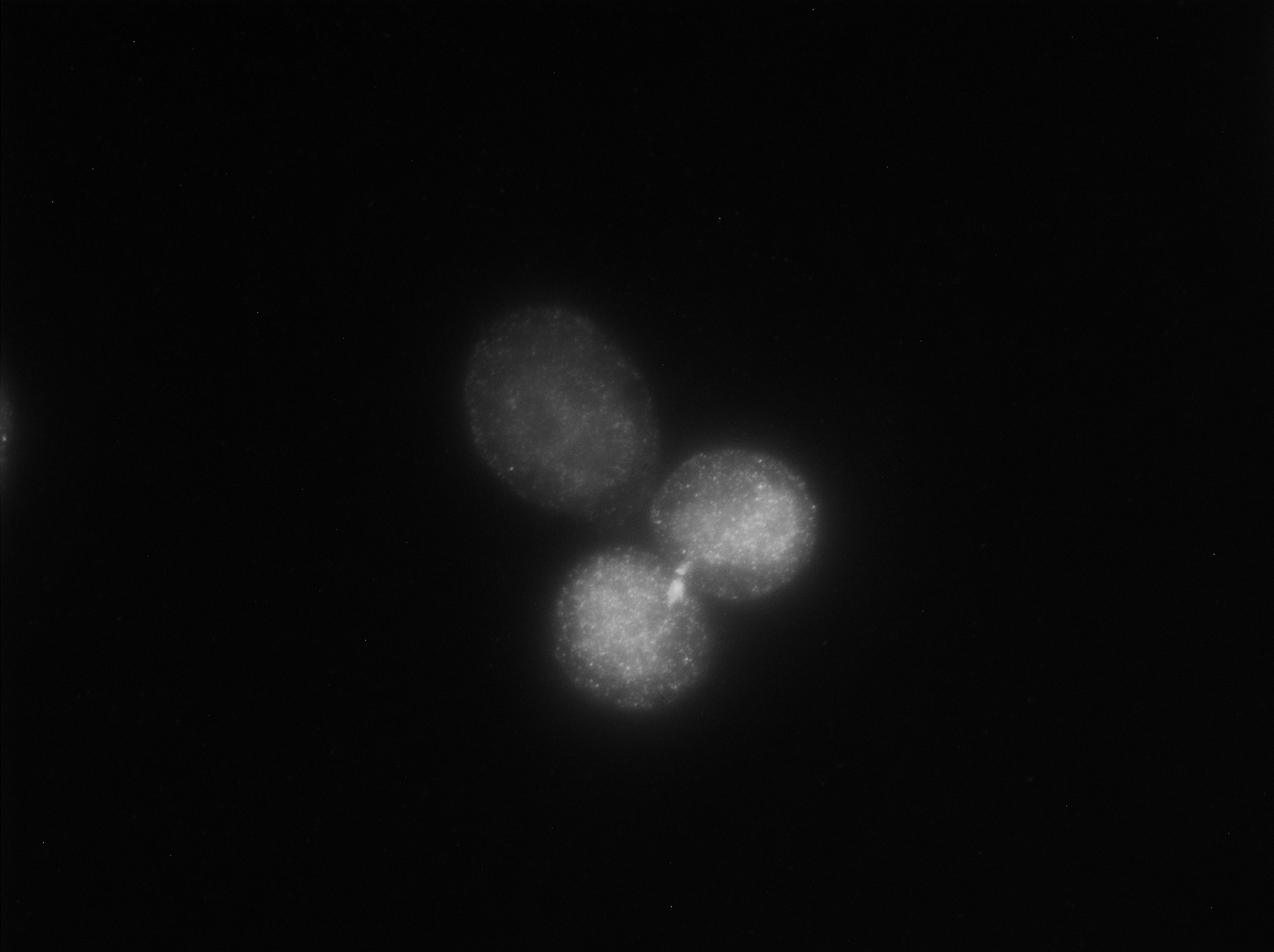

Supplement: Supplementary file 4 — Source data Fig. 2 part 1 [file 44319_2026_742_MOESM4_ESM.zip › Figure 2 Part 1/Fig 2c Cas9 Hela rGT335 atubulin/Cas9 GT335recomb atub 3-24-25 R1 ET5.Project Maximum Z_XY1743102526_Z0_T0_C1.tif]

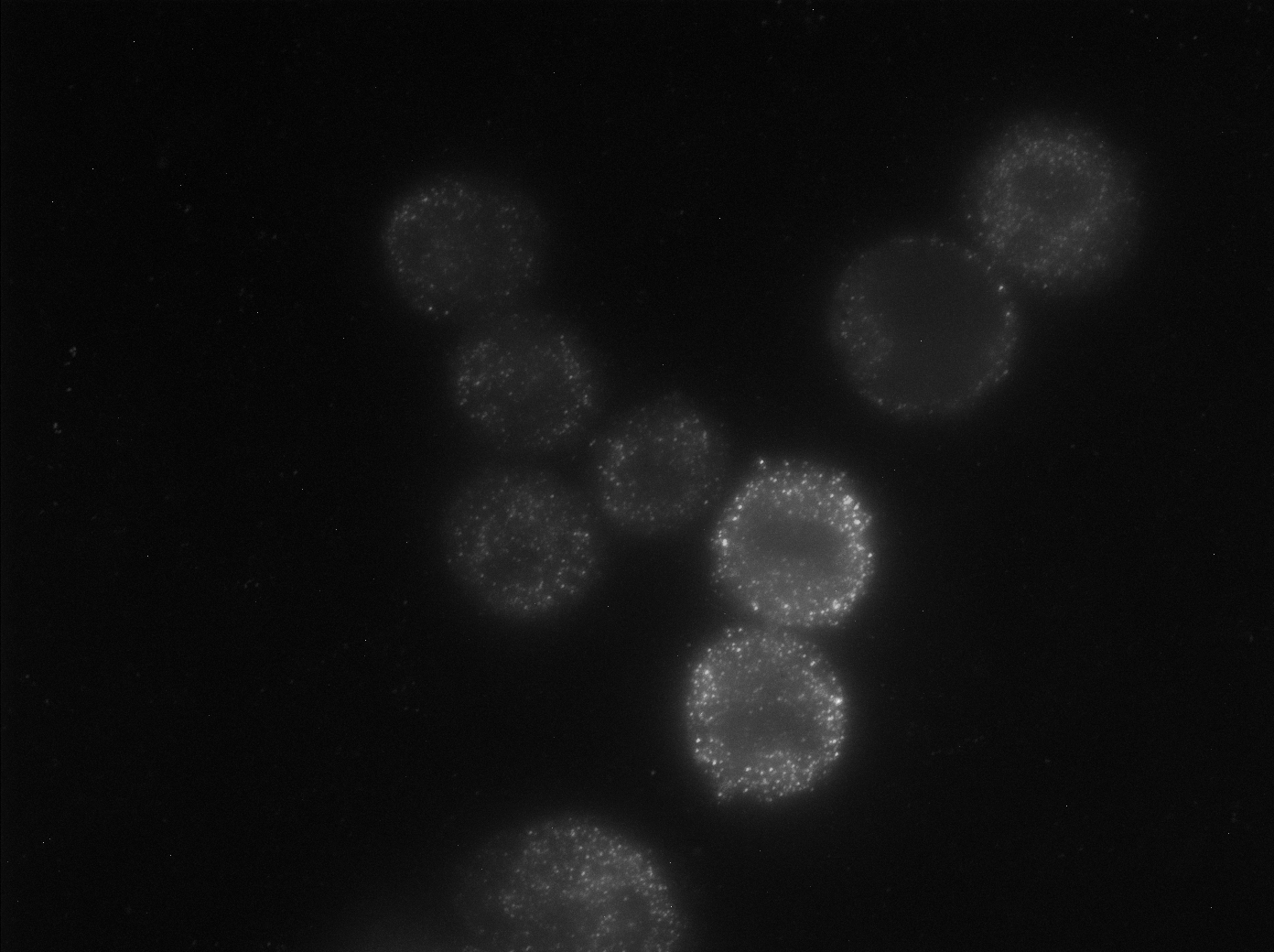

Supplement: Supplementary file 4 — Source data Fig. 2 part 1 [file 44319_2026_742_MOESM4_ESM.zip › Figure 2 Part 1/Fig 2c Cas9 Hela rGT335 atubulin/Cas9 GT335recomb atub 3-24-25 R3 ET5.Project Maximum Z_XY1743454833_Z0_T0_C1.tif]

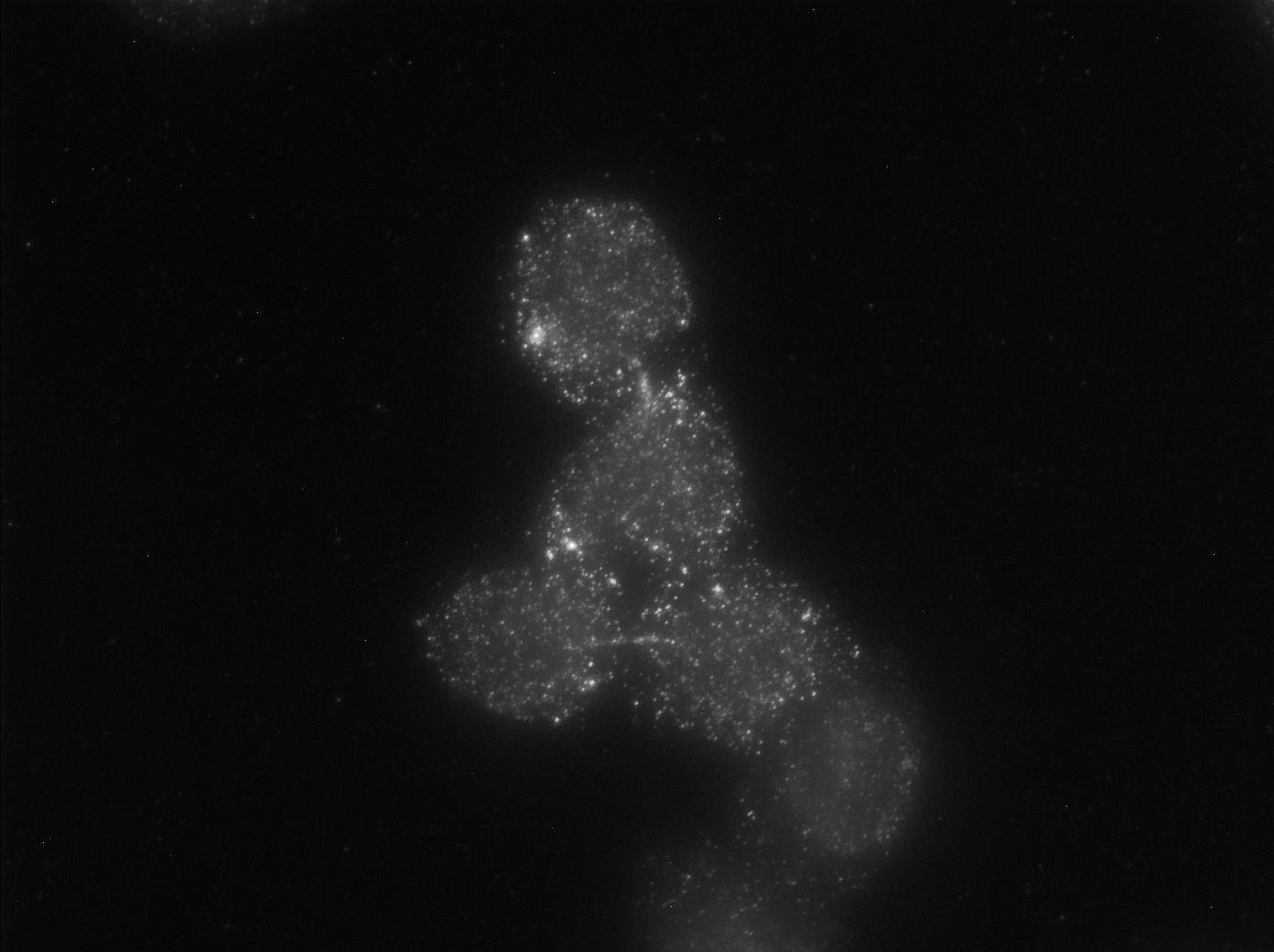

Supplement: Supplementary file 4 — Source data Fig. 2 part 1 [file 44319_2026_742_MOESM4_ESM.zip › Figure 2 Part 1/Fig 2c Cas9 Hela rGT335 atubulin/Cas9 GT335recomb atub 3-24-25 R3 LT7LT8.Project Maximum Z_XY1743452825_Z0_T0_C1.tif]

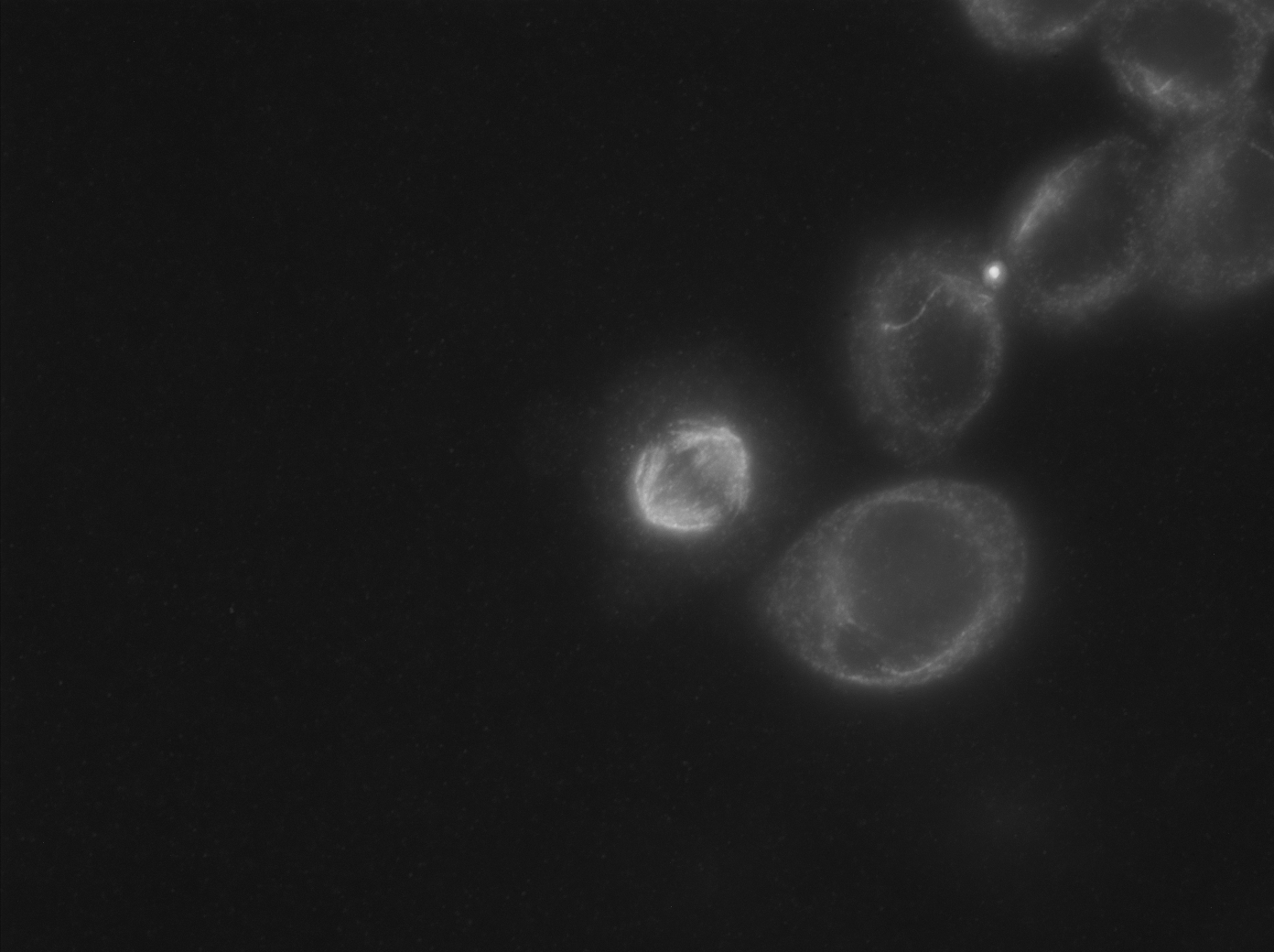

Supplement: Supplementary file 4 — Source data Fig. 2 part 1 [file 44319_2026_742_MOESM4_ESM.zip › Figure 2 Part 1/Fig 2c Cas9 Hela rGT335 atubulin/Cas9 GT335recomb atub 3-24-25 R3 M4 PA5.Project Maximum Z_XY1743453144_Z0_T0_C2.tif]

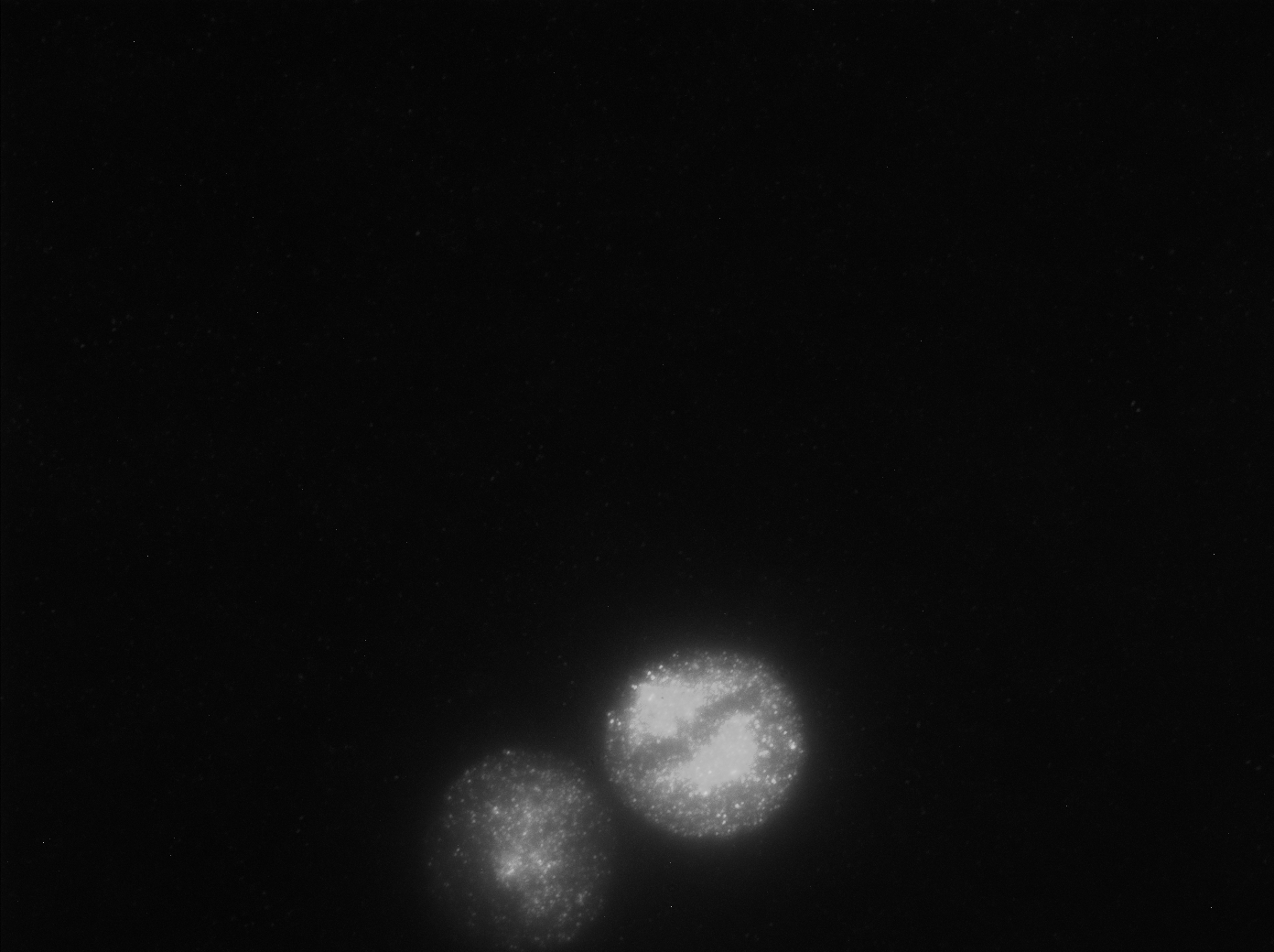

Supplement: Supplementary file 4 — Source data Fig. 2 part 1 [file 44319_2026_742_MOESM4_ESM.zip › Figure 2 Part 1/Fig 2c Cas9 Hela rGT335 atubulin/Cas9 GT335recomb atub 3-24-25 R1 M5.Project Maximum Z_XY1743104305_Z0_T0_C1.tif]

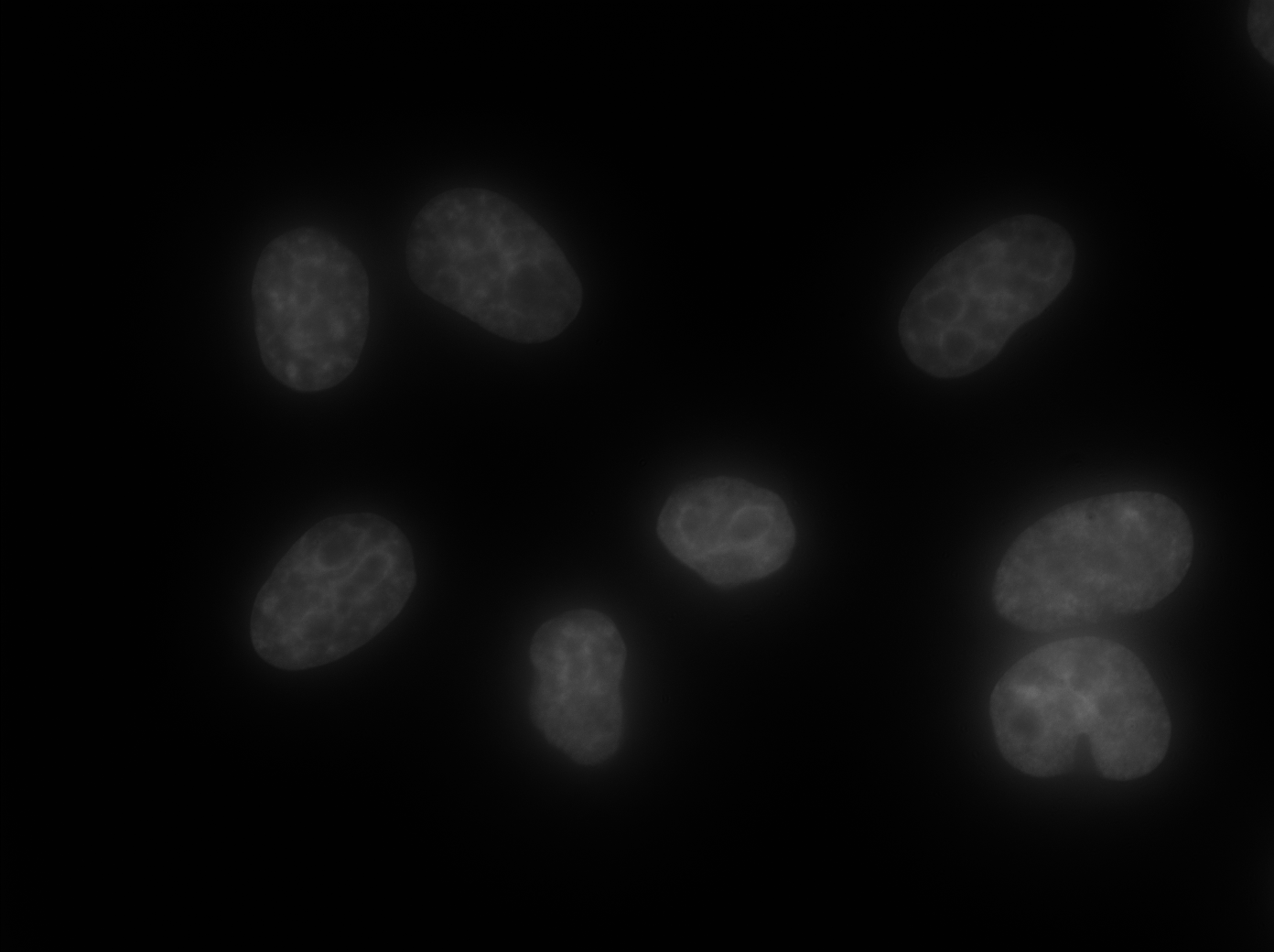

Supplement: Supplementary file 4 — Source data Fig. 2 part 1 [file 44319_2026_742_MOESM4_ESM.zip › Figure 2 Part 1/Fig 2c Cas9 Hela rGT335 atubulin/Cas9 GT335recomb atub 3-24-25 R1 LT3.Project Maximum Z_XY1743100808_Z0_T0_C0.tif]

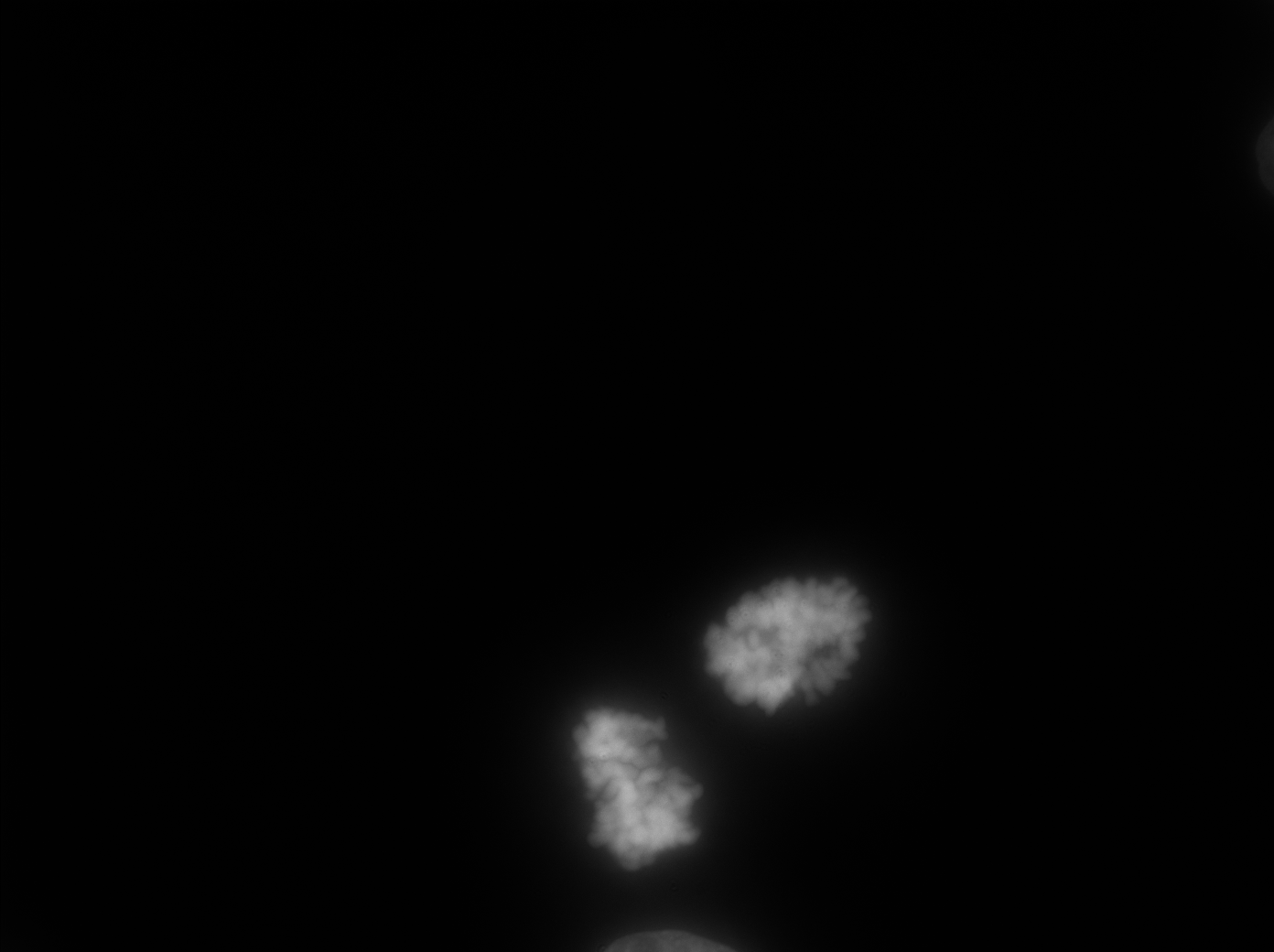

Supplement: Supplementary file 4 — Source data Fig. 2 part 1 [file 44319_2026_742_MOESM4_ESM.zip › Figure 2 Part 1/Fig 2c Cas9 Hela rGT335 atubulin/Cas9 GT335recomb atub 3-24-25 R2 M5M6.Project Maximum Z_XY1743446381_Z0_T0_C0.tif]

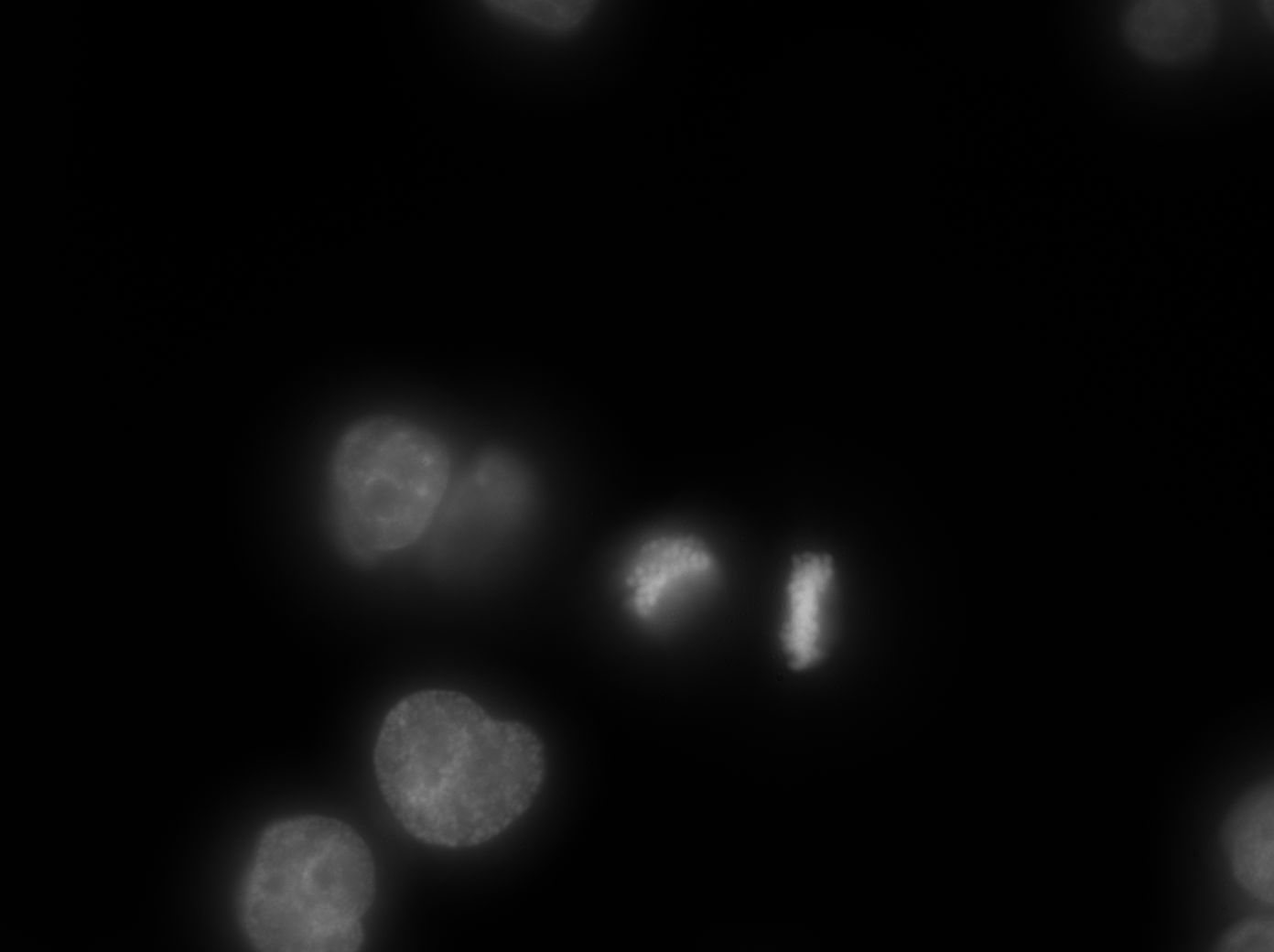

Supplement: Supplementary file 4 — Source data Fig. 2 part 1 [file 44319_2026_742_MOESM4_ESM.zip › Figure 2 Part 1/Fig 2c Cas9 Hela rGT335 atubulin/Cas9 GT335recomb atub 3-24-25 R3 ET2.Project Maximum Z_XY1743451921_Z0_T0_C0.tif]

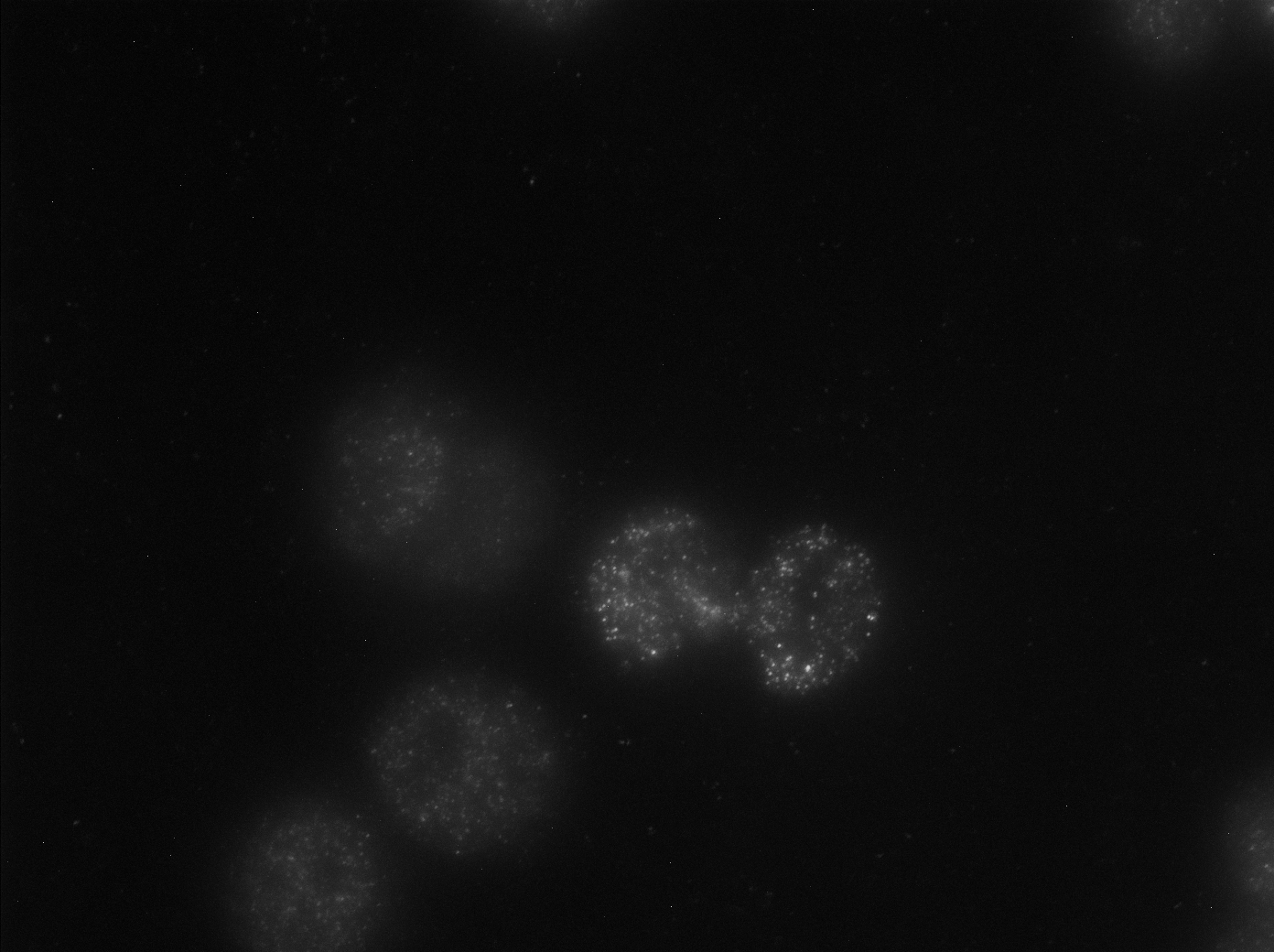

Supplement: Supplementary file 4 — Source data Fig. 2 part 1 [file 44319_2026_742_MOESM4_ESM.zip › Figure 2 Part 1/Fig 2c Cas9 Hela rGT335 atubulin/Cas9 GT335recomb atub 3-24-25 R3 ET2.Project Maximum Z_XY1743451921_Z0_T0_C1.tif]

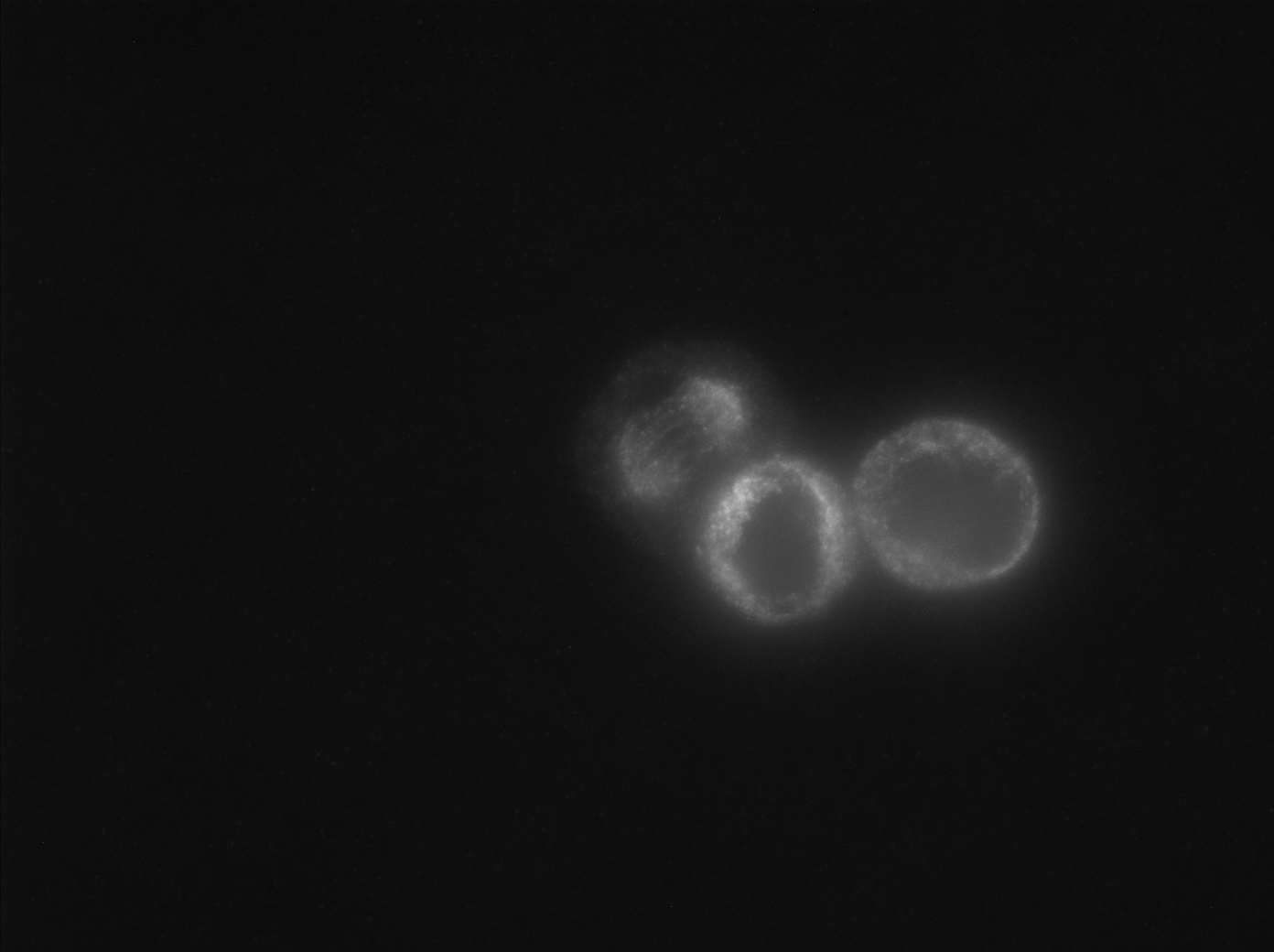

Supplement: Supplementary file 4 — Source data Fig. 2 part 1 [file 44319_2026_742_MOESM4_ESM.zip › Figure 2 Part 1/Fig 2c Cas9 Hela rGT335 atubulin/Cas9 GT335recomb atub 3-24-25 R1 M6.Project Maximum Z_XY1743104509_Z0_T0_C2.tif]

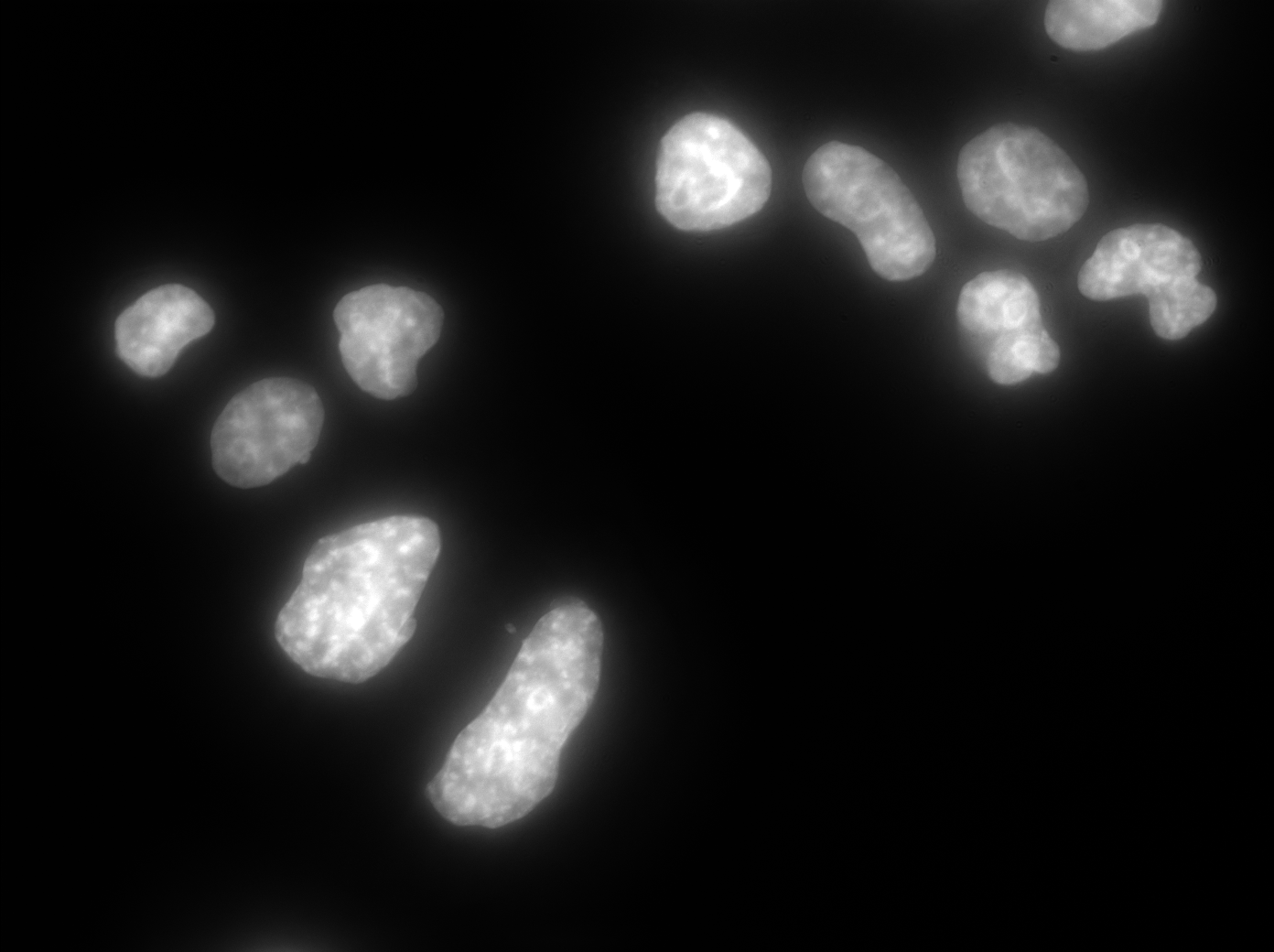

Supplement: Supplementary file 5 — Source data Fig. 2 part 2 [file 44319_2026_742_MOESM5_ESM.zip › Figure 2 Part 2/Fig 2e WT Hela acetylated a tubulin atubulin/actub-atub 8-14-24 R2 LT3 PA1.Project Maximum Z_XY1724689811_Z0_T0_C0.tif]
