# Supplementary figures and images for: TPGS1 regulates central spindle microtubule glutamylation and remodeling during telophase and abscission (part 3 of 36)
Source: EMBO Rep. 2026 Mar 23;27(8):1944–63. doi: 10.1038/s44319-026-00742-3 (PMC13121839; doi:10.1038/s44319-026-00742-3)

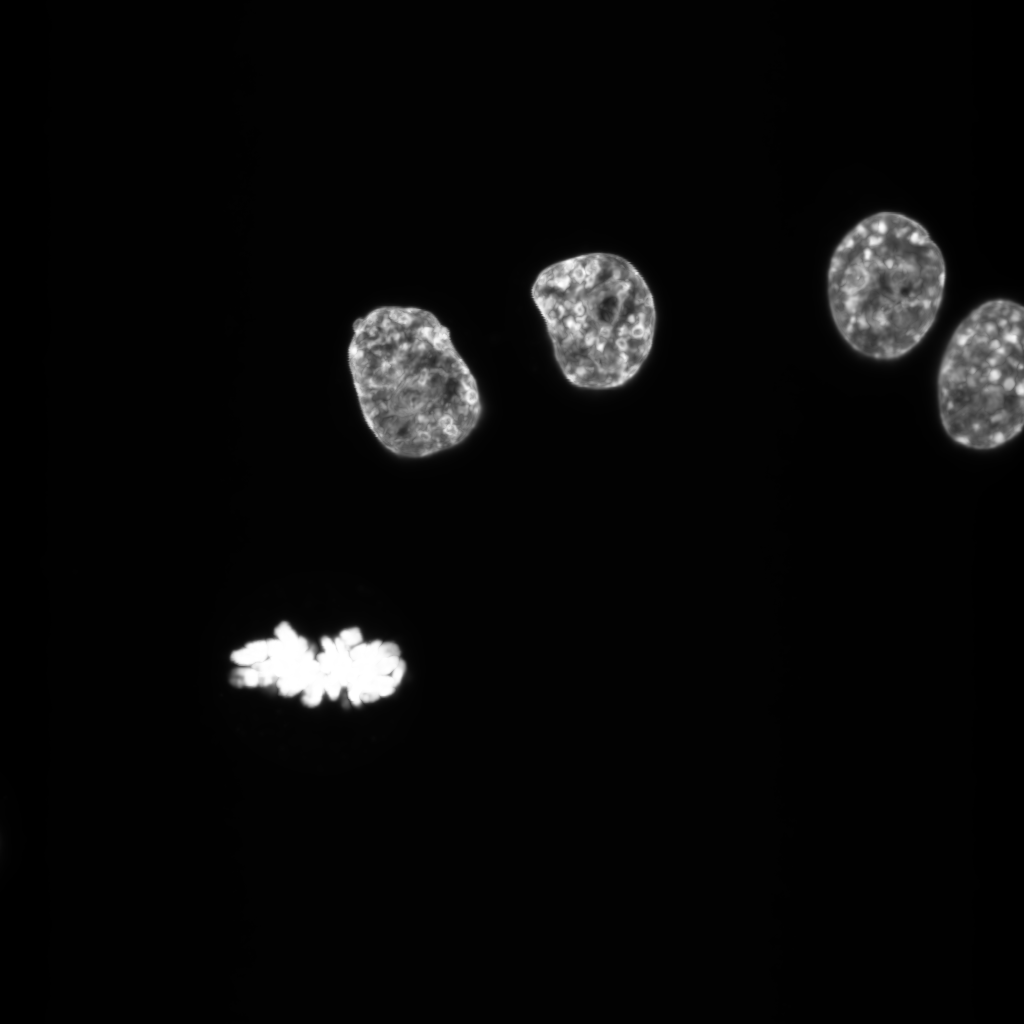

Supplement: Supplementary file 4 — Source data Fig. 2 part 1 [file 44319_2026_742_MOESM4_ESM.zip › Figure 2 Part 1/Fig 2a rgt335 acetylated tubulin control confocal/GTacLT-MaxIP_DAPI.tif]

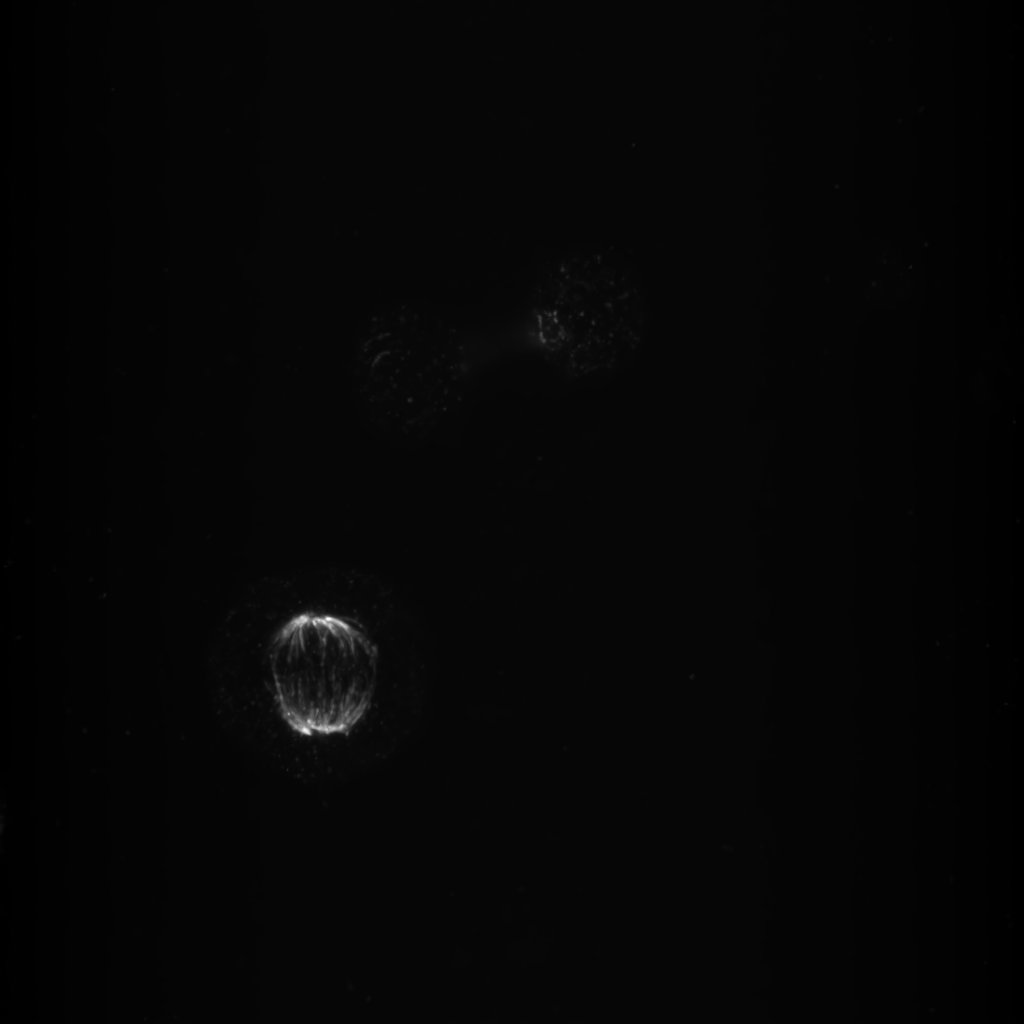

Supplement: Supplementary file 4 — Source data Fig. 2 part 1 [file 44319_2026_742_MOESM4_ESM.zip › Figure 2 Part 1/Fig 2a rgt335 acetylated tubulin control confocal/GTacM-MaxIP_594.tif]

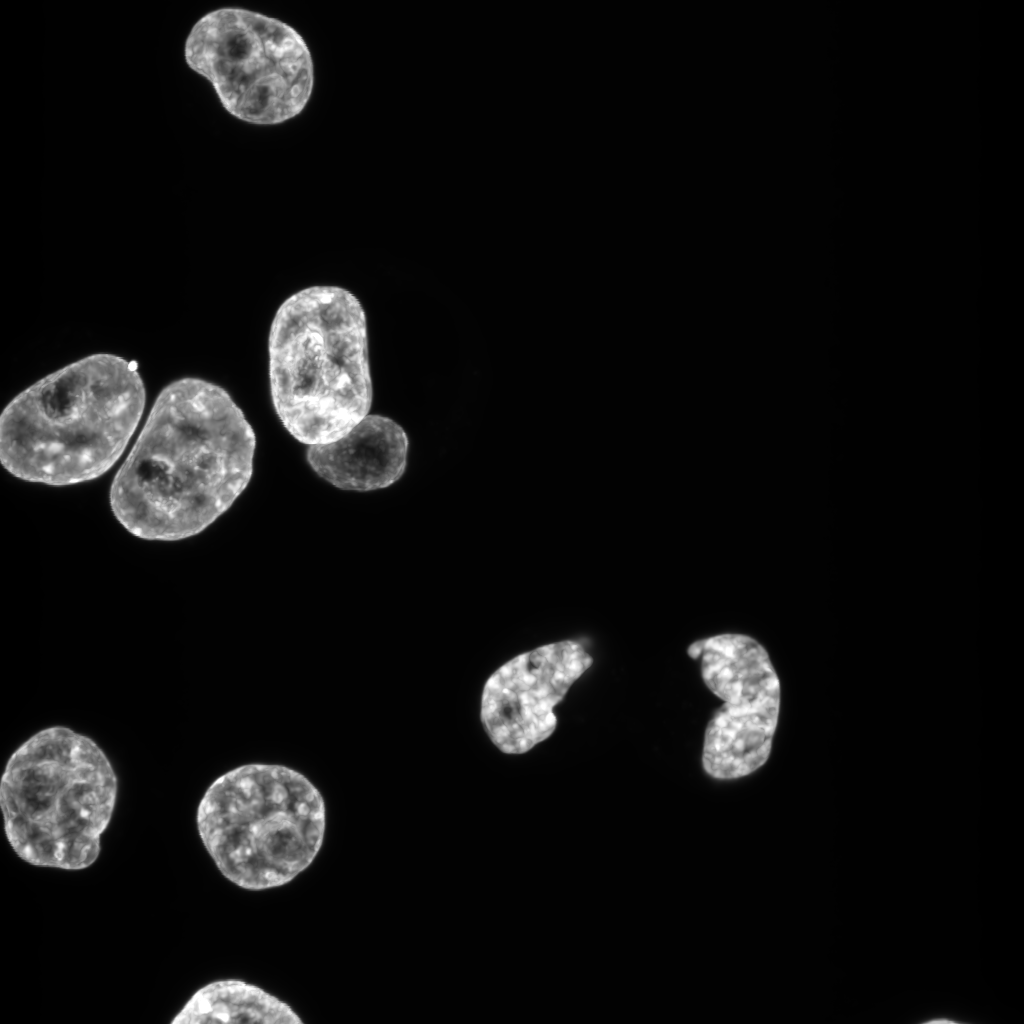

Supplement: Supplementary file 4 — Source data Fig. 2 part 1 [file 44319_2026_742_MOESM4_ESM.zip › Figure 2 Part 1/Fig 2a rgt335 acetylated tubulin control confocal/GTacET2-MaxIP_DAPI.tif]

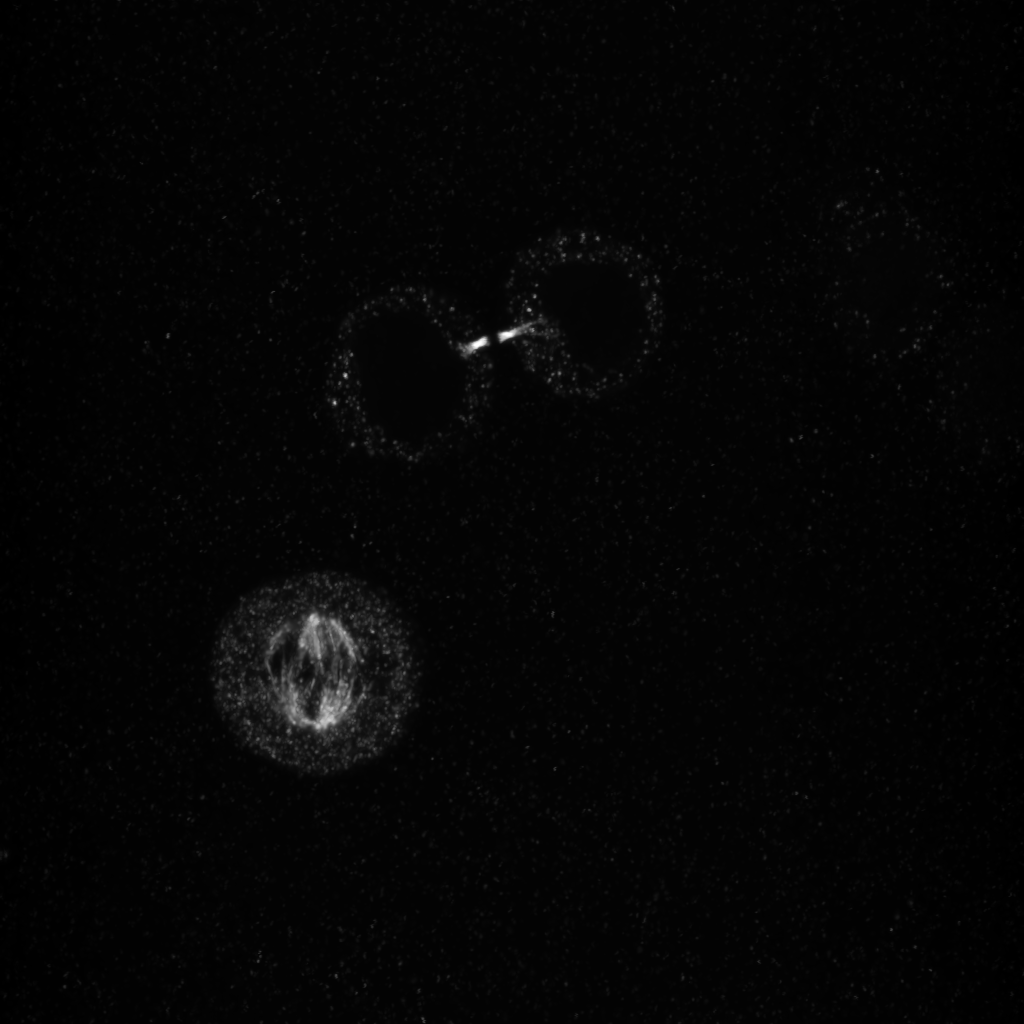

Supplement: Supplementary file 4 — Source data Fig. 2 part 1 [file 44319_2026_742_MOESM4_ESM.zip › Figure 2 Part 1/Fig 2a rgt335 acetylated tubulin control confocal/GTacLT-MaxIP_488.tif]

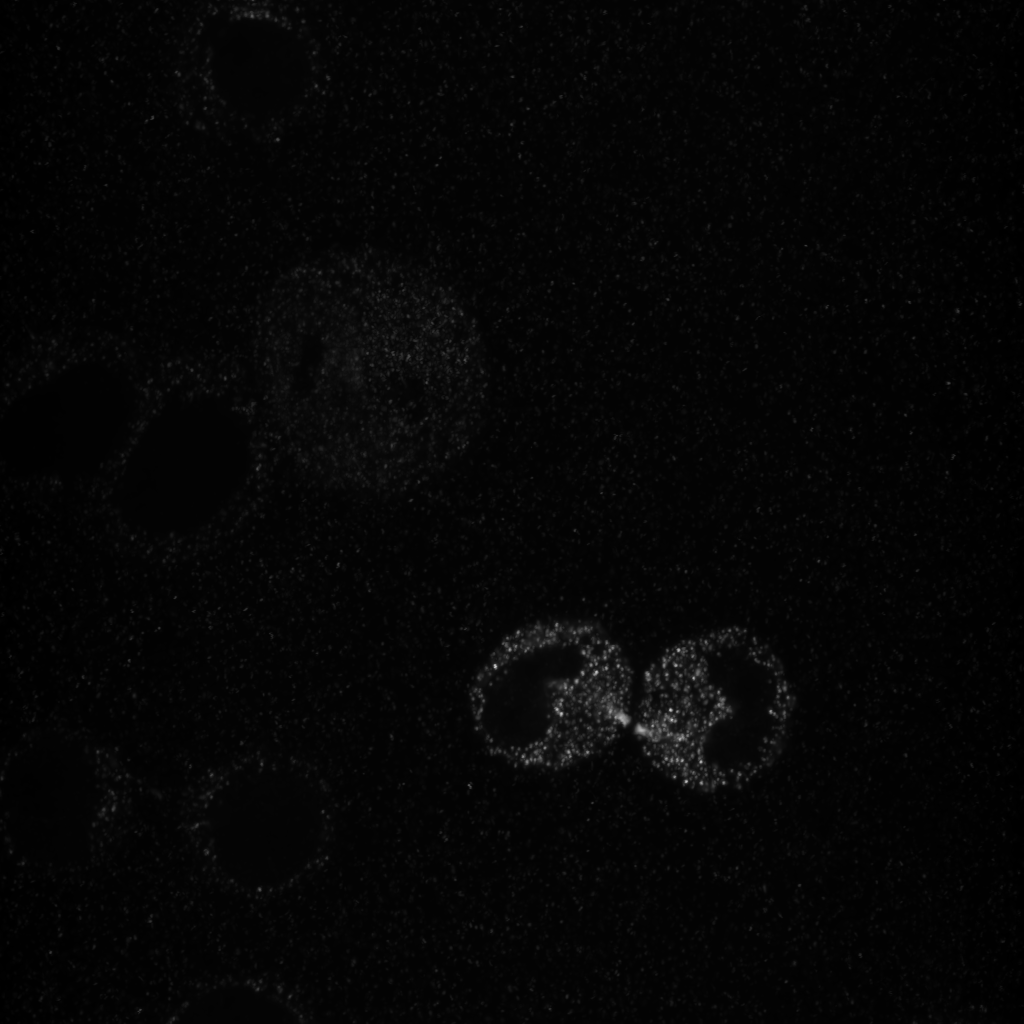

Supplement: Supplementary file 4 — Source data Fig. 2 part 1 [file 44319_2026_742_MOESM4_ESM.zip › Figure 2 Part 1/Fig 2a rgt335 acetylated tubulin control confocal/GTacET2-MaxIP_488.tif]

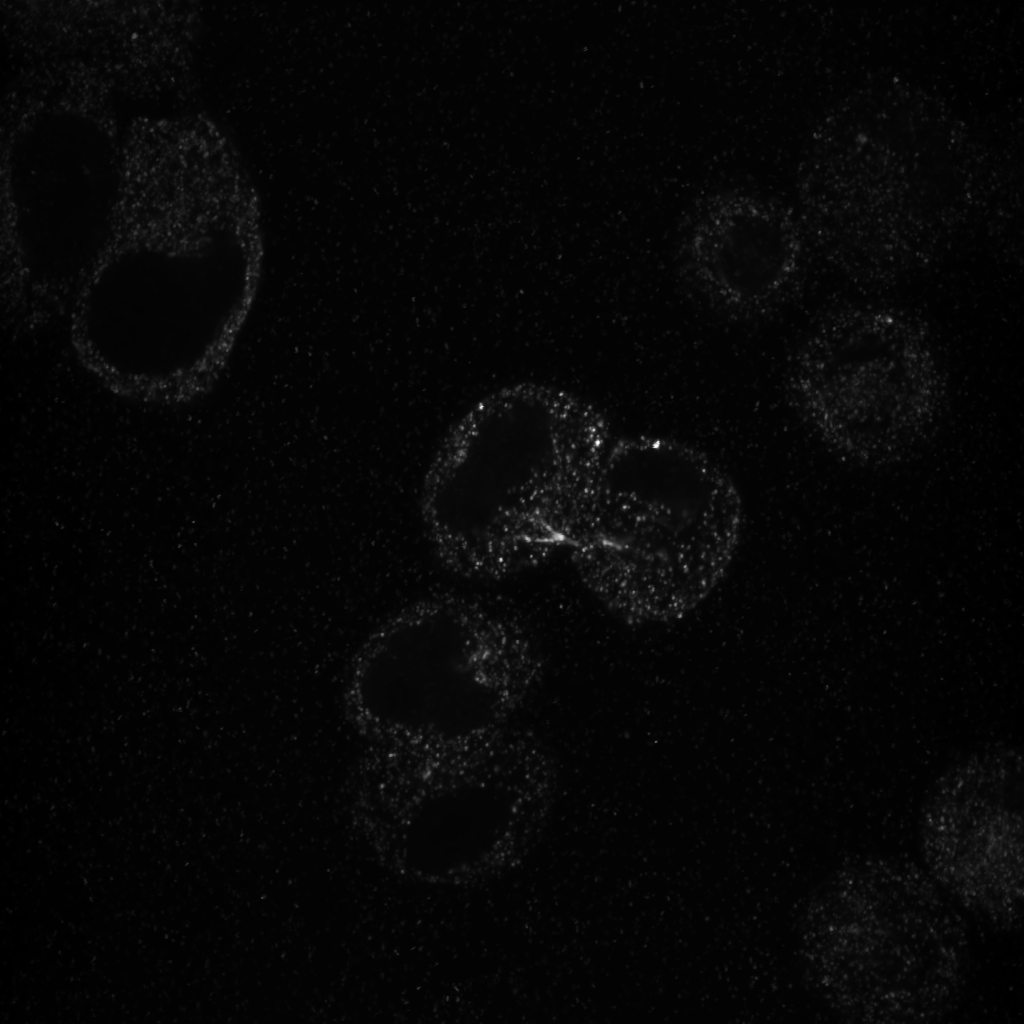

Supplement: Supplementary file 4 — Source data Fig. 2 part 1 [file 44319_2026_742_MOESM4_ESM.zip › Figure 2 Part 1/Fig 2a rgt335 acetylated tubulin control confocal/GTacPA1002-MaxIP_488.tif]

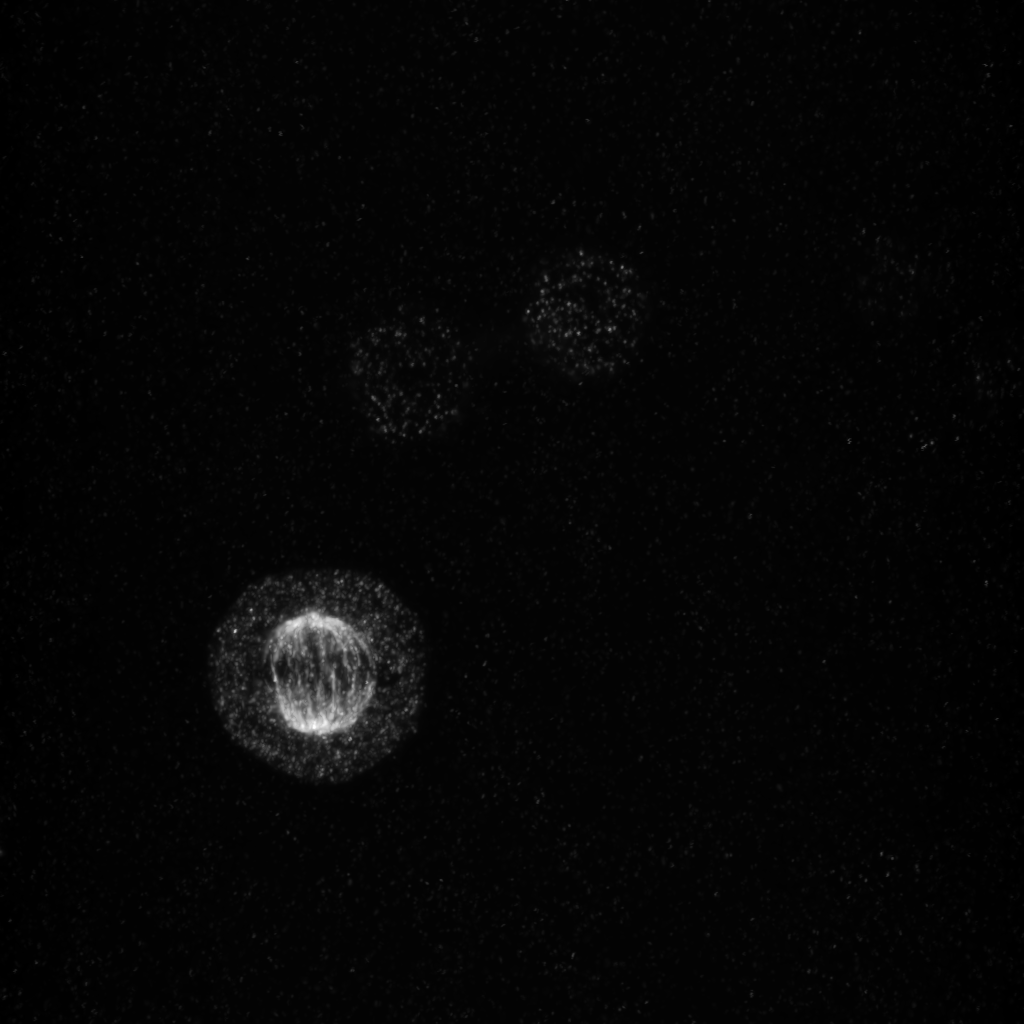

Supplement: Supplementary file 4 — Source data Fig. 2 part 1 [file 44319_2026_742_MOESM4_ESM.zip › Figure 2 Part 1/Fig 2a rgt335 acetylated tubulin control confocal/GTacM-MaxIP_488.tif]

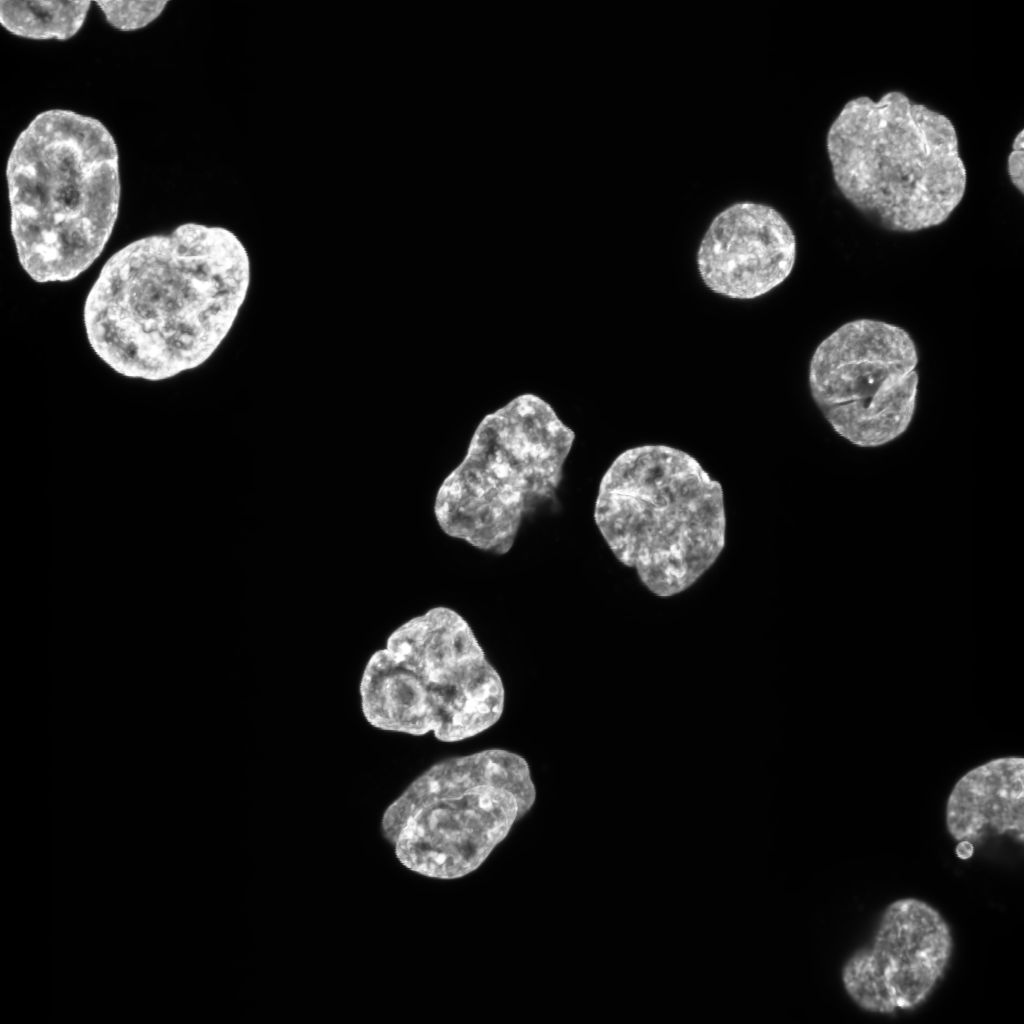

Supplement: Supplementary file 4 — Source data Fig. 2 part 1 [file 44319_2026_742_MOESM4_ESM.zip › Figure 2 Part 1/Fig 2a rgt335 acetylated tubulin control confocal/GTacPA1002-MaxIP_DAPI.tif]

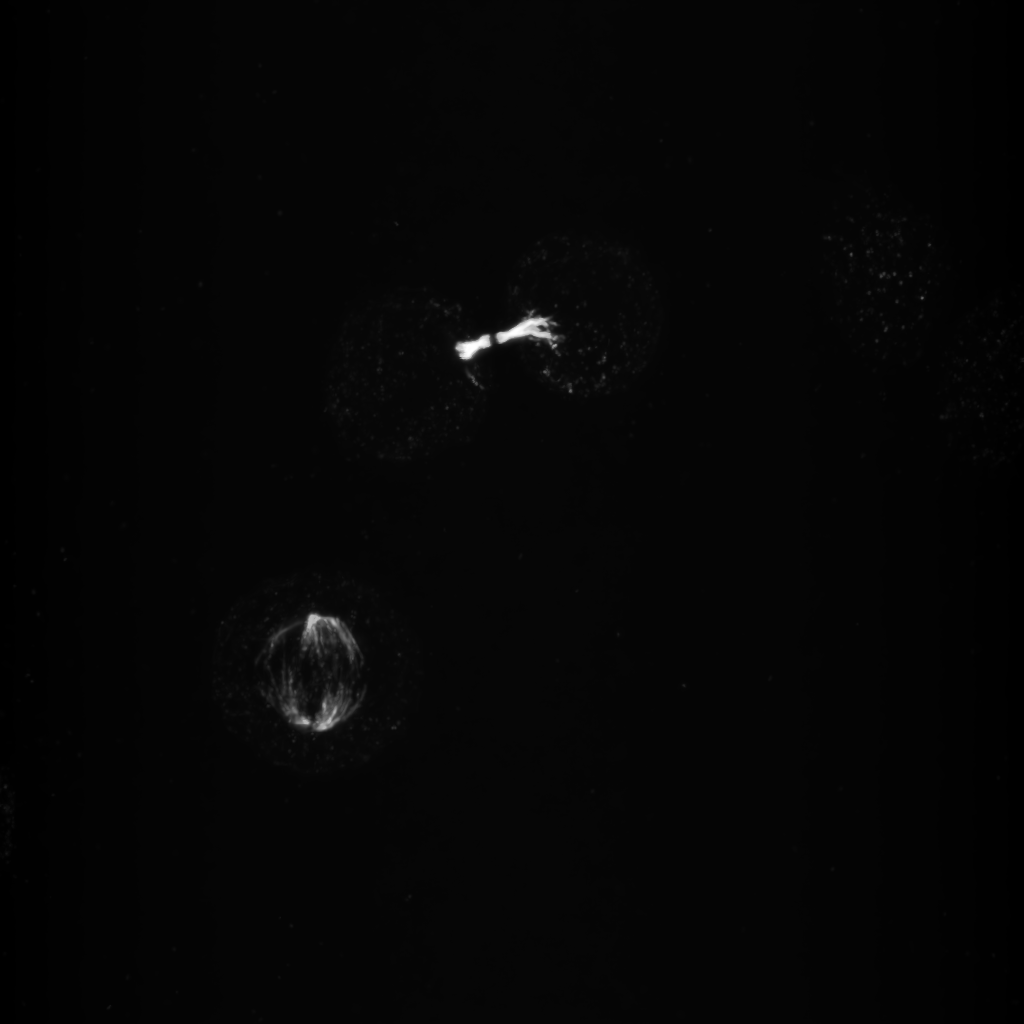

Supplement: Supplementary file 4 — Source data Fig. 2 part 1 [file 44319_2026_742_MOESM4_ESM.zip › Figure 2 Part 1/Fig 2a rgt335 acetylated tubulin control confocal/GTacLT-MaxIP_594.tif]

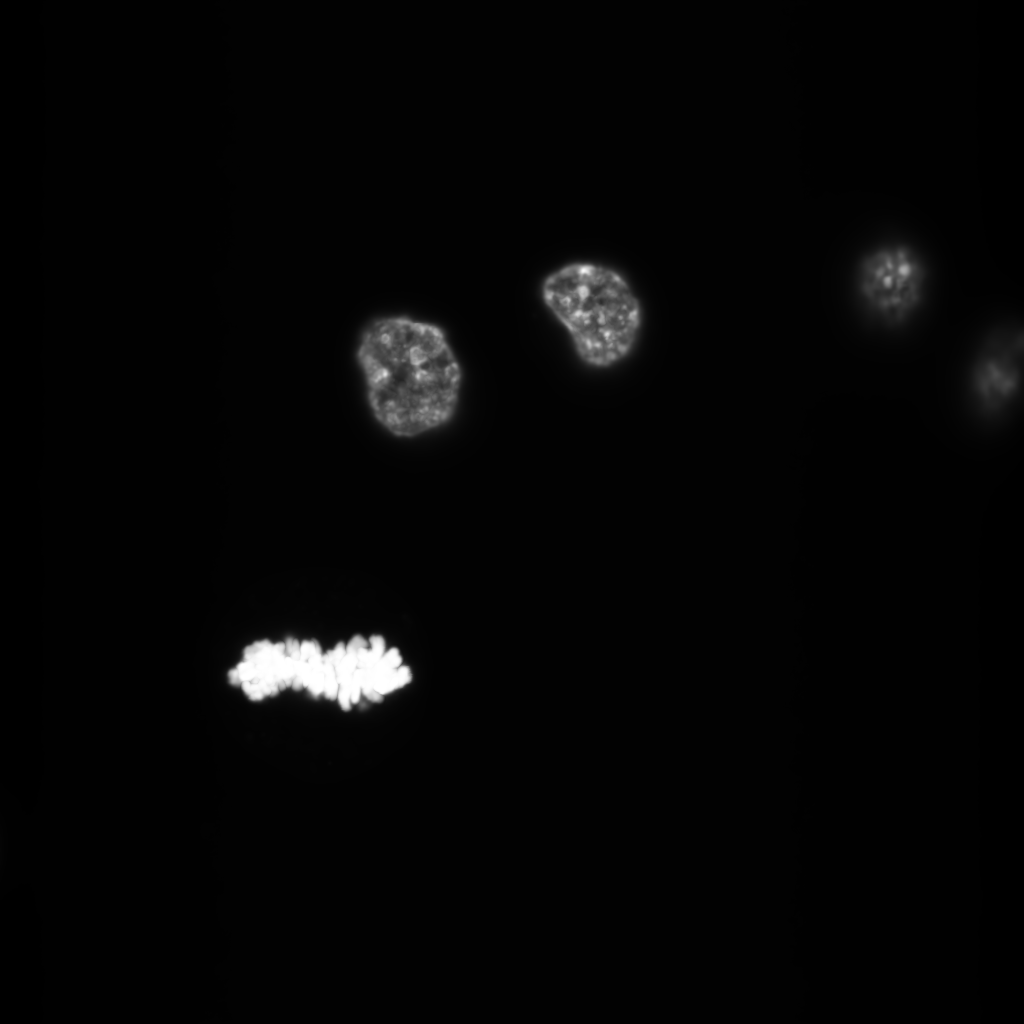

Supplement: Supplementary file 4 — Source data Fig. 2 part 1 [file 44319_2026_742_MOESM4_ESM.zip › Figure 2 Part 1/Fig 2a rgt335 acetylated tubulin control confocal/GTacM-MaxIP_DAPI.tif]

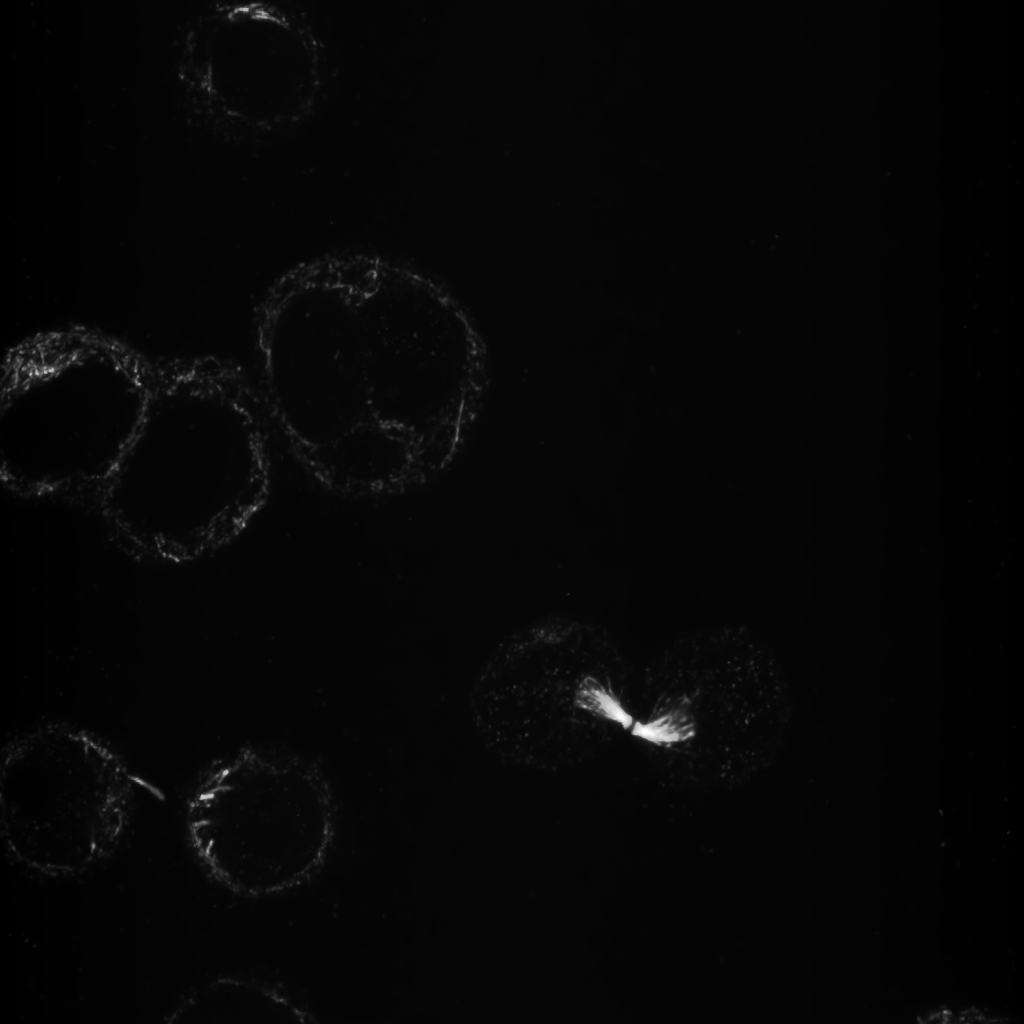

Supplement: Supplementary file 4 — Source data Fig. 2 part 1 [file 44319_2026_742_MOESM4_ESM.zip › Figure 2 Part 1/Fig 2a rgt335 acetylated tubulin control confocal/GTacET2-MaxIP_594.tif]

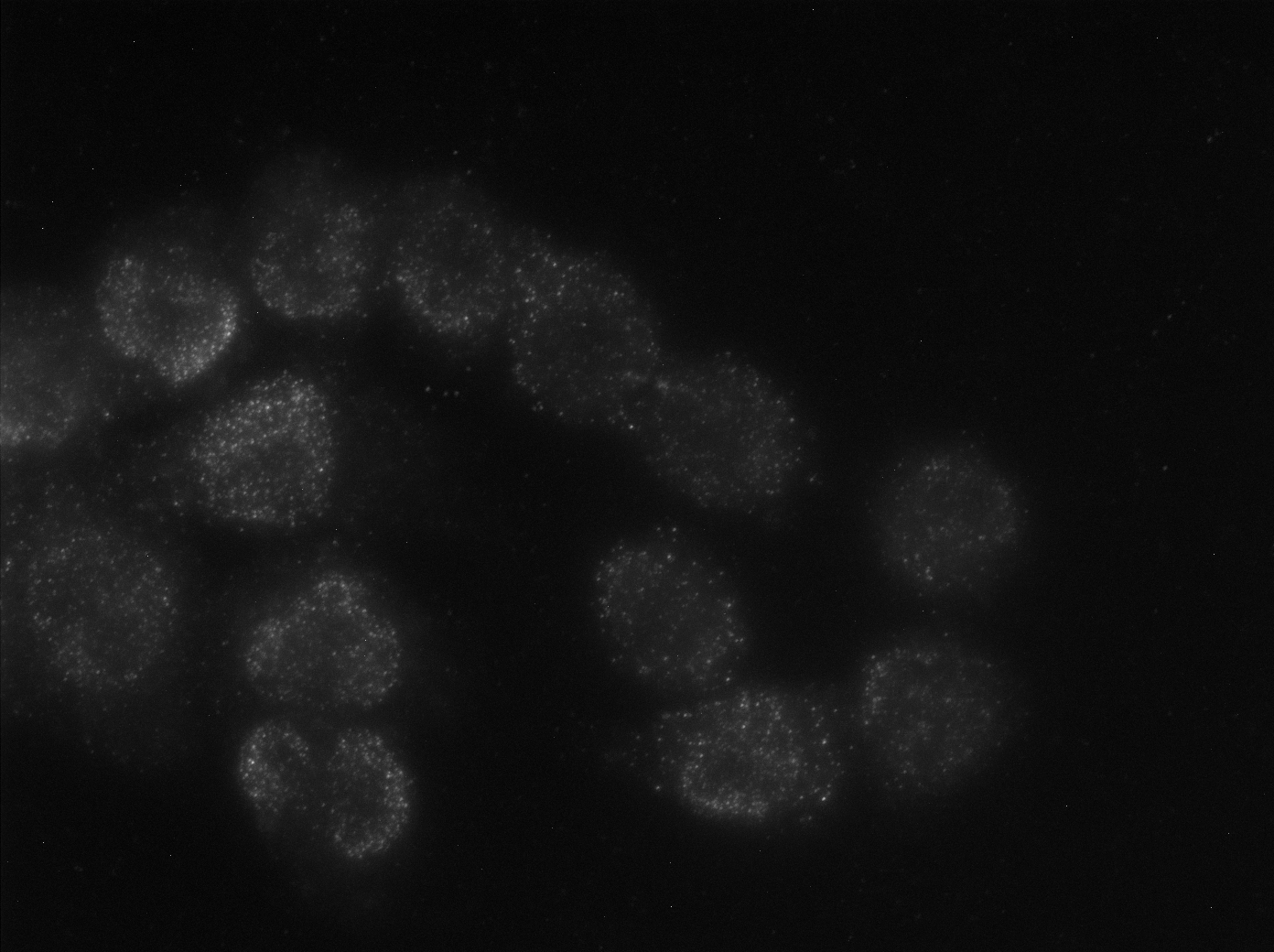

Supplement: Supplementary file 4 — Source data Fig. 2 part 1 [file 44319_2026_742_MOESM4_ESM.zip › Figure 2 Part 1/Fig 2c Cas9 Hela rGT335 atubulin/Cas9 GT335recomb atub 3-24-25 R3 LT4 PA2.Project Maximum Z_XY1743451709_Z0_T0_C1.tif]

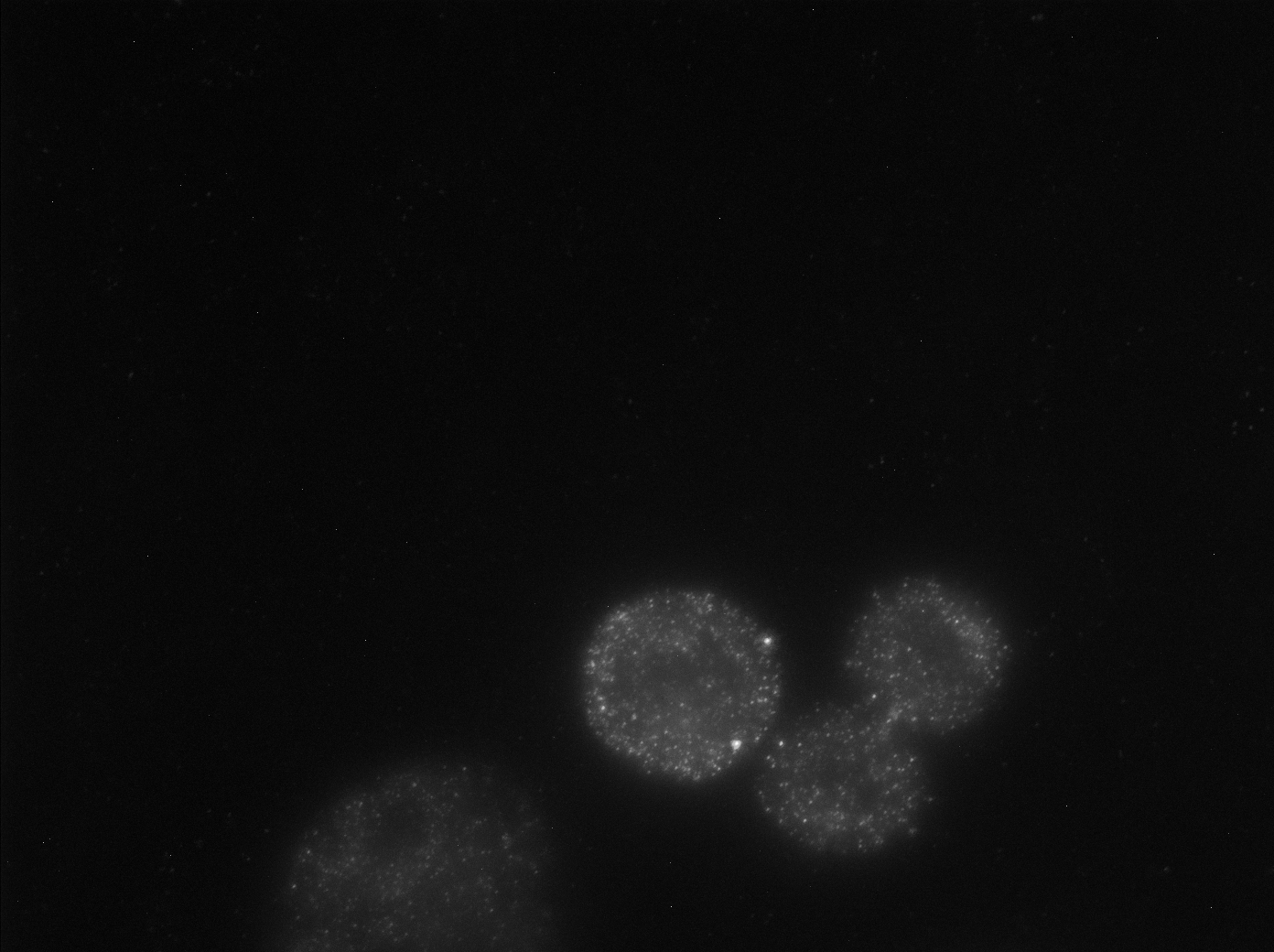

Supplement: Supplementary file 4 — Source data Fig. 2 part 1 [file 44319_2026_742_MOESM4_ESM.zip › Figure 2 Part 1/Fig 2c Cas9 Hela rGT335 atubulin/Cas9 GT335recomb atub 3-24-25 R3 ET6 M10.Project Maximum Z_XY1743454994_Z0_T0_C1.tif]

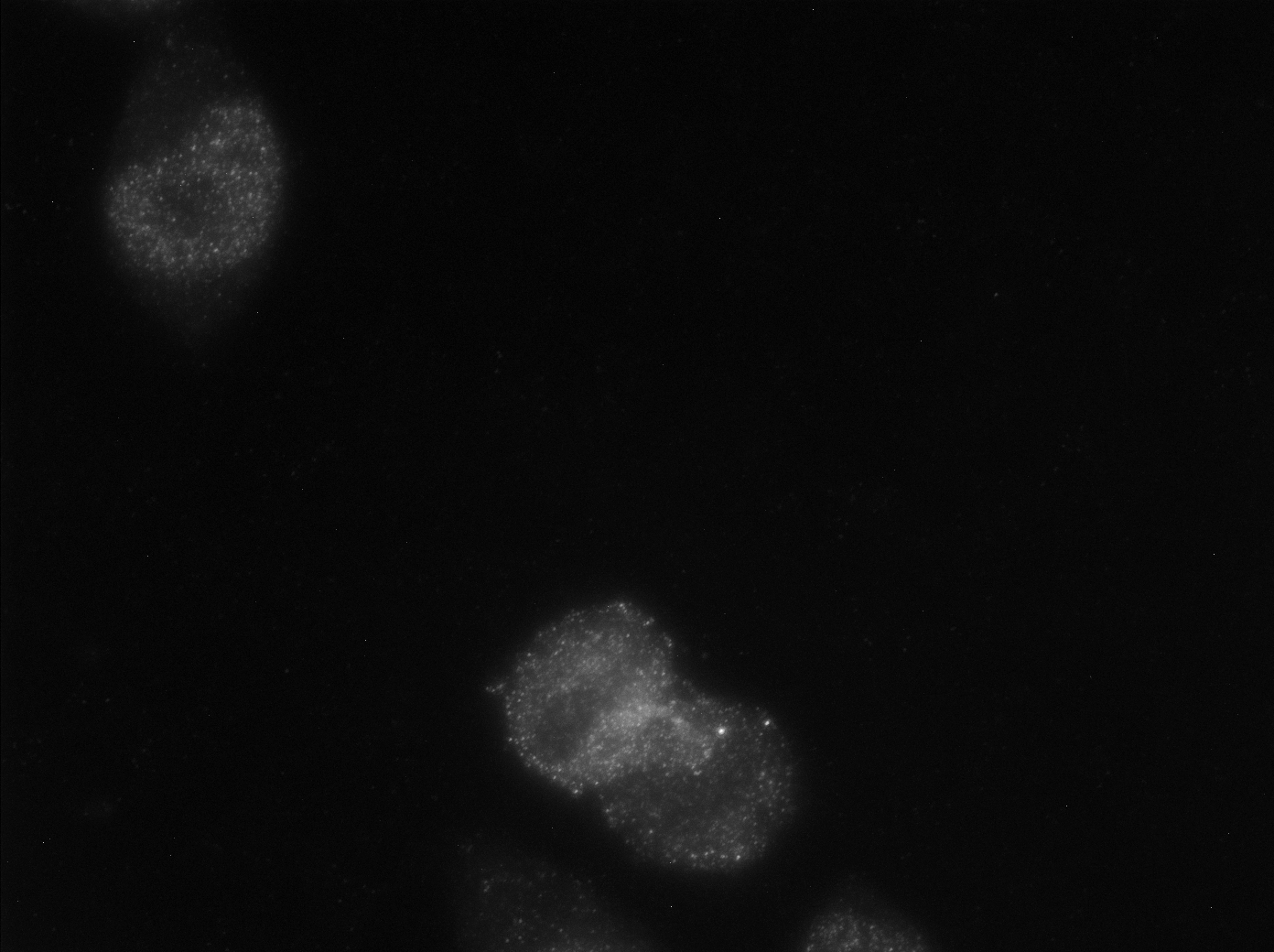

Supplement: Supplementary file 4 — Source data Fig. 2 part 1 [file 44319_2026_742_MOESM4_ESM.zip › Figure 2 Part 1/Fig 2c Cas9 Hela rGT335 atubulin/Cas9 GT335recomb atub 3-24-25 R2 ET5.Project Maximum Z_XY1743443077_Z0_T0_C1.tif]

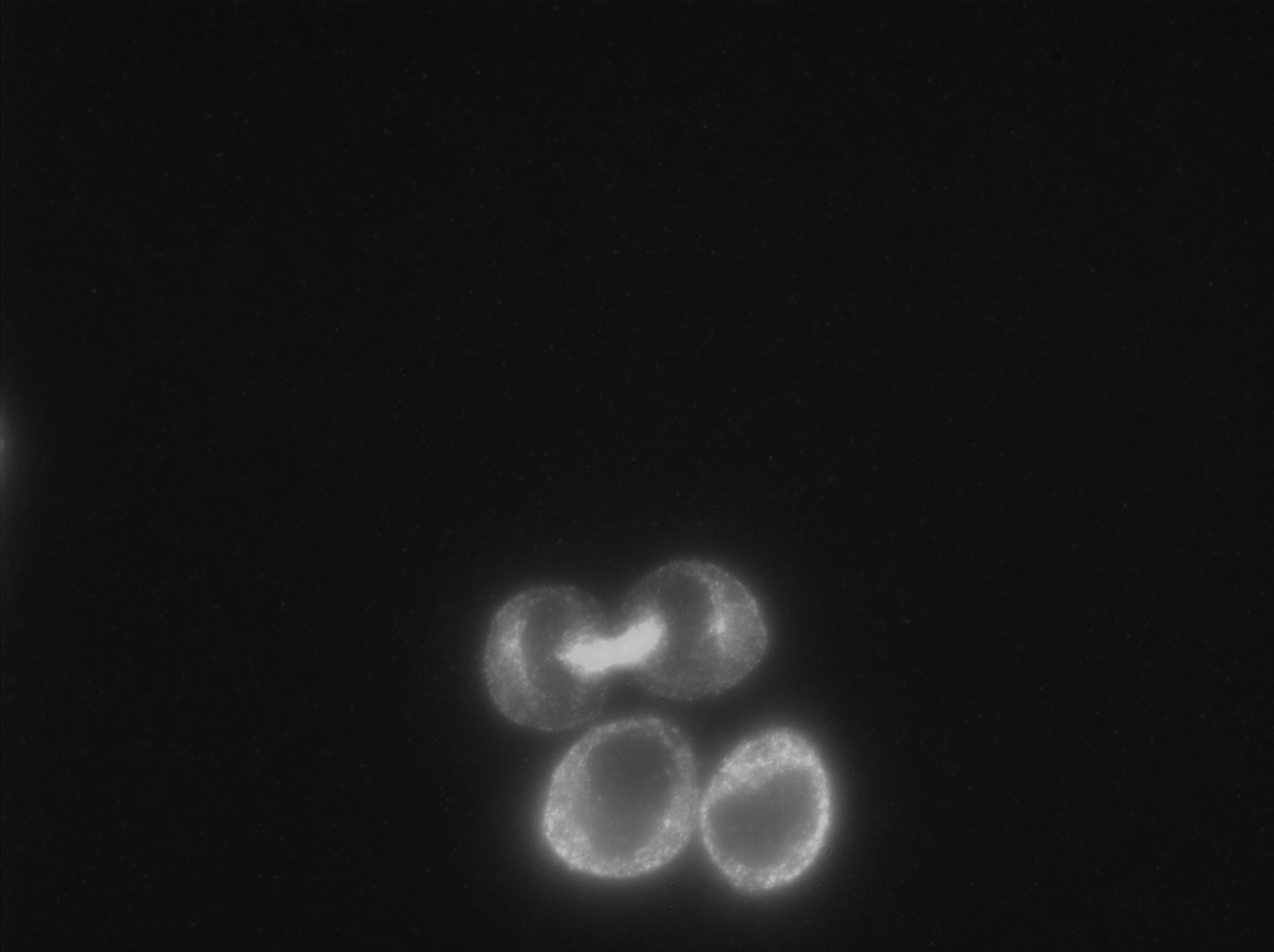

Supplement: Supplementary file 4 — Source data Fig. 2 part 1 [file 44319_2026_742_MOESM4_ESM.zip › Figure 2 Part 1/Fig 2c Cas9 Hela rGT335 atubulin/Cas9 GT335recomb atub 3-24-25 R1 ET8.Project Maximum Z_XY1743102842_Z0_T0_C2.tif]

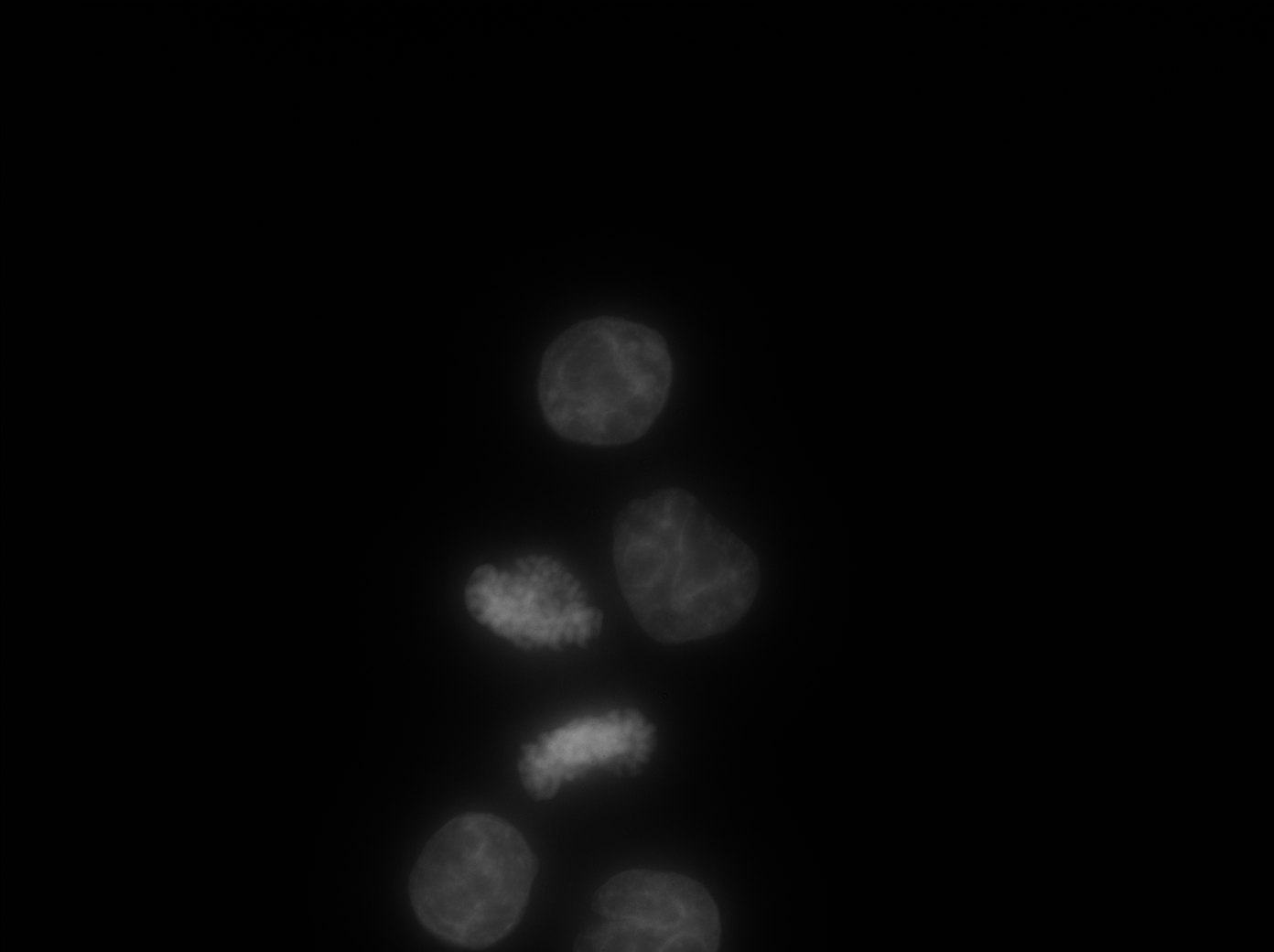

Supplement: Supplementary file 4 — Source data Fig. 2 part 1 [file 44319_2026_742_MOESM4_ESM.zip › Figure 2 Part 1/Fig 2c Cas9 Hela rGT335 atubulin/Cas9 GT335recomb atub 3-24-25 R2 ET7.Project Maximum Z_XY1743444621_Z0_T0_C0.tif]

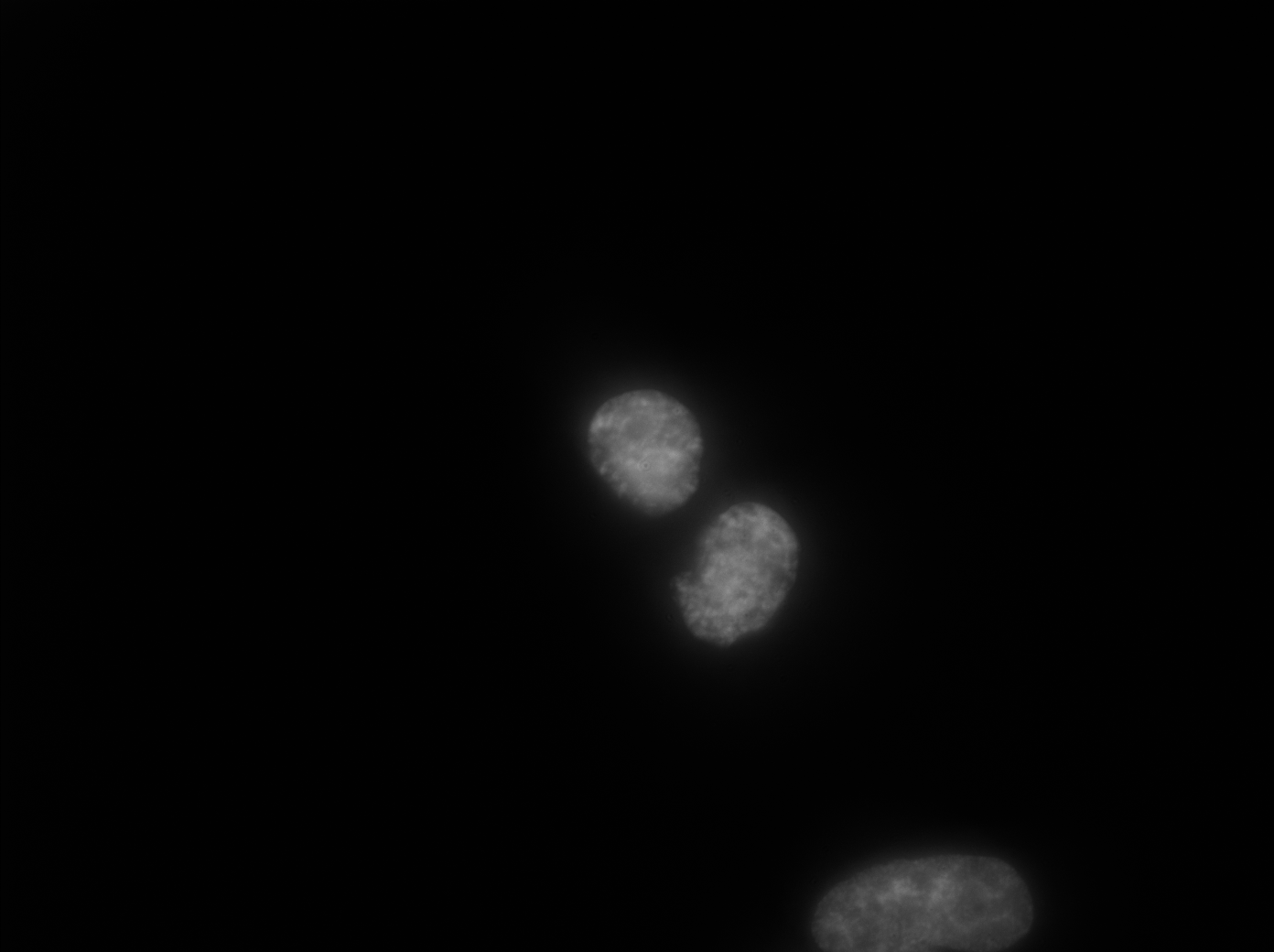

Supplement: Supplementary file 4 — Source data Fig. 2 part 1 [file 44319_2026_742_MOESM4_ESM.zip › Figure 2 Part 1/Fig 2c Cas9 Hela rGT335 atubulin/Cas9 GT335recomb atub 3-24-25 R2 ET3.Project Maximum Z_XY1743440248_Z0_T0_C0.tif]

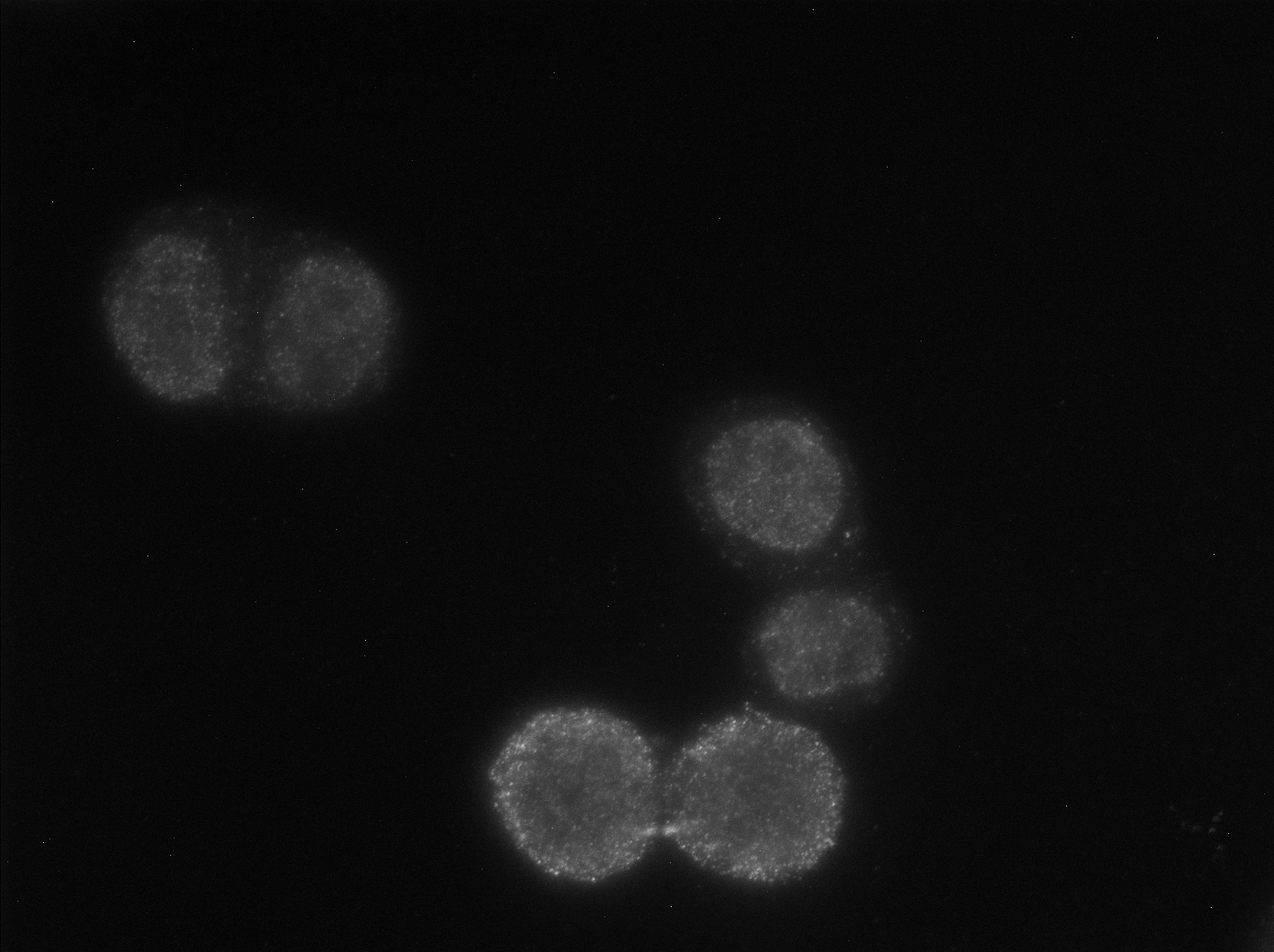

Supplement: Supplementary file 4 — Source data Fig. 2 part 1 [file 44319_2026_742_MOESM4_ESM.zip › Figure 2 Part 1/Fig 2c Cas9 Hela rGT335 atubulin/Cas9 GT335recomb atub 3-24-25 R3 ET4.Project Maximum Z_XY1743453805_Z0_T0_C1.tif]

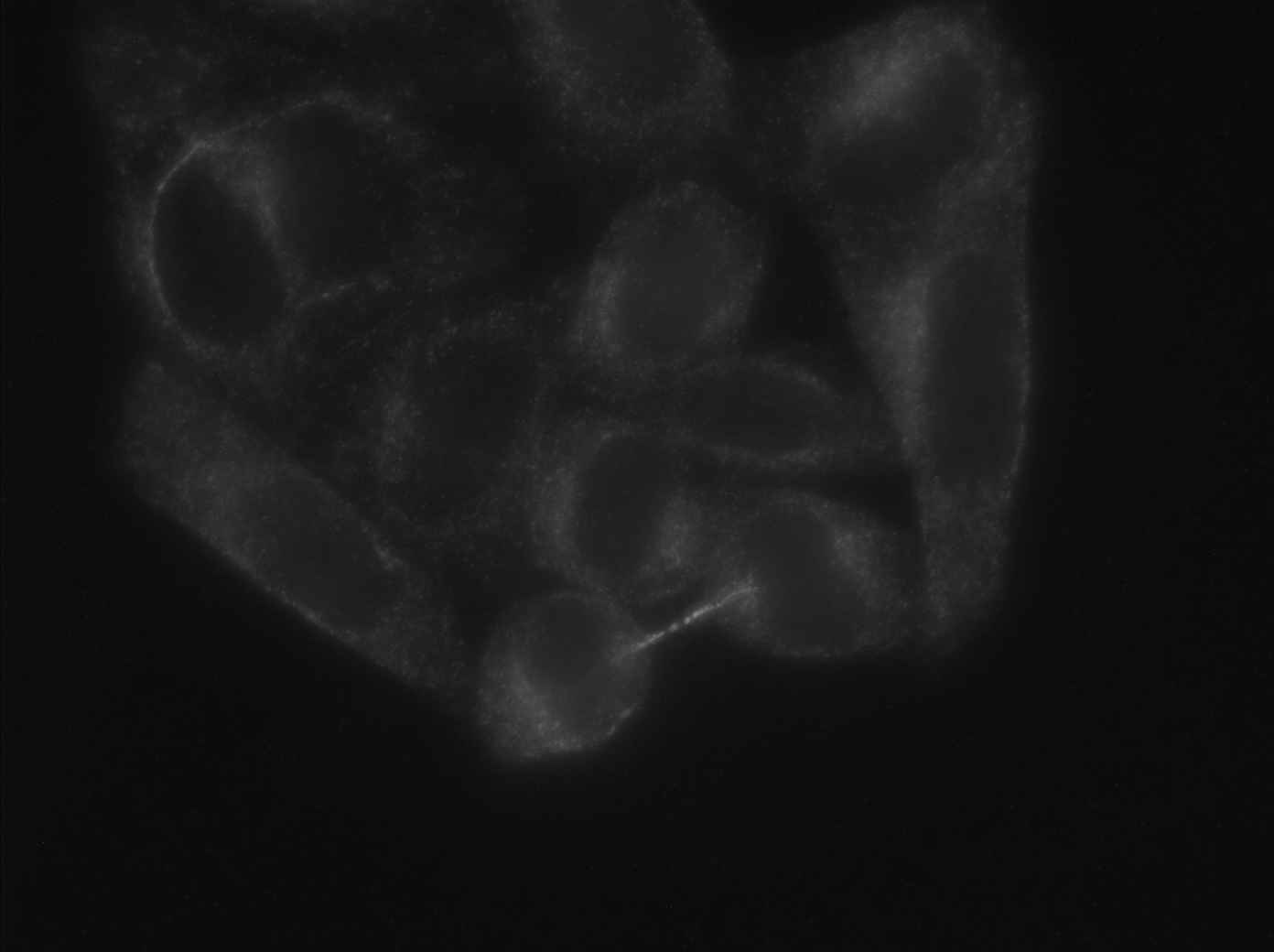

Supplement: Supplementary file 4 — Source data Fig. 2 part 1 [file 44319_2026_742_MOESM4_ESM.zip › Figure 2 Part 1/Fig 2c Cas9 Hela rGT335 atubulin/Cas9 GT335recomb atub 3-24-25 R1 LT1.Project Maximum Z_XY1743100595_Z0_T0_C2.tif]

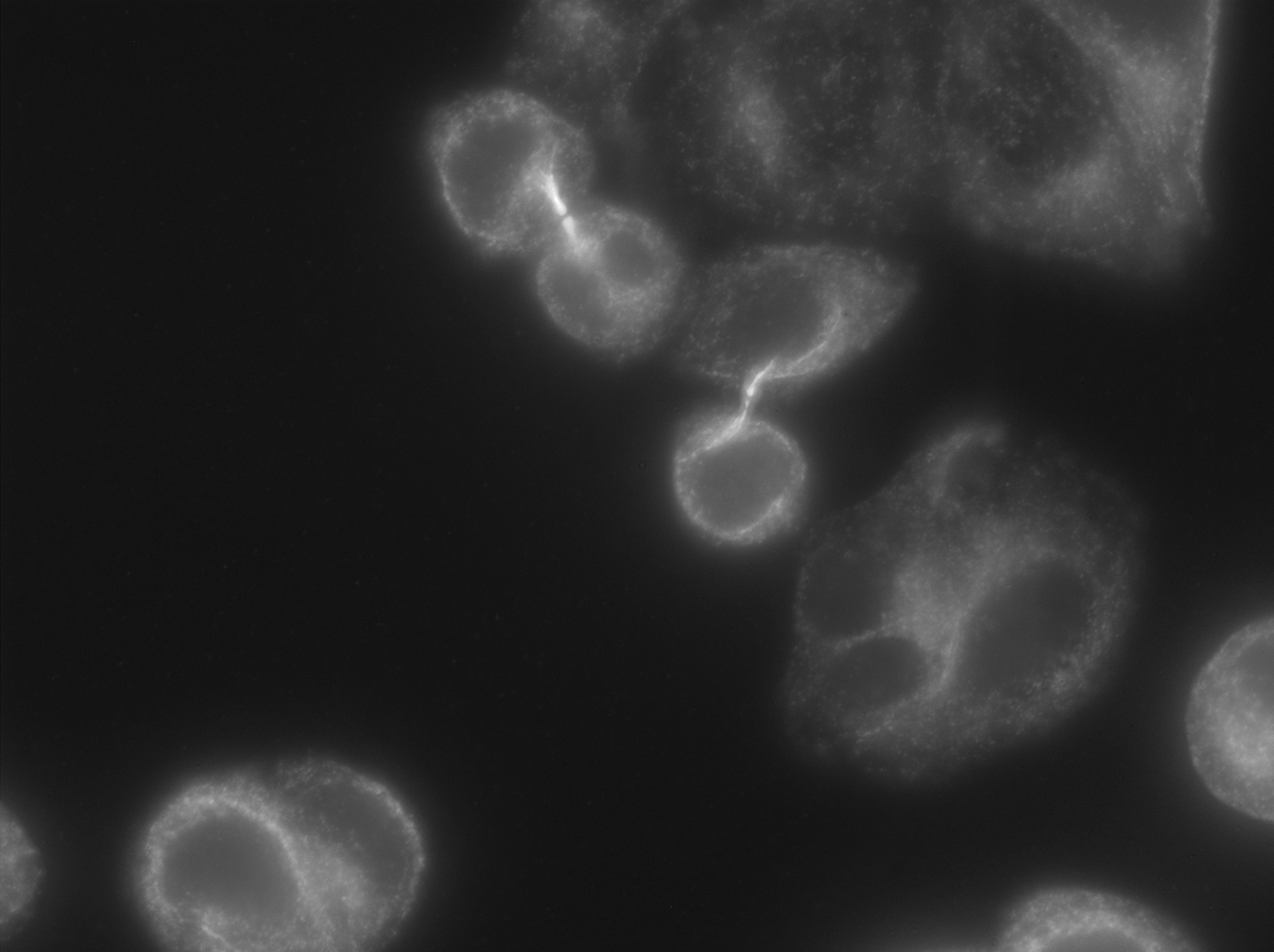

Supplement: Supplementary file 4 — Source data Fig. 2 part 1 [file 44319_2026_742_MOESM4_ESM.zip › Figure 2 Part 1/Fig 2c Cas9 Hela rGT335 atubulin/Cas9 GT335recomb atub 3-24-25 R3 LT10LT11.Project Maximum Z_XY1743455704_Z0_T0_C2.tif]

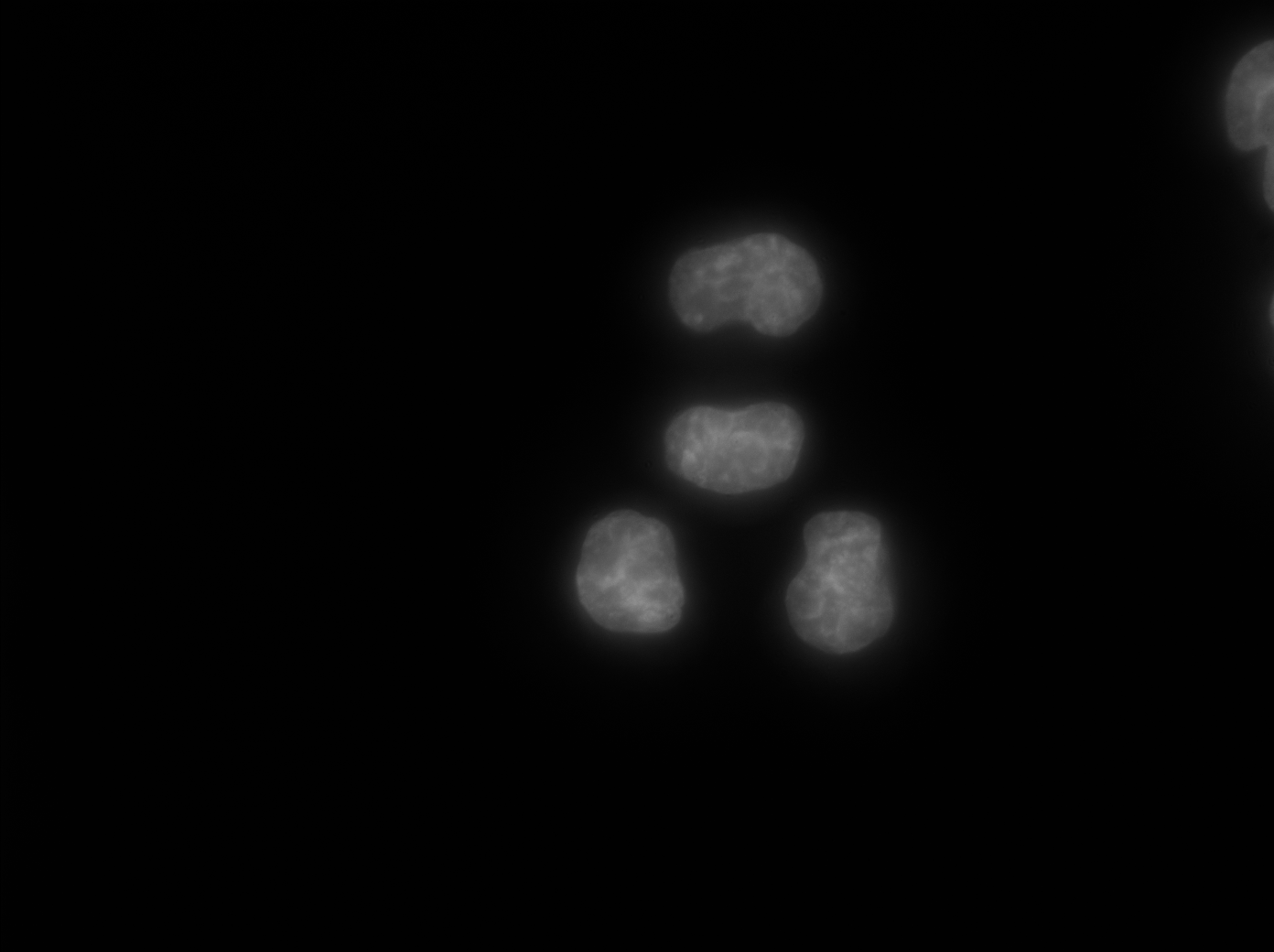

Supplement: Supplementary file 4 — Source data Fig. 2 part 1 [file 44319_2026_742_MOESM4_ESM.zip › Figure 2 Part 1/Fig 2c Cas9 Hela rGT335 atubulin/Cas9 GT335recomb atub 3-24-25 R1 LT10.Project Maximum Z_XY1743102059_Z0_T0_C0.tif]

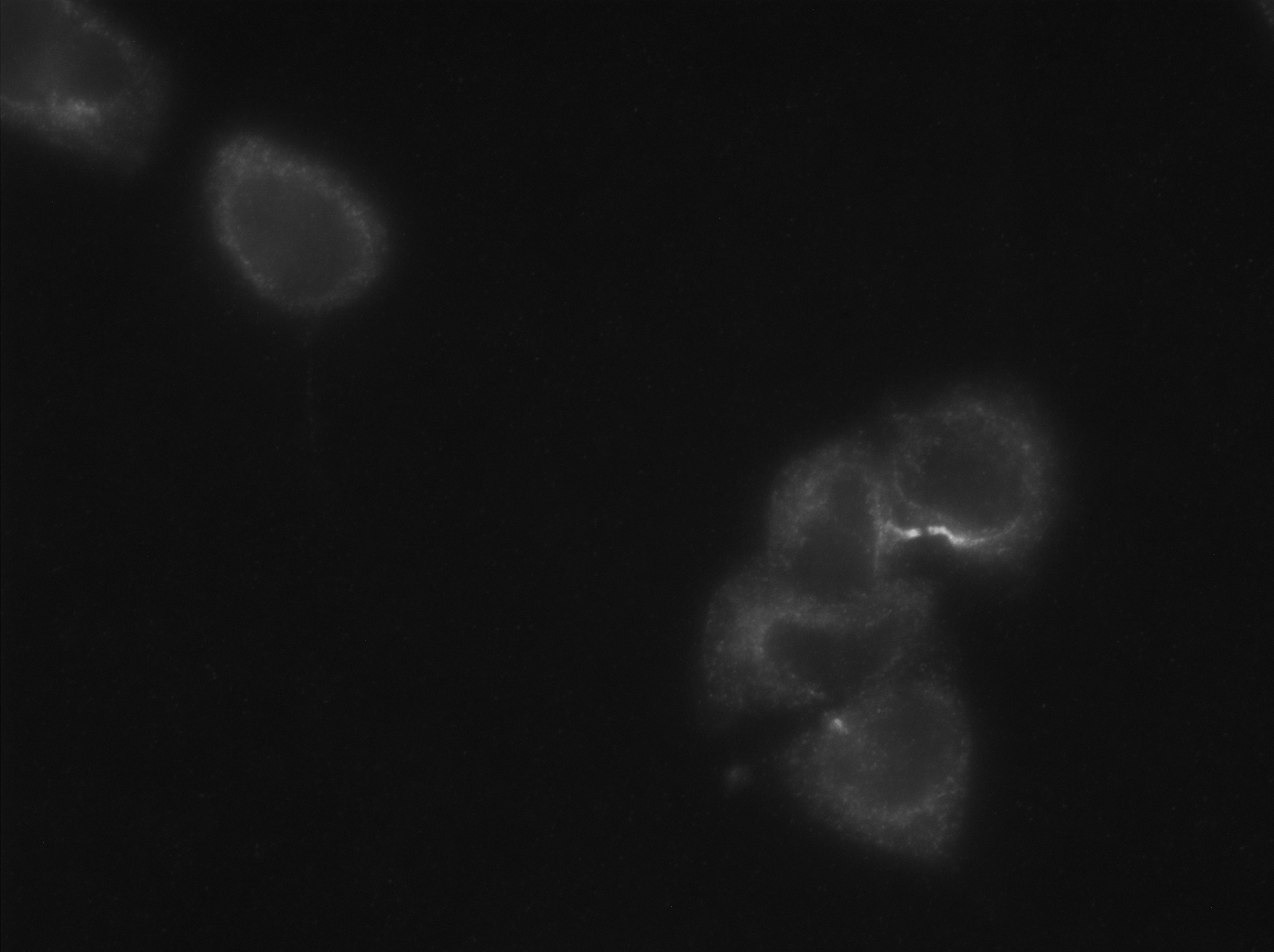

Supplement: Supplementary file 4 — Source data Fig. 2 part 1 [file 44319_2026_742_MOESM4_ESM.zip › Figure 2 Part 1/Fig 2c Cas9 Hela rGT335 atubulin/Cas9 GT335recomb atub 3-24-25 R1 LT4 PA5.Project Maximum Z_XY1743101109_Z0_T0_C2.tif]

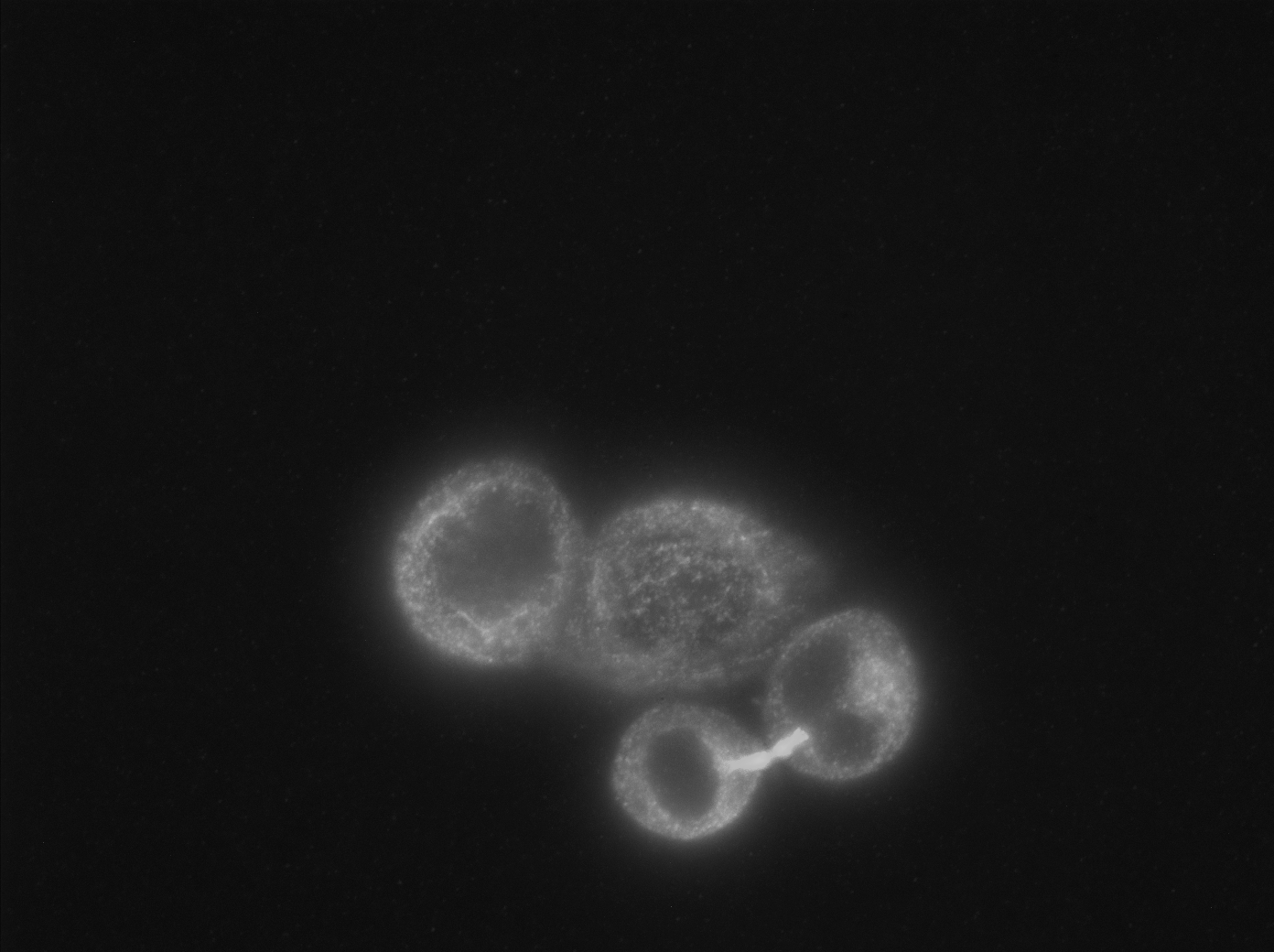

Supplement: Supplementary file 4 — Source data Fig. 2 part 1 [file 44319_2026_742_MOESM4_ESM.zip › Figure 2 Part 1/Fig 2c Cas9 Hela rGT335 atubulin/Cas9 GT335recomb atub 3-24-25 R1 ET7.Project Maximum Z_XY1743102735_Z0_T0_C2.tif]

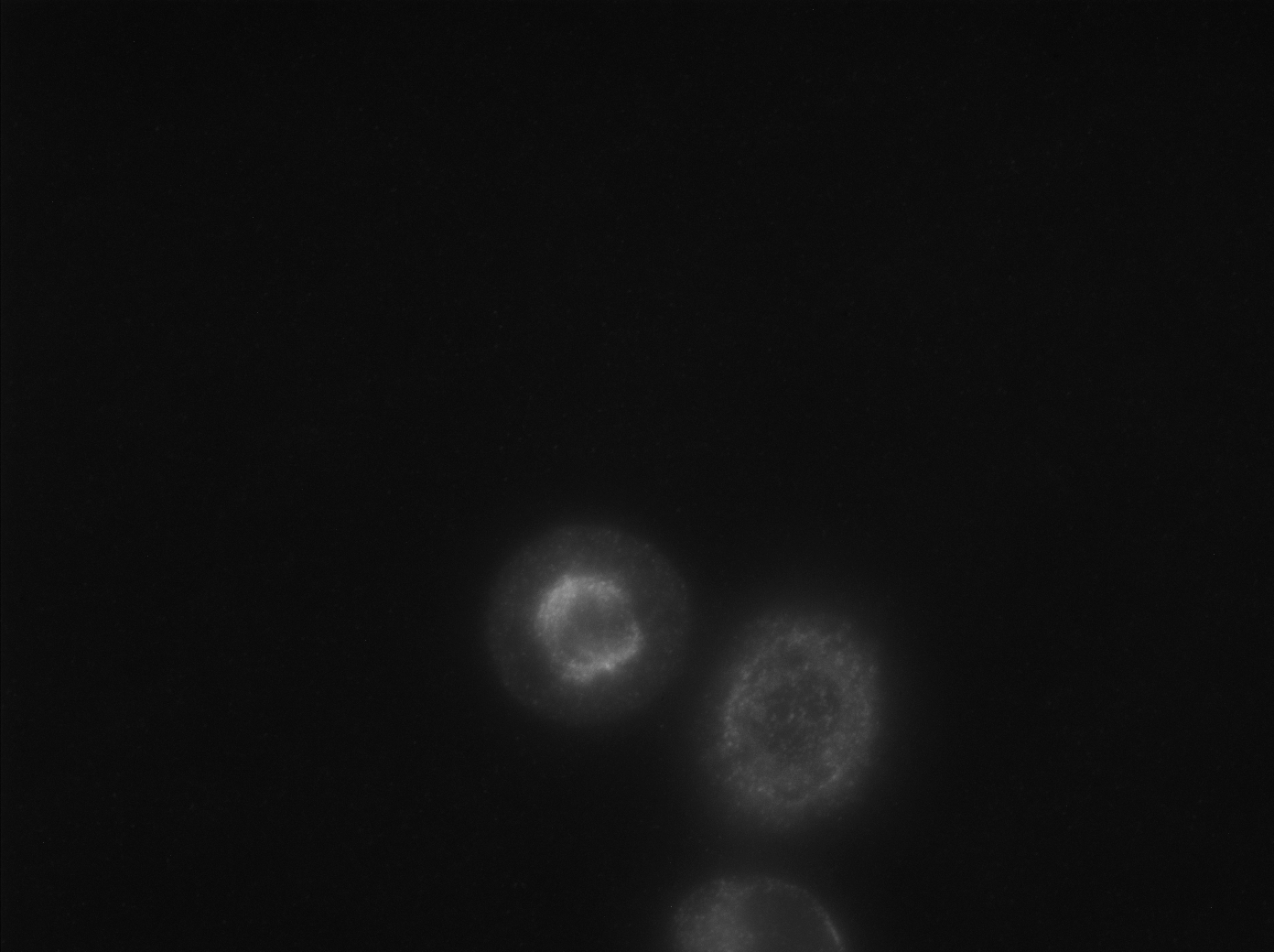

Supplement: Supplementary file 4 — Source data Fig. 2 part 1 [file 44319_2026_742_MOESM4_ESM.zip › Figure 2 Part 1/Fig 2c Cas9 Hela rGT335 atubulin/Cas9 GT335recomb atub 3-24-25 R1 M10.Project Maximum Z_XY1743105878_Z0_T0_C2.tif]

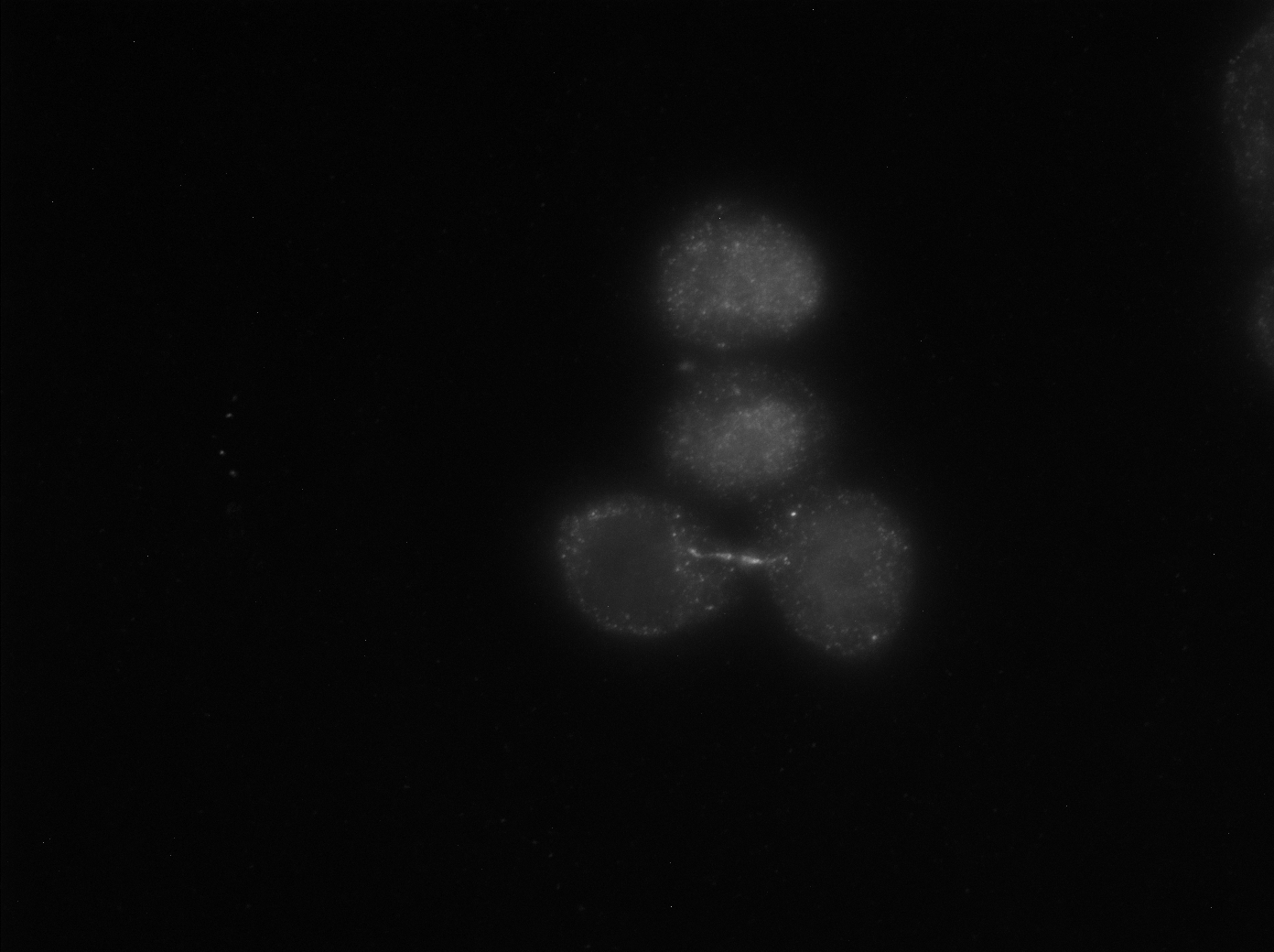

Supplement: Supplementary file 4 — Source data Fig. 2 part 1 [file 44319_2026_742_MOESM4_ESM.zip › Figure 2 Part 1/Fig 2c Cas9 Hela rGT335 atubulin/Cas9 GT335recomb atub 3-24-25 R1 LT10.Project Maximum Z_XY1743102059_Z0_T0_C1.tif]

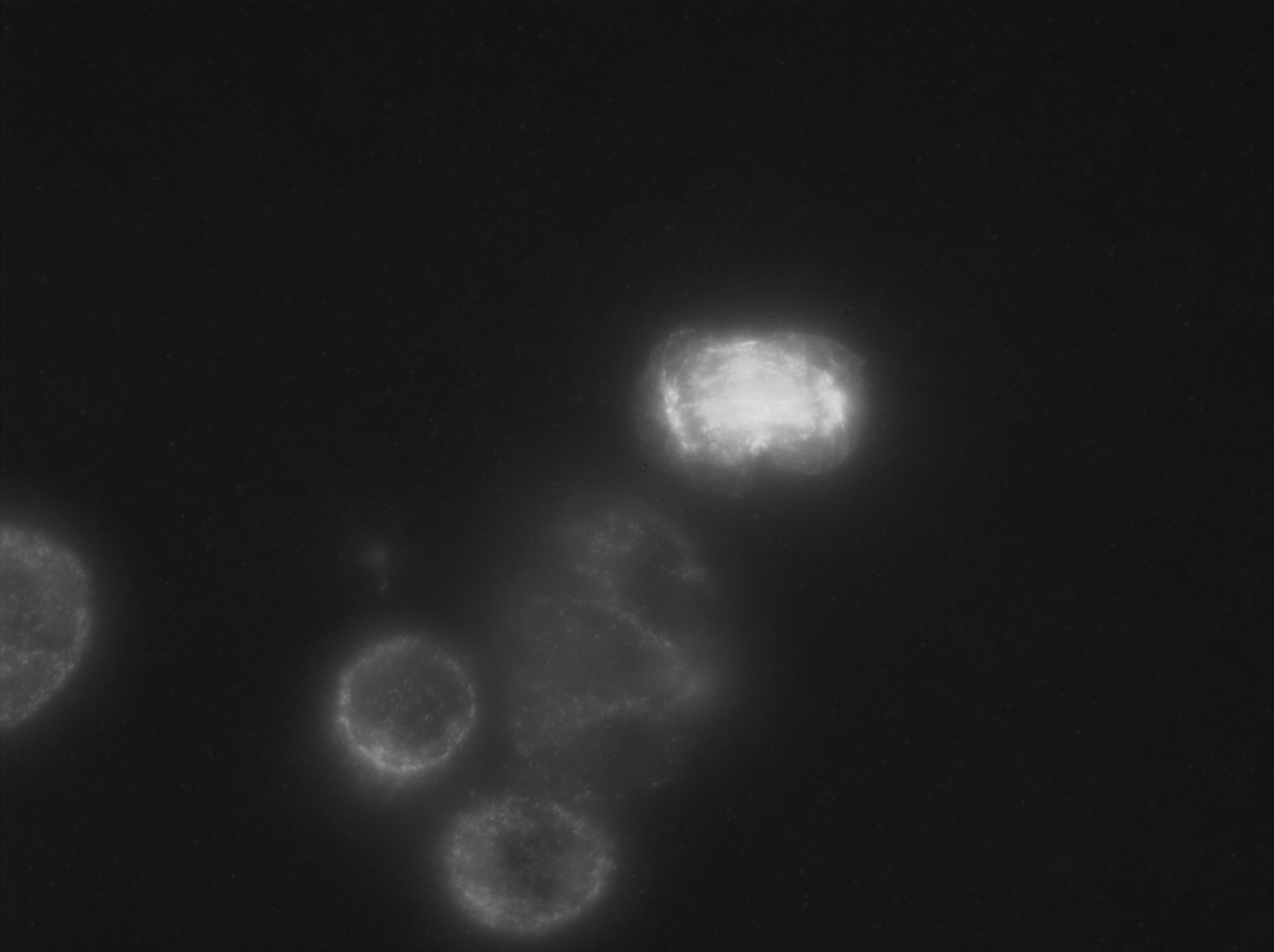

Supplement: Supplementary file 4 — Source data Fig. 2 part 1 [file 44319_2026_742_MOESM4_ESM.zip › Figure 2 Part 1/Fig 2c Cas9 Hela rGT335 atubulin/Cas9 GT335recomb atub 3-24-25 R3 preET3.Project Maximum Z_XY1743453338_Z0_T0_C2.tif]

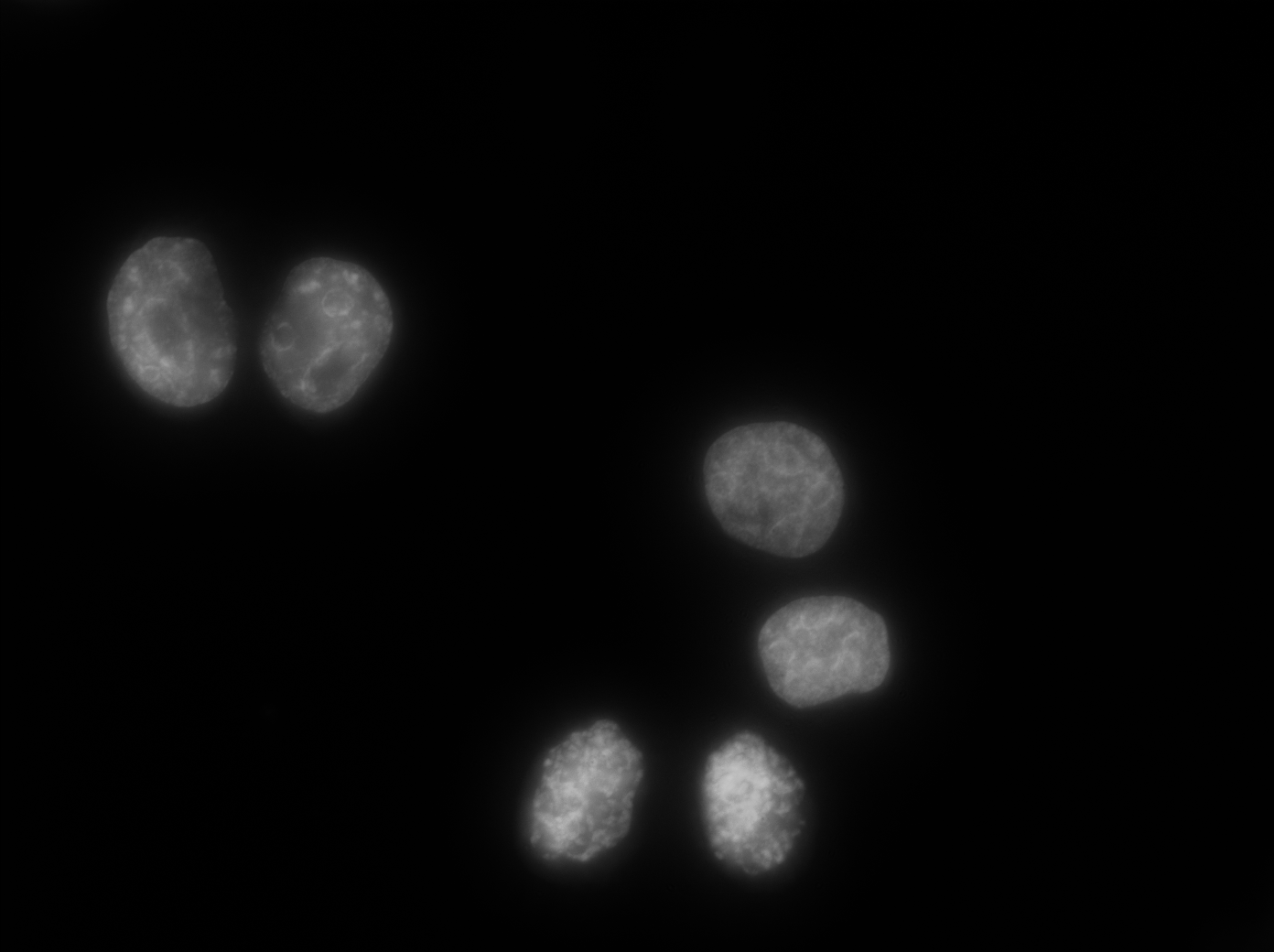

Supplement: Supplementary file 4 — Source data Fig. 2 part 1 [file 44319_2026_742_MOESM4_ESM.zip › Figure 2 Part 1/Fig 2c Cas9 Hela rGT335 atubulin/Cas9 GT335recomb atub 3-24-25 R3 ET4.Project Maximum Z_XY1743453805_Z0_T0_C0.tif]

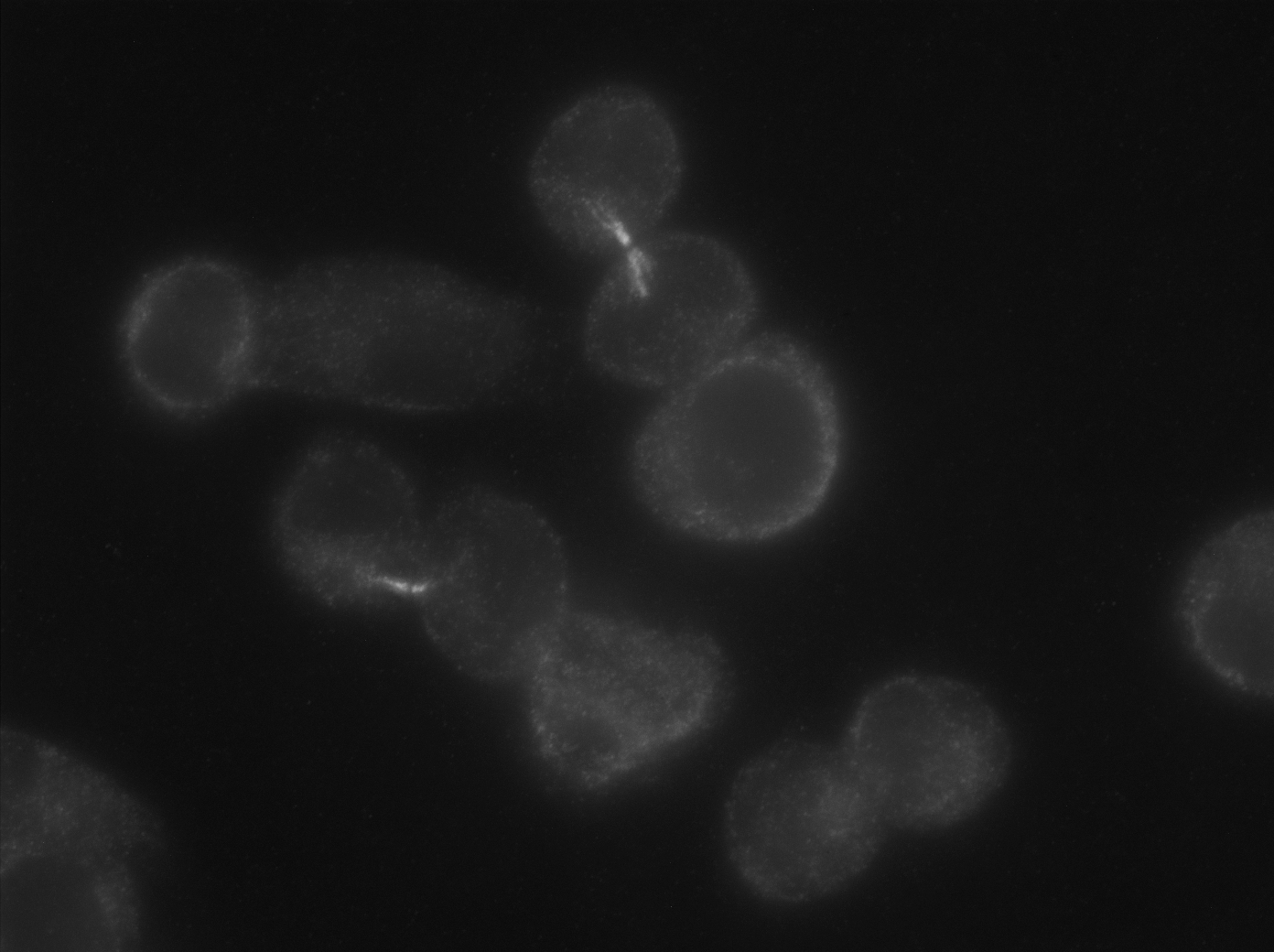

Supplement: Supplementary file 4 — Source data Fig. 2 part 1 [file 44319_2026_742_MOESM4_ESM.zip › Figure 2 Part 1/Fig 2c Cas9 Hela rGT335 atubulin/Cas9 GT335recomb atub 3-24-25 R1 ET1ET2.Project Maximum Z_XY1743101008_Z0_T0_C2.tif]

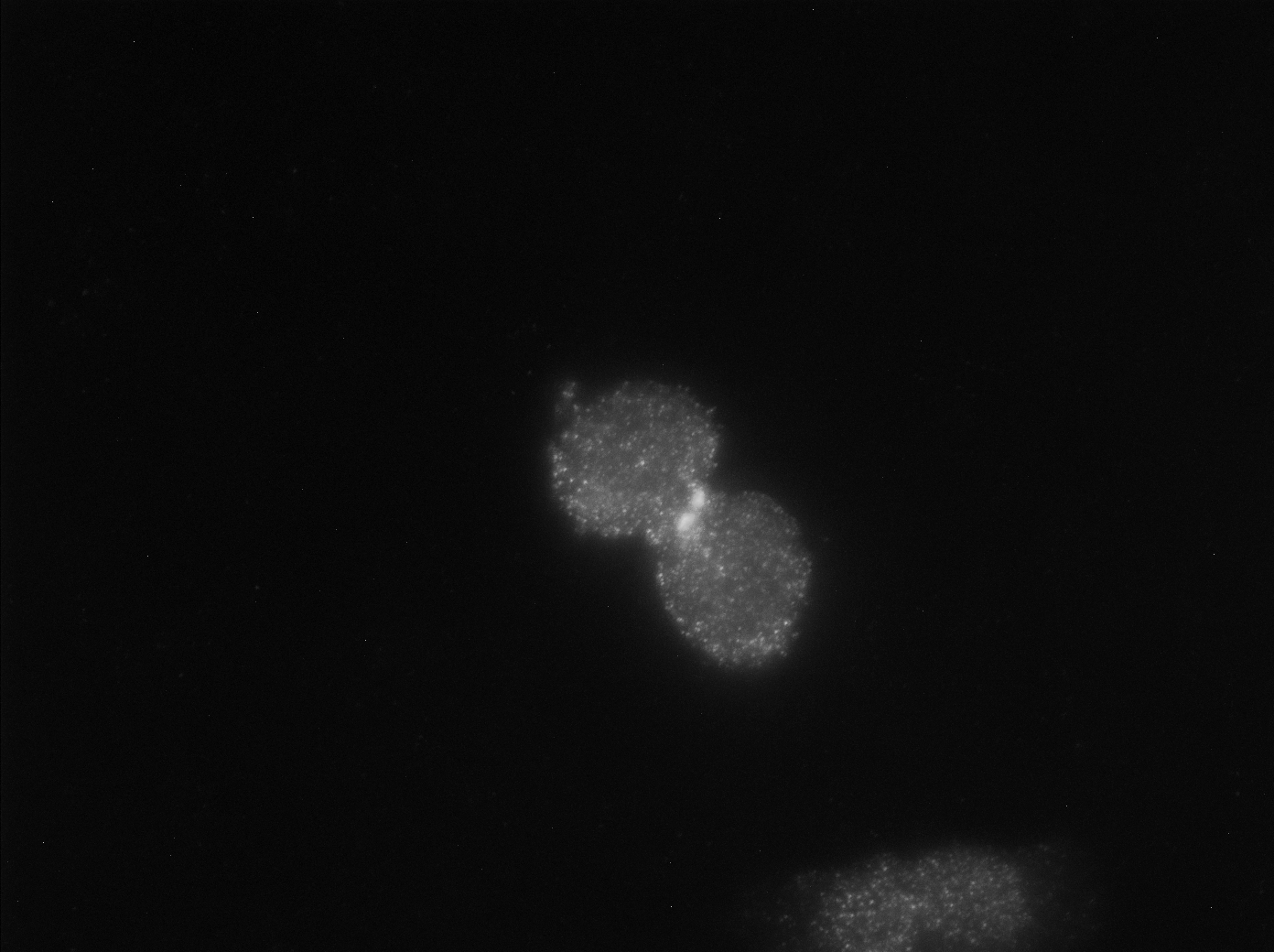

Supplement: Supplementary file 4 — Source data Fig. 2 part 1 [file 44319_2026_742_MOESM4_ESM.zip › Figure 2 Part 1/Fig 2c Cas9 Hela rGT335 atubulin/Cas9 GT335recomb atub 3-24-25 R2 ET3.Project Maximum Z_XY1743440248_Z0_T0_C1.tif]

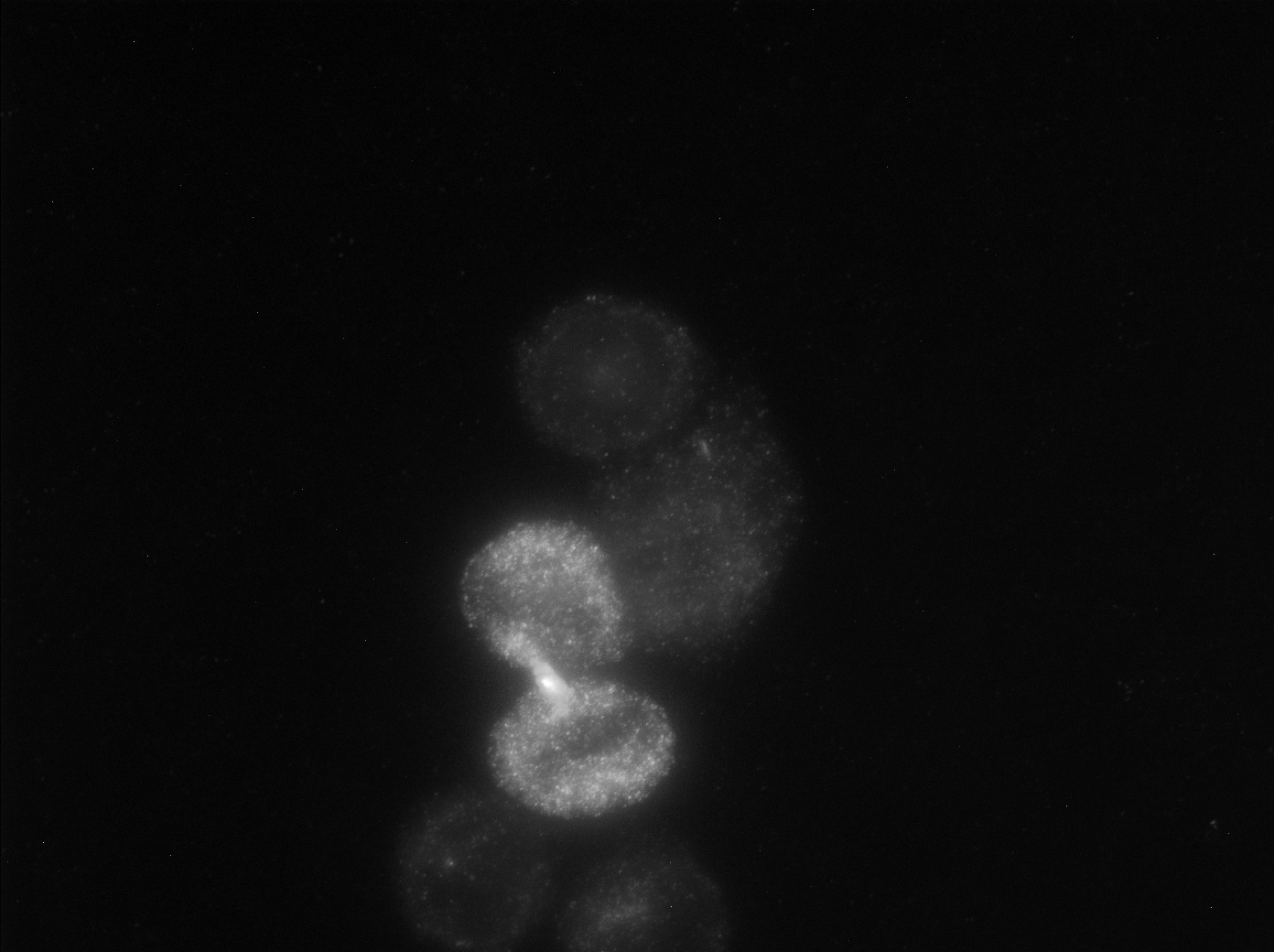

Supplement: Supplementary file 4 — Source data Fig. 2 part 1 [file 44319_2026_742_MOESM4_ESM.zip › Figure 2 Part 1/Fig 2c Cas9 Hela rGT335 atubulin/Cas9 GT335recomb atub 3-24-25 R2 ET7.Project Maximum Z_XY1743444621_Z0_T0_C1.tif]

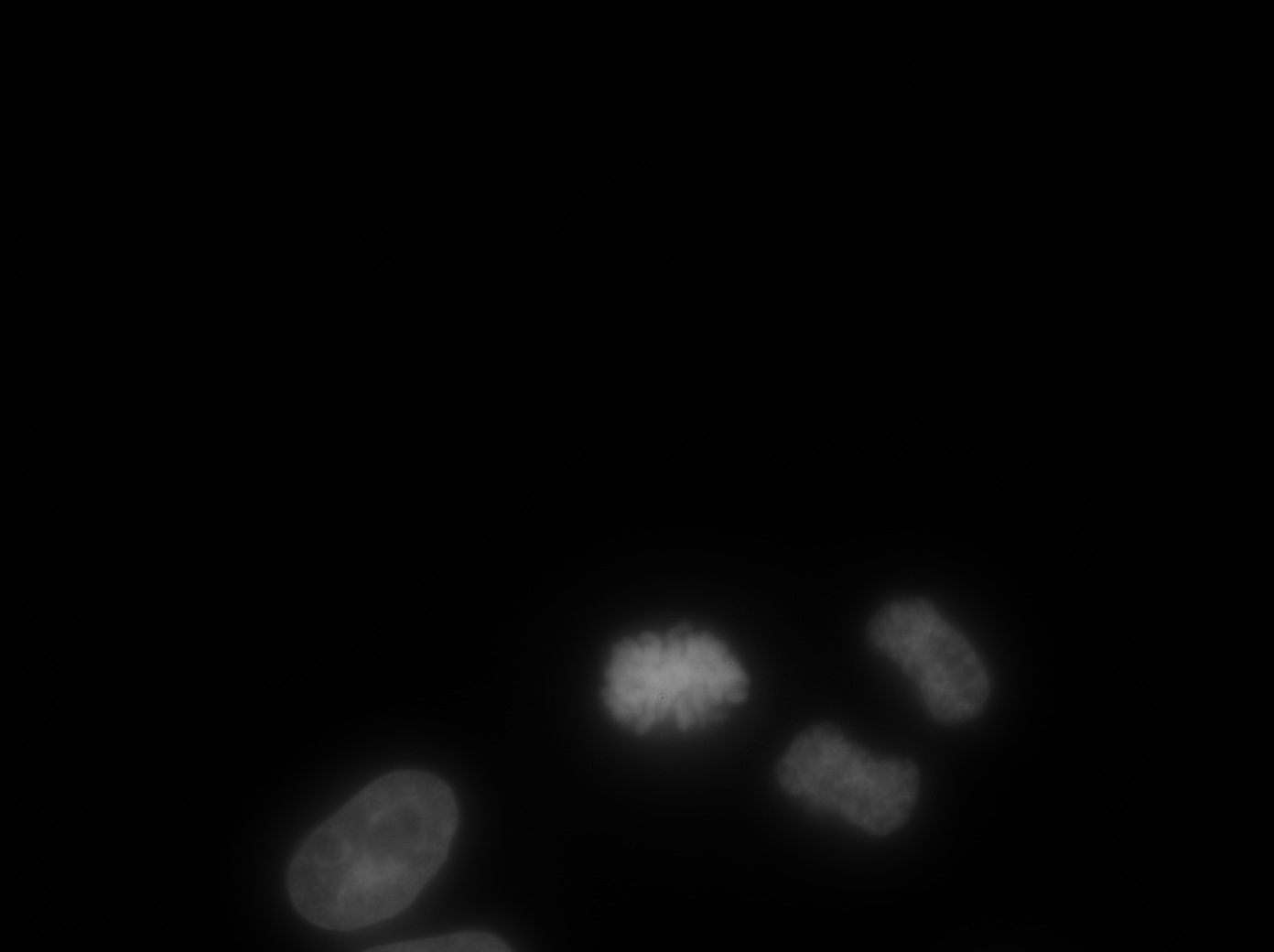

Supplement: Supplementary file 4 — Source data Fig. 2 part 1 [file 44319_2026_742_MOESM4_ESM.zip › Figure 2 Part 1/Fig 2c Cas9 Hela rGT335 atubulin/Cas9 GT335recomb atub 3-24-25 R3 ET6 M10.Project Maximum Z_XY1743454994_Z0_T0_C0.tif]

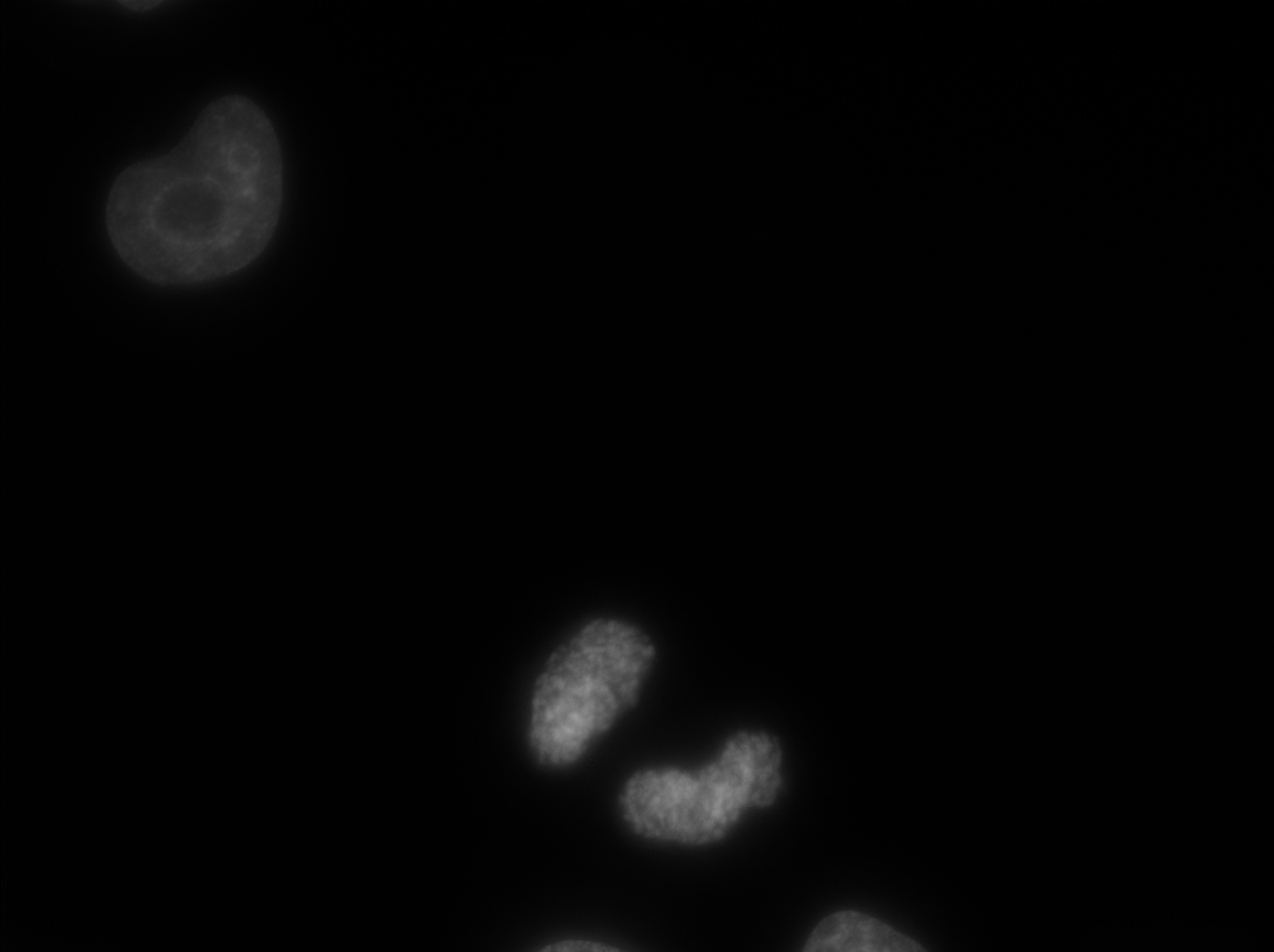

Supplement: Supplementary file 4 — Source data Fig. 2 part 1 [file 44319_2026_742_MOESM4_ESM.zip › Figure 2 Part 1/Fig 2c Cas9 Hela rGT335 atubulin/Cas9 GT335recomb atub 3-24-25 R2 ET5.Project Maximum Z_XY1743443077_Z0_T0_C0.tif]

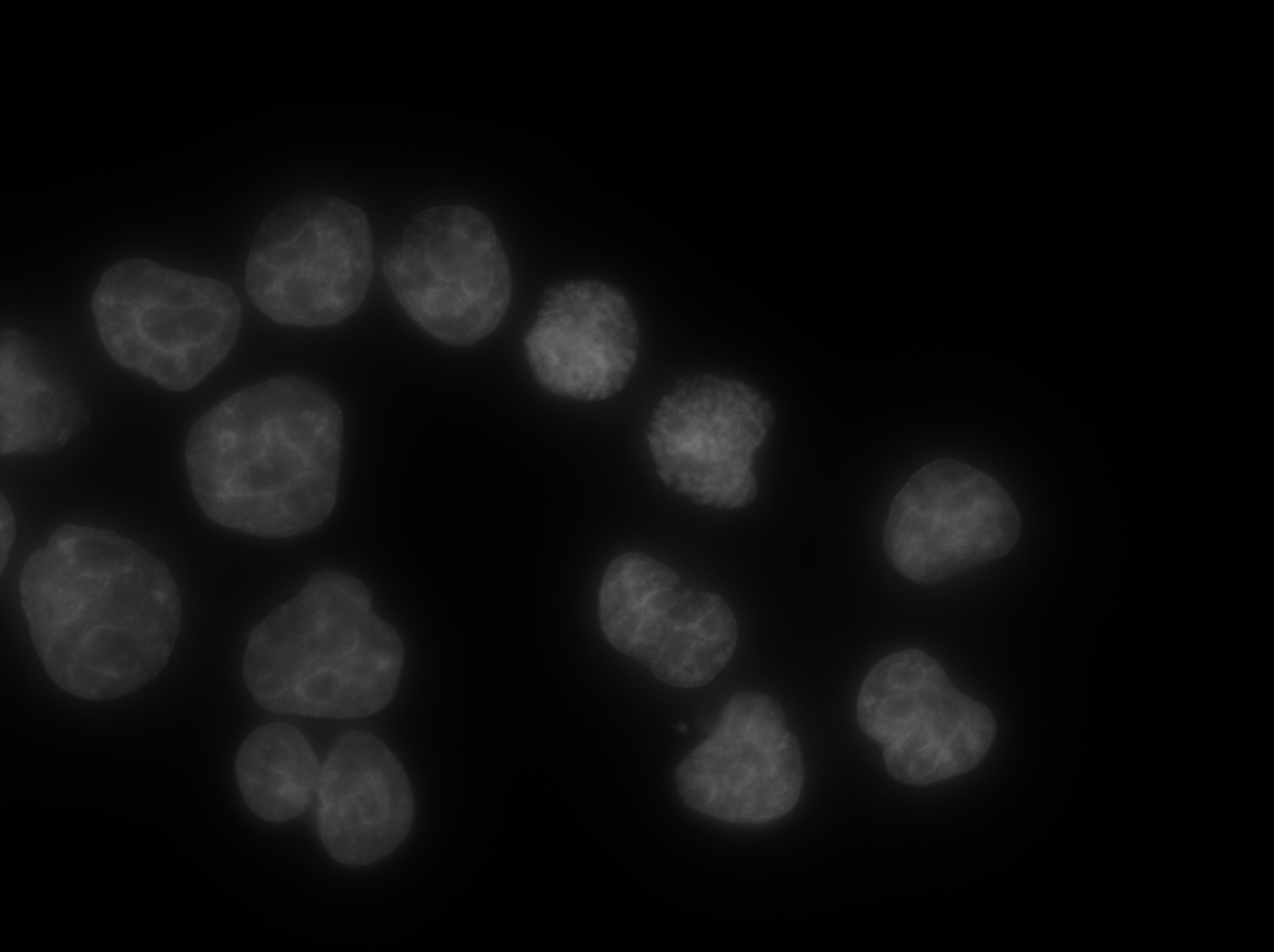

Supplement: Supplementary file 4 — Source data Fig. 2 part 1 [file 44319_2026_742_MOESM4_ESM.zip › Figure 2 Part 1/Fig 2c Cas9 Hela rGT335 atubulin/Cas9 GT335recomb atub 3-24-25 R3 LT4 PA2.Project Maximum Z_XY1743451709_Z0_T0_C0.tif]

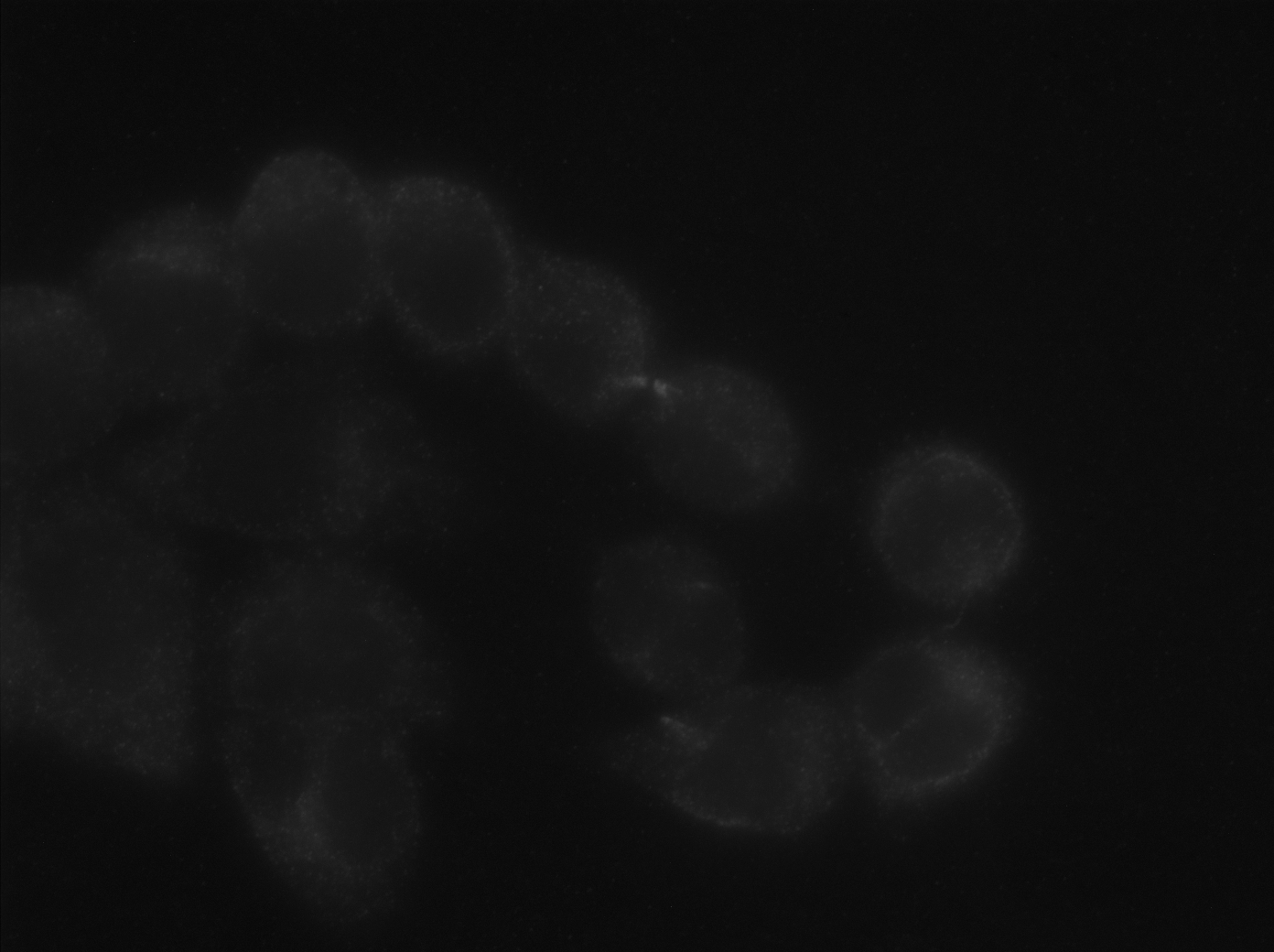

Supplement: Supplementary file 4 — Source data Fig. 2 part 1 [file 44319_2026_742_MOESM4_ESM.zip › Figure 2 Part 1/Fig 2c Cas9 Hela rGT335 atubulin/Cas9 GT335recomb atub 3-24-25 R3 LT4 PA2.Project Maximum Z_XY1743451709_Z0_T0_C2.tif]

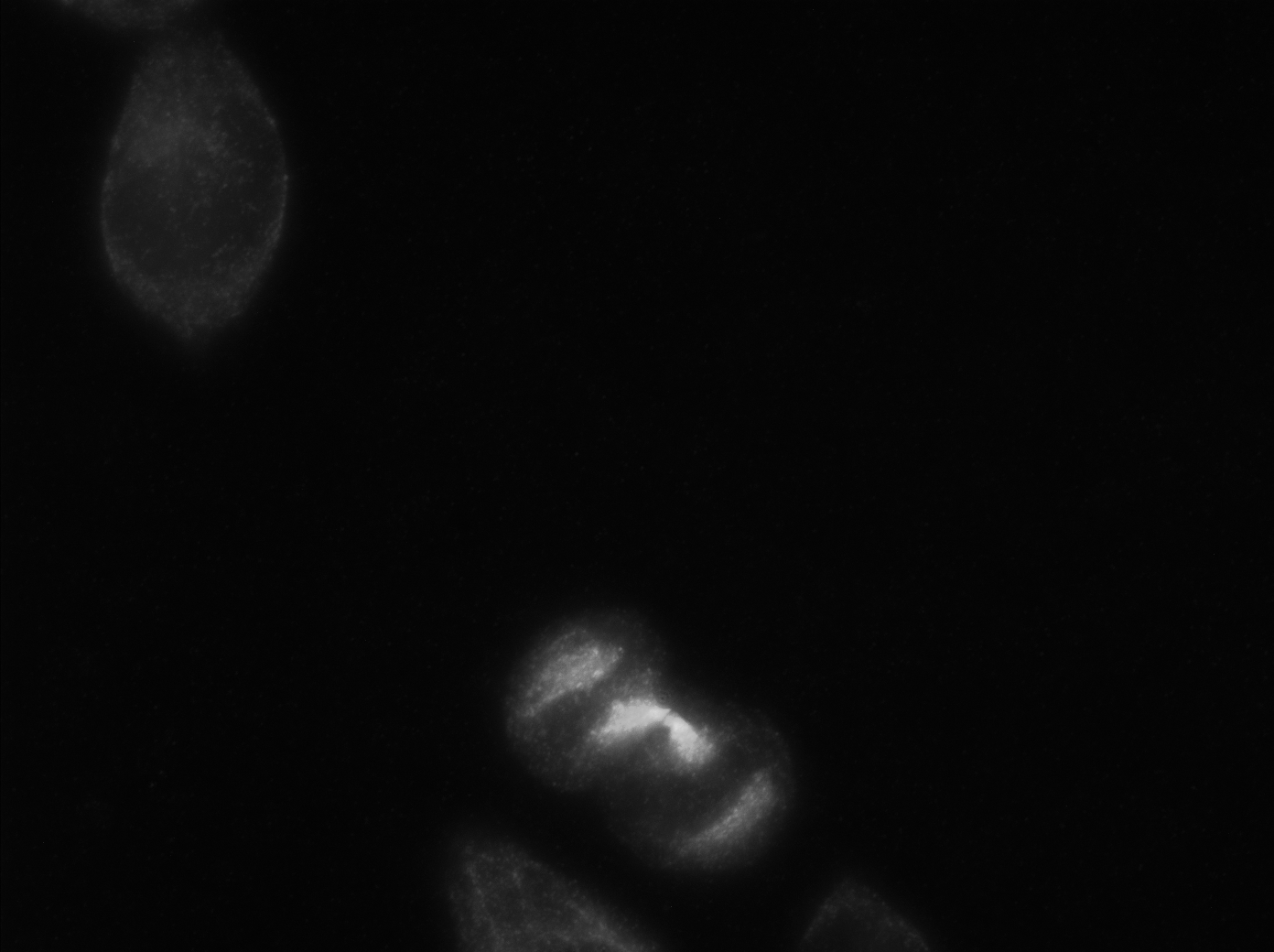

Supplement: Supplementary file 4 — Source data Fig. 2 part 1 [file 44319_2026_742_MOESM4_ESM.zip › Figure 2 Part 1/Fig 2c Cas9 Hela rGT335 atubulin/Cas9 GT335recomb atub 3-24-25 R2 ET5.Project Maximum Z_XY1743443077_Z0_T0_C2.tif]

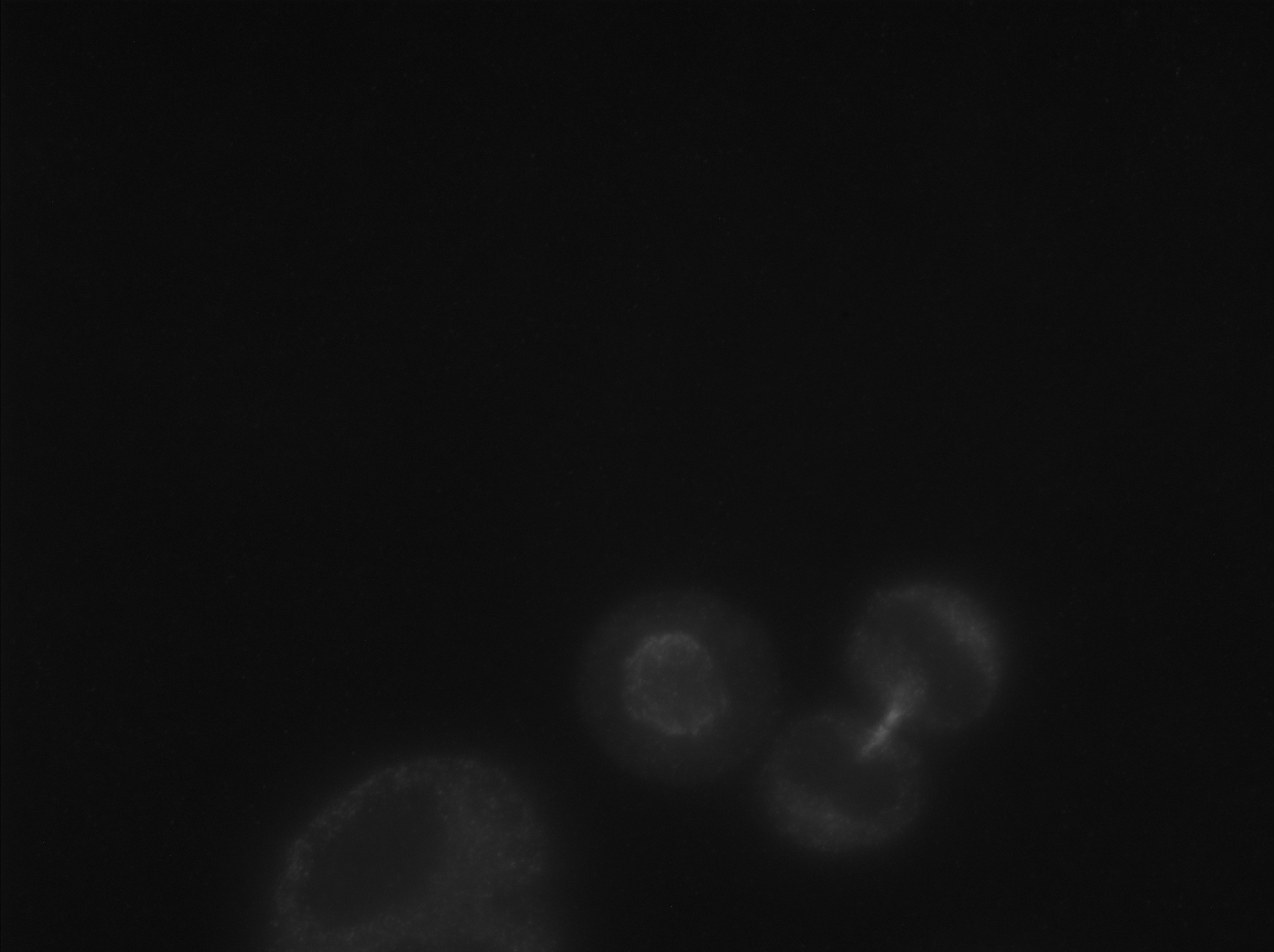

Supplement: Supplementary file 4 — Source data Fig. 2 part 1 [file 44319_2026_742_MOESM4_ESM.zip › Figure 2 Part 1/Fig 2c Cas9 Hela rGT335 atubulin/Cas9 GT335recomb atub 3-24-25 R3 ET6 M10.Project Maximum Z_XY1743454994_Z0_T0_C2.tif]

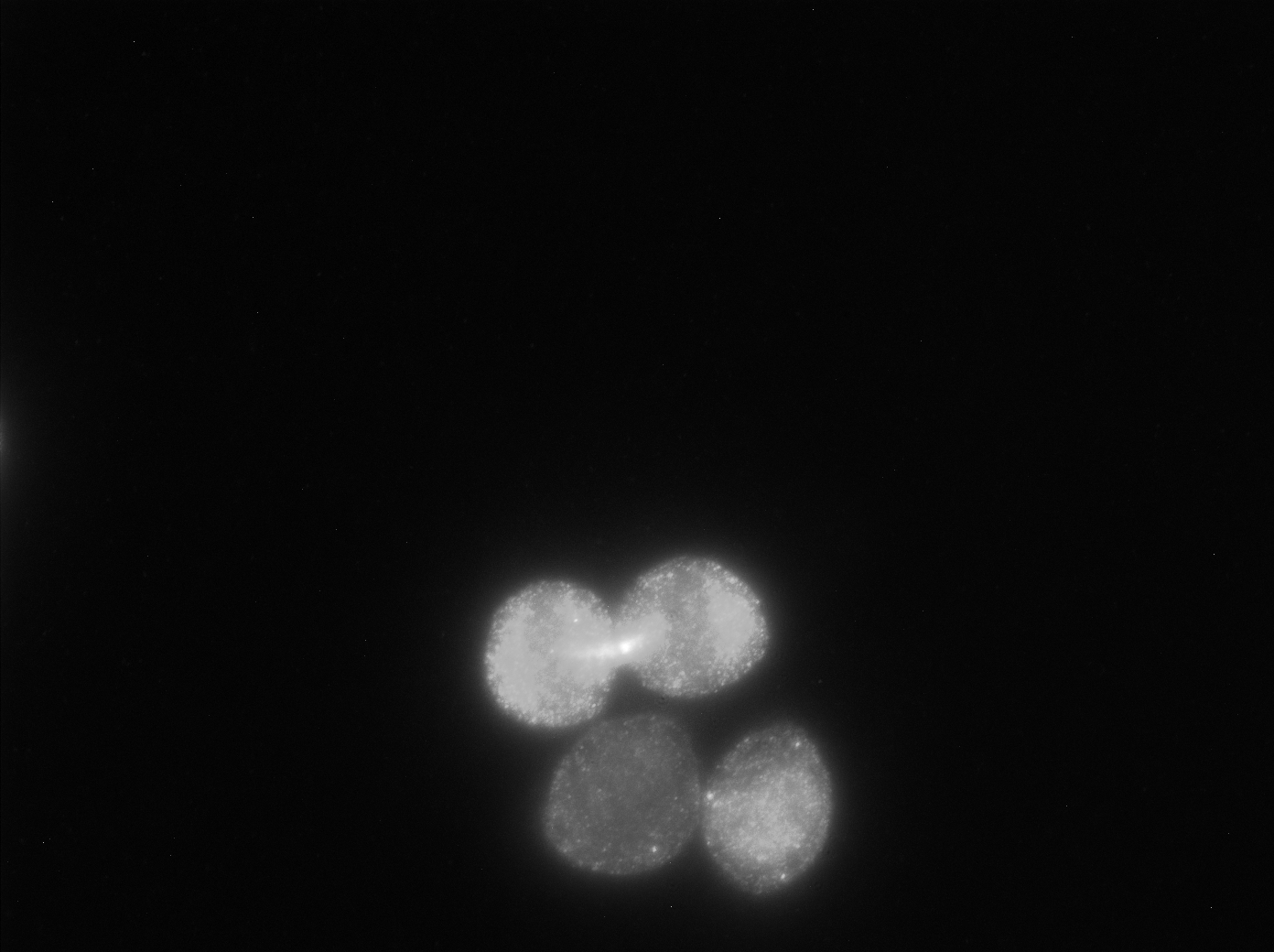

Supplement: Supplementary file 4 — Source data Fig. 2 part 1 [file 44319_2026_742_MOESM4_ESM.zip › Figure 2 Part 1/Fig 2c Cas9 Hela rGT335 atubulin/Cas9 GT335recomb atub 3-24-25 R1 ET8.Project Maximum Z_XY1743102842_Z0_T0_C1.tif]

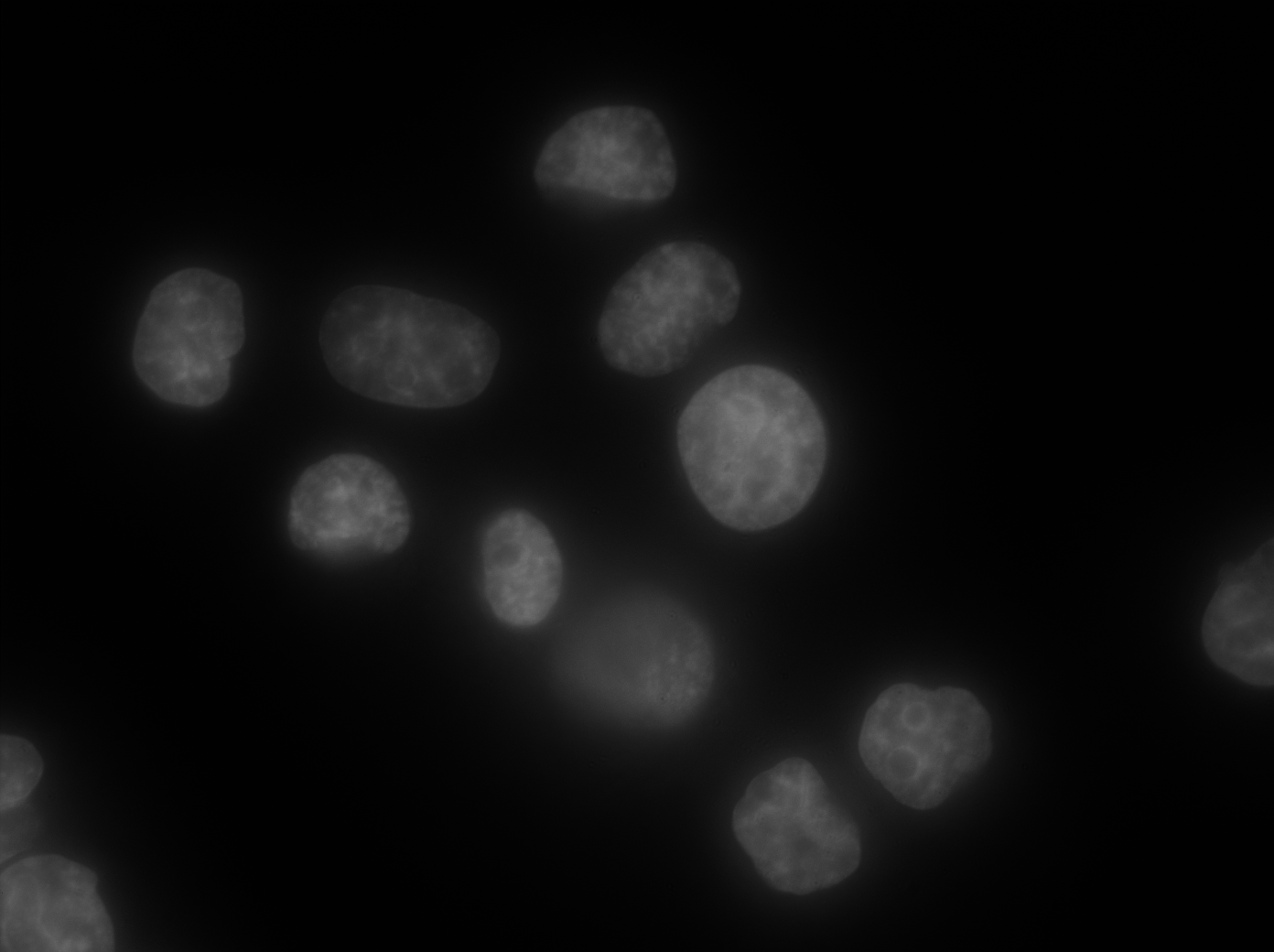

Supplement: Supplementary file 4 — Source data Fig. 2 part 1 [file 44319_2026_742_MOESM4_ESM.zip › Figure 2 Part 1/Fig 2c Cas9 Hela rGT335 atubulin/Cas9 GT335recomb atub 3-24-25 R1 ET1ET2.Project Maximum Z_XY1743101008_Z0_T0_C0.tif]

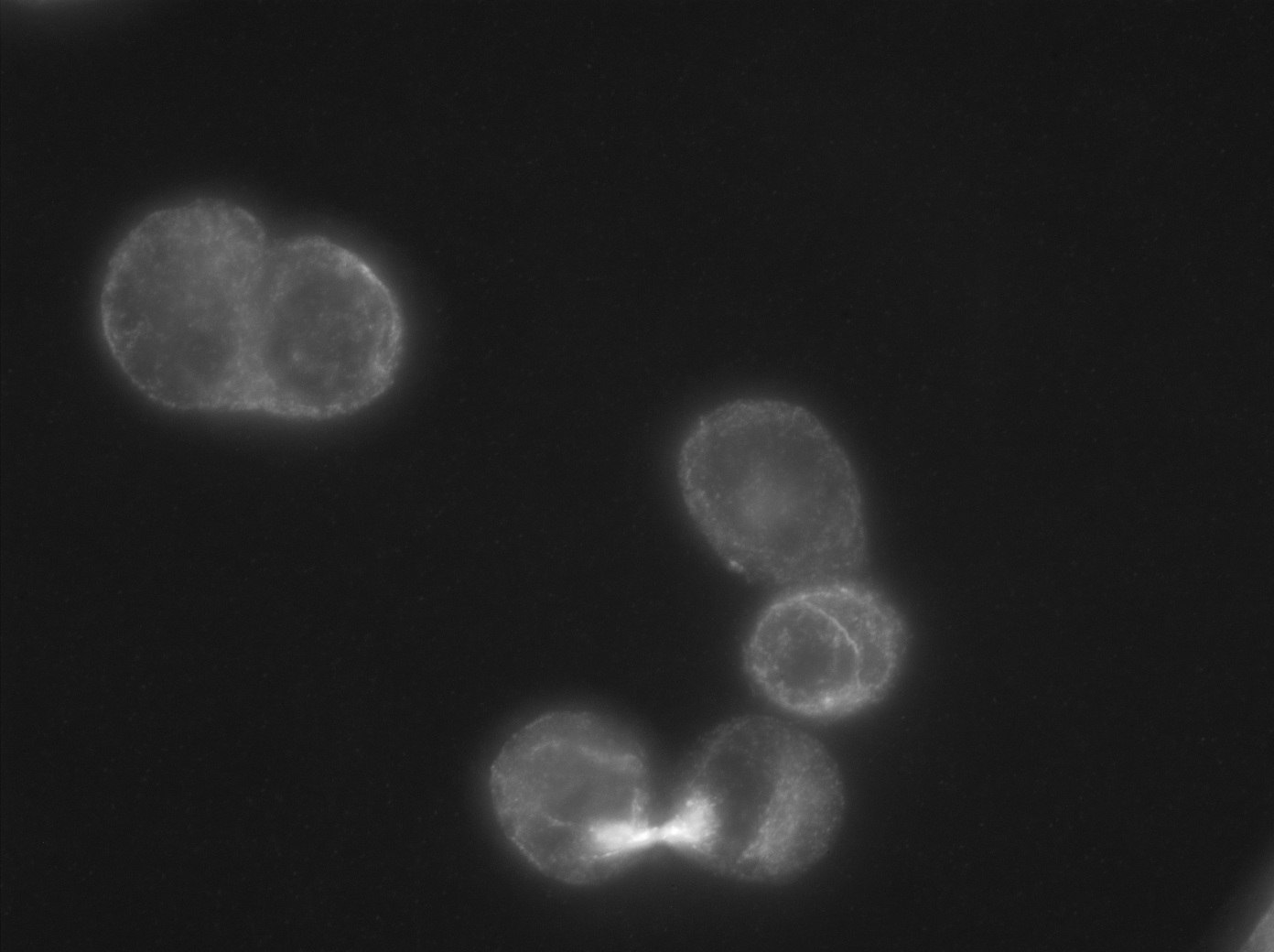

Supplement: Supplementary file 4 — Source data Fig. 2 part 1 [file 44319_2026_742_MOESM4_ESM.zip › Figure 2 Part 1/Fig 2c Cas9 Hela rGT335 atubulin/Cas9 GT335recomb atub 3-24-25 R3 ET4.Project Maximum Z_XY1743453805_Z0_T0_C2.tif]

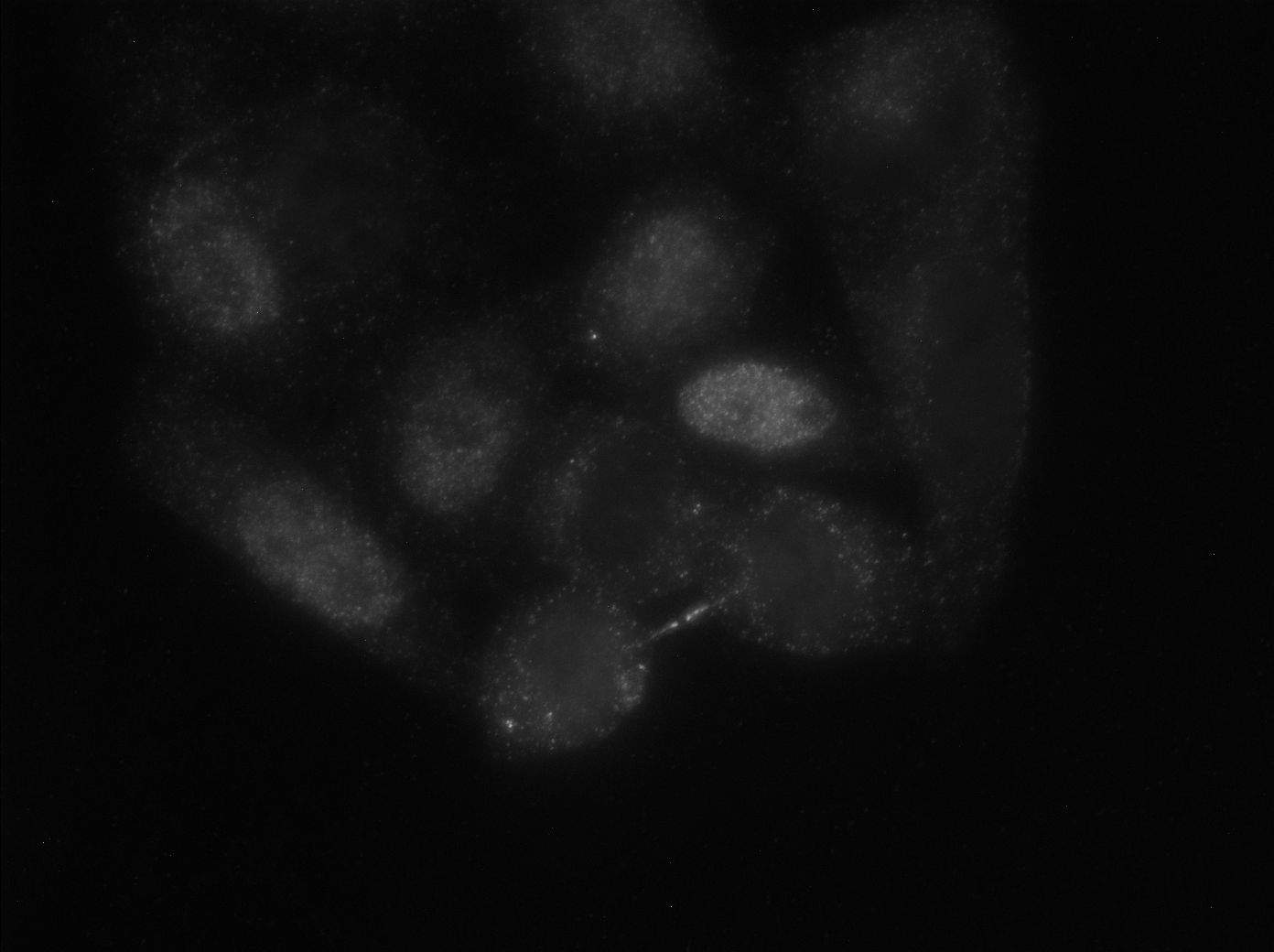

Supplement: Supplementary file 4 — Source data Fig. 2 part 1 [file 44319_2026_742_MOESM4_ESM.zip › Figure 2 Part 1/Fig 2c Cas9 Hela rGT335 atubulin/Cas9 GT335recomb atub 3-24-25 R1 LT1.Project Maximum Z_XY1743100595_Z0_T0_C1.tif]

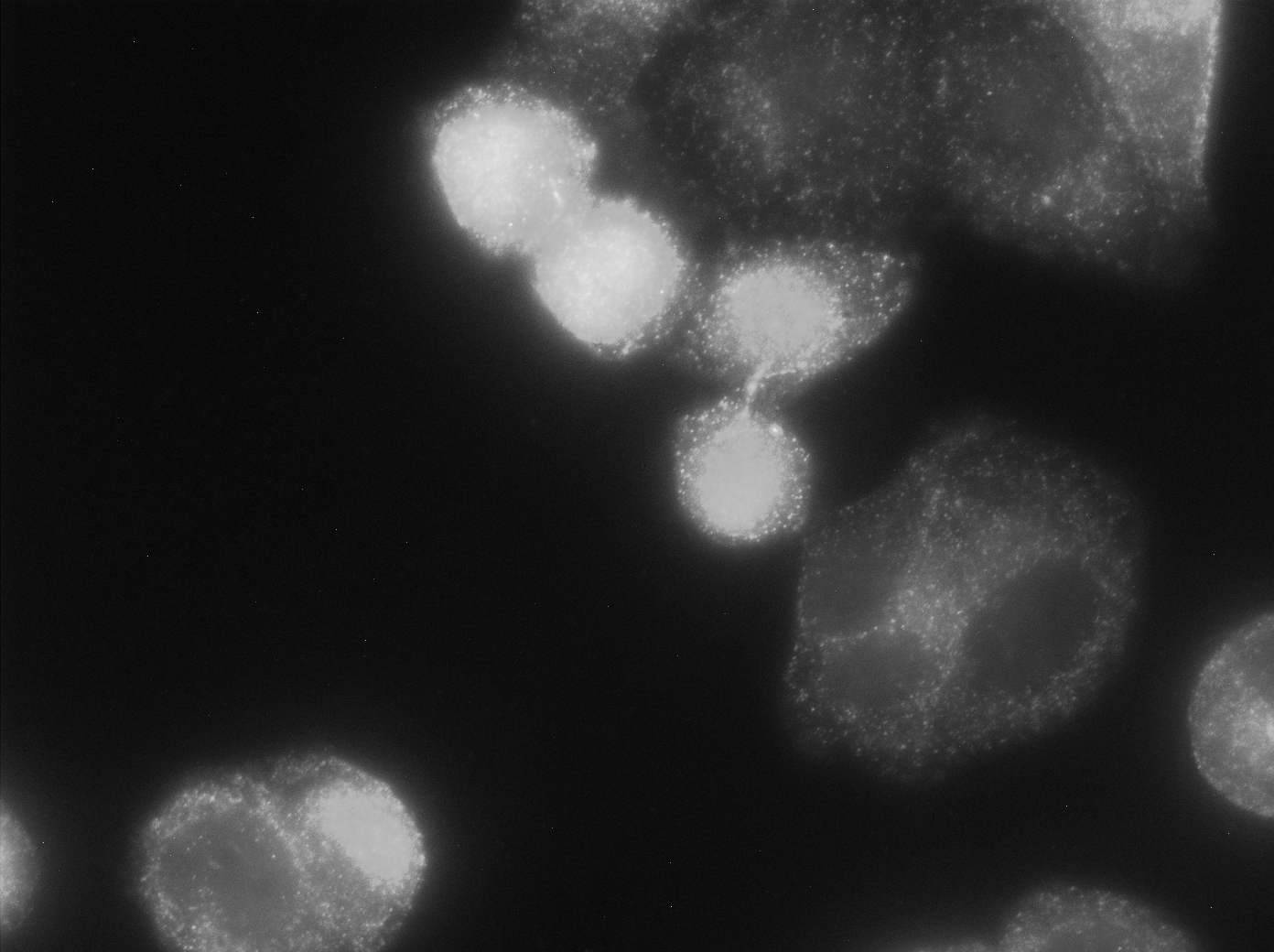

Supplement: Supplementary file 4 — Source data Fig. 2 part 1 [file 44319_2026_742_MOESM4_ESM.zip › Figure 2 Part 1/Fig 2c Cas9 Hela rGT335 atubulin/Cas9 GT335recomb atub 3-24-25 R3 LT10LT11.Project Maximum Z_XY1743455704_Z0_T0_C1.tif]

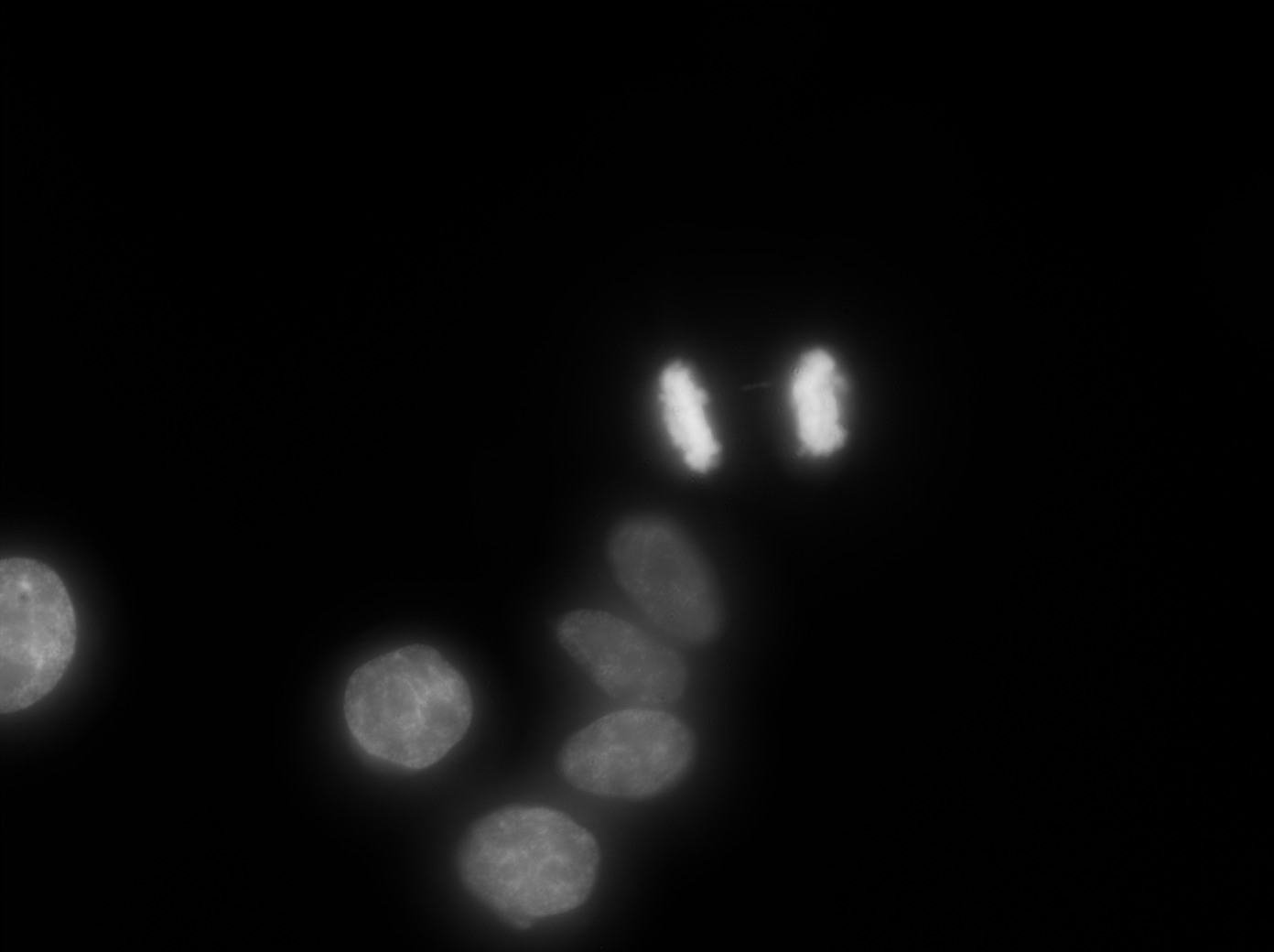

Supplement: Supplementary file 4 — Source data Fig. 2 part 1 [file 44319_2026_742_MOESM4_ESM.zip › Figure 2 Part 1/Fig 2c Cas9 Hela rGT335 atubulin/Cas9 GT335recomb atub 3-24-25 R3 preET3.Project Maximum Z_XY1743453338_Z0_T0_C0.tif]

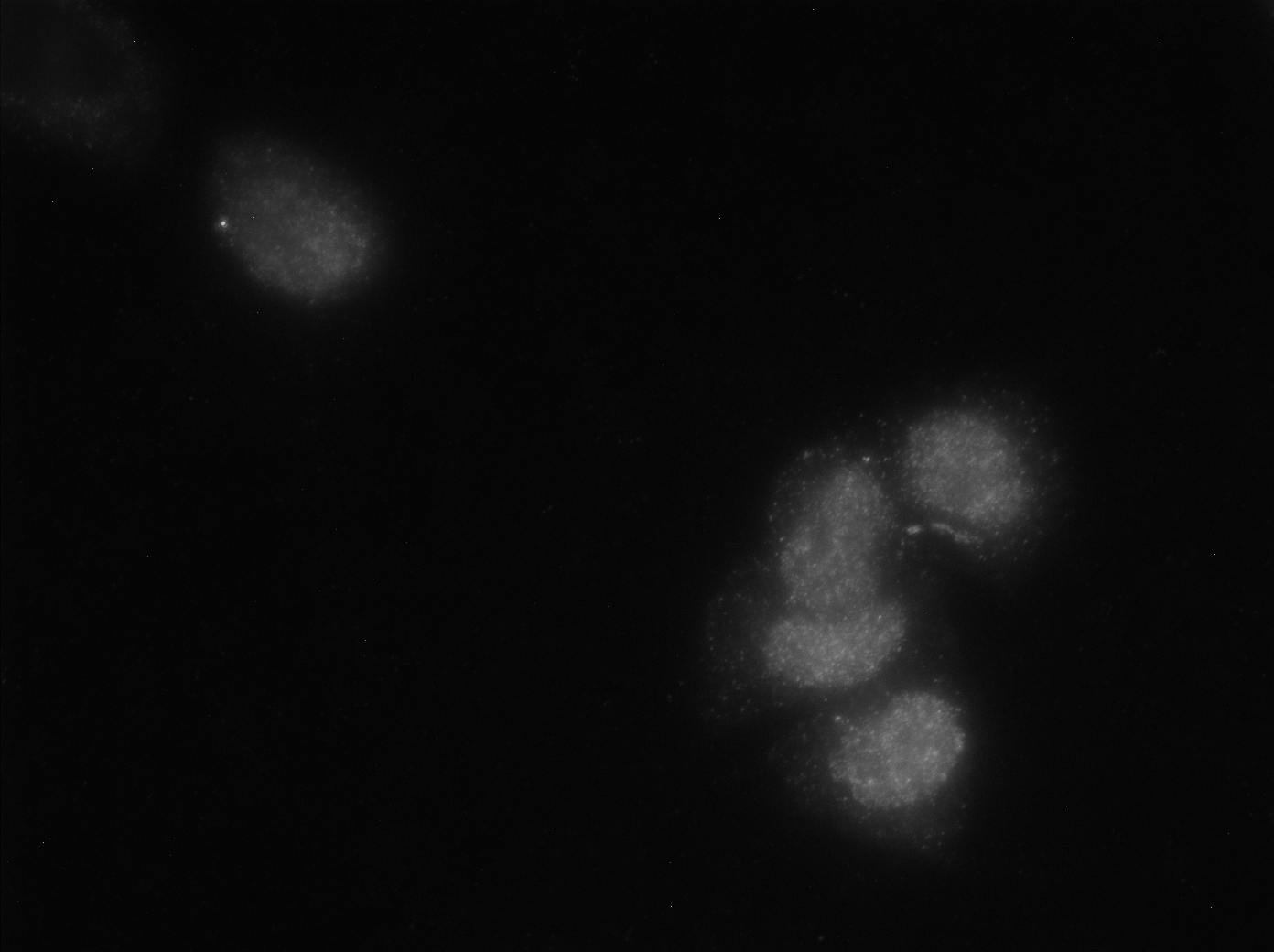

Supplement: Supplementary file 4 — Source data Fig. 2 part 1 [file 44319_2026_742_MOESM4_ESM.zip › Figure 2 Part 1/Fig 2c Cas9 Hela rGT335 atubulin/Cas9 GT335recomb atub 3-24-25 R1 LT4 PA5.Project Maximum Z_XY1743101109_Z0_T0_C1.tif]

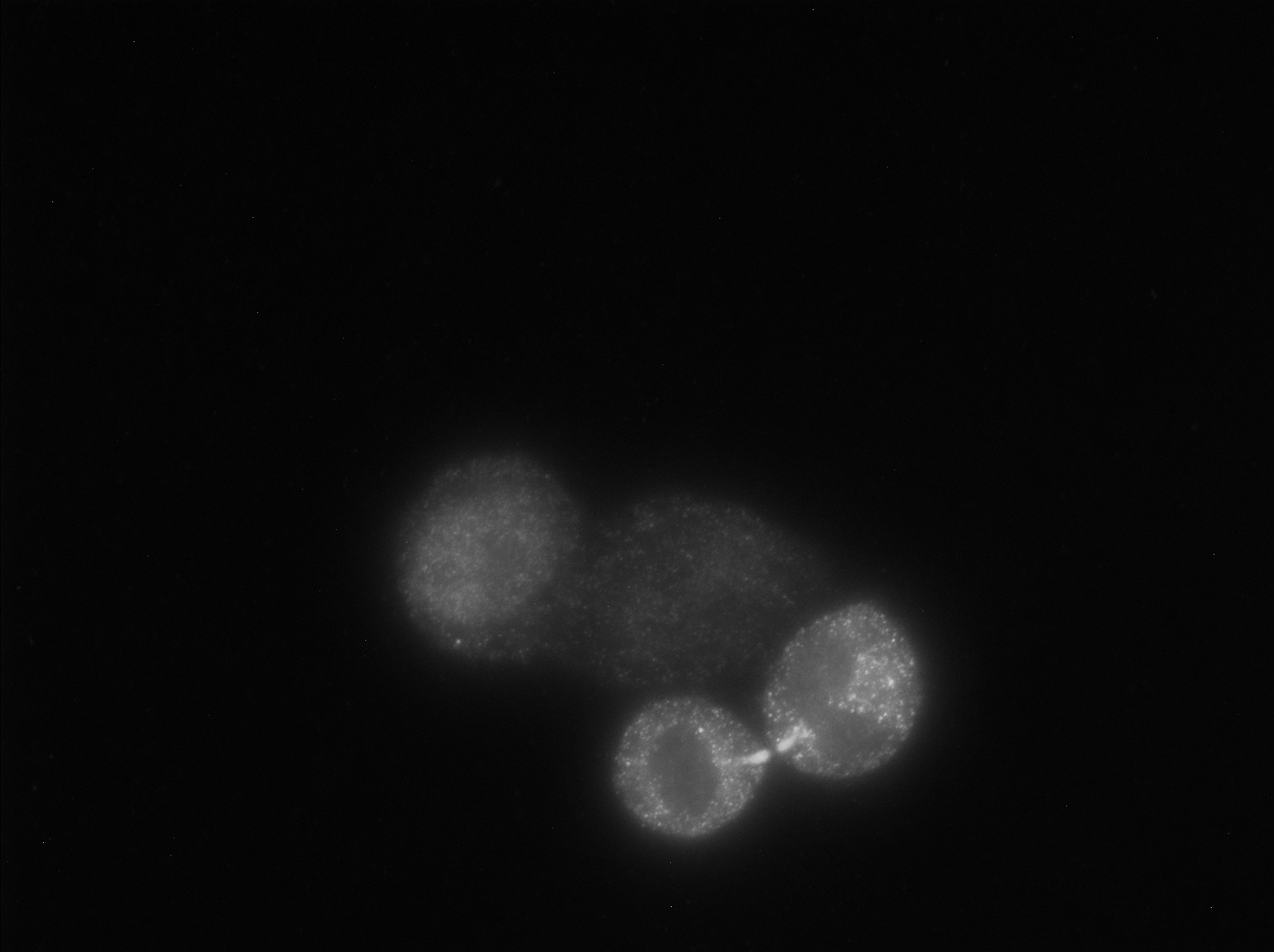

Supplement: Supplementary file 4 — Source data Fig. 2 part 1 [file 44319_2026_742_MOESM4_ESM.zip › Figure 2 Part 1/Fig 2c Cas9 Hela rGT335 atubulin/Cas9 GT335recomb atub 3-24-25 R1 ET7.Project Maximum Z_XY1743102735_Z0_T0_C1.tif]

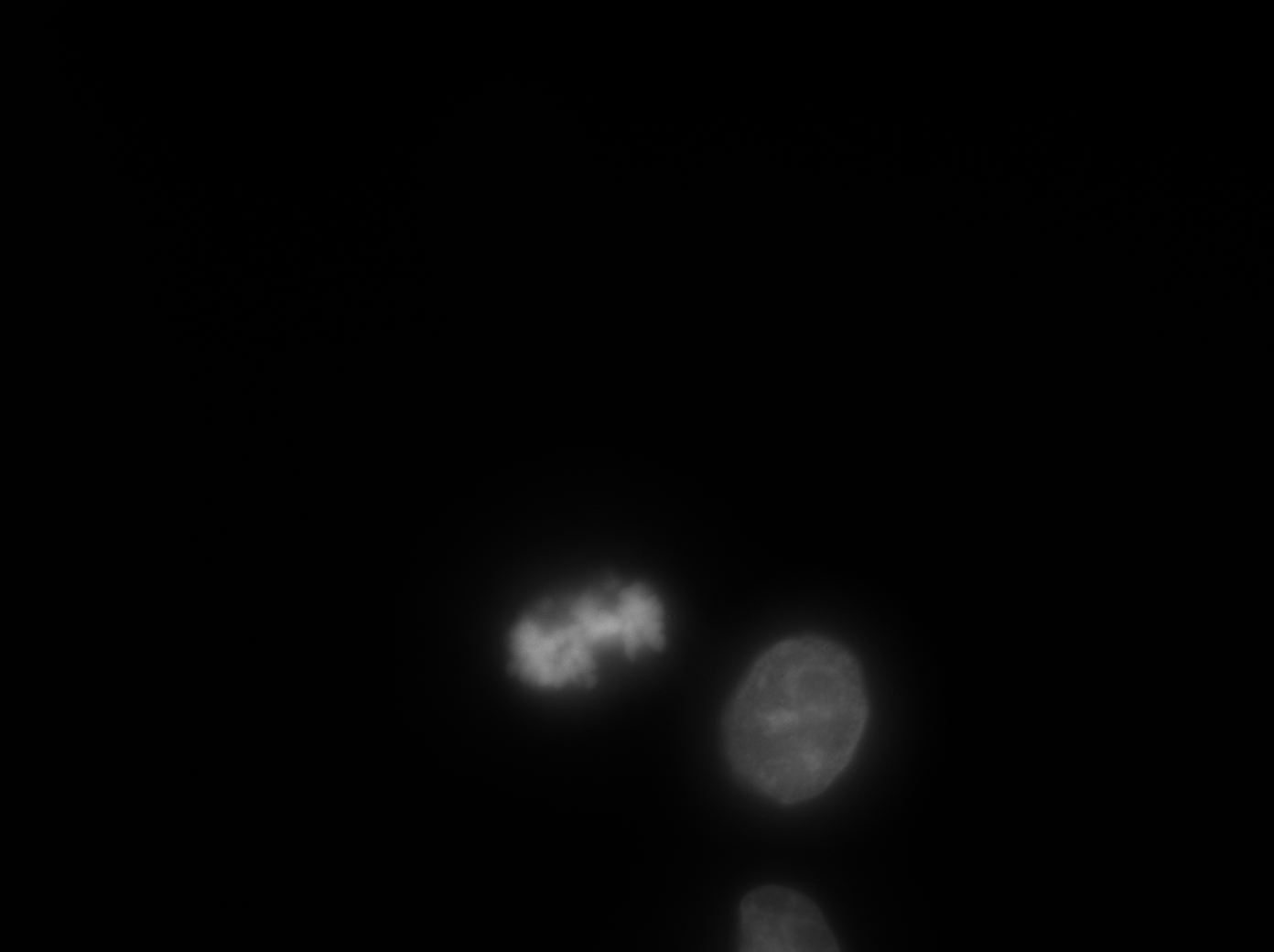

Supplement: Supplementary file 4 — Source data Fig. 2 part 1 [file 44319_2026_742_MOESM4_ESM.zip › Figure 2 Part 1/Fig 2c Cas9 Hela rGT335 atubulin/Cas9 GT335recomb atub 3-24-25 R1 M10.Project Maximum Z_XY1743105878_Z0_T0_C0.tif]

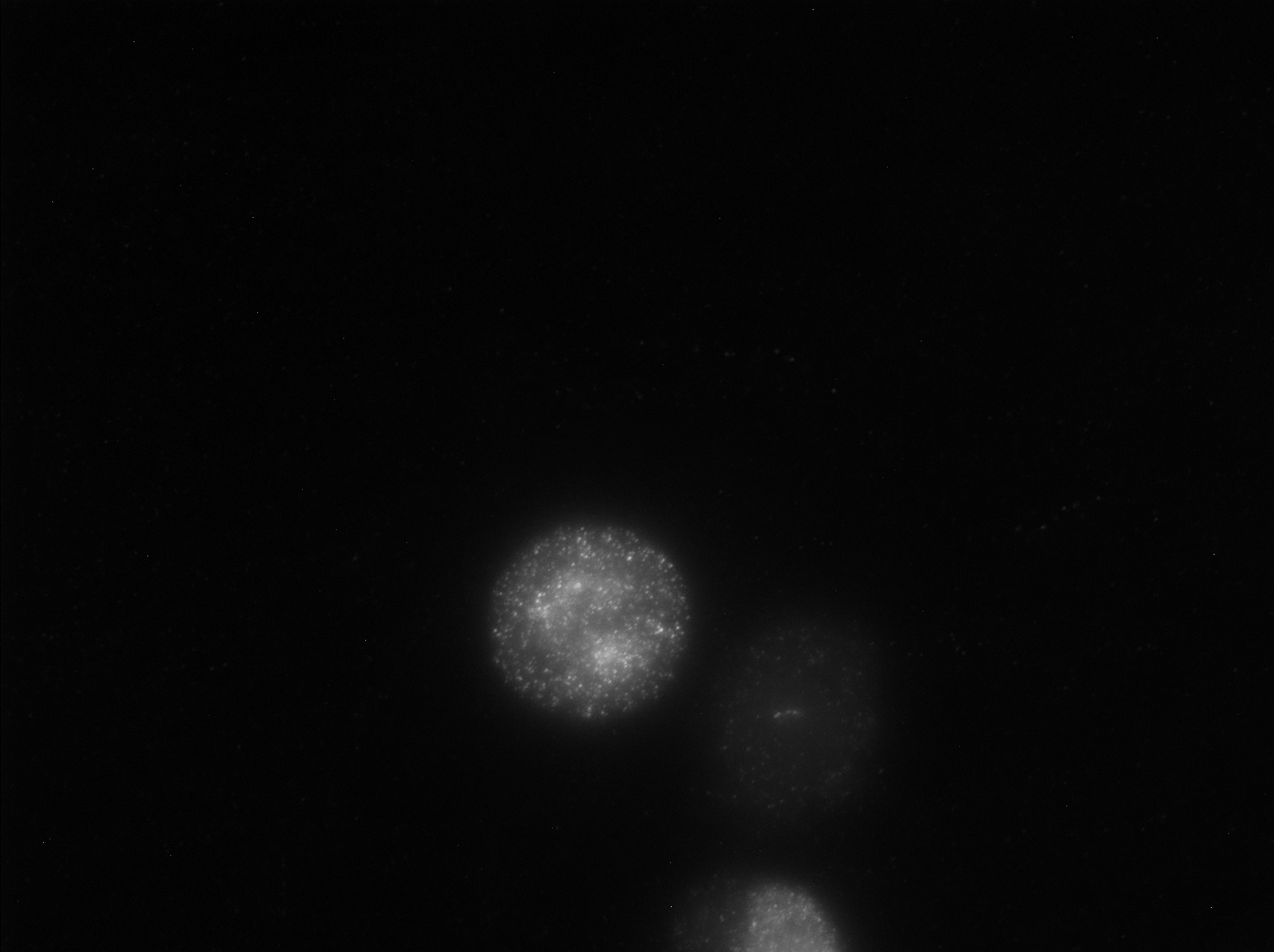

Supplement: Supplementary file 4 — Source data Fig. 2 part 1 [file 44319_2026_742_MOESM4_ESM.zip › Figure 2 Part 1/Fig 2c Cas9 Hela rGT335 atubulin/Cas9 GT335recomb atub 3-24-25 R1 M10.Project Maximum Z_XY1743105878_Z0_T0_C1.tif]

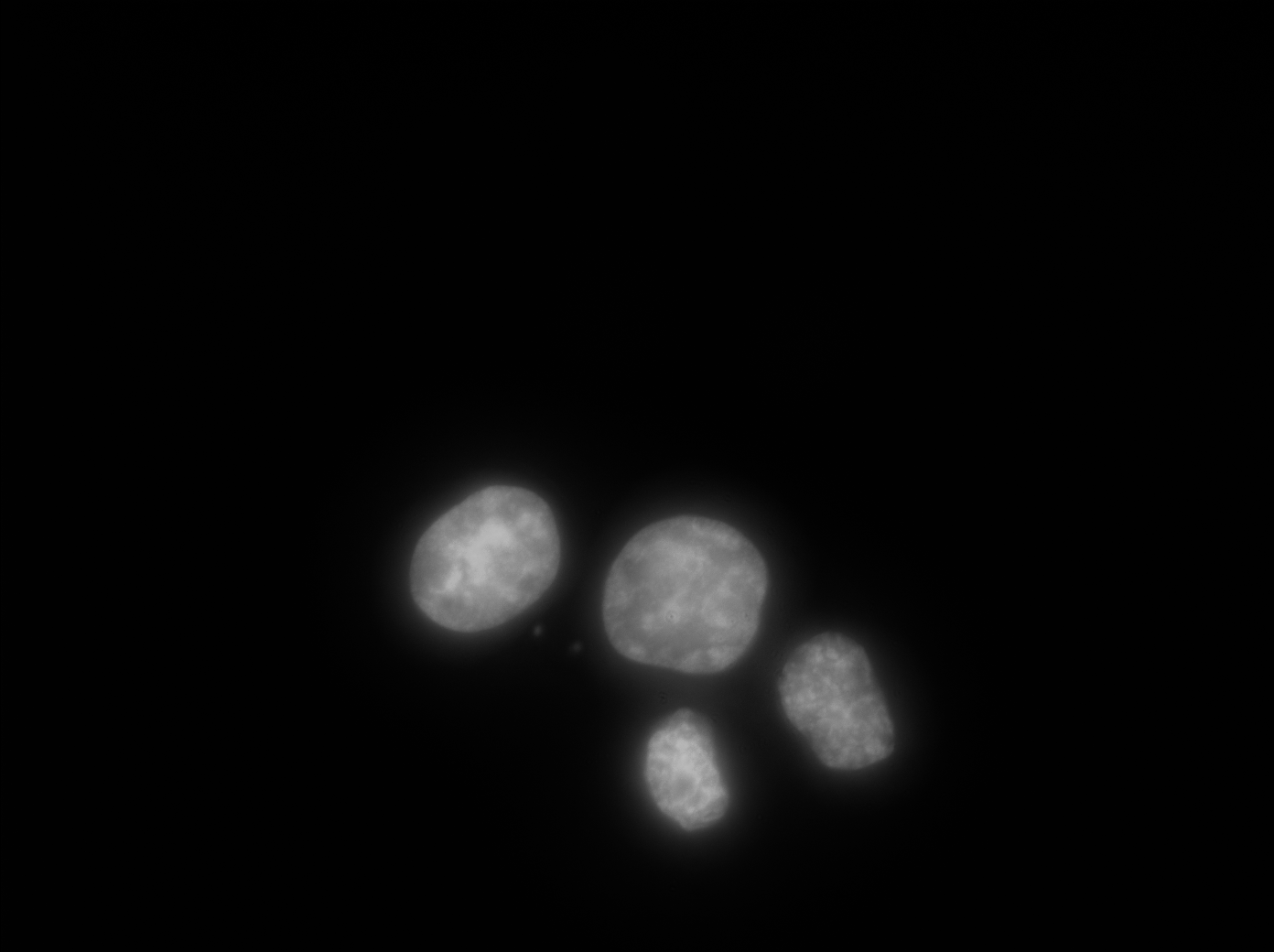

Supplement: Supplementary file 4 — Source data Fig. 2 part 1 [file 44319_2026_742_MOESM4_ESM.zip › Figure 2 Part 1/Fig 2c Cas9 Hela rGT335 atubulin/Cas9 GT335recomb atub 3-24-25 R1 ET7.Project Maximum Z_XY1743102735_Z0_T0_C0.tif]

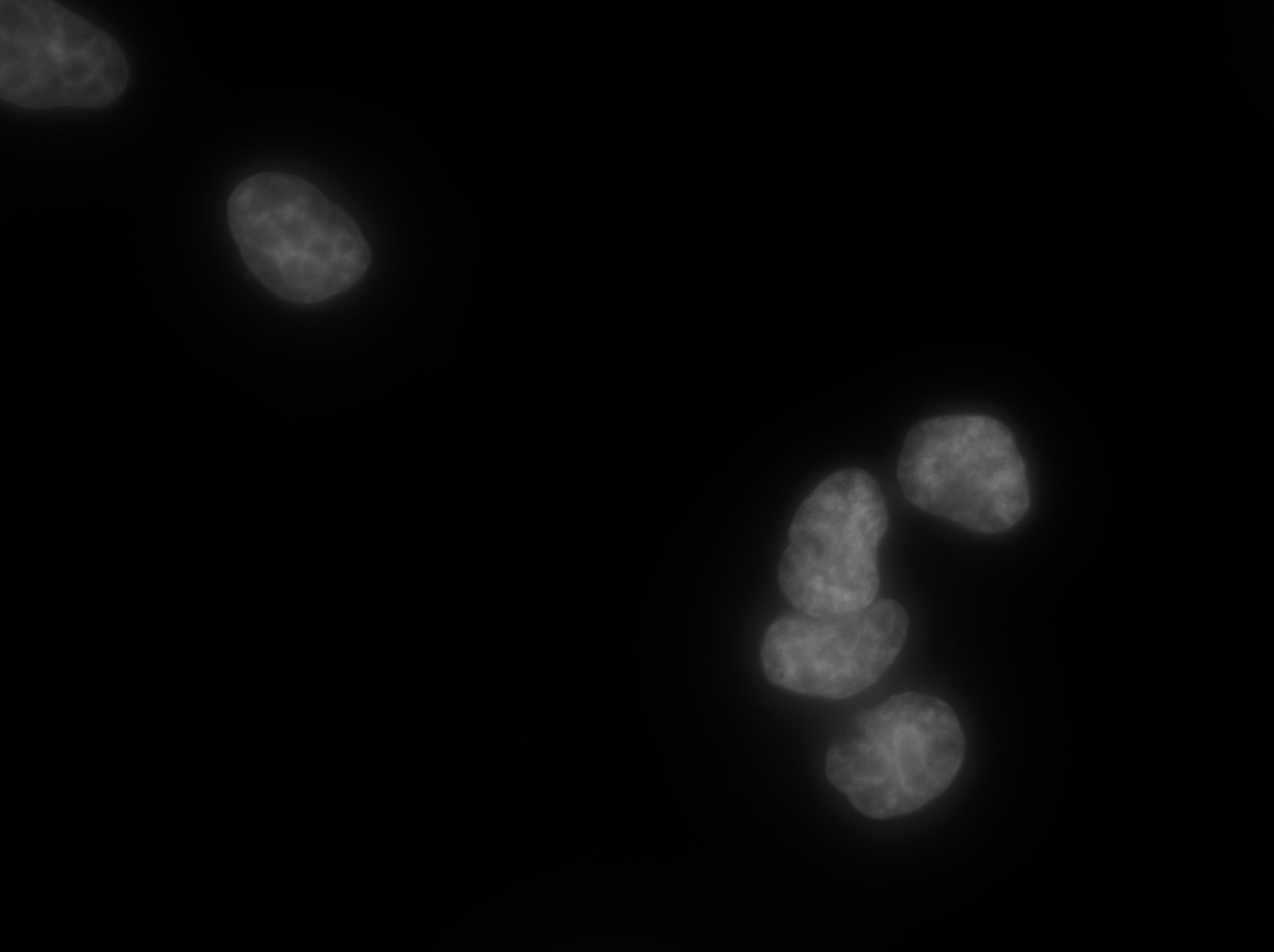

Supplement: Supplementary file 4 — Source data Fig. 2 part 1 [file 44319_2026_742_MOESM4_ESM.zip › Figure 2 Part 1/Fig 2c Cas9 Hela rGT335 atubulin/Cas9 GT335recomb atub 3-24-25 R1 LT4 PA5.Project Maximum Z_XY1743101109_Z0_T0_C0.tif]

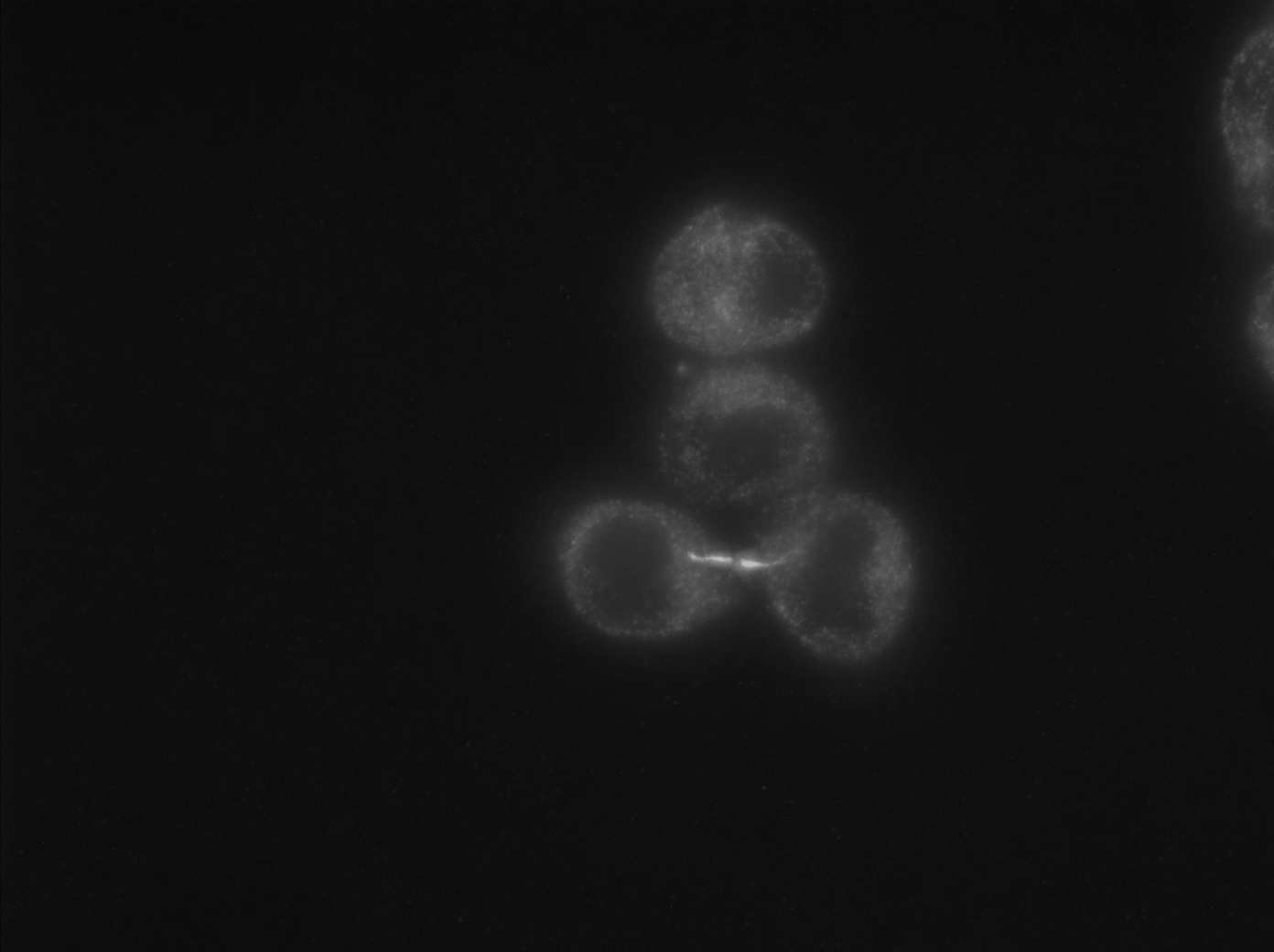

Supplement: Supplementary file 4 — Source data Fig. 2 part 1 [file 44319_2026_742_MOESM4_ESM.zip › Figure 2 Part 1/Fig 2c Cas9 Hela rGT335 atubulin/Cas9 GT335recomb atub 3-24-25 R1 LT10.Project Maximum Z_XY1743102059_Z0_T0_C2.tif]

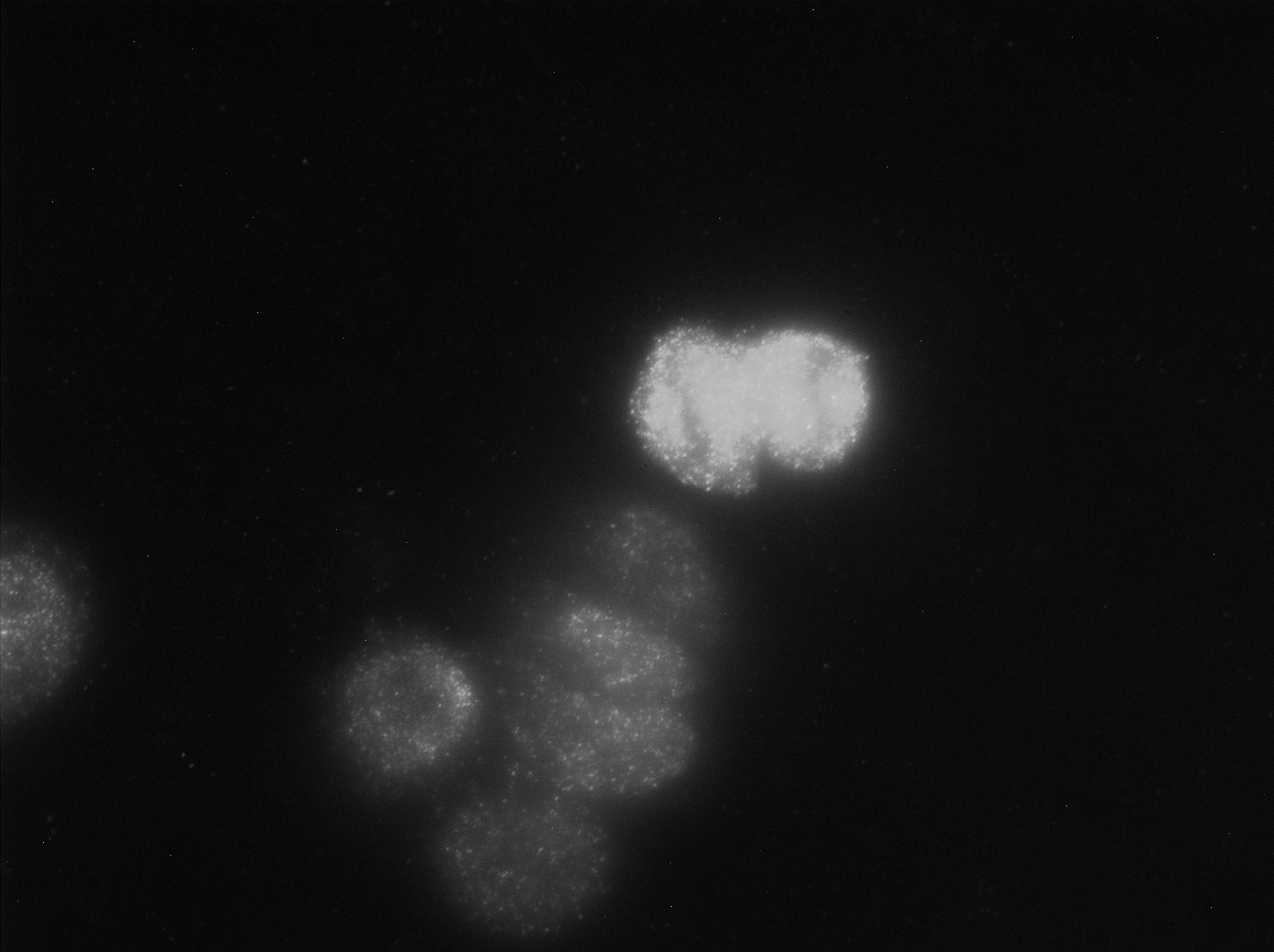

Supplement: Supplementary file 4 — Source data Fig. 2 part 1 [file 44319_2026_742_MOESM4_ESM.zip › Figure 2 Part 1/Fig 2c Cas9 Hela rGT335 atubulin/Cas9 GT335recomb atub 3-24-25 R3 preET3.Project Maximum Z_XY1743453338_Z0_T0_C1.tif]

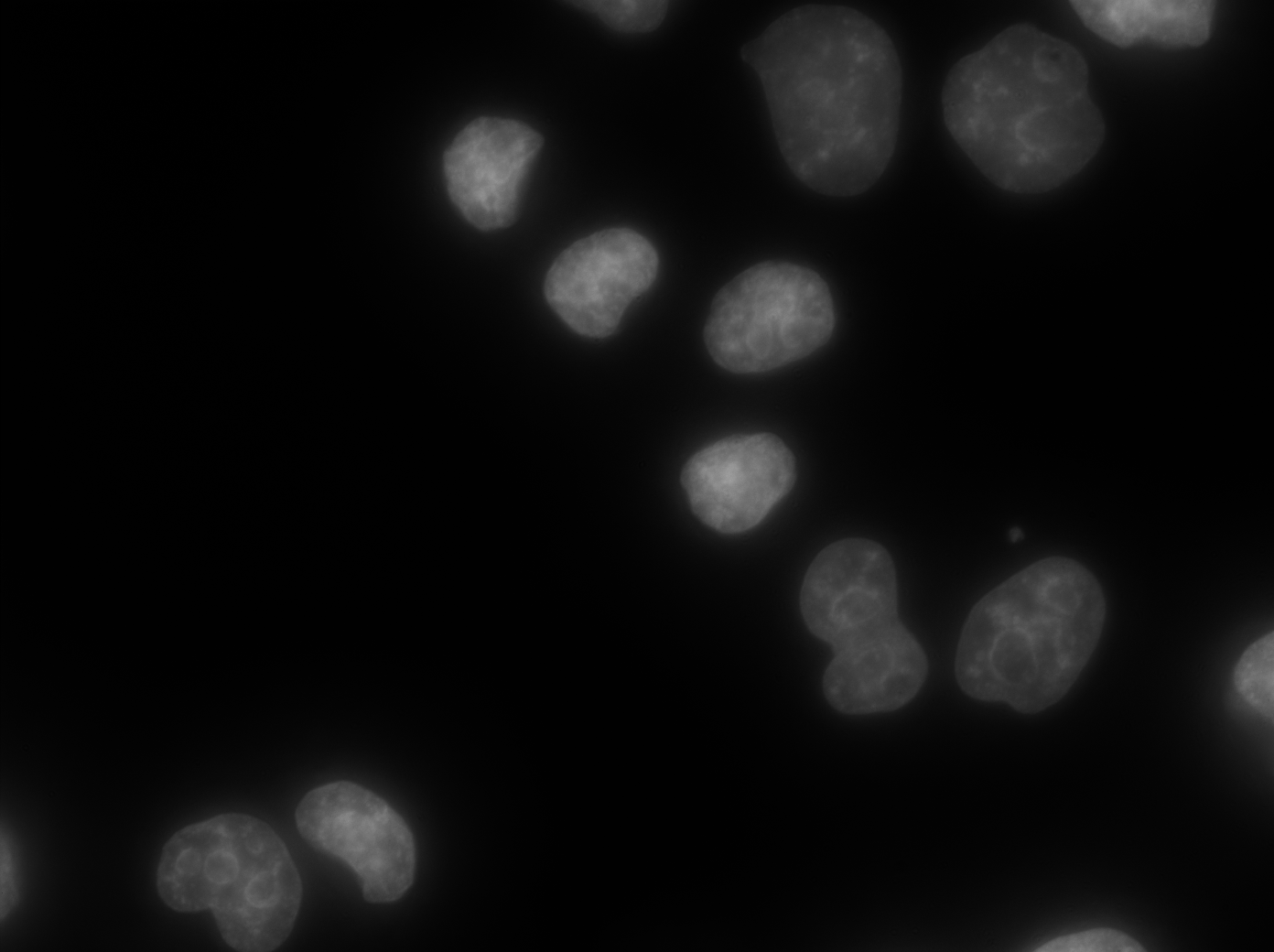

Supplement: Supplementary file 4 — Source data Fig. 2 part 1 [file 44319_2026_742_MOESM4_ESM.zip › Figure 2 Part 1/Fig 2c Cas9 Hela rGT335 atubulin/Cas9 GT335recomb atub 3-24-25 R3 LT10LT11.Project Maximum Z_XY1743455704_Z0_T0_C0.tif]

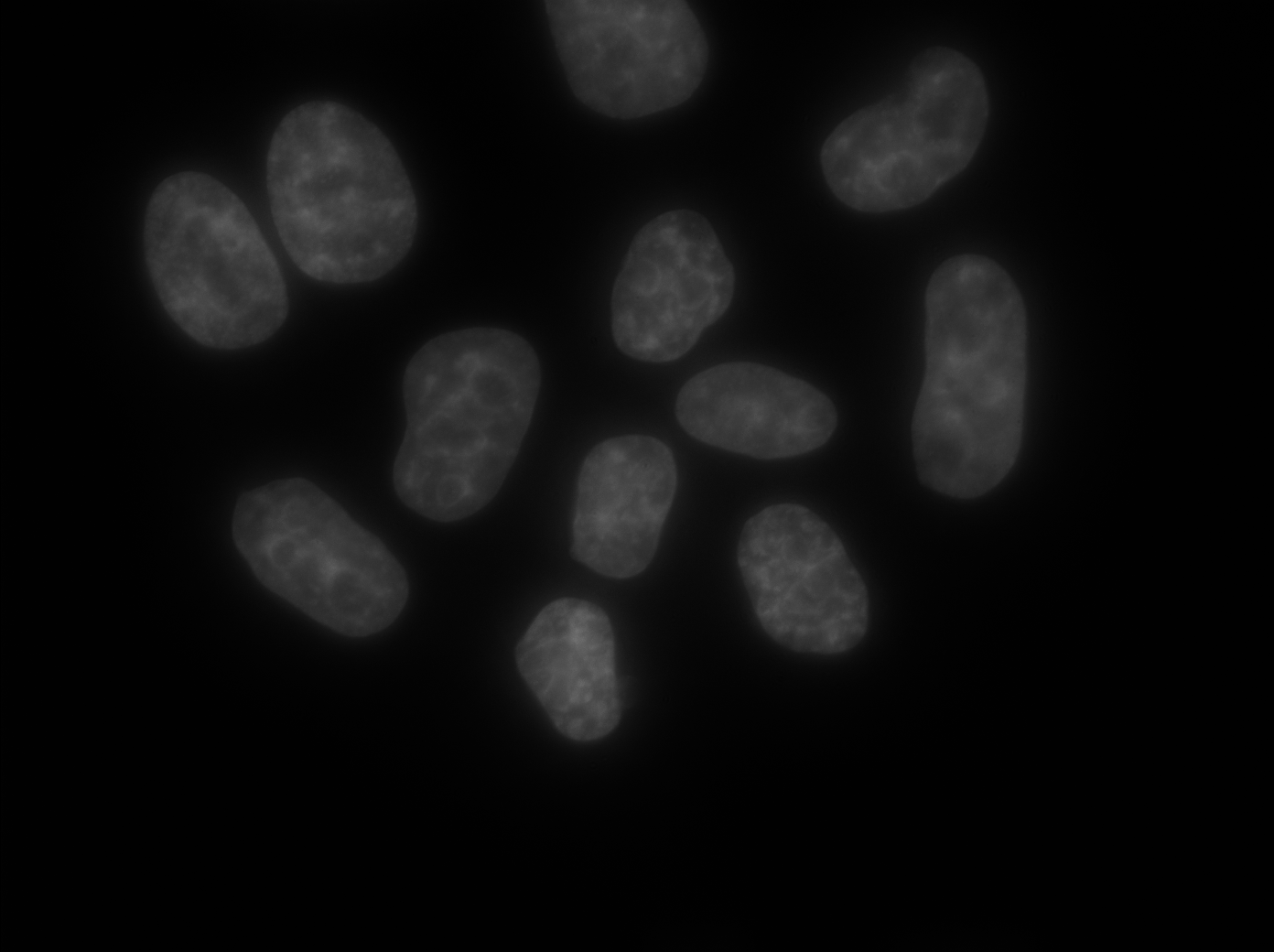

Supplement: Supplementary file 4 — Source data Fig. 2 part 1 [file 44319_2026_742_MOESM4_ESM.zip › Figure 2 Part 1/Fig 2c Cas9 Hela rGT335 atubulin/Cas9 GT335recomb atub 3-24-25 R1 LT1.Project Maximum Z_XY1743100595_Z0_T0_C0.tif]

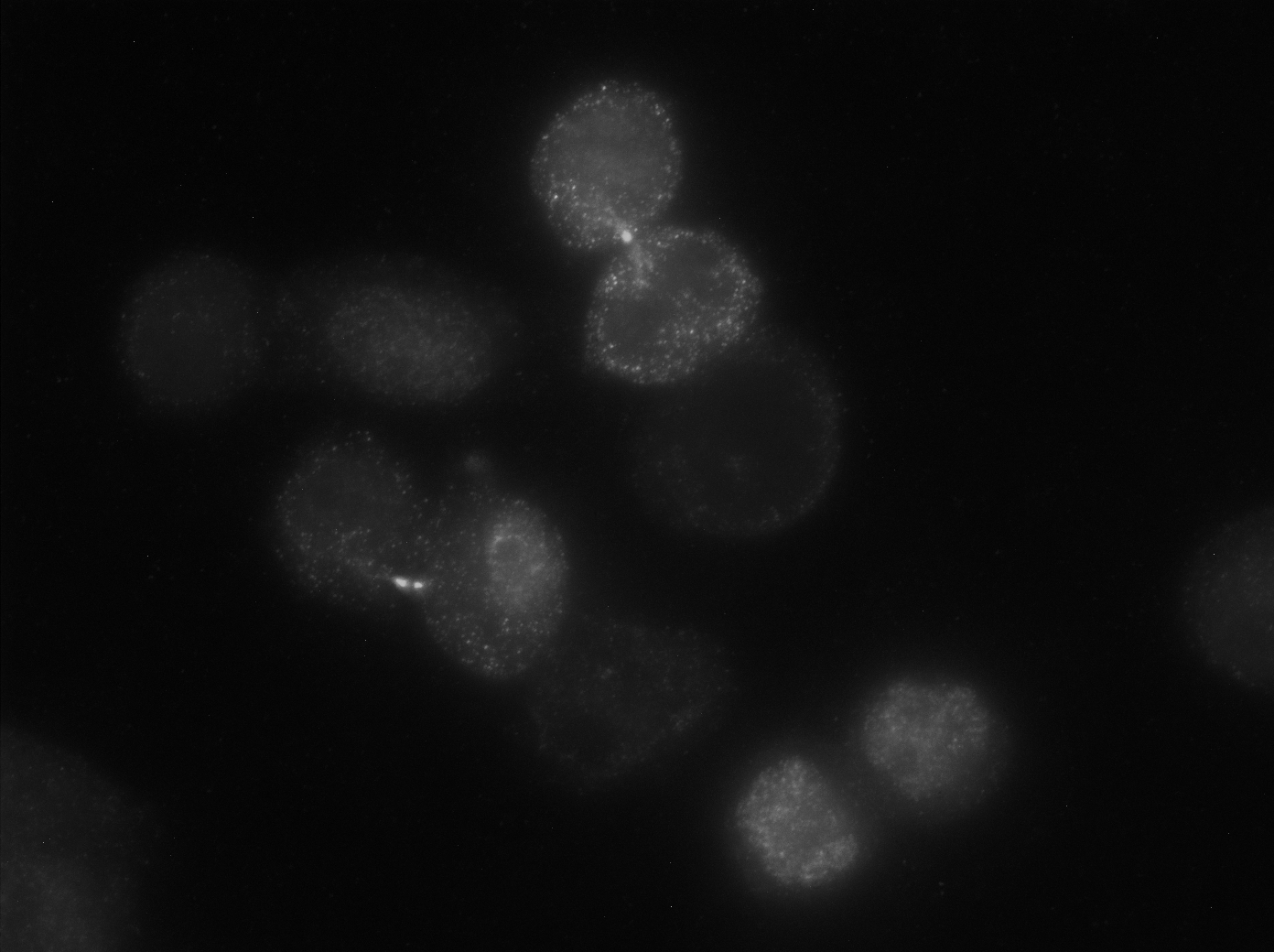

Supplement: Supplementary file 4 — Source data Fig. 2 part 1 [file 44319_2026_742_MOESM4_ESM.zip › Figure 2 Part 1/Fig 2c Cas9 Hela rGT335 atubulin/Cas9 GT335recomb atub 3-24-25 R1 ET1ET2.Project Maximum Z_XY1743101008_Z0_T0_C1.tif]

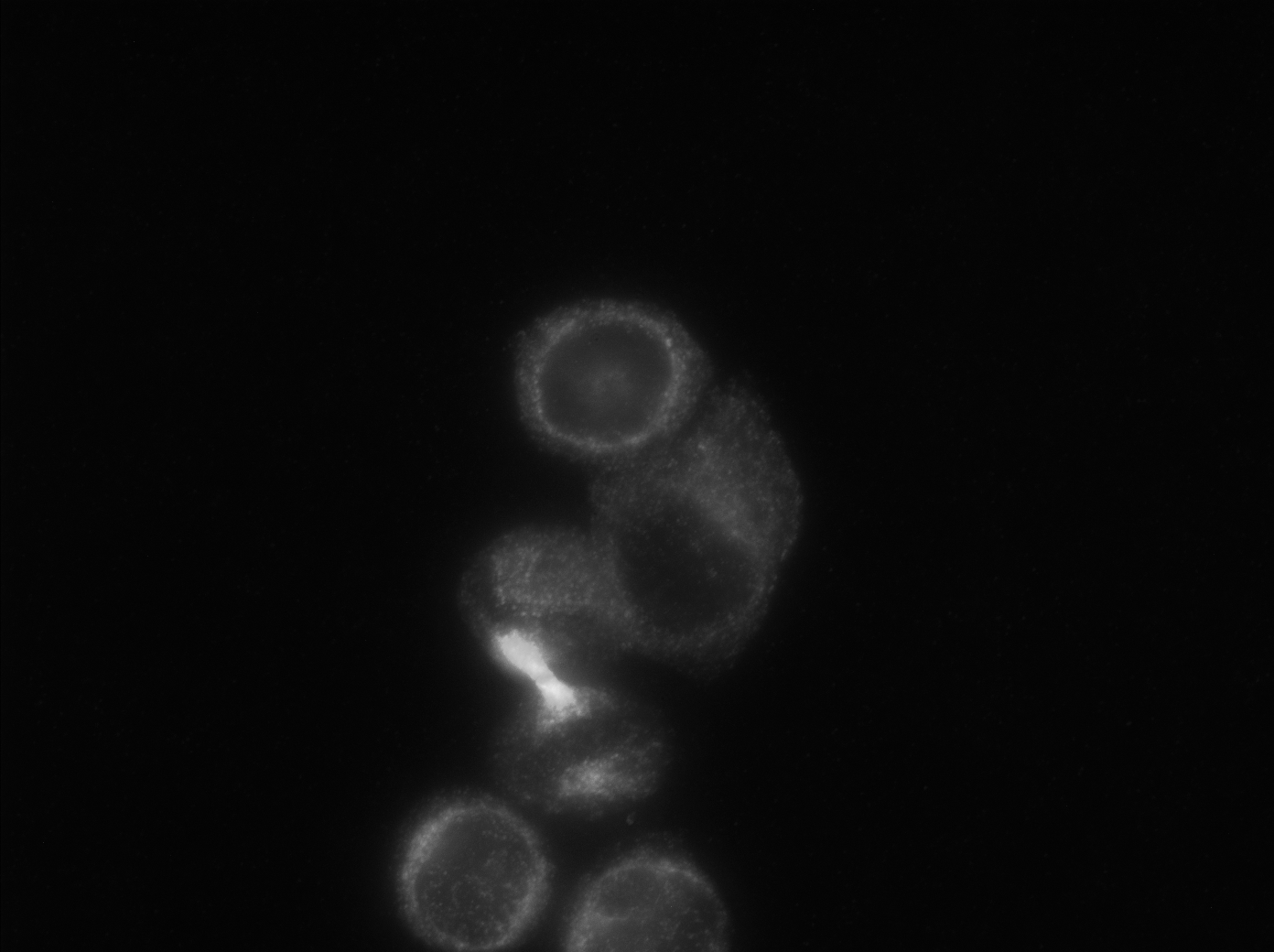

Supplement: Supplementary file 4 — Source data Fig. 2 part 1 [file 44319_2026_742_MOESM4_ESM.zip › Figure 2 Part 1/Fig 2c Cas9 Hela rGT335 atubulin/Cas9 GT335recomb atub 3-24-25 R2 ET7.Project Maximum Z_XY1743444621_Z0_T0_C2.tif]

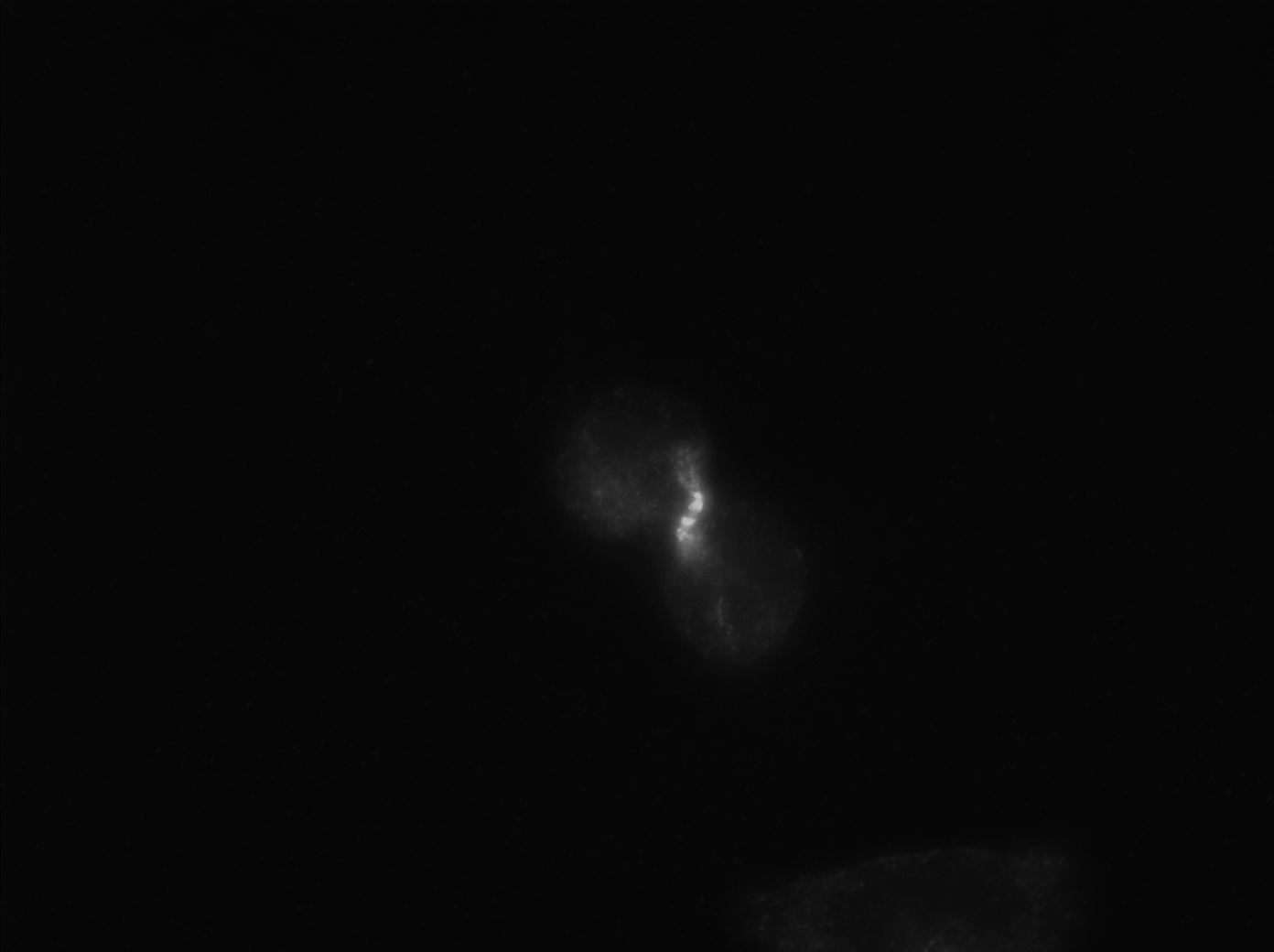

Supplement: Supplementary file 4 — Source data Fig. 2 part 1 [file 44319_2026_742_MOESM4_ESM.zip › Figure 2 Part 1/Fig 2c Cas9 Hela rGT335 atubulin/Cas9 GT335recomb atub 3-24-25 R2 ET3.Project Maximum Z_XY1743440248_Z0_T0_C2.tif]

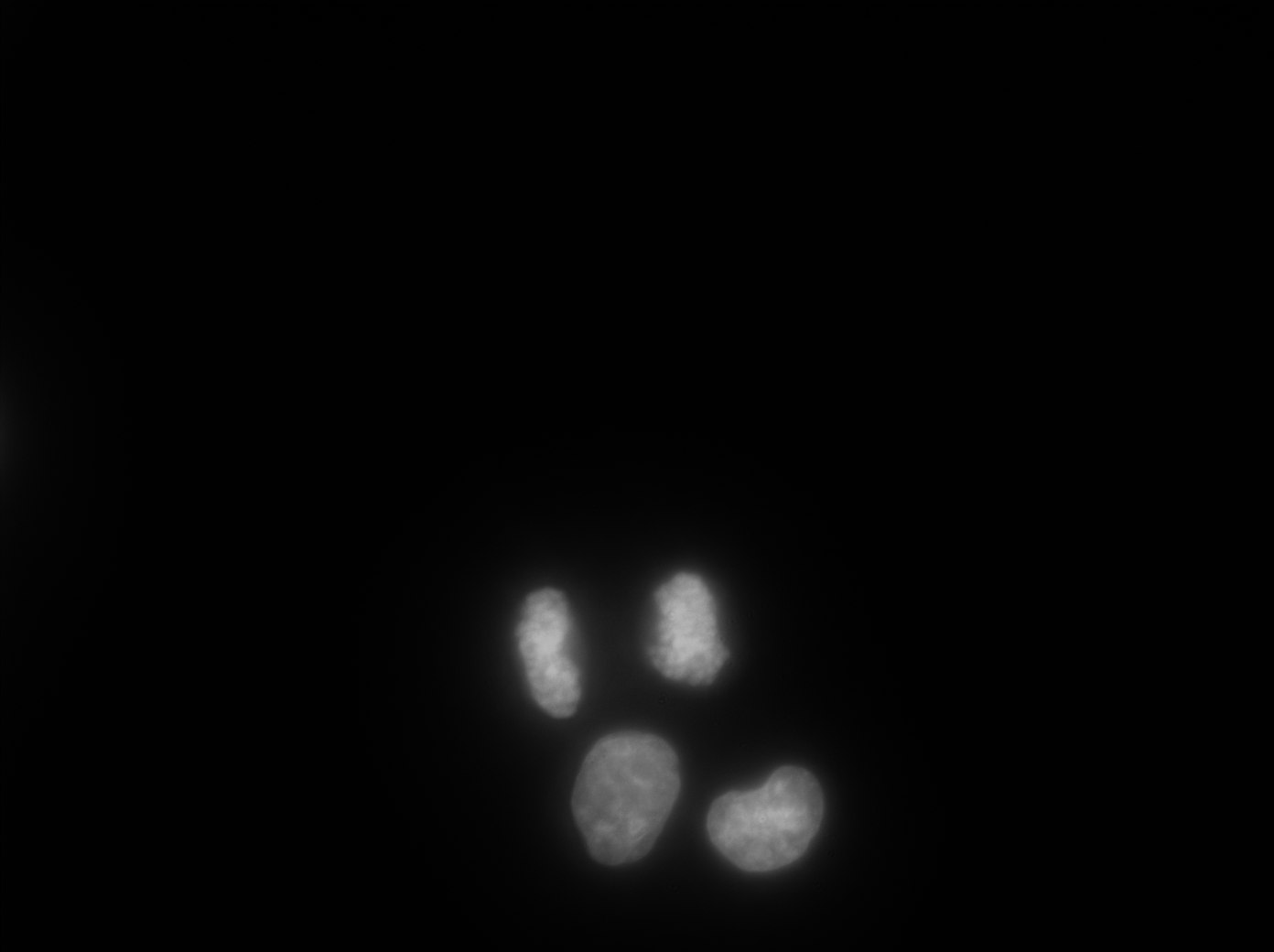

Supplement: Supplementary file 4 — Source data Fig. 2 part 1 [file 44319_2026_742_MOESM4_ESM.zip › Figure 2 Part 1/Fig 2c Cas9 Hela rGT335 atubulin/Cas9 GT335recomb atub 3-24-25 R1 ET8.Project Maximum Z_XY1743102842_Z0_T0_C0.tif]

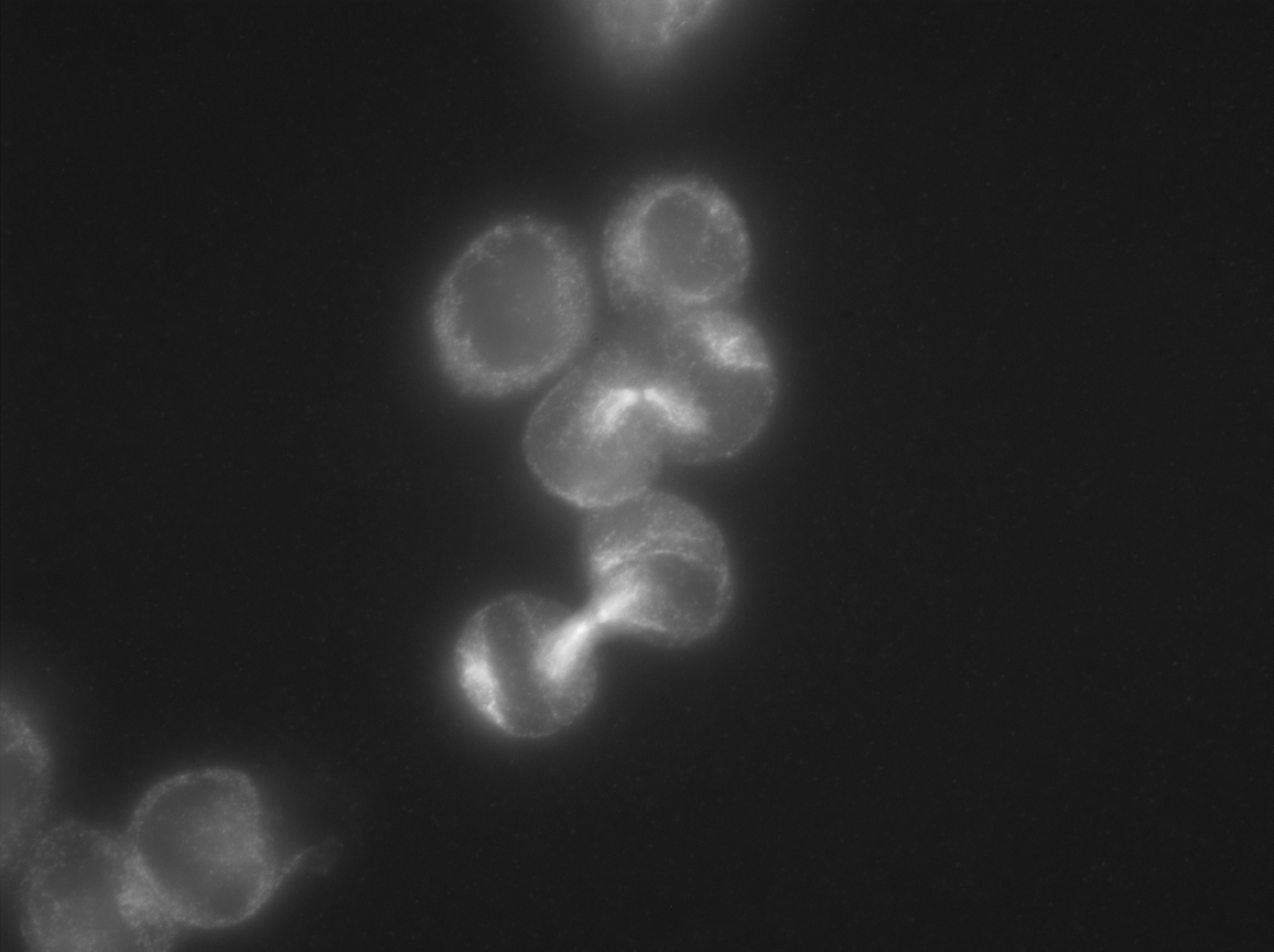

Supplement: Supplementary file 4 — Source data Fig. 2 part 1 [file 44319_2026_742_MOESM4_ESM.zip › Figure 2 Part 1/Fig 2c Cas9 Hela rGT335 atubulin/Cas9 GT335recomb atub 3-24-25 R3 ET9ET10.Project Maximum Z_XY1743455471_Z0_T0_C2.tif]

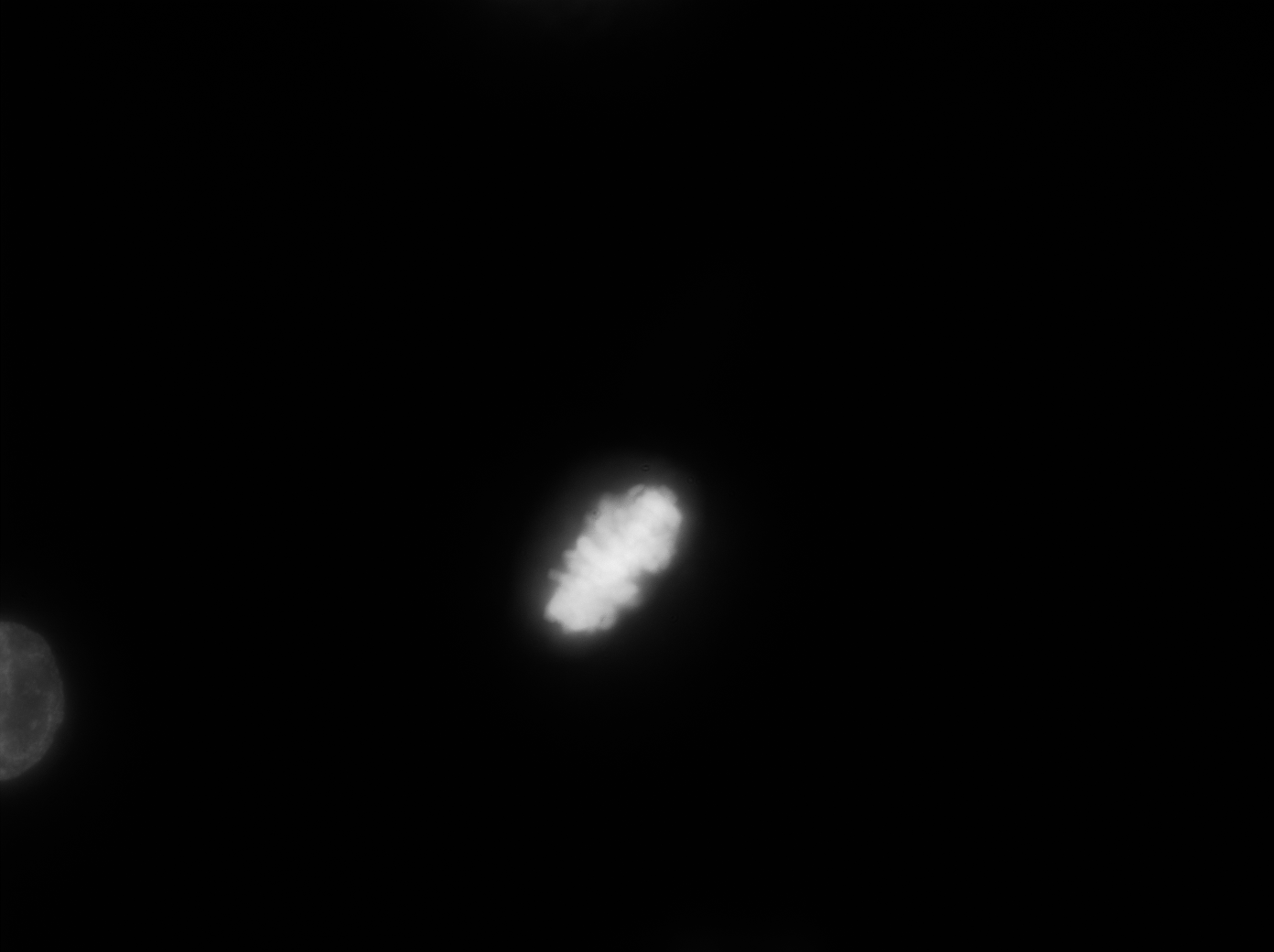

Supplement: Supplementary file 4 — Source data Fig. 2 part 1 [file 44319_2026_742_MOESM4_ESM.zip › Figure 2 Part 1/Fig 2c Cas9 Hela rGT335 atubulin/Cas9 GT335recomb atub 3-24-25 R2 M1.Project Maximum Z_XY1743440680_Z0_T0_C0.tif]

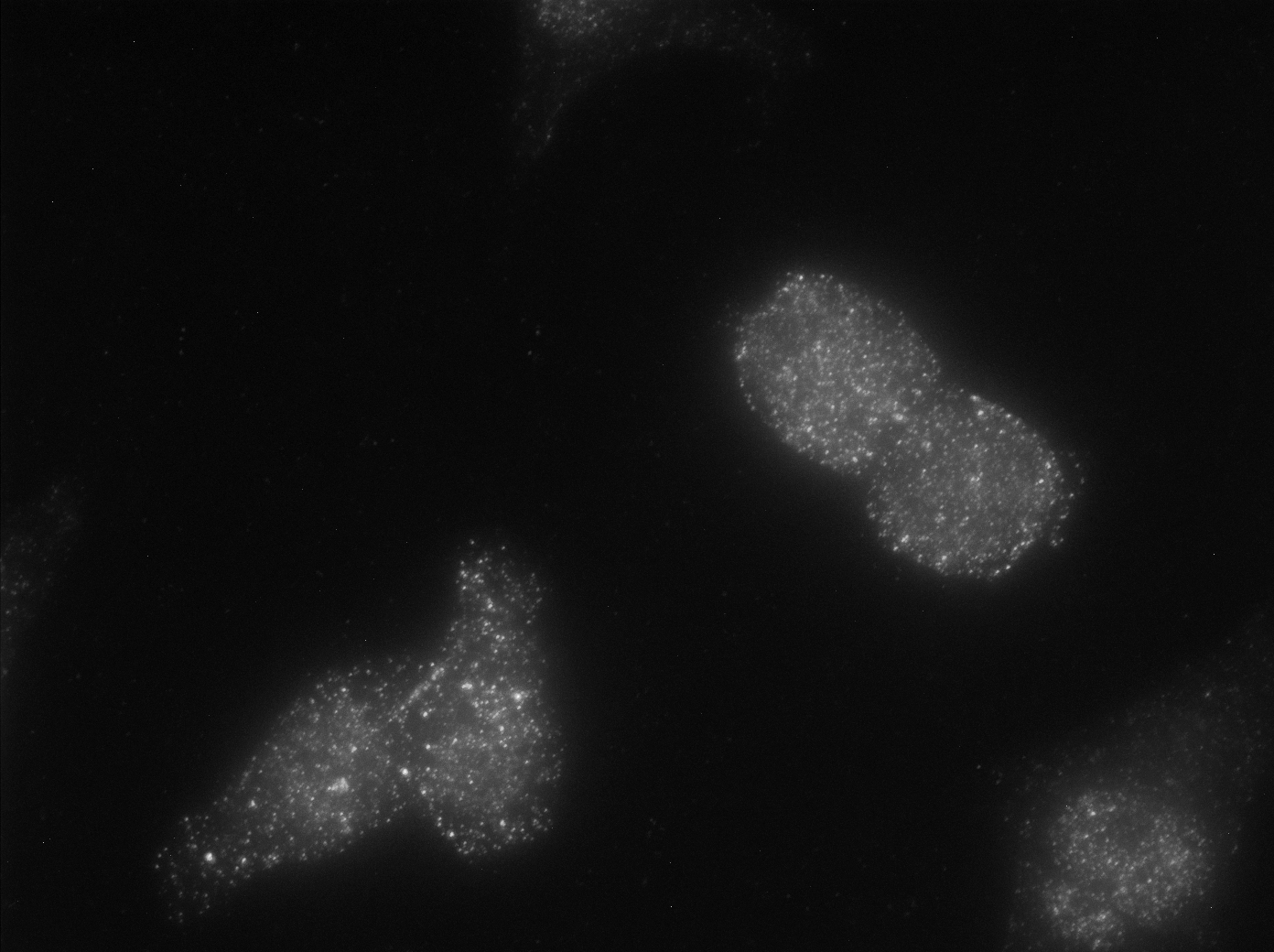

Supplement: Supplementary file 4 — Source data Fig. 2 part 1 [file 44319_2026_742_MOESM4_ESM.zip › Figure 2 Part 1/Fig 2c Cas9 Hela rGT335 atubulin/Cas9 GT335recomb atub 3-24-25 R2 LT1LT2.Project Maximum Z_XY1743439366_Z0_T0_C1.tif]

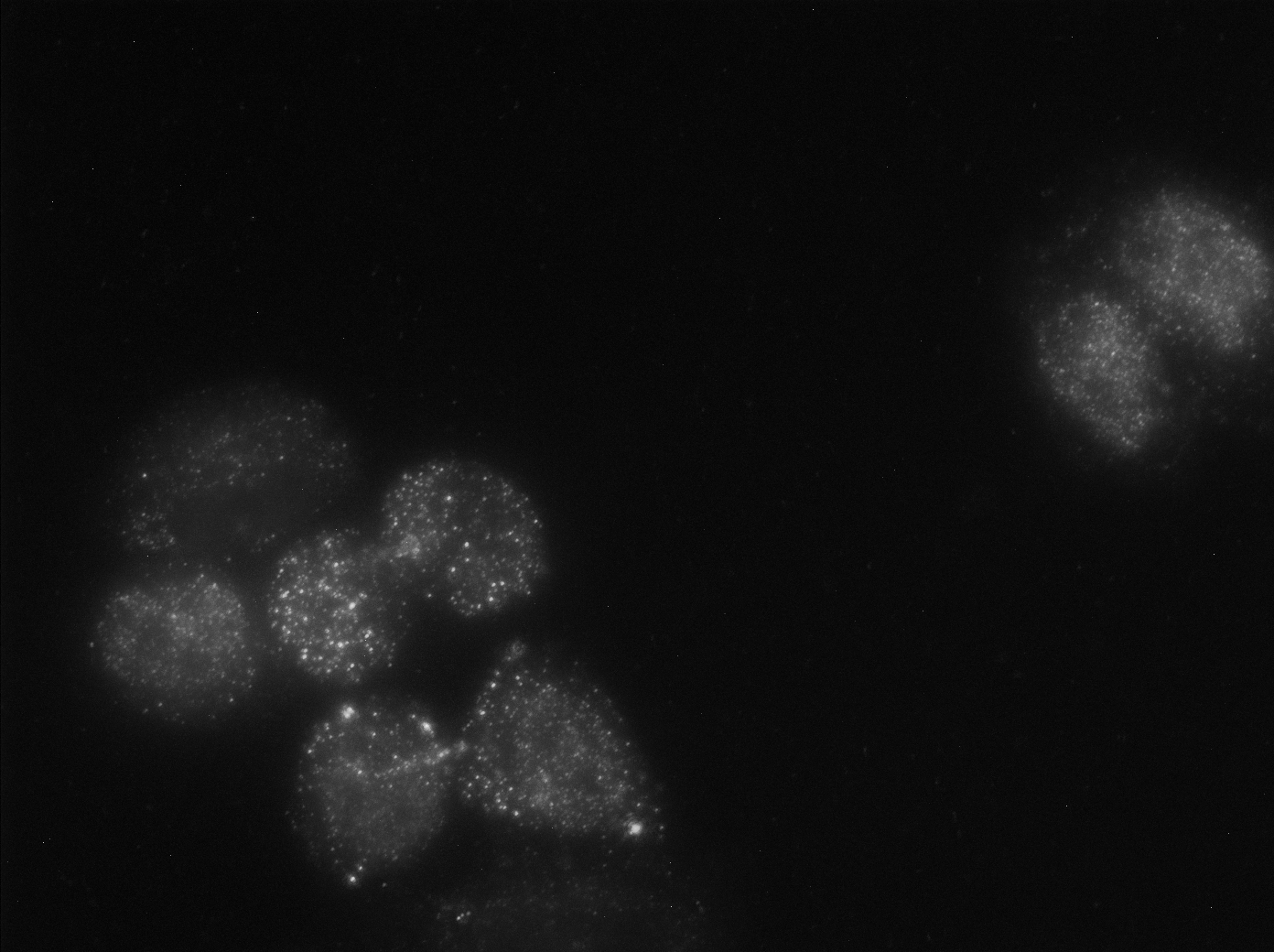

Supplement: Supplementary file 4 — Source data Fig. 2 part 1 [file 44319_2026_742_MOESM4_ESM.zip › Figure 2 Part 1/Fig 2c Cas9 Hela rGT335 atubulin/Cas9 GT335recomb atub 3-24-25 R3 LT1 ET1.Project Maximum Z_XY1743451093_Z0_T0_C1.tif]

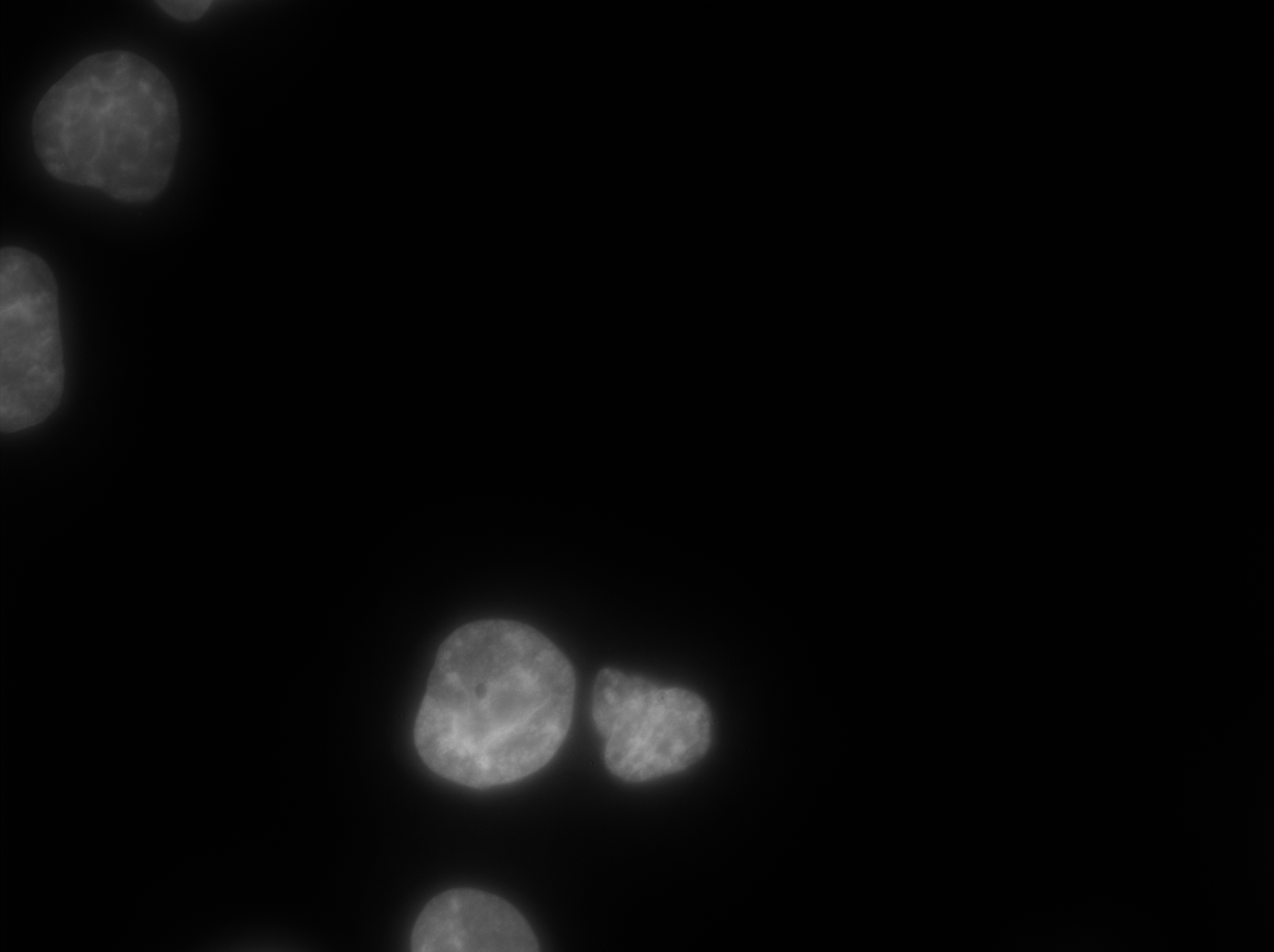

Supplement: Supplementary file 4 — Source data Fig. 2 part 1 [file 44319_2026_742_MOESM4_ESM.zip › Figure 2 Part 1/Fig 2c Cas9 Hela rGT335 atubulin/Cas9 GT335recomb atub 3-24-25 R1 PA8.Project Maximum Z_XY1743103480_Z0_T0_C0.tif]

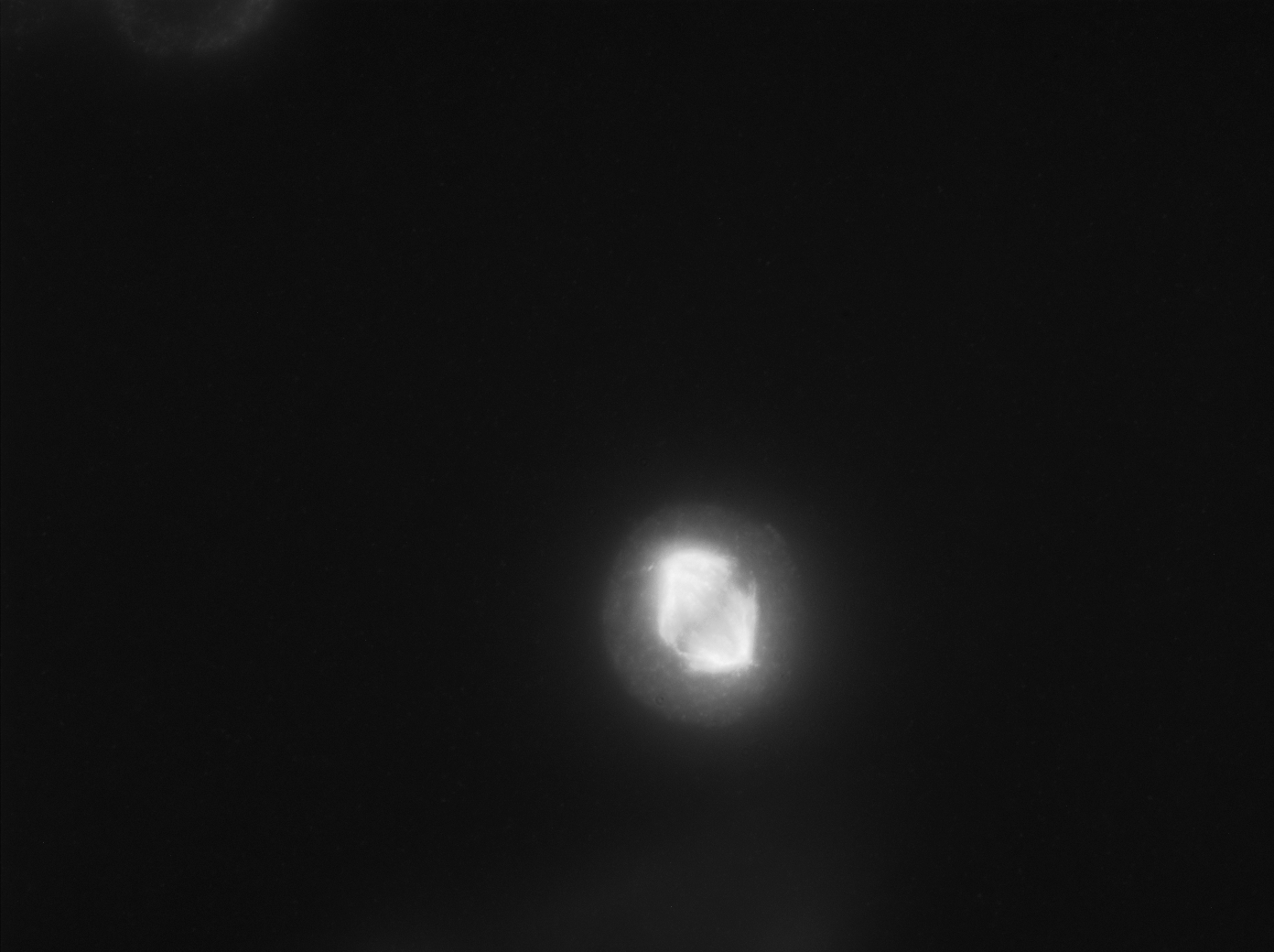

Supplement: Supplementary file 4 — Source data Fig. 2 part 1 [file 44319_2026_742_MOESM4_ESM.zip › Figure 2 Part 1/Fig 2c Cas9 Hela rGT335 atubulin/Cas9 GT335recomb atub 3-24-25 R3 M1.Project Maximum Z_XY1743451455_Z0_T0_C2.tif]

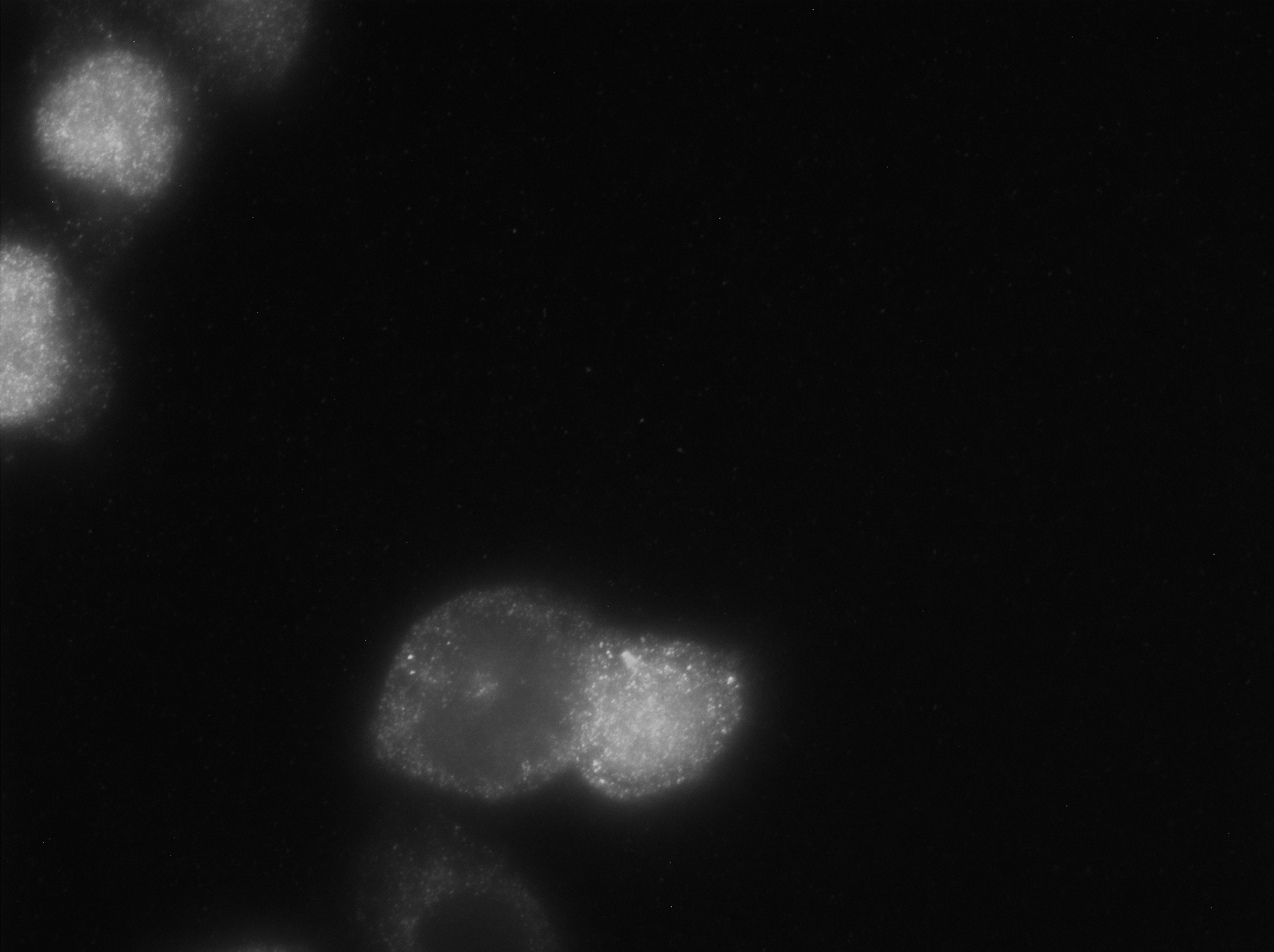

Supplement: Supplementary file 4 — Source data Fig. 2 part 1 [file 44319_2026_742_MOESM4_ESM.zip › Figure 2 Part 1/Fig 2c Cas9 Hela rGT335 atubulin/Cas9 GT335recomb atub 3-24-25 R1 PA8.Project Maximum Z_XY1743103480_Z0_T0_C1.tif]

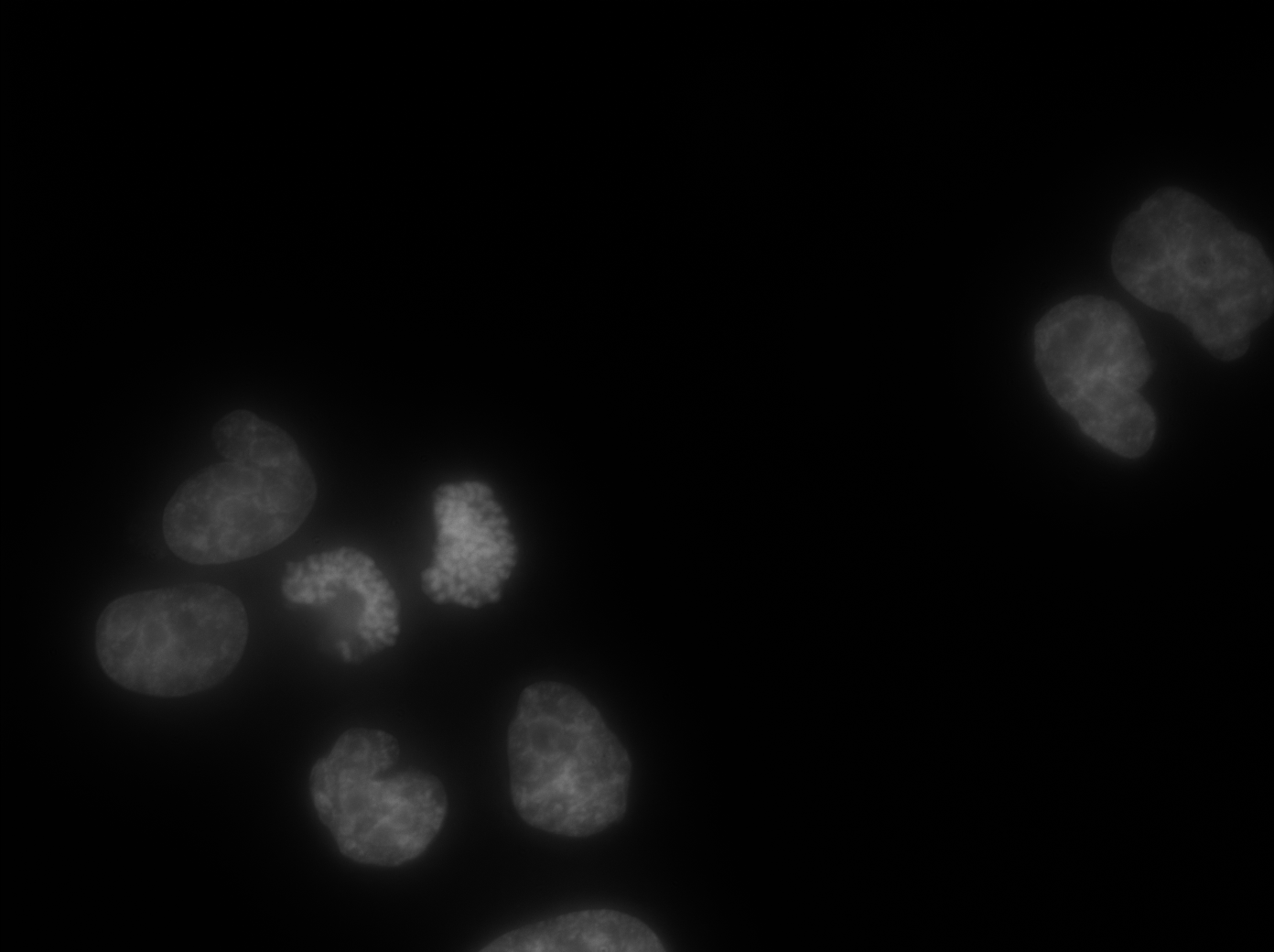

Supplement: Supplementary file 4 — Source data Fig. 2 part 1 [file 44319_2026_742_MOESM4_ESM.zip › Figure 2 Part 1/Fig 2c Cas9 Hela rGT335 atubulin/Cas9 GT335recomb atub 3-24-25 R3 LT1 ET1.Project Maximum Z_XY1743451093_Z0_T0_C0.tif]

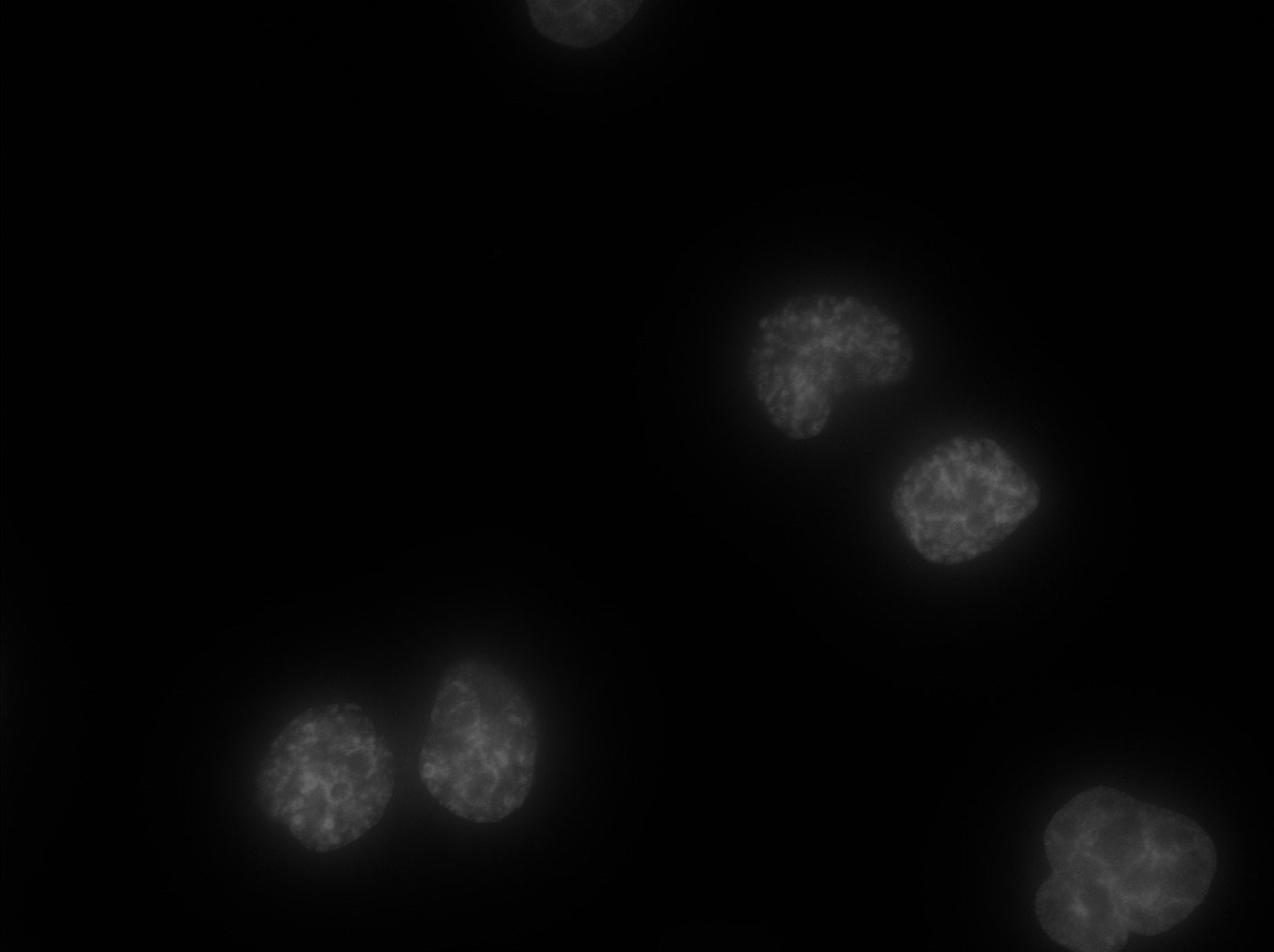

Supplement: Supplementary file 4 — Source data Fig. 2 part 1 [file 44319_2026_742_MOESM4_ESM.zip › Figure 2 Part 1/Fig 2c Cas9 Hela rGT335 atubulin/Cas9 GT335recomb atub 3-24-25 R2 LT1LT2.Project Maximum Z_XY1743439366_Z0_T0_C0.tif]

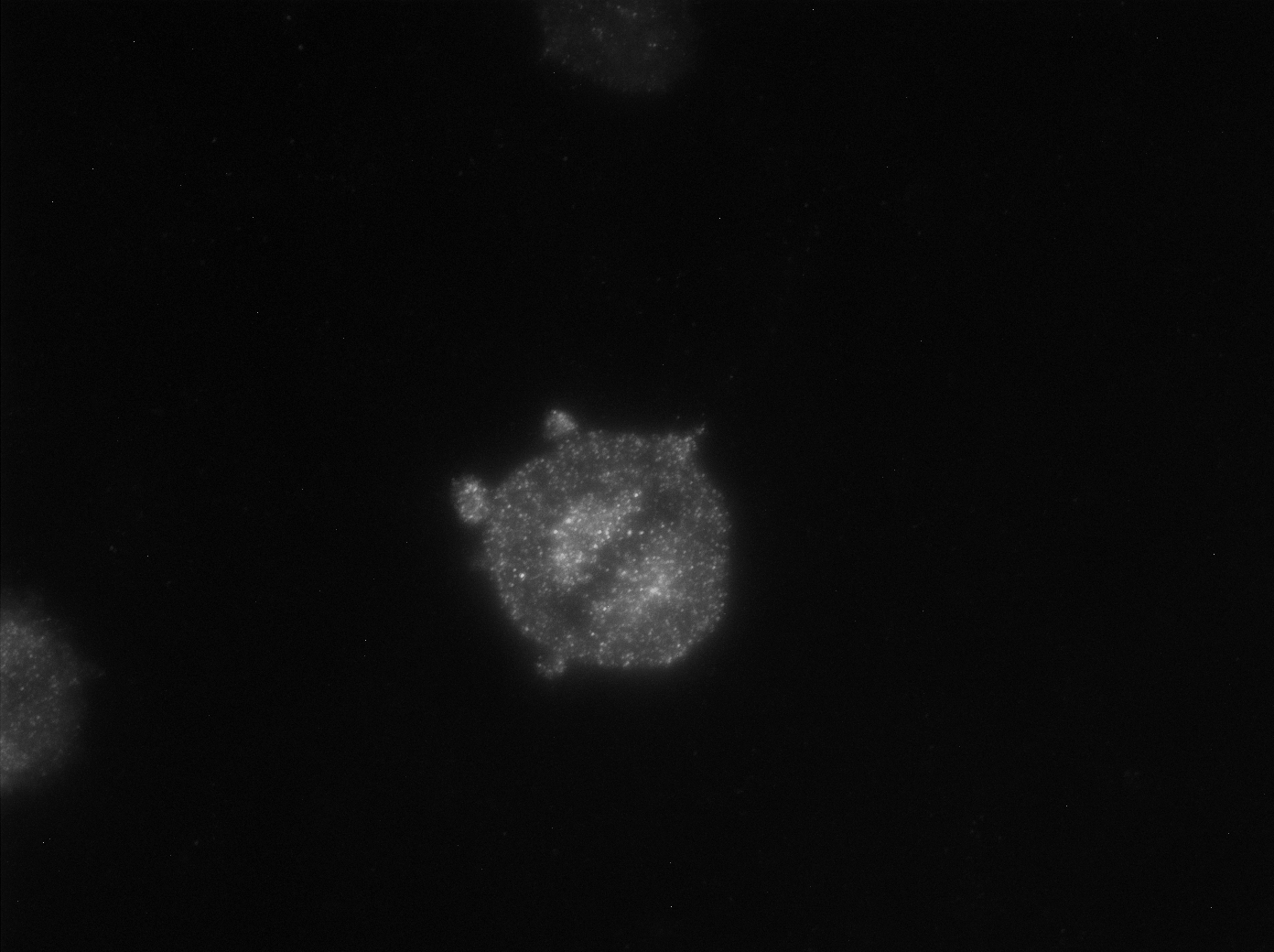

Supplement: Supplementary file 4 — Source data Fig. 2 part 1 [file 44319_2026_742_MOESM4_ESM.zip › Figure 2 Part 1/Fig 2c Cas9 Hela rGT335 atubulin/Cas9 GT335recomb atub 3-24-25 R2 M1.Project Maximum Z_XY1743440680_Z0_T0_C1.tif]

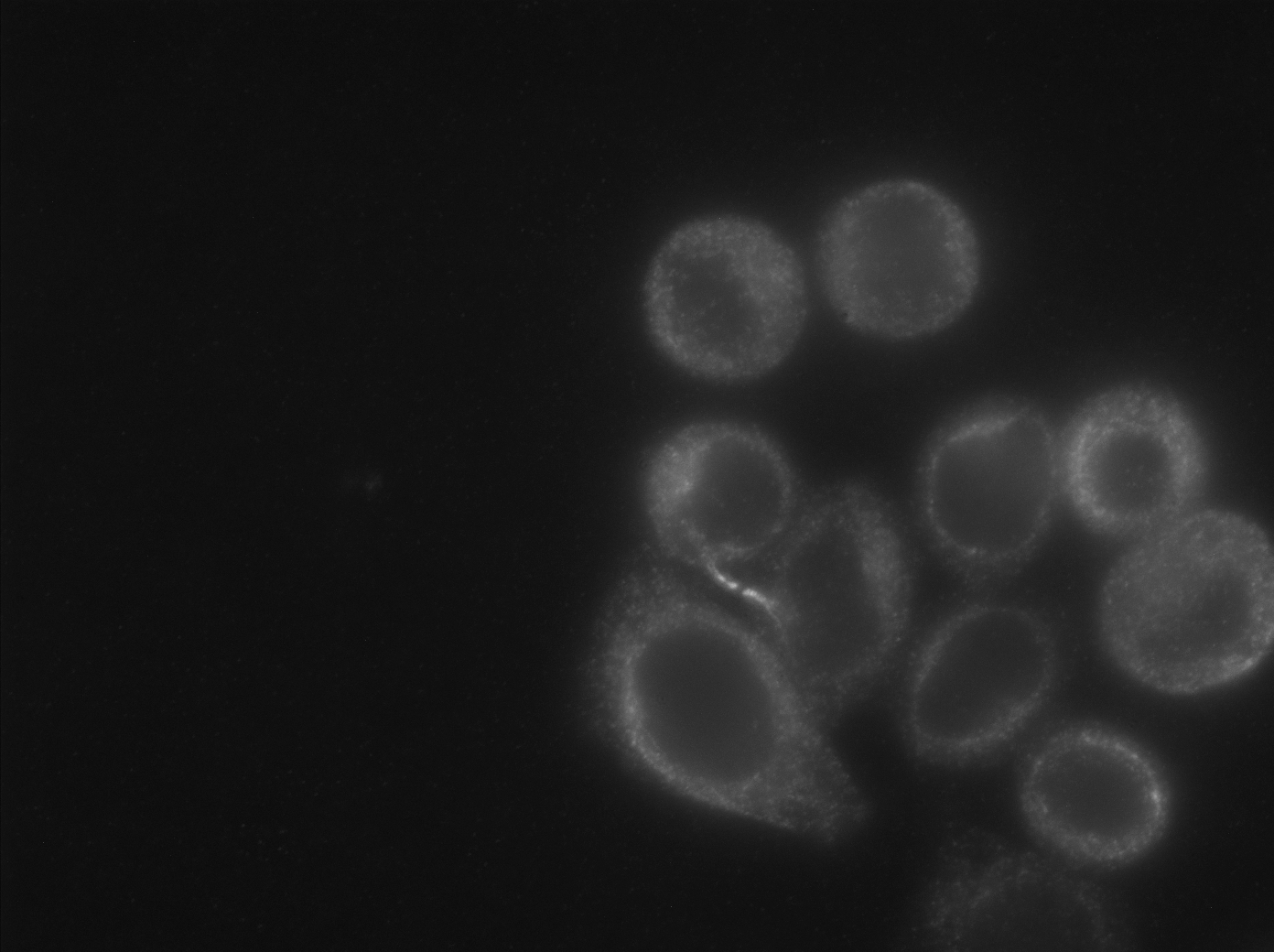

Supplement: Supplementary file 4 — Source data Fig. 2 part 1 [file 44319_2026_742_MOESM4_ESM.zip › Figure 2 Part 1/Fig 2c Cas9 Hela rGT335 atubulin/Cas9 GT335recomb atub 3-24-25 R1 LT6.Project Maximum Z_XY1743101300_Z0_T0_C2.tif]

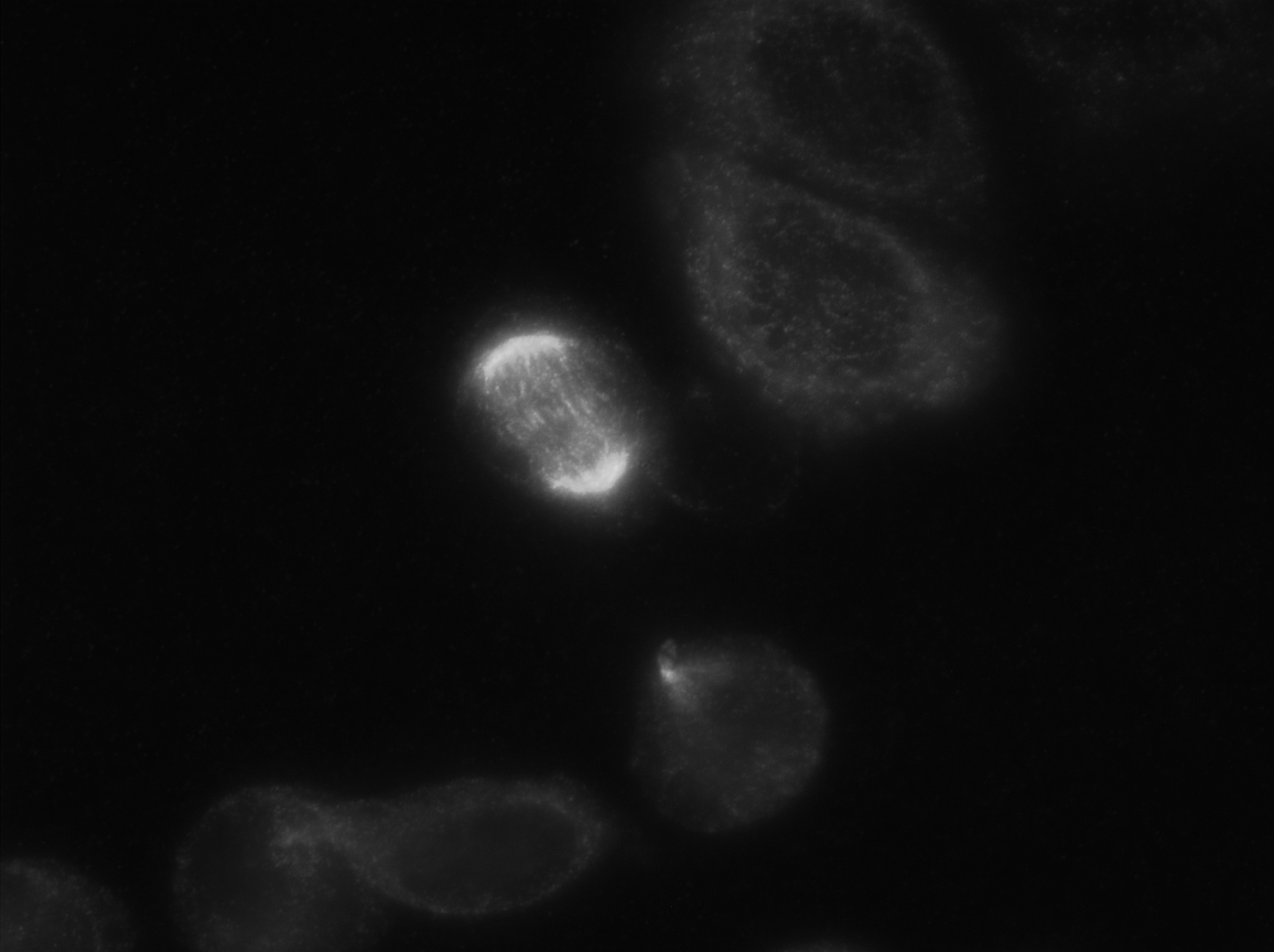

Supplement: Supplementary file 4 — Source data Fig. 2 part 1 [file 44319_2026_742_MOESM4_ESM.zip › Figure 2 Part 1/Fig 2c Cas9 Hela rGT335 atubulin/Cas9 GT335recomb atub 3-24-25 R2 PA2 A2.Project Maximum Z_XY1743440822_Z0_T0_C2.tif]

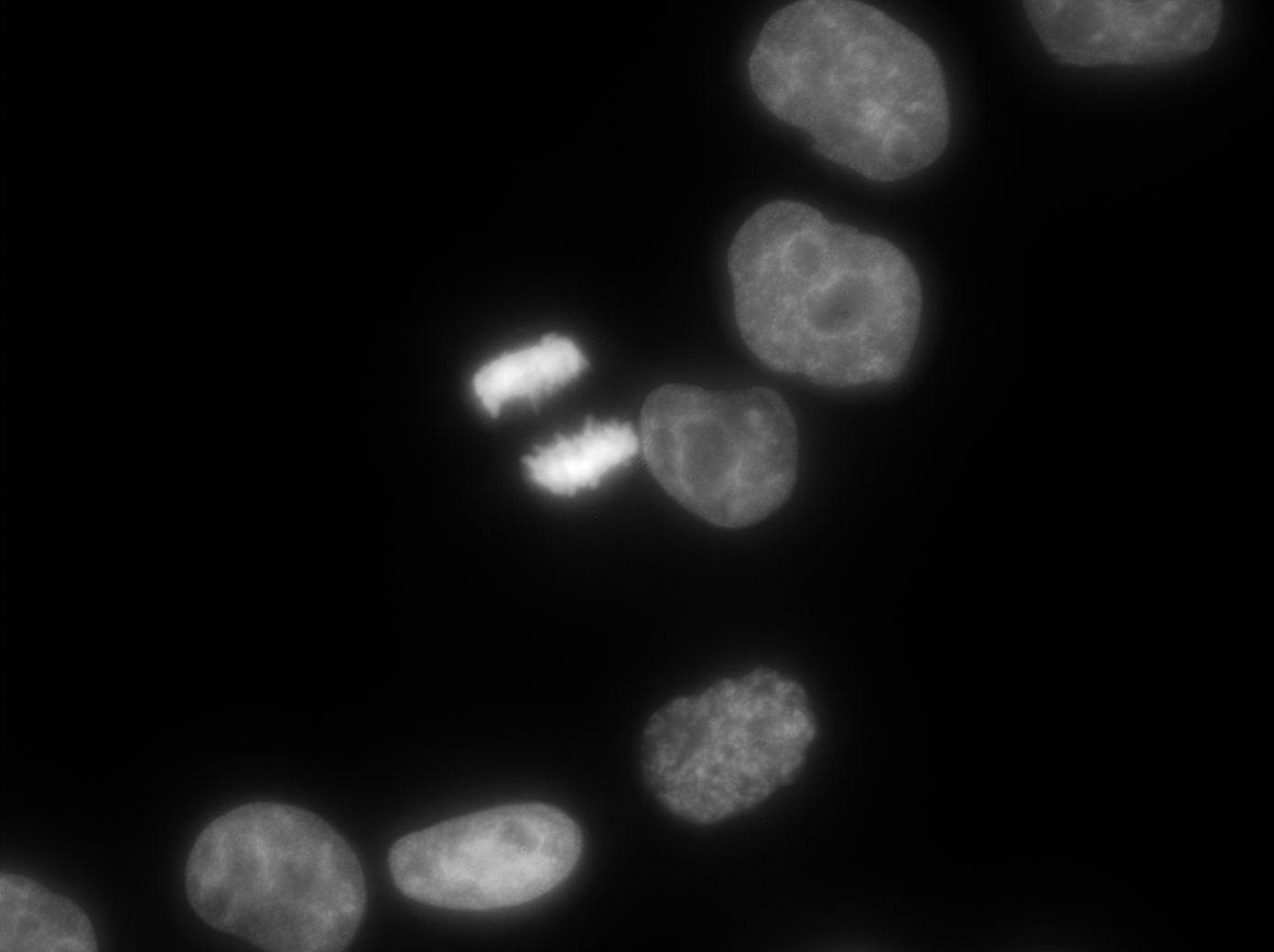

Supplement: Supplementary file 4 — Source data Fig. 2 part 1 [file 44319_2026_742_MOESM4_ESM.zip › Figure 2 Part 1/Fig 2c Cas9 Hela rGT335 atubulin/Cas9 GT335recomb atub 3-24-25 R2 PA2 A2.Project Maximum Z_XY1743440822_Z0_T0_C0.tif]

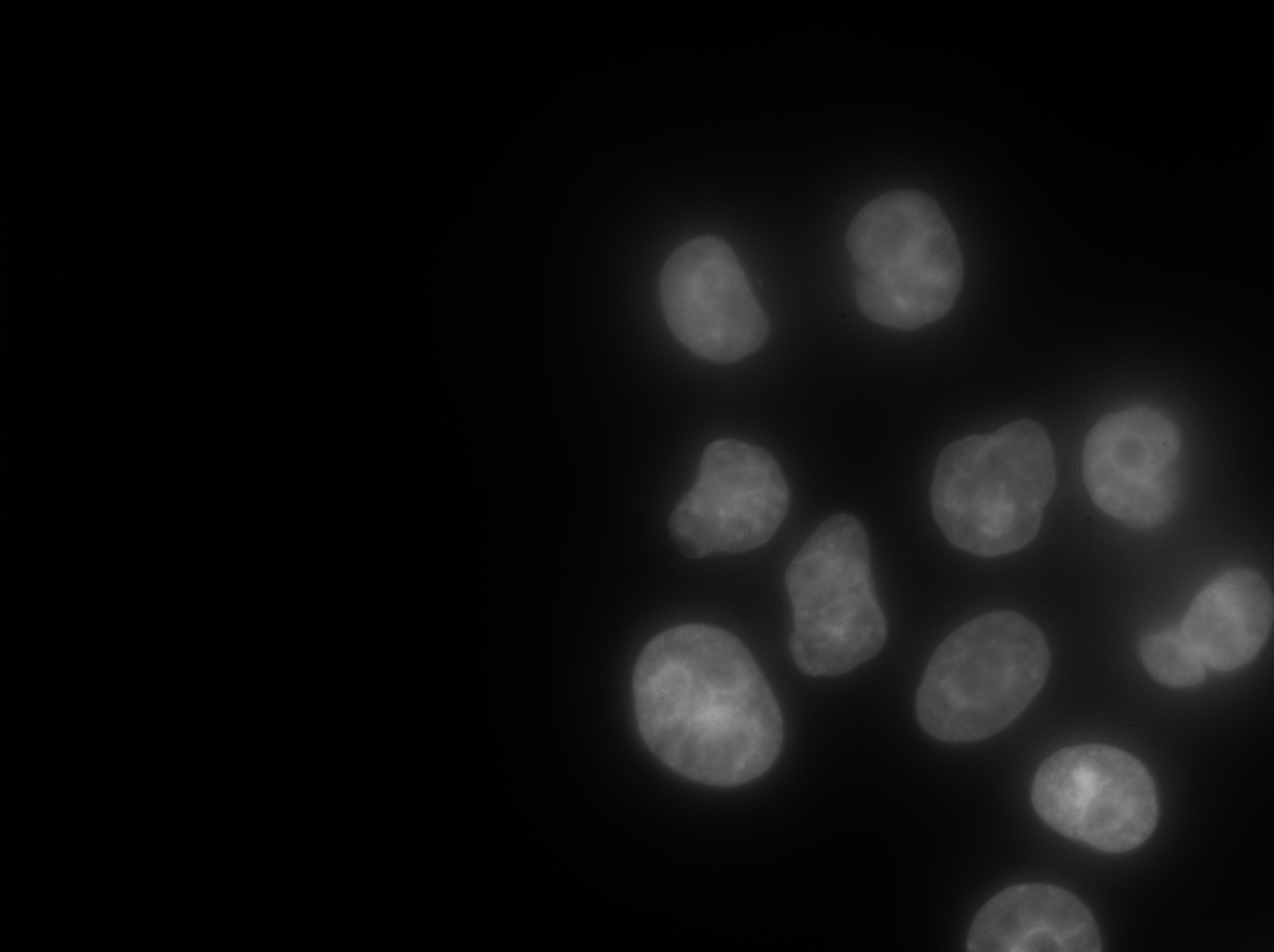

Supplement: Supplementary file 4 — Source data Fig. 2 part 1 [file 44319_2026_742_MOESM4_ESM.zip › Figure 2 Part 1/Fig 2c Cas9 Hela rGT335 atubulin/Cas9 GT335recomb atub 3-24-25 R1 LT6.Project Maximum Z_XY1743101300_Z0_T0_C0.tif]

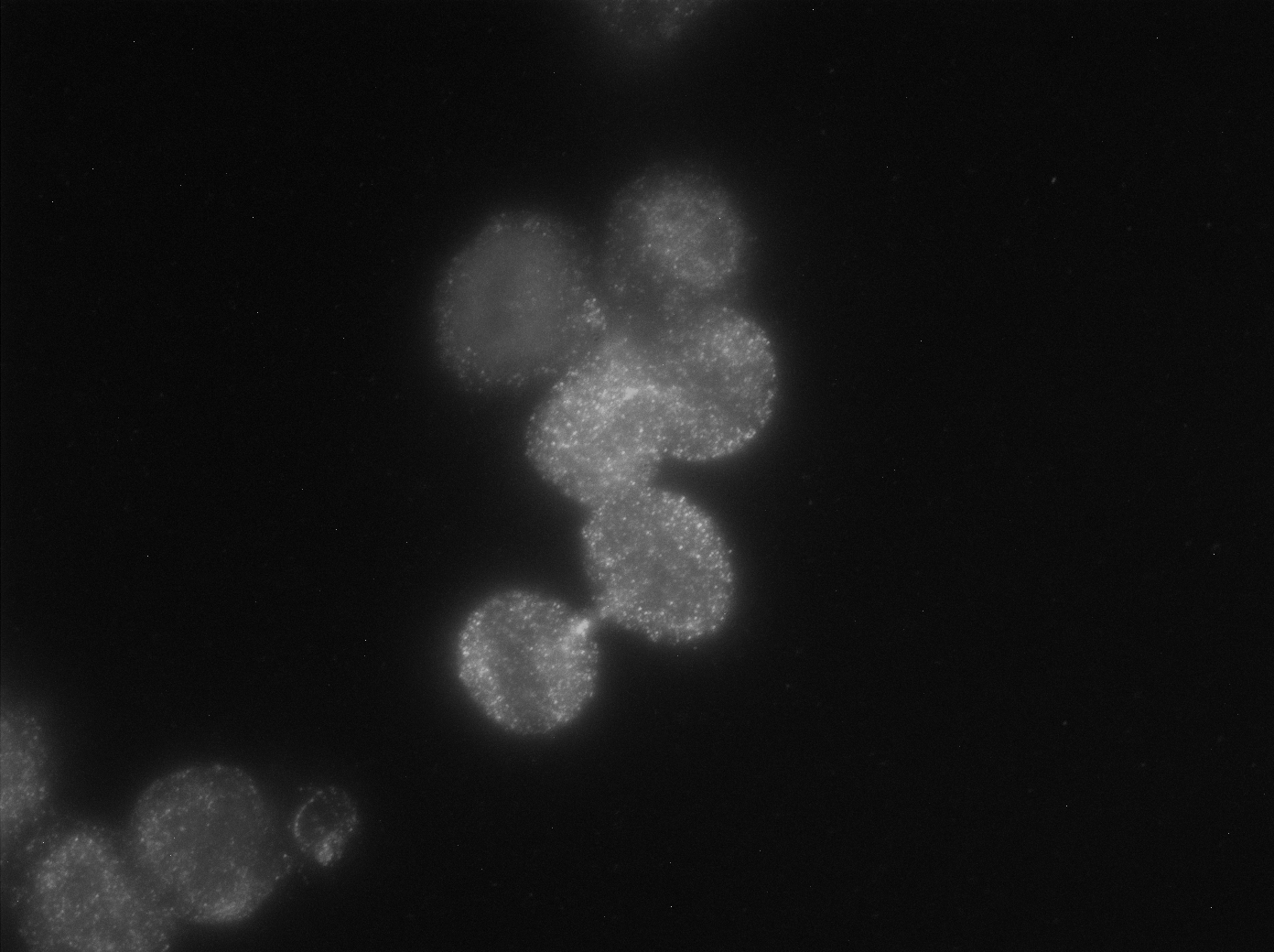

Supplement: Supplementary file 4 — Source data Fig. 2 part 1 [file 44319_2026_742_MOESM4_ESM.zip › Figure 2 Part 1/Fig 2c Cas9 Hela rGT335 atubulin/Cas9 GT335recomb atub 3-24-25 R3 ET9ET10.Project Maximum Z_XY1743455471_Z0_T0_C1.tif]

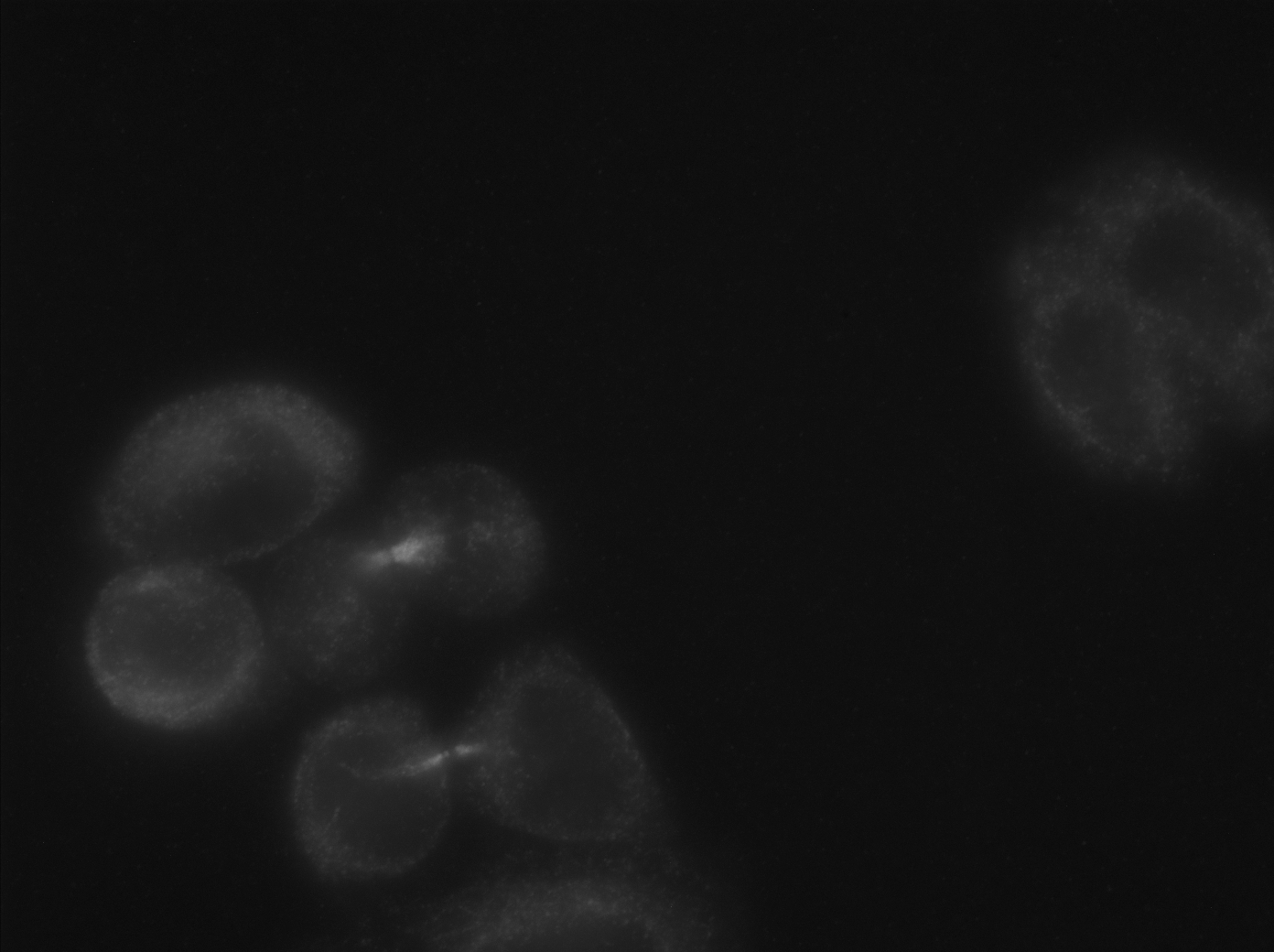

Supplement: Supplementary file 4 — Source data Fig. 2 part 1 [file 44319_2026_742_MOESM4_ESM.zip › Figure 2 Part 1/Fig 2c Cas9 Hela rGT335 atubulin/Cas9 GT335recomb atub 3-24-25 R3 LT1 ET1.Project Maximum Z_XY1743451093_Z0_T0_C2.tif]

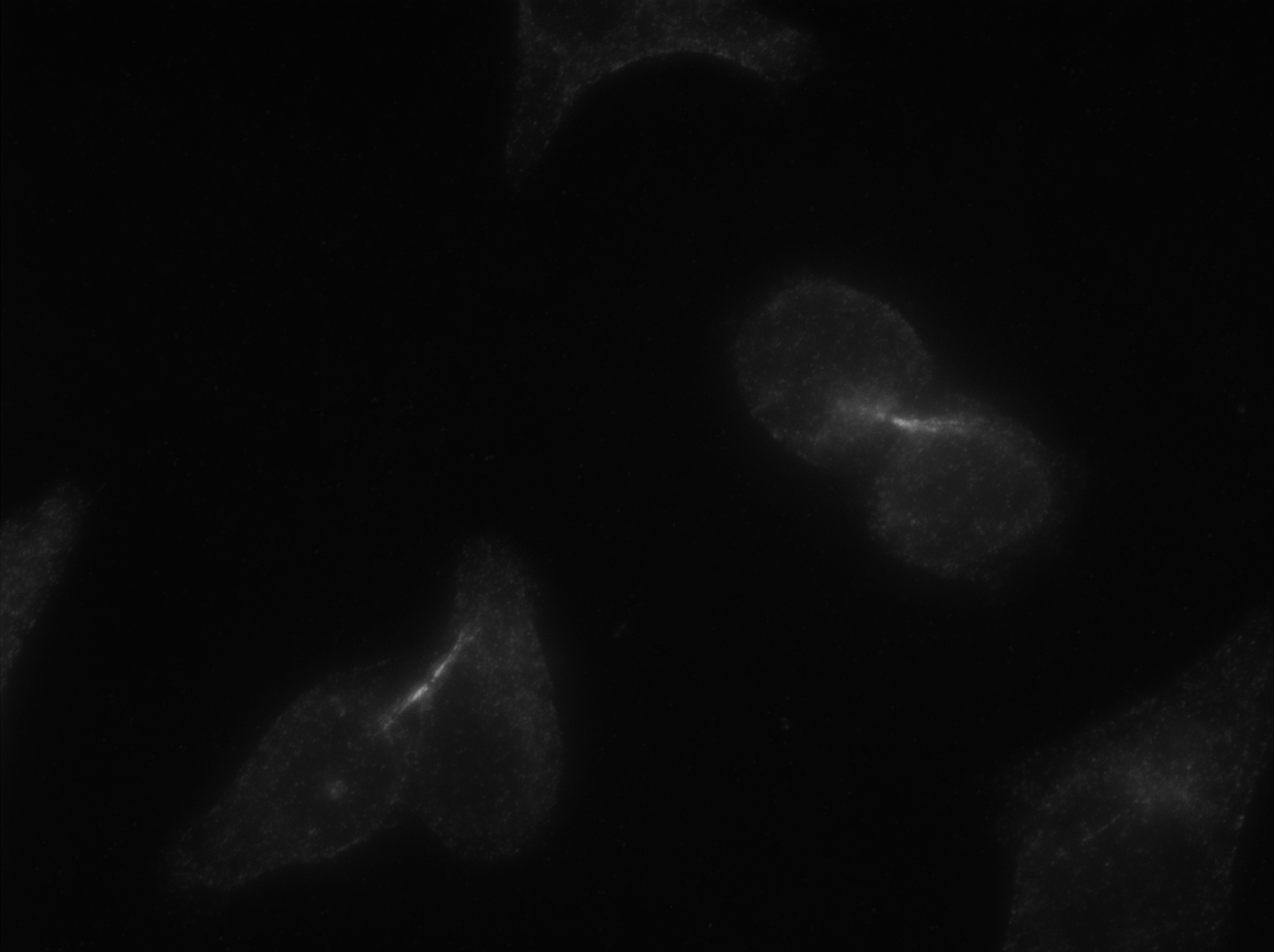

Supplement: Supplementary file 4 — Source data Fig. 2 part 1 [file 44319_2026_742_MOESM4_ESM.zip › Figure 2 Part 1/Fig 2c Cas9 Hela rGT335 atubulin/Cas9 GT335recomb atub 3-24-25 R2 LT1LT2.Project Maximum Z_XY1743439366_Z0_T0_C2.tif]

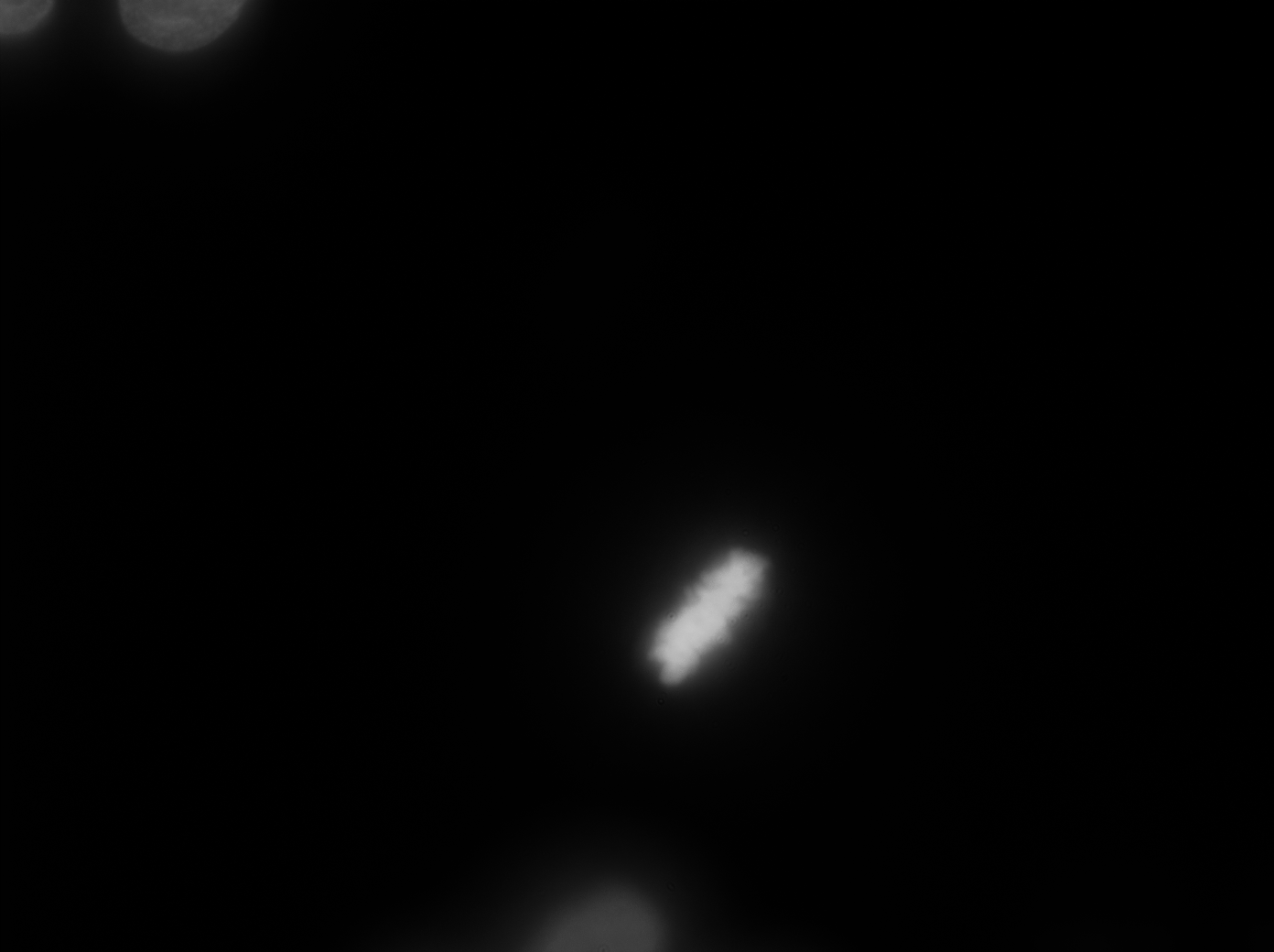

Supplement: Supplementary file 4 — Source data Fig. 2 part 1 [file 44319_2026_742_MOESM4_ESM.zip › Figure 2 Part 1/Fig 2c Cas9 Hela rGT335 atubulin/Cas9 GT335recomb atub 3-24-25 R3 M1.Project Maximum Z_XY1743451455_Z0_T0_C0.tif]

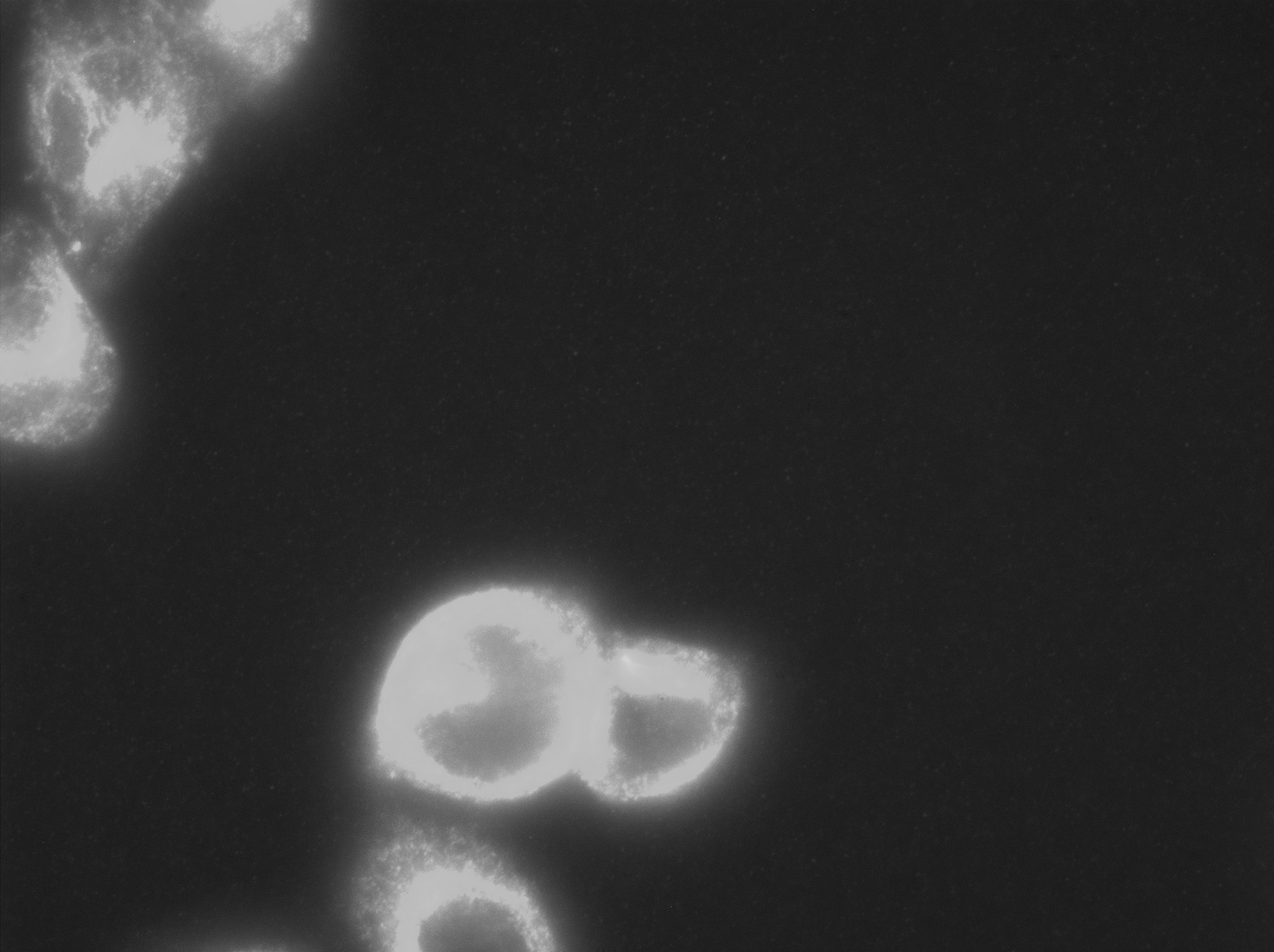

Supplement: Supplementary file 4 — Source data Fig. 2 part 1 [file 44319_2026_742_MOESM4_ESM.zip › Figure 2 Part 1/Fig 2c Cas9 Hela rGT335 atubulin/Cas9 GT335recomb atub 3-24-25 R1 PA8.Project Maximum Z_XY1743103480_Z0_T0_C2.tif]

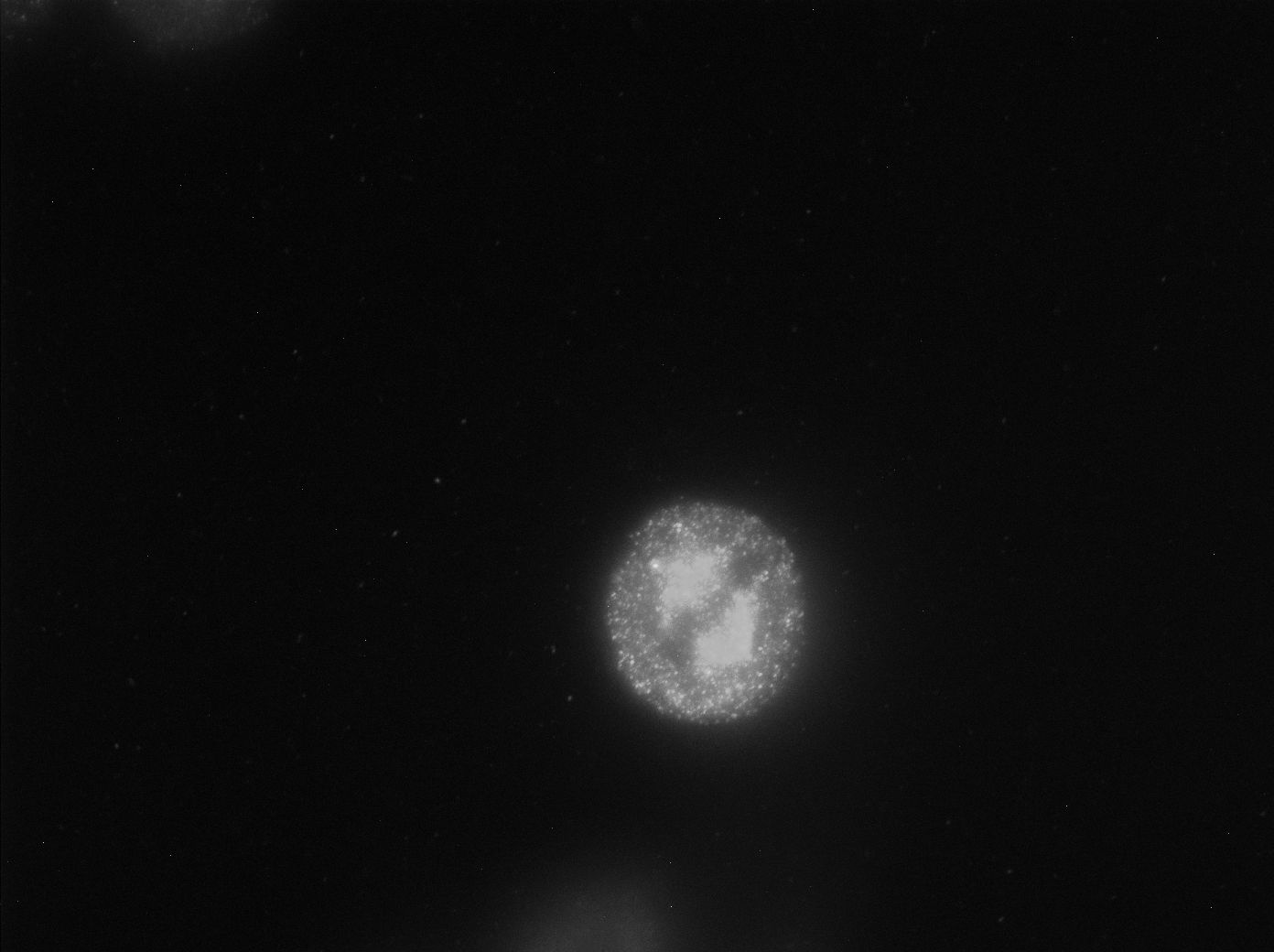

Supplement: Supplementary file 4 — Source data Fig. 2 part 1 [file 44319_2026_742_MOESM4_ESM.zip › Figure 2 Part 1/Fig 2c Cas9 Hela rGT335 atubulin/Cas9 GT335recomb atub 3-24-25 R3 M1.Project Maximum Z_XY1743451455_Z0_T0_C1.tif]

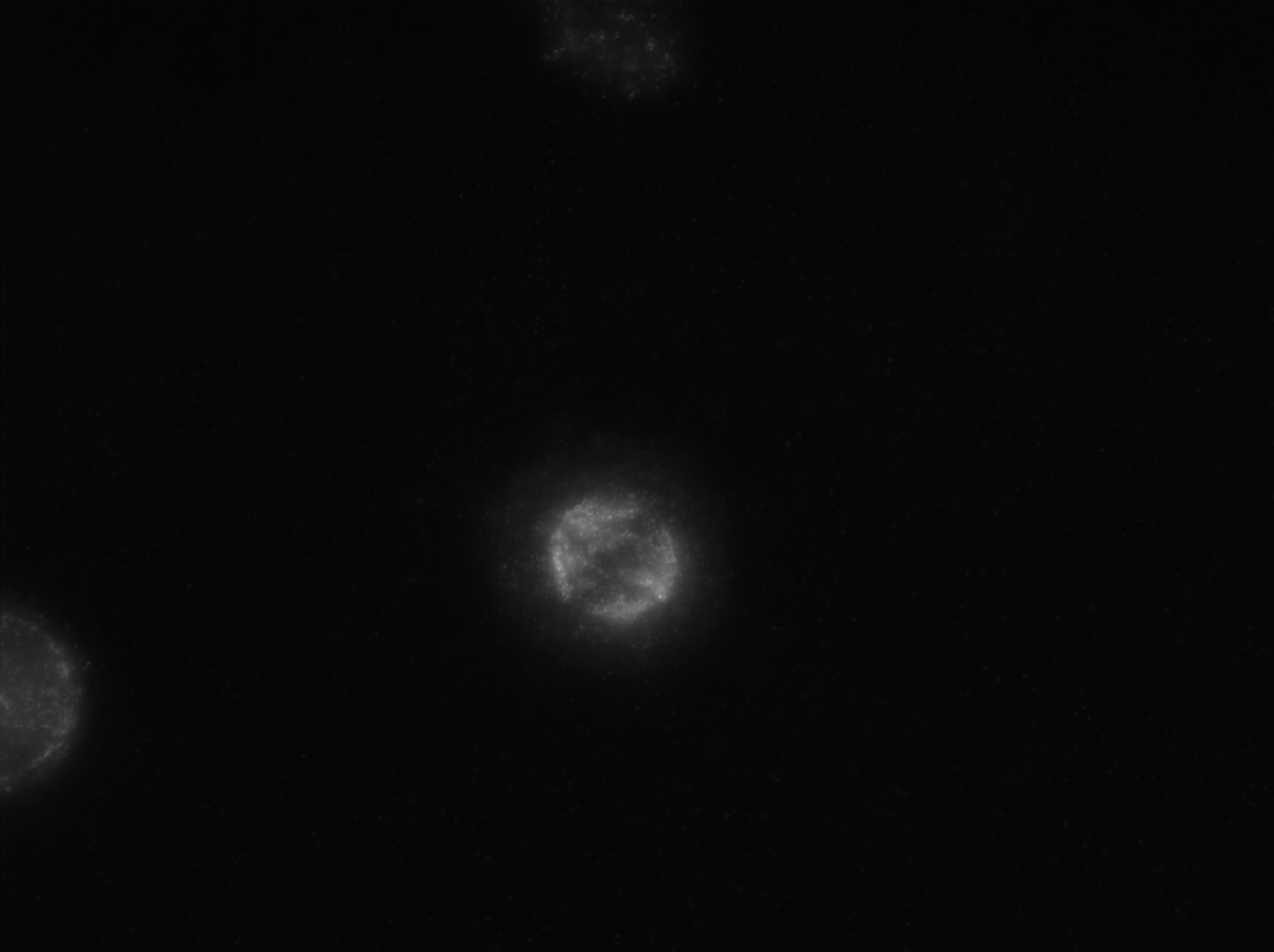

Supplement: Supplementary file 4 — Source data Fig. 2 part 1 [file 44319_2026_742_MOESM4_ESM.zip › Figure 2 Part 1/Fig 2c Cas9 Hela rGT335 atubulin/Cas9 GT335recomb atub 3-24-25 R2 M1.Project Maximum Z_XY1743440680_Z0_T0_C2.tif]

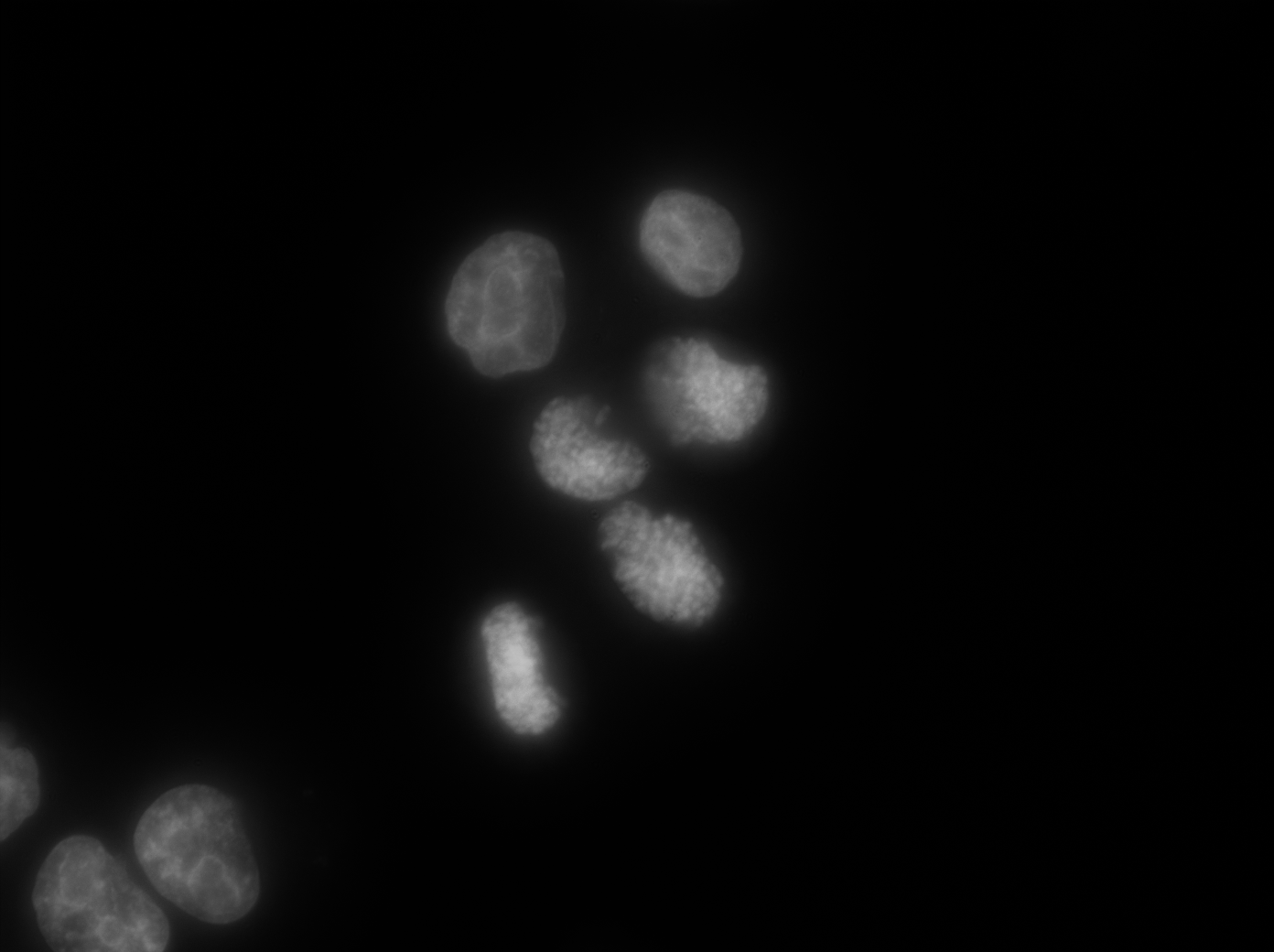

Supplement: Supplementary file 4 — Source data Fig. 2 part 1 [file 44319_2026_742_MOESM4_ESM.zip › Figure 2 Part 1/Fig 2c Cas9 Hela rGT335 atubulin/Cas9 GT335recomb atub 3-24-25 R3 ET9ET10.Project Maximum Z_XY1743455471_Z0_T0_C0.tif]

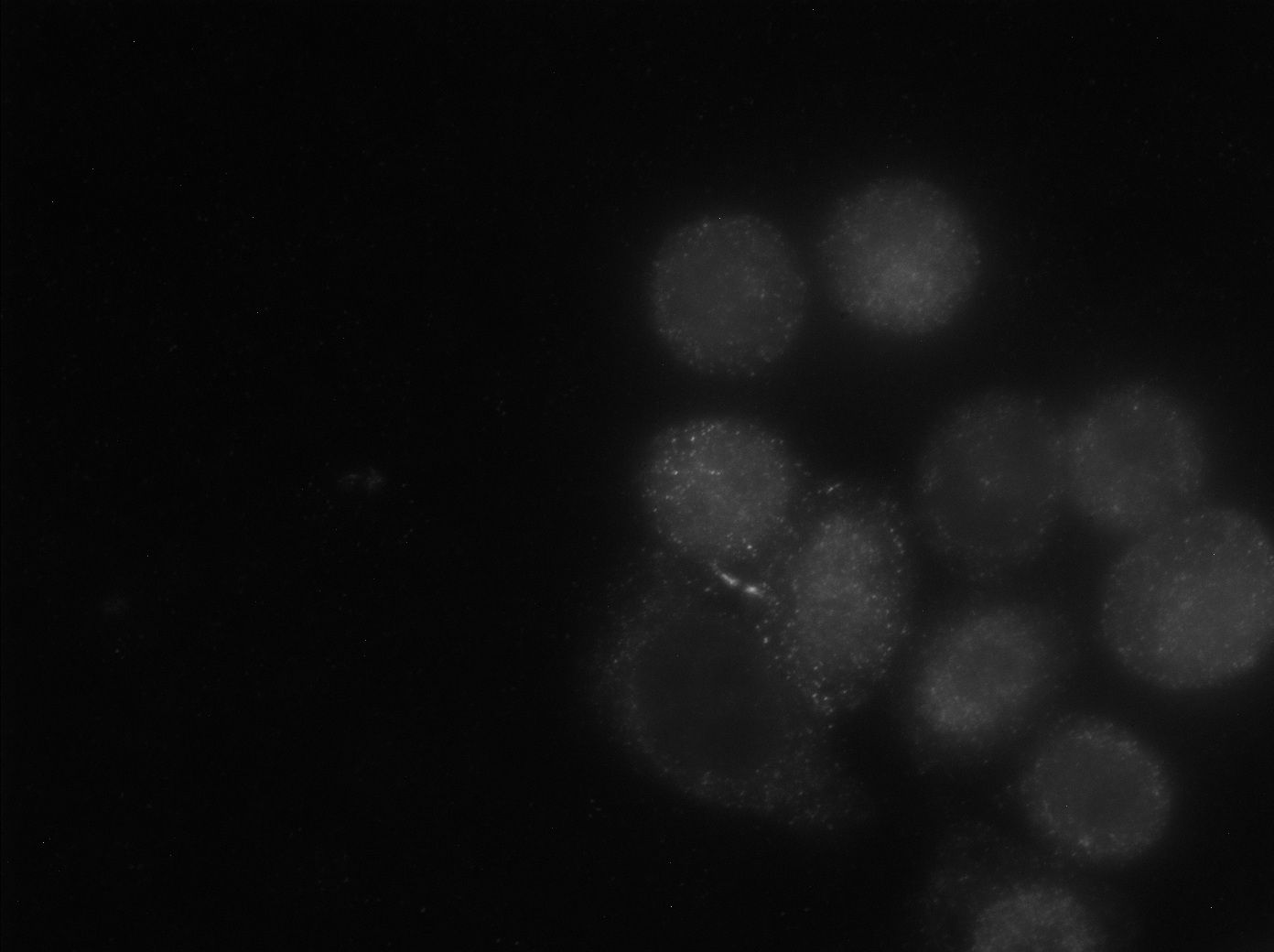

Supplement: Supplementary file 4 — Source data Fig. 2 part 1 [file 44319_2026_742_MOESM4_ESM.zip › Figure 2 Part 1/Fig 2c Cas9 Hela rGT335 atubulin/Cas9 GT335recomb atub 3-24-25 R1 LT6.Project Maximum Z_XY1743101300_Z0_T0_C1.tif]

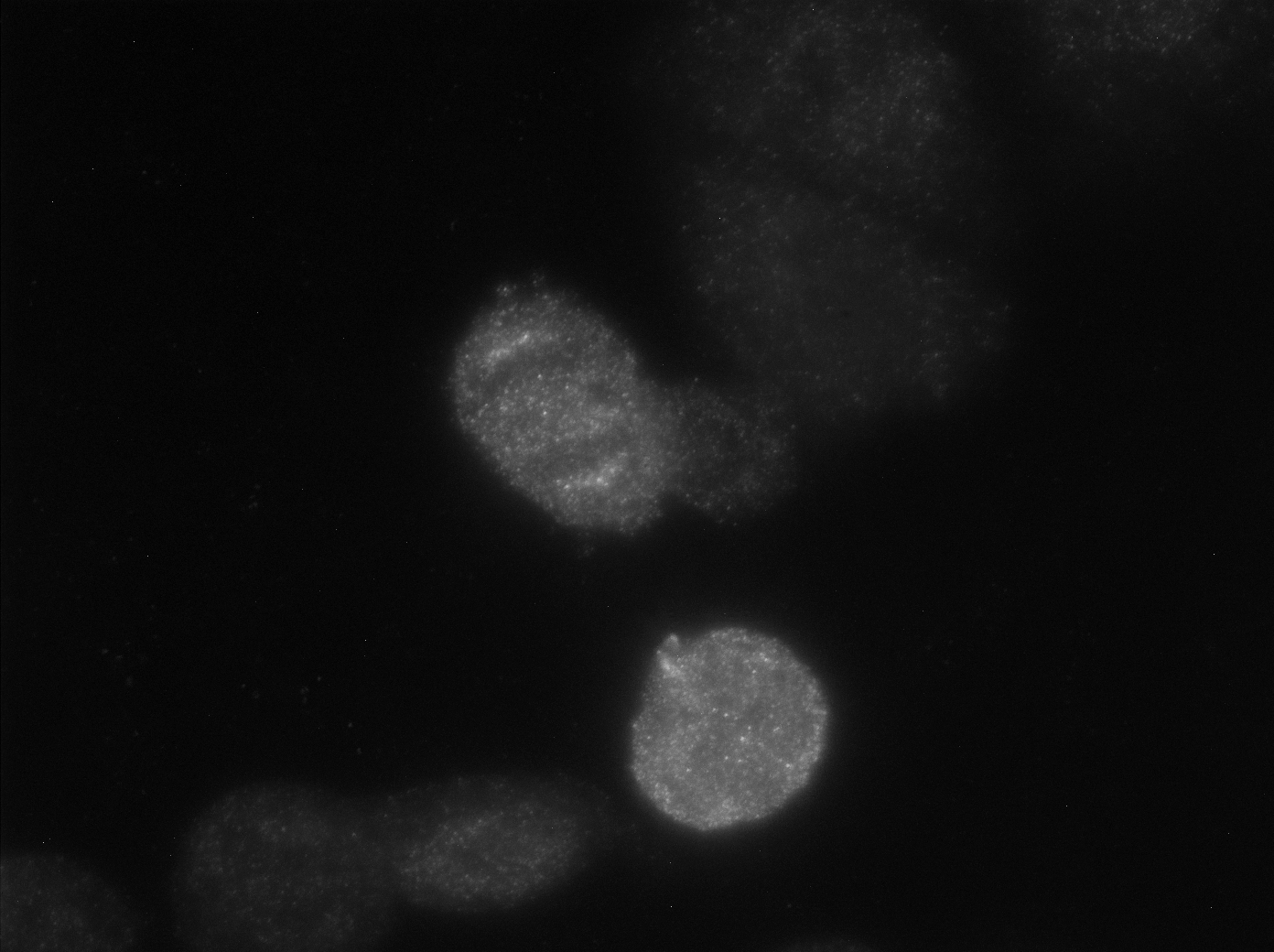

Supplement: Supplementary file 4 — Source data Fig. 2 part 1 [file 44319_2026_742_MOESM4_ESM.zip › Figure 2 Part 1/Fig 2c Cas9 Hela rGT335 atubulin/Cas9 GT335recomb atub 3-24-25 R2 PA2 A2.Project Maximum Z_XY1743440822_Z0_T0_C1.tif]

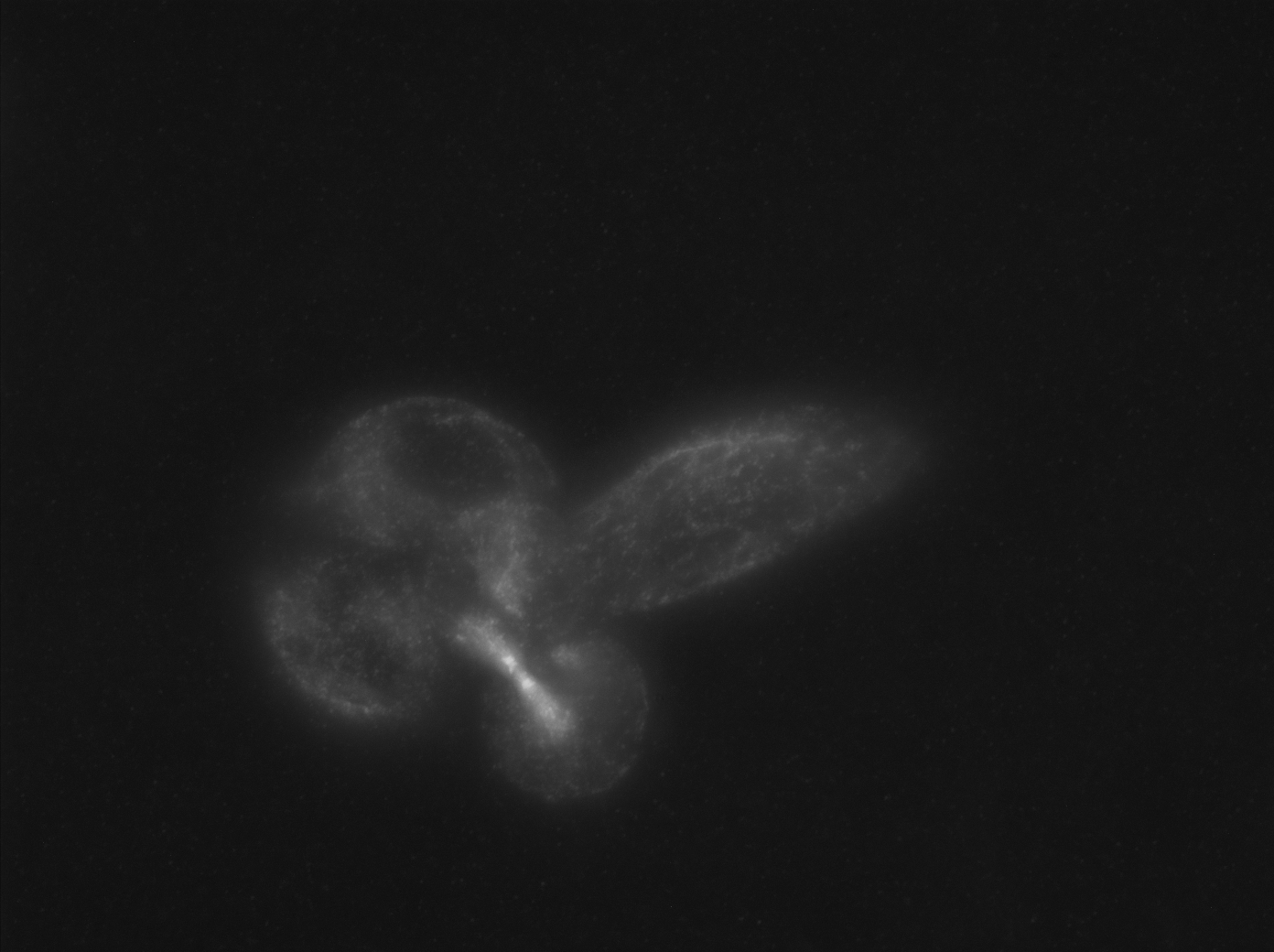

Supplement: Supplementary file 4 — Source data Fig. 2 part 1 [file 44319_2026_742_MOESM4_ESM.zip › Figure 2 Part 1/Fig 2c Cas9 Hela rGT335 atubulin/Cas9 GT335recomb atub 3-24-25 R3 ET3.Project Maximum Z_XY1743452941_Z0_T0_C2.tif]

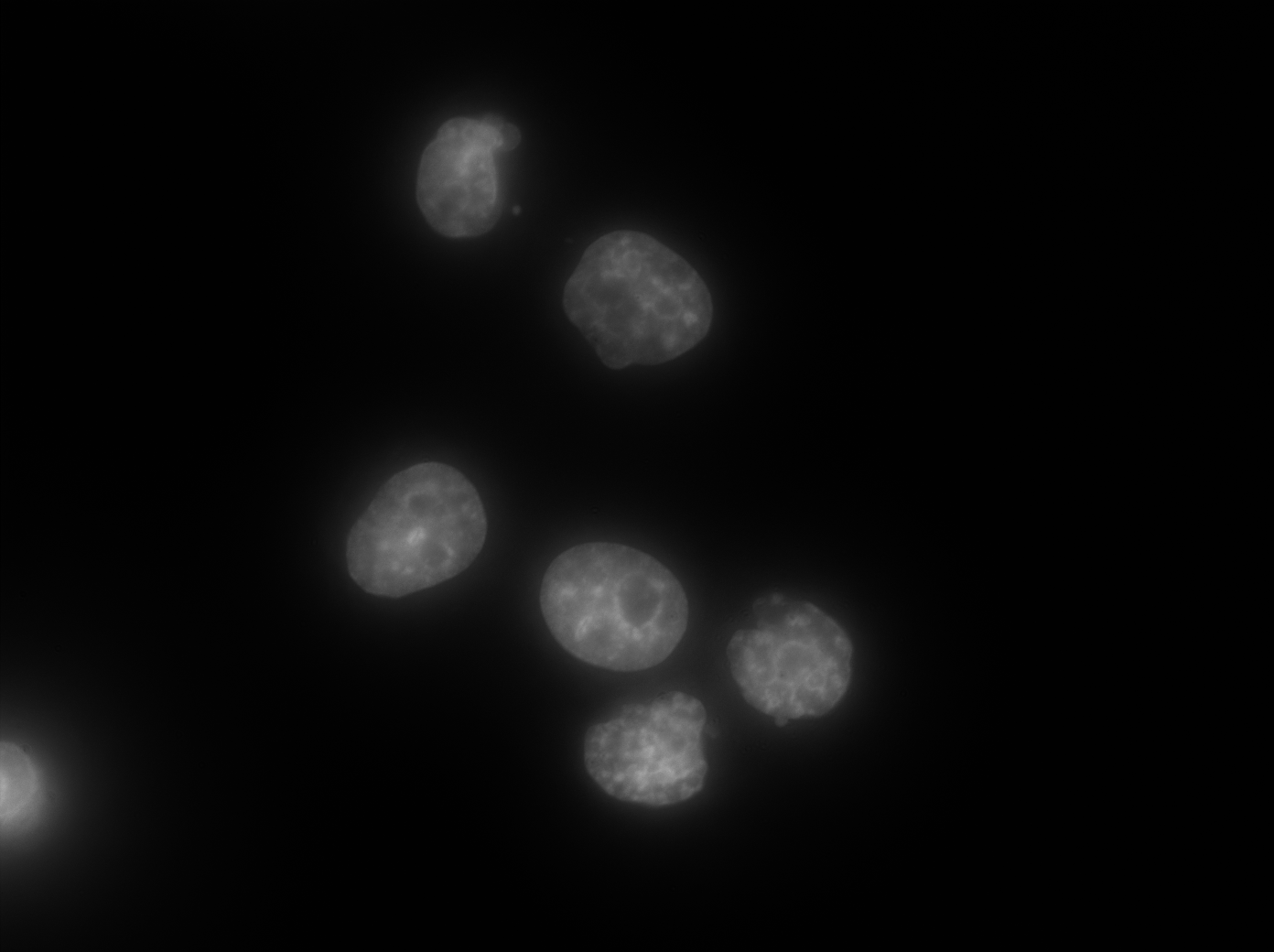

Supplement: Supplementary file 4 — Source data Fig. 2 part 1 [file 44319_2026_742_MOESM4_ESM.zip › Figure 2 Part 1/Fig 2c Cas9 Hela rGT335 atubulin/Cas9 GT335recomb atub 3-24-25 R1 LT9.Project Maximum Z_XY1743101906_Z0_T0_C0.tif]

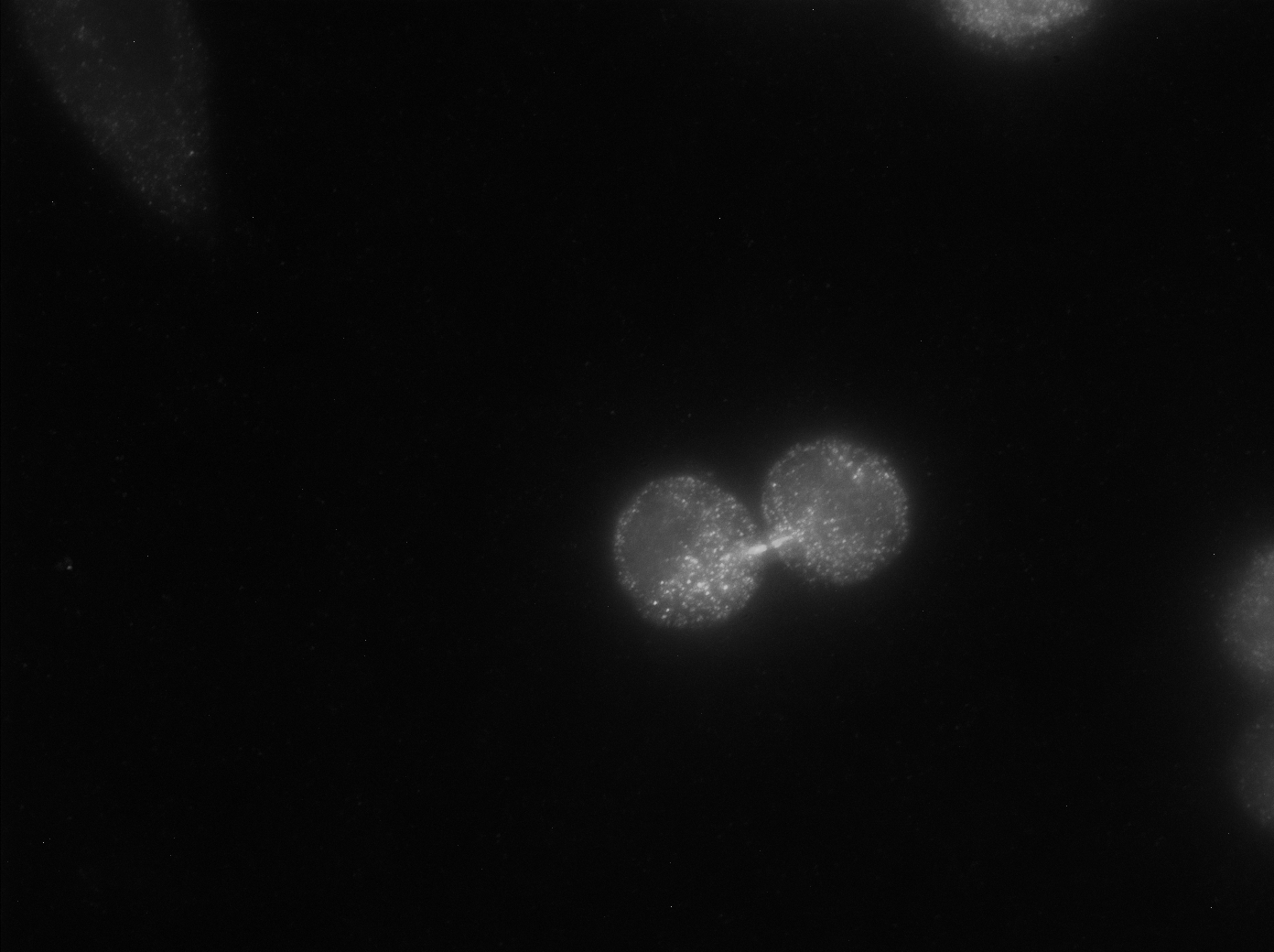

Supplement: Supplementary file 4 — Source data Fig. 2 part 1 [file 44319_2026_742_MOESM4_ESM.zip › Figure 2 Part 1/Fig 2c Cas9 Hela rGT335 atubulin/Cas9 GT335recomb atub 3-24-25 R1 ET3.Project Maximum Z_XY1743101375_Z0_T0_C1.tif]

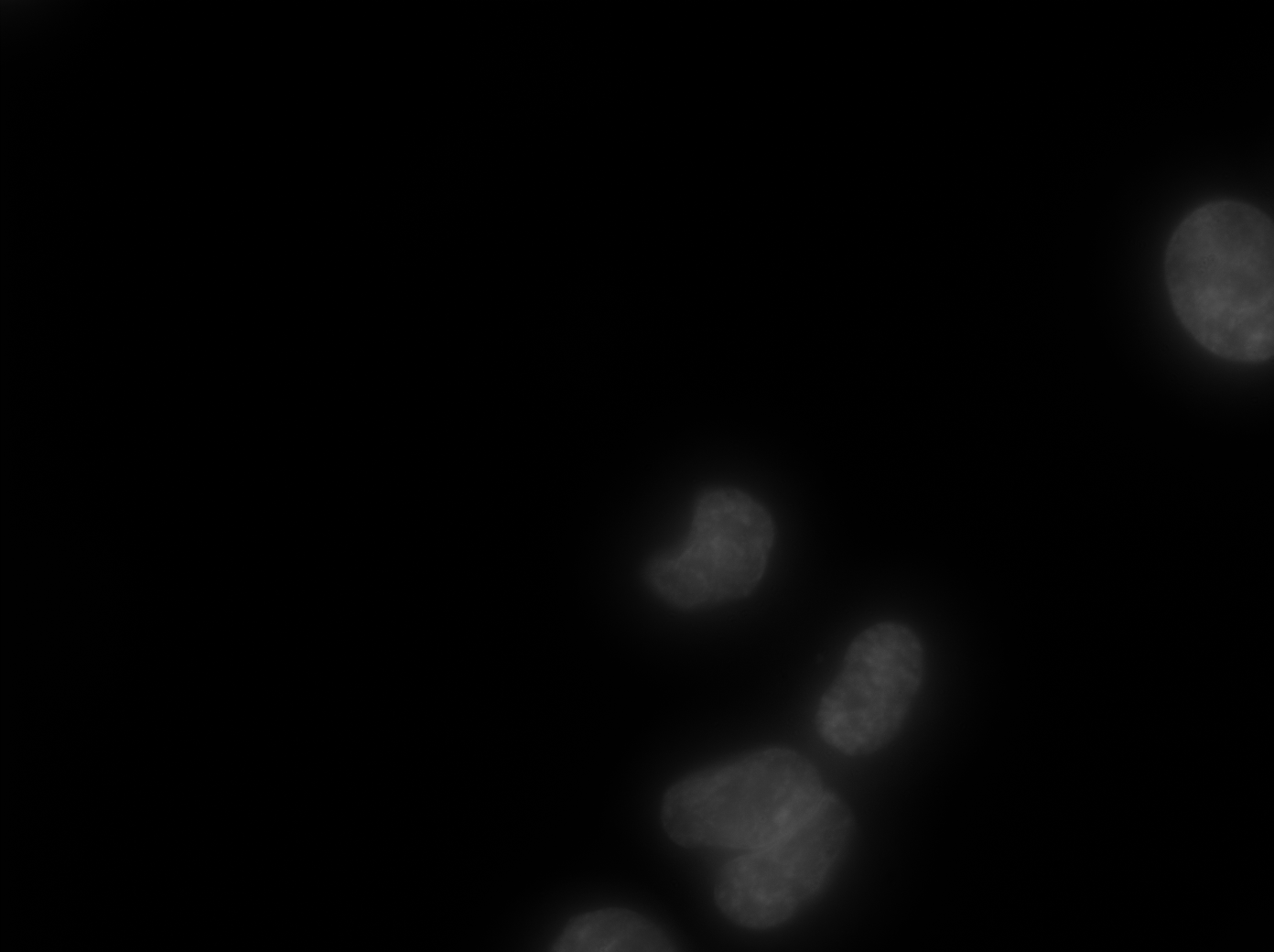

Supplement: Supplementary file 4 — Source data Fig. 2 part 1 [file 44319_2026_742_MOESM4_ESM.zip › Figure 2 Part 1/Fig 2c Cas9 Hela rGT335 atubulin/Cas9 GT335recomb atub 3-24-25 R3 PA10.Project Maximum Z_XY1743454681_Z0_T0_C0.tif]

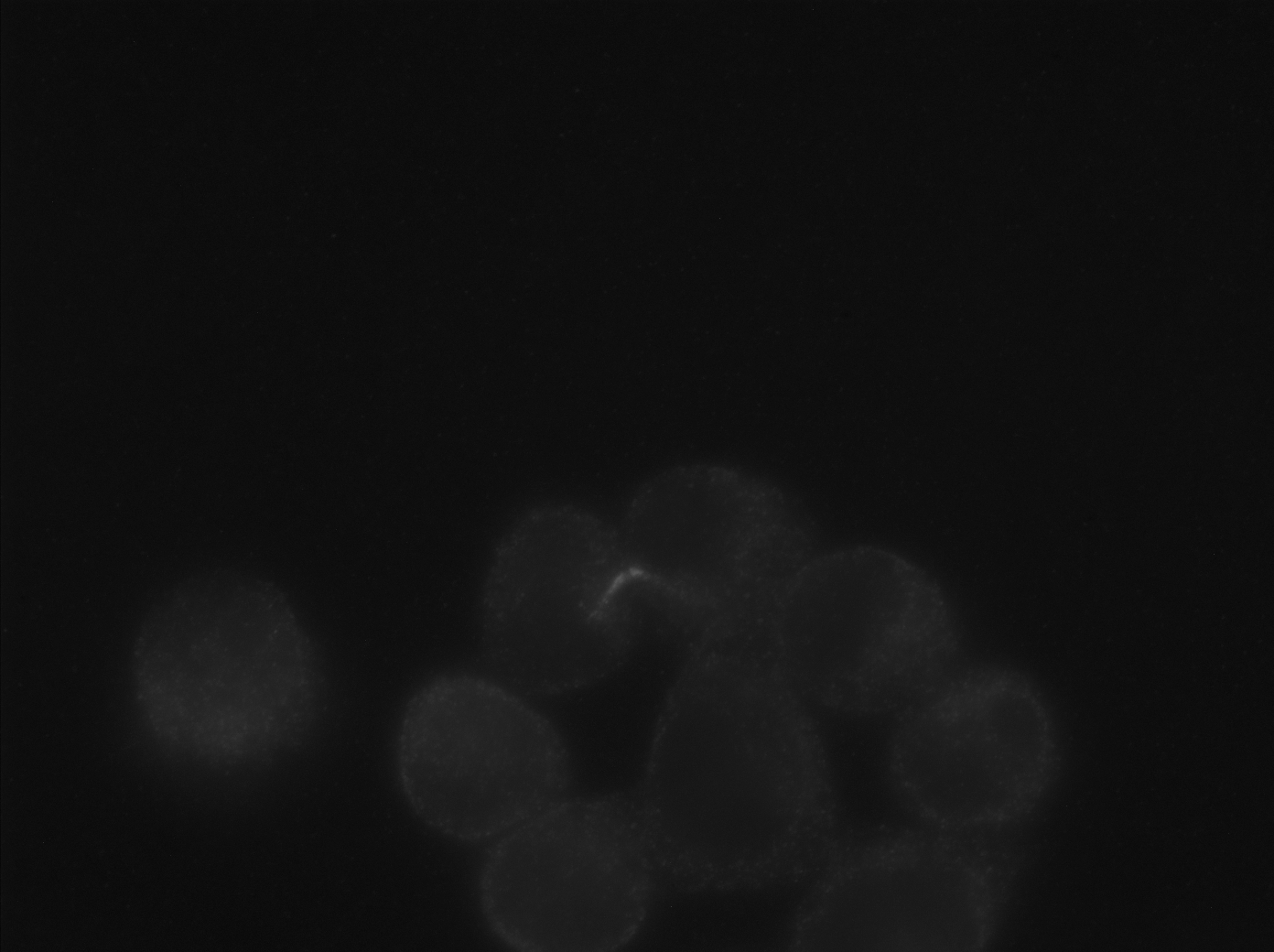

Supplement: Supplementary file 4 — Source data Fig. 2 part 1 [file 44319_2026_742_MOESM4_ESM.zip › Figure 2 Part 1/Fig 2c Cas9 Hela rGT335 atubulin/Cas9 GT335recomb atub 3-24-25 R3 LT3.Project Maximum Z_XY1743451368_Z0_T0_C2.tif]

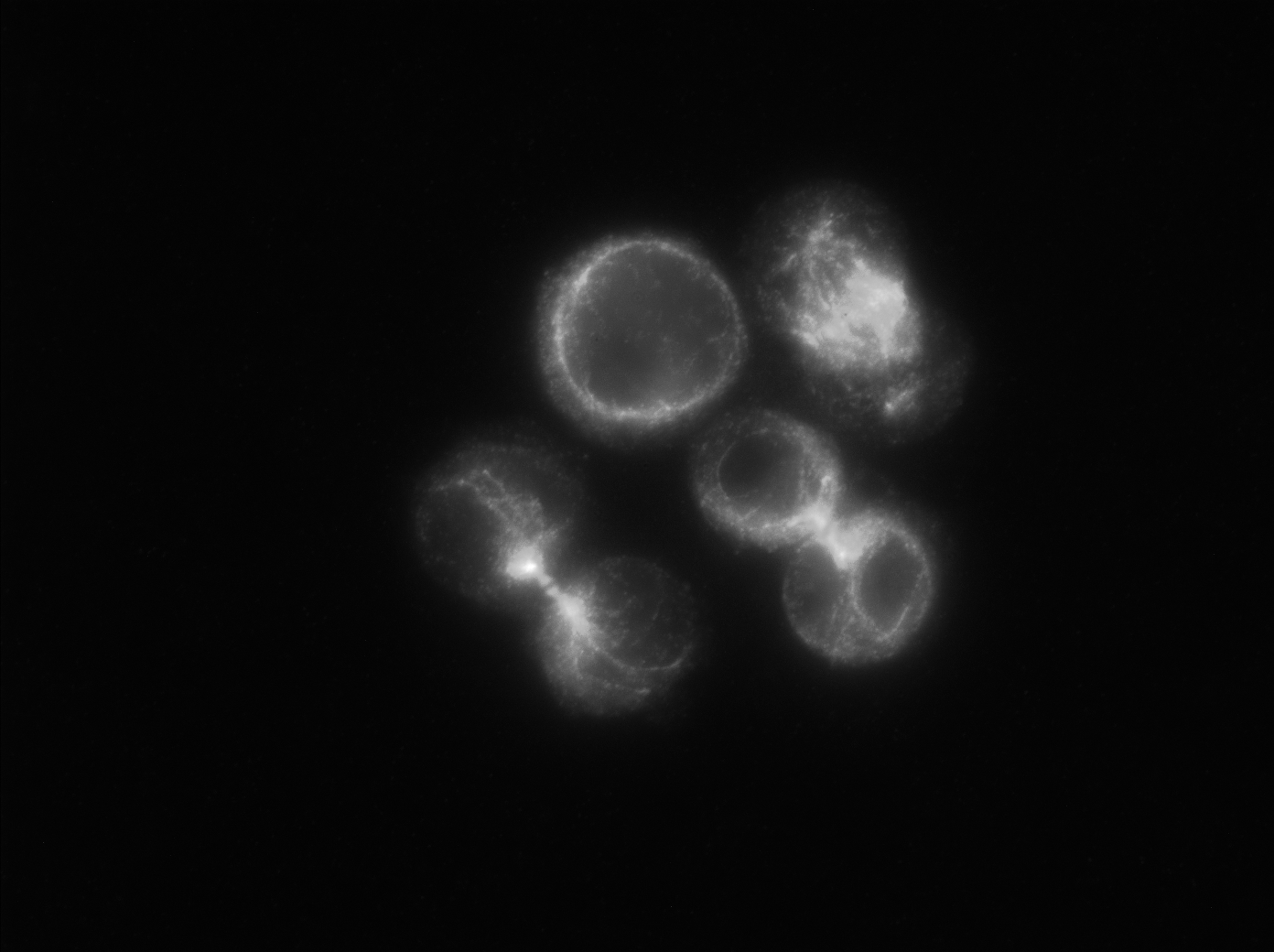

Supplement: Supplementary file 4 — Source data Fig. 2 part 1 [file 44319_2026_742_MOESM4_ESM.zip › Figure 2 Part 1/Fig 2c Cas9 Hela rGT335 atubulin/Cas9 GT335recomb atub 3-24-25 R2 A6.Project Maximum Z_XY1743446093_Z0_T0_C2.tif]

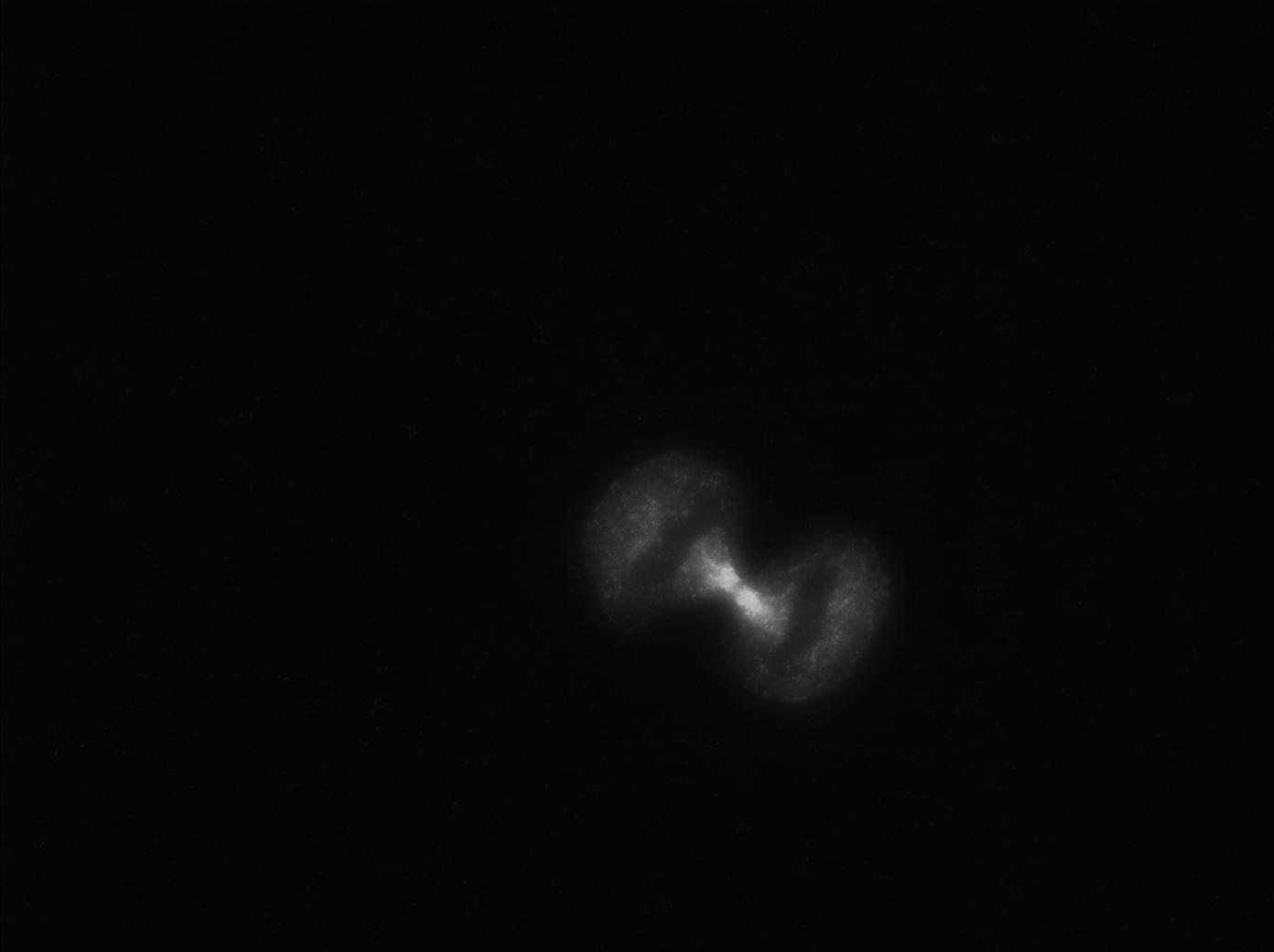

Supplement: Supplementary file 4 — Source data Fig. 2 part 1 [file 44319_2026_742_MOESM4_ESM.zip › Figure 2 Part 1/Fig 2c Cas9 Hela rGT335 atubulin/Cas9 GT335recomb atub 3-24-25 R2 ET1.Project Maximum Z_XY1743439798_Z0_T0_C2.tif]

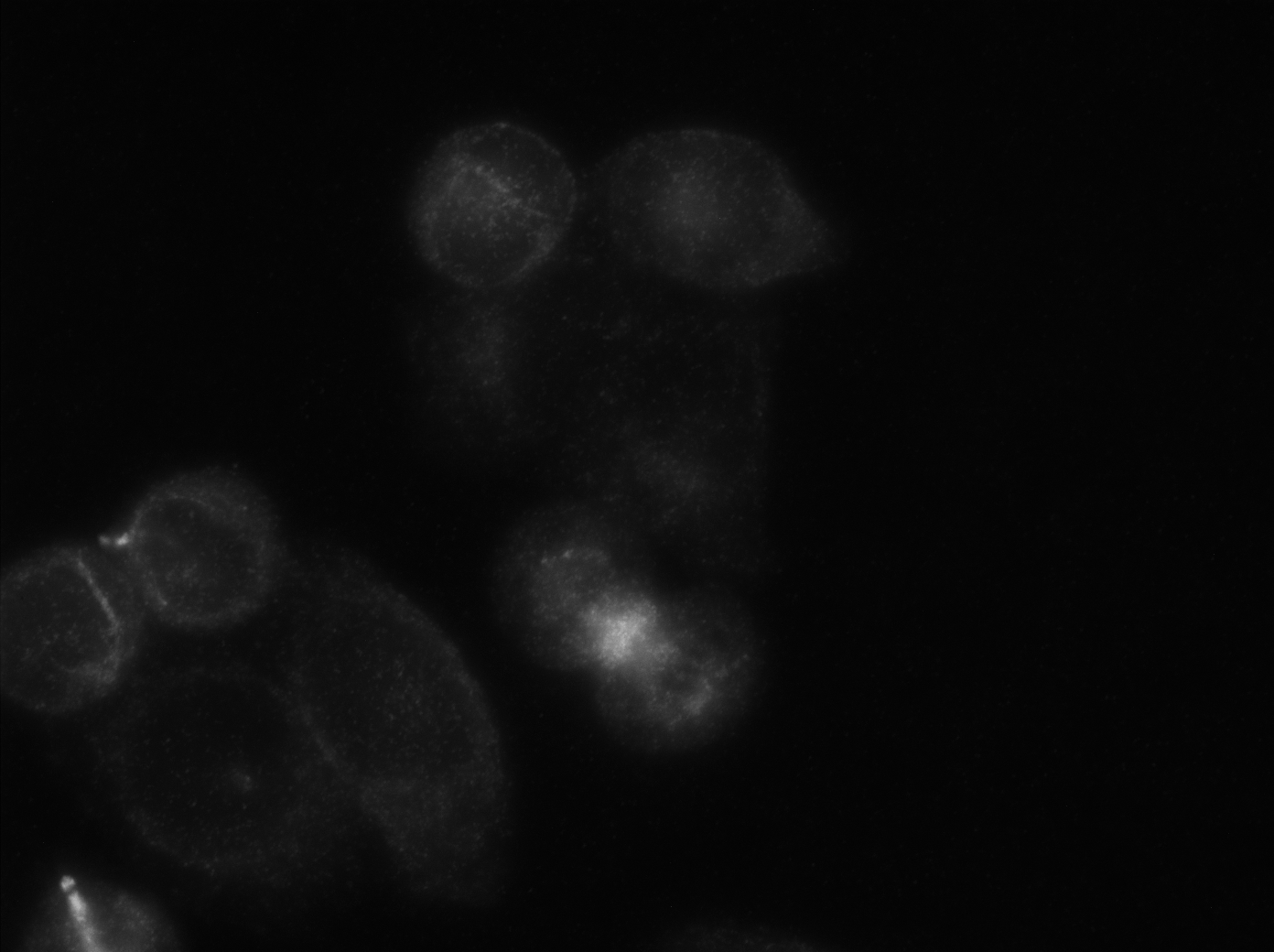

Supplement: Supplementary file 4 — Source data Fig. 2 part 1 [file 44319_2026_742_MOESM4_ESM.zip › Figure 2 Part 1/Fig 2c Cas9 Hela rGT335 atubulin/Cas9 GT335recomb atub 3-24-25 R2 preET2.Project Maximum Z_XY1743447696_Z0_T0_C2.tif]

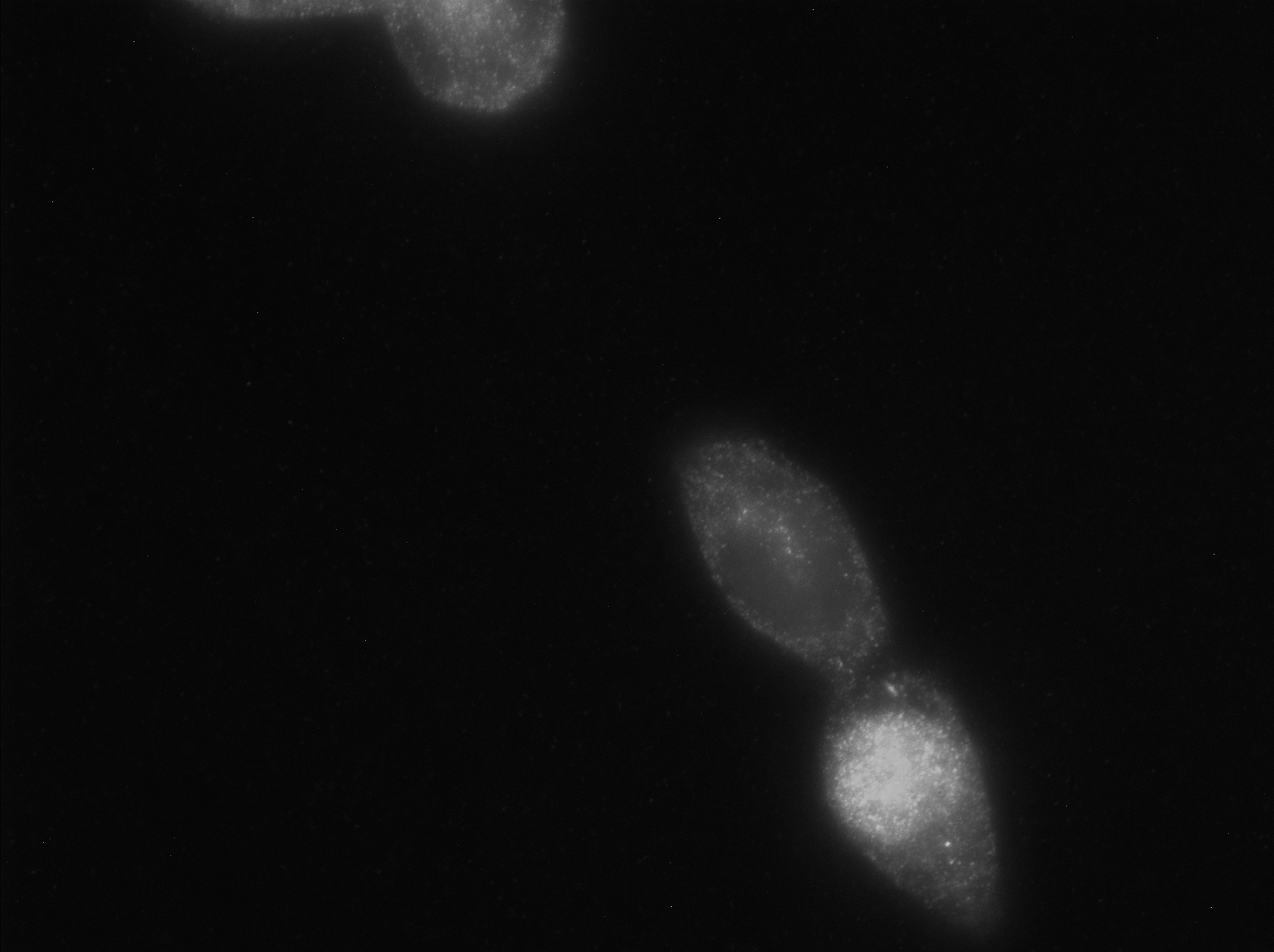

Supplement: Supplementary file 4 — Source data Fig. 2 part 1 [file 44319_2026_742_MOESM4_ESM.zip › Figure 2 Part 1/Fig 2c Cas9 Hela rGT335 atubulin/Cas9 GT335recomb atub 3-24-25 R1 PA9.Project Maximum Z_XY1743103585_Z0_T0_C1.tif]

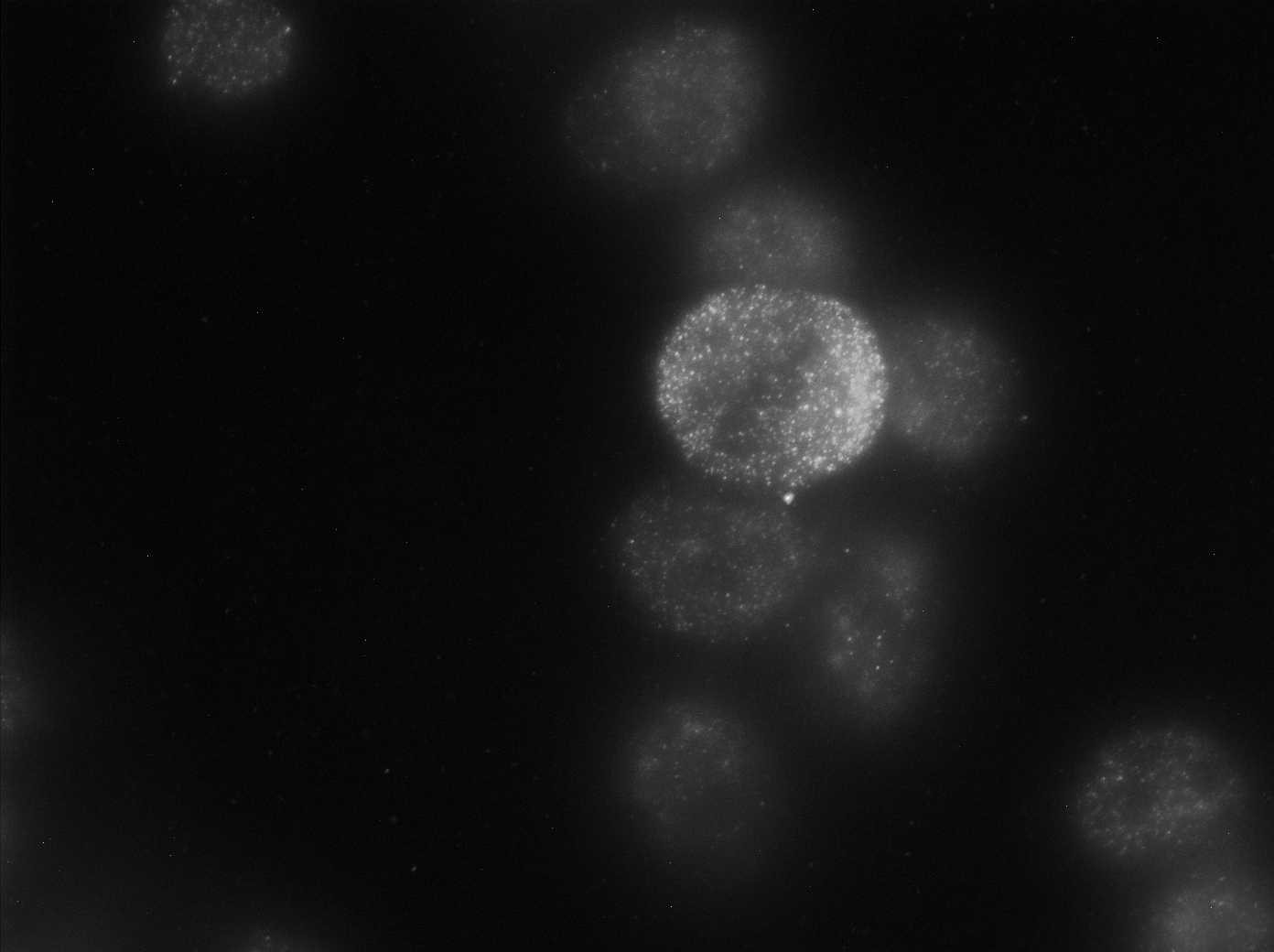

Supplement: Supplementary file 4 — Source data Fig. 2 part 1 [file 44319_2026_742_MOESM4_ESM.zip › Figure 2 Part 1/Fig 2c Cas9 Hela rGT335 atubulin/Cas9 GT335recomb atub 3-24-25 R3 M9.Project Maximum Z_XY1743454469_Z0_T0_C1.tif]

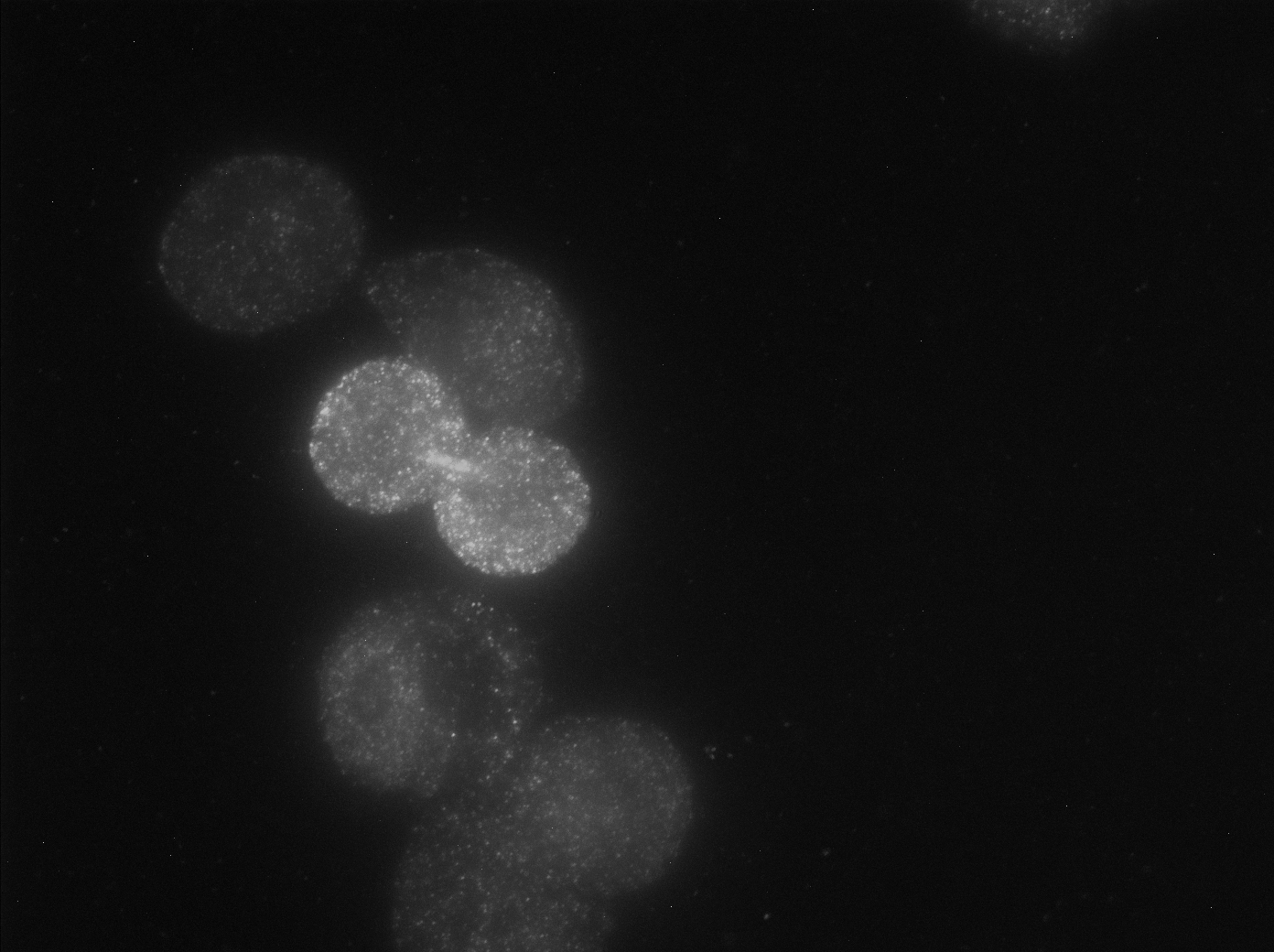

Supplement: Supplementary file 4 — Source data Fig. 2 part 1 [file 44319_2026_742_MOESM4_ESM.zip › Figure 2 Part 1/Fig 2c Cas9 Hela rGT335 atubulin/Cas9 GT335recomb atub 3-24-25 R3 ET8.Project Maximum Z_XY1743455361_Z0_T0_C1.tif]

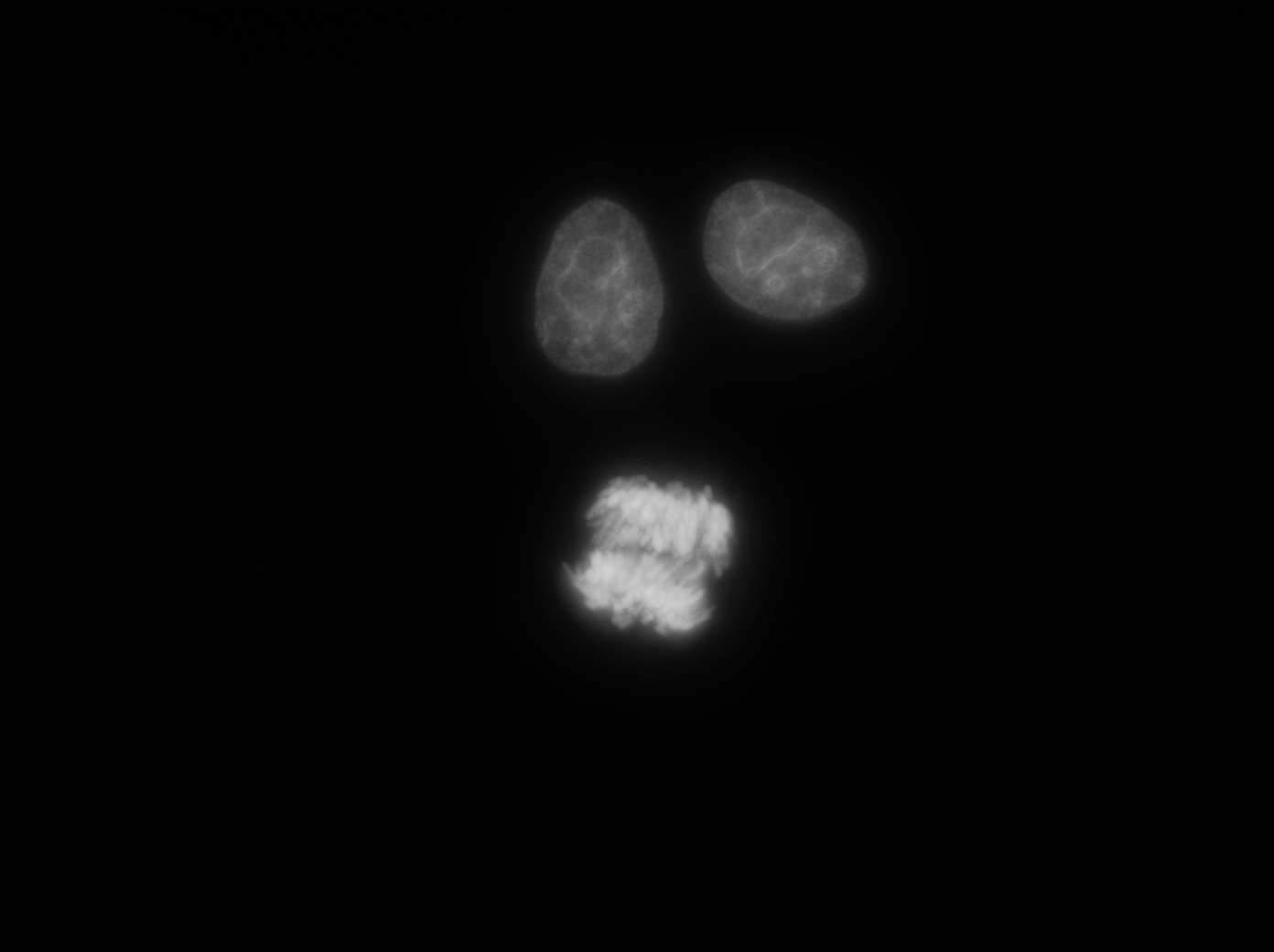

Supplement: Supplementary file 4 — Source data Fig. 2 part 1 [file 44319_2026_742_MOESM4_ESM.zip › Figure 2 Part 1/Fig 2c Cas9 Hela rGT335 atubulin/Cas9 GT335recomb atub 3-24-25 R2 A3.Project Maximum Z_XY1743442497_Z0_T0_C0.tif]

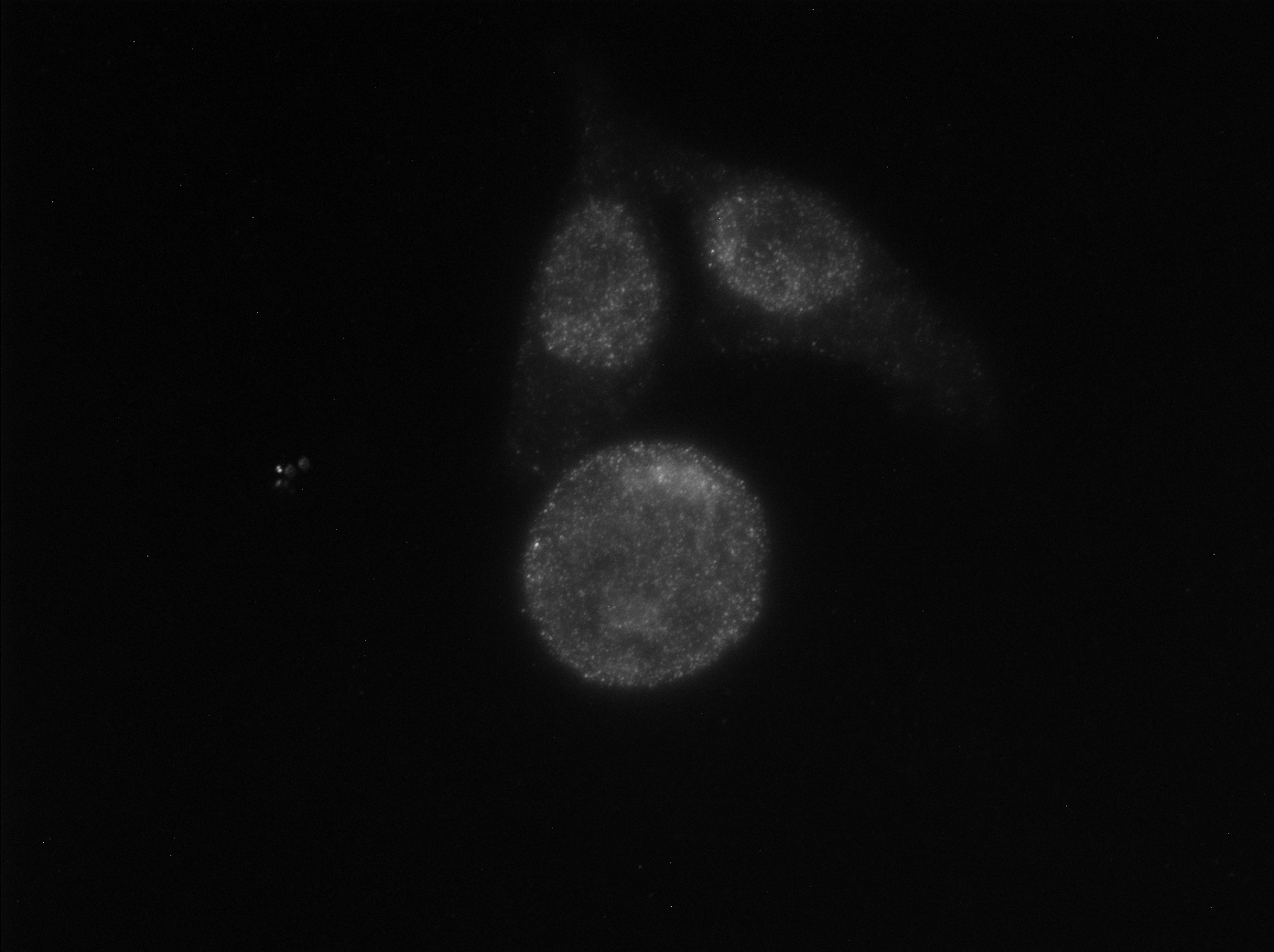

Supplement: Supplementary file 4 — Source data Fig. 2 part 1 [file 44319_2026_742_MOESM4_ESM.zip › Figure 2 Part 1/Fig 2c Cas9 Hela rGT335 atubulin/Cas9 GT335recomb atub 3-24-25 R2 A3.Project Maximum Z_XY1743442497_Z0_T0_C1.tif]

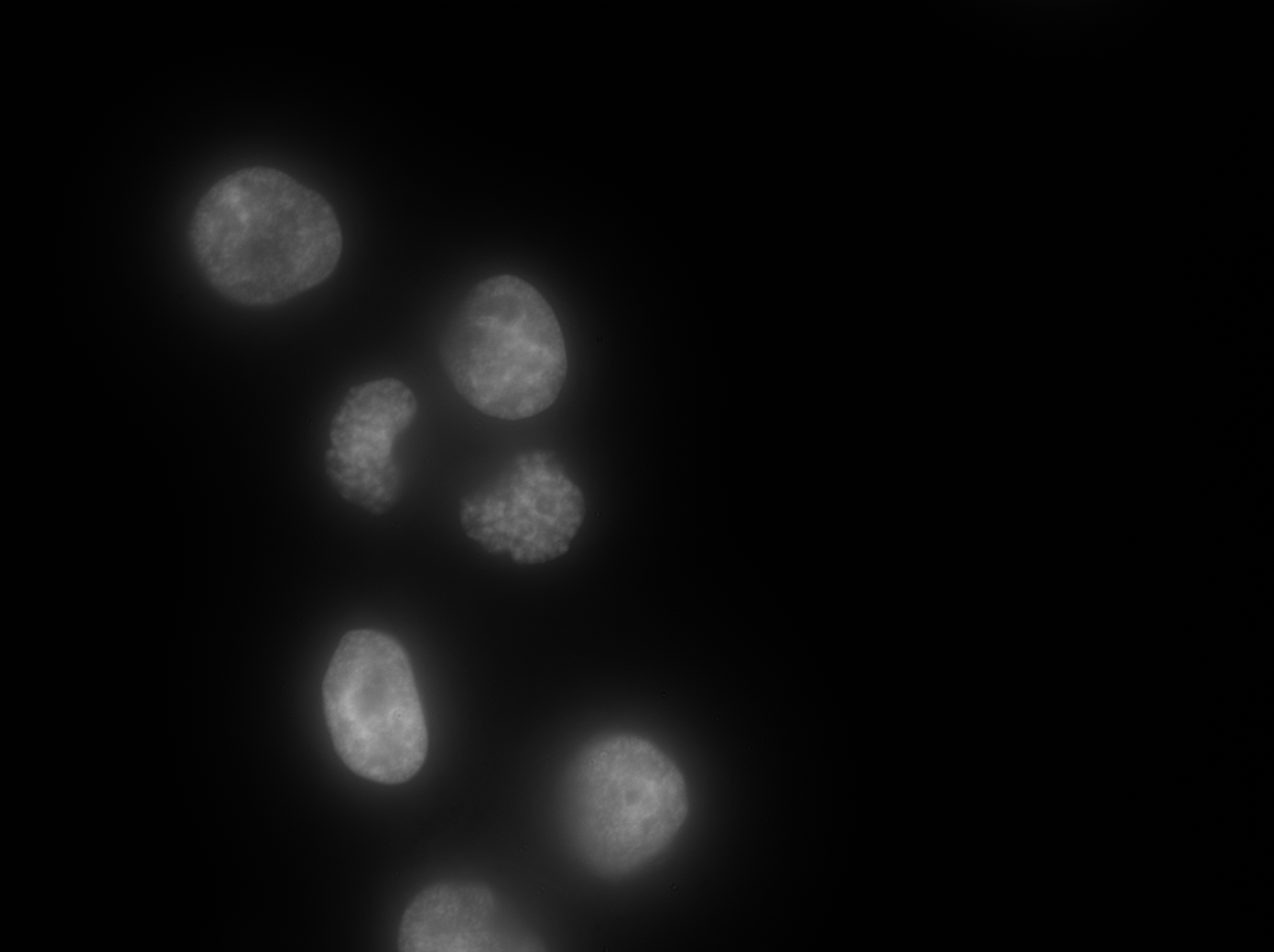

Supplement: Supplementary file 4 — Source data Fig. 2 part 1 [file 44319_2026_742_MOESM4_ESM.zip › Figure 2 Part 1/Fig 2c Cas9 Hela rGT335 atubulin/Cas9 GT335recomb atub 3-24-25 R3 ET8.Project Maximum Z_XY1743455361_Z0_T0_C0.tif]

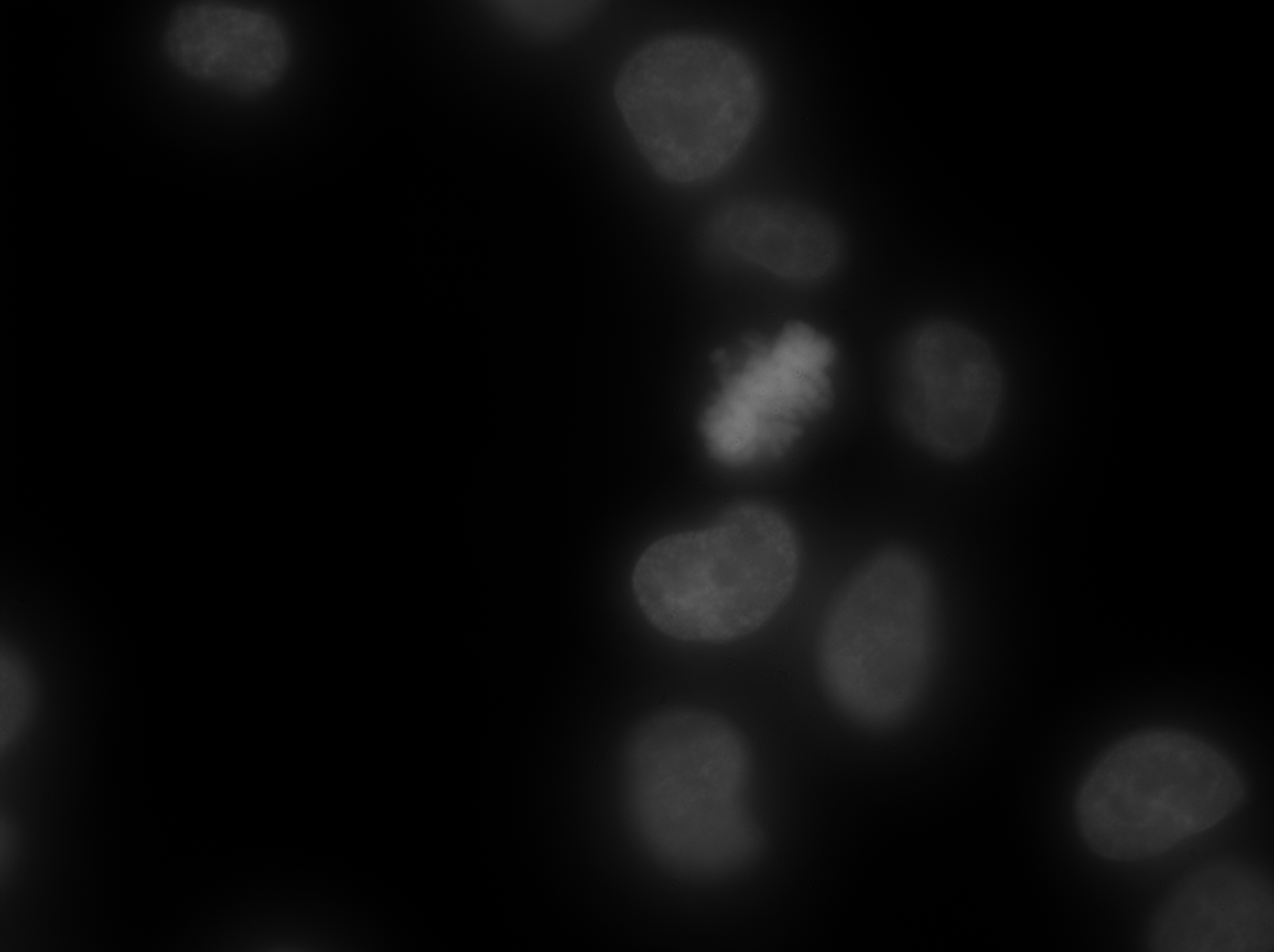

Supplement: Supplementary file 4 — Source data Fig. 2 part 1 [file 44319_2026_742_MOESM4_ESM.zip › Figure 2 Part 1/Fig 2c Cas9 Hela rGT335 atubulin/Cas9 GT335recomb atub 3-24-25 R3 M9.Project Maximum Z_XY1743454469_Z0_T0_C0.tif]

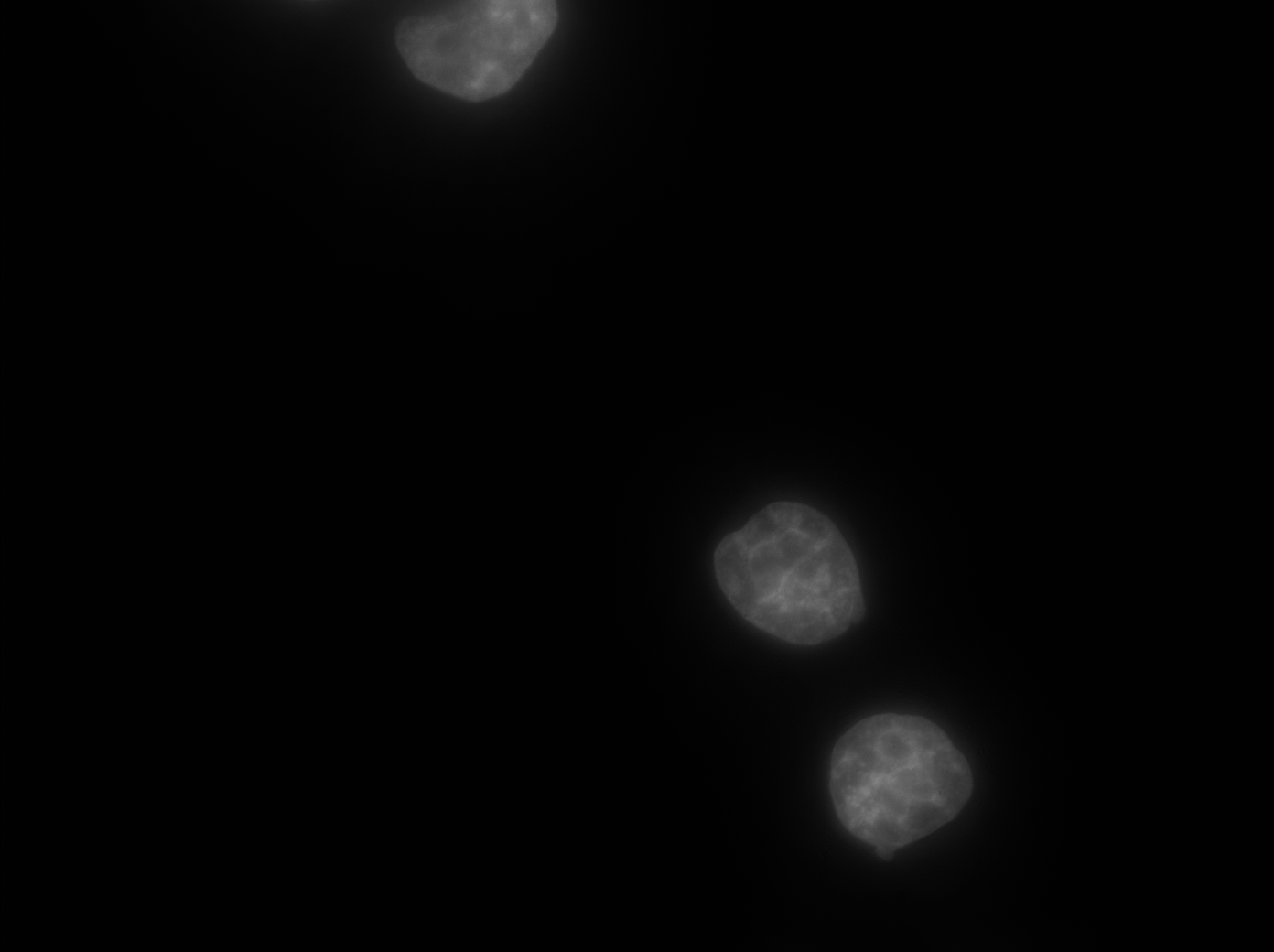

Supplement: Supplementary file 4 — Source data Fig. 2 part 1 [file 44319_2026_742_MOESM4_ESM.zip › Figure 2 Part 1/Fig 2c Cas9 Hela rGT335 atubulin/Cas9 GT335recomb atub 3-24-25 R1 PA9.Project Maximum Z_XY1743103585_Z0_T0_C0.tif]

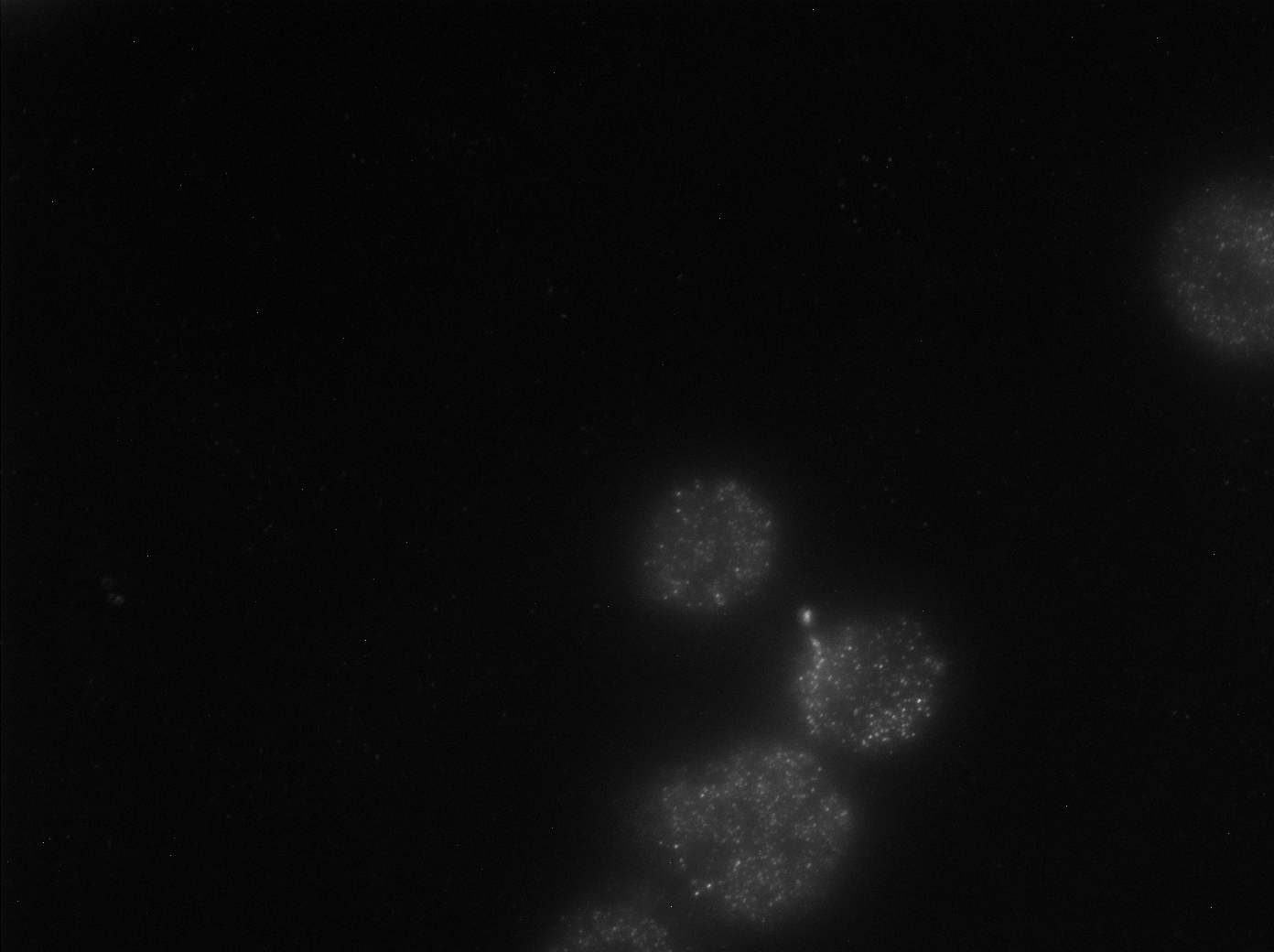

Supplement: Supplementary file 4 — Source data Fig. 2 part 1 [file 44319_2026_742_MOESM4_ESM.zip › Figure 2 Part 1/Fig 2c Cas9 Hela rGT335 atubulin/Cas9 GT335recomb atub 3-24-25 R3 PA10.Project Maximum Z_XY1743454681_Z0_T0_C1.tif]

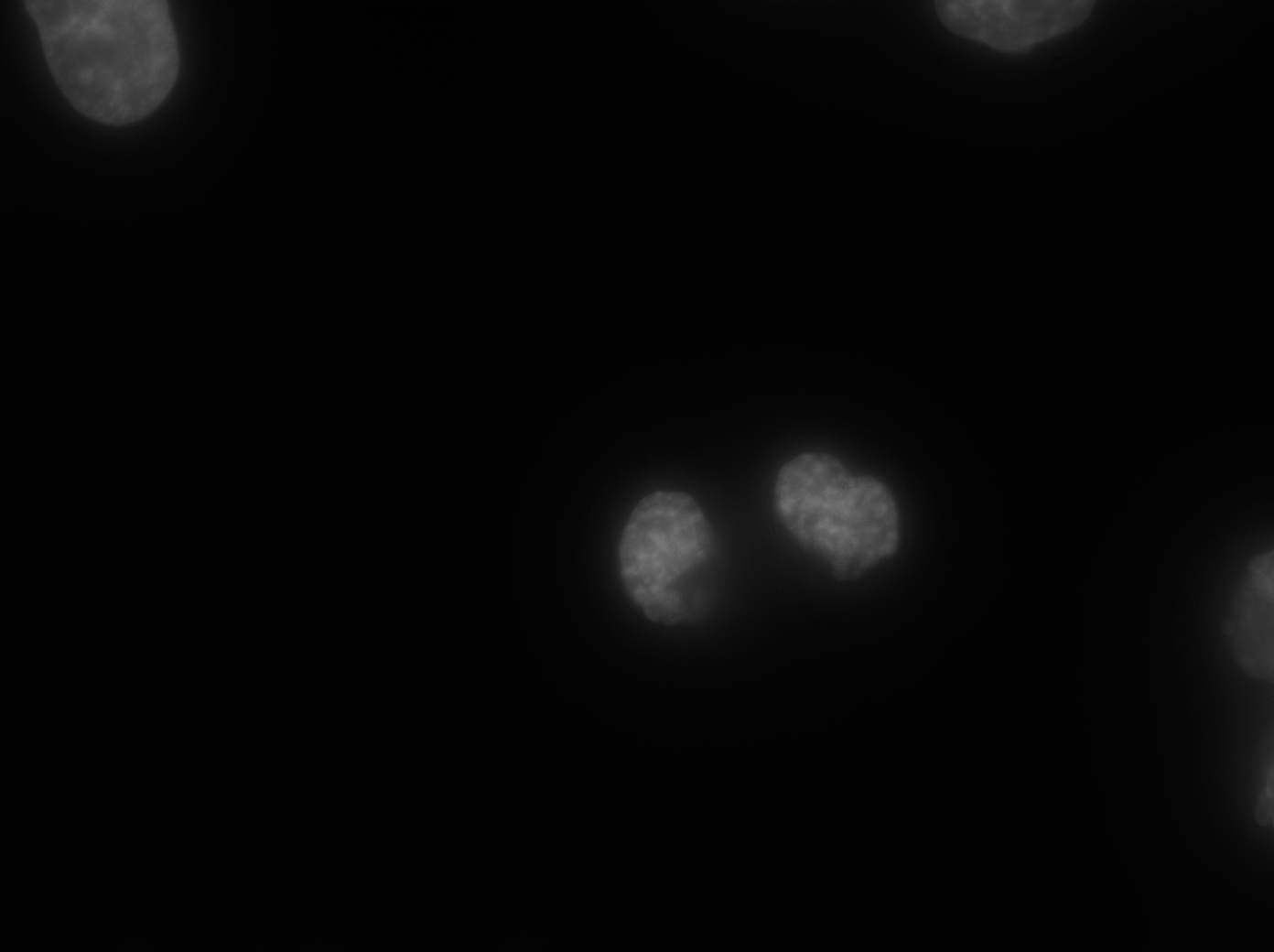

Supplement: Supplementary file 4 — Source data Fig. 2 part 1 [file 44319_2026_742_MOESM4_ESM.zip › Figure 2 Part 1/Fig 2c Cas9 Hela rGT335 atubulin/Cas9 GT335recomb atub 3-24-25 R1 ET3.Project Maximum Z_XY1743101375_Z0_T0_C0.tif]

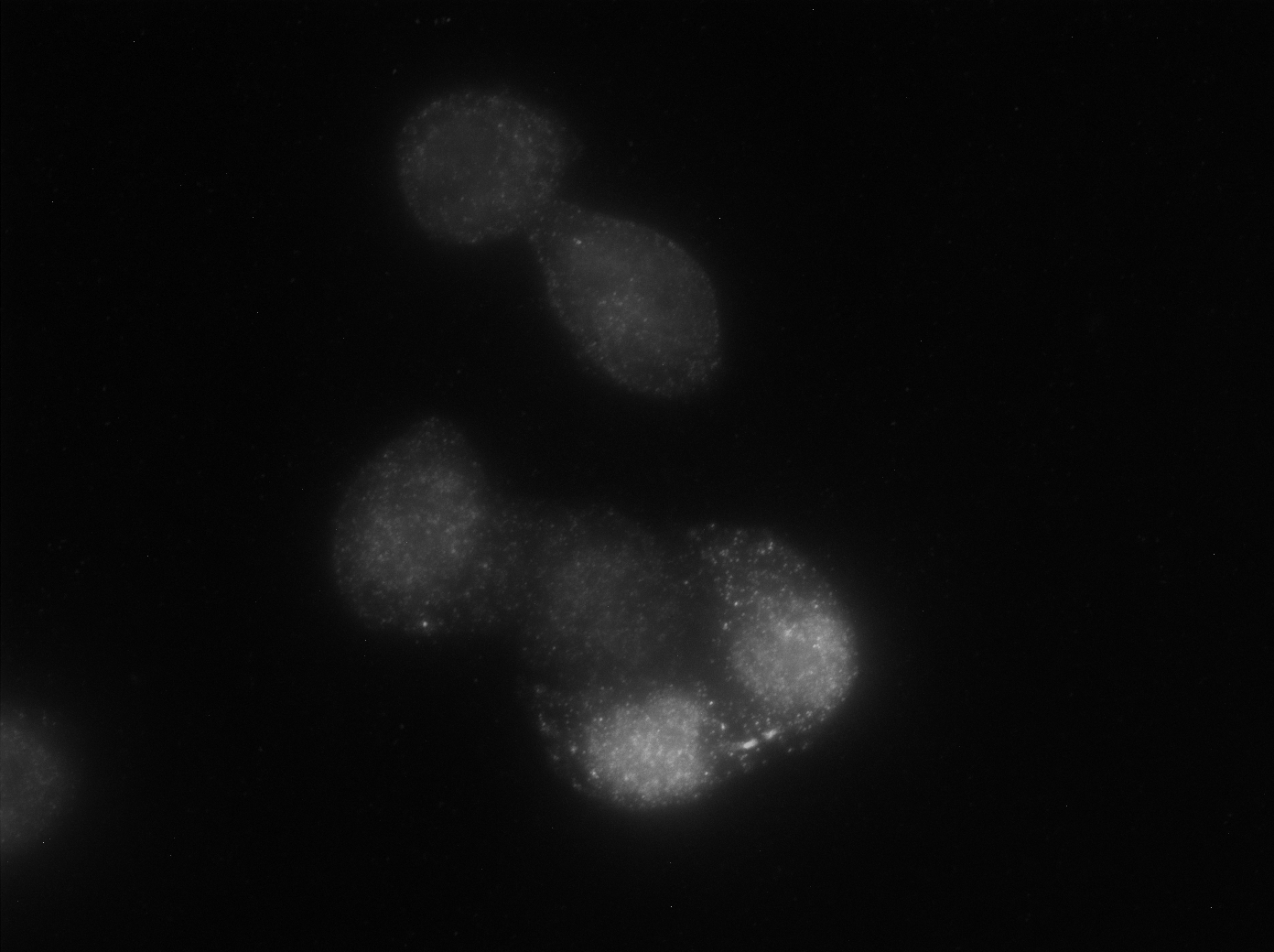

Supplement: Supplementary file 4 — Source data Fig. 2 part 1 [file 44319_2026_742_MOESM4_ESM.zip › Figure 2 Part 1/Fig 2c Cas9 Hela rGT335 atubulin/Cas9 GT335recomb atub 3-24-25 R1 LT9.Project Maximum Z_XY1743101906_Z0_T0_C1.tif]

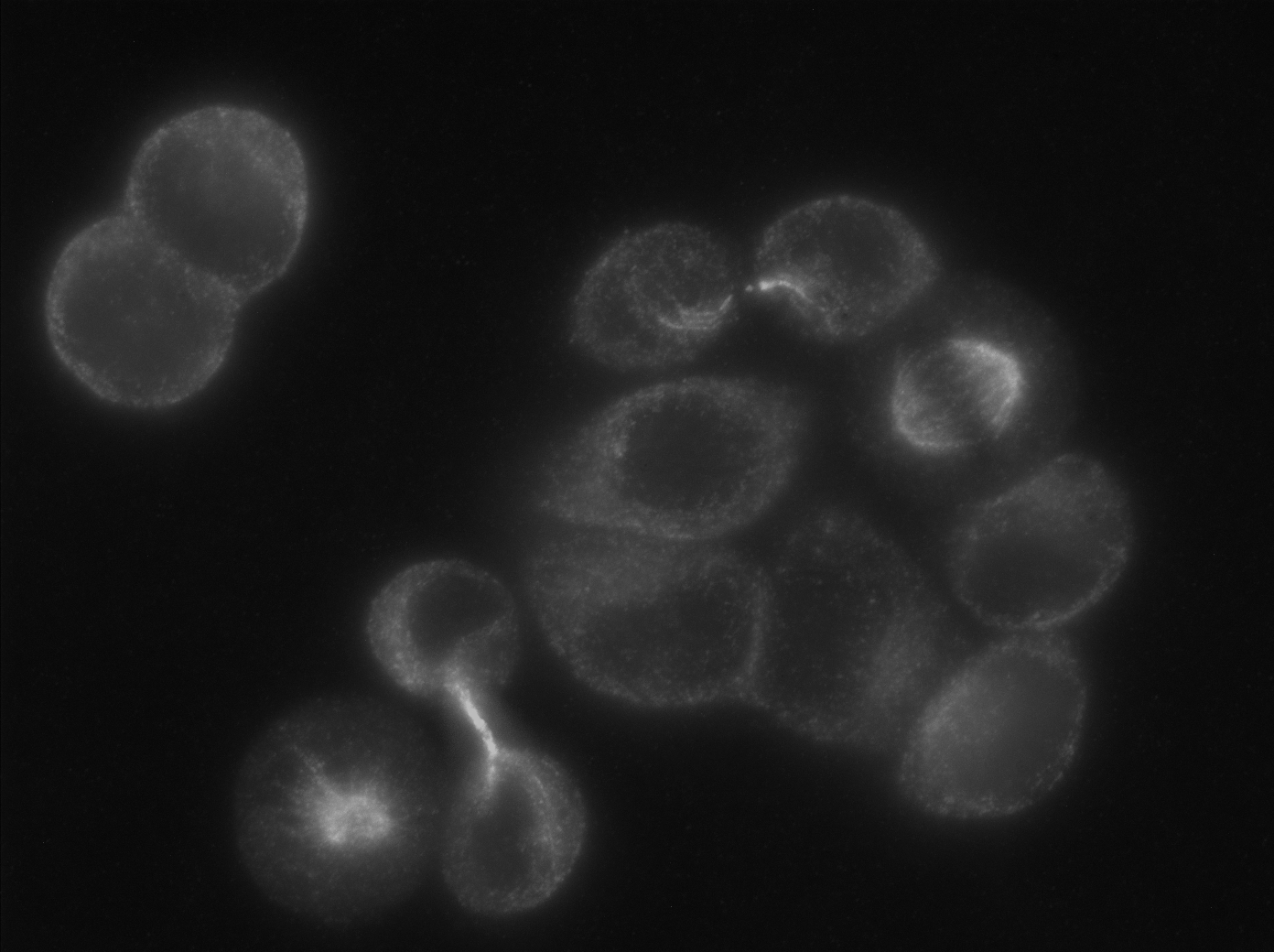

Supplement: Supplementary file 4 — Source data Fig. 2 part 1 [file 44319_2026_742_MOESM4_ESM.zip › Figure 2 Part 1/Fig 2c Cas9 Hela rGT335 atubulin/Cas9 GT335recomb atub 3-24-25 R1 PA1 M1.Project Maximum Z_XY1742835552_Z0_T0_C2.tif]
