# Supplementary figures and images for: TPGS1 regulates central spindle microtubule glutamylation and remodeling during telophase and abscission (part 5 of 36)
Source: EMBO Rep. 2026 Mar 23;27(8):1944–63. doi: 10.1038/s44319-026-00742-3 (PMC13121839; doi:10.1038/s44319-026-00742-3)

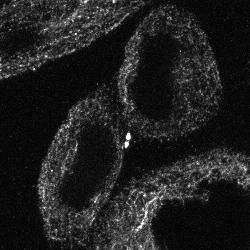

Supplement: Supplementary file 6 — Source data Fig. 2 part 3 [file 44319_2026_742_MOESM6_ESM.zip › Figure 2 Part 3/Fig 2b polye acetylated tubulin confocal/PolyE-actub PA actub Full ns.tif]

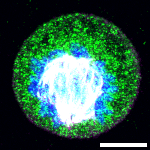

Supplement: Supplementary file 6 — Source data Fig. 2 part 3 [file 44319_2026_742_MOESM6_ESM.zip › Figure 2 Part 3/Fig 2b polye acetylated tubulin confocal/PolyE-actub M RGB Full sc10.png]

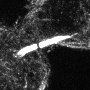

Supplement: Supplementary file 6 — Source data Fig. 2 part 3 [file 44319_2026_742_MOESM6_ESM.zip › Figure 2 Part 3/Fig 2b polye acetylated tubulin confocal/PolyE-actub LT actub crop ns.jpg]

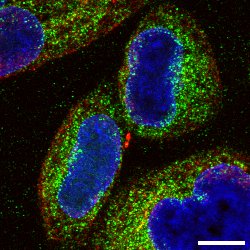

Supplement: Supplementary file 6 — Source data Fig. 2 part 3 [file 44319_2026_742_MOESM6_ESM.zip › Figure 2 Part 3/Fig 2b polye acetylated tubulin confocal/PolyE-actub PA RGB Full sc10 v2.jpg]

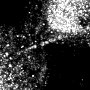

Supplement: Supplementary file 6 — Source data Fig. 2 part 3 [file 44319_2026_742_MOESM6_ESM.zip › Figure 2 Part 3/Fig 2b polye acetylated tubulin confocal/PolyE-actub LT polye crop ns.jpg]

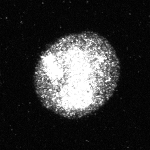

Supplement: Supplementary file 6 — Source data Fig. 2 part 3 [file 44319_2026_742_MOESM6_ESM.zip › Figure 2 Part 3/Fig 2b polye acetylated tubulin confocal/PolyE-actub PM RGP Full ns v2.tif]

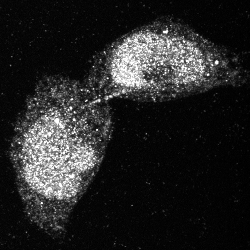

Supplement: Supplementary file 6 — Source data Fig. 2 part 3 [file 44319_2026_742_MOESM6_ESM.zip › Figure 2 Part 3/Fig 2b polye acetylated tubulin confocal/PolyE-actub LT RGB Full sc10.tif]

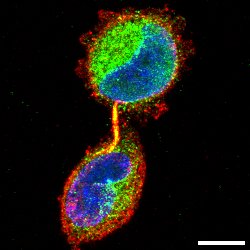

Supplement: Supplementary file 6 — Source data Fig. 2 part 3 [file 44319_2026_742_MOESM6_ESM.zip › Figure 2 Part 3/Fig 2b polye acetylated tubulin confocal/PolyE-actub ET RGB Full sc10 v2.jpg]

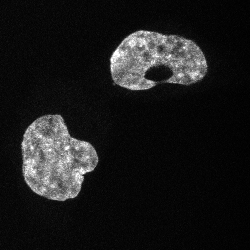

Supplement: Supplementary file 6 — Source data Fig. 2 part 3 [file 44319_2026_742_MOESM6_ESM.zip › Figure 2 Part 3/Fig 2b polye acetylated tubulin confocal/PolyE-actub LT DAPI Full ns.tif]

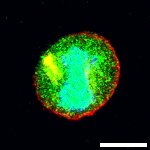

Supplement: Supplementary file 6 — Source data Fig. 2 part 3 [file 44319_2026_742_MOESM6_ESM.zip › Figure 2 Part 3/Fig 2b polye acetylated tubulin confocal/PolyE-actub PM RGP Full ns v2.jpg]

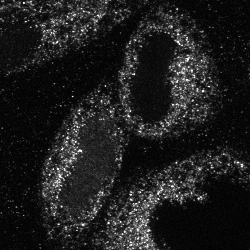

Supplement: Supplementary file 6 — Source data Fig. 2 part 3 [file 44319_2026_742_MOESM6_ESM.zip › Figure 2 Part 3/Fig 2b polye acetylated tubulin confocal/PolyE-actub PA polye Full sc10.tif]

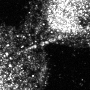

Supplement: Supplementary file 6 — Source data Fig. 2 part 3 [file 44319_2026_742_MOESM6_ESM.zip › Figure 2 Part 3/Fig 2b polye acetylated tubulin confocal/PolyE-actub LT polye crop ns.tif]

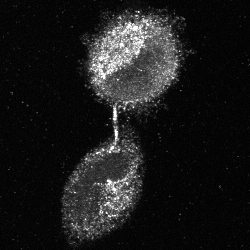

Supplement: Supplementary file 6 — Source data Fig. 2 part 3 [file 44319_2026_742_MOESM6_ESM.zip › Figure 2 Part 3/Fig 2b polye acetylated tubulin confocal/PolyE-actub ET polye Full ns.tif]

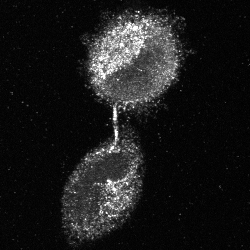

Supplement: Supplementary file 6 — Source data Fig. 2 part 3 [file 44319_2026_742_MOESM6_ESM.zip › Figure 2 Part 3/Fig 2b polye acetylated tubulin confocal/PolyE-actub ET RGB Full sc10 v2.tif]

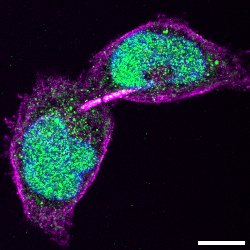

Supplement: Supplementary file 6 — Source data Fig. 2 part 3 [file 44319_2026_742_MOESM6_ESM.zip › Figure 2 Part 3/Fig 2b polye acetylated tubulin confocal/PolyE-actub LT RGB Full sc10.png]

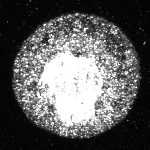

Supplement: Supplementary file 6 — Source data Fig. 2 part 3 [file 44319_2026_742_MOESM6_ESM.zip › Figure 2 Part 3/Fig 2b polye acetylated tubulin confocal/PolyE-actub M polye Full sc10.tif]

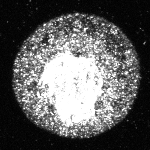

Supplement: Supplementary file 6 — Source data Fig. 2 part 3 [file 44319_2026_742_MOESM6_ESM.zip › Figure 2 Part 3/Fig 2b polye acetylated tubulin confocal/PolyE-actub M RGB Full sc10.tif]

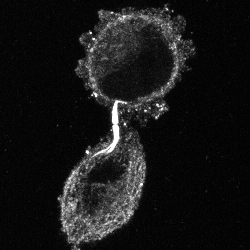

Supplement: Supplementary file 6 — Source data Fig. 2 part 3 [file 44319_2026_742_MOESM6_ESM.zip › Figure 2 Part 3/Fig 2b polye acetylated tubulin confocal/PolyE-actub ET actub Full ns.tif]

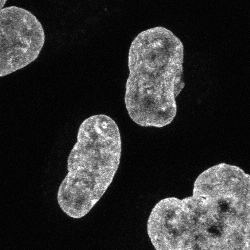

Supplement: Supplementary file 6 — Source data Fig. 2 part 3 [file 44319_2026_742_MOESM6_ESM.zip › Figure 2 Part 3/Fig 2b polye acetylated tubulin confocal/PolyE-actub PA DAPI Full sc10.tif]

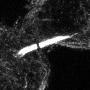

Supplement: Supplementary file 6 — Source data Fig. 2 part 3 [file 44319_2026_742_MOESM6_ESM.zip › Figure 2 Part 3/Fig 2b polye acetylated tubulin confocal/PolyE-actub LT actub crop ns.tif]

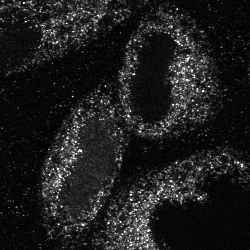

Supplement: Supplementary file 6 — Source data Fig. 2 part 3 [file 44319_2026_742_MOESM6_ESM.zip › Figure 2 Part 3/Fig 2b polye acetylated tubulin confocal/PolyE-actub PA RGB Full sc10 v2.tif]

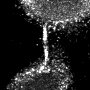

Supplement: Supplementary file 6 — Source data Fig. 2 part 3 [file 44319_2026_742_MOESM6_ESM.zip › Figure 2 Part 3/Fig 2b polye acetylated tubulin confocal/PolyE-actub ET polye crop ns.jpg]

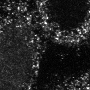

Supplement: Supplementary file 6 — Source data Fig. 2 part 3 [file 44319_2026_742_MOESM6_ESM.zip › Figure 2 Part 3/Fig 2b polye acetylated tubulin confocal/PolyE-actub PA polye crop ns.tif]

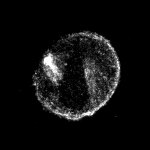

Supplement: Supplementary file 6 — Source data Fig. 2 part 3 [file 44319_2026_742_MOESM6_ESM.zip › Figure 2 Part 3/Fig 2b polye acetylated tubulin confocal/PolyE-actub PM actub Full ns.jpg]

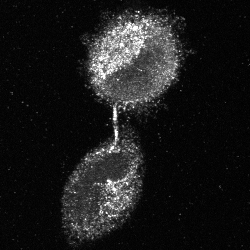

Supplement: Supplementary file 6 — Source data Fig. 2 part 3 [file 44319_2026_742_MOESM6_ESM.zip › Figure 2 Part 3/Fig 2b polye acetylated tubulin confocal/PolyE-actub ET RGB Full sc10.tif]

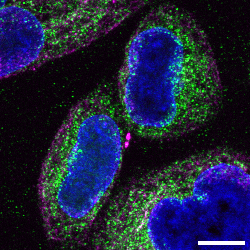

Supplement: Supplementary file 6 — Source data Fig. 2 part 3 [file 44319_2026_742_MOESM6_ESM.zip › Figure 2 Part 3/Fig 2b polye acetylated tubulin confocal/PolyE-actub PA RGB Full sc10.png]

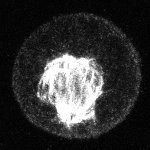

Supplement: Supplementary file 6 — Source data Fig. 2 part 3 [file 44319_2026_742_MOESM6_ESM.zip › Figure 2 Part 3/Fig 2b polye acetylated tubulin confocal/PolyE-actub M actub Full ns.tif]

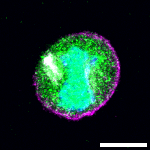

Supplement: Supplementary file 6 — Source data Fig. 2 part 3 [file 44319_2026_742_MOESM6_ESM.zip › Figure 2 Part 3/Fig 2b polye acetylated tubulin confocal/PolyE-actub PM RGP Full ns.png]

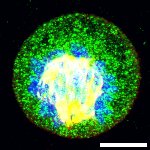

Supplement: Supplementary file 6 — Source data Fig. 2 part 3 [file 44319_2026_742_MOESM6_ESM.zip › Figure 2 Part 3/Fig 2b polye acetylated tubulin confocal/PolyE-actub M RGB Full sc10 v2.jpg]

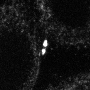

Supplement: Supplementary file 6 — Source data Fig. 2 part 3 [file 44319_2026_742_MOESM6_ESM.zip › Figure 2 Part 3/Fig 2b polye acetylated tubulin confocal/PolyE-actub PA actub crop ns.tif]

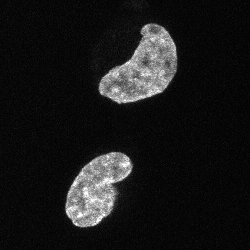

Supplement: Supplementary file 6 — Source data Fig. 2 part 3 [file 44319_2026_742_MOESM6_ESM.zip › Figure 2 Part 3/Fig 2b polye acetylated tubulin confocal/PolyE-actub ET DAPI Full ns.tif]

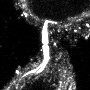

Supplement: Supplementary file 6 — Source data Fig. 2 part 3 [file 44319_2026_742_MOESM6_ESM.zip › Figure 2 Part 3/Fig 2b polye acetylated tubulin confocal/PolyE-actub ET actub crop ns.jpg]

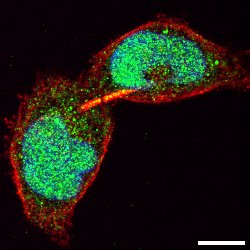

Supplement: Supplementary file 6 — Source data Fig. 2 part 3 [file 44319_2026_742_MOESM6_ESM.zip › Figure 2 Part 3/Fig 2b polye acetylated tubulin confocal/PolyE-actub LT RGB Full sc10 v2.jpg]

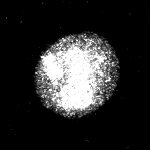

Supplement: Supplementary file 6 — Source data Fig. 2 part 3 [file 44319_2026_742_MOESM6_ESM.zip › Figure 2 Part 3/Fig 2b polye acetylated tubulin confocal/PolyE-actub PM polye Full ns.jpg]

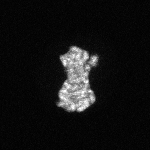

Supplement: Supplementary file 6 — Source data Fig. 2 part 3 [file 44319_2026_742_MOESM6_ESM.zip › Figure 2 Part 3/Fig 2b polye acetylated tubulin confocal/PolyE-actub PM DAPI Full ns.tif]

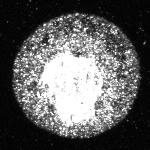

Supplement: Supplementary file 6 — Source data Fig. 2 part 3 [file 44319_2026_742_MOESM6_ESM.zip › Figure 2 Part 3/Fig 2b polye acetylated tubulin confocal/PolyE-actub M RGB Full sc10 v2.tif]

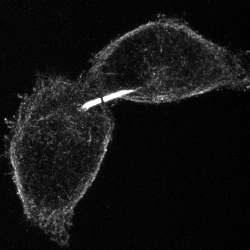

Supplement: Supplementary file 6 — Source data Fig. 2 part 3 [file 44319_2026_742_MOESM6_ESM.zip › Figure 2 Part 3/Fig 2b polye acetylated tubulin confocal/PolyE-actub LT actub Full ns.tif]

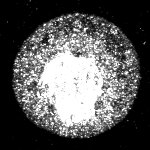

Supplement: Supplementary file 6 — Source data Fig. 2 part 3 [file 44319_2026_742_MOESM6_ESM.zip › Figure 2 Part 3/Fig 2b polye acetylated tubulin confocal/PolyE-actub M polye Full ns.jpg]

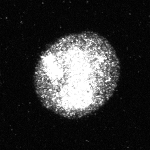

Supplement: Supplementary file 6 — Source data Fig. 2 part 3 [file 44319_2026_742_MOESM6_ESM.zip › Figure 2 Part 3/Fig 2b polye acetylated tubulin confocal/PolyE-actub PM polye Full ns.tif]

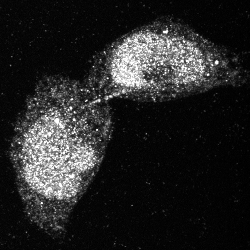

Supplement: Supplementary file 6 — Source data Fig. 2 part 3 [file 44319_2026_742_MOESM6_ESM.zip › Figure 2 Part 3/Fig 2b polye acetylated tubulin confocal/PolyE-actub LT RGB Full sc10 v2.tif]

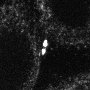

Supplement: Supplementary file 6 — Source data Fig. 2 part 3 [file 44319_2026_742_MOESM6_ESM.zip › Figure 2 Part 3/Fig 2b polye acetylated tubulin confocal/PolyE-actub PA actub crop ns.jpg]

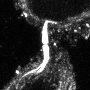

Supplement: Supplementary file 6 — Source data Fig. 2 part 3 [file 44319_2026_742_MOESM6_ESM.zip › Figure 2 Part 3/Fig 2b polye acetylated tubulin confocal/PolyE-actub ET actub crop ns.tif]

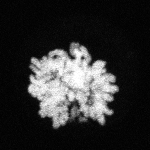

Supplement: Supplementary file 6 — Source data Fig. 2 part 3 [file 44319_2026_742_MOESM6_ESM.zip › Figure 2 Part 3/Fig 2b polye acetylated tubulin confocal/PolyE-actub M DAPI Full sc10.tif]

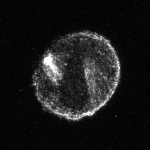

Supplement: Supplementary file 6 — Source data Fig. 2 part 3 [file 44319_2026_742_MOESM6_ESM.zip › Figure 2 Part 3/Fig 2b polye acetylated tubulin confocal/PolyE-actub PM actub Full ns.tif]

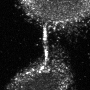

Supplement: Supplementary file 6 — Source data Fig. 2 part 3 [file 44319_2026_742_MOESM6_ESM.zip › Figure 2 Part 3/Fig 2b polye acetylated tubulin confocal/PolyE-actub ET polye crop ns.tif]

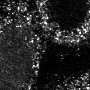

Supplement: Supplementary file 6 — Source data Fig. 2 part 3 [file 44319_2026_742_MOESM6_ESM.zip › Figure 2 Part 3/Fig 2b polye acetylated tubulin confocal/PolyE-actub PA polye crop ns.jpg]

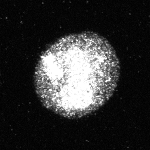

Supplement: Supplementary file 6 — Source data Fig. 2 part 3 [file 44319_2026_742_MOESM6_ESM.zip › Figure 2 Part 3/Fig 2b polye acetylated tubulin confocal/PolyE-actub PM RGP Full ns.tif]

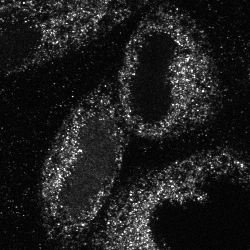

Supplement: Supplementary file 6 — Source data Fig. 2 part 3 [file 44319_2026_742_MOESM6_ESM.zip › Figure 2 Part 3/Fig 2b polye acetylated tubulin confocal/PolyE-actub PA RGB Full sc10.tif]

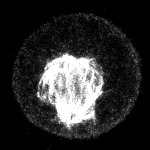

Supplement: Supplementary file 6 — Source data Fig. 2 part 3 [file 44319_2026_742_MOESM6_ESM.zip › Figure 2 Part 3/Fig 2b polye acetylated tubulin confocal/PolyE-actub M actub Full ns.jpg]

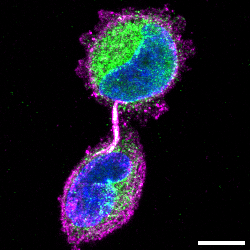

Supplement: Supplementary file 6 — Source data Fig. 2 part 3 [file 44319_2026_742_MOESM6_ESM.zip › Figure 2 Part 3/Fig 2b polye acetylated tubulin confocal/PolyE-actub ET RGB Full sc10.png]

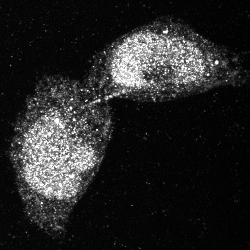

Supplement: Supplementary file 6 — Source data Fig. 2 part 3 [file 44319_2026_742_MOESM6_ESM.zip › Figure 2 Part 3/Fig 2b polye acetylated tubulin confocal/PolyE-actub LT polye Full ns.tif]

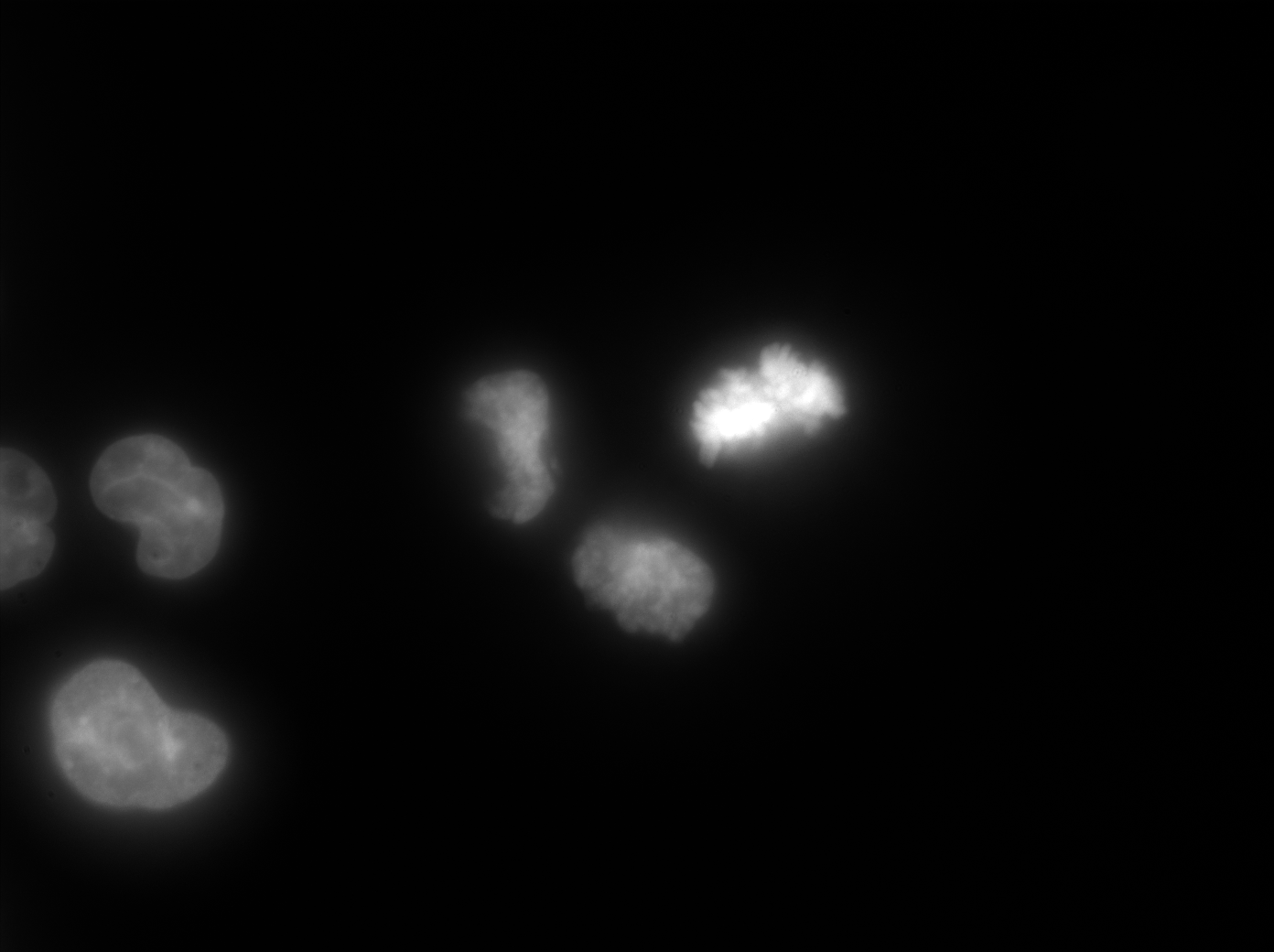

Supplement: Supplementary file 7 — Source data Fig. 2 part 4 [file 44319_2026_742_MOESM7_ESM.zip › Figure 2 Part 4/Fig 2d polye atubulin/WT PolyE-atub 8-14-24 R2 ET10 M7.Project Maximum Z_XY1723838292_Z0_T0_C0.tif]

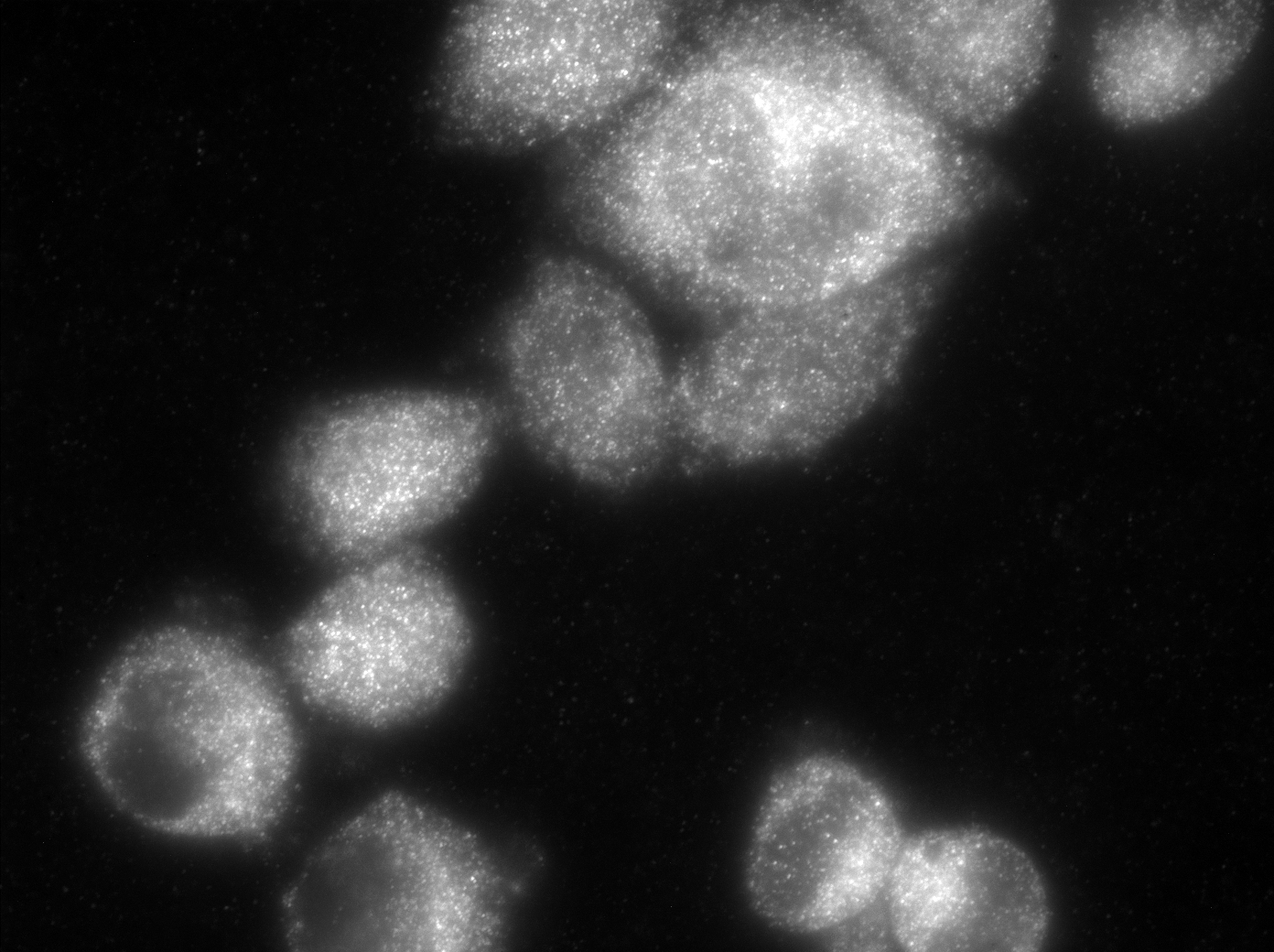

Supplement: Supplementary file 7 — Source data Fig. 2 part 4 [file 44319_2026_742_MOESM7_ESM.zip › Figure 2 Part 4/Fig 2d polye atubulin/WT PolyE-atub 8-14-24 R1 LT8LT9.Project Maximum Z_XY1723760012_Z0_T0_C2.tif]

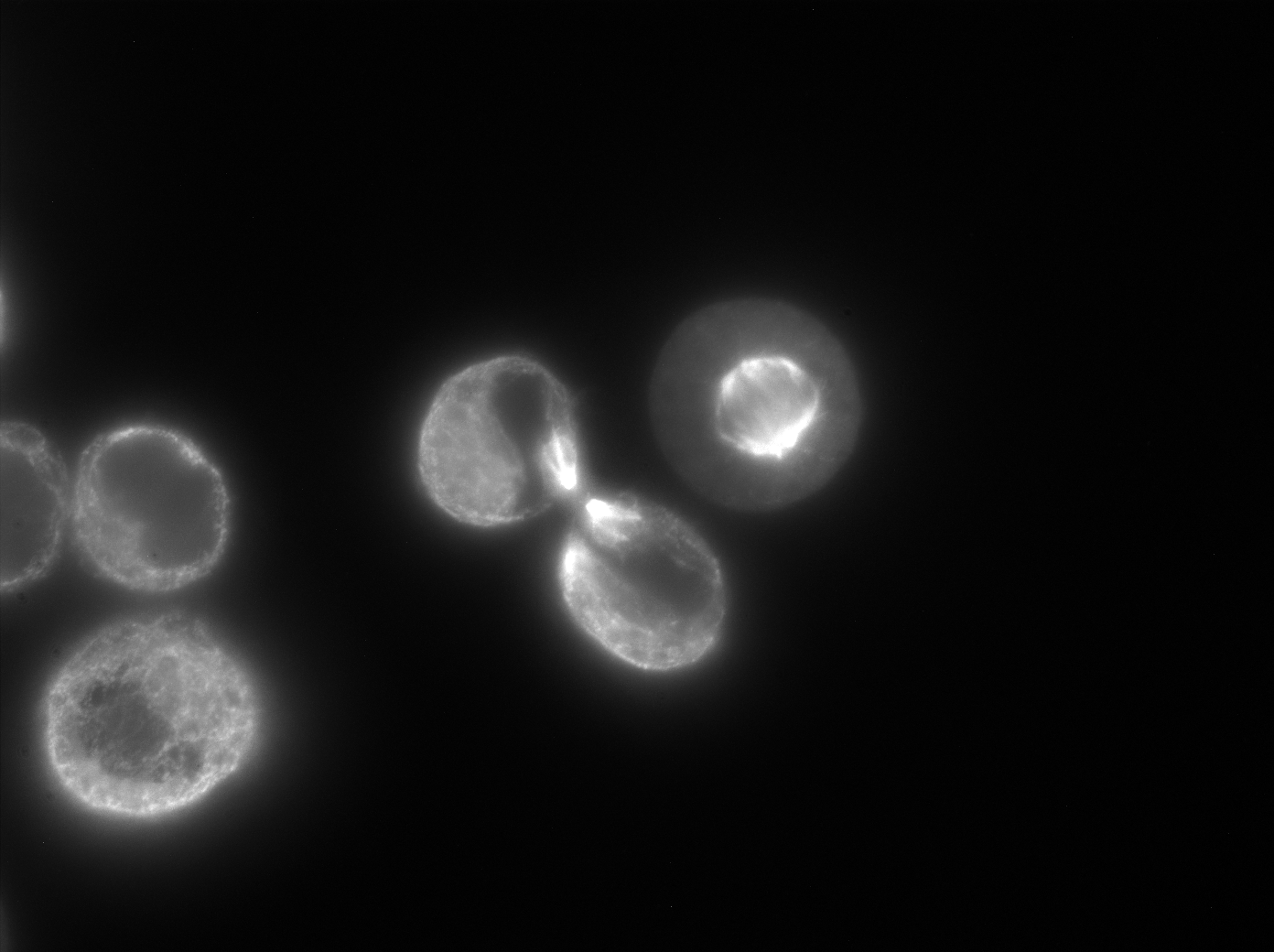

Supplement: Supplementary file 7 — Source data Fig. 2 part 4 [file 44319_2026_742_MOESM7_ESM.zip › Figure 2 Part 4/Fig 2d polye atubulin/WT PolyE-atub 8-14-24 R2 ET10 M7.Project Maximum Z_XY1723838292_Z0_T0_C1.tif]

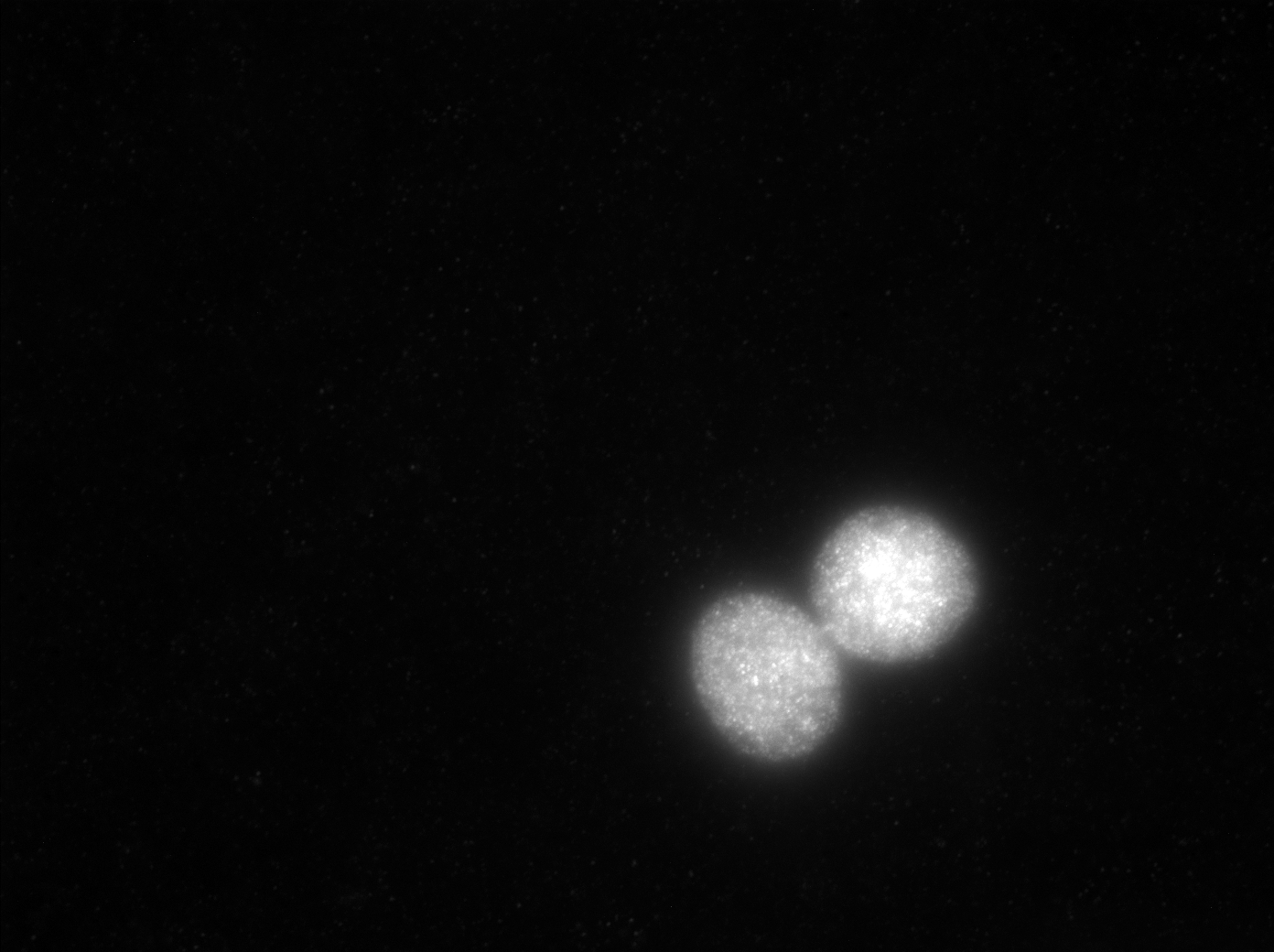

Supplement: Supplementary file 7 — Source data Fig. 2 part 4 [file 44319_2026_742_MOESM7_ESM.zip › Figure 2 Part 4/Fig 2d polye atubulin/WT PolyE-atub 8-14-24 R1 ET10.Project Maximum Z_XY1723759495_Z0_T0_C2.tif]

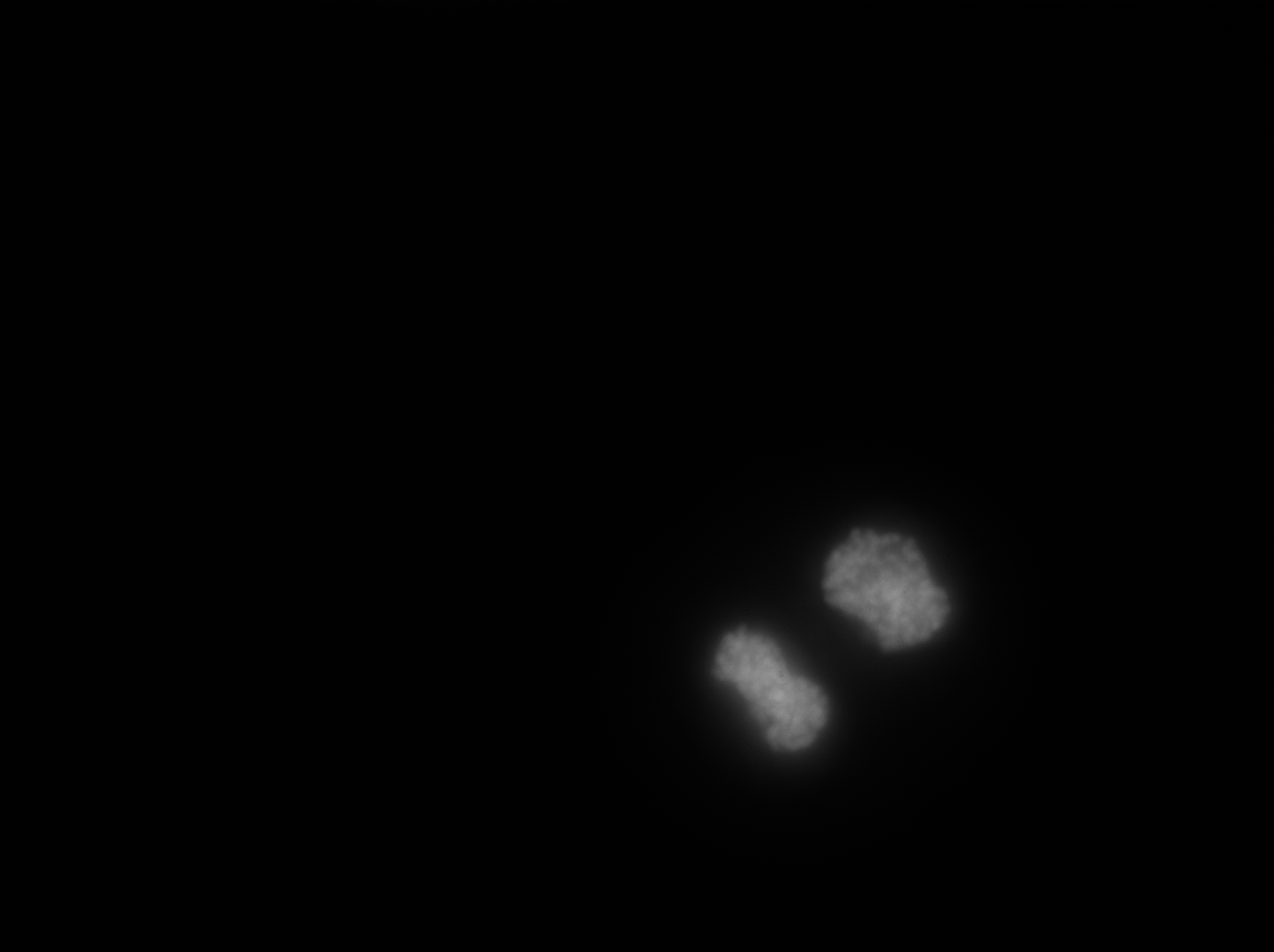

Supplement: Supplementary file 7 — Source data Fig. 2 part 4 [file 44319_2026_742_MOESM7_ESM.zip › Figure 2 Part 4/Fig 2d polye atubulin/WT PolyE-atub 8-14-24 R1 ET10.Project Maximum Z_XY1723759495_Z0_T0_C0.tif]

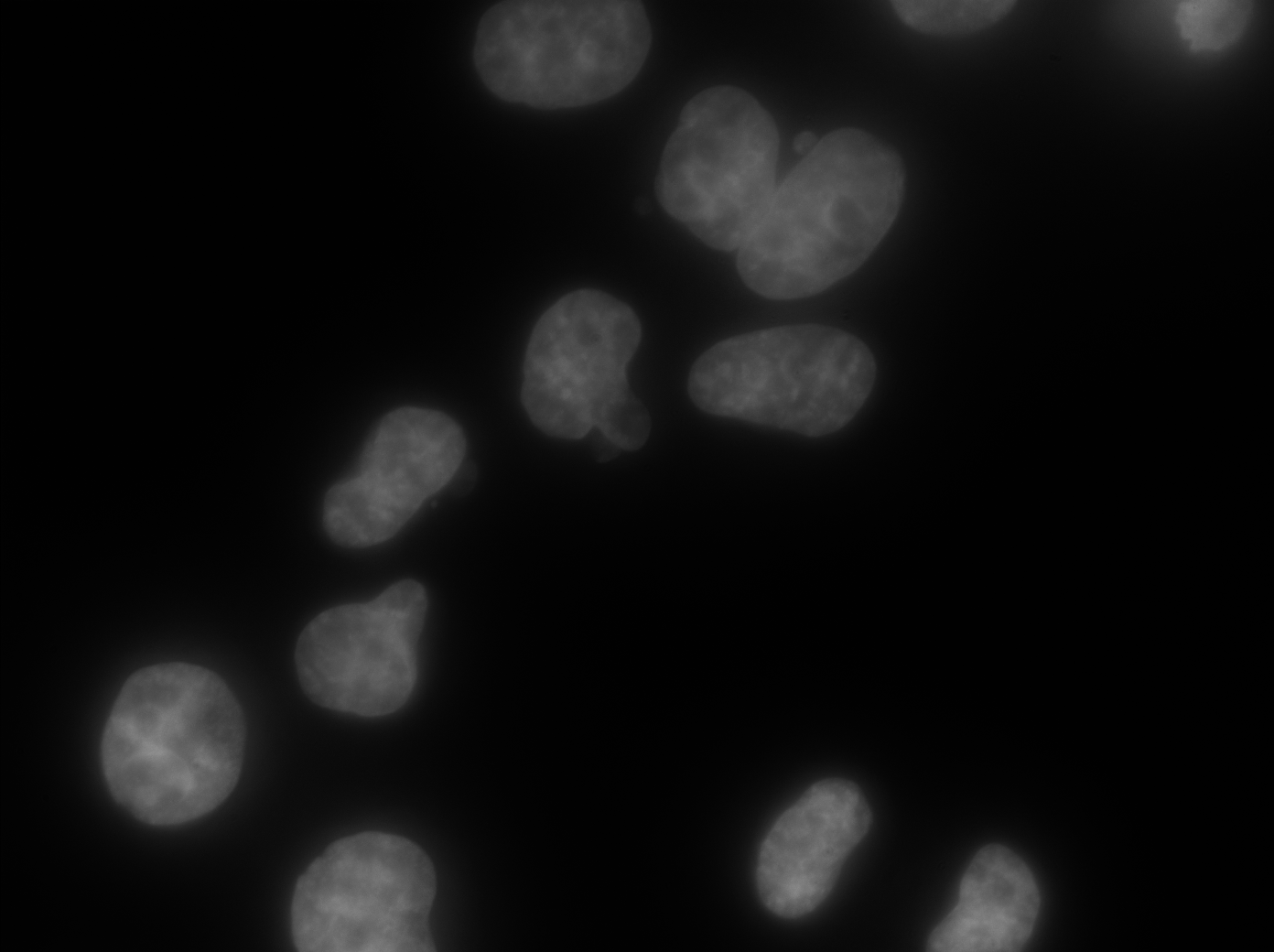

Supplement: Supplementary file 7 — Source data Fig. 2 part 4 [file 44319_2026_742_MOESM7_ESM.zip › Figure 2 Part 4/Fig 2d polye atubulin/WT PolyE-atub 8-14-24 R1 LT8LT9.Project Maximum Z_XY1723760012_Z0_T0_C0.tif]

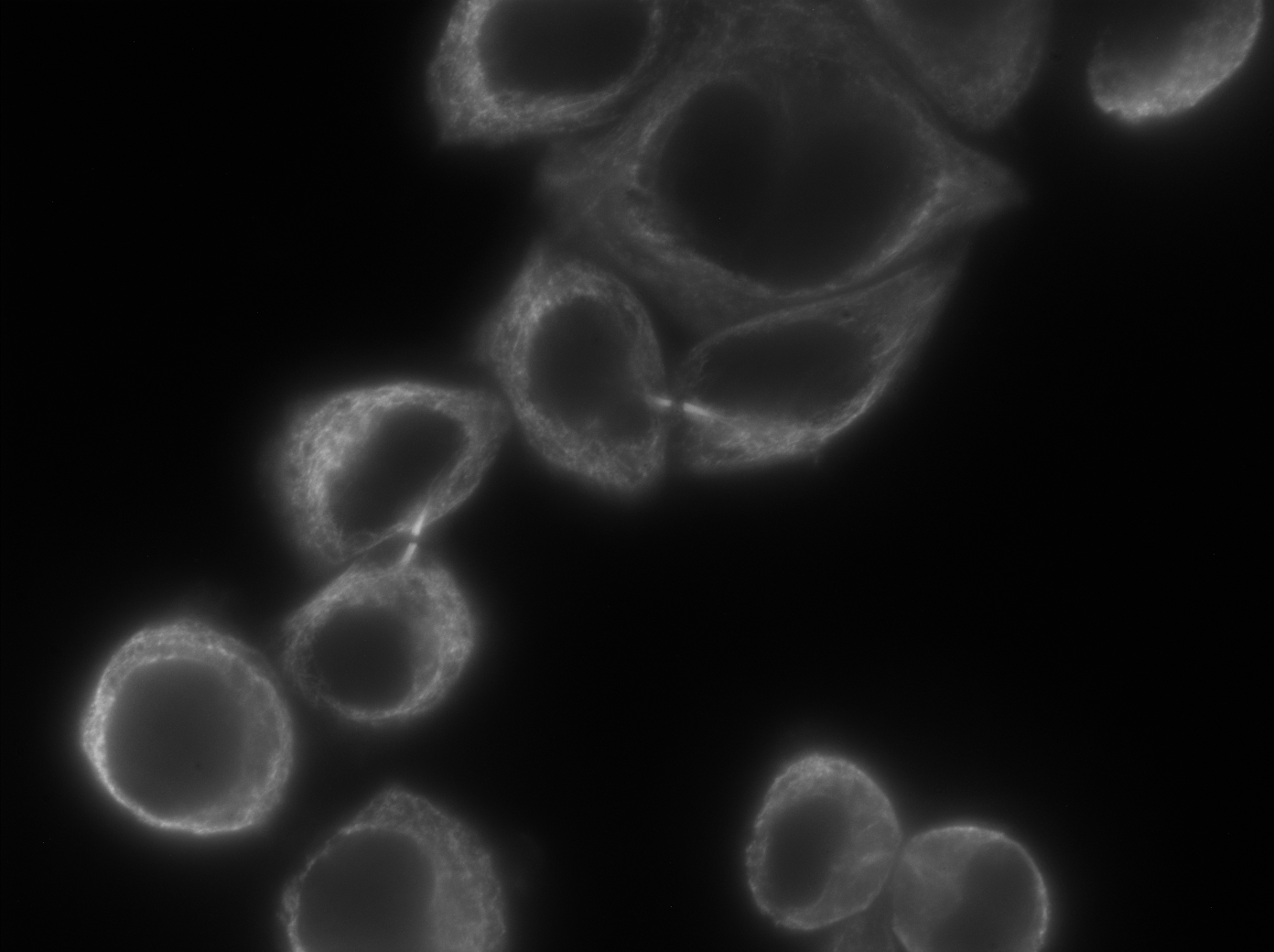

Supplement: Supplementary file 7 — Source data Fig. 2 part 4 [file 44319_2026_742_MOESM7_ESM.zip › Figure 2 Part 4/Fig 2d polye atubulin/WT PolyE-atub 8-14-24 R1 LT8LT9.Project Maximum Z_XY1723760012_Z0_T0_C1.tif]

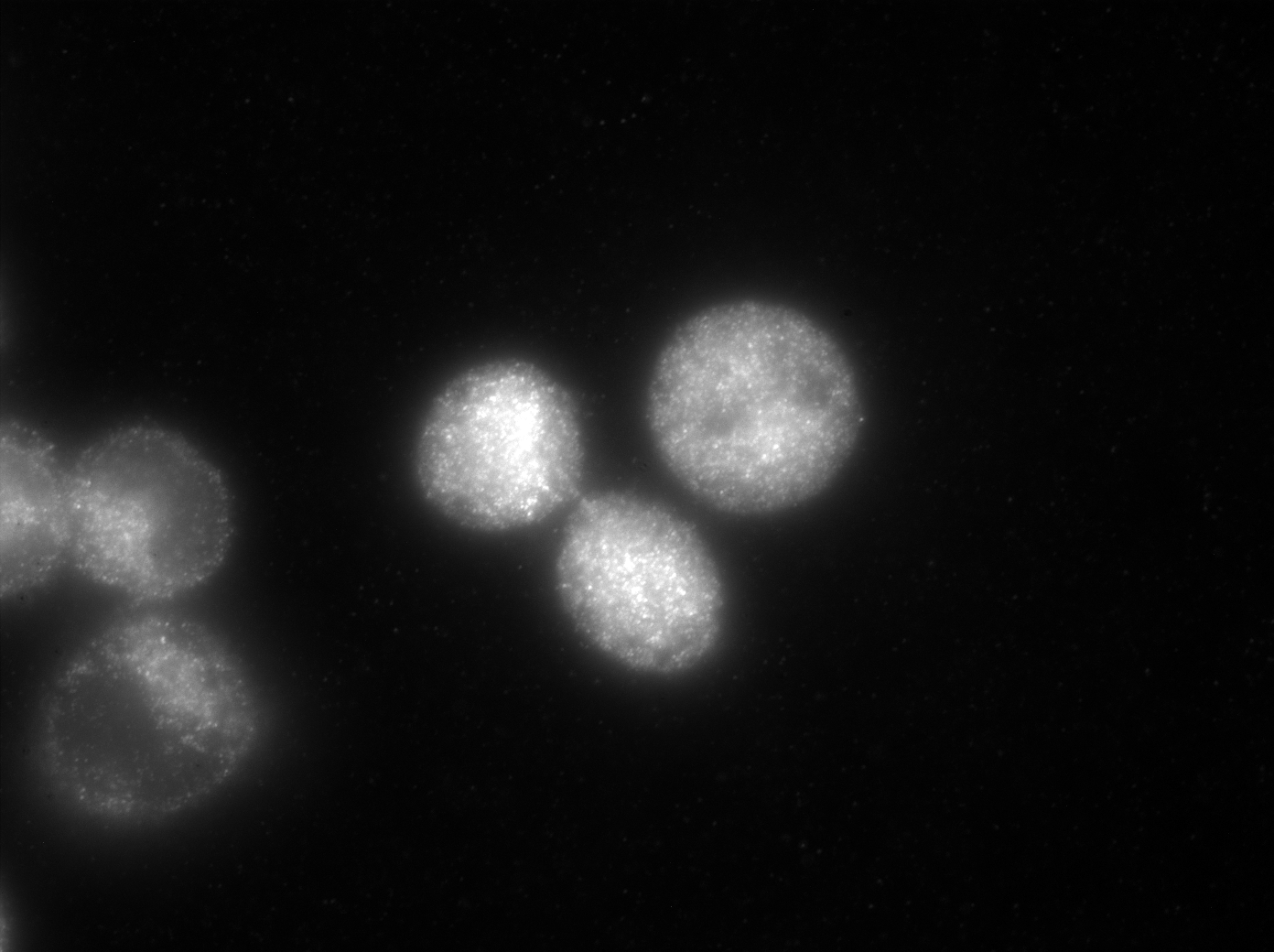

Supplement: Supplementary file 7 — Source data Fig. 2 part 4 [file 44319_2026_742_MOESM7_ESM.zip › Figure 2 Part 4/Fig 2d polye atubulin/WT PolyE-atub 8-14-24 R2 ET10 M7.Project Maximum Z_XY1723838292_Z0_T0_C2.tif]

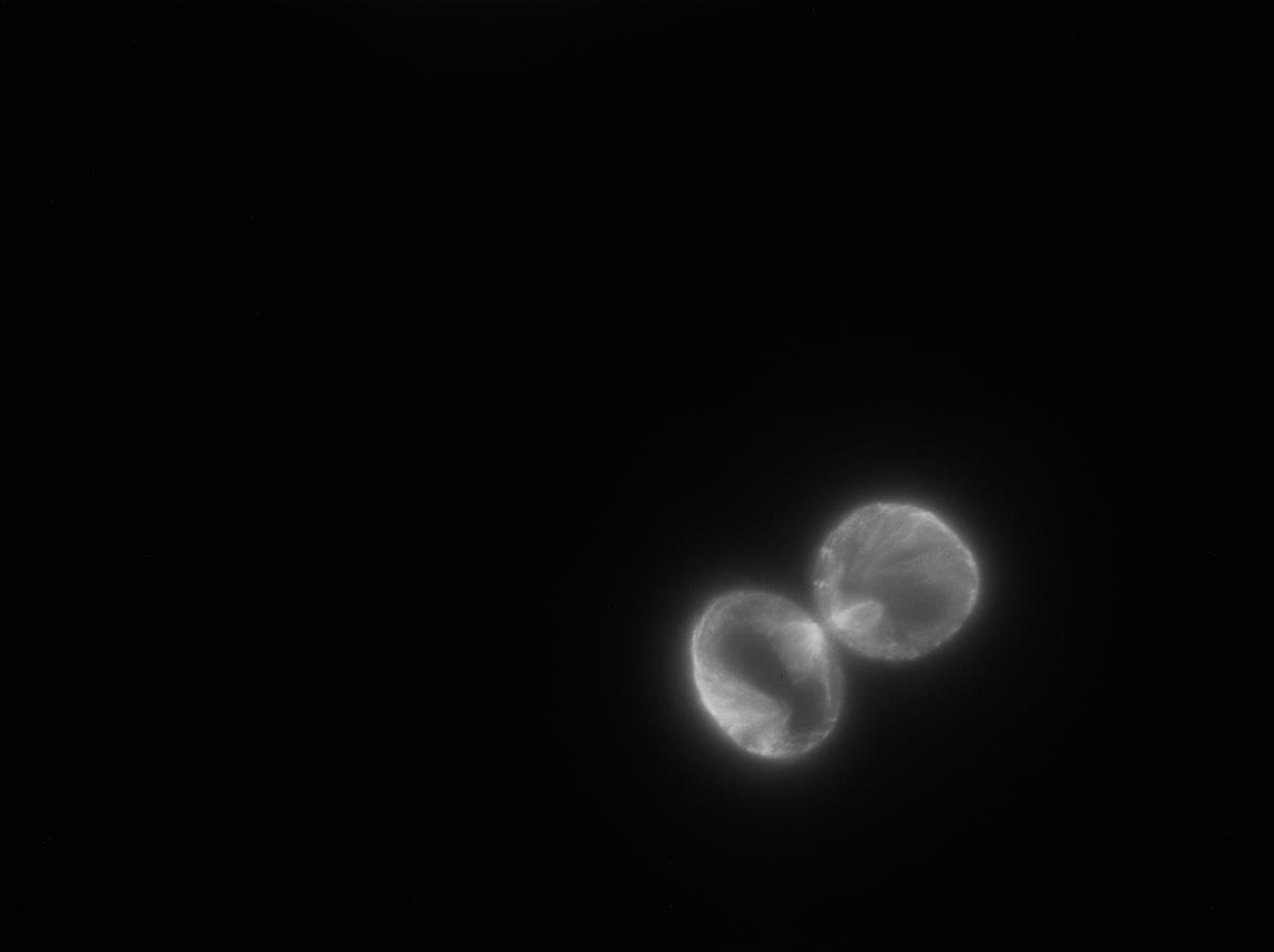

Supplement: Supplementary file 7 — Source data Fig. 2 part 4 [file 44319_2026_742_MOESM7_ESM.zip › Figure 2 Part 4/Fig 2d polye atubulin/WT PolyE-atub 8-14-24 R1 ET10.Project Maximum Z_XY1723759495_Z0_T0_C1.tif]

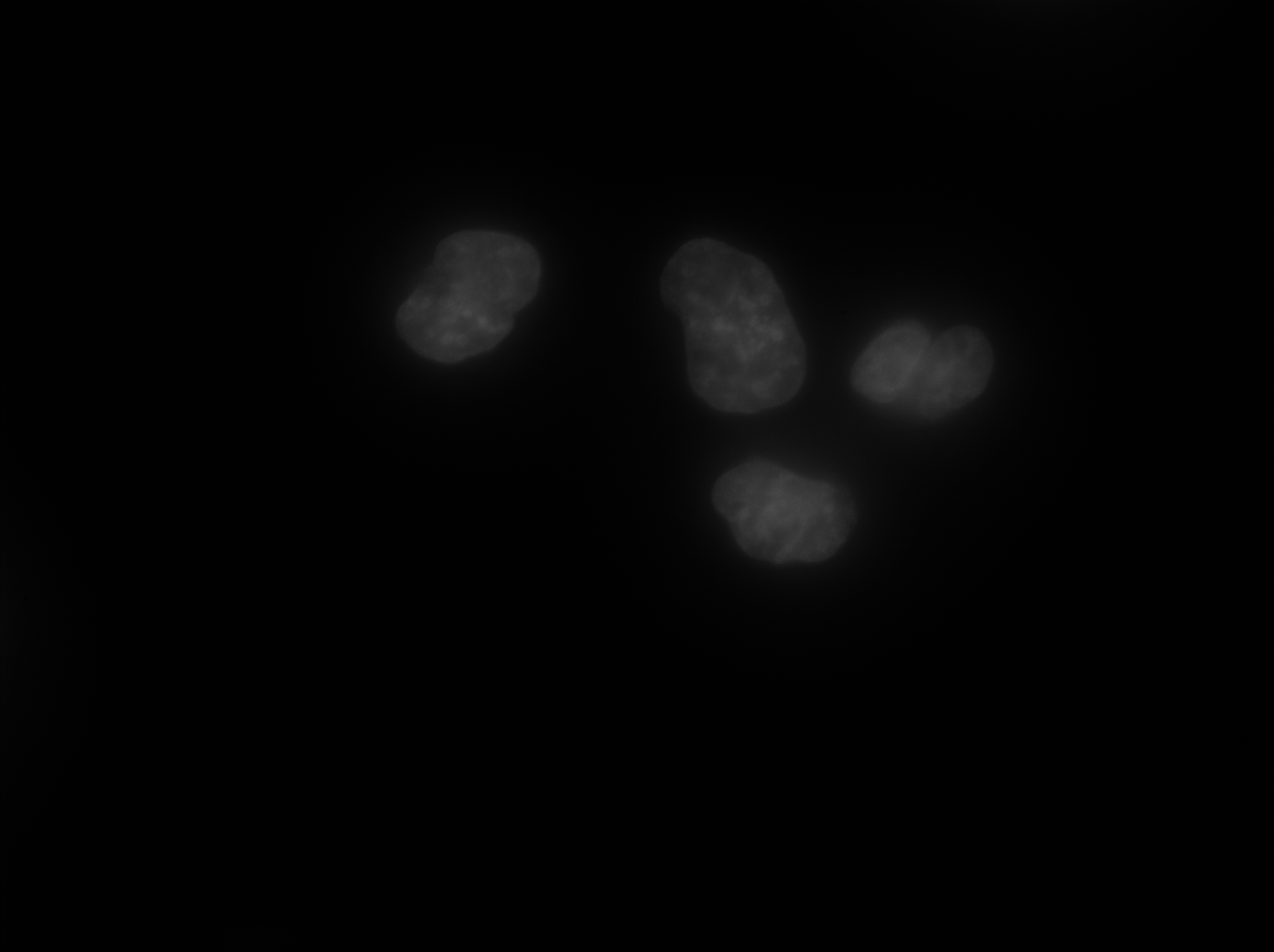

Supplement: Supplementary file 7 — Source data Fig. 2 part 4 [file 44319_2026_742_MOESM7_ESM.zip › Figure 2 Part 4/Fig 2d polye atubulin/WT PolyE-atub 8-14-24 R1 LT1.Project Maximum Z_XY1723756056_Z0_T0_C0.tif]

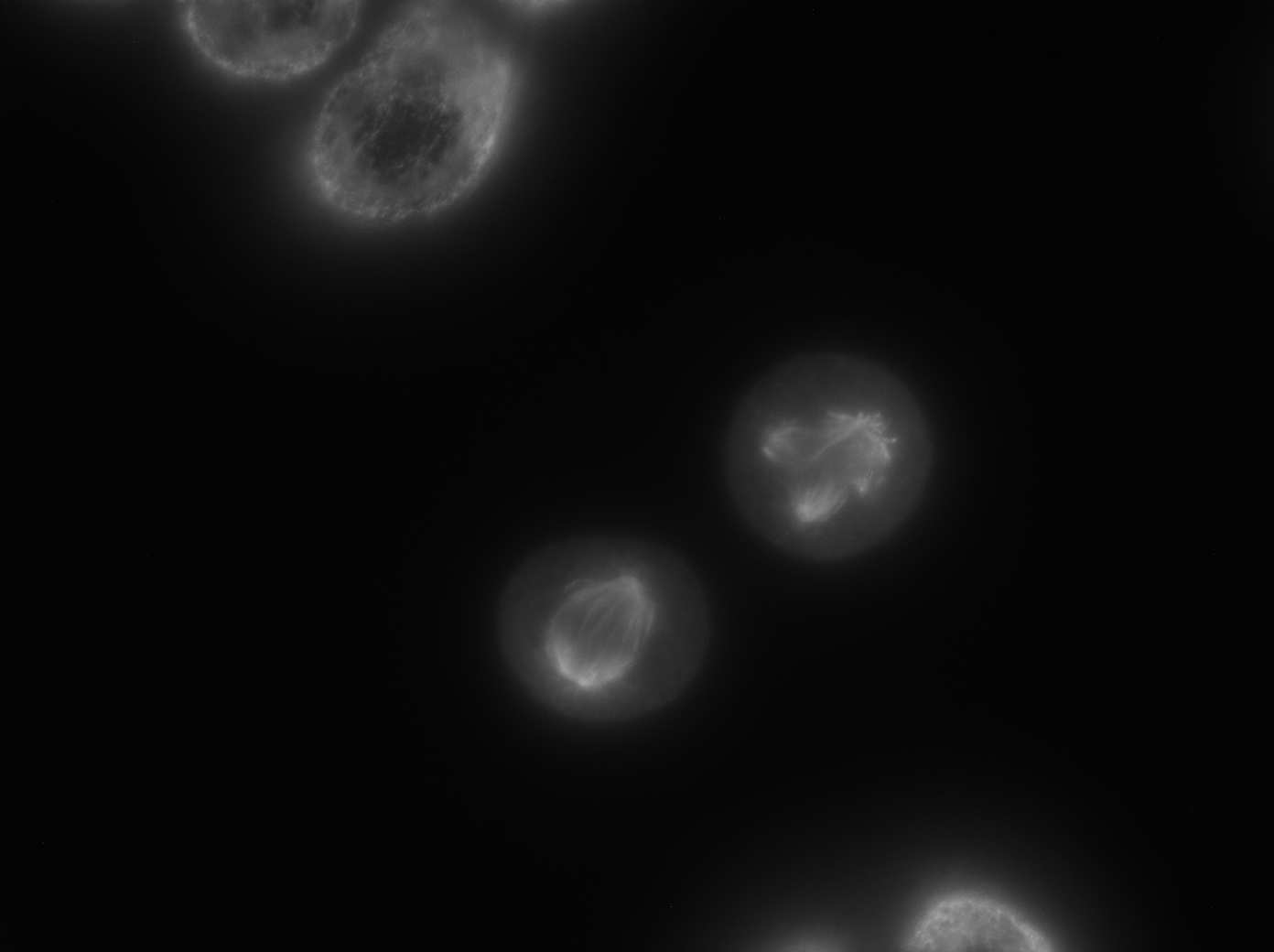

Supplement: Supplementary file 7 — Source data Fig. 2 part 4 [file 44319_2026_742_MOESM7_ESM.zip › Figure 2 Part 4/Fig 2d polye atubulin/WT PolyE-atub 8-14-24 R1 M1.Project Maximum Z_XY1723755847_Z0_T0_C1.tif]

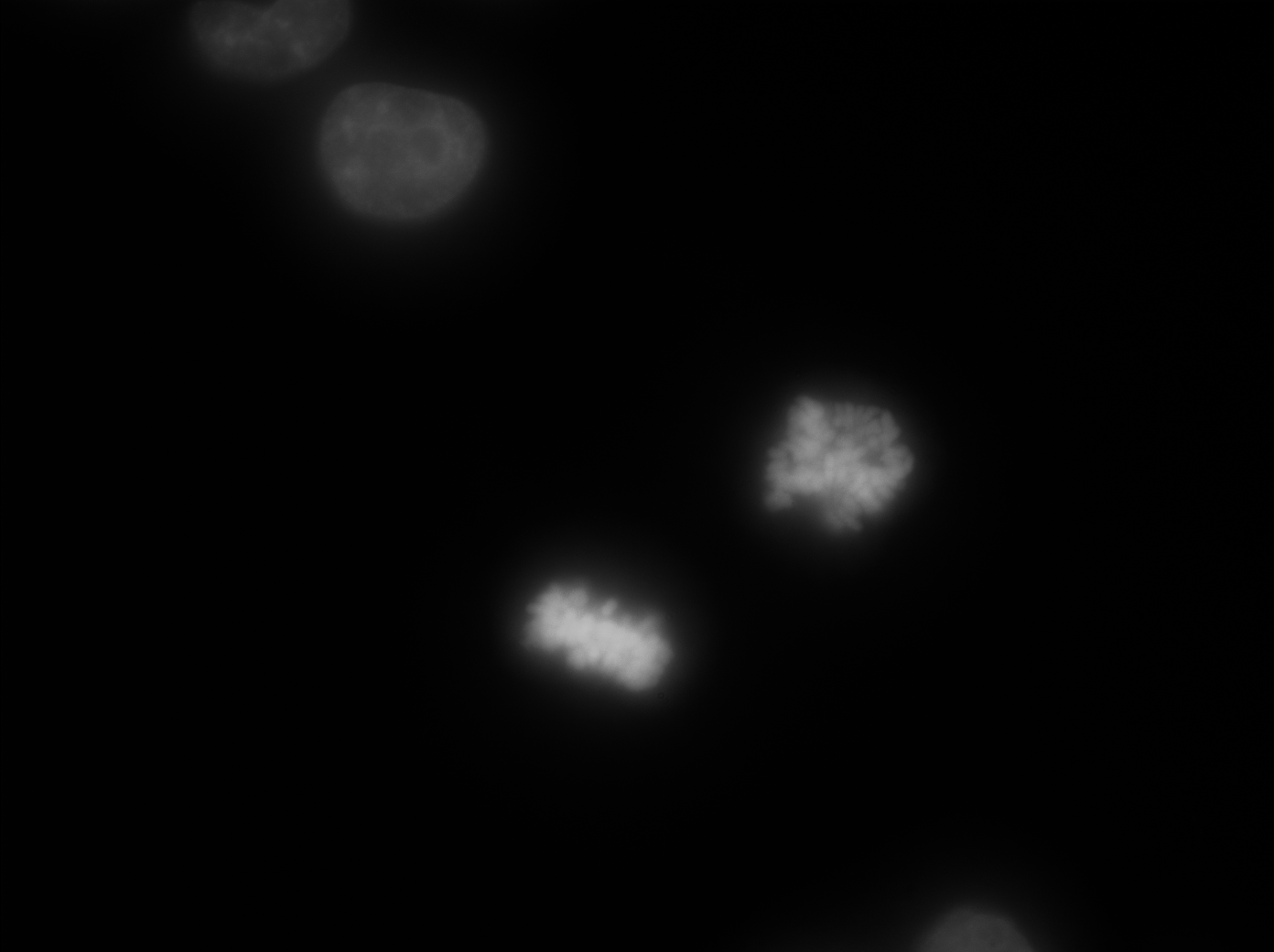

Supplement: Supplementary file 7 — Source data Fig. 2 part 4 [file 44319_2026_742_MOESM7_ESM.zip › Figure 2 Part 4/Fig 2d polye atubulin/WT PolyE-atub 8-14-24 R1 M1.Project Maximum Z_XY1723755847_Z0_T0_C0.tif]

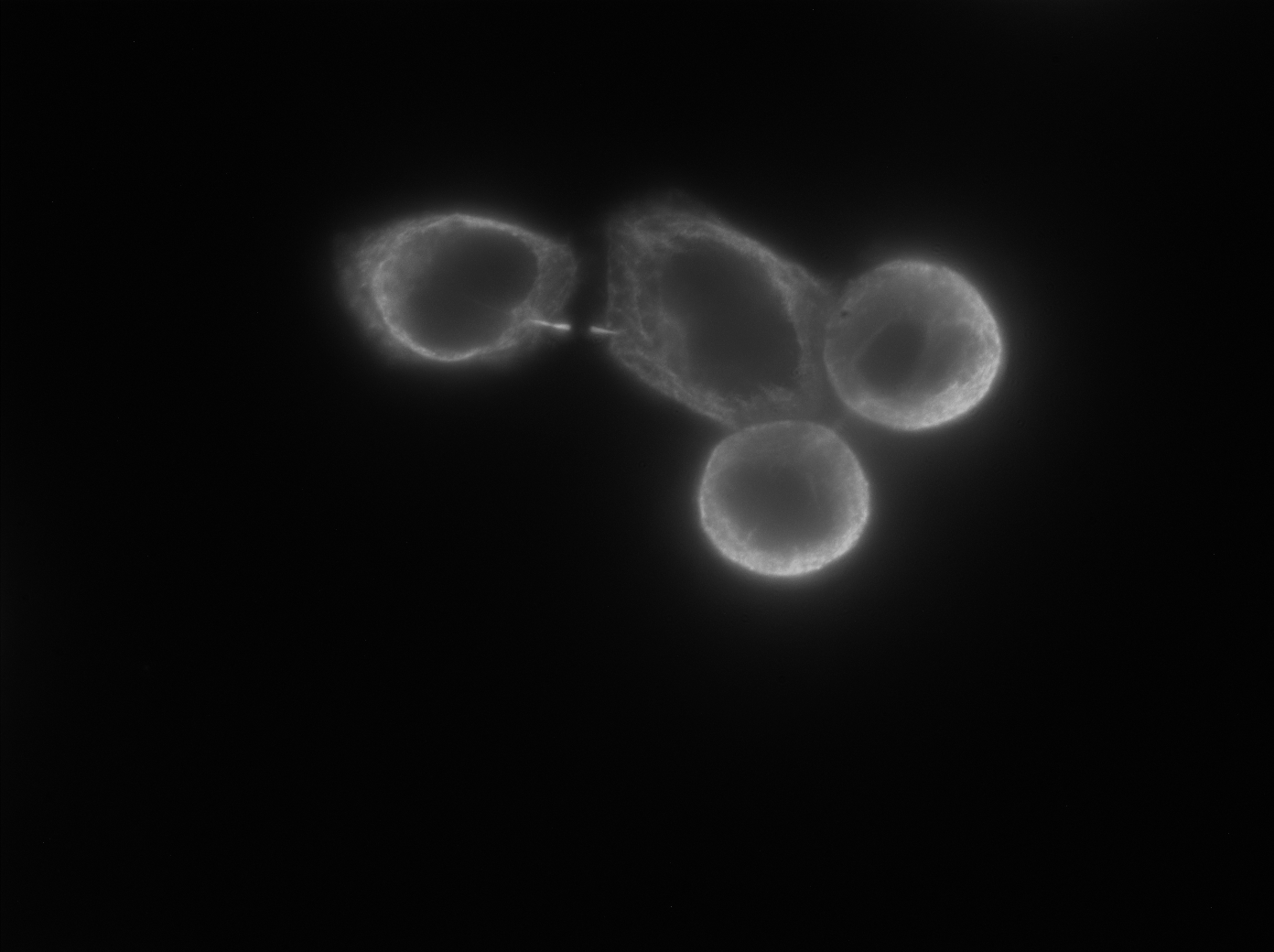

Supplement: Supplementary file 7 — Source data Fig. 2 part 4 [file 44319_2026_742_MOESM7_ESM.zip › Figure 2 Part 4/Fig 2d polye atubulin/WT PolyE-atub 8-14-24 R1 LT1.Project Maximum Z_XY1723756056_Z0_T0_C1.tif]

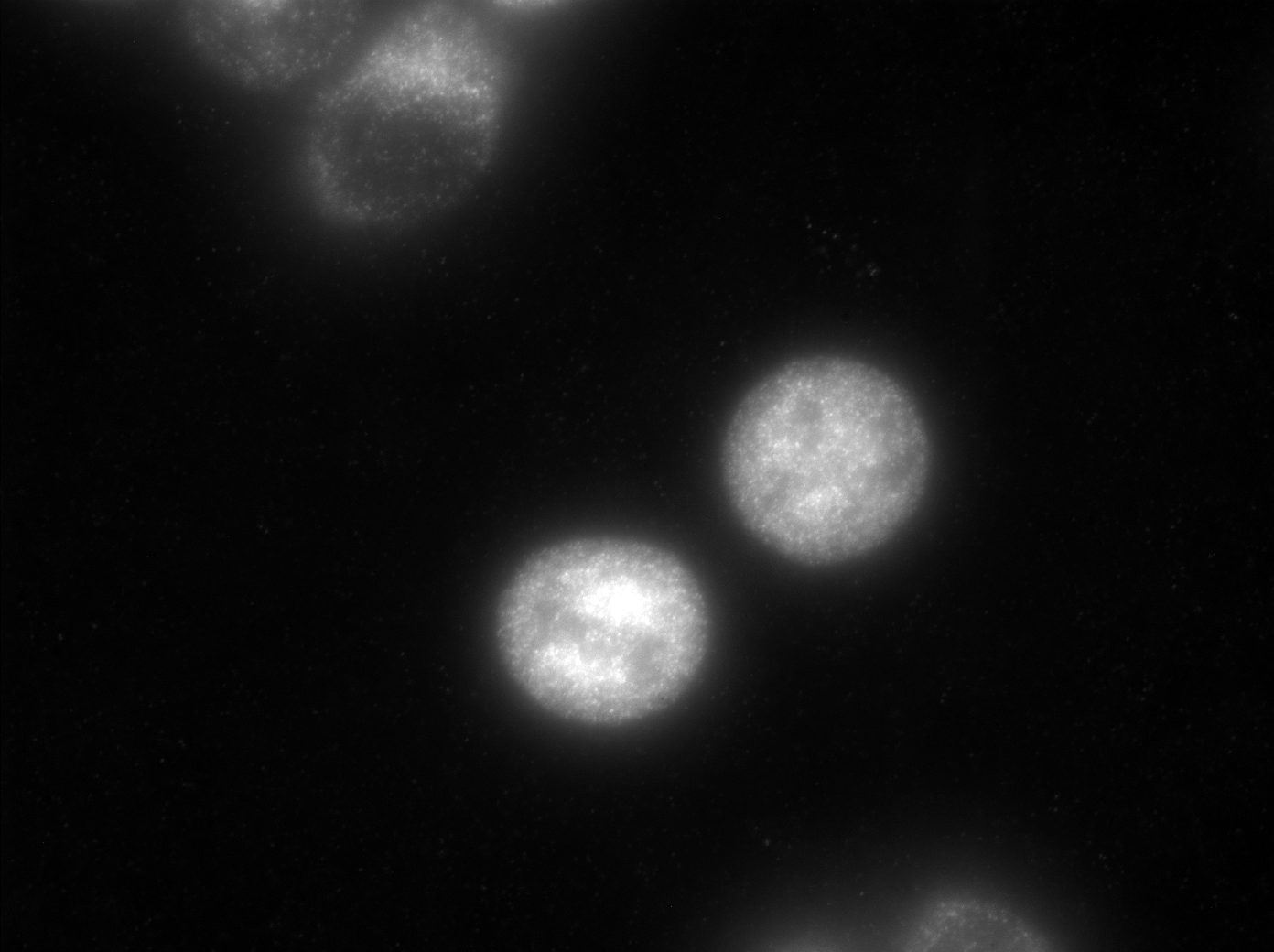

Supplement: Supplementary file 7 — Source data Fig. 2 part 4 [file 44319_2026_742_MOESM7_ESM.zip › Figure 2 Part 4/Fig 2d polye atubulin/WT PolyE-atub 8-14-24 R1 M1.Project Maximum Z_XY1723755847_Z0_T0_C2.tif]

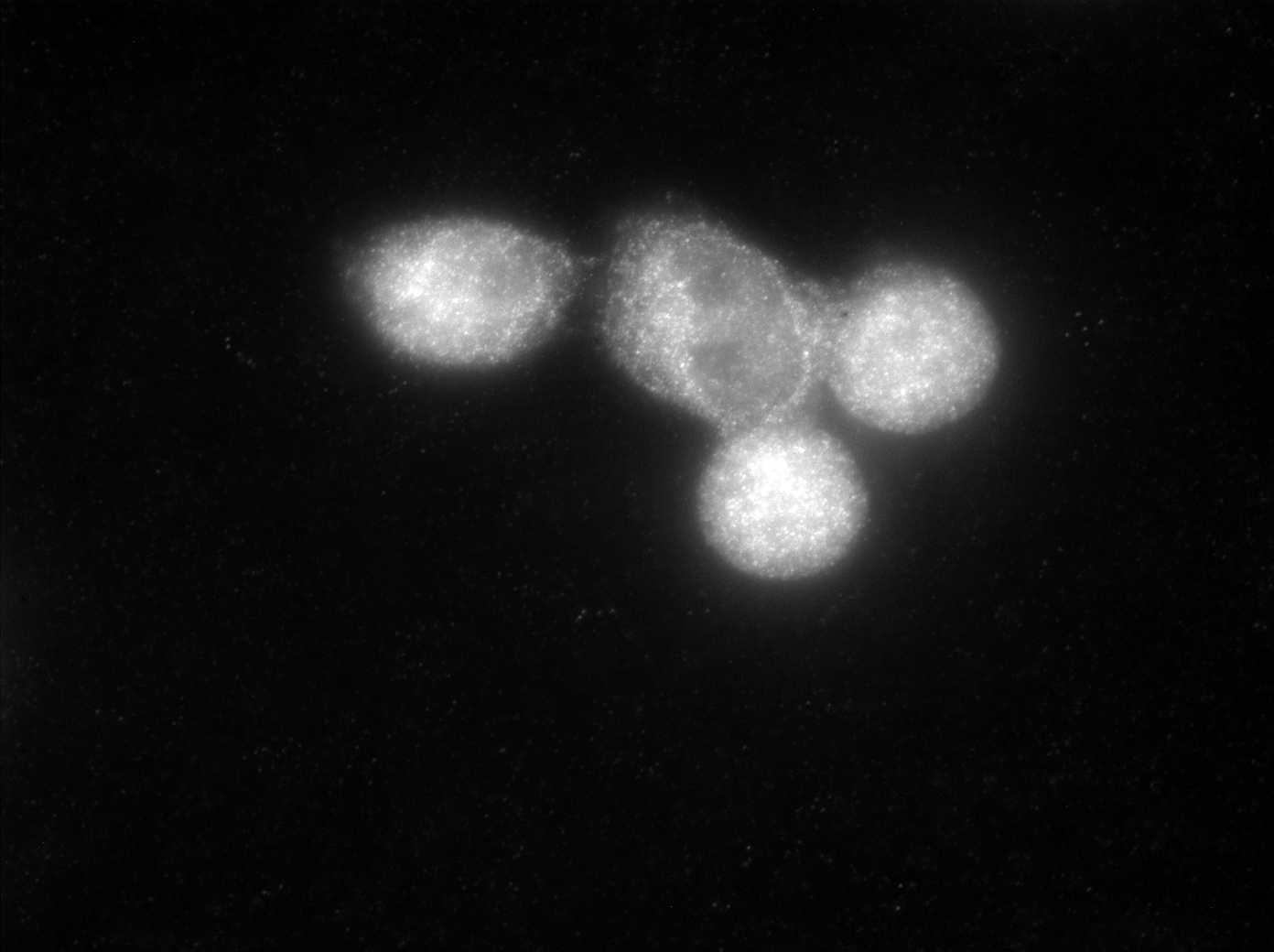

Supplement: Supplementary file 7 — Source data Fig. 2 part 4 [file 44319_2026_742_MOESM7_ESM.zip › Figure 2 Part 4/Fig 2d polye atubulin/WT PolyE-atub 8-14-24 R1 LT1.Project Maximum Z_XY1723756056_Z0_T0_C2.tif]

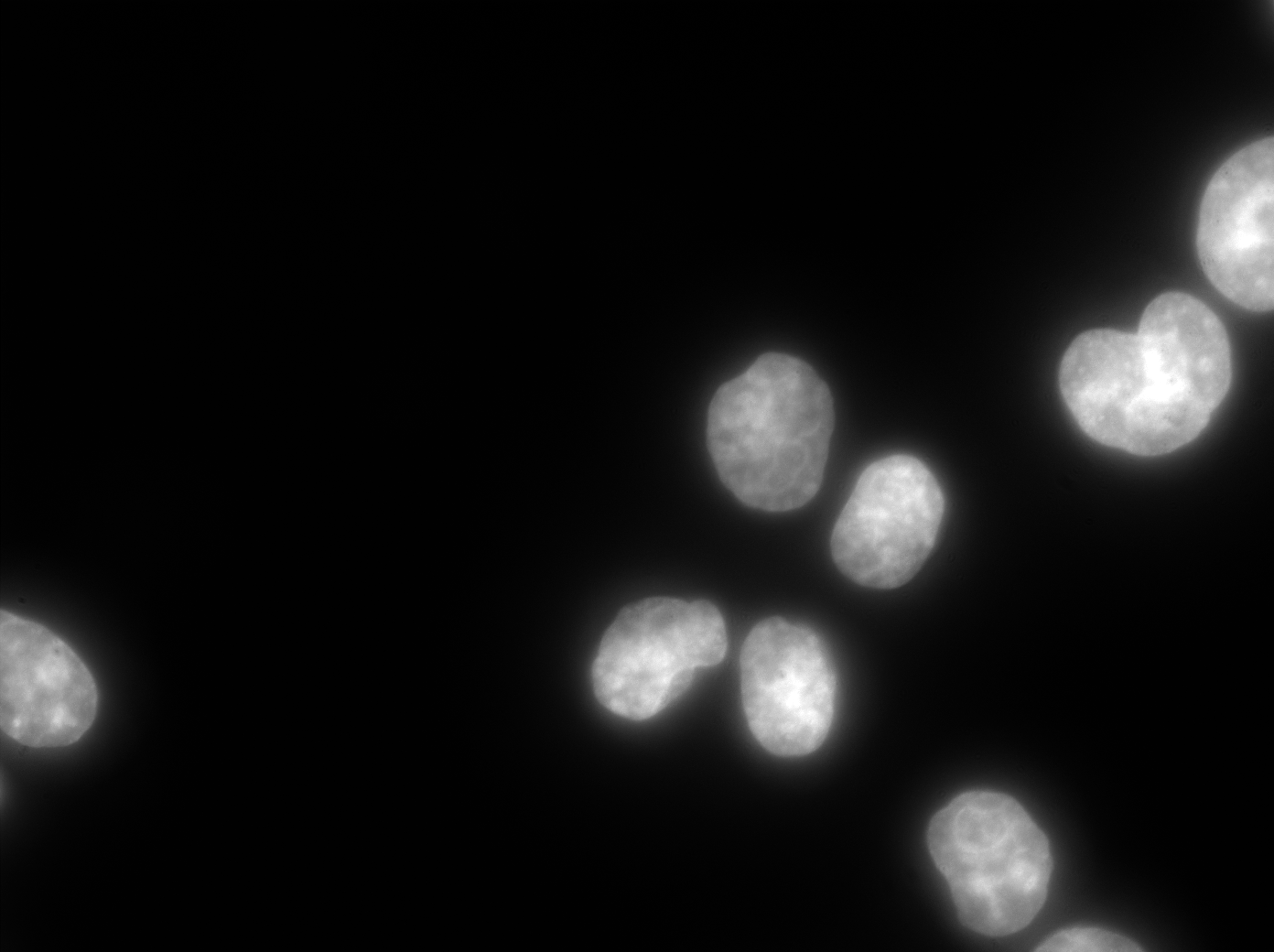

Supplement: Supplementary file 7 — Source data Fig. 2 part 4 [file 44319_2026_742_MOESM7_ESM.zip › Figure 2 Part 4/Fig 2d polye atubulin/WT PolyE-atub 8-14-24 R2 ET3ET4.Project Maximum Z_XY1723834195_Z0_T0_C0.tif]

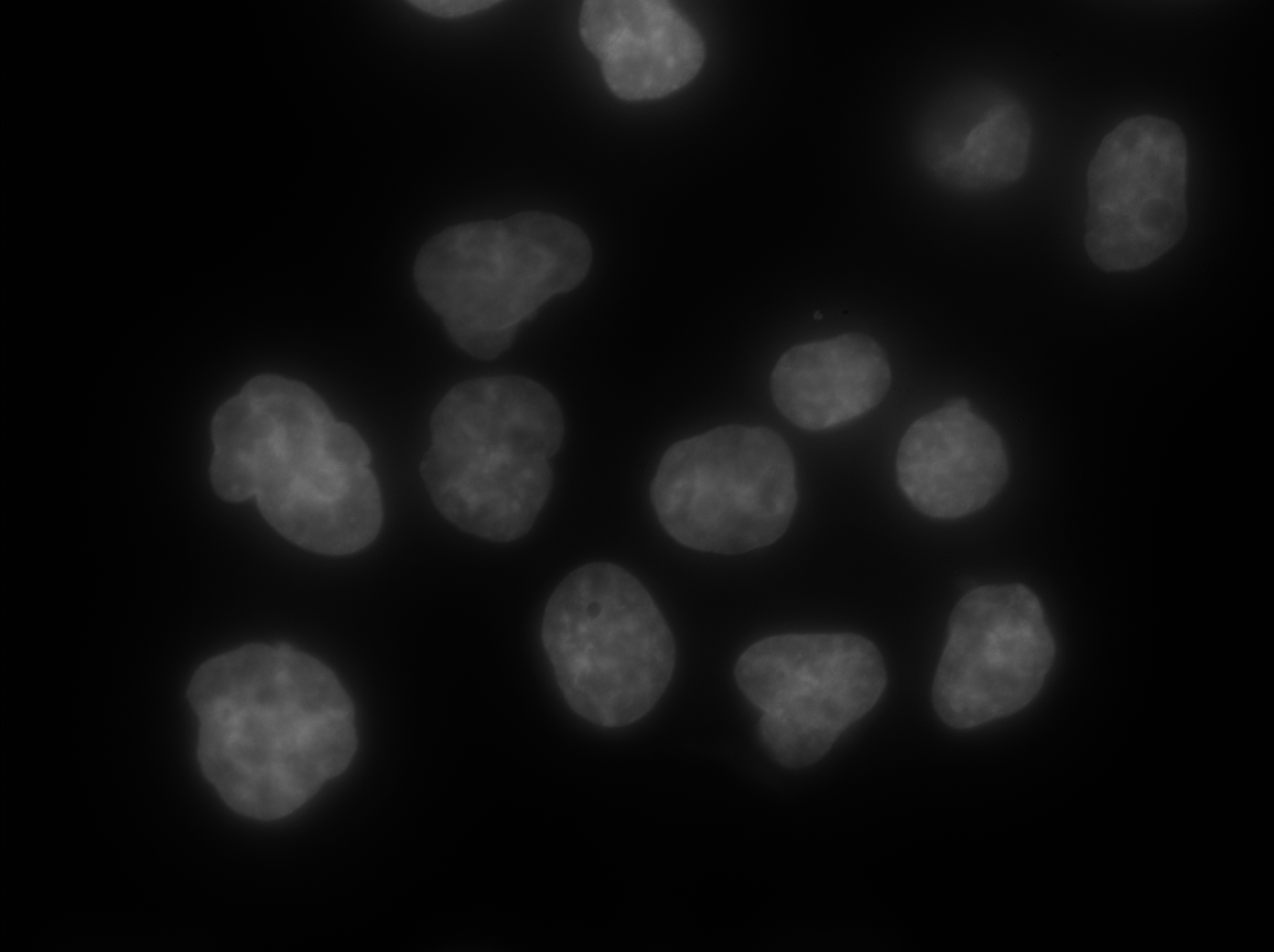

Supplement: Supplementary file 7 — Source data Fig. 2 part 4 [file 44319_2026_742_MOESM7_ESM.zip › Figure 2 Part 4/Fig 2d polye atubulin/WT PolyE-atub 8-14-24 R1 PA9PA10.Project Maximum Z_XY1723761363_Z0_T0_C0.tif]

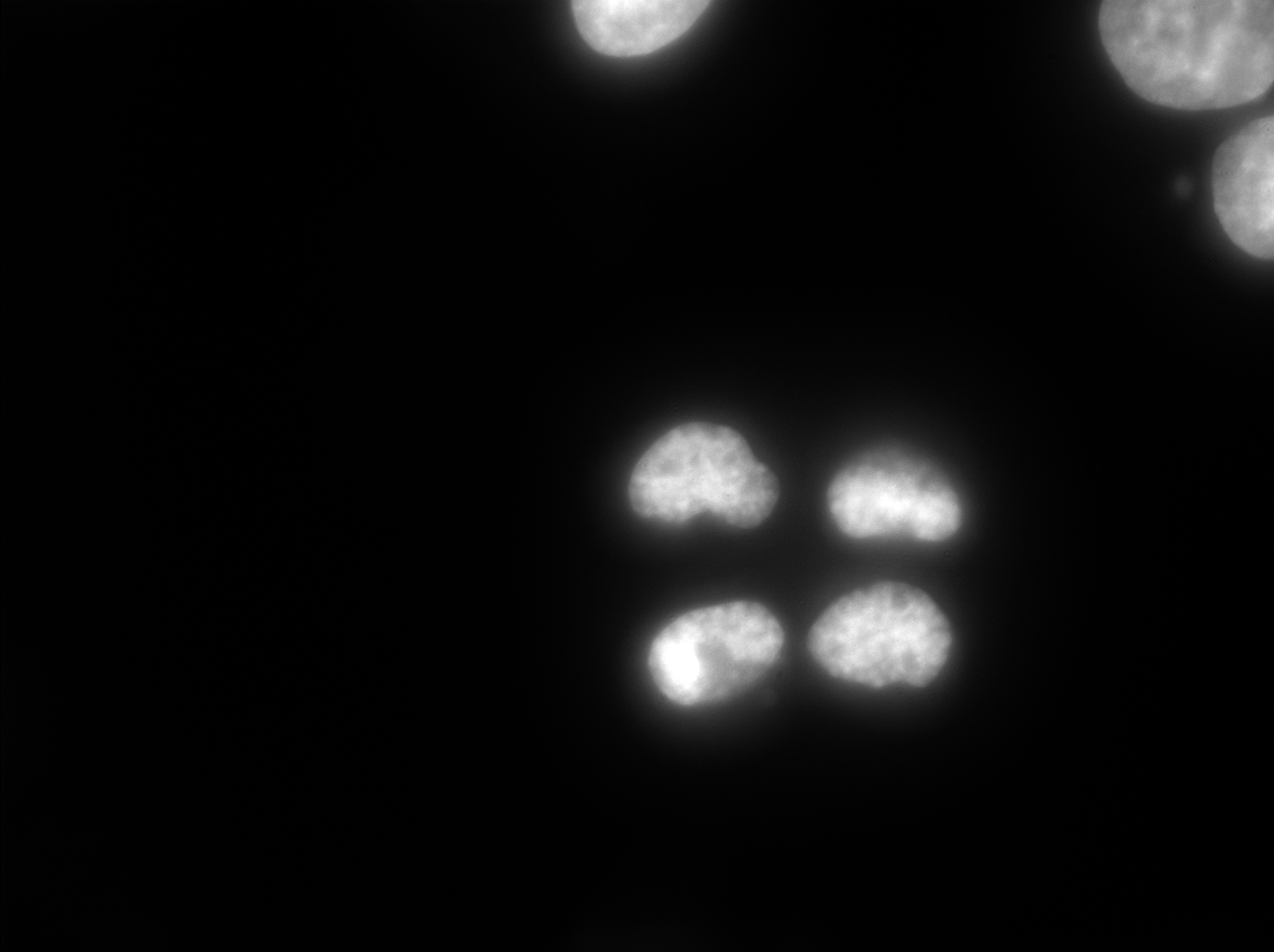

Supplement: Supplementary file 7 — Source data Fig. 2 part 4 [file 44319_2026_742_MOESM7_ESM.zip › Figure 2 Part 4/Fig 2d polye atubulin/WT PolyE-atub 8-14-24 R2 ET6ET7.Project Maximum Z_XY1723836630_Z0_T0_C0.tif]

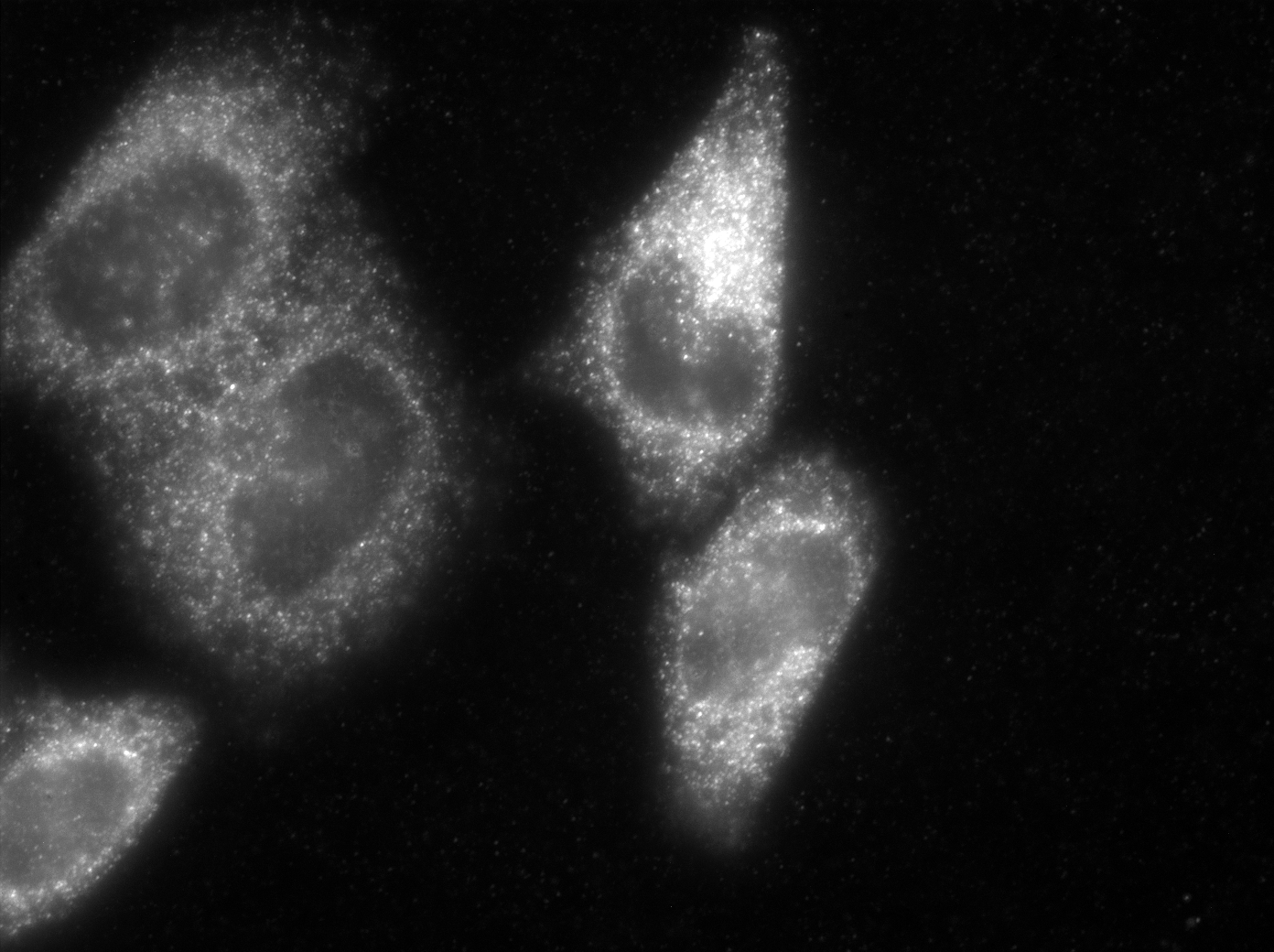

Supplement: Supplementary file 7 — Source data Fig. 2 part 4 [file 44319_2026_742_MOESM7_ESM.zip › Figure 2 Part 4/Fig 2d polye atubulin/WT PolyE-atub 8-14-24 R1 LT6.Project Maximum Z_XY1723758789_Z0_T0_C2.tif]

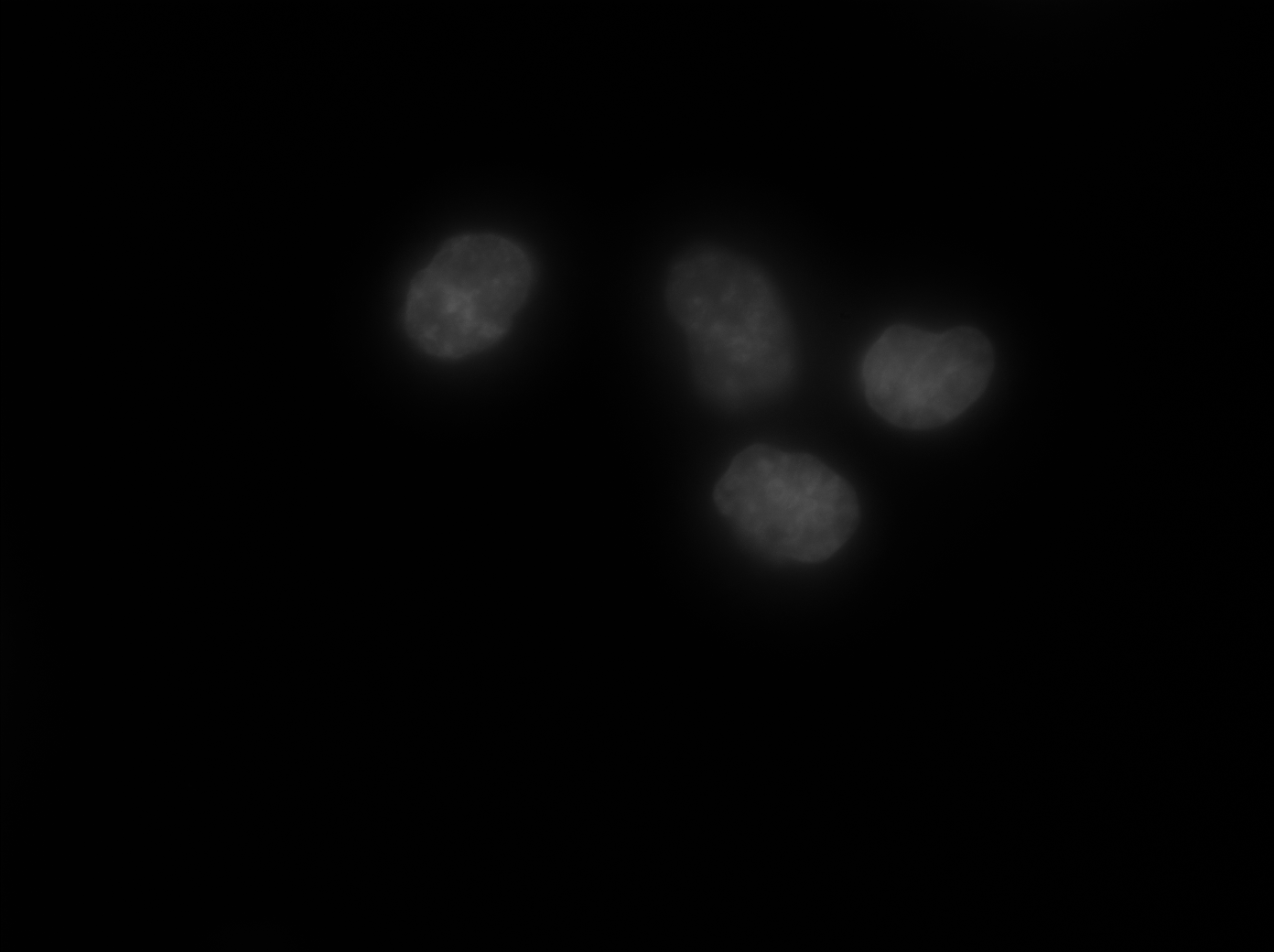

Supplement: Supplementary file 7 — Source data Fig. 2 part 4 [file 44319_2026_742_MOESM7_ESM.zip › Figure 2 Part 4/Fig 2d polye atubulin/WT PolyE-atub 8-14-24 R1 ET1.Project Maximum Z_XY1723755979_Z0_T0_C0.tif]

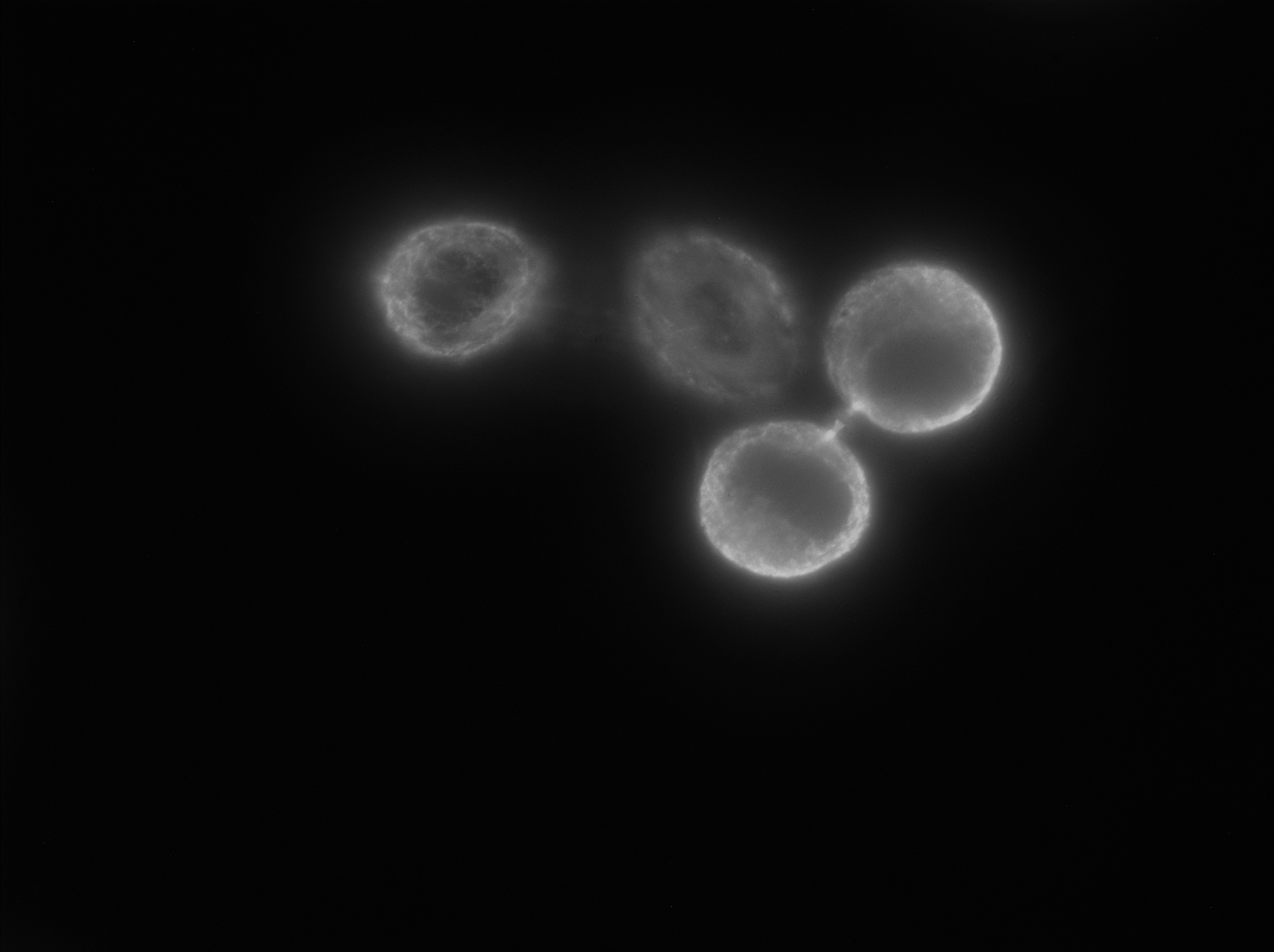

Supplement: Supplementary file 7 — Source data Fig. 2 part 4 [file 44319_2026_742_MOESM7_ESM.zip › Figure 2 Part 4/Fig 2d polye atubulin/WT PolyE-atub 8-14-24 R1 ET1.Project Maximum Z_XY1723755979_Z0_T0_C1.tif]

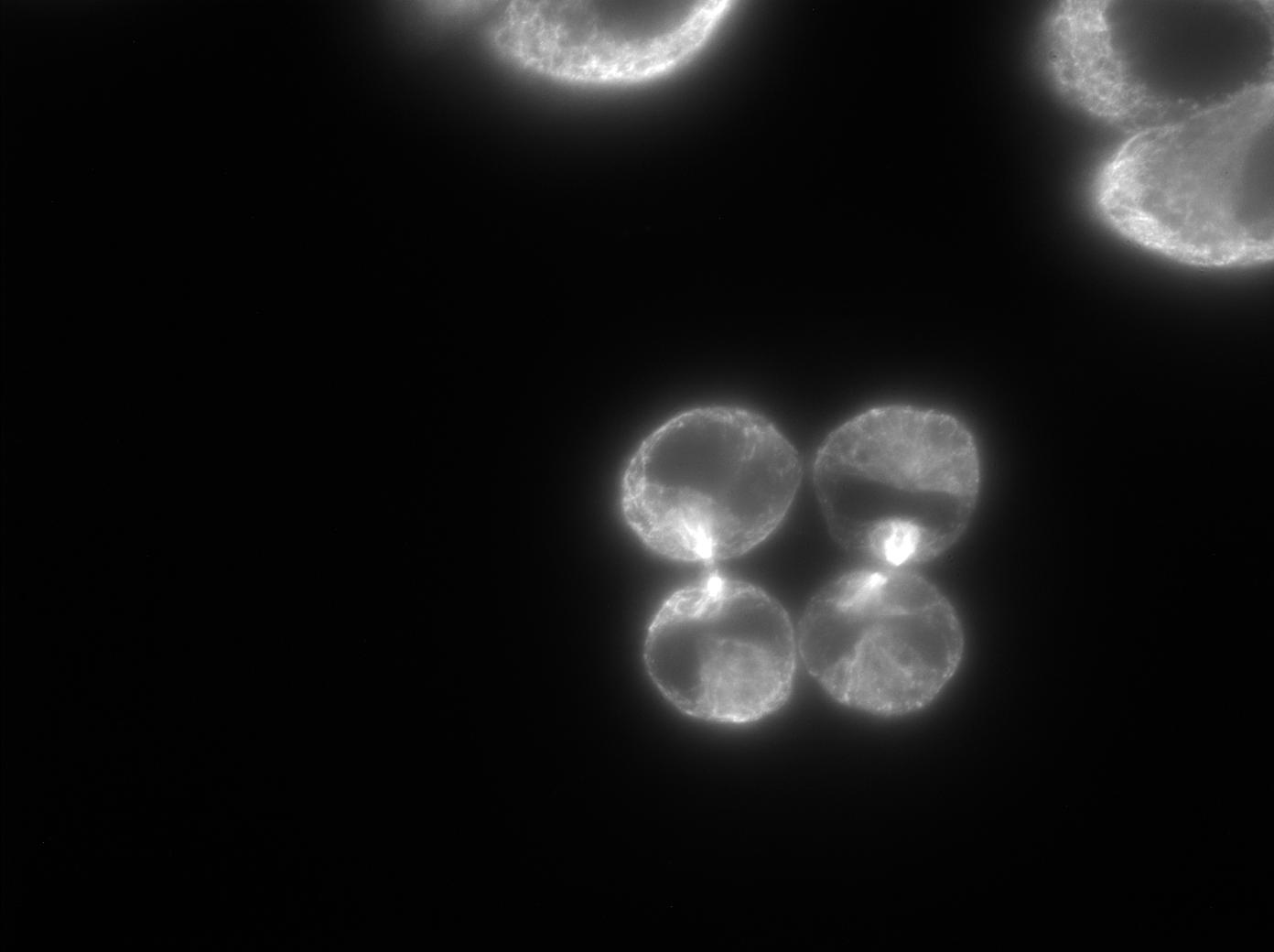

Supplement: Supplementary file 7 — Source data Fig. 2 part 4 [file 44319_2026_742_MOESM7_ESM.zip › Figure 2 Part 4/Fig 2d polye atubulin/WT PolyE-atub 8-14-24 R2 ET6ET7.Project Maximum Z_XY1723836630_Z0_T0_C1.tif]

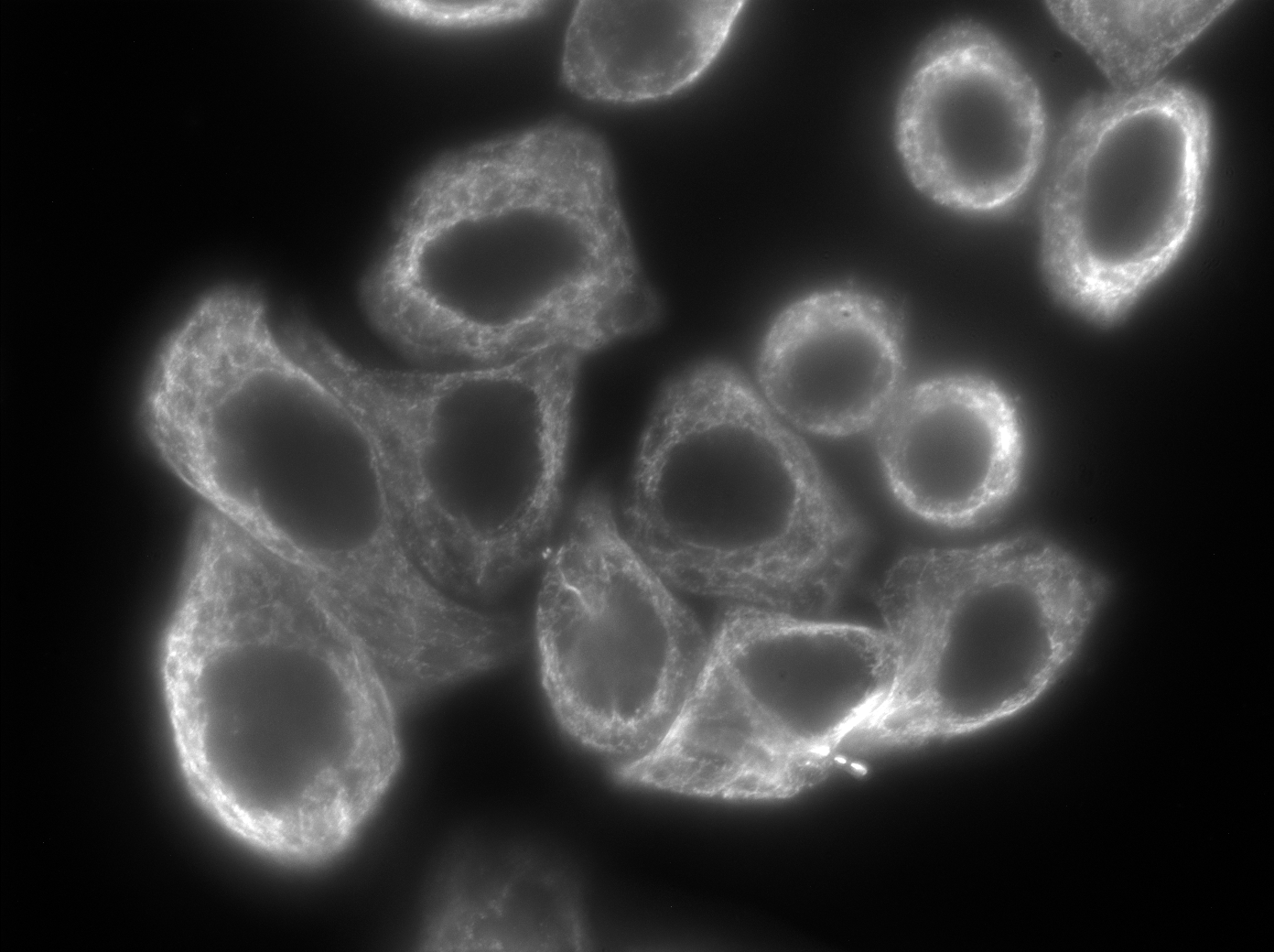

Supplement: Supplementary file 7 — Source data Fig. 2 part 4 [file 44319_2026_742_MOESM7_ESM.zip › Figure 2 Part 4/Fig 2d polye atubulin/WT PolyE-atub 8-14-24 R1 PA9PA10.Project Maximum Z_XY1723761363_Z0_T0_C1.tif]

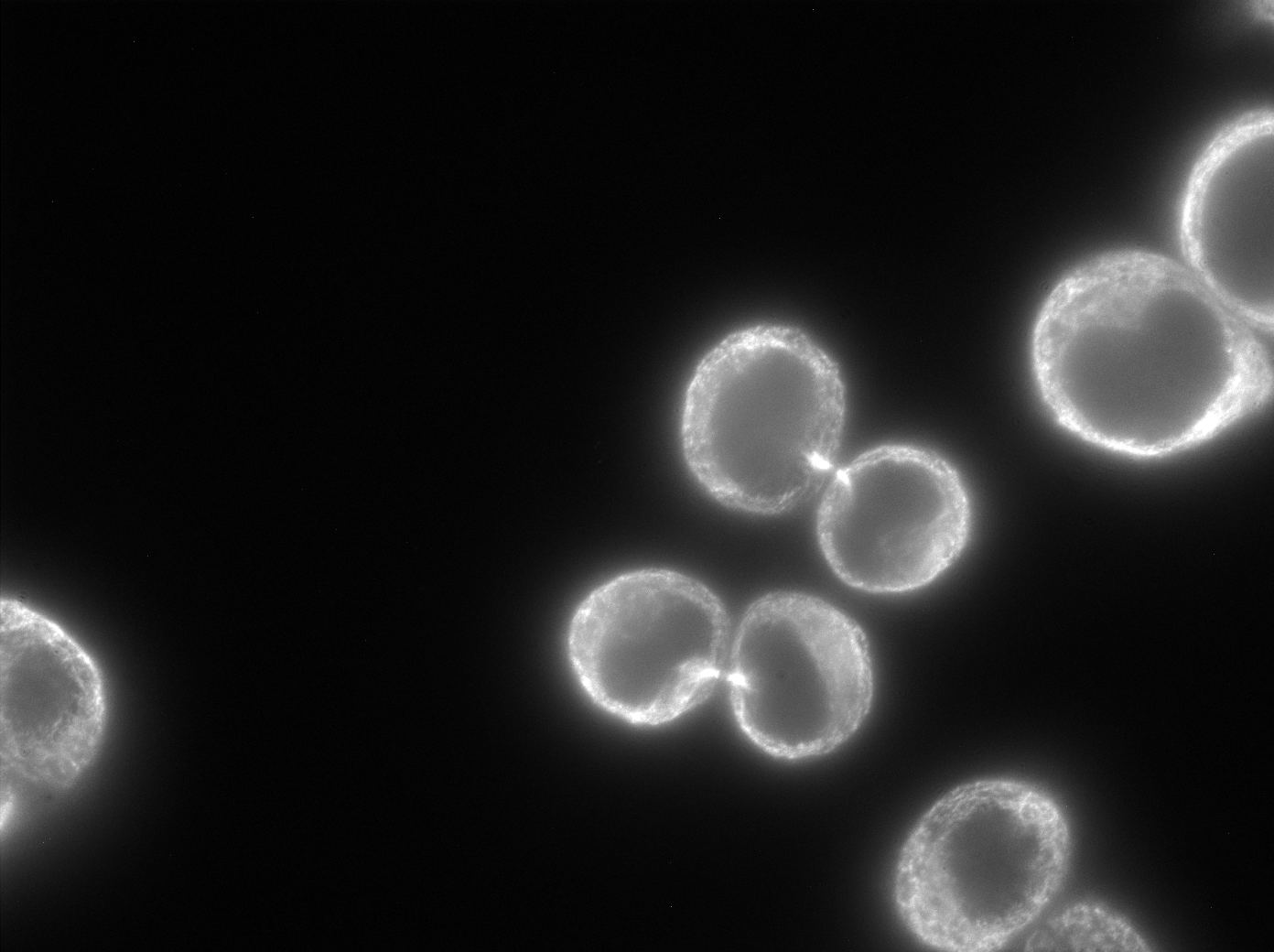

Supplement: Supplementary file 7 — Source data Fig. 2 part 4 [file 44319_2026_742_MOESM7_ESM.zip › Figure 2 Part 4/Fig 2d polye atubulin/WT PolyE-atub 8-14-24 R2 ET3ET4.Project Maximum Z_XY1723834195_Z0_T0_C1.tif]

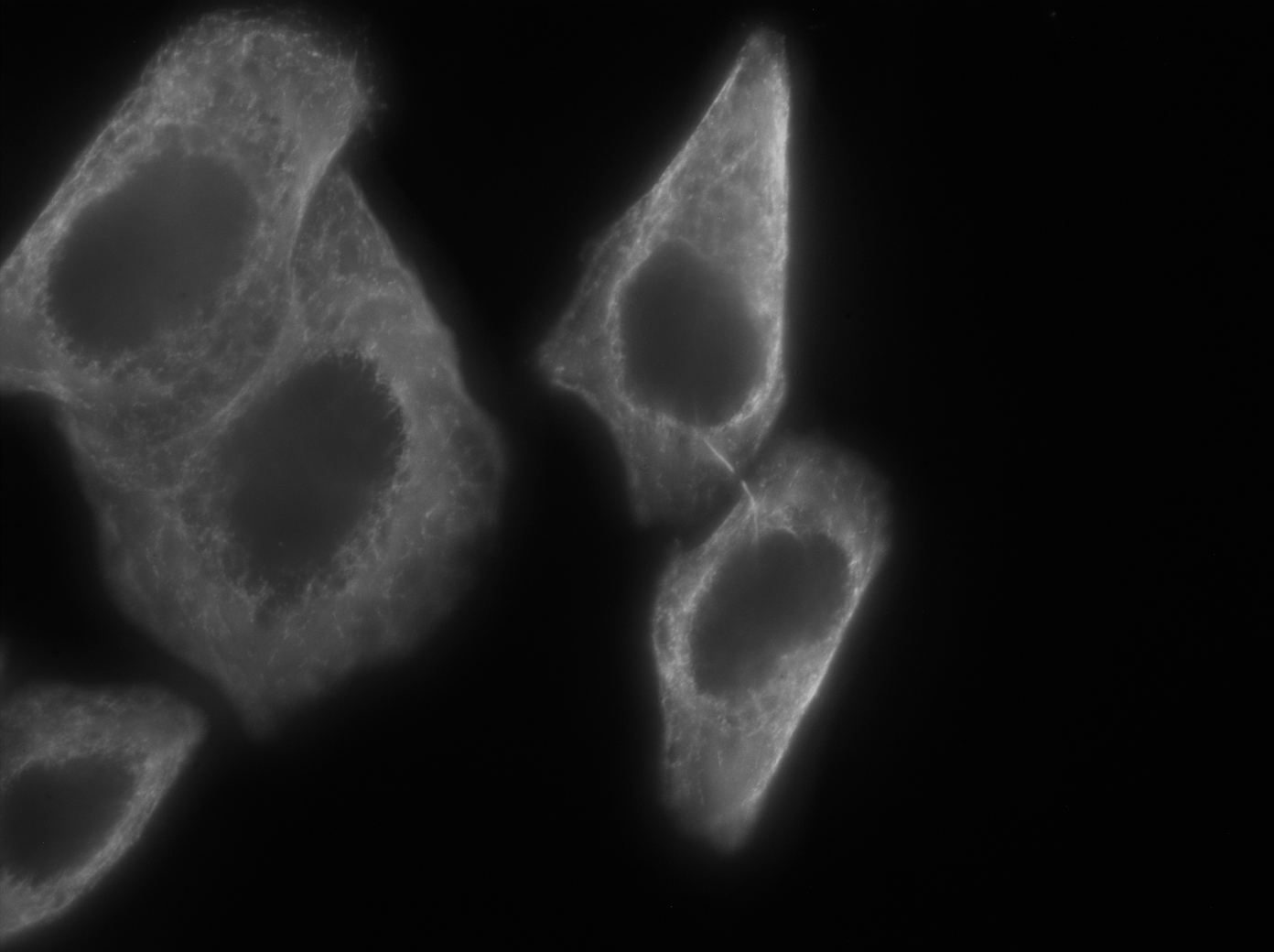

Supplement: Supplementary file 7 — Source data Fig. 2 part 4 [file 44319_2026_742_MOESM7_ESM.zip › Figure 2 Part 4/Fig 2d polye atubulin/WT PolyE-atub 8-14-24 R1 LT6.Project Maximum Z_XY1723758789_Z0_T0_C1.tif]

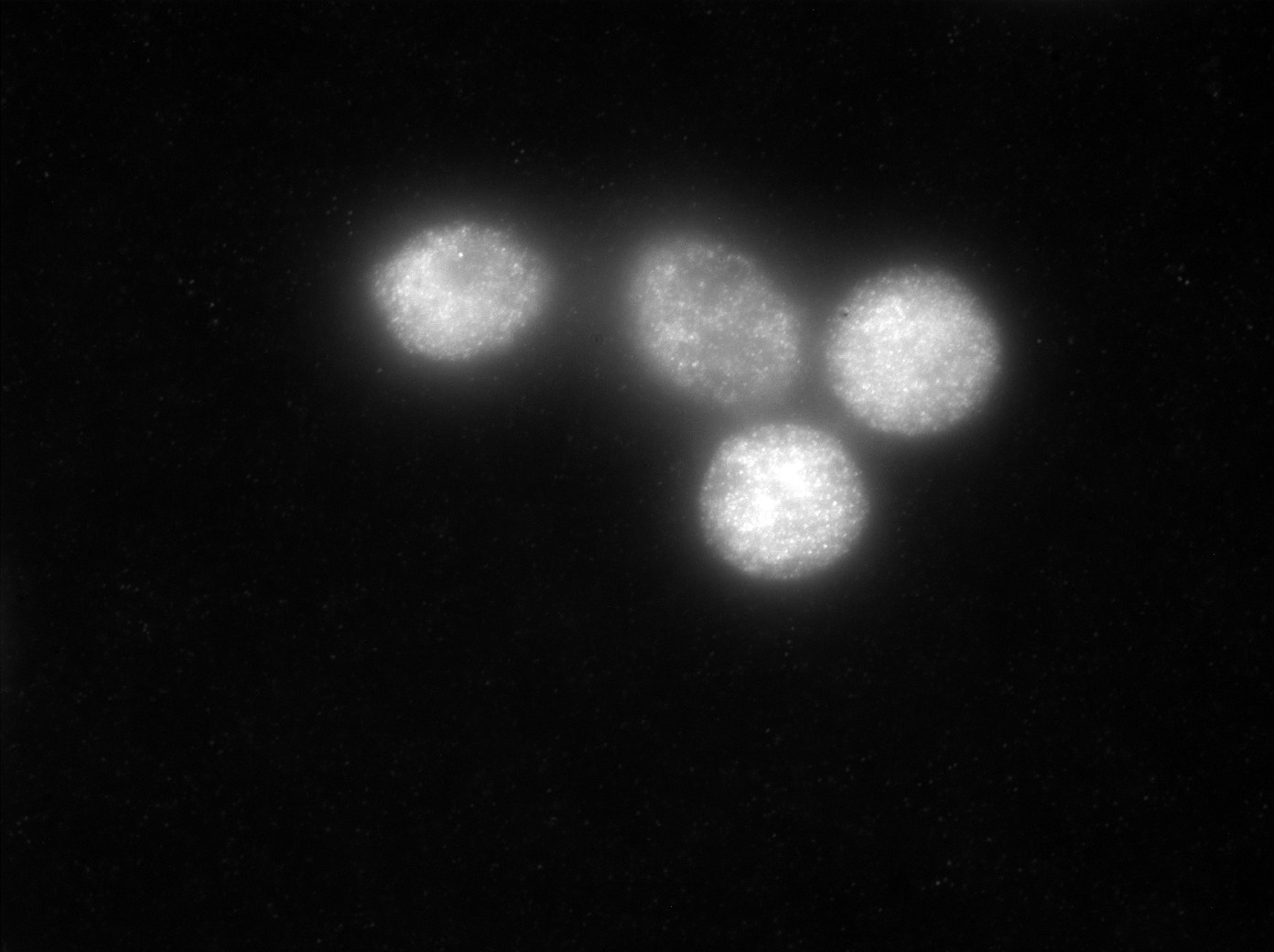

Supplement: Supplementary file 7 — Source data Fig. 2 part 4 [file 44319_2026_742_MOESM7_ESM.zip › Figure 2 Part 4/Fig 2d polye atubulin/WT PolyE-atub 8-14-24 R1 ET1.Project Maximum Z_XY1723755979_Z0_T0_C2.tif]

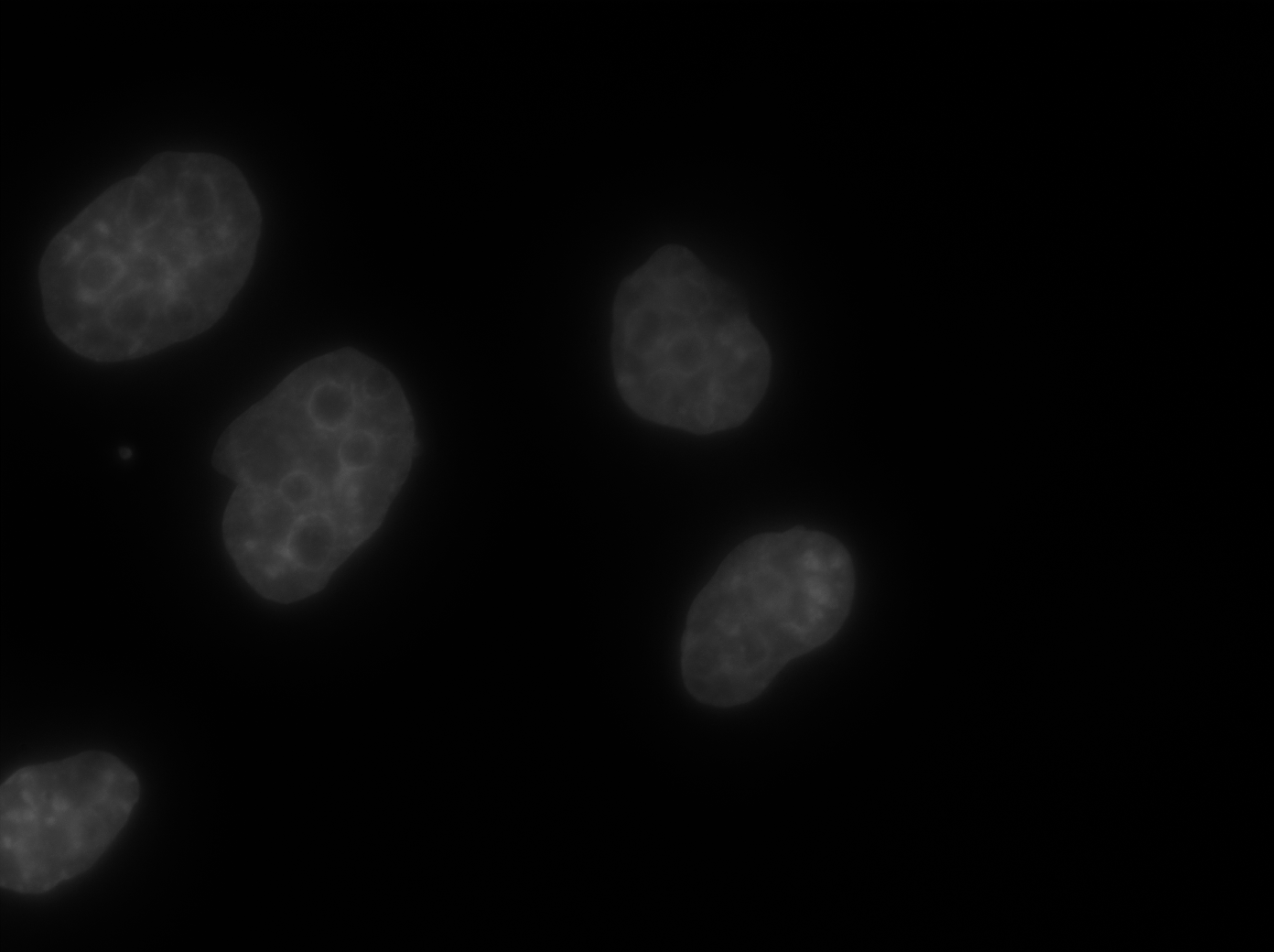

Supplement: Supplementary file 7 — Source data Fig. 2 part 4 [file 44319_2026_742_MOESM7_ESM.zip › Figure 2 Part 4/Fig 2d polye atubulin/WT PolyE-atub 8-14-24 R1 LT6.Project Maximum Z_XY1723758789_Z0_T0_C0.tif]

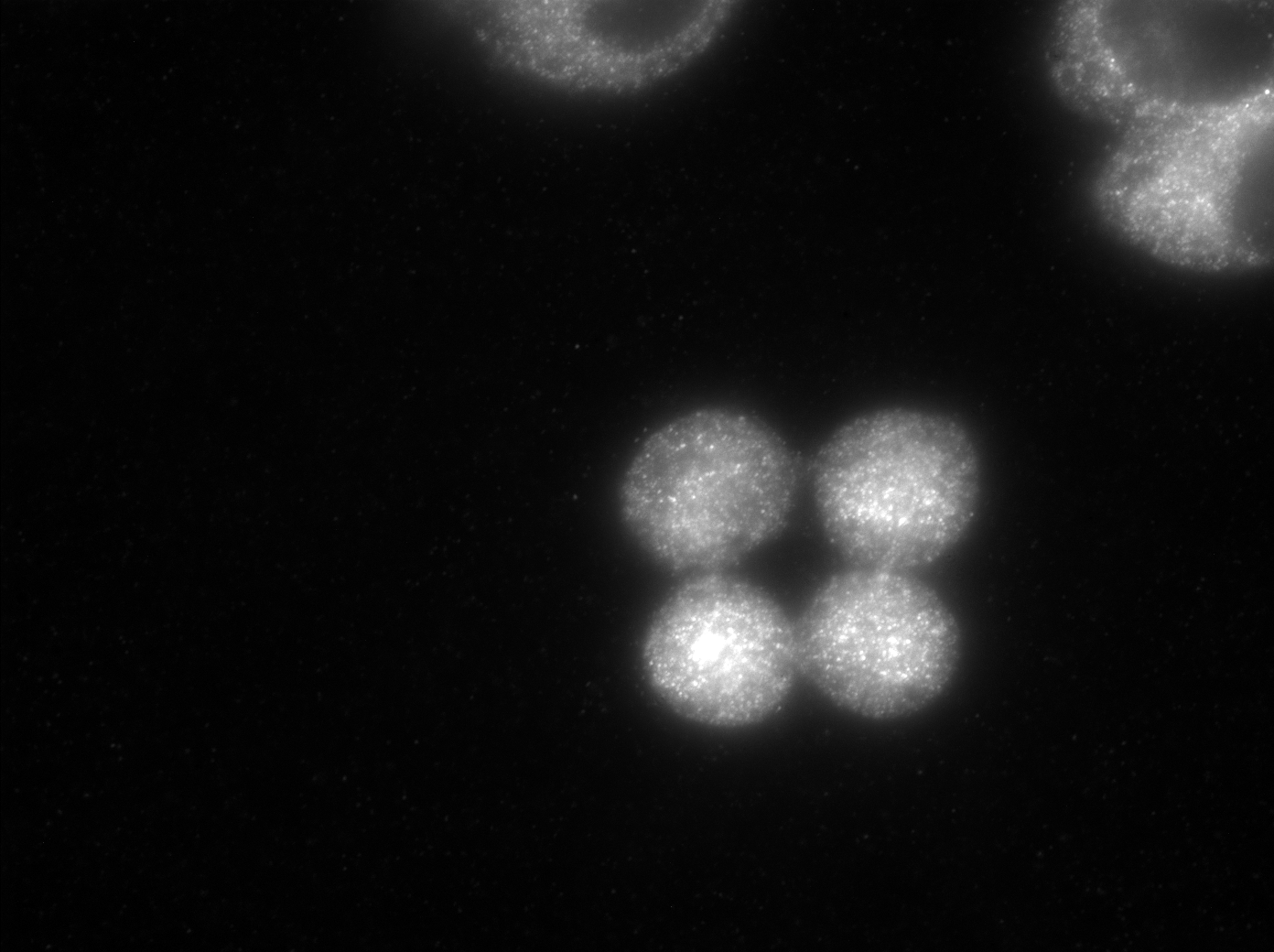

Supplement: Supplementary file 7 — Source data Fig. 2 part 4 [file 44319_2026_742_MOESM7_ESM.zip › Figure 2 Part 4/Fig 2d polye atubulin/WT PolyE-atub 8-14-24 R2 ET6ET7.Project Maximum Z_XY1723836630_Z0_T0_C2.tif]

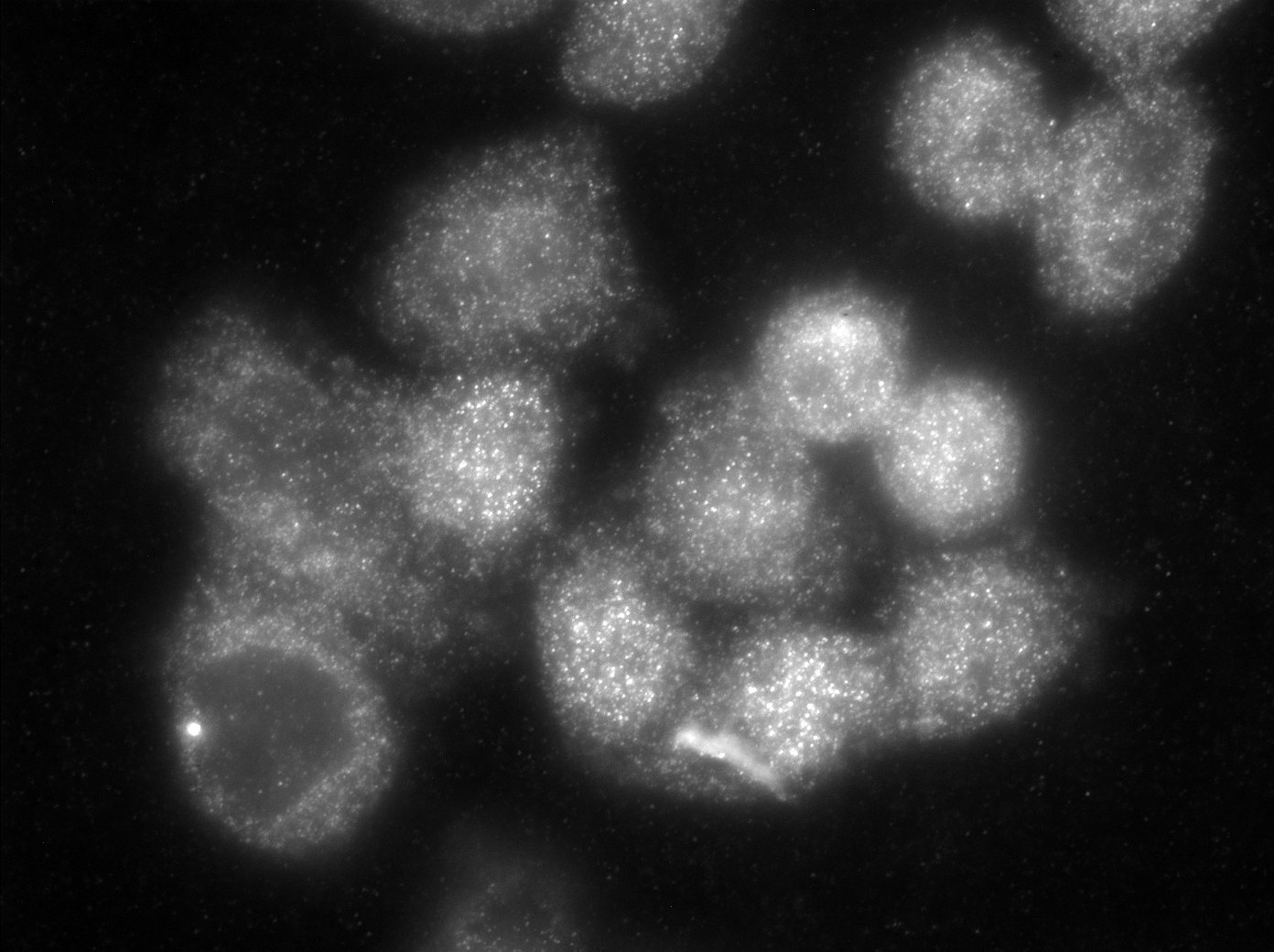

Supplement: Supplementary file 7 — Source data Fig. 2 part 4 [file 44319_2026_742_MOESM7_ESM.zip › Figure 2 Part 4/Fig 2d polye atubulin/WT PolyE-atub 8-14-24 R1 PA9PA10.Project Maximum Z_XY1723761363_Z0_T0_C2.tif]

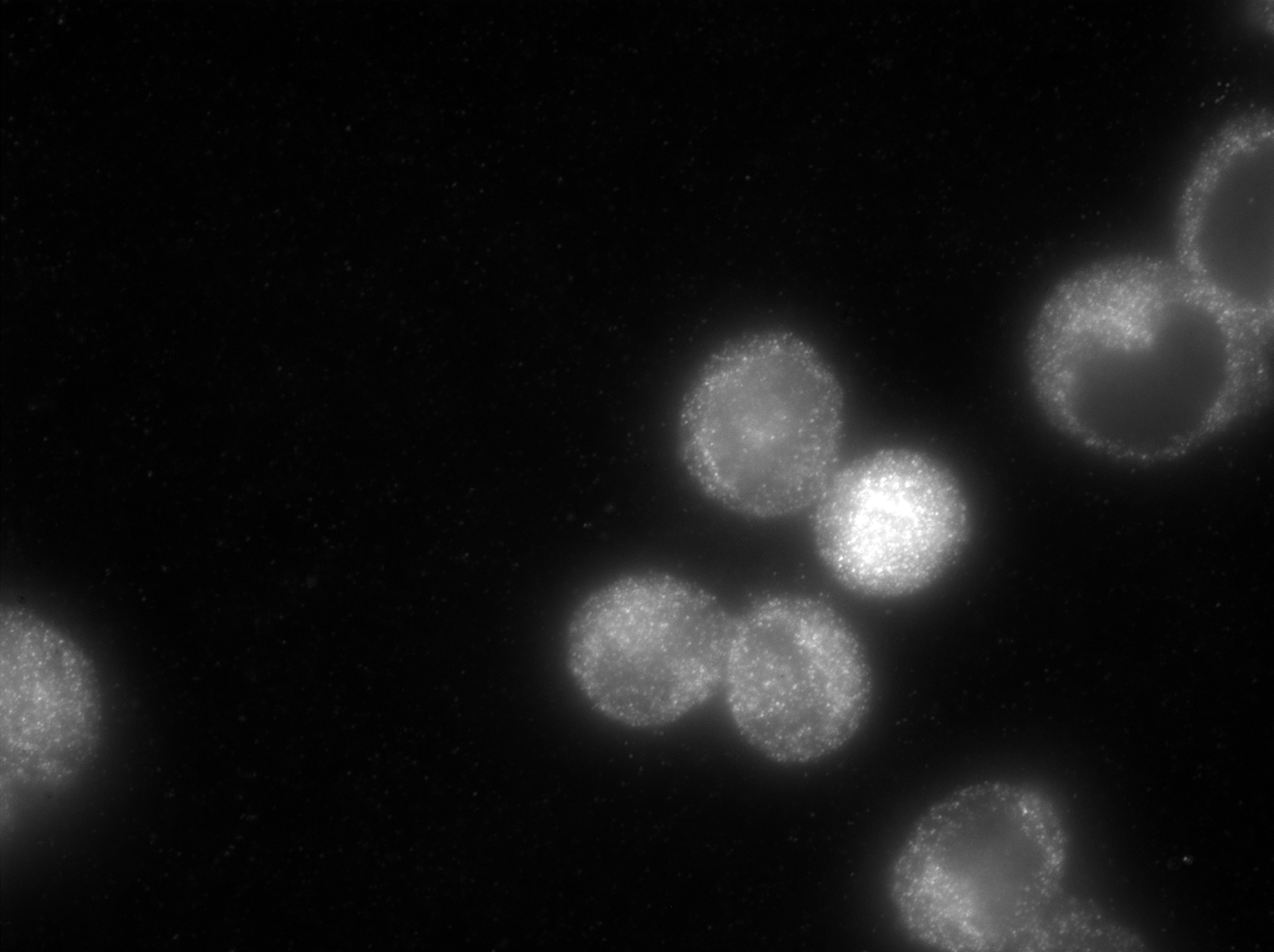

Supplement: Supplementary file 7 — Source data Fig. 2 part 4 [file 44319_2026_742_MOESM7_ESM.zip › Figure 2 Part 4/Fig 2d polye atubulin/WT PolyE-atub 8-14-24 R2 ET3ET4.Project Maximum Z_XY1723834195_Z0_T0_C2.tif]

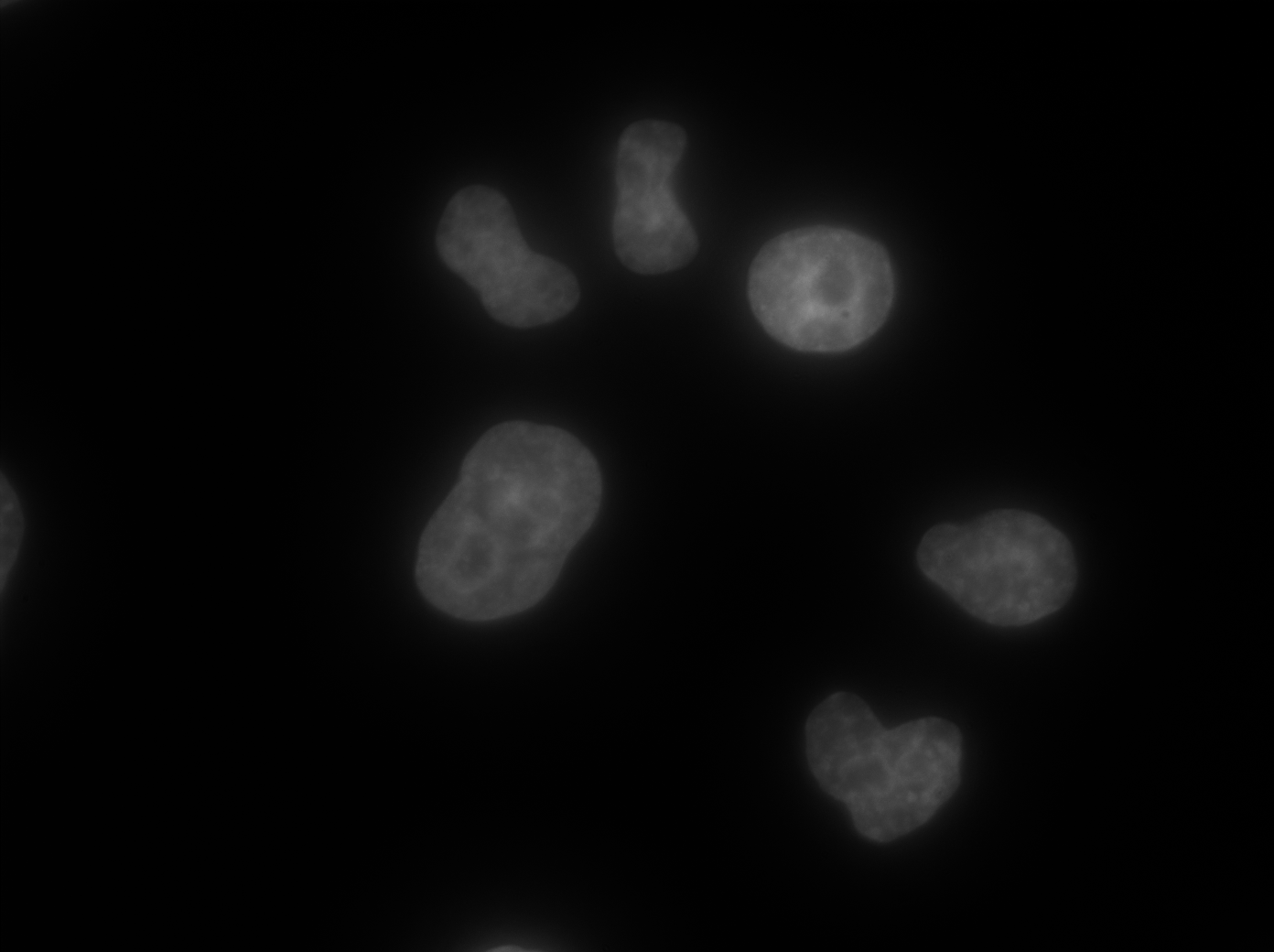

Supplement: Supplementary file 7 — Source data Fig. 2 part 4 [file 44319_2026_742_MOESM7_ESM.zip › Figure 2 Part 4/Fig 2d polye atubulin/WT PolyE-atub 8-14-24 R1 ET7 PA3.Project Maximum Z_XY1723758121_Z0_T0_C0.tif]

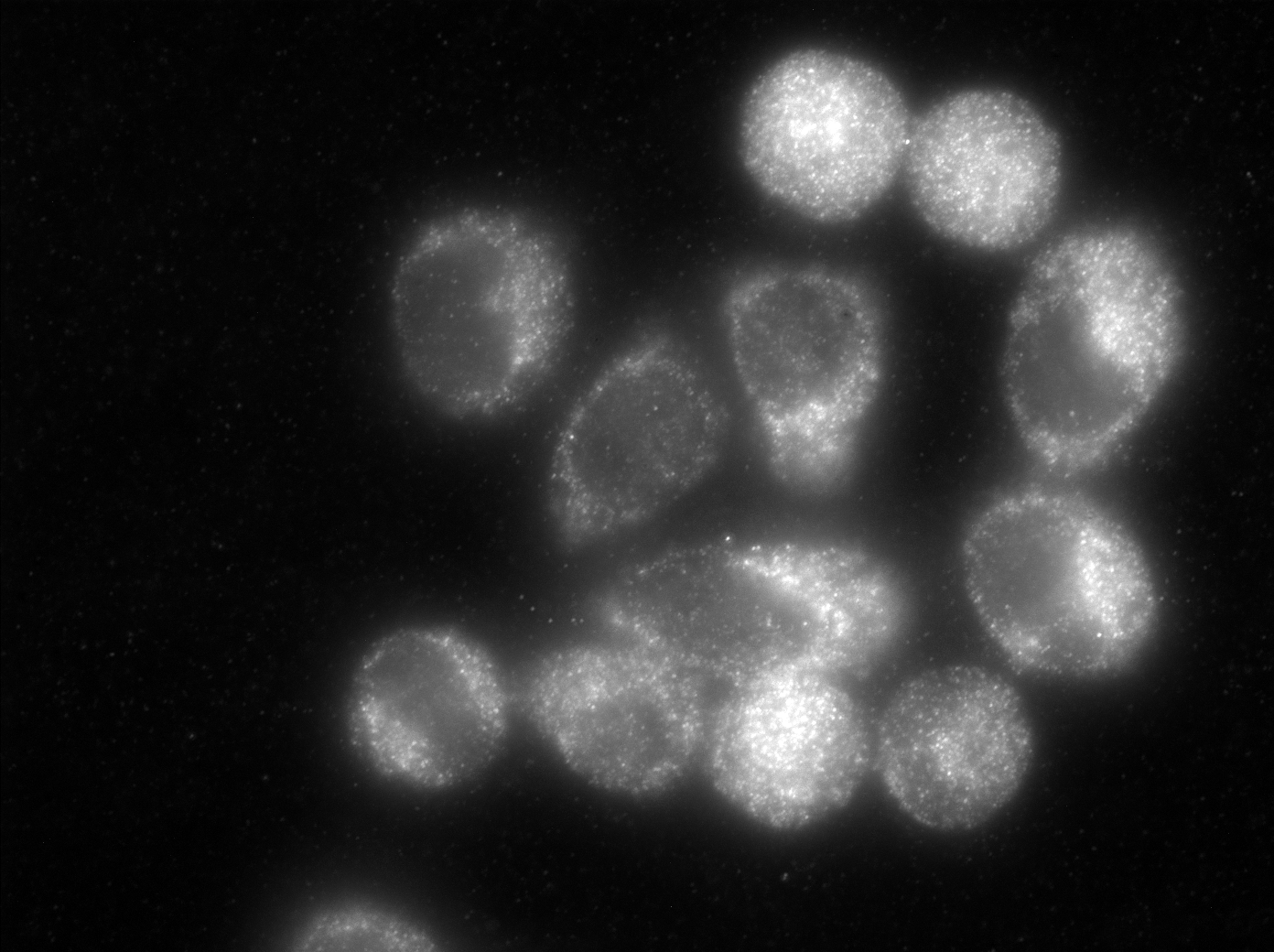

Supplement: Supplementary file 7 — Source data Fig. 2 part 4 [file 44319_2026_742_MOESM7_ESM.zip › Figure 2 Part 4/Fig 2d polye atubulin/WT PolyE-atub 8-14-24 R1 LT7 ET8ET9.Project Maximum Z_XY1723758965_Z0_T0_C2.tif]

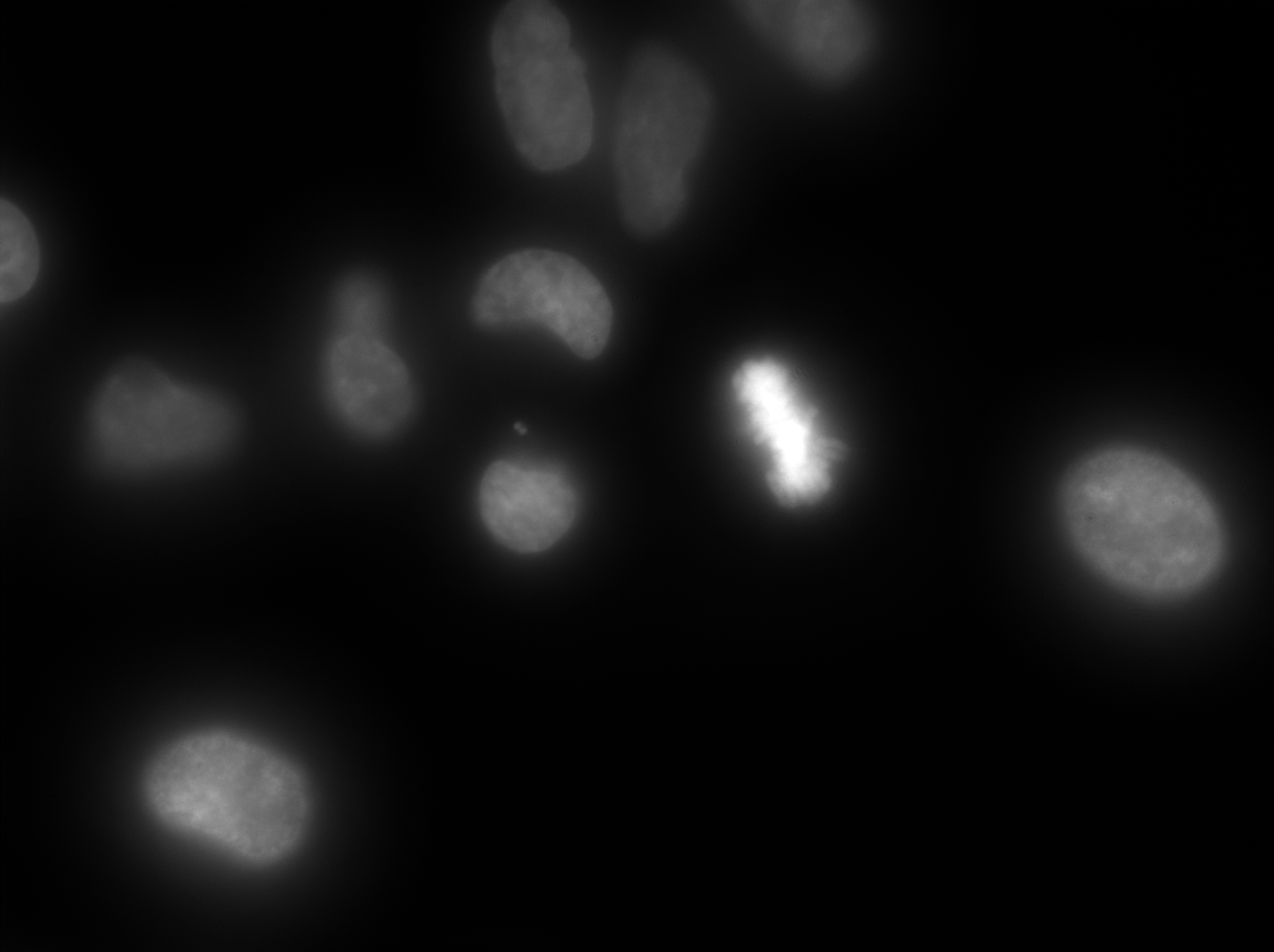

Supplement: Supplementary file 7 — Source data Fig. 2 part 4 [file 44319_2026_742_MOESM7_ESM.zip › Figure 2 Part 4/Fig 2d polye atubulin/WT PolyE-atub 8-14-24 R2 ET9M6.Project Maximum Z_XY1723837940_Z0_T0_C0.tif]

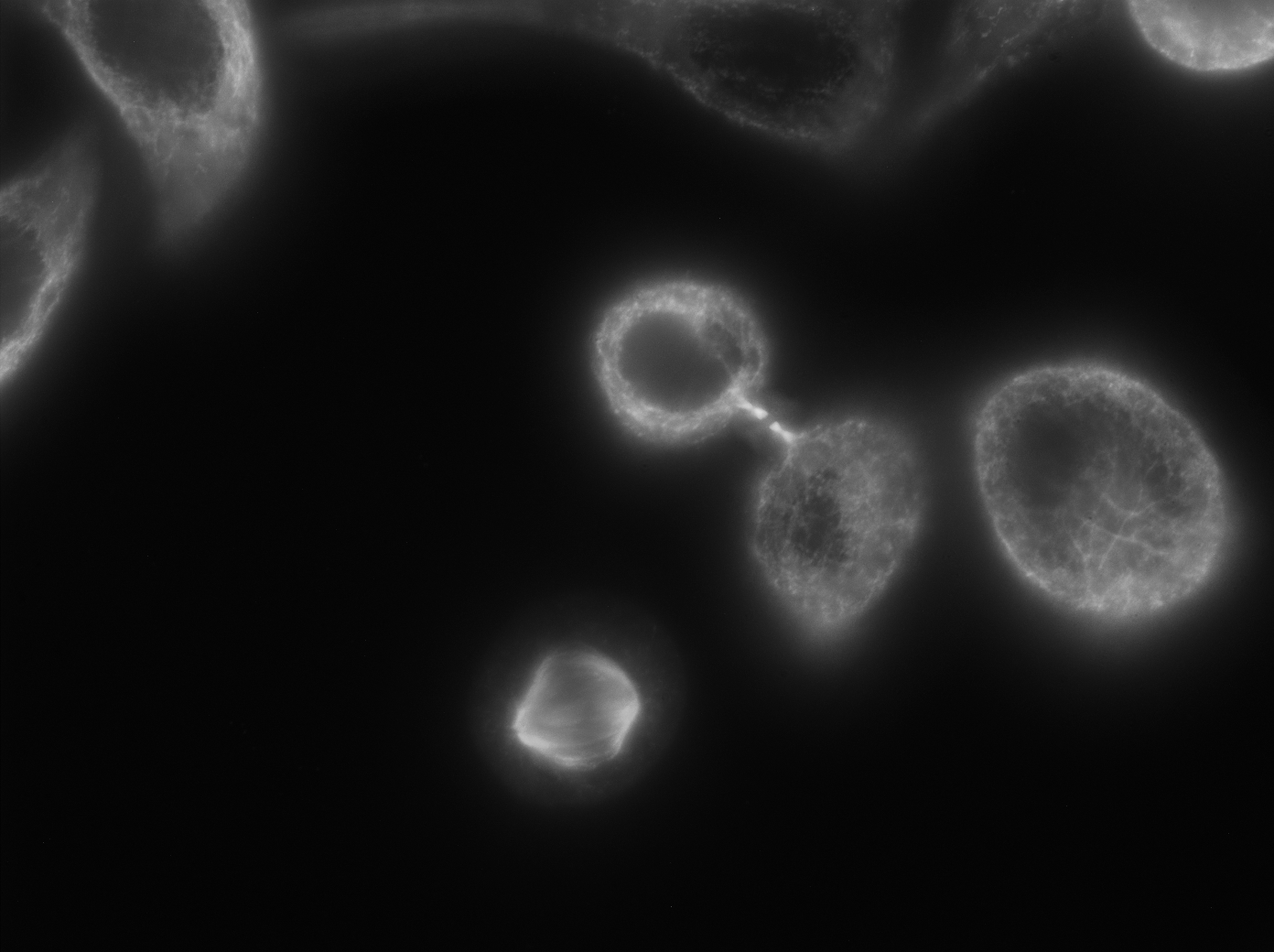

Supplement: Supplementary file 7 — Source data Fig. 2 part 4 [file 44319_2026_742_MOESM7_ESM.zip › Figure 2 Part 4/Fig 2d polye atubulin/WT PolyE-atub 8-14-24 R1 LT10 M7.Project Maximum Z_XY1723760525_Z0_T0_C1.tif]

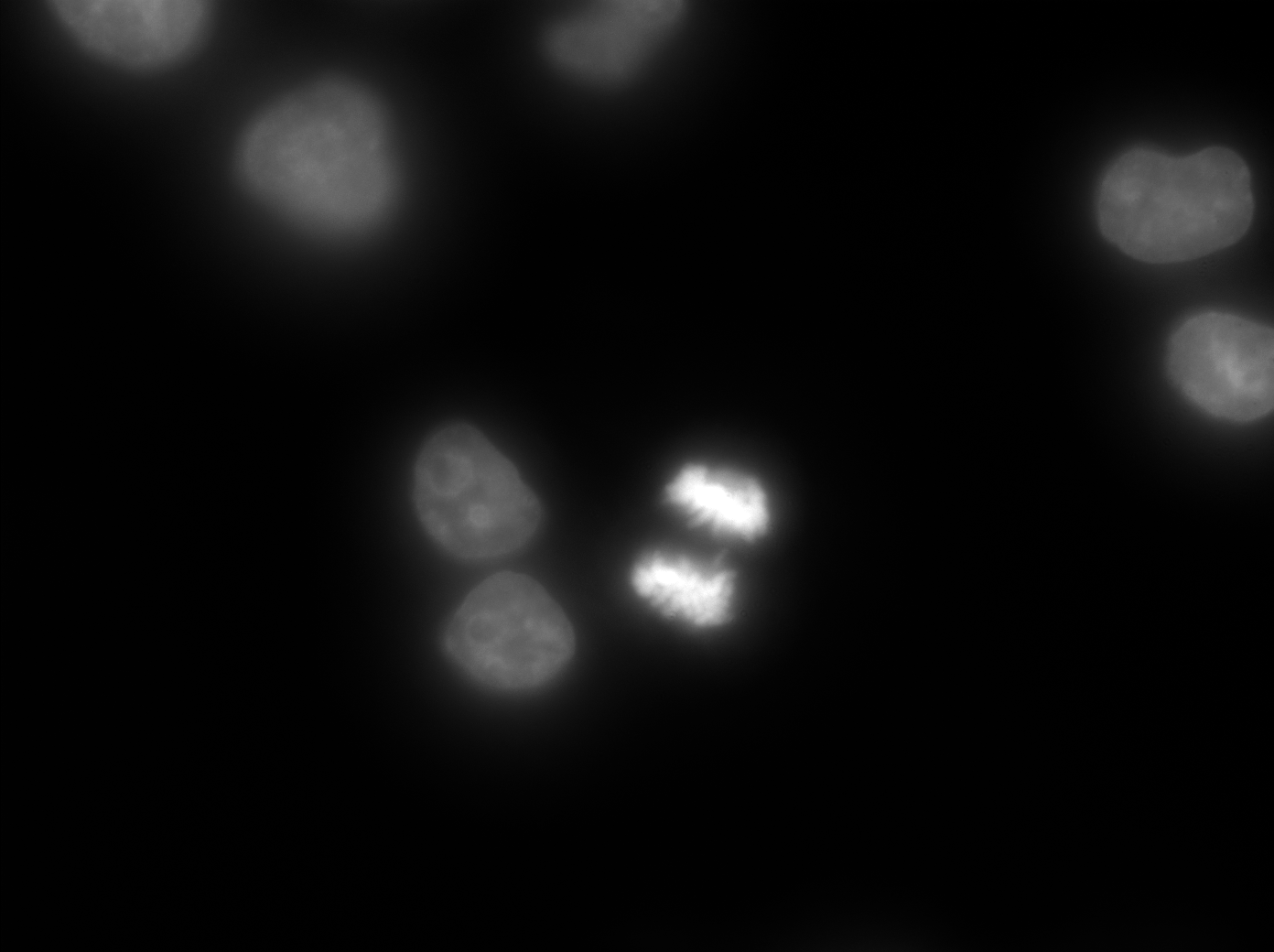

Supplement: Supplementary file 7 — Source data Fig. 2 part 4 [file 44319_2026_742_MOESM7_ESM.zip › Figure 2 Part 4/Fig 2d polye atubulin/WT PolyE-atub 8-14-24 R2 A1.Project Maximum Z_XY1723837650_Z0_T0_C0.tif]

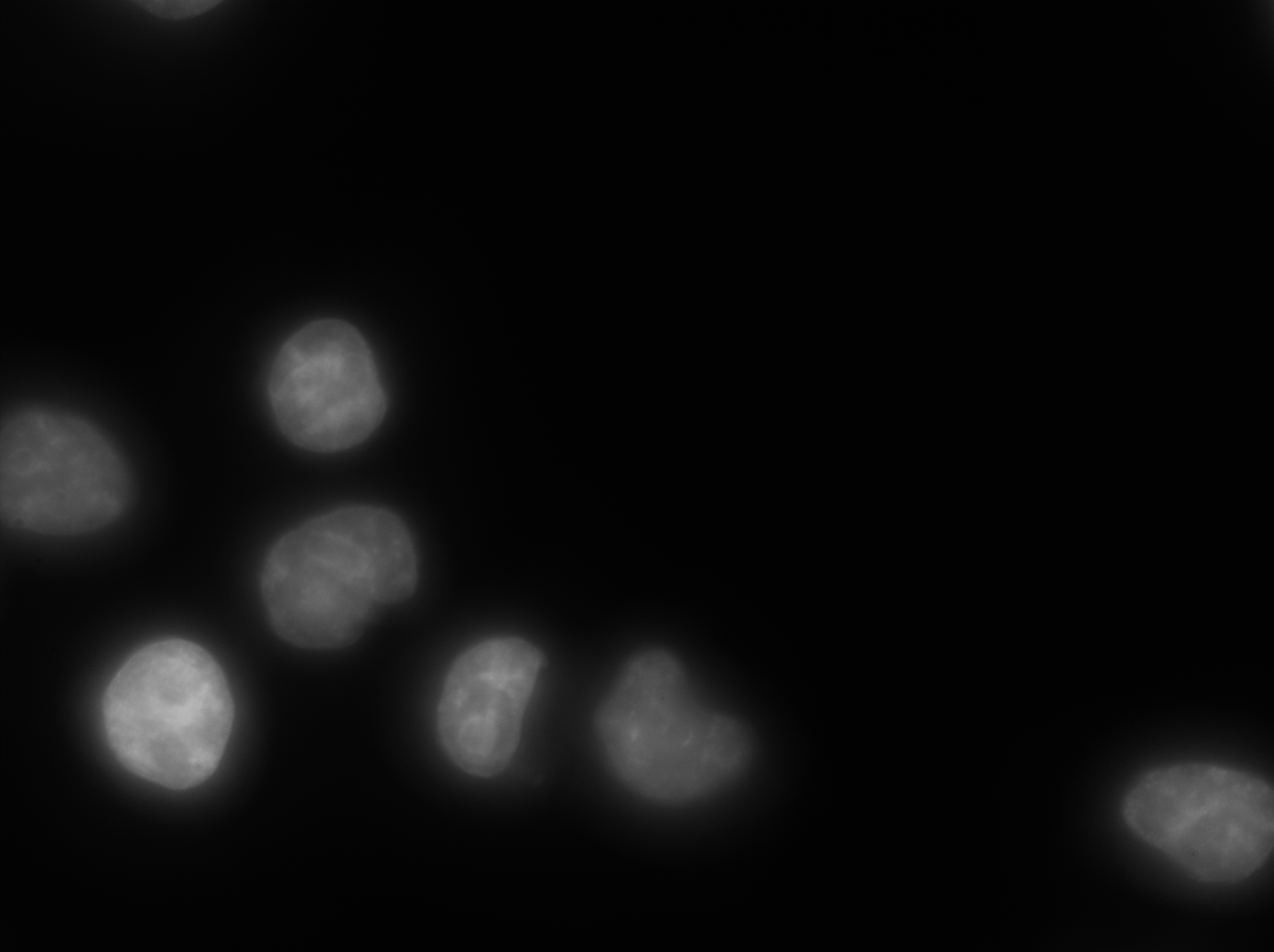

Supplement: Supplementary file 7 — Source data Fig. 2 part 4 [file 44319_2026_742_MOESM7_ESM.zip › Figure 2 Part 4/Fig 2d polye atubulin/WT PolyE-atub 8-14-24 R1 PA8.Project Maximum Z_XY1723761161_Z0_T0_C0.tif]

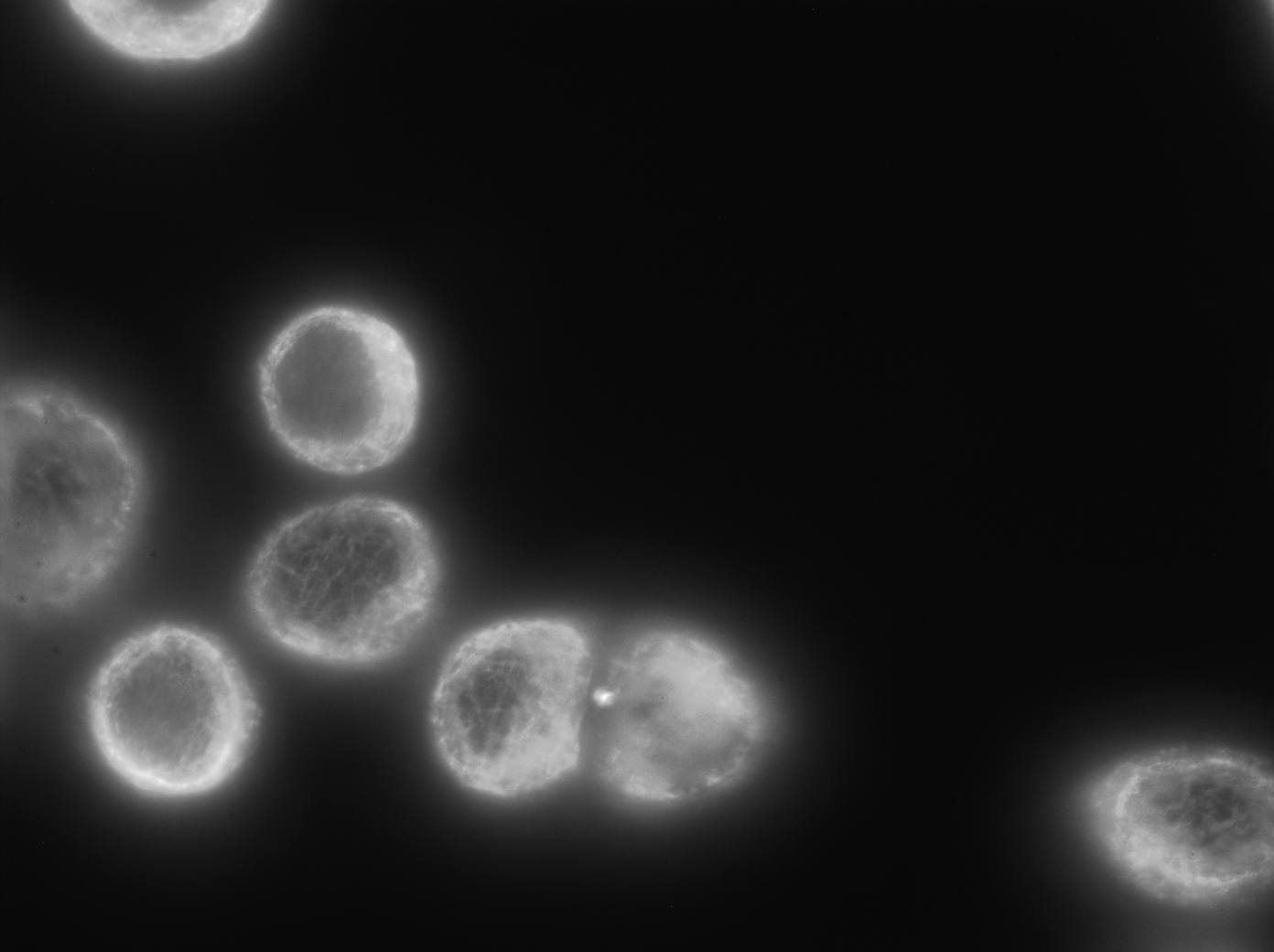

Supplement: Supplementary file 7 — Source data Fig. 2 part 4 [file 44319_2026_742_MOESM7_ESM.zip › Figure 2 Part 4/Fig 2d polye atubulin/WT PolyE-atub 8-14-24 R1 PA8.Project Maximum Z_XY1723761161_Z0_T0_C1.tif]

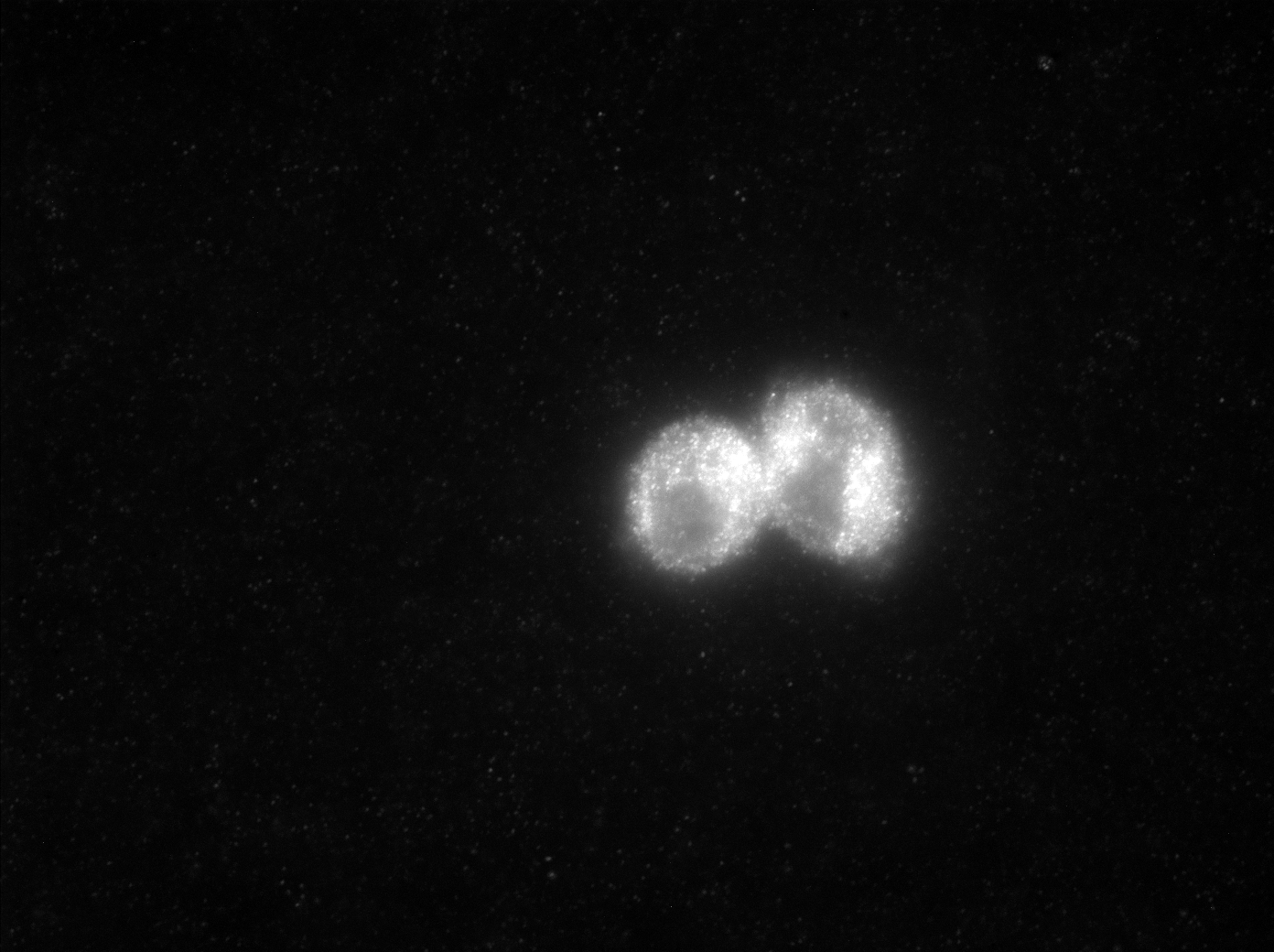

Supplement: Supplementary file 7 — Source data Fig. 2 part 4 [file 44319_2026_742_MOESM7_ESM.zip › Figure 2 Part 4/Fig 2d polye atubulin/WT PolyE-atub 8-14-24 R1 ET3.Project Maximum Z_XY1723756294_Z0_T0_C2.tif]

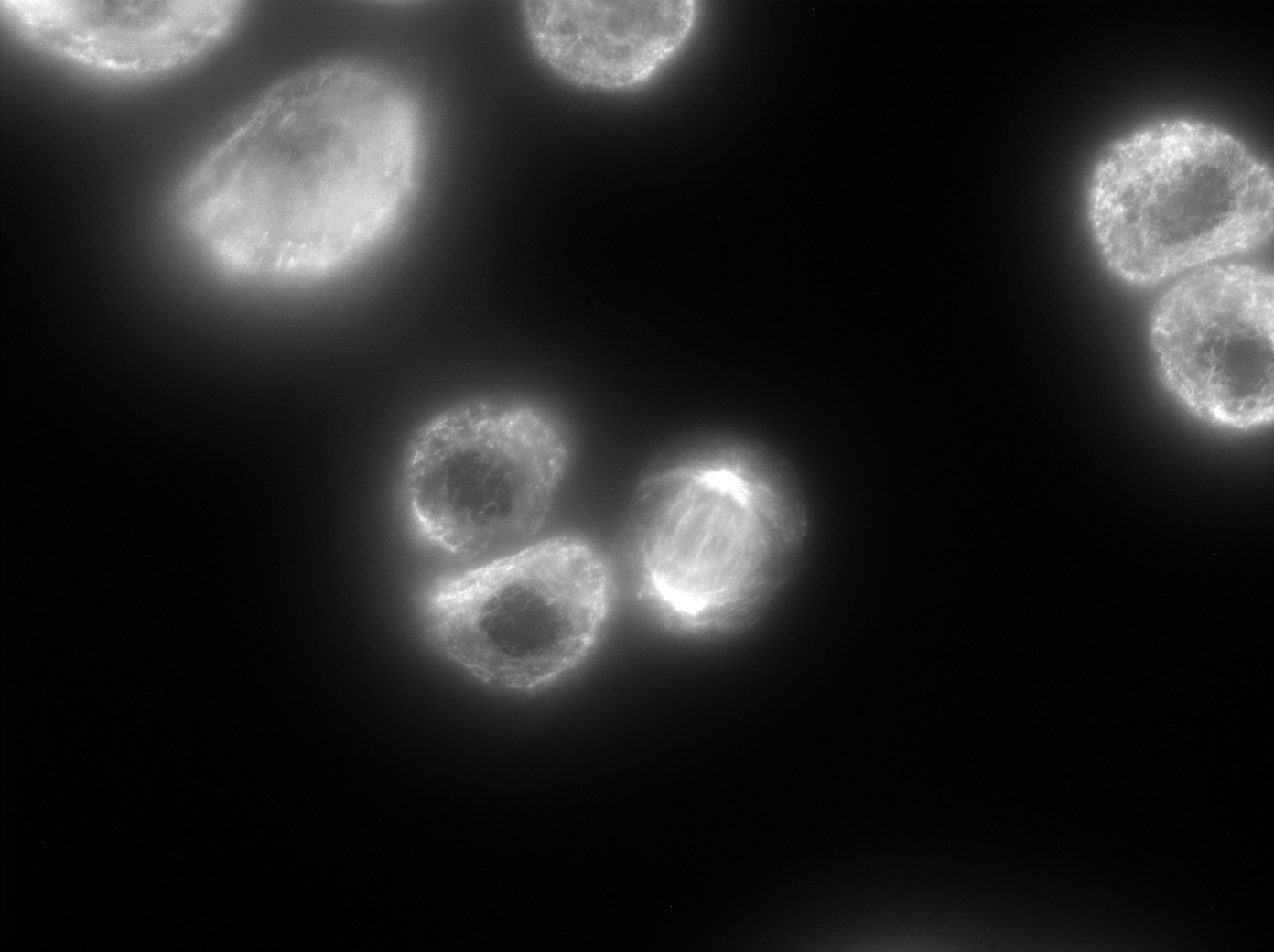

Supplement: Supplementary file 7 — Source data Fig. 2 part 4 [file 44319_2026_742_MOESM7_ESM.zip › Figure 2 Part 4/Fig 2d polye atubulin/WT PolyE-atub 8-14-24 R2 A1.Project Maximum Z_XY1723837650_Z0_T0_C1.tif]

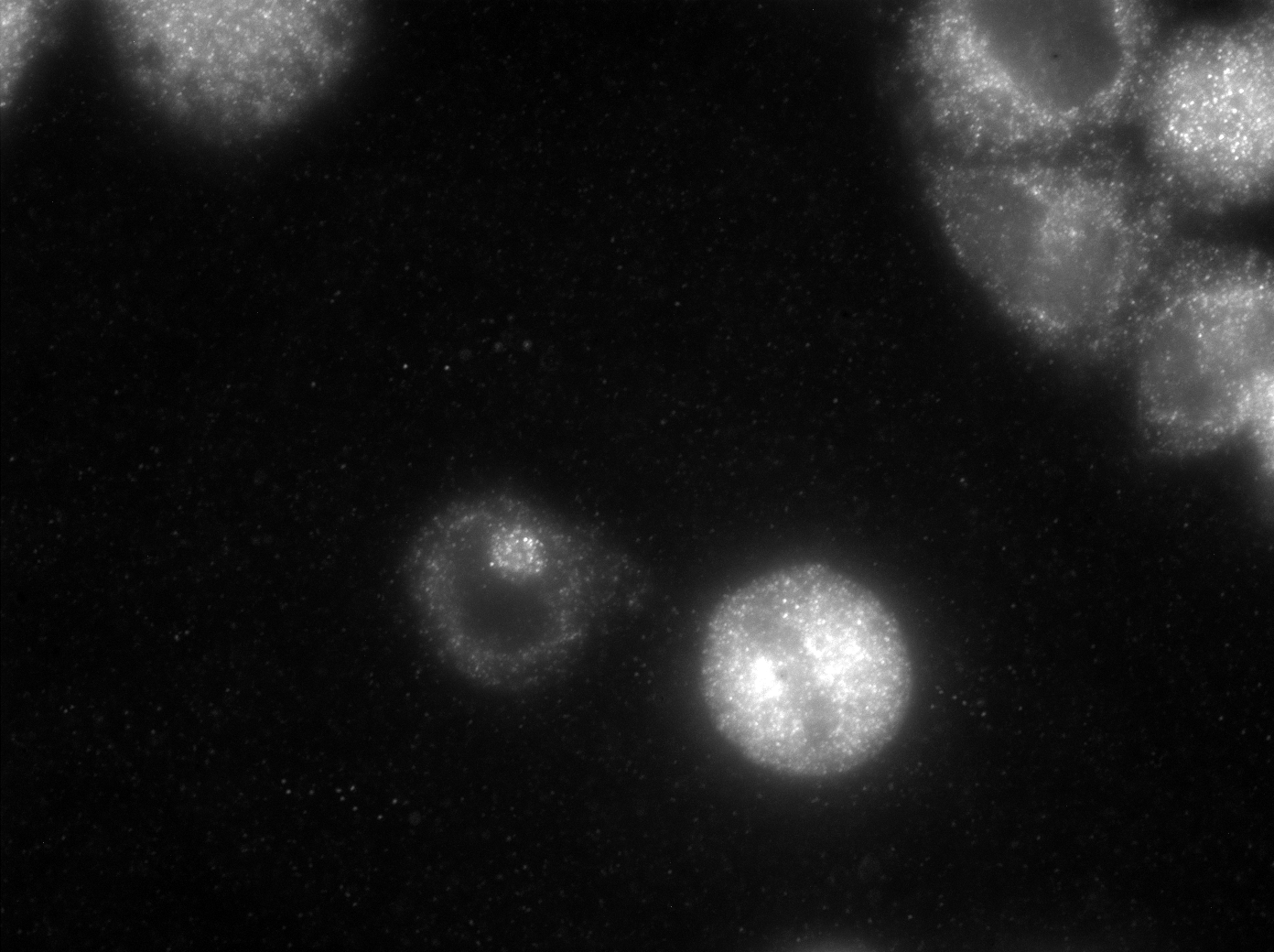

Supplement: Supplementary file 7 — Source data Fig. 2 part 4 [file 44319_2026_742_MOESM7_ESM.zip › Figure 2 Part 4/Fig 2d polye atubulin/WT PolyE-atub 8-14-24 R1 M6.Project Maximum Z_XY1723760417_Z0_T0_C2.tif]

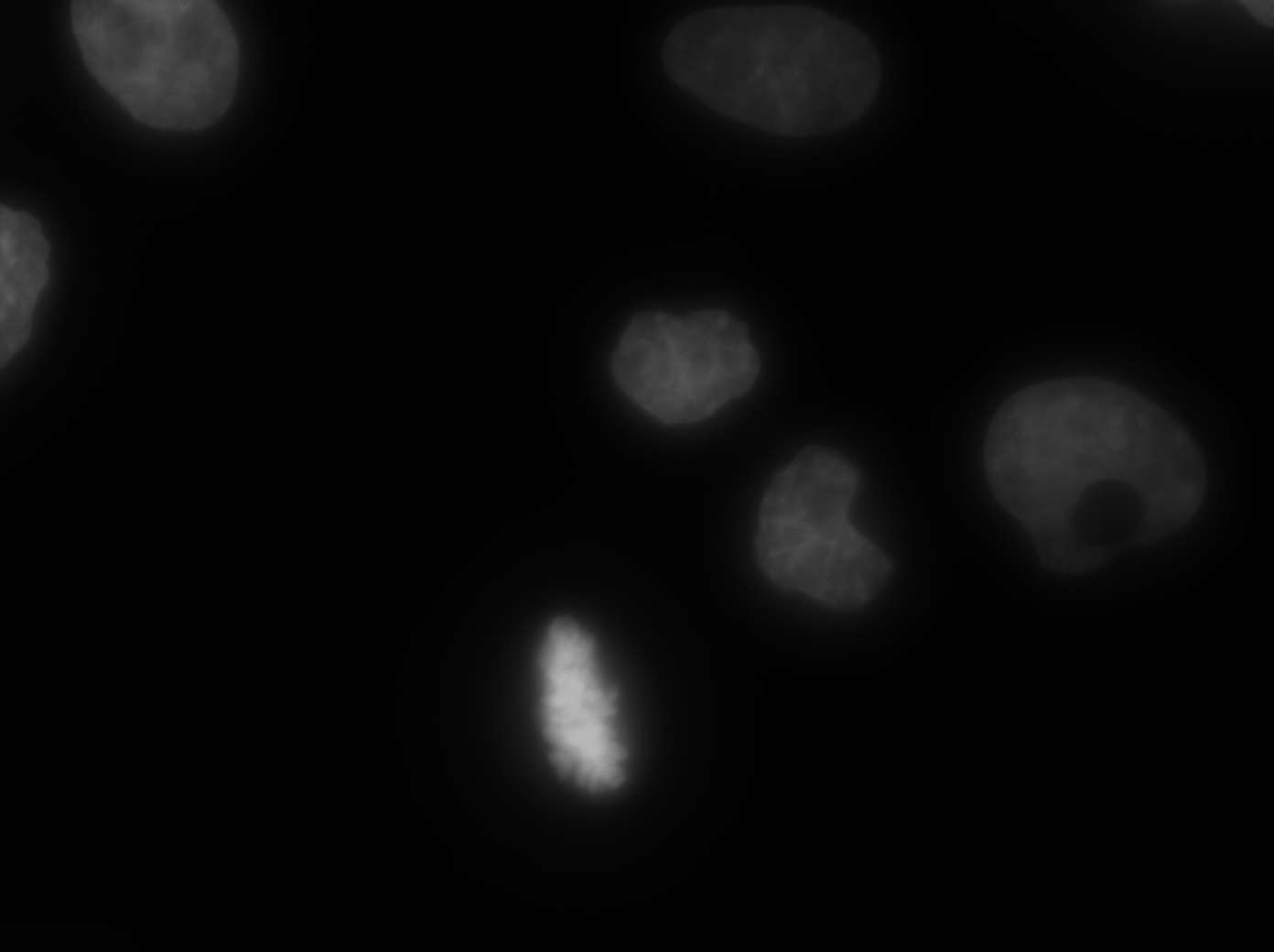

Supplement: Supplementary file 7 — Source data Fig. 2 part 4 [file 44319_2026_742_MOESM7_ESM.zip › Figure 2 Part 4/Fig 2d polye atubulin/WT PolyE-atub 8-14-24 R1 LT10 M7.Project Maximum Z_XY1723760525_Z0_T0_C0.tif]

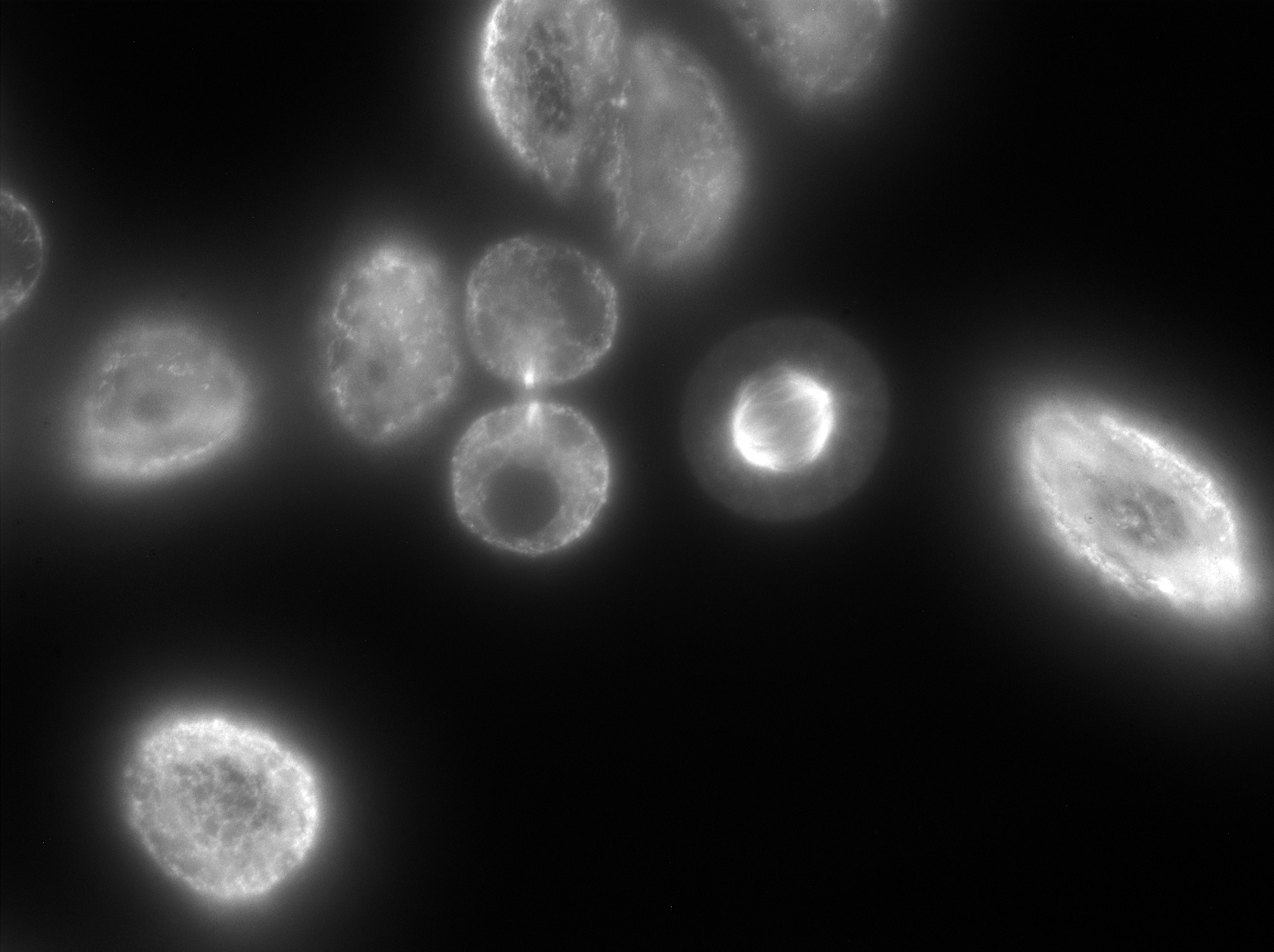

Supplement: Supplementary file 7 — Source data Fig. 2 part 4 [file 44319_2026_742_MOESM7_ESM.zip › Figure 2 Part 4/Fig 2d polye atubulin/WT PolyE-atub 8-14-24 R2 ET9M6.Project Maximum Z_XY1723837940_Z0_T0_C1.tif]

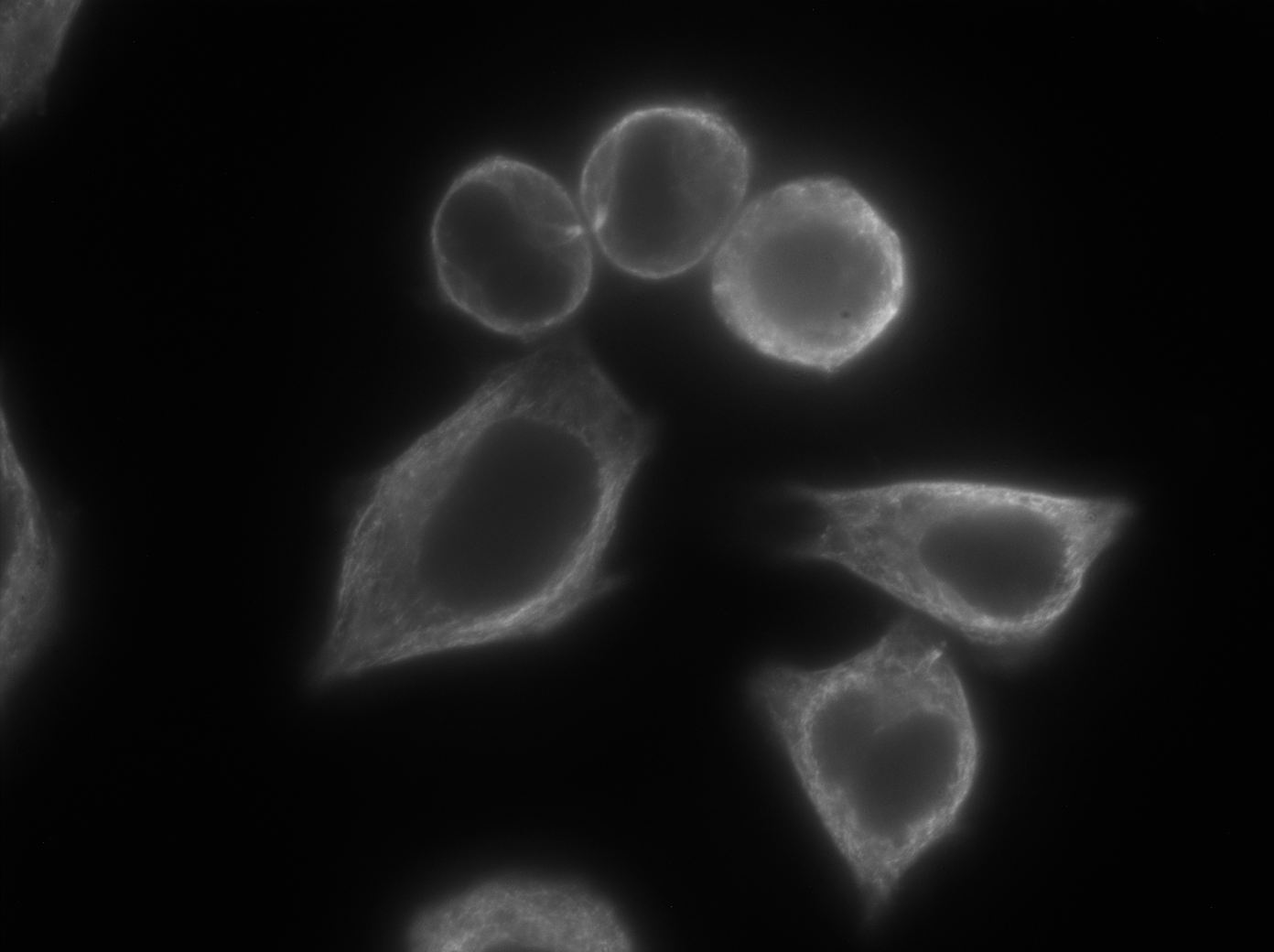

Supplement: Supplementary file 7 — Source data Fig. 2 part 4 [file 44319_2026_742_MOESM7_ESM.zip › Figure 2 Part 4/Fig 2d polye atubulin/WT PolyE-atub 8-14-24 R1 ET7 PA3.Project Maximum Z_XY1723758121_Z0_T0_C1.tif]

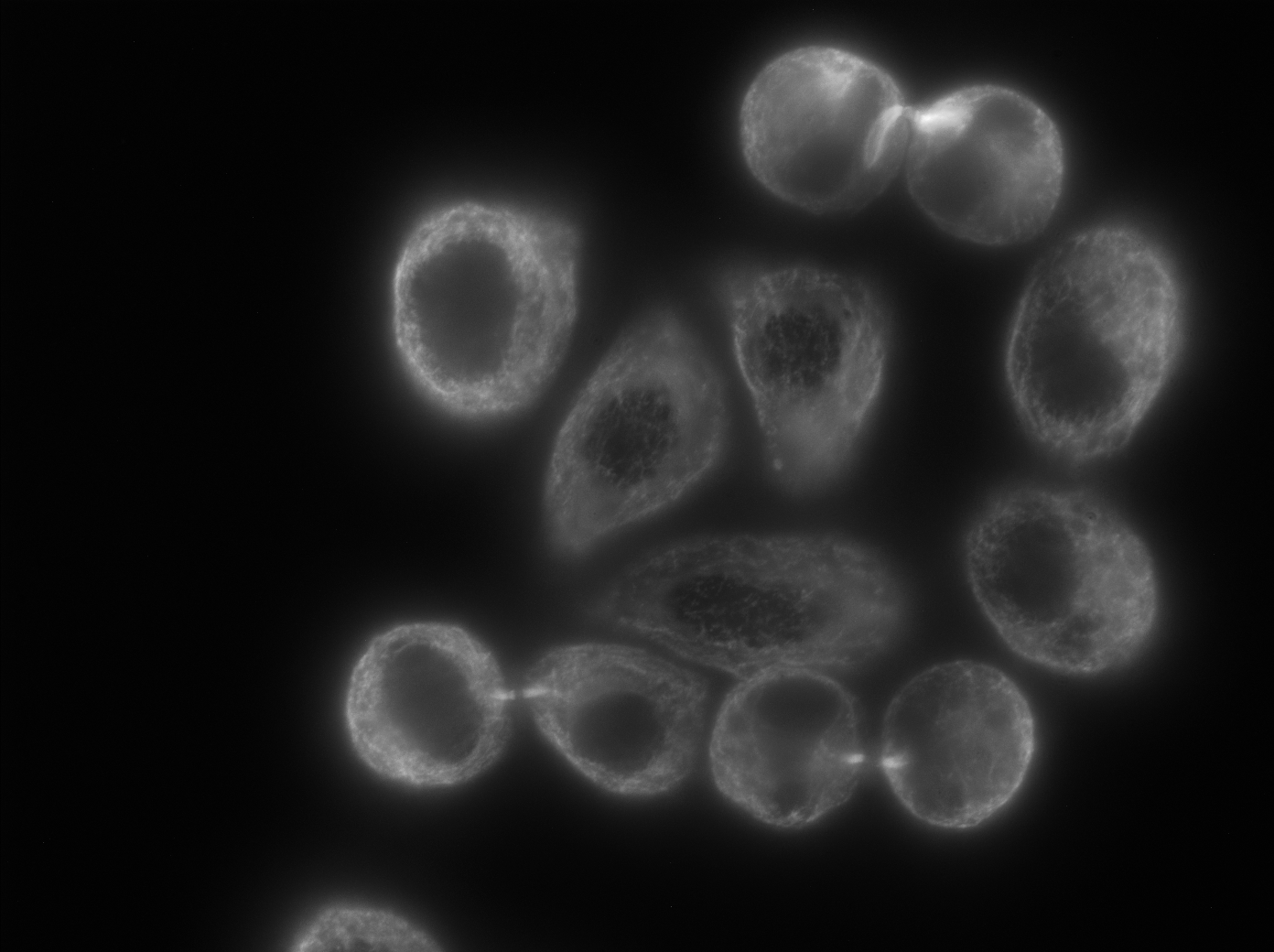

Supplement: Supplementary file 7 — Source data Fig. 2 part 4 [file 44319_2026_742_MOESM7_ESM.zip › Figure 2 Part 4/Fig 2d polye atubulin/WT PolyE-atub 8-14-24 R1 LT7 ET8ET9.Project Maximum Z_XY1723758965_Z0_T0_C1.tif]

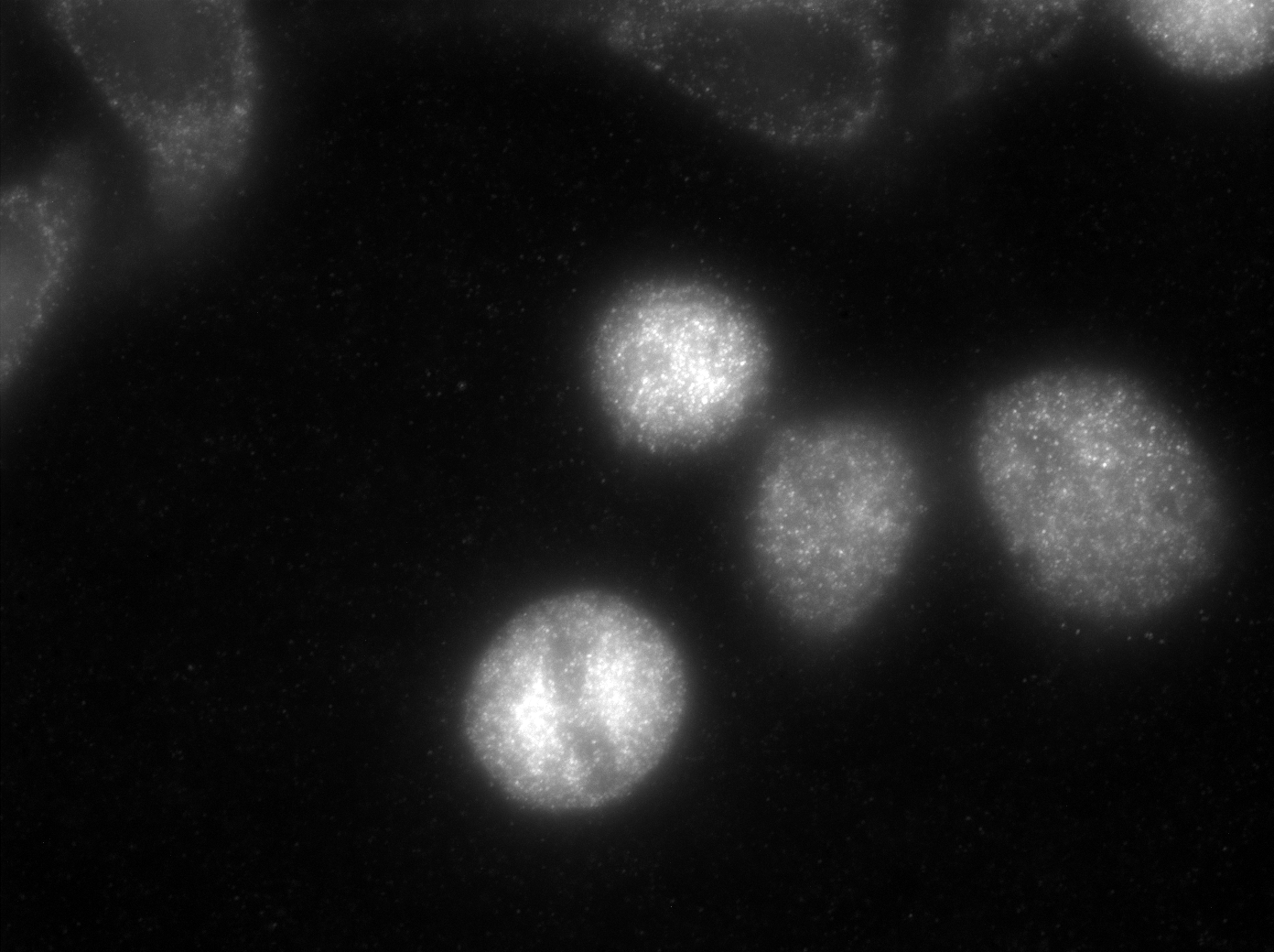

Supplement: Supplementary file 7 — Source data Fig. 2 part 4 [file 44319_2026_742_MOESM7_ESM.zip › Figure 2 Part 4/Fig 2d polye atubulin/WT PolyE-atub 8-14-24 R1 LT10 M7.Project Maximum Z_XY1723760525_Z0_T0_C2.tif]

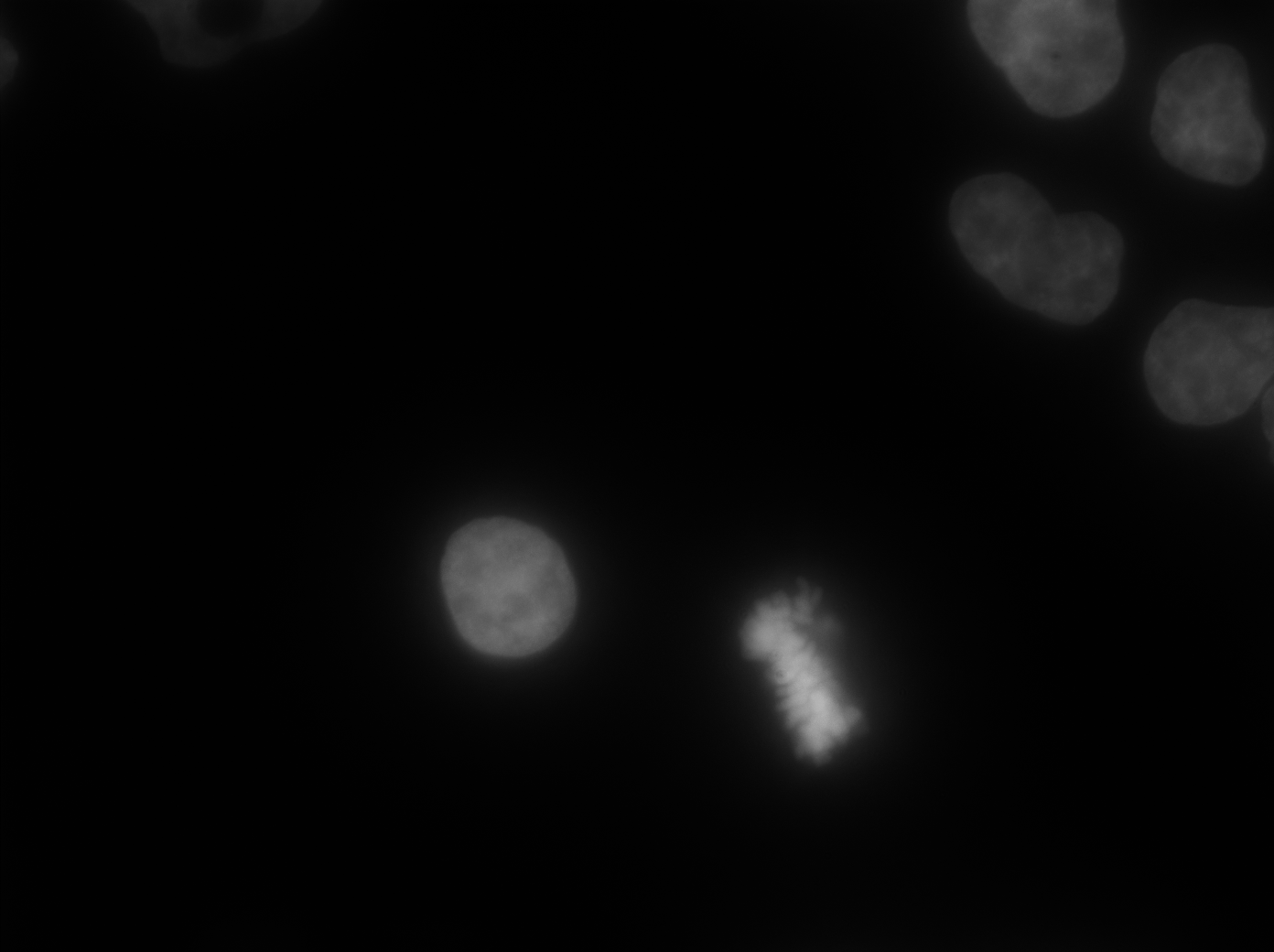

Supplement: Supplementary file 7 — Source data Fig. 2 part 4 [file 44319_2026_742_MOESM7_ESM.zip › Figure 2 Part 4/Fig 2d polye atubulin/WT PolyE-atub 8-14-24 R1 M6.Project Maximum Z_XY1723760417_Z0_T0_C0.tif]

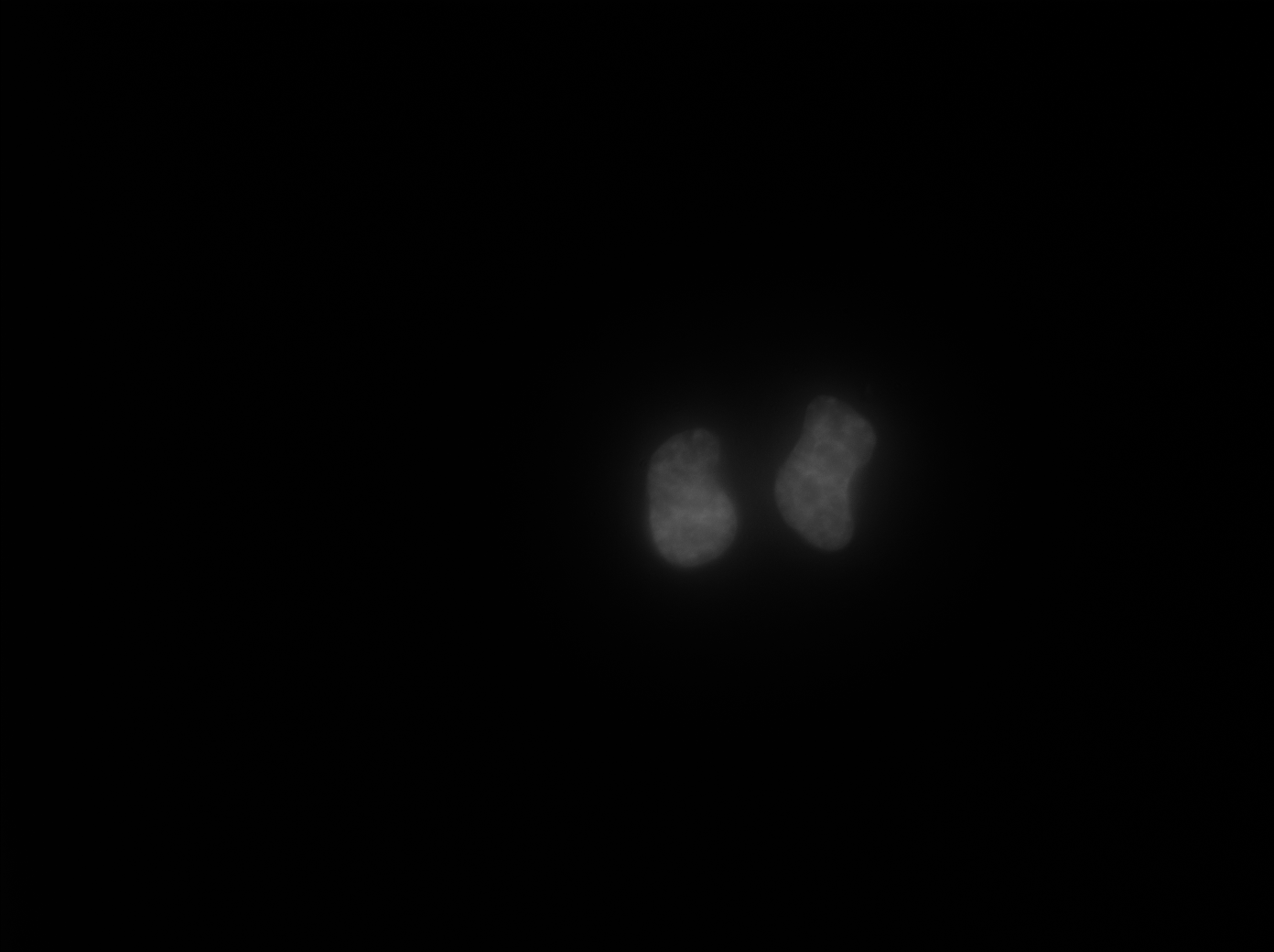

Supplement: Supplementary file 7 — Source data Fig. 2 part 4 [file 44319_2026_742_MOESM7_ESM.zip › Figure 2 Part 4/Fig 2d polye atubulin/WT PolyE-atub 8-14-24 R1 ET3.Project Maximum Z_XY1723756294_Z0_T0_C0.tif]

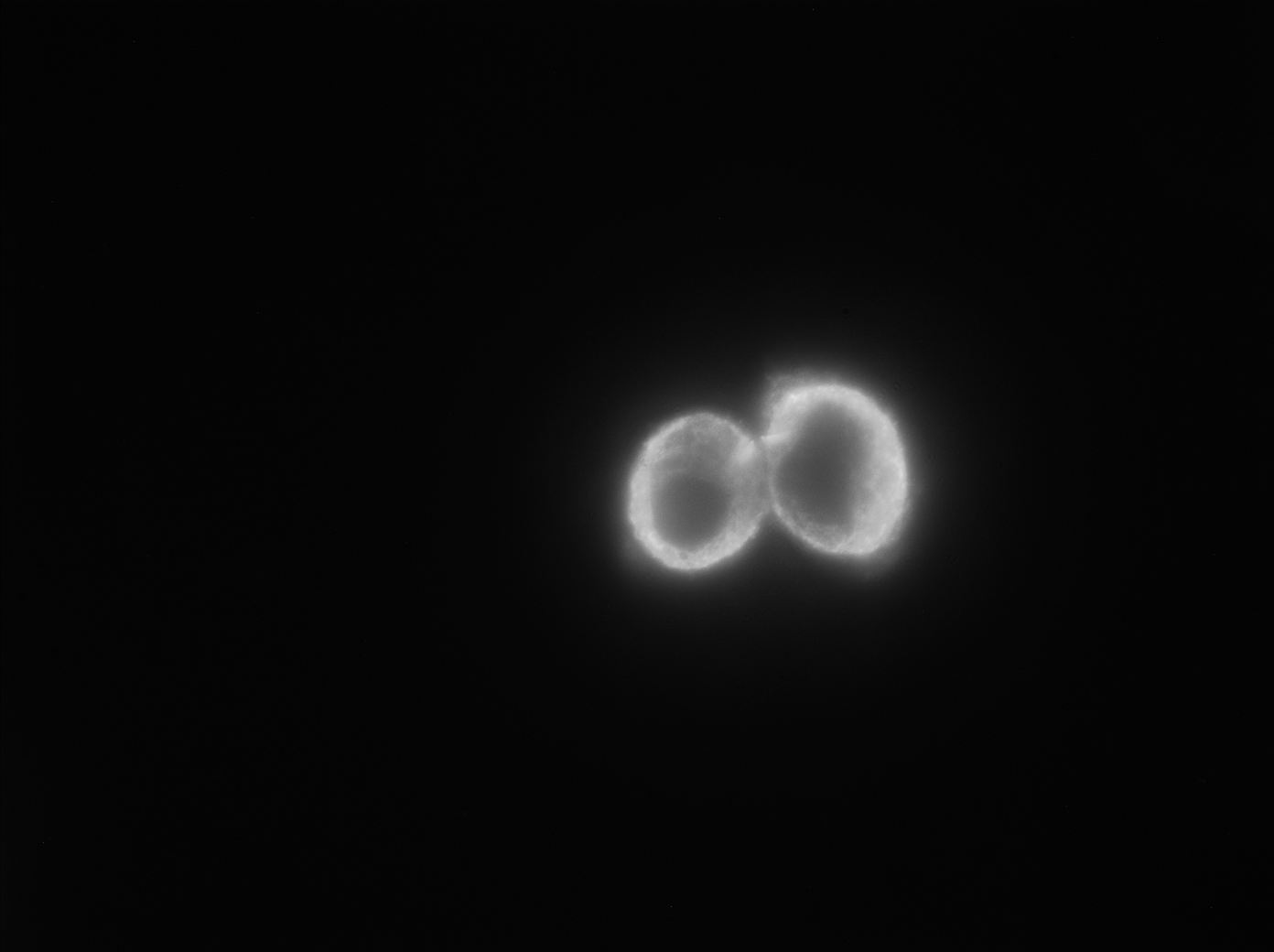

Supplement: Supplementary file 7 — Source data Fig. 2 part 4 [file 44319_2026_742_MOESM7_ESM.zip › Figure 2 Part 4/Fig 2d polye atubulin/WT PolyE-atub 8-14-24 R1 ET3.Project Maximum Z_XY1723756294_Z0_T0_C1.tif]

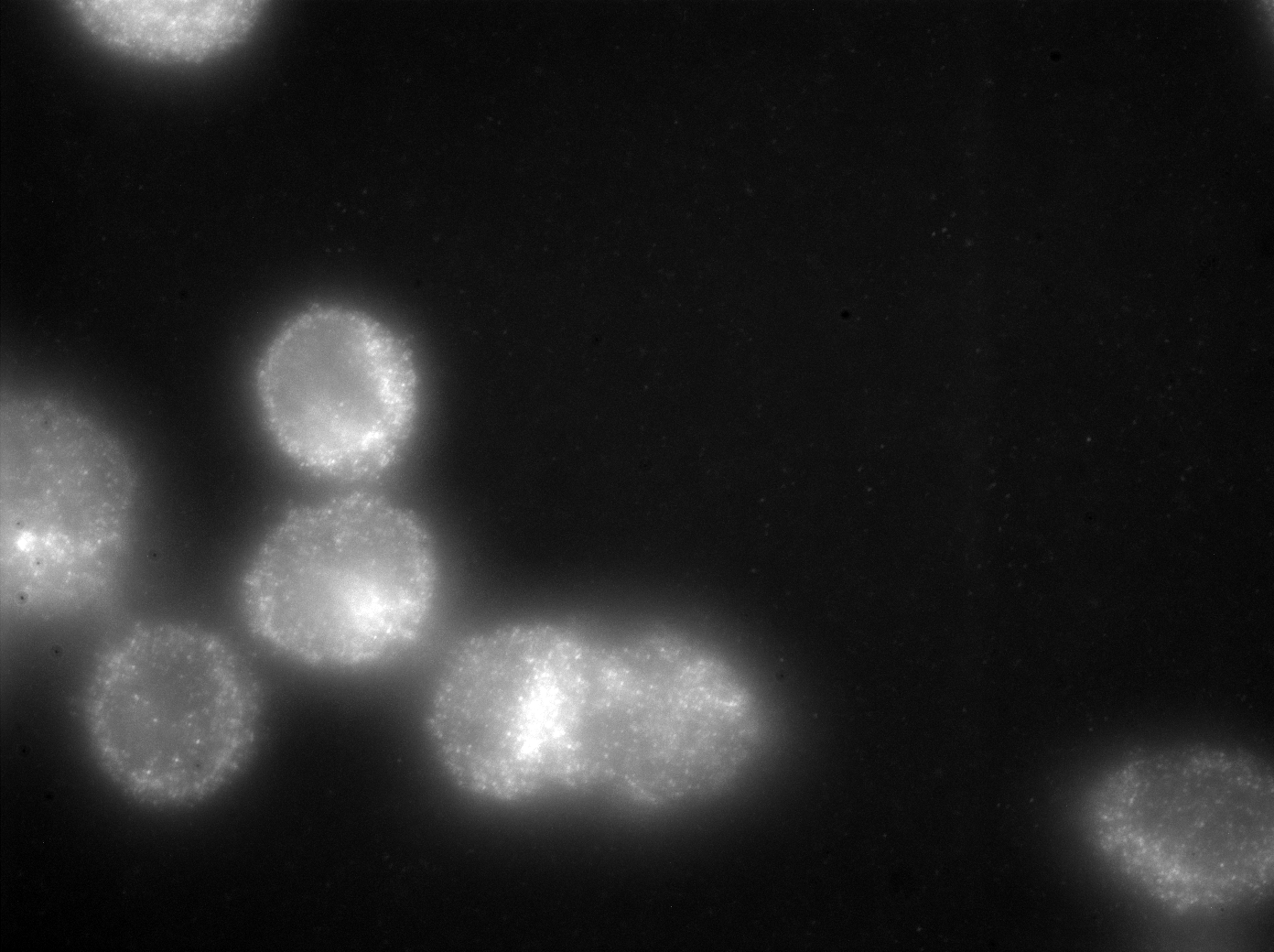

Supplement: Supplementary file 7 — Source data Fig. 2 part 4 [file 44319_2026_742_MOESM7_ESM.zip › Figure 2 Part 4/Fig 2d polye atubulin/WT PolyE-atub 8-14-24 R1 PA8.Project Maximum Z_XY1723761161_Z0_T0_C2.tif]
