# Supplementary figures and images for: TPGS1 regulates central spindle microtubule glutamylation and remodeling during telophase and abscission (part 6 of 36)
Source: EMBO Rep. 2026 Mar 23;27(8):1944–63. doi: 10.1038/s44319-026-00742-3 (PMC13121839; doi:10.1038/s44319-026-00742-3)

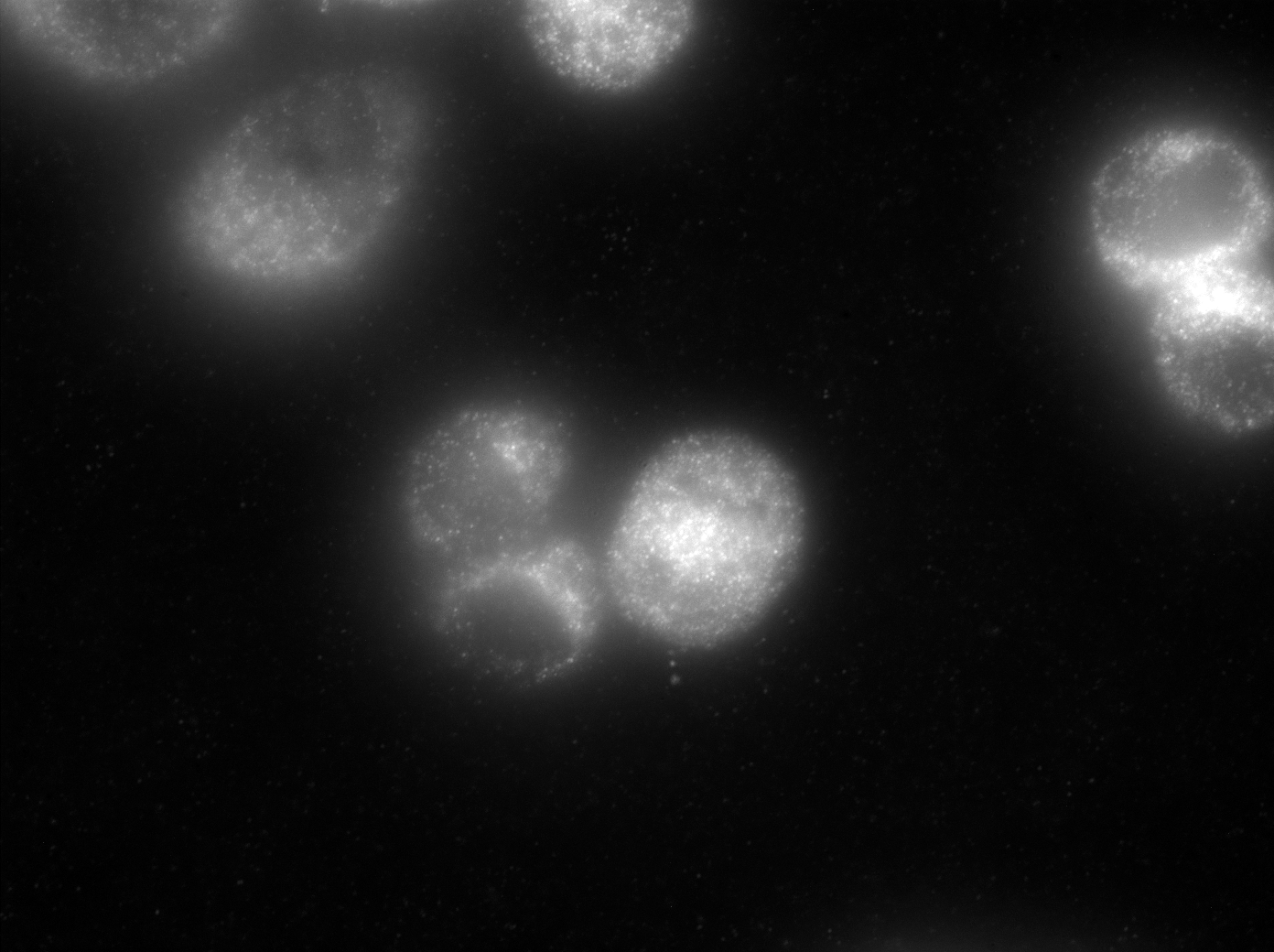

Supplement: Supplementary file 7 — Source data Fig. 2 part 4 [file 44319_2026_742_MOESM7_ESM.zip › Figure 2 Part 4/Fig 2d polye atubulin/WT PolyE-atub 8-14-24 R2 A1.Project Maximum Z_XY1723837650_Z0_T0_C2.tif]

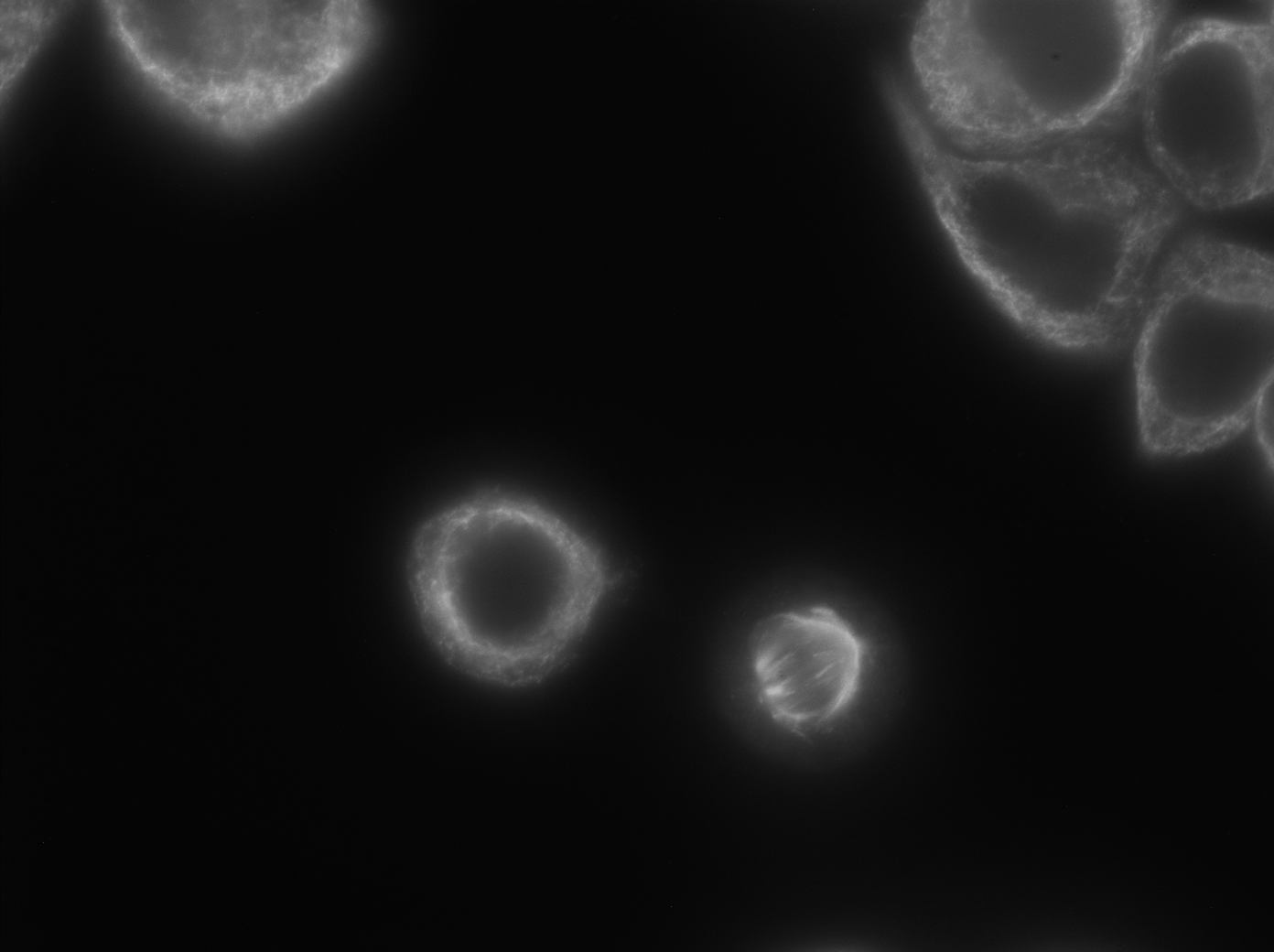

Supplement: Supplementary file 7 — Source data Fig. 2 part 4 [file 44319_2026_742_MOESM7_ESM.zip › Figure 2 Part 4/Fig 2d polye atubulin/WT PolyE-atub 8-14-24 R1 M6.Project Maximum Z_XY1723760417_Z0_T0_C1.tif]

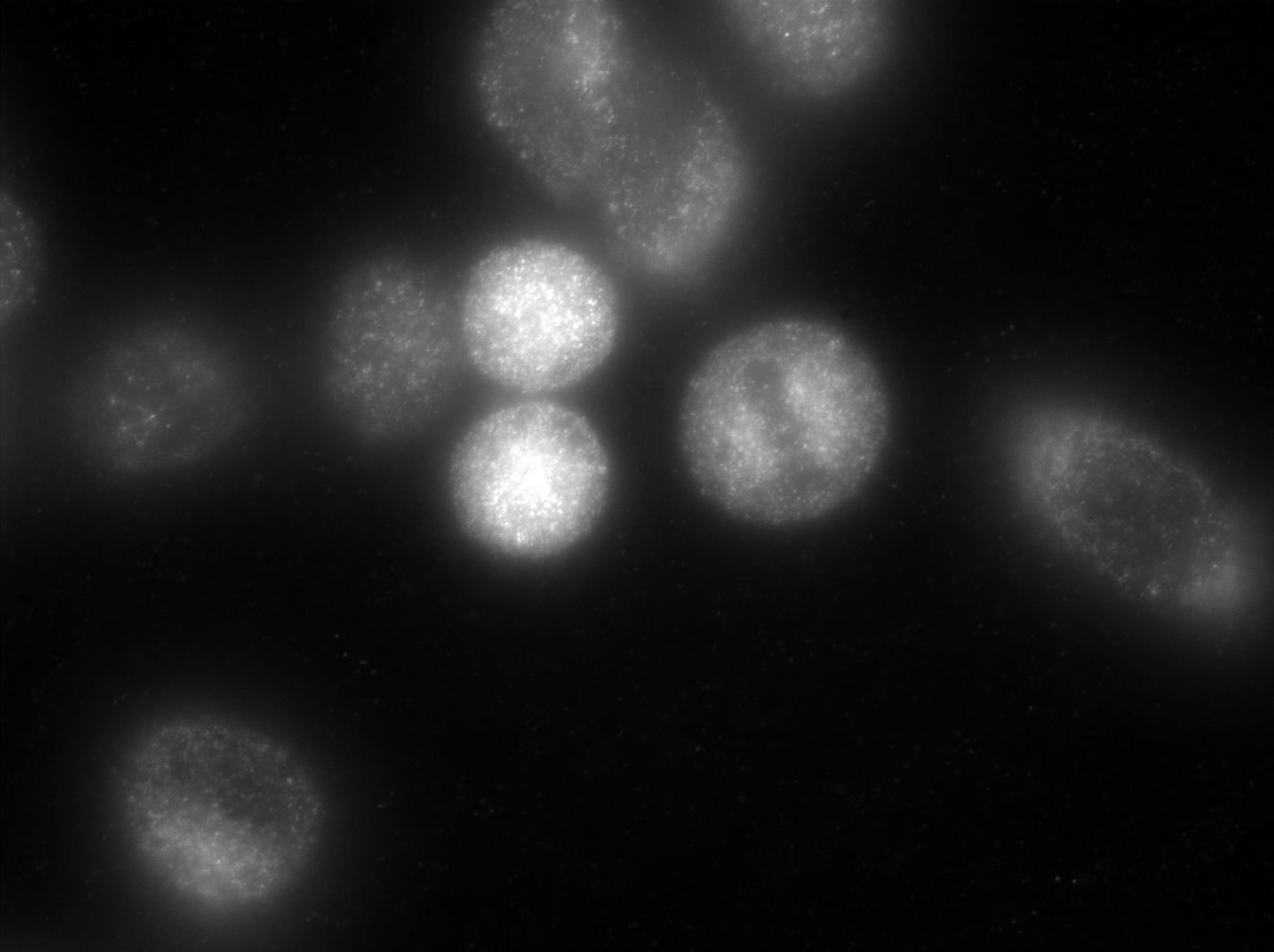

Supplement: Supplementary file 7 — Source data Fig. 2 part 4 [file 44319_2026_742_MOESM7_ESM.zip › Figure 2 Part 4/Fig 2d polye atubulin/WT PolyE-atub 8-14-24 R2 ET9M6.Project Maximum Z_XY1723837940_Z0_T0_C2.tif]

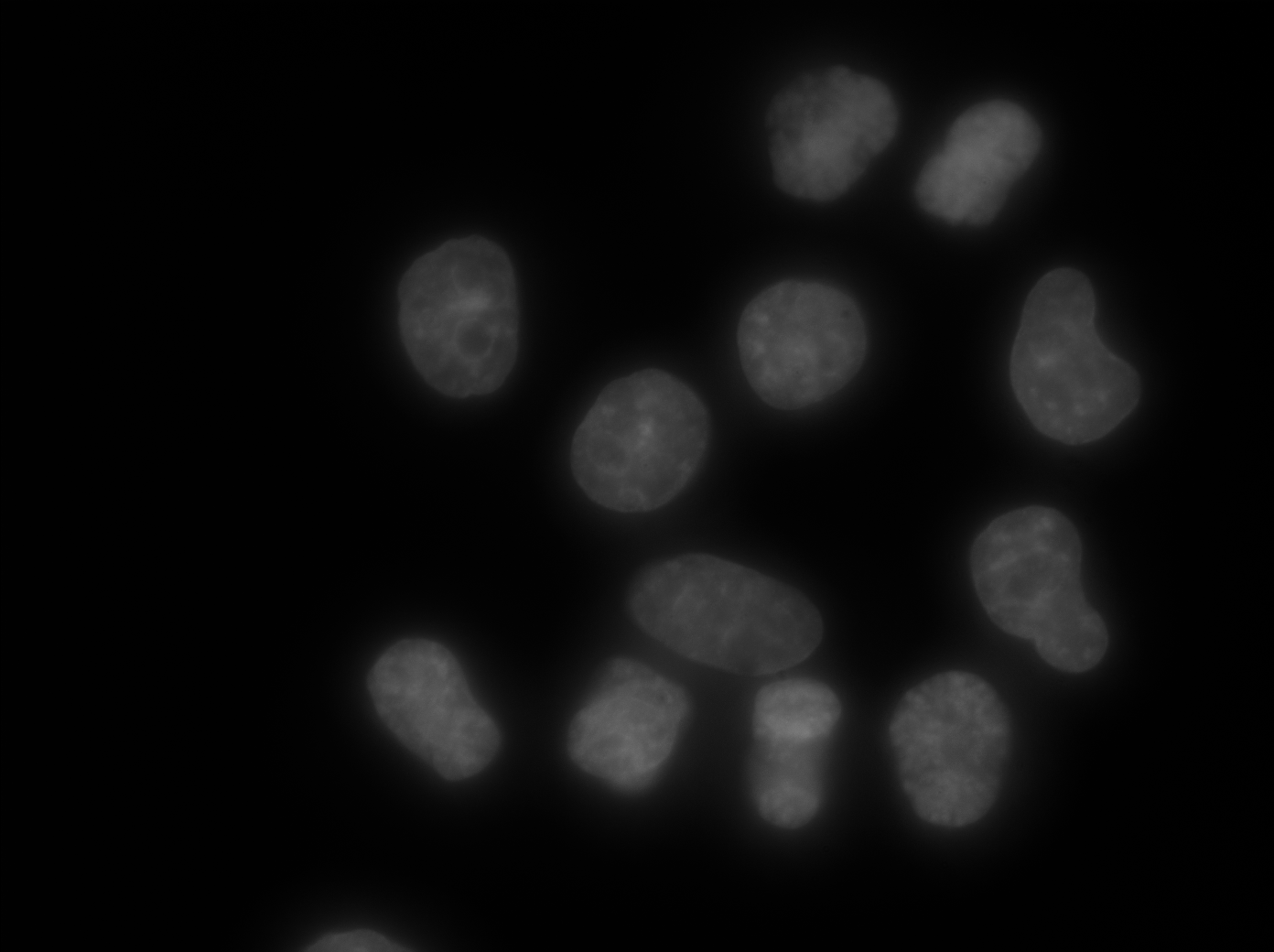

Supplement: Supplementary file 7 — Source data Fig. 2 part 4 [file 44319_2026_742_MOESM7_ESM.zip › Figure 2 Part 4/Fig 2d polye atubulin/WT PolyE-atub 8-14-24 R1 LT7 ET8ET9.Project Maximum Z_XY1723758965_Z0_T0_C0.tif]

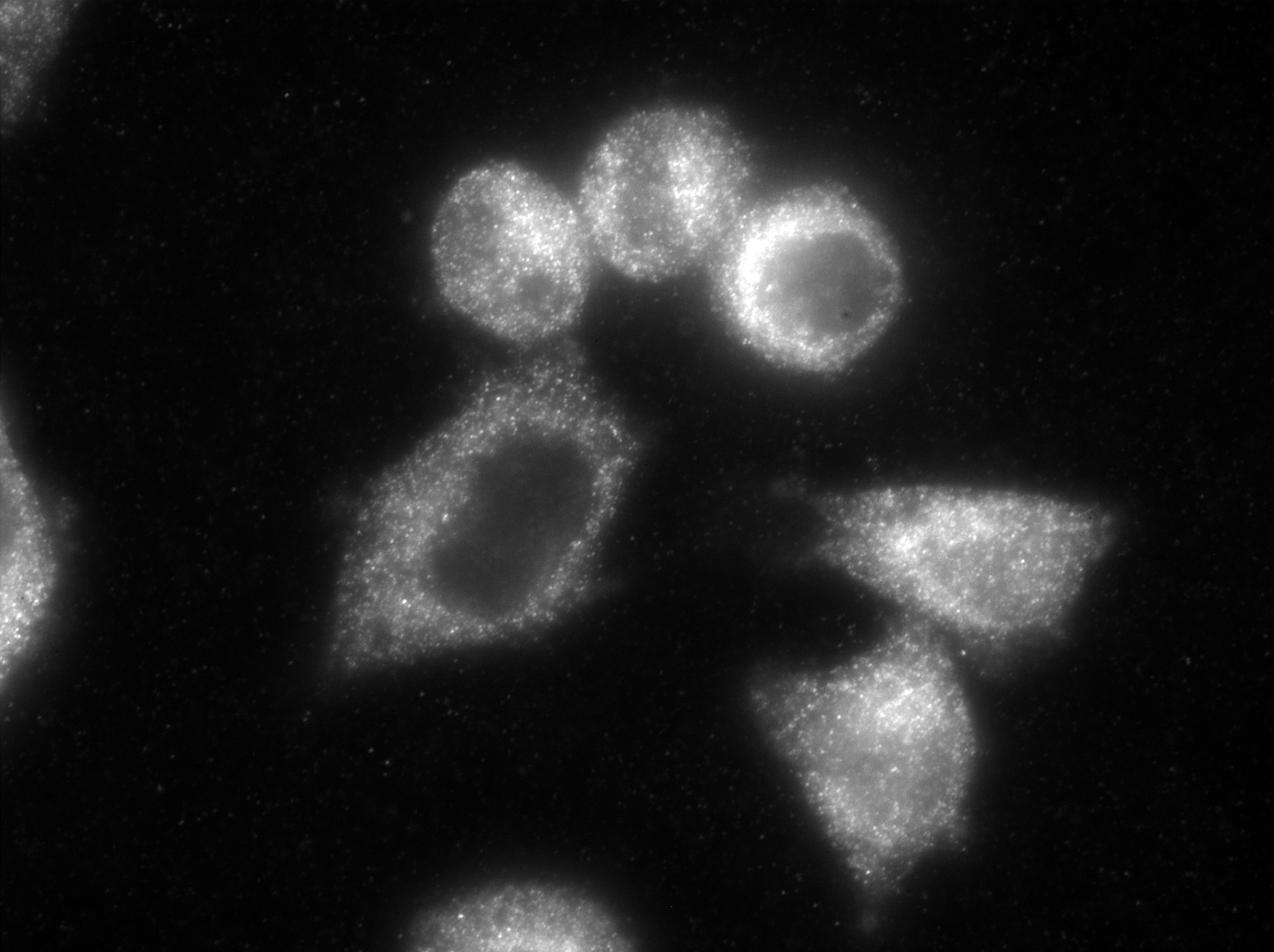

Supplement: Supplementary file 7 — Source data Fig. 2 part 4 [file 44319_2026_742_MOESM7_ESM.zip › Figure 2 Part 4/Fig 2d polye atubulin/WT PolyE-atub 8-14-24 R1 ET7 PA3.Project Maximum Z_XY1723758121_Z0_T0_C2.tif]

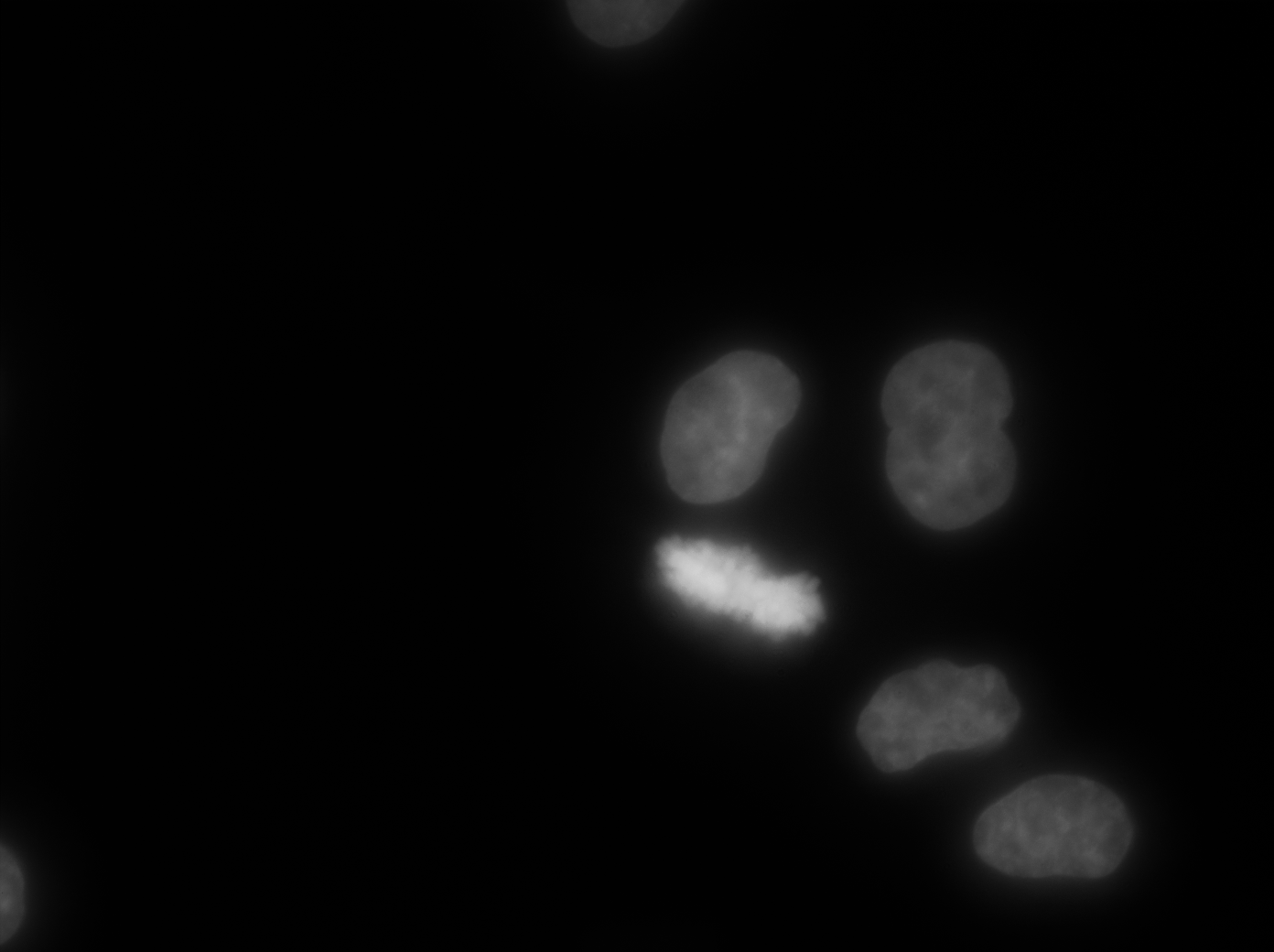

Supplement: Supplementary file 7 — Source data Fig. 2 part 4 [file 44319_2026_742_MOESM7_ESM.zip › Figure 2 Part 4/Fig 2d polye atubulin/WT PolyE-atub 8-14-24 R1 M2.Project Maximum Z_XY1723759215_Z0_T0_C0.tif]

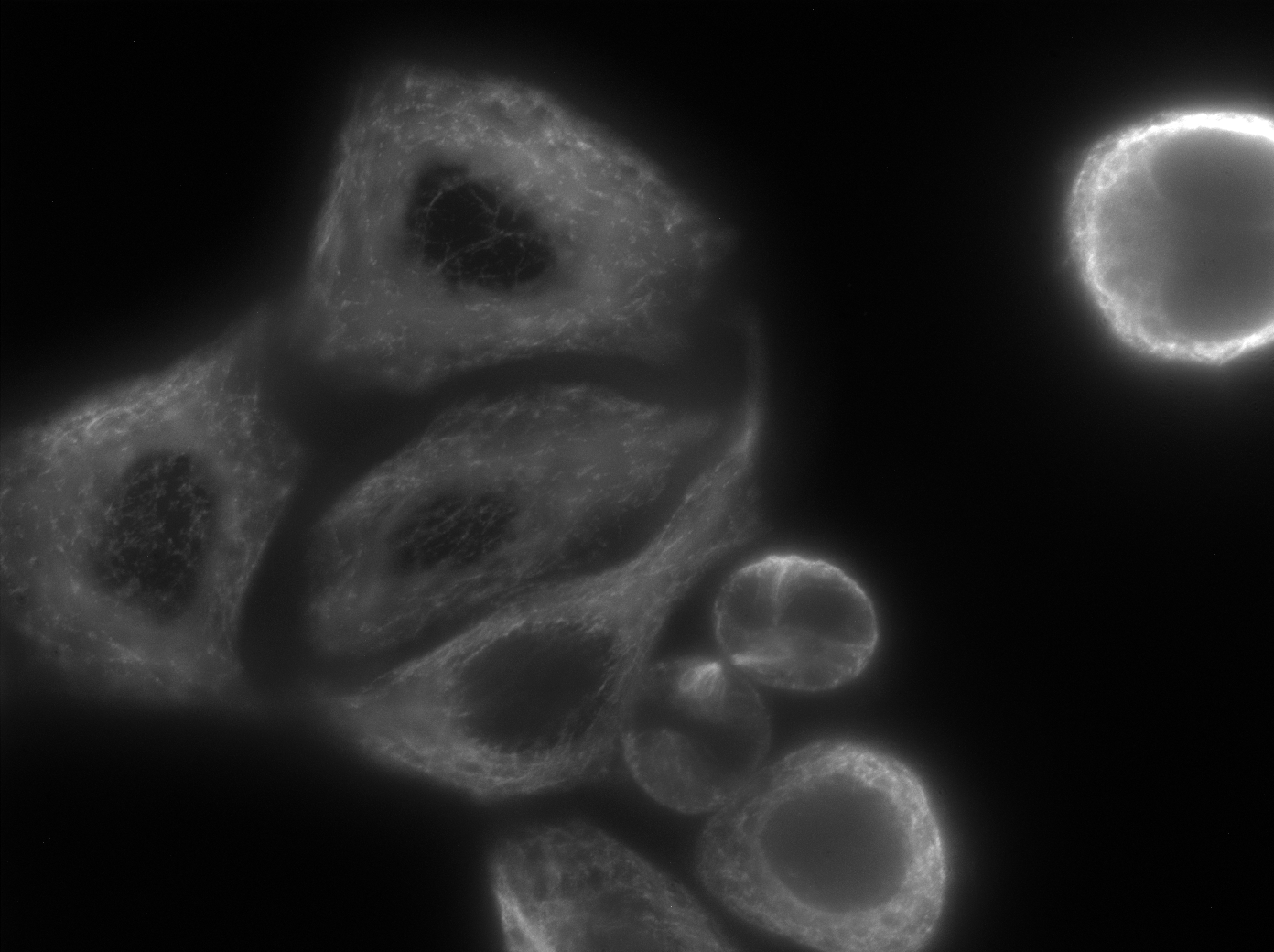

Supplement: Supplementary file 7 — Source data Fig. 2 part 4 [file 44319_2026_742_MOESM7_ESM.zip › Figure 2 Part 4/Fig 2d polye atubulin/WT PolyE-atub 8-14-24 R2 ET1.Project Maximum Z_XY1723832482_Z0_T0_C1.tif]

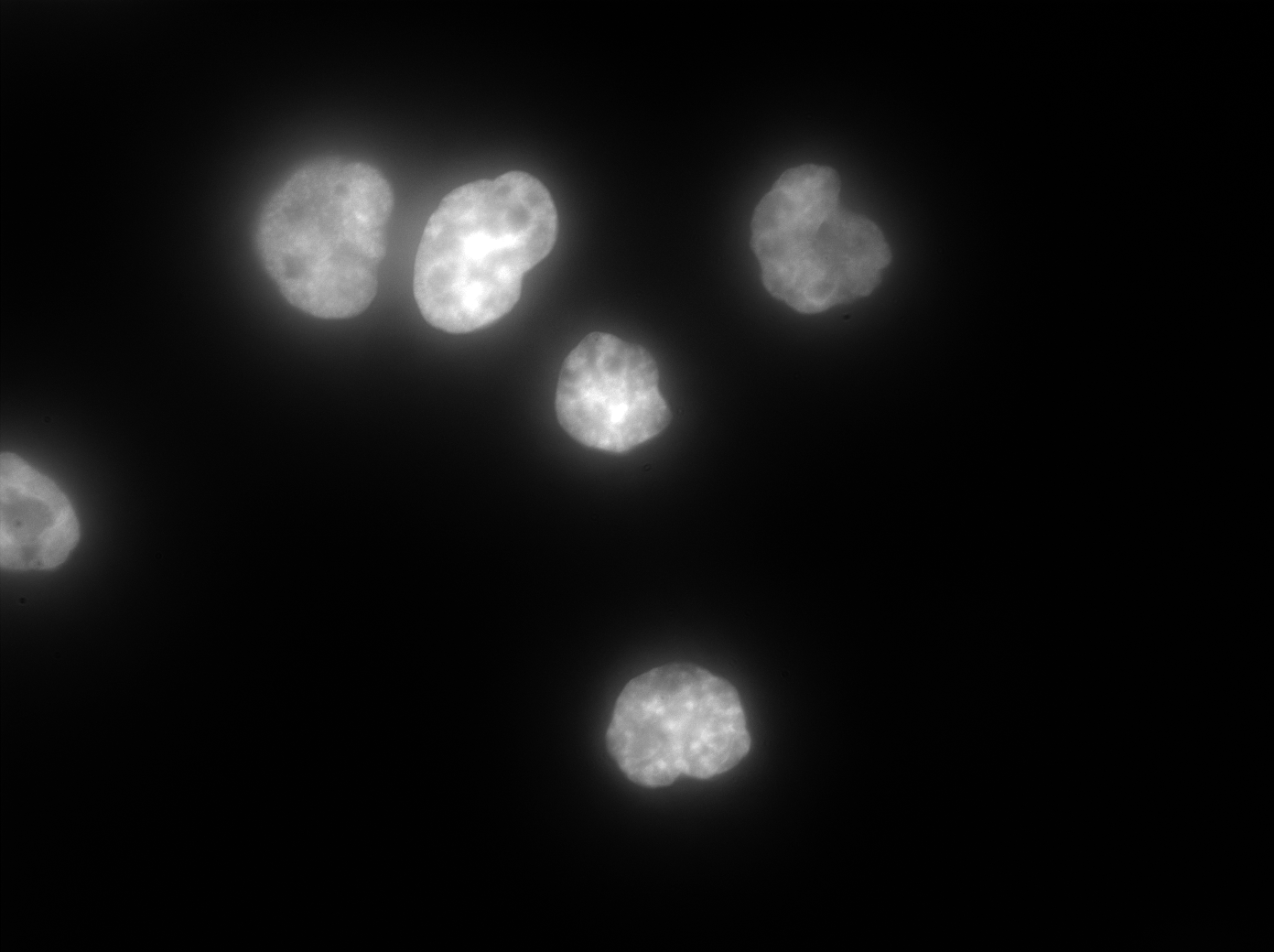

Supplement: Supplementary file 7 — Source data Fig. 2 part 4 [file 44319_2026_742_MOESM7_ESM.zip › Figure 2 Part 4/Fig 2d polye atubulin/WT PolyE-atub 8-14-24 R2 LT3.Project Maximum Z_XY1723834528_Z0_T0_C0.tif]

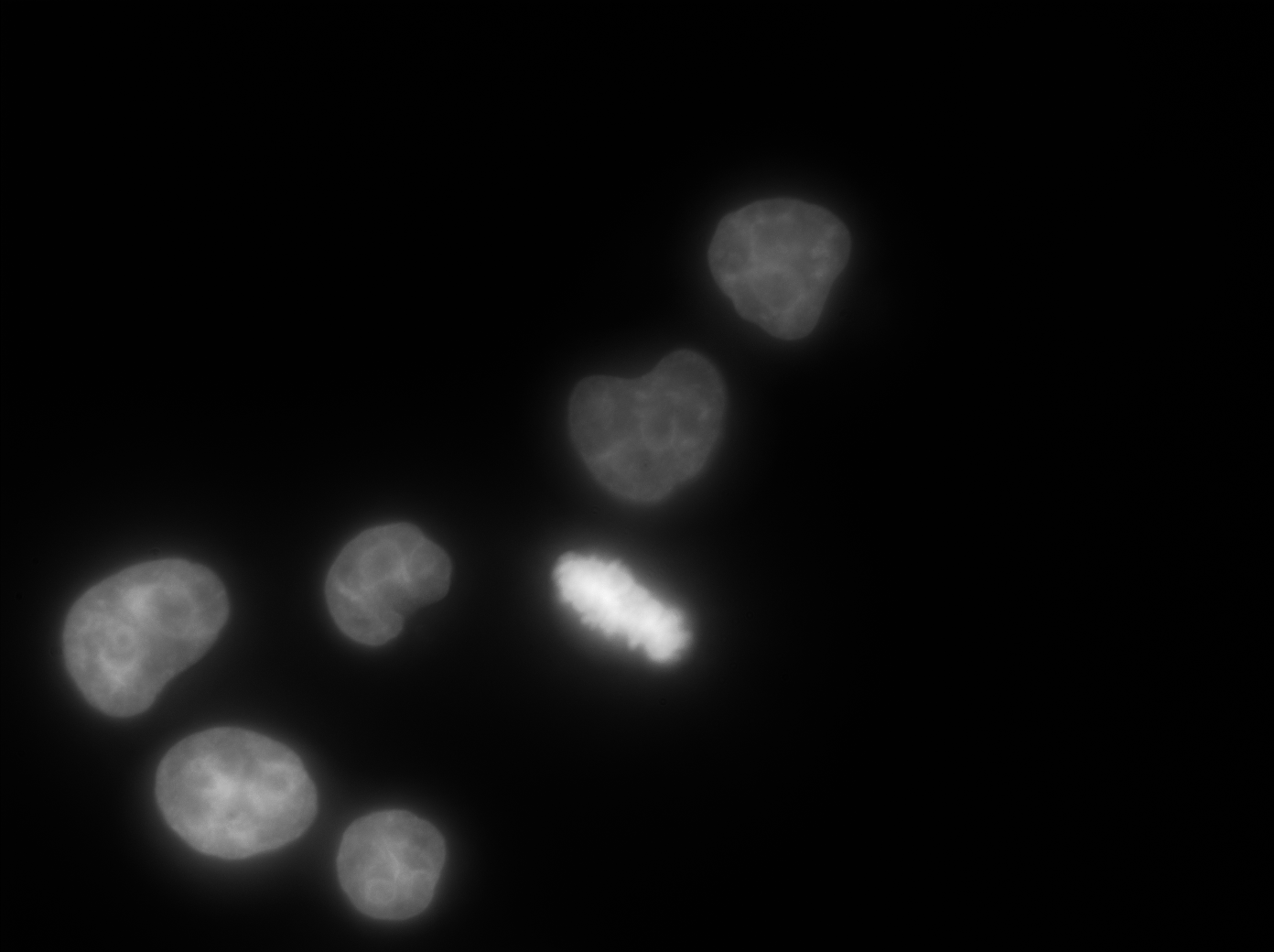

Supplement: Supplementary file 7 — Source data Fig. 2 part 4 [file 44319_2026_742_MOESM7_ESM.zip › Figure 2 Part 4/Fig 2d polye atubulin/WT PolyE-atub 8-14-24 R1 M3 PA5.Project Maximum Z_XY1723759662_Z0_T0_C0.tif]

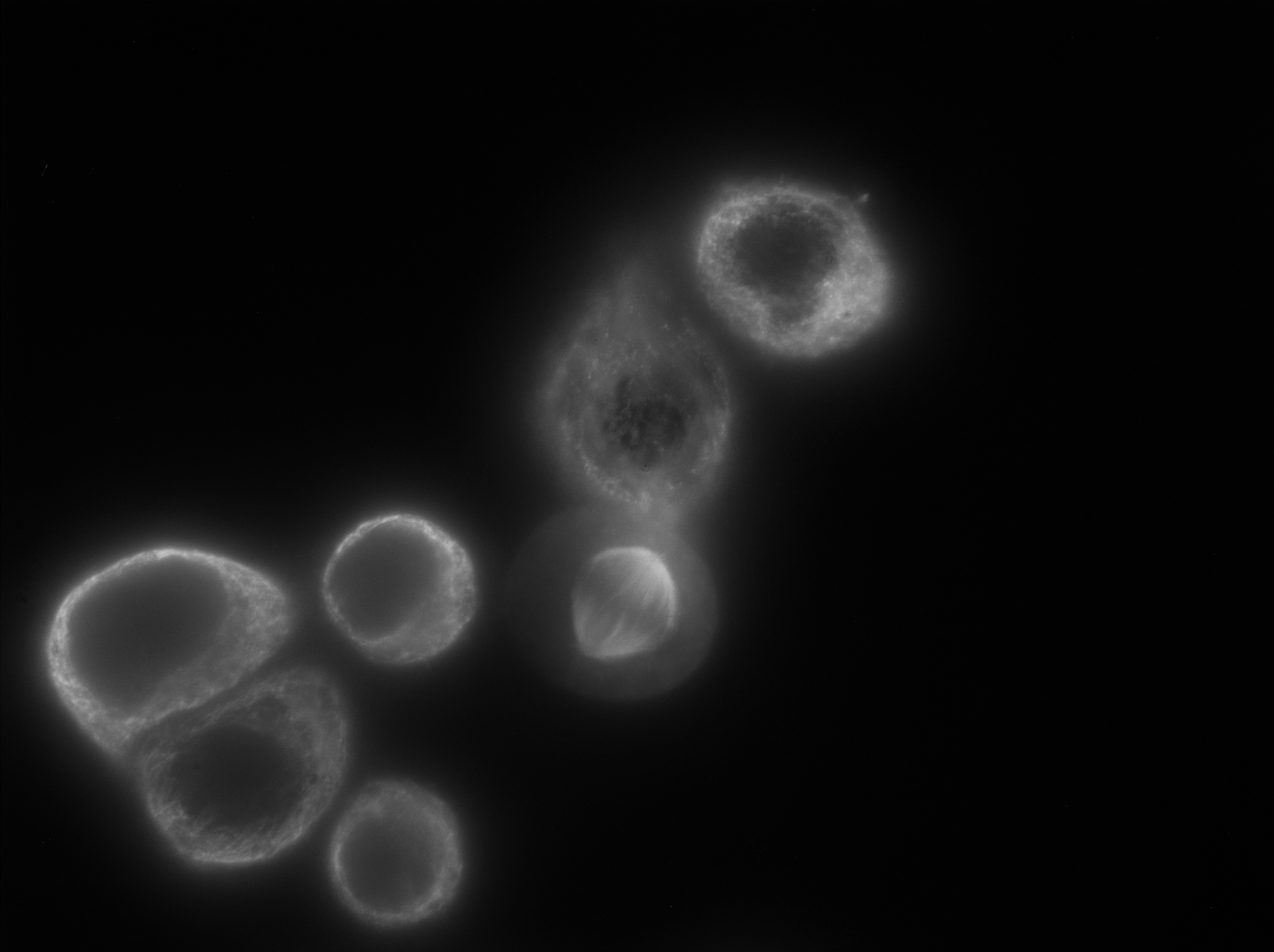

Supplement: Supplementary file 7 — Source data Fig. 2 part 4 [file 44319_2026_742_MOESM7_ESM.zip › Figure 2 Part 4/Fig 2d polye atubulin/WT PolyE-atub 8-14-24 R1 M3 PA5.Project Maximum Z_XY1723759662_Z0_T0_C1.tif]

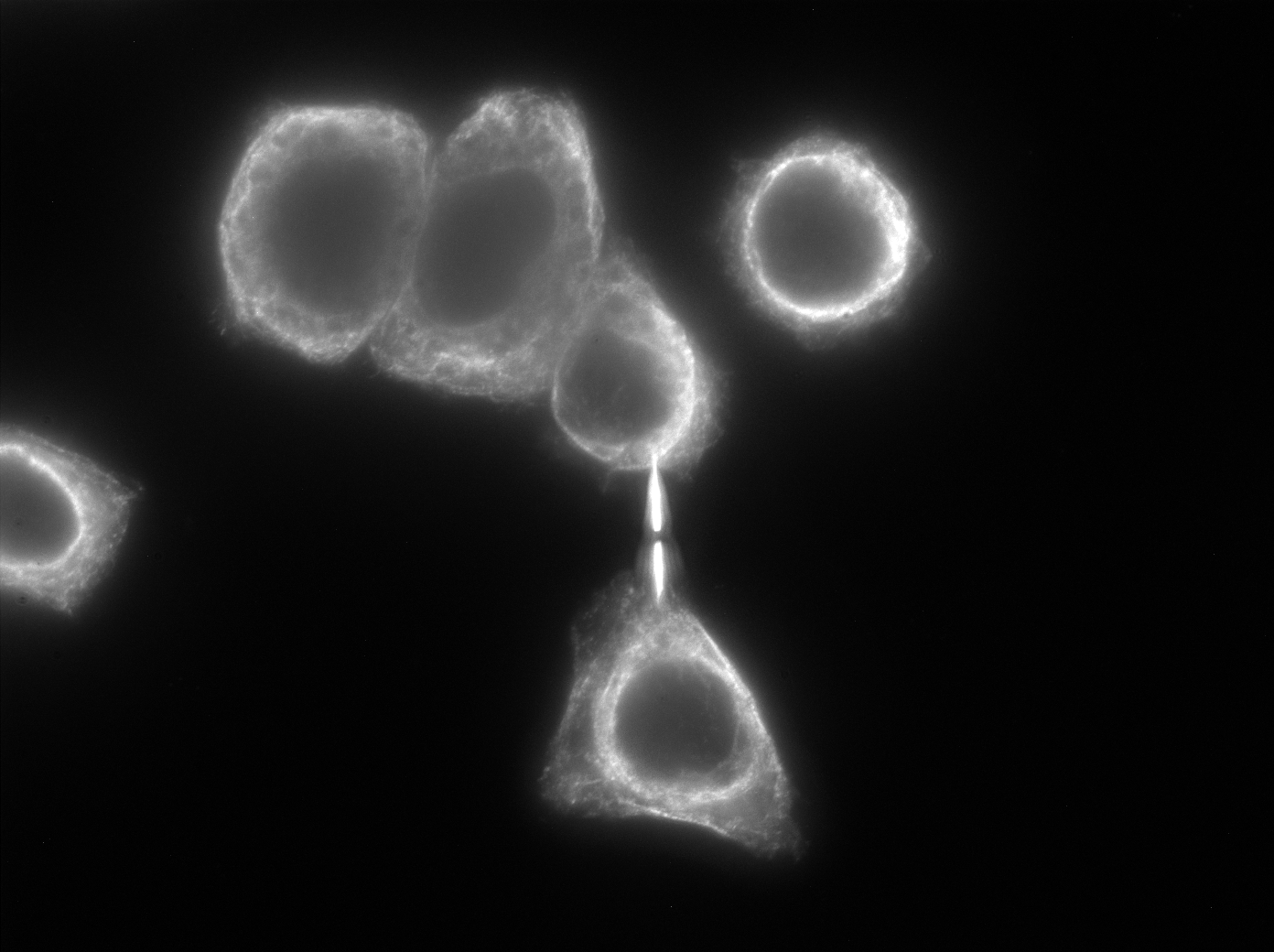

Supplement: Supplementary file 7 — Source data Fig. 2 part 4 [file 44319_2026_742_MOESM7_ESM.zip › Figure 2 Part 4/Fig 2d polye atubulin/WT PolyE-atub 8-14-24 R2 LT3.Project Maximum Z_XY1723834528_Z0_T0_C1.tif]

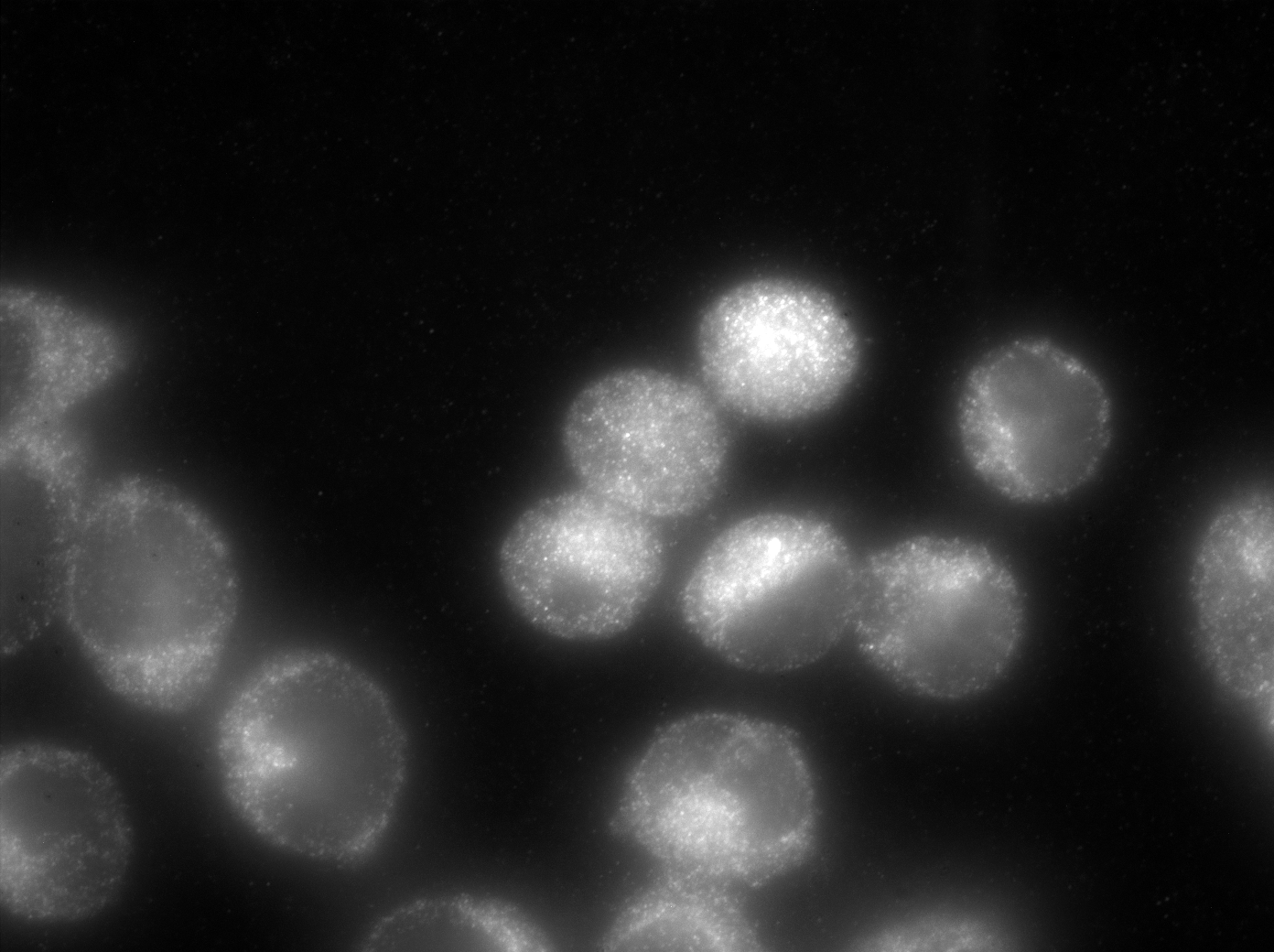

Supplement: Supplementary file 7 — Source data Fig. 2 part 4 [file 44319_2026_742_MOESM7_ESM.zip › Figure 2 Part 4/Fig 2d polye atubulin/WT PolyE-atub 8-14-24 R1 ET2 PA1.Project Maximum Z_XY1723756179_Z0_T0_C2.tif]

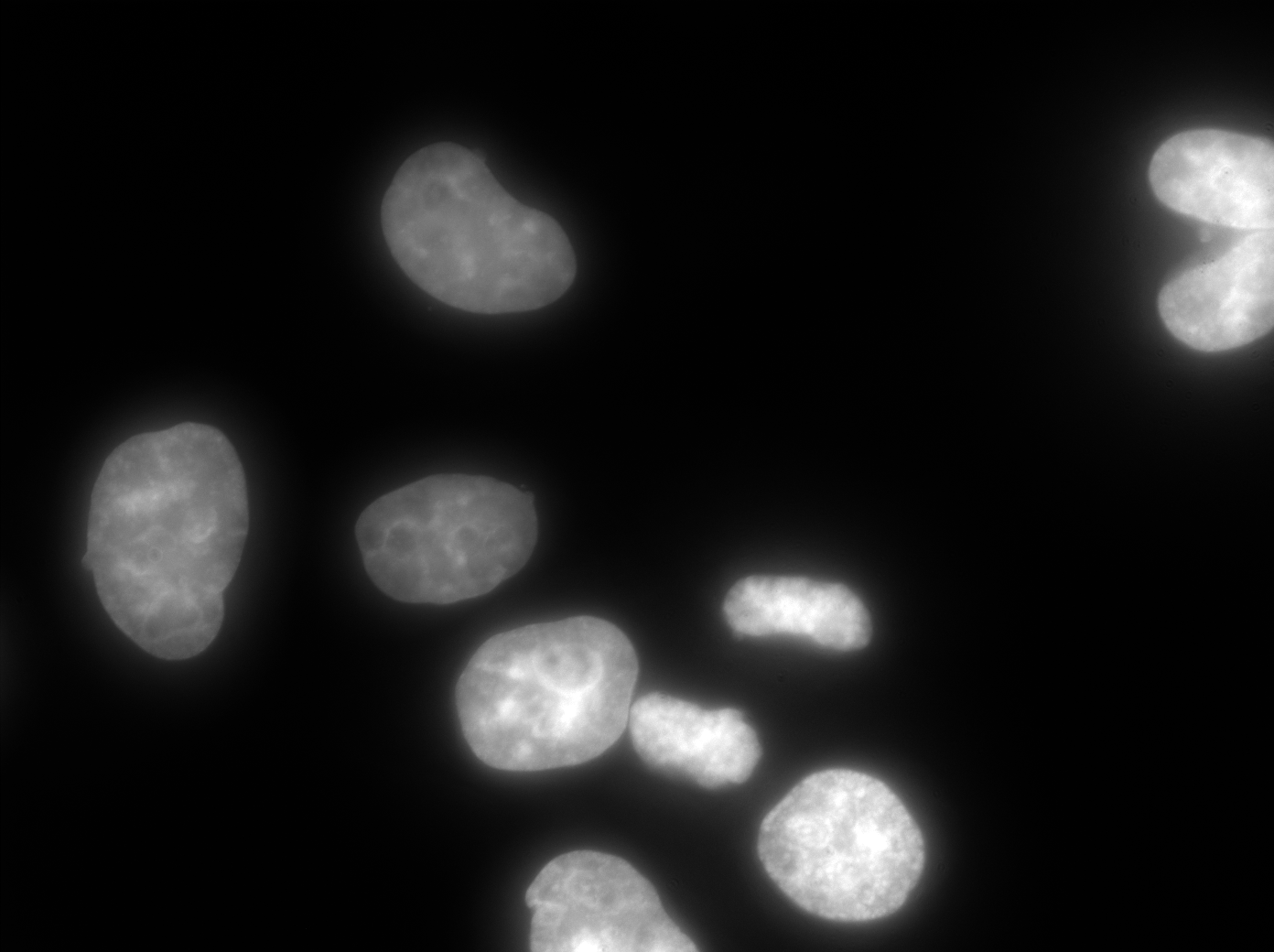

Supplement: Supplementary file 7 — Source data Fig. 2 part 4 [file 44319_2026_742_MOESM7_ESM.zip › Figure 2 Part 4/Fig 2d polye atubulin/WT PolyE-atub 8-14-24 R2 ET1.Project Maximum Z_XY1723832482_Z0_T0_C0.tif]

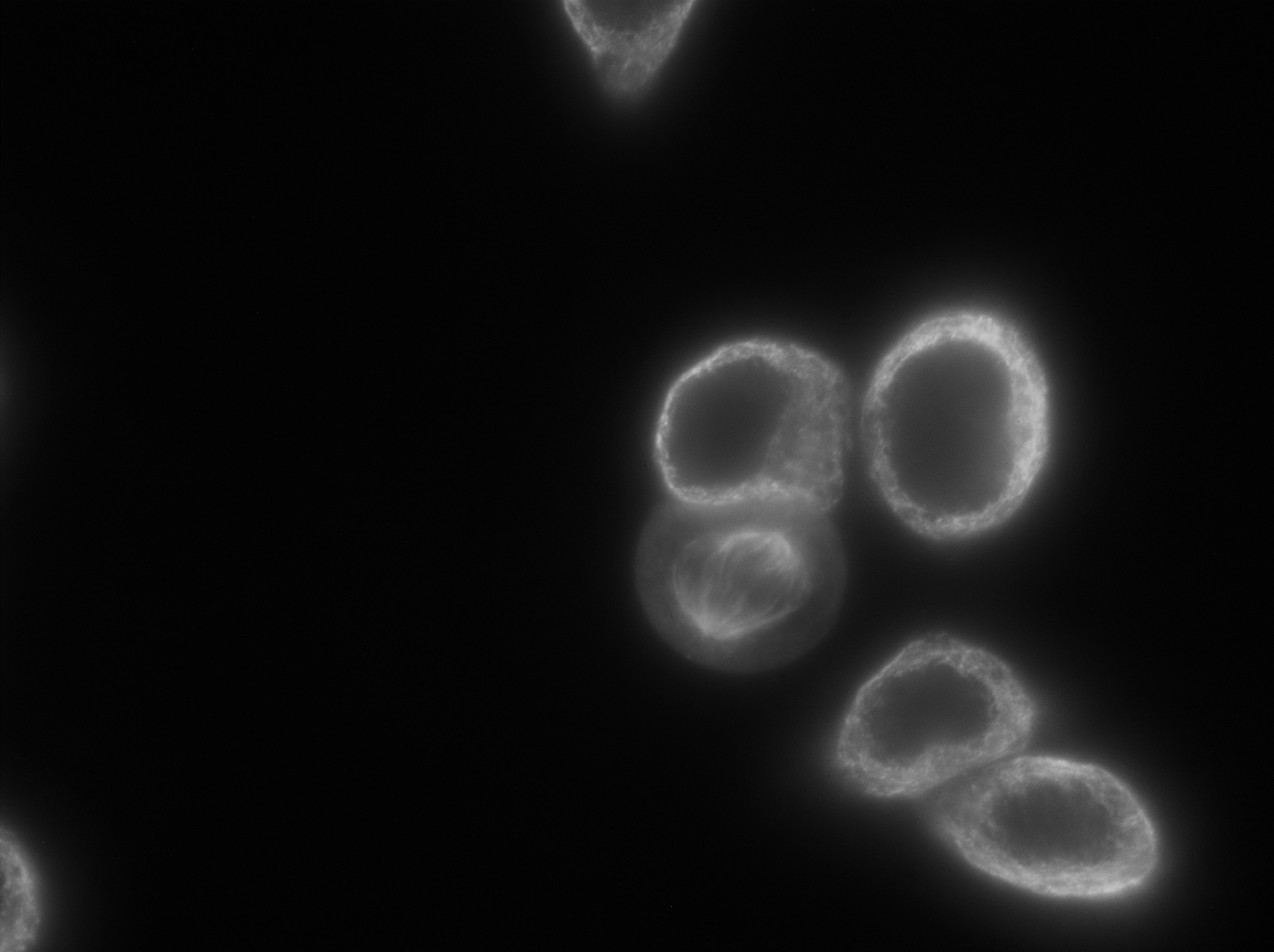

Supplement: Supplementary file 7 — Source data Fig. 2 part 4 [file 44319_2026_742_MOESM7_ESM.zip › Figure 2 Part 4/Fig 2d polye atubulin/WT PolyE-atub 8-14-24 R1 M2.Project Maximum Z_XY1723759215_Z0_T0_C1.tif]

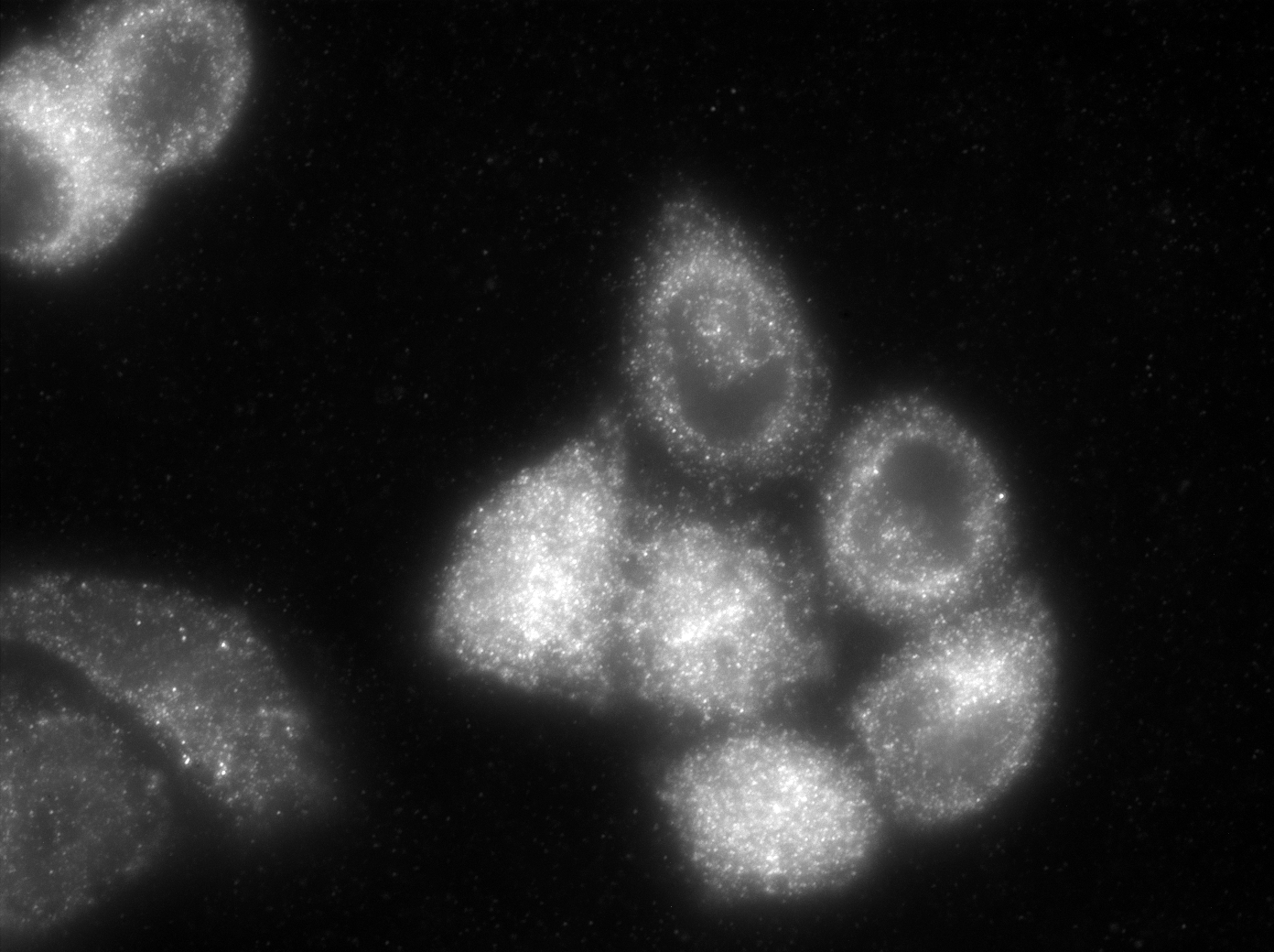

Supplement: Supplementary file 7 — Source data Fig. 2 part 4 [file 44319_2026_742_MOESM7_ESM.zip › Figure 2 Part 4/Fig 2d polye atubulin/WT PolyE-atub 8-14-24 R2 LT1 PA1.Project Maximum Z_XY1723834071_Z0_T0_C2.tif]

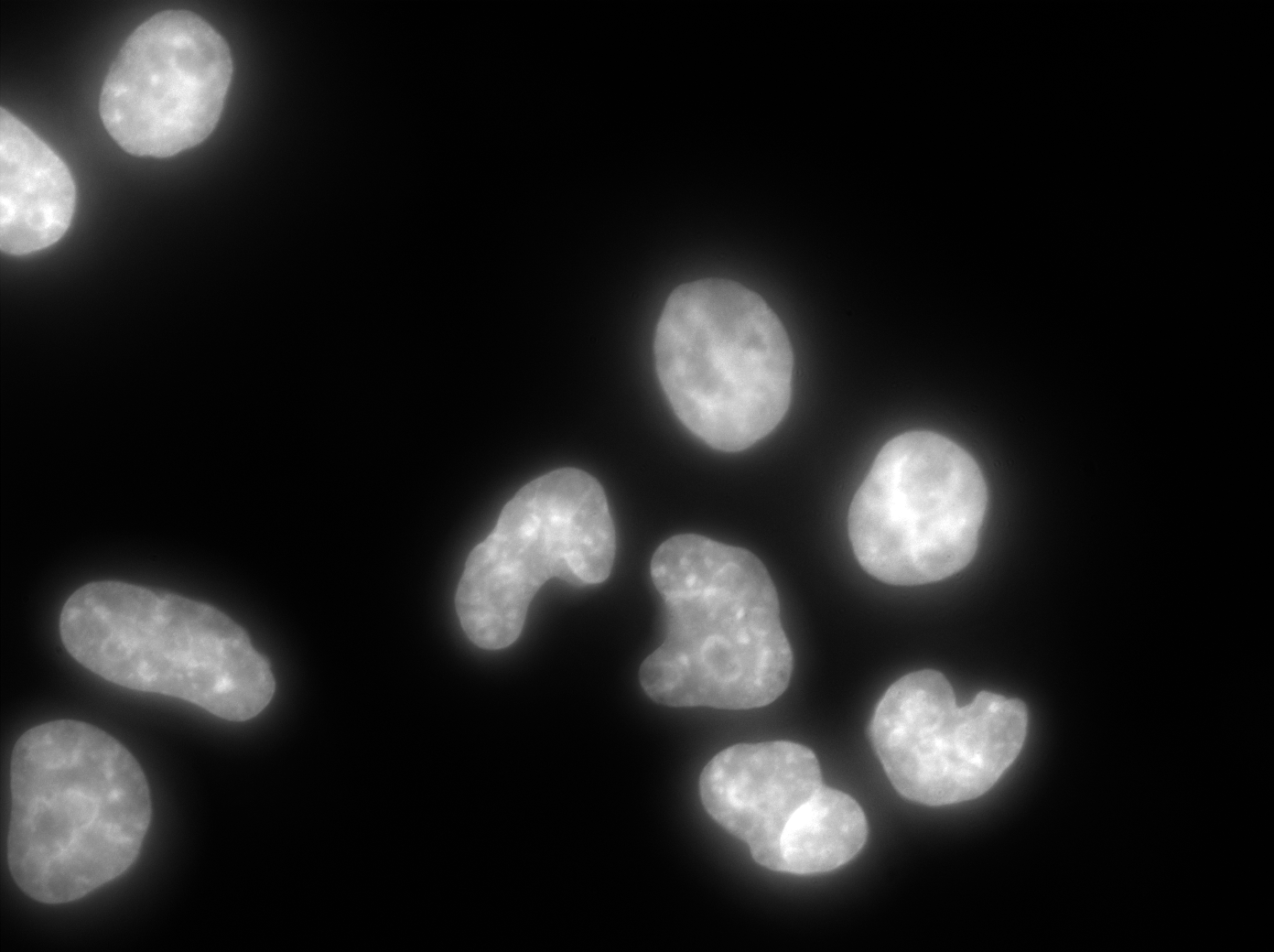

Supplement: Supplementary file 7 — Source data Fig. 2 part 4 [file 44319_2026_742_MOESM7_ESM.zip › Figure 2 Part 4/Fig 2d polye atubulin/WT PolyE-atub 8-14-24 R2 LT1 PA1.Project Maximum Z_XY1723834071_Z0_T0_C0.tif]

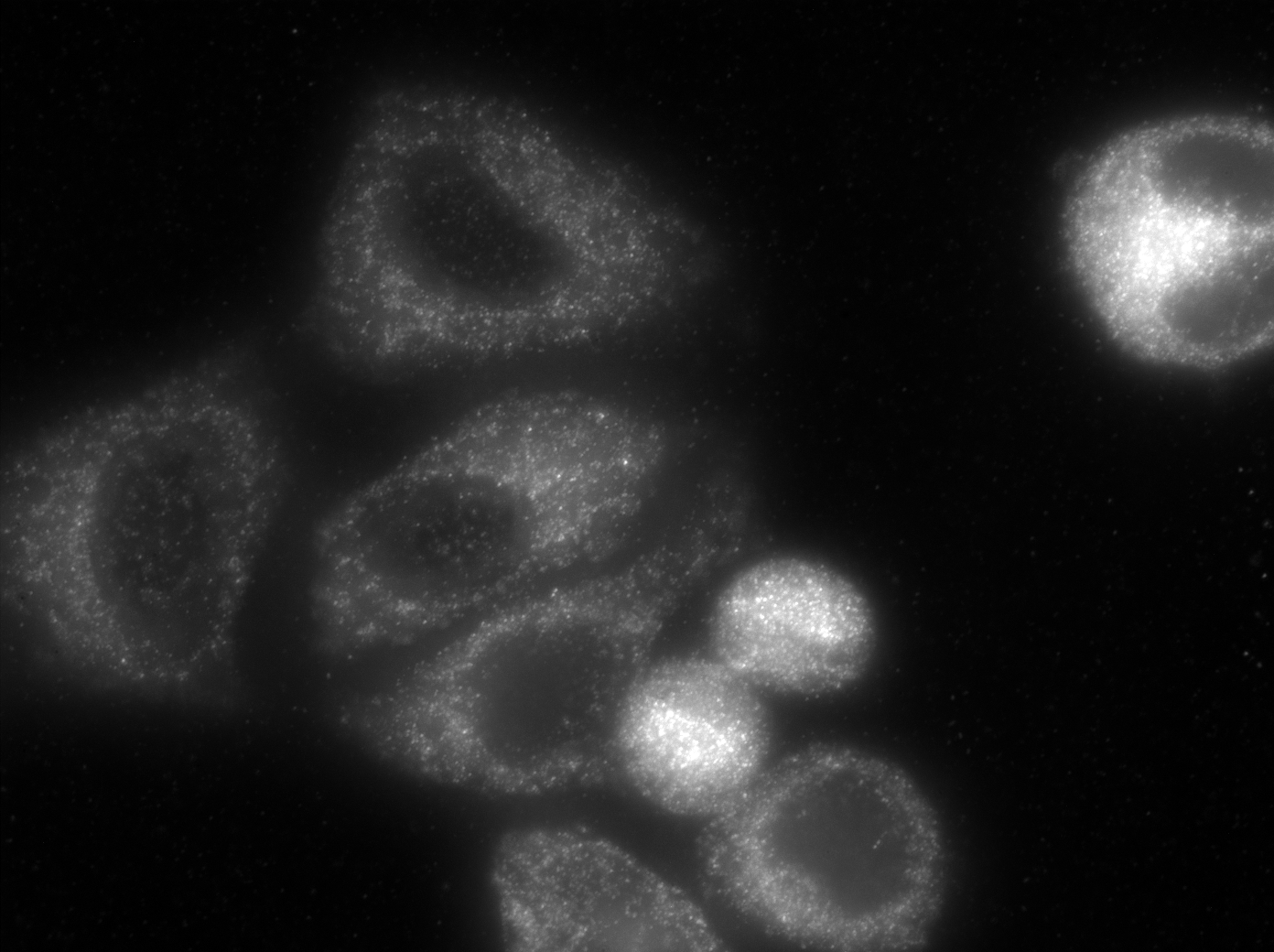

Supplement: Supplementary file 7 — Source data Fig. 2 part 4 [file 44319_2026_742_MOESM7_ESM.zip › Figure 2 Part 4/Fig 2d polye atubulin/WT PolyE-atub 8-14-24 R2 ET1.Project Maximum Z_XY1723832482_Z0_T0_C2.tif]

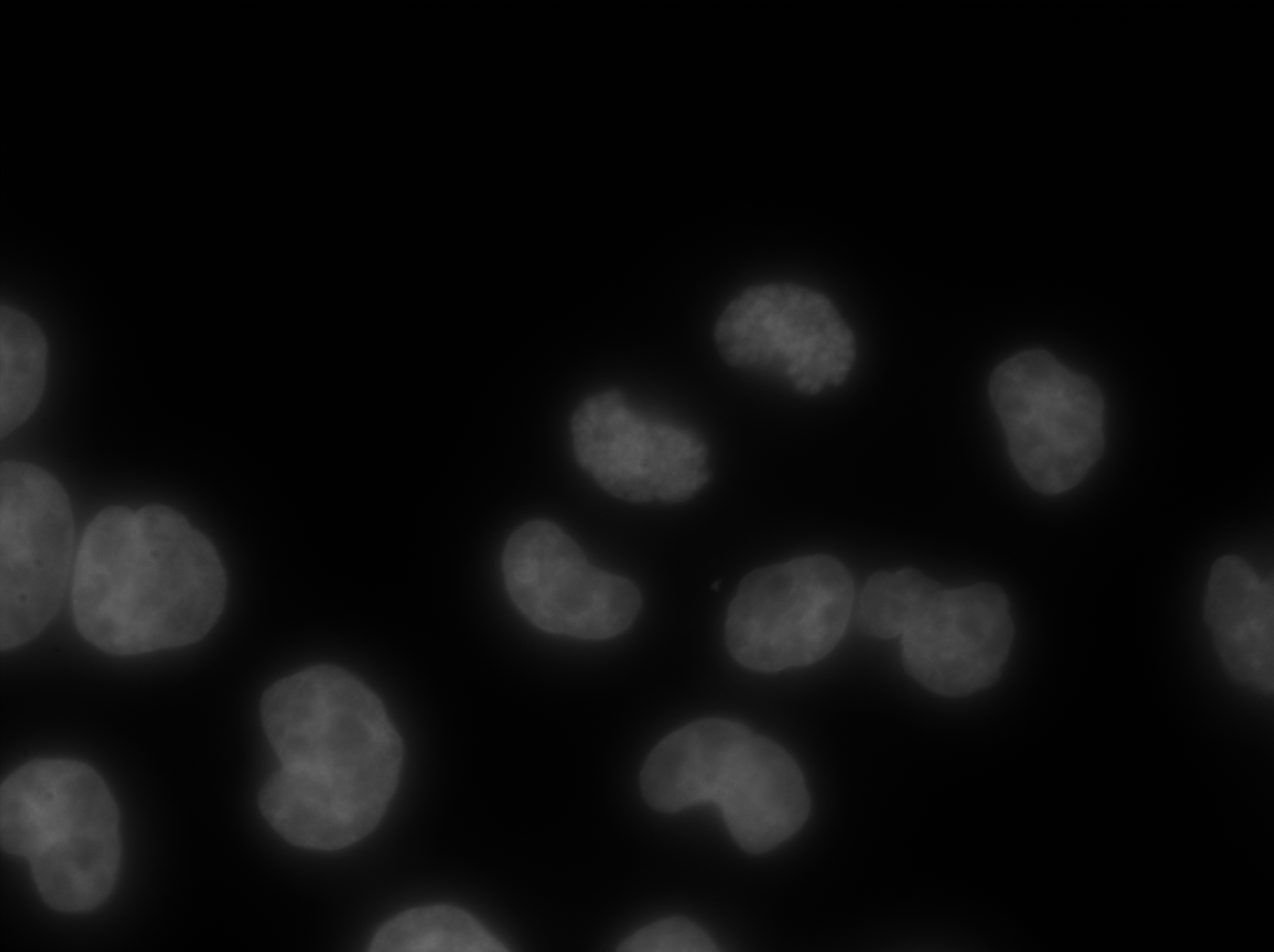

Supplement: Supplementary file 7 — Source data Fig. 2 part 4 [file 44319_2026_742_MOESM7_ESM.zip › Figure 2 Part 4/Fig 2d polye atubulin/WT PolyE-atub 8-14-24 R1 ET2 PA1.Project Maximum Z_XY1723756179_Z0_T0_C0.tif]

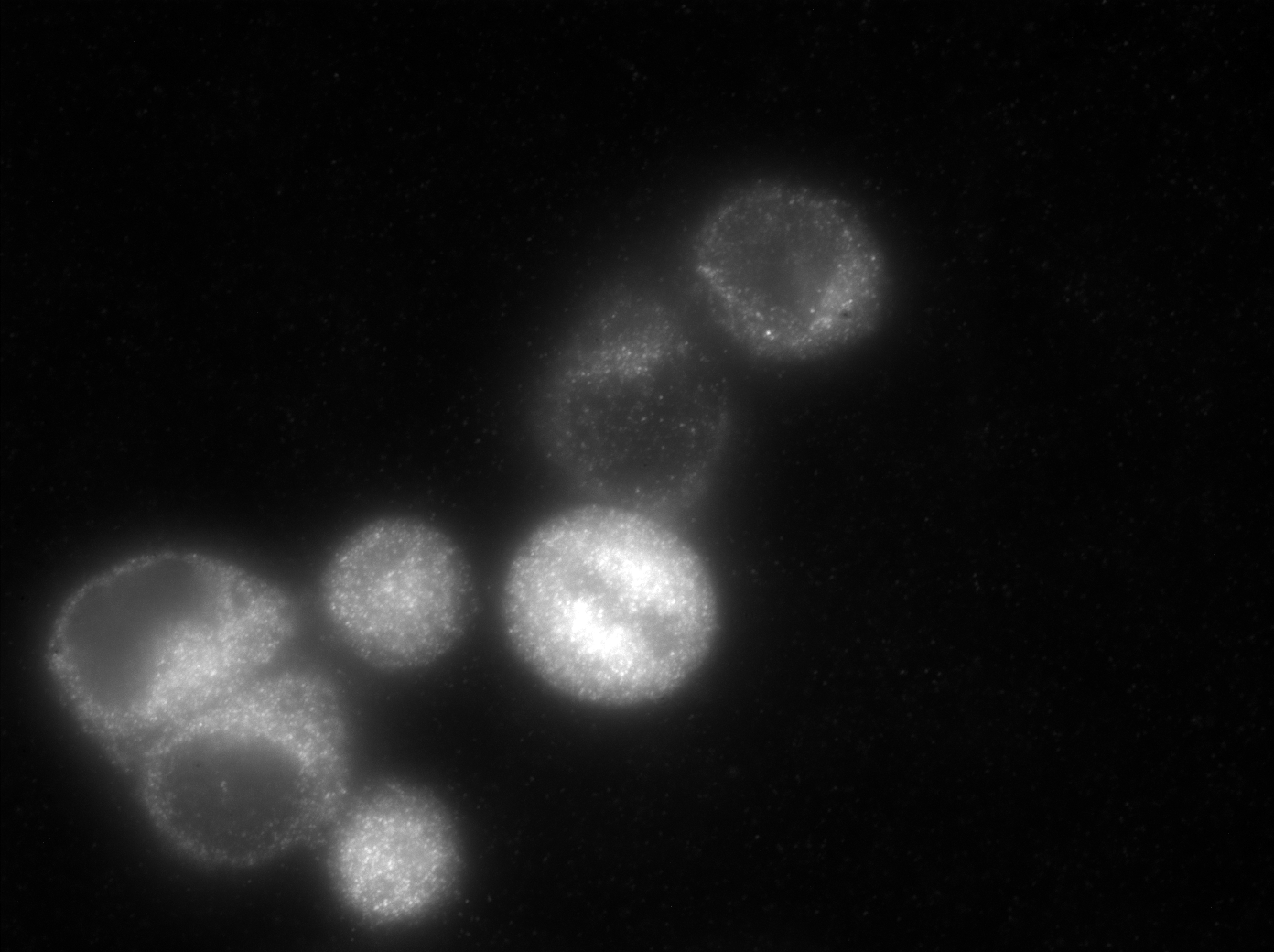

Supplement: Supplementary file 7 — Source data Fig. 2 part 4 [file 44319_2026_742_MOESM7_ESM.zip › Figure 2 Part 4/Fig 2d polye atubulin/WT PolyE-atub 8-14-24 R1 M3 PA5.Project Maximum Z_XY1723759662_Z0_T0_C2.tif]

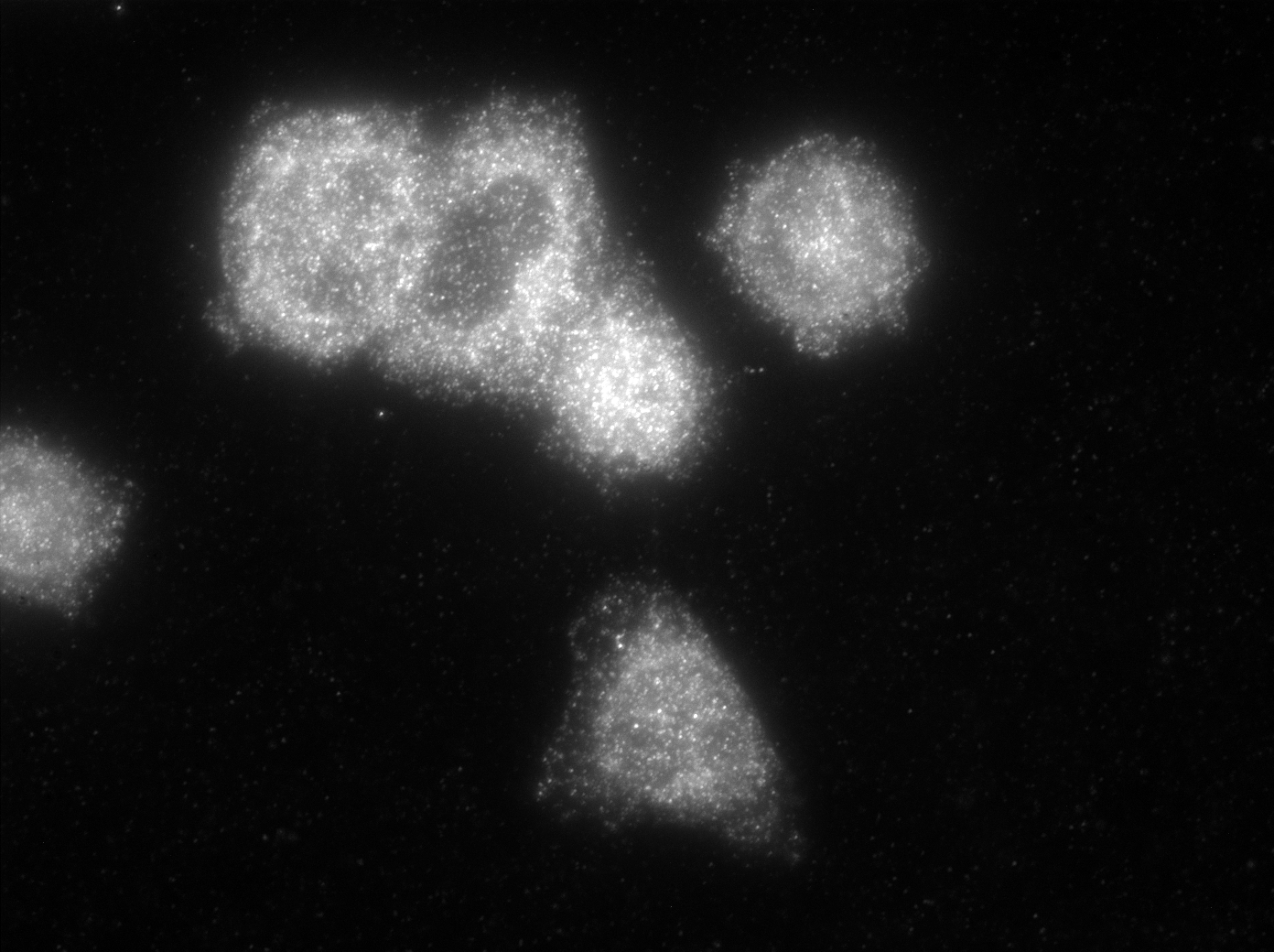

Supplement: Supplementary file 7 — Source data Fig. 2 part 4 [file 44319_2026_742_MOESM7_ESM.zip › Figure 2 Part 4/Fig 2d polye atubulin/WT PolyE-atub 8-14-24 R2 LT3.Project Maximum Z_XY1723834528_Z0_T0_C2.tif]

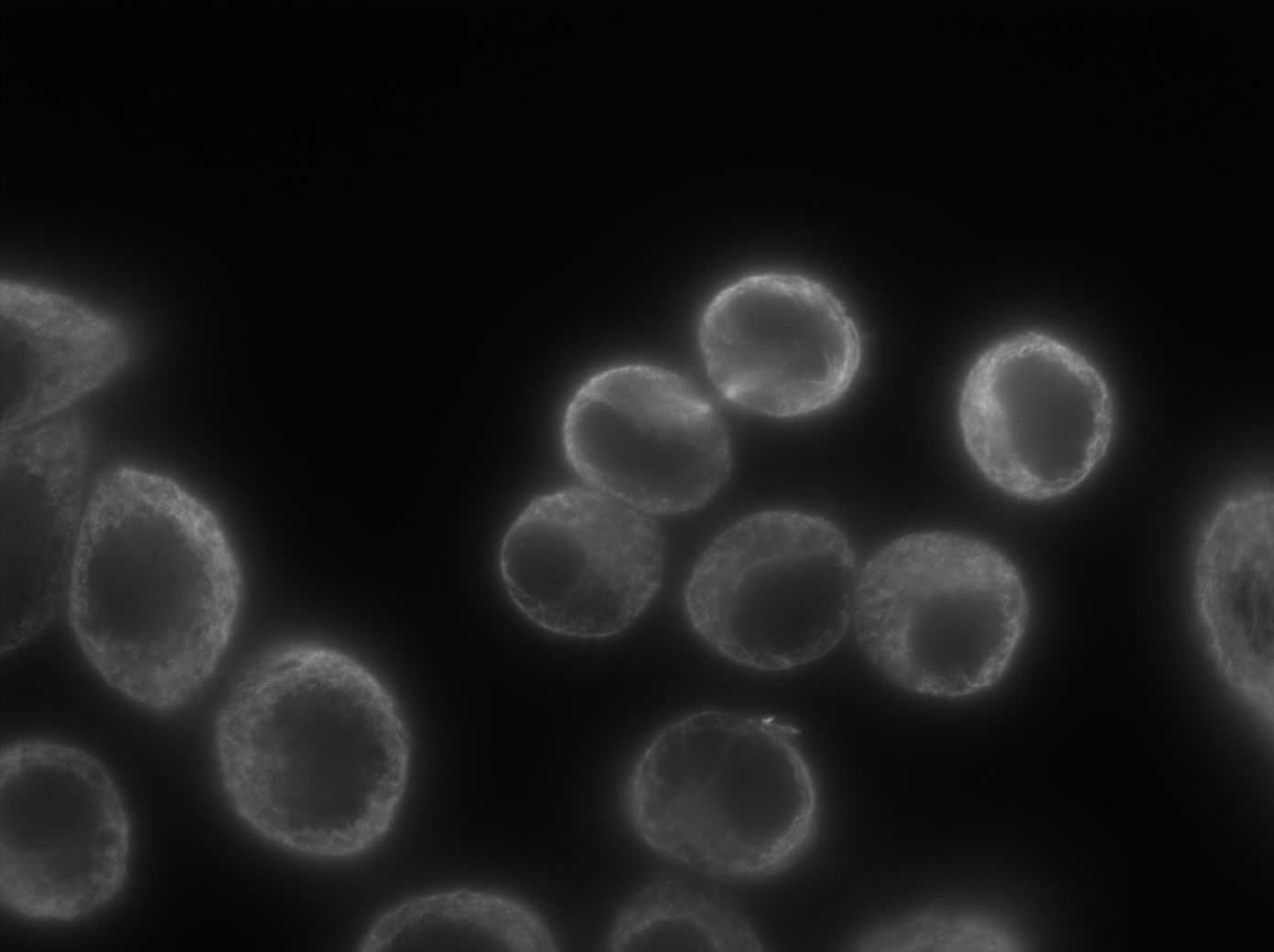

Supplement: Supplementary file 7 — Source data Fig. 2 part 4 [file 44319_2026_742_MOESM7_ESM.zip › Figure 2 Part 4/Fig 2d polye atubulin/WT PolyE-atub 8-14-24 R1 ET2 PA1.Project Maximum Z_XY1723756179_Z0_T0_C1.tif]

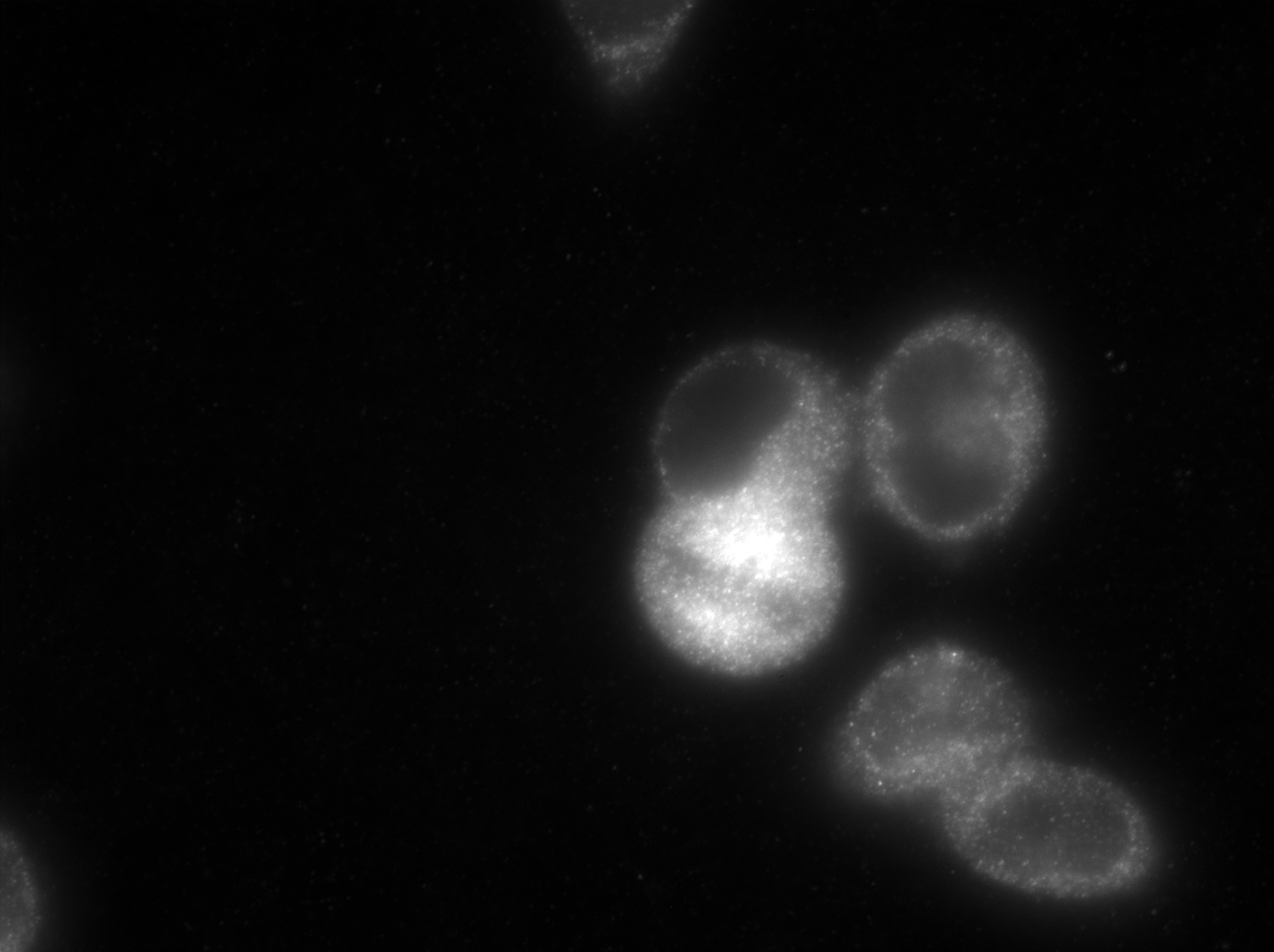

Supplement: Supplementary file 7 — Source data Fig. 2 part 4 [file 44319_2026_742_MOESM7_ESM.zip › Figure 2 Part 4/Fig 2d polye atubulin/WT PolyE-atub 8-14-24 R1 M2.Project Maximum Z_XY1723759215_Z0_T0_C2.tif]

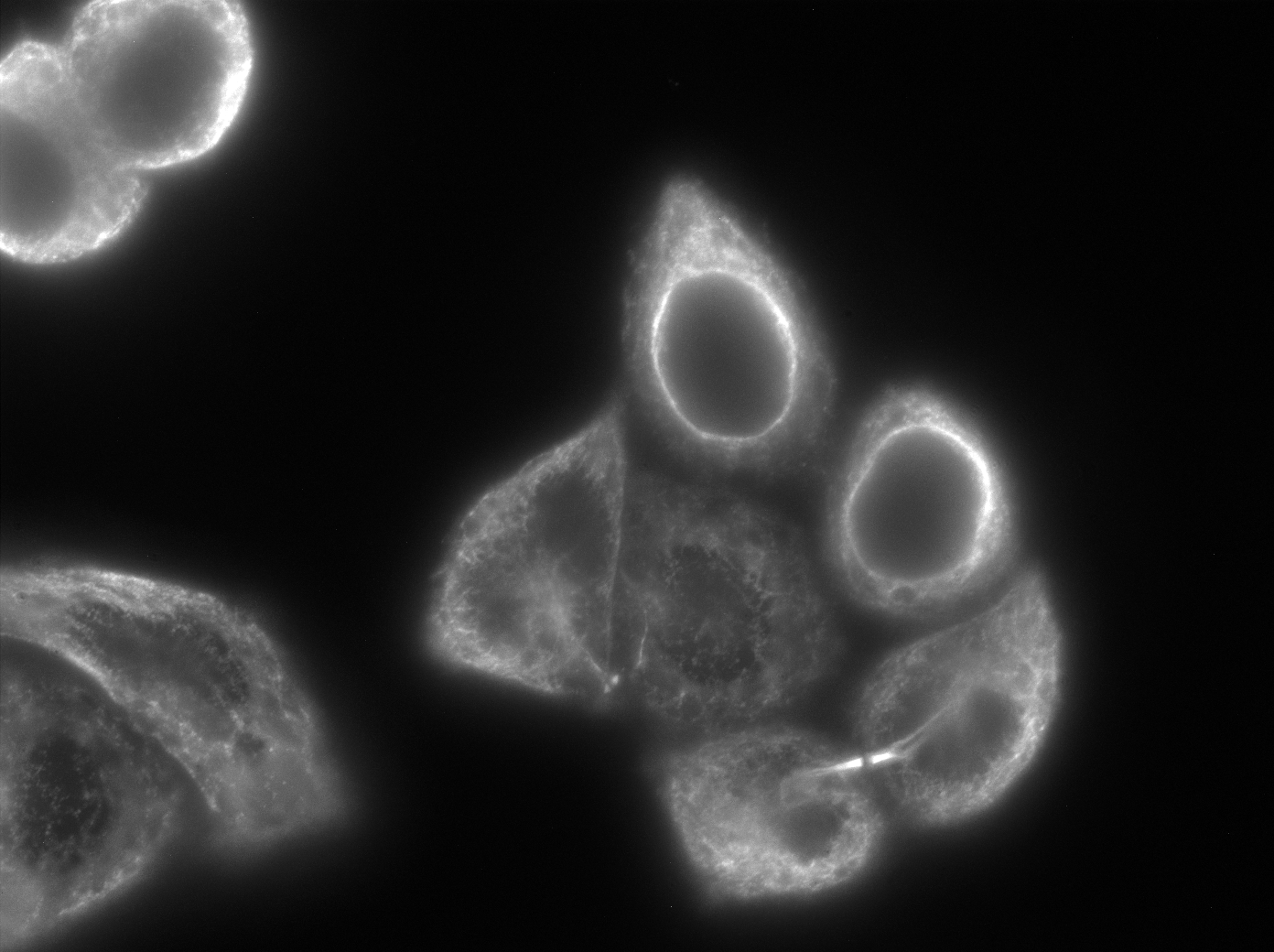

Supplement: Supplementary file 7 — Source data Fig. 2 part 4 [file 44319_2026_742_MOESM7_ESM.zip › Figure 2 Part 4/Fig 2d polye atubulin/WT PolyE-atub 8-14-24 R2 LT1 PA1.Project Maximum Z_XY1723834071_Z0_T0_C1.tif]

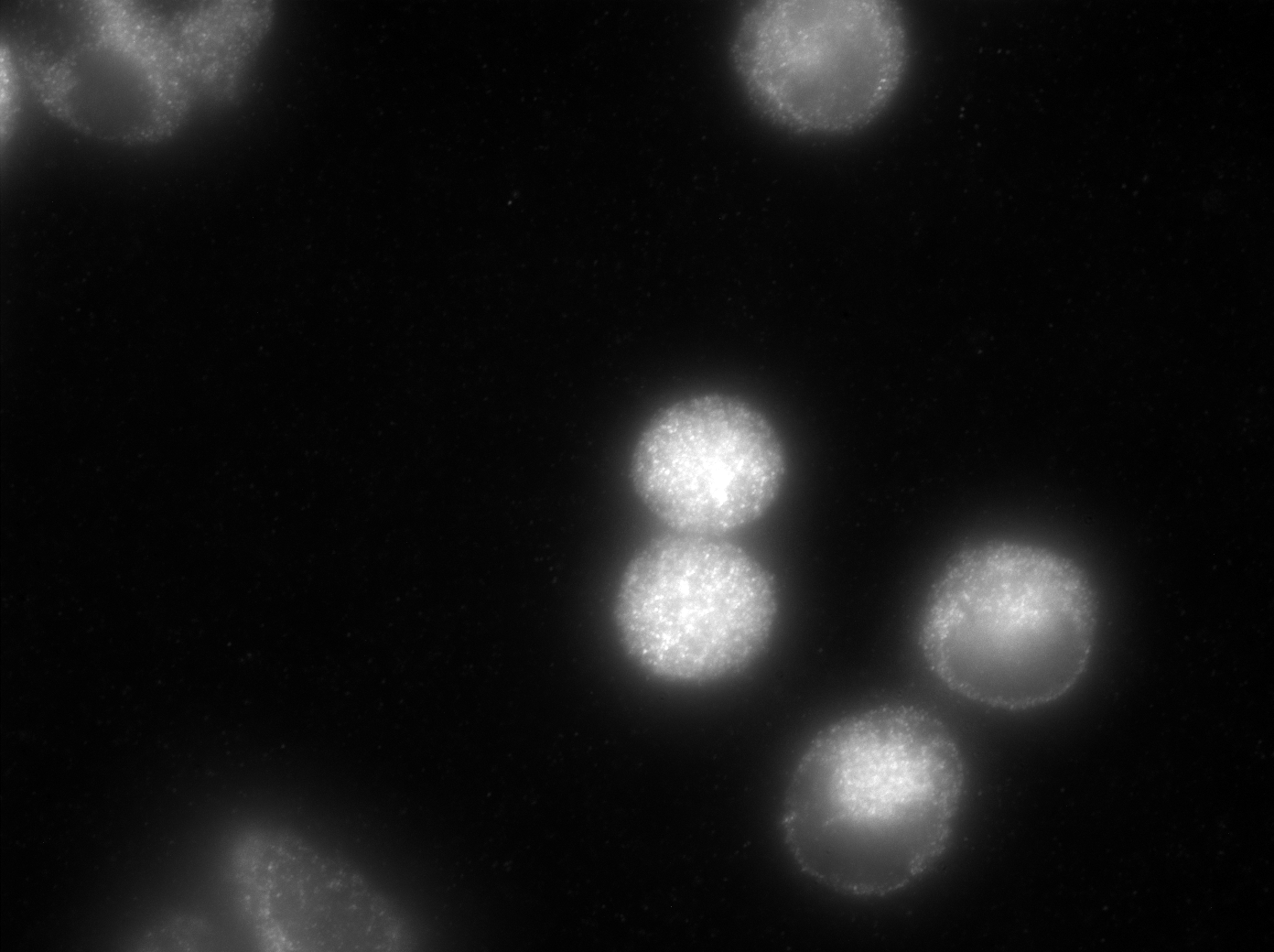

Supplement: Supplementary file 7 — Source data Fig. 2 part 4 [file 44319_2026_742_MOESM7_ESM.zip › Figure 2 Part 4/Fig 2d polye atubulin/WT PolyE-atub 8-14-24 R1 ET4.Project Maximum Z_XY1723756492_Z0_T0_C2.tif]

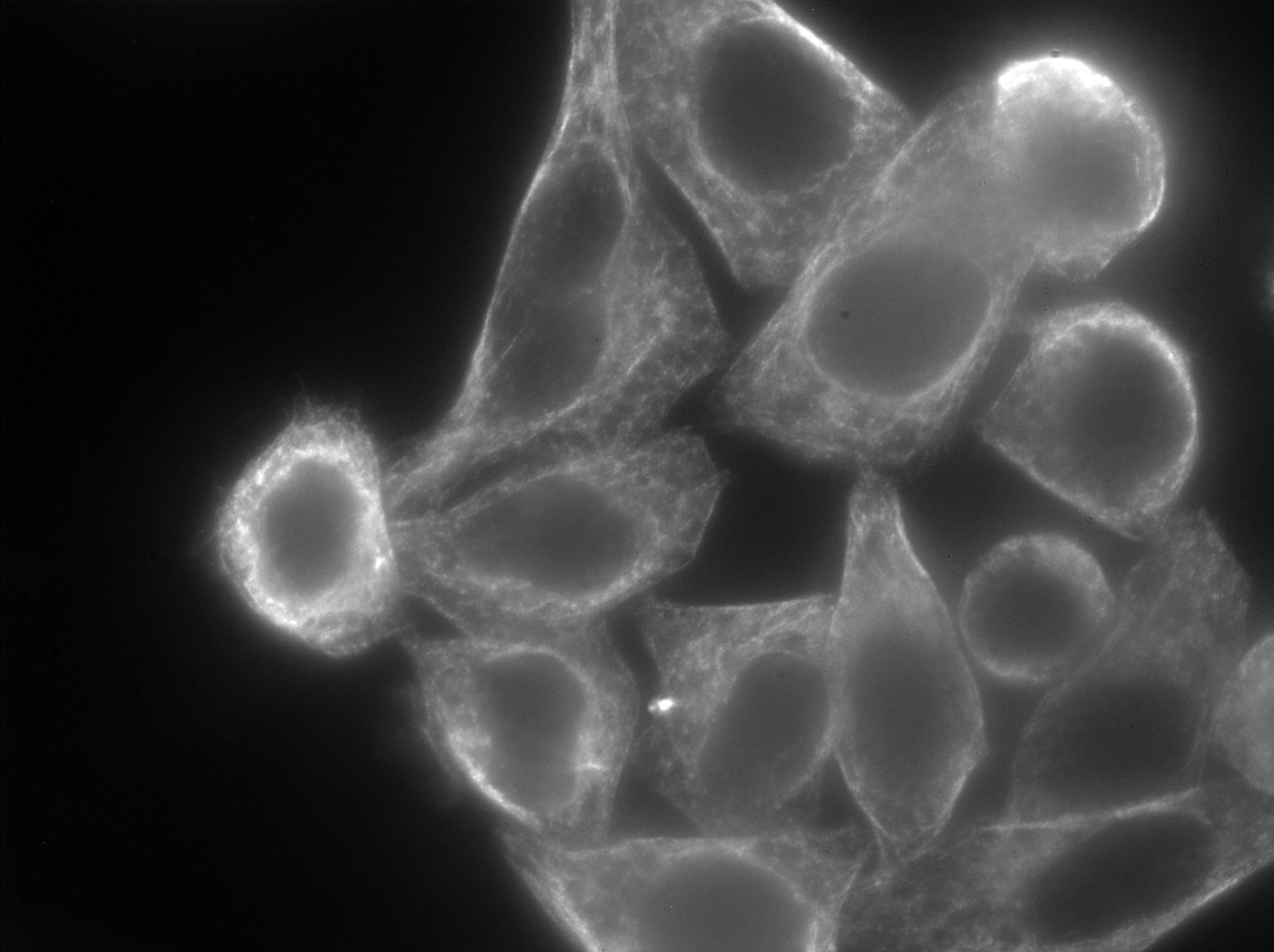

Supplement: Supplementary file 7 — Source data Fig. 2 part 4 [file 44319_2026_742_MOESM7_ESM.zip › Figure 2 Part 4/Fig 2d polye atubulin/WT PolyE-atub 8-14-24 R1 PA7.Project Maximum Z_XY1723761053_Z0_T0_C1.tif]

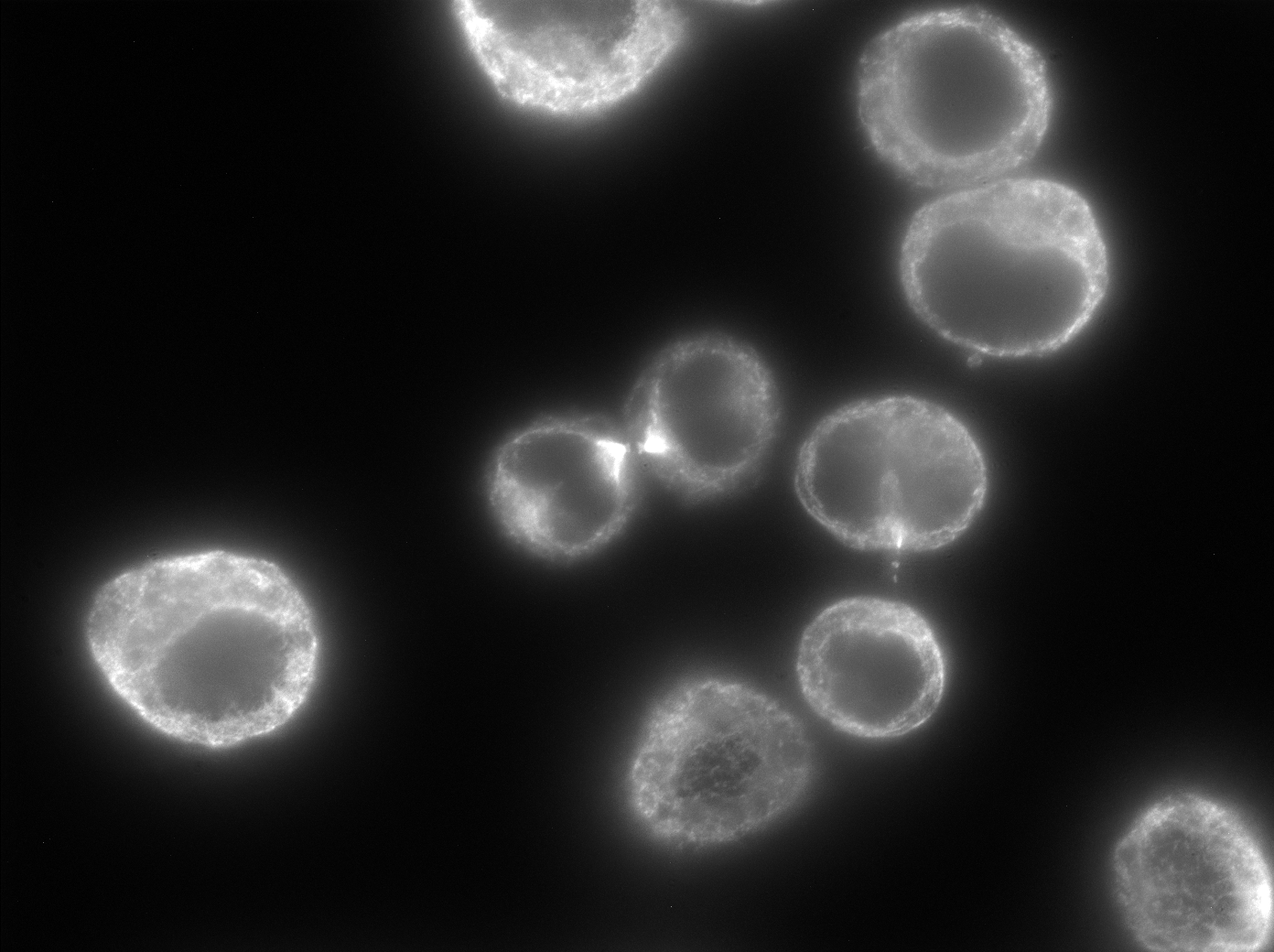

Supplement: Supplementary file 7 — Source data Fig. 2 part 4 [file 44319_2026_742_MOESM7_ESM.zip › Figure 2 Part 4/Fig 2d polye atubulin/WT PolyE-atub 8-14-24 R2 ET5 PA2.Project Maximum Z_XY1723834667_Z0_T0_C1.tif]

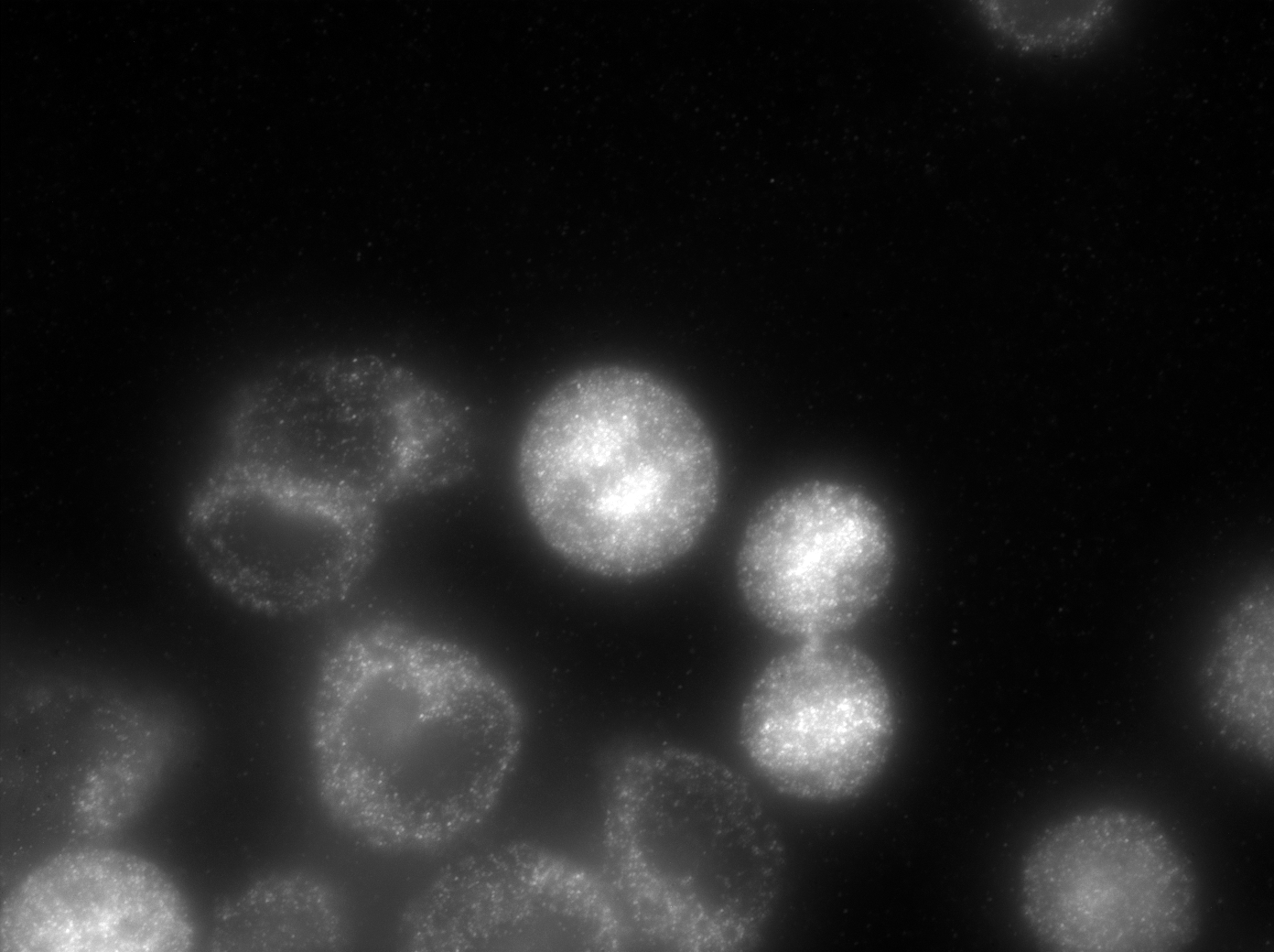

Supplement: Supplementary file 7 — Source data Fig. 2 part 4 [file 44319_2026_742_MOESM7_ESM.zip › Figure 2 Part 4/Fig 2d polye atubulin/WT PolyE-atub 8-14-24 R2 ET2 M1.Project Maximum Z_XY1723832643_Z0_T0_C2.tif]

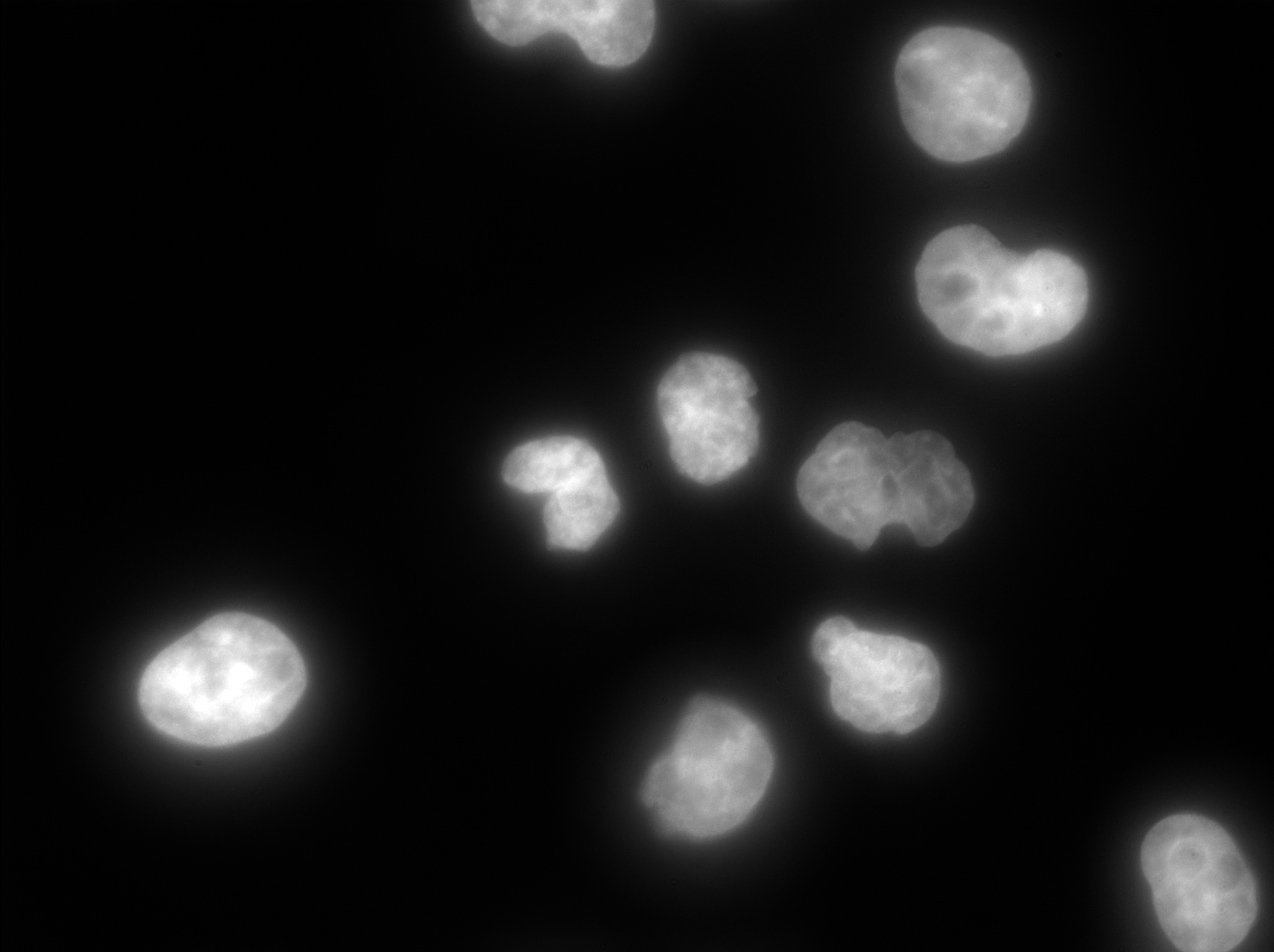

Supplement: Supplementary file 7 — Source data Fig. 2 part 4 [file 44319_2026_742_MOESM7_ESM.zip › Figure 2 Part 4/Fig 2d polye atubulin/WT PolyE-atub 8-14-24 R2 ET5 PA2.Project Maximum Z_XY1723834667_Z0_T0_C0.tif]

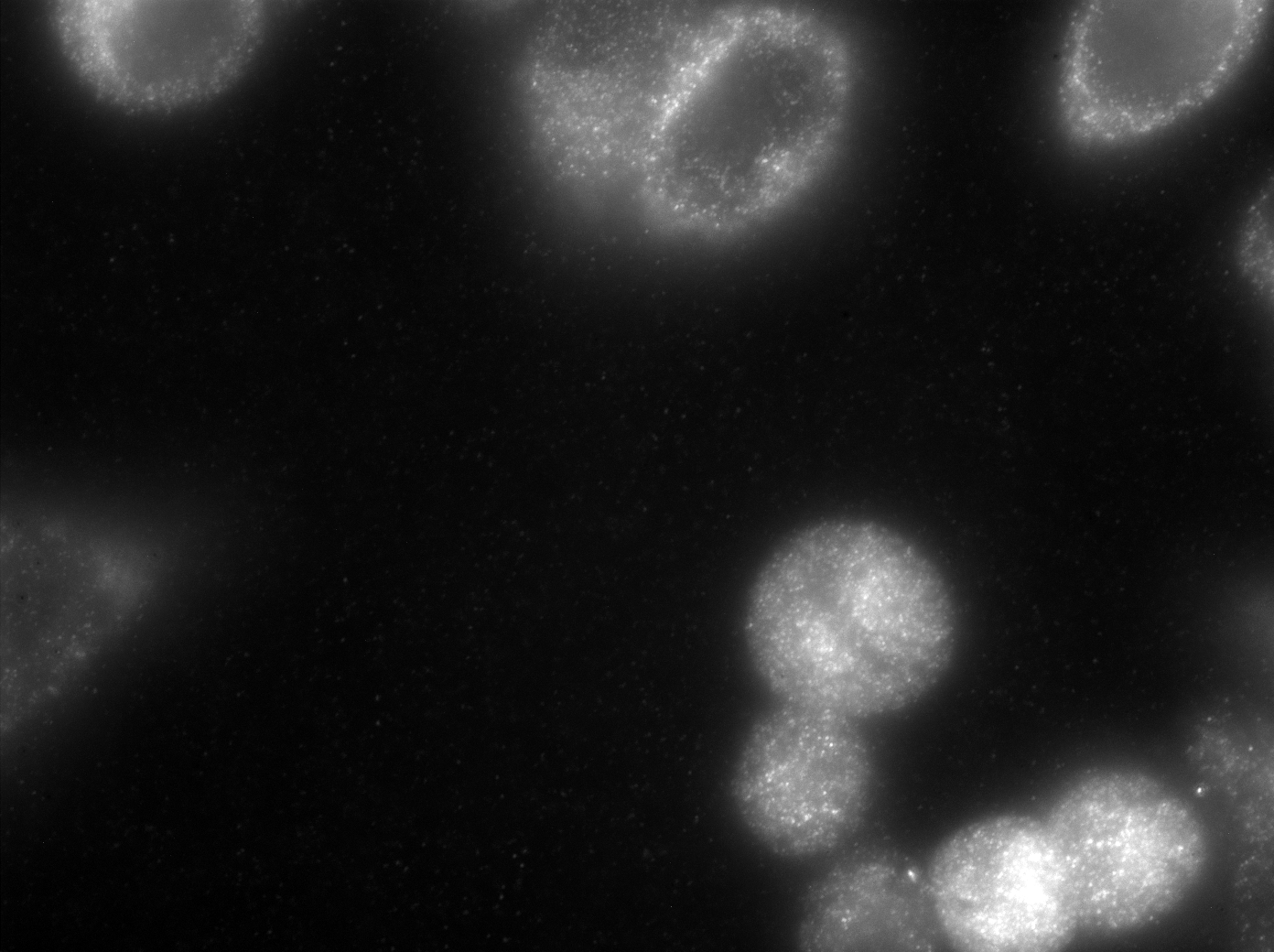

Supplement: Supplementary file 7 — Source data Fig. 2 part 4 [file 44319_2026_742_MOESM7_ESM.zip › Figure 2 Part 4/Fig 2d polye atubulin/WT PolyE-atub 8-14-24 R1 M8.Project Maximum Z_XY1723761273_Z0_T0_C2.tif]

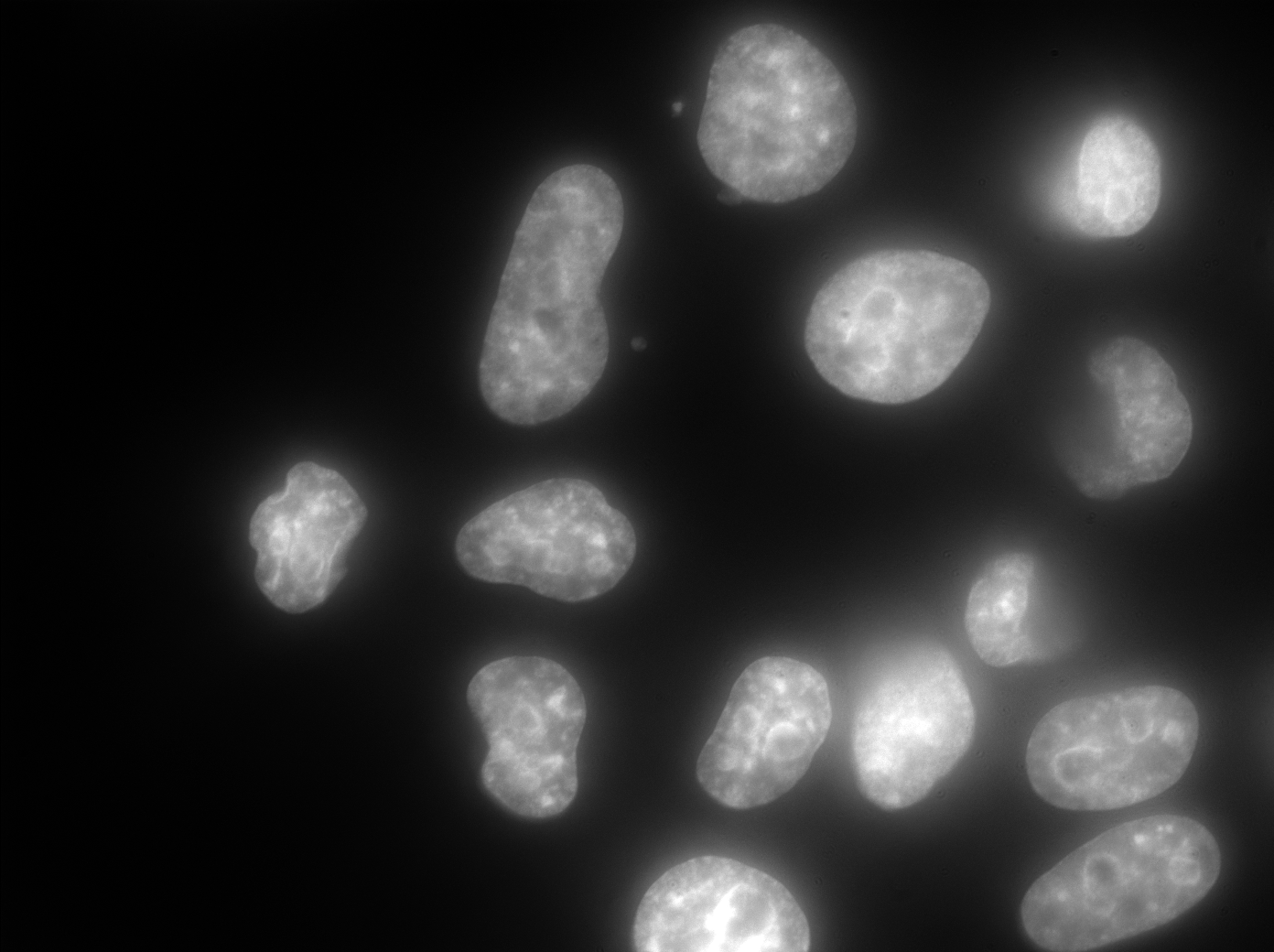

Supplement: Supplementary file 7 — Source data Fig. 2 part 4 [file 44319_2026_742_MOESM7_ESM.zip › Figure 2 Part 4/Fig 2d polye atubulin/WT PolyE-atub 8-14-24 R1 PA7.Project Maximum Z_XY1723761053_Z0_T0_C0.tif]

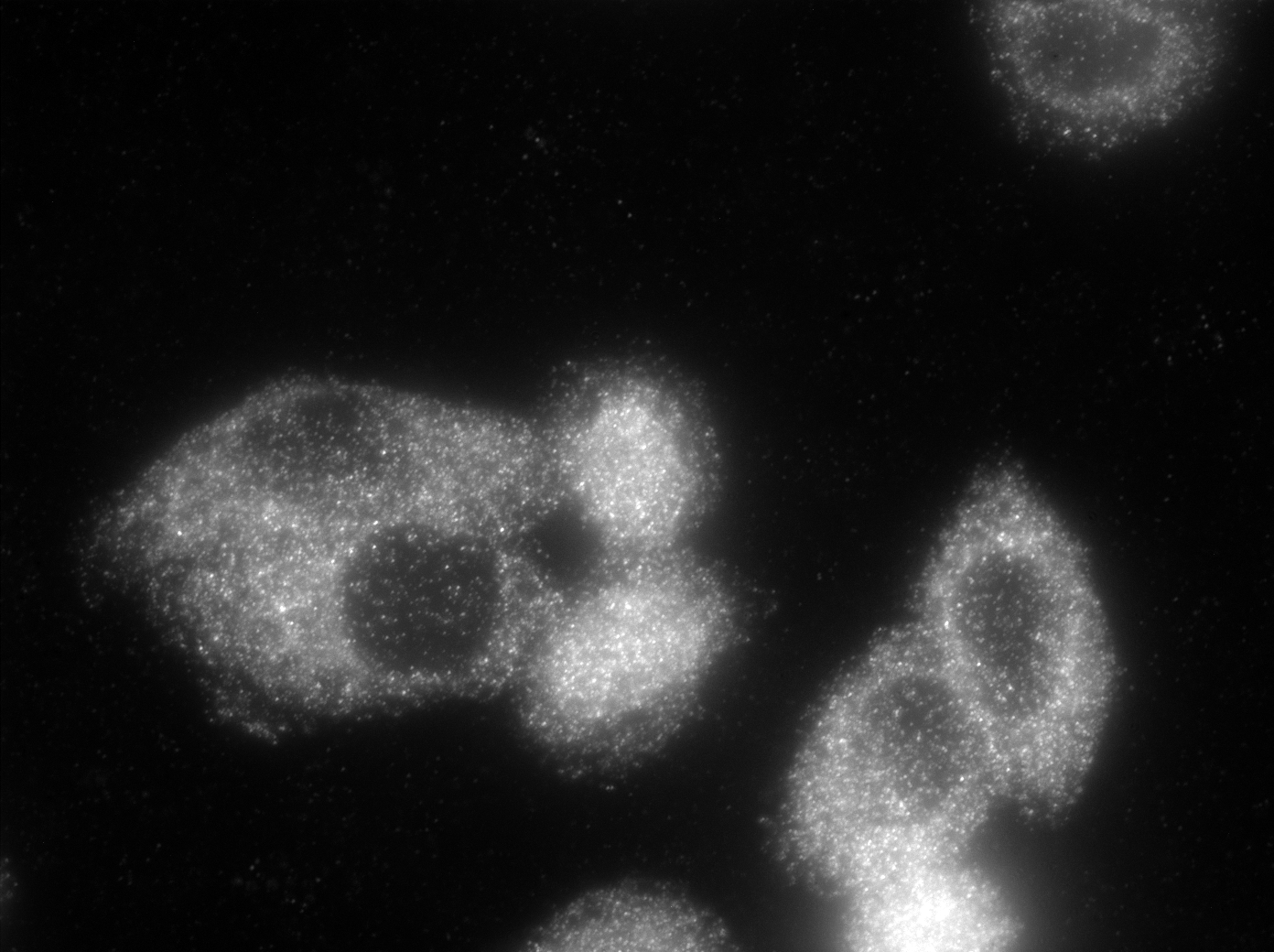

Supplement: Supplementary file 7 — Source data Fig. 2 part 4 [file 44319_2026_742_MOESM7_ESM.zip › Figure 2 Part 4/Fig 2d polye atubulin/WT PolyE-atub 8-14-24 R2 LT2.Project Maximum Z_XY1723834298_Z0_T0_C2.tif]

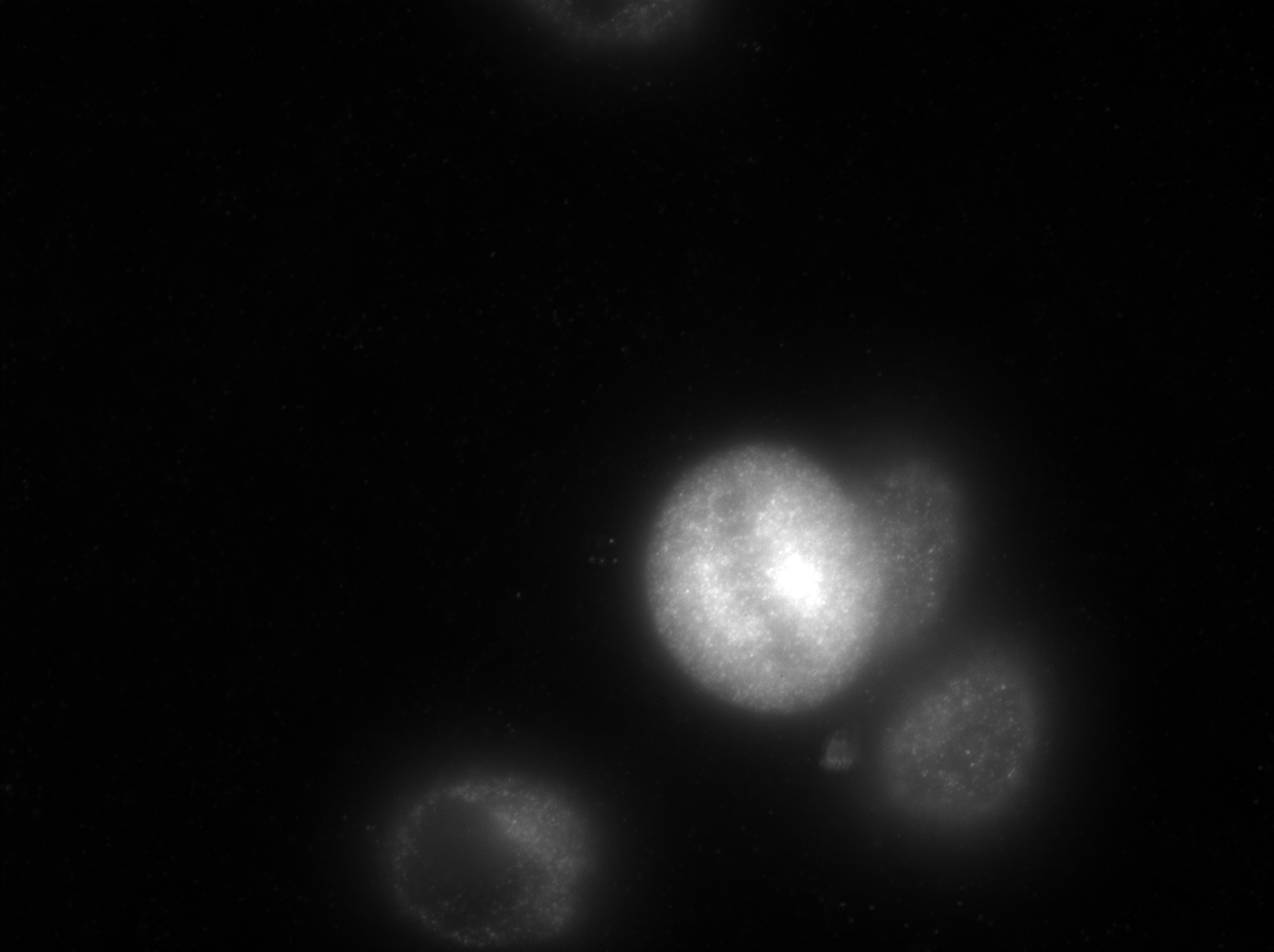

Supplement: Supplementary file 7 — Source data Fig. 2 part 4 [file 44319_2026_742_MOESM7_ESM.zip › Figure 2 Part 4/Fig 2d polye atubulin/WT PolyE-atub 8-14-24 R1 M4.Project Maximum Z_XY1723759870_Z0_T0_C2.tif]

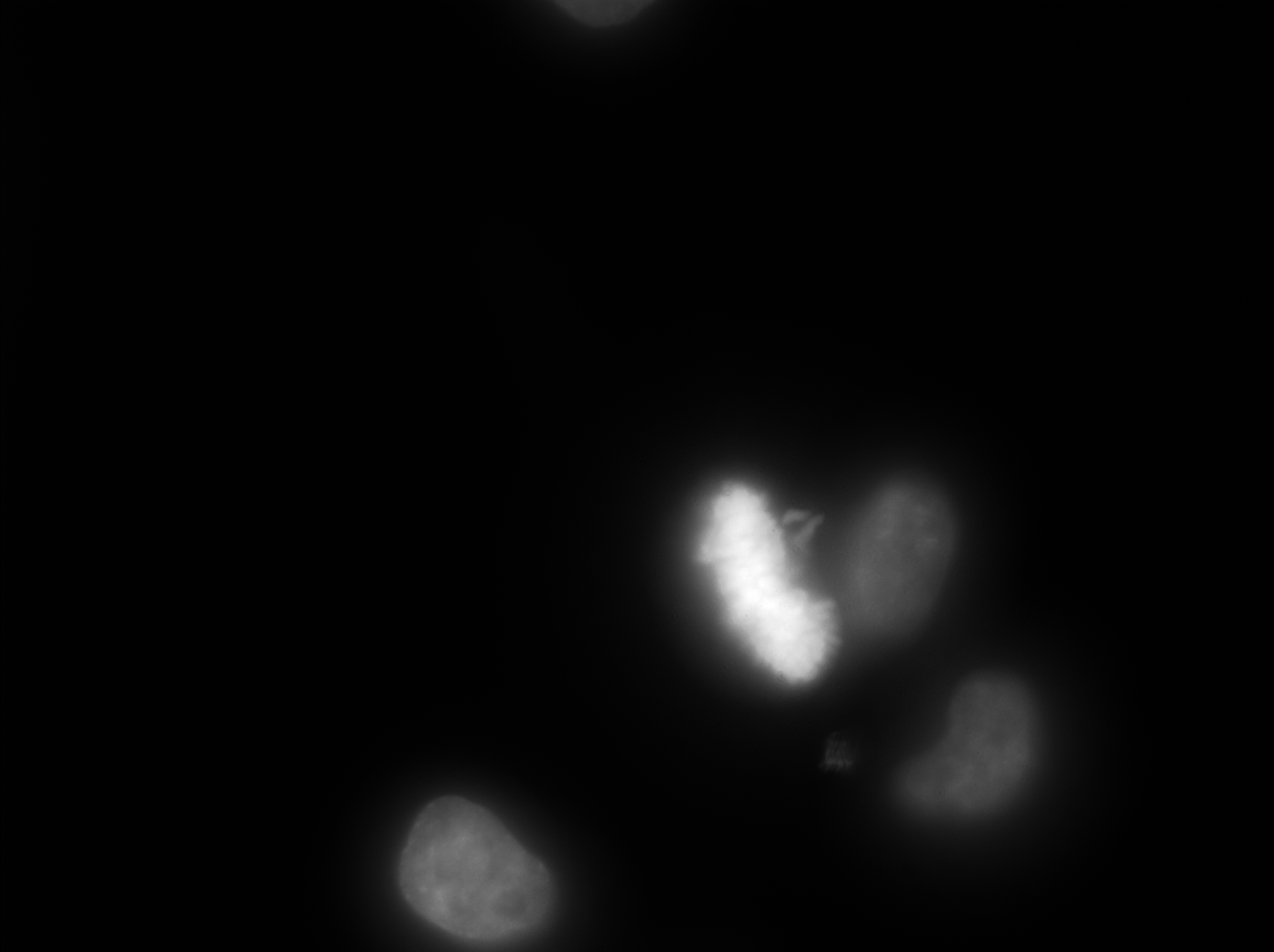

Supplement: Supplementary file 7 — Source data Fig. 2 part 4 [file 44319_2026_742_MOESM7_ESM.zip › Figure 2 Part 4/Fig 2d polye atubulin/WT PolyE-atub 8-14-24 R1 M4.Project Maximum Z_XY1723759870_Z0_T0_C0.tif]

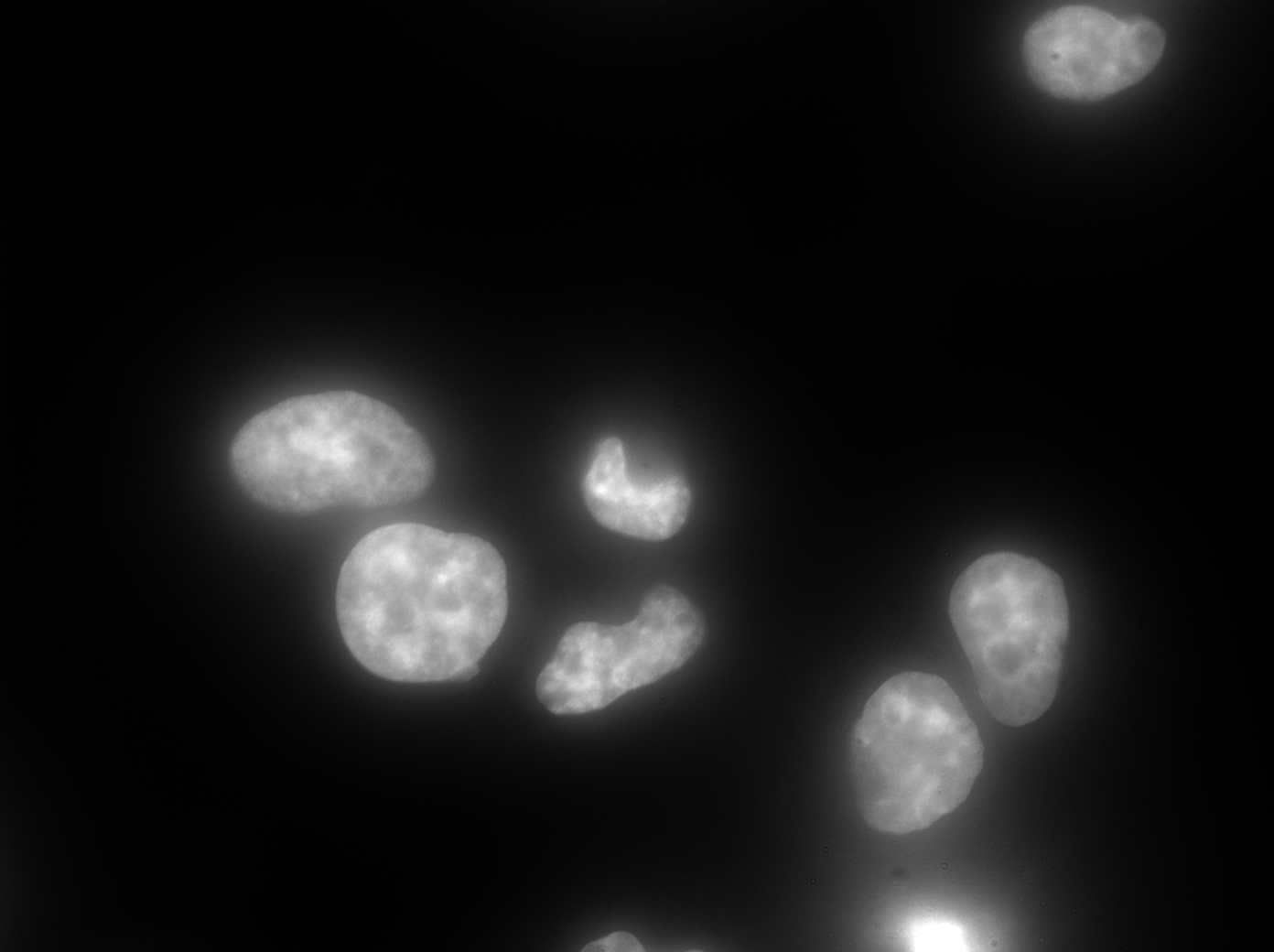

Supplement: Supplementary file 7 — Source data Fig. 2 part 4 [file 44319_2026_742_MOESM7_ESM.zip › Figure 2 Part 4/Fig 2d polye atubulin/WT PolyE-atub 8-14-24 R2 LT2.Project Maximum Z_XY1723834298_Z0_T0_C0.tif]

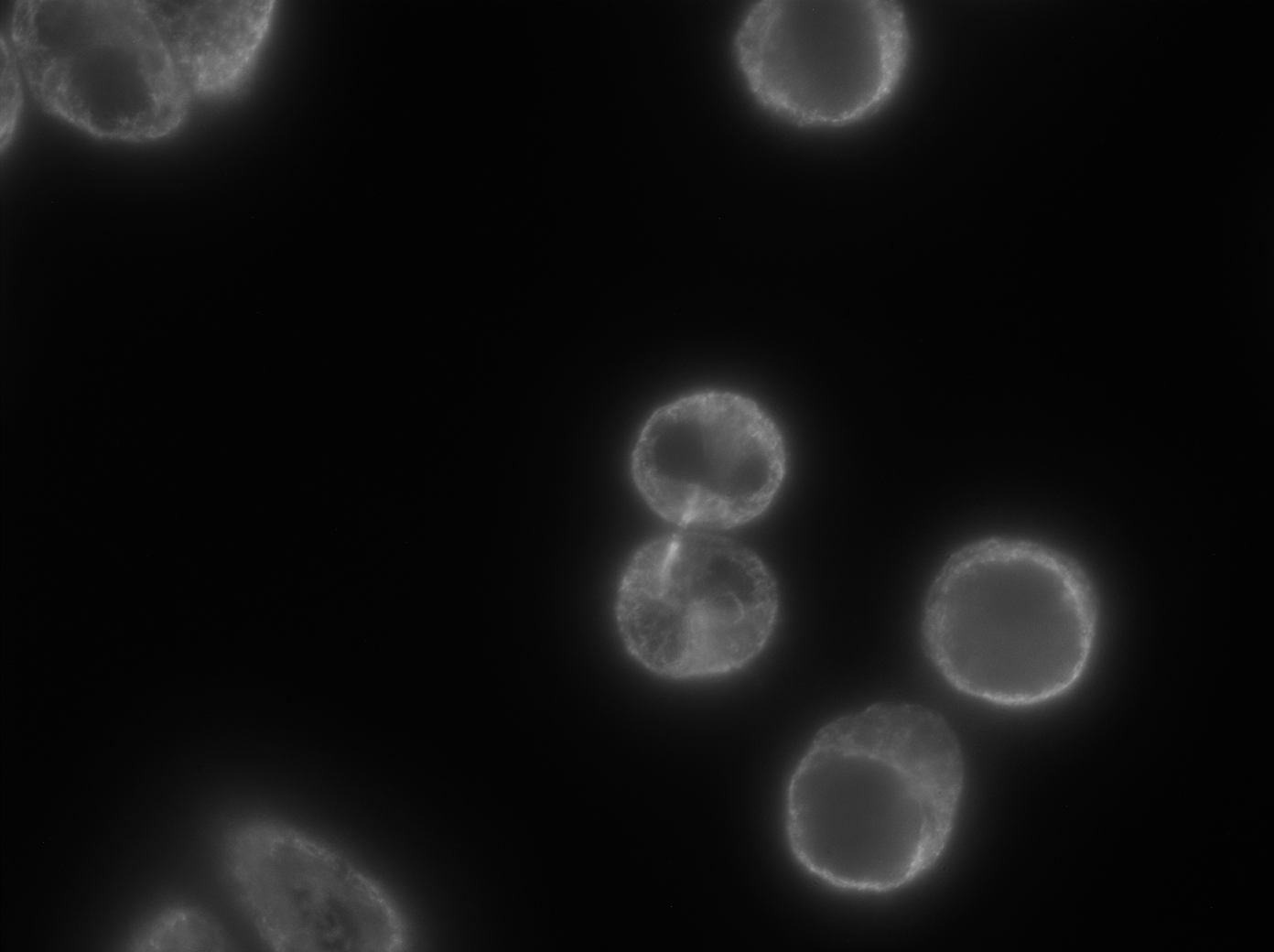

Supplement: Supplementary file 7 — Source data Fig. 2 part 4 [file 44319_2026_742_MOESM7_ESM.zip › Figure 2 Part 4/Fig 2d polye atubulin/WT PolyE-atub 8-14-24 R1 ET4.Project Maximum Z_XY1723756492_Z0_T0_C1.tif]

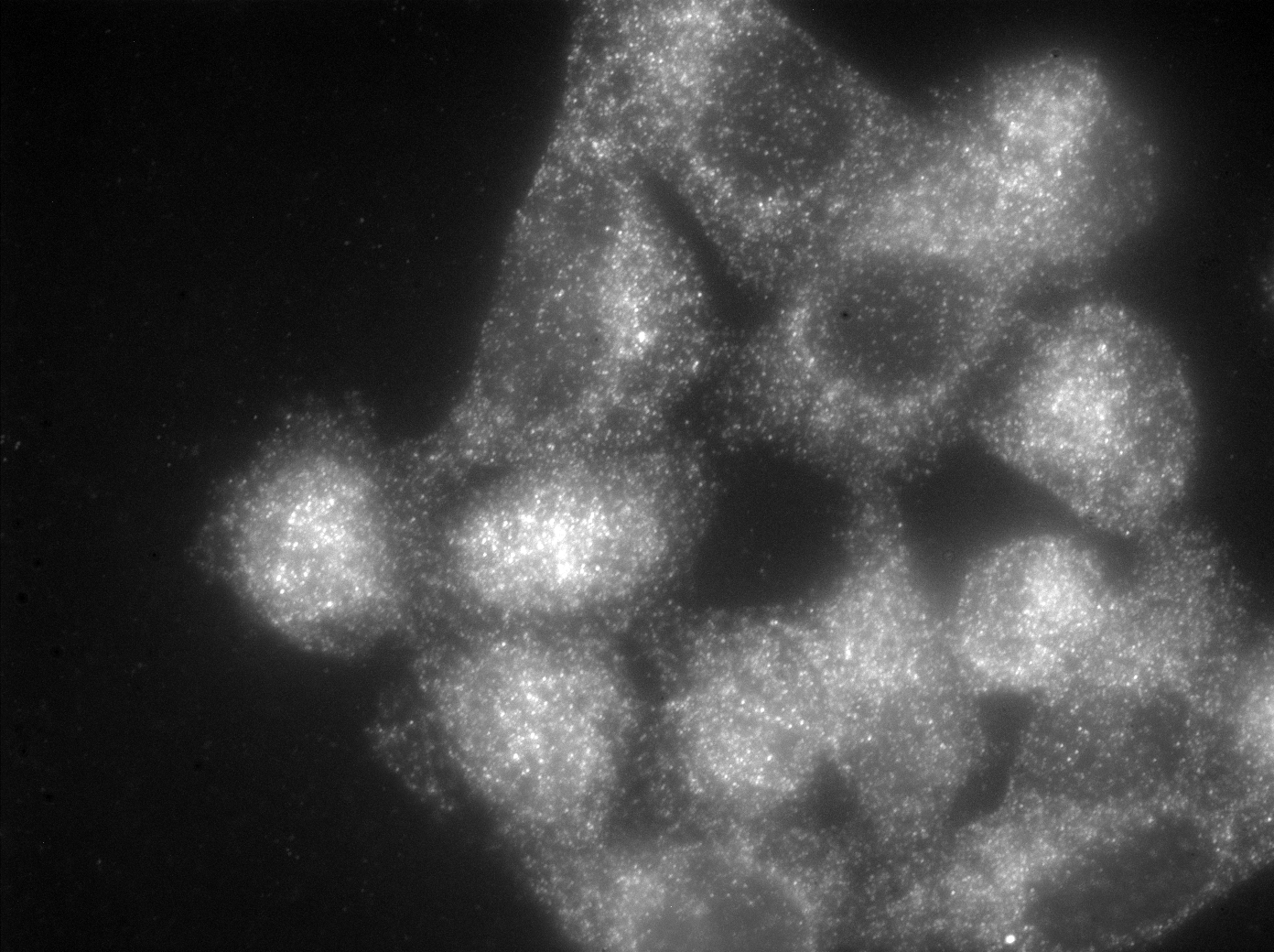

Supplement: Supplementary file 7 — Source data Fig. 2 part 4 [file 44319_2026_742_MOESM7_ESM.zip › Figure 2 Part 4/Fig 2d polye atubulin/WT PolyE-atub 8-14-24 R1 PA7.Project Maximum Z_XY1723761053_Z0_T0_C2.tif]

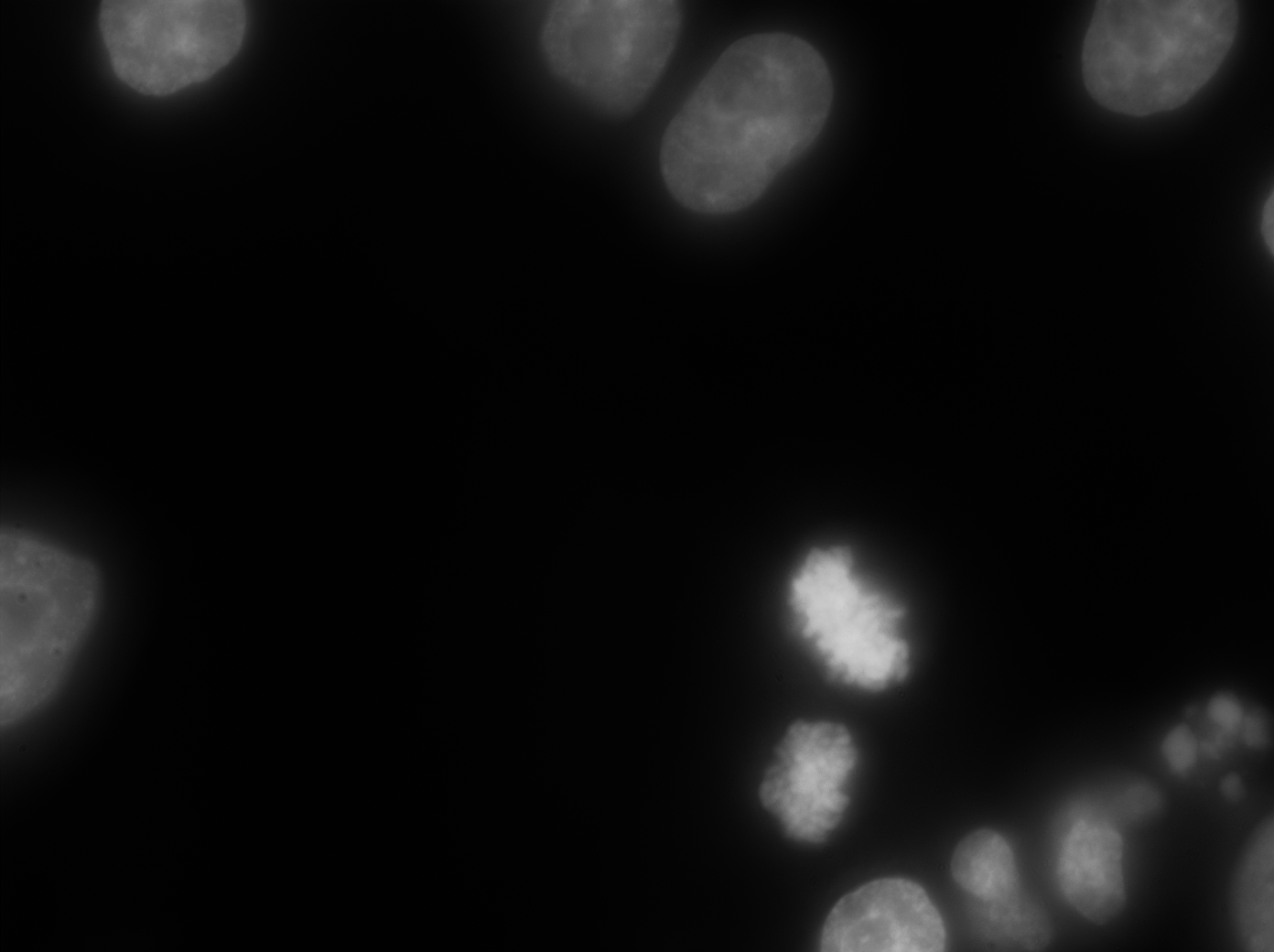

Supplement: Supplementary file 7 — Source data Fig. 2 part 4 [file 44319_2026_742_MOESM7_ESM.zip › Figure 2 Part 4/Fig 2d polye atubulin/WT PolyE-atub 8-14-24 R1 M8.Project Maximum Z_XY1723761273_Z0_T0_C0.tif]

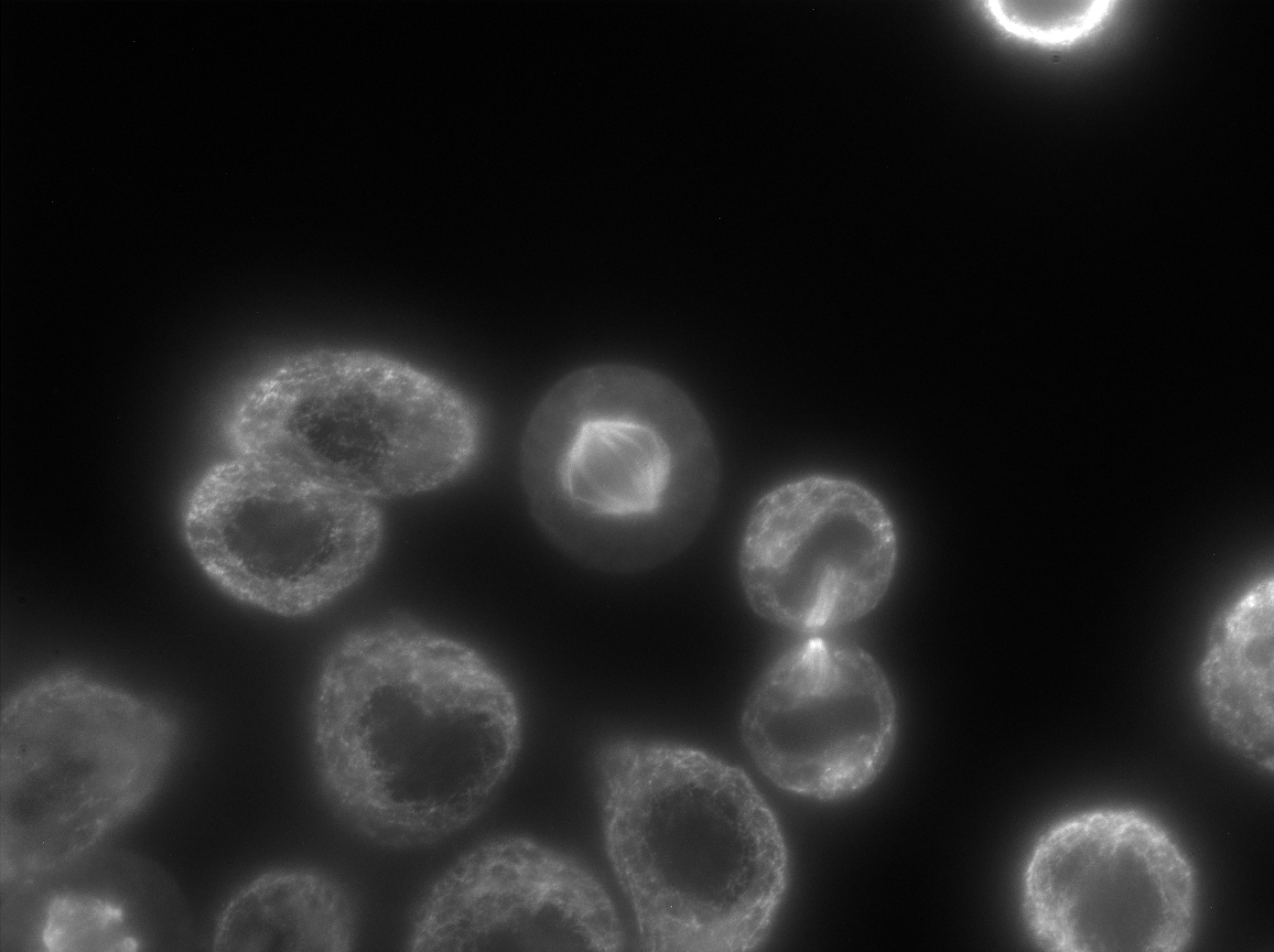

Supplement: Supplementary file 7 — Source data Fig. 2 part 4 [file 44319_2026_742_MOESM7_ESM.zip › Figure 2 Part 4/Fig 2d polye atubulin/WT PolyE-atub 8-14-24 R2 ET2 M1.Project Maximum Z_XY1723832643_Z0_T0_C1.tif]

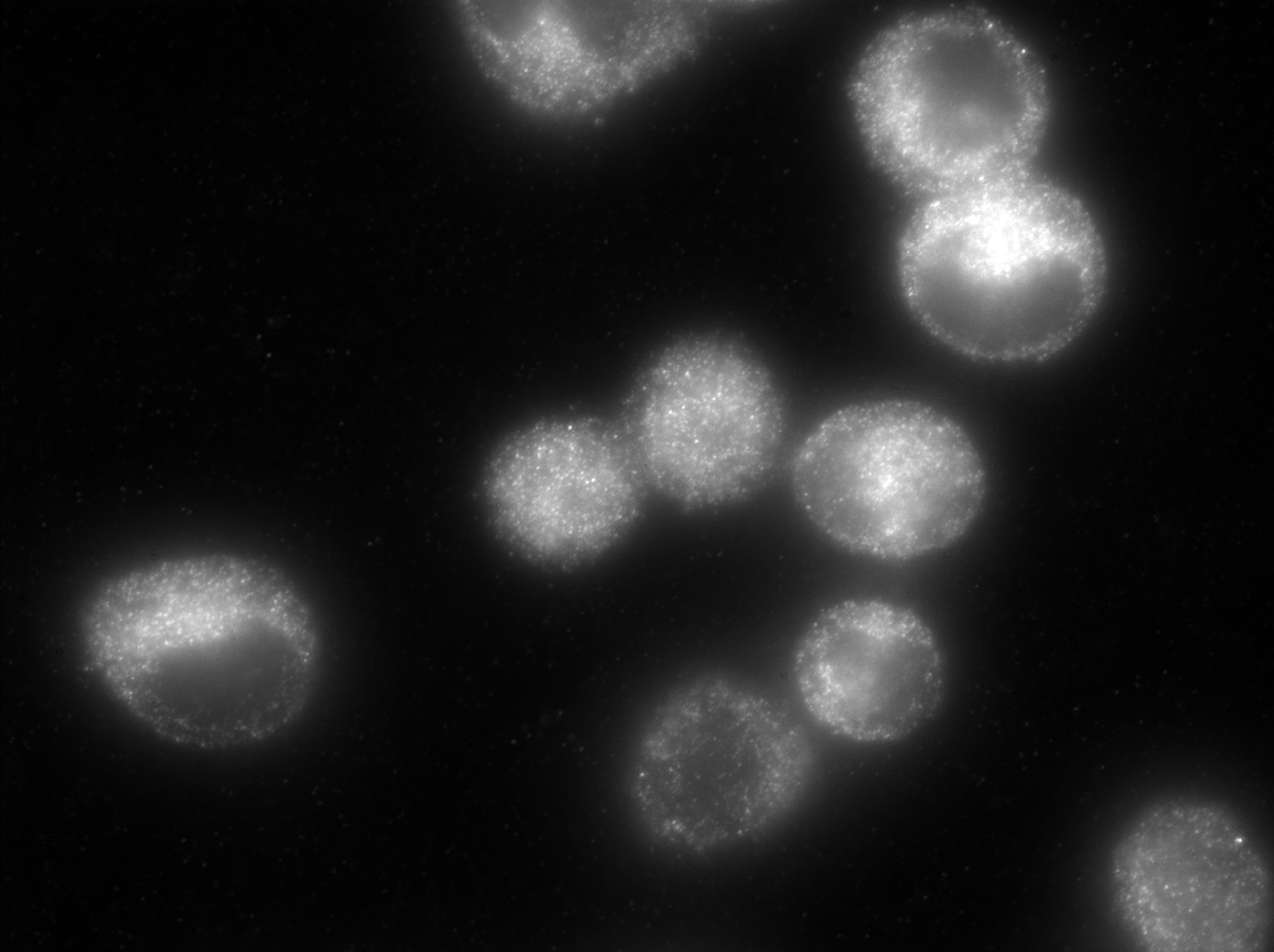

Supplement: Supplementary file 7 — Source data Fig. 2 part 4 [file 44319_2026_742_MOESM7_ESM.zip › Figure 2 Part 4/Fig 2d polye atubulin/WT PolyE-atub 8-14-24 R2 ET5 PA2.Project Maximum Z_XY1723834667_Z0_T0_C2.tif]

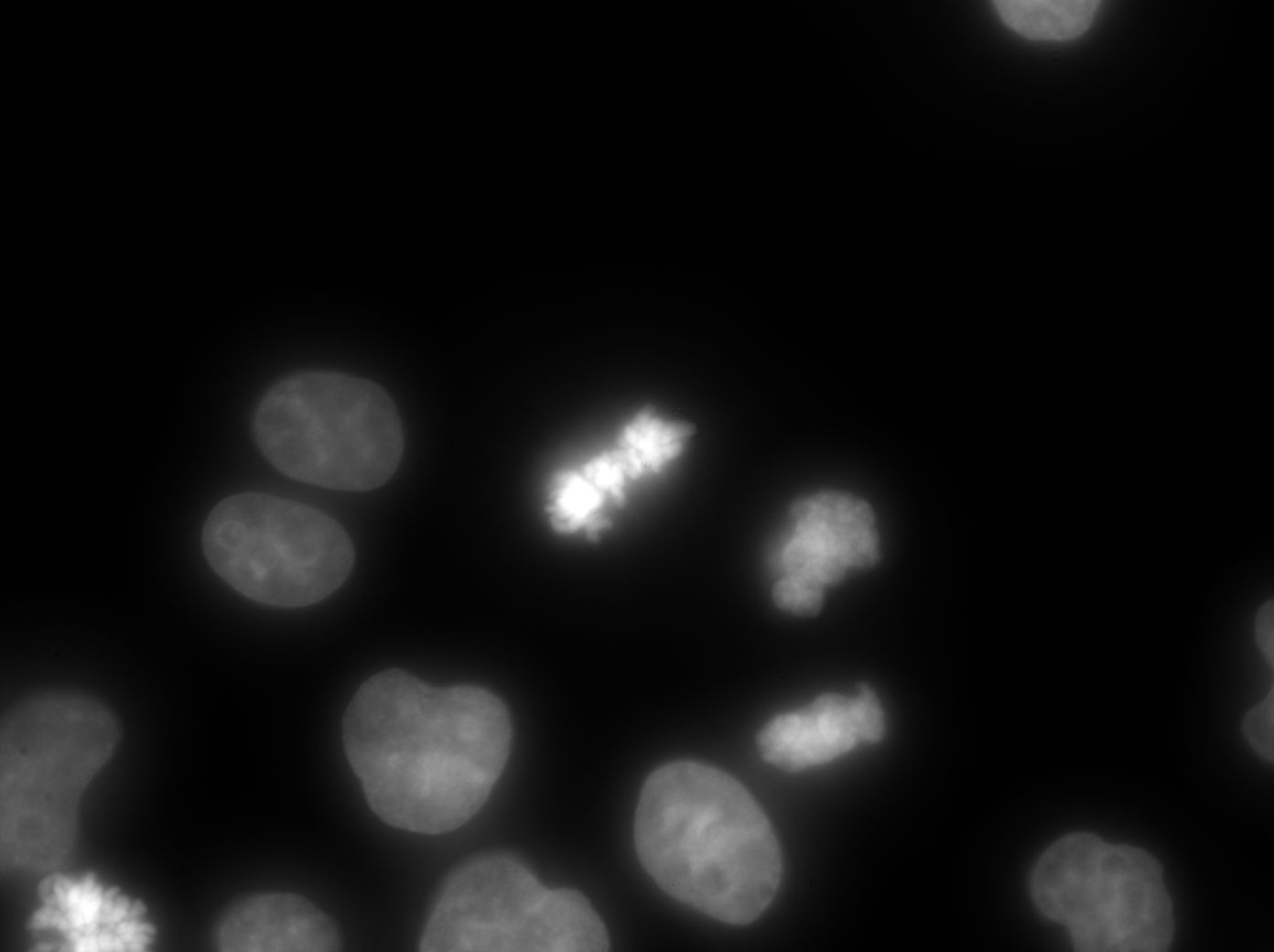

Supplement: Supplementary file 7 — Source data Fig. 2 part 4 [file 44319_2026_742_MOESM7_ESM.zip › Figure 2 Part 4/Fig 2d polye atubulin/WT PolyE-atub 8-14-24 R2 ET2 M1.Project Maximum Z_XY1723832643_Z0_T0_C0.tif]

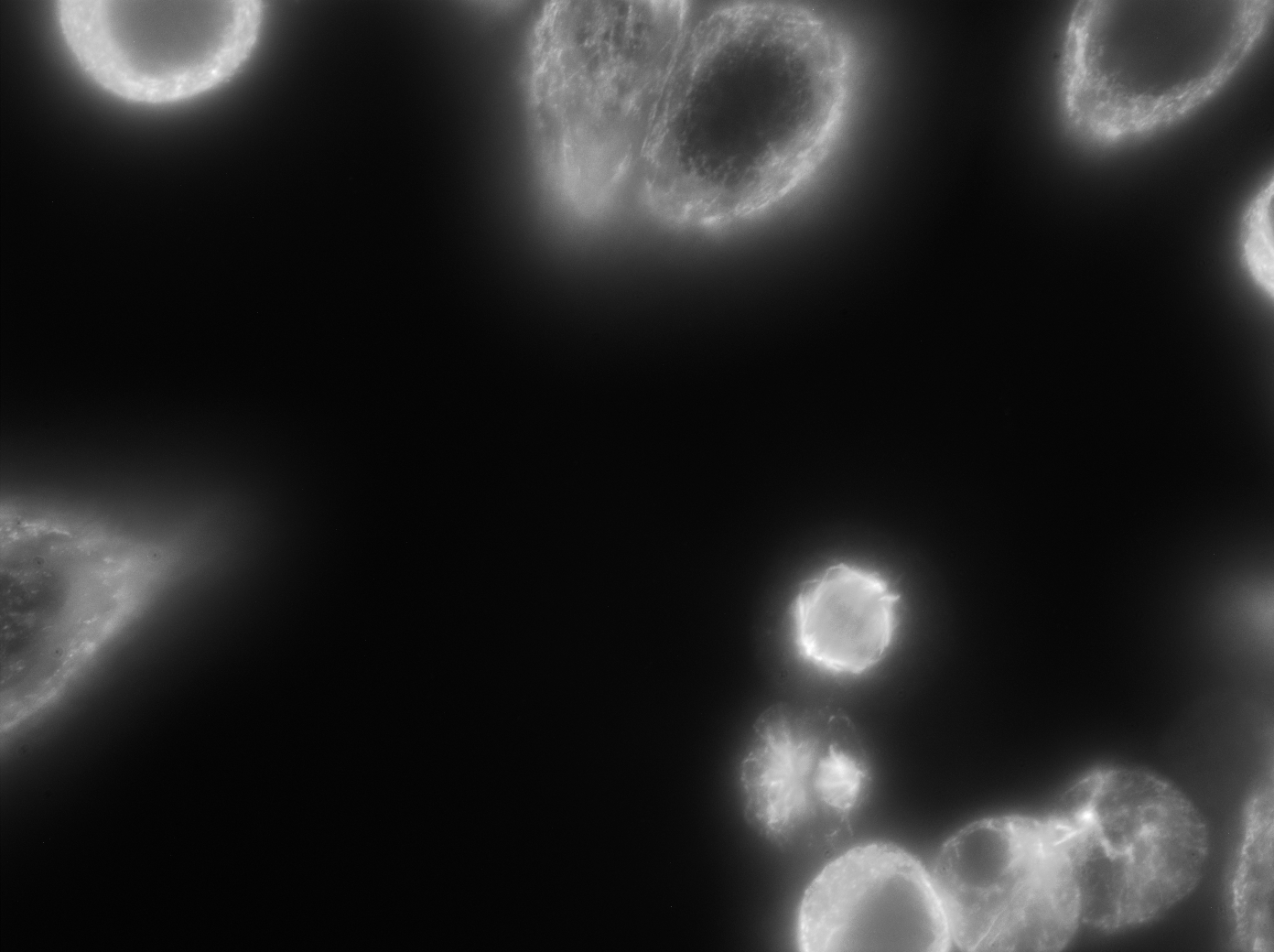

Supplement: Supplementary file 7 — Source data Fig. 2 part 4 [file 44319_2026_742_MOESM7_ESM.zip › Figure 2 Part 4/Fig 2d polye atubulin/WT PolyE-atub 8-14-24 R1 M8.Project Maximum Z_XY1723761273_Z0_T0_C1.tif]

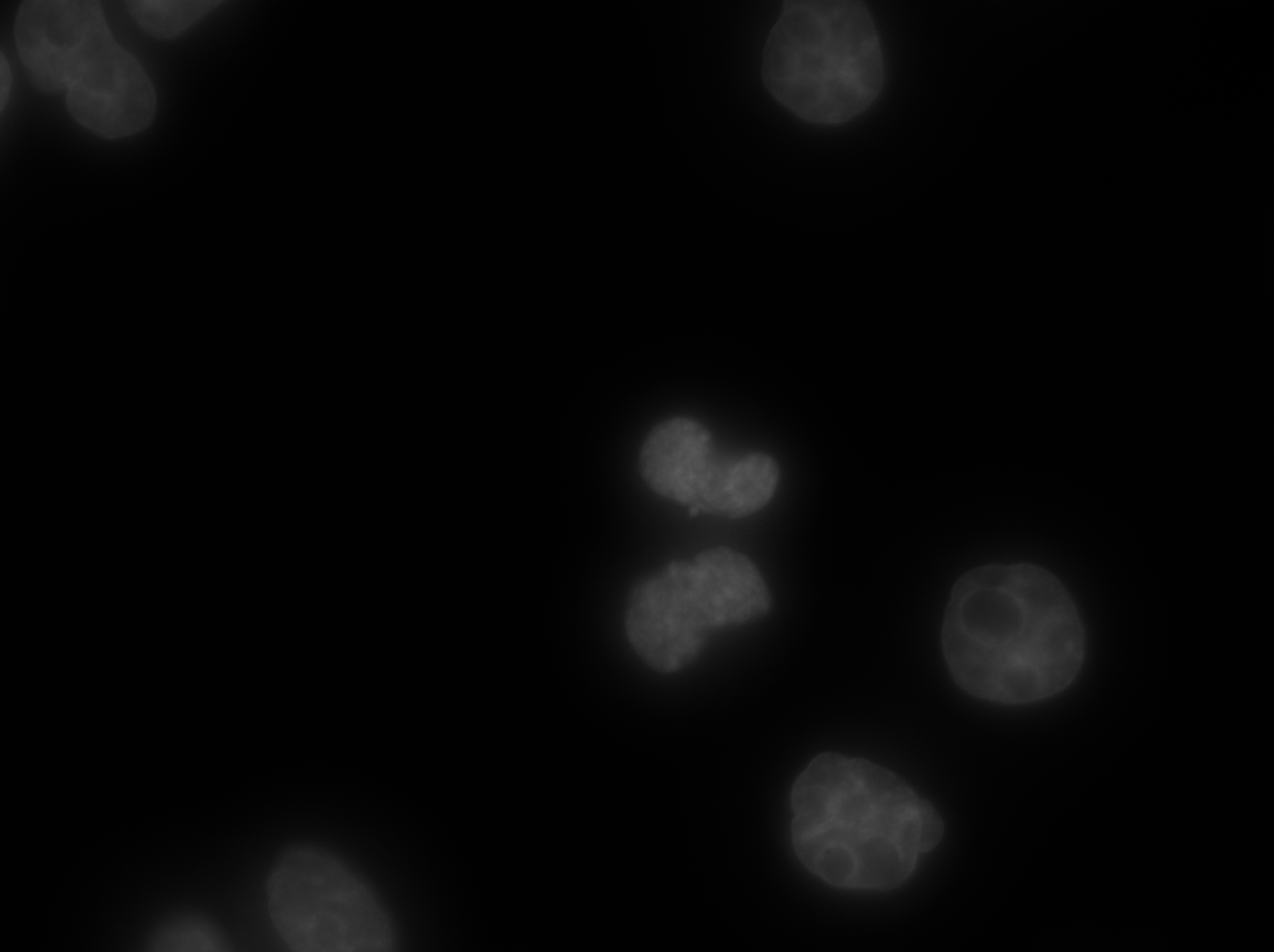

Supplement: Supplementary file 7 — Source data Fig. 2 part 4 [file 44319_2026_742_MOESM7_ESM.zip › Figure 2 Part 4/Fig 2d polye atubulin/WT PolyE-atub 8-14-24 R1 ET4.Project Maximum Z_XY1723756492_Z0_T0_C0.tif]

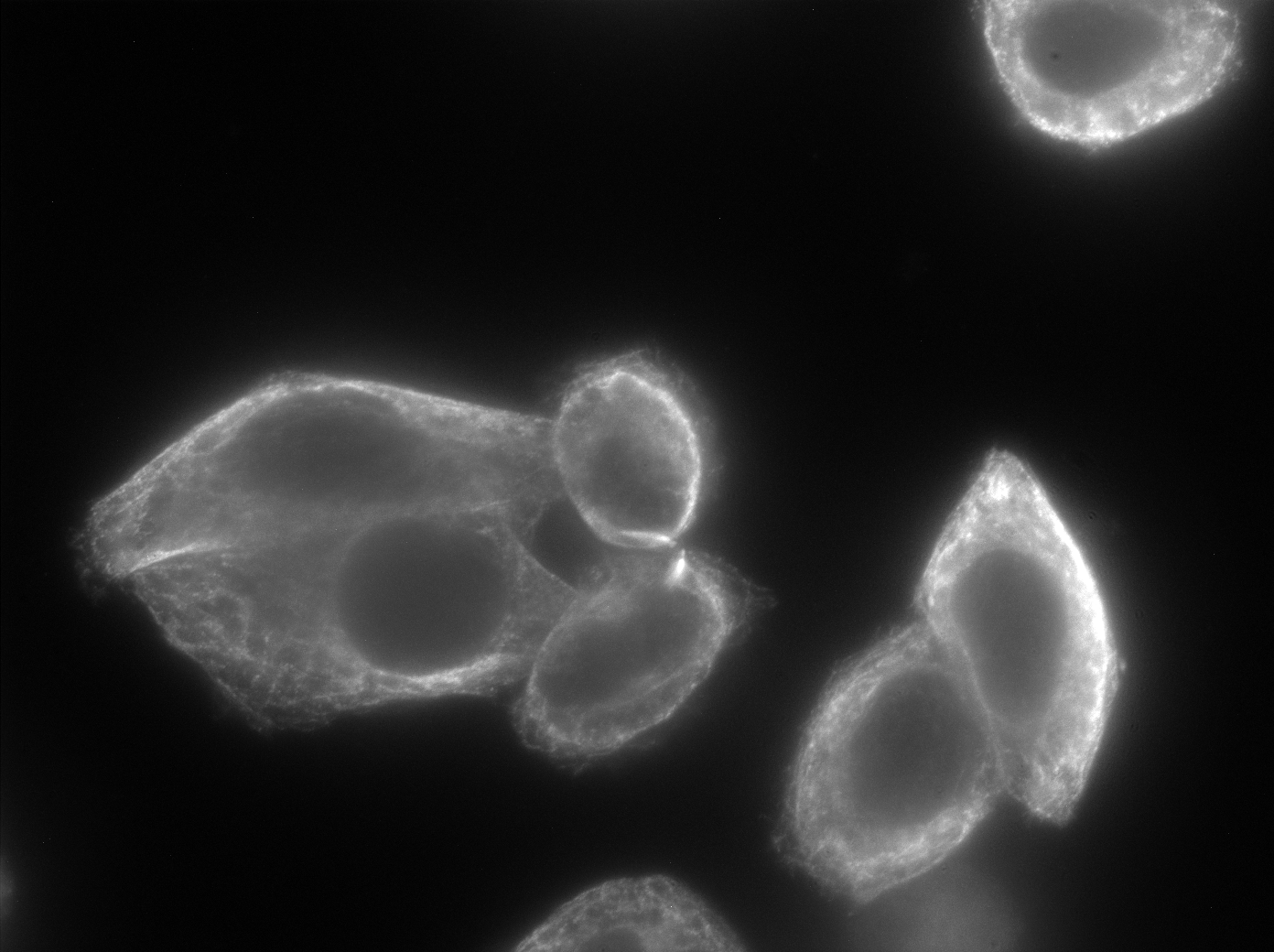

Supplement: Supplementary file 7 — Source data Fig. 2 part 4 [file 44319_2026_742_MOESM7_ESM.zip › Figure 2 Part 4/Fig 2d polye atubulin/WT PolyE-atub 8-14-24 R2 LT2.Project Maximum Z_XY1723834298_Z0_T0_C1.tif]

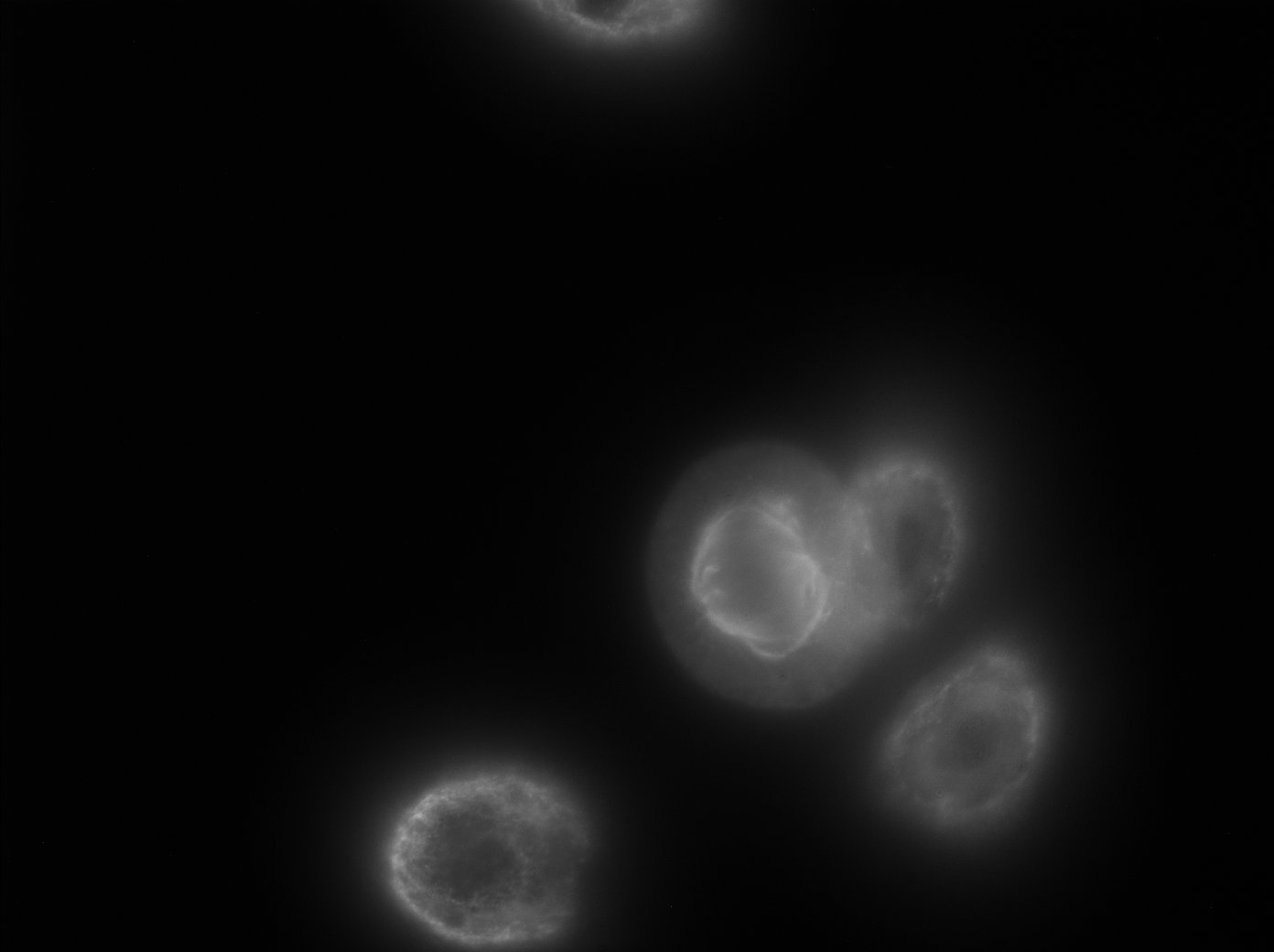

Supplement: Supplementary file 7 — Source data Fig. 2 part 4 [file 44319_2026_742_MOESM7_ESM.zip › Figure 2 Part 4/Fig 2d polye atubulin/WT PolyE-atub 8-14-24 R1 M4.Project Maximum Z_XY1723759870_Z0_T0_C1.tif]

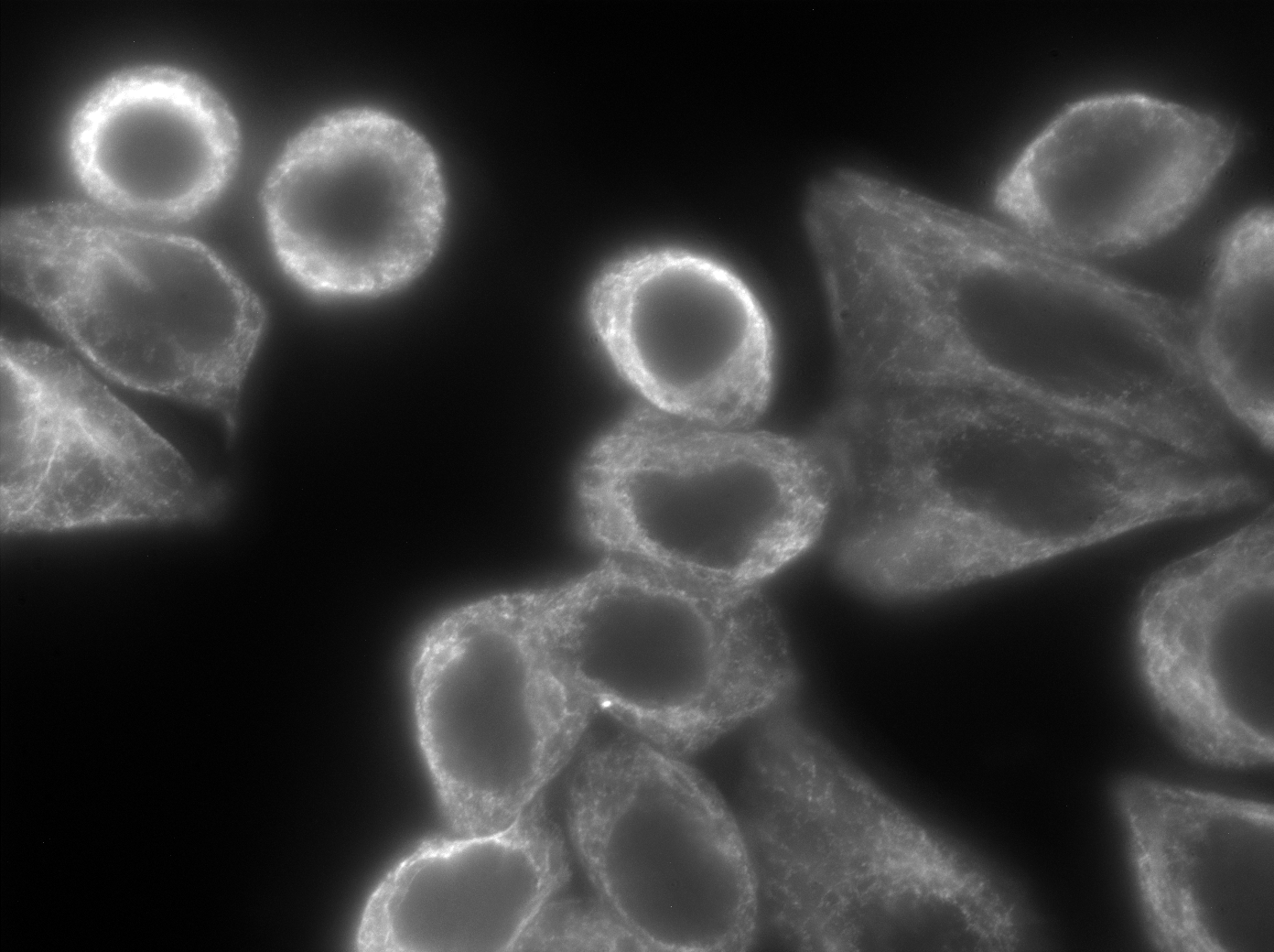

Supplement: Supplementary file 7 — Source data Fig. 2 part 4 [file 44319_2026_742_MOESM7_ESM.zip › Figure 2 Part 4/Fig 2d polye atubulin/WT PolyE-atub 8-14-24 R1 PA6.Project Maximum Z_XY1723760848_Z0_T0_C1.tif]

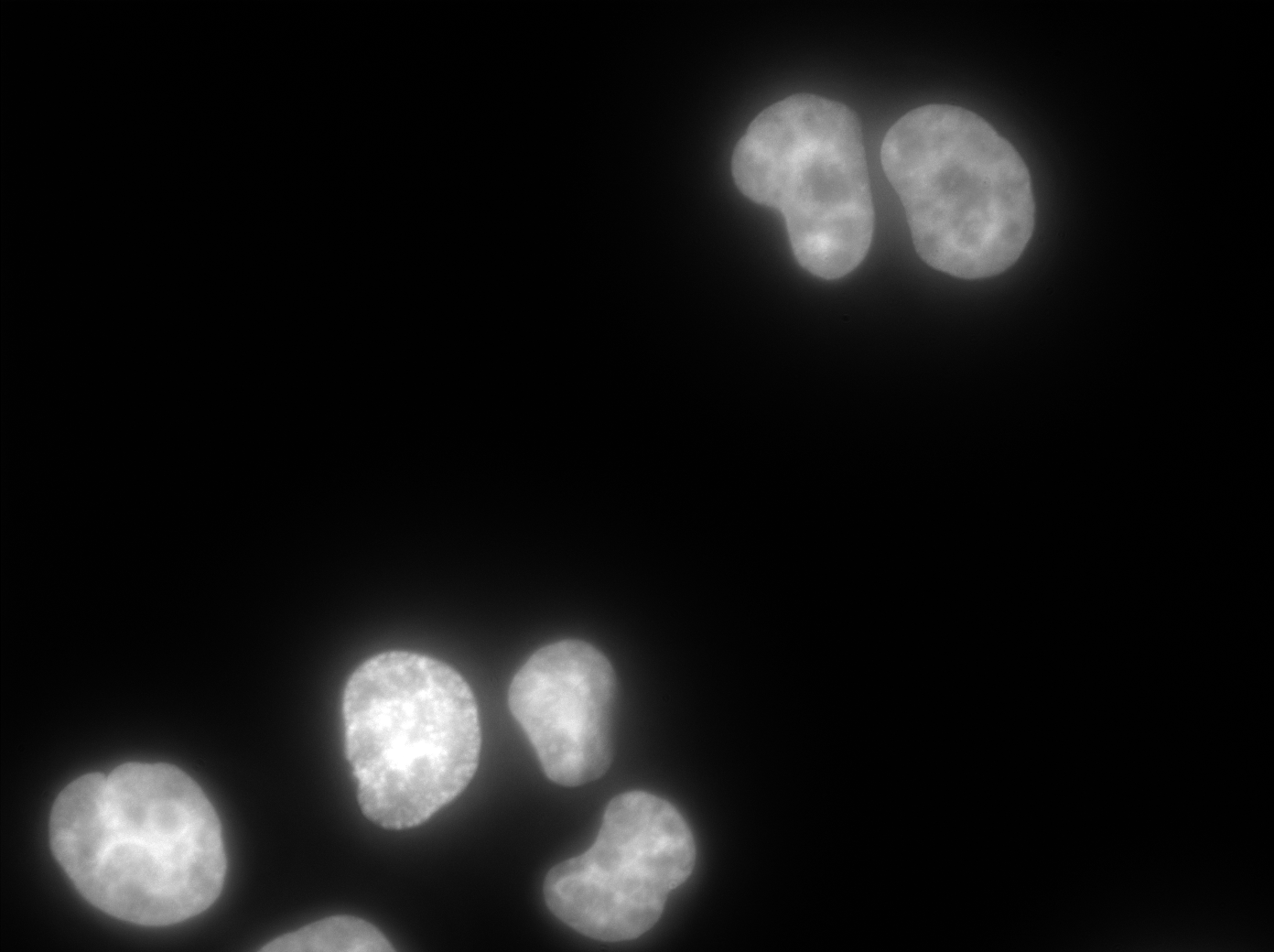

Supplement: Supplementary file 7 — Source data Fig. 2 part 4 [file 44319_2026_742_MOESM7_ESM.zip › Figure 2 Part 4/Fig 2d polye atubulin/WT PolyE-atub 8-14-24 R2 ET8 PA8.Project Maximum Z_XY1723837557_Z0_T0_C0.tif]

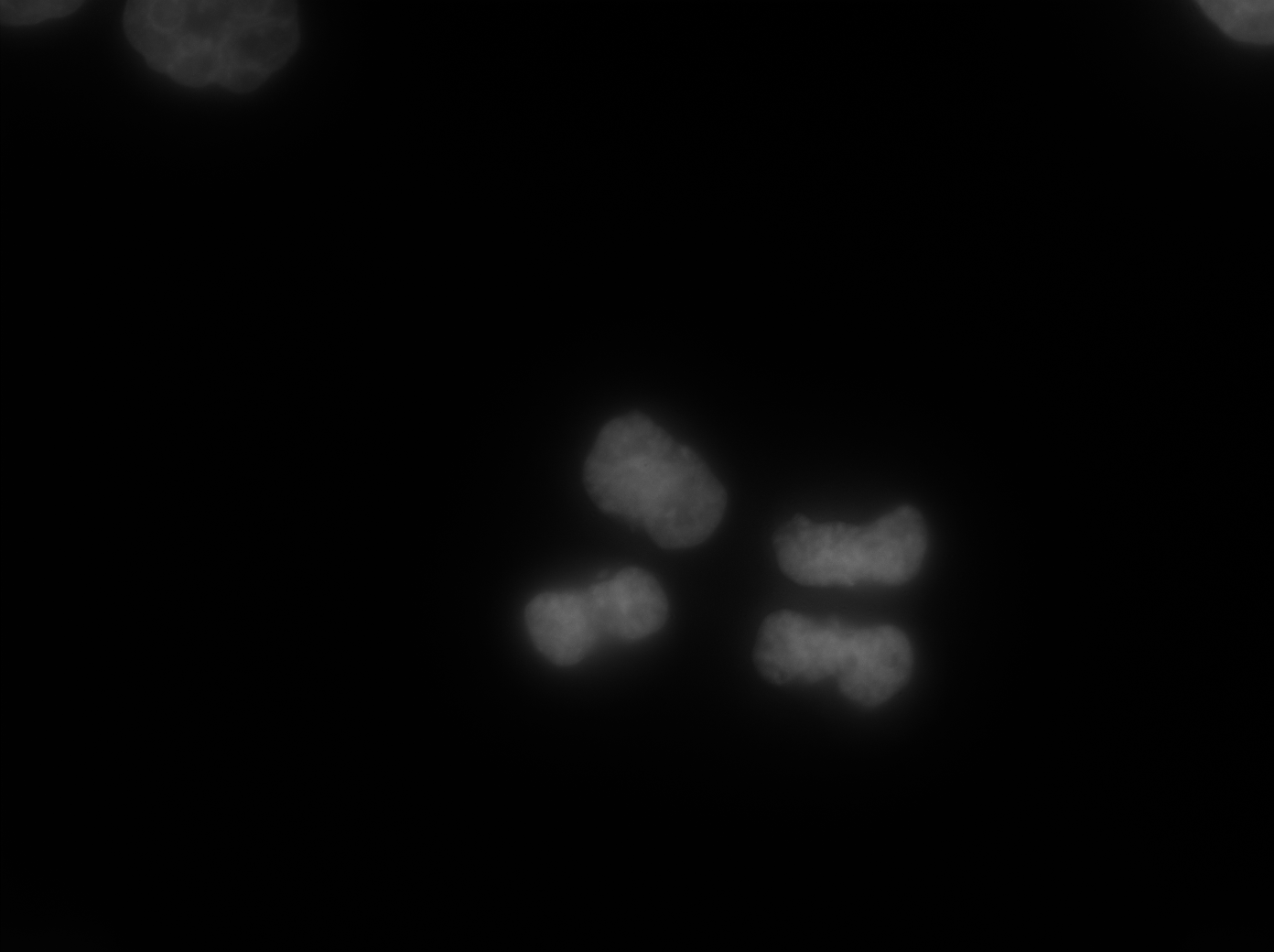

Supplement: Supplementary file 7 — Source data Fig. 2 part 4 [file 44319_2026_742_MOESM7_ESM.zip › Figure 2 Part 4/Fig 2d polye atubulin/WT PolyE-atub 8-14-24 R1 ET5ET6.Project Maximum Z_XY1723757811_Z0_T0_C0.tif]

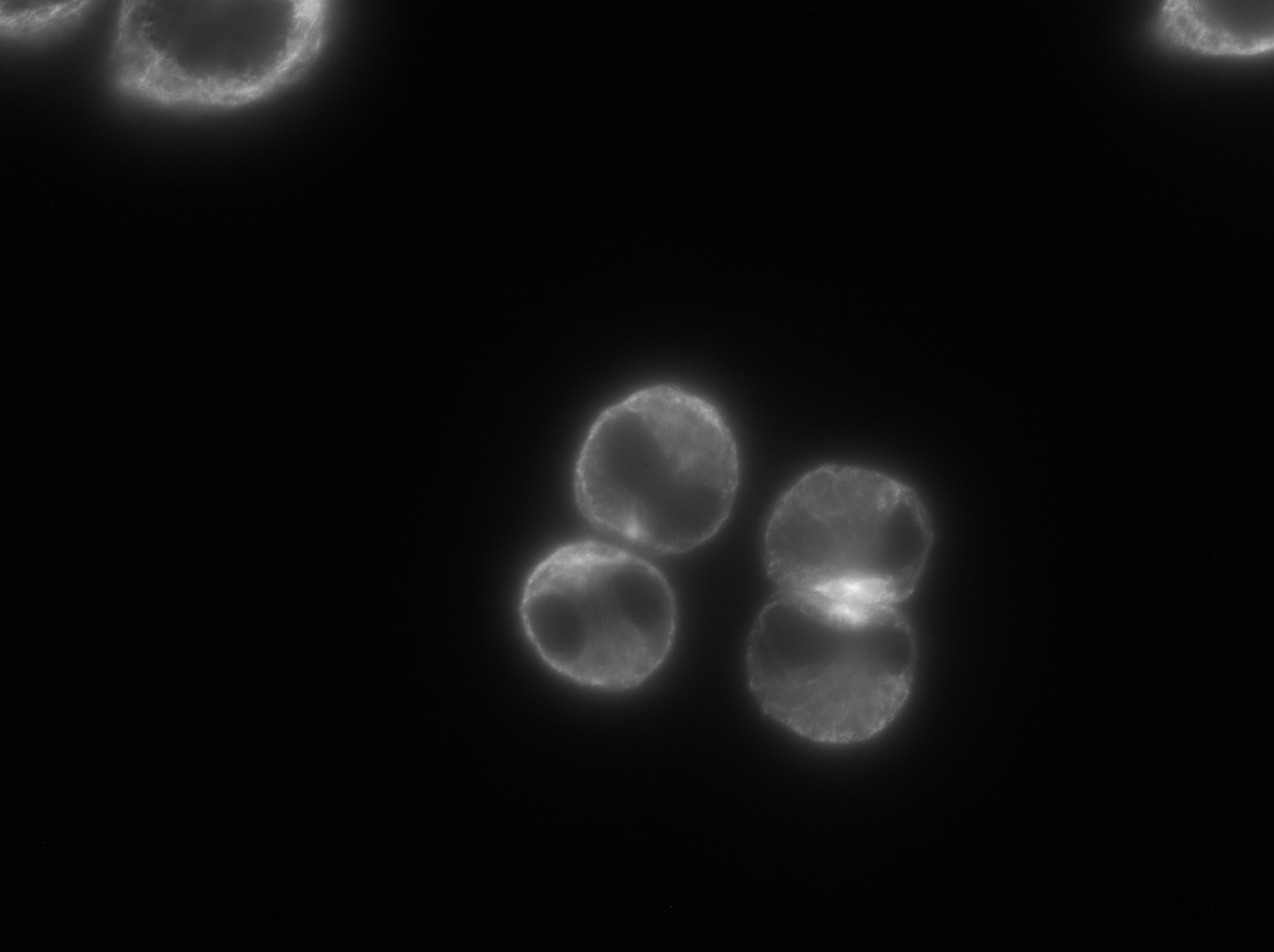

Supplement: Supplementary file 7 — Source data Fig. 2 part 4 [file 44319_2026_742_MOESM7_ESM.zip › Figure 2 Part 4/Fig 2d polye atubulin/WT PolyE-atub 8-14-24 R1 ET5ET6.Project Maximum Z_XY1723757811_Z0_T0_C1.tif]

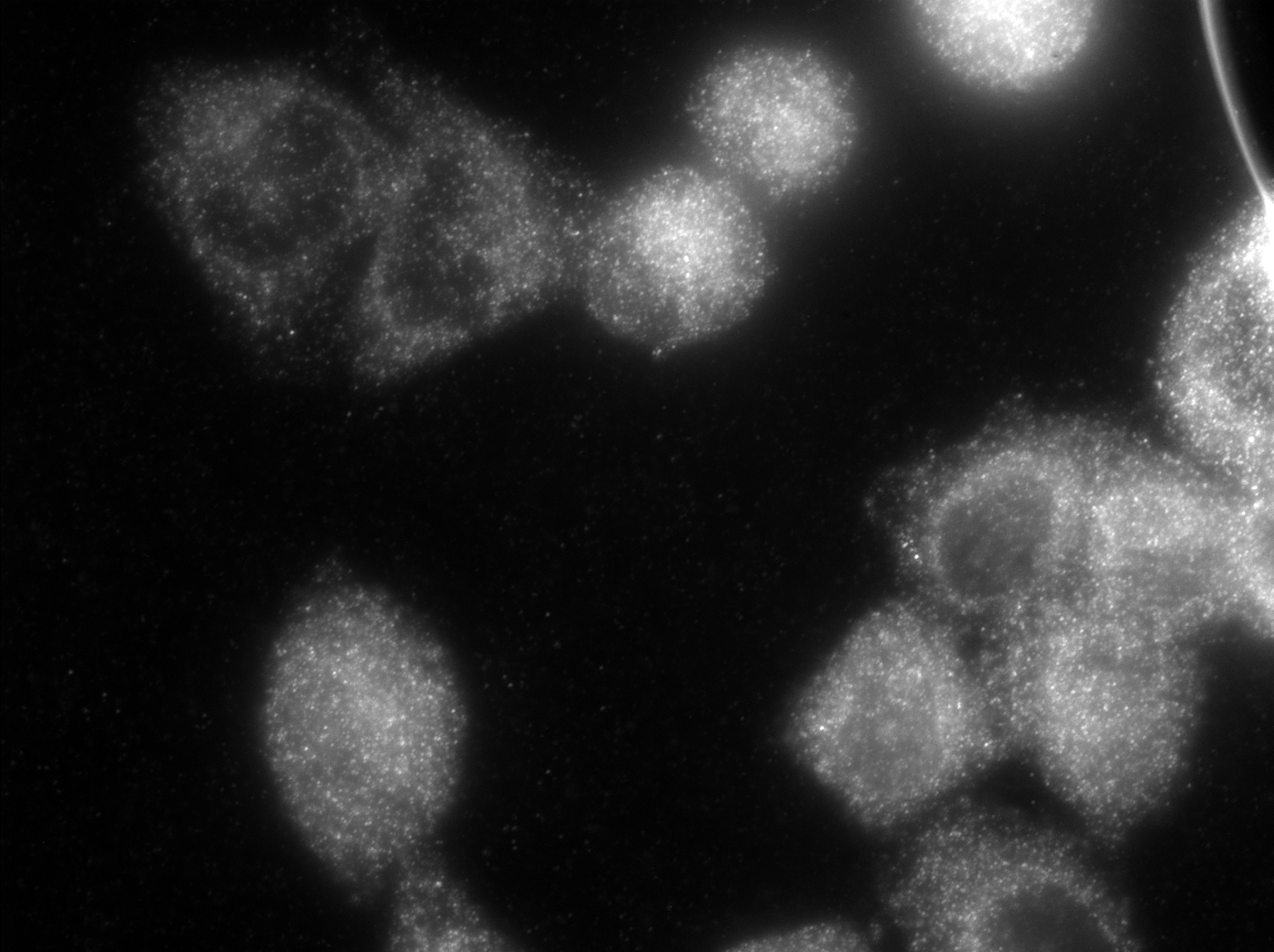

Supplement: Supplementary file 7 — Source data Fig. 2 part 4 [file 44319_2026_742_MOESM7_ESM.zip › Figure 2 Part 4/Fig 2d polye atubulin/WT PolyE-atub 8-14-24 R1 LT5 PA4.Project Maximum Z_XY1723758562_Z0_T0_C2.tif]

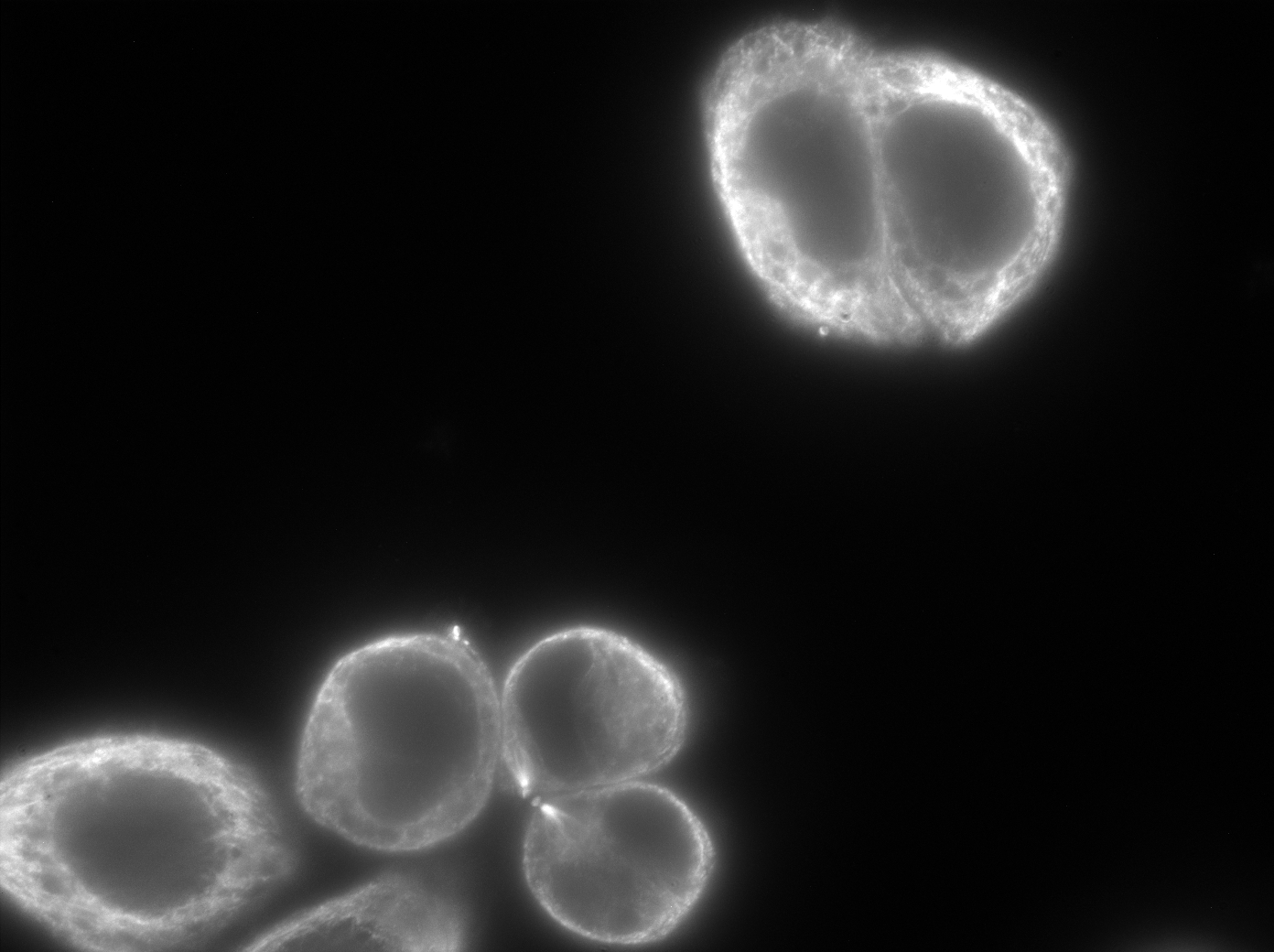

Supplement: Supplementary file 7 — Source data Fig. 2 part 4 [file 44319_2026_742_MOESM7_ESM.zip › Figure 2 Part 4/Fig 2d polye atubulin/WT PolyE-atub 8-14-24 R2 ET8 PA8.Project Maximum Z_XY1723837557_Z0_T0_C1.tif]

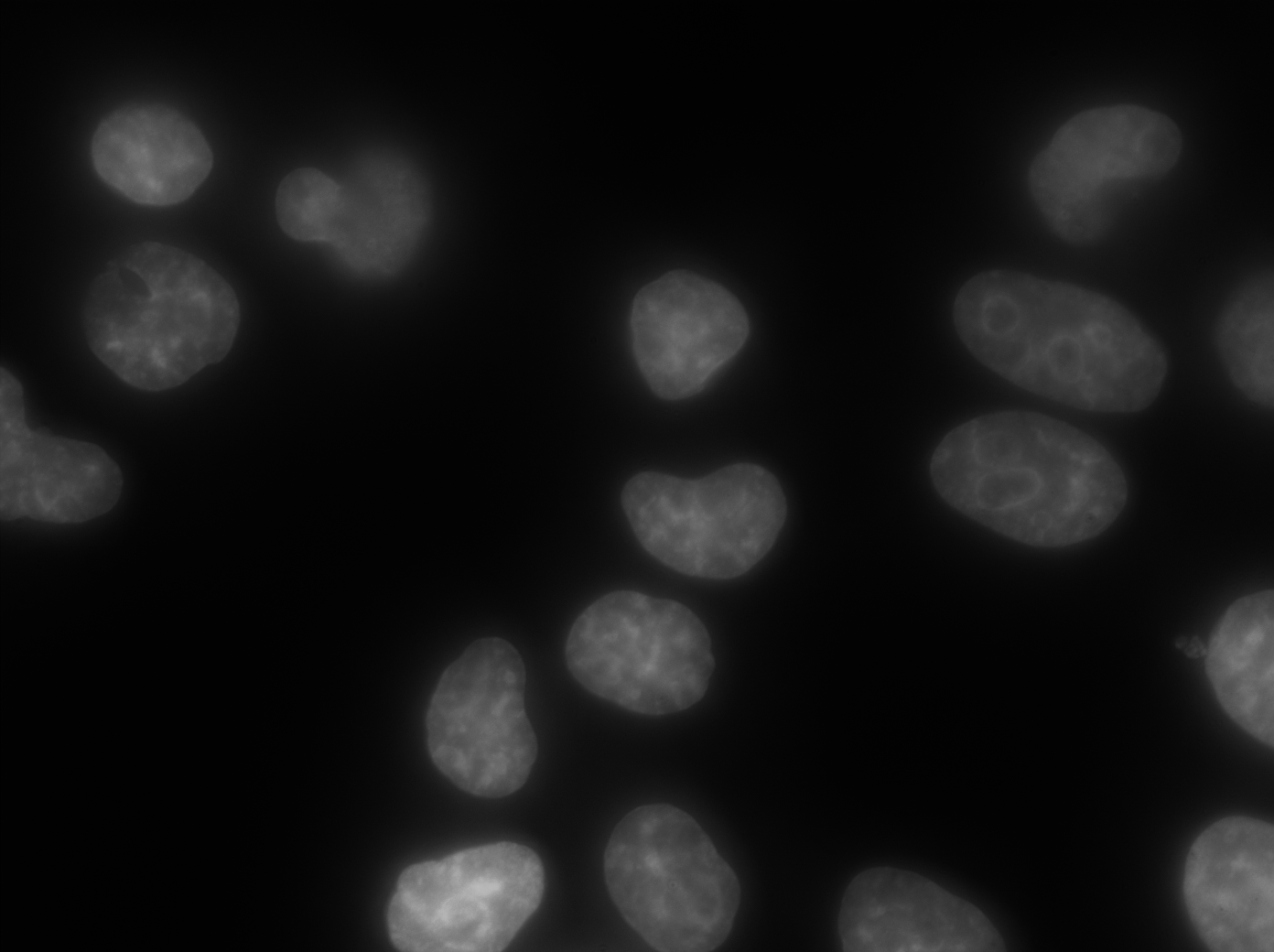

Supplement: Supplementary file 7 — Source data Fig. 2 part 4 [file 44319_2026_742_MOESM7_ESM.zip › Figure 2 Part 4/Fig 2d polye atubulin/WT PolyE-atub 8-14-24 R1 PA6.Project Maximum Z_XY1723760848_Z0_T0_C0.tif]

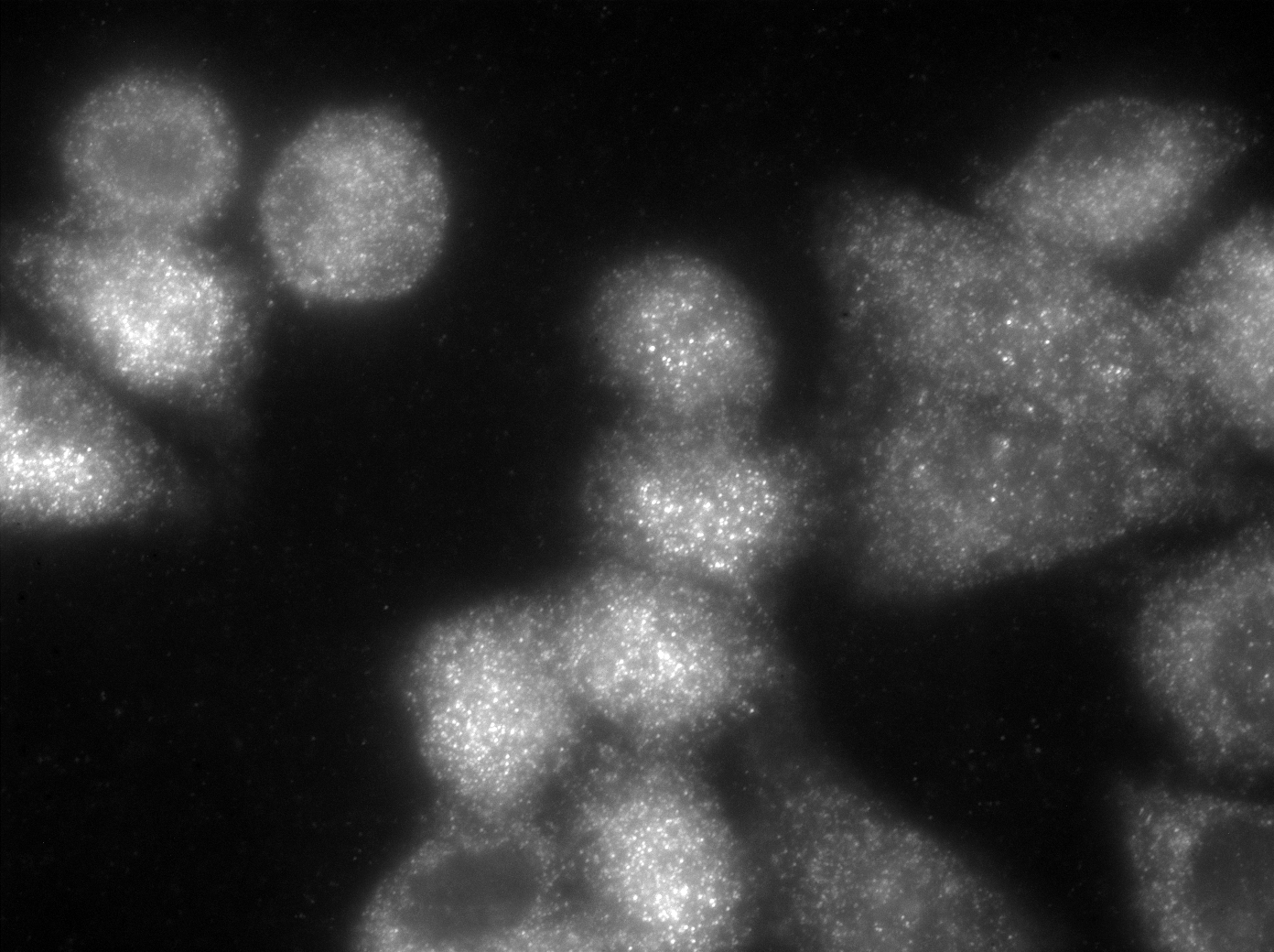

Supplement: Supplementary file 7 — Source data Fig. 2 part 4 [file 44319_2026_742_MOESM7_ESM.zip › Figure 2 Part 4/Fig 2d polye atubulin/WT PolyE-atub 8-14-24 R1 PA6.Project Maximum Z_XY1723760848_Z0_T0_C2.tif]

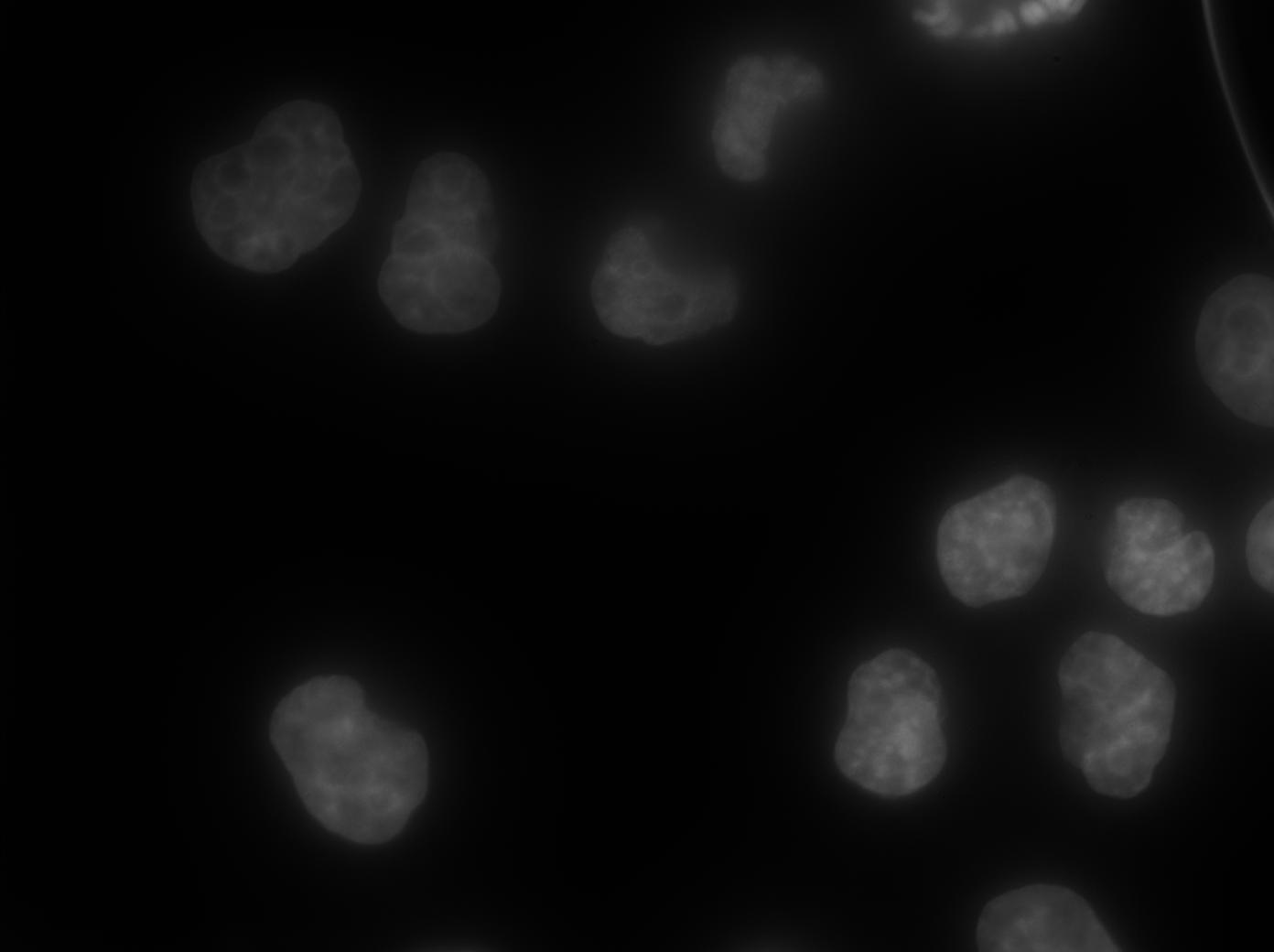

Supplement: Supplementary file 7 — Source data Fig. 2 part 4 [file 44319_2026_742_MOESM7_ESM.zip › Figure 2 Part 4/Fig 2d polye atubulin/WT PolyE-atub 8-14-24 R1 LT5 PA4.Project Maximum Z_XY1723758562_Z0_T0_C0.tif]

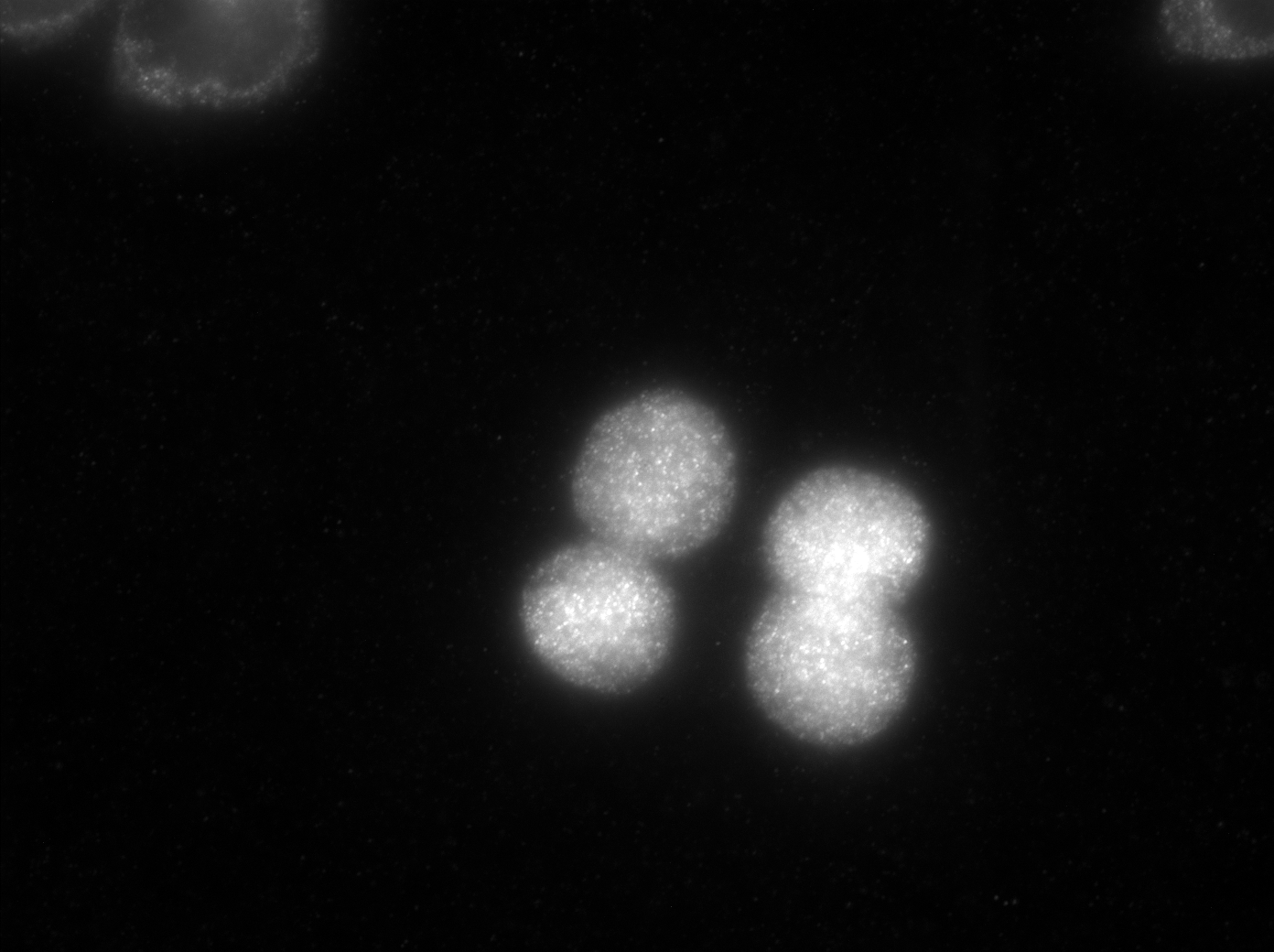

Supplement: Supplementary file 7 — Source data Fig. 2 part 4 [file 44319_2026_742_MOESM7_ESM.zip › Figure 2 Part 4/Fig 2d polye atubulin/WT PolyE-atub 8-14-24 R1 ET5ET6.Project Maximum Z_XY1723757811_Z0_T0_C2.tif]

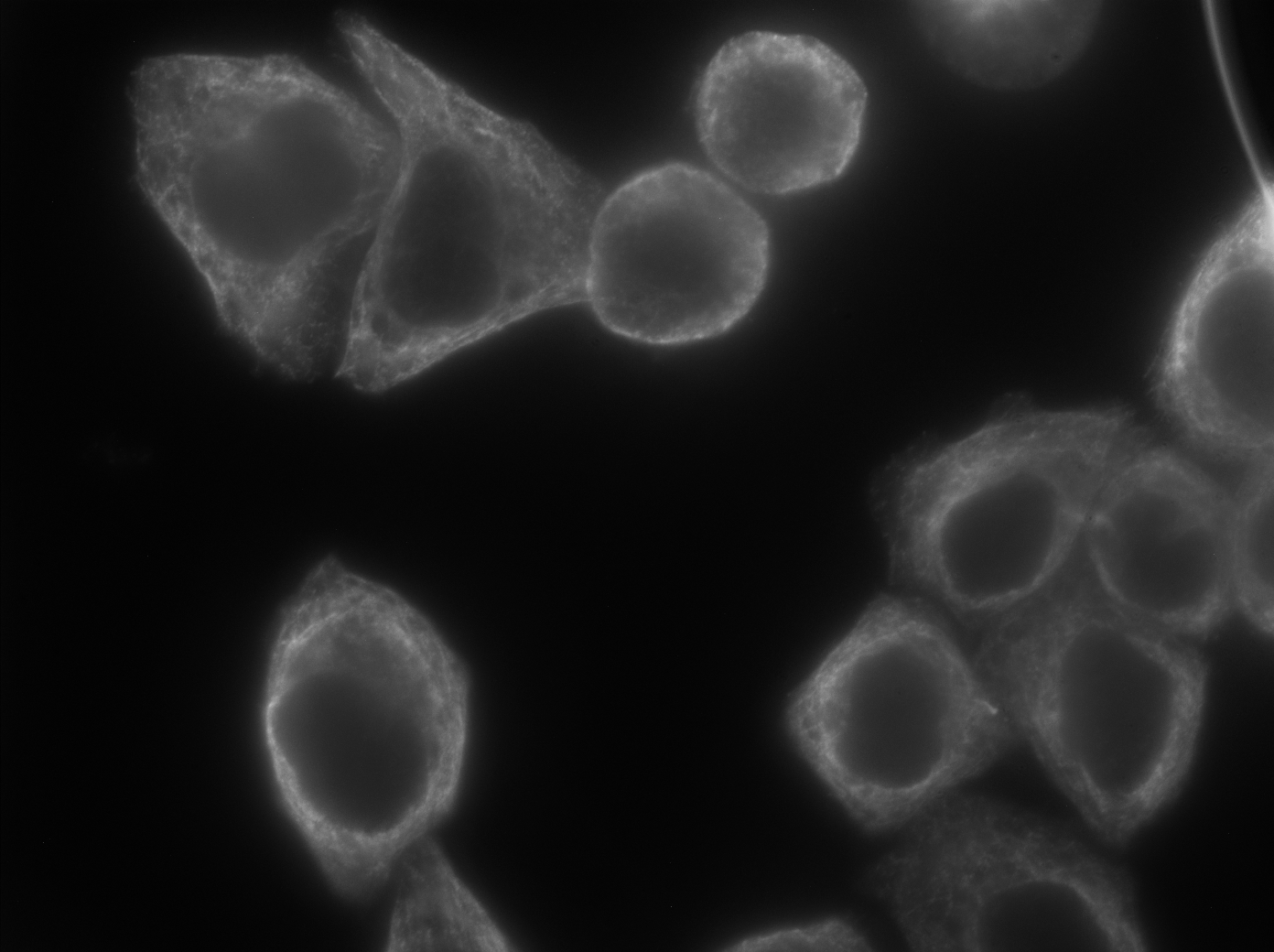

Supplement: Supplementary file 7 — Source data Fig. 2 part 4 [file 44319_2026_742_MOESM7_ESM.zip › Figure 2 Part 4/Fig 2d polye atubulin/WT PolyE-atub 8-14-24 R1 LT5 PA4.Project Maximum Z_XY1723758562_Z0_T0_C1.tif]

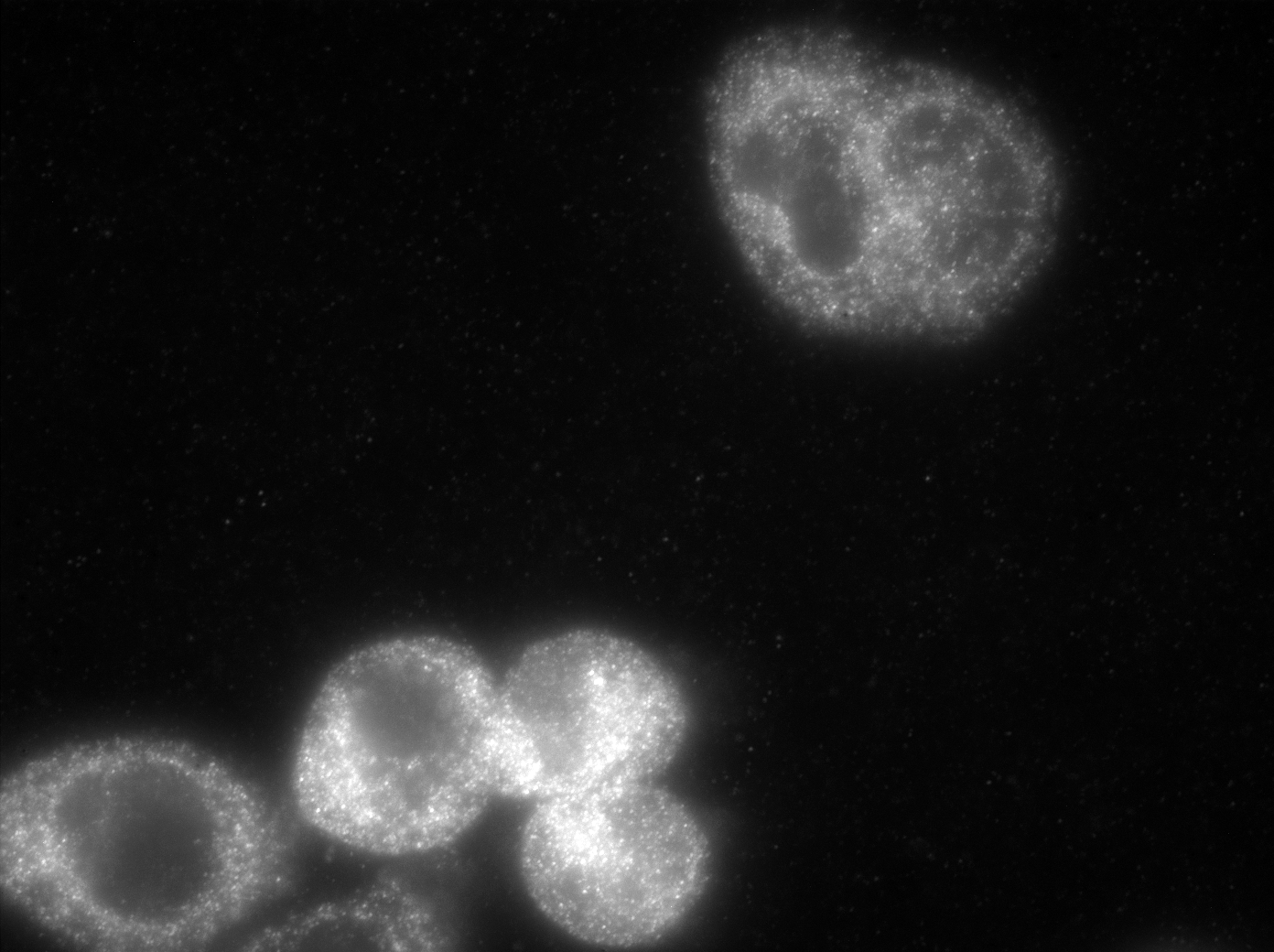

Supplement: Supplementary file 7 — Source data Fig. 2 part 4 [file 44319_2026_742_MOESM7_ESM.zip › Figure 2 Part 4/Fig 2d polye atubulin/WT PolyE-atub 8-14-24 R2 ET8 PA8.Project Maximum Z_XY1723837557_Z0_T0_C2.tif]

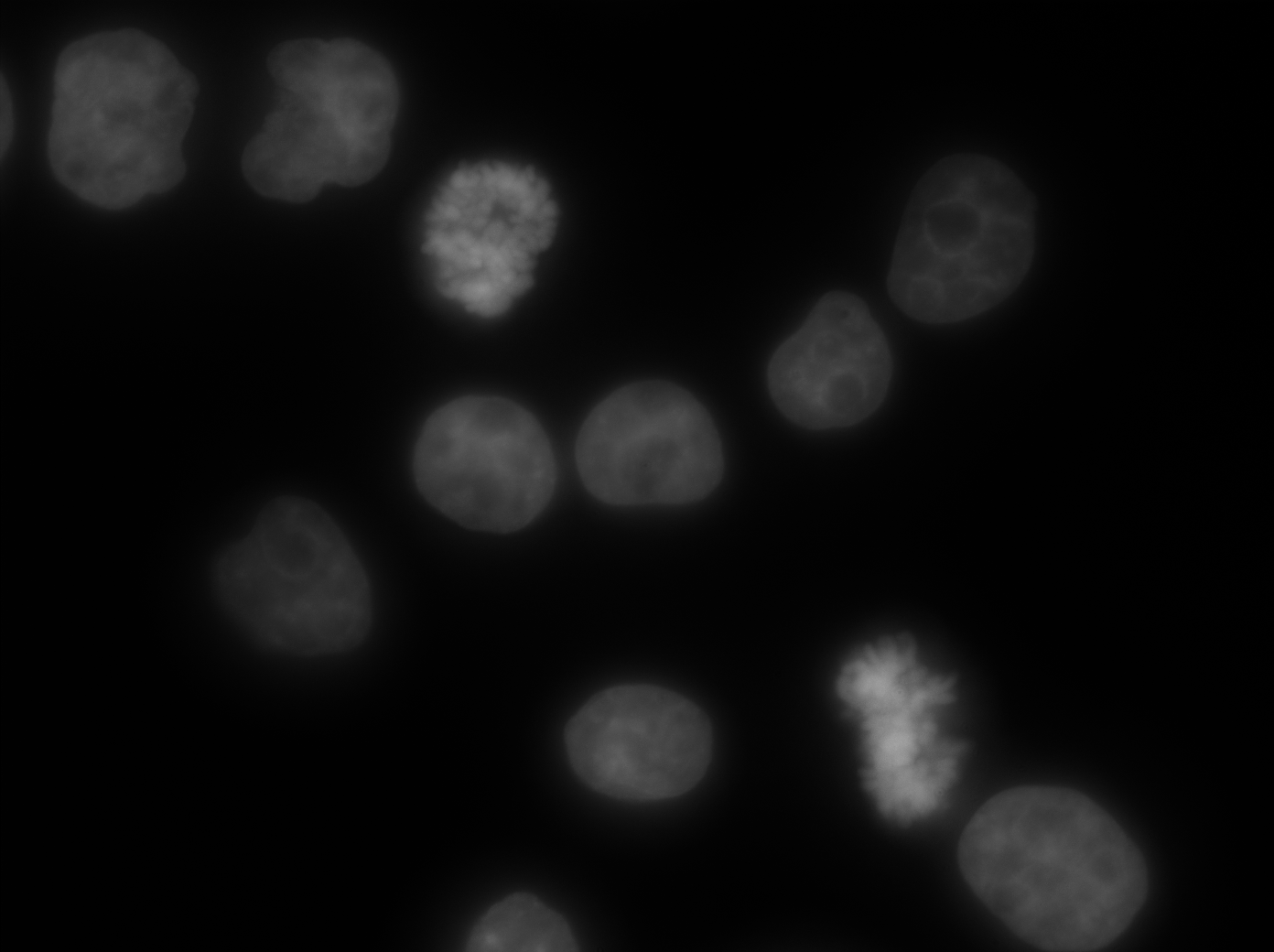

Supplement: Supplementary file 7 — Source data Fig. 2 part 4 [file 44319_2026_742_MOESM7_ESM.zip › Figure 2 Part 4/Fig 2d polye atubulin/WT PolyE-atub 8-14-24 R1 M9M10.Project Maximum Z_XY1723761763_Z0_T0_C0.tif]

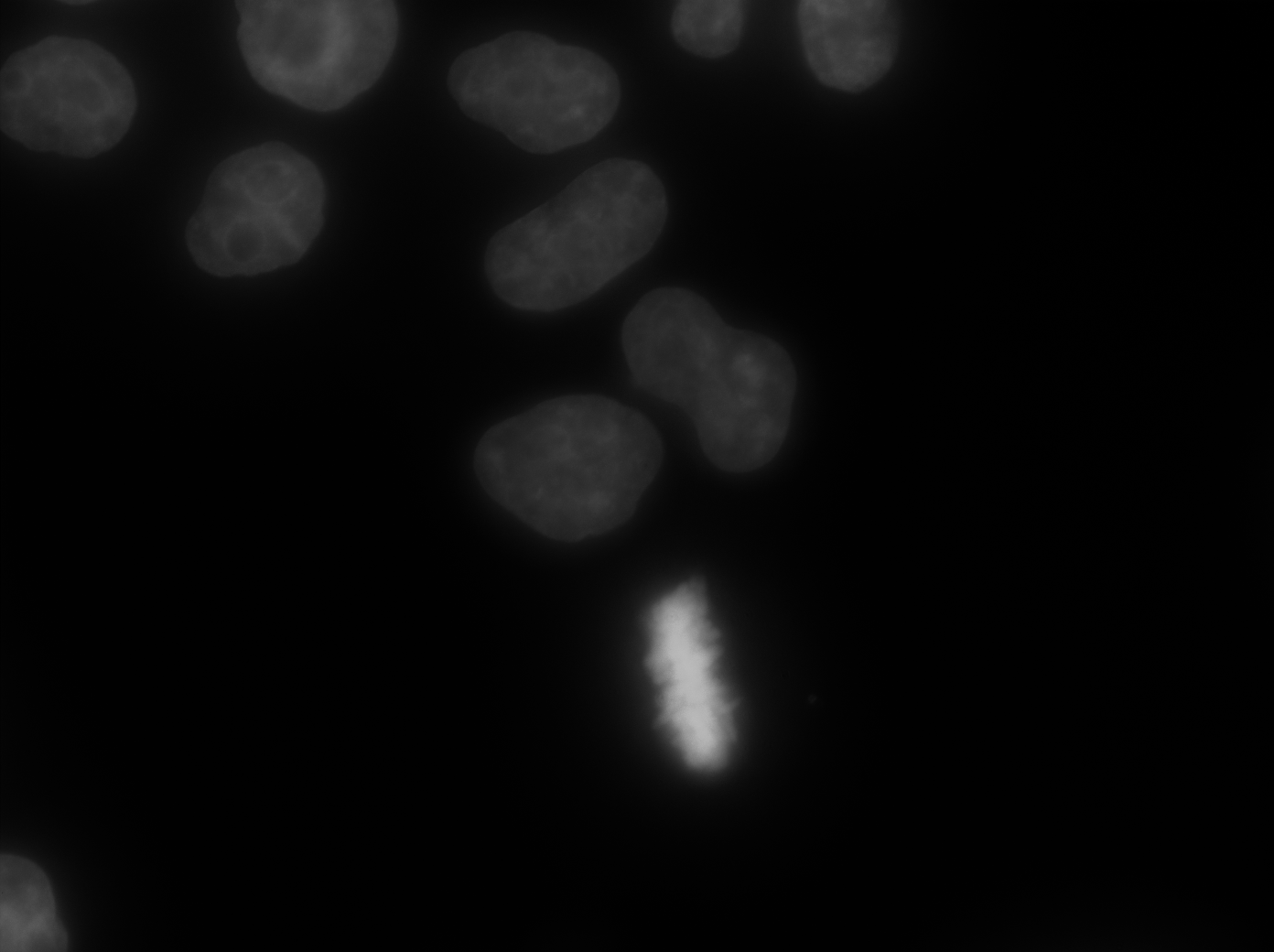

Supplement: Supplementary file 7 — Source data Fig. 2 part 4 [file 44319_2026_742_MOESM7_ESM.zip › Figure 2 Part 4/Fig 2d polye atubulin/WT PolyE-atub 8-14-24 R1 M5.Project Maximum Z_XY1723760230_Z0_T0_C0.tif]

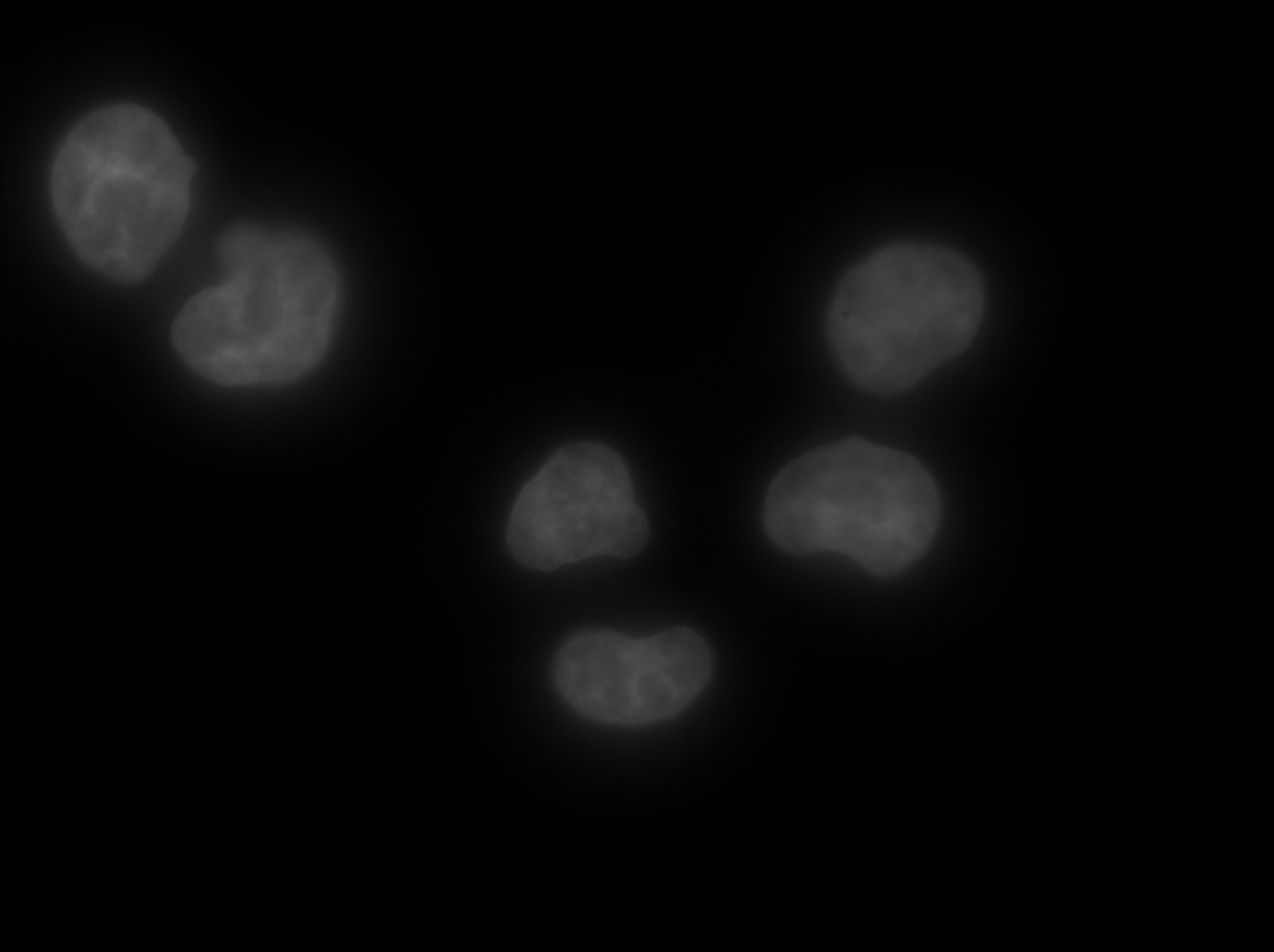

Supplement: Supplementary file 7 — Source data Fig. 2 part 4 [file 44319_2026_742_MOESM7_ESM.zip › Figure 2 Part 4/Fig 2d polye atubulin/WT PolyE-atub 8-14-24 R1 PA2.Project Maximum Z_XY1723757705_Z0_T0_C0.tif]

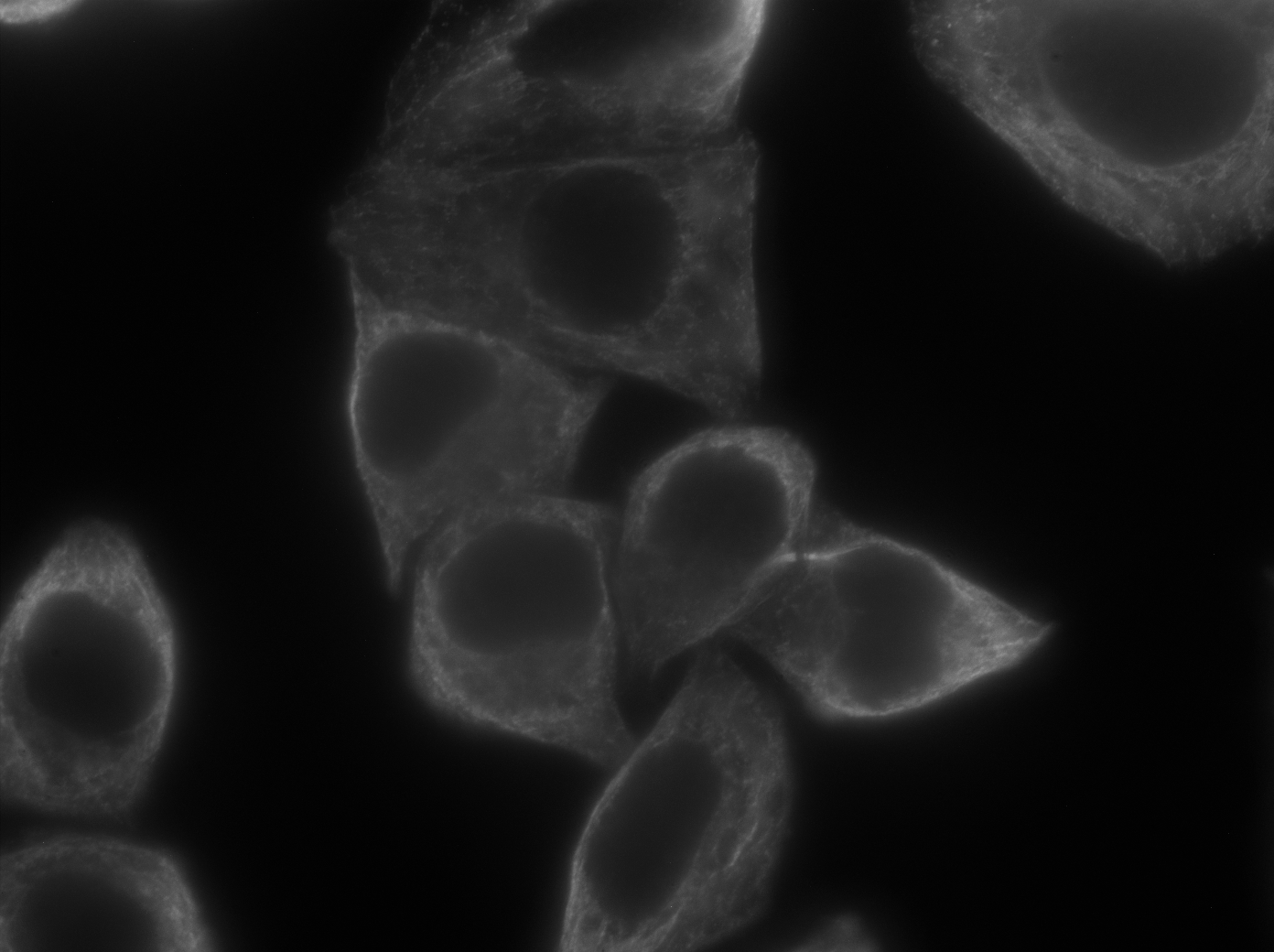

Supplement: Supplementary file 7 — Source data Fig. 2 part 4 [file 44319_2026_742_MOESM7_ESM.zip › Figure 2 Part 4/Fig 2d polye atubulin/WT PolyE-atub 8-14-24 R1 LT4.Project Maximum Z_XY1723758306_Z0_T0_C1.tif]

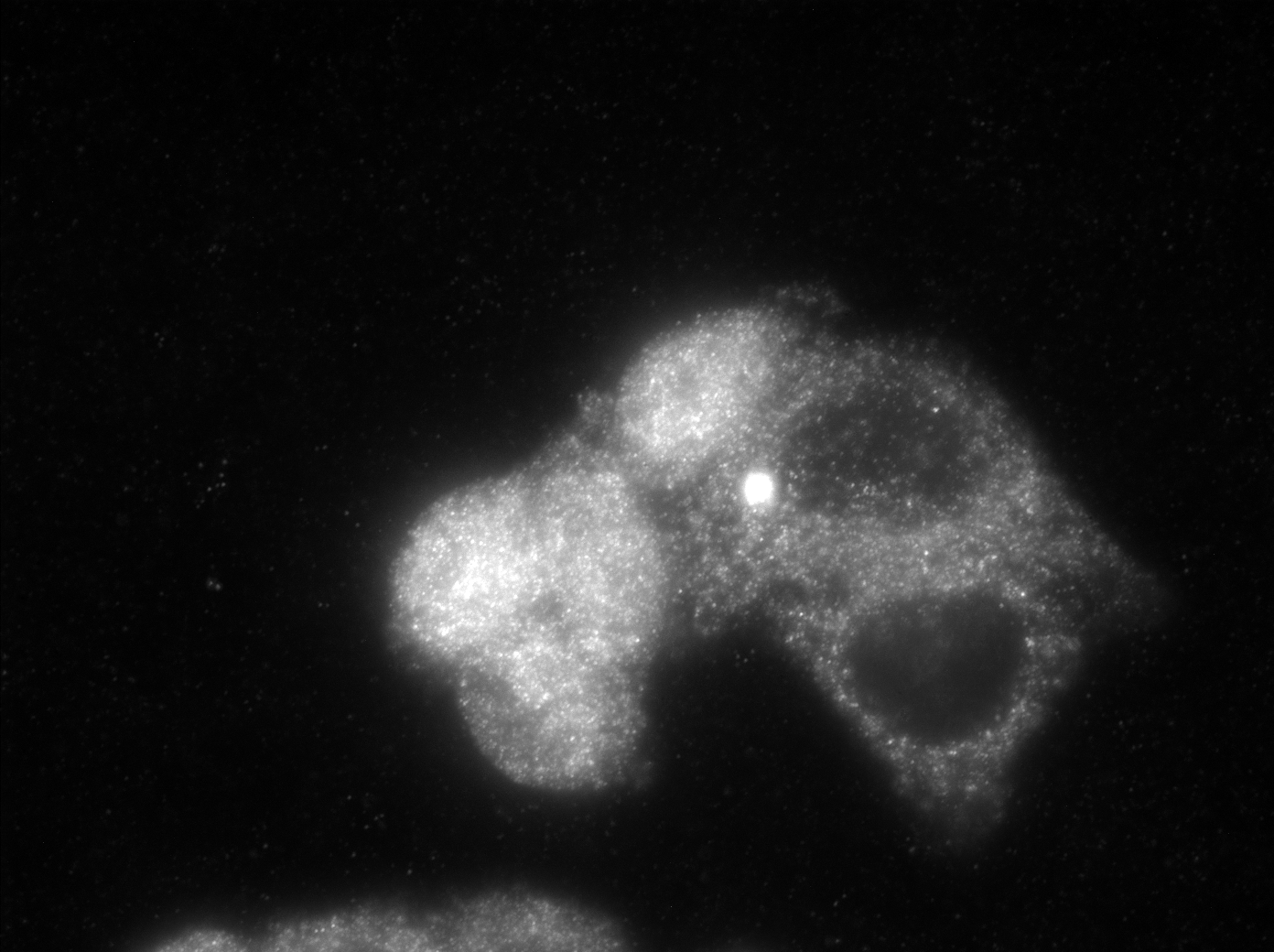

Supplement: Supplementary file 7 — Source data Fig. 2 part 4 [file 44319_2026_742_MOESM7_ESM.zip › Figure 2 Part 4/Fig 2d polye atubulin/WT PolyE-atub 8-14-24 R1 LT2LT3.Project Maximum Z_XY1723757968_Z0_T0_C2.tif]

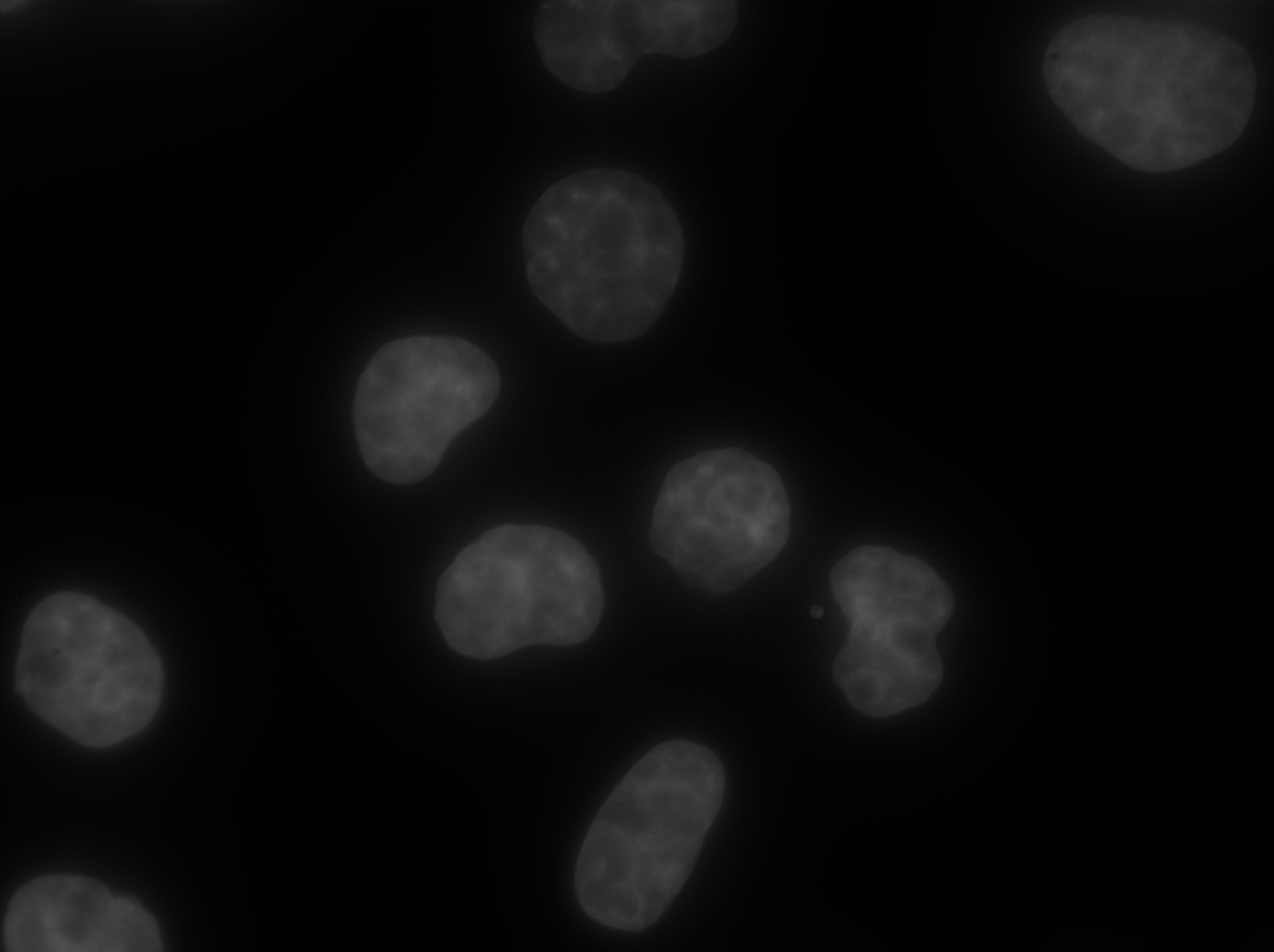

Supplement: Supplementary file 7 — Source data Fig. 2 part 4 [file 44319_2026_742_MOESM7_ESM.zip › Figure 2 Part 4/Fig 2d polye atubulin/WT PolyE-atub 8-14-24 R1 LT4.Project Maximum Z_XY1723758306_Z0_T0_C0.tif]

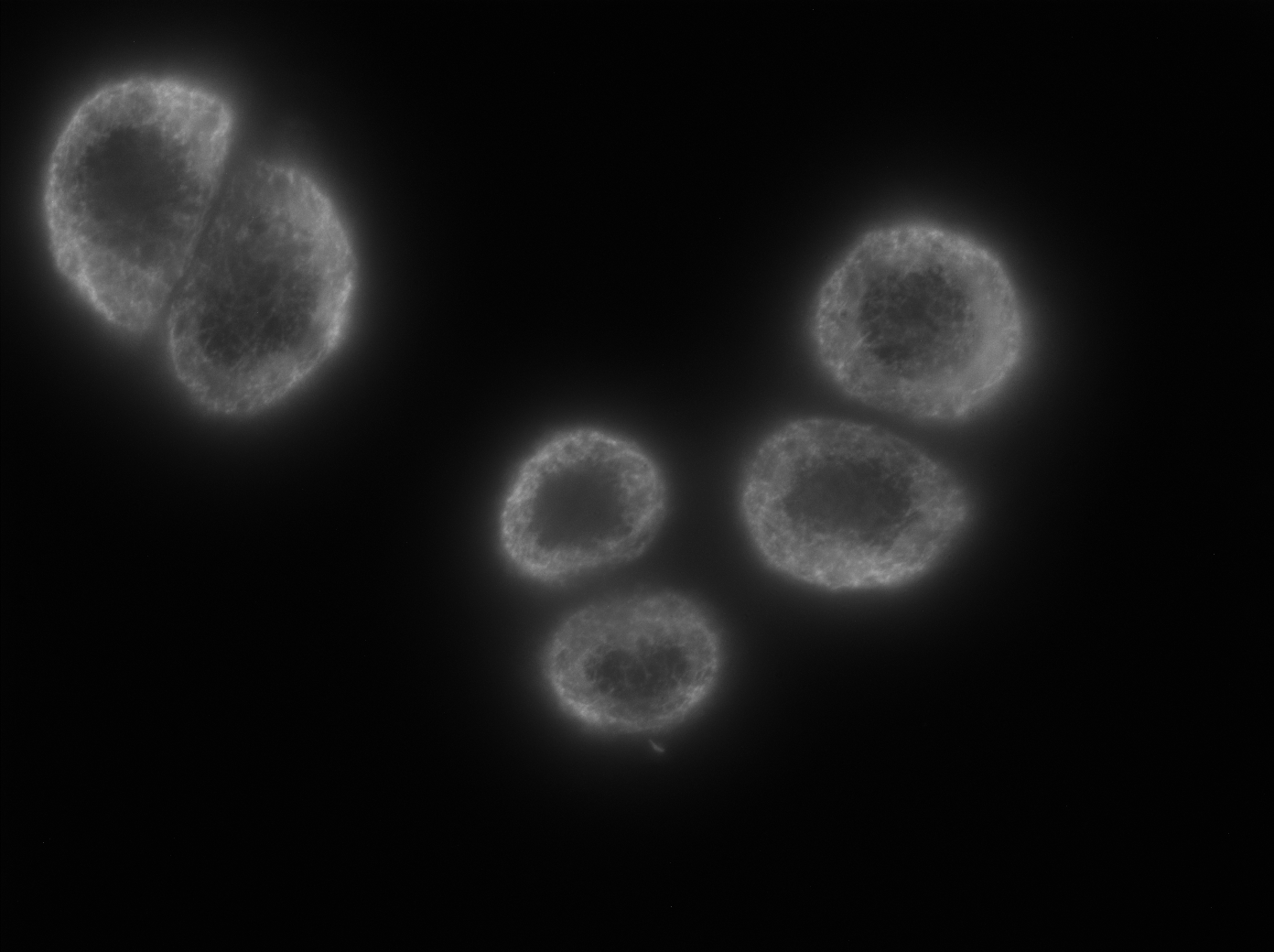

Supplement: Supplementary file 7 — Source data Fig. 2 part 4 [file 44319_2026_742_MOESM7_ESM.zip › Figure 2 Part 4/Fig 2d polye atubulin/WT PolyE-atub 8-14-24 R1 PA2.Project Maximum Z_XY1723757705_Z0_T0_C1.tif]

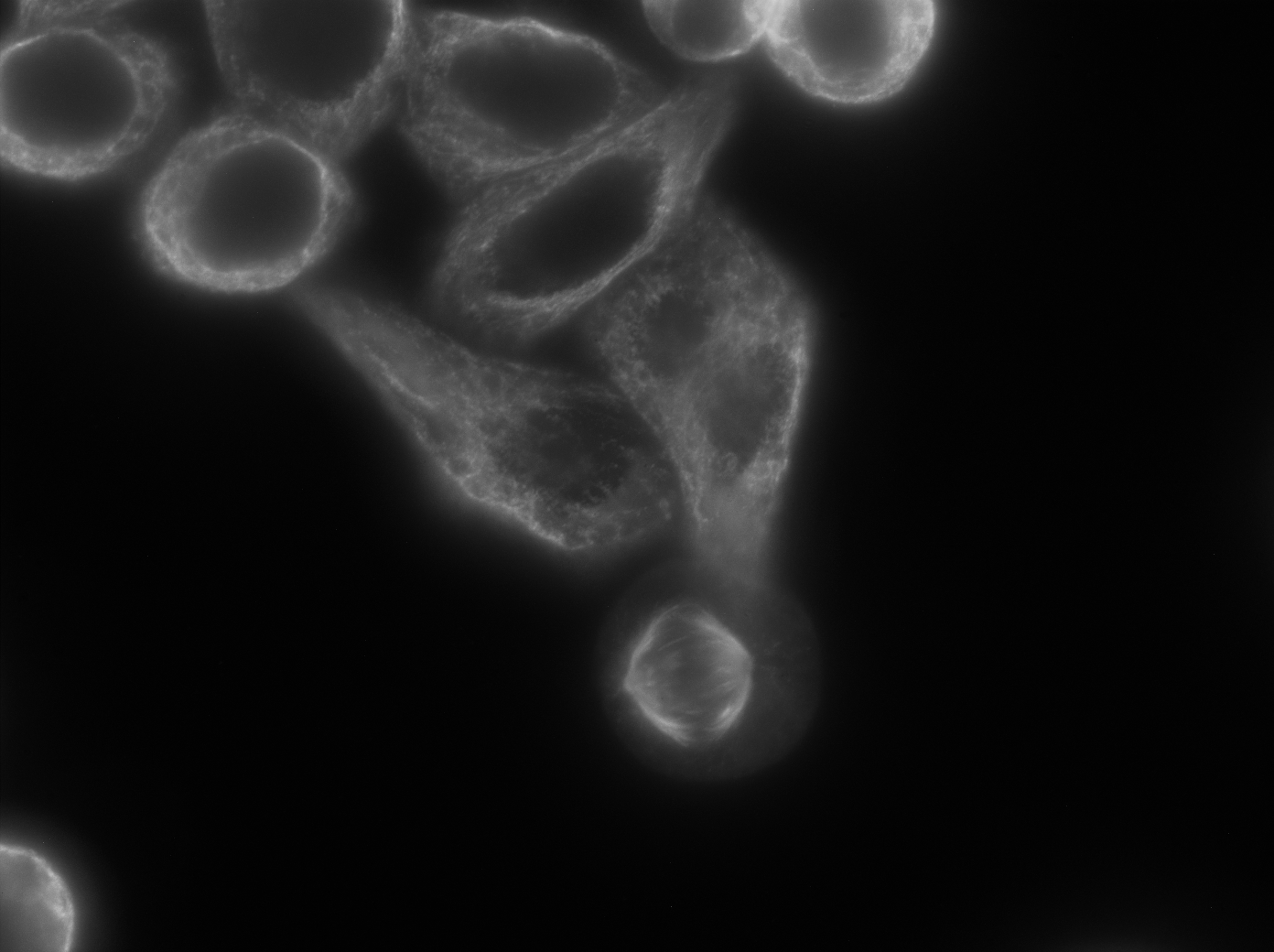

Supplement: Supplementary file 7 — Source data Fig. 2 part 4 [file 44319_2026_742_MOESM7_ESM.zip › Figure 2 Part 4/Fig 2d polye atubulin/WT PolyE-atub 8-14-24 R1 M5.Project Maximum Z_XY1723760230_Z0_T0_C1.tif]

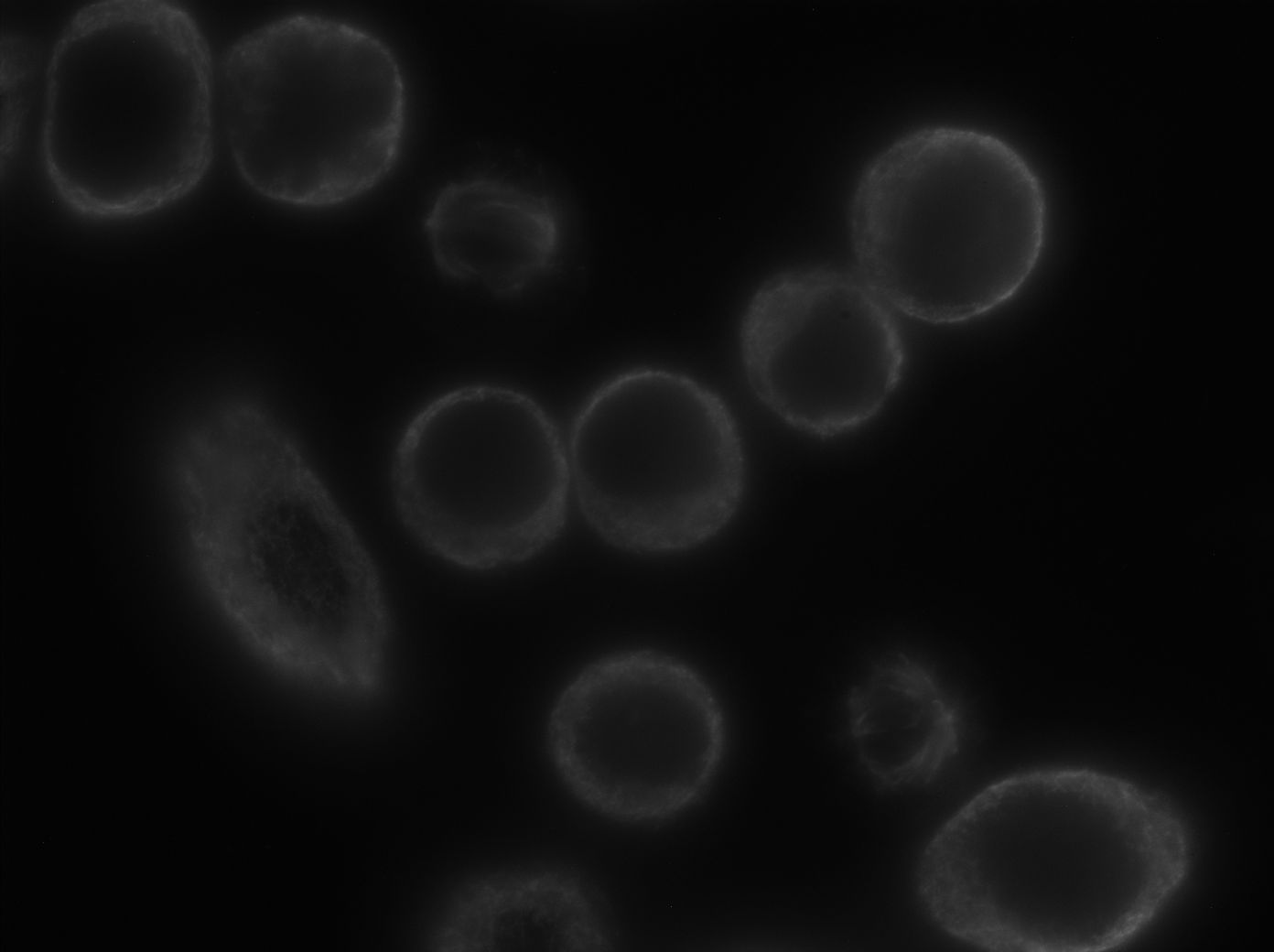

Supplement: Supplementary file 7 — Source data Fig. 2 part 4 [file 44319_2026_742_MOESM7_ESM.zip › Figure 2 Part 4/Fig 2d polye atubulin/WT PolyE-atub 8-14-24 R1 M9M10.Project Maximum Z_XY1723761763_Z0_T0_C1.tif]

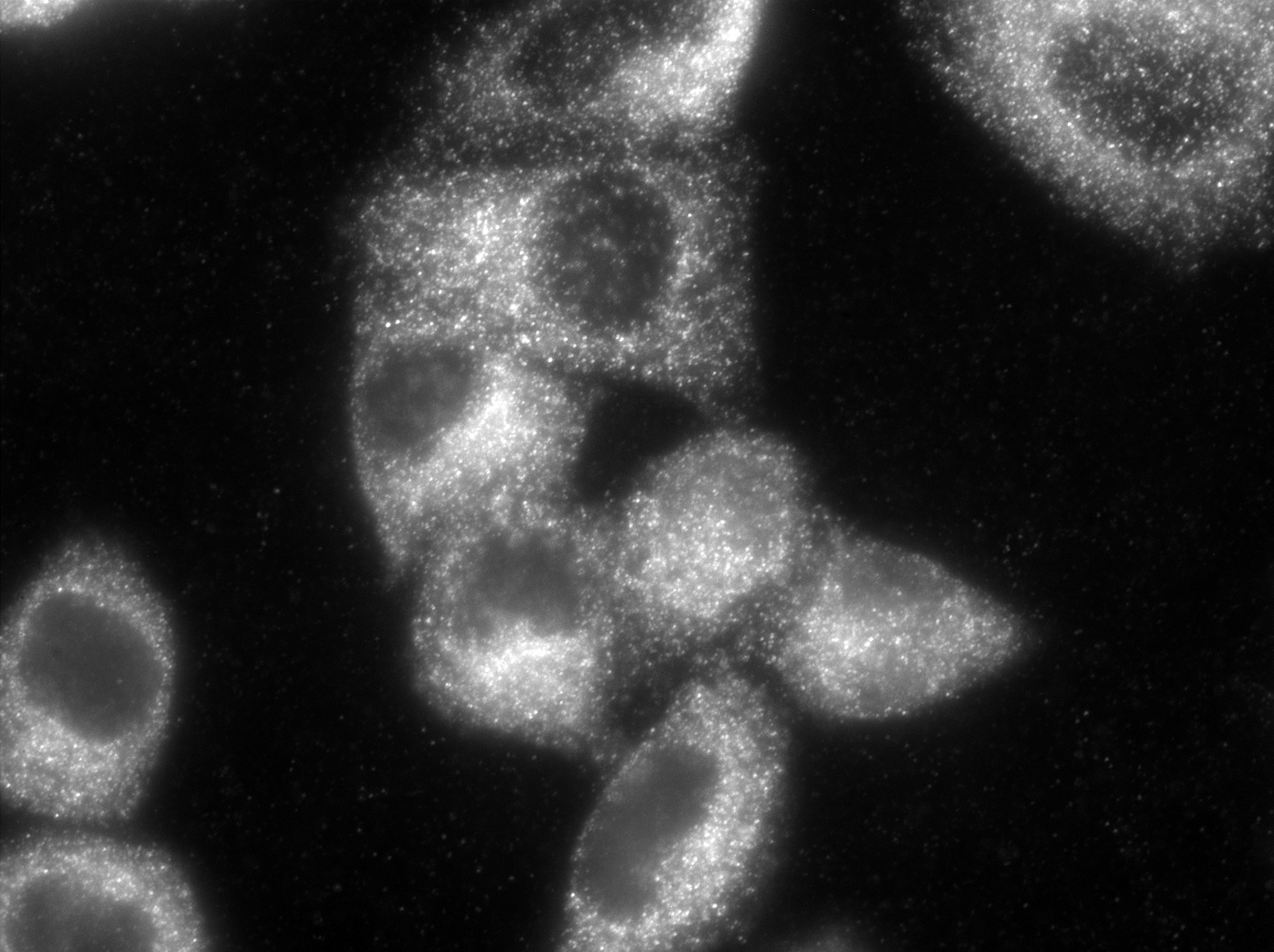

Supplement: Supplementary file 7 — Source data Fig. 2 part 4 [file 44319_2026_742_MOESM7_ESM.zip › Figure 2 Part 4/Fig 2d polye atubulin/WT PolyE-atub 8-14-24 R1 LT4.Project Maximum Z_XY1723758306_Z0_T0_C2.tif]

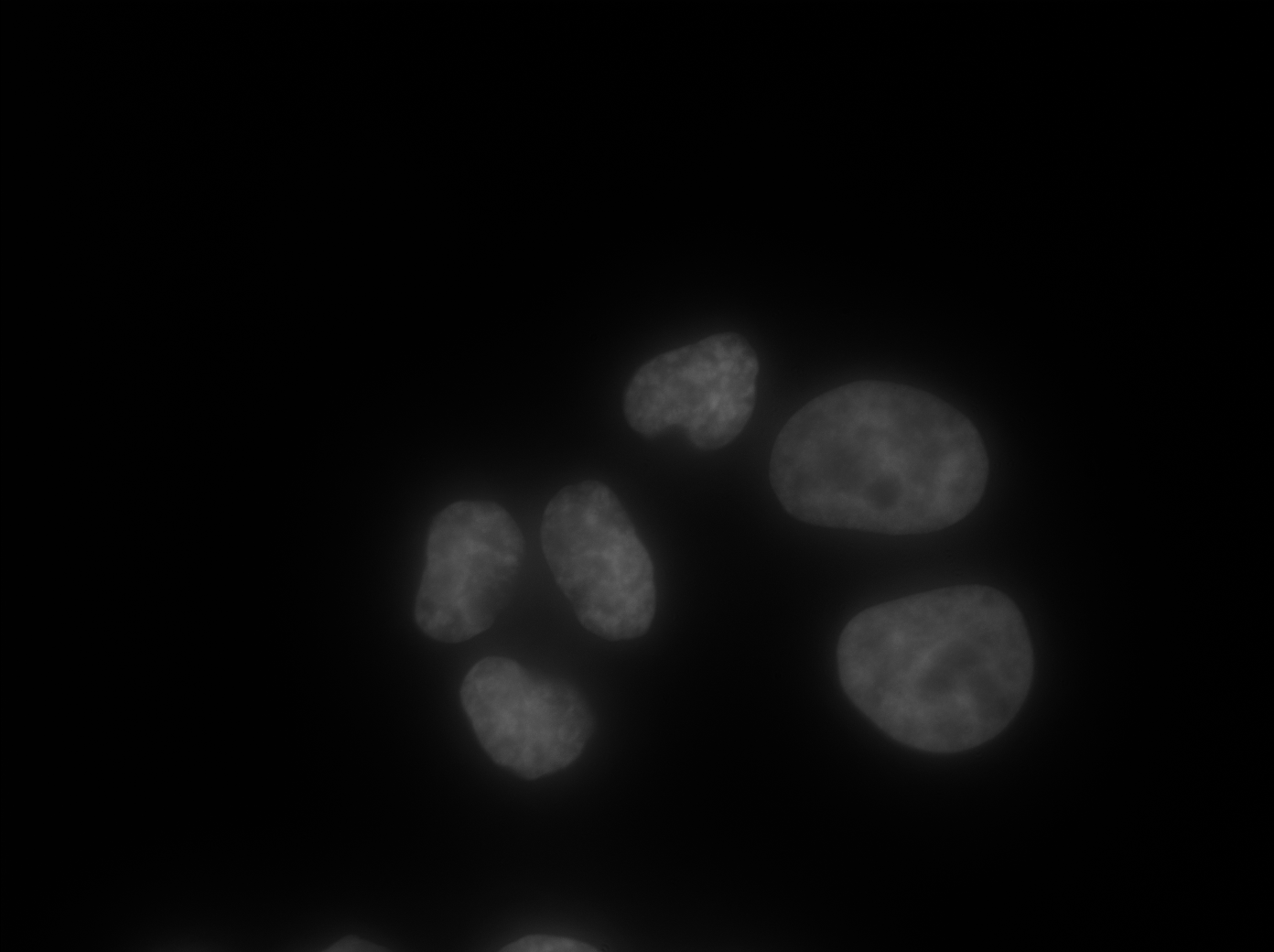

Supplement: Supplementary file 7 — Source data Fig. 2 part 4 [file 44319_2026_742_MOESM7_ESM.zip › Figure 2 Part 4/Fig 2d polye atubulin/WT PolyE-atub 8-14-24 R1 LT2LT3.Project Maximum Z_XY1723757968_Z0_T0_C0.tif]

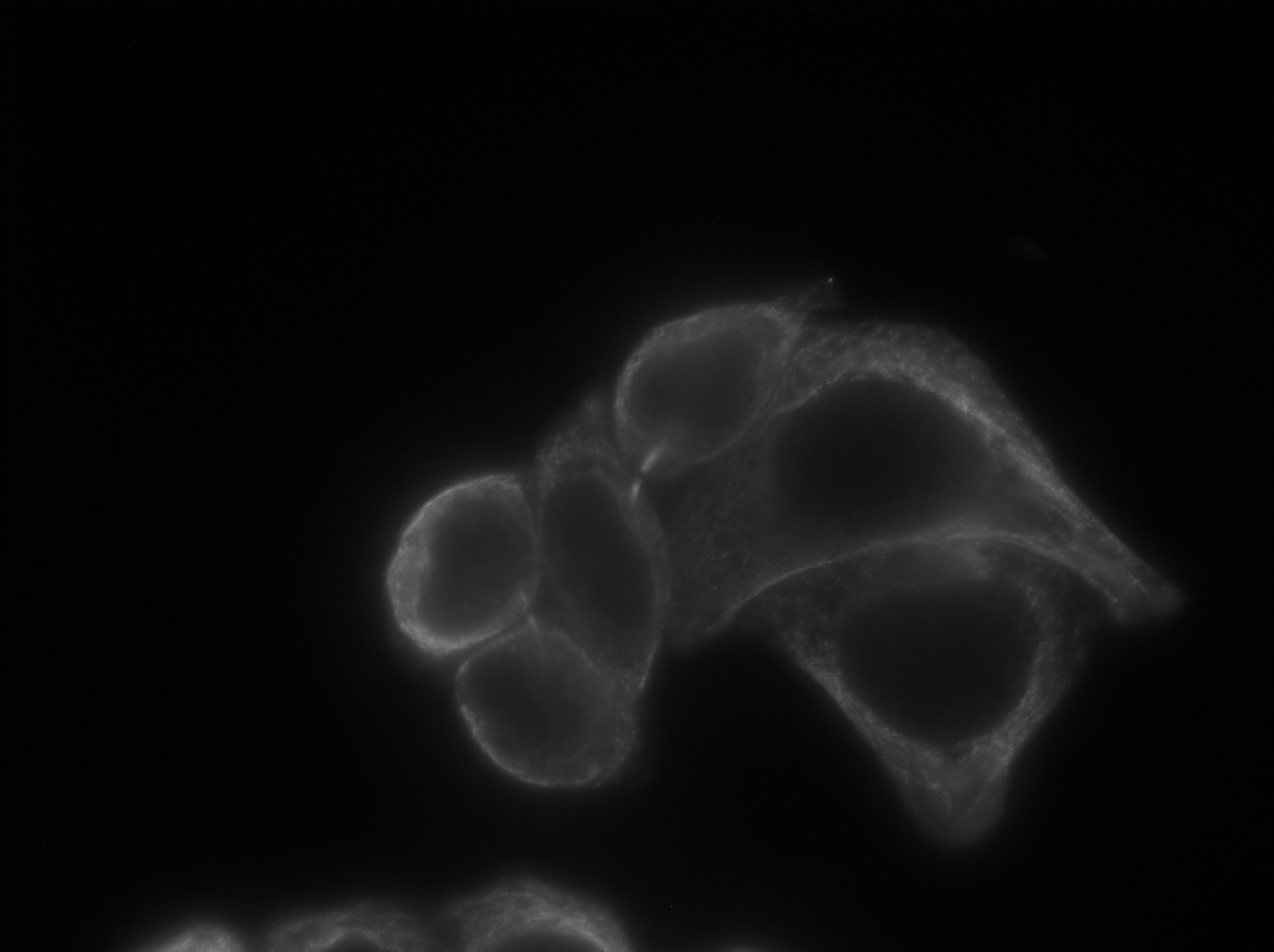

Supplement: Supplementary file 7 — Source data Fig. 2 part 4 [file 44319_2026_742_MOESM7_ESM.zip › Figure 2 Part 4/Fig 2d polye atubulin/WT PolyE-atub 8-14-24 R1 LT2LT3.Project Maximum Z_XY1723757968_Z0_T0_C1.tif]

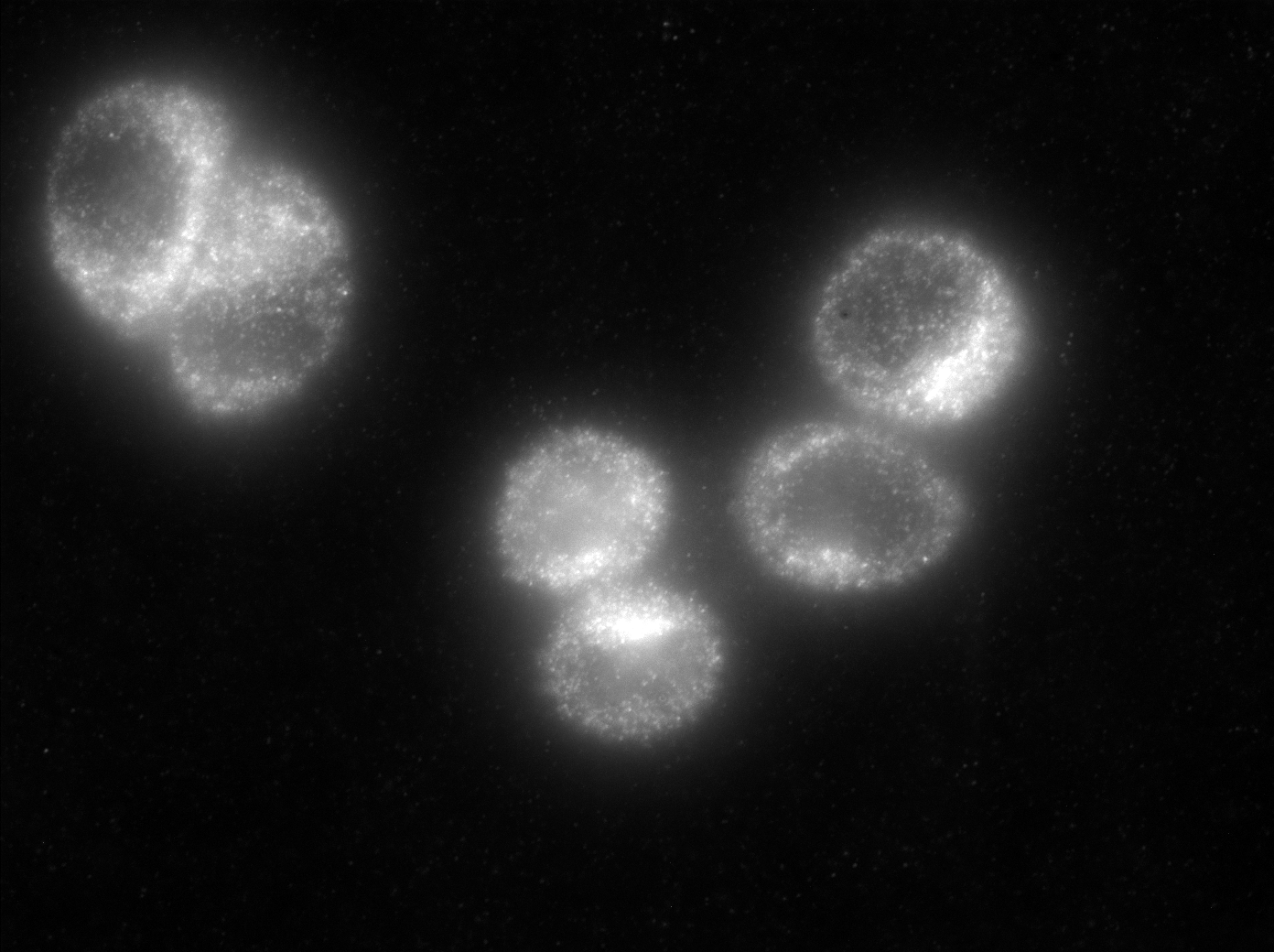

Supplement: Supplementary file 7 — Source data Fig. 2 part 4 [file 44319_2026_742_MOESM7_ESM.zip › Figure 2 Part 4/Fig 2d polye atubulin/WT PolyE-atub 8-14-24 R1 PA2.Project Maximum Z_XY1723757705_Z0_T0_C2.tif]

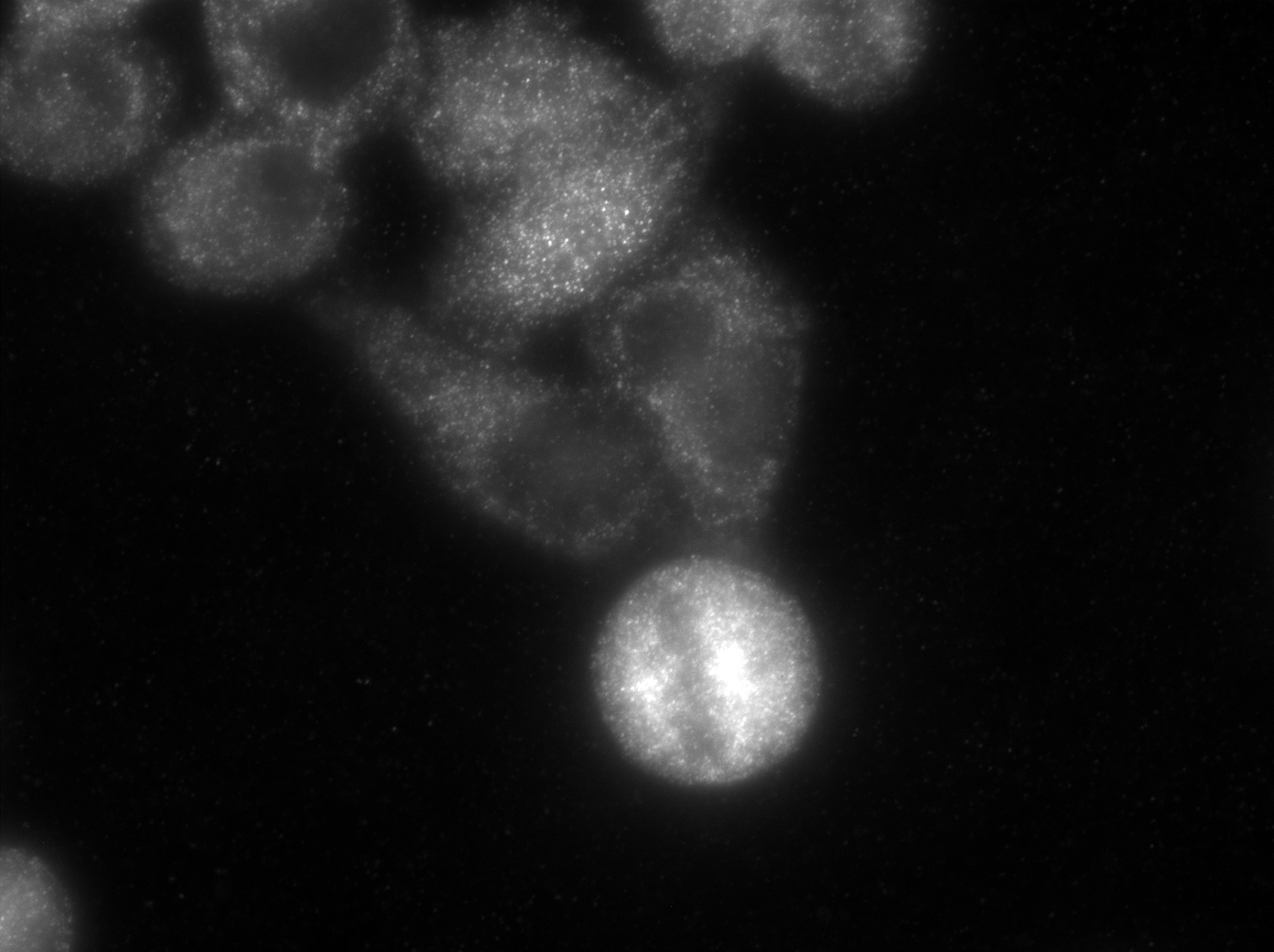

Supplement: Supplementary file 7 — Source data Fig. 2 part 4 [file 44319_2026_742_MOESM7_ESM.zip › Figure 2 Part 4/Fig 2d polye atubulin/WT PolyE-atub 8-14-24 R1 M5.Project Maximum Z_XY1723760230_Z0_T0_C2.tif]

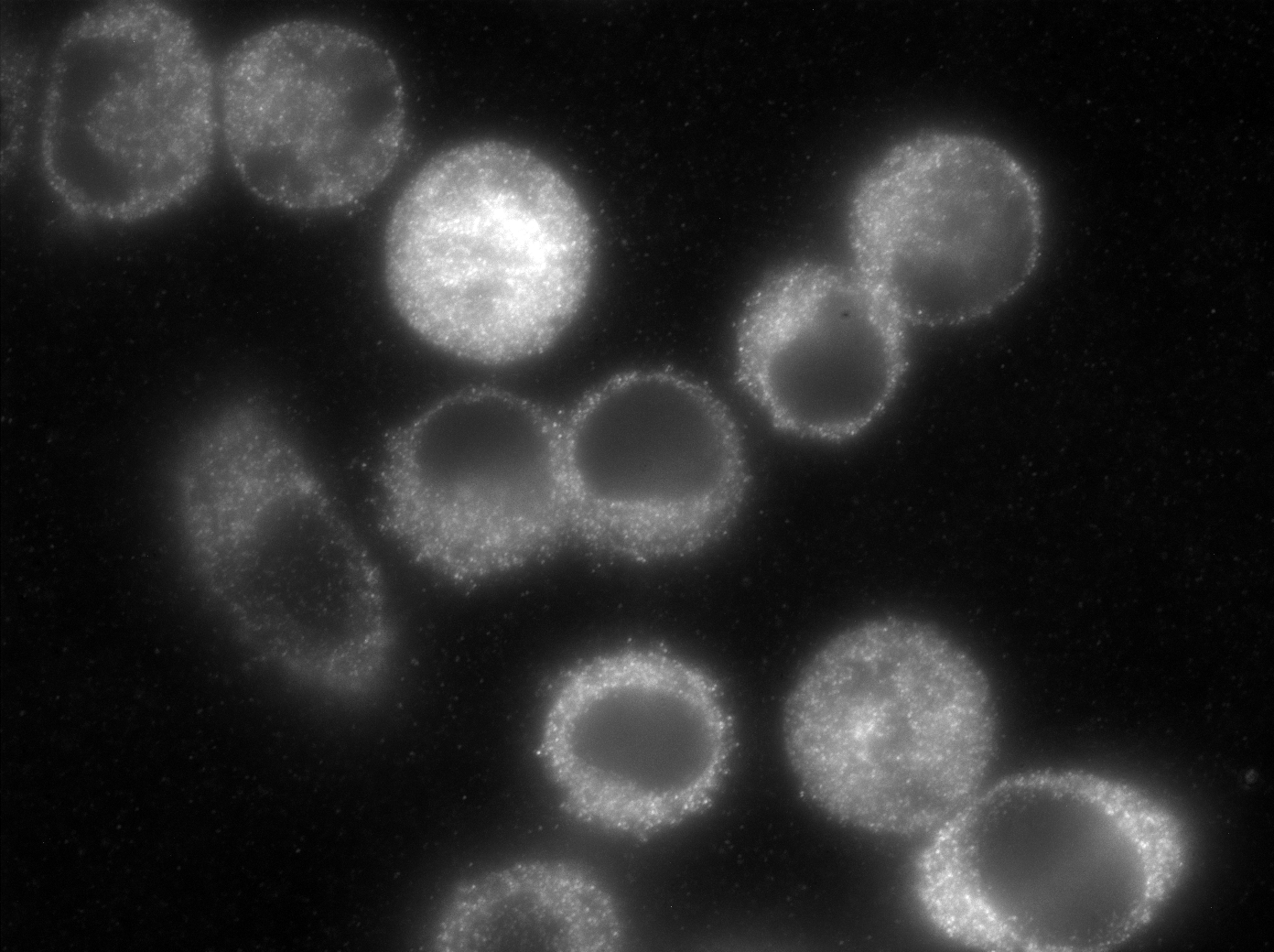

Supplement: Supplementary file 7 — Source data Fig. 2 part 4 [file 44319_2026_742_MOESM7_ESM.zip › Figure 2 Part 4/Fig 2d polye atubulin/WT PolyE-atub 8-14-24 R1 M9M10.Project Maximum Z_XY1723761763_Z0_T0_C2.tif]

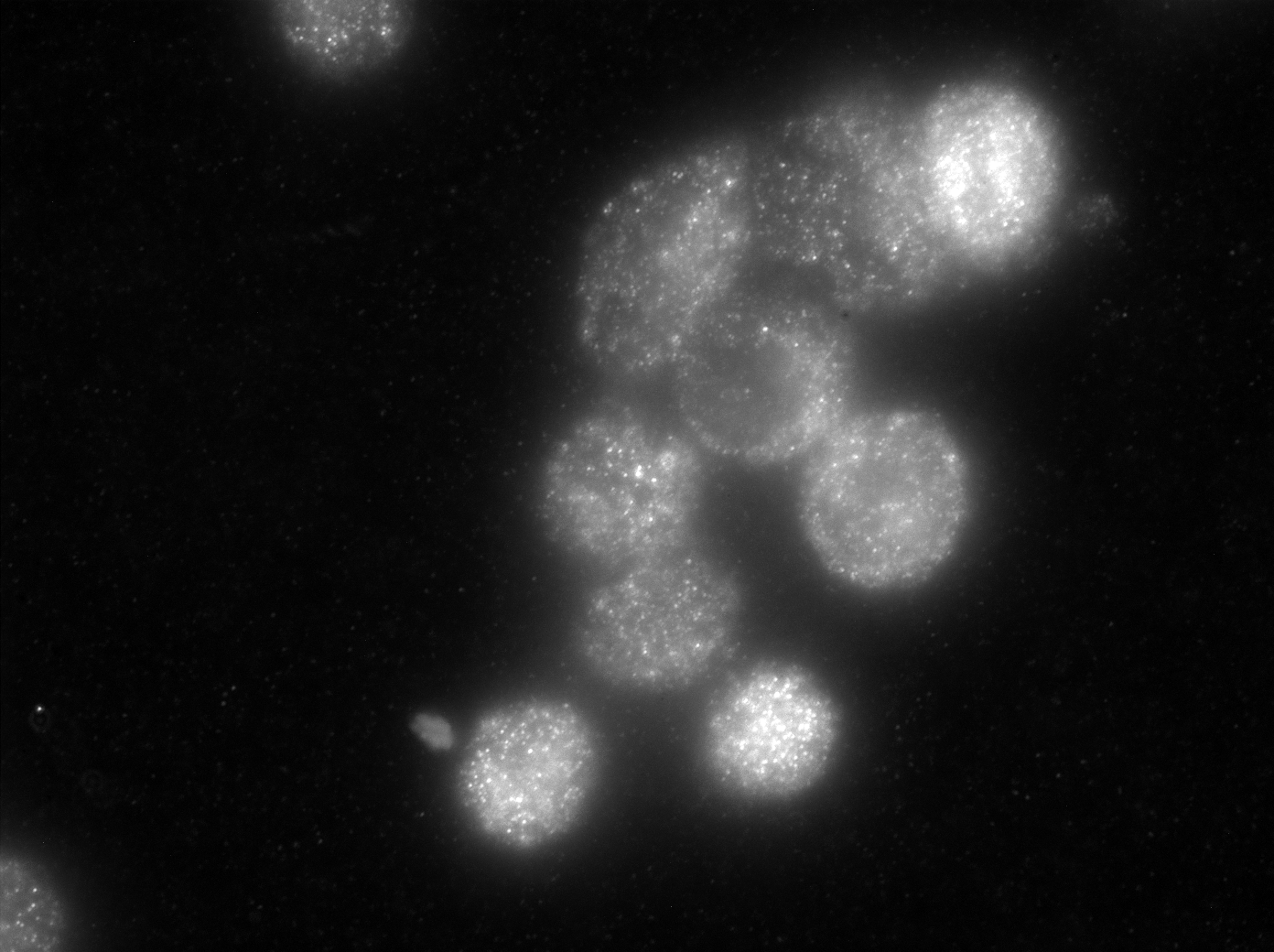

Supplement: Supplementary file 8 — Source data Fig. 2 part 5 [file 44319_2026_742_MOESM8_ESM.zip › Figure 2 Part 5/Fig 2d polye atubulin part 2/WT PolyE-atub 8-14-24 R3 PA4PA5.Project Maximum Z_XY1723842045_Z0_T0_C2.tif]

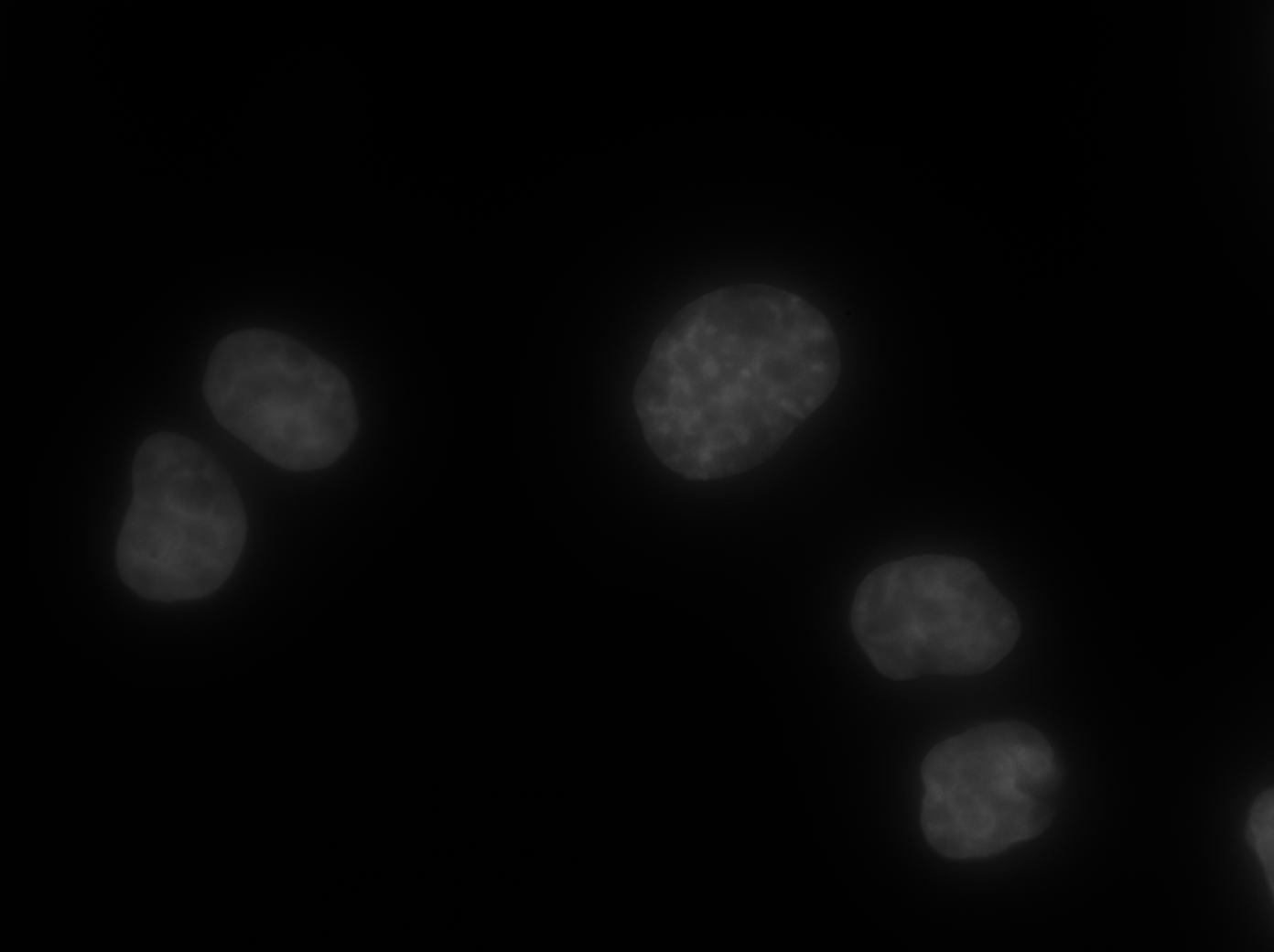

Supplement: Supplementary file 8 — Source data Fig. 2 part 5 [file 44319_2026_742_MOESM8_ESM.zip › Figure 2 Part 5/Fig 2d polye atubulin part 2/WT PolyE-atub 8-14-24 R2 PA9.Project Maximum Z_XY1723838117_Z0_T0_C0.tif]

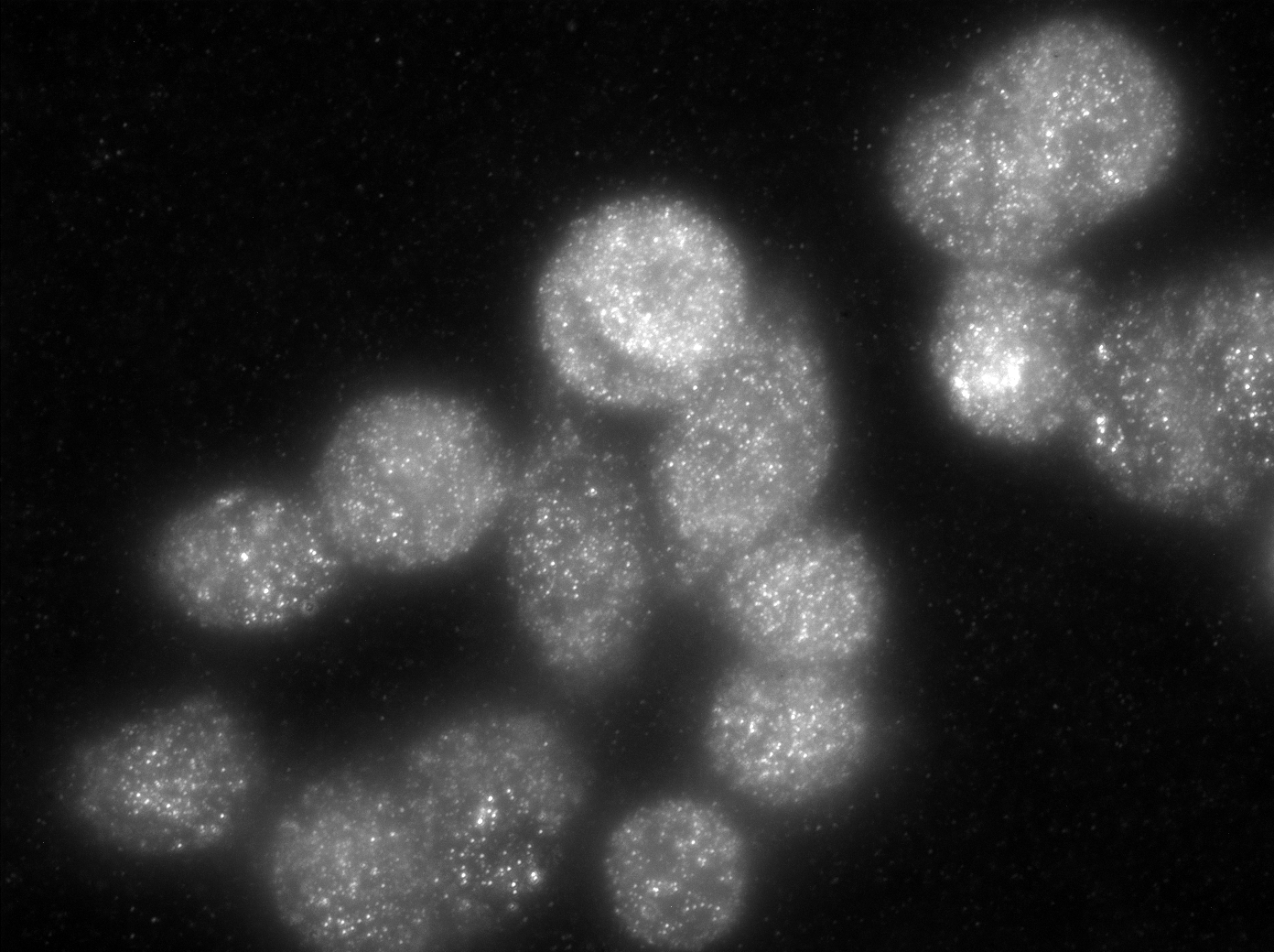

Supplement: Supplementary file 8 — Source data Fig. 2 part 5 [file 44319_2026_742_MOESM8_ESM.zip › Figure 2 Part 5/Fig 2d polye atubulin part 2/WT PolyE-atub 8-14-24 R3 PA6 M3.Project Maximum Z_XY1723842779_Z0_T0_C2.tif]

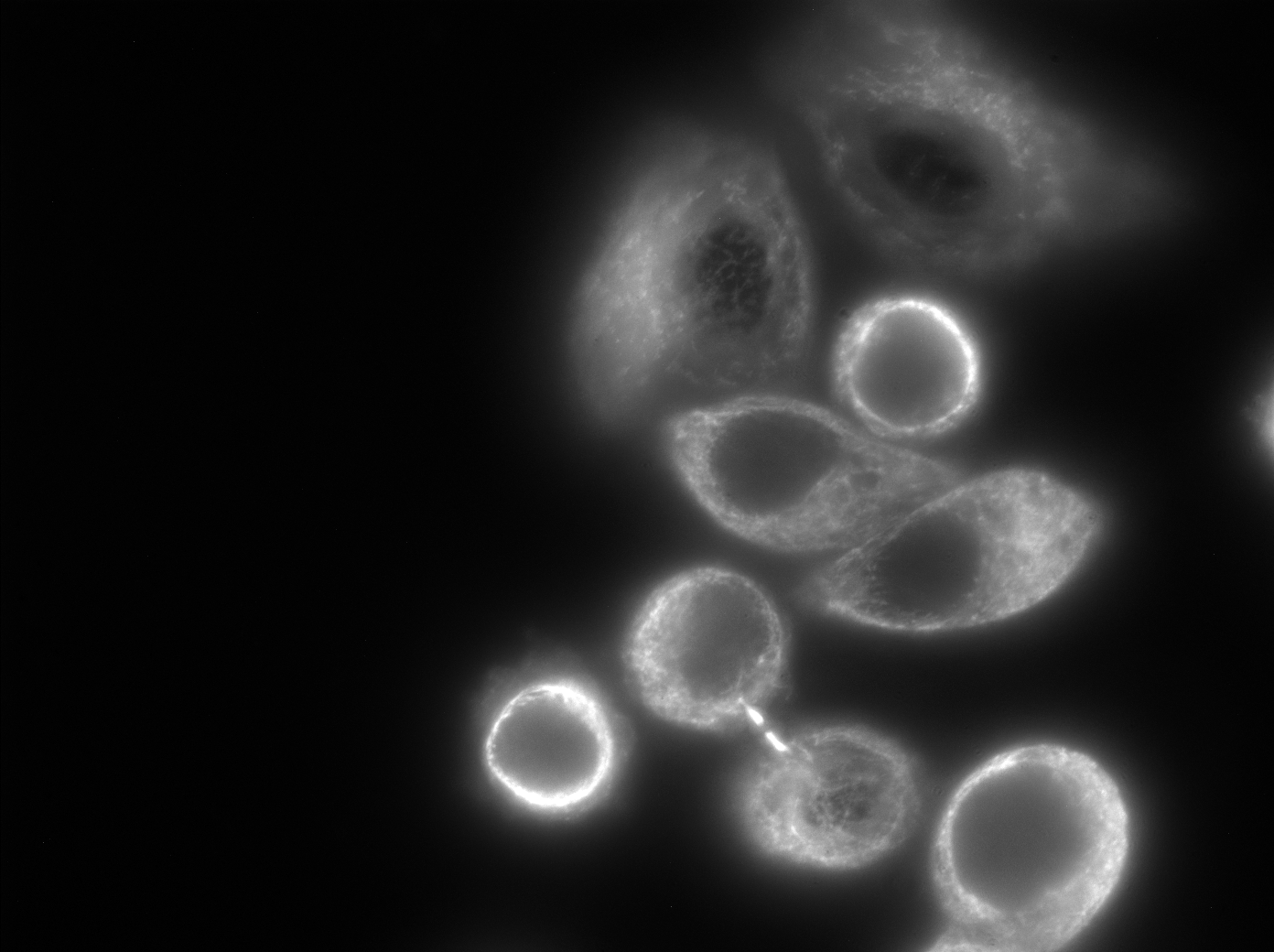

Supplement: Supplementary file 8 — Source data Fig. 2 part 5 [file 44319_2026_742_MOESM8_ESM.zip › Figure 2 Part 5/Fig 2d polye atubulin part 2/WT PolyE-atub 8-14-24 R3 LT7.Project Maximum Z_XY1723843635_Z0_T0_C1.tif]

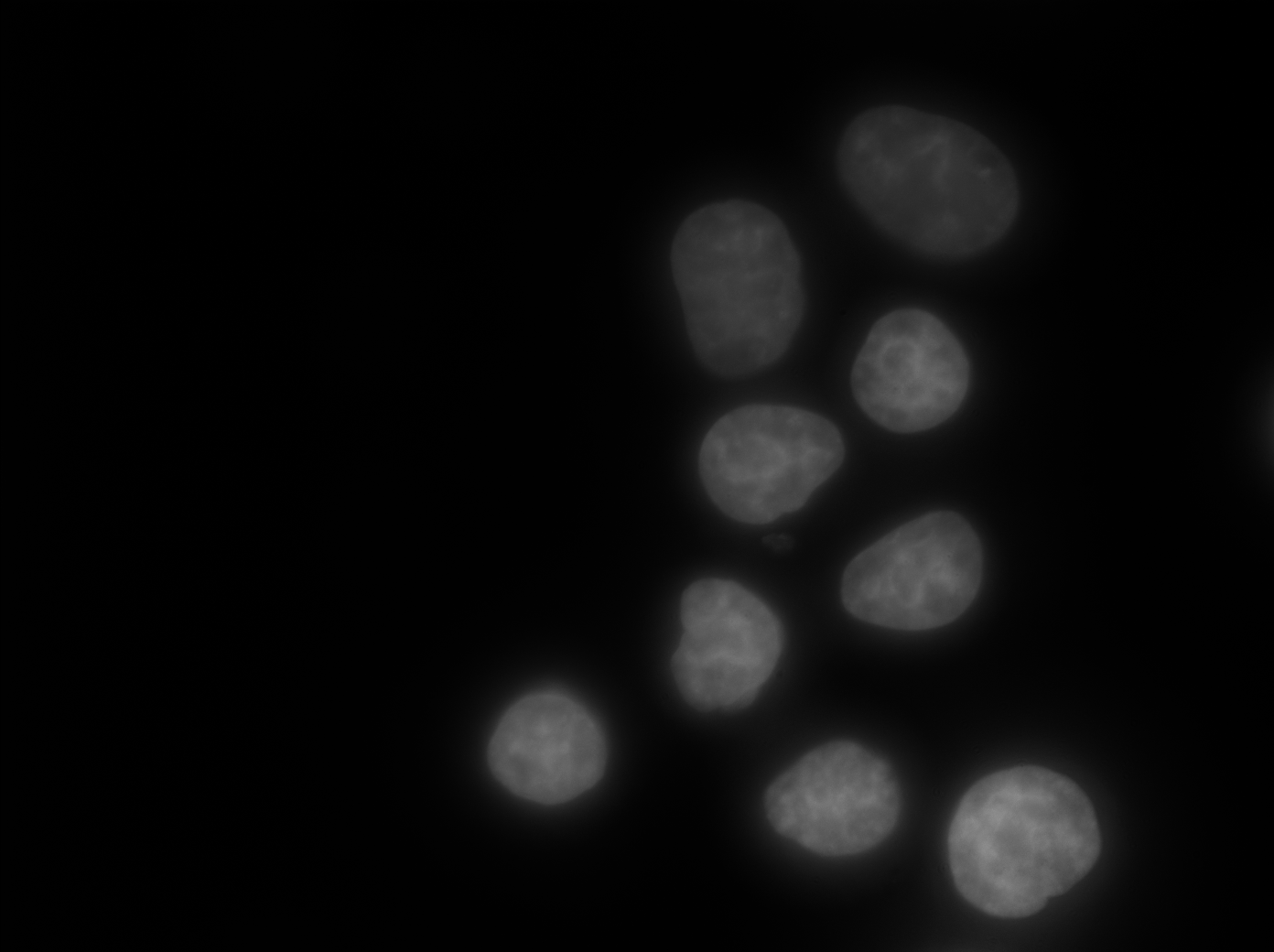

Supplement: Supplementary file 8 — Source data Fig. 2 part 5 [file 44319_2026_742_MOESM8_ESM.zip › Figure 2 Part 5/Fig 2d polye atubulin part 2/WT PolyE-atub 8-14-24 R3 LT7.Project Maximum Z_XY1723843635_Z0_T0_C0.tif]

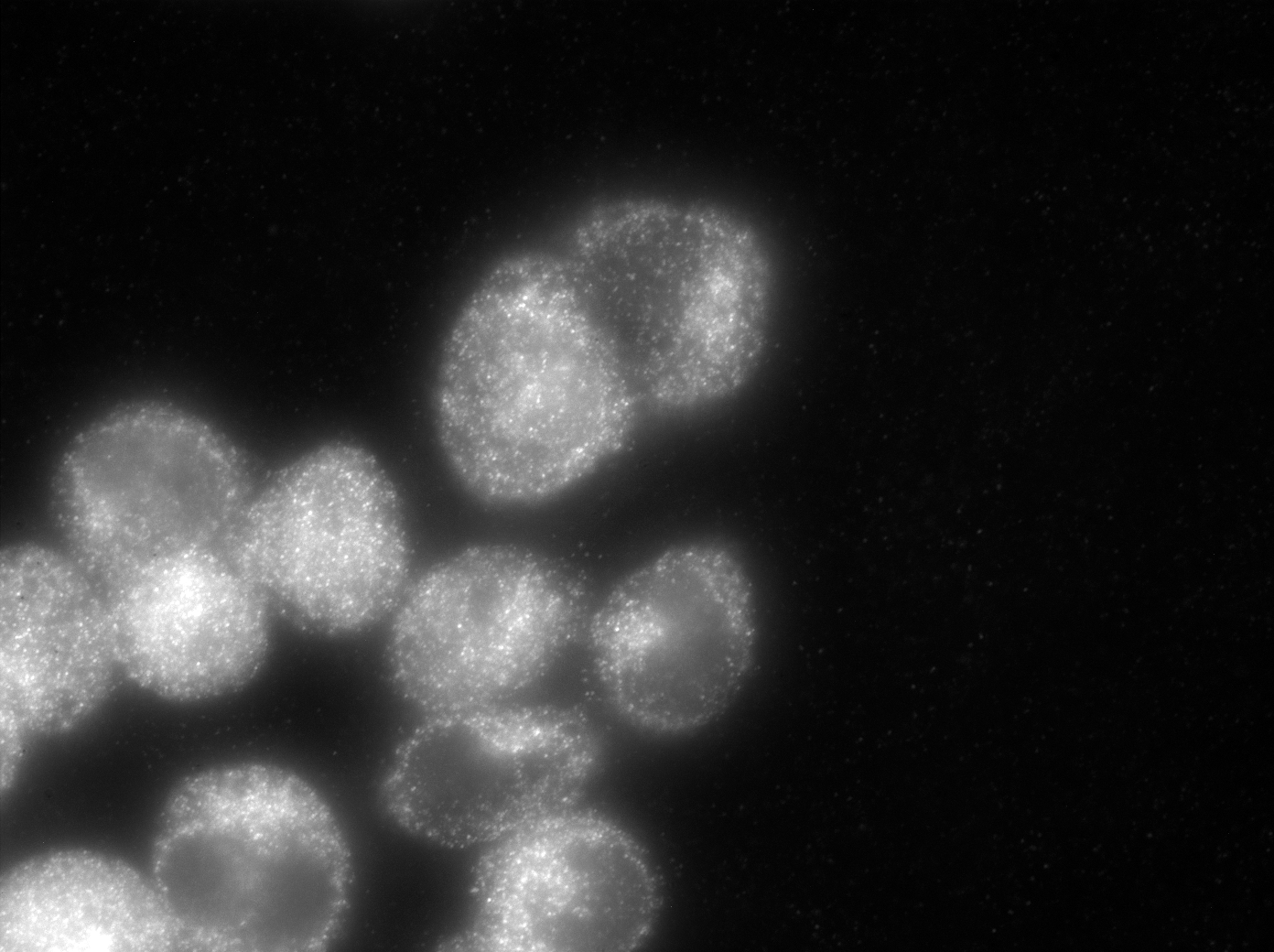

Supplement: Supplementary file 8 — Source data Fig. 2 part 5 [file 44319_2026_742_MOESM8_ESM.zip › Figure 2 Part 5/Fig 2d polye atubulin part 2/WT PolyE-atub 8-14-24 R2 LT4 PA3.Project Maximum Z_XY1723834883_Z0_T0_C2.tif]

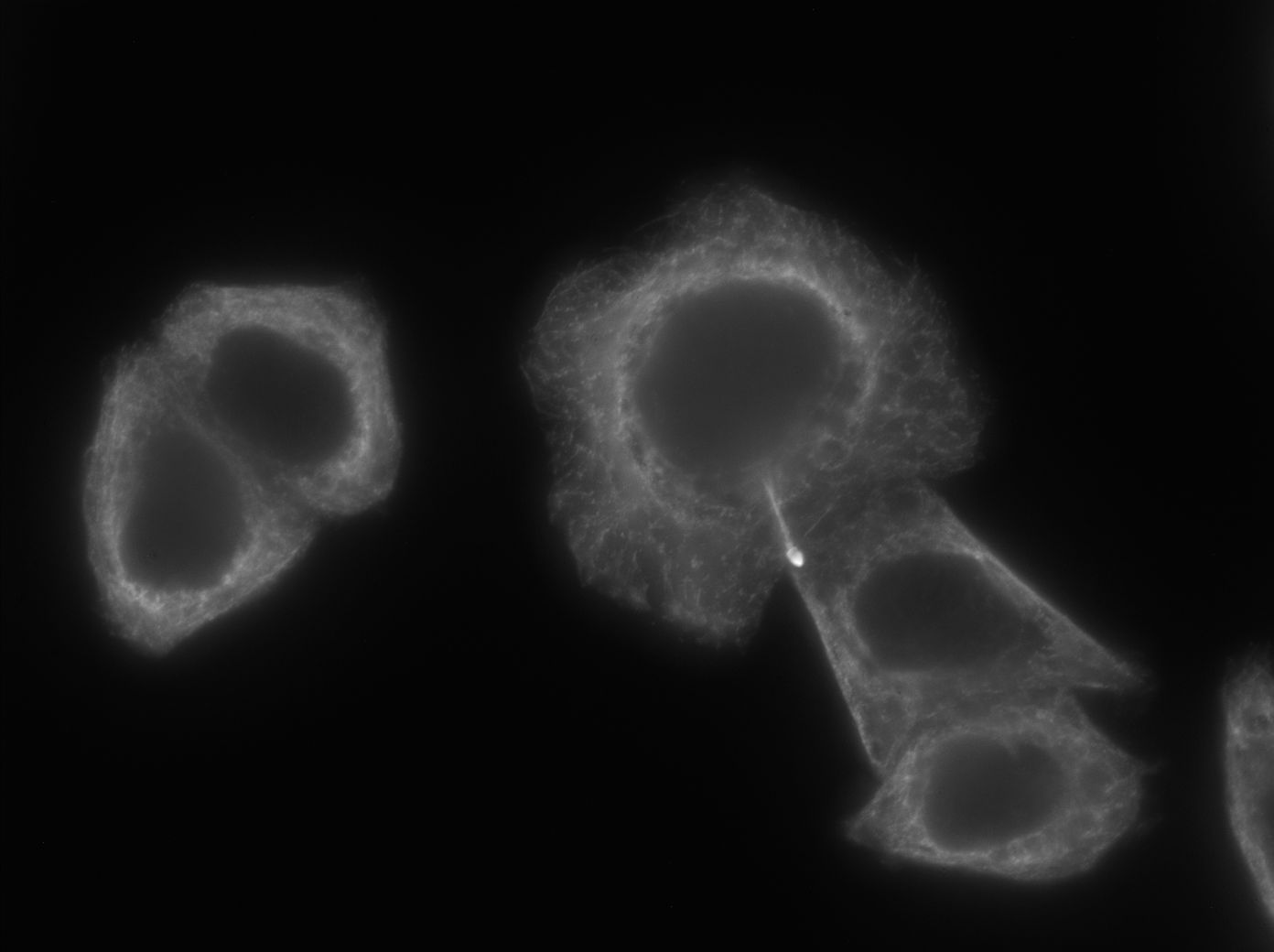

Supplement: Supplementary file 8 — Source data Fig. 2 part 5 [file 44319_2026_742_MOESM8_ESM.zip › Figure 2 Part 5/Fig 2d polye atubulin part 2/WT PolyE-atub 8-14-24 R2 PA9.Project Maximum Z_XY1723838117_Z0_T0_C1.tif]

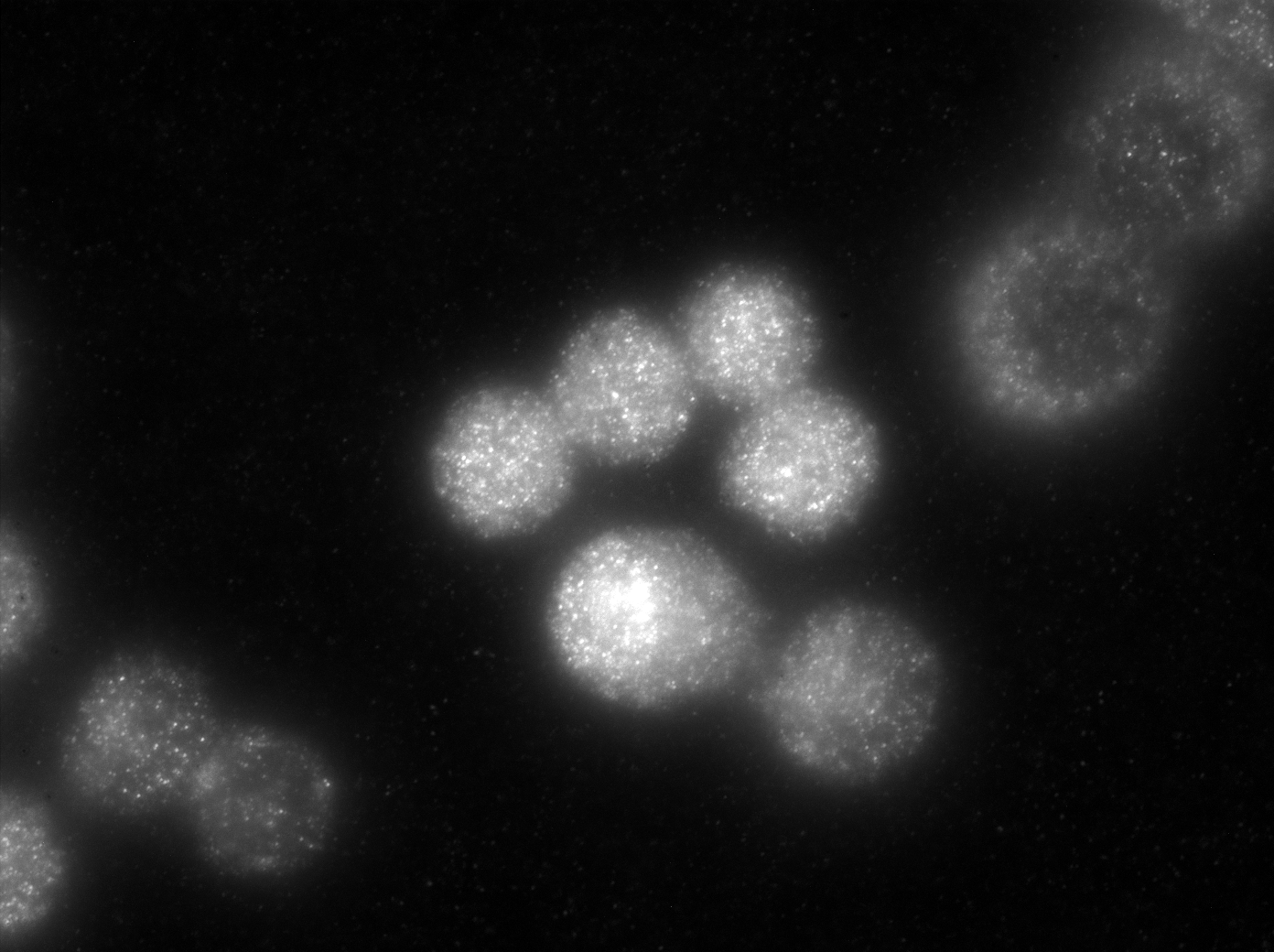

Supplement: Supplementary file 8 — Source data Fig. 2 part 5 [file 44319_2026_742_MOESM8_ESM.zip › Figure 2 Part 5/Fig 2d polye atubulin part 2/WT PolyE-atub 8-14-24 R3 ET3ET4.Project Maximum Z_XY1723842279_Z0_T0_C2.tif]

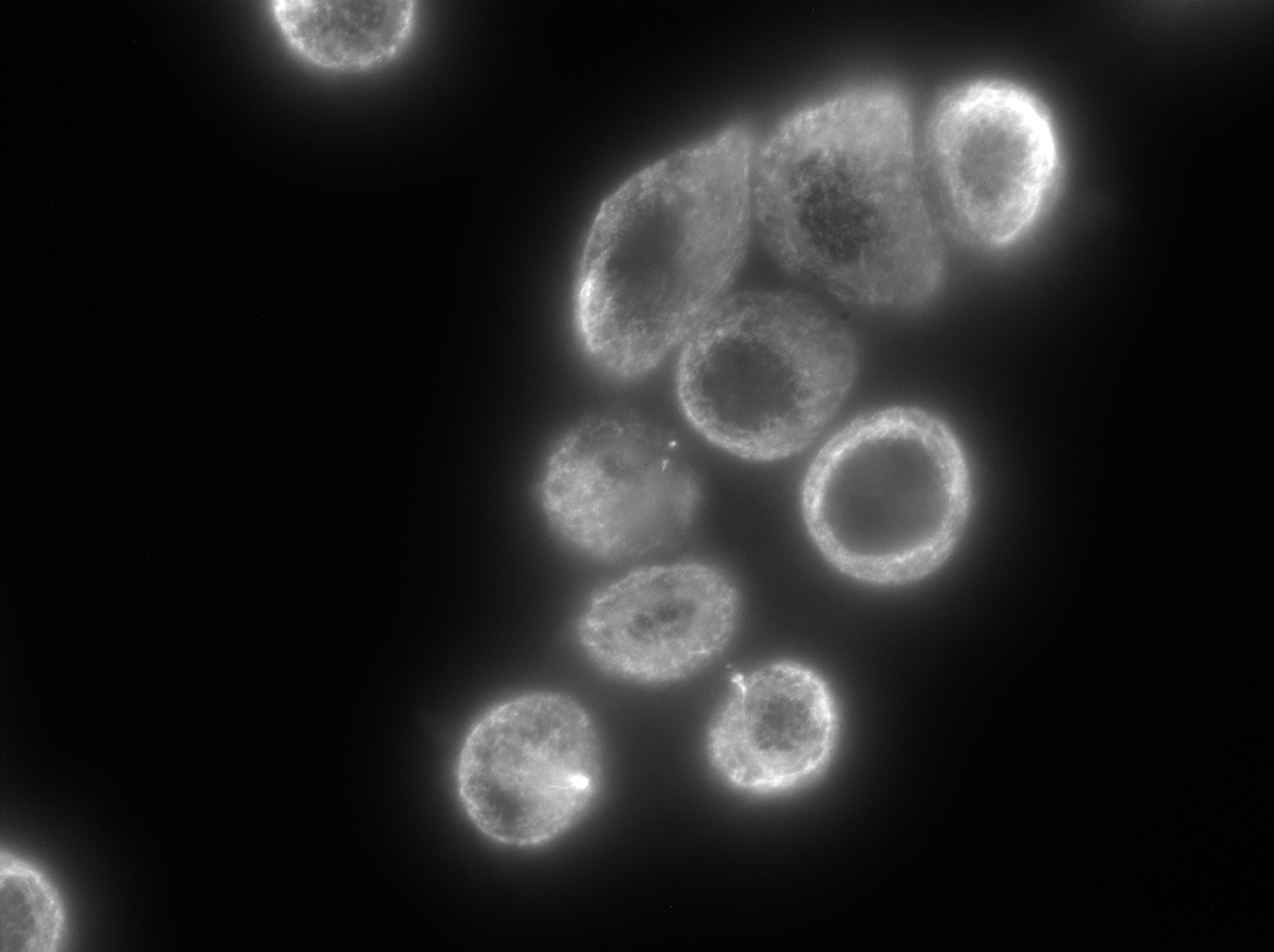

Supplement: Supplementary file 8 — Source data Fig. 2 part 5 [file 44319_2026_742_MOESM8_ESM.zip › Figure 2 Part 5/Fig 2d polye atubulin part 2/WT PolyE-atub 8-14-24 R3 PA4PA5.Project Maximum Z_XY1723842045_Z0_T0_C1.tif]

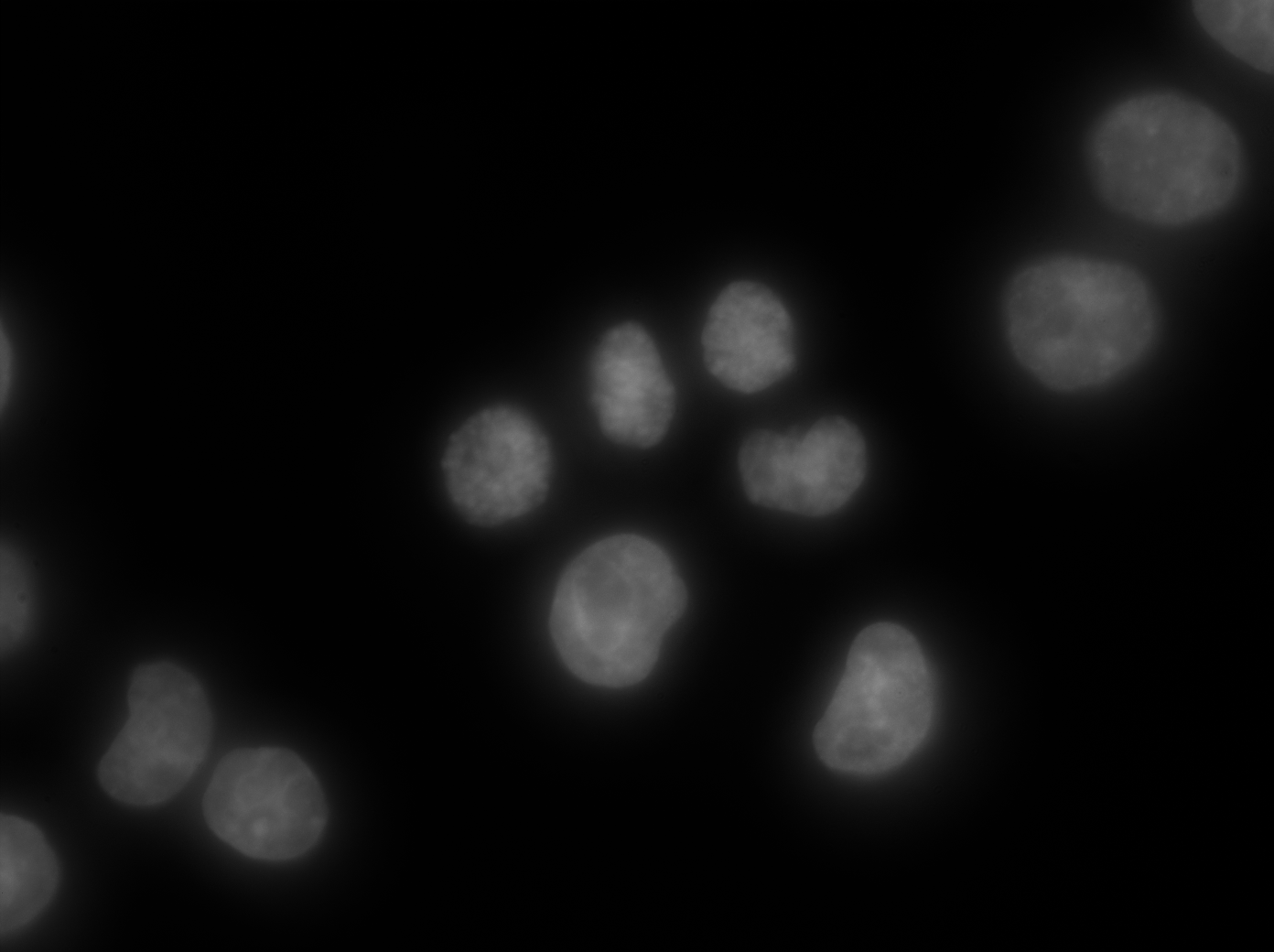

Supplement: Supplementary file 8 — Source data Fig. 2 part 5 [file 44319_2026_742_MOESM8_ESM.zip › Figure 2 Part 5/Fig 2d polye atubulin part 2/WT PolyE-atub 8-14-24 R3 ET3ET4.Project Maximum Z_XY1723842279_Z0_T0_C0.tif]

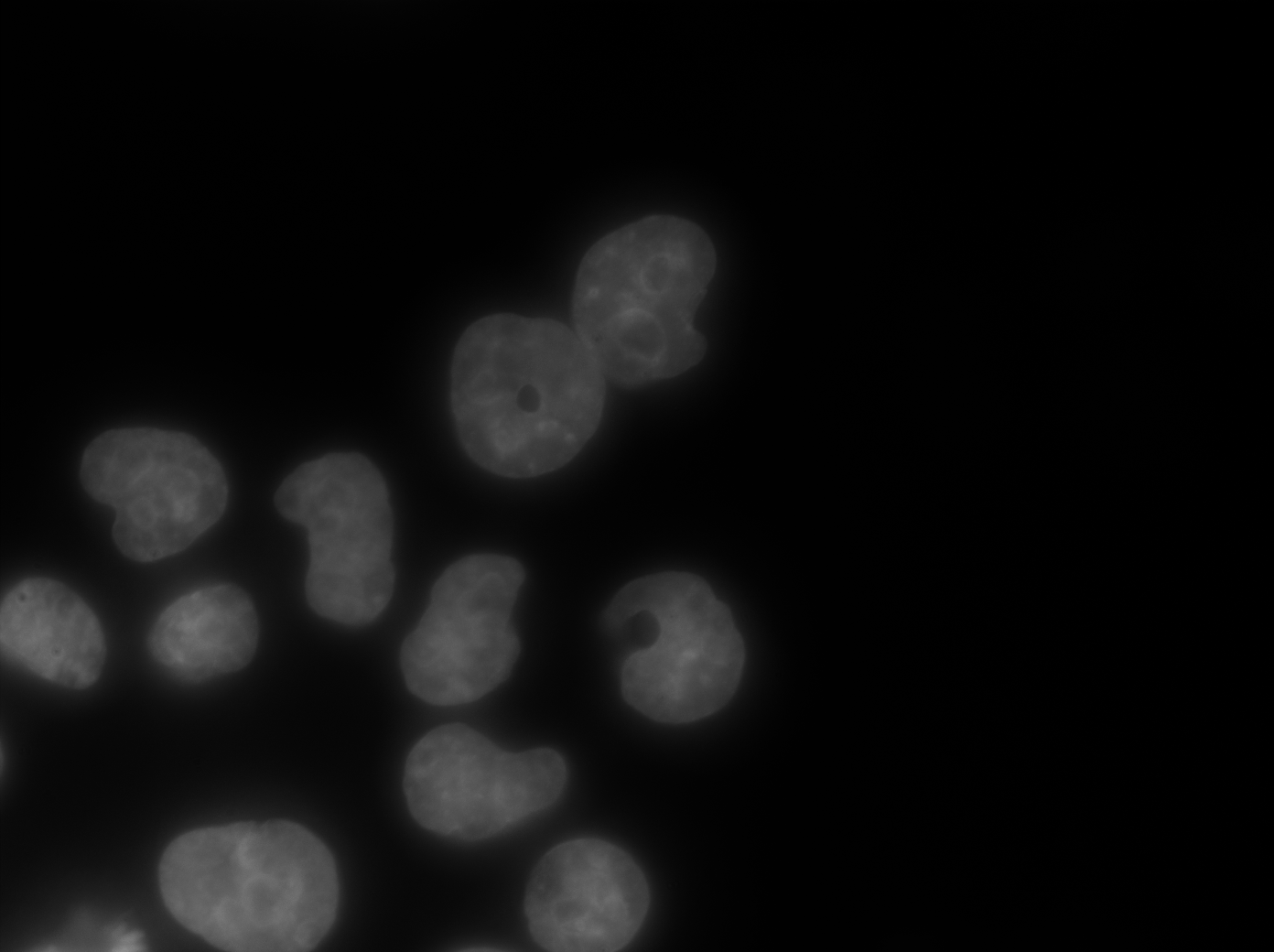

Supplement: Supplementary file 8 — Source data Fig. 2 part 5 [file 44319_2026_742_MOESM8_ESM.zip › Figure 2 Part 5/Fig 2d polye atubulin part 2/WT PolyE-atub 8-14-24 R2 LT4 PA3.Project Maximum Z_XY1723834883_Z0_T0_C0.tif]

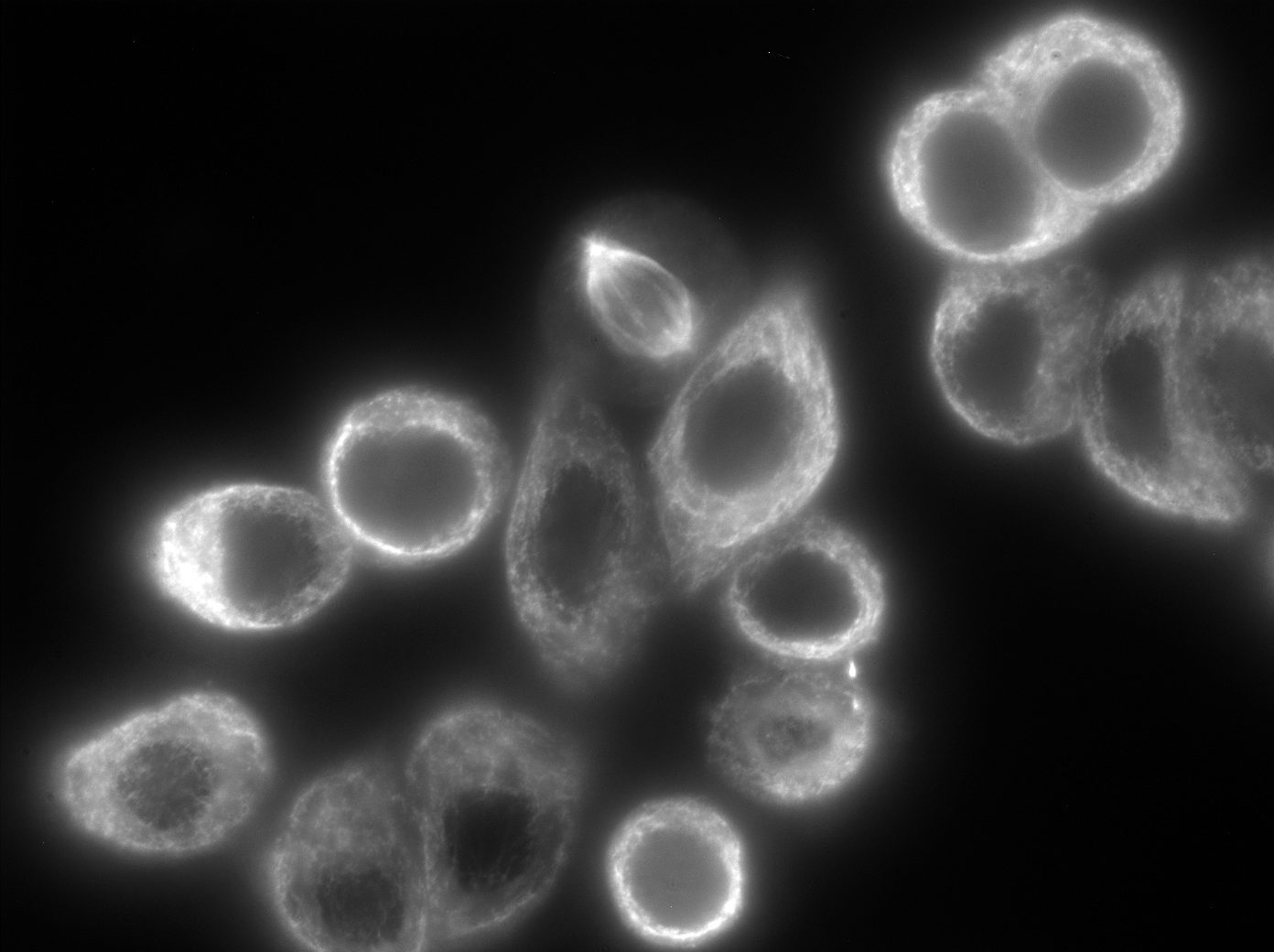

Supplement: Supplementary file 8 — Source data Fig. 2 part 5 [file 44319_2026_742_MOESM8_ESM.zip › Figure 2 Part 5/Fig 2d polye atubulin part 2/WT PolyE-atub 8-14-24 R3 PA6 M3.Project Maximum Z_XY1723842779_Z0_T0_C1.tif]

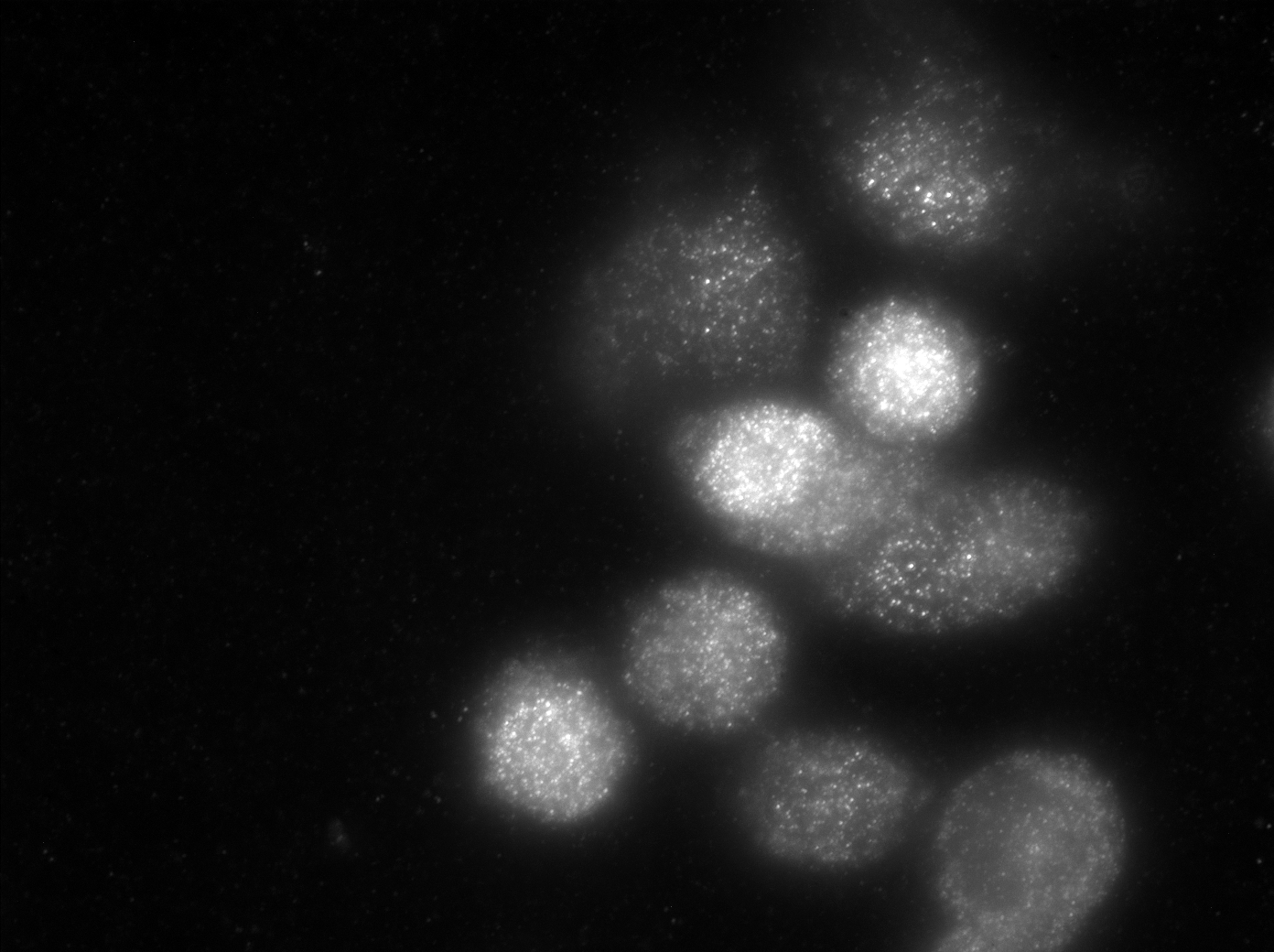

Supplement: Supplementary file 8 — Source data Fig. 2 part 5 [file 44319_2026_742_MOESM8_ESM.zip › Figure 2 Part 5/Fig 2d polye atubulin part 2/WT PolyE-atub 8-14-24 R3 LT7.Project Maximum Z_XY1723843635_Z0_T0_C2.tif]

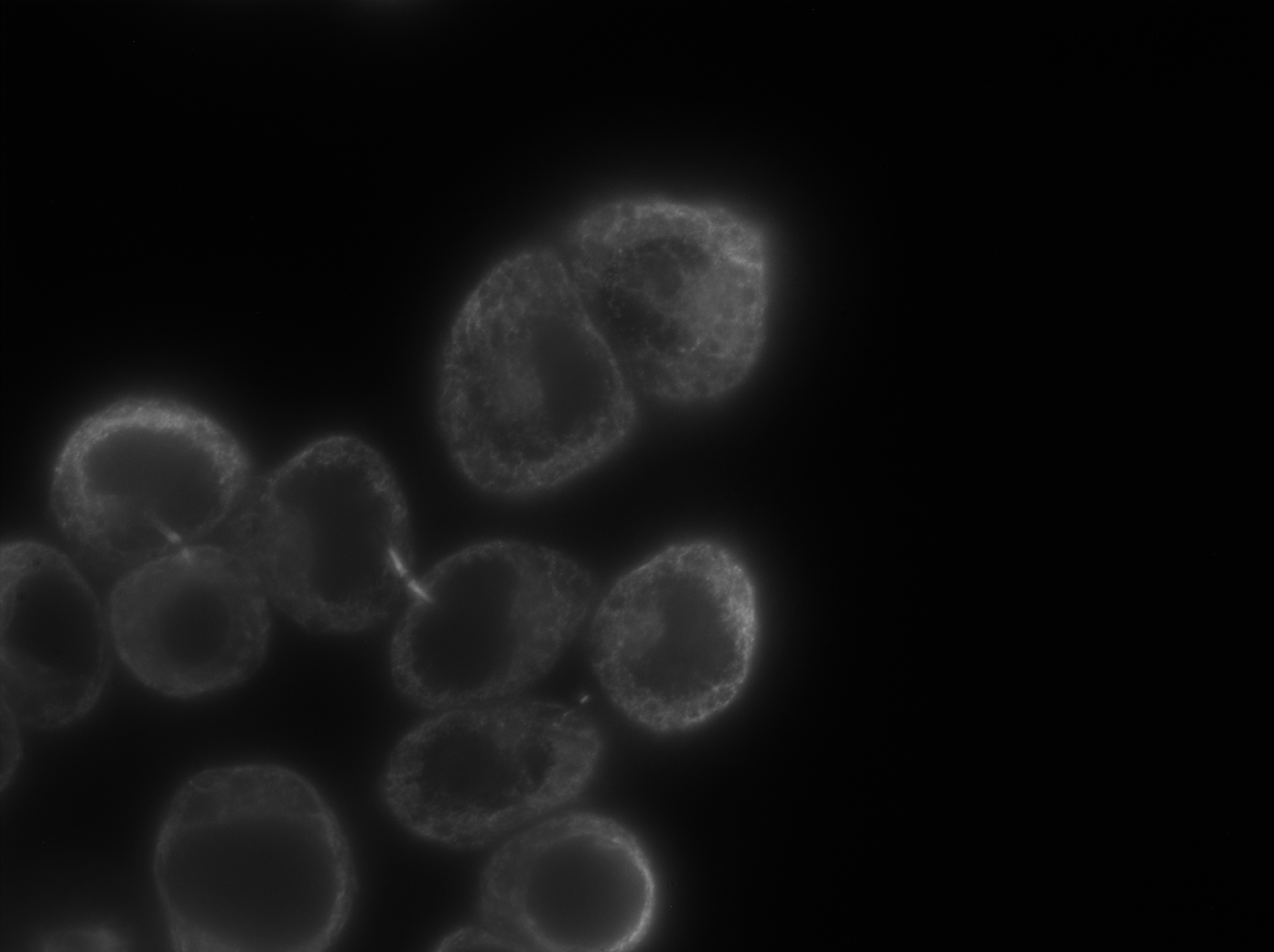

Supplement: Supplementary file 8 — Source data Fig. 2 part 5 [file 44319_2026_742_MOESM8_ESM.zip › Figure 2 Part 5/Fig 2d polye atubulin part 2/WT PolyE-atub 8-14-24 R2 LT4 PA3.Project Maximum Z_XY1723834883_Z0_T0_C1.tif]

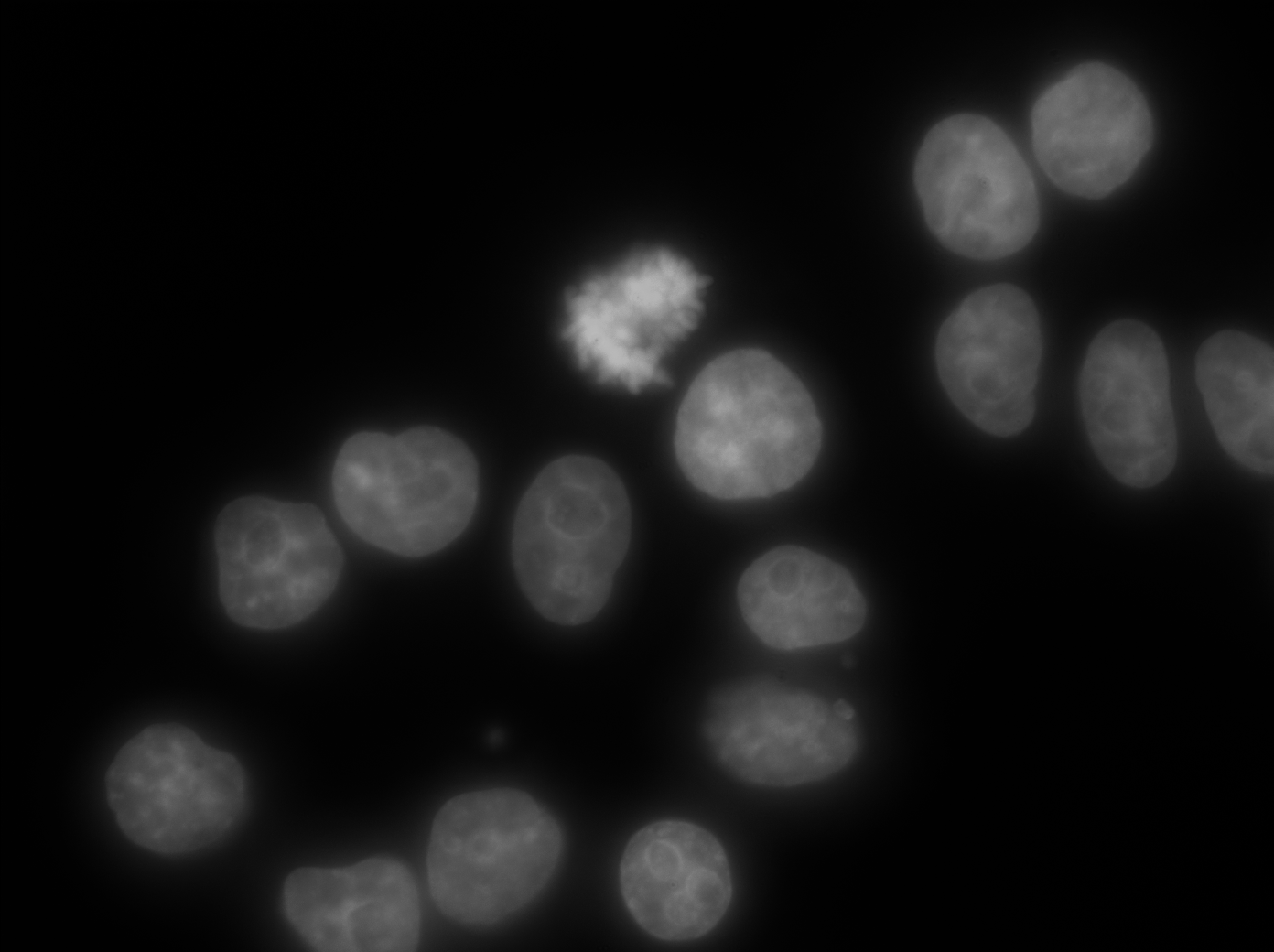

Supplement: Supplementary file 8 — Source data Fig. 2 part 5 [file 44319_2026_742_MOESM8_ESM.zip › Figure 2 Part 5/Fig 2d polye atubulin part 2/WT PolyE-atub 8-14-24 R3 PA6 M3.Project Maximum Z_XY1723842779_Z0_T0_C0.tif]

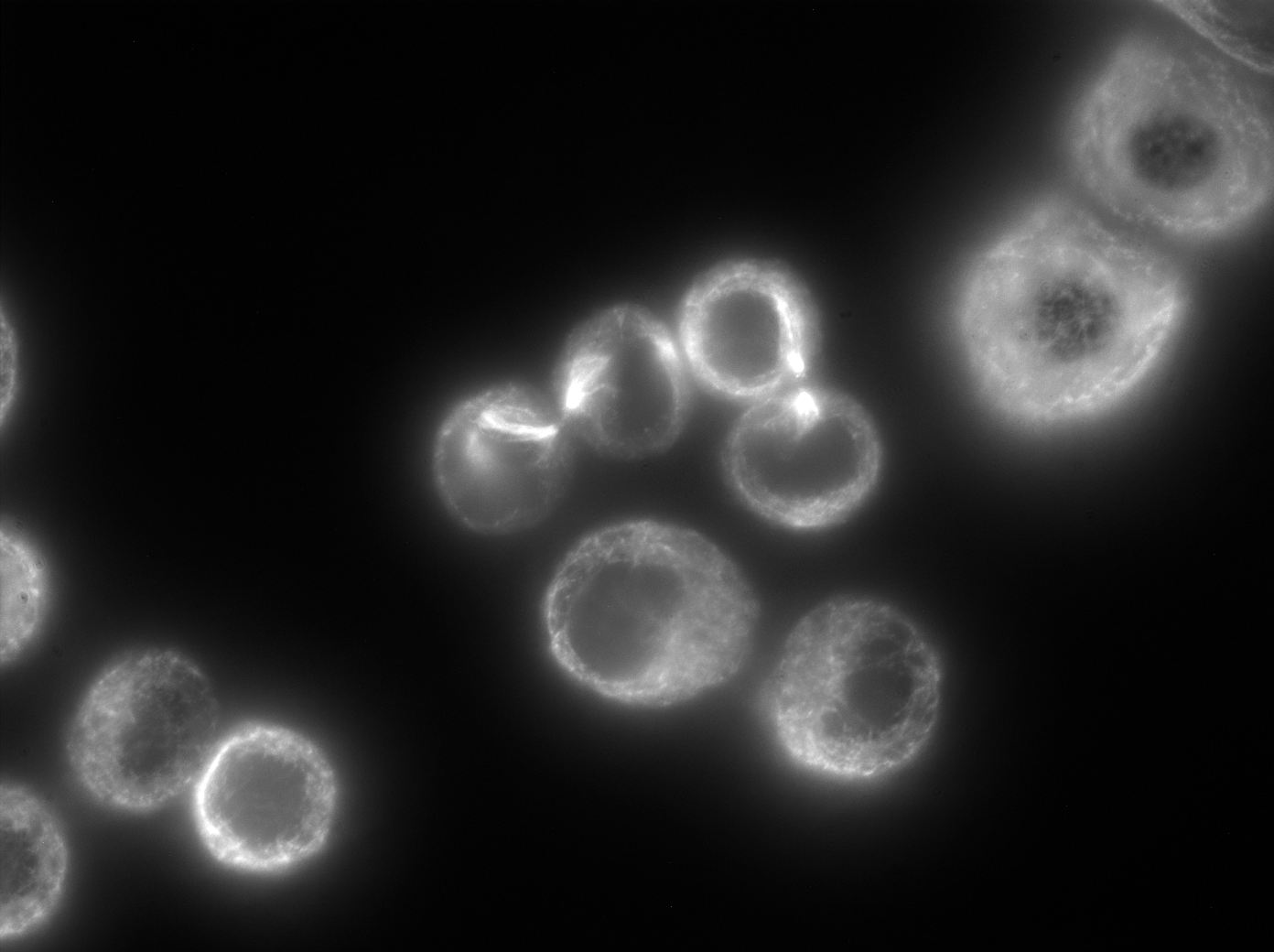

Supplement: Supplementary file 8 — Source data Fig. 2 part 5 [file 44319_2026_742_MOESM8_ESM.zip › Figure 2 Part 5/Fig 2d polye atubulin part 2/WT PolyE-atub 8-14-24 R3 ET3ET4.Project Maximum Z_XY1723842279_Z0_T0_C1.tif]

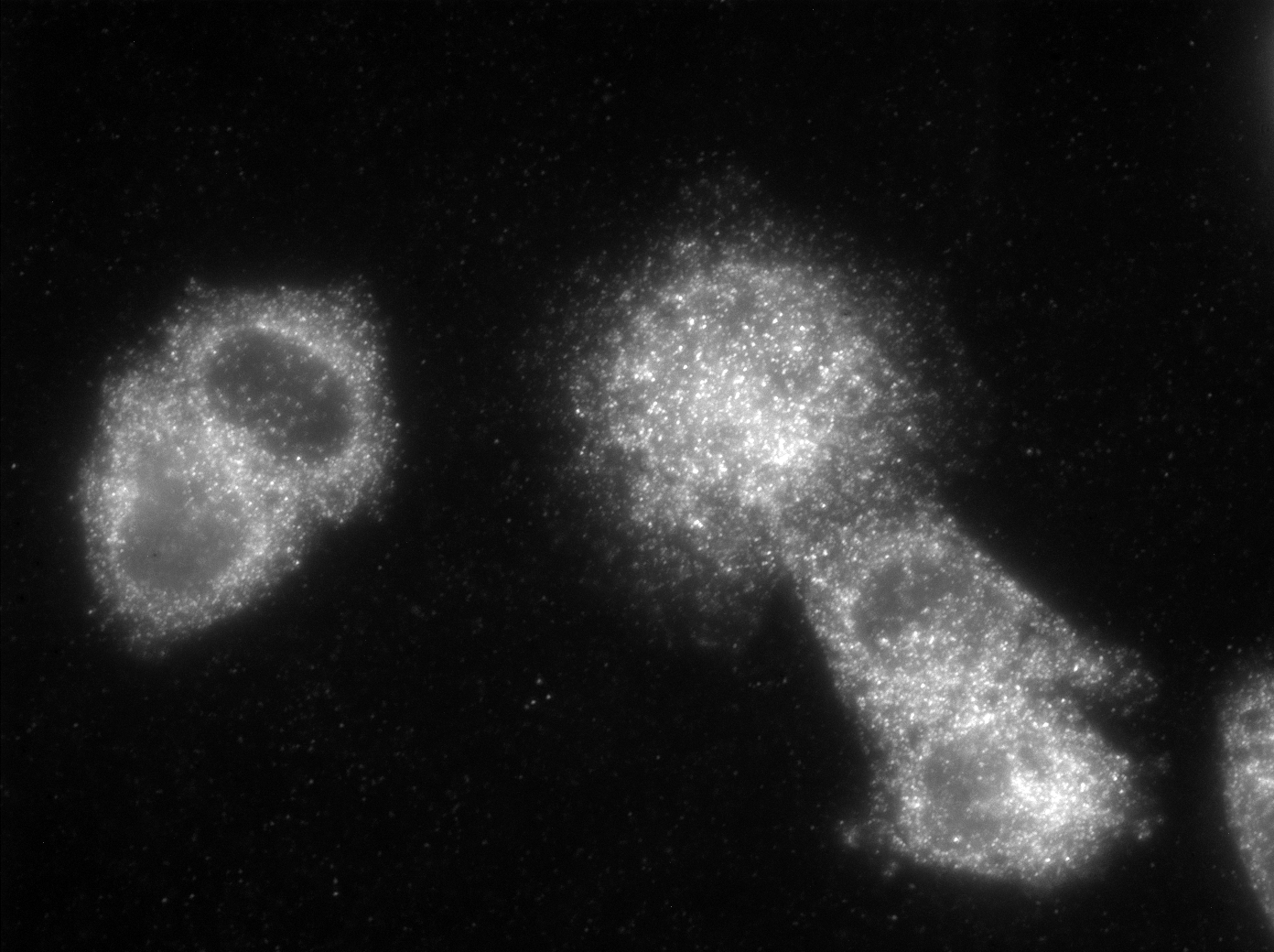

Supplement: Supplementary file 8 — Source data Fig. 2 part 5 [file 44319_2026_742_MOESM8_ESM.zip › Figure 2 Part 5/Fig 2d polye atubulin part 2/WT PolyE-atub 8-14-24 R2 PA9.Project Maximum Z_XY1723838117_Z0_T0_C2.tif]

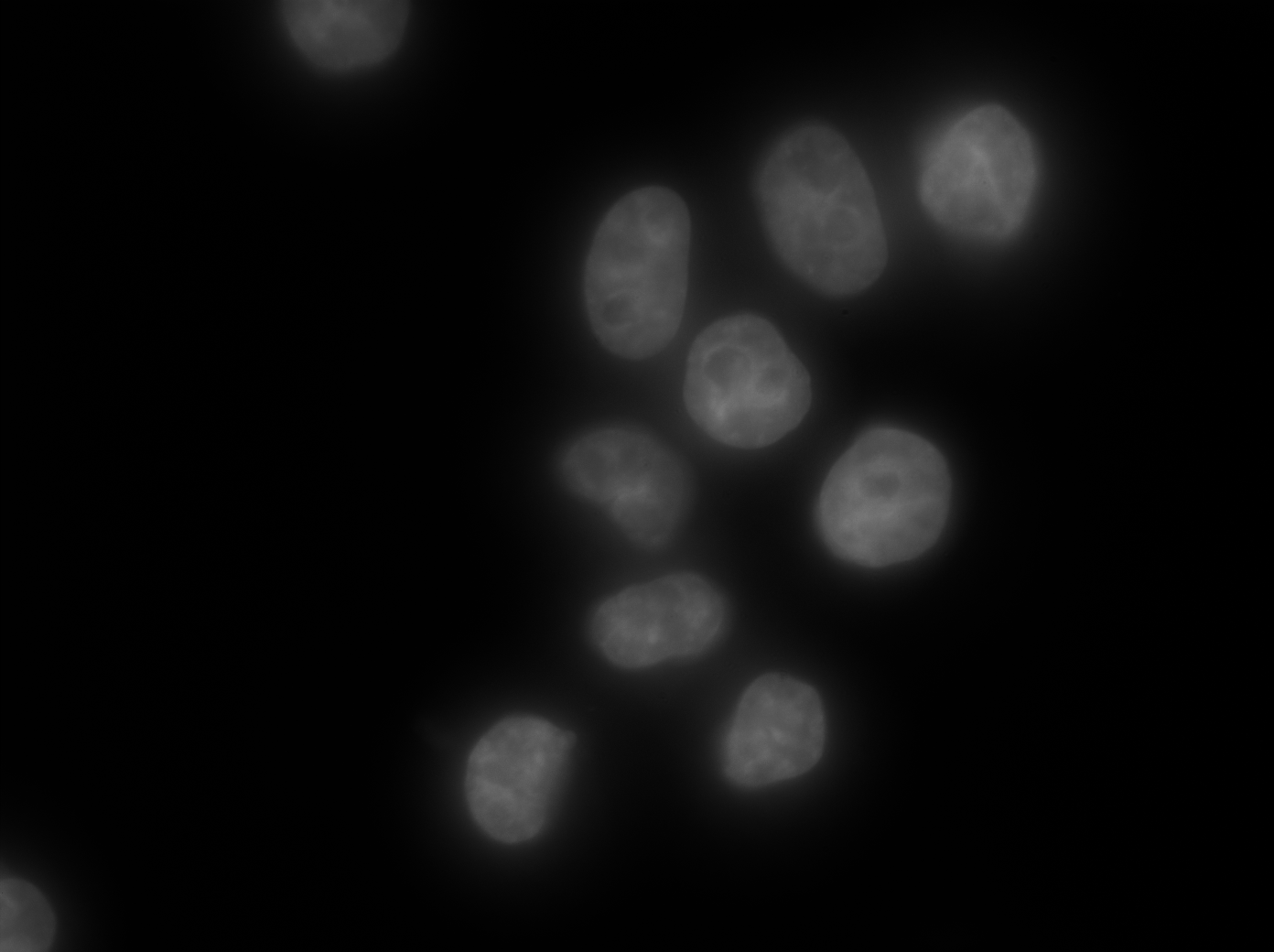

Supplement: Supplementary file 8 — Source data Fig. 2 part 5 [file 44319_2026_742_MOESM8_ESM.zip › Figure 2 Part 5/Fig 2d polye atubulin part 2/WT PolyE-atub 8-14-24 R3 PA4PA5.Project Maximum Z_XY1723842045_Z0_T0_C0.tif]

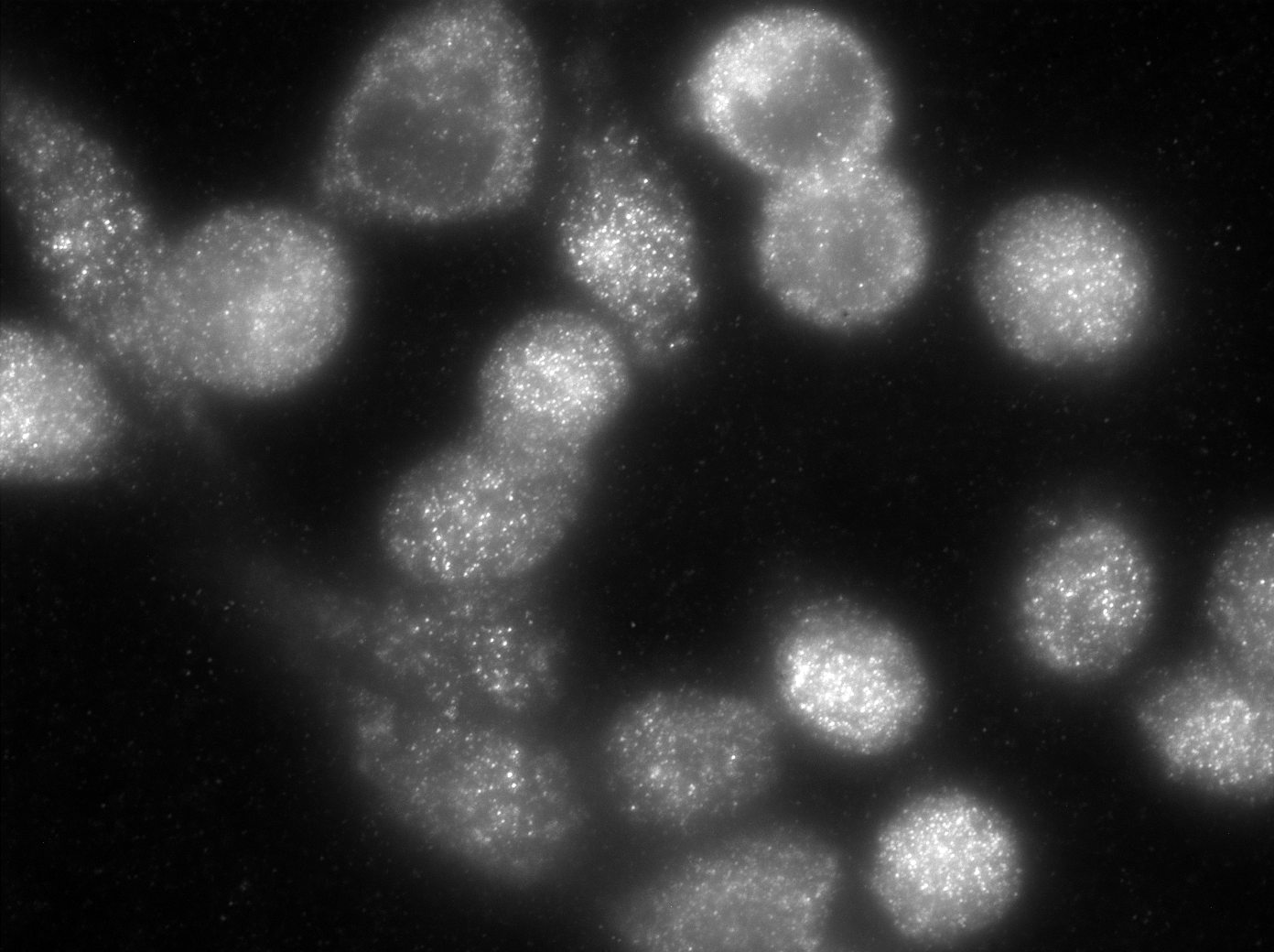

Supplement: Supplementary file 8 — Source data Fig. 2 part 5 [file 44319_2026_742_MOESM8_ESM.zip › Figure 2 Part 5/Fig 2d polye atubulin part 2/WT PolyE-atub 8-14-24 R3 LT9 PA9.Project Maximum Z_XY1723843870_Z0_T0_C2.tif]

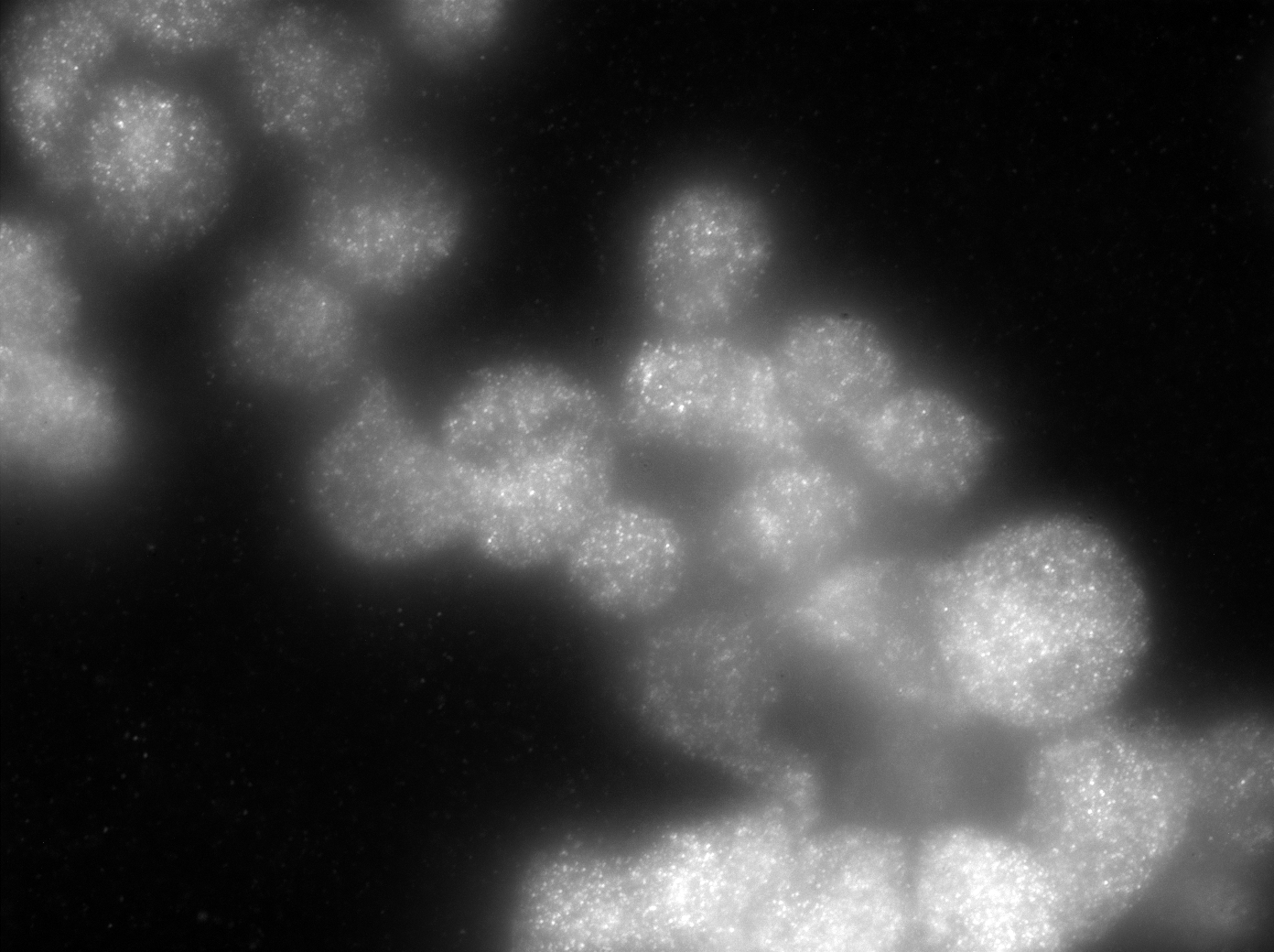

Supplement: Supplementary file 8 — Source data Fig. 2 part 5 [file 44319_2026_742_MOESM8_ESM.zip › Figure 2 Part 5/Fig 2d polye atubulin part 2/WT PolyE-atub 8-14-24 R3 M7M8.Project Maximum Z_XY1723845881_Z0_T0_C2.tif]

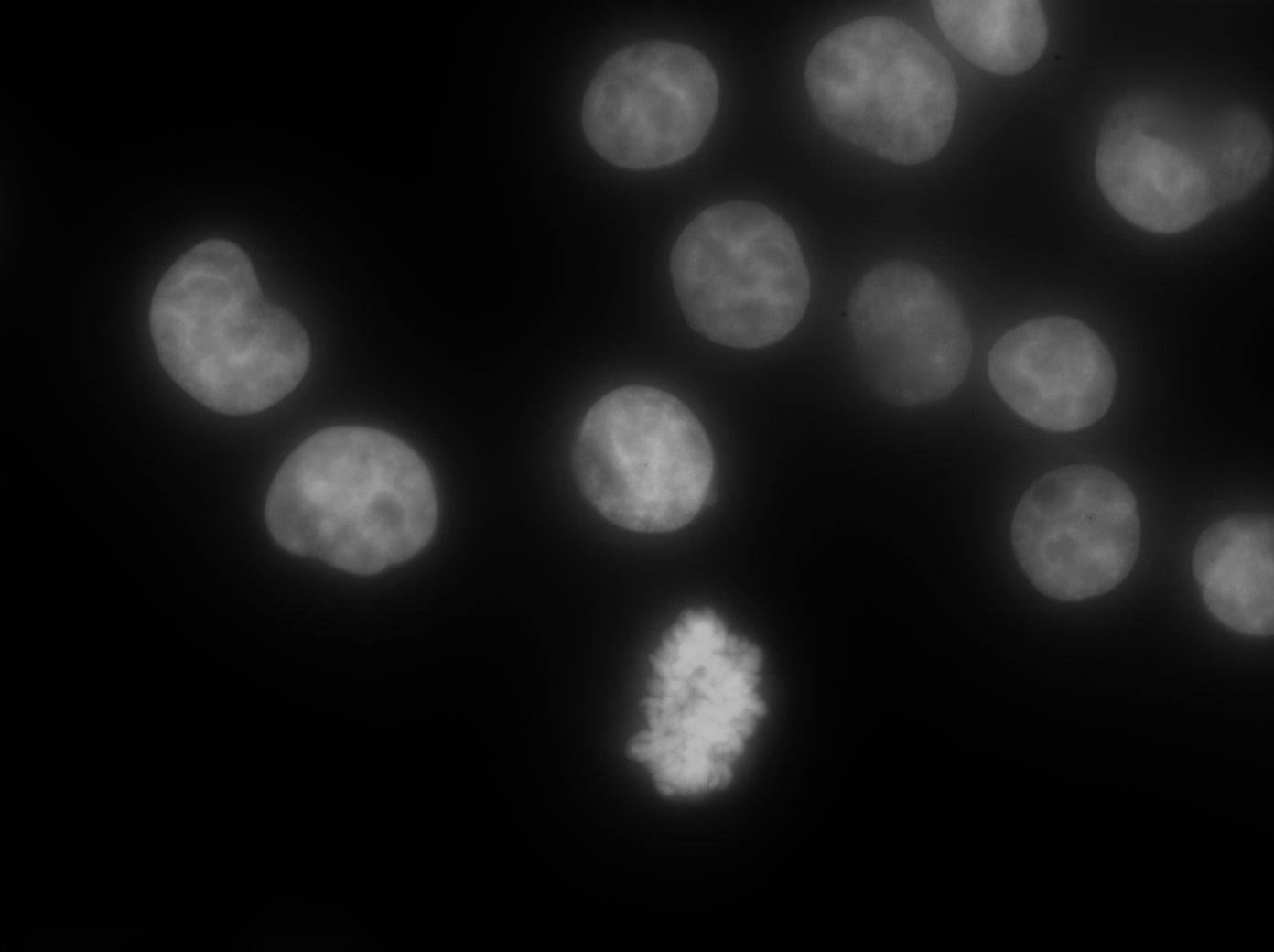

Supplement: Supplementary file 8 — Source data Fig. 2 part 5 [file 44319_2026_742_MOESM8_ESM.zip › Figure 2 Part 5/Fig 2d polye atubulin part 2/WT PolyE-atub 8-14-24 R3 M1.Project Maximum Z_XY1723841228_Z0_T0_C0.tif]

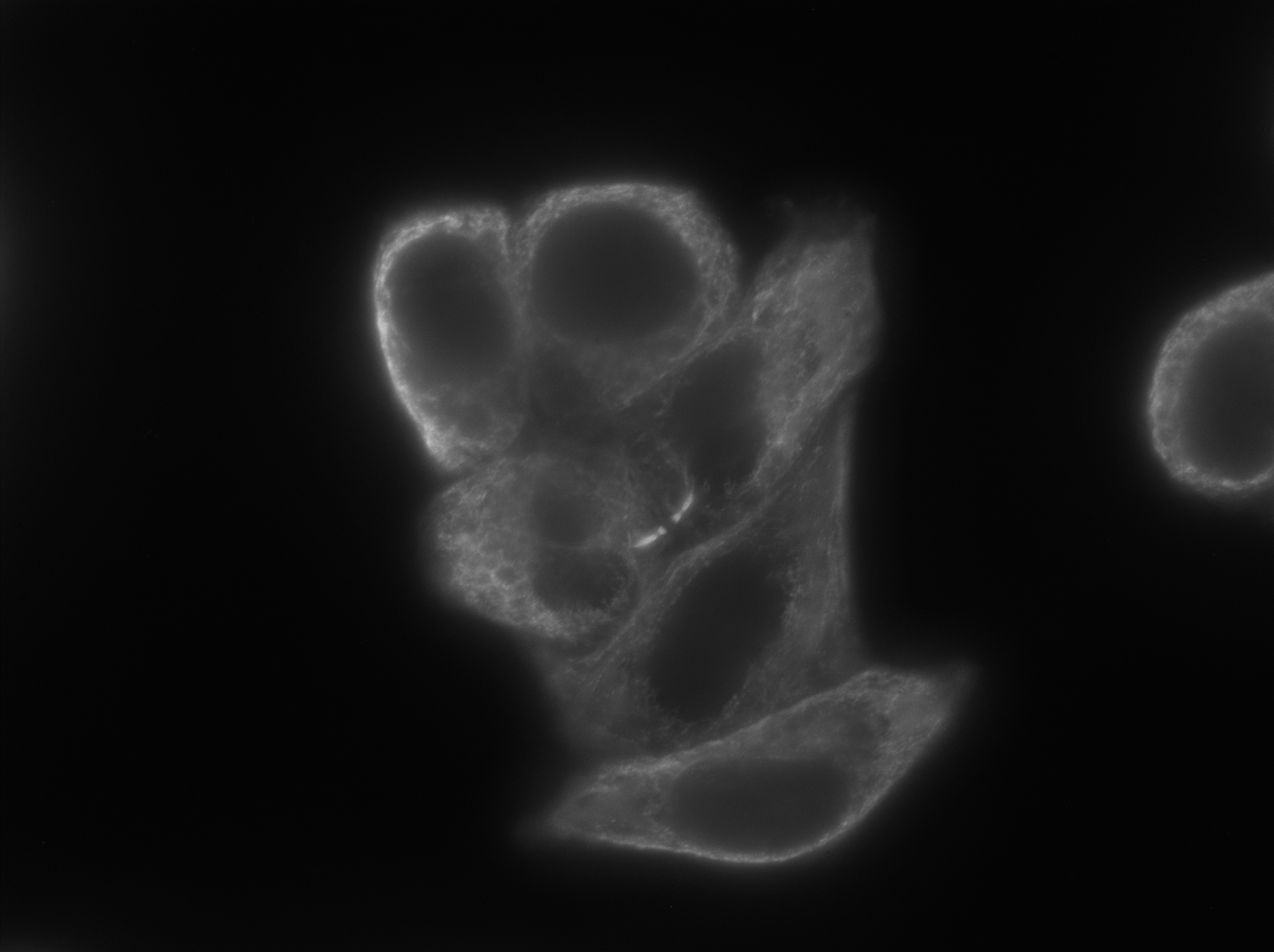

Supplement: Supplementary file 8 — Source data Fig. 2 part 5 [file 44319_2026_742_MOESM8_ESM.zip › Figure 2 Part 5/Fig 2d polye atubulin part 2/WT PolyE-atub 8-14-24 R2 LT10.Project Maximum Z_XY1723837856_Z0_T0_C1.tif]

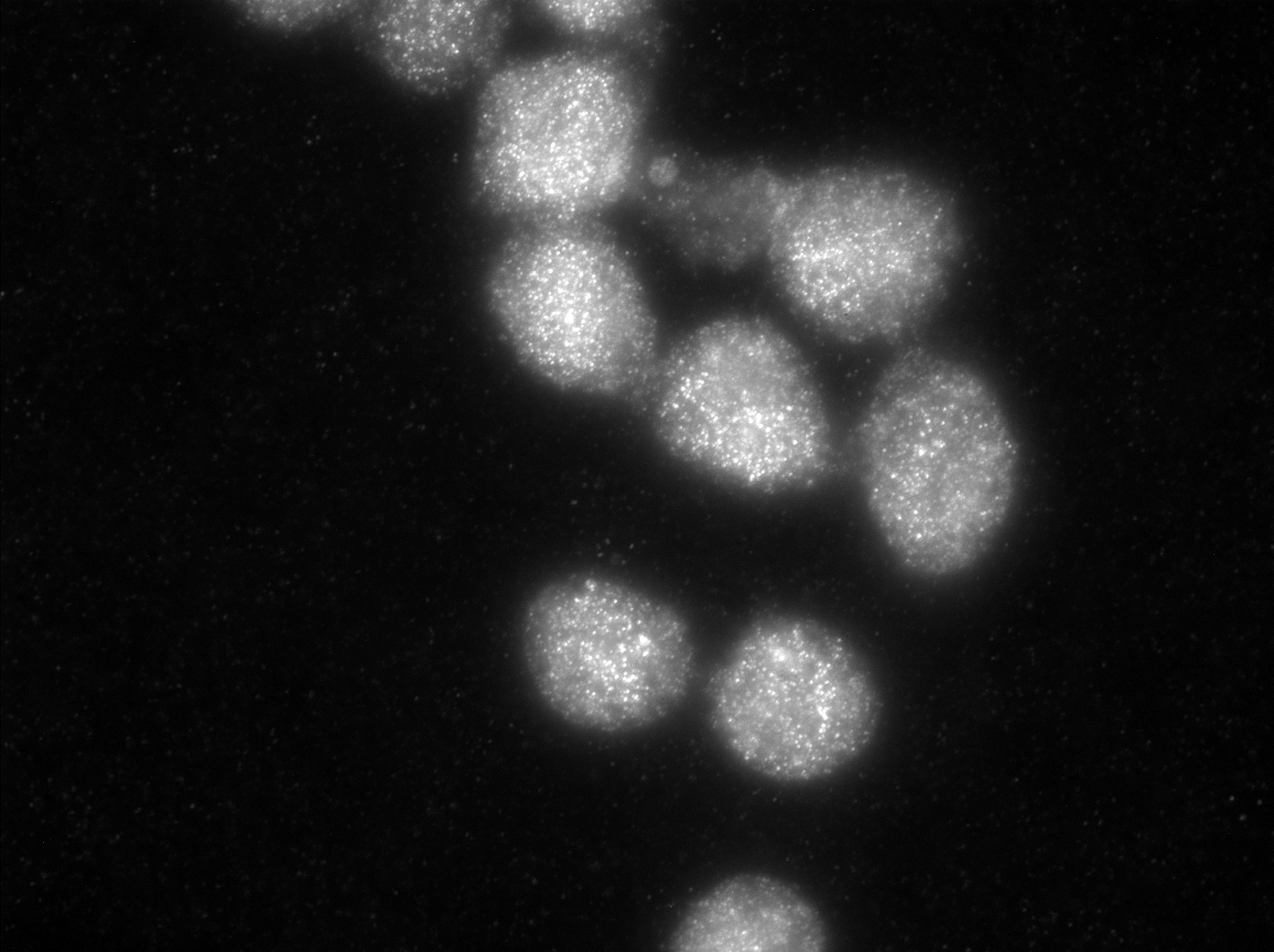

Supplement: Supplementary file 8 — Source data Fig. 2 part 5 [file 44319_2026_742_MOESM8_ESM.zip › Figure 2 Part 5/Fig 2d polye atubulin part 2/WT PolyE-atub 8-14-24 R3 ET7 PA7.Project Maximum Z_XY1723843057_Z0_T0_C2.tif]

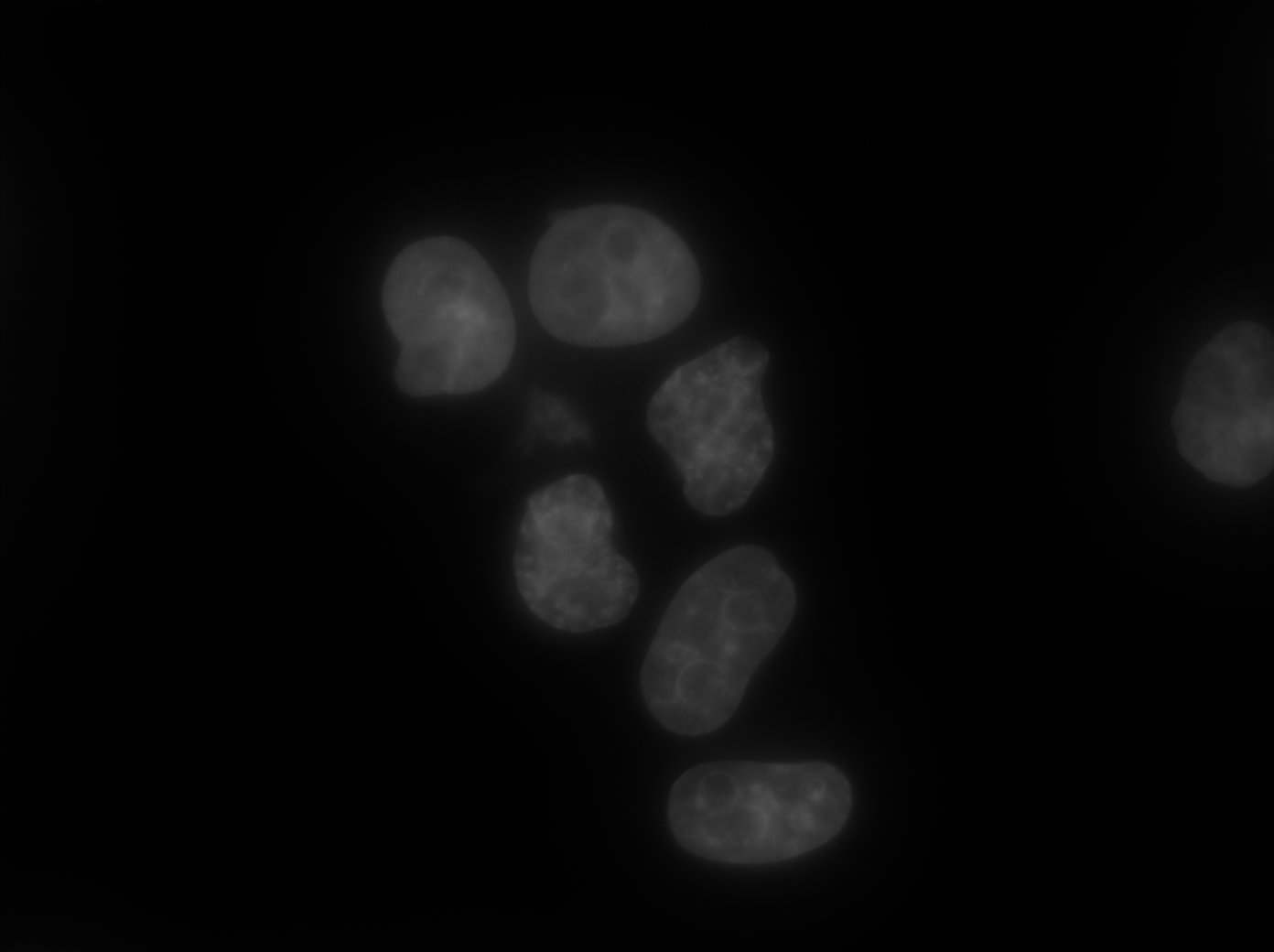

Supplement: Supplementary file 8 — Source data Fig. 2 part 5 [file 44319_2026_742_MOESM8_ESM.zip › Figure 2 Part 5/Fig 2d polye atubulin part 2/WT PolyE-atub 8-14-24 R2 LT10.Project Maximum Z_XY1723837856_Z0_T0_C0.tif]

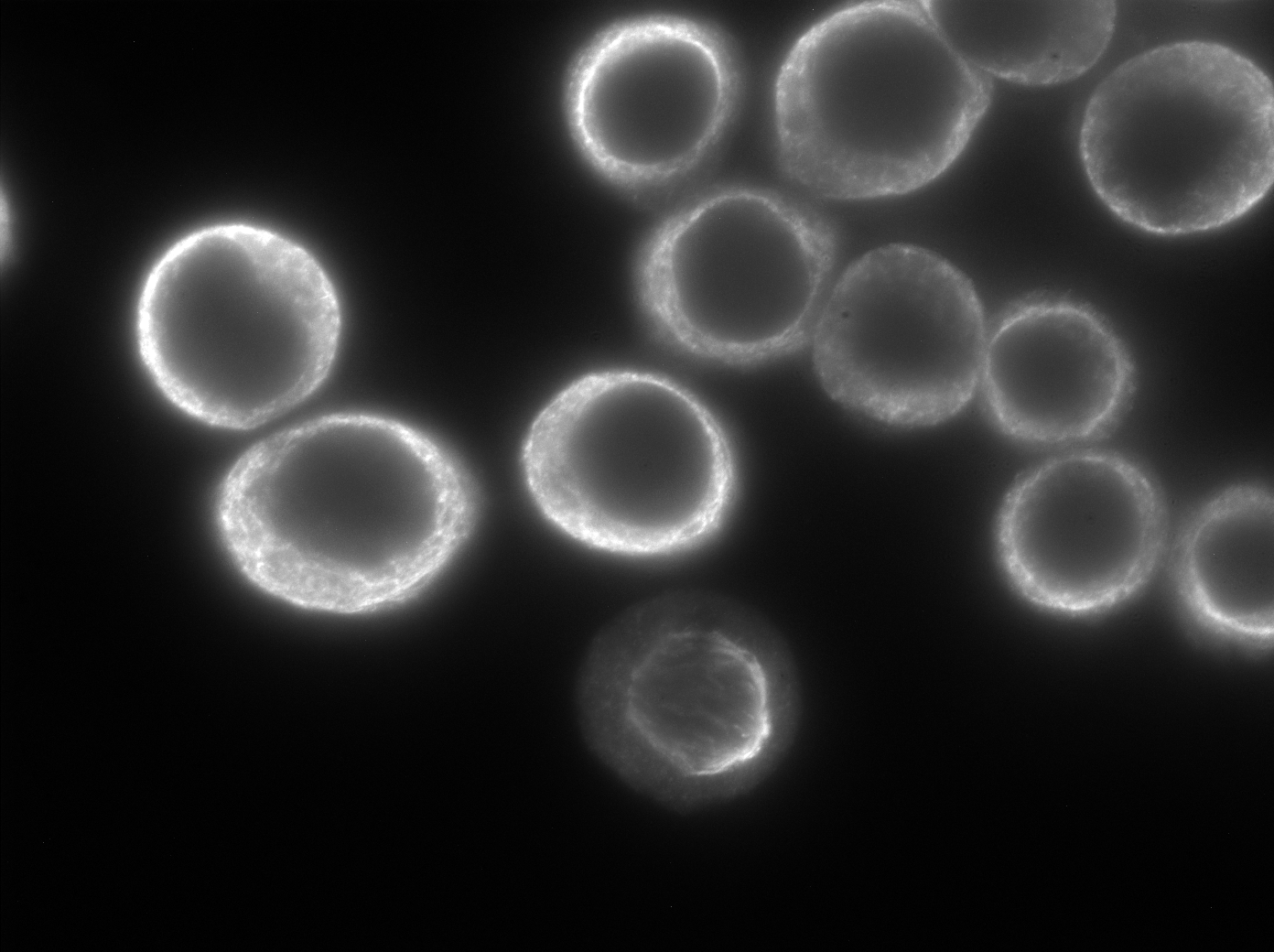

Supplement: Supplementary file 8 — Source data Fig. 2 part 5 [file 44319_2026_742_MOESM8_ESM.zip › Figure 2 Part 5/Fig 2d polye atubulin part 2/WT PolyE-atub 8-14-24 R3 M1.Project Maximum Z_XY1723841228_Z0_T0_C1.tif]

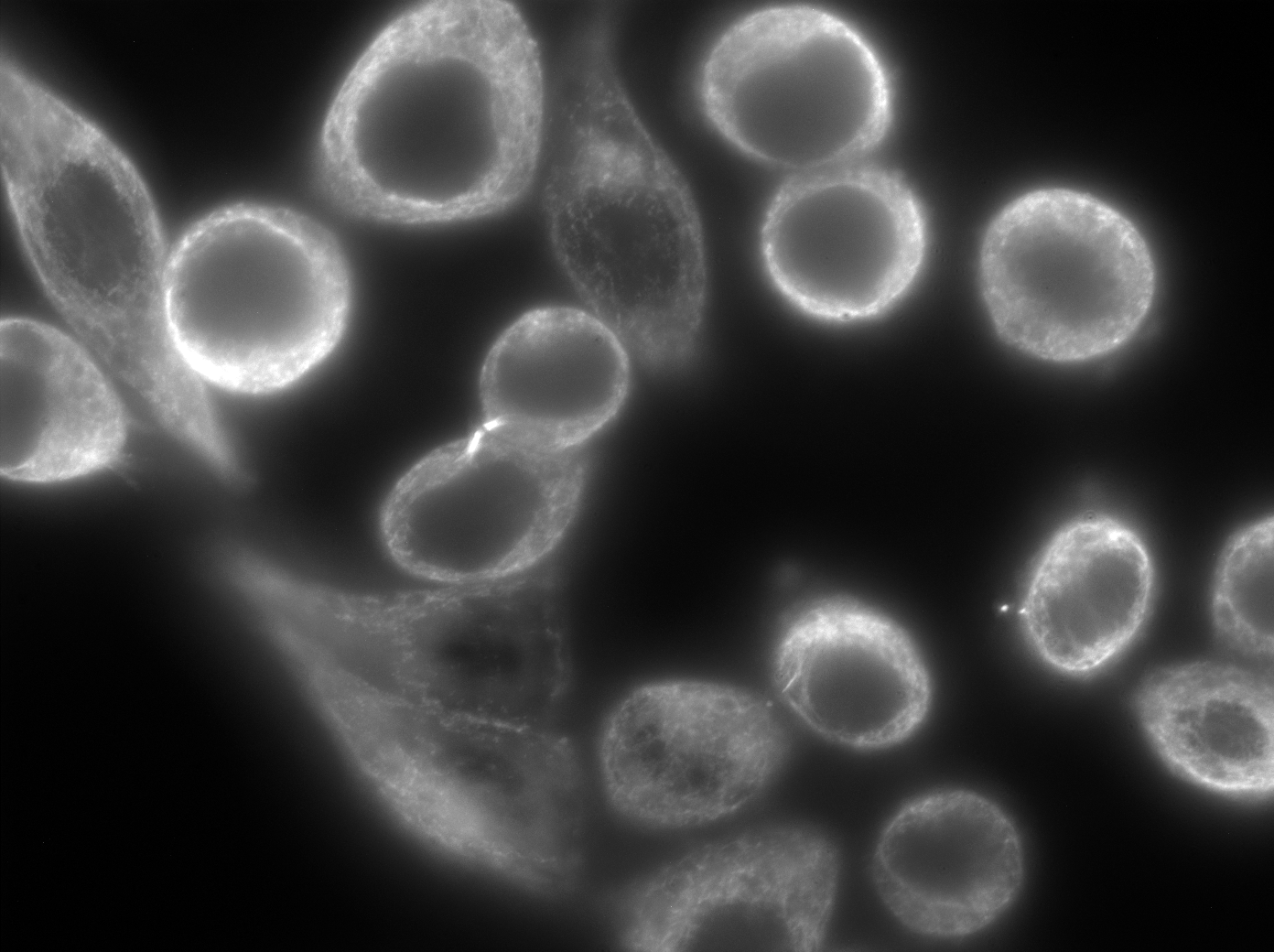

Supplement: Supplementary file 8 — Source data Fig. 2 part 5 [file 44319_2026_742_MOESM8_ESM.zip › Figure 2 Part 5/Fig 2d polye atubulin part 2/WT PolyE-atub 8-14-24 R3 LT9 PA9.Project Maximum Z_XY1723843870_Z0_T0_C1.tif]

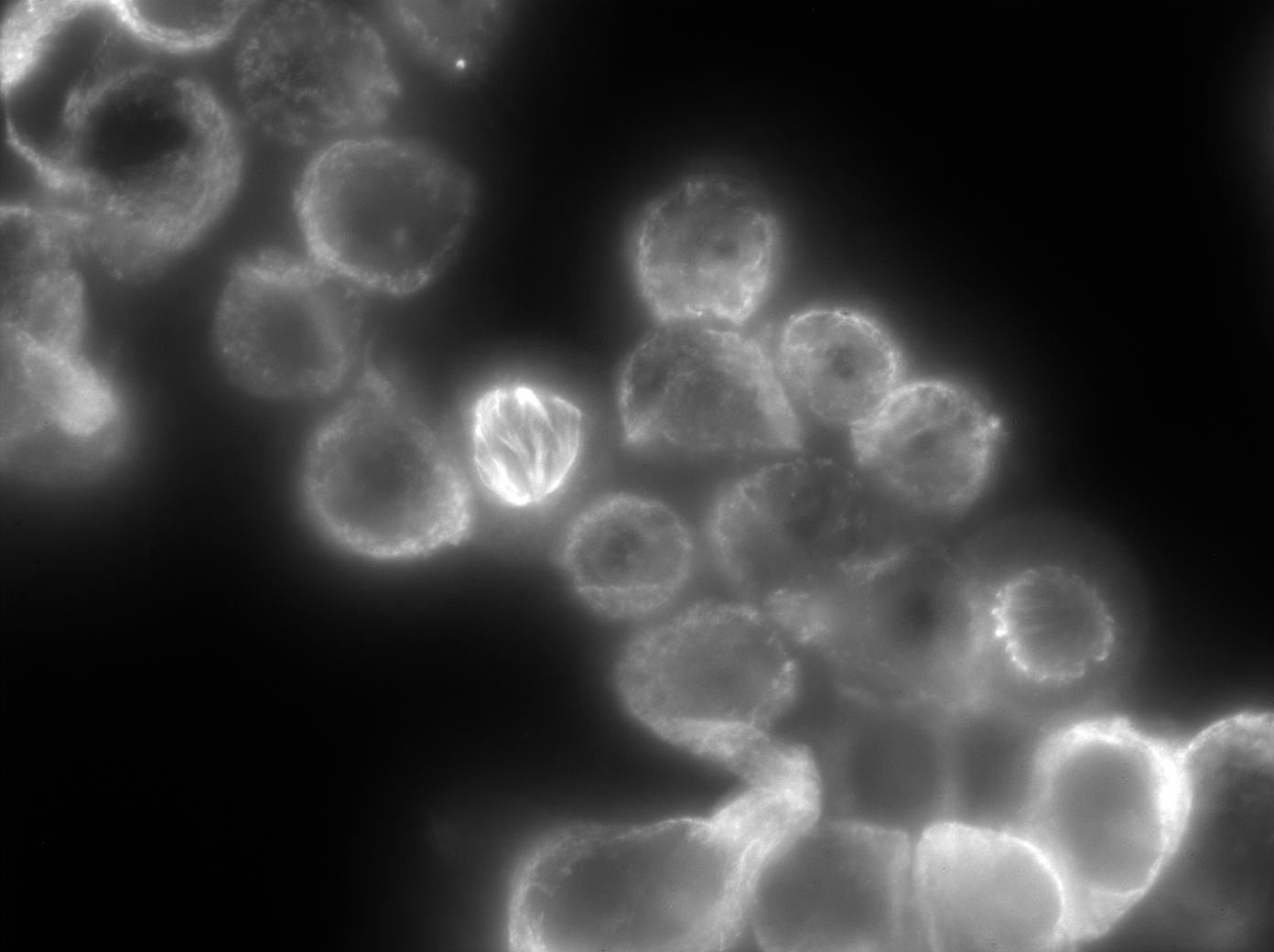

Supplement: Supplementary file 8 — Source data Fig. 2 part 5 [file 44319_2026_742_MOESM8_ESM.zip › Figure 2 Part 5/Fig 2d polye atubulin part 2/WT PolyE-atub 8-14-24 R3 M7M8.Project Maximum Z_XY1723845881_Z0_T0_C1.tif]

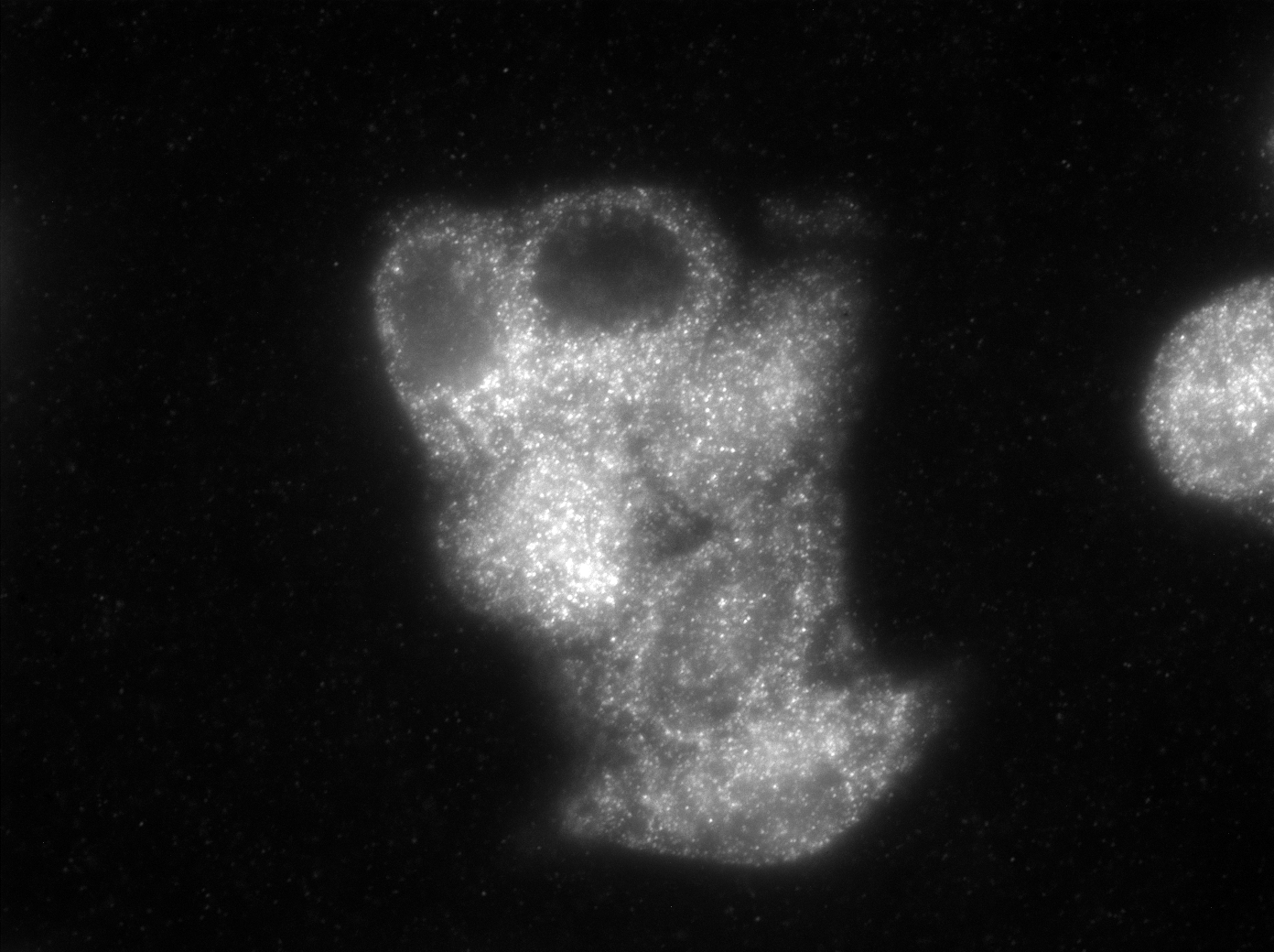

Supplement: Supplementary file 8 — Source data Fig. 2 part 5 [file 44319_2026_742_MOESM8_ESM.zip › Figure 2 Part 5/Fig 2d polye atubulin part 2/WT PolyE-atub 8-14-24 R2 LT10.Project Maximum Z_XY1723837856_Z0_T0_C2.tif]

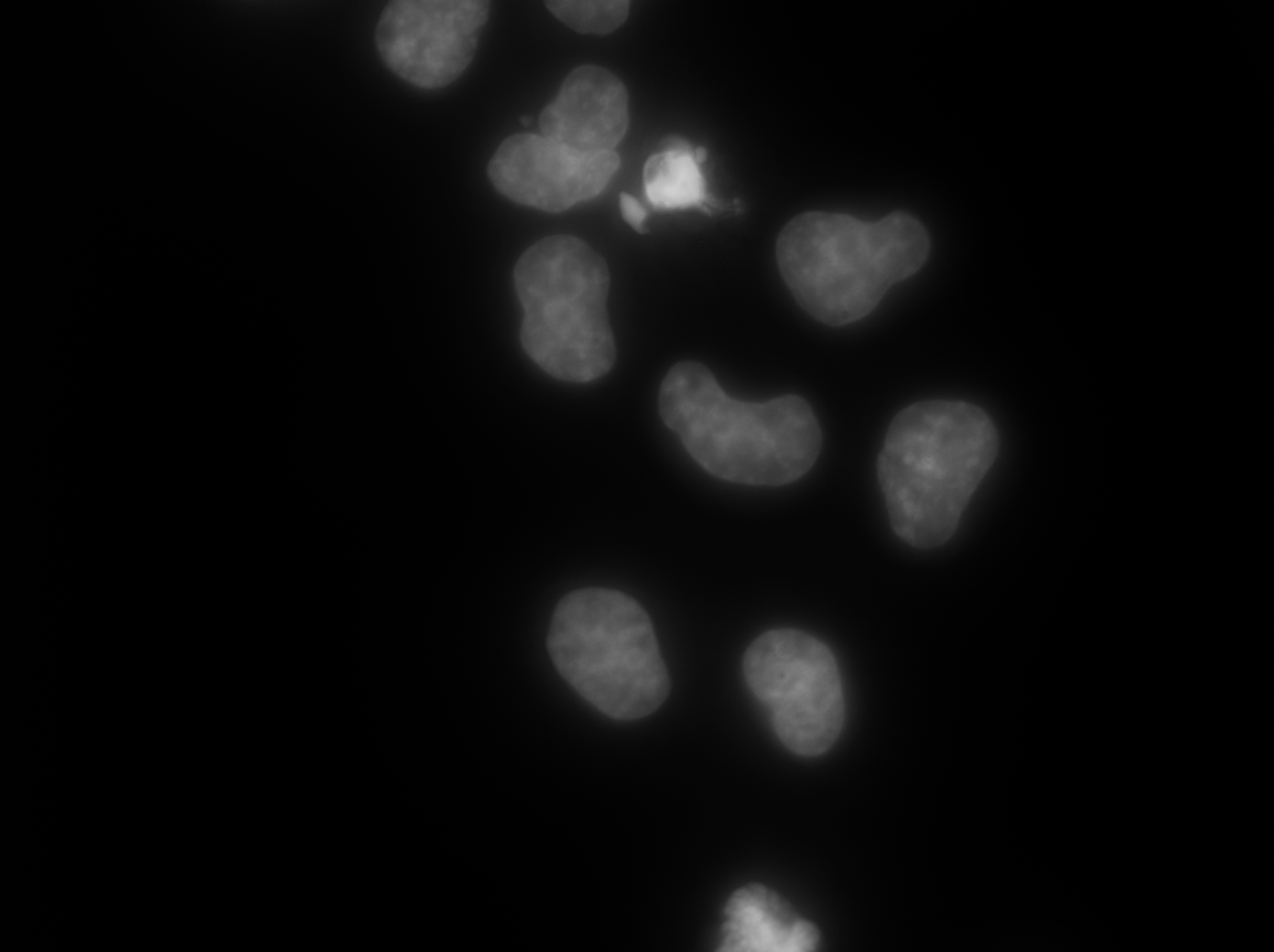

Supplement: Supplementary file 8 — Source data Fig. 2 part 5 [file 44319_2026_742_MOESM8_ESM.zip › Figure 2 Part 5/Fig 2d polye atubulin part 2/WT PolyE-atub 8-14-24 R3 ET7 PA7.Project Maximum Z_XY1723843057_Z0_T0_C0.tif]
