# Supplementary figures and images for: TPGS1 regulates central spindle microtubule glutamylation and remodeling during telophase and abscission (part 8 of 36)
Source: EMBO Rep. 2026 Mar 23;27(8):1944–63. doi: 10.1038/s44319-026-00742-3 (PMC13121839; doi:10.1038/s44319-026-00742-3)

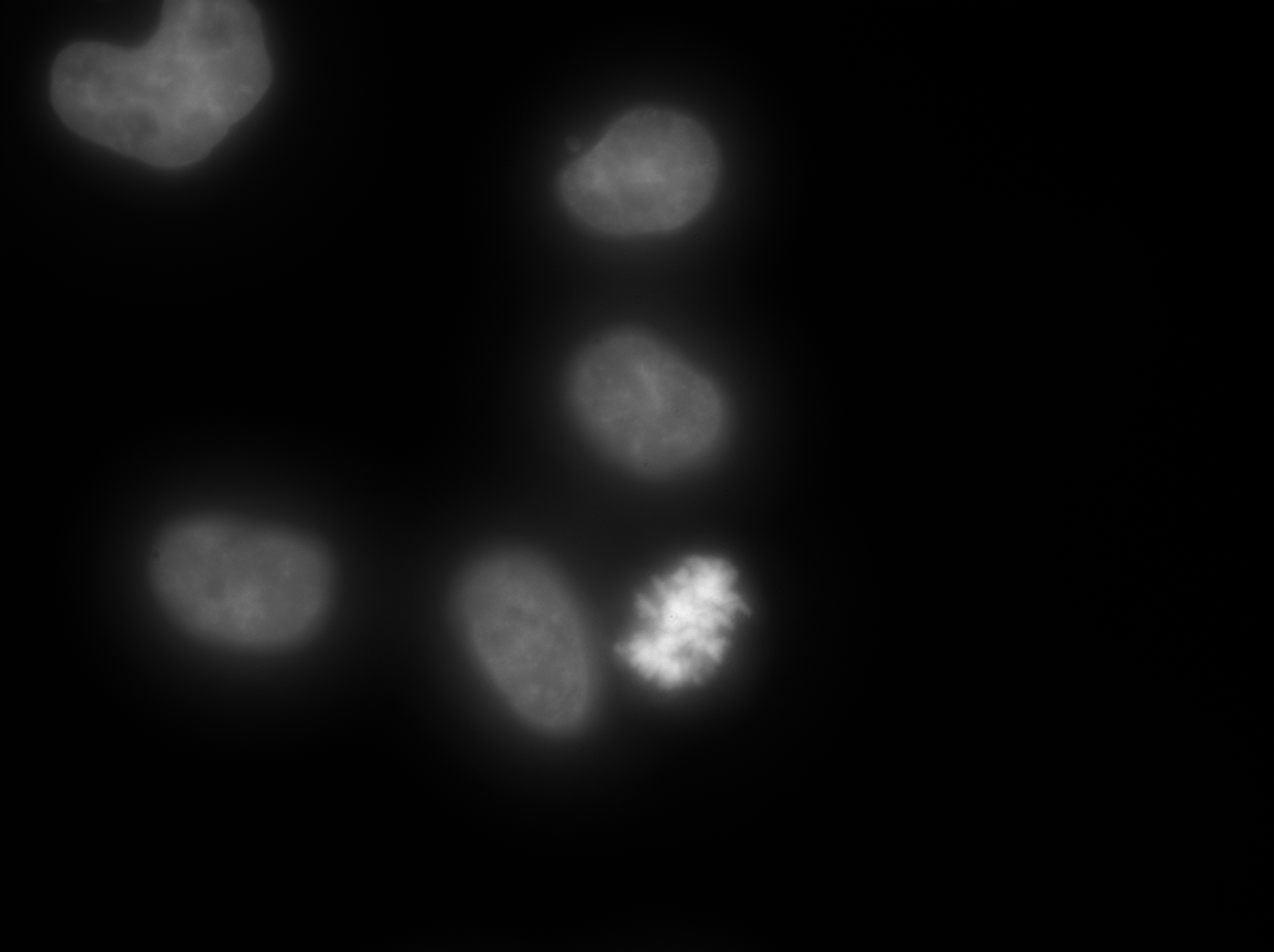

Supplement: Supplementary file 8 — Source data Fig. 2 part 5 [file 44319_2026_742_MOESM8_ESM.zip › Figure 2 Part 5/Fig 2d polye atubulin part 2/WT PolyE-atub 8-14-24 R2 M9.Project Maximum Z_XY1723838859_Z0_T0_C0.tif]

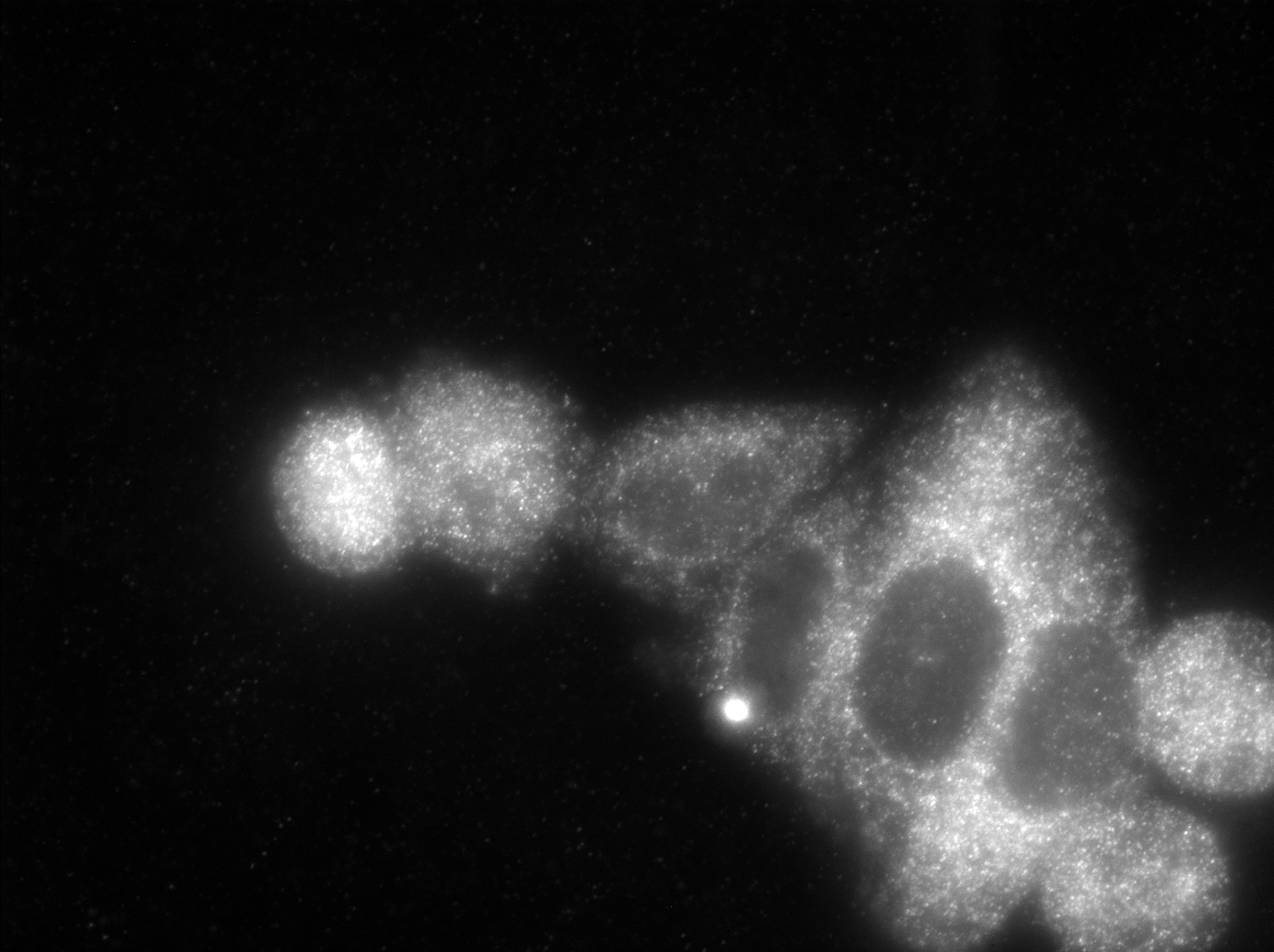

Supplement: Supplementary file 8 — Source data Fig. 2 part 5 [file 44319_2026_742_MOESM8_ESM.zip › Figure 2 Part 5/Fig 2d polye atubulin part 2/WT PolyE-atub 8-14-24 R2 LT9.Project Maximum Z_XY1723836948_Z0_T0_C2.tif]

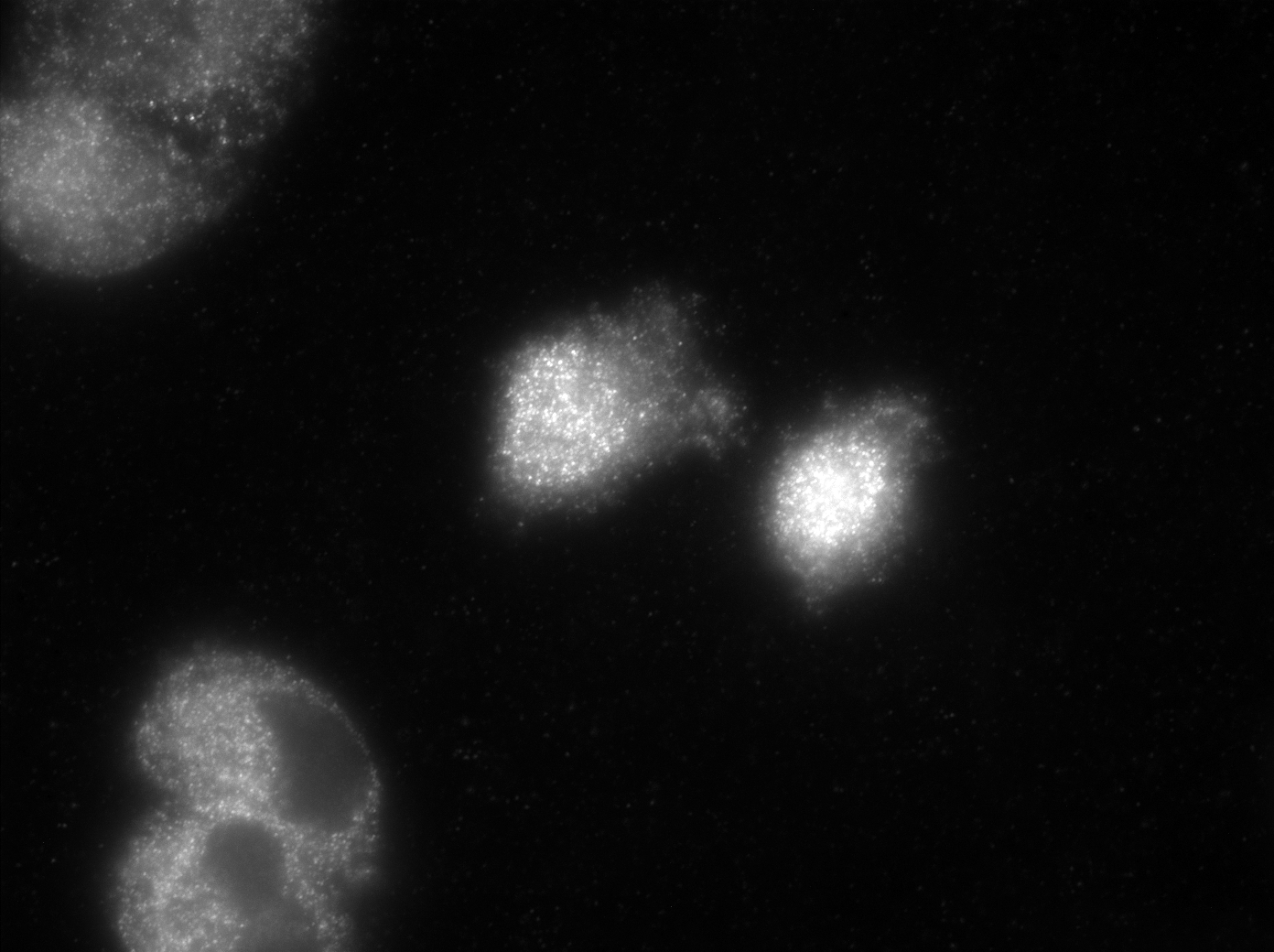

Supplement: Supplementary file 8 — Source data Fig. 2 part 5 [file 44319_2026_742_MOESM8_ESM.zip › Figure 2 Part 5/Fig 2d polye atubulin part 2/WT PolyE-atub 8-14-24 R2 PA5.Project Maximum Z_XY1723835442_Z0_T0_C2.tif]

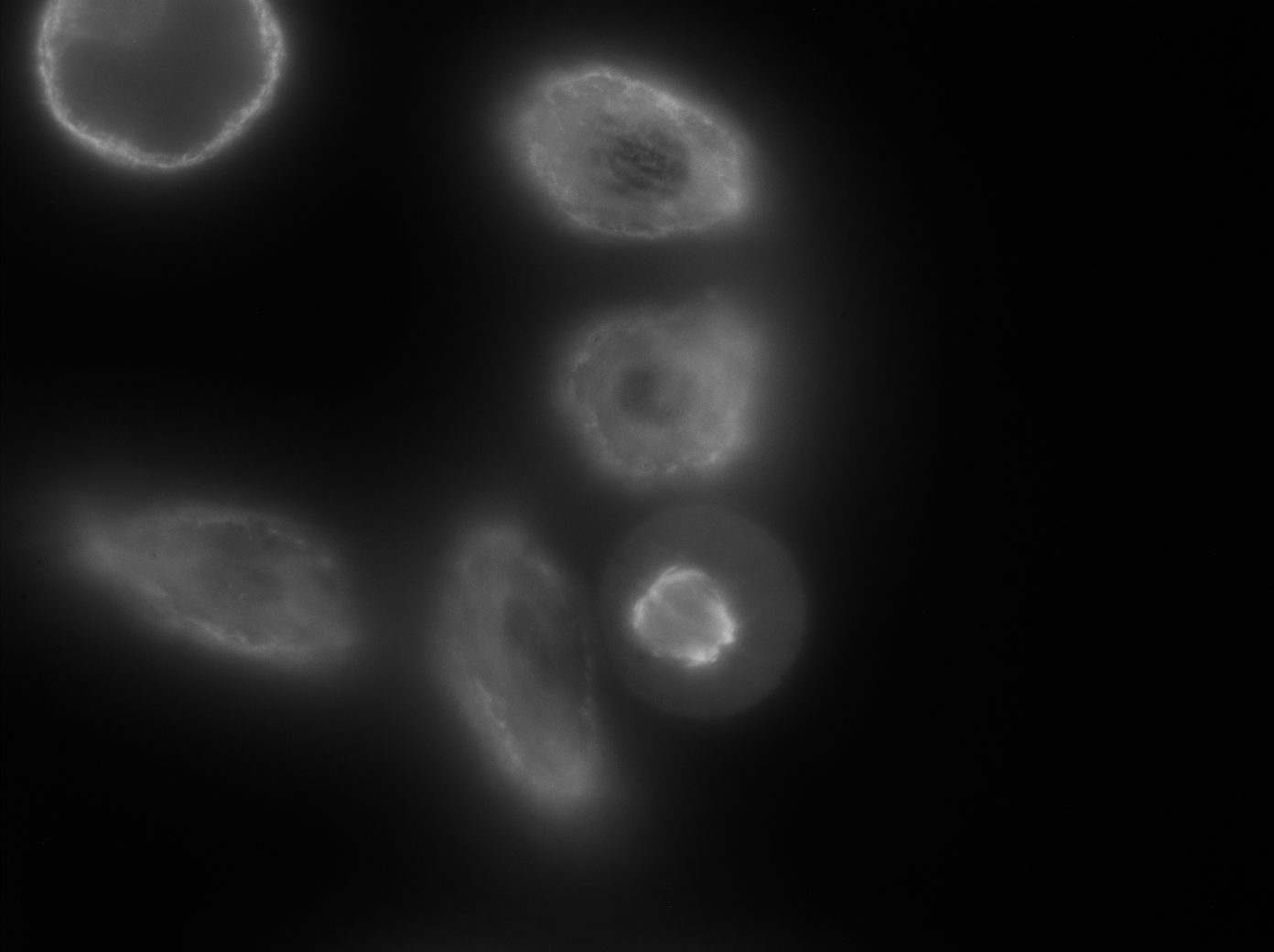

Supplement: Supplementary file 8 — Source data Fig. 2 part 5 [file 44319_2026_742_MOESM8_ESM.zip › Figure 2 Part 5/Fig 2d polye atubulin part 2/WT PolyE-atub 8-14-24 R2 M9.Project Maximum Z_XY1723838859_Z0_T0_C1.tif]

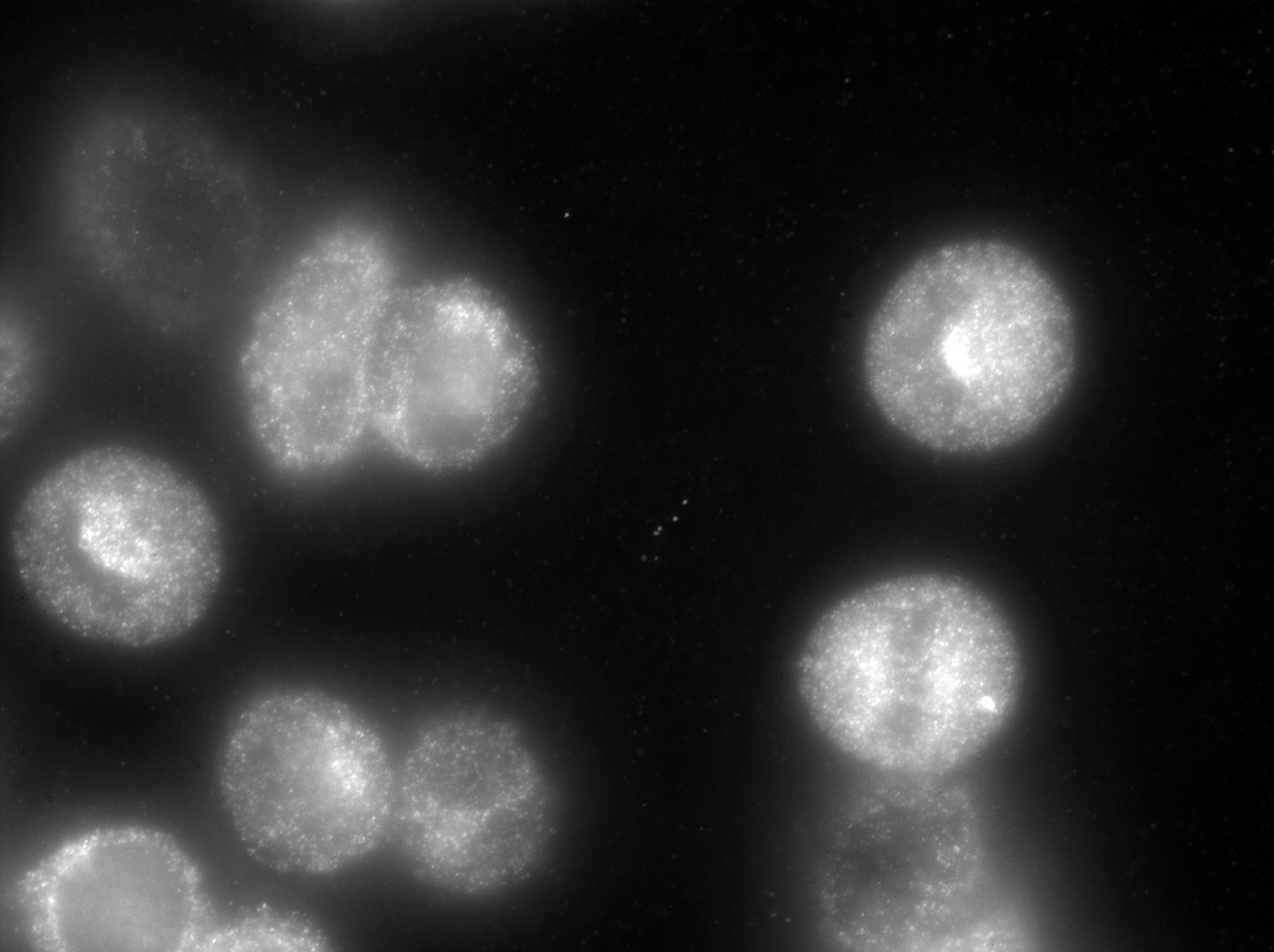

Supplement: Supplementary file 8 — Source data Fig. 2 part 5 [file 44319_2026_742_MOESM8_ESM.zip › Figure 2 Part 5/Fig 2d polye atubulin part 2/WT PolyE-atub 8-14-24 R2 M5.Project Maximum Z_XY1723837319_Z0_T0_C2.tif]

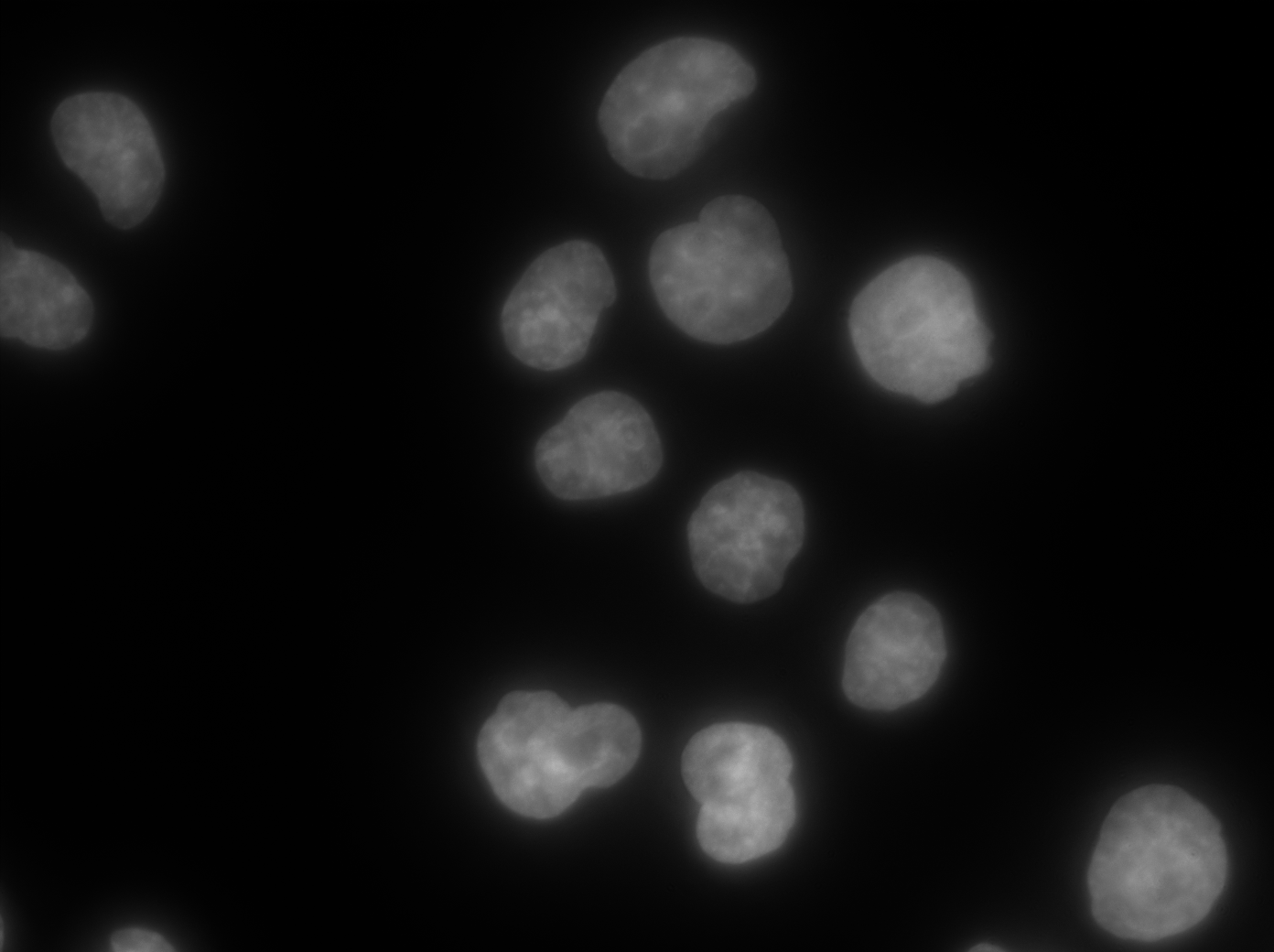

Supplement: Supplementary file 8 — Source data Fig. 2 part 5 [file 44319_2026_742_MOESM8_ESM.zip › Figure 2 Part 5/Fig 2d polye atubulin part 2/WT PolyE-atub 8-14-24 R3 LT6.Project Maximum Z_XY1723843473_Z0_T0_C0.tif]

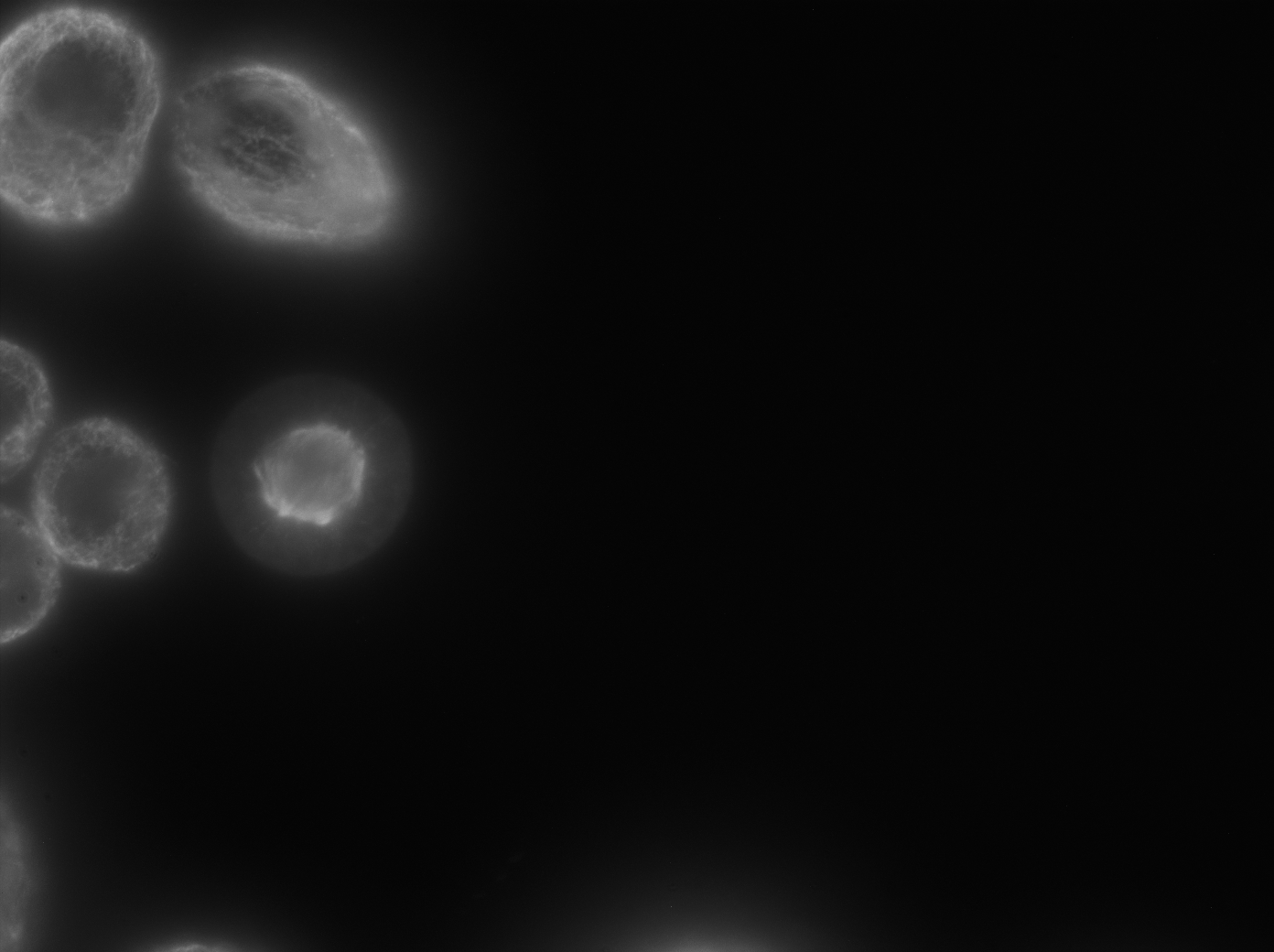

Supplement: Supplementary file 8 — Source data Fig. 2 part 5 [file 44319_2026_742_MOESM8_ESM.zip › Figure 2 Part 5/Fig 2d polye atubulin part 2/WT PolyE-atub 8-14-24 R2 M4.Project Maximum Z_XY1723837179_Z0_T0_C1.tif]

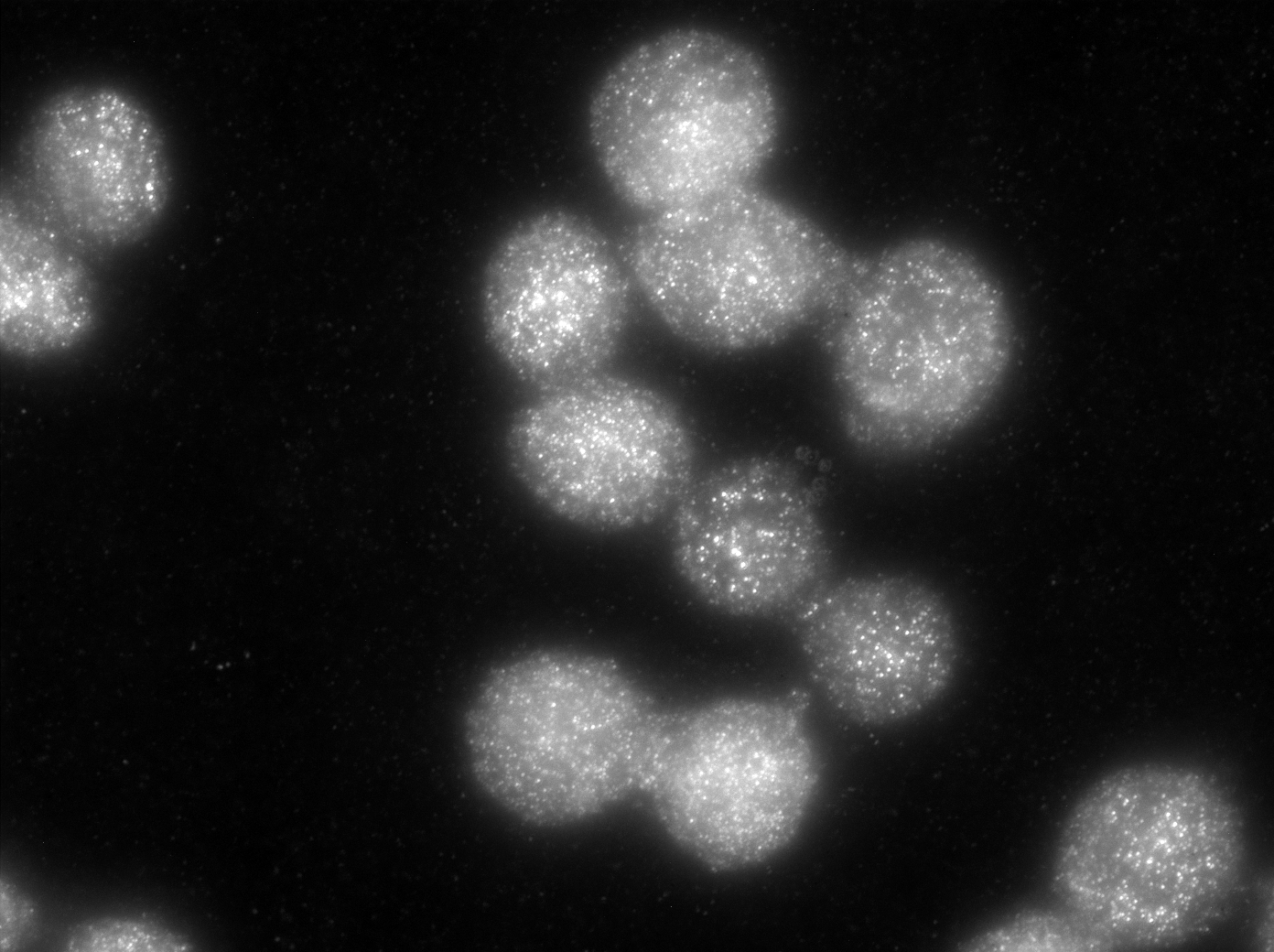

Supplement: Supplementary file 8 — Source data Fig. 2 part 5 [file 44319_2026_742_MOESM8_ESM.zip › Figure 2 Part 5/Fig 2d polye atubulin part 2/WT PolyE-atub 8-14-24 R3 LT6.Project Maximum Z_XY1723843473_Z0_T0_C2.tif]

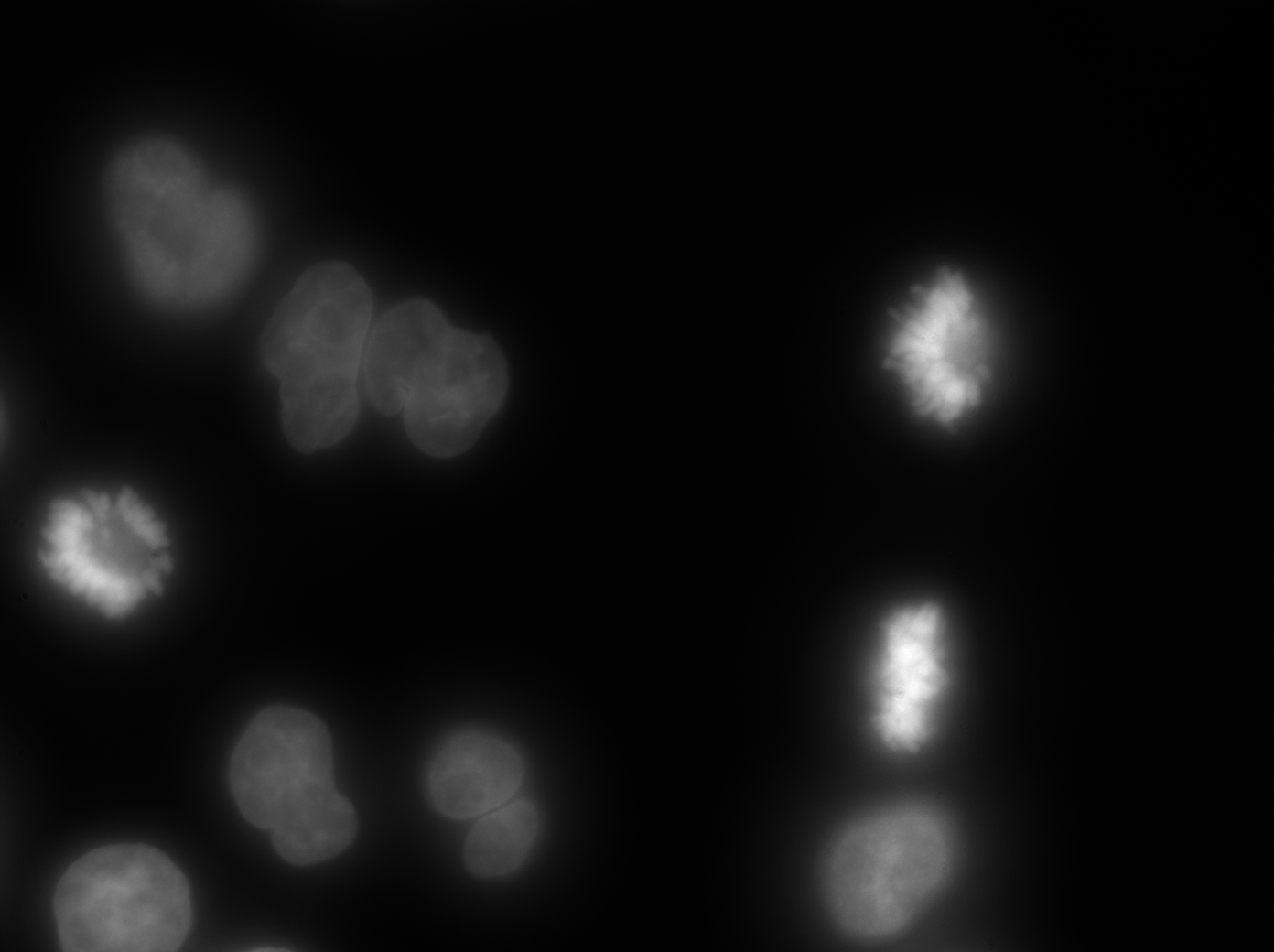

Supplement: Supplementary file 8 — Source data Fig. 2 part 5 [file 44319_2026_742_MOESM8_ESM.zip › Figure 2 Part 5/Fig 2d polye atubulin part 2/WT PolyE-atub 8-14-24 R2 M5.Project Maximum Z_XY1723837319_Z0_T0_C0.tif]

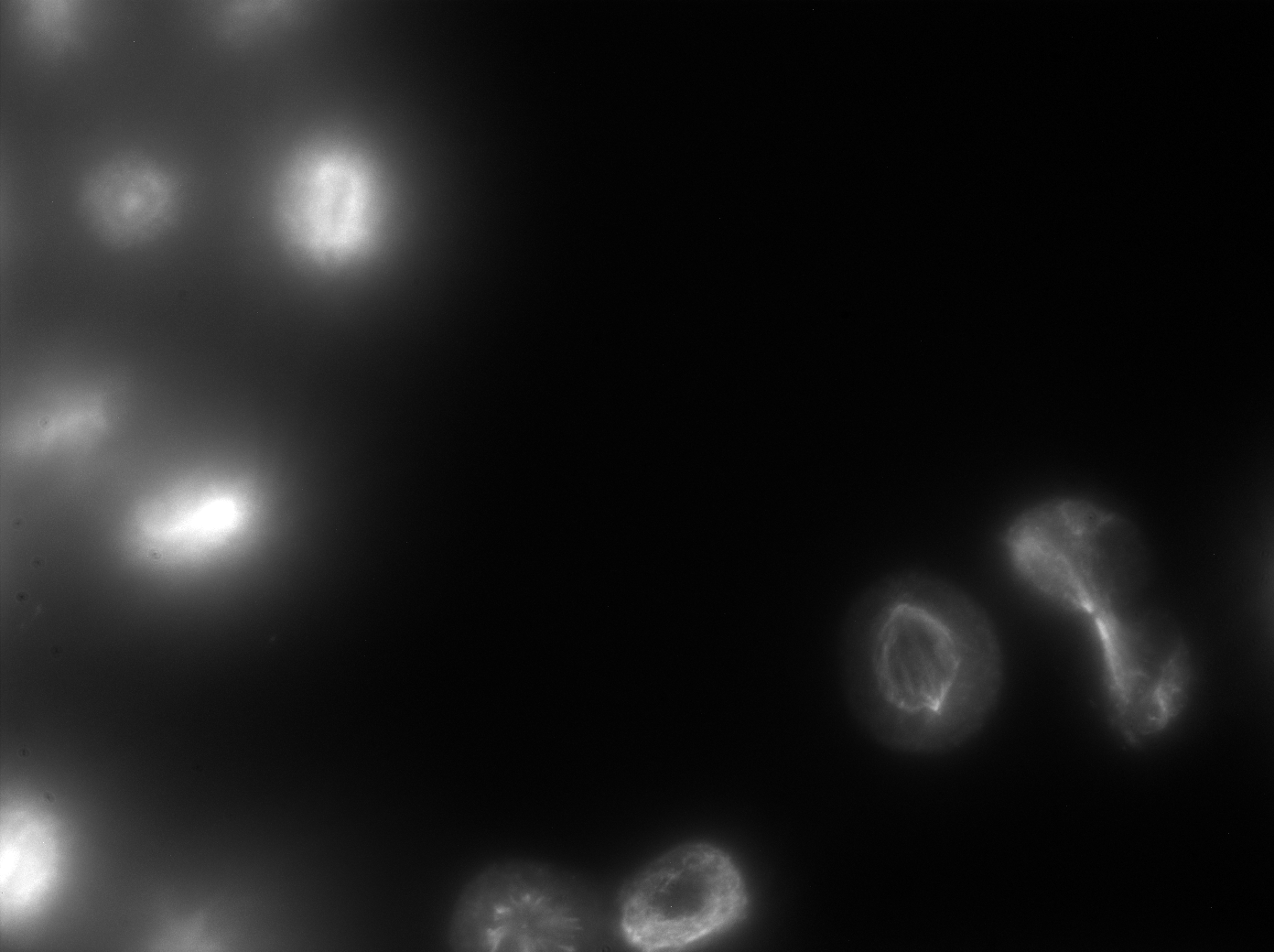

Supplement: Supplementary file 8 — Source data Fig. 2 part 5 [file 44319_2026_742_MOESM8_ESM.zip › Figure 2 Part 5/Fig 2d polye atubulin part 2/WT PolyE-atub 8-14-24 R3 M9.Project Maximum Z_XY1723846152_Z0_T0_C1.tif]

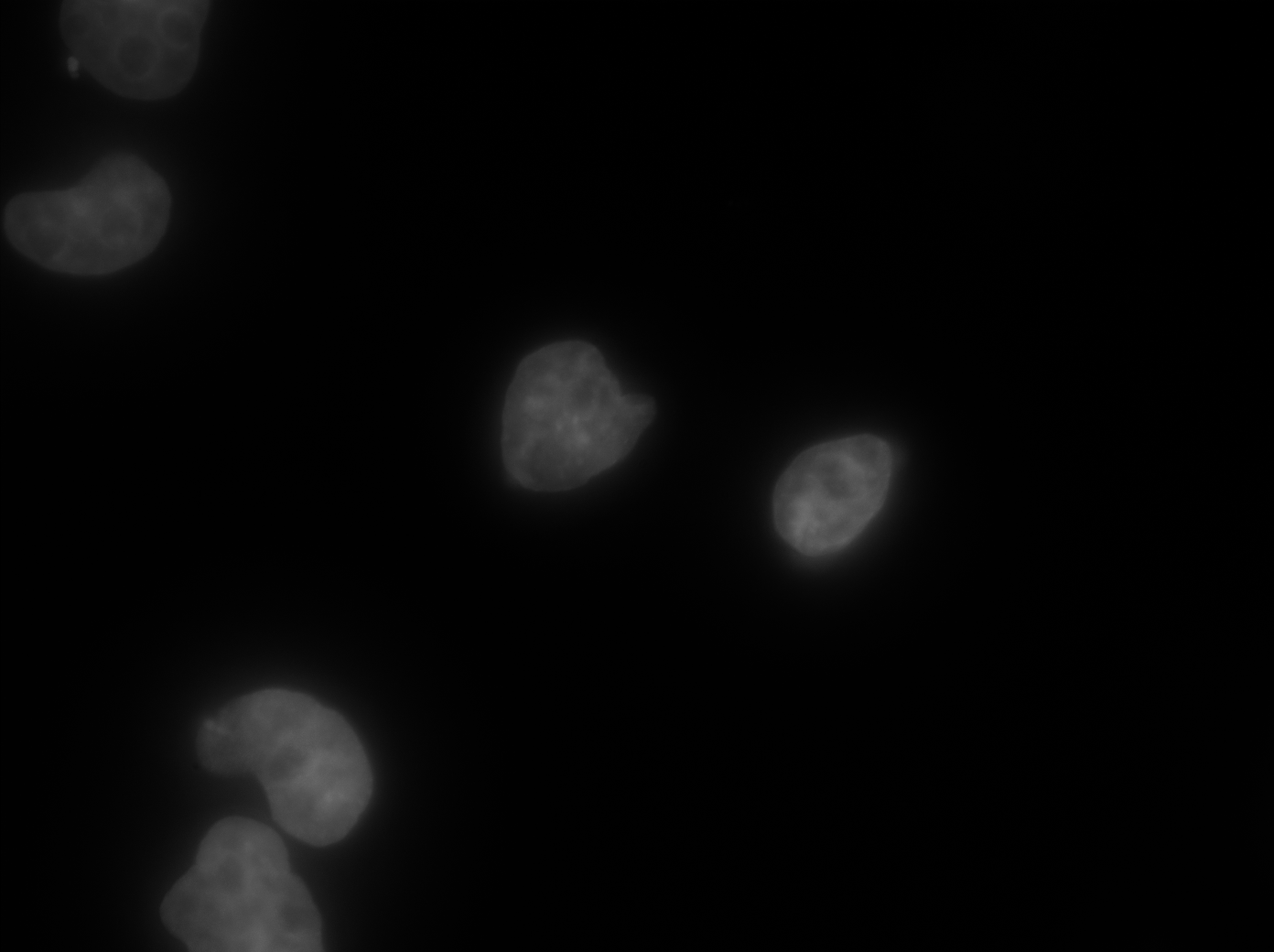

Supplement: Supplementary file 8 — Source data Fig. 2 part 5 [file 44319_2026_742_MOESM8_ESM.zip › Figure 2 Part 5/Fig 2d polye atubulin part 2/WT PolyE-atub 8-14-24 R2 PA5.Project Maximum Z_XY1723835442_Z0_T0_C0.tif]

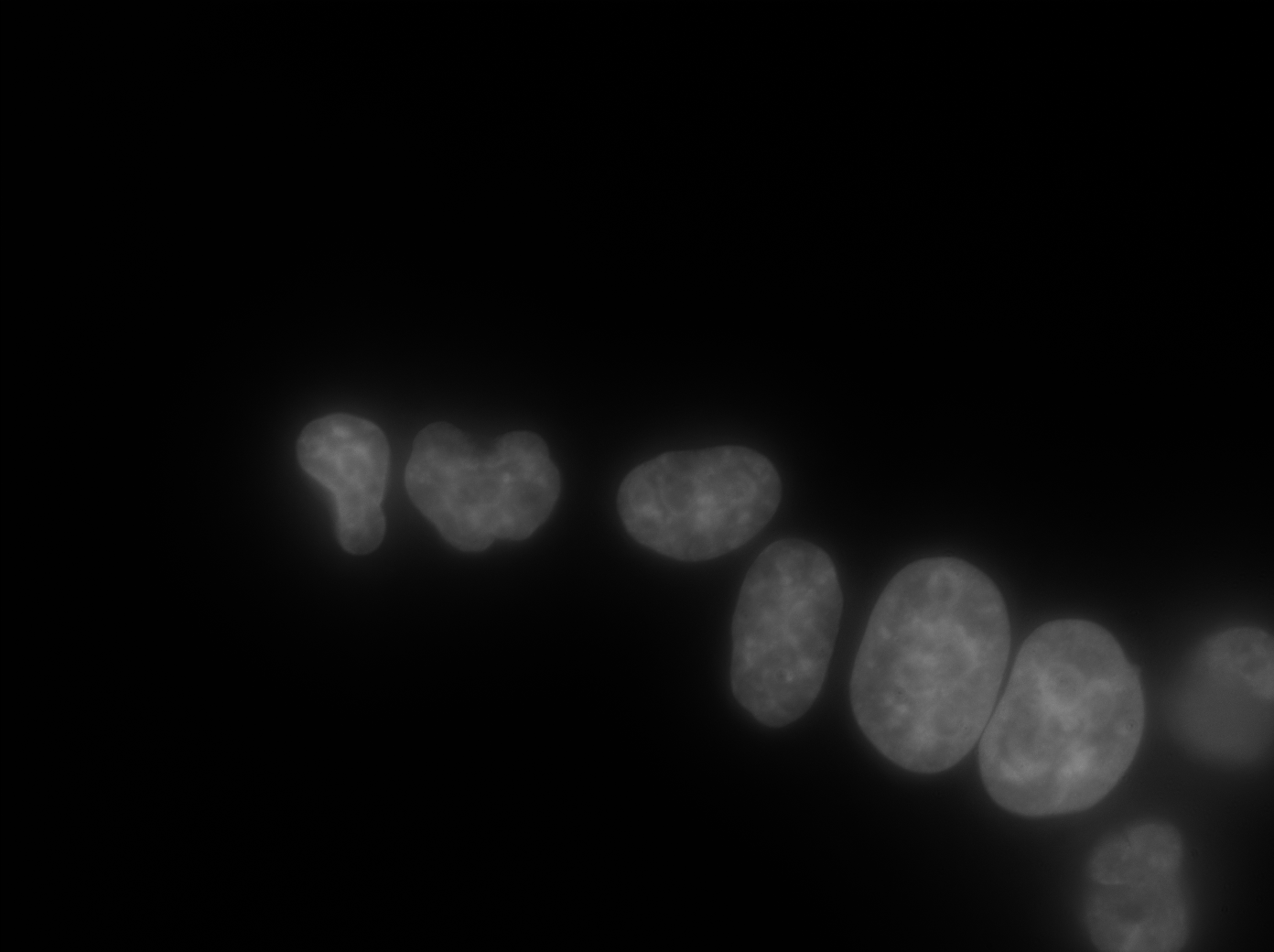

Supplement: Supplementary file 8 — Source data Fig. 2 part 5 [file 44319_2026_742_MOESM8_ESM.zip › Figure 2 Part 5/Fig 2d polye atubulin part 2/WT PolyE-atub 8-14-24 R2 LT9.Project Maximum Z_XY1723836948_Z0_T0_C0.tif]

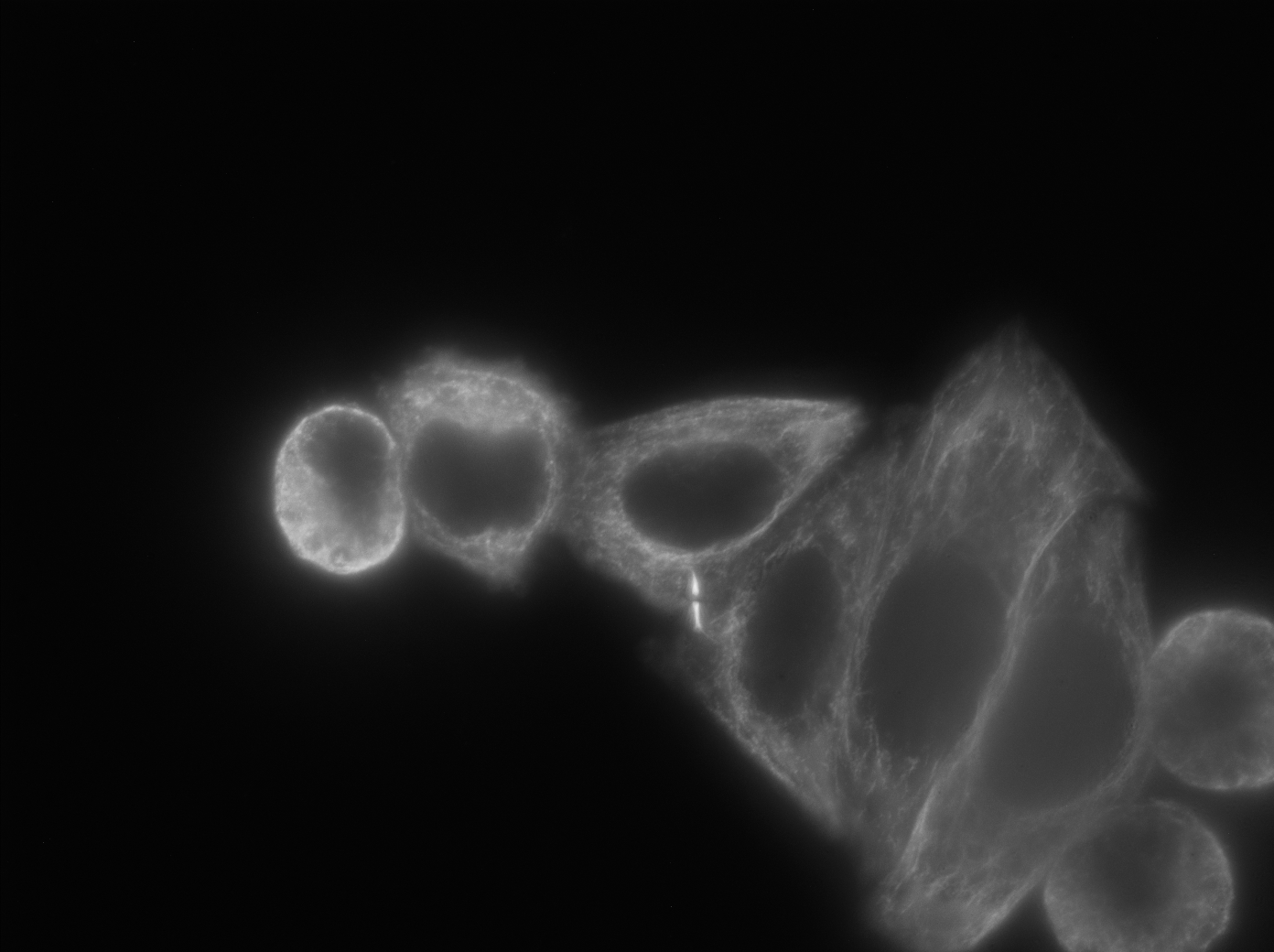

Supplement: Supplementary file 8 — Source data Fig. 2 part 5 [file 44319_2026_742_MOESM8_ESM.zip › Figure 2 Part 5/Fig 2d polye atubulin part 2/WT PolyE-atub 8-14-24 R2 LT9.Project Maximum Z_XY1723836948_Z0_T0_C1.tif]

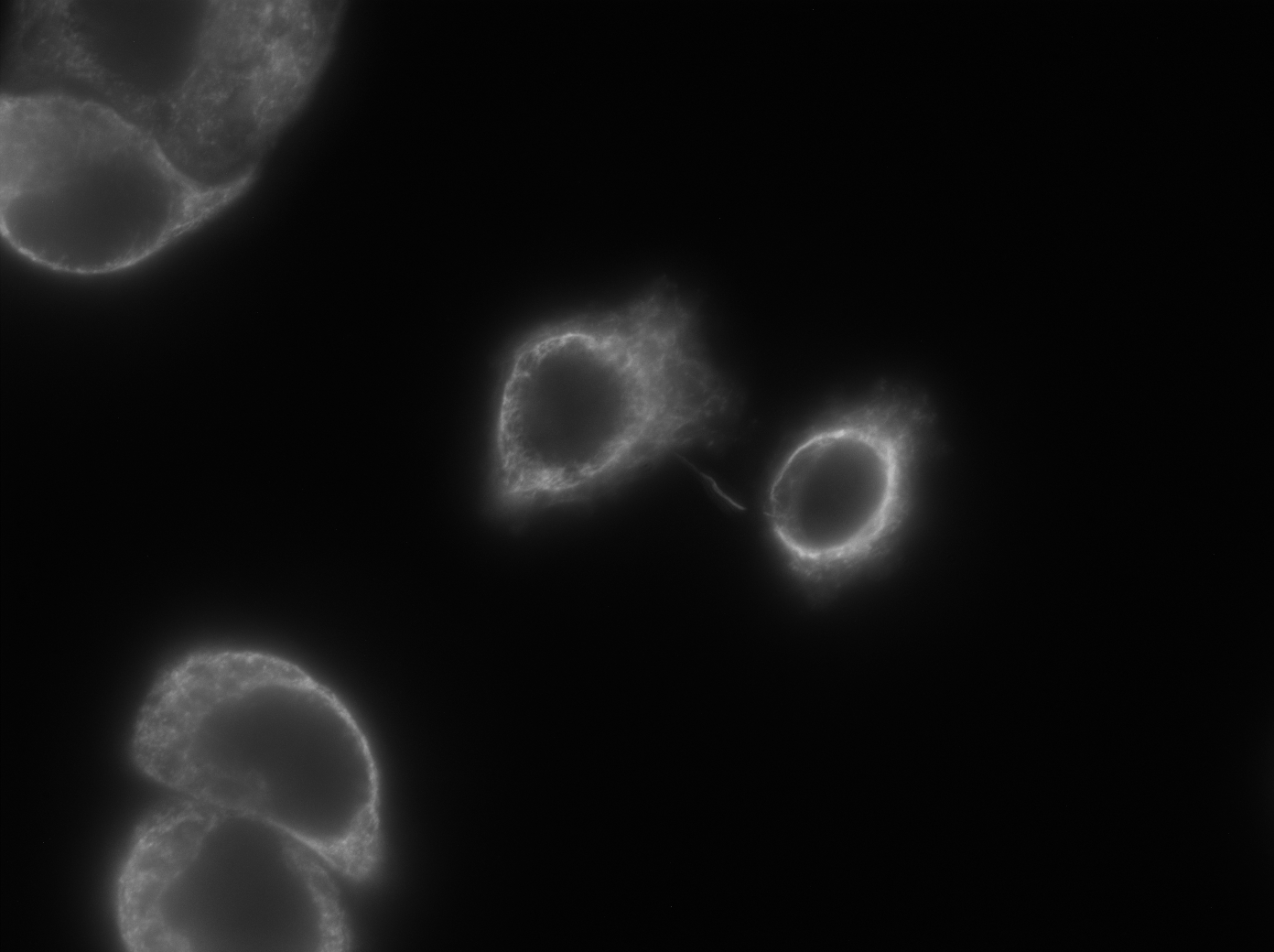

Supplement: Supplementary file 8 — Source data Fig. 2 part 5 [file 44319_2026_742_MOESM8_ESM.zip › Figure 2 Part 5/Fig 2d polye atubulin part 2/WT PolyE-atub 8-14-24 R2 PA5.Project Maximum Z_XY1723835442_Z0_T0_C1.tif]

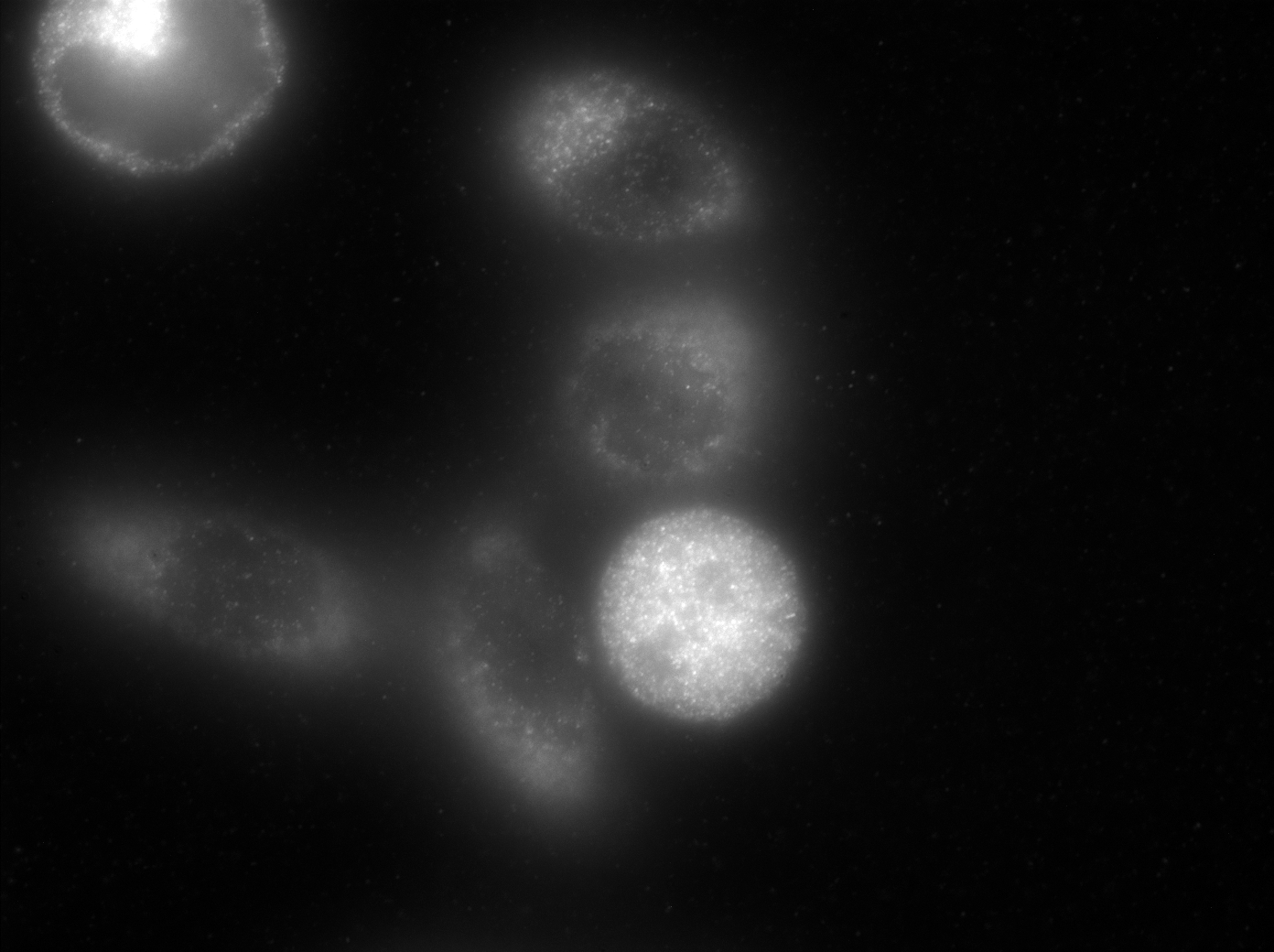

Supplement: Supplementary file 8 — Source data Fig. 2 part 5 [file 44319_2026_742_MOESM8_ESM.zip › Figure 2 Part 5/Fig 2d polye atubulin part 2/WT PolyE-atub 8-14-24 R2 M9.Project Maximum Z_XY1723838859_Z0_T0_C2.tif]

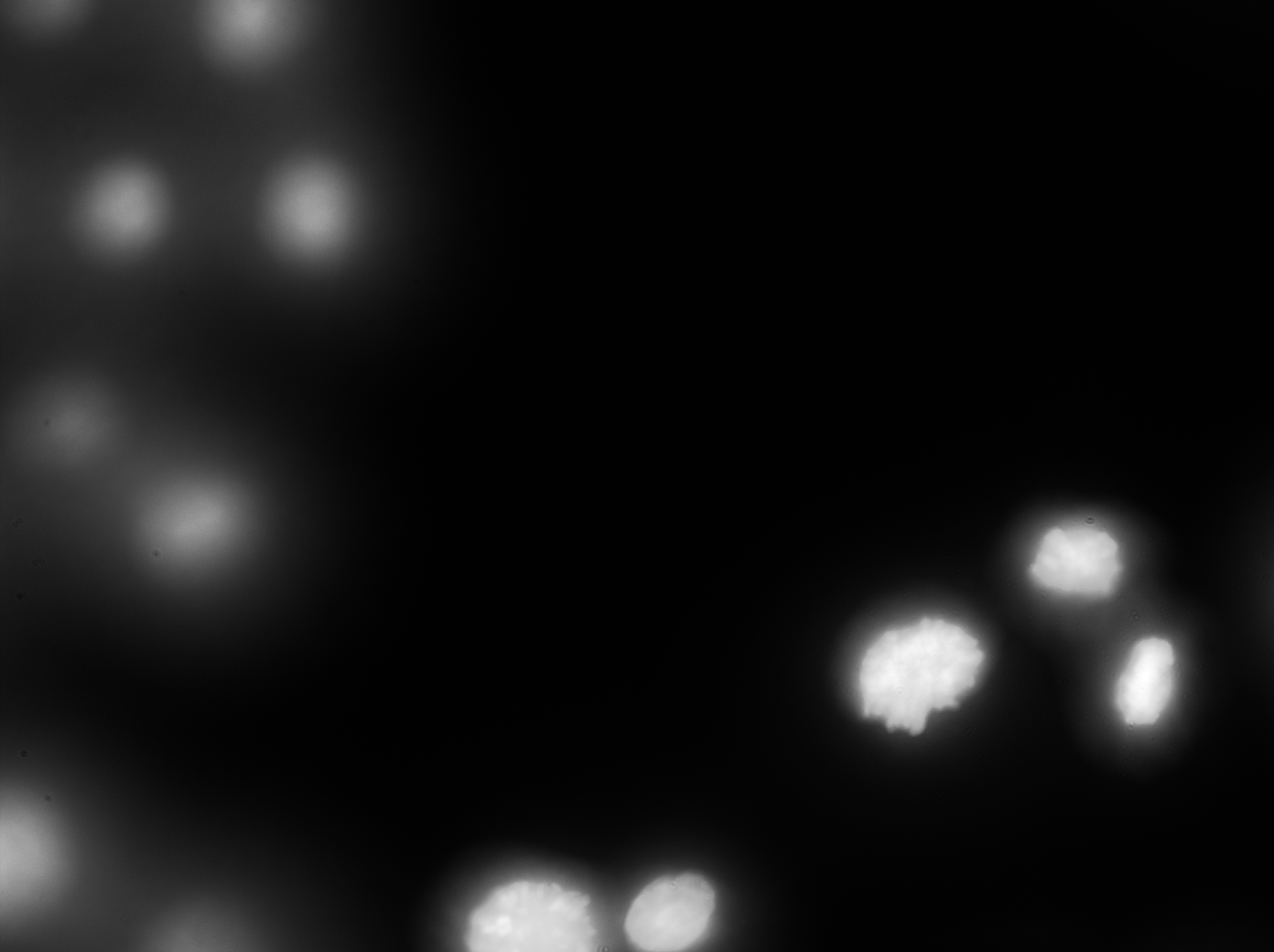

Supplement: Supplementary file 8 — Source data Fig. 2 part 5 [file 44319_2026_742_MOESM8_ESM.zip › Figure 2 Part 5/Fig 2d polye atubulin part 2/WT PolyE-atub 8-14-24 R3 M9.Project Maximum Z_XY1723846152_Z0_T0_C0.tif]

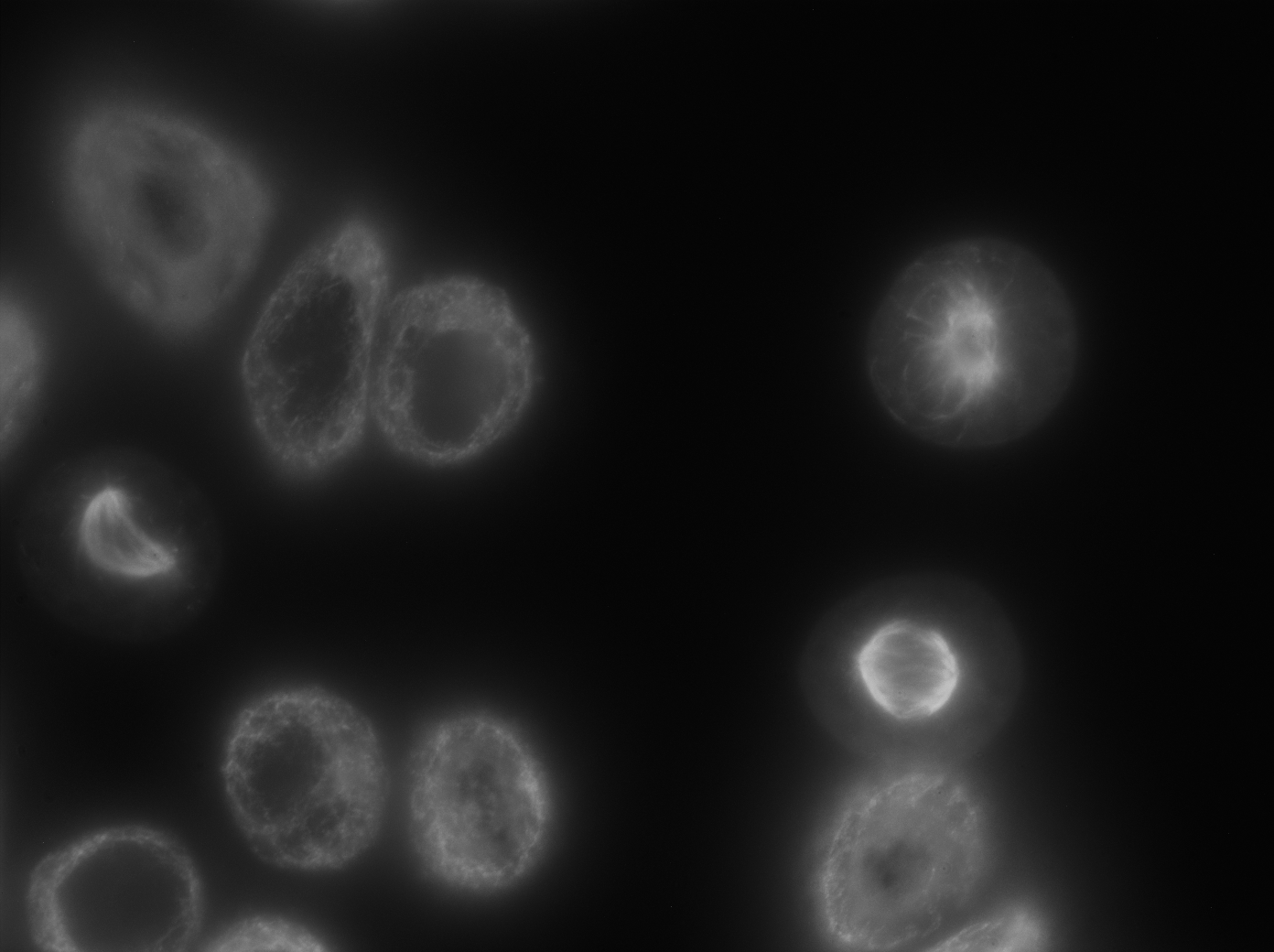

Supplement: Supplementary file 8 — Source data Fig. 2 part 5 [file 44319_2026_742_MOESM8_ESM.zip › Figure 2 Part 5/Fig 2d polye atubulin part 2/WT PolyE-atub 8-14-24 R2 M5.Project Maximum Z_XY1723837319_Z0_T0_C1.tif]

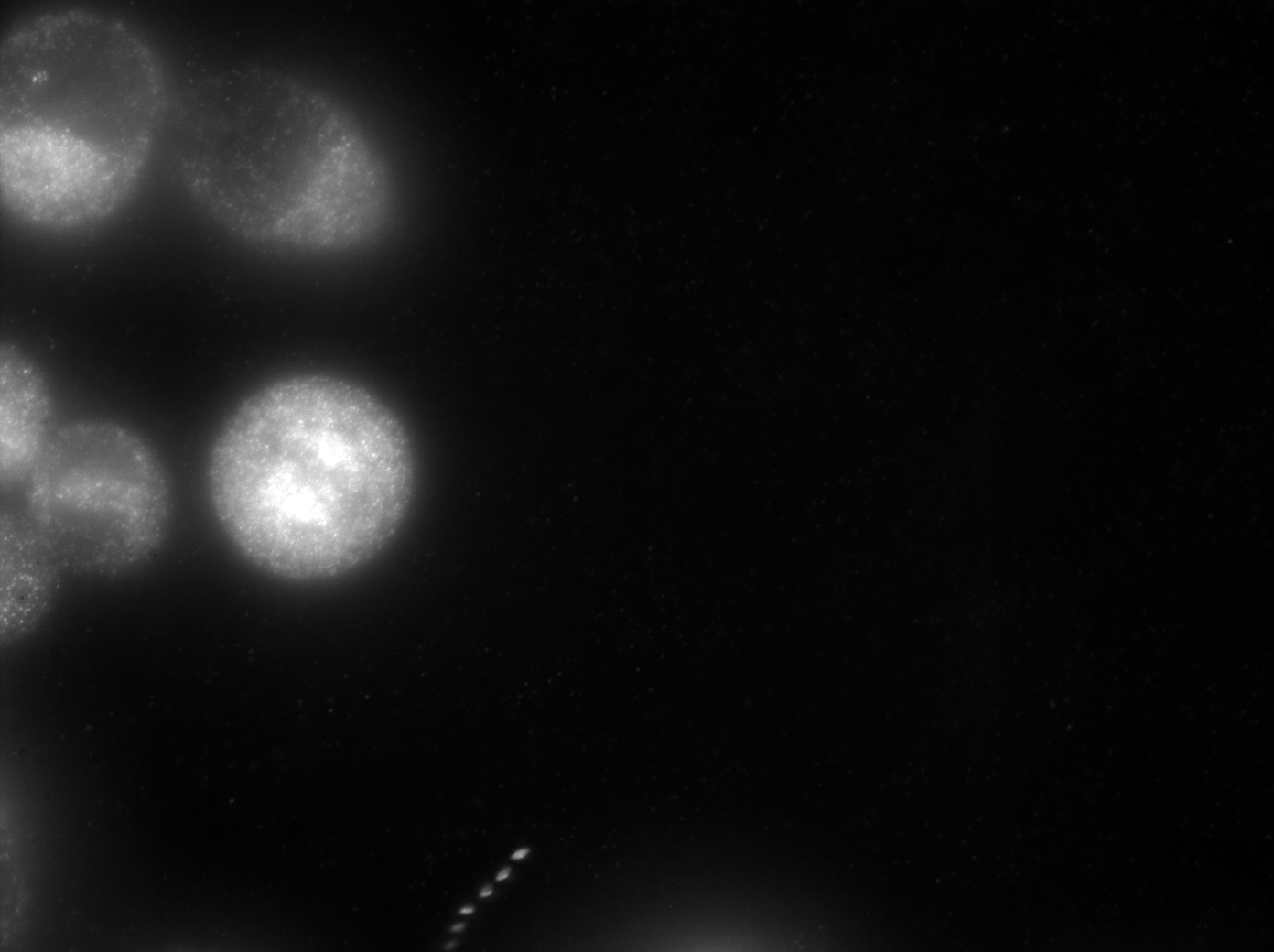

Supplement: Supplementary file 8 — Source data Fig. 2 part 5 [file 44319_2026_742_MOESM8_ESM.zip › Figure 2 Part 5/Fig 2d polye atubulin part 2/WT PolyE-atub 8-14-24 R2 M4.Project Maximum Z_XY1723837179_Z0_T0_C2.tif]

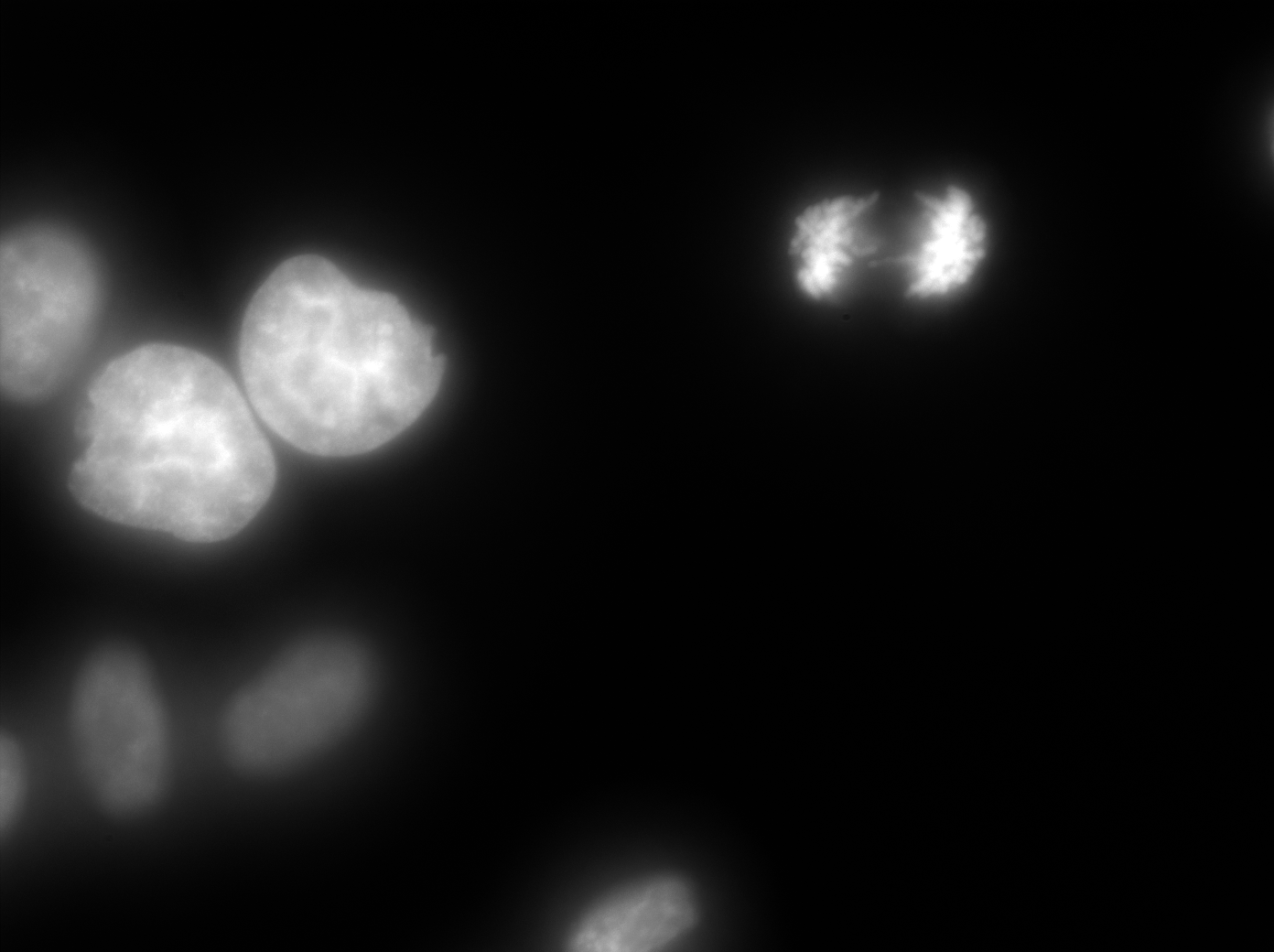

Supplement: Supplementary file 9 — Source data Fig. 2 part 6 [file 44319_2026_742_MOESM9_ESM.zip › Figure 2 Part 6/Fig 2fg Control Hela rGT335 acetylated tubulin/Anaphase/Cas9 actub rGT335 9-8-25 R3 A3.Project Maximum Z_XY1757366260_Z0_T0_C0.tif]

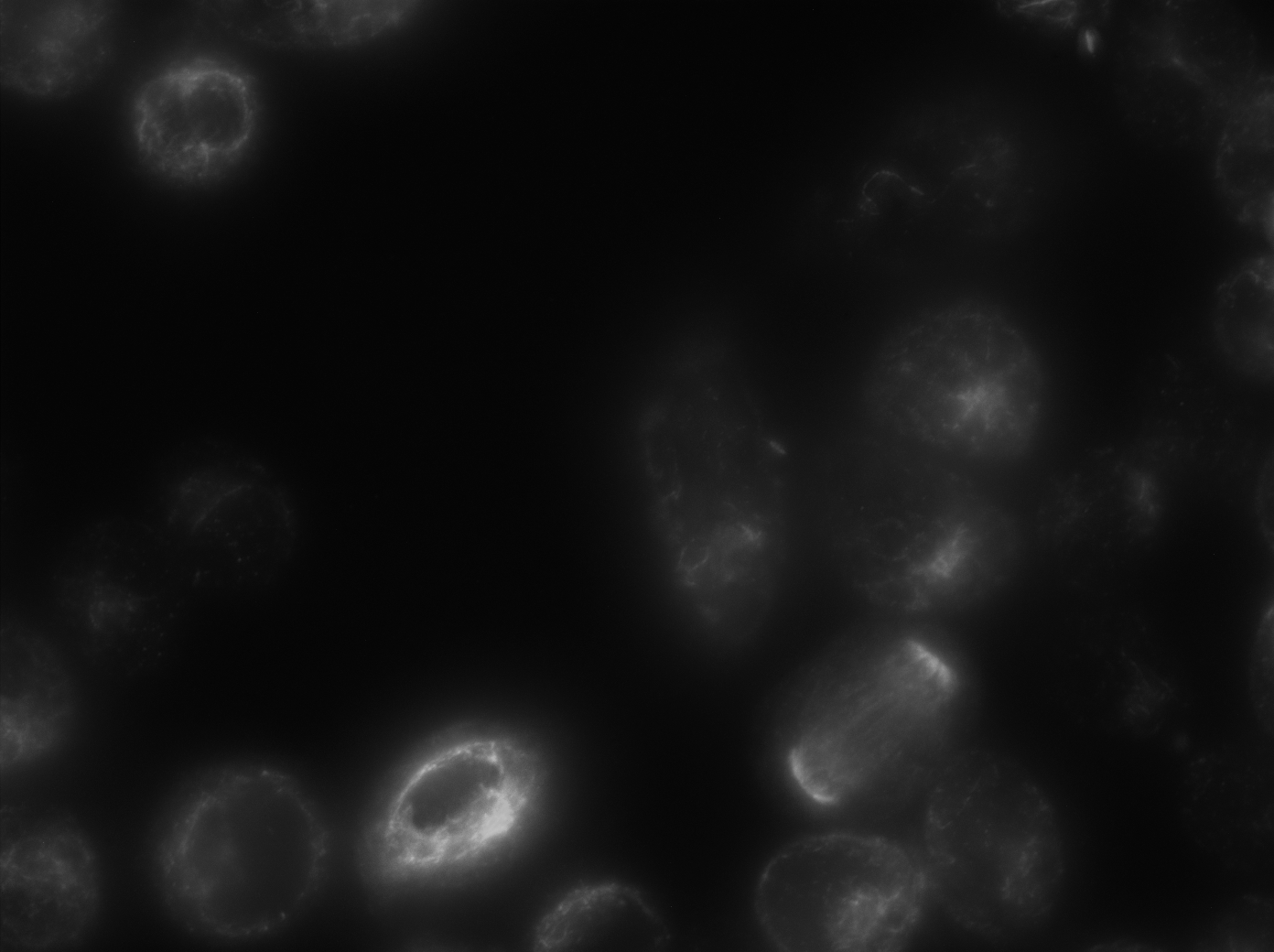

Supplement: Supplementary file 9 — Source data Fig. 2 part 6 [file 44319_2026_742_MOESM9_ESM.zip › Figure 2 Part 6/Fig 2fg Control Hela rGT335 acetylated tubulin/Anaphase/Cas9 actub rGT335 9-8-25 R2 A7.Project Maximum Z_XY1757361610_Z0_T0_C2.tif]

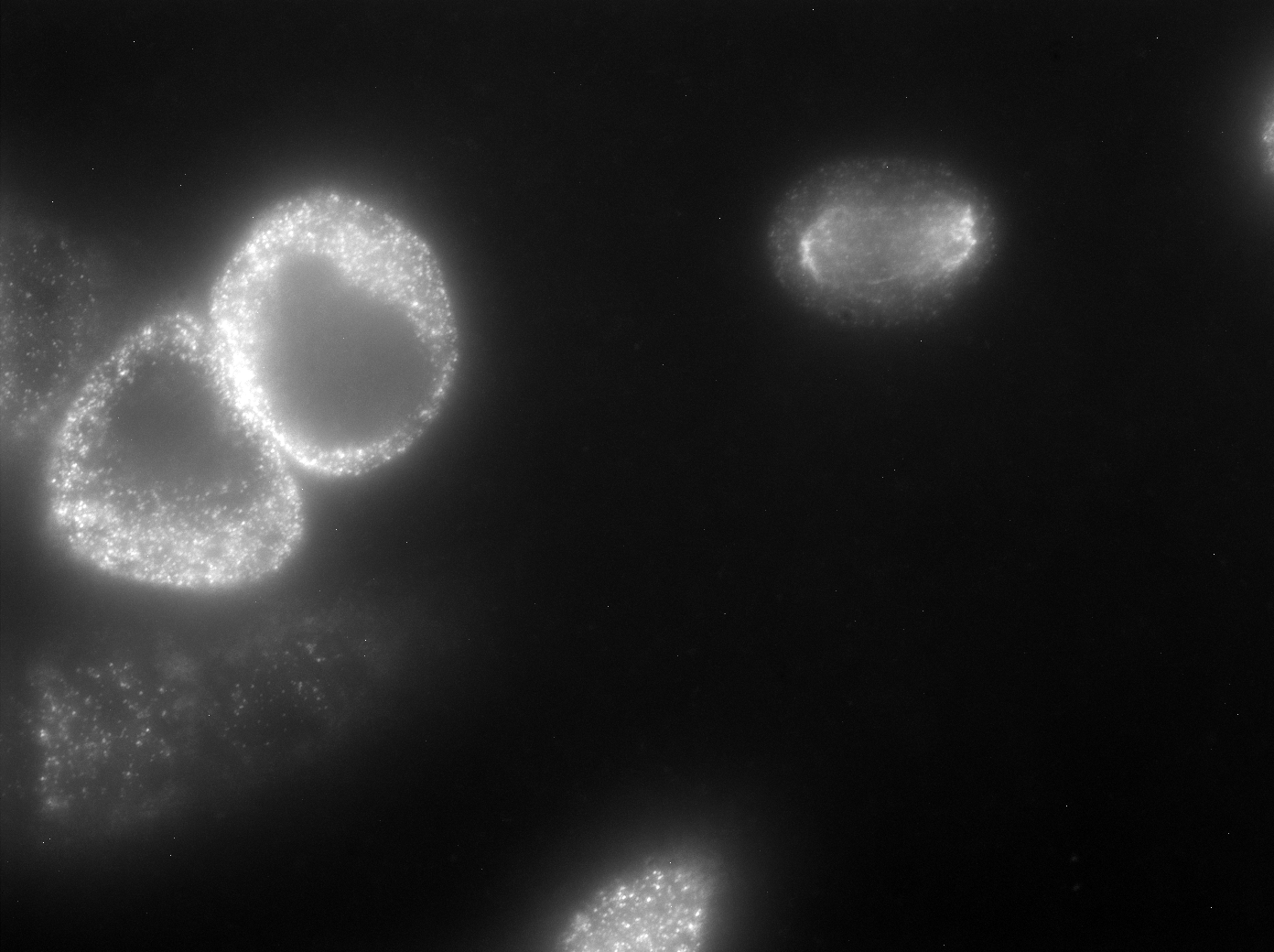

Supplement: Supplementary file 9 — Source data Fig. 2 part 6 [file 44319_2026_742_MOESM9_ESM.zip › Figure 2 Part 6/Fig 2fg Control Hela rGT335 acetylated tubulin/Anaphase/Cas9 actub rGT335 9-8-25 R3 A3.Project Maximum Z_XY1757366260_Z0_T0_C1.tif]

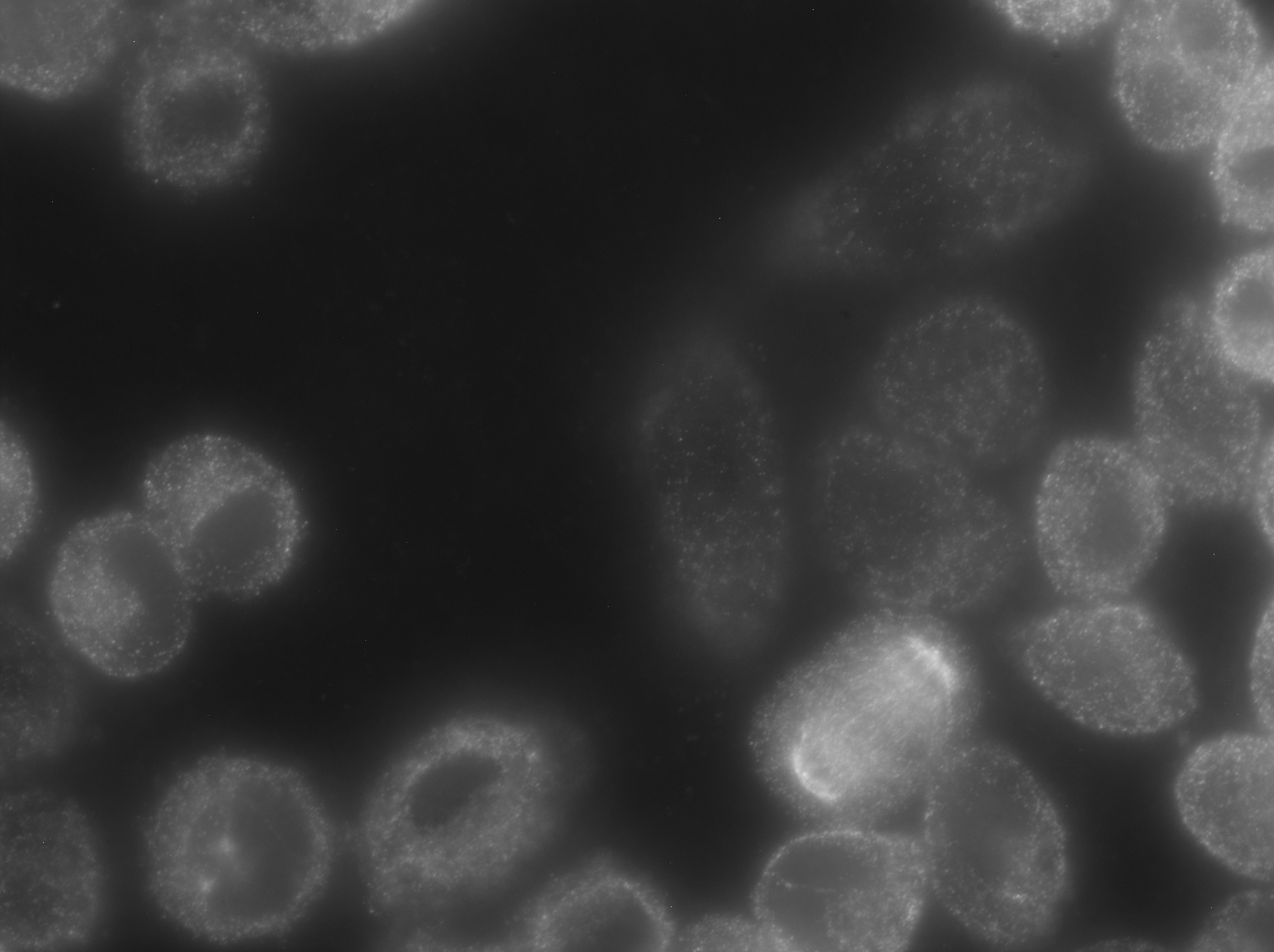

Supplement: Supplementary file 9 — Source data Fig. 2 part 6 [file 44319_2026_742_MOESM9_ESM.zip › Figure 2 Part 6/Fig 2fg Control Hela rGT335 acetylated tubulin/Anaphase/Cas9 actub rGT335 9-8-25 R2 A7.Project Maximum Z_XY1757361610_Z0_T0_C1.tif]

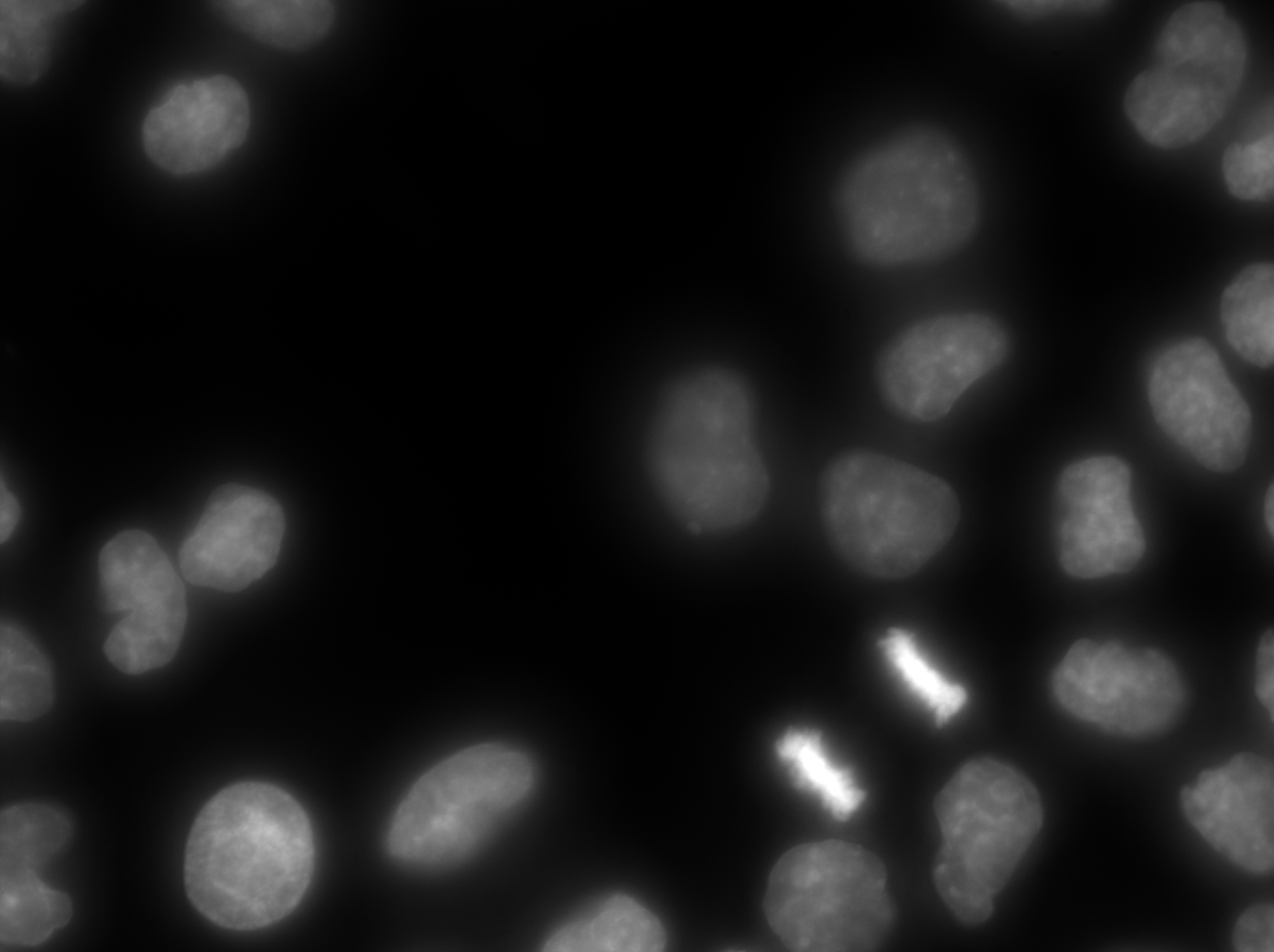

Supplement: Supplementary file 9 — Source data Fig. 2 part 6 [file 44319_2026_742_MOESM9_ESM.zip › Figure 2 Part 6/Fig 2fg Control Hela rGT335 acetylated tubulin/Anaphase/Cas9 actub rGT335 9-8-25 R2 A7.Project Maximum Z_XY1757361610_Z0_T0_C0.tif]

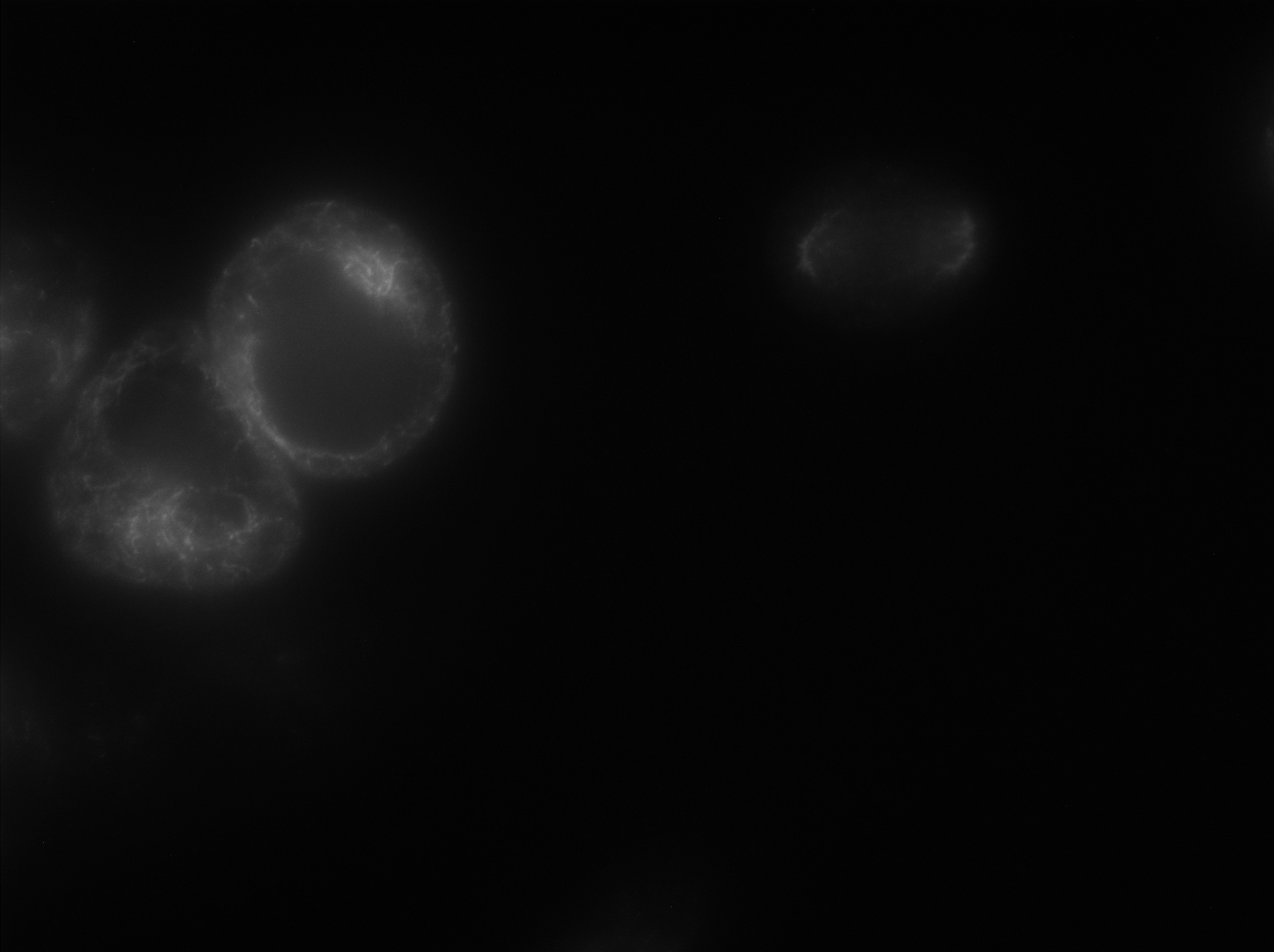

Supplement: Supplementary file 9 — Source data Fig. 2 part 6 [file 44319_2026_742_MOESM9_ESM.zip › Figure 2 Part 6/Fig 2fg Control Hela rGT335 acetylated tubulin/Anaphase/Cas9 actub rGT335 9-8-25 R3 A3.Project Maximum Z_XY1757366260_Z0_T0_C2.tif]

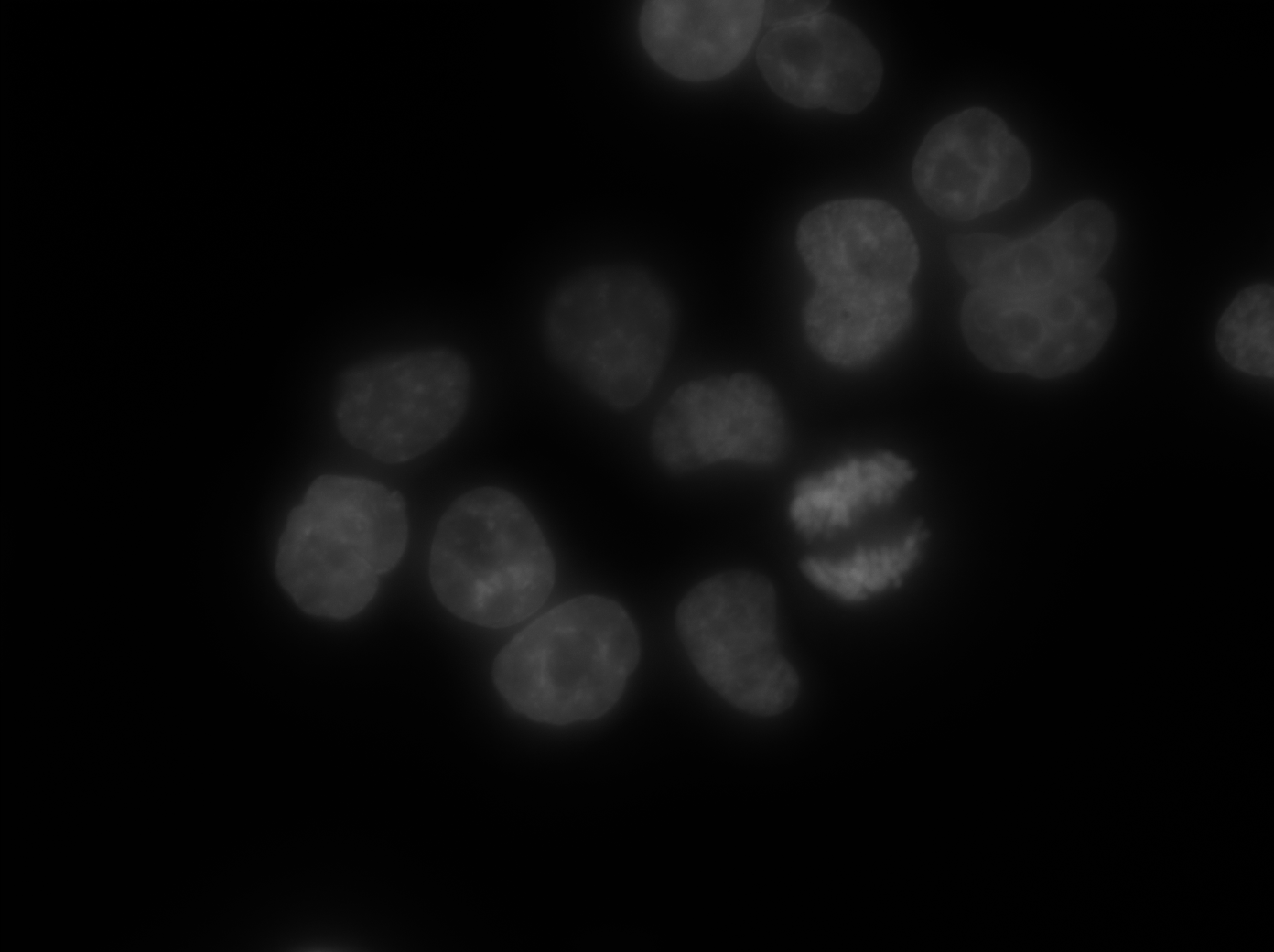

Supplement: Supplementary file 9 — Source data Fig. 2 part 6 [file 44319_2026_742_MOESM9_ESM.zip › Figure 2 Part 6/Fig 2fg Control Hela rGT335 acetylated tubulin/Anaphase/Cas9 actub rGT335 9-8-25 R1 A7.Project Maximum Z_XY1757356335_Z0_T0_C0.tif]

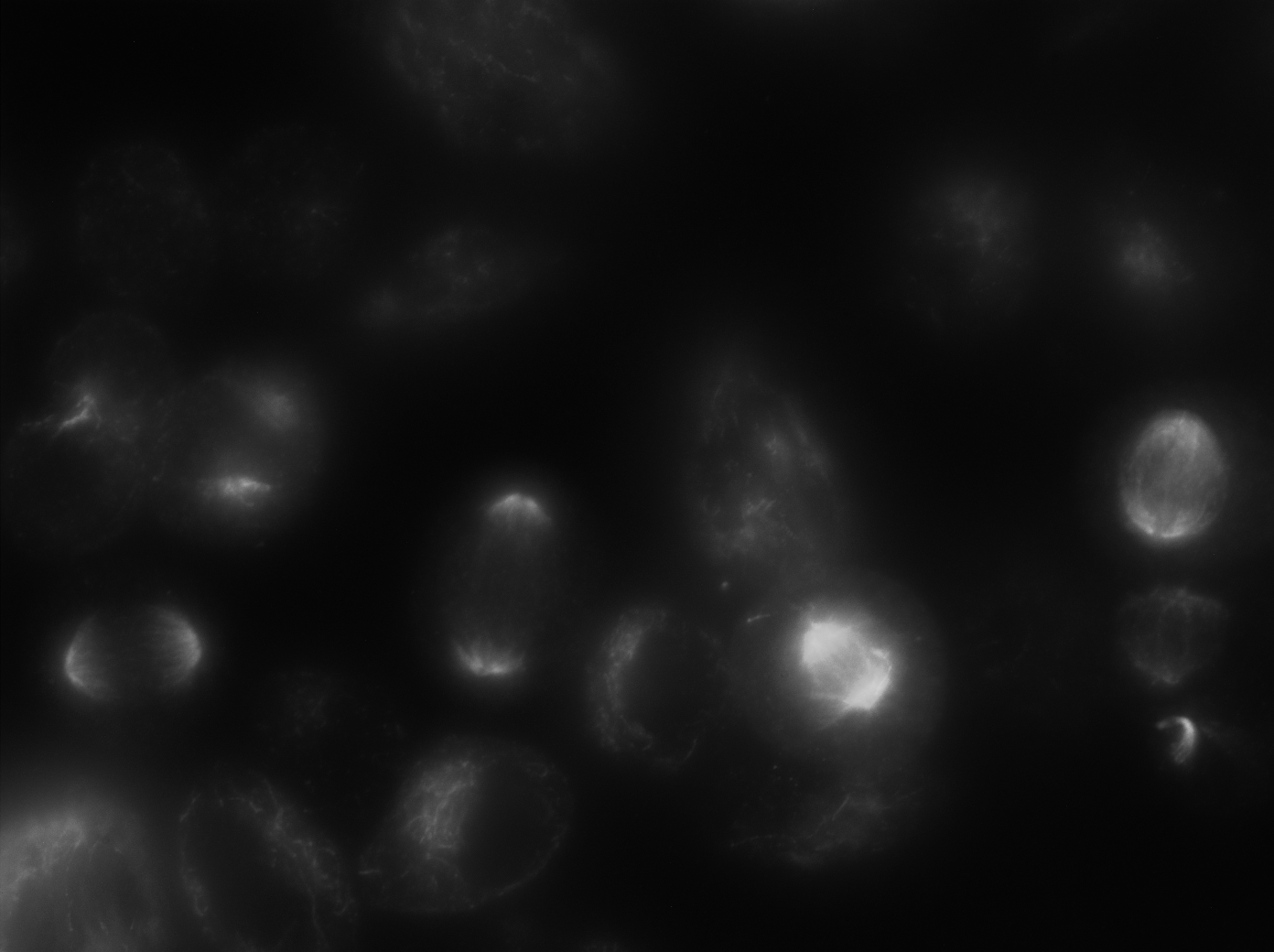

Supplement: Supplementary file 9 — Source data Fig. 2 part 6 [file 44319_2026_742_MOESM9_ESM.zip › Figure 2 Part 6/Fig 2fg Control Hela rGT335 acetylated tubulin/Anaphase/Cas9 actub rGT335 9-8-25 R2 A10A11 M10.Project Maximum Z_XY1757362500_Z0_T0_C2.tif]

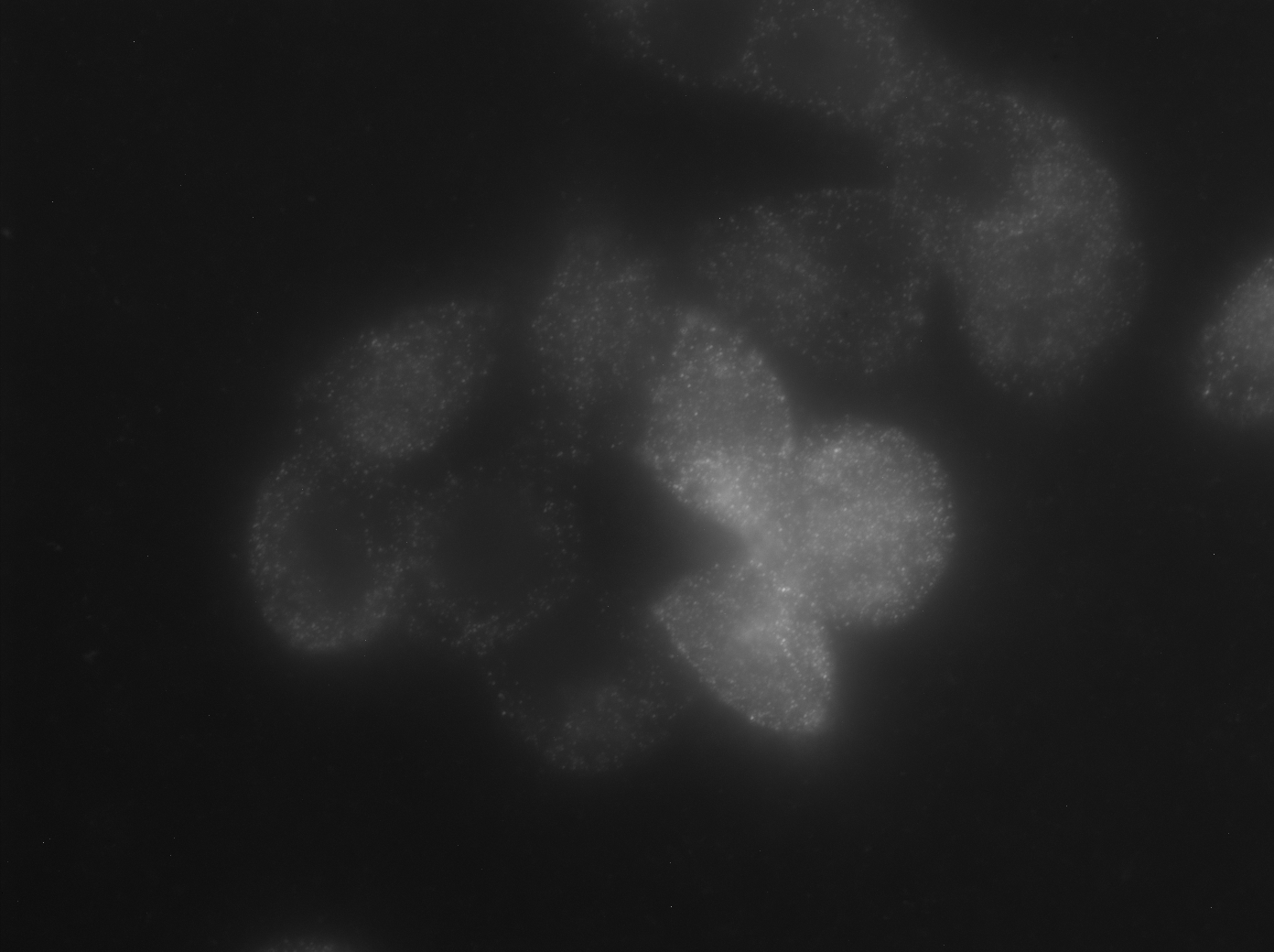

Supplement: Supplementary file 9 — Source data Fig. 2 part 6 [file 44319_2026_742_MOESM9_ESM.zip › Figure 2 Part 6/Fig 2fg Control Hela rGT335 acetylated tubulin/Anaphase/Cas9 actub rGT335 9-8-25 R1 A7.Project Maximum Z_XY1757356335_Z0_T0_C1.tif]

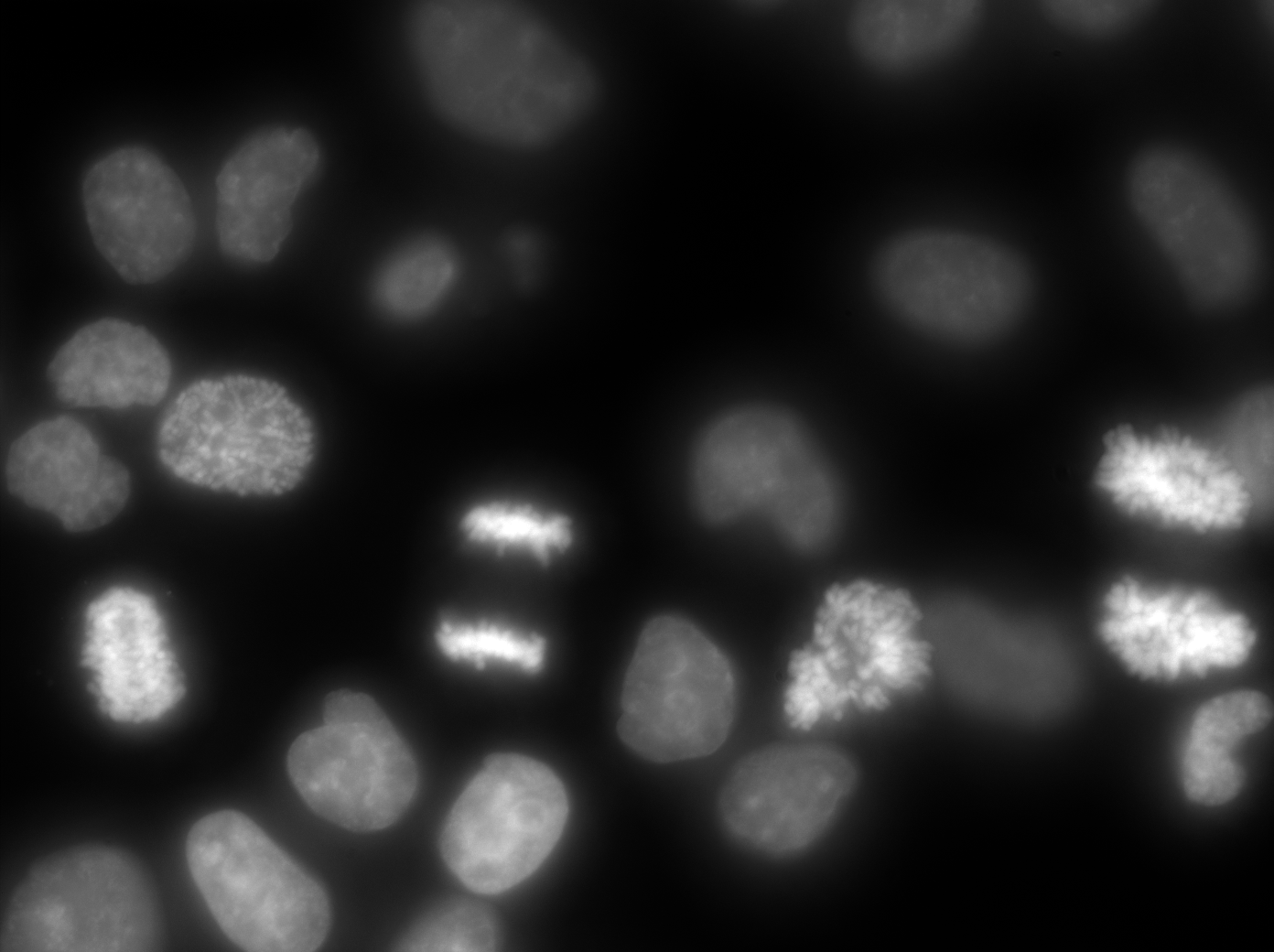

Supplement: Supplementary file 9 — Source data Fig. 2 part 6 [file 44319_2026_742_MOESM9_ESM.zip › Figure 2 Part 6/Fig 2fg Control Hela rGT335 acetylated tubulin/Anaphase/Cas9 actub rGT335 9-8-25 R2 A10A11 M10.Project Maximum Z_XY1757362500_Z0_T0_C0.tif]

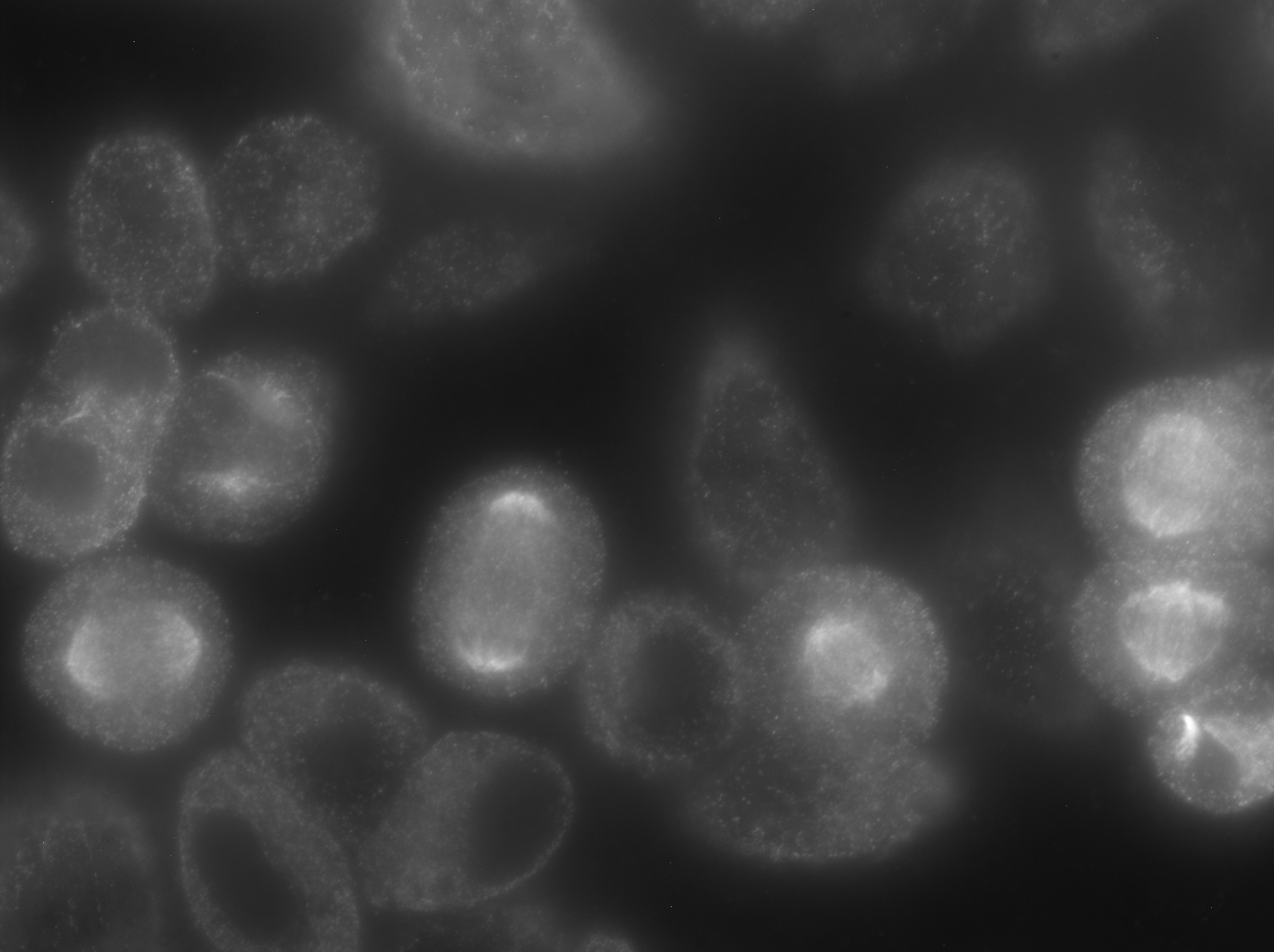

Supplement: Supplementary file 9 — Source data Fig. 2 part 6 [file 44319_2026_742_MOESM9_ESM.zip › Figure 2 Part 6/Fig 2fg Control Hela rGT335 acetylated tubulin/Anaphase/Cas9 actub rGT335 9-8-25 R2 A10A11 M10.Project Maximum Z_XY1757362500_Z0_T0_C1.tif]

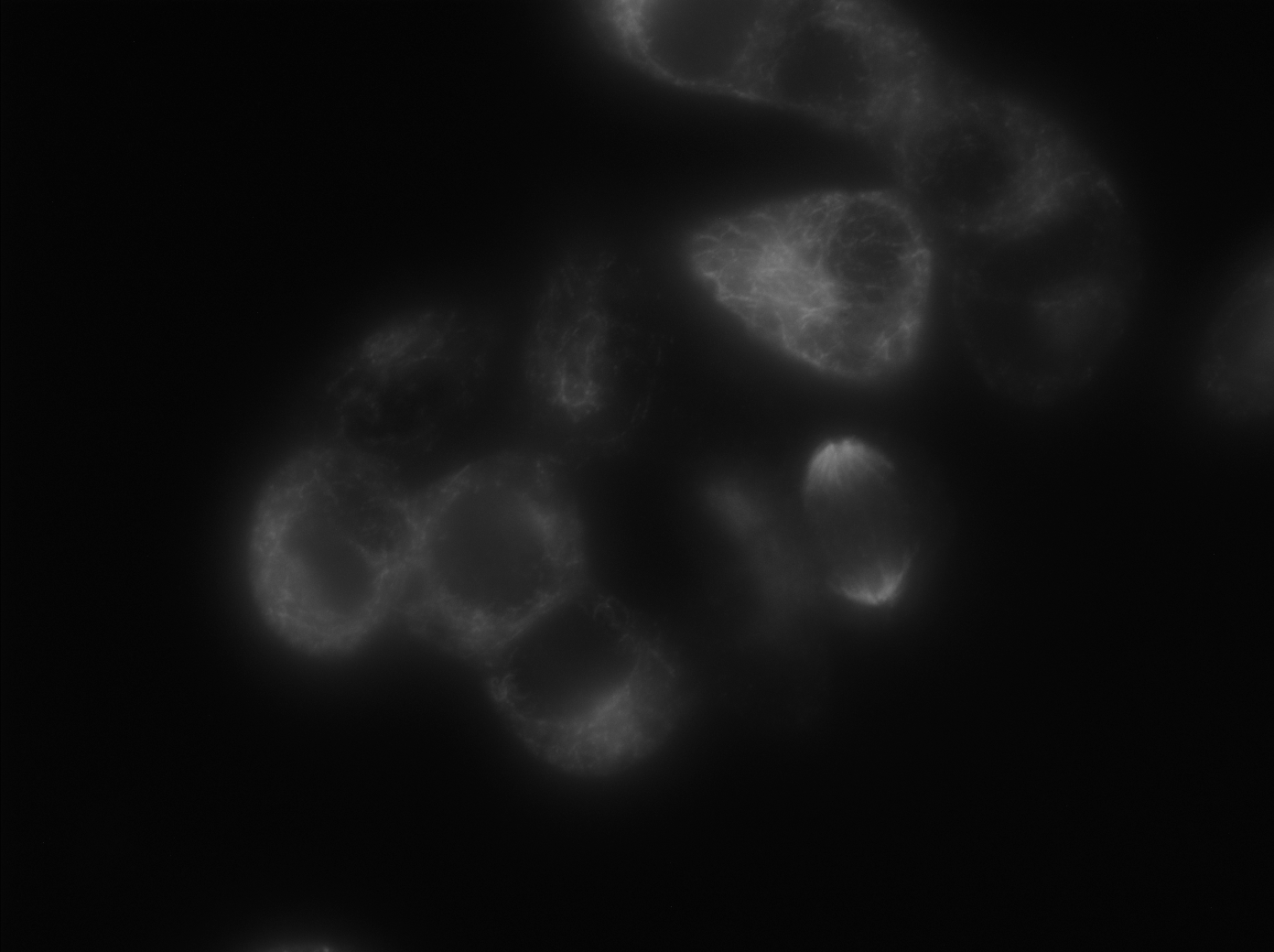

Supplement: Supplementary file 9 — Source data Fig. 2 part 6 [file 44319_2026_742_MOESM9_ESM.zip › Figure 2 Part 6/Fig 2fg Control Hela rGT335 acetylated tubulin/Anaphase/Cas9 actub rGT335 9-8-25 R1 A7.Project Maximum Z_XY1757356335_Z0_T0_C2.tif]

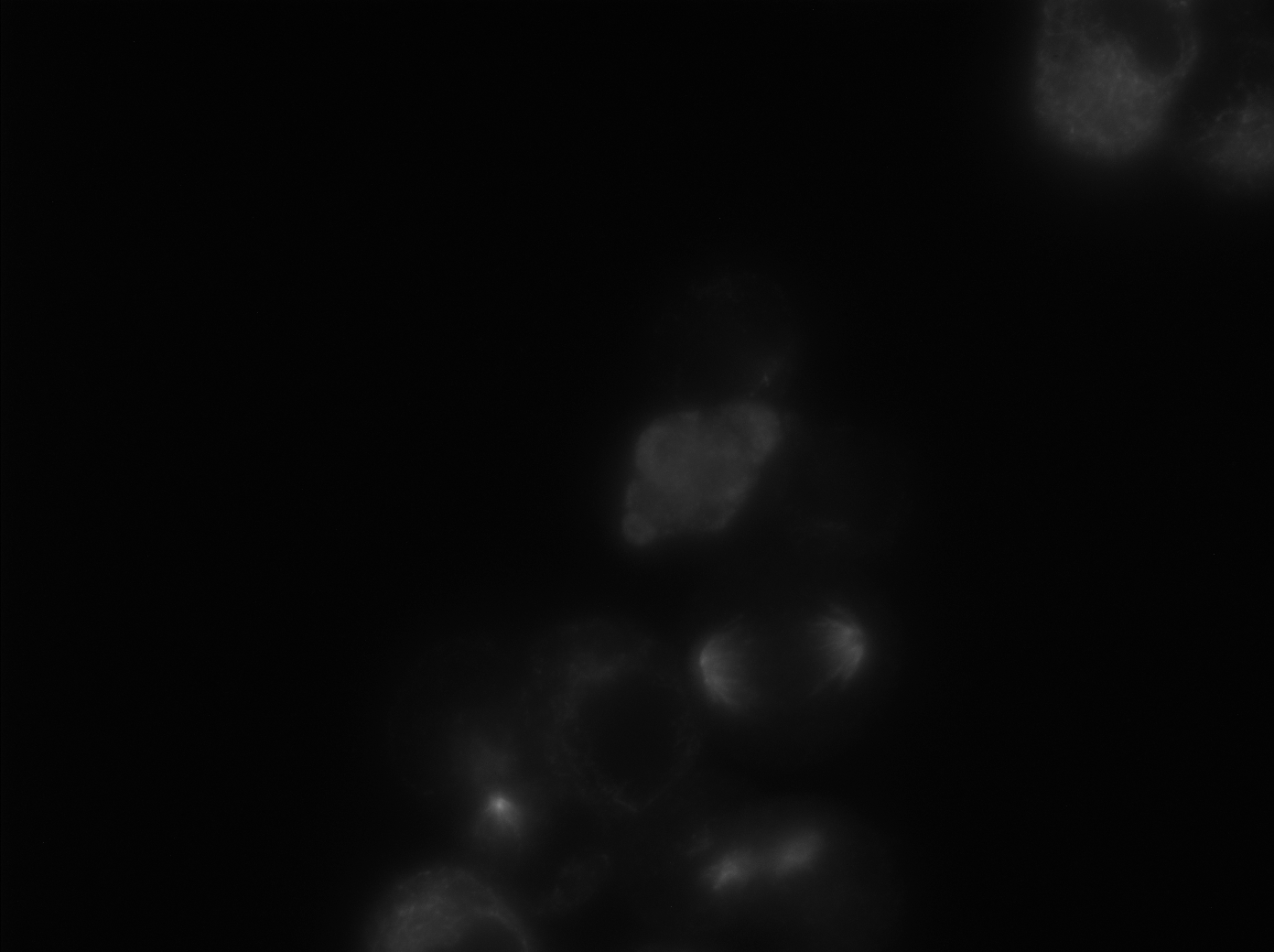

Supplement: Supplementary file 9 — Source data Fig. 2 part 6 [file 44319_2026_742_MOESM9_ESM.zip › Figure 2 Part 6/Fig 2fg Control Hela rGT335 acetylated tubulin/Anaphase/Cas9 actub rGT335 9-8-25 R1 A10.Project Maximum Z_XY1757358159_Z0_T0_C2.tif]

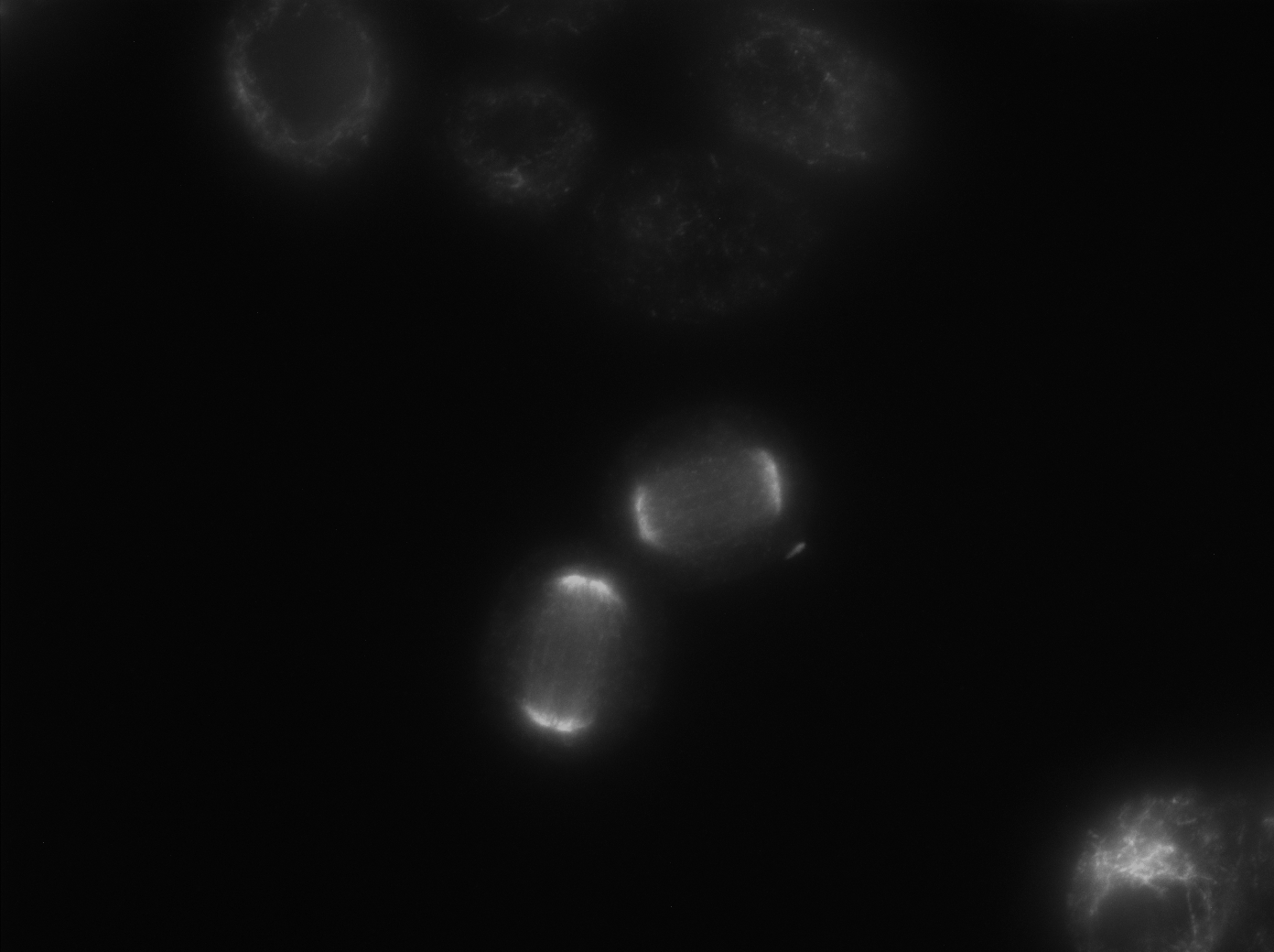

Supplement: Supplementary file 9 — Source data Fig. 2 part 6 [file 44319_2026_742_MOESM9_ESM.zip › Figure 2 Part 6/Fig 2fg Control Hela rGT335 acetylated tubulin/Anaphase/Cas9 actub rGT335 9-8-25 R3 A4A5.Project Maximum Z_XY1757363463_Z0_T0_C2.tif]

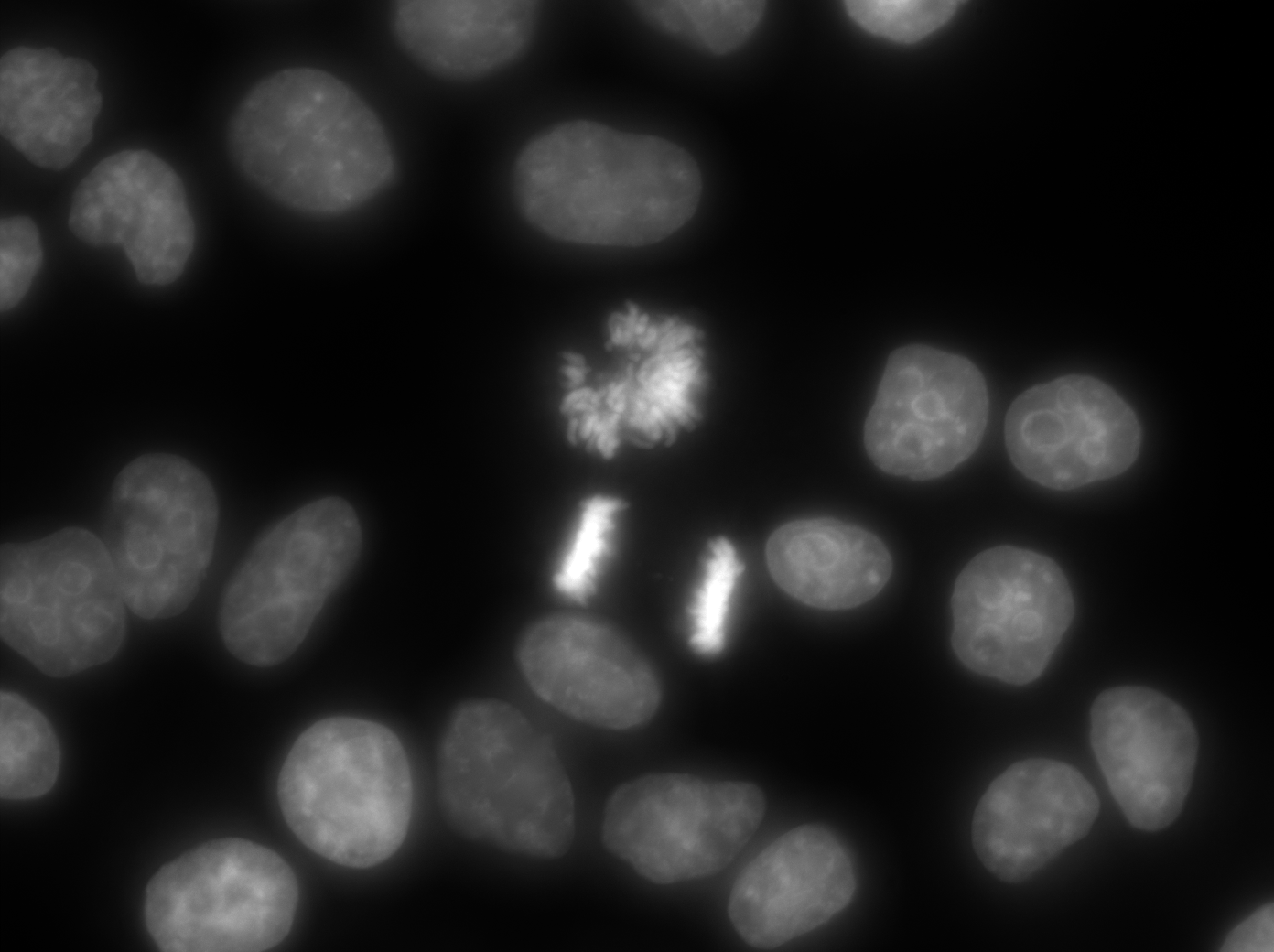

Supplement: Supplementary file 9 — Source data Fig. 2 part 6 [file 44319_2026_742_MOESM9_ESM.zip › Figure 2 Part 6/Fig 2fg Control Hela rGT335 acetylated tubulin/Anaphase/Cas9 actub rGT335 9-8-25 R2 A6.Project Maximum Z_XY1757361053_Z0_T0_C0.tif]

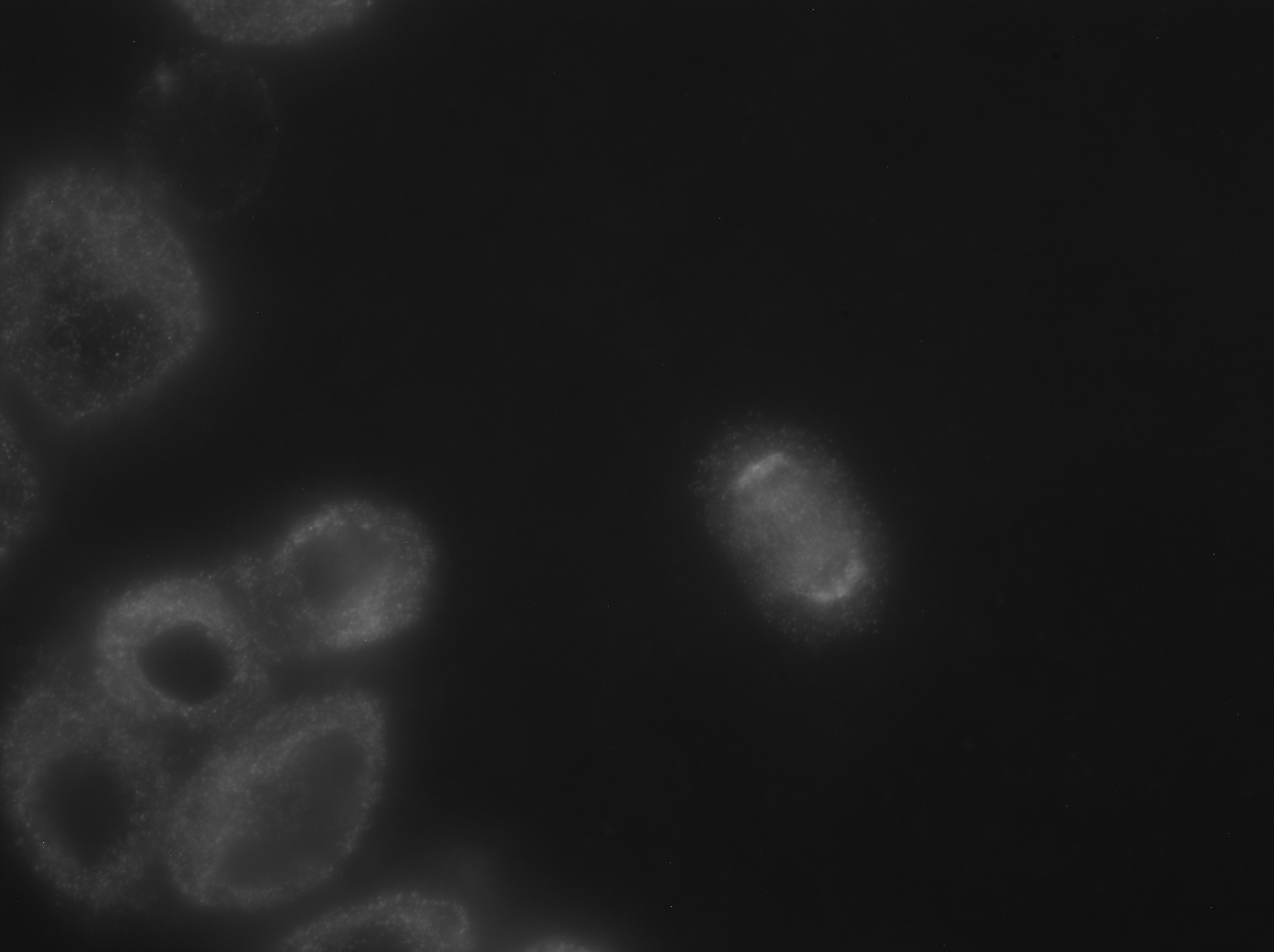

Supplement: Supplementary file 9 — Source data Fig. 2 part 6 [file 44319_2026_742_MOESM9_ESM.zip › Figure 2 Part 6/Fig 2fg Control Hela rGT335 acetylated tubulin/Anaphase/Cas9 actub rGT335 9-8-25 R2 A5.Project Maximum Z_XY1757360924_Z0_T0_C1.tif]

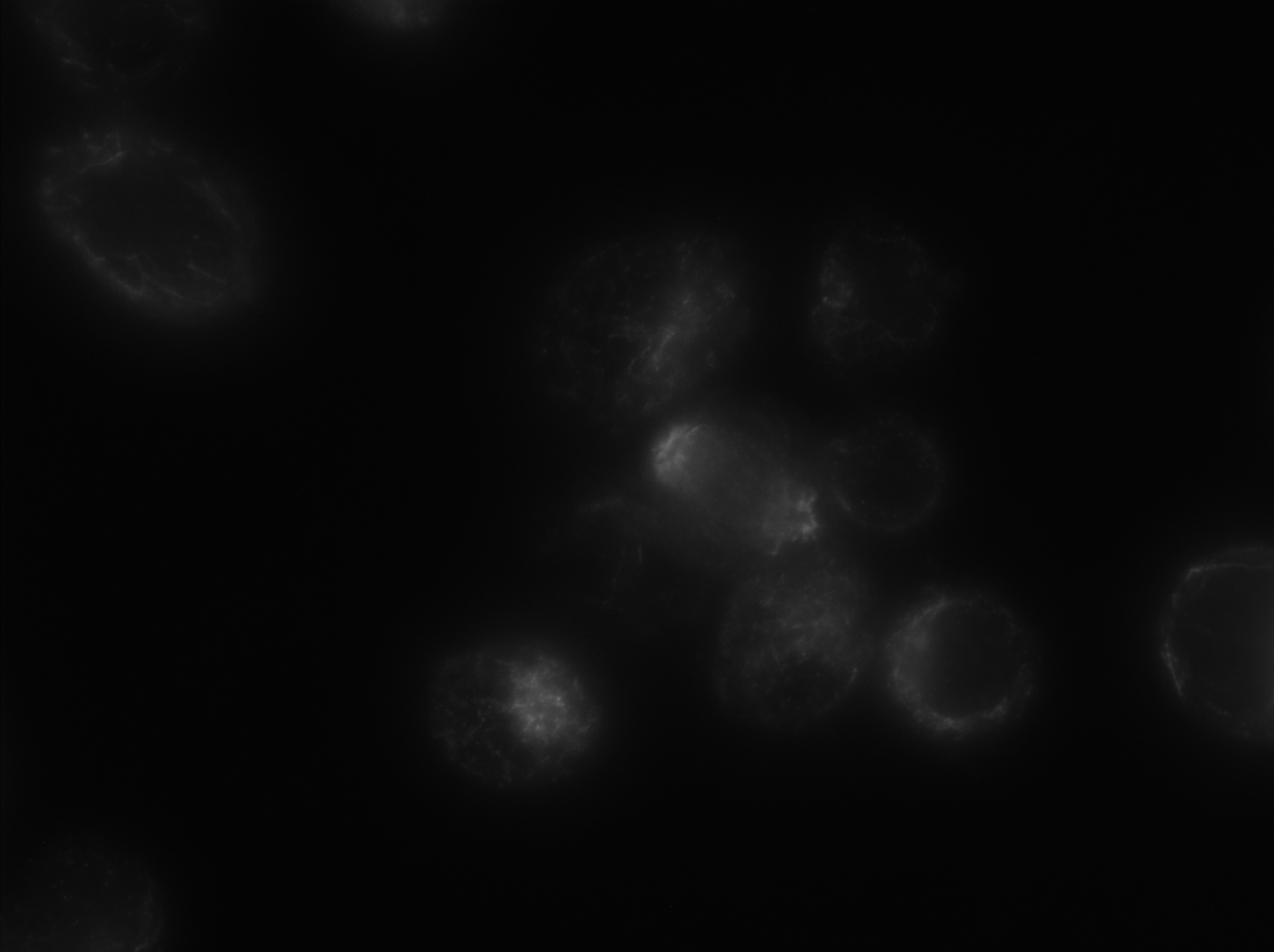

Supplement: Supplementary file 9 — Source data Fig. 2 part 6 [file 44319_2026_742_MOESM9_ESM.zip › Figure 2 Part 6/Fig 2fg Control Hela rGT335 acetylated tubulin/Anaphase/Cas9 actub rGT335 9-8-25 R2 A8.Project Maximum Z_XY1757361749_Z0_T0_C2.tif]

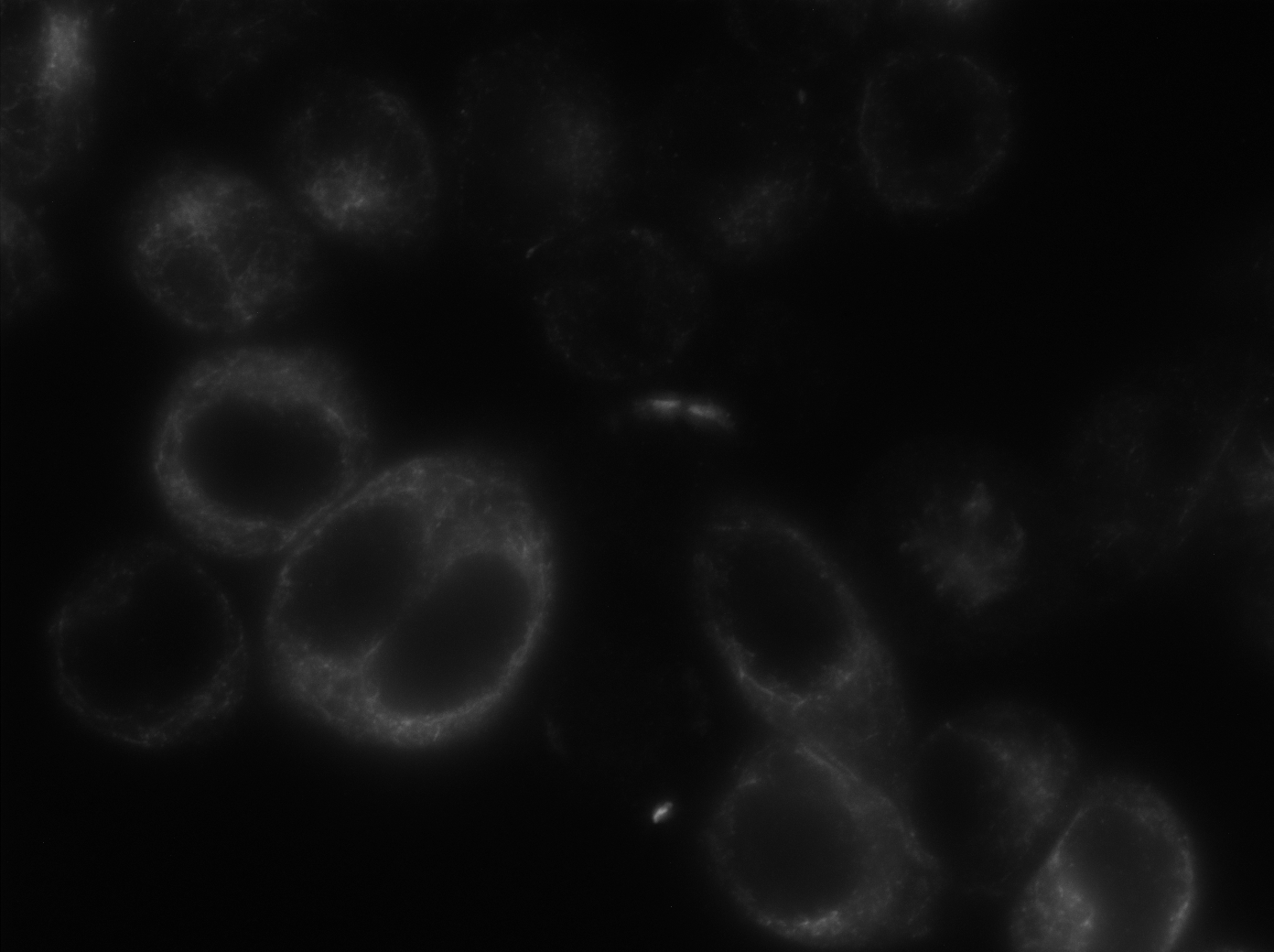

Supplement: Supplementary file 9 — Source data Fig. 2 part 6 [file 44319_2026_742_MOESM9_ESM.zip › Figure 2 Part 6/Fig 2fg Control Hela rGT335 acetylated tubulin/Anaphase/Cas9 actub rGT335 9-8-25 R3 A7.Project Maximum Z_XY1757368647_Z0_T0_C2.tif]

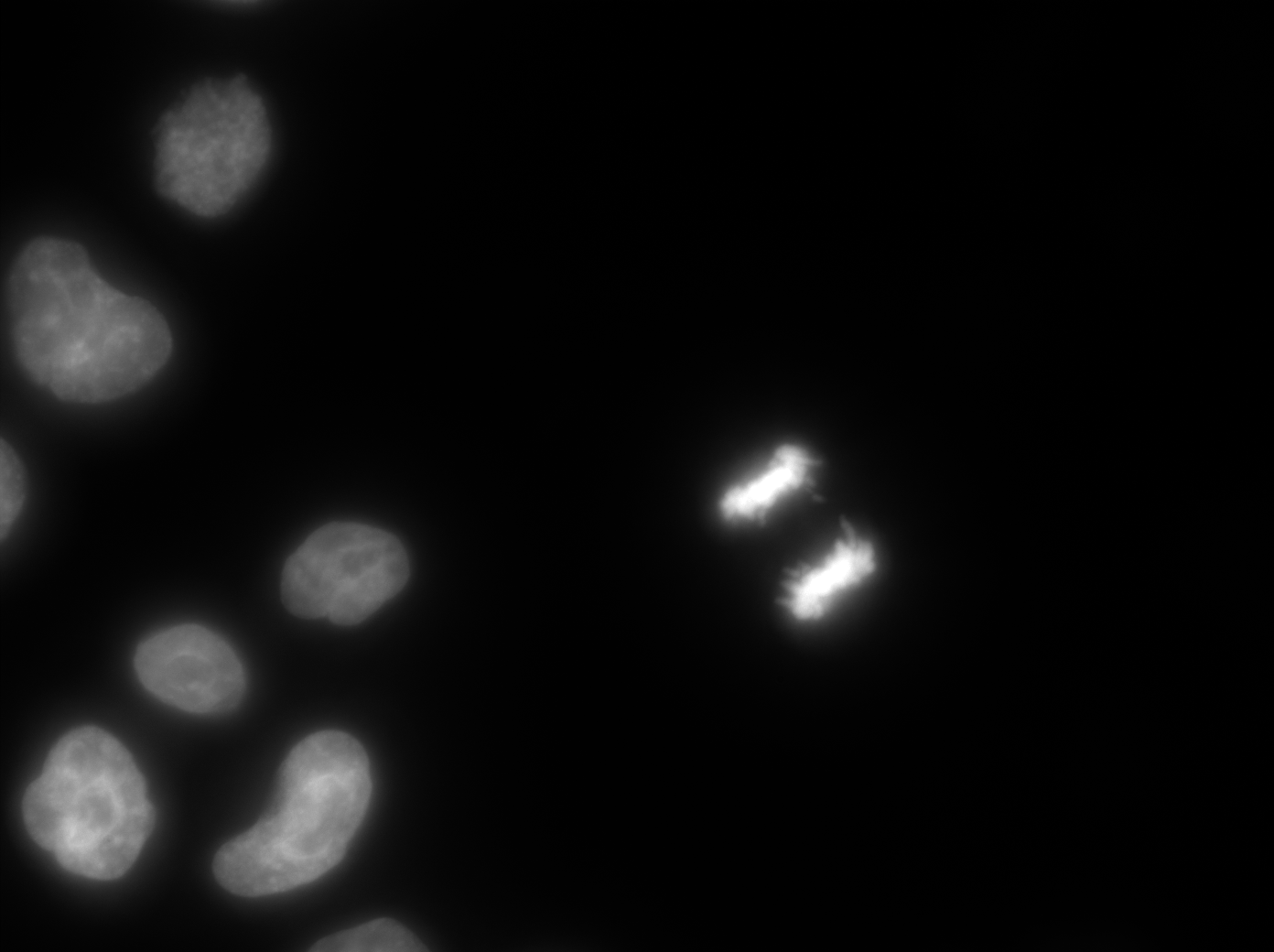

Supplement: Supplementary file 9 — Source data Fig. 2 part 6 [file 44319_2026_742_MOESM9_ESM.zip › Figure 2 Part 6/Fig 2fg Control Hela rGT335 acetylated tubulin/Anaphase/Cas9 actub rGT335 9-8-25 R2 A5.Project Maximum Z_XY1757360924_Z0_T0_C0.tif]

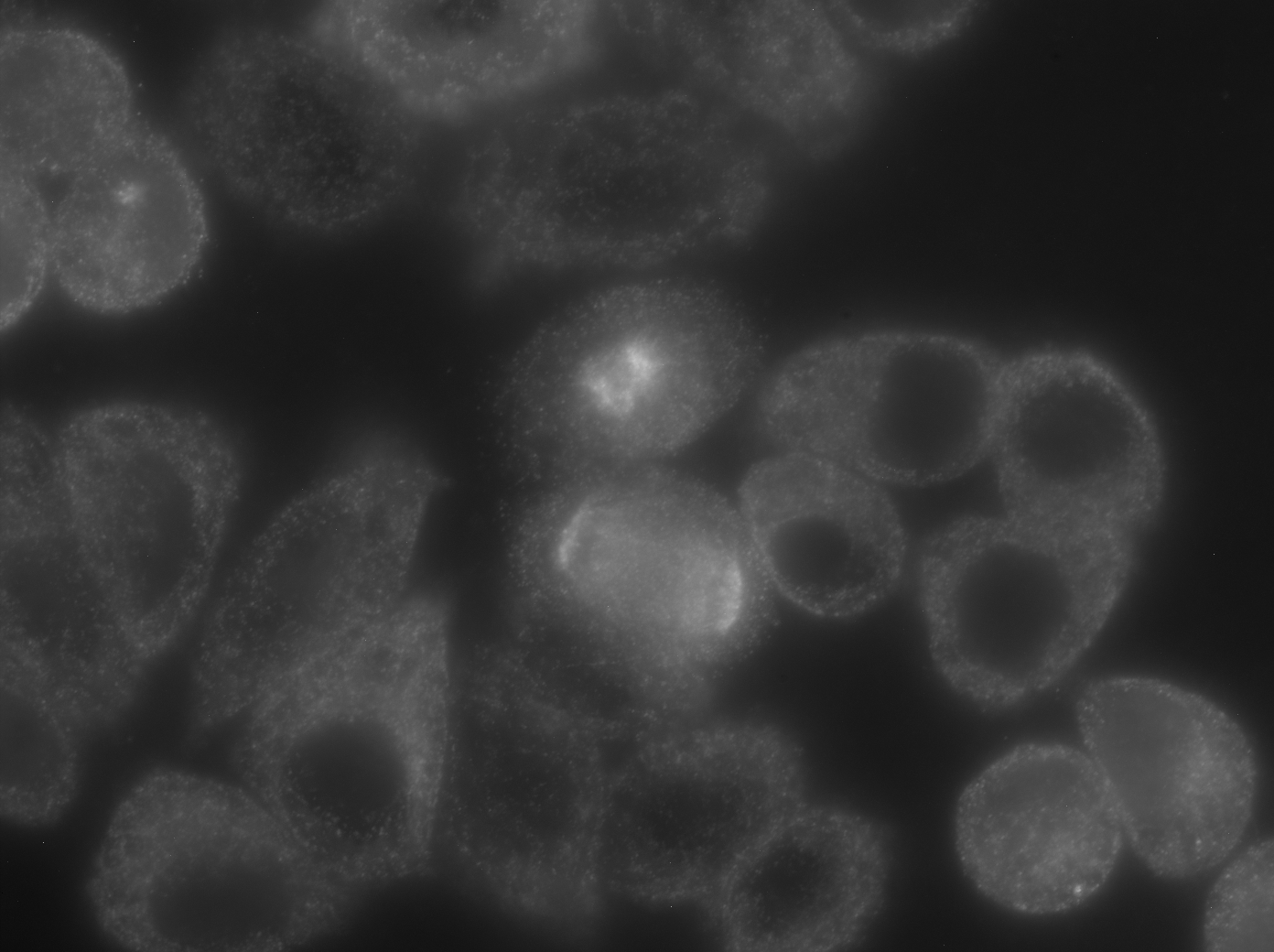

Supplement: Supplementary file 9 — Source data Fig. 2 part 6 [file 44319_2026_742_MOESM9_ESM.zip › Figure 2 Part 6/Fig 2fg Control Hela rGT335 acetylated tubulin/Anaphase/Cas9 actub rGT335 9-8-25 R2 A6.Project Maximum Z_XY1757361053_Z0_T0_C1.tif]

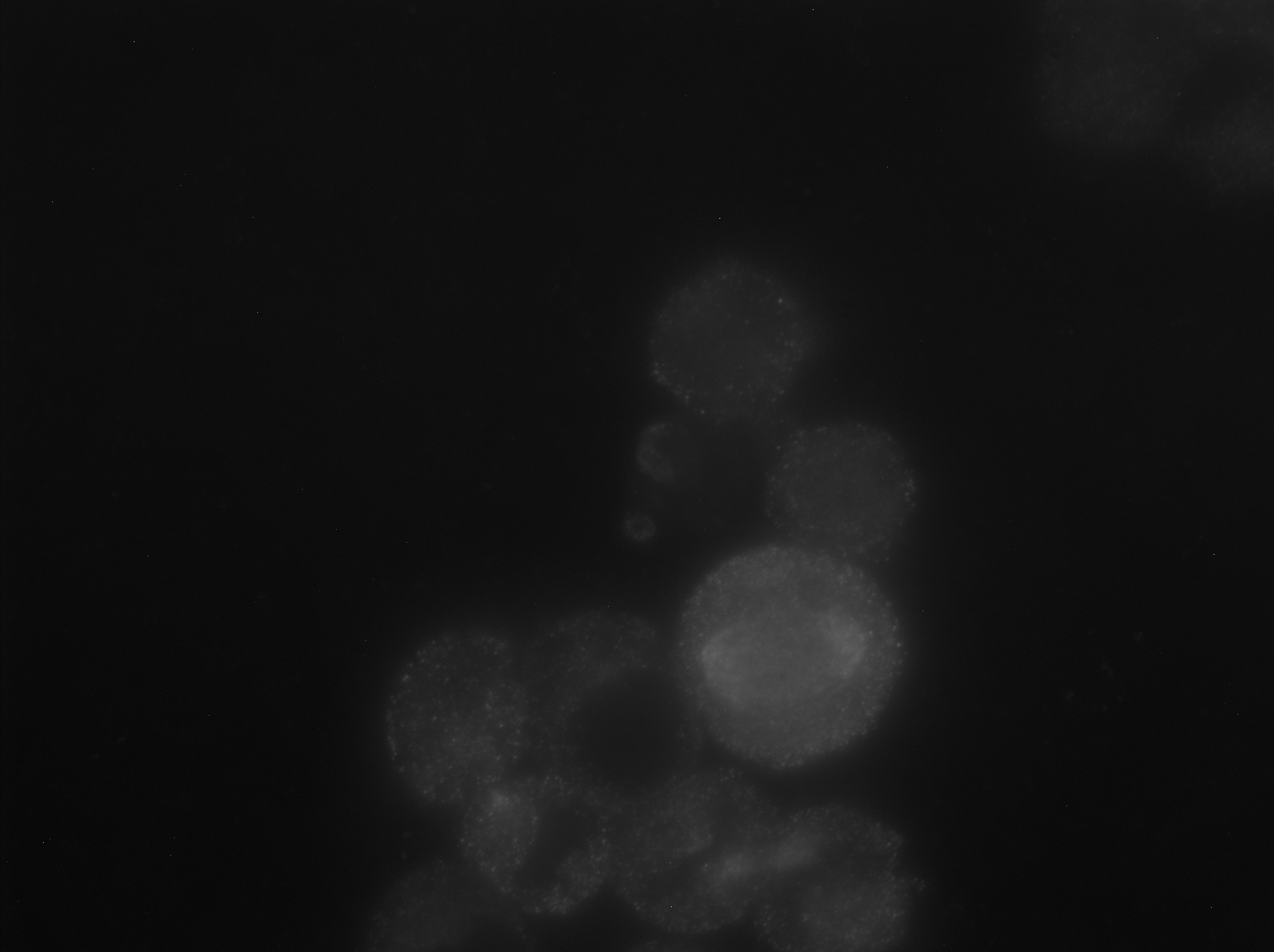

Supplement: Supplementary file 9 — Source data Fig. 2 part 6 [file 44319_2026_742_MOESM9_ESM.zip › Figure 2 Part 6/Fig 2fg Control Hela rGT335 acetylated tubulin/Anaphase/Cas9 actub rGT335 9-8-25 R1 A10.Project Maximum Z_XY1757358159_Z0_T0_C1.tif]

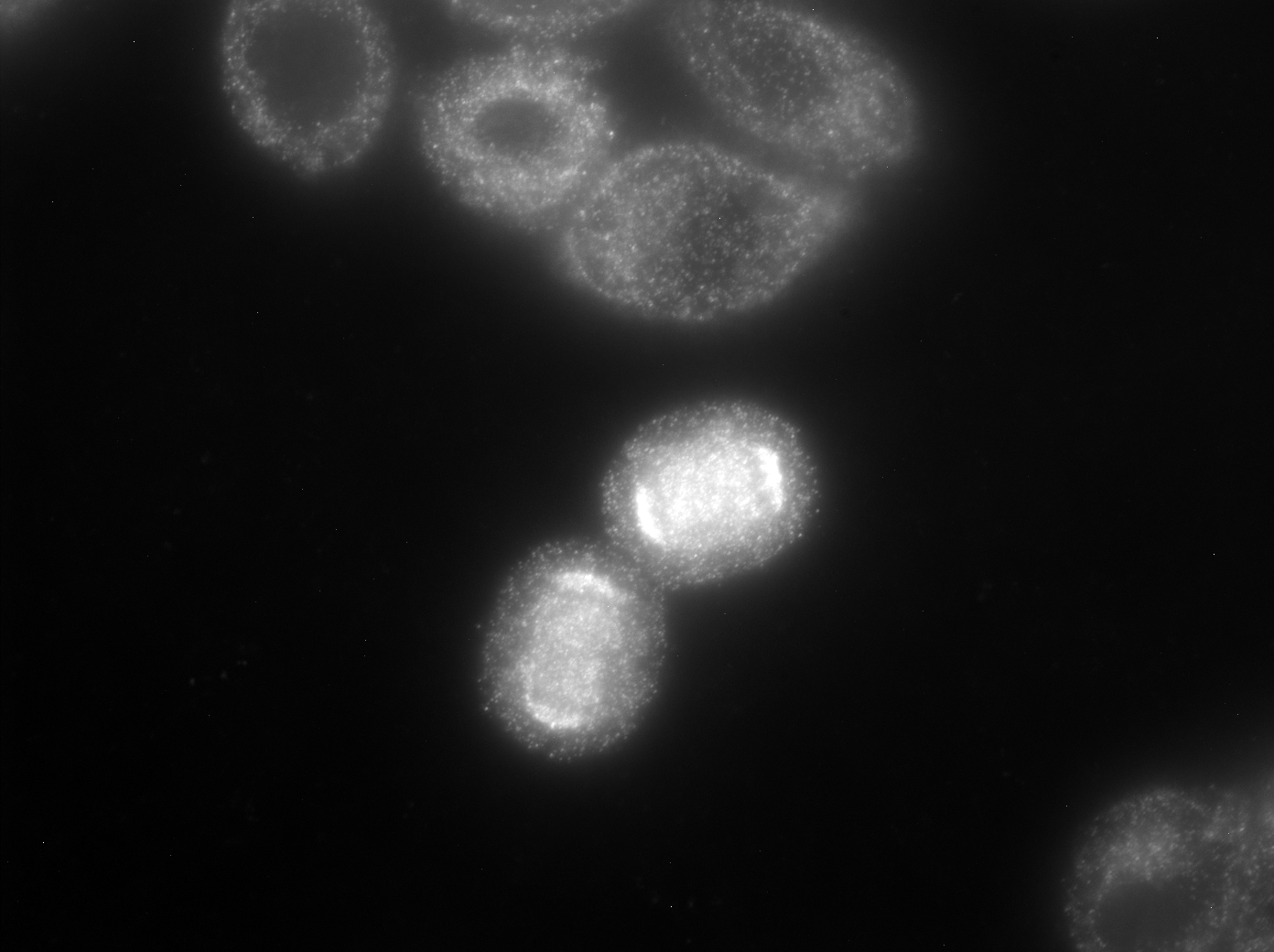

Supplement: Supplementary file 9 — Source data Fig. 2 part 6 [file 44319_2026_742_MOESM9_ESM.zip › Figure 2 Part 6/Fig 2fg Control Hela rGT335 acetylated tubulin/Anaphase/Cas9 actub rGT335 9-8-25 R3 A4A5.Project Maximum Z_XY1757363463_Z0_T0_C1.tif]

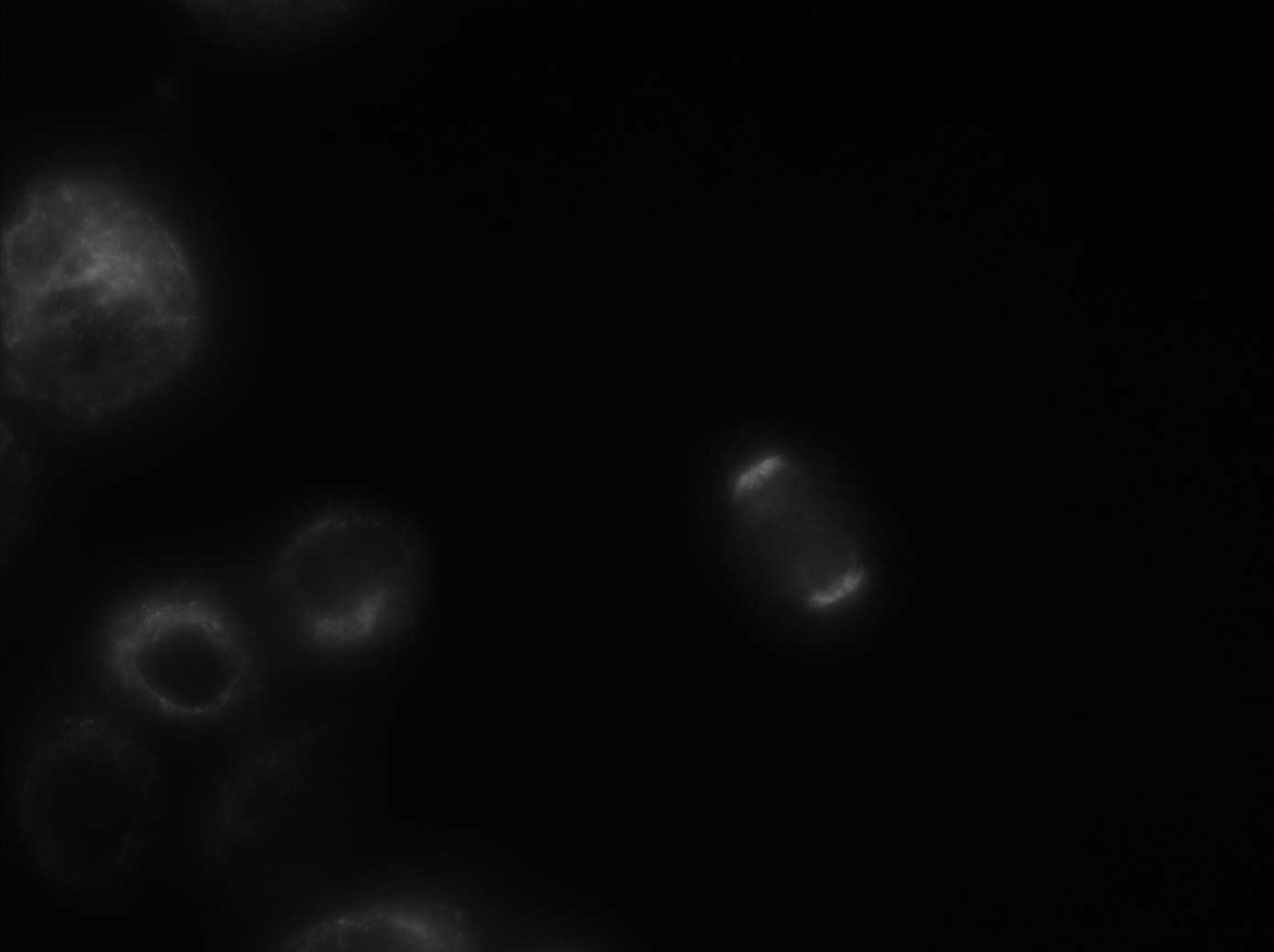

Supplement: Supplementary file 9 — Source data Fig. 2 part 6 [file 44319_2026_742_MOESM9_ESM.zip › Figure 2 Part 6/Fig 2fg Control Hela rGT335 acetylated tubulin/Anaphase/Cas9 actub rGT335 9-8-25 R2 A5.Project Maximum Z_XY1757360924_Z0_T0_C2.tif]

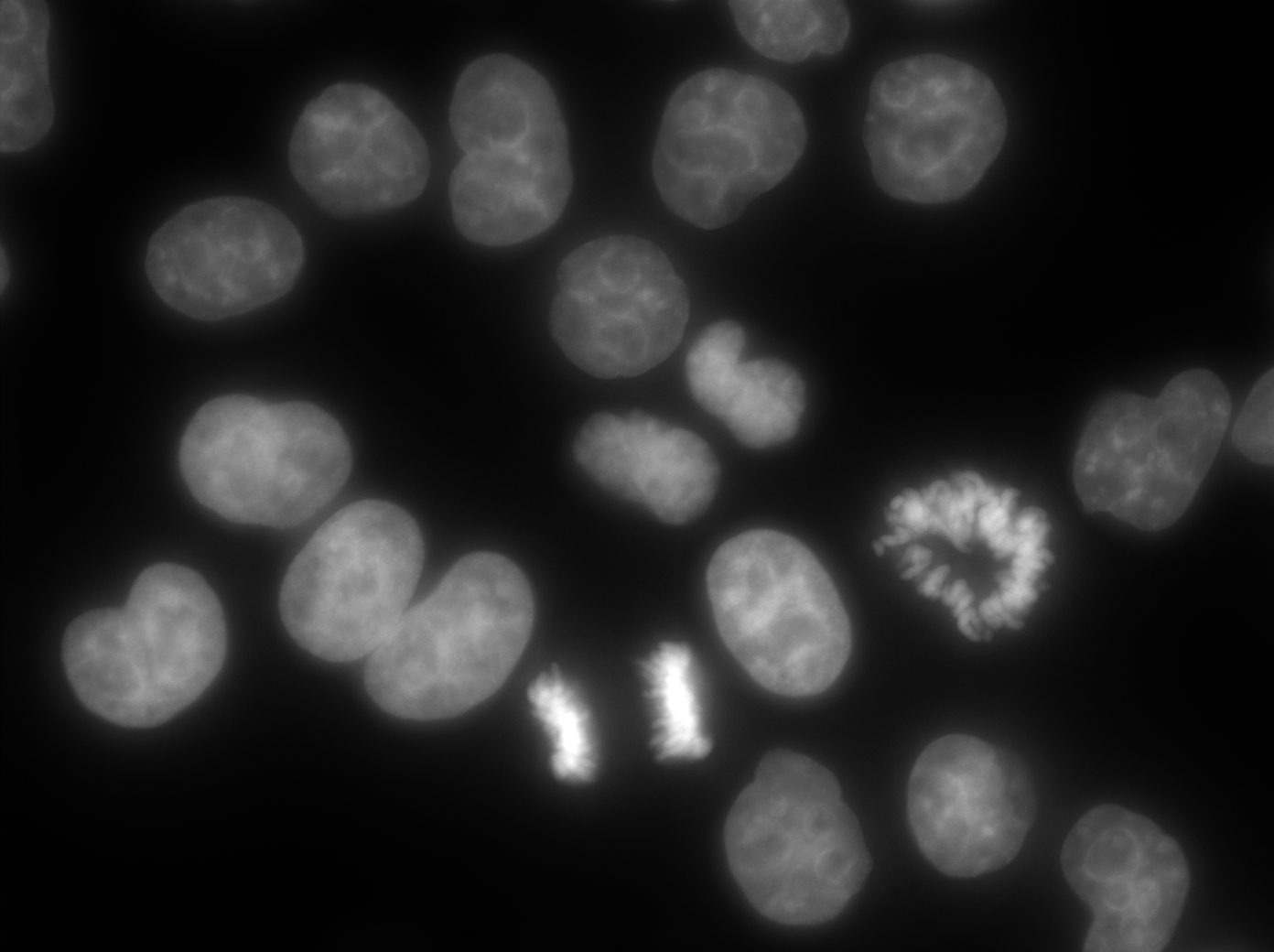

Supplement: Supplementary file 9 — Source data Fig. 2 part 6 [file 44319_2026_742_MOESM9_ESM.zip › Figure 2 Part 6/Fig 2fg Control Hela rGT335 acetylated tubulin/Anaphase/Cas9 actub rGT335 9-8-25 R3 A7.Project Maximum Z_XY1757368647_Z0_T0_C0.tif]

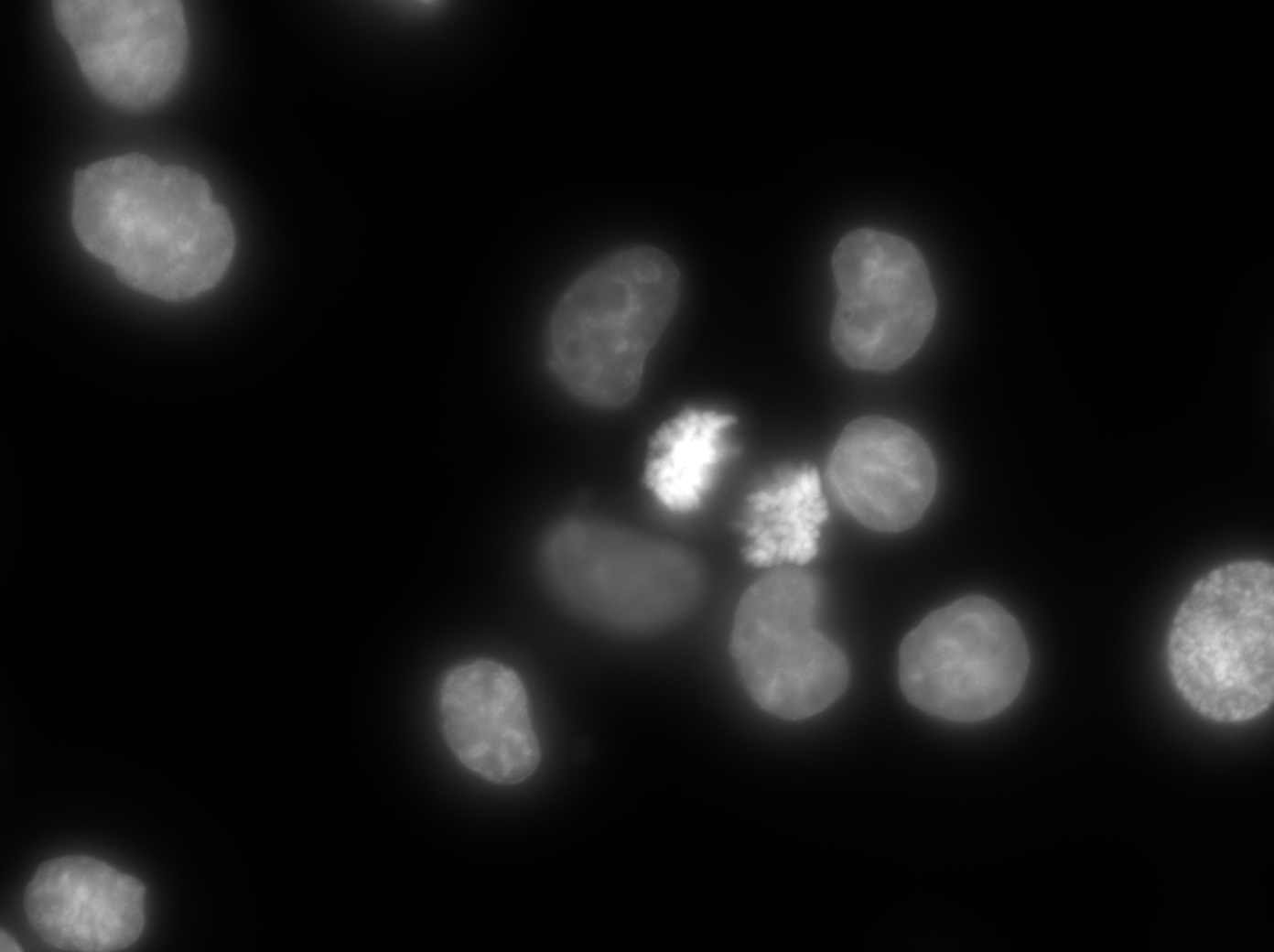

Supplement: Supplementary file 9 — Source data Fig. 2 part 6 [file 44319_2026_742_MOESM9_ESM.zip › Figure 2 Part 6/Fig 2fg Control Hela rGT335 acetylated tubulin/Anaphase/Cas9 actub rGT335 9-8-25 R2 A8.Project Maximum Z_XY1757361749_Z0_T0_C0.tif]

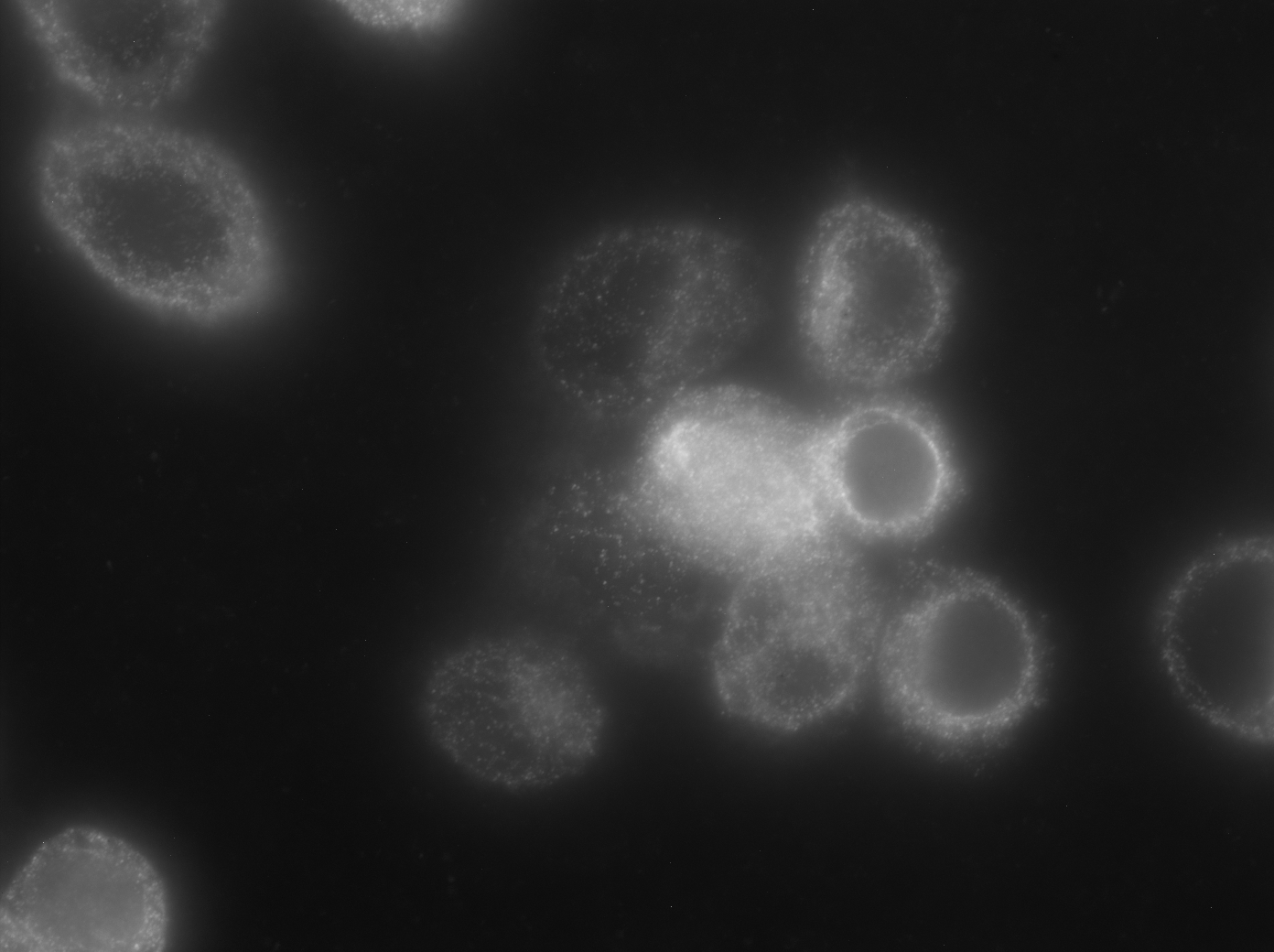

Supplement: Supplementary file 9 — Source data Fig. 2 part 6 [file 44319_2026_742_MOESM9_ESM.zip › Figure 2 Part 6/Fig 2fg Control Hela rGT335 acetylated tubulin/Anaphase/Cas9 actub rGT335 9-8-25 R2 A8.Project Maximum Z_XY1757361749_Z0_T0_C1.tif]

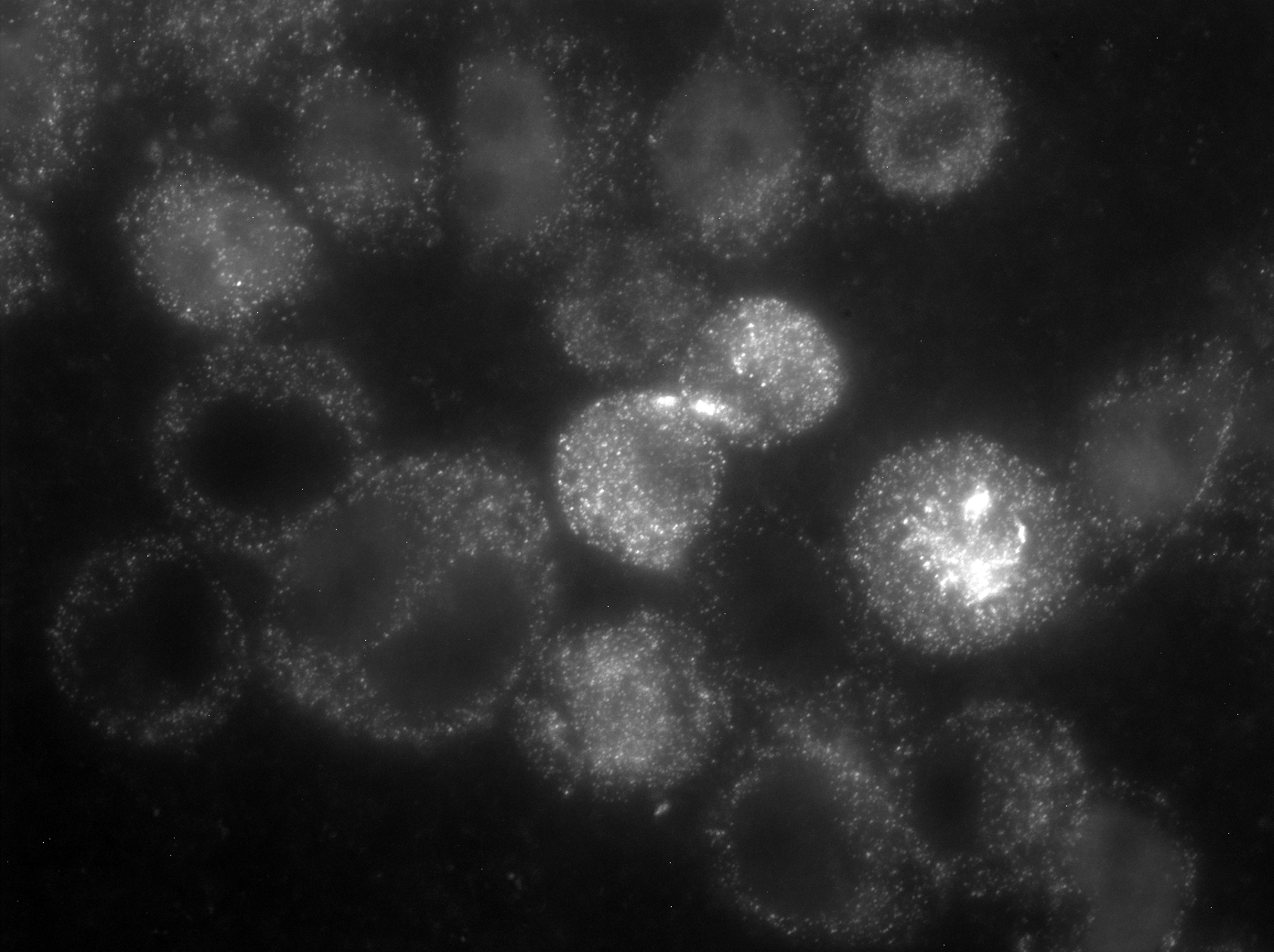

Supplement: Supplementary file 9 — Source data Fig. 2 part 6 [file 44319_2026_742_MOESM9_ESM.zip › Figure 2 Part 6/Fig 2fg Control Hela rGT335 acetylated tubulin/Anaphase/Cas9 actub rGT335 9-8-25 R3 A7.Project Maximum Z_XY1757368647_Z0_T0_C1.tif]

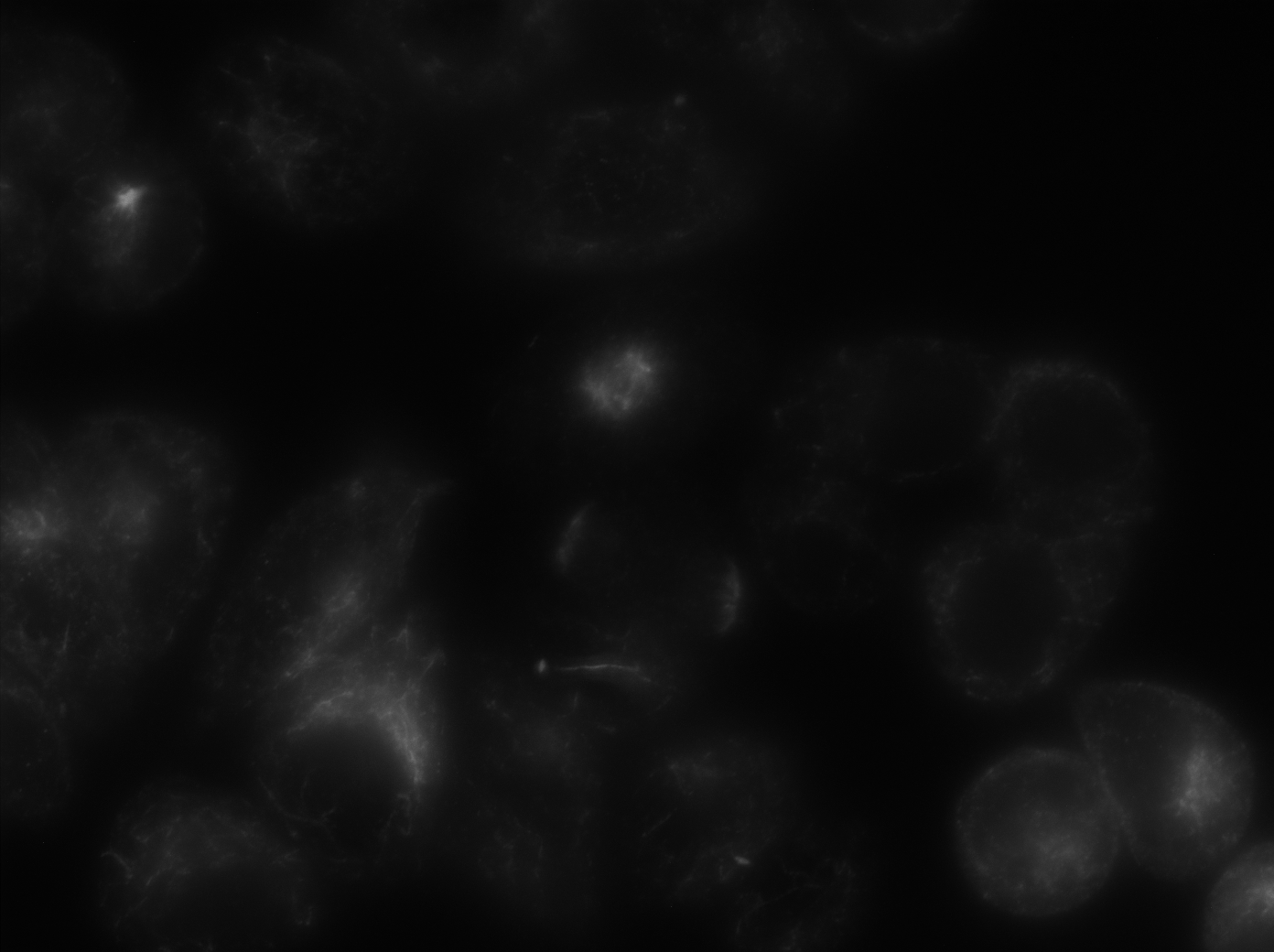

Supplement: Supplementary file 9 — Source data Fig. 2 part 6 [file 44319_2026_742_MOESM9_ESM.zip › Figure 2 Part 6/Fig 2fg Control Hela rGT335 acetylated tubulin/Anaphase/Cas9 actub rGT335 9-8-25 R2 A6.Project Maximum Z_XY1757361053_Z0_T0_C2.tif]

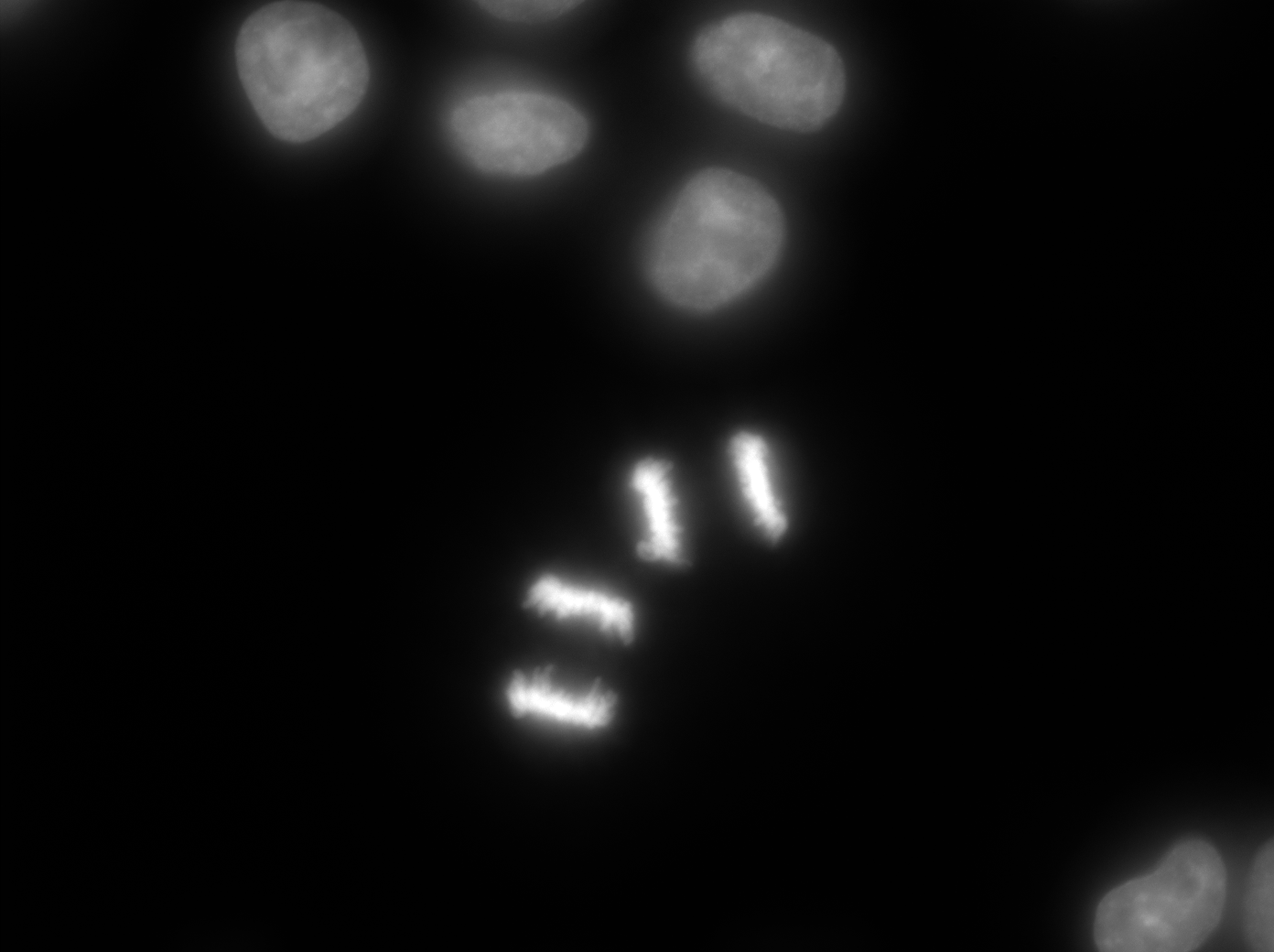

Supplement: Supplementary file 9 — Source data Fig. 2 part 6 [file 44319_2026_742_MOESM9_ESM.zip › Figure 2 Part 6/Fig 2fg Control Hela rGT335 acetylated tubulin/Anaphase/Cas9 actub rGT335 9-8-25 R3 A4A5.Project Maximum Z_XY1757363463_Z0_T0_C0.tif]

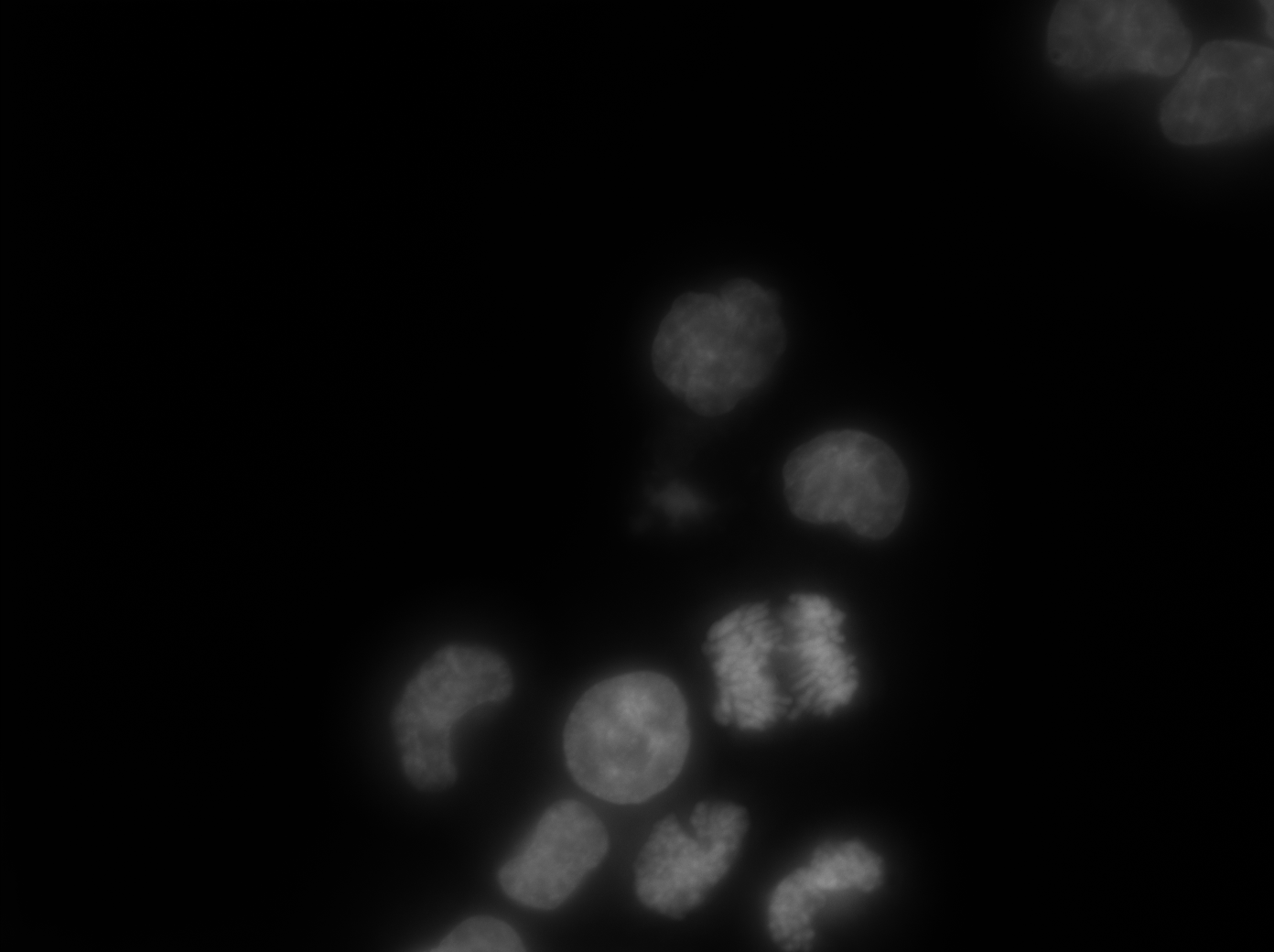

Supplement: Supplementary file 9 — Source data Fig. 2 part 6 [file 44319_2026_742_MOESM9_ESM.zip › Figure 2 Part 6/Fig 2fg Control Hela rGT335 acetylated tubulin/Anaphase/Cas9 actub rGT335 9-8-25 R1 A10.Project Maximum Z_XY1757358159_Z0_T0_C0.tif]

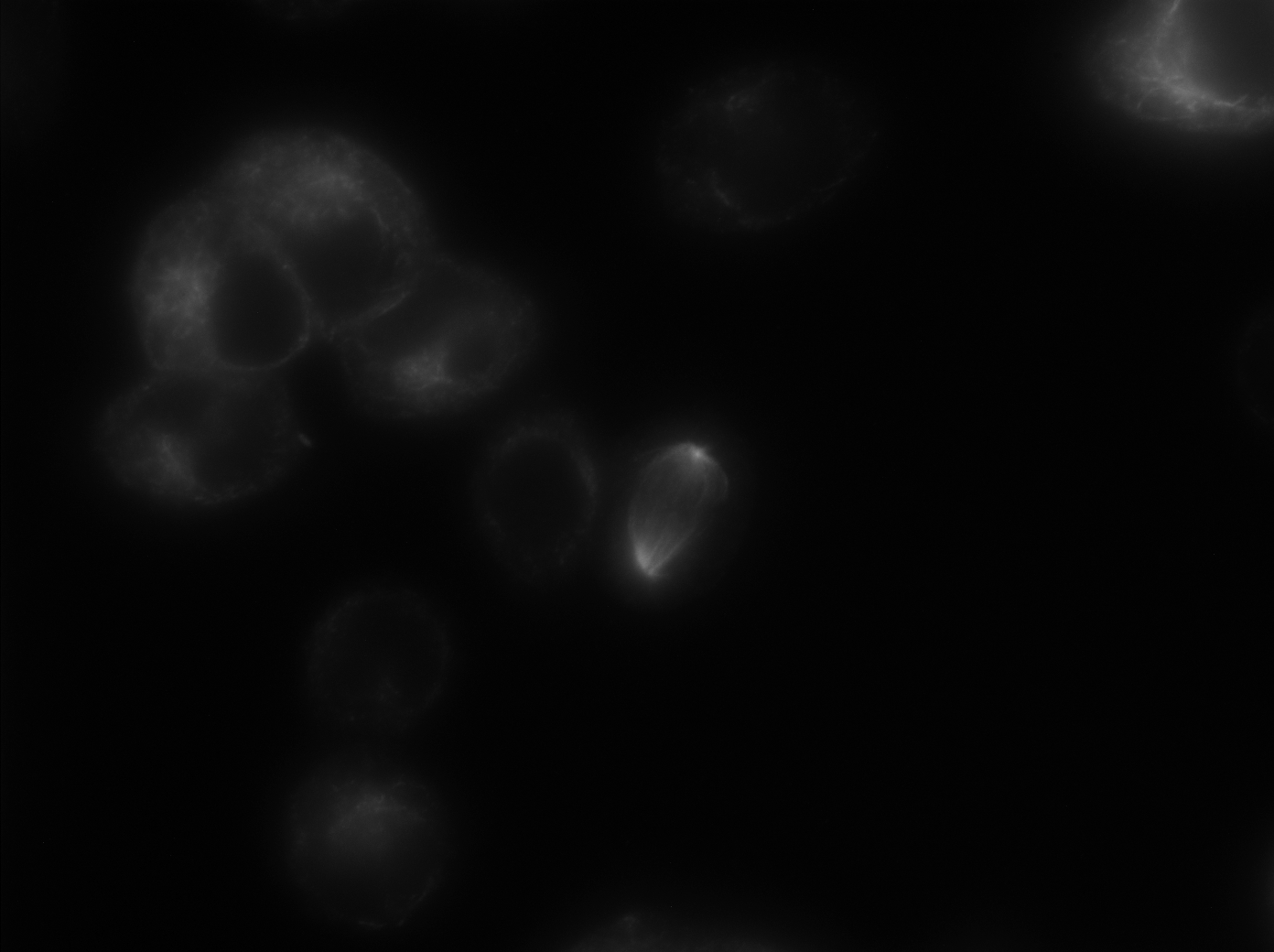

Supplement: Supplementary file 9 — Source data Fig. 2 part 6 [file 44319_2026_742_MOESM9_ESM.zip › Figure 2 Part 6/Fig 2fg Control Hela rGT335 acetylated tubulin/Anaphase/Cas9 actub rGT335 9-8-25 R1 A5.Project Maximum Z_XY1757355139_Z0_T0_C2.tif]

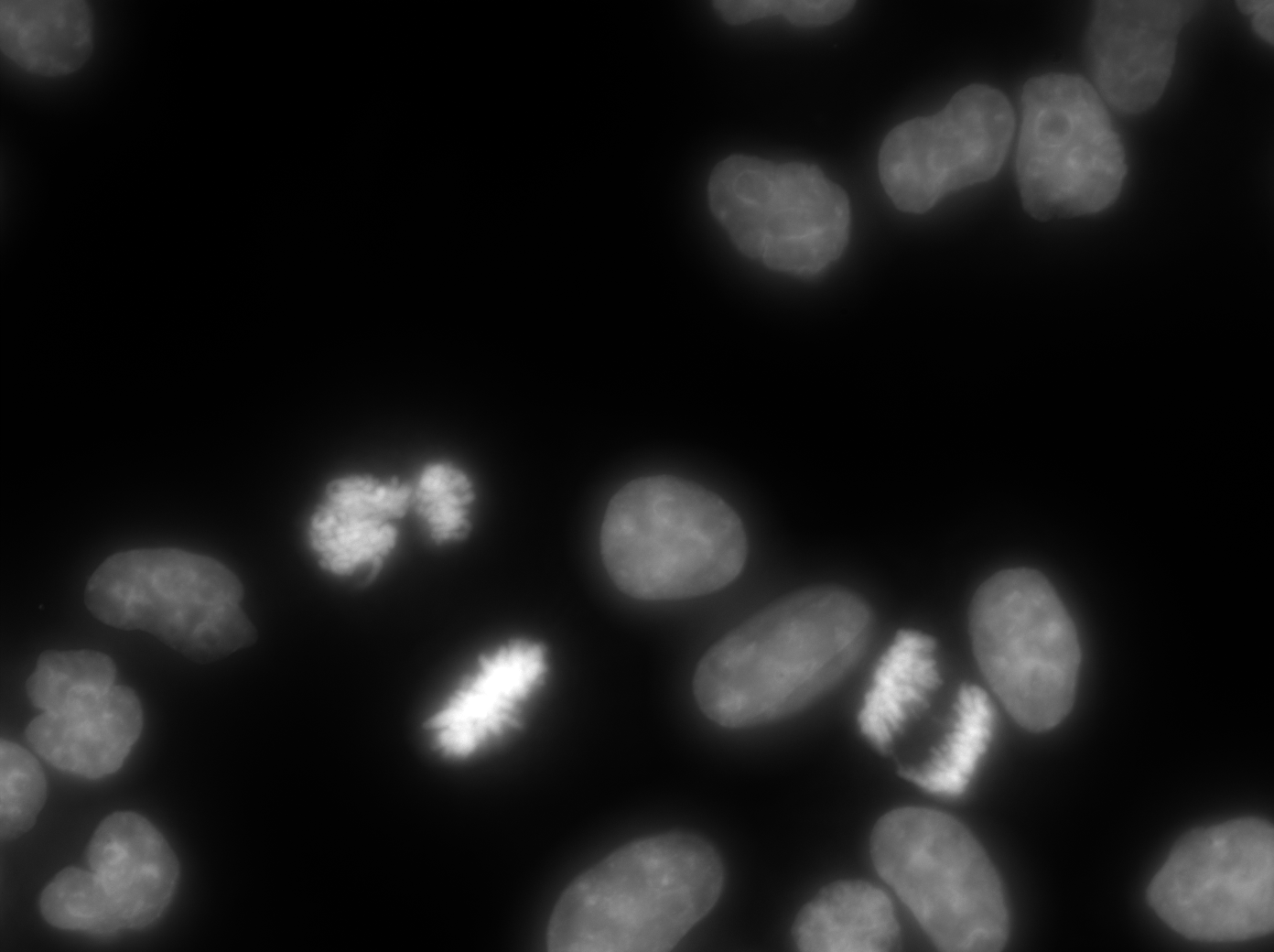

Supplement: Supplementary file 9 — Source data Fig. 2 part 6 [file 44319_2026_742_MOESM9_ESM.zip › Figure 2 Part 6/Fig 2fg Control Hela rGT335 acetylated tubulin/Anaphase/Cas9 actub rGT335 9-8-25 R2 A1 M1.Project Maximum Z_XY1757360040_Z0_T0_C0.tif]

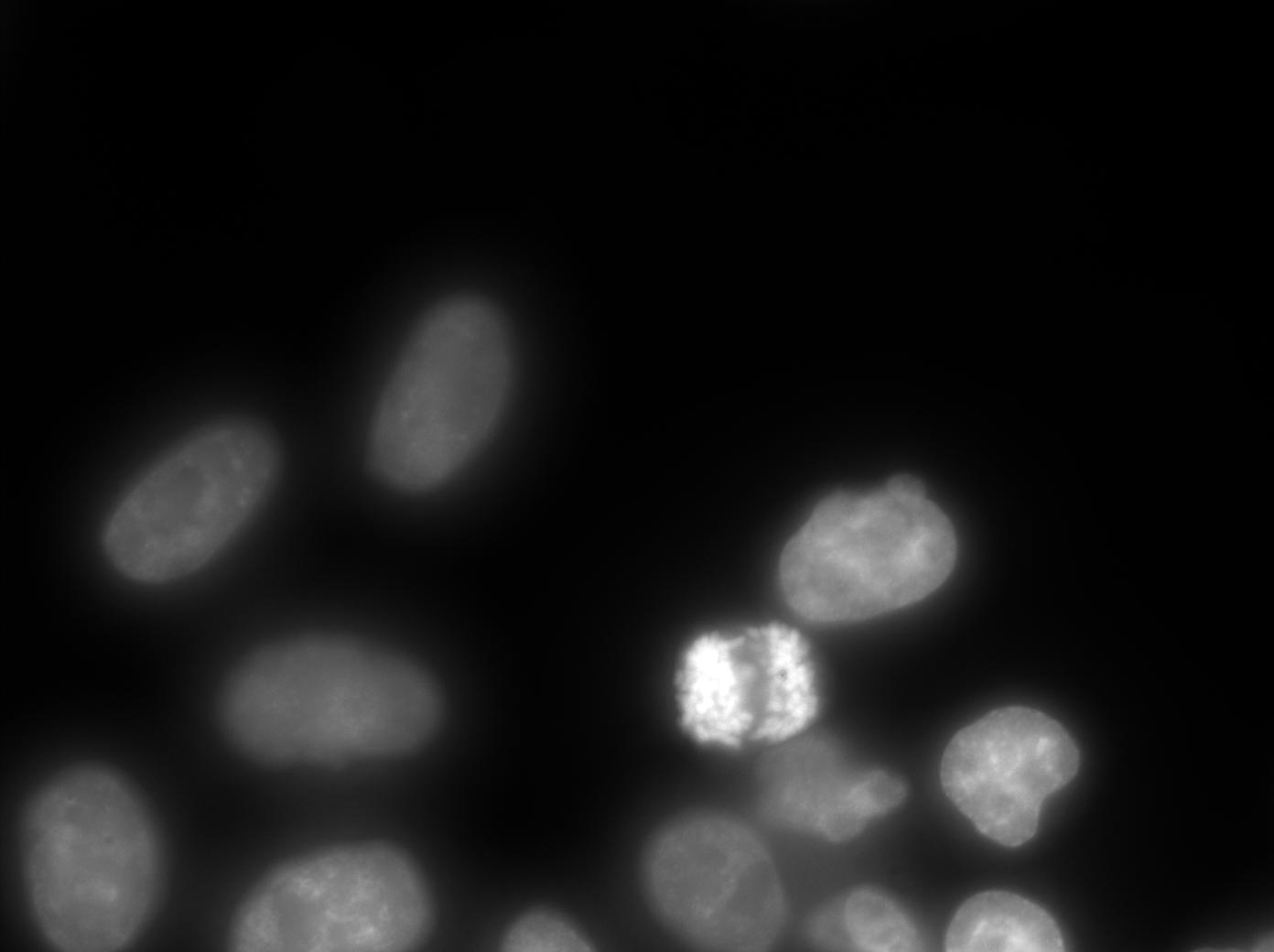

Supplement: Supplementary file 9 — Source data Fig. 2 part 6 [file 44319_2026_742_MOESM9_ESM.zip › Figure 2 Part 6/Fig 2fg Control Hela rGT335 acetylated tubulin/Anaphase/Cas9 actub rGT335 9-8-25 R3 A10.Project Maximum Z_XY1757369402_Z0_T0_C0.tif]

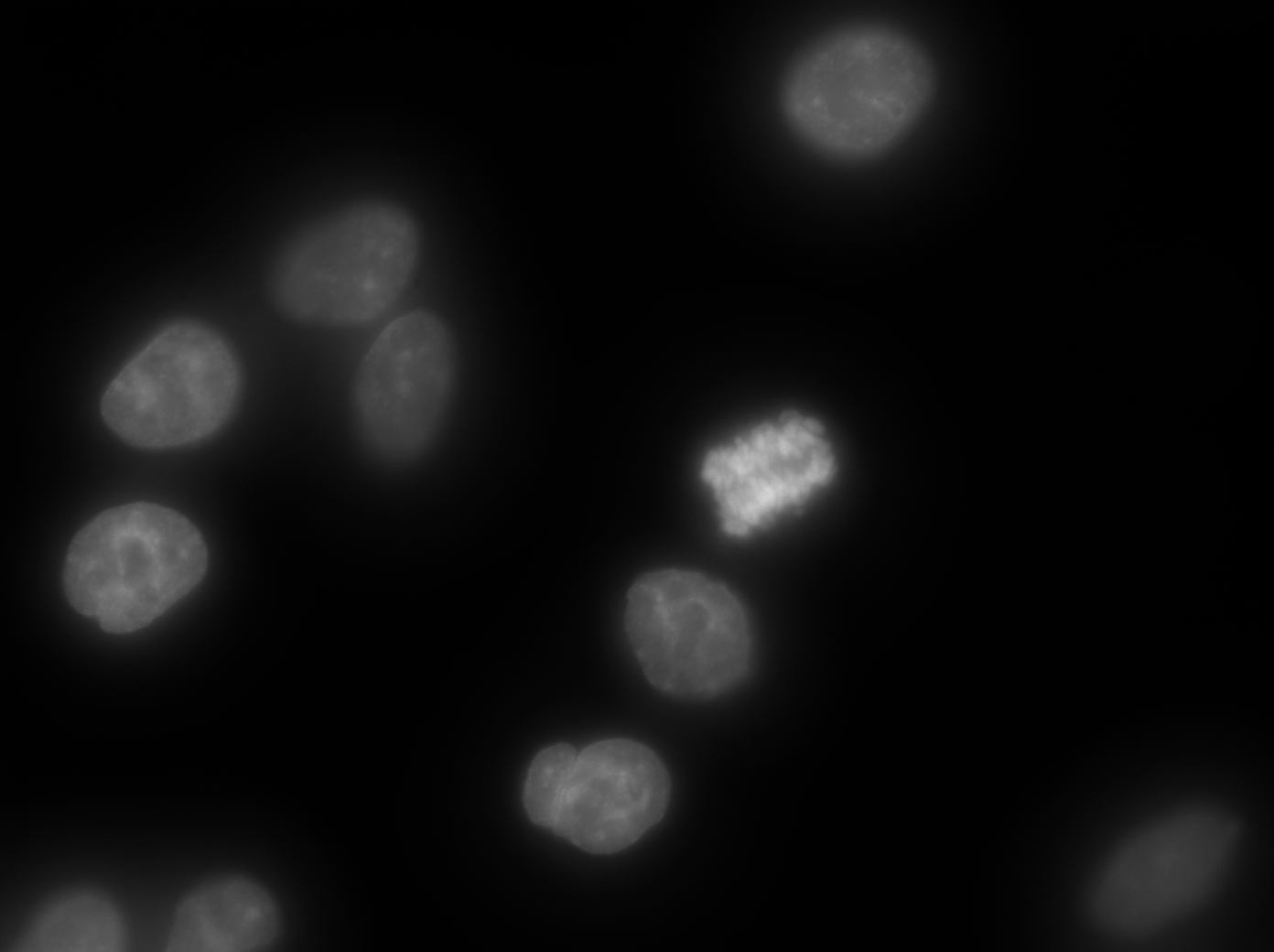

Supplement: Supplementary file 9 — Source data Fig. 2 part 6 [file 44319_2026_742_MOESM9_ESM.zip › Figure 2 Part 6/Fig 2fg Control Hela rGT335 acetylated tubulin/Anaphase/Cas9 actub rGT335 9-8-25 R1 A1 EX.Project Maximum Z_XY1757350637_Z0_T0_C0.tif]

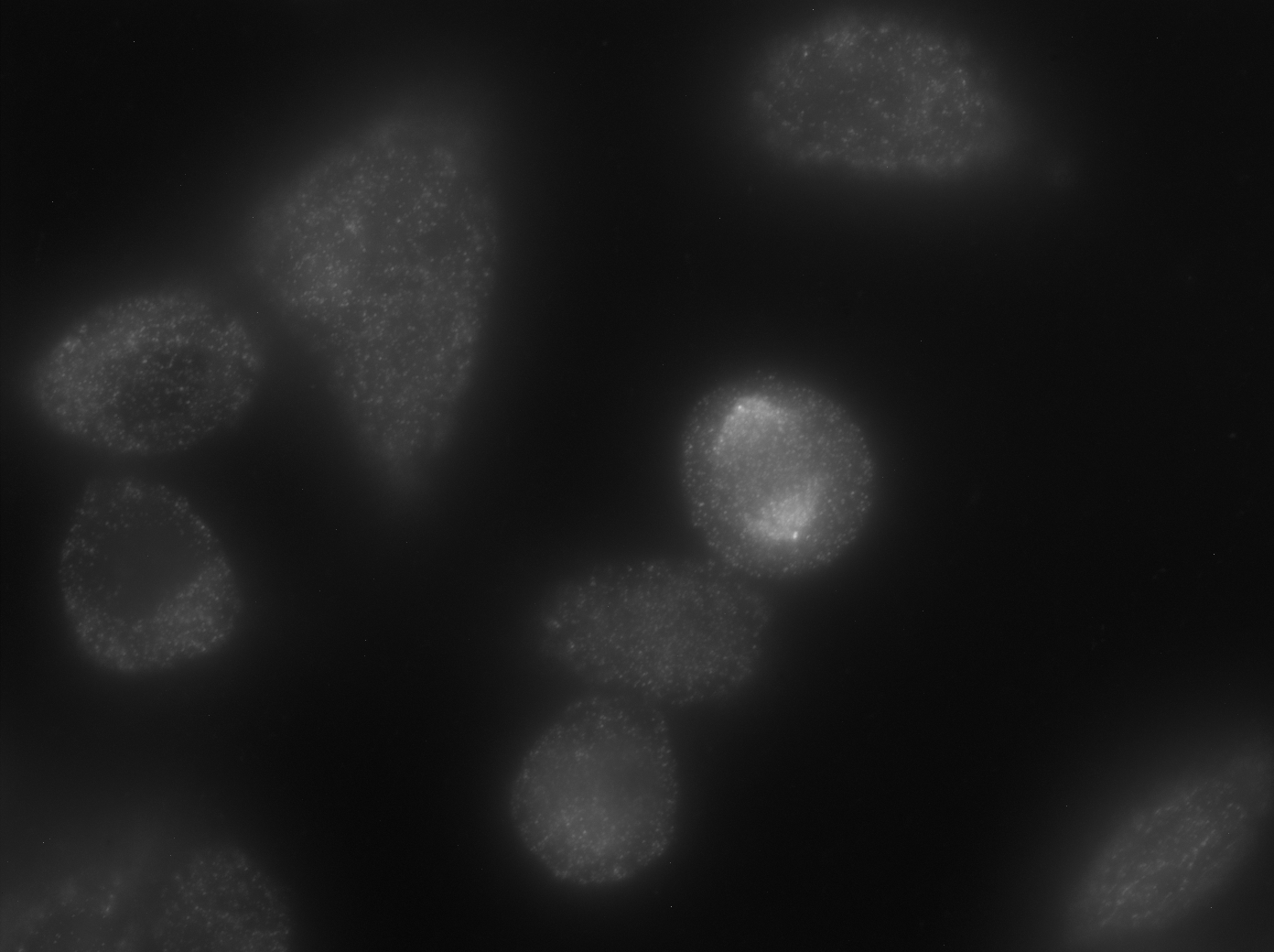

Supplement: Supplementary file 9 — Source data Fig. 2 part 6 [file 44319_2026_742_MOESM9_ESM.zip › Figure 2 Part 6/Fig 2fg Control Hela rGT335 acetylated tubulin/Anaphase/Cas9 actub rGT335 9-8-25 R1 A1 EX.Project Maximum Z_XY1757350637_Z0_T0_C1.tif]

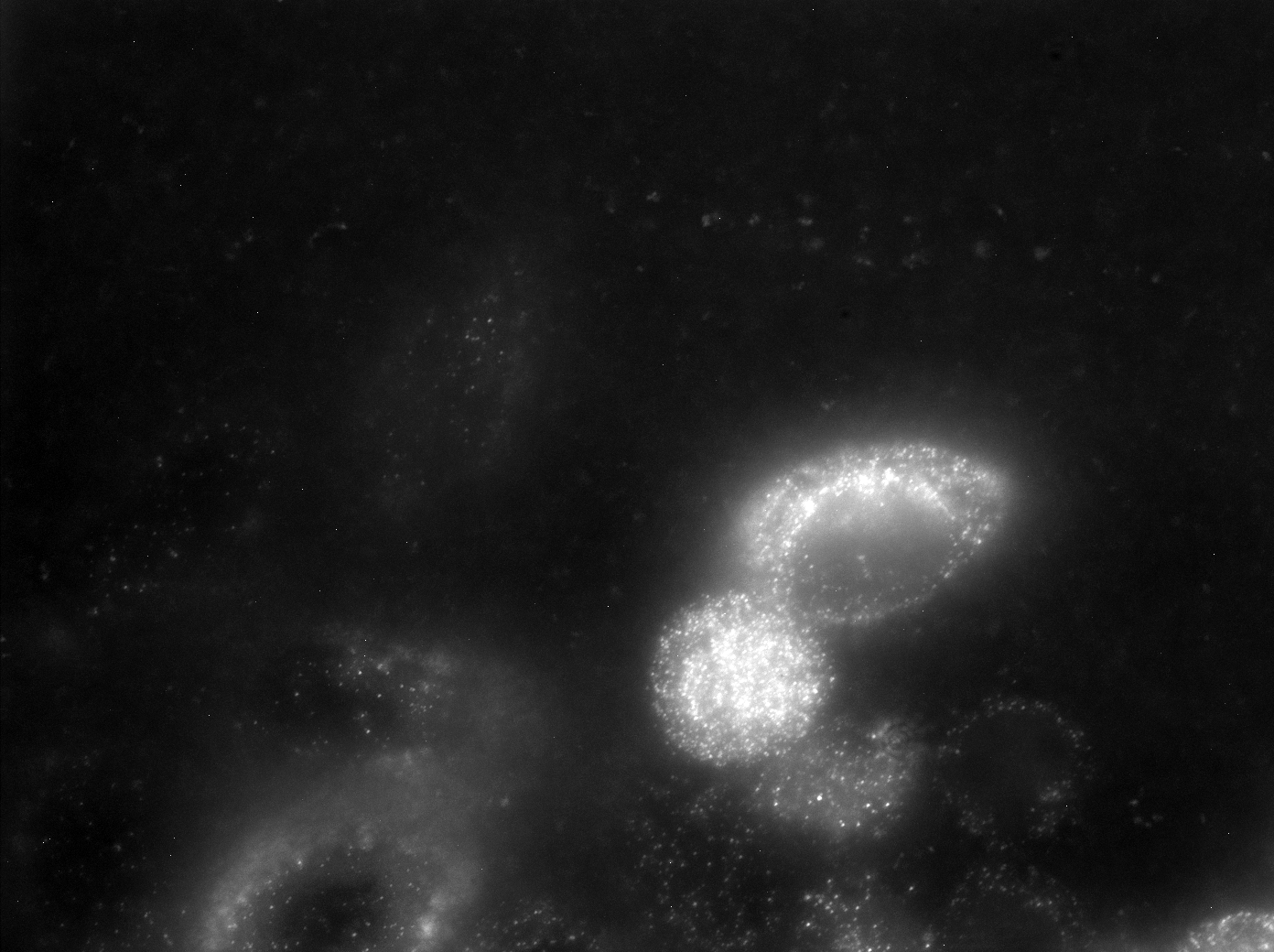

Supplement: Supplementary file 9 — Source data Fig. 2 part 6 [file 44319_2026_742_MOESM9_ESM.zip › Figure 2 Part 6/Fig 2fg Control Hela rGT335 acetylated tubulin/Anaphase/Cas9 actub rGT335 9-8-25 R3 A10.Project Maximum Z_XY1757369402_Z0_T0_C1.tif]

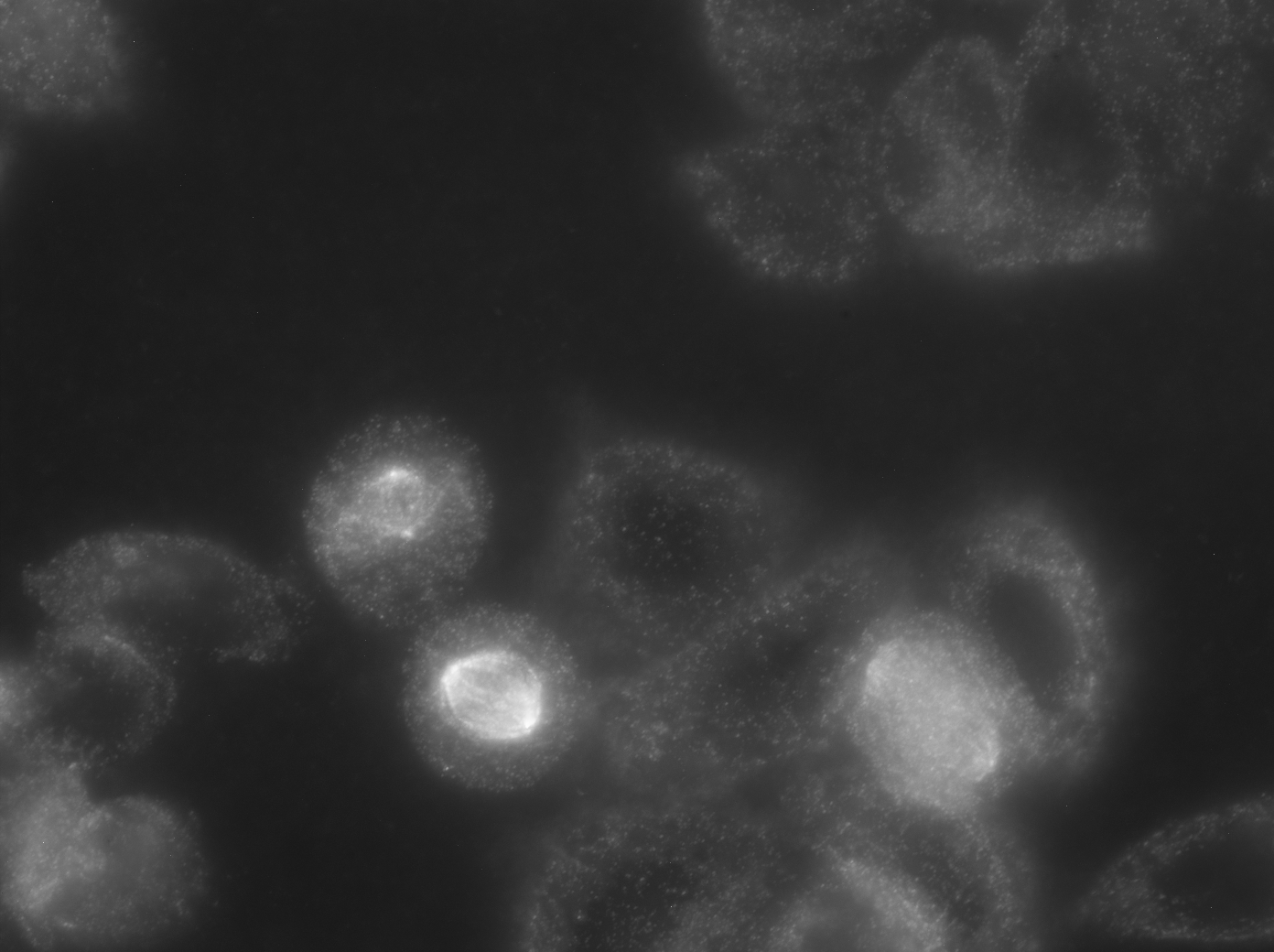

Supplement: Supplementary file 9 — Source data Fig. 2 part 6 [file 44319_2026_742_MOESM9_ESM.zip › Figure 2 Part 6/Fig 2fg Control Hela rGT335 acetylated tubulin/Anaphase/Cas9 actub rGT335 9-8-25 R2 A1 M1.Project Maximum Z_XY1757360040_Z0_T0_C1.tif]

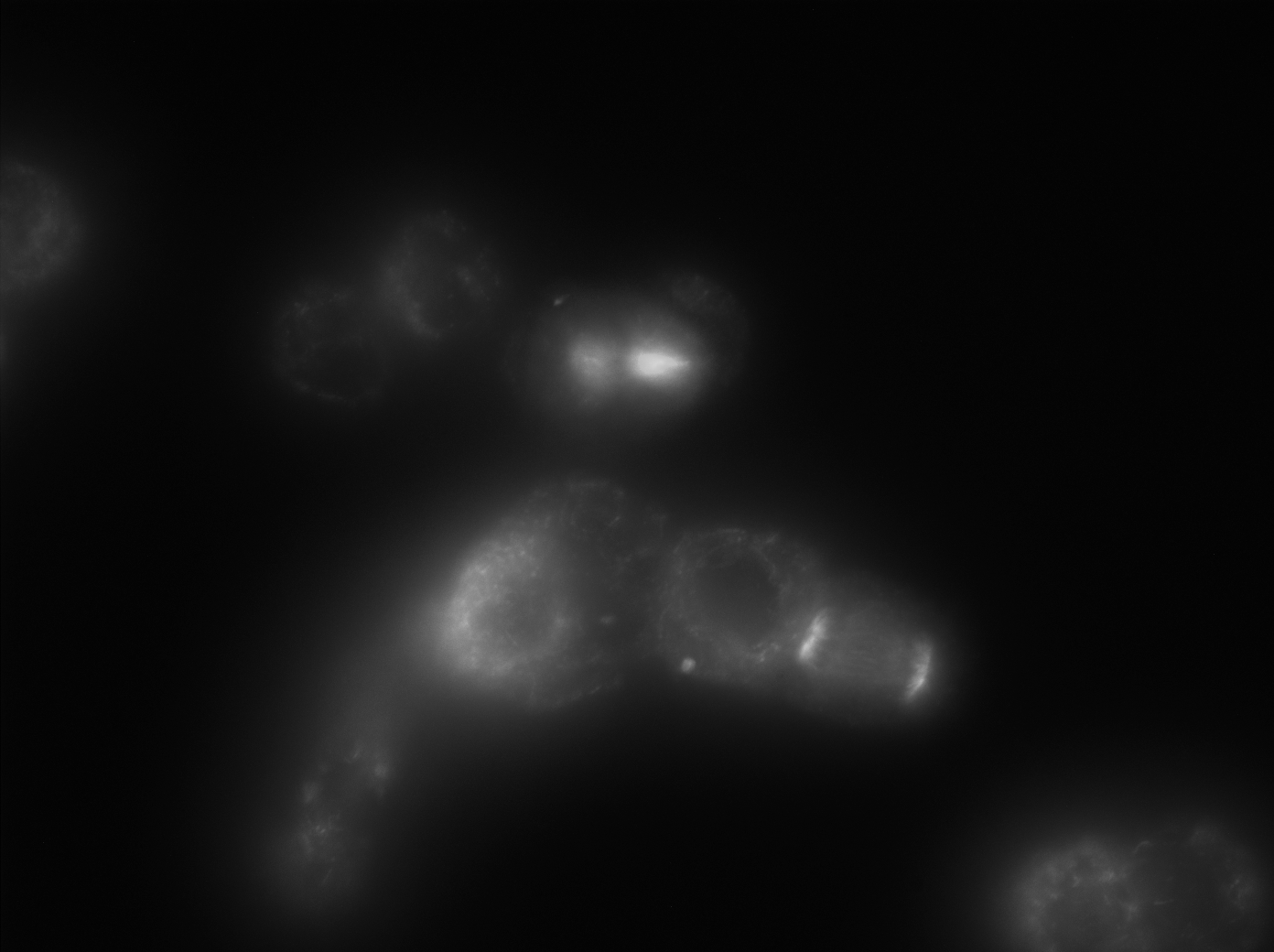

Supplement: Supplementary file 9 — Source data Fig. 2 part 6 [file 44319_2026_742_MOESM9_ESM.zip › Figure 2 Part 6/Fig 2fg Control Hela rGT335 acetylated tubulin/Anaphase/Cas9 actub rGT335 9-8-25 R1 A2 EX.Project Maximum Z_XY1757351774_Z0_T0_C2.tif]

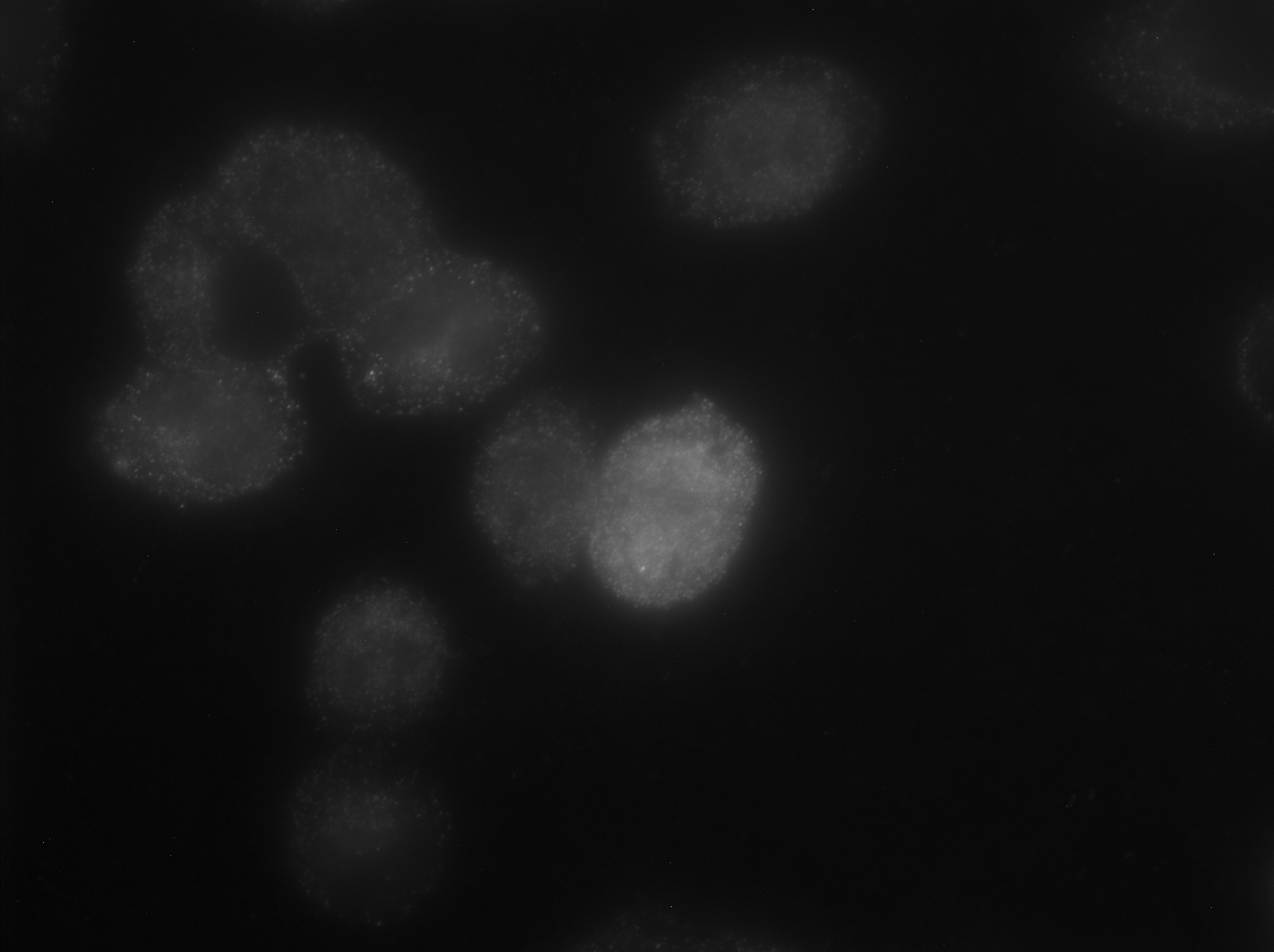

Supplement: Supplementary file 9 — Source data Fig. 2 part 6 [file 44319_2026_742_MOESM9_ESM.zip › Figure 2 Part 6/Fig 2fg Control Hela rGT335 acetylated tubulin/Anaphase/Cas9 actub rGT335 9-8-25 R1 A5.Project Maximum Z_XY1757355139_Z0_T0_C1.tif]

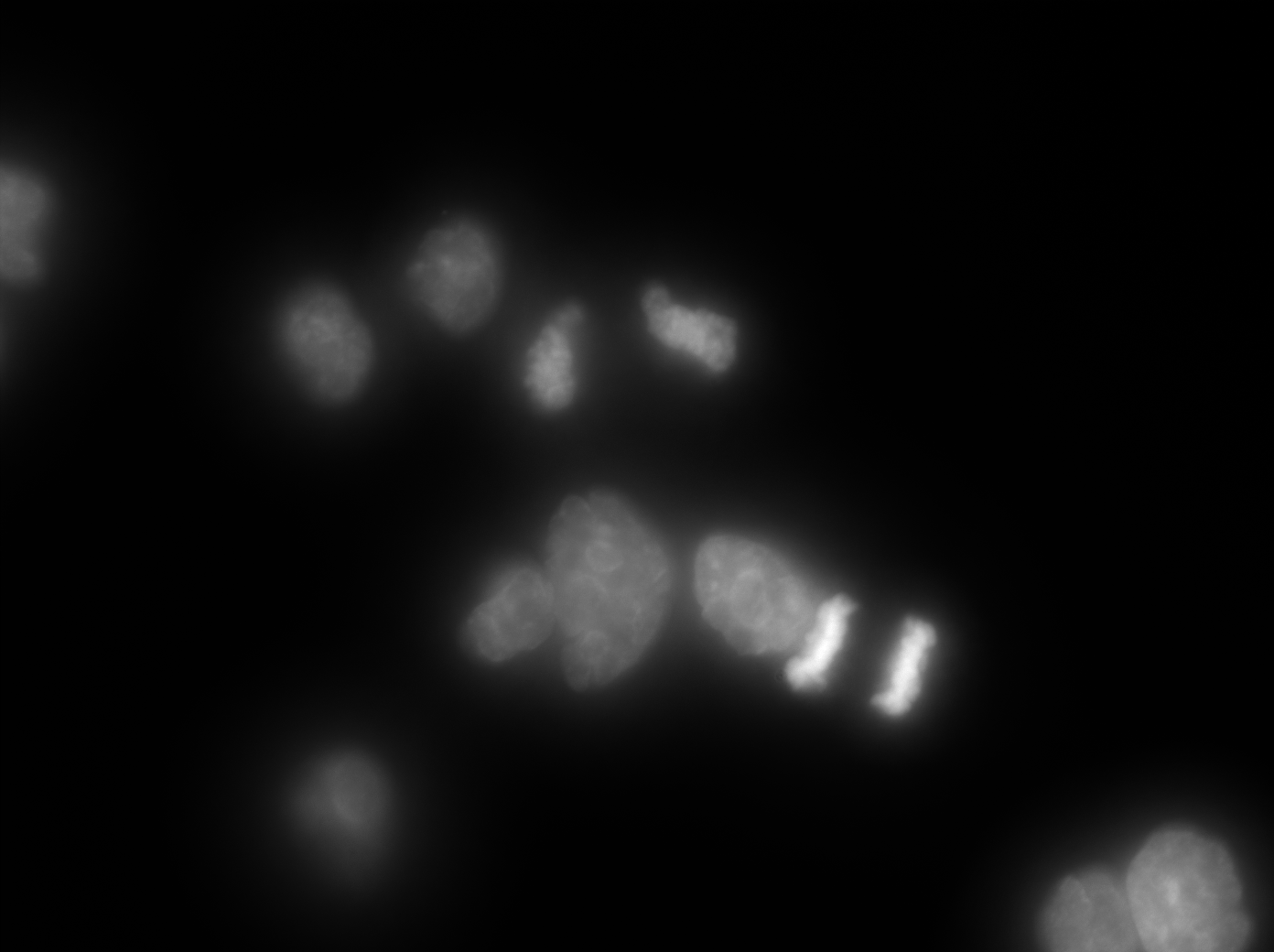

Supplement: Supplementary file 9 — Source data Fig. 2 part 6 [file 44319_2026_742_MOESM9_ESM.zip › Figure 2 Part 6/Fig 2fg Control Hela rGT335 acetylated tubulin/Anaphase/Cas9 actub rGT335 9-8-25 R1 A2 EX.Project Maximum Z_XY1757351774_Z0_T0_C0.tif]

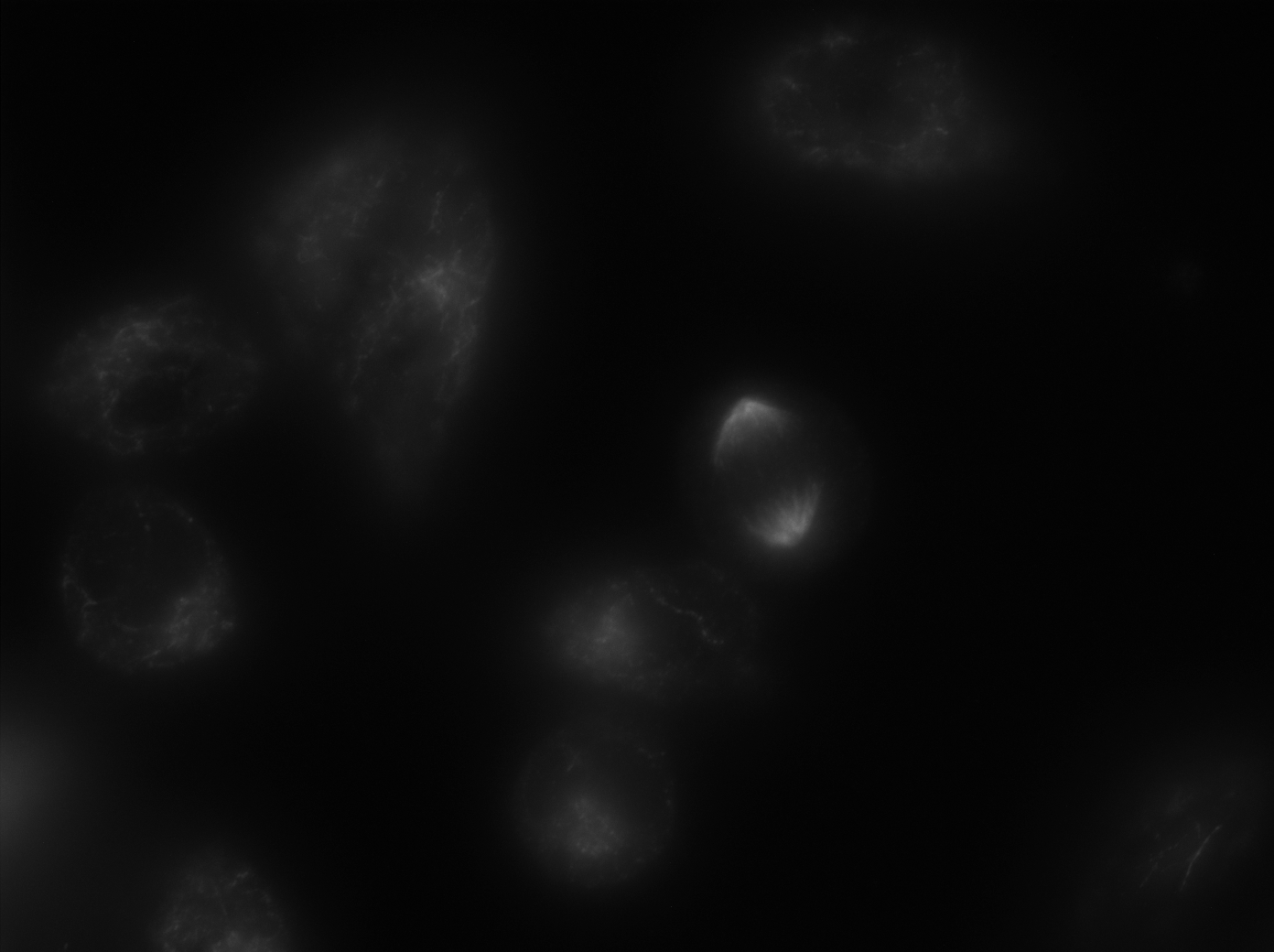

Supplement: Supplementary file 9 — Source data Fig. 2 part 6 [file 44319_2026_742_MOESM9_ESM.zip › Figure 2 Part 6/Fig 2fg Control Hela rGT335 acetylated tubulin/Anaphase/Cas9 actub rGT335 9-8-25 R1 A1 EX.Project Maximum Z_XY1757350637_Z0_T0_C2.tif]

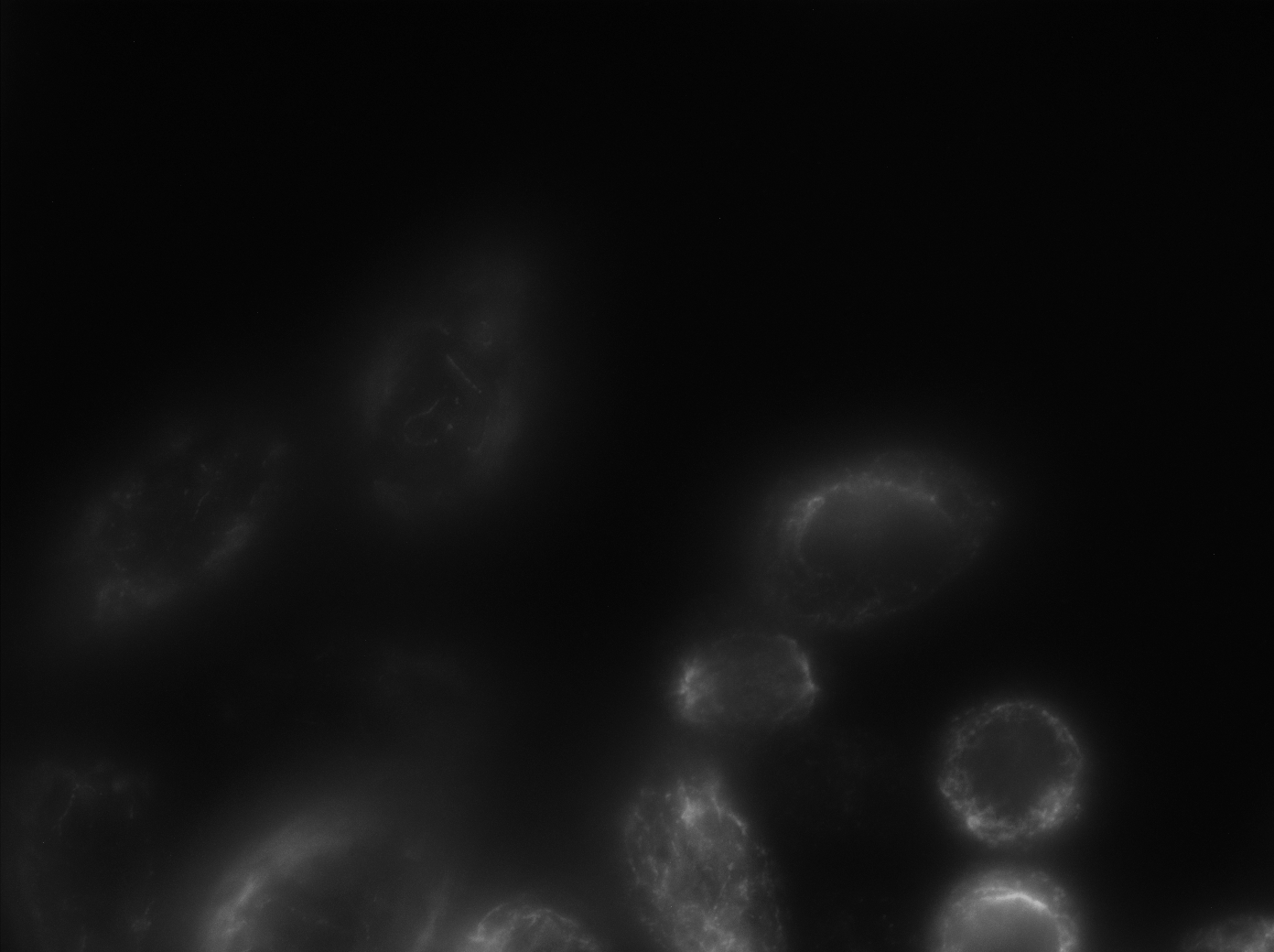

Supplement: Supplementary file 9 — Source data Fig. 2 part 6 [file 44319_2026_742_MOESM9_ESM.zip › Figure 2 Part 6/Fig 2fg Control Hela rGT335 acetylated tubulin/Anaphase/Cas9 actub rGT335 9-8-25 R3 A10.Project Maximum Z_XY1757369402_Z0_T0_C2.tif]

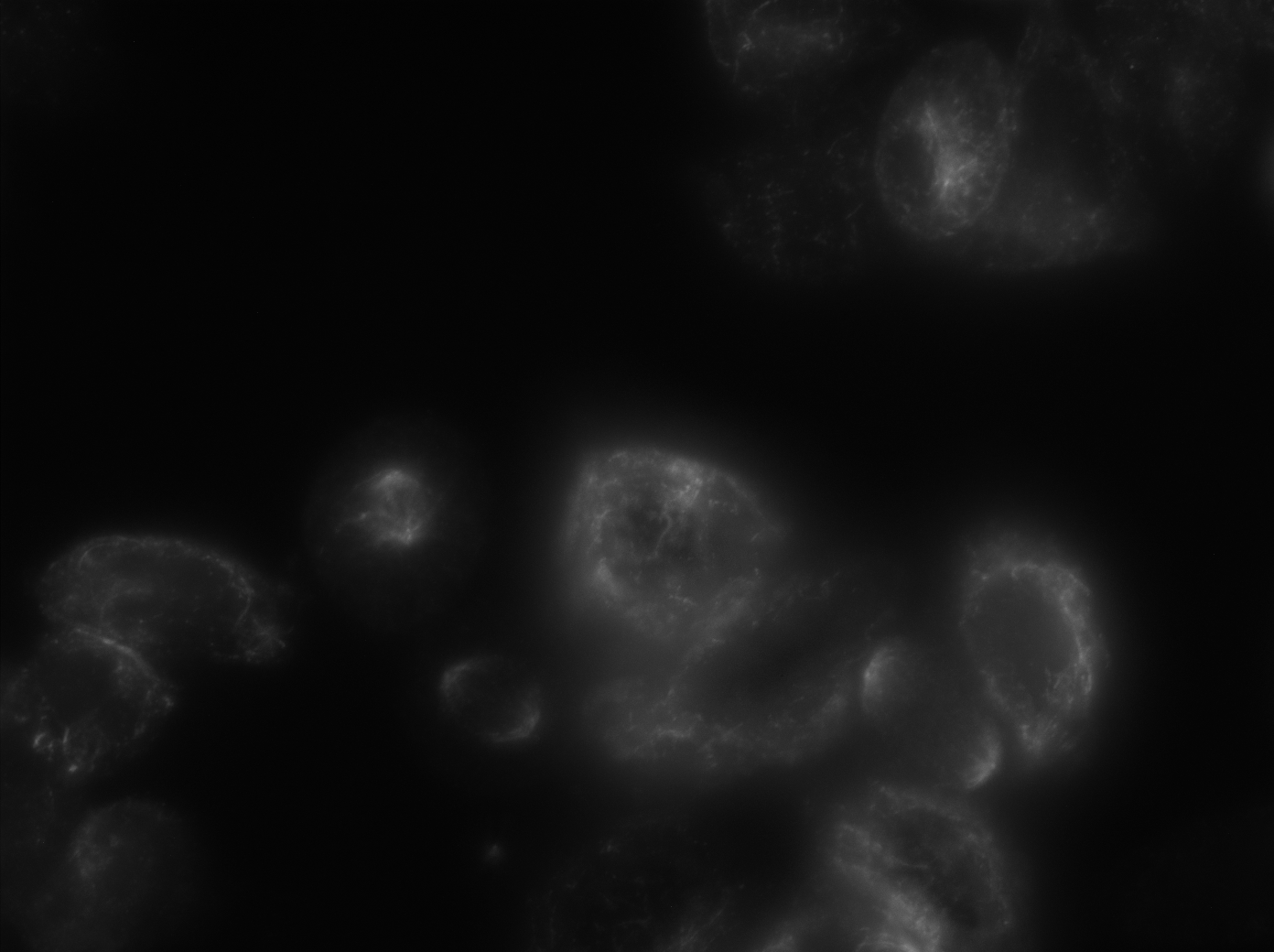

Supplement: Supplementary file 9 — Source data Fig. 2 part 6 [file 44319_2026_742_MOESM9_ESM.zip › Figure 2 Part 6/Fig 2fg Control Hela rGT335 acetylated tubulin/Anaphase/Cas9 actub rGT335 9-8-25 R2 A1 M1.Project Maximum Z_XY1757360040_Z0_T0_C2.tif]

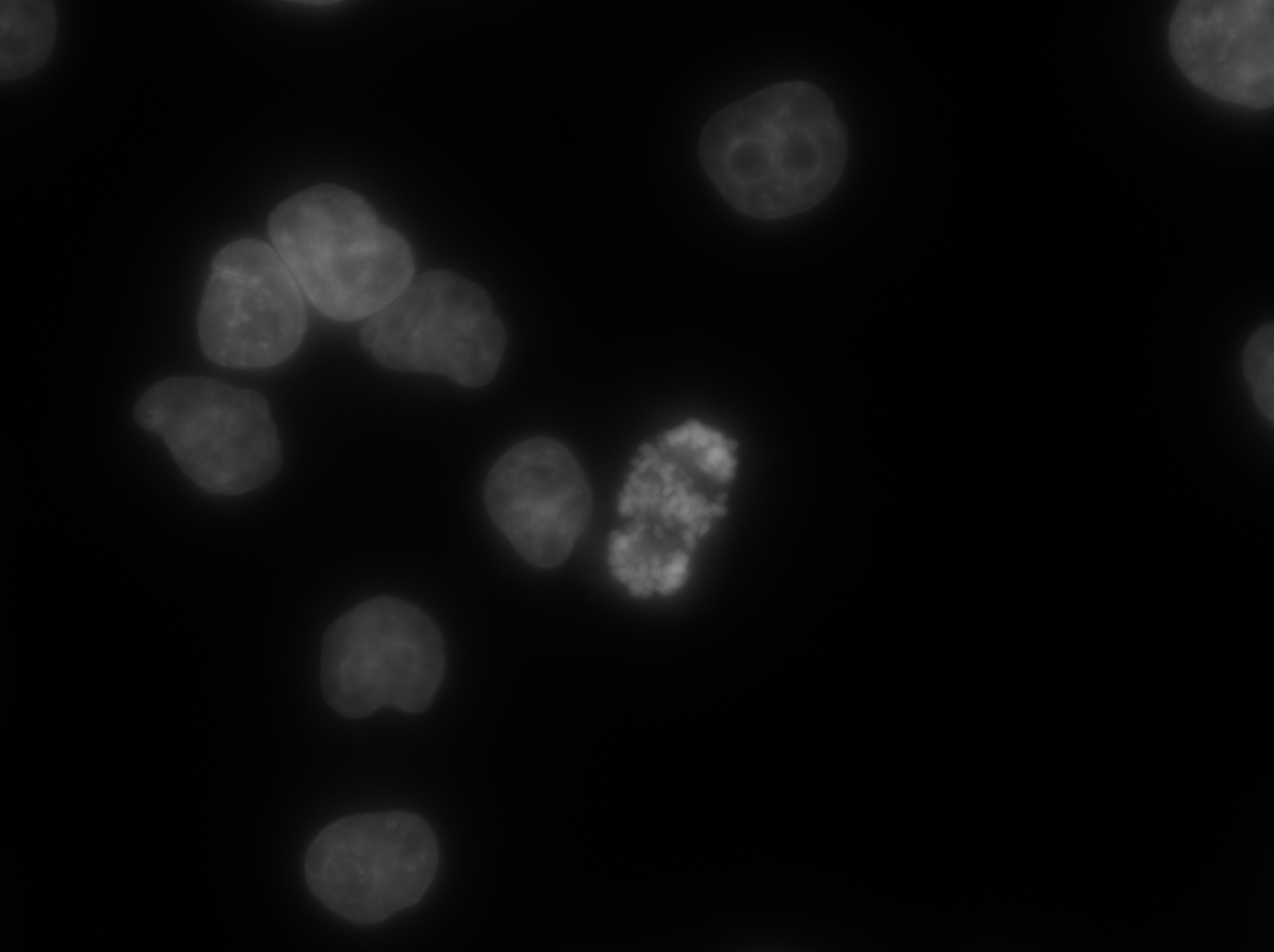

Supplement: Supplementary file 9 — Source data Fig. 2 part 6 [file 44319_2026_742_MOESM9_ESM.zip › Figure 2 Part 6/Fig 2fg Control Hela rGT335 acetylated tubulin/Anaphase/Cas9 actub rGT335 9-8-25 R1 A5.Project Maximum Z_XY1757355139_Z0_T0_C0.tif]

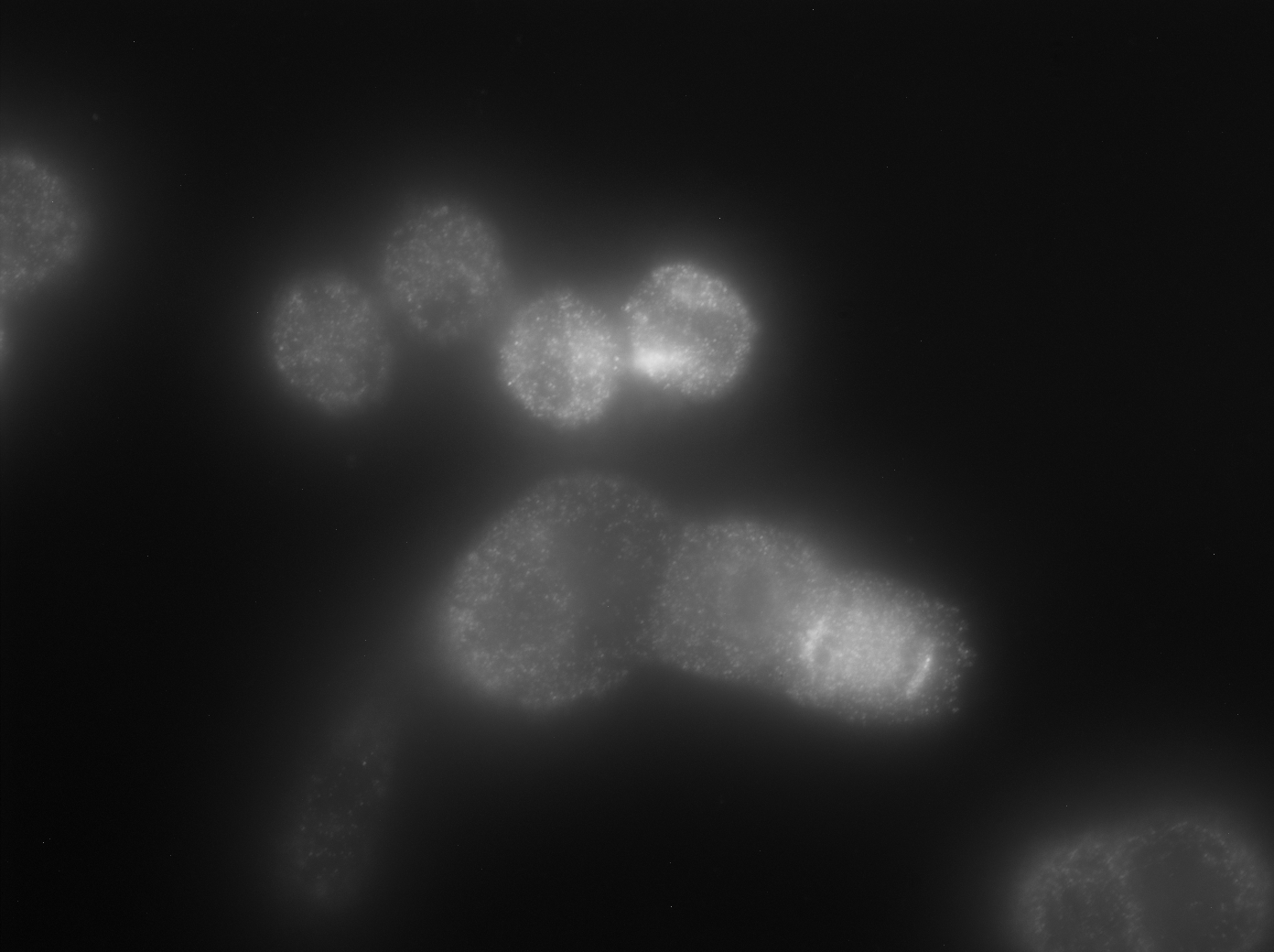

Supplement: Supplementary file 9 — Source data Fig. 2 part 6 [file 44319_2026_742_MOESM9_ESM.zip › Figure 2 Part 6/Fig 2fg Control Hela rGT335 acetylated tubulin/Anaphase/Cas9 actub rGT335 9-8-25 R1 A2 EX.Project Maximum Z_XY1757351774_Z0_T0_C1.tif]

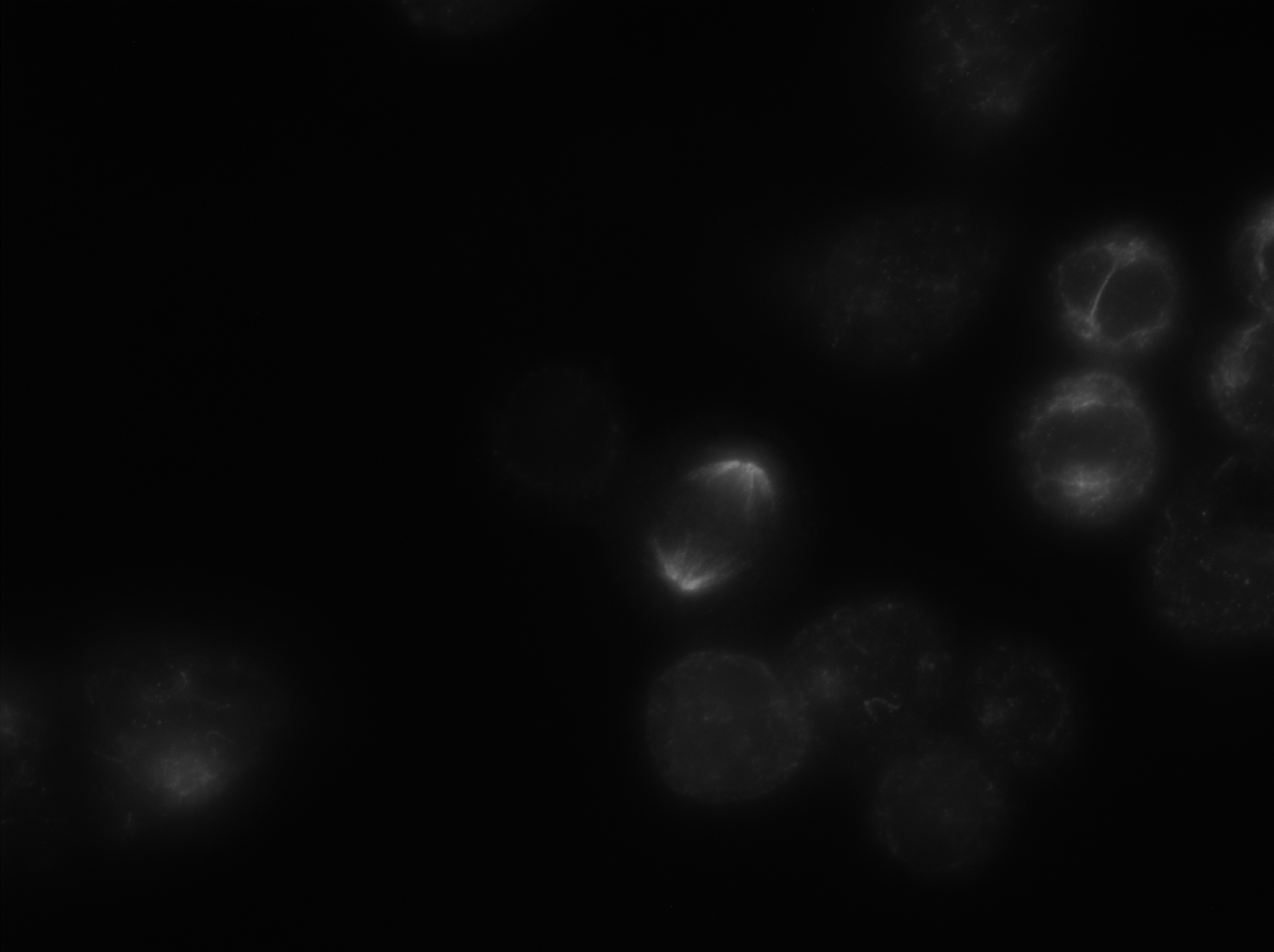

Supplement: Supplementary file 9 — Source data Fig. 2 part 6 [file 44319_2026_742_MOESM9_ESM.zip › Figure 2 Part 6/Fig 2fg Control Hela rGT335 acetylated tubulin/Anaphase/Cas9 actub rGT335 9-8-25 R2 A3.Project Maximum Z_XY1757360420_Z0_T0_C2.tif]

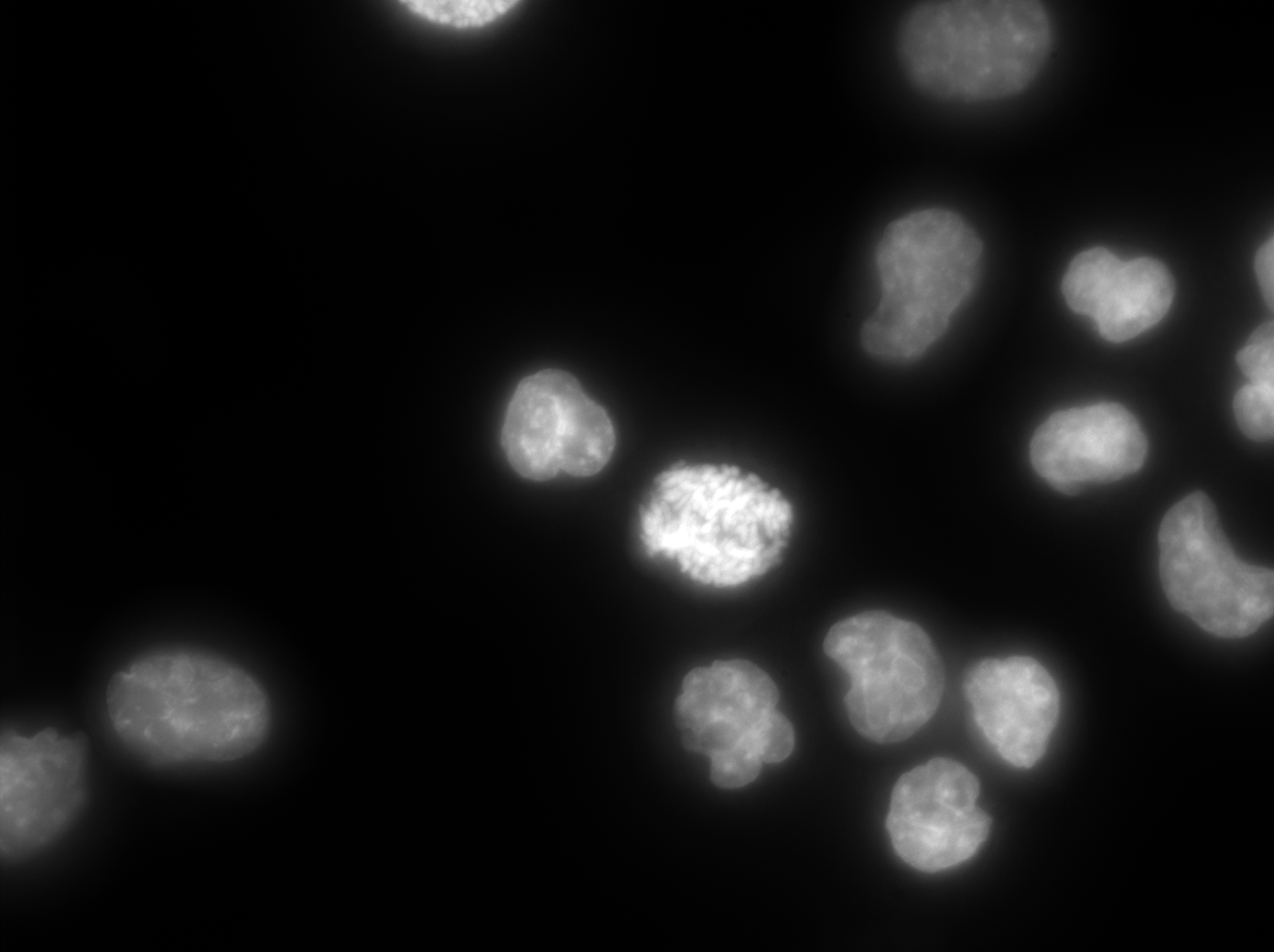

Supplement: Supplementary file 9 — Source data Fig. 2 part 6 [file 44319_2026_742_MOESM9_ESM.zip › Figure 2 Part 6/Fig 2fg Control Hela rGT335 acetylated tubulin/Anaphase/Cas9 actub rGT335 9-8-25 R2 A3.Project Maximum Z_XY1757360420_Z0_T0_C0.tif]

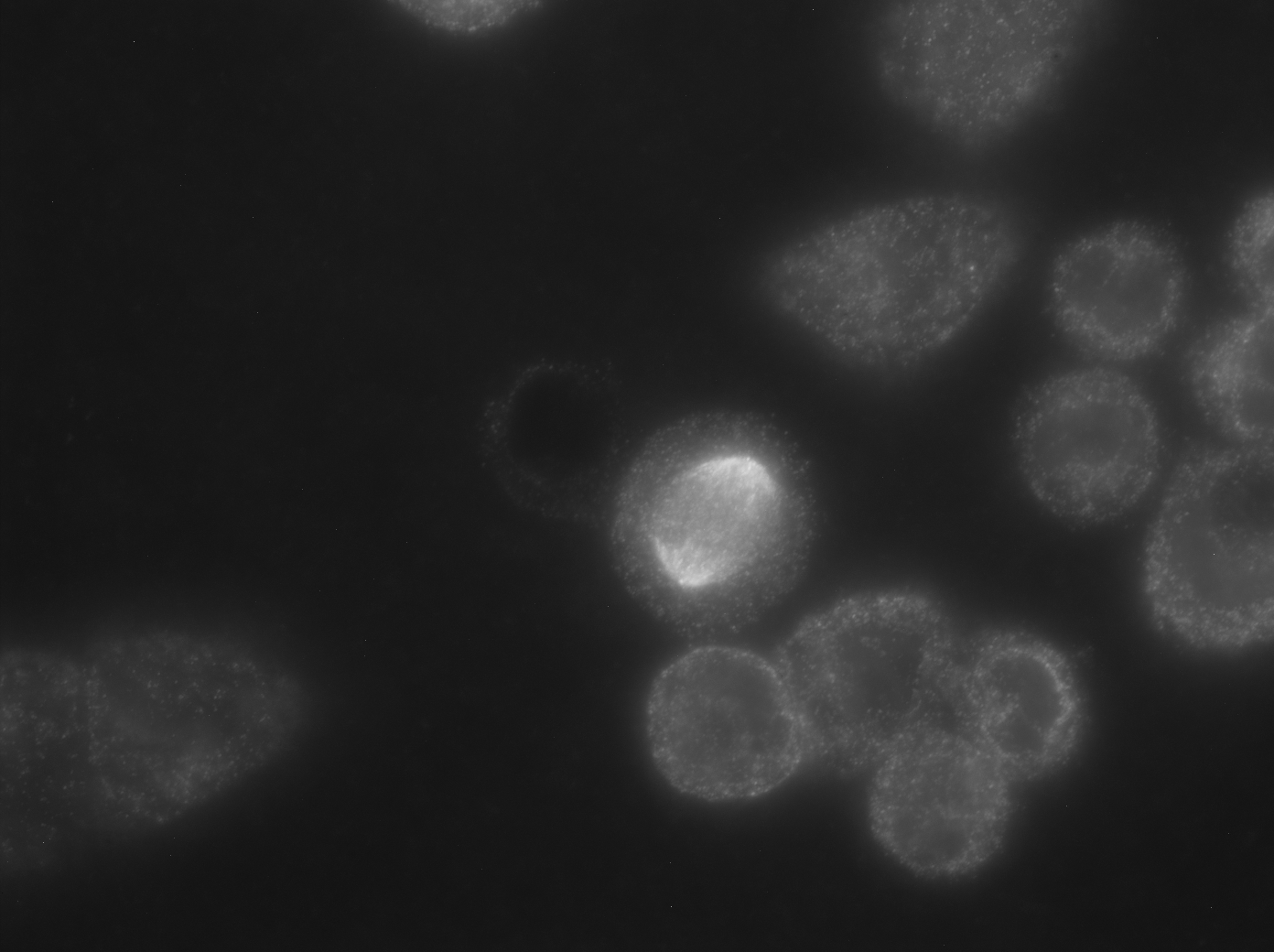

Supplement: Supplementary file 9 — Source data Fig. 2 part 6 [file 44319_2026_742_MOESM9_ESM.zip › Figure 2 Part 6/Fig 2fg Control Hela rGT335 acetylated tubulin/Anaphase/Cas9 actub rGT335 9-8-25 R2 A3.Project Maximum Z_XY1757360420_Z0_T0_C1.tif]

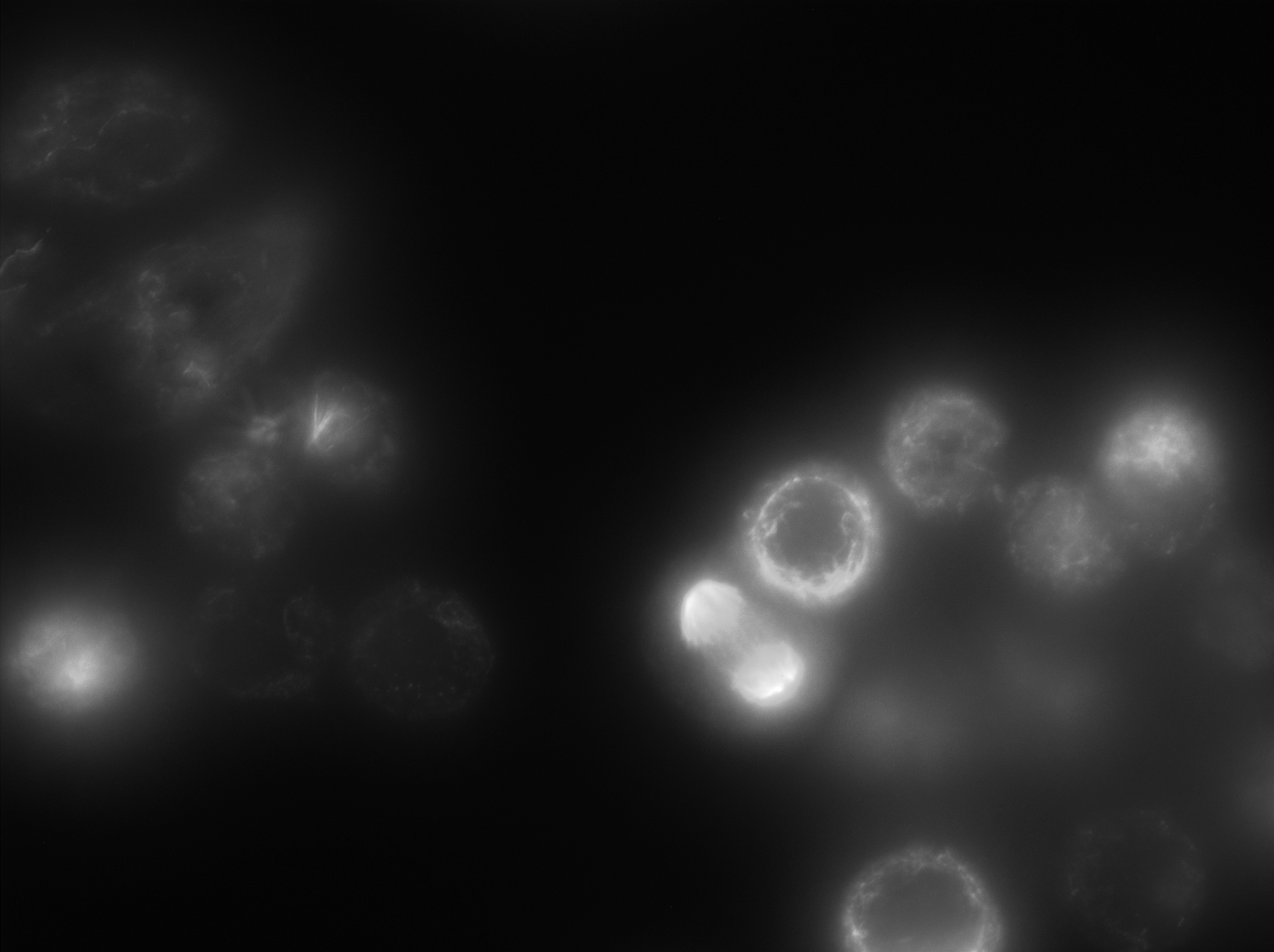

Supplement: Supplementary file 9 — Source data Fig. 2 part 6 [file 44319_2026_742_MOESM9_ESM.zip › Figure 2 Part 6/Fig 2fg Control Hela rGT335 acetylated tubulin/Anaphase/Cas9 actub rGT335 9-8-25 R1 A3.Project Maximum Z_XY1757352025_Z0_T0_C2.tif]

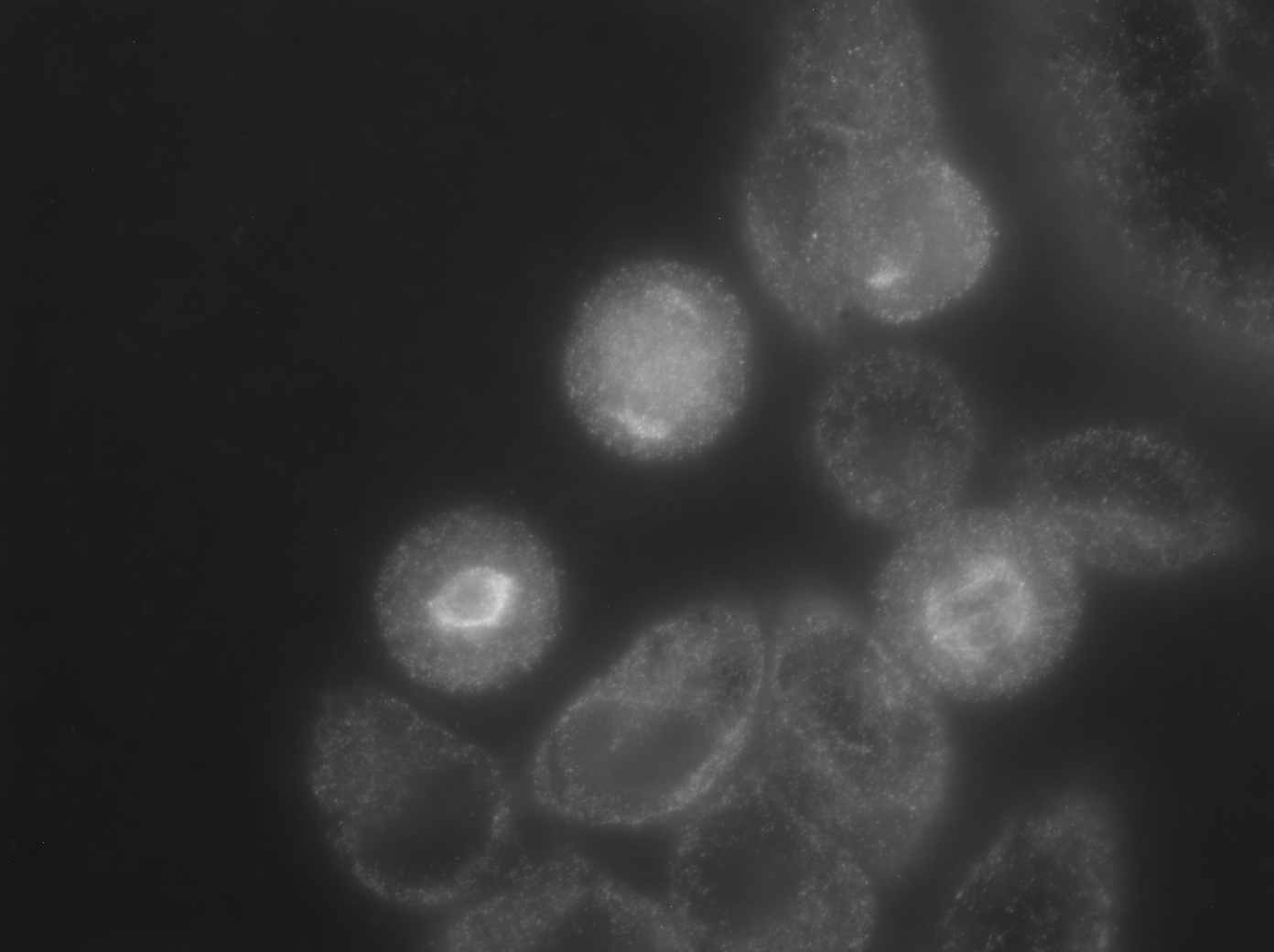

Supplement: Supplementary file 9 — Source data Fig. 2 part 6 [file 44319_2026_742_MOESM9_ESM.zip › Figure 2 Part 6/Fig 2fg Control Hela rGT335 acetylated tubulin/Anaphase/Cas9 actub rGT335 9-8-25 R2 A4 M2.Project Maximum Z_XY1757360557_Z0_T0_C1.tif]

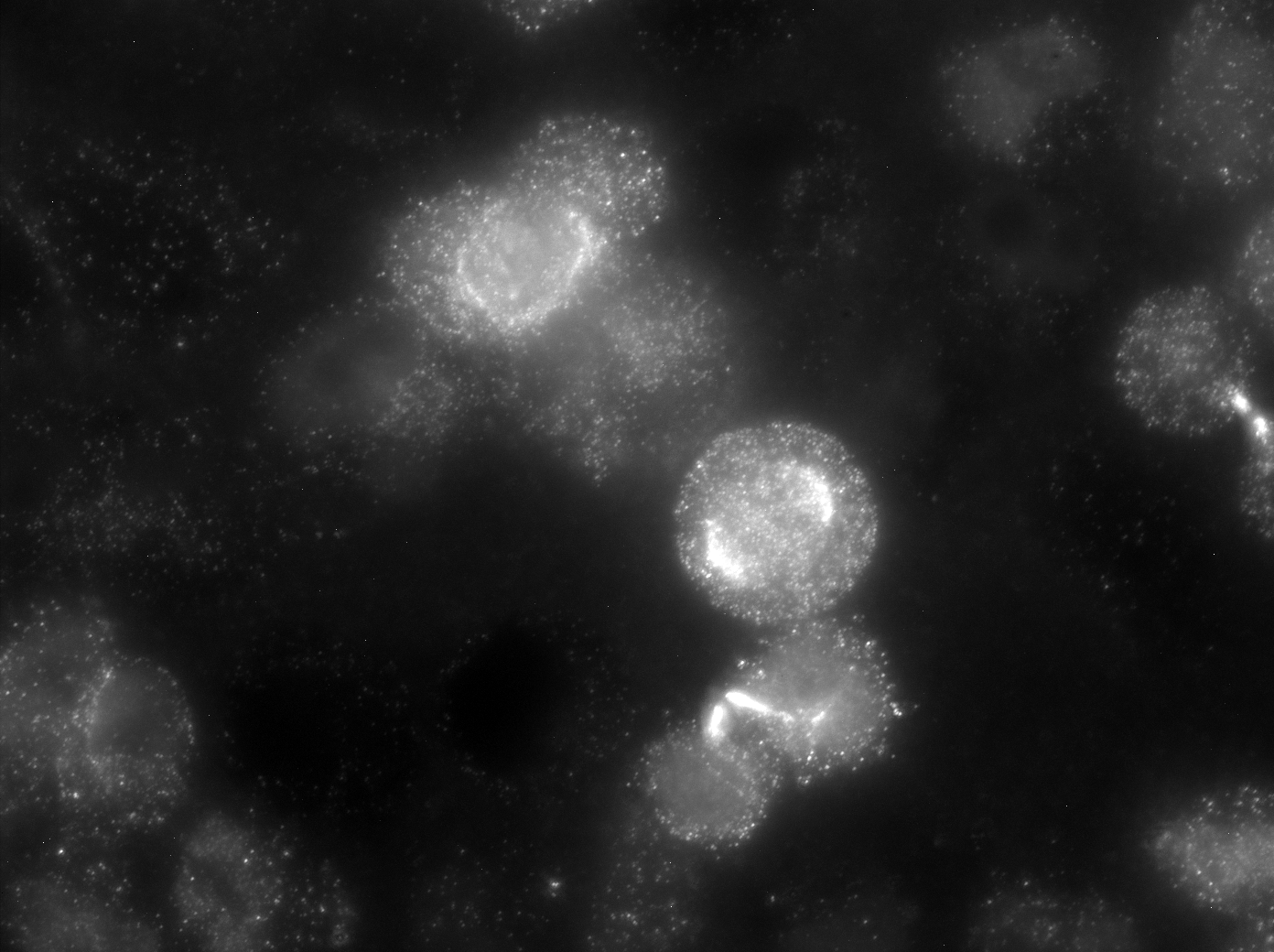

Supplement: Supplementary file 9 — Source data Fig. 2 part 6 [file 44319_2026_742_MOESM9_ESM.zip › Figure 2 Part 6/Fig 2fg Control Hela rGT335 acetylated tubulin/Anaphase/Cas9 actub rGT335 9-8-25 R3 A8.Project Maximum Z_XY1757368874_Z0_T0_C1.tif]

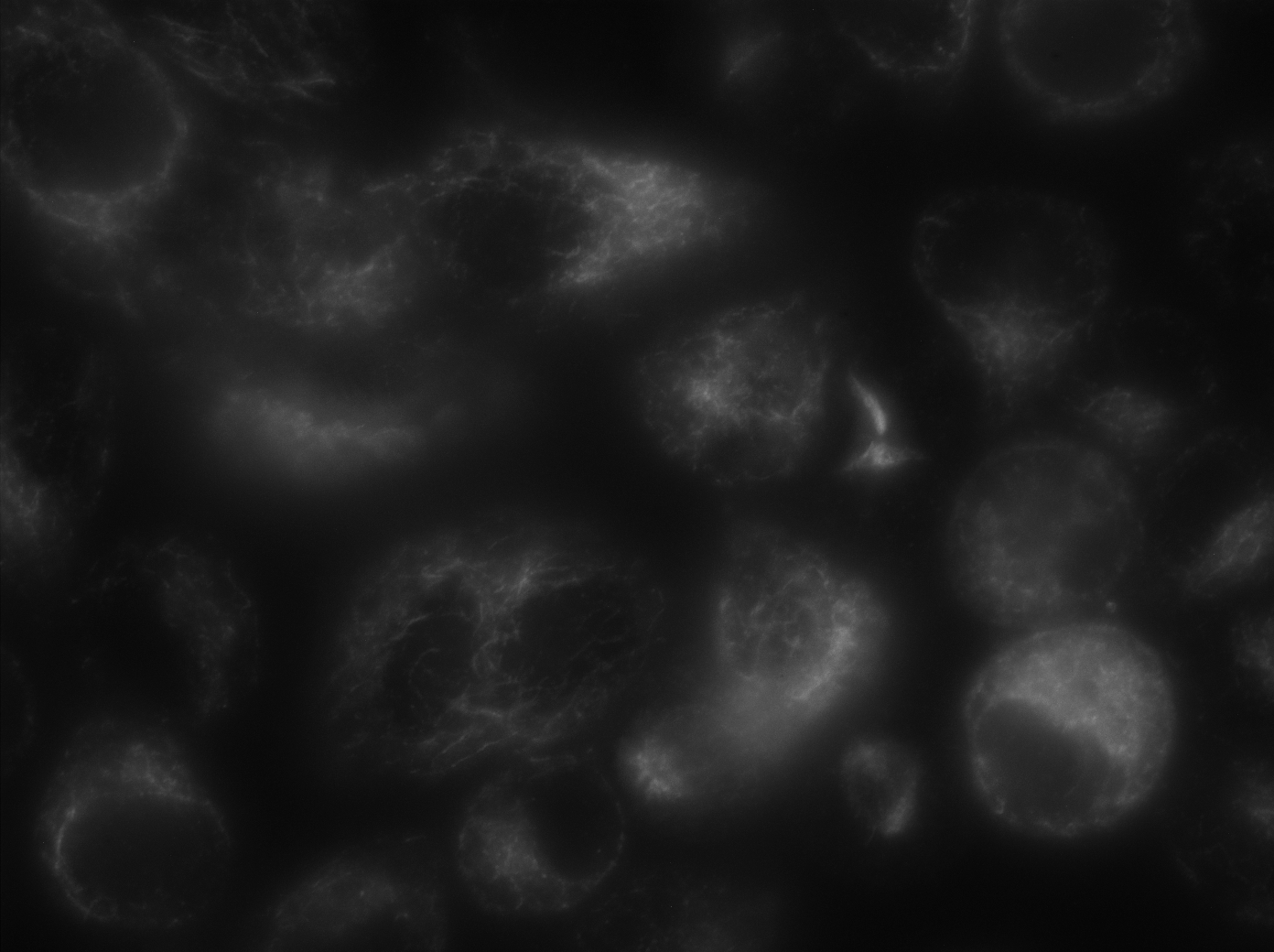

Supplement: Supplementary file 9 — Source data Fig. 2 part 6 [file 44319_2026_742_MOESM9_ESM.zip › Figure 2 Part 6/Fig 2fg Control Hela rGT335 acetylated tubulin/Anaphase/Cas9 actub rGT335 9-8-25 R3 A6.Project Maximum Z_XY1757367515_Z0_T0_C2.tif]

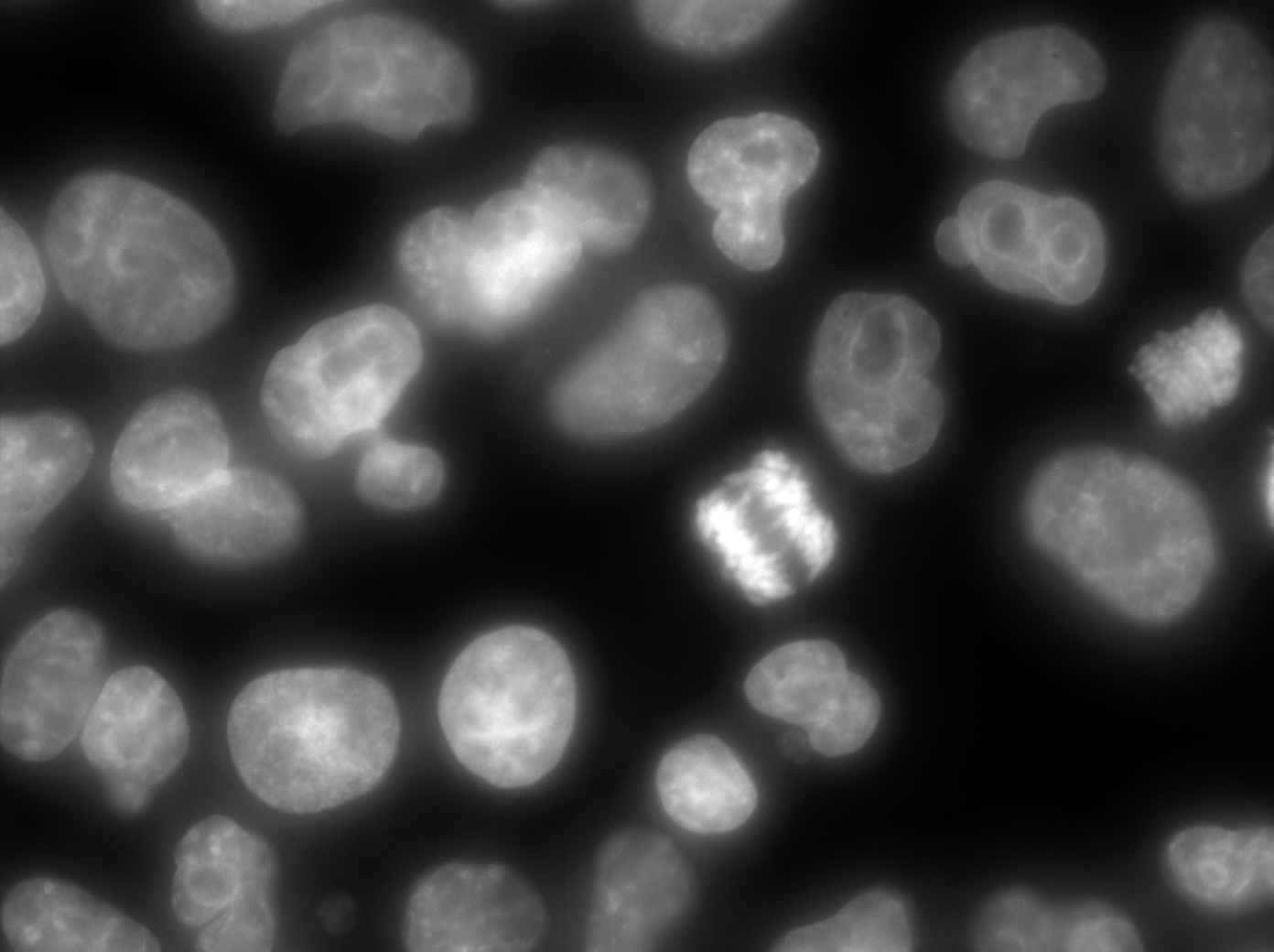

Supplement: Supplementary file 9 — Source data Fig. 2 part 6 [file 44319_2026_742_MOESM9_ESM.zip › Figure 2 Part 6/Fig 2fg Control Hela rGT335 acetylated tubulin/Anaphase/Cas9 actub rGT335 9-8-25 R3 A8.Project Maximum Z_XY1757368874_Z0_T0_C0.tif]

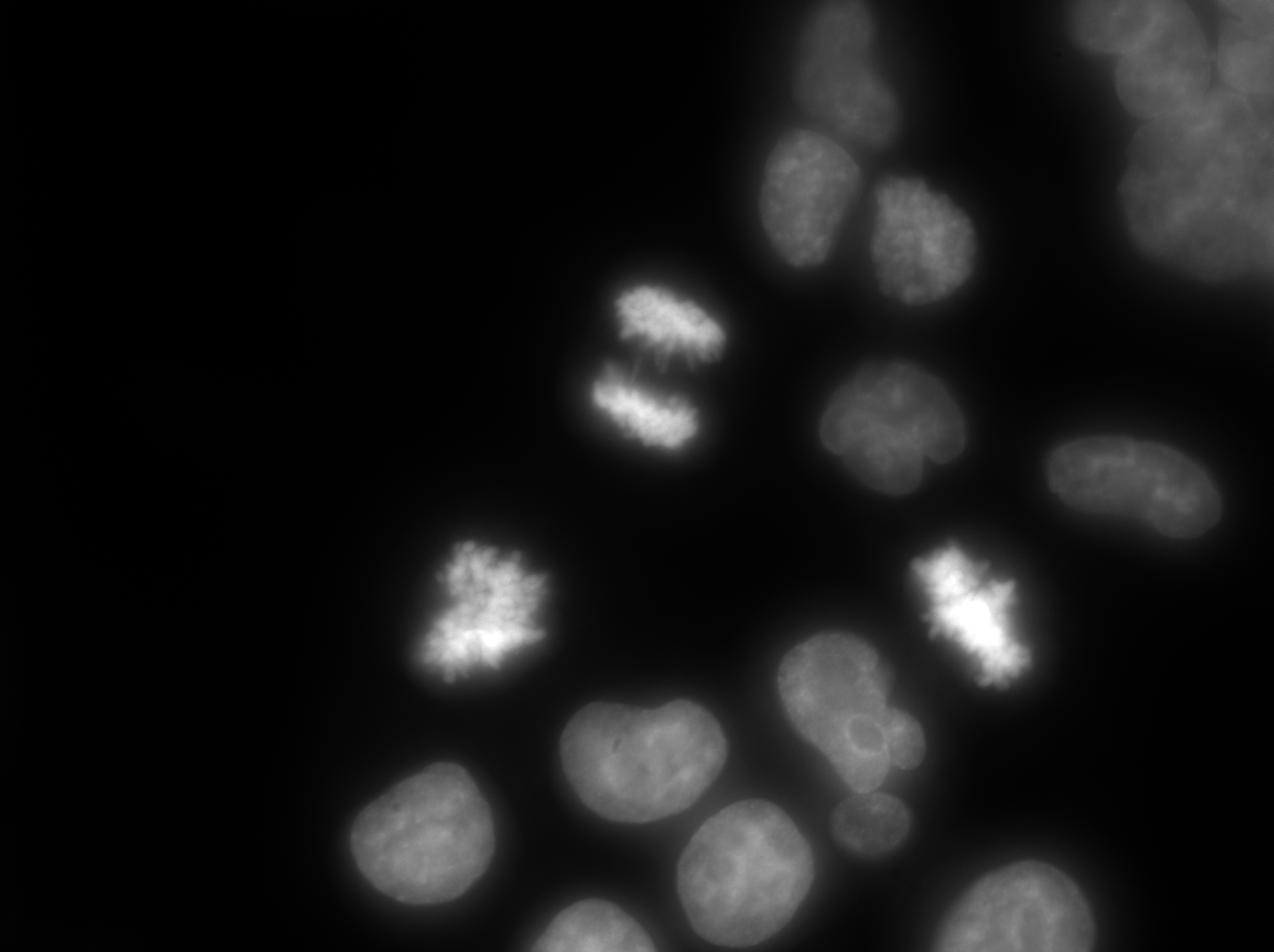

Supplement: Supplementary file 9 — Source data Fig. 2 part 6 [file 44319_2026_742_MOESM9_ESM.zip › Figure 2 Part 6/Fig 2fg Control Hela rGT335 acetylated tubulin/Anaphase/Cas9 actub rGT335 9-8-25 R2 A4 M2.Project Maximum Z_XY1757360557_Z0_T0_C0.tif]

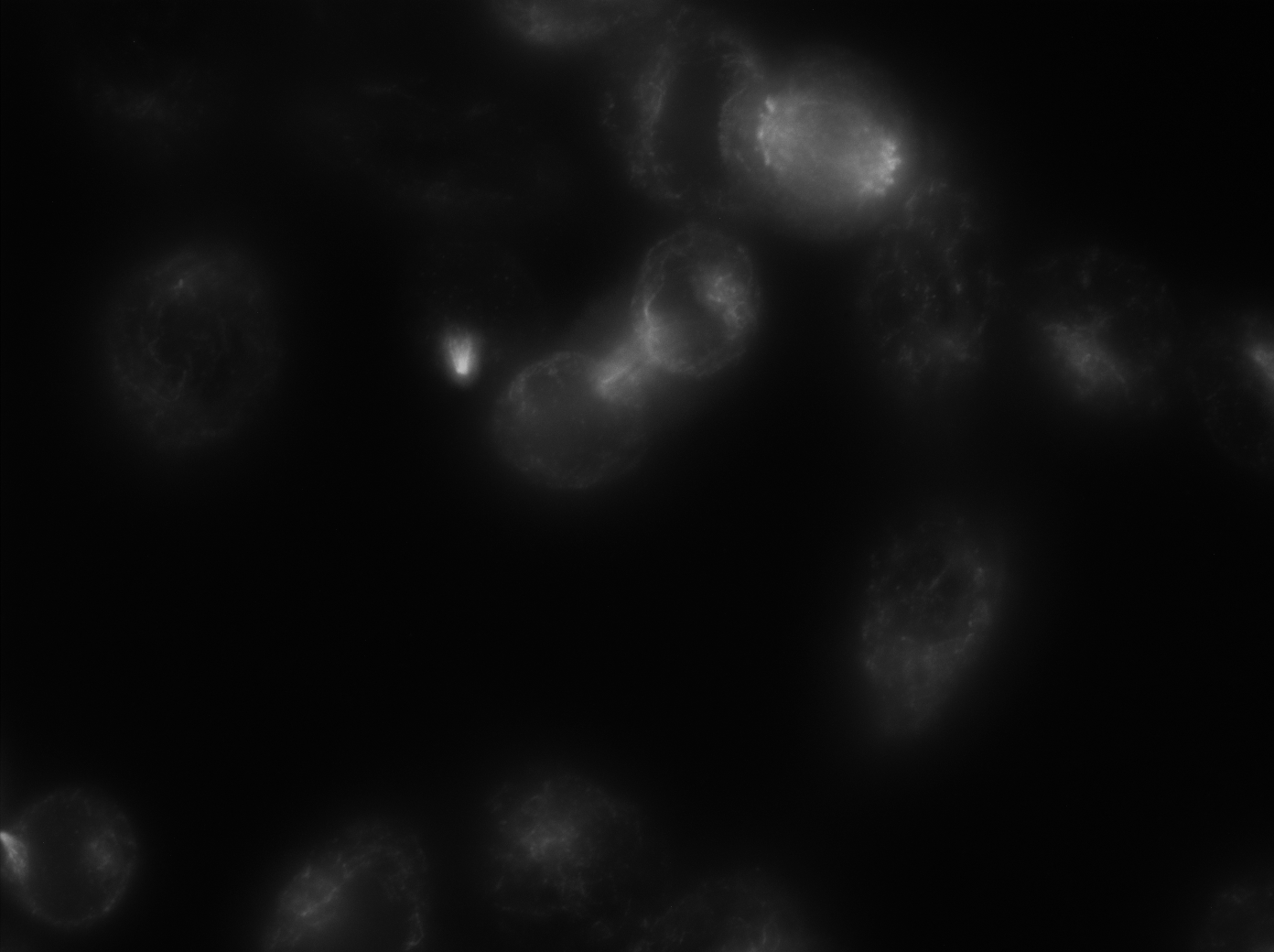

Supplement: Supplementary file 9 — Source data Fig. 2 part 6 [file 44319_2026_742_MOESM9_ESM.zip › Figure 2 Part 6/Fig 2fg Control Hela rGT335 acetylated tubulin/Anaphase/Cas9 actub rGT335 9-8-25 R2 A9.Project Maximum Z_XY1757362363_Z0_T0_C2.tif]

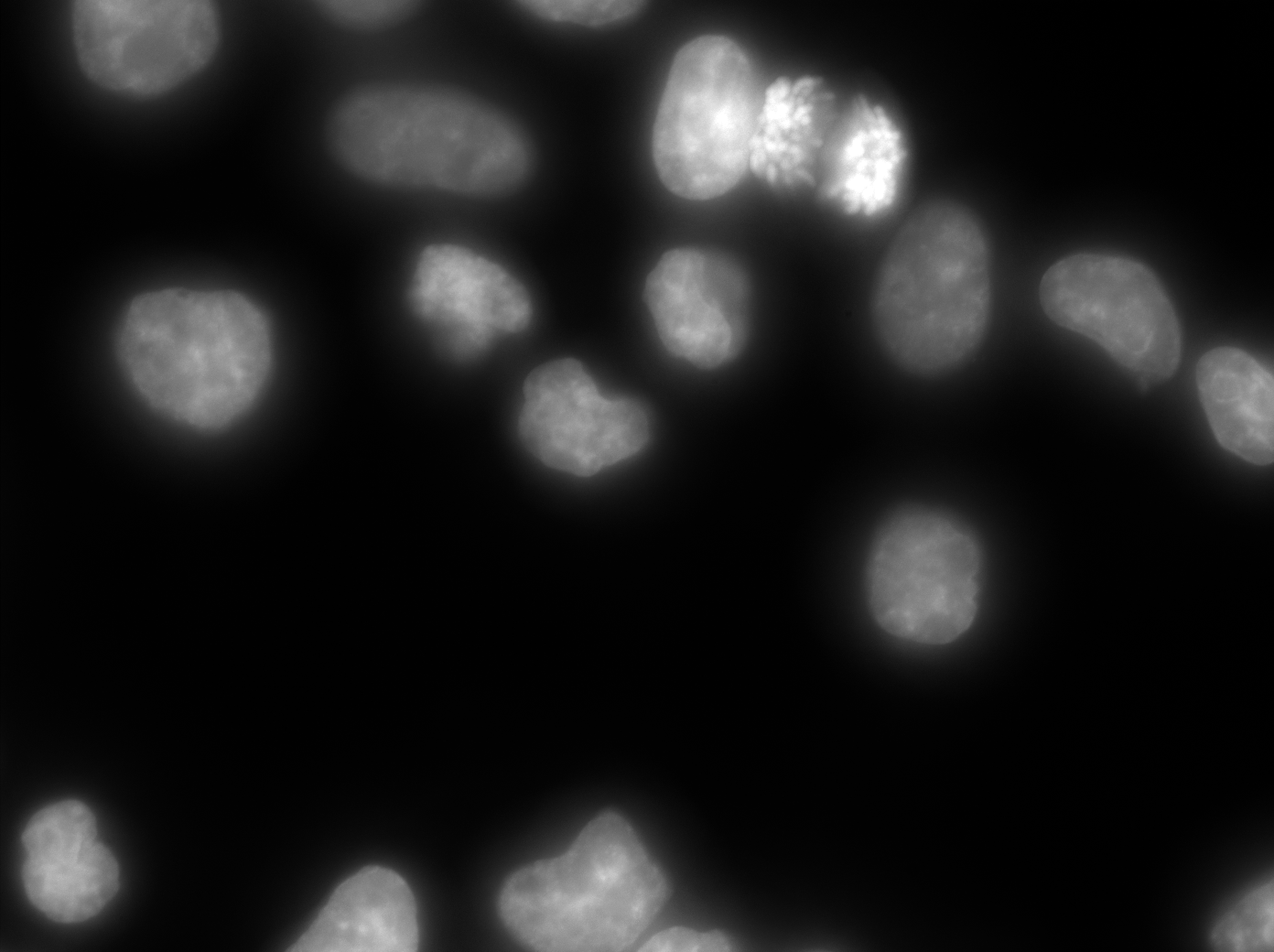

Supplement: Supplementary file 9 — Source data Fig. 2 part 6 [file 44319_2026_742_MOESM9_ESM.zip › Figure 2 Part 6/Fig 2fg Control Hela rGT335 acetylated tubulin/Anaphase/Cas9 actub rGT335 9-8-25 R2 A9.Project Maximum Z_XY1757362363_Z0_T0_C0.tif]

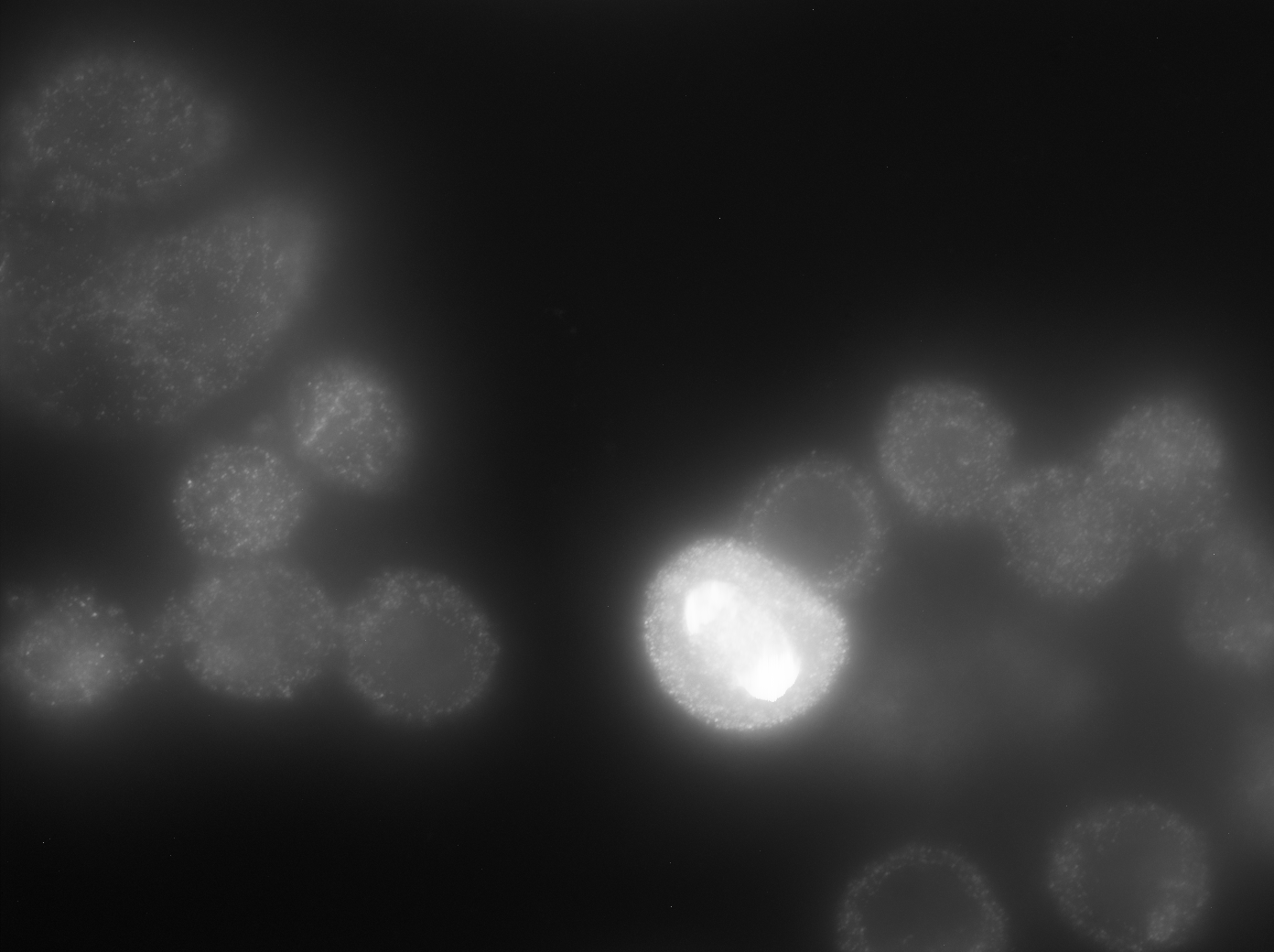

Supplement: Supplementary file 9 — Source data Fig. 2 part 6 [file 44319_2026_742_MOESM9_ESM.zip › Figure 2 Part 6/Fig 2fg Control Hela rGT335 acetylated tubulin/Anaphase/Cas9 actub rGT335 9-8-25 R1 A3.Project Maximum Z_XY1757352025_Z0_T0_C1.tif]

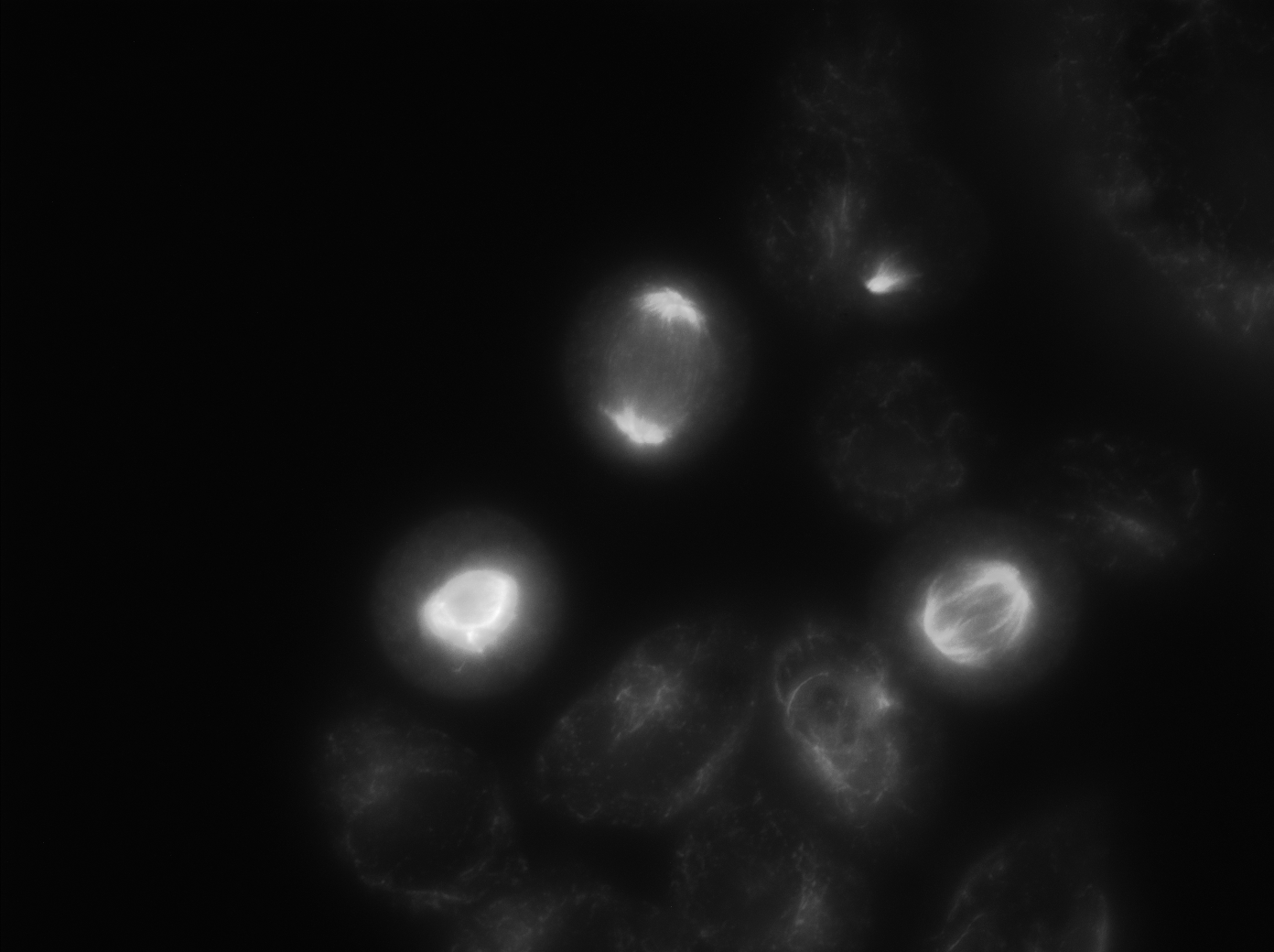

Supplement: Supplementary file 9 — Source data Fig. 2 part 6 [file 44319_2026_742_MOESM9_ESM.zip › Figure 2 Part 6/Fig 2fg Control Hela rGT335 acetylated tubulin/Anaphase/Cas9 actub rGT335 9-8-25 R2 A4 M2.Project Maximum Z_XY1757360557_Z0_T0_C2.tif]

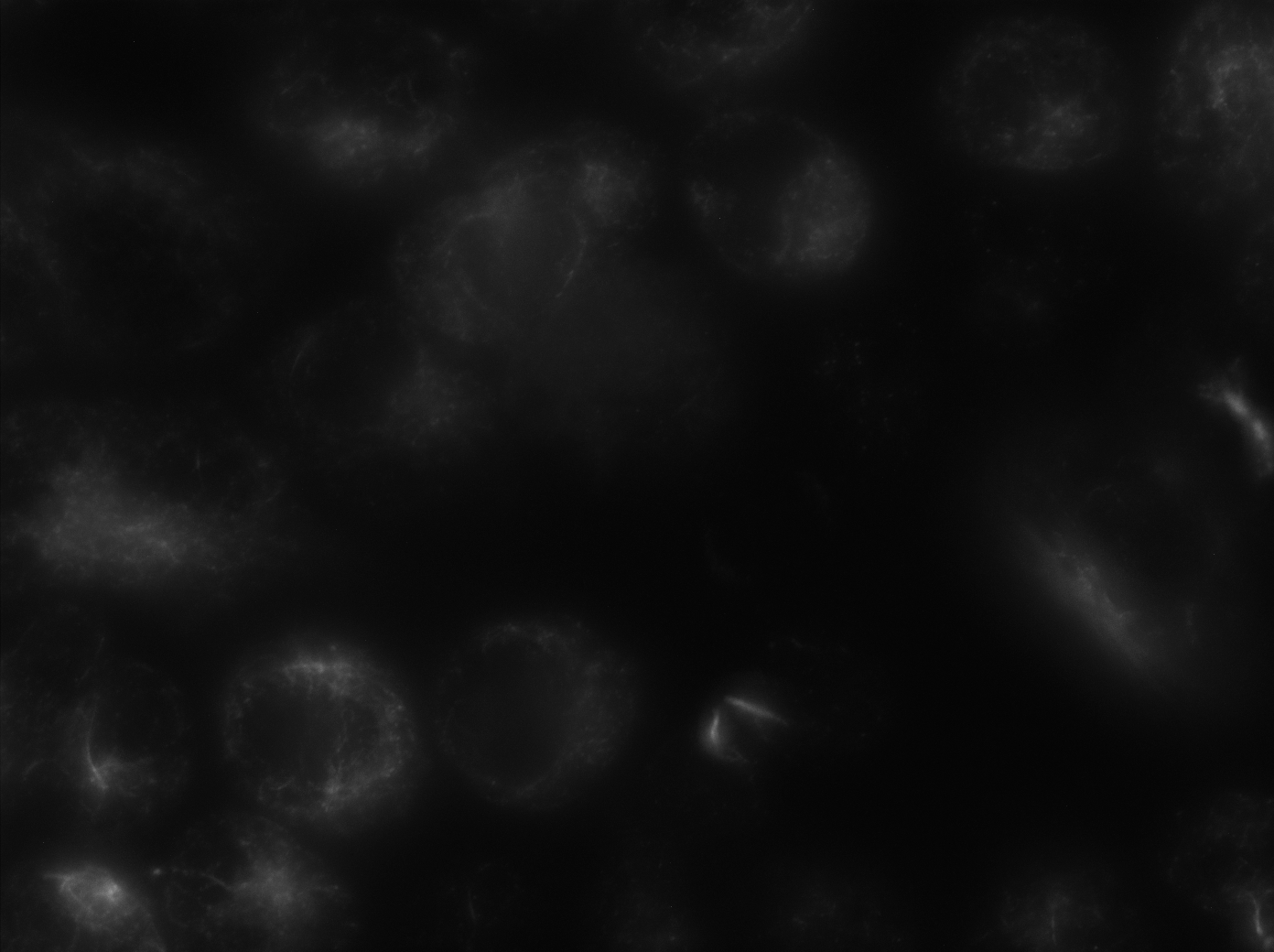

Supplement: Supplementary file 9 — Source data Fig. 2 part 6 [file 44319_2026_742_MOESM9_ESM.zip › Figure 2 Part 6/Fig 2fg Control Hela rGT335 acetylated tubulin/Anaphase/Cas9 actub rGT335 9-8-25 R3 A8.Project Maximum Z_XY1757368874_Z0_T0_C2.tif]

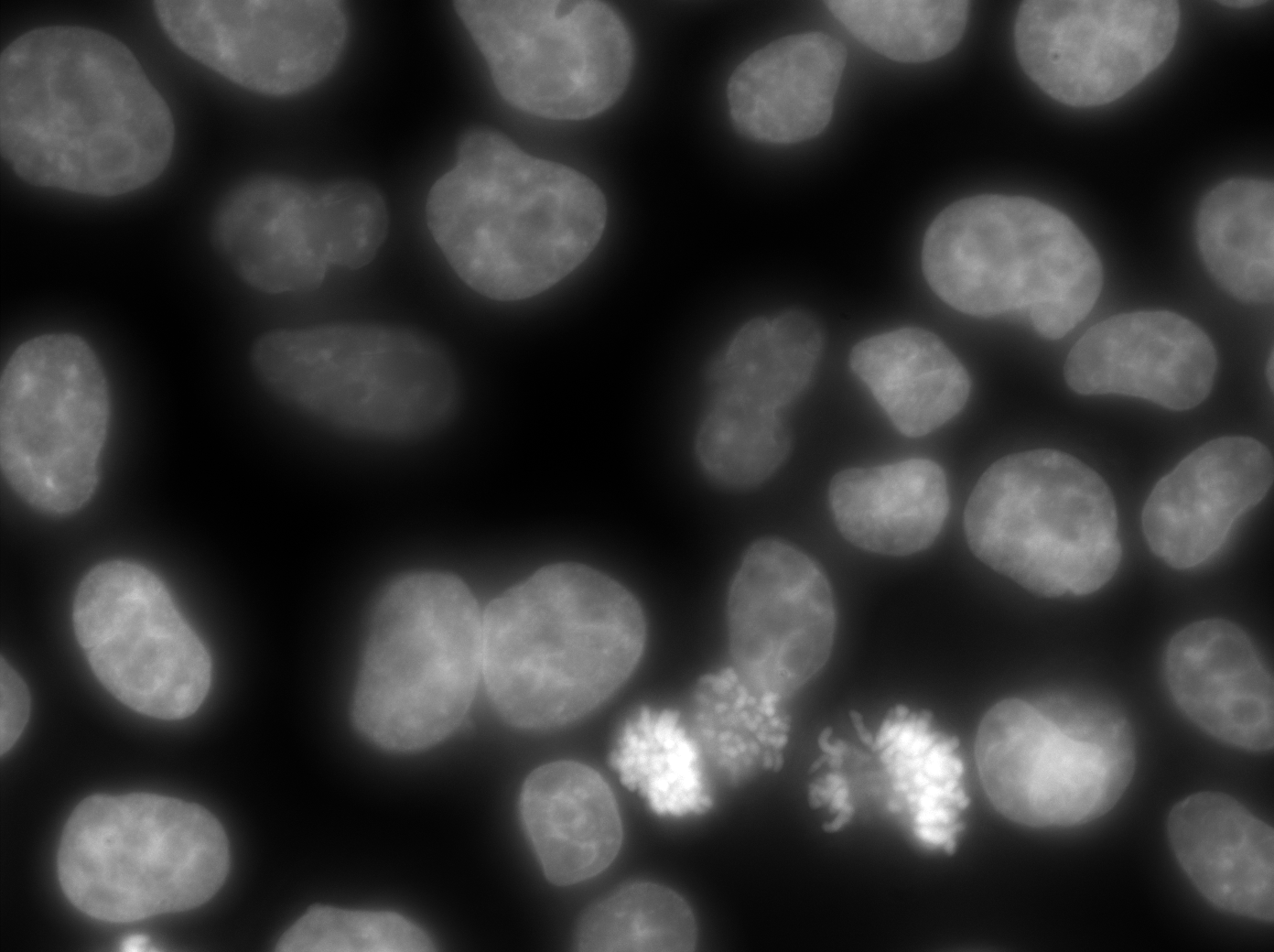

Supplement: Supplementary file 9 — Source data Fig. 2 part 6 [file 44319_2026_742_MOESM9_ESM.zip › Figure 2 Part 6/Fig 2fg Control Hela rGT335 acetylated tubulin/Anaphase/Cas9 actub rGT335 9-8-25 R3 A6.Project Maximum Z_XY1757367515_Z0_T0_C0.tif]

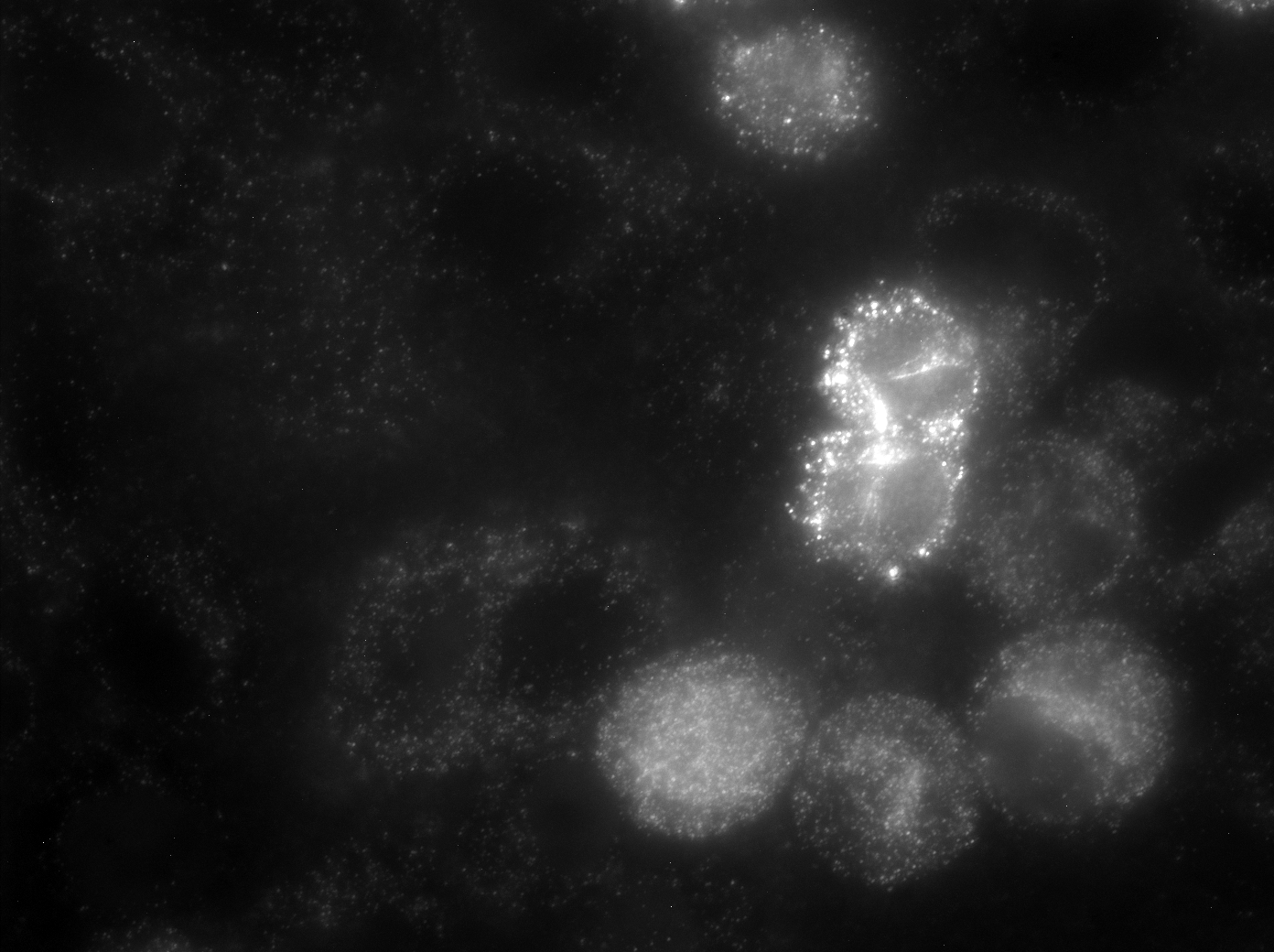

Supplement: Supplementary file 9 — Source data Fig. 2 part 6 [file 44319_2026_742_MOESM9_ESM.zip › Figure 2 Part 6/Fig 2fg Control Hela rGT335 acetylated tubulin/Anaphase/Cas9 actub rGT335 9-8-25 R3 A6.Project Maximum Z_XY1757367515_Z0_T0_C1.tif]

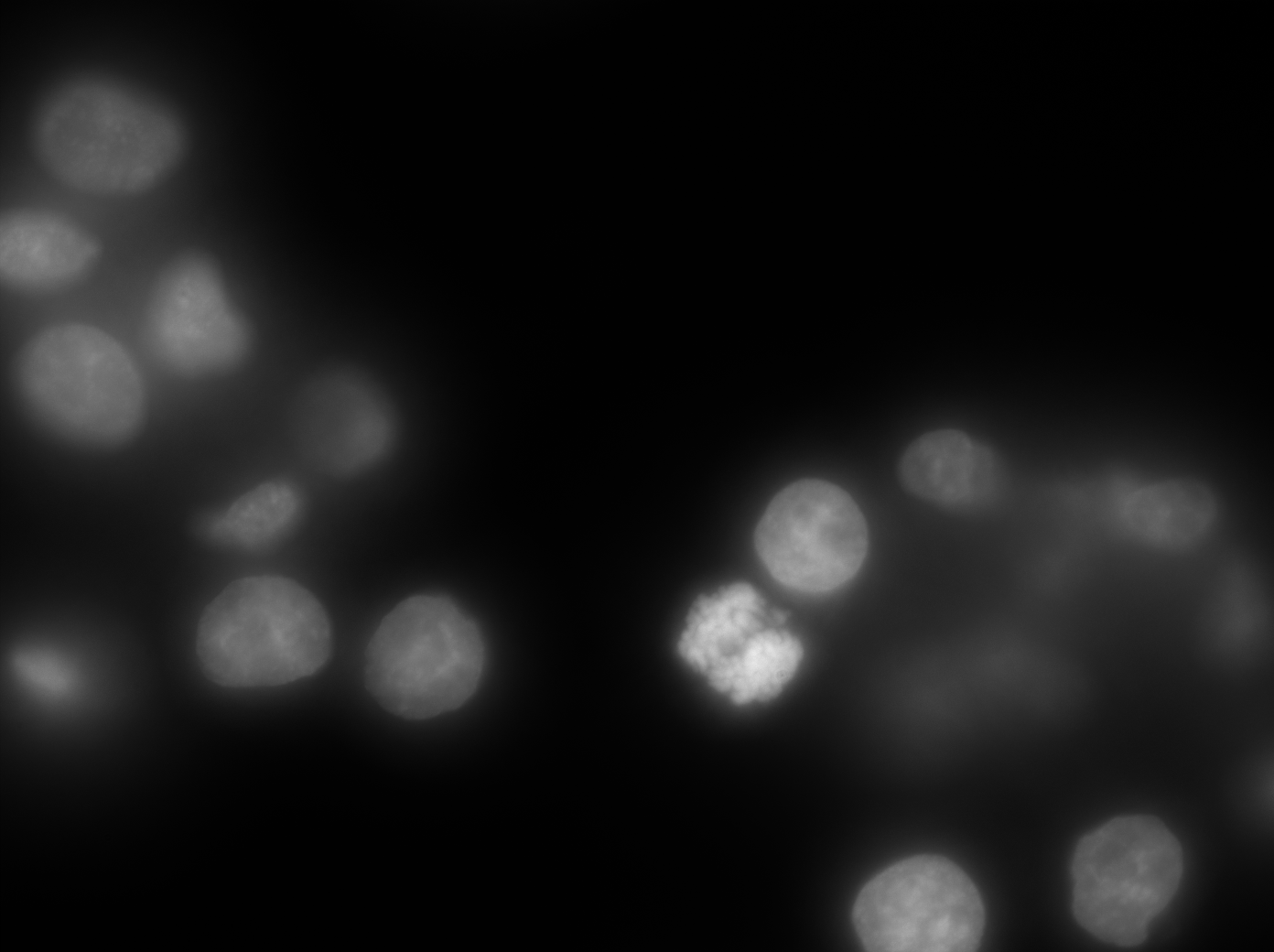

Supplement: Supplementary file 9 — Source data Fig. 2 part 6 [file 44319_2026_742_MOESM9_ESM.zip › Figure 2 Part 6/Fig 2fg Control Hela rGT335 acetylated tubulin/Anaphase/Cas9 actub rGT335 9-8-25 R1 A3.Project Maximum Z_XY1757352025_Z0_T0_C0.tif]

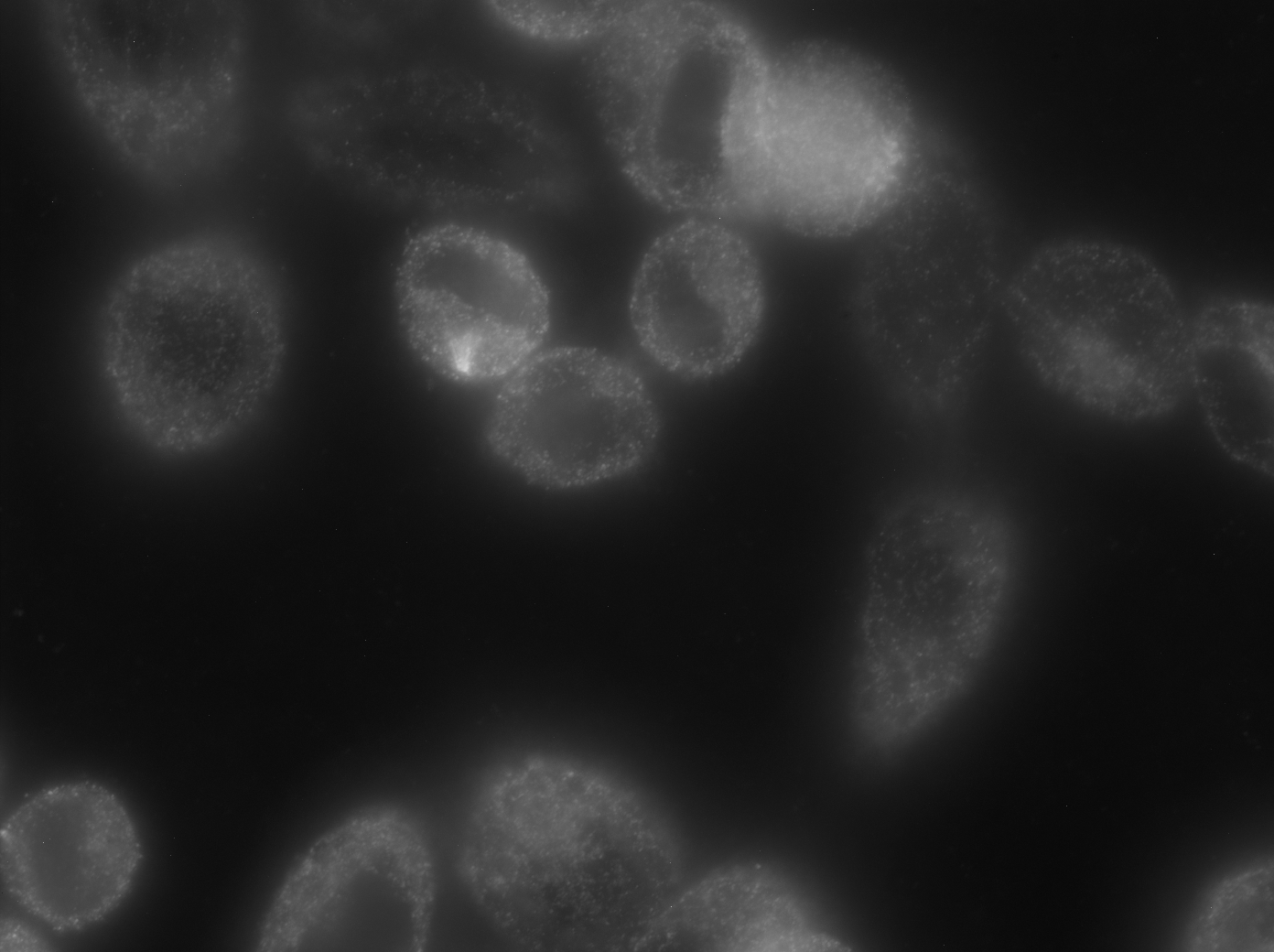

Supplement: Supplementary file 9 — Source data Fig. 2 part 6 [file 44319_2026_742_MOESM9_ESM.zip › Figure 2 Part 6/Fig 2fg Control Hela rGT335 acetylated tubulin/Anaphase/Cas9 actub rGT335 9-8-25 R2 A9.Project Maximum Z_XY1757362363_Z0_T0_C1.tif]

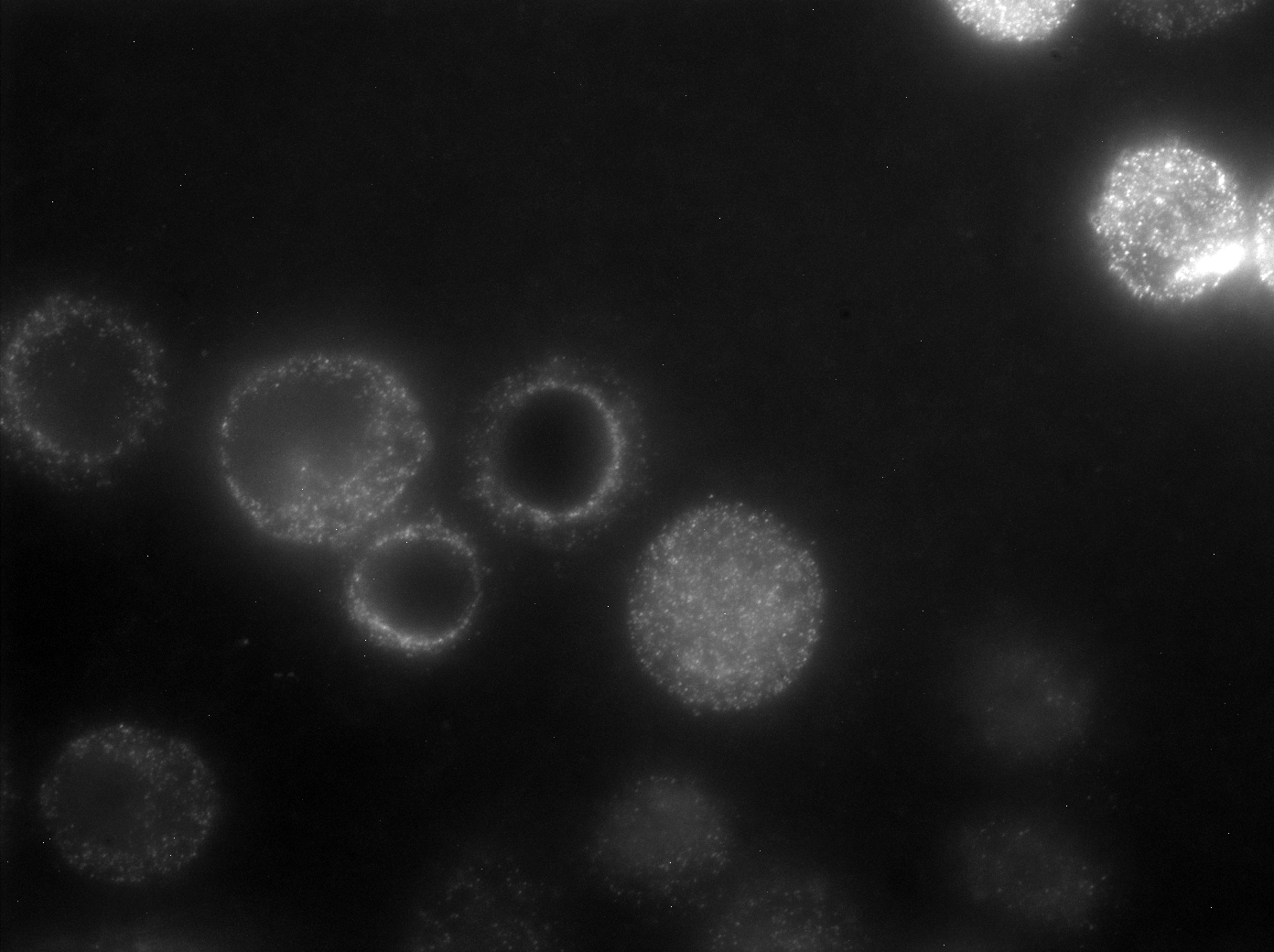

Supplement: Supplementary file 9 — Source data Fig. 2 part 6 [file 44319_2026_742_MOESM9_ESM.zip › Figure 2 Part 6/Fig 2fg Control Hela rGT335 acetylated tubulin/Anaphase/Cas9 actub rGT335 9-8-25 R3 A9.Project Maximum Z_XY1757369097_Z0_T0_C1.tif]

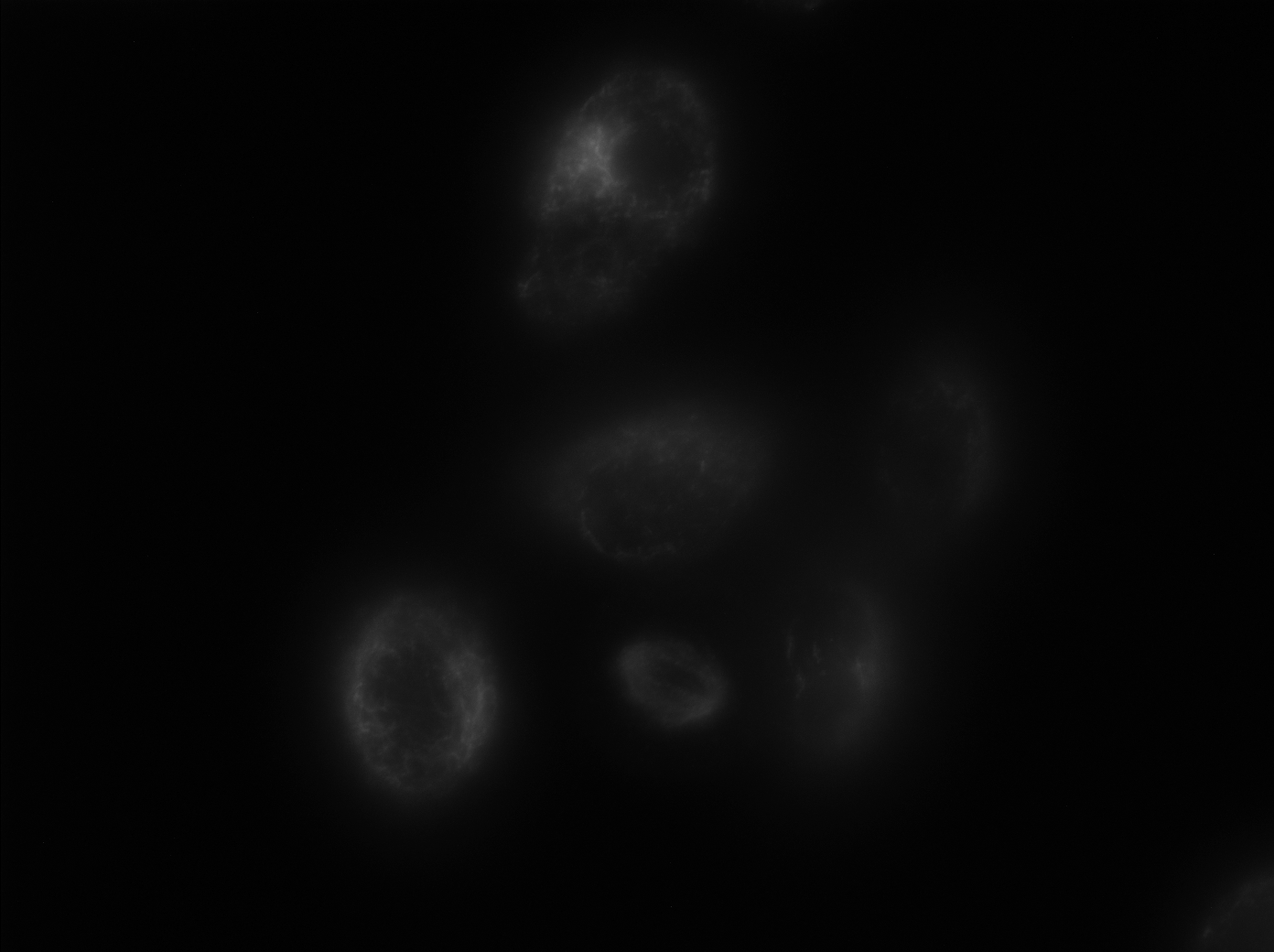

Supplement: Supplementary file 9 — Source data Fig. 2 part 6 [file 44319_2026_742_MOESM9_ESM.zip › Figure 2 Part 6/Fig 2fg Control Hela rGT335 acetylated tubulin/Anaphase/Cas9 actub rGT335 9-8-25 R1 A6.Project Maximum Z_XY1757355240_Z0_T0_C2.tif]

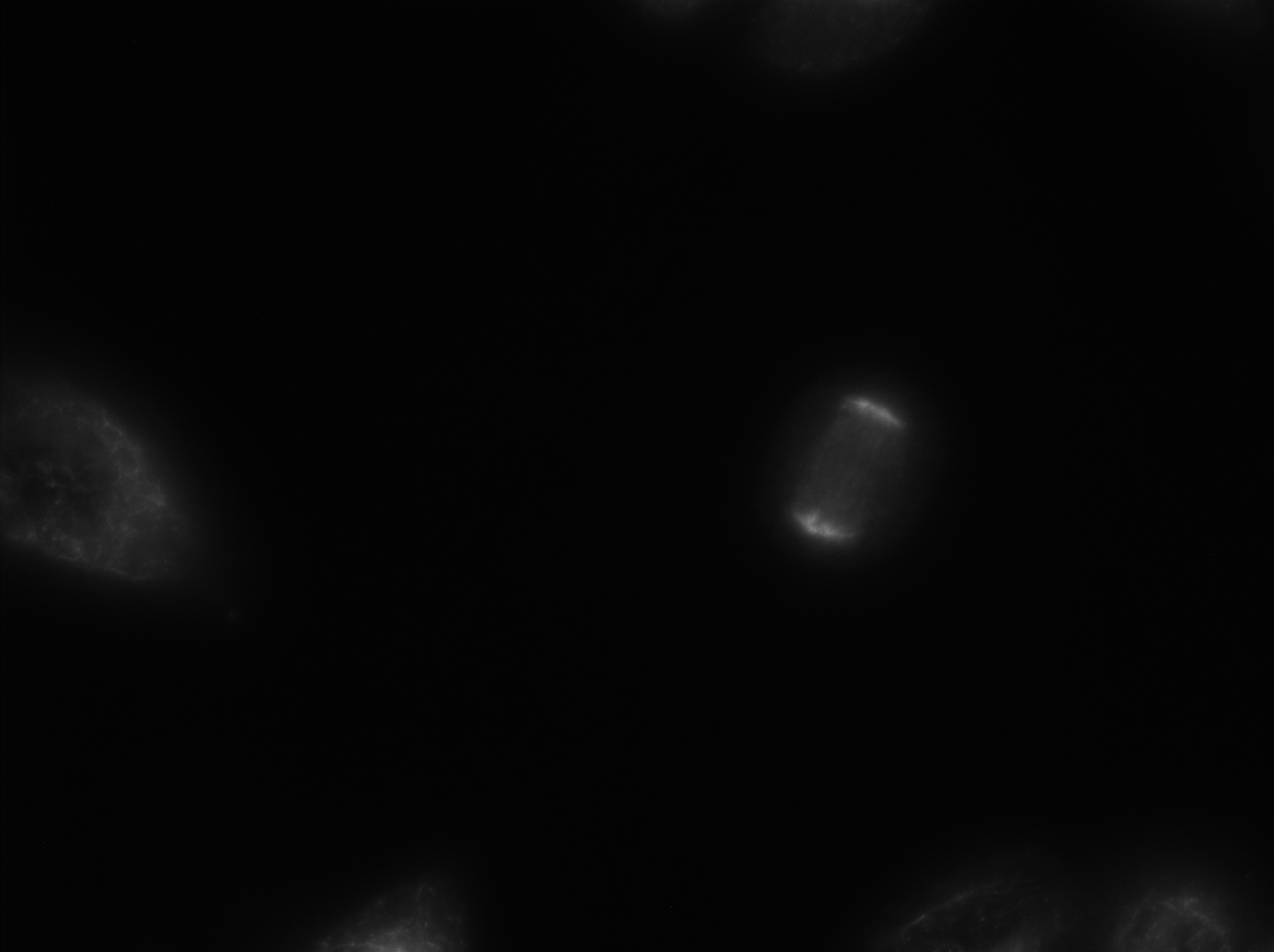

Supplement: Supplementary file 9 — Source data Fig. 2 part 6 [file 44319_2026_742_MOESM9_ESM.zip › Figure 2 Part 6/Fig 2fg Control Hela rGT335 acetylated tubulin/Anaphase/Cas9 actub rGT335 9-8-25 R2 A2.Project Maximum Z_XY1757360168_Z0_T0_C2.tif]

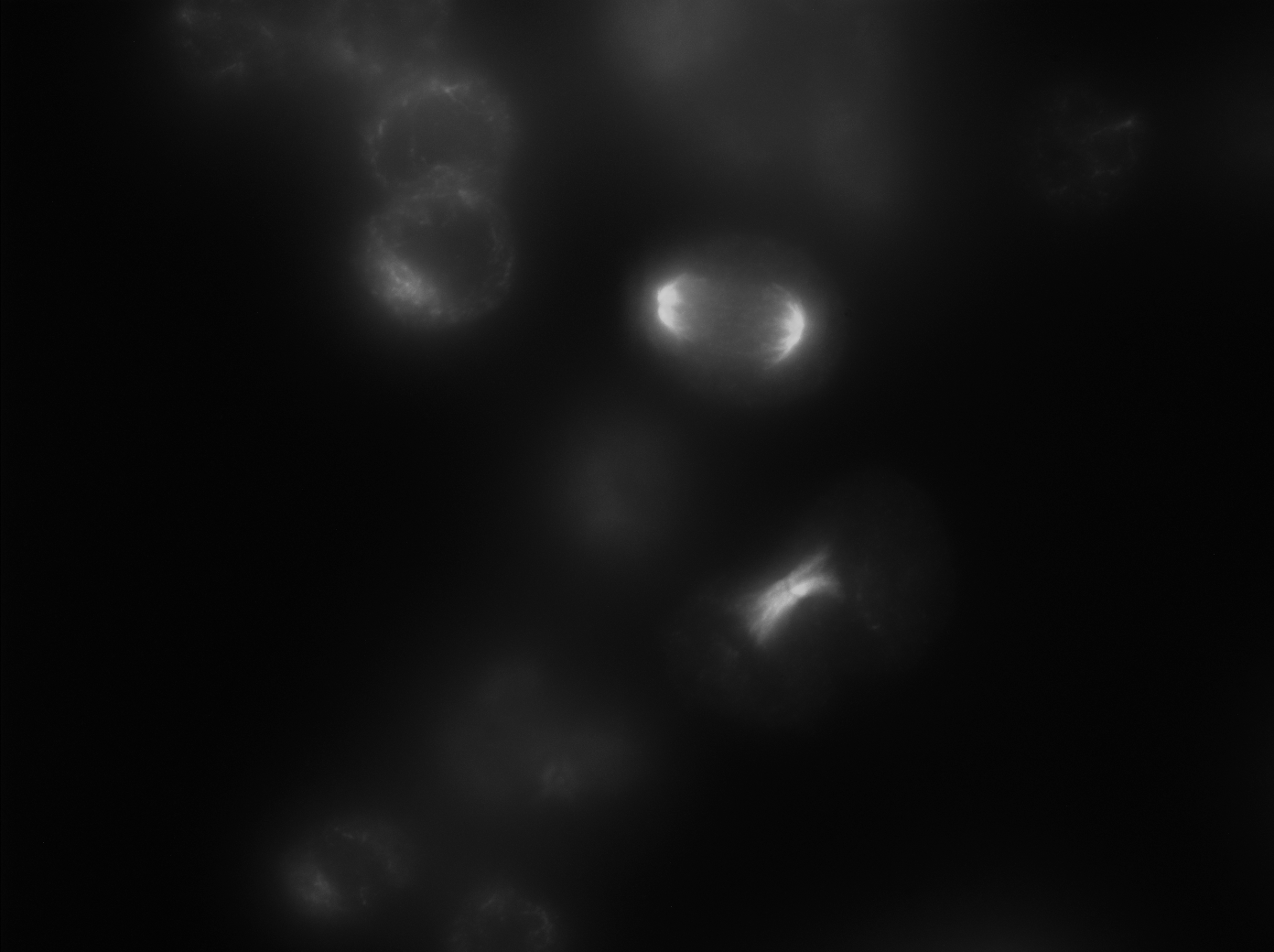

Supplement: Supplementary file 9 — Source data Fig. 2 part 6 [file 44319_2026_742_MOESM9_ESM.zip › Figure 2 Part 6/Fig 2fg Control Hela rGT335 acetylated tubulin/Anaphase/Cas9 actub rGT335 9-8-25 R1 A4 LFI1.Project Maximum Z_XY1757352232_Z0_T0_C2.tif]

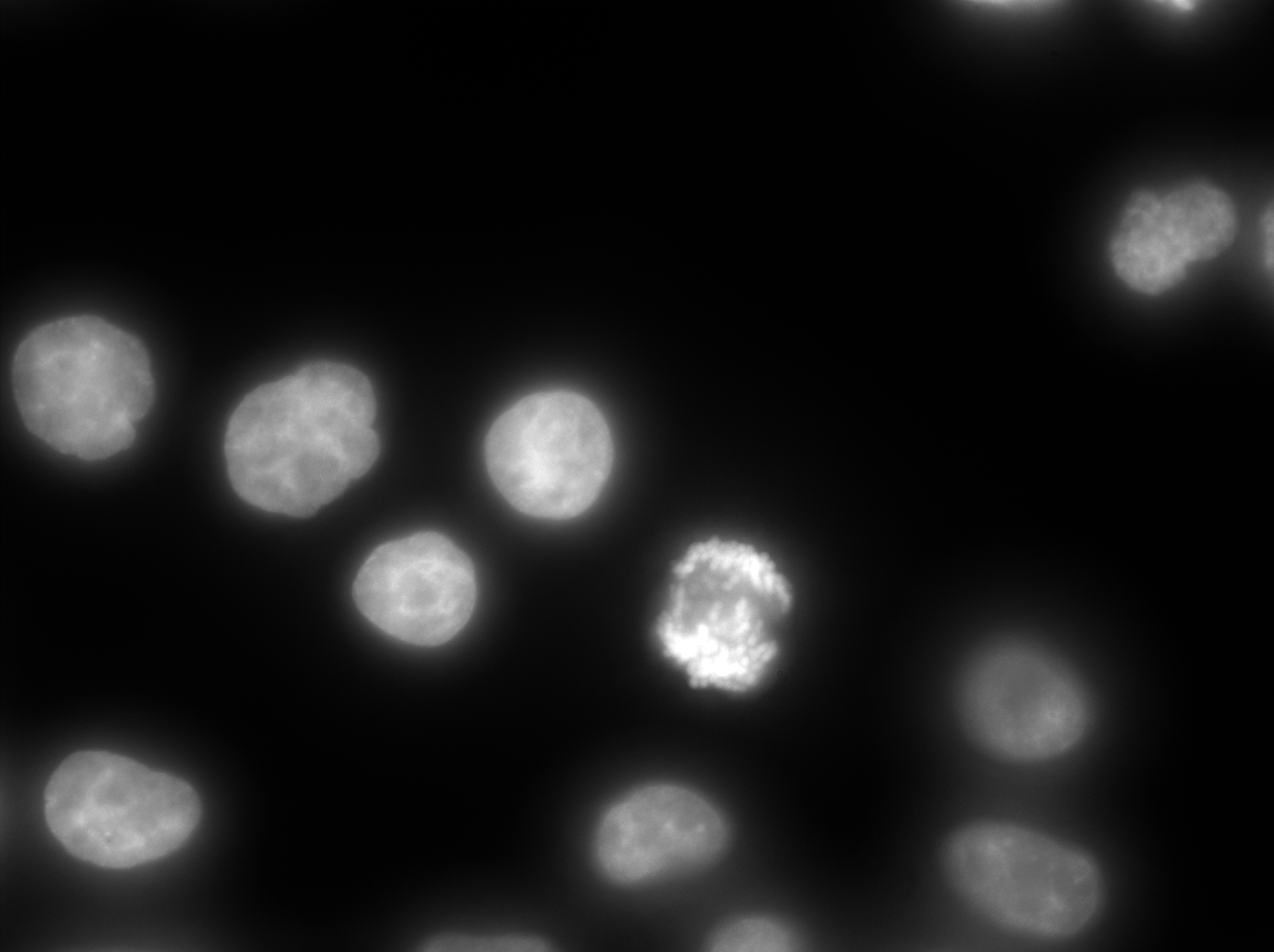

Supplement: Supplementary file 9 — Source data Fig. 2 part 6 [file 44319_2026_742_MOESM9_ESM.zip › Figure 2 Part 6/Fig 2fg Control Hela rGT335 acetylated tubulin/Anaphase/Cas9 actub rGT335 9-8-25 R3 A9.Project Maximum Z_XY1757369097_Z0_T0_C0.tif]

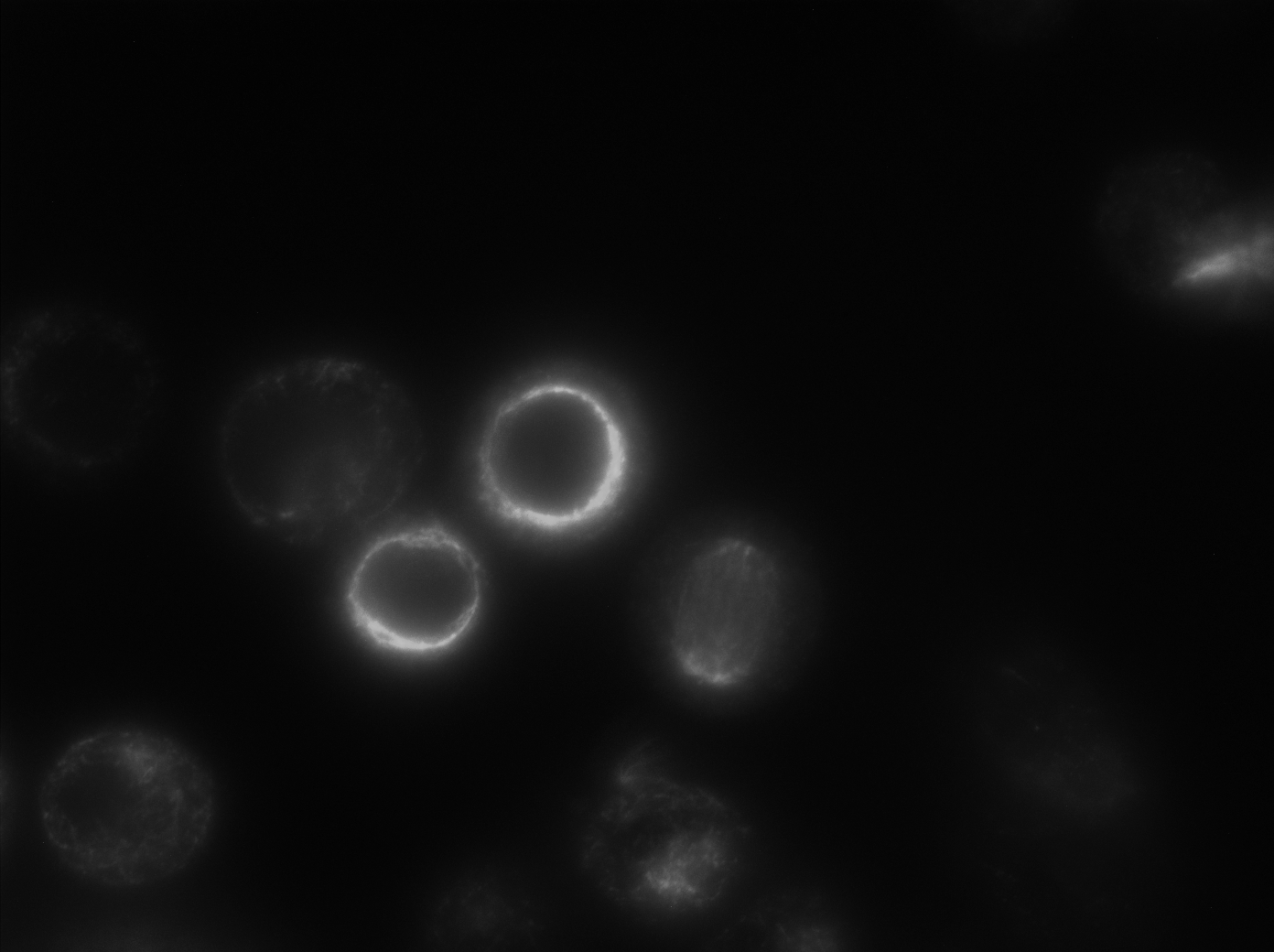

Supplement: Supplementary file 9 — Source data Fig. 2 part 6 [file 44319_2026_742_MOESM9_ESM.zip › Figure 2 Part 6/Fig 2fg Control Hela rGT335 acetylated tubulin/Anaphase/Cas9 actub rGT335 9-8-25 R3 A9.Project Maximum Z_XY1757369097_Z0_T0_C2.tif]

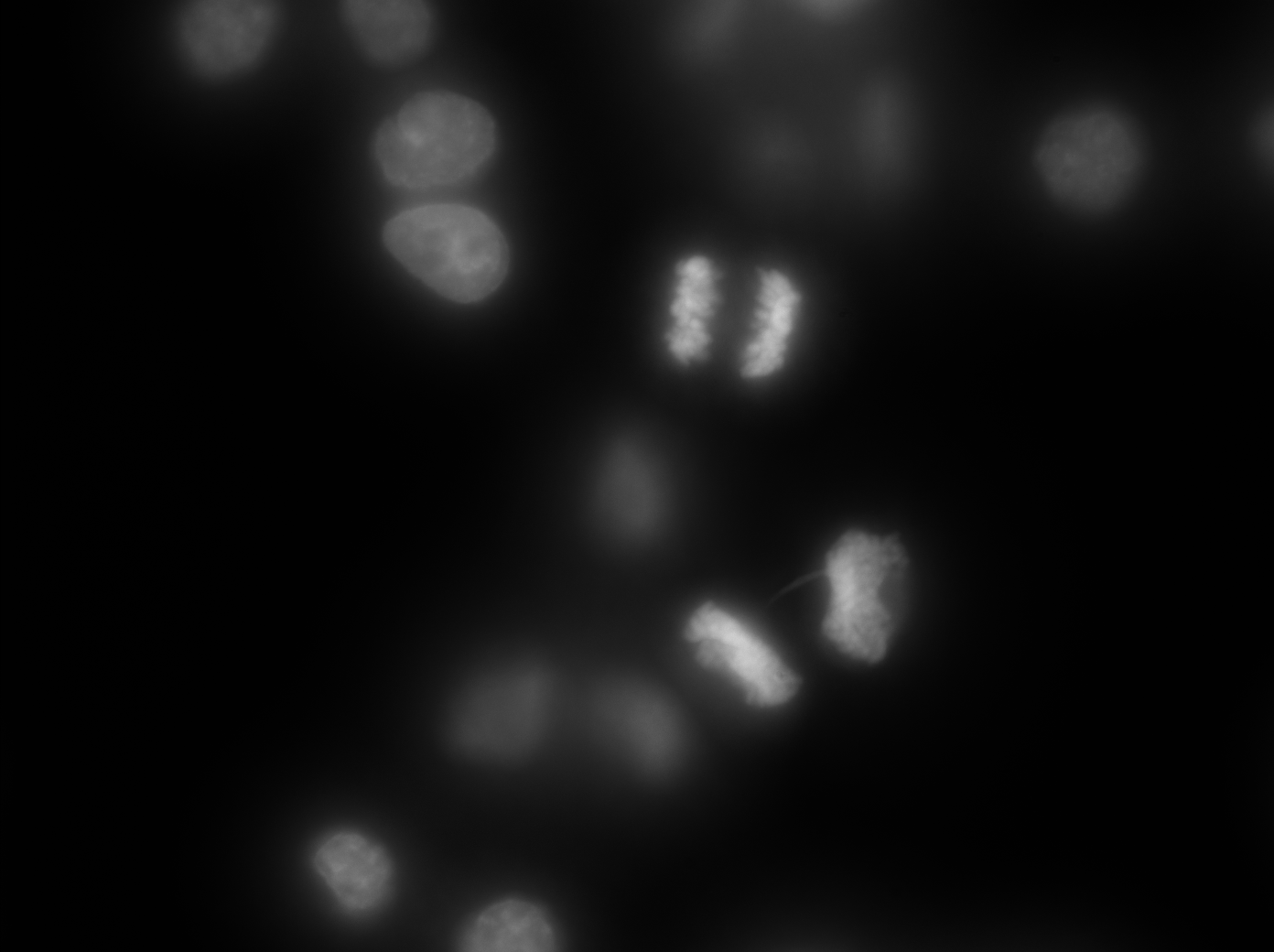

Supplement: Supplementary file 9 — Source data Fig. 2 part 6 [file 44319_2026_742_MOESM9_ESM.zip › Figure 2 Part 6/Fig 2fg Control Hela rGT335 acetylated tubulin/Anaphase/Cas9 actub rGT335 9-8-25 R1 A4 LFI1.Project Maximum Z_XY1757352232_Z0_T0_C0.tif]

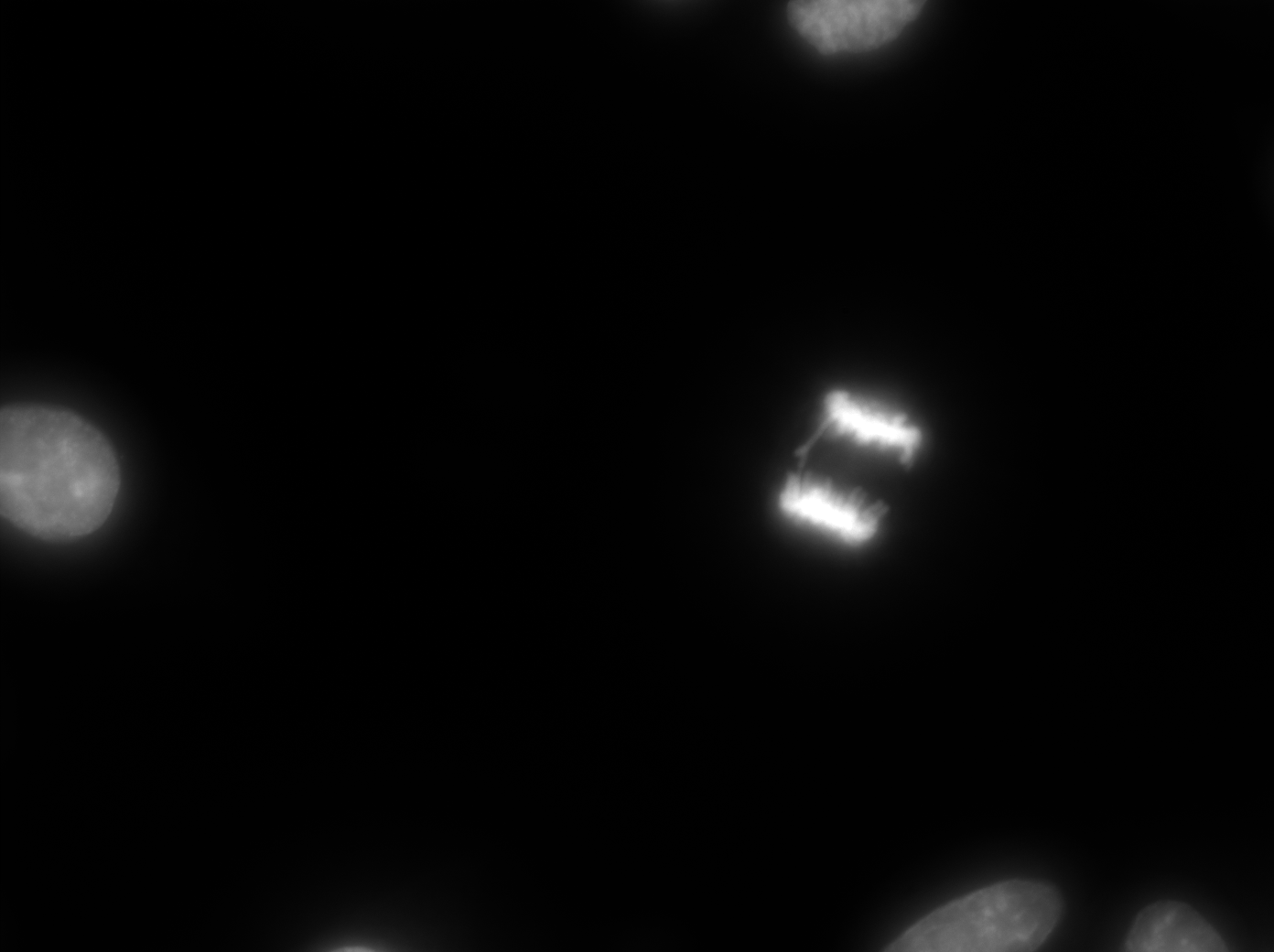

Supplement: Supplementary file 9 — Source data Fig. 2 part 6 [file 44319_2026_742_MOESM9_ESM.zip › Figure 2 Part 6/Fig 2fg Control Hela rGT335 acetylated tubulin/Anaphase/Cas9 actub rGT335 9-8-25 R2 A2.Project Maximum Z_XY1757360168_Z0_T0_C0.tif]

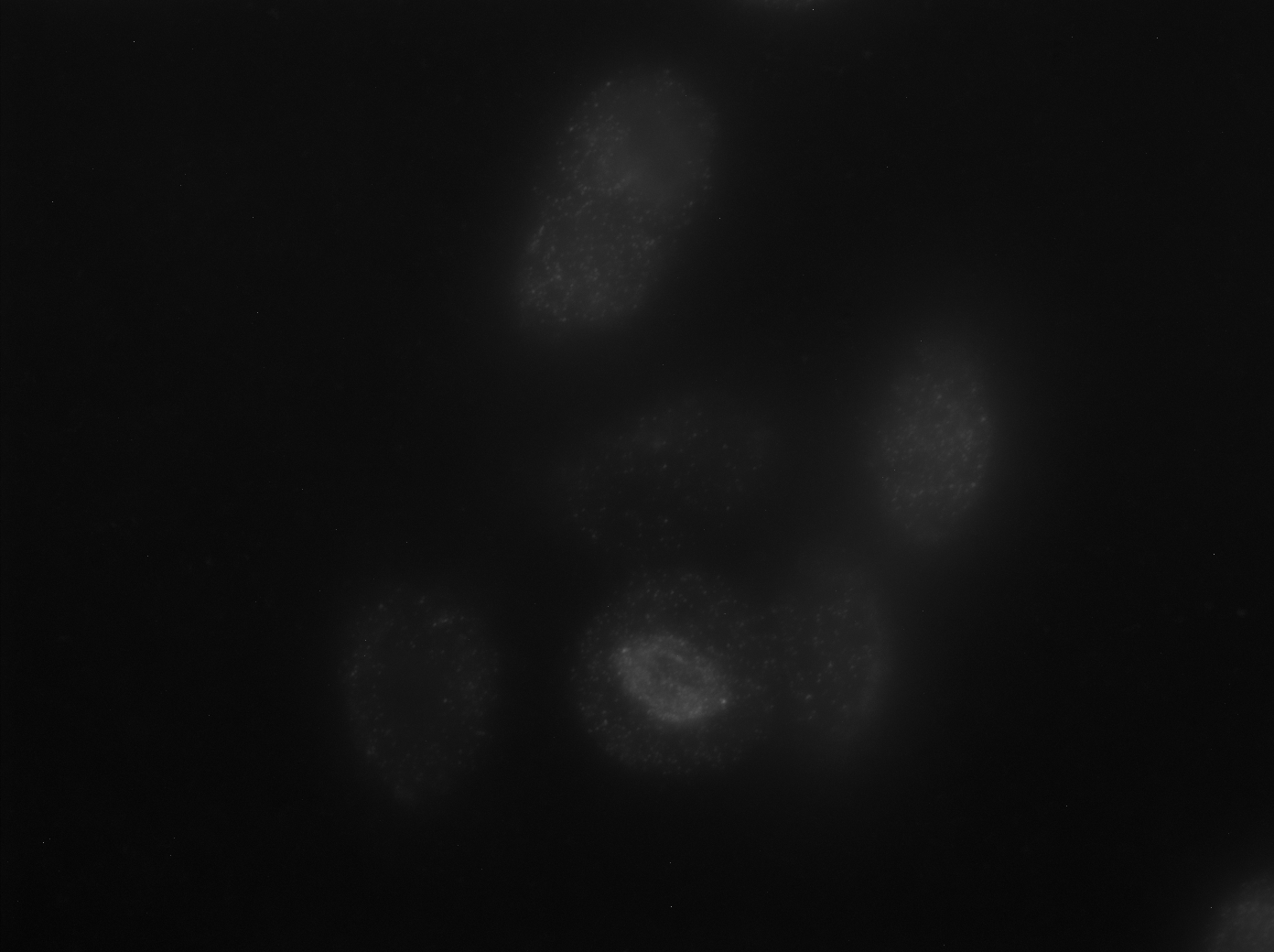

Supplement: Supplementary file 9 — Source data Fig. 2 part 6 [file 44319_2026_742_MOESM9_ESM.zip › Figure 2 Part 6/Fig 2fg Control Hela rGT335 acetylated tubulin/Anaphase/Cas9 actub rGT335 9-8-25 R1 A6.Project Maximum Z_XY1757355240_Z0_T0_C1.tif]

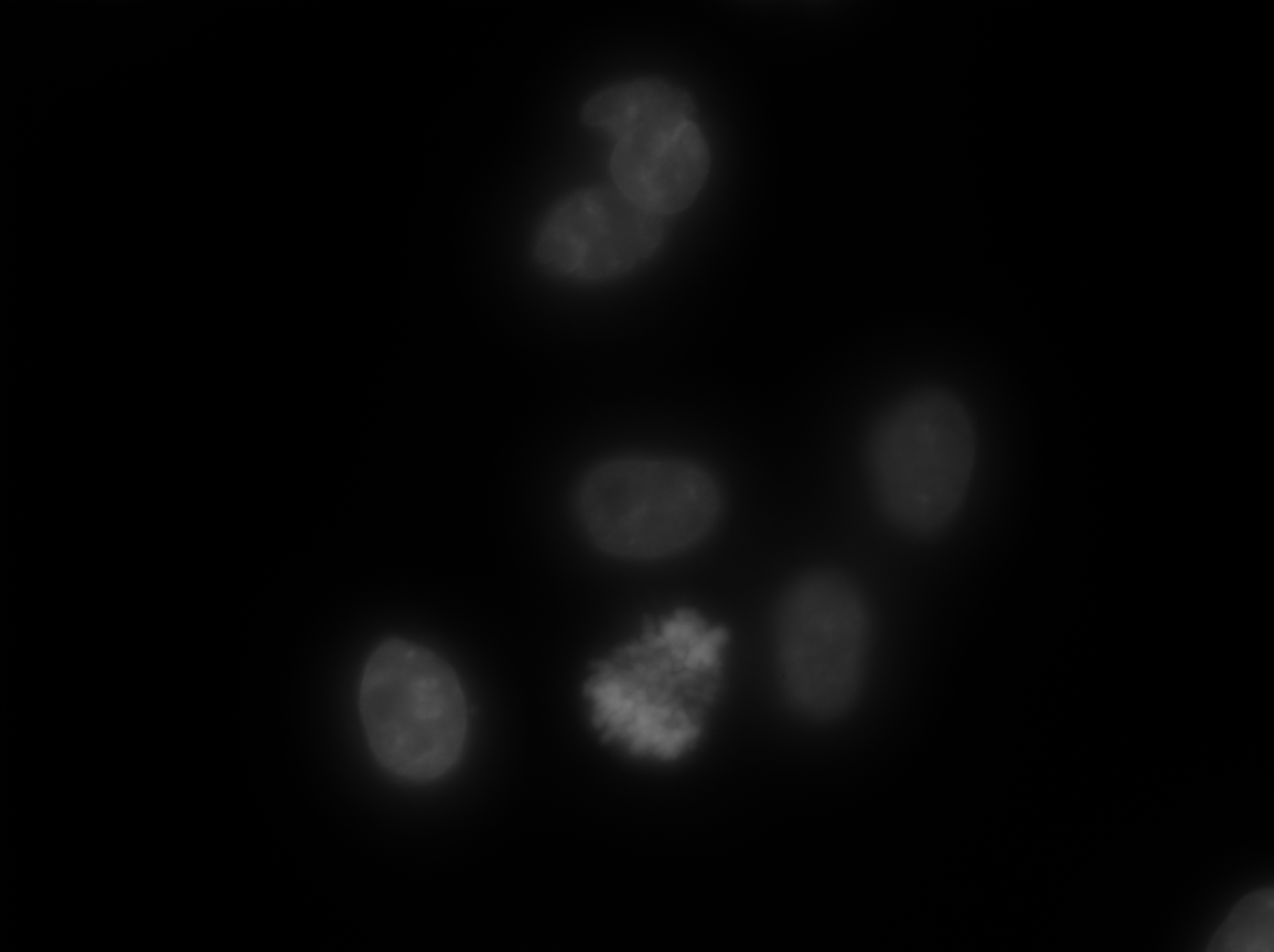

Supplement: Supplementary file 9 — Source data Fig. 2 part 6 [file 44319_2026_742_MOESM9_ESM.zip › Figure 2 Part 6/Fig 2fg Control Hela rGT335 acetylated tubulin/Anaphase/Cas9 actub rGT335 9-8-25 R1 A6.Project Maximum Z_XY1757355240_Z0_T0_C0.tif]

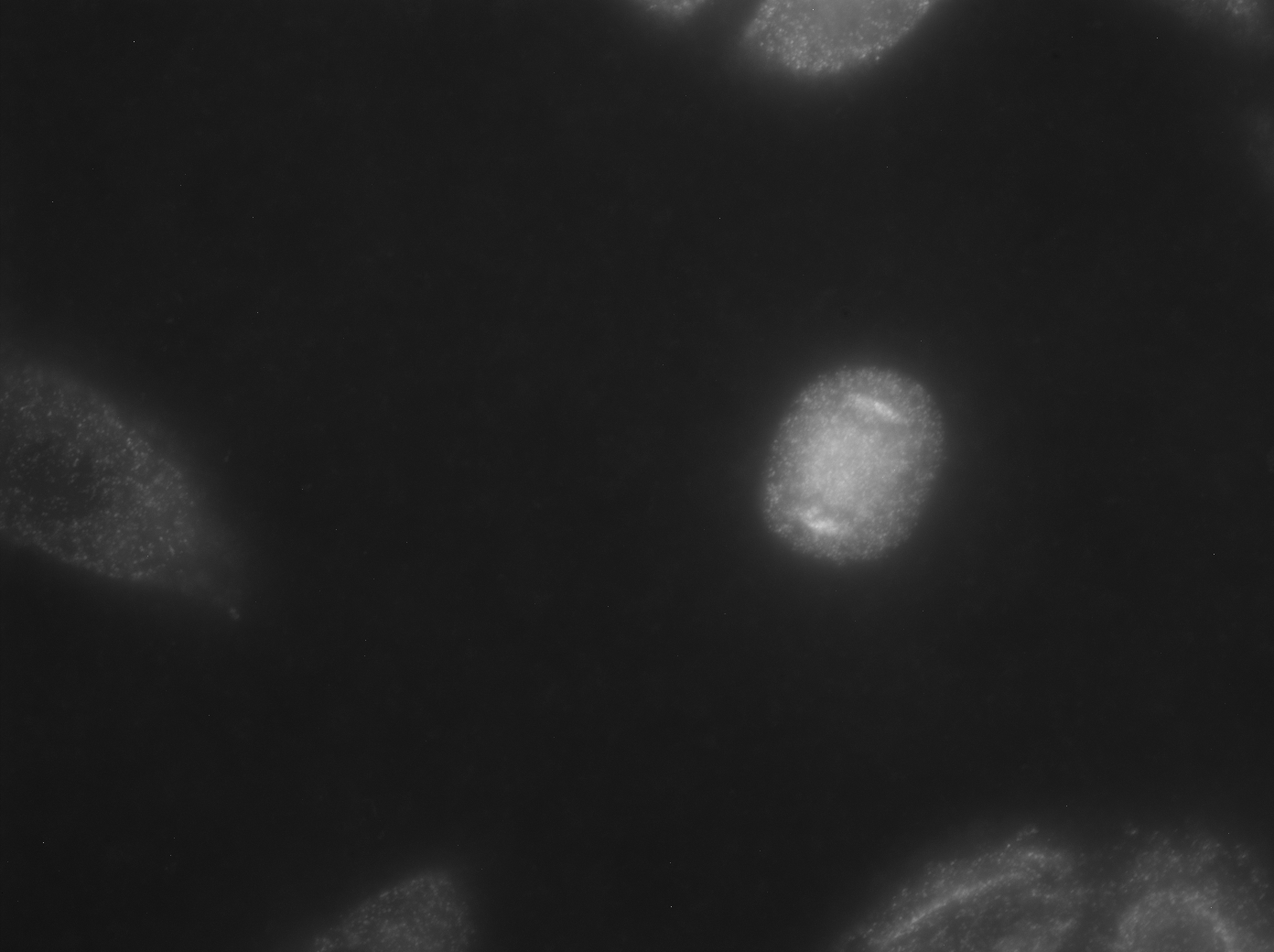

Supplement: Supplementary file 9 — Source data Fig. 2 part 6 [file 44319_2026_742_MOESM9_ESM.zip › Figure 2 Part 6/Fig 2fg Control Hela rGT335 acetylated tubulin/Anaphase/Cas9 actub rGT335 9-8-25 R2 A2.Project Maximum Z_XY1757360168_Z0_T0_C1.tif]

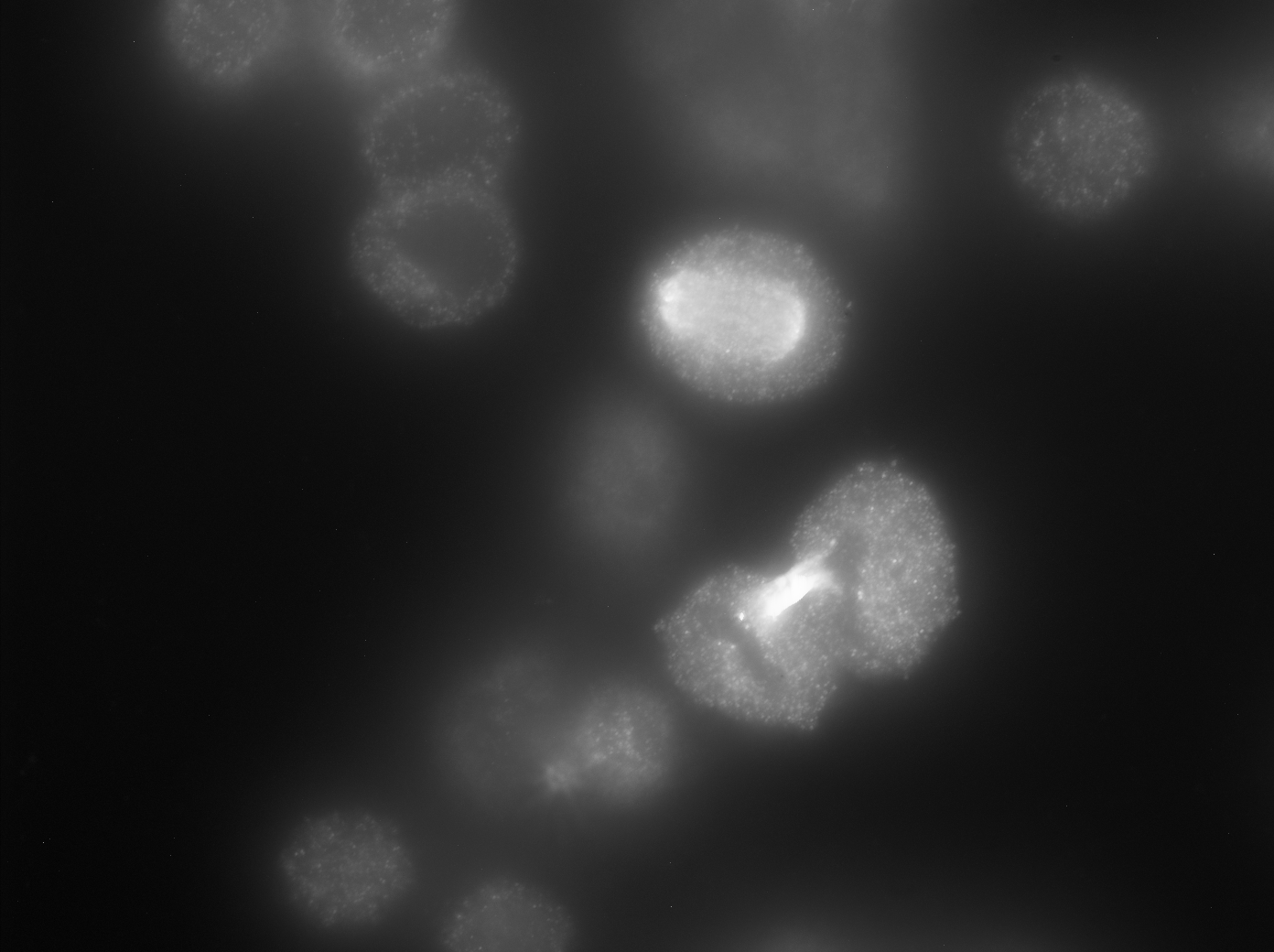

Supplement: Supplementary file 9 — Source data Fig. 2 part 6 [file 44319_2026_742_MOESM9_ESM.zip › Figure 2 Part 6/Fig 2fg Control Hela rGT335 acetylated tubulin/Anaphase/Cas9 actub rGT335 9-8-25 R1 A4 LFI1.Project Maximum Z_XY1757352232_Z0_T0_C1.tif]

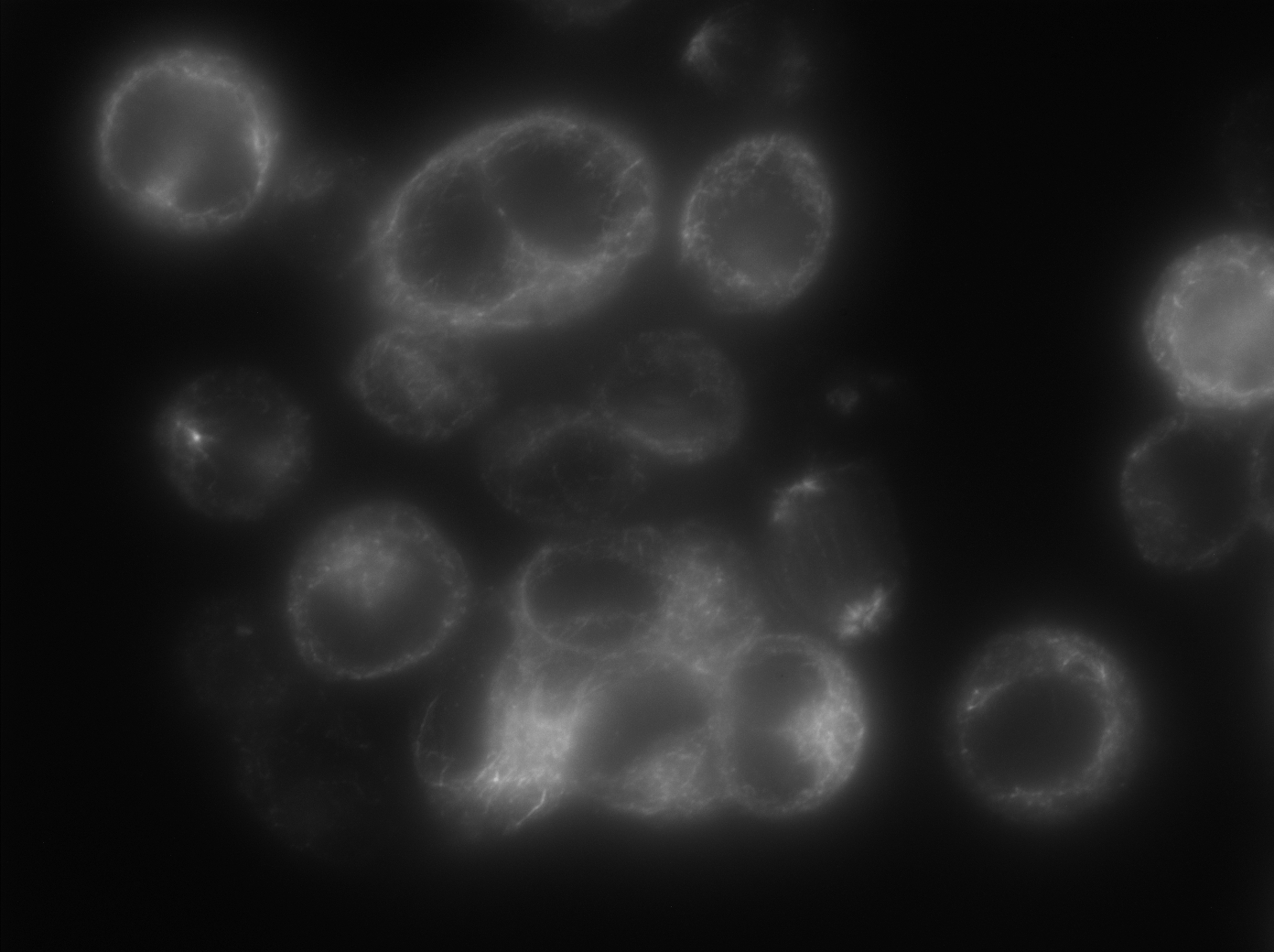

Supplement: Supplementary file 9 — Source data Fig. 2 part 6 [file 44319_2026_742_MOESM9_ESM.zip › Figure 2 Part 6/Fig 2fg Control Hela rGT335 acetylated tubulin/Anaphase/Cas9 actub rGT335 9-8-25 R1 A9.Project Maximum Z_XY1757357993_Z0_T0_C2.tif]

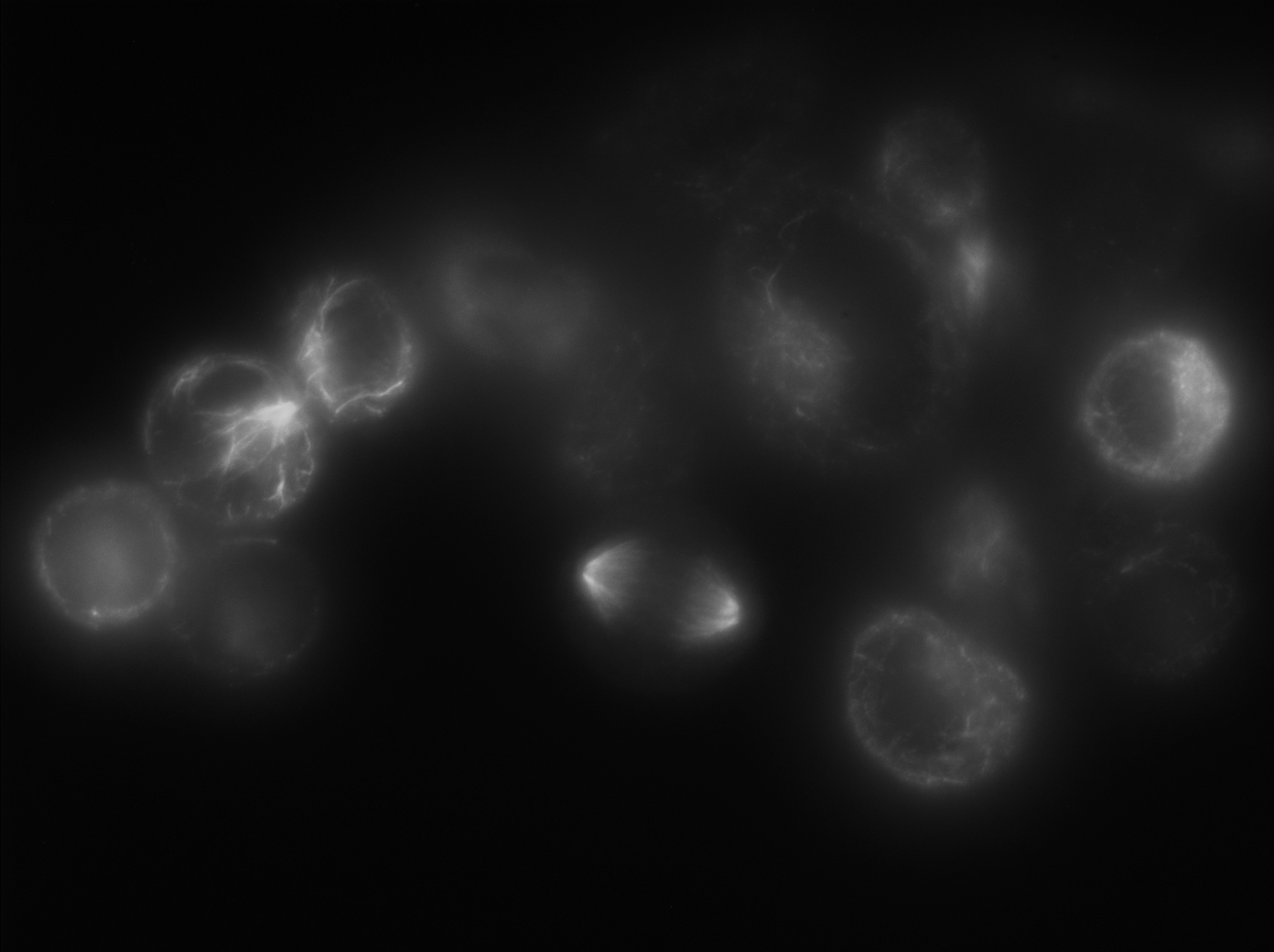

Supplement: Supplementary file 9 — Source data Fig. 2 part 6 [file 44319_2026_742_MOESM9_ESM.zip › Figure 2 Part 6/Fig 2fg Control Hela rGT335 acetylated tubulin/Anaphase/Cas9 actub rGT335 9-8-25 R1 A8 EX.Project Maximum Z_XY1757357377_Z0_T0_C2.tif]

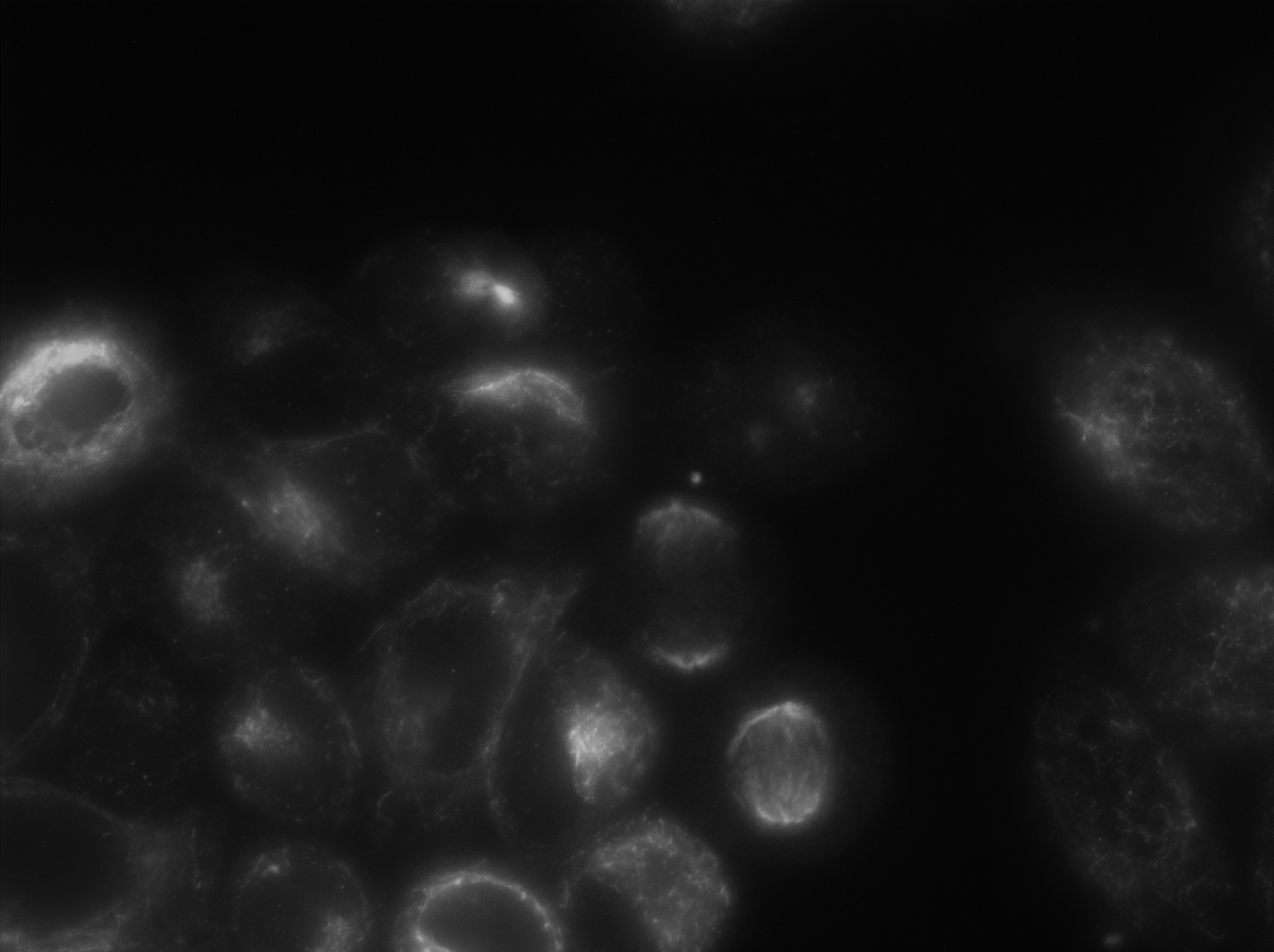

Supplement: Supplementary file 9 — Source data Fig. 2 part 6 [file 44319_2026_742_MOESM9_ESM.zip › Figure 2 Part 6/Fig 2fg Control Hela rGT335 acetylated tubulin/Anaphase/Cas9 actub rGT335 9-8-25 R3 A2 M2.Project Maximum Z_XY1757365779_Z0_T0_C2.tif]

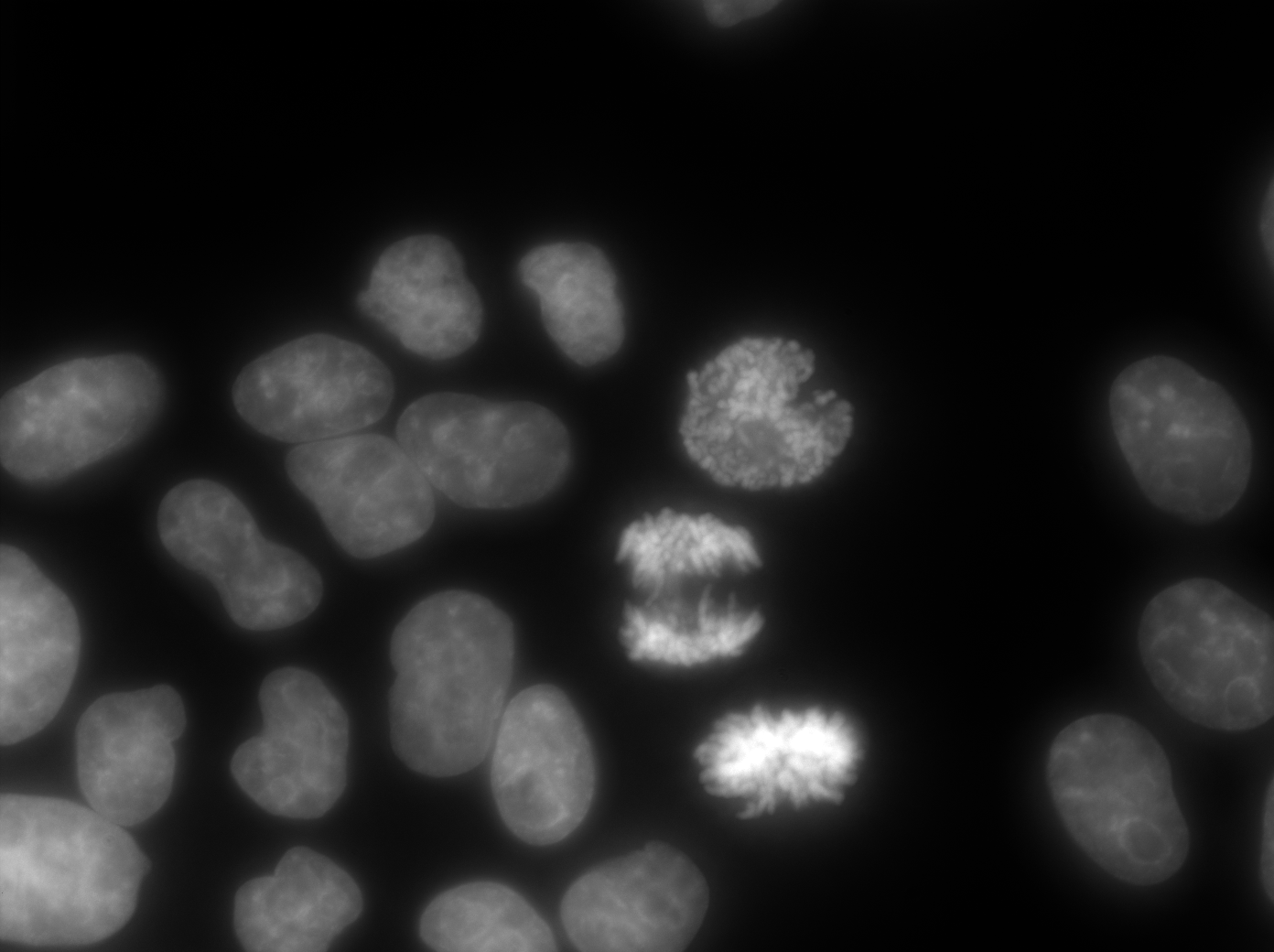

Supplement: Supplementary file 9 — Source data Fig. 2 part 6 [file 44319_2026_742_MOESM9_ESM.zip › Figure 2 Part 6/Fig 2fg Control Hela rGT335 acetylated tubulin/Anaphase/Cas9 actub rGT335 9-8-25 R3 A2 M2.Project Maximum Z_XY1757365779_Z0_T0_C0.tif]

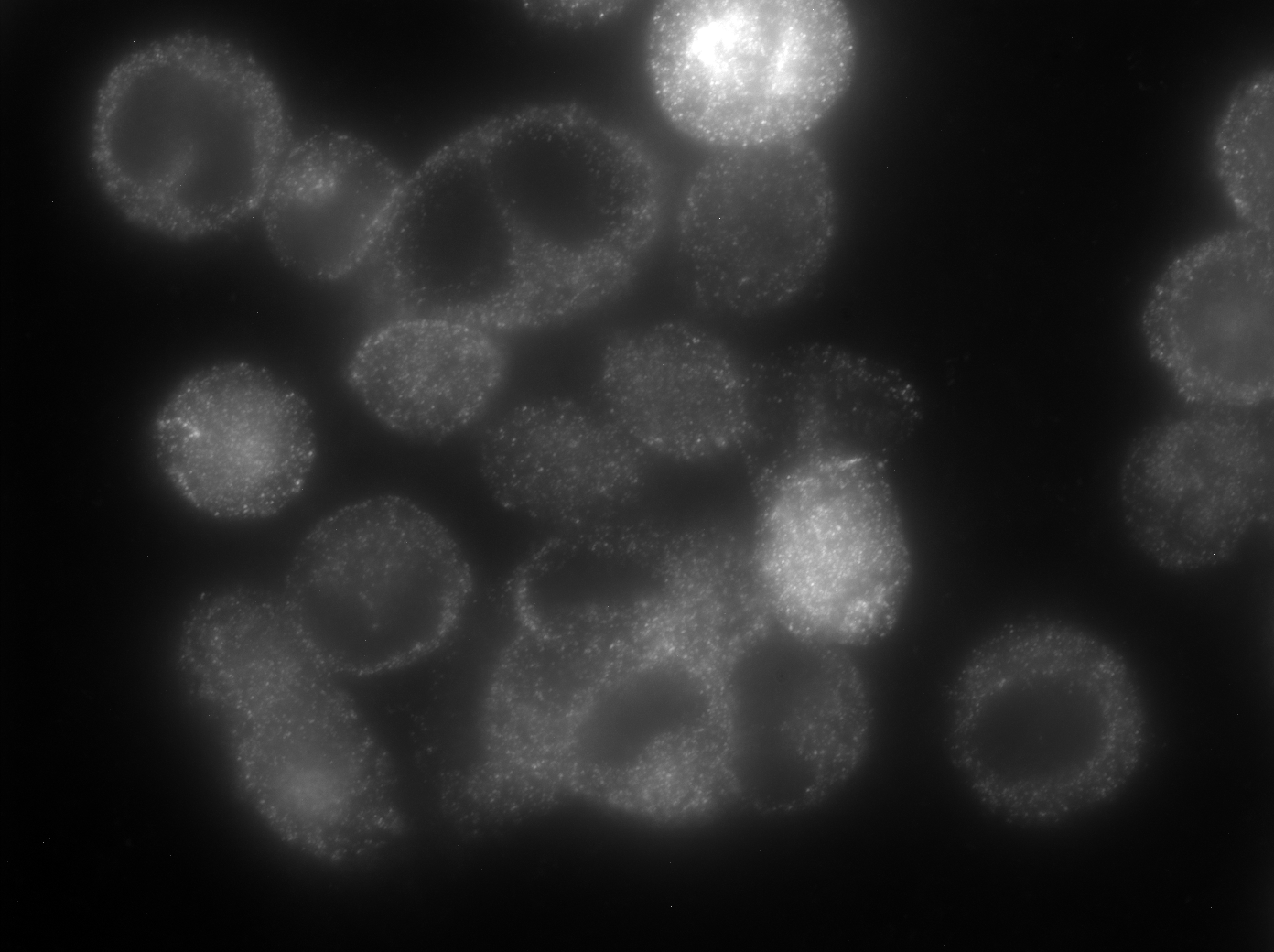

Supplement: Supplementary file 9 — Source data Fig. 2 part 6 [file 44319_2026_742_MOESM9_ESM.zip › Figure 2 Part 6/Fig 2fg Control Hela rGT335 acetylated tubulin/Anaphase/Cas9 actub rGT335 9-8-25 R1 A9.Project Maximum Z_XY1757357993_Z0_T0_C1.tif]

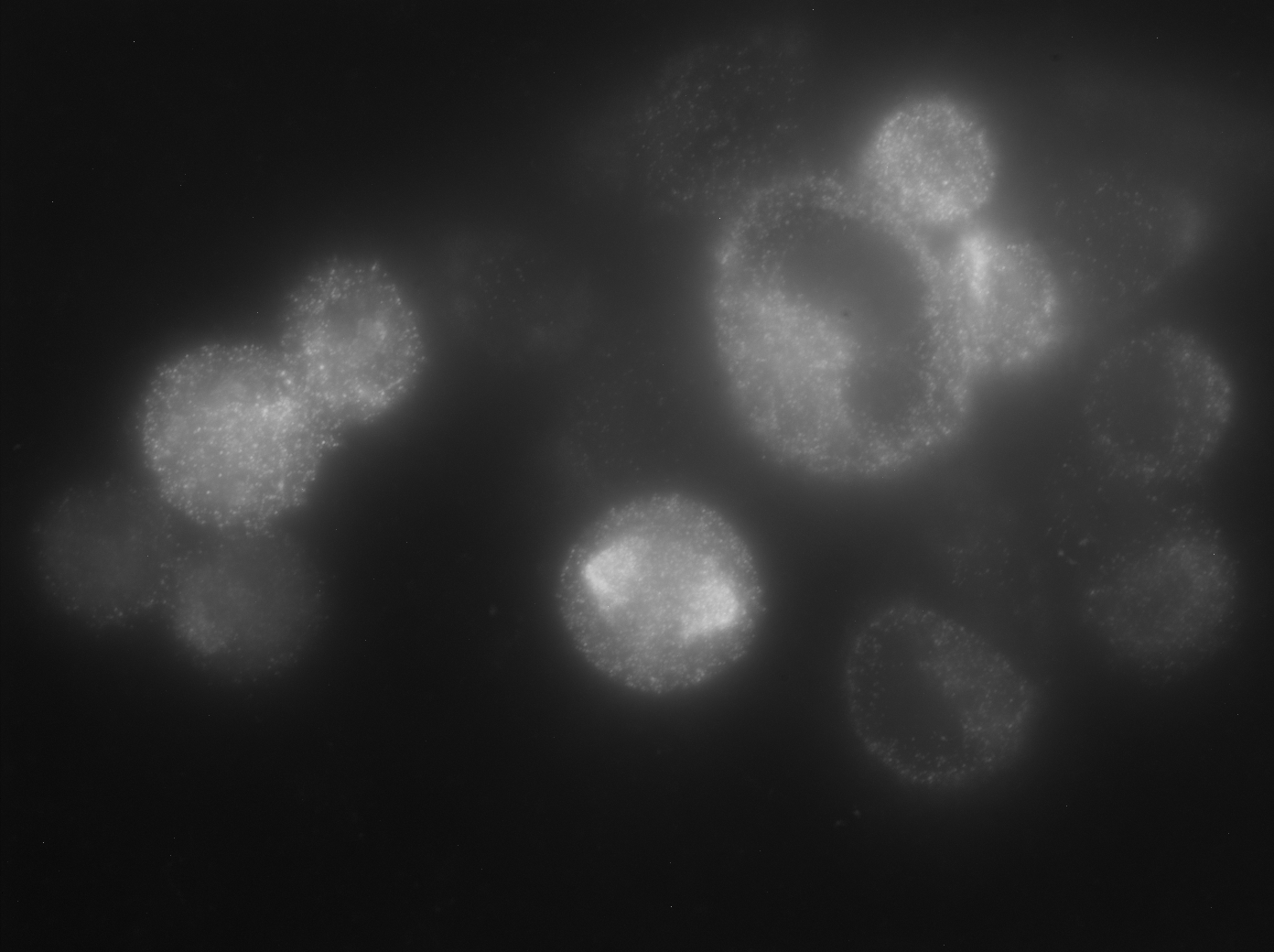

Supplement: Supplementary file 9 — Source data Fig. 2 part 6 [file 44319_2026_742_MOESM9_ESM.zip › Figure 2 Part 6/Fig 2fg Control Hela rGT335 acetylated tubulin/Anaphase/Cas9 actub rGT335 9-8-25 R1 A8 EX.Project Maximum Z_XY1757357377_Z0_T0_C1.tif]

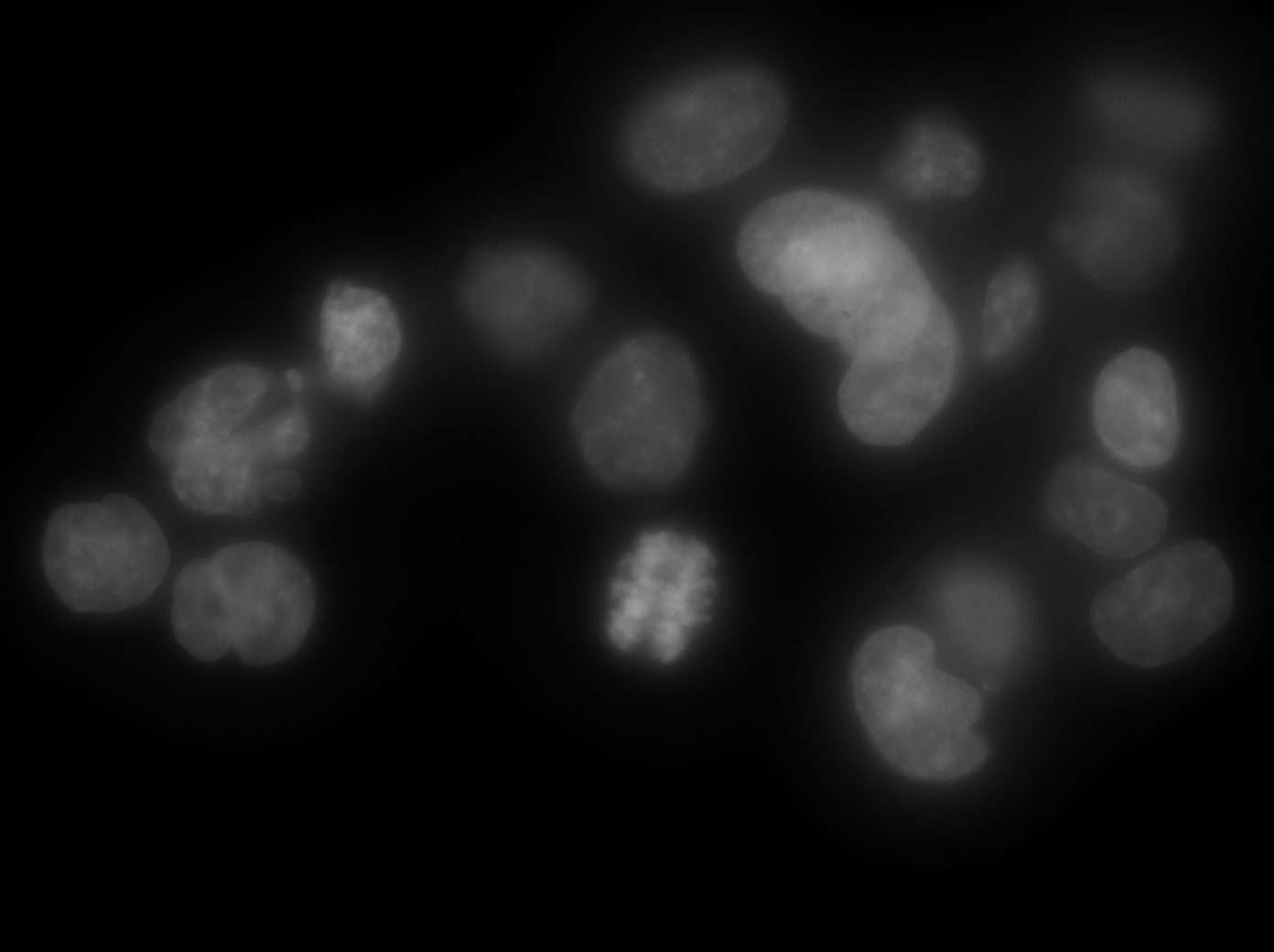

Supplement: Supplementary file 9 — Source data Fig. 2 part 6 [file 44319_2026_742_MOESM9_ESM.zip › Figure 2 Part 6/Fig 2fg Control Hela rGT335 acetylated tubulin/Anaphase/Cas9 actub rGT335 9-8-25 R1 A8 EX.Project Maximum Z_XY1757357377_Z0_T0_C0.tif]
