# Supplementary figures and images for: TPGS1 regulates central spindle microtubule glutamylation and remodeling during telophase and abscission (part 7 of 36)
Source: EMBO Rep. 2026 Mar 23;27(8):1944–63. doi: 10.1038/s44319-026-00742-3 (PMC13121839; doi:10.1038/s44319-026-00742-3)

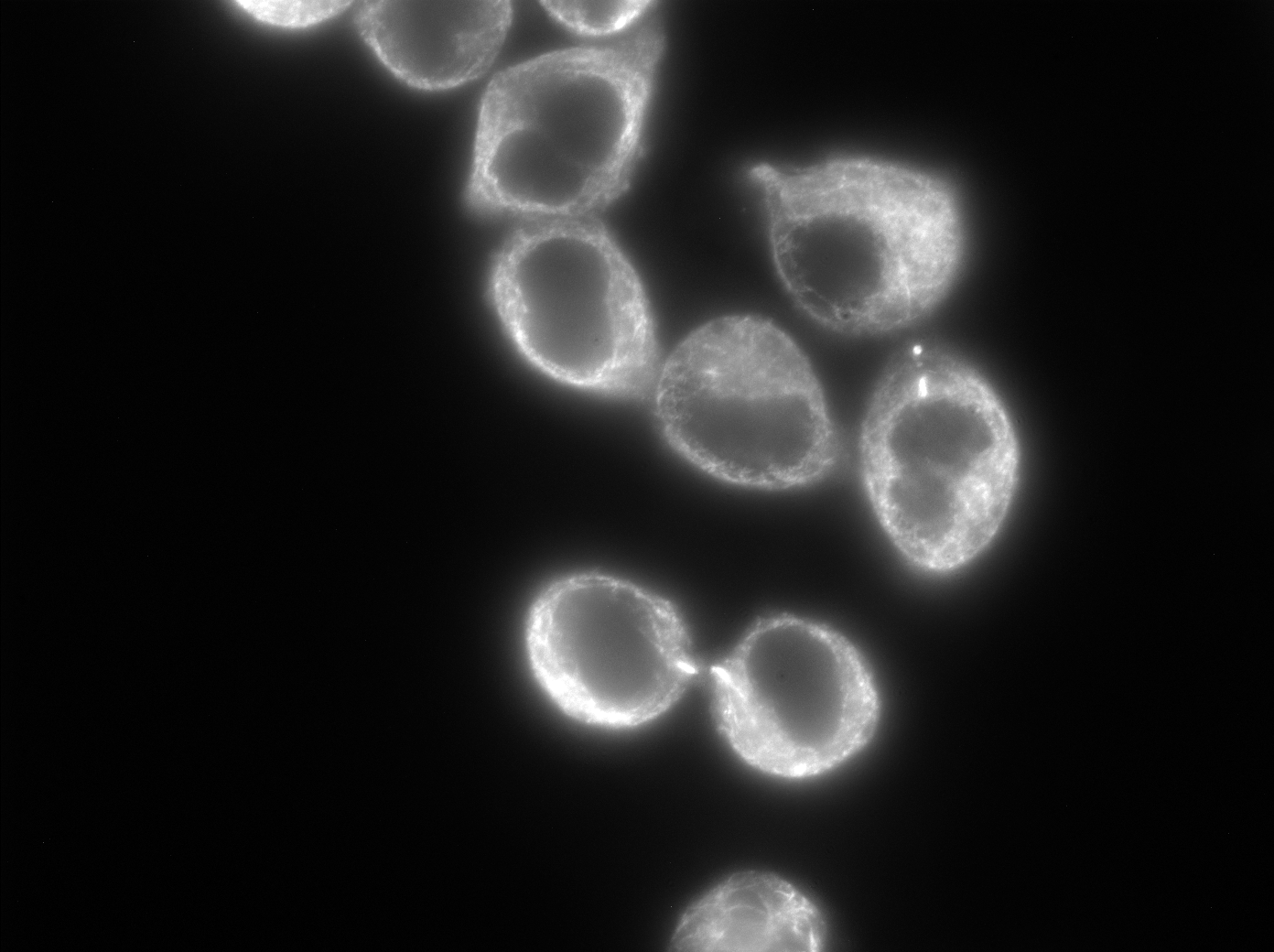

Supplement: Supplementary file 8 — Source data Fig. 2 part 5 [file 44319_2026_742_MOESM8_ESM.zip › Figure 2 Part 5/Fig 2d polye atubulin part 2/WT PolyE-atub 8-14-24 R3 ET7 PA7.Project Maximum Z_XY1723843057_Z0_T0_C1.tif]

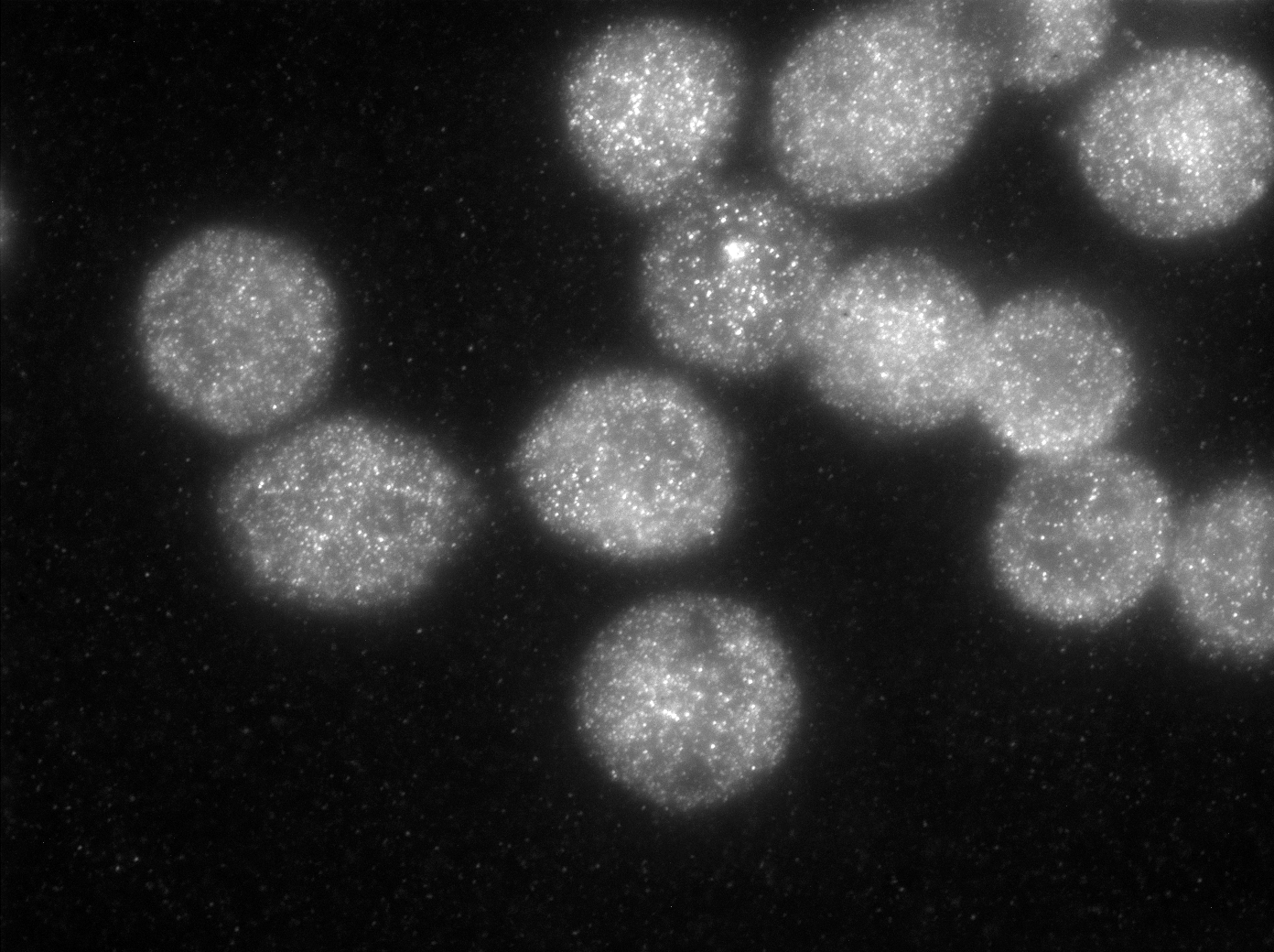

Supplement: Supplementary file 8 — Source data Fig. 2 part 5 [file 44319_2026_742_MOESM8_ESM.zip › Figure 2 Part 5/Fig 2d polye atubulin part 2/WT PolyE-atub 8-14-24 R3 M1.Project Maximum Z_XY1723841228_Z0_T0_C2.tif]

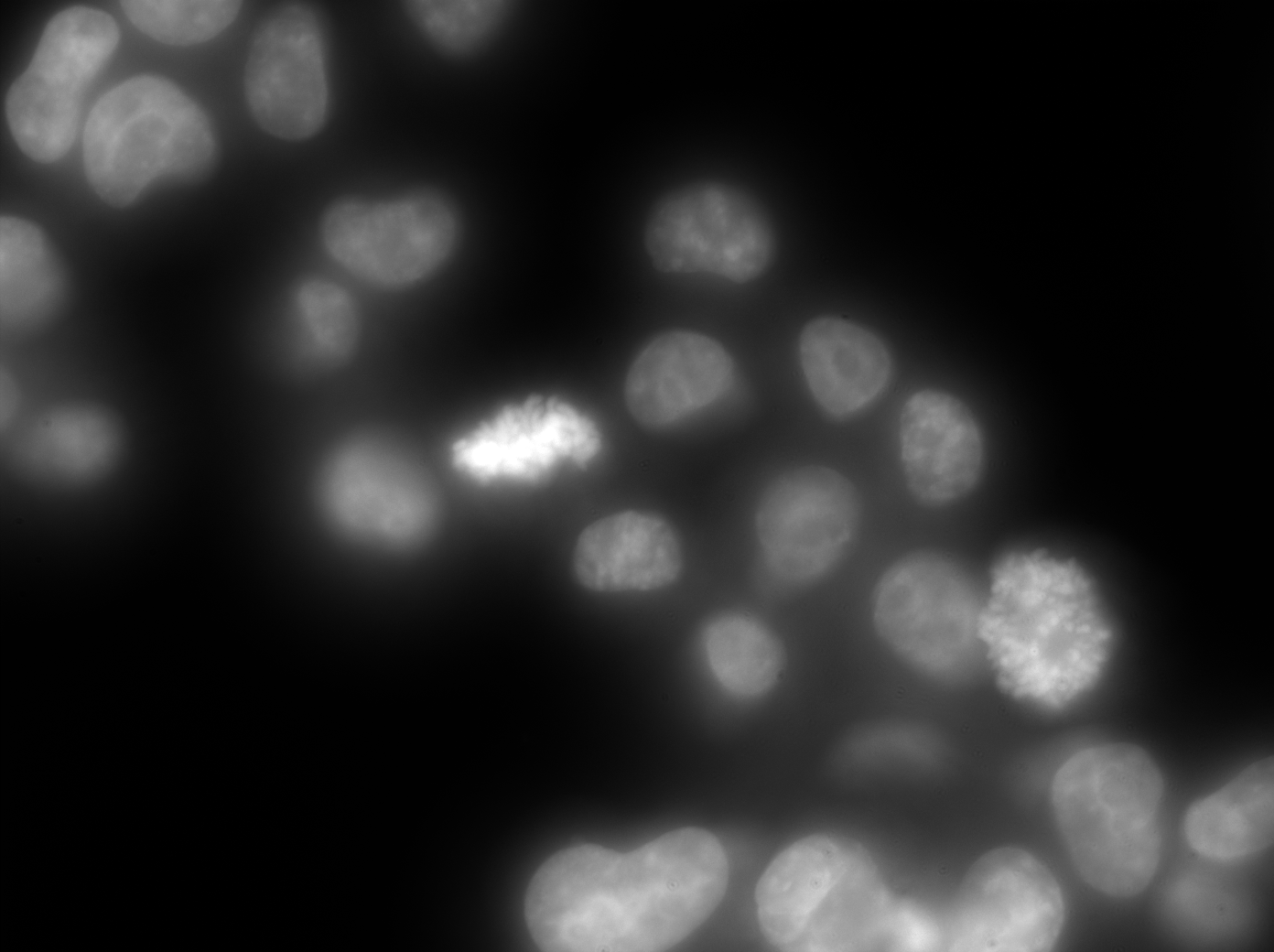

Supplement: Supplementary file 8 — Source data Fig. 2 part 5 [file 44319_2026_742_MOESM8_ESM.zip › Figure 2 Part 5/Fig 2d polye atubulin part 2/WT PolyE-atub 8-14-24 R3 M7M8.Project Maximum Z_XY1723845881_Z0_T0_C0.tif]

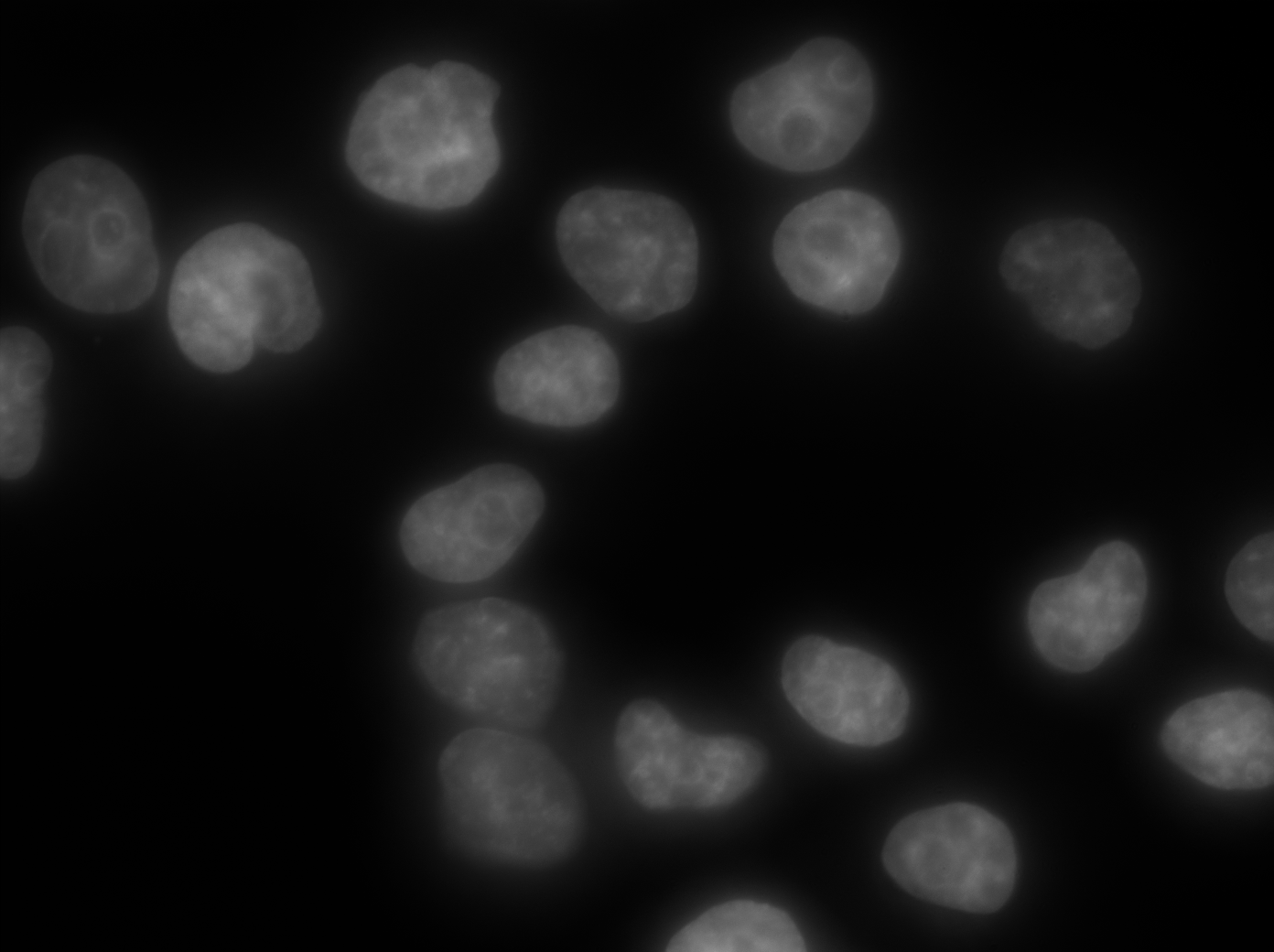

Supplement: Supplementary file 8 — Source data Fig. 2 part 5 [file 44319_2026_742_MOESM8_ESM.zip › Figure 2 Part 5/Fig 2d polye atubulin part 2/WT PolyE-atub 8-14-24 R3 LT9 PA9.Project Maximum Z_XY1723843870_Z0_T0_C0.tif]

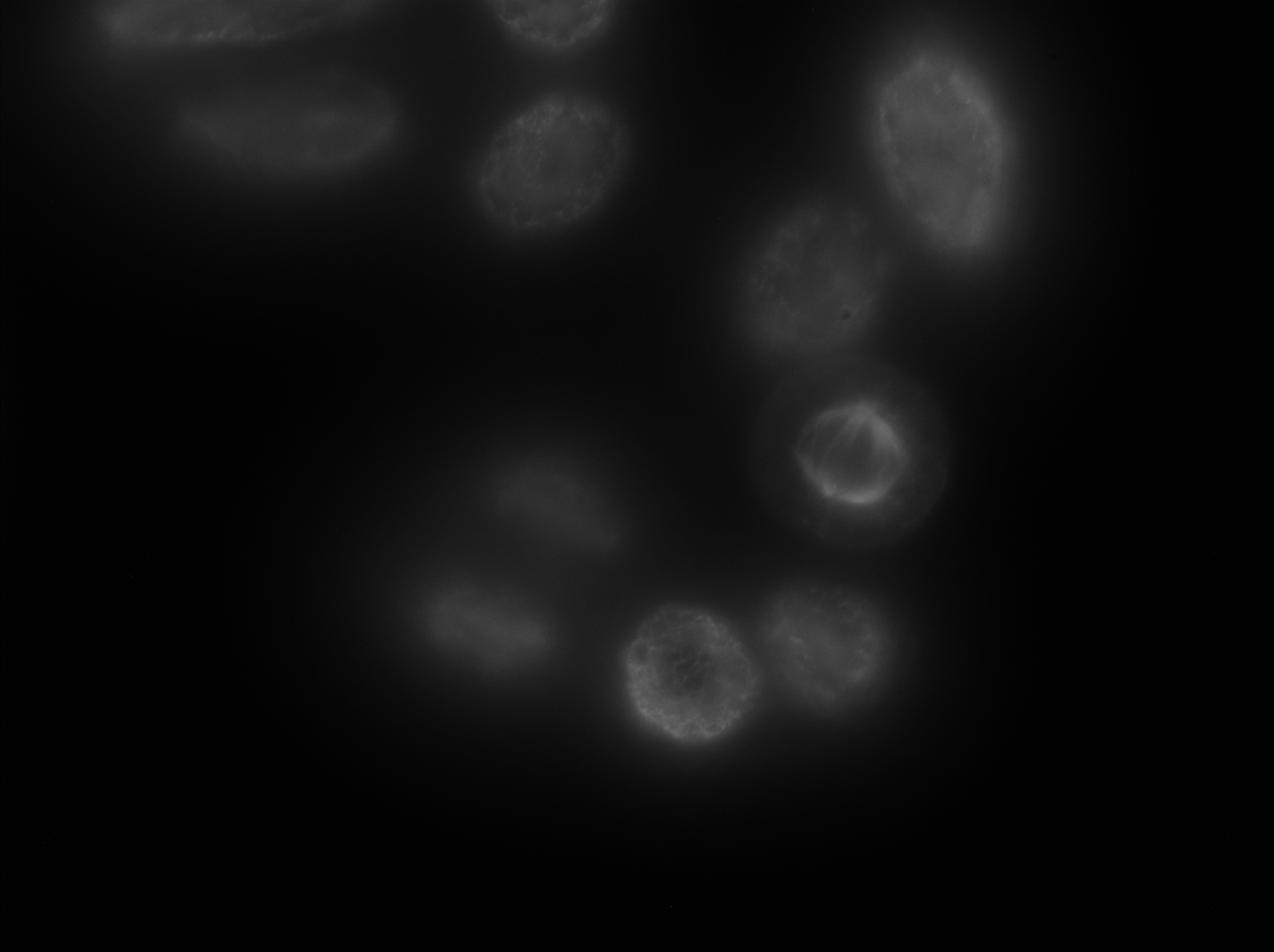

Supplement: Supplementary file 8 — Source data Fig. 2 part 5 [file 44319_2026_742_MOESM8_ESM.zip › Figure 2 Part 5/Fig 2d polye atubulin part 2/WT PolyE-atub 8-14-24 R2 M2.Project Maximum Z_XY1723834393_Z0_T0_C1.tif]

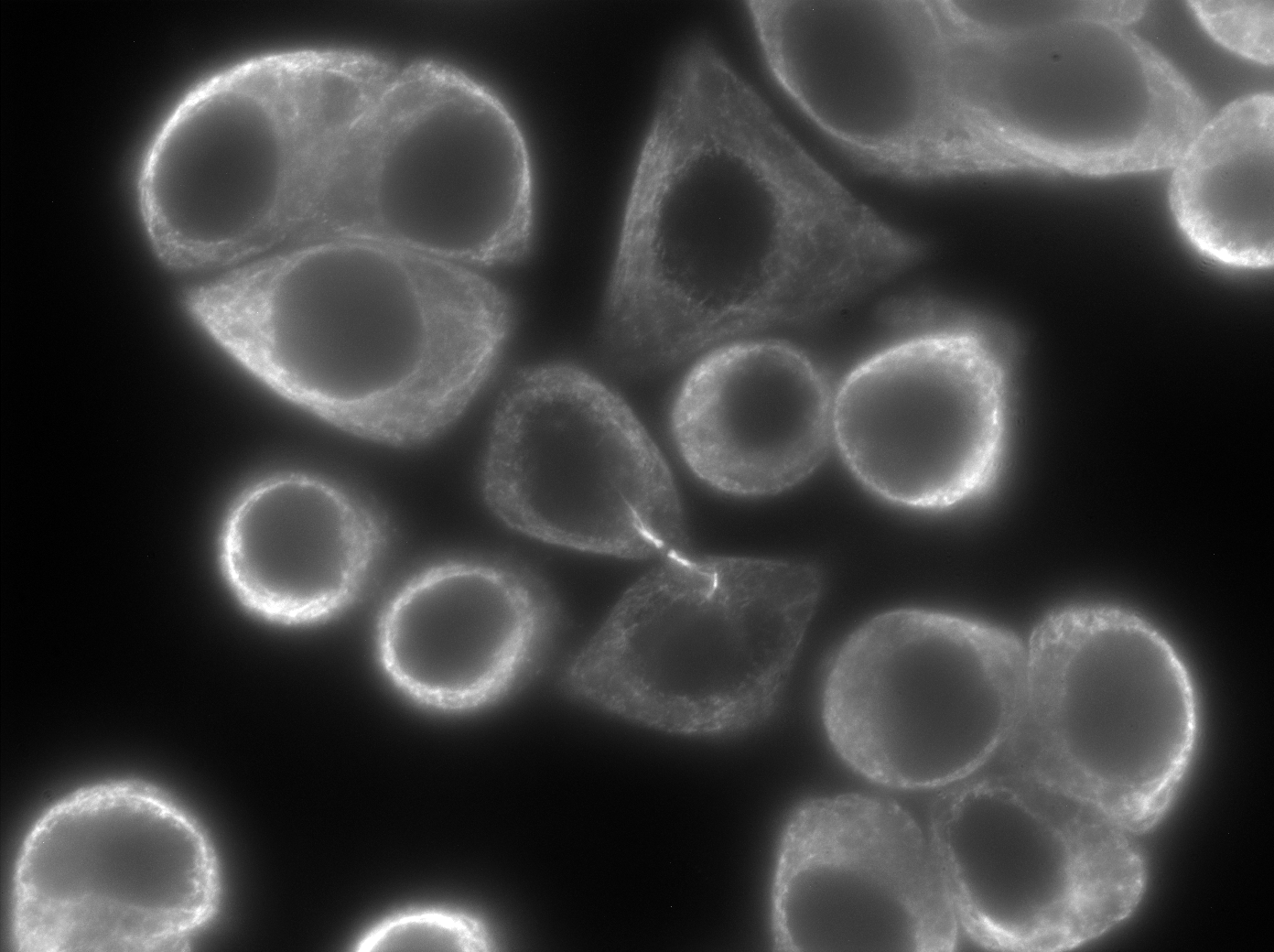

Supplement: Supplementary file 8 — Source data Fig. 2 part 5 [file 44319_2026_742_MOESM8_ESM.zip › Figure 2 Part 5/Fig 2d polye atubulin part 2/WT PolyE-atub 8-14-24 R3 LT1.Project Maximum Z_XY1723841584_Z0_T0_C1.tif]

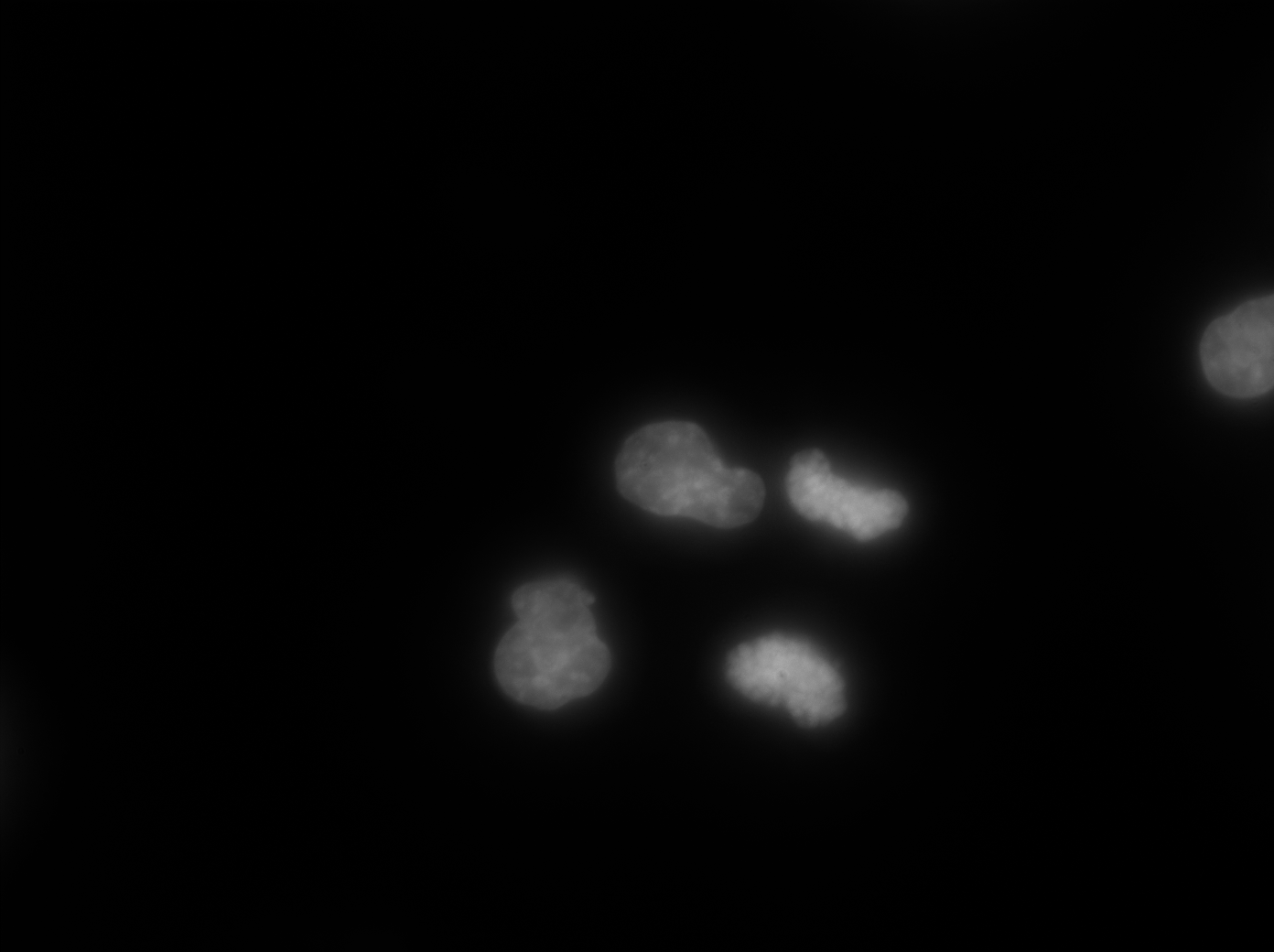

Supplement: Supplementary file 8 — Source data Fig. 2 part 5 [file 44319_2026_742_MOESM8_ESM.zip › Figure 2 Part 5/Fig 2d polye atubulin part 2/WT PolyE-atub 8-14-24 R3 ET6 LT3.Project Maximum Z_XY1723842893_Z0_T0_C0.tif]

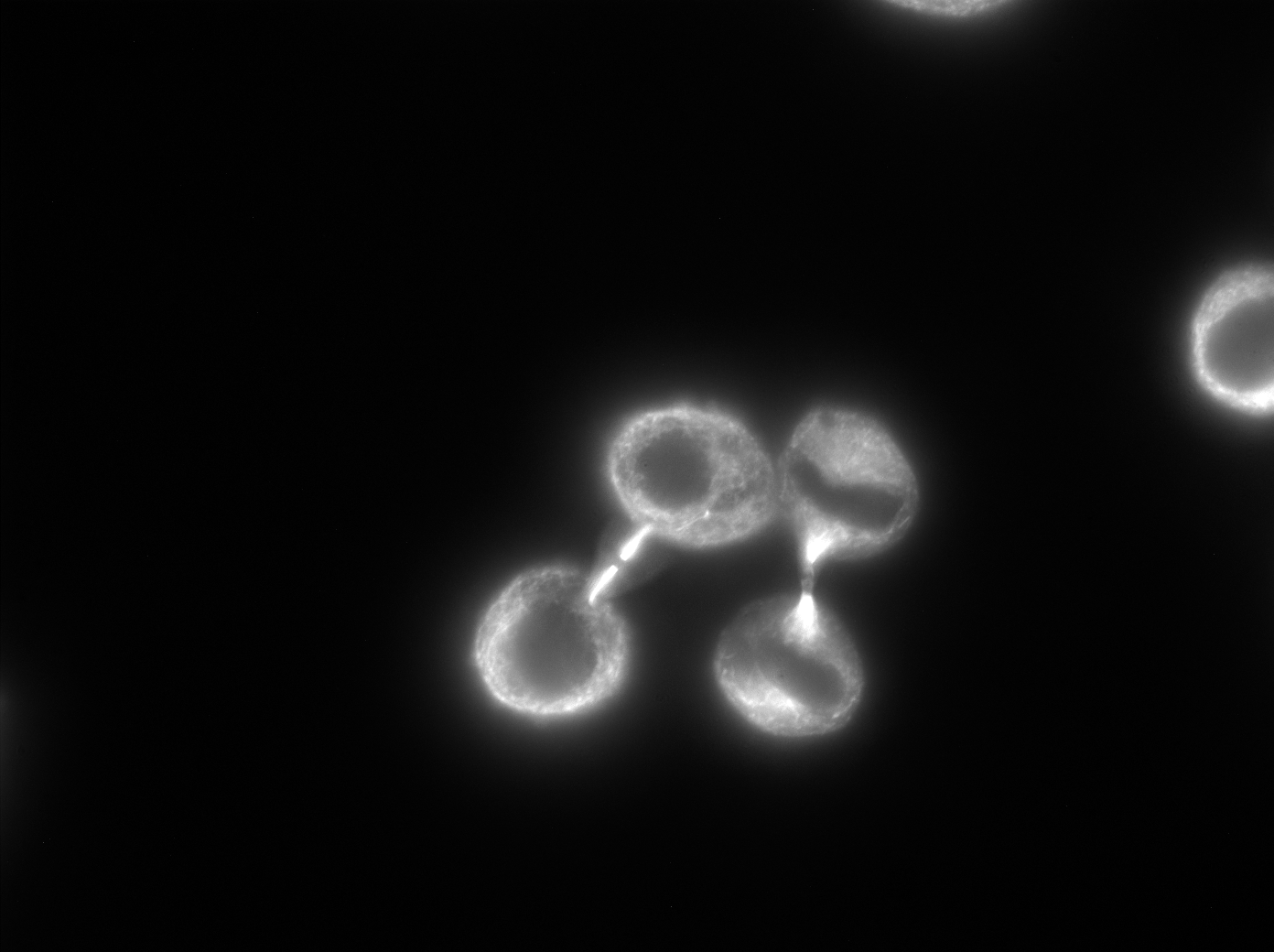

Supplement: Supplementary file 8 — Source data Fig. 2 part 5 [file 44319_2026_742_MOESM8_ESM.zip › Figure 2 Part 5/Fig 2d polye atubulin part 2/WT PolyE-atub 8-14-24 R3 ET6 LT3.Project Maximum Z_XY1723842893_Z0_T0_C1.tif]

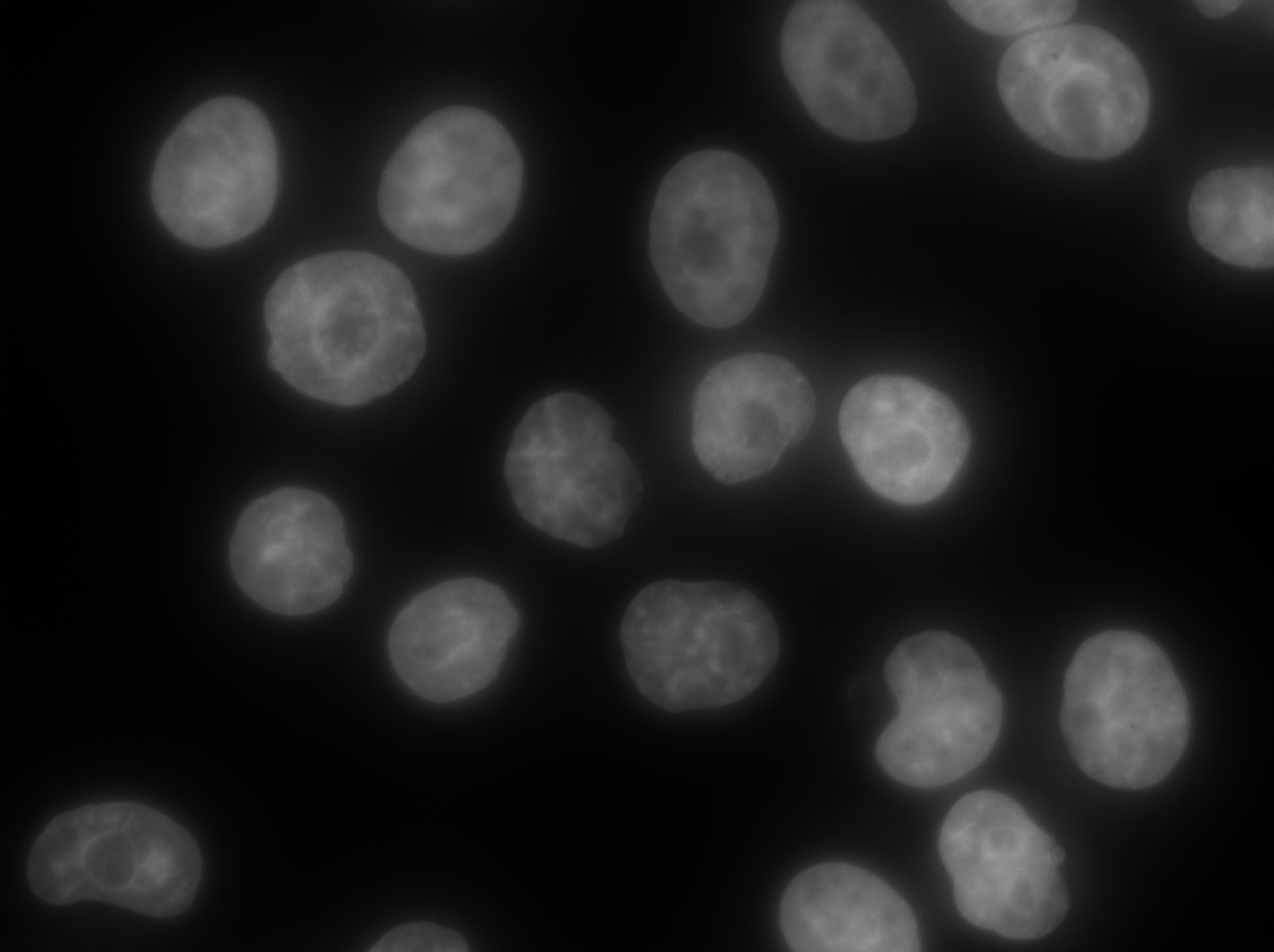

Supplement: Supplementary file 8 — Source data Fig. 2 part 5 [file 44319_2026_742_MOESM8_ESM.zip › Figure 2 Part 5/Fig 2d polye atubulin part 2/WT PolyE-atub 8-14-24 R3 LT1.Project Maximum Z_XY1723841584_Z0_T0_C0.tif]

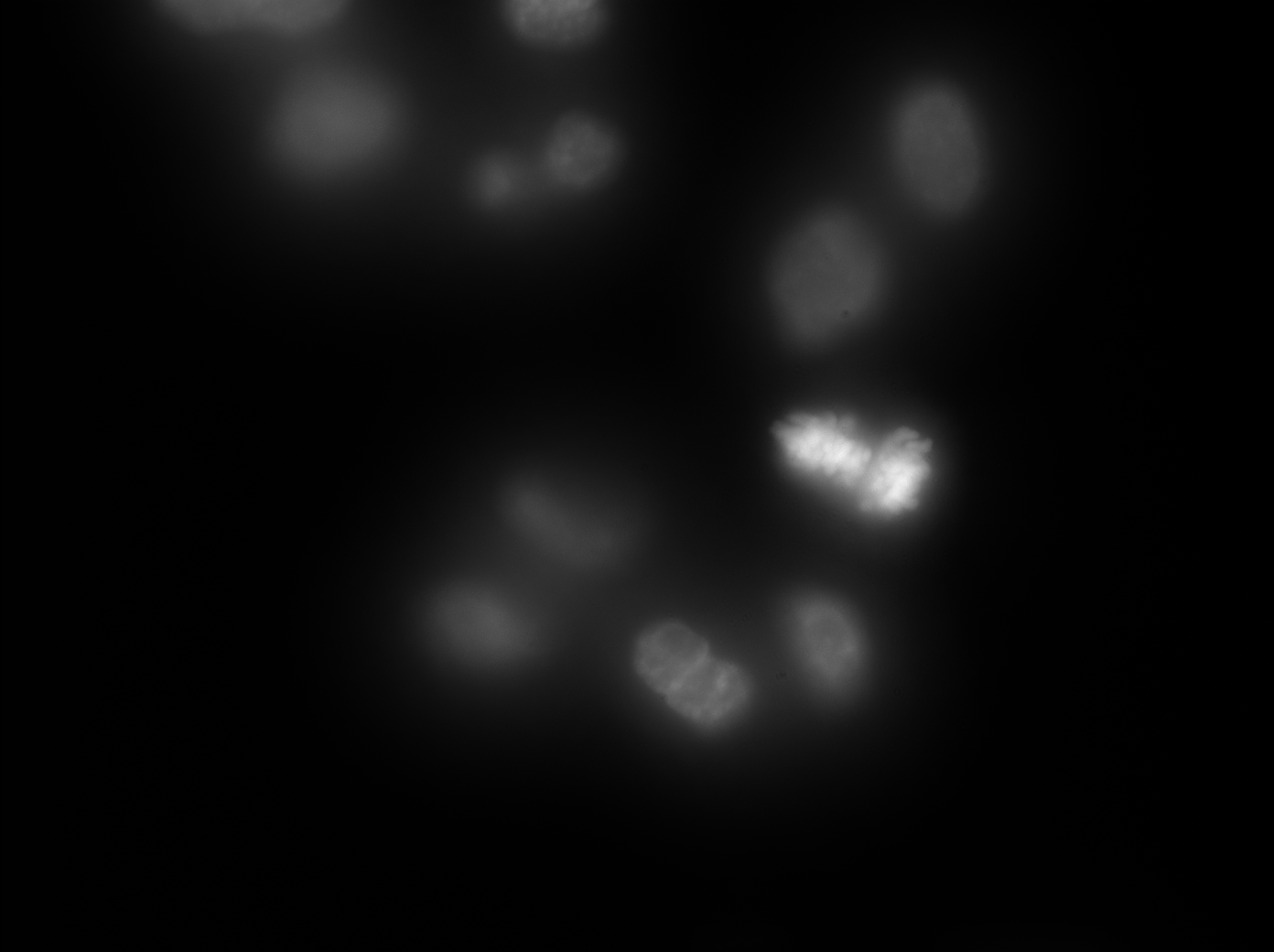

Supplement: Supplementary file 8 — Source data Fig. 2 part 5 [file 44319_2026_742_MOESM8_ESM.zip › Figure 2 Part 5/Fig 2d polye atubulin part 2/WT PolyE-atub 8-14-24 R2 M2.Project Maximum Z_XY1723834393_Z0_T0_C0.tif]

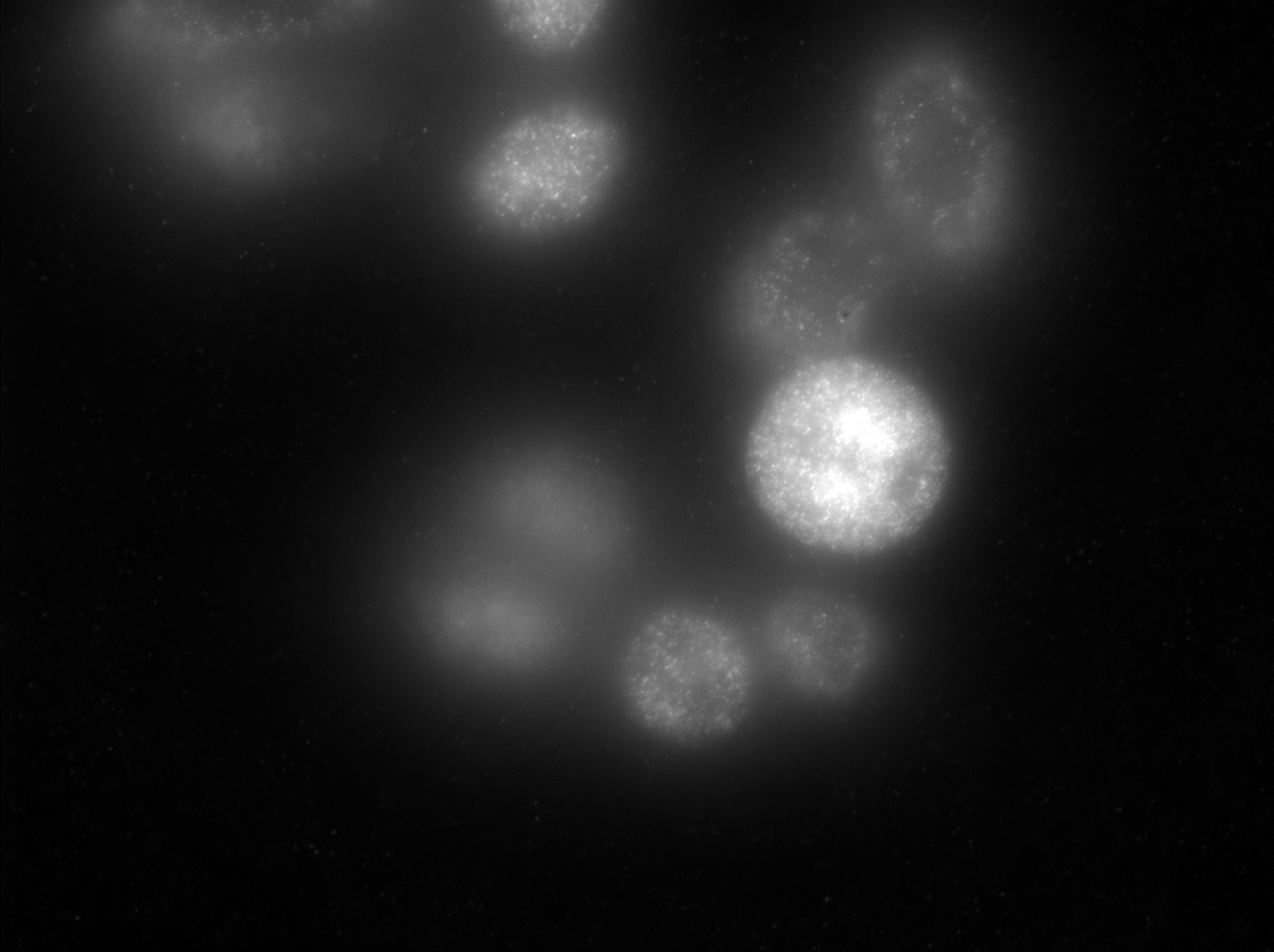

Supplement: Supplementary file 8 — Source data Fig. 2 part 5 [file 44319_2026_742_MOESM8_ESM.zip › Figure 2 Part 5/Fig 2d polye atubulin part 2/WT PolyE-atub 8-14-24 R2 M2.Project Maximum Z_XY1723834393_Z0_T0_C2.tif]

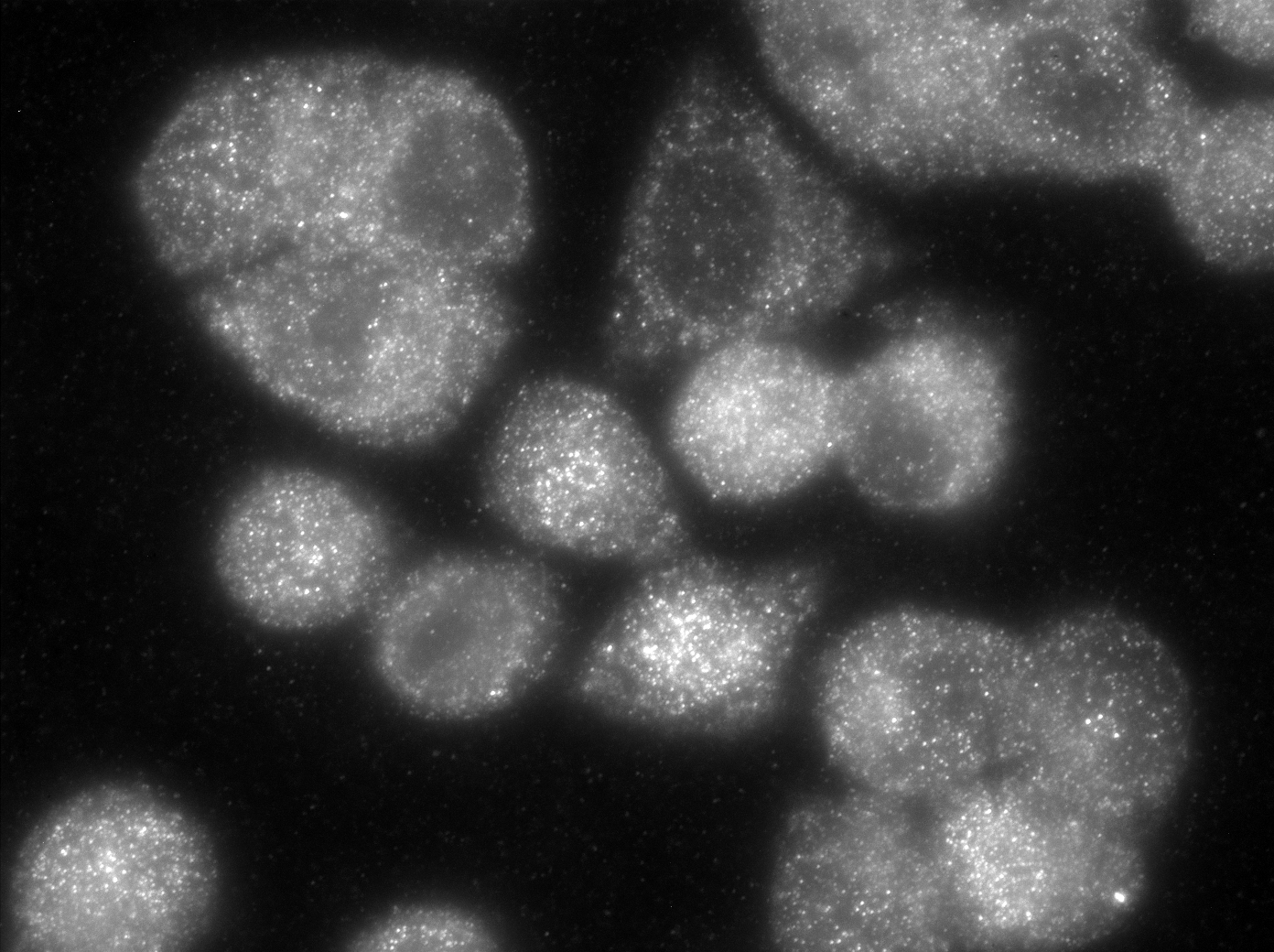

Supplement: Supplementary file 8 — Source data Fig. 2 part 5 [file 44319_2026_742_MOESM8_ESM.zip › Figure 2 Part 5/Fig 2d polye atubulin part 2/WT PolyE-atub 8-14-24 R3 LT1.Project Maximum Z_XY1723841584_Z0_T0_C2.tif]

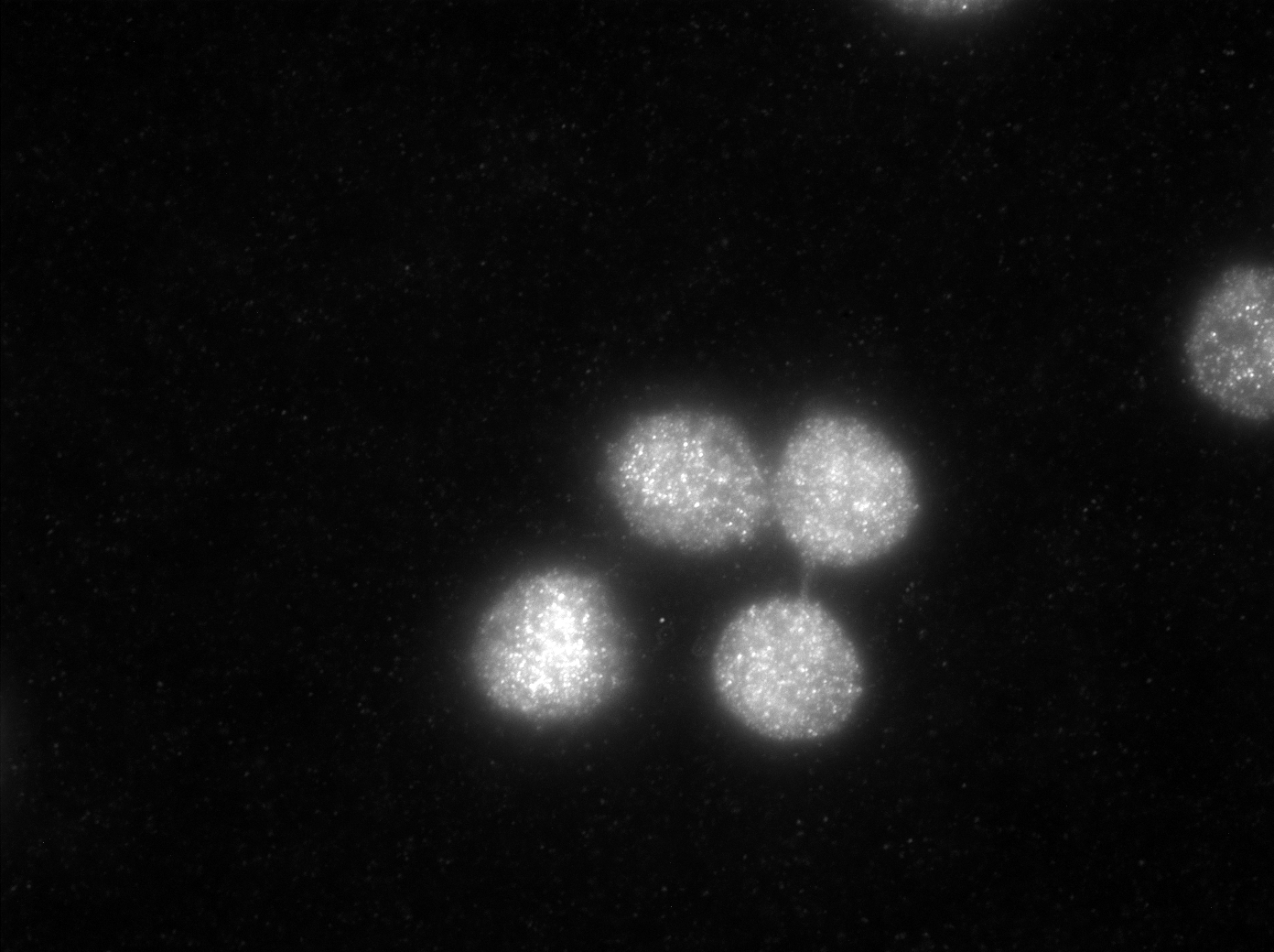

Supplement: Supplementary file 8 — Source data Fig. 2 part 5 [file 44319_2026_742_MOESM8_ESM.zip › Figure 2 Part 5/Fig 2d polye atubulin part 2/WT PolyE-atub 8-14-24 R3 ET6 LT3.Project Maximum Z_XY1723842893_Z0_T0_C2.tif]

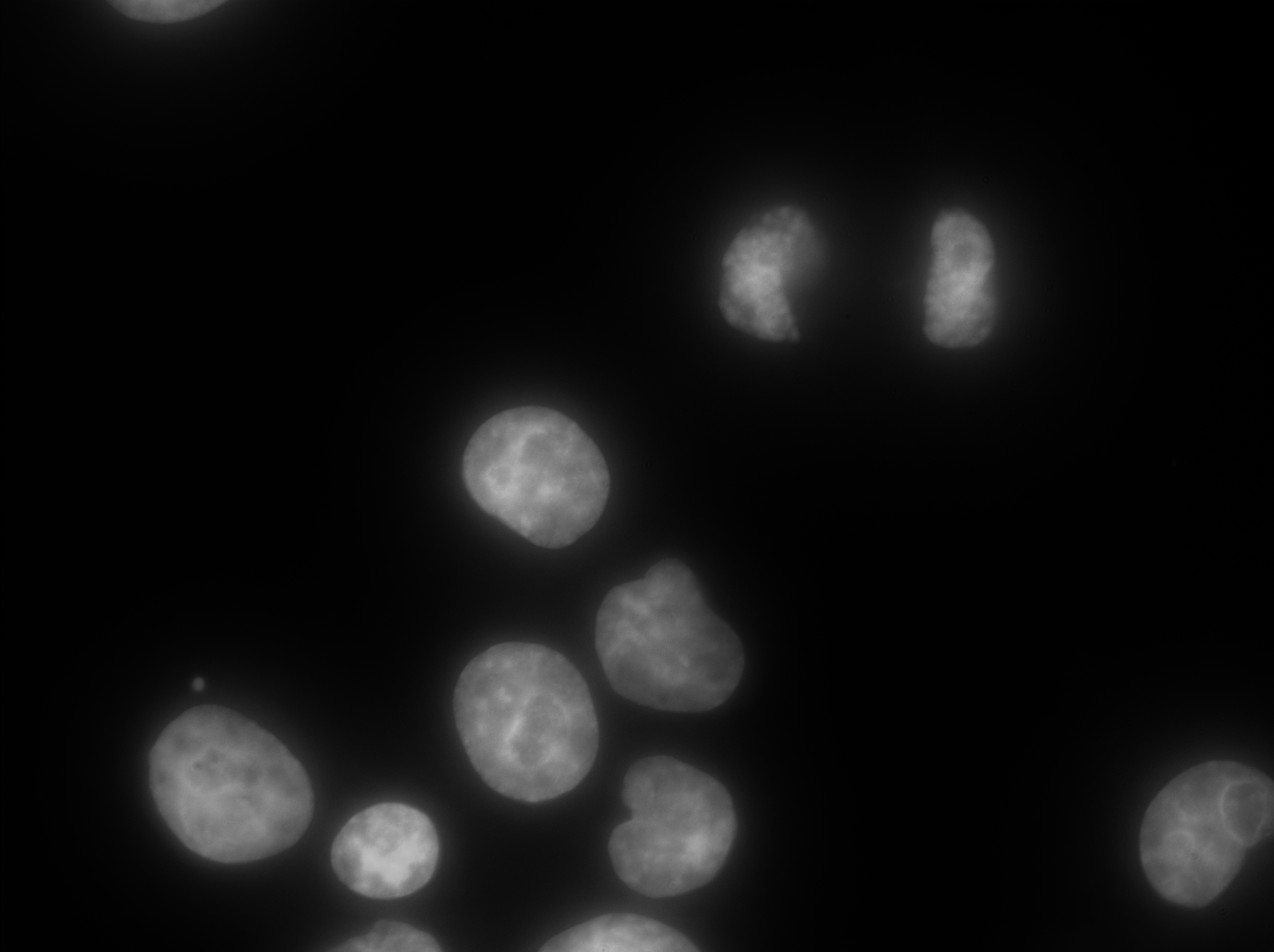

Supplement: Supplementary file 8 — Source data Fig. 2 part 5 [file 44319_2026_742_MOESM8_ESM.zip › Figure 2 Part 5/Fig 2d polye atubulin part 2/WT PolyE-atub 8-14-24 R3 PA1.Project Maximum Z_XY1723841105_Z0_T0_C0.tif]

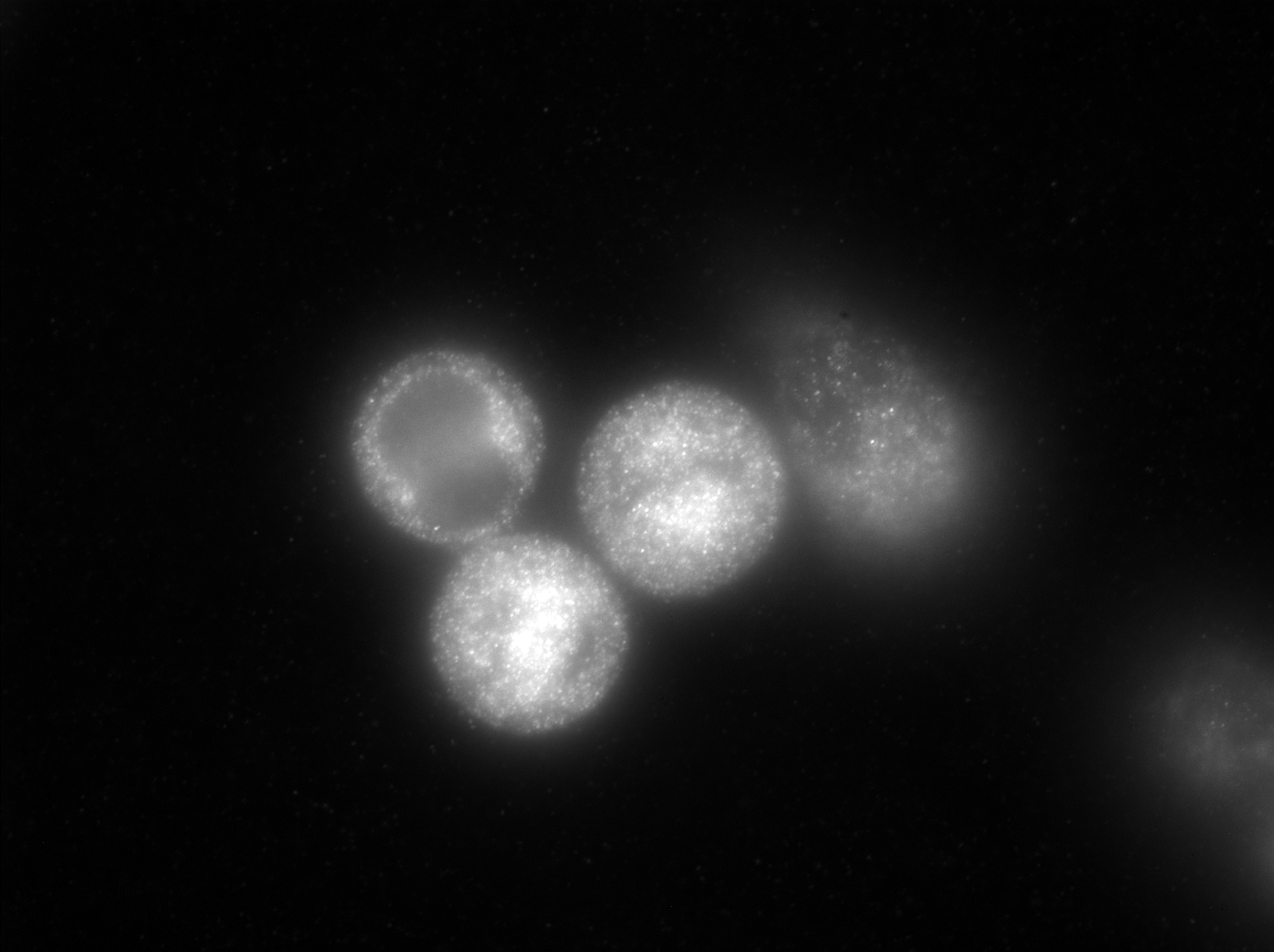

Supplement: Supplementary file 8 — Source data Fig. 2 part 5 [file 44319_2026_742_MOESM8_ESM.zip › Figure 2 Part 5/Fig 2d polye atubulin part 2/WT PolyE-atub 8-14-24 R2 M10.Project Maximum Z_XY1723839028_Z0_T0_C2.tif]

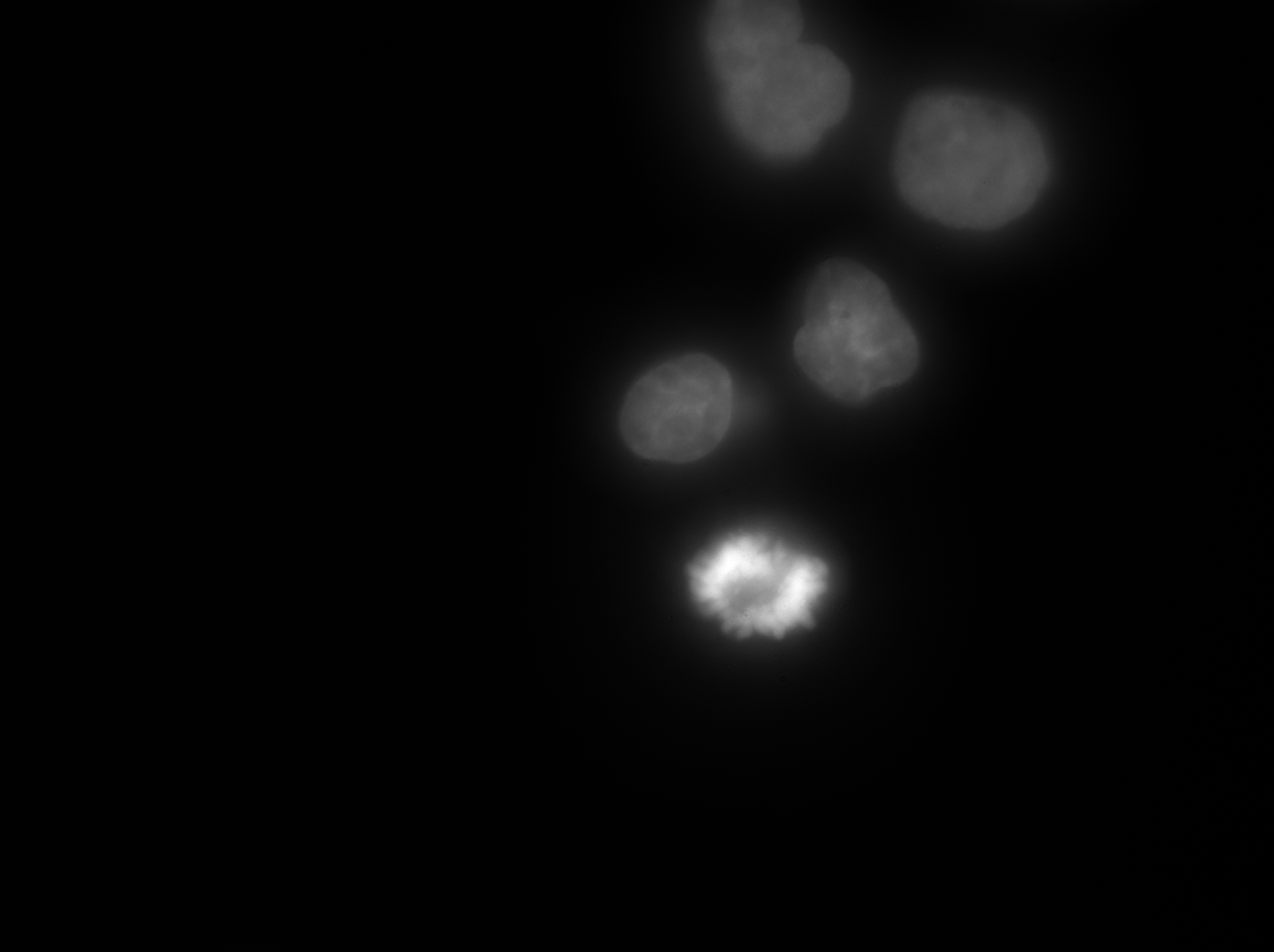

Supplement: Supplementary file 8 — Source data Fig. 2 part 5 [file 44319_2026_742_MOESM8_ESM.zip › Figure 2 Part 5/Fig 2d polye atubulin part 2/WT PolyE-atub 8-14-24 R2 M3.Project Maximum Z_XY1723837033_Z0_T0_C0.tif]

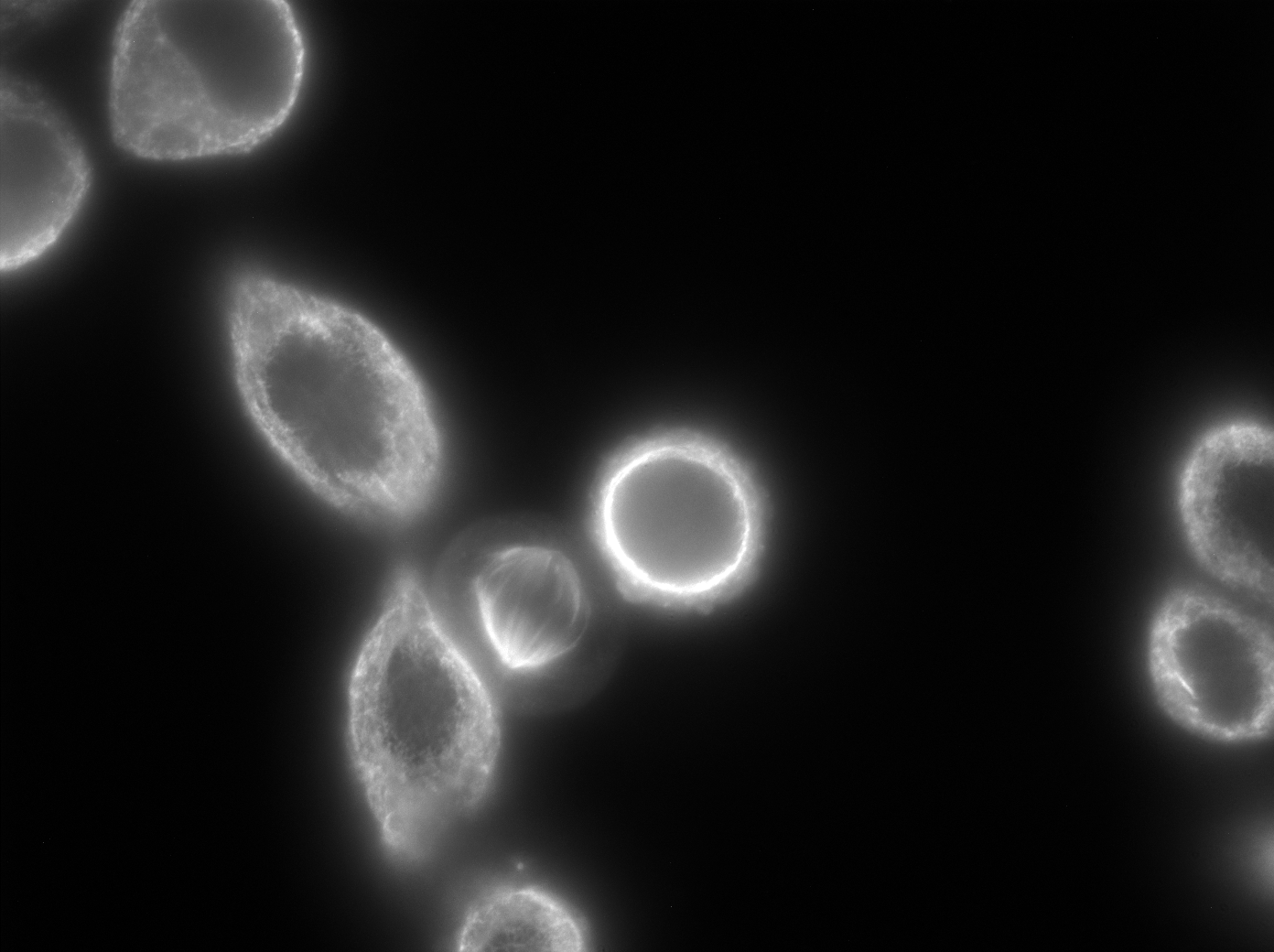

Supplement: Supplementary file 8 — Source data Fig. 2 part 5 [file 44319_2026_742_MOESM8_ESM.zip › Figure 2 Part 5/Fig 2d polye atubulin part 2/WT PolyE-atub 8-14-24 R3 M6.Project Maximum Z_XY1723845259_Z0_T0_C1.tif]

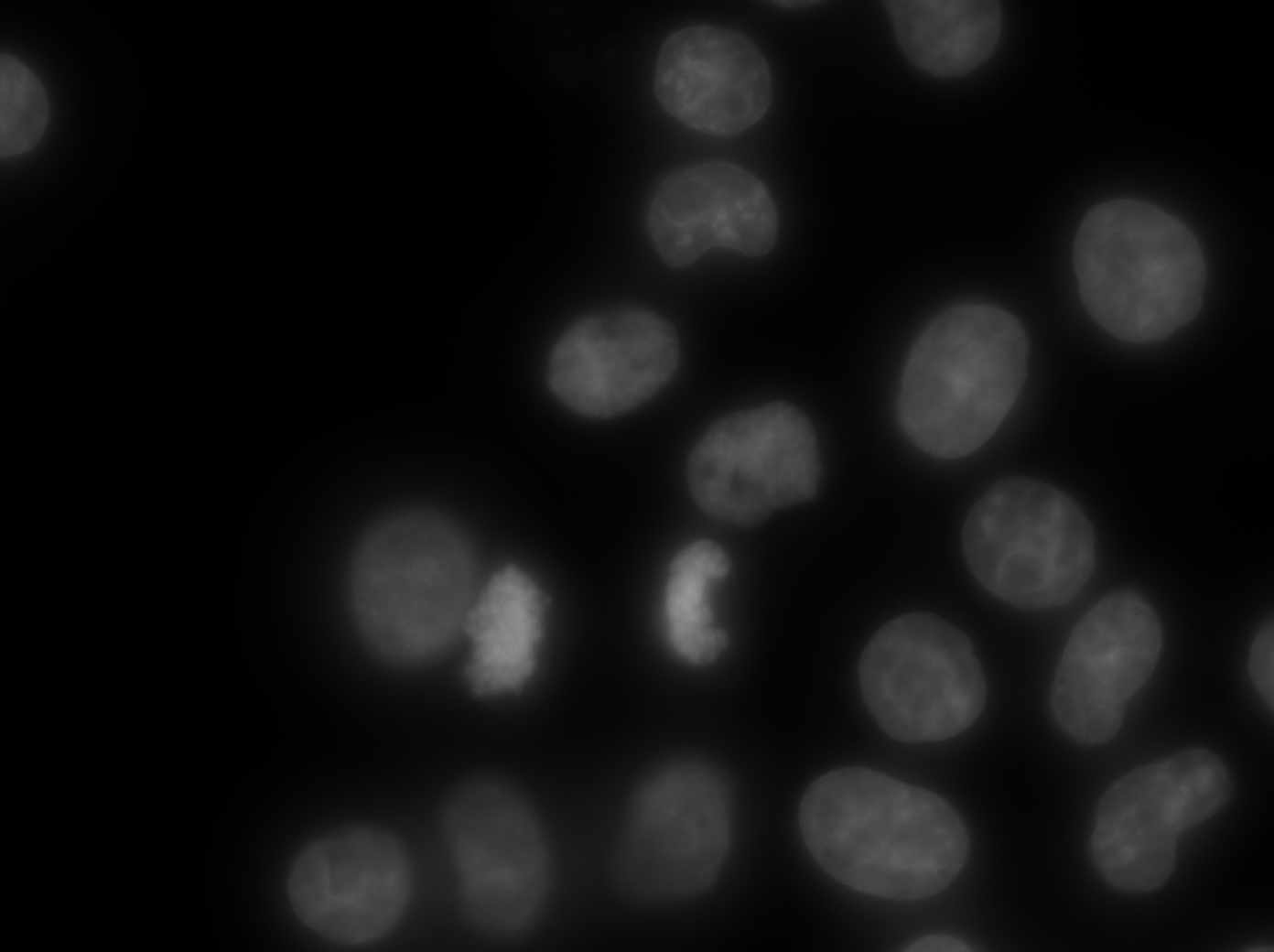

Supplement: Supplementary file 8 — Source data Fig. 2 part 5 [file 44319_2026_742_MOESM8_ESM.zip › Figure 2 Part 5/Fig 2d polye atubulin part 2/WT PolyE-atub 8-14-24 R3 ET10ET11.Project Maximum Z_XY1723845138_Z0_T0_C0.tif]

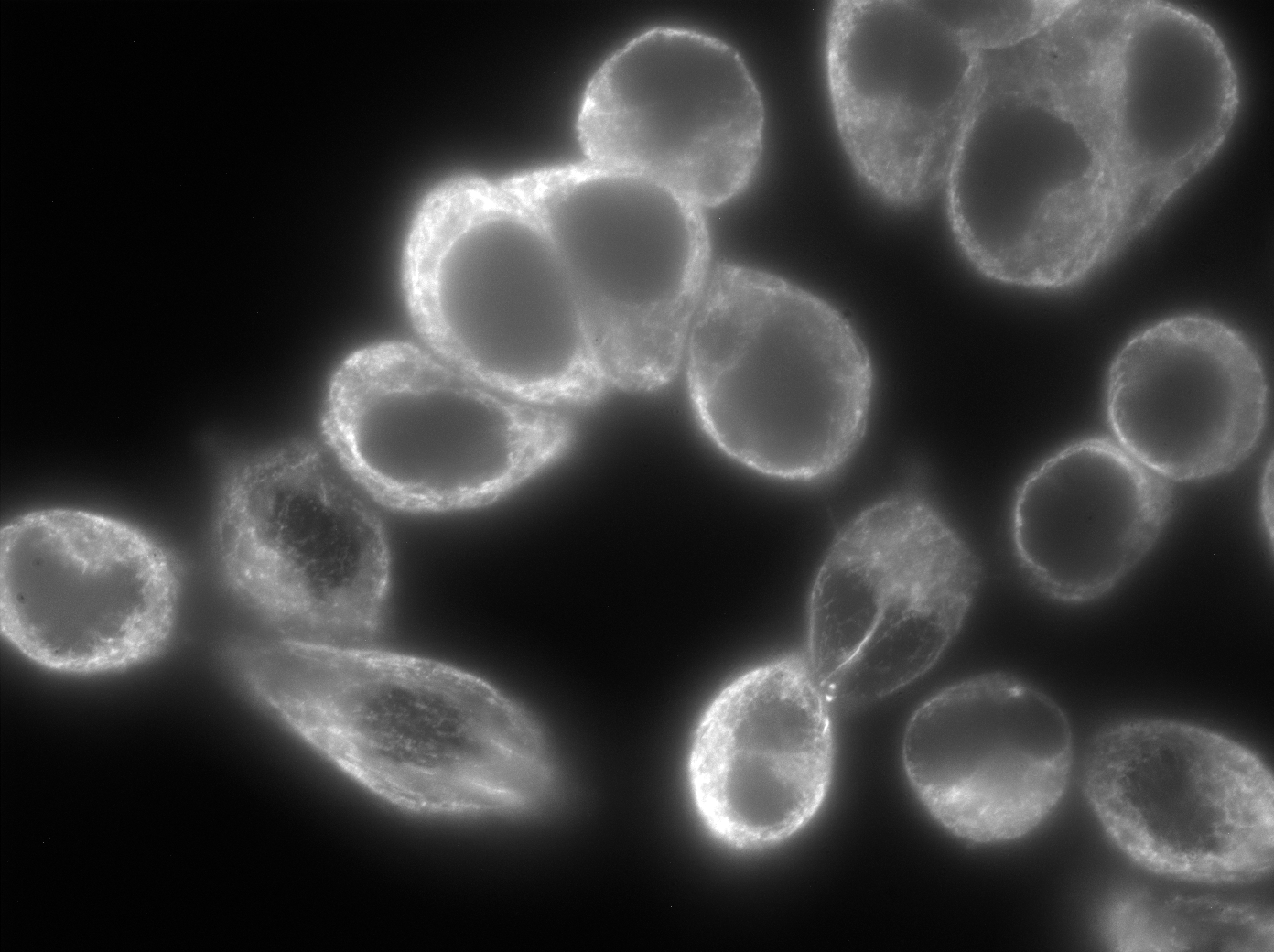

Supplement: Supplementary file 8 — Source data Fig. 2 part 5 [file 44319_2026_742_MOESM8_ESM.zip › Figure 2 Part 5/Fig 2d polye atubulin part 2/WT PolyE-atub 8-14-24 R3 PA2.Project Maximum Z_XY1723841333_Z0_T0_C1.tif]

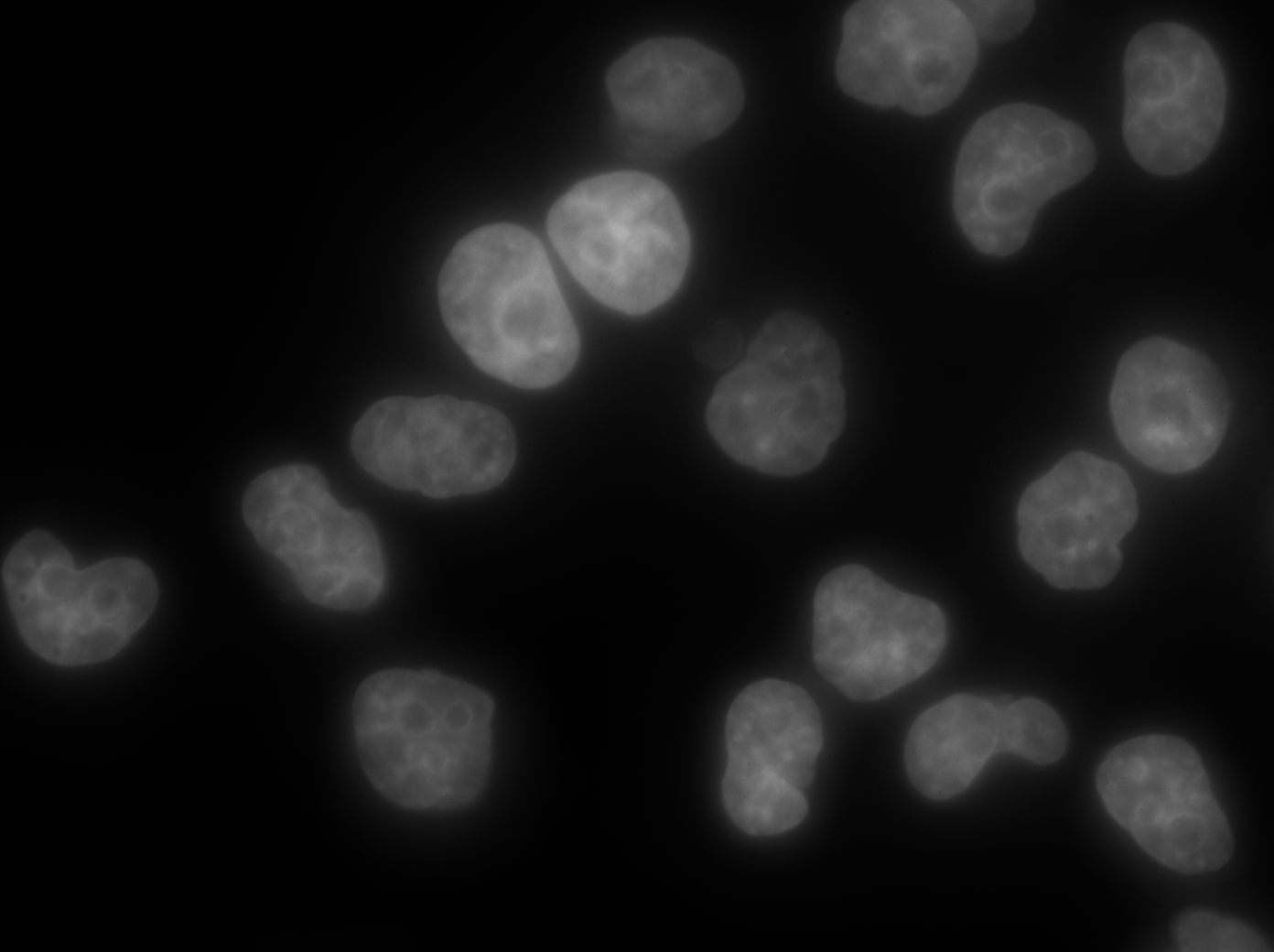

Supplement: Supplementary file 8 — Source data Fig. 2 part 5 [file 44319_2026_742_MOESM8_ESM.zip › Figure 2 Part 5/Fig 2d polye atubulin part 2/WT PolyE-atub 8-14-24 R3 PA2.Project Maximum Z_XY1723841333_Z0_T0_C0.tif]

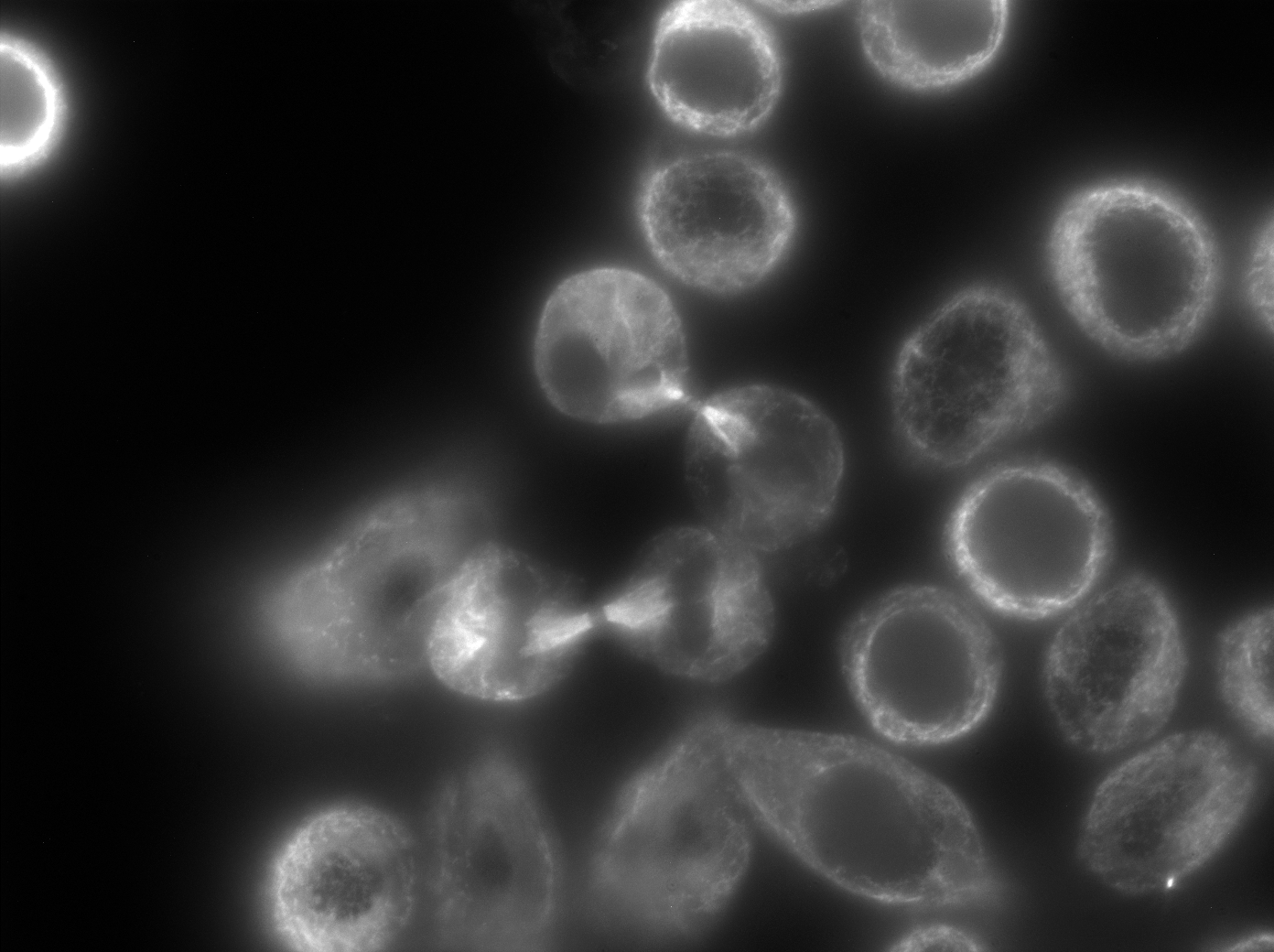

Supplement: Supplementary file 8 — Source data Fig. 2 part 5 [file 44319_2026_742_MOESM8_ESM.zip › Figure 2 Part 5/Fig 2d polye atubulin part 2/WT PolyE-atub 8-14-24 R3 ET10ET11.Project Maximum Z_XY1723845138_Z0_T0_C1.tif]

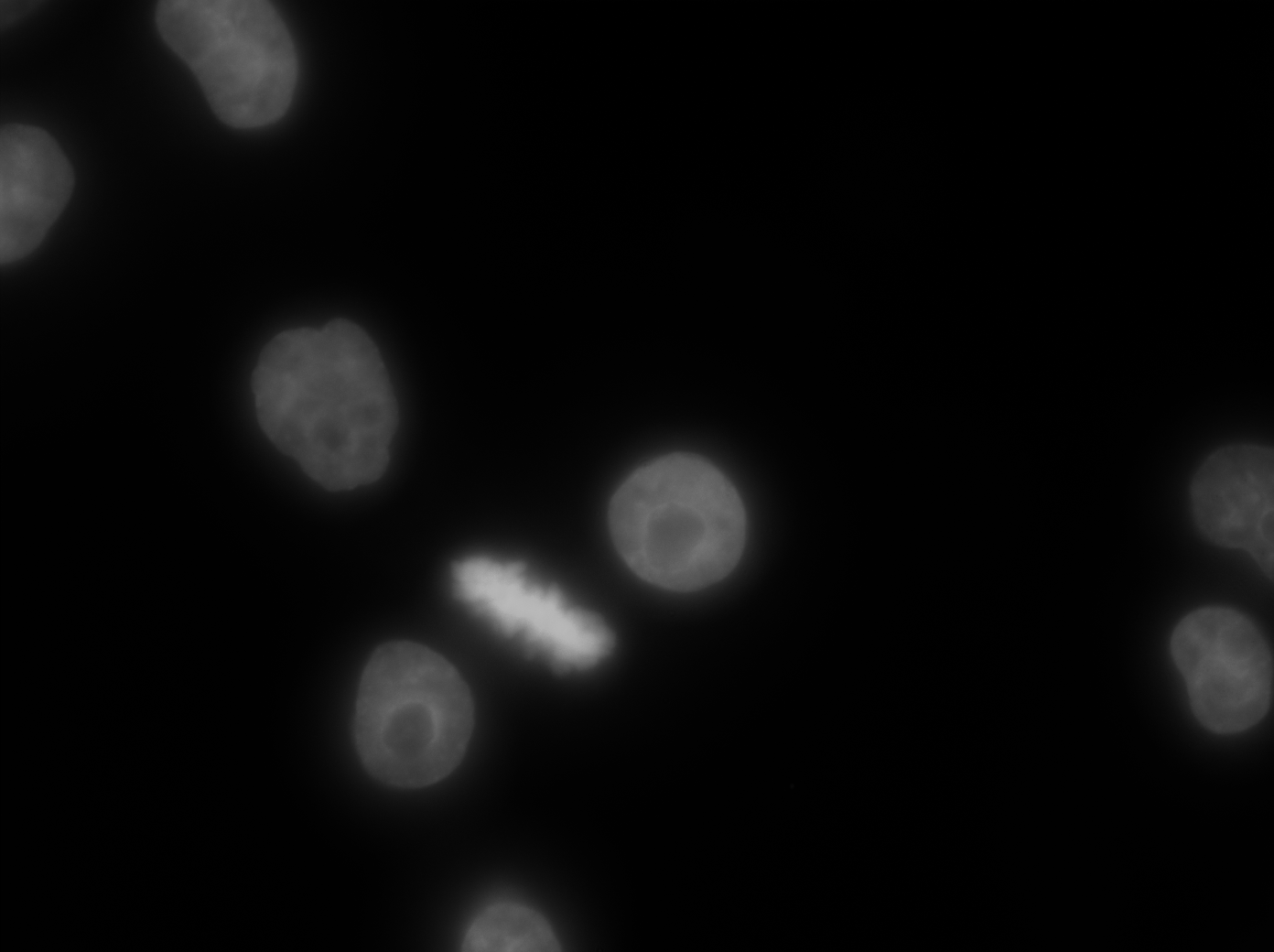

Supplement: Supplementary file 8 — Source data Fig. 2 part 5 [file 44319_2026_742_MOESM8_ESM.zip › Figure 2 Part 5/Fig 2d polye atubulin part 2/WT PolyE-atub 8-14-24 R3 M6.Project Maximum Z_XY1723845259_Z0_T0_C0.tif]

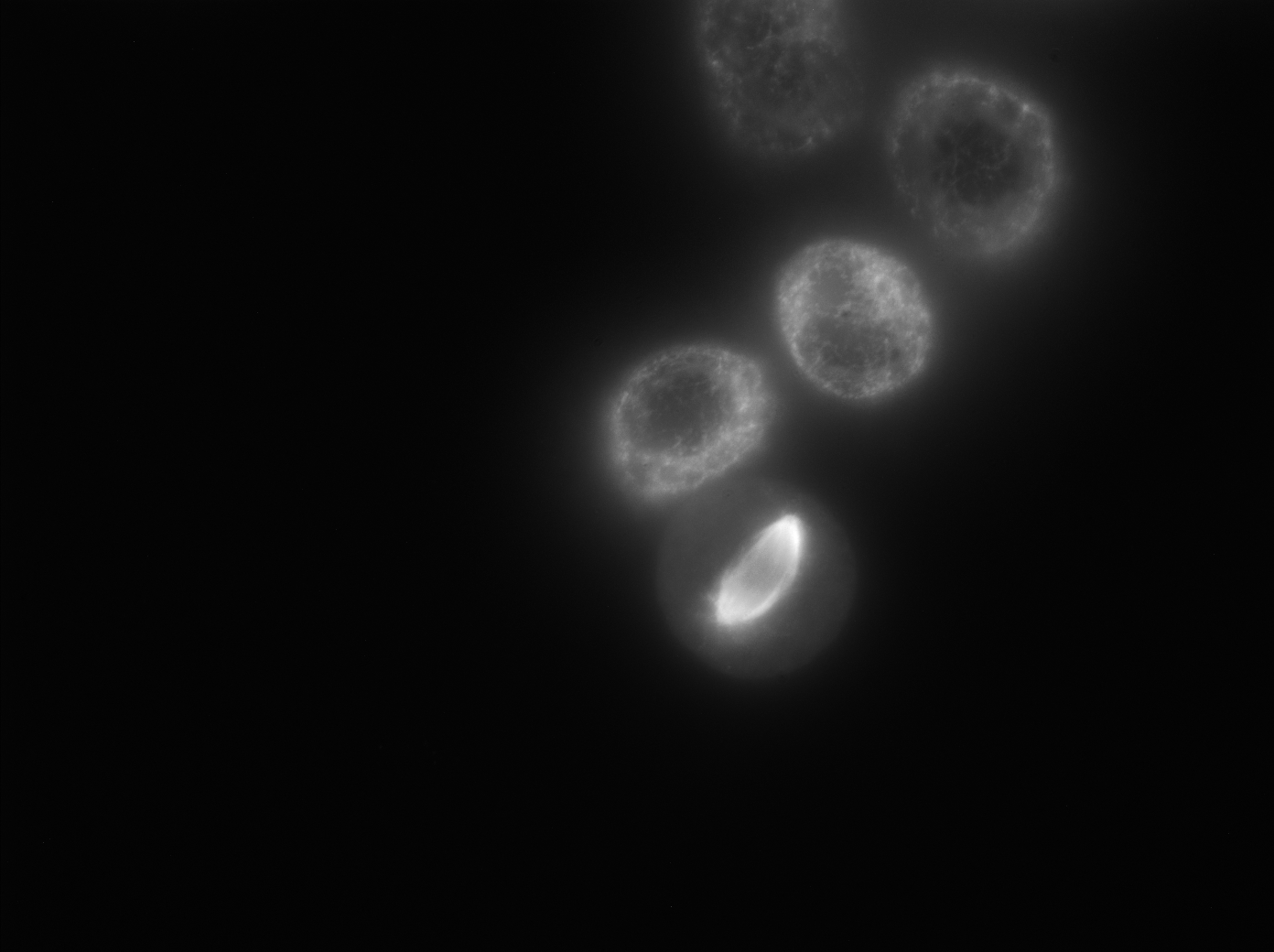

Supplement: Supplementary file 8 — Source data Fig. 2 part 5 [file 44319_2026_742_MOESM8_ESM.zip › Figure 2 Part 5/Fig 2d polye atubulin part 2/WT PolyE-atub 8-14-24 R2 M3.Project Maximum Z_XY1723837033_Z0_T0_C1.tif]

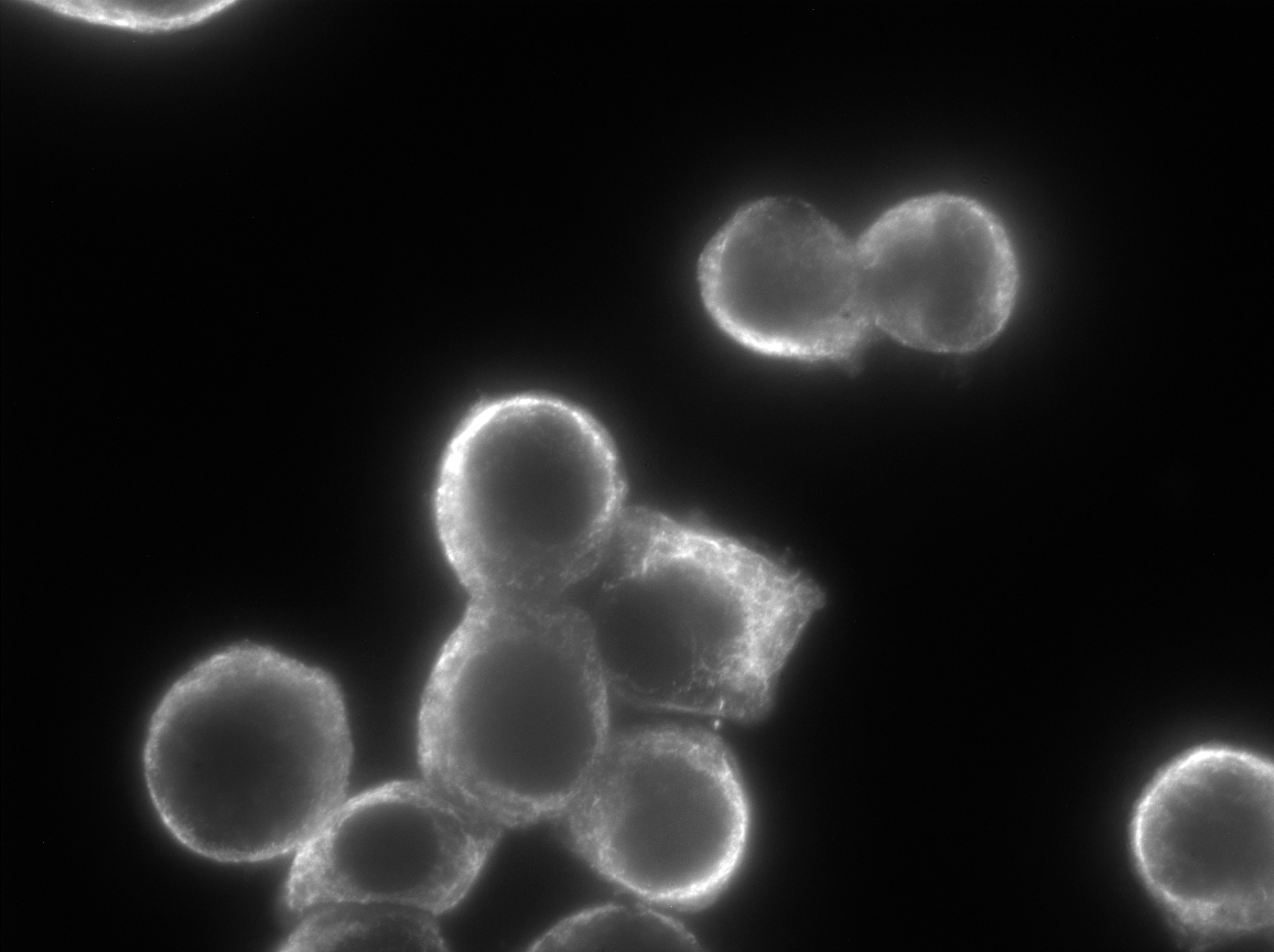

Supplement: Supplementary file 8 — Source data Fig. 2 part 5 [file 44319_2026_742_MOESM8_ESM.zip › Figure 2 Part 5/Fig 2d polye atubulin part 2/WT PolyE-atub 8-14-24 R3 PA1.Project Maximum Z_XY1723841105_Z0_T0_C1.tif]

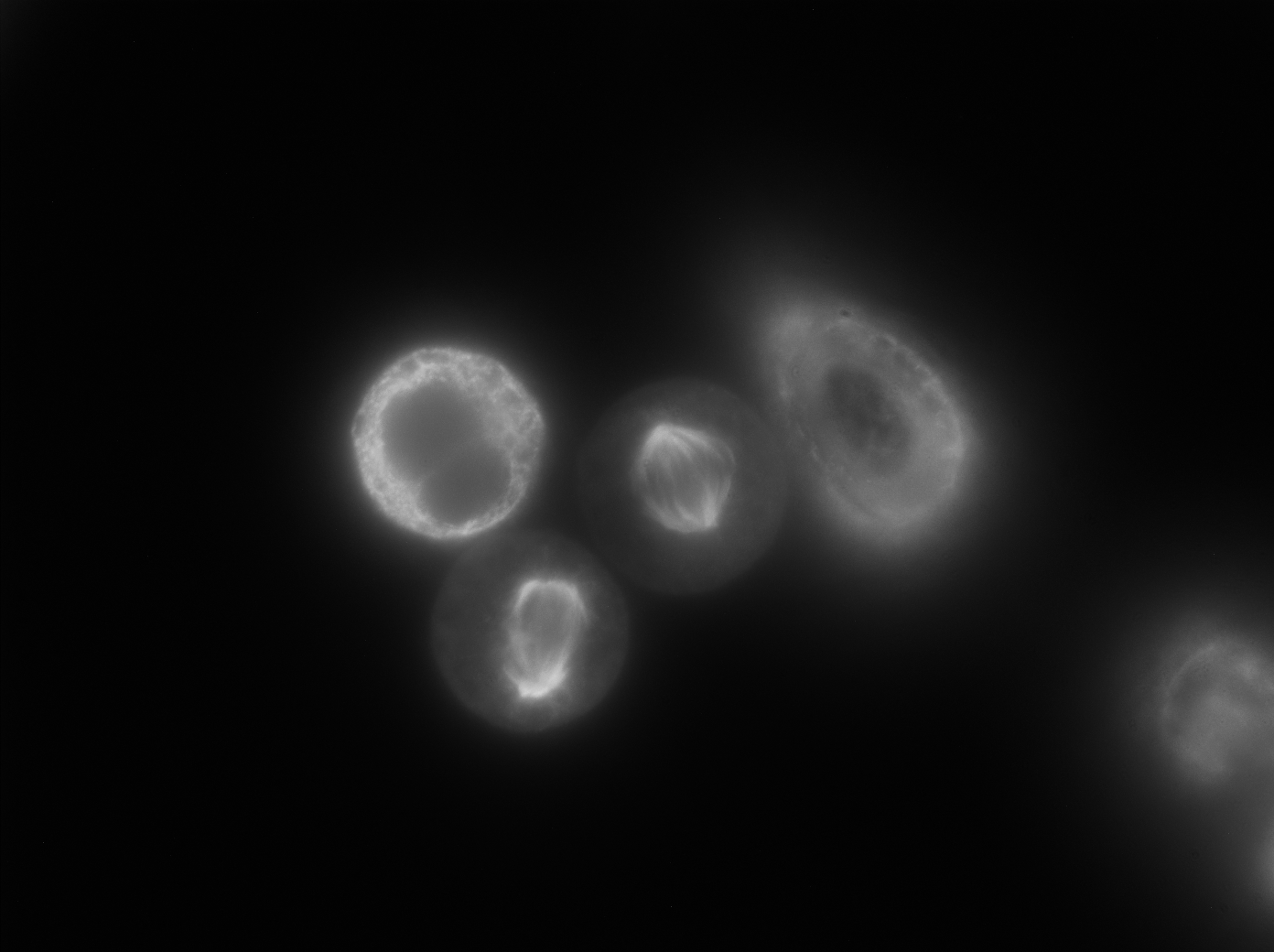

Supplement: Supplementary file 8 — Source data Fig. 2 part 5 [file 44319_2026_742_MOESM8_ESM.zip › Figure 2 Part 5/Fig 2d polye atubulin part 2/WT PolyE-atub 8-14-24 R2 M10.Project Maximum Z_XY1723839028_Z0_T0_C1.tif]

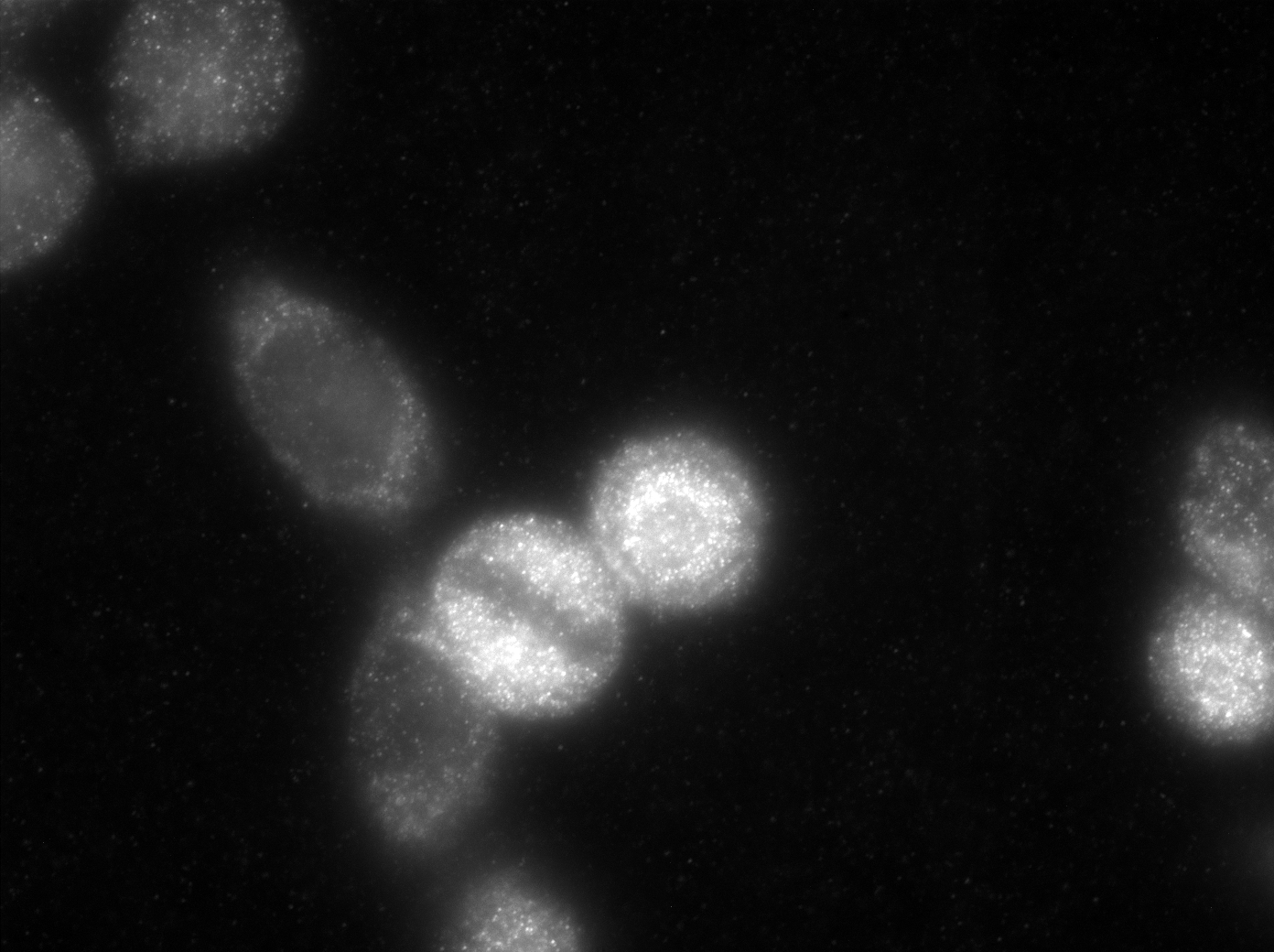

Supplement: Supplementary file 8 — Source data Fig. 2 part 5 [file 44319_2026_742_MOESM8_ESM.zip › Figure 2 Part 5/Fig 2d polye atubulin part 2/WT PolyE-atub 8-14-24 R3 M6.Project Maximum Z_XY1723845259_Z0_T0_C2.tif]

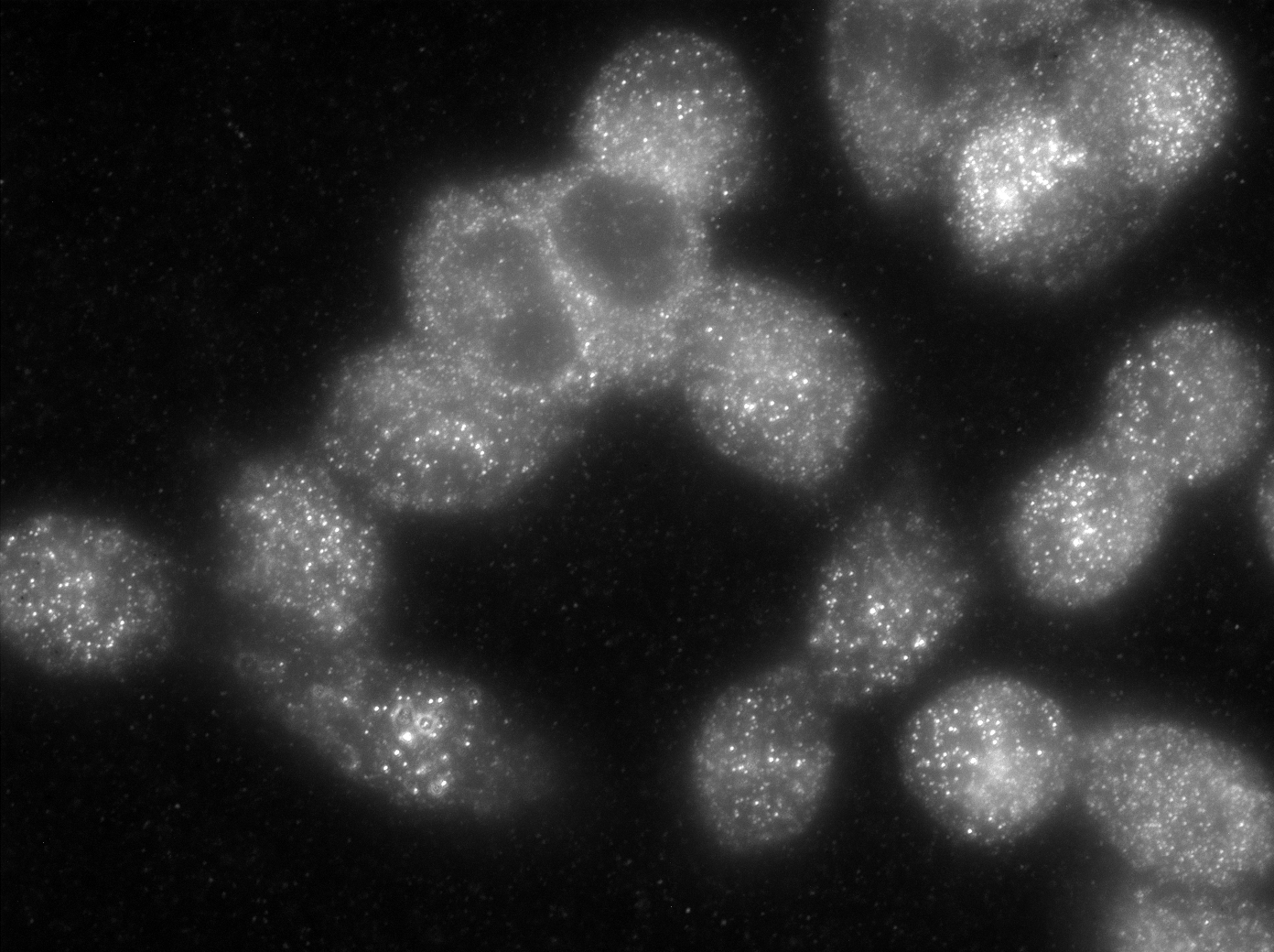

Supplement: Supplementary file 8 — Source data Fig. 2 part 5 [file 44319_2026_742_MOESM8_ESM.zip › Figure 2 Part 5/Fig 2d polye atubulin part 2/WT PolyE-atub 8-14-24 R3 PA2.Project Maximum Z_XY1723841333_Z0_T0_C2.tif]

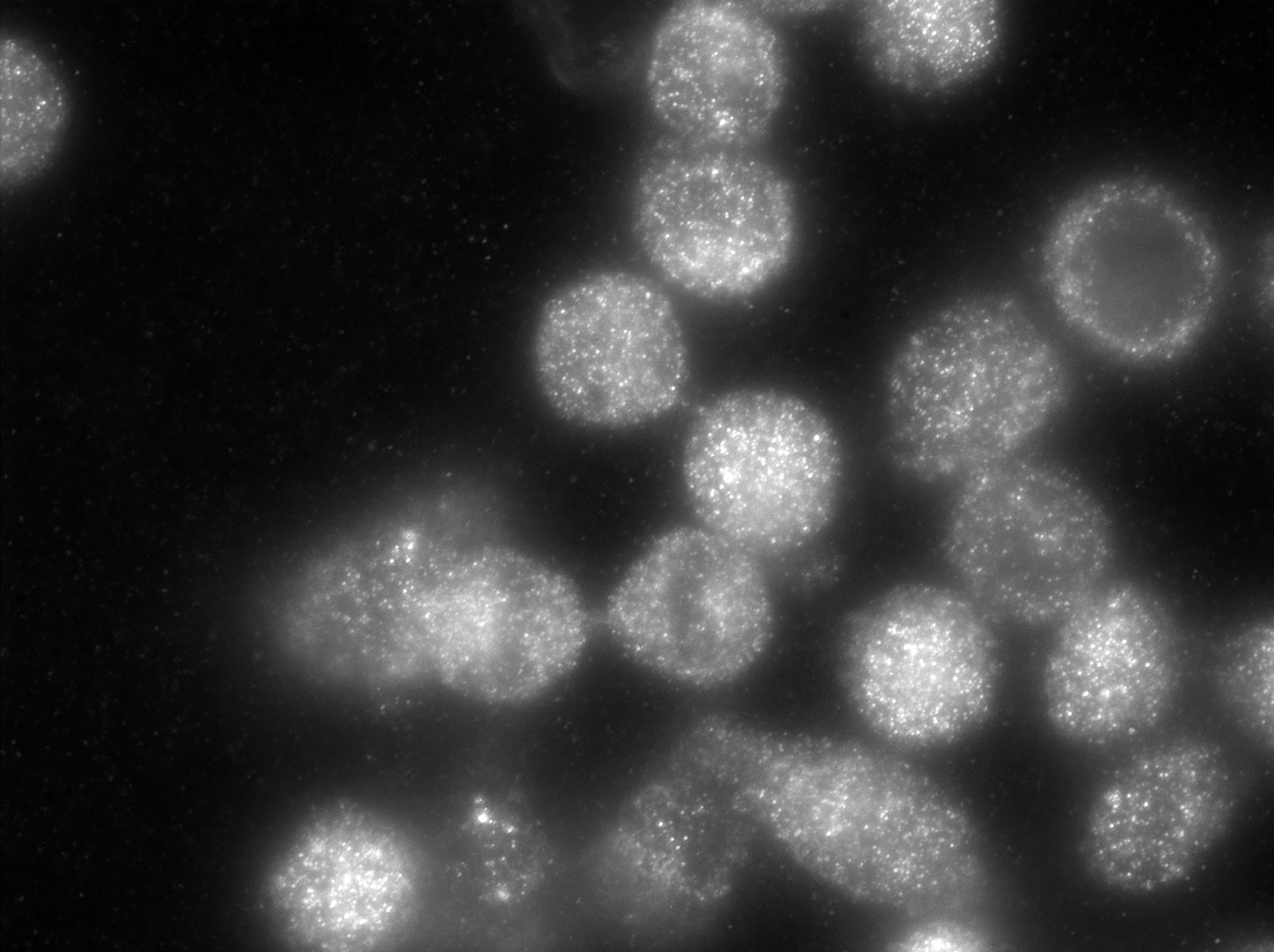

Supplement: Supplementary file 8 — Source data Fig. 2 part 5 [file 44319_2026_742_MOESM8_ESM.zip › Figure 2 Part 5/Fig 2d polye atubulin part 2/WT PolyE-atub 8-14-24 R3 ET10ET11.Project Maximum Z_XY1723845138_Z0_T0_C2.tif]

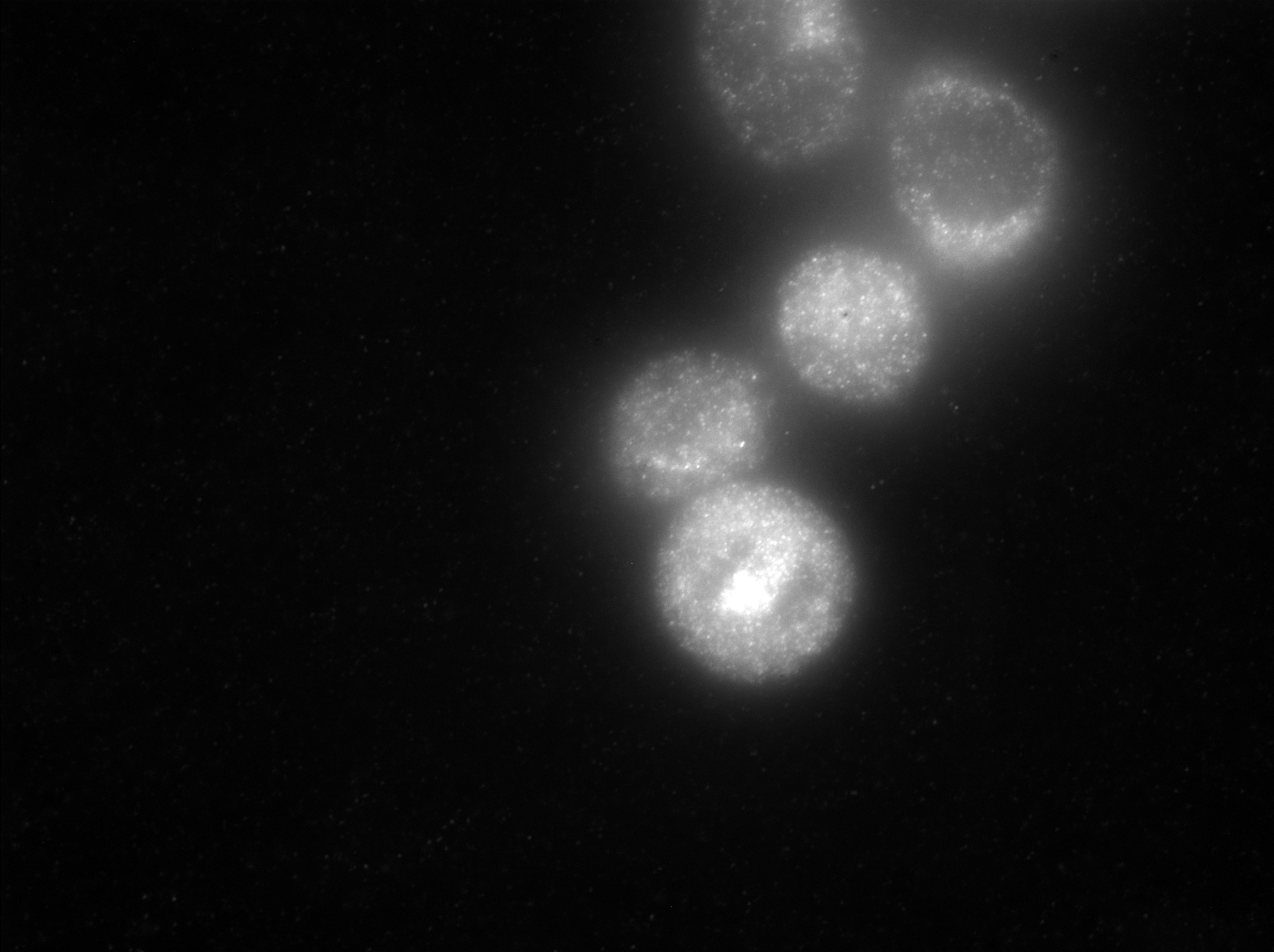

Supplement: Supplementary file 8 — Source data Fig. 2 part 5 [file 44319_2026_742_MOESM8_ESM.zip › Figure 2 Part 5/Fig 2d polye atubulin part 2/WT PolyE-atub 8-14-24 R2 M3.Project Maximum Z_XY1723837033_Z0_T0_C2.tif]

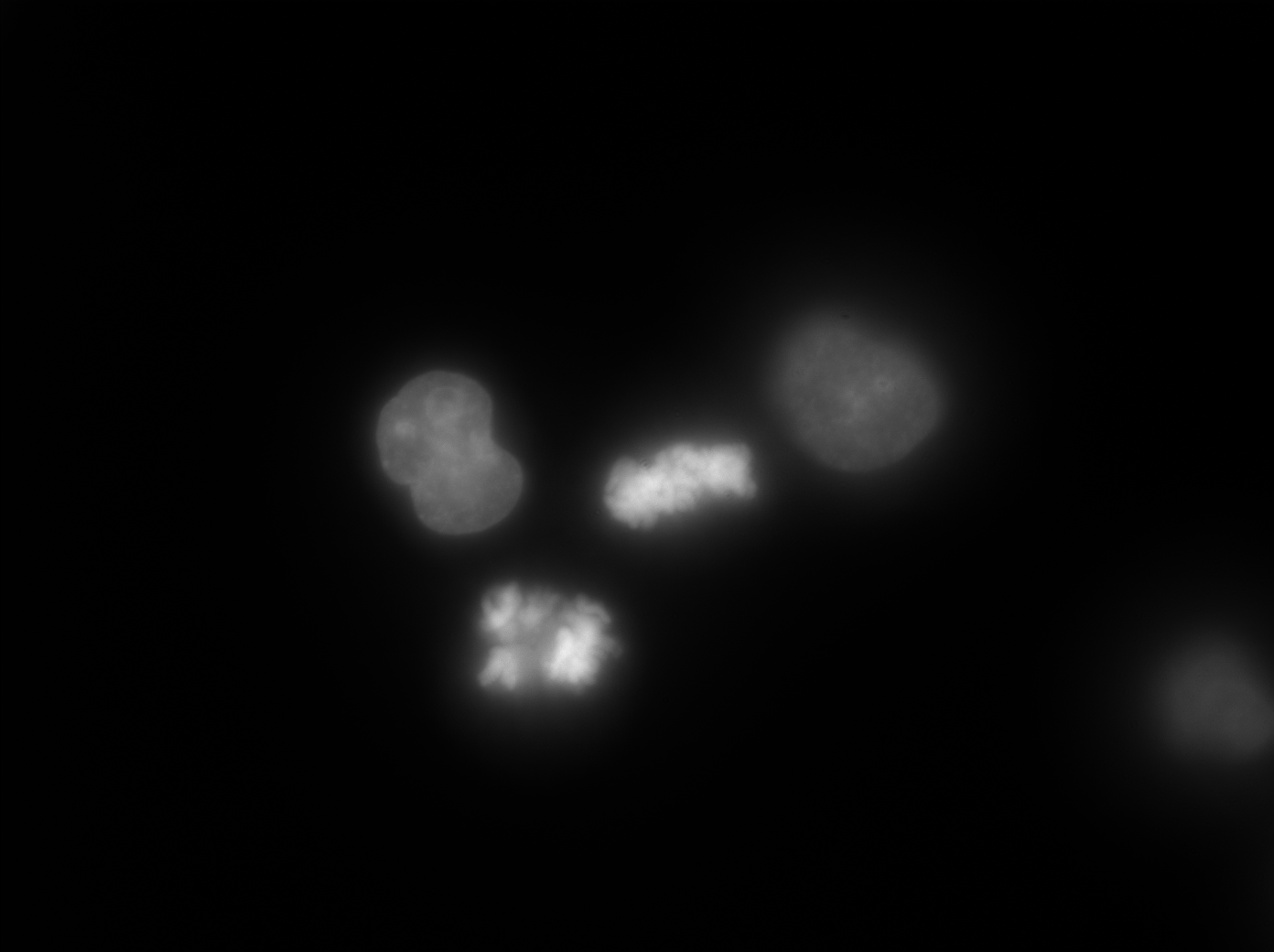

Supplement: Supplementary file 8 — Source data Fig. 2 part 5 [file 44319_2026_742_MOESM8_ESM.zip › Figure 2 Part 5/Fig 2d polye atubulin part 2/WT PolyE-atub 8-14-24 R2 M10.Project Maximum Z_XY1723839028_Z0_T0_C0.tif]

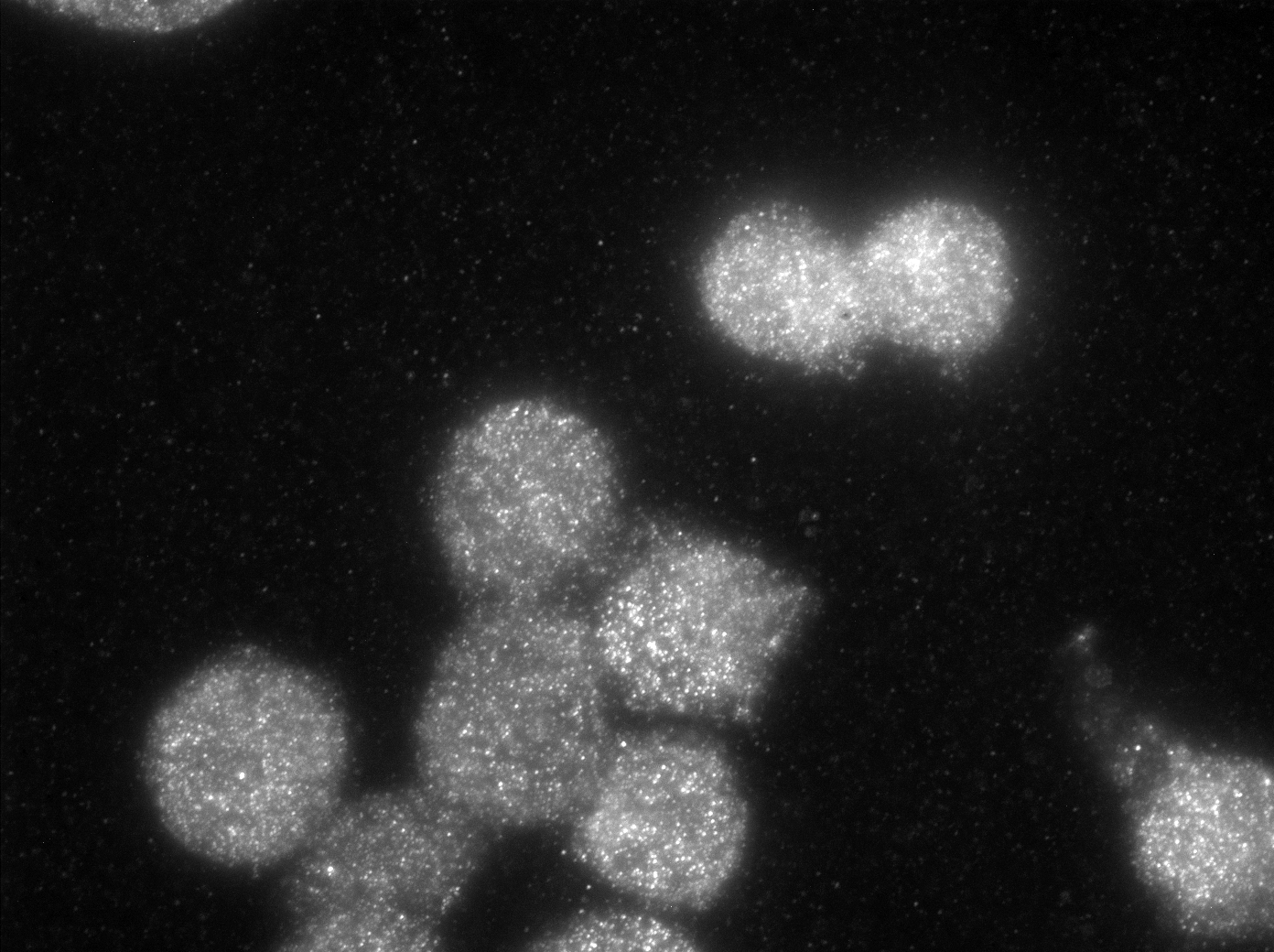

Supplement: Supplementary file 8 — Source data Fig. 2 part 5 [file 44319_2026_742_MOESM8_ESM.zip › Figure 2 Part 5/Fig 2d polye atubulin part 2/WT PolyE-atub 8-14-24 R3 PA1.Project Maximum Z_XY1723841105_Z0_T0_C2.tif]

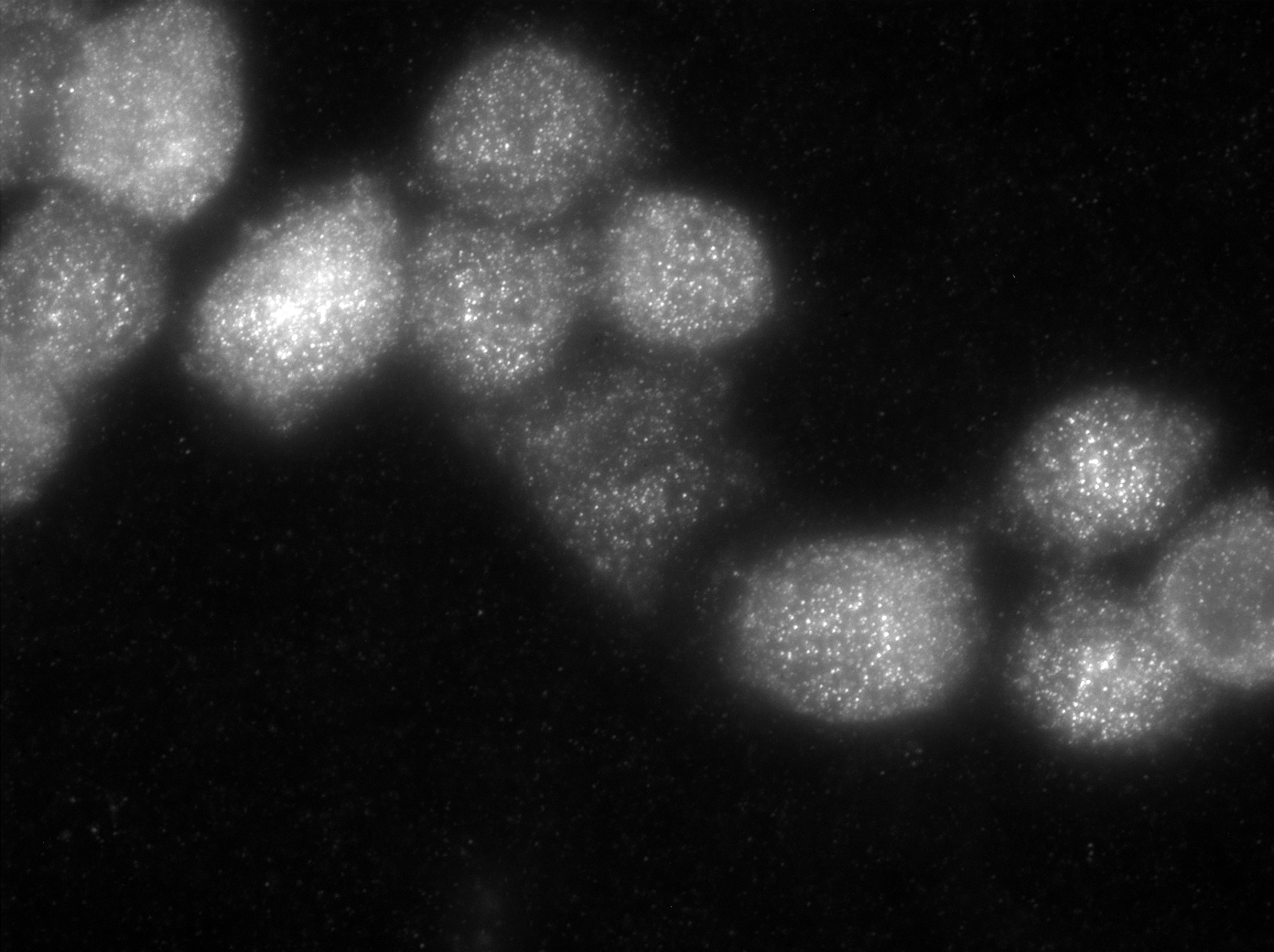

Supplement: Supplementary file 8 — Source data Fig. 2 part 5 [file 44319_2026_742_MOESM8_ESM.zip › Figure 2 Part 5/Fig 2d polye atubulin part 2/WT PolyE-atub 8-14-24 R3 PA8.Project Maximum Z_XY1723843244_Z0_T0_C2.tif]

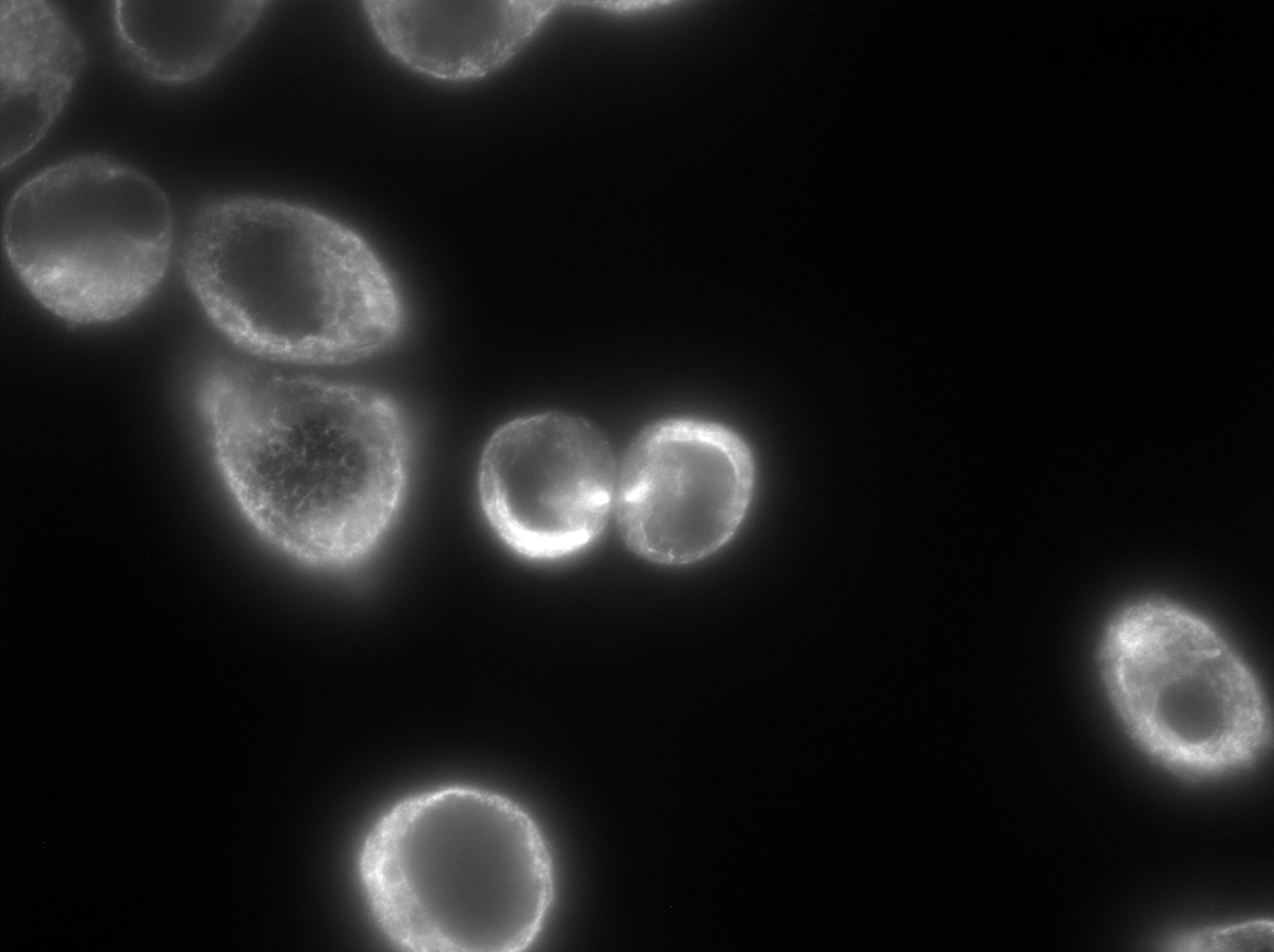

Supplement: Supplementary file 8 — Source data Fig. 2 part 5 [file 44319_2026_742_MOESM8_ESM.zip › Figure 2 Part 5/Fig 2d polye atubulin part 2/WT PolyE-atub 8-14-24 R3 ET1.Project Maximum Z_XY1723841453_Z0_T0_C1.tif]

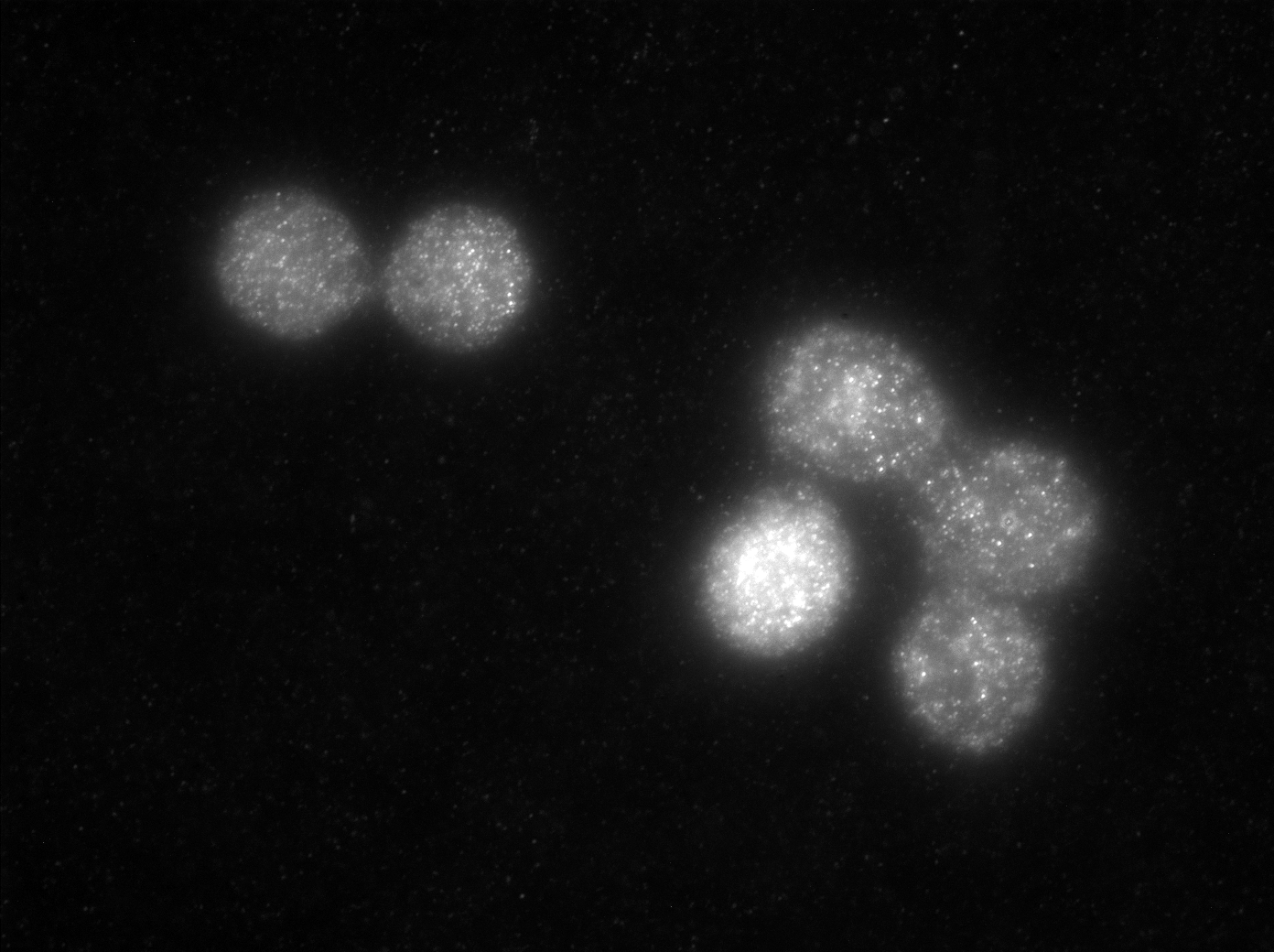

Supplement: Supplementary file 8 — Source data Fig. 2 part 5 [file 44319_2026_742_MOESM8_ESM.zip › Figure 2 Part 5/Fig 2d polye atubulin part 2/WT PolyE-atub 8-14-24 R3 ET2.Project Maximum Z_XY1723841789_Z0_T0_C2.tif]

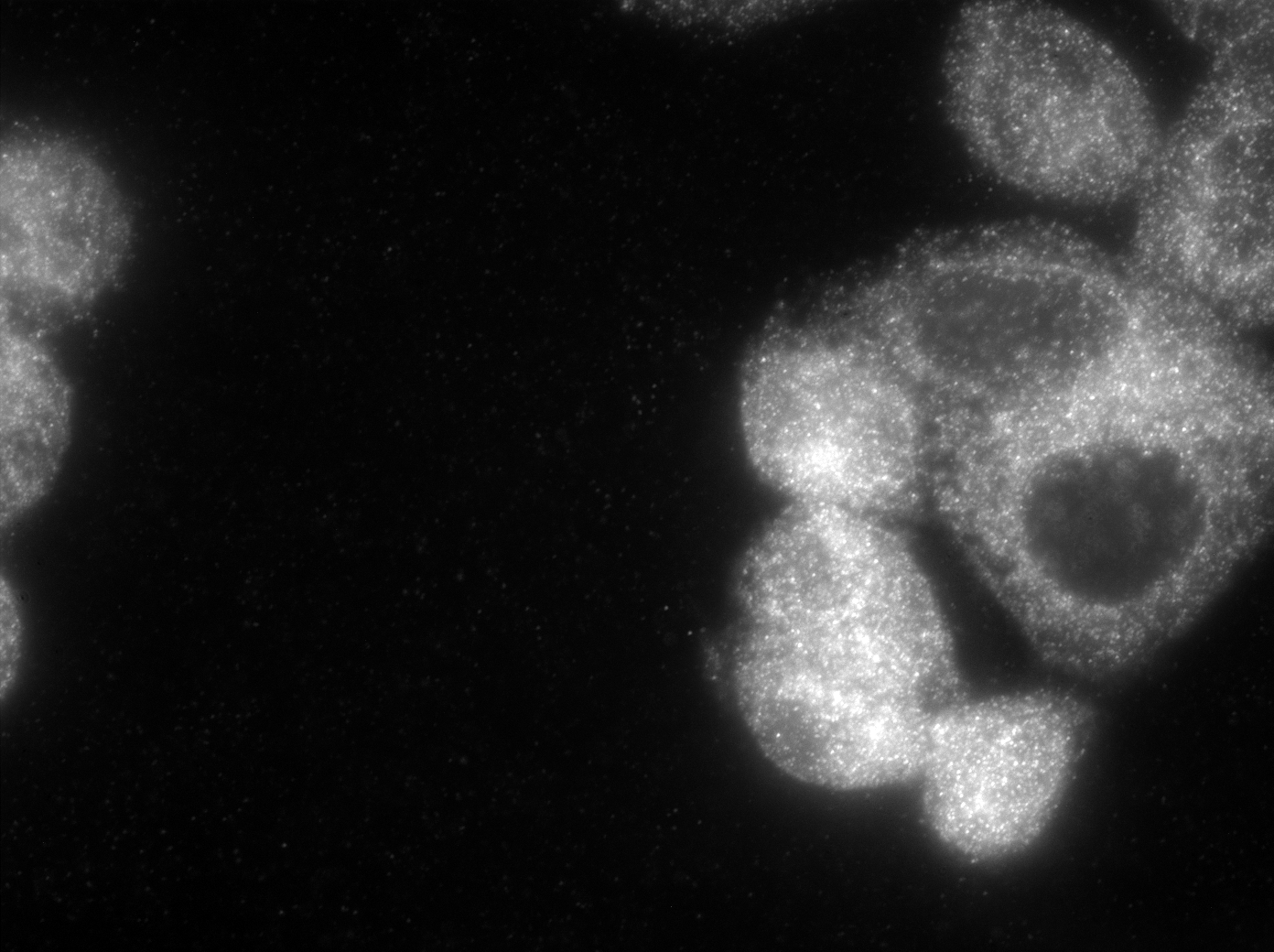

Supplement: Supplementary file 8 — Source data Fig. 2 part 5 [file 44319_2026_742_MOESM8_ESM.zip › Figure 2 Part 5/Fig 2d polye atubulin part 2/WT PolyE-atub 8-14-24 R2 LT5LT6.Project Maximum Z_XY1723835311_Z0_T0_C2.tif]

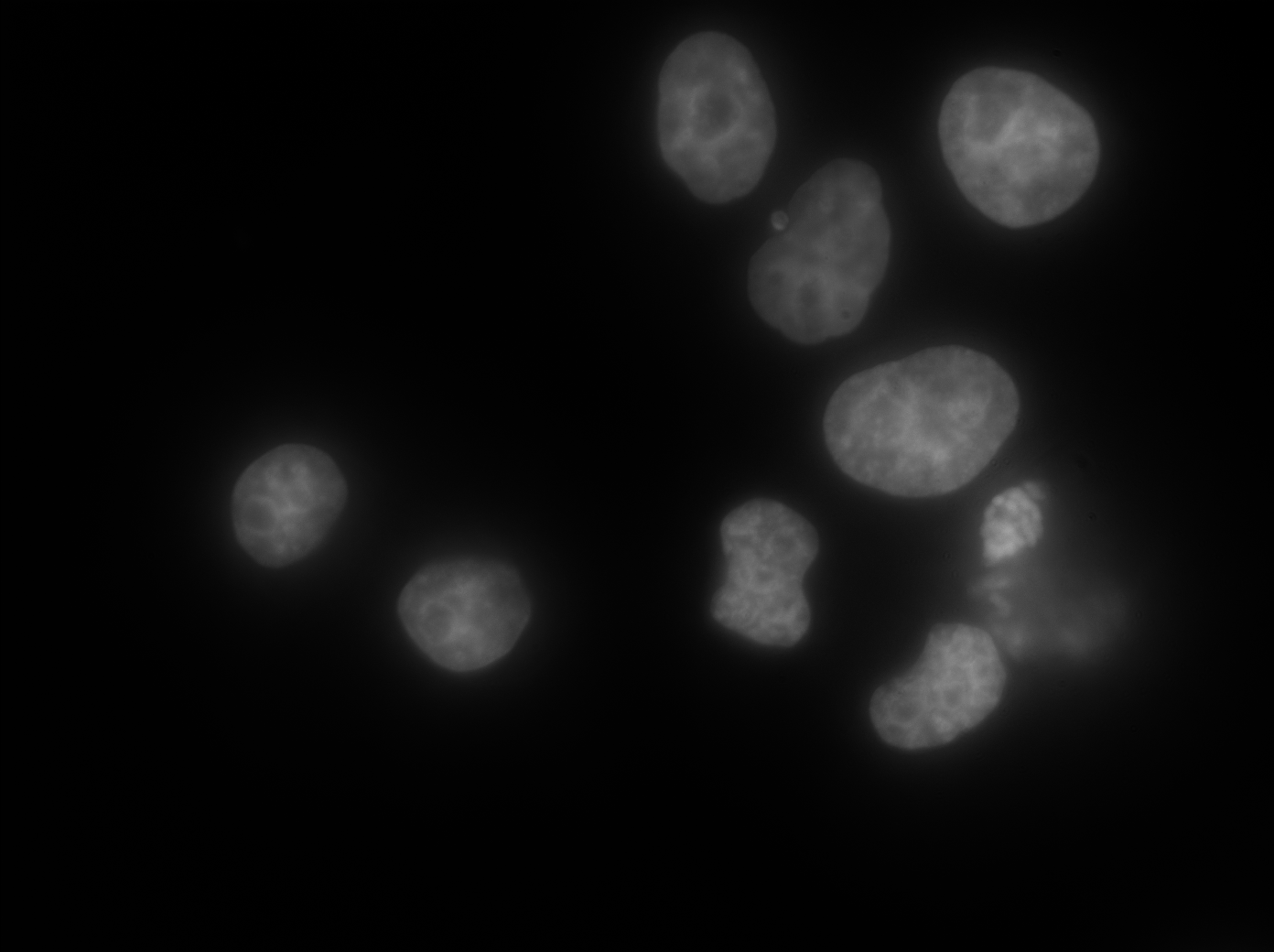

Supplement: Supplementary file 8 — Source data Fig. 2 part 5 [file 44319_2026_742_MOESM8_ESM.zip › Figure 2 Part 5/Fig 2d polye atubulin part 2/WT PolyE-atub 8-14-24 R3 LT8.Project Maximum Z_XY1723843728_Z0_T0_C0.tif]

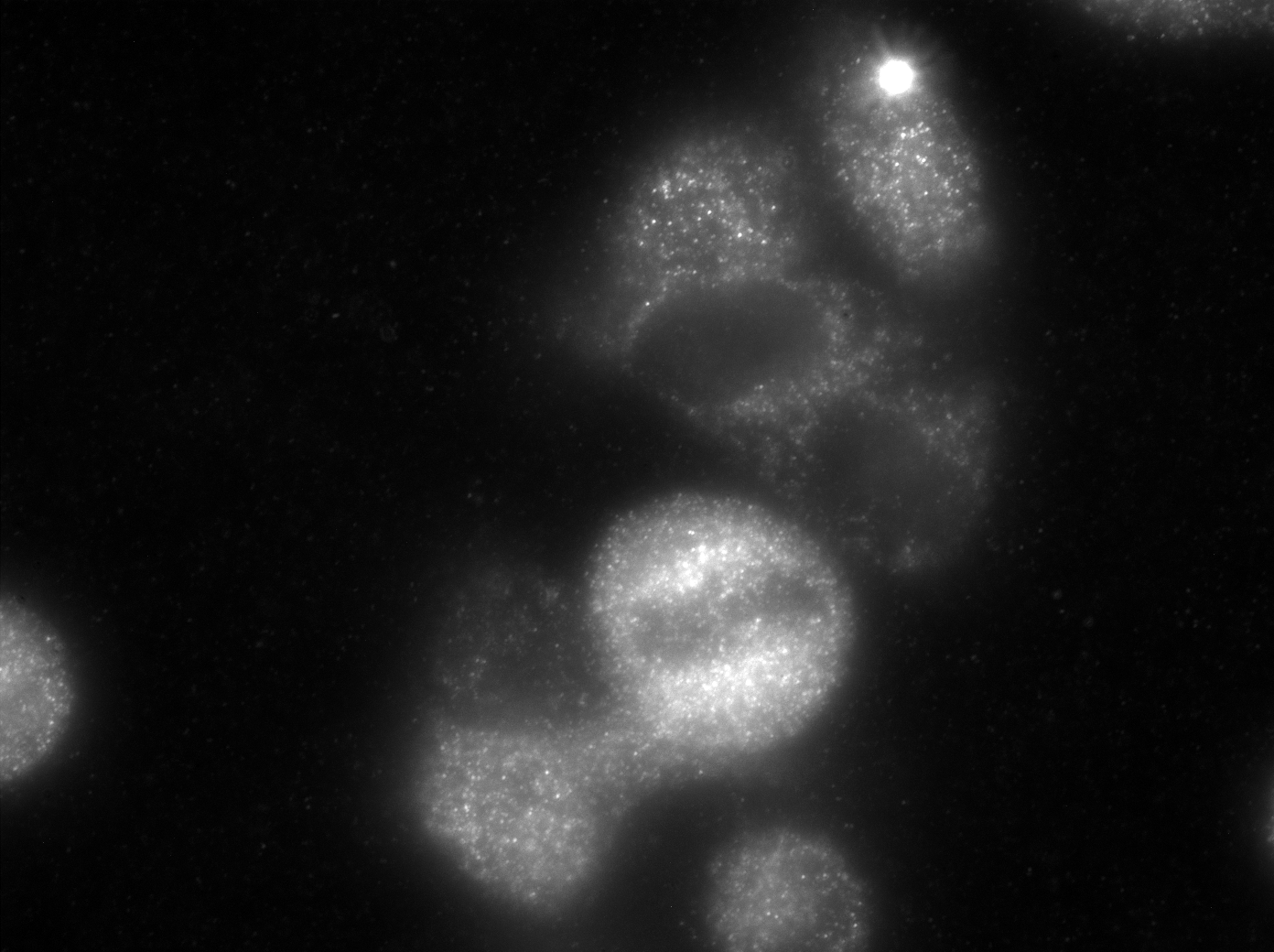

Supplement: Supplementary file 8 — Source data Fig. 2 part 5 [file 44319_2026_742_MOESM8_ESM.zip › Figure 2 Part 5/Fig 2d polye atubulin part 2/WT PolyE-atub 8-14-24 R3 M5.Project Maximum Z_XY1723844950_Z0_T0_C2.tif]

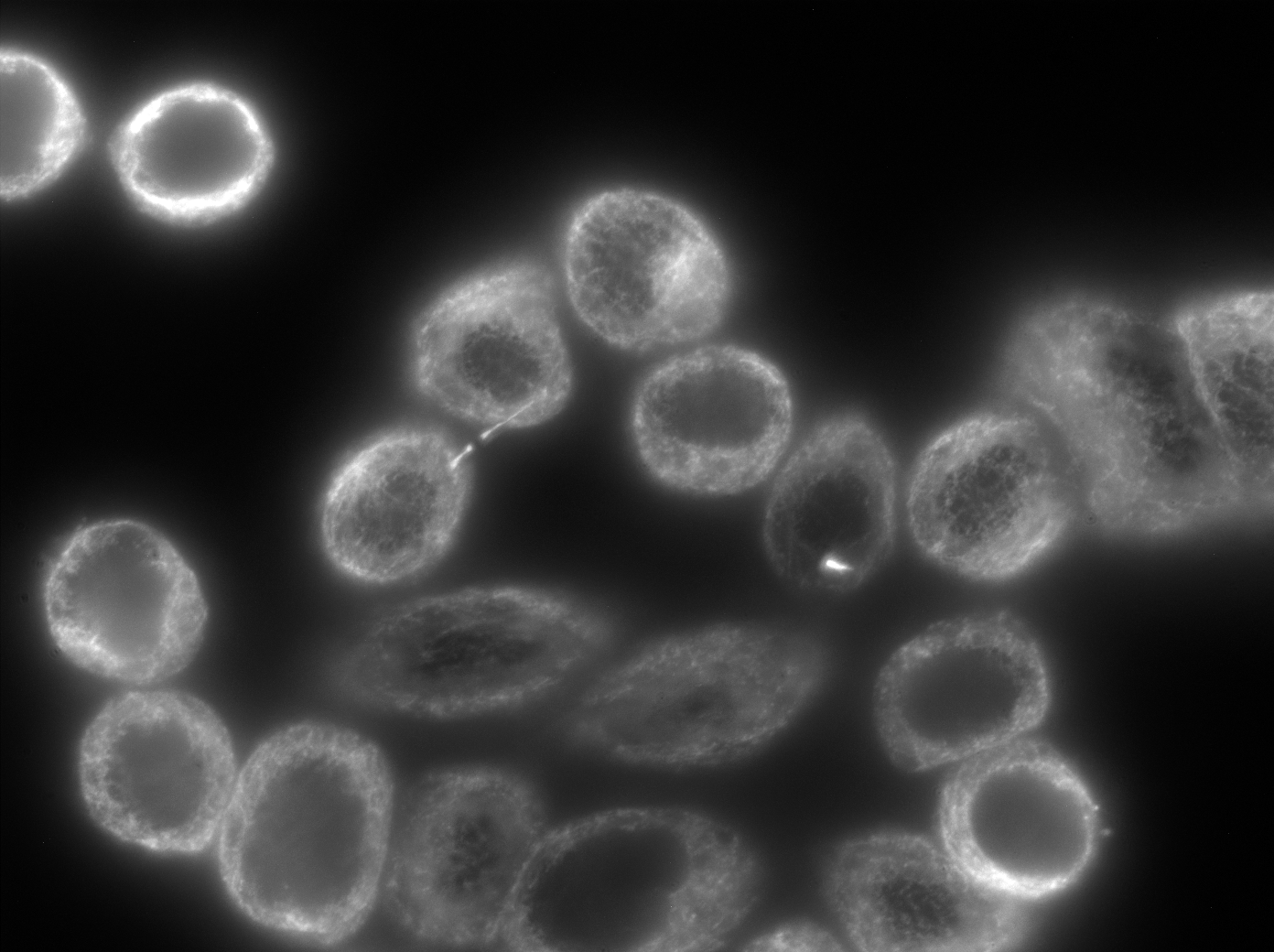

Supplement: Supplementary file 8 — Source data Fig. 2 part 5 [file 44319_2026_742_MOESM8_ESM.zip › Figure 2 Part 5/Fig 2d polye atubulin part 2/WT PolyE-atub 8-14-24 R3 LT2 PA3.Project Maximum Z_XY1723841895_Z0_T0_C1.tif]

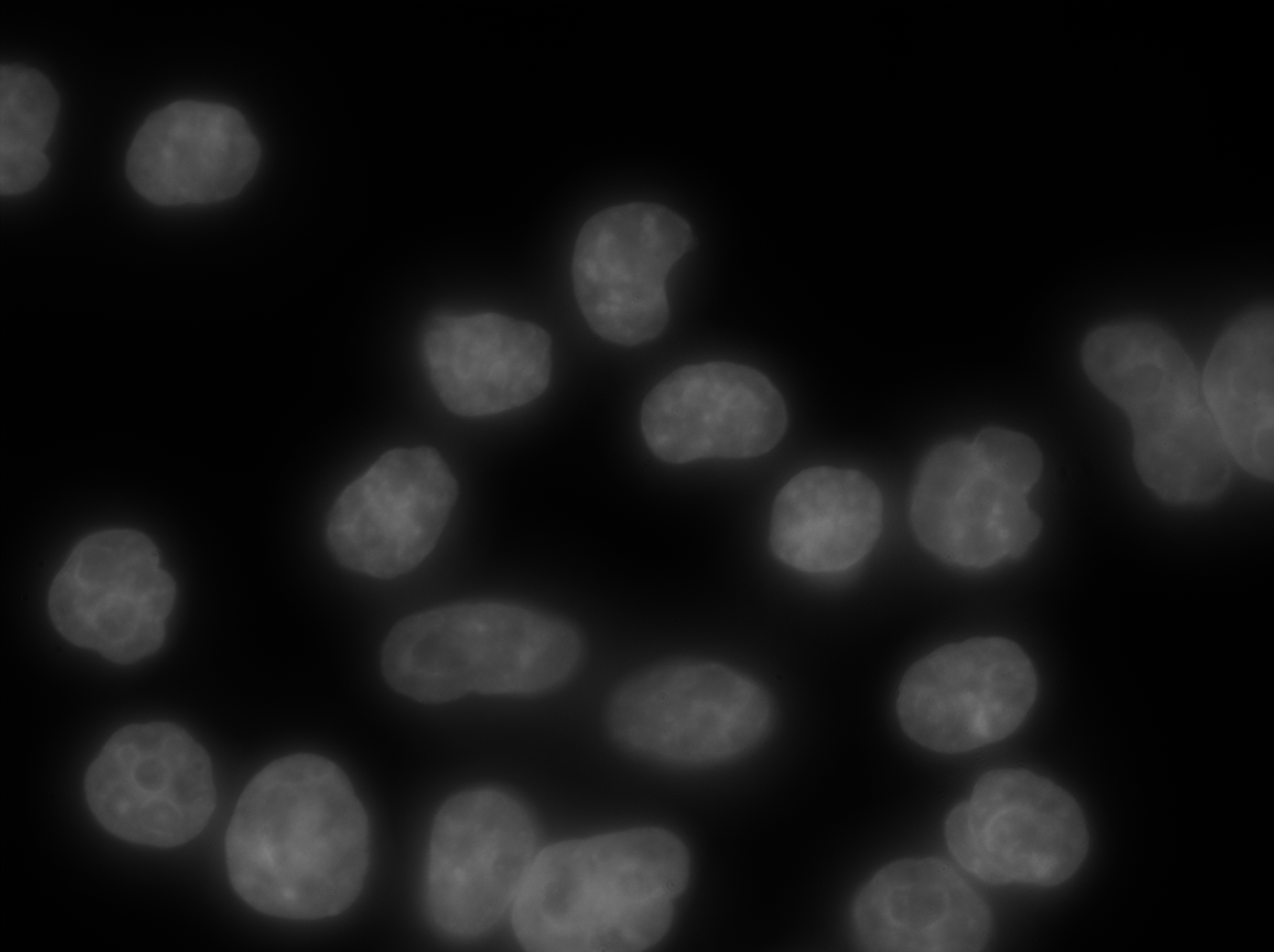

Supplement: Supplementary file 8 — Source data Fig. 2 part 5 [file 44319_2026_742_MOESM8_ESM.zip › Figure 2 Part 5/Fig 2d polye atubulin part 2/WT PolyE-atub 8-14-24 R3 LT2 PA3.Project Maximum Z_XY1723841895_Z0_T0_C0.tif]

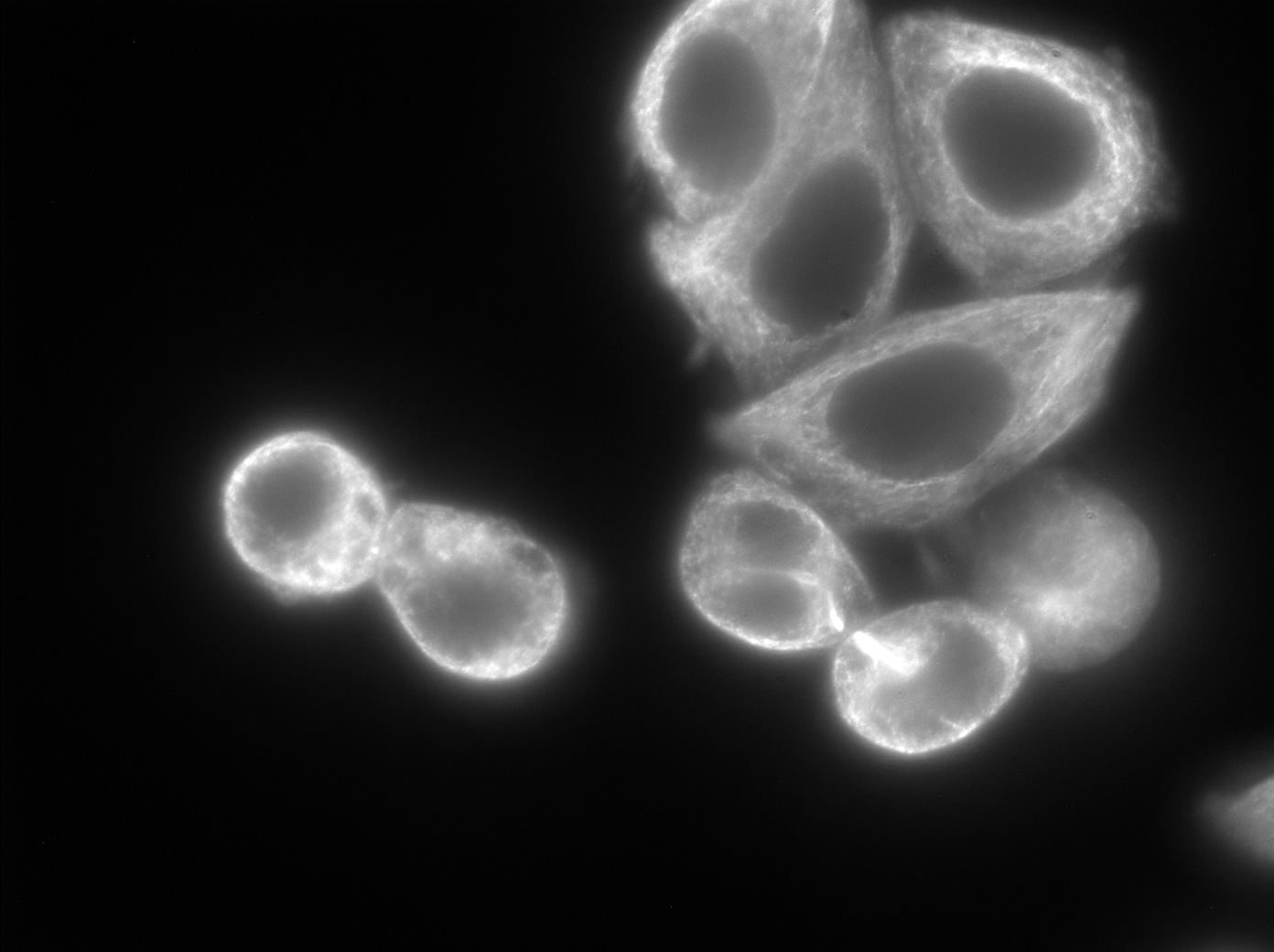

Supplement: Supplementary file 8 — Source data Fig. 2 part 5 [file 44319_2026_742_MOESM8_ESM.zip › Figure 2 Part 5/Fig 2d polye atubulin part 2/WT PolyE-atub 8-14-24 R3 LT8.Project Maximum Z_XY1723843728_Z0_T0_C1.tif]

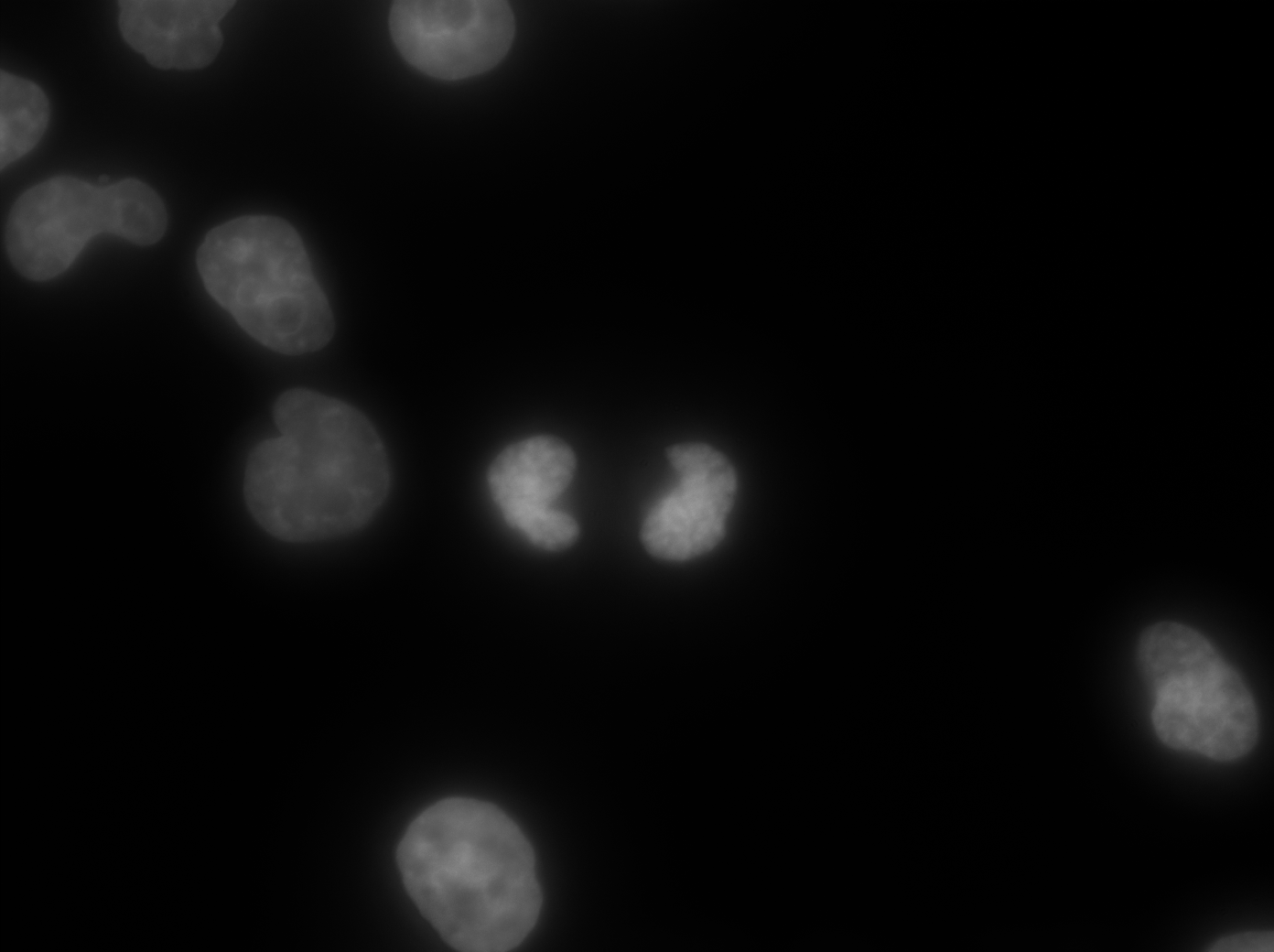

Supplement: Supplementary file 8 — Source data Fig. 2 part 5 [file 44319_2026_742_MOESM8_ESM.zip › Figure 2 Part 5/Fig 2d polye atubulin part 2/WT PolyE-atub 8-14-24 R3 ET1.Project Maximum Z_XY1723841453_Z0_T0_C0.tif]

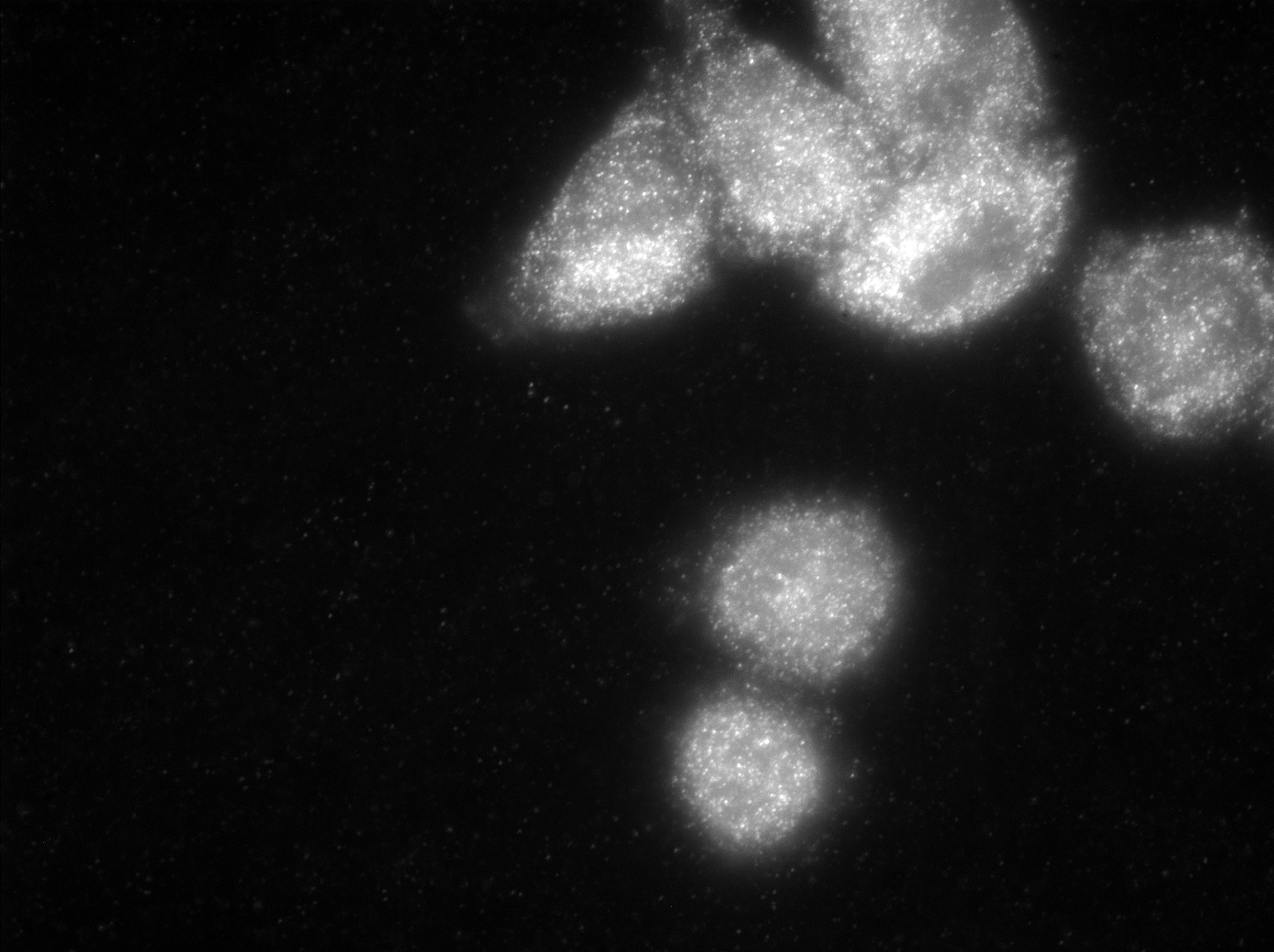

Supplement: Supplementary file 8 — Source data Fig. 2 part 5 [file 44319_2026_742_MOESM8_ESM.zip › Figure 2 Part 5/Fig 2d polye atubulin part 2/WT PolyE-atub 8-14-24 R2 PA6.Project Maximum Z_XY1723836482_Z0_T0_C2.tif]

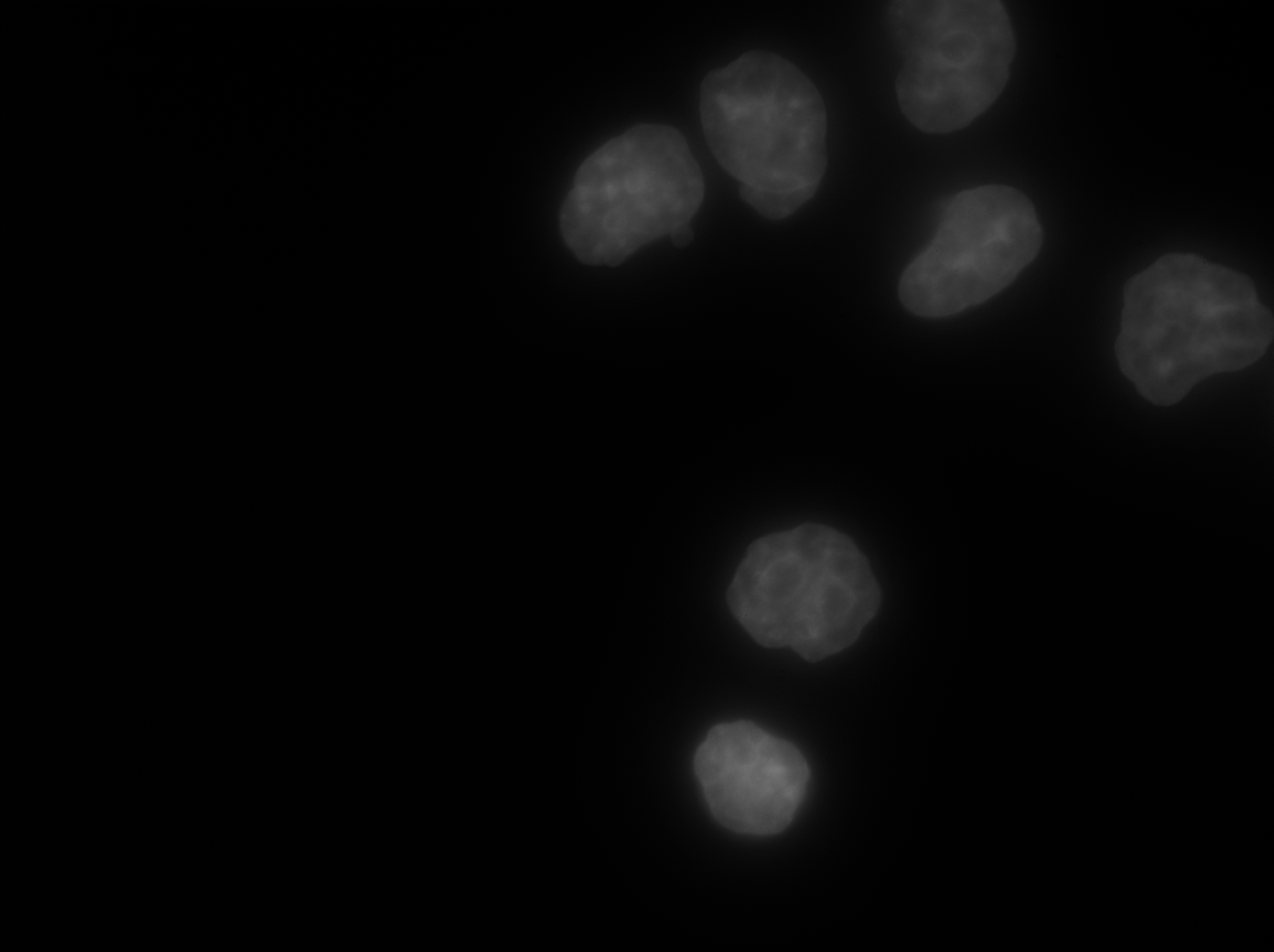

Supplement: Supplementary file 8 — Source data Fig. 2 part 5 [file 44319_2026_742_MOESM8_ESM.zip › Figure 2 Part 5/Fig 2d polye atubulin part 2/WT PolyE-atub 8-14-24 R2 PA6.Project Maximum Z_XY1723836482_Z0_T0_C0.tif]

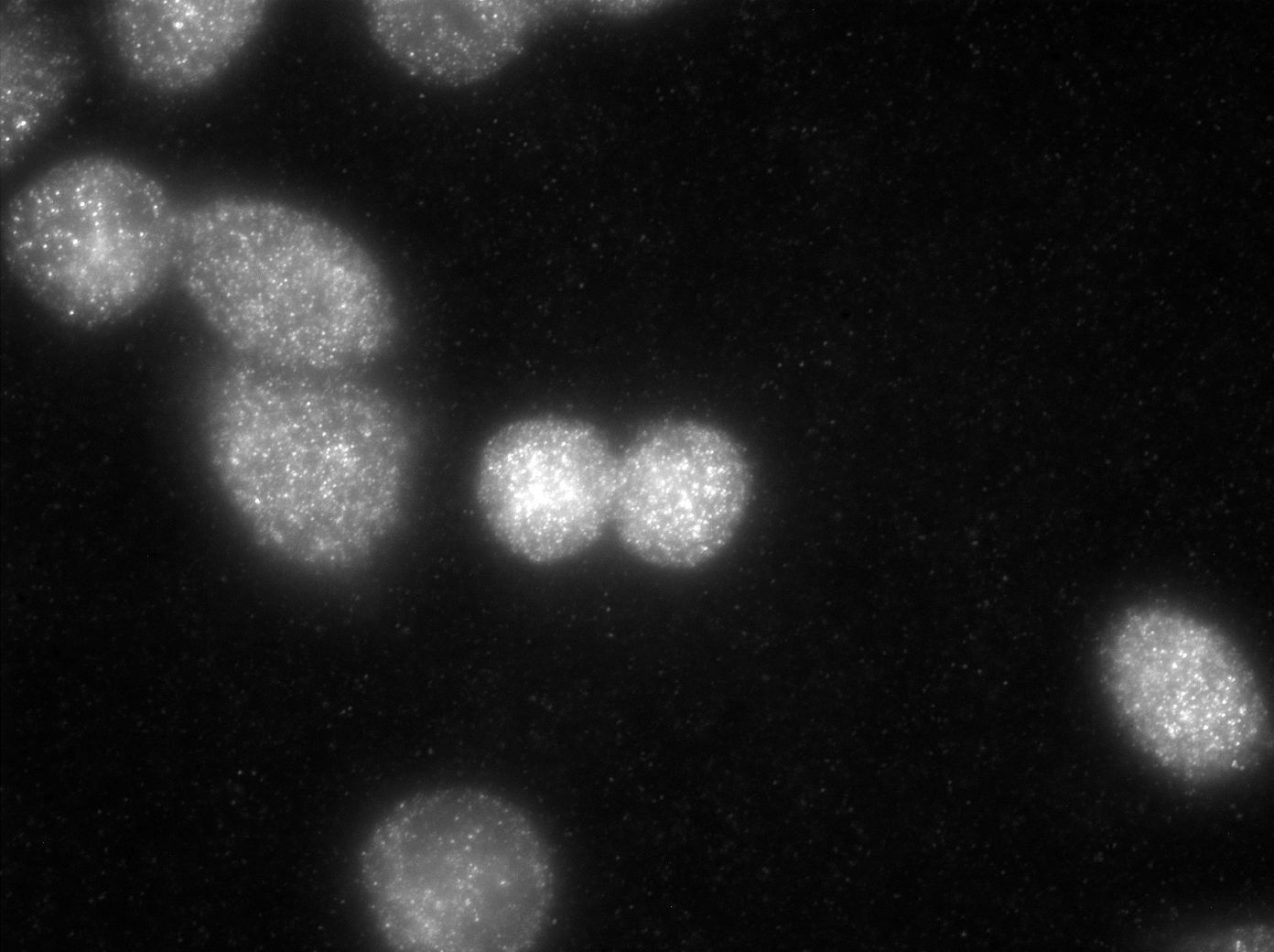

Supplement: Supplementary file 8 — Source data Fig. 2 part 5 [file 44319_2026_742_MOESM8_ESM.zip › Figure 2 Part 5/Fig 2d polye atubulin part 2/WT PolyE-atub 8-14-24 R3 ET1.Project Maximum Z_XY1723841453_Z0_T0_C2.tif]

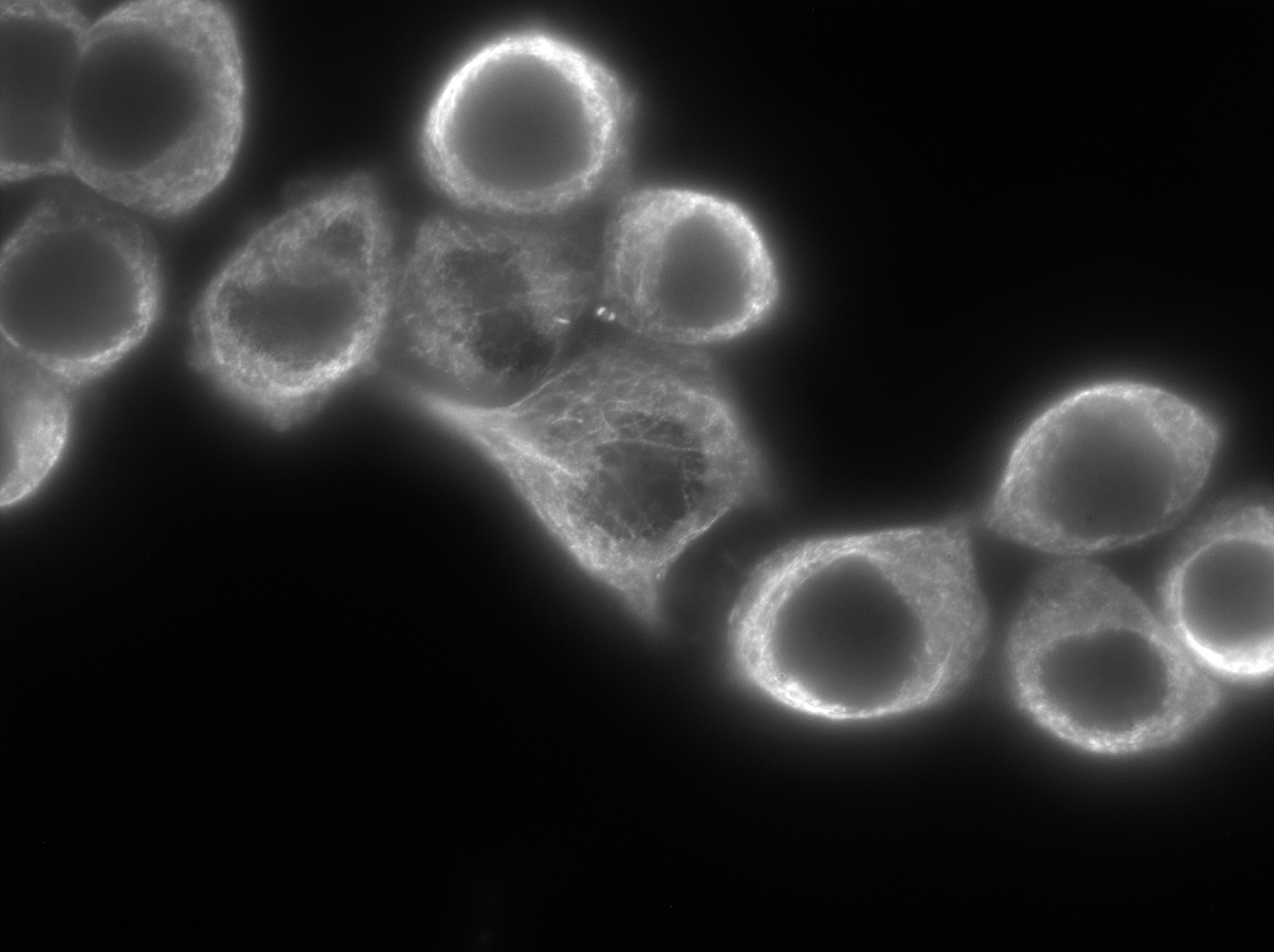

Supplement: Supplementary file 8 — Source data Fig. 2 part 5 [file 44319_2026_742_MOESM8_ESM.zip › Figure 2 Part 5/Fig 2d polye atubulin part 2/WT PolyE-atub 8-14-24 R3 PA8.Project Maximum Z_XY1723843244_Z0_T0_C1.tif]

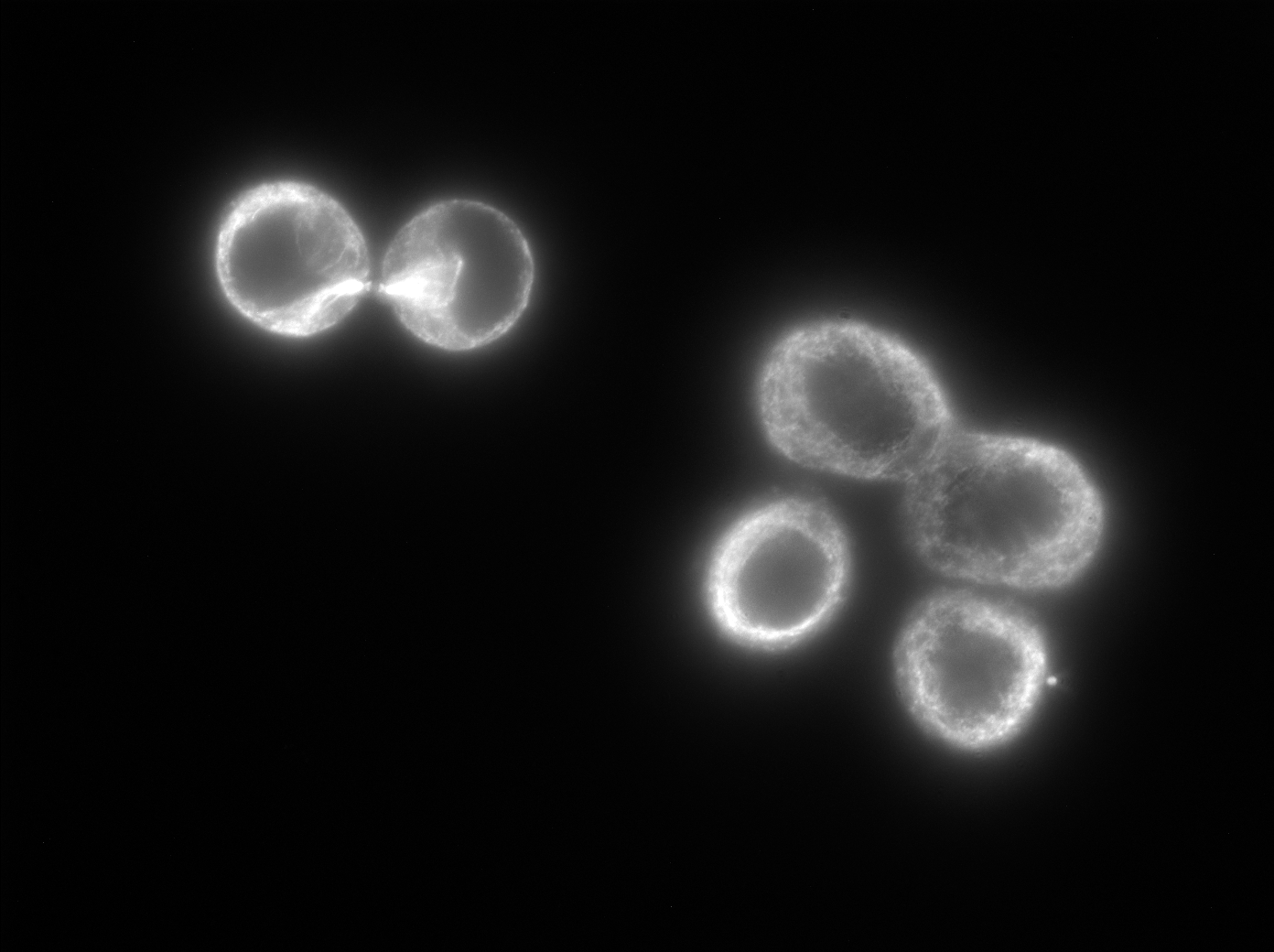

Supplement: Supplementary file 8 — Source data Fig. 2 part 5 [file 44319_2026_742_MOESM8_ESM.zip › Figure 2 Part 5/Fig 2d polye atubulin part 2/WT PolyE-atub 8-14-24 R3 ET2.Project Maximum Z_XY1723841789_Z0_T0_C1.tif]

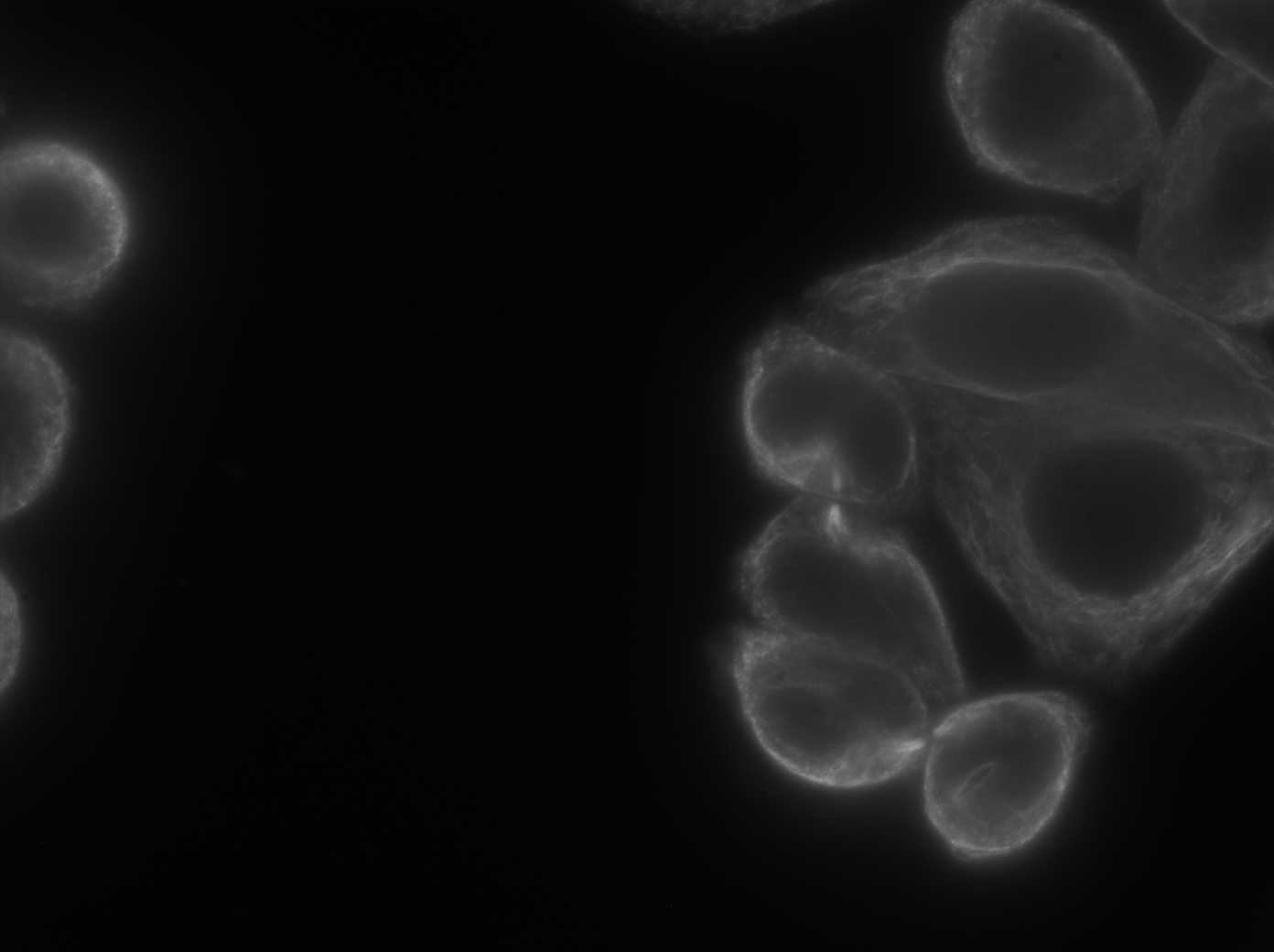

Supplement: Supplementary file 8 — Source data Fig. 2 part 5 [file 44319_2026_742_MOESM8_ESM.zip › Figure 2 Part 5/Fig 2d polye atubulin part 2/WT PolyE-atub 8-14-24 R2 LT5LT6.Project Maximum Z_XY1723835311_Z0_T0_C1.tif]

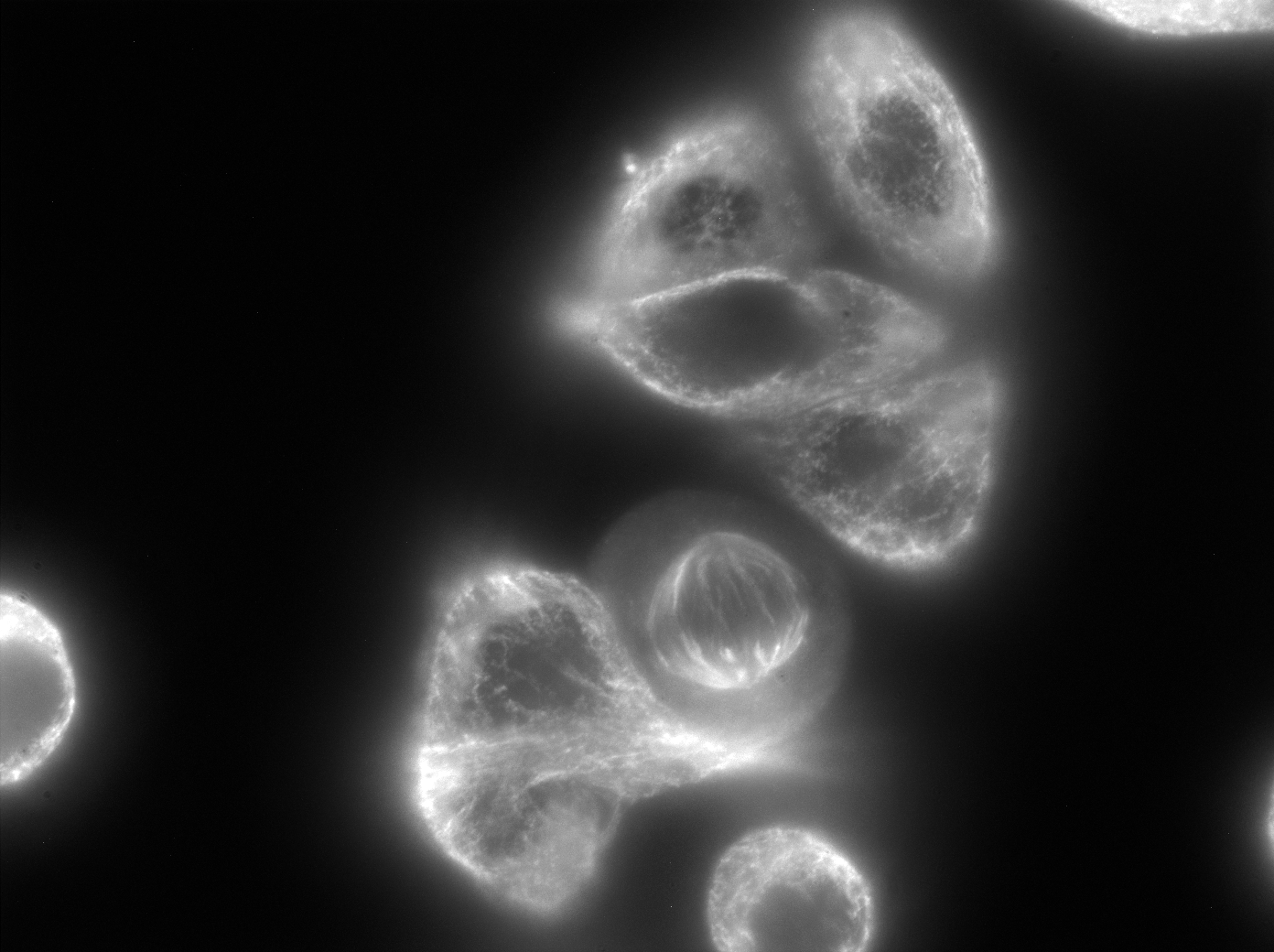

Supplement: Supplementary file 8 — Source data Fig. 2 part 5 [file 44319_2026_742_MOESM8_ESM.zip › Figure 2 Part 5/Fig 2d polye atubulin part 2/WT PolyE-atub 8-14-24 R3 M5.Project Maximum Z_XY1723844950_Z0_T0_C1.tif]

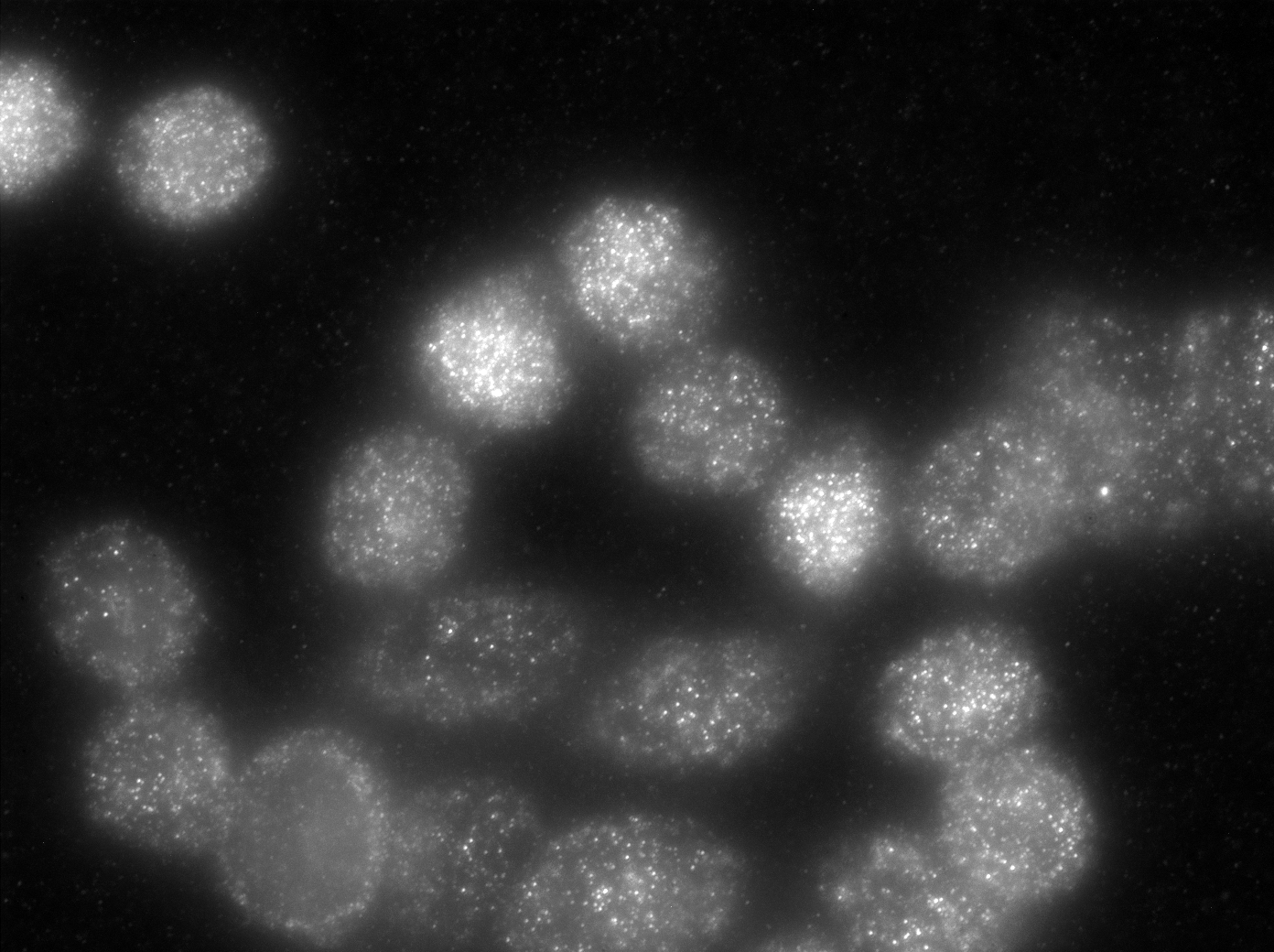

Supplement: Supplementary file 8 — Source data Fig. 2 part 5 [file 44319_2026_742_MOESM8_ESM.zip › Figure 2 Part 5/Fig 2d polye atubulin part 2/WT PolyE-atub 8-14-24 R3 LT2 PA3.Project Maximum Z_XY1723841895_Z0_T0_C2.tif]

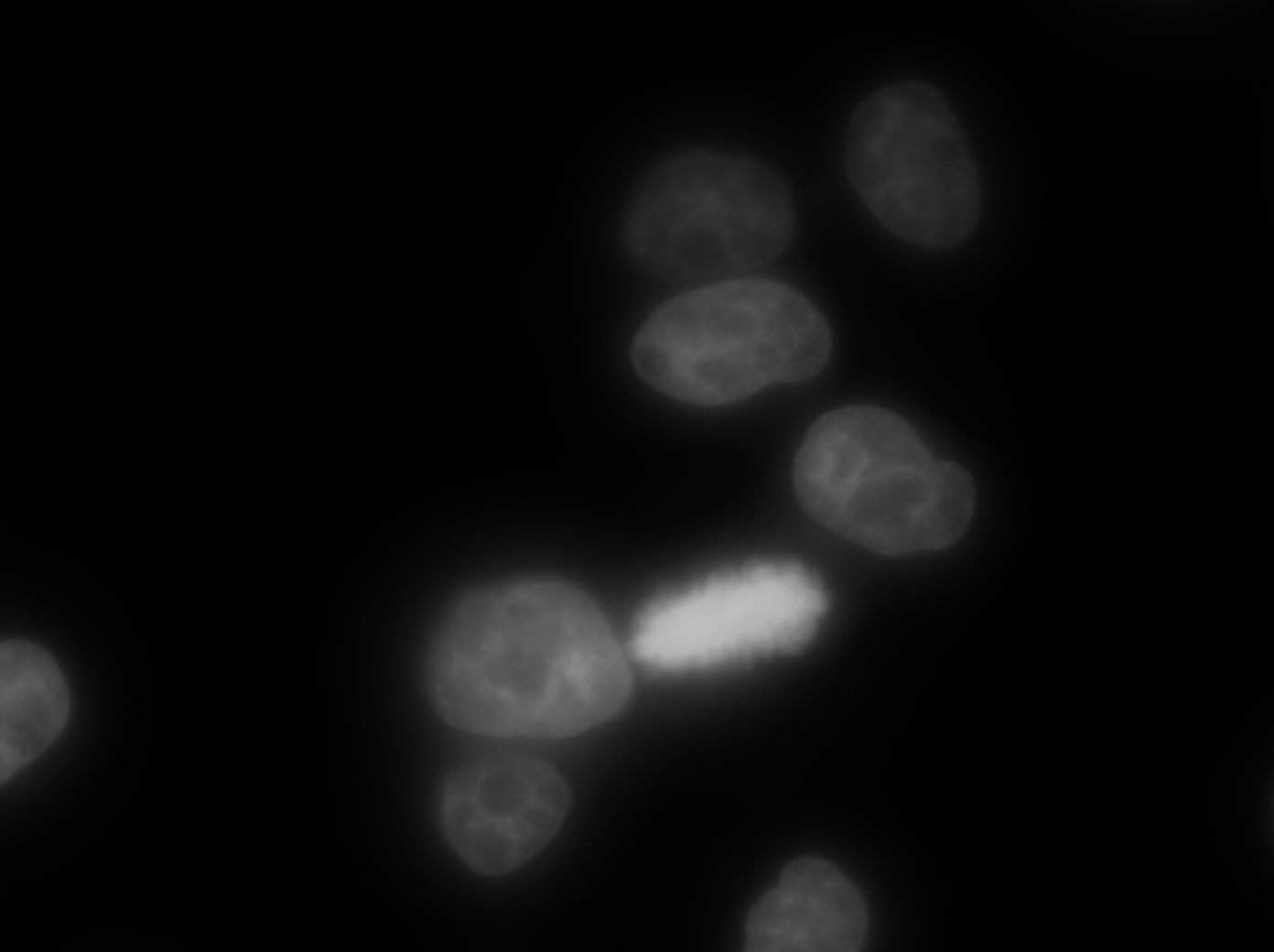

Supplement: Supplementary file 8 — Source data Fig. 2 part 5 [file 44319_2026_742_MOESM8_ESM.zip › Figure 2 Part 5/Fig 2d polye atubulin part 2/WT PolyE-atub 8-14-24 R3 M5.Project Maximum Z_XY1723844950_Z0_T0_C0.tif]

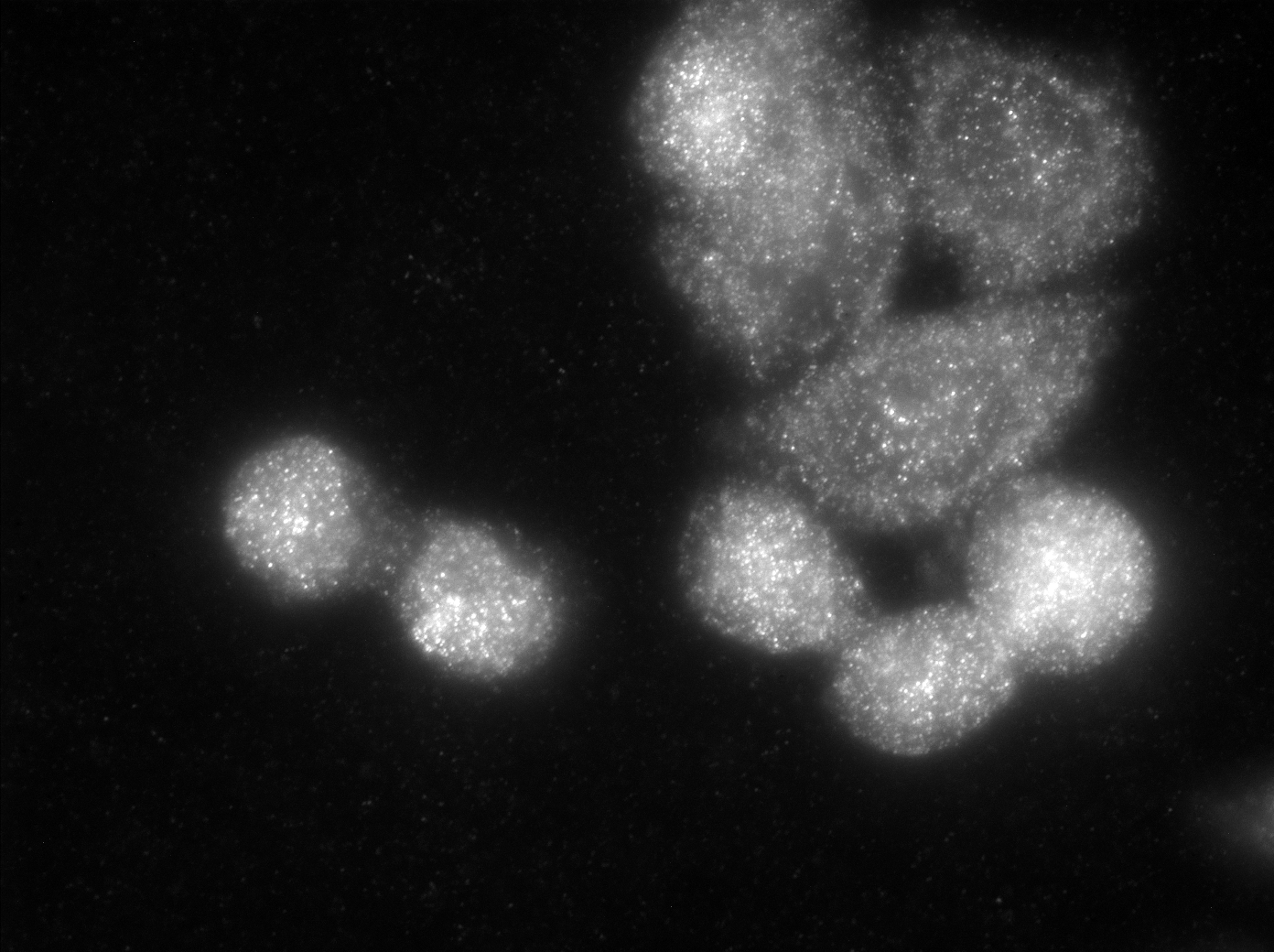

Supplement: Supplementary file 8 — Source data Fig. 2 part 5 [file 44319_2026_742_MOESM8_ESM.zip › Figure 2 Part 5/Fig 2d polye atubulin part 2/WT PolyE-atub 8-14-24 R3 LT8.Project Maximum Z_XY1723843728_Z0_T0_C2.tif]

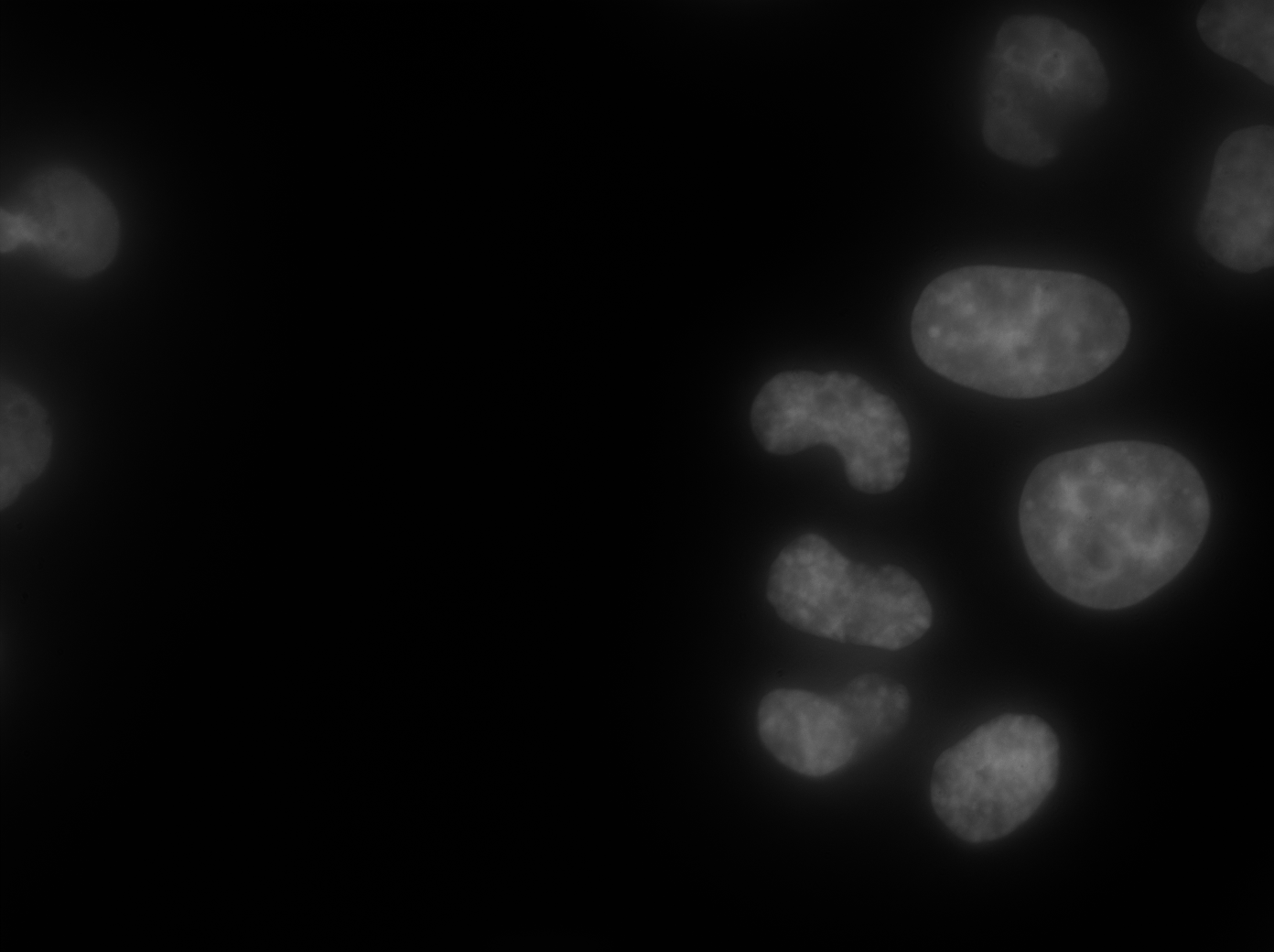

Supplement: Supplementary file 8 — Source data Fig. 2 part 5 [file 44319_2026_742_MOESM8_ESM.zip › Figure 2 Part 5/Fig 2d polye atubulin part 2/WT PolyE-atub 8-14-24 R2 LT5LT6.Project Maximum Z_XY1723835311_Z0_T0_C0.tif]

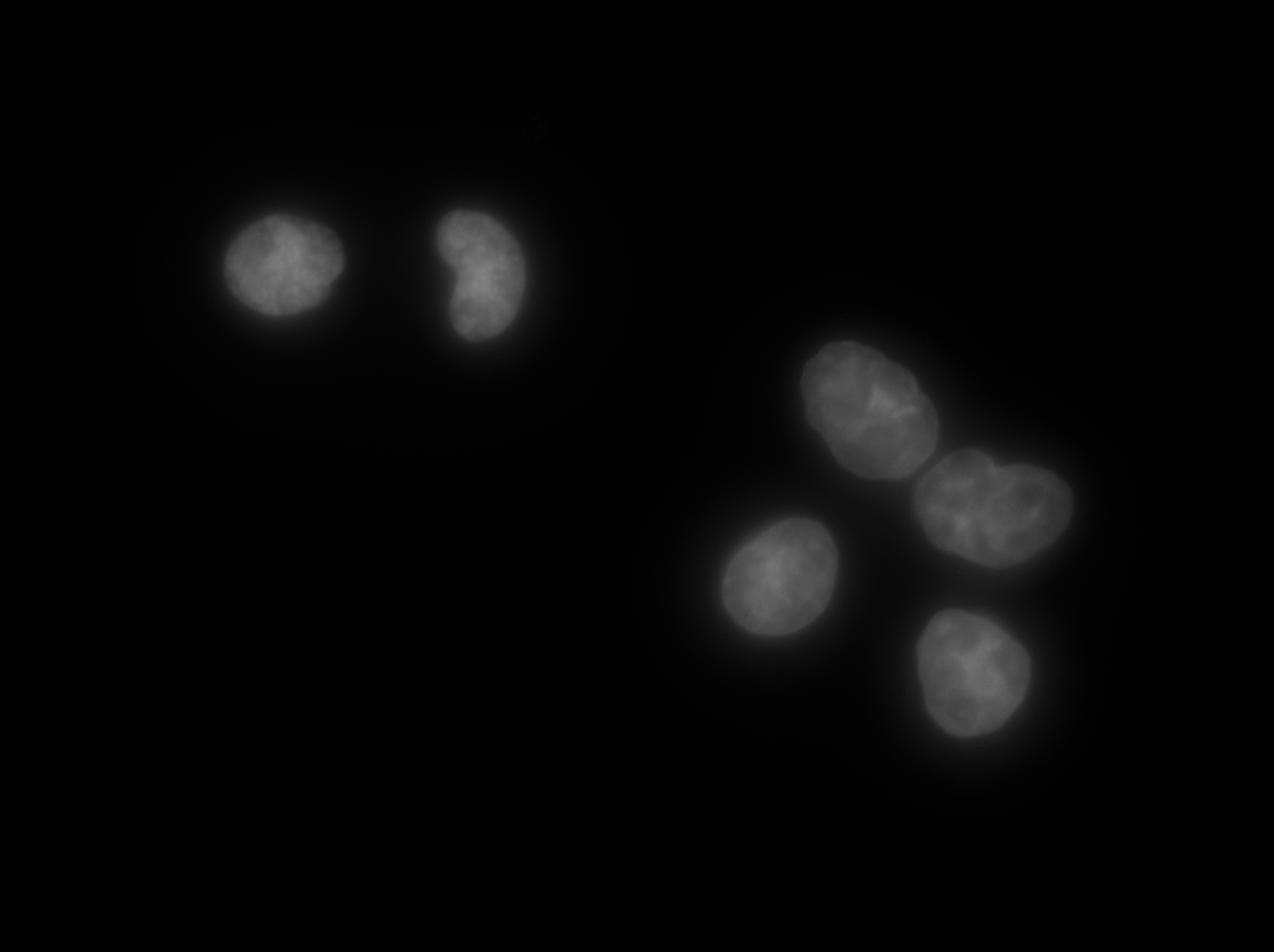

Supplement: Supplementary file 8 — Source data Fig. 2 part 5 [file 44319_2026_742_MOESM8_ESM.zip › Figure 2 Part 5/Fig 2d polye atubulin part 2/WT PolyE-atub 8-14-24 R3 ET2.Project Maximum Z_XY1723841789_Z0_T0_C0.tif]

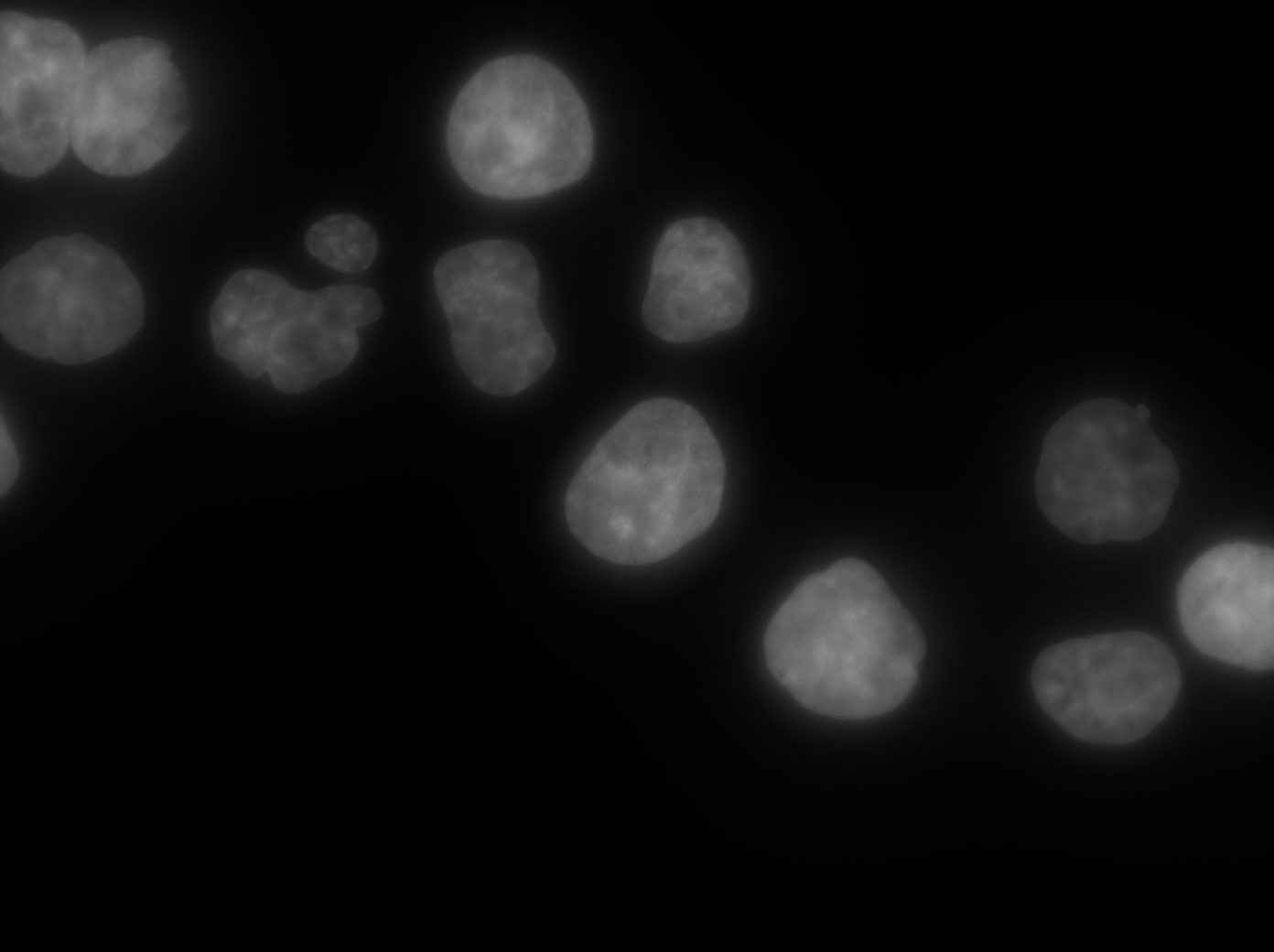

Supplement: Supplementary file 8 — Source data Fig. 2 part 5 [file 44319_2026_742_MOESM8_ESM.zip › Figure 2 Part 5/Fig 2d polye atubulin part 2/WT PolyE-atub 8-14-24 R3 PA8.Project Maximum Z_XY1723843244_Z0_T0_C0.tif]

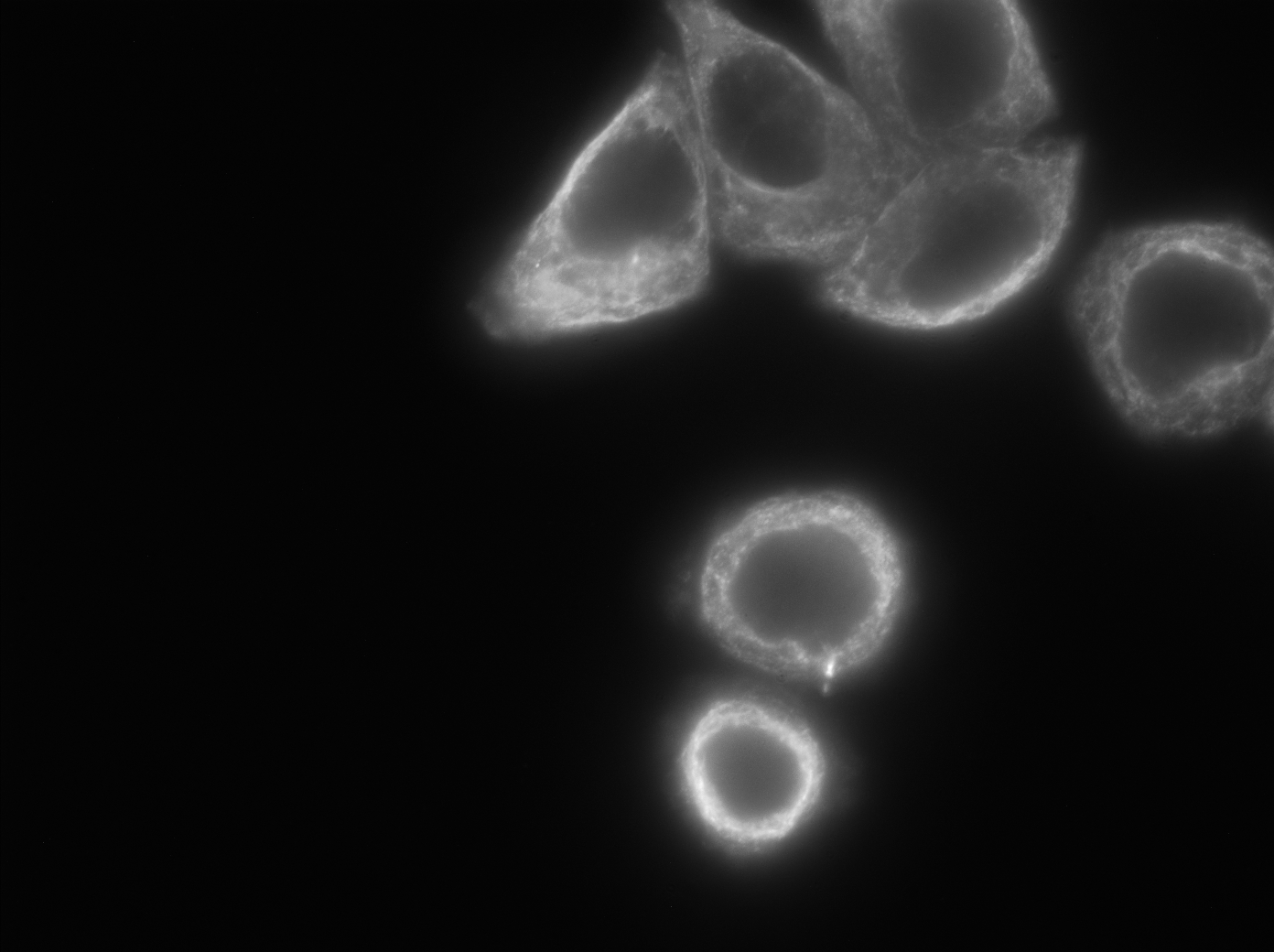

Supplement: Supplementary file 8 — Source data Fig. 2 part 5 [file 44319_2026_742_MOESM8_ESM.zip › Figure 2 Part 5/Fig 2d polye atubulin part 2/WT PolyE-atub 8-14-24 R2 PA6.Project Maximum Z_XY1723836482_Z0_T0_C1.tif]

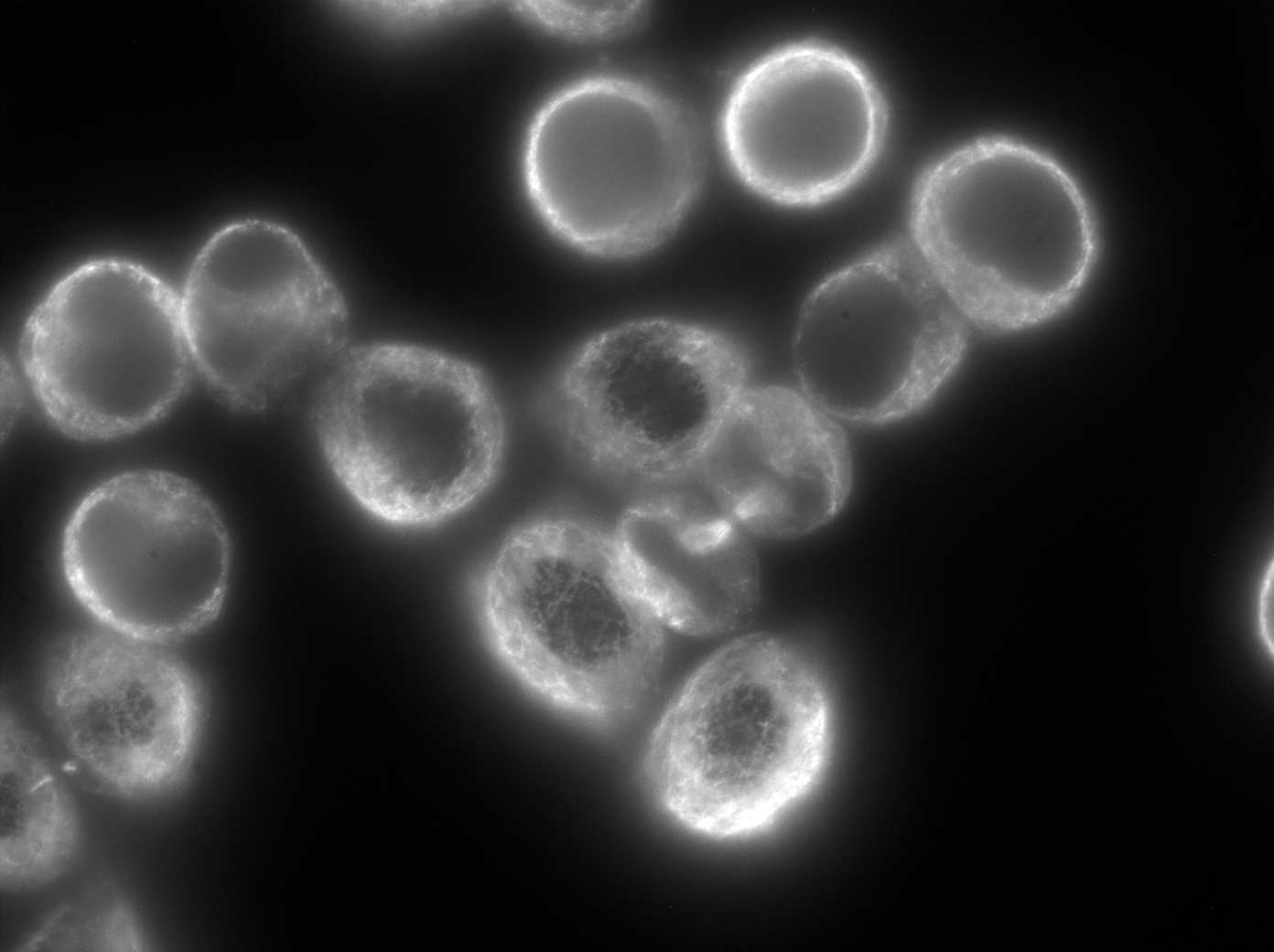

Supplement: Supplementary file 8 — Source data Fig. 2 part 5 [file 44319_2026_742_MOESM8_ESM.zip › Figure 2 Part 5/Fig 2d polye atubulin part 2/WT PolyE-atub 8-14-24 R3 ET5.Project Maximum Z_XY1723842447_Z0_T0_C1.tif]

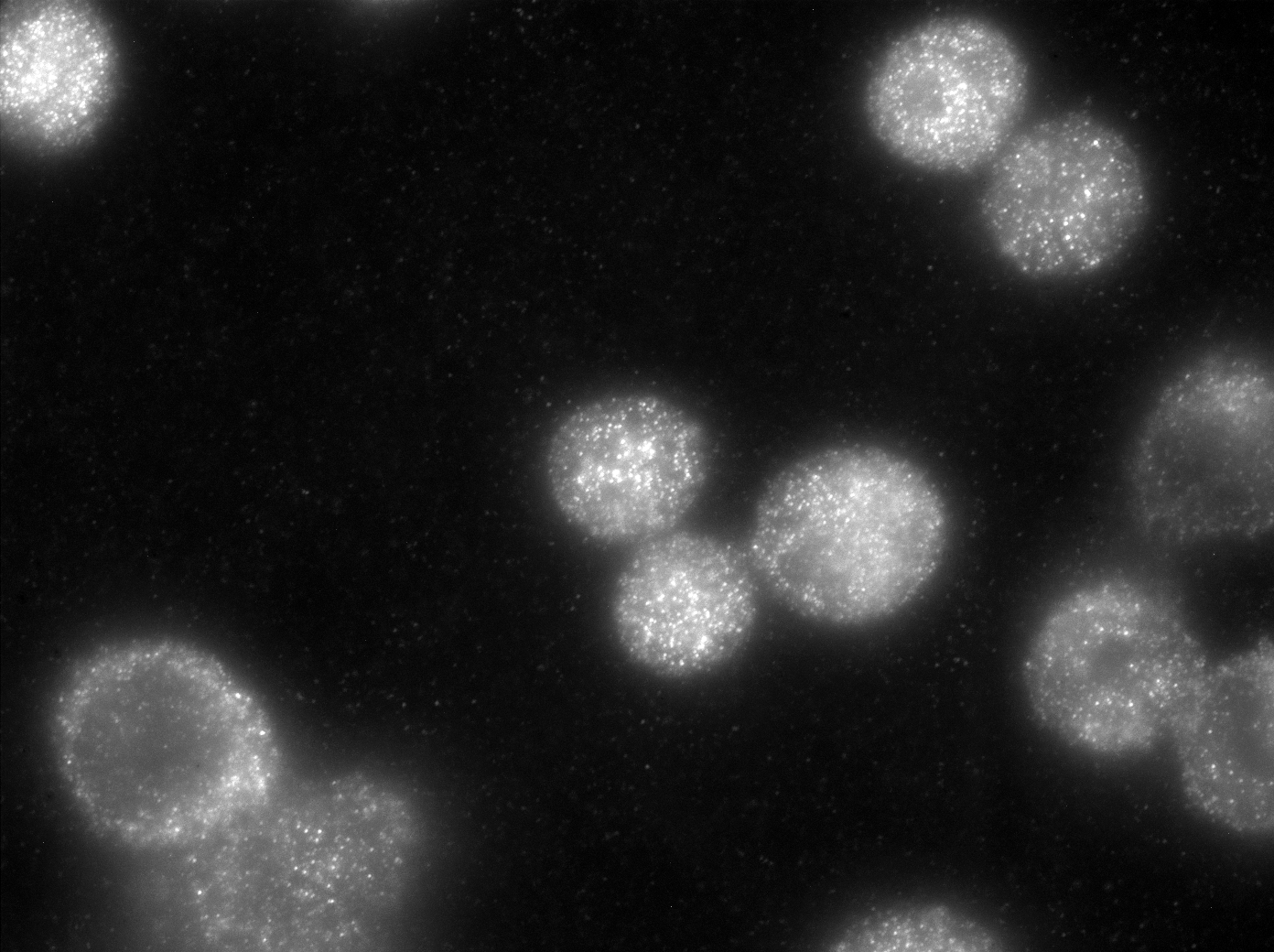

Supplement: Supplementary file 8 — Source data Fig. 2 part 5 [file 44319_2026_742_MOESM8_ESM.zip › Figure 2 Part 5/Fig 2d polye atubulin part 2/WT PolyE-atub 8-14-24 R3 ET9.Project Maximum Z_XY1723844323_Z0_T0_C2.tif]

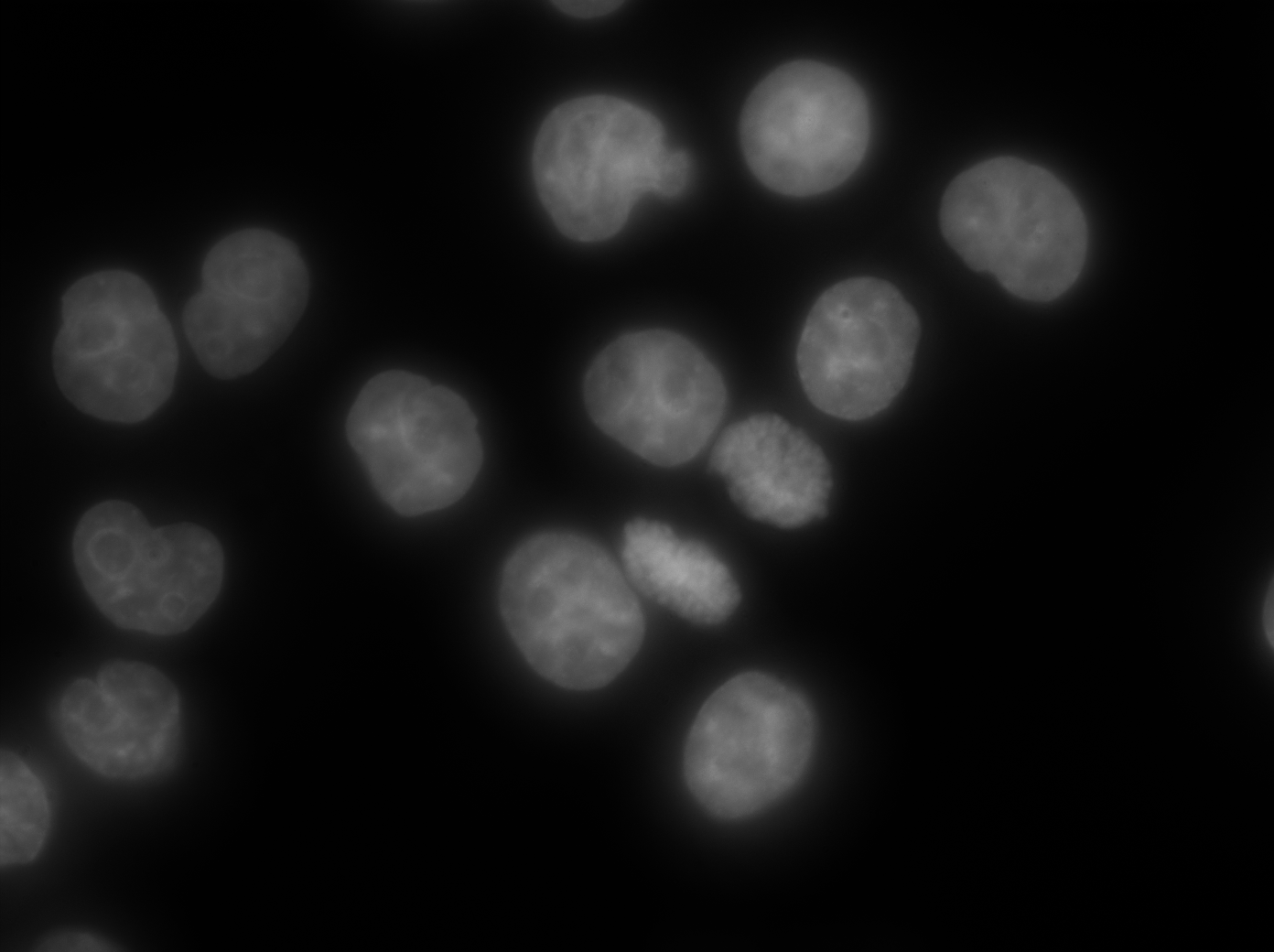

Supplement: Supplementary file 8 — Source data Fig. 2 part 5 [file 44319_2026_742_MOESM8_ESM.zip › Figure 2 Part 5/Fig 2d polye atubulin part 2/WT PolyE-atub 8-14-24 R3 ET5.Project Maximum Z_XY1723842447_Z0_T0_C0.tif]

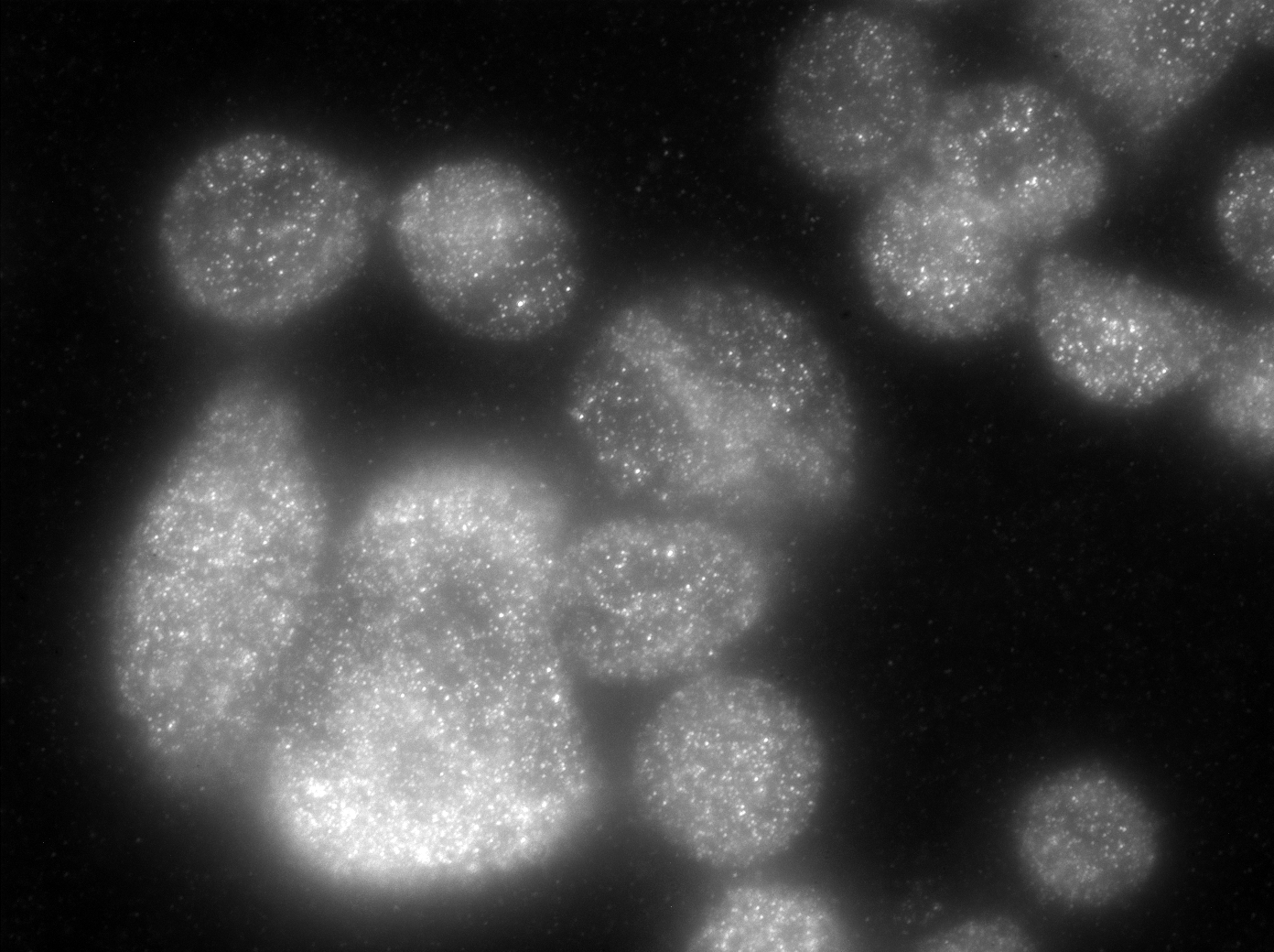

Supplement: Supplementary file 8 — Source data Fig. 2 part 5 [file 44319_2026_742_MOESM8_ESM.zip › Figure 2 Part 5/Fig 2d polye atubulin part 2/WT PolyE-atub 8-14-24 R3 LT4.Project Maximum Z_XY1723843164_Z0_T0_C2.tif]

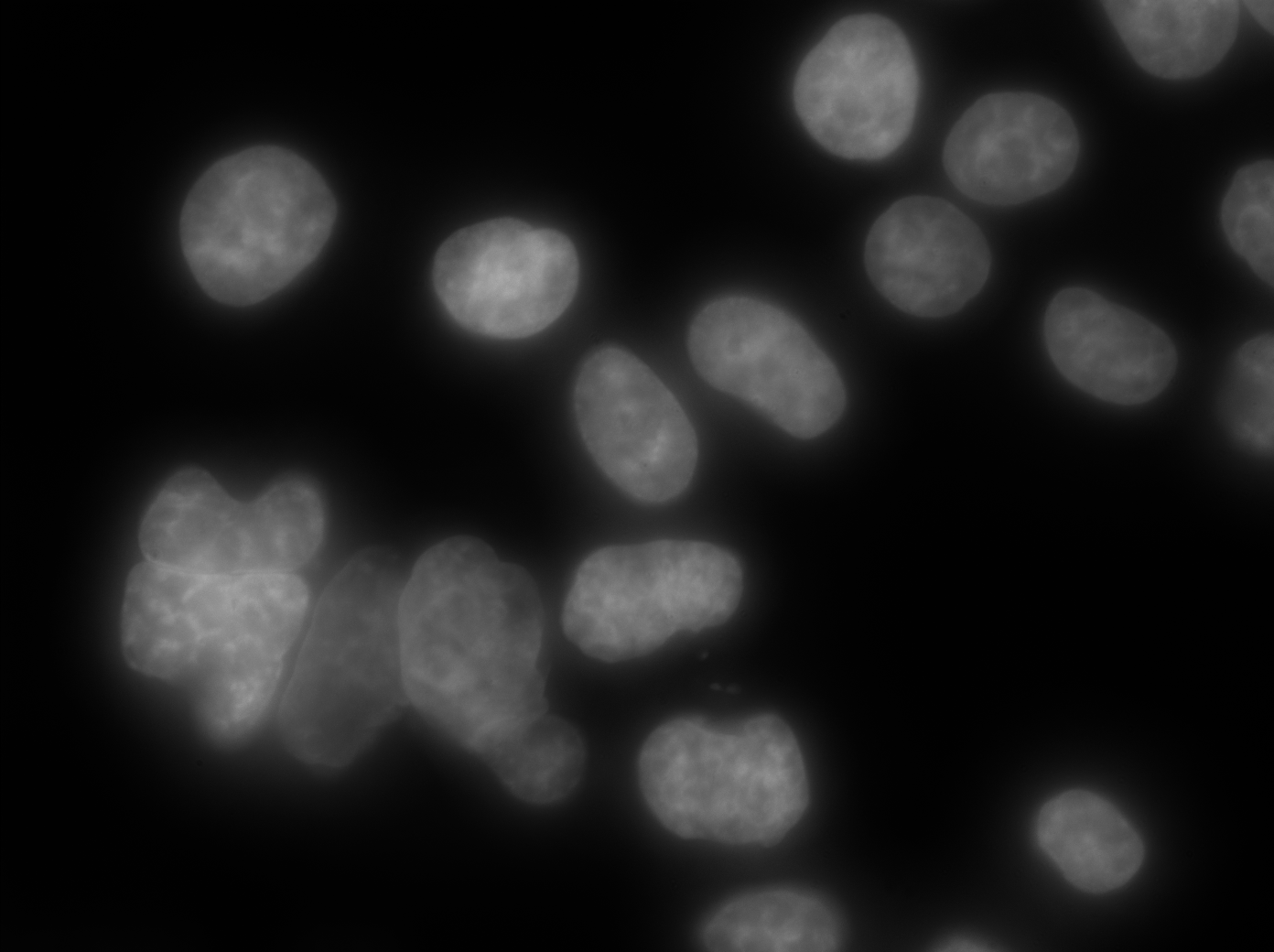

Supplement: Supplementary file 8 — Source data Fig. 2 part 5 [file 44319_2026_742_MOESM8_ESM.zip › Figure 2 Part 5/Fig 2d polye atubulin part 2/WT PolyE-atub 8-14-24 R3 LT4.Project Maximum Z_XY1723843164_Z0_T0_C0.tif]

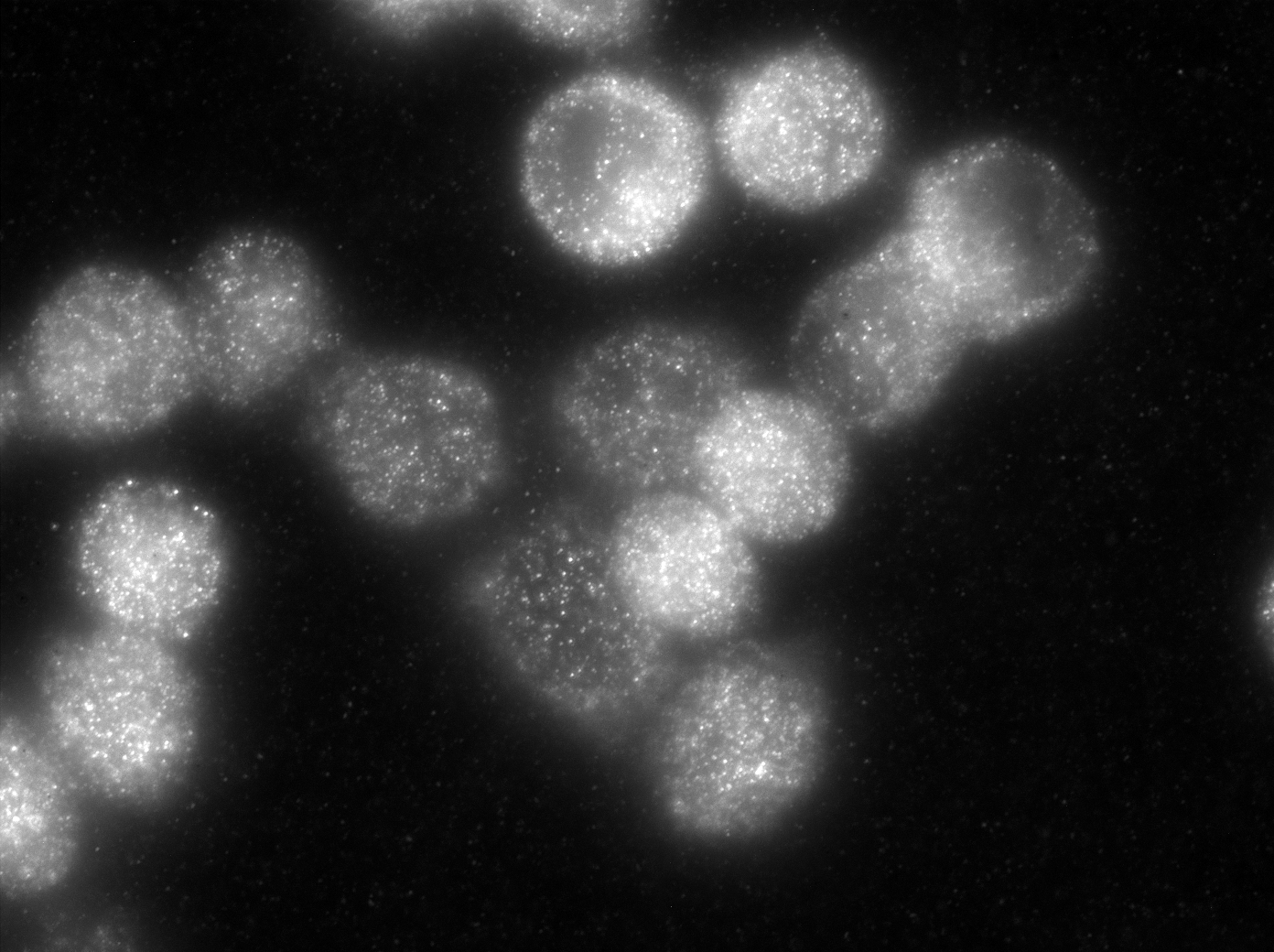

Supplement: Supplementary file 8 — Source data Fig. 2 part 5 [file 44319_2026_742_MOESM8_ESM.zip › Figure 2 Part 5/Fig 2d polye atubulin part 2/WT PolyE-atub 8-14-24 R3 ET5.Project Maximum Z_XY1723842447_Z0_T0_C2.tif]

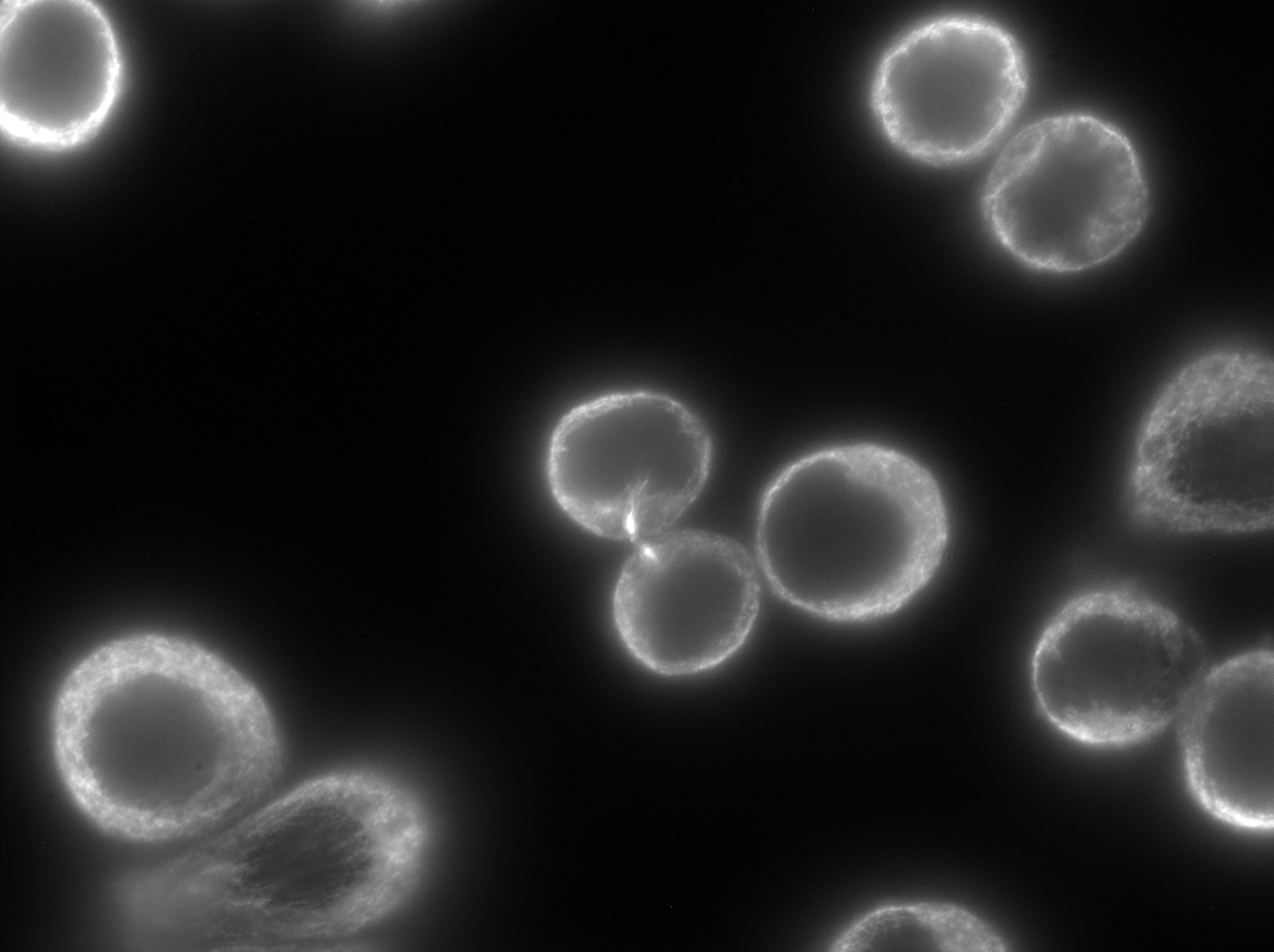

Supplement: Supplementary file 8 — Source data Fig. 2 part 5 [file 44319_2026_742_MOESM8_ESM.zip › Figure 2 Part 5/Fig 2d polye atubulin part 2/WT PolyE-atub 8-14-24 R3 ET9.Project Maximum Z_XY1723844323_Z0_T0_C1.tif]

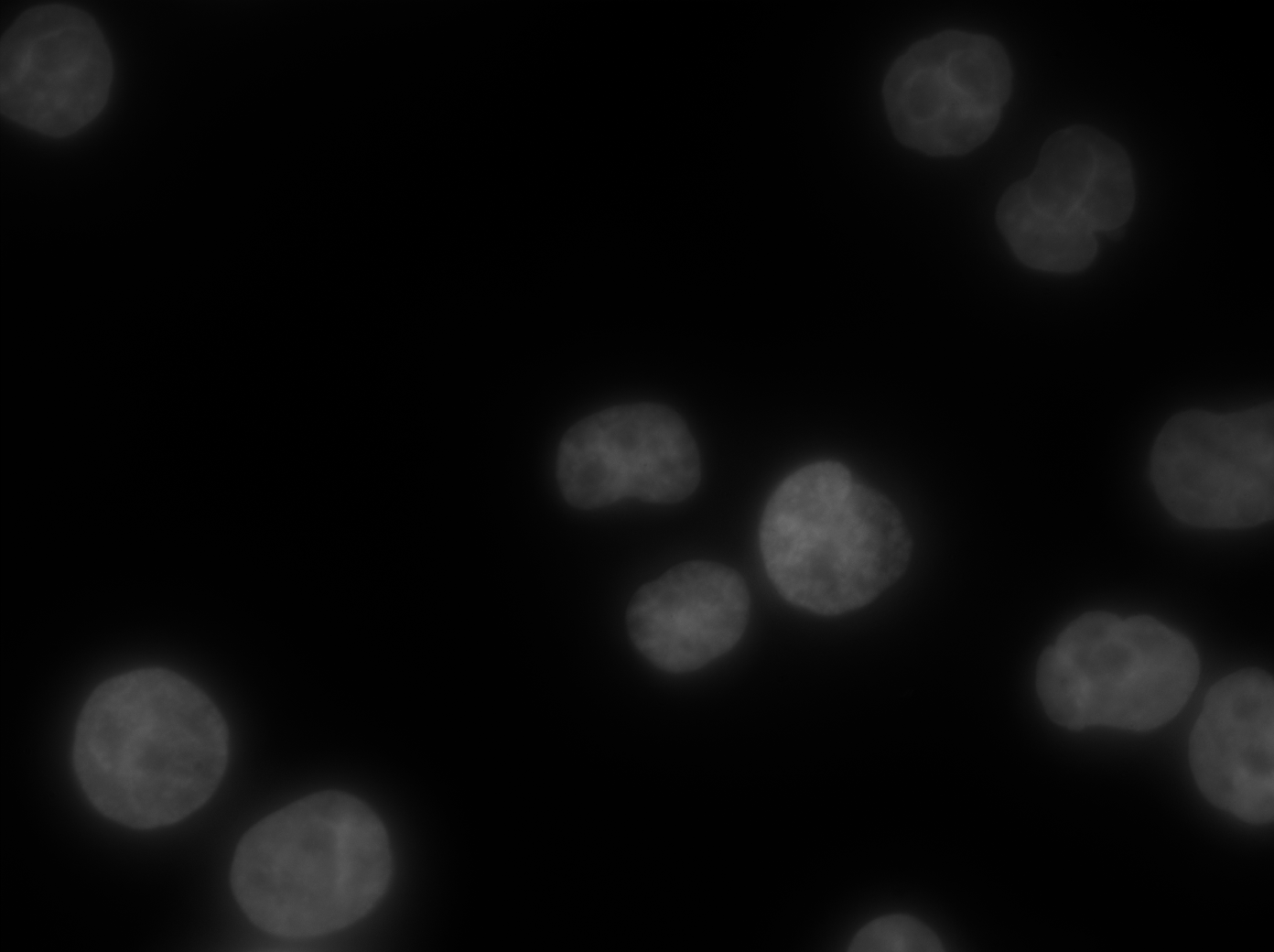

Supplement: Supplementary file 8 — Source data Fig. 2 part 5 [file 44319_2026_742_MOESM8_ESM.zip › Figure 2 Part 5/Fig 2d polye atubulin part 2/WT PolyE-atub 8-14-24 R3 ET9.Project Maximum Z_XY1723844323_Z0_T0_C0.tif]

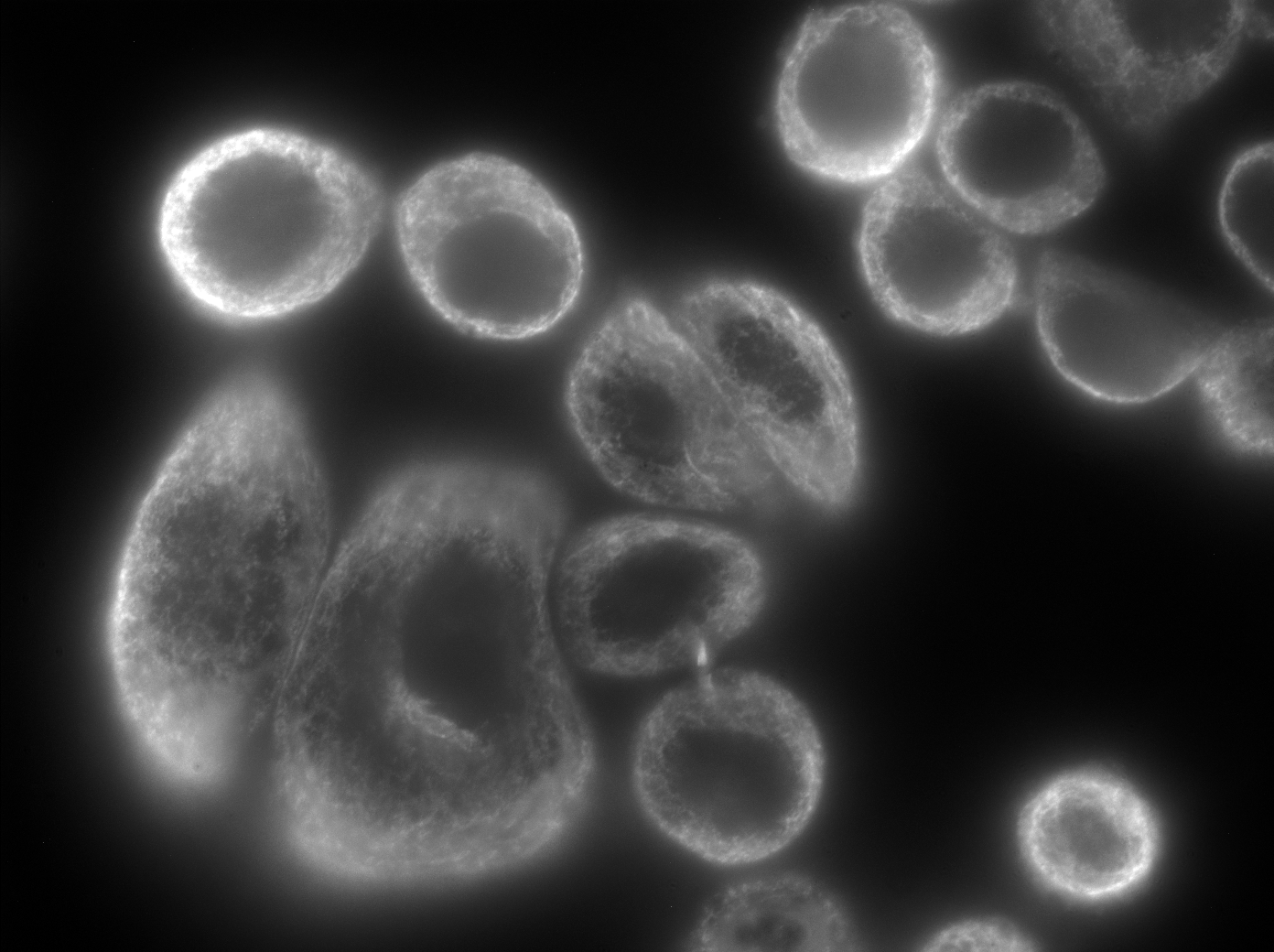

Supplement: Supplementary file 8 — Source data Fig. 2 part 5 [file 44319_2026_742_MOESM8_ESM.zip › Figure 2 Part 5/Fig 2d polye atubulin part 2/WT PolyE-atub 8-14-24 R3 LT4.Project Maximum Z_XY1723843164_Z0_T0_C1.tif]

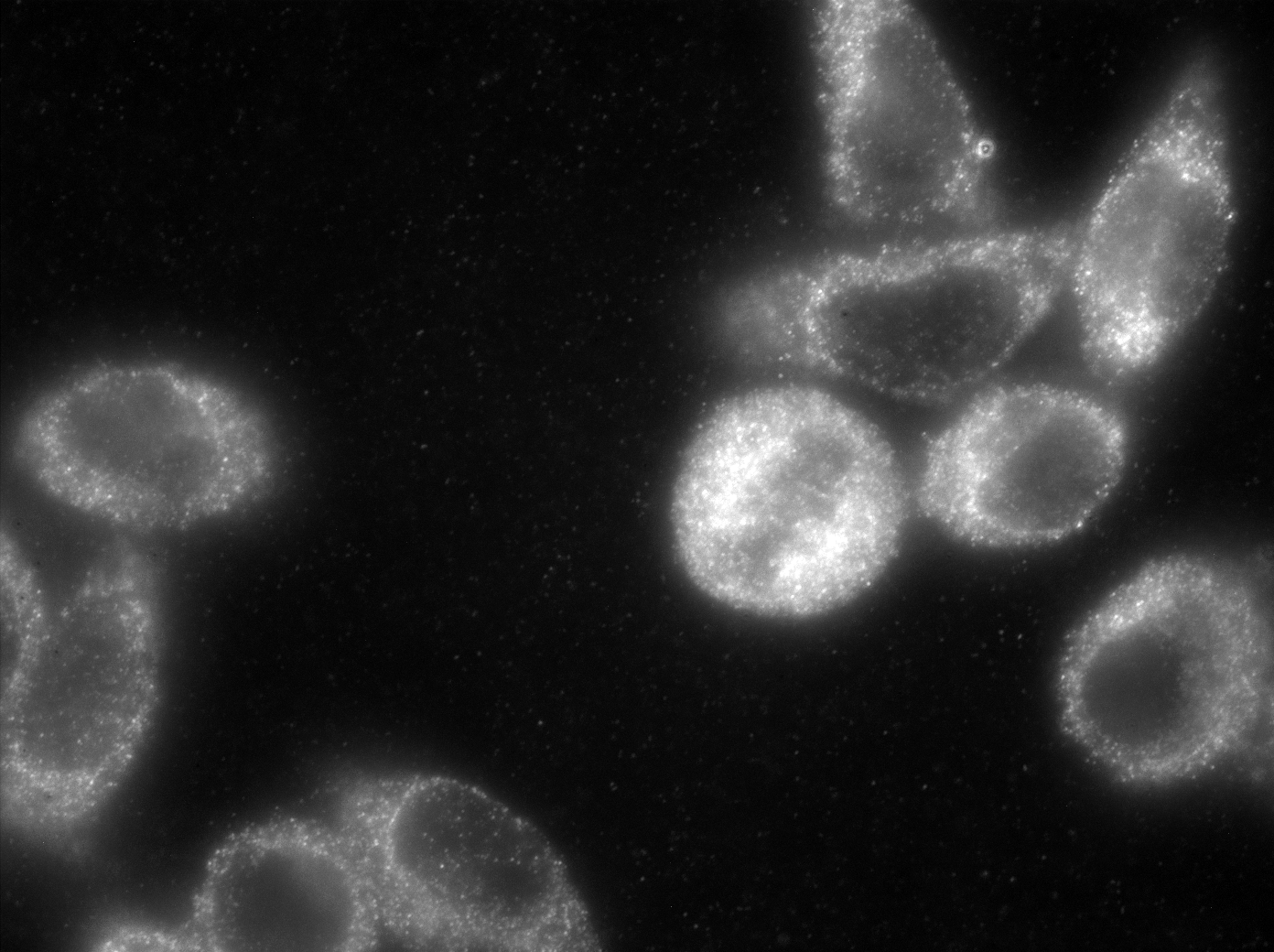

Supplement: Supplementary file 8 — Source data Fig. 2 part 5 [file 44319_2026_742_MOESM8_ESM.zip › Figure 2 Part 5/Fig 2d polye atubulin part 2/WT PolyE-atub 8-14-24 R2 M8.Project Maximum Z_XY1723838633_Z0_T0_C2.tif]

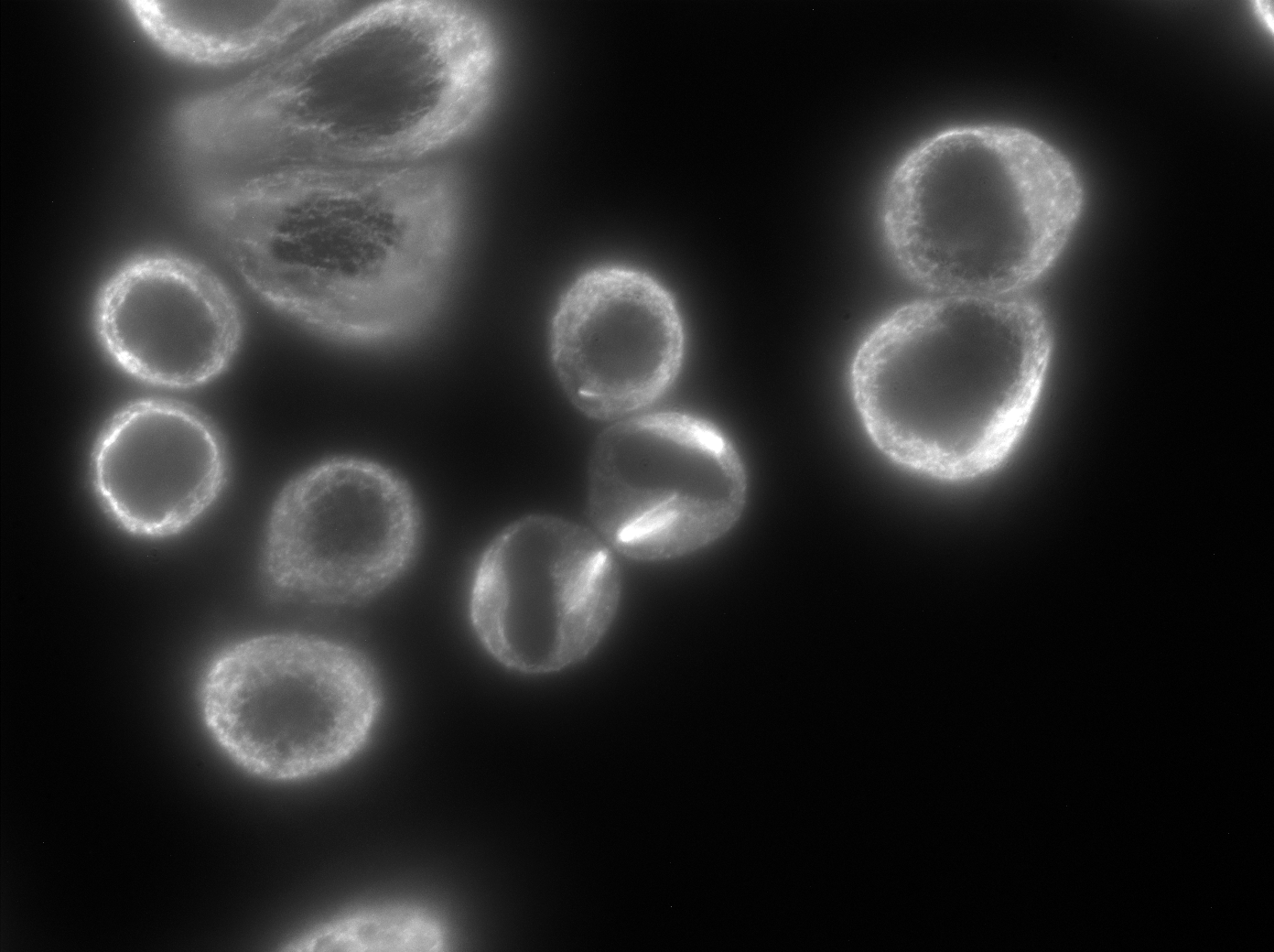

Supplement: Supplementary file 8 — Source data Fig. 2 part 5 [file 44319_2026_742_MOESM8_ESM.zip › Figure 2 Part 5/Fig 2d polye atubulin part 2/WT PolyE-atub 8-14-24 R3 ET8.Project Maximum Z_XY1723844245_Z0_T0_C1.tif]

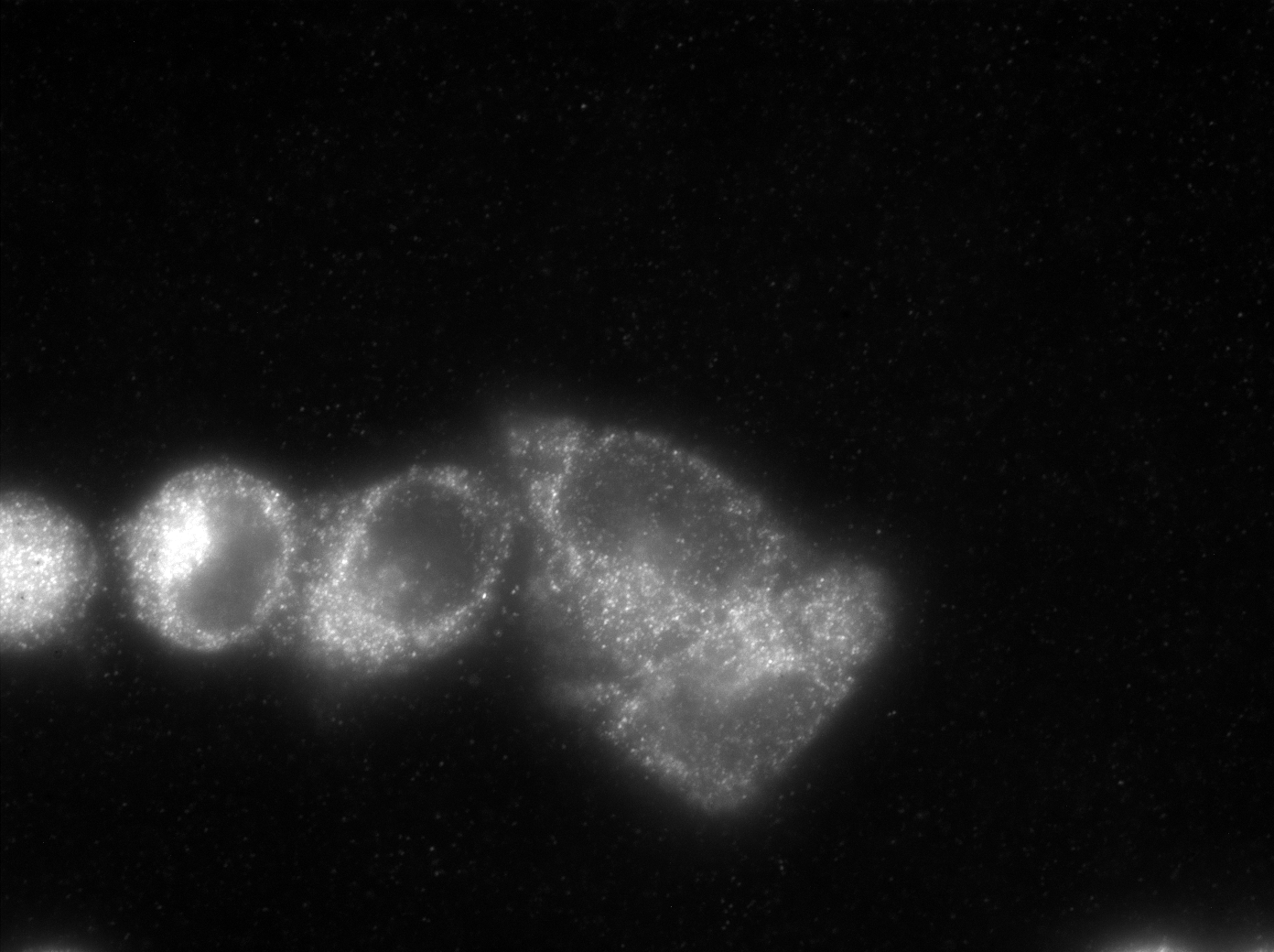

Supplement: Supplementary file 8 — Source data Fig. 2 part 5 [file 44319_2026_742_MOESM8_ESM.zip › Figure 2 Part 5/Fig 2d polye atubulin part 2/WT PolyE-atub 8-14-24 R2 PA4.Project Maximum Z_XY1723835053_Z0_T0_C2.tif]

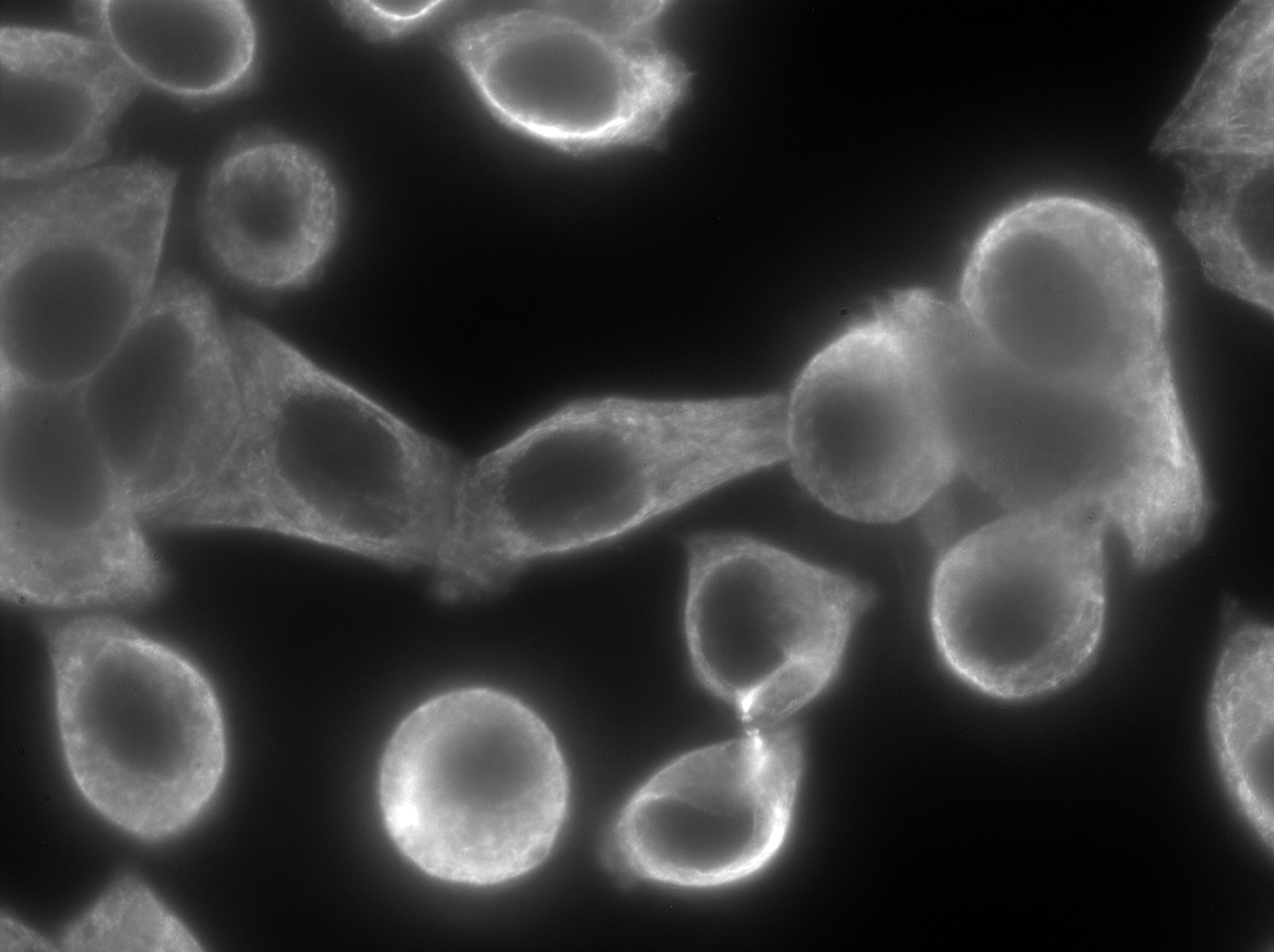

Supplement: Supplementary file 8 — Source data Fig. 2 part 5 [file 44319_2026_742_MOESM8_ESM.zip › Figure 2 Part 5/Fig 2d polye atubulin part 2/WT PolyE-atub 8-14-24 R3 LT10.Project Maximum Z_XY1723844042_Z0_T0_C1.tif]

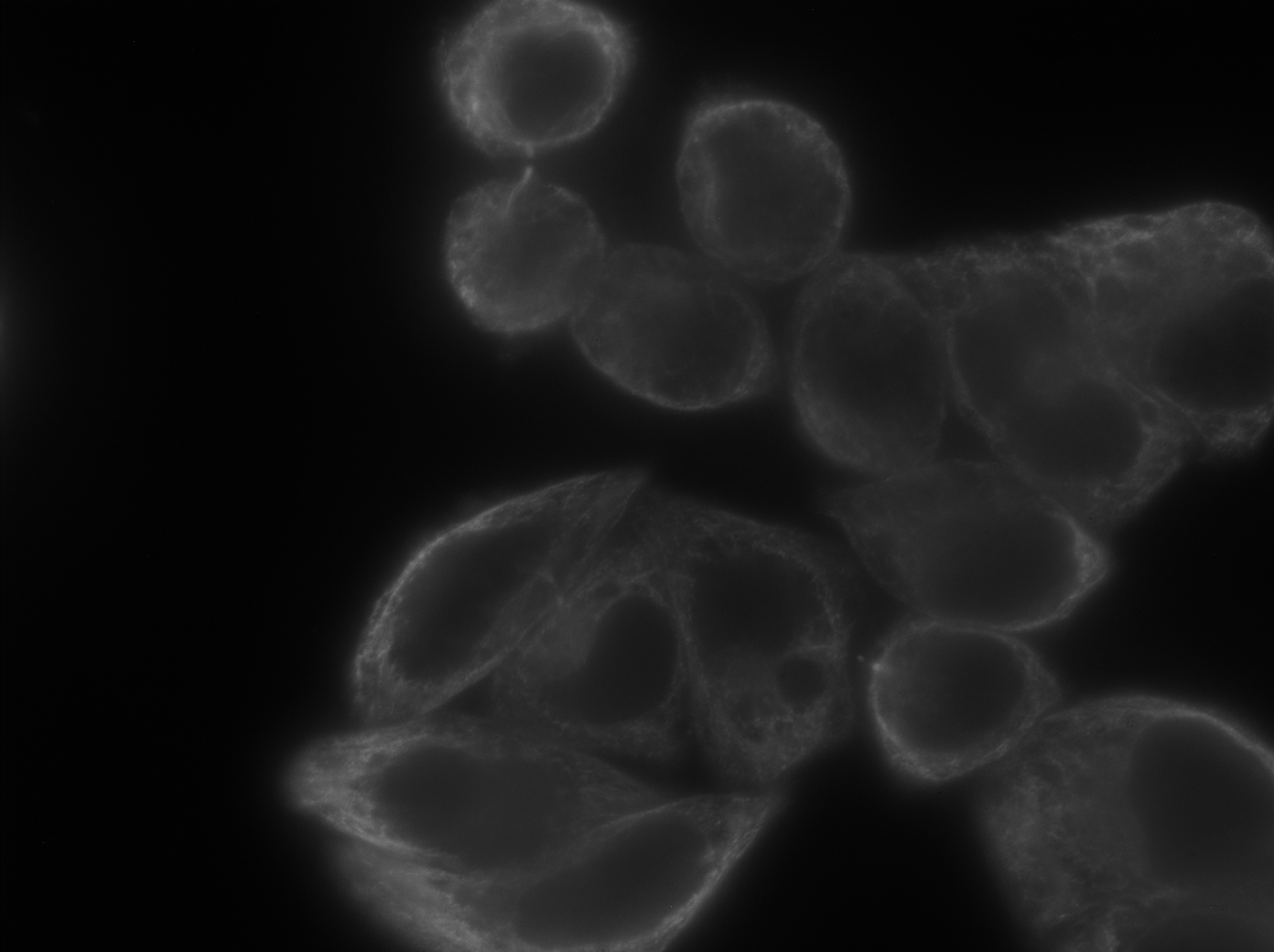

Supplement: Supplementary file 8 — Source data Fig. 2 part 5 [file 44319_2026_742_MOESM8_ESM.zip › Figure 2 Part 5/Fig 2d polye atubulin part 2/WT PolyE-atub 8-14-24 R2 PA10.Project Maximum Z_XY1723838433_Z0_T0_C1.tif]

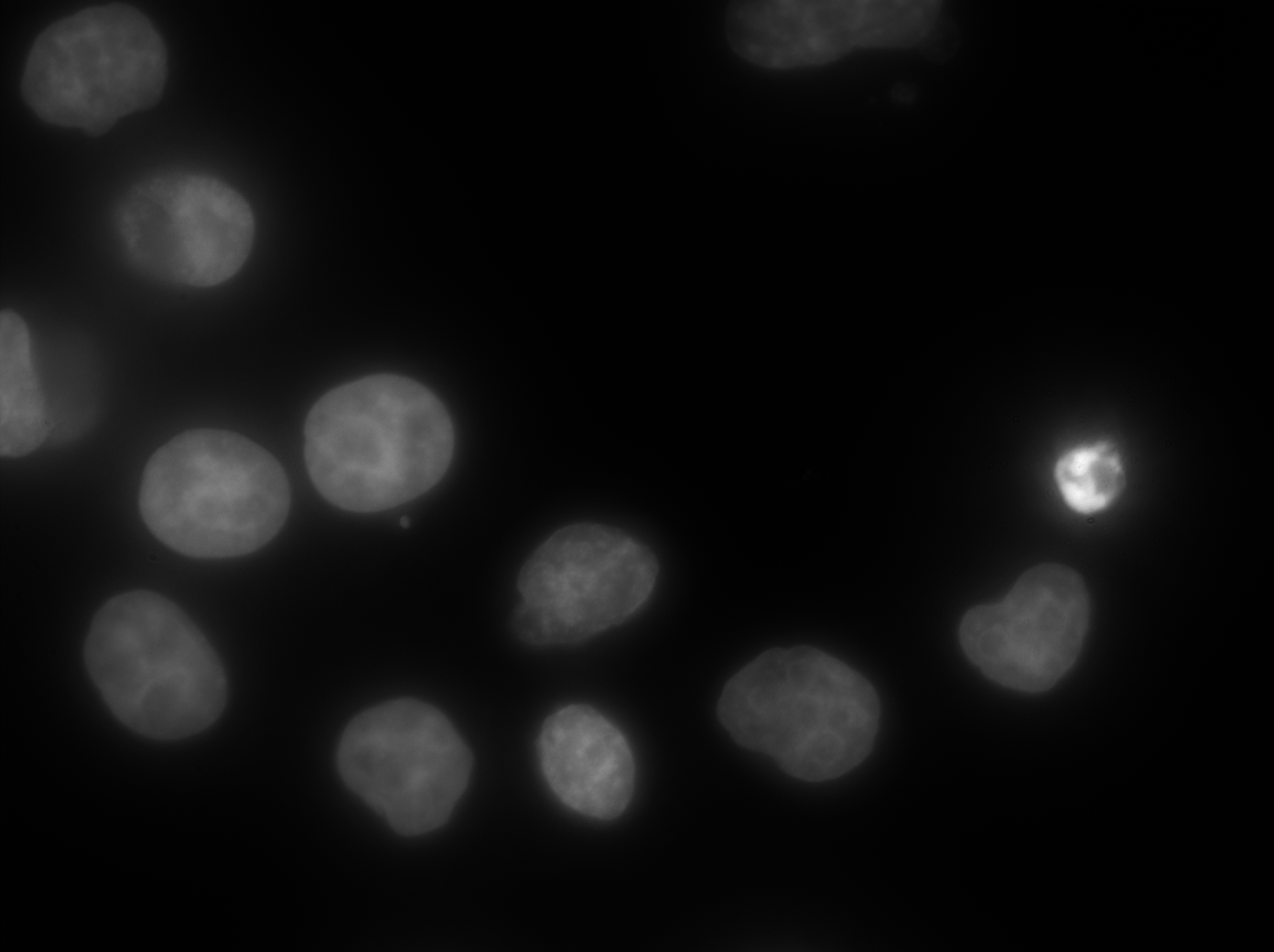

Supplement: Supplementary file 8 — Source data Fig. 2 part 5 [file 44319_2026_742_MOESM8_ESM.zip › Figure 2 Part 5/Fig 2d polye atubulin part 2/WT PolyE-atub 8-14-24 R2 LT7LT8.Project Maximum Z_XY1723836343_Z0_T0_C0.tif]

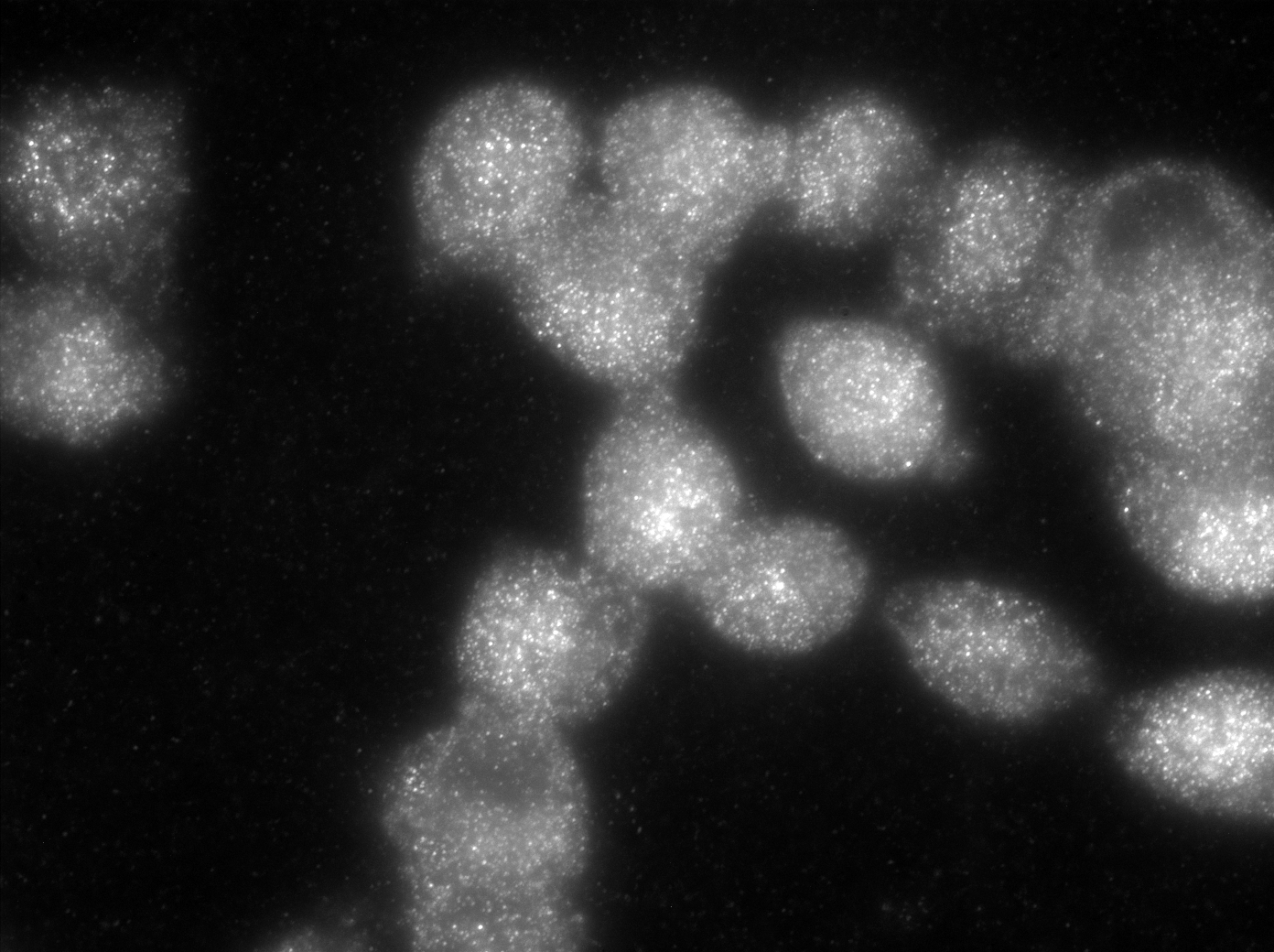

Supplement: Supplementary file 8 — Source data Fig. 2 part 5 [file 44319_2026_742_MOESM8_ESM.zip › Figure 2 Part 5/Fig 2d polye atubulin part 2/WT PolyE-atub 8-14-24 R3 PA10.Project Maximum Z_XY1723845723_Z0_T0_C2.tif]

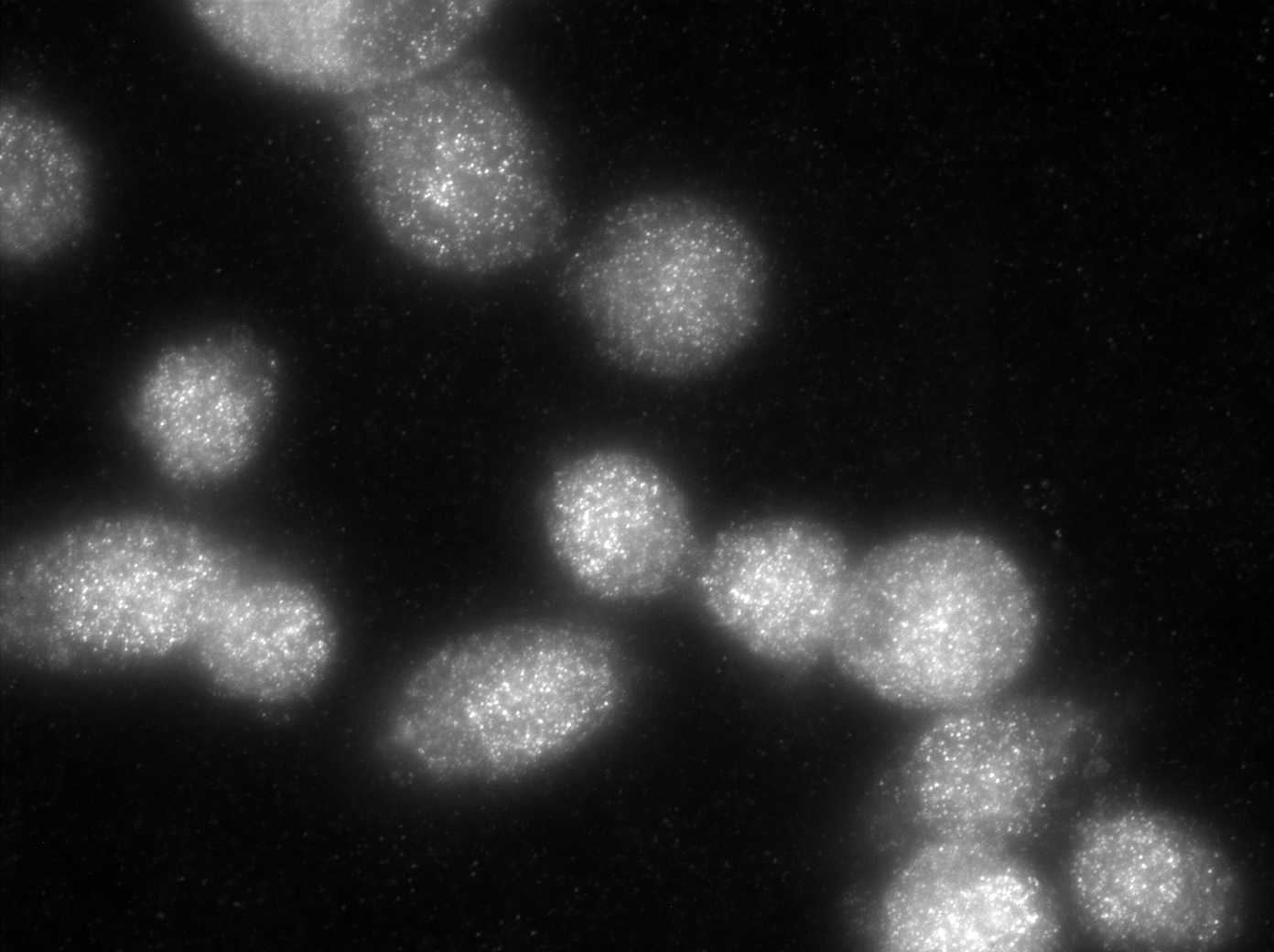

Supplement: Supplementary file 8 — Source data Fig. 2 part 5 [file 44319_2026_742_MOESM8_ESM.zip › Figure 2 Part 5/Fig 2d polye atubulin part 2/WT PolyE-atub 8-14-24 R3 LT5 M4.Project Maximum Z_XY1723843361_Z0_T0_C2.tif]

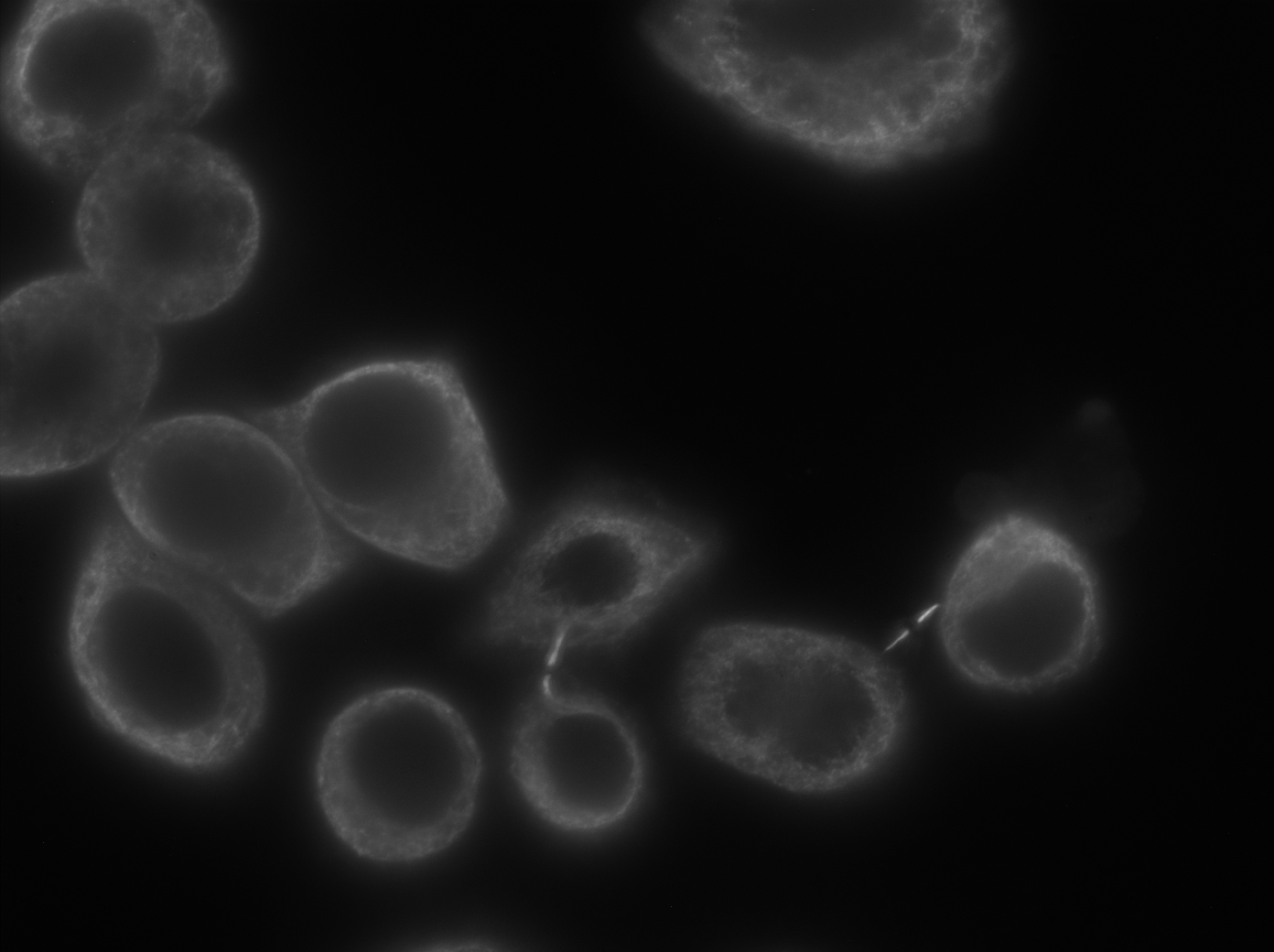

Supplement: Supplementary file 8 — Source data Fig. 2 part 5 [file 44319_2026_742_MOESM8_ESM.zip › Figure 2 Part 5/Fig 2d polye atubulin part 2/WT PolyE-atub 8-14-24 R2 LT7LT8.Project Maximum Z_XY1723836343_Z0_T0_C1.tif]

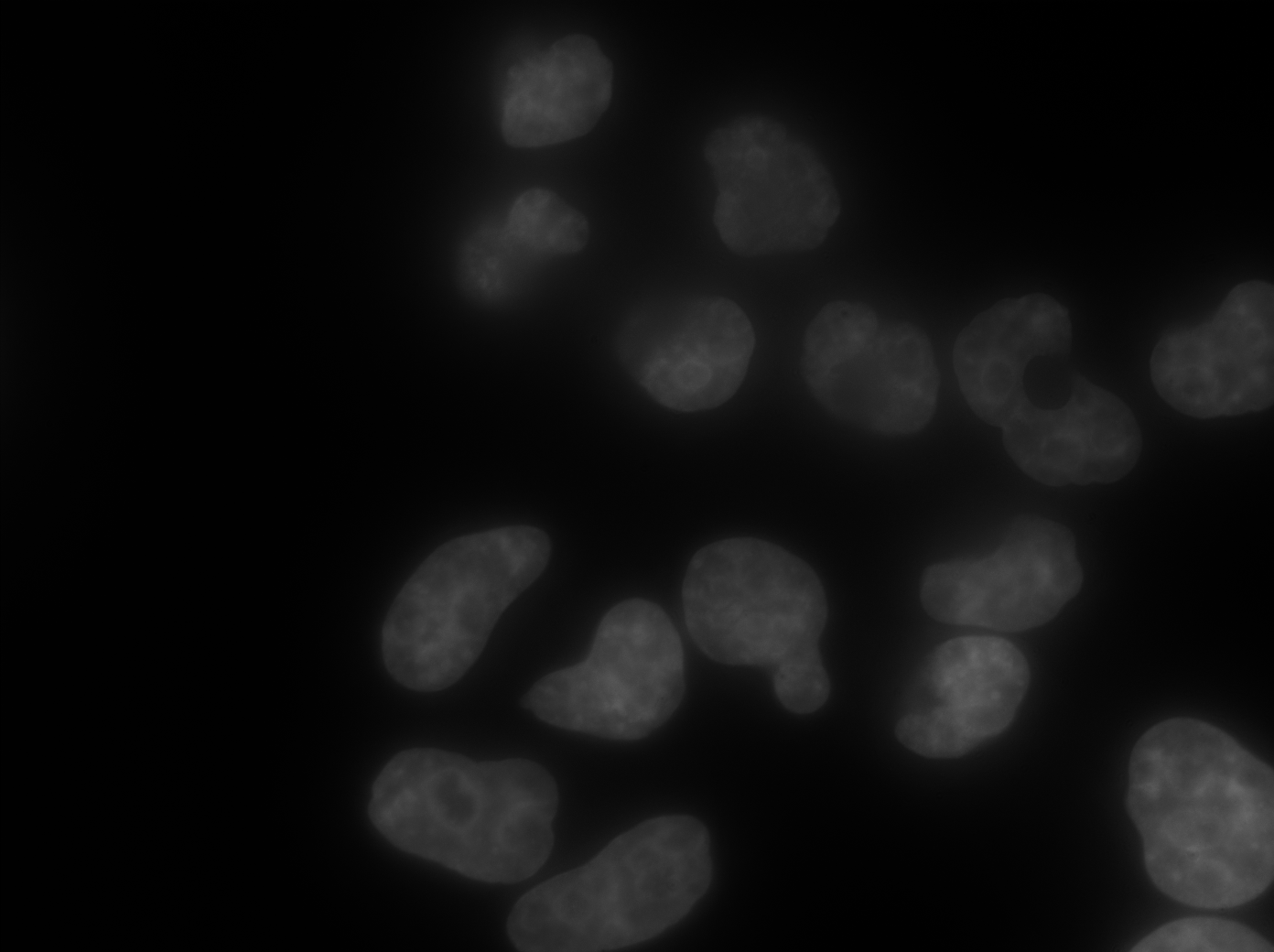

Supplement: Supplementary file 8 — Source data Fig. 2 part 5 [file 44319_2026_742_MOESM8_ESM.zip › Figure 2 Part 5/Fig 2d polye atubulin part 2/WT PolyE-atub 8-14-24 R2 PA10.Project Maximum Z_XY1723838433_Z0_T0_C0.tif]

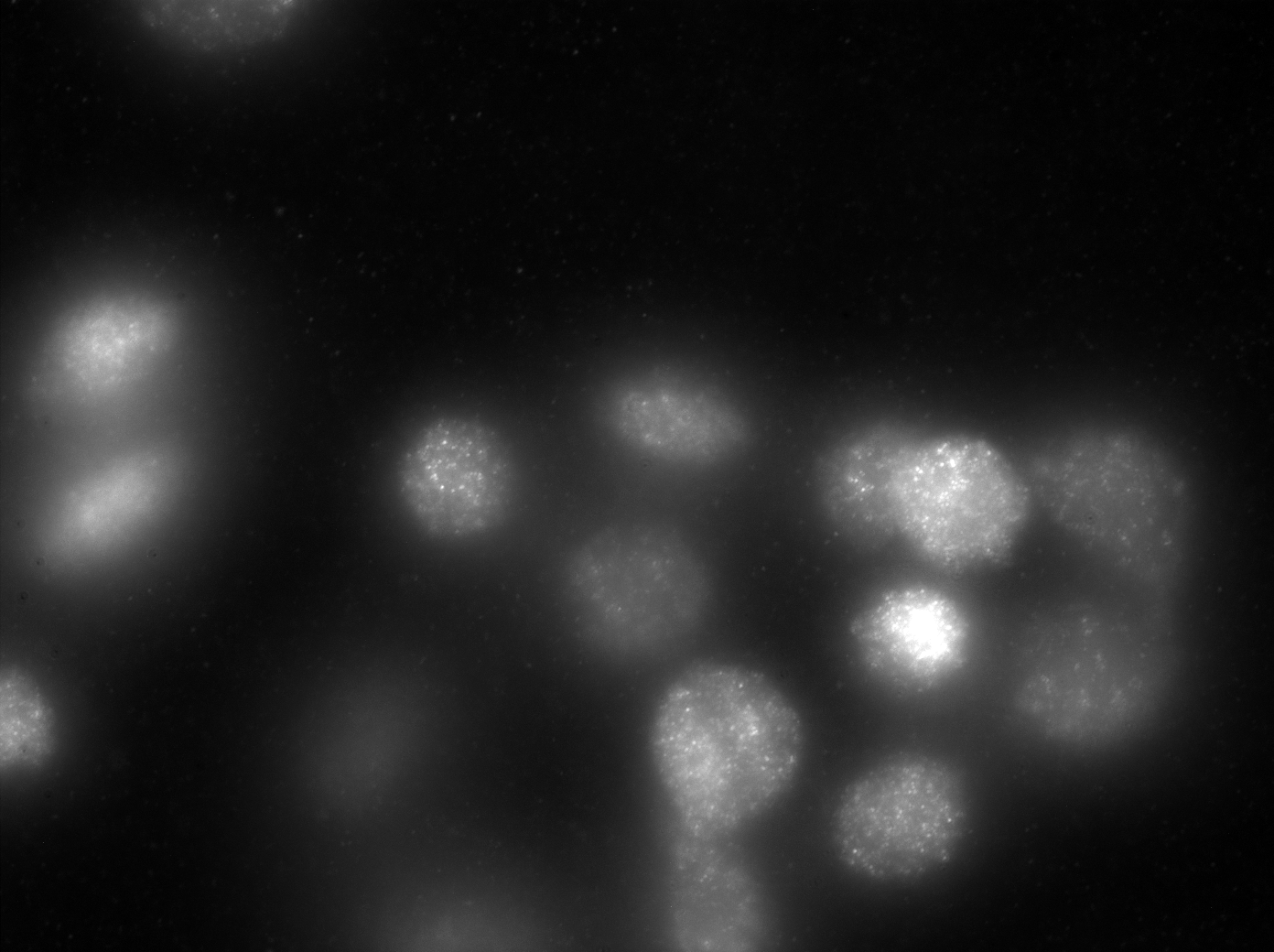

Supplement: Supplementary file 8 — Source data Fig. 2 part 5 [file 44319_2026_742_MOESM8_ESM.zip › Figure 2 Part 5/Fig 2d polye atubulin part 2/WT PolyE-atub 8-14-24 R3 M10.Project Maximum Z_XY1723846282_Z0_T0_C2.tif]

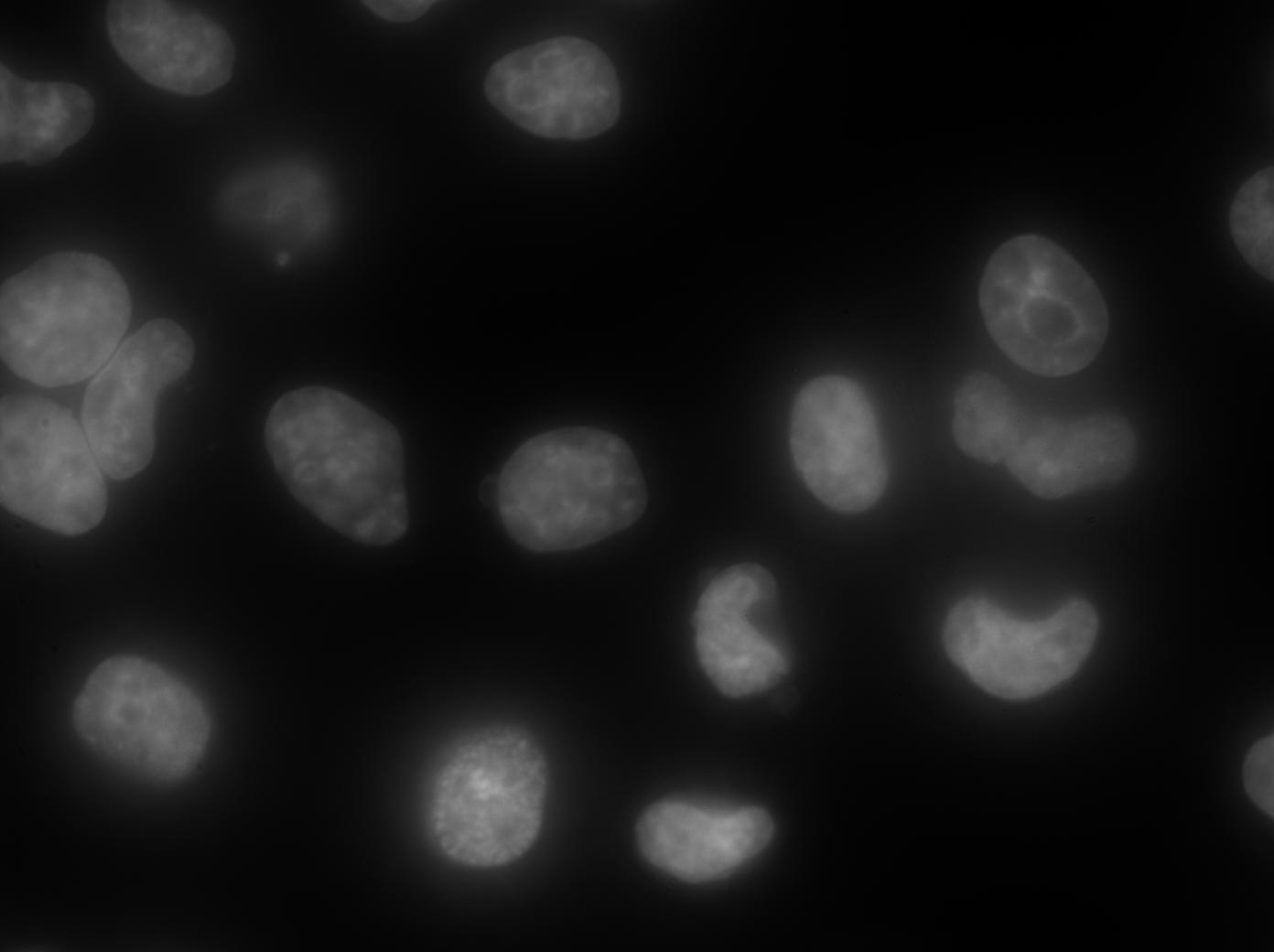

Supplement: Supplementary file 8 — Source data Fig. 2 part 5 [file 44319_2026_742_MOESM8_ESM.zip › Figure 2 Part 5/Fig 2d polye atubulin part 2/WT PolyE-atub 8-14-24 R3 LT10.Project Maximum Z_XY1723844042_Z0_T0_C0.tif]

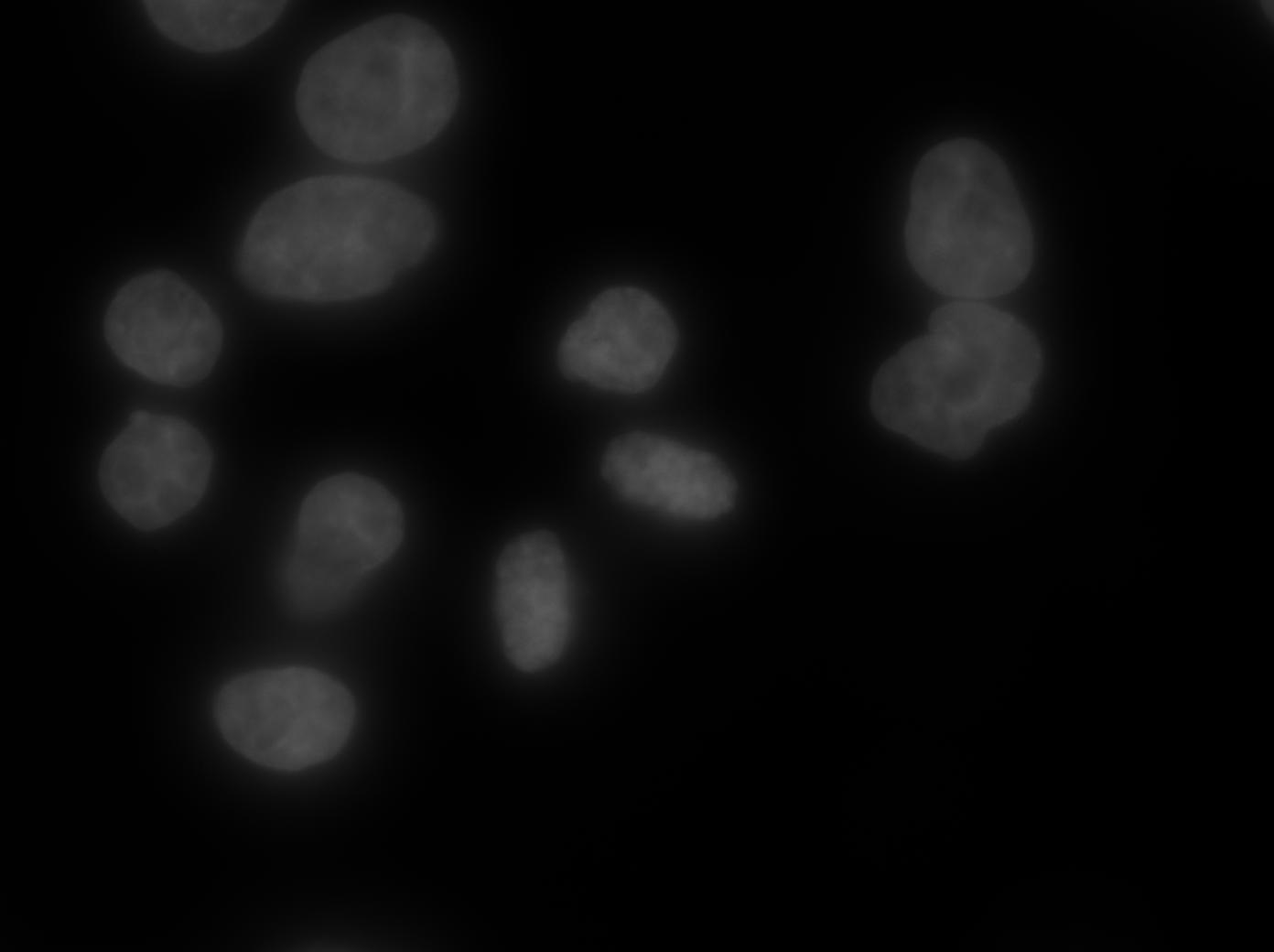

Supplement: Supplementary file 8 — Source data Fig. 2 part 5 [file 44319_2026_742_MOESM8_ESM.zip › Figure 2 Part 5/Fig 2d polye atubulin part 2/WT PolyE-atub 8-14-24 R3 ET8.Project Maximum Z_XY1723844245_Z0_T0_C0.tif]

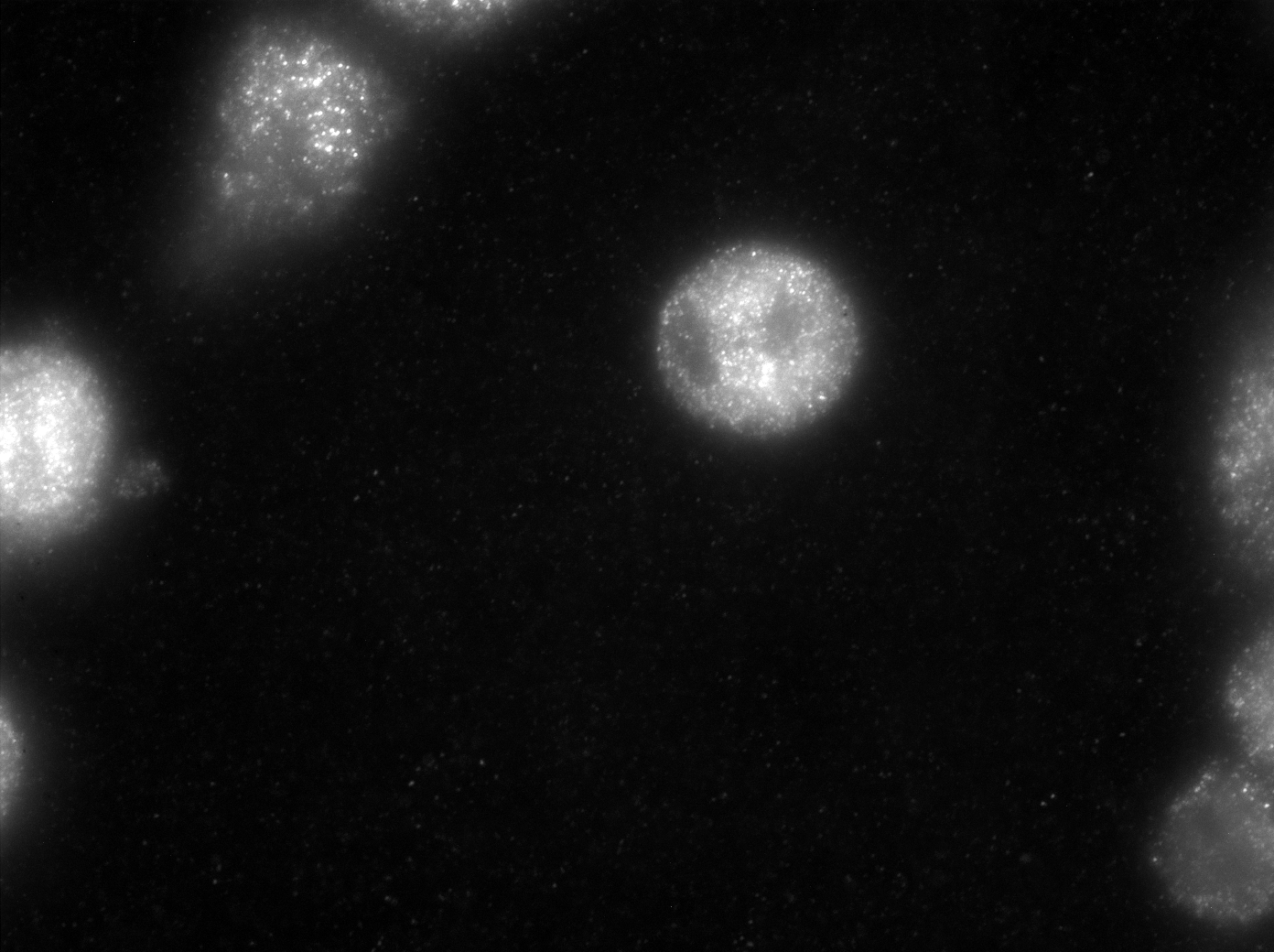

Supplement: Supplementary file 8 — Source data Fig. 2 part 5 [file 44319_2026_742_MOESM8_ESM.zip › Figure 2 Part 5/Fig 2d polye atubulin part 2/WT PolyE-atub 8-14-24 R3 M2.Project Maximum Z_XY1723842130_Z0_T0_C2.tif]

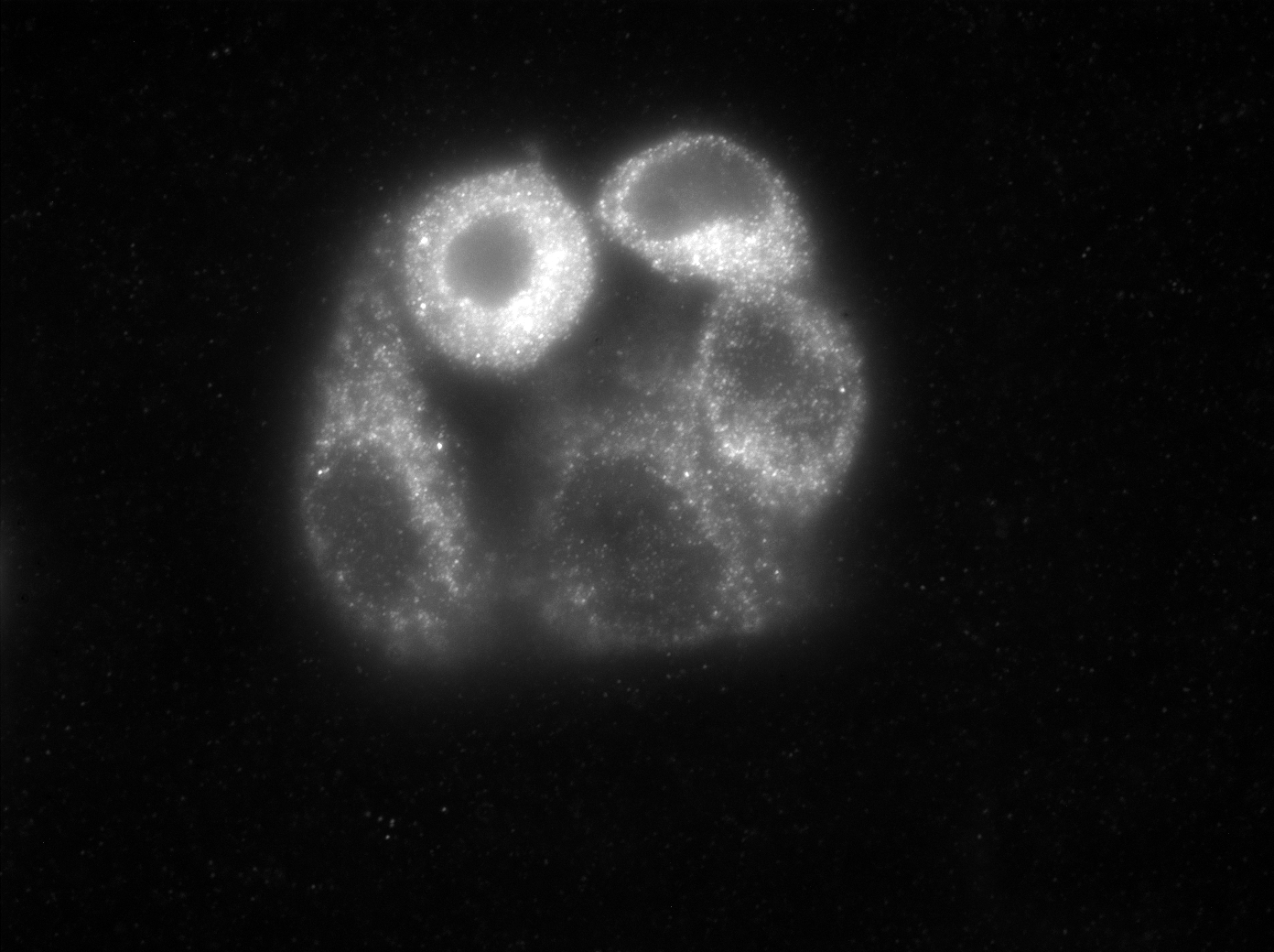

Supplement: Supplementary file 8 — Source data Fig. 2 part 5 [file 44319_2026_742_MOESM8_ESM.zip › Figure 2 Part 5/Fig 2d polye atubulin part 2/WT PolyE-atub 8-14-24 R2 PA7.Project Maximum Z_XY1723836799_Z0_T0_C2.tif]

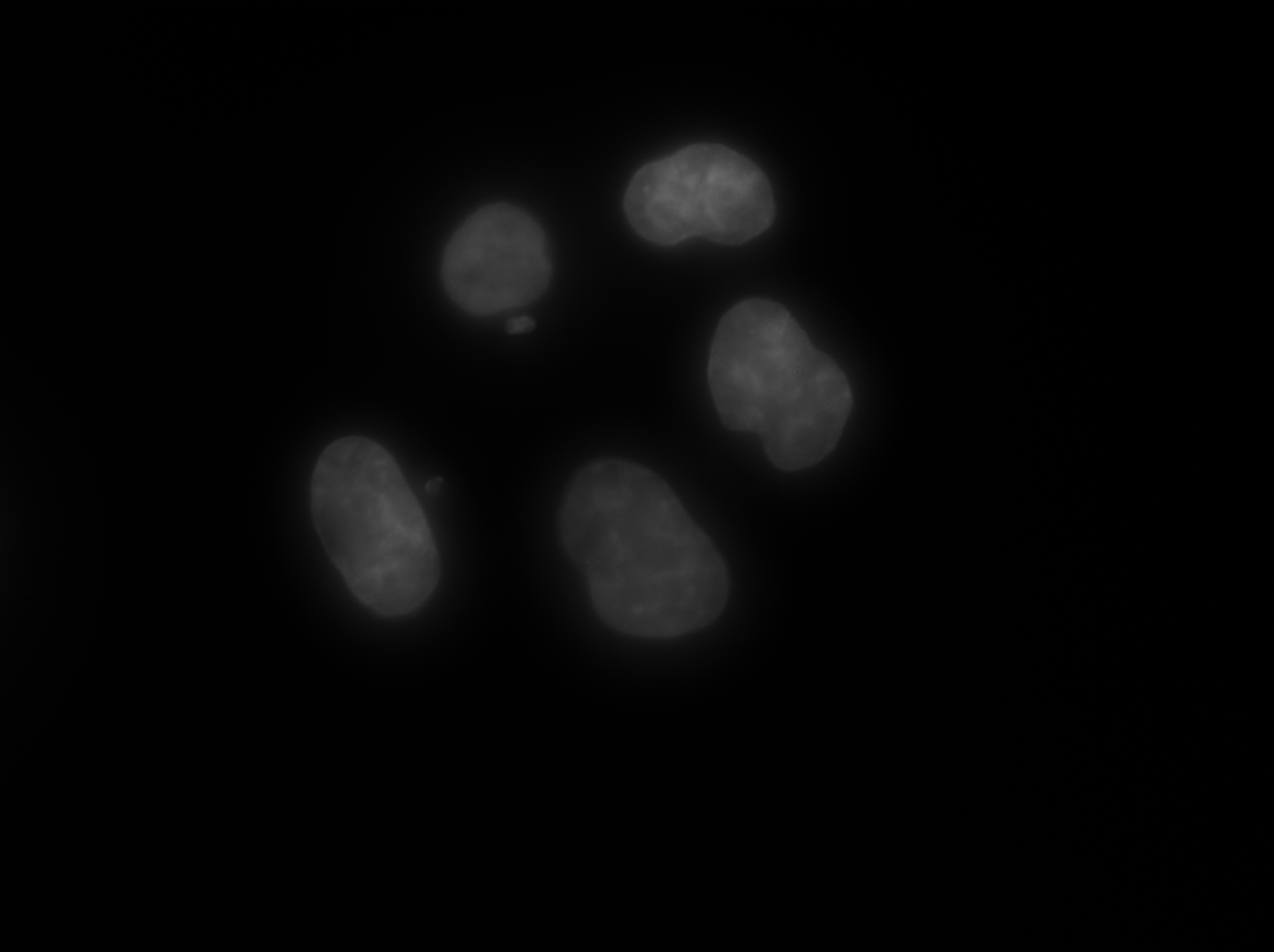

Supplement: Supplementary file 8 — Source data Fig. 2 part 5 [file 44319_2026_742_MOESM8_ESM.zip › Figure 2 Part 5/Fig 2d polye atubulin part 2/WT PolyE-atub 8-14-24 R2 PA7.Project Maximum Z_XY1723836799_Z0_T0_C0.tif]

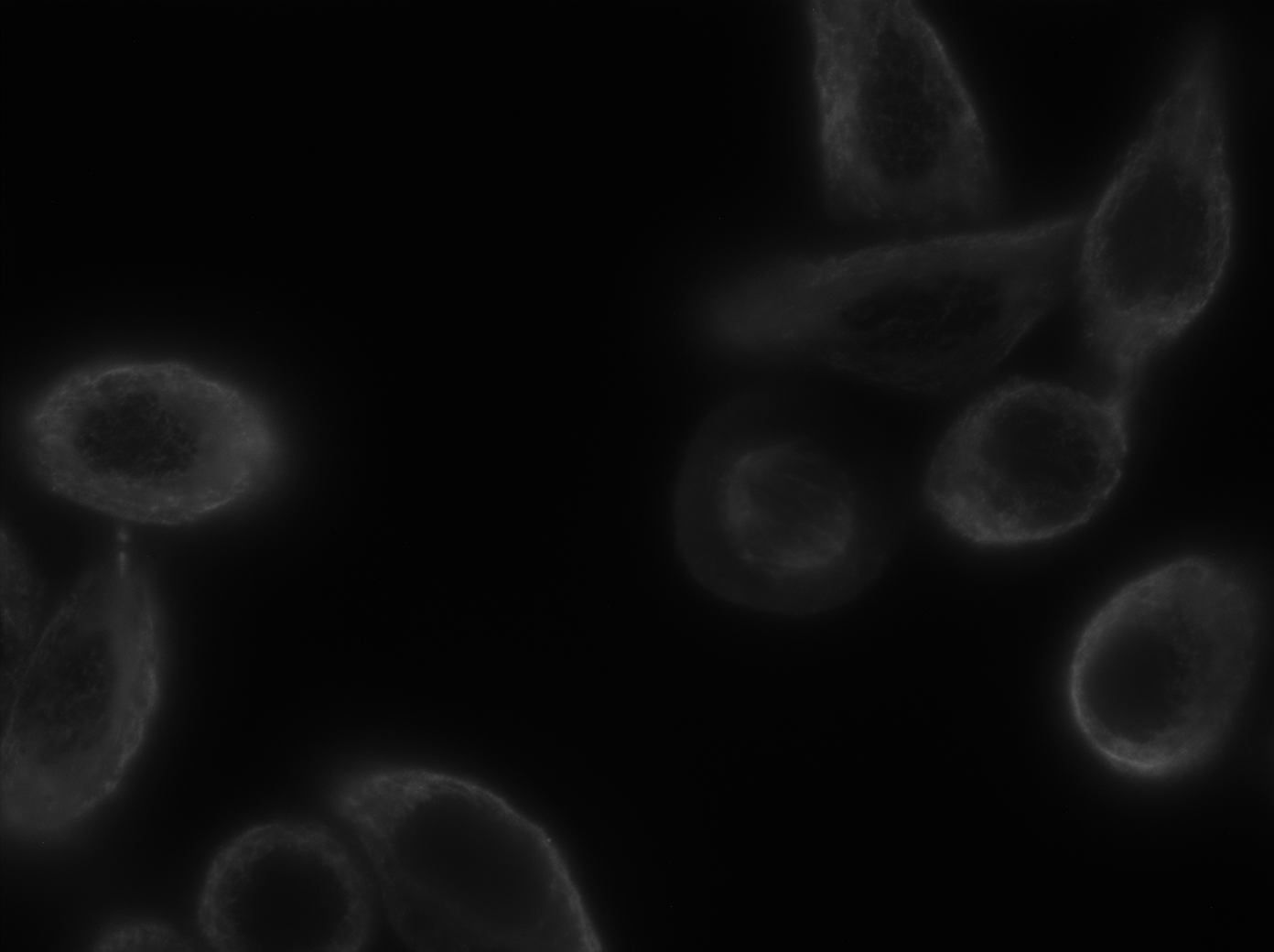

Supplement: Supplementary file 8 — Source data Fig. 2 part 5 [file 44319_2026_742_MOESM8_ESM.zip › Figure 2 Part 5/Fig 2d polye atubulin part 2/WT PolyE-atub 8-14-24 R2 M8.Project Maximum Z_XY1723838633_Z0_T0_C1.tif]

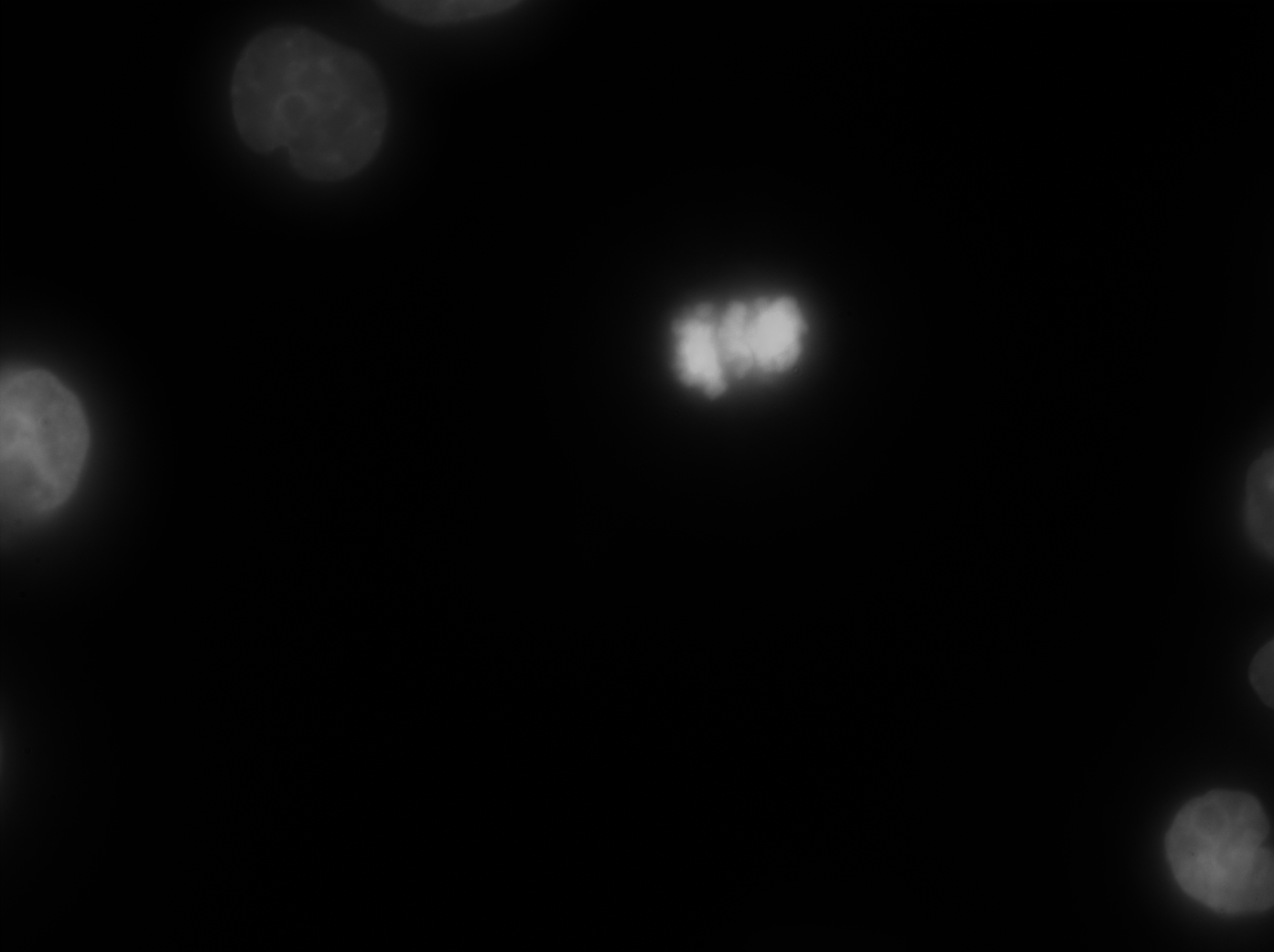

Supplement: Supplementary file 8 — Source data Fig. 2 part 5 [file 44319_2026_742_MOESM8_ESM.zip › Figure 2 Part 5/Fig 2d polye atubulin part 2/WT PolyE-atub 8-14-24 R3 M2.Project Maximum Z_XY1723842130_Z0_T0_C0.tif]

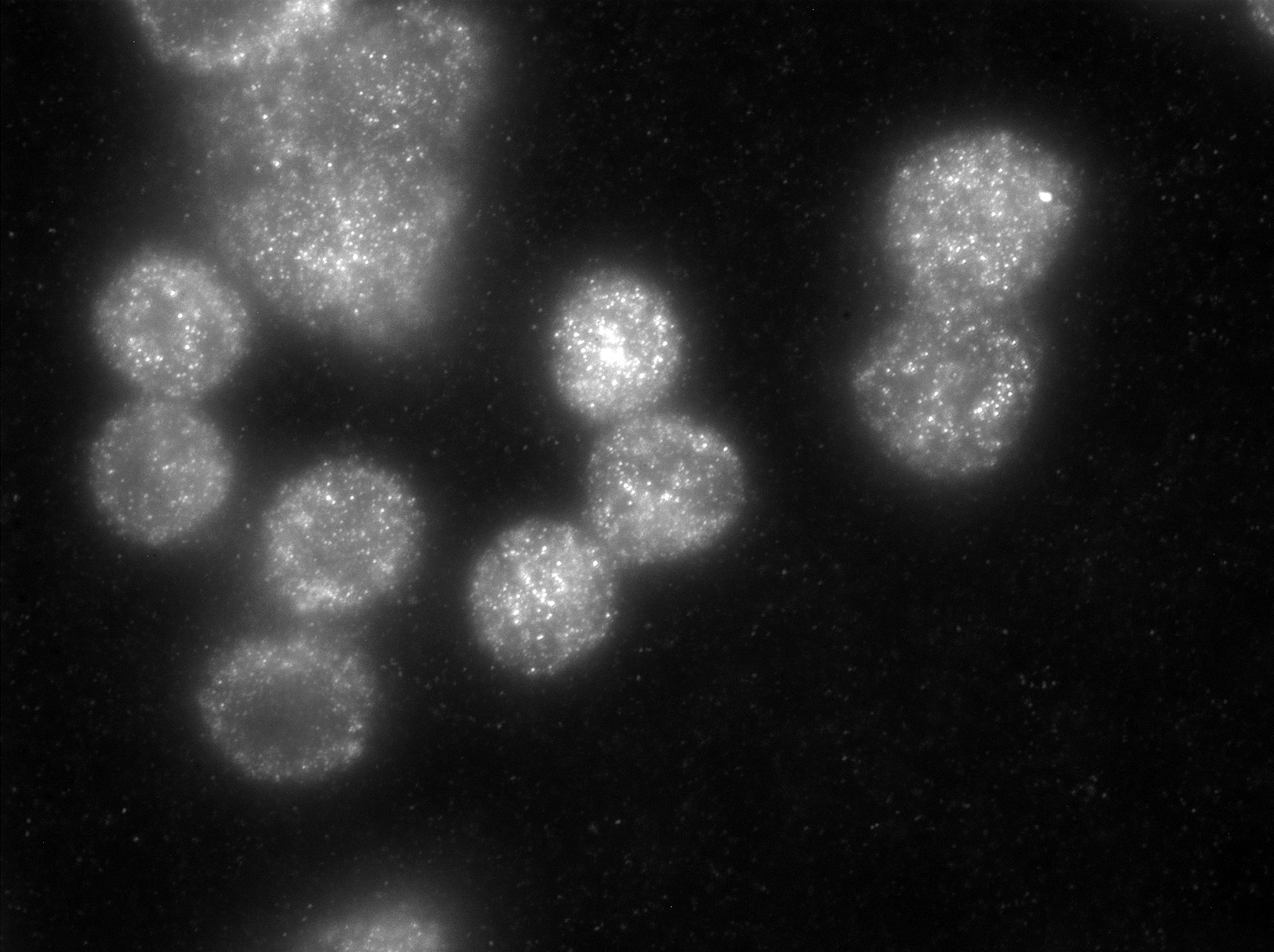

Supplement: Supplementary file 8 — Source data Fig. 2 part 5 [file 44319_2026_742_MOESM8_ESM.zip › Figure 2 Part 5/Fig 2d polye atubulin part 2/WT PolyE-atub 8-14-24 R3 ET8.Project Maximum Z_XY1723844245_Z0_T0_C2.tif]

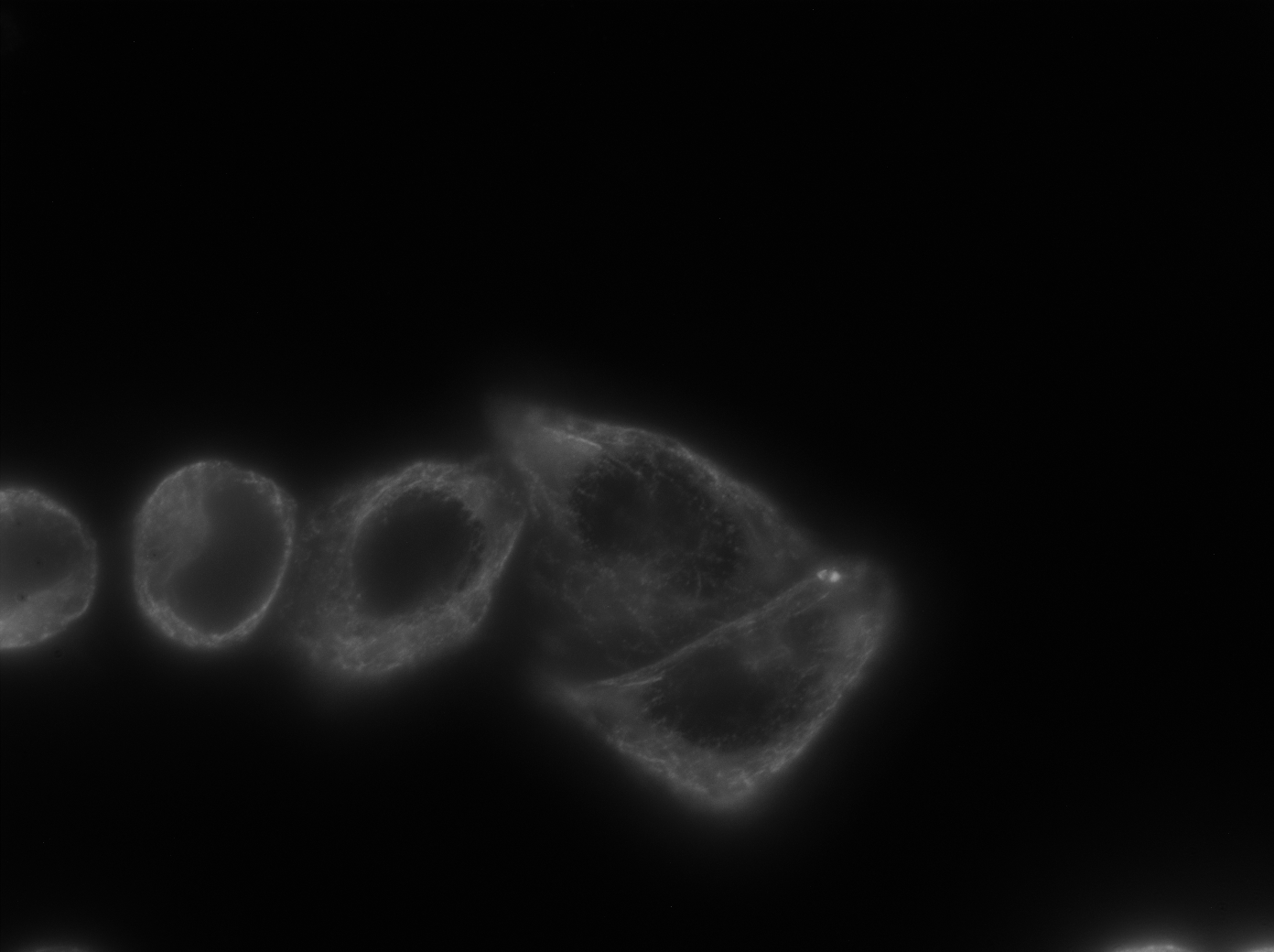

Supplement: Supplementary file 8 — Source data Fig. 2 part 5 [file 44319_2026_742_MOESM8_ESM.zip › Figure 2 Part 5/Fig 2d polye atubulin part 2/WT PolyE-atub 8-14-24 R2 PA4.Project Maximum Z_XY1723835053_Z0_T0_C1.tif]

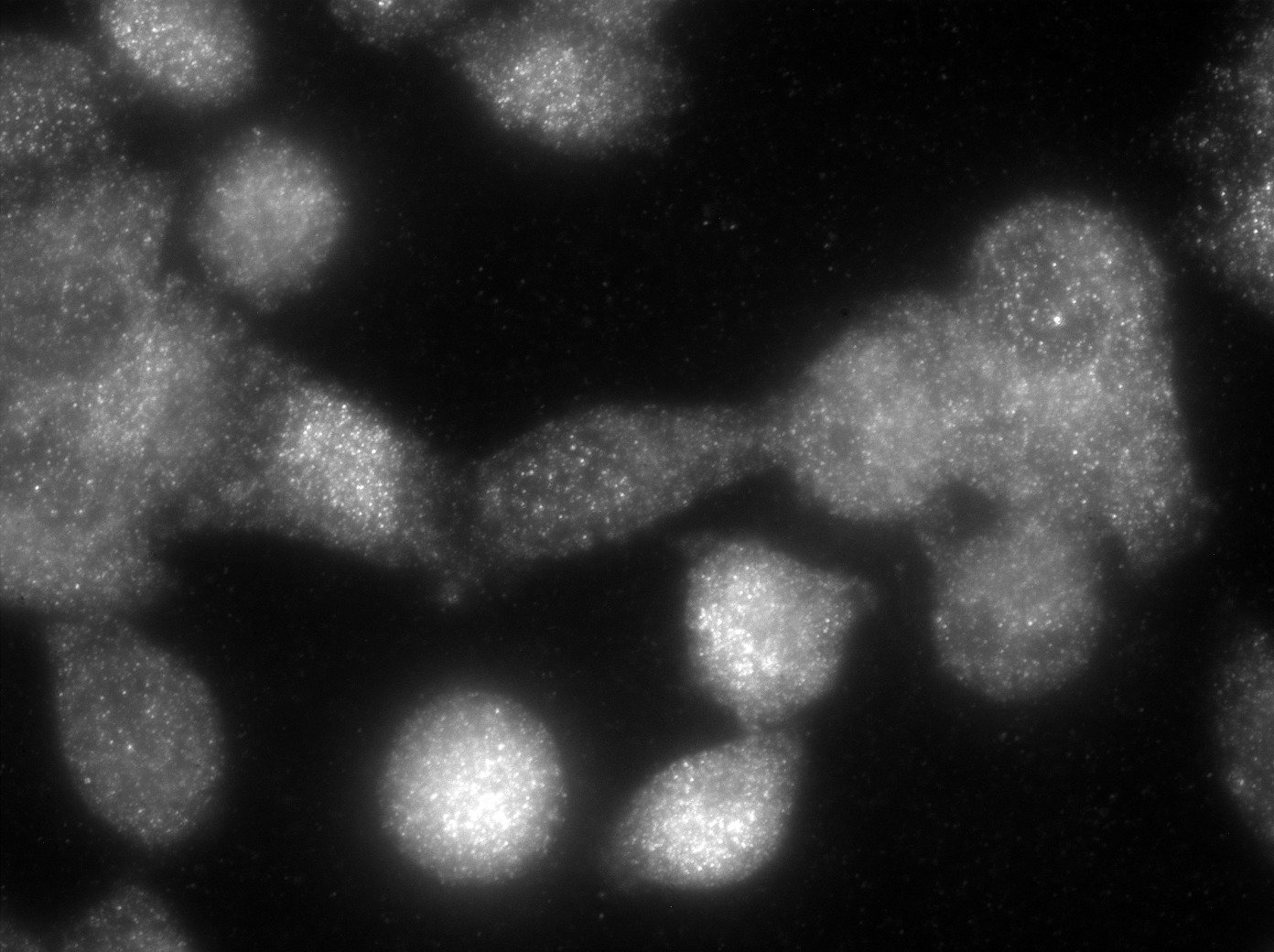

Supplement: Supplementary file 8 — Source data Fig. 2 part 5 [file 44319_2026_742_MOESM8_ESM.zip › Figure 2 Part 5/Fig 2d polye atubulin part 2/WT PolyE-atub 8-14-24 R3 LT10.Project Maximum Z_XY1723844042_Z0_T0_C2.tif]

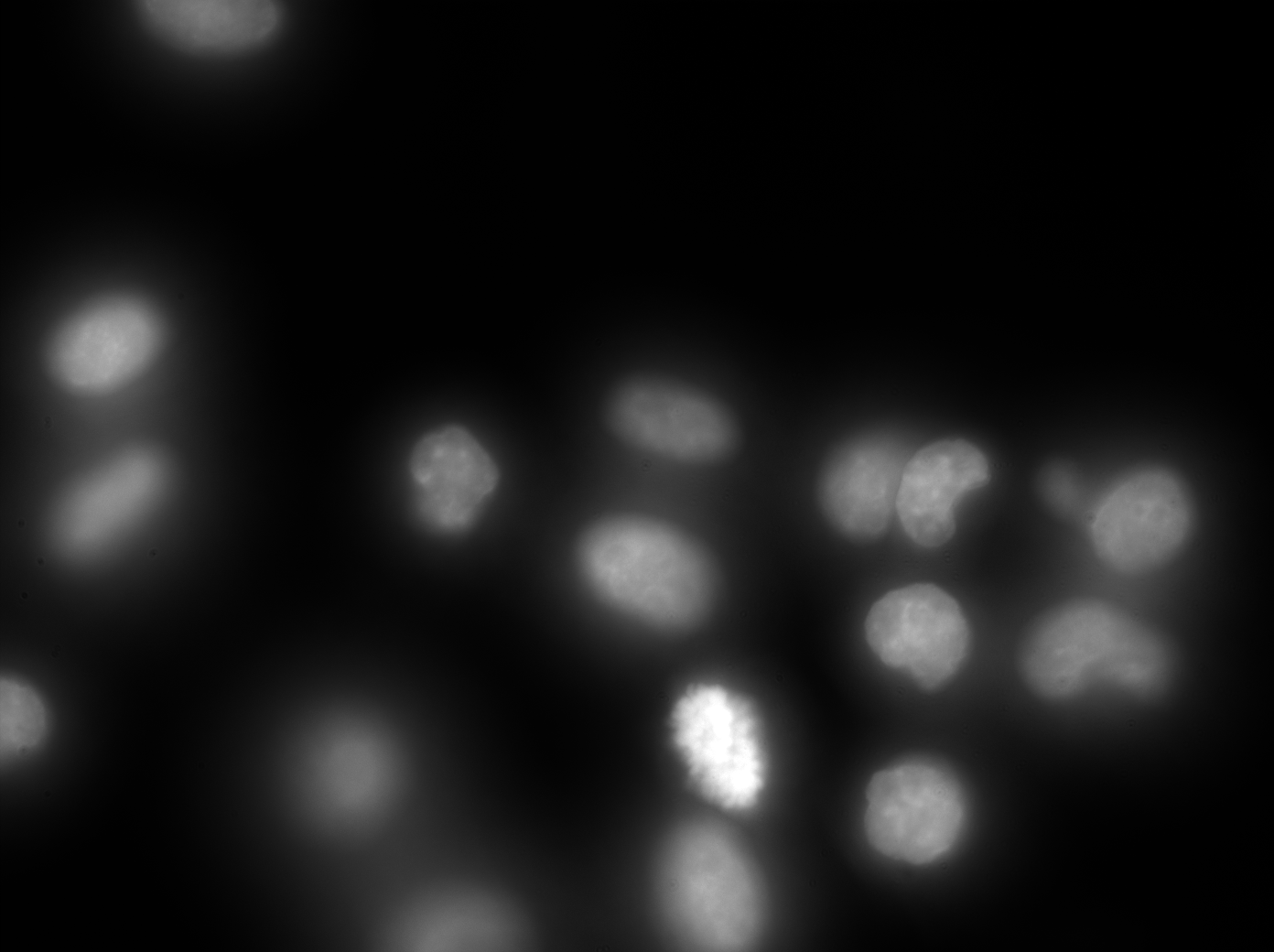

Supplement: Supplementary file 8 — Source data Fig. 2 part 5 [file 44319_2026_742_MOESM8_ESM.zip › Figure 2 Part 5/Fig 2d polye atubulin part 2/WT PolyE-atub 8-14-24 R3 M10.Project Maximum Z_XY1723846282_Z0_T0_C0.tif]

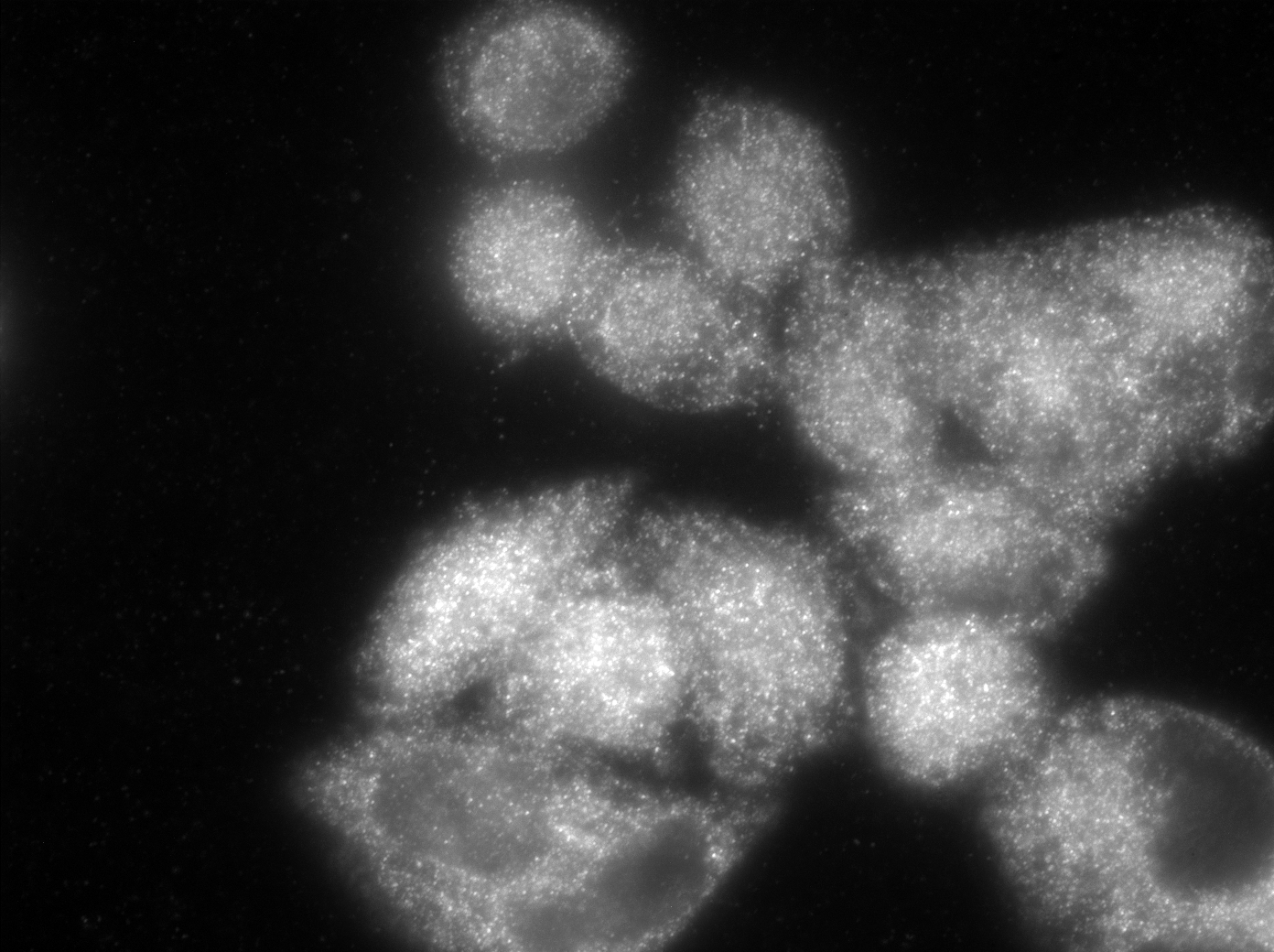

Supplement: Supplementary file 8 — Source data Fig. 2 part 5 [file 44319_2026_742_MOESM8_ESM.zip › Figure 2 Part 5/Fig 2d polye atubulin part 2/WT PolyE-atub 8-14-24 R2 PA10.Project Maximum Z_XY1723838433_Z0_T0_C2.tif]

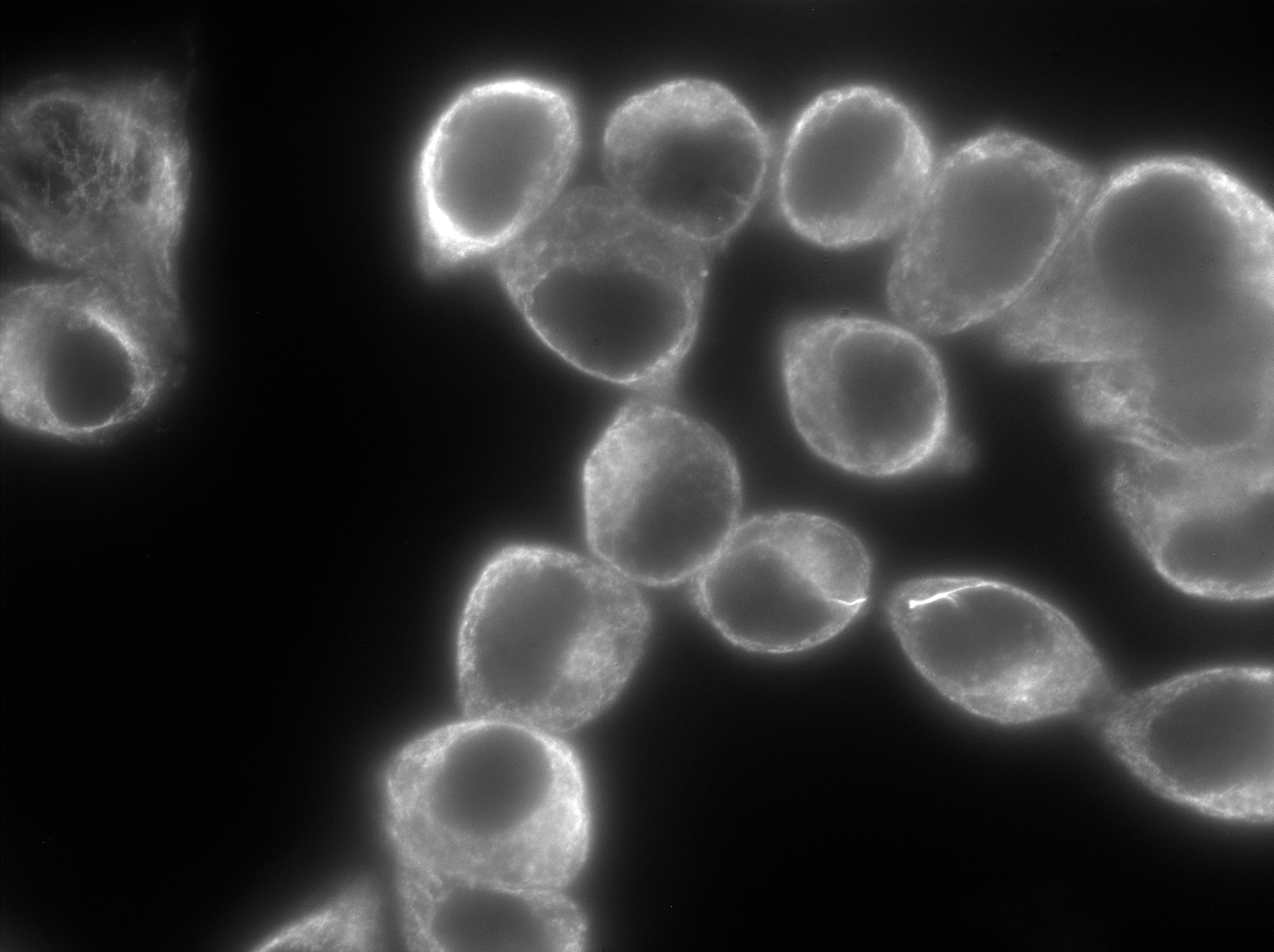

Supplement: Supplementary file 8 — Source data Fig. 2 part 5 [file 44319_2026_742_MOESM8_ESM.zip › Figure 2 Part 5/Fig 2d polye atubulin part 2/WT PolyE-atub 8-14-24 R3 PA10.Project Maximum Z_XY1723845723_Z0_T0_C1.tif]

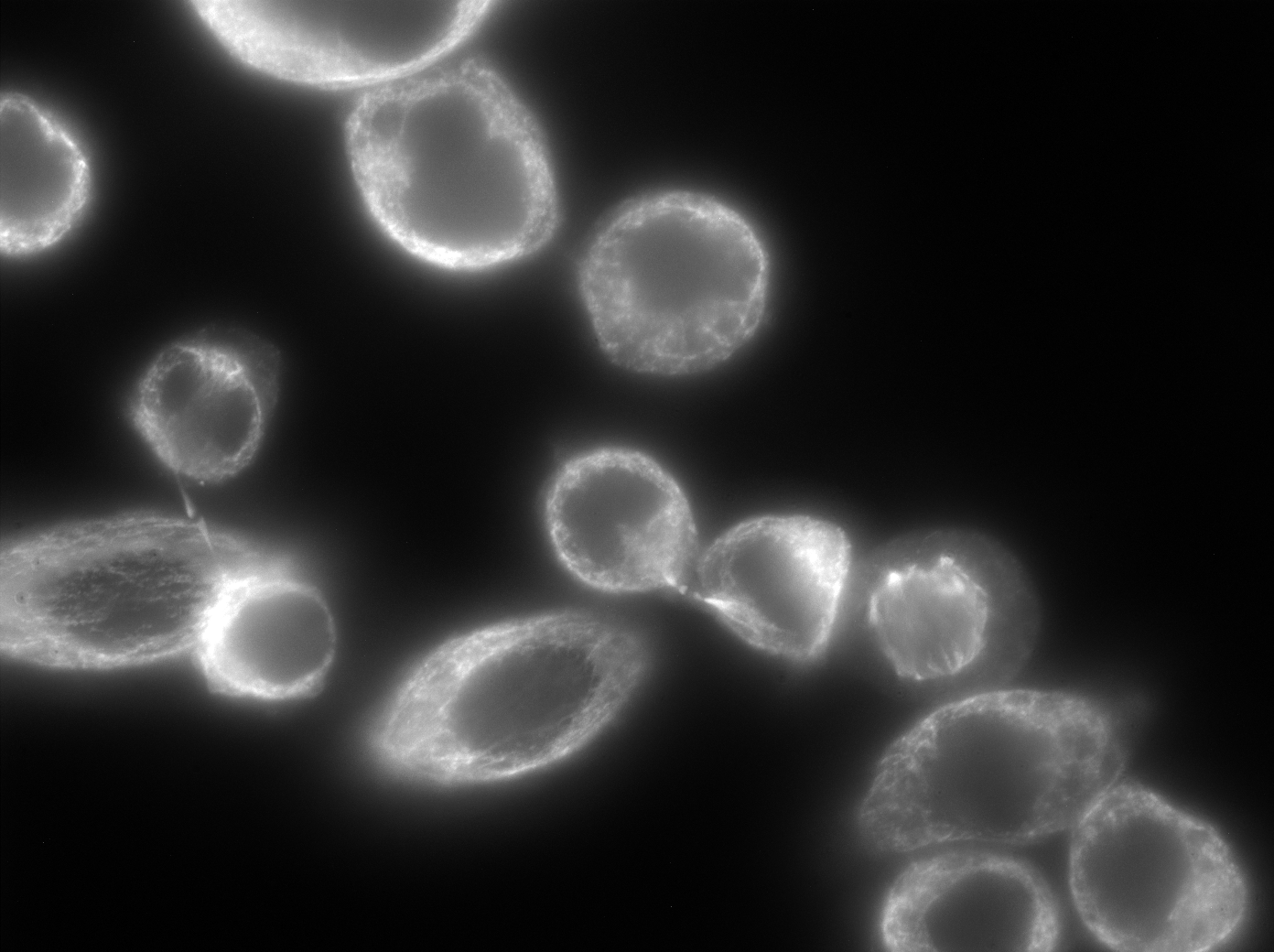

Supplement: Supplementary file 8 — Source data Fig. 2 part 5 [file 44319_2026_742_MOESM8_ESM.zip › Figure 2 Part 5/Fig 2d polye atubulin part 2/WT PolyE-atub 8-14-24 R3 LT5 M4.Project Maximum Z_XY1723843361_Z0_T0_C1.tif]

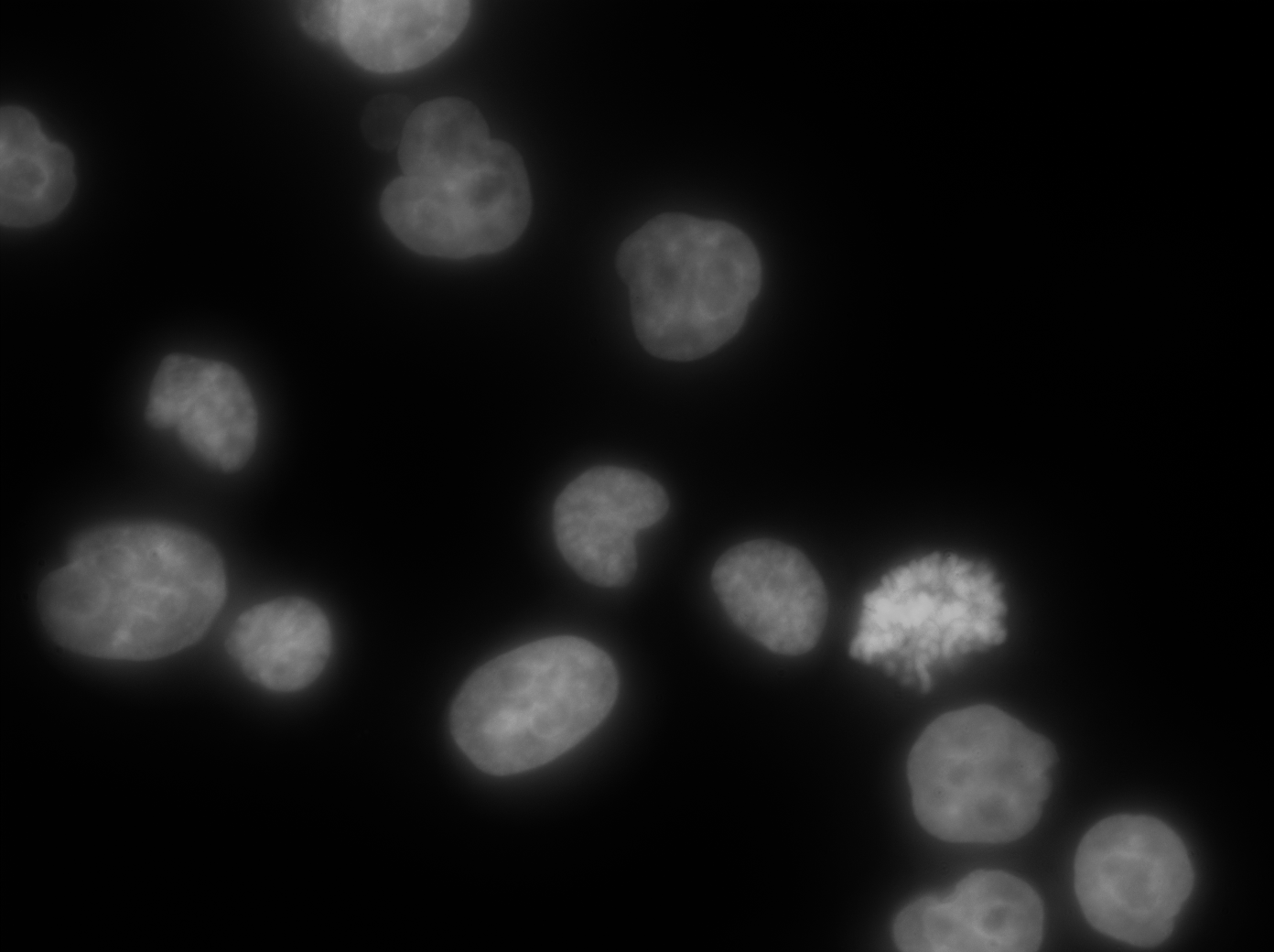

Supplement: Supplementary file 8 — Source data Fig. 2 part 5 [file 44319_2026_742_MOESM8_ESM.zip › Figure 2 Part 5/Fig 2d polye atubulin part 2/WT PolyE-atub 8-14-24 R3 LT5 M4.Project Maximum Z_XY1723843361_Z0_T0_C0.tif]

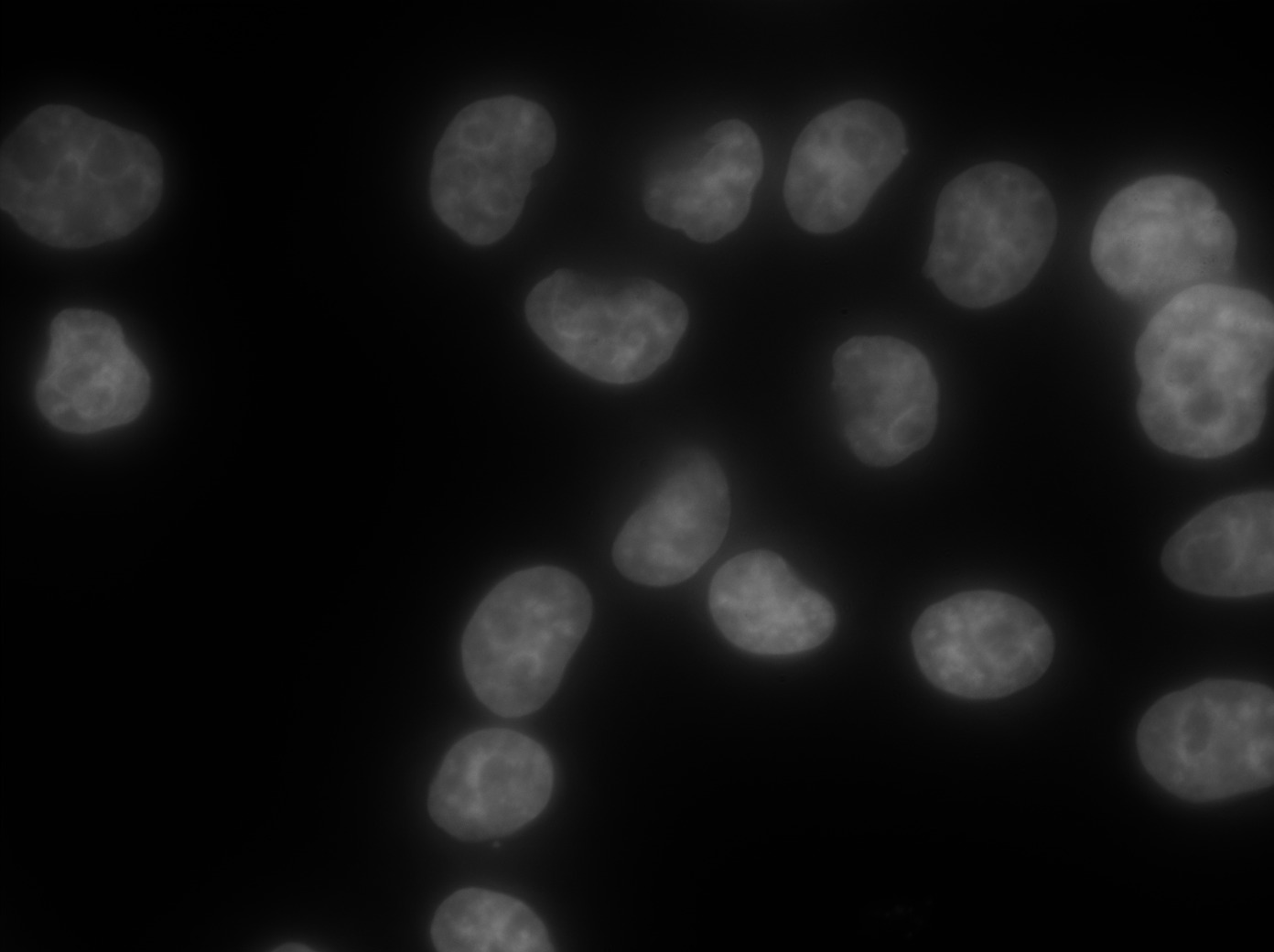

Supplement: Supplementary file 8 — Source data Fig. 2 part 5 [file 44319_2026_742_MOESM8_ESM.zip › Figure 2 Part 5/Fig 2d polye atubulin part 2/WT PolyE-atub 8-14-24 R3 PA10.Project Maximum Z_XY1723845723_Z0_T0_C0.tif]

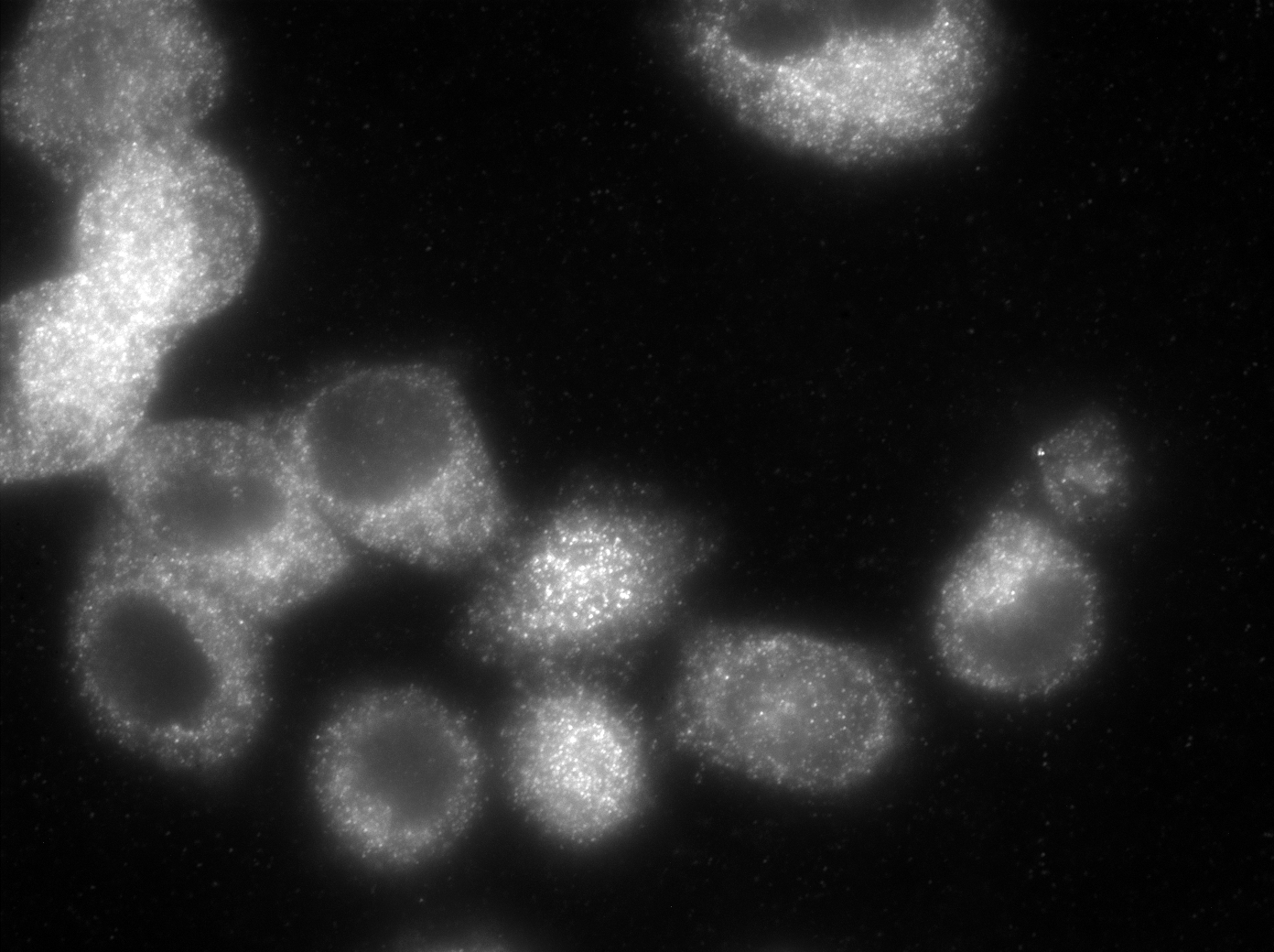

Supplement: Supplementary file 8 — Source data Fig. 2 part 5 [file 44319_2026_742_MOESM8_ESM.zip › Figure 2 Part 5/Fig 2d polye atubulin part 2/WT PolyE-atub 8-14-24 R2 LT7LT8.Project Maximum Z_XY1723836343_Z0_T0_C2.tif]

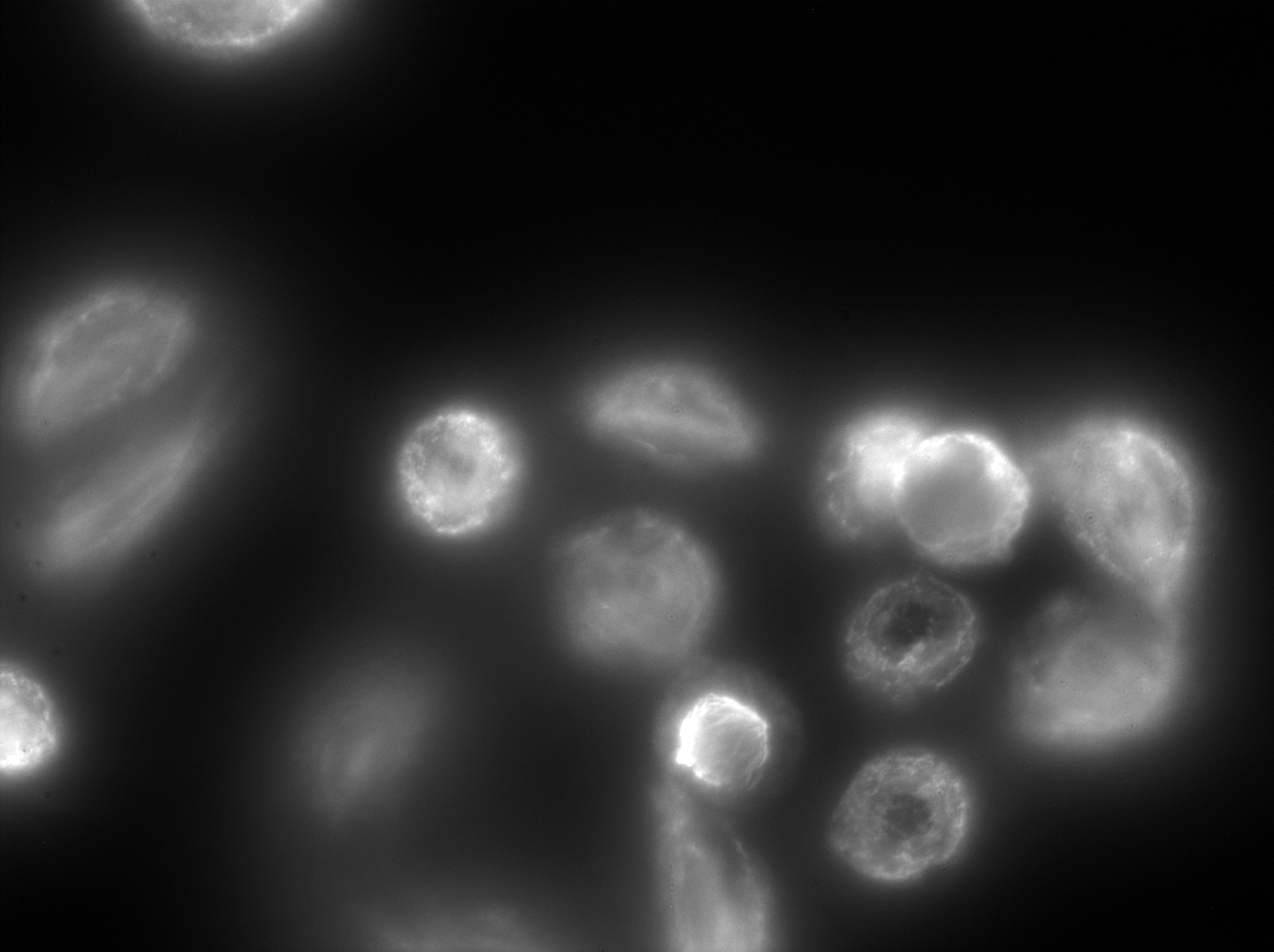

Supplement: Supplementary file 8 — Source data Fig. 2 part 5 [file 44319_2026_742_MOESM8_ESM.zip › Figure 2 Part 5/Fig 2d polye atubulin part 2/WT PolyE-atub 8-14-24 R3 M10.Project Maximum Z_XY1723846282_Z0_T0_C1.tif]

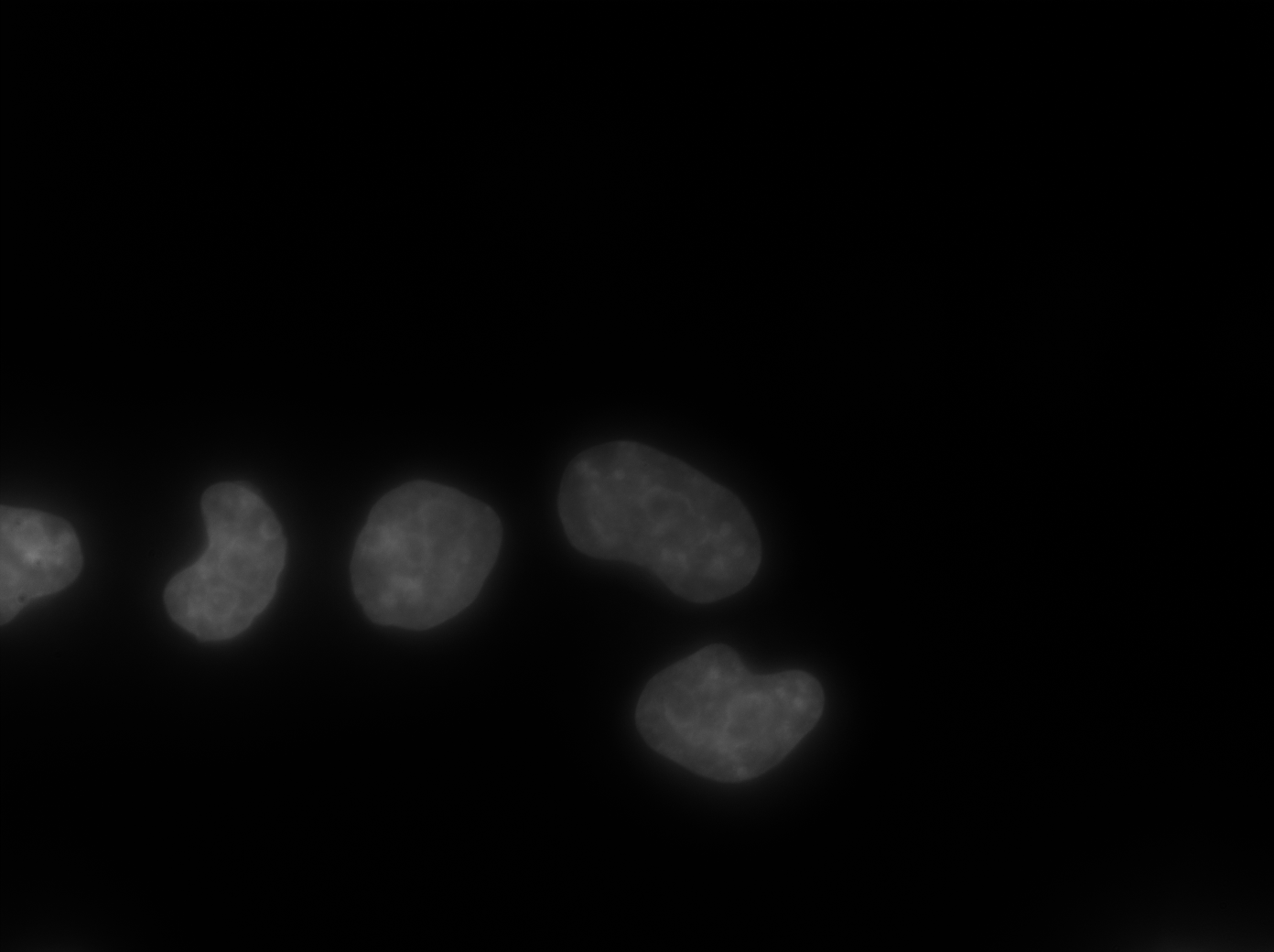

Supplement: Supplementary file 8 — Source data Fig. 2 part 5 [file 44319_2026_742_MOESM8_ESM.zip › Figure 2 Part 5/Fig 2d polye atubulin part 2/WT PolyE-atub 8-14-24 R2 PA4.Project Maximum Z_XY1723835053_Z0_T0_C0.tif]

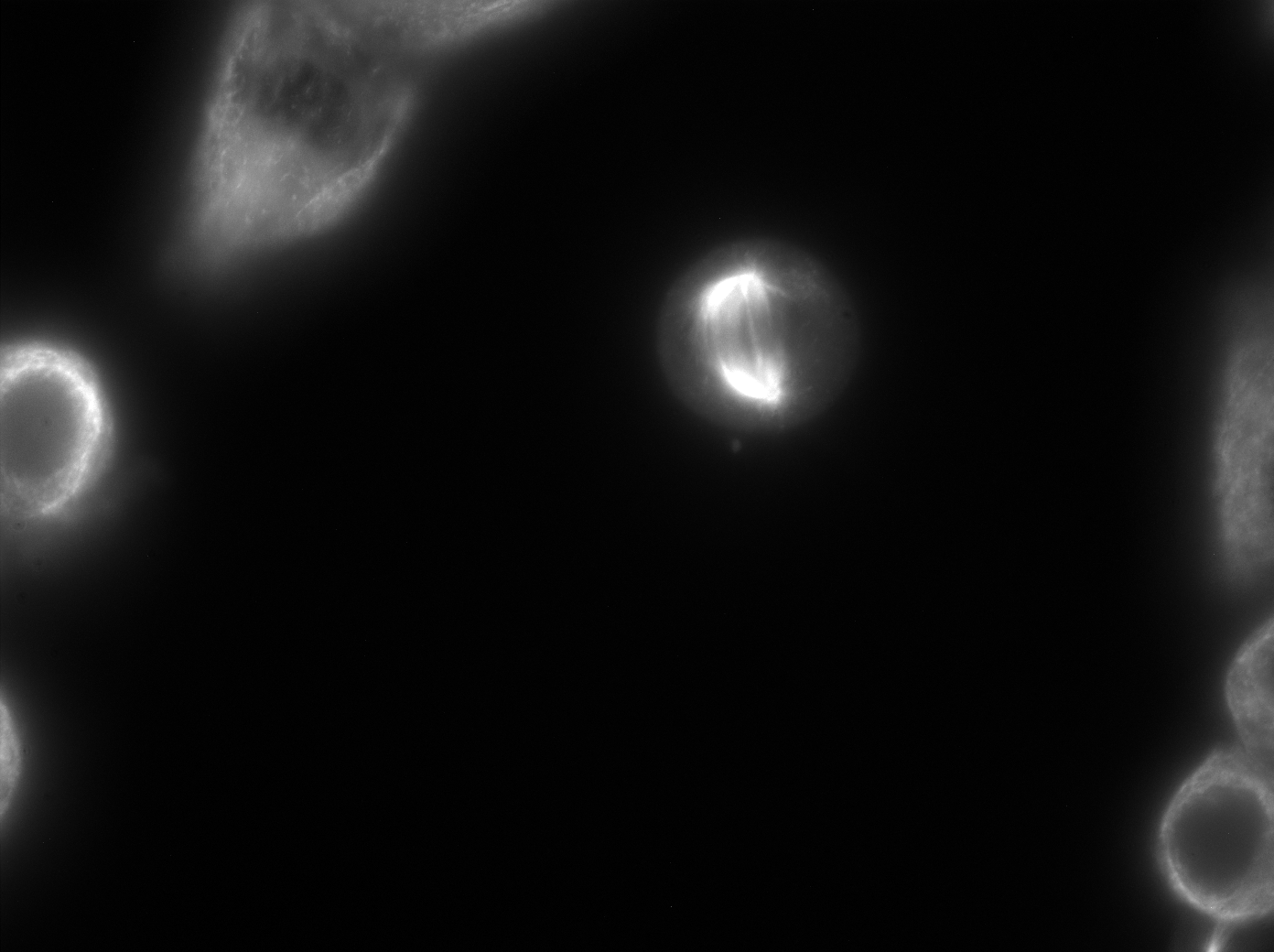

Supplement: Supplementary file 8 — Source data Fig. 2 part 5 [file 44319_2026_742_MOESM8_ESM.zip › Figure 2 Part 5/Fig 2d polye atubulin part 2/WT PolyE-atub 8-14-24 R3 M2.Project Maximum Z_XY1723842130_Z0_T0_C1.tif]

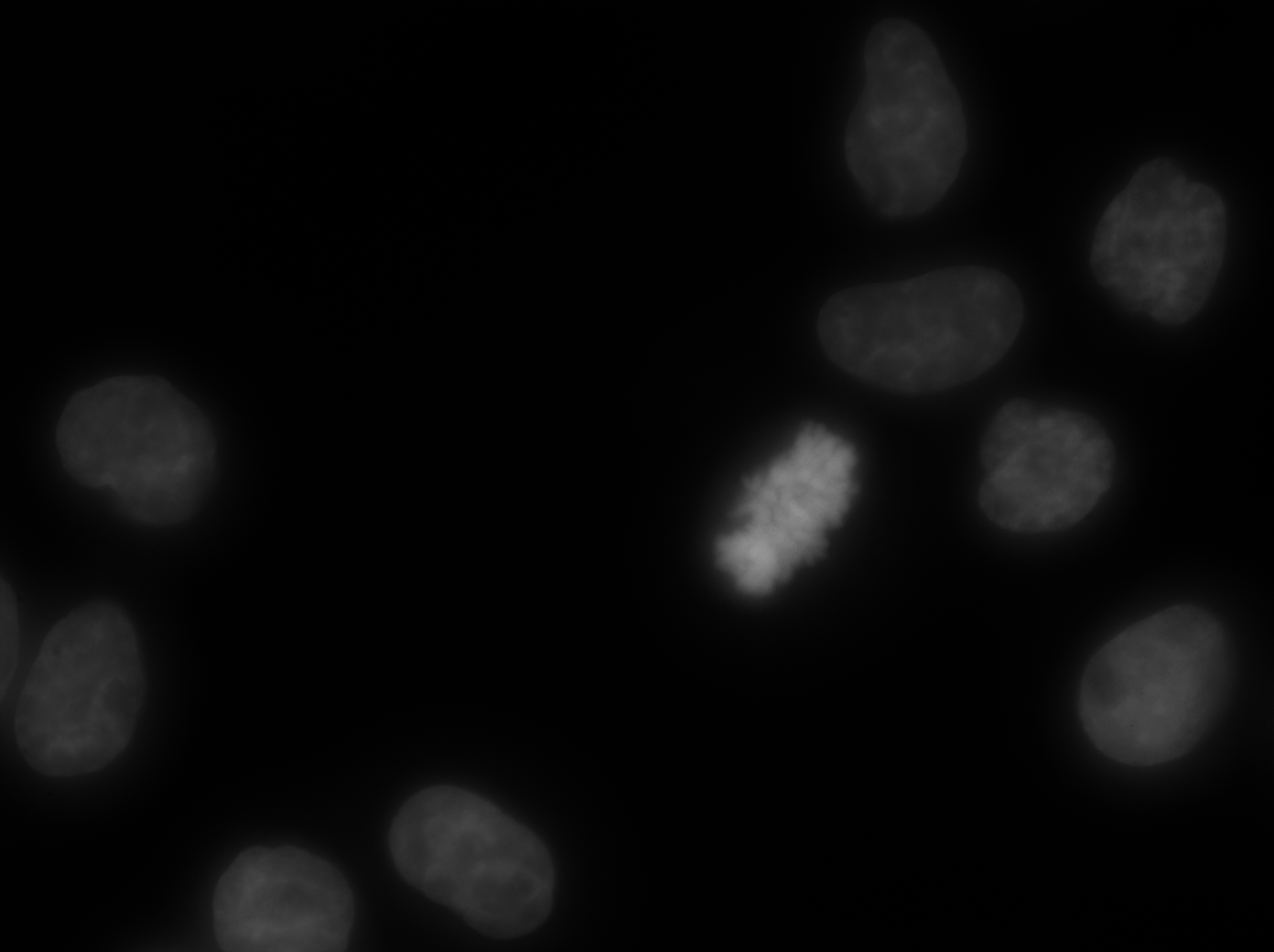

Supplement: Supplementary file 8 — Source data Fig. 2 part 5 [file 44319_2026_742_MOESM8_ESM.zip › Figure 2 Part 5/Fig 2d polye atubulin part 2/WT PolyE-atub 8-14-24 R2 M8.Project Maximum Z_XY1723838633_Z0_T0_C0.tif]

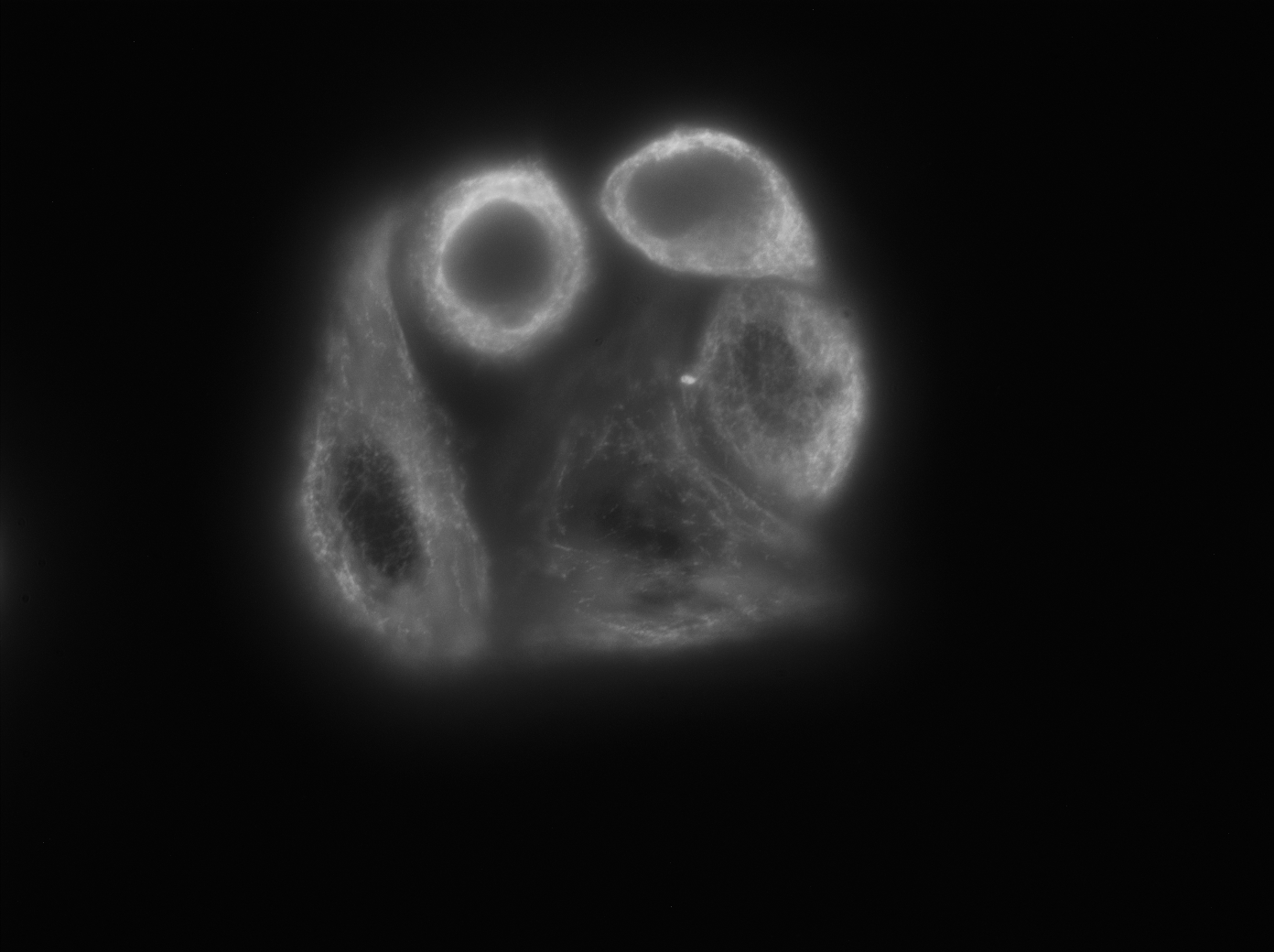

Supplement: Supplementary file 8 — Source data Fig. 2 part 5 [file 44319_2026_742_MOESM8_ESM.zip › Figure 2 Part 5/Fig 2d polye atubulin part 2/WT PolyE-atub 8-14-24 R2 PA7.Project Maximum Z_XY1723836799_Z0_T0_C1.tif]

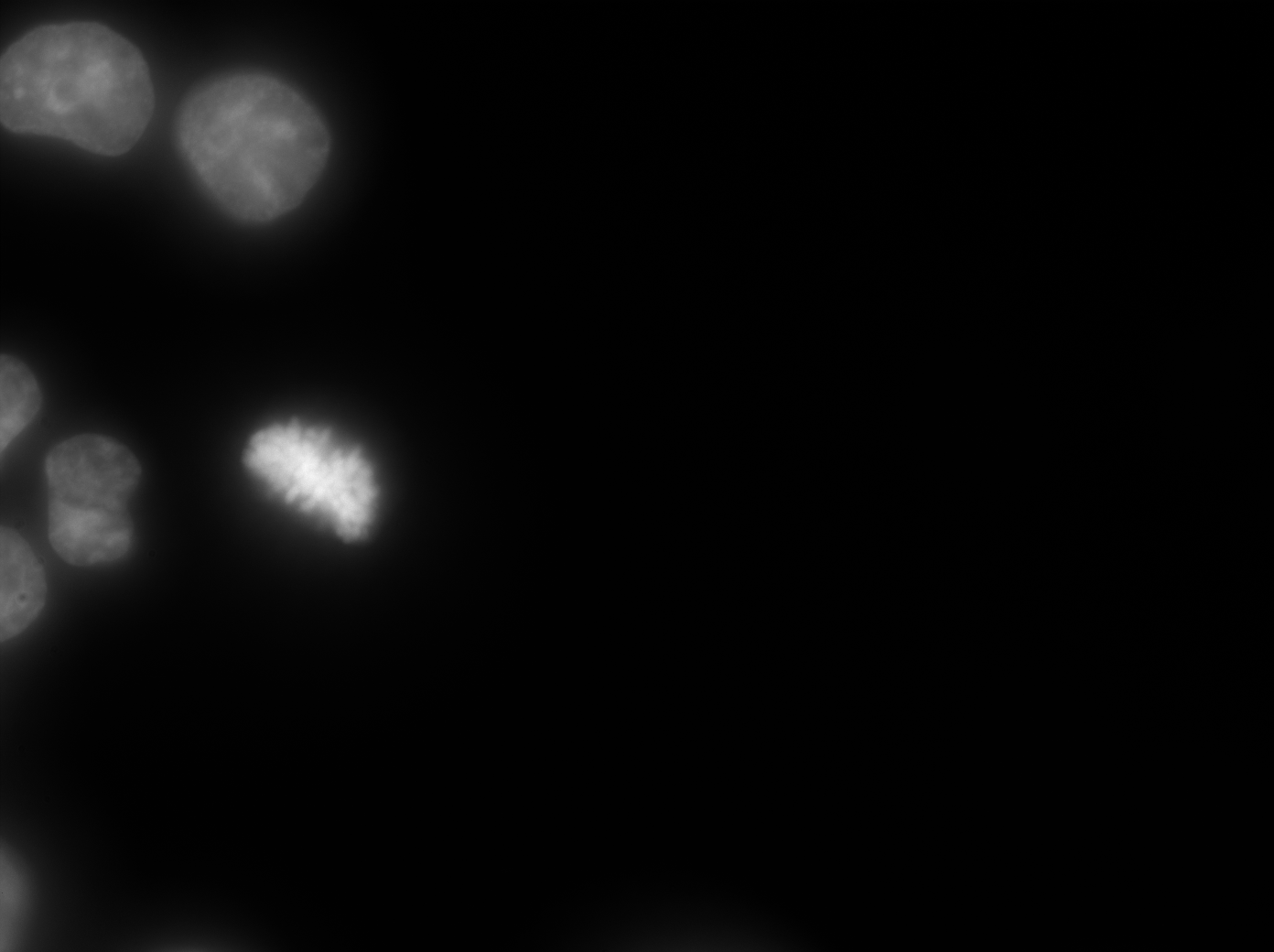

Supplement: Supplementary file 8 — Source data Fig. 2 part 5 [file 44319_2026_742_MOESM8_ESM.zip › Figure 2 Part 5/Fig 2d polye atubulin part 2/WT PolyE-atub 8-14-24 R2 M4.Project Maximum Z_XY1723837179_Z0_T0_C0.tif]

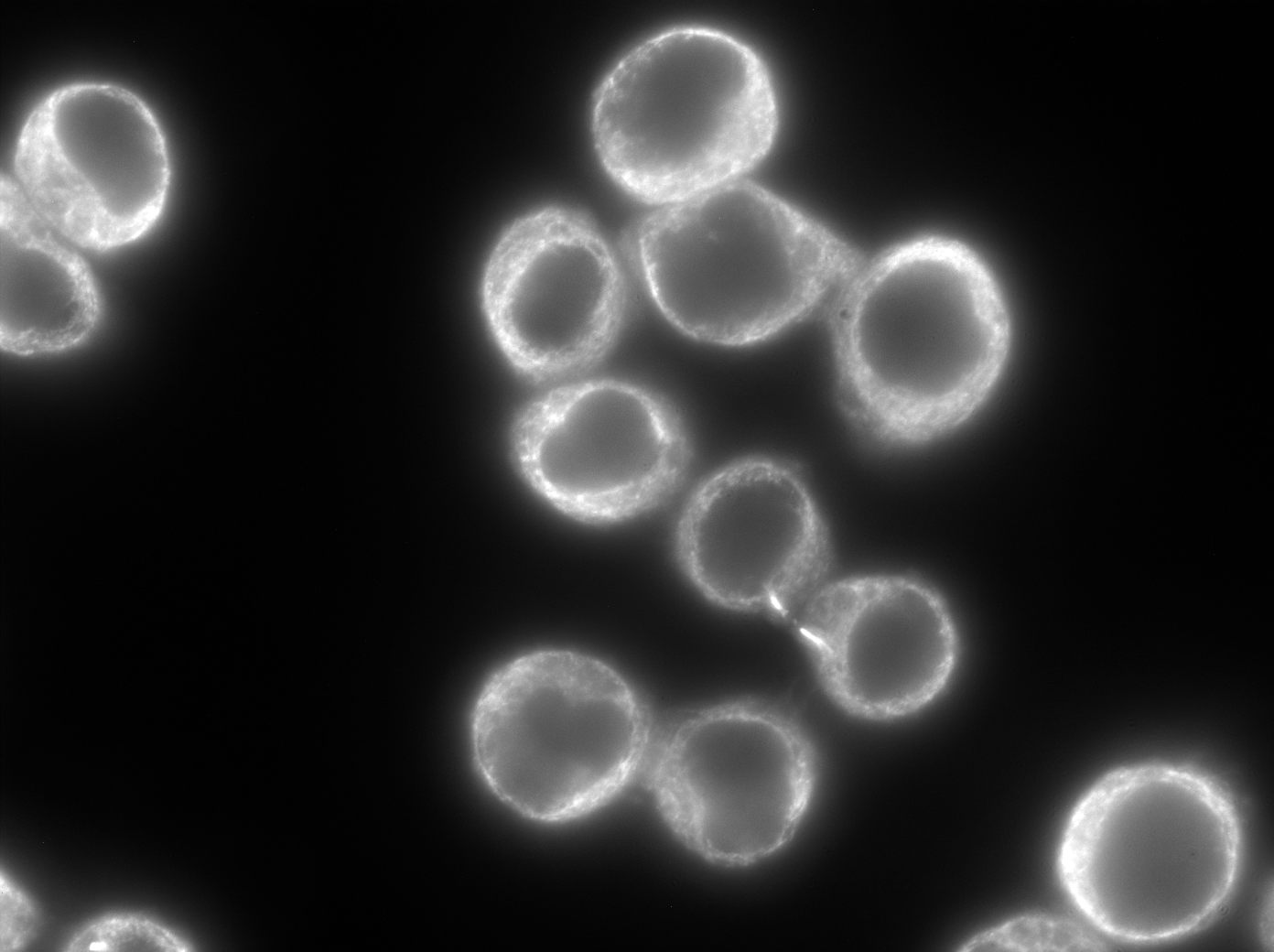

Supplement: Supplementary file 8 — Source data Fig. 2 part 5 [file 44319_2026_742_MOESM8_ESM.zip › Figure 2 Part 5/Fig 2d polye atubulin part 2/WT PolyE-atub 8-14-24 R3 LT6.Project Maximum Z_XY1723843473_Z0_T0_C1.tif]

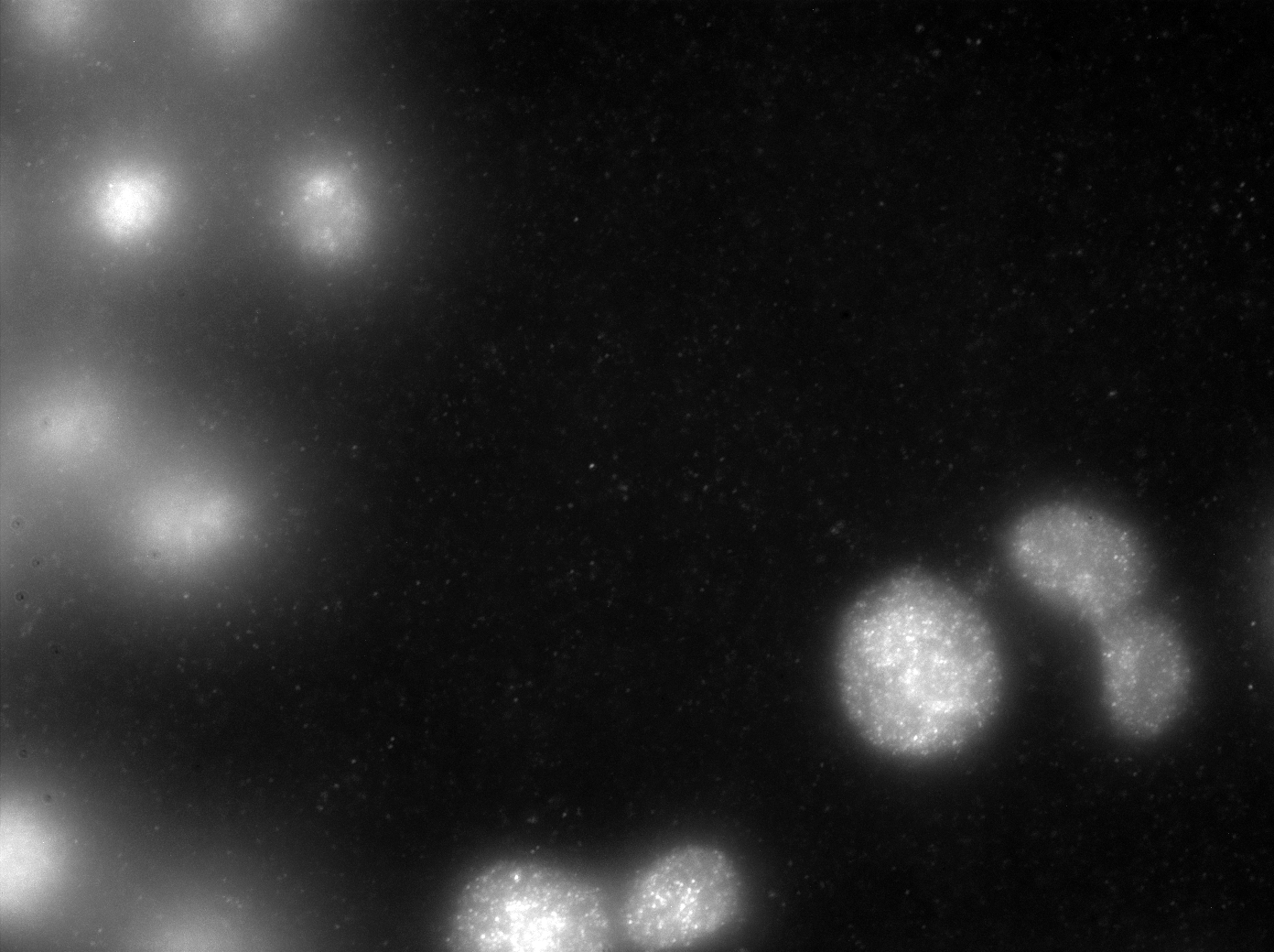

Supplement: Supplementary file 8 — Source data Fig. 2 part 5 [file 44319_2026_742_MOESM8_ESM.zip › Figure 2 Part 5/Fig 2d polye atubulin part 2/WT PolyE-atub 8-14-24 R3 M9.Project Maximum Z_XY1723846152_Z0_T0_C2.tif]
