# Supplementary figures and images for: TPGS1 regulates central spindle microtubule glutamylation and remodeling during telophase and abscission (part 9 of 36)
Source: EMBO Rep. 2026 Mar 23;27(8):1944–63. doi: 10.1038/s44319-026-00742-3 (PMC13121839; doi:10.1038/s44319-026-00742-3)

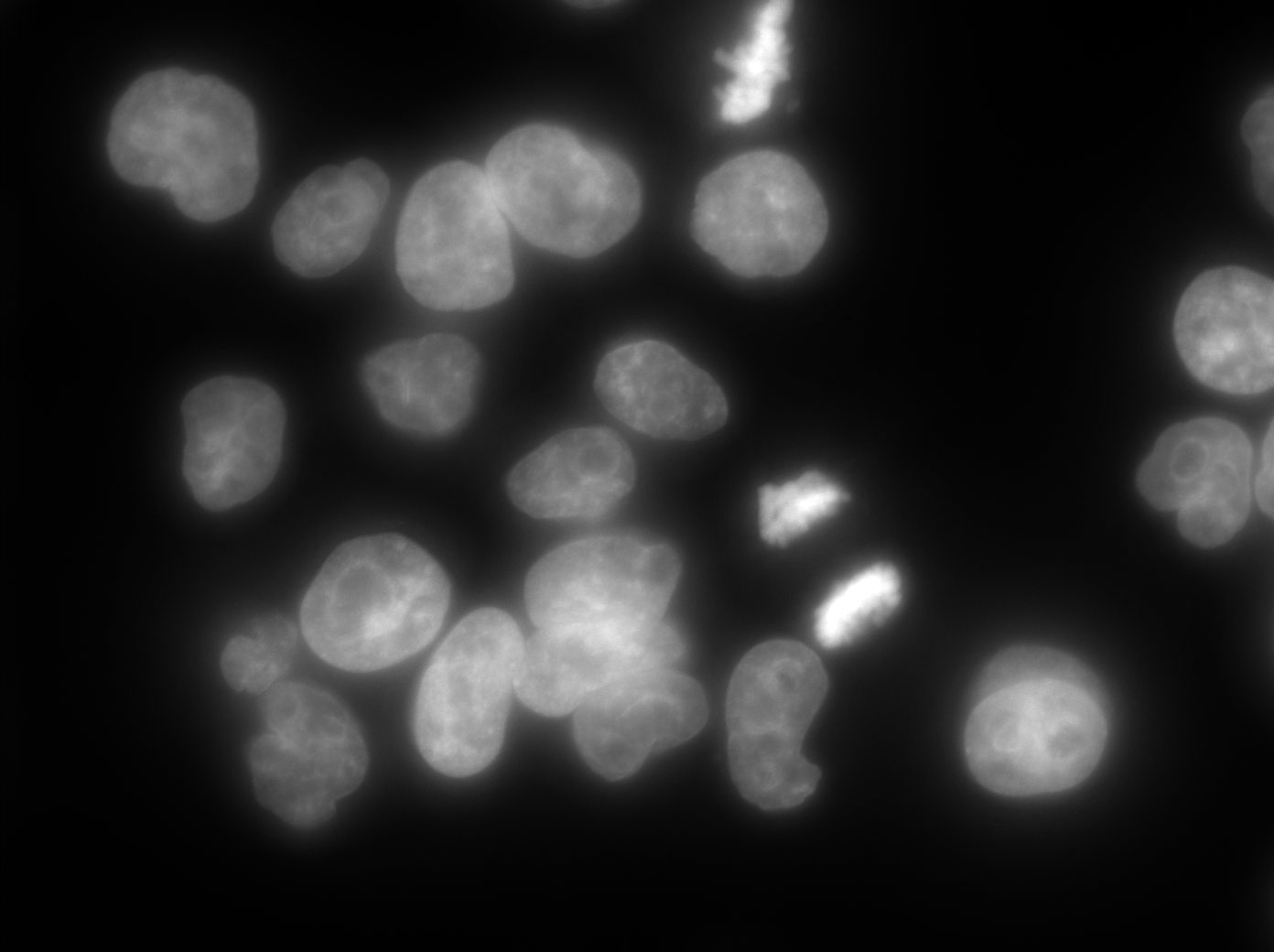

Supplement: Supplementary file 9 — Source data Fig. 2 part 6 [file 44319_2026_742_MOESM9_ESM.zip › Figure 2 Part 6/Fig 2fg Control Hela rGT335 acetylated tubulin/Anaphase/Cas9 actub rGT335 9-8-25 R1 A9.Project Maximum Z_XY1757357993_Z0_T0_C0.tif]

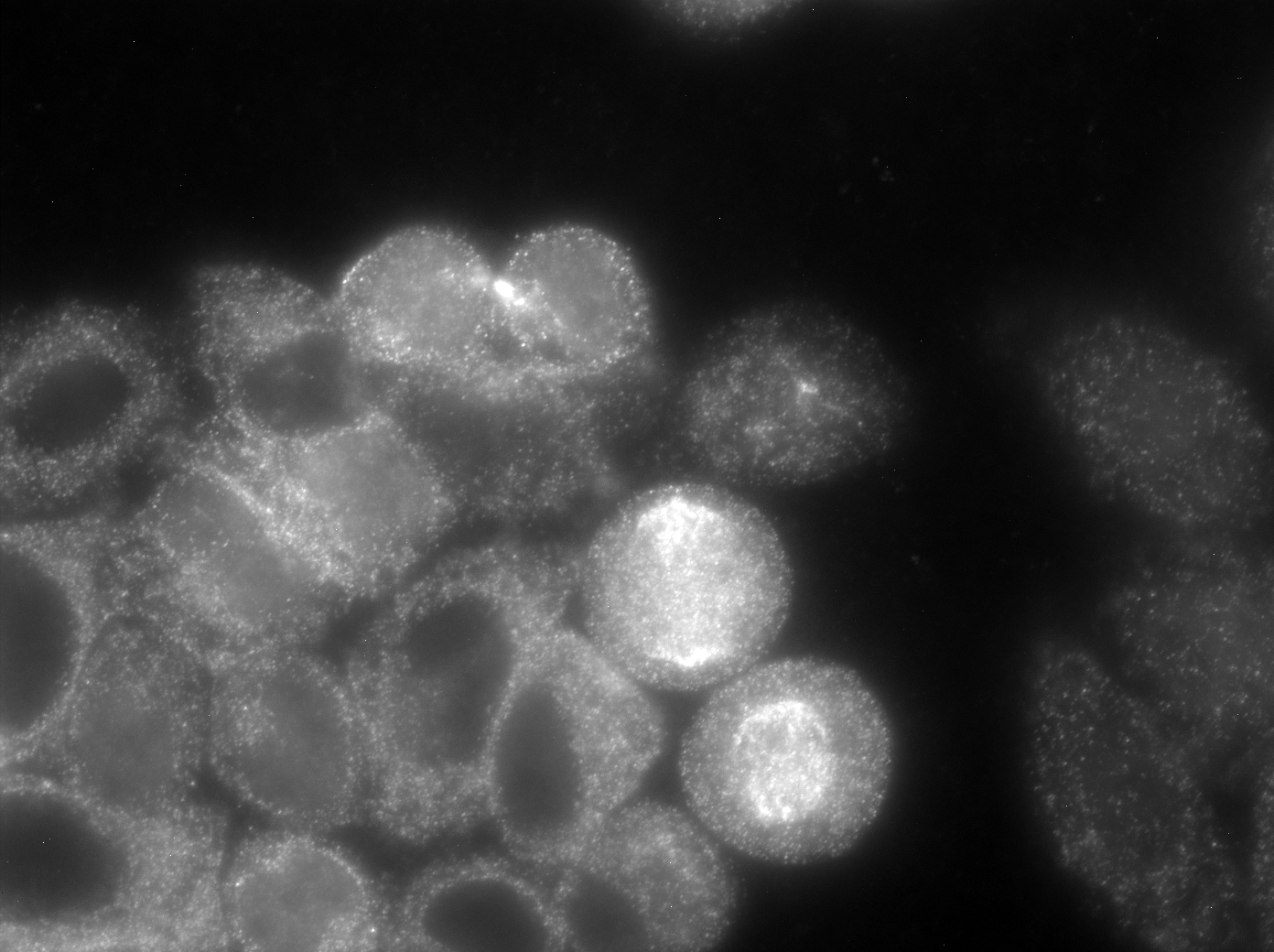

Supplement: Supplementary file 9 — Source data Fig. 2 part 6 [file 44319_2026_742_MOESM9_ESM.zip › Figure 2 Part 6/Fig 2fg Control Hela rGT335 acetylated tubulin/Anaphase/Cas9 actub rGT335 9-8-25 R3 A2 M2.Project Maximum Z_XY1757365779_Z0_T0_C1.tif]

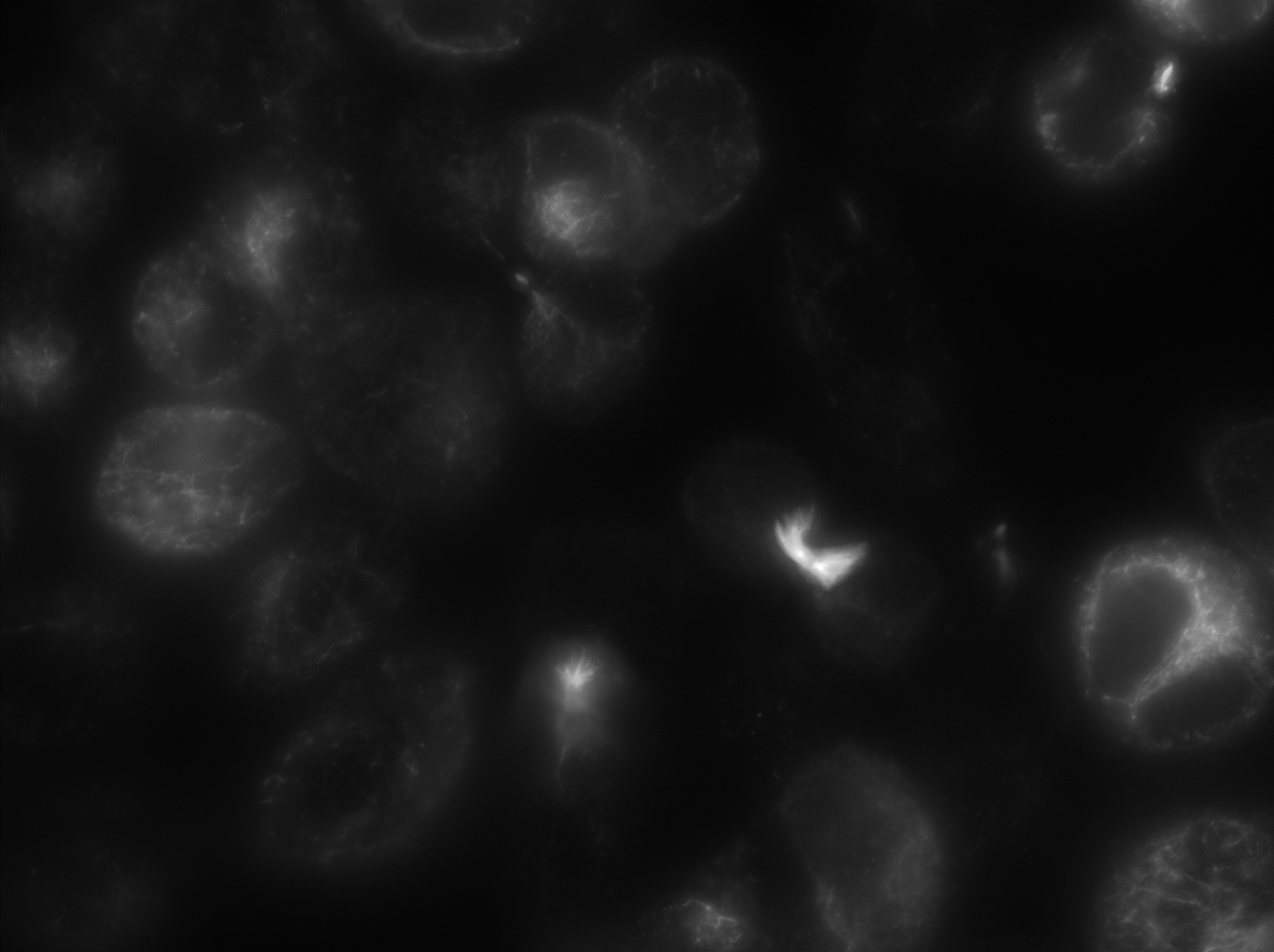

Supplement: Supplementary file 9 — Source data Fig. 2 part 6 [file 44319_2026_742_MOESM9_ESM.zip › Figure 2 Part 6/Fig 2fg Control Hela rGT335 acetylated tubulin/ET/Cas9 actub rGT335 9-8-25 R2 ET8.Project Maximum Z_XY1757362625_Z0_T0_C2.tif]

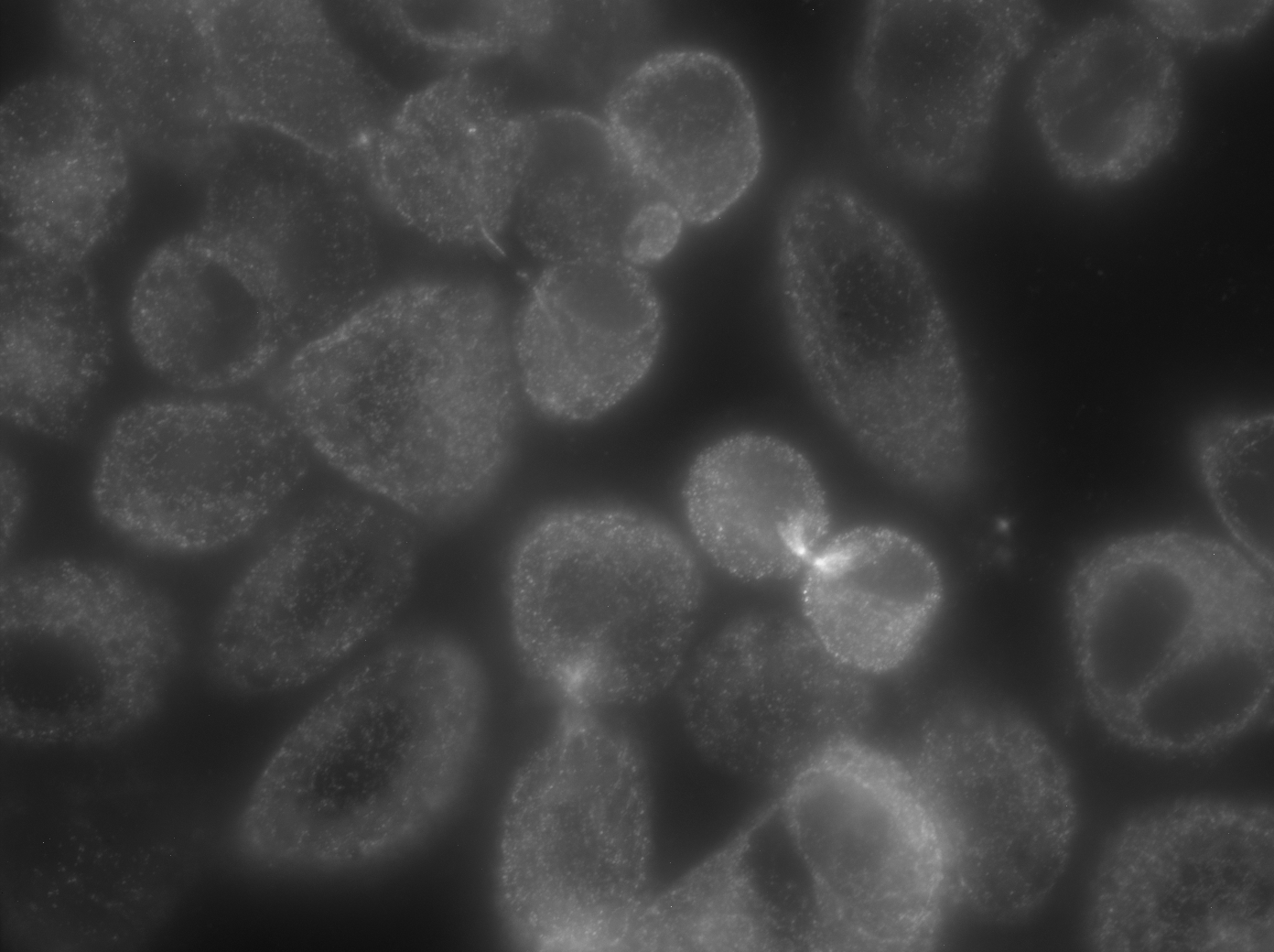

Supplement: Supplementary file 9 — Source data Fig. 2 part 6 [file 44319_2026_742_MOESM9_ESM.zip › Figure 2 Part 6/Fig 2fg Control Hela rGT335 acetylated tubulin/ET/Cas9 actub rGT335 9-8-25 R2 ET8.Project Maximum Z_XY1757362625_Z0_T0_C1.tif]

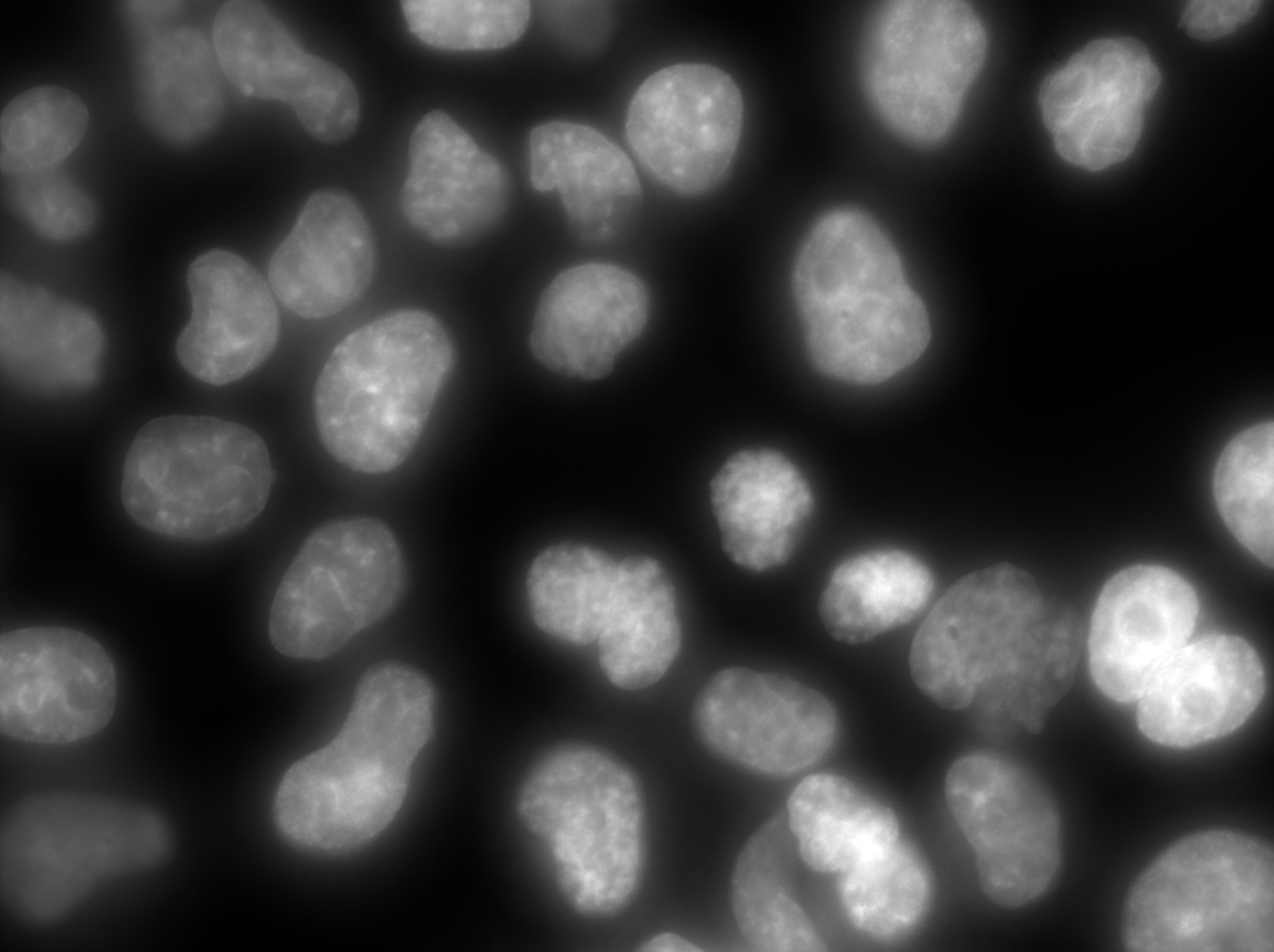

Supplement: Supplementary file 9 — Source data Fig. 2 part 6 [file 44319_2026_742_MOESM9_ESM.zip › Figure 2 Part 6/Fig 2fg Control Hela rGT335 acetylated tubulin/ET/Cas9 actub rGT335 9-8-25 R2 ET8.Project Maximum Z_XY1757362625_Z0_T0_C0.tif]

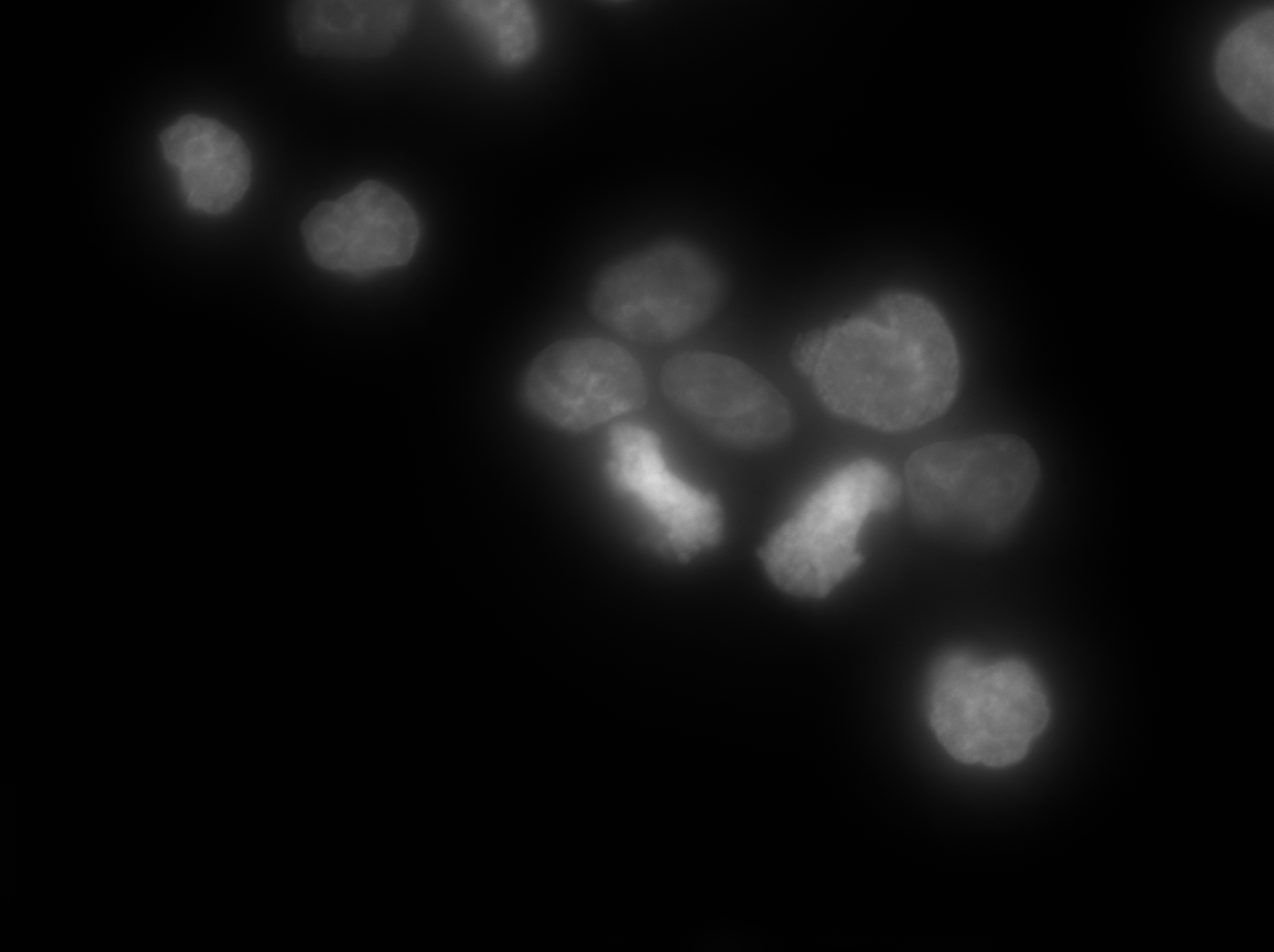

Supplement: Supplementary file 9 — Source data Fig. 2 part 6 [file 44319_2026_742_MOESM9_ESM.zip › Figure 2 Part 6/Fig 2fg Control Hela rGT335 acetylated tubulin/ET/Cas9 actub rGT335 9-8-25 R1 ET5.Project Maximum Z_XY1757352365_Z0_T0_C0.tif]

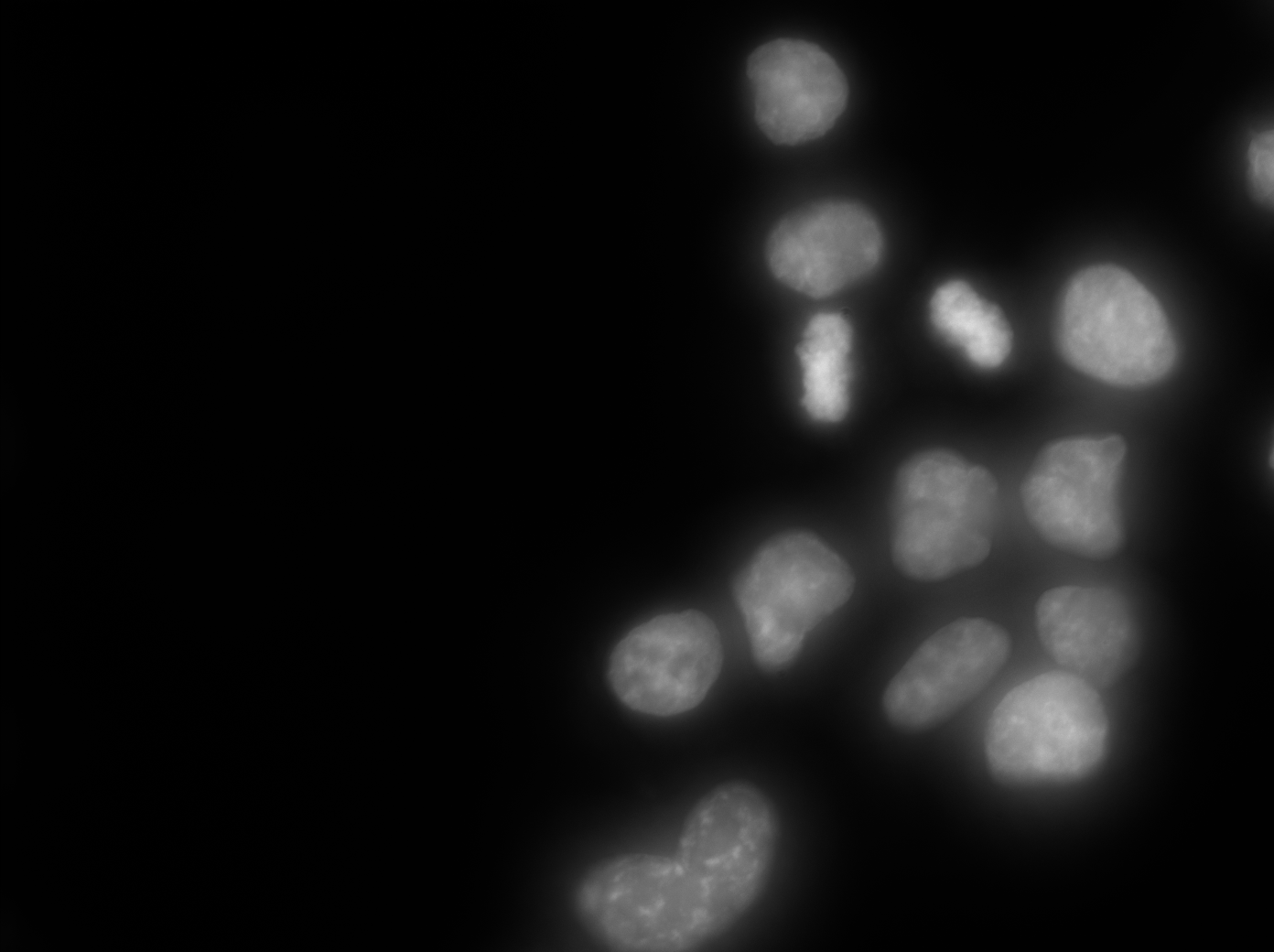

Supplement: Supplementary file 9 — Source data Fig. 2 part 6 [file 44319_2026_742_MOESM9_ESM.zip › Figure 2 Part 6/Fig 2fg Control Hela rGT335 acetylated tubulin/ET/Cas9 actub rGT335 9-8-25 R1 ET6.Project Maximum Z_XY1757353337_Z0_T0_C0.tif]

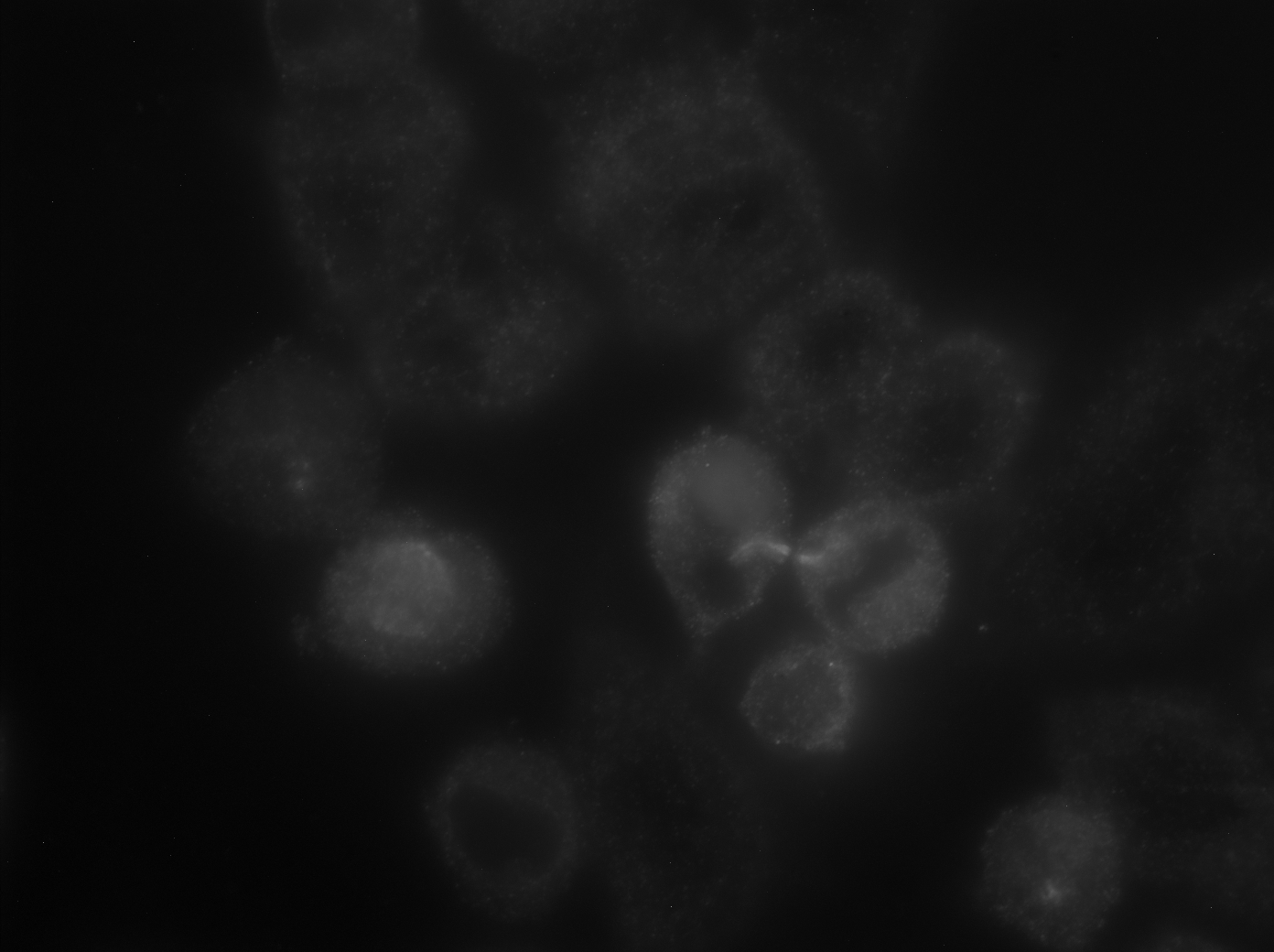

Supplement: Supplementary file 9 — Source data Fig. 2 part 6 [file 44319_2026_742_MOESM9_ESM.zip › Figure 2 Part 6/Fig 2fg Control Hela rGT335 acetylated tubulin/ET/Cas9 actub rGT335 9-8-25 R2 ET10.Project Maximum Z_XY1757363113_Z0_T0_C1.tif]

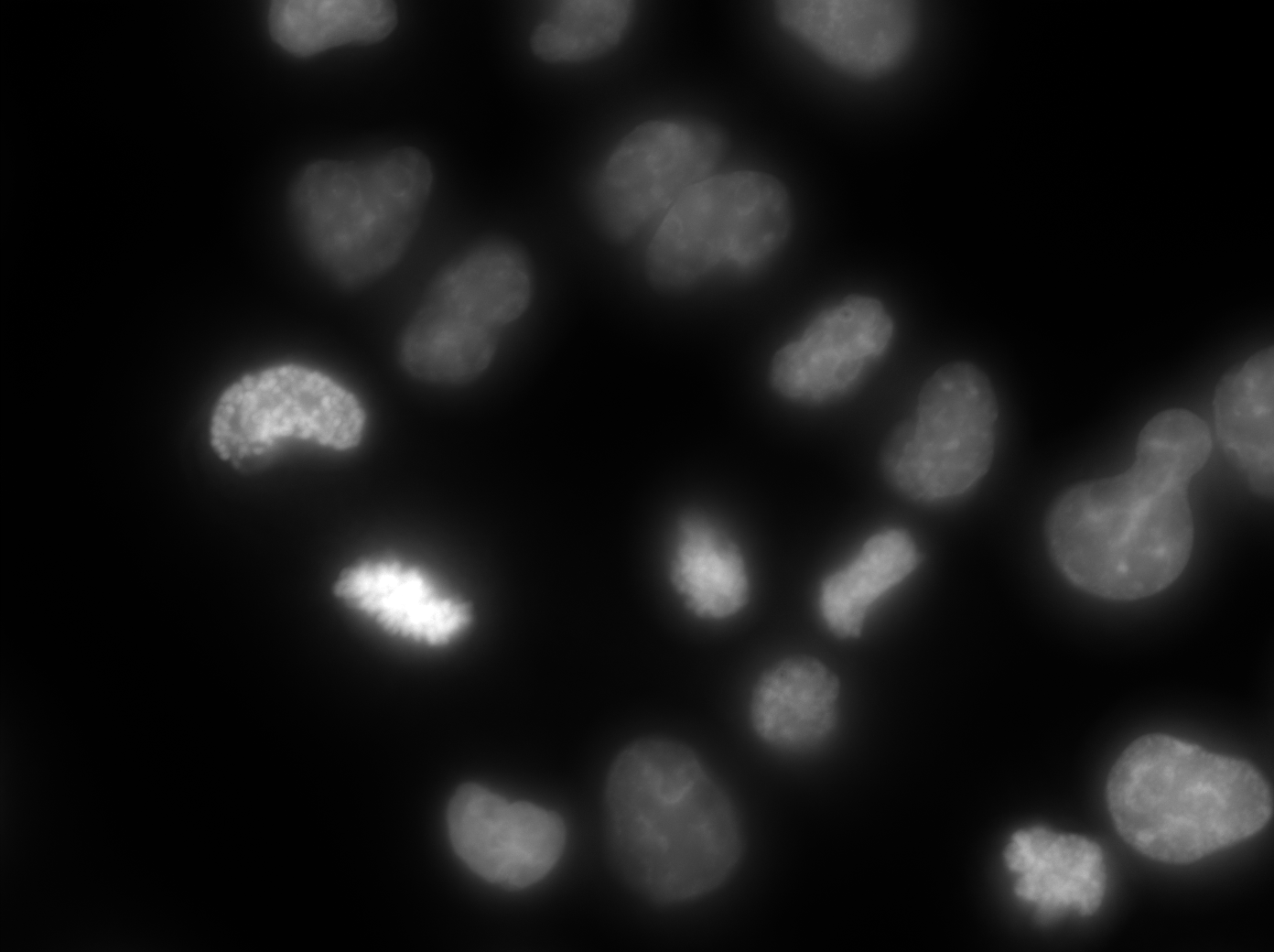

Supplement: Supplementary file 9 — Source data Fig. 2 part 6 [file 44319_2026_742_MOESM9_ESM.zip › Figure 2 Part 6/Fig 2fg Control Hela rGT335 acetylated tubulin/ET/Cas9 actub rGT335 9-8-25 R2 ET10.Project Maximum Z_XY1757363113_Z0_T0_C0.tif]

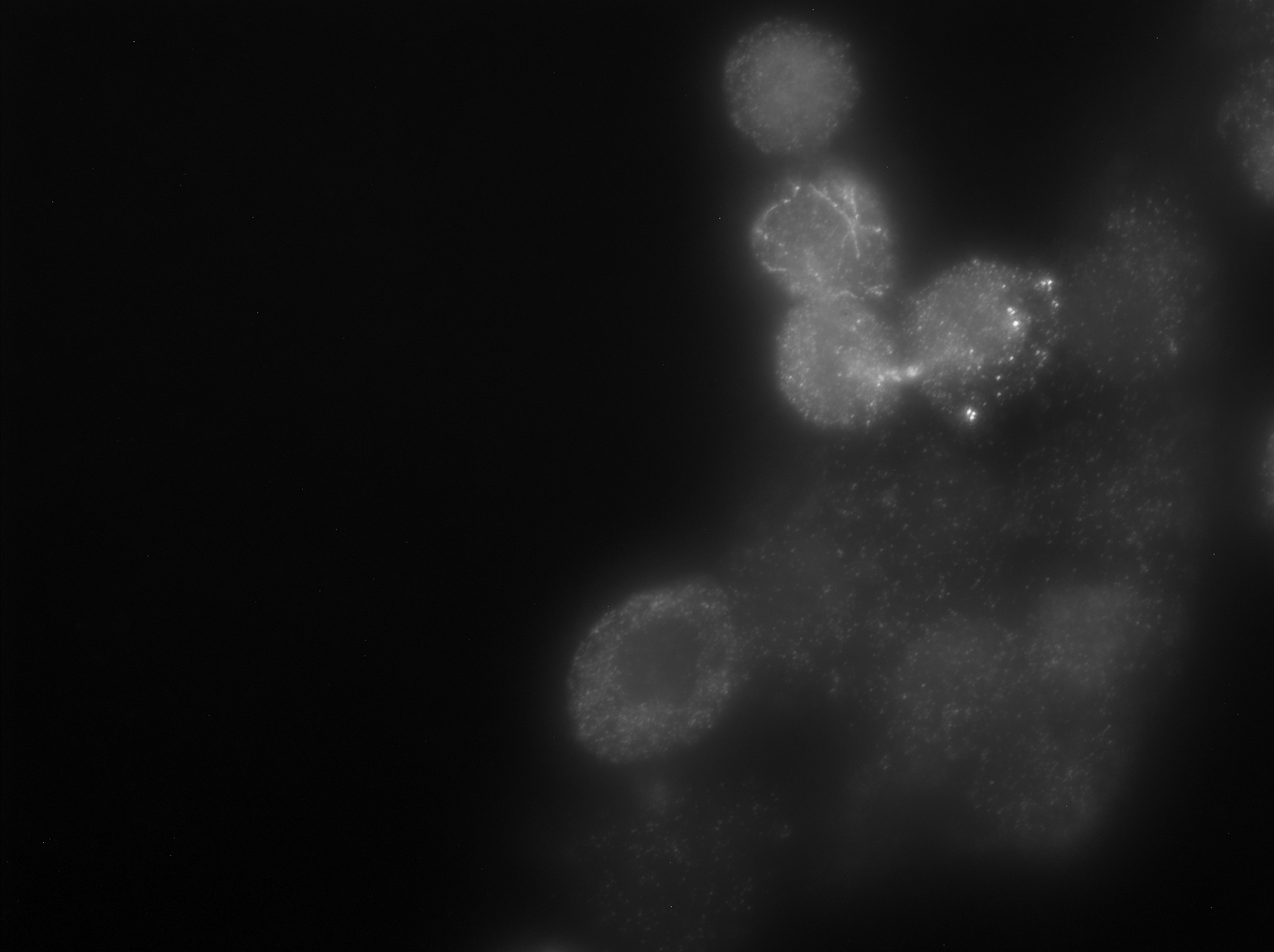

Supplement: Supplementary file 9 — Source data Fig. 2 part 6 [file 44319_2026_742_MOESM9_ESM.zip › Figure 2 Part 6/Fig 2fg Control Hela rGT335 acetylated tubulin/ET/Cas9 actub rGT335 9-8-25 R1 ET6.Project Maximum Z_XY1757353337_Z0_T0_C1.tif]

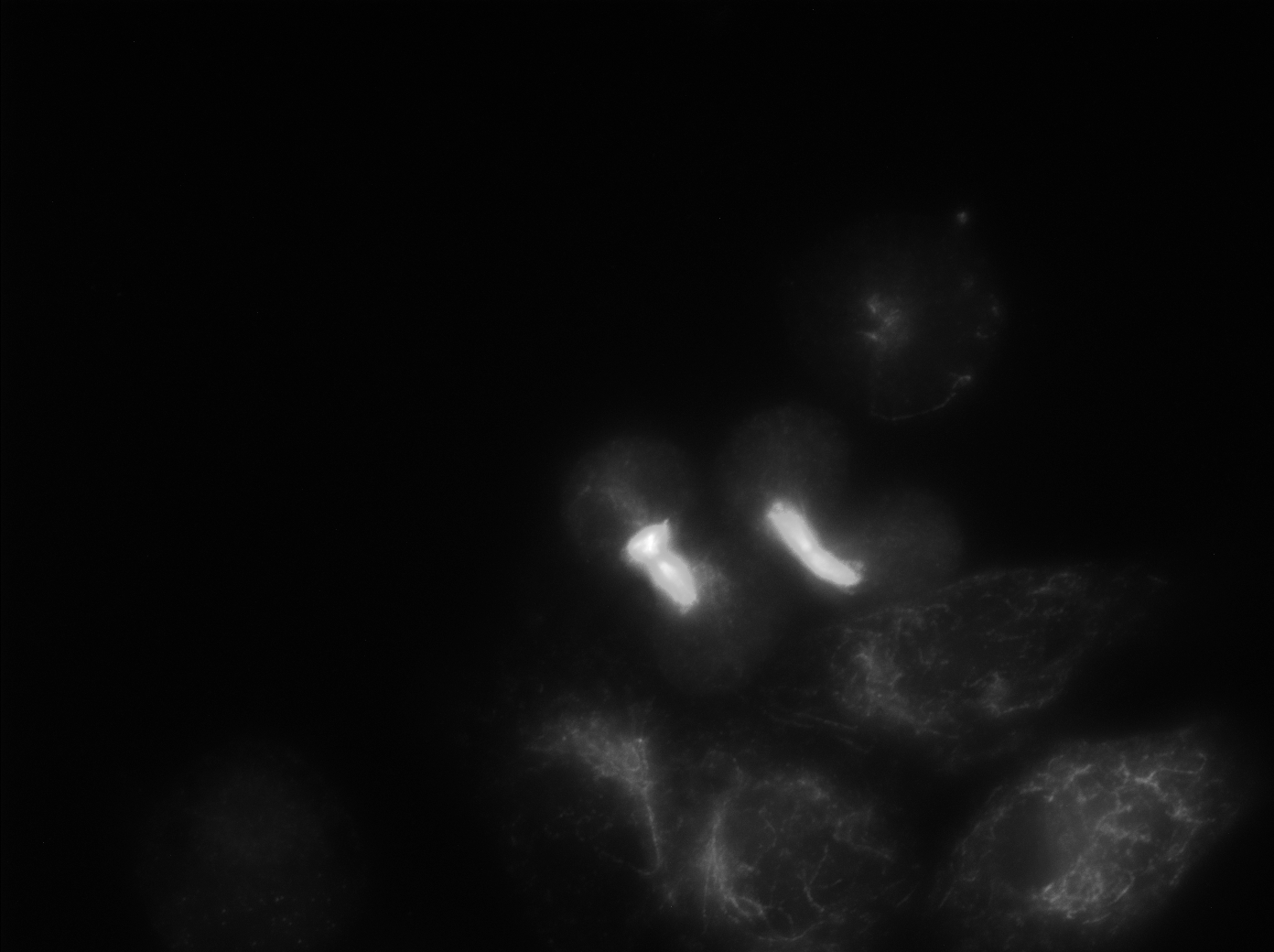

Supplement: Supplementary file 9 — Source data Fig. 2 part 6 [file 44319_2026_742_MOESM9_ESM.zip › Figure 2 Part 6/Fig 2fg Control Hela rGT335 acetylated tubulin/ET/Cas9 actub rGT335 9-8-25 R3 ET2ET3.Project Maximum Z_XY1757364815_Z0_T0_C2.tif]

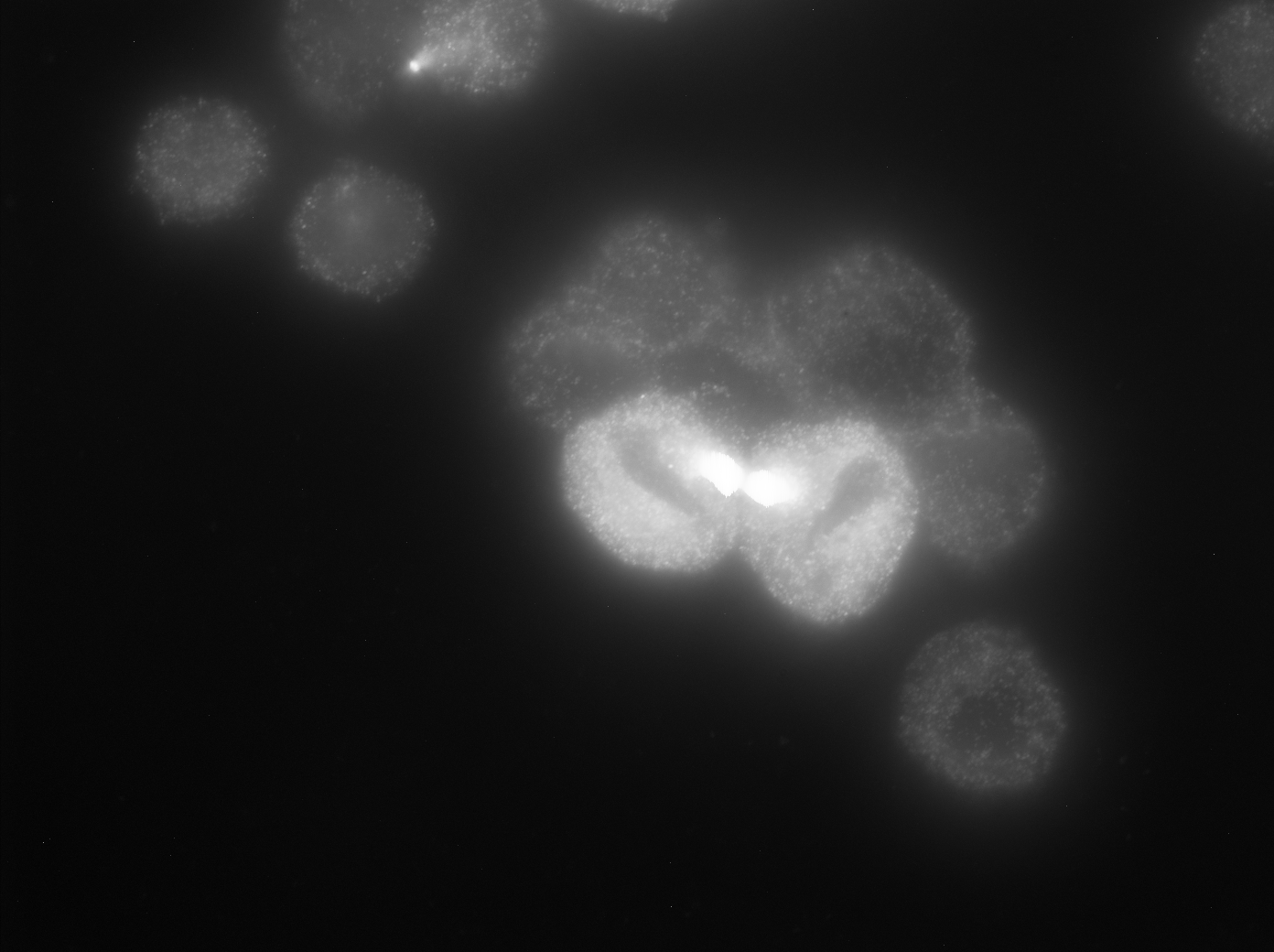

Supplement: Supplementary file 9 — Source data Fig. 2 part 6 [file 44319_2026_742_MOESM9_ESM.zip › Figure 2 Part 6/Fig 2fg Control Hela rGT335 acetylated tubulin/ET/Cas9 actub rGT335 9-8-25 R1 ET5.Project Maximum Z_XY1757352365_Z0_T0_C1.tif]

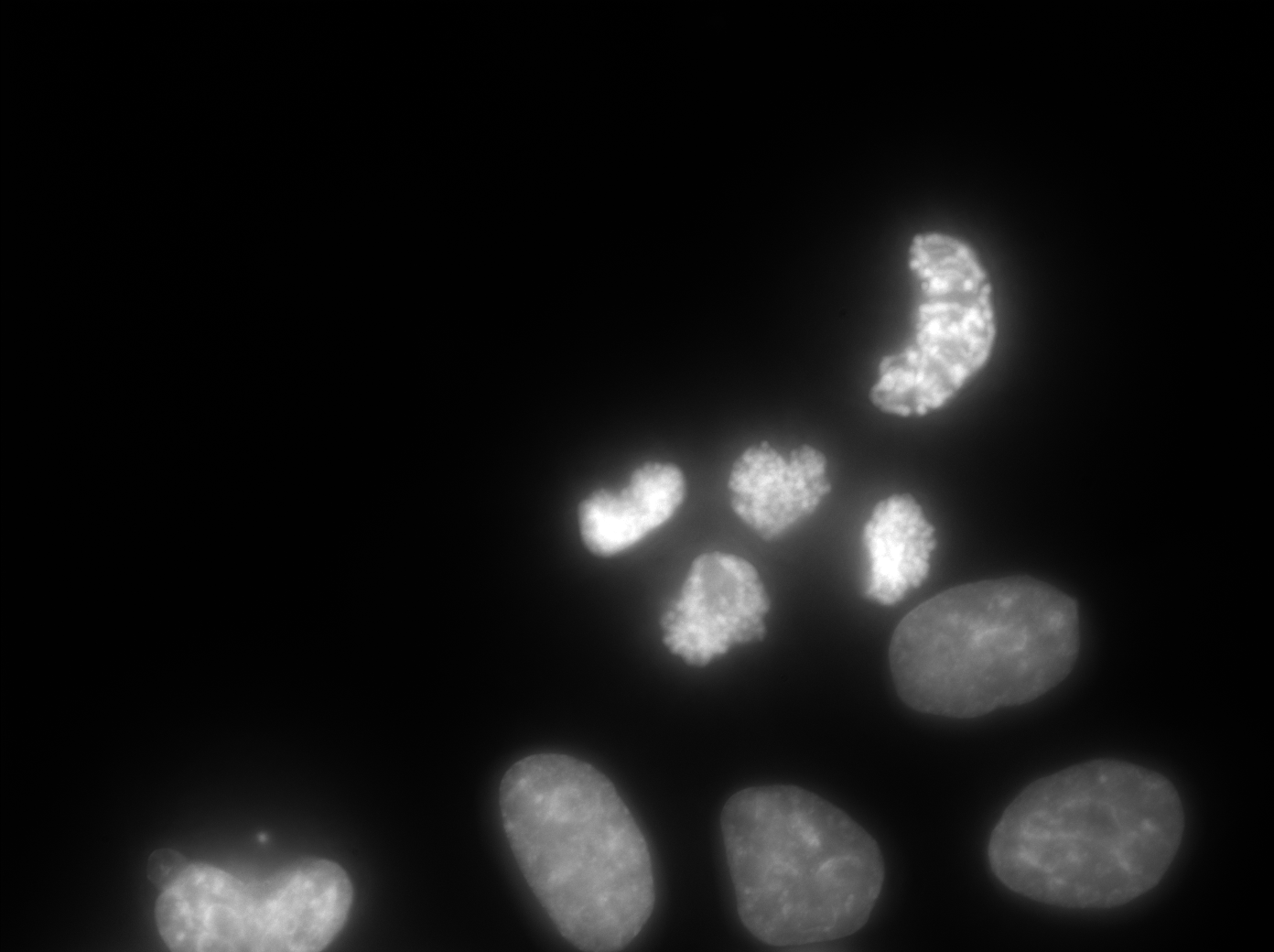

Supplement: Supplementary file 9 — Source data Fig. 2 part 6 [file 44319_2026_742_MOESM9_ESM.zip › Figure 2 Part 6/Fig 2fg Control Hela rGT335 acetylated tubulin/ET/Cas9 actub rGT335 9-8-25 R3 ET2ET3.Project Maximum Z_XY1757364815_Z0_T0_C0.tif]

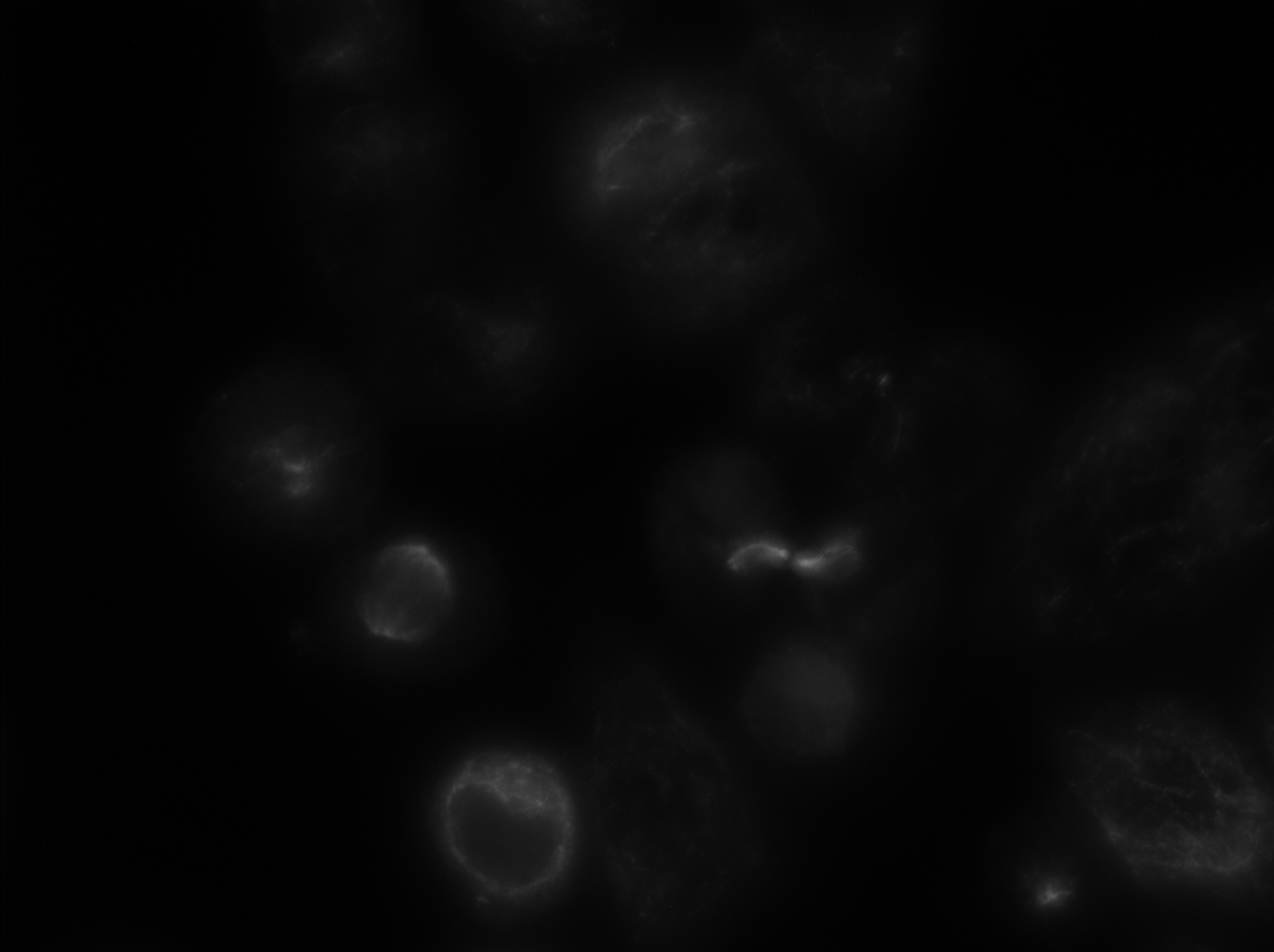

Supplement: Supplementary file 9 — Source data Fig. 2 part 6 [file 44319_2026_742_MOESM9_ESM.zip › Figure 2 Part 6/Fig 2fg Control Hela rGT335 acetylated tubulin/ET/Cas9 actub rGT335 9-8-25 R2 ET10.Project Maximum Z_XY1757363113_Z0_T0_C2.tif]

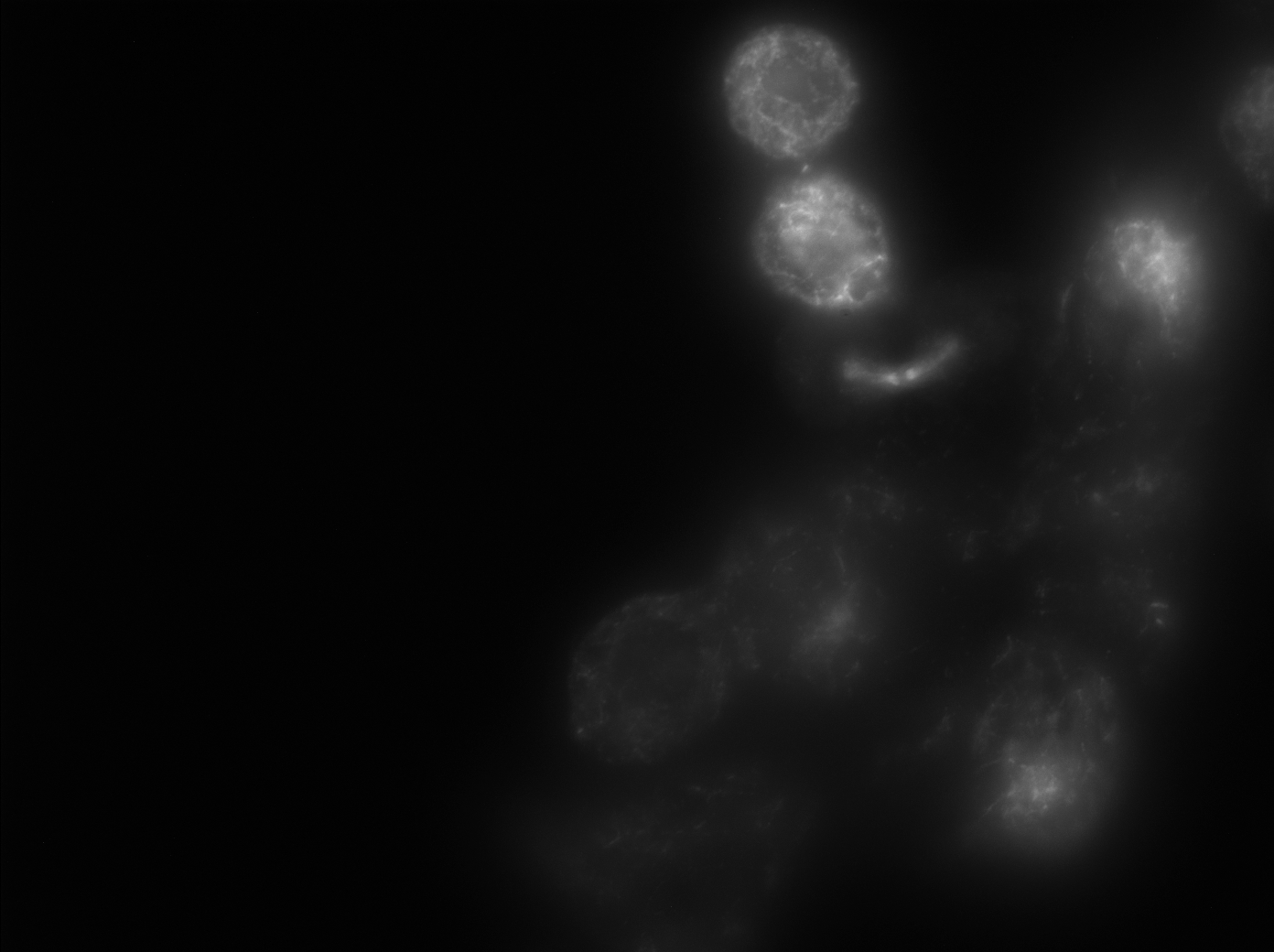

Supplement: Supplementary file 9 — Source data Fig. 2 part 6 [file 44319_2026_742_MOESM9_ESM.zip › Figure 2 Part 6/Fig 2fg Control Hela rGT335 acetylated tubulin/ET/Cas9 actub rGT335 9-8-25 R1 ET6.Project Maximum Z_XY1757353337_Z0_T0_C2.tif]

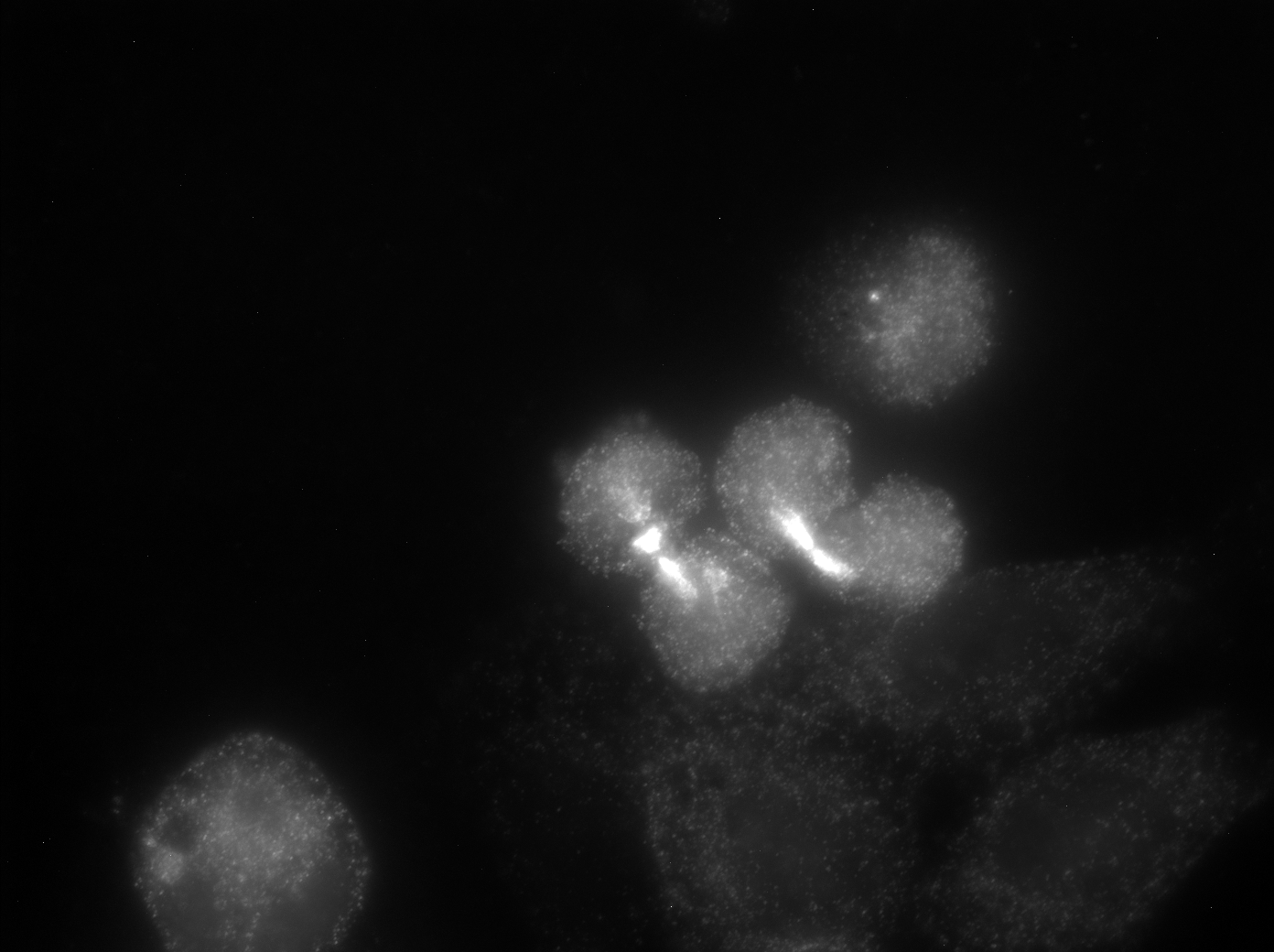

Supplement: Supplementary file 9 — Source data Fig. 2 part 6 [file 44319_2026_742_MOESM9_ESM.zip › Figure 2 Part 6/Fig 2fg Control Hela rGT335 acetylated tubulin/ET/Cas9 actub rGT335 9-8-25 R3 ET2ET3.Project Maximum Z_XY1757364815_Z0_T0_C1.tif]

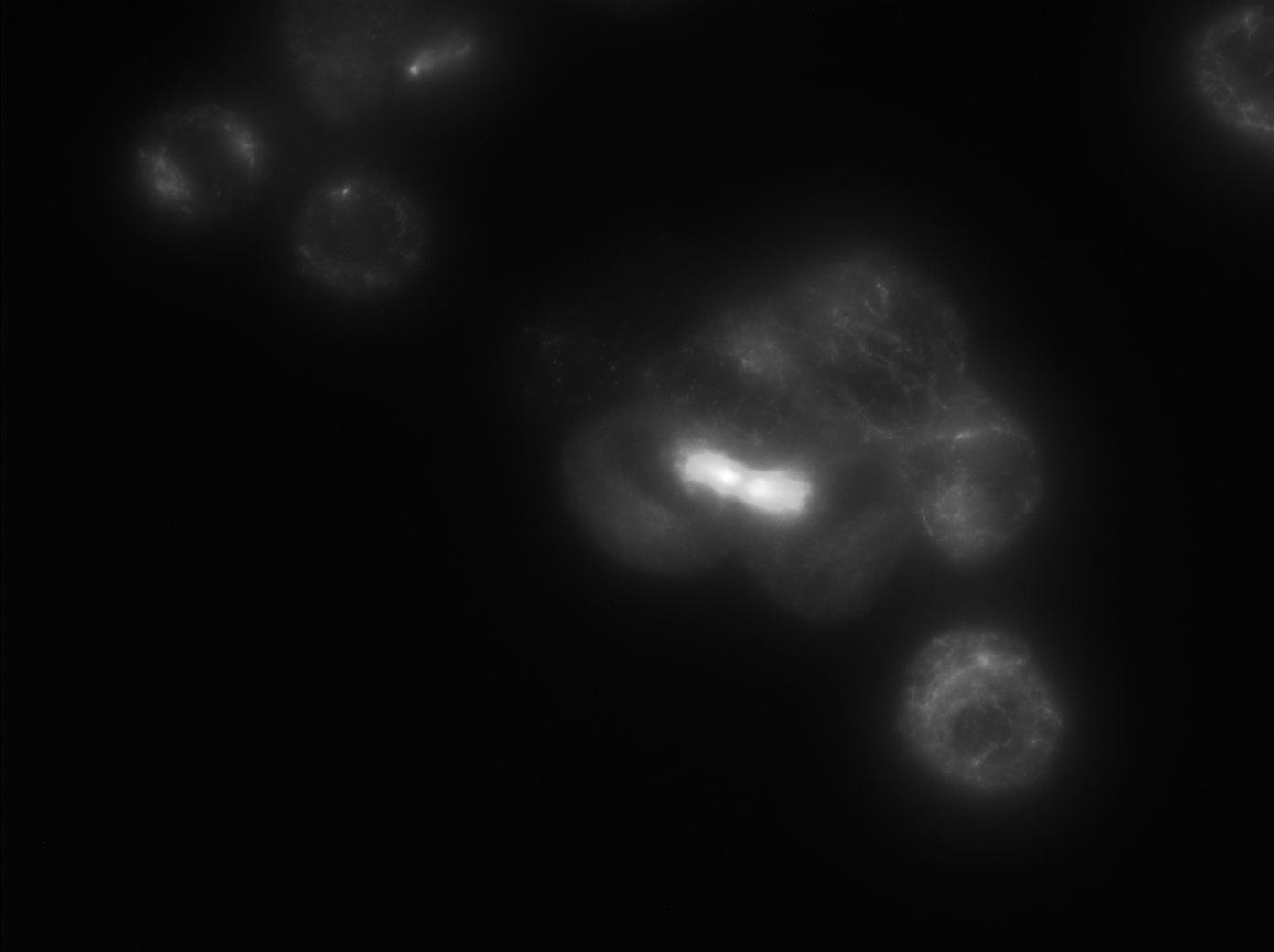

Supplement: Supplementary file 9 — Source data Fig. 2 part 6 [file 44319_2026_742_MOESM9_ESM.zip › Figure 2 Part 6/Fig 2fg Control Hela rGT335 acetylated tubulin/ET/Cas9 actub rGT335 9-8-25 R1 ET5.Project Maximum Z_XY1757352365_Z0_T0_C2.tif]

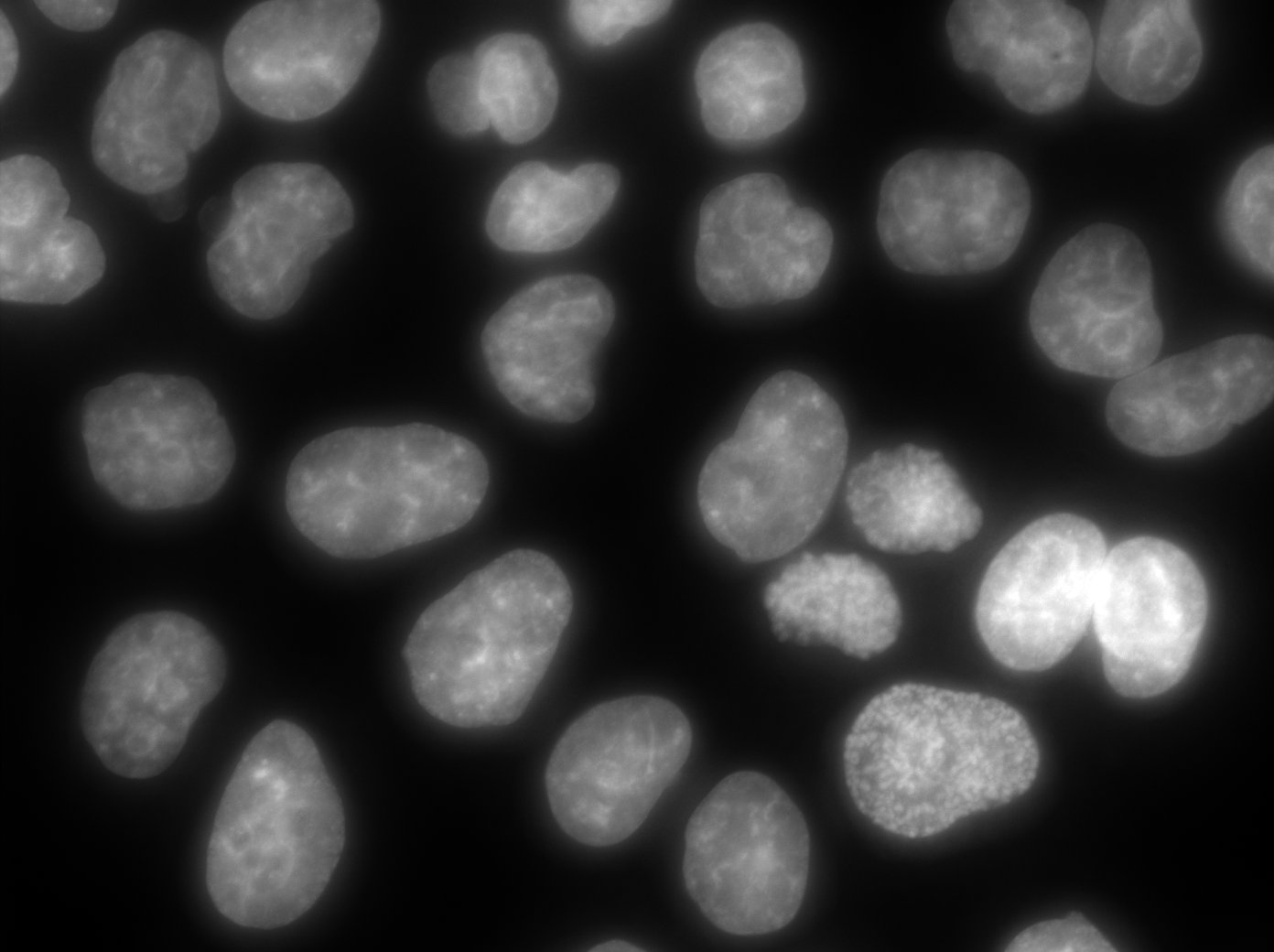

Supplement: Supplementary file 9 — Source data Fig. 2 part 6 [file 44319_2026_742_MOESM9_ESM.zip › Figure 2 Part 6/Fig 2fg Control Hela rGT335 acetylated tubulin/ET/Cas9 actub rGT335 9-8-25 R3 ET4.Project Maximum Z_XY1757365064_Z0_T0_C0.tif]

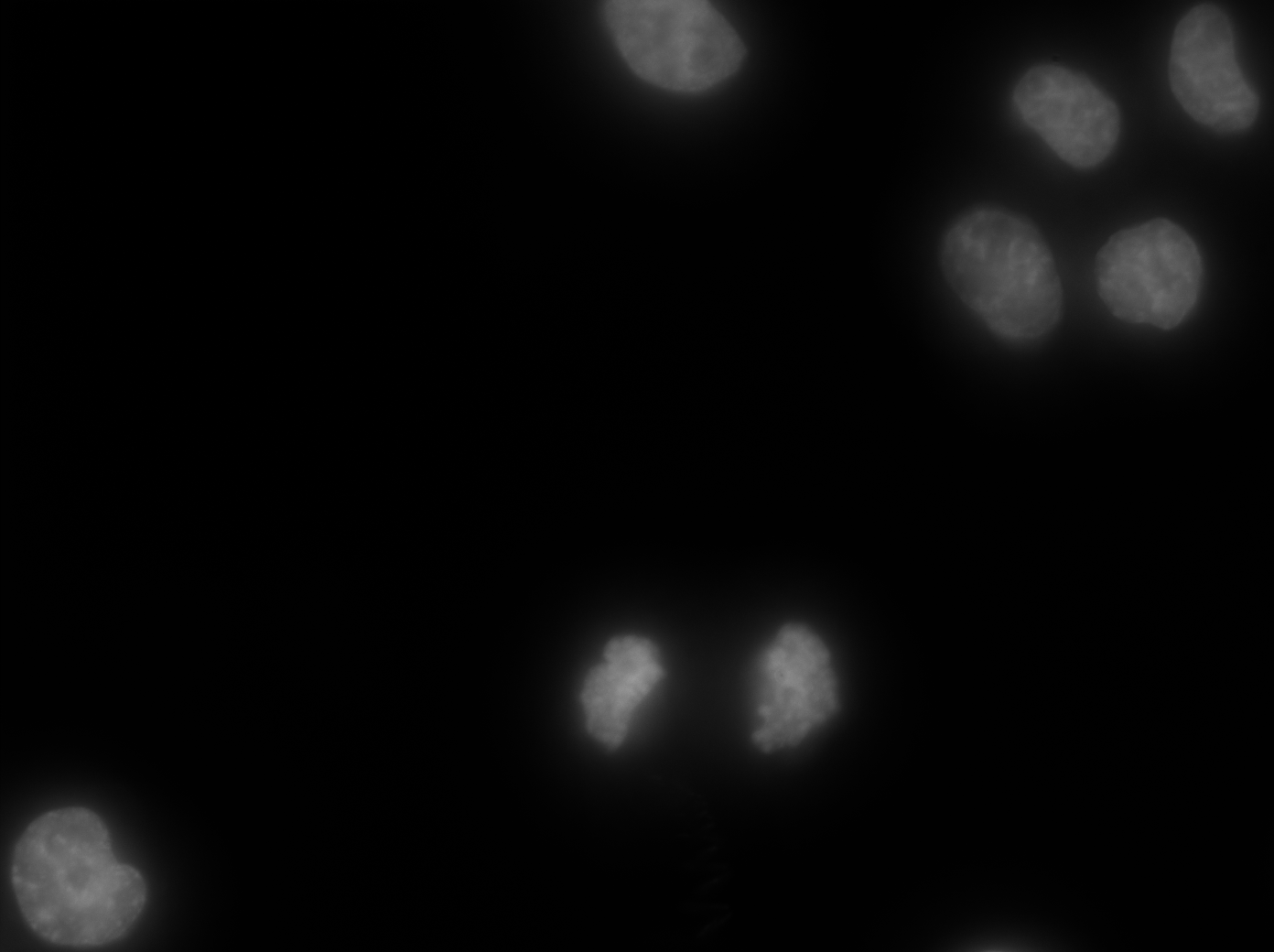

Supplement: Supplementary file 9 — Source data Fig. 2 part 6 [file 44319_2026_742_MOESM9_ESM.zip › Figure 2 Part 6/Fig 2fg Control Hela rGT335 acetylated tubulin/ET/Cas9 actub rGT335 9-8-25 R1 ET1 EX.Project Maximum Z_XY1757350852_Z0_T0_C0.tif]

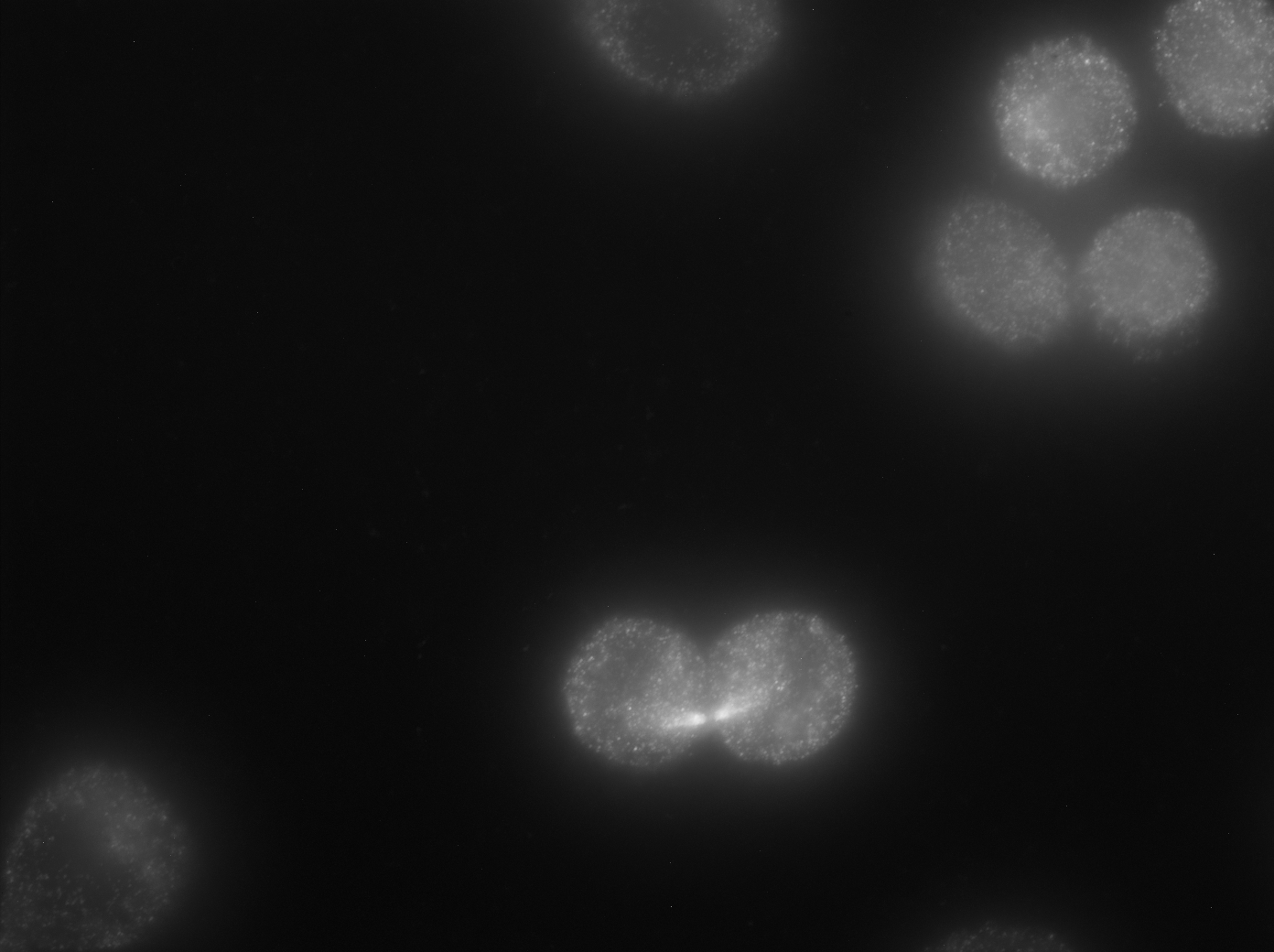

Supplement: Supplementary file 9 — Source data Fig. 2 part 6 [file 44319_2026_742_MOESM9_ESM.zip › Figure 2 Part 6/Fig 2fg Control Hela rGT335 acetylated tubulin/ET/Cas9 actub rGT335 9-8-25 R1 ET1 EX.Project Maximum Z_XY1757350852_Z0_T0_C1.tif]

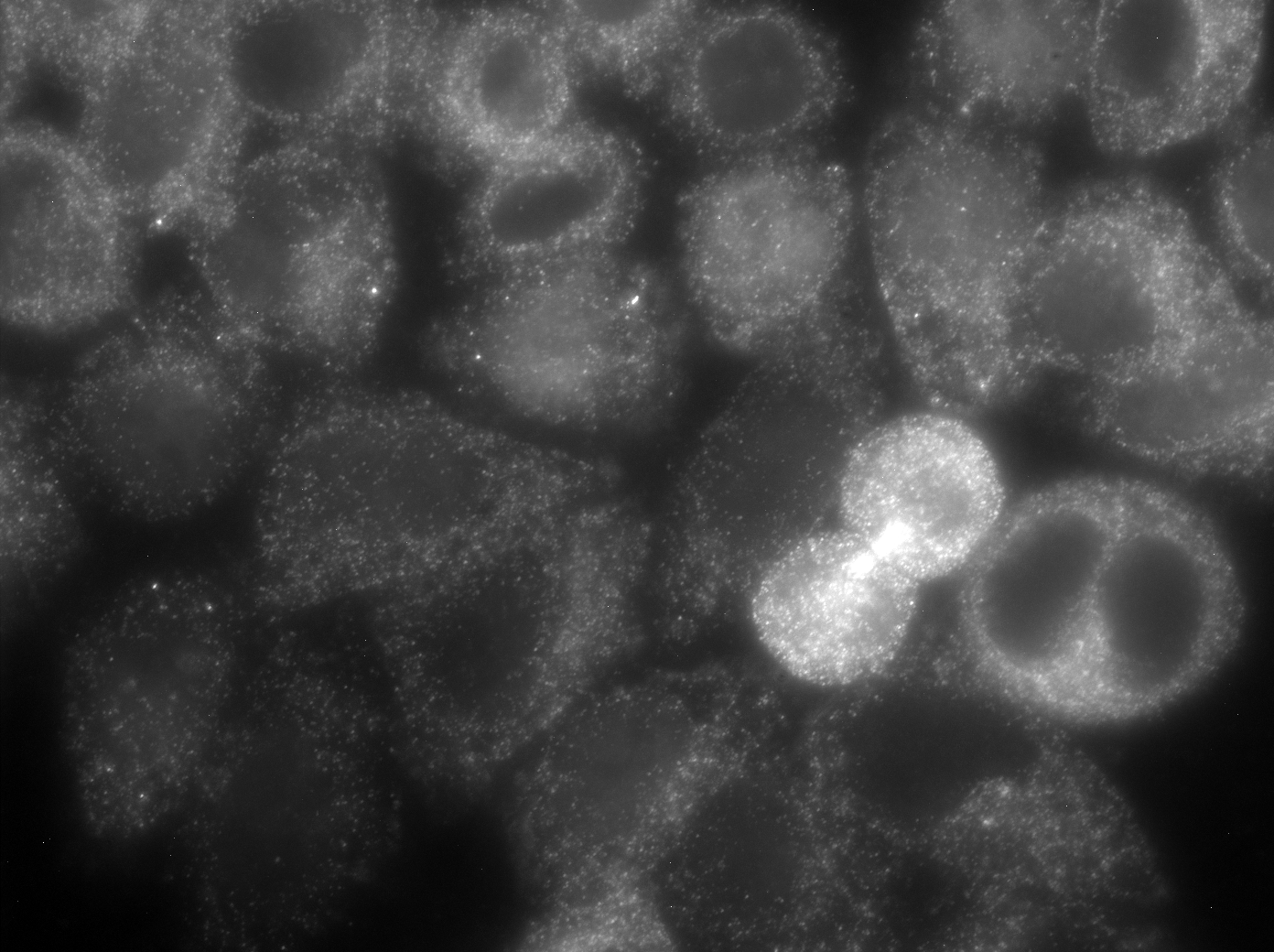

Supplement: Supplementary file 9 — Source data Fig. 2 part 6 [file 44319_2026_742_MOESM9_ESM.zip › Figure 2 Part 6/Fig 2fg Control Hela rGT335 acetylated tubulin/ET/Cas9 actub rGT335 9-8-25 R3 ET4.Project Maximum Z_XY1757365064_Z0_T0_C1.tif]

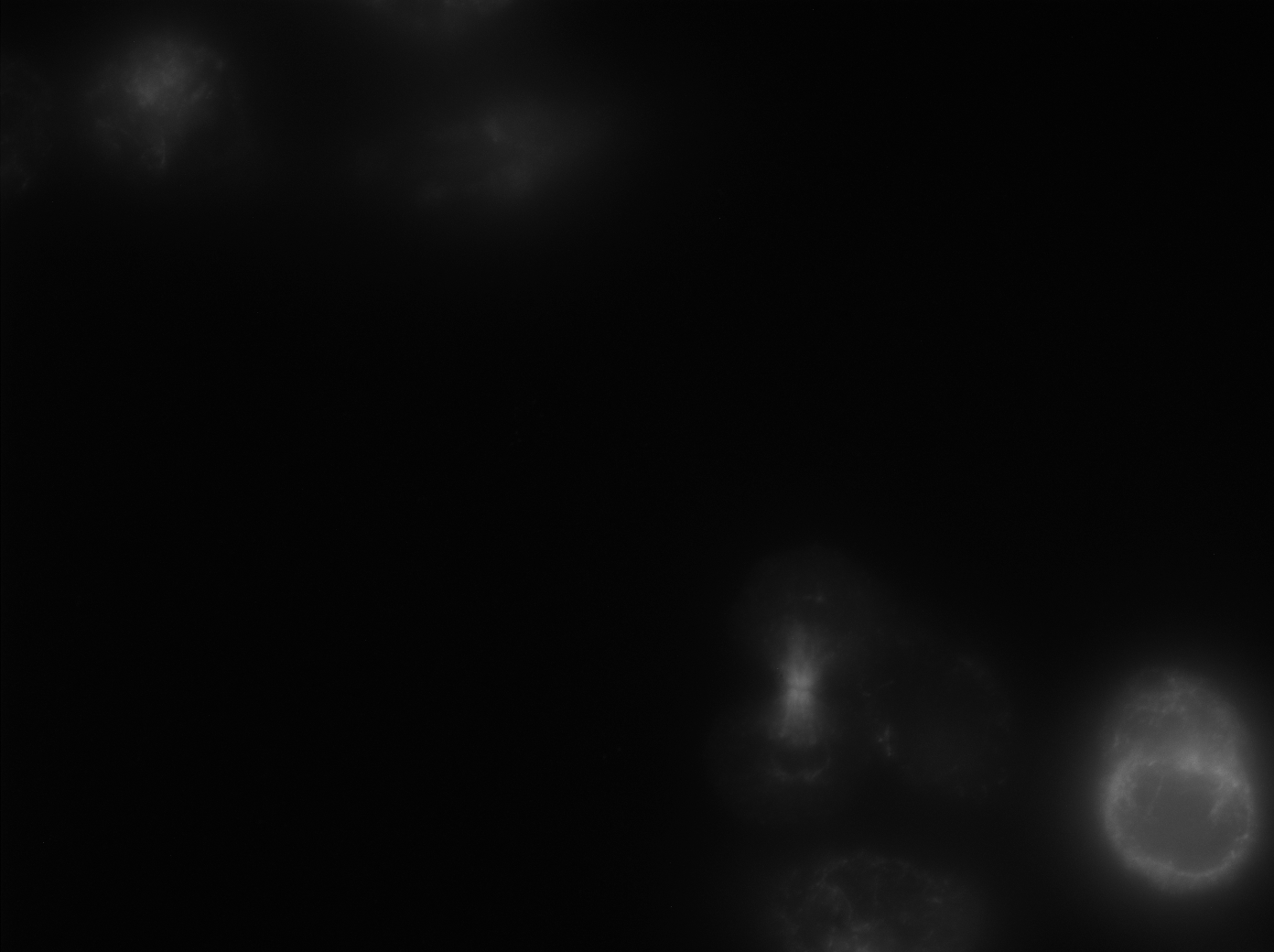

Supplement: Supplementary file 9 — Source data Fig. 2 part 6 [file 44319_2026_742_MOESM9_ESM.zip › Figure 2 Part 6/Fig 2fg Control Hela rGT335 acetylated tubulin/ET/Cas9 actub rGT335 9-8-25 R1 ET10.Project Maximum Z_XY1757354707_Z0_T0_C2.tif]

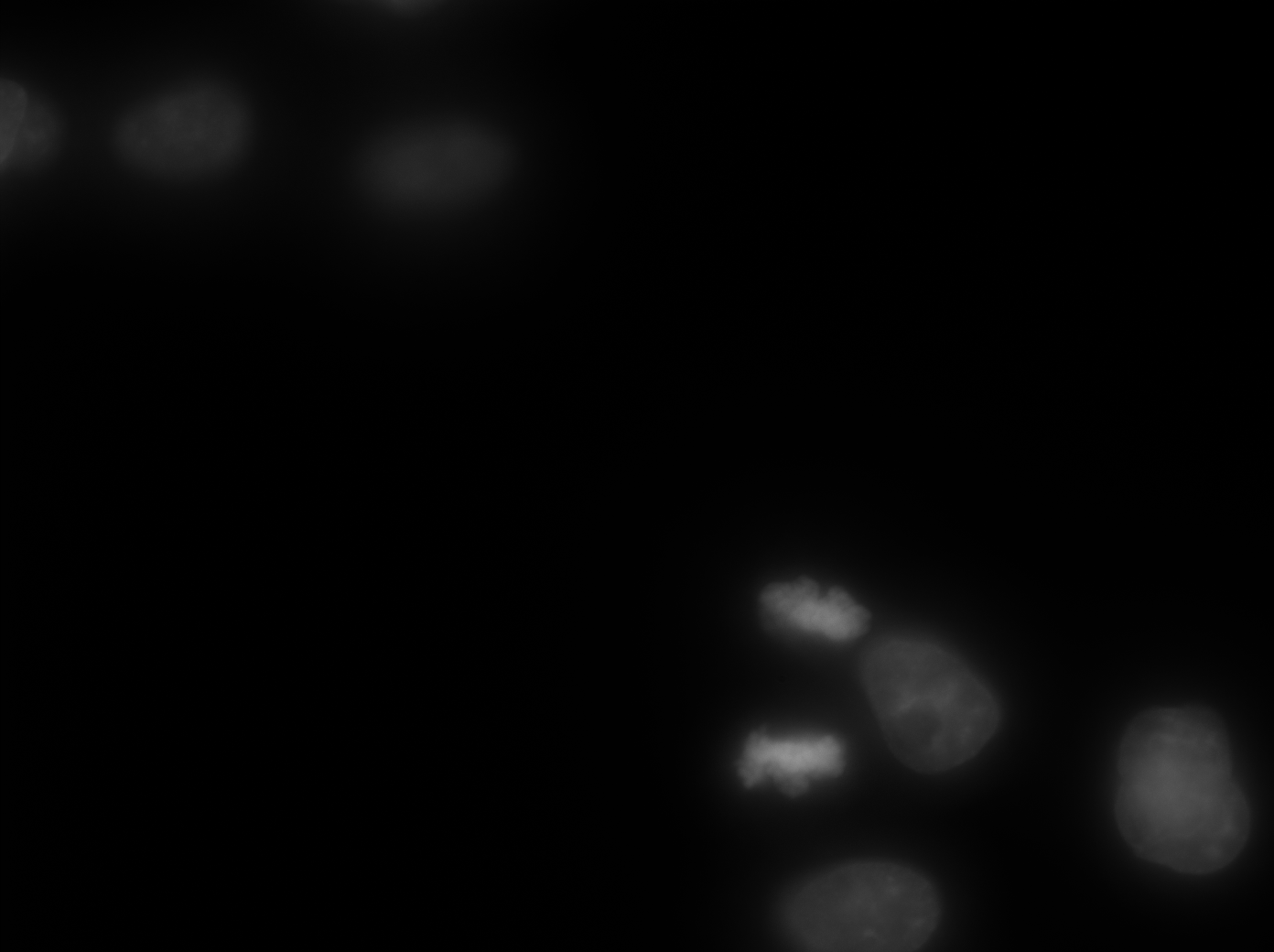

Supplement: Supplementary file 9 — Source data Fig. 2 part 6 [file 44319_2026_742_MOESM9_ESM.zip › Figure 2 Part 6/Fig 2fg Control Hela rGT335 acetylated tubulin/ET/Cas9 actub rGT335 9-8-25 R1 ET10.Project Maximum Z_XY1757354707_Z0_T0_C0.tif]

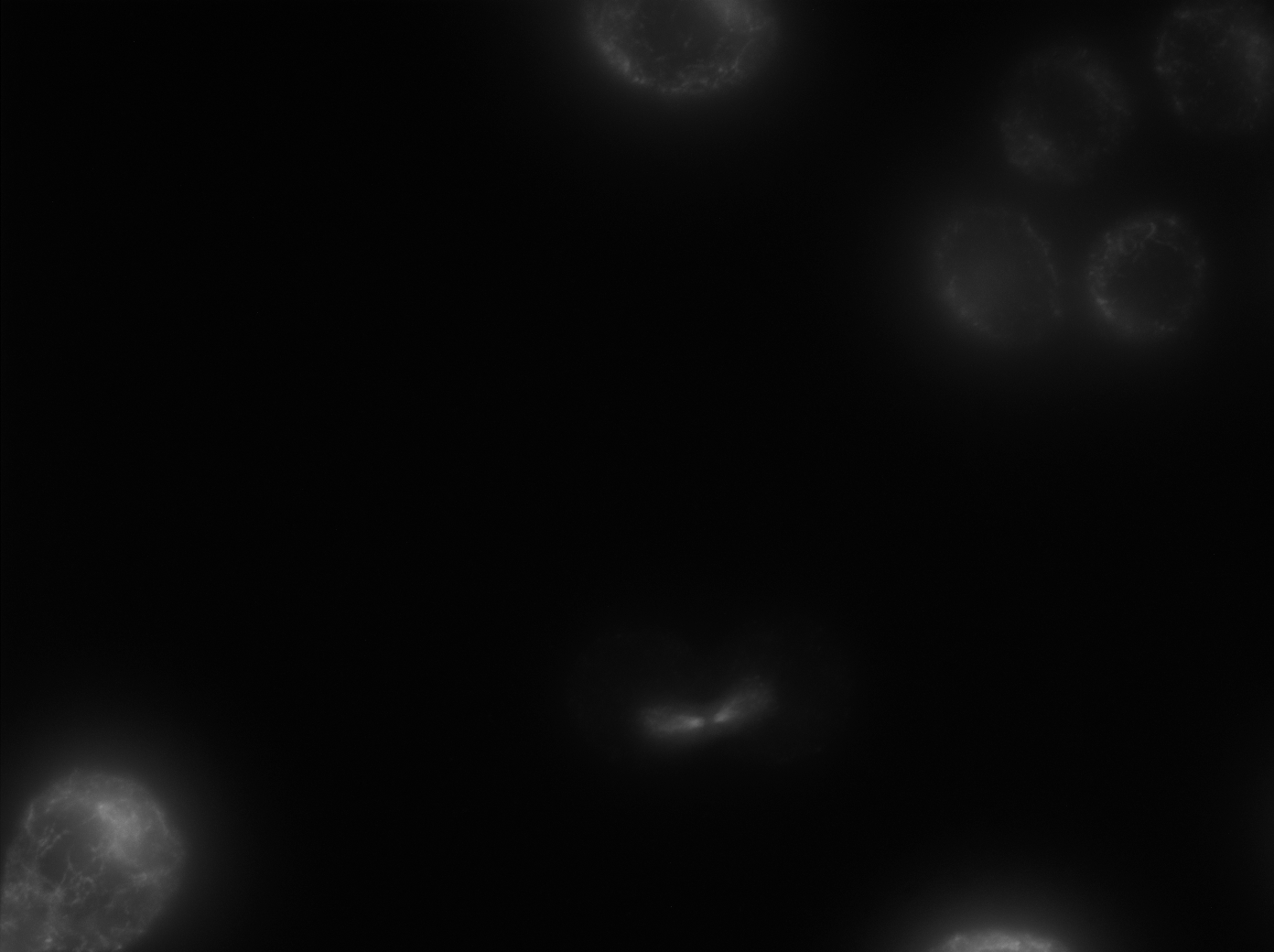

Supplement: Supplementary file 9 — Source data Fig. 2 part 6 [file 44319_2026_742_MOESM9_ESM.zip › Figure 2 Part 6/Fig 2fg Control Hela rGT335 acetylated tubulin/ET/Cas9 actub rGT335 9-8-25 R1 ET1 EX.Project Maximum Z_XY1757350852_Z0_T0_C2.tif]

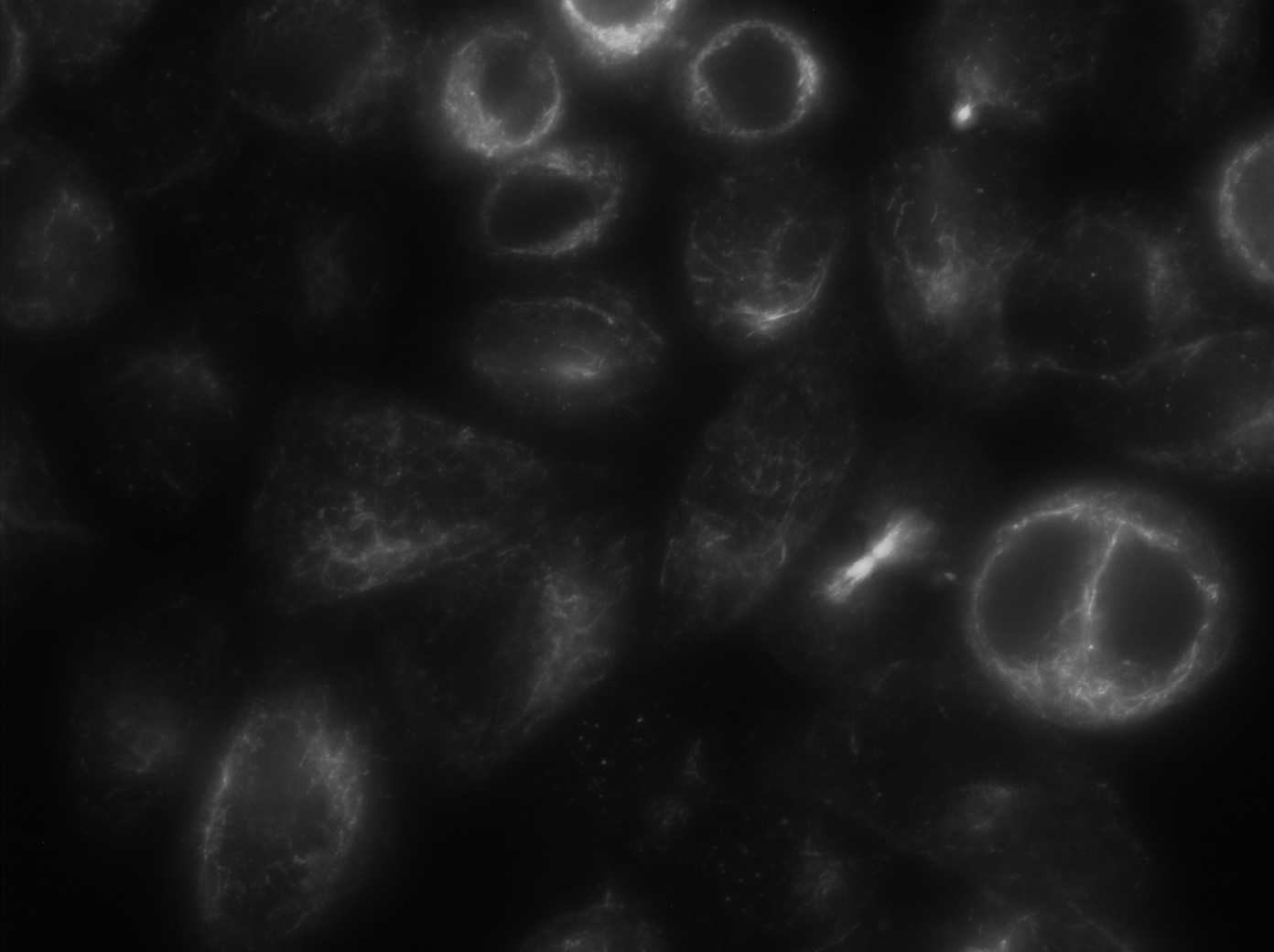

Supplement: Supplementary file 9 — Source data Fig. 2 part 6 [file 44319_2026_742_MOESM9_ESM.zip › Figure 2 Part 6/Fig 2fg Control Hela rGT335 acetylated tubulin/ET/Cas9 actub rGT335 9-8-25 R3 ET4.Project Maximum Z_XY1757365064_Z0_T0_C2.tif]

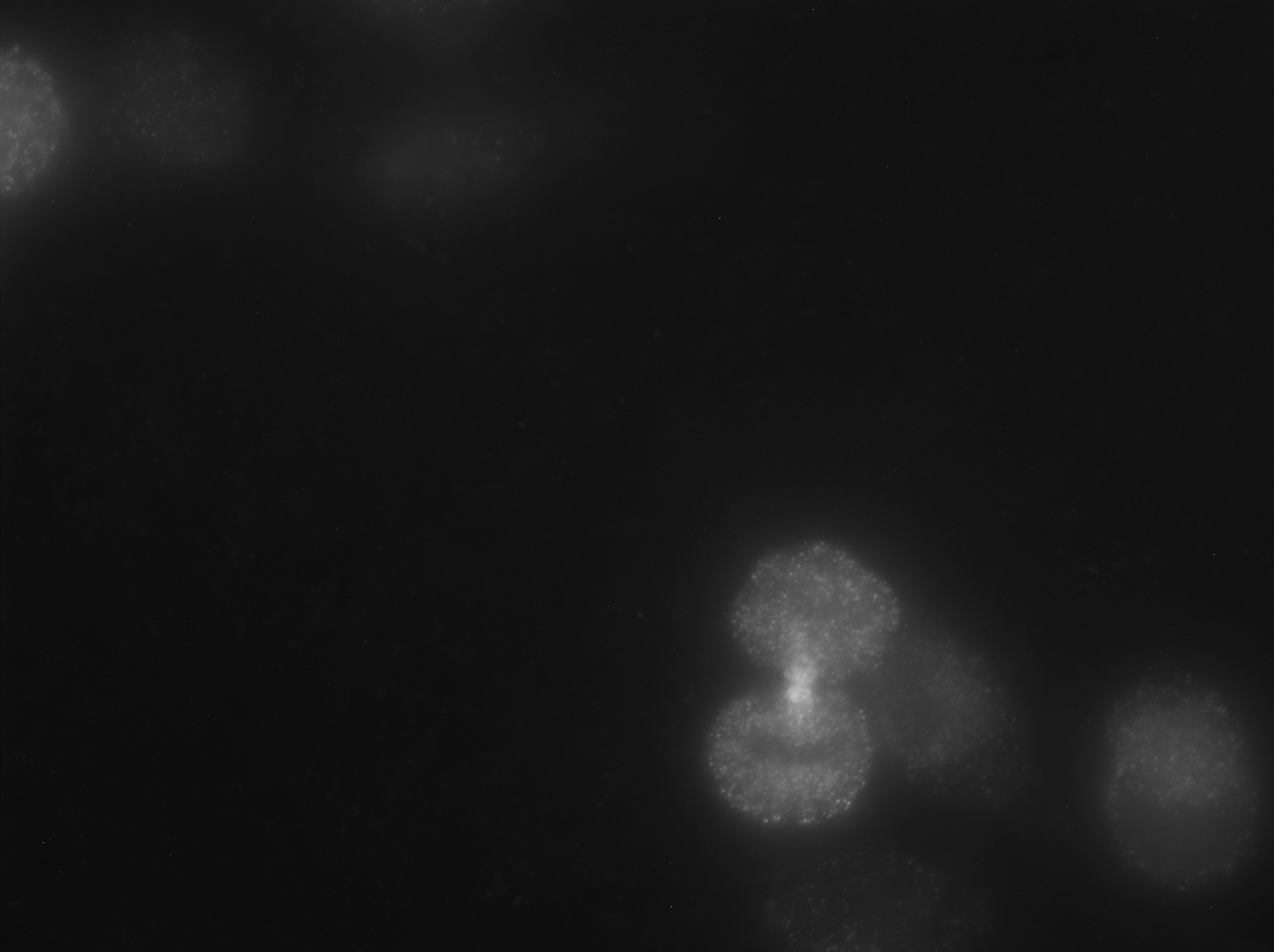

Supplement: Supplementary file 9 — Source data Fig. 2 part 6 [file 44319_2026_742_MOESM9_ESM.zip › Figure 2 Part 6/Fig 2fg Control Hela rGT335 acetylated tubulin/ET/Cas9 actub rGT335 9-8-25 R1 ET10.Project Maximum Z_XY1757354707_Z0_T0_C1.tif]

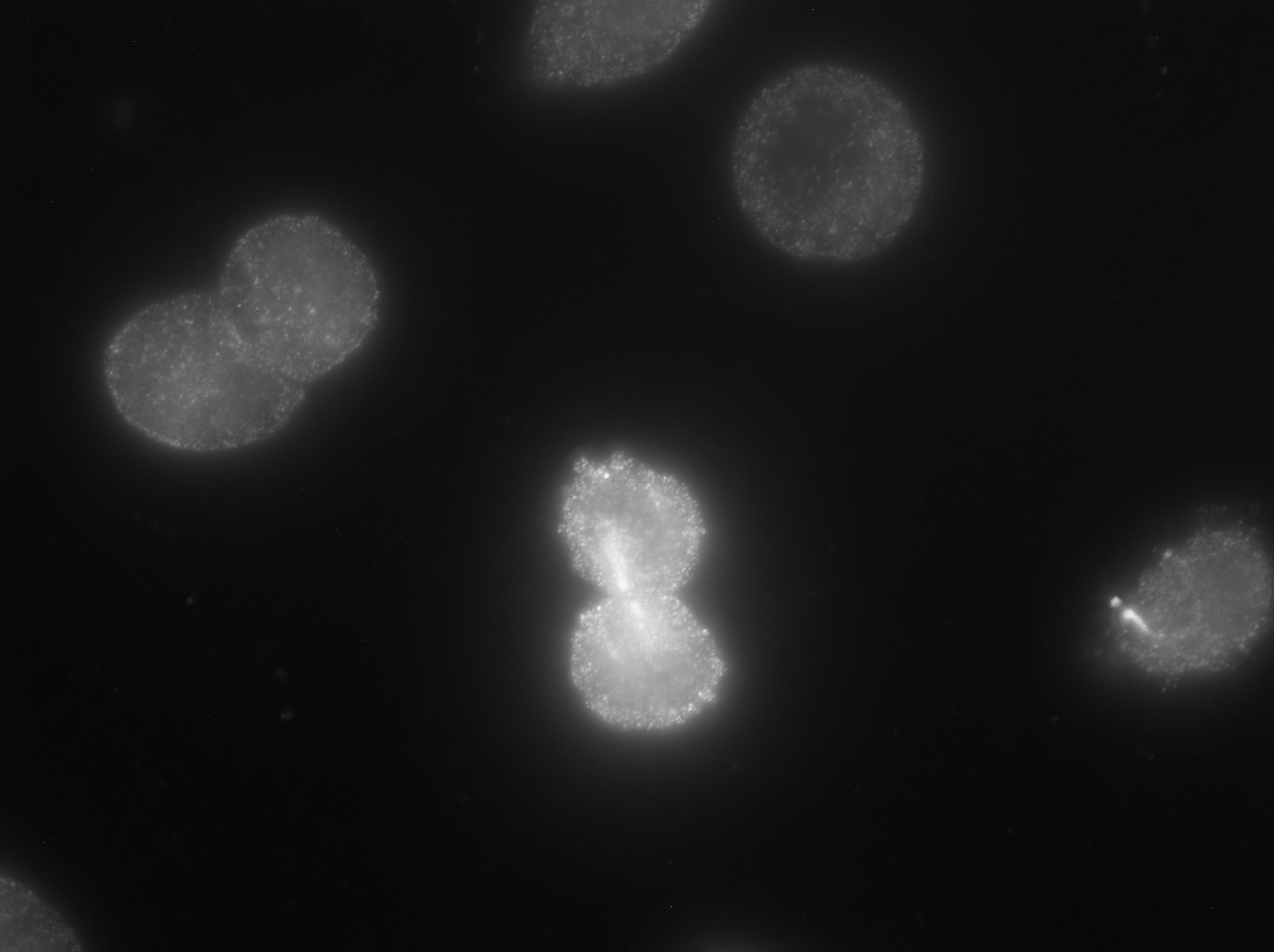

Supplement: Supplementary file 9 — Source data Fig. 2 part 6 [file 44319_2026_742_MOESM9_ESM.zip › Figure 2 Part 6/Fig 2fg Control Hela rGT335 acetylated tubulin/ET/Cas9 actub rGT335 9-8-25 R1 ET2.Project Maximum Z_XY1757350955_Z0_T0_C1.tif]

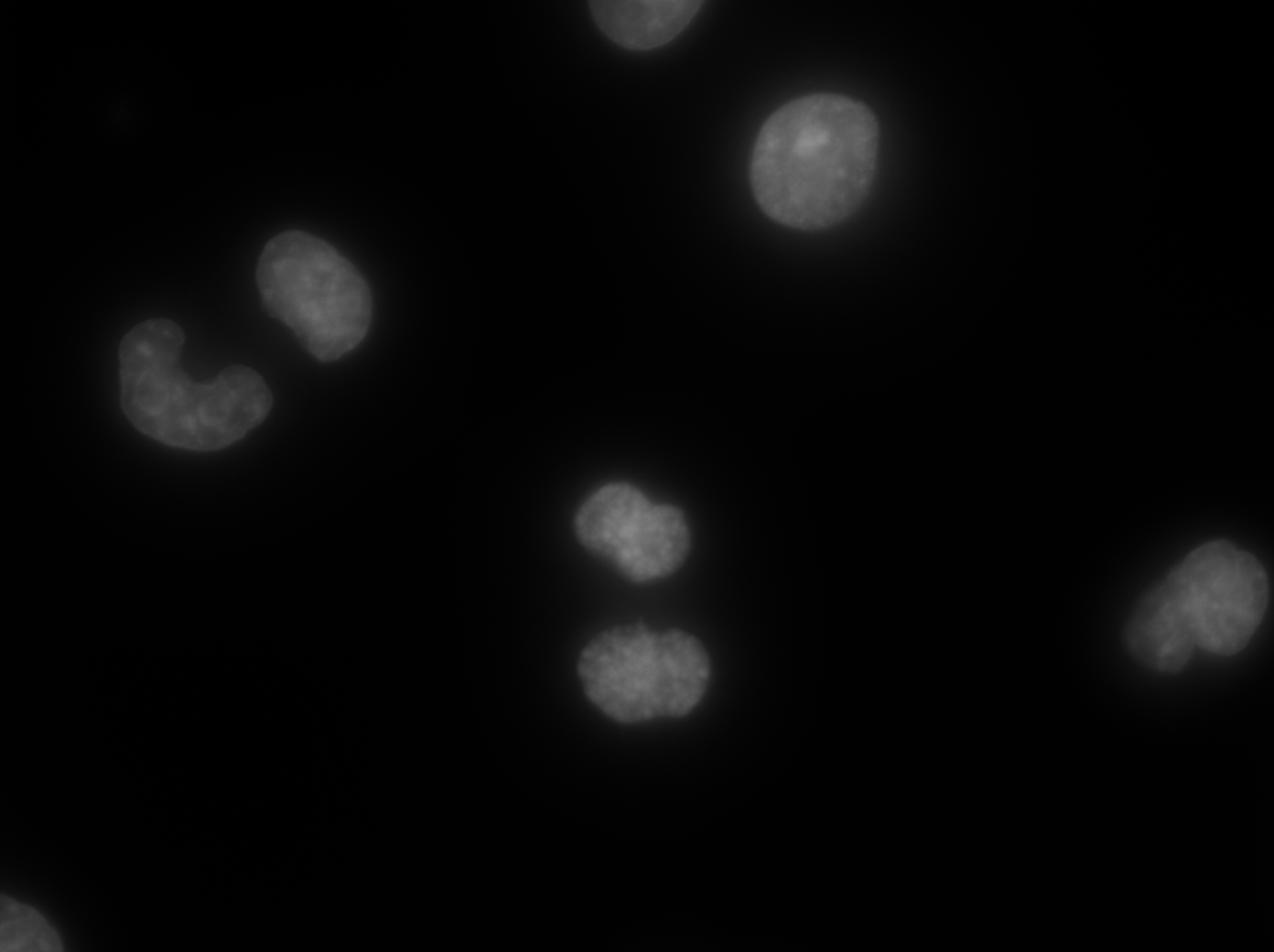

Supplement: Supplementary file 9 — Source data Fig. 2 part 6 [file 44319_2026_742_MOESM9_ESM.zip › Figure 2 Part 6/Fig 2fg Control Hela rGT335 acetylated tubulin/ET/Cas9 actub rGT335 9-8-25 R1 ET2.Project Maximum Z_XY1757350955_Z0_T0_C0.tif]

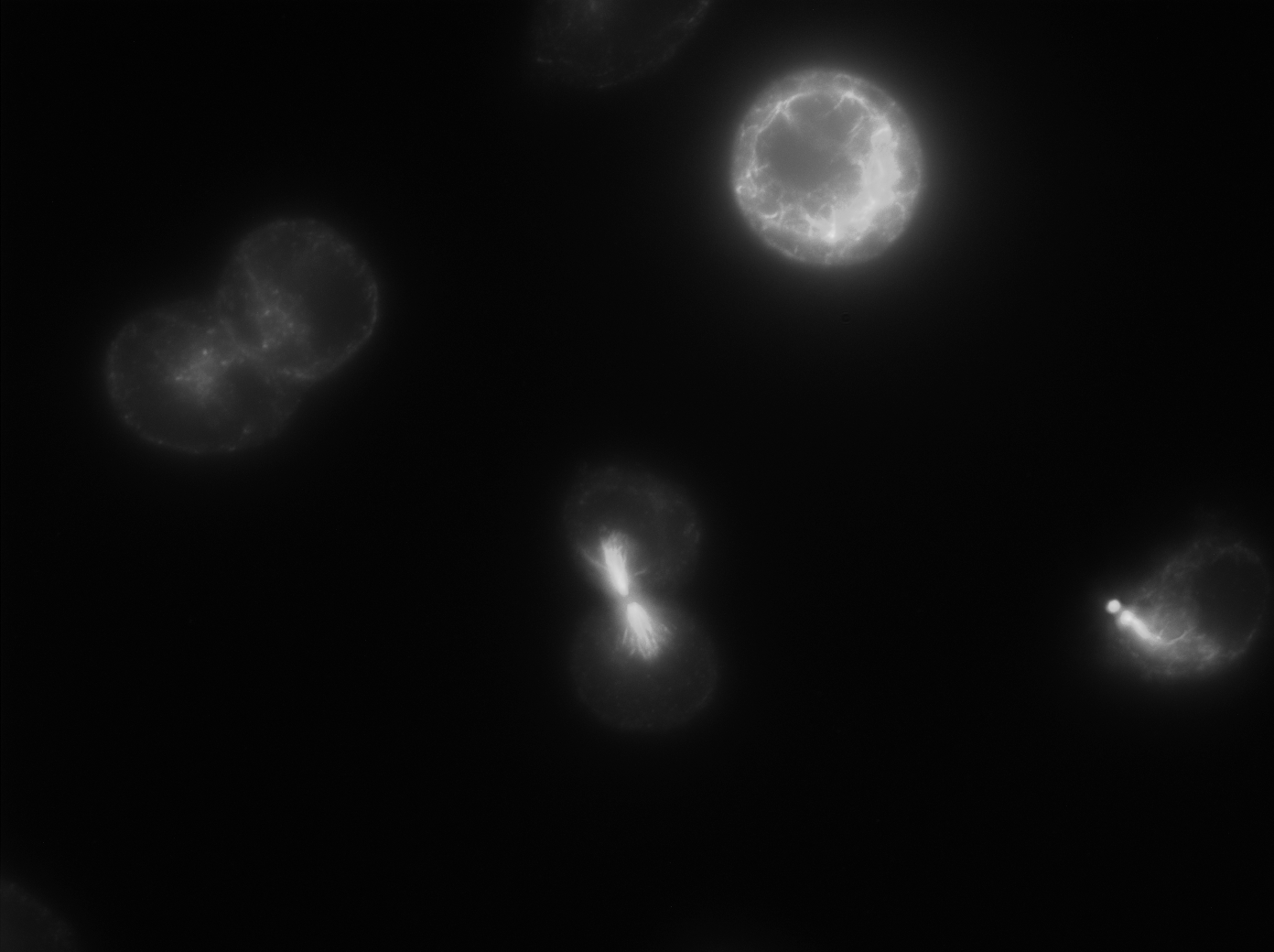

Supplement: Supplementary file 9 — Source data Fig. 2 part 6 [file 44319_2026_742_MOESM9_ESM.zip › Figure 2 Part 6/Fig 2fg Control Hela rGT335 acetylated tubulin/ET/Cas9 actub rGT335 9-8-25 R1 ET2.Project Maximum Z_XY1757350955_Z0_T0_C2.tif]

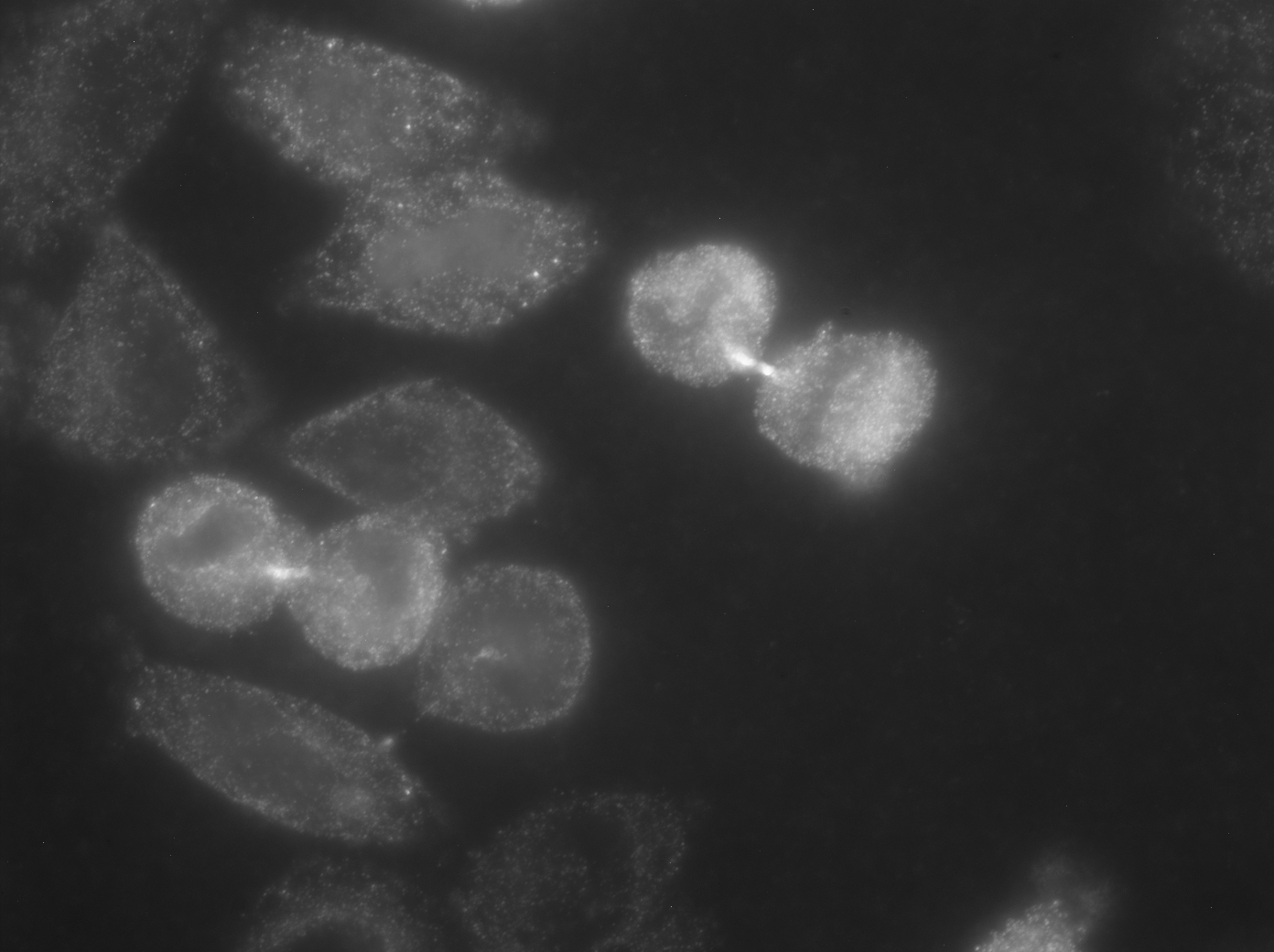

Supplement: Supplementary file 9 — Source data Fig. 2 part 6 [file 44319_2026_742_MOESM9_ESM.zip › Figure 2 Part 6/Fig 2fg Control Hela rGT335 acetylated tubulin/ET/Cas9 actub rGT335 9-8-25 R2 ET1ET2.Project Maximum Z_XY1757359584_Z0_T0_C1.tif]

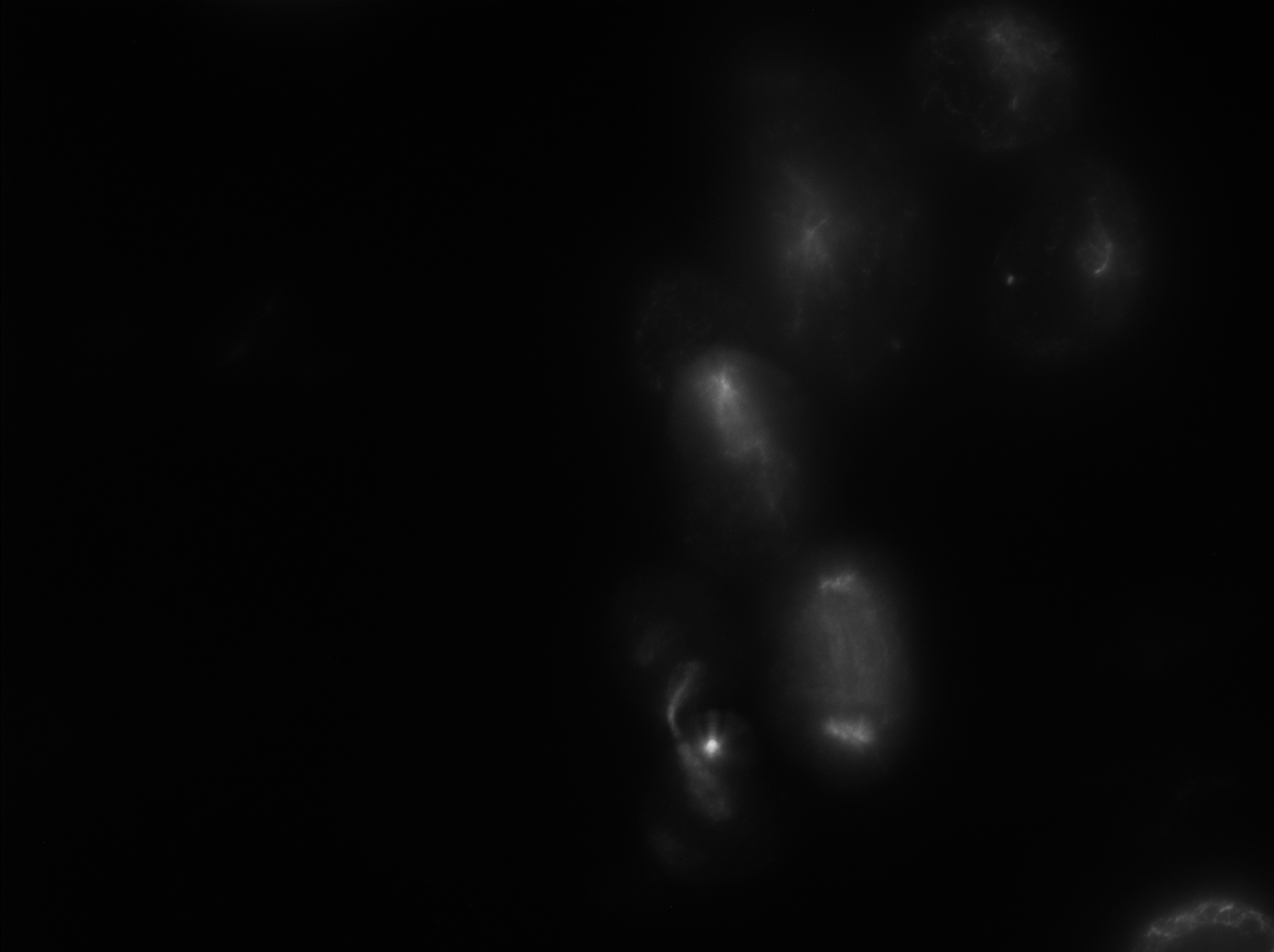

Supplement: Supplementary file 9 — Source data Fig. 2 part 6 [file 44319_2026_742_MOESM9_ESM.zip › Figure 2 Part 6/Fig 2fg Control Hela rGT335 acetylated tubulin/ET/Cas9 actub rGT335 9-8-25 R2 ET7 FI6.Project Maximum Z_XY1757362111_Z0_T0_C2.tif]

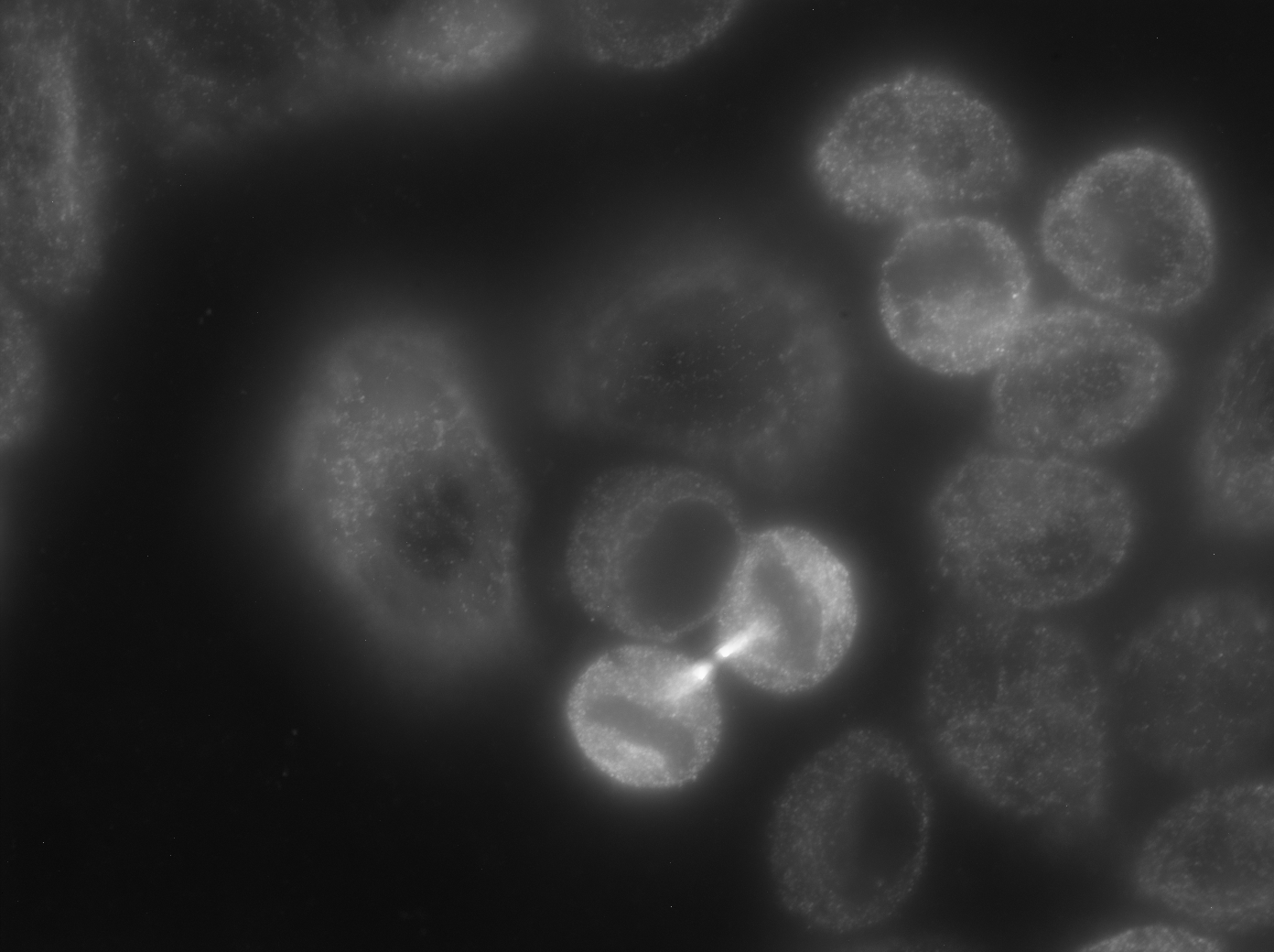

Supplement: Supplementary file 9 — Source data Fig. 2 part 6 [file 44319_2026_742_MOESM9_ESM.zip › Figure 2 Part 6/Fig 2fg Control Hela rGT335 acetylated tubulin/ET/Cas9 actub rGT335 9-8-25 R2 ET6.Project Maximum Z_XY1757361986_Z0_T0_C1.tif]

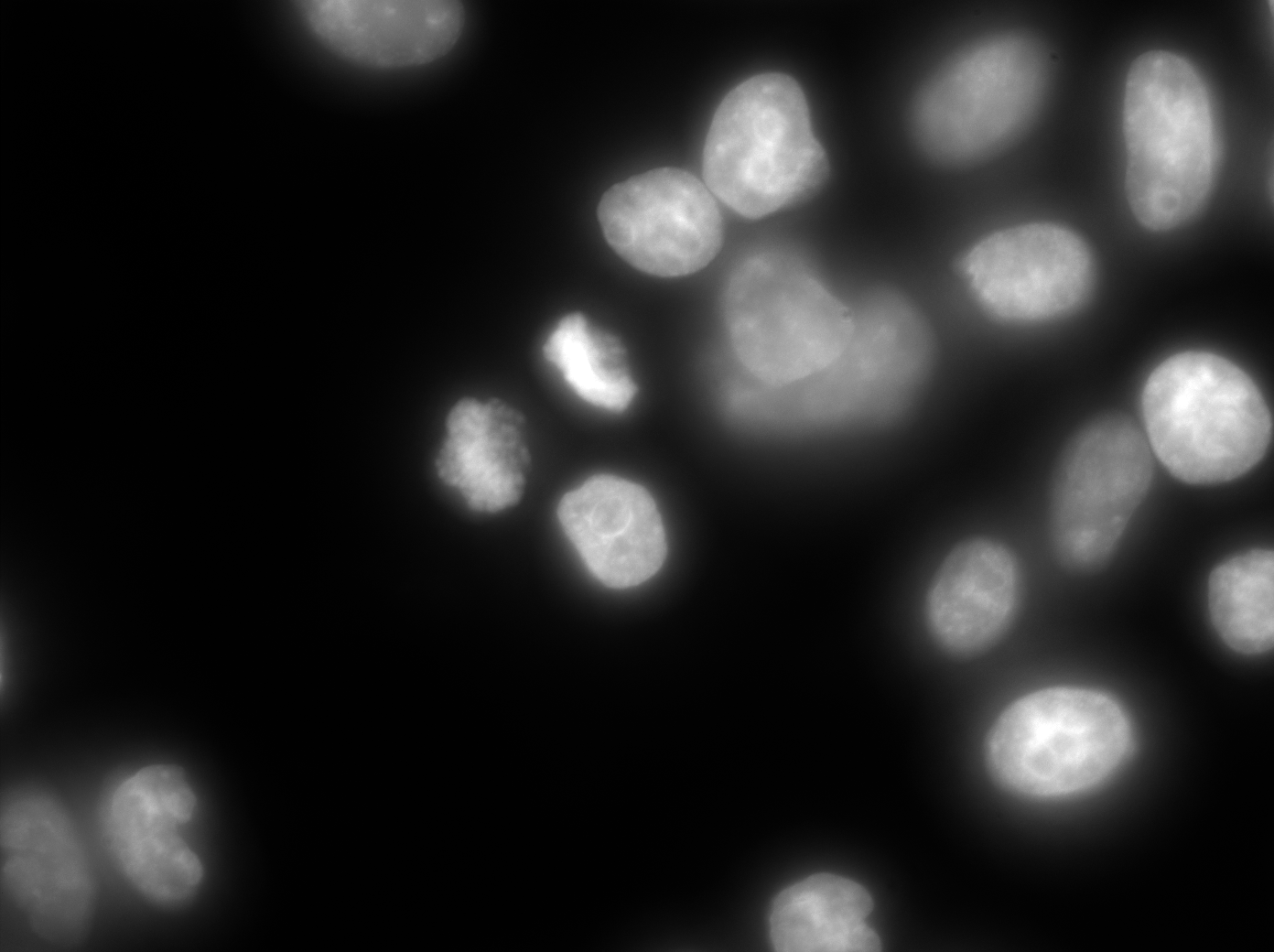

Supplement: Supplementary file 9 — Source data Fig. 2 part 6 [file 44319_2026_742_MOESM9_ESM.zip › Figure 2 Part 6/Fig 2fg Control Hela rGT335 acetylated tubulin/ET/Cas9 actub rGT335 9-8-25 R2 ET9.Project Maximum Z_XY1757362947_Z0_T0_C0.tif]

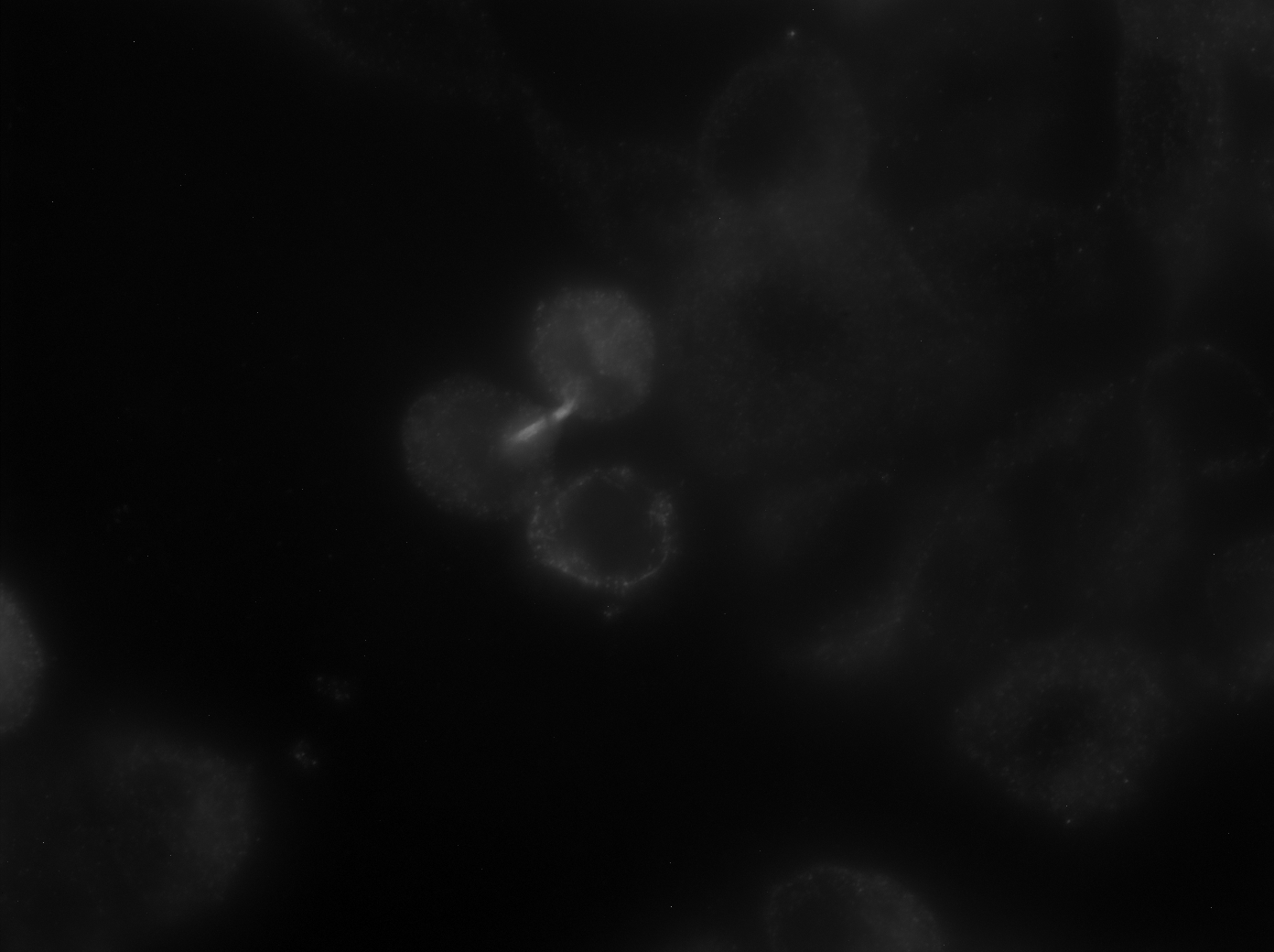

Supplement: Supplementary file 9 — Source data Fig. 2 part 6 [file 44319_2026_742_MOESM9_ESM.zip › Figure 2 Part 6/Fig 2fg Control Hela rGT335 acetylated tubulin/ET/Cas9 actub rGT335 9-8-25 R2 ET9.Project Maximum Z_XY1757362947_Z0_T0_C1.tif]

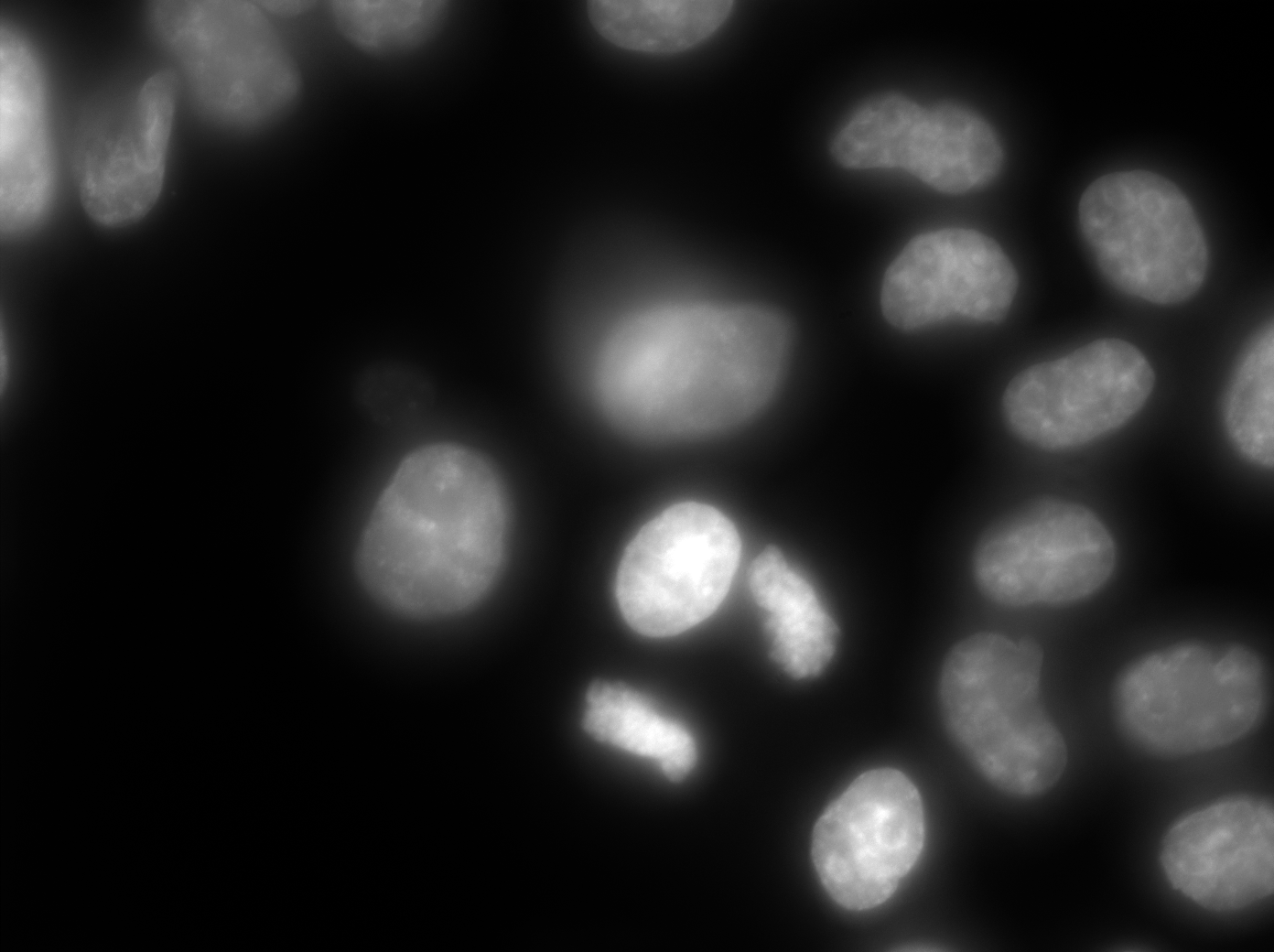

Supplement: Supplementary file 9 — Source data Fig. 2 part 6 [file 44319_2026_742_MOESM9_ESM.zip › Figure 2 Part 6/Fig 2fg Control Hela rGT335 acetylated tubulin/ET/Cas9 actub rGT335 9-8-25 R2 ET6.Project Maximum Z_XY1757361986_Z0_T0_C0.tif]

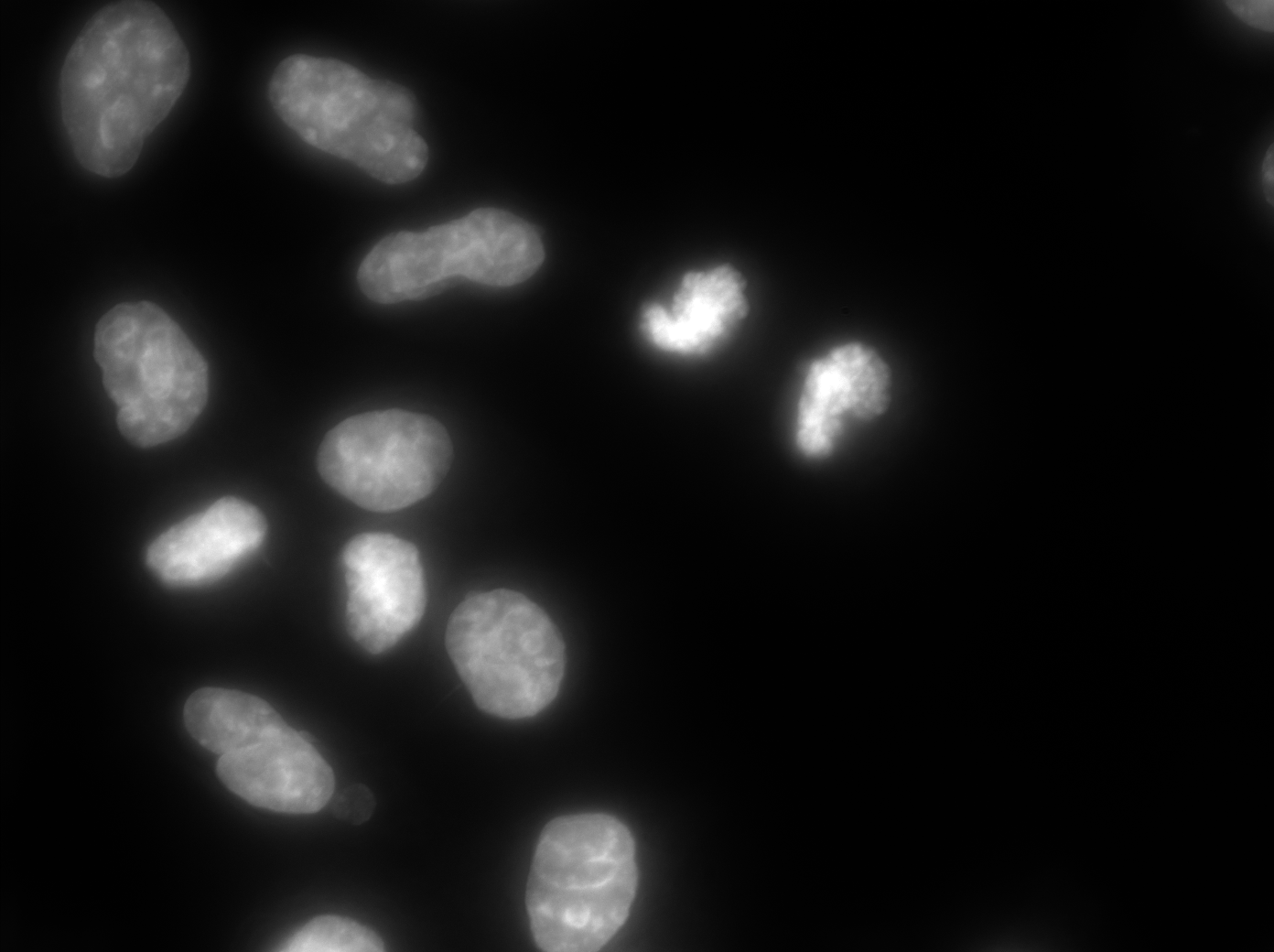

Supplement: Supplementary file 9 — Source data Fig. 2 part 6 [file 44319_2026_742_MOESM9_ESM.zip › Figure 2 Part 6/Fig 2fg Control Hela rGT335 acetylated tubulin/ET/Cas9 actub rGT335 9-8-25 R2 ET1ET2.Project Maximum Z_XY1757359584_Z0_T0_C0.tif]

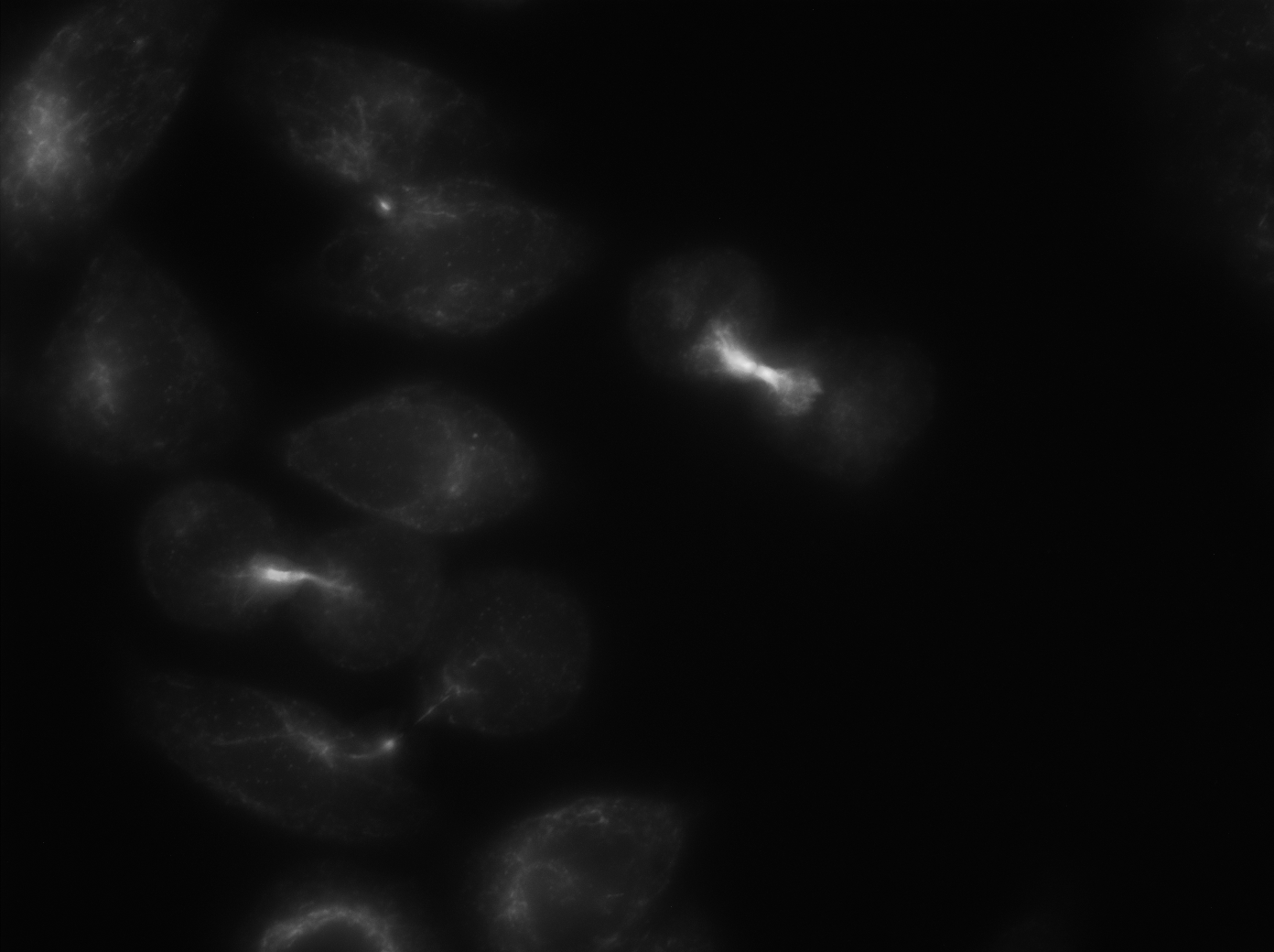

Supplement: Supplementary file 9 — Source data Fig. 2 part 6 [file 44319_2026_742_MOESM9_ESM.zip › Figure 2 Part 6/Fig 2fg Control Hela rGT335 acetylated tubulin/ET/Cas9 actub rGT335 9-8-25 R2 ET1ET2.Project Maximum Z_XY1757359584_Z0_T0_C2.tif]

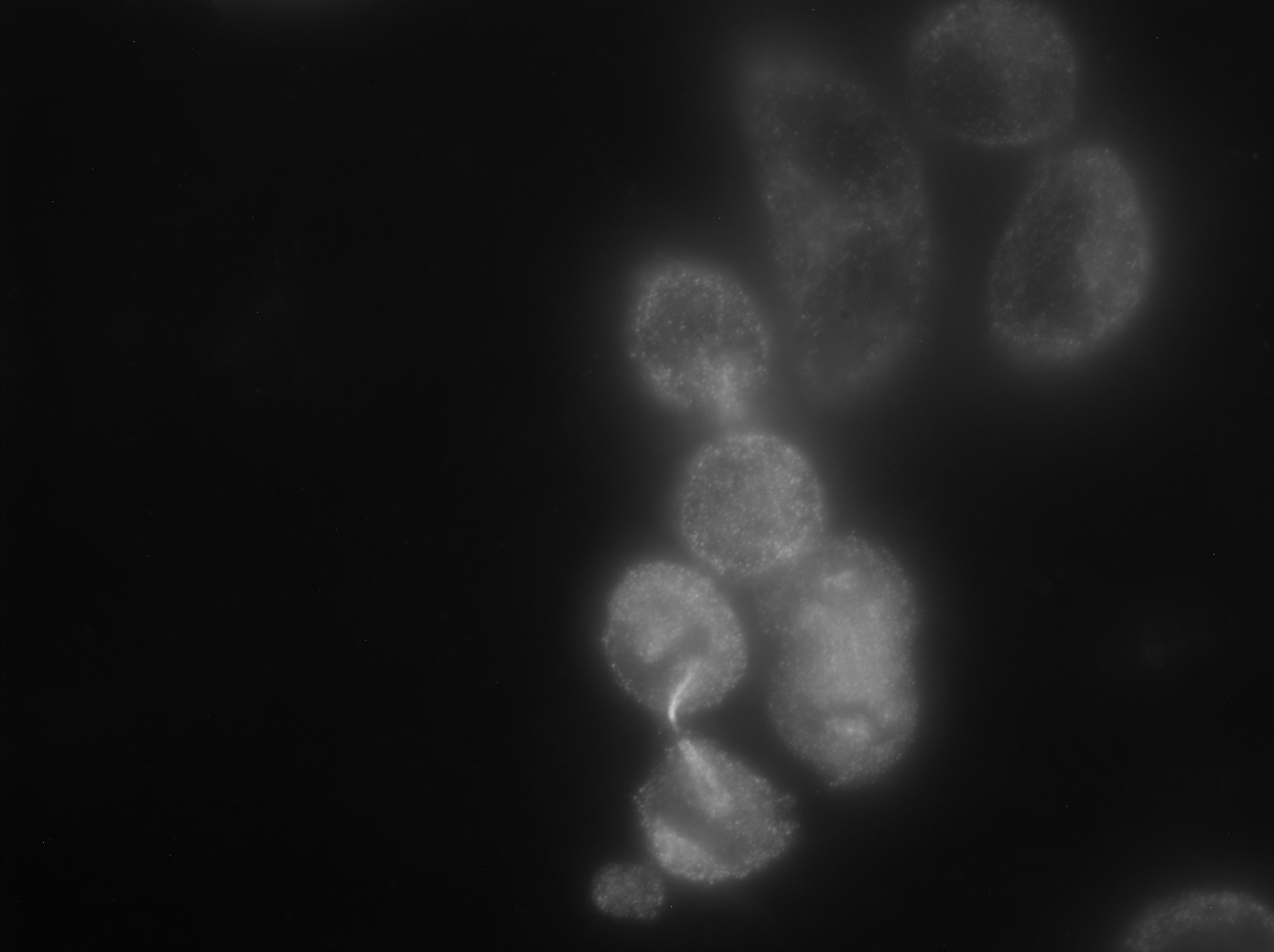

Supplement: Supplementary file 9 — Source data Fig. 2 part 6 [file 44319_2026_742_MOESM9_ESM.zip › Figure 2 Part 6/Fig 2fg Control Hela rGT335 acetylated tubulin/ET/Cas9 actub rGT335 9-8-25 R2 ET7 FI6.Project Maximum Z_XY1757362111_Z0_T0_C1.tif]

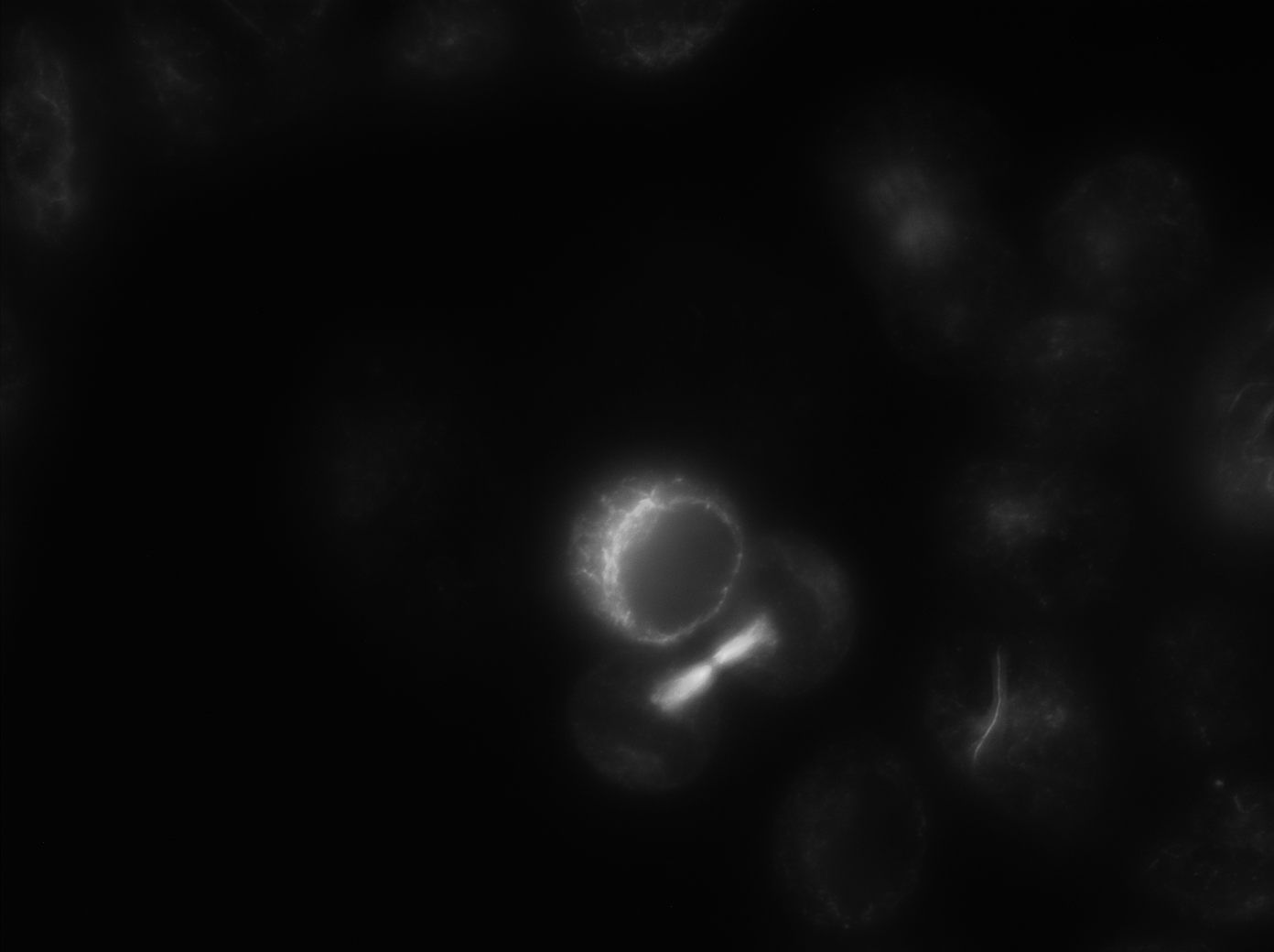

Supplement: Supplementary file 9 — Source data Fig. 2 part 6 [file 44319_2026_742_MOESM9_ESM.zip › Figure 2 Part 6/Fig 2fg Control Hela rGT335 acetylated tubulin/ET/Cas9 actub rGT335 9-8-25 R2 ET6.Project Maximum Z_XY1757361986_Z0_T0_C2.tif]

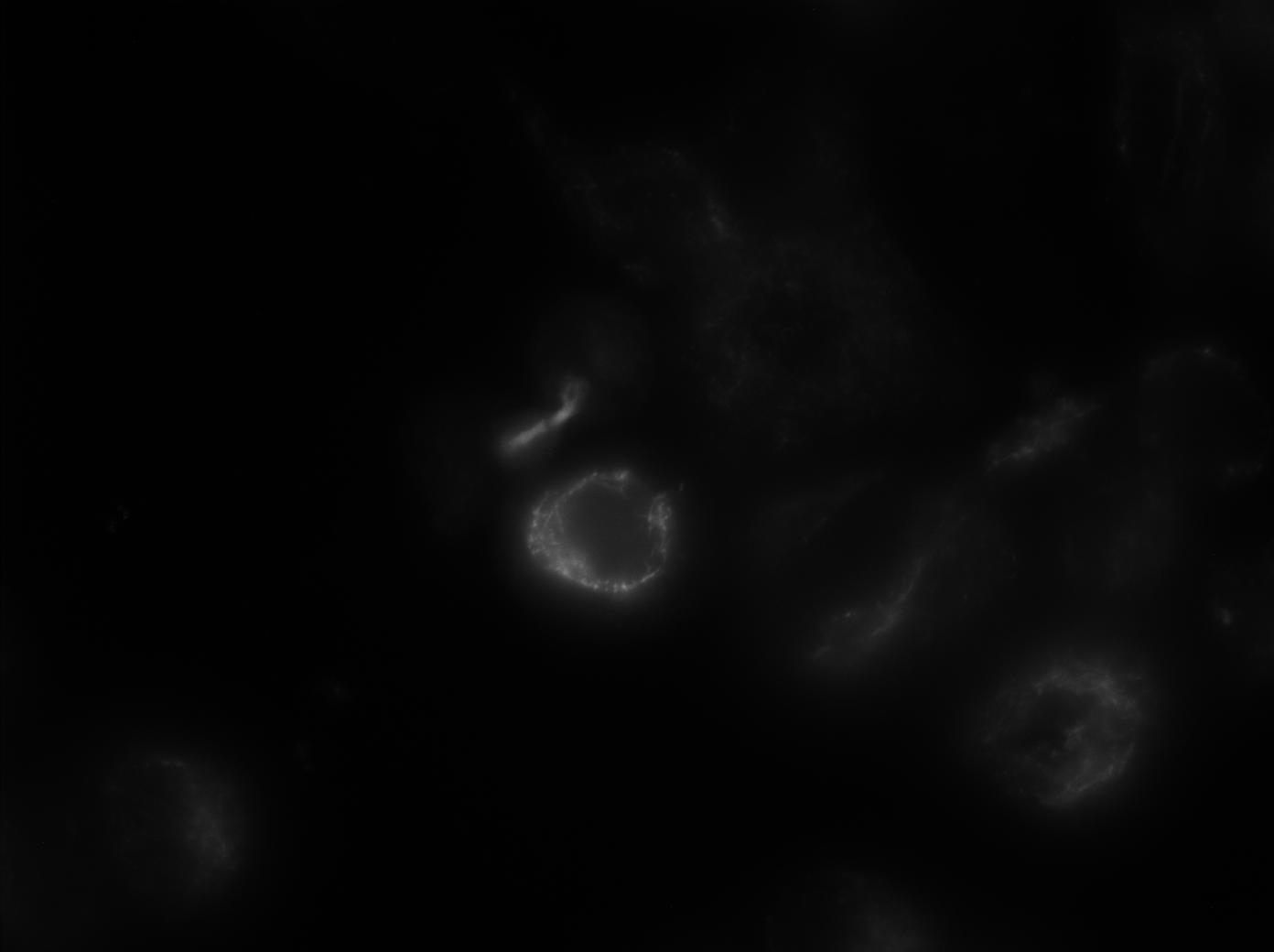

Supplement: Supplementary file 9 — Source data Fig. 2 part 6 [file 44319_2026_742_MOESM9_ESM.zip › Figure 2 Part 6/Fig 2fg Control Hela rGT335 acetylated tubulin/ET/Cas9 actub rGT335 9-8-25 R2 ET9.Project Maximum Z_XY1757362947_Z0_T0_C2.tif]

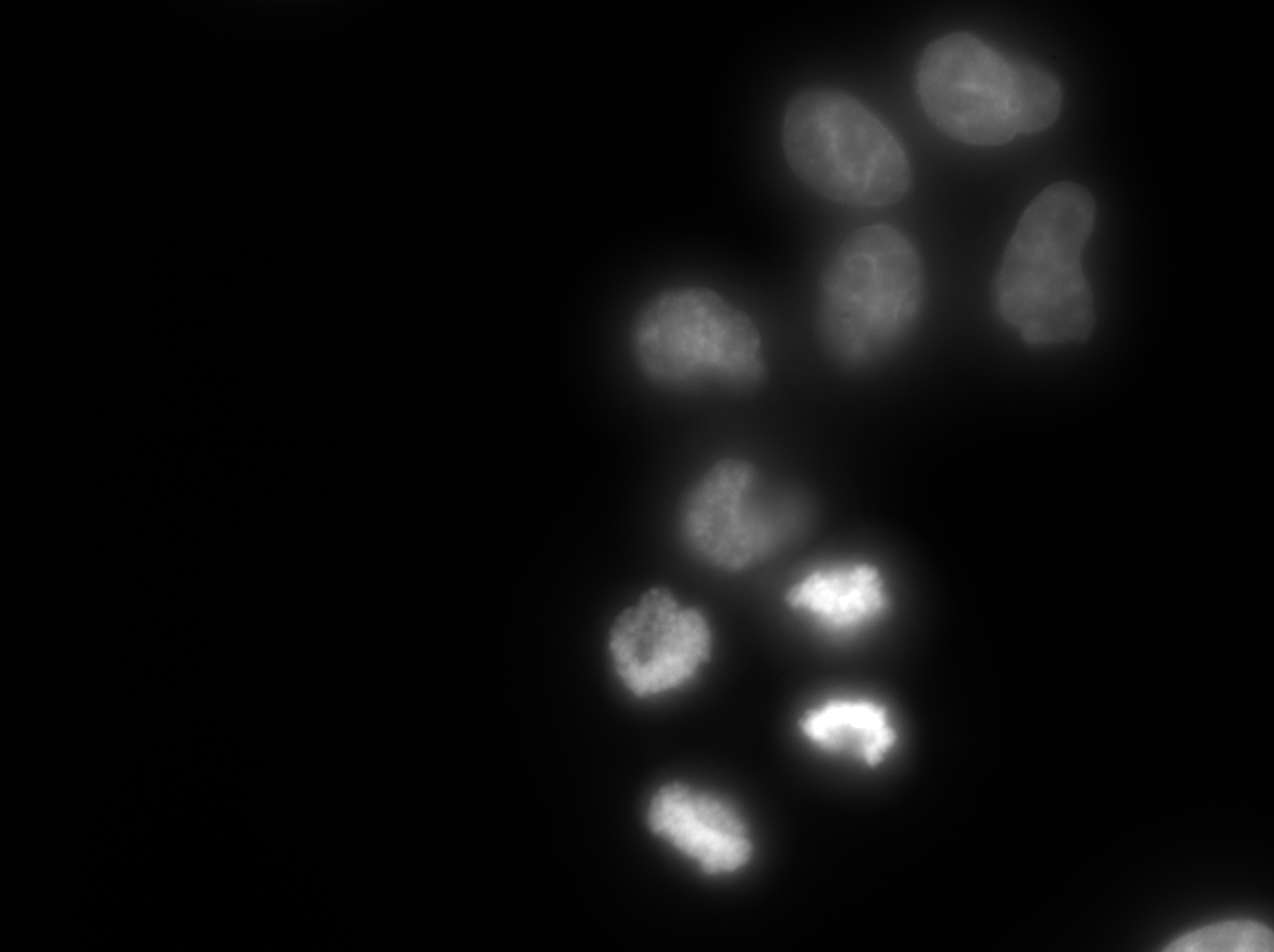

Supplement: Supplementary file 9 — Source data Fig. 2 part 6 [file 44319_2026_742_MOESM9_ESM.zip › Figure 2 Part 6/Fig 2fg Control Hela rGT335 acetylated tubulin/ET/Cas9 actub rGT335 9-8-25 R2 ET7 FI6.Project Maximum Z_XY1757362111_Z0_T0_C0.tif]

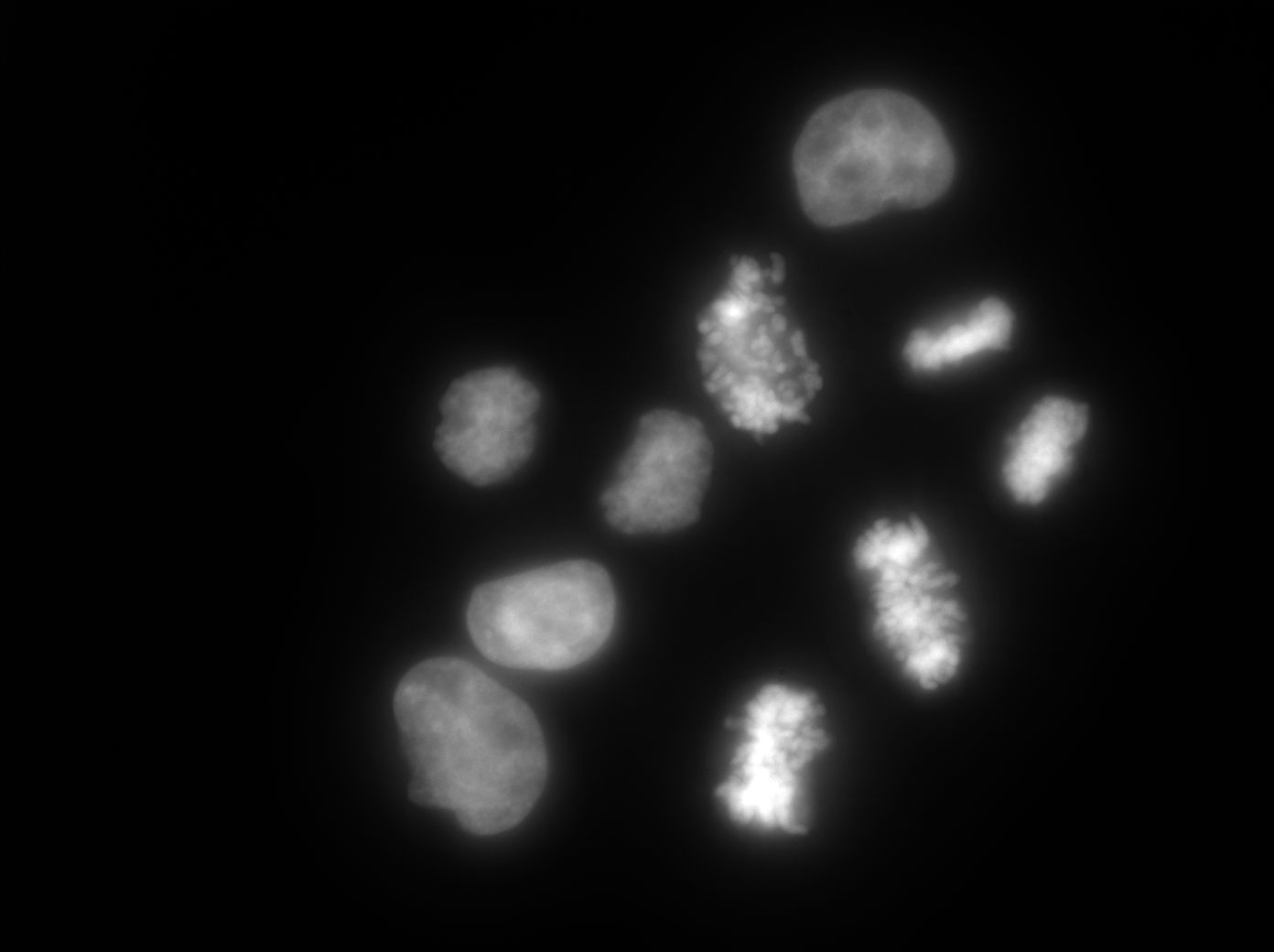

Supplement: Supplementary file 9 — Source data Fig. 2 part 6 [file 44319_2026_742_MOESM9_ESM.zip › Figure 2 Part 6/Fig 2fg Control Hela rGT335 acetylated tubulin/ET/Cas9 actub rGT335 9-8-25 R3 ET9ET10 M3M4.Project Maximum Z_XY1757366049_Z0_T0_C0.tif]

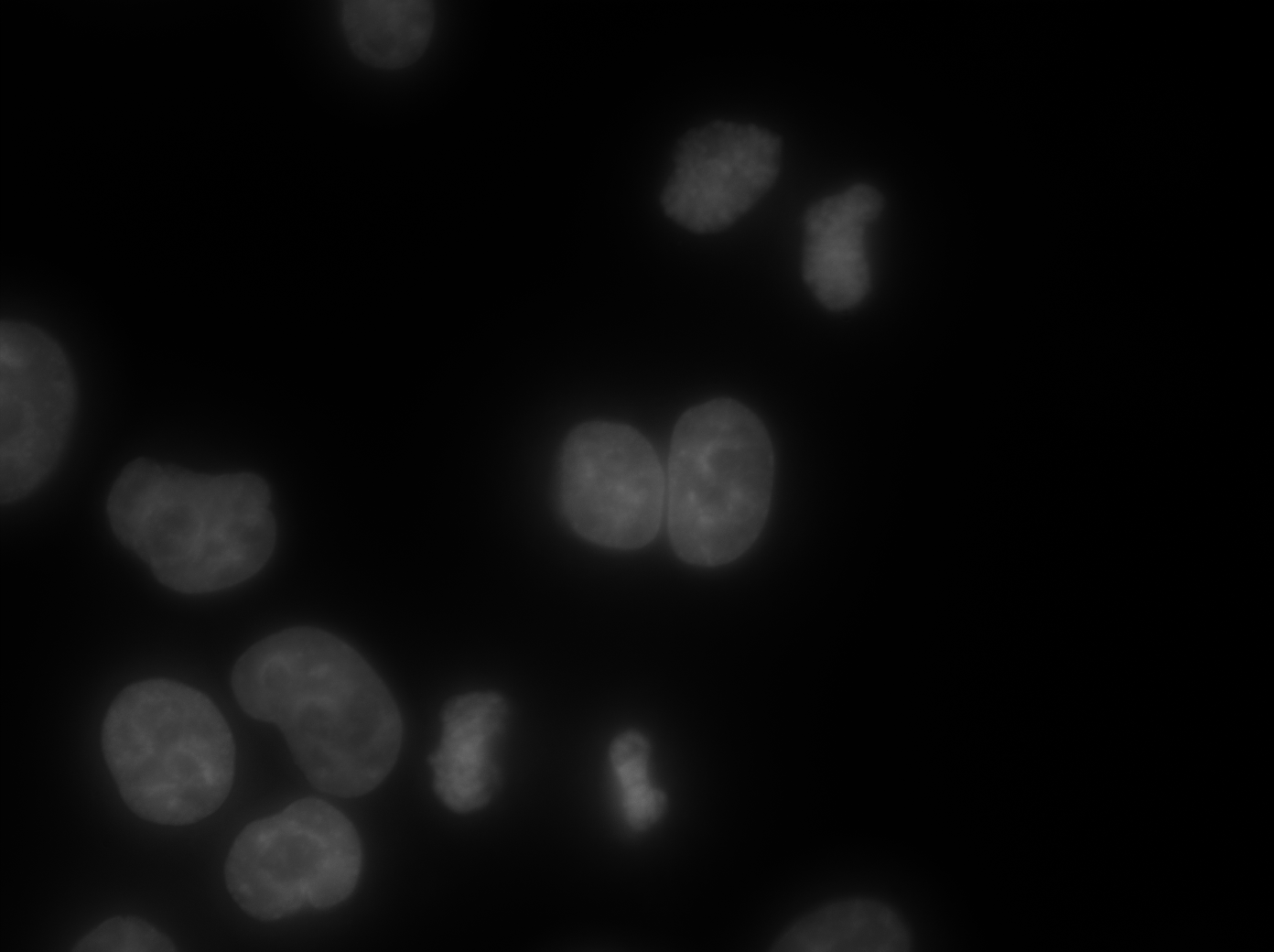

Supplement: Supplementary file 9 — Source data Fig. 2 part 6 [file 44319_2026_742_MOESM9_ESM.zip › Figure 2 Part 6/Fig 2fg Control Hela rGT335 acetylated tubulin/ET/Cas9 actub rGT335 9-8-25 R1 ET9.Project Maximum Z_XY1757354338_Z0_T0_C0.tif]

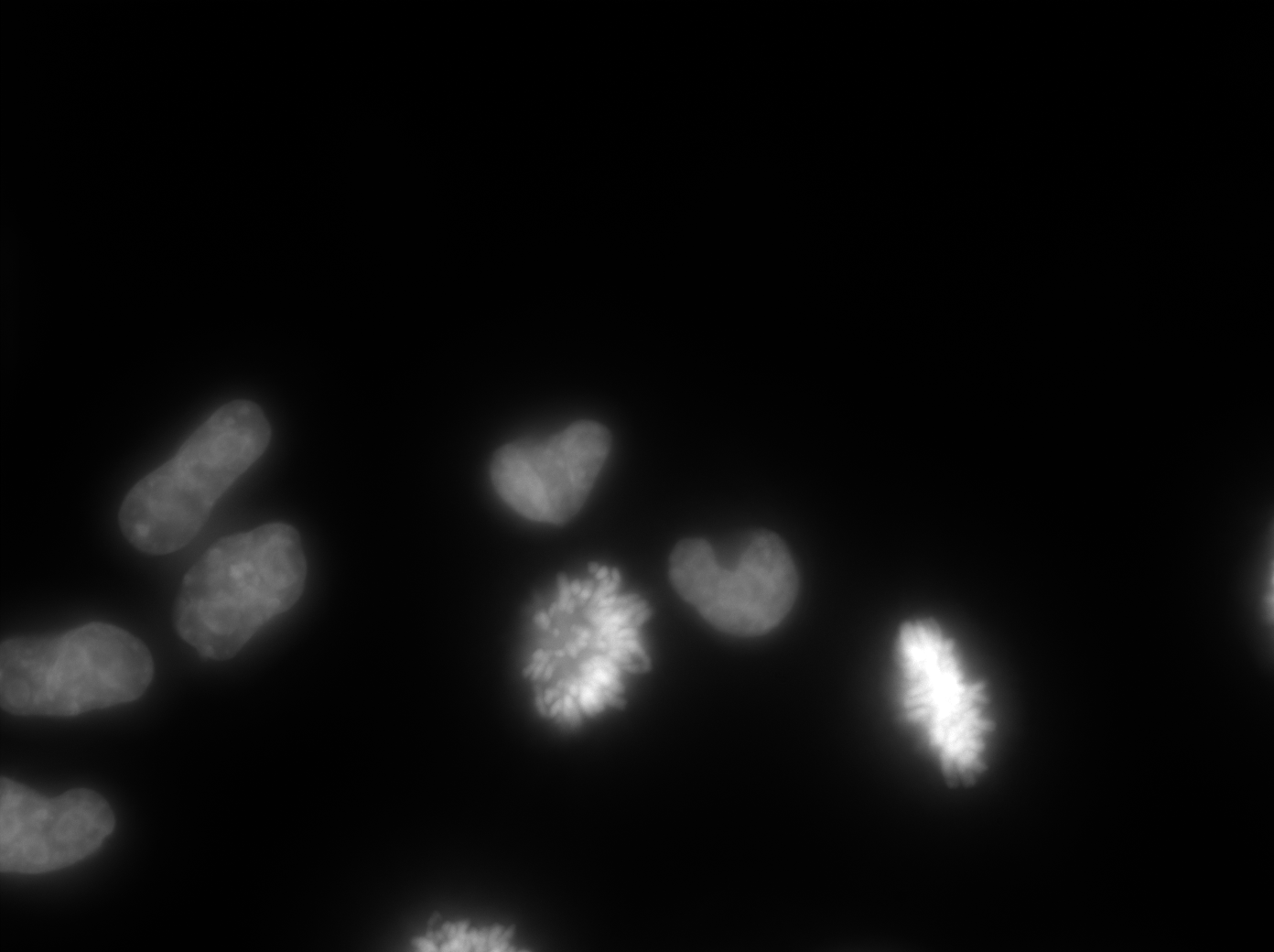

Supplement: Supplementary file 9 — Source data Fig. 2 part 6 [file 44319_2026_742_MOESM9_ESM.zip › Figure 2 Part 6/Fig 2fg Control Hela rGT335 acetylated tubulin/ET/Cas9 actub rGT335 9-8-25 R2 ET5 M5.Project Maximum Z_XY1757361480_Z0_T0_C0.tif]

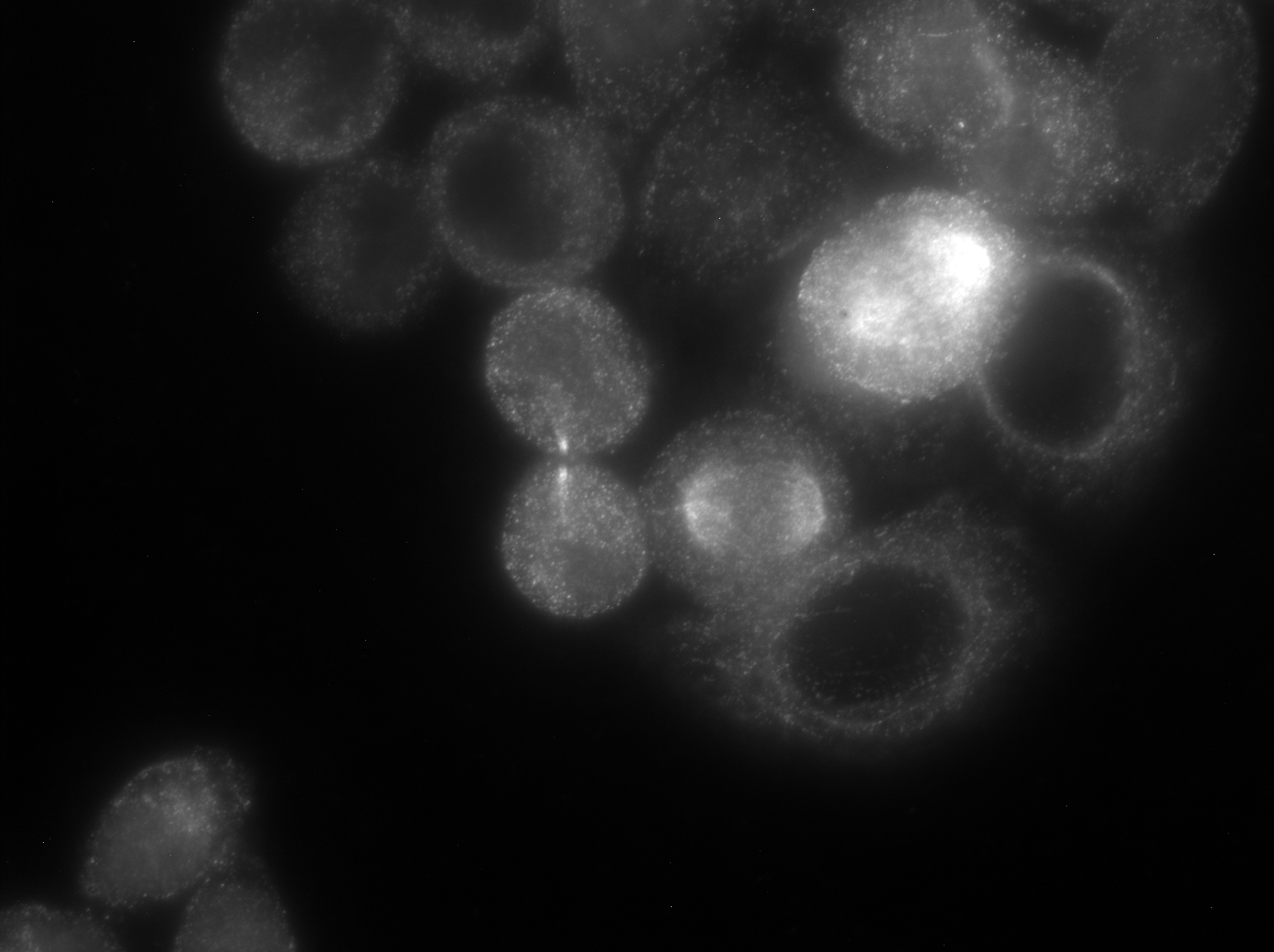

Supplement: Supplementary file 9 — Source data Fig. 2 part 6 [file 44319_2026_742_MOESM9_ESM.zip › Figure 2 Part 6/Fig 2fg Control Hela rGT335 acetylated tubulin/ET/Cas9 actub rGT335 9-8-25 R3 A1 ET1 M1.Project Maximum Z_XY1757364698_Z0_T0_C1.tif]

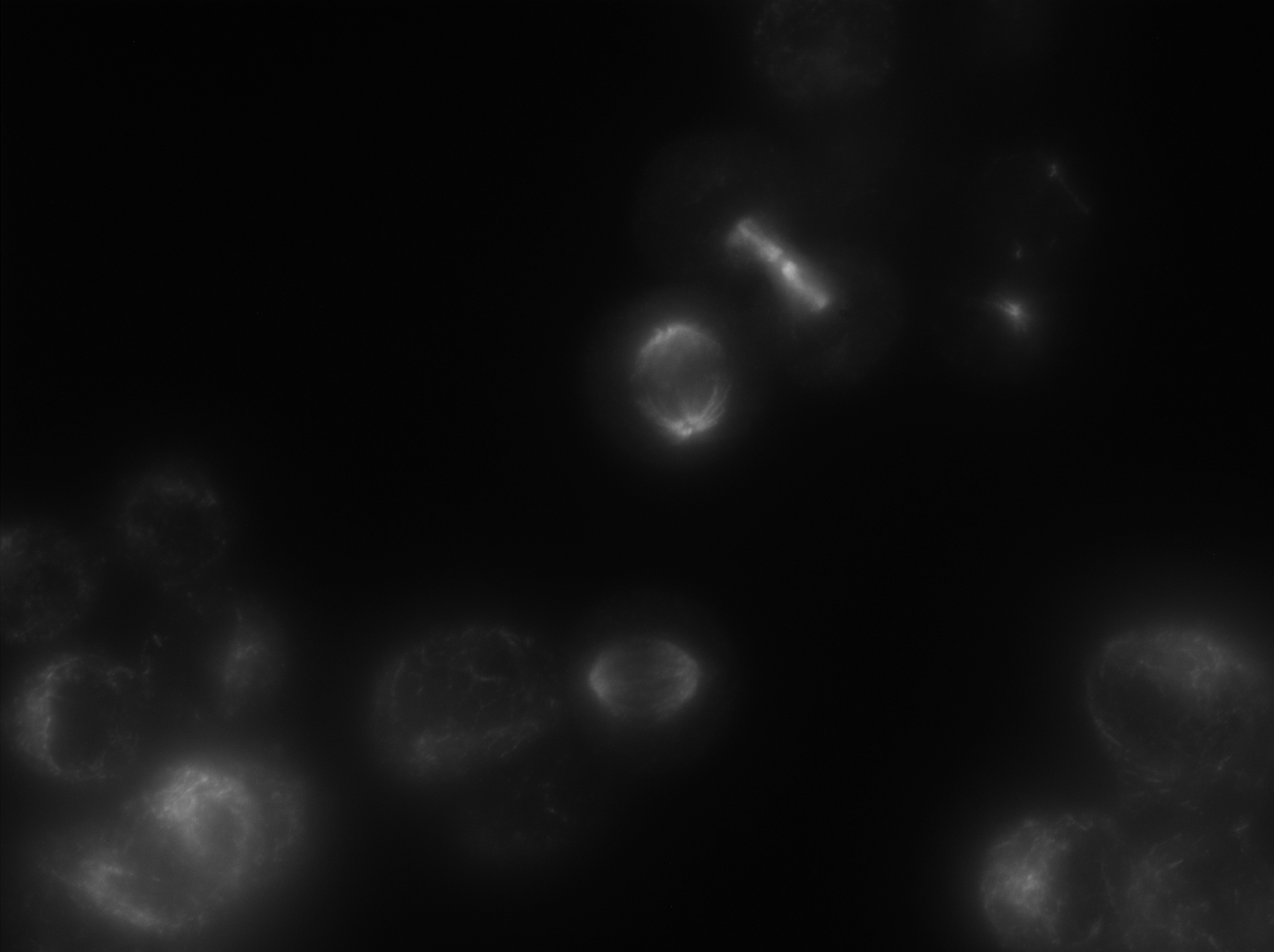

Supplement: Supplementary file 9 — Source data Fig. 2 part 6 [file 44319_2026_742_MOESM9_ESM.zip › Figure 2 Part 6/Fig 2fg Control Hela rGT335 acetylated tubulin/ET/Cas9 actub rGT335 9-8-25 R1 ET8 M4M5.Project Maximum Z_XY1757354024_Z0_T0_C2.tif]

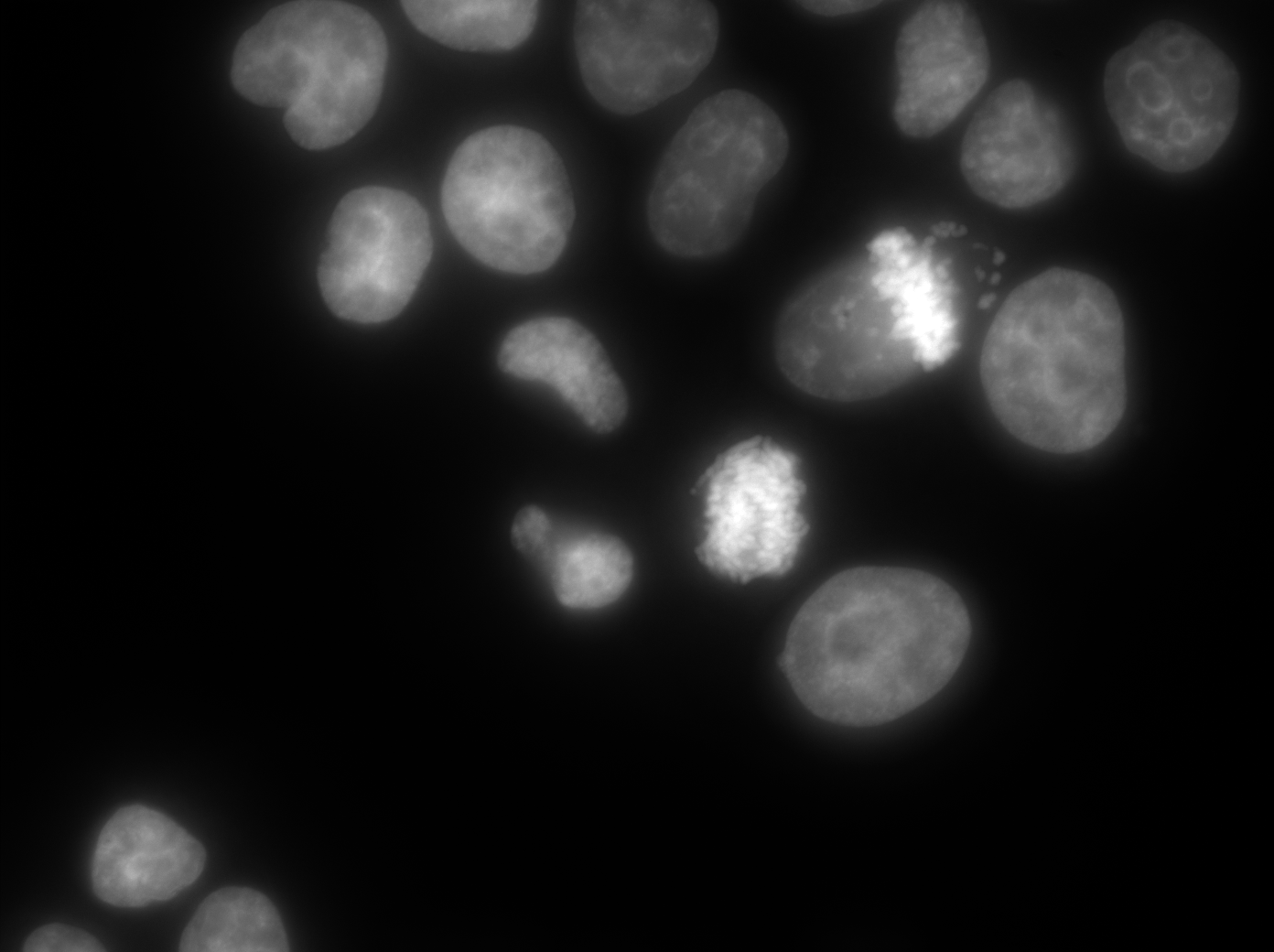

Supplement: Supplementary file 9 — Source data Fig. 2 part 6 [file 44319_2026_742_MOESM9_ESM.zip › Figure 2 Part 6/Fig 2fg Control Hela rGT335 acetylated tubulin/ET/Cas9 actub rGT335 9-8-25 R3 A1 ET1 M1.Project Maximum Z_XY1757364698_Z0_T0_C0.tif]

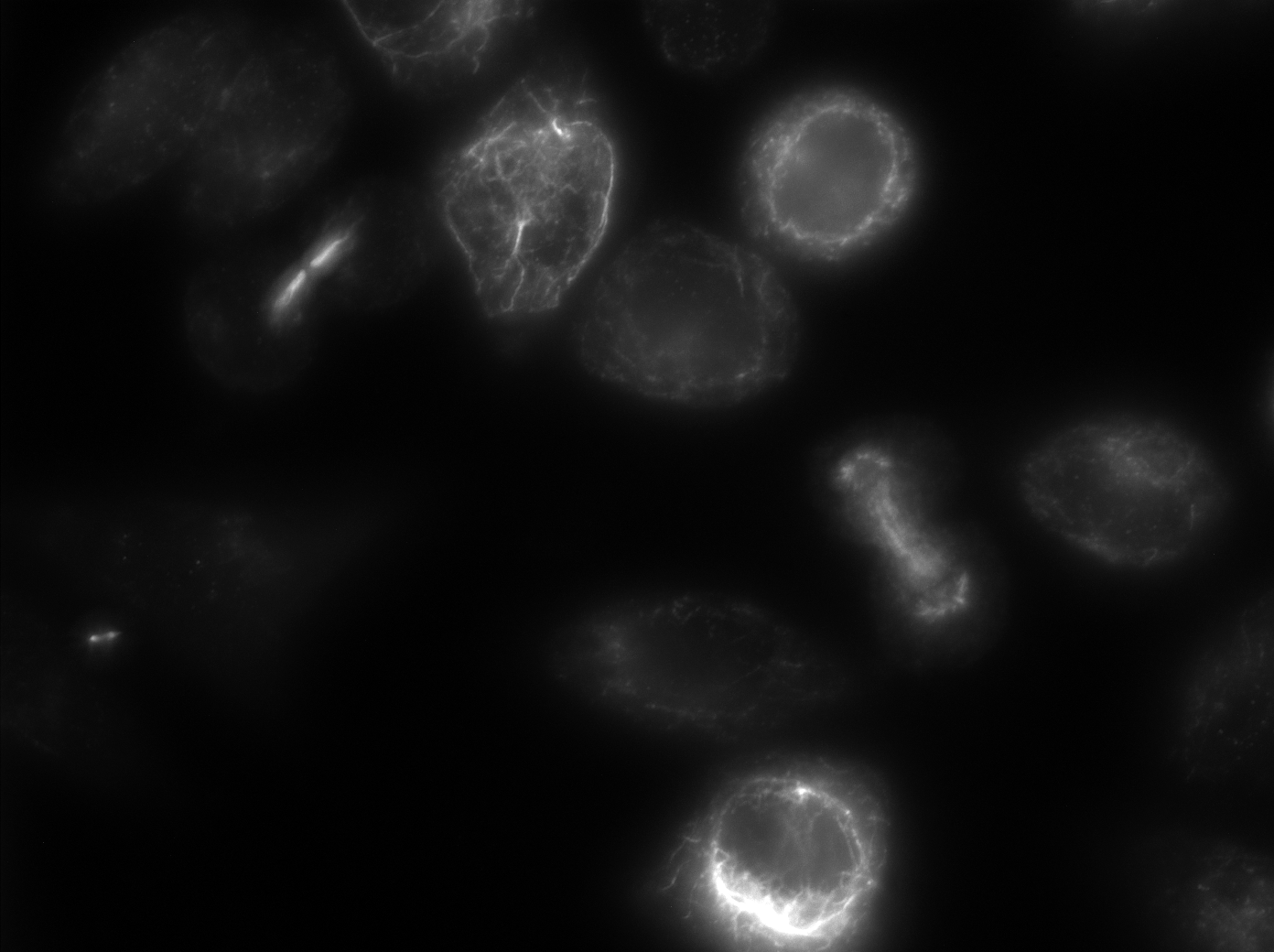

Supplement: Supplementary file 9 — Source data Fig. 2 part 6 [file 44319_2026_742_MOESM9_ESM.zip › Figure 2 Part 6/Fig 2fg Control Hela rGT335 acetylated tubulin/ET/Cas9 actub rGT335 9-8-25 R2 FI2 ET3.Project Maximum Z_XY1757359877_Z0_T0_C2.tif]

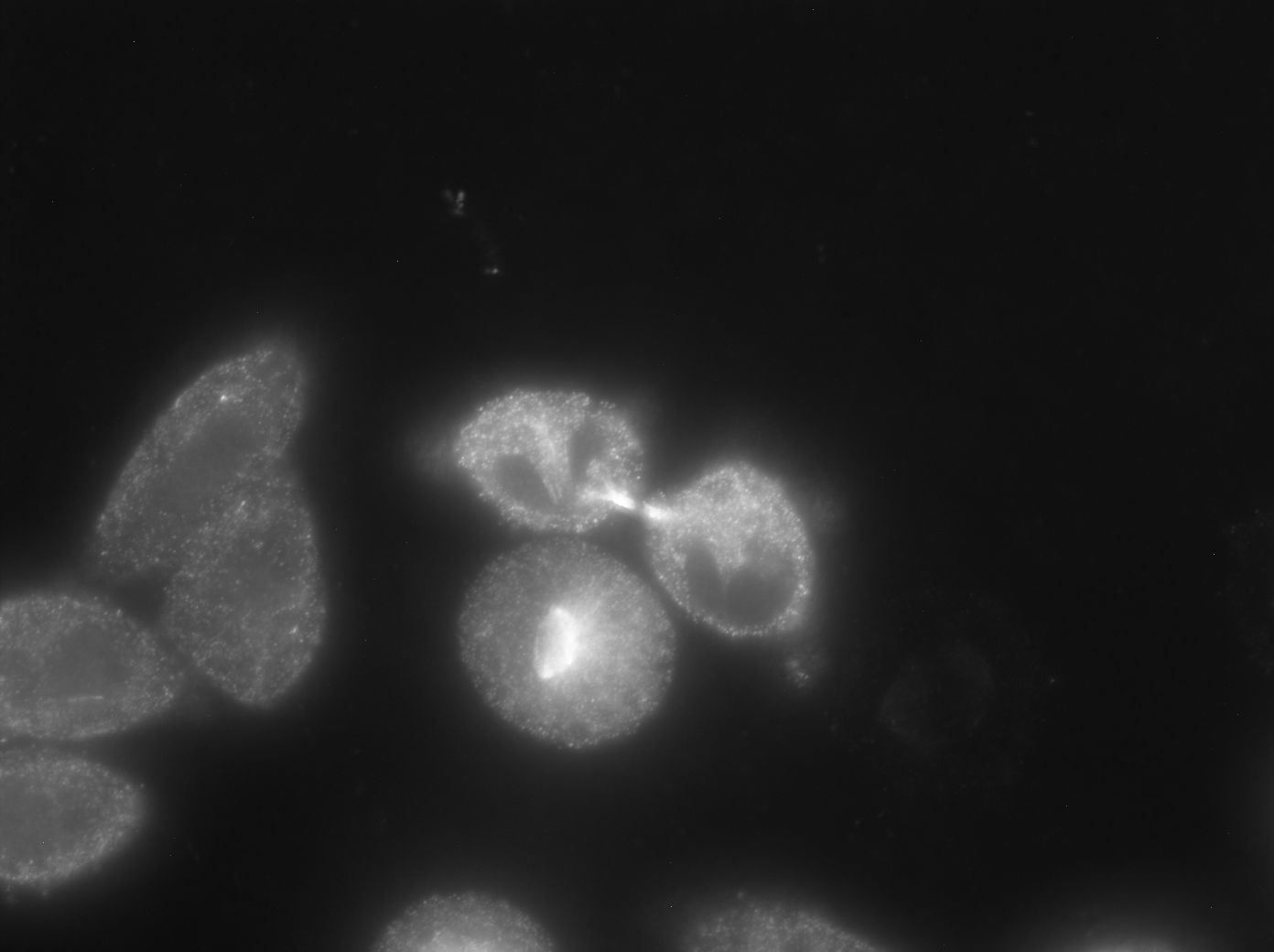

Supplement: Supplementary file 9 — Source data Fig. 2 part 6 [file 44319_2026_742_MOESM9_ESM.zip › Figure 2 Part 6/Fig 2fg Control Hela rGT335 acetylated tubulin/ET/Cas9 actub rGT335 9-8-25 R2 ET5 M5.Project Maximum Z_XY1757361480_Z0_T0_C1.tif]

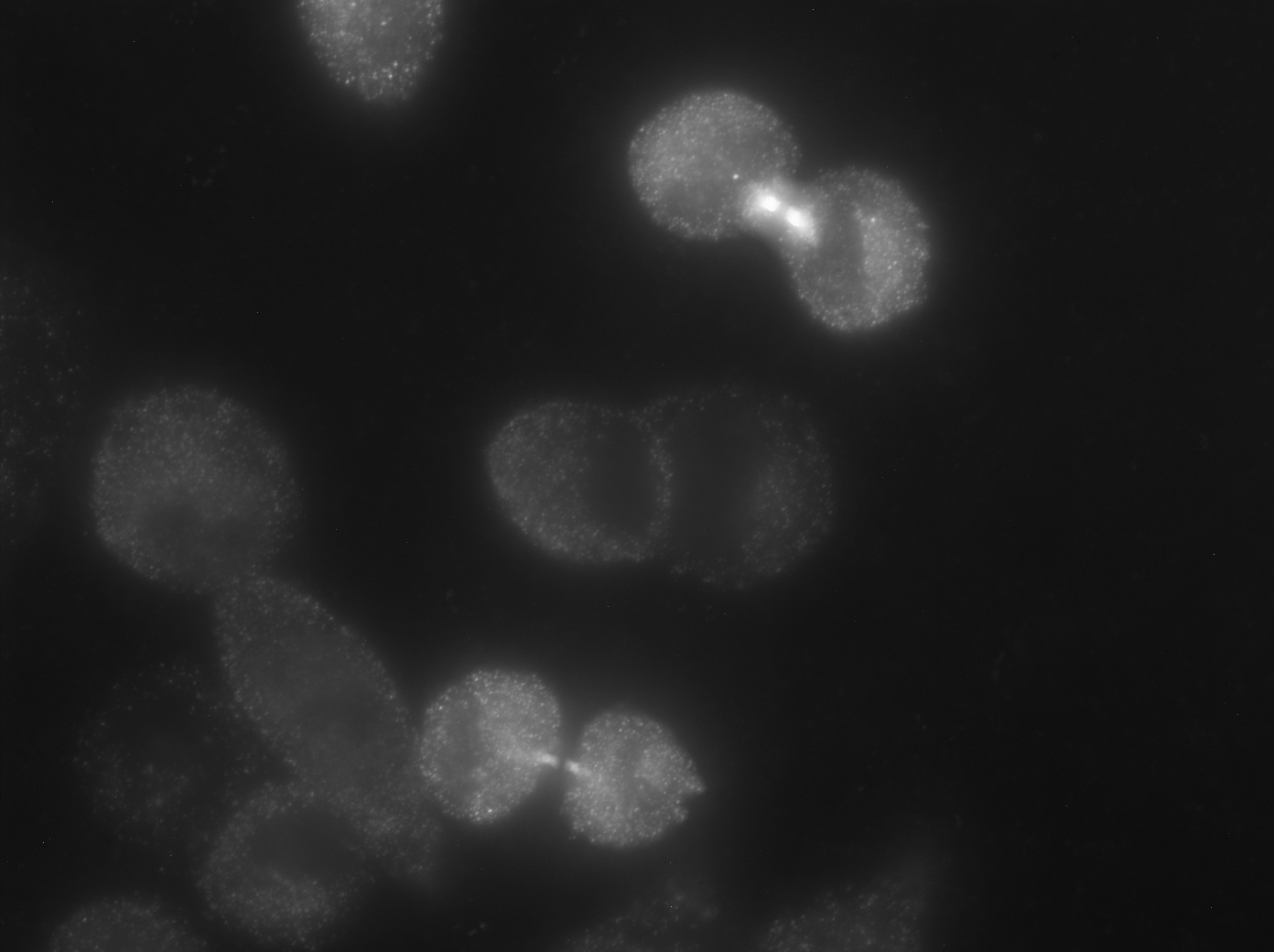

Supplement: Supplementary file 9 — Source data Fig. 2 part 6 [file 44319_2026_742_MOESM9_ESM.zip › Figure 2 Part 6/Fig 2fg Control Hela rGT335 acetylated tubulin/ET/Cas9 actub rGT335 9-8-25 R1 ET9.Project Maximum Z_XY1757354338_Z0_T0_C1.tif]

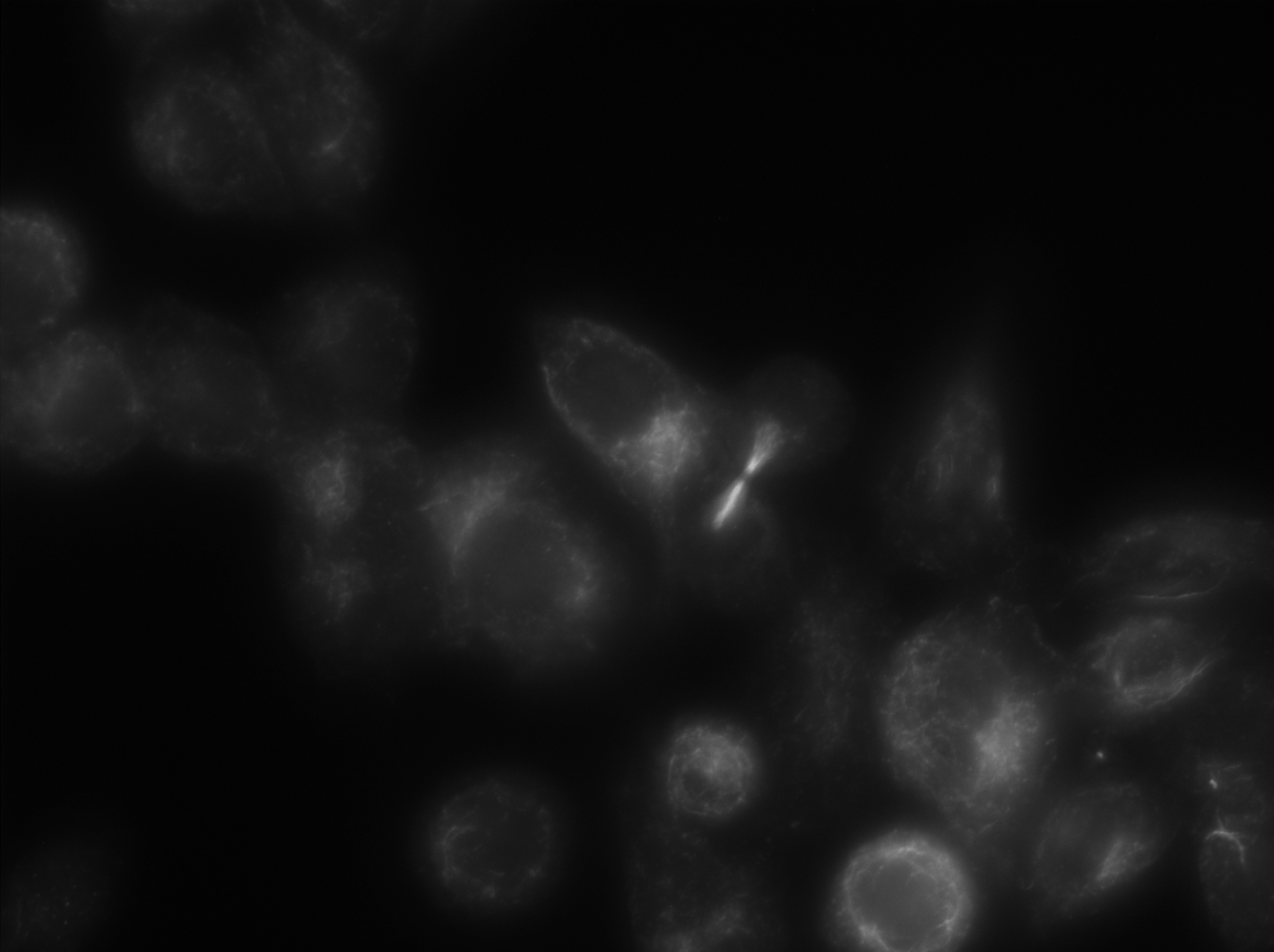

Supplement: Supplementary file 9 — Source data Fig. 2 part 6 [file 44319_2026_742_MOESM9_ESM.zip › Figure 2 Part 6/Fig 2fg Control Hela rGT335 acetylated tubulin/ET/Cas9 actub rGT335 9-8-25 R1 ET7.Project Maximum Z_XY1757353588_Z0_T0_C2.tif]

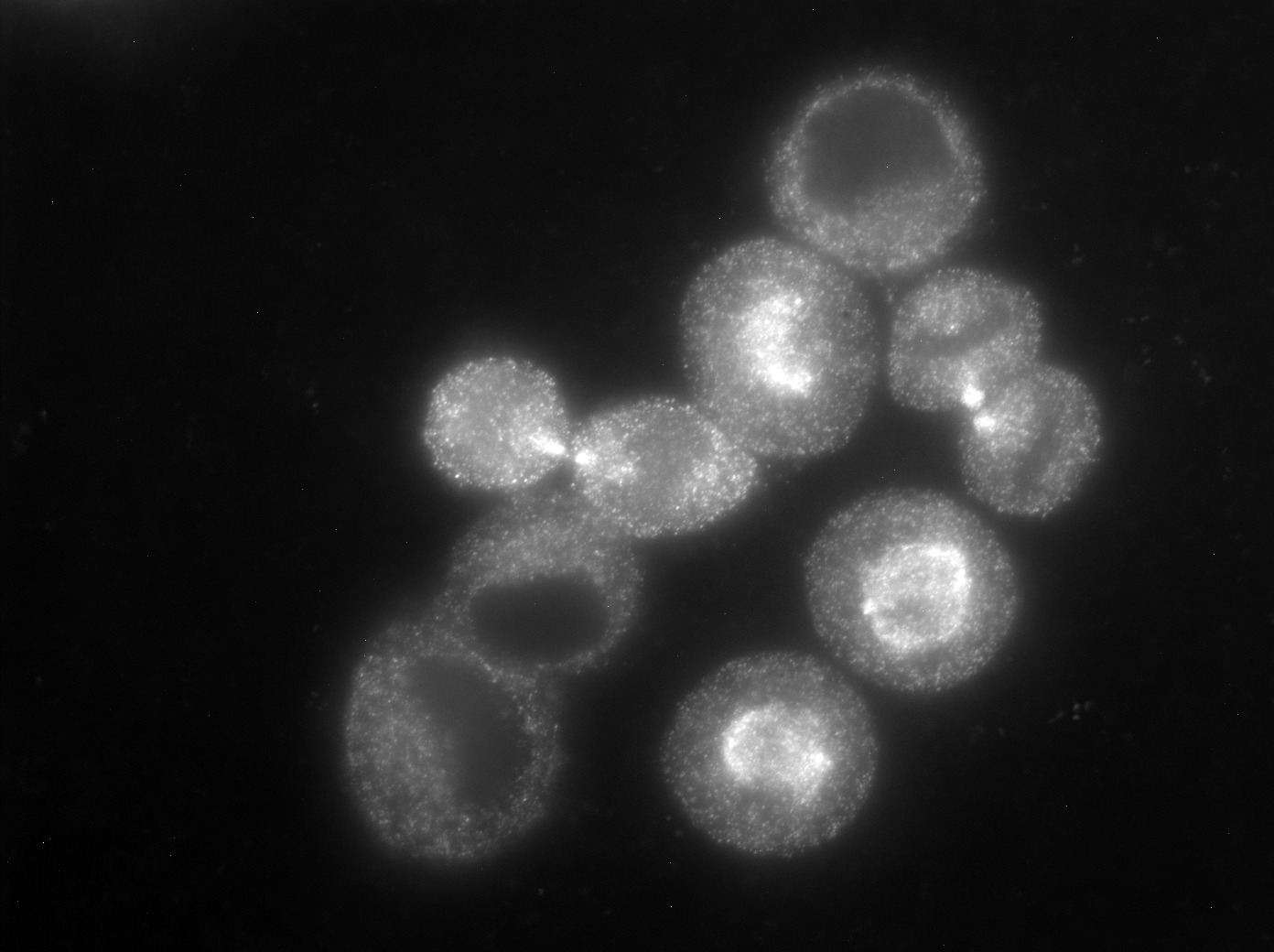

Supplement: Supplementary file 9 — Source data Fig. 2 part 6 [file 44319_2026_742_MOESM9_ESM.zip › Figure 2 Part 6/Fig 2fg Control Hela rGT335 acetylated tubulin/ET/Cas9 actub rGT335 9-8-25 R3 ET9ET10 M3M4.Project Maximum Z_XY1757366049_Z0_T0_C1.tif]

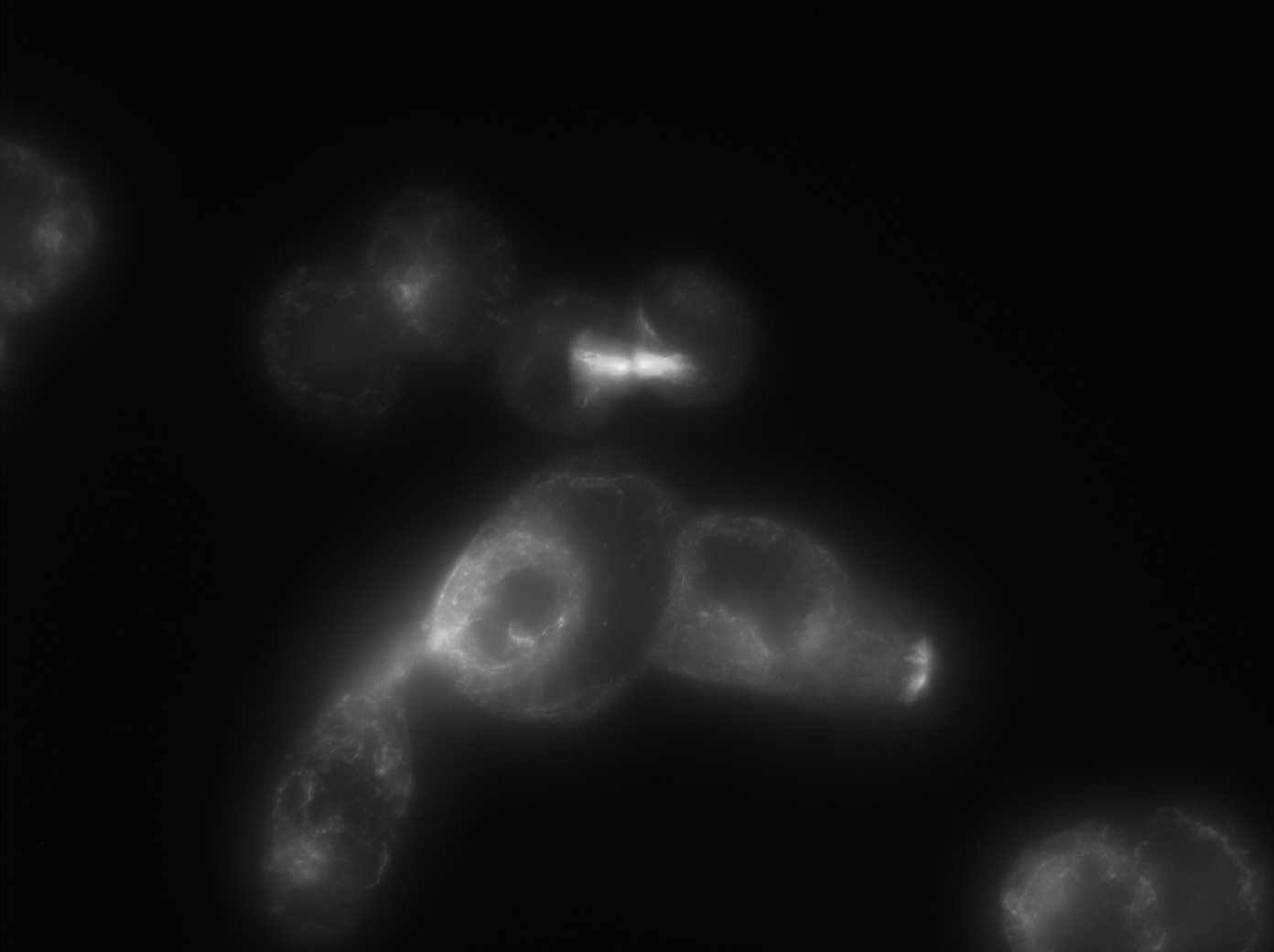

Supplement: Supplementary file 9 — Source data Fig. 2 part 6 [file 44319_2026_742_MOESM9_ESM.zip › Figure 2 Part 6/Fig 2fg Control Hela rGT335 acetylated tubulin/ET/Cas9 actub rGT335 9-8-25 R1 ET4.Project Maximum Z_XY1757351866_Z0_T0_C2.tif]

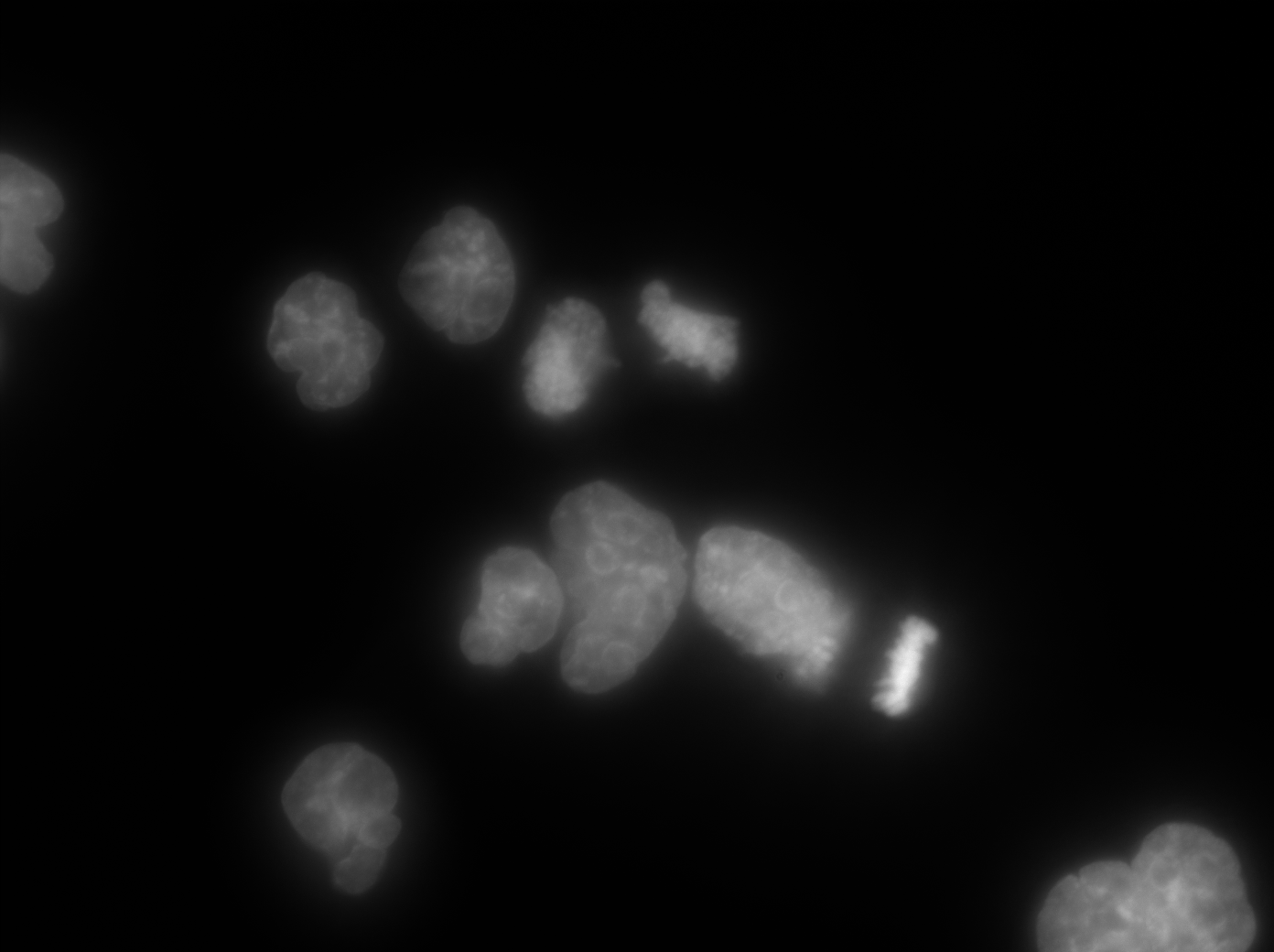

Supplement: Supplementary file 9 — Source data Fig. 2 part 6 [file 44319_2026_742_MOESM9_ESM.zip › Figure 2 Part 6/Fig 2fg Control Hela rGT335 acetylated tubulin/ET/Cas9 actub rGT335 9-8-25 R1 ET4.Project Maximum Z_XY1757351866_Z0_T0_C0.tif]

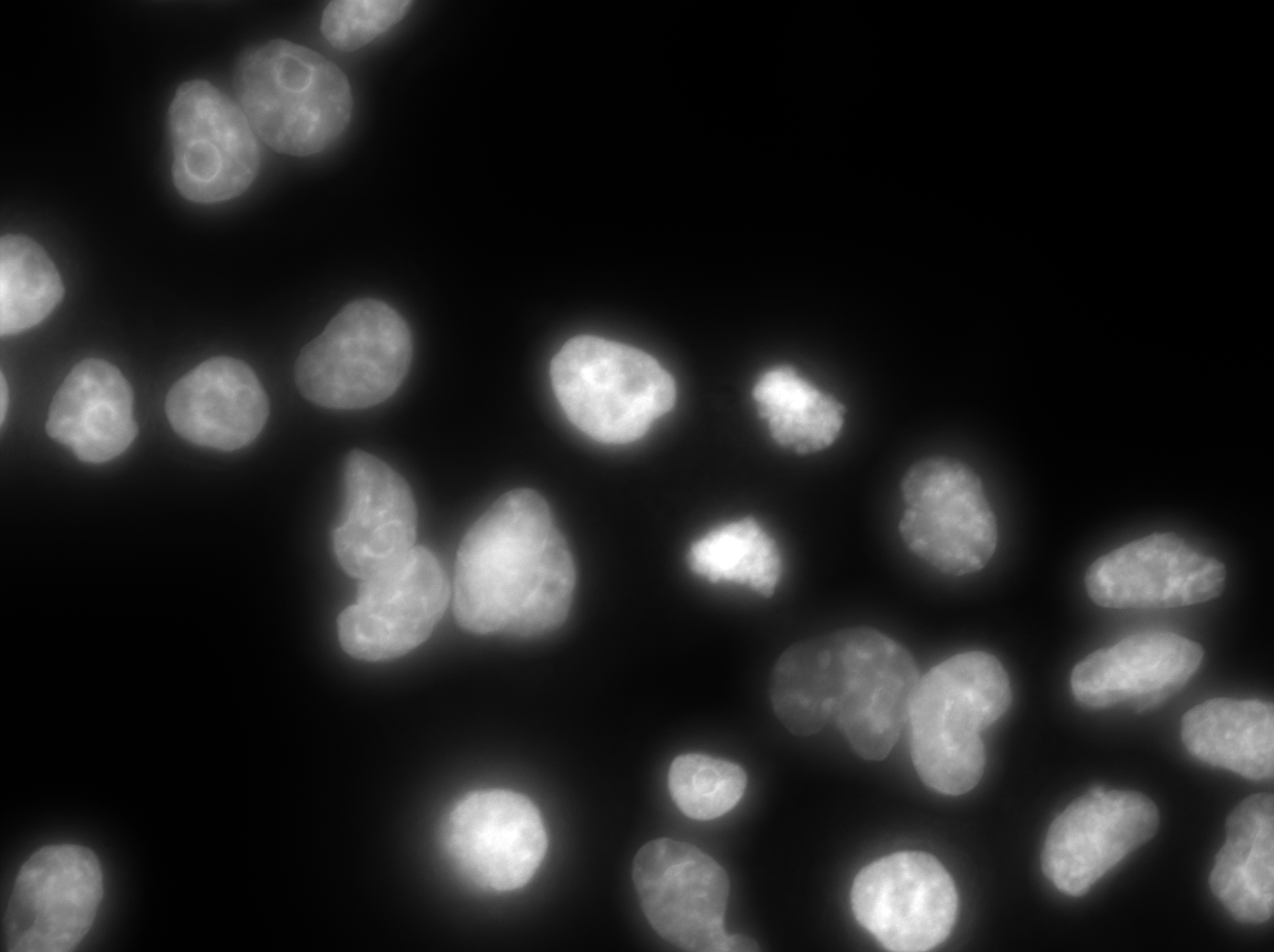

Supplement: Supplementary file 9 — Source data Fig. 2 part 6 [file 44319_2026_742_MOESM9_ESM.zip › Figure 2 Part 6/Fig 2fg Control Hela rGT335 acetylated tubulin/ET/Cas9 actub rGT335 9-8-25 R1 ET7.Project Maximum Z_XY1757353588_Z0_T0_C0.tif]

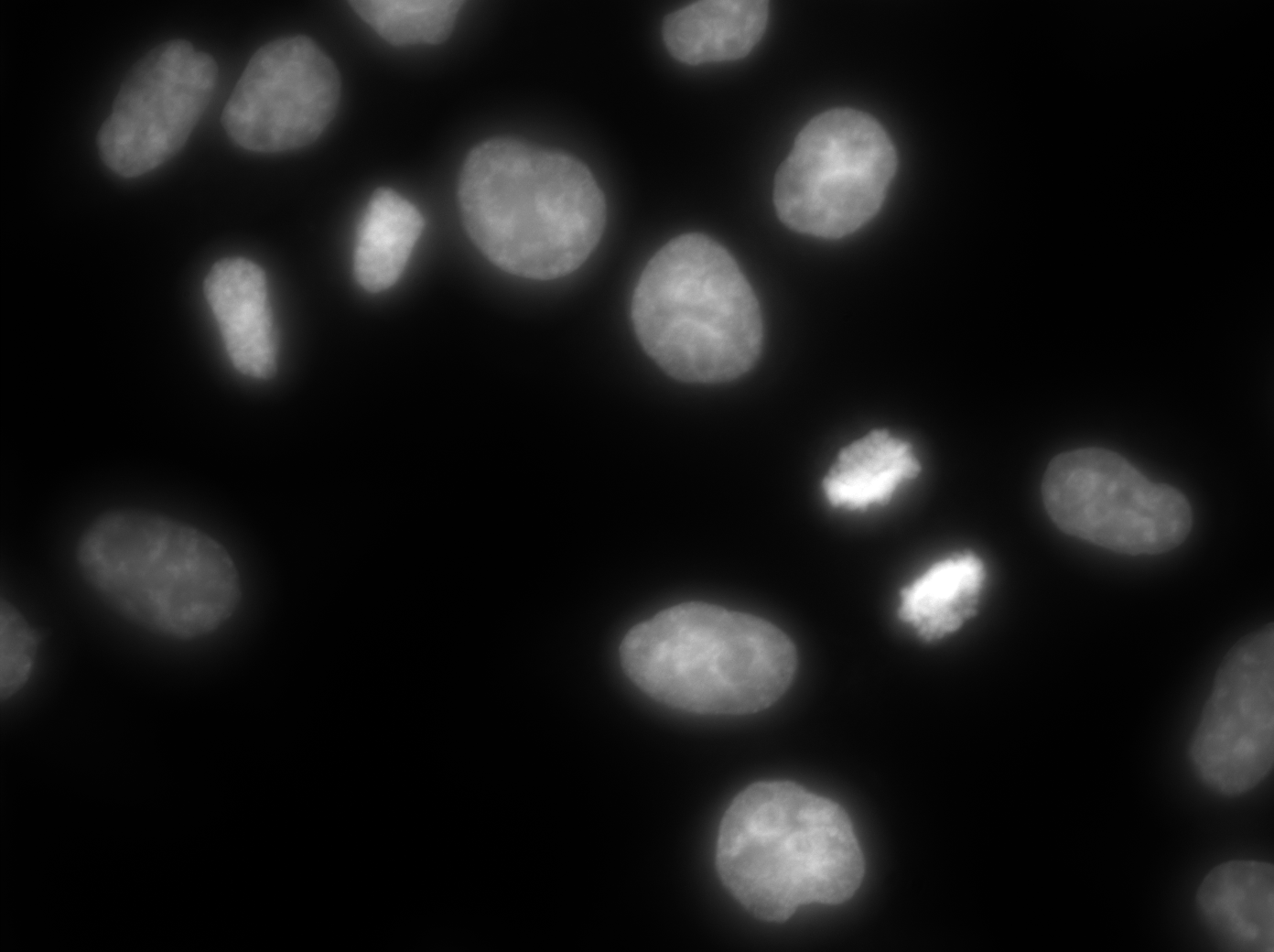

Supplement: Supplementary file 9 — Source data Fig. 2 part 6 [file 44319_2026_742_MOESM9_ESM.zip › Figure 2 Part 6/Fig 2fg Control Hela rGT335 acetylated tubulin/ET/Cas9 actub rGT335 9-8-25 R2 FI2 ET3.Project Maximum Z_XY1757359877_Z0_T0_C0.tif]

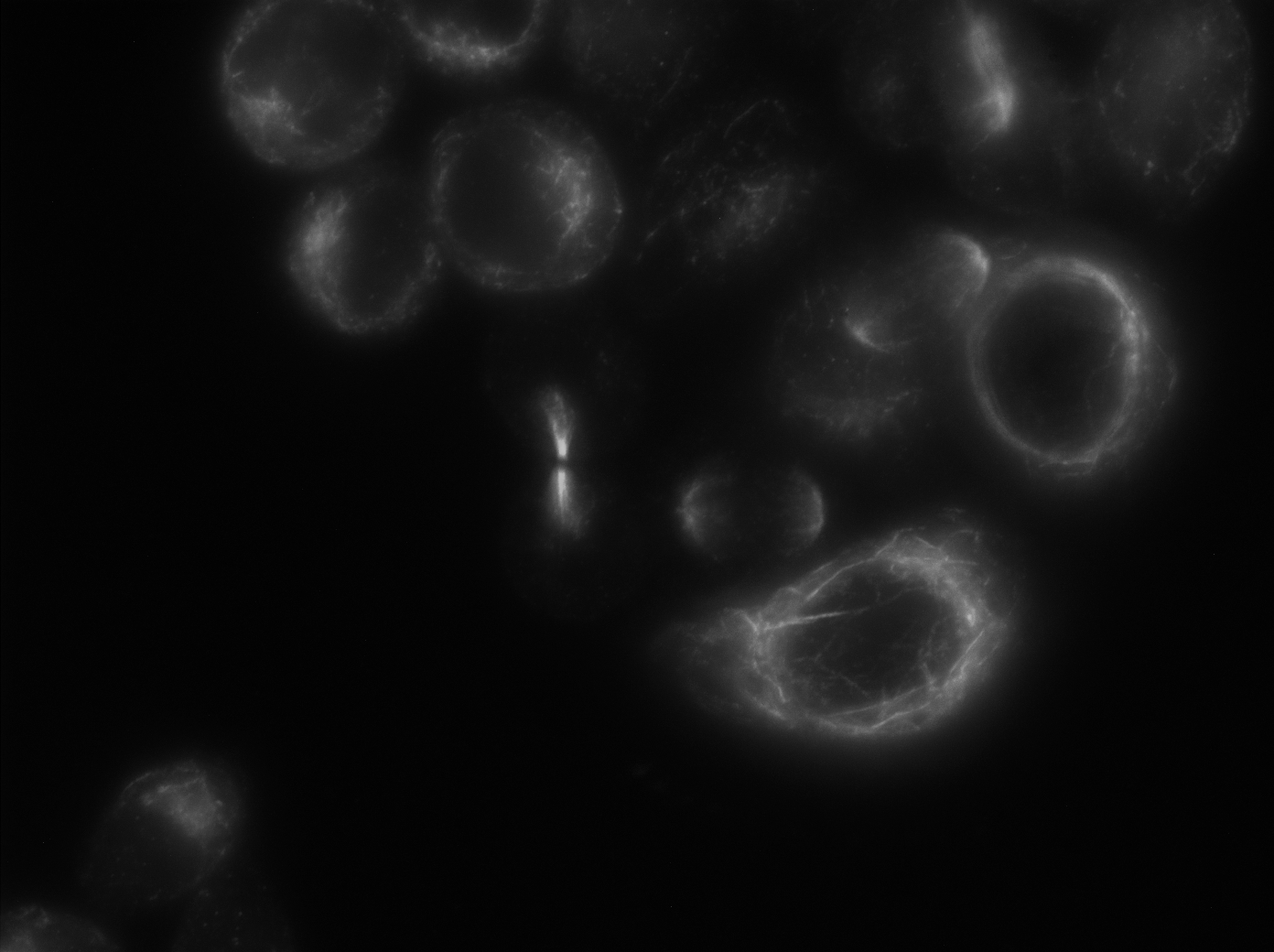

Supplement: Supplementary file 9 — Source data Fig. 2 part 6 [file 44319_2026_742_MOESM9_ESM.zip › Figure 2 Part 6/Fig 2fg Control Hela rGT335 acetylated tubulin/ET/Cas9 actub rGT335 9-8-25 R3 A1 ET1 M1.Project Maximum Z_XY1757364698_Z0_T0_C2.tif]

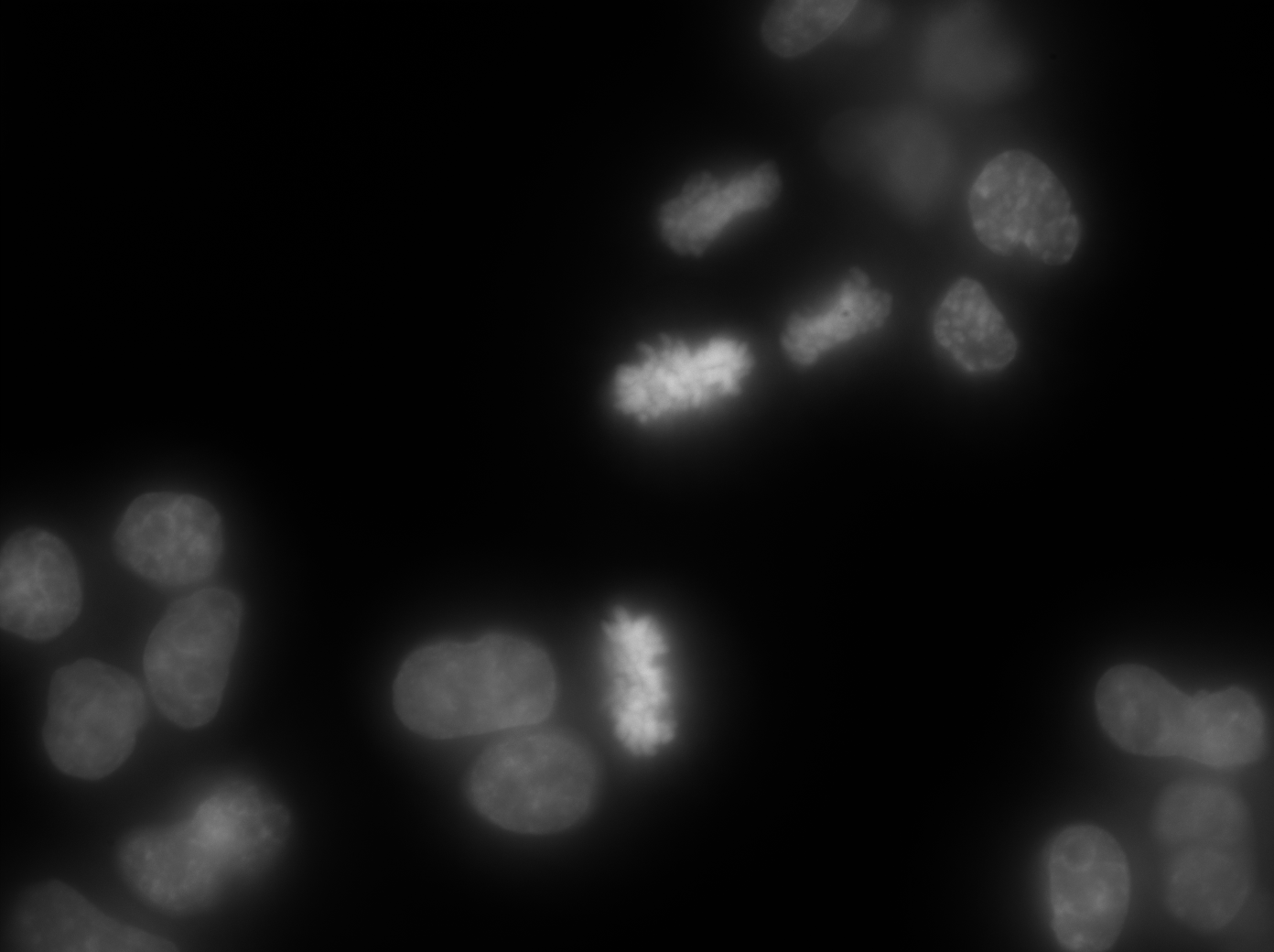

Supplement: Supplementary file 9 — Source data Fig. 2 part 6 [file 44319_2026_742_MOESM9_ESM.zip › Figure 2 Part 6/Fig 2fg Control Hela rGT335 acetylated tubulin/ET/Cas9 actub rGT335 9-8-25 R1 ET8 M4M5.Project Maximum Z_XY1757354024_Z0_T0_C0.tif]

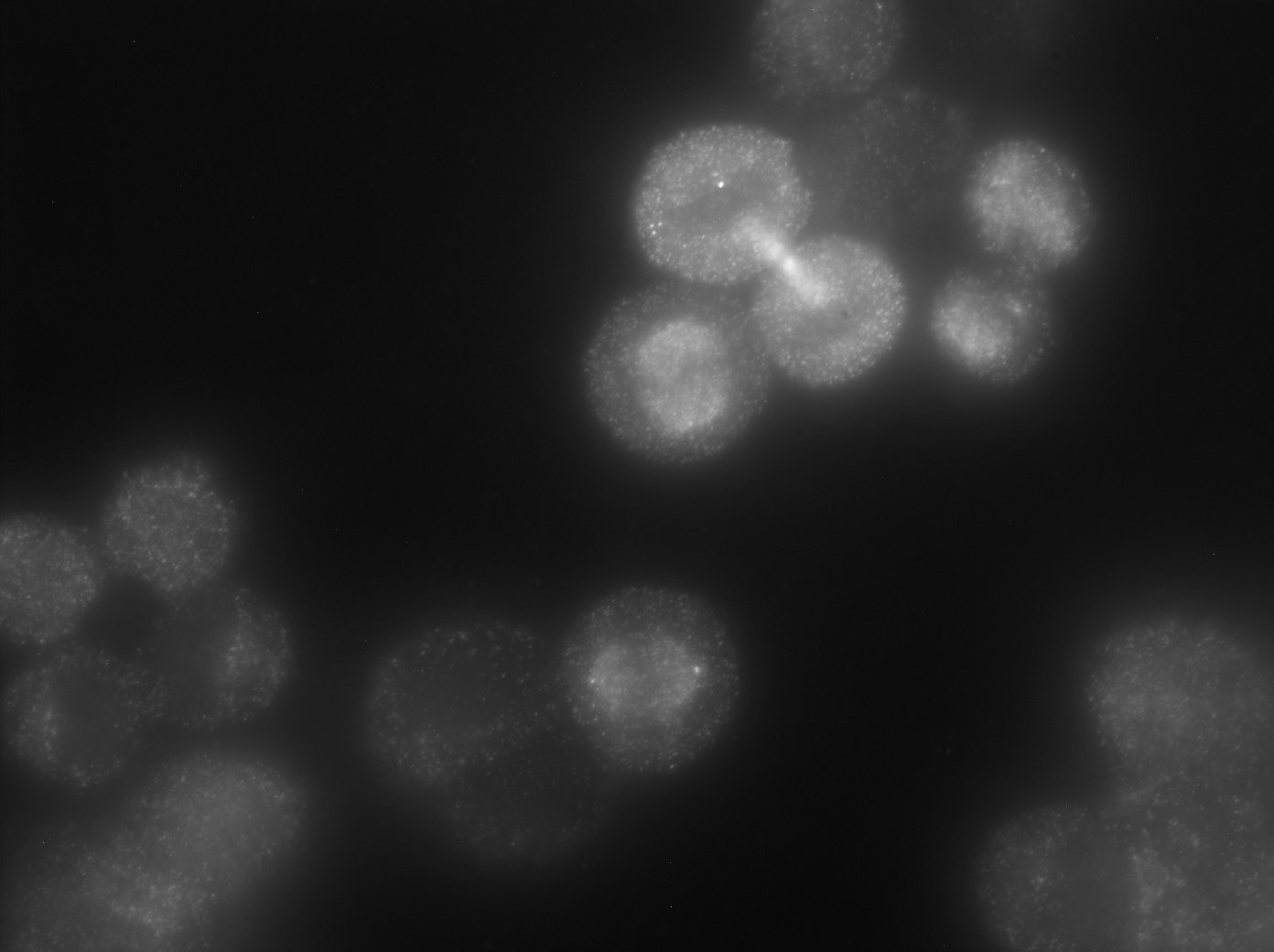

Supplement: Supplementary file 9 — Source data Fig. 2 part 6 [file 44319_2026_742_MOESM9_ESM.zip › Figure 2 Part 6/Fig 2fg Control Hela rGT335 acetylated tubulin/ET/Cas9 actub rGT335 9-8-25 R1 ET8 M4M5.Project Maximum Z_XY1757354024_Z0_T0_C1.tif]

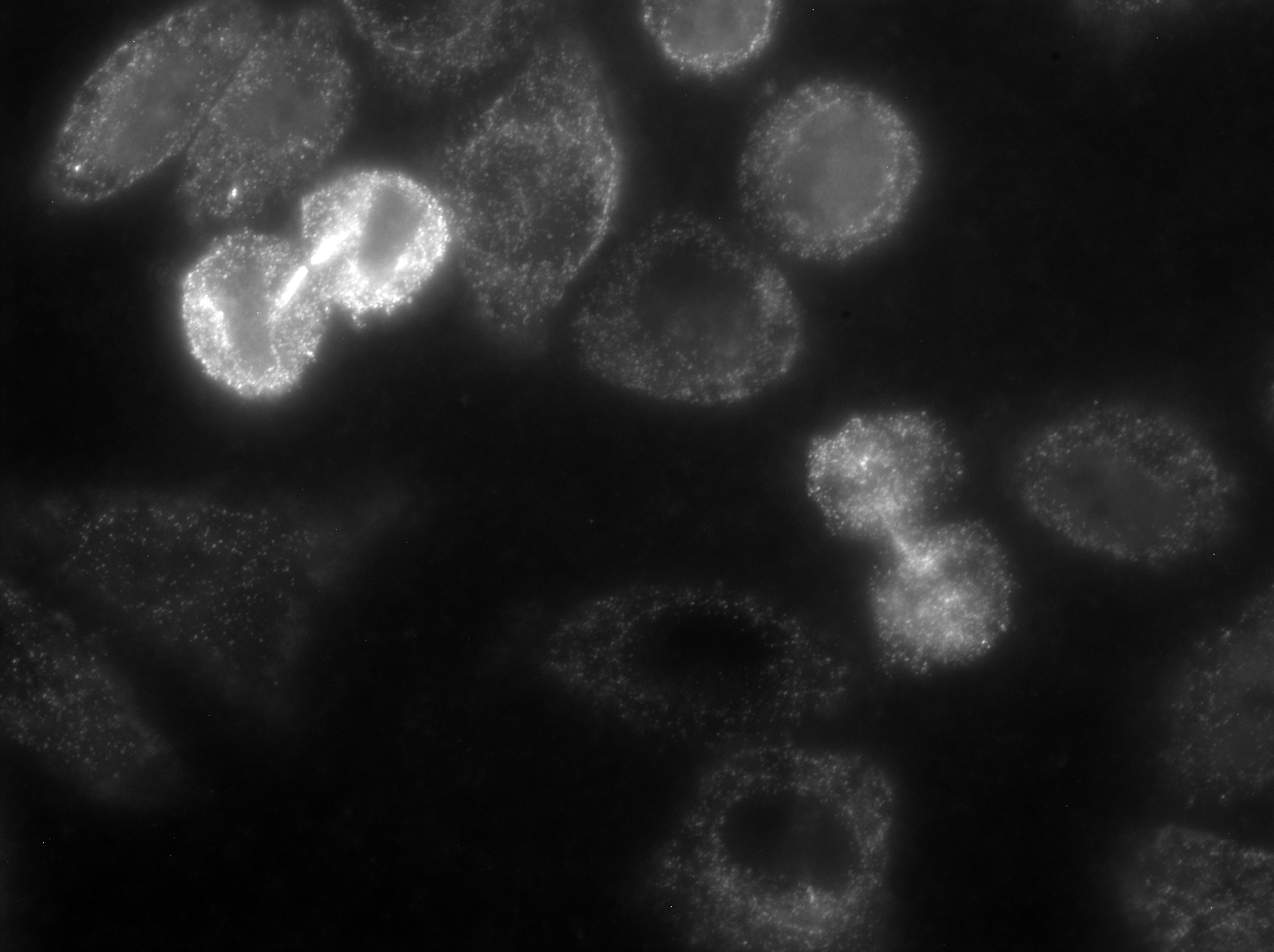

Supplement: Supplementary file 9 — Source data Fig. 2 part 6 [file 44319_2026_742_MOESM9_ESM.zip › Figure 2 Part 6/Fig 2fg Control Hela rGT335 acetylated tubulin/ET/Cas9 actub rGT335 9-8-25 R2 FI2 ET3.Project Maximum Z_XY1757359877_Z0_T0_C1.tif]

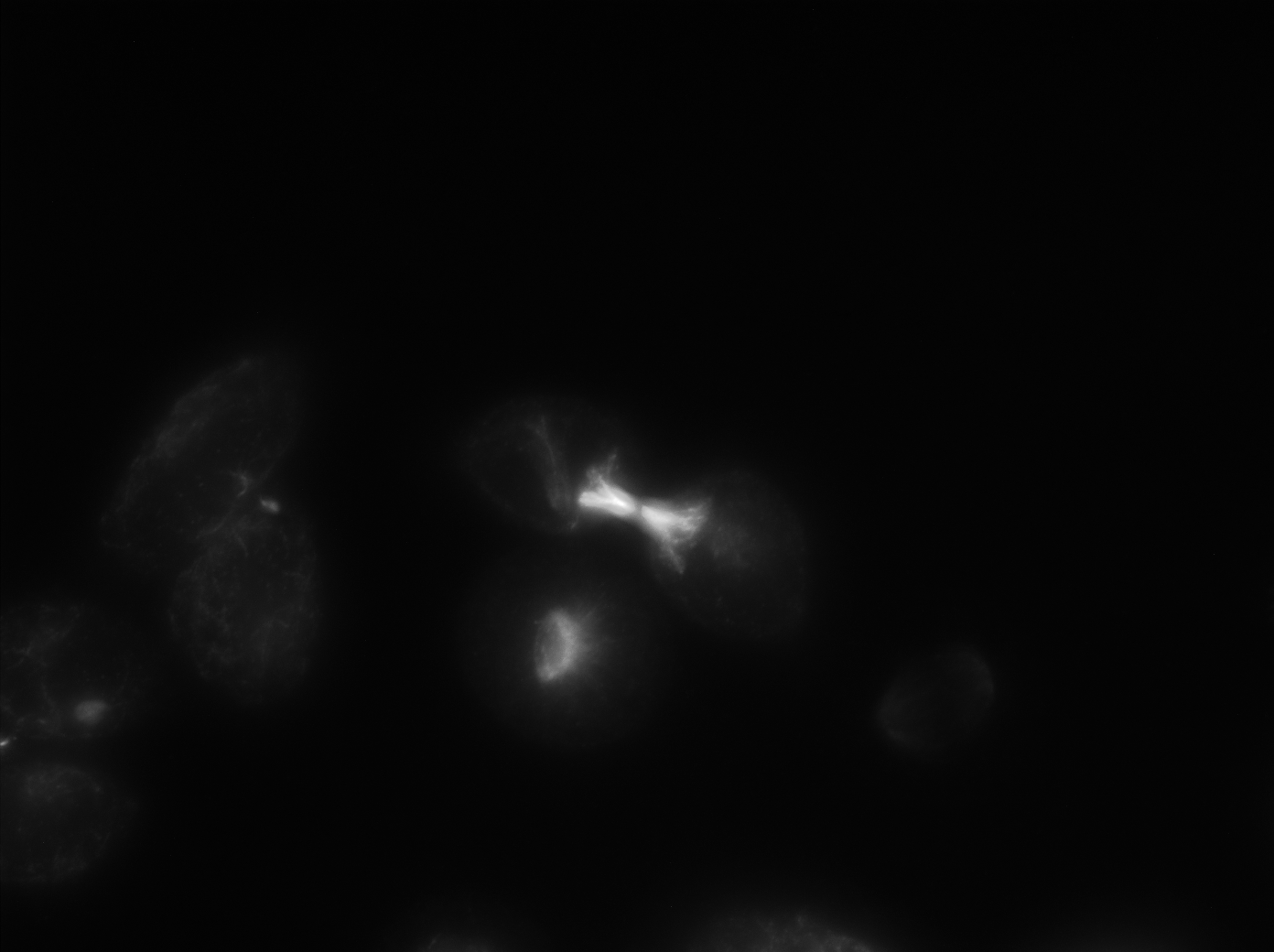

Supplement: Supplementary file 9 — Source data Fig. 2 part 6 [file 44319_2026_742_MOESM9_ESM.zip › Figure 2 Part 6/Fig 2fg Control Hela rGT335 acetylated tubulin/ET/Cas9 actub rGT335 9-8-25 R2 ET5 M5.Project Maximum Z_XY1757361480_Z0_T0_C2.tif]

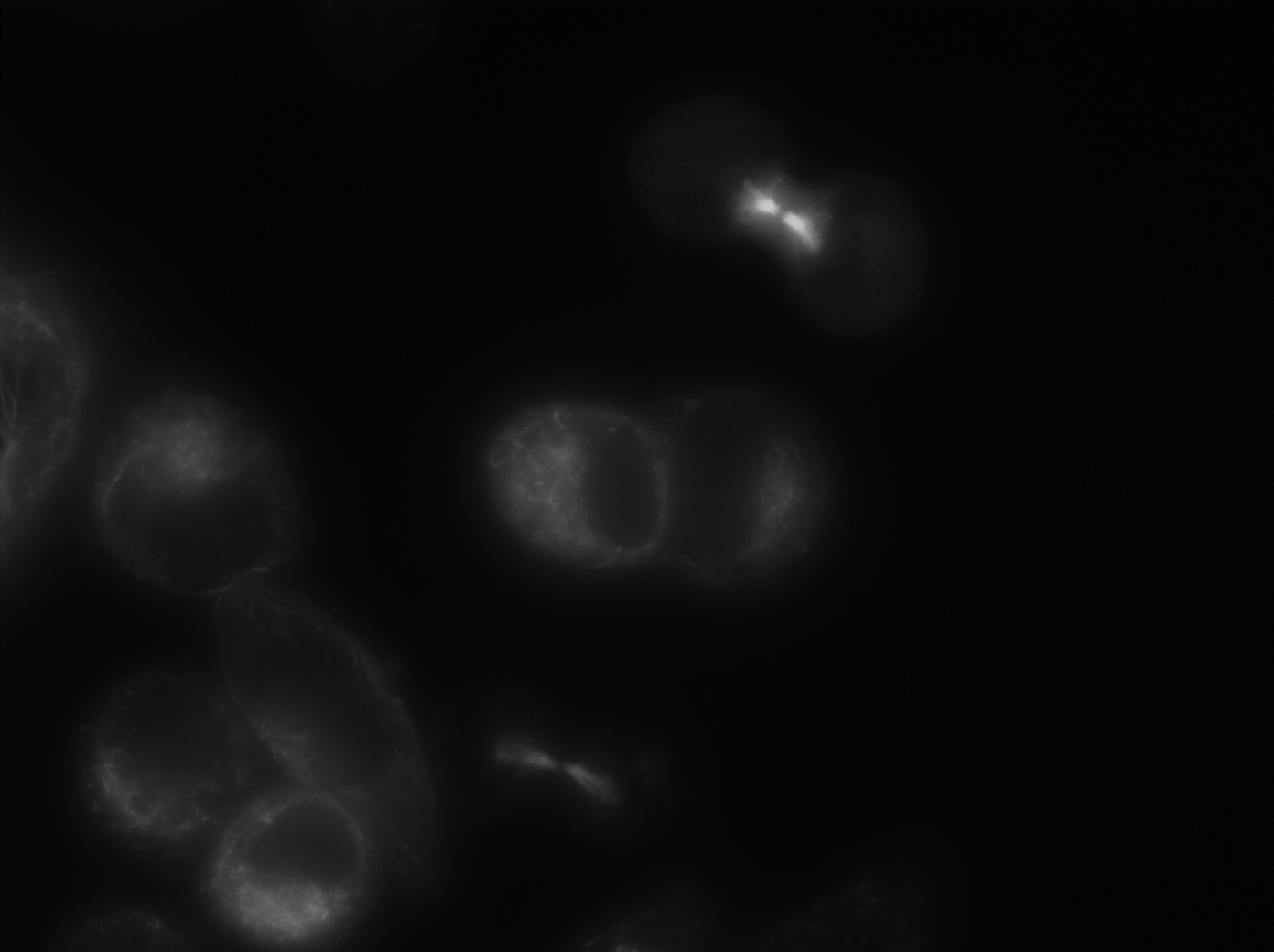

Supplement: Supplementary file 9 — Source data Fig. 2 part 6 [file 44319_2026_742_MOESM9_ESM.zip › Figure 2 Part 6/Fig 2fg Control Hela rGT335 acetylated tubulin/ET/Cas9 actub rGT335 9-8-25 R1 ET9.Project Maximum Z_XY1757354338_Z0_T0_C2.tif]

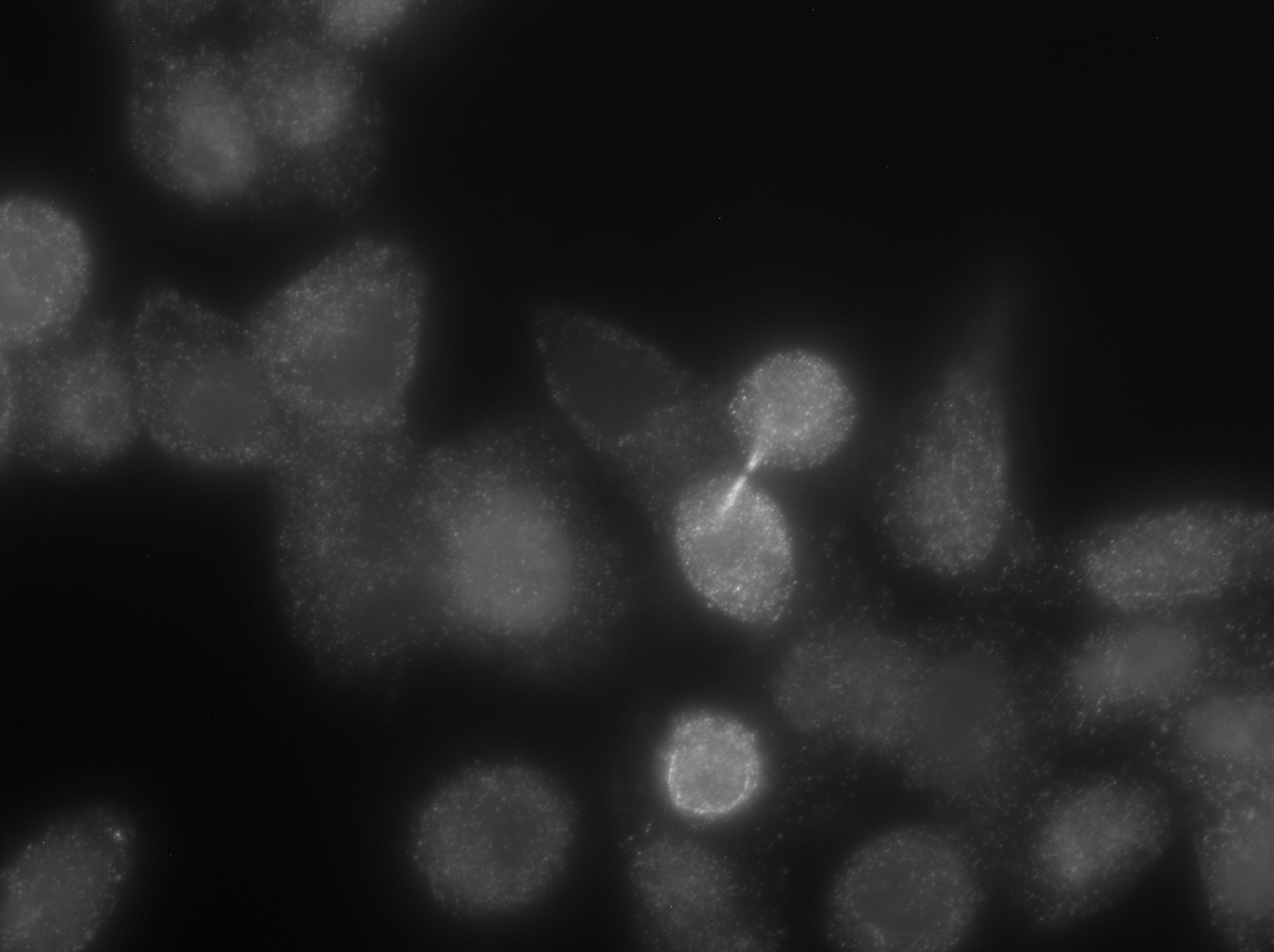

Supplement: Supplementary file 9 — Source data Fig. 2 part 6 [file 44319_2026_742_MOESM9_ESM.zip › Figure 2 Part 6/Fig 2fg Control Hela rGT335 acetylated tubulin/ET/Cas9 actub rGT335 9-8-25 R1 ET7.Project Maximum Z_XY1757353588_Z0_T0_C1.tif]

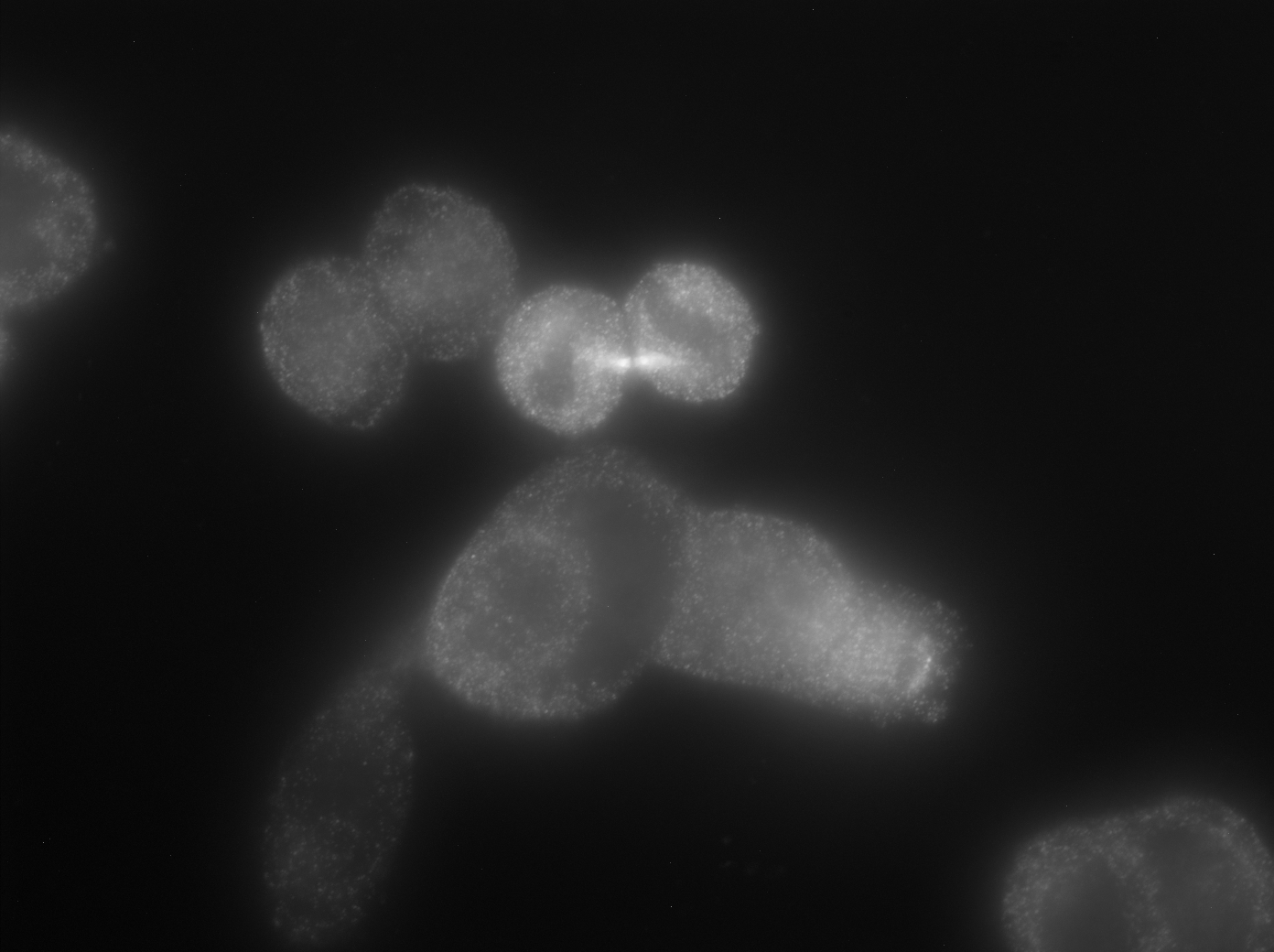

Supplement: Supplementary file 9 — Source data Fig. 2 part 6 [file 44319_2026_742_MOESM9_ESM.zip › Figure 2 Part 6/Fig 2fg Control Hela rGT335 acetylated tubulin/ET/Cas9 actub rGT335 9-8-25 R1 ET4.Project Maximum Z_XY1757351866_Z0_T0_C1.tif]

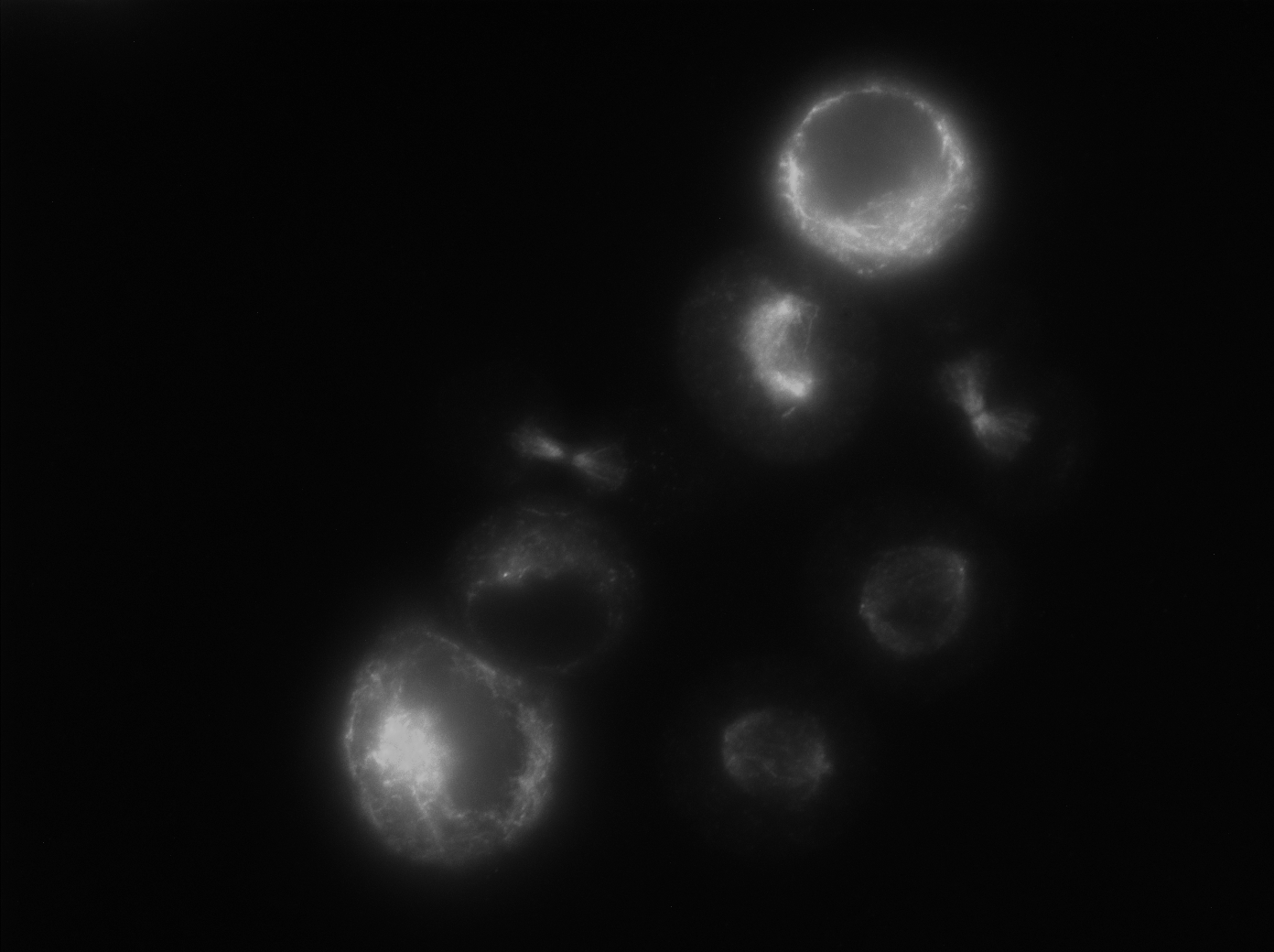

Supplement: Supplementary file 9 — Source data Fig. 2 part 6 [file 44319_2026_742_MOESM9_ESM.zip › Figure 2 Part 6/Fig 2fg Control Hela rGT335 acetylated tubulin/ET/Cas9 actub rGT335 9-8-25 R3 ET9ET10 M3M4.Project Maximum Z_XY1757366049_Z0_T0_C2.tif]

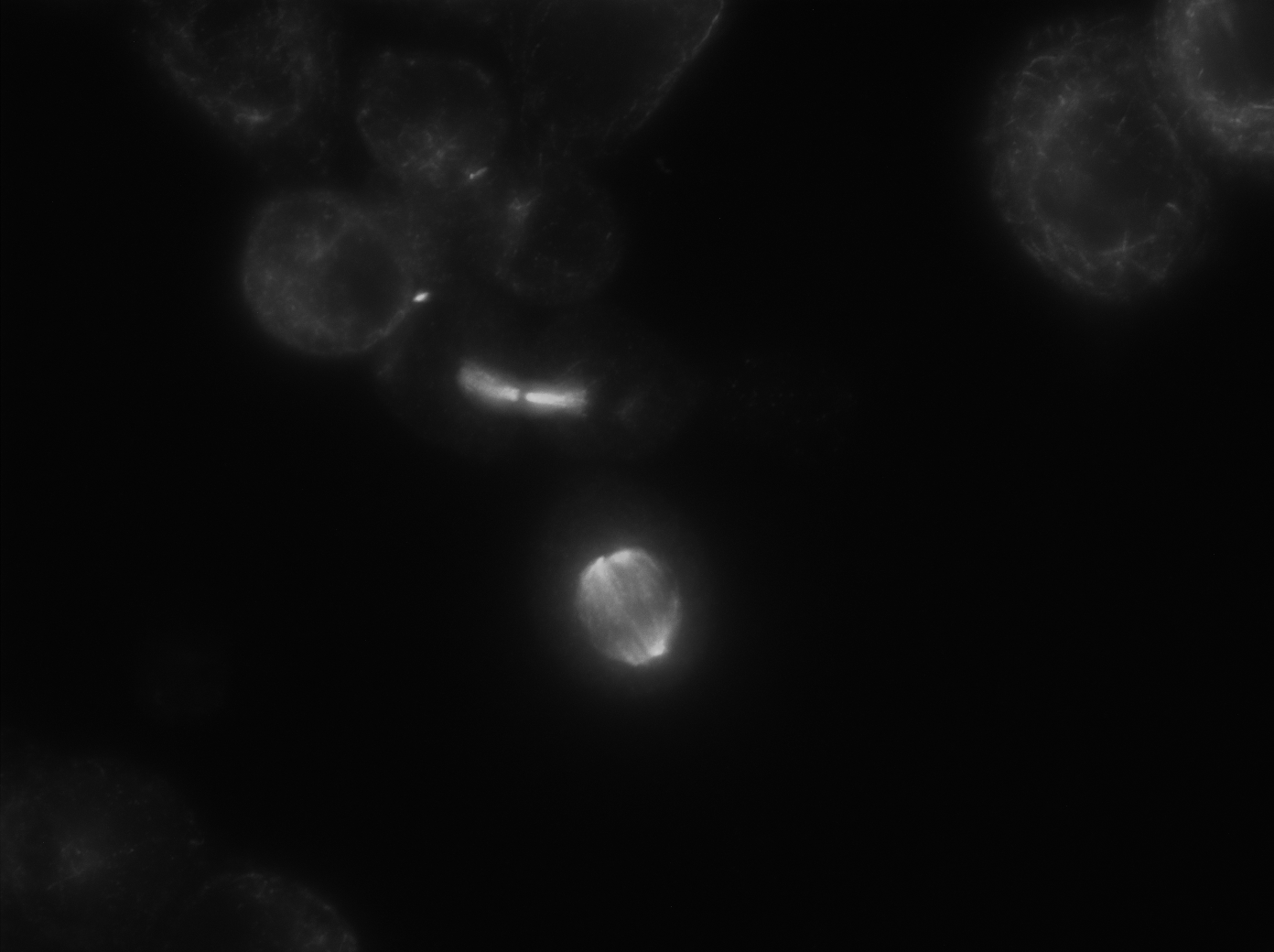

Supplement: Supplementary file 9 — Source data Fig. 2 part 6 [file 44319_2026_742_MOESM9_ESM.zip › Figure 2 Part 6/Fig 2fg Control Hela rGT335 acetylated tubulin/ET/Cas9 actub rGT335 9-8-25 R2 ET4 M3.Project Maximum Z_XY1757360796_Z0_T0_C2.tif]

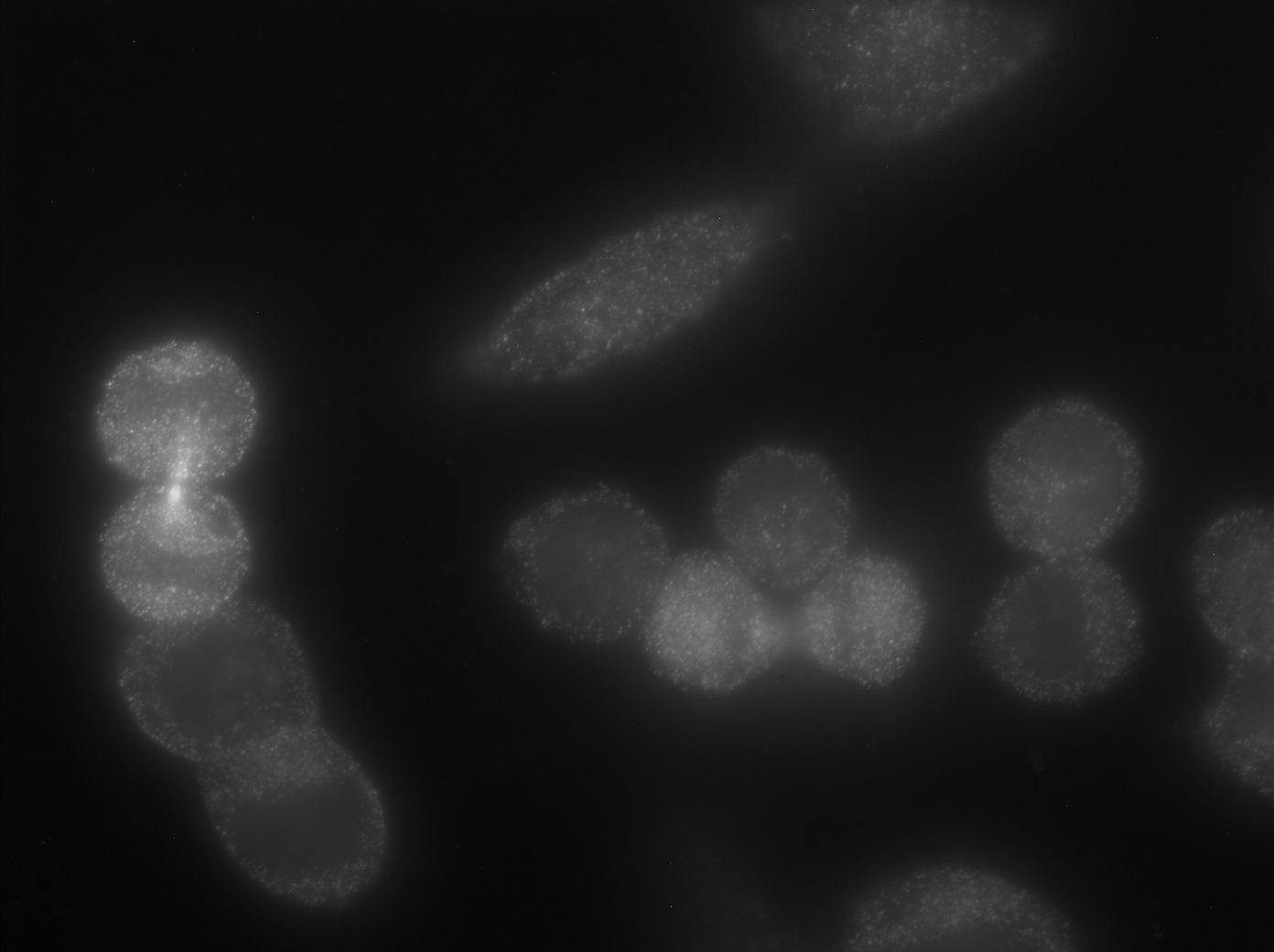

Supplement: Supplementary file 9 — Source data Fig. 2 part 6 [file 44319_2026_742_MOESM9_ESM.zip › Figure 2 Part 6/Fig 2fg Control Hela rGT335 acetylated tubulin/ET/Cas9 actub rGT335 9-8-25 R1 ET3 EX.Project Maximum Z_XY1757351356_Z0_T0_C1.tif]

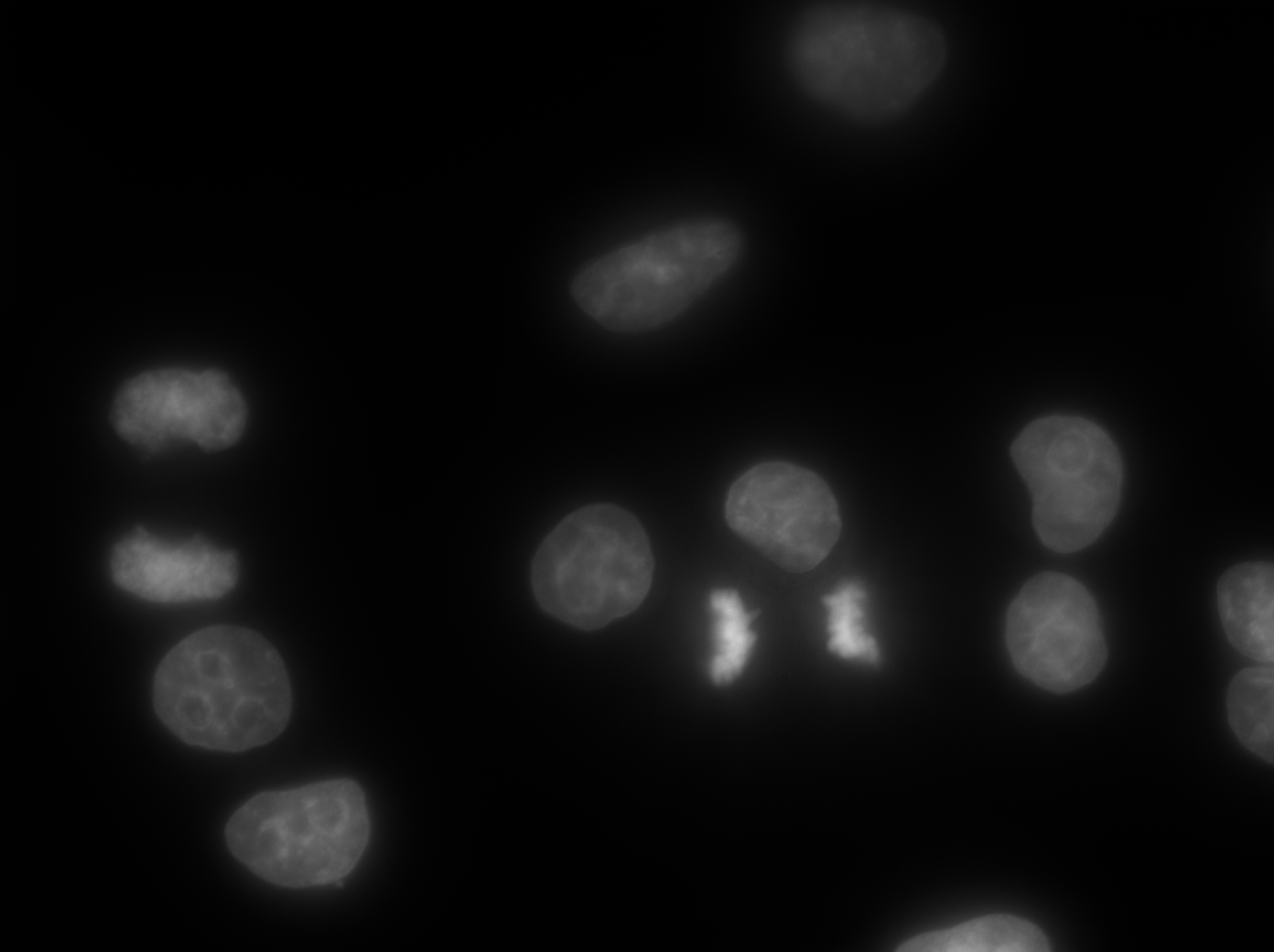

Supplement: Supplementary file 9 — Source data Fig. 2 part 6 [file 44319_2026_742_MOESM9_ESM.zip › Figure 2 Part 6/Fig 2fg Control Hela rGT335 acetylated tubulin/ET/Cas9 actub rGT335 9-8-25 R1 ET3 EX.Project Maximum Z_XY1757351356_Z0_T0_C0.tif]

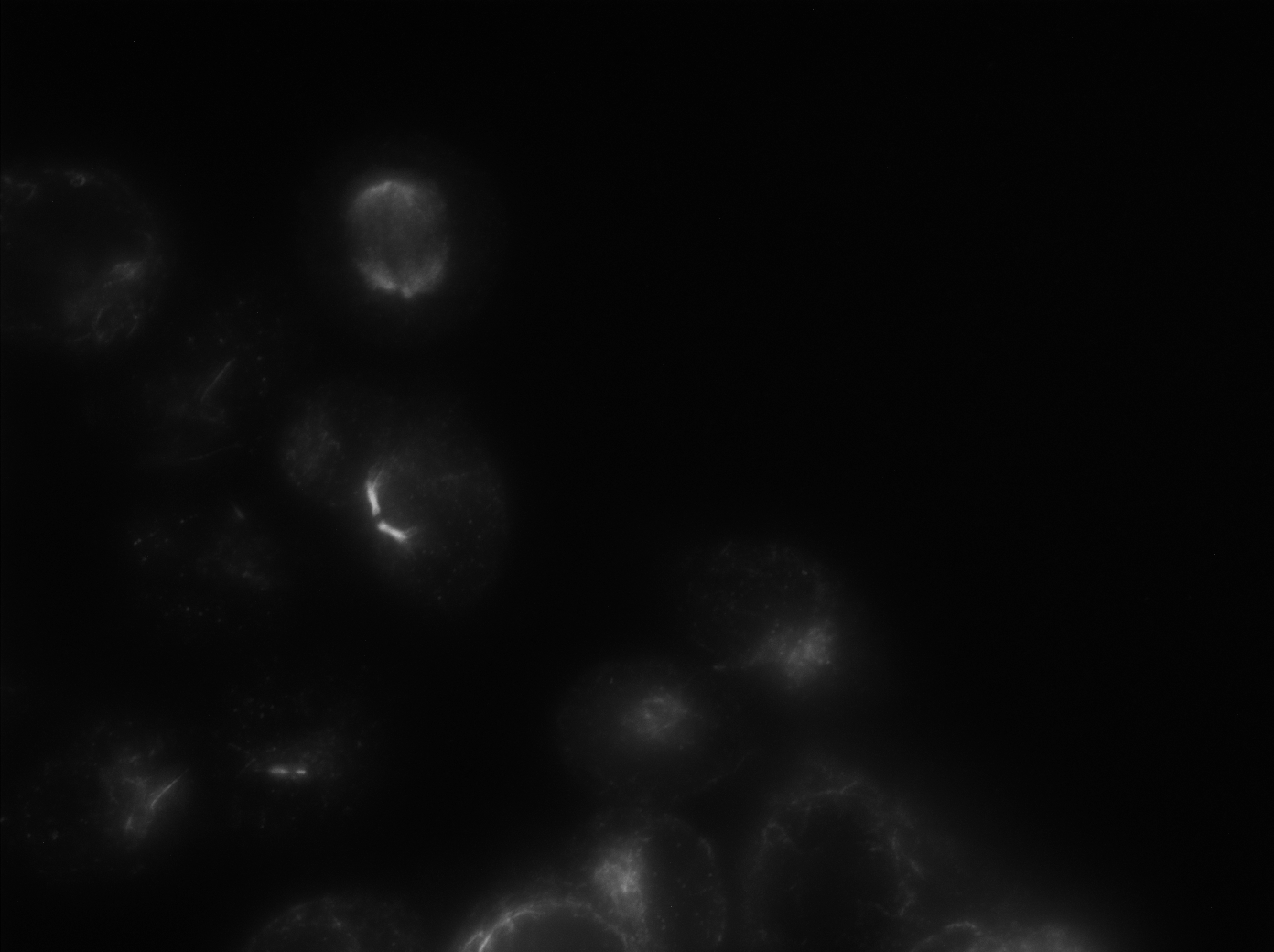

Supplement: Supplementary file 9 — Source data Fig. 2 part 6 [file 44319_2026_742_MOESM9_ESM.zip › Figure 2 Part 6/Fig 2fg Control Hela rGT335 acetylated tubulin/ET/Cas9 actub rGT335 9-8-25 R3 M8 ET11.Project Maximum Z_XY1757367702_Z0_T0_C2.tif]

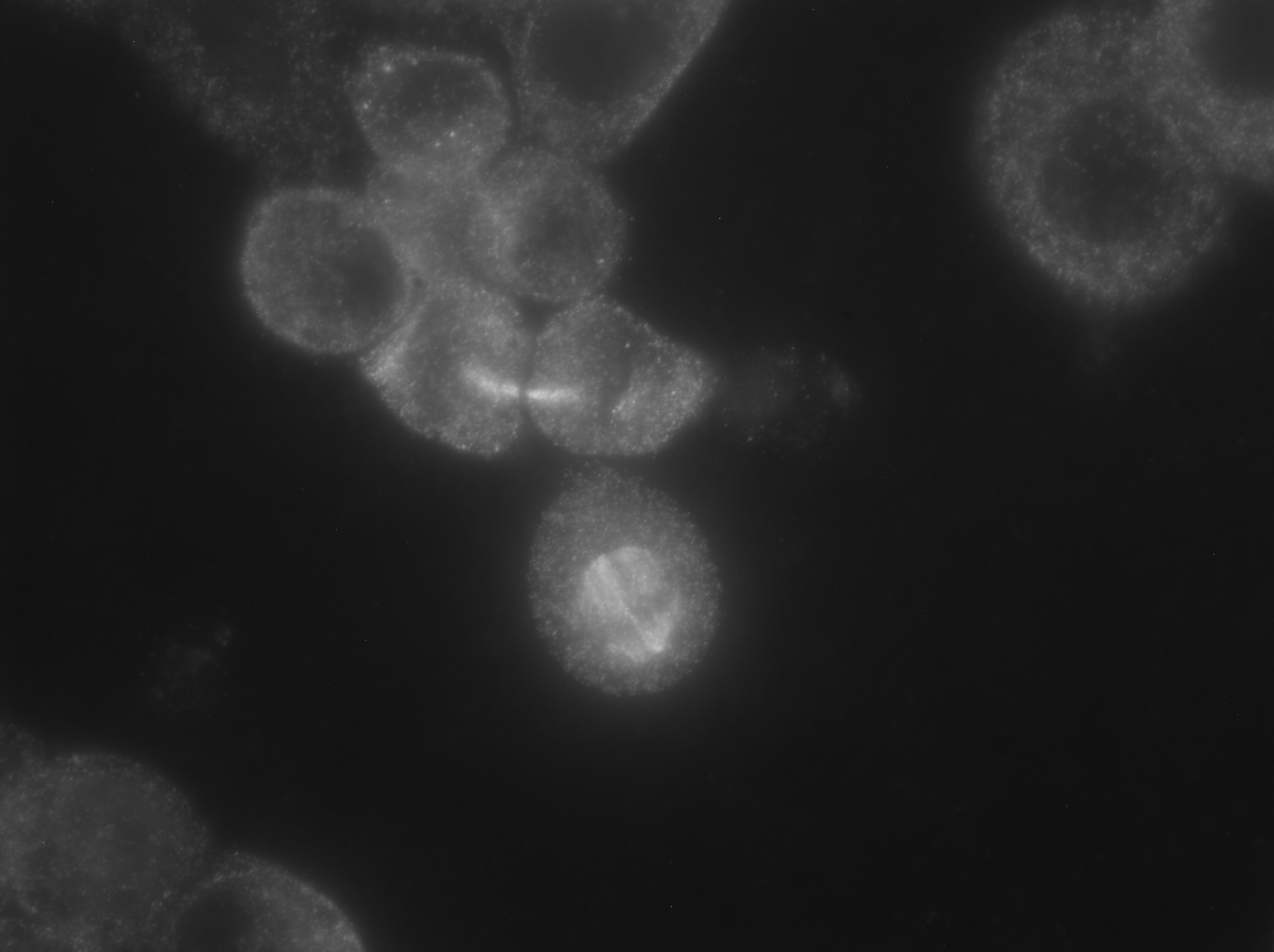

Supplement: Supplementary file 9 — Source data Fig. 2 part 6 [file 44319_2026_742_MOESM9_ESM.zip › Figure 2 Part 6/Fig 2fg Control Hela rGT335 acetylated tubulin/ET/Cas9 actub rGT335 9-8-25 R2 ET4 M3.Project Maximum Z_XY1757360796_Z0_T0_C1.tif]

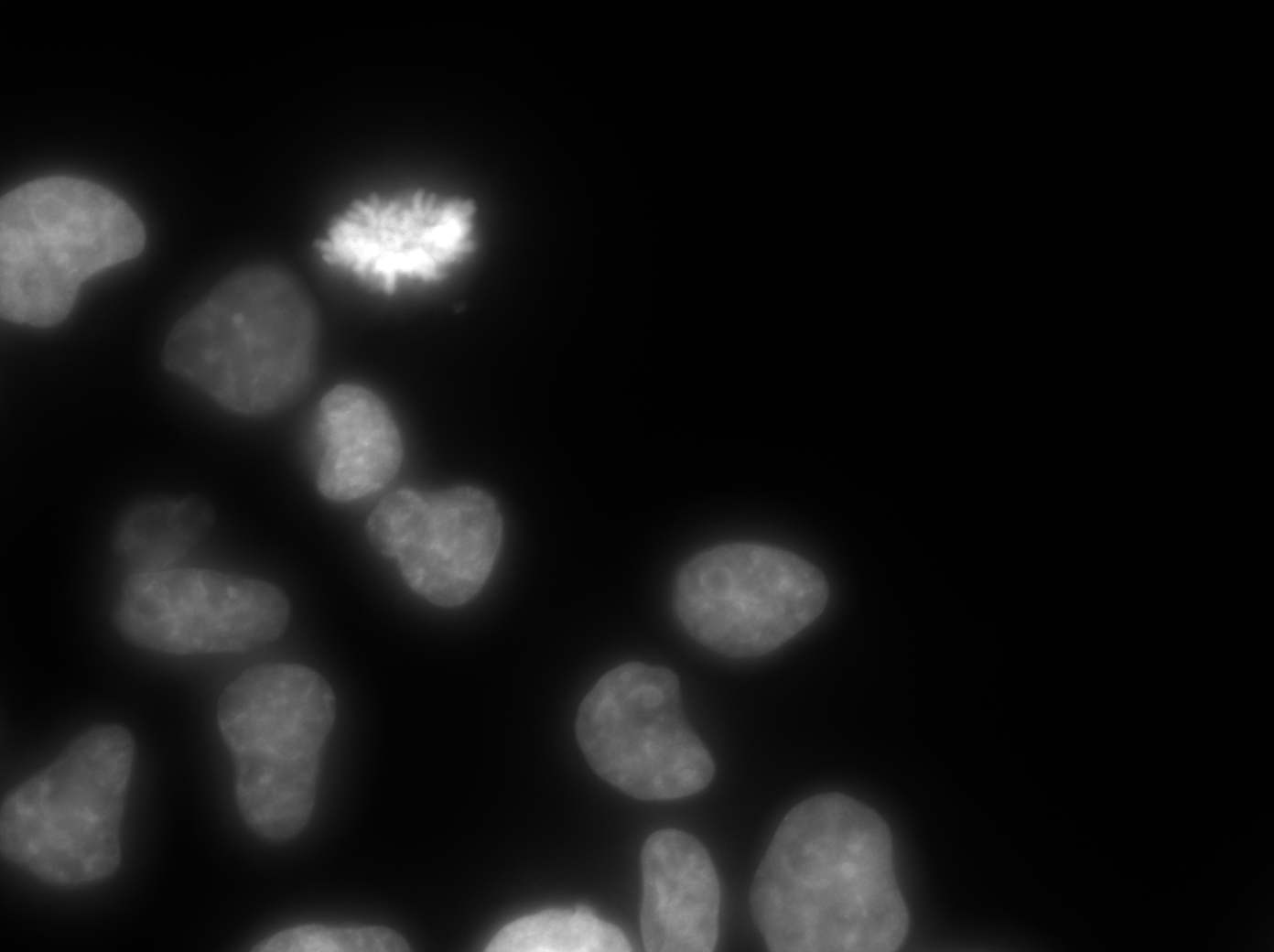

Supplement: Supplementary file 9 — Source data Fig. 2 part 6 [file 44319_2026_742_MOESM9_ESM.zip › Figure 2 Part 6/Fig 2fg Control Hela rGT335 acetylated tubulin/ET/Cas9 actub rGT335 9-8-25 R3 M8 ET11.Project Maximum Z_XY1757367702_Z0_T0_C0.tif]

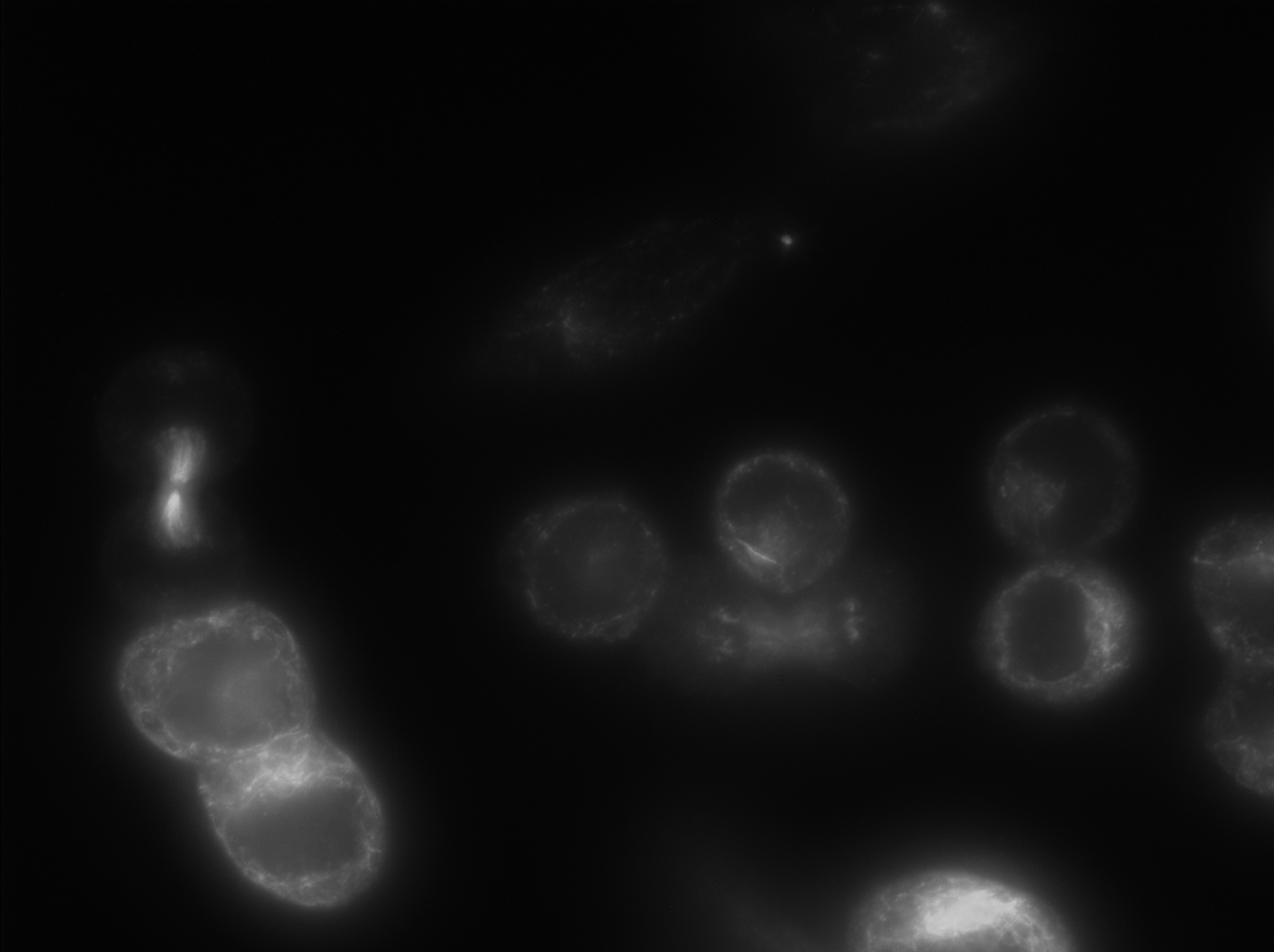

Supplement: Supplementary file 9 — Source data Fig. 2 part 6 [file 44319_2026_742_MOESM9_ESM.zip › Figure 2 Part 6/Fig 2fg Control Hela rGT335 acetylated tubulin/ET/Cas9 actub rGT335 9-8-25 R1 ET3 EX.Project Maximum Z_XY1757351356_Z0_T0_C2.tif]

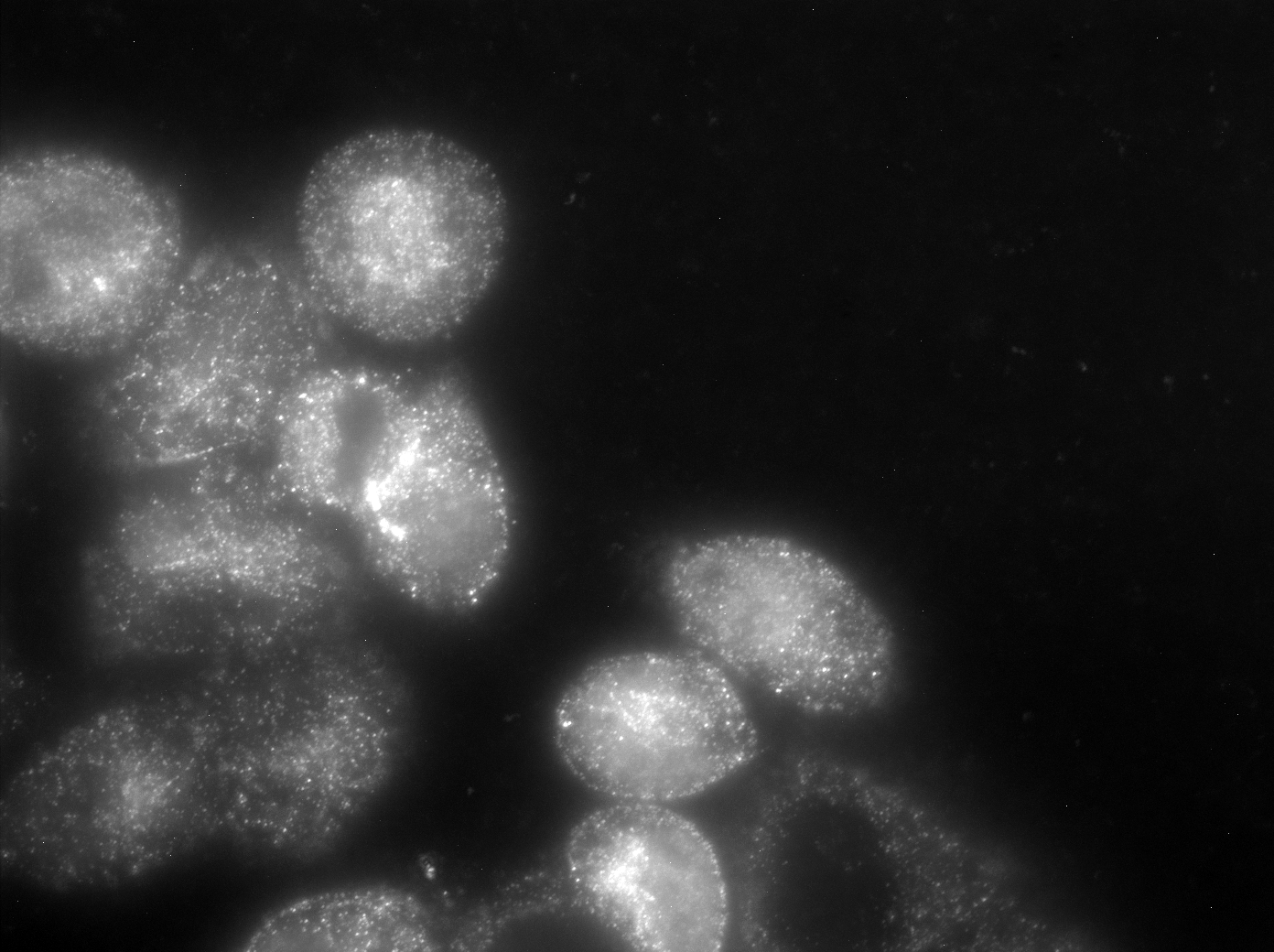

Supplement: Supplementary file 9 — Source data Fig. 2 part 6 [file 44319_2026_742_MOESM9_ESM.zip › Figure 2 Part 6/Fig 2fg Control Hela rGT335 acetylated tubulin/ET/Cas9 actub rGT335 9-8-25 R3 M8 ET11.Project Maximum Z_XY1757367702_Z0_T0_C1.tif]

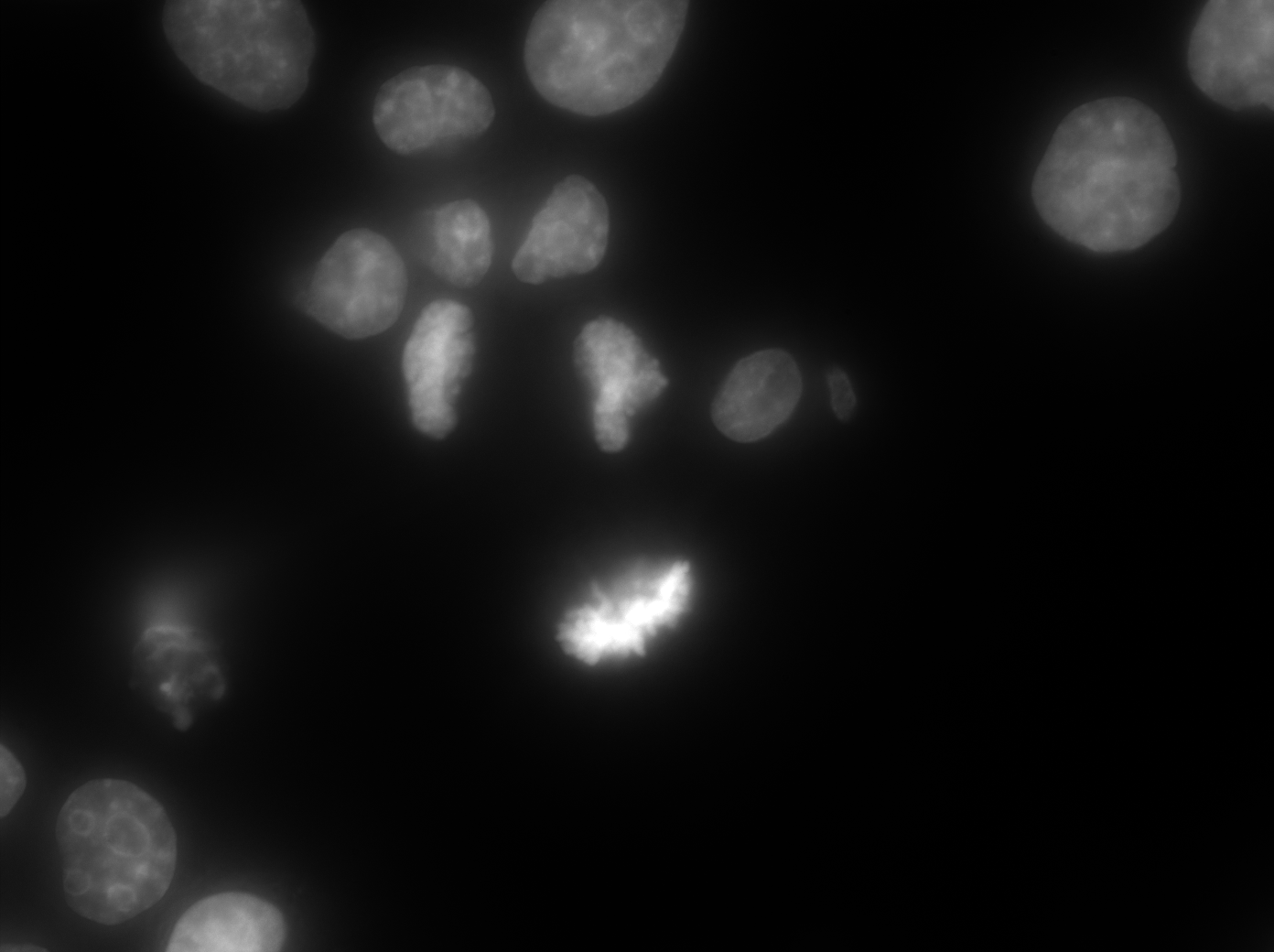

Supplement: Supplementary file 9 — Source data Fig. 2 part 6 [file 44319_2026_742_MOESM9_ESM.zip › Figure 2 Part 6/Fig 2fg Control Hela rGT335 acetylated tubulin/ET/Cas9 actub rGT335 9-8-25 R2 ET4 M3.Project Maximum Z_XY1757360796_Z0_T0_C0.tif]

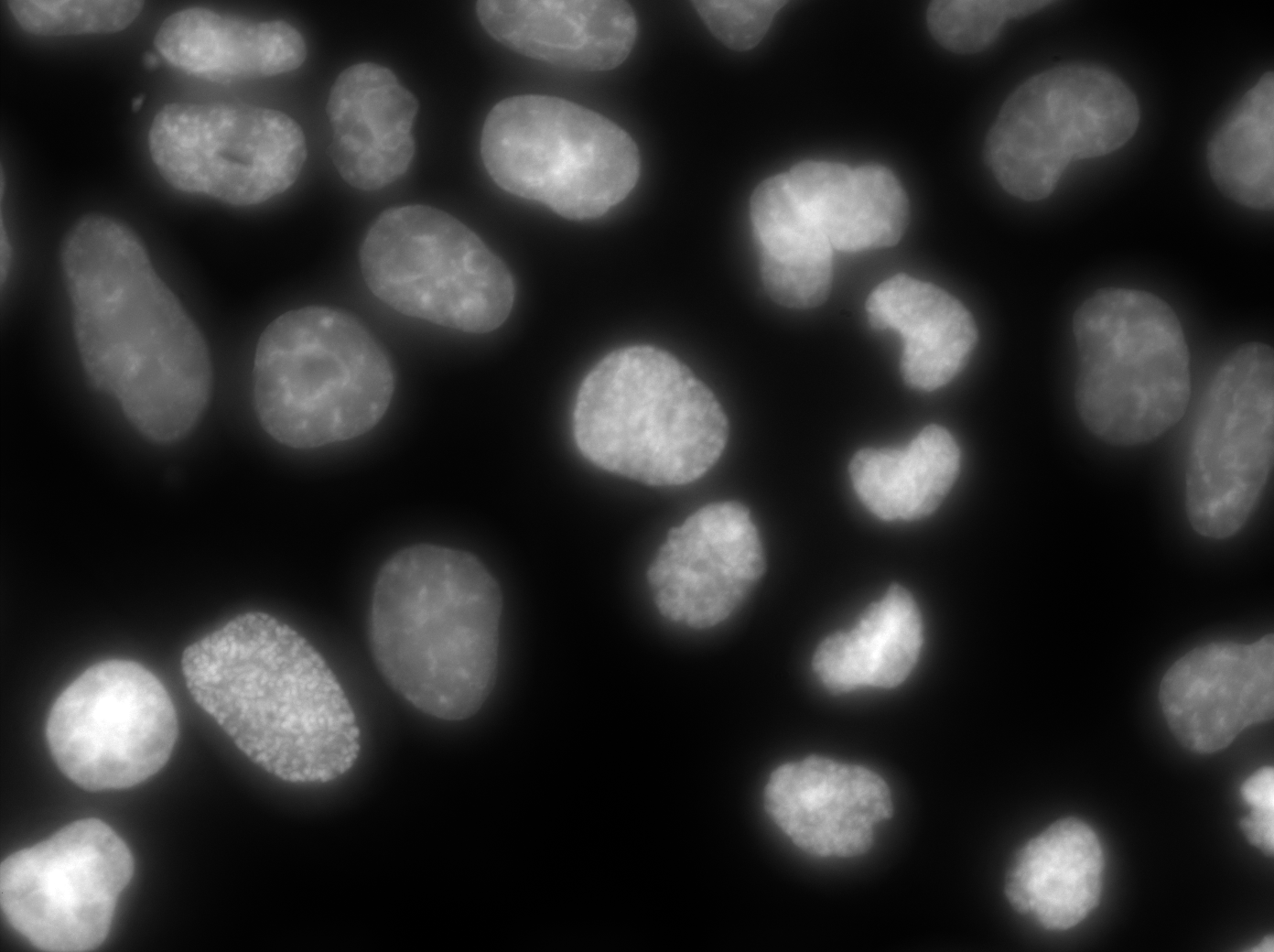

Supplement: Supplementary file 9 — Source data Fig. 2 part 6 [file 44319_2026_742_MOESM9_ESM.zip › Figure 2 Part 6/Fig 2fg Control Hela rGT335 acetylated tubulin/ET/Cas9 actub rGT335 9-8-25 R3 ET5-8.Project Maximum Z_XY1757365224_Z0_T0_C0.tif]

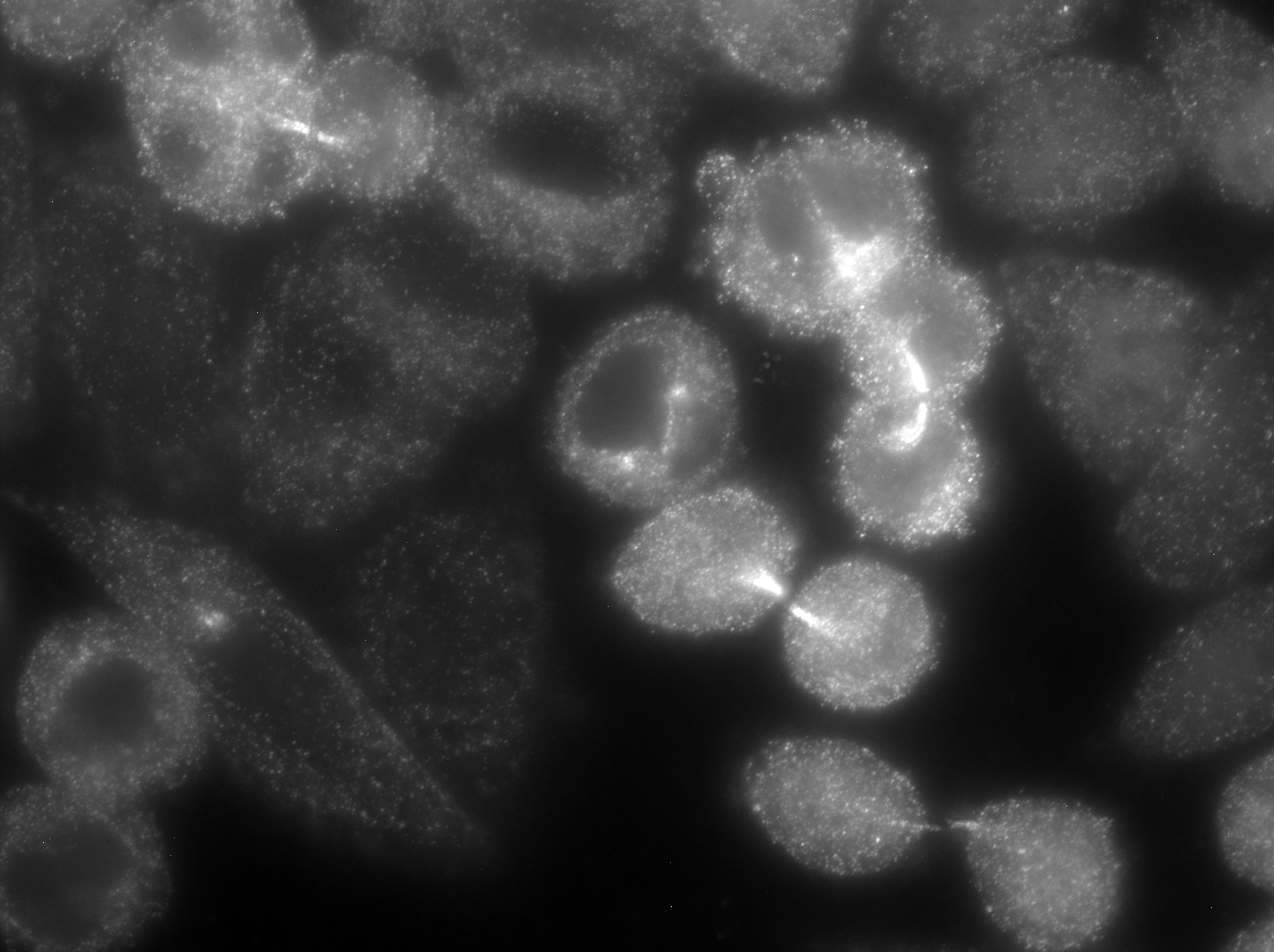

Supplement: Supplementary file 9 — Source data Fig. 2 part 6 [file 44319_2026_742_MOESM9_ESM.zip › Figure 2 Part 6/Fig 2fg Control Hela rGT335 acetylated tubulin/ET/Cas9 actub rGT335 9-8-25 R3 ET5-8.Project Maximum Z_XY1757365224_Z0_T0_C1.tif]

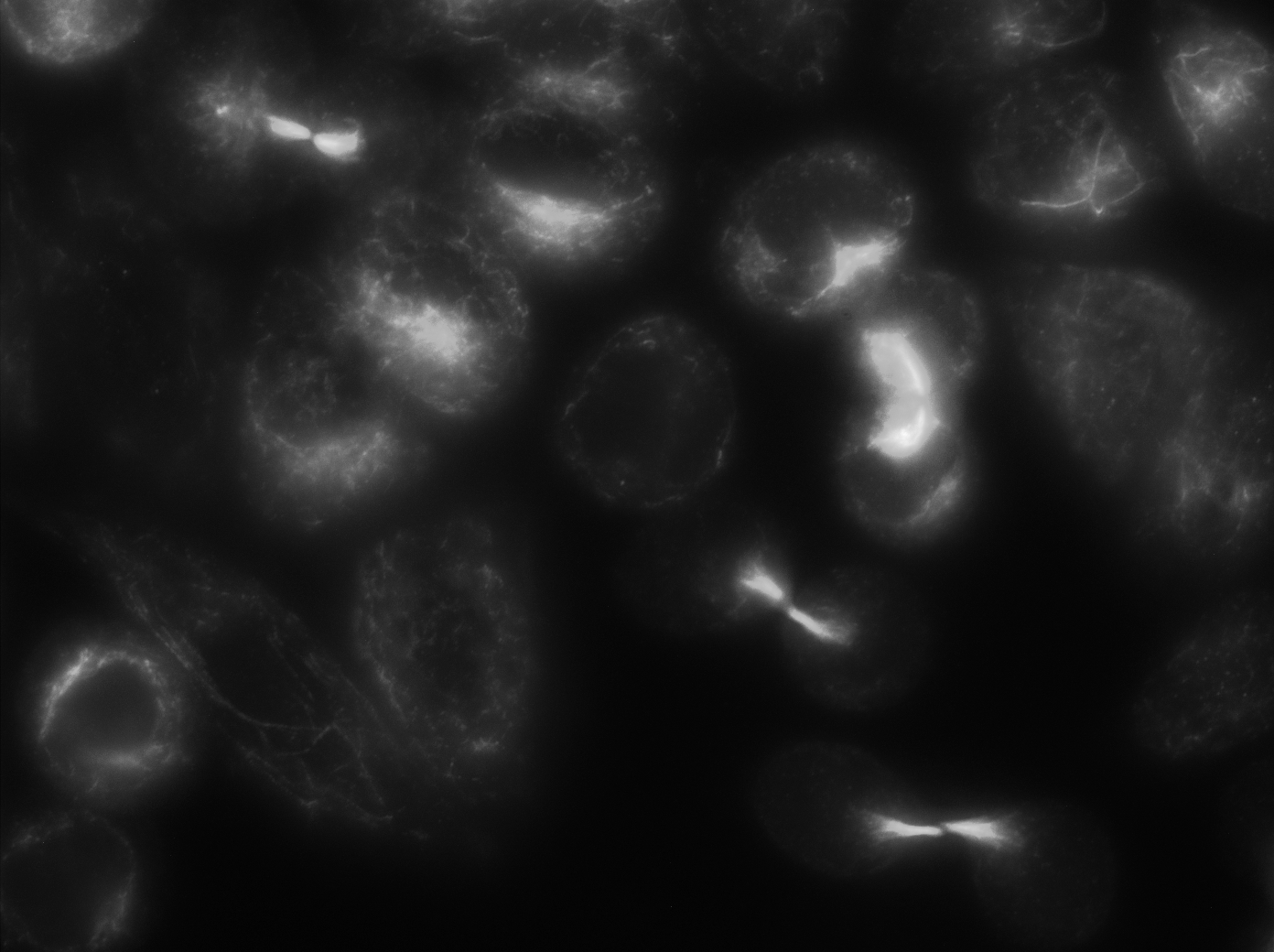

Supplement: Supplementary file 9 — Source data Fig. 2 part 6 [file 44319_2026_742_MOESM9_ESM.zip › Figure 2 Part 6/Fig 2fg Control Hela rGT335 acetylated tubulin/ET/Cas9 actub rGT335 9-8-25 R3 ET5-8.Project Maximum Z_XY1757365224_Z0_T0_C2.tif]

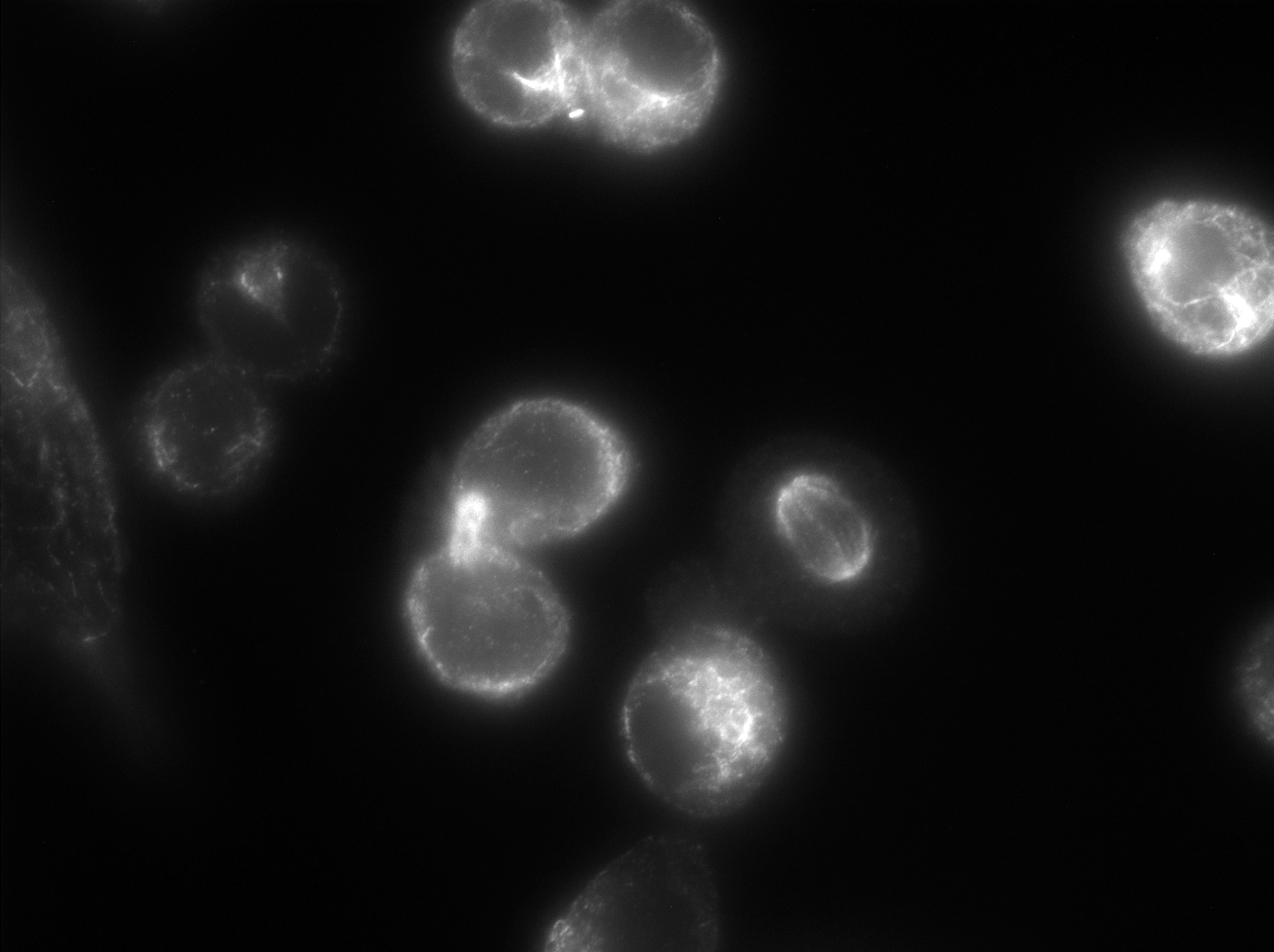

Supplement: Supplementary file 10 — Source data Fig. 2 part 7 [file 44319_2026_742_MOESM10_ESM.zip › Figure 2 Part 7/Fig 2fg Control Hela rGT335 acetylated tubulin part 2/Metaphase/Cas9 actub rGT335 9-8-25 R1 M2 EX.Project Maximum Z_XY1757351046_Z0_T0_C2.tif]

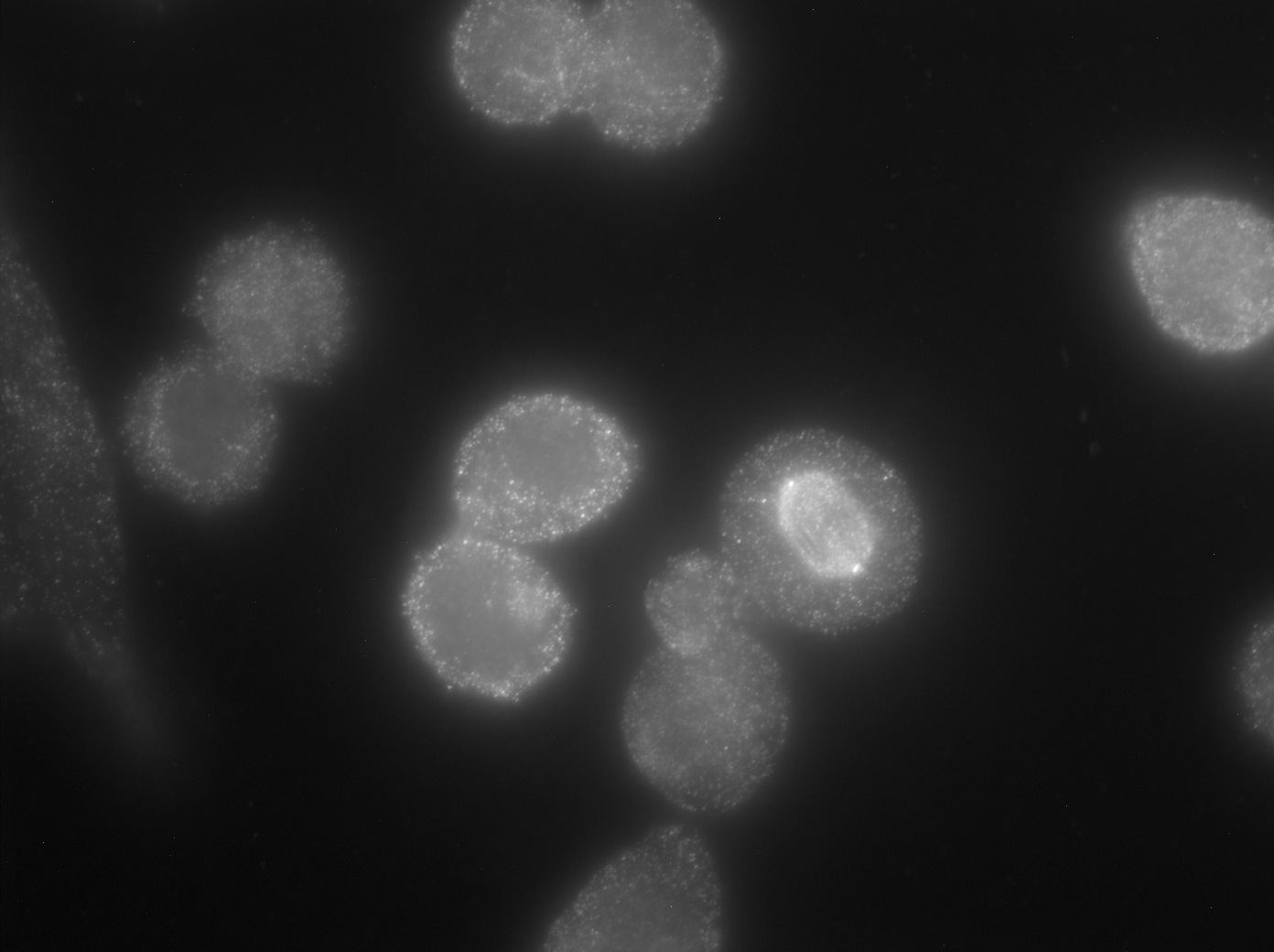

Supplement: Supplementary file 10 — Source data Fig. 2 part 7 [file 44319_2026_742_MOESM10_ESM.zip › Figure 2 Part 7/Fig 2fg Control Hela rGT335 acetylated tubulin part 2/Metaphase/Cas9 actub rGT335 9-8-25 R1 M2 EX.Project Maximum Z_XY1757351046_Z0_T0_C1.tif]

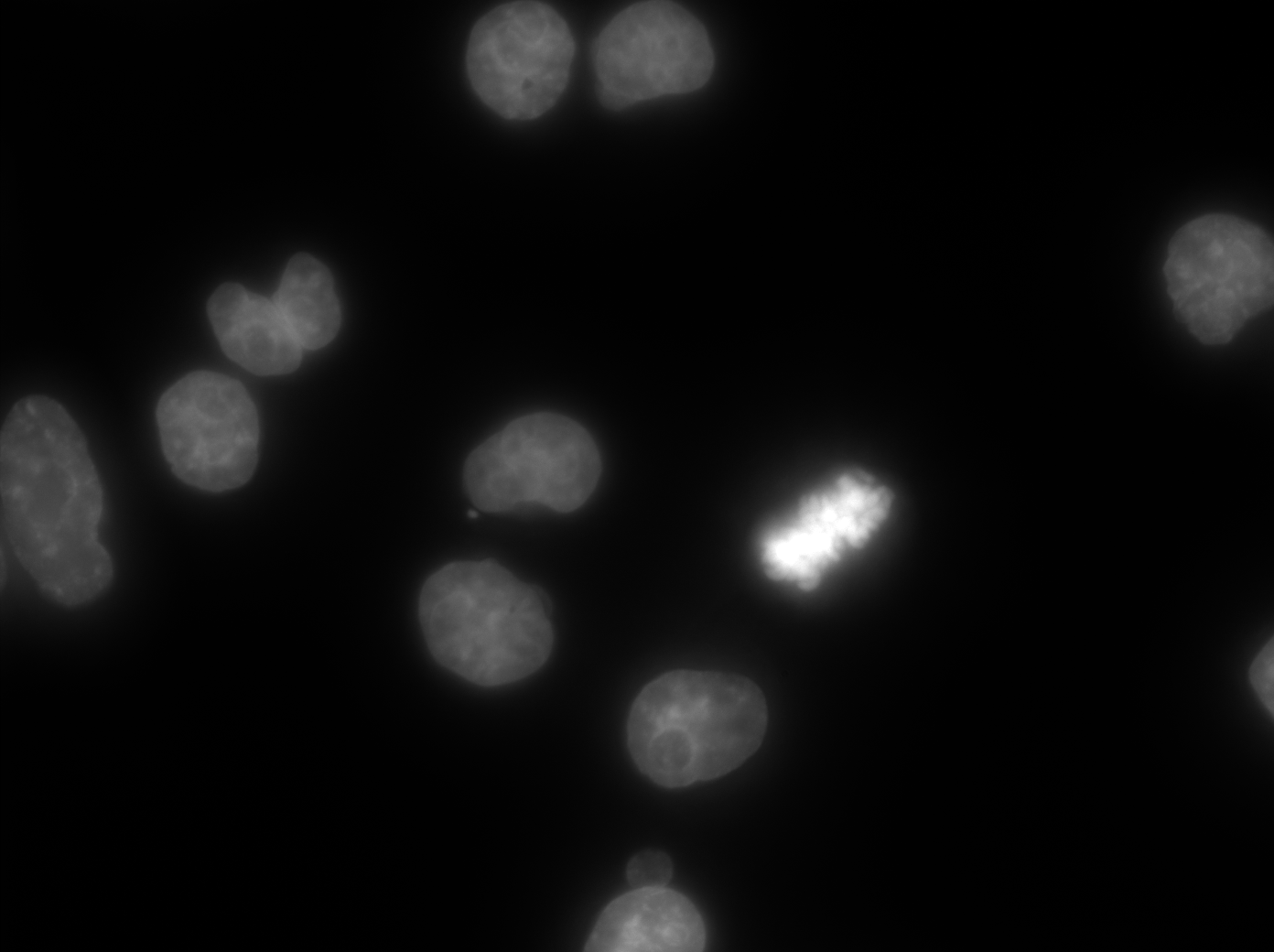

Supplement: Supplementary file 10 — Source data Fig. 2 part 7 [file 44319_2026_742_MOESM10_ESM.zip › Figure 2 Part 7/Fig 2fg Control Hela rGT335 acetylated tubulin part 2/Metaphase/Cas9 actub rGT335 9-8-25 R1 M2 EX.Project Maximum Z_XY1757351046_Z0_T0_C0.tif]

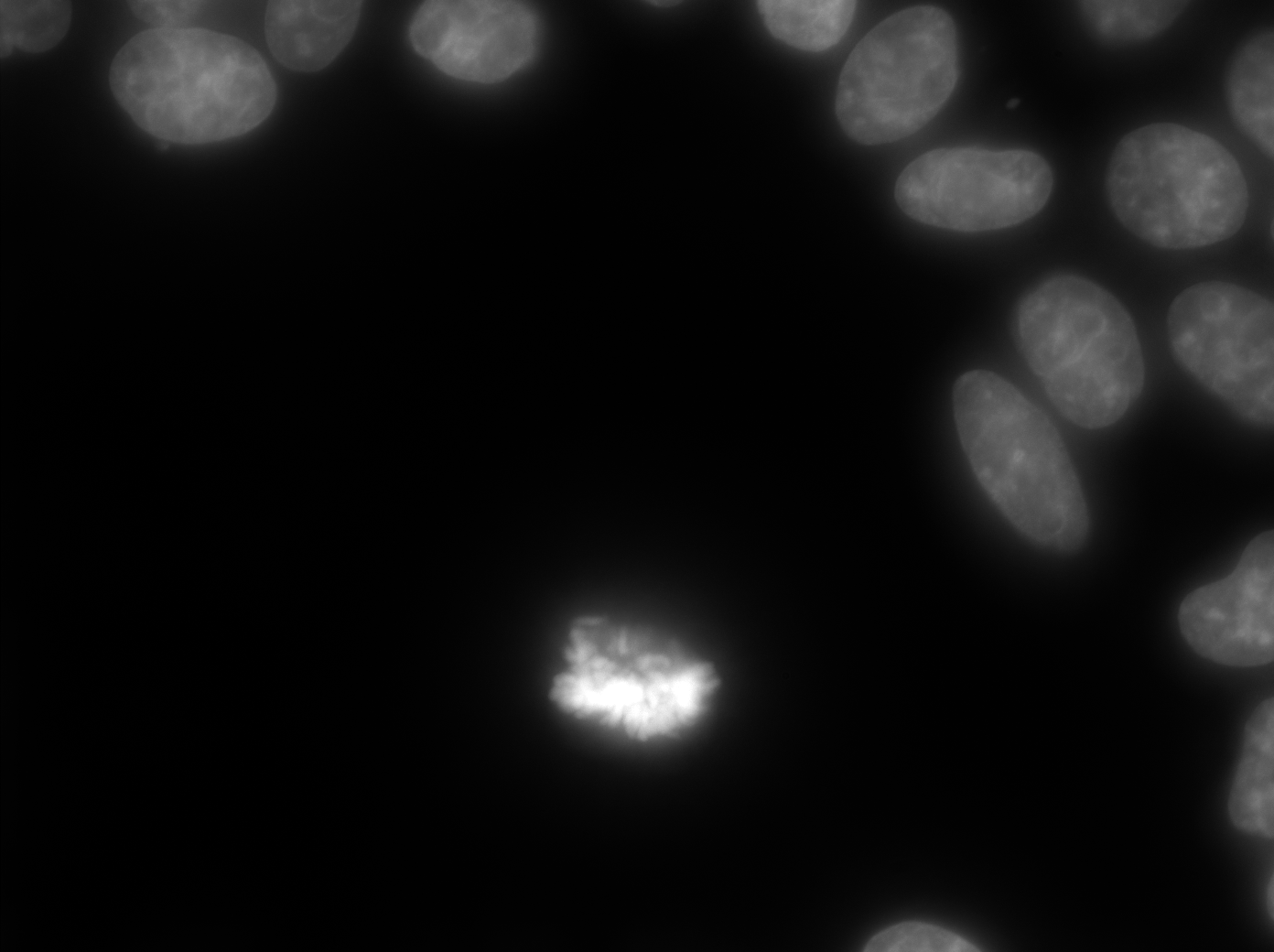

Supplement: Supplementary file 10 — Source data Fig. 2 part 7 [file 44319_2026_742_MOESM10_ESM.zip › Figure 2 Part 7/Fig 2fg Control Hela rGT335 acetylated tubulin part 2/Metaphase/Cas9 actub rGT335 9-8-25 R3 M10.Project Maximum Z_XY1757368750_Z0_T0_C0.tif]

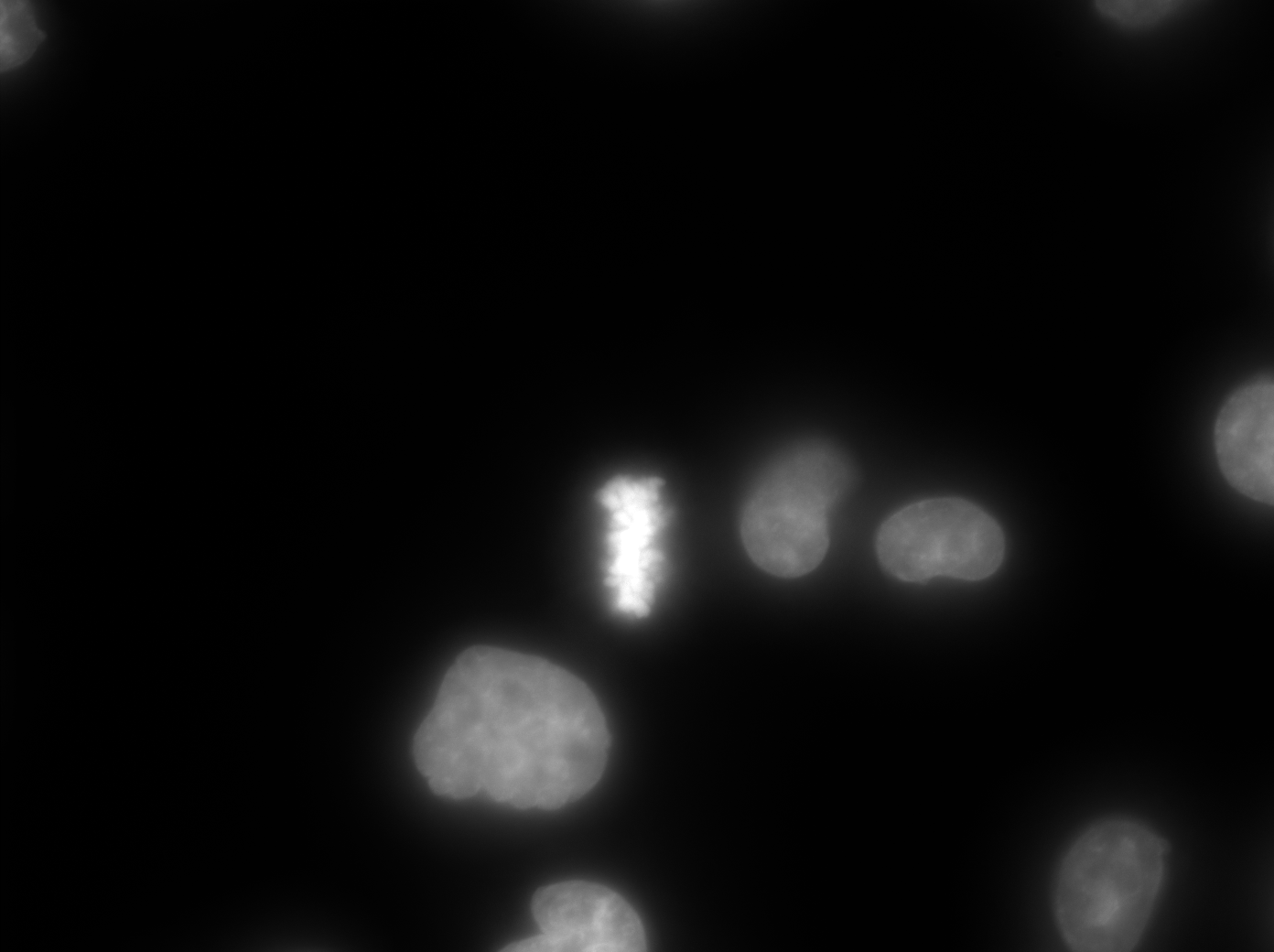

Supplement: Supplementary file 10 — Source data Fig. 2 part 7 [file 44319_2026_742_MOESM10_ESM.zip › Figure 2 Part 7/Fig 2fg Control Hela rGT335 acetylated tubulin part 2/Metaphase/Cas9 actub rGT335 9-8-25 R1 M1 EX.Project Maximum Z_XY1757350739_Z0_T0_C0.tif]

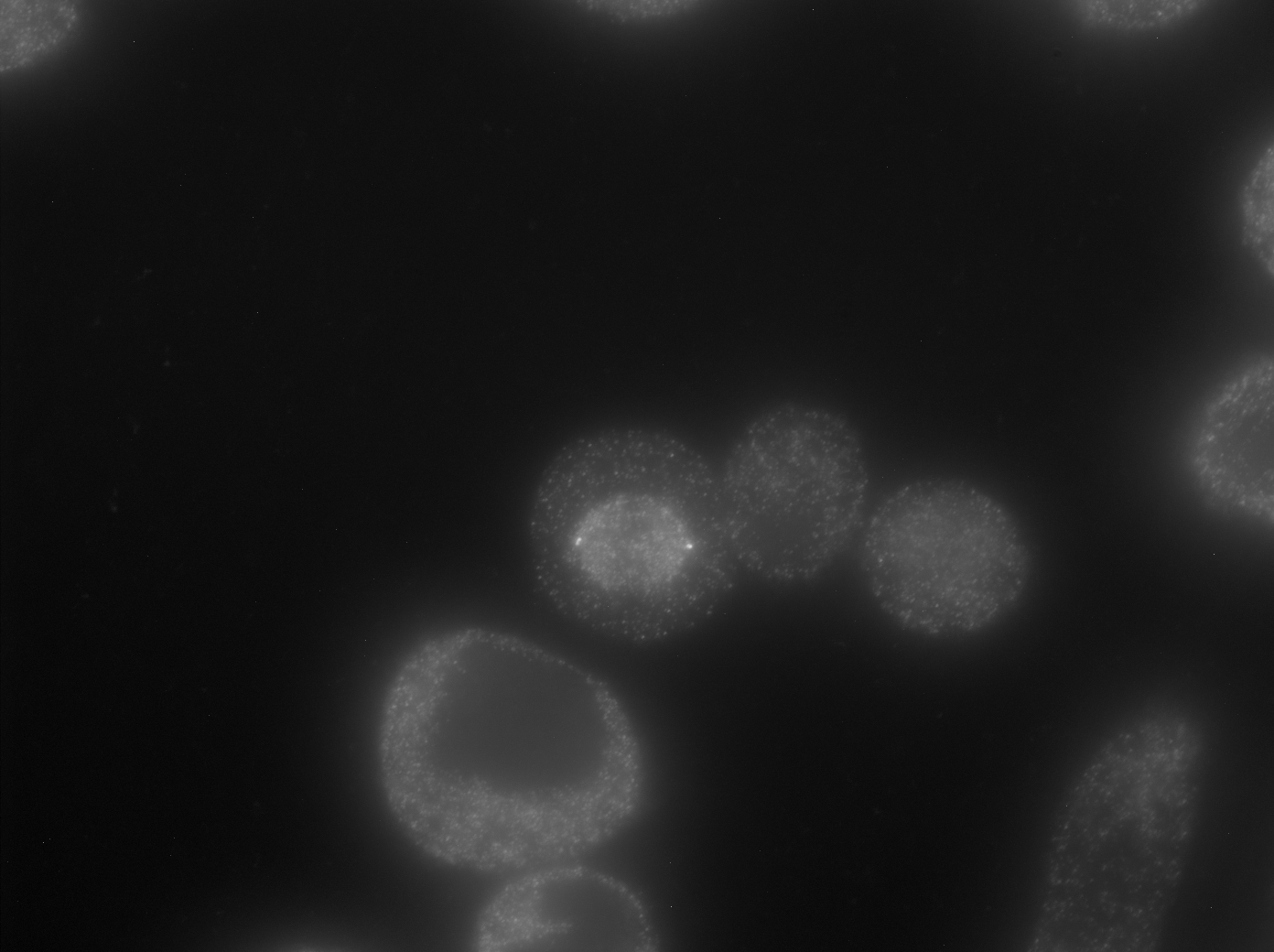

Supplement: Supplementary file 10 — Source data Fig. 2 part 7 [file 44319_2026_742_MOESM10_ESM.zip › Figure 2 Part 7/Fig 2fg Control Hela rGT335 acetylated tubulin part 2/Metaphase/Cas9 actub rGT335 9-8-25 R1 M1 EX.Project Maximum Z_XY1757350739_Z0_T0_C1.tif]

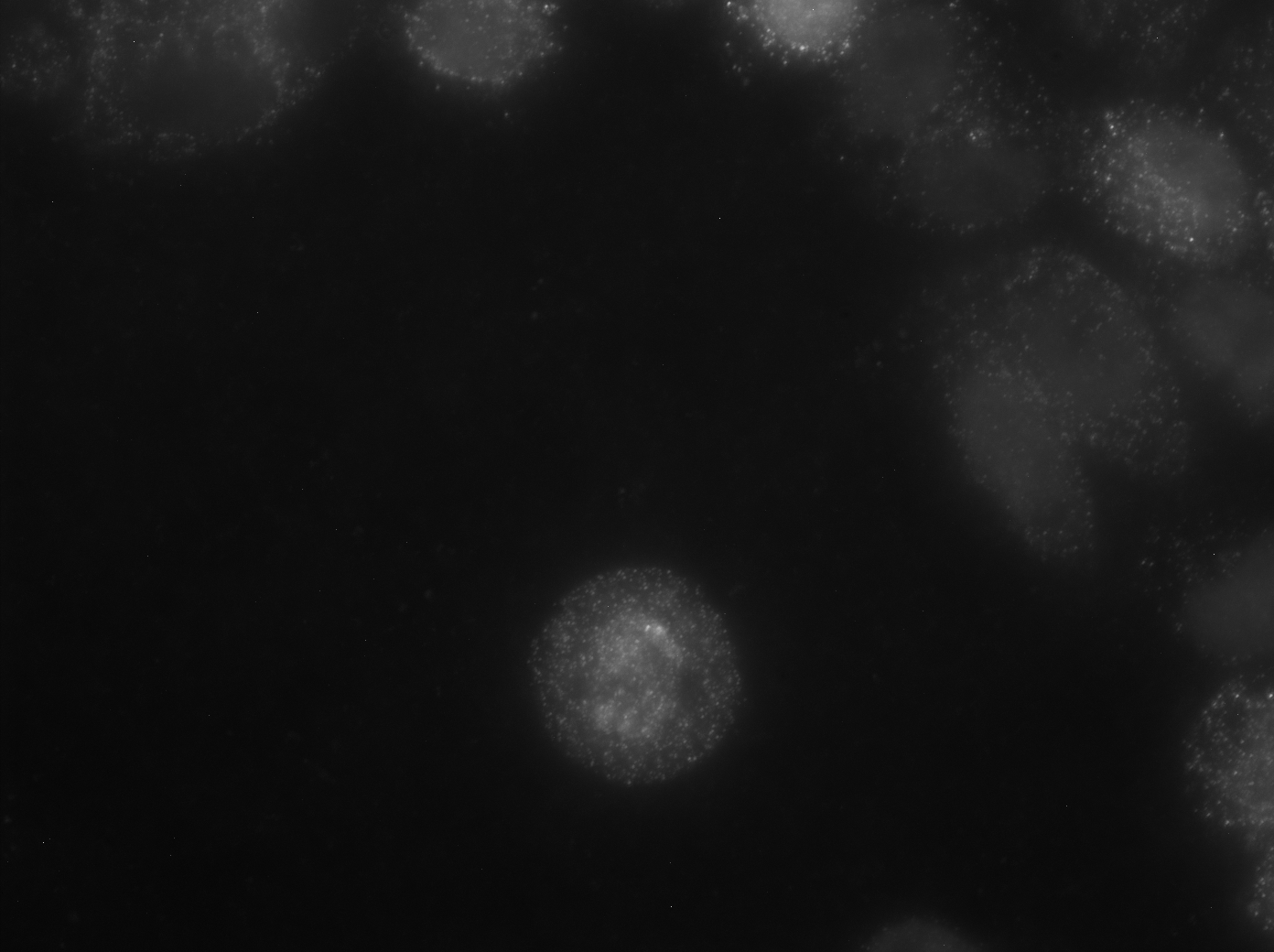

Supplement: Supplementary file 10 — Source data Fig. 2 part 7 [file 44319_2026_742_MOESM10_ESM.zip › Figure 2 Part 7/Fig 2fg Control Hela rGT335 acetylated tubulin part 2/Metaphase/Cas9 actub rGT335 9-8-25 R3 M10.Project Maximum Z_XY1757368750_Z0_T0_C1.tif]

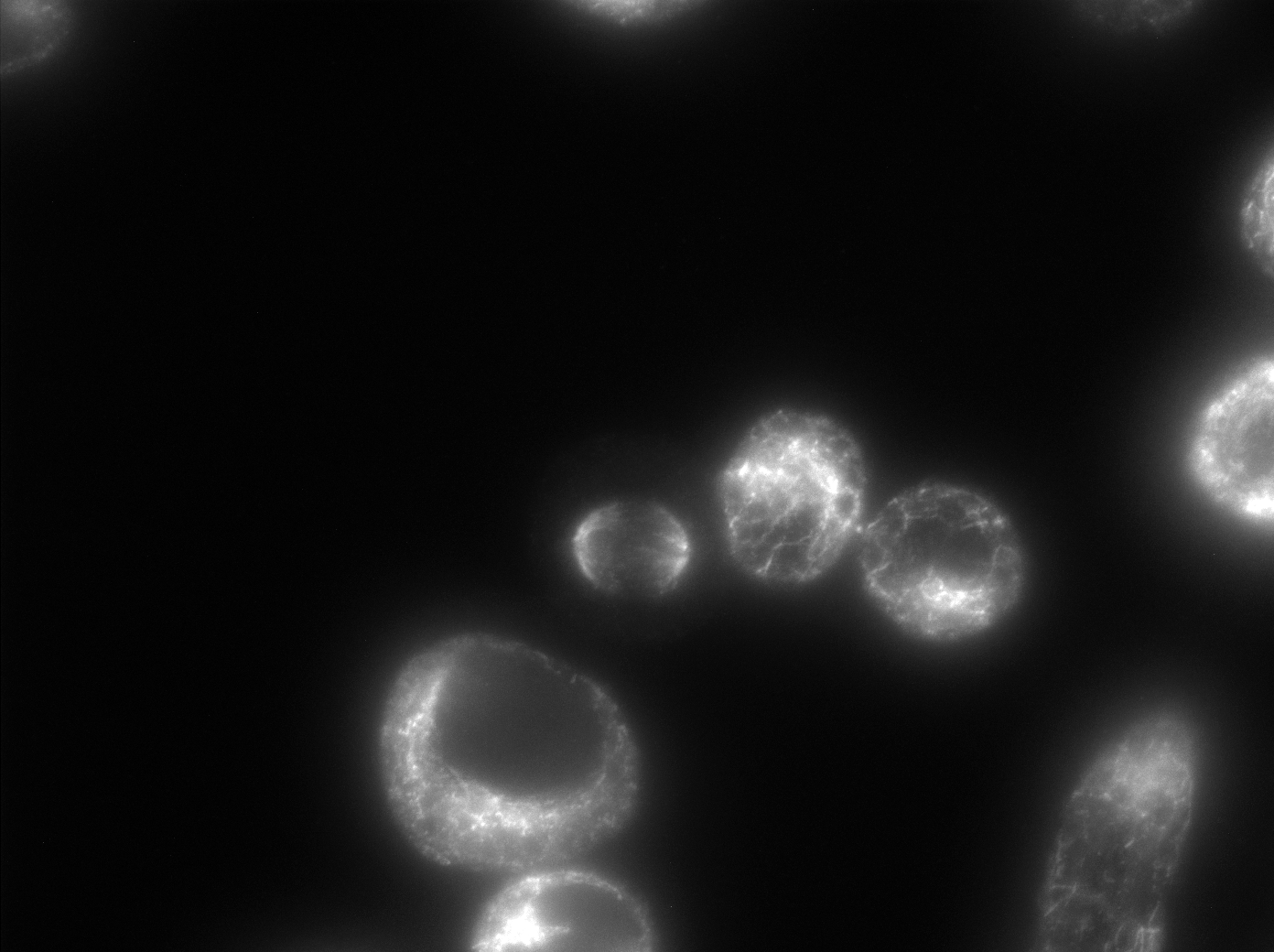

Supplement: Supplementary file 10 — Source data Fig. 2 part 7 [file 44319_2026_742_MOESM10_ESM.zip › Figure 2 Part 7/Fig 2fg Control Hela rGT335 acetylated tubulin part 2/Metaphase/Cas9 actub rGT335 9-8-25 R1 M1 EX.Project Maximum Z_XY1757350739_Z0_T0_C2.tif]

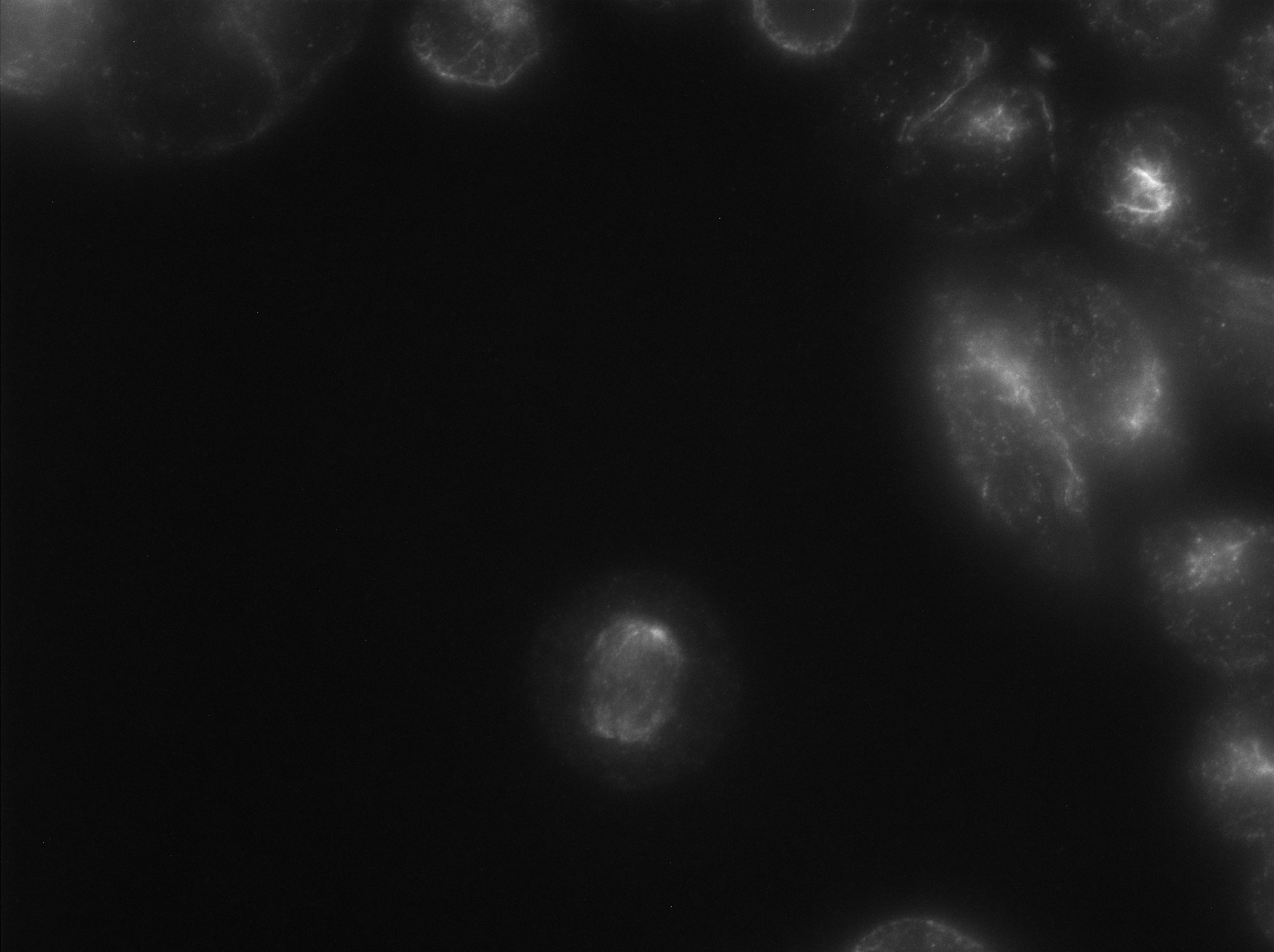

Supplement: Supplementary file 10 — Source data Fig. 2 part 7 [file 44319_2026_742_MOESM10_ESM.zip › Figure 2 Part 7/Fig 2fg Control Hela rGT335 acetylated tubulin part 2/Metaphase/Cas9 actub rGT335 9-8-25 R3 M10.Project Maximum Z_XY1757368750_Z0_T0_C2.tif]

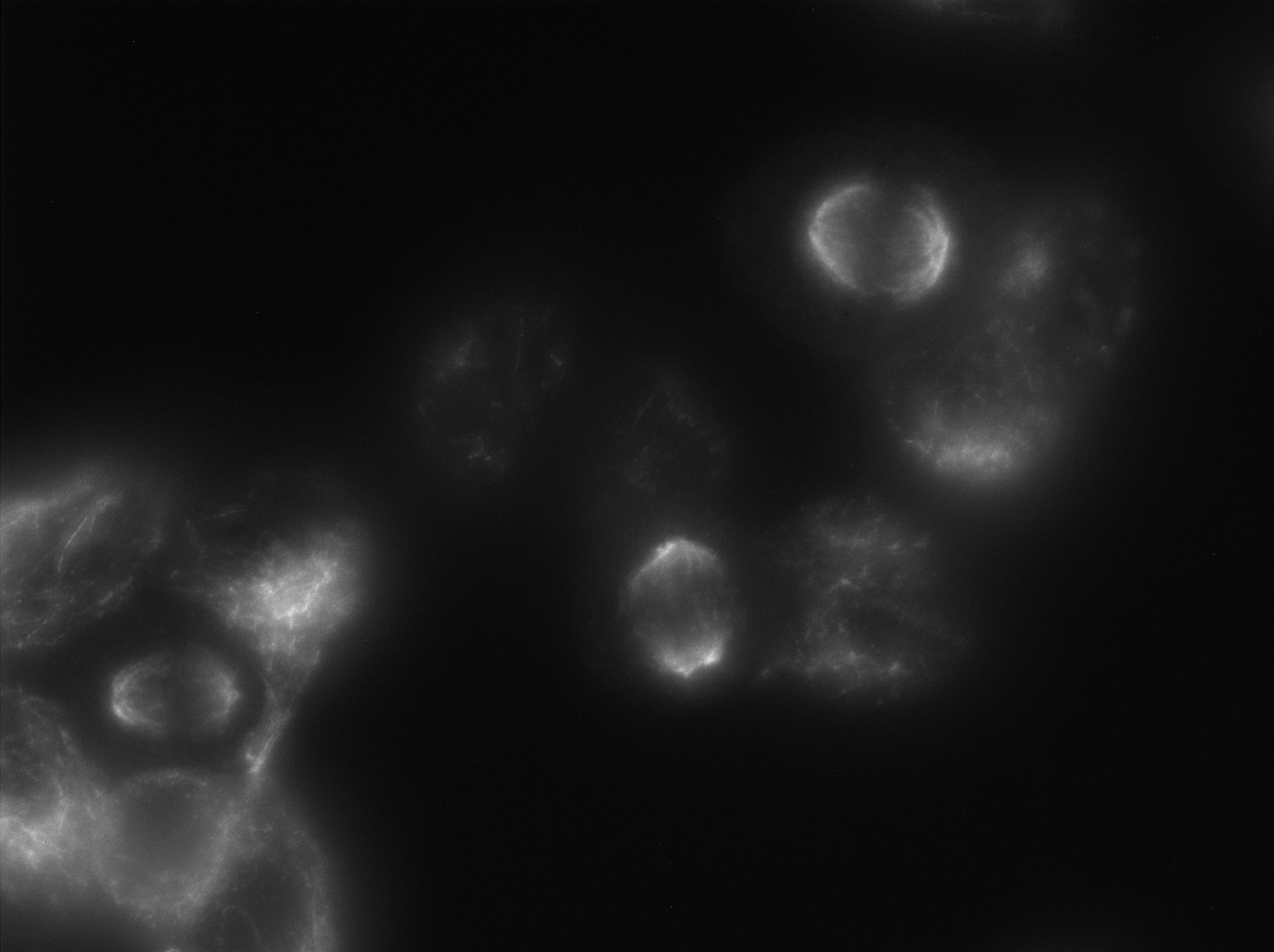

Supplement: Supplementary file 10 — Source data Fig. 2 part 7 [file 44319_2026_742_MOESM10_ESM.zip › Figure 2 Part 7/Fig 2fg Control Hela rGT335 acetylated tubulin part 2/Metaphase/Cas9 actub rGT335 9-8-25 R1 M9M10.Project Maximum Z_XY1757355609_Z0_T0_C2.tif]

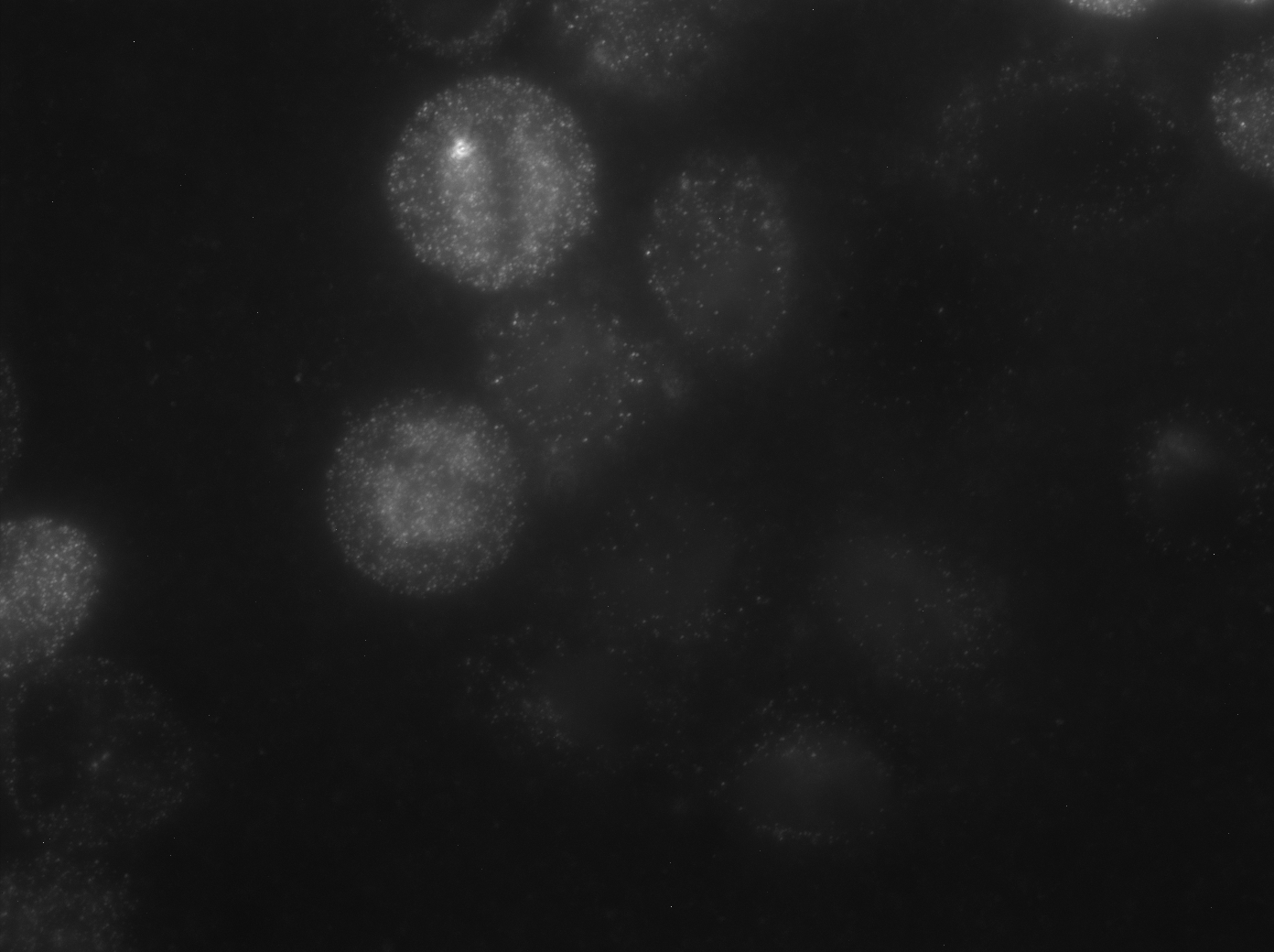

Supplement: Supplementary file 10 — Source data Fig. 2 part 7 [file 44319_2026_742_MOESM10_ESM.zip › Figure 2 Part 7/Fig 2fg Control Hela rGT335 acetylated tubulin part 2/Metaphase/Cas9 actub rGT335 9-8-25 R3 M6M7.Project Maximum Z_XY1757367401_Z0_T0_C1.tif]

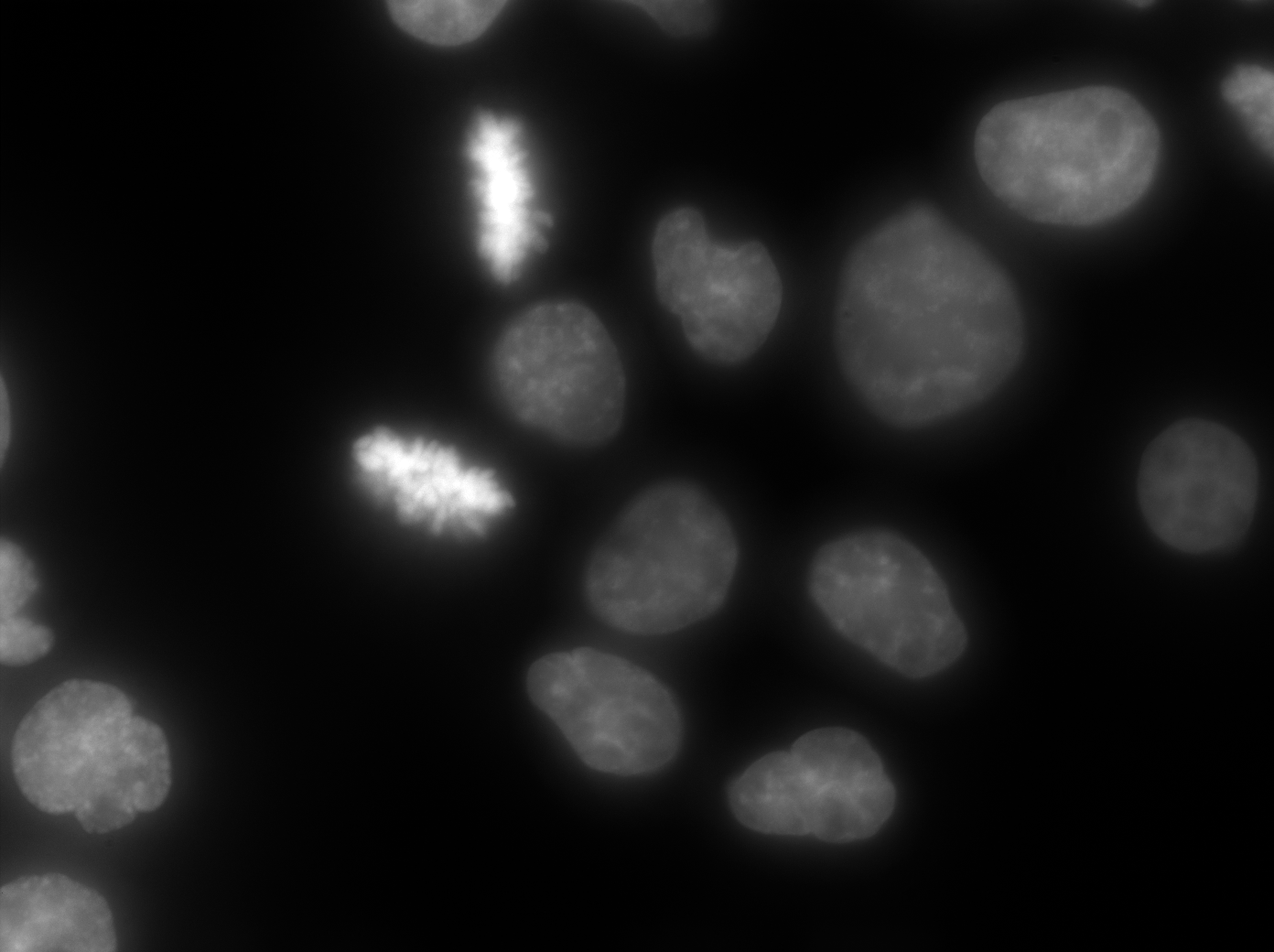

Supplement: Supplementary file 10 — Source data Fig. 2 part 7 [file 44319_2026_742_MOESM10_ESM.zip › Figure 2 Part 7/Fig 2fg Control Hela rGT335 acetylated tubulin part 2/Metaphase/Cas9 actub rGT335 9-8-25 R3 M6M7.Project Maximum Z_XY1757367401_Z0_T0_C0.tif]

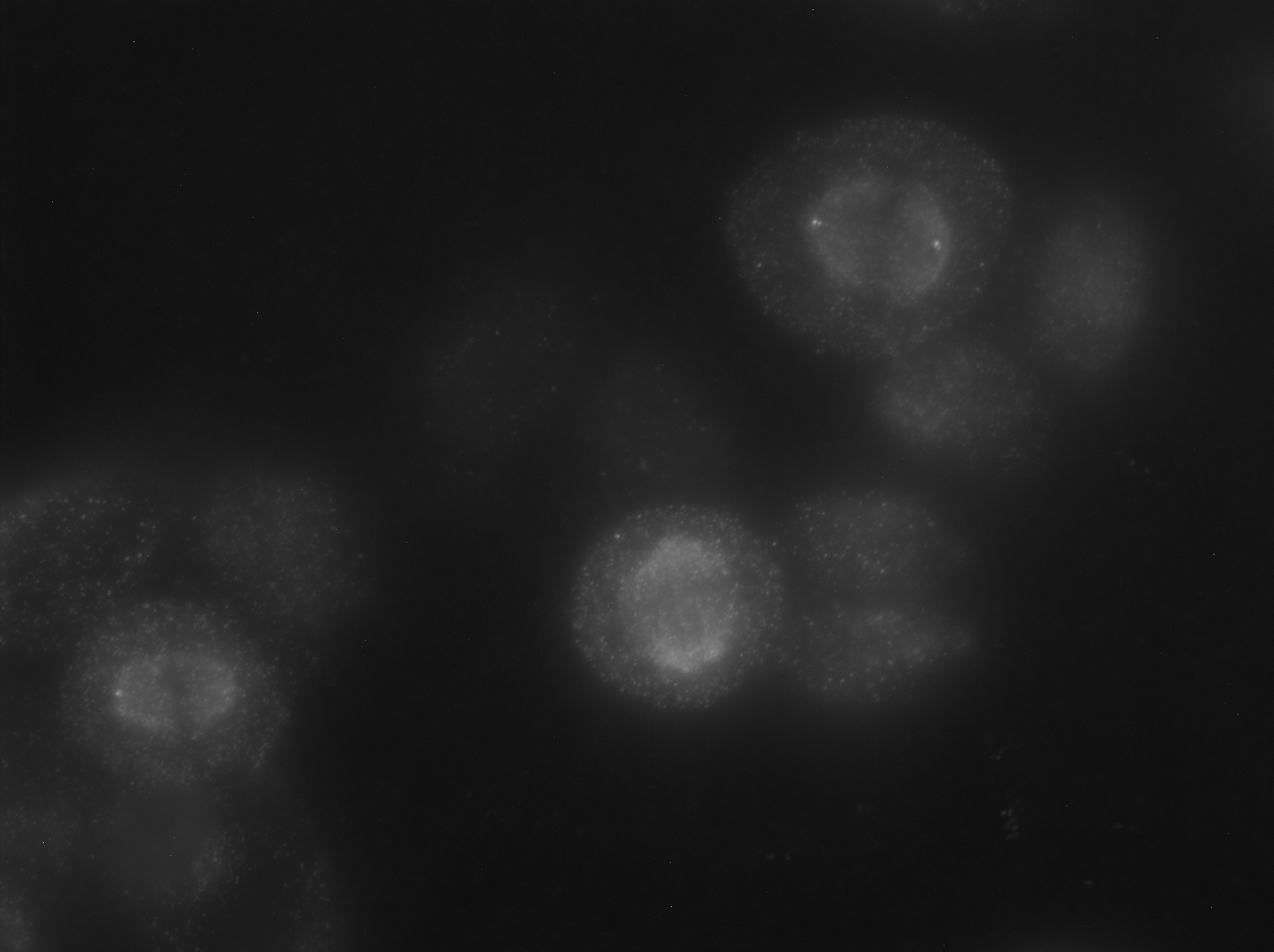

Supplement: Supplementary file 10 — Source data Fig. 2 part 7 [file 44319_2026_742_MOESM10_ESM.zip › Figure 2 Part 7/Fig 2fg Control Hela rGT335 acetylated tubulin part 2/Metaphase/Cas9 actub rGT335 9-8-25 R1 M9M10.Project Maximum Z_XY1757355609_Z0_T0_C1.tif]

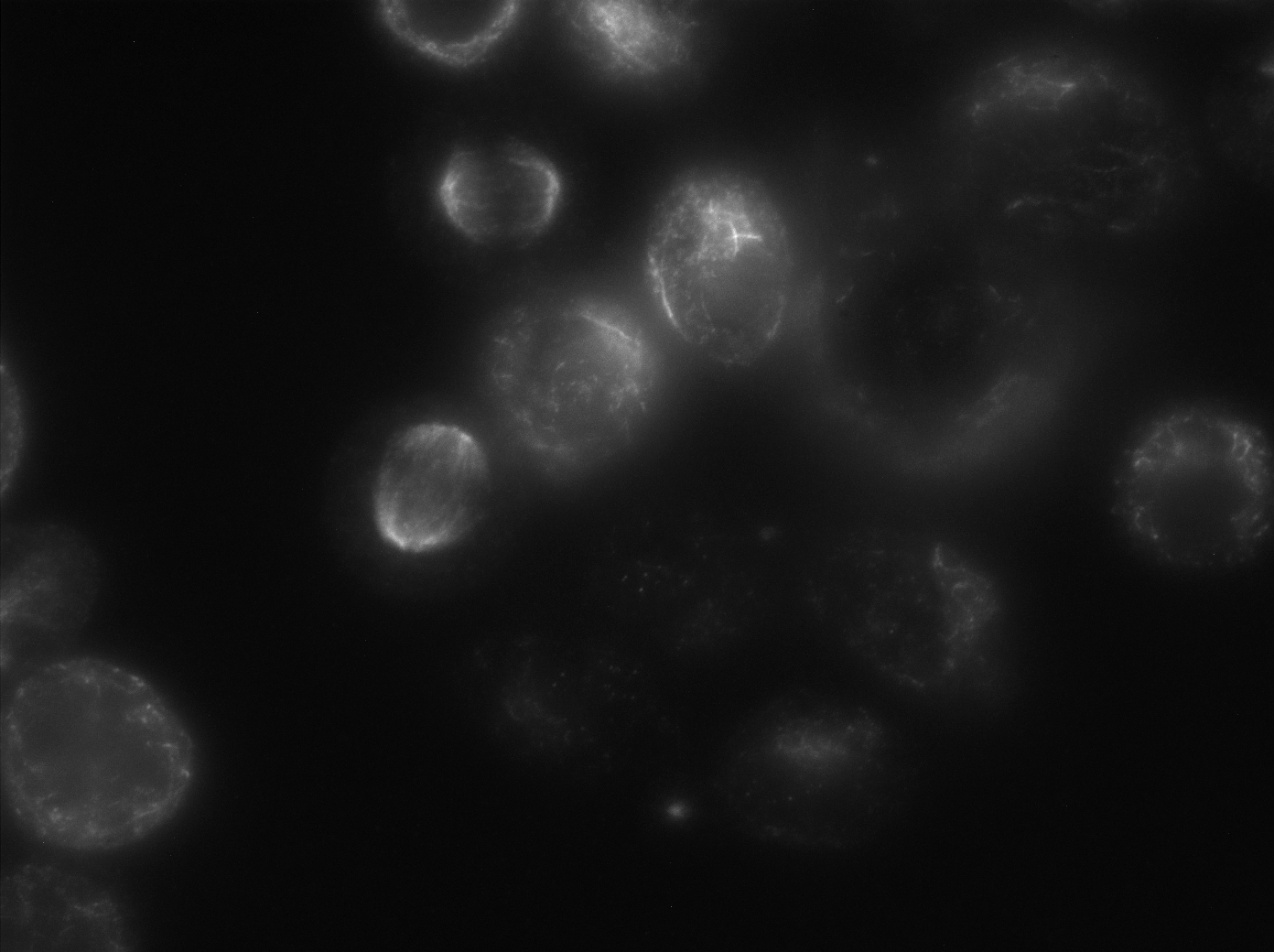

Supplement: Supplementary file 10 — Source data Fig. 2 part 7 [file 44319_2026_742_MOESM10_ESM.zip › Figure 2 Part 7/Fig 2fg Control Hela rGT335 acetylated tubulin part 2/Metaphase/Cas9 actub rGT335 9-8-25 R3 M6M7.Project Maximum Z_XY1757367401_Z0_T0_C2.tif]

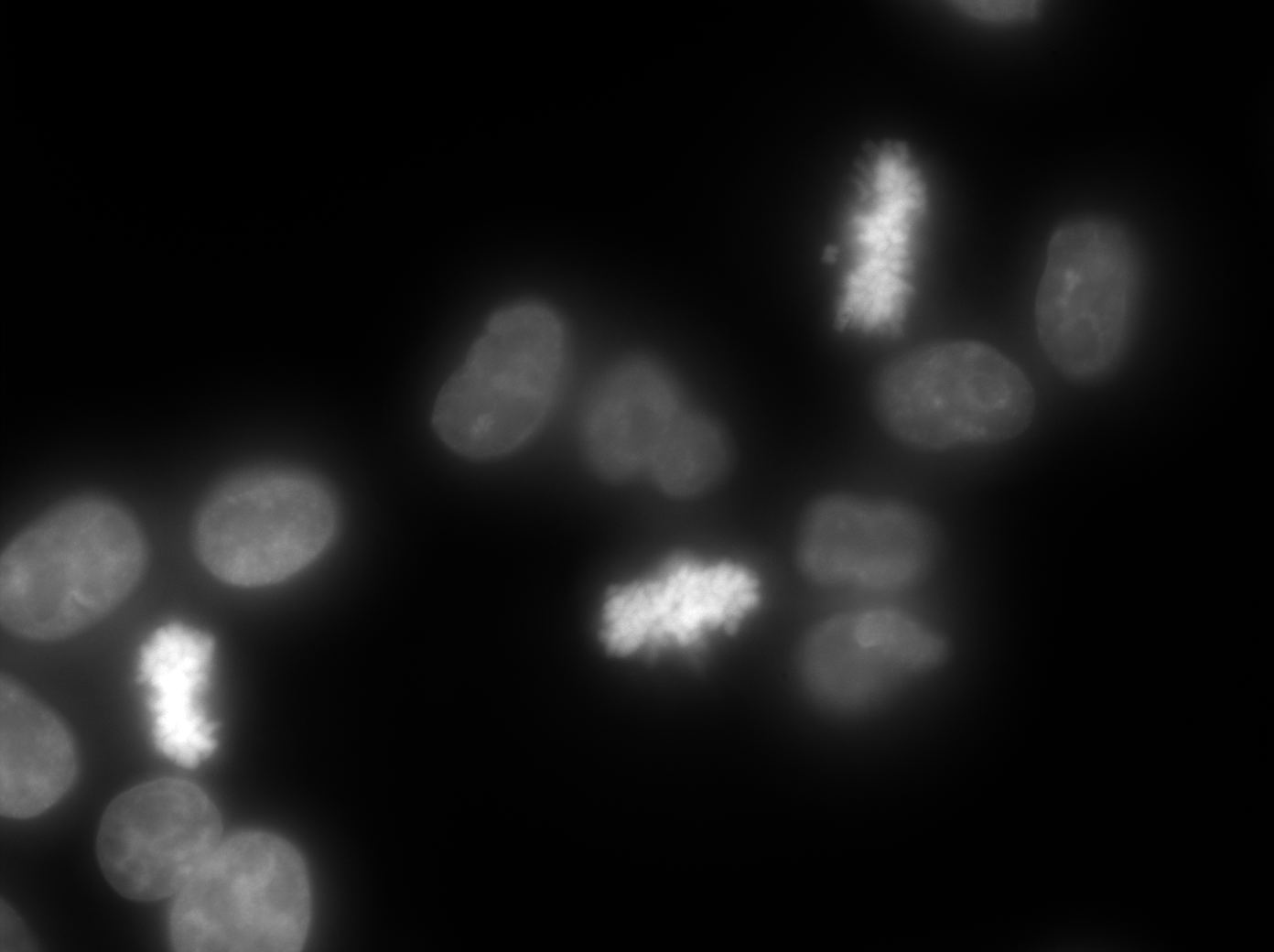

Supplement: Supplementary file 10 — Source data Fig. 2 part 7 [file 44319_2026_742_MOESM10_ESM.zip › Figure 2 Part 7/Fig 2fg Control Hela rGT335 acetylated tubulin part 2/Metaphase/Cas9 actub rGT335 9-8-25 R1 M9M10.Project Maximum Z_XY1757355609_Z0_T0_C0.tif]

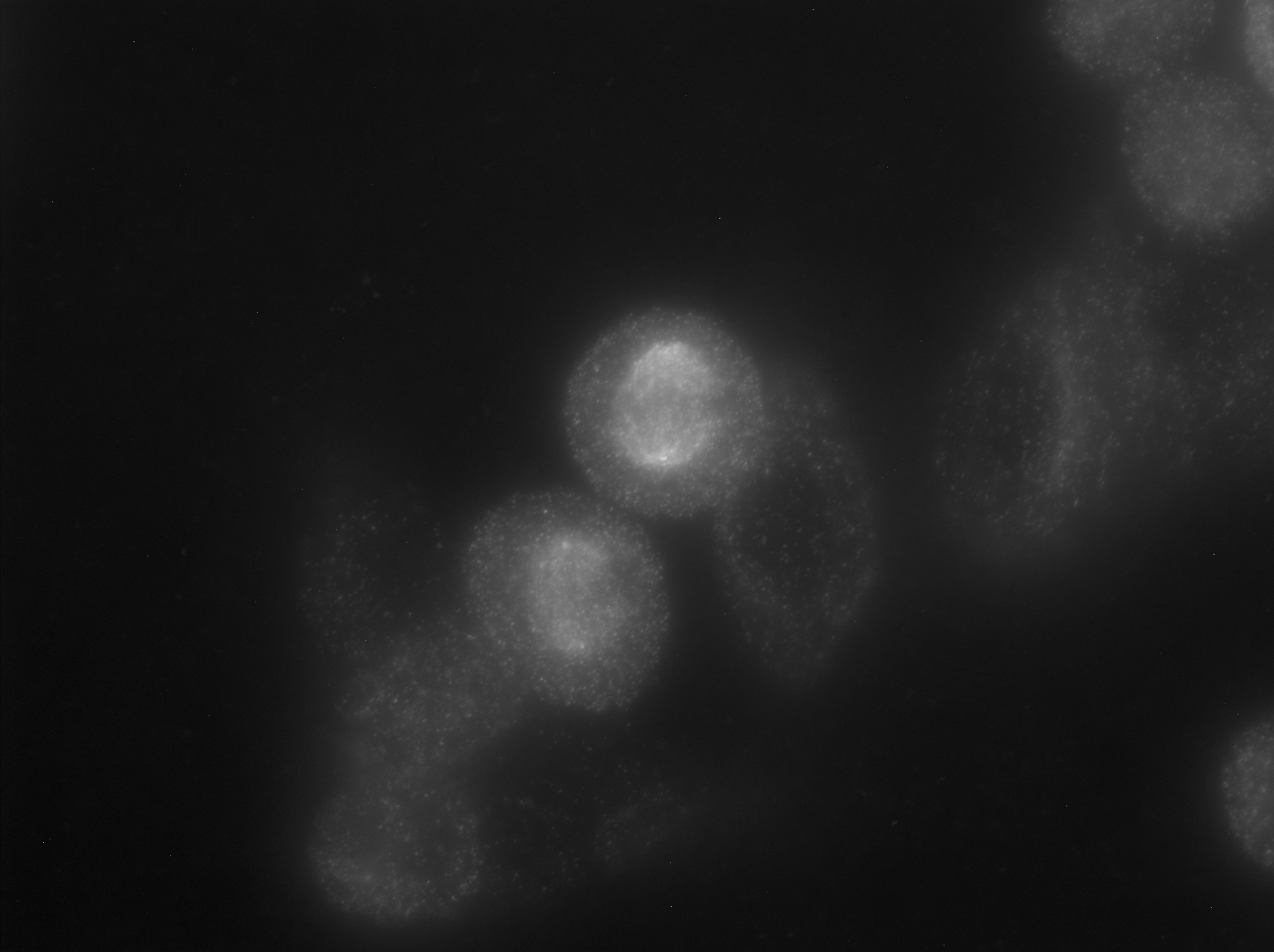

Supplement: Supplementary file 10 — Source data Fig. 2 part 7 [file 44319_2026_742_MOESM10_ESM.zip › Figure 2 Part 7/Fig 2fg Control Hela rGT335 acetylated tubulin part 2/Metaphase/Cas9 actub rGT335 9-8-25 R1 M7M8.Project Maximum Z_XY1757355008_Z0_T0_C1.tif]

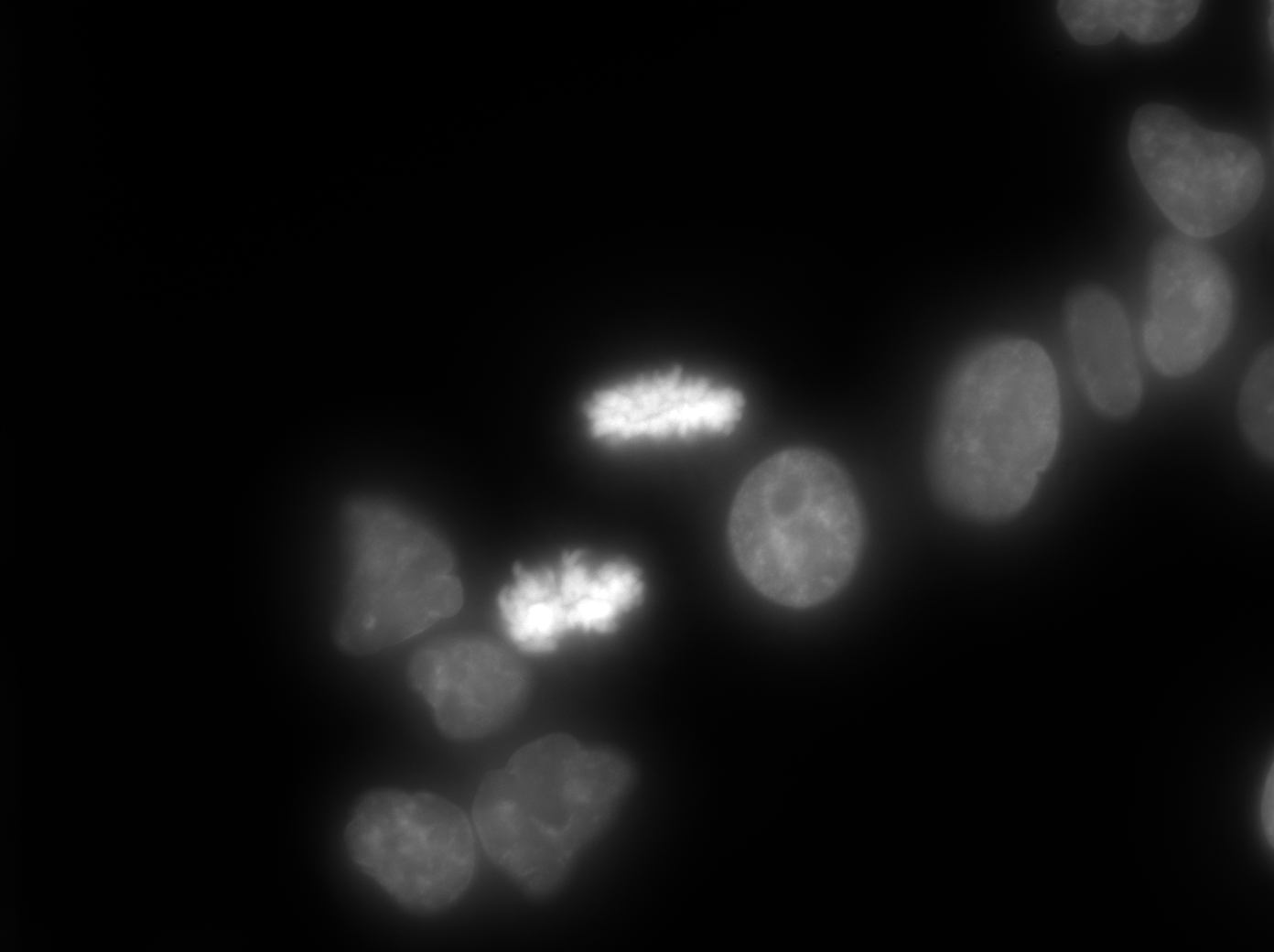

Supplement: Supplementary file 10 — Source data Fig. 2 part 7 [file 44319_2026_742_MOESM10_ESM.zip › Figure 2 Part 7/Fig 2fg Control Hela rGT335 acetylated tubulin part 2/Metaphase/Cas9 actub rGT335 9-8-25 R1 M7M8.Project Maximum Z_XY1757355008_Z0_T0_C0.tif]

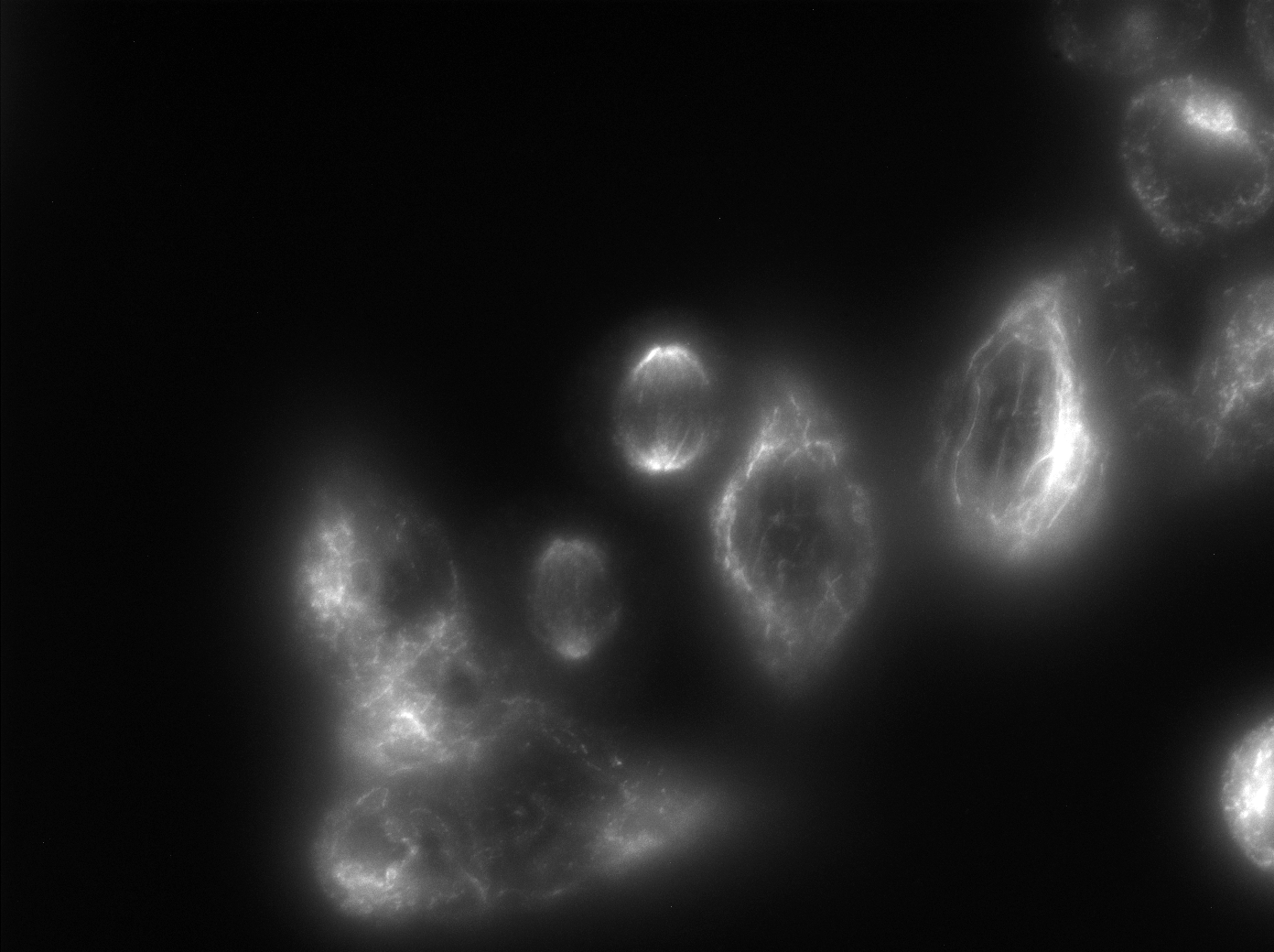

Supplement: Supplementary file 10 — Source data Fig. 2 part 7 [file 44319_2026_742_MOESM10_ESM.zip › Figure 2 Part 7/Fig 2fg Control Hela rGT335 acetylated tubulin part 2/Metaphase/Cas9 actub rGT335 9-8-25 R1 M7M8.Project Maximum Z_XY1757355008_Z0_T0_C2.tif]

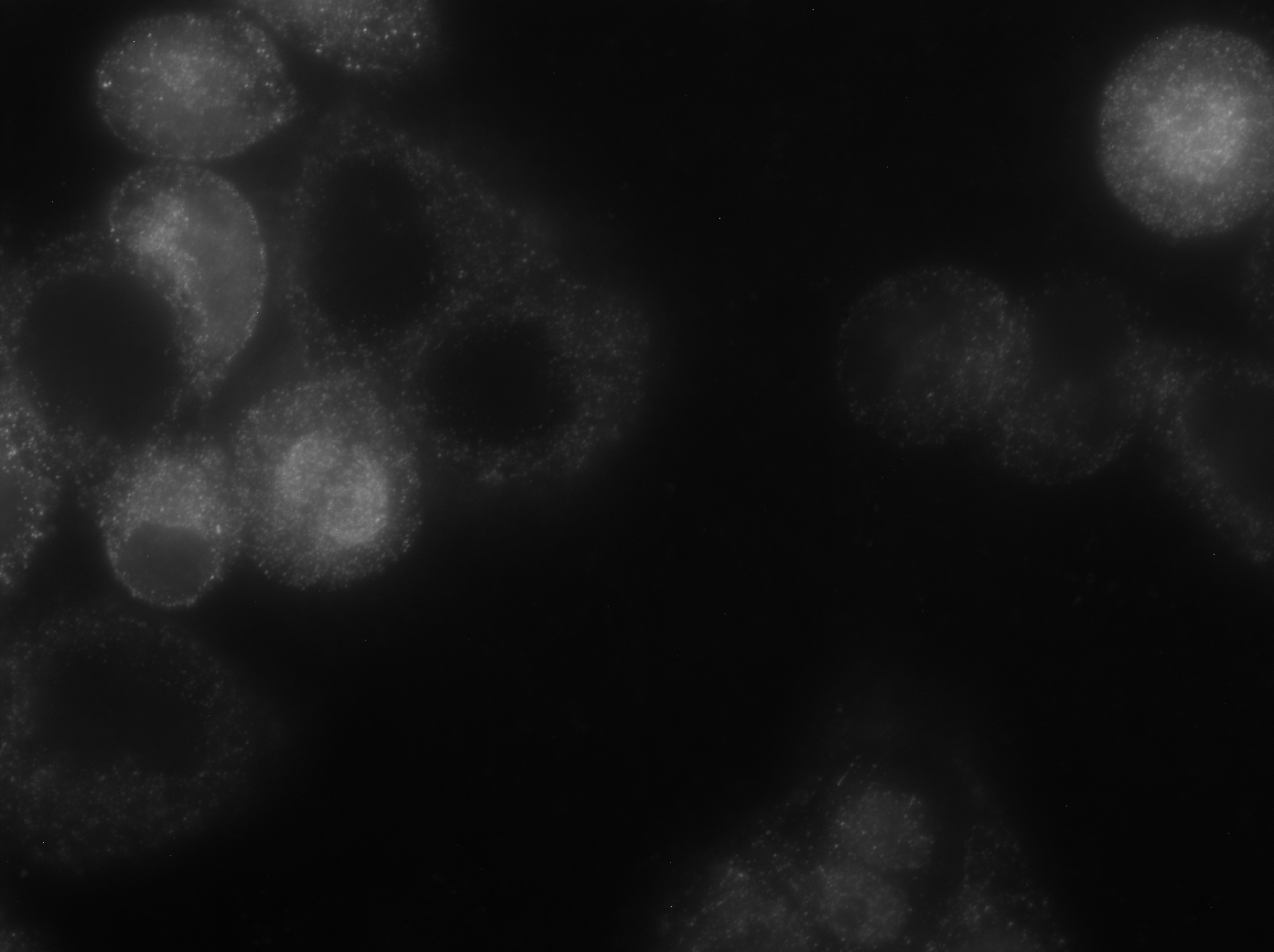

Supplement: Supplementary file 10 — Source data Fig. 2 part 7 [file 44319_2026_742_MOESM10_ESM.zip › Figure 2 Part 7/Fig 2fg Control Hela rGT335 acetylated tubulin part 2/Metaphase/Cas9 actub rGT335 9-8-25 R3 M9.Project Maximum Z_XY1757368504_Z0_T0_C1.tif]

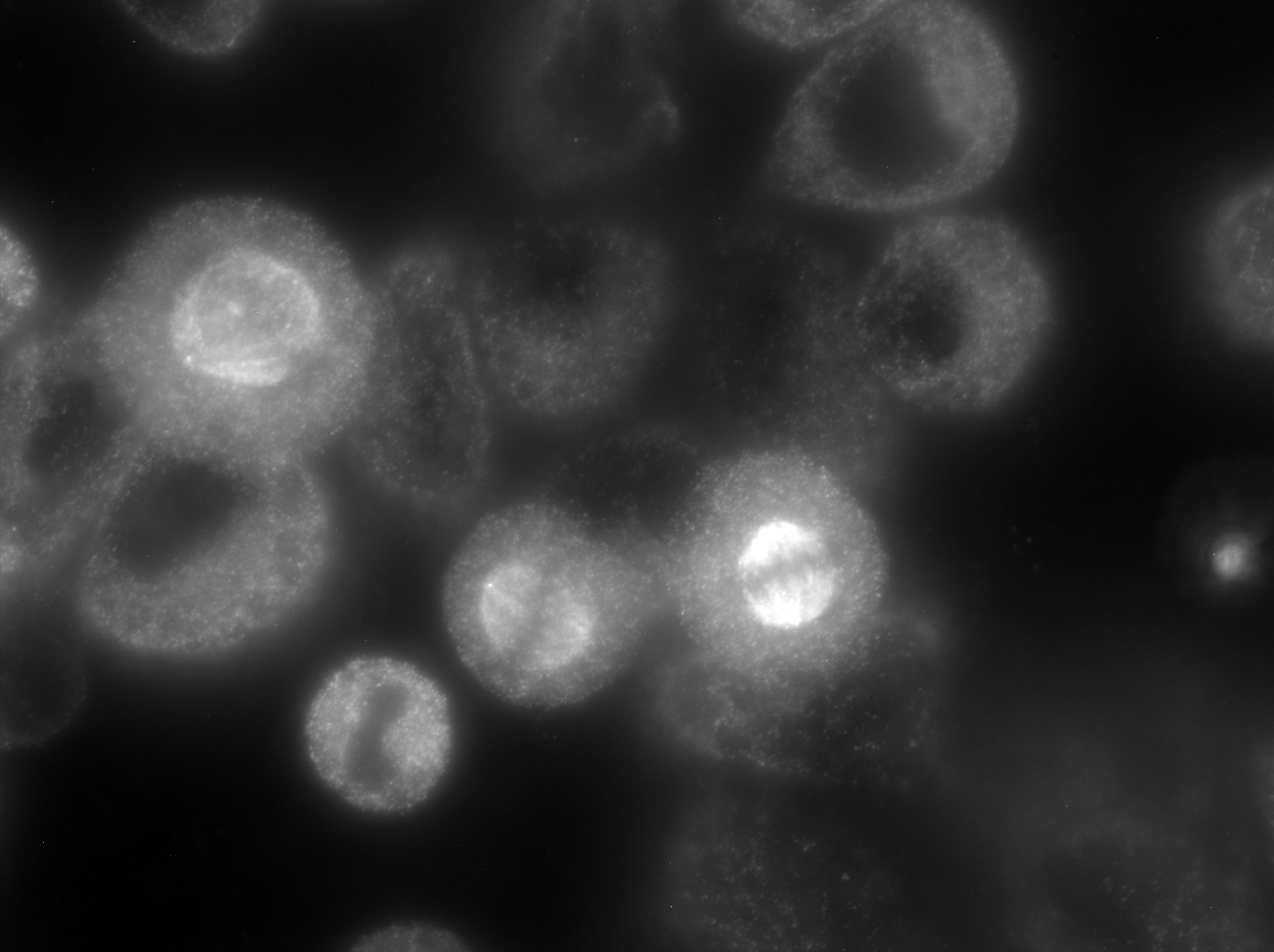

Supplement: Supplementary file 10 — Source data Fig. 2 part 7 [file 44319_2026_742_MOESM10_ESM.zip › Figure 2 Part 7/Fig 2fg Control Hela rGT335 acetylated tubulin part 2/Metaphase/Cas9 actub rGT335 9-8-25 R2 M8M9.Project Maximum Z_XY1757362229_Z0_T0_C1.tif]

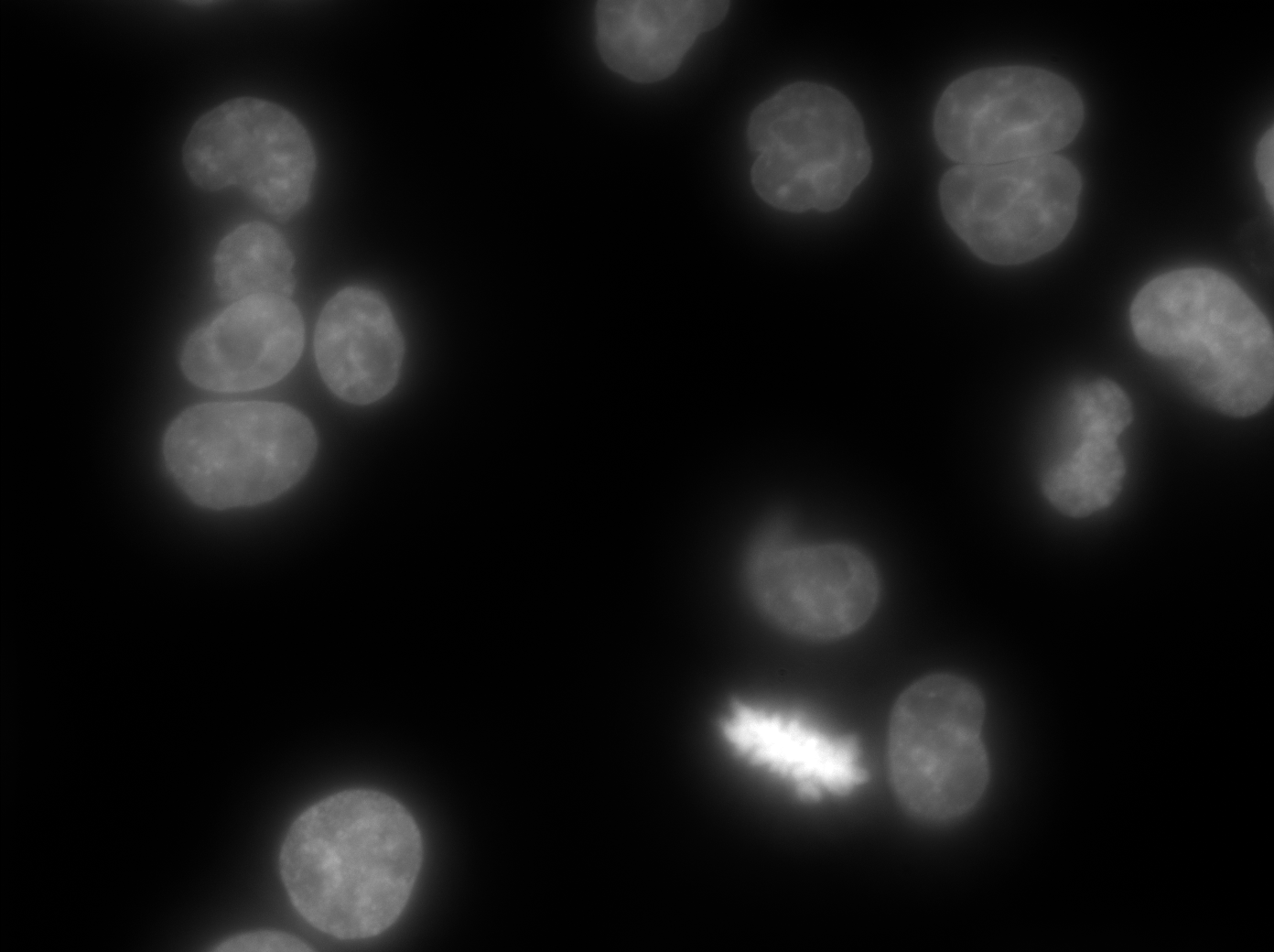

Supplement: Supplementary file 10 — Source data Fig. 2 part 7 [file 44319_2026_742_MOESM10_ESM.zip › Figure 2 Part 7/Fig 2fg Control Hela rGT335 acetylated tubulin part 2/Metaphase/Cas9 actub rGT335 9-8-25 R1 M6.Project Maximum Z_XY1757354221_Z0_T0_C0.tif]

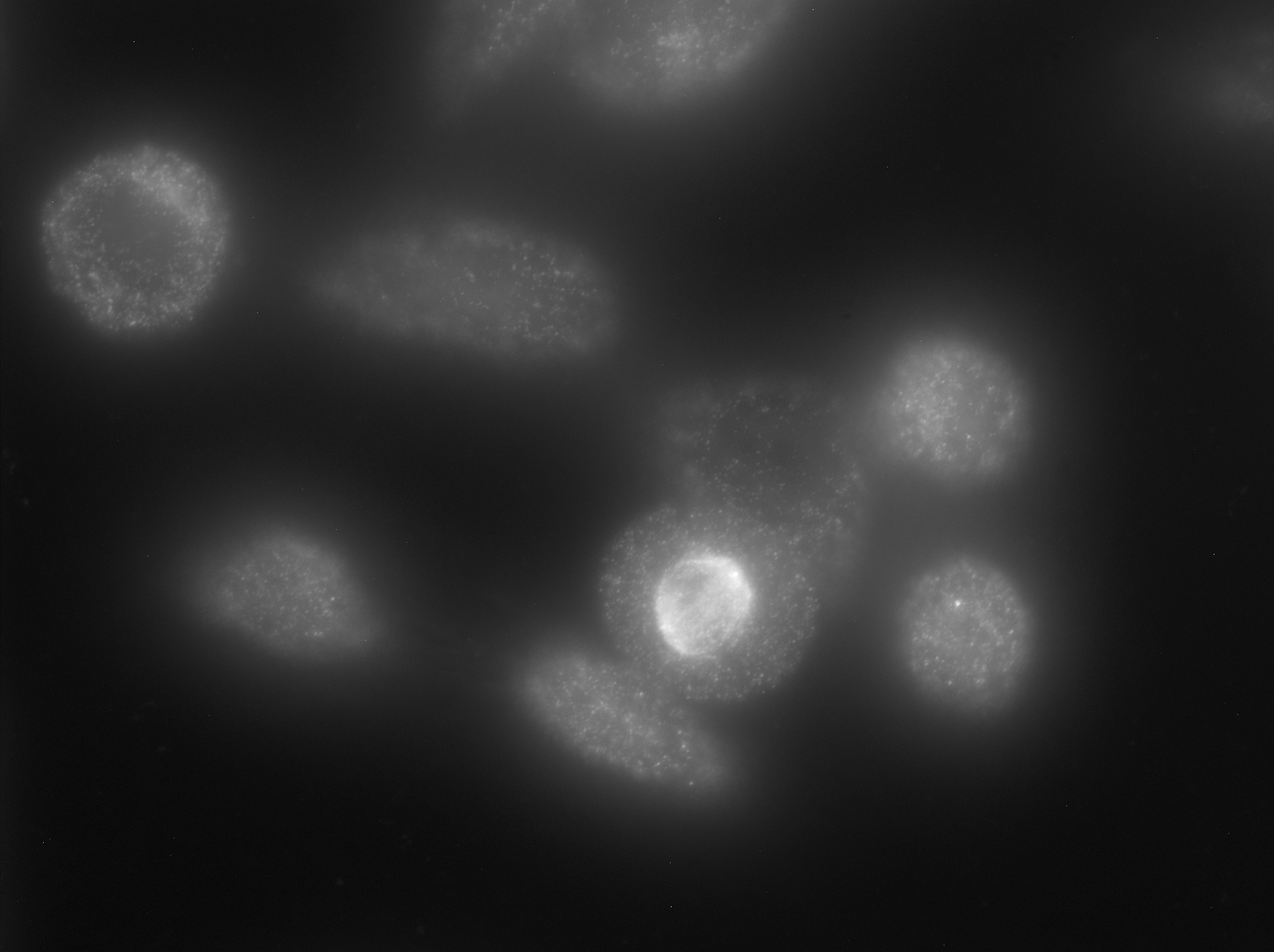

Supplement: Supplementary file 10 — Source data Fig. 2 part 7 [file 44319_2026_742_MOESM10_ESM.zip › Figure 2 Part 7/Fig 2fg Control Hela rGT335 acetylated tubulin part 2/Metaphase/Cas9 actub rGT335 9-8-25 R1 M3.Project Maximum Z_XY1757351500_Z0_T0_C1.tif]

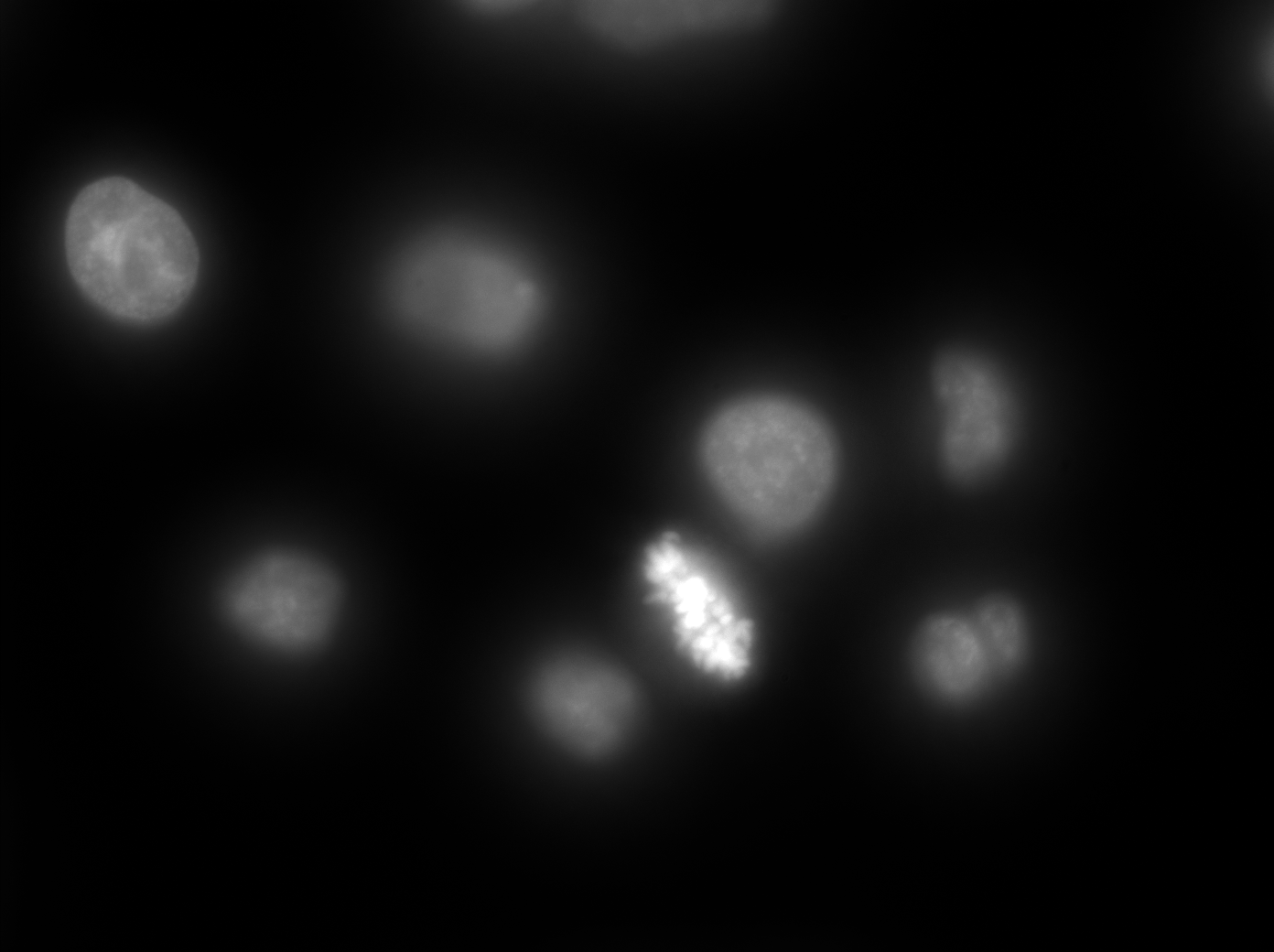

Supplement: Supplementary file 10 — Source data Fig. 2 part 7 [file 44319_2026_742_MOESM10_ESM.zip › Figure 2 Part 7/Fig 2fg Control Hela rGT335 acetylated tubulin part 2/Metaphase/Cas9 actub rGT335 9-8-25 R1 M3.Project Maximum Z_XY1757351500_Z0_T0_C0.tif]
