# Supplementary figures and images for: TPGS1 regulates central spindle microtubule glutamylation and remodeling during telophase and abscission (part 11 of 36)
Source: EMBO Rep. 2026 Mar 23;27(8):1944–63. doi: 10.1038/s44319-026-00742-3 (PMC13121839; doi:10.1038/s44319-026-00742-3)

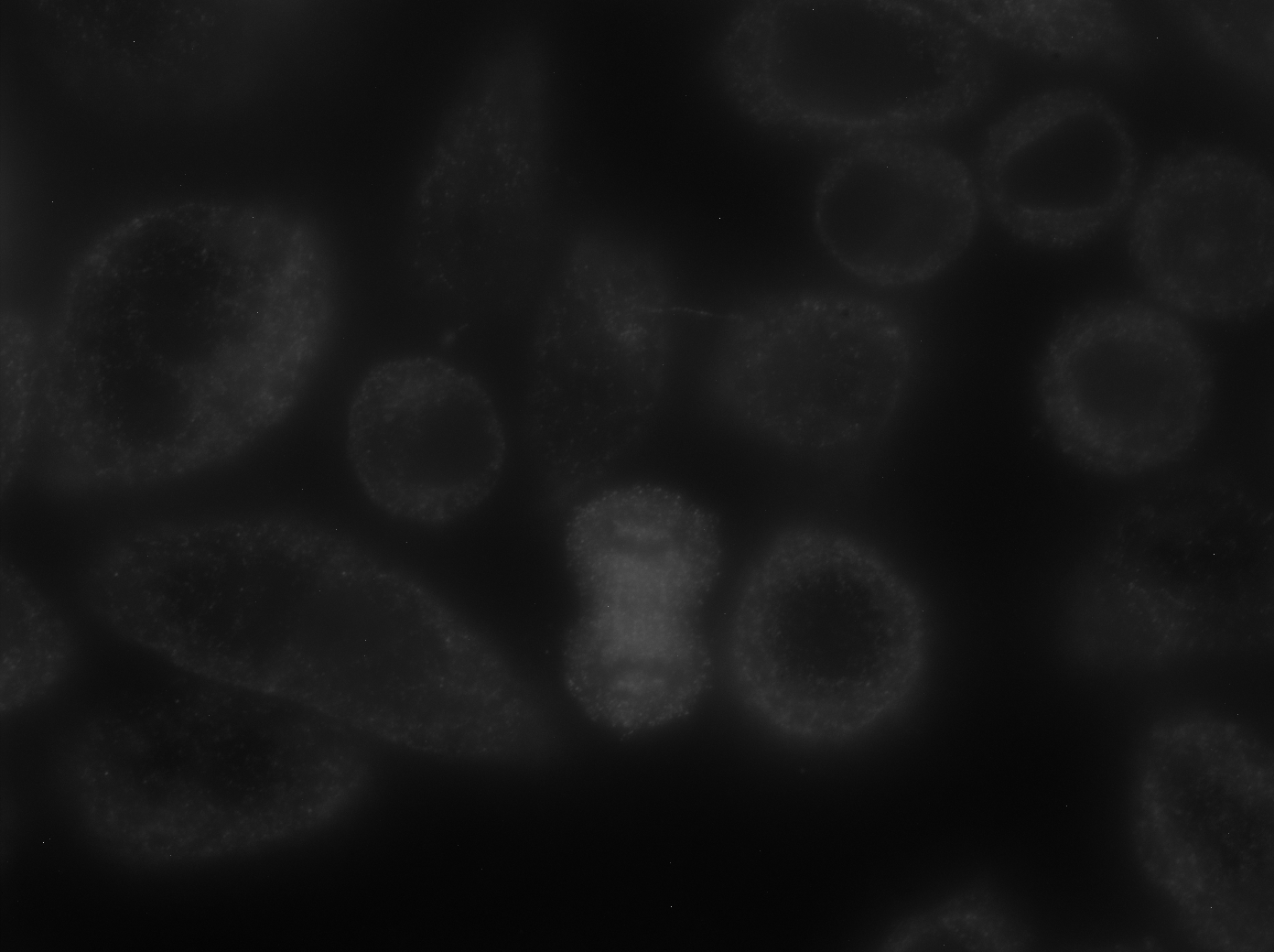

Supplement: Supplementary file 10 — Source data Fig. 2 part 7 [file 44319_2026_742_MOESM10_ESM.zip › Figure 2 Part 7/Fig 2fg Control Hela rGT335 acetylated tubulin part 2/Furrow Ingression/Cas9 actub rGT335 9-8-25 R3 FI8.Project Maximum Z_XY1757366668_Z0_T0_C1.tif]

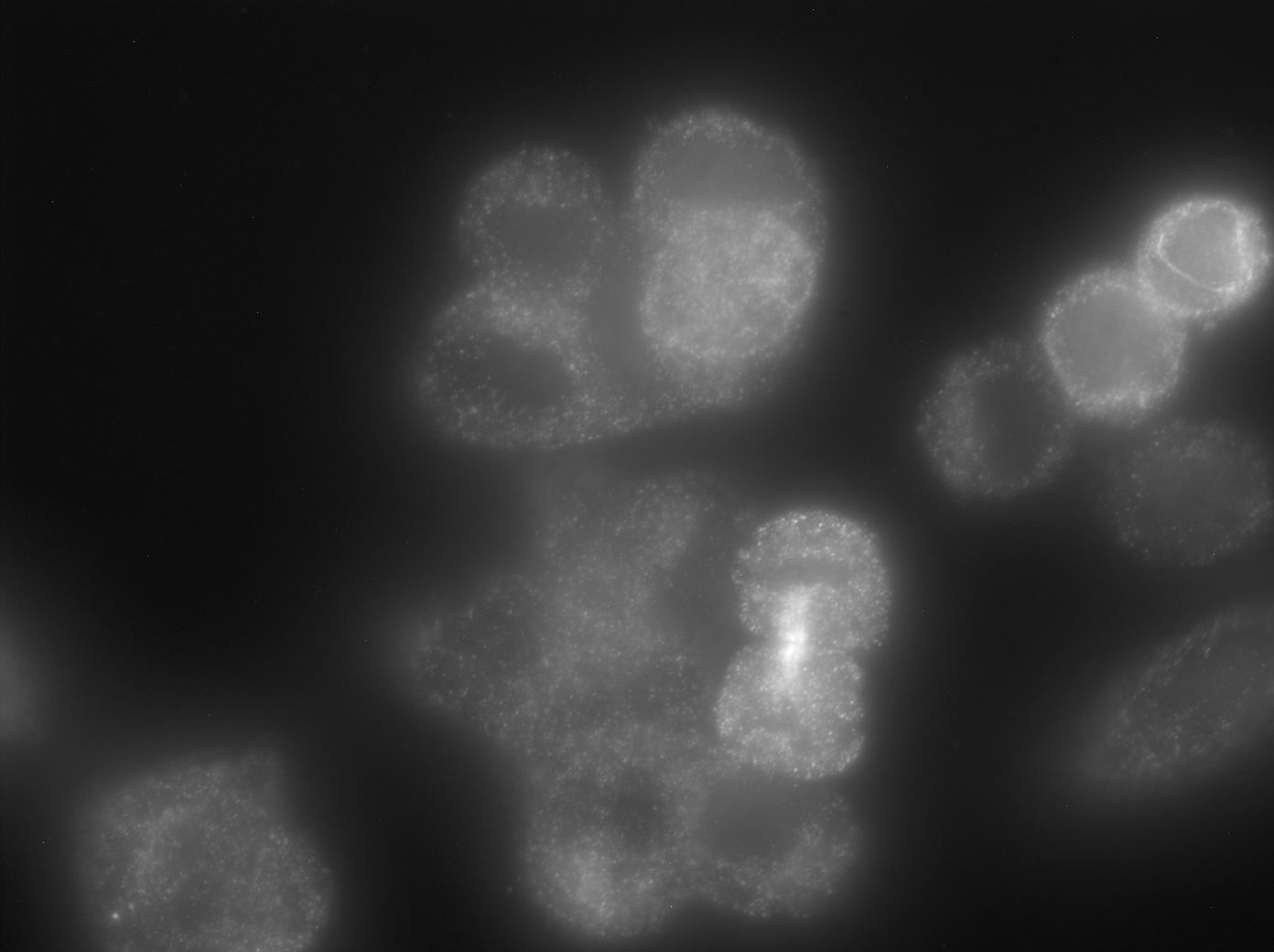

Supplement: Supplementary file 10 — Source data Fig. 2 part 7 [file 44319_2026_742_MOESM10_ESM.zip › Figure 2 Part 7/Fig 2fg Control Hela rGT335 acetylated tubulin part 2/Furrow Ingression/Cas9 actub rGT335 9-8-25 R1 FI5.Project Maximum Z_XY1757353899_Z0_T0_C1.tif]

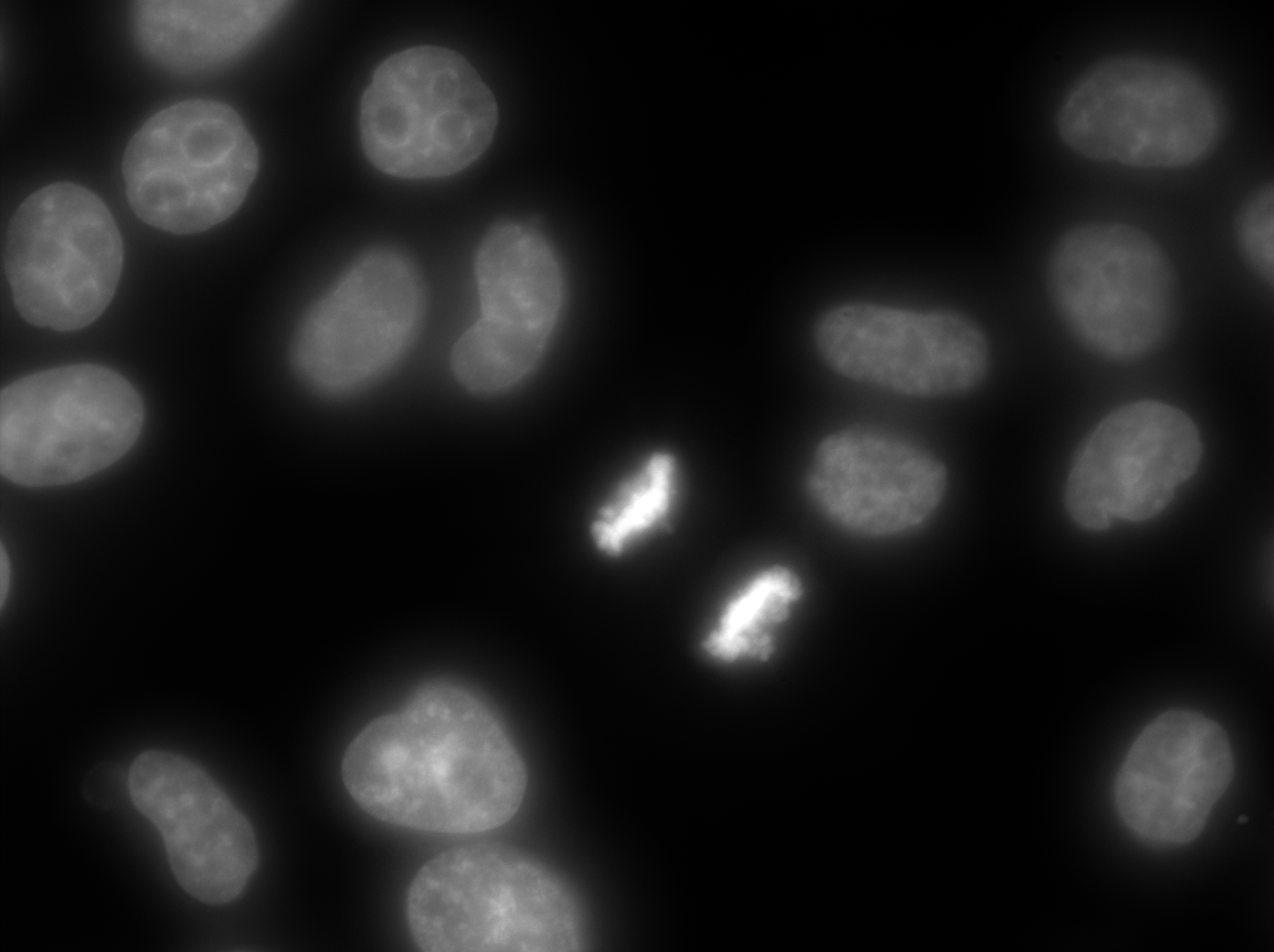

Supplement: Supplementary file 10 — Source data Fig. 2 part 7 [file 44319_2026_742_MOESM10_ESM.zip › Figure 2 Part 7/Fig 2fg Control Hela rGT335 acetylated tubulin part 2/Furrow Ingression/Cas9 actub rGT335 9-8-25 R3 FI6.Project Maximum Z_XY1757366392_Z0_T0_C0.tif]

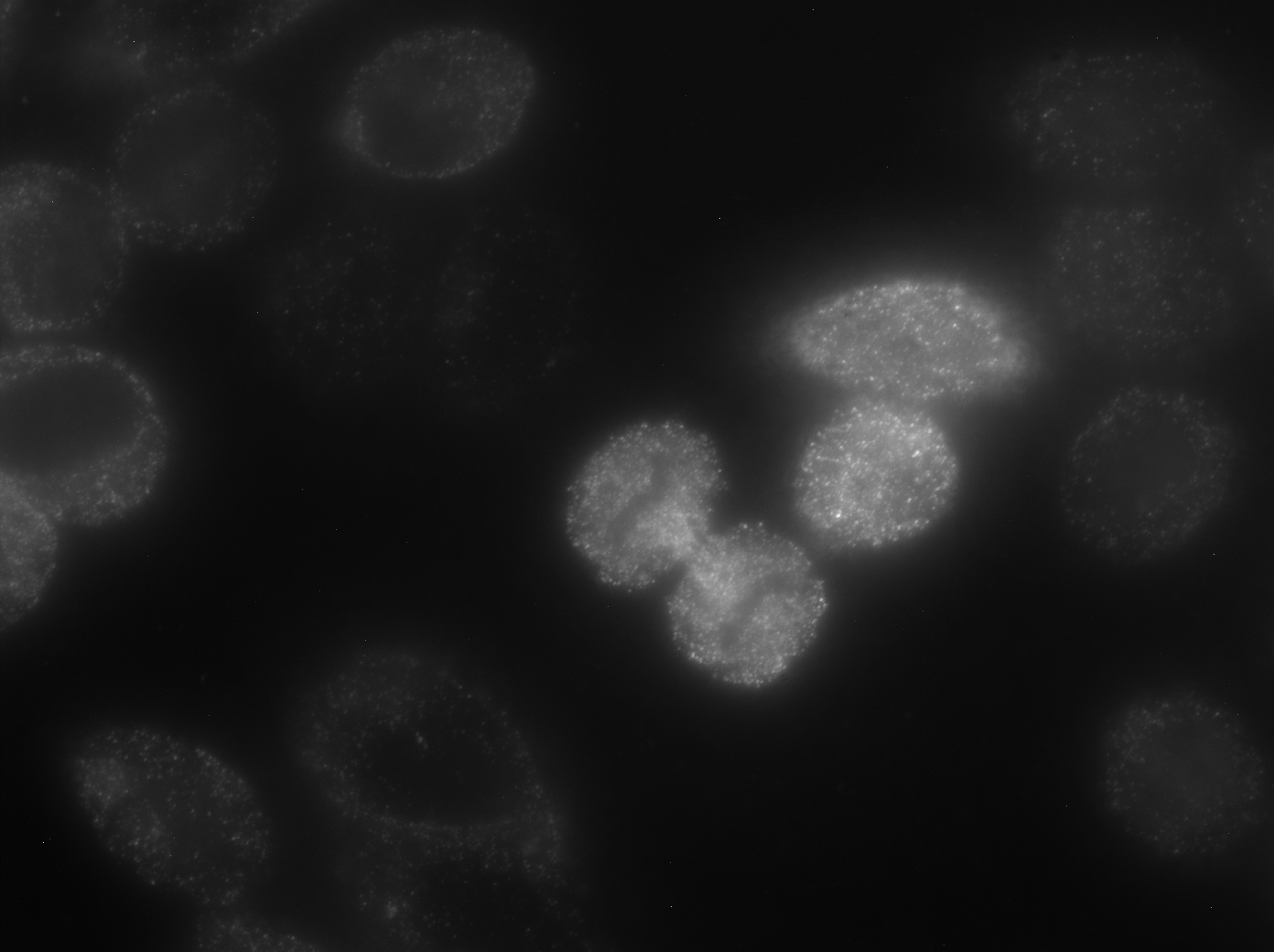

Supplement: Supplementary file 10 — Source data Fig. 2 part 7 [file 44319_2026_742_MOESM10_ESM.zip › Figure 2 Part 7/Fig 2fg Control Hela rGT335 acetylated tubulin part 2/Furrow Ingression/Cas9 actub rGT335 9-8-25 R3 FI6.Project Maximum Z_XY1757366392_Z0_T0_C1.tif]

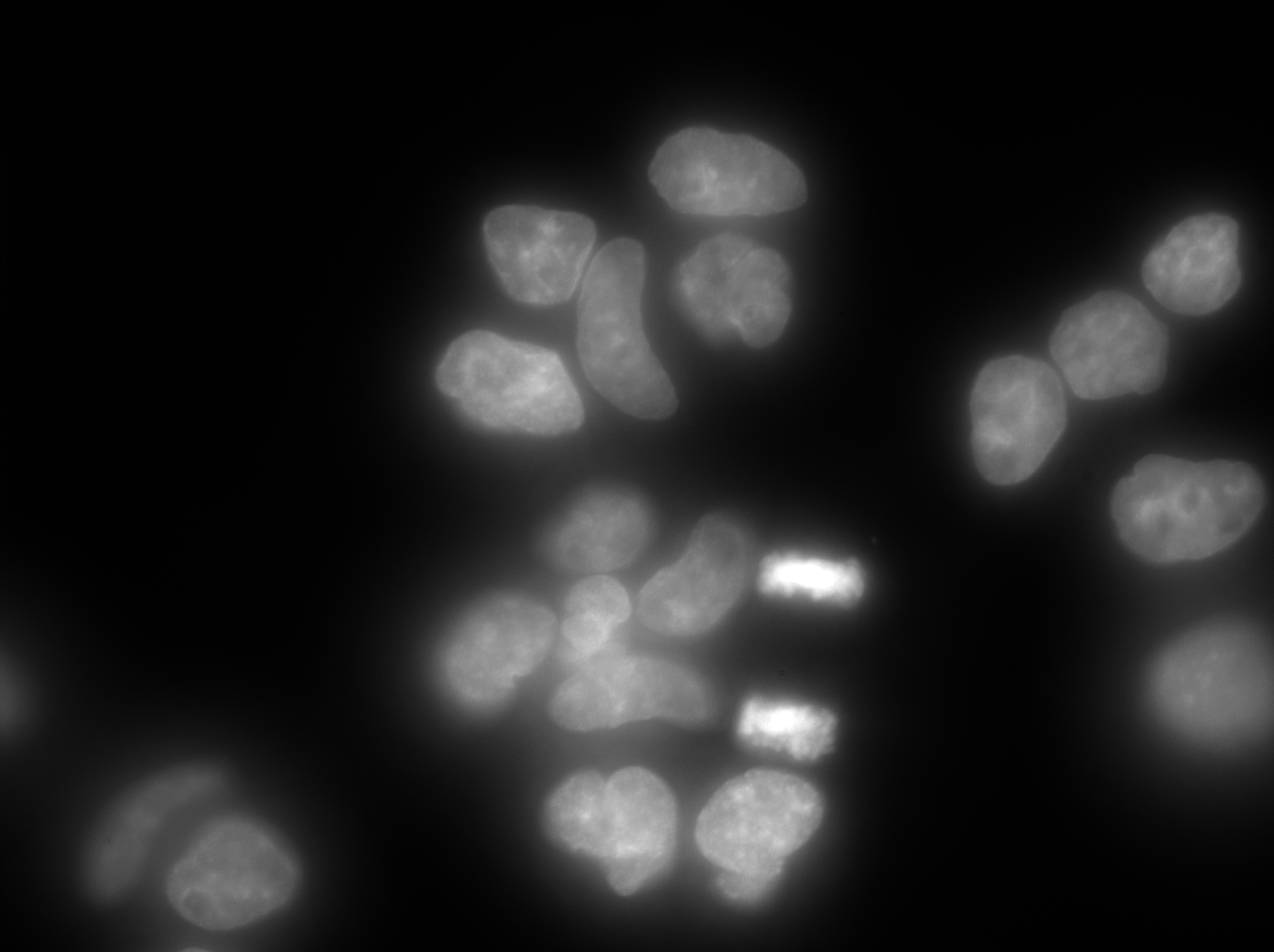

Supplement: Supplementary file 10 — Source data Fig. 2 part 7 [file 44319_2026_742_MOESM10_ESM.zip › Figure 2 Part 7/Fig 2fg Control Hela rGT335 acetylated tubulin part 2/Furrow Ingression/Cas9 actub rGT335 9-8-25 R1 FI5.Project Maximum Z_XY1757353899_Z0_T0_C0.tif]

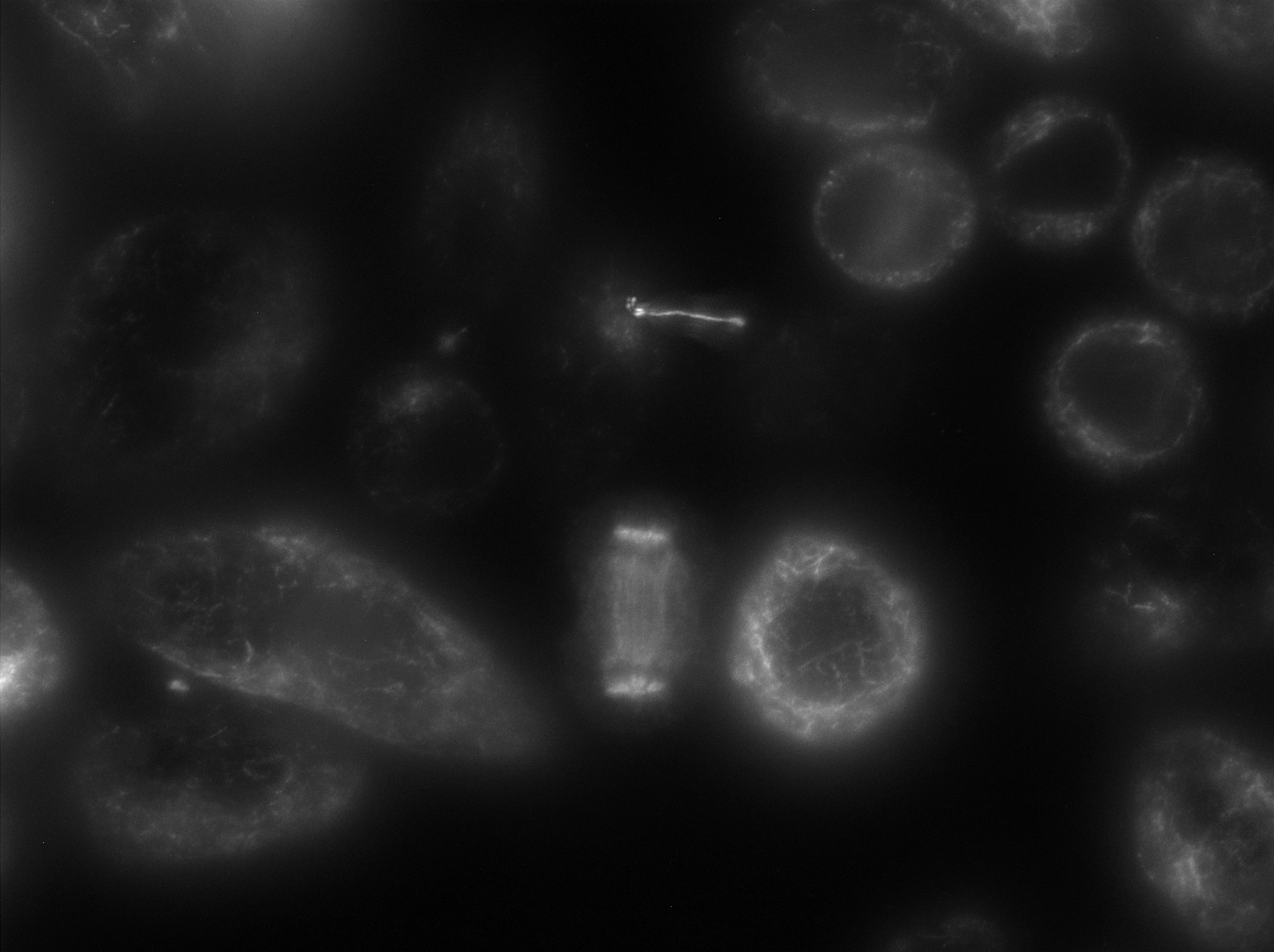

Supplement: Supplementary file 10 — Source data Fig. 2 part 7 [file 44319_2026_742_MOESM10_ESM.zip › Figure 2 Part 7/Fig 2fg Control Hela rGT335 acetylated tubulin part 2/Furrow Ingression/Cas9 actub rGT335 9-8-25 R3 FI8.Project Maximum Z_XY1757366668_Z0_T0_C2.tif]

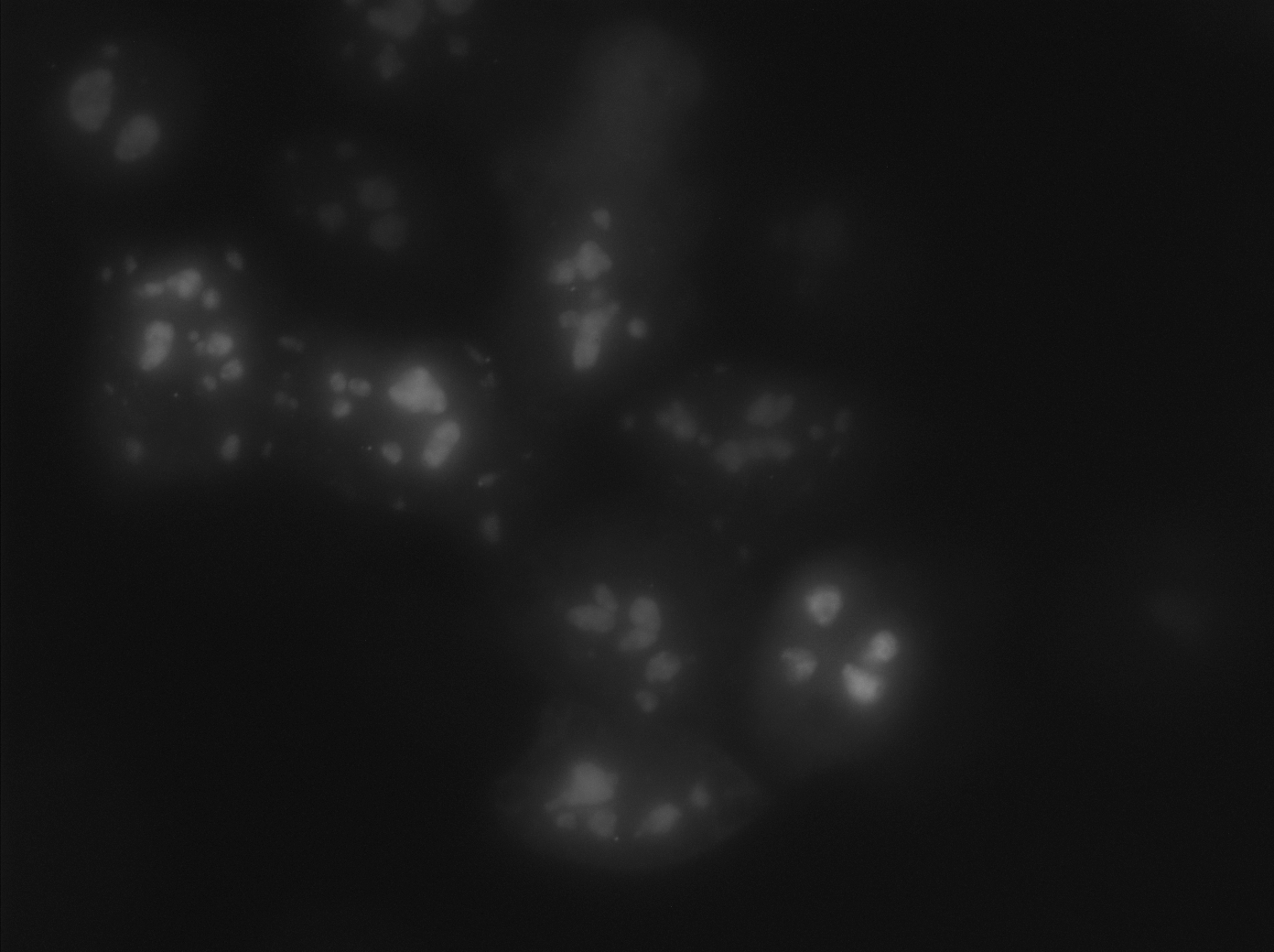

Supplement: Supplementary file 11 — Source data Fig. 3 part 1 [file 44319_2026_742_MOESM11_ESM.zip › Figure 3 Part 1/Fig 3b-e TTLL screen/TTLL4-YFPy I1.Project Maximum Z_XY1679075087_Z0_T0_C1.tif]

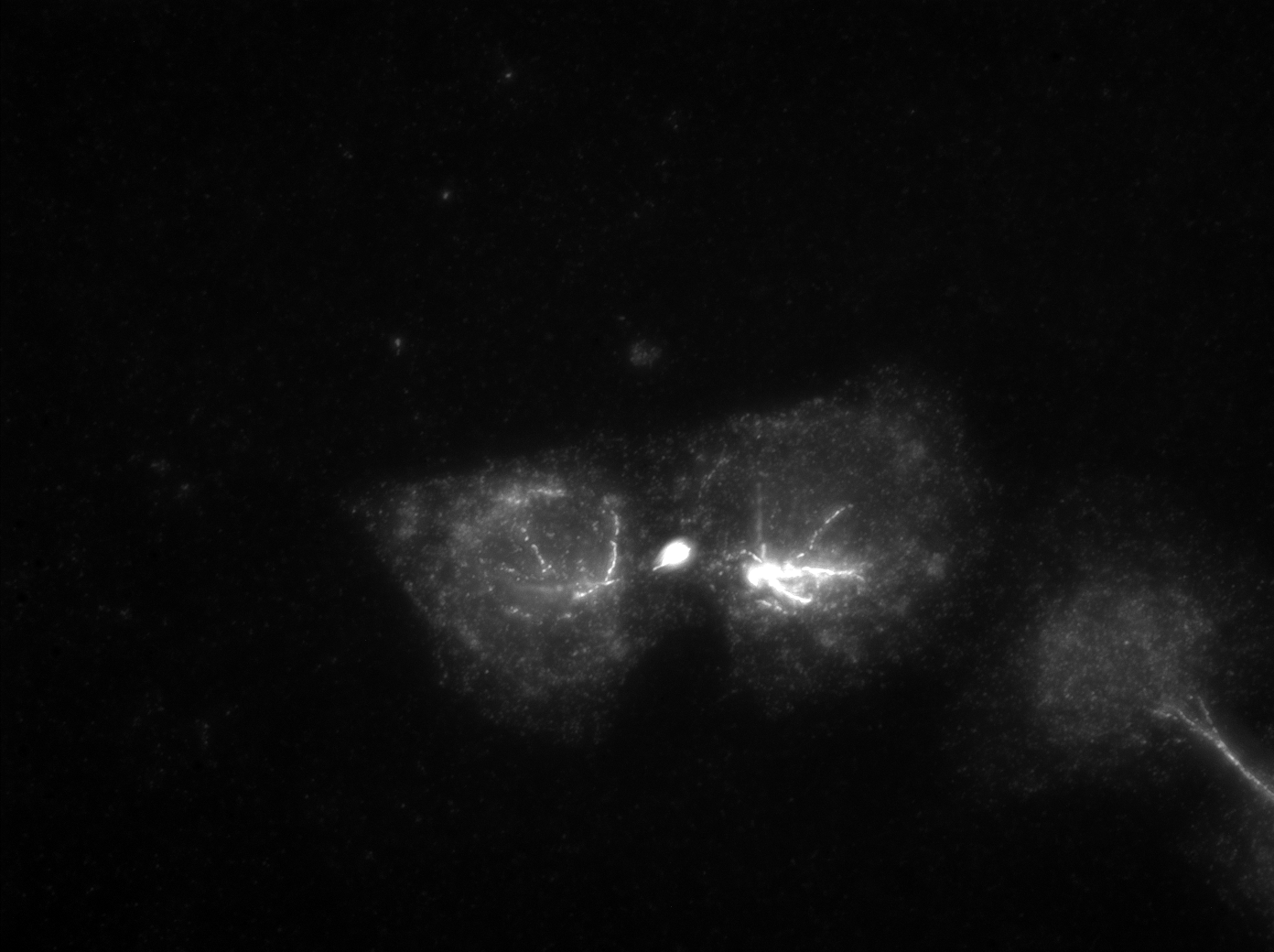

Supplement: Supplementary file 11 — Source data Fig. 3 part 1 [file 44319_2026_742_MOESM11_ESM.zip › Figure 3 Part 1/Fig 3b-e TTLL screen/TTLL1-GFP A3 I10.Project Maximum Z_XY1679695716_Z0_T0_C2.tif]

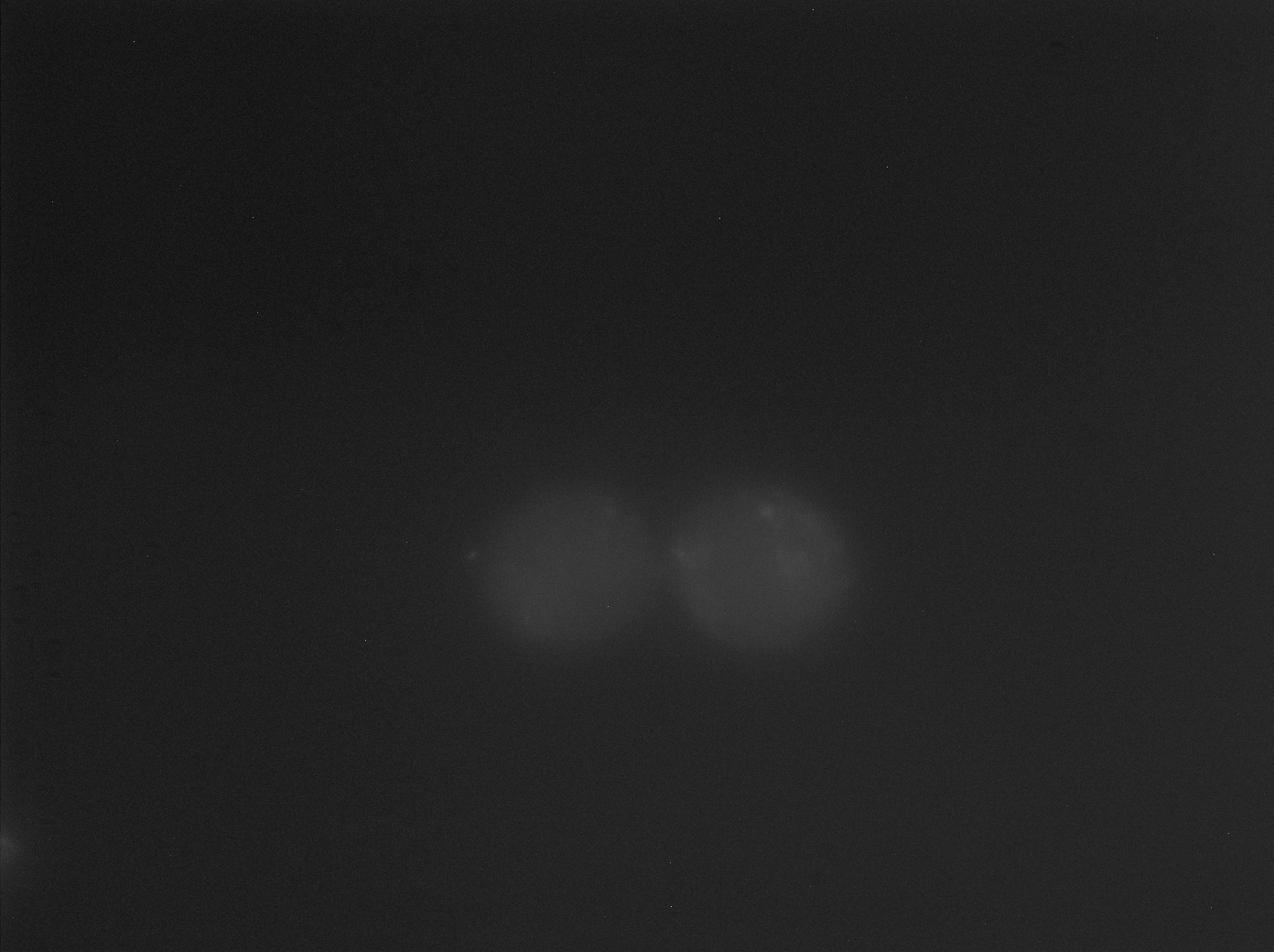

Supplement: Supplementary file 11 — Source data Fig. 3 part 1 [file 44319_2026_742_MOESM11_ESM.zip › Figure 3 Part 1/Fig 3b-e TTLL screen/TTLL1-GFP A3 I2.Project Maximum Z_XY1674673788_Z0_T0_C1.tif]

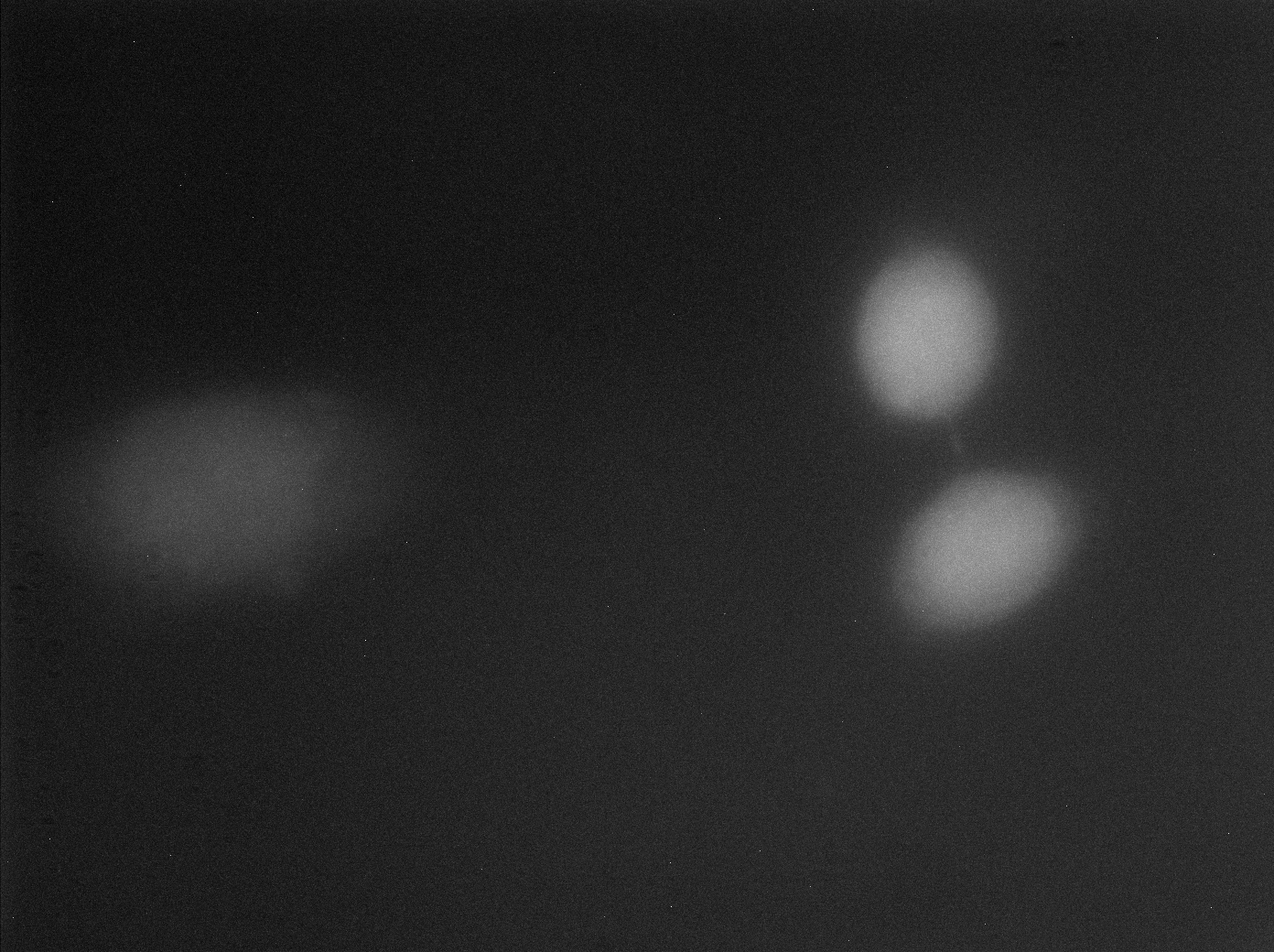

Supplement: Supplementary file 11 — Source data Fig. 3 part 1 [file 44319_2026_742_MOESM11_ESM.zip › Figure 3 Part 1/Fig 3b-e TTLL screen/TTLL1-GFP R1 I2.Project Maximum Z_XY1674162792_Z0_T0_C1.tif]

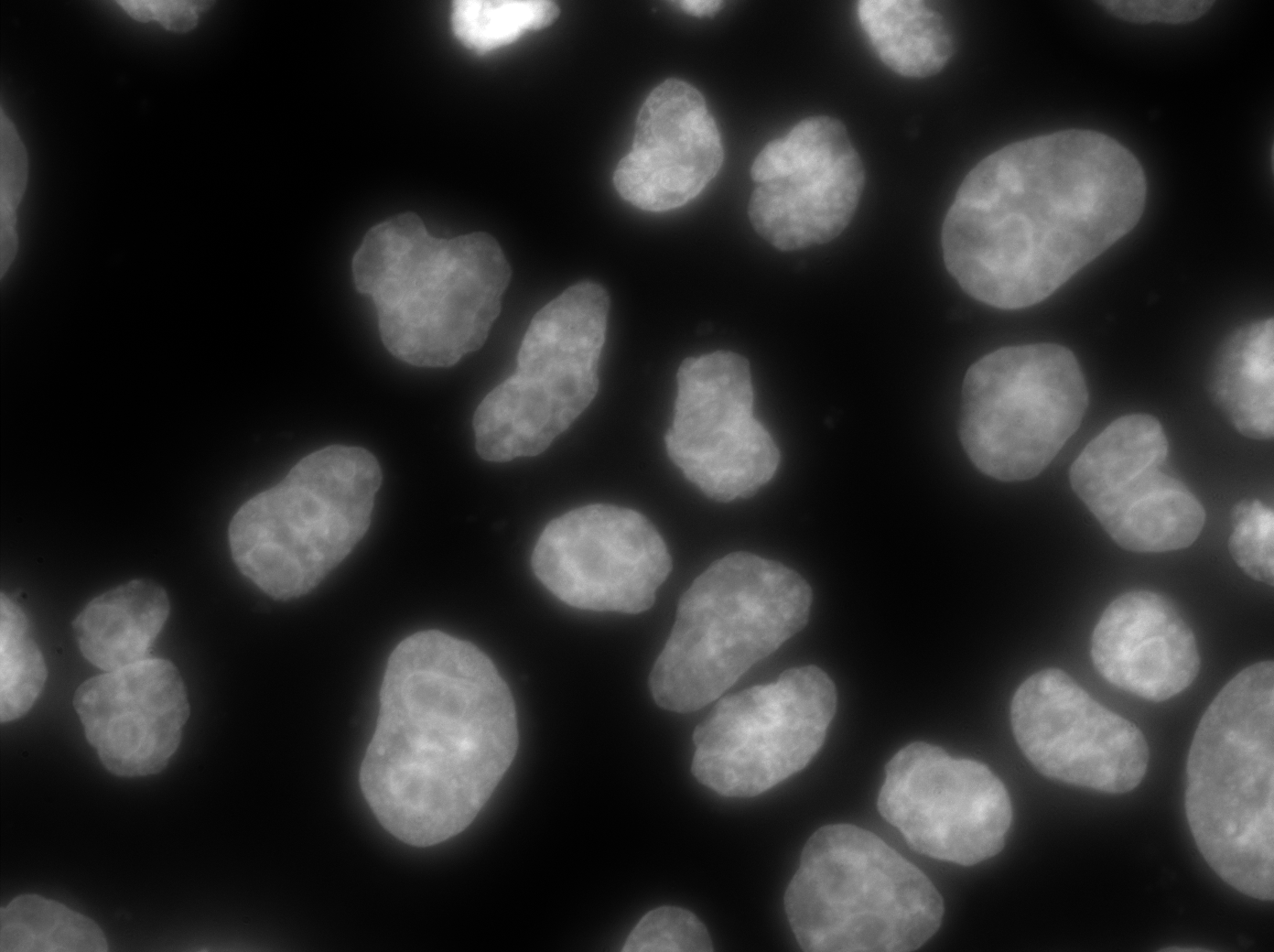

Supplement: Supplementary file 11 — Source data Fig. 3 part 1 [file 44319_2026_742_MOESM11_ESM.zip › Figure 3 Part 1/Fig 3b-e TTLL screen/TTLL1-GFP A3 I3 - 1.Project Maximum Z_XY1679694543_Z0_T0_C0.tif]

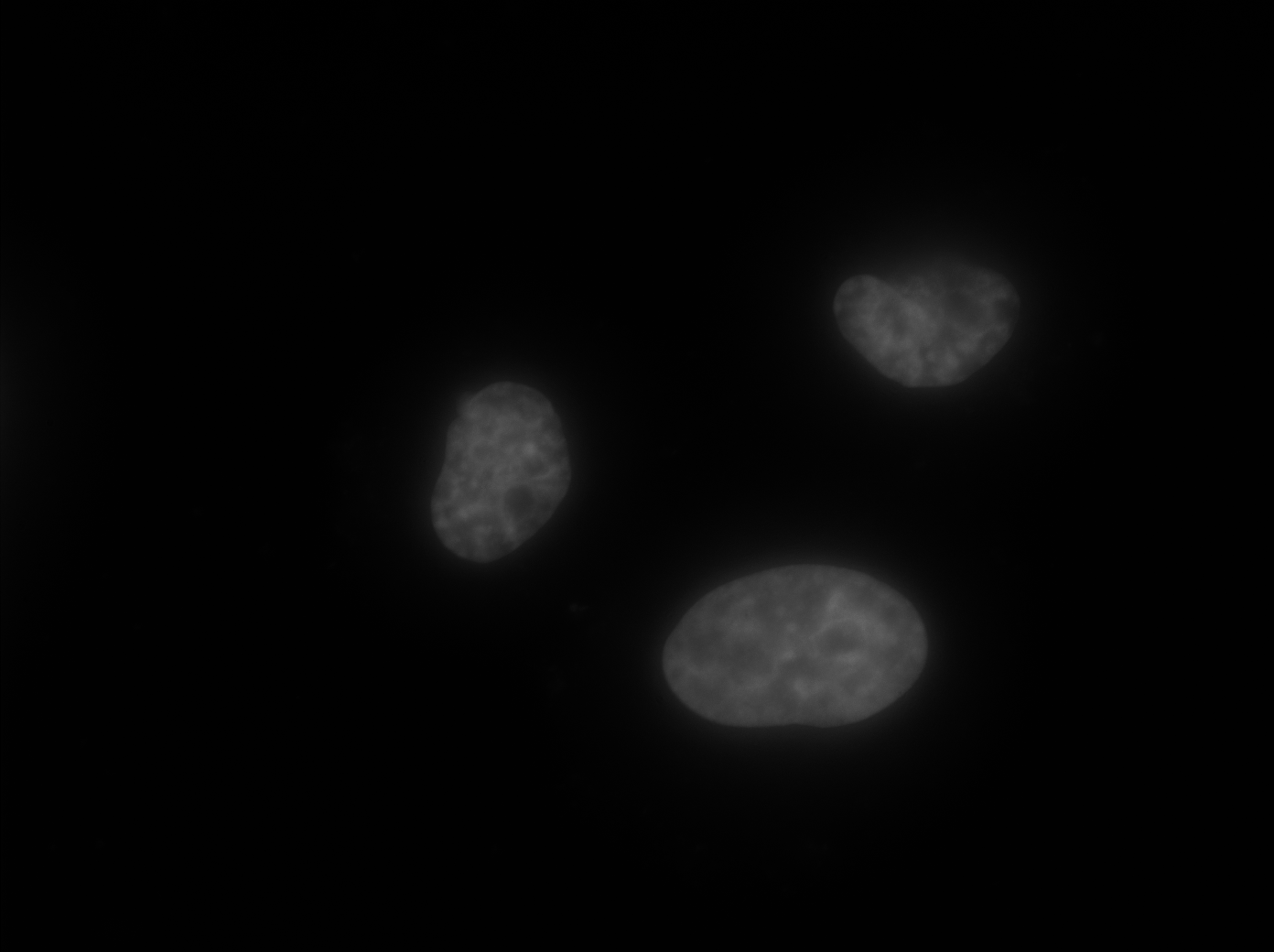

Supplement: Supplementary file 11 — Source data Fig. 3 part 1 [file 44319_2026_742_MOESM11_ESM.zip › Figure 3 Part 1/Fig 3b-e TTLL screen/EYFP MB I7.Project Maximum Z_XY1663878776_Z0_T0_C0.tif]

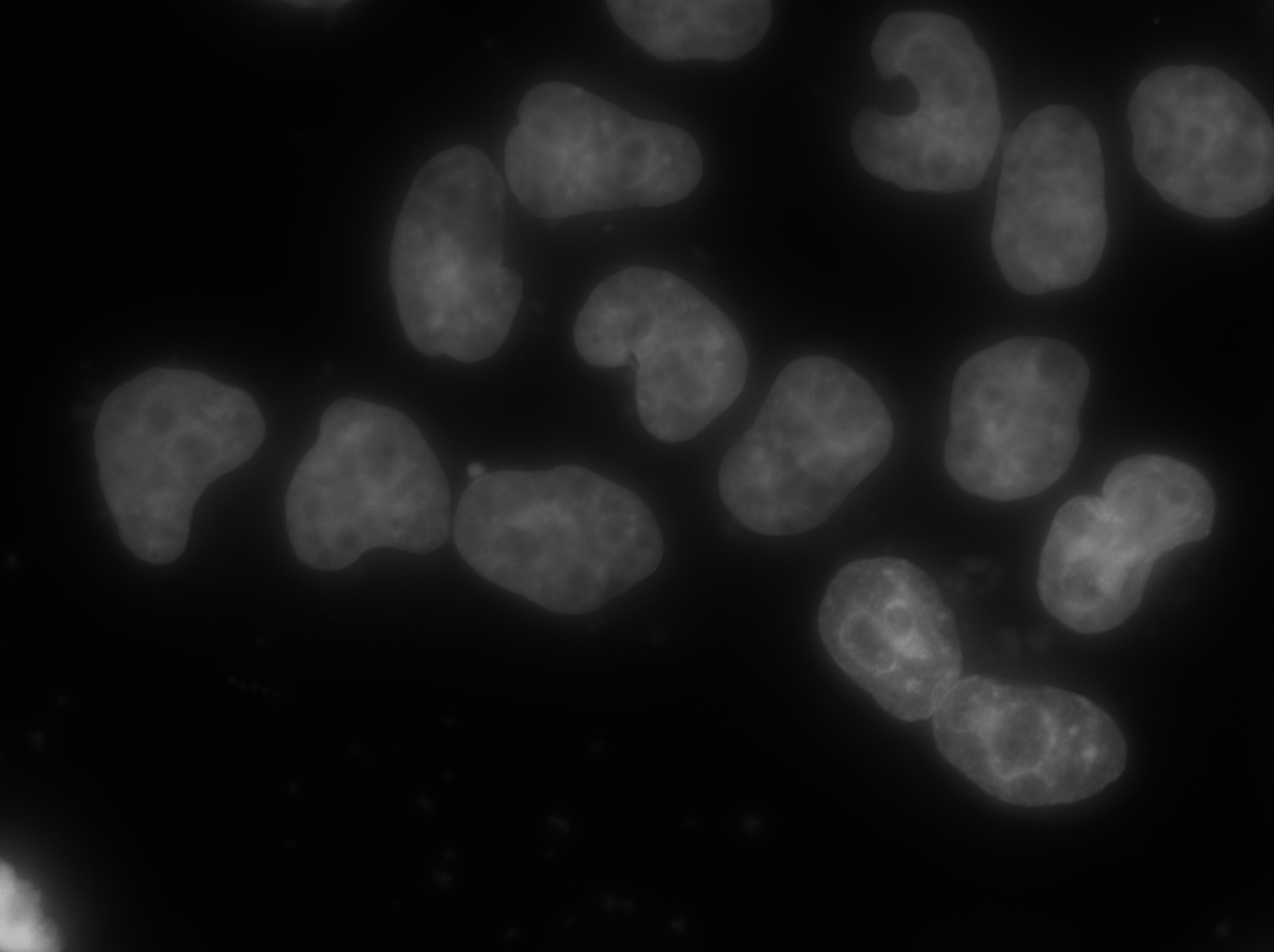

Supplement: Supplementary file 11 — Source data Fig. 3 part 1 [file 44319_2026_742_MOESM11_ESM.zip › Figure 3 Part 1/Fig 3b-e TTLL screen/TTLL1-GFP A3 I19.Project Maximum Z_XY1679698158_Z0_T0_C0.tif]

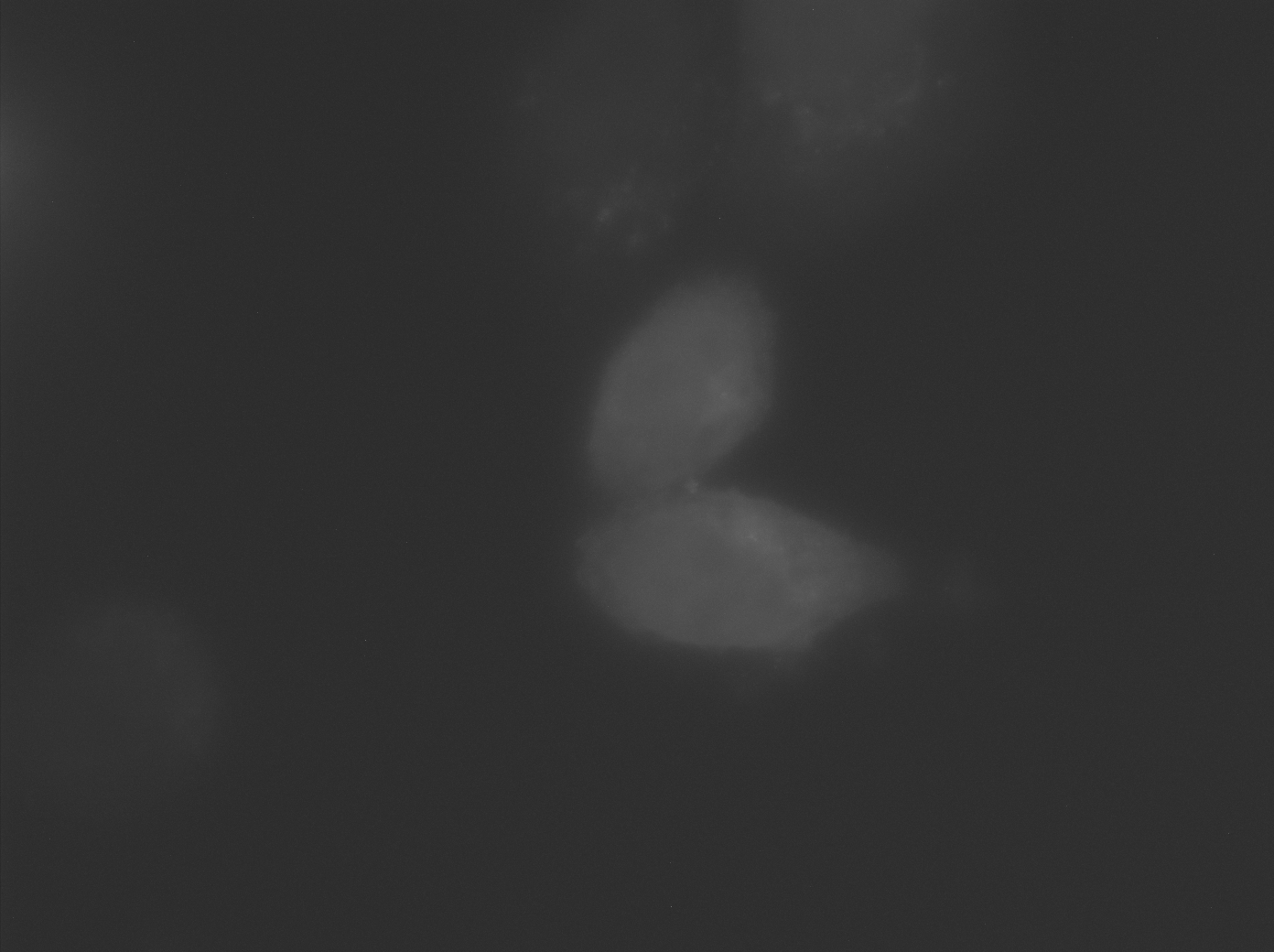

Supplement: Supplementary file 11 — Source data Fig. 3 part 1 [file 44319_2026_742_MOESM11_ESM.zip › Figure 3 Part 1/Fig 3b-e TTLL screen/TTLL1-GFP A4 I11.Project Maximum Z_XY1675964964_Z0_T0_C1.tif]

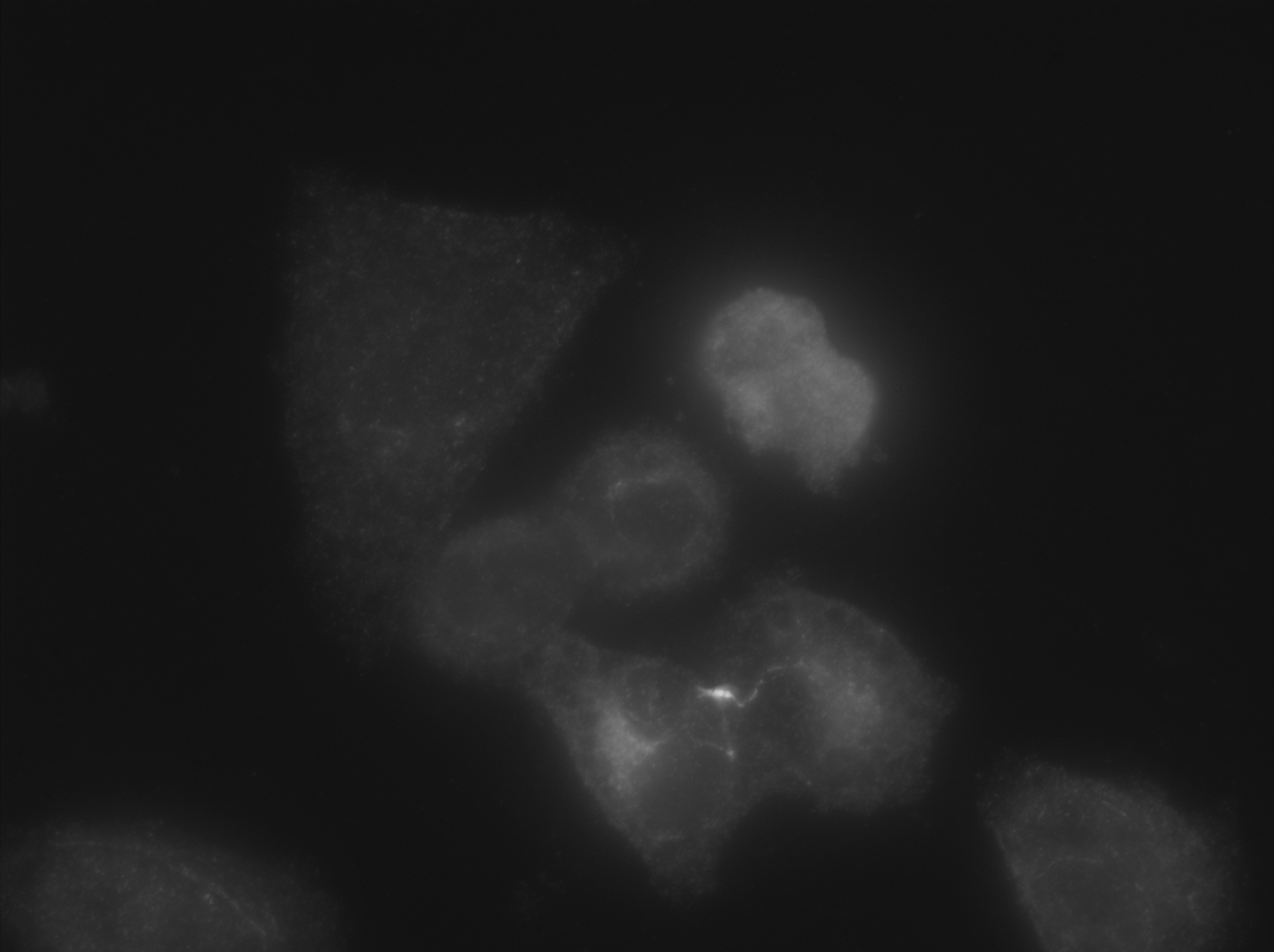

Supplement: Supplementary file 11 — Source data Fig. 3 part 1 [file 44319_2026_742_MOESM11_ESM.zip › Figure 3 Part 1/Fig 3b-e TTLL screen/TTLL1-GFPy I3.Project Maximum Z_XY1679086703_Z0_T0_C2.tif]

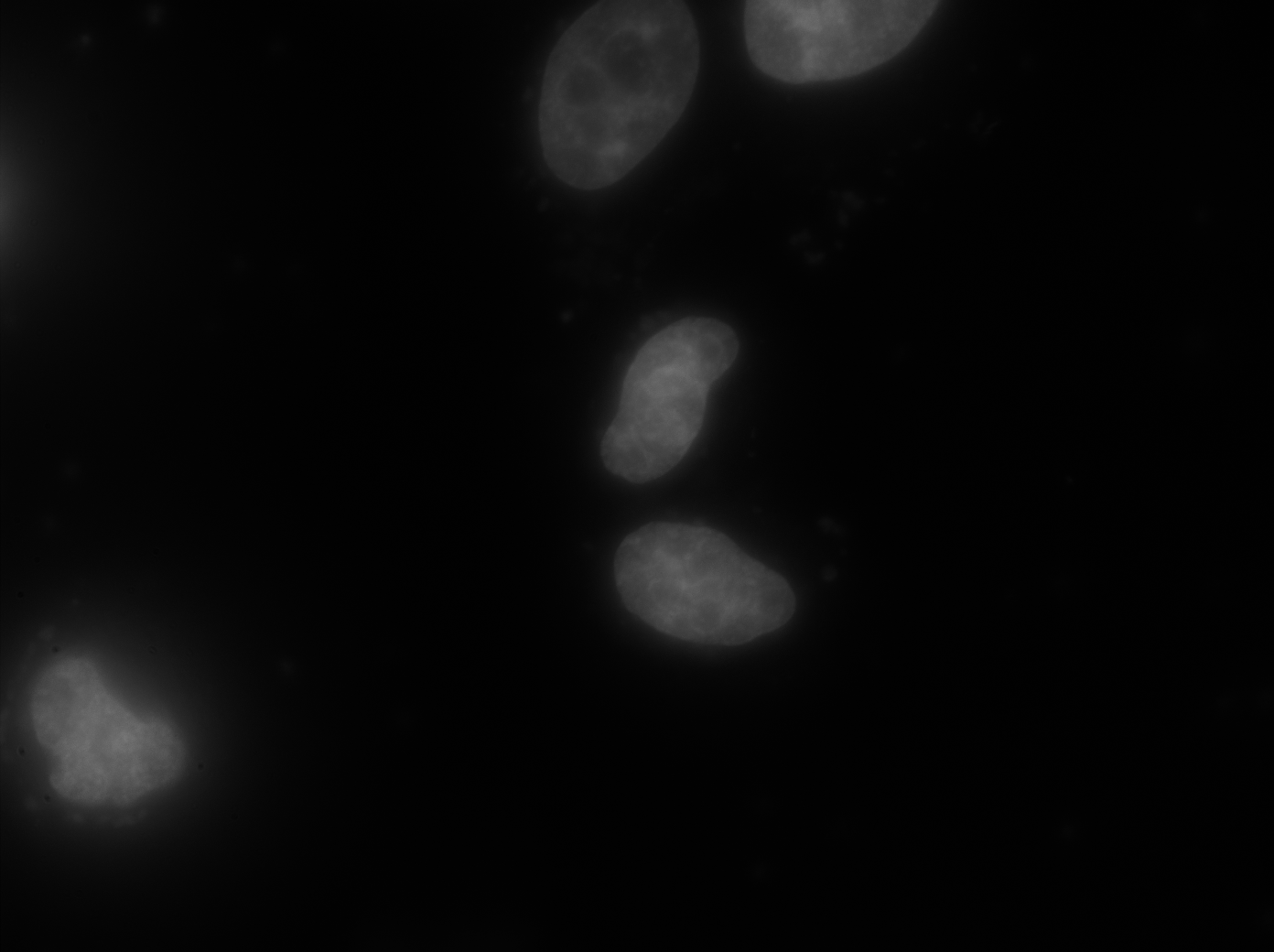

Supplement: Supplementary file 11 — Source data Fig. 3 part 1 [file 44319_2026_742_MOESM11_ESM.zip › Figure 3 Part 1/Fig 3b-e TTLL screen/TTLL1-GFP A4 I11.Project Maximum Z_XY1675964964_Z0_T0_C0.tif]

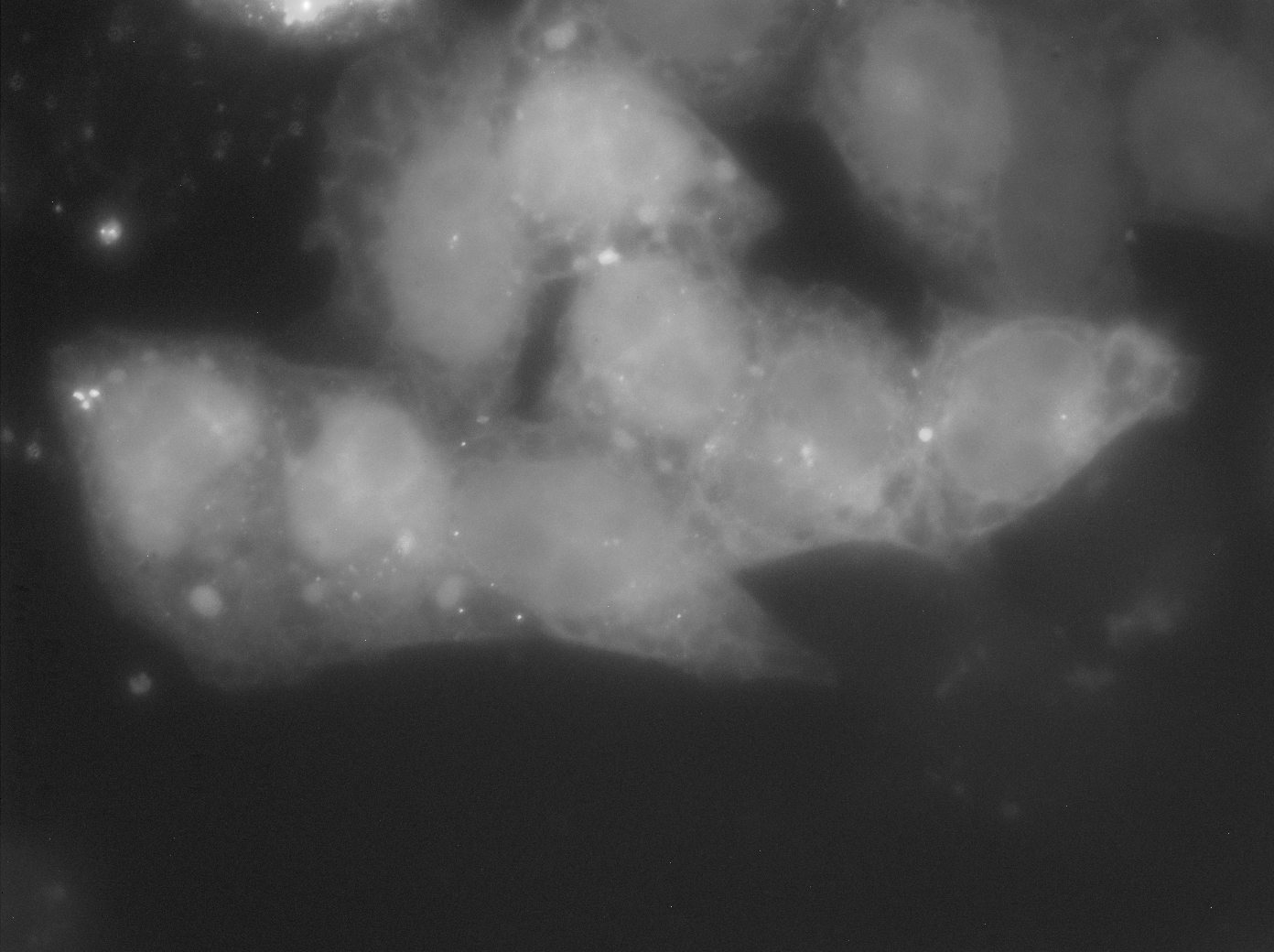

Supplement: Supplementary file 11 — Source data Fig. 3 part 1 [file 44319_2026_742_MOESM11_ESM.zip › Figure 3 Part 1/Fig 3b-e TTLL screen/TTLL1-GFP A3 I19.Project Maximum Z_XY1679698158_Z0_T0_C1.tif]

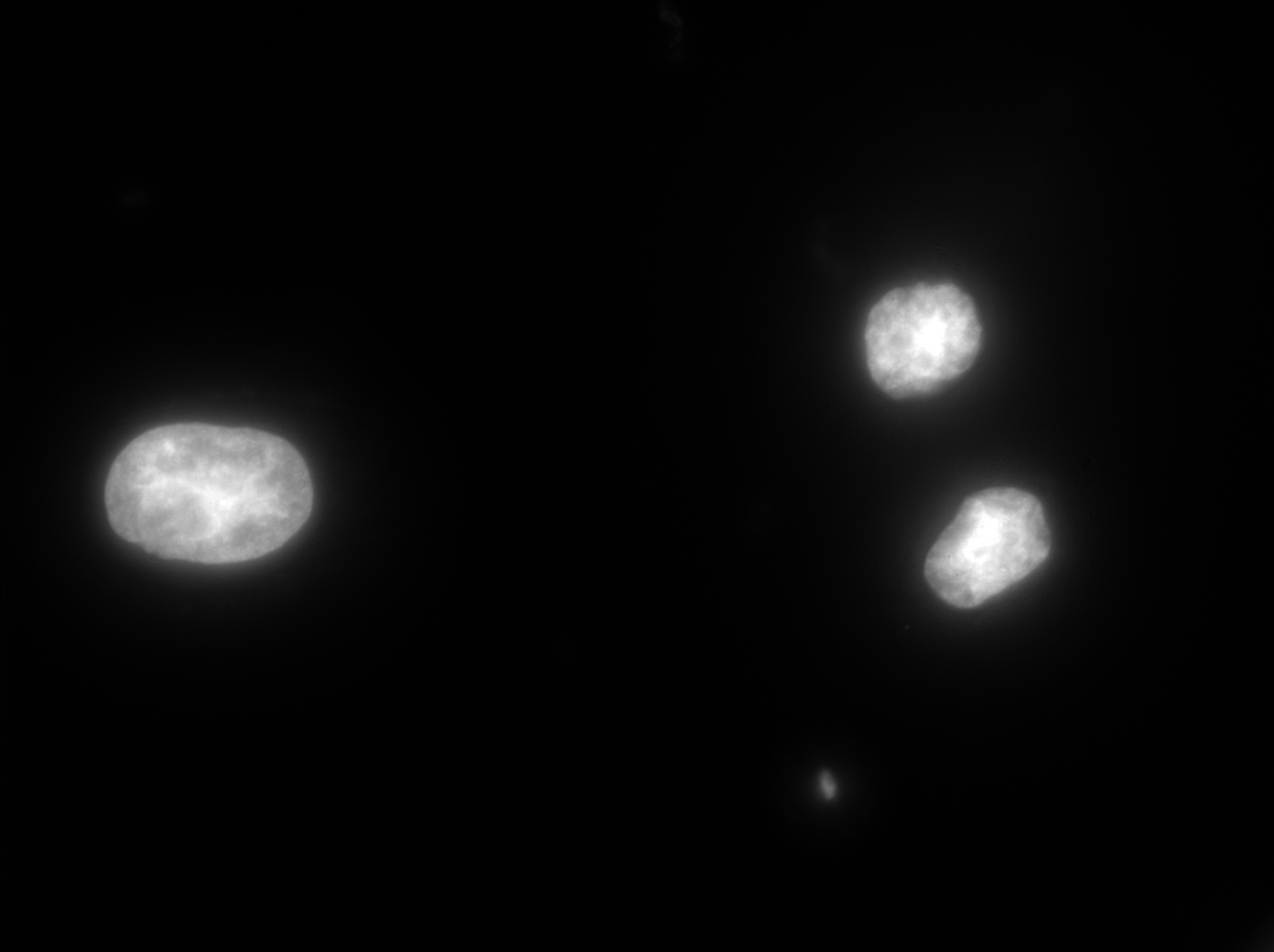

Supplement: Supplementary file 11 — Source data Fig. 3 part 1 [file 44319_2026_742_MOESM11_ESM.zip › Figure 3 Part 1/Fig 3b-e TTLL screen/TTLL1-GFP R1 I2.Project Maximum Z_XY1674162792_Z0_T0_C0.tif]

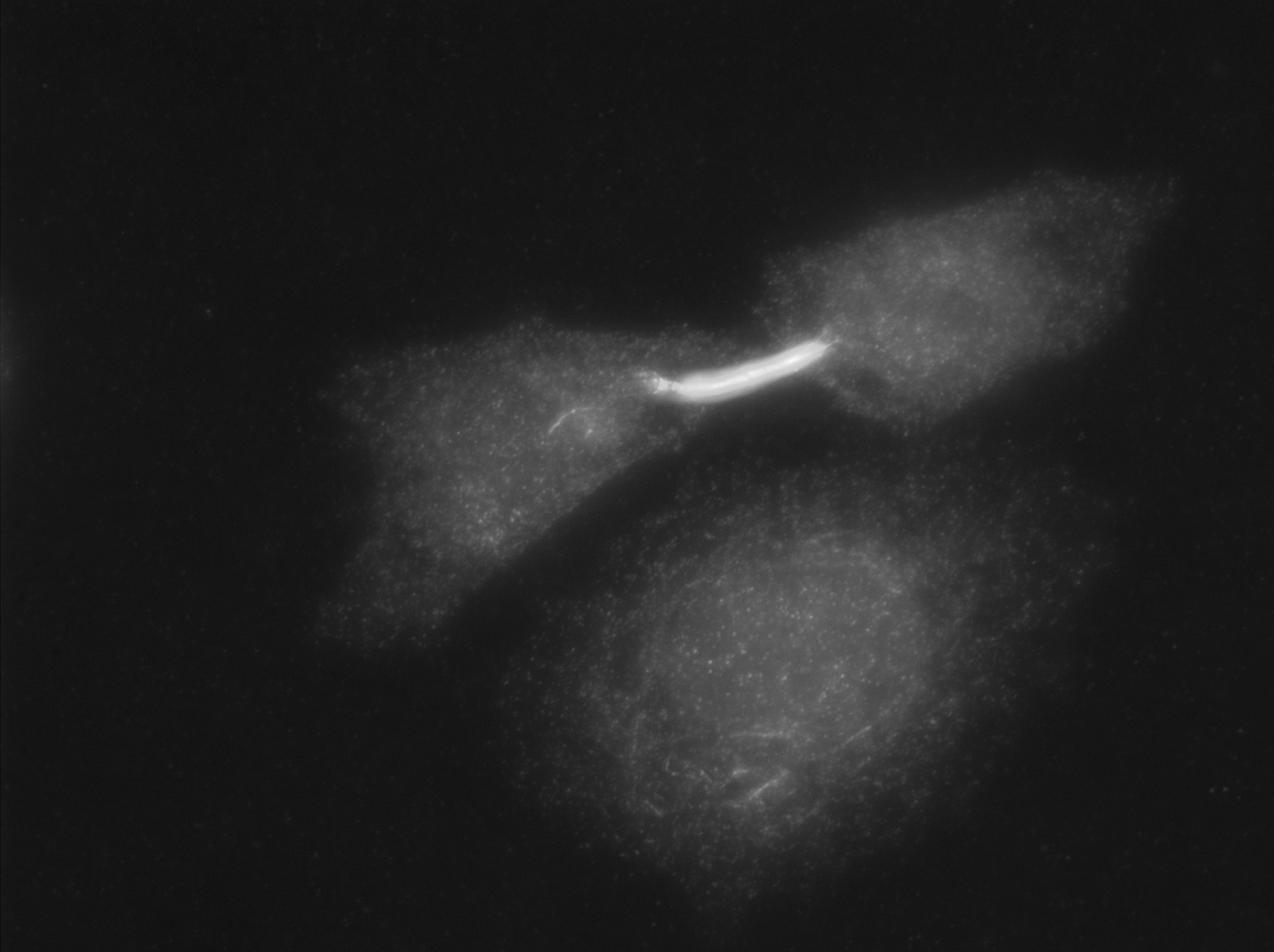

Supplement: Supplementary file 11 — Source data Fig. 3 part 1 [file 44319_2026_742_MOESM11_ESM.zip › Figure 3 Part 1/Fig 3b-e TTLL screen/EYFP MB I7.Project Maximum Z_XY1663878776_Z0_T0_C1.tif]

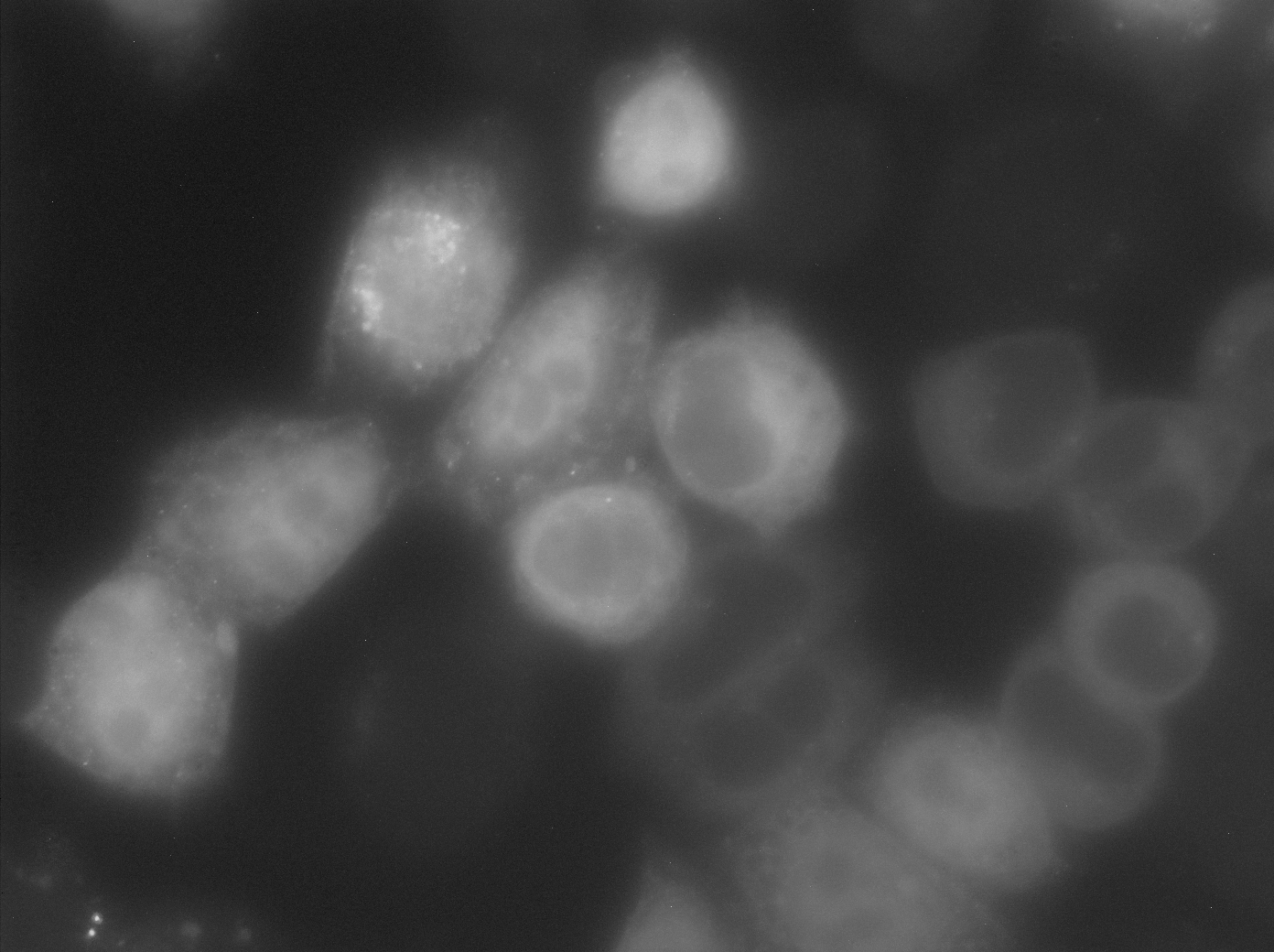

Supplement: Supplementary file 11 — Source data Fig. 3 part 1 [file 44319_2026_742_MOESM11_ESM.zip › Figure 3 Part 1/Fig 3b-e TTLL screen/TTLL1-GFP A3 I3 - 1.Project Maximum Z_XY1679694543_Z0_T0_C1.tif]

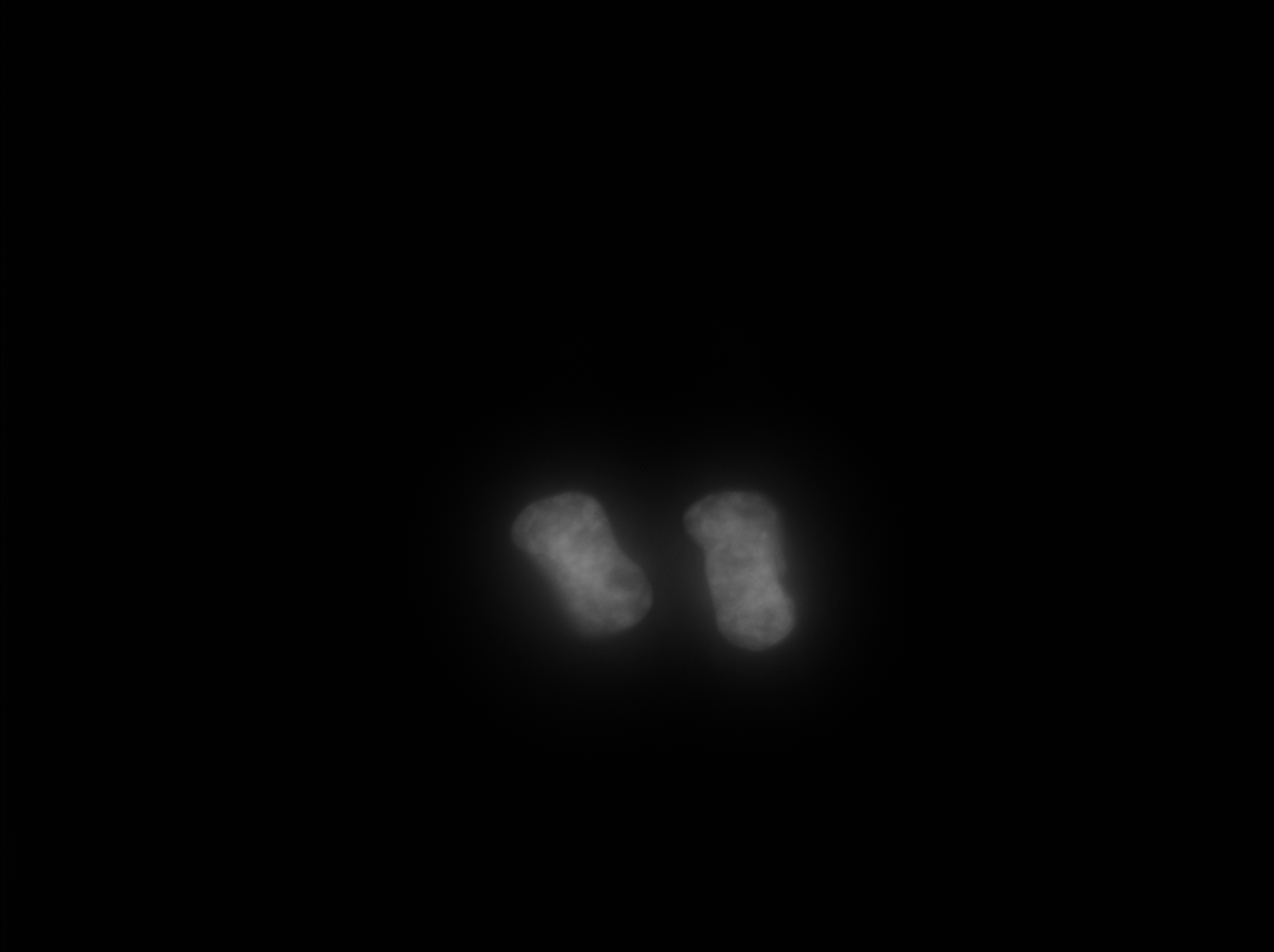

Supplement: Supplementary file 11 — Source data Fig. 3 part 1 [file 44319_2026_742_MOESM11_ESM.zip › Figure 3 Part 1/Fig 3b-e TTLL screen/TTLL1-GFP A3 I2.Project Maximum Z_XY1674673788_Z0_T0_C0.tif]

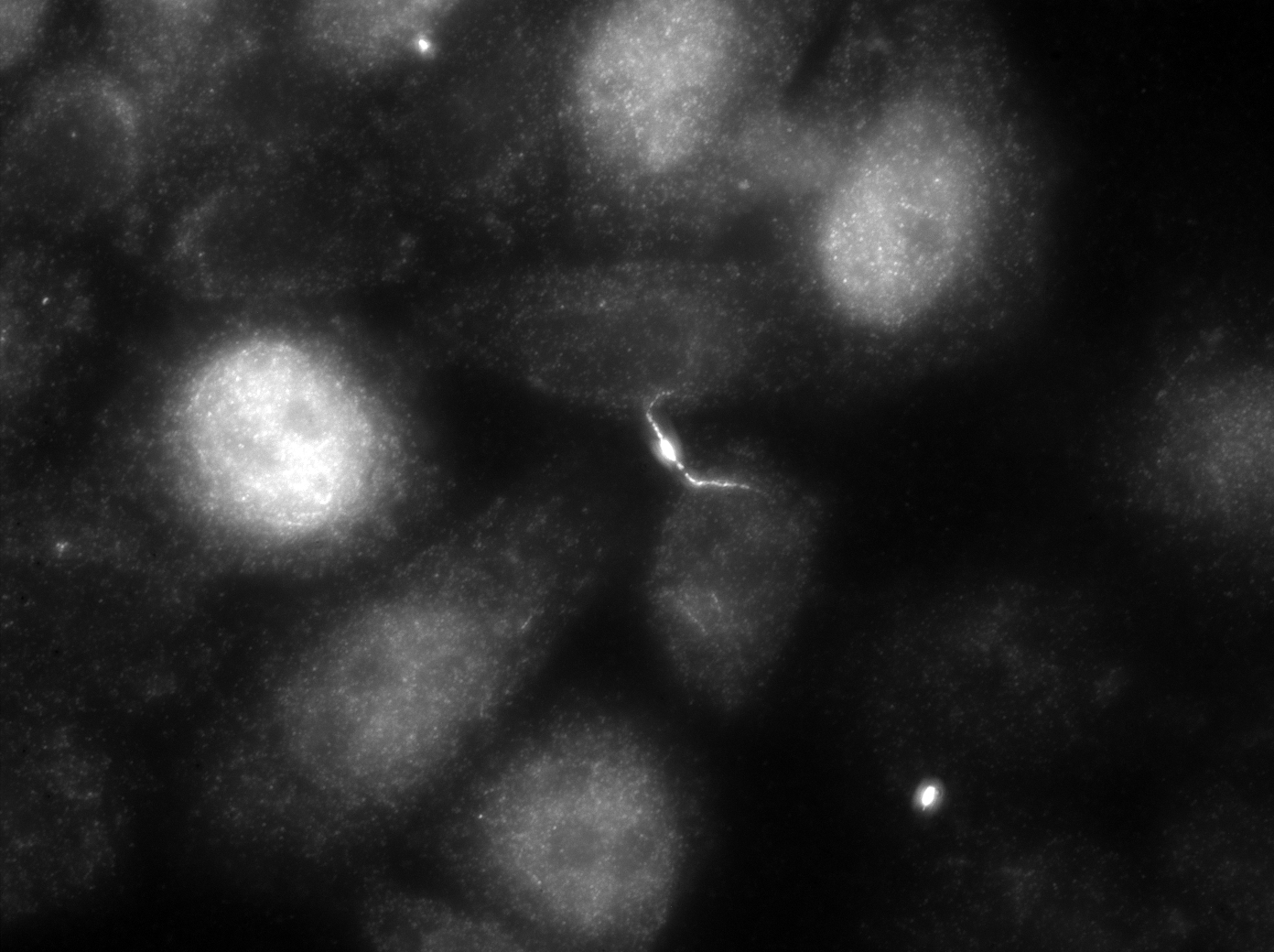

Supplement: Supplementary file 11 — Source data Fig. 3 part 1 [file 44319_2026_742_MOESM11_ESM.zip › Figure 3 Part 1/Fig 3b-e TTLL screen/TTLL1-GFP A3 I12.Project Maximum Z_XY1679695977_Z0_T0_C2.tif]

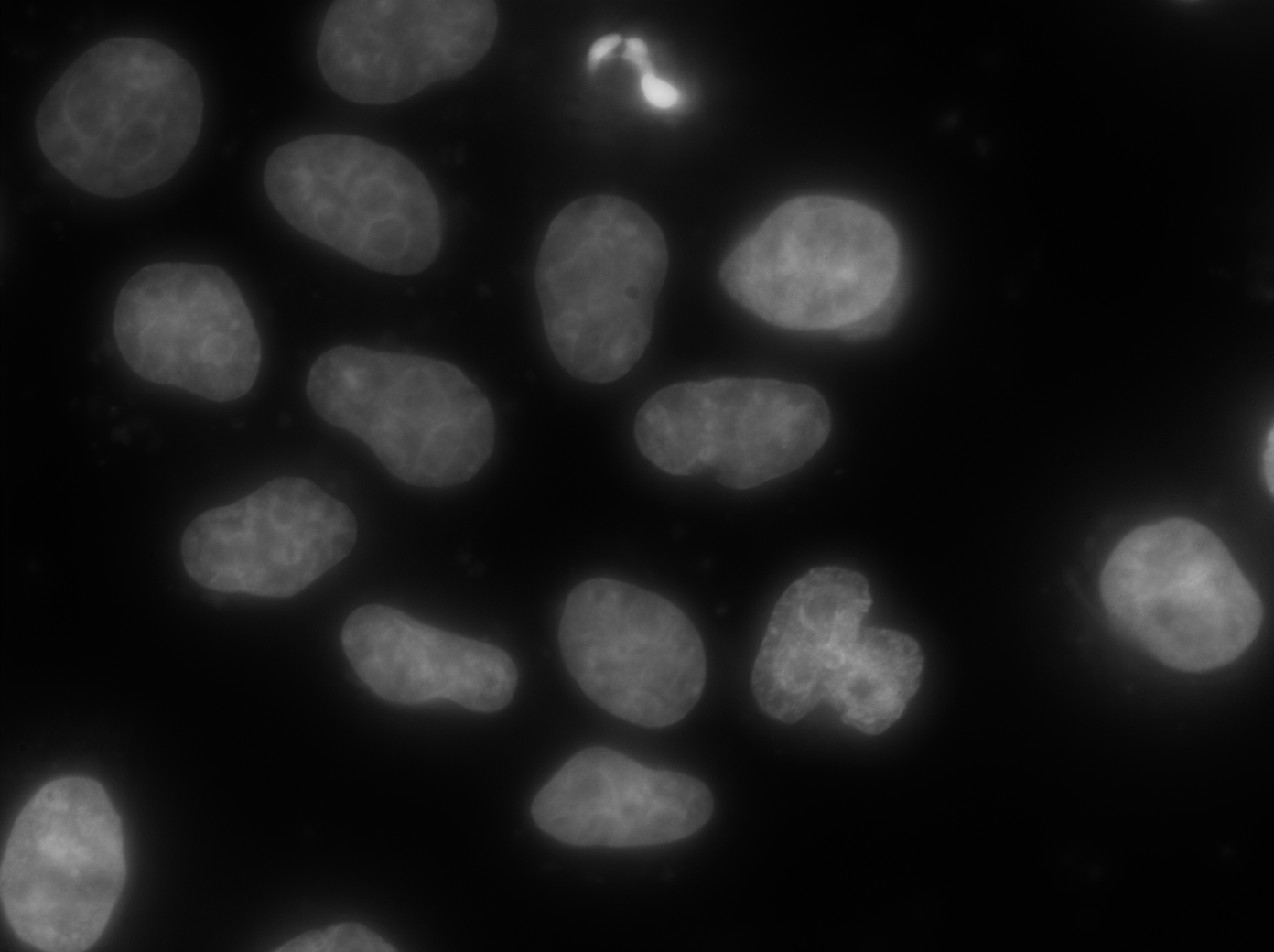

Supplement: Supplementary file 11 — Source data Fig. 3 part 1 [file 44319_2026_742_MOESM11_ESM.zip › Figure 3 Part 1/Fig 3b-e TTLL screen/TTLL4-YFPy I1.Project Maximum Z_XY1679075087_Z0_T0_C0.tif]

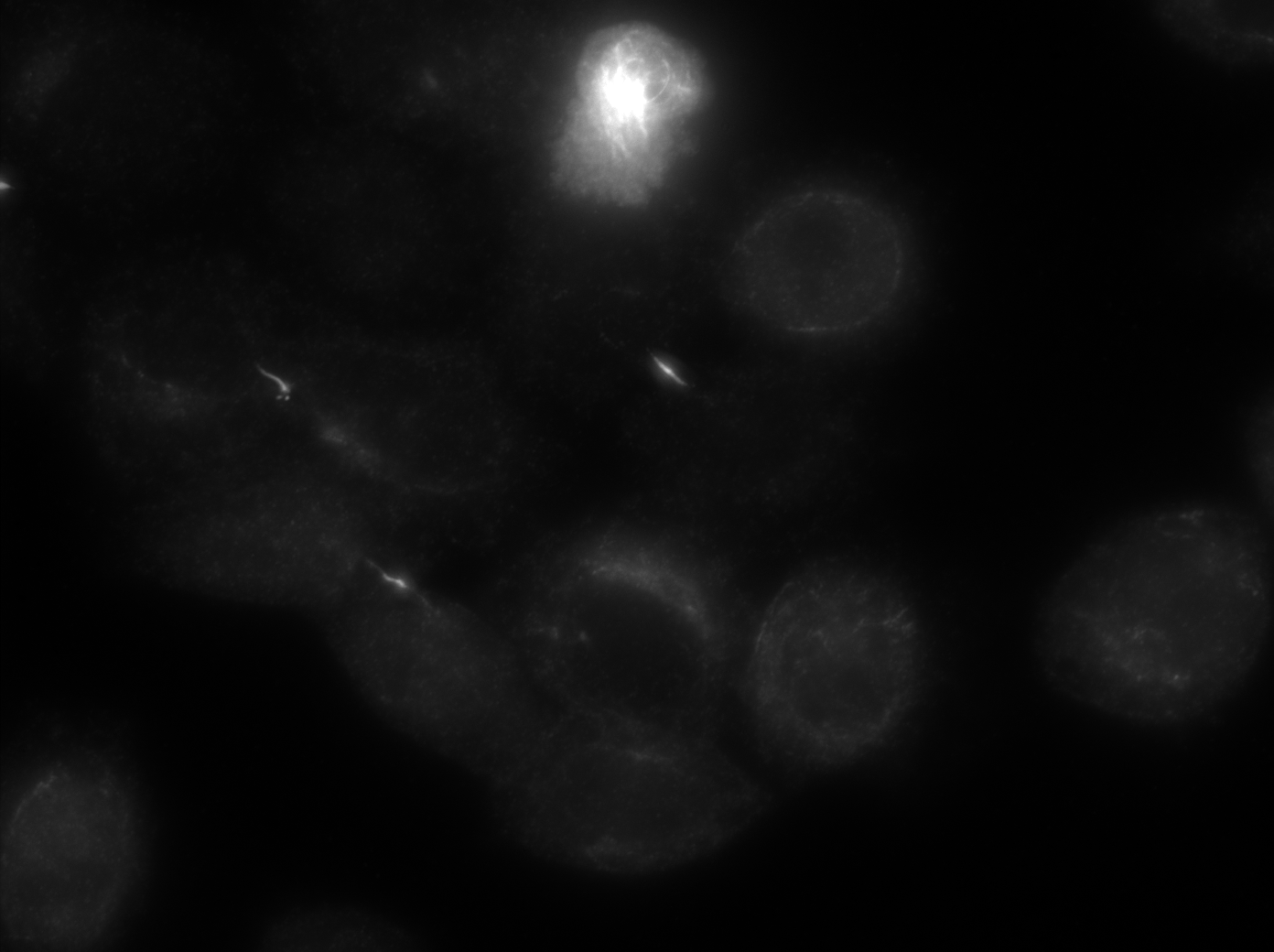

Supplement: Supplementary file 11 — Source data Fig. 3 part 1 [file 44319_2026_742_MOESM11_ESM.zip › Figure 3 Part 1/Fig 3b-e TTLL screen/TTLL4-YFPy I1.Project Maximum Z_XY1679075087_Z0_T0_C2.tif]

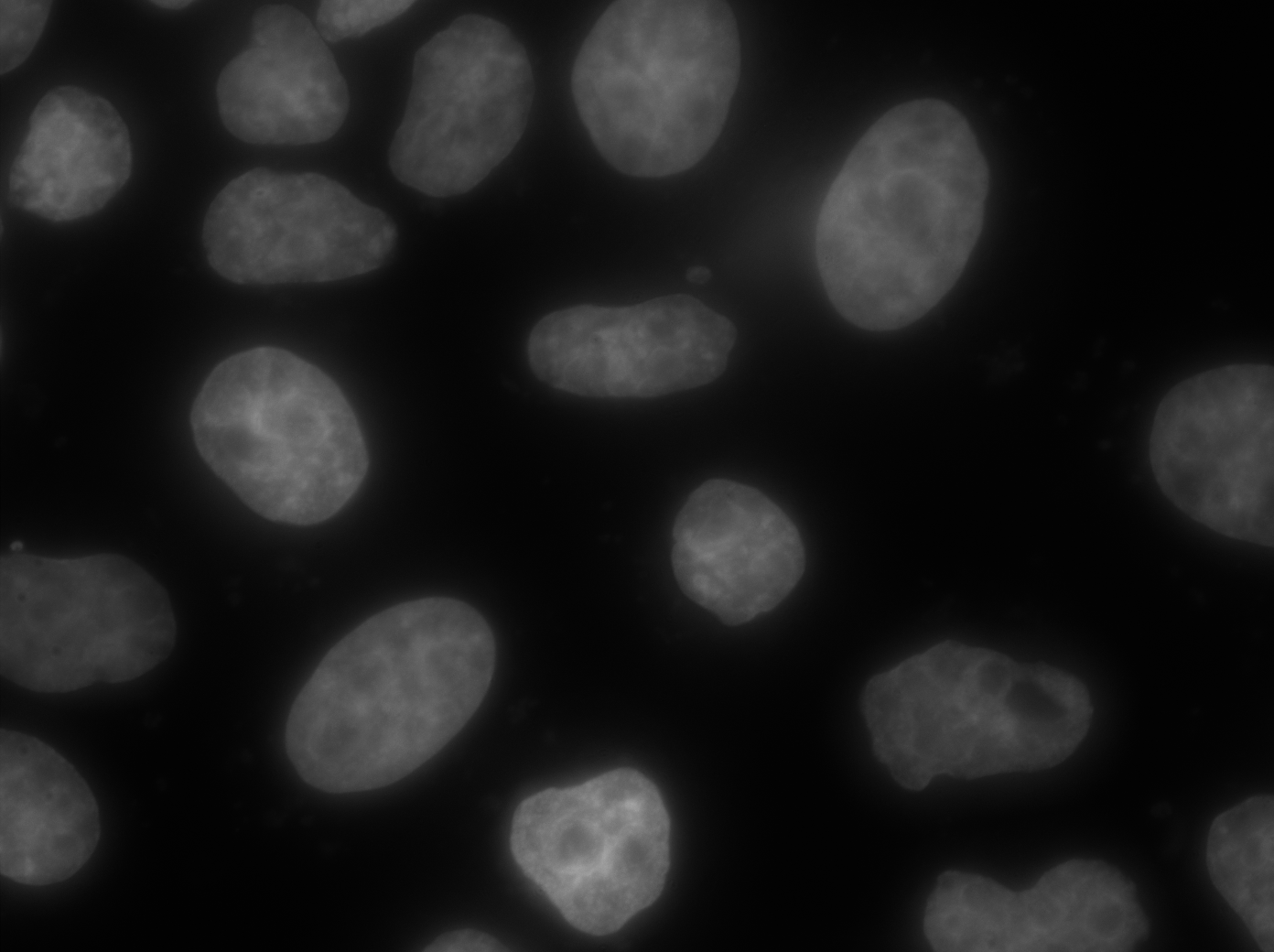

Supplement: Supplementary file 11 — Source data Fig. 3 part 1 [file 44319_2026_742_MOESM11_ESM.zip › Figure 3 Part 1/Fig 3b-e TTLL screen/TTLL1-GFP A3 I12.Project Maximum Z_XY1679695977_Z0_T0_C0.tif]

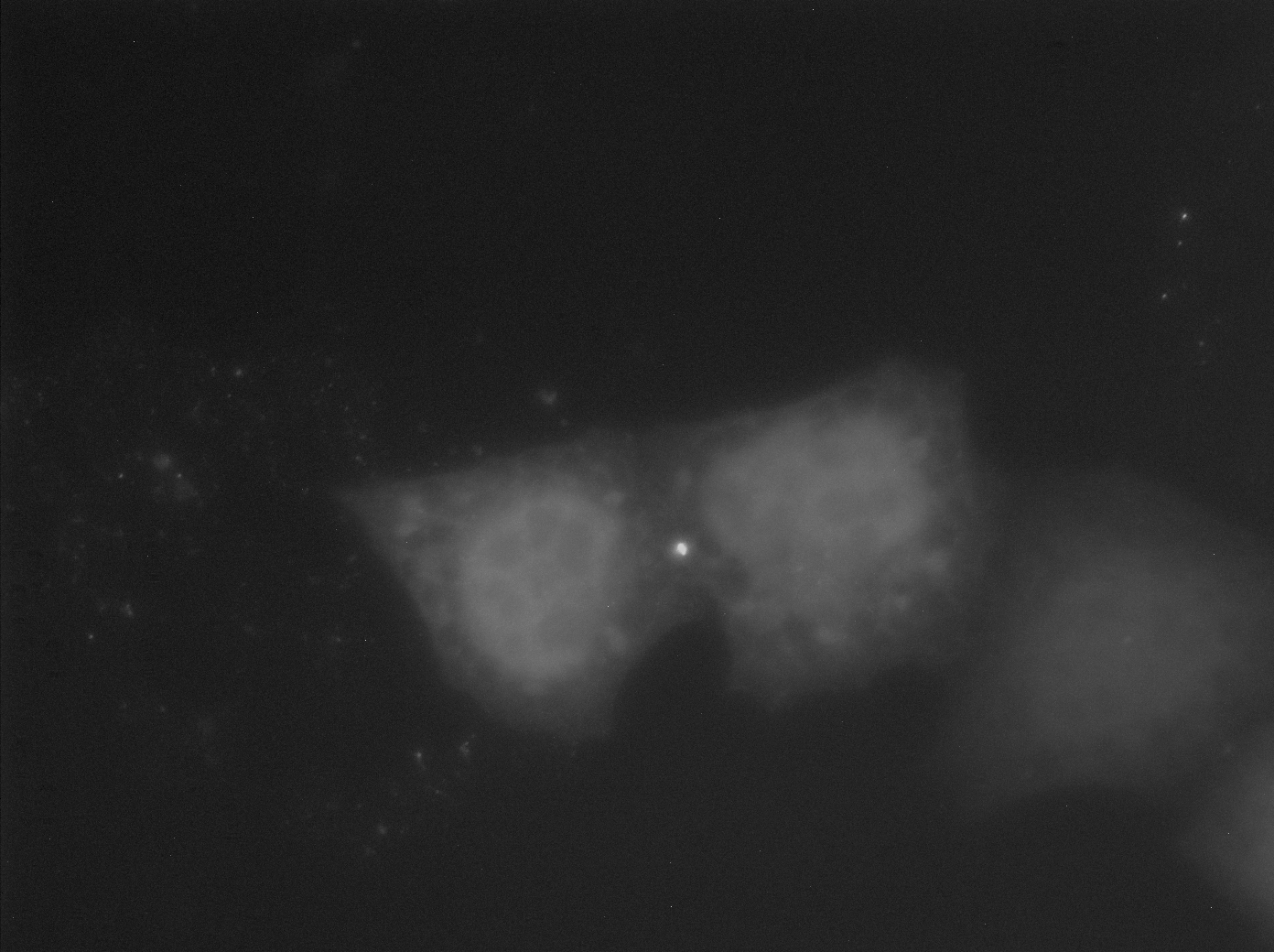

Supplement: Supplementary file 11 — Source data Fig. 3 part 1 [file 44319_2026_742_MOESM11_ESM.zip › Figure 3 Part 1/Fig 3b-e TTLL screen/TTLL1-GFP A3 I10.Project Maximum Z_XY1679695716_Z0_T0_C1.tif]

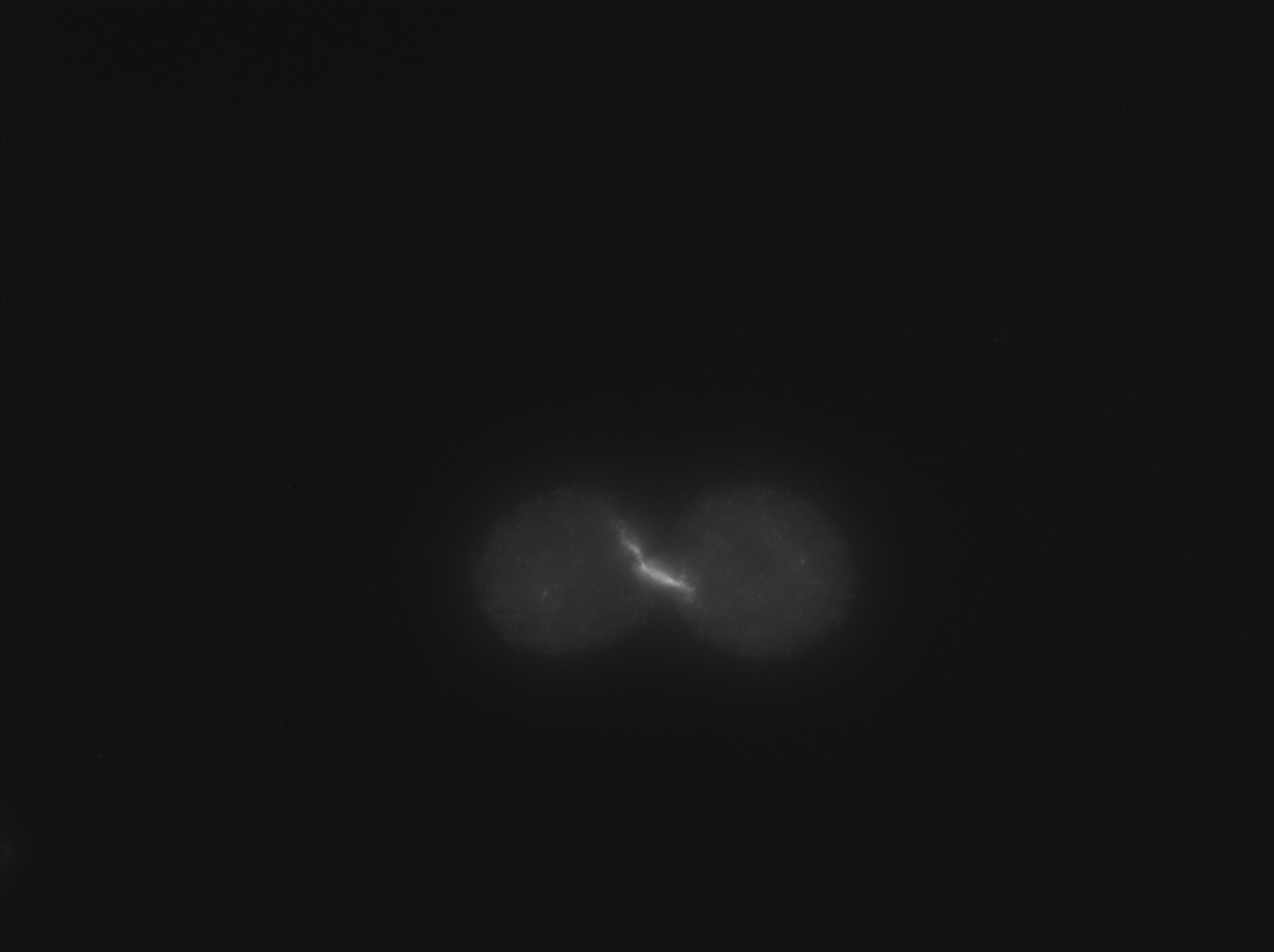

Supplement: Supplementary file 11 — Source data Fig. 3 part 1 [file 44319_2026_742_MOESM11_ESM.zip › Figure 3 Part 1/Fig 3b-e TTLL screen/TTLL1-GFP A3 I2.Project Maximum Z_XY1674673788_Z0_T0_C2.tif]

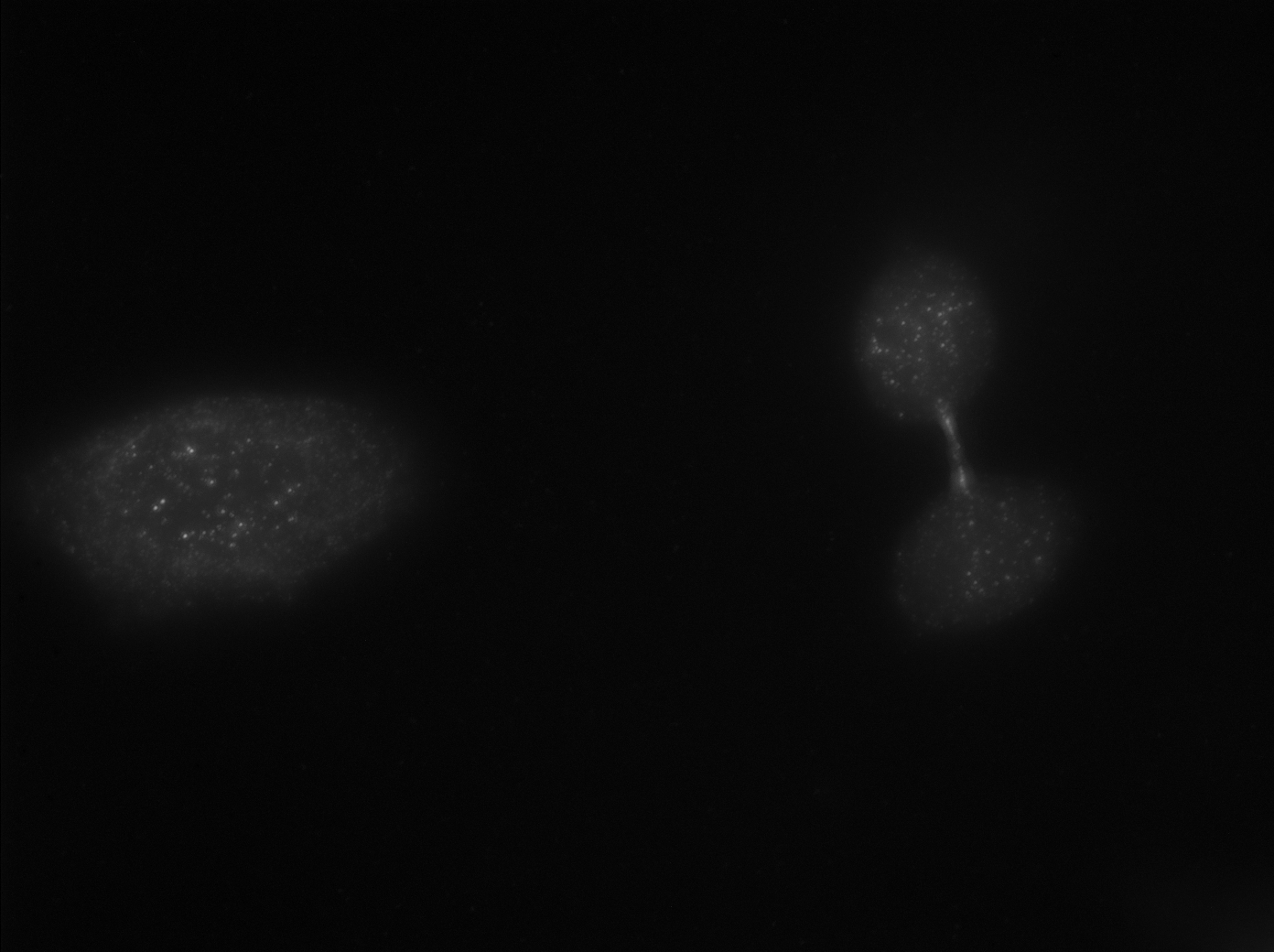

Supplement: Supplementary file 11 — Source data Fig. 3 part 1 [file 44319_2026_742_MOESM11_ESM.zip › Figure 3 Part 1/Fig 3b-e TTLL screen/TTLL1-GFP R1 I2.Project Maximum Z_XY1674162792_Z0_T0_C2.tif]

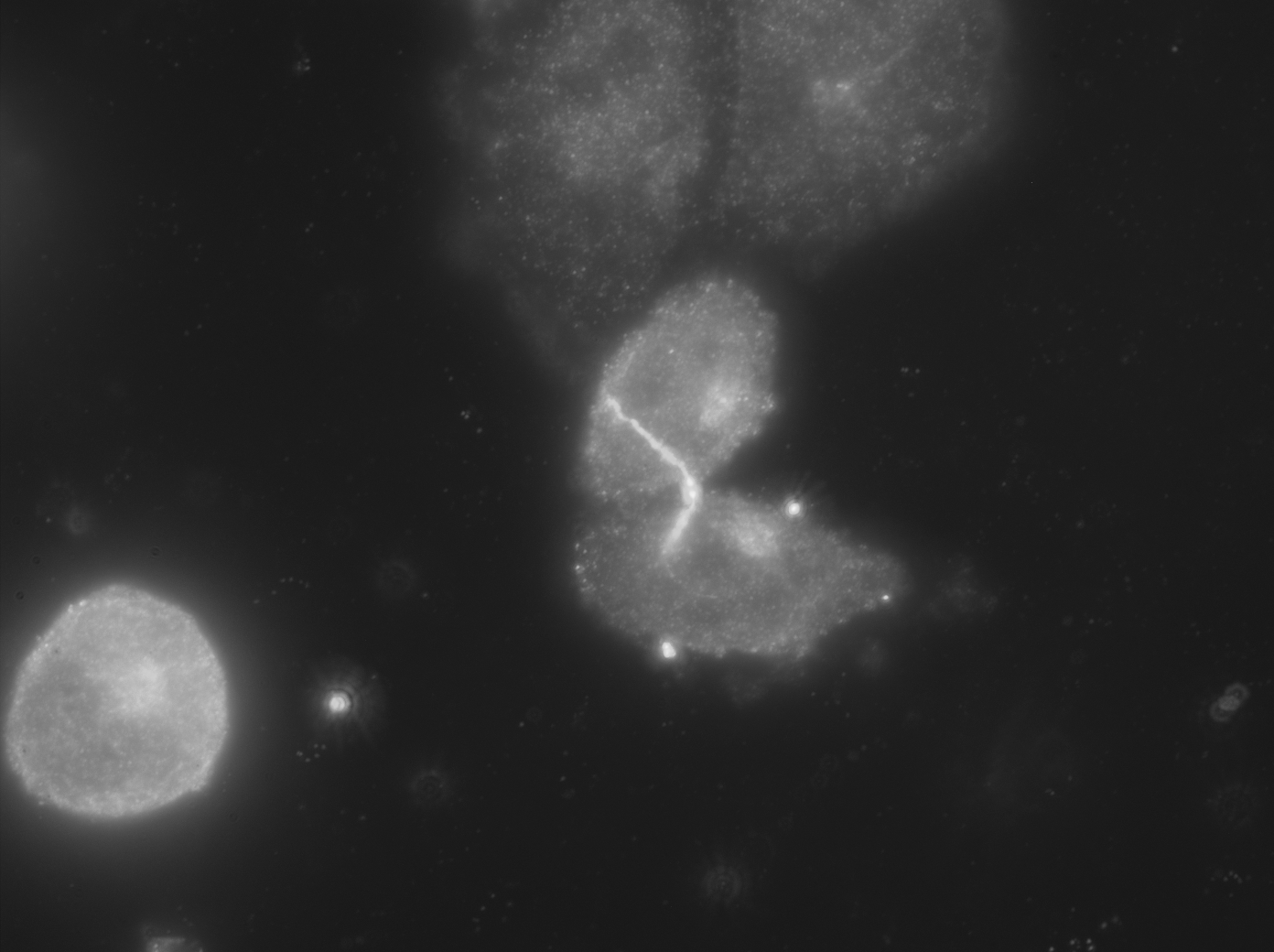

Supplement: Supplementary file 11 — Source data Fig. 3 part 1 [file 44319_2026_742_MOESM11_ESM.zip › Figure 3 Part 1/Fig 3b-e TTLL screen/TTLL1-GFP A4 I11.Project Maximum Z_XY1675964964_Z0_T0_C2.tif]

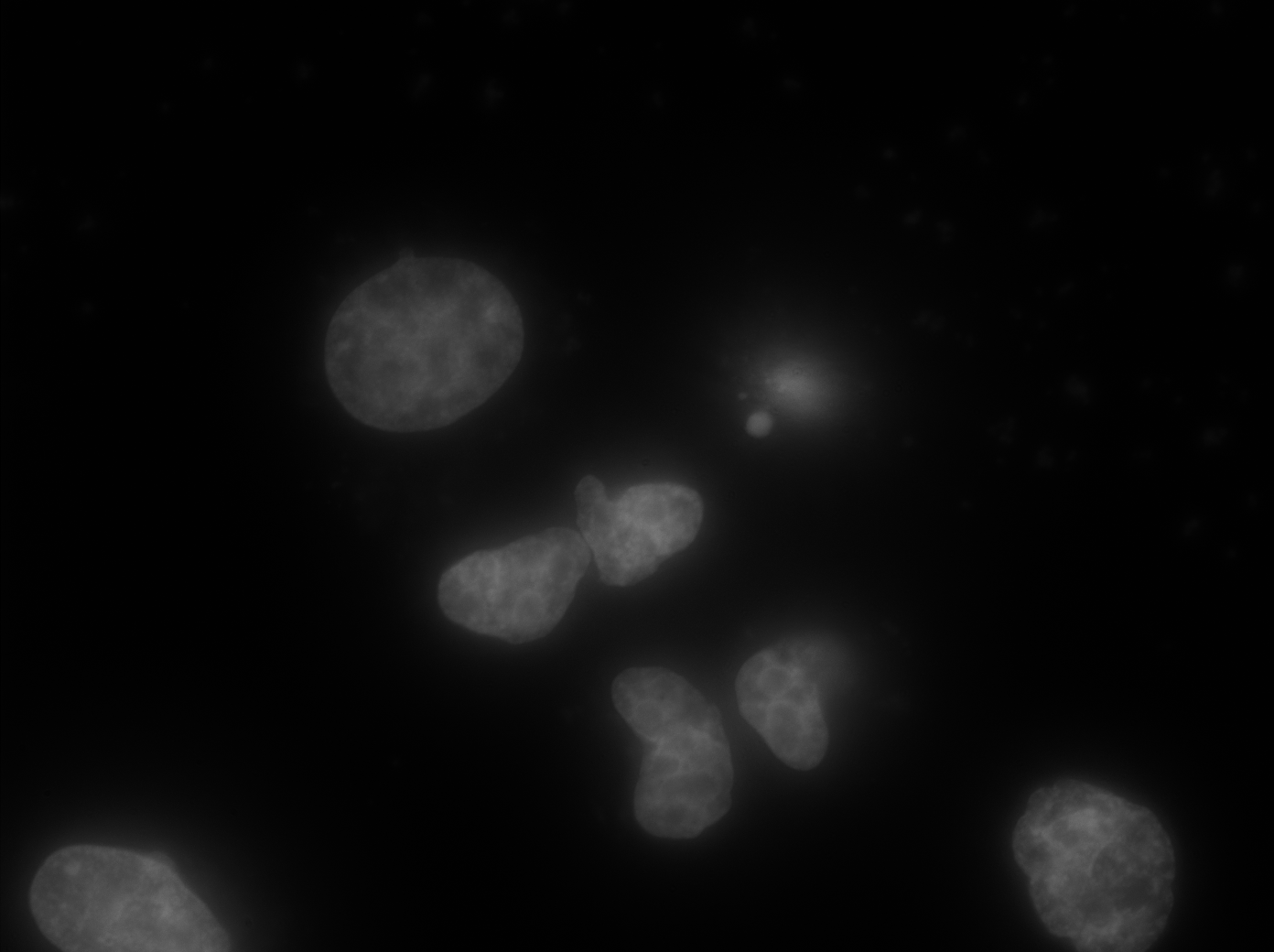

Supplement: Supplementary file 11 — Source data Fig. 3 part 1 [file 44319_2026_742_MOESM11_ESM.zip › Figure 3 Part 1/Fig 3b-e TTLL screen/TTLL1-GFPy I3.Project Maximum Z_XY1679086703_Z0_T0_C0.tif]

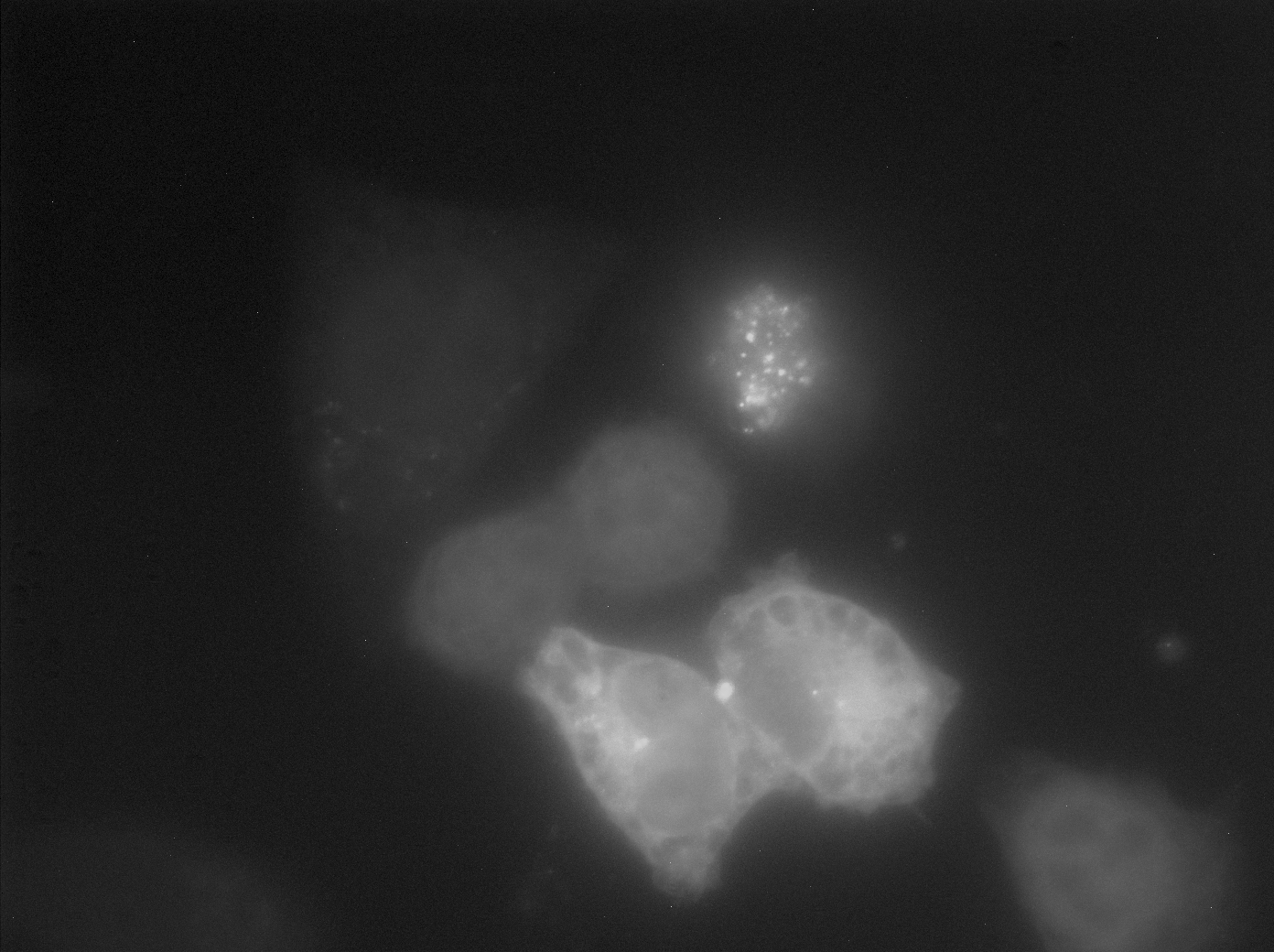

Supplement: Supplementary file 11 — Source data Fig. 3 part 1 [file 44319_2026_742_MOESM11_ESM.zip › Figure 3 Part 1/Fig 3b-e TTLL screen/TTLL1-GFPy I3.Project Maximum Z_XY1679086703_Z0_T0_C1.tif]

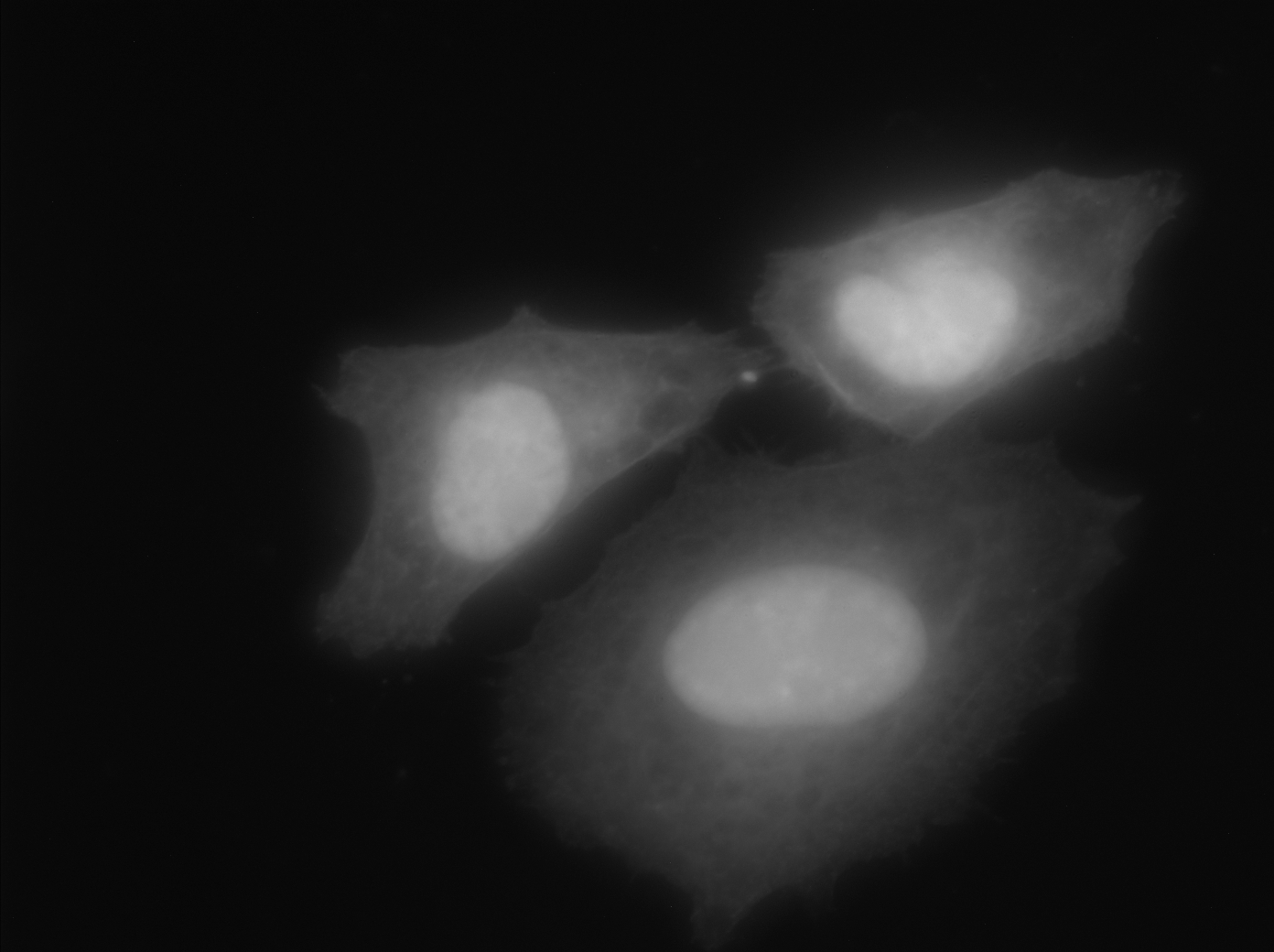

Supplement: Supplementary file 11 — Source data Fig. 3 part 1 [file 44319_2026_742_MOESM11_ESM.zip › Figure 3 Part 1/Fig 3b-e TTLL screen/EYFP MB I7.Project Maximum Z_XY1663878776_Z0_T0_C2.tif]

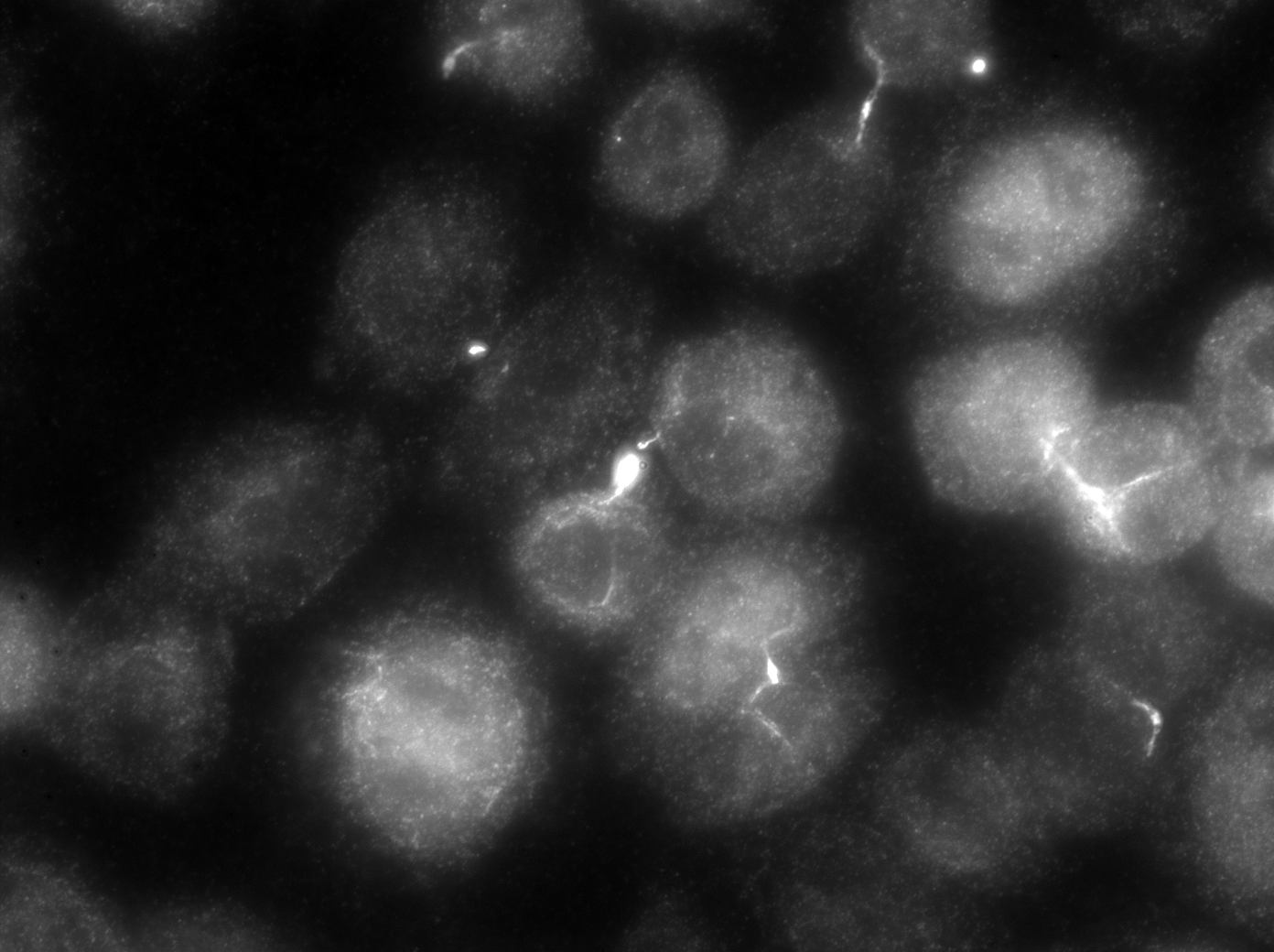

Supplement: Supplementary file 11 — Source data Fig. 3 part 1 [file 44319_2026_742_MOESM11_ESM.zip › Figure 3 Part 1/Fig 3b-e TTLL screen/TTLL1-GFP A3 I3 - 1.Project Maximum Z_XY1679694543_Z0_T0_C2.tif]

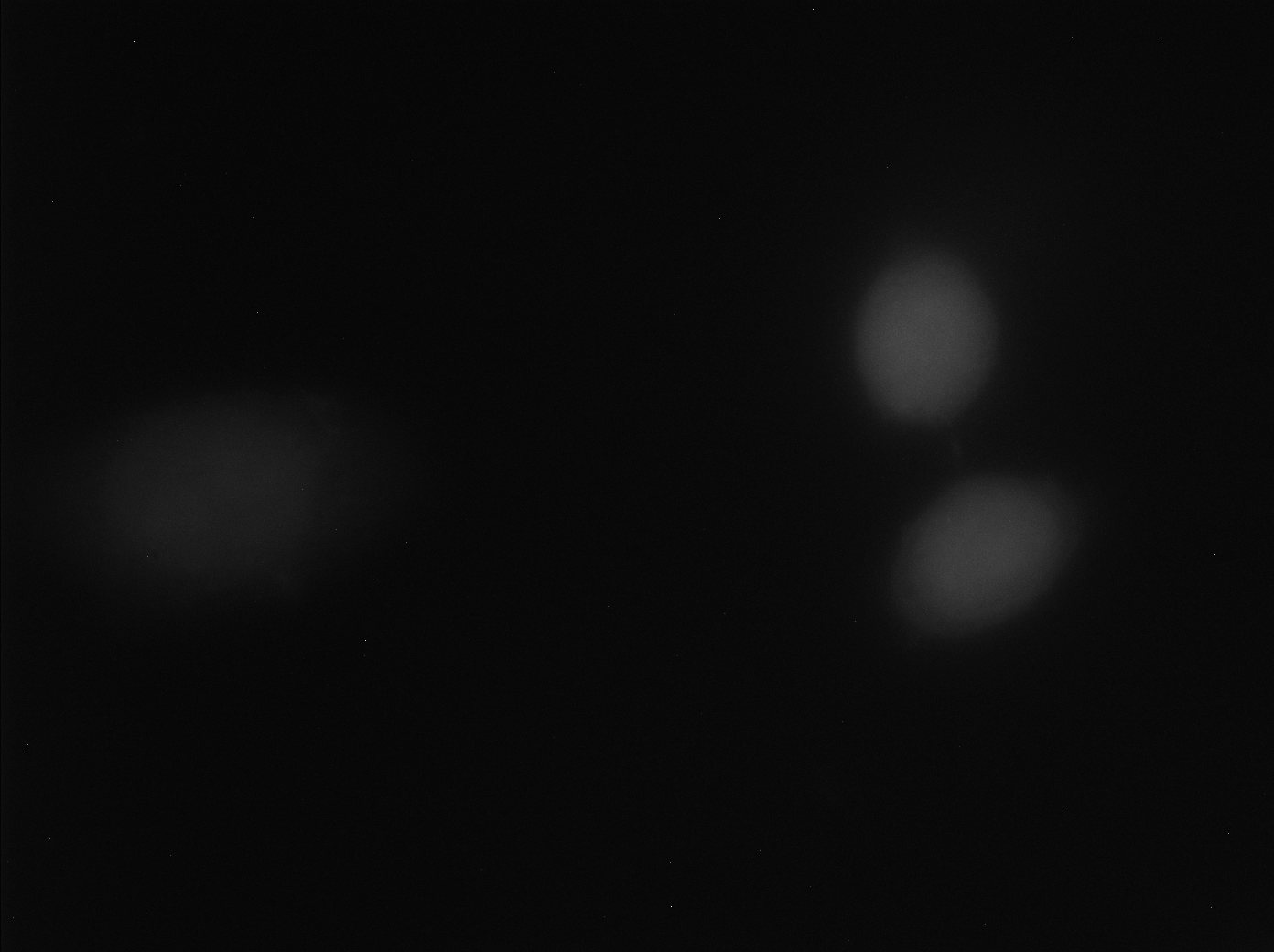

Supplement: Supplementary file 11 — Source data Fig. 3 part 1 [file 44319_2026_742_MOESM11_ESM.zip › Figure 3 Part 1/Fig 3b-e TTLL screen/TTLL1-GFP R1 I2.Project Maximum Z_XY1674162792_Z0_T0_C3.tif]

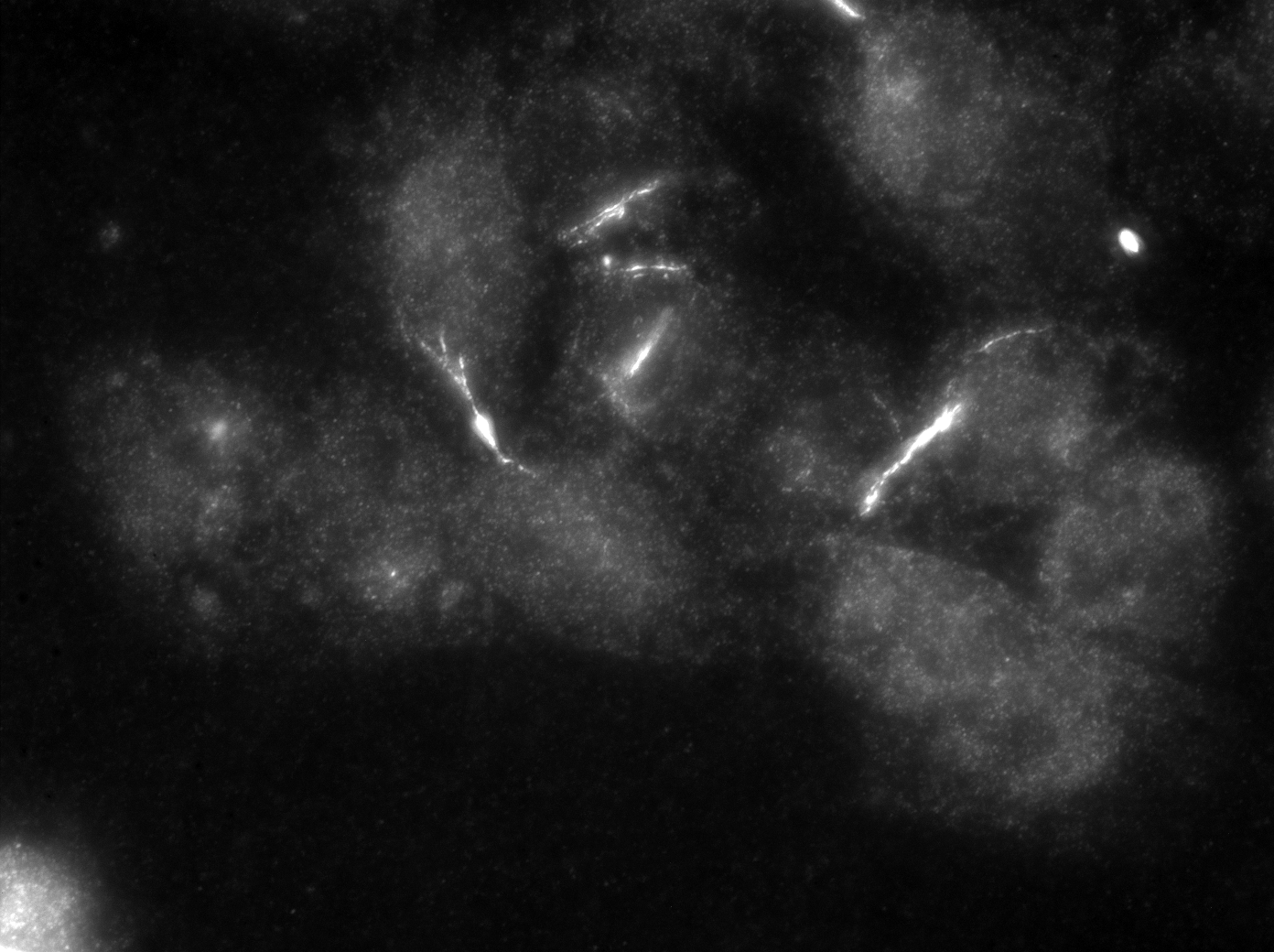

Supplement: Supplementary file 11 — Source data Fig. 3 part 1 [file 44319_2026_742_MOESM11_ESM.zip › Figure 3 Part 1/Fig 3b-e TTLL screen/TTLL1-GFP A3 I19.Project Maximum Z_XY1679698158_Z0_T0_C2.tif]

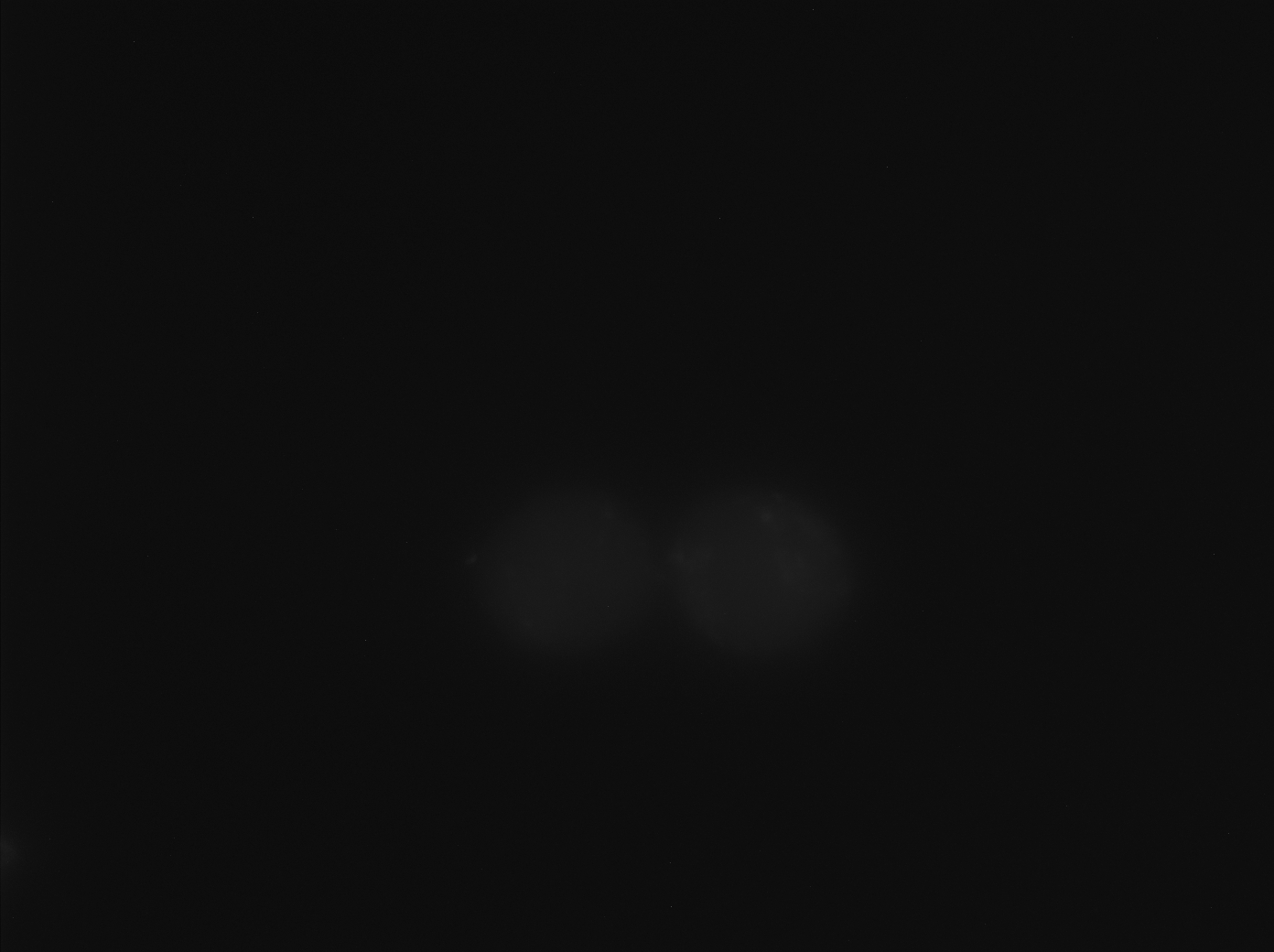

Supplement: Supplementary file 11 — Source data Fig. 3 part 1 [file 44319_2026_742_MOESM11_ESM.zip › Figure 3 Part 1/Fig 3b-e TTLL screen/TTLL1-GFP A3 I2.Project Maximum Z_XY1674673788_Z0_T0_C3.tif]

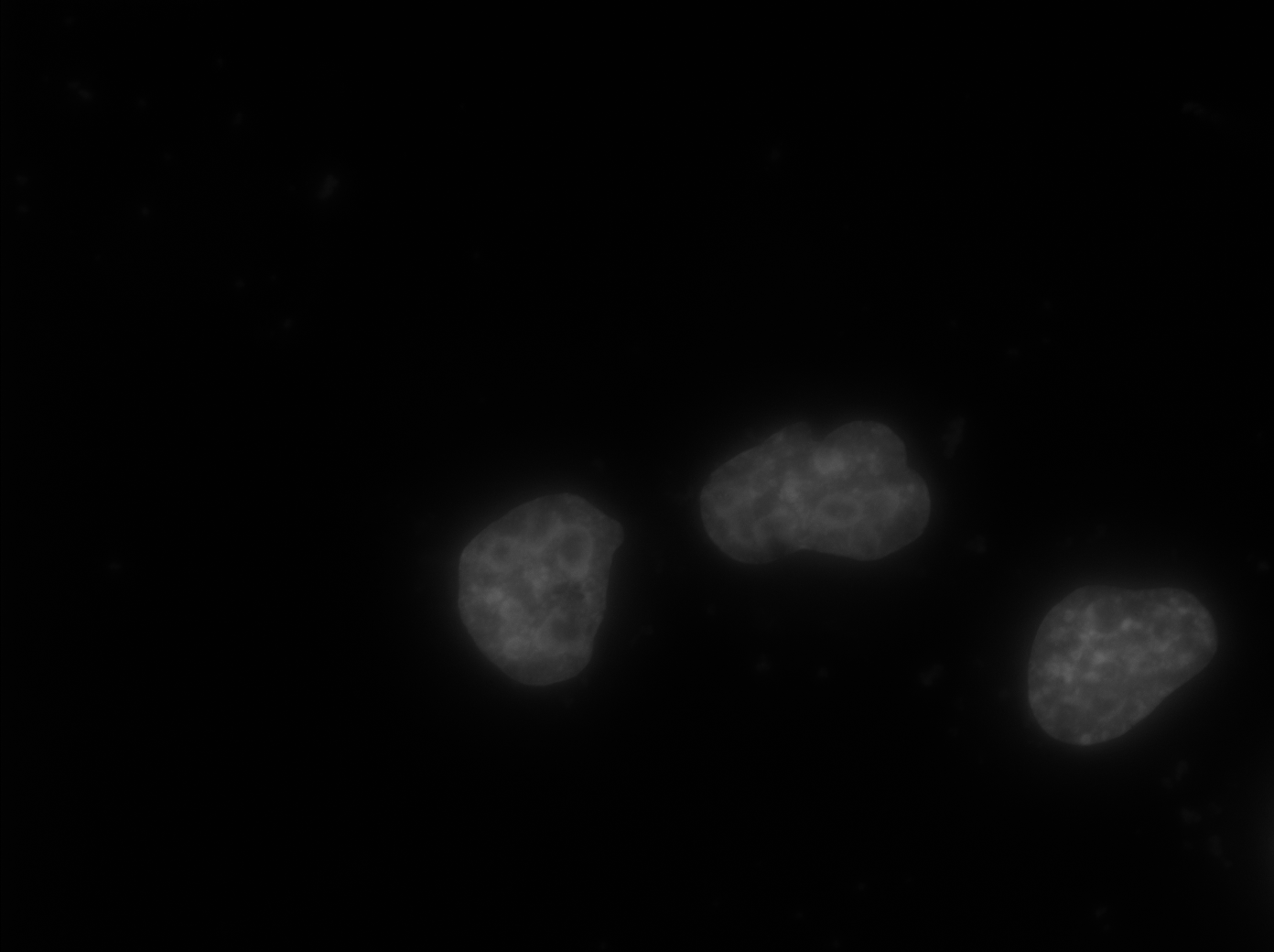

Supplement: Supplementary file 11 — Source data Fig. 3 part 1 [file 44319_2026_742_MOESM11_ESM.zip › Figure 3 Part 1/Fig 3b-e TTLL screen/TTLL1-GFP A3 I10.Project Maximum Z_XY1679695716_Z0_T0_C0.tif]

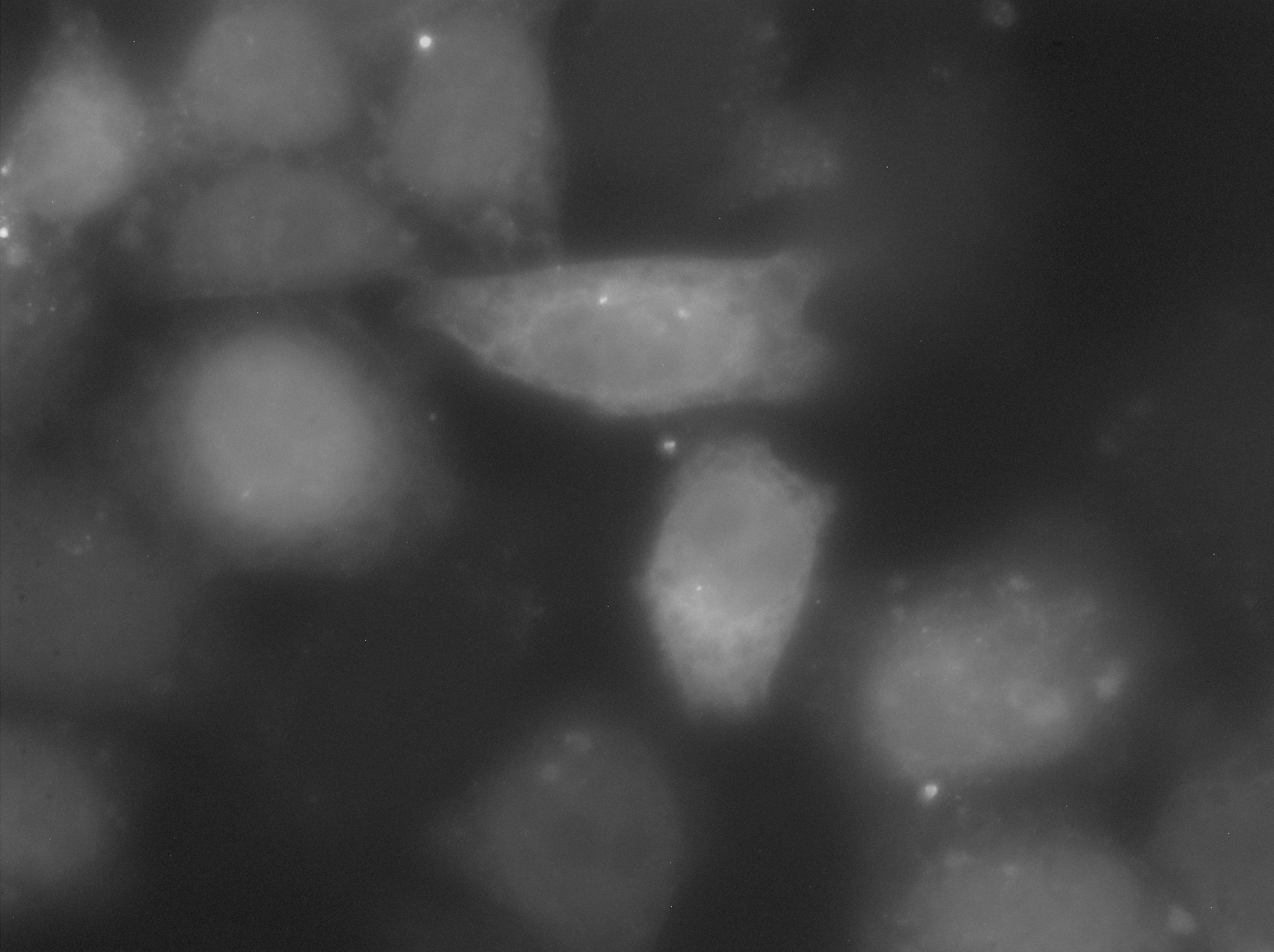

Supplement: Supplementary file 11 — Source data Fig. 3 part 1 [file 44319_2026_742_MOESM11_ESM.zip › Figure 3 Part 1/Fig 3b-e TTLL screen/TTLL1-GFP A3 I12.Project Maximum Z_XY1679695977_Z0_T0_C1.tif]

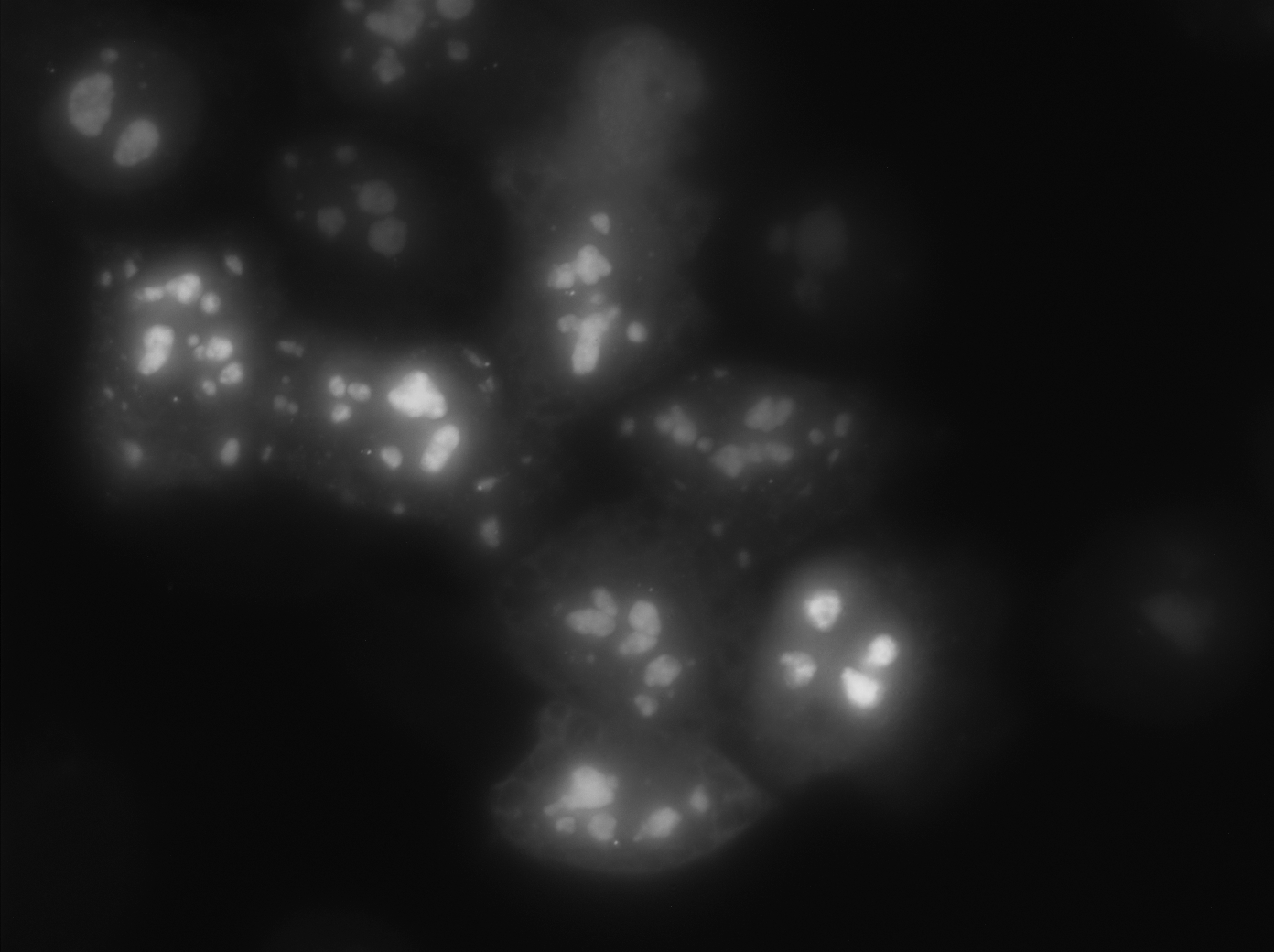

Supplement: Supplementary file 11 — Source data Fig. 3 part 1 [file 44319_2026_742_MOESM11_ESM.zip › Figure 3 Part 1/Fig 3b-e TTLL screen/TTLL4-YFPy I1.Project Maximum Z_XY1679075087_Z0_T0_C3.tif]

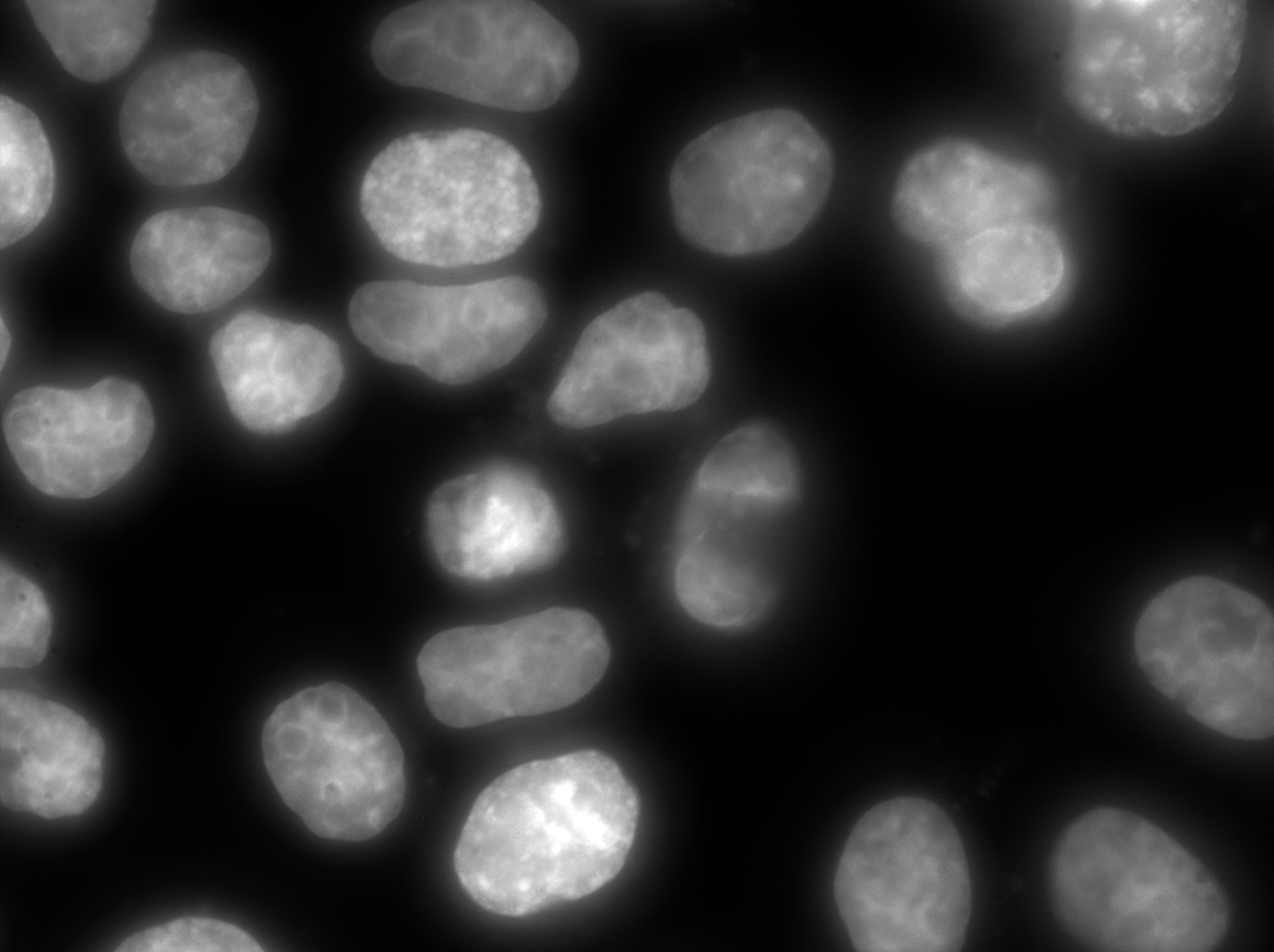

Supplement: Supplementary file 11 — Source data Fig. 3 part 1 [file 44319_2026_742_MOESM11_ESM.zip › Figure 3 Part 1/Fig 3b-e TTLL screen/TTLL1-GFP A3 I15.Project Maximum Z_XY1679697290_Z0_T0_C0.tif]

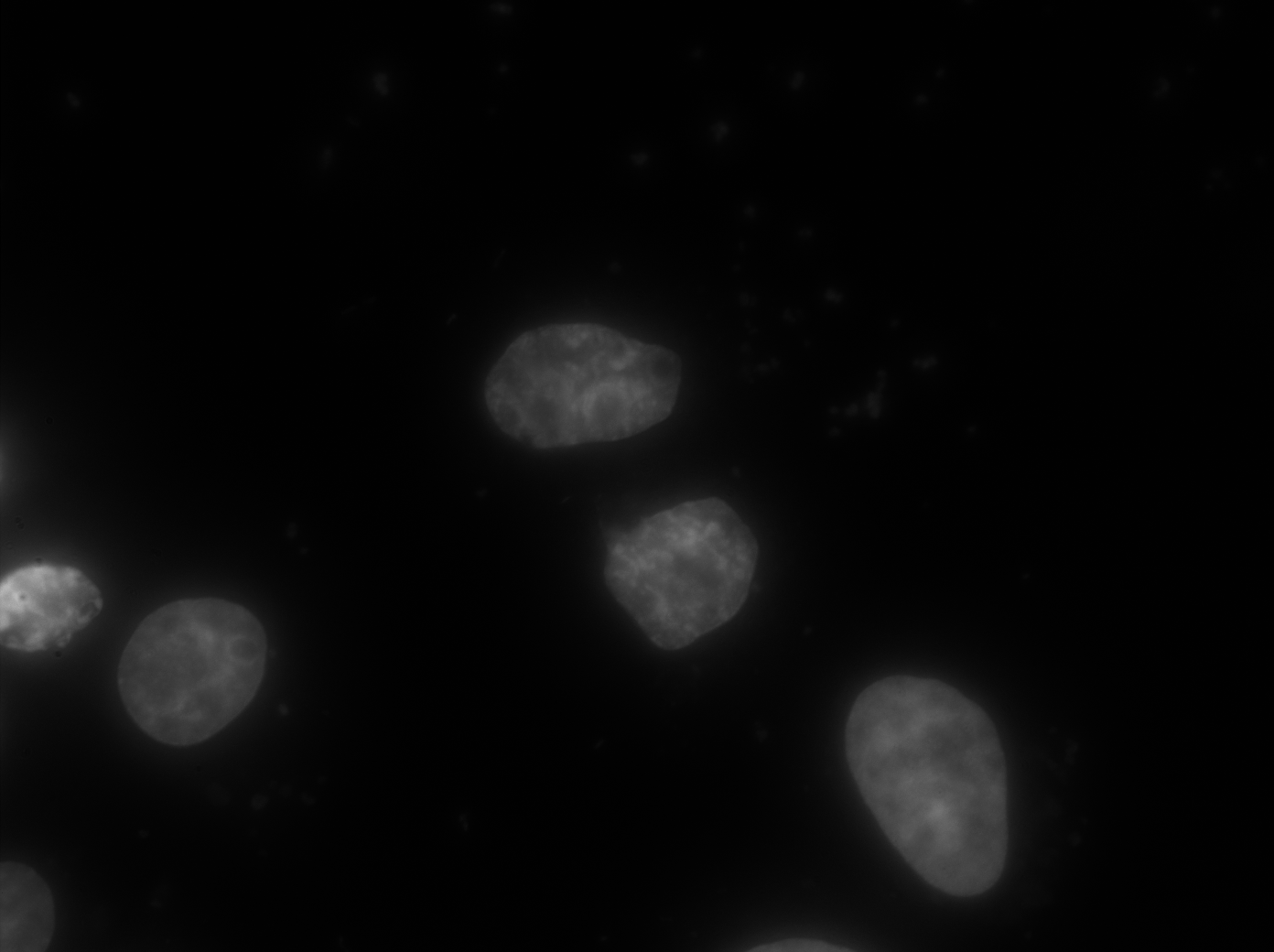

Supplement: Supplementary file 11 — Source data Fig. 3 part 1 [file 44319_2026_742_MOESM11_ESM.zip › Figure 3 Part 1/Fig 3b-e TTLL screen/TTLL1-GFP A4 I7.Project Maximum Z_XY1675962740_Z0_T0_C0.tif]

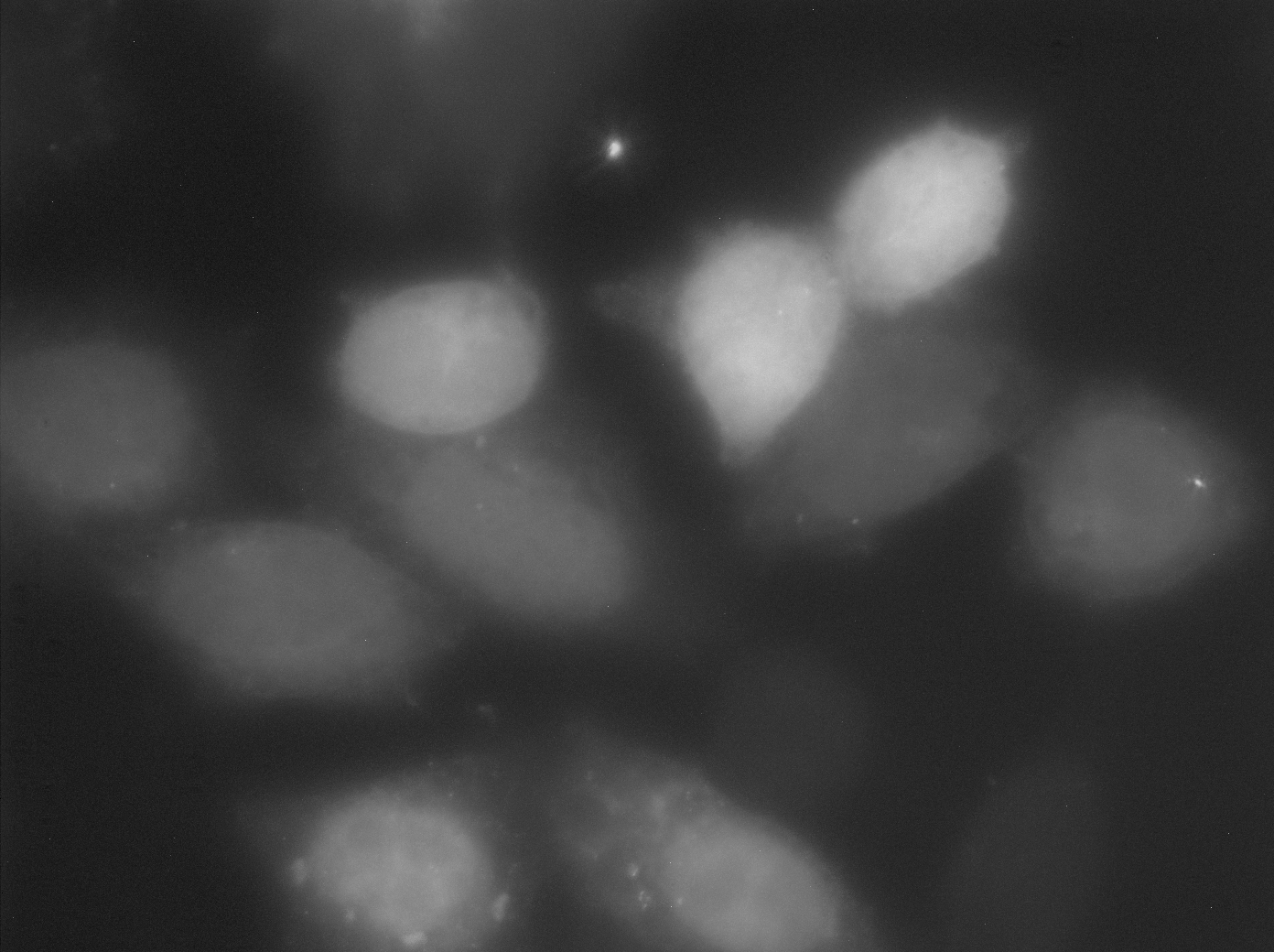

Supplement: Supplementary file 11 — Source data Fig. 3 part 1 [file 44319_2026_742_MOESM11_ESM.zip › Figure 3 Part 1/Fig 3b-e TTLL screen/TTLL1-GFP A3 I5.Project Maximum Z_XY1679694938_Z0_T0_C1.tif]

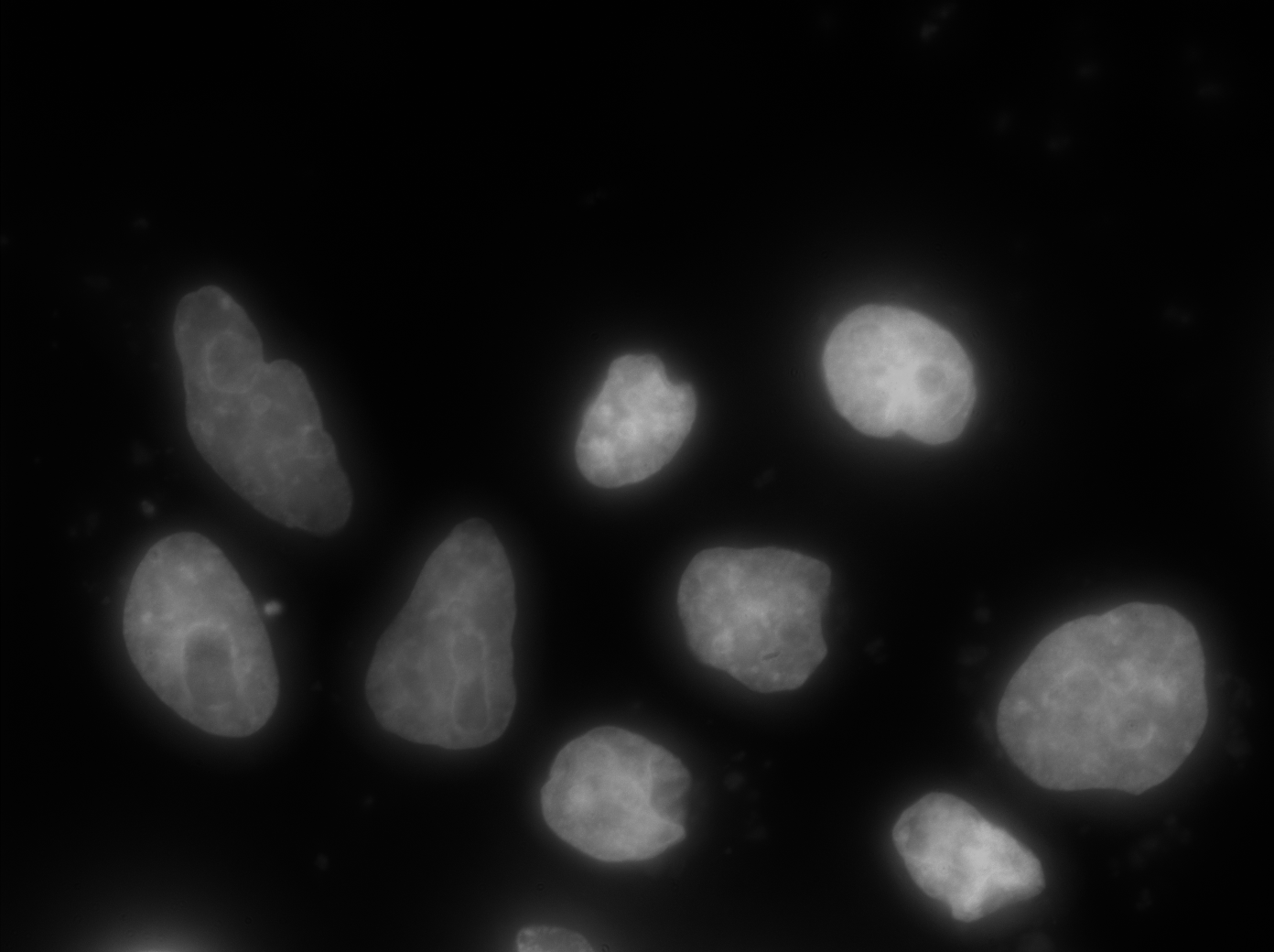

Supplement: Supplementary file 11 — Source data Fig. 3 part 1 [file 44319_2026_742_MOESM11_ESM.zip › Figure 3 Part 1/Fig 3b-e TTLL screen/TTLL4-YFPy I19.Project Maximum Z_XY1679338016_Z0_T0_C0.tif]

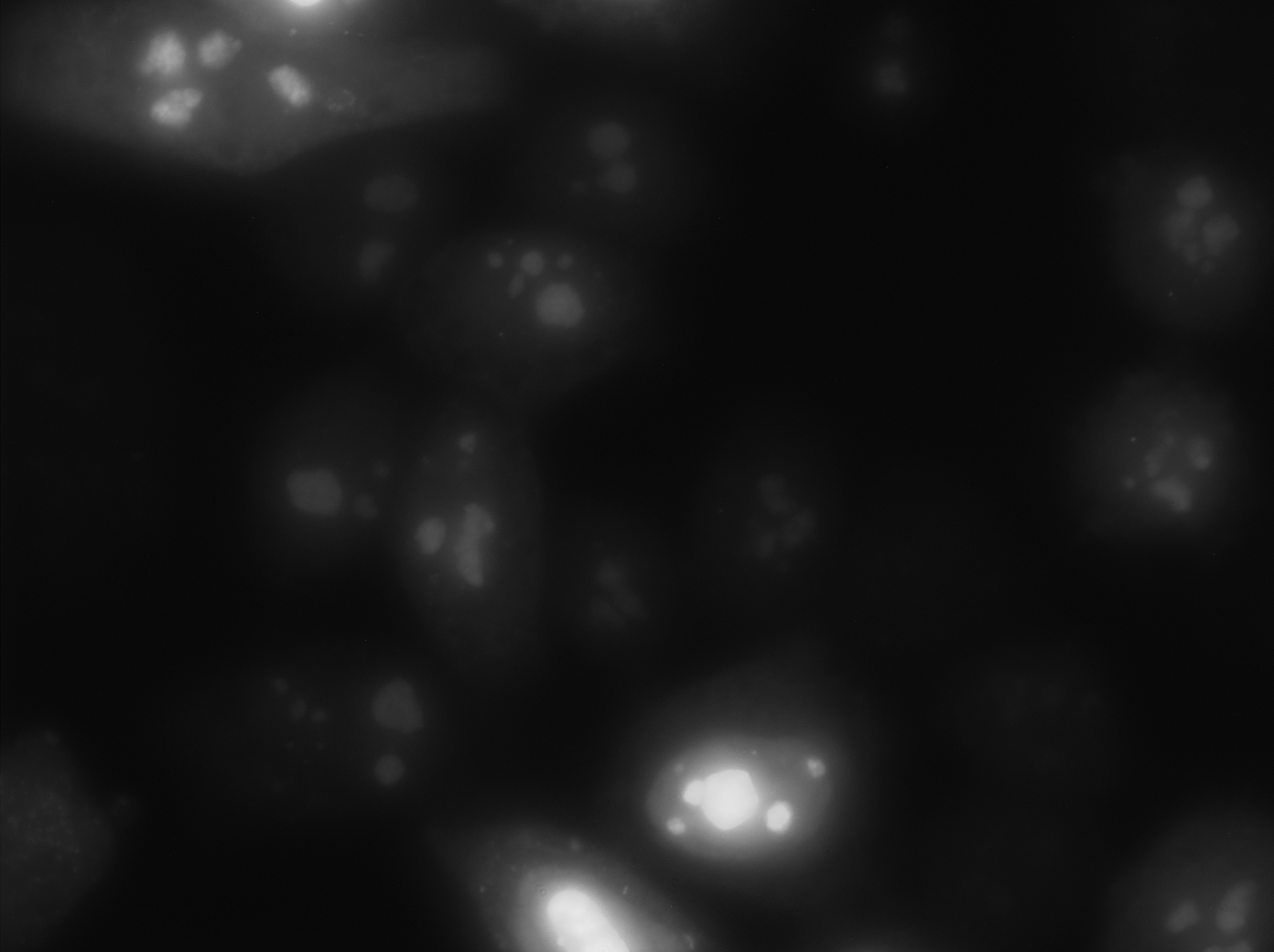

Supplement: Supplementary file 11 — Source data Fig. 3 part 1 [file 44319_2026_742_MOESM11_ESM.zip › Figure 3 Part 1/Fig 3b-e TTLL screen/TTLL4-YFPy I7.Project Maximum Z_XY1679076541_Z0_T0_C2.tif]

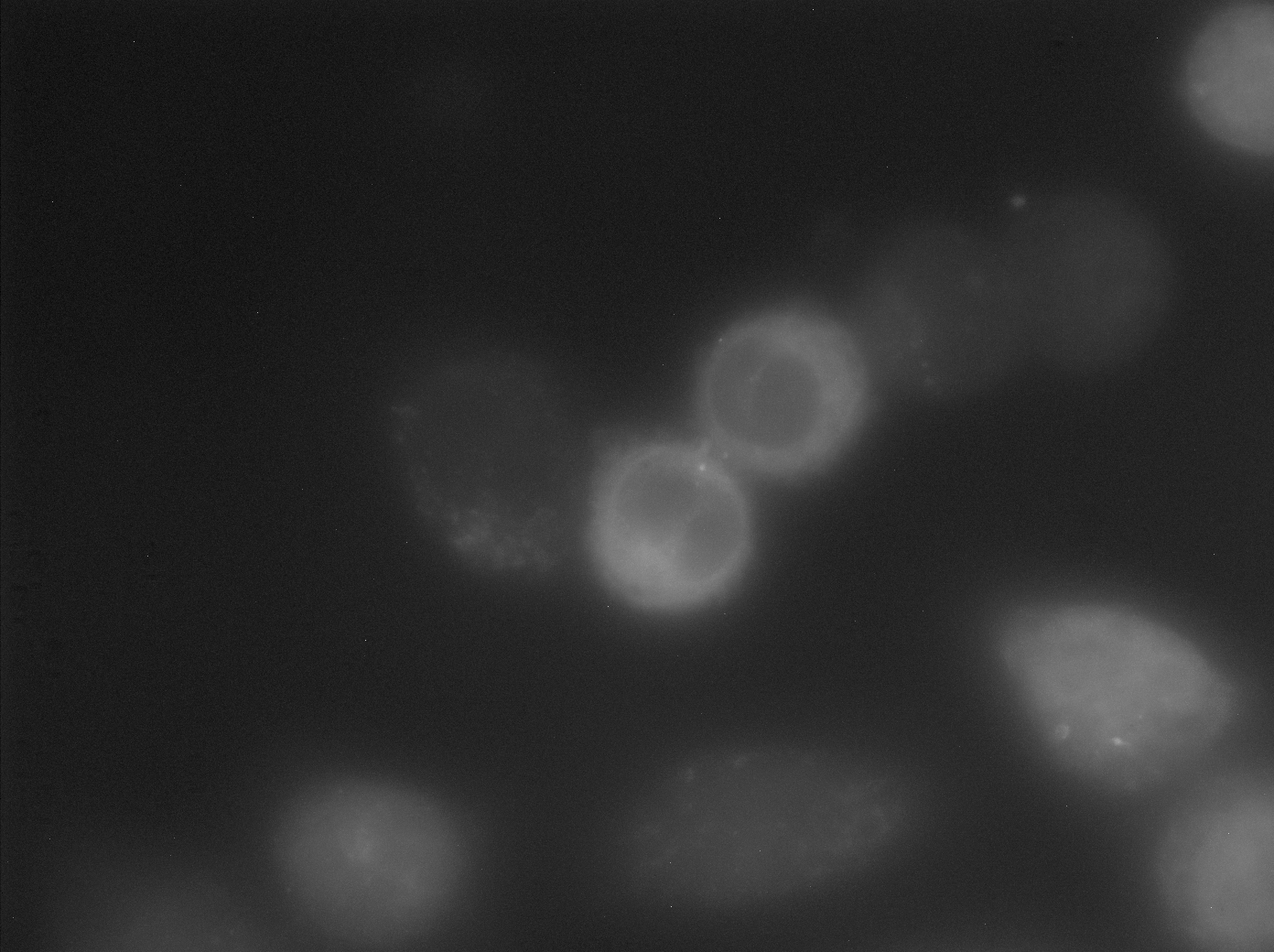

Supplement: Supplementary file 11 — Source data Fig. 3 part 1 [file 44319_2026_742_MOESM11_ESM.zip › Figure 3 Part 1/Fig 3b-e TTLL screen/TTLL1-GFP A3 I18.Project Maximum Z_XY1679697973_Z0_T0_C1.tif]

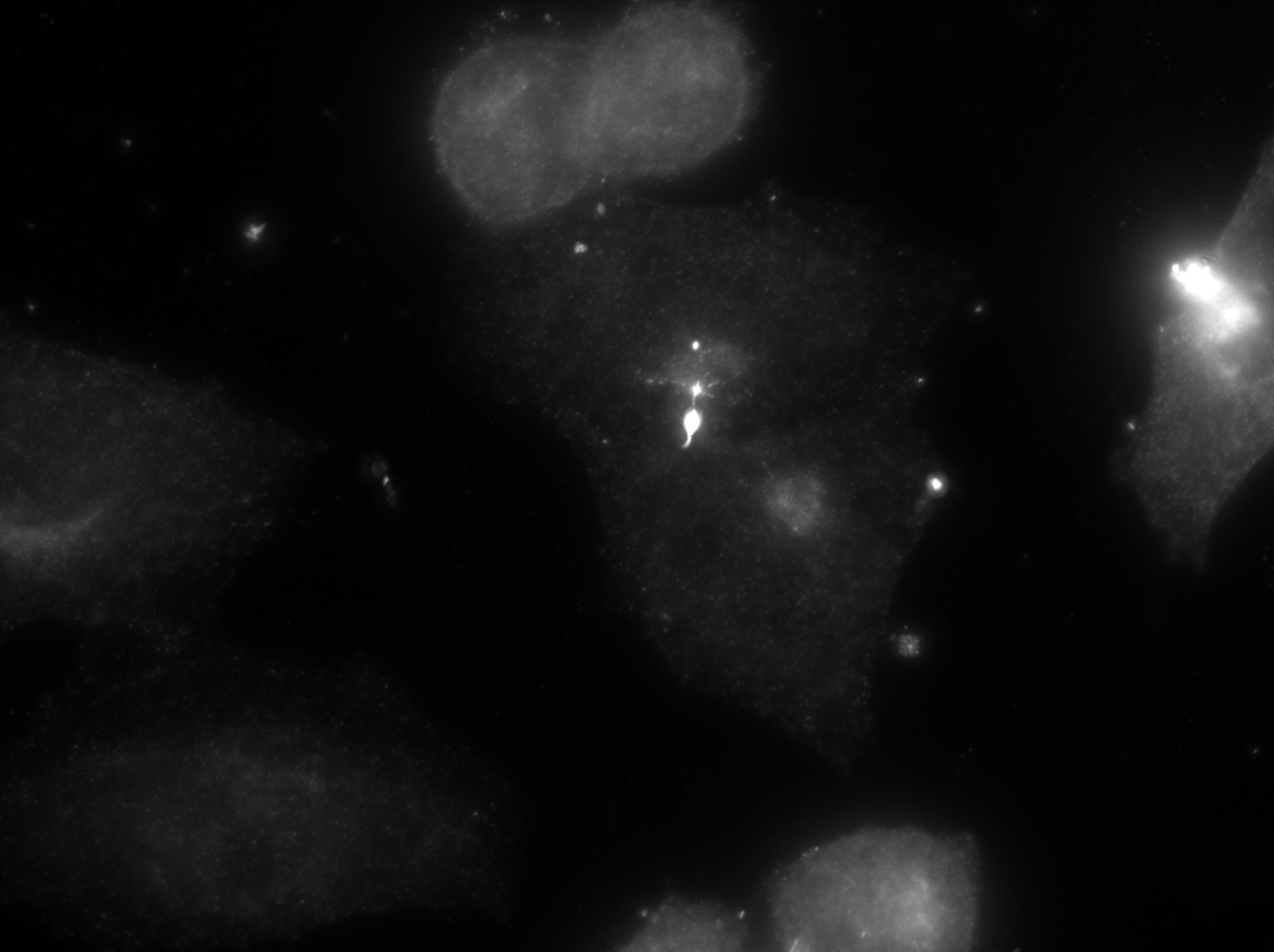

Supplement: Supplementary file 11 — Source data Fig. 3 part 1 [file 44319_2026_742_MOESM11_ESM.zip › Figure 3 Part 1/Fig 3b-e TTLL screen/TTLL1-GFP A4 I6.Project Maximum Z_XY1675962442_Z0_T0_C2.tif]

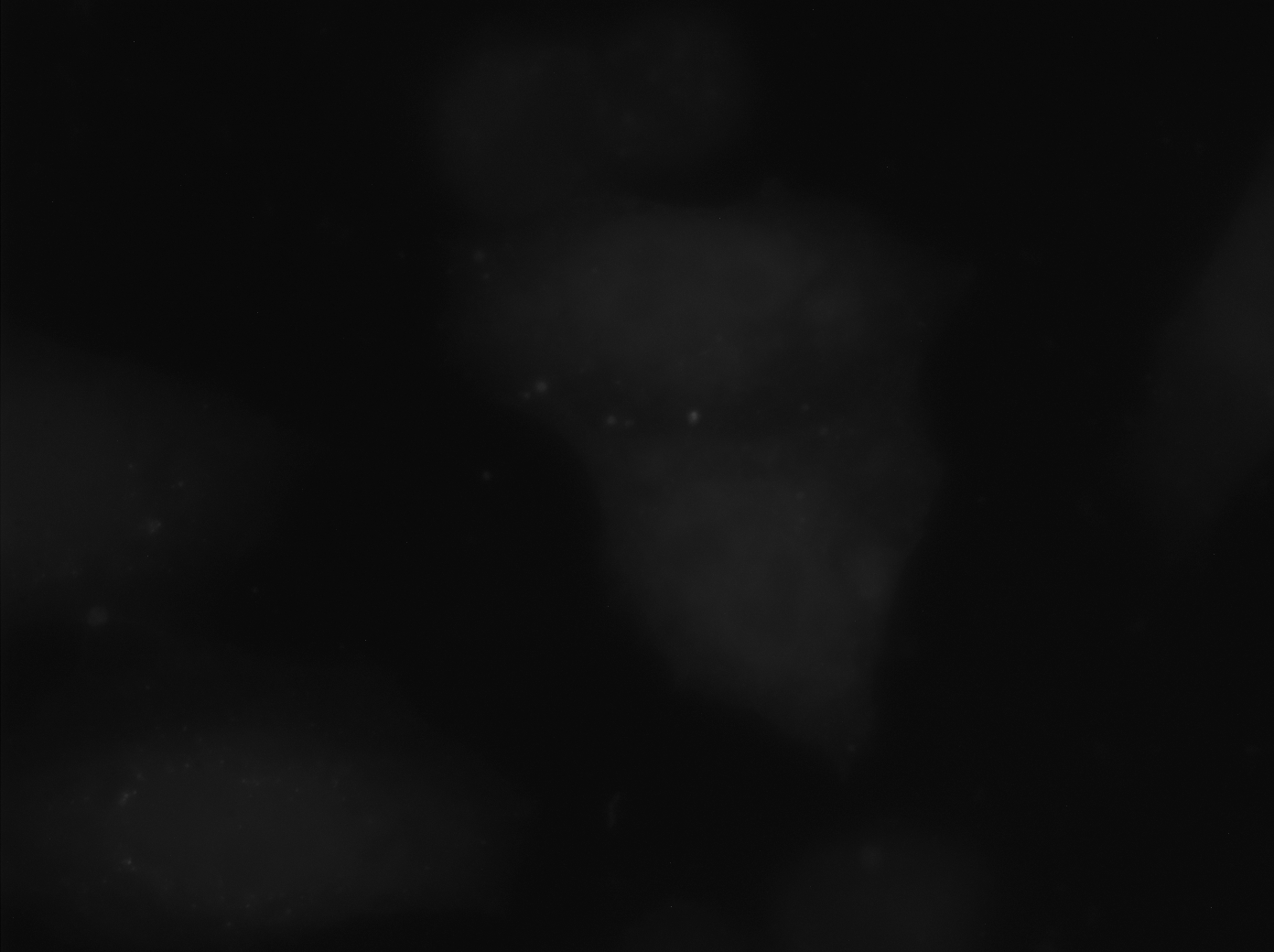

Supplement: Supplementary file 11 — Source data Fig. 3 part 1 [file 44319_2026_742_MOESM11_ESM.zip › Figure 3 Part 1/Fig 3b-e TTLL screen/TTLL1-GFP A4 I6.Project Maximum Z_XY1675962442_Z0_T0_C3.tif]

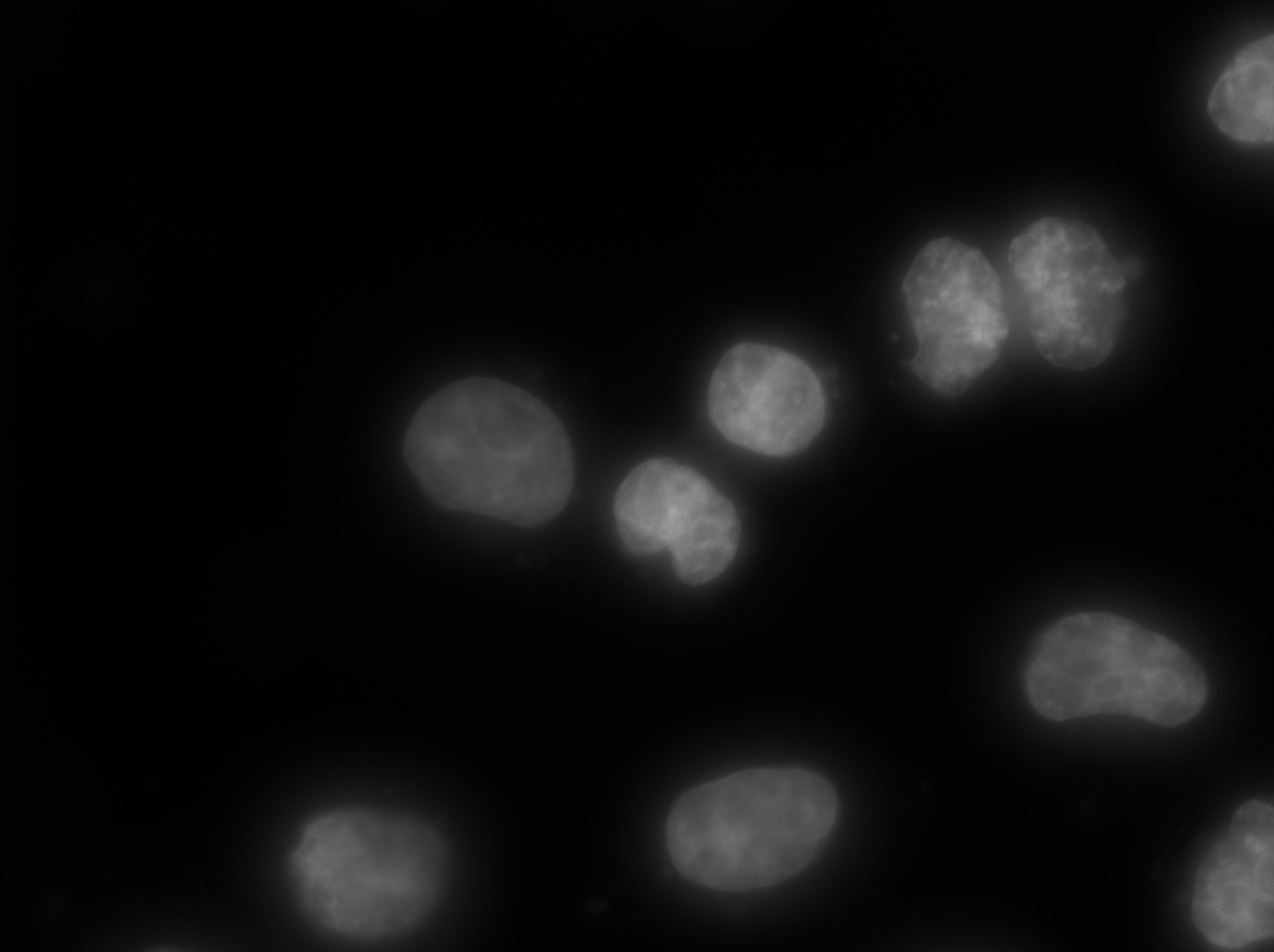

Supplement: Supplementary file 11 — Source data Fig. 3 part 1 [file 44319_2026_742_MOESM11_ESM.zip › Figure 3 Part 1/Fig 3b-e TTLL screen/TTLL1-GFP A3 I18.Project Maximum Z_XY1679697973_Z0_T0_C0.tif]

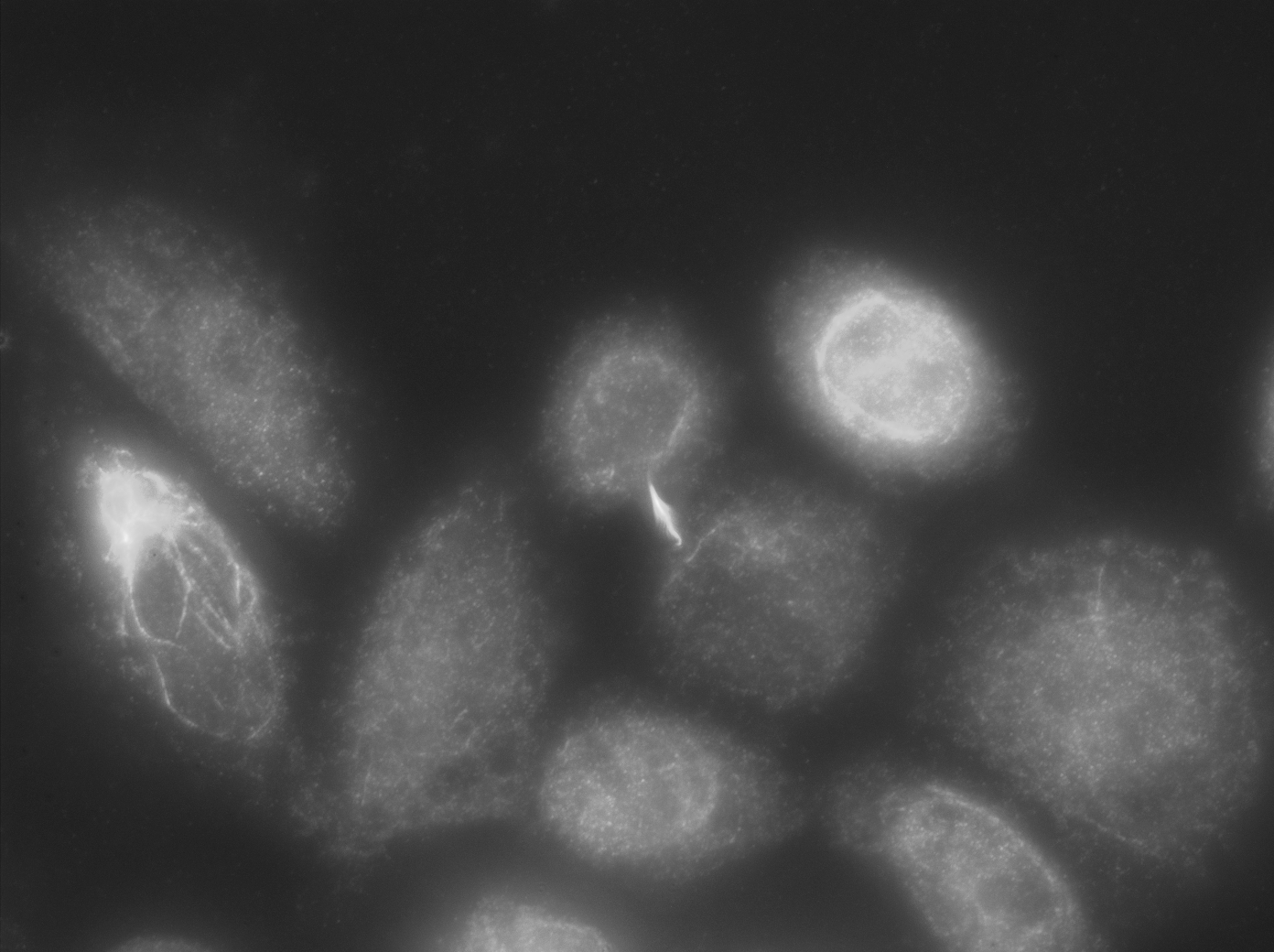

Supplement: Supplementary file 11 — Source data Fig. 3 part 1 [file 44319_2026_742_MOESM11_ESM.zip › Figure 3 Part 1/Fig 3b-e TTLL screen/TTLL4-YFPy I19.Project Maximum Z_XY1679338016_Z0_T0_C1.tif]

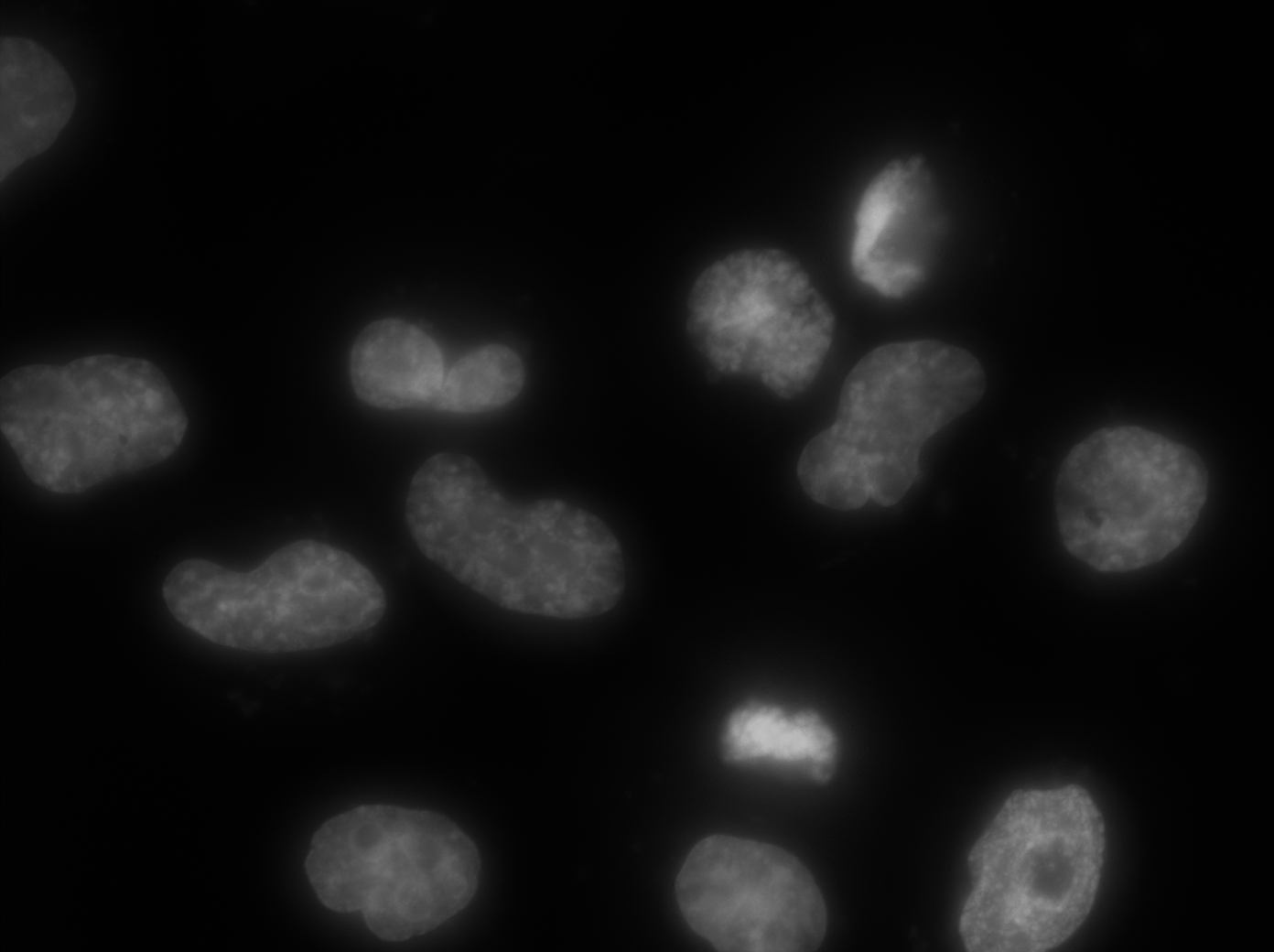

Supplement: Supplementary file 11 — Source data Fig. 3 part 1 [file 44319_2026_742_MOESM11_ESM.zip › Figure 3 Part 1/Fig 3b-e TTLL screen/TTLL1-GFP A3 I5.Project Maximum Z_XY1679694938_Z0_T0_C0.tif]

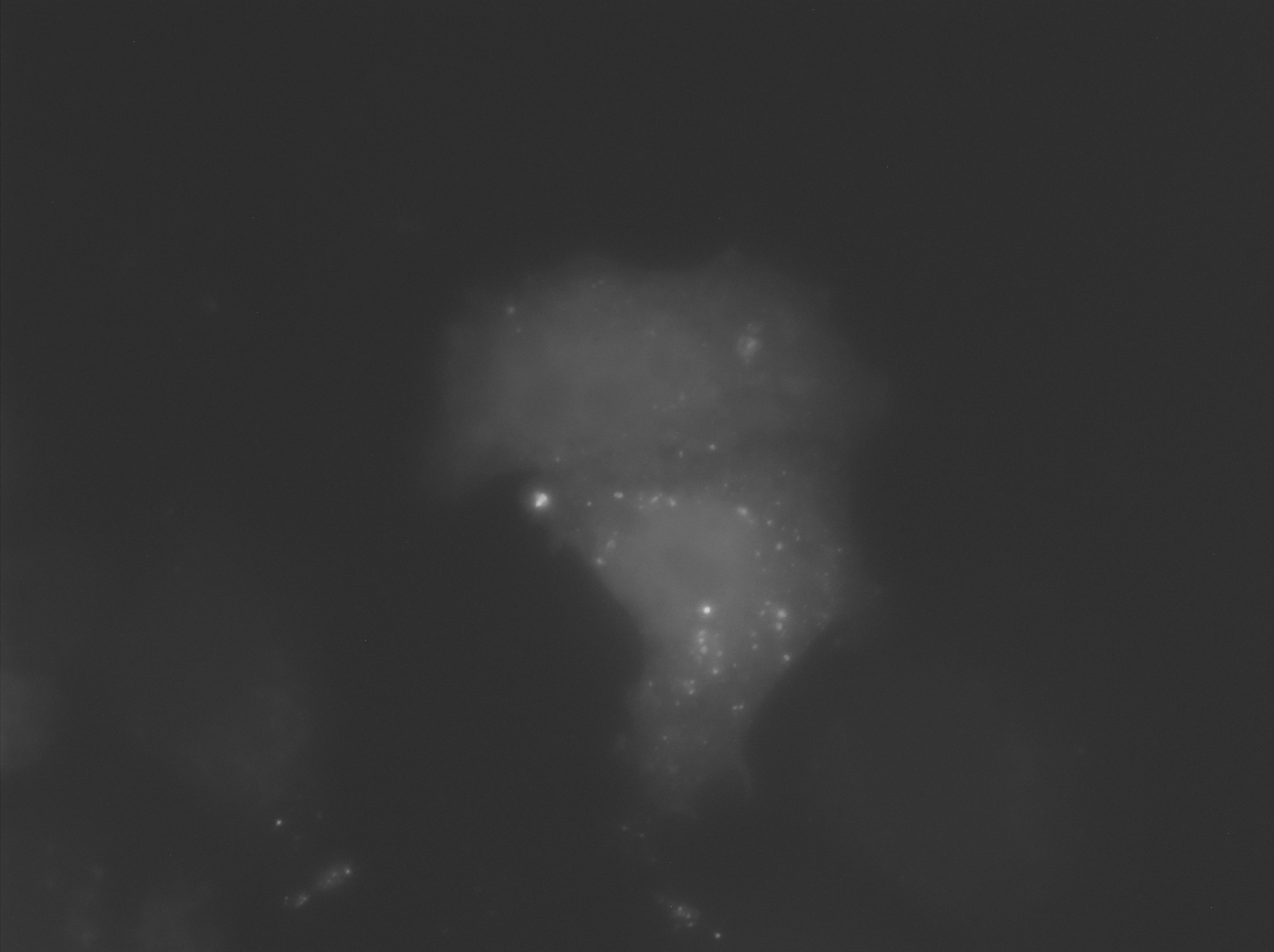

Supplement: Supplementary file 11 — Source data Fig. 3 part 1 [file 44319_2026_742_MOESM11_ESM.zip › Figure 3 Part 1/Fig 3b-e TTLL screen/TTLL1-GFP A4 I7.Project Maximum Z_XY1675962740_Z0_T0_C1.tif]

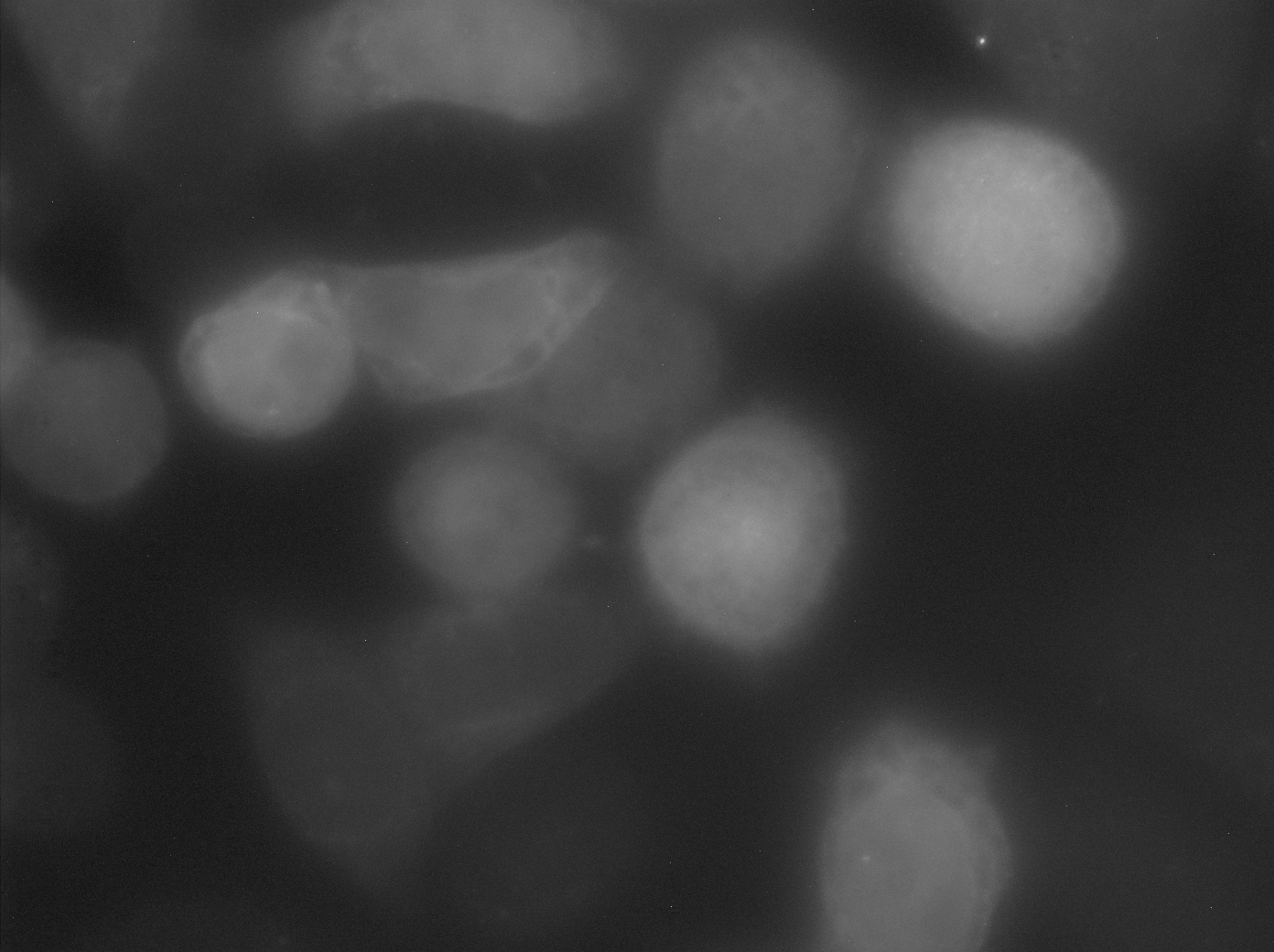

Supplement: Supplementary file 11 — Source data Fig. 3 part 1 [file 44319_2026_742_MOESM11_ESM.zip › Figure 3 Part 1/Fig 3b-e TTLL screen/TTLL1-GFP A3 I15.Project Maximum Z_XY1679697290_Z0_T0_C1.tif]

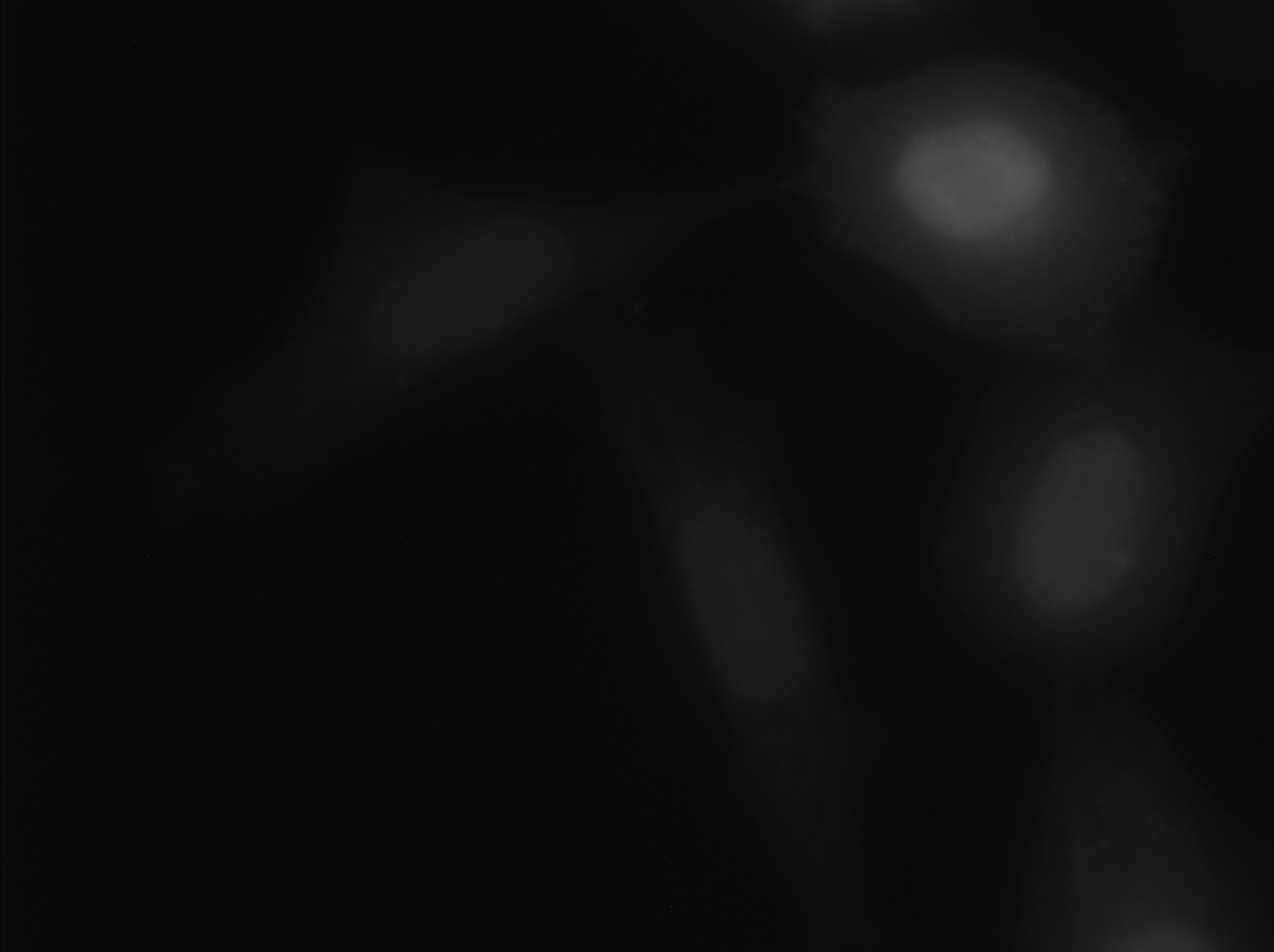

Supplement: Supplementary file 11 — Source data Fig. 3 part 1 [file 44319_2026_742_MOESM11_ESM.zip › Figure 3 Part 1/Fig 3b-e TTLL screen/EYFP MB I3.Project Maximum Z_XY1663875225_Z0_T0_C2.tif]

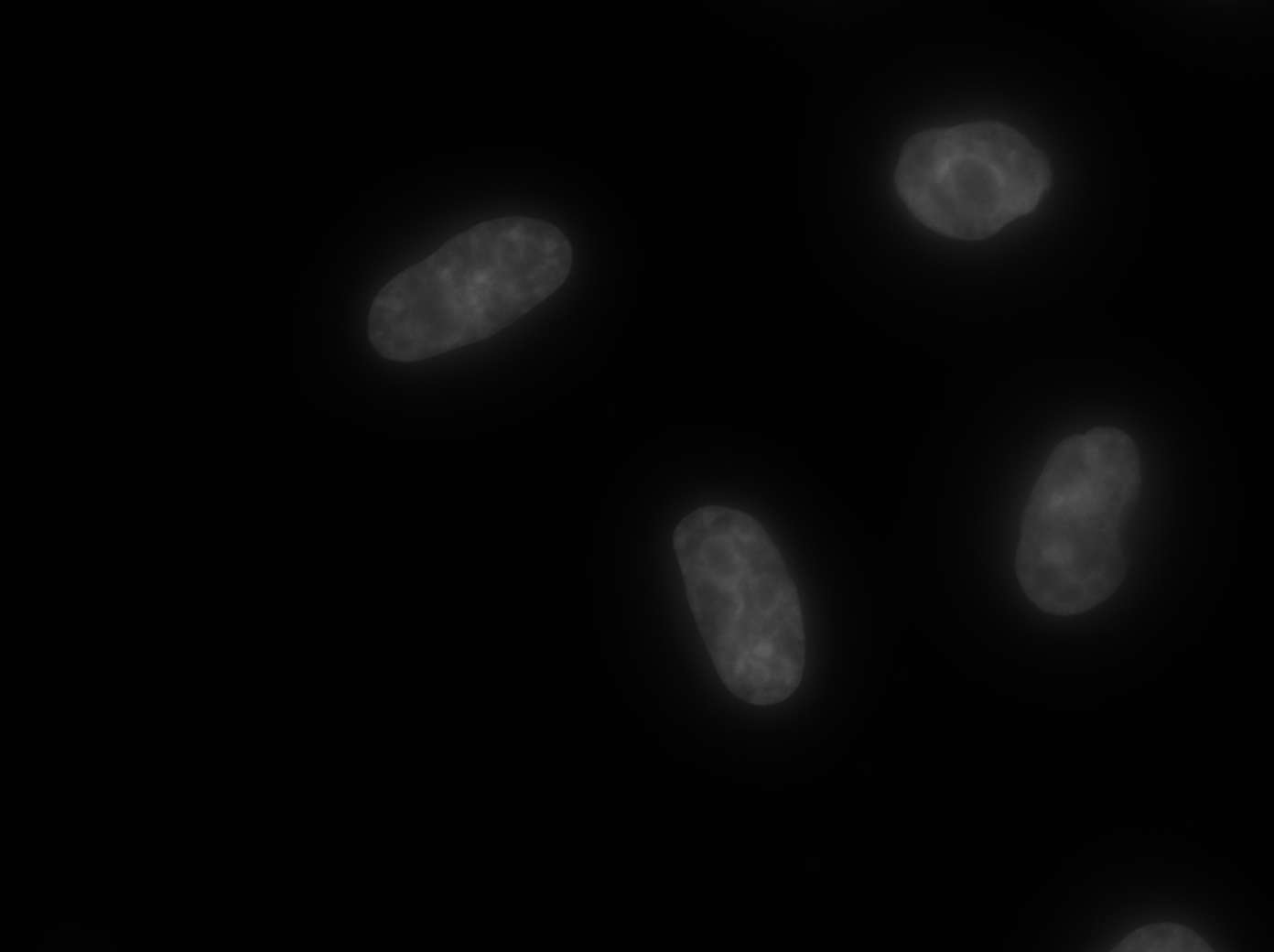

Supplement: Supplementary file 11 — Source data Fig. 3 part 1 [file 44319_2026_742_MOESM11_ESM.zip › Figure 3 Part 1/Fig 3b-e TTLL screen/EYFP MB I3.Project Maximum Z_XY1663875225_Z0_T0_C0.tif]

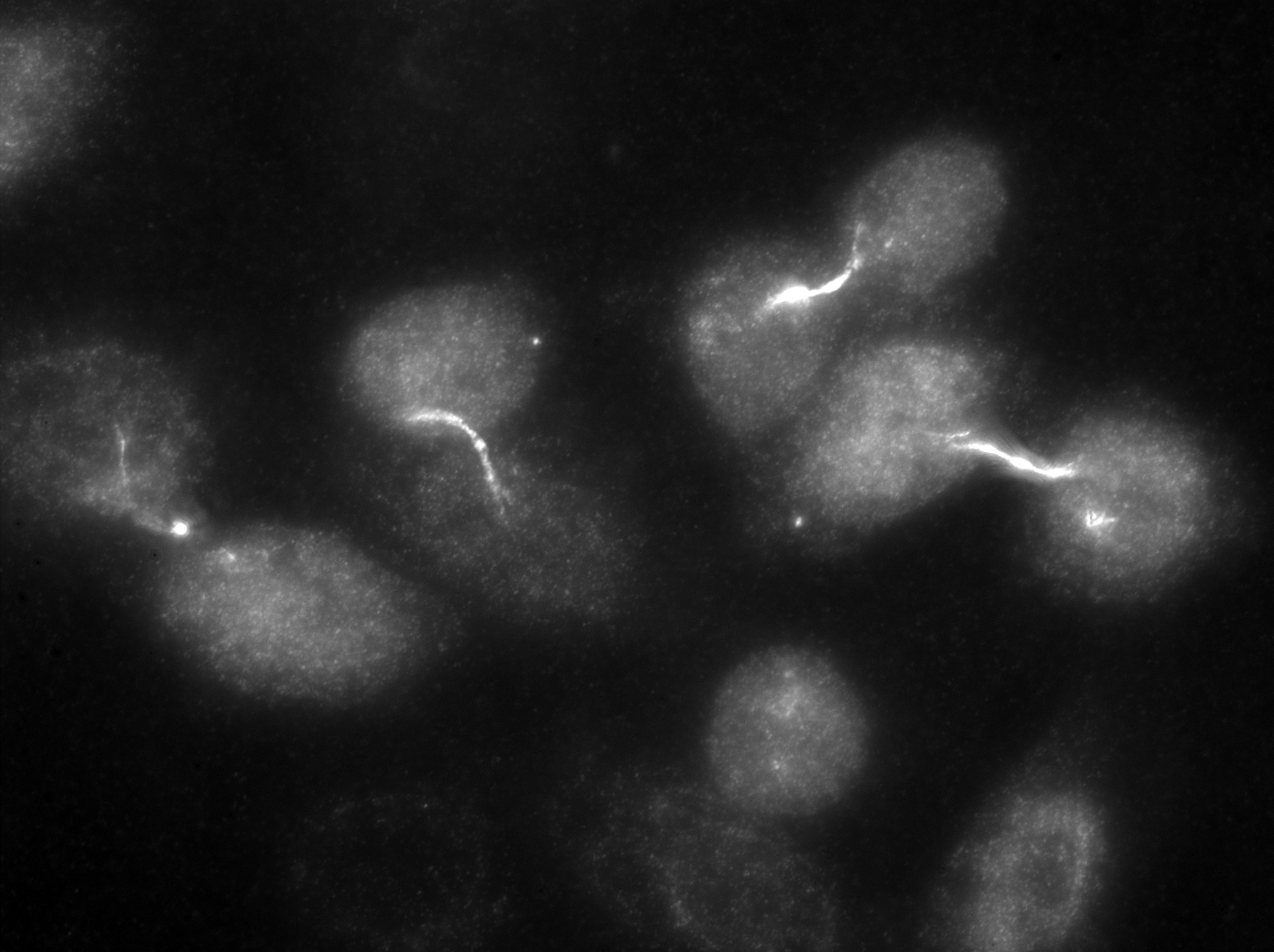

Supplement: Supplementary file 11 — Source data Fig. 3 part 1 [file 44319_2026_742_MOESM11_ESM.zip › Figure 3 Part 1/Fig 3b-e TTLL screen/TTLL1-GFP A3 I5.Project Maximum Z_XY1679694938_Z0_T0_C2.tif]

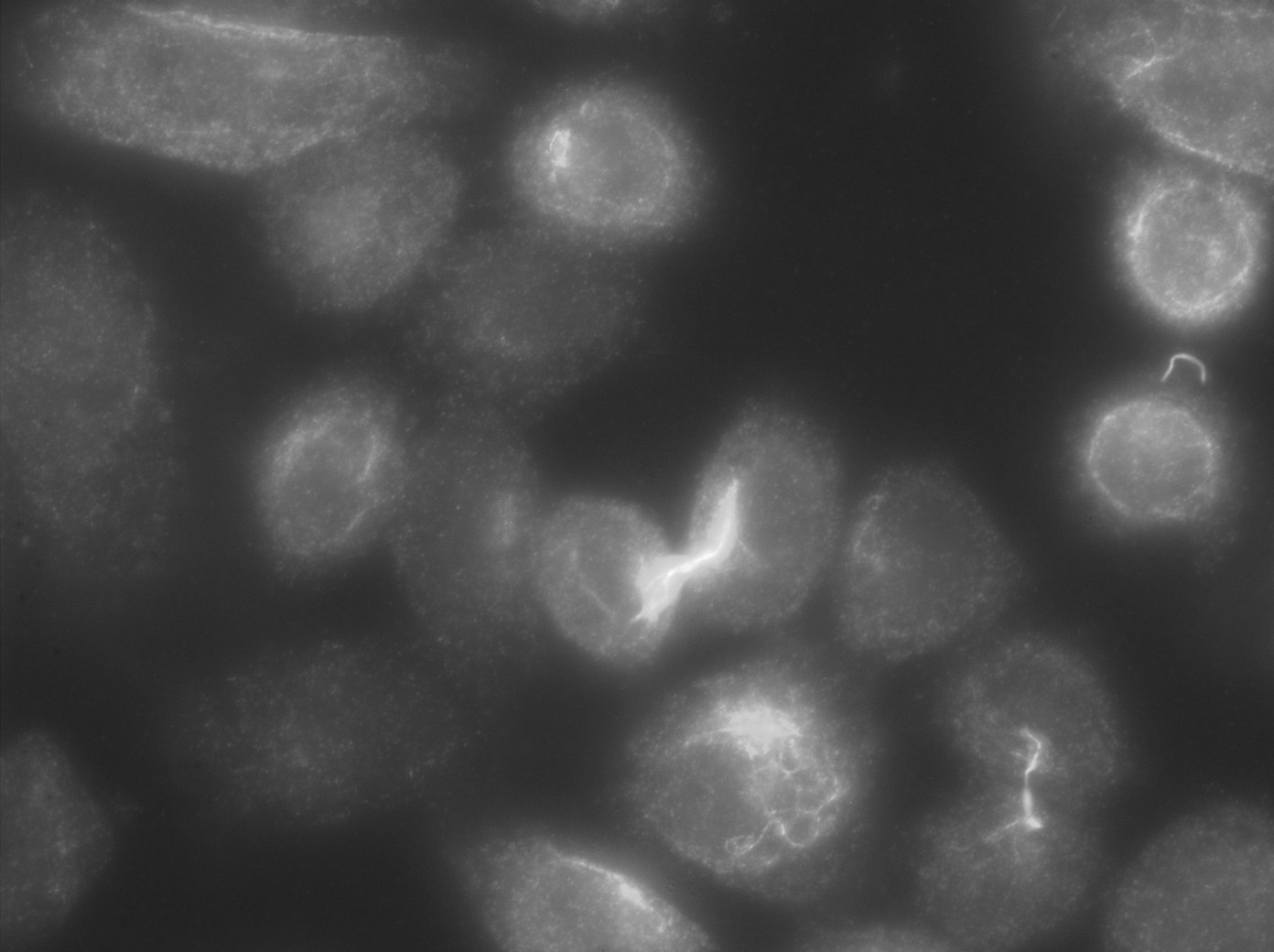

Supplement: Supplementary file 11 — Source data Fig. 3 part 1 [file 44319_2026_742_MOESM11_ESM.zip › Figure 3 Part 1/Fig 3b-e TTLL screen/TTLL4-YFPy I7.Project Maximum Z_XY1679076541_Z0_T0_C1.tif]

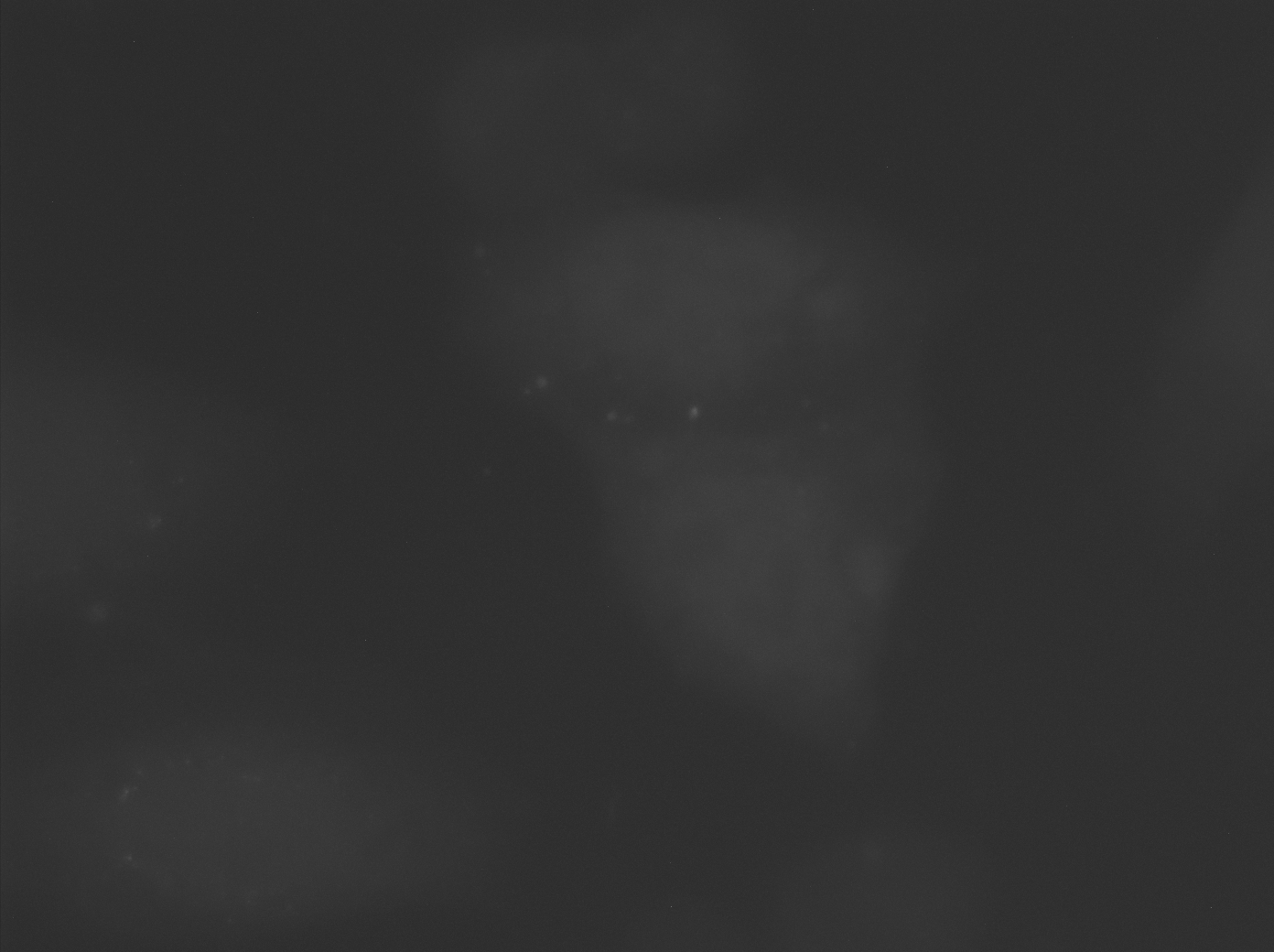

Supplement: Supplementary file 11 — Source data Fig. 3 part 1 [file 44319_2026_742_MOESM11_ESM.zip › Figure 3 Part 1/Fig 3b-e TTLL screen/TTLL1-GFP A4 I6.Project Maximum Z_XY1675962442_Z0_T0_C1.tif]

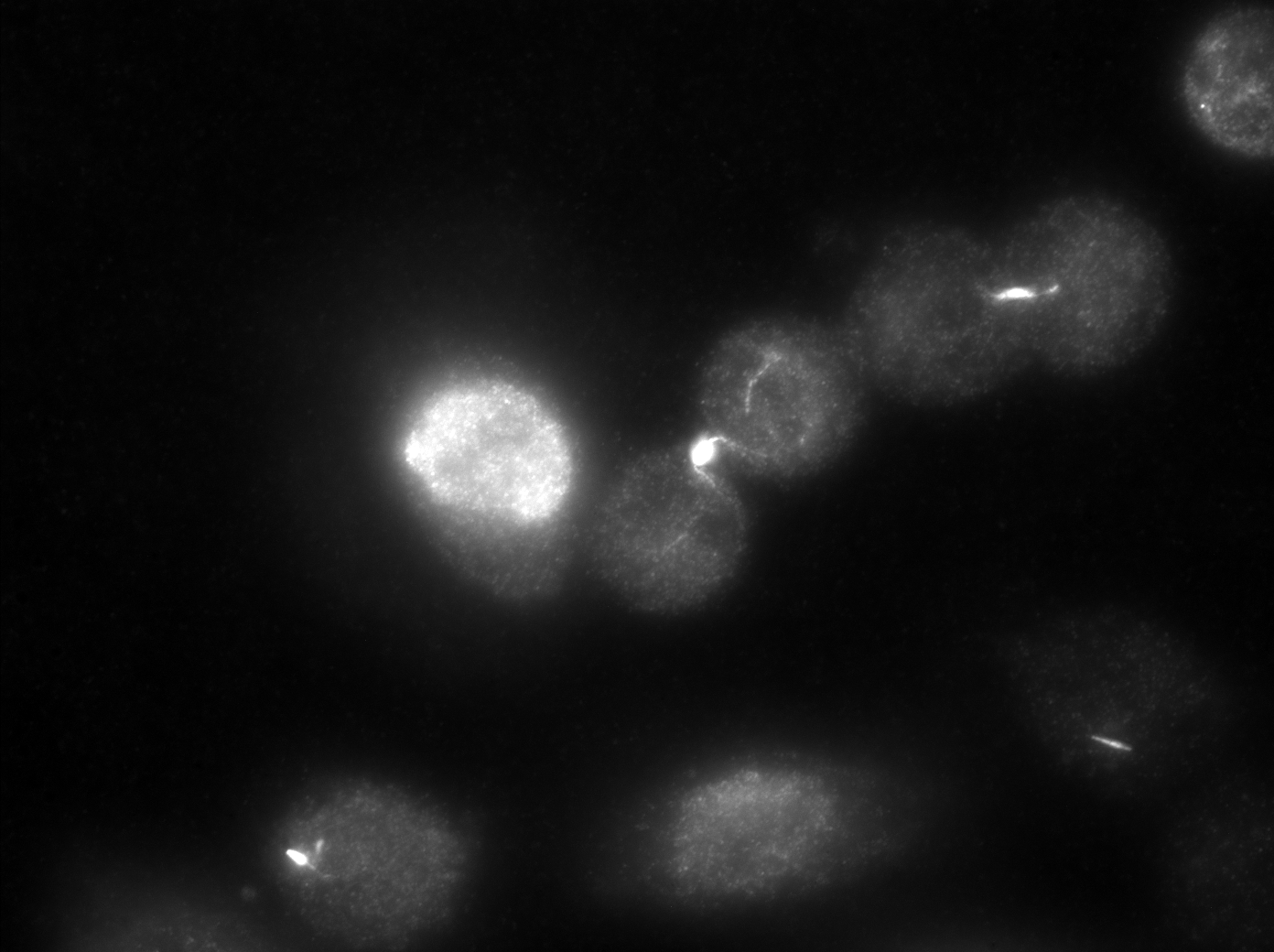

Supplement: Supplementary file 11 — Source data Fig. 3 part 1 [file 44319_2026_742_MOESM11_ESM.zip › Figure 3 Part 1/Fig 3b-e TTLL screen/TTLL1-GFP A3 I18.Project Maximum Z_XY1679697973_Z0_T0_C2.tif]

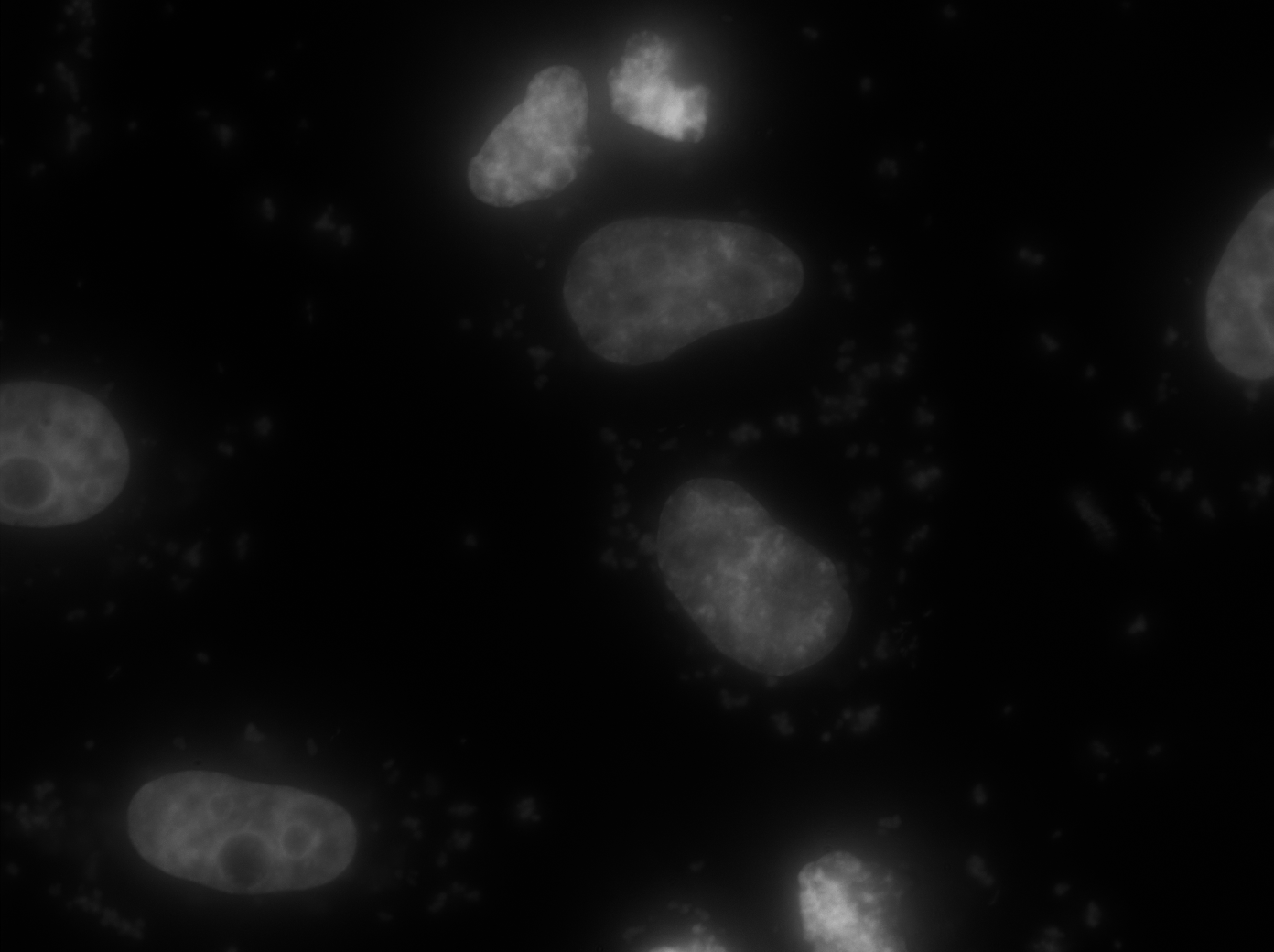

Supplement: Supplementary file 11 — Source data Fig. 3 part 1 [file 44319_2026_742_MOESM11_ESM.zip › Figure 3 Part 1/Fig 3b-e TTLL screen/TTLL1-GFP A4 I6.Project Maximum Z_XY1675962442_Z0_T0_C0.tif]

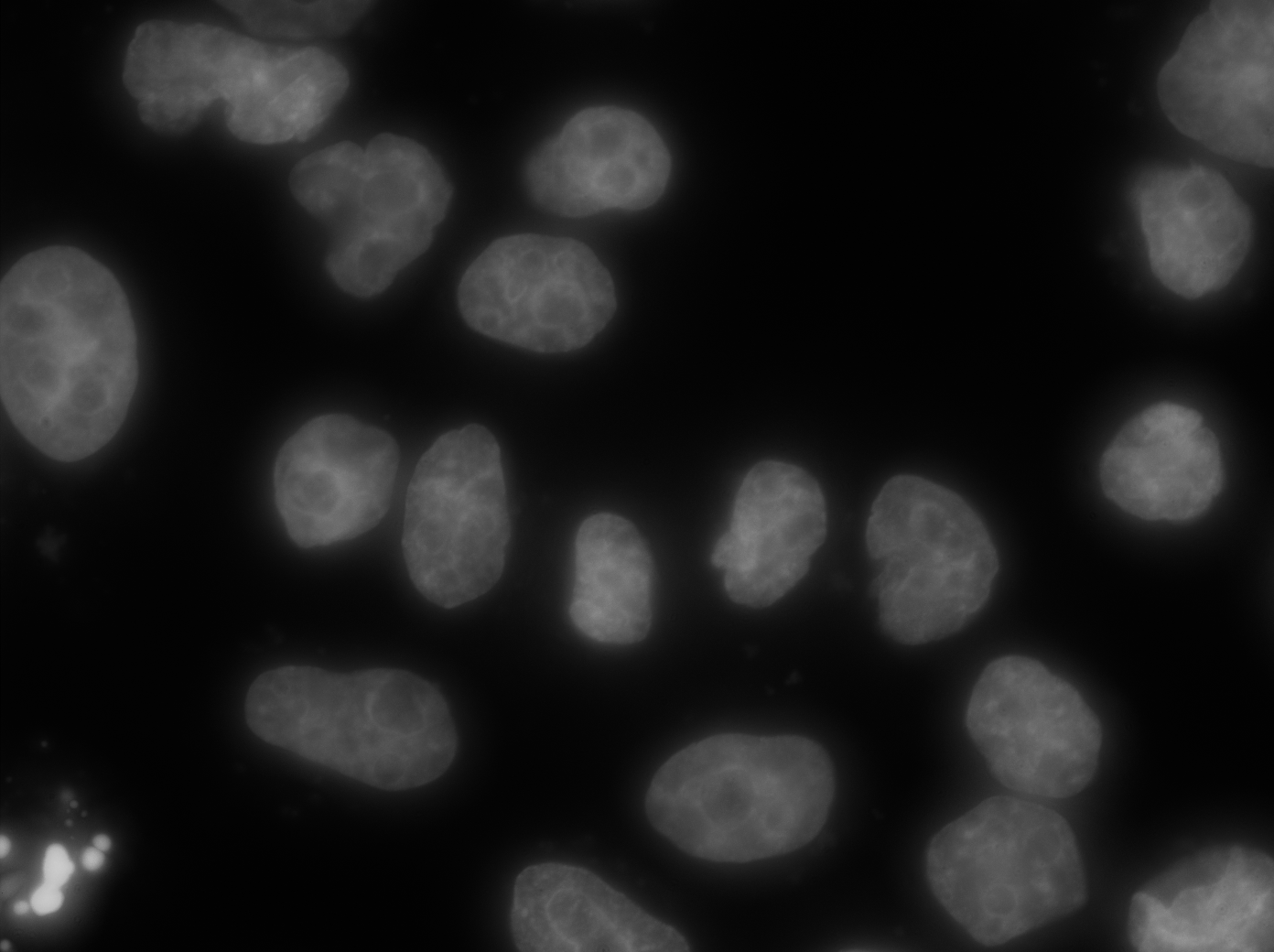

Supplement: Supplementary file 11 — Source data Fig. 3 part 1 [file 44319_2026_742_MOESM11_ESM.zip › Figure 3 Part 1/Fig 3b-e TTLL screen/TTLL4-YFPy I7.Project Maximum Z_XY1679076541_Z0_T0_C0.tif]

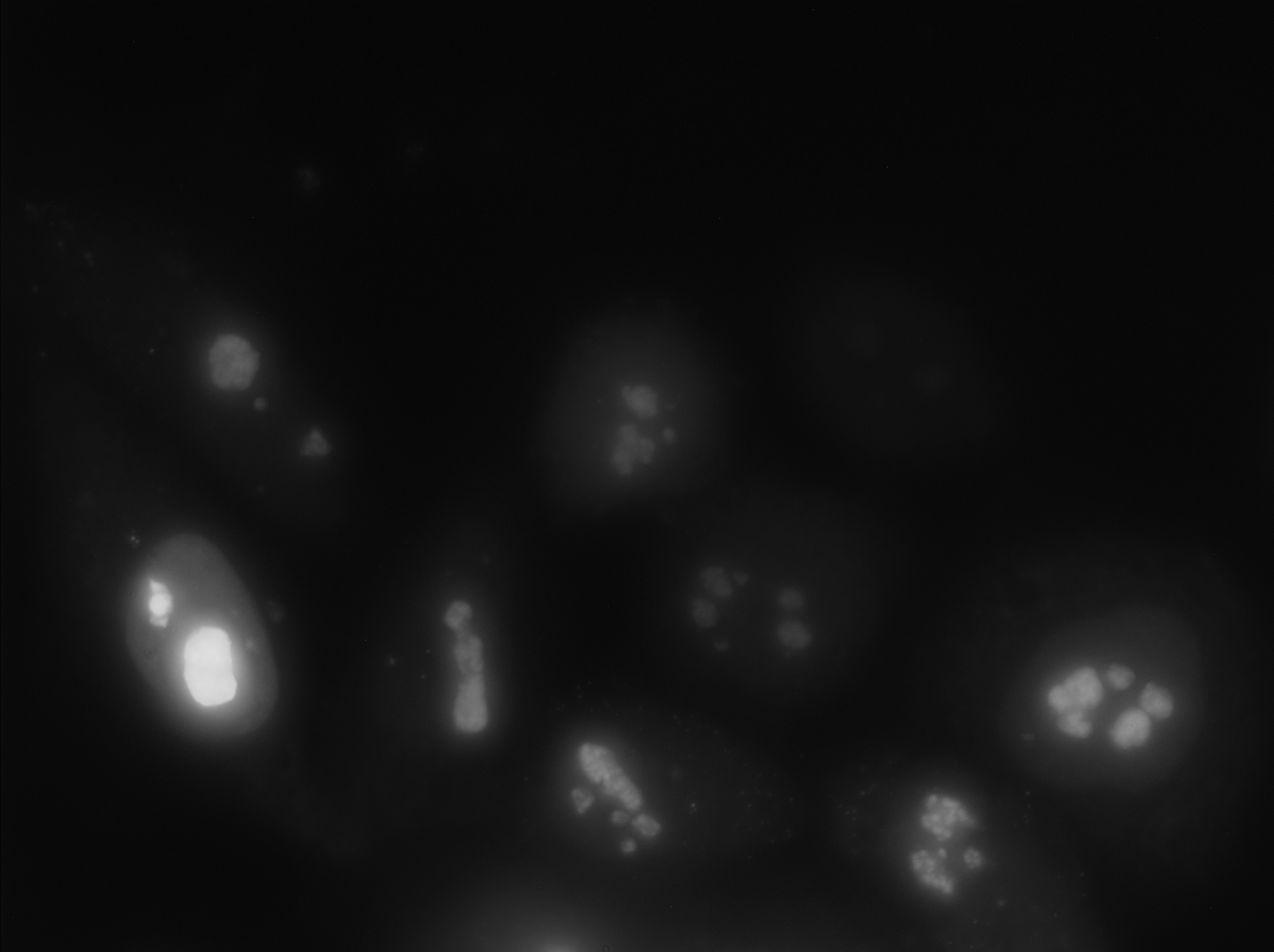

Supplement: Supplementary file 11 — Source data Fig. 3 part 1 [file 44319_2026_742_MOESM11_ESM.zip › Figure 3 Part 1/Fig 3b-e TTLL screen/TTLL4-YFPy I19.Project Maximum Z_XY1679338016_Z0_T0_C2.tif]

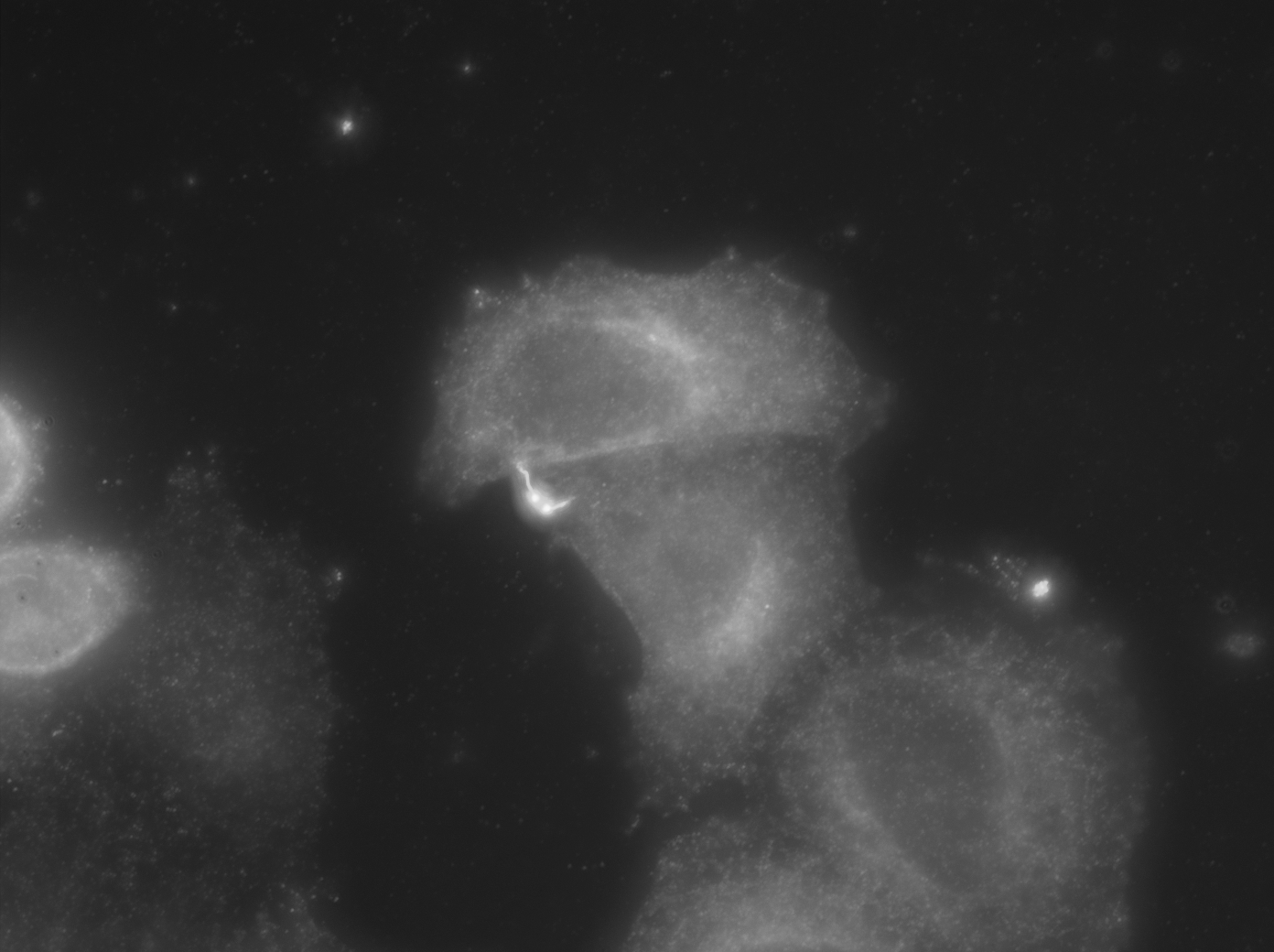

Supplement: Supplementary file 11 — Source data Fig. 3 part 1 [file 44319_2026_742_MOESM11_ESM.zip › Figure 3 Part 1/Fig 3b-e TTLL screen/TTLL1-GFP A4 I7.Project Maximum Z_XY1675962740_Z0_T0_C2.tif]

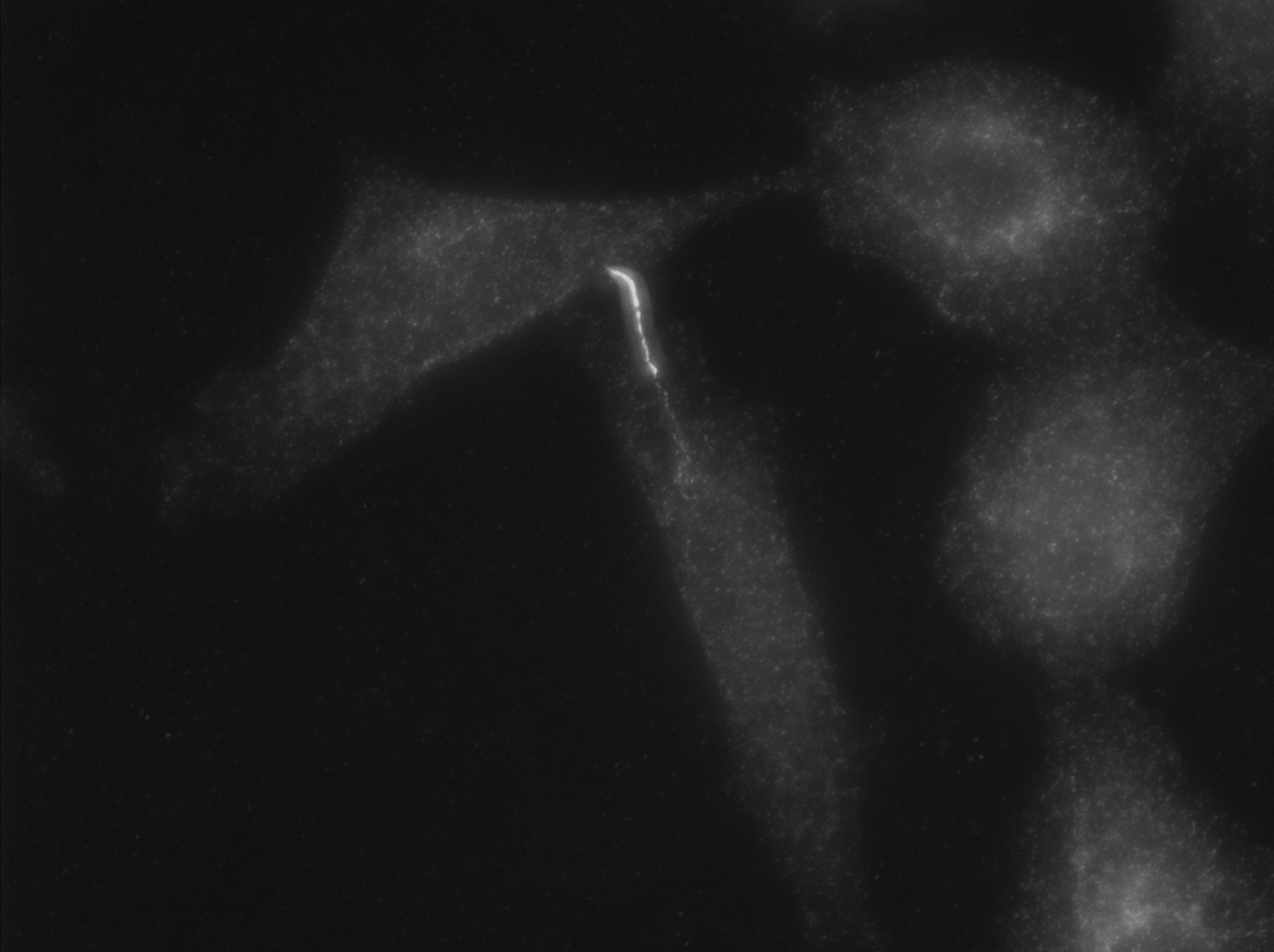

Supplement: Supplementary file 11 — Source data Fig. 3 part 1 [file 44319_2026_742_MOESM11_ESM.zip › Figure 3 Part 1/Fig 3b-e TTLL screen/EYFP MB I3.Project Maximum Z_XY1663875225_Z0_T0_C1.tif]

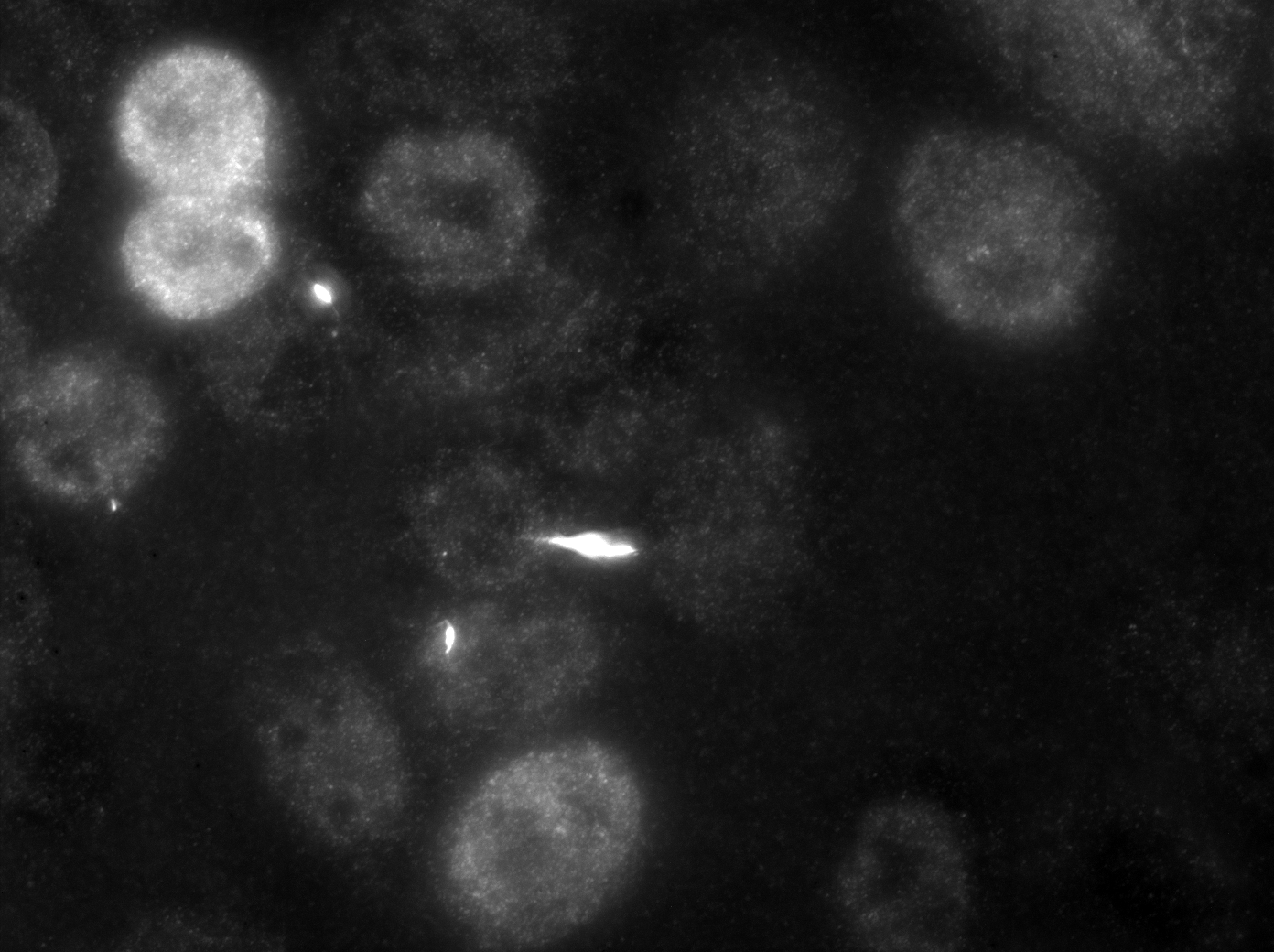

Supplement: Supplementary file 11 — Source data Fig. 3 part 1 [file 44319_2026_742_MOESM11_ESM.zip › Figure 3 Part 1/Fig 3b-e TTLL screen/TTLL1-GFP A3 I15.Project Maximum Z_XY1679697290_Z0_T0_C2.tif]

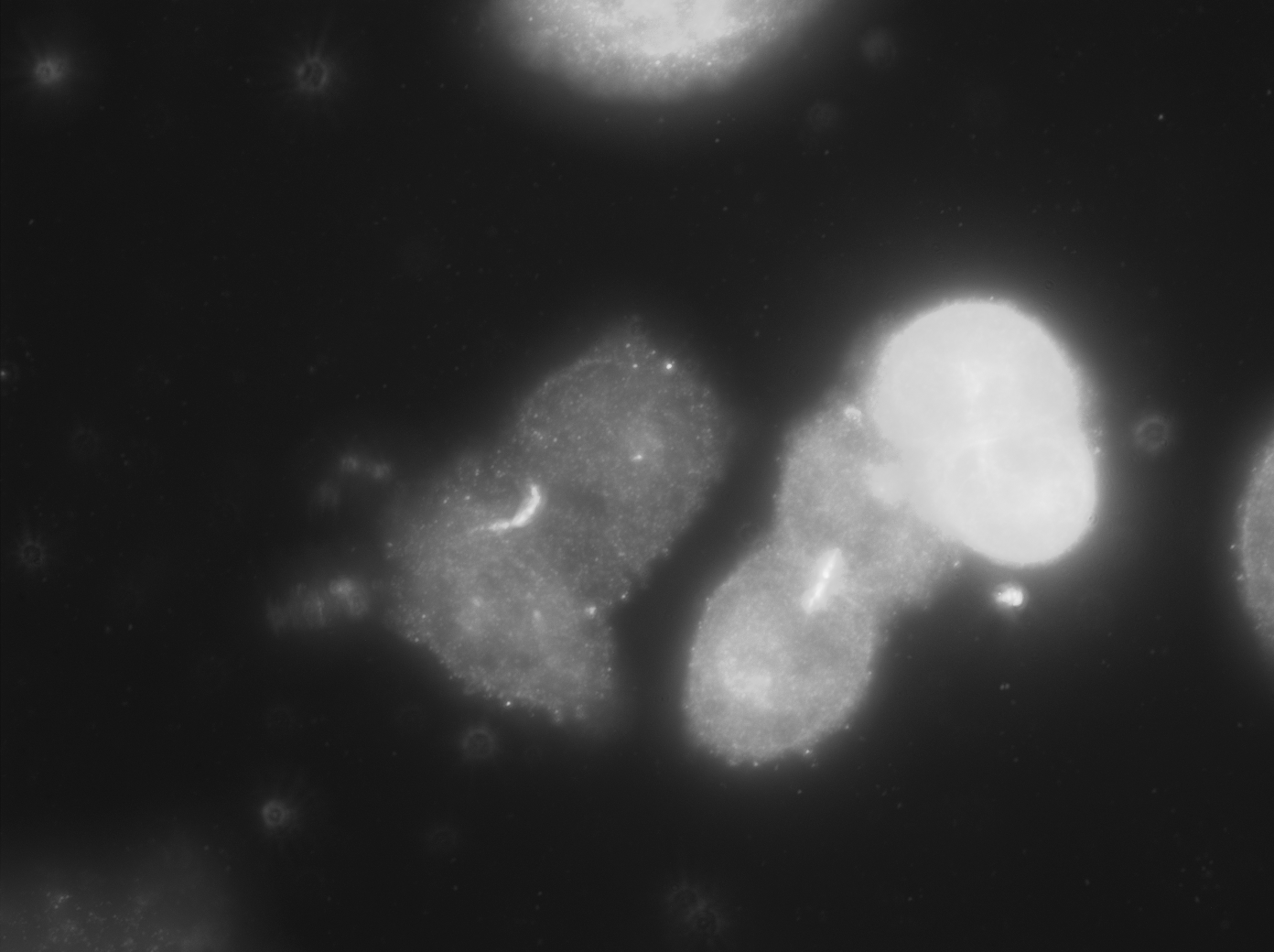

Supplement: Supplementary file 11 — Source data Fig. 3 part 1 [file 44319_2026_742_MOESM11_ESM.zip › Figure 3 Part 1/Fig 3b-e TTLL screen/TTLL1-GFP A4 I8.Project Maximum Z_XY1675962918_Z0_T0_C2.tif]

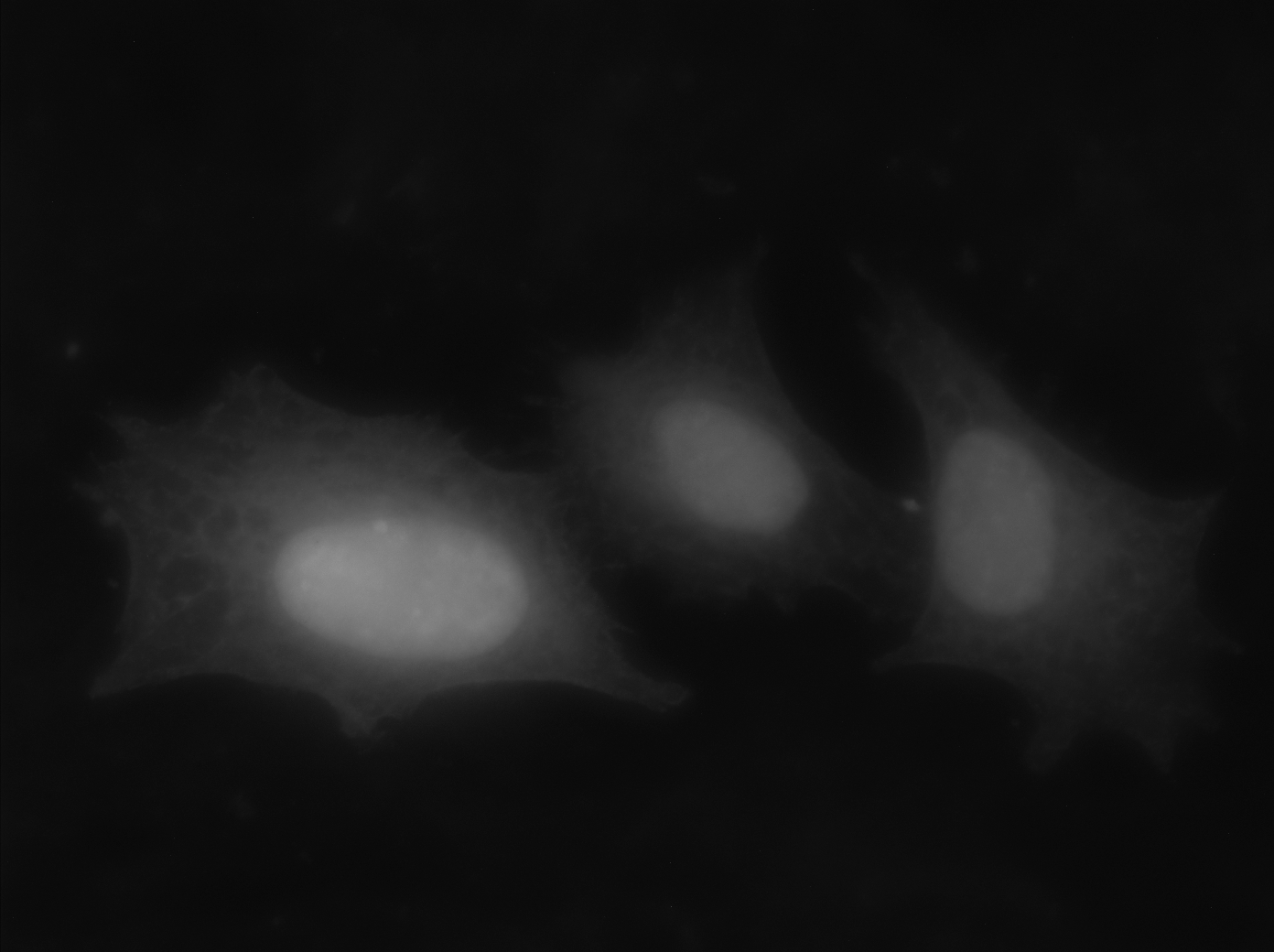

Supplement: Supplementary file 11 — Source data Fig. 3 part 1 [file 44319_2026_742_MOESM11_ESM.zip › Figure 3 Part 1/Fig 3b-e TTLL screen/EYFP MB I1.Project Maximum Z_XY1663874451_Z0_T0_C2.tif]

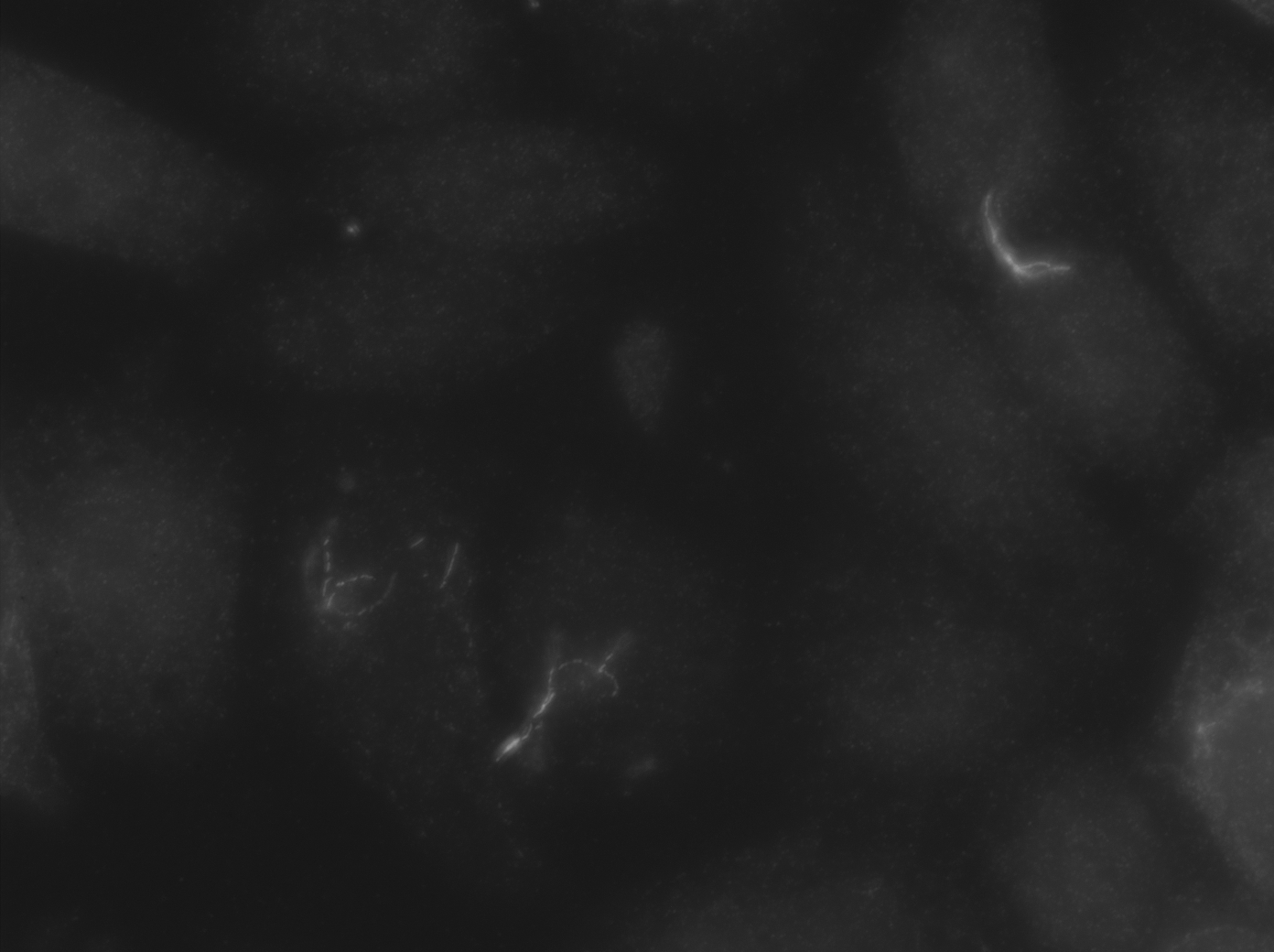

Supplement: Supplementary file 11 — Source data Fig. 3 part 1 [file 44319_2026_742_MOESM11_ESM.zip › Figure 3 Part 1/Fig 3b-e TTLL screen/TTLL4-YFPy I3.Project Maximum Z_XY1679075765_Z0_T0_C2.tif]

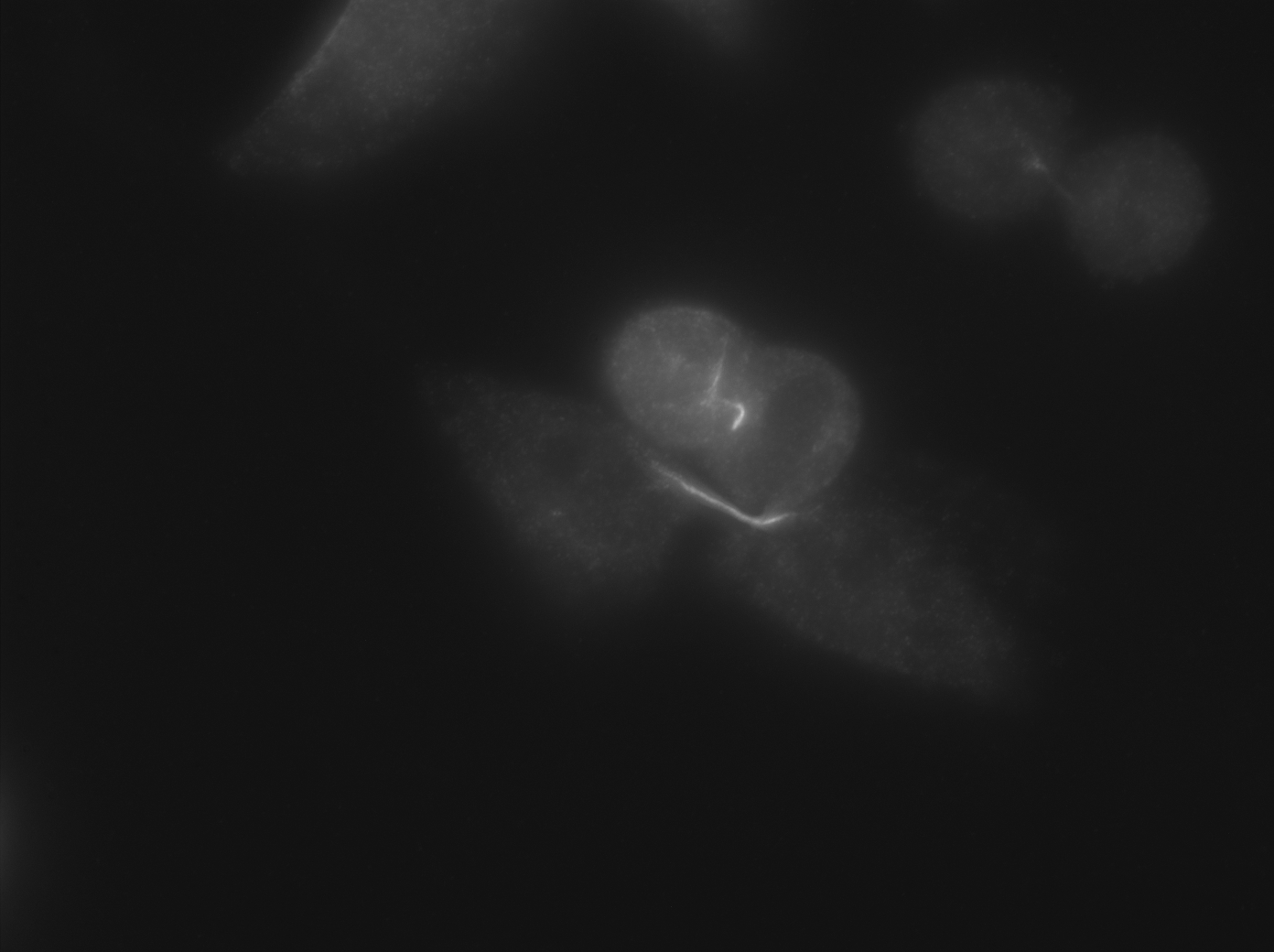

Supplement: Supplementary file 11 — Source data Fig. 3 part 1 [file 44319_2026_742_MOESM11_ESM.zip › Figure 3 Part 1/Fig 3b-e TTLL screen/TTLL1-GFPy I1.Project Maximum Z_XY1679086158_Z0_T0_C2.tif]

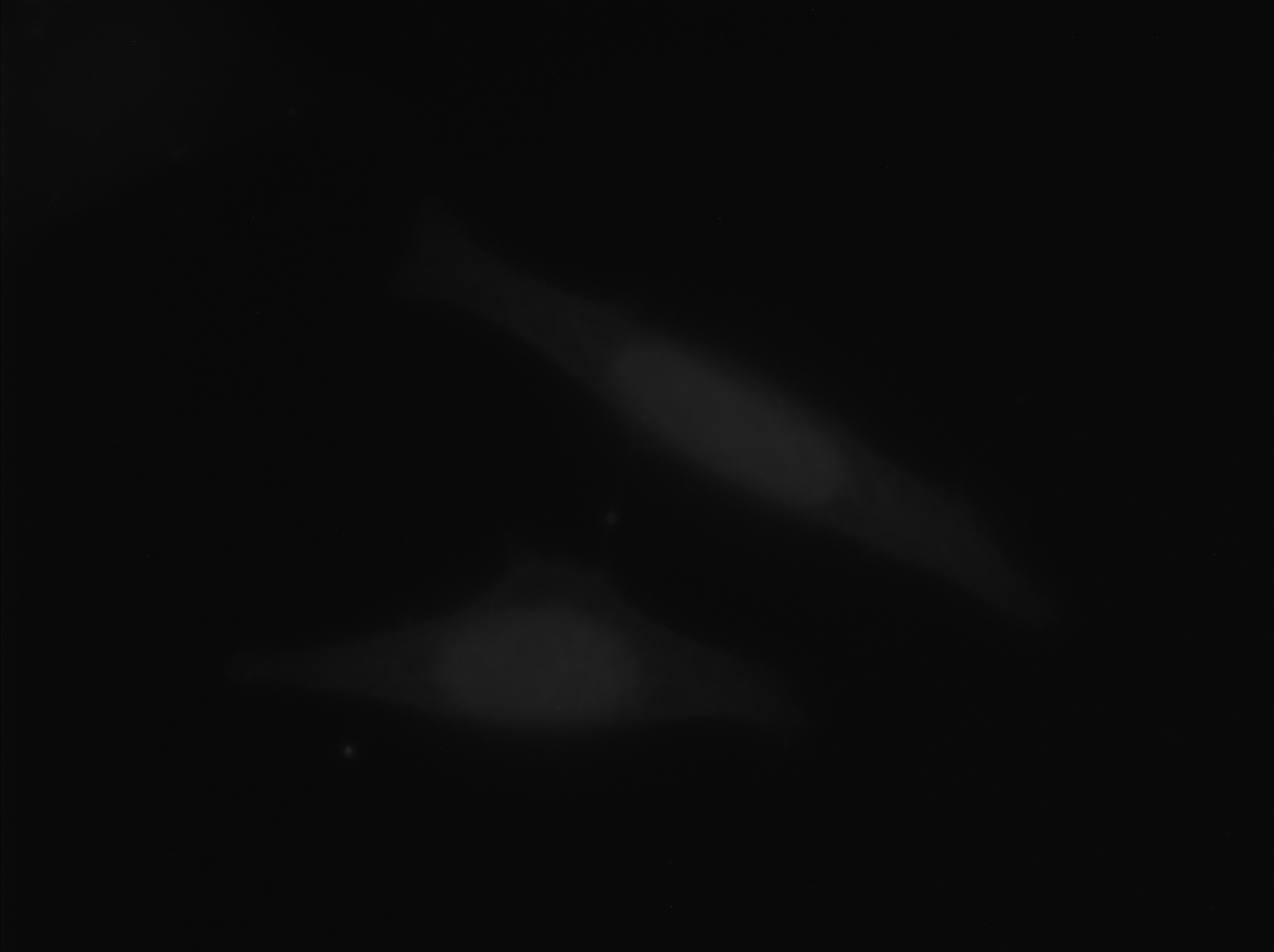

Supplement: Supplementary file 11 — Source data Fig. 3 part 1 [file 44319_2026_742_MOESM11_ESM.zip › Figure 3 Part 1/Fig 3b-e TTLL screen/EYFP MB I2.Project Maximum Z_XY1663875048_Z0_T0_C2.tif]

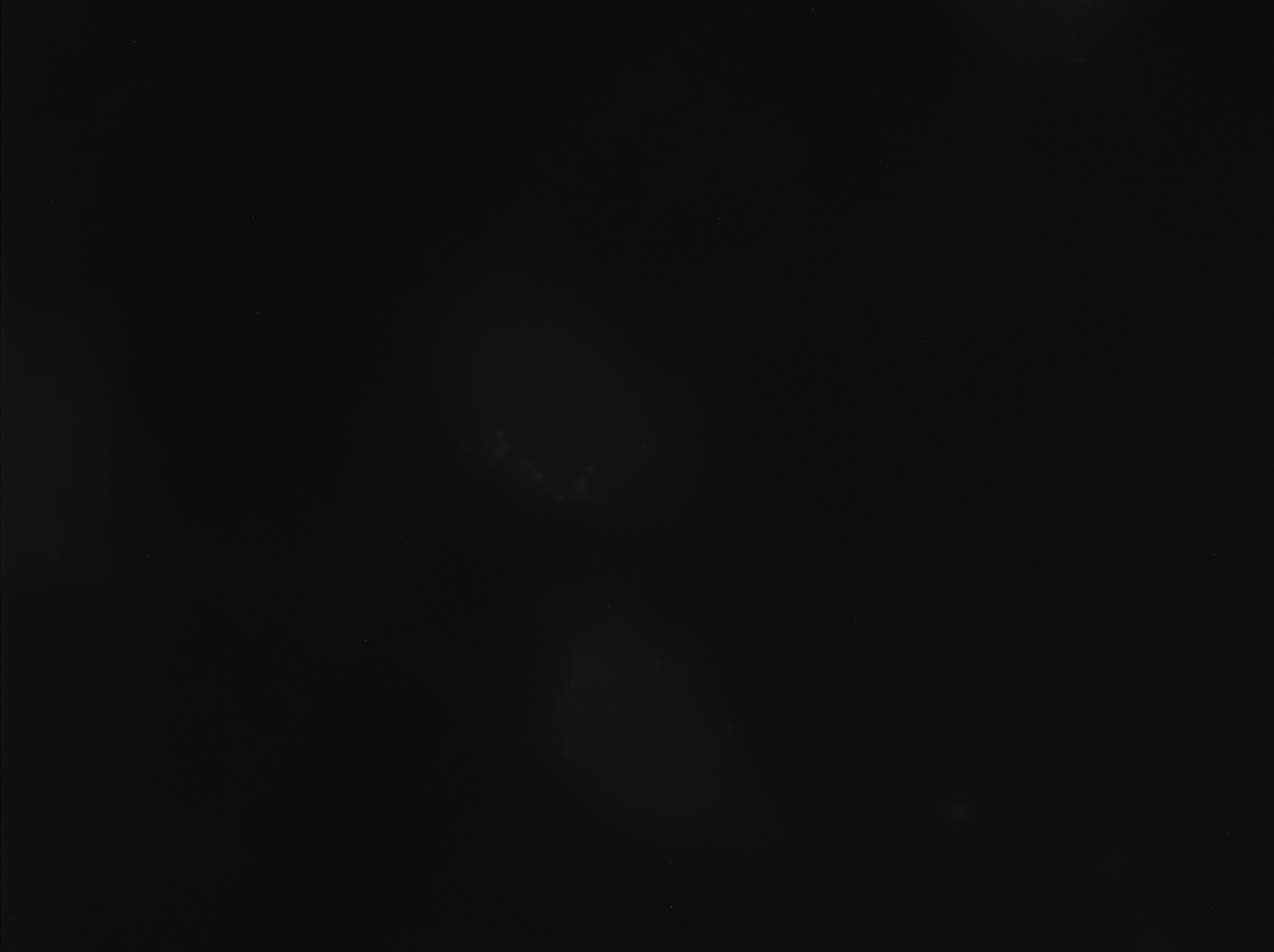

Supplement: Supplementary file 11 — Source data Fig. 3 part 1 [file 44319_2026_742_MOESM11_ESM.zip › Figure 3 Part 1/Fig 3b-e TTLL screen/TTLL1-GFP A3 I3.Project Maximum Z_XY1674674061_Z0_T0_C3.tif]

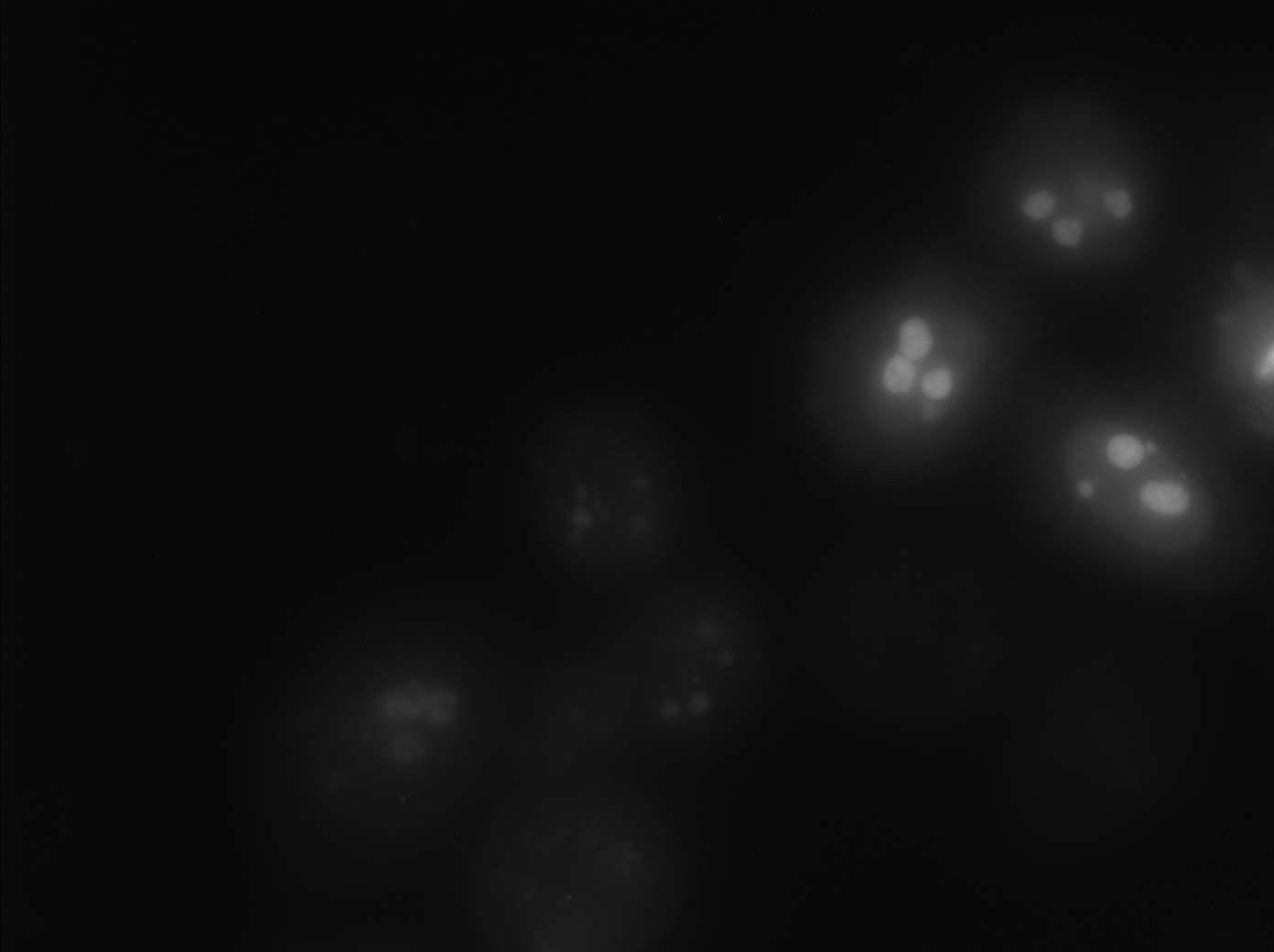

Supplement: Supplementary file 11 — Source data Fig. 3 part 1 [file 44319_2026_742_MOESM11_ESM.zip › Figure 3 Part 1/Fig 3b-e TTLL screen/TTLL4-YFPy I16.Project Maximum Z_XY1679337550_Z0_T0_C2.tif]

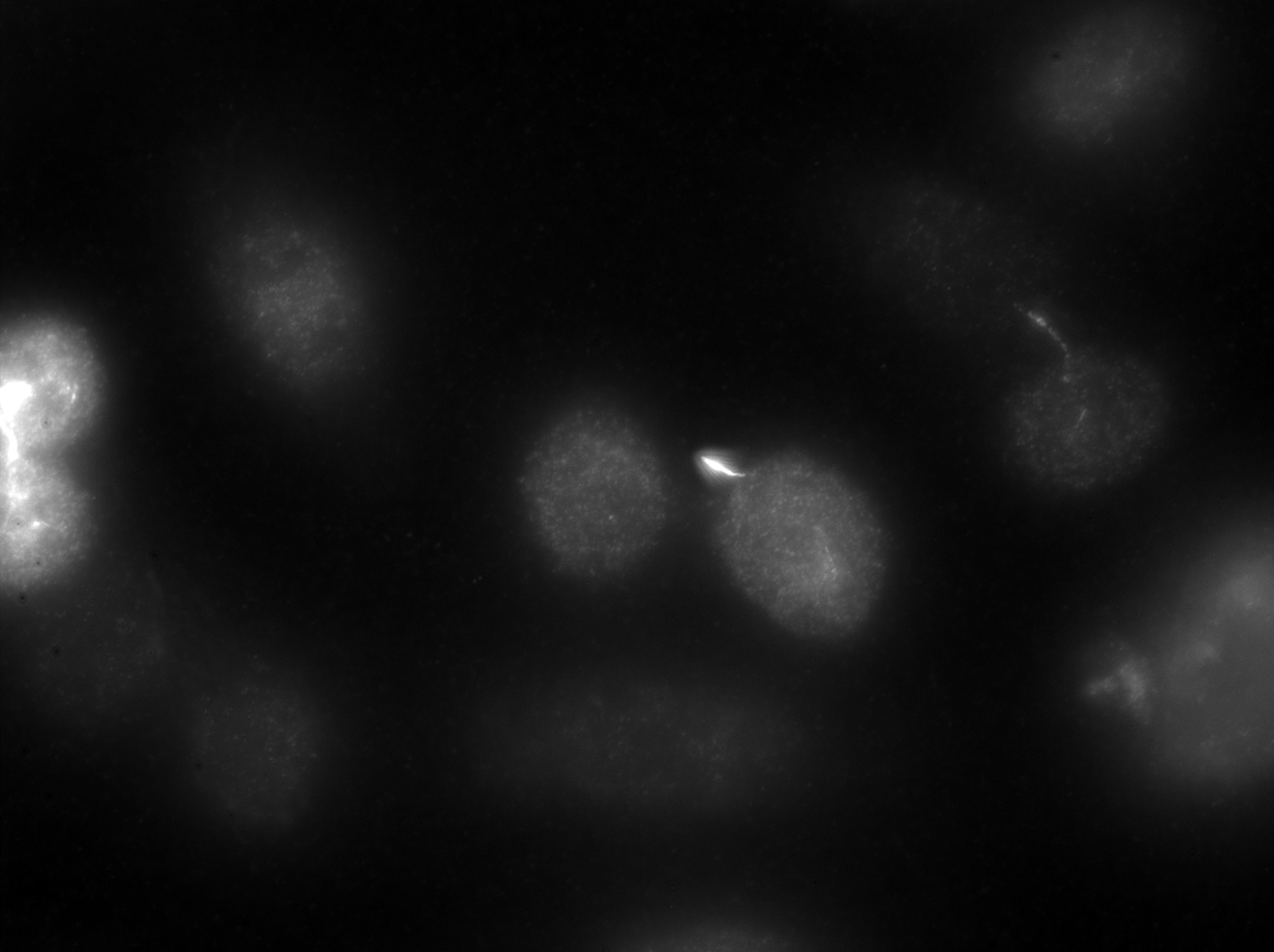

Supplement: Supplementary file 11 — Source data Fig. 3 part 1 [file 44319_2026_742_MOESM11_ESM.zip › Figure 3 Part 1/Fig 3b-e TTLL screen/TTLL1-GFP A3 I7.Project Maximum Z_XY1679695246_Z0_T0_C2.tif]

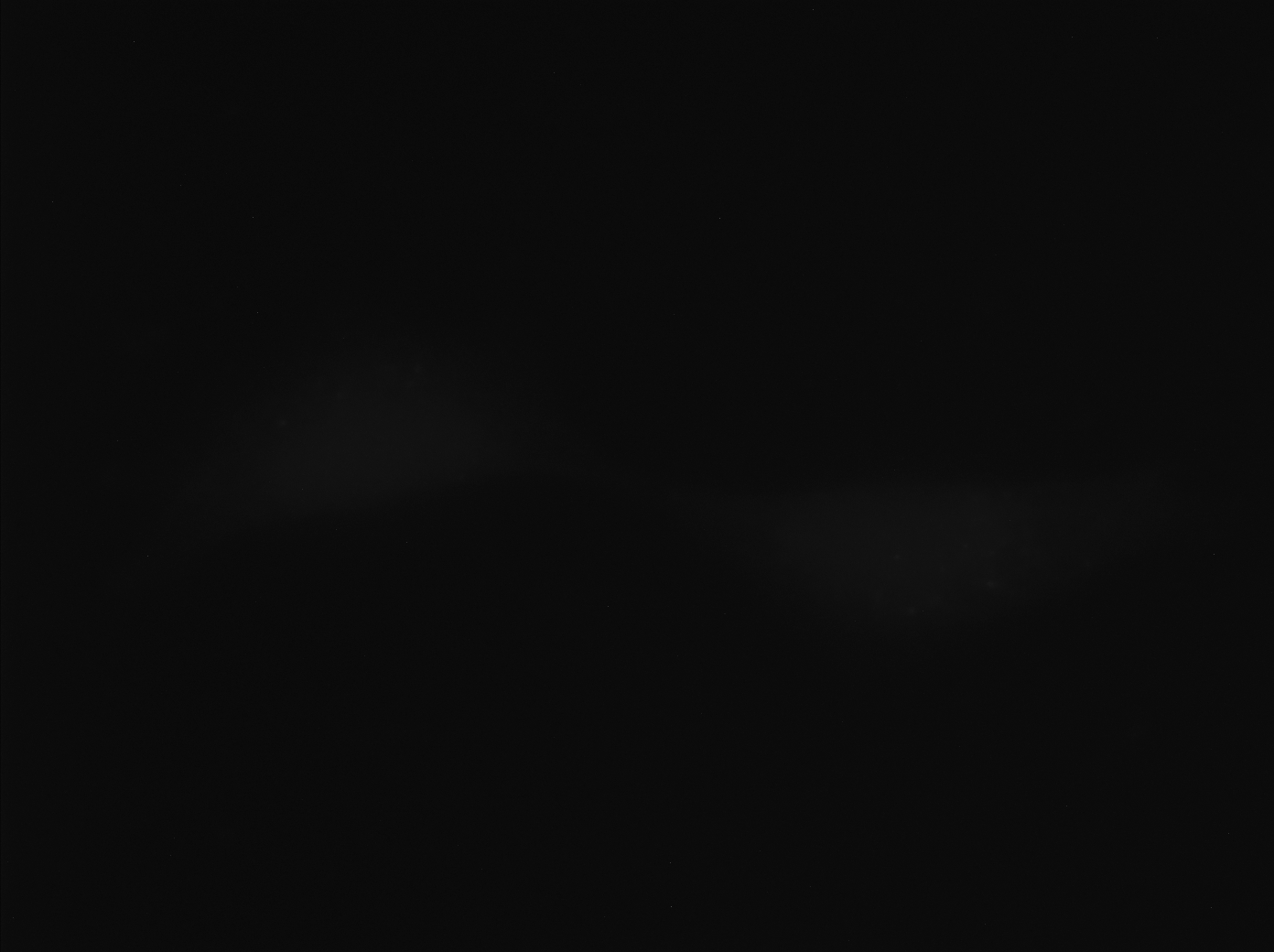

Supplement: Supplementary file 11 — Source data Fig. 3 part 1 [file 44319_2026_742_MOESM11_ESM.zip › Figure 3 Part 1/Fig 3b-e TTLL screen/EYFP MB light I5.Project Maximum Z_XY1663875722_Z0_T0_C2.tif]

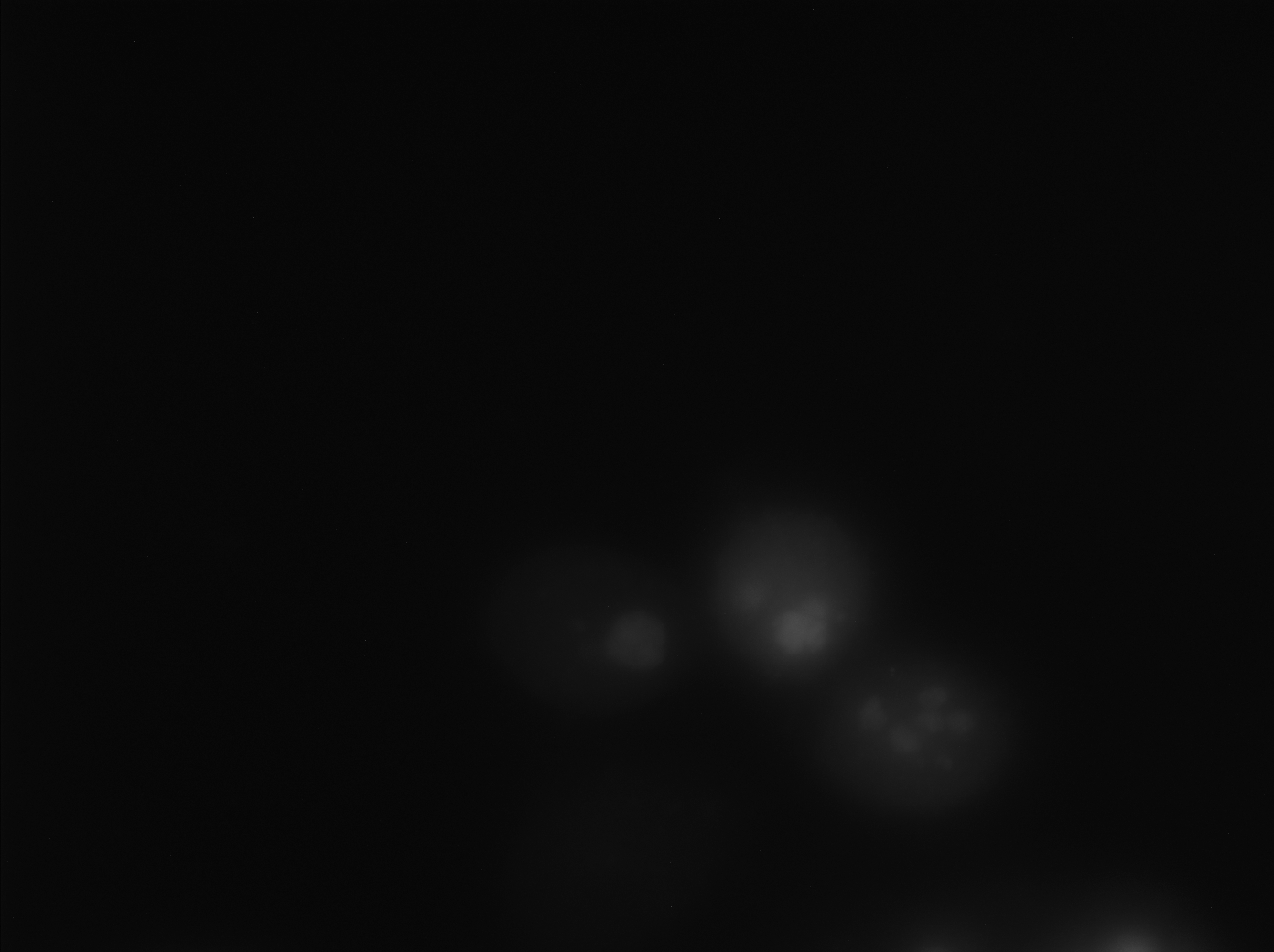

Supplement: Supplementary file 11 — Source data Fig. 3 part 1 [file 44319_2026_742_MOESM11_ESM.zip › Figure 3 Part 1/Fig 3b-e TTLL screen/TTLL4-YFPy I9.Project Maximum Z_XY1679081878_Z0_T0_C2.tif]

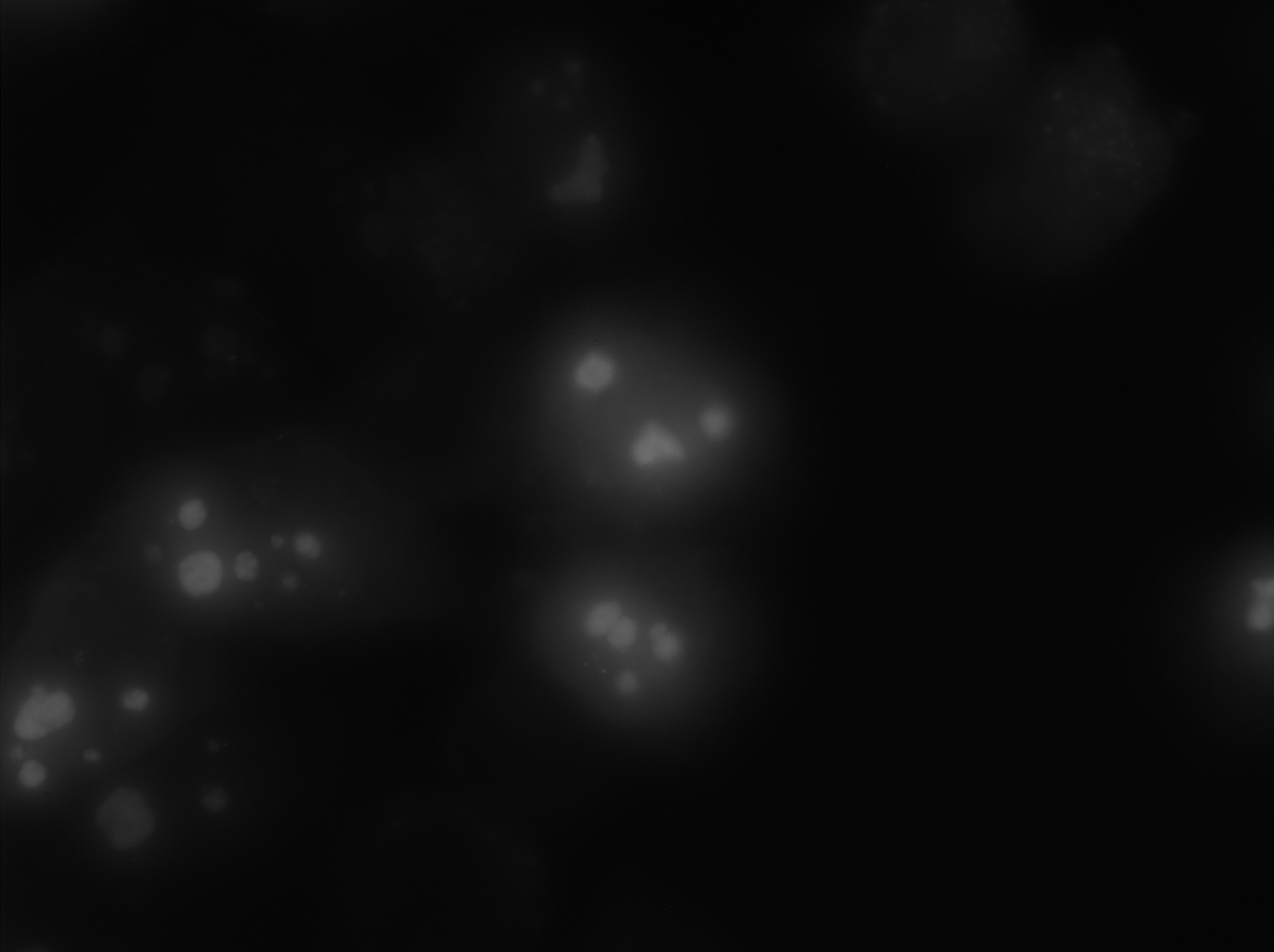

Supplement: Supplementary file 11 — Source data Fig. 3 part 1 [file 44319_2026_742_MOESM11_ESM.zip › Figure 3 Part 1/Fig 3b-e TTLL screen/TTLL4-YFPy I6.Project Maximum Z_XY1679076411_Z0_T0_C2.tif]

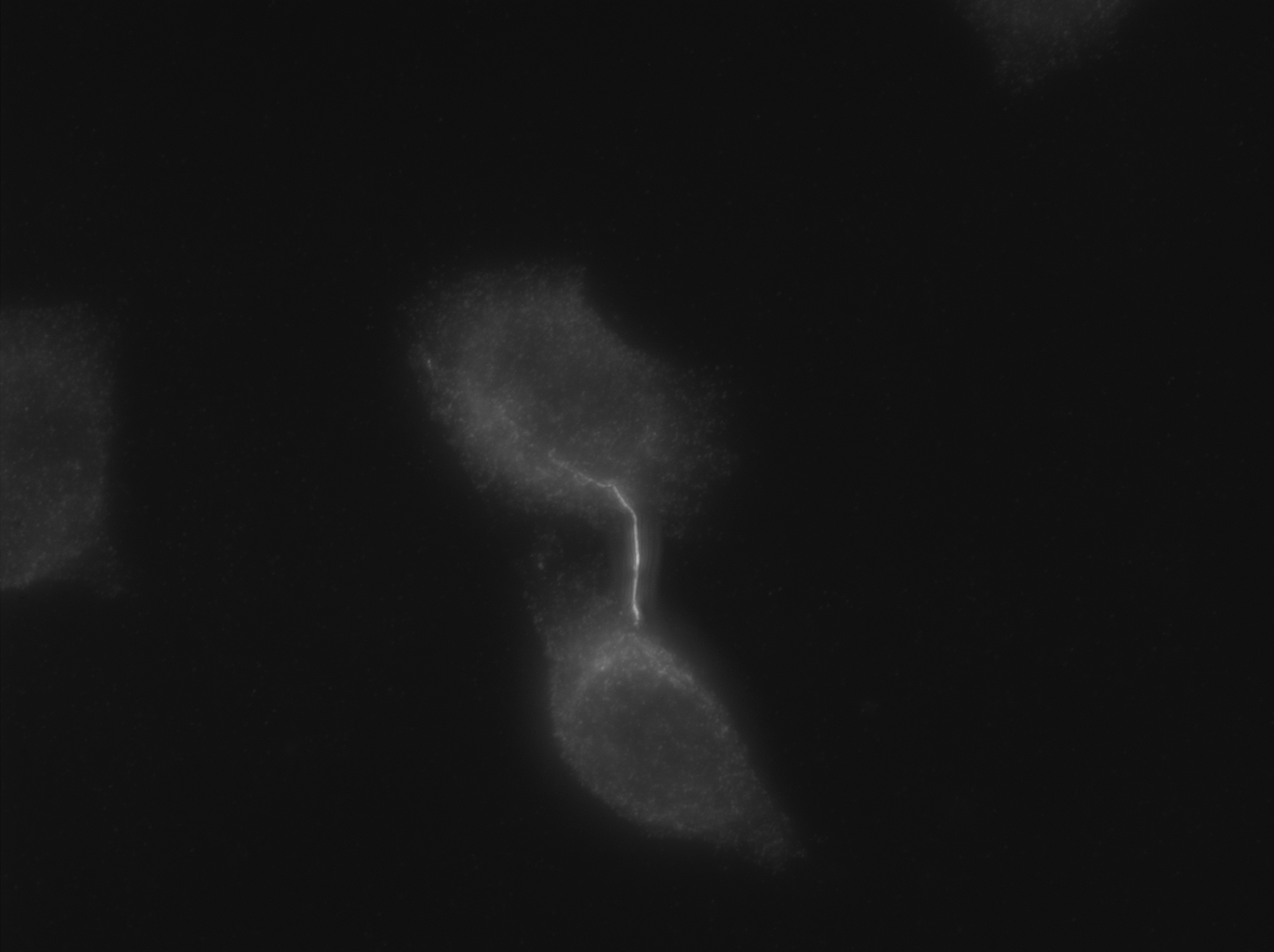

Supplement: Supplementary file 11 — Source data Fig. 3 part 1 [file 44319_2026_742_MOESM11_ESM.zip › Figure 3 Part 1/Fig 3b-e TTLL screen/TTLL1-GFP A3 I3.Project Maximum Z_XY1674674061_Z0_T0_C2.tif]

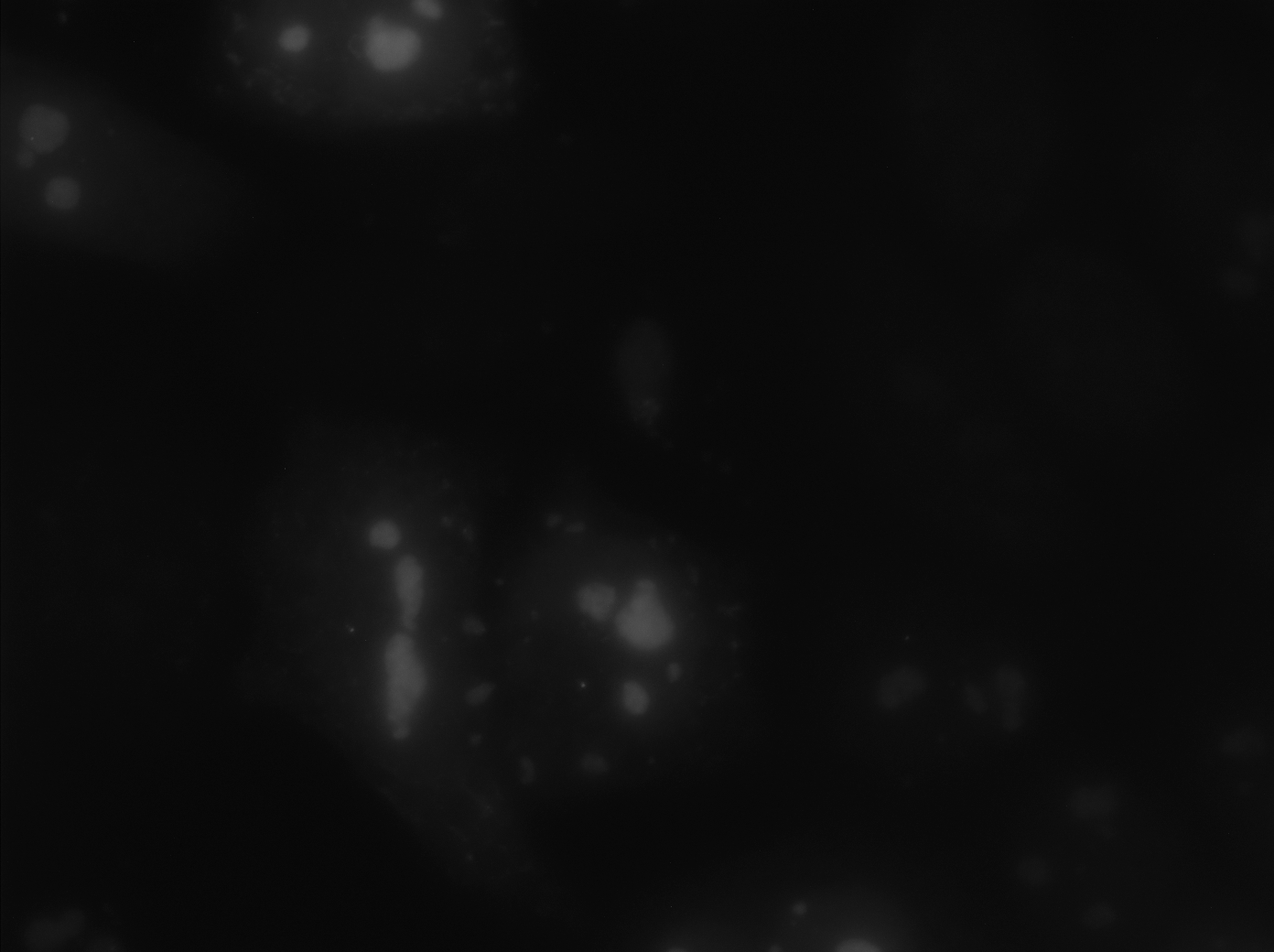

Supplement: Supplementary file 11 — Source data Fig. 3 part 1 [file 44319_2026_742_MOESM11_ESM.zip › Figure 3 Part 1/Fig 3b-e TTLL screen/TTLL4-YFPy I3.Project Maximum Z_XY1679075765_Z0_T0_C3.tif]

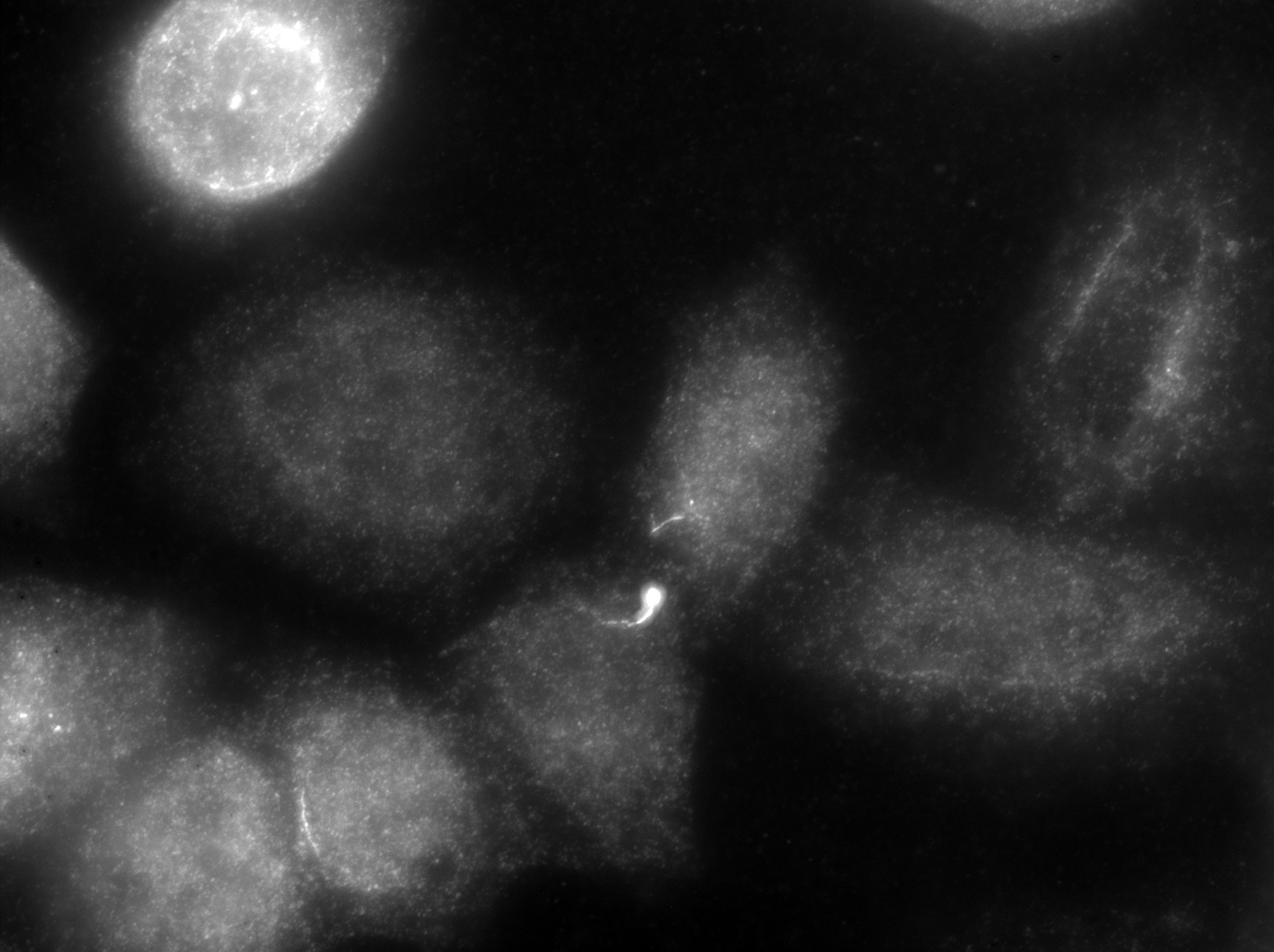

Supplement: Supplementary file 11 — Source data Fig. 3 part 1 [file 44319_2026_742_MOESM11_ESM.zip › Figure 3 Part 1/Fig 3b-e TTLL screen/TTLL1-GFP A3 I9.Project Maximum Z_XY1679695580_Z0_T0_C2.tif]

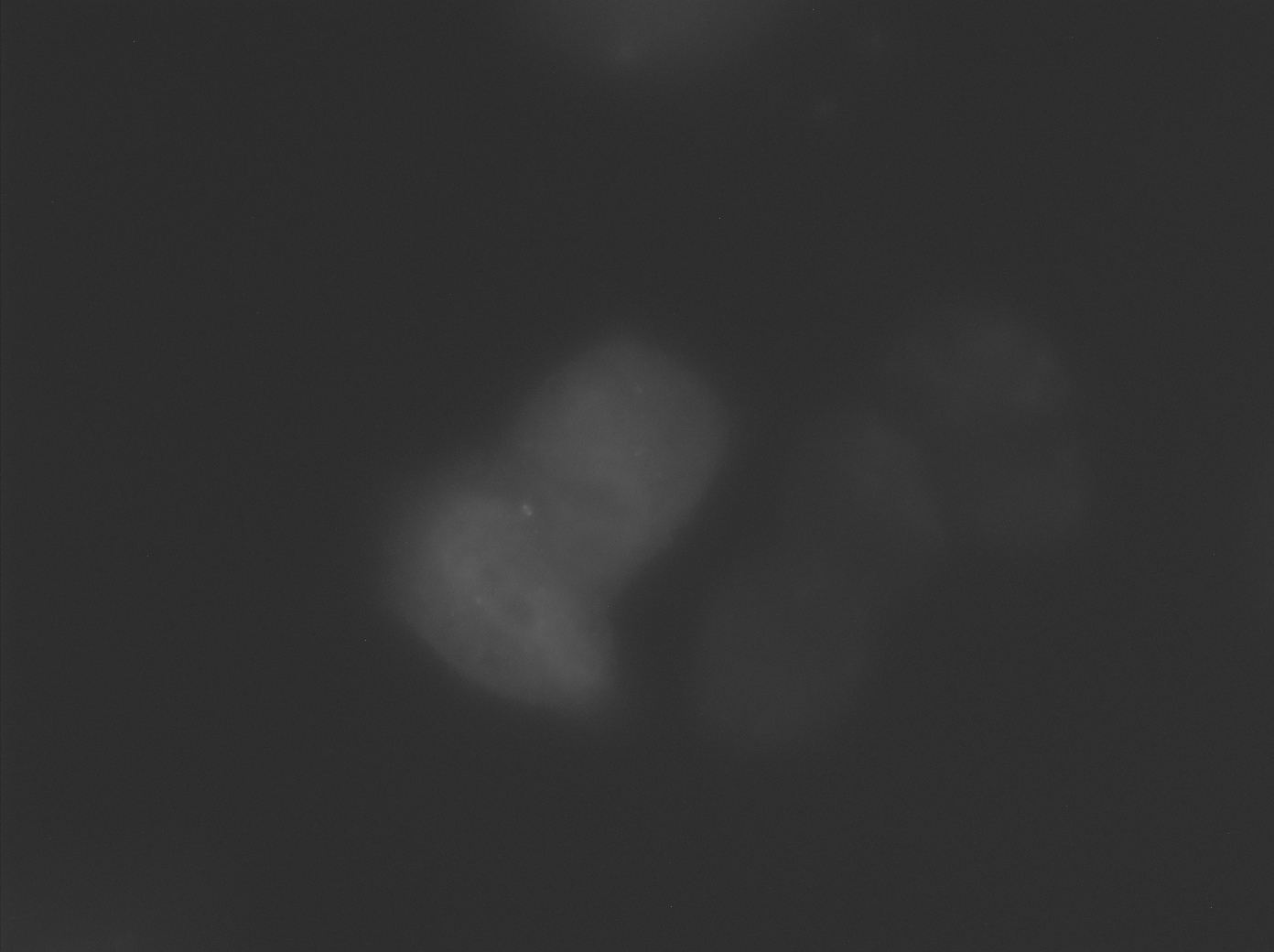

Supplement: Supplementary file 11 — Source data Fig. 3 part 1 [file 44319_2026_742_MOESM11_ESM.zip › Figure 3 Part 1/Fig 3b-e TTLL screen/TTLL1-GFP A4 I8.Project Maximum Z_XY1675962918_Z0_T0_C1.tif]

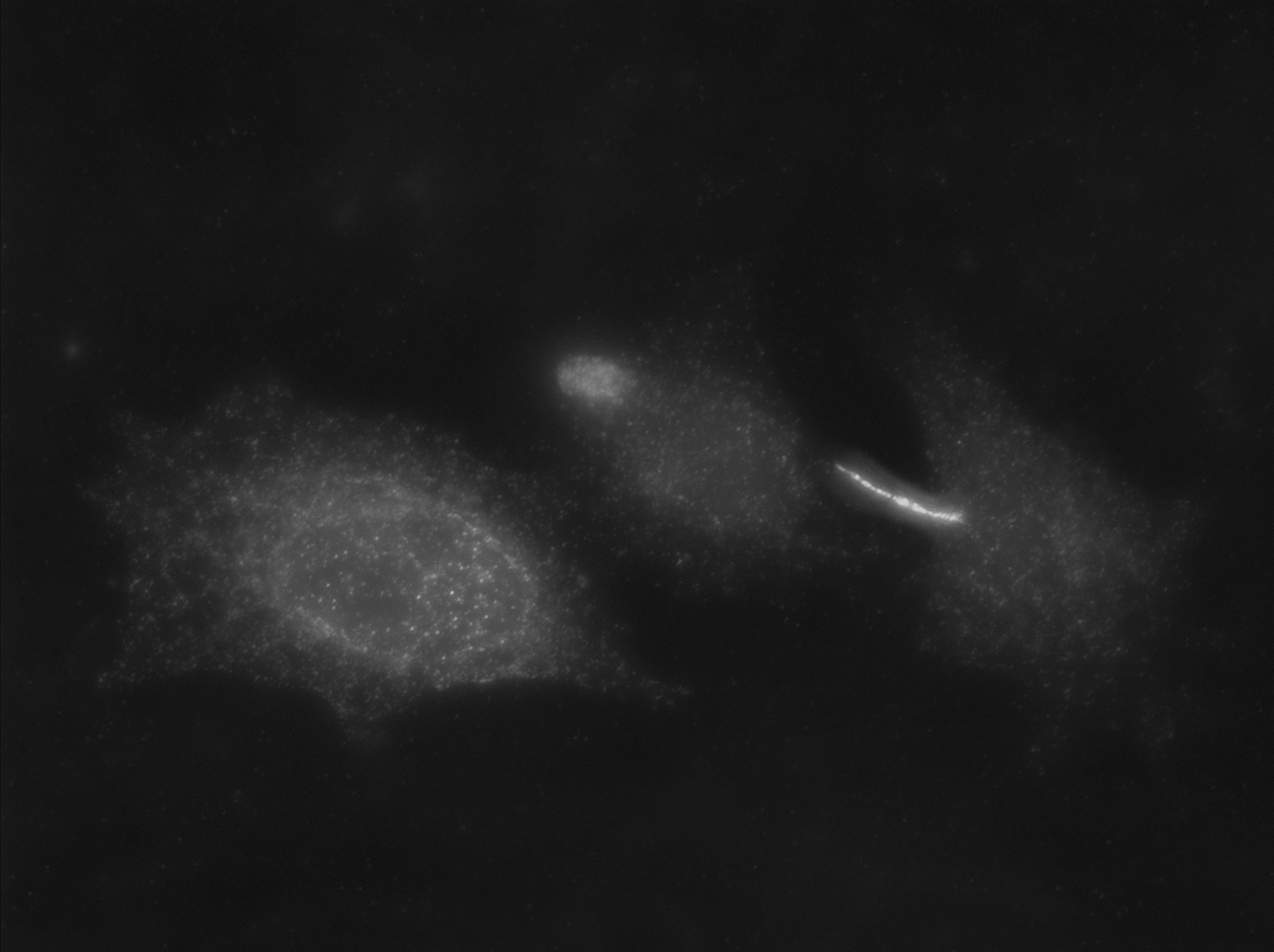

Supplement: Supplementary file 11 — Source data Fig. 3 part 1 [file 44319_2026_742_MOESM11_ESM.zip › Figure 3 Part 1/Fig 3b-e TTLL screen/EYFP MB I1.Project Maximum Z_XY1663874451_Z0_T0_C1.tif]

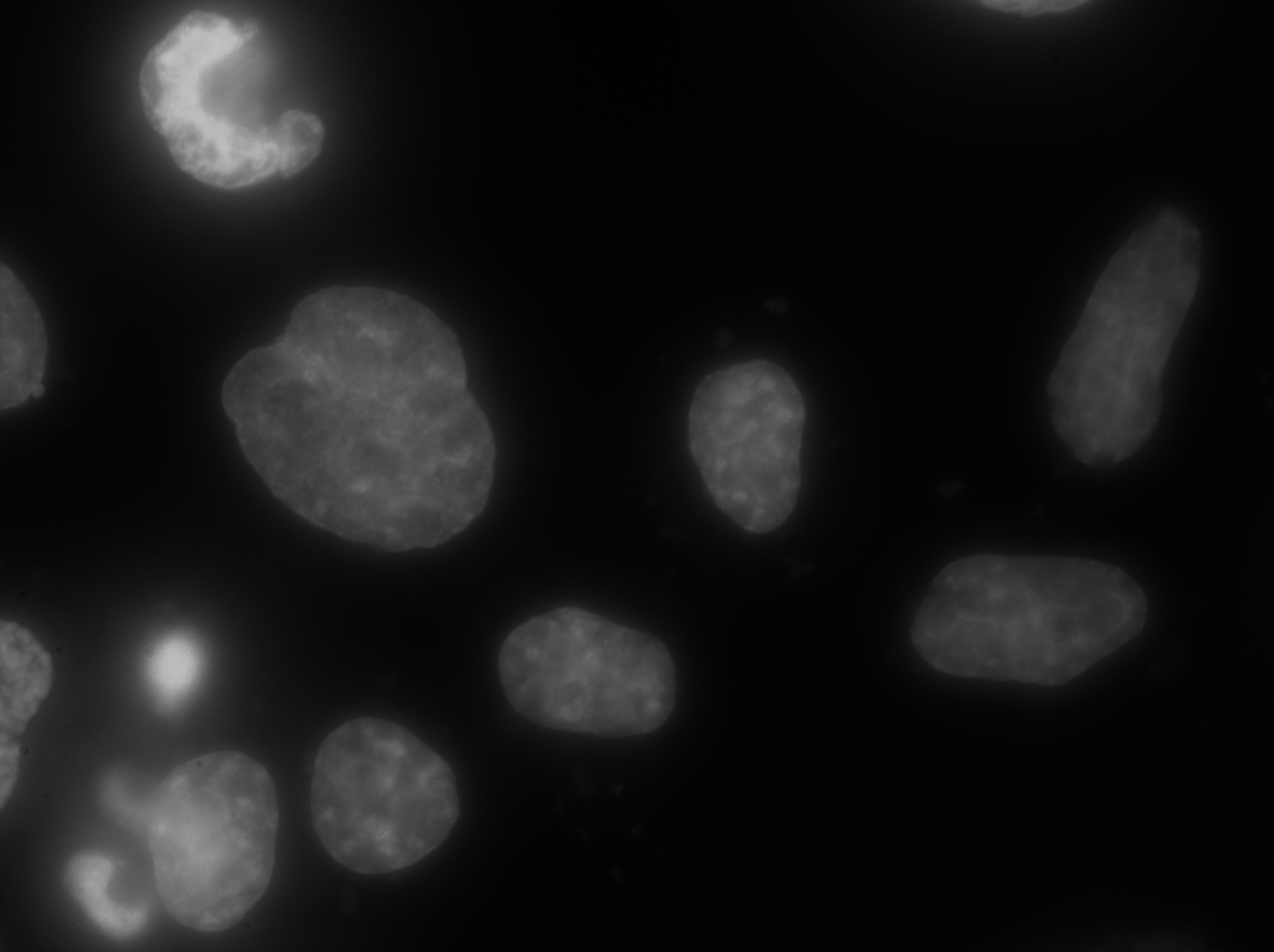

Supplement: Supplementary file 11 — Source data Fig. 3 part 1 [file 44319_2026_742_MOESM11_ESM.zip › Figure 3 Part 1/Fig 3b-e TTLL screen/TTLL1-GFP A3 I9.Project Maximum Z_XY1679695580_Z0_T0_C0.tif]

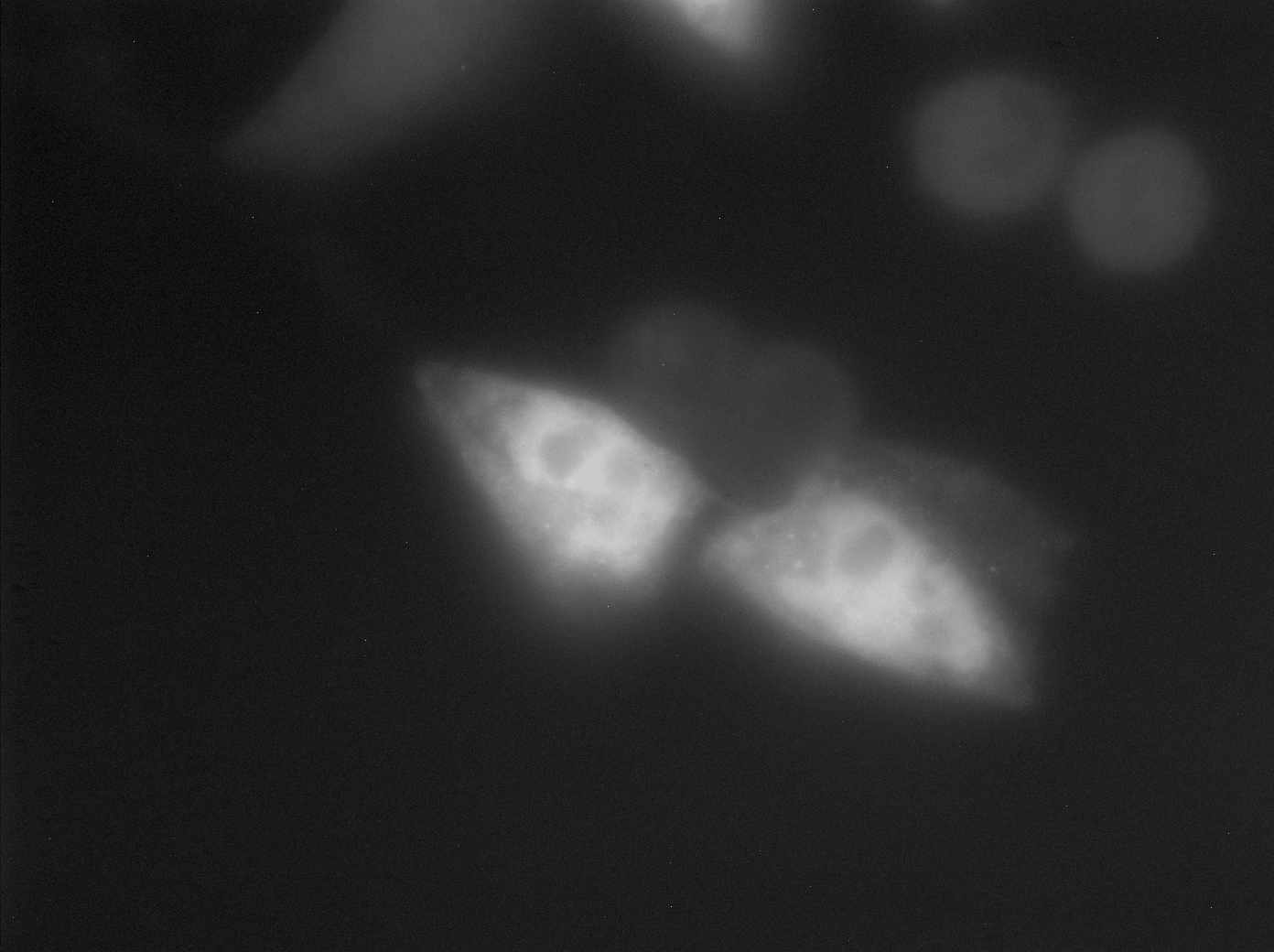

Supplement: Supplementary file 11 — Source data Fig. 3 part 1 [file 44319_2026_742_MOESM11_ESM.zip › Figure 3 Part 1/Fig 3b-e TTLL screen/TTLL1-GFPy I1.Project Maximum Z_XY1679086158_Z0_T0_C1.tif]

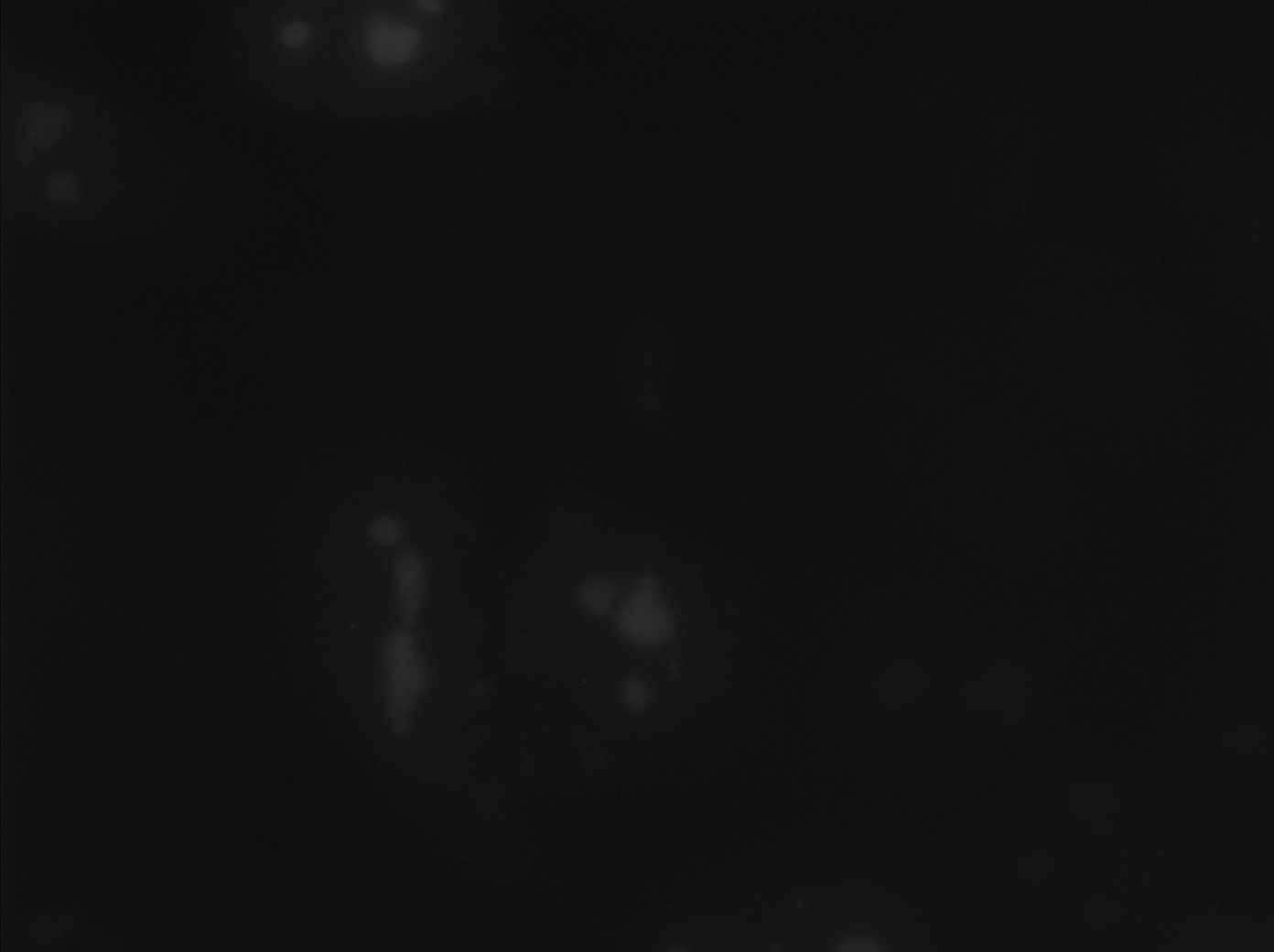

Supplement: Supplementary file 11 — Source data Fig. 3 part 1 [file 44319_2026_742_MOESM11_ESM.zip › Figure 3 Part 1/Fig 3b-e TTLL screen/TTLL4-YFPy I3.Project Maximum Z_XY1679075765_Z0_T0_C1.tif]

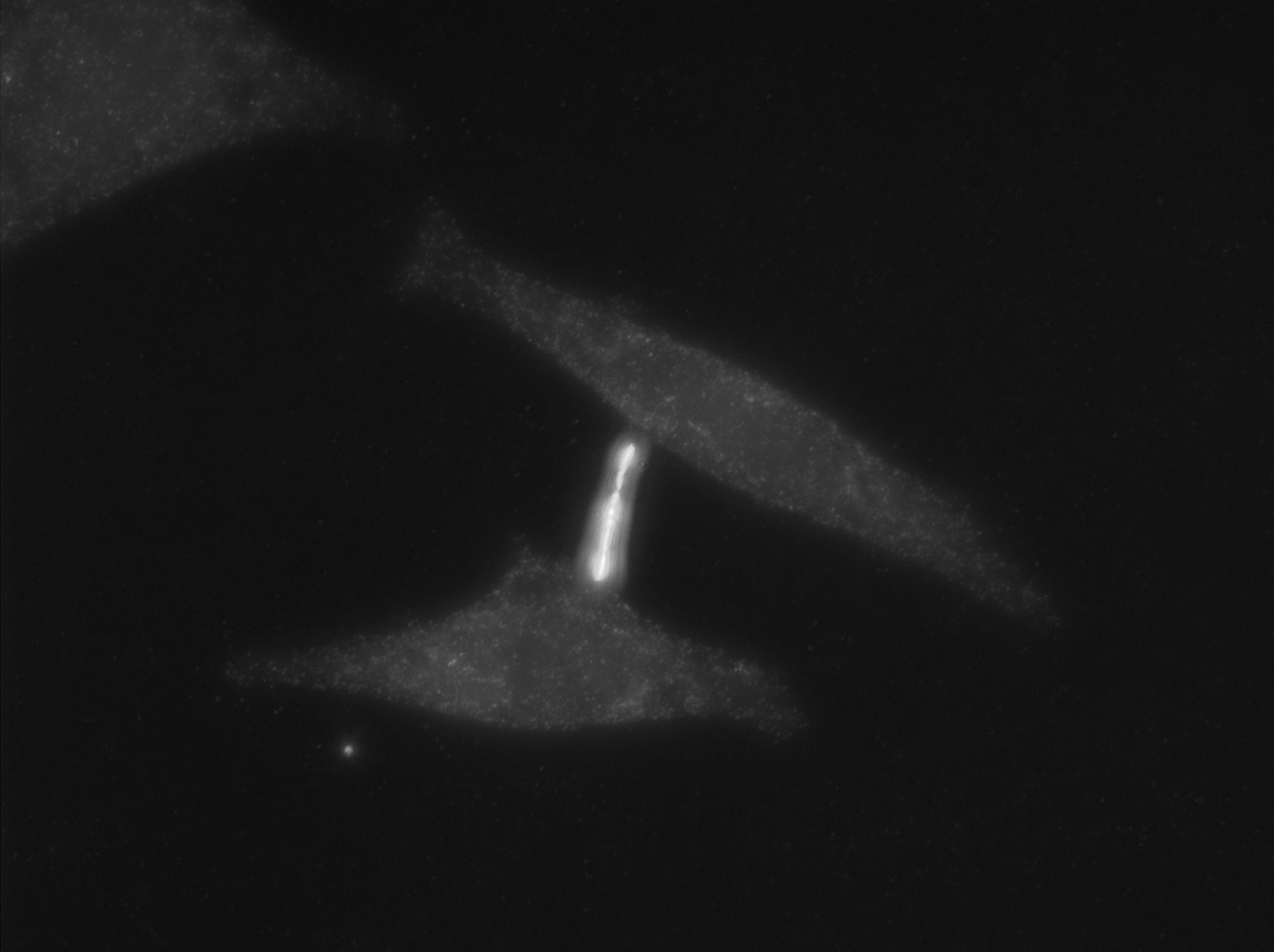

Supplement: Supplementary file 11 — Source data Fig. 3 part 1 [file 44319_2026_742_MOESM11_ESM.zip › Figure 3 Part 1/Fig 3b-e TTLL screen/EYFP MB I2.Project Maximum Z_XY1663875048_Z0_T0_C1.tif]

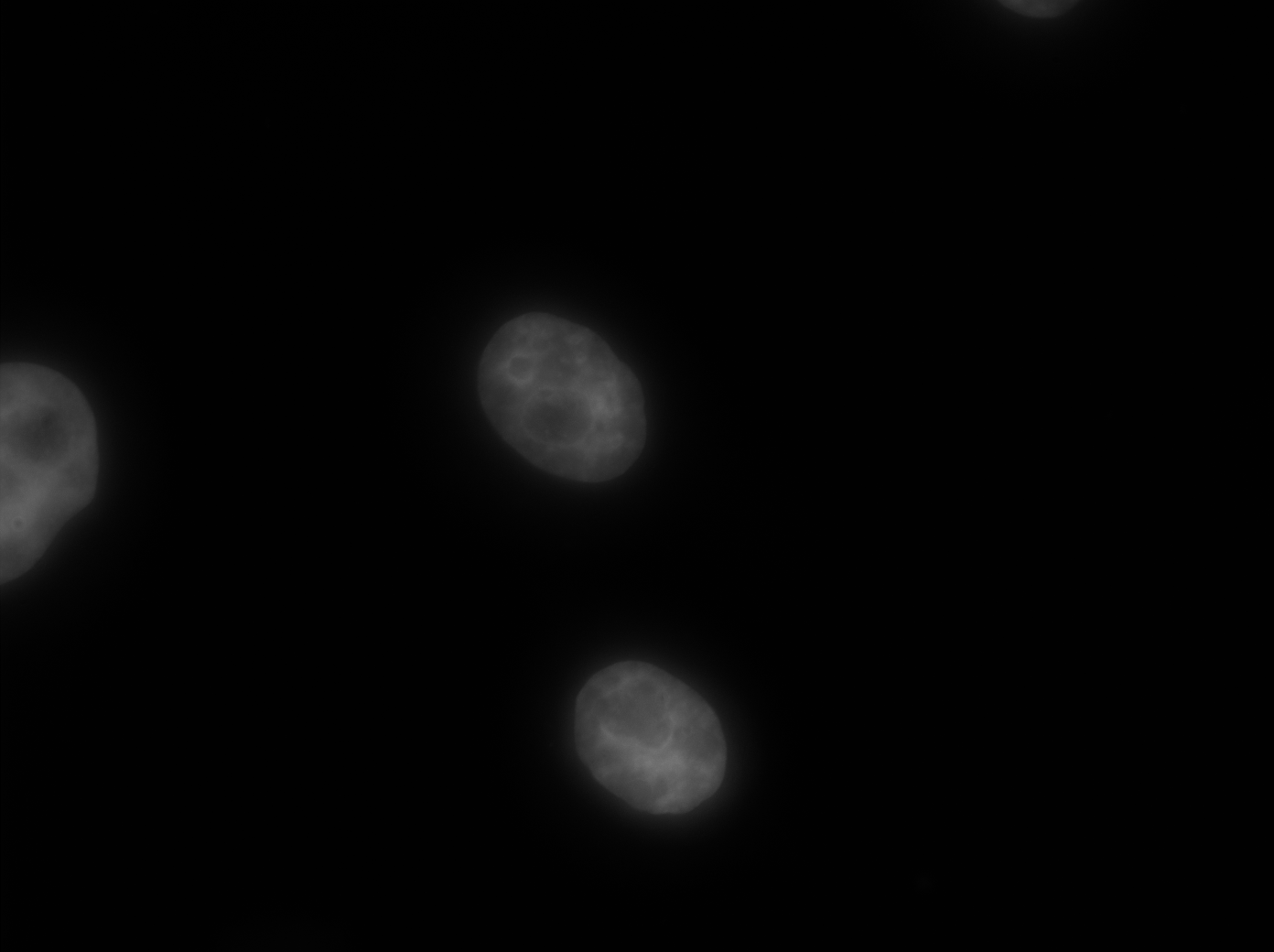

Supplement: Supplementary file 11 — Source data Fig. 3 part 1 [file 44319_2026_742_MOESM11_ESM.zip › Figure 3 Part 1/Fig 3b-e TTLL screen/TTLL1-GFP A3 I3.Project Maximum Z_XY1674674061_Z0_T0_C0.tif]

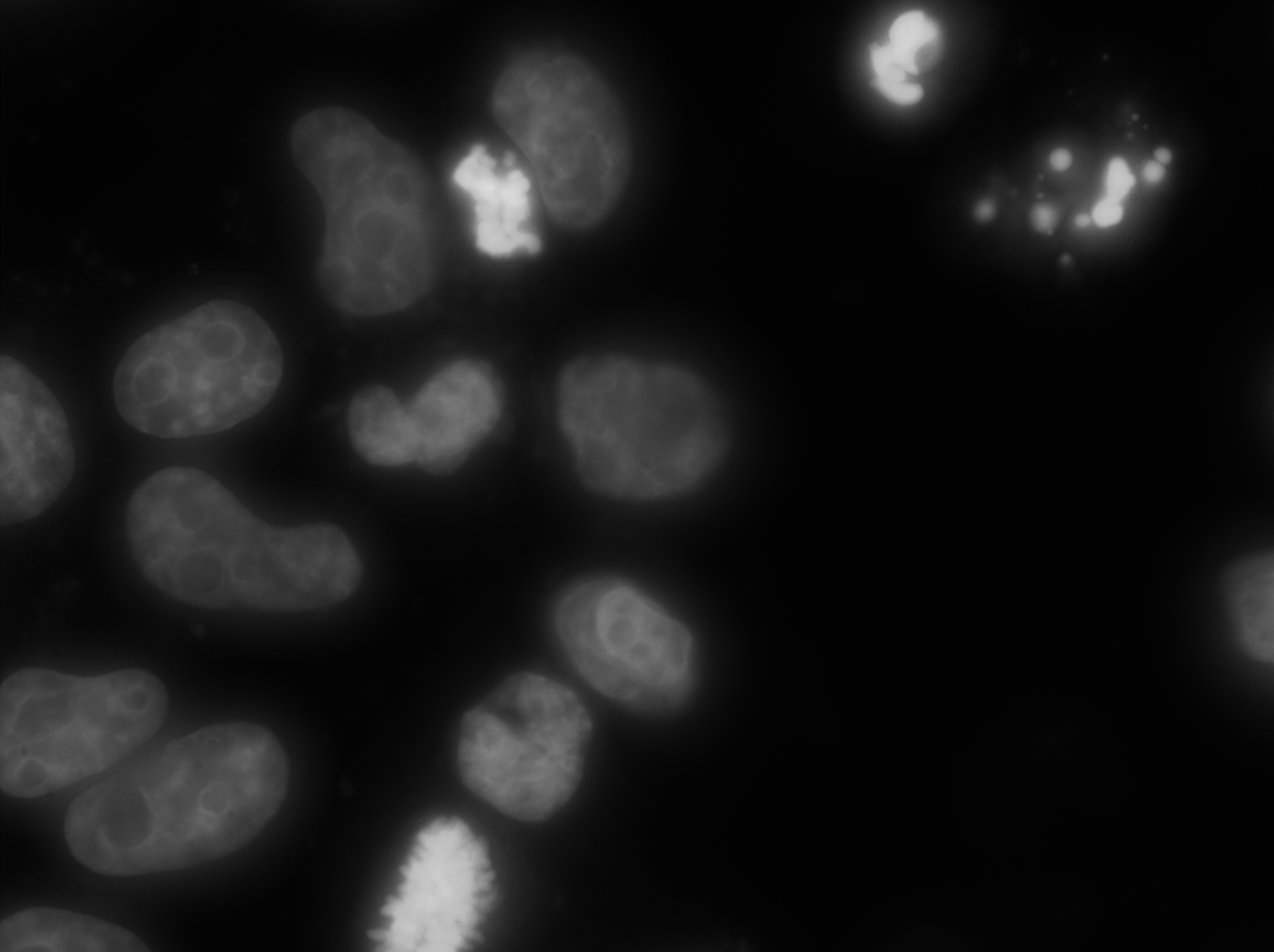

Supplement: Supplementary file 11 — Source data Fig. 3 part 1 [file 44319_2026_742_MOESM11_ESM.zip › Figure 3 Part 1/Fig 3b-e TTLL screen/TTLL4-YFPy I6.Project Maximum Z_XY1679076411_Z0_T0_C0.tif]

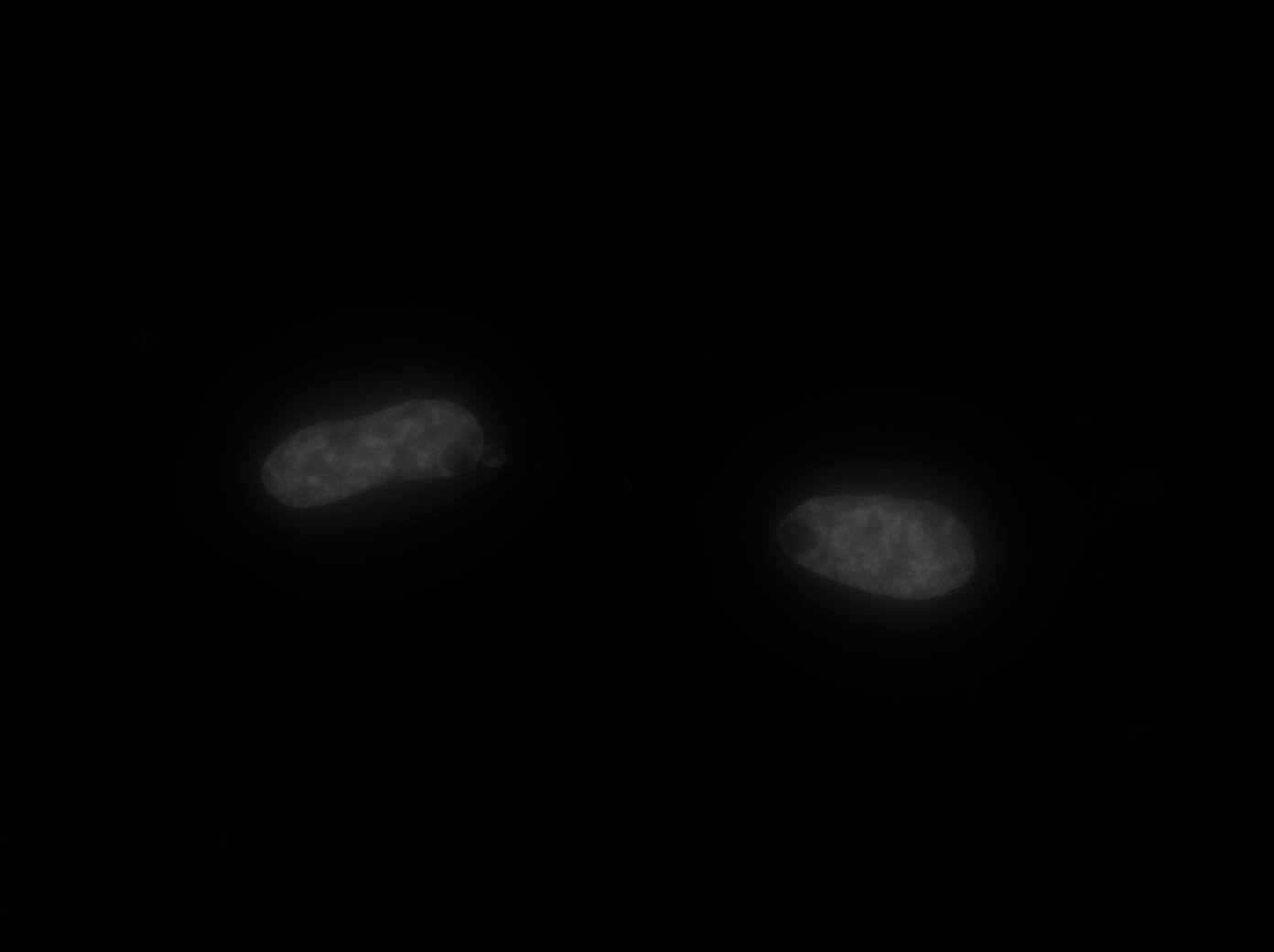

Supplement: Supplementary file 11 — Source data Fig. 3 part 1 [file 44319_2026_742_MOESM11_ESM.zip › Figure 3 Part 1/Fig 3b-e TTLL screen/EYFP MB light I5.Project Maximum Z_XY1663875722_Z0_T0_C0.tif]

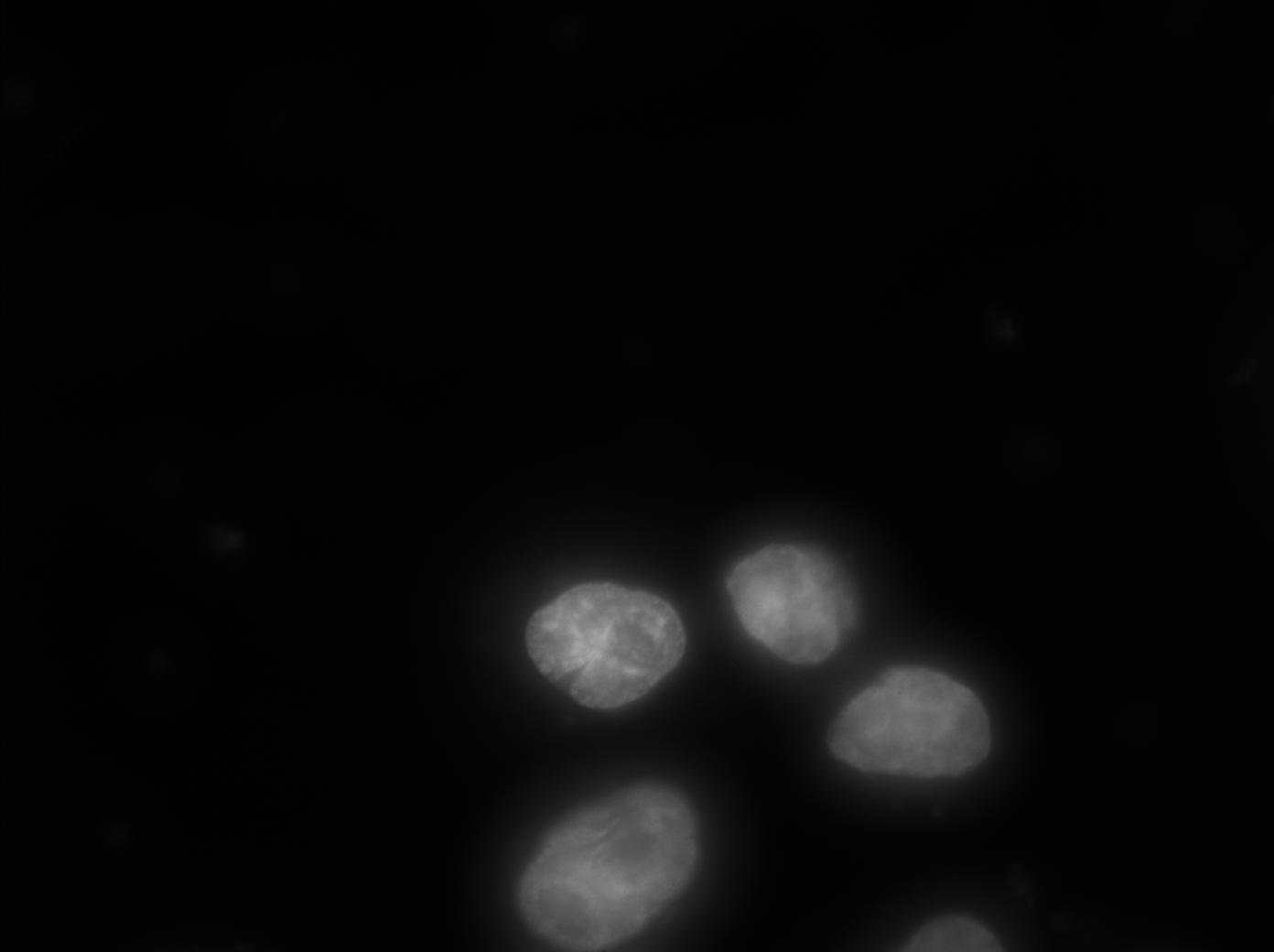

Supplement: Supplementary file 11 — Source data Fig. 3 part 1 [file 44319_2026_742_MOESM11_ESM.zip › Figure 3 Part 1/Fig 3b-e TTLL screen/TTLL4-YFPy I9.Project Maximum Z_XY1679081878_Z0_T0_C0.tif]

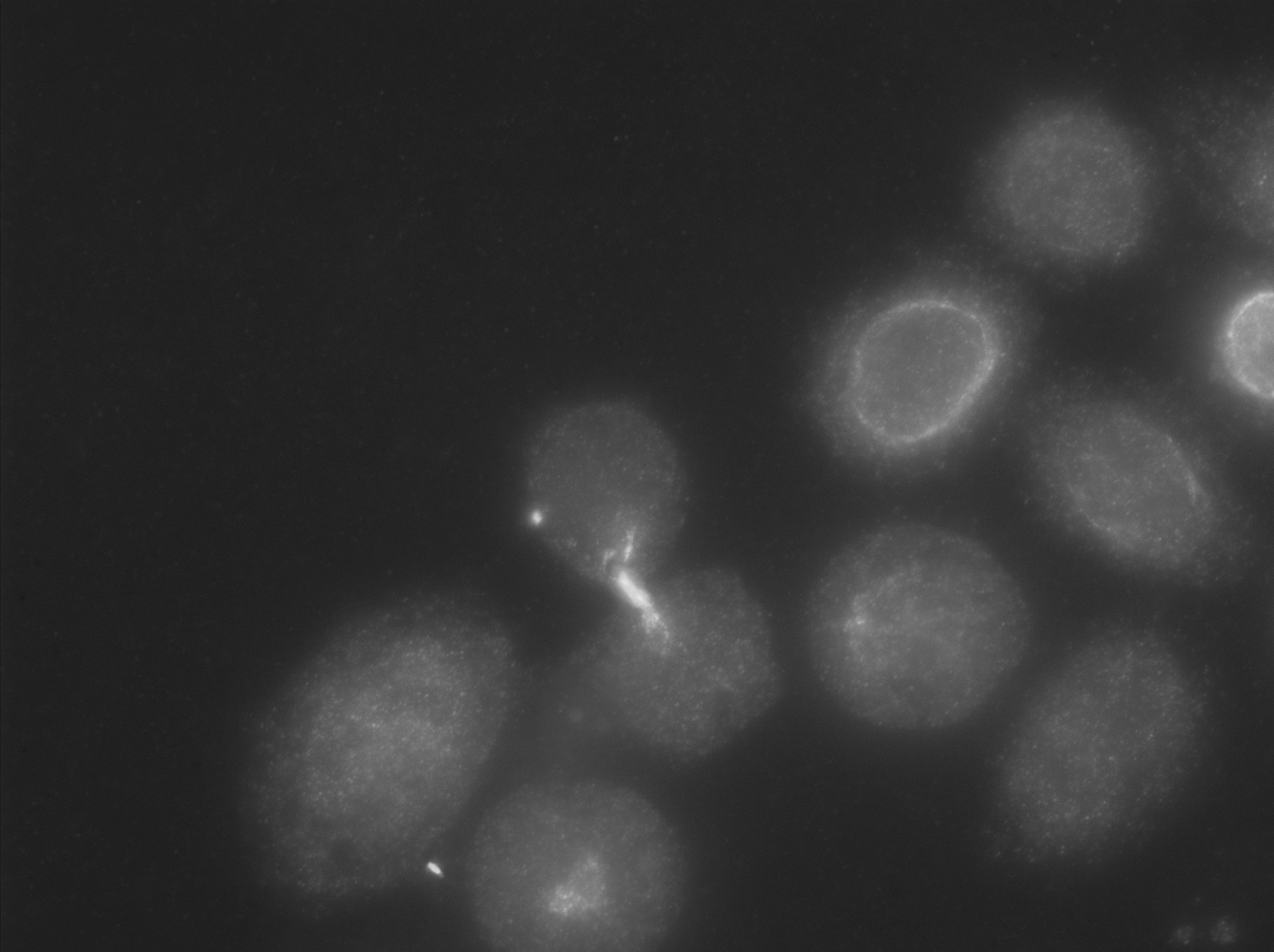

Supplement: Supplementary file 11 — Source data Fig. 3 part 1 [file 44319_2026_742_MOESM11_ESM.zip › Figure 3 Part 1/Fig 3b-e TTLL screen/TTLL4-YFPy I16.Project Maximum Z_XY1679337550_Z0_T0_C1.tif]

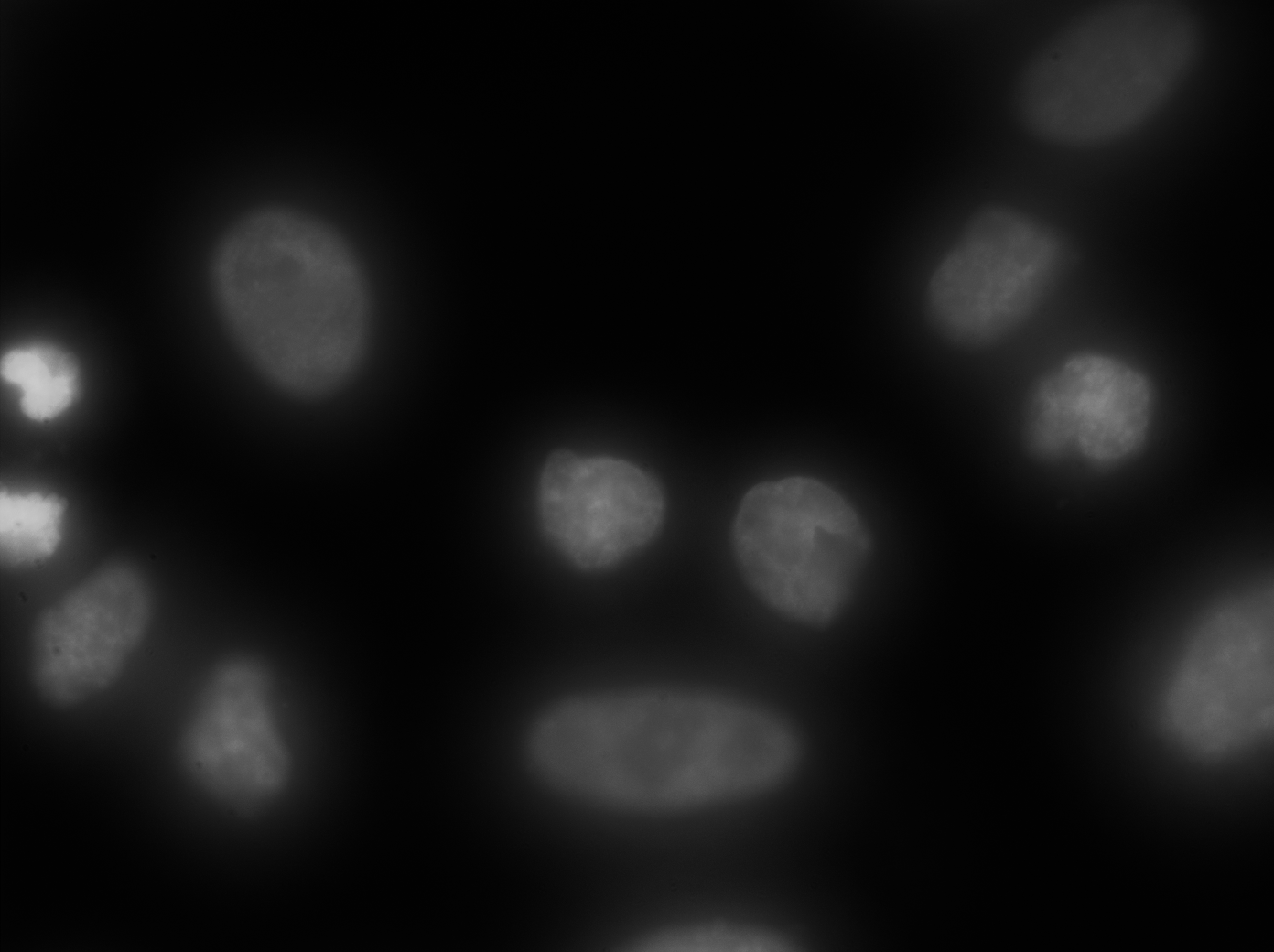

Supplement: Supplementary file 11 — Source data Fig. 3 part 1 [file 44319_2026_742_MOESM11_ESM.zip › Figure 3 Part 1/Fig 3b-e TTLL screen/TTLL1-GFP A3 I7.Project Maximum Z_XY1679695246_Z0_T0_C0.tif]

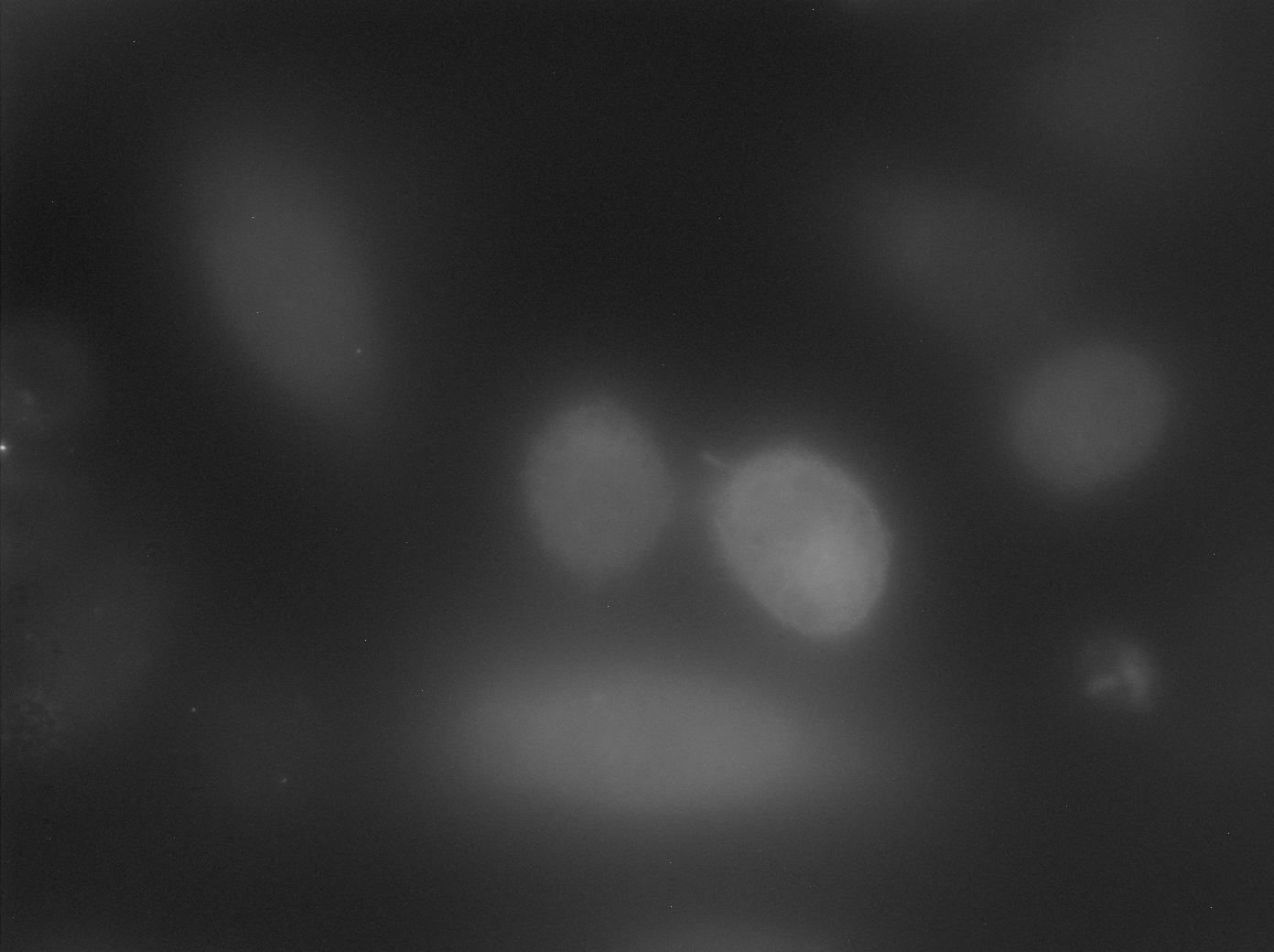

Supplement: Supplementary file 11 — Source data Fig. 3 part 1 [file 44319_2026_742_MOESM11_ESM.zip › Figure 3 Part 1/Fig 3b-e TTLL screen/TTLL1-GFP A3 I7.Project Maximum Z_XY1679695246_Z0_T0_C1.tif]

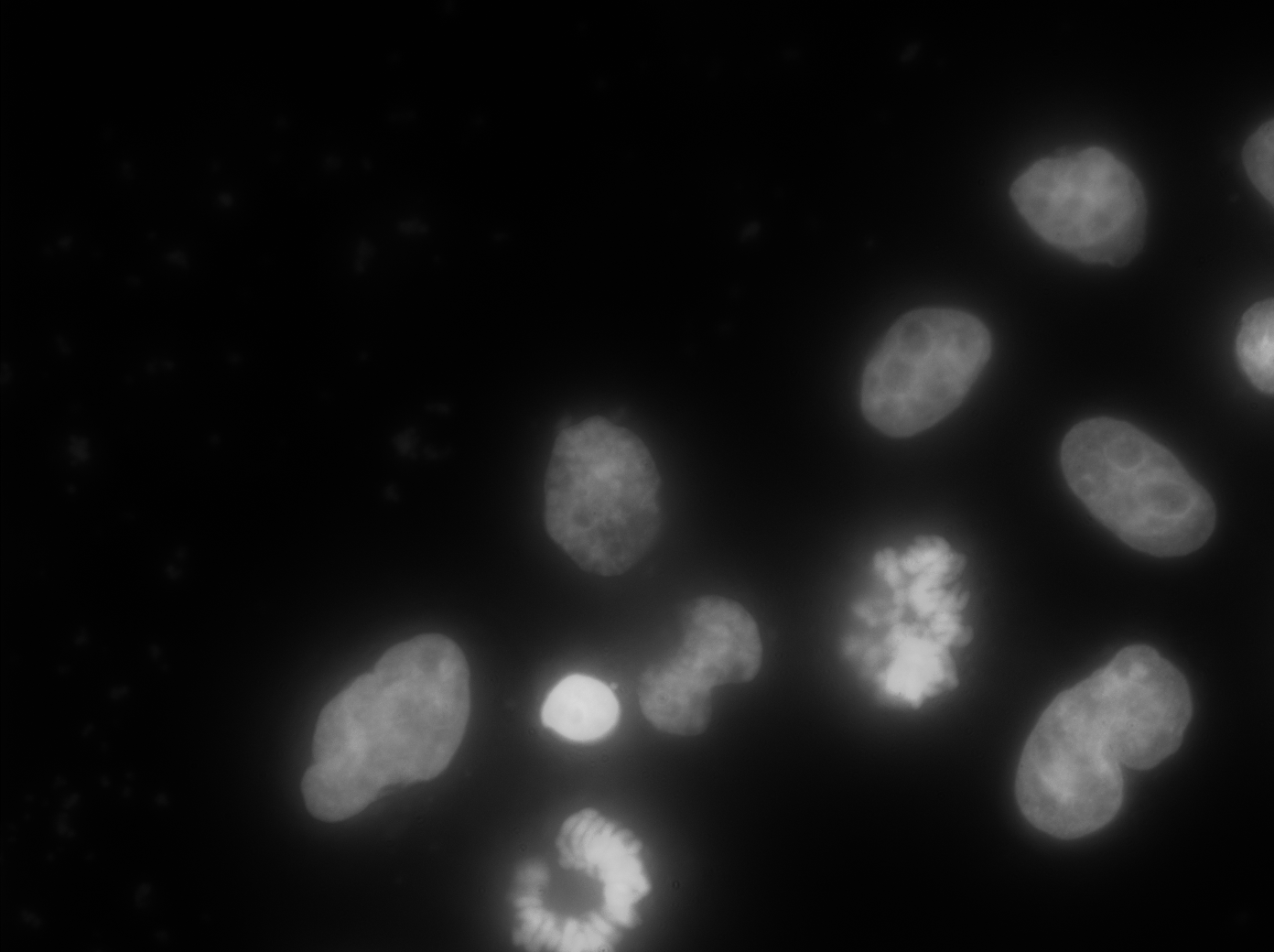

Supplement: Supplementary file 11 — Source data Fig. 3 part 1 [file 44319_2026_742_MOESM11_ESM.zip › Figure 3 Part 1/Fig 3b-e TTLL screen/TTLL4-YFPy I16.Project Maximum Z_XY1679337550_Z0_T0_C0.tif]

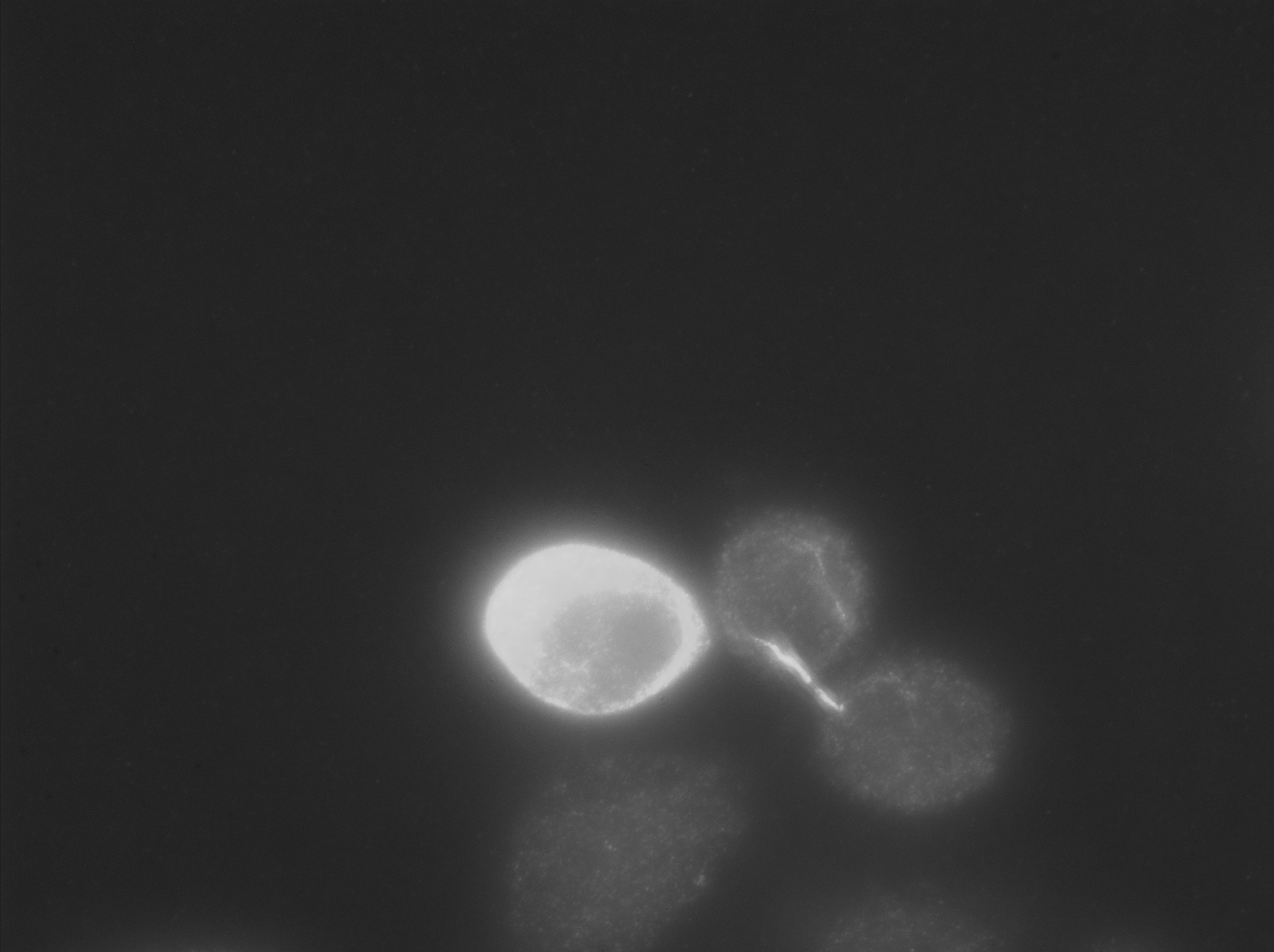

Supplement: Supplementary file 11 — Source data Fig. 3 part 1 [file 44319_2026_742_MOESM11_ESM.zip › Figure 3 Part 1/Fig 3b-e TTLL screen/TTLL4-YFPy I9.Project Maximum Z_XY1679081878_Z0_T0_C1.tif]

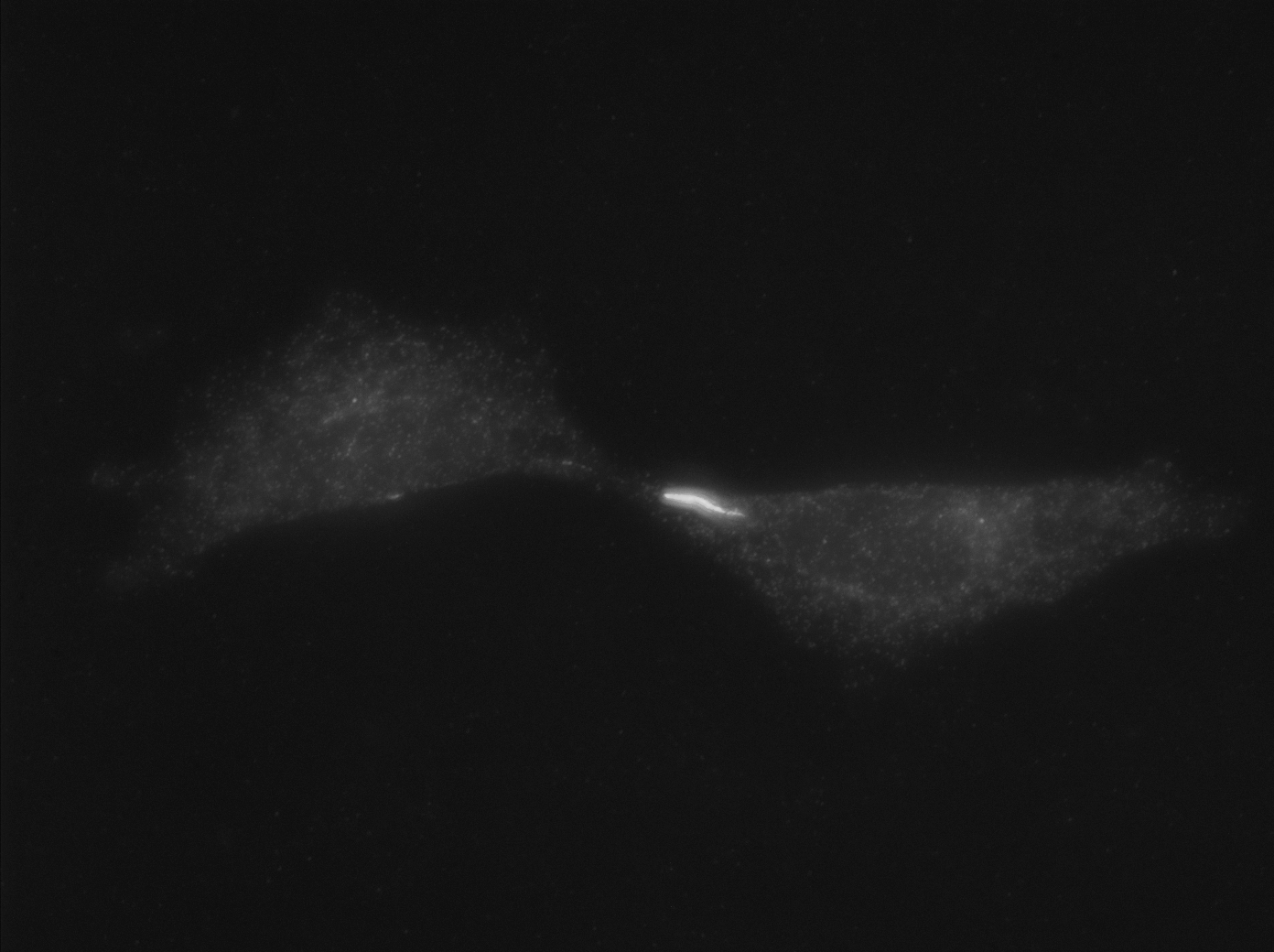

Supplement: Supplementary file 11 — Source data Fig. 3 part 1 [file 44319_2026_742_MOESM11_ESM.zip › Figure 3 Part 1/Fig 3b-e TTLL screen/EYFP MB light I5.Project Maximum Z_XY1663875722_Z0_T0_C1.tif]

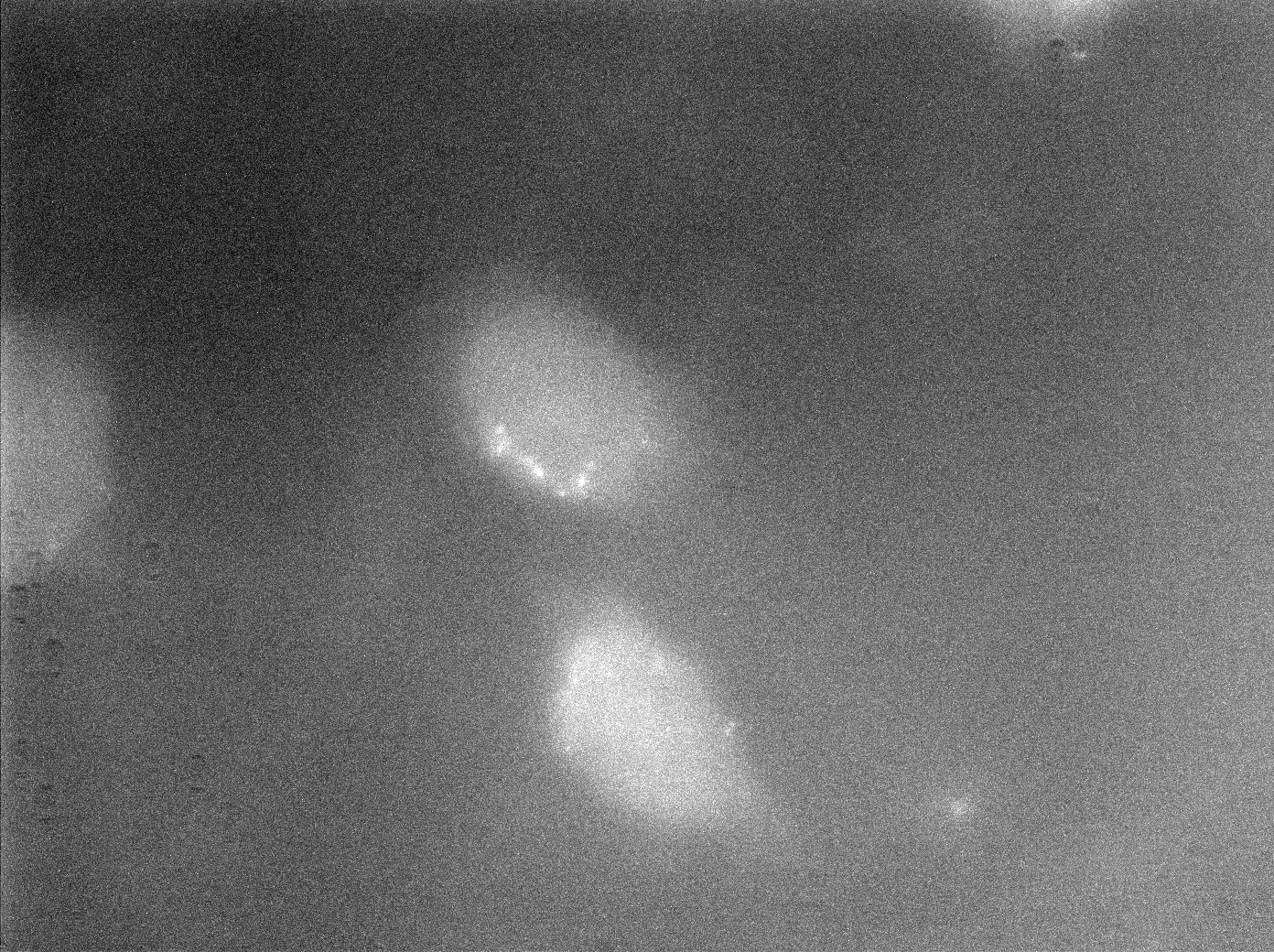

Supplement: Supplementary file 11 — Source data Fig. 3 part 1 [file 44319_2026_742_MOESM11_ESM.zip › Figure 3 Part 1/Fig 3b-e TTLL screen/TTLL1-GFP A3 I3.Project Maximum Z_XY1674674061_Z0_T0_C1.tif]

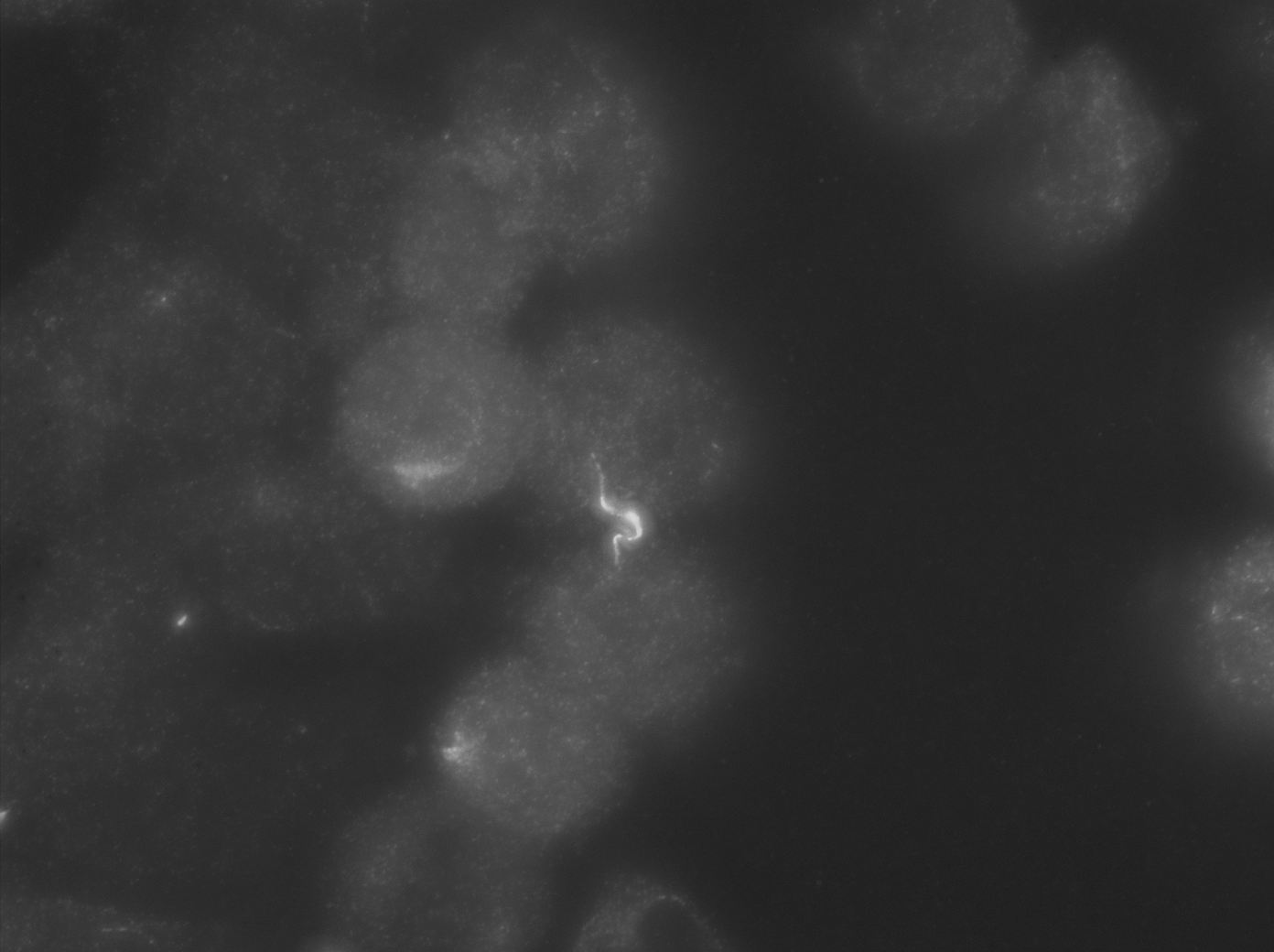

Supplement: Supplementary file 11 — Source data Fig. 3 part 1 [file 44319_2026_742_MOESM11_ESM.zip › Figure 3 Part 1/Fig 3b-e TTLL screen/TTLL4-YFPy I6.Project Maximum Z_XY1679076411_Z0_T0_C1.tif]

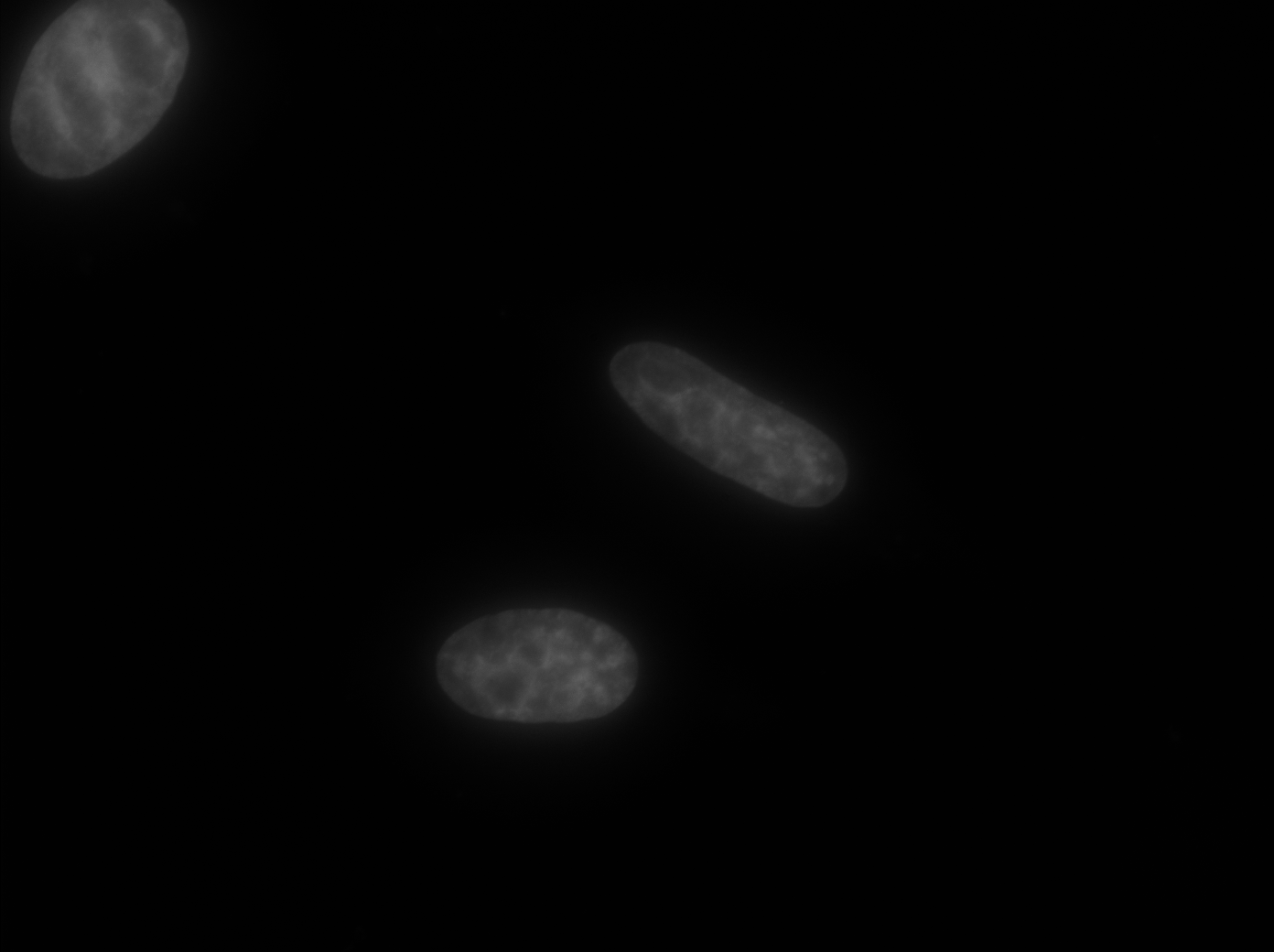

Supplement: Supplementary file 11 — Source data Fig. 3 part 1 [file 44319_2026_742_MOESM11_ESM.zip › Figure 3 Part 1/Fig 3b-e TTLL screen/EYFP MB I2.Project Maximum Z_XY1663875048_Z0_T0_C0.tif]

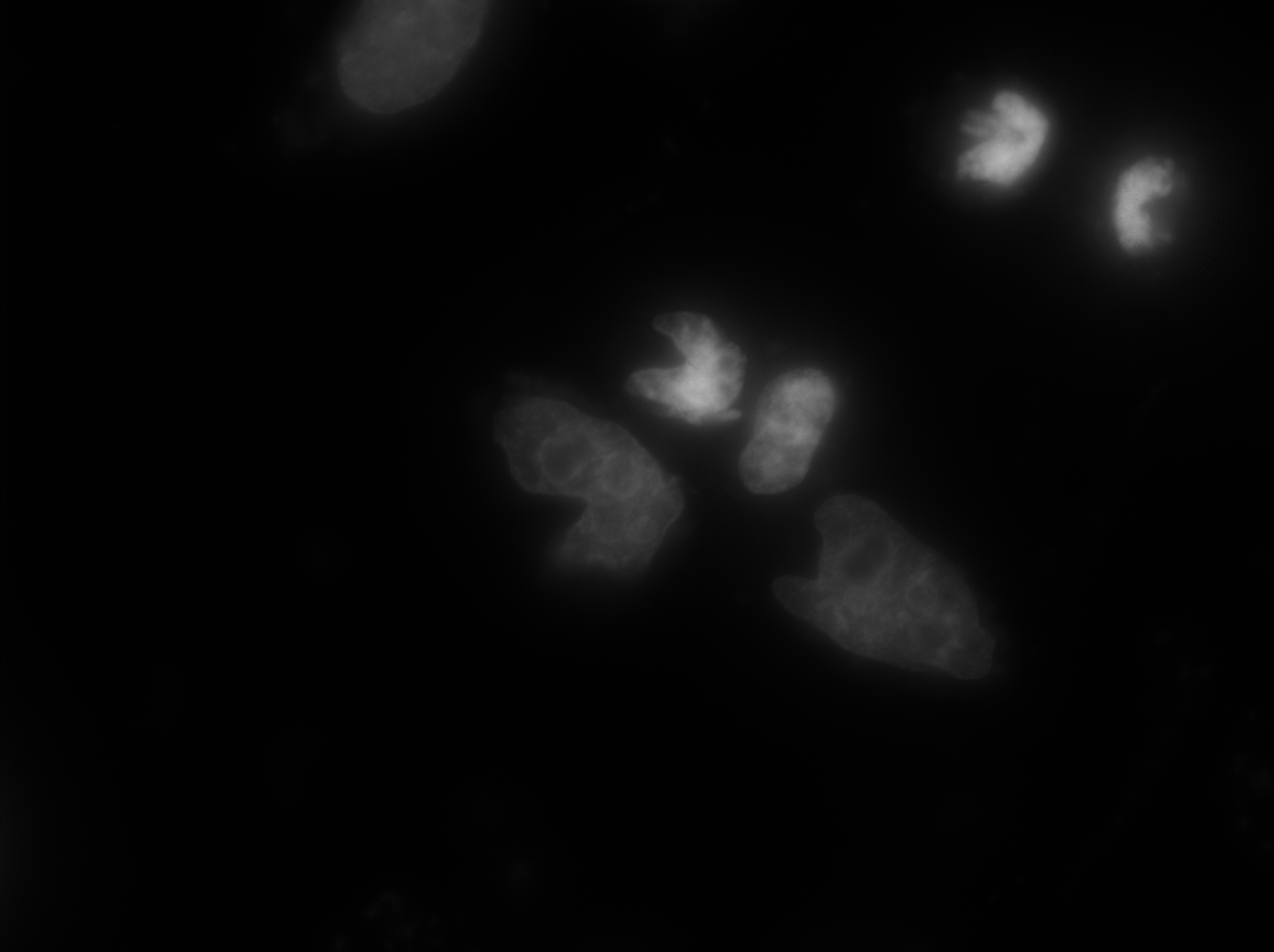

Supplement: Supplementary file 11 — Source data Fig. 3 part 1 [file 44319_2026_742_MOESM11_ESM.zip › Figure 3 Part 1/Fig 3b-e TTLL screen/TTLL1-GFPy I1.Project Maximum Z_XY1679086158_Z0_T0_C0.tif]

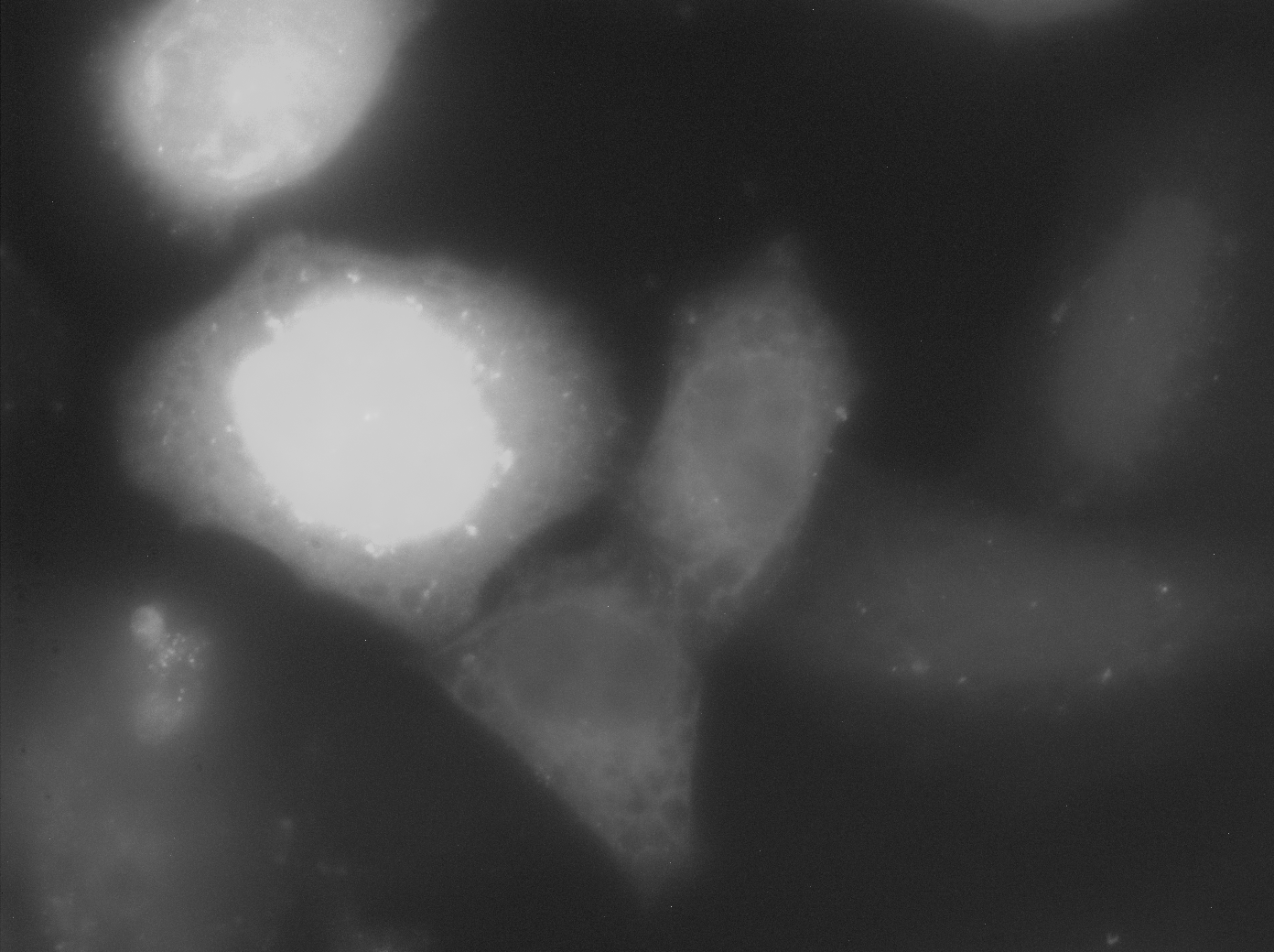

Supplement: Supplementary file 11 — Source data Fig. 3 part 1 [file 44319_2026_742_MOESM11_ESM.zip › Figure 3 Part 1/Fig 3b-e TTLL screen/TTLL1-GFP A3 I9.Project Maximum Z_XY1679695580_Z0_T0_C1.tif]

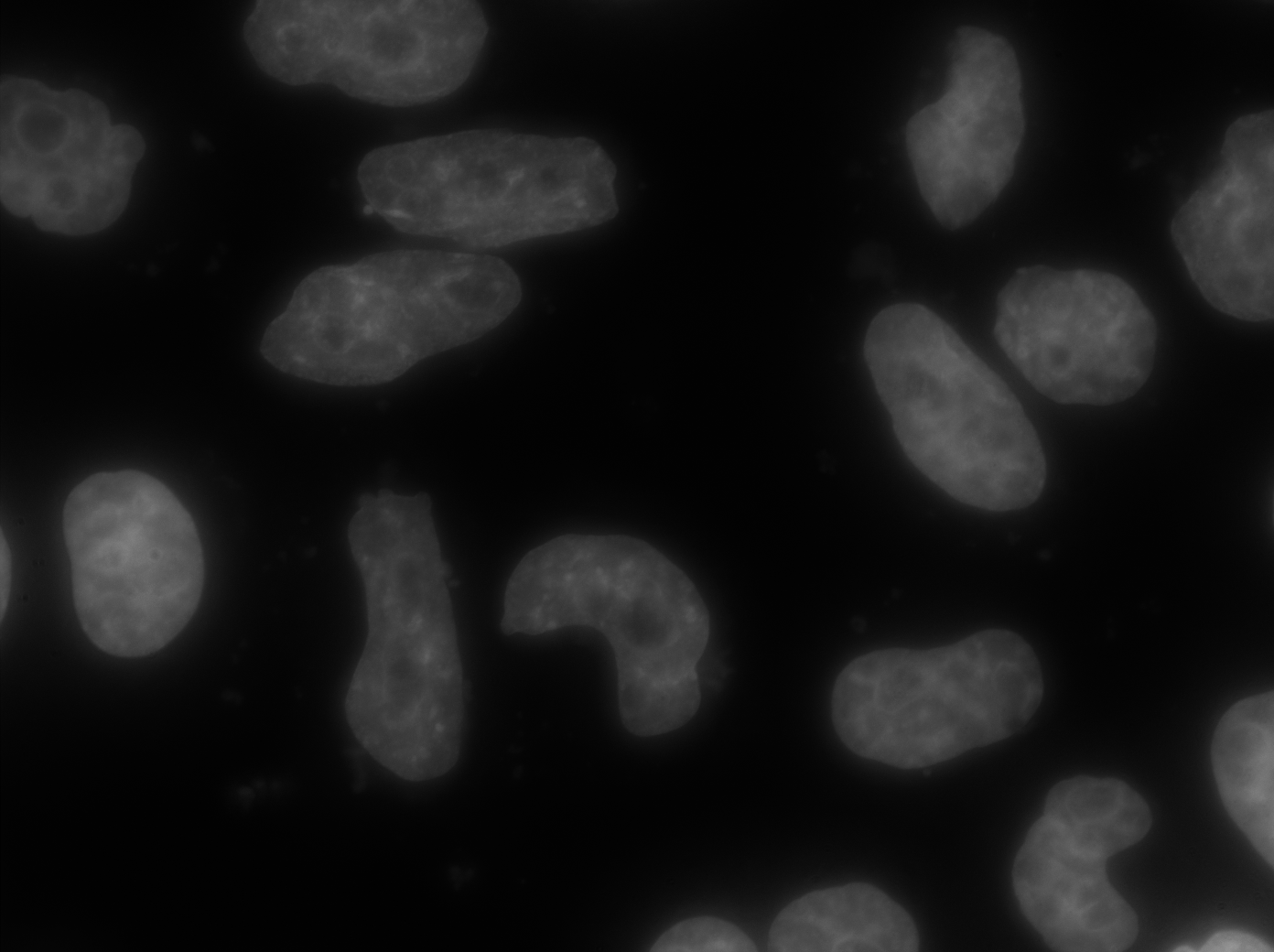

Supplement: Supplementary file 11 — Source data Fig. 3 part 1 [file 44319_2026_742_MOESM11_ESM.zip › Figure 3 Part 1/Fig 3b-e TTLL screen/TTLL4-YFPy I3.Project Maximum Z_XY1679075765_Z0_T0_C0.tif]
