# Supplementary figures and images for: TPGS1 regulates central spindle microtubule glutamylation and remodeling during telophase and abscission (part 10 of 36)
Source: EMBO Rep. 2026 Mar 23;27(8):1944–63. doi: 10.1038/s44319-026-00742-3 (PMC13121839; doi:10.1038/s44319-026-00742-3)

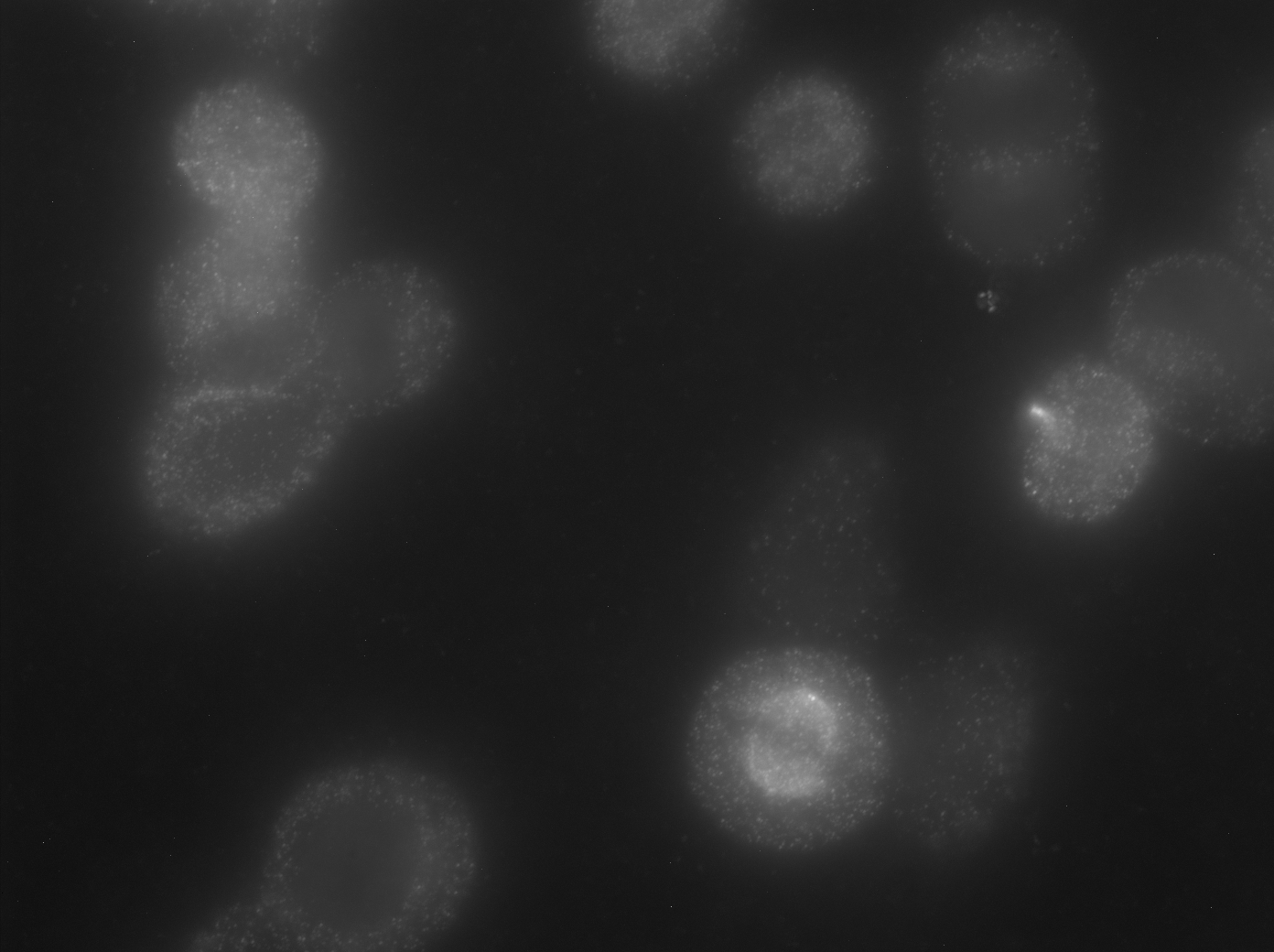

Supplement: Supplementary file 10 — Source data Fig. 2 part 7 [file 44319_2026_742_MOESM10_ESM.zip › Figure 2 Part 7/Fig 2fg Control Hela rGT335 acetylated tubulin part 2/Metaphase/Cas9 actub rGT335 9-8-25 R1 M6.Project Maximum Z_XY1757354221_Z0_T0_C1.tif]

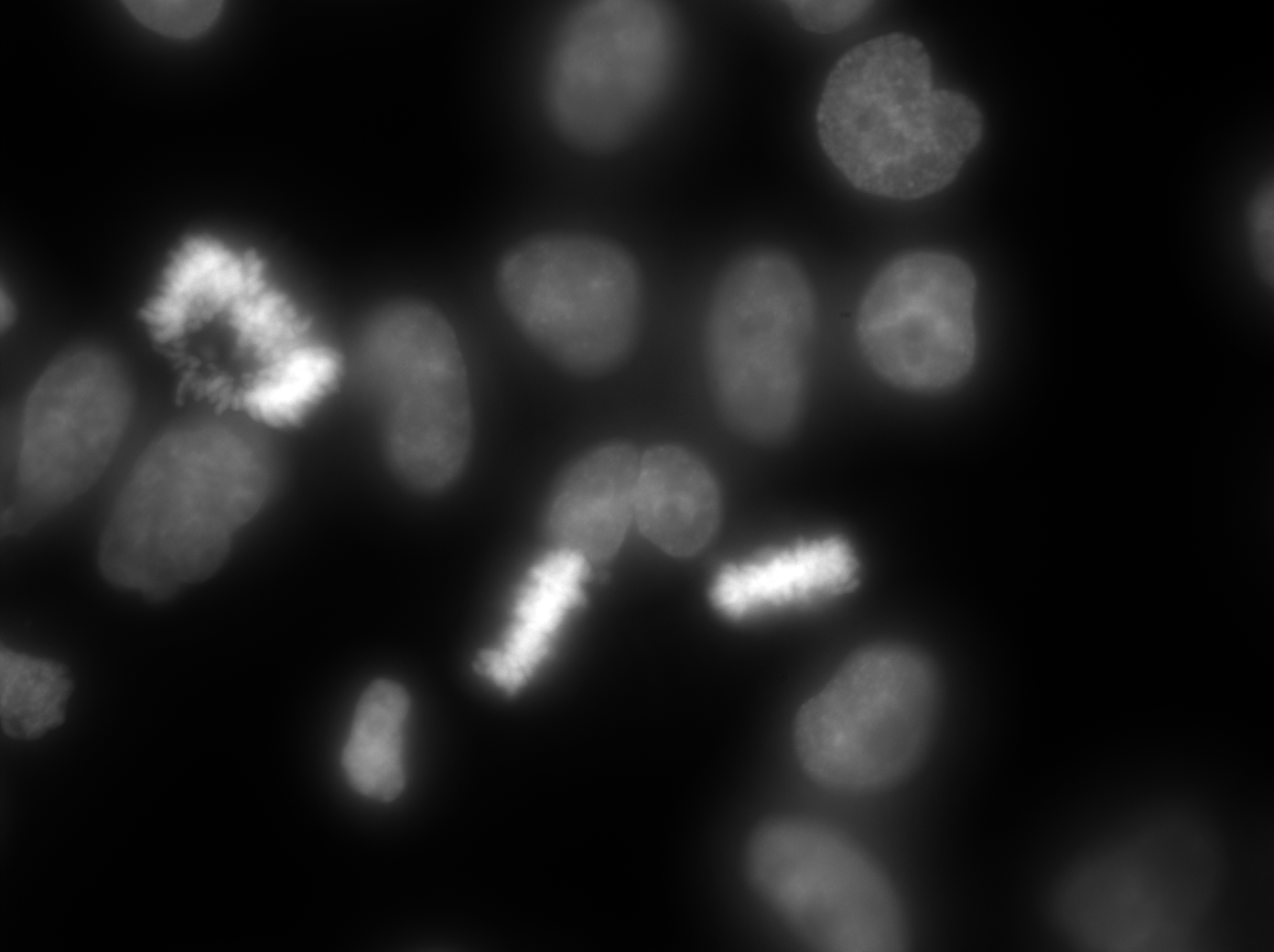

Supplement: Supplementary file 10 — Source data Fig. 2 part 7 [file 44319_2026_742_MOESM10_ESM.zip › Figure 2 Part 7/Fig 2fg Control Hela rGT335 acetylated tubulin part 2/Metaphase/Cas9 actub rGT335 9-8-25 R2 M8M9.Project Maximum Z_XY1757362229_Z0_T0_C0.tif]

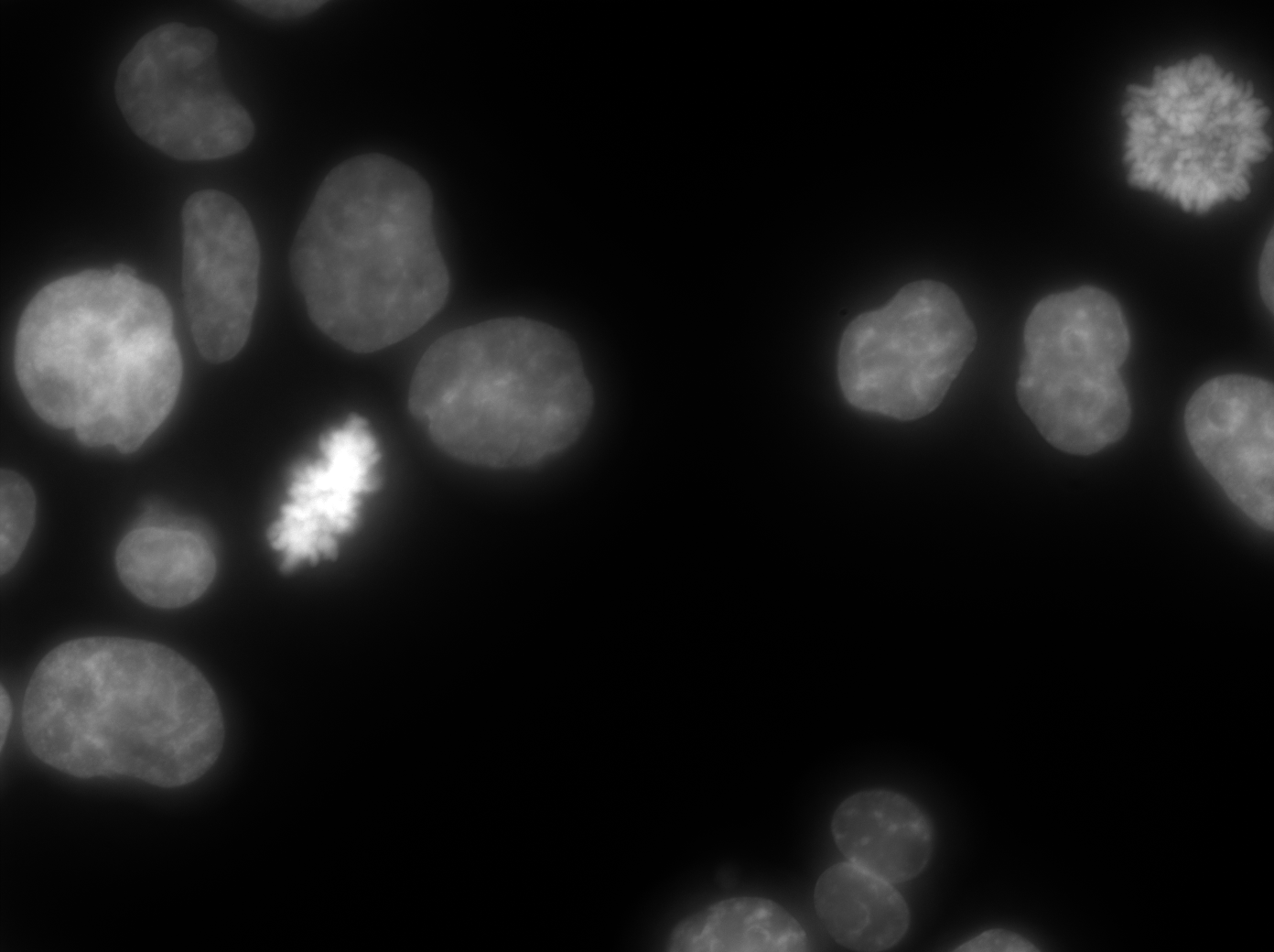

Supplement: Supplementary file 10 — Source data Fig. 2 part 7 [file 44319_2026_742_MOESM10_ESM.zip › Figure 2 Part 7/Fig 2fg Control Hela rGT335 acetylated tubulin part 2/Metaphase/Cas9 actub rGT335 9-8-25 R3 M9.Project Maximum Z_XY1757368504_Z0_T0_C0.tif]

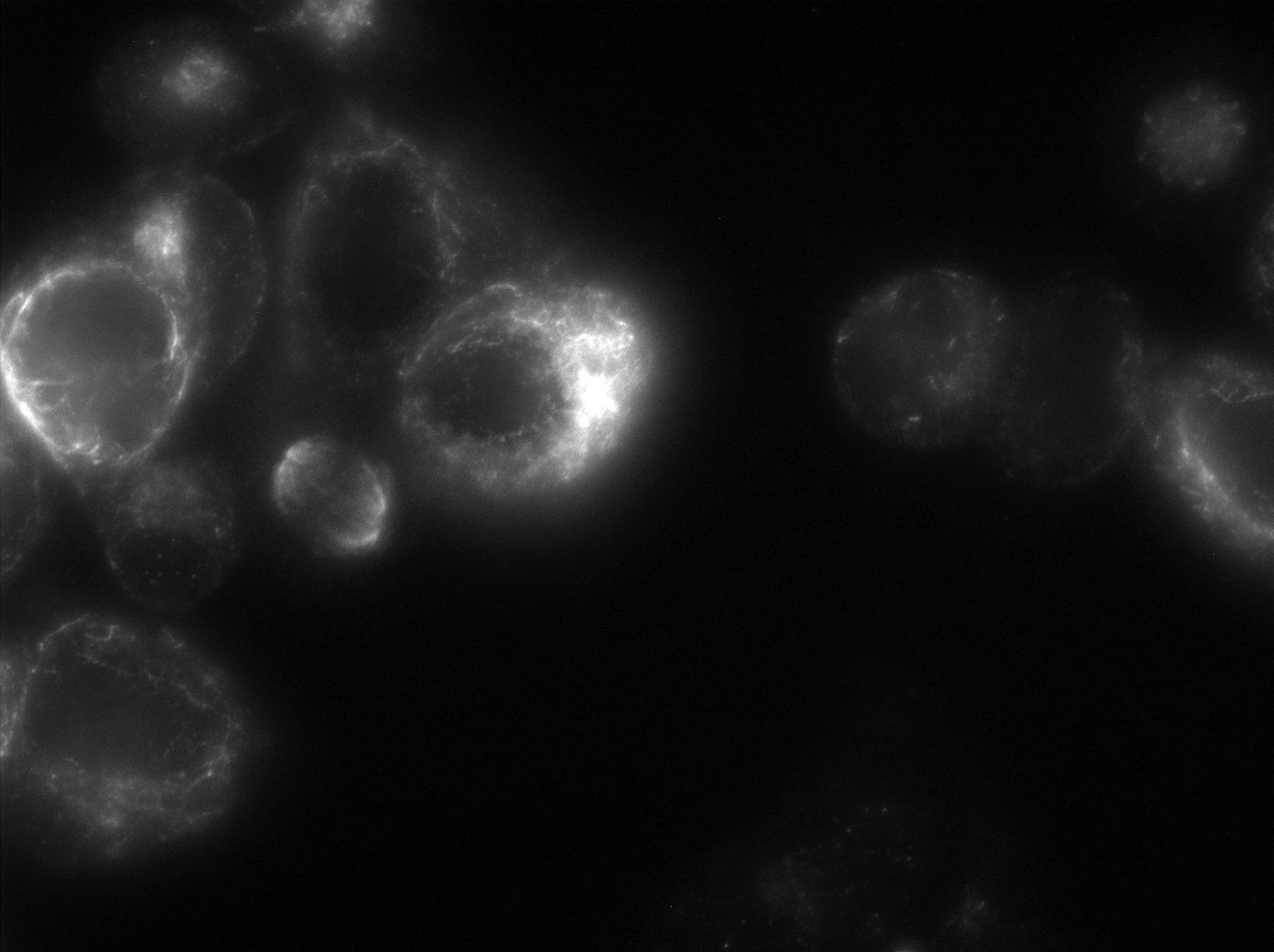

Supplement: Supplementary file 10 — Source data Fig. 2 part 7 [file 44319_2026_742_MOESM10_ESM.zip › Figure 2 Part 7/Fig 2fg Control Hela rGT335 acetylated tubulin part 2/Metaphase/Cas9 actub rGT335 9-8-25 R3 M9.Project Maximum Z_XY1757368504_Z0_T0_C2.tif]

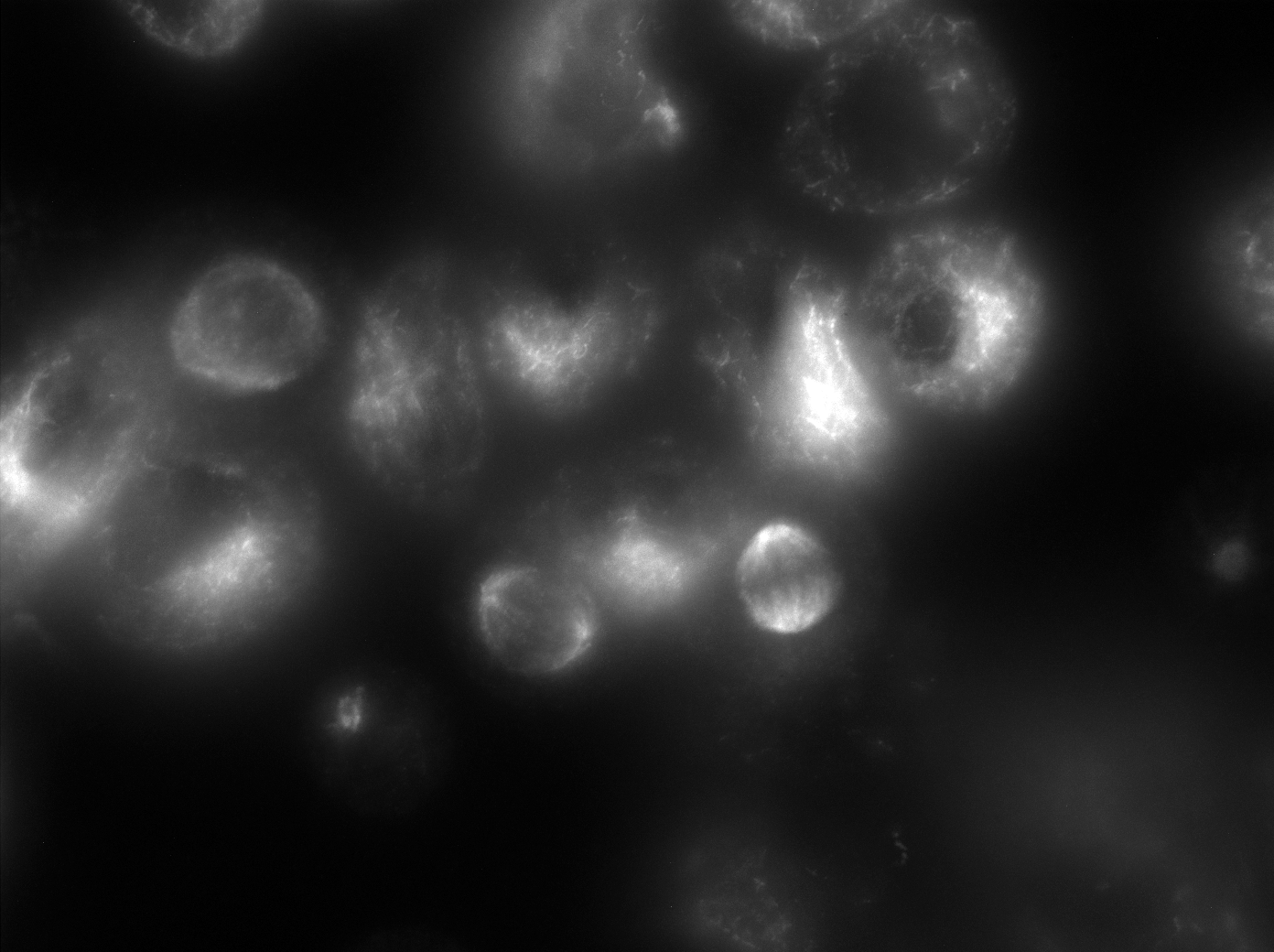

Supplement: Supplementary file 10 — Source data Fig. 2 part 7 [file 44319_2026_742_MOESM10_ESM.zip › Figure 2 Part 7/Fig 2fg Control Hela rGT335 acetylated tubulin part 2/Metaphase/Cas9 actub rGT335 9-8-25 R2 M8M9.Project Maximum Z_XY1757362229_Z0_T0_C2.tif]

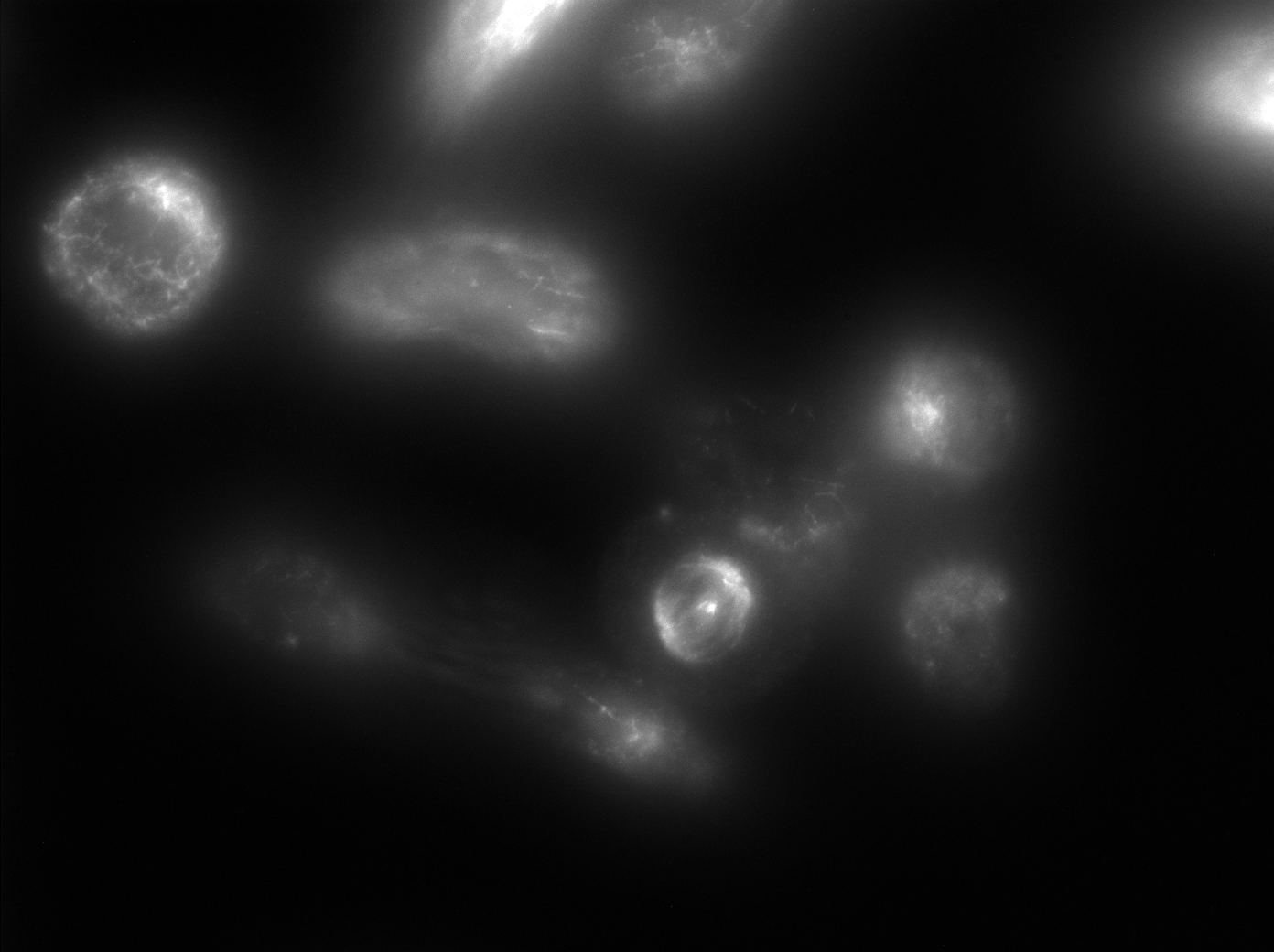

Supplement: Supplementary file 10 — Source data Fig. 2 part 7 [file 44319_2026_742_MOESM10_ESM.zip › Figure 2 Part 7/Fig 2fg Control Hela rGT335 acetylated tubulin part 2/Metaphase/Cas9 actub rGT335 9-8-25 R1 M3.Project Maximum Z_XY1757351500_Z0_T0_C2.tif]

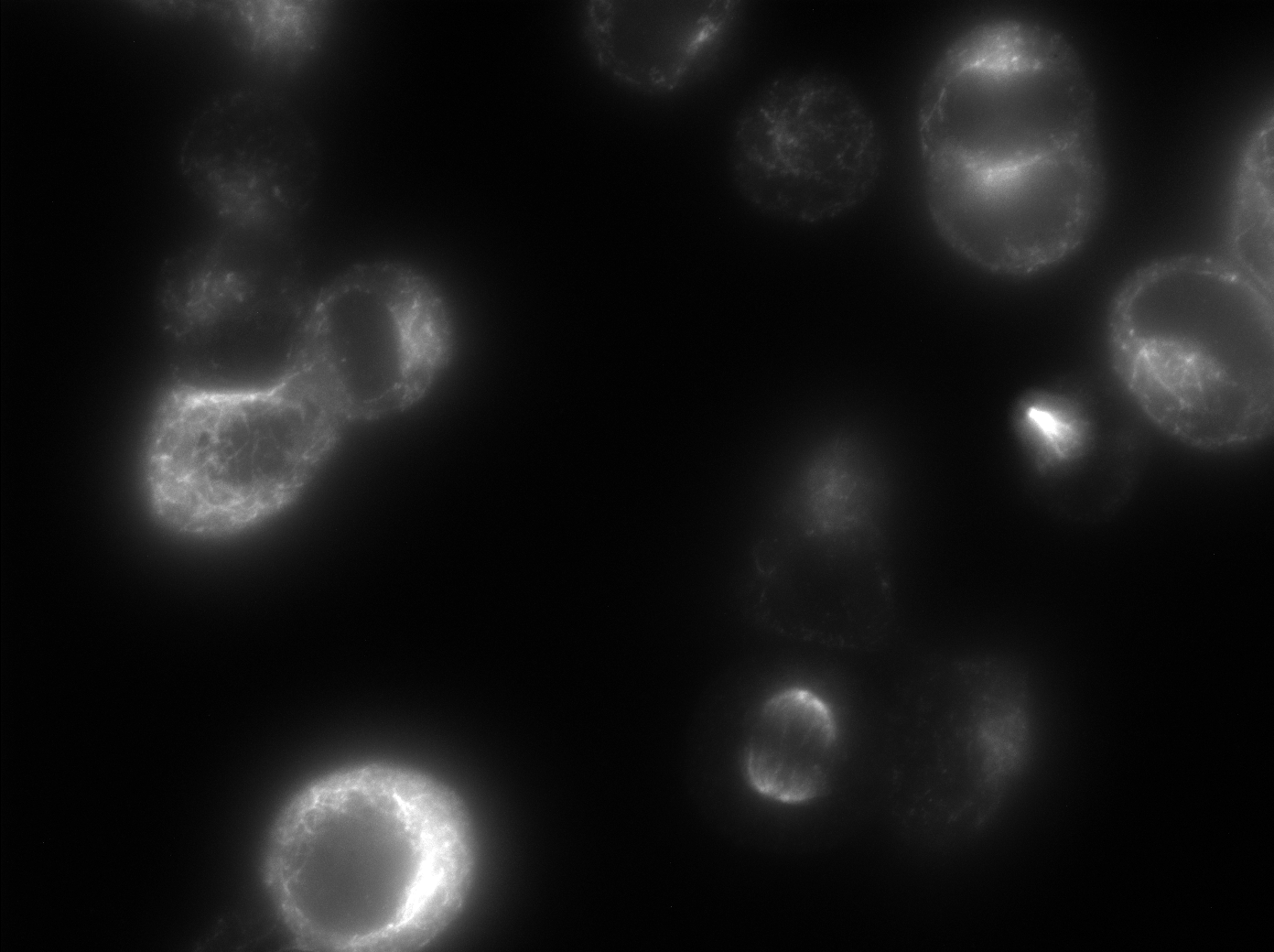

Supplement: Supplementary file 10 — Source data Fig. 2 part 7 [file 44319_2026_742_MOESM10_ESM.zip › Figure 2 Part 7/Fig 2fg Control Hela rGT335 acetylated tubulin part 2/Metaphase/Cas9 actub rGT335 9-8-25 R1 M6.Project Maximum Z_XY1757354221_Z0_T0_C2.tif]

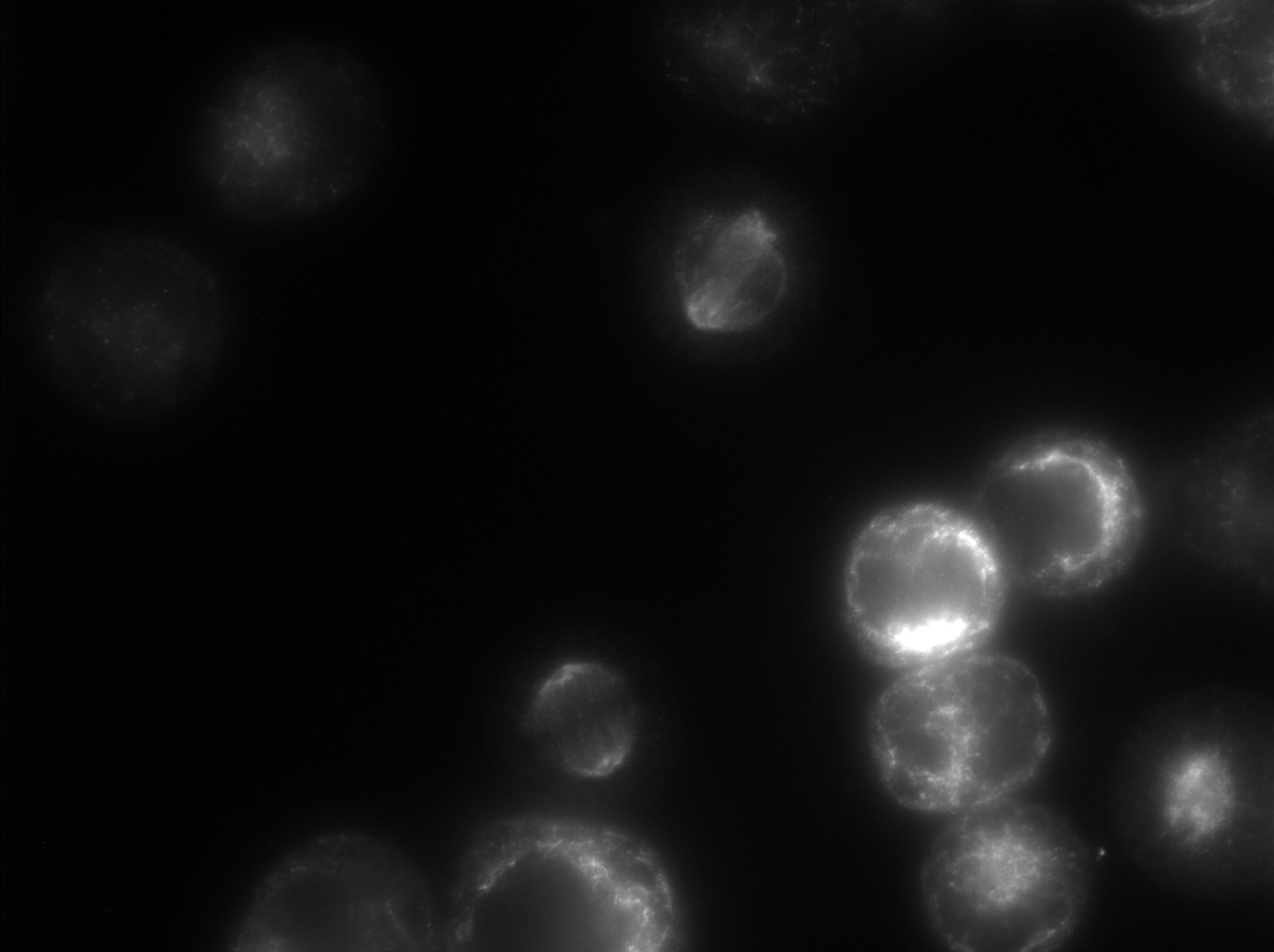

Supplement: Supplementary file 10 — Source data Fig. 2 part 7 [file 44319_2026_742_MOESM10_ESM.zip › Figure 2 Part 7/Fig 2fg Control Hela rGT335 acetylated tubulin part 2/Metaphase/Cas9 actub rGT335 9-8-25 R2 M6M7.Project Maximum Z_XY1757361876_Z0_T0_C2.tif]

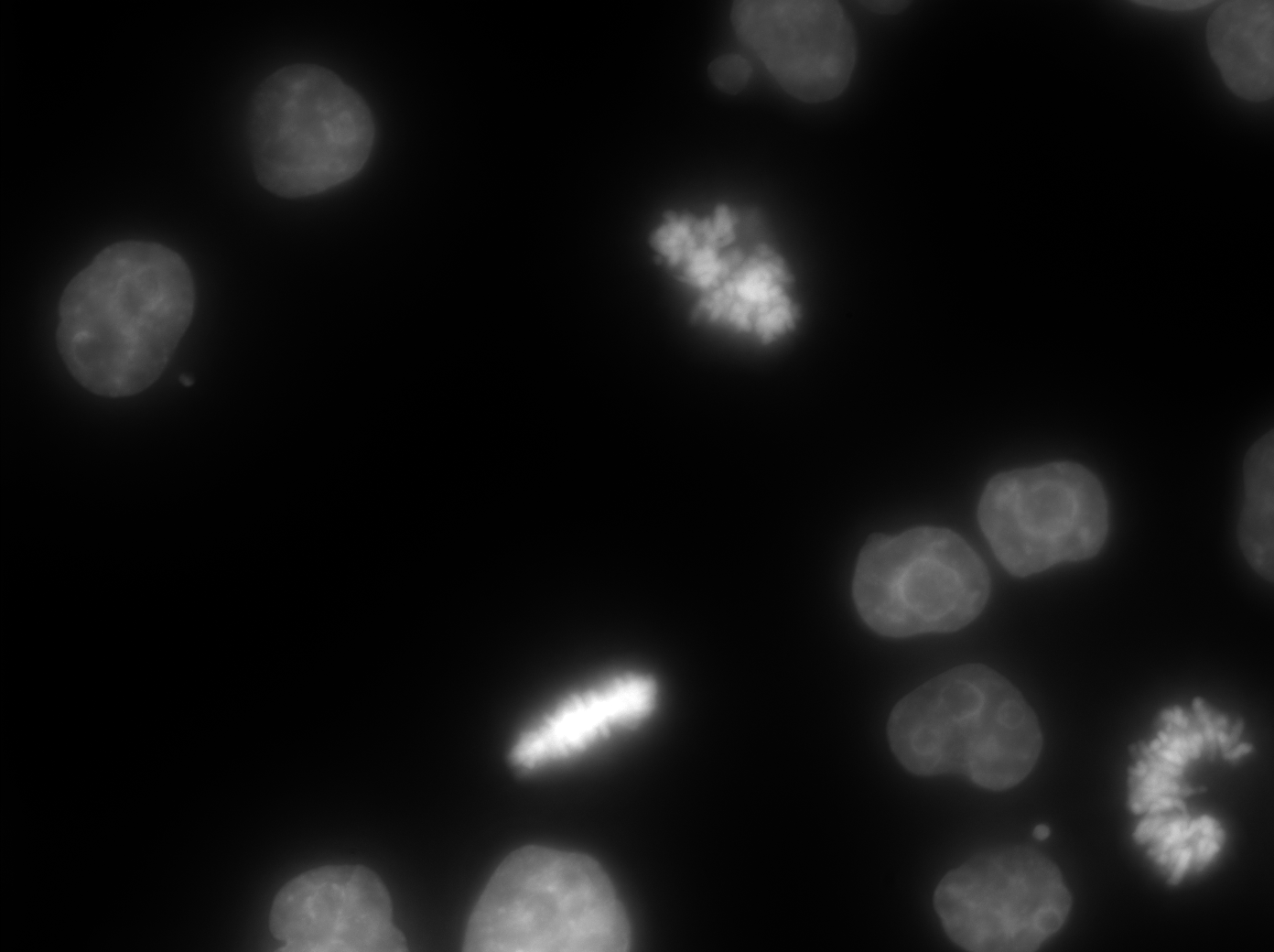

Supplement: Supplementary file 10 — Source data Fig. 2 part 7 [file 44319_2026_742_MOESM10_ESM.zip › Figure 2 Part 7/Fig 2fg Control Hela rGT335 acetylated tubulin part 2/Metaphase/Cas9 actub rGT335 9-8-25 R2 M6M7.Project Maximum Z_XY1757361876_Z0_T0_C0.tif]

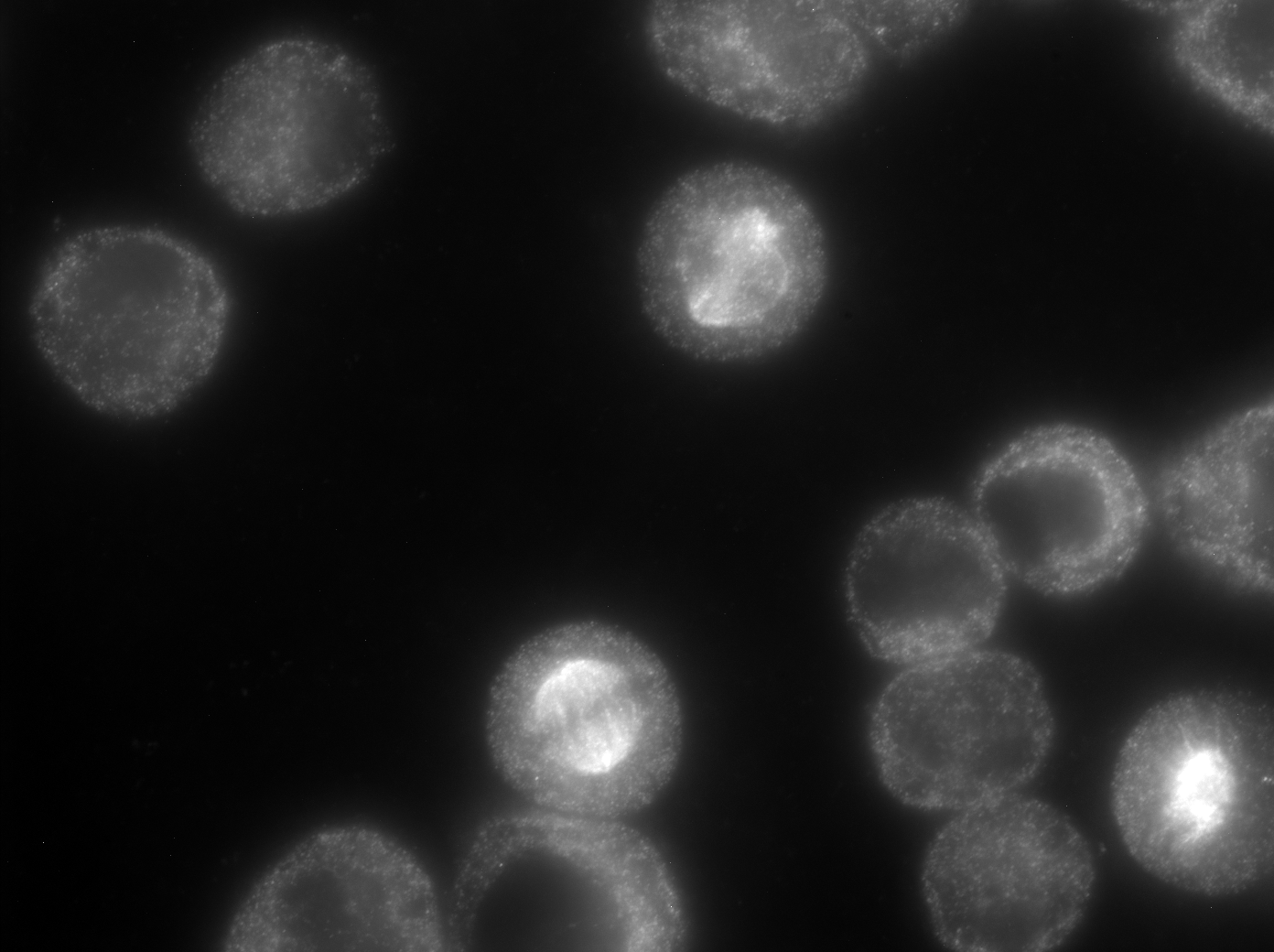

Supplement: Supplementary file 10 — Source data Fig. 2 part 7 [file 44319_2026_742_MOESM10_ESM.zip › Figure 2 Part 7/Fig 2fg Control Hela rGT335 acetylated tubulin part 2/Metaphase/Cas9 actub rGT335 9-8-25 R2 M6M7.Project Maximum Z_XY1757361876_Z0_T0_C1.tif]

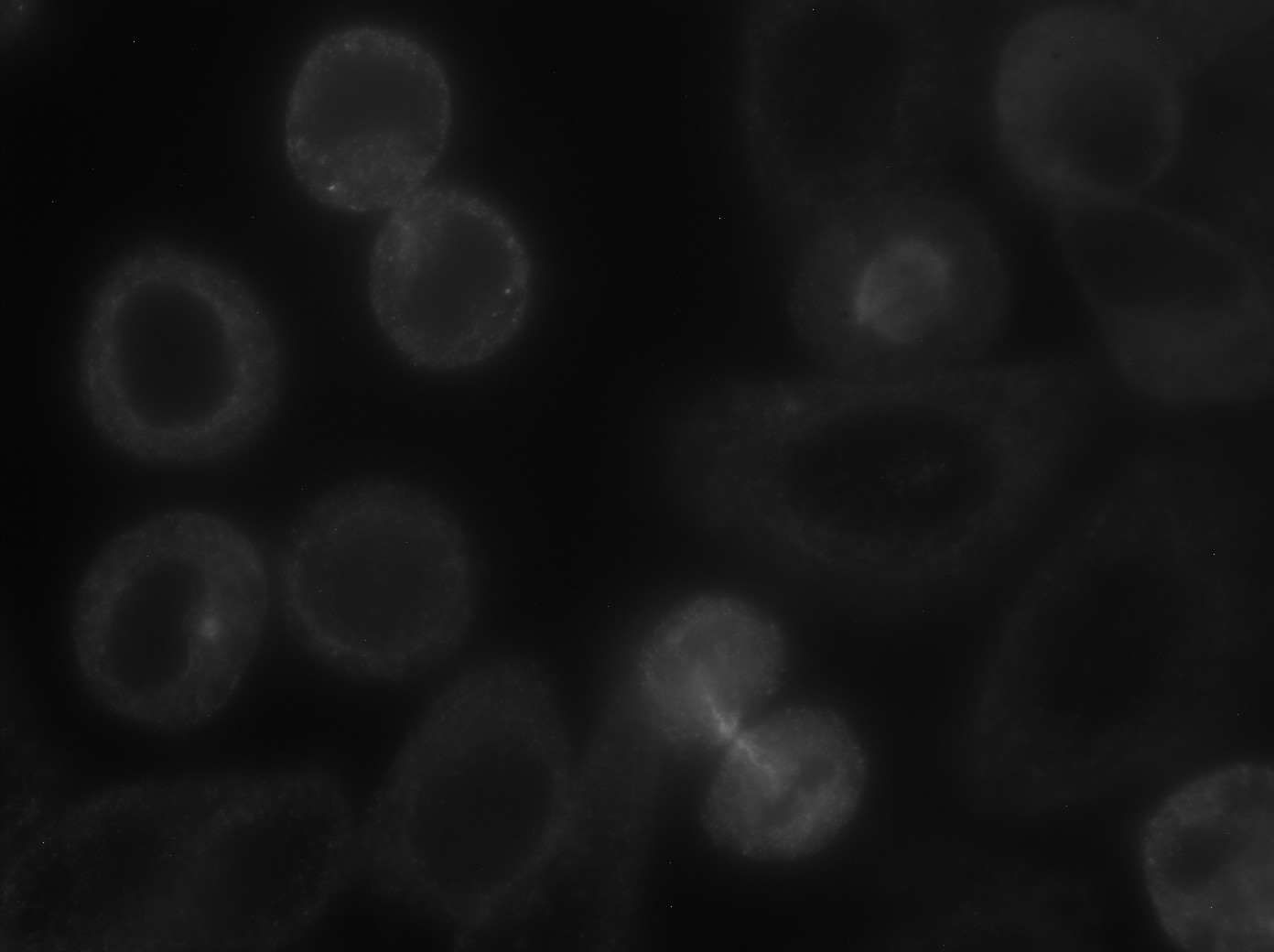

Supplement: Supplementary file 10 — Source data Fig. 2 part 7 [file 44319_2026_742_MOESM10_ESM.zip › Figure 2 Part 7/Fig 2fg Control Hela rGT335 acetylated tubulin part 2/Furrow Ingression/Cas9 actub rGT335 9-8-25 R3 FI9 M5.Project Maximum Z_XY1757366799_Z0_T0_C1.tif]

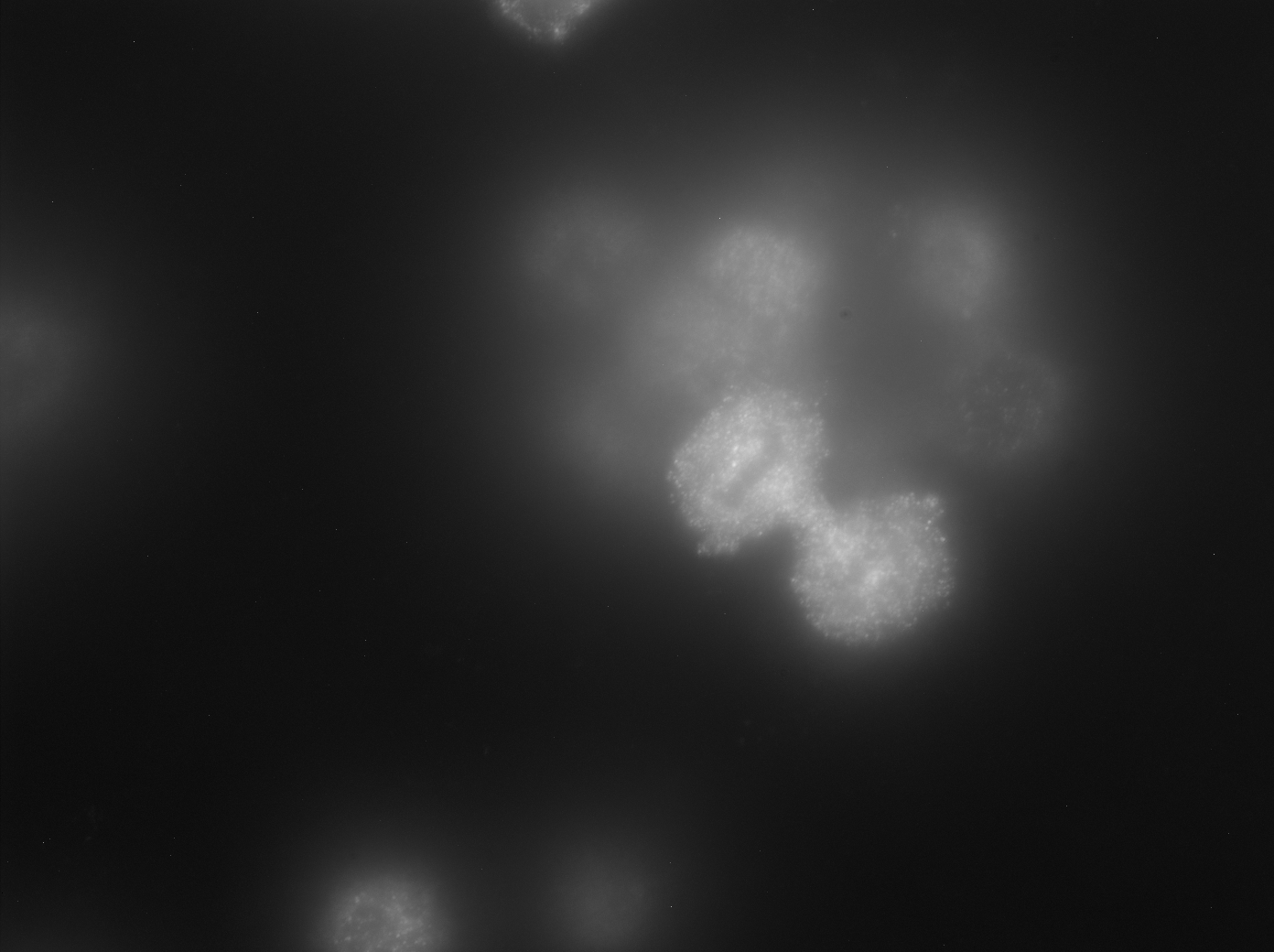

Supplement: Supplementary file 10 — Source data Fig. 2 part 7 [file 44319_2026_742_MOESM10_ESM.zip › Figure 2 Part 7/Fig 2fg Control Hela rGT335 acetylated tubulin part 2/Furrow Ingression/Cas9 actub rGT335 9-8-25 R1 FI4 EX.Project Maximum Z_XY1757352882_Z0_T0_C1.tif]

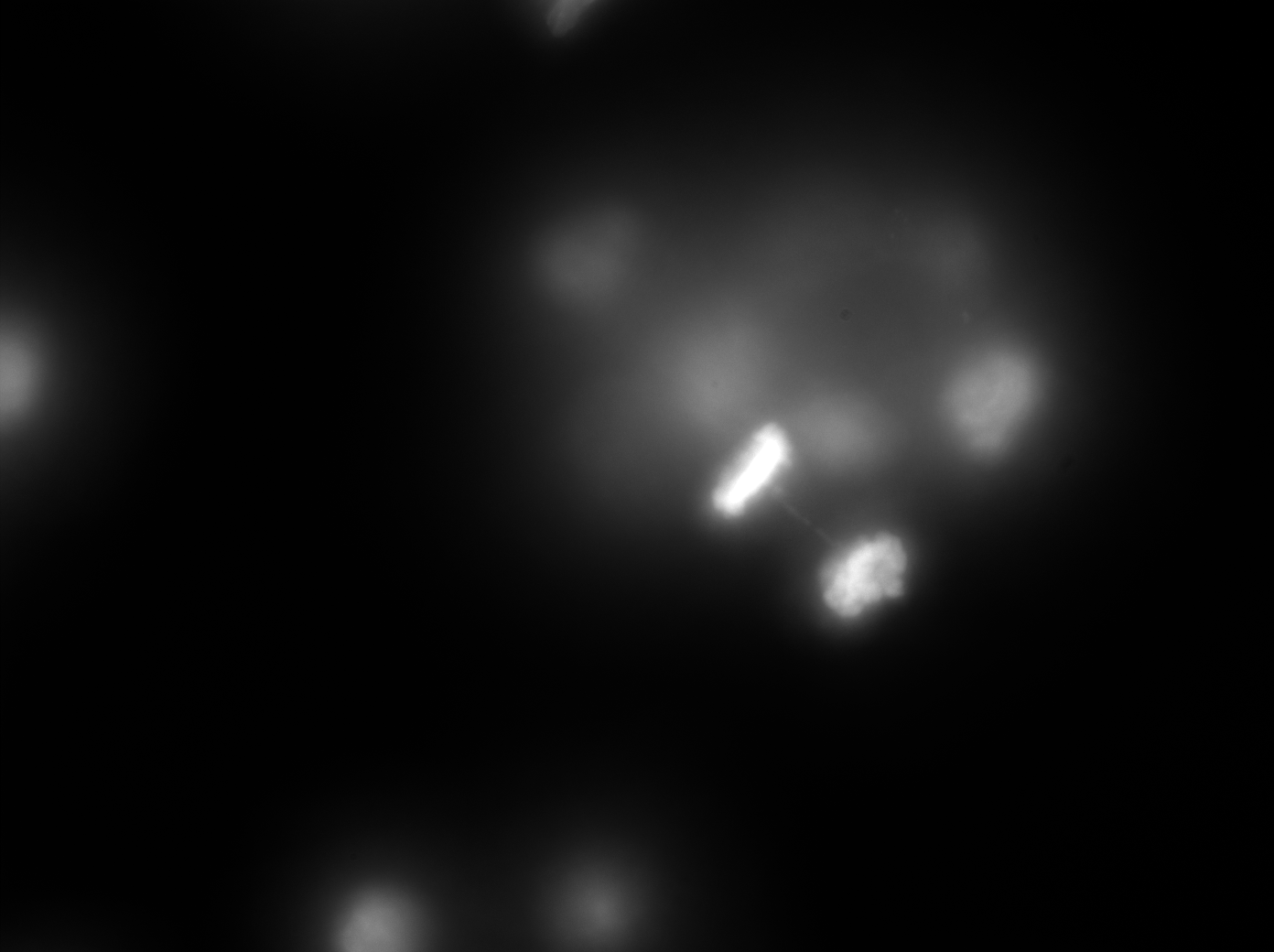

Supplement: Supplementary file 10 — Source data Fig. 2 part 7 [file 44319_2026_742_MOESM10_ESM.zip › Figure 2 Part 7/Fig 2fg Control Hela rGT335 acetylated tubulin part 2/Furrow Ingression/Cas9 actub rGT335 9-8-25 R1 FI4 EX.Project Maximum Z_XY1757352882_Z0_T0_C0.tif]

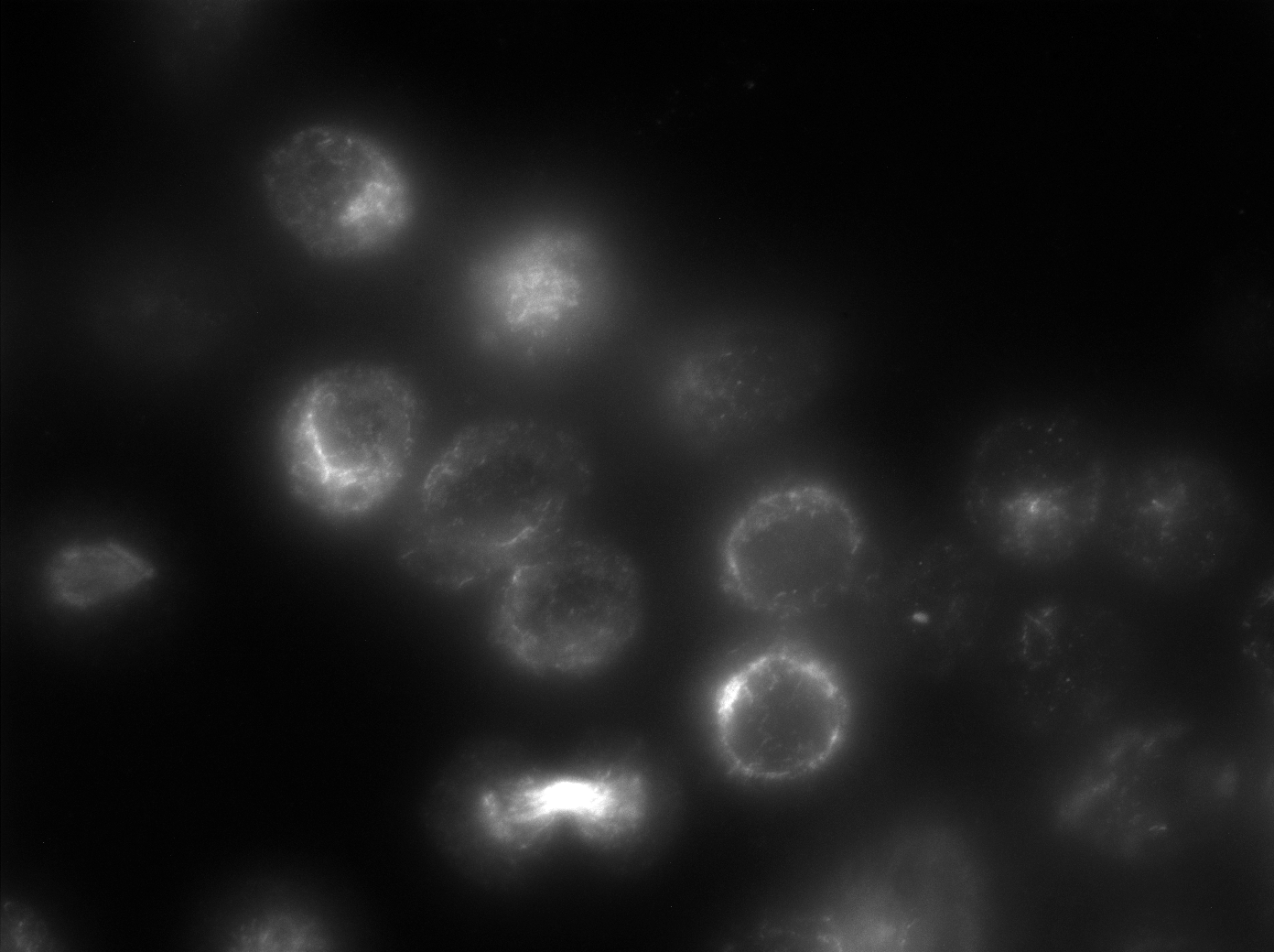

Supplement: Supplementary file 10 — Source data Fig. 2 part 7 [file 44319_2026_742_MOESM10_ESM.zip › Figure 2 Part 7/Fig 2fg Control Hela rGT335 acetylated tubulin part 2/Furrow Ingression/Cas9 actub rGT335 9-8-25 R2 FI4.Project Maximum Z_XY1757360709_Z0_T0_C2.tif]

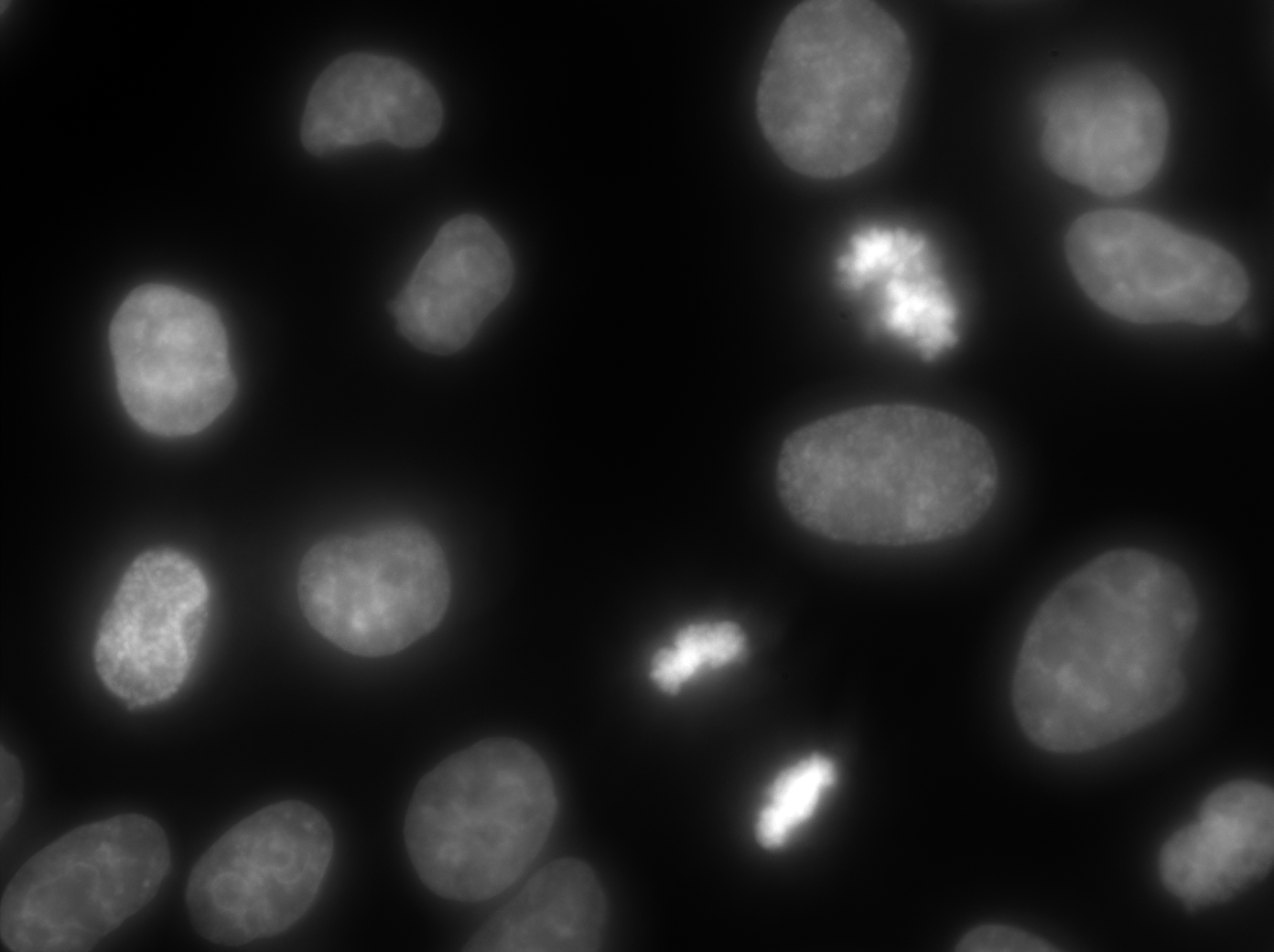

Supplement: Supplementary file 10 — Source data Fig. 2 part 7 [file 44319_2026_742_MOESM10_ESM.zip › Figure 2 Part 7/Fig 2fg Control Hela rGT335 acetylated tubulin part 2/Furrow Ingression/Cas9 actub rGT335 9-8-25 R3 FI9 M5.Project Maximum Z_XY1757366799_Z0_T0_C0.tif]

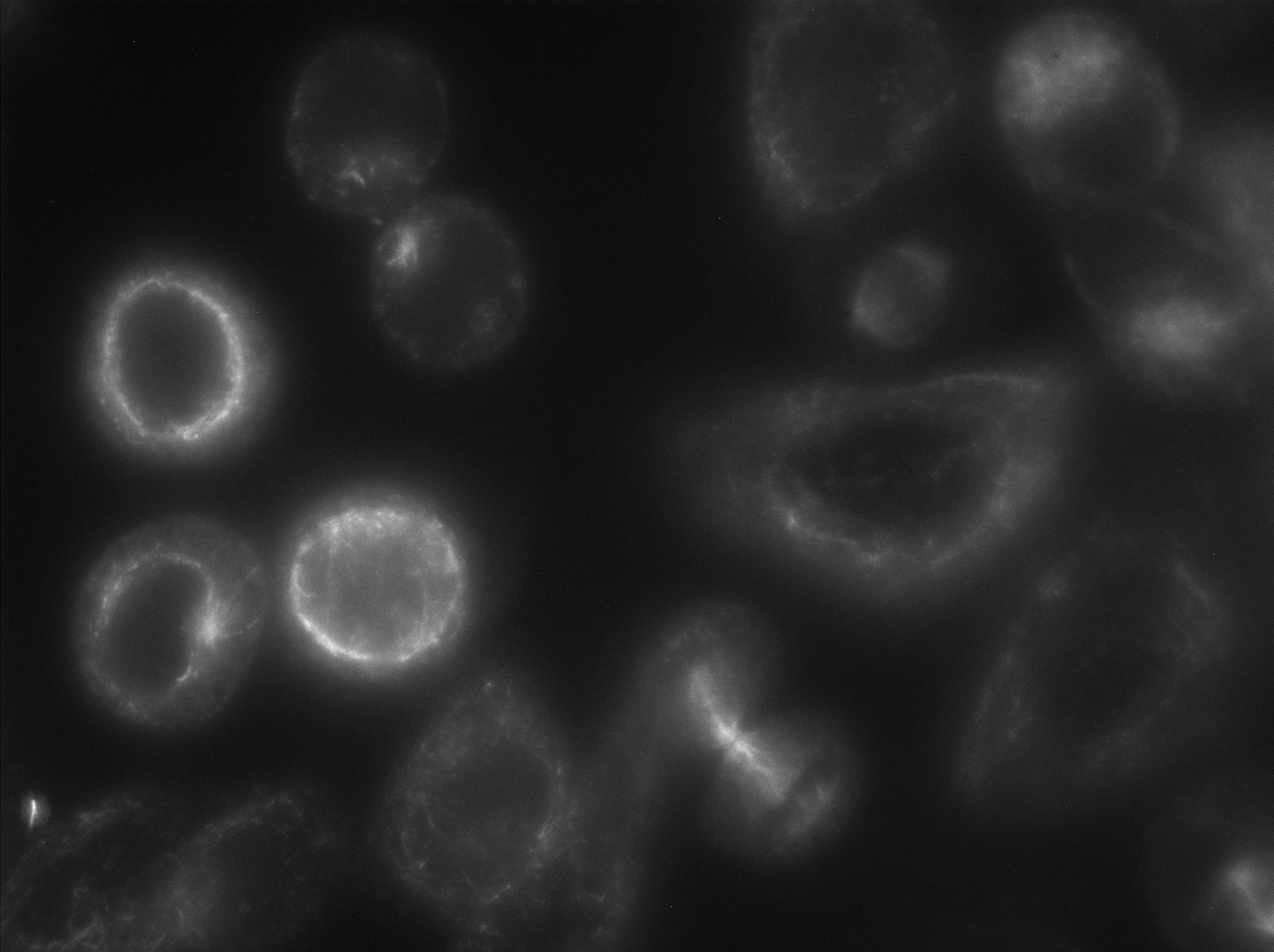

Supplement: Supplementary file 10 — Source data Fig. 2 part 7 [file 44319_2026_742_MOESM10_ESM.zip › Figure 2 Part 7/Fig 2fg Control Hela rGT335 acetylated tubulin part 2/Furrow Ingression/Cas9 actub rGT335 9-8-25 R3 FI9 M5.Project Maximum Z_XY1757366799_Z0_T0_C2.tif]

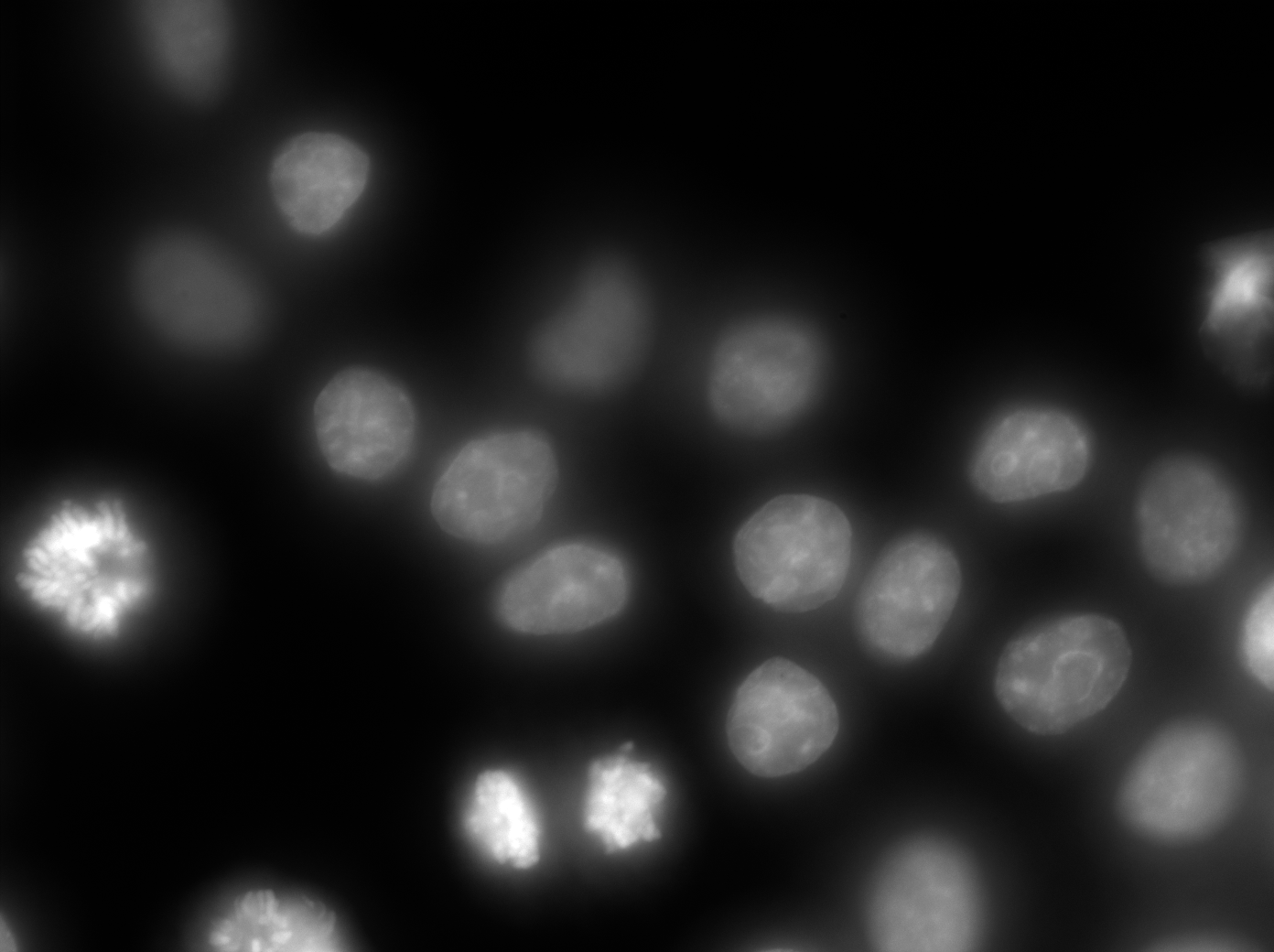

Supplement: Supplementary file 10 — Source data Fig. 2 part 7 [file 44319_2026_742_MOESM10_ESM.zip › Figure 2 Part 7/Fig 2fg Control Hela rGT335 acetylated tubulin part 2/Furrow Ingression/Cas9 actub rGT335 9-8-25 R2 FI4.Project Maximum Z_XY1757360709_Z0_T0_C0.tif]

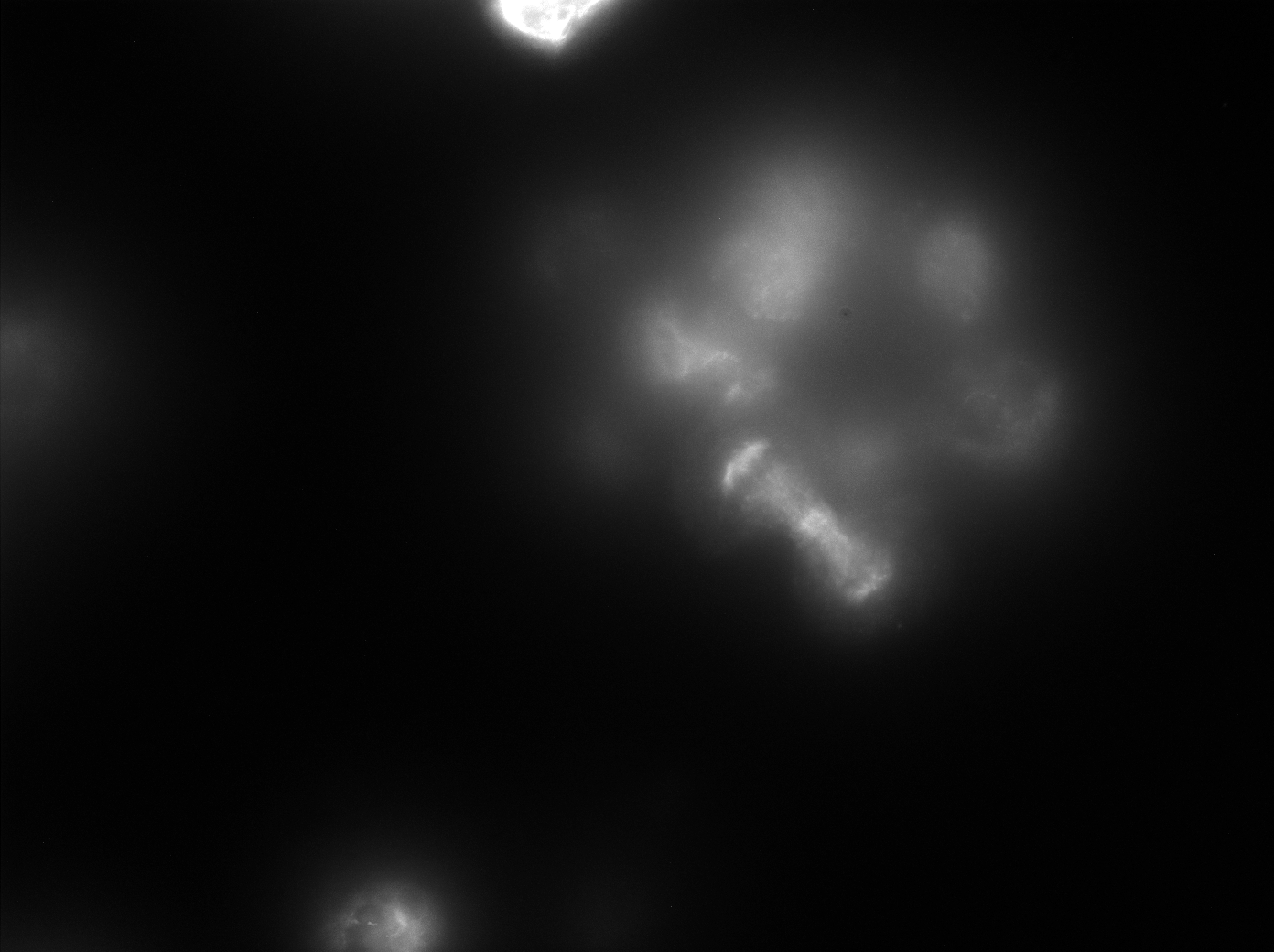

Supplement: Supplementary file 10 — Source data Fig. 2 part 7 [file 44319_2026_742_MOESM10_ESM.zip › Figure 2 Part 7/Fig 2fg Control Hela rGT335 acetylated tubulin part 2/Furrow Ingression/Cas9 actub rGT335 9-8-25 R1 FI4 EX.Project Maximum Z_XY1757352882_Z0_T0_C2.tif]

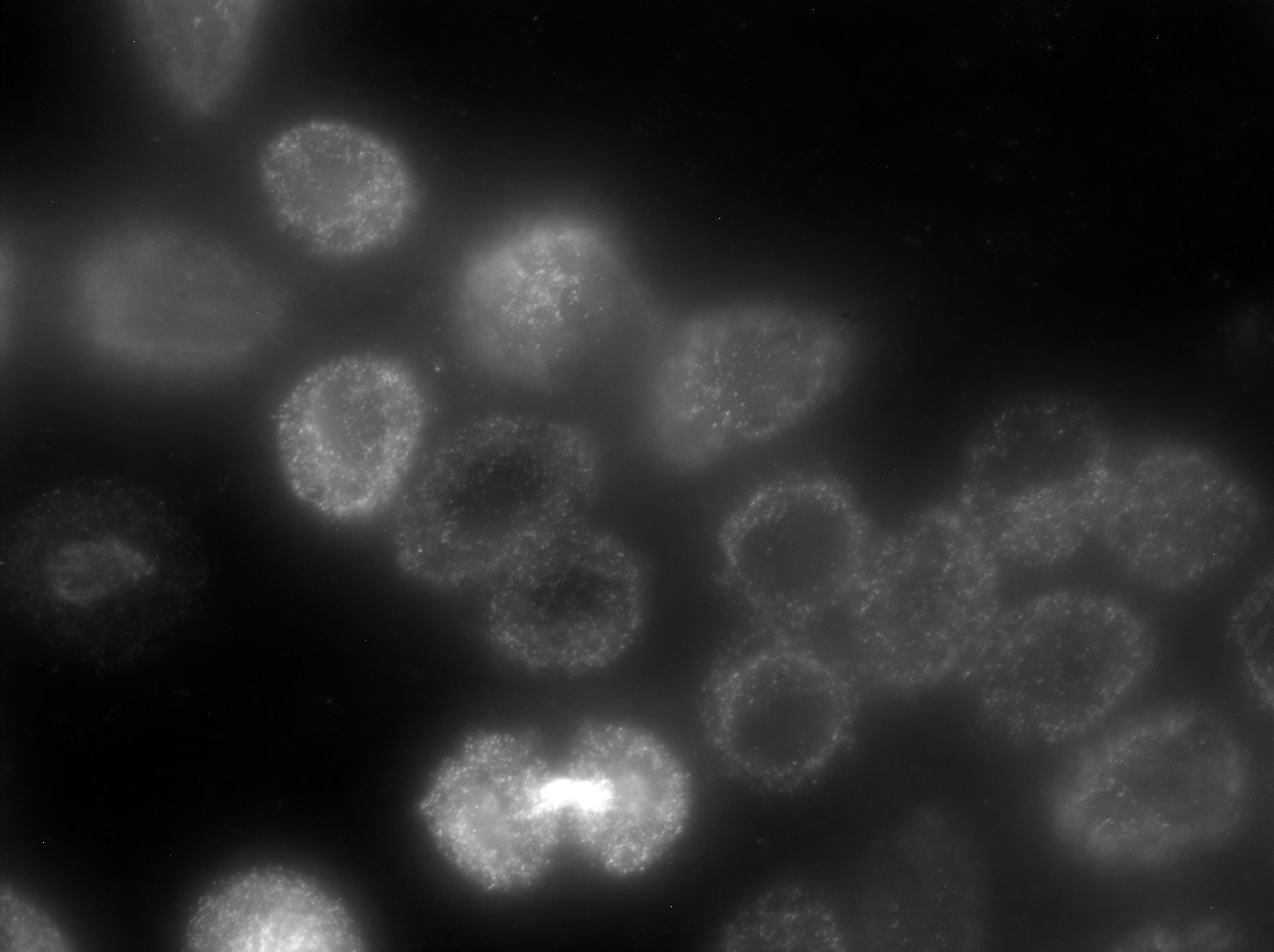

Supplement: Supplementary file 10 — Source data Fig. 2 part 7 [file 44319_2026_742_MOESM10_ESM.zip › Figure 2 Part 7/Fig 2fg Control Hela rGT335 acetylated tubulin part 2/Furrow Ingression/Cas9 actub rGT335 9-8-25 R2 FI4.Project Maximum Z_XY1757360709_Z0_T0_C1.tif]

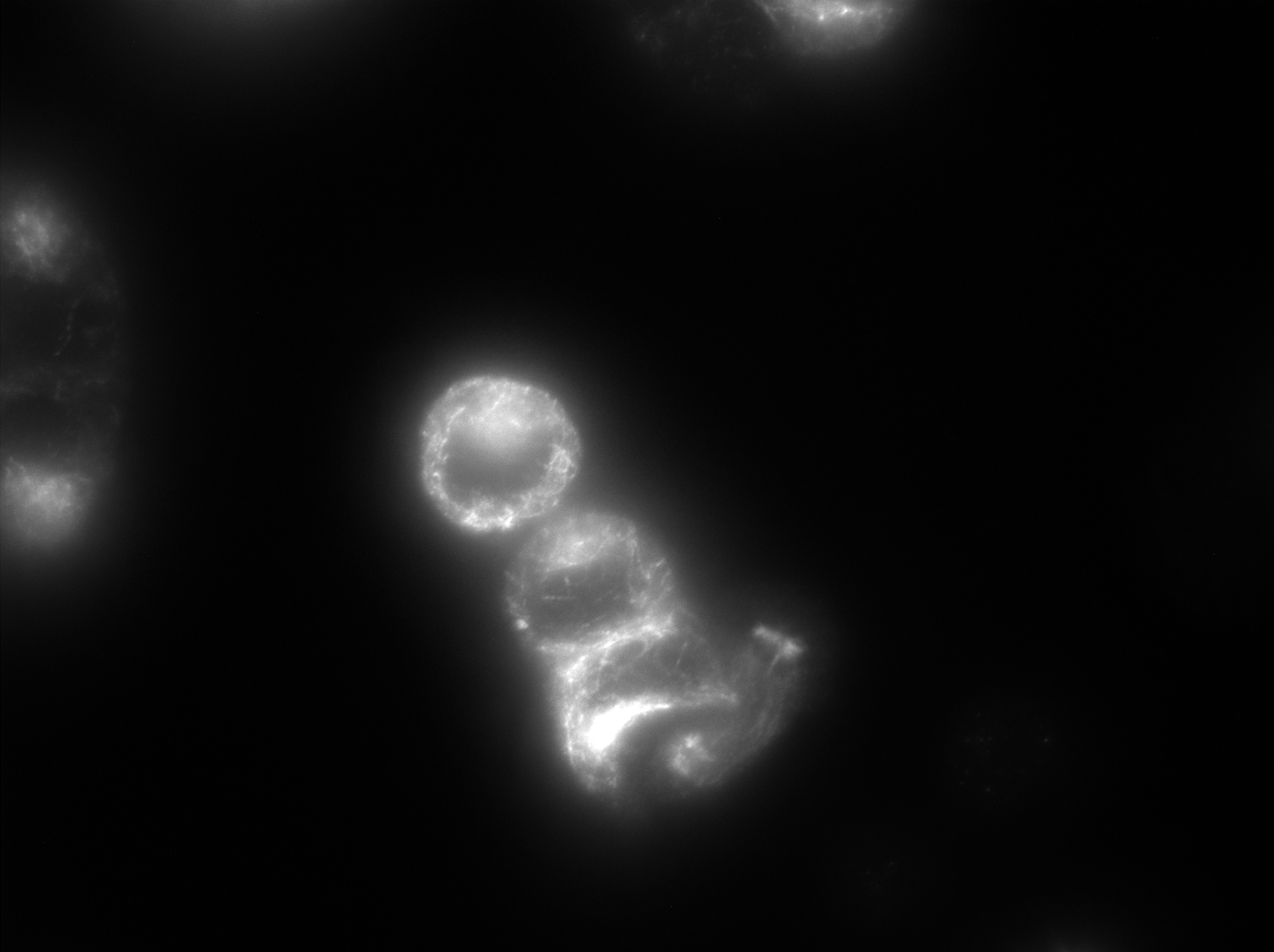

Supplement: Supplementary file 10 — Source data Fig. 2 part 7 [file 44319_2026_742_MOESM10_ESM.zip › Figure 2 Part 7/Fig 2fg Control Hela rGT335 acetylated tubulin part 2/Furrow Ingression/Cas9 actub rGT335 9-8-25 R1 FI3.Project Maximum Z_XY1757352722_Z0_T0_C2.tif]

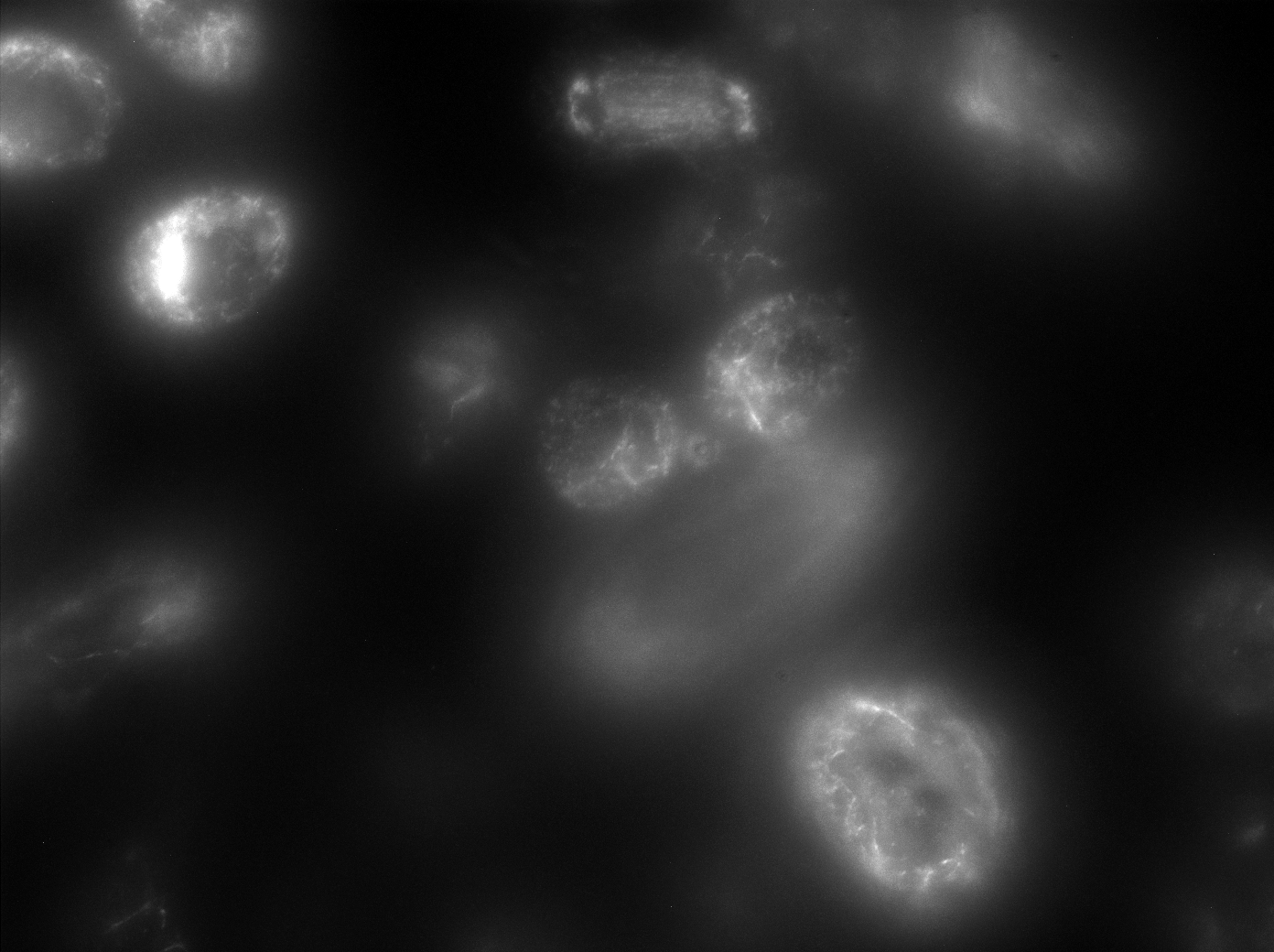

Supplement: Supplementary file 10 — Source data Fig. 2 part 7 [file 44319_2026_742_MOESM10_ESM.zip › Figure 2 Part 7/Fig 2fg Control Hela rGT335 acetylated tubulin part 2/Furrow Ingression/Cas9 actub rGT335 9-8-25 R3 FI7.Project Maximum Z_XY1757366561_Z0_T0_C2.tif]

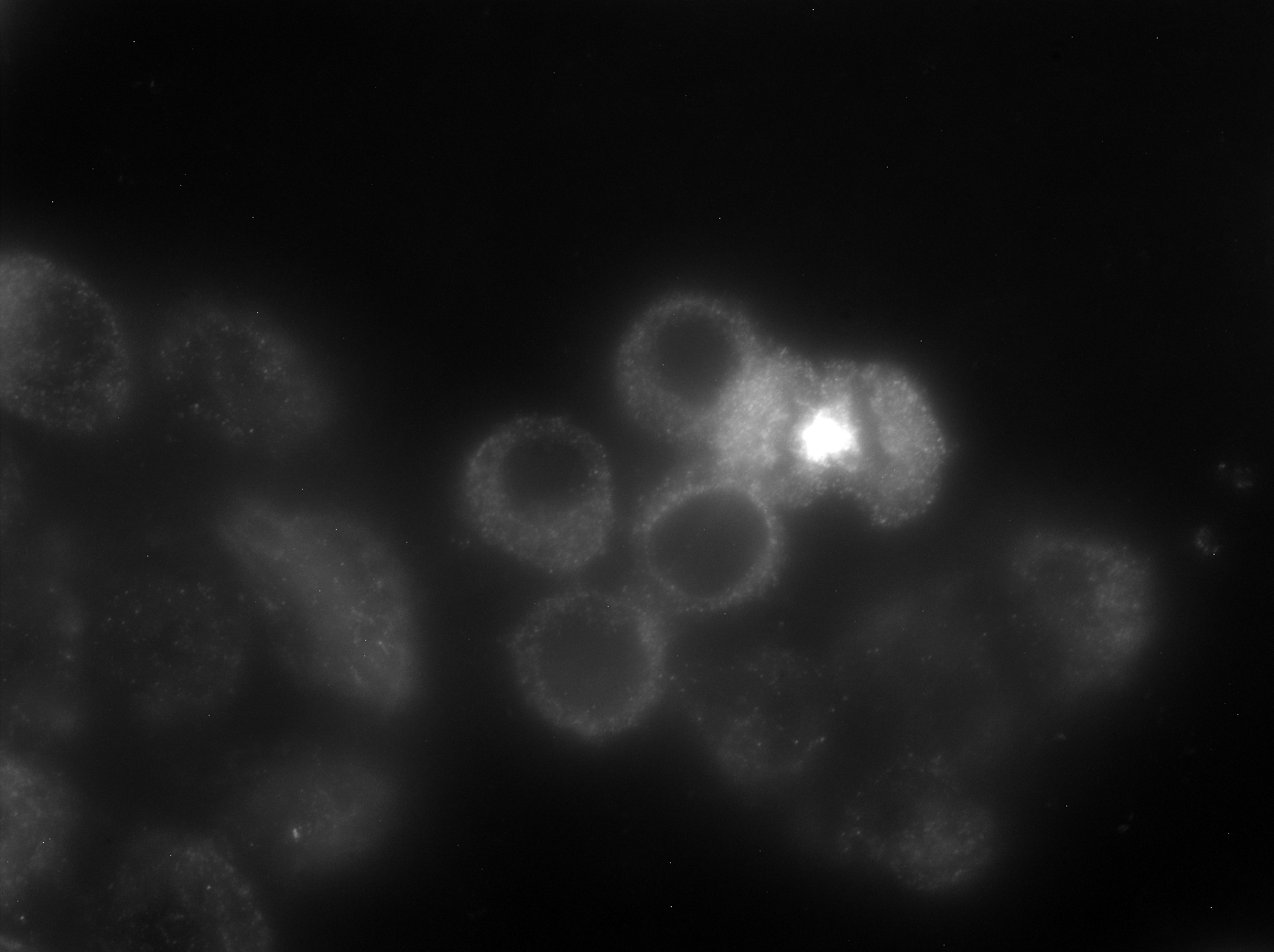

Supplement: Supplementary file 10 — Source data Fig. 2 part 7 [file 44319_2026_742_MOESM10_ESM.zip › Figure 2 Part 7/Fig 2fg Control Hela rGT335 acetylated tubulin part 2/Furrow Ingression/Cas9 actub rGT335 9-8-25 R2 FI8.Project Maximum Z_XY1757362863_Z0_T0_C1.tif]

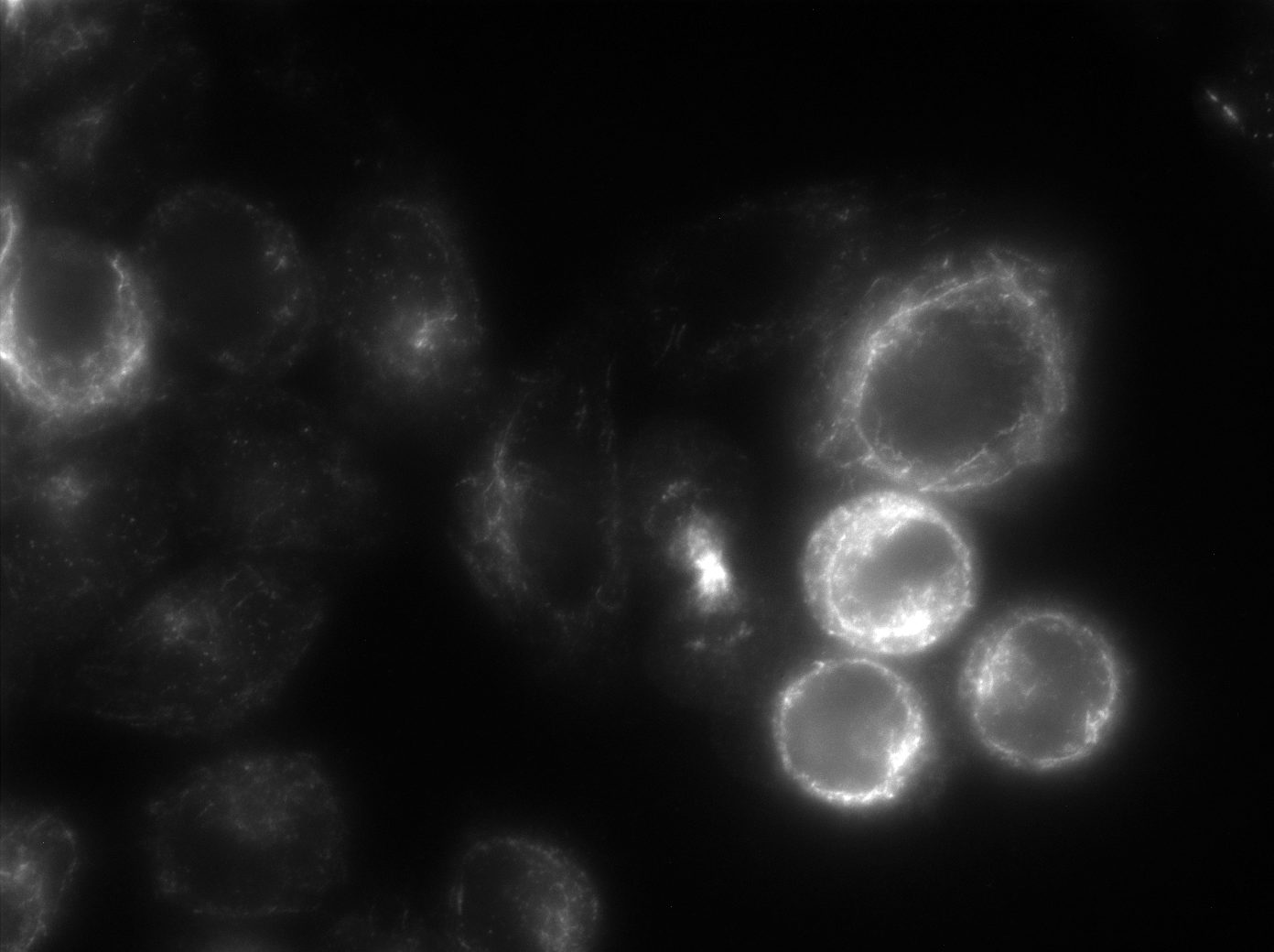

Supplement: Supplementary file 10 — Source data Fig. 2 part 7 [file 44319_2026_742_MOESM10_ESM.zip › Figure 2 Part 7/Fig 2fg Control Hela rGT335 acetylated tubulin part 2/Furrow Ingression/Cas9 actub rGT335 9-8-25 R3 FI5.Project Maximum Z_XY1757365932_Z0_T0_C2.tif]

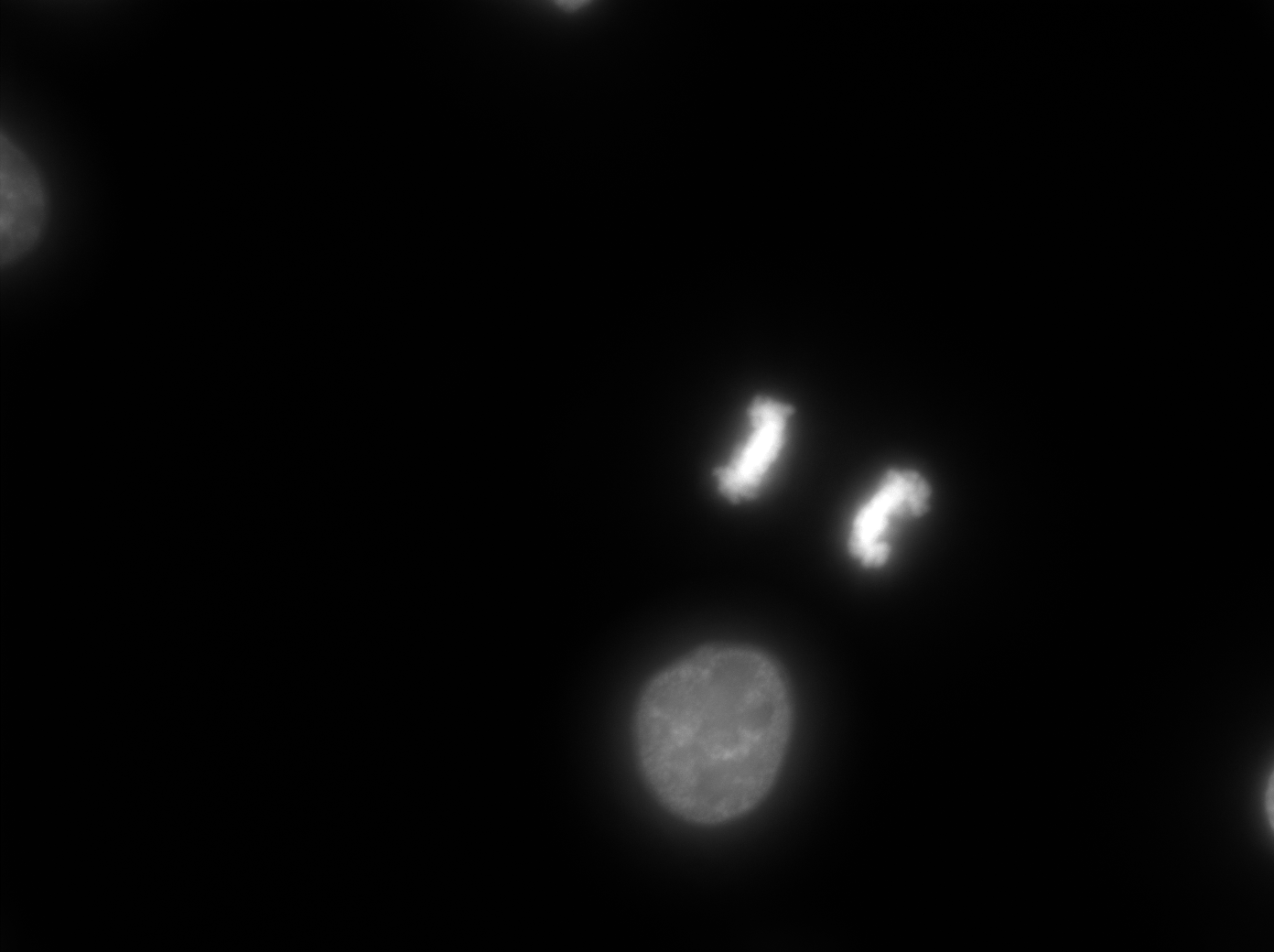

Supplement: Supplementary file 10 — Source data Fig. 2 part 7 [file 44319_2026_742_MOESM10_ESM.zip › Figure 2 Part 7/Fig 2fg Control Hela rGT335 acetylated tubulin part 2/Furrow Ingression/Cas9 actub rGT335 9-8-25 R1 FI6.Project Maximum Z_XY1757354454_Z0_T0_C0.tif]

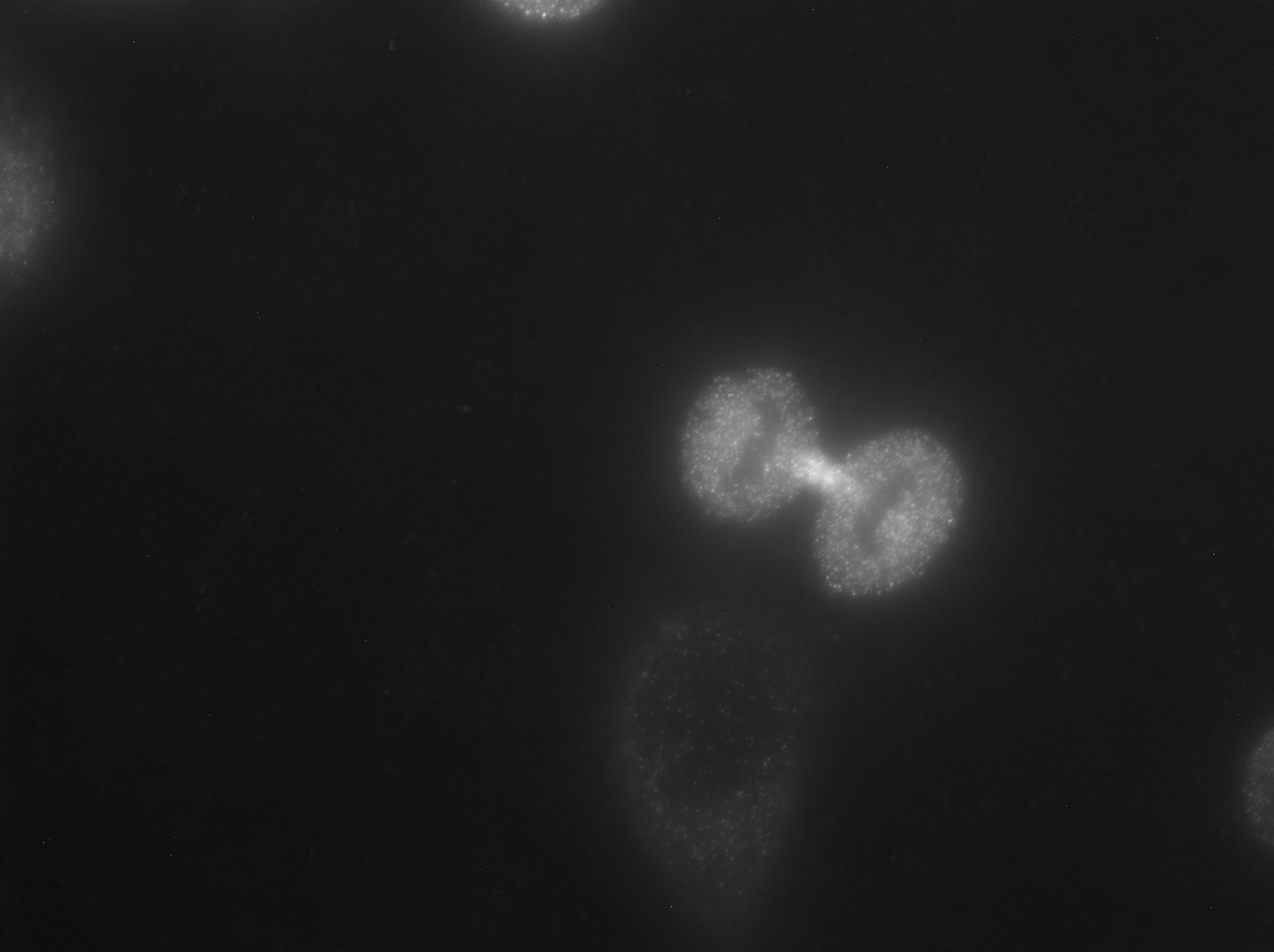

Supplement: Supplementary file 10 — Source data Fig. 2 part 7 [file 44319_2026_742_MOESM10_ESM.zip › Figure 2 Part 7/Fig 2fg Control Hela rGT335 acetylated tubulin part 2/Furrow Ingression/Cas9 actub rGT335 9-8-25 R1 FI6.Project Maximum Z_XY1757354454_Z0_T0_C1.tif]

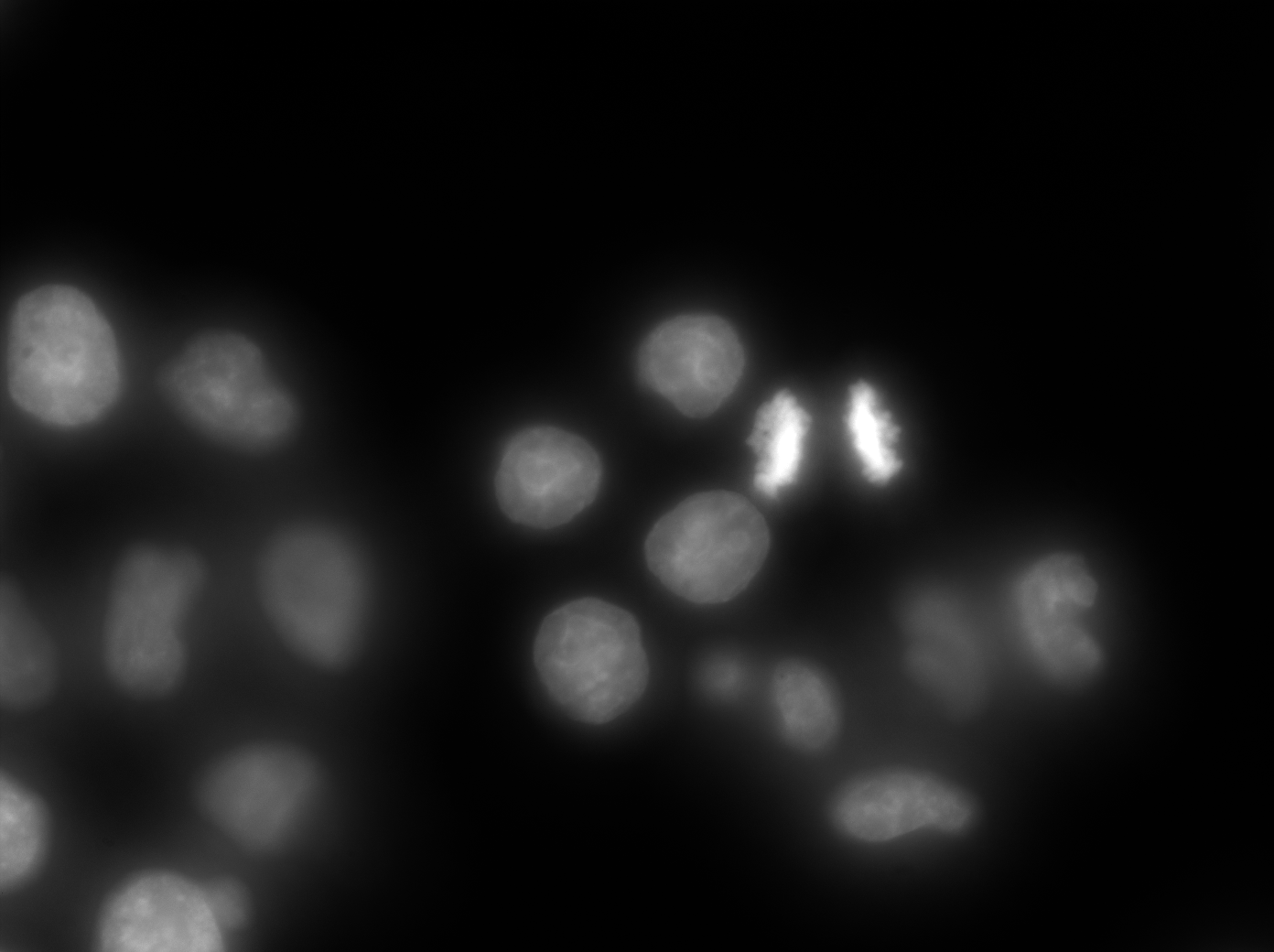

Supplement: Supplementary file 10 — Source data Fig. 2 part 7 [file 44319_2026_742_MOESM10_ESM.zip › Figure 2 Part 7/Fig 2fg Control Hela rGT335 acetylated tubulin part 2/Furrow Ingression/Cas9 actub rGT335 9-8-25 R2 FI8.Project Maximum Z_XY1757362863_Z0_T0_C0.tif]

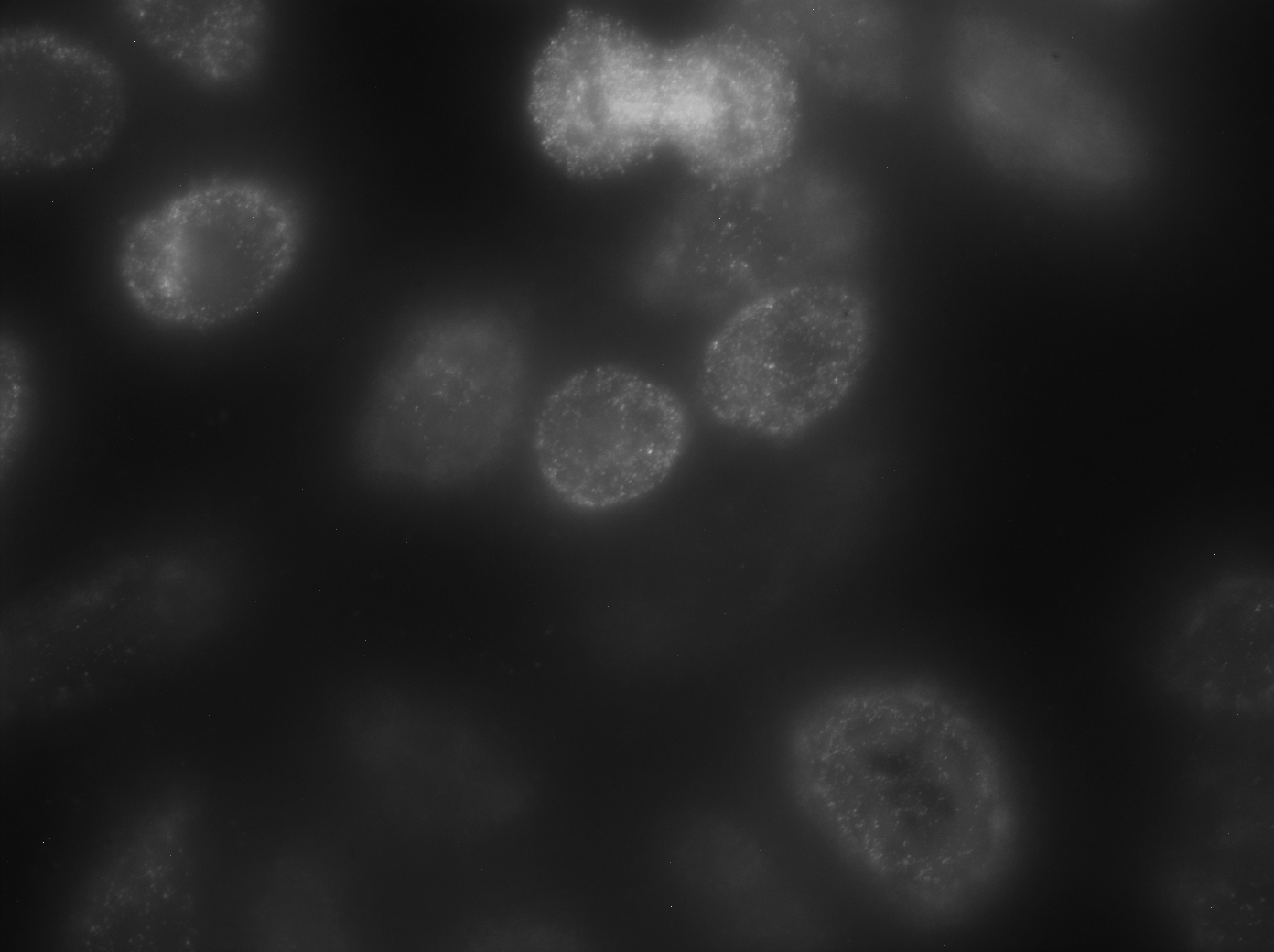

Supplement: Supplementary file 10 — Source data Fig. 2 part 7 [file 44319_2026_742_MOESM10_ESM.zip › Figure 2 Part 7/Fig 2fg Control Hela rGT335 acetylated tubulin part 2/Furrow Ingression/Cas9 actub rGT335 9-8-25 R3 FI7.Project Maximum Z_XY1757366561_Z0_T0_C1.tif]

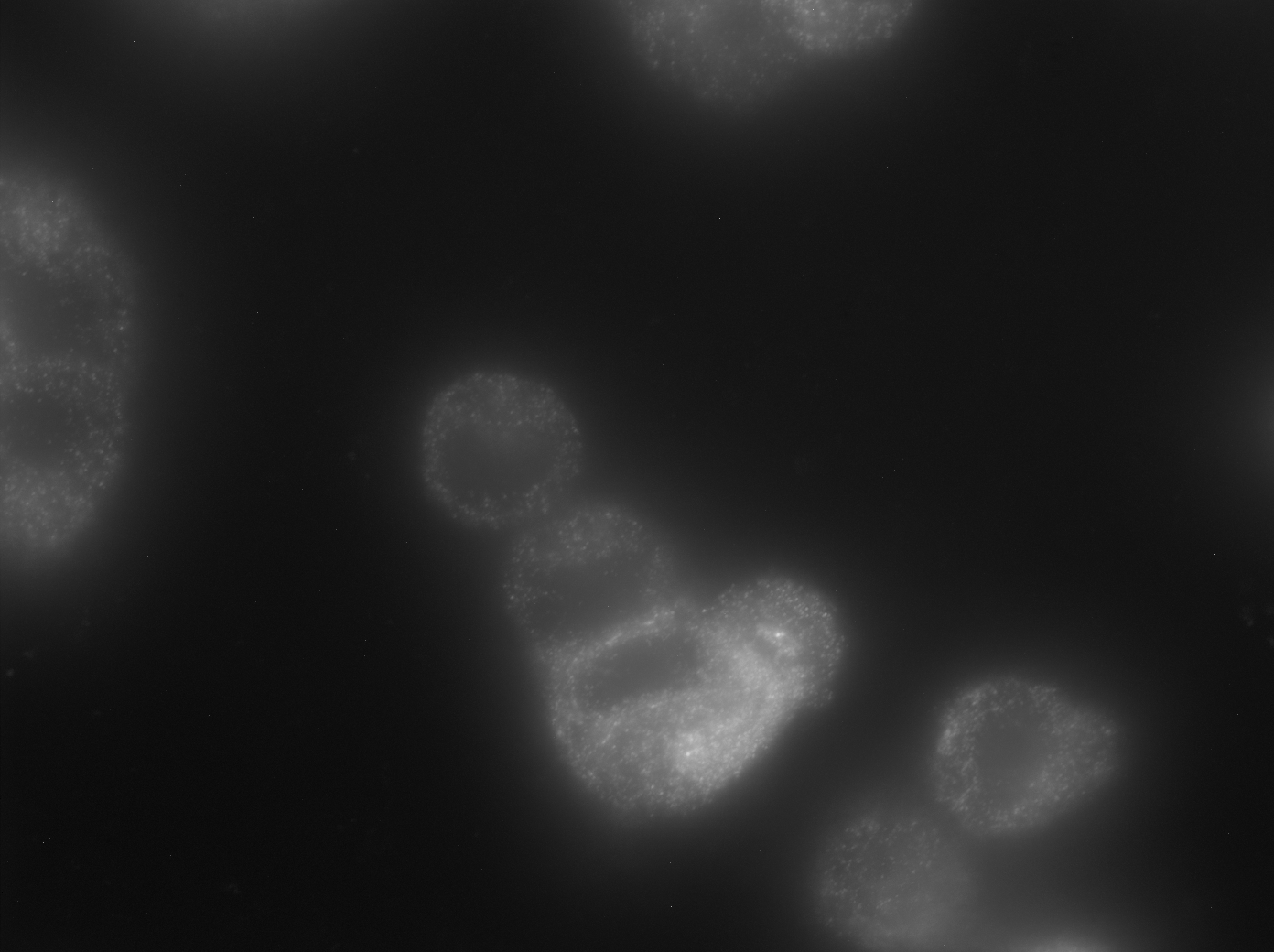

Supplement: Supplementary file 10 — Source data Fig. 2 part 7 [file 44319_2026_742_MOESM10_ESM.zip › Figure 2 Part 7/Fig 2fg Control Hela rGT335 acetylated tubulin part 2/Furrow Ingression/Cas9 actub rGT335 9-8-25 R1 FI3.Project Maximum Z_XY1757352722_Z0_T0_C1.tif]

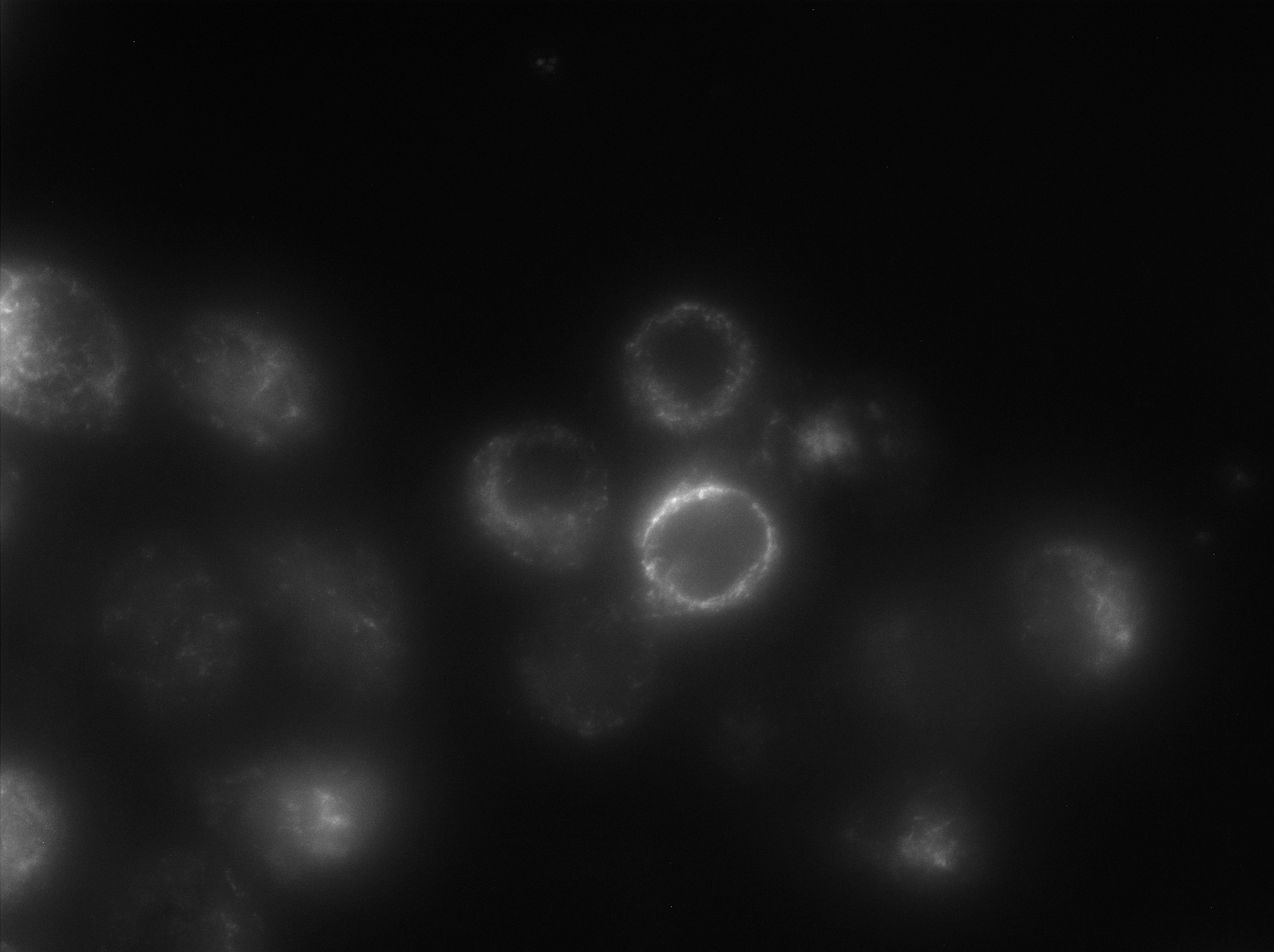

Supplement: Supplementary file 10 — Source data Fig. 2 part 7 [file 44319_2026_742_MOESM10_ESM.zip › Figure 2 Part 7/Fig 2fg Control Hela rGT335 acetylated tubulin part 2/Furrow Ingression/Cas9 actub rGT335 9-8-25 R2 FI8.Project Maximum Z_XY1757362863_Z0_T0_C2.tif]

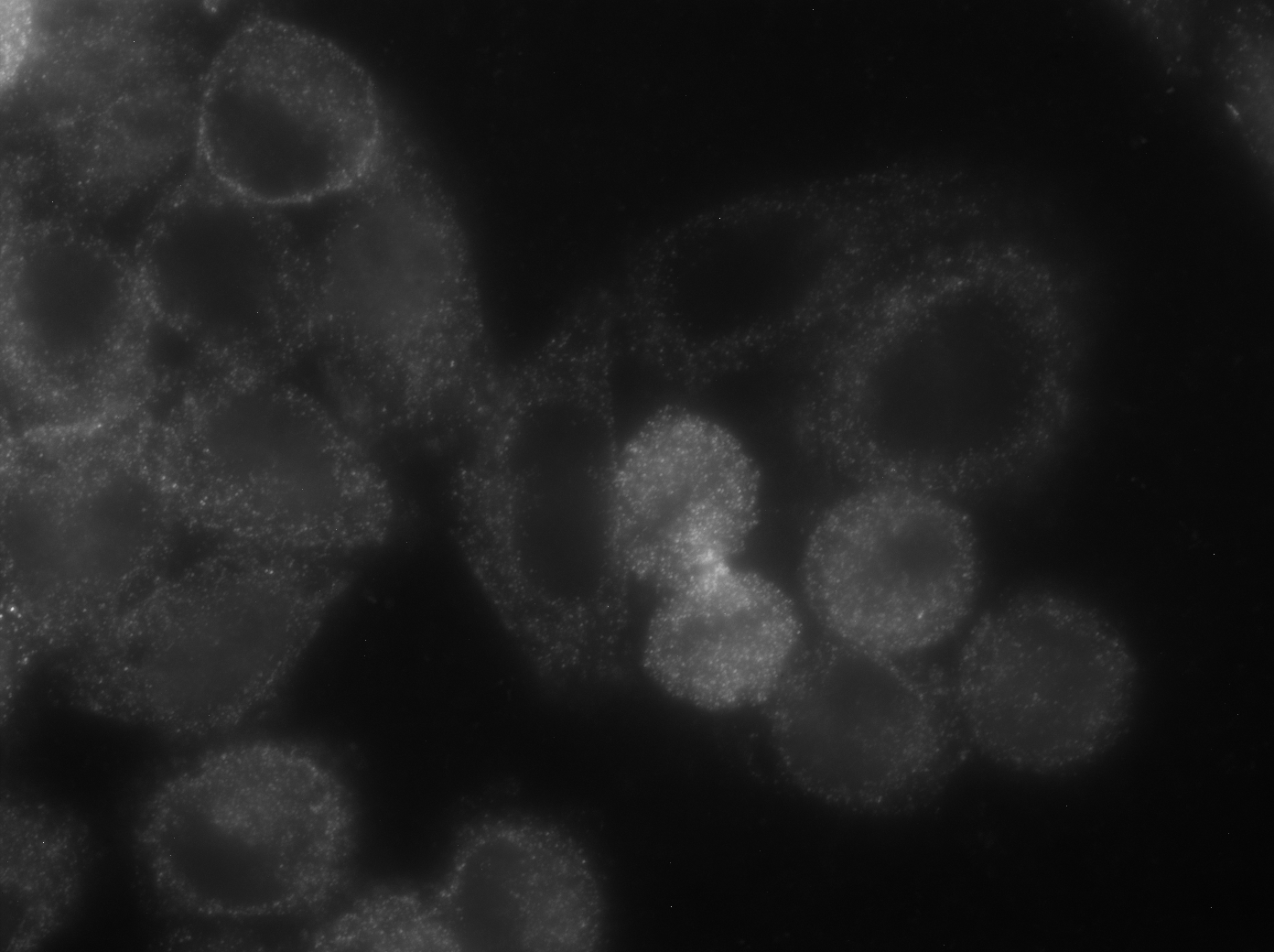

Supplement: Supplementary file 10 — Source data Fig. 2 part 7 [file 44319_2026_742_MOESM10_ESM.zip › Figure 2 Part 7/Fig 2fg Control Hela rGT335 acetylated tubulin part 2/Furrow Ingression/Cas9 actub rGT335 9-8-25 R3 FI5.Project Maximum Z_XY1757365932_Z0_T0_C1.tif]

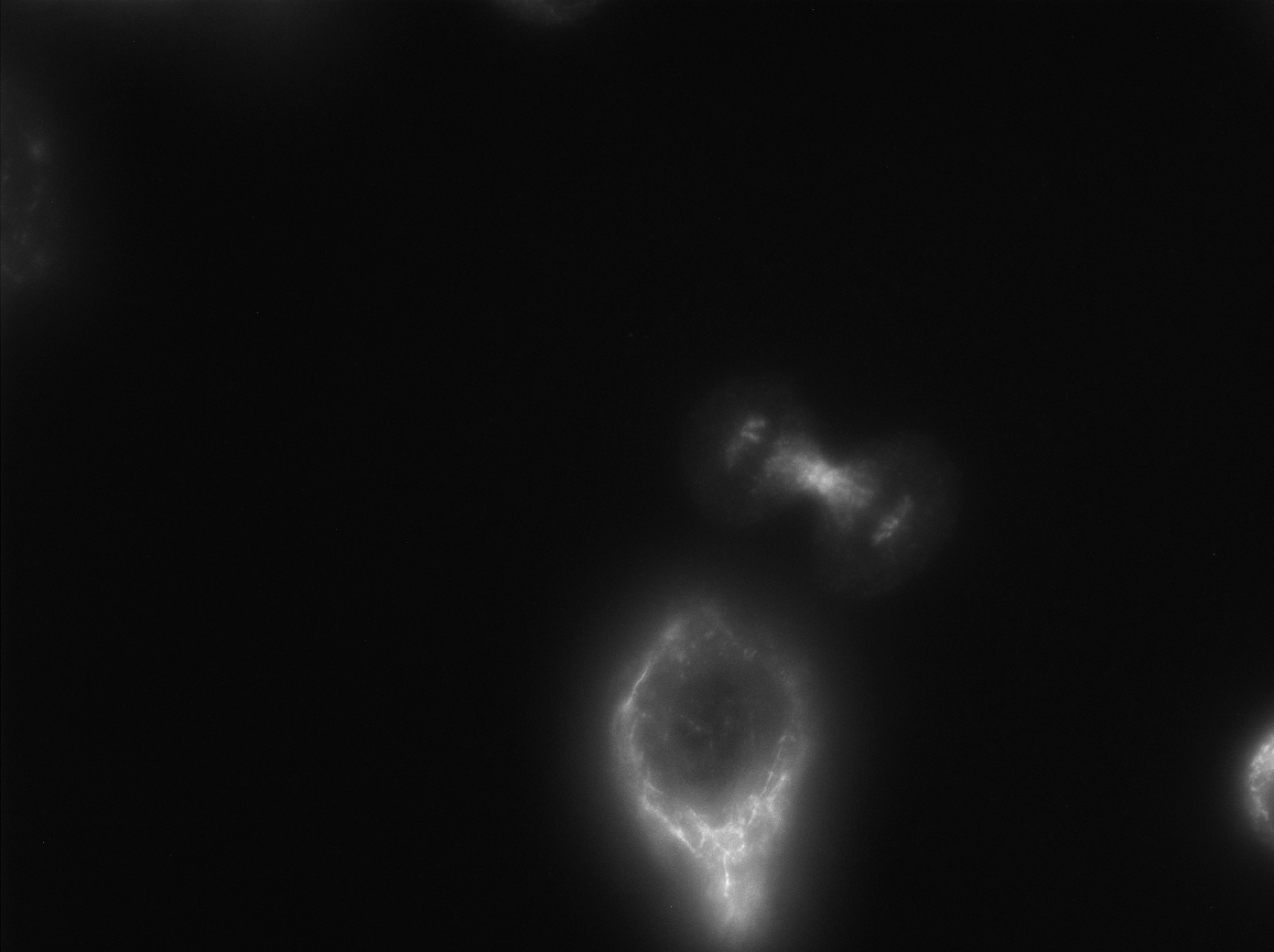

Supplement: Supplementary file 10 — Source data Fig. 2 part 7 [file 44319_2026_742_MOESM10_ESM.zip › Figure 2 Part 7/Fig 2fg Control Hela rGT335 acetylated tubulin part 2/Furrow Ingression/Cas9 actub rGT335 9-8-25 R1 FI6.Project Maximum Z_XY1757354454_Z0_T0_C2.tif]

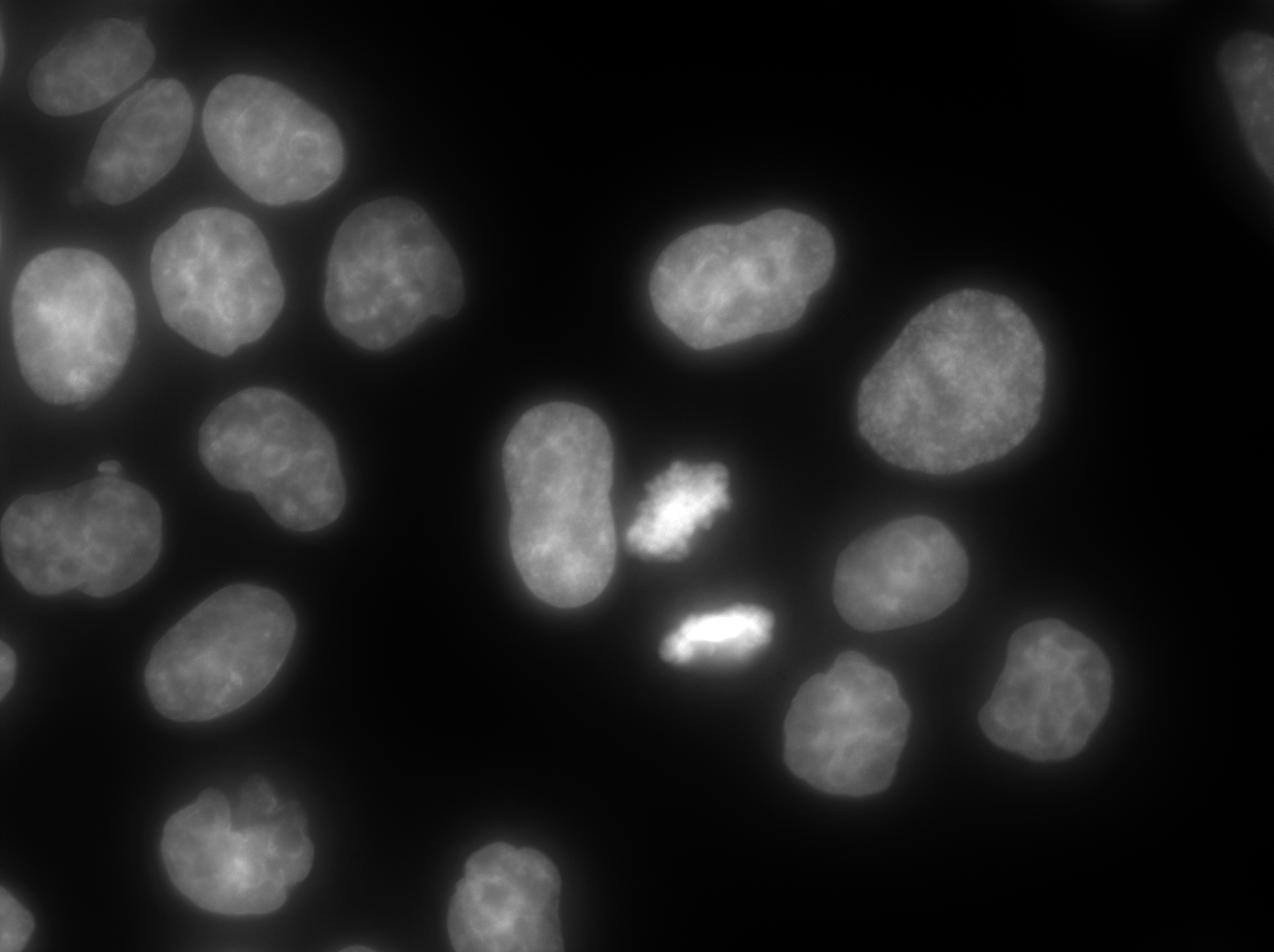

Supplement: Supplementary file 10 — Source data Fig. 2 part 7 [file 44319_2026_742_MOESM10_ESM.zip › Figure 2 Part 7/Fig 2fg Control Hela rGT335 acetylated tubulin part 2/Furrow Ingression/Cas9 actub rGT335 9-8-25 R3 FI5.Project Maximum Z_XY1757365932_Z0_T0_C0.tif]

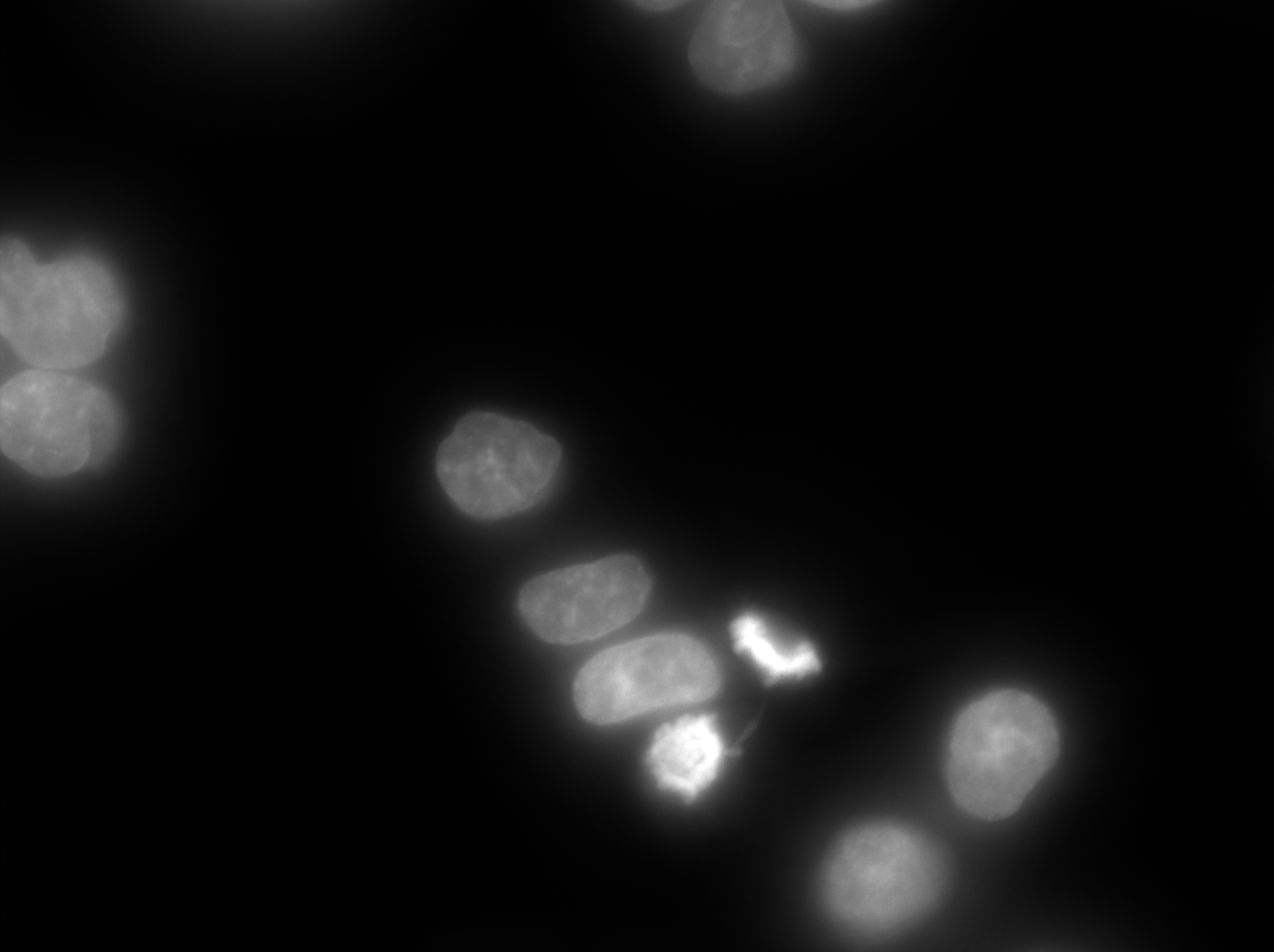

Supplement: Supplementary file 10 — Source data Fig. 2 part 7 [file 44319_2026_742_MOESM10_ESM.zip › Figure 2 Part 7/Fig 2fg Control Hela rGT335 acetylated tubulin part 2/Furrow Ingression/Cas9 actub rGT335 9-8-25 R1 FI3.Project Maximum Z_XY1757352722_Z0_T0_C0.tif]

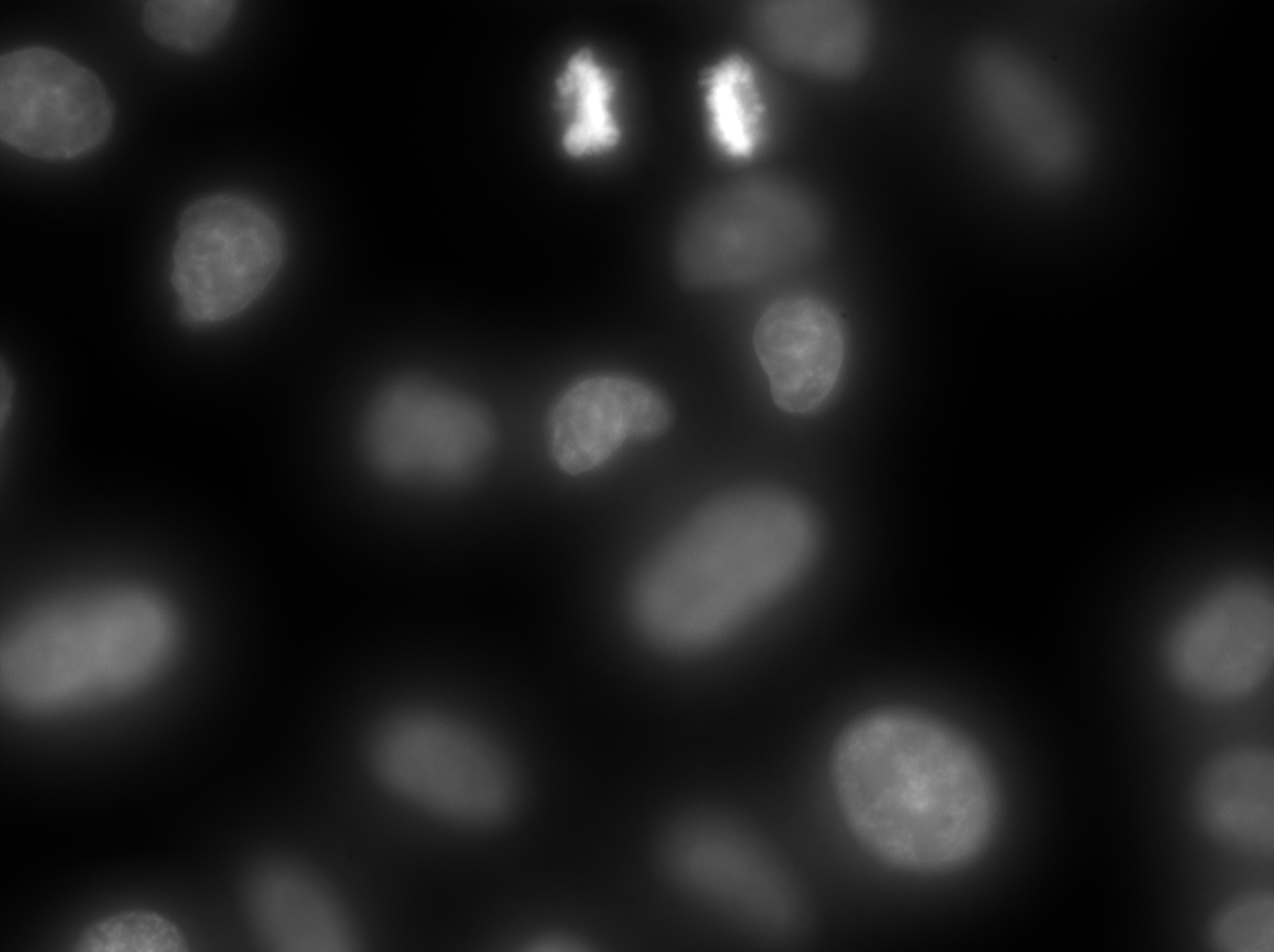

Supplement: Supplementary file 10 — Source data Fig. 2 part 7 [file 44319_2026_742_MOESM10_ESM.zip › Figure 2 Part 7/Fig 2fg Control Hela rGT335 acetylated tubulin part 2/Furrow Ingression/Cas9 actub rGT335 9-8-25 R3 FI7.Project Maximum Z_XY1757366561_Z0_T0_C0.tif]

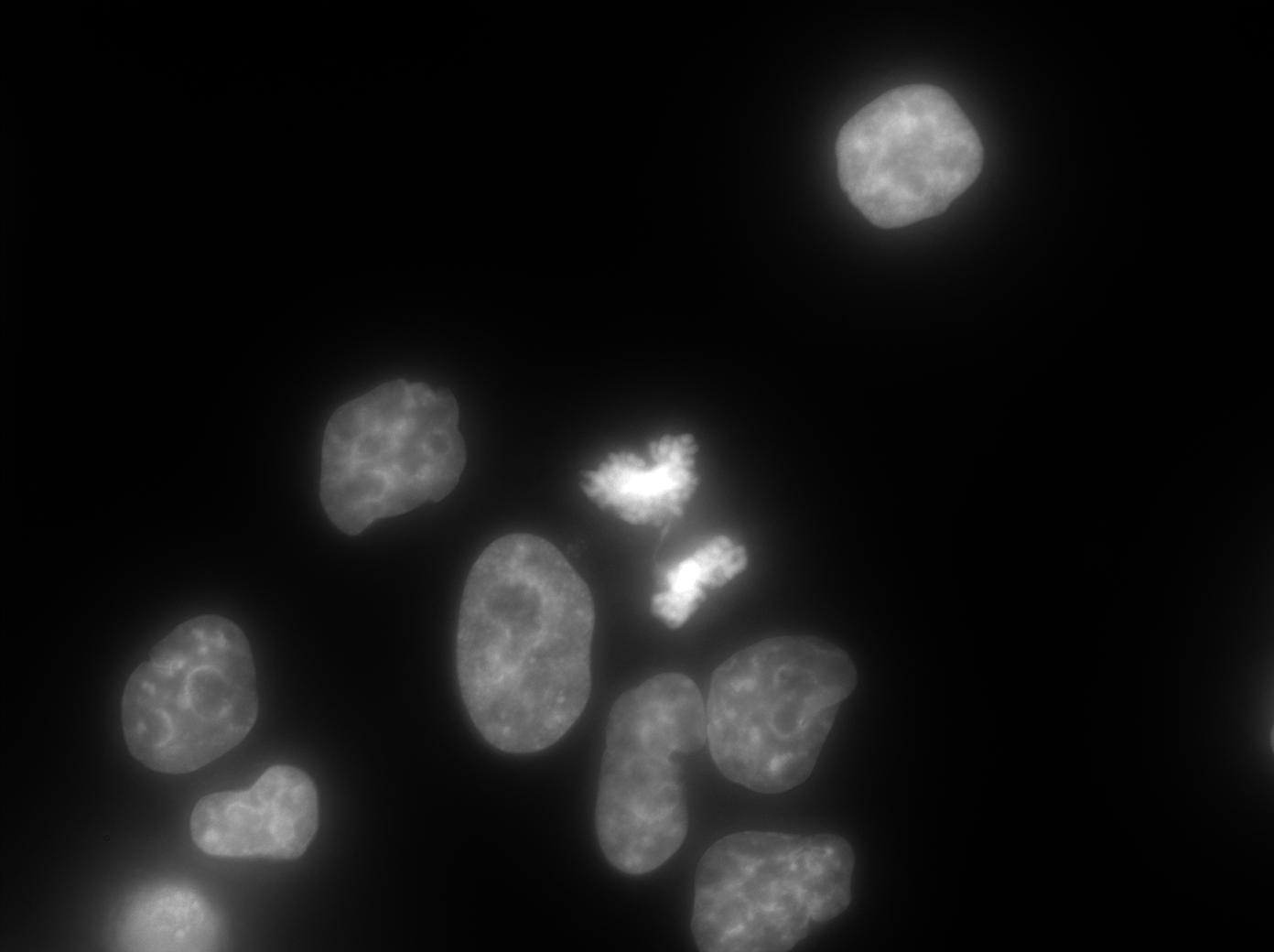

Supplement: Supplementary file 10 — Source data Fig. 2 part 7 [file 44319_2026_742_MOESM10_ESM.zip › Figure 2 Part 7/Fig 2fg Control Hela rGT335 acetylated tubulin part 2/Furrow Ingression/Cas9 actub rGT335 9-8-25 R1 FI11 chrbridge.Project Maximum Z_XY1757357644_Z0_T0_C0.tif]

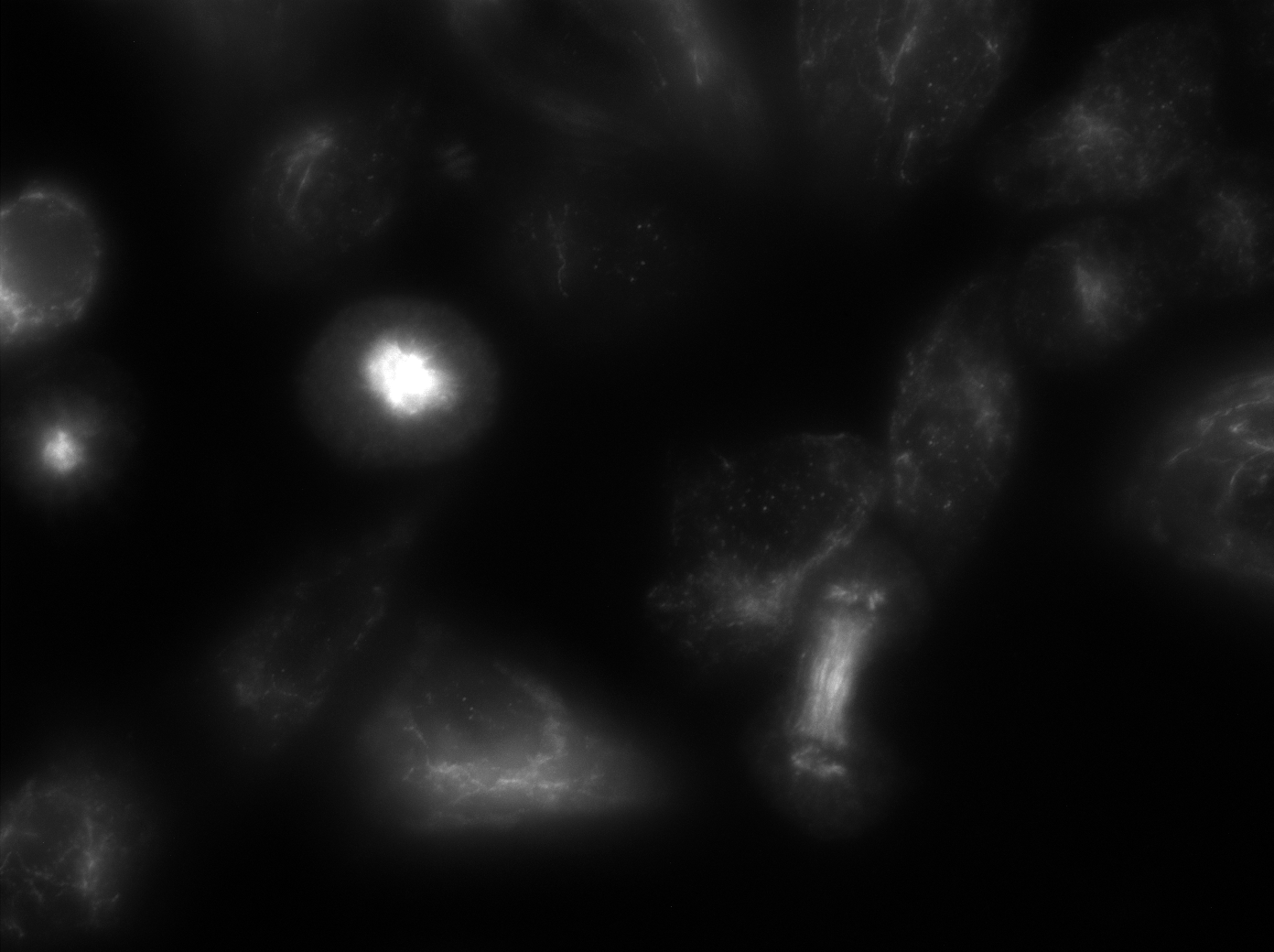

Supplement: Supplementary file 10 — Source data Fig. 2 part 7 [file 44319_2026_742_MOESM10_ESM.zip › Figure 2 Part 7/Fig 2fg Control Hela rGT335 acetylated tubulin part 2/Furrow Ingression/Cas9 actub rGT335 9-8-25 R2 FI1.Project Maximum Z_XY1757359718_Z0_T0_C2.tif]

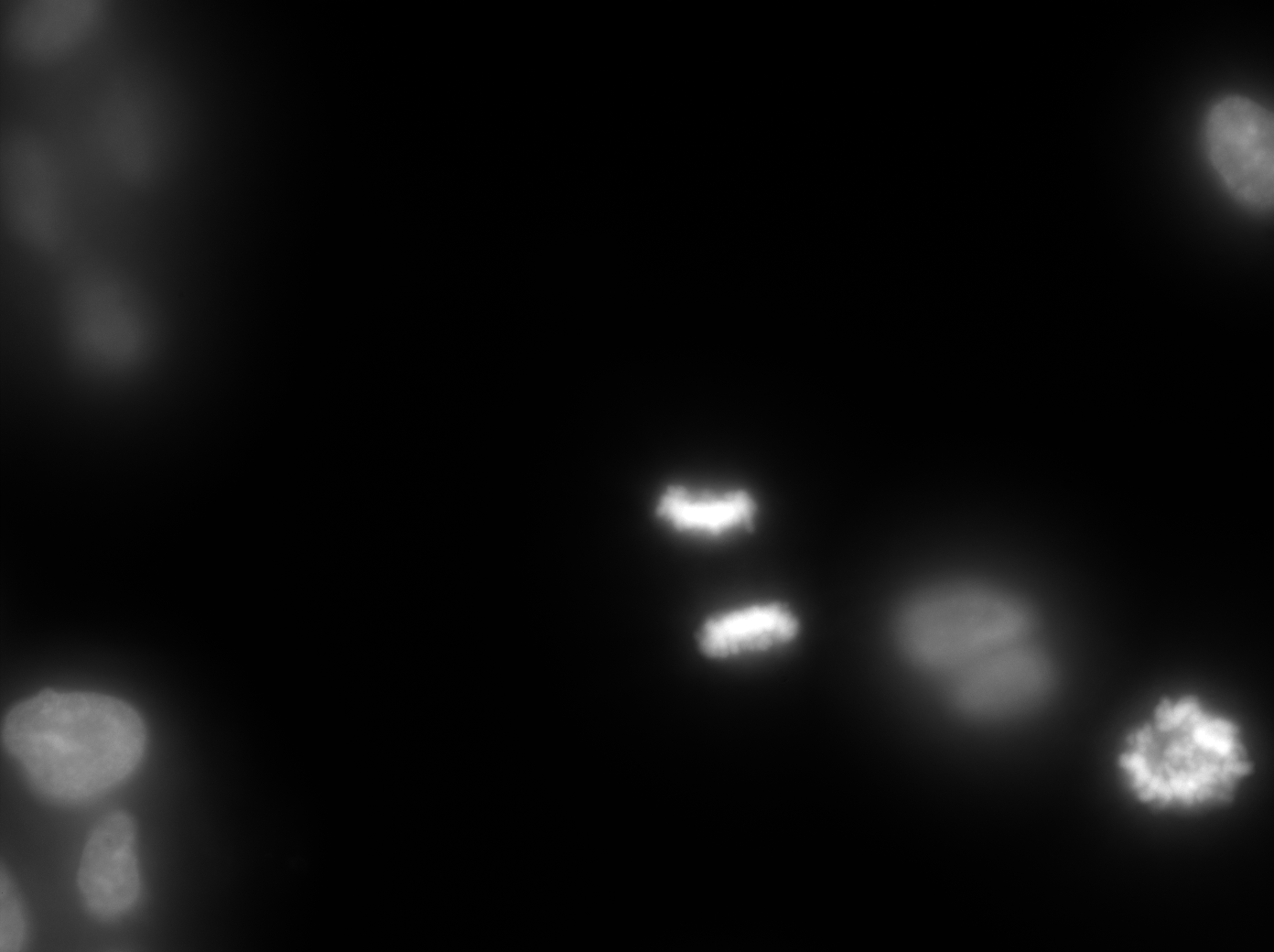

Supplement: Supplementary file 10 — Source data Fig. 2 part 7 [file 44319_2026_742_MOESM10_ESM.zip › Figure 2 Part 7/Fig 2fg Control Hela rGT335 acetylated tubulin part 2/Furrow Ingression/Cas9 actub rGT335 9-8-25 R1 FI2 EX.Project Maximum Z_XY1757351654_Z0_T0_C0.tif]

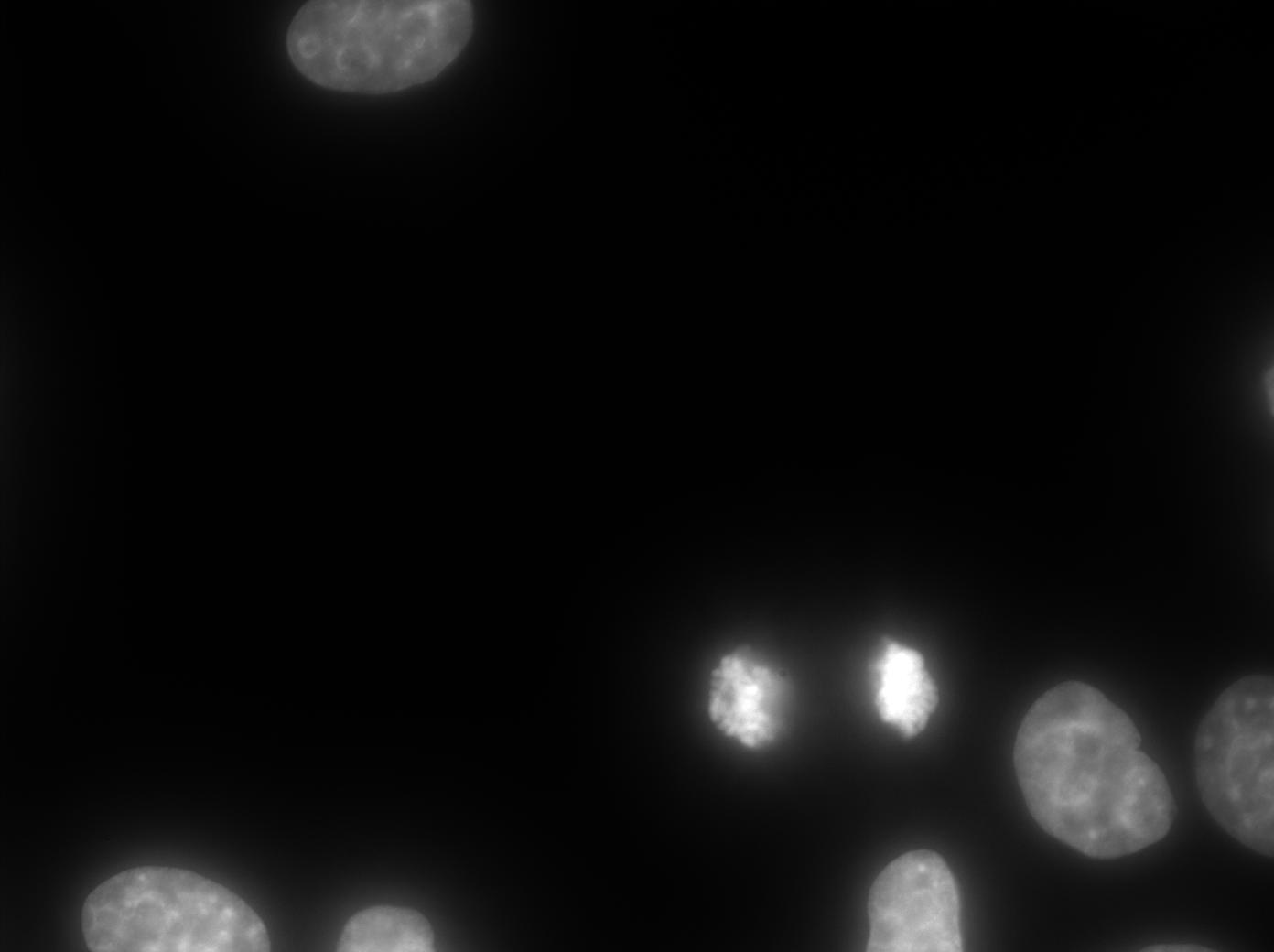

Supplement: Supplementary file 10 — Source data Fig. 2 part 7 [file 44319_2026_742_MOESM10_ESM.zip › Figure 2 Part 7/Fig 2fg Control Hela rGT335 acetylated tubulin part 2/Furrow Ingression/Cas9 actub rGT335 9-8-25 R3 FI3.Project Maximum Z_XY1757365539_Z0_T0_C0.tif]

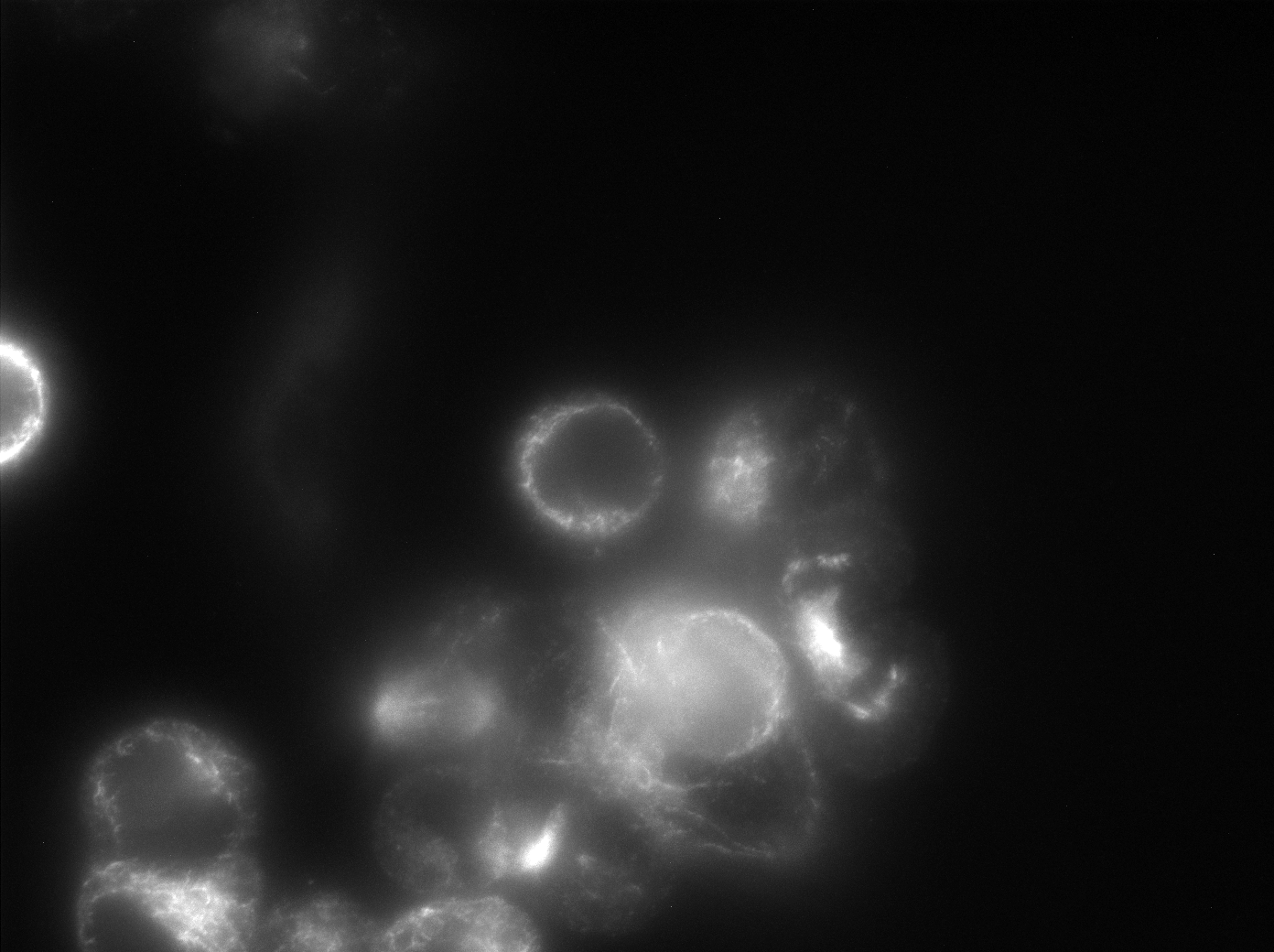

Supplement: Supplementary file 10 — Source data Fig. 2 part 7 [file 44319_2026_742_MOESM10_ESM.zip › Figure 2 Part 7/Fig 2fg Control Hela rGT335 acetylated tubulin part 2/Furrow Ingression/Cas9 actub rGT335 9-8-25 R1 FI8.Project Maximum Z_XY1757355943_Z0_T0_C2.tif]

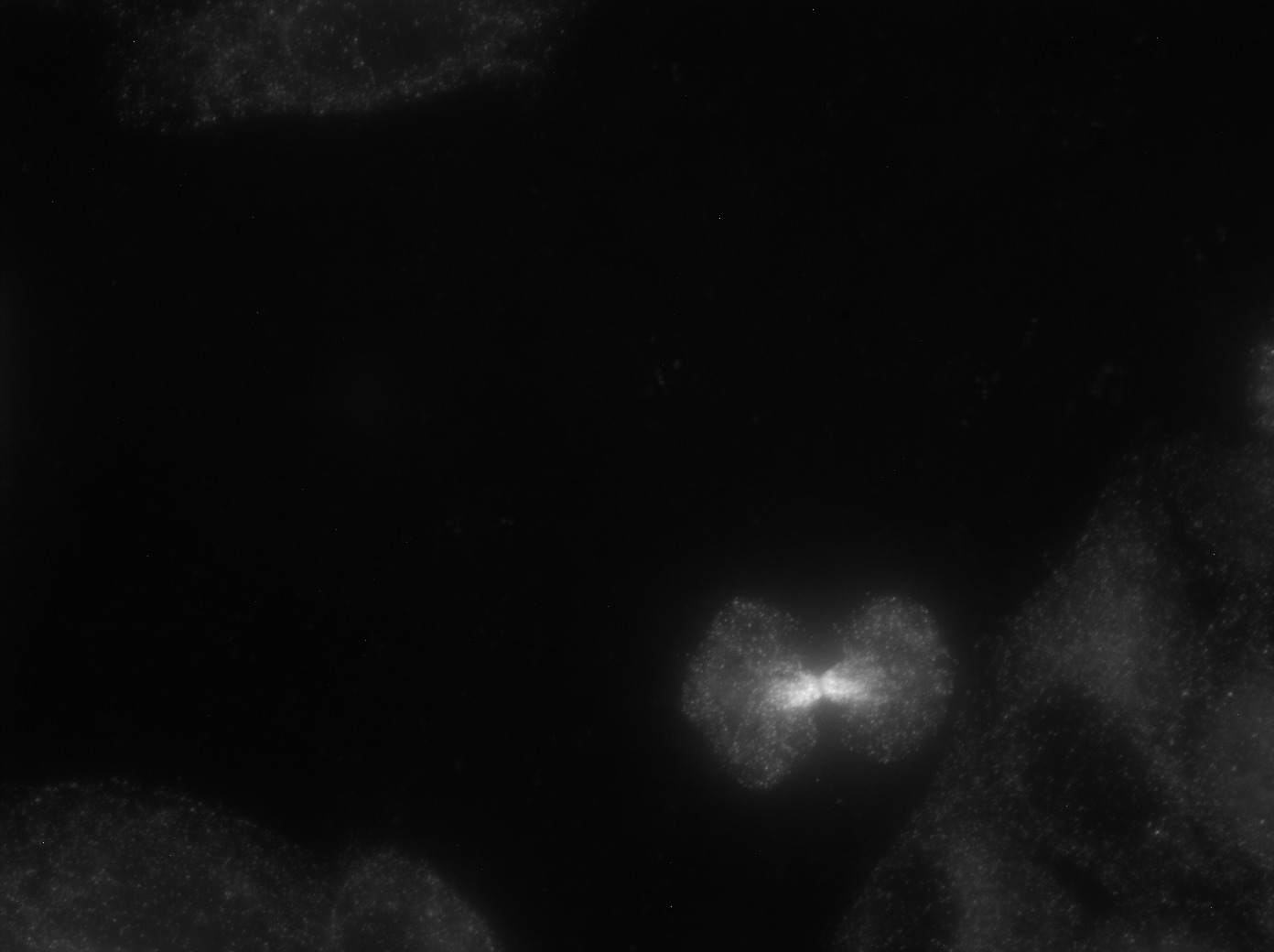

Supplement: Supplementary file 10 — Source data Fig. 2 part 7 [file 44319_2026_742_MOESM10_ESM.zip › Figure 2 Part 7/Fig 2fg Control Hela rGT335 acetylated tubulin part 2/Furrow Ingression/Cas9 actub rGT335 9-8-25 R3 FI3.Project Maximum Z_XY1757365539_Z0_T0_C1.tif]

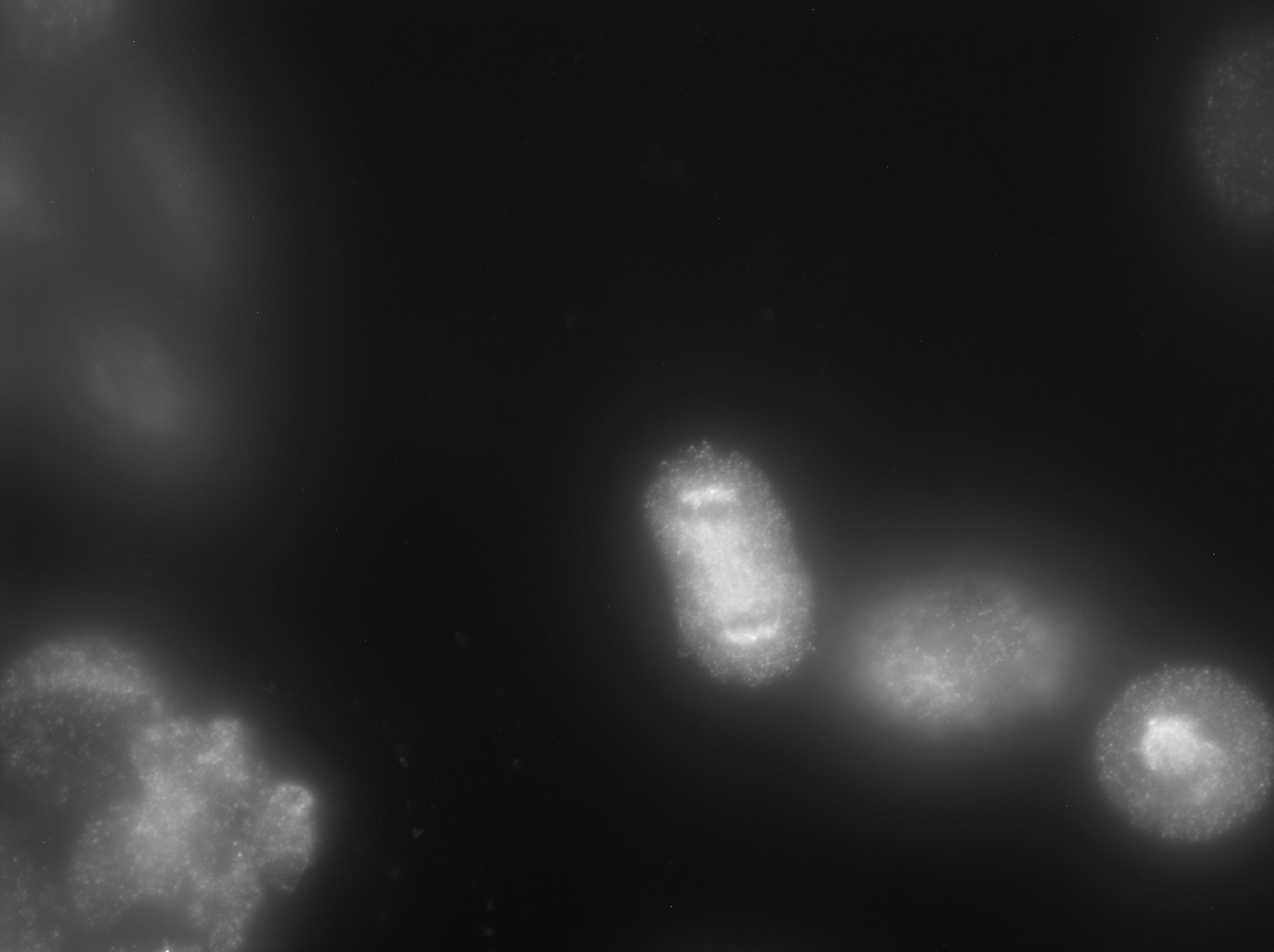

Supplement: Supplementary file 10 — Source data Fig. 2 part 7 [file 44319_2026_742_MOESM10_ESM.zip › Figure 2 Part 7/Fig 2fg Control Hela rGT335 acetylated tubulin part 2/Furrow Ingression/Cas9 actub rGT335 9-8-25 R1 FI2 EX.Project Maximum Z_XY1757351654_Z0_T0_C1.tif]

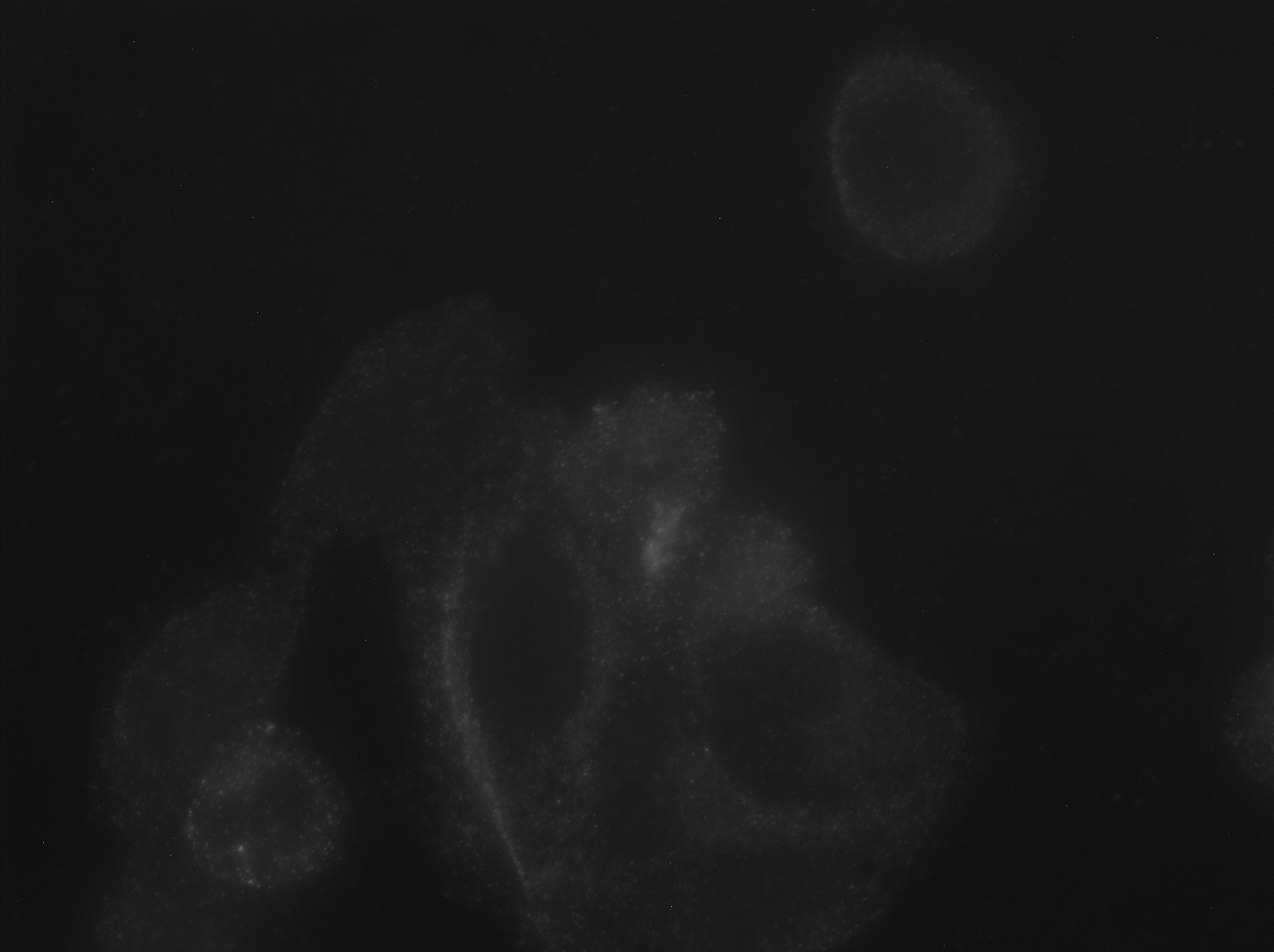

Supplement: Supplementary file 10 — Source data Fig. 2 part 7 [file 44319_2026_742_MOESM10_ESM.zip › Figure 2 Part 7/Fig 2fg Control Hela rGT335 acetylated tubulin part 2/Furrow Ingression/Cas9 actub rGT335 9-8-25 R1 FI11 chrbridge.Project Maximum Z_XY1757357644_Z0_T0_C1.tif]

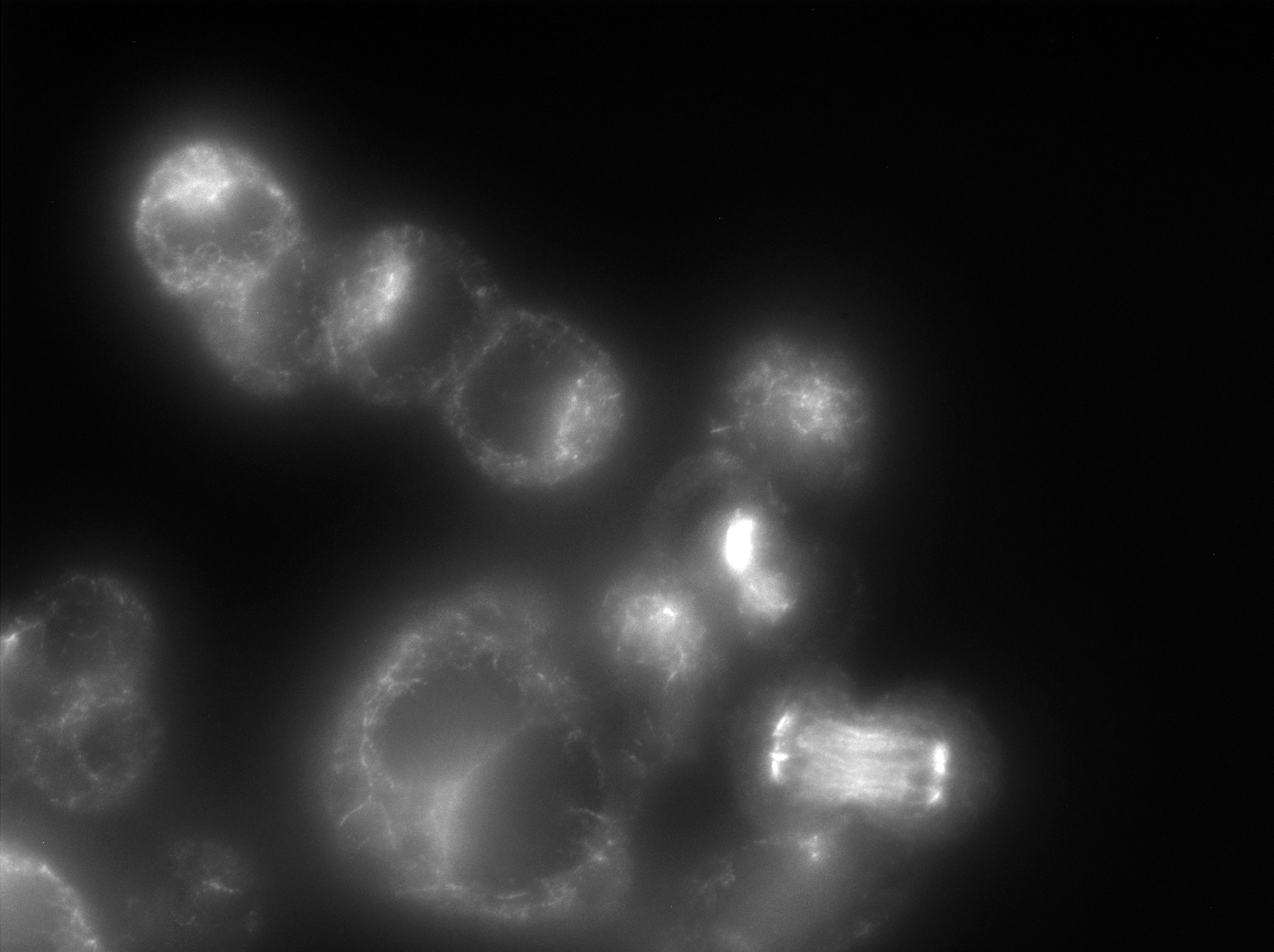

Supplement: Supplementary file 10 — Source data Fig. 2 part 7 [file 44319_2026_742_MOESM10_ESM.zip › Figure 2 Part 7/Fig 2fg Control Hela rGT335 acetylated tubulin part 2/Furrow Ingression/Cas9 actub rGT335 9-8-25 R1 FI12.Project Maximum Z_XY1757357885_Z0_T0_C2.tif]

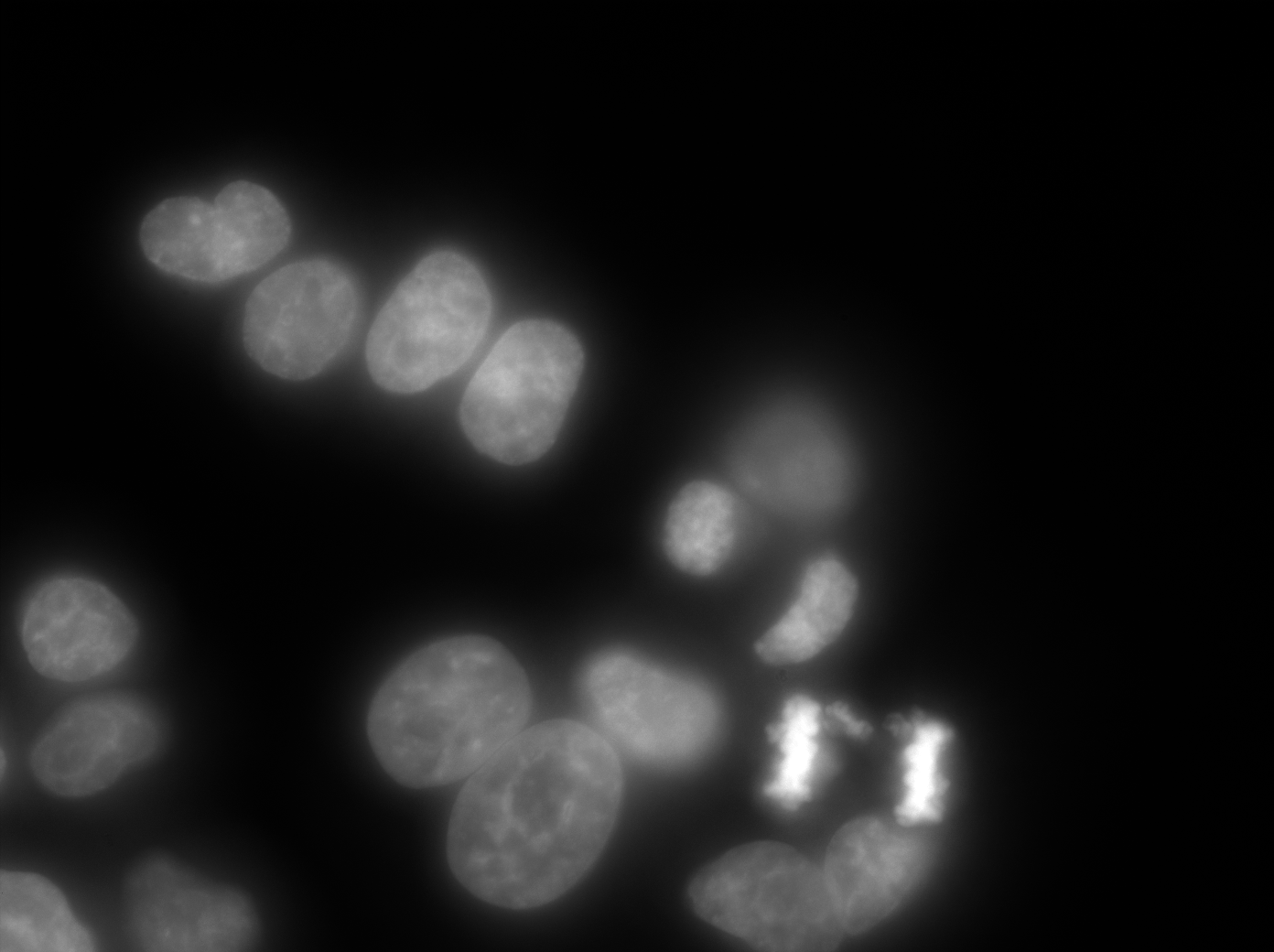

Supplement: Supplementary file 10 — Source data Fig. 2 part 7 [file 44319_2026_742_MOESM10_ESM.zip › Figure 2 Part 7/Fig 2fg Control Hela rGT335 acetylated tubulin part 2/Furrow Ingression/Cas9 actub rGT335 9-8-25 R1 FI12.Project Maximum Z_XY1757357885_Z0_T0_C0.tif]

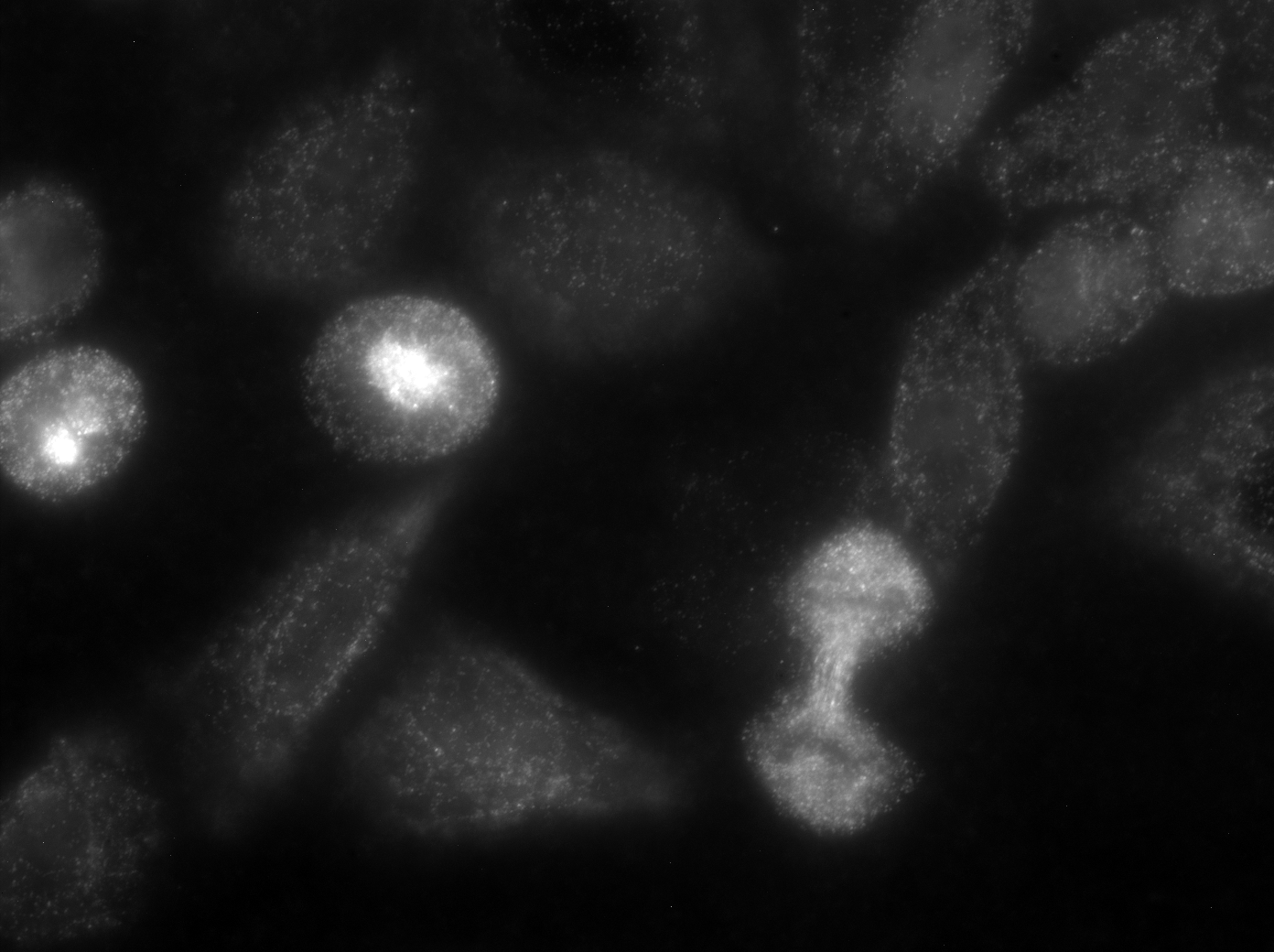

Supplement: Supplementary file 10 — Source data Fig. 2 part 7 [file 44319_2026_742_MOESM10_ESM.zip › Figure 2 Part 7/Fig 2fg Control Hela rGT335 acetylated tubulin part 2/Furrow Ingression/Cas9 actub rGT335 9-8-25 R2 FI1.Project Maximum Z_XY1757359718_Z0_T0_C1.tif]

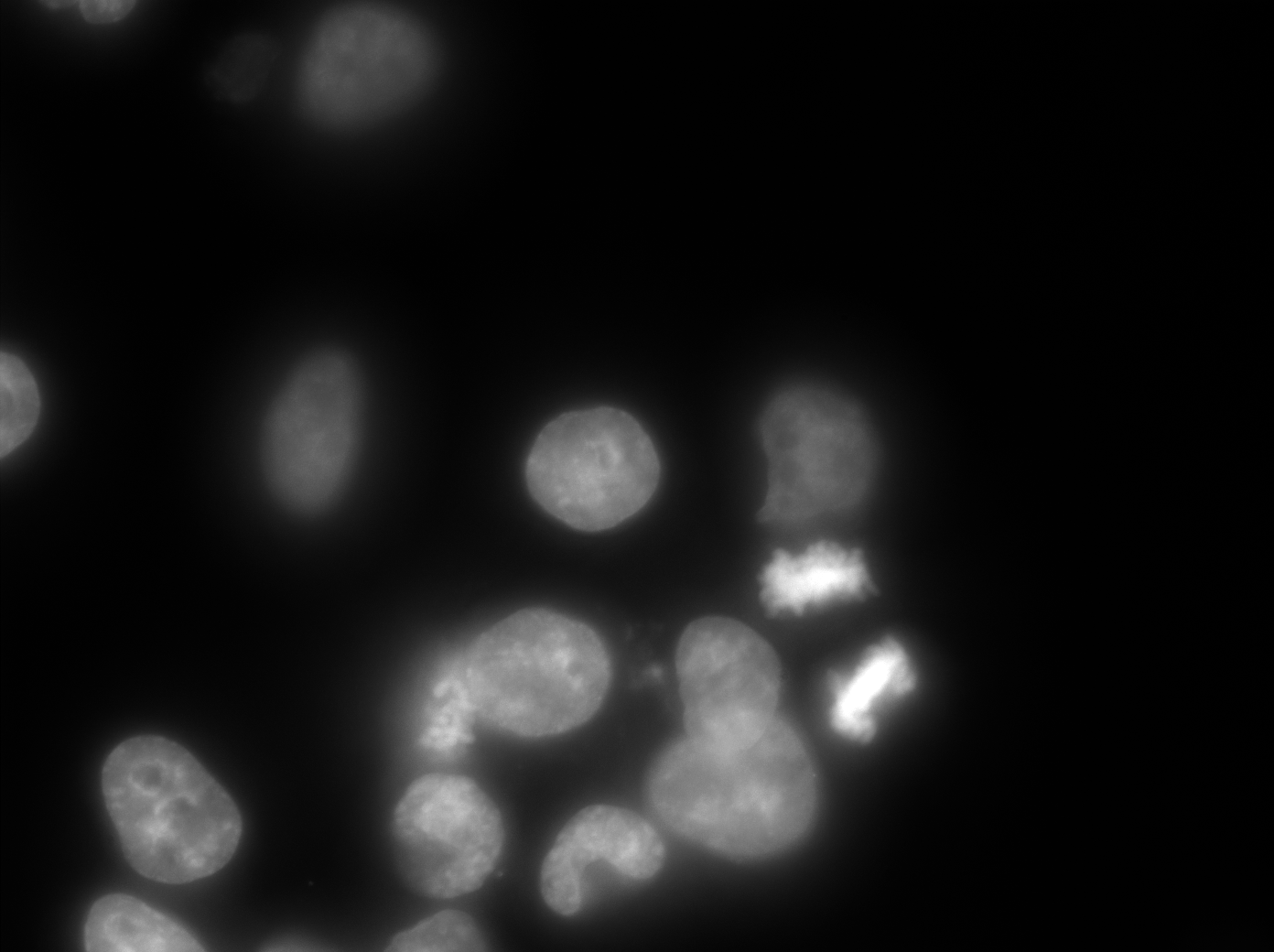

Supplement: Supplementary file 10 — Source data Fig. 2 part 7 [file 44319_2026_742_MOESM10_ESM.zip › Figure 2 Part 7/Fig 2fg Control Hela rGT335 acetylated tubulin part 2/Furrow Ingression/Cas9 actub rGT335 9-8-25 R1 FI8.Project Maximum Z_XY1757355943_Z0_T0_C0.tif]

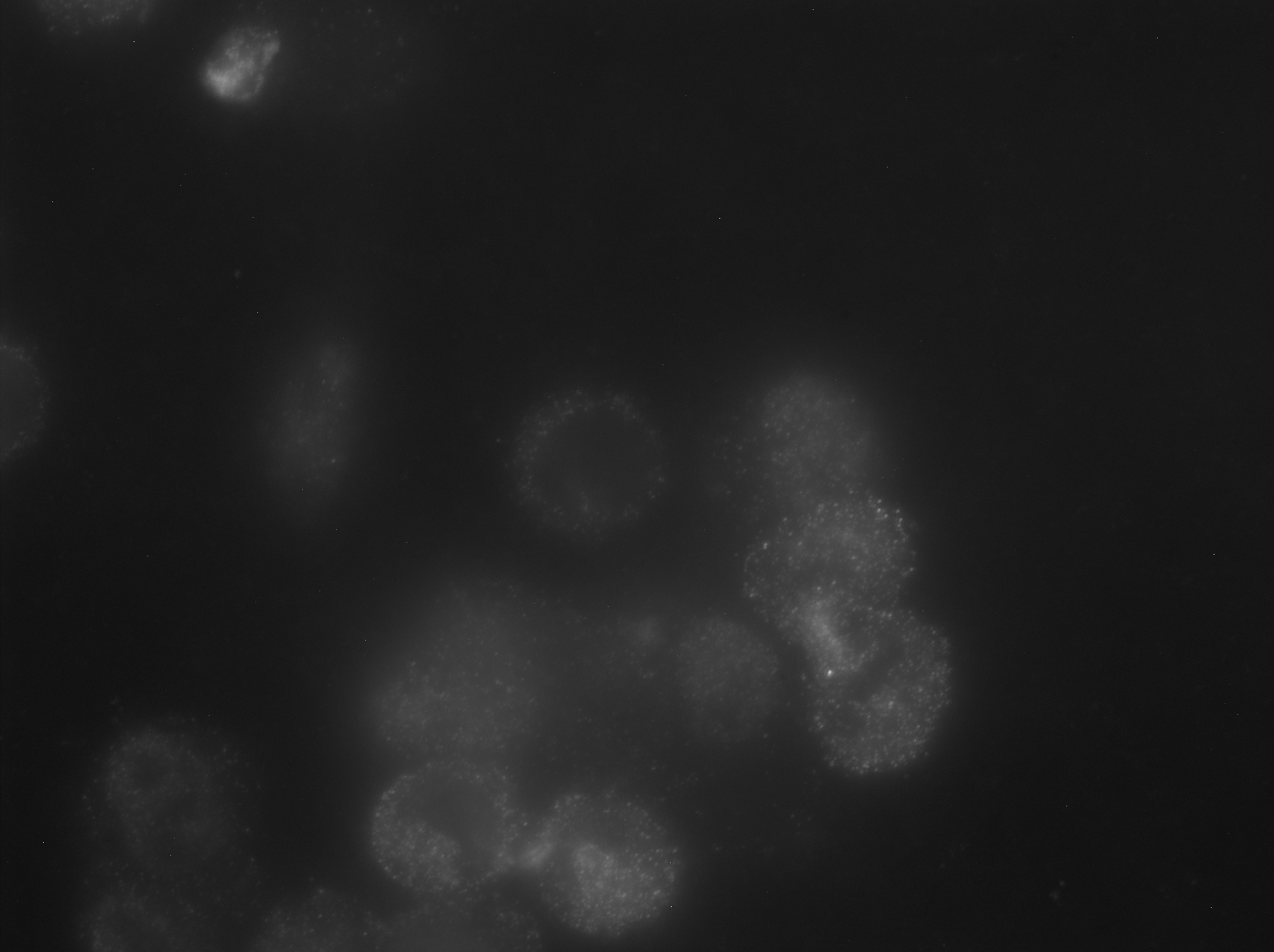

Supplement: Supplementary file 10 — Source data Fig. 2 part 7 [file 44319_2026_742_MOESM10_ESM.zip › Figure 2 Part 7/Fig 2fg Control Hela rGT335 acetylated tubulin part 2/Furrow Ingression/Cas9 actub rGT335 9-8-25 R1 FI8.Project Maximum Z_XY1757355943_Z0_T0_C1.tif]

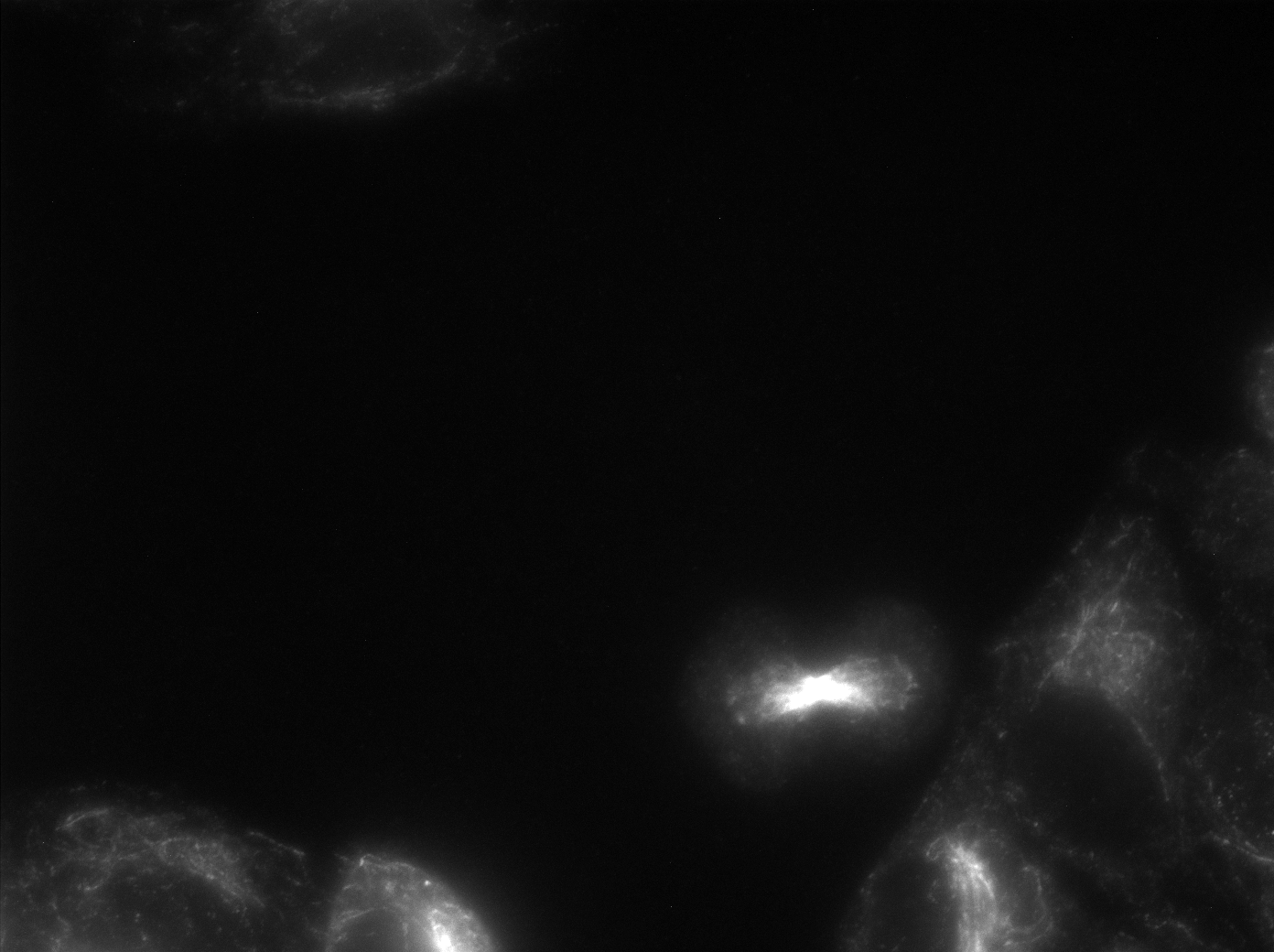

Supplement: Supplementary file 10 — Source data Fig. 2 part 7 [file 44319_2026_742_MOESM10_ESM.zip › Figure 2 Part 7/Fig 2fg Control Hela rGT335 acetylated tubulin part 2/Furrow Ingression/Cas9 actub rGT335 9-8-25 R3 FI3.Project Maximum Z_XY1757365539_Z0_T0_C2.tif]

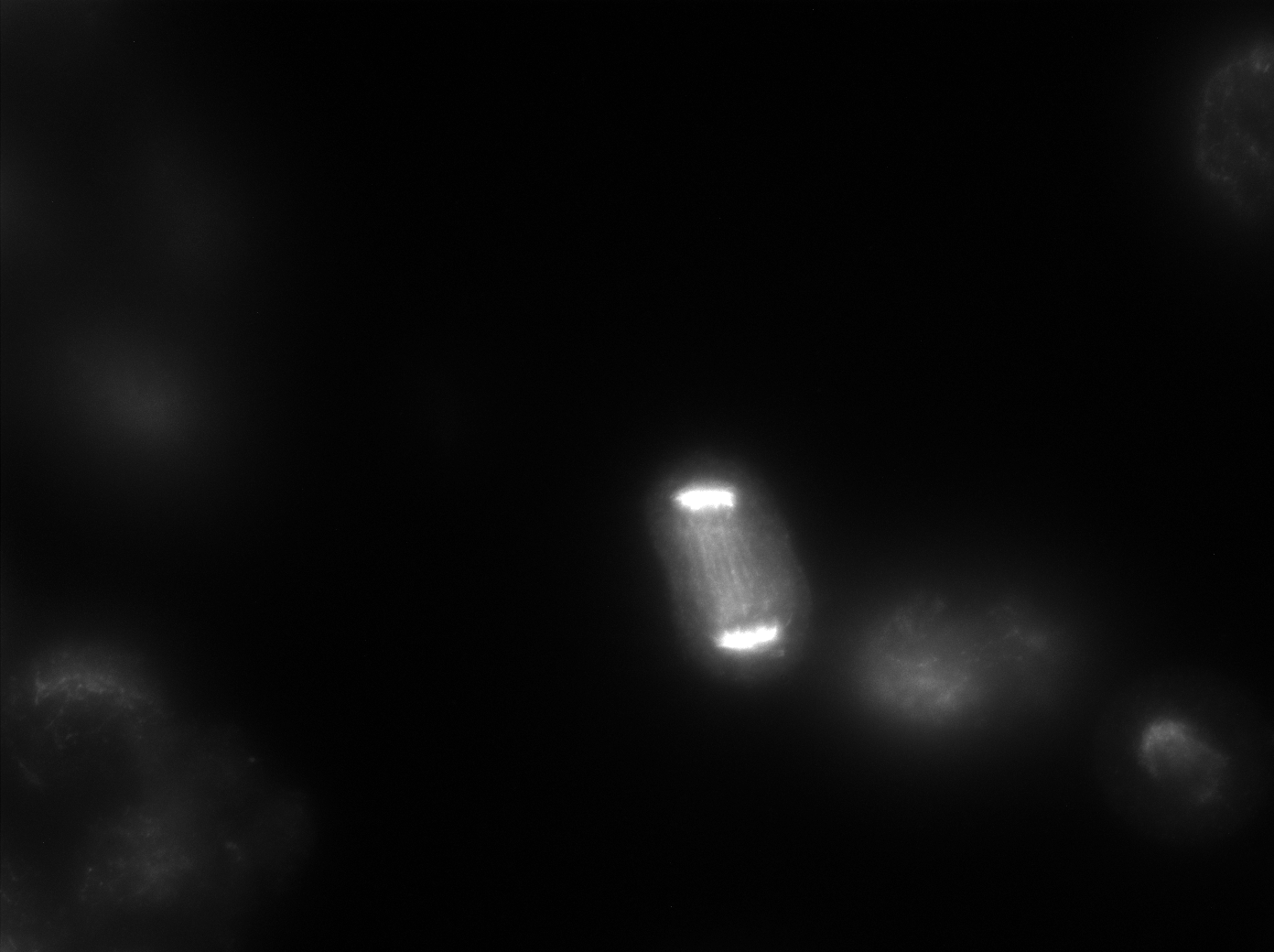

Supplement: Supplementary file 10 — Source data Fig. 2 part 7 [file 44319_2026_742_MOESM10_ESM.zip › Figure 2 Part 7/Fig 2fg Control Hela rGT335 acetylated tubulin part 2/Furrow Ingression/Cas9 actub rGT335 9-8-25 R1 FI2 EX.Project Maximum Z_XY1757351654_Z0_T0_C2.tif]

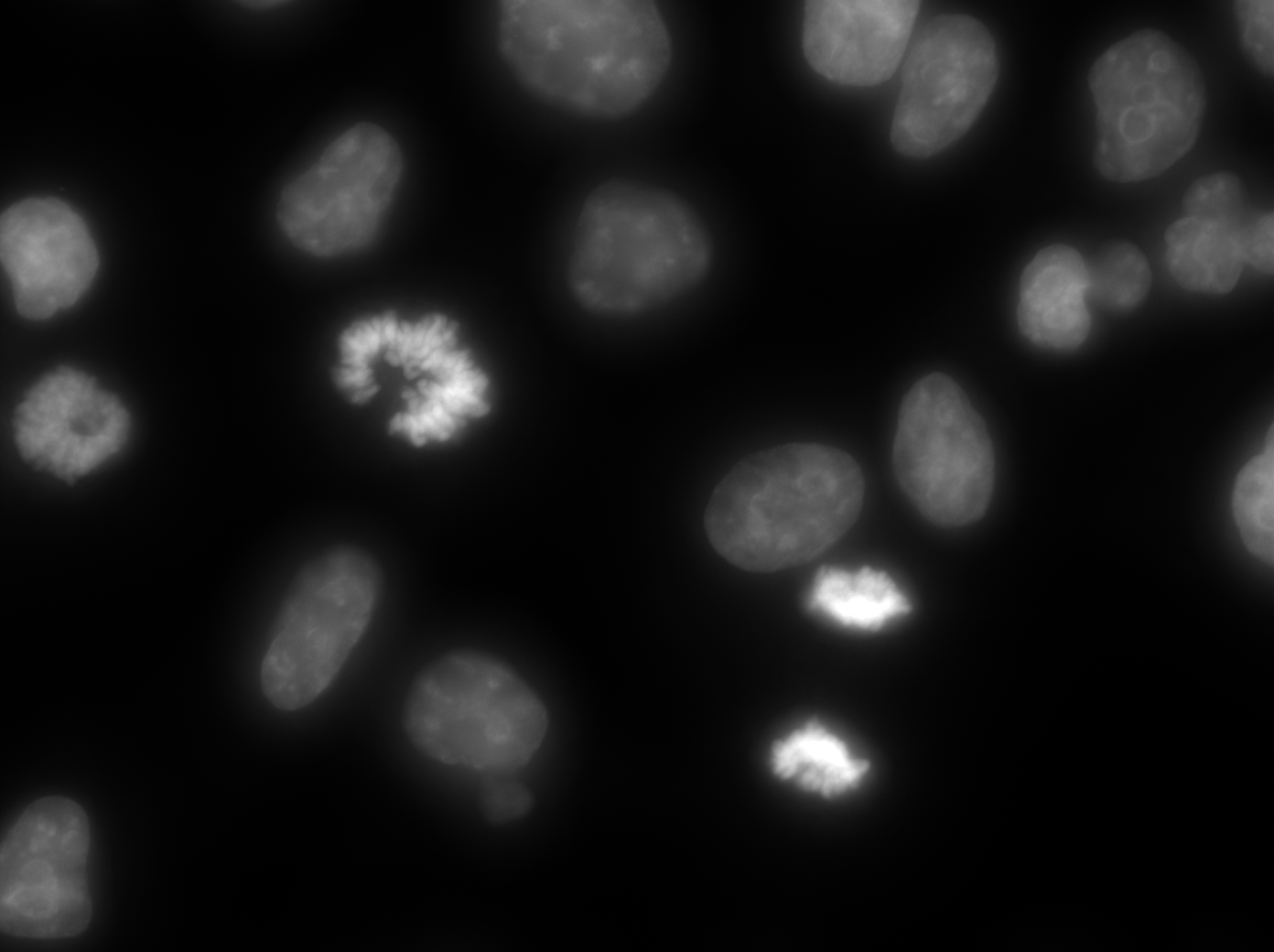

Supplement: Supplementary file 10 — Source data Fig. 2 part 7 [file 44319_2026_742_MOESM10_ESM.zip › Figure 2 Part 7/Fig 2fg Control Hela rGT335 acetylated tubulin part 2/Furrow Ingression/Cas9 actub rGT335 9-8-25 R2 FI1.Project Maximum Z_XY1757359718_Z0_T0_C0.tif]

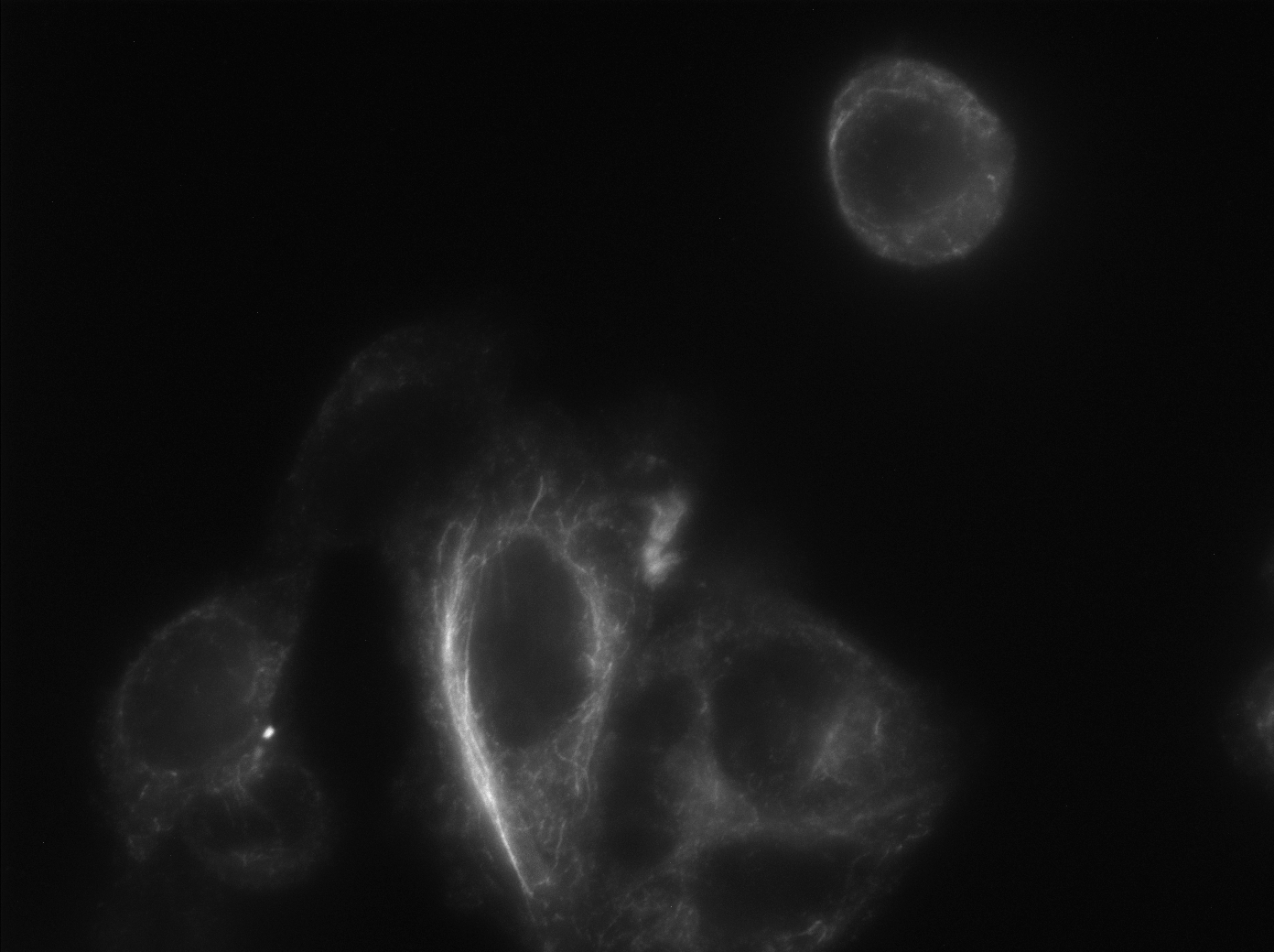

Supplement: Supplementary file 10 — Source data Fig. 2 part 7 [file 44319_2026_742_MOESM10_ESM.zip › Figure 2 Part 7/Fig 2fg Control Hela rGT335 acetylated tubulin part 2/Furrow Ingression/Cas9 actub rGT335 9-8-25 R1 FI11 chrbridge.Project Maximum Z_XY1757357644_Z0_T0_C2.tif]

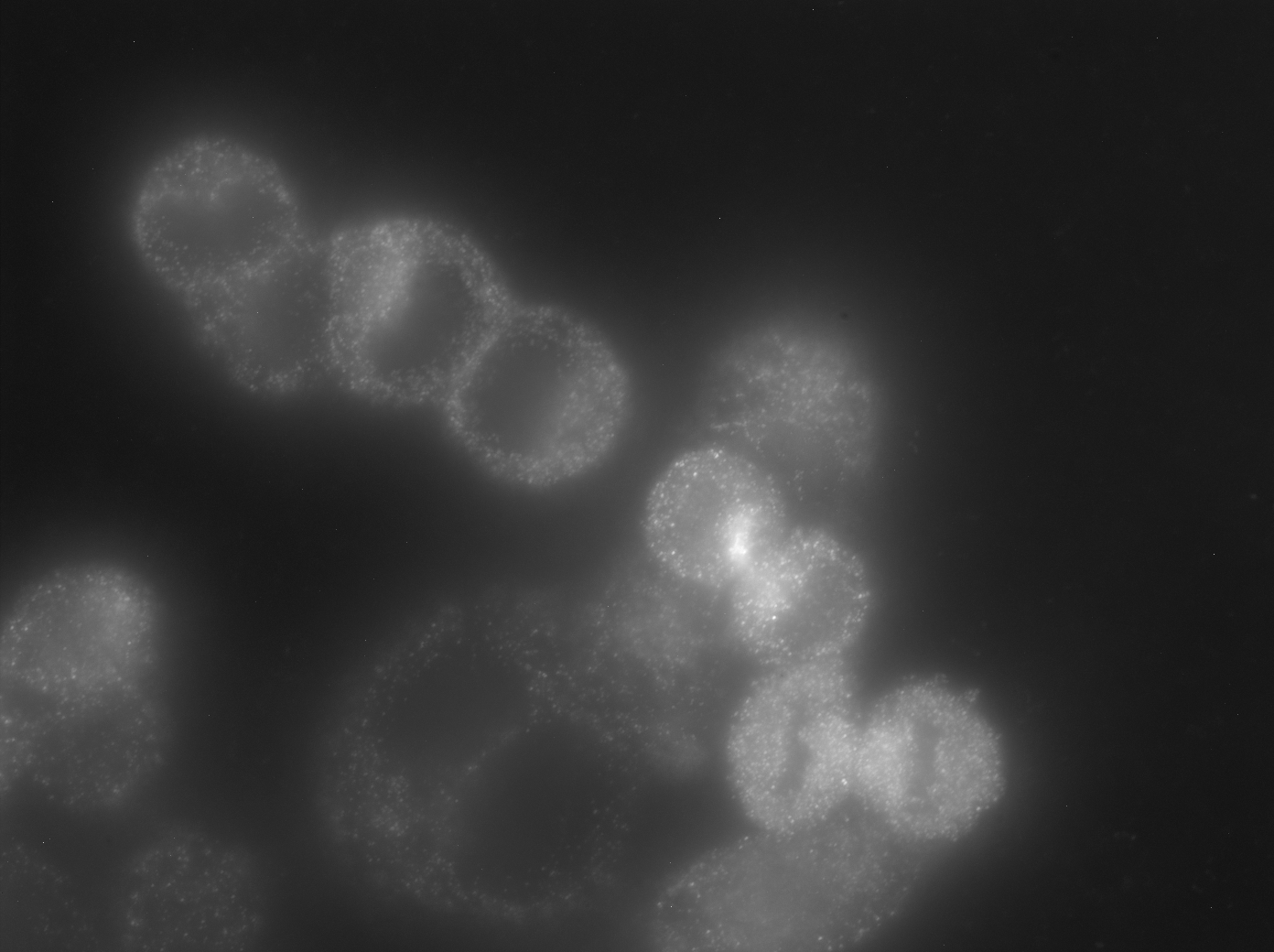

Supplement: Supplementary file 10 — Source data Fig. 2 part 7 [file 44319_2026_742_MOESM10_ESM.zip › Figure 2 Part 7/Fig 2fg Control Hela rGT335 acetylated tubulin part 2/Furrow Ingression/Cas9 actub rGT335 9-8-25 R1 FI12.Project Maximum Z_XY1757357885_Z0_T0_C1.tif]

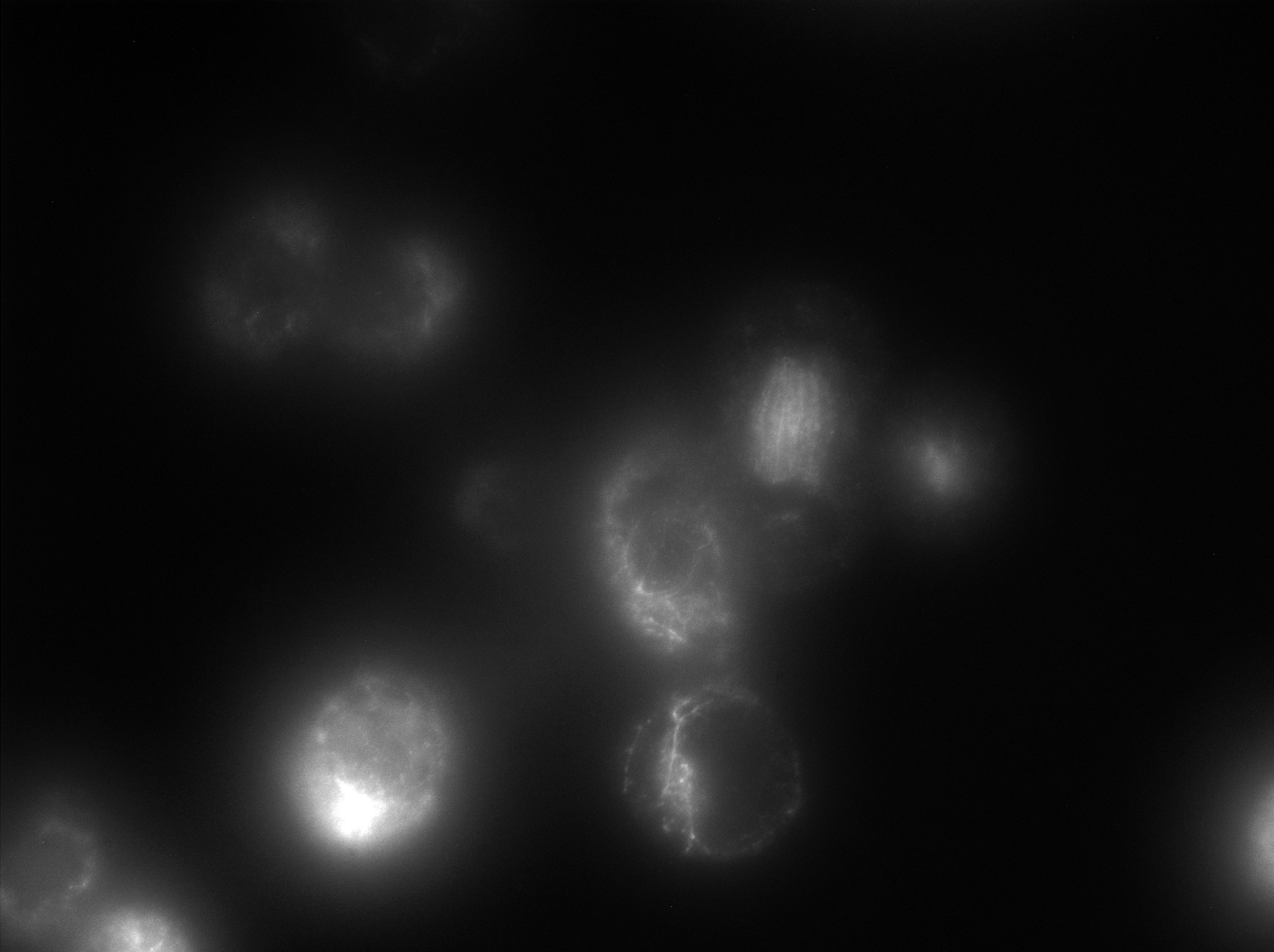

Supplement: Supplementary file 10 — Source data Fig. 2 part 7 [file 44319_2026_742_MOESM10_ESM.zip › Figure 2 Part 7/Fig 2fg Control Hela rGT335 acetylated tubulin part 2/Furrow Ingression/Cas9 actub rGT335 9-8-25 R1 FI10 EX.Project Maximum Z_XY1757357762_Z0_T0_C2.tif]

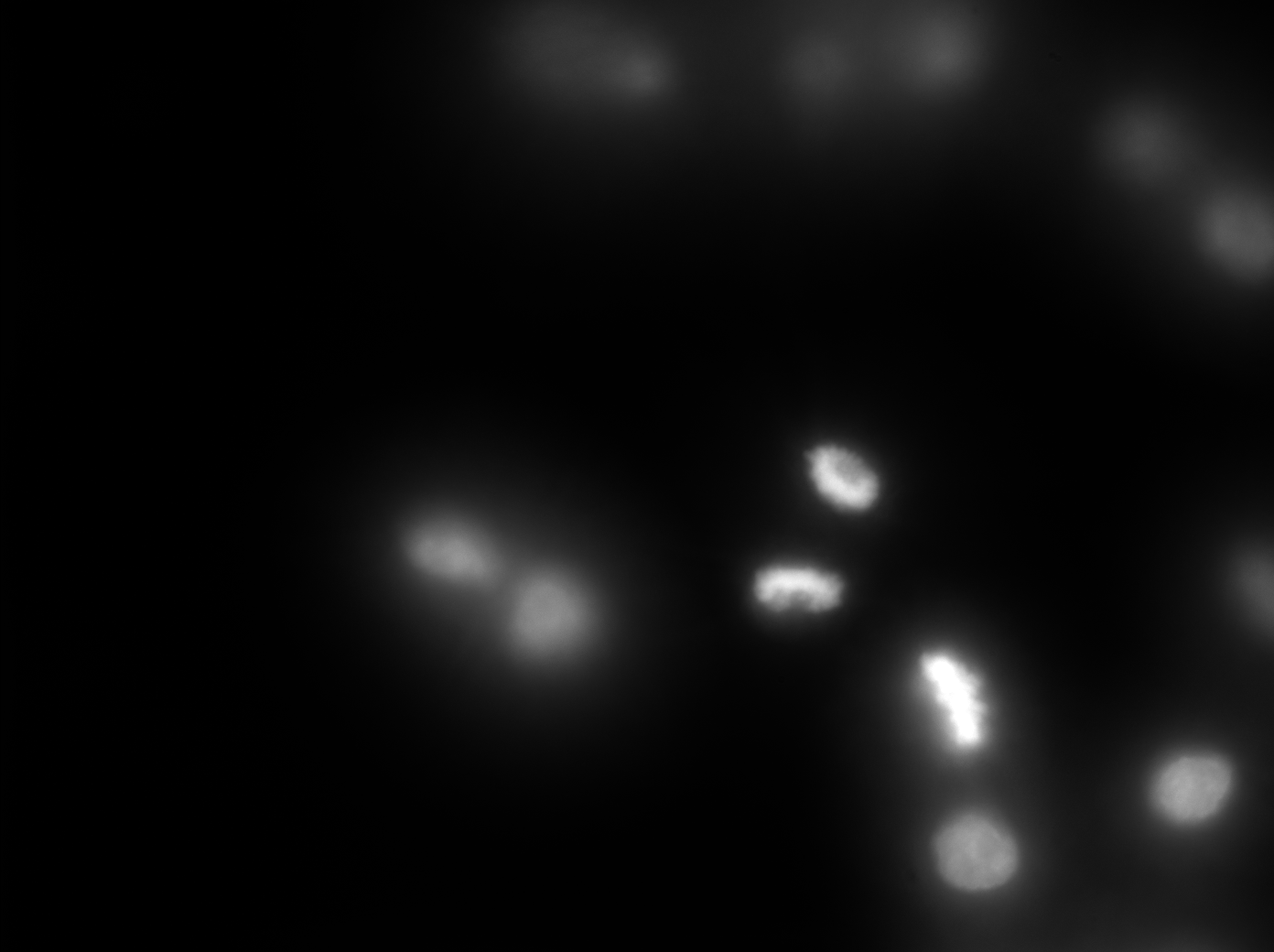

Supplement: Supplementary file 10 — Source data Fig. 2 part 7 [file 44319_2026_742_MOESM10_ESM.zip › Figure 2 Part 7/Fig 2fg Control Hela rGT335 acetylated tubulin part 2/Furrow Ingression/Cas9 actub rGT335 9-8-25 R1 FI7.Project Maximum Z_XY1757353457_Z0_T0_C0.tif]

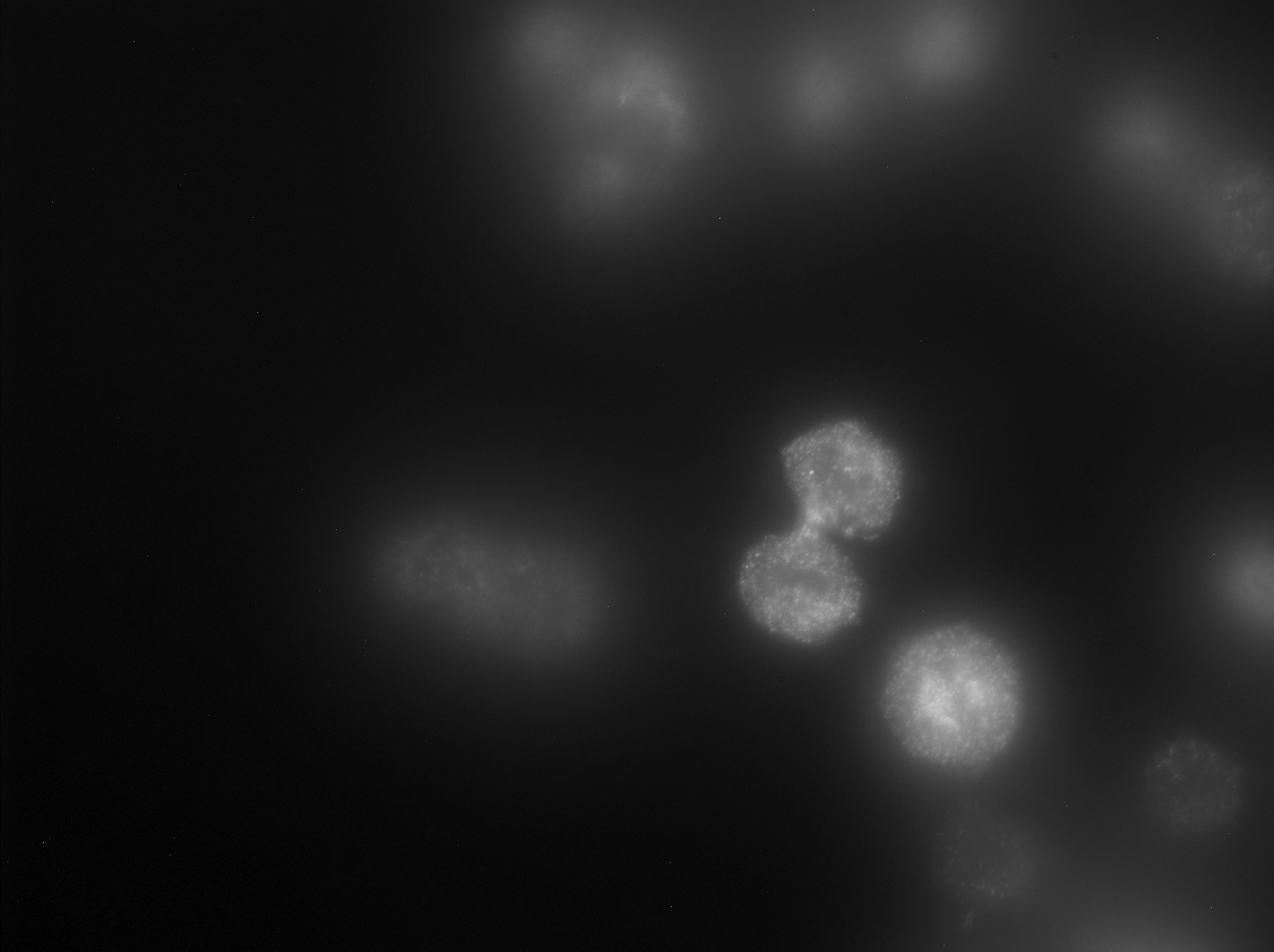

Supplement: Supplementary file 10 — Source data Fig. 2 part 7 [file 44319_2026_742_MOESM10_ESM.zip › Figure 2 Part 7/Fig 2fg Control Hela rGT335 acetylated tubulin part 2/Furrow Ingression/Cas9 actub rGT335 9-8-25 R1 FI7.Project Maximum Z_XY1757353457_Z0_T0_C1.tif]

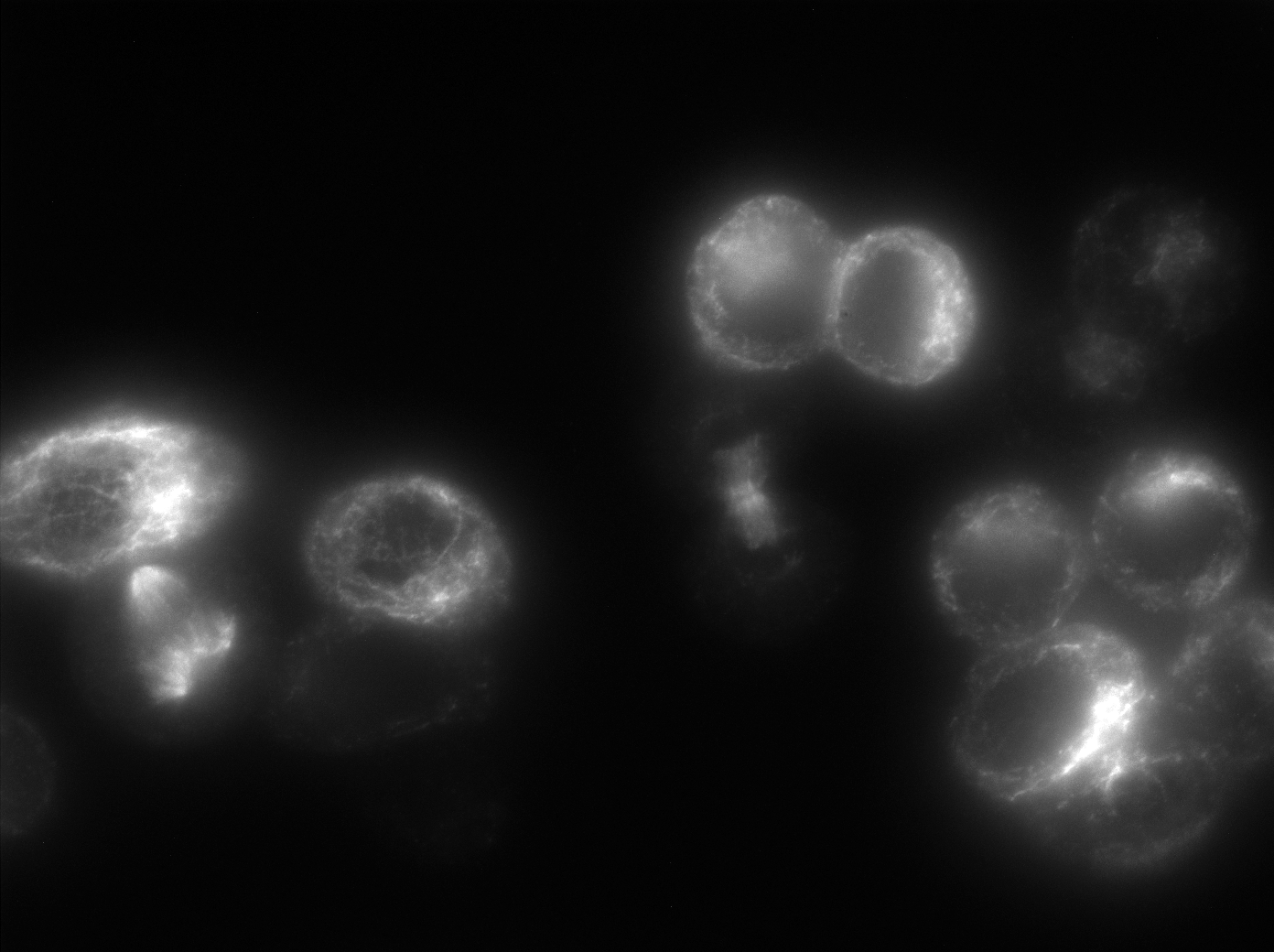

Supplement: Supplementary file 10 — Source data Fig. 2 part 7 [file 44319_2026_742_MOESM10_ESM.zip › Figure 2 Part 7/Fig 2fg Control Hela rGT335 acetylated tubulin part 2/Furrow Ingression/Cas9 actub rGT335 9-8-25 R1 FI9.Project Maximum Z_XY1757357227_Z0_T0_C2.tif]

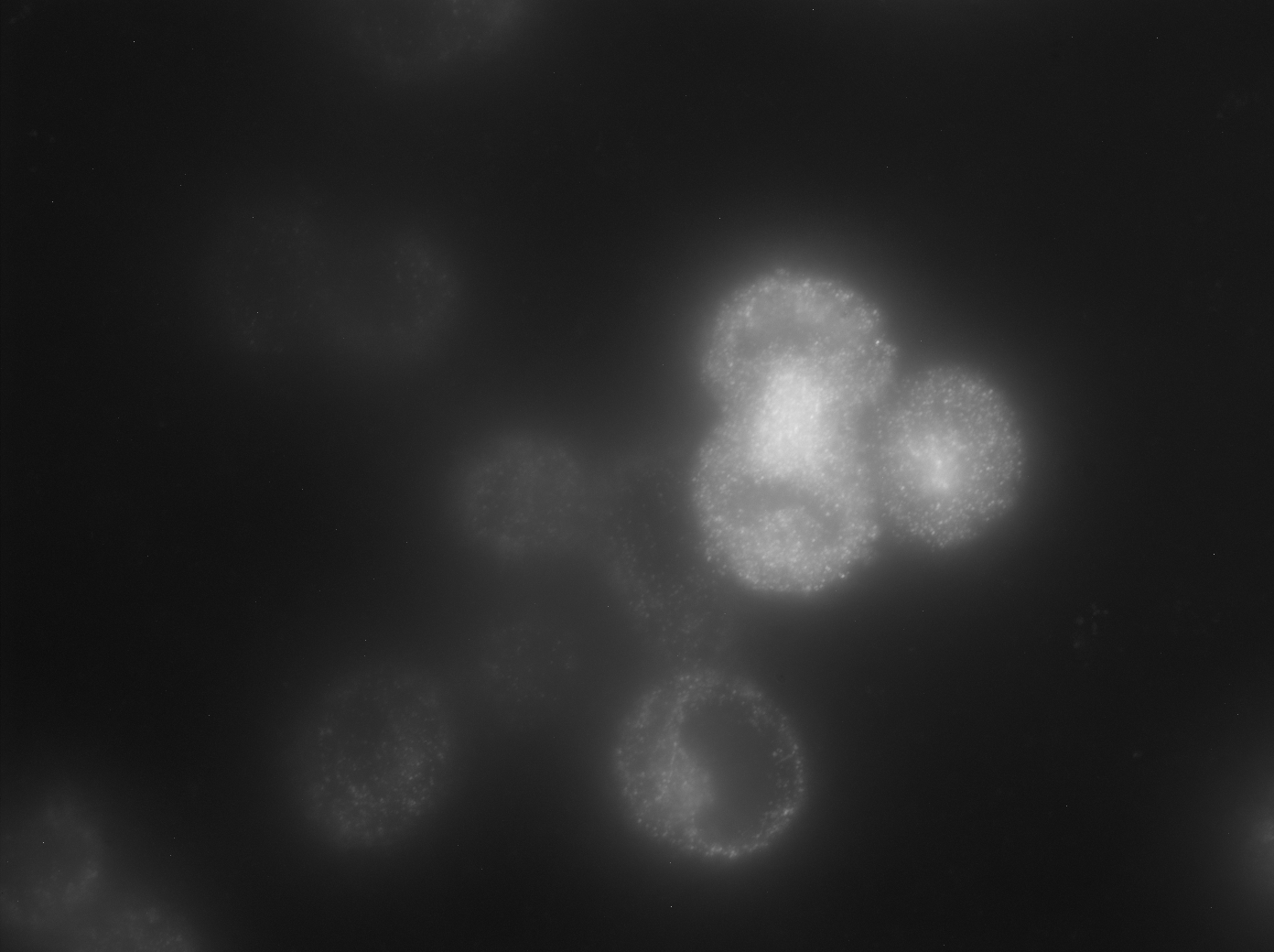

Supplement: Supplementary file 10 — Source data Fig. 2 part 7 [file 44319_2026_742_MOESM10_ESM.zip › Figure 2 Part 7/Fig 2fg Control Hela rGT335 acetylated tubulin part 2/Furrow Ingression/Cas9 actub rGT335 9-8-25 R1 FI10 EX.Project Maximum Z_XY1757357762_Z0_T0_C1.tif]

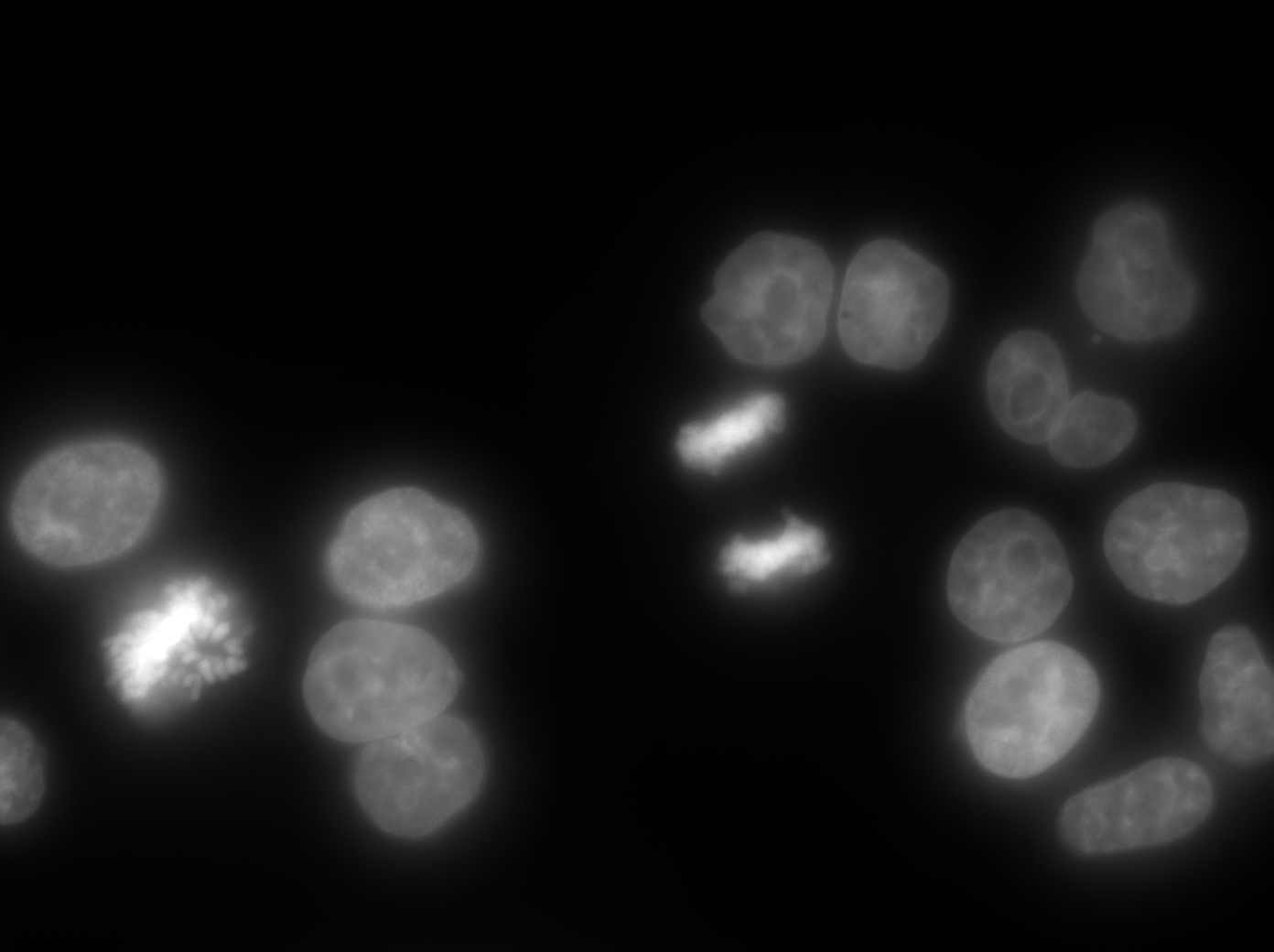

Supplement: Supplementary file 10 — Source data Fig. 2 part 7 [file 44319_2026_742_MOESM10_ESM.zip › Figure 2 Part 7/Fig 2fg Control Hela rGT335 acetylated tubulin part 2/Furrow Ingression/Cas9 actub rGT335 9-8-25 R1 FI9.Project Maximum Z_XY1757357227_Z0_T0_C0.tif]

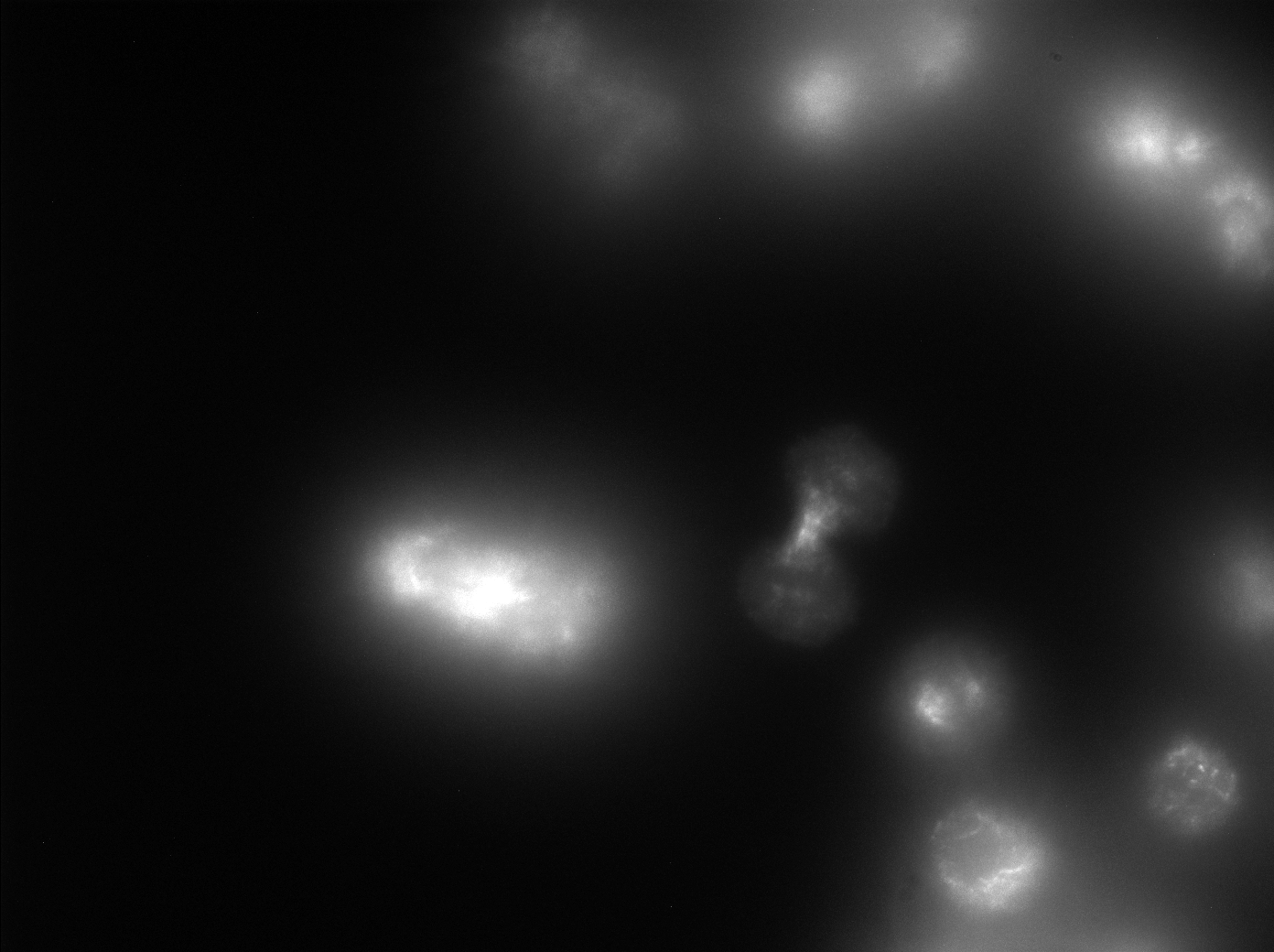

Supplement: Supplementary file 10 — Source data Fig. 2 part 7 [file 44319_2026_742_MOESM10_ESM.zip › Figure 2 Part 7/Fig 2fg Control Hela rGT335 acetylated tubulin part 2/Furrow Ingression/Cas9 actub rGT335 9-8-25 R1 FI7.Project Maximum Z_XY1757353457_Z0_T0_C2.tif]

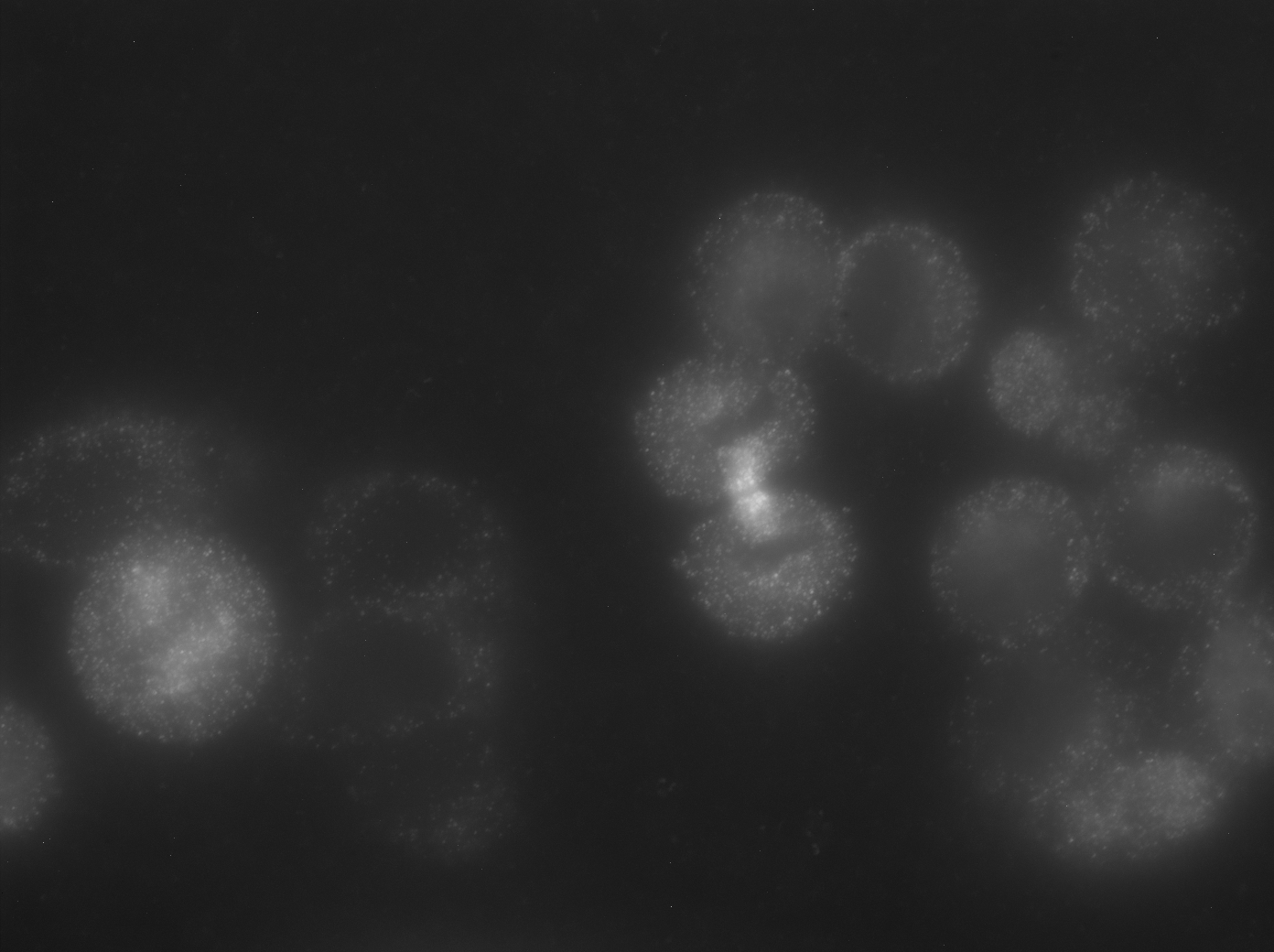

Supplement: Supplementary file 10 — Source data Fig. 2 part 7 [file 44319_2026_742_MOESM10_ESM.zip › Figure 2 Part 7/Fig 2fg Control Hela rGT335 acetylated tubulin part 2/Furrow Ingression/Cas9 actub rGT335 9-8-25 R1 FI9.Project Maximum Z_XY1757357227_Z0_T0_C1.tif]

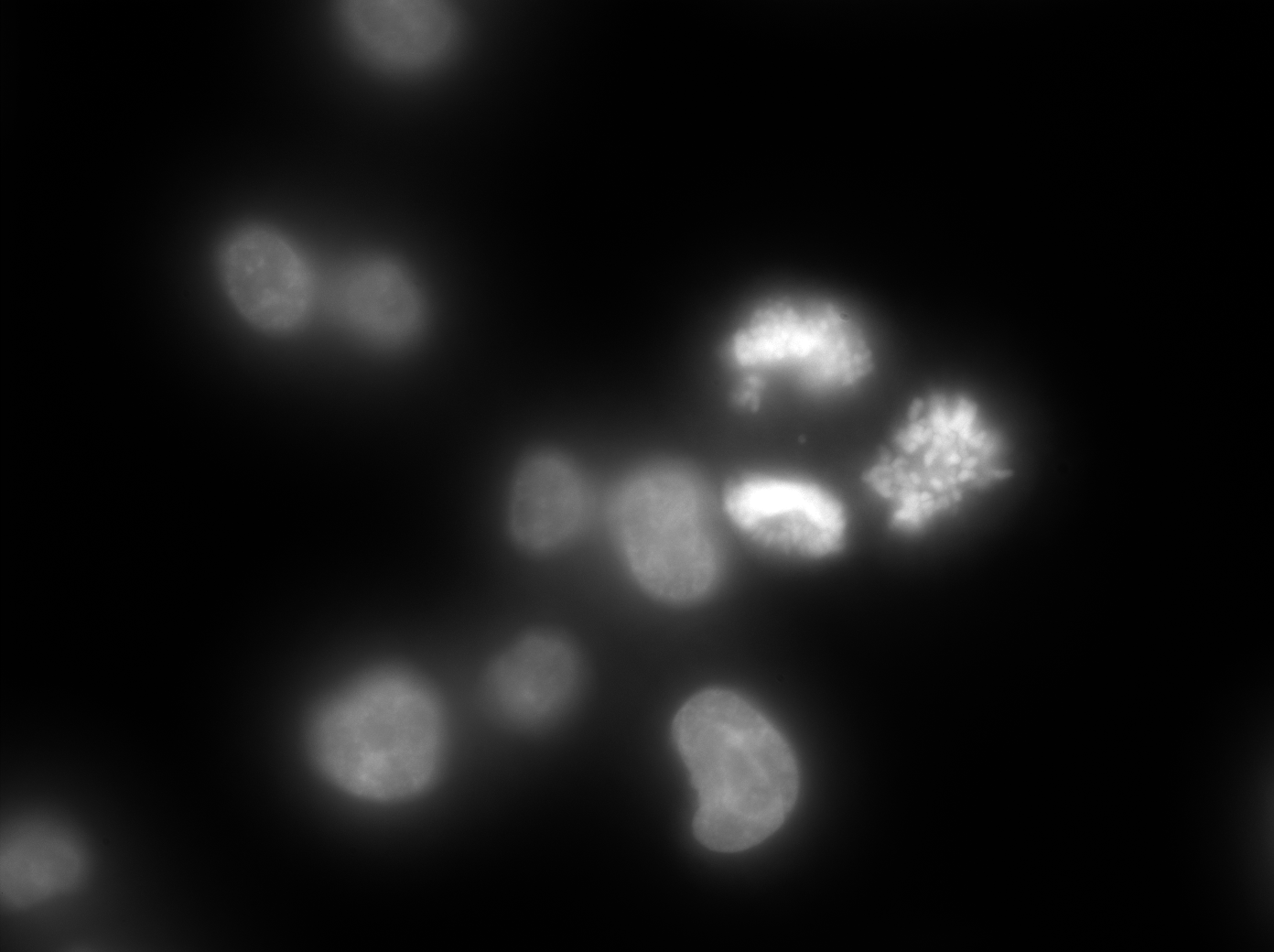

Supplement: Supplementary file 10 — Source data Fig. 2 part 7 [file 44319_2026_742_MOESM10_ESM.zip › Figure 2 Part 7/Fig 2fg Control Hela rGT335 acetylated tubulin part 2/Furrow Ingression/Cas9 actub rGT335 9-8-25 R1 FI10 EX.Project Maximum Z_XY1757357762_Z0_T0_C0.tif]

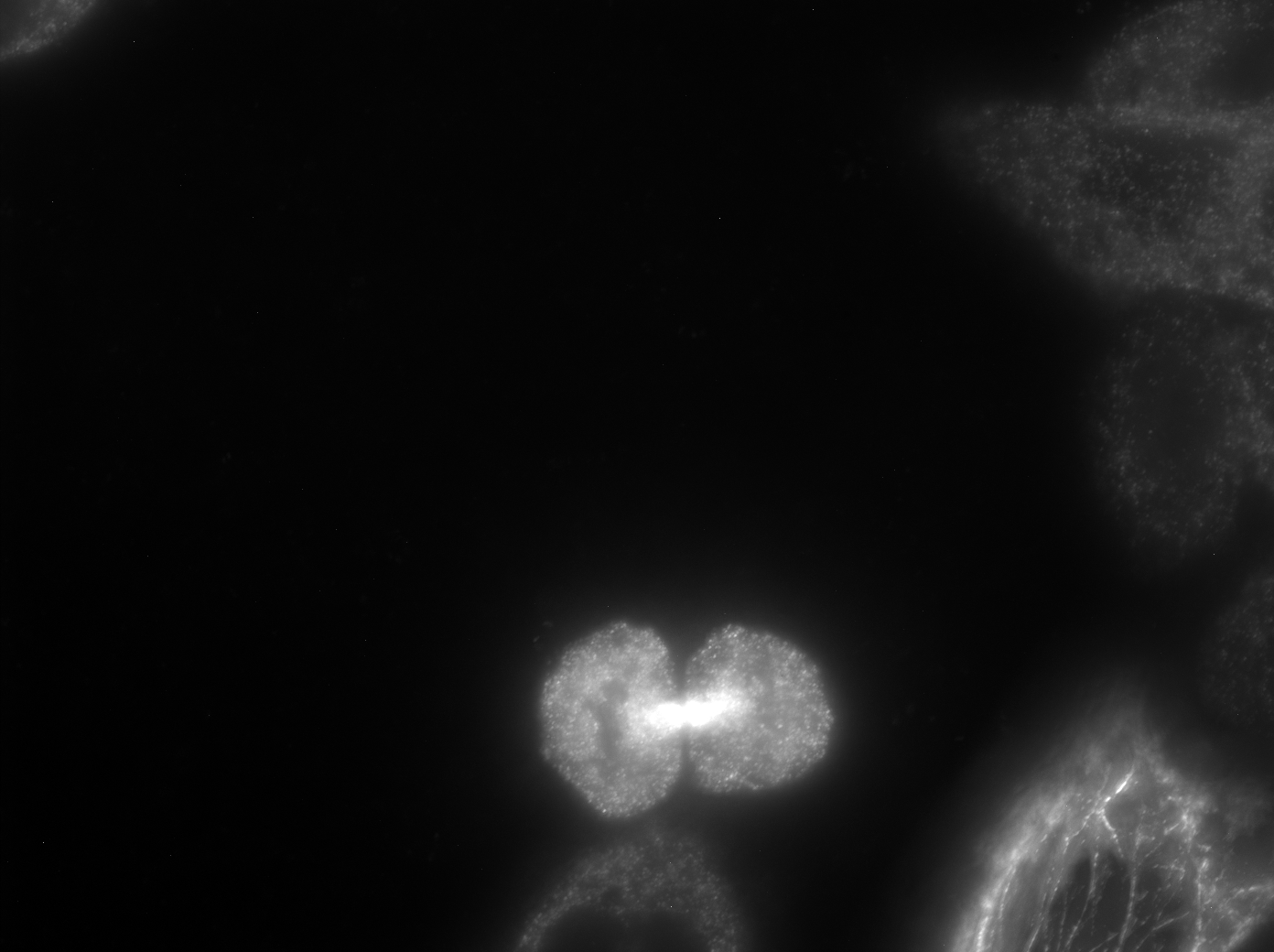

Supplement: Supplementary file 10 — Source data Fig. 2 part 7 [file 44319_2026_742_MOESM10_ESM.zip › Figure 2 Part 7/Fig 2fg Control Hela rGT335 acetylated tubulin part 2/Furrow Ingression/Cas9 actub rGT335 9-8-25 R2 FI10.Project Maximum Z_XY1757363561_Z0_T0_C1.tif]

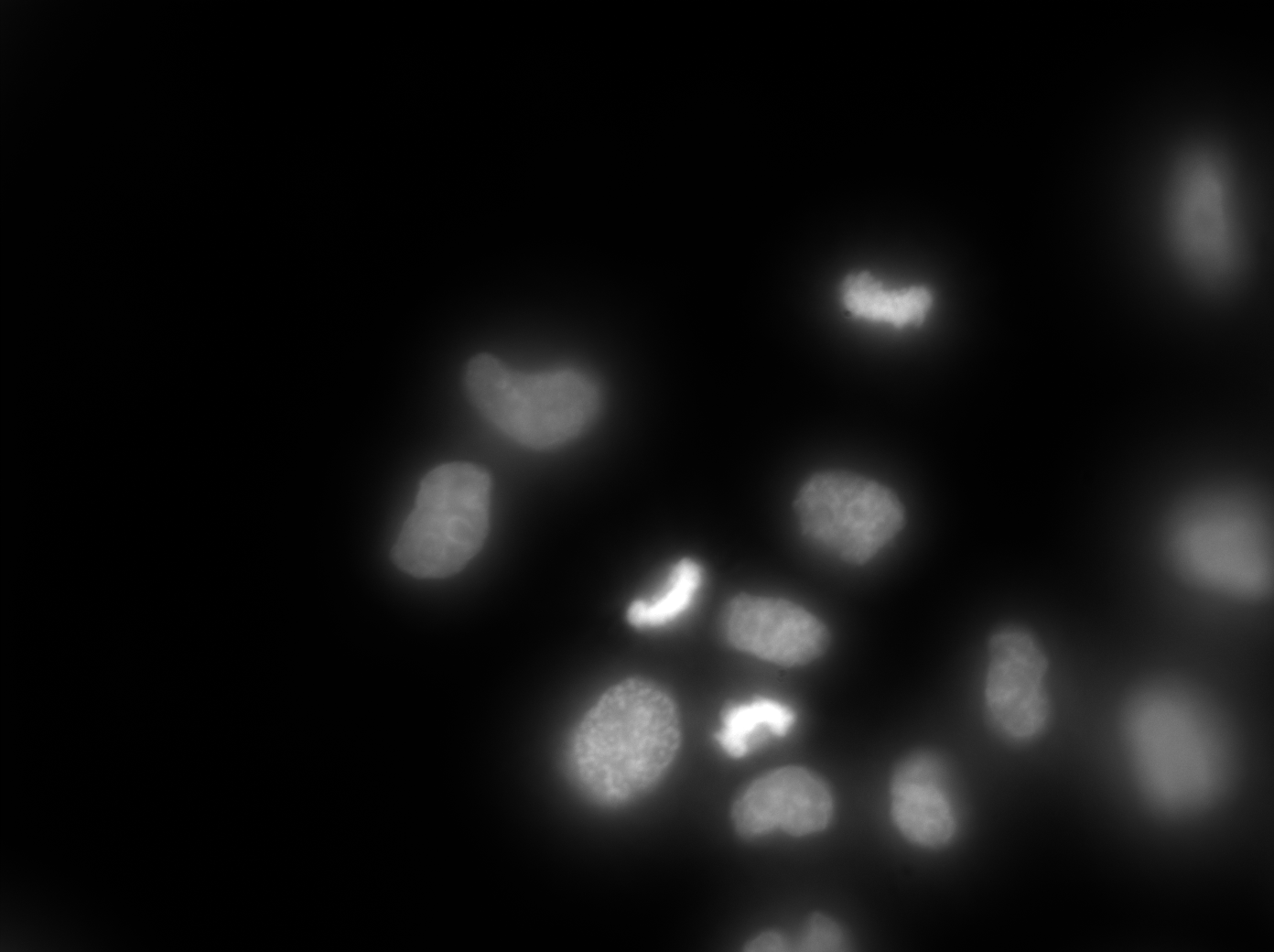

Supplement: Supplementary file 10 — Source data Fig. 2 part 7 [file 44319_2026_742_MOESM10_ESM.zip › Figure 2 Part 7/Fig 2fg Control Hela rGT335 acetylated tubulin part 2/Furrow Ingression/Cas9 actub rGT335 9-8-25 R2 FI9.Project Maximum Z_XY1757363314_Z0_T0_C0.tif]

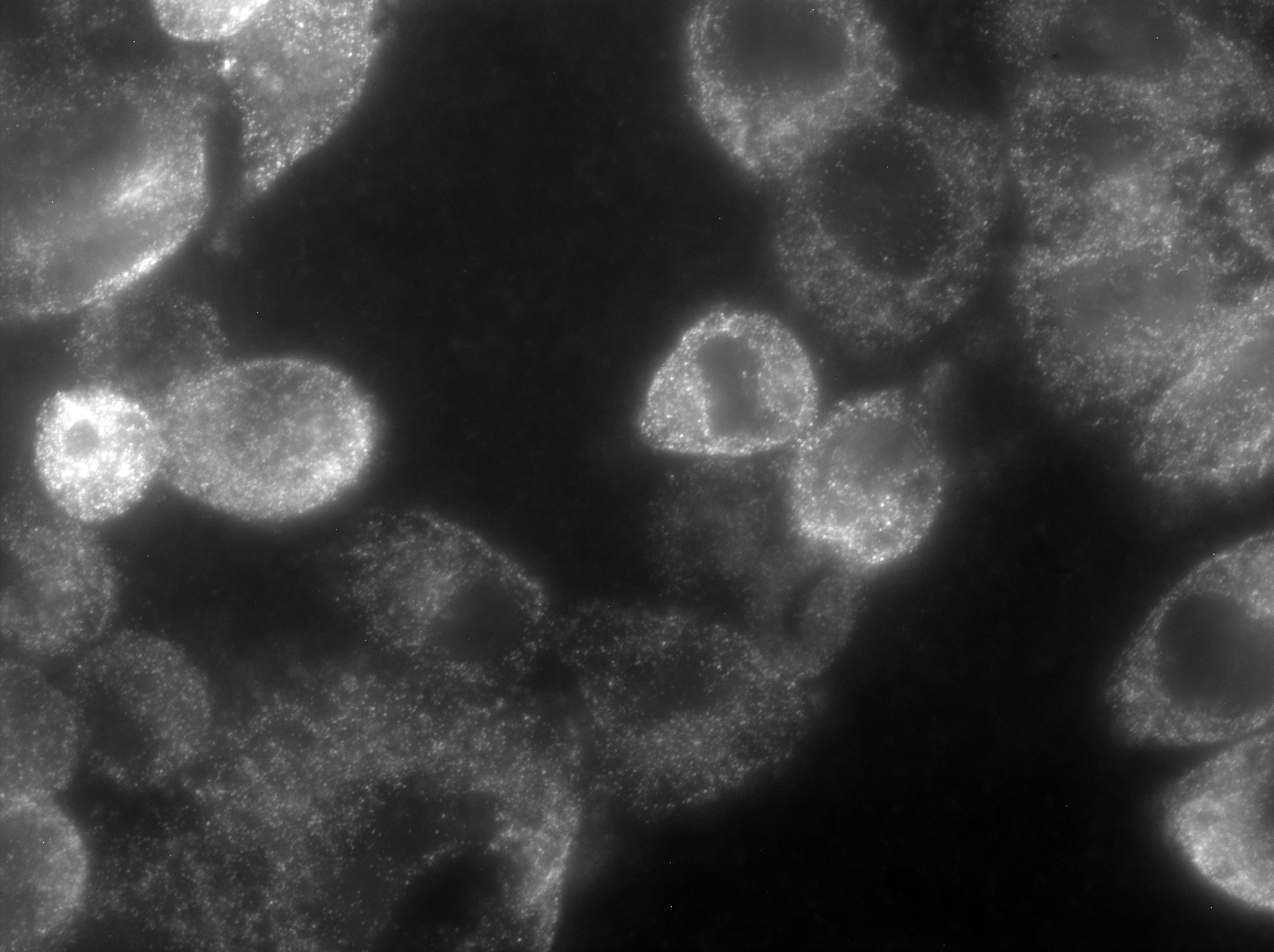

Supplement: Supplementary file 10 — Source data Fig. 2 part 7 [file 44319_2026_742_MOESM10_ESM.zip › Figure 2 Part 7/Fig 2fg Control Hela rGT335 acetylated tubulin part 2/Furrow Ingression/Cas9 actub rGT335 9-8-25 R2 FI3.Project Maximum Z_XY1757360294_Z0_T0_C1.tif]

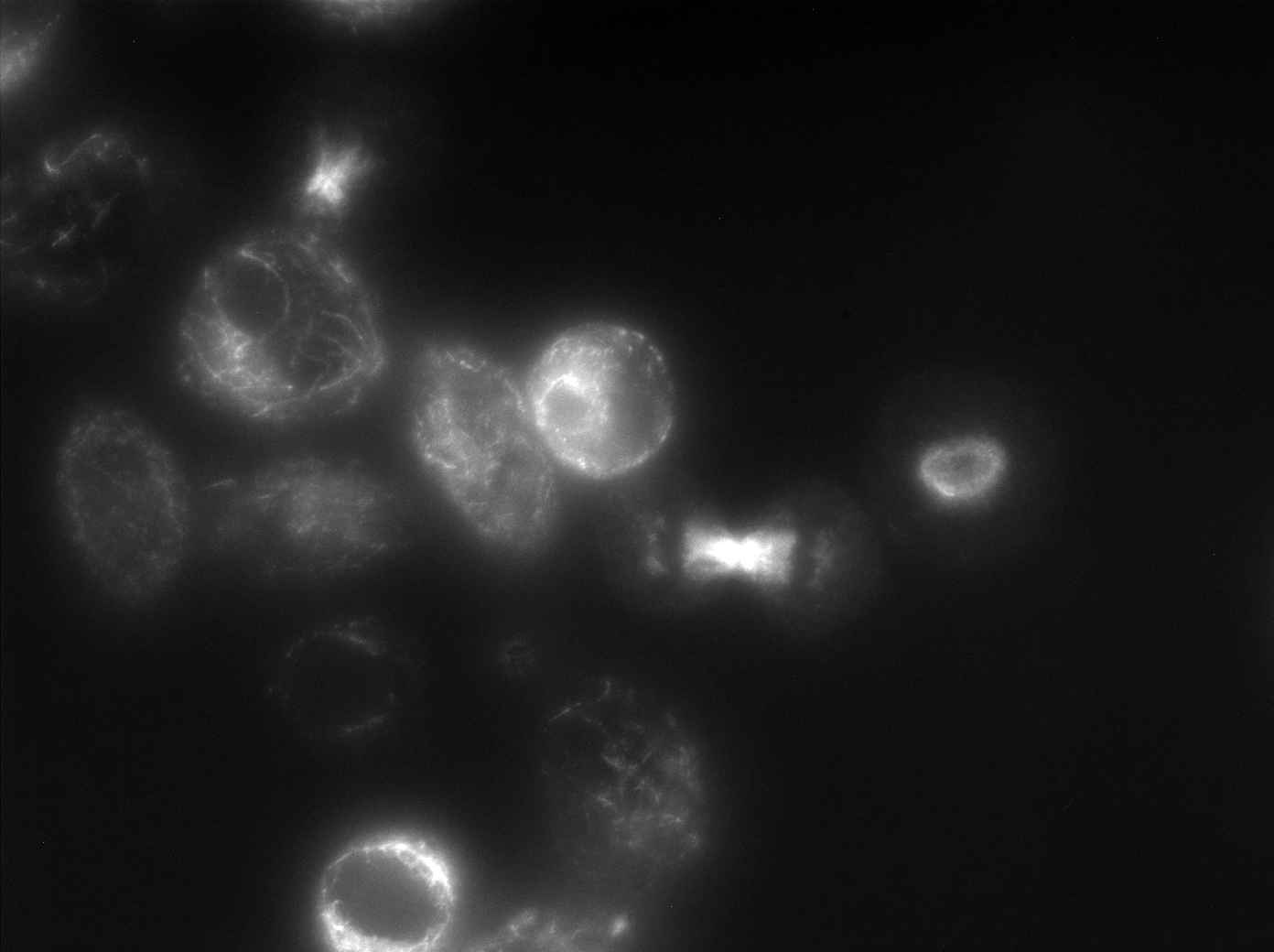

Supplement: Supplementary file 10 — Source data Fig. 2 part 7 [file 44319_2026_742_MOESM10_ESM.zip › Figure 2 Part 7/Fig 2fg Control Hela rGT335 acetylated tubulin part 2/Furrow Ingression/Cas9 actub rGT335 9-8-25 R3 FI10.Project Maximum Z_XY1757366916_Z0_T0_C2.tif]

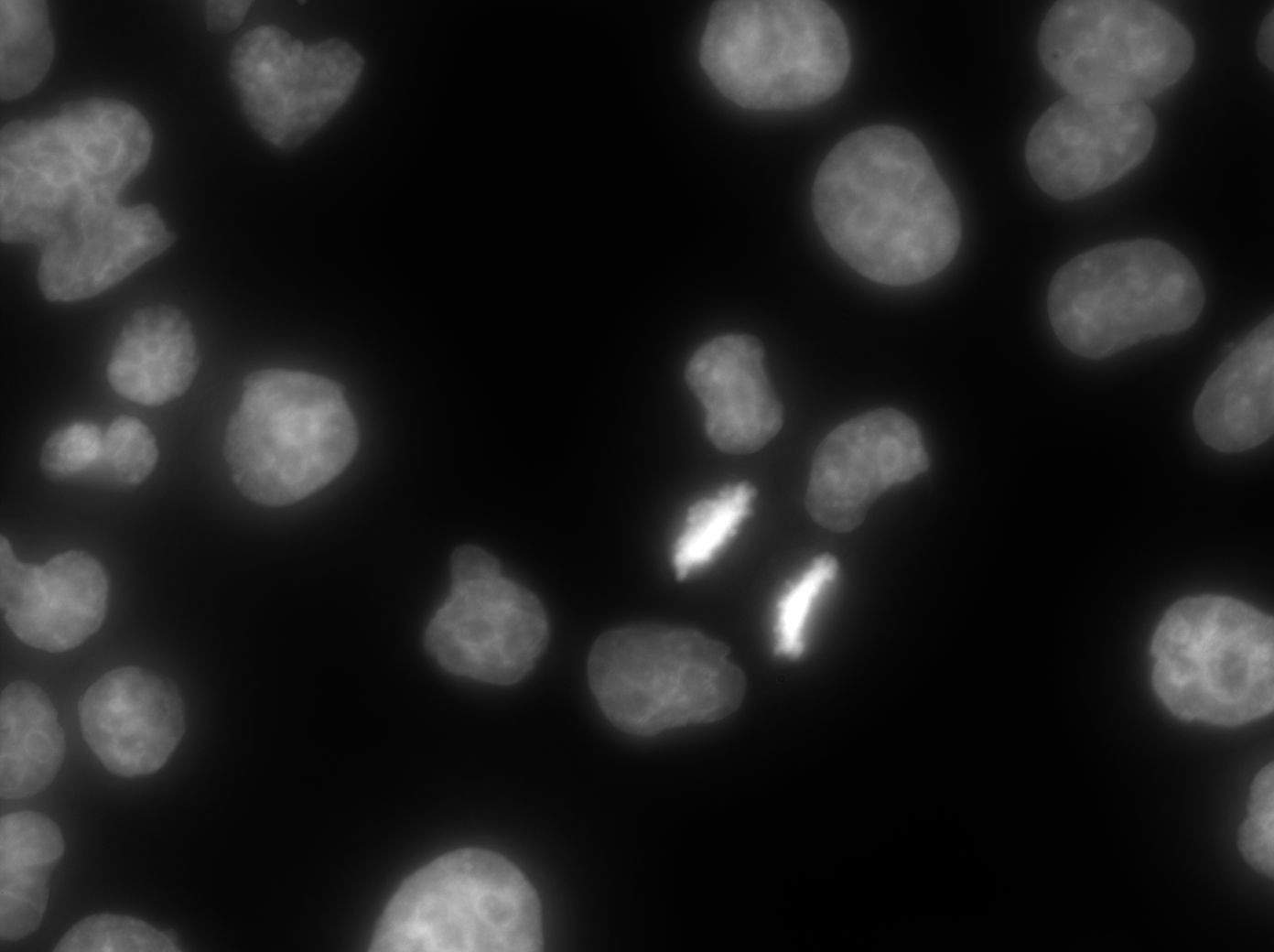

Supplement: Supplementary file 10 — Source data Fig. 2 part 7 [file 44319_2026_742_MOESM10_ESM.zip › Figure 2 Part 7/Fig 2fg Control Hela rGT335 acetylated tubulin part 2/Furrow Ingression/Cas9 actub rGT335 9-8-25 R2 FI3.Project Maximum Z_XY1757360294_Z0_T0_C0.tif]

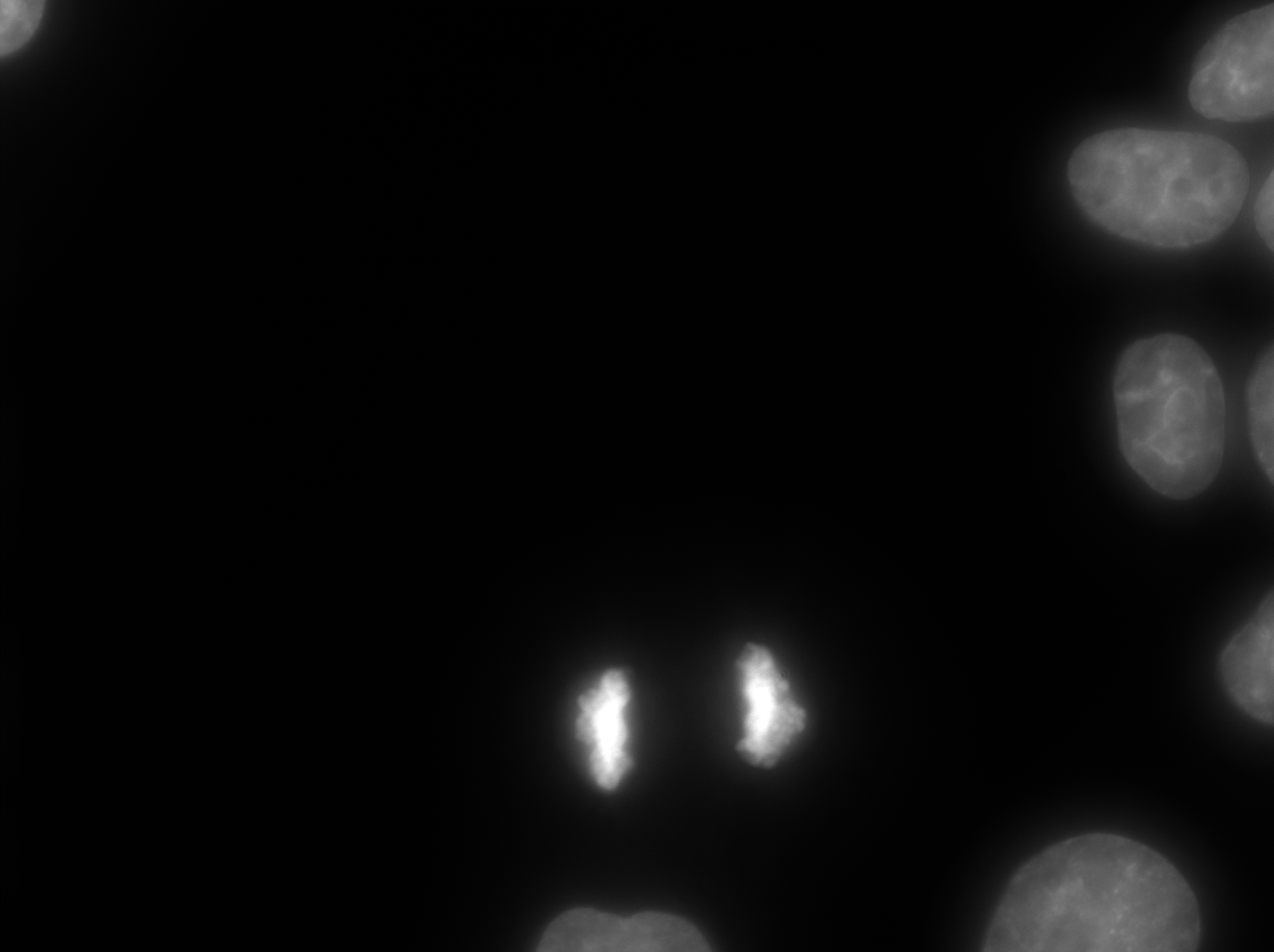

Supplement: Supplementary file 10 — Source data Fig. 2 part 7 [file 44319_2026_742_MOESM10_ESM.zip › Figure 2 Part 7/Fig 2fg Control Hela rGT335 acetylated tubulin part 2/Furrow Ingression/Cas9 actub rGT335 9-8-25 R2 FI10.Project Maximum Z_XY1757363561_Z0_T0_C0.tif]

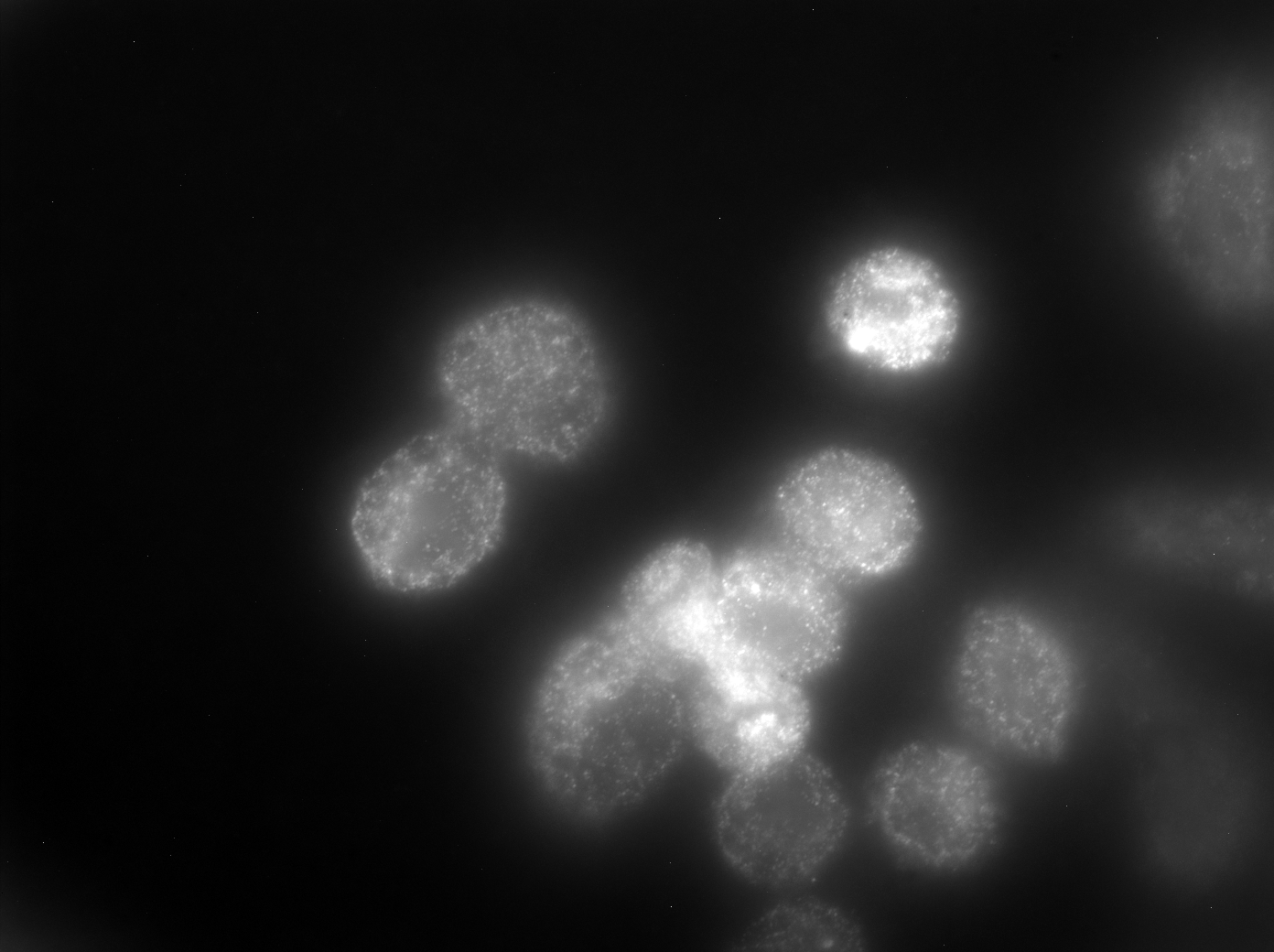

Supplement: Supplementary file 10 — Source data Fig. 2 part 7 [file 44319_2026_742_MOESM10_ESM.zip › Figure 2 Part 7/Fig 2fg Control Hela rGT335 acetylated tubulin part 2/Furrow Ingression/Cas9 actub rGT335 9-8-25 R2 FI9.Project Maximum Z_XY1757363314_Z0_T0_C1.tif]

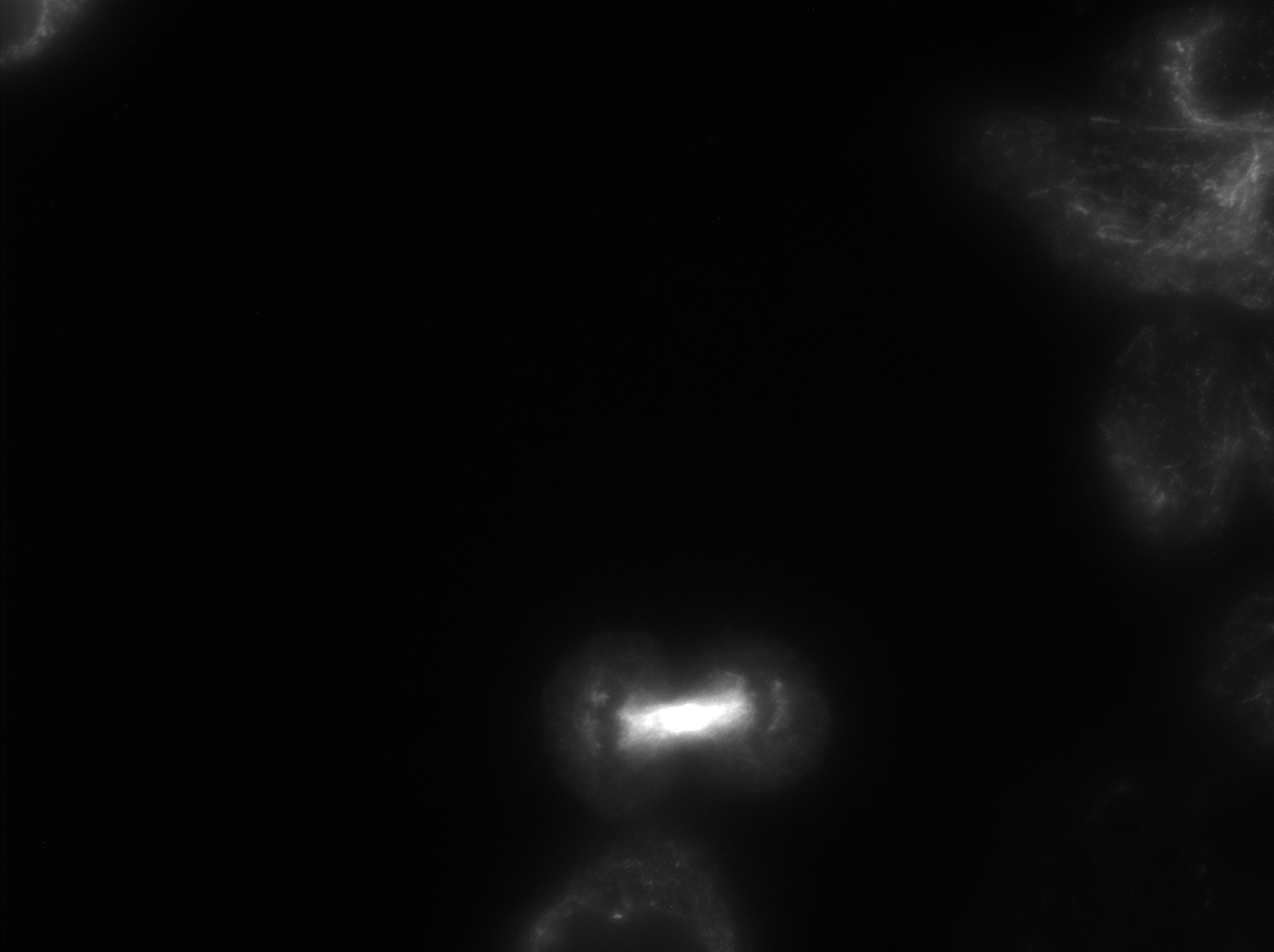

Supplement: Supplementary file 10 — Source data Fig. 2 part 7 [file 44319_2026_742_MOESM10_ESM.zip › Figure 2 Part 7/Fig 2fg Control Hela rGT335 acetylated tubulin part 2/Furrow Ingression/Cas9 actub rGT335 9-8-25 R2 FI10.Project Maximum Z_XY1757363561_Z0_T0_C2.tif]

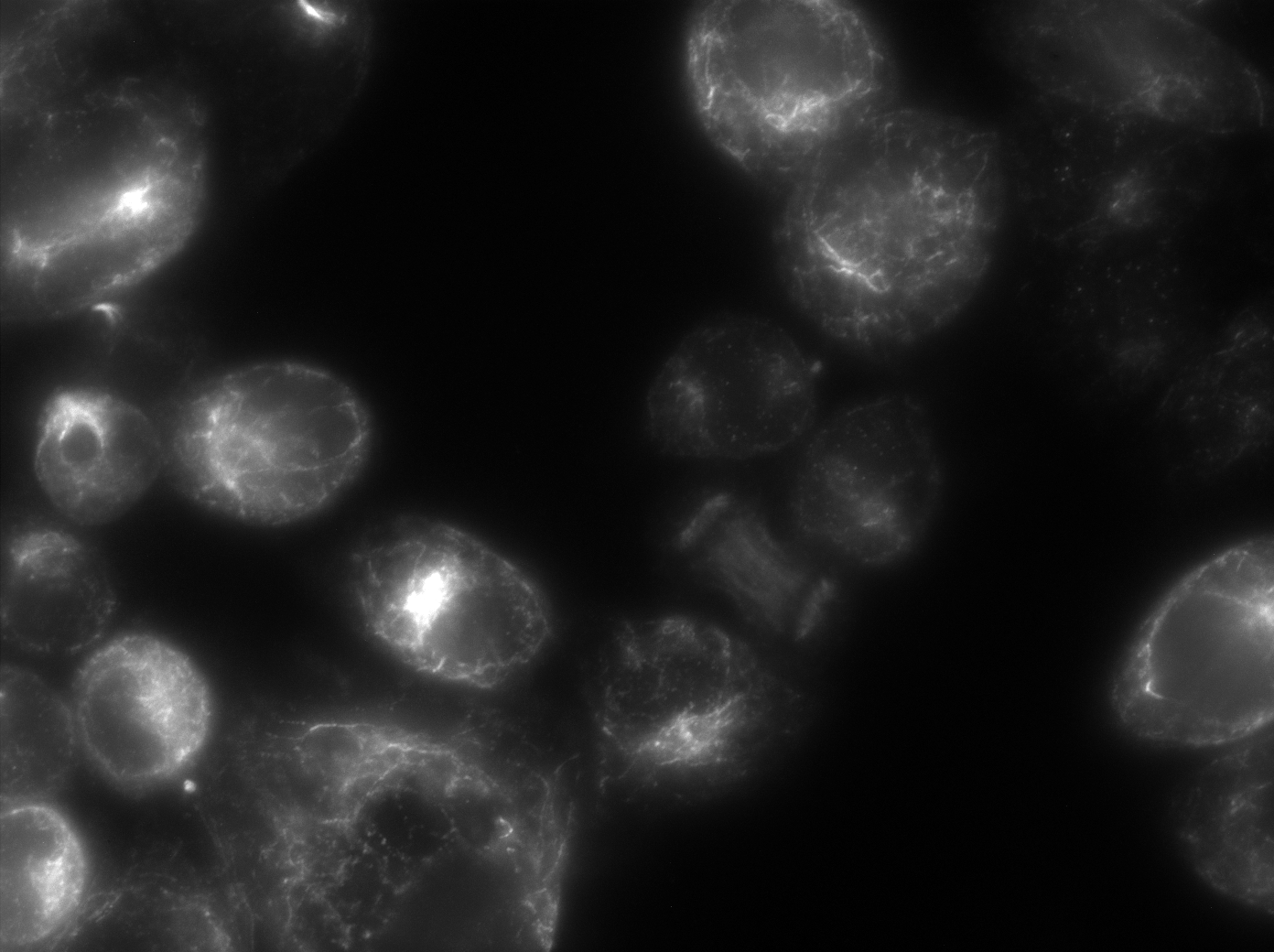

Supplement: Supplementary file 10 — Source data Fig. 2 part 7 [file 44319_2026_742_MOESM10_ESM.zip › Figure 2 Part 7/Fig 2fg Control Hela rGT335 acetylated tubulin part 2/Furrow Ingression/Cas9 actub rGT335 9-8-25 R2 FI3.Project Maximum Z_XY1757360294_Z0_T0_C2.tif]

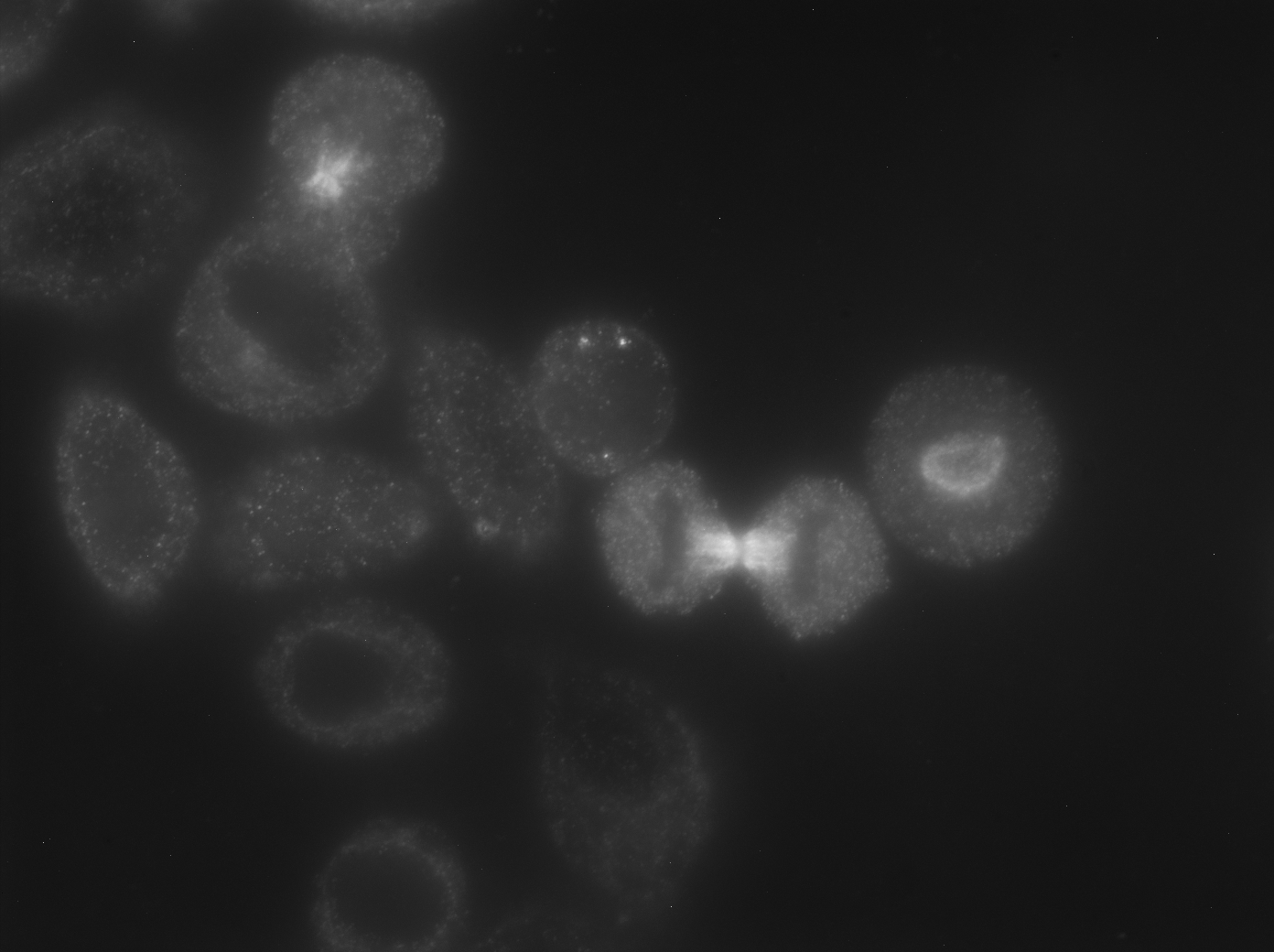

Supplement: Supplementary file 10 — Source data Fig. 2 part 7 [file 44319_2026_742_MOESM10_ESM.zip › Figure 2 Part 7/Fig 2fg Control Hela rGT335 acetylated tubulin part 2/Furrow Ingression/Cas9 actub rGT335 9-8-25 R3 FI10.Project Maximum Z_XY1757366916_Z0_T0_C1.tif]

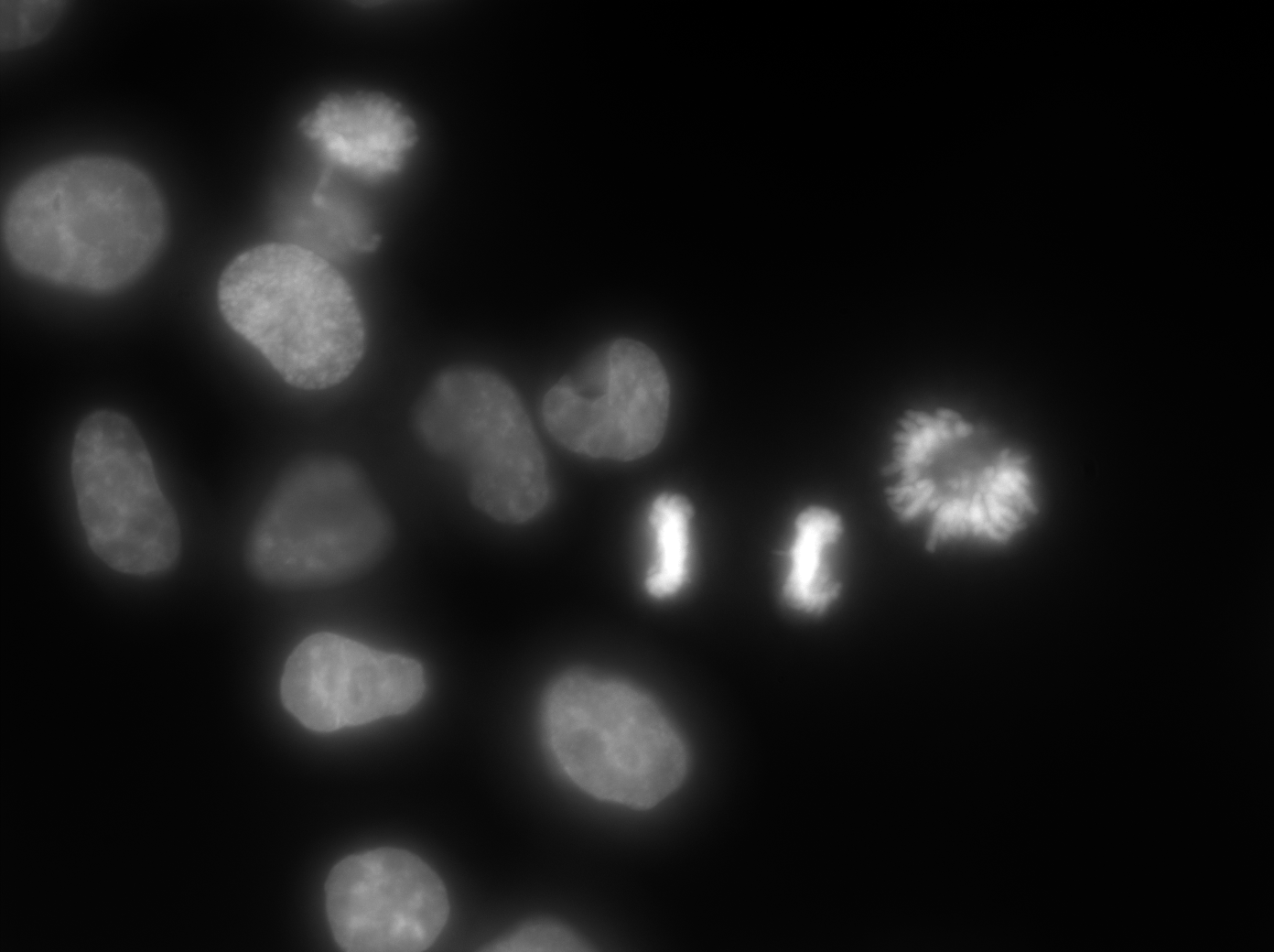

Supplement: Supplementary file 10 — Source data Fig. 2 part 7 [file 44319_2026_742_MOESM10_ESM.zip › Figure 2 Part 7/Fig 2fg Control Hela rGT335 acetylated tubulin part 2/Furrow Ingression/Cas9 actub rGT335 9-8-25 R3 FI10.Project Maximum Z_XY1757366916_Z0_T0_C0.tif]

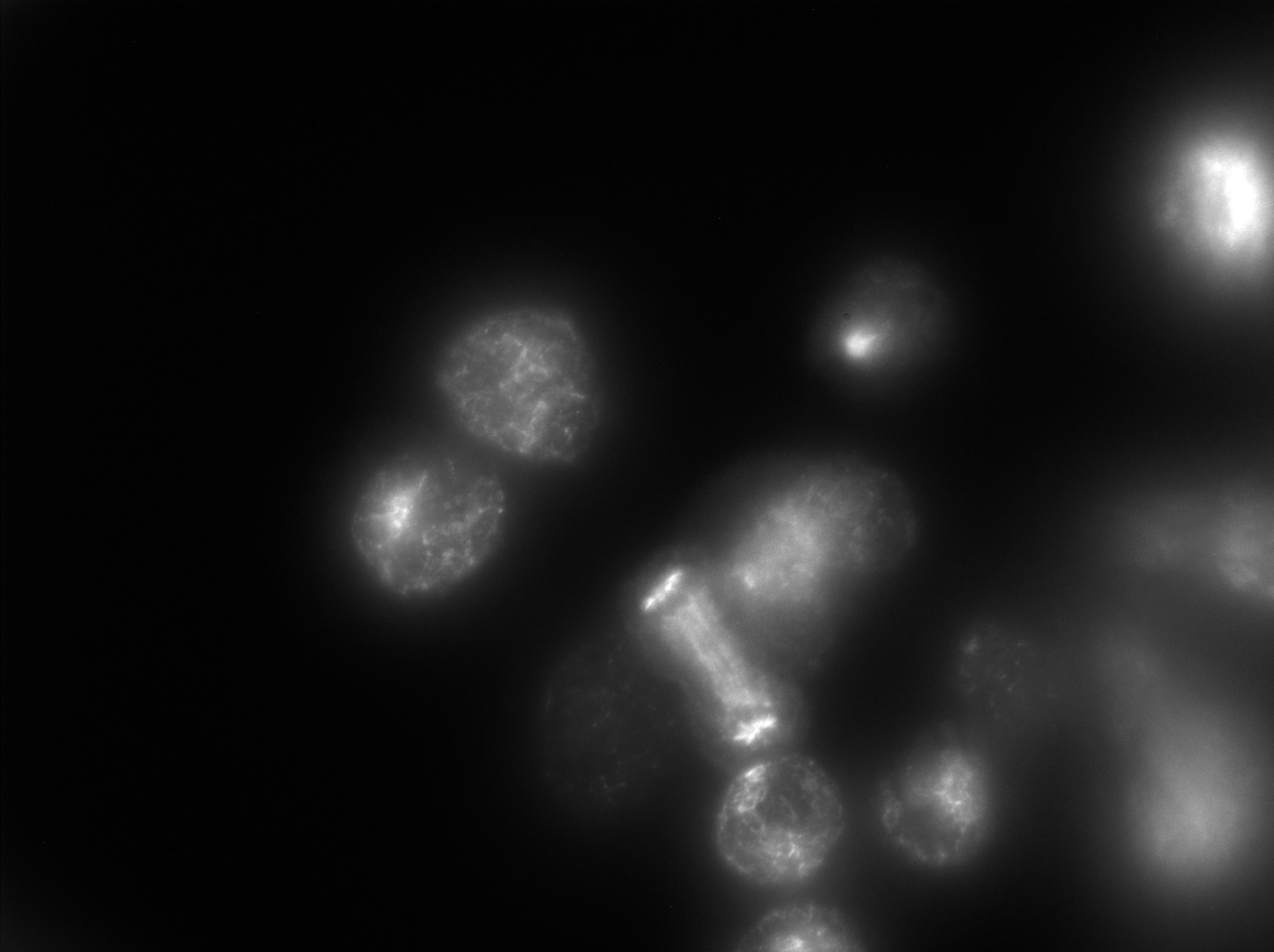

Supplement: Supplementary file 10 — Source data Fig. 2 part 7 [file 44319_2026_742_MOESM10_ESM.zip › Figure 2 Part 7/Fig 2fg Control Hela rGT335 acetylated tubulin part 2/Furrow Ingression/Cas9 actub rGT335 9-8-25 R2 FI9.Project Maximum Z_XY1757363314_Z0_T0_C2.tif]

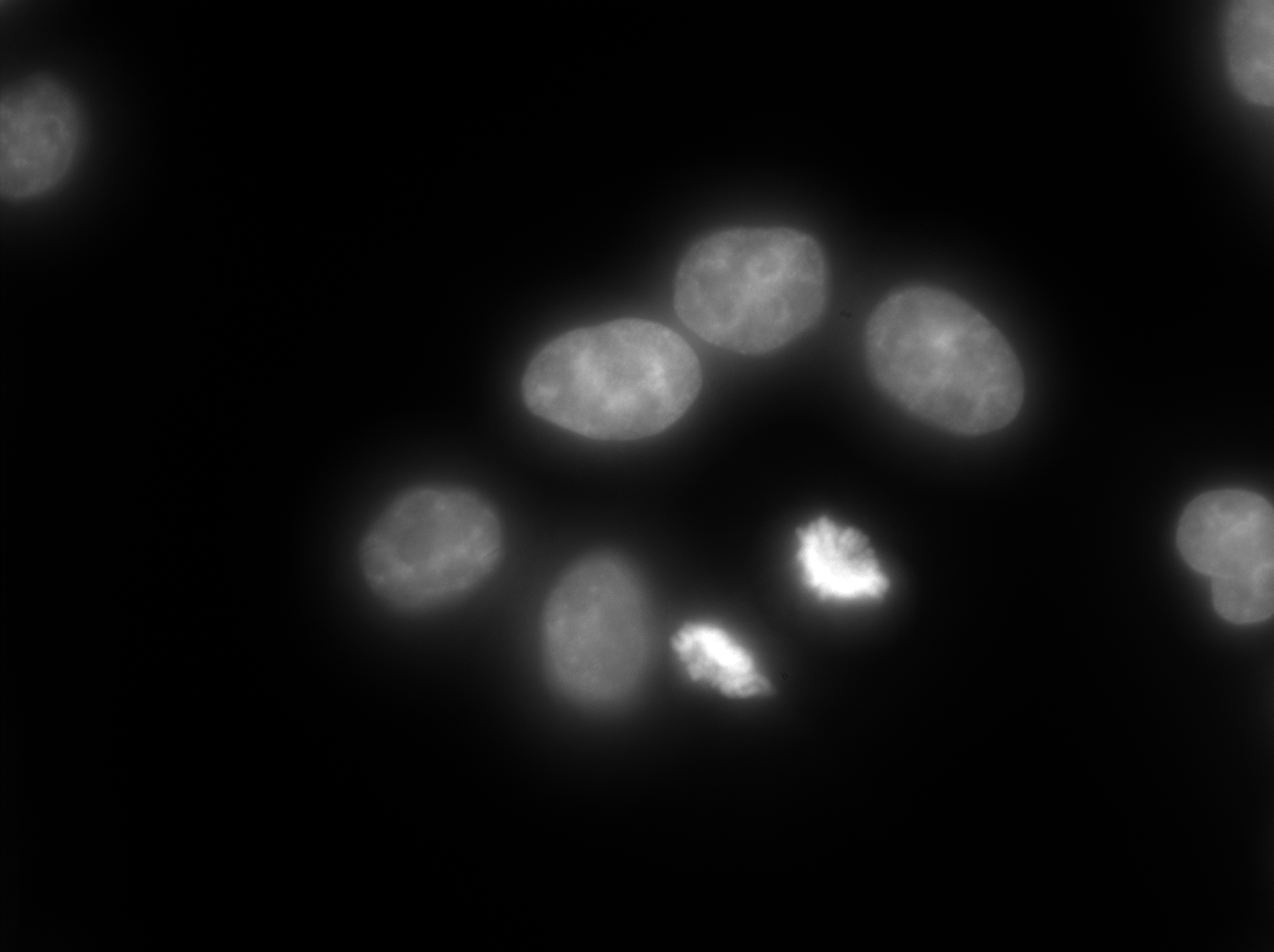

Supplement: Supplementary file 10 — Source data Fig. 2 part 7 [file 44319_2026_742_MOESM10_ESM.zip › Figure 2 Part 7/Fig 2fg Control Hela rGT335 acetylated tubulin part 2/Furrow Ingression/Cas9 actub rGT335 9-8-25 R3 FI11.Project Maximum Z_XY1757367064_Z0_T0_C0.tif]

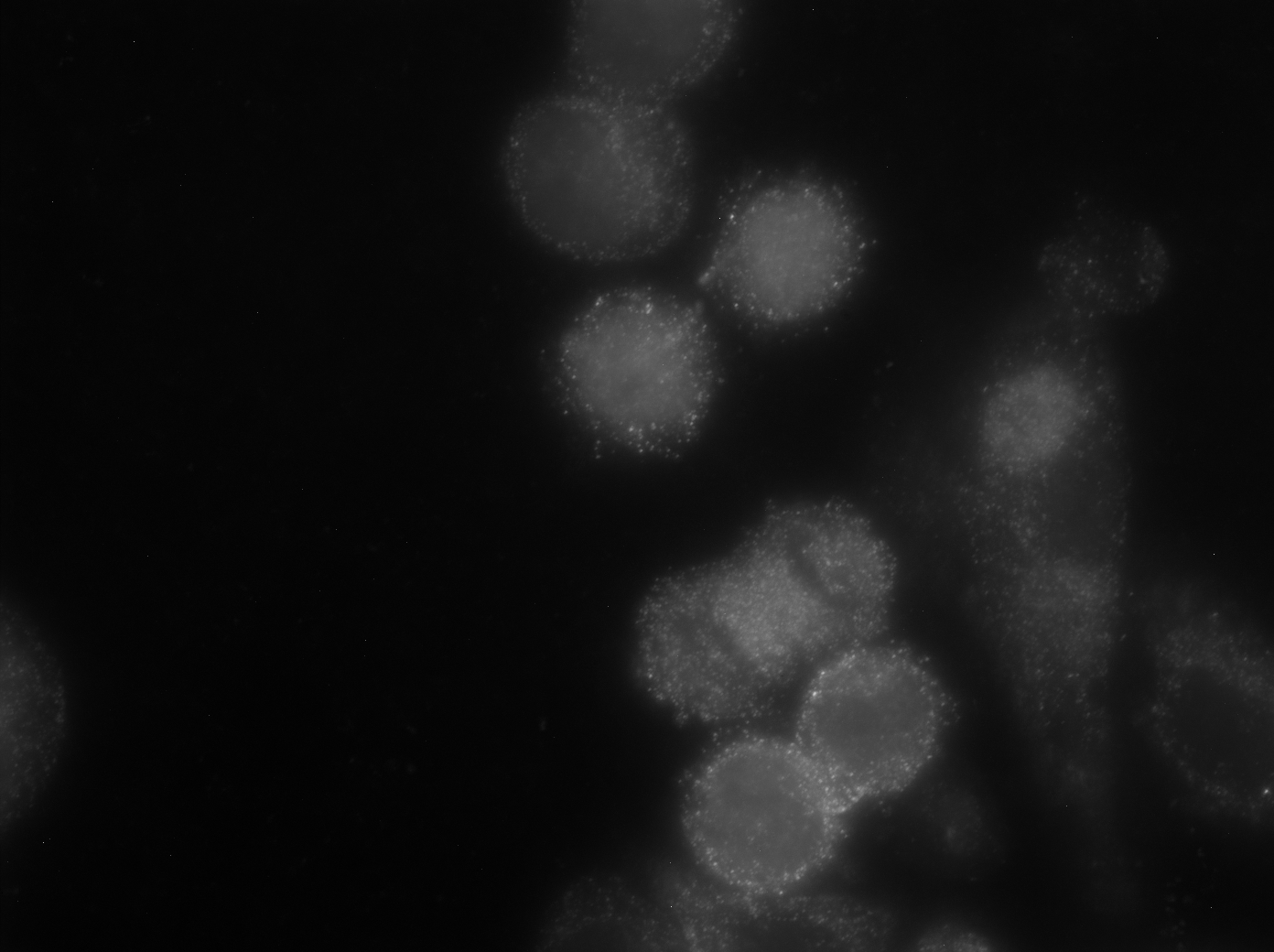

Supplement: Supplementary file 10 — Source data Fig. 2 part 7 [file 44319_2026_742_MOESM10_ESM.zip › Figure 2 Part 7/Fig 2fg Control Hela rGT335 acetylated tubulin part 2/Furrow Ingression/Cas9 actub rGT335 9-8-25 R3 FI4.Project Maximum Z_XY1757365645_Z0_T0_C1.tif]

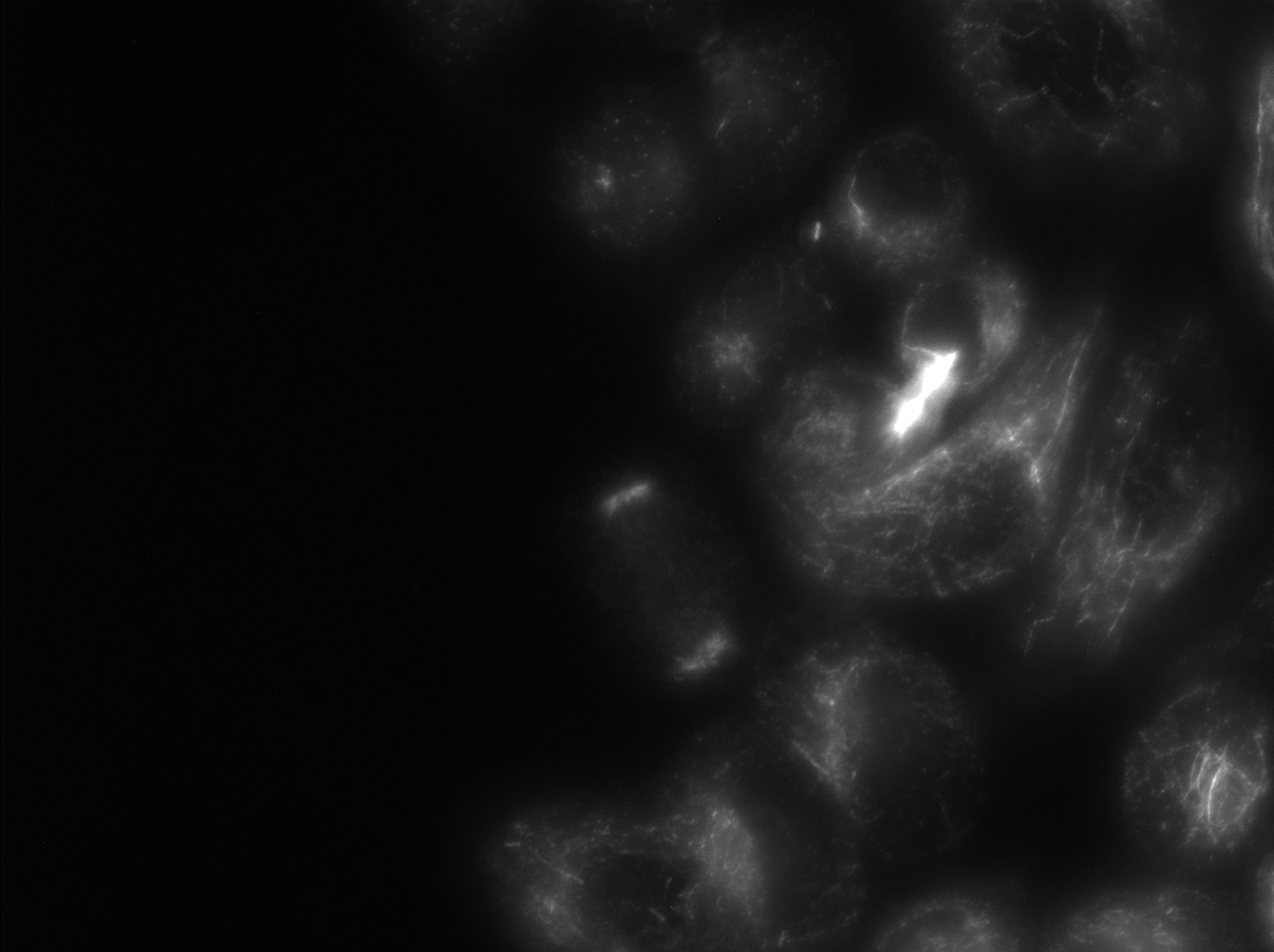

Supplement: Supplementary file 10 — Source data Fig. 2 part 7 [file 44319_2026_742_MOESM10_ESM.zip › Figure 2 Part 7/Fig 2fg Control Hela rGT335 acetylated tubulin part 2/Furrow Ingression/Cas9 actub rGT335 9-8-25 R3 FI1.Project Maximum Z_XY1757364948_Z0_T0_C2.tif]

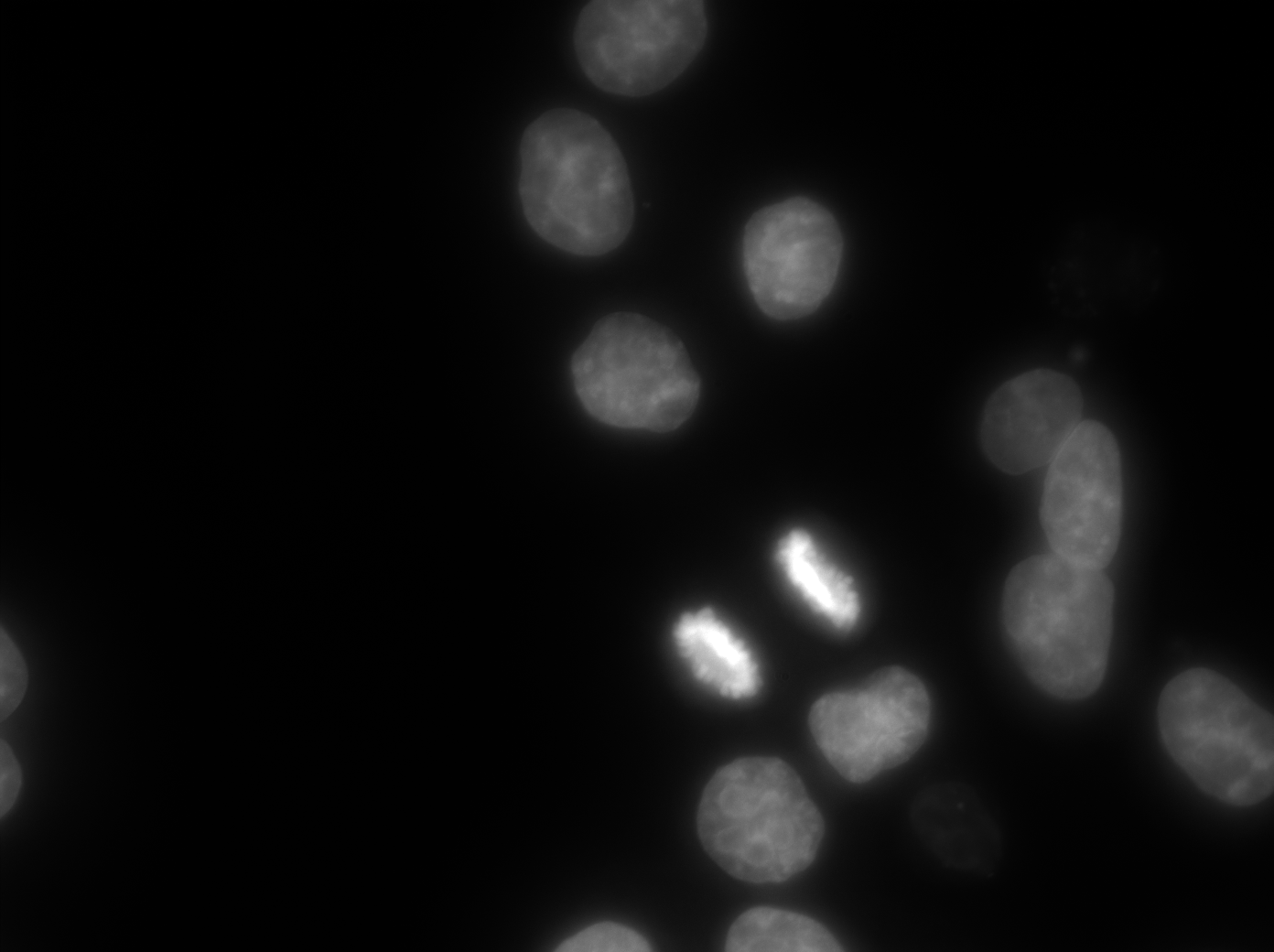

Supplement: Supplementary file 10 — Source data Fig. 2 part 7 [file 44319_2026_742_MOESM10_ESM.zip › Figure 2 Part 7/Fig 2fg Control Hela rGT335 acetylated tubulin part 2/Furrow Ingression/Cas9 actub rGT335 9-8-25 R3 FI4.Project Maximum Z_XY1757365645_Z0_T0_C0.tif]

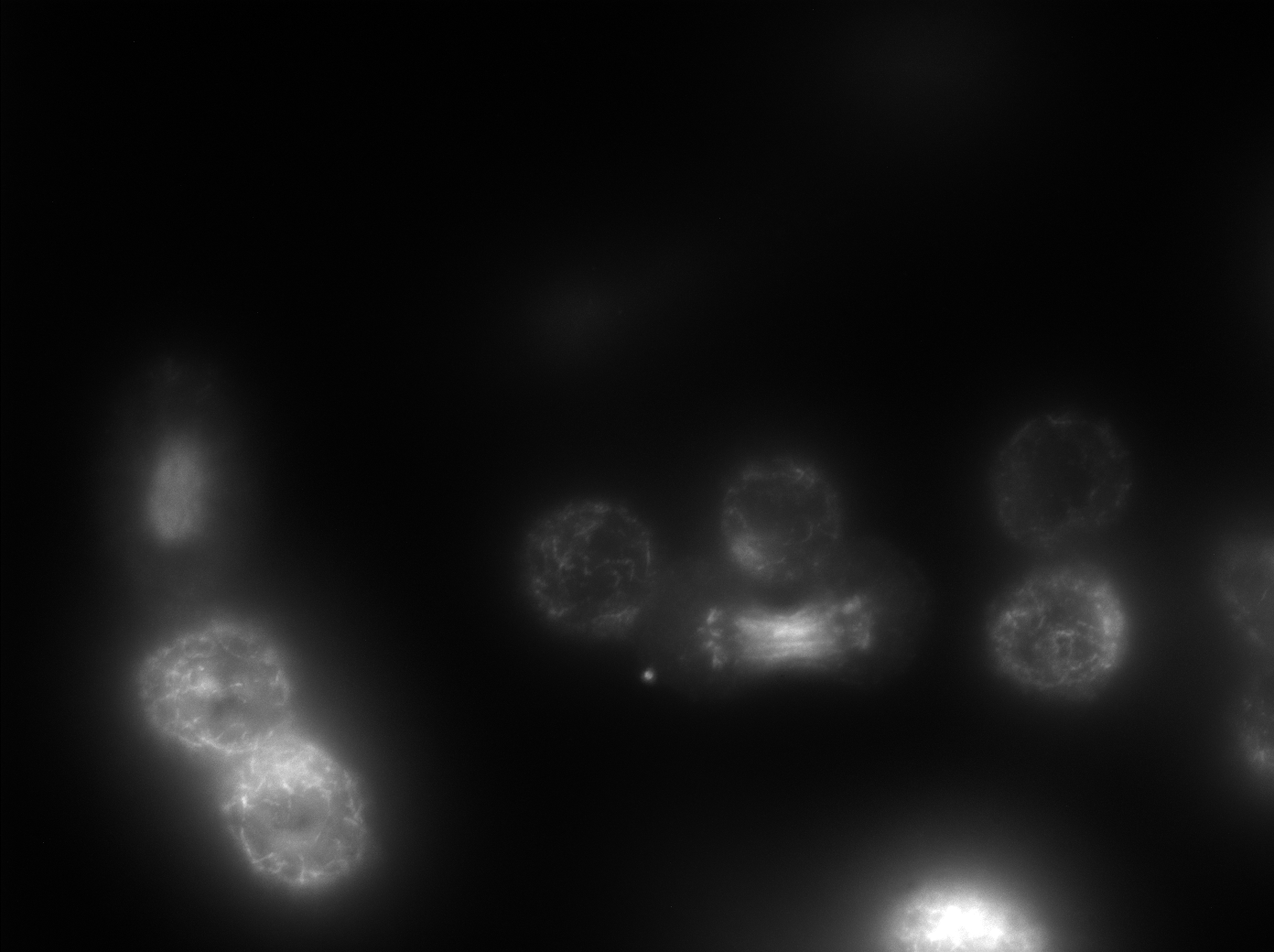

Supplement: Supplementary file 10 — Source data Fig. 2 part 7 [file 44319_2026_742_MOESM10_ESM.zip › Figure 2 Part 7/Fig 2fg Control Hela rGT335 acetylated tubulin part 2/Furrow Ingression/Cas9 actub rGT335 9-8-25 R1 FI1 EX.Project Maximum Z_XY1757351247_Z0_T0_C2.tif]

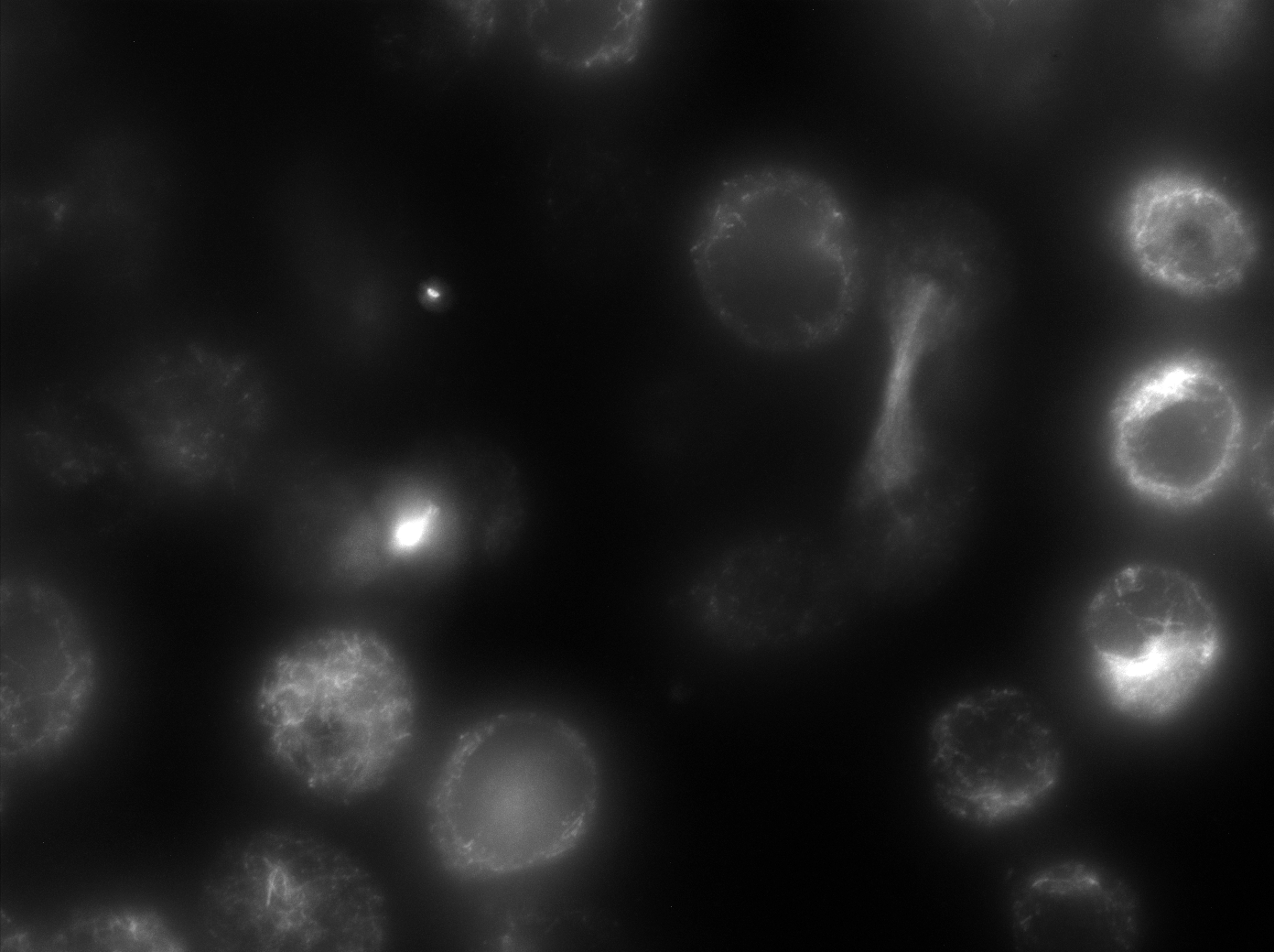

Supplement: Supplementary file 10 — Source data Fig. 2 part 7 [file 44319_2026_742_MOESM10_ESM.zip › Figure 2 Part 7/Fig 2fg Control Hela rGT335 acetylated tubulin part 2/Furrow Ingression/Cas9 actub rGT335 9-8-25 R2 FI7.Project Maximum Z_XY1757362749_Z0_T0_C2.tif]

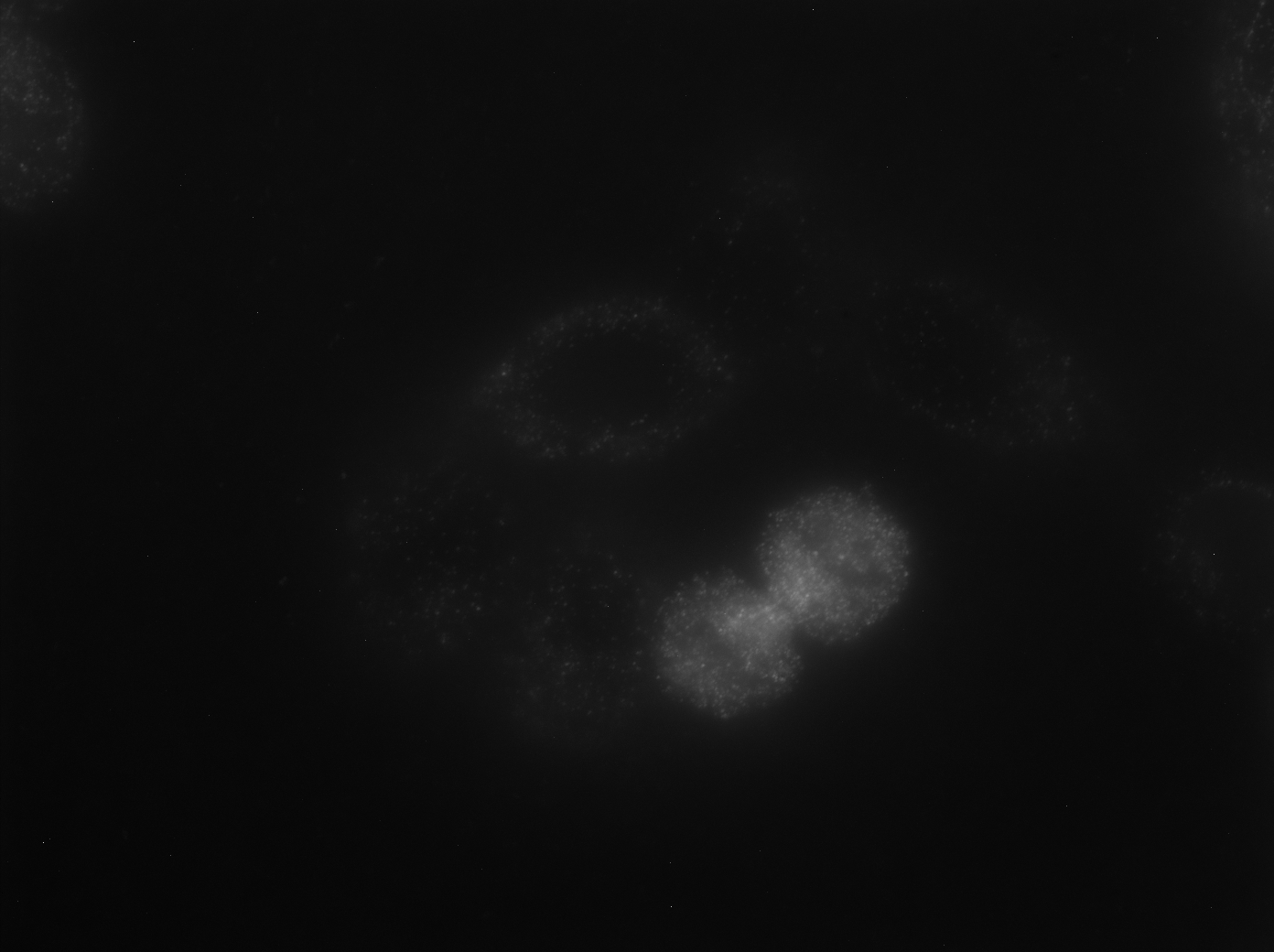

Supplement: Supplementary file 10 — Source data Fig. 2 part 7 [file 44319_2026_742_MOESM10_ESM.zip › Figure 2 Part 7/Fig 2fg Control Hela rGT335 acetylated tubulin part 2/Furrow Ingression/Cas9 actub rGT335 9-8-25 R3 FI11.Project Maximum Z_XY1757367064_Z0_T0_C1.tif]

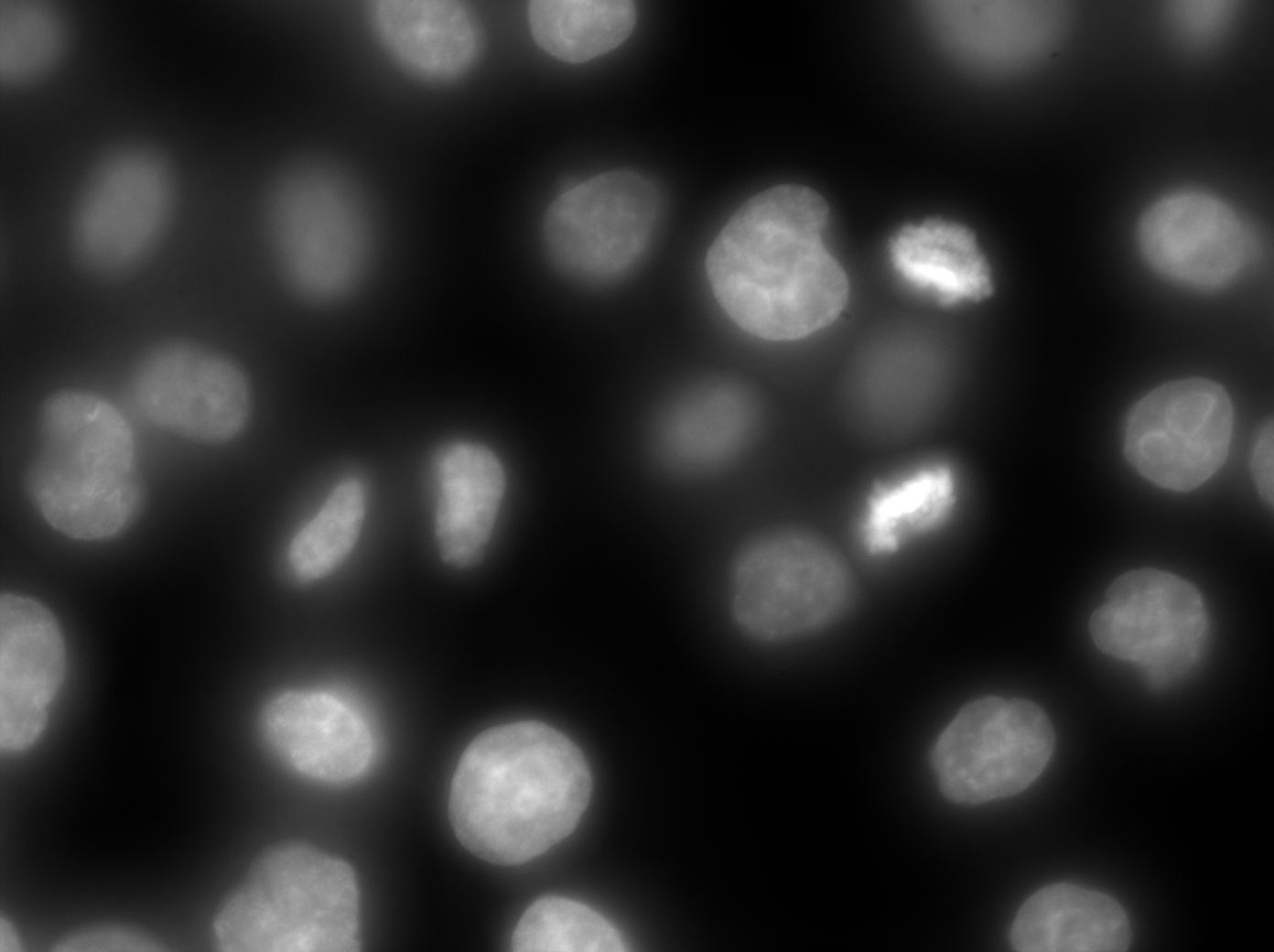

Supplement: Supplementary file 10 — Source data Fig. 2 part 7 [file 44319_2026_742_MOESM10_ESM.zip › Figure 2 Part 7/Fig 2fg Control Hela rGT335 acetylated tubulin part 2/Furrow Ingression/Cas9 actub rGT335 9-8-25 R2 FI7.Project Maximum Z_XY1757362749_Z0_T0_C0.tif]

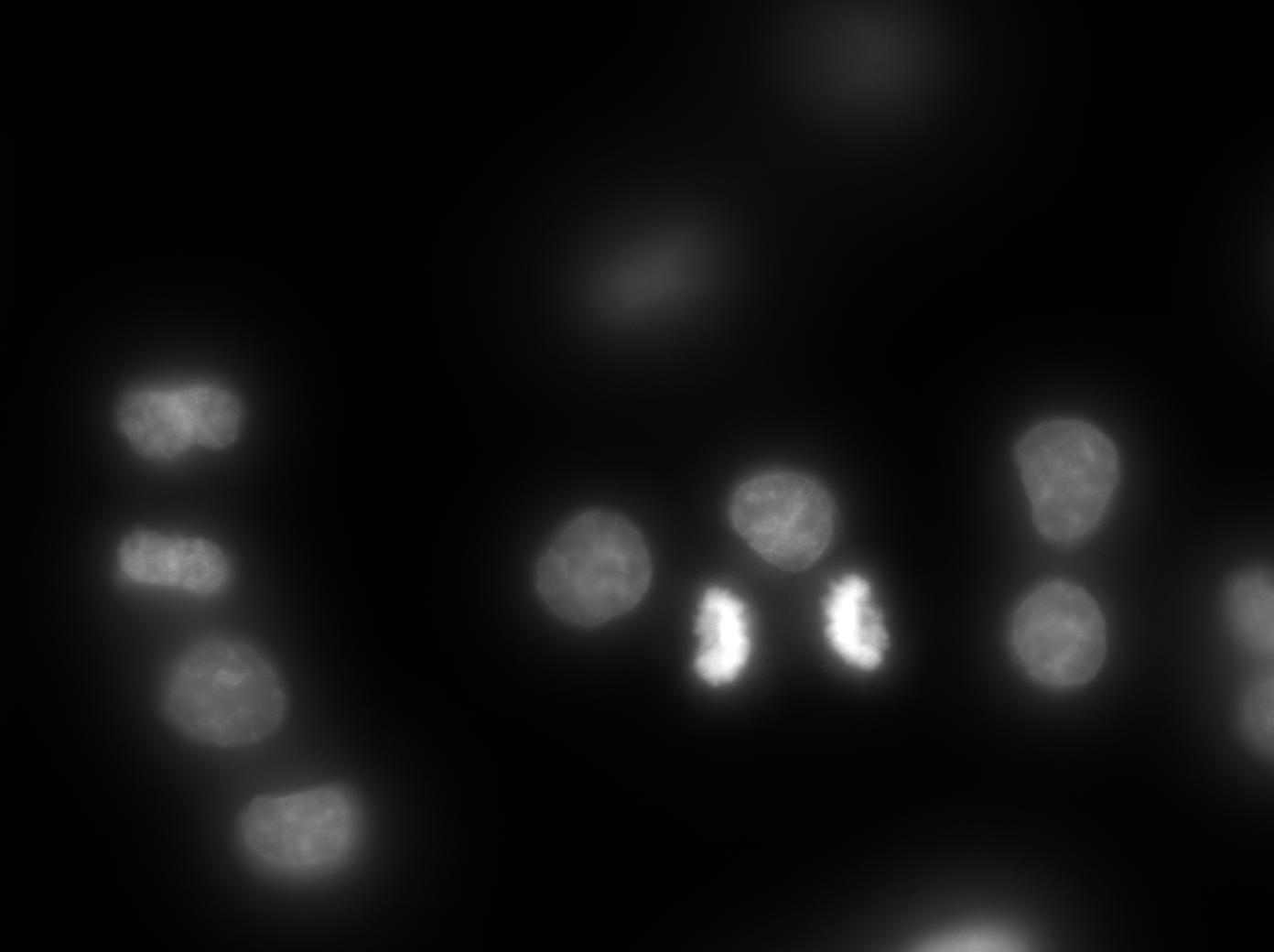

Supplement: Supplementary file 10 — Source data Fig. 2 part 7 [file 44319_2026_742_MOESM10_ESM.zip › Figure 2 Part 7/Fig 2fg Control Hela rGT335 acetylated tubulin part 2/Furrow Ingression/Cas9 actub rGT335 9-8-25 R1 FI1 EX.Project Maximum Z_XY1757351247_Z0_T0_C0.tif]

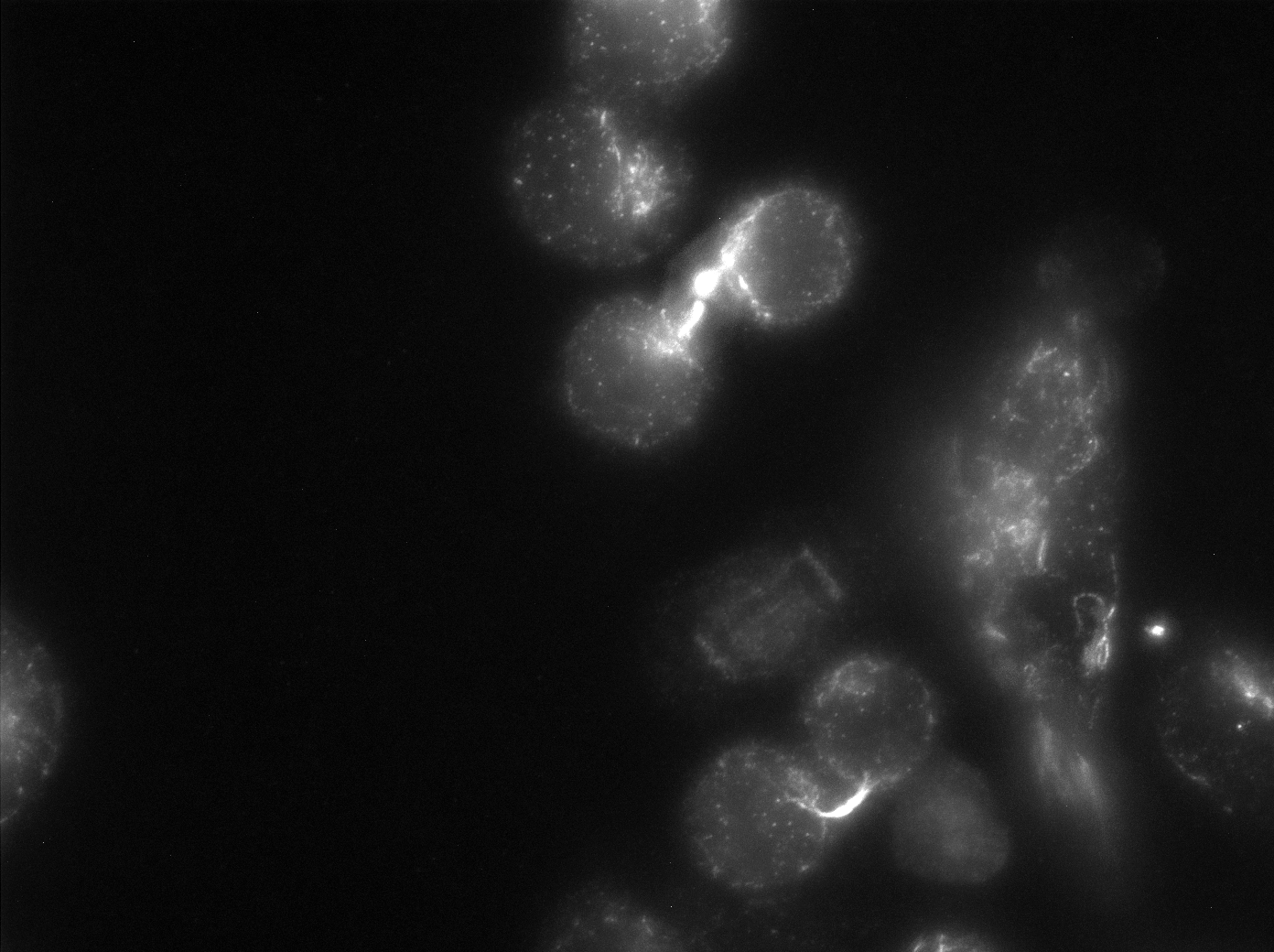

Supplement: Supplementary file 10 — Source data Fig. 2 part 7 [file 44319_2026_742_MOESM10_ESM.zip › Figure 2 Part 7/Fig 2fg Control Hela rGT335 acetylated tubulin part 2/Furrow Ingression/Cas9 actub rGT335 9-8-25 R3 FI4.Project Maximum Z_XY1757365645_Z0_T0_C2.tif]

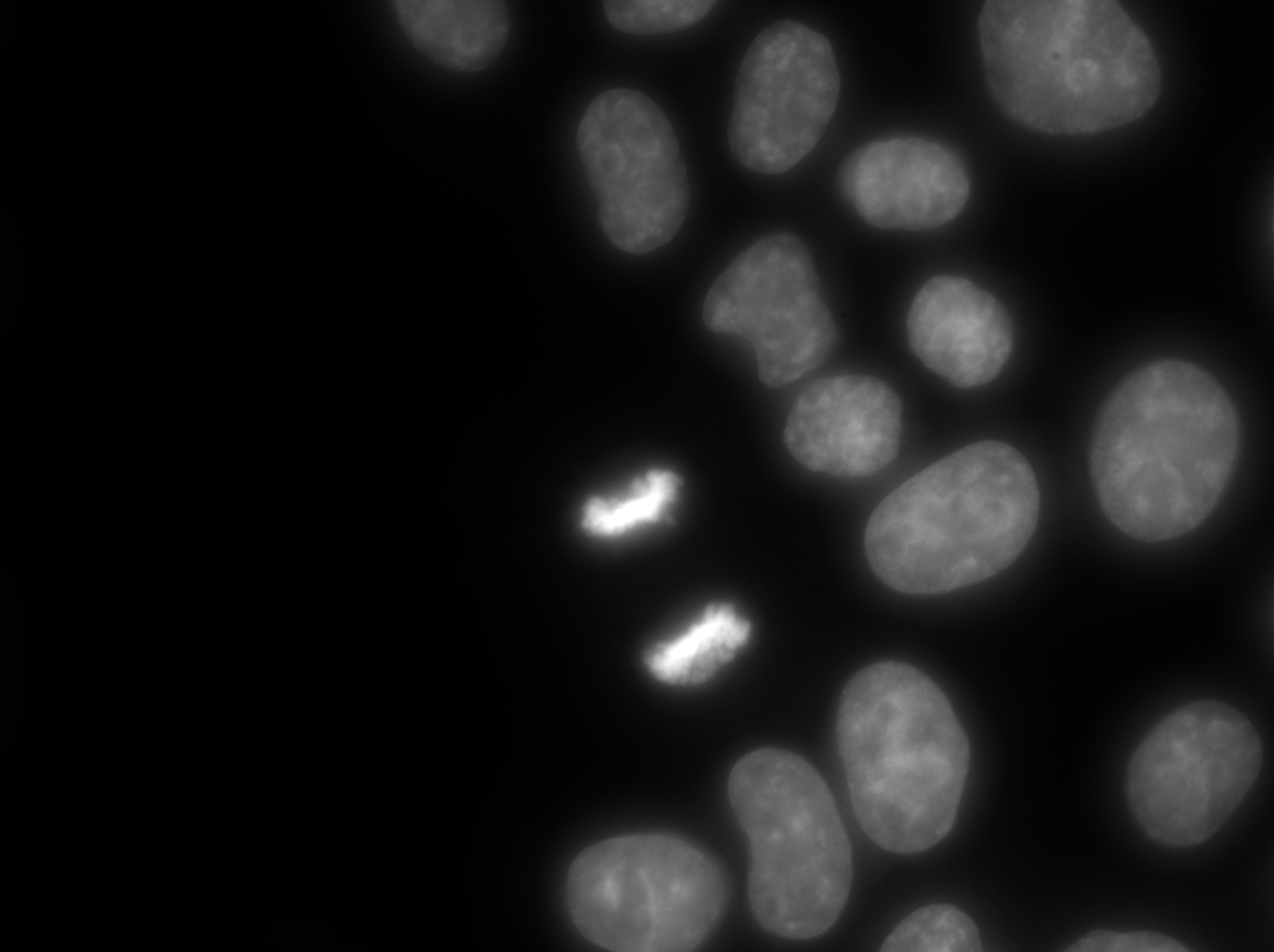

Supplement: Supplementary file 10 — Source data Fig. 2 part 7 [file 44319_2026_742_MOESM10_ESM.zip › Figure 2 Part 7/Fig 2fg Control Hela rGT335 acetylated tubulin part 2/Furrow Ingression/Cas9 actub rGT335 9-8-25 R3 FI1.Project Maximum Z_XY1757364948_Z0_T0_C0.tif]

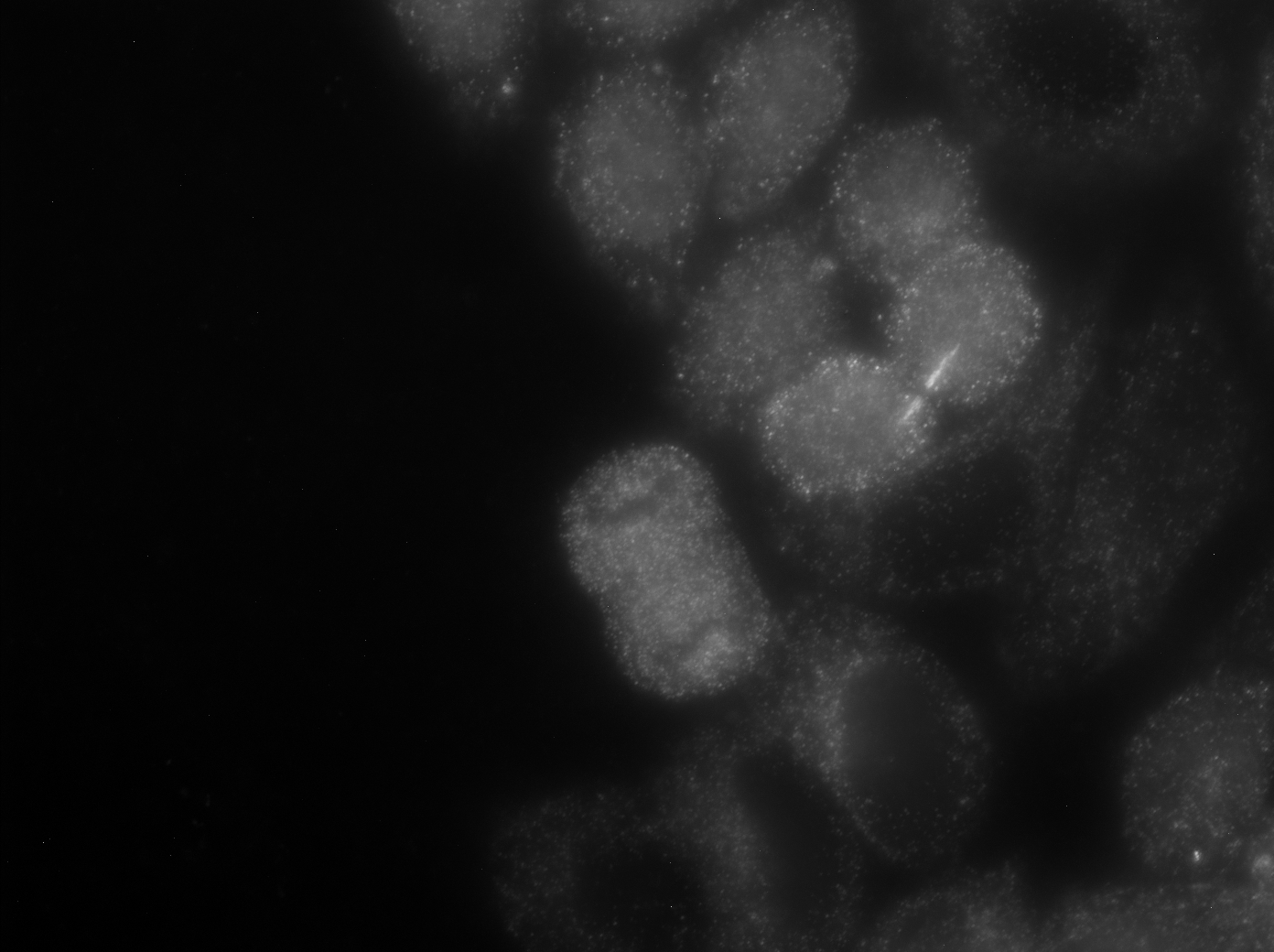

Supplement: Supplementary file 10 — Source data Fig. 2 part 7 [file 44319_2026_742_MOESM10_ESM.zip › Figure 2 Part 7/Fig 2fg Control Hela rGT335 acetylated tubulin part 2/Furrow Ingression/Cas9 actub rGT335 9-8-25 R3 FI1.Project Maximum Z_XY1757364948_Z0_T0_C1.tif]

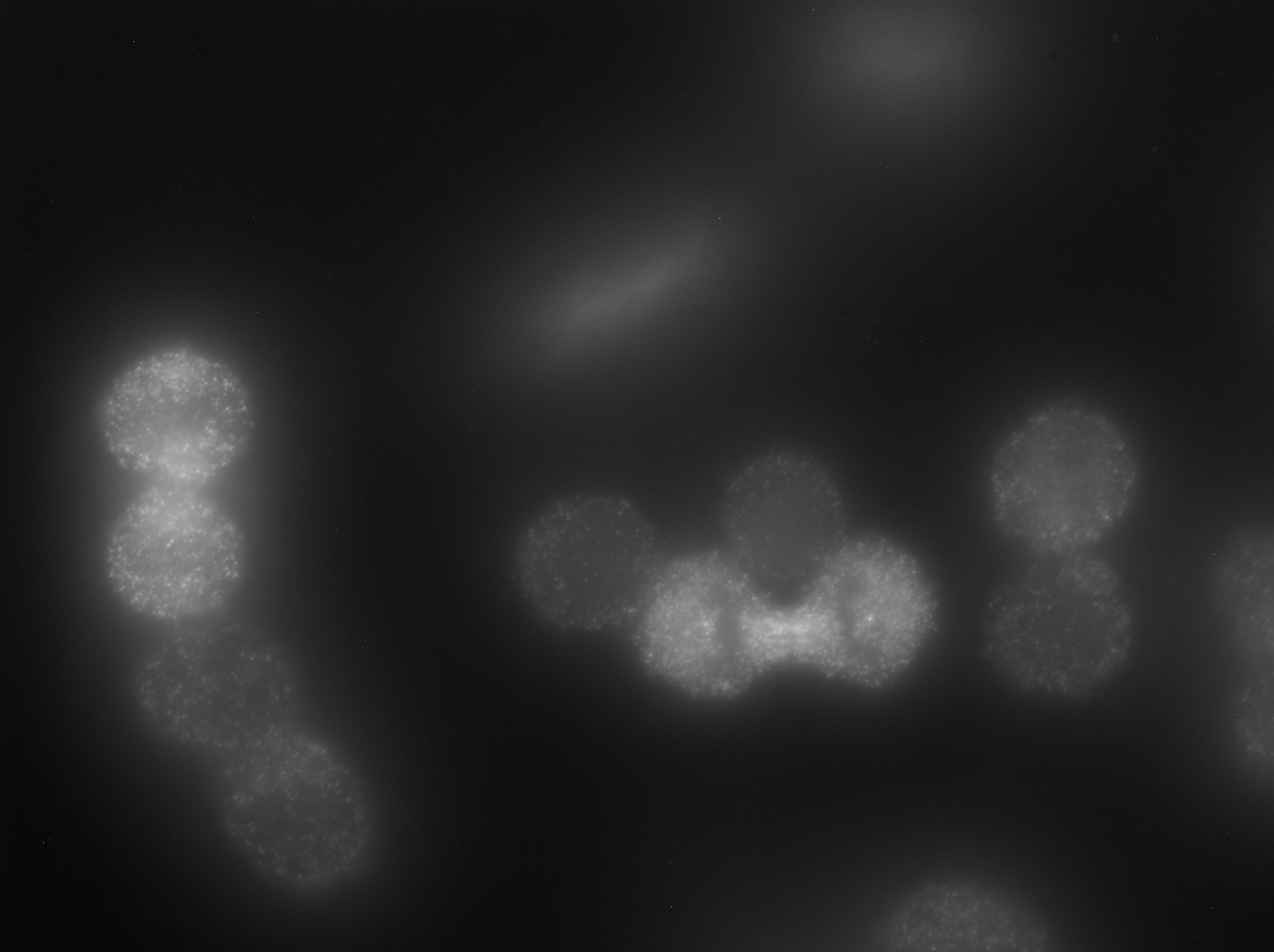

Supplement: Supplementary file 10 — Source data Fig. 2 part 7 [file 44319_2026_742_MOESM10_ESM.zip › Figure 2 Part 7/Fig 2fg Control Hela rGT335 acetylated tubulin part 2/Furrow Ingression/Cas9 actub rGT335 9-8-25 R1 FI1 EX.Project Maximum Z_XY1757351247_Z0_T0_C1.tif]

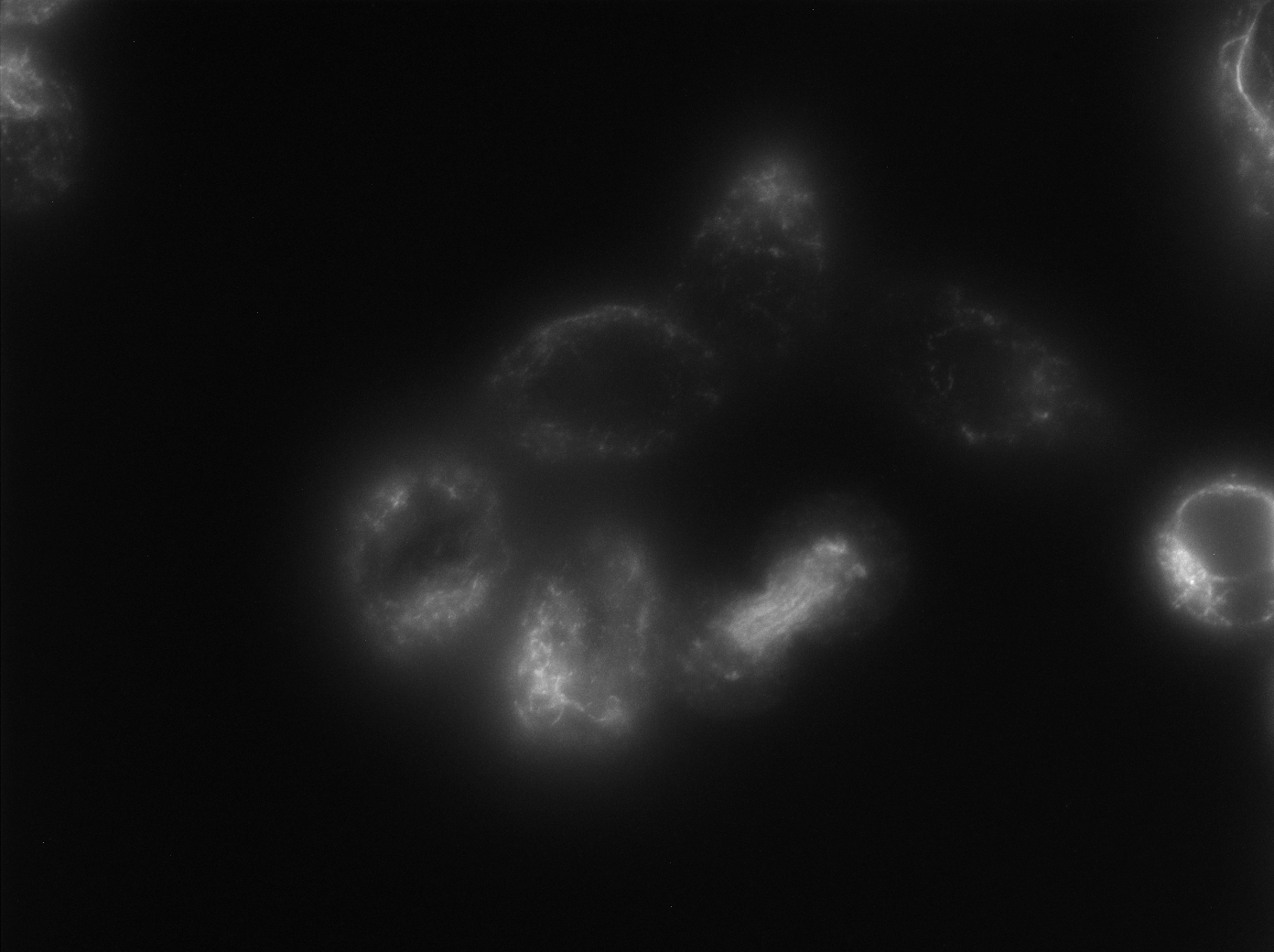

Supplement: Supplementary file 10 — Source data Fig. 2 part 7 [file 44319_2026_742_MOESM10_ESM.zip › Figure 2 Part 7/Fig 2fg Control Hela rGT335 acetylated tubulin part 2/Furrow Ingression/Cas9 actub rGT335 9-8-25 R3 FI11.Project Maximum Z_XY1757367064_Z0_T0_C2.tif]

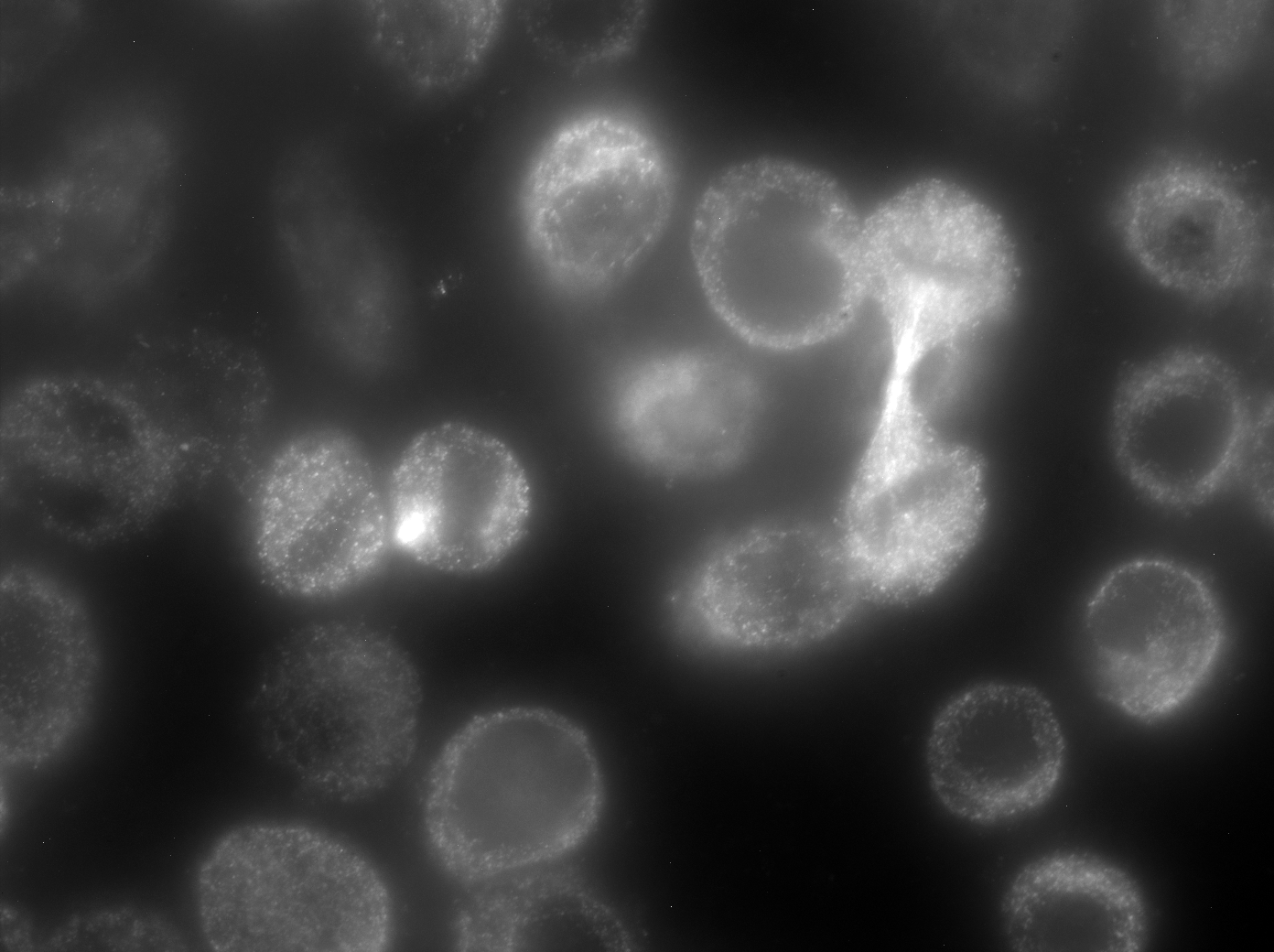

Supplement: Supplementary file 10 — Source data Fig. 2 part 7 [file 44319_2026_742_MOESM10_ESM.zip › Figure 2 Part 7/Fig 2fg Control Hela rGT335 acetylated tubulin part 2/Furrow Ingression/Cas9 actub rGT335 9-8-25 R2 FI7.Project Maximum Z_XY1757362749_Z0_T0_C1.tif]

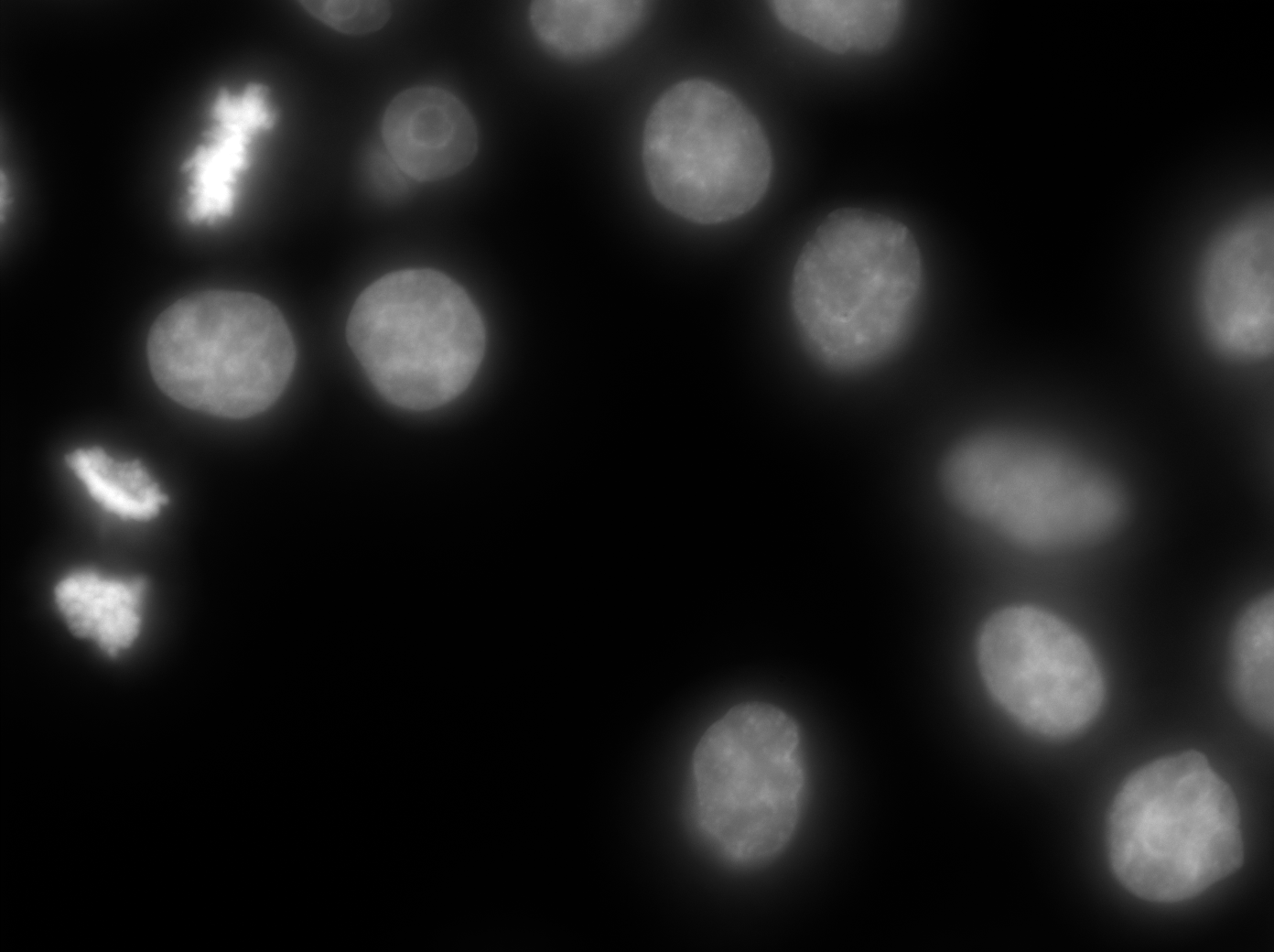

Supplement: Supplementary file 10 — Source data Fig. 2 part 7 [file 44319_2026_742_MOESM10_ESM.zip › Figure 2 Part 7/Fig 2fg Control Hela rGT335 acetylated tubulin part 2/Furrow Ingression/Cas9 actub rGT335 9-8-25 R2 FI5 M4.Project Maximum Z_XY1757361339_Z0_T0_C0.tif]

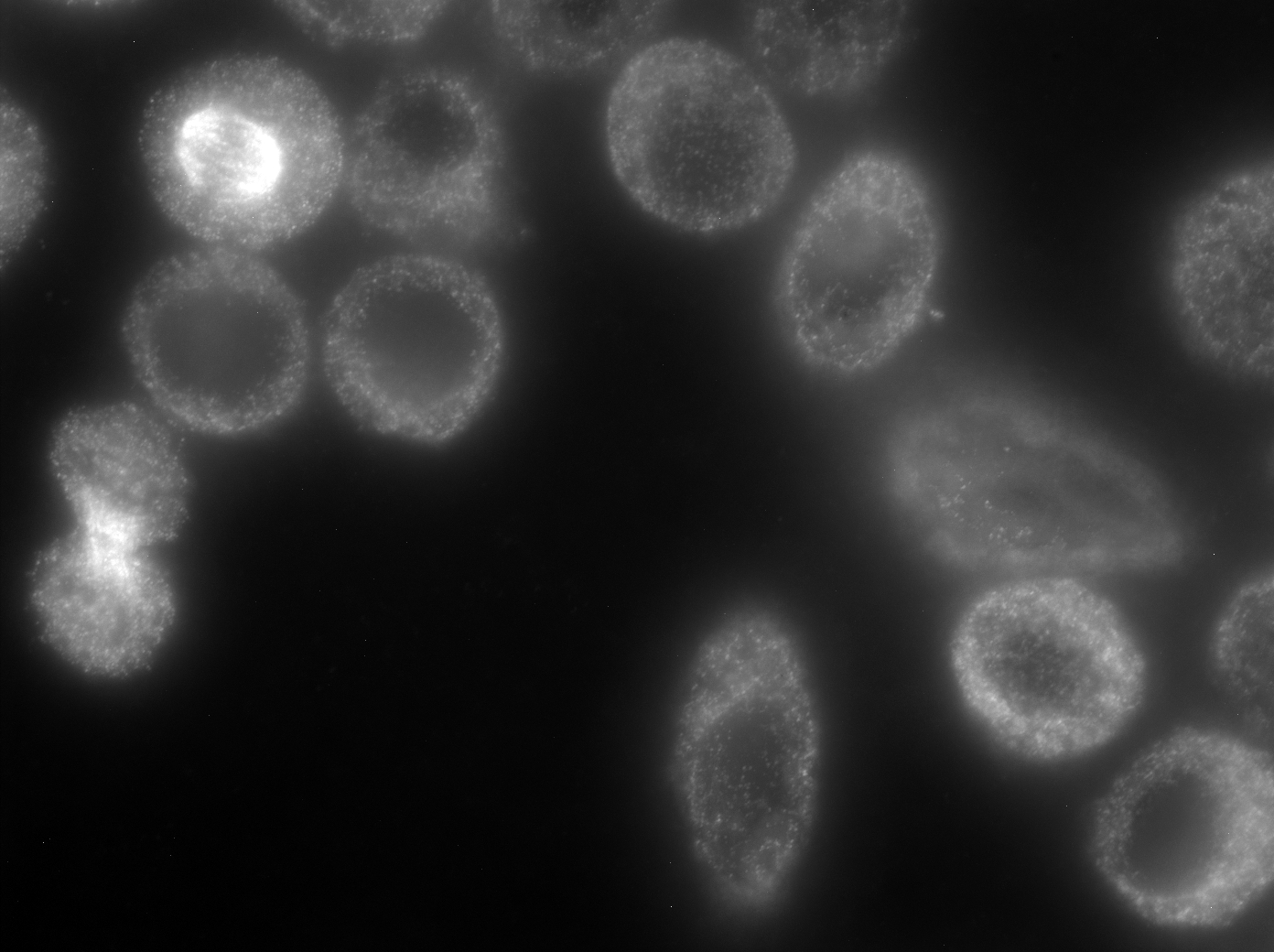

Supplement: Supplementary file 10 — Source data Fig. 2 part 7 [file 44319_2026_742_MOESM10_ESM.zip › Figure 2 Part 7/Fig 2fg Control Hela rGT335 acetylated tubulin part 2/Furrow Ingression/Cas9 actub rGT335 9-8-25 R2 FI5 M4.Project Maximum Z_XY1757361339_Z0_T0_C1.tif]

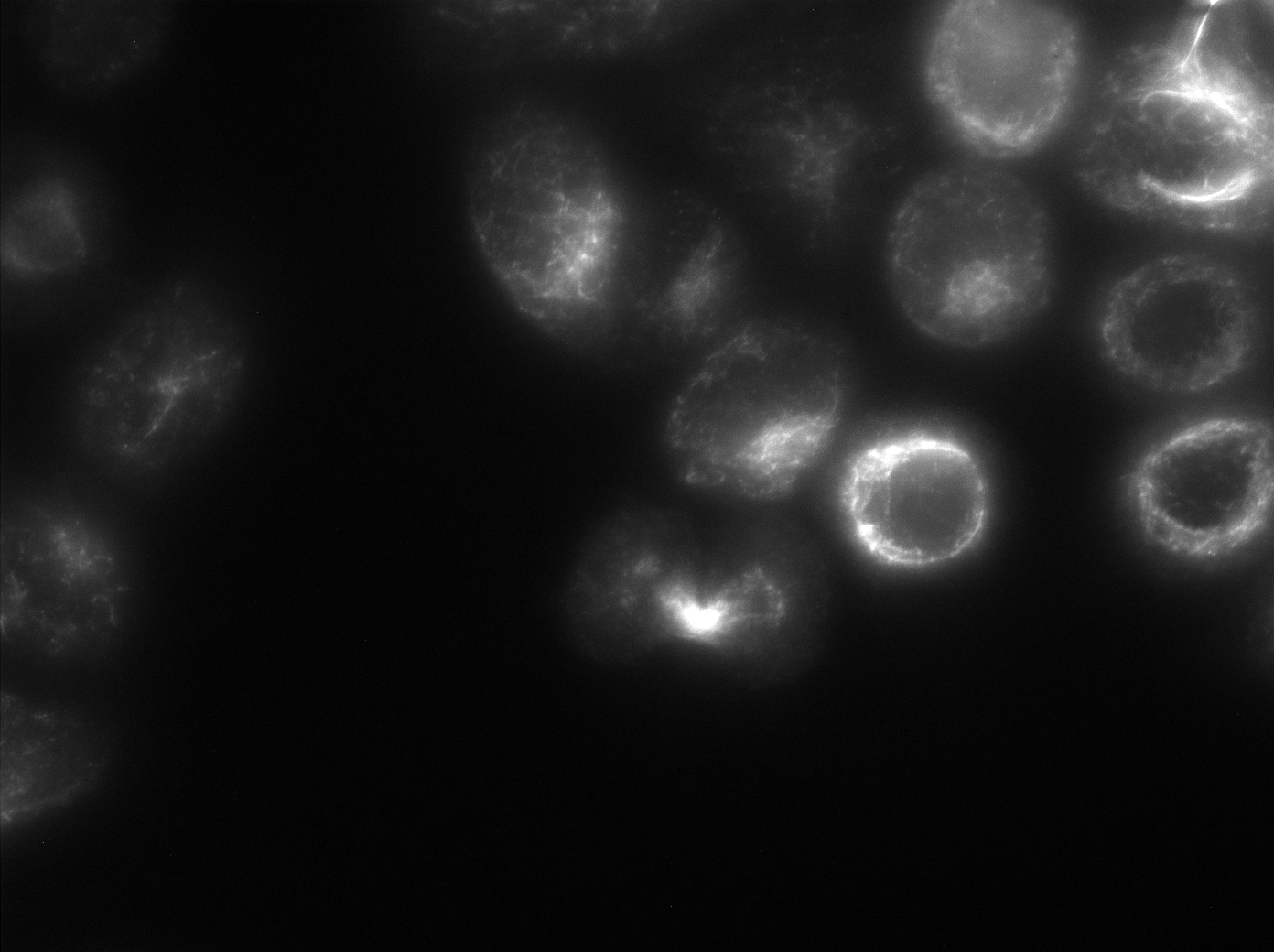

Supplement: Supplementary file 10 — Source data Fig. 2 part 7 [file 44319_2026_742_MOESM10_ESM.zip › Figure 2 Part 7/Fig 2fg Control Hela rGT335 acetylated tubulin part 2/Furrow Ingression/Cas9 actub rGT335 9-8-25 R3 FI2.Project Maximum Z_XY1757365408_Z0_T0_C2.tif]

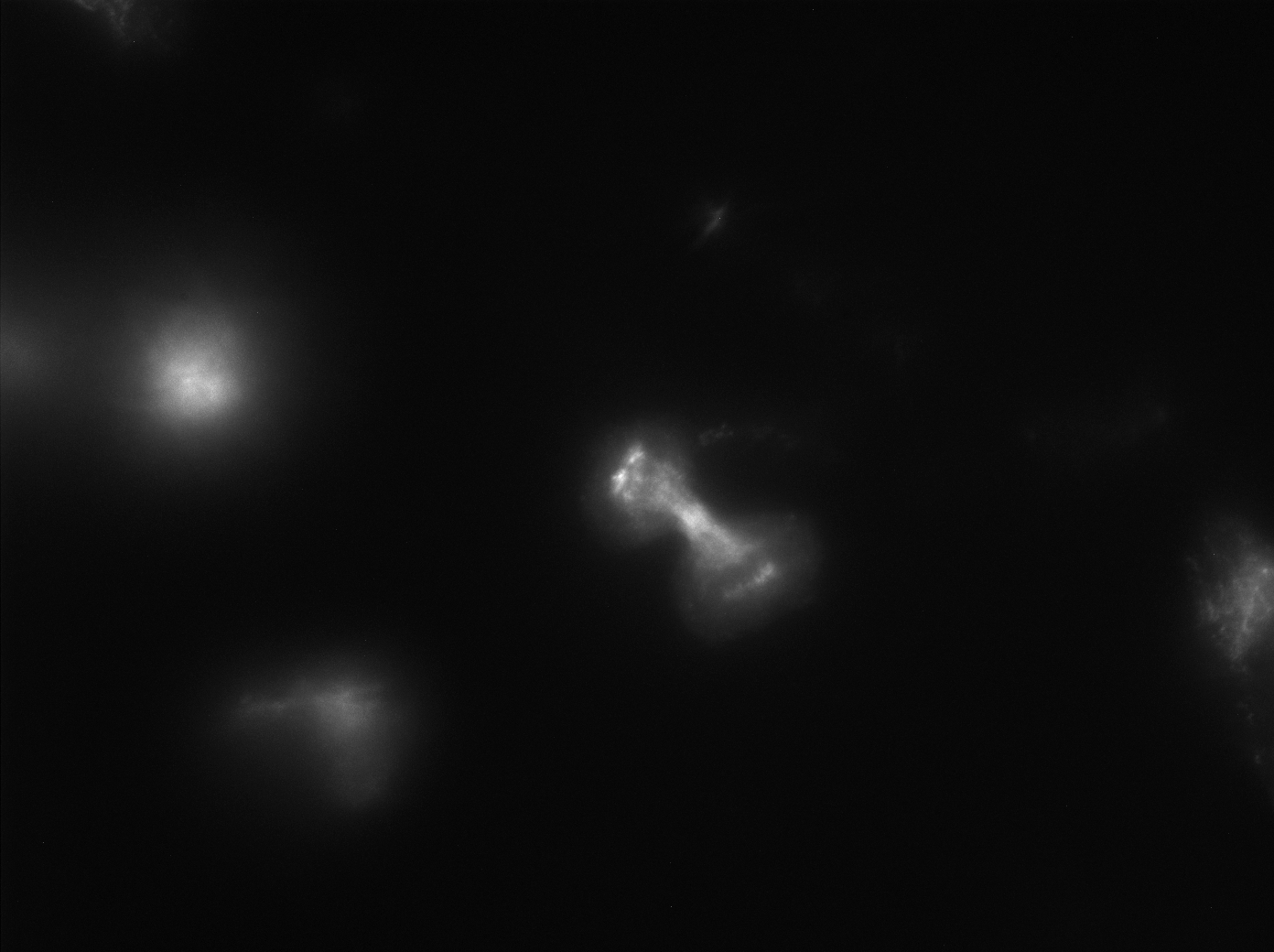

Supplement: Supplementary file 10 — Source data Fig. 2 part 7 [file 44319_2026_742_MOESM10_ESM.zip › Figure 2 Part 7/Fig 2fg Control Hela rGT335 acetylated tubulin part 2/Furrow Ingression/Cas9 actub rGT335 9-8-25 R1 LFI2.Project Maximum Z_XY1757353171_Z0_T0_C2.tif]

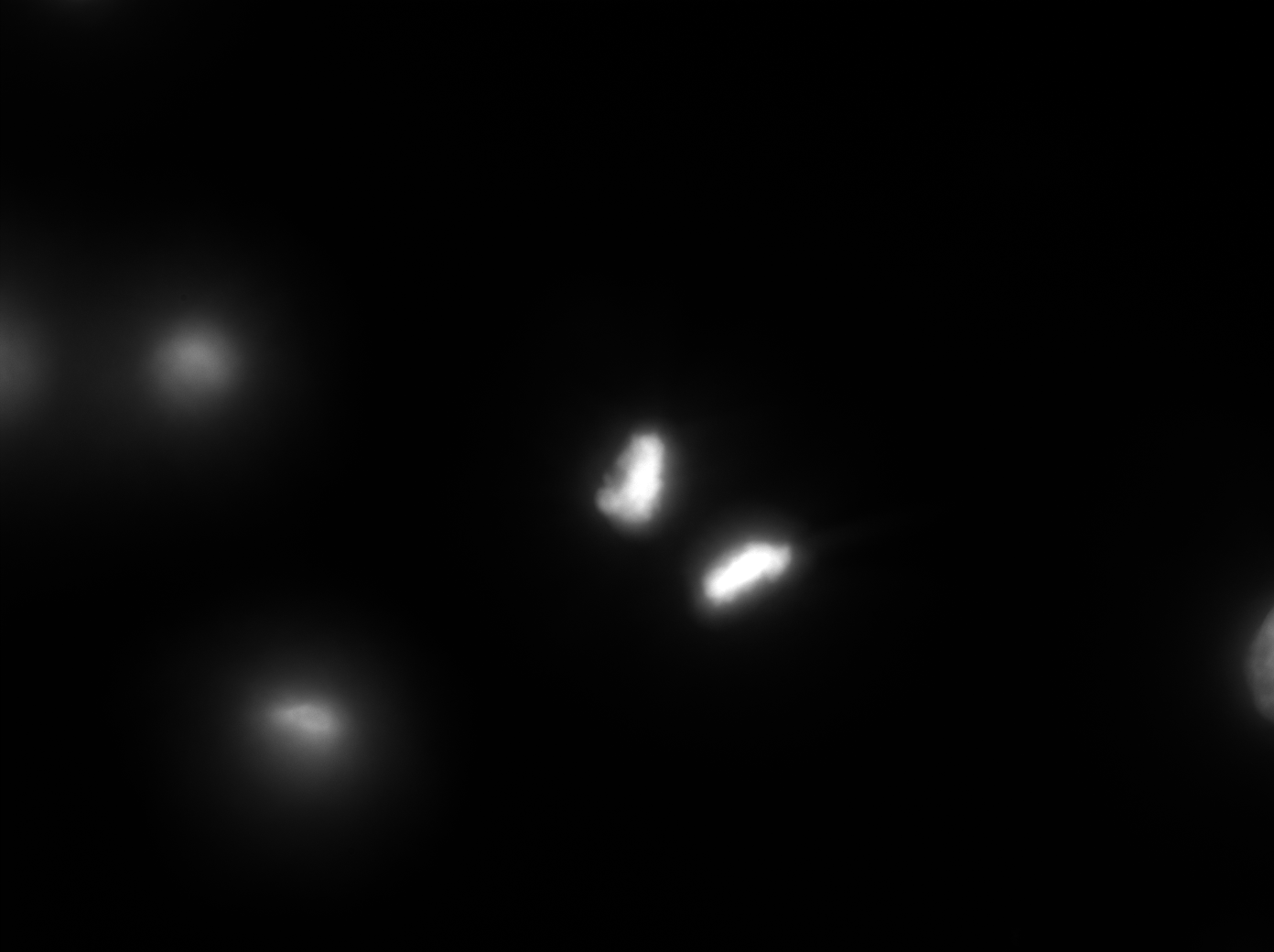

Supplement: Supplementary file 10 — Source data Fig. 2 part 7 [file 44319_2026_742_MOESM10_ESM.zip › Figure 2 Part 7/Fig 2fg Control Hela rGT335 acetylated tubulin part 2/Furrow Ingression/Cas9 actub rGT335 9-8-25 R1 LFI2.Project Maximum Z_XY1757353171_Z0_T0_C0.tif]

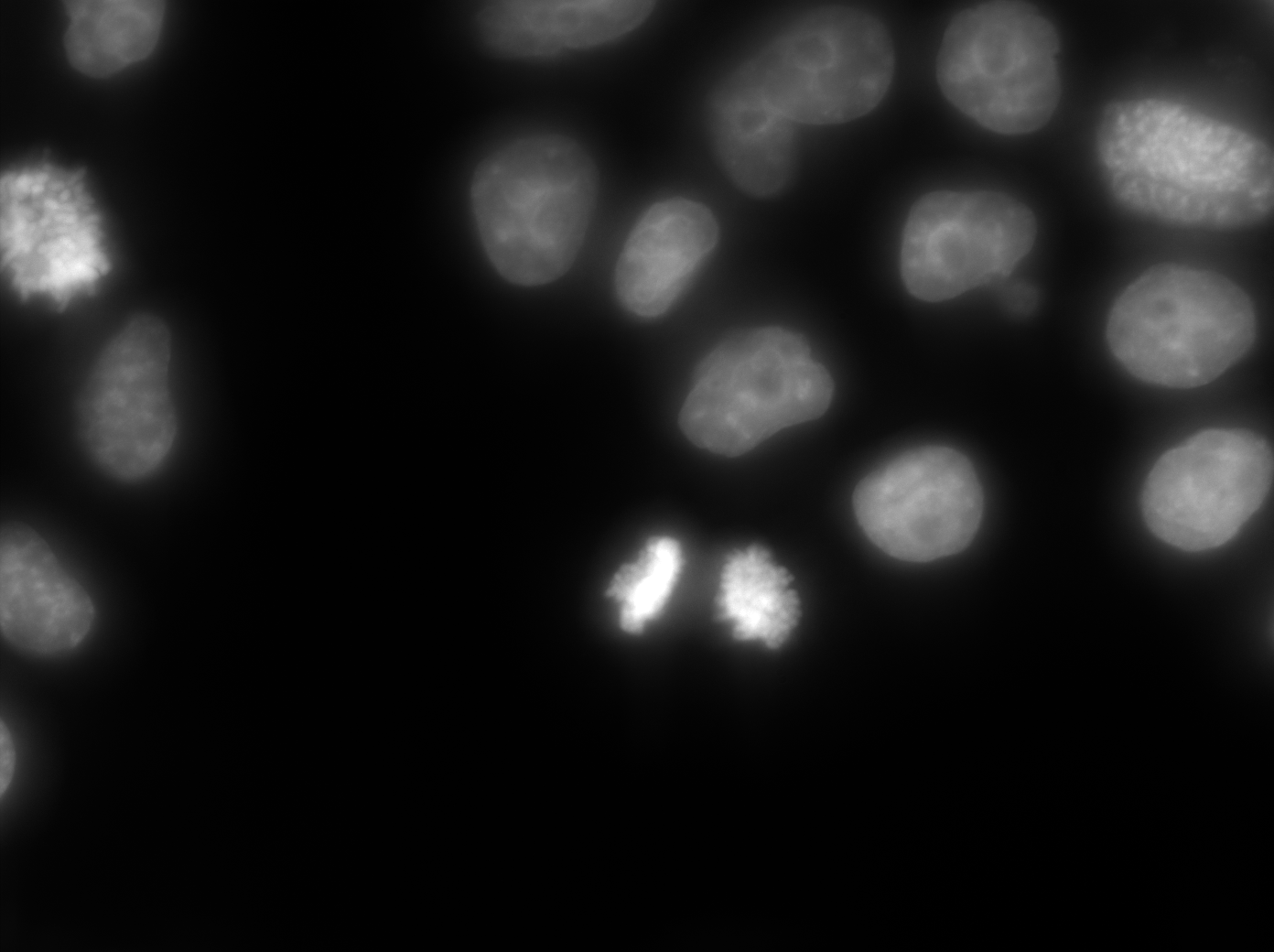

Supplement: Supplementary file 10 — Source data Fig. 2 part 7 [file 44319_2026_742_MOESM10_ESM.zip › Figure 2 Part 7/Fig 2fg Control Hela rGT335 acetylated tubulin part 2/Furrow Ingression/Cas9 actub rGT335 9-8-25 R3 FI2.Project Maximum Z_XY1757365408_Z0_T0_C0.tif]

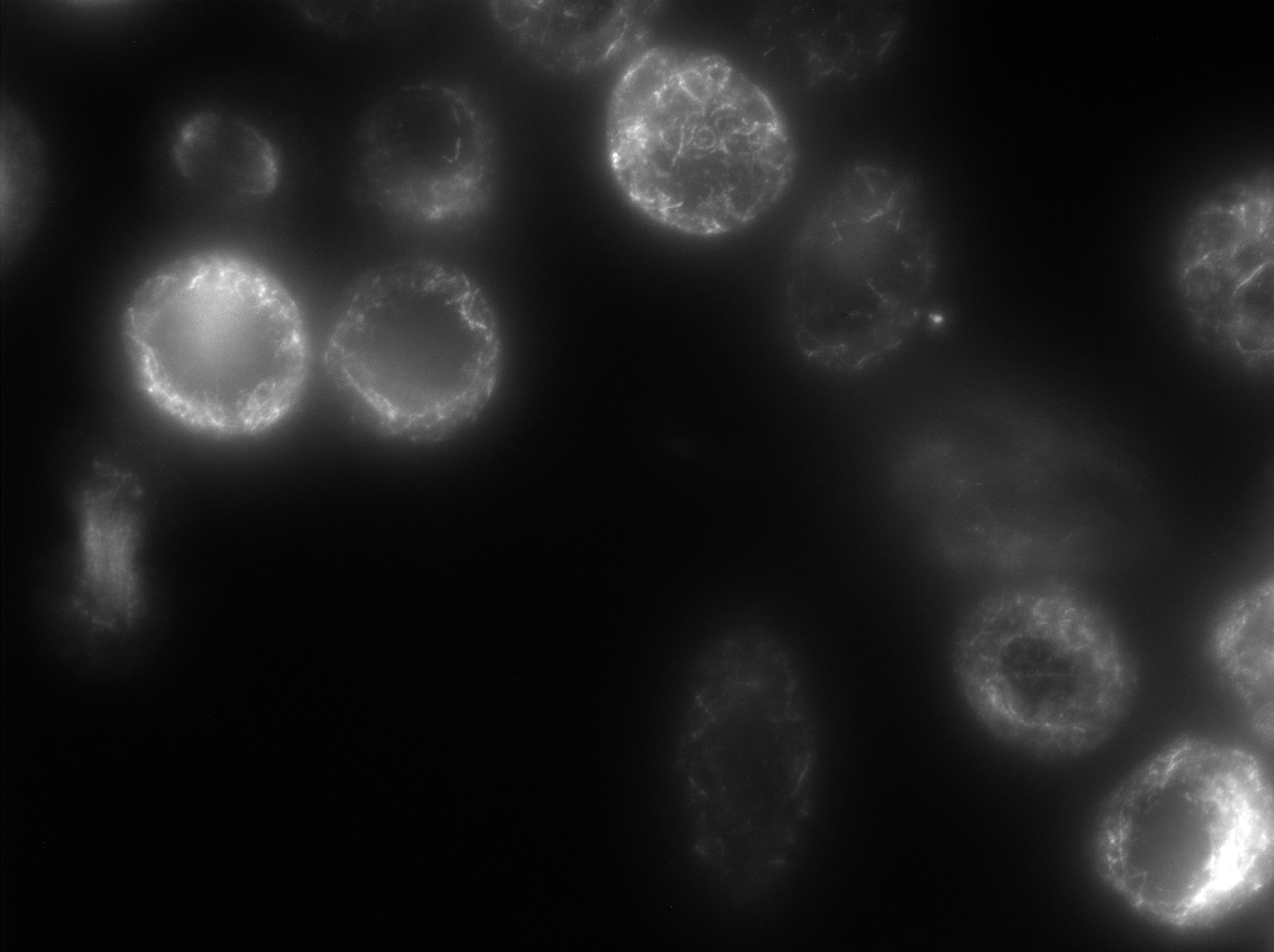

Supplement: Supplementary file 10 — Source data Fig. 2 part 7 [file 44319_2026_742_MOESM10_ESM.zip › Figure 2 Part 7/Fig 2fg Control Hela rGT335 acetylated tubulin part 2/Furrow Ingression/Cas9 actub rGT335 9-8-25 R2 FI5 M4.Project Maximum Z_XY1757361339_Z0_T0_C2.tif]

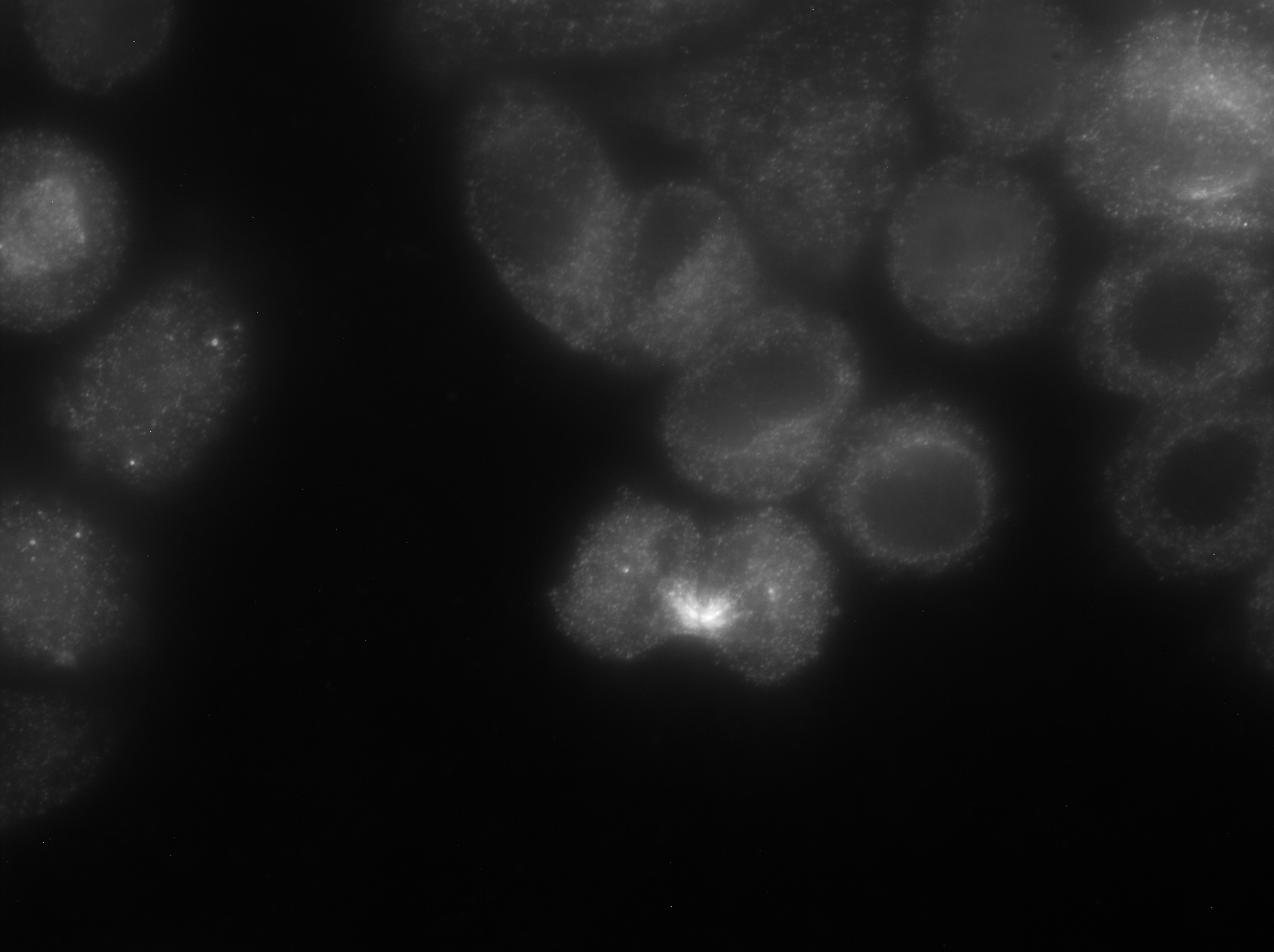

Supplement: Supplementary file 10 — Source data Fig. 2 part 7 [file 44319_2026_742_MOESM10_ESM.zip › Figure 2 Part 7/Fig 2fg Control Hela rGT335 acetylated tubulin part 2/Furrow Ingression/Cas9 actub rGT335 9-8-25 R3 FI2.Project Maximum Z_XY1757365408_Z0_T0_C1.tif]

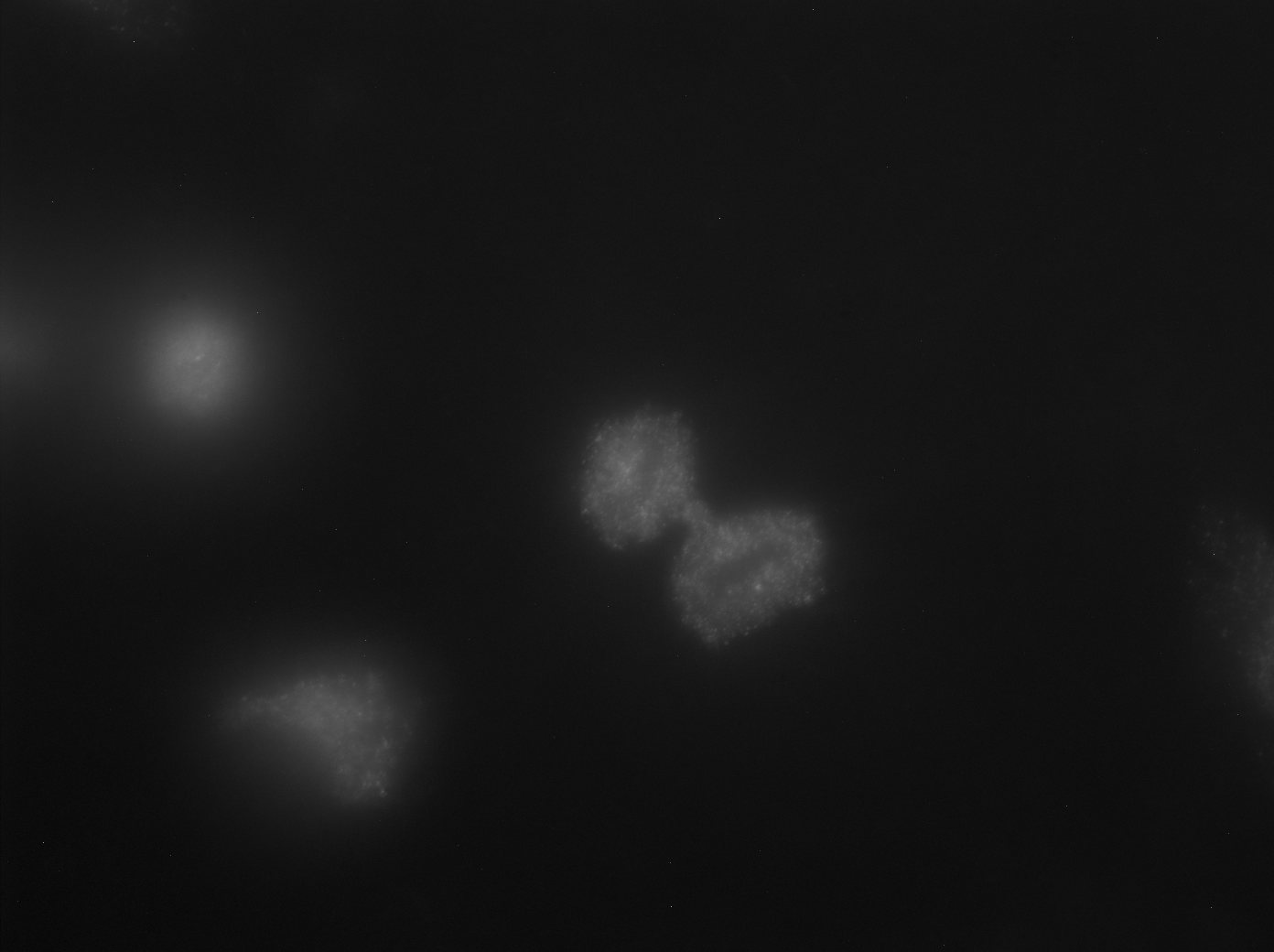

Supplement: Supplementary file 10 — Source data Fig. 2 part 7 [file 44319_2026_742_MOESM10_ESM.zip › Figure 2 Part 7/Fig 2fg Control Hela rGT335 acetylated tubulin part 2/Furrow Ingression/Cas9 actub rGT335 9-8-25 R1 LFI2.Project Maximum Z_XY1757353171_Z0_T0_C1.tif]

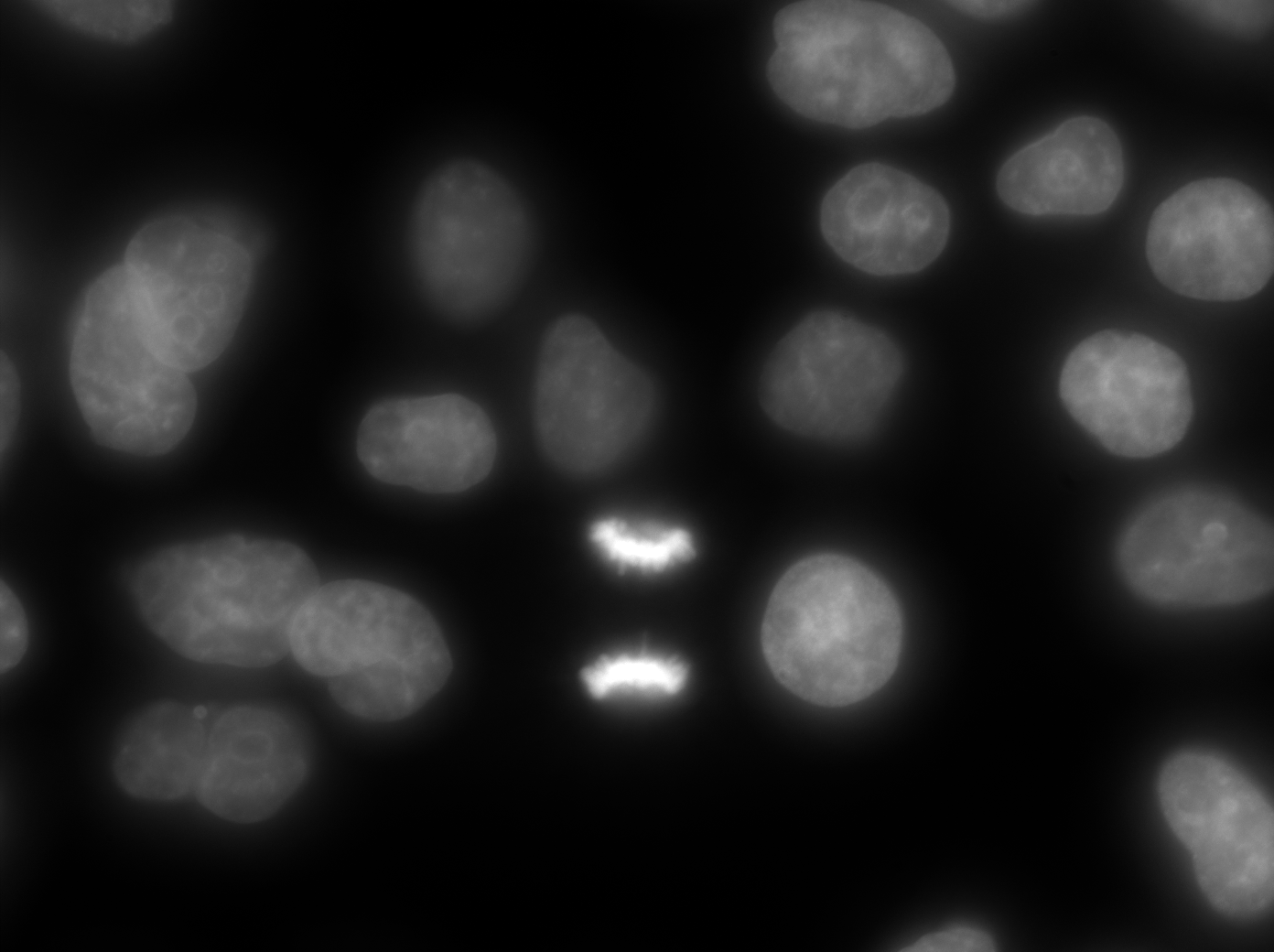

Supplement: Supplementary file 10 — Source data Fig. 2 part 7 [file 44319_2026_742_MOESM10_ESM.zip › Figure 2 Part 7/Fig 2fg Control Hela rGT335 acetylated tubulin part 2/Furrow Ingression/Cas9 actub rGT335 9-8-25 R3 FI8.Project Maximum Z_XY1757366668_Z0_T0_C0.tif]

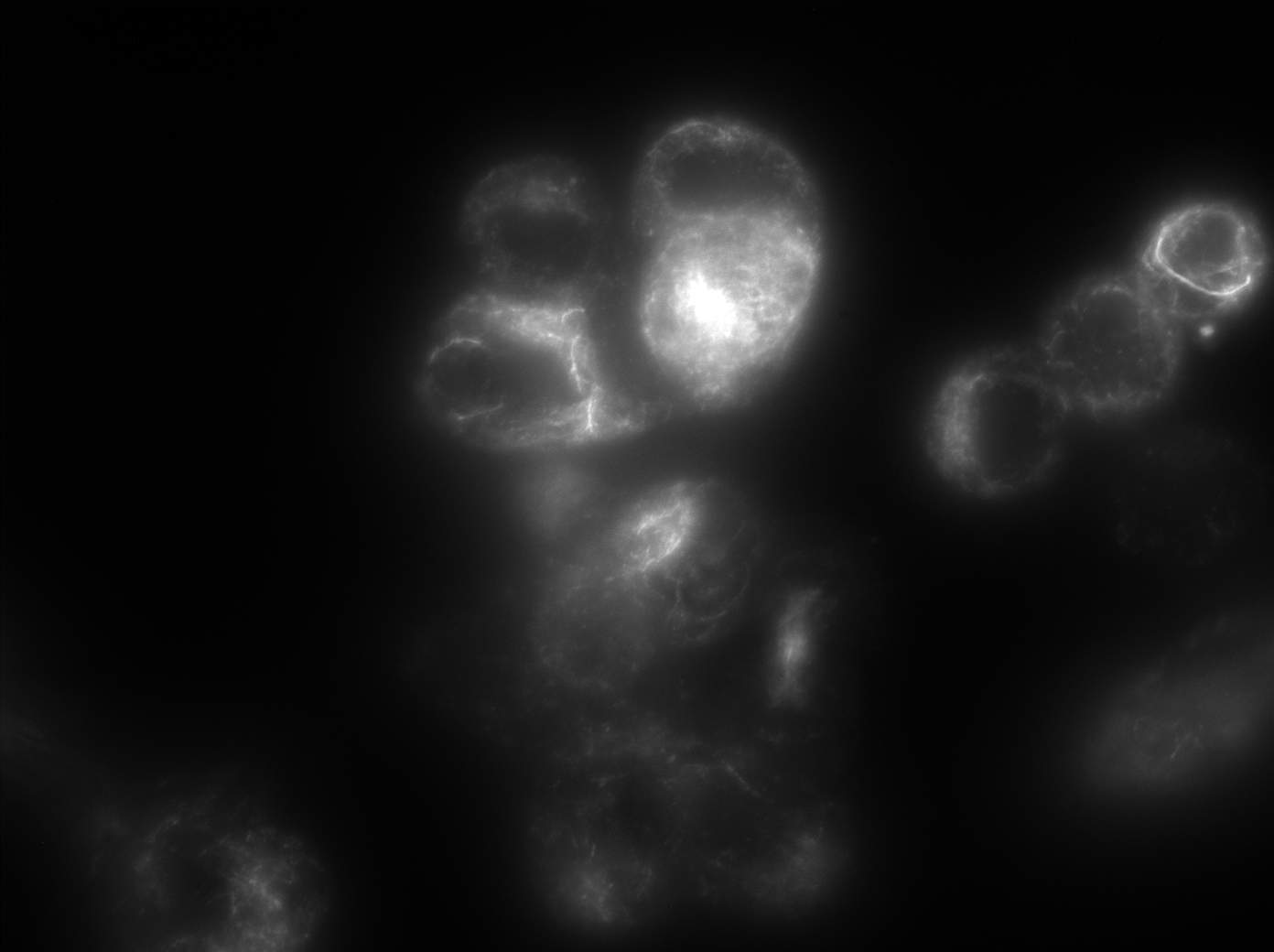

Supplement: Supplementary file 10 — Source data Fig. 2 part 7 [file 44319_2026_742_MOESM10_ESM.zip › Figure 2 Part 7/Fig 2fg Control Hela rGT335 acetylated tubulin part 2/Furrow Ingression/Cas9 actub rGT335 9-8-25 R1 FI5.Project Maximum Z_XY1757353899_Z0_T0_C2.tif]

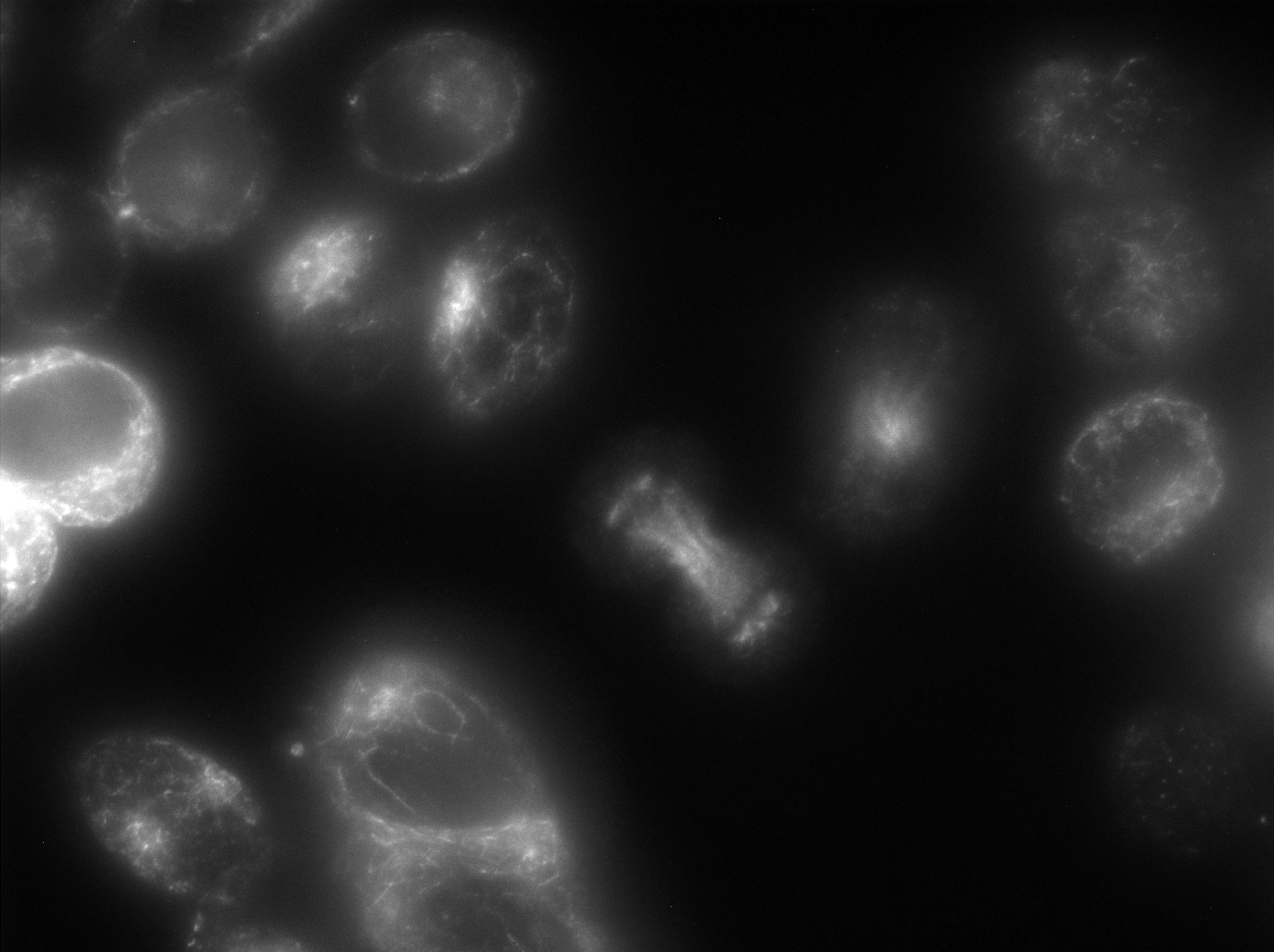

Supplement: Supplementary file 10 — Source data Fig. 2 part 7 [file 44319_2026_742_MOESM10_ESM.zip › Figure 2 Part 7/Fig 2fg Control Hela rGT335 acetylated tubulin part 2/Furrow Ingression/Cas9 actub rGT335 9-8-25 R3 FI6.Project Maximum Z_XY1757366392_Z0_T0_C2.tif]
