# Supplementary figures and images for: TPGS1 regulates central spindle microtubule glutamylation and remodeling during telophase and abscission (part 13 of 36)
Source: EMBO Rep. 2026 Mar 23;27(8):1944–63. doi: 10.1038/s44319-026-00742-3 (PMC13121839; doi:10.1038/s44319-026-00742-3)

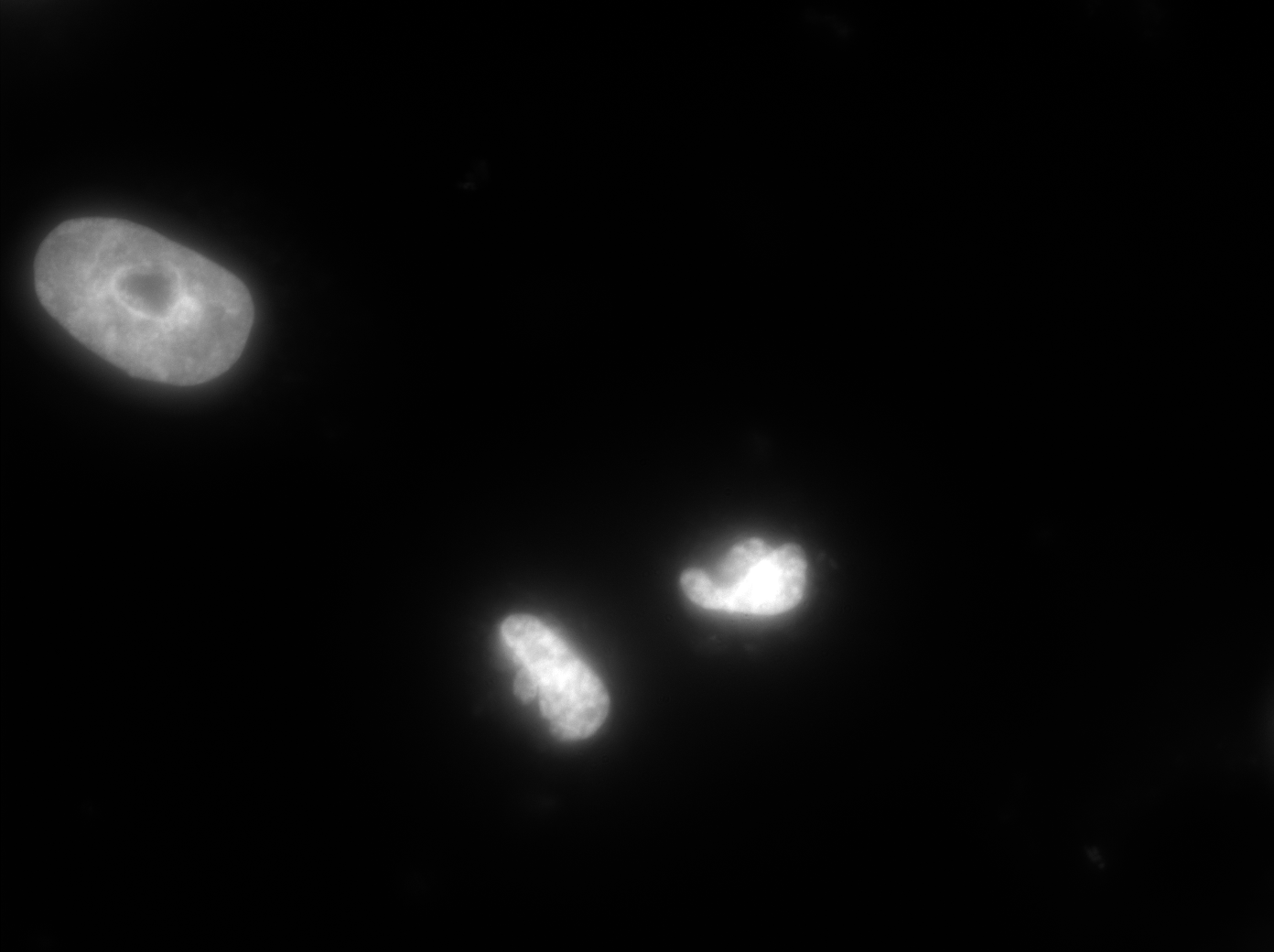

Supplement: Supplementary file 11 — Source data Fig. 3 part 1 [file 44319_2026_742_MOESM11_ESM.zip › Figure 3 Part 1/Fig 3b-e TTLL screen/TTLL1-GFP R1 I5.Project Maximum Z_XY1674164172_Z0_T0_C0.tif]

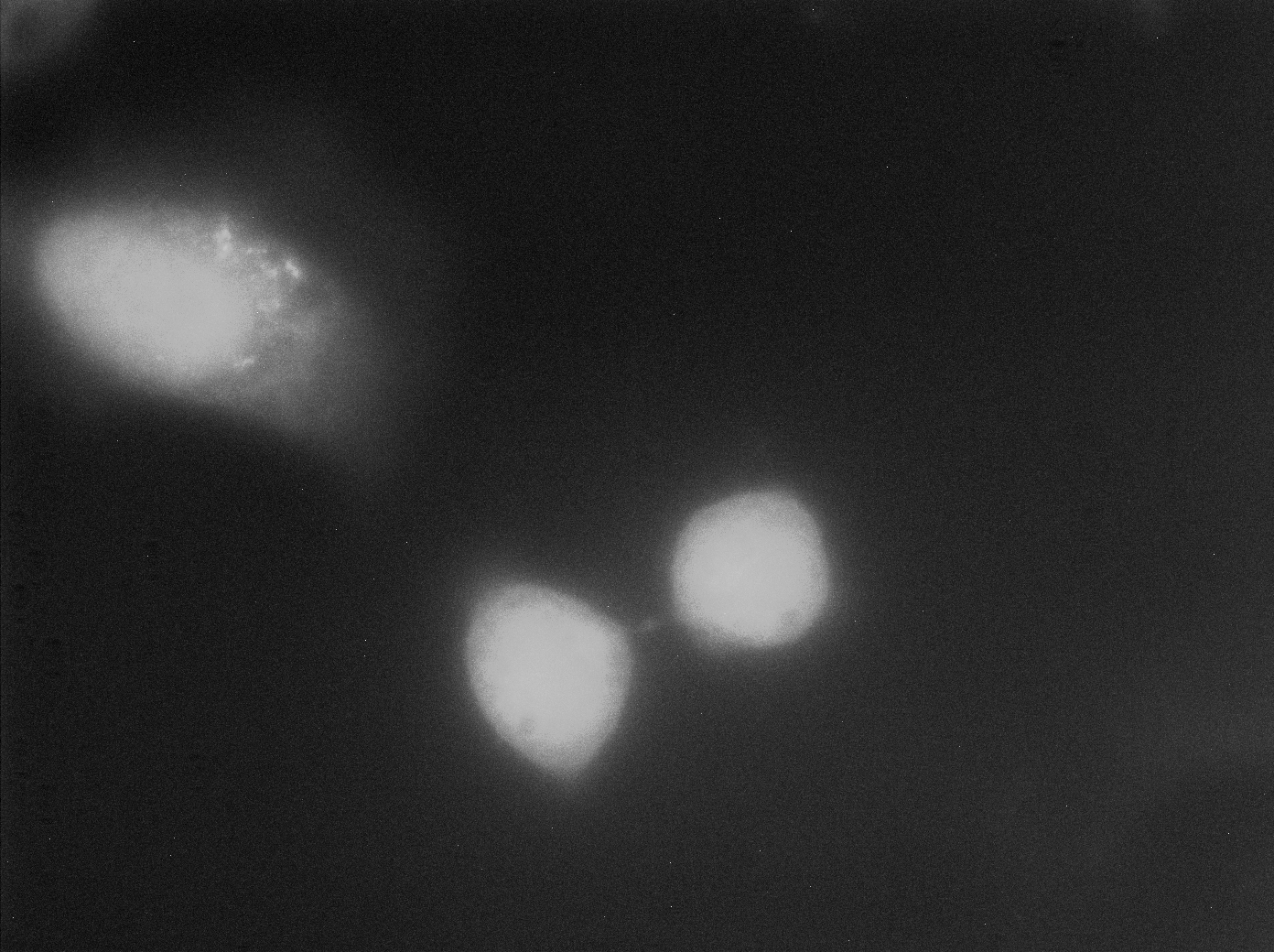

Supplement: Supplementary file 11 — Source data Fig. 3 part 1 [file 44319_2026_742_MOESM11_ESM.zip › Figure 3 Part 1/Fig 3b-e TTLL screen/TTLL1-GFP R1 I5.Project Maximum Z_XY1674164172_Z0_T0_C1.tif]

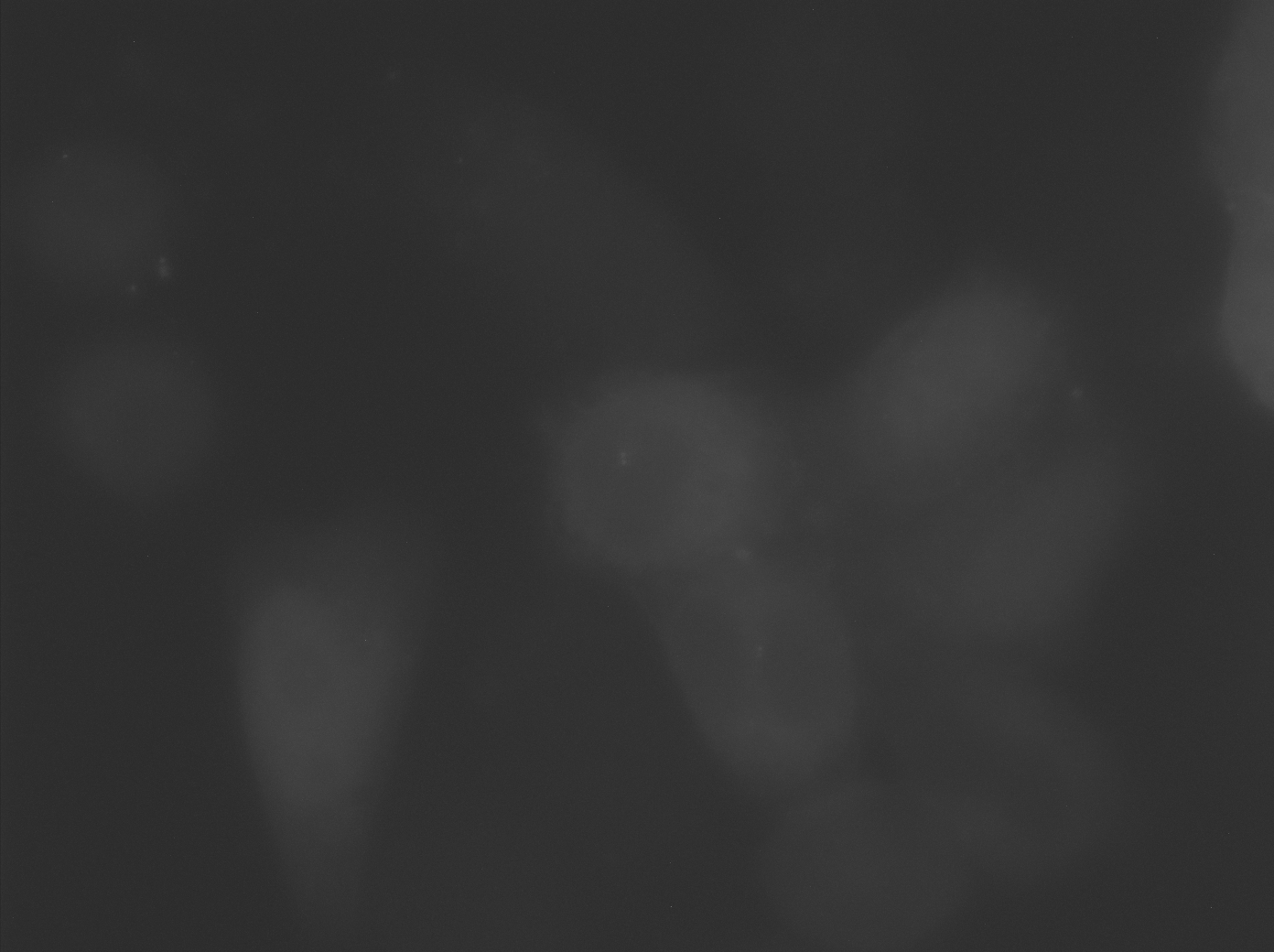

Supplement: Supplementary file 11 — Source data Fig. 3 part 1 [file 44319_2026_742_MOESM11_ESM.zip › Figure 3 Part 1/Fig 3b-e TTLL screen/TTLL1-GFP A4 I9.Project Maximum Z_XY1675963165_Z0_T0_C1.tif]

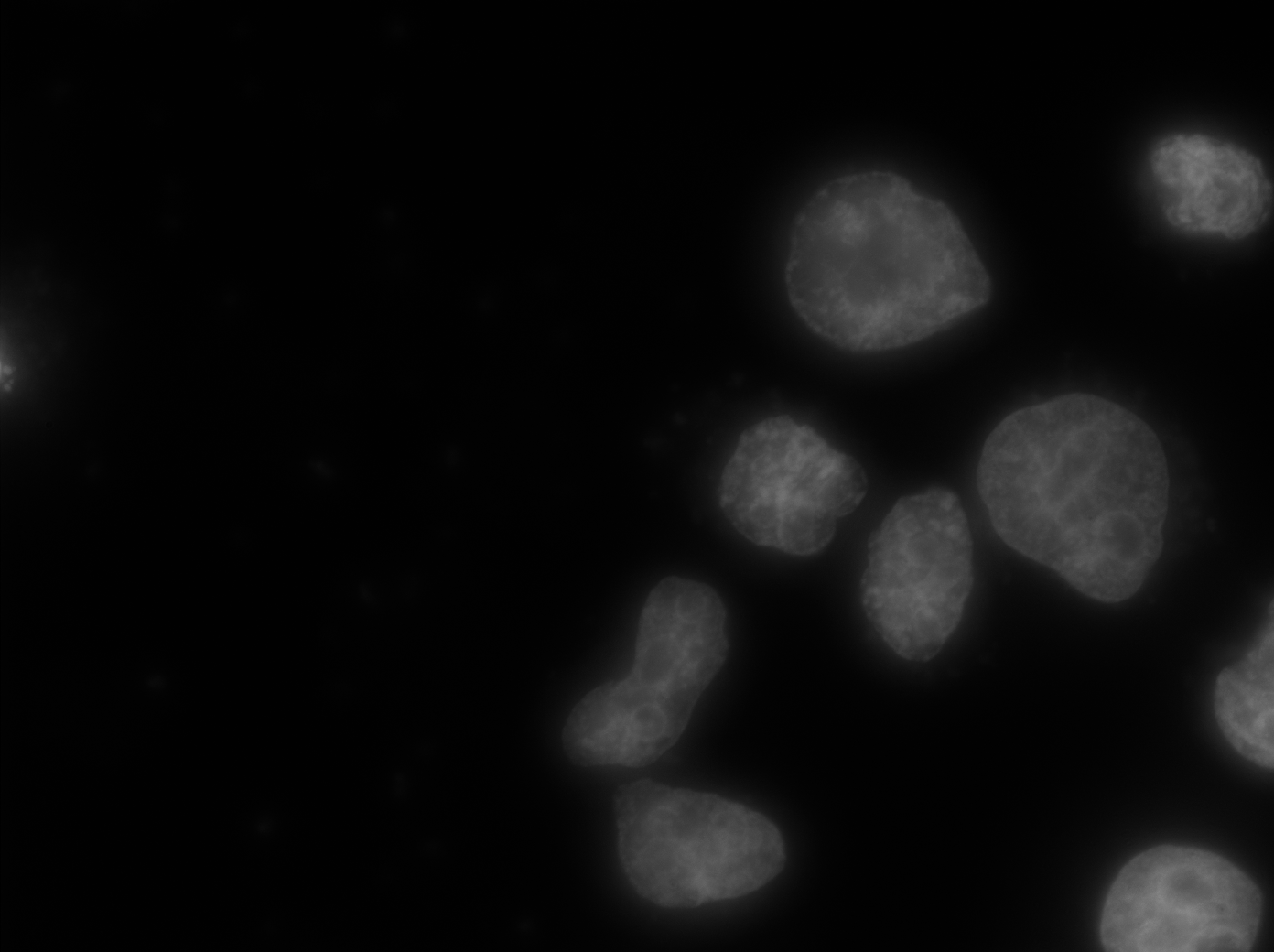

Supplement: Supplementary file 11 — Source data Fig. 3 part 1 [file 44319_2026_742_MOESM11_ESM.zip › Figure 3 Part 1/Fig 3b-e TTLL screen/TTLL1-GFPy I2.Project Maximum Z_XY1679086410_Z0_T0_C0.tif]

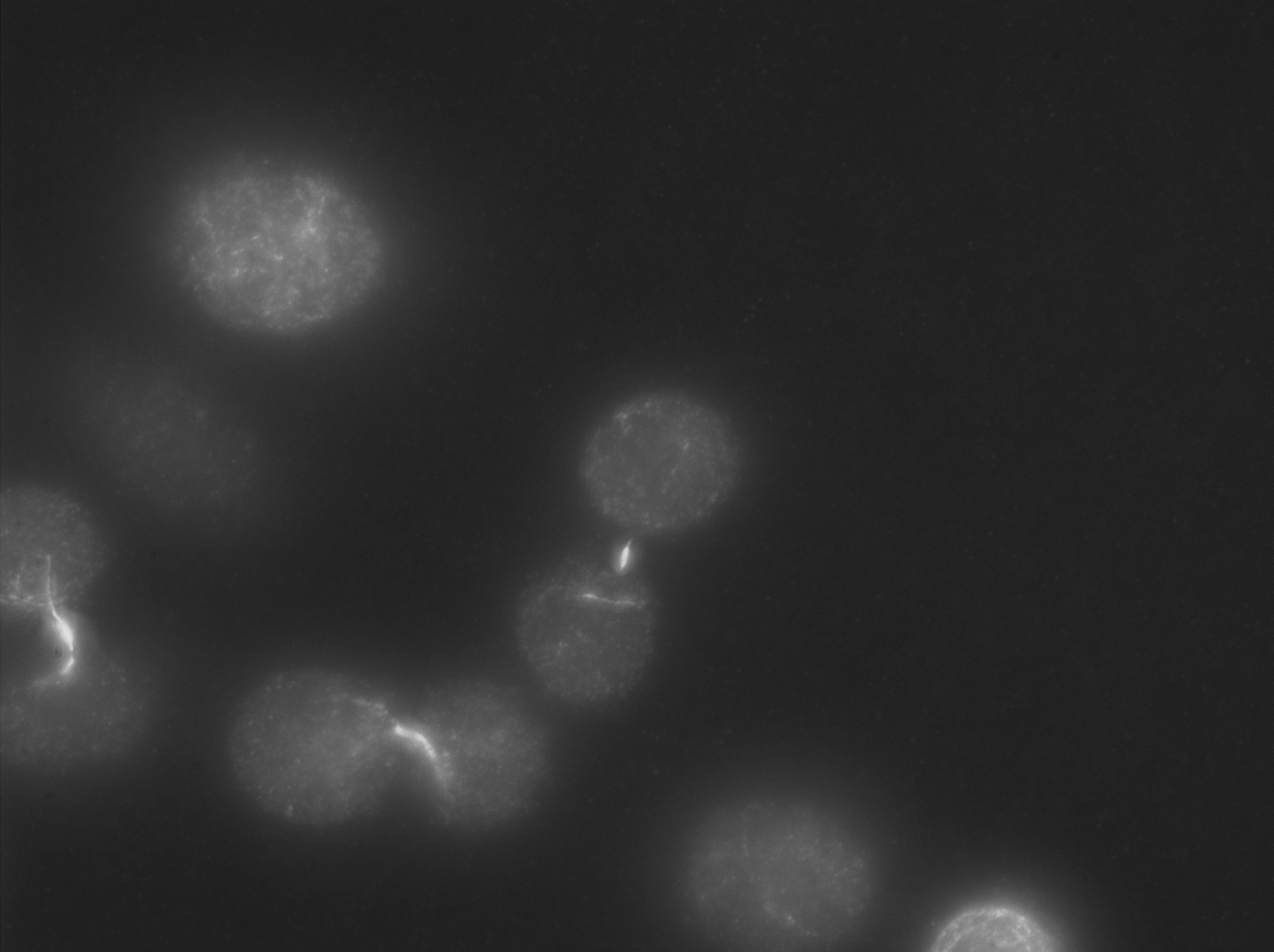

Supplement: Supplementary file 11 — Source data Fig. 3 part 1 [file 44319_2026_742_MOESM11_ESM.zip › Figure 3 Part 1/Fig 3b-e TTLL screen/TTLL4-YFPy I13.Project Maximum Z_XY1679337113_Z0_T0_C1.tif]

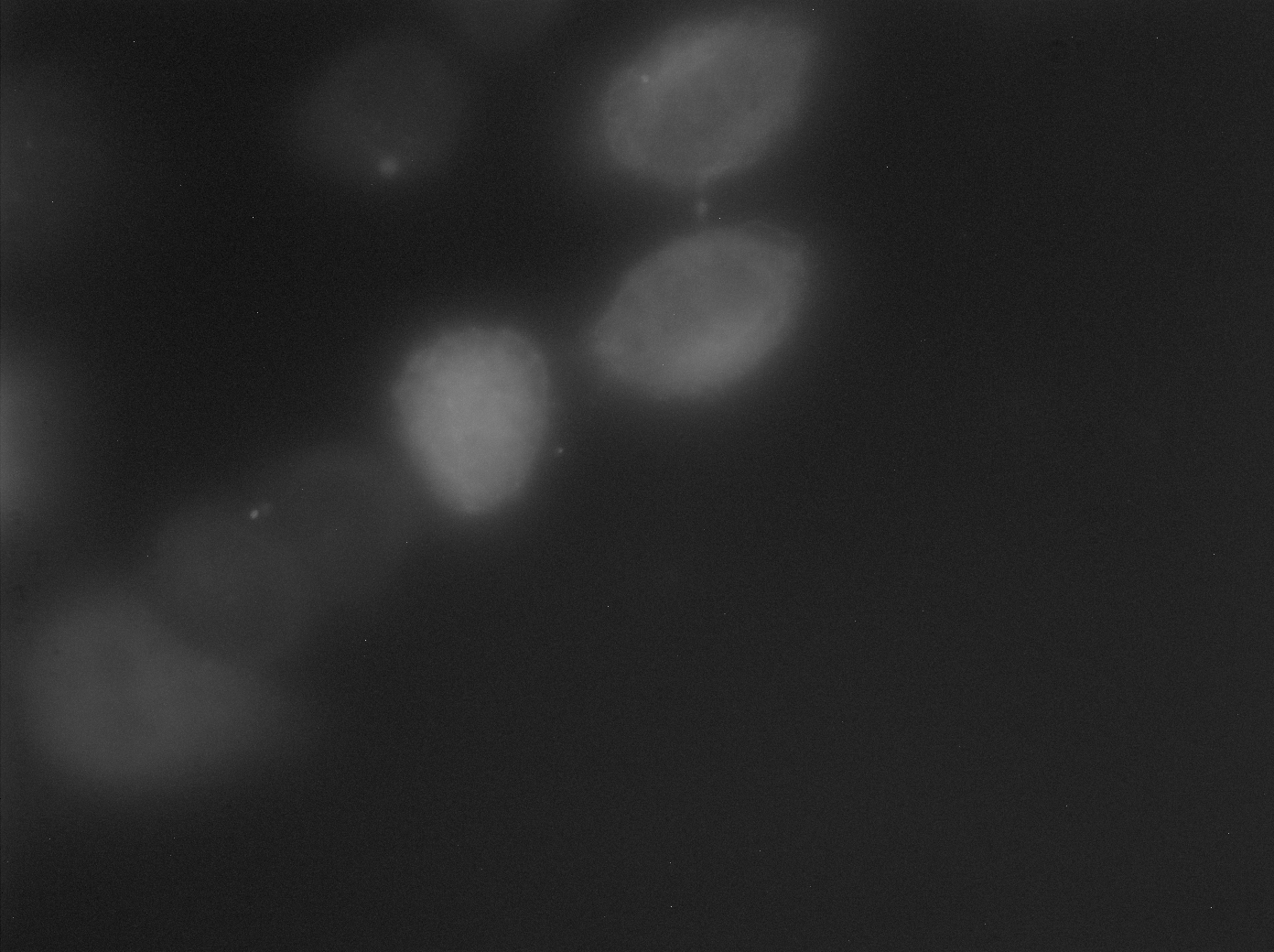

Supplement: Supplementary file 11 — Source data Fig. 3 part 1 [file 44319_2026_742_MOESM11_ESM.zip › Figure 3 Part 1/Fig 3b-e TTLL screen/TTLL1-GFP A3 I17.Project Maximum Z_XY1679697836_Z0_T0_C1.tif]

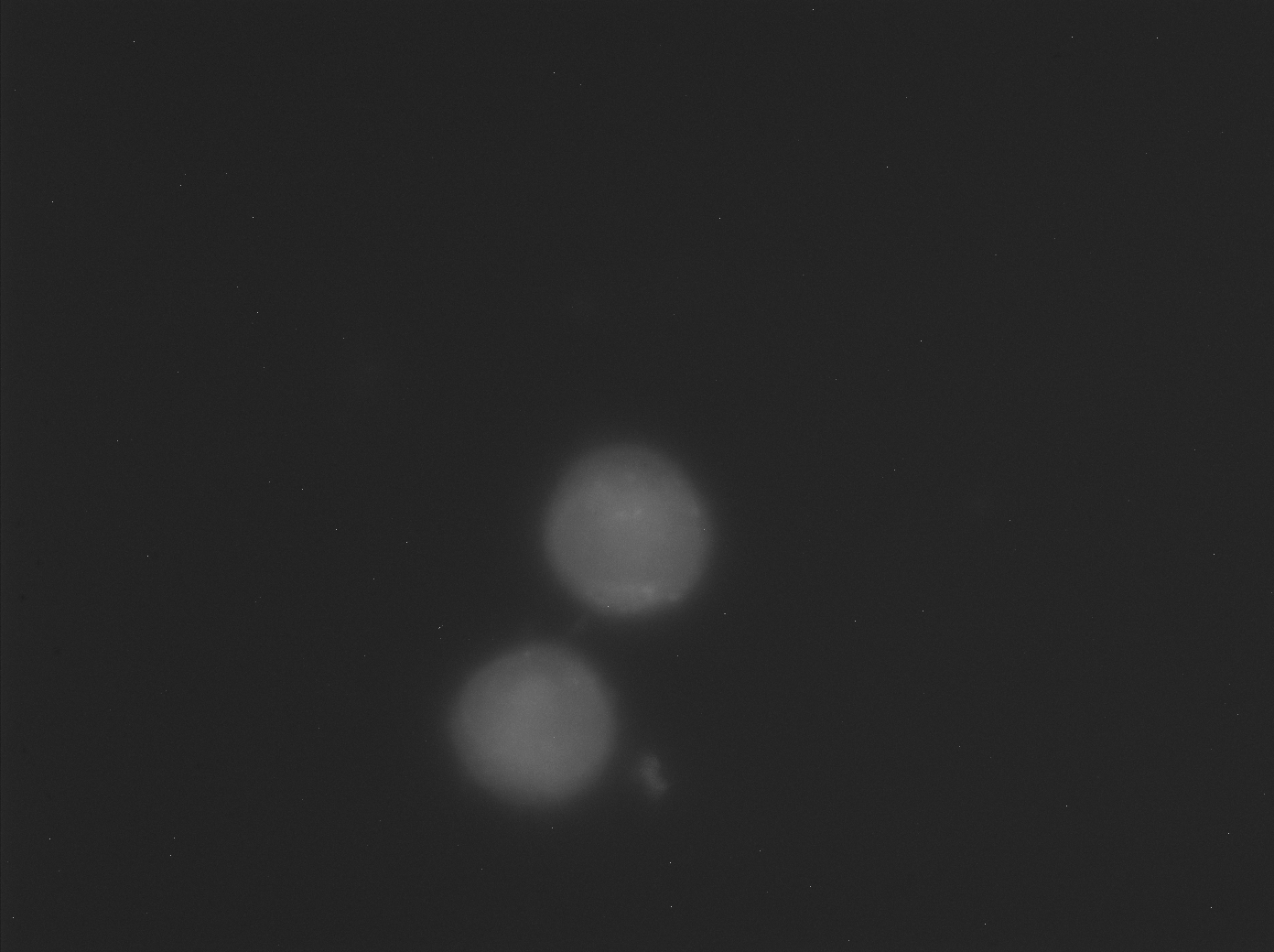

Supplement: Supplementary file 12 — Source data Fig. 3 part 2 [file 44319_2026_742_MOESM12_ESM.zip › Figure 3 Part 2/Fig 3b-e TTLL screen part 2/TTLL6-YFP R1 I3.Project Maximum Z_XY1663277237_Z0_T0_C2.tif]

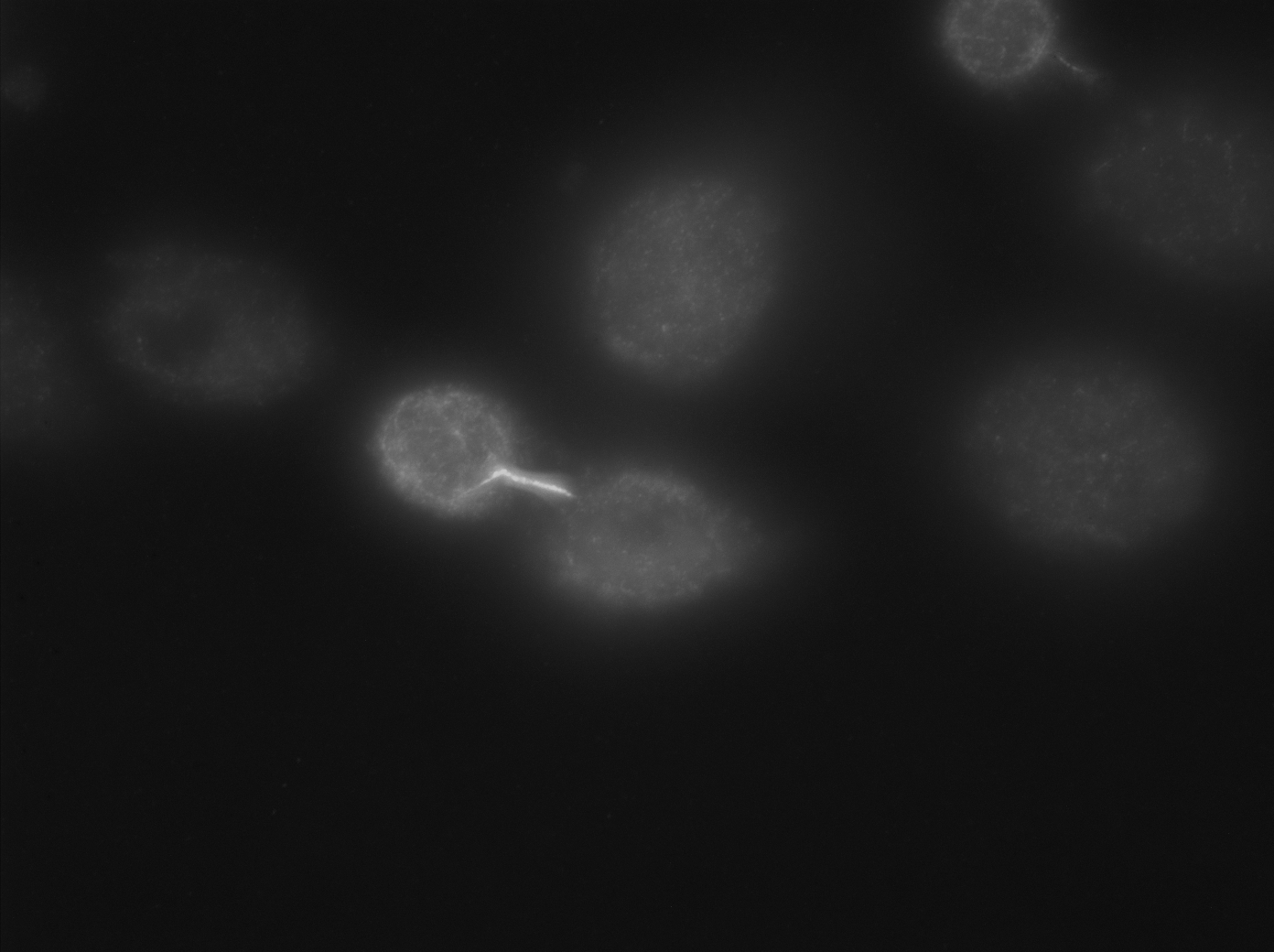

Supplement: Supplementary file 12 — Source data Fig. 3 part 2 [file 44319_2026_742_MOESM12_ESM.zip › Figure 3 Part 2/Fig 3b-e TTLL screen part 2/TTLL7-YFPy I12.Project Maximum Z_XY1679089567_Z0_T0_C1.tif]

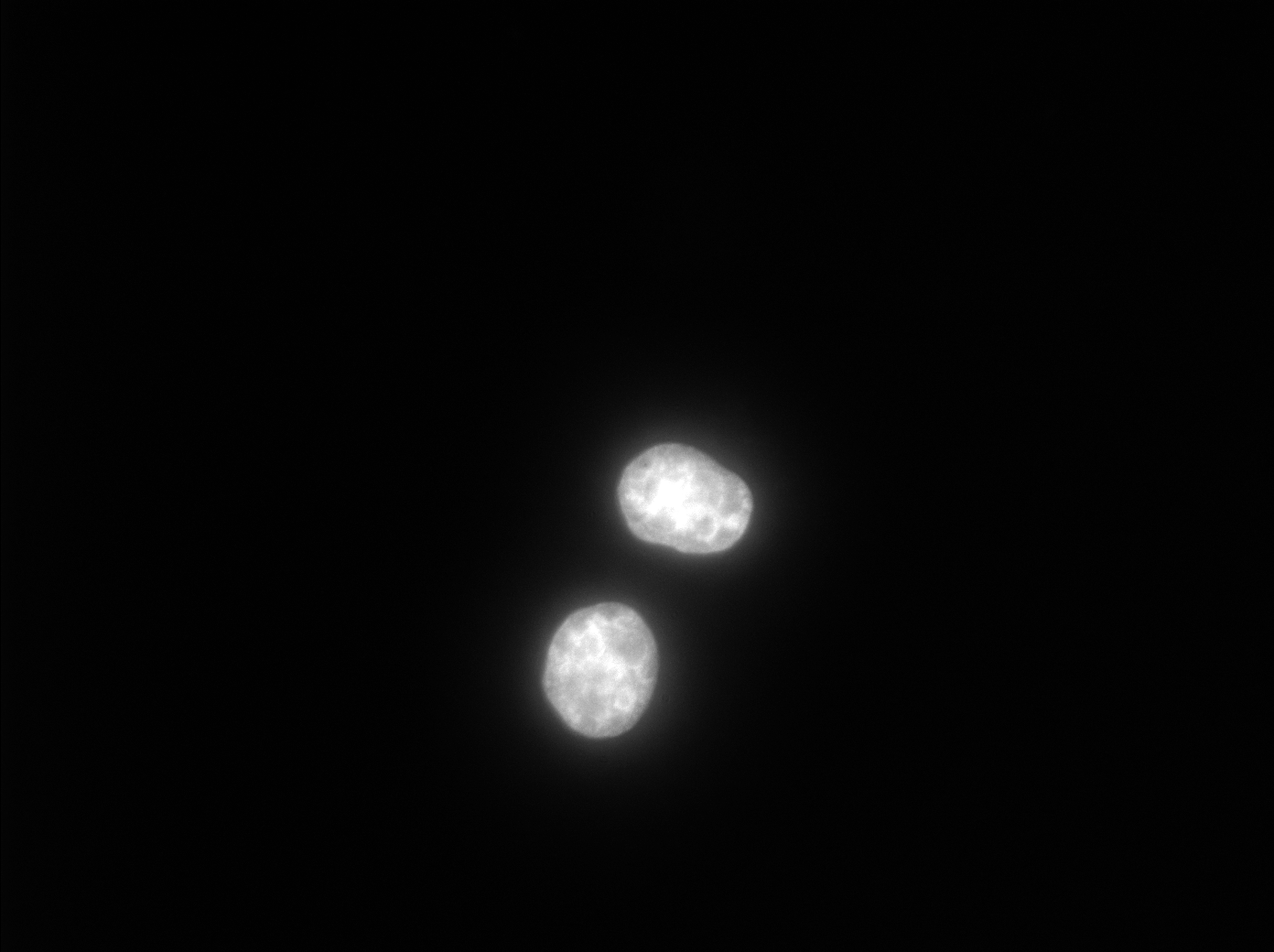

Supplement: Supplementary file 12 — Source data Fig. 3 part 2 [file 44319_2026_742_MOESM12_ESM.zip › Figure 3 Part 2/Fig 3b-e TTLL screen part 2/TTLL6-YFP R1 I5 low int.Project Maximum Z_XY1661548370_Z0_T0_C0.tif]

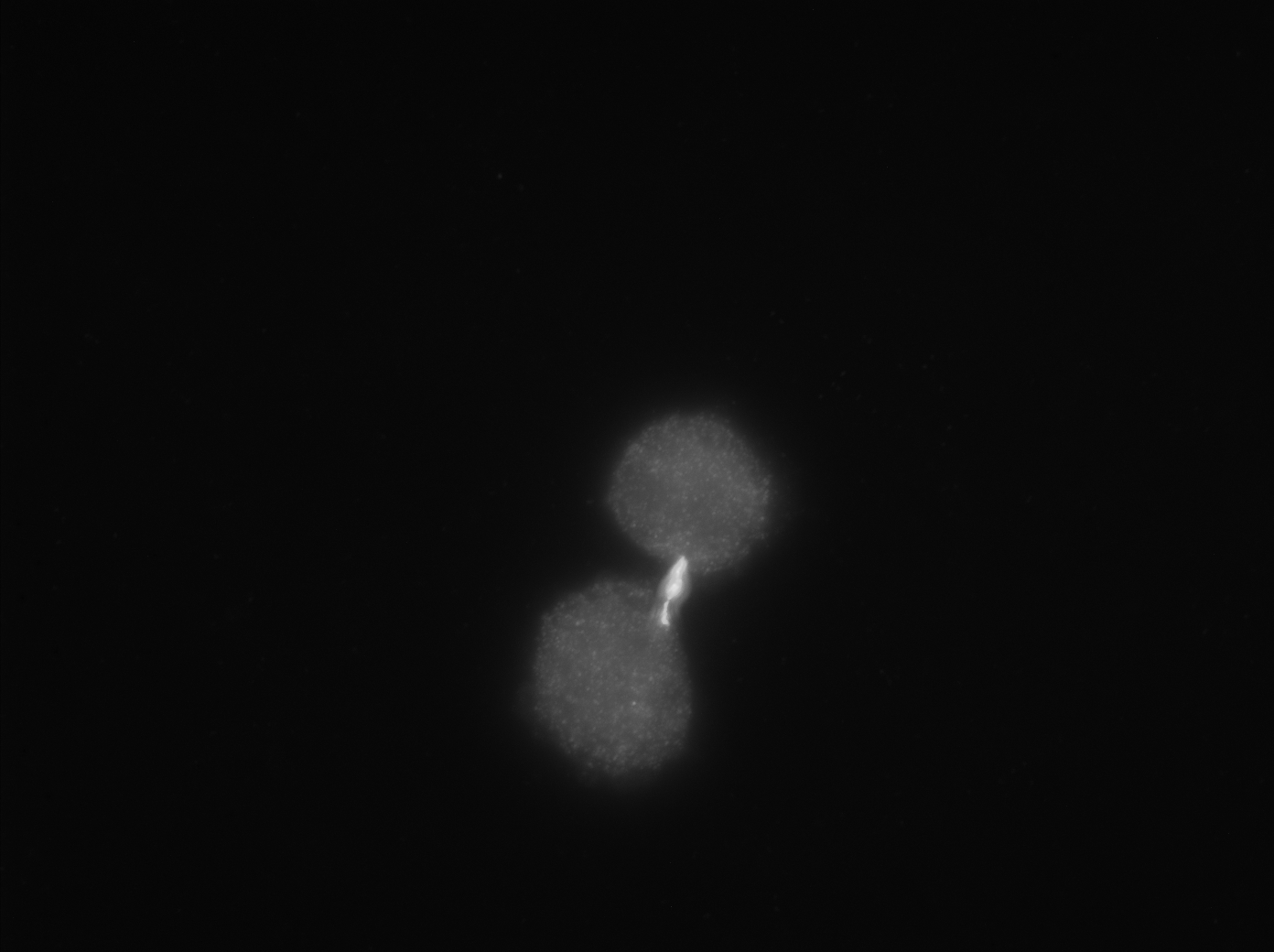

Supplement: Supplementary file 12 — Source data Fig. 3 part 2 [file 44319_2026_742_MOESM12_ESM.zip › Figure 3 Part 2/Fig 3b-e TTLL screen part 2/TTLL6-YFP R1 I5 low int.Project Maximum Z_XY1661548370_Z0_T0_C1.tif]

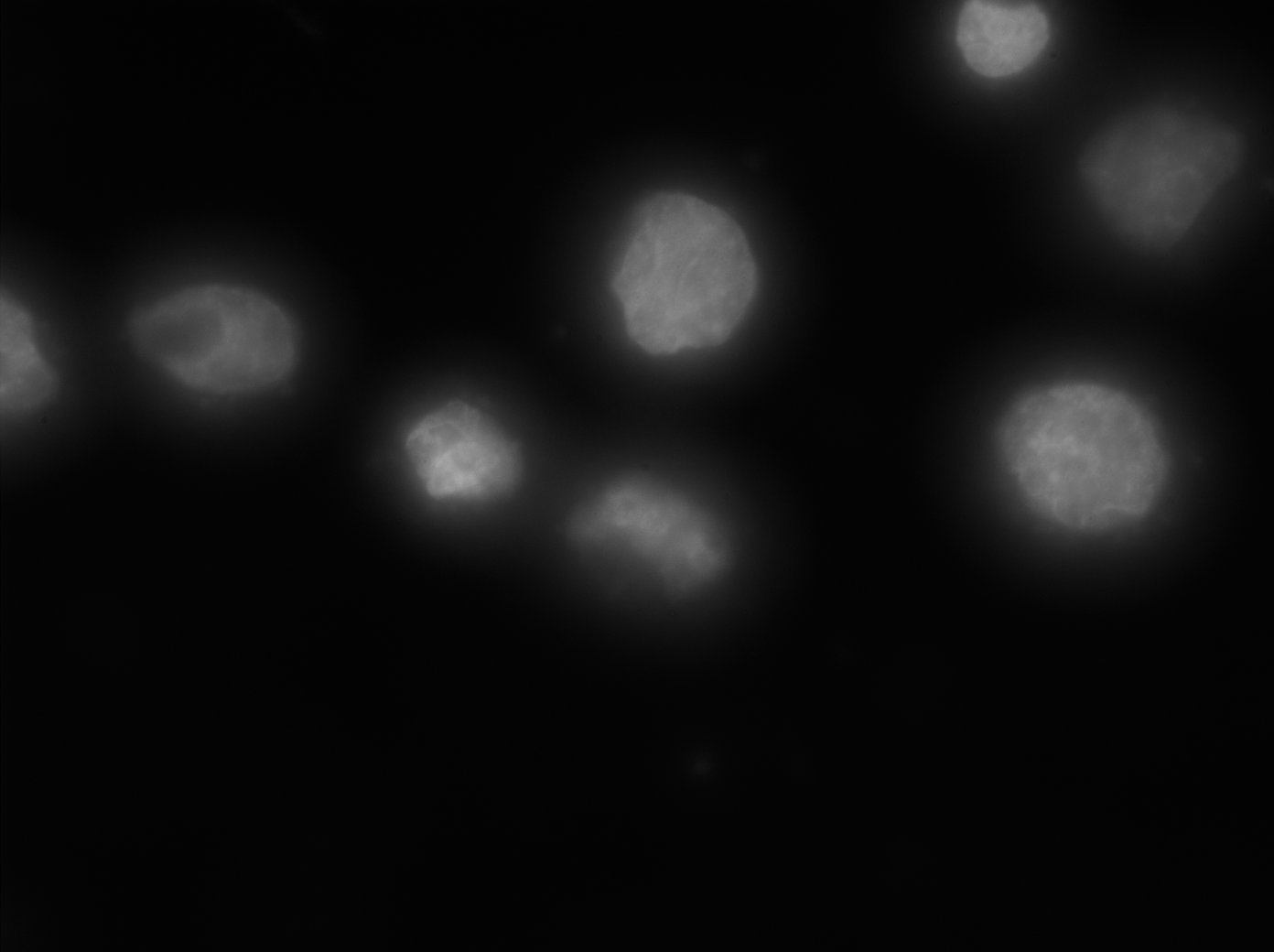

Supplement: Supplementary file 12 — Source data Fig. 3 part 2 [file 44319_2026_742_MOESM12_ESM.zip › Figure 3 Part 2/Fig 3b-e TTLL screen part 2/TTLL7-YFPy I12.Project Maximum Z_XY1679089567_Z0_T0_C0.tif]

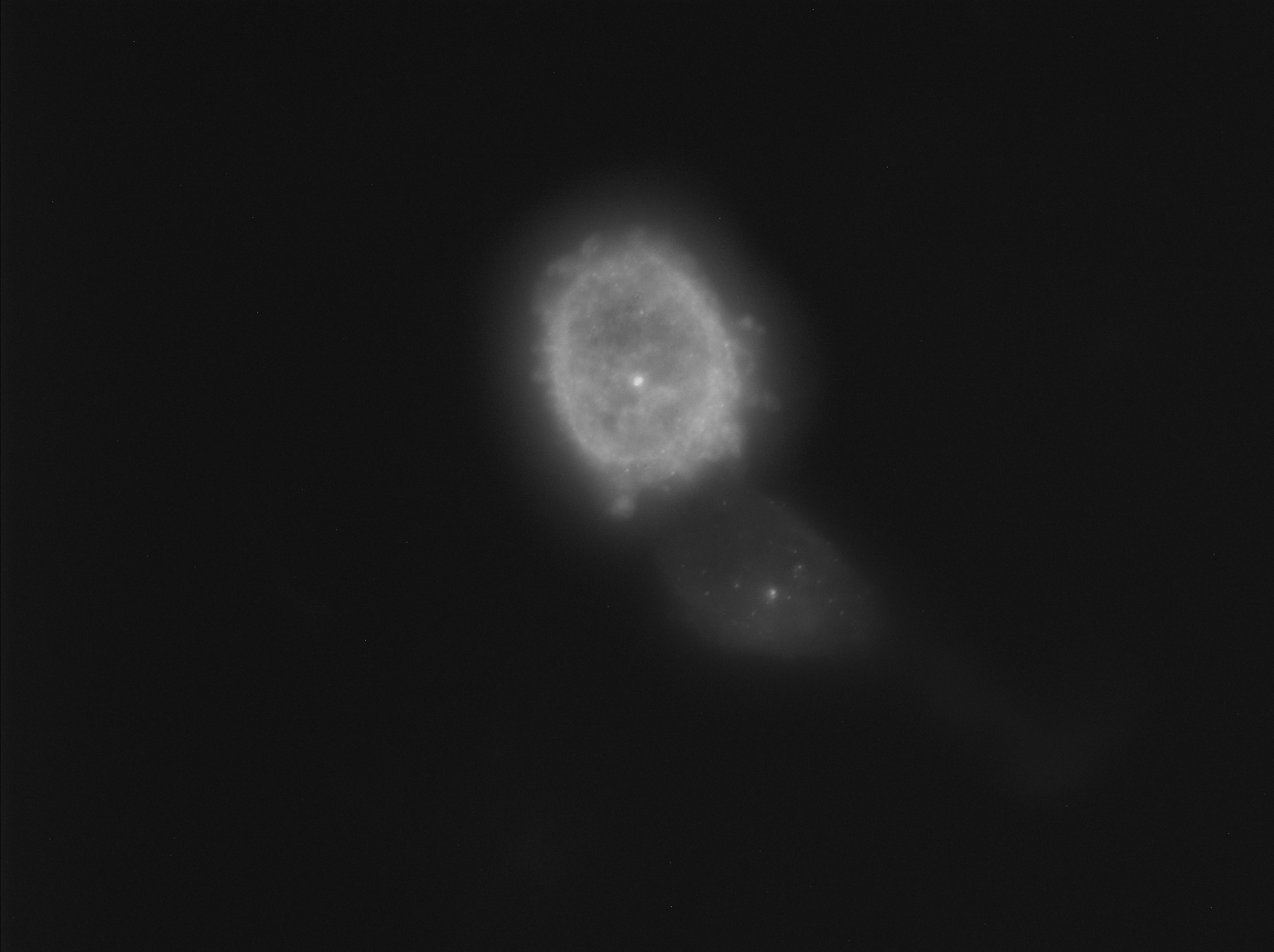

Supplement: Supplementary file 12 — Source data Fig. 3 part 2 [file 44319_2026_742_MOESM12_ESM.zip › Figure 3 Part 2/Fig 3b-e TTLL screen part 2/TTLL5-YFPy I19.Project Maximum Z_XY1679341498_Z0_T0_C2.tif]

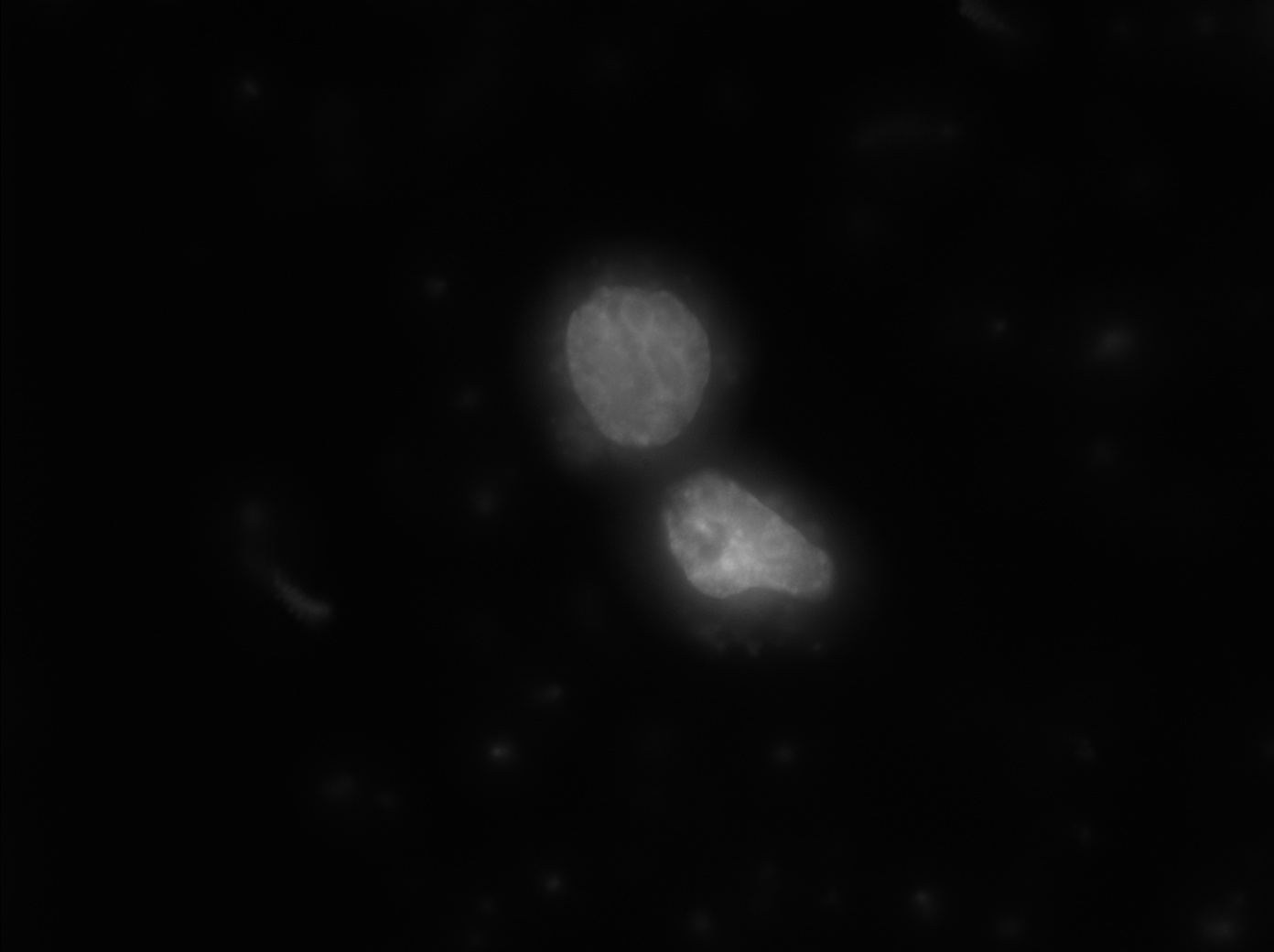

Supplement: Supplementary file 12 — Source data Fig. 3 part 2 [file 44319_2026_742_MOESM12_ESM.zip › Figure 3 Part 2/Fig 3b-e TTLL screen part 2/TTLL5-YFPy I19.Project Maximum Z_XY1679341498_Z0_T0_C0.tif]

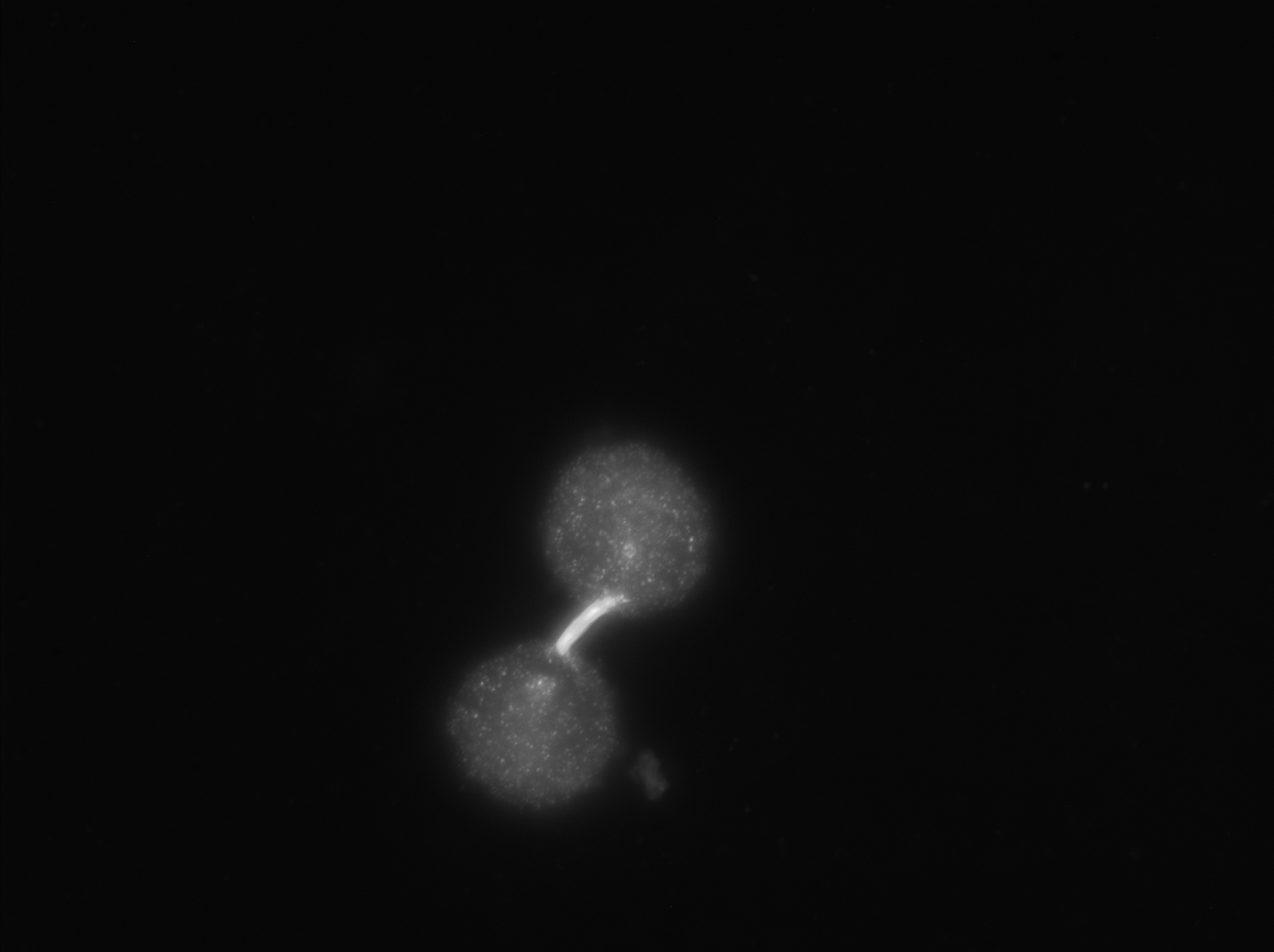

Supplement: Supplementary file 12 — Source data Fig. 3 part 2 [file 44319_2026_742_MOESM12_ESM.zip › Figure 3 Part 2/Fig 3b-e TTLL screen part 2/TTLL6-YFP R1 I3.Project Maximum Z_XY1663277237_Z0_T0_C1.tif]

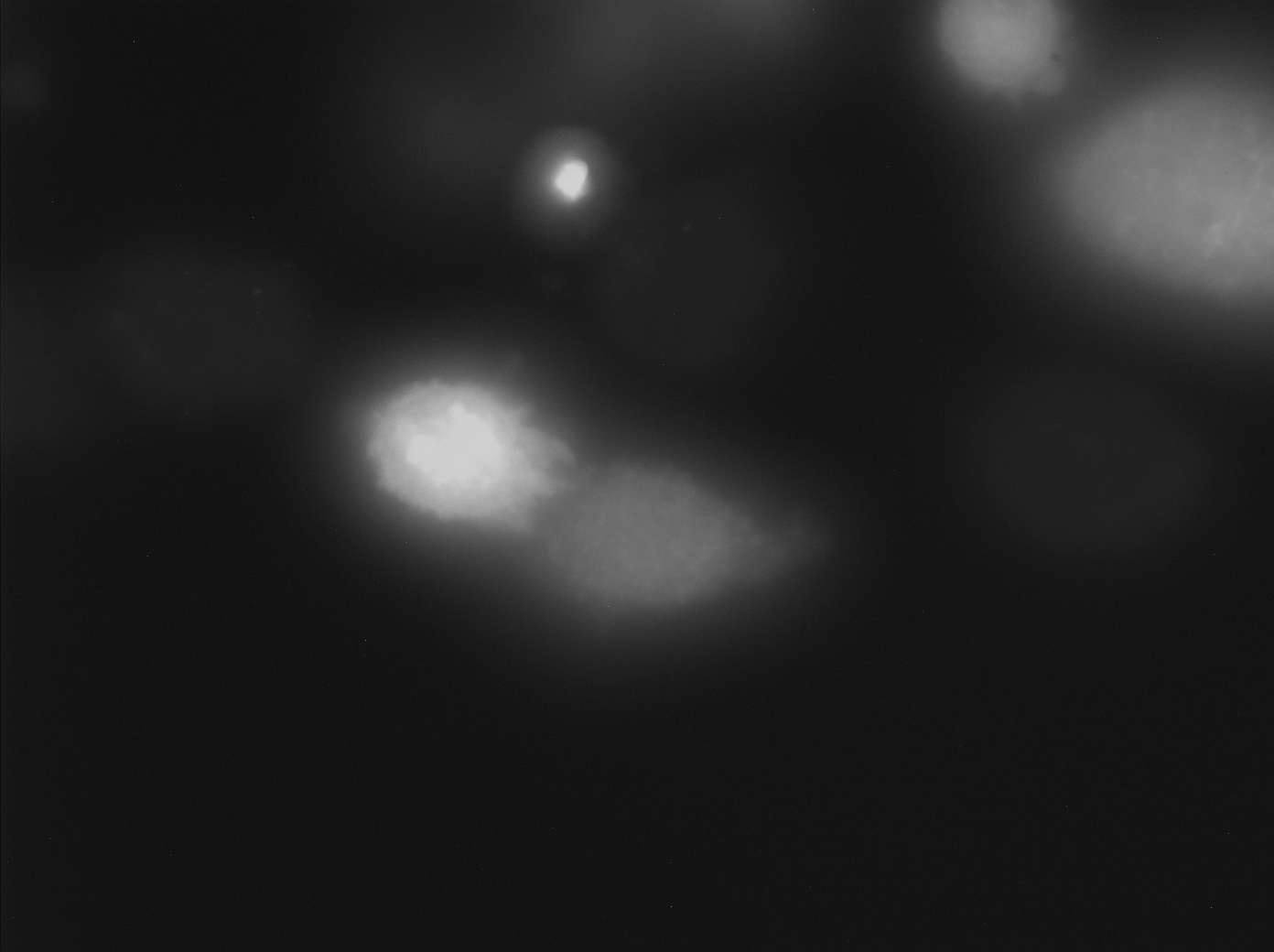

Supplement: Supplementary file 12 — Source data Fig. 3 part 2 [file 44319_2026_742_MOESM12_ESM.zip › Figure 3 Part 2/Fig 3b-e TTLL screen part 2/TTLL7-YFPy I12.Project Maximum Z_XY1679089567_Z0_T0_C2.tif]

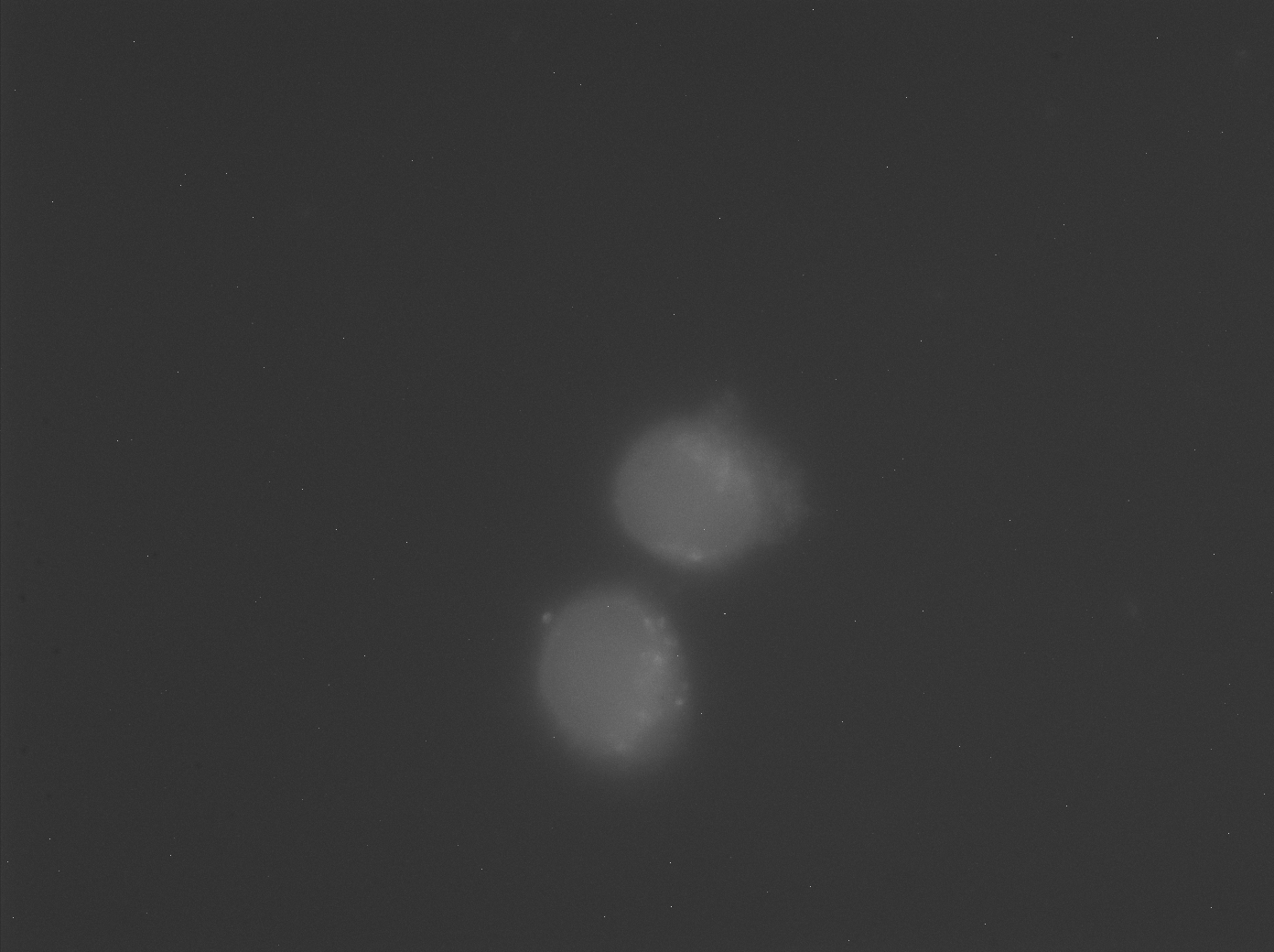

Supplement: Supplementary file 12 — Source data Fig. 3 part 2 [file 44319_2026_742_MOESM12_ESM.zip › Figure 3 Part 2/Fig 3b-e TTLL screen part 2/TTLL6-YFP R1 I5 low int.Project Maximum Z_XY1661548370_Z0_T0_C2.tif]

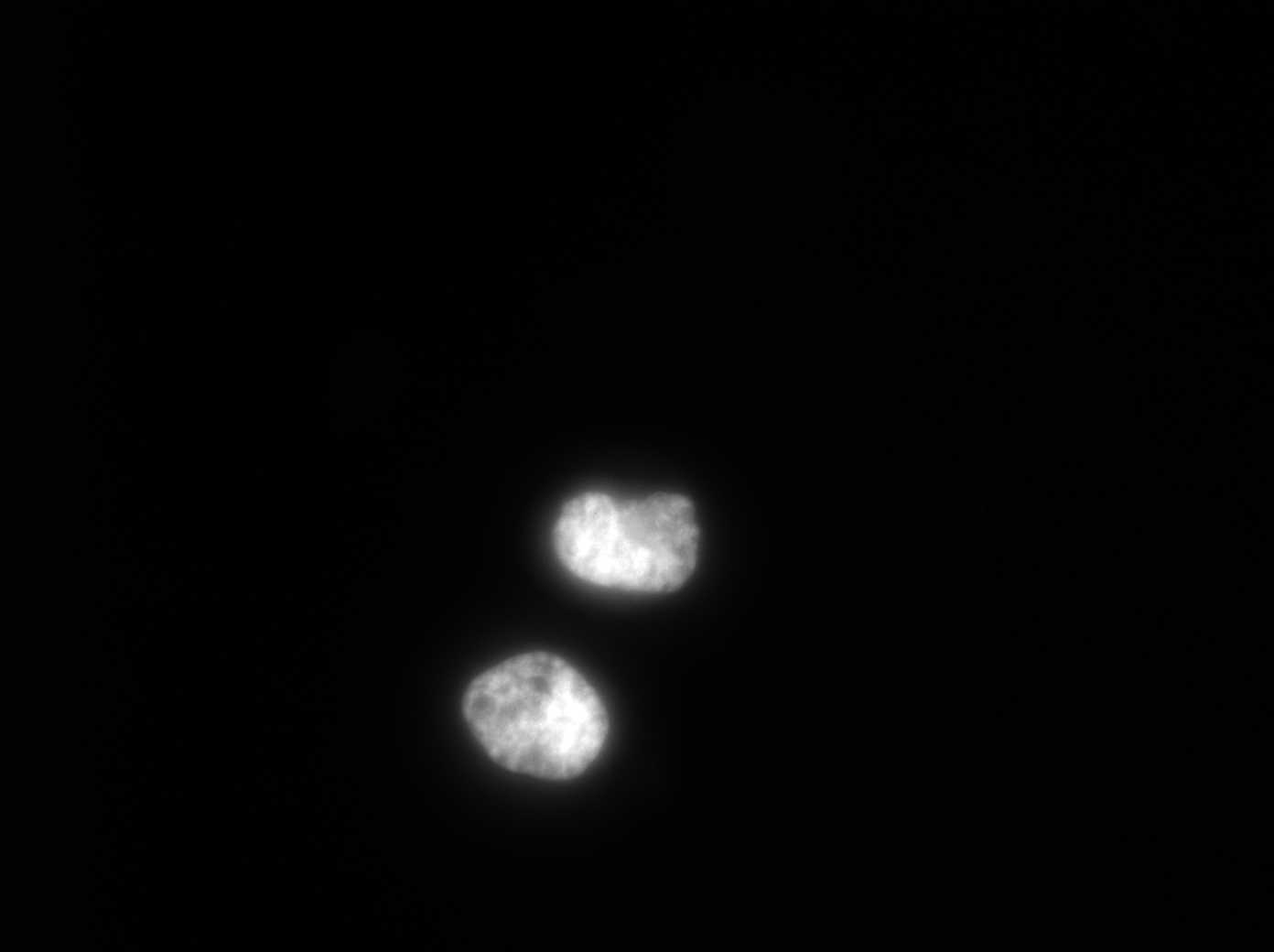

Supplement: Supplementary file 12 — Source data Fig. 3 part 2 [file 44319_2026_742_MOESM12_ESM.zip › Figure 3 Part 2/Fig 3b-e TTLL screen part 2/TTLL6-YFP R1 I3.Project Maximum Z_XY1663277237_Z0_T0_C0.tif]

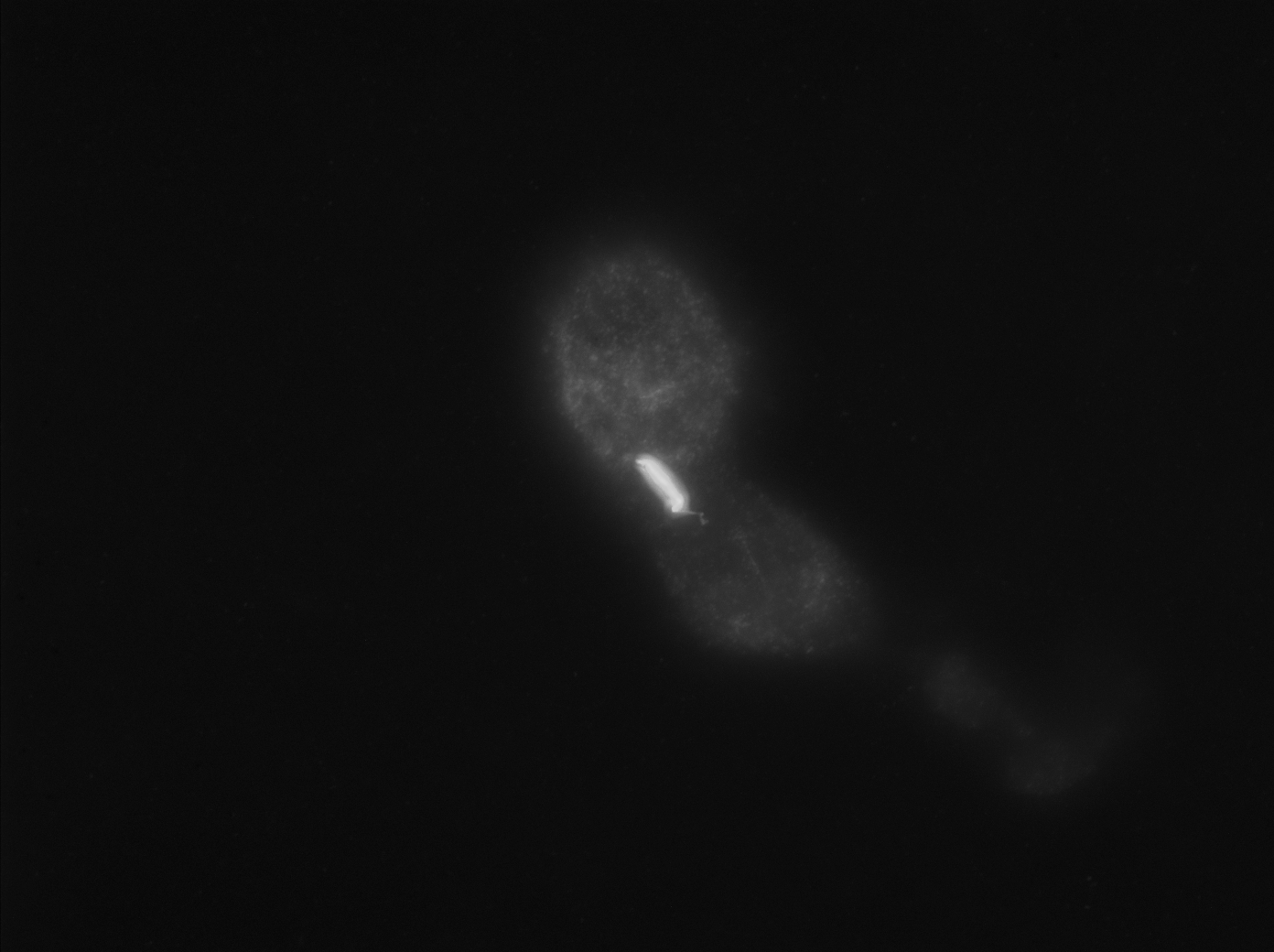

Supplement: Supplementary file 12 — Source data Fig. 3 part 2 [file 44319_2026_742_MOESM12_ESM.zip › Figure 3 Part 2/Fig 3b-e TTLL screen part 2/TTLL5-YFPy I19.Project Maximum Z_XY1679341498_Z0_T0_C1.tif]

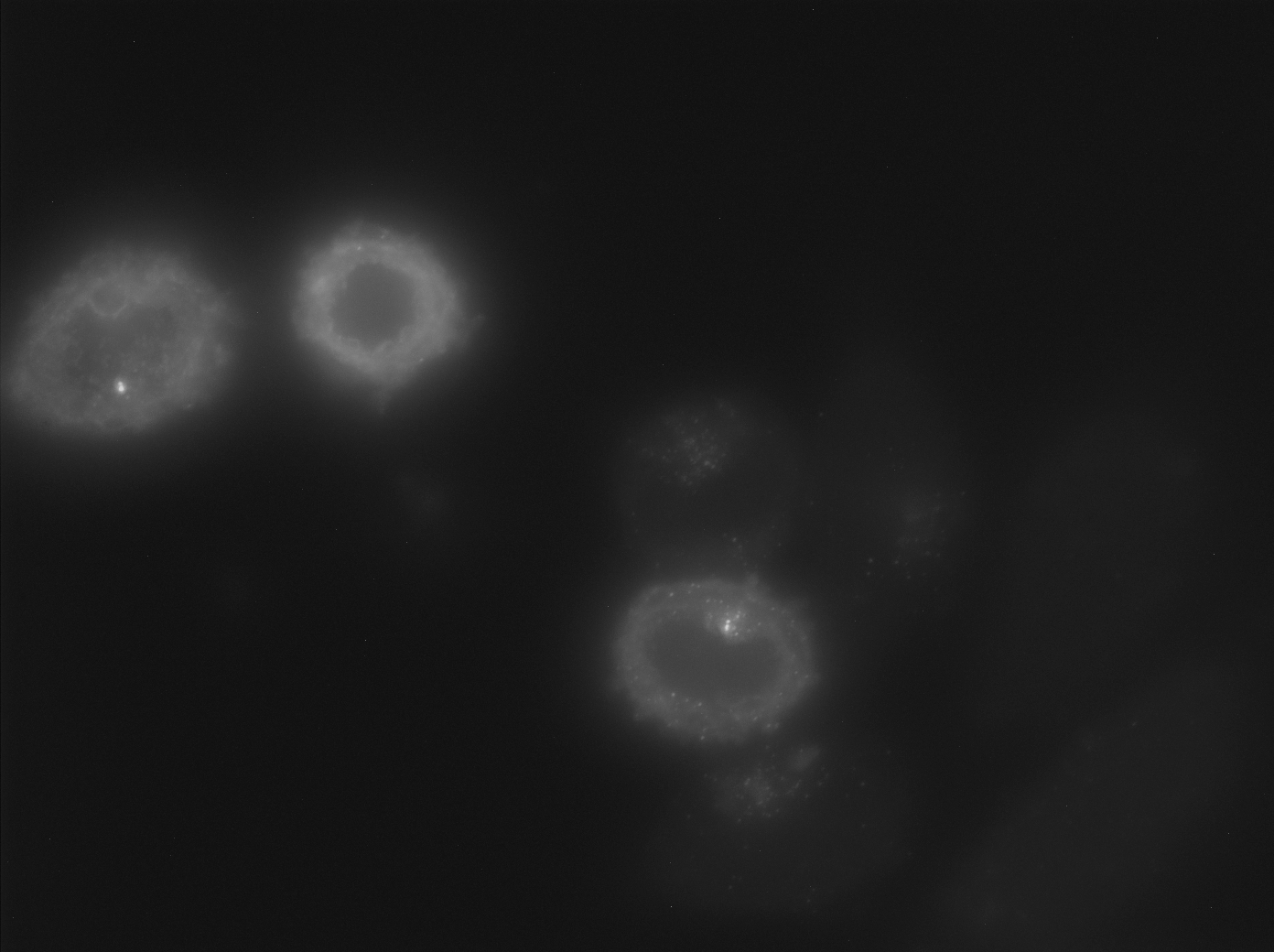

Supplement: Supplementary file 12 — Source data Fig. 3 part 2 [file 44319_2026_742_MOESM12_ESM.zip › Figure 3 Part 2/Fig 3b-e TTLL screen part 2/TTLL5-YFPy I6.Project Maximum Z_XY1679084596_Z0_T0_C2.tif]

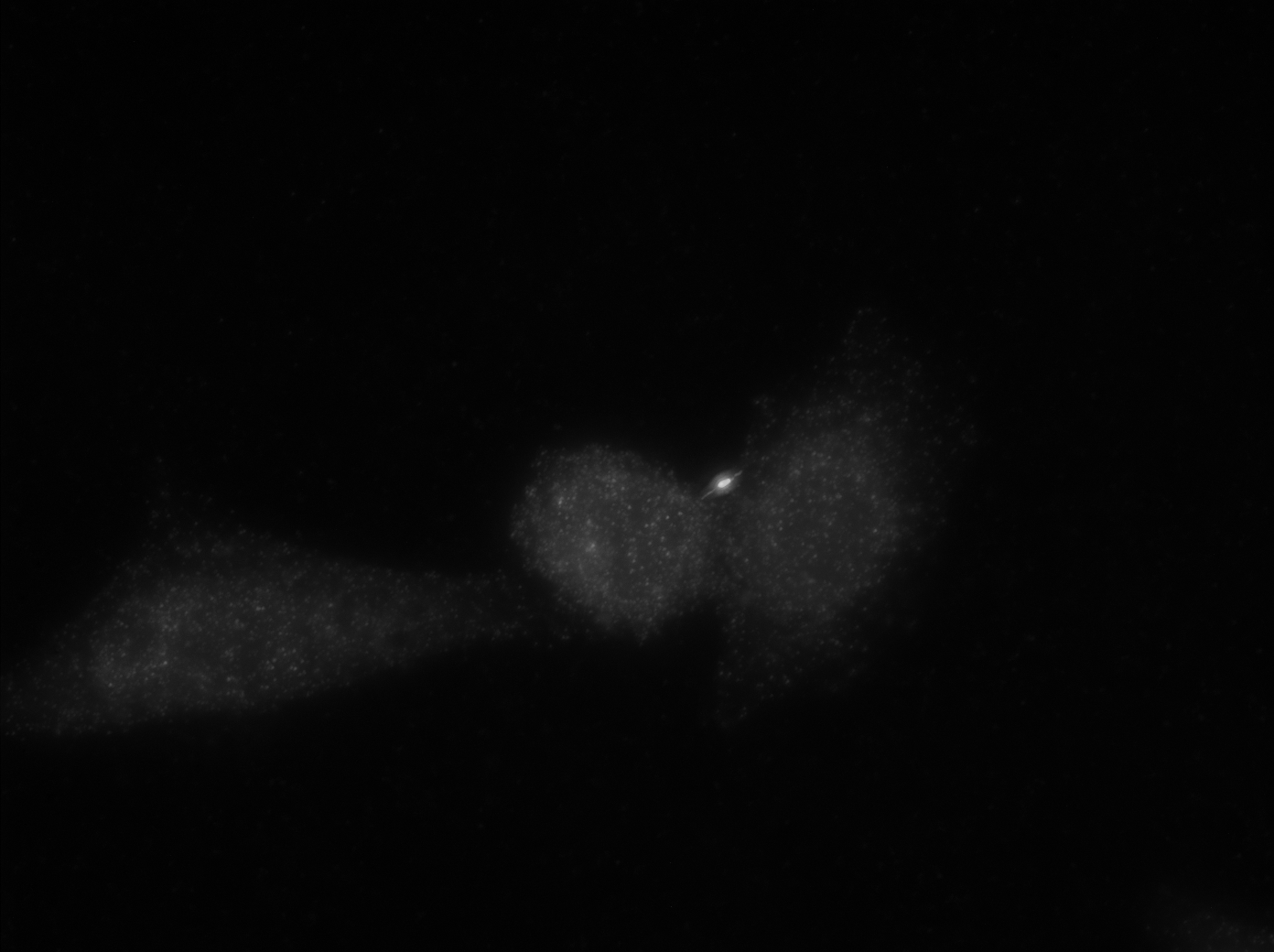

Supplement: Supplementary file 12 — Source data Fig. 3 part 2 [file 44319_2026_742_MOESM12_ESM.zip › Figure 3 Part 2/Fig 3b-e TTLL screen part 2/TTLL6-YFP R1 I2 C2 med.Project Maximum Z_XY1661791814_Z0_T0_C1.tif]

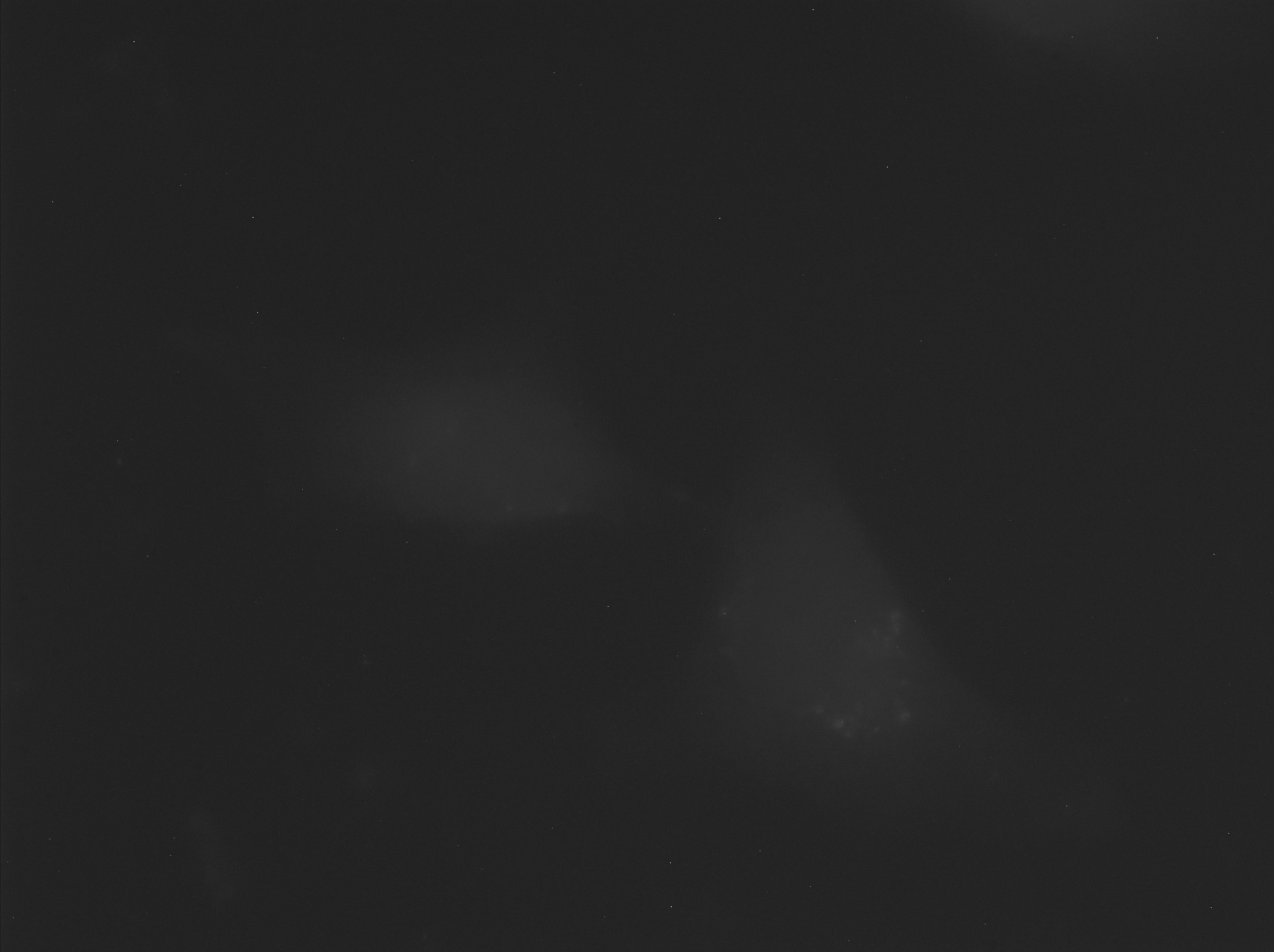

Supplement: Supplementary file 12 — Source data Fig. 3 part 2 [file 44319_2026_742_MOESM12_ESM.zip › Figure 3 Part 2/Fig 3b-e TTLL screen part 2/TTLL6-YFP MB light I1.Project Maximum Z_XY1663880365_Z0_T0_C2.tif]

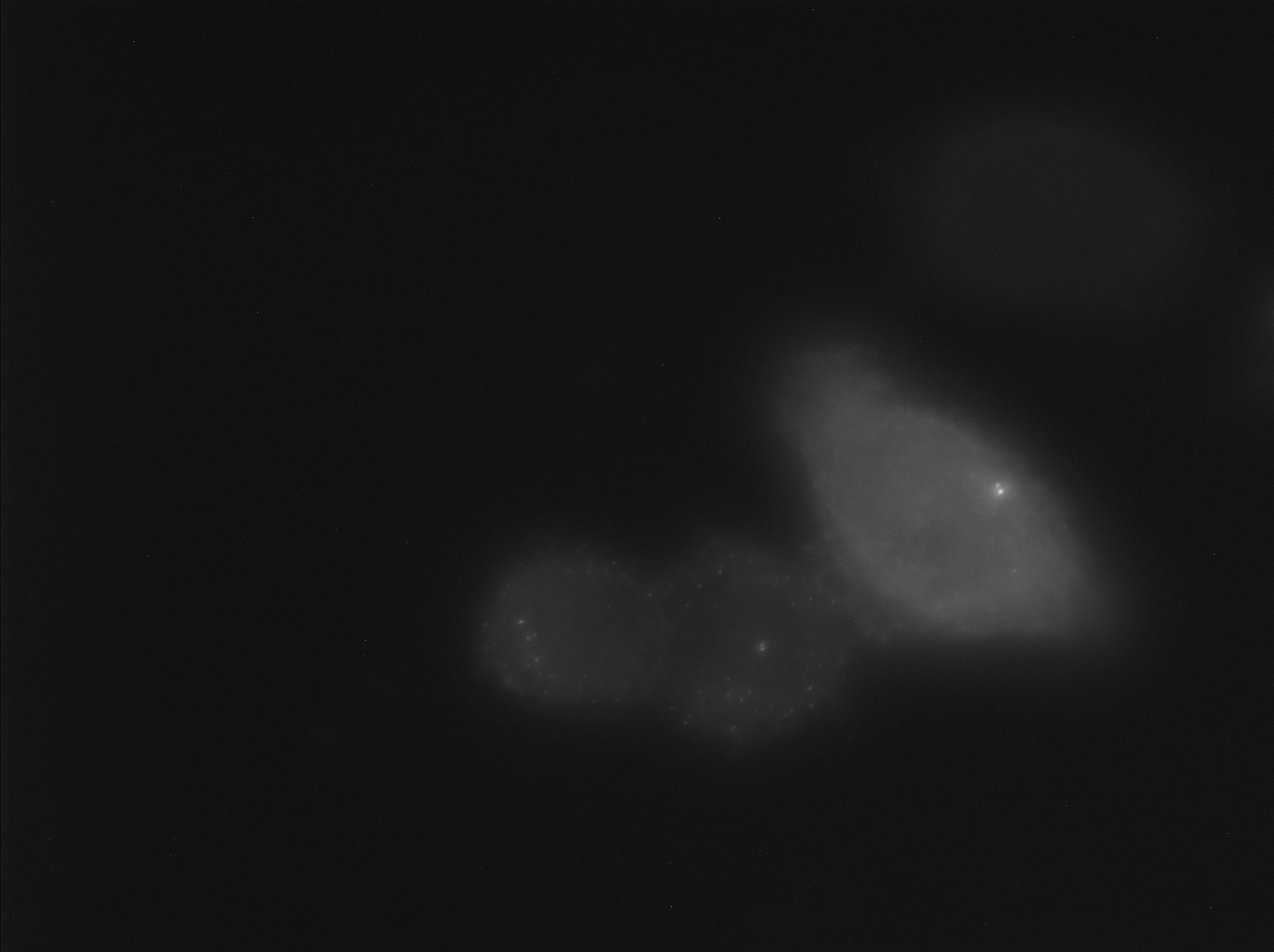

Supplement: Supplementary file 12 — Source data Fig. 3 part 2 [file 44319_2026_742_MOESM12_ESM.zip › Figure 3 Part 2/Fig 3b-e TTLL screen part 2/TTLL5-YFPy I8.Project Maximum Z_XY1679085074_Z0_T0_C2.tif]

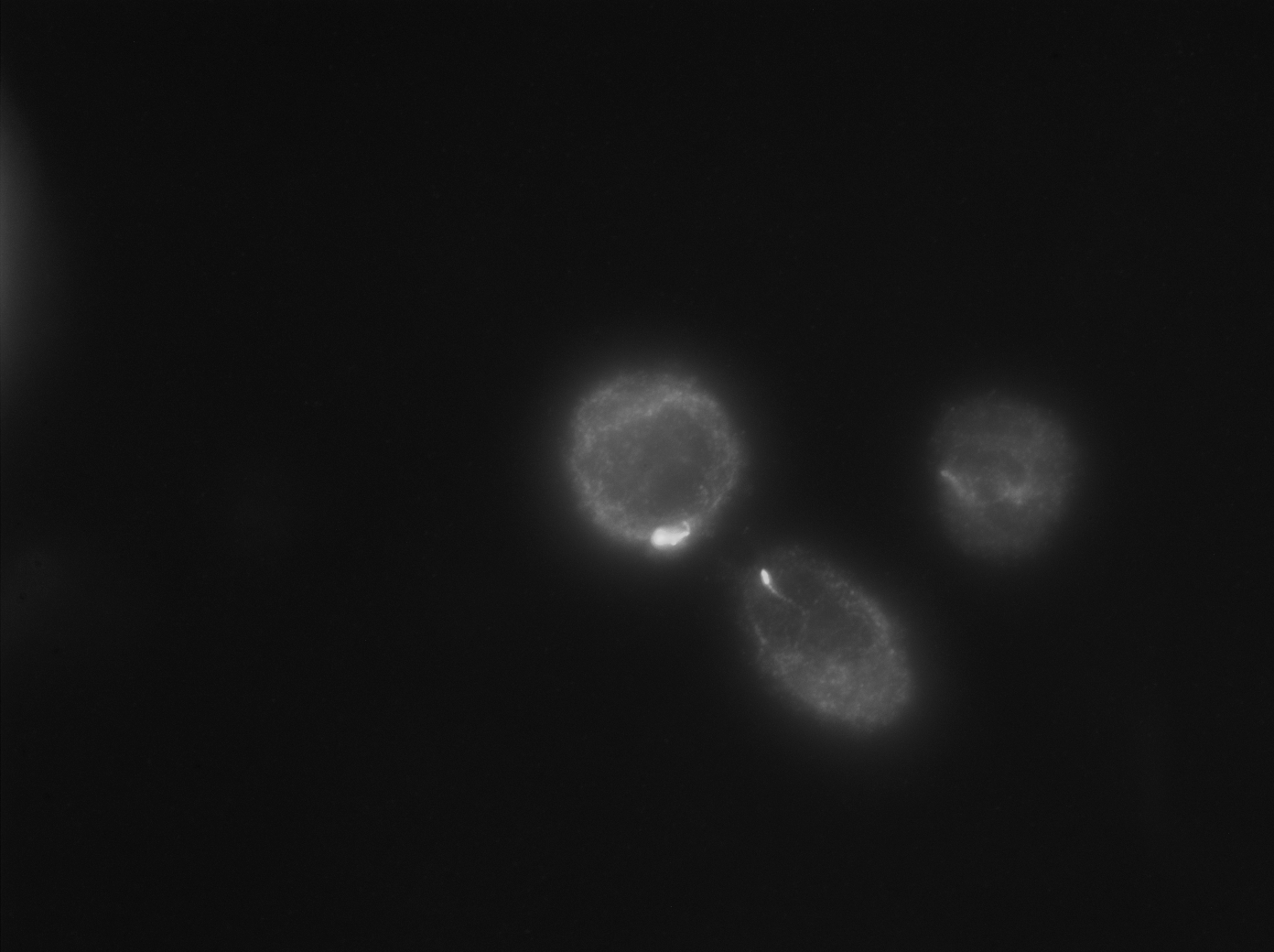

Supplement: Supplementary file 12 — Source data Fig. 3 part 2 [file 44319_2026_742_MOESM12_ESM.zip › Figure 3 Part 2/Fig 3b-e TTLL screen part 2/TTLL5-YFPy I5.Project Maximum Z_XY1679084454_Z0_T0_C1.tif]

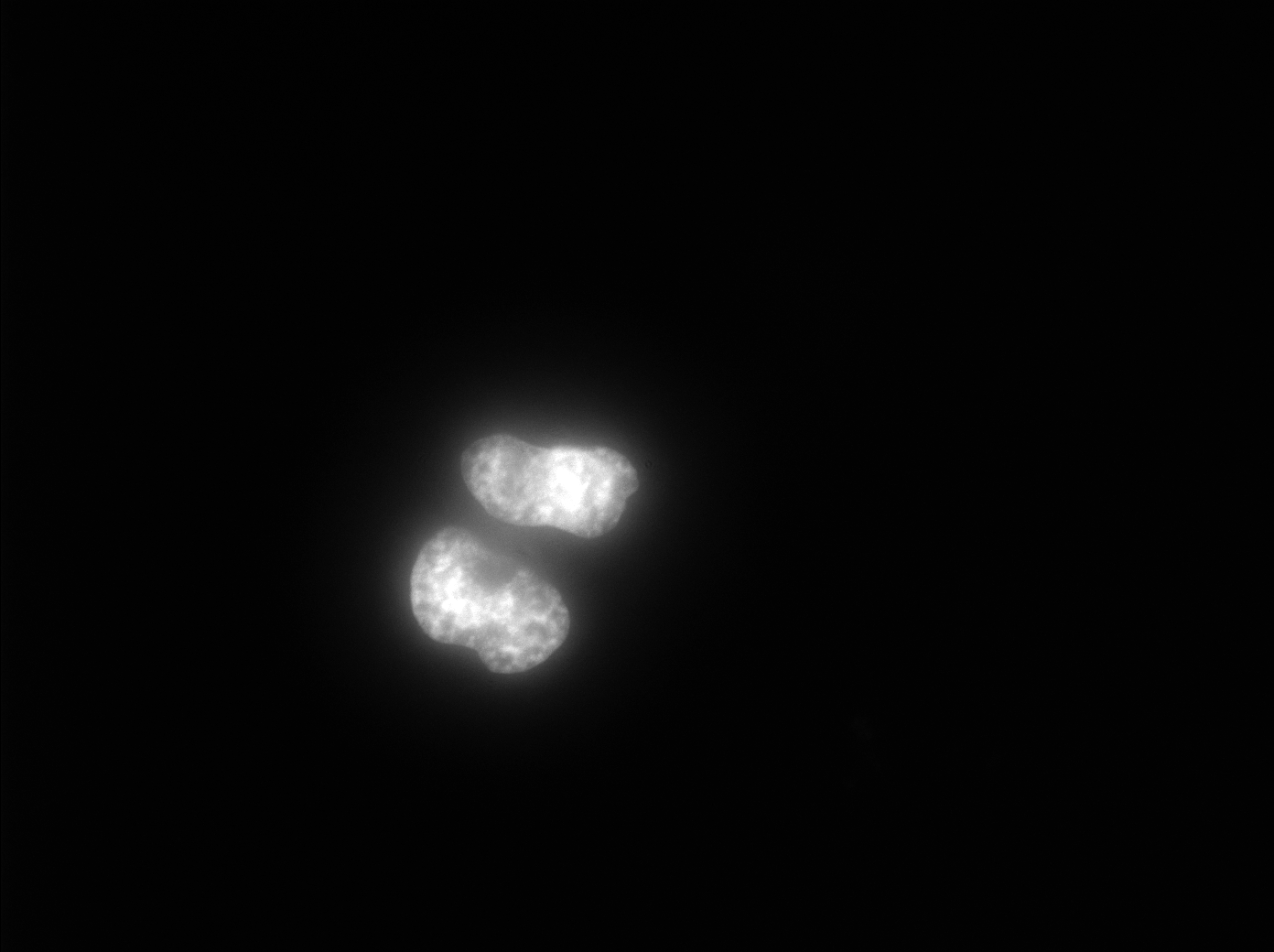

Supplement: Supplementary file 12 — Source data Fig. 3 part 2 [file 44319_2026_742_MOESM12_ESM.zip › Figure 3 Part 2/Fig 3b-e TTLL screen part 2/TTLL6-YFP R1 I4 low int.Project Maximum Z_XY1661547983_Z0_T0_C0.tif]

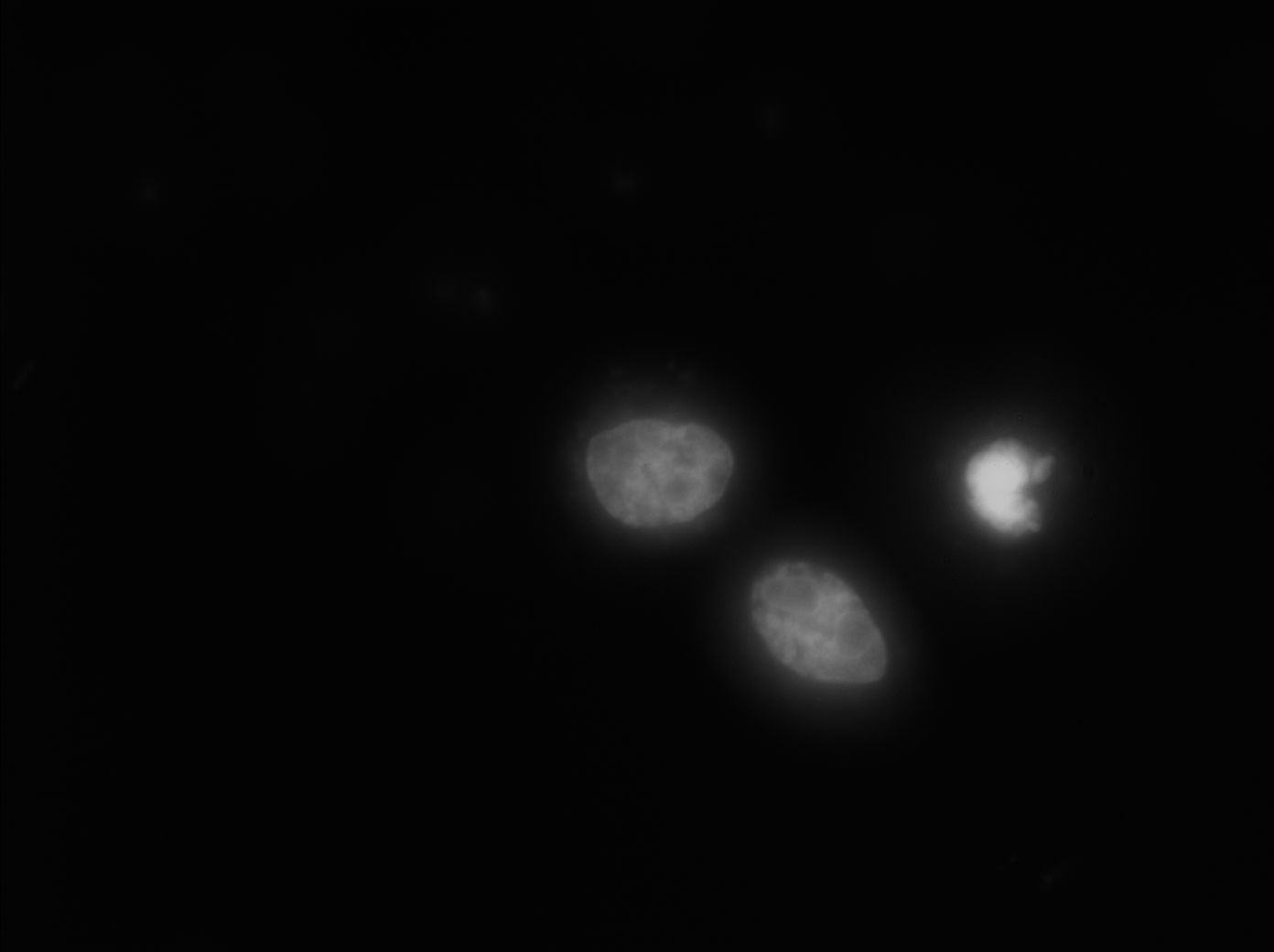

Supplement: Supplementary file 12 — Source data Fig. 3 part 2 [file 44319_2026_742_MOESM12_ESM.zip › Figure 3 Part 2/Fig 3b-e TTLL screen part 2/TTLL5-YFPy I5.Project Maximum Z_XY1679084454_Z0_T0_C0.tif]

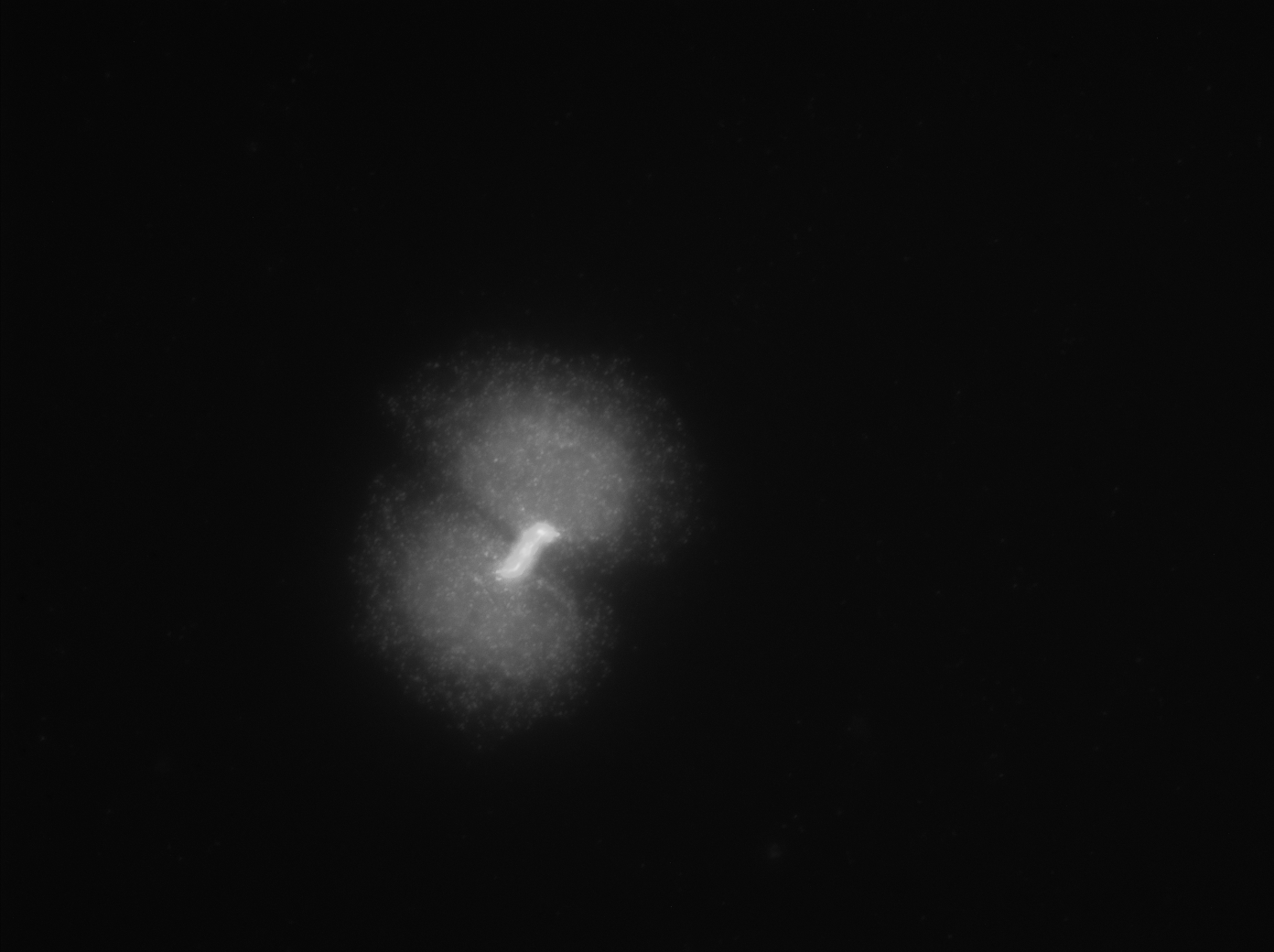

Supplement: Supplementary file 12 — Source data Fig. 3 part 2 [file 44319_2026_742_MOESM12_ESM.zip › Figure 3 Part 2/Fig 3b-e TTLL screen part 2/TTLL6-YFP R1 I4 low int.Project Maximum Z_XY1661547983_Z0_T0_C1.tif]

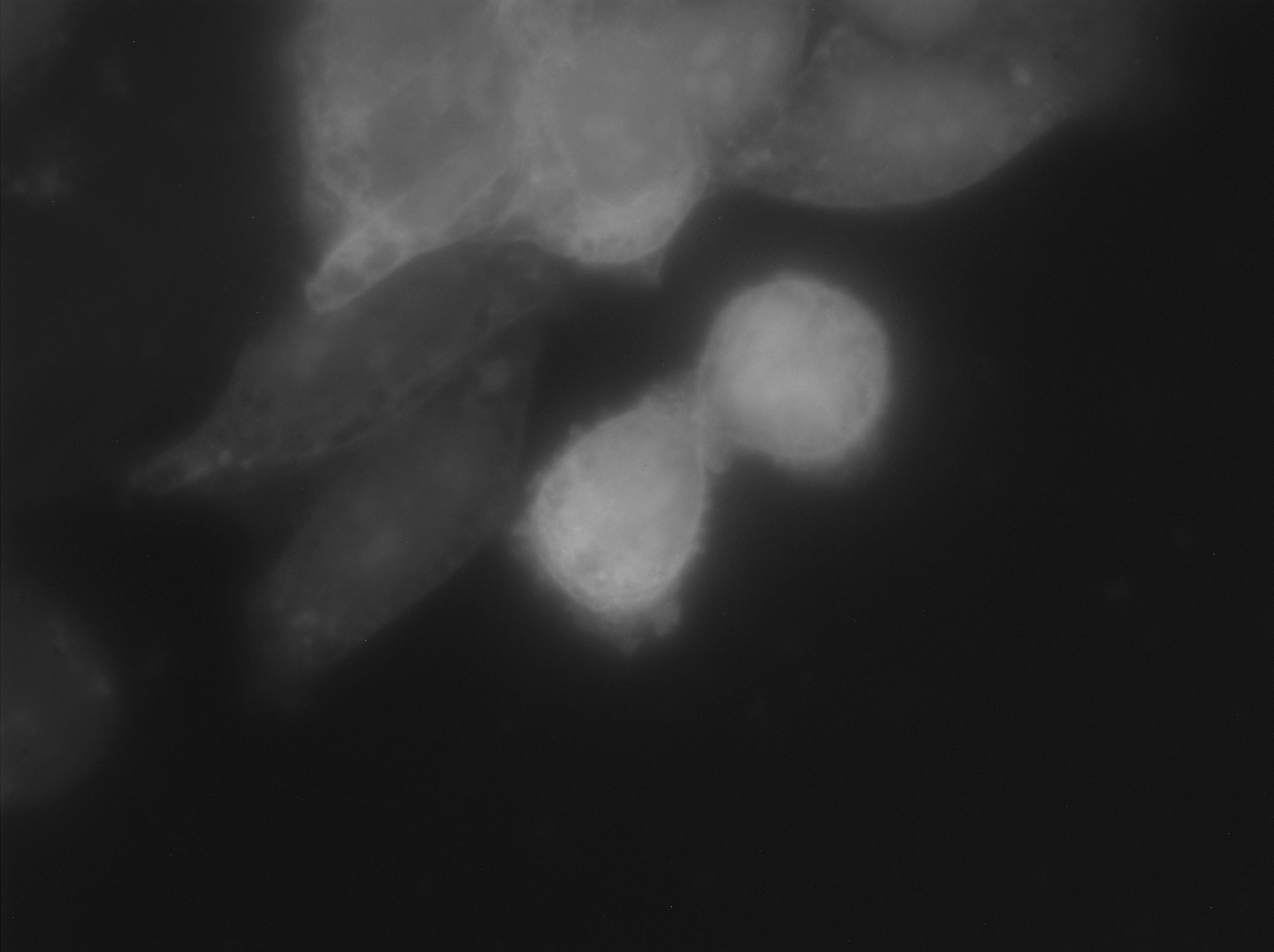

Supplement: Supplementary file 12 — Source data Fig. 3 part 2 [file 44319_2026_742_MOESM12_ESM.zip › Figure 3 Part 2/Fig 3b-e TTLL screen part 2/TTLL7-YFPy I9.Project Maximum Z_XY1679088970_Z0_T0_C2.tif]

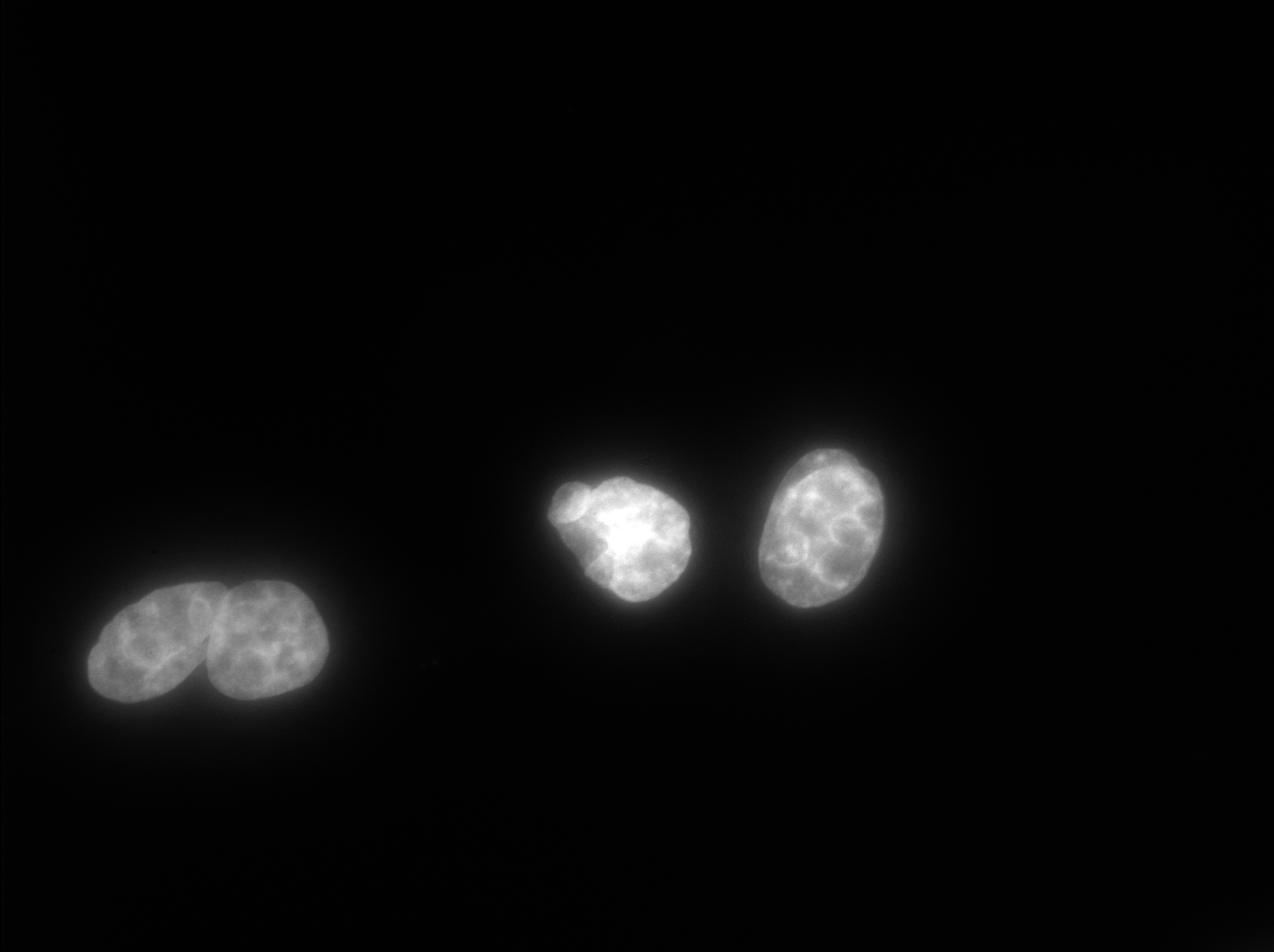

Supplement: Supplementary file 12 — Source data Fig. 3 part 2 [file 44319_2026_742_MOESM12_ESM.zip › Figure 3 Part 2/Fig 3b-e TTLL screen part 2/TTLL6-YFP R1 I2 C2 med.Project Maximum Z_XY1661791814_Z0_T0_C0.tif]

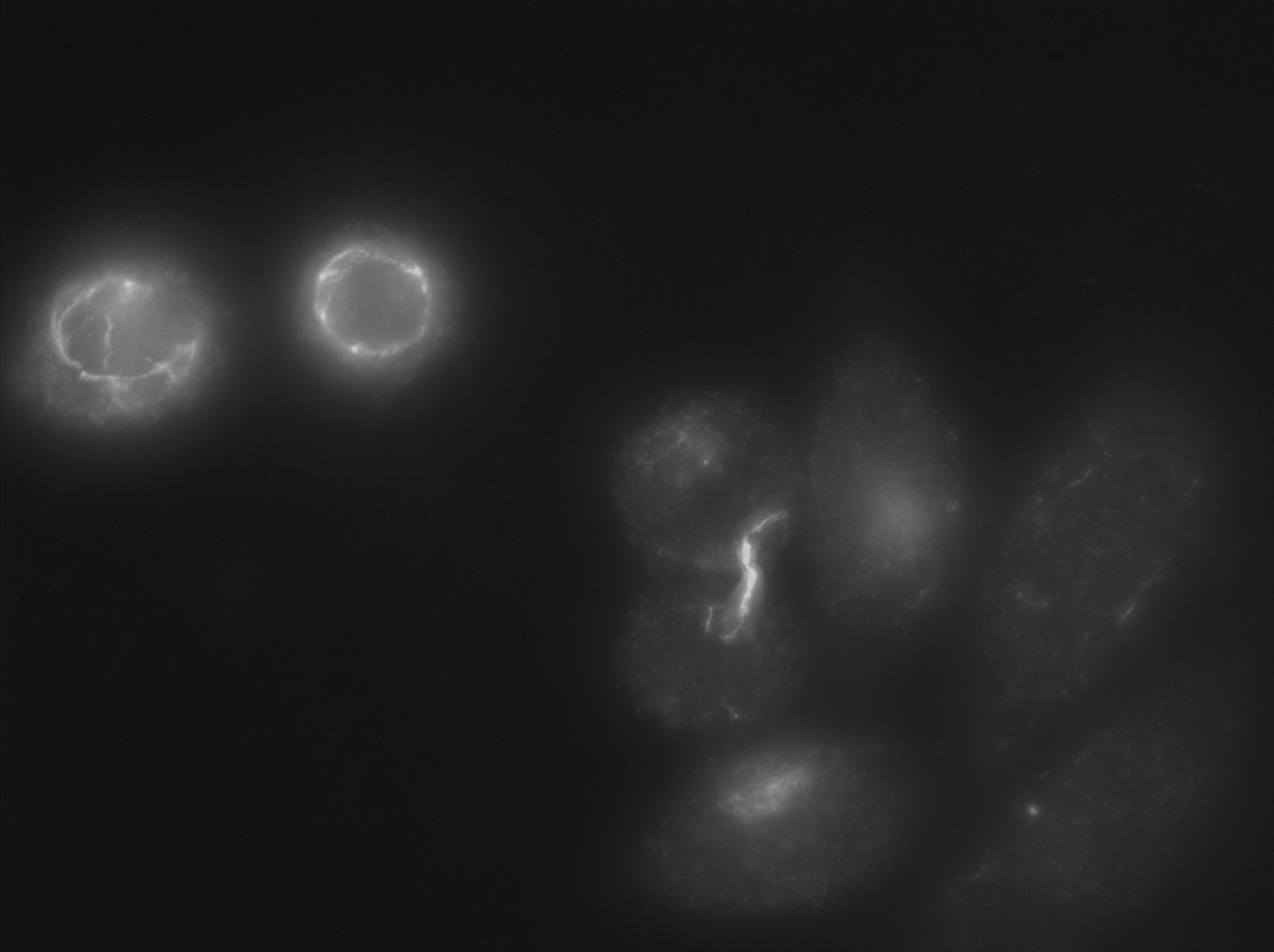

Supplement: Supplementary file 12 — Source data Fig. 3 part 2 [file 44319_2026_742_MOESM12_ESM.zip › Figure 3 Part 2/Fig 3b-e TTLL screen part 2/TTLL5-YFPy I6.Project Maximum Z_XY1679084596_Z0_T0_C1.tif]

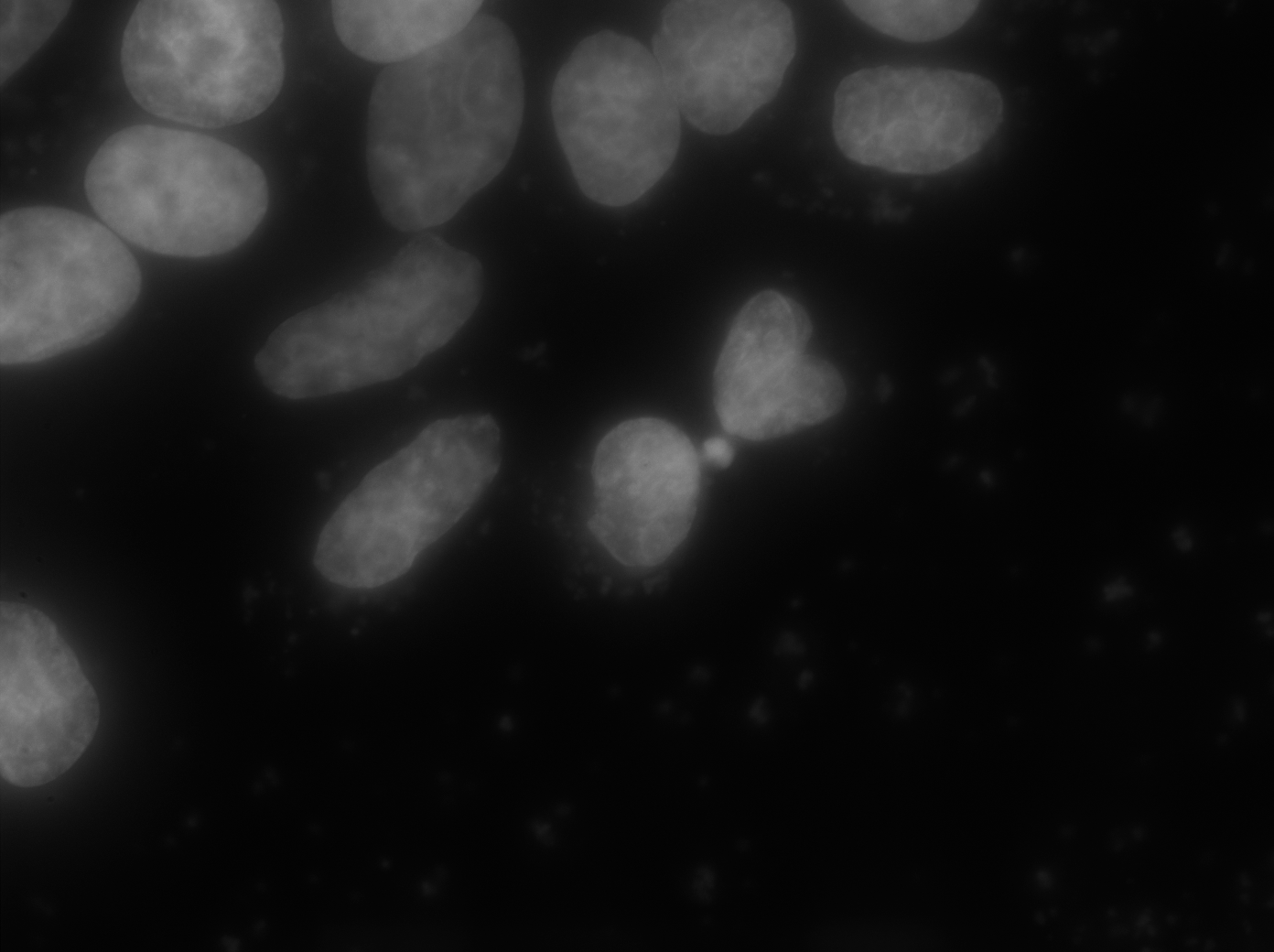

Supplement: Supplementary file 12 — Source data Fig. 3 part 2 [file 44319_2026_742_MOESM12_ESM.zip › Figure 3 Part 2/Fig 3b-e TTLL screen part 2/TTLL7-YFPy I9.Project Maximum Z_XY1679088970_Z0_T0_C0.tif]

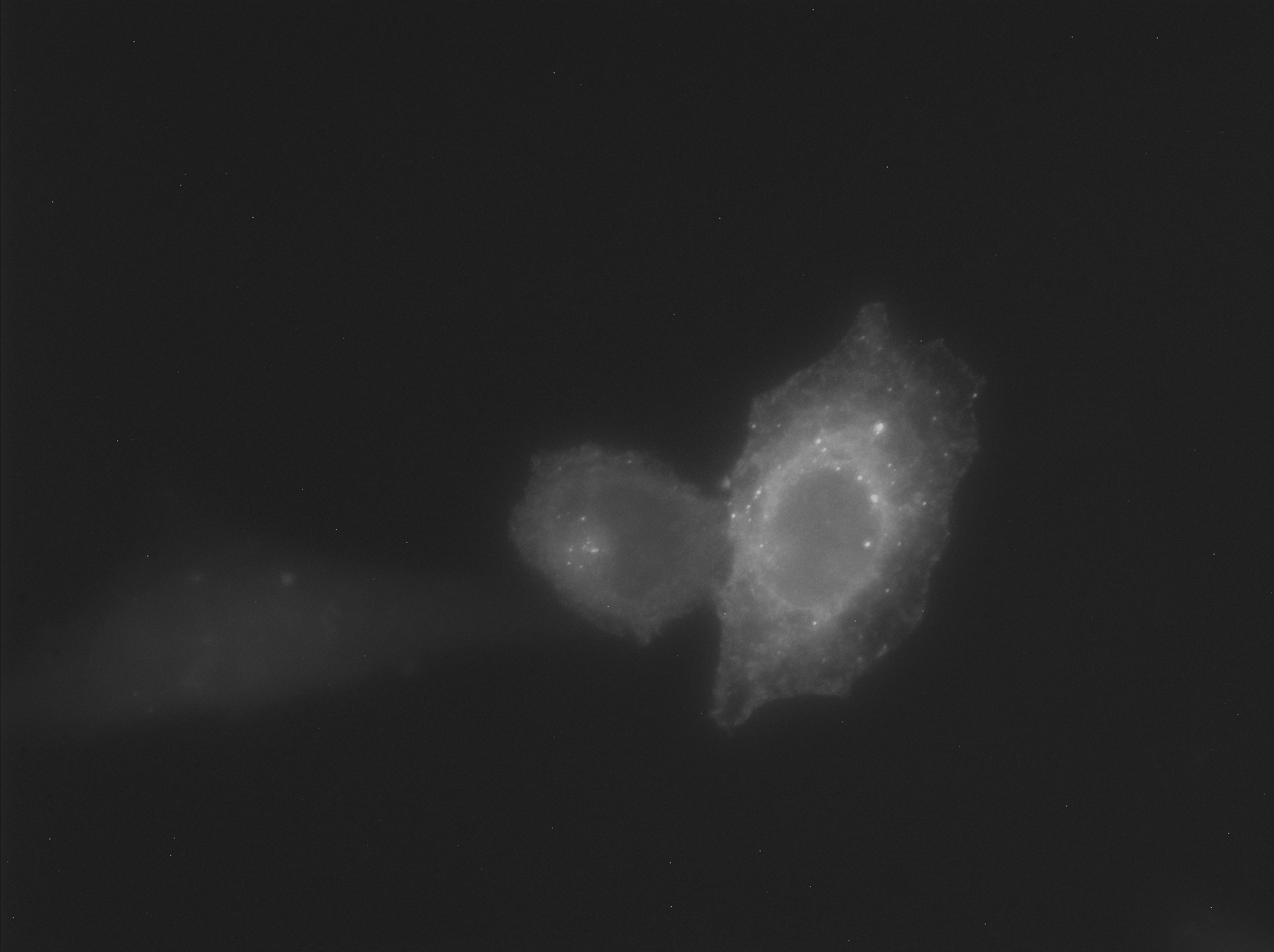

Supplement: Supplementary file 12 — Source data Fig. 3 part 2 [file 44319_2026_742_MOESM12_ESM.zip › Figure 3 Part 2/Fig 3b-e TTLL screen part 2/TTLL6-YFP R1 I2 C2 med.Project Maximum Z_XY1661791814_Z0_T0_C2.tif]

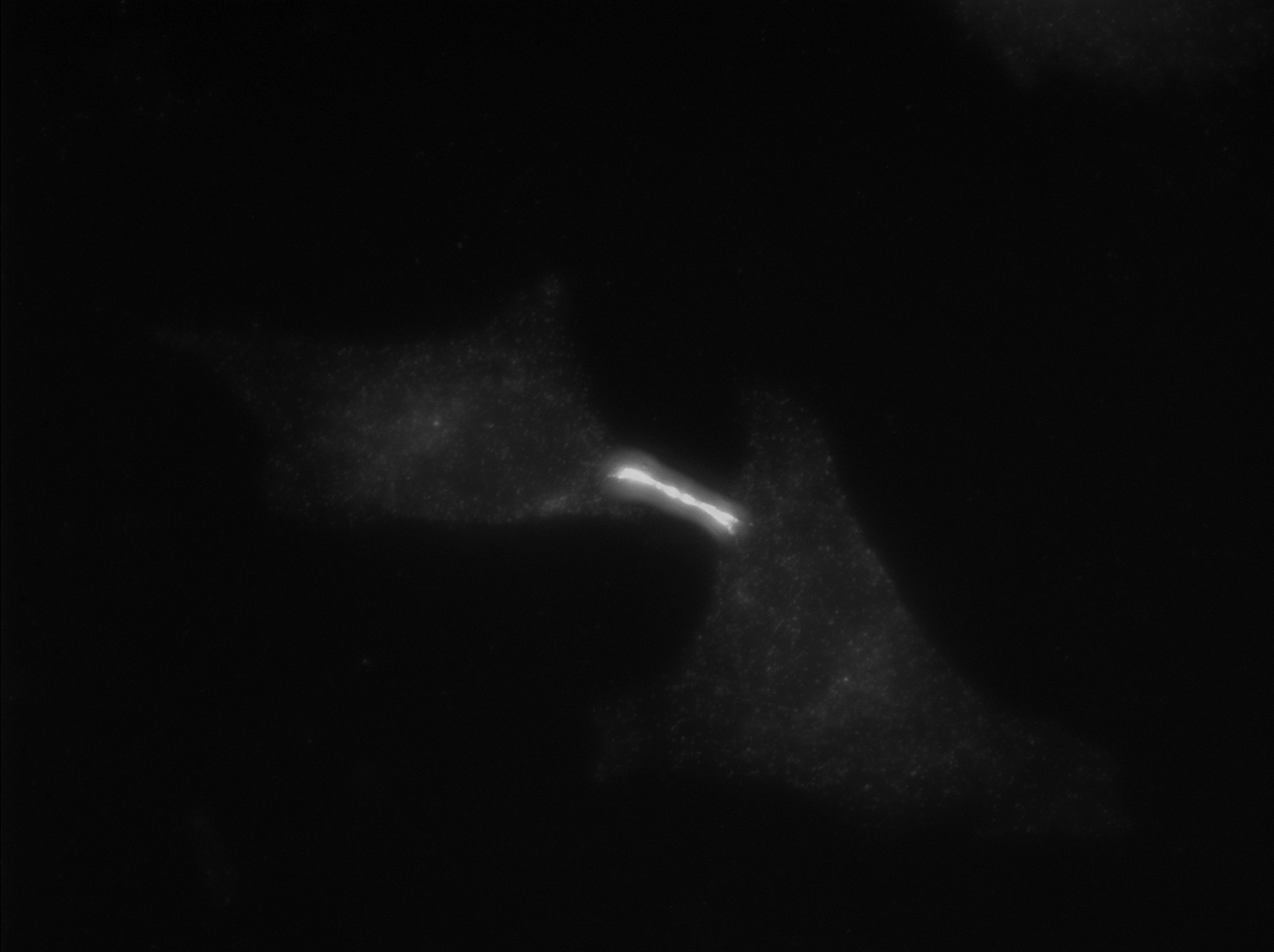

Supplement: Supplementary file 12 — Source data Fig. 3 part 2 [file 44319_2026_742_MOESM12_ESM.zip › Figure 3 Part 2/Fig 3b-e TTLL screen part 2/TTLL6-YFP MB light I1.Project Maximum Z_XY1663880365_Z0_T0_C1.tif]

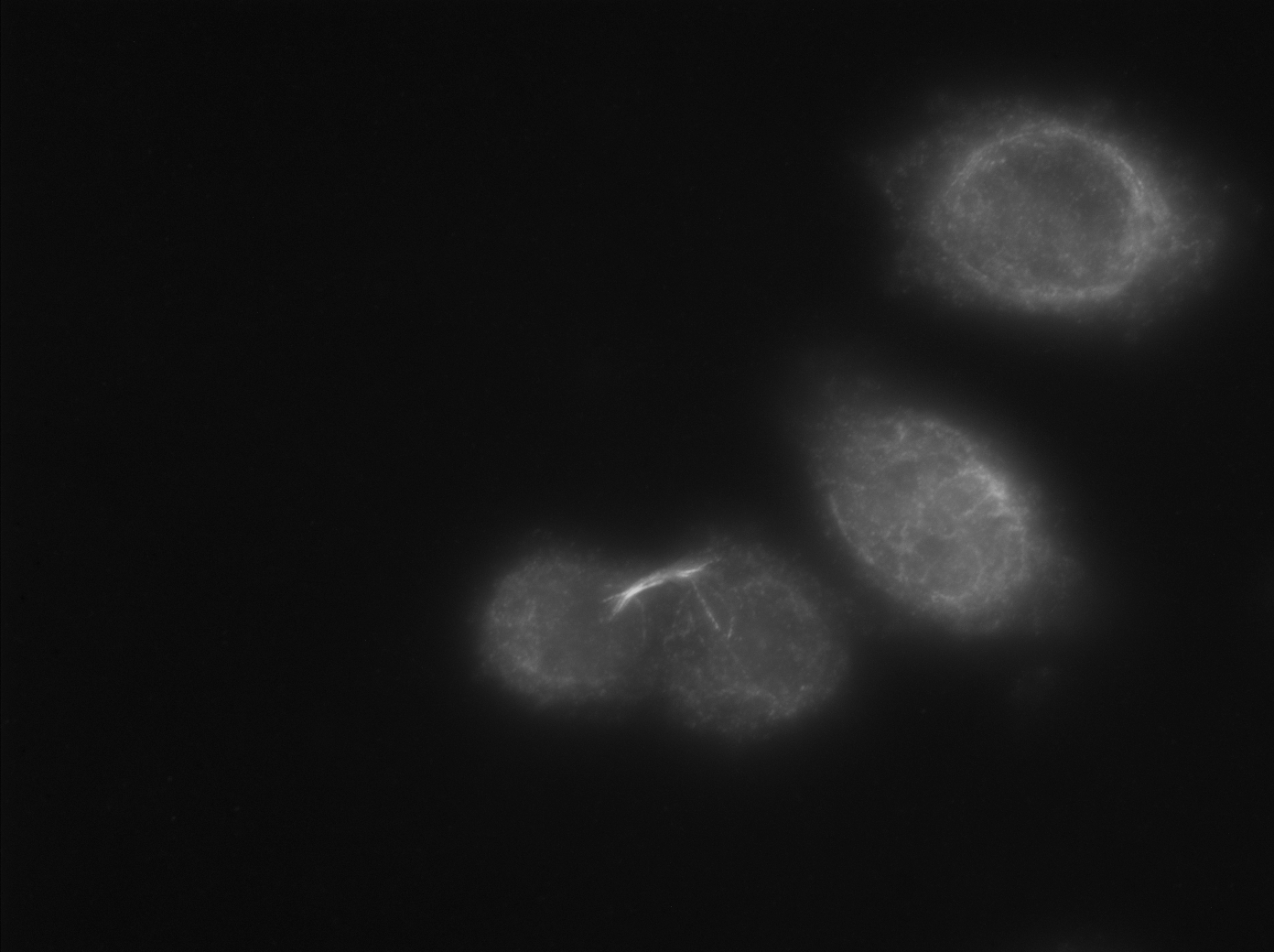

Supplement: Supplementary file 12 — Source data Fig. 3 part 2 [file 44319_2026_742_MOESM12_ESM.zip › Figure 3 Part 2/Fig 3b-e TTLL screen part 2/TTLL5-YFPy I8.Project Maximum Z_XY1679085074_Z0_T0_C1.tif]

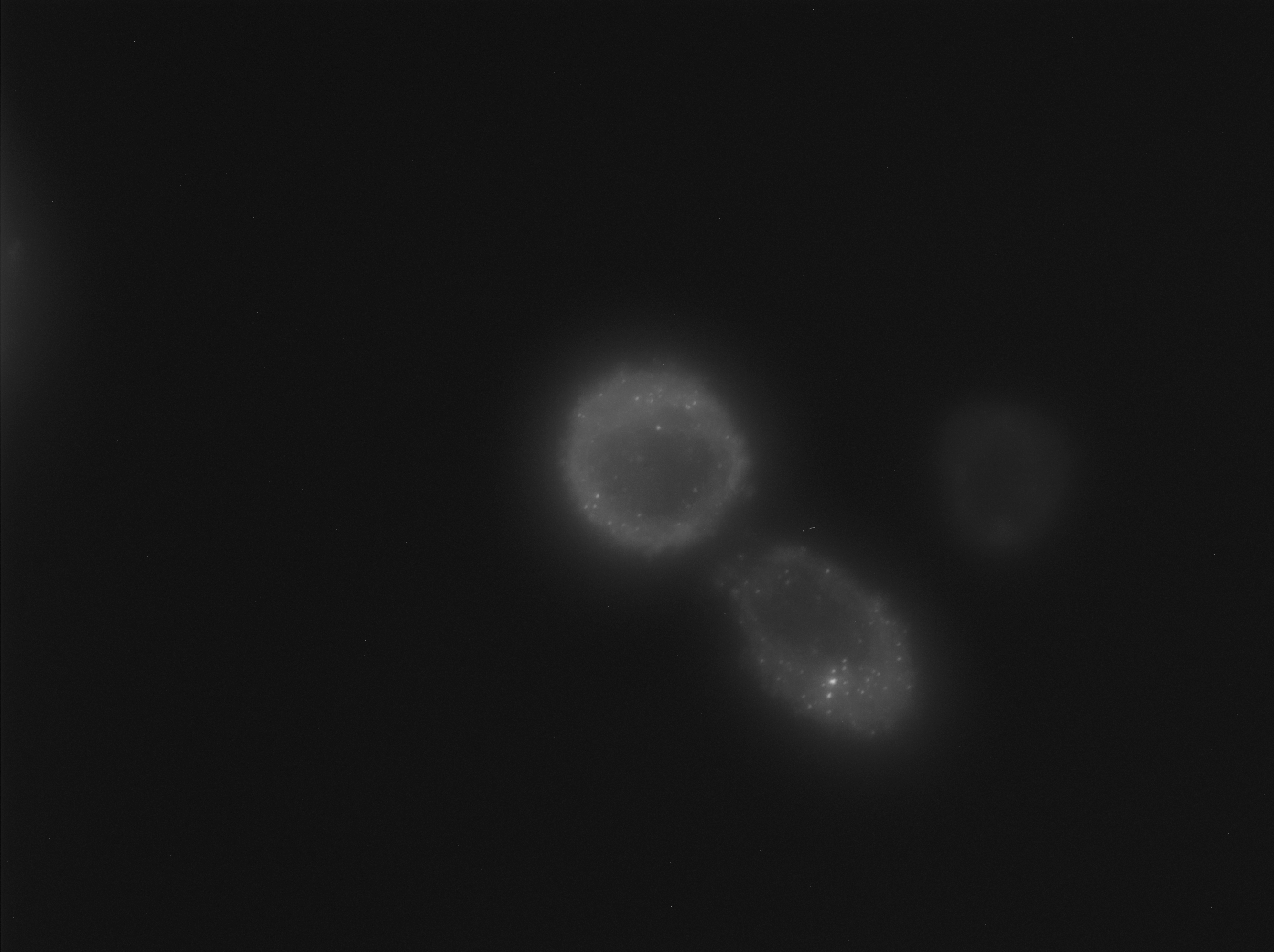

Supplement: Supplementary file 12 — Source data Fig. 3 part 2 [file 44319_2026_742_MOESM12_ESM.zip › Figure 3 Part 2/Fig 3b-e TTLL screen part 2/TTLL5-YFPy I5.Project Maximum Z_XY1679084454_Z0_T0_C2.tif]

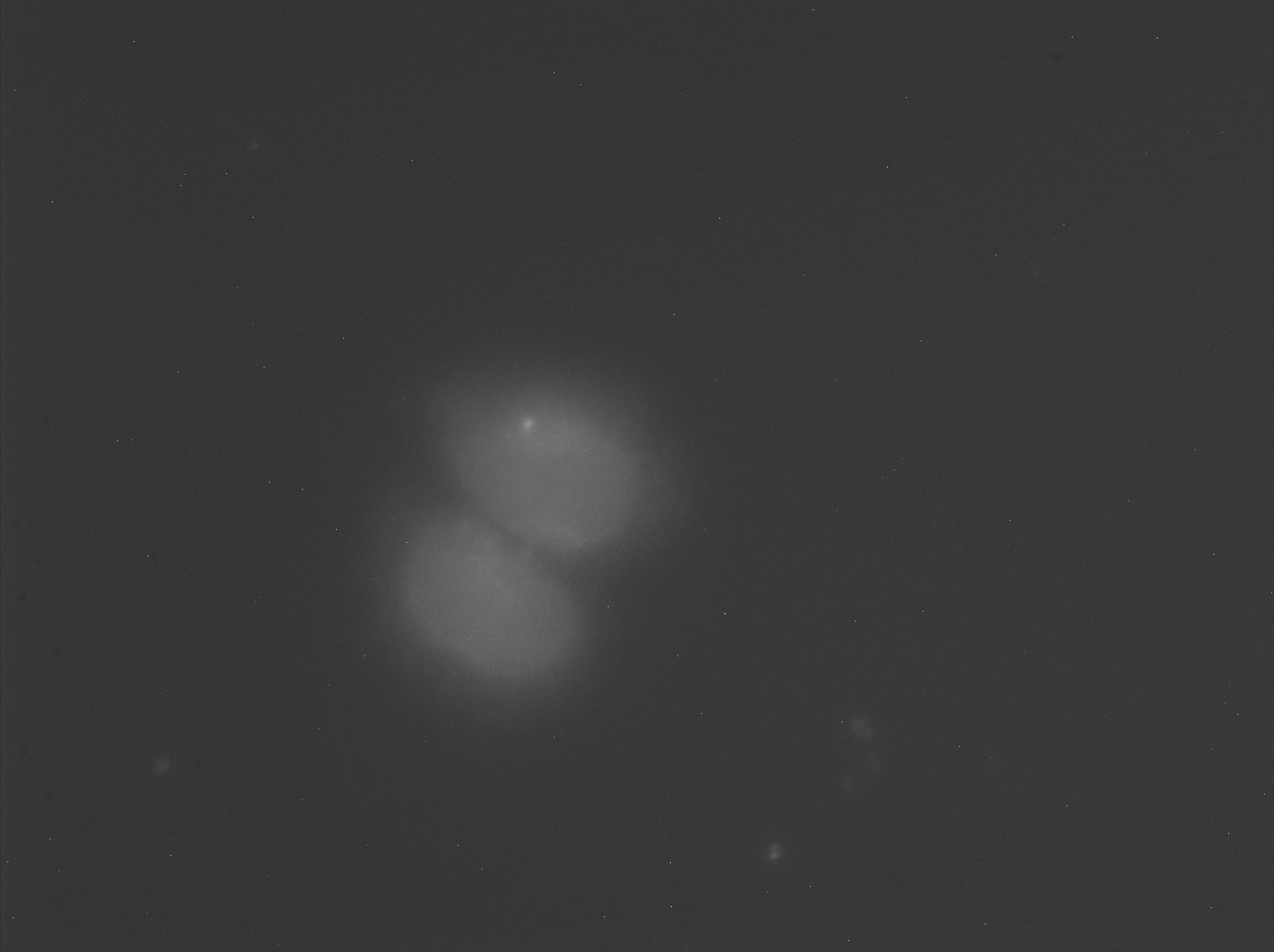

Supplement: Supplementary file 12 — Source data Fig. 3 part 2 [file 44319_2026_742_MOESM12_ESM.zip › Figure 3 Part 2/Fig 3b-e TTLL screen part 2/TTLL6-YFP R1 I4 low int.Project Maximum Z_XY1661547983_Z0_T0_C2.tif]

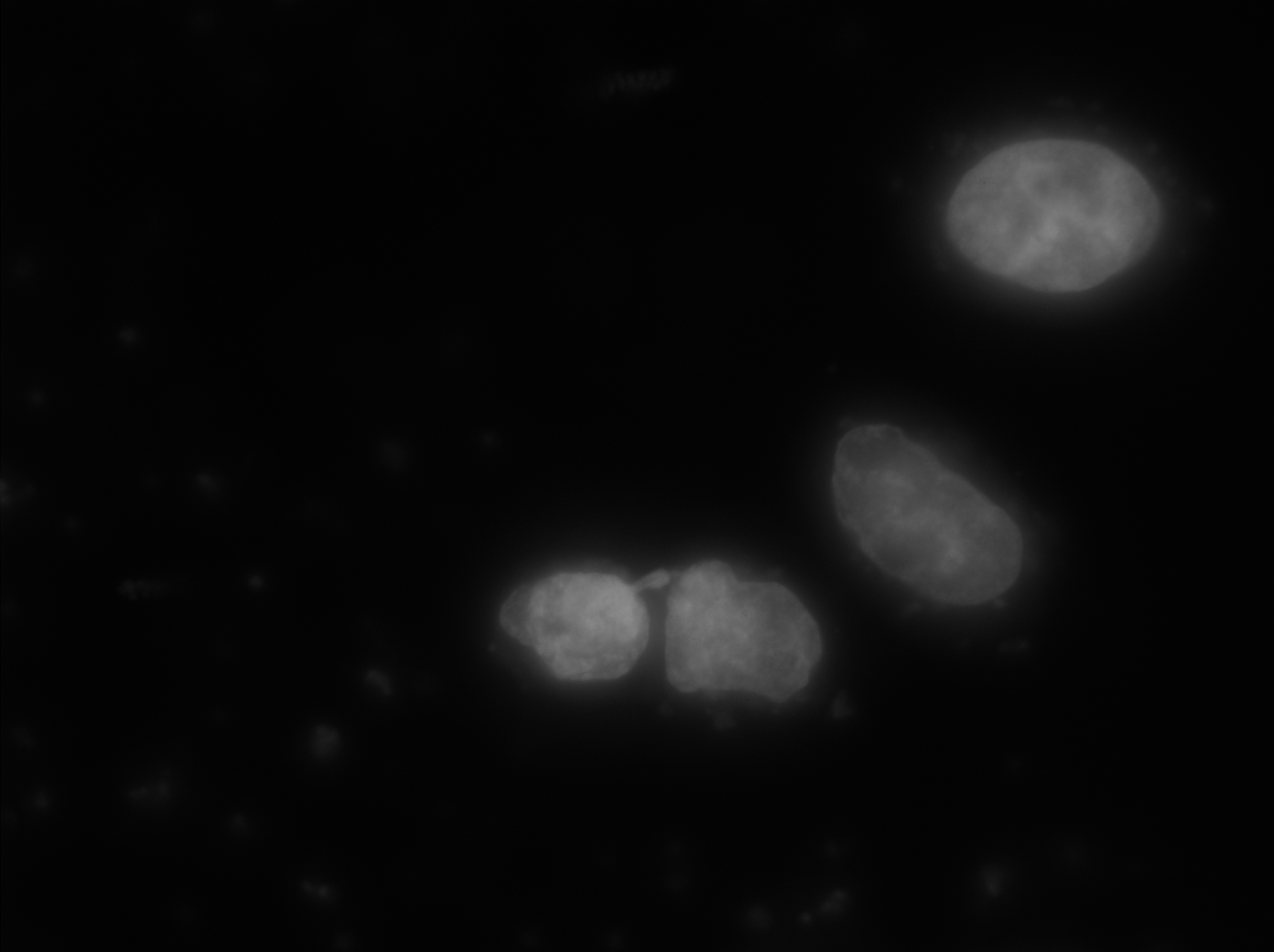

Supplement: Supplementary file 12 — Source data Fig. 3 part 2 [file 44319_2026_742_MOESM12_ESM.zip › Figure 3 Part 2/Fig 3b-e TTLL screen part 2/TTLL5-YFPy I8.Project Maximum Z_XY1679085074_Z0_T0_C0.tif]

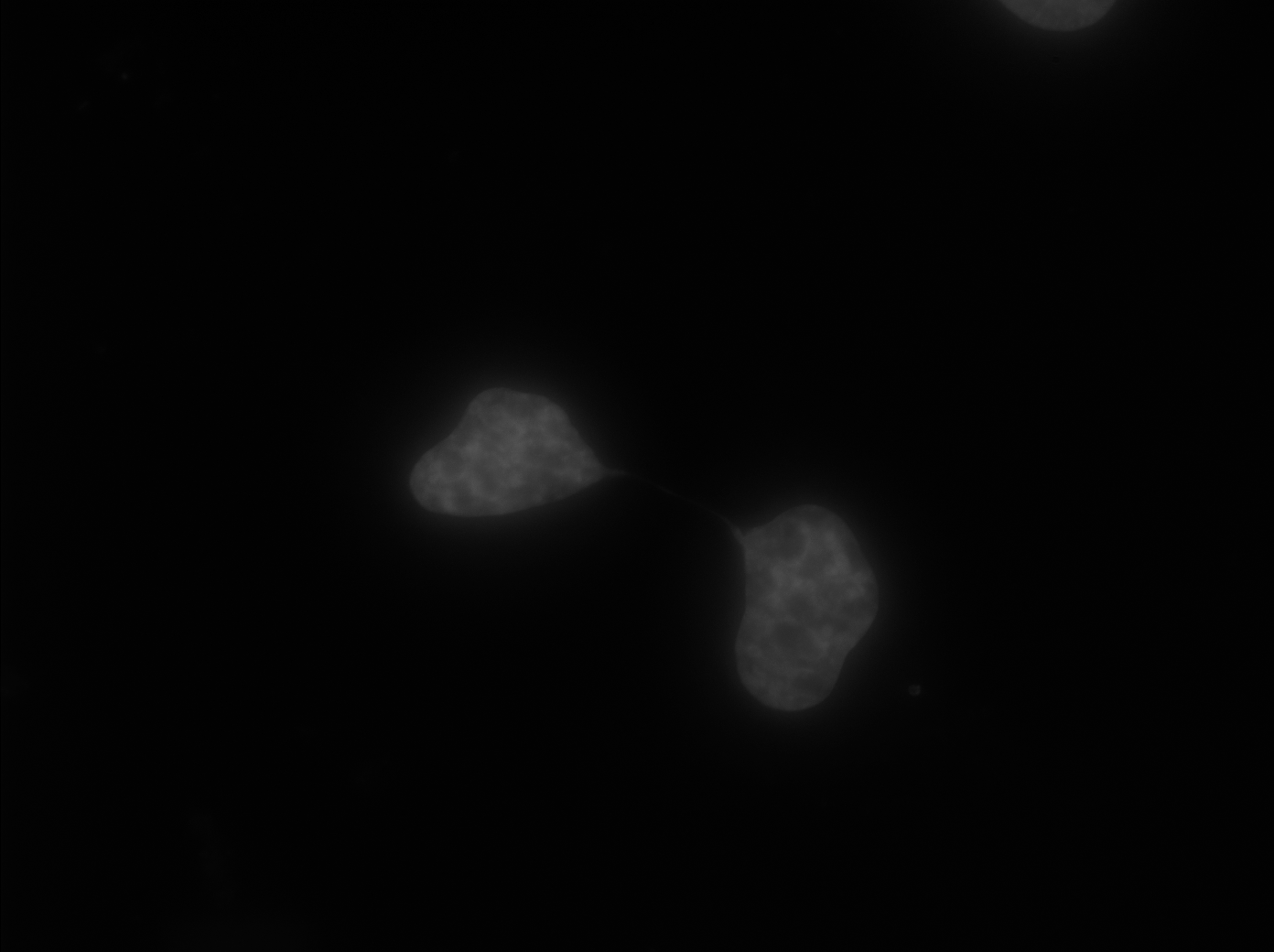

Supplement: Supplementary file 12 — Source data Fig. 3 part 2 [file 44319_2026_742_MOESM12_ESM.zip › Figure 3 Part 2/Fig 3b-e TTLL screen part 2/TTLL6-YFP MB light I1.Project Maximum Z_XY1663880365_Z0_T0_C0.tif]

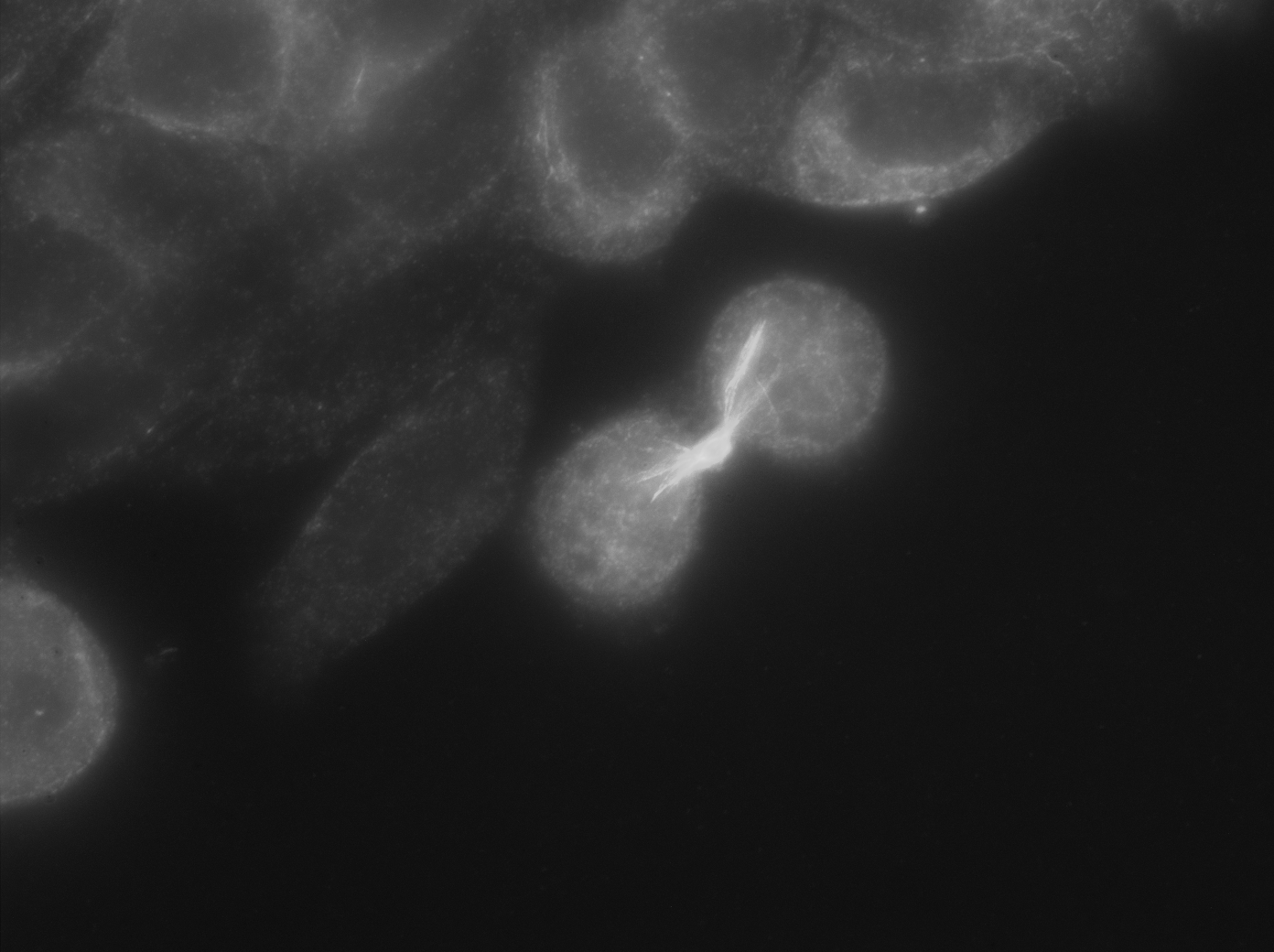

Supplement: Supplementary file 12 — Source data Fig. 3 part 2 [file 44319_2026_742_MOESM12_ESM.zip › Figure 3 Part 2/Fig 3b-e TTLL screen part 2/TTLL7-YFPy I9.Project Maximum Z_XY1679088970_Z0_T0_C1.tif]

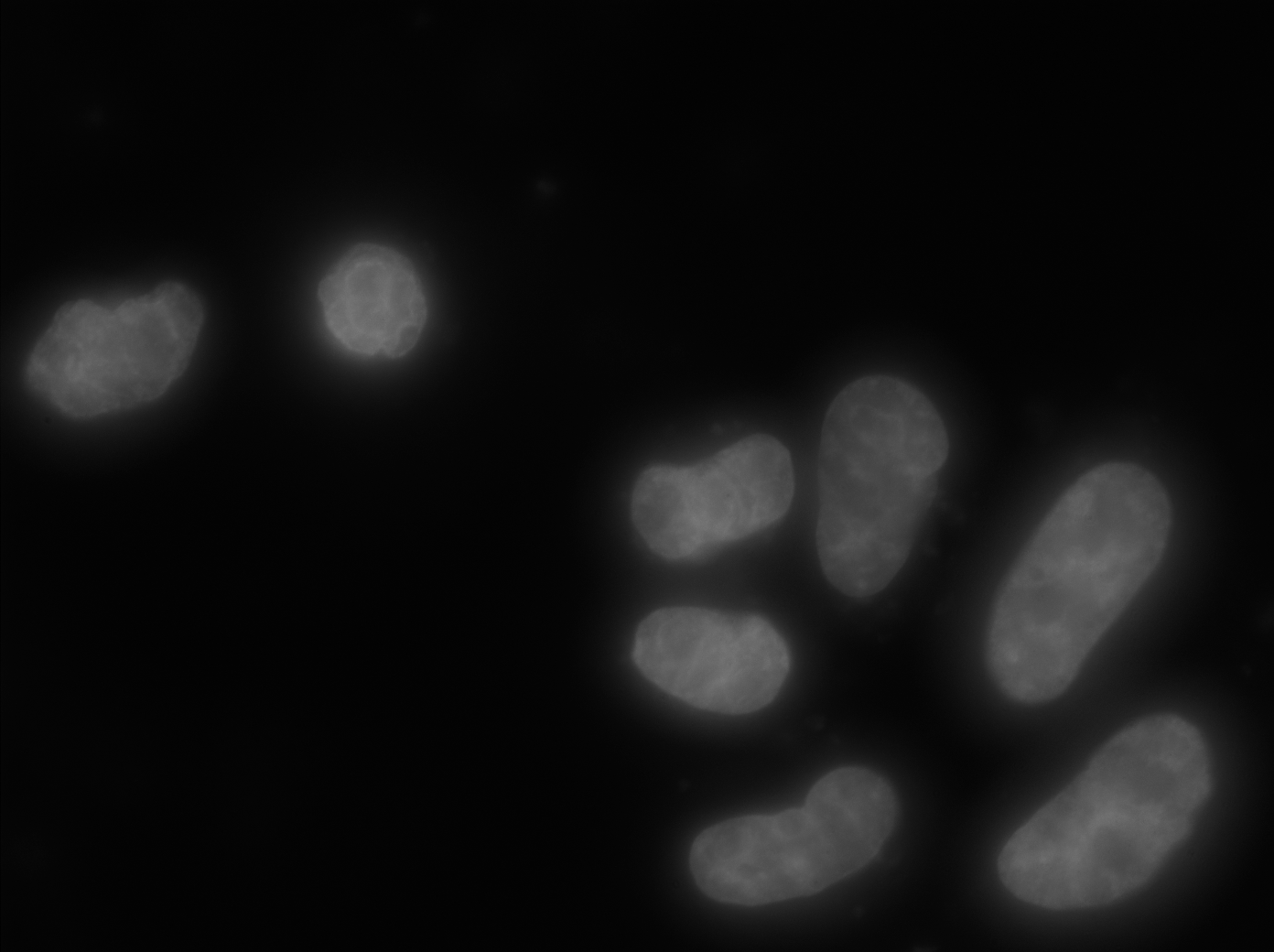

Supplement: Supplementary file 12 — Source data Fig. 3 part 2 [file 44319_2026_742_MOESM12_ESM.zip › Figure 3 Part 2/Fig 3b-e TTLL screen part 2/TTLL5-YFPy I6.Project Maximum Z_XY1679084596_Z0_T0_C0.tif]

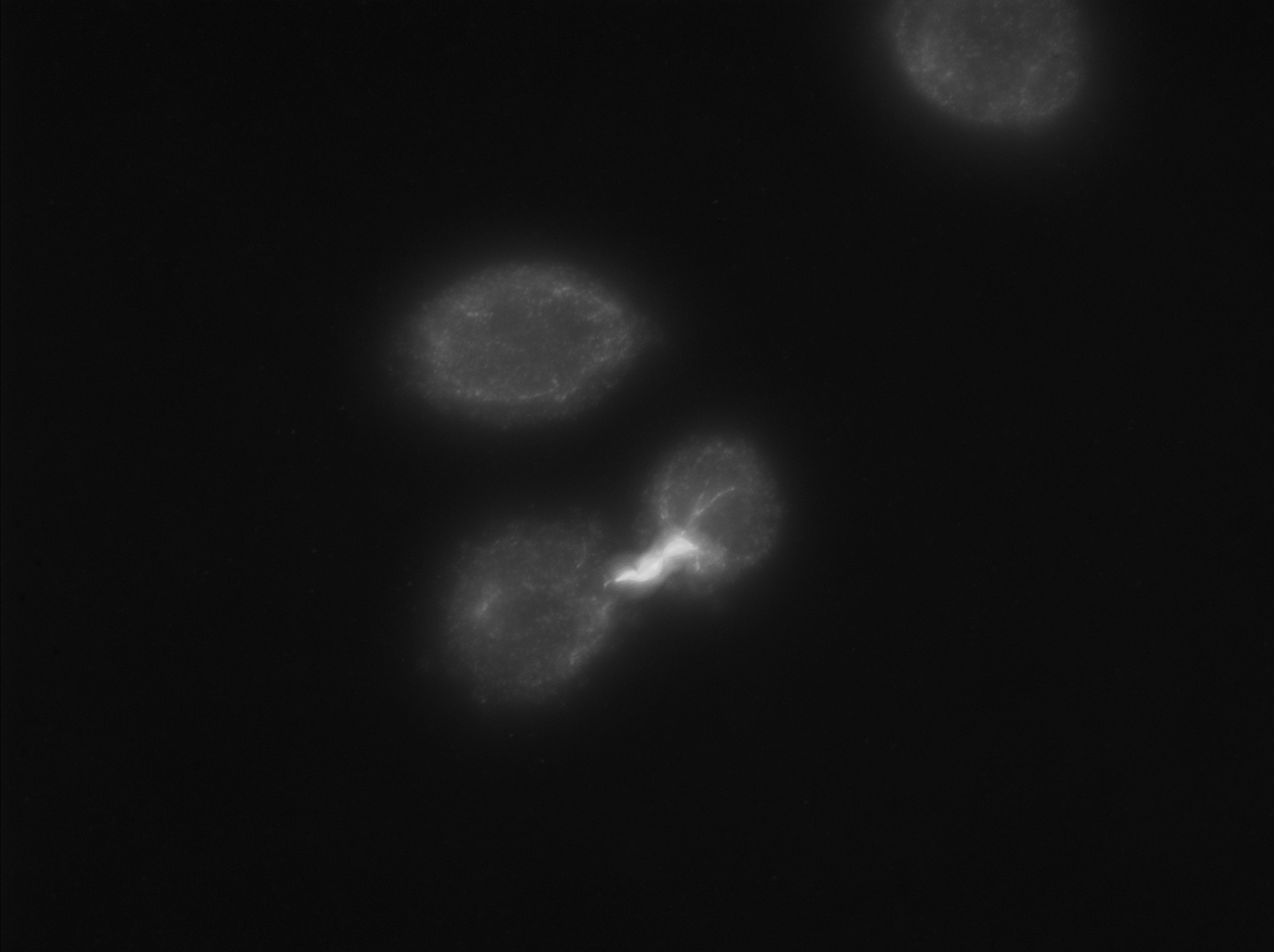

Supplement: Supplementary file 12 — Source data Fig. 3 part 2 [file 44319_2026_742_MOESM12_ESM.zip › Figure 3 Part 2/Fig 3b-e TTLL screen part 2/TTLL5-YFPy I20.Project Maximum Z_XY1679341653_Z0_T0_C1.tif]

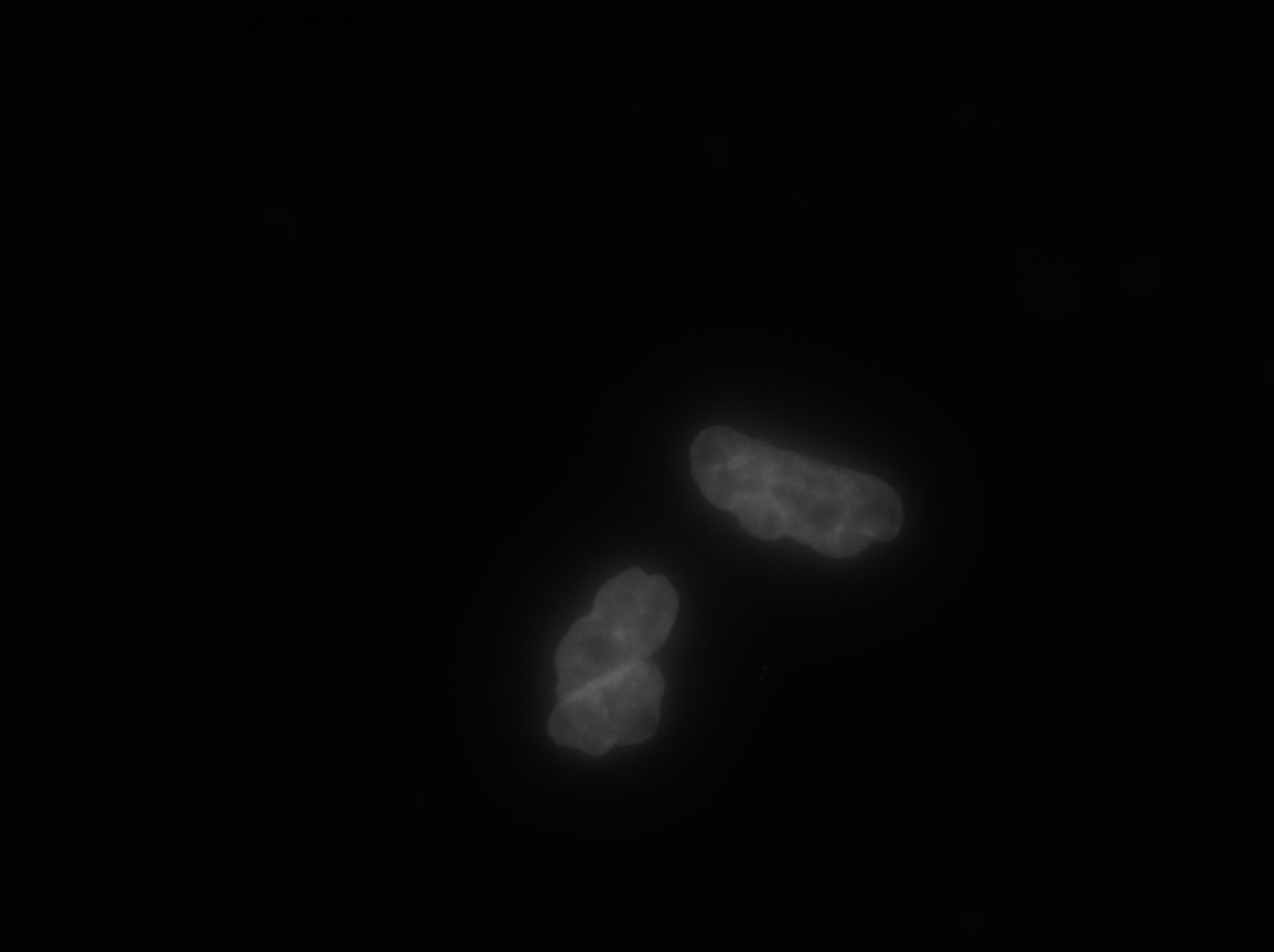

Supplement: Supplementary file 12 — Source data Fig. 3 part 2 [file 44319_2026_742_MOESM12_ESM.zip › Figure 3 Part 2/Fig 3b-e TTLL screen part 2/TTLL6-YFP SS I1.Project Maximum Z_XY1663881583_Z0_T0_C0.tif]

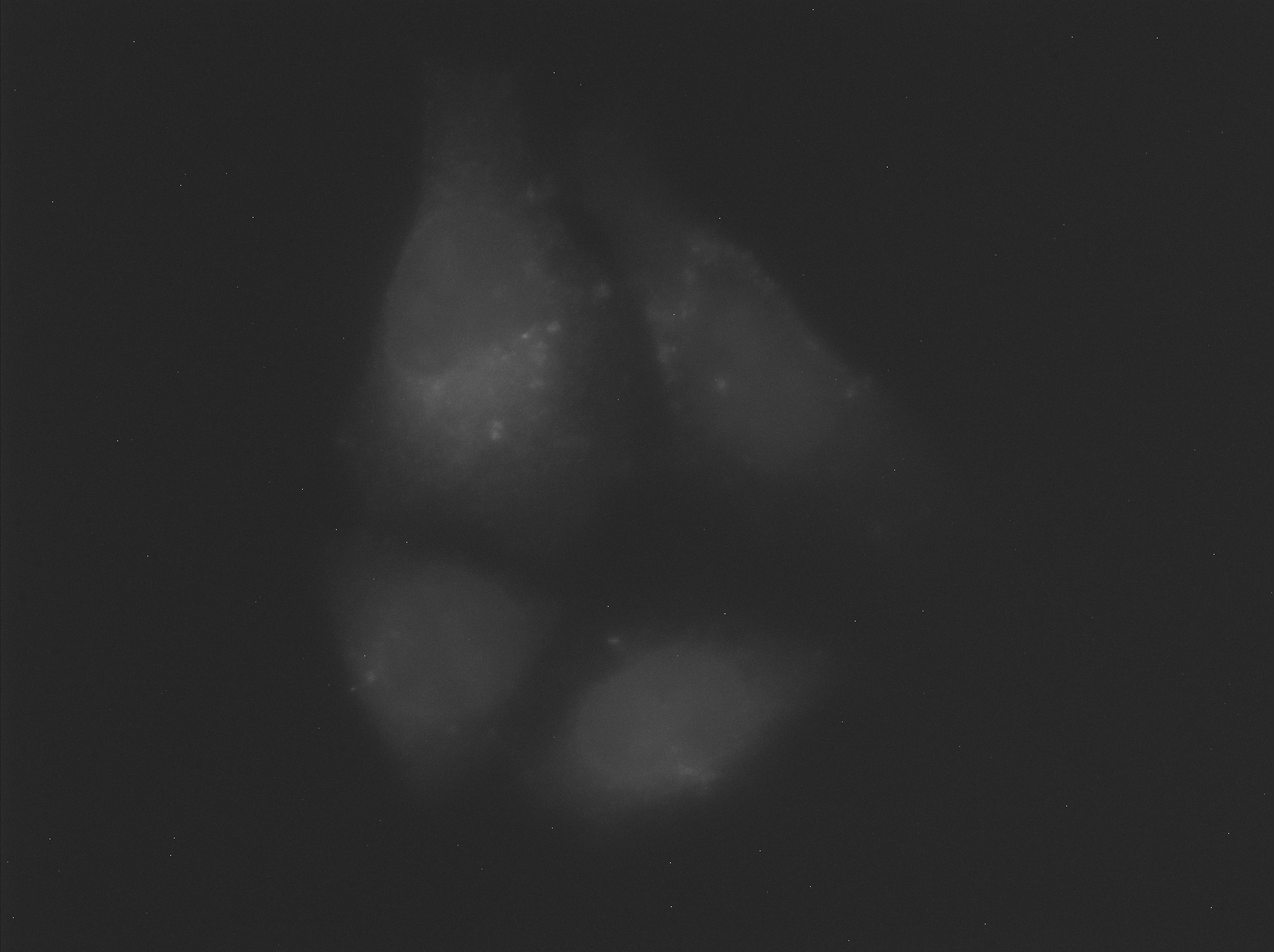

Supplement: Supplementary file 12 — Source data Fig. 3 part 2 [file 44319_2026_742_MOESM12_ESM.zip › Figure 3 Part 2/Fig 3b-e TTLL screen part 2/TTLL6-YFP R1 I1 C2.Project Maximum Z_XY1661791366_Z0_T0_C2.tif]

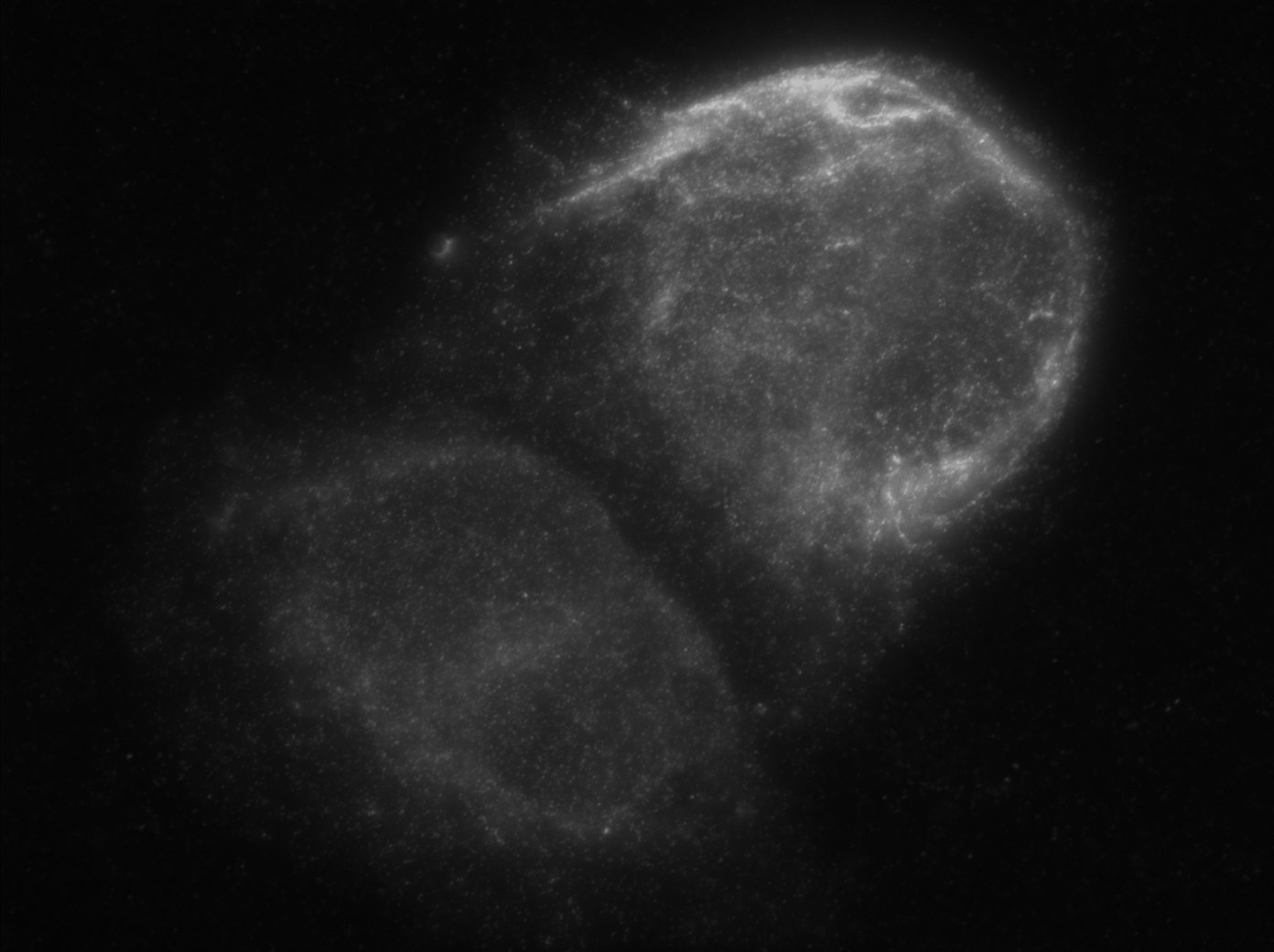

Supplement: Supplementary file 12 — Source data Fig. 3 part 2 [file 44319_2026_742_MOESM12_ESM.zip › Figure 3 Part 2/Fig 3b-e TTLL screen part 2/TTLL6-YFP R1 T5 hiigh.Project Maximum Z_XY1661551952_Z0_T0_C1.tif]

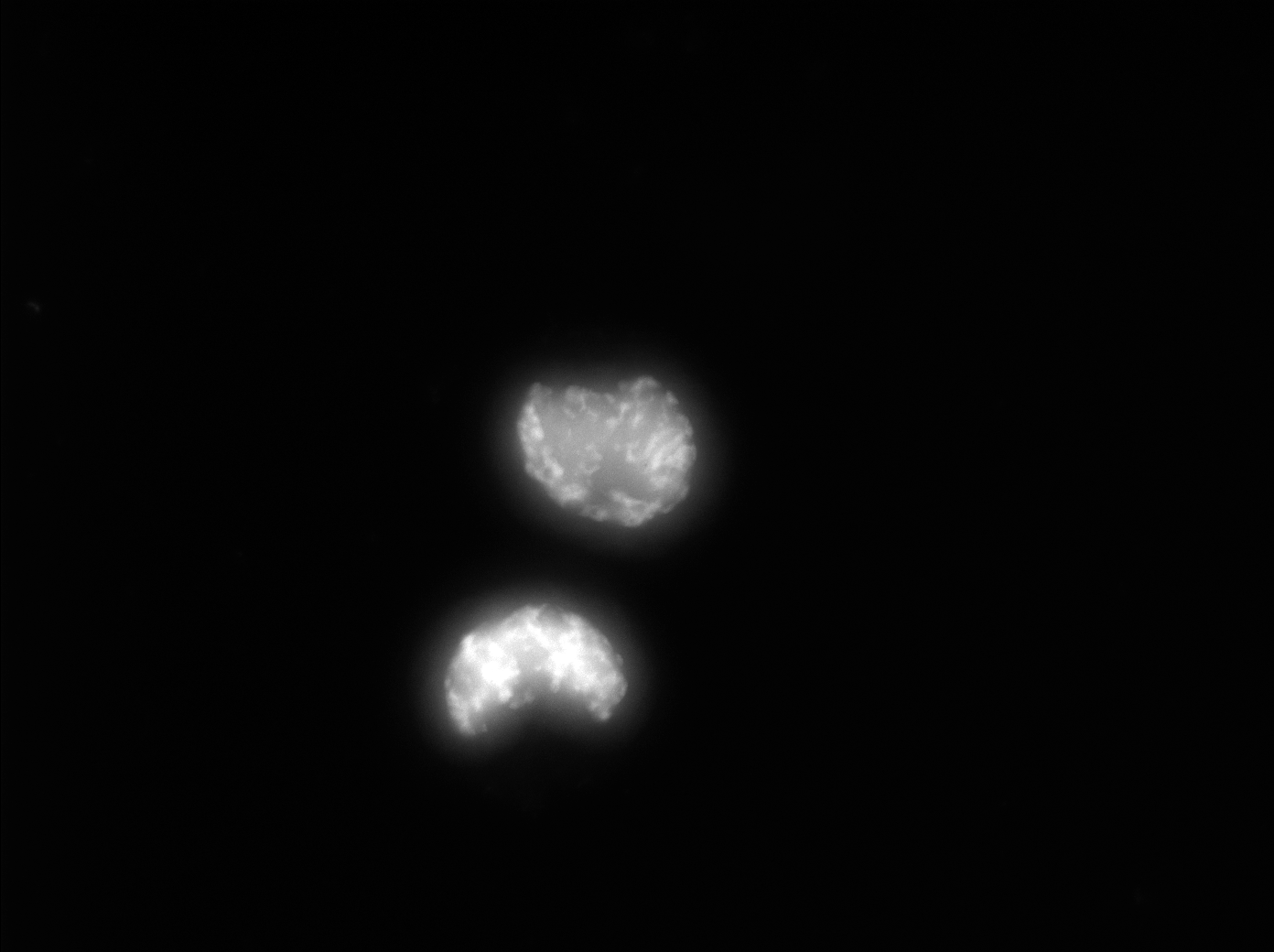

Supplement: Supplementary file 12 — Source data Fig. 3 part 2 [file 44319_2026_742_MOESM12_ESM.zip › Figure 3 Part 2/Fig 3b-e TTLL screen part 2/TTLL6-YFP R1 I2.Project Maximum Z_XY1663275354_Z0_T0_C0.tif]

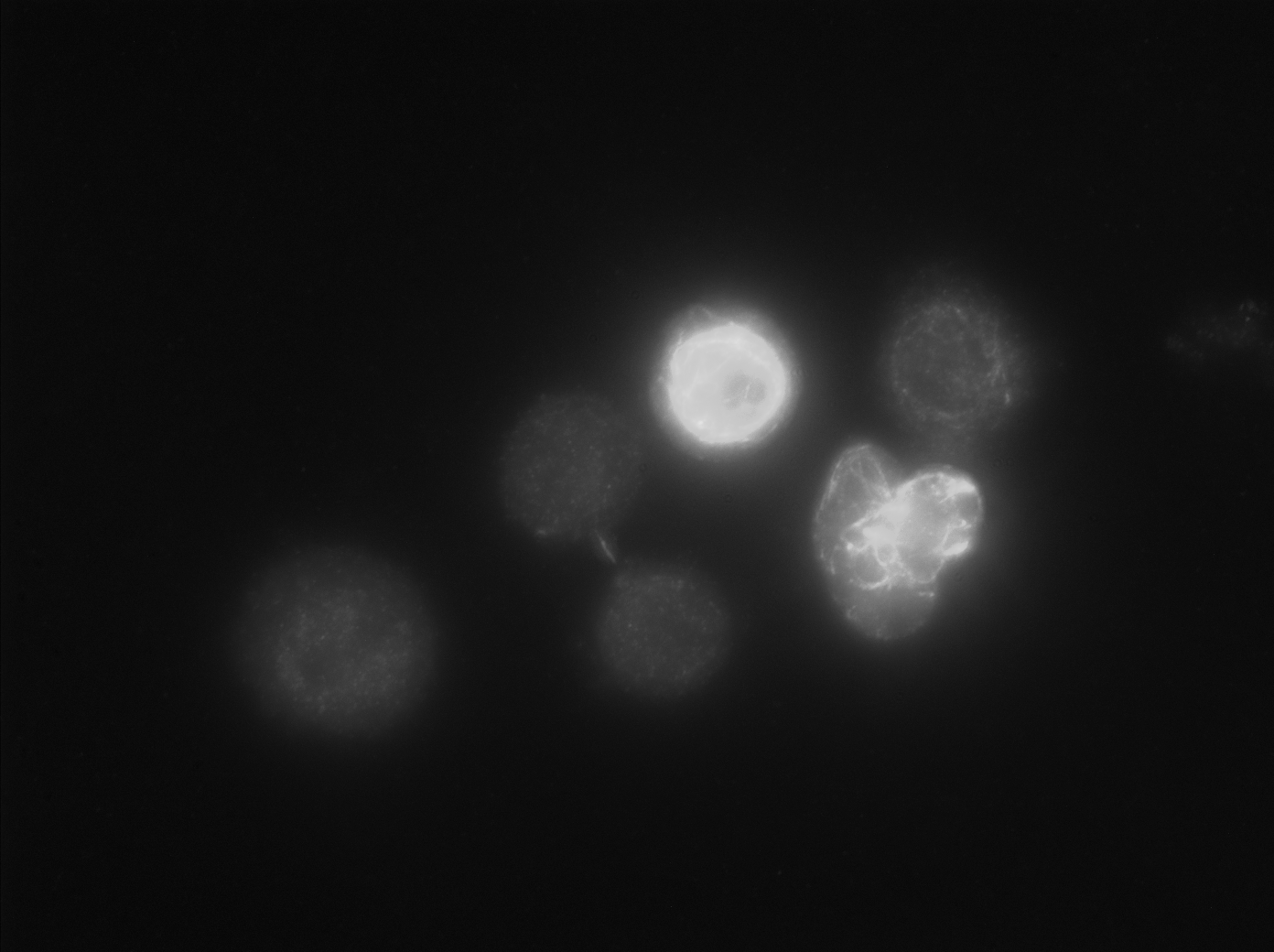

Supplement: Supplementary file 12 — Source data Fig. 3 part 2 [file 44319_2026_742_MOESM12_ESM.zip › Figure 3 Part 2/Fig 3b-e TTLL screen part 2/TTLL7-YFPy I2.Project Maximum Z_XY1679087175_Z0_T0_C1.tif]

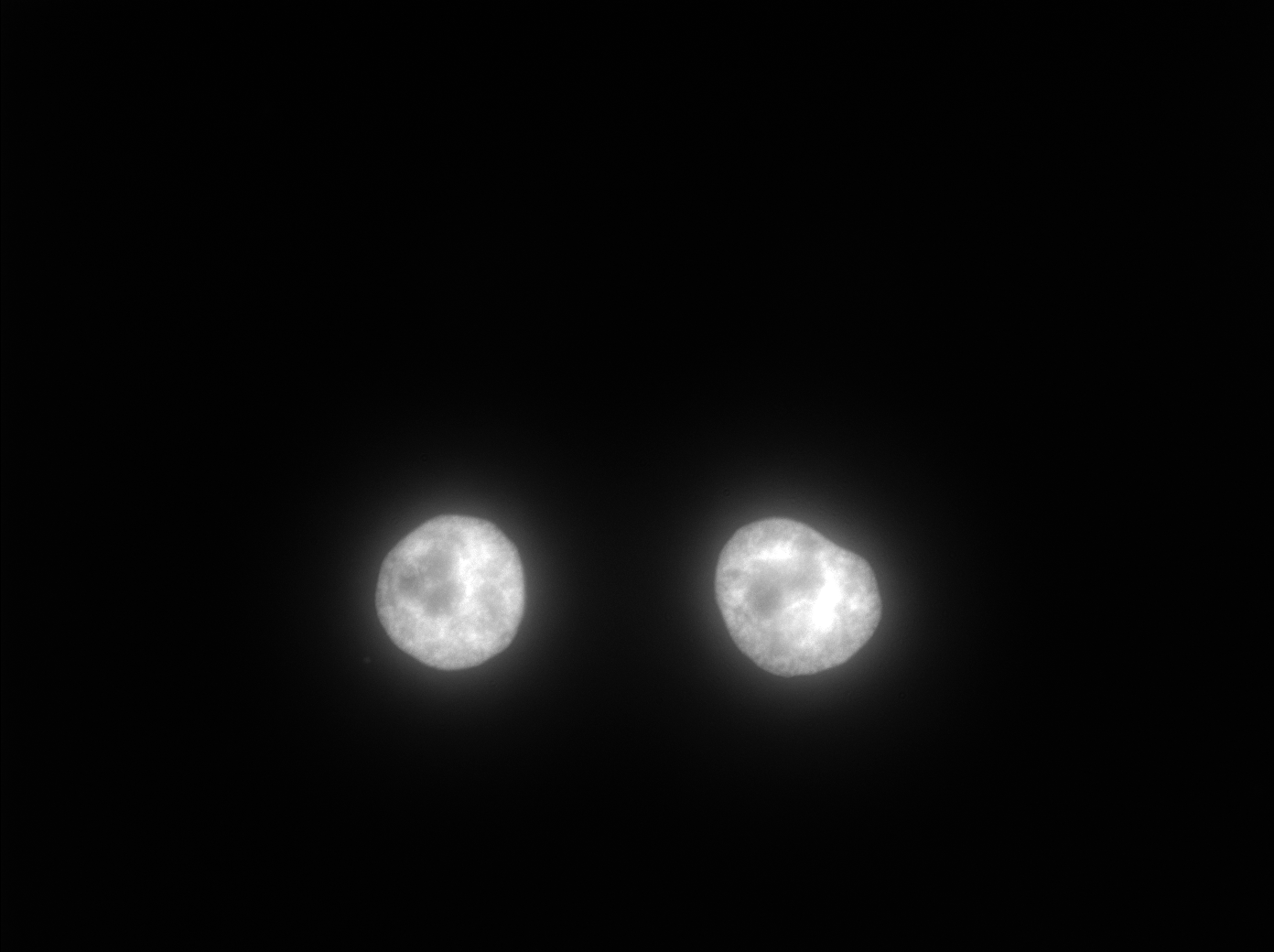

Supplement: Supplementary file 12 — Source data Fig. 3 part 2 [file 44319_2026_742_MOESM12_ESM.zip › Figure 3 Part 2/Fig 3b-e TTLL screen part 2/TTLL6-YFP R1 T3 high int.Project Maximum Z_XY1661549582_Z0_T0_C0.tif]

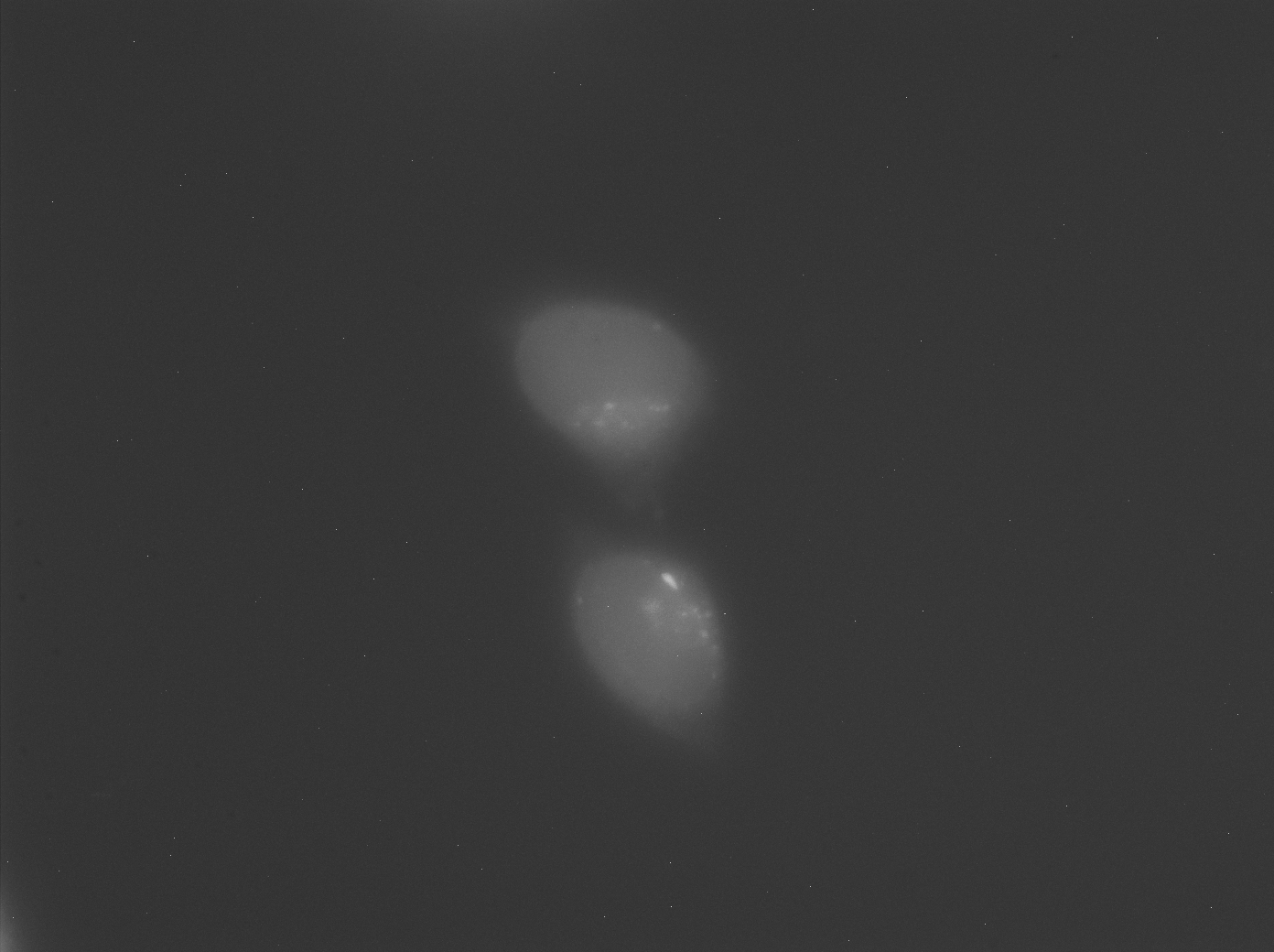

Supplement: Supplementary file 12 — Source data Fig. 3 part 2 [file 44319_2026_742_MOESM12_ESM.zip › Figure 3 Part 2/Fig 3b-e TTLL screen part 2/TTLL6-YFP R1 I1 low int.Project Maximum Z_XY1661545829_Z0_T0_C2.tif]

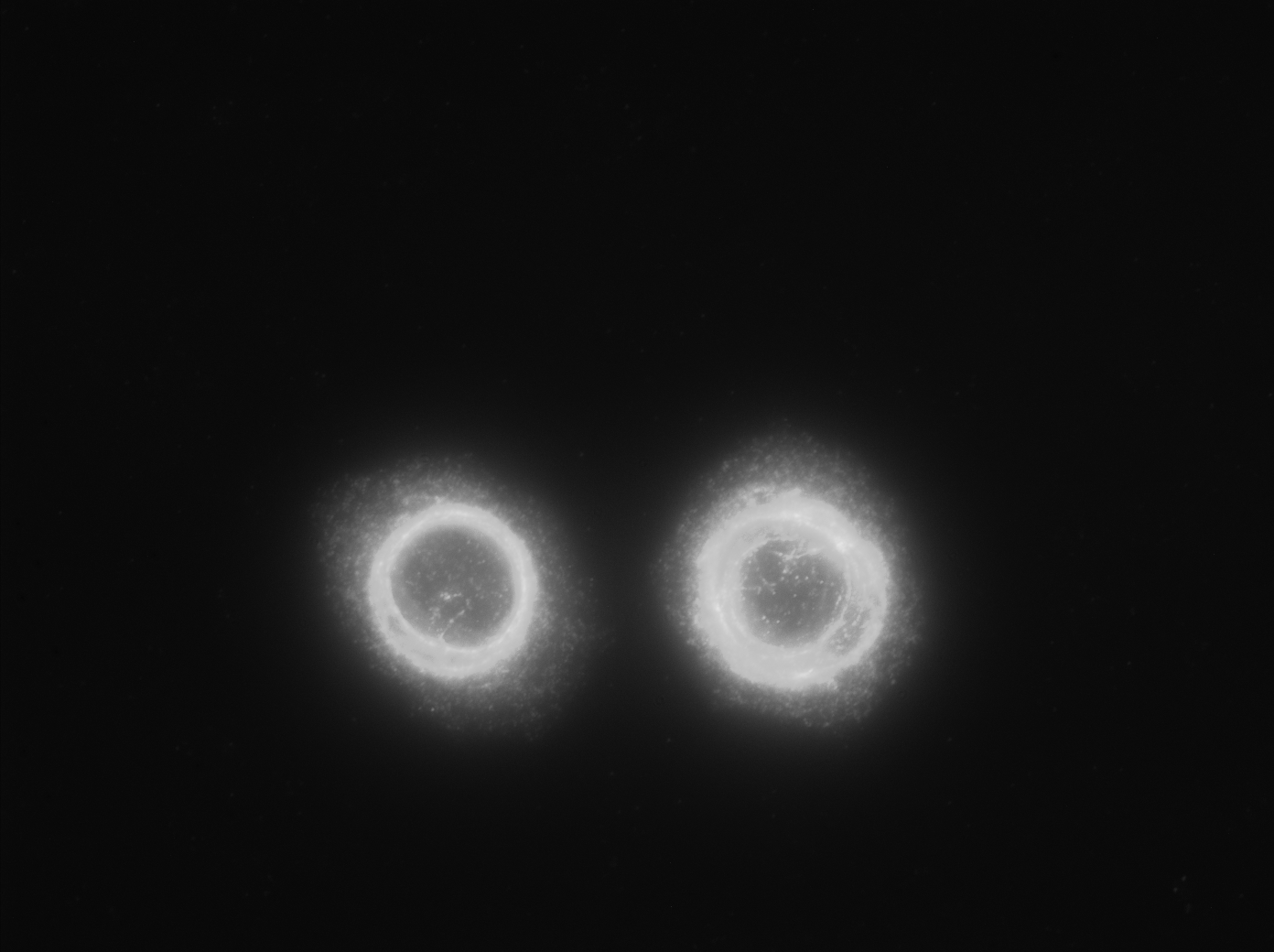

Supplement: Supplementary file 12 — Source data Fig. 3 part 2 [file 44319_2026_742_MOESM12_ESM.zip › Figure 3 Part 2/Fig 3b-e TTLL screen part 2/TTLL6-YFP R1 T3 high int.Project Maximum Z_XY1661549582_Z0_T0_C1.tif]

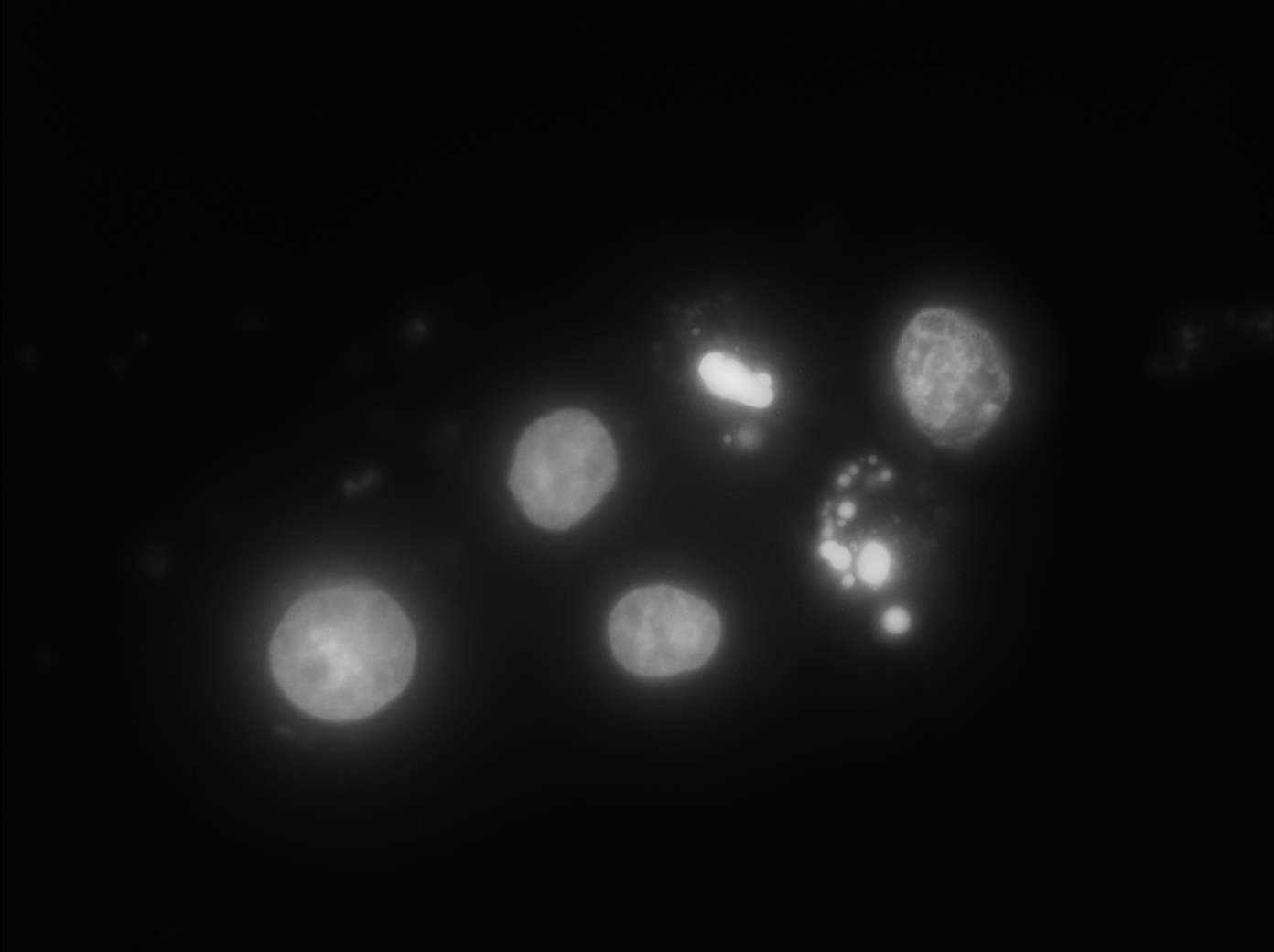

Supplement: Supplementary file 12 — Source data Fig. 3 part 2 [file 44319_2026_742_MOESM12_ESM.zip › Figure 3 Part 2/Fig 3b-e TTLL screen part 2/TTLL7-YFPy I2.Project Maximum Z_XY1679087175_Z0_T0_C0.tif]

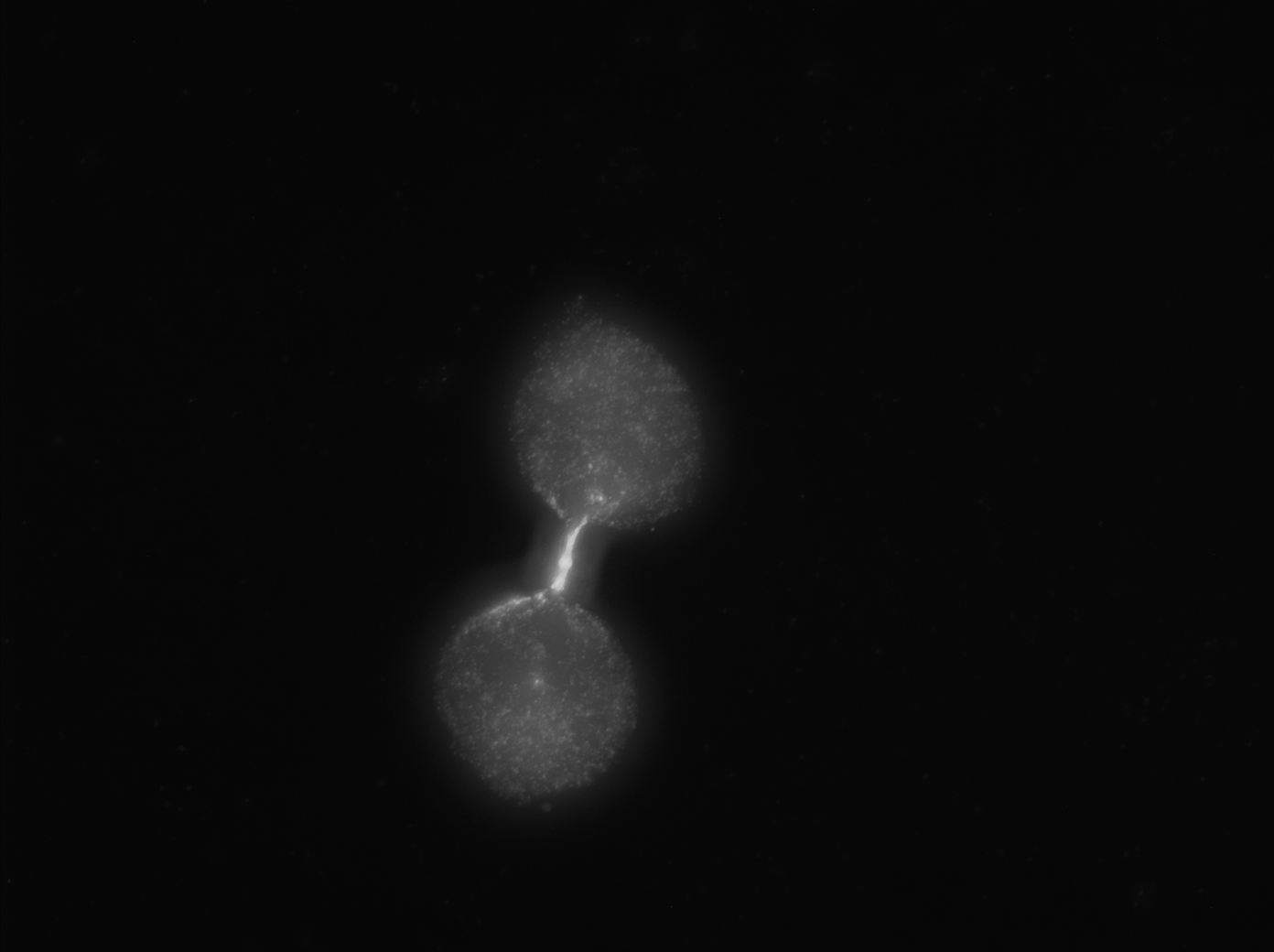

Supplement: Supplementary file 12 — Source data Fig. 3 part 2 [file 44319_2026_742_MOESM12_ESM.zip › Figure 3 Part 2/Fig 3b-e TTLL screen part 2/TTLL6-YFP R1 I2.Project Maximum Z_XY1663275354_Z0_T0_C1.tif]

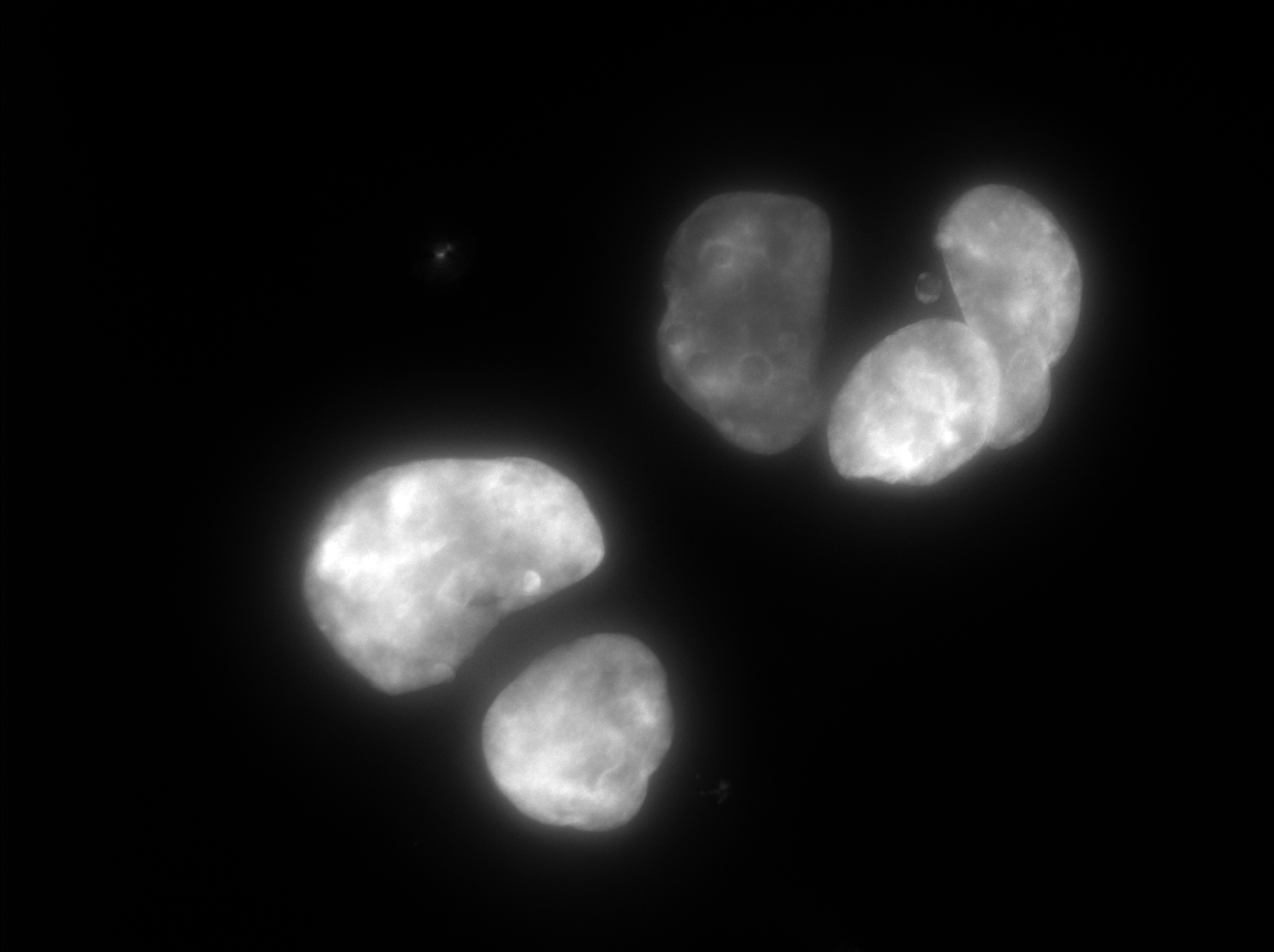

Supplement: Supplementary file 12 — Source data Fig. 3 part 2 [file 44319_2026_742_MOESM12_ESM.zip › Figure 3 Part 2/Fig 3b-e TTLL screen part 2/TTLL6-YFP R1 T5 hiigh.Project Maximum Z_XY1661551952_Z0_T0_C0.tif]

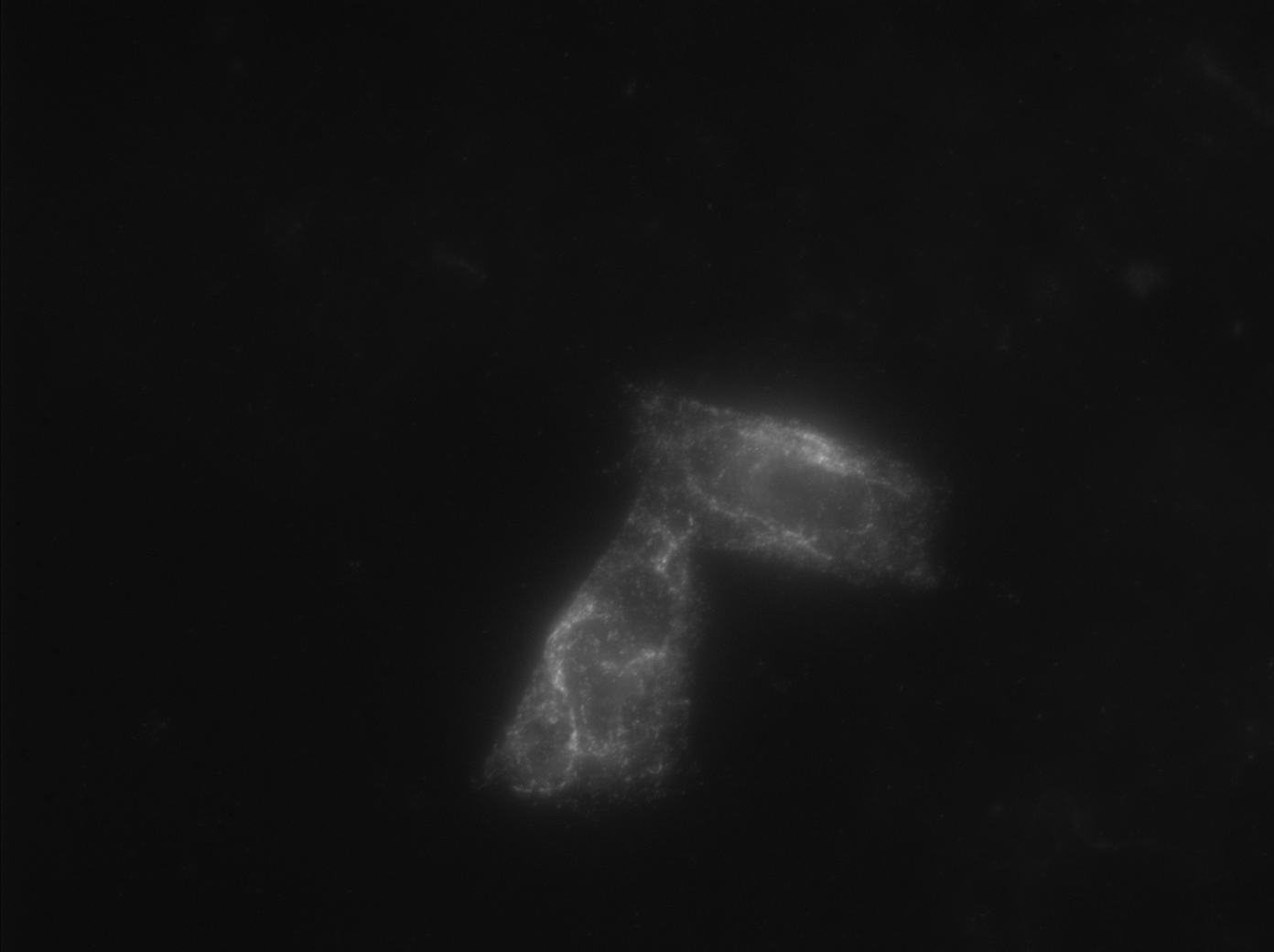

Supplement: Supplementary file 12 — Source data Fig. 3 part 2 [file 44319_2026_742_MOESM12_ESM.zip › Figure 3 Part 2/Fig 3b-e TTLL screen part 2/TTLL6-YFP SS I1.Project Maximum Z_XY1663881583_Z0_T0_C1.tif]

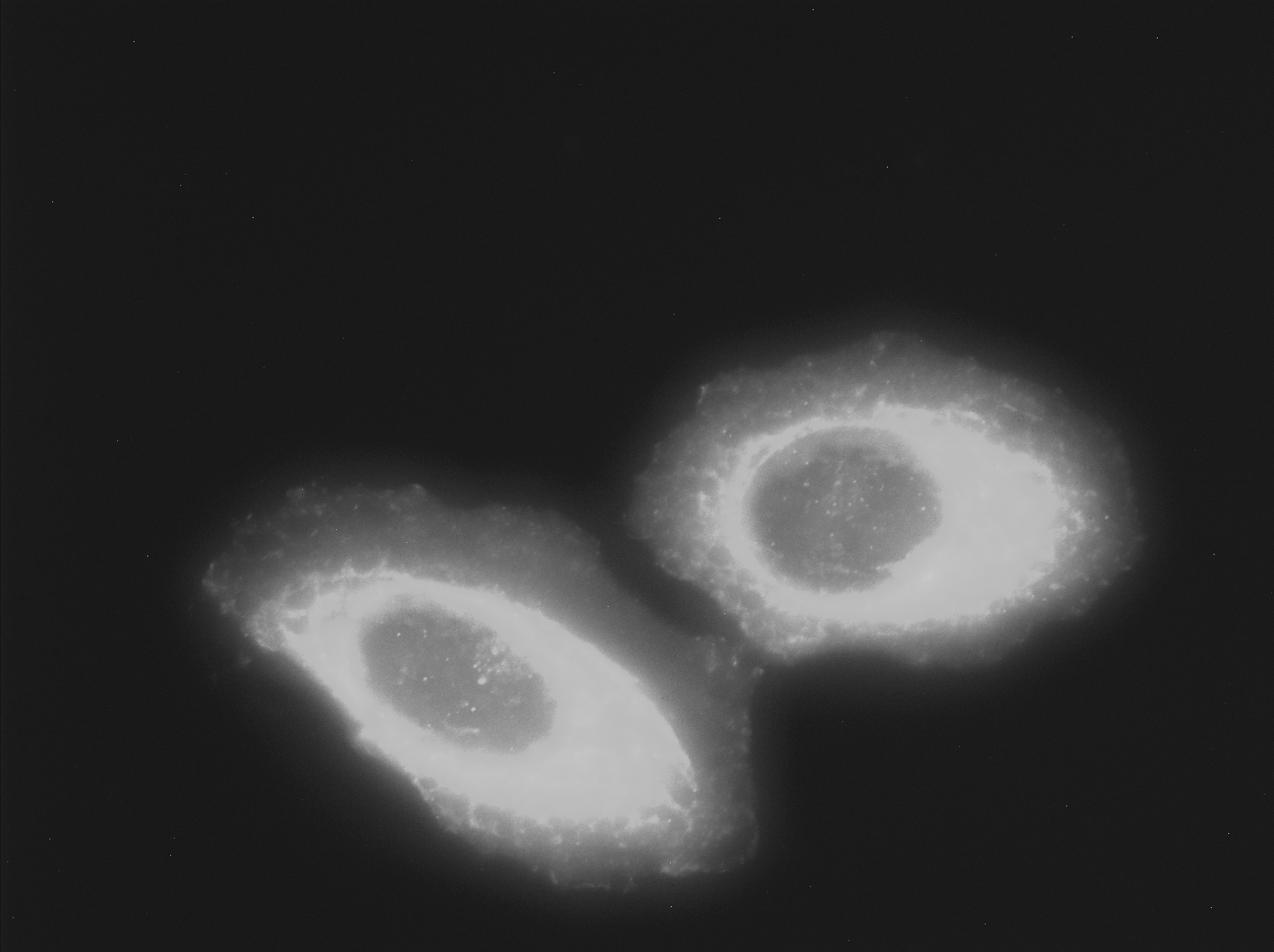

Supplement: Supplementary file 12 — Source data Fig. 3 part 2 [file 44319_2026_742_MOESM12_ESM.zip › Figure 3 Part 2/Fig 3b-e TTLL screen part 2/TTLL6-YFP R1 T1.Project Maximum Z_XY1661548775_Z0_T0_C2.tif]

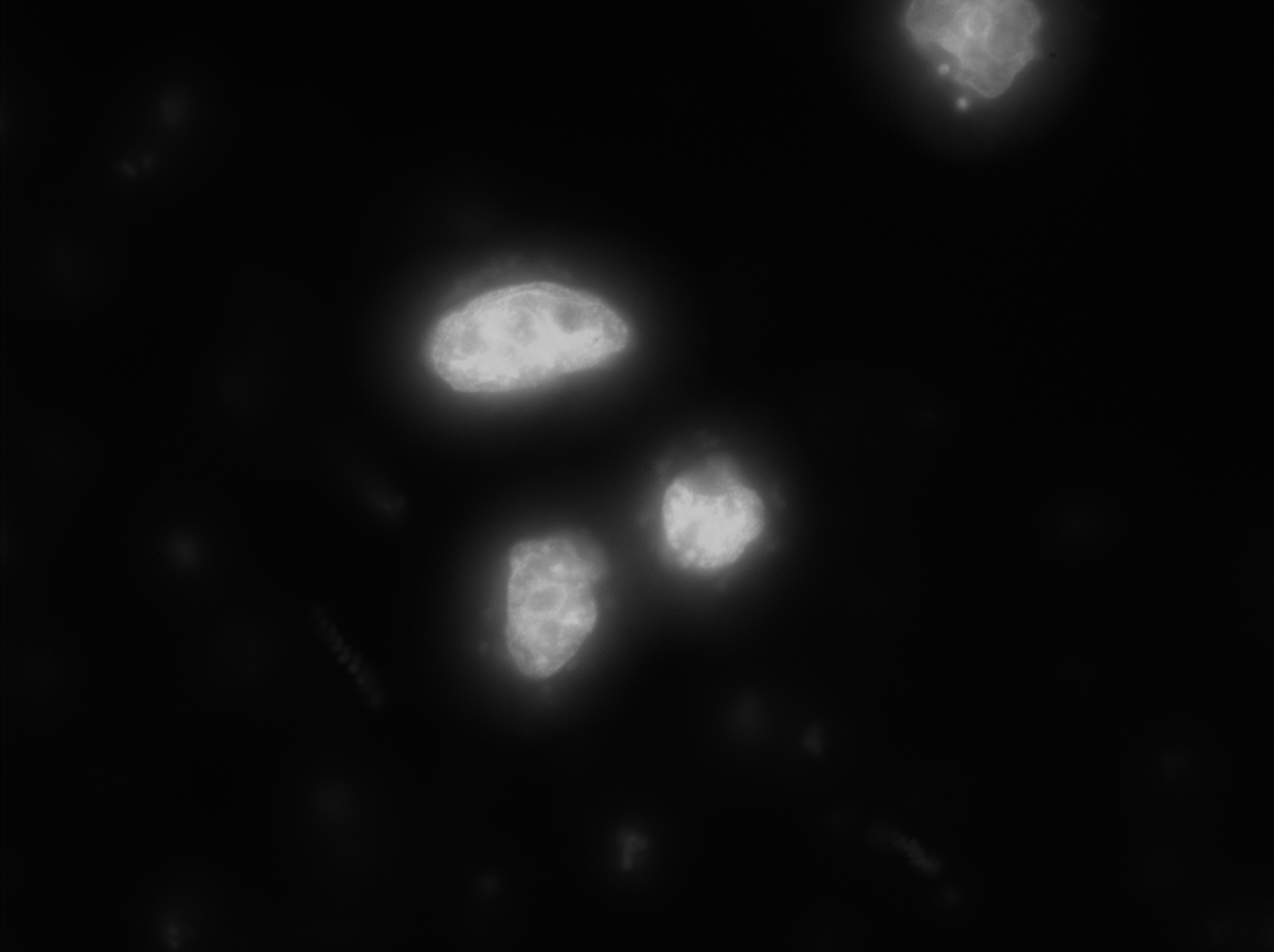

Supplement: Supplementary file 12 — Source data Fig. 3 part 2 [file 44319_2026_742_MOESM12_ESM.zip › Figure 3 Part 2/Fig 3b-e TTLL screen part 2/TTLL5-YFPy I20.Project Maximum Z_XY1679341653_Z0_T0_C0.tif]

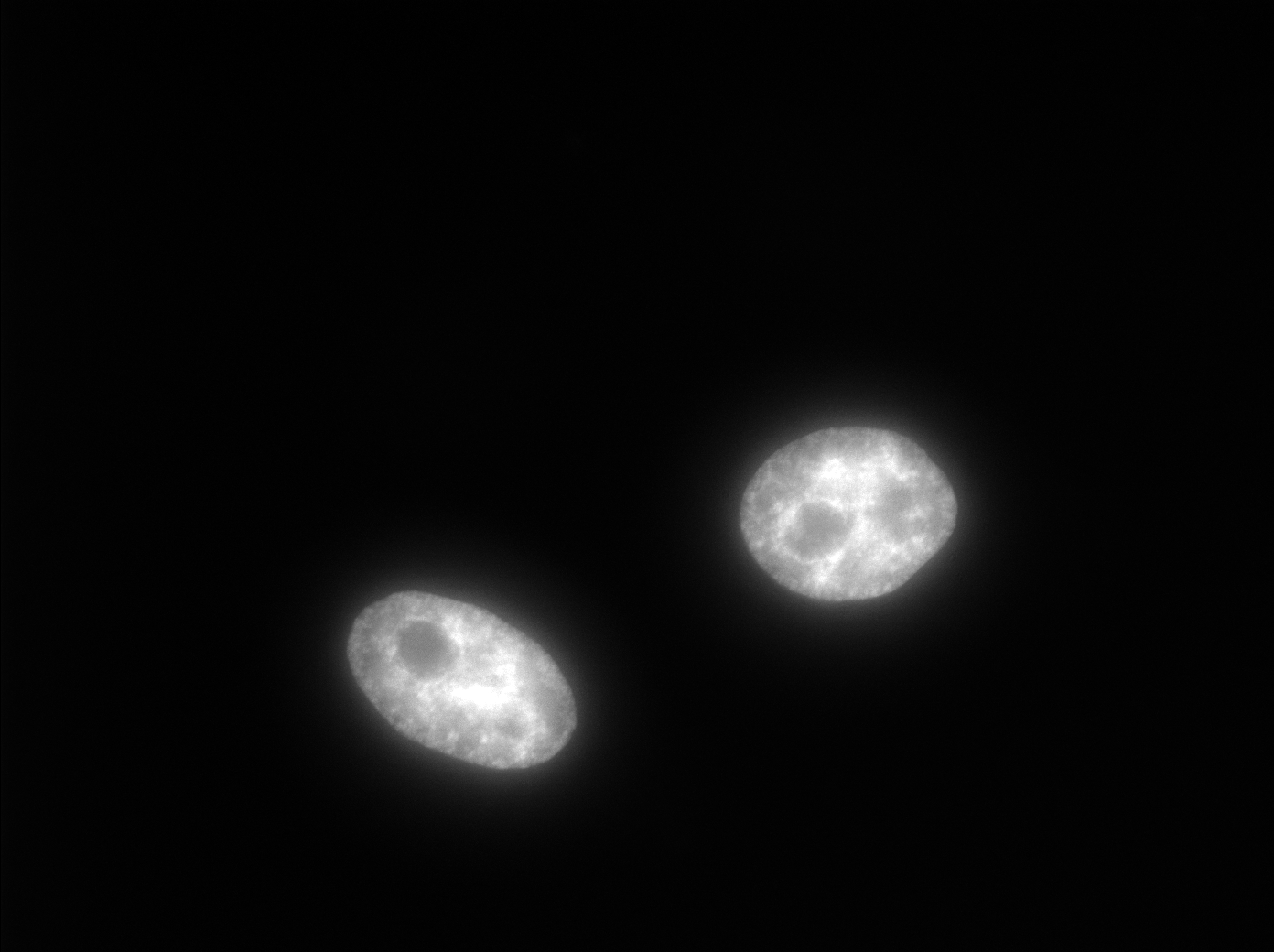

Supplement: Supplementary file 12 — Source data Fig. 3 part 2 [file 44319_2026_742_MOESM12_ESM.zip › Figure 3 Part 2/Fig 3b-e TTLL screen part 2/TTLL6-YFP R1 T1.Project Maximum Z_XY1661548775_Z0_T0_C0.tif]

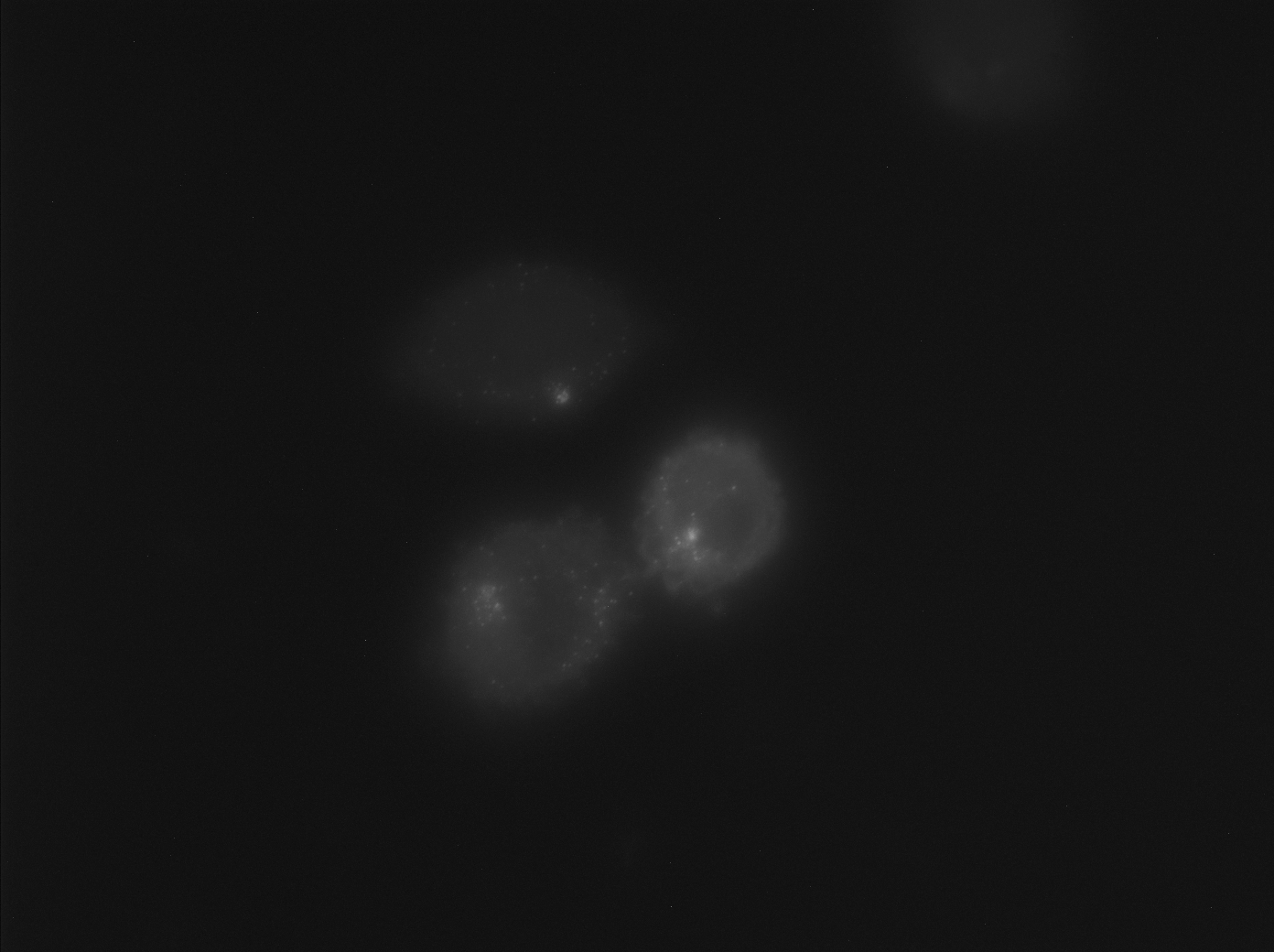

Supplement: Supplementary file 12 — Source data Fig. 3 part 2 [file 44319_2026_742_MOESM12_ESM.zip › Figure 3 Part 2/Fig 3b-e TTLL screen part 2/TTLL5-YFPy I20.Project Maximum Z_XY1679341653_Z0_T0_C2.tif]

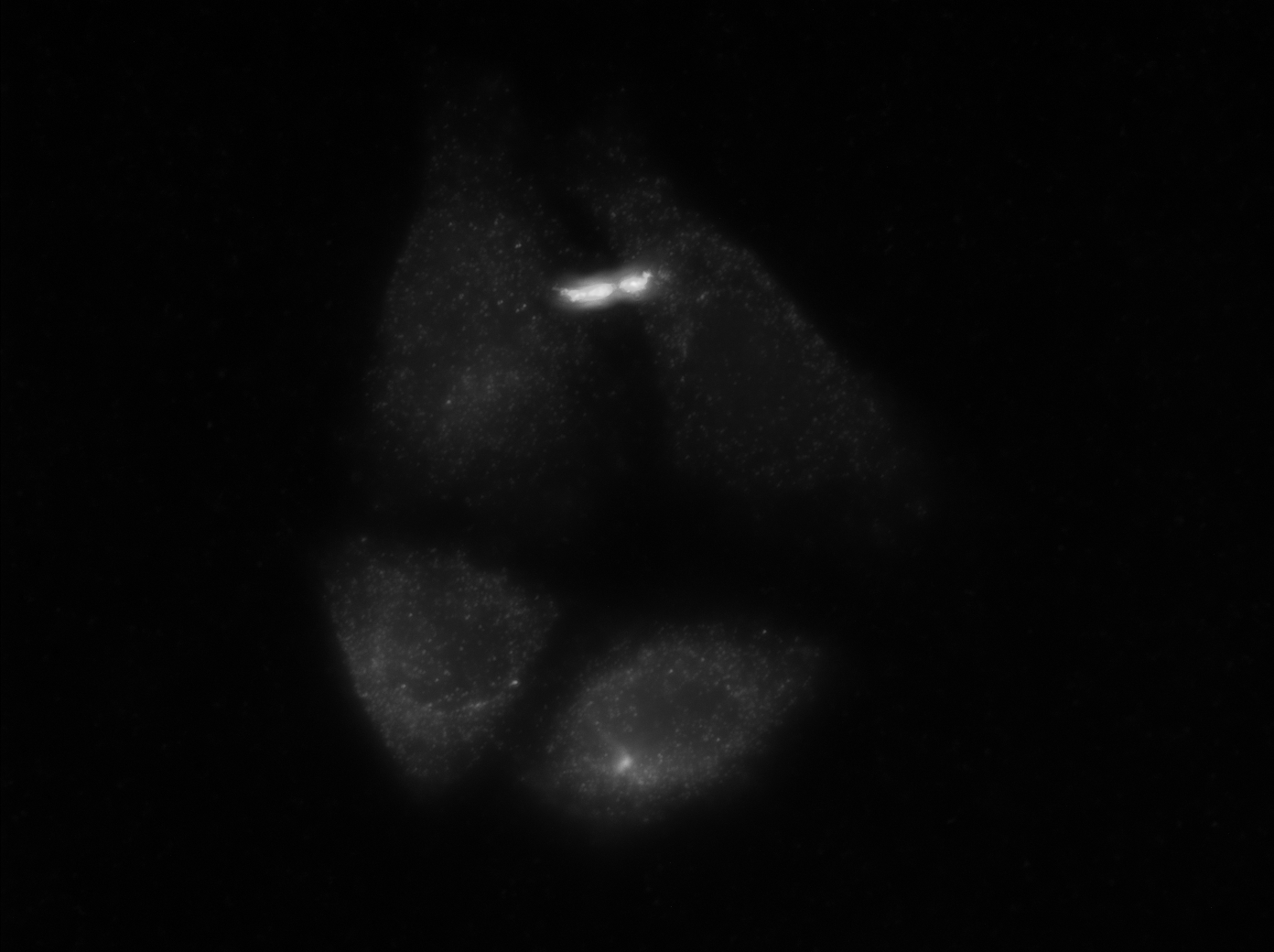

Supplement: Supplementary file 12 — Source data Fig. 3 part 2 [file 44319_2026_742_MOESM12_ESM.zip › Figure 3 Part 2/Fig 3b-e TTLL screen part 2/TTLL6-YFP R1 I1 C2.Project Maximum Z_XY1661791366_Z0_T0_C1.tif]

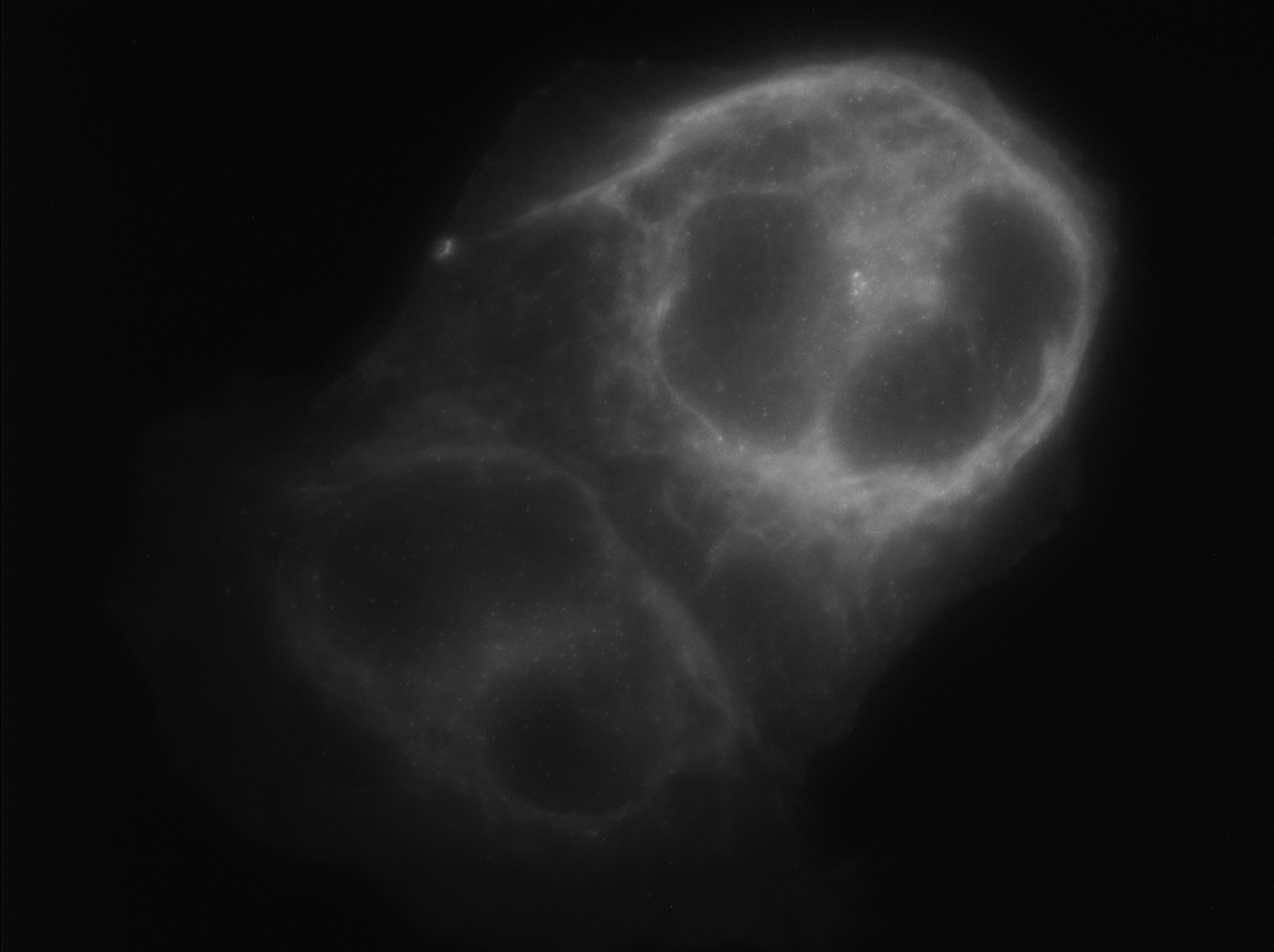

Supplement: Supplementary file 12 — Source data Fig. 3 part 2 [file 44319_2026_742_MOESM12_ESM.zip › Figure 3 Part 2/Fig 3b-e TTLL screen part 2/TTLL6-YFP R1 T5 hiigh.Project Maximum Z_XY1661551952_Z0_T0_C2.tif]

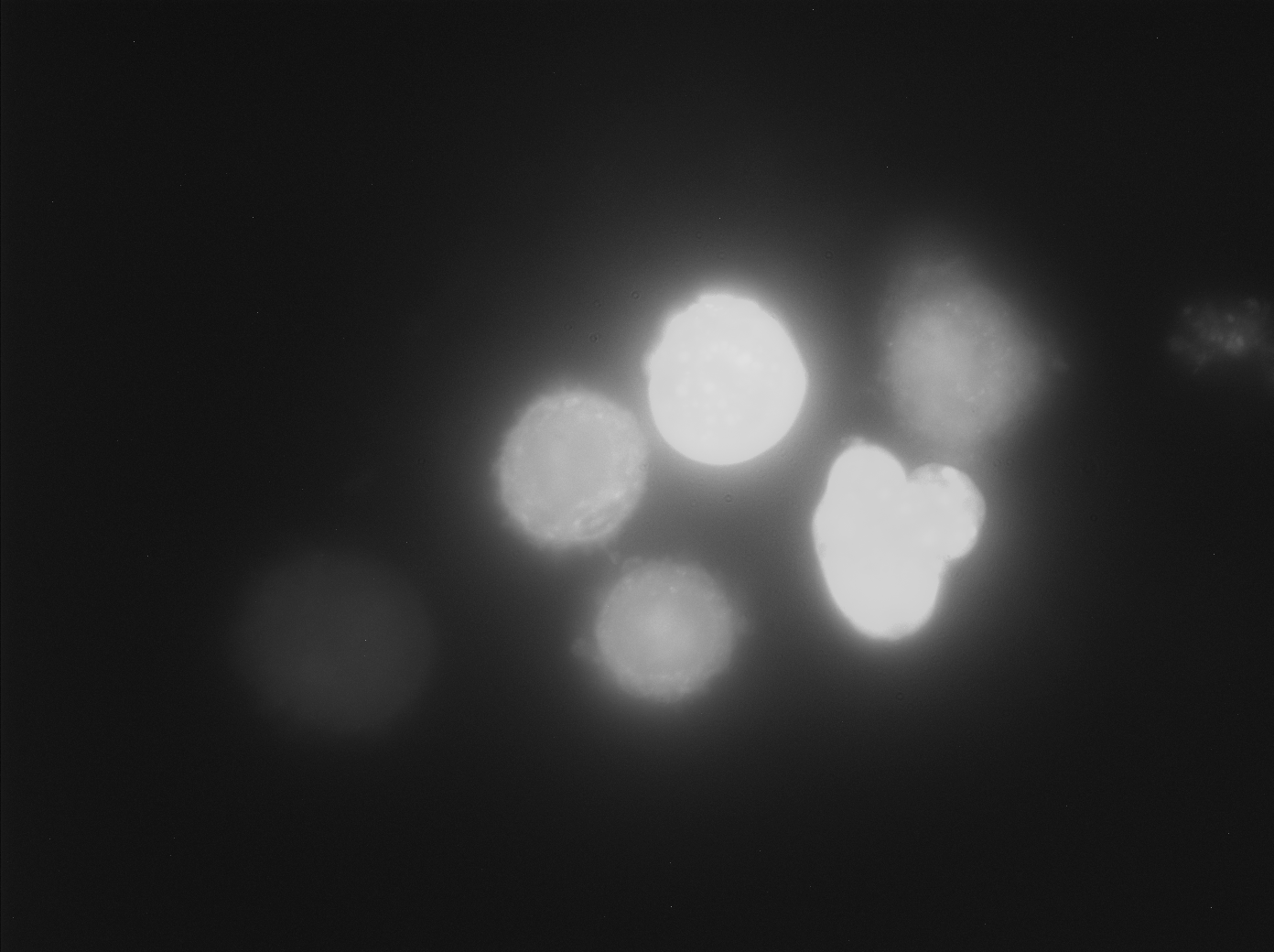

Supplement: Supplementary file 12 — Source data Fig. 3 part 2 [file 44319_2026_742_MOESM12_ESM.zip › Figure 3 Part 2/Fig 3b-e TTLL screen part 2/TTLL7-YFPy I2.Project Maximum Z_XY1679087175_Z0_T0_C2.tif]

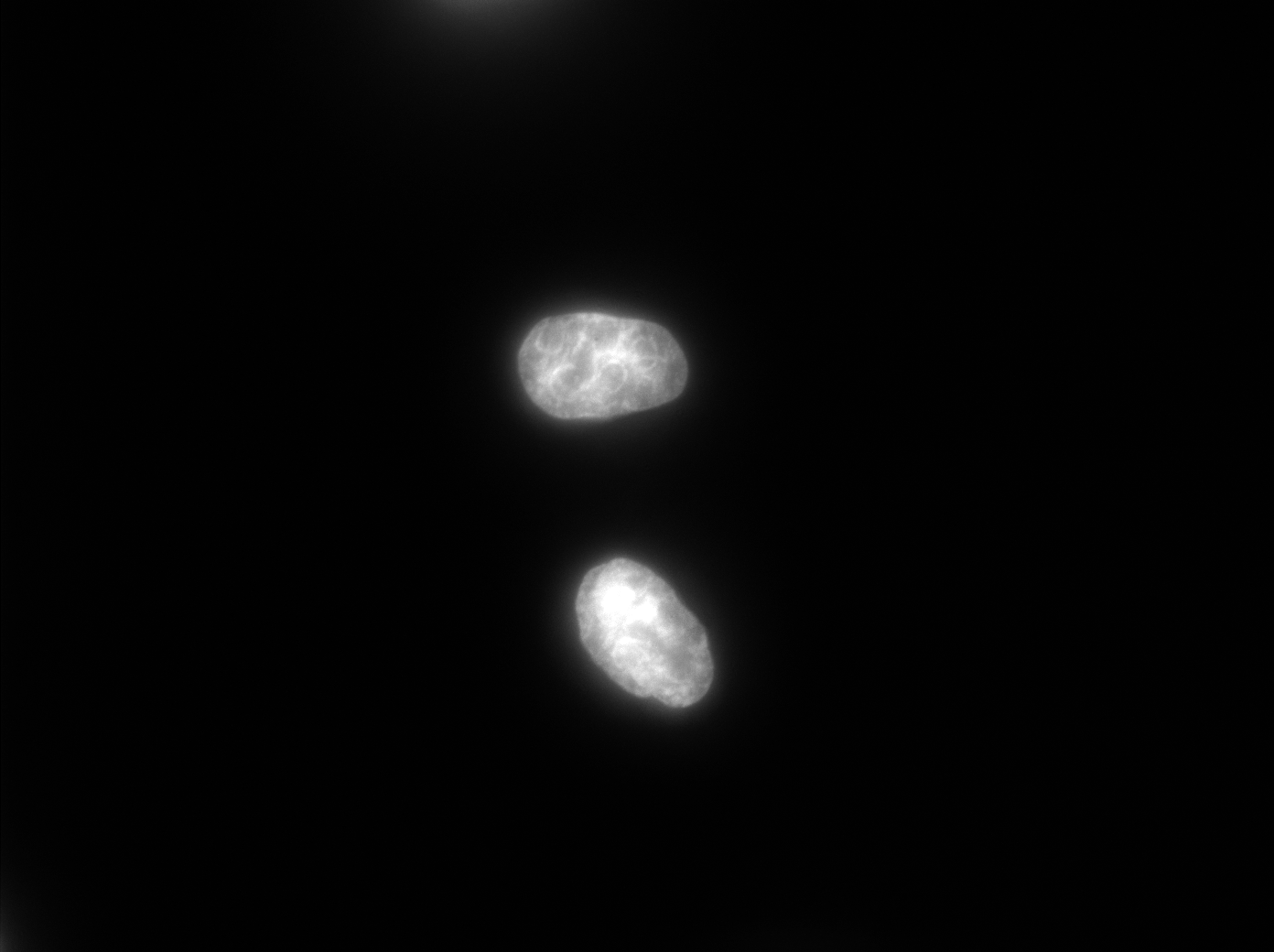

Supplement: Supplementary file 12 — Source data Fig. 3 part 2 [file 44319_2026_742_MOESM12_ESM.zip › Figure 3 Part 2/Fig 3b-e TTLL screen part 2/TTLL6-YFP R1 I1 low int.Project Maximum Z_XY1661545829_Z0_T0_C0.tif]

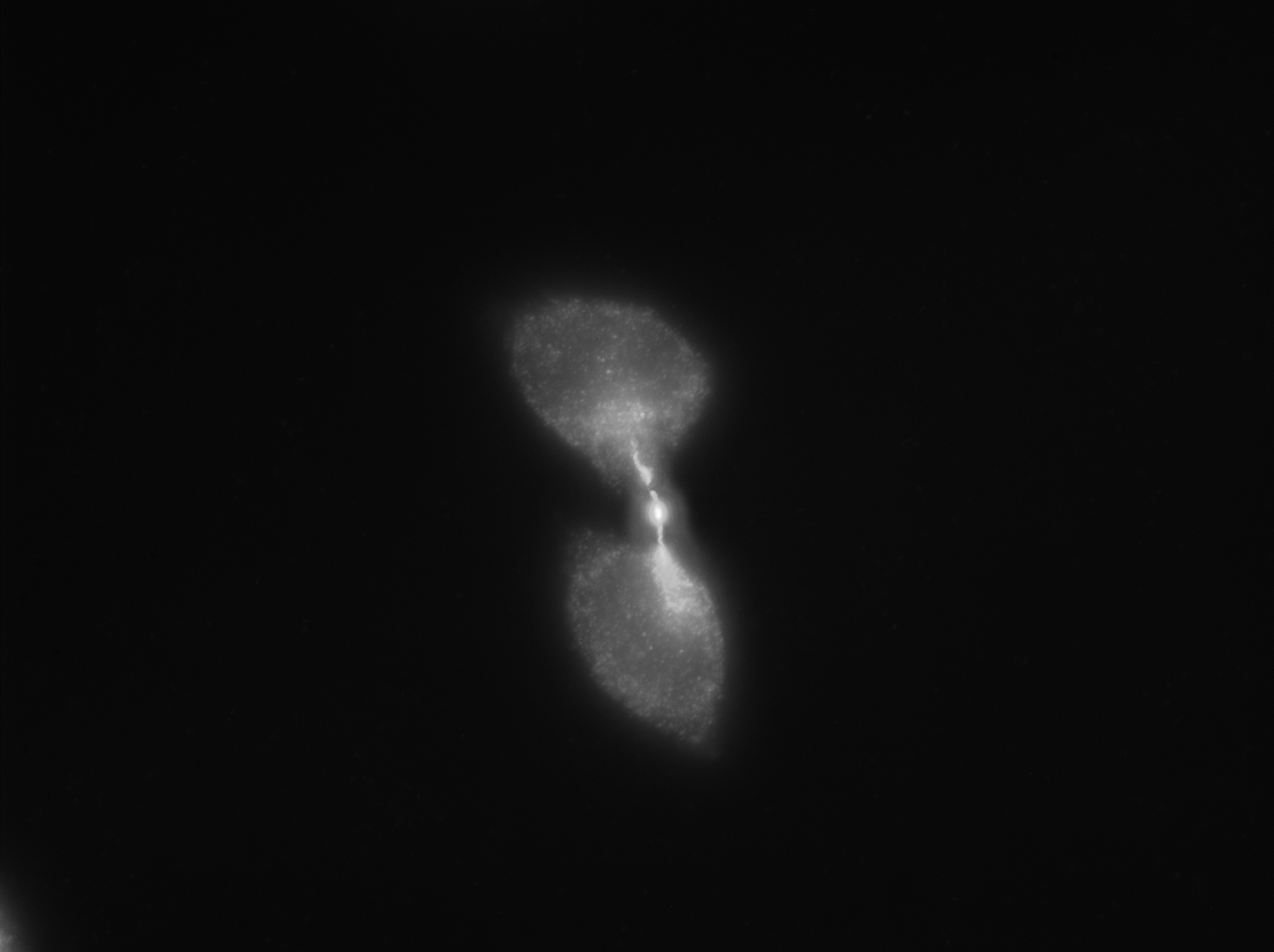

Supplement: Supplementary file 12 — Source data Fig. 3 part 2 [file 44319_2026_742_MOESM12_ESM.zip › Figure 3 Part 2/Fig 3b-e TTLL screen part 2/TTLL6-YFP R1 I1 low int.Project Maximum Z_XY1661545829_Z0_T0_C1.tif]

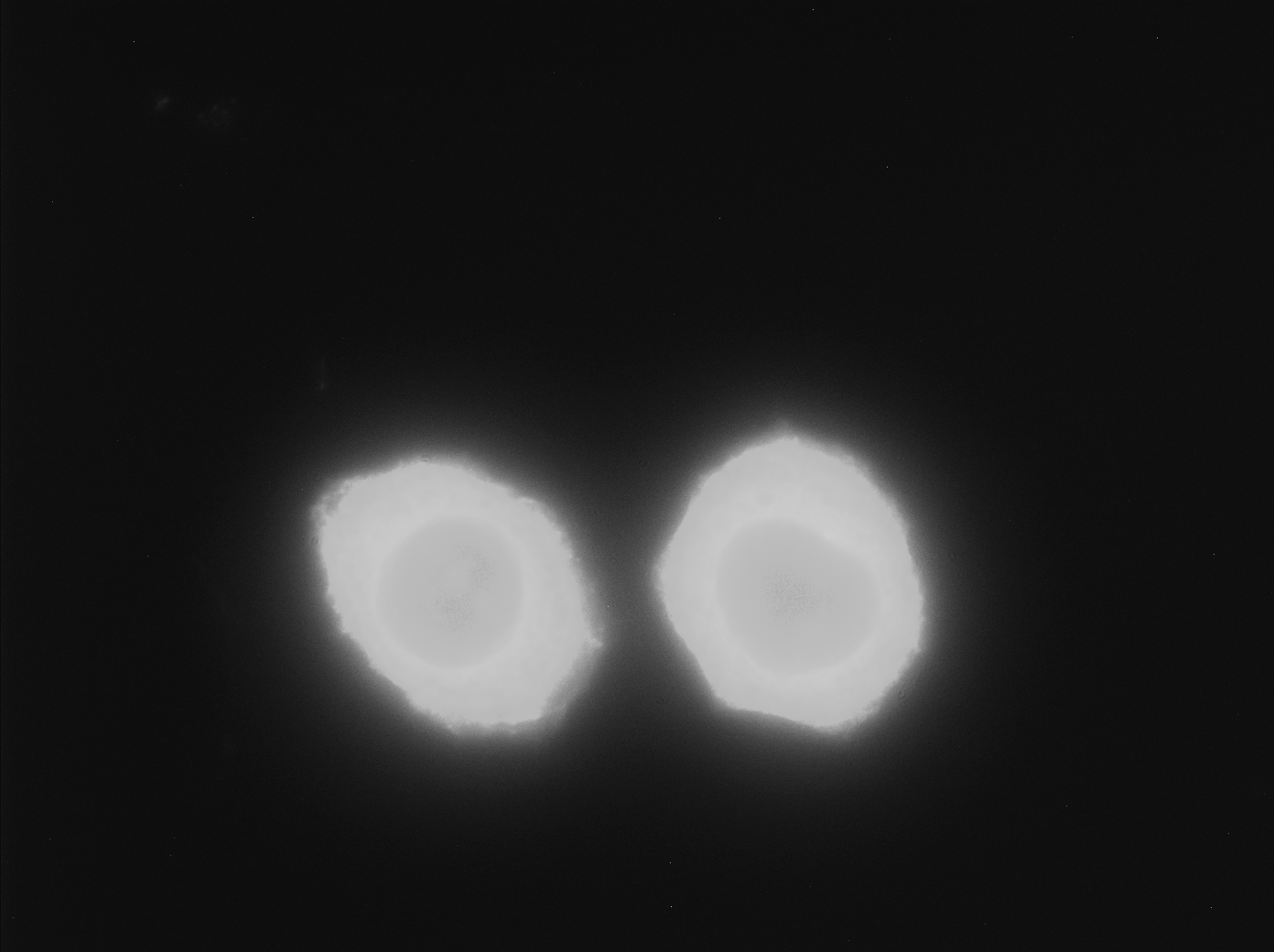

Supplement: Supplementary file 12 — Source data Fig. 3 part 2 [file 44319_2026_742_MOESM12_ESM.zip › Figure 3 Part 2/Fig 3b-e TTLL screen part 2/TTLL6-YFP R1 T3 high int.Project Maximum Z_XY1661549582_Z0_T0_C2.tif]

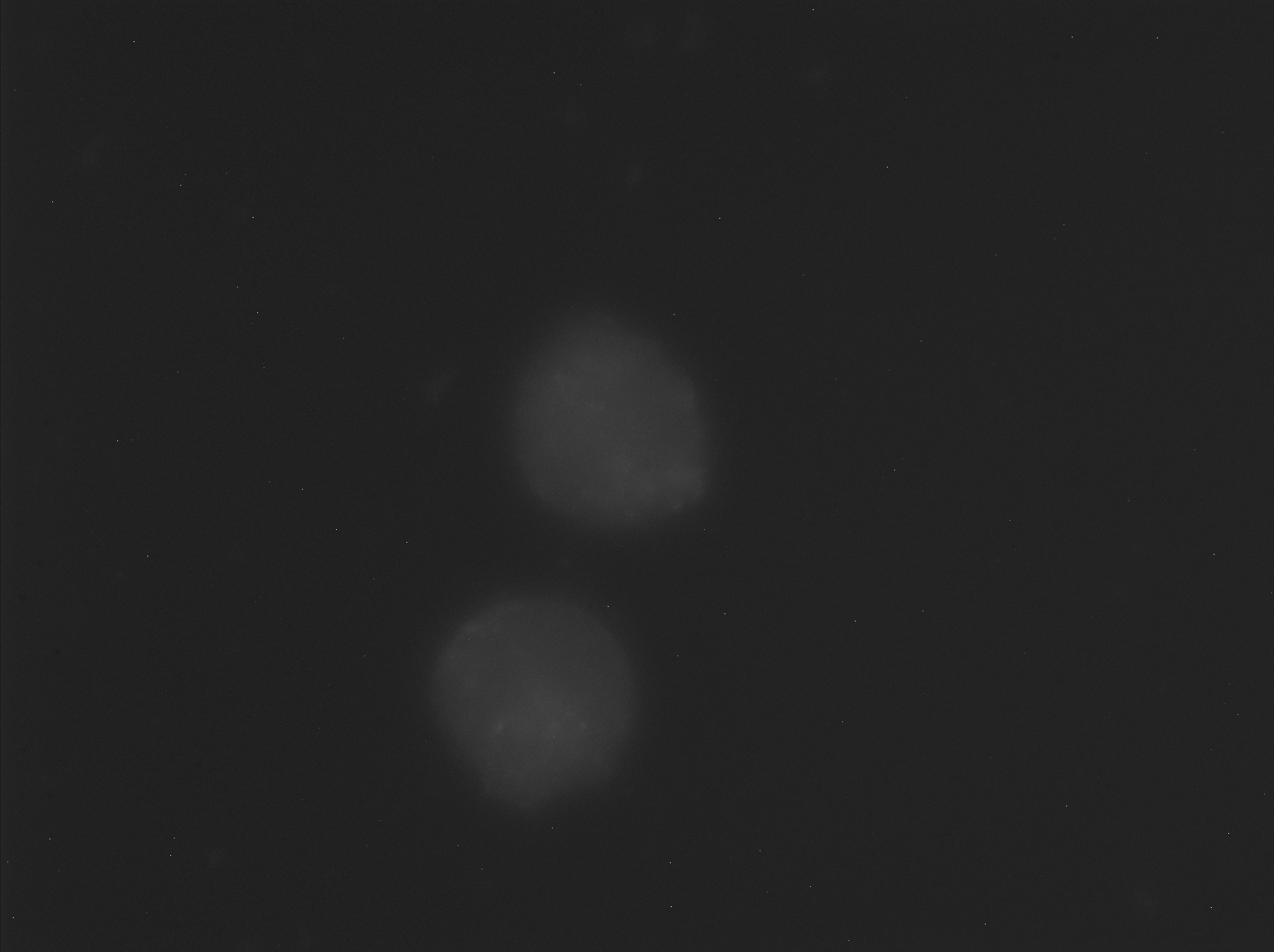

Supplement: Supplementary file 12 — Source data Fig. 3 part 2 [file 44319_2026_742_MOESM12_ESM.zip › Figure 3 Part 2/Fig 3b-e TTLL screen part 2/TTLL6-YFP R1 I2.Project Maximum Z_XY1663275354_Z0_T0_C2.tif]

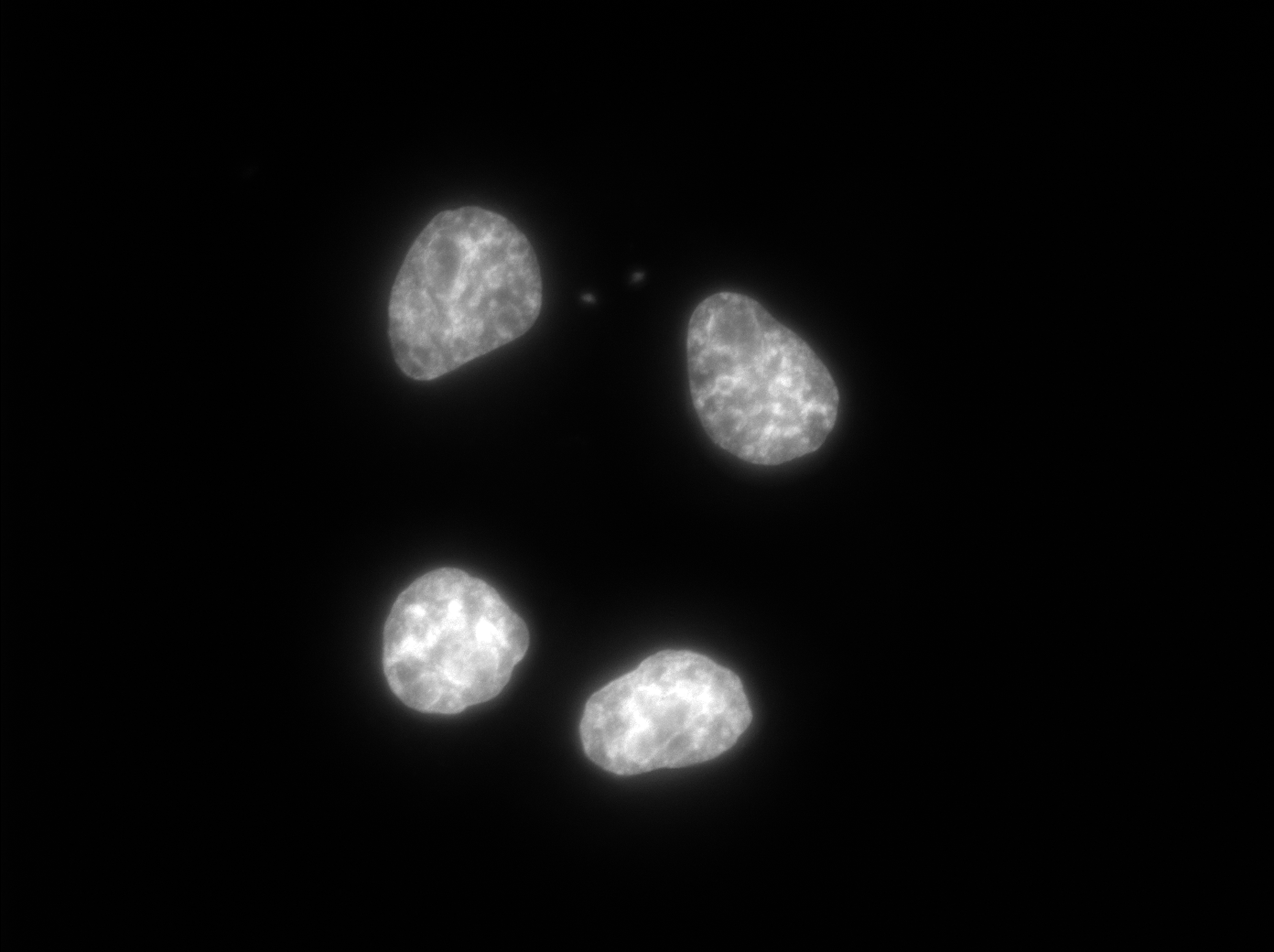

Supplement: Supplementary file 12 — Source data Fig. 3 part 2 [file 44319_2026_742_MOESM12_ESM.zip › Figure 3 Part 2/Fig 3b-e TTLL screen part 2/TTLL6-YFP R1 I1 C2.Project Maximum Z_XY1661791366_Z0_T0_C0.tif]

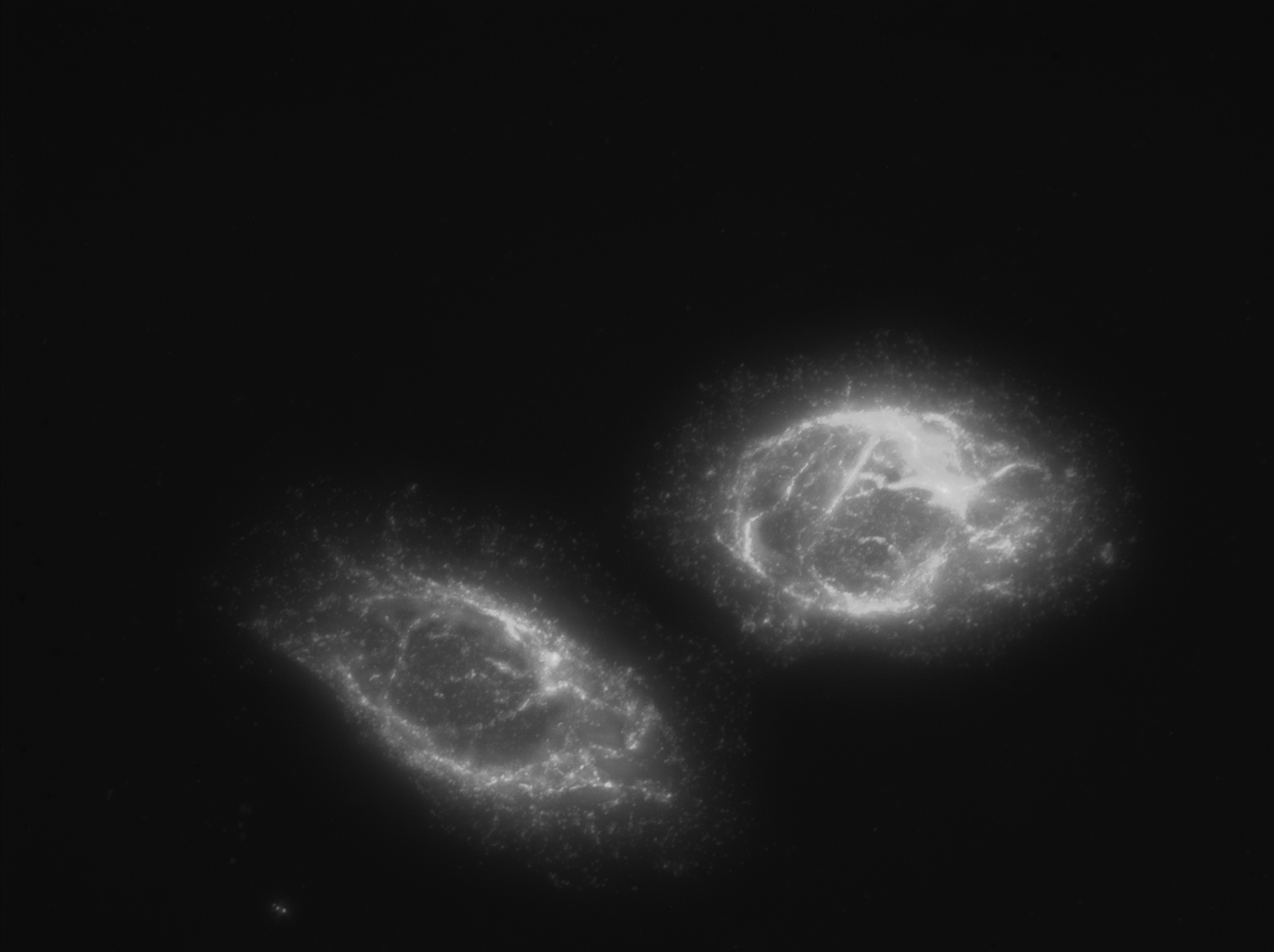

Supplement: Supplementary file 12 — Source data Fig. 3 part 2 [file 44319_2026_742_MOESM12_ESM.zip › Figure 3 Part 2/Fig 3b-e TTLL screen part 2/TTLL6-YFP R1 T1.Project Maximum Z_XY1661548775_Z0_T0_C1.tif]

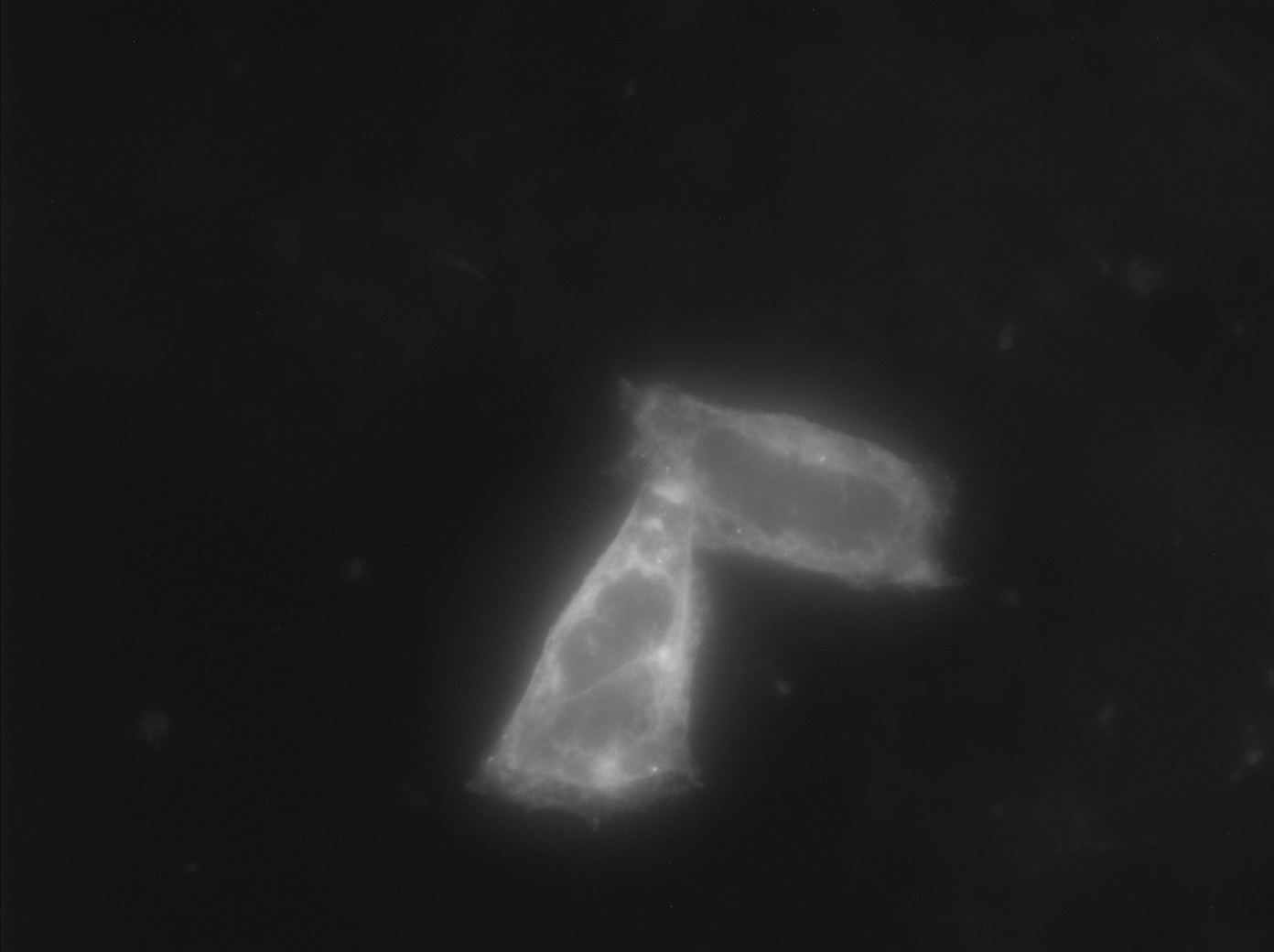

Supplement: Supplementary file 12 — Source data Fig. 3 part 2 [file 44319_2026_742_MOESM12_ESM.zip › Figure 3 Part 2/Fig 3b-e TTLL screen part 2/TTLL6-YFP SS I1.Project Maximum Z_XY1663881583_Z0_T0_C2.tif]

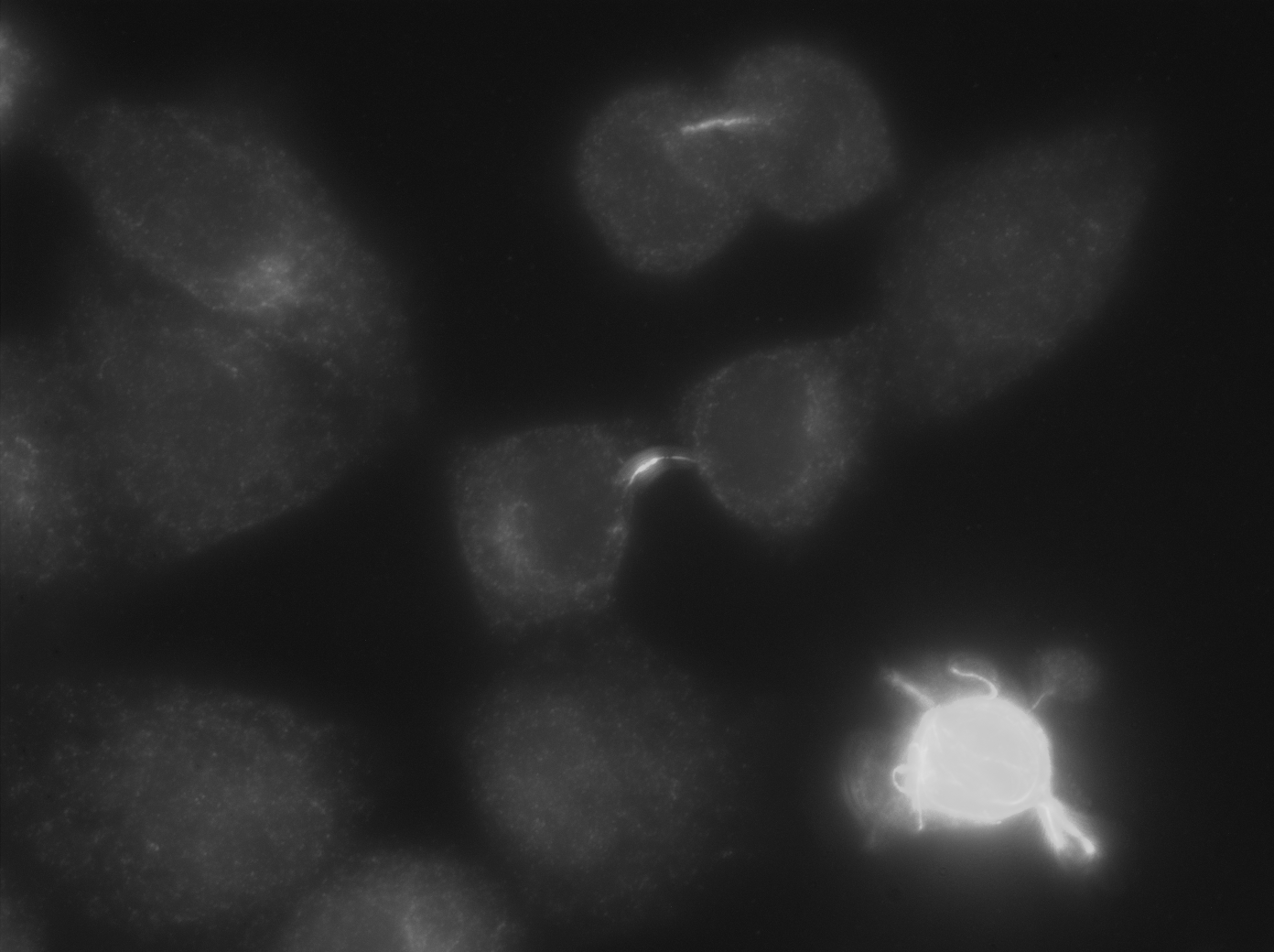

Supplement: Supplementary file 12 — Source data Fig. 3 part 2 [file 44319_2026_742_MOESM12_ESM.zip › Figure 3 Part 2/Fig 3b-e TTLL screen part 2/TTLL7-YFPy I14.Project Maximum Z_XY1679090342_Z0_T0_C1.tif]

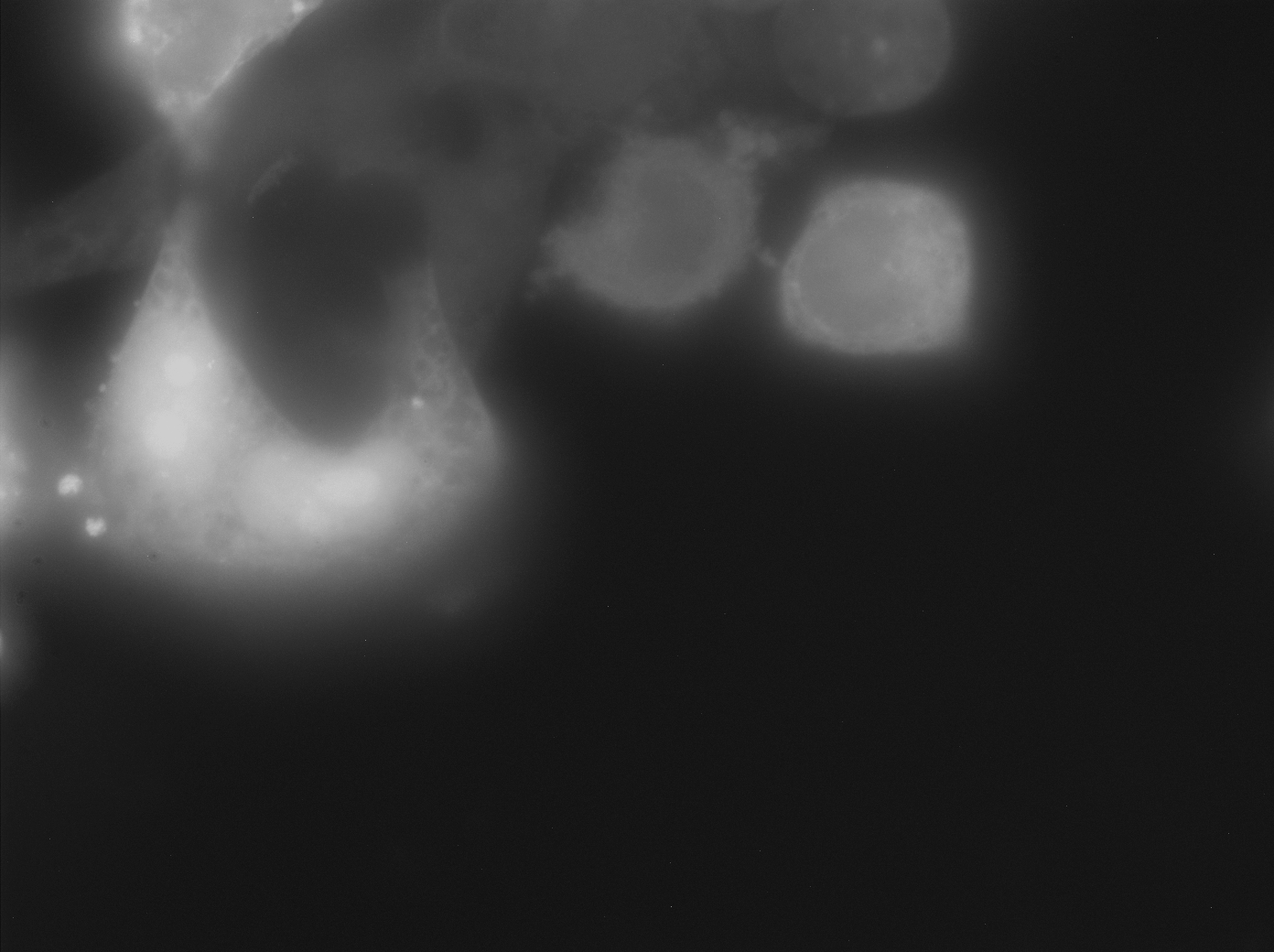

Supplement: Supplementary file 12 — Source data Fig. 3 part 2 [file 44319_2026_742_MOESM12_ESM.zip › Figure 3 Part 2/Fig 3b-e TTLL screen part 2/TTLL7-YFPy I11.Project Maximum Z_XY1679089317_Z0_T0_C2.tif]

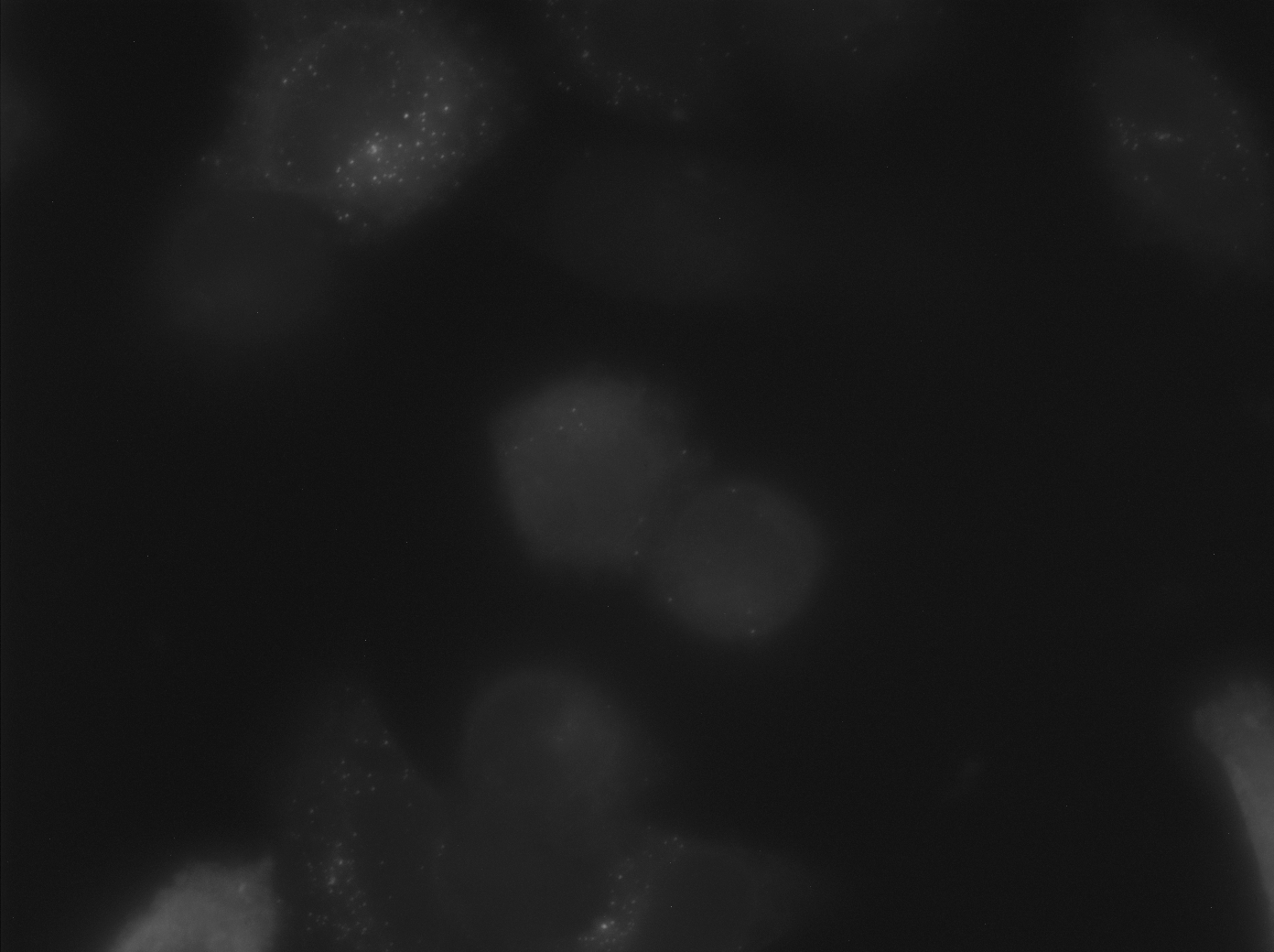

Supplement: Supplementary file 12 — Source data Fig. 3 part 2 [file 44319_2026_742_MOESM12_ESM.zip › Figure 3 Part 2/Fig 3b-e TTLL screen part 2/TTLL5-YFPy I11.Project Maximum Z_XY1679340264_Z0_T0_C2.tif]

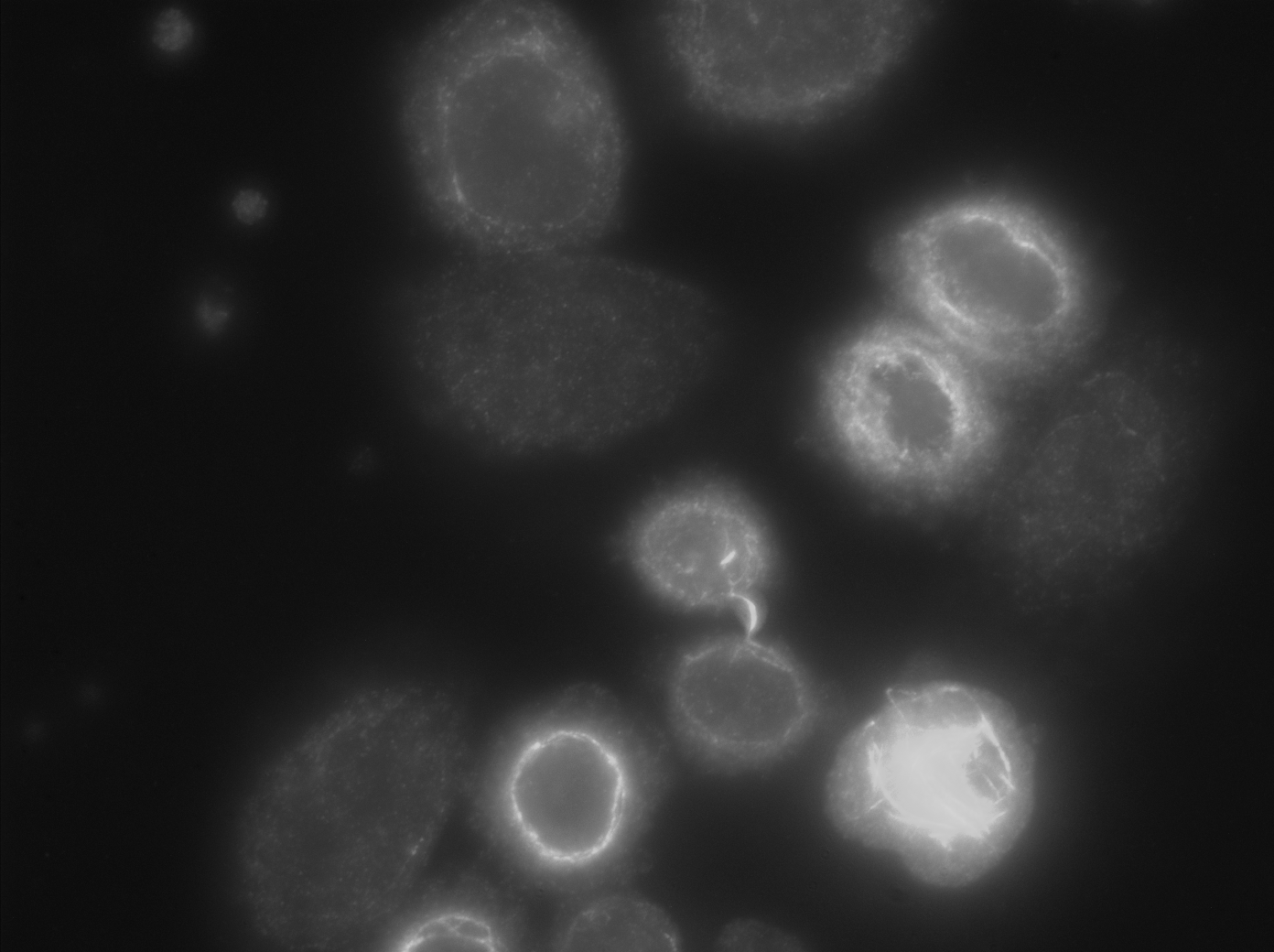

Supplement: Supplementary file 12 — Source data Fig. 3 part 2 [file 44319_2026_742_MOESM12_ESM.zip › Figure 3 Part 2/Fig 3b-e TTLL screen part 2/TTLL7-YFPy I19.Project Maximum Z_XY1679091282_Z0_T0_C1.tif]

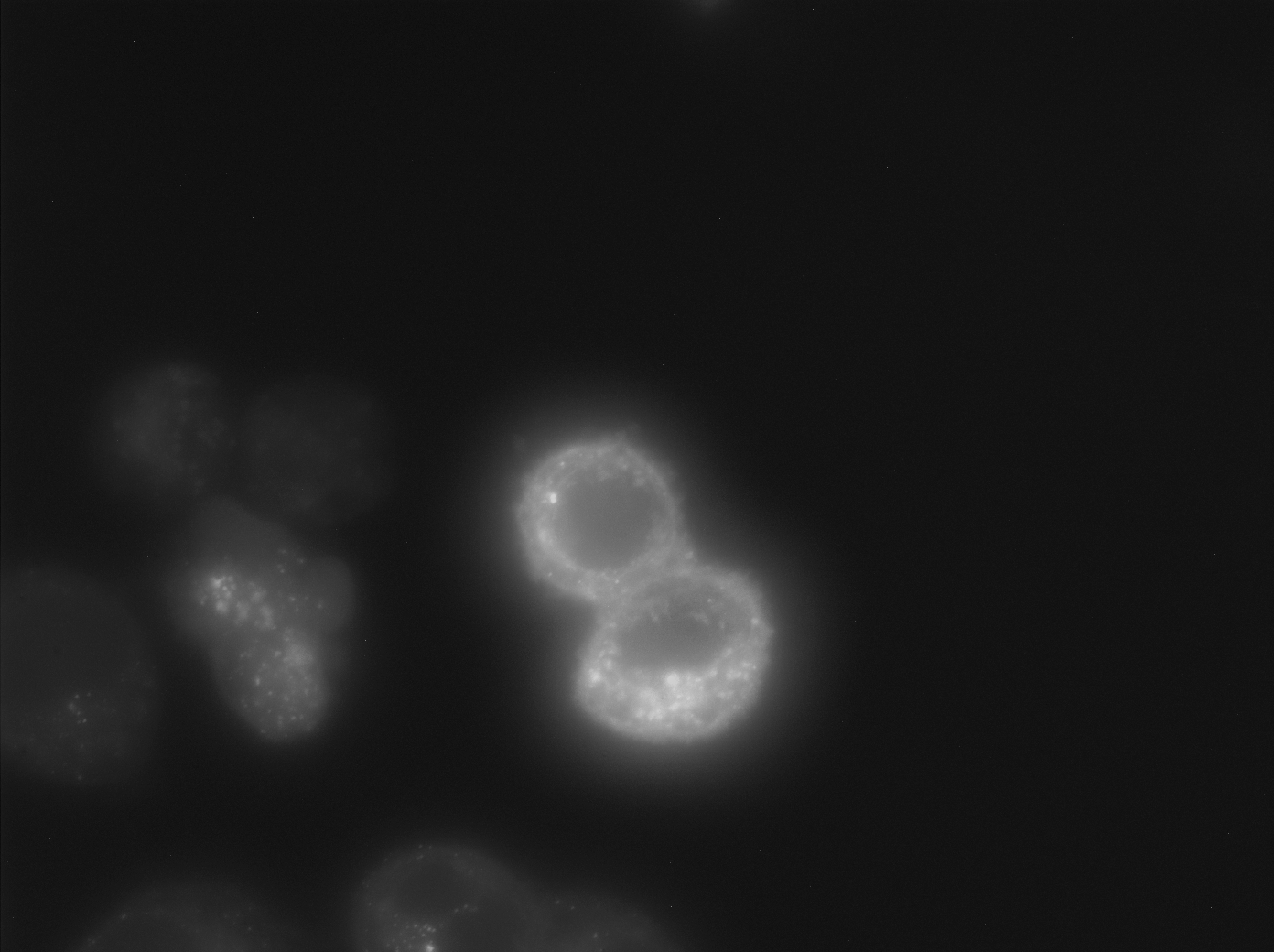

Supplement: Supplementary file 12 — Source data Fig. 3 part 2 [file 44319_2026_742_MOESM12_ESM.zip › Figure 3 Part 2/Fig 3b-e TTLL screen part 2/TTLL5-YFPy I1.Project Maximum Z_XY1679082481_Z0_T0_C2.tif]

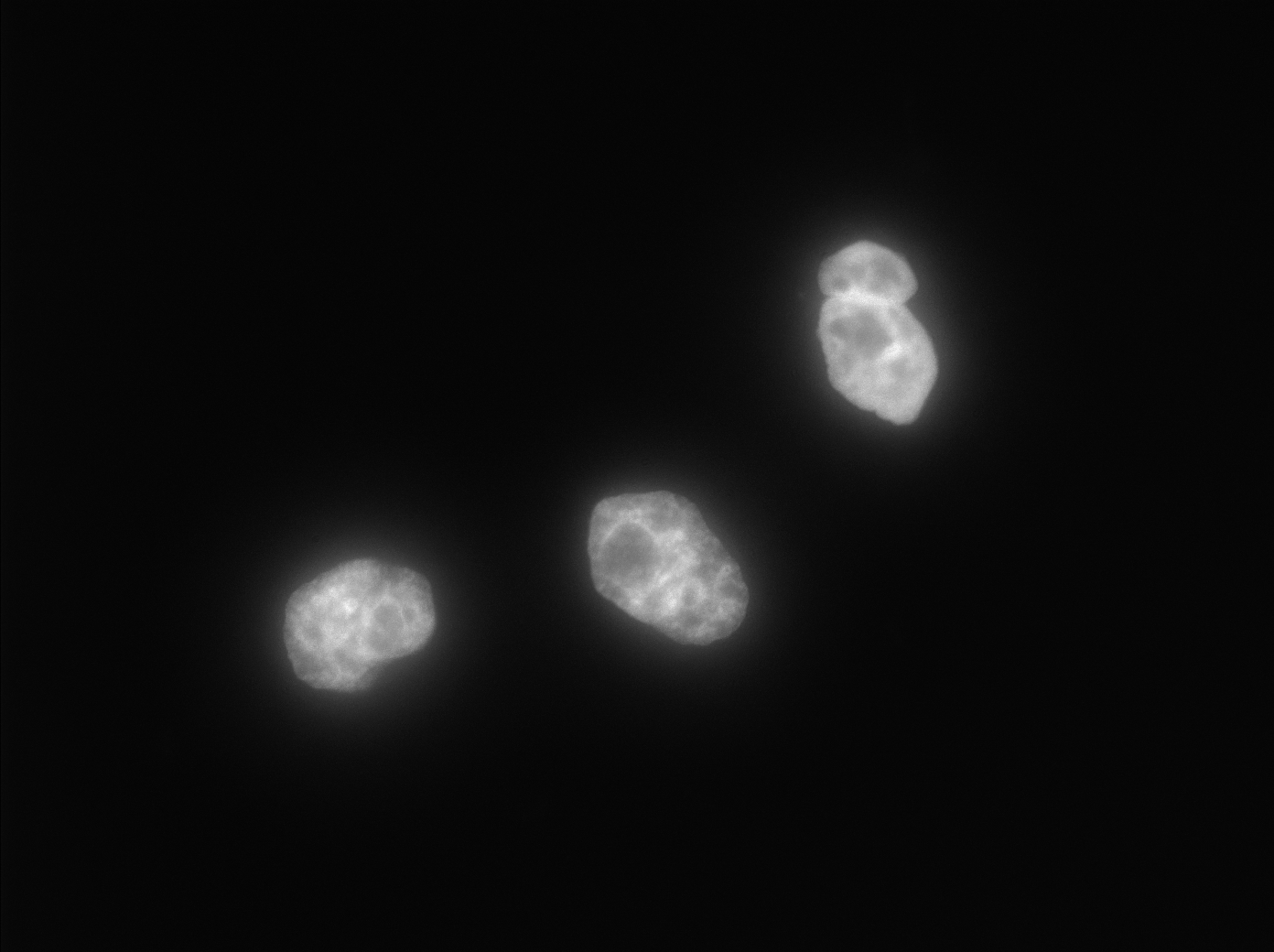

Supplement: Supplementary file 12 — Source data Fig. 3 part 2 [file 44319_2026_742_MOESM12_ESM.zip › Figure 3 Part 2/Fig 3b-e TTLL screen part 2/TTLL6-YFP R1 I5 w T high.Project Maximum Z_XY1661550001_Z0_T0_C0.tif]

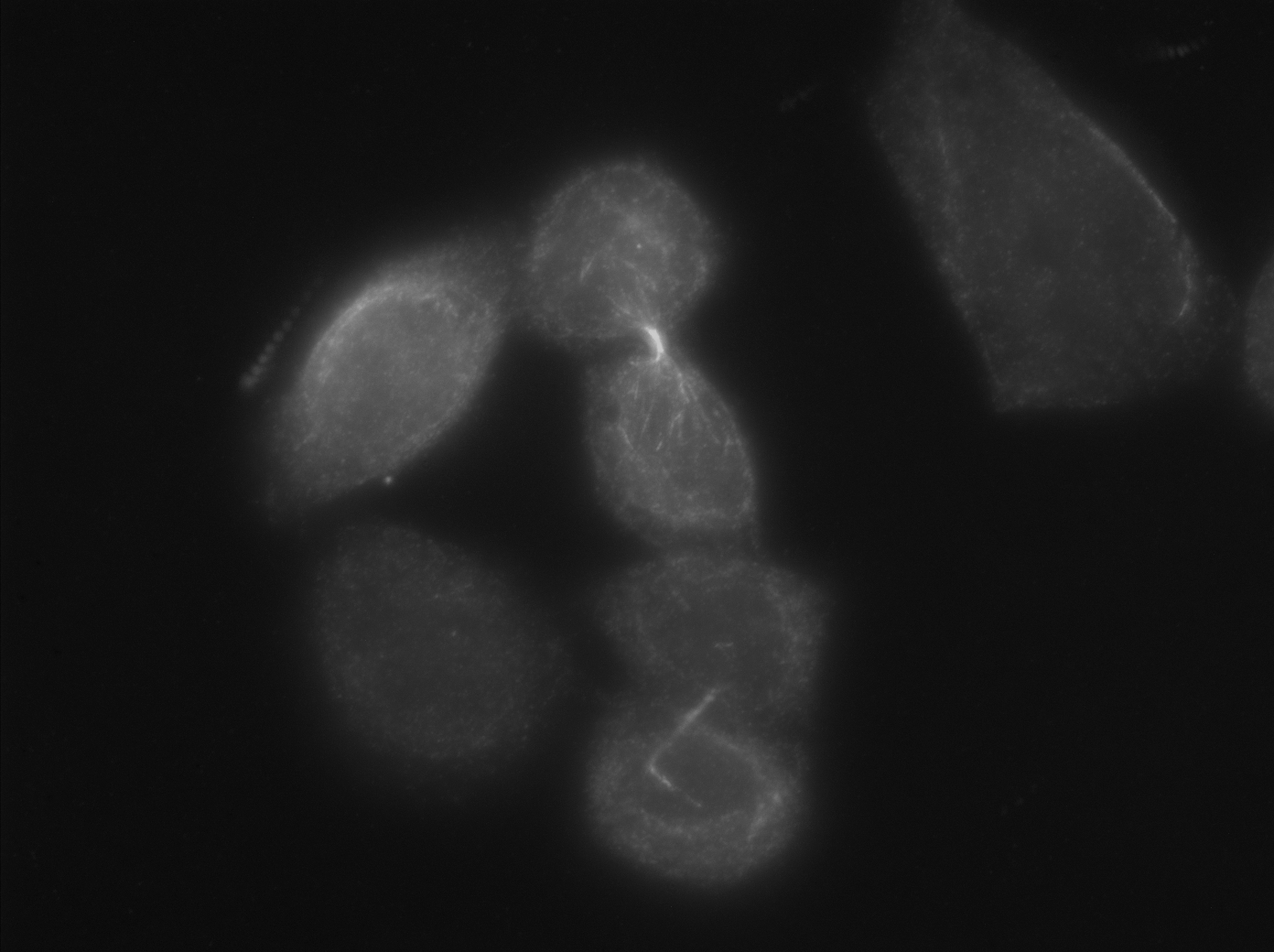

Supplement: Supplementary file 12 — Source data Fig. 3 part 2 [file 44319_2026_742_MOESM12_ESM.zip › Figure 3 Part 2/Fig 3b-e TTLL screen part 2/TTLL5-YFPy I2.Project Maximum Z_XY1679082686_Z0_T0_C1.tif]

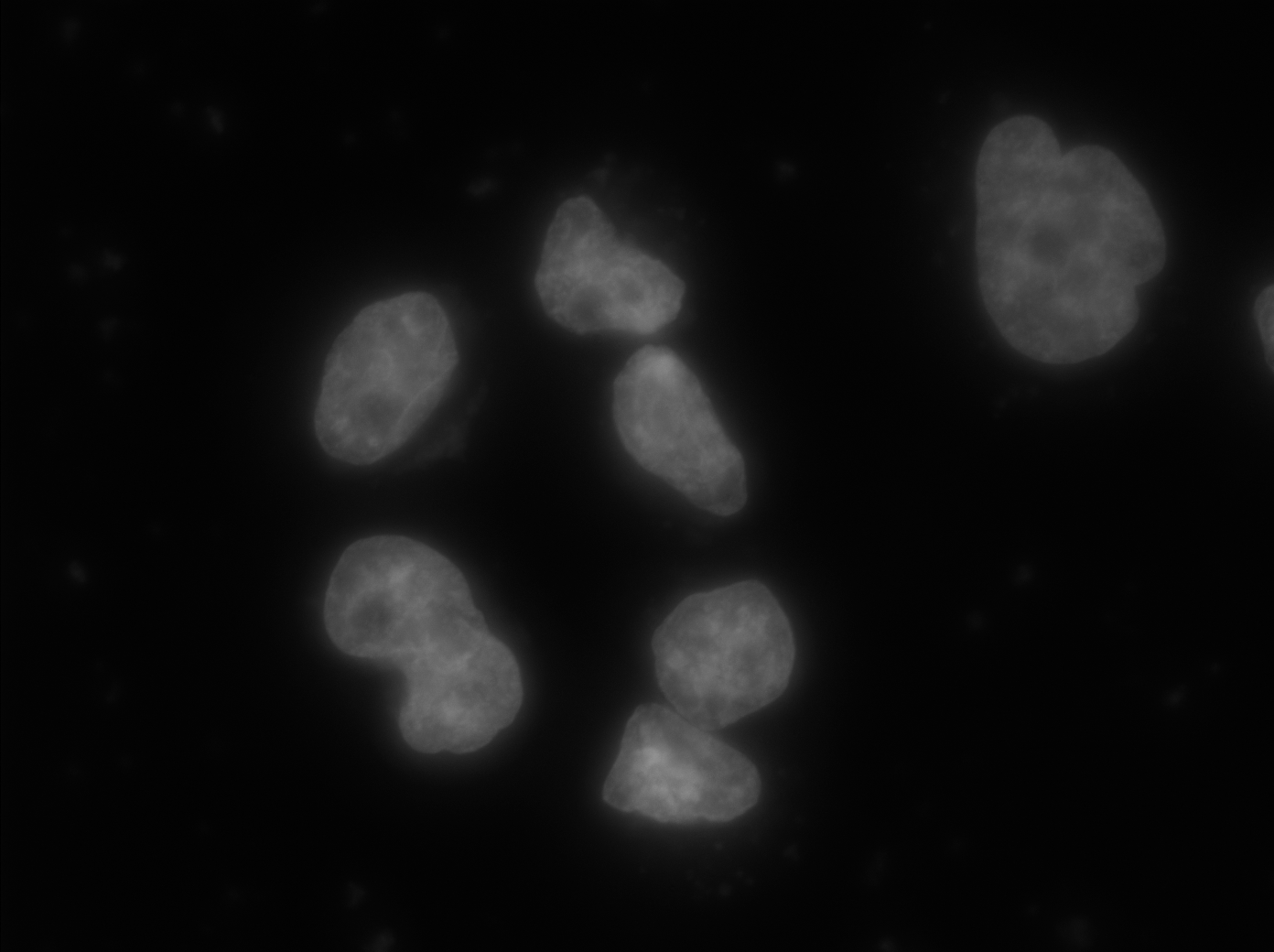

Supplement: Supplementary file 12 — Source data Fig. 3 part 2 [file 44319_2026_742_MOESM12_ESM.zip › Figure 3 Part 2/Fig 3b-e TTLL screen part 2/TTLL5-YFPy I2.Project Maximum Z_XY1679082686_Z0_T0_C0.tif]

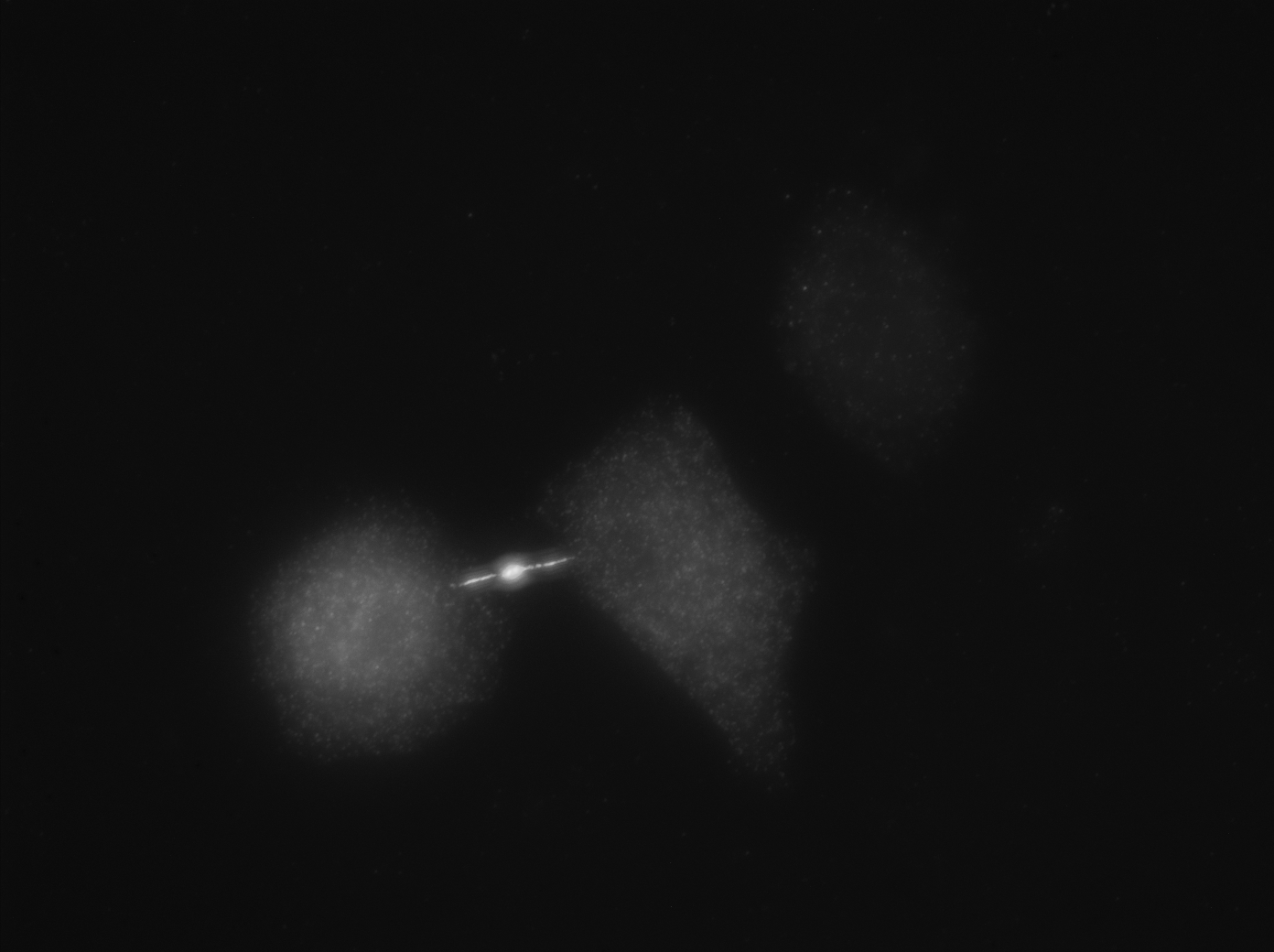

Supplement: Supplementary file 12 — Source data Fig. 3 part 2 [file 44319_2026_742_MOESM12_ESM.zip › Figure 3 Part 2/Fig 3b-e TTLL screen part 2/TTLL6-YFP R1 I5 w T high.Project Maximum Z_XY1661550001_Z0_T0_C1.tif]

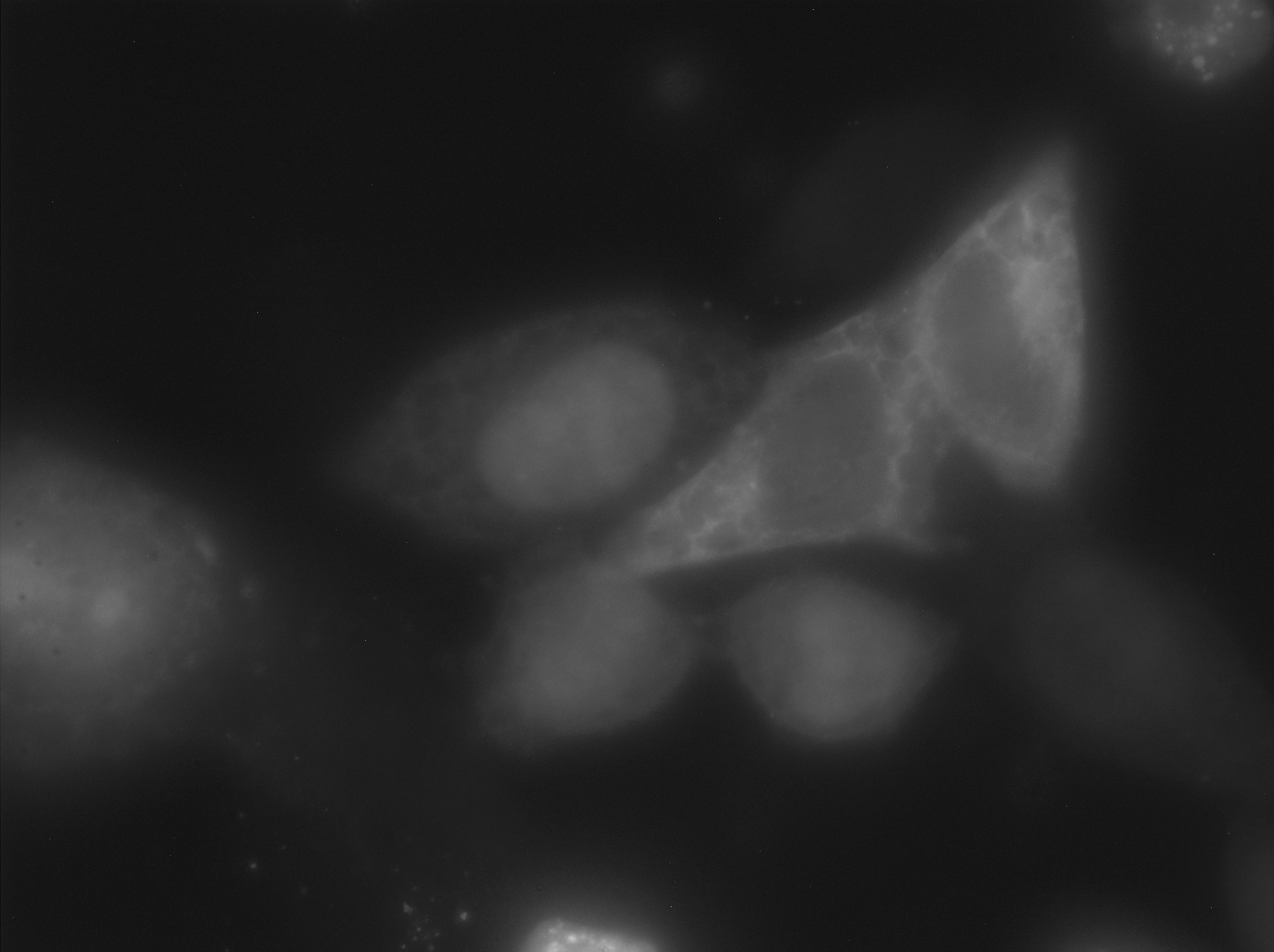

Supplement: Supplementary file 12 — Source data Fig. 3 part 2 [file 44319_2026_742_MOESM12_ESM.zip › Figure 3 Part 2/Fig 3b-e TTLL screen part 2/TTLL7-YFPy I7.Project Maximum Z_XY1679088493_Z0_T0_C2.tif]

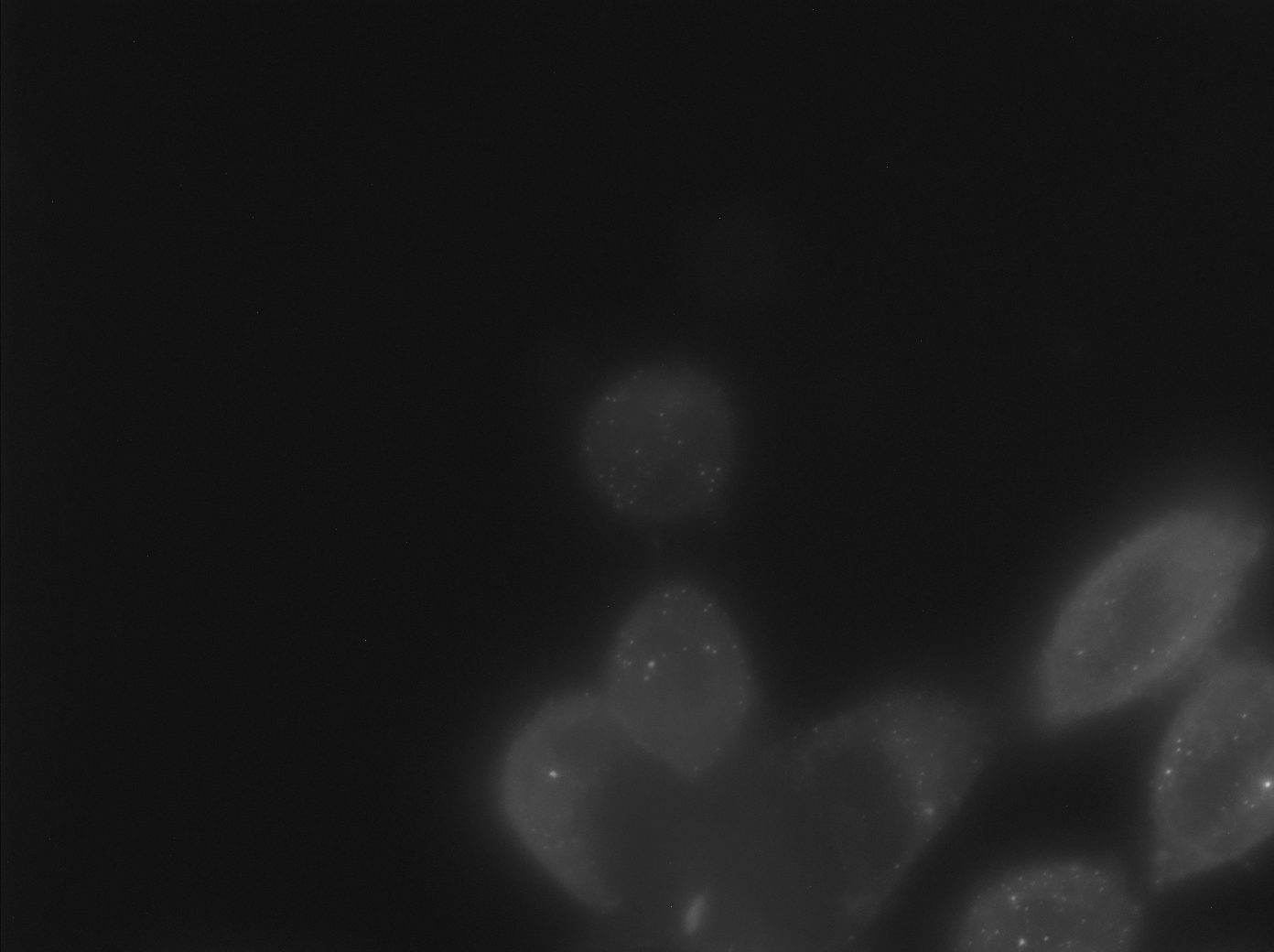

Supplement: Supplementary file 12 — Source data Fig. 3 part 2 [file 44319_2026_742_MOESM12_ESM.zip › Figure 3 Part 2/Fig 3b-e TTLL screen part 2/TTLL5-YFPy I18.Project Maximum Z_XY1679341389_Z0_T0_C2.tif]

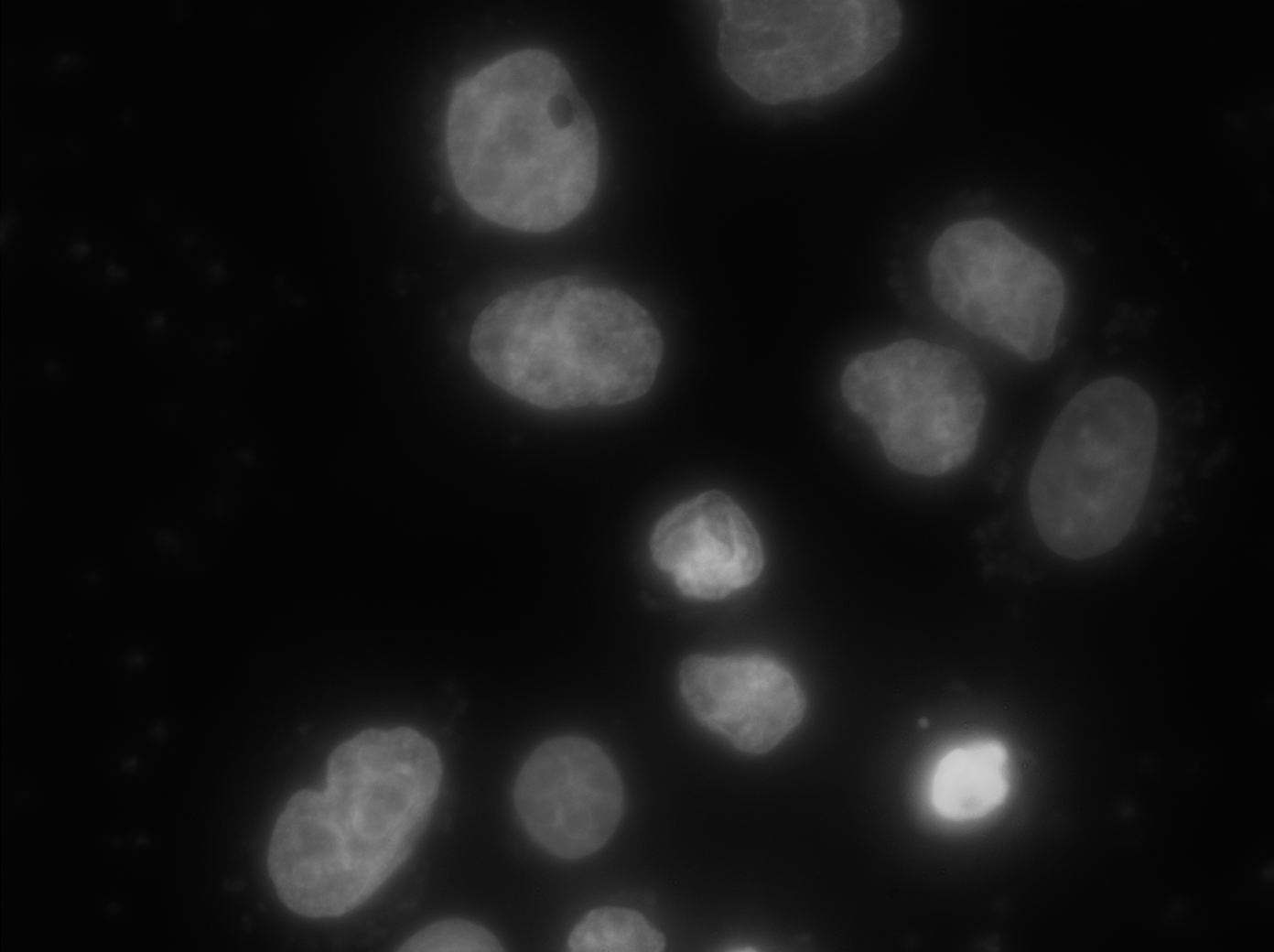

Supplement: Supplementary file 12 — Source data Fig. 3 part 2 [file 44319_2026_742_MOESM12_ESM.zip › Figure 3 Part 2/Fig 3b-e TTLL screen part 2/TTLL7-YFPy I19.Project Maximum Z_XY1679091282_Z0_T0_C0.tif]

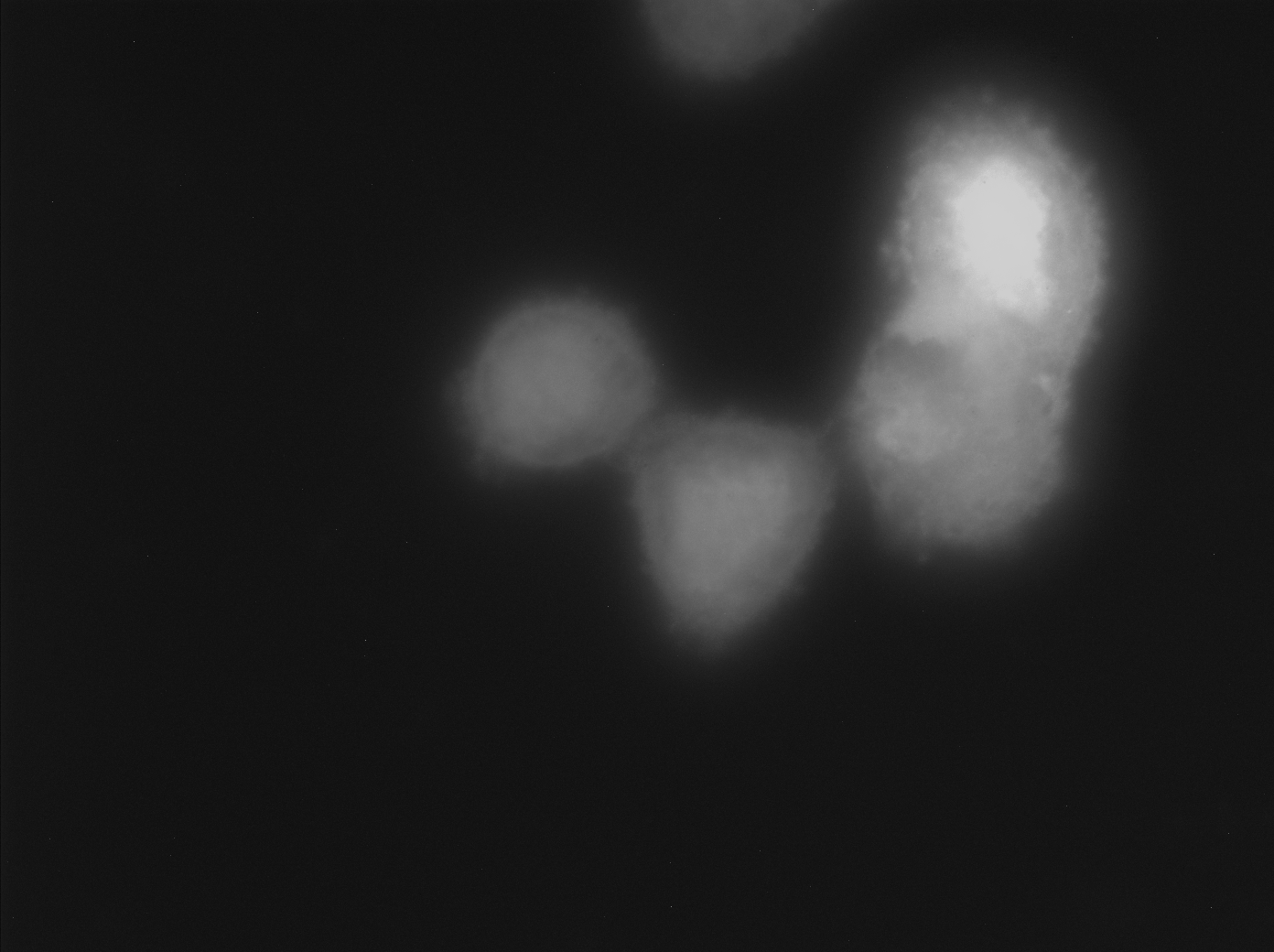

Supplement: Supplementary file 12 — Source data Fig. 3 part 2 [file 44319_2026_742_MOESM12_ESM.zip › Figure 3 Part 2/Fig 3b-e TTLL screen part 2/TTLL7-YFPy I13.Project Maximum Z_XY1679090168_Z0_T0_C2.tif]

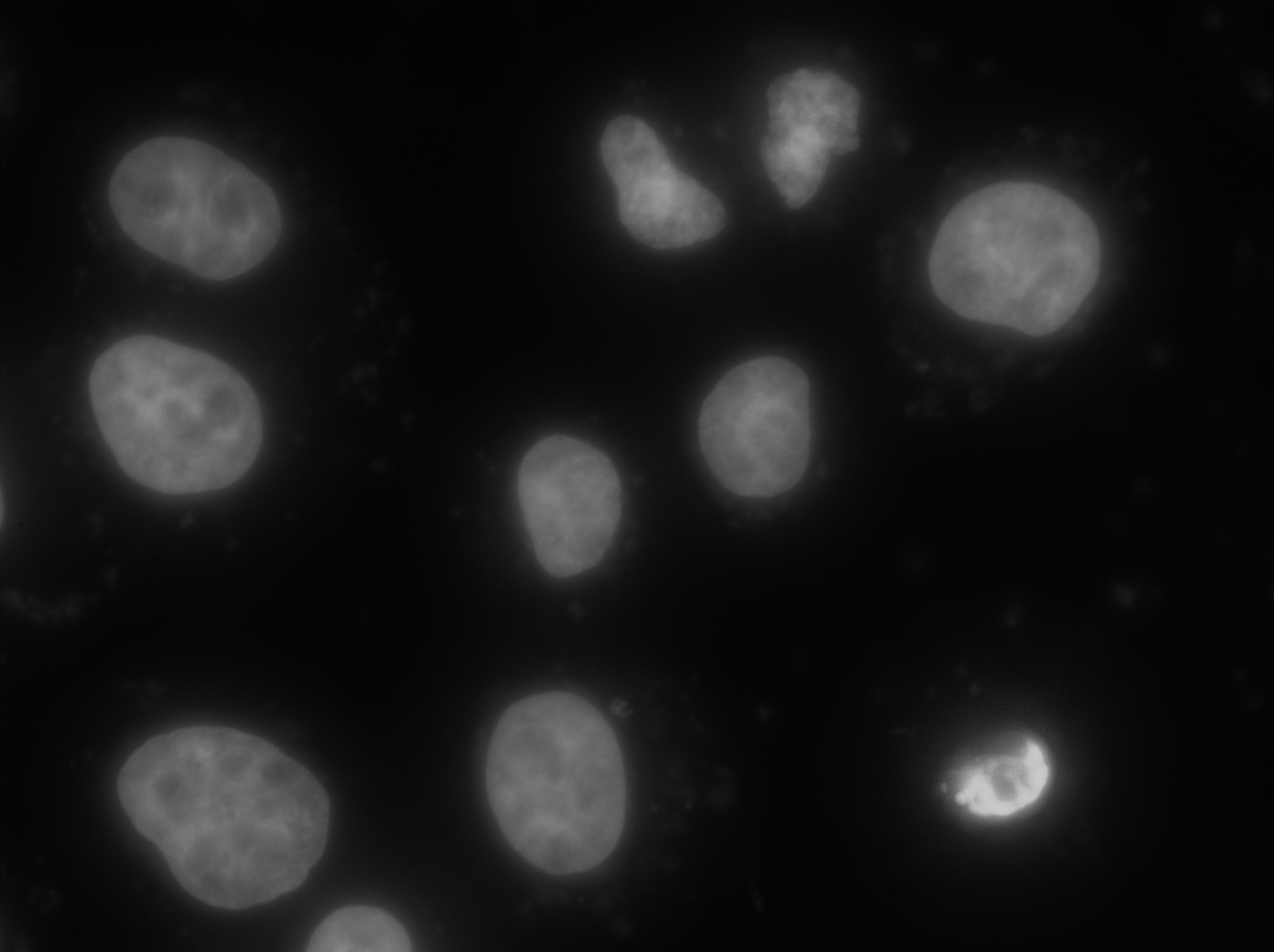

Supplement: Supplementary file 12 — Source data Fig. 3 part 2 [file 44319_2026_742_MOESM12_ESM.zip › Figure 3 Part 2/Fig 3b-e TTLL screen part 2/TTLL7-YFPy I14.Project Maximum Z_XY1679090342_Z0_T0_C0.tif]

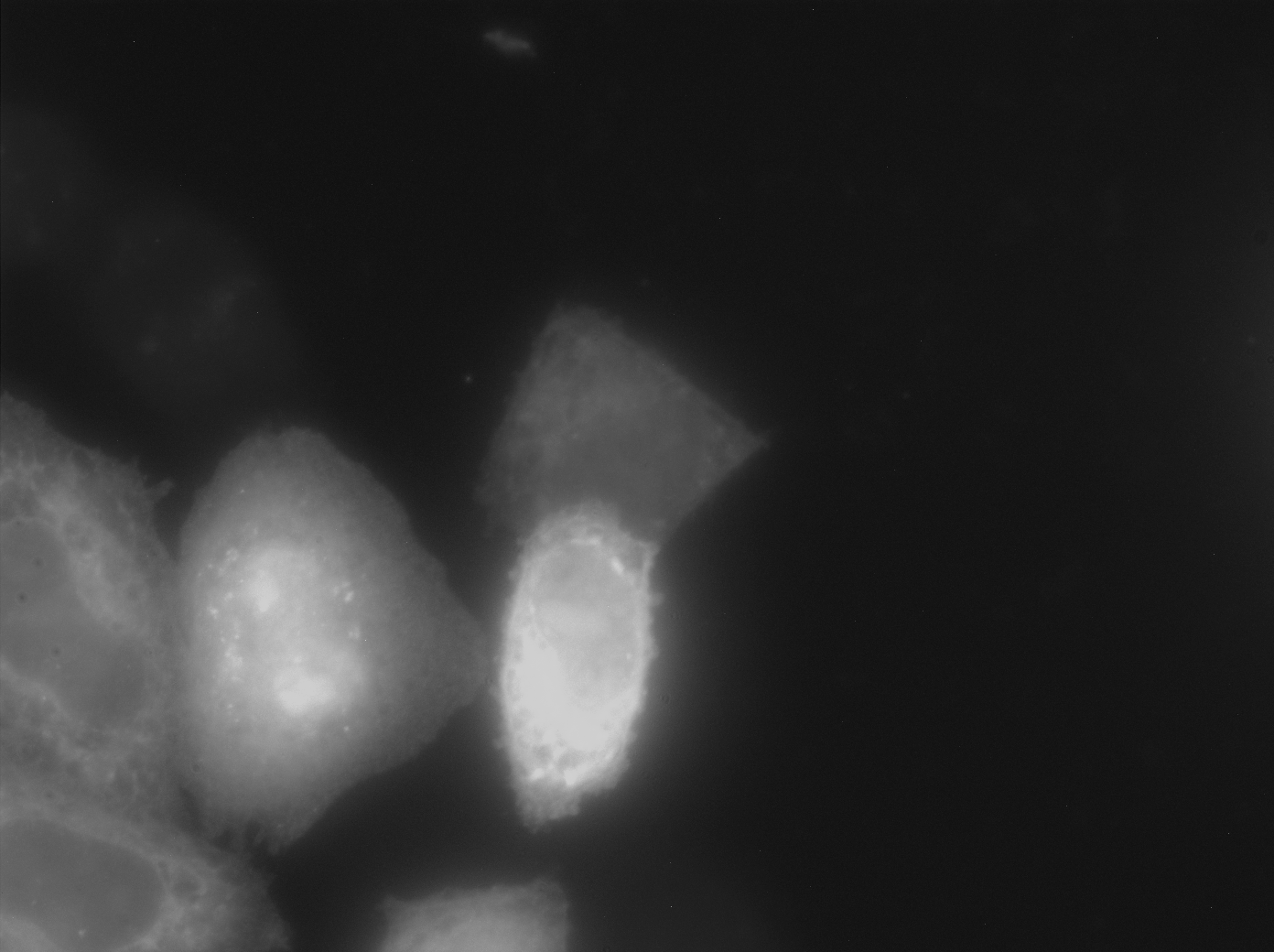

Supplement: Supplementary file 12 — Source data Fig. 3 part 2 [file 44319_2026_742_MOESM12_ESM.zip › Figure 3 Part 2/Fig 3b-e TTLL screen part 2/TTLL7-YFPy I16.Project Maximum Z_XY1679090685_Z0_T0_C2.tif]

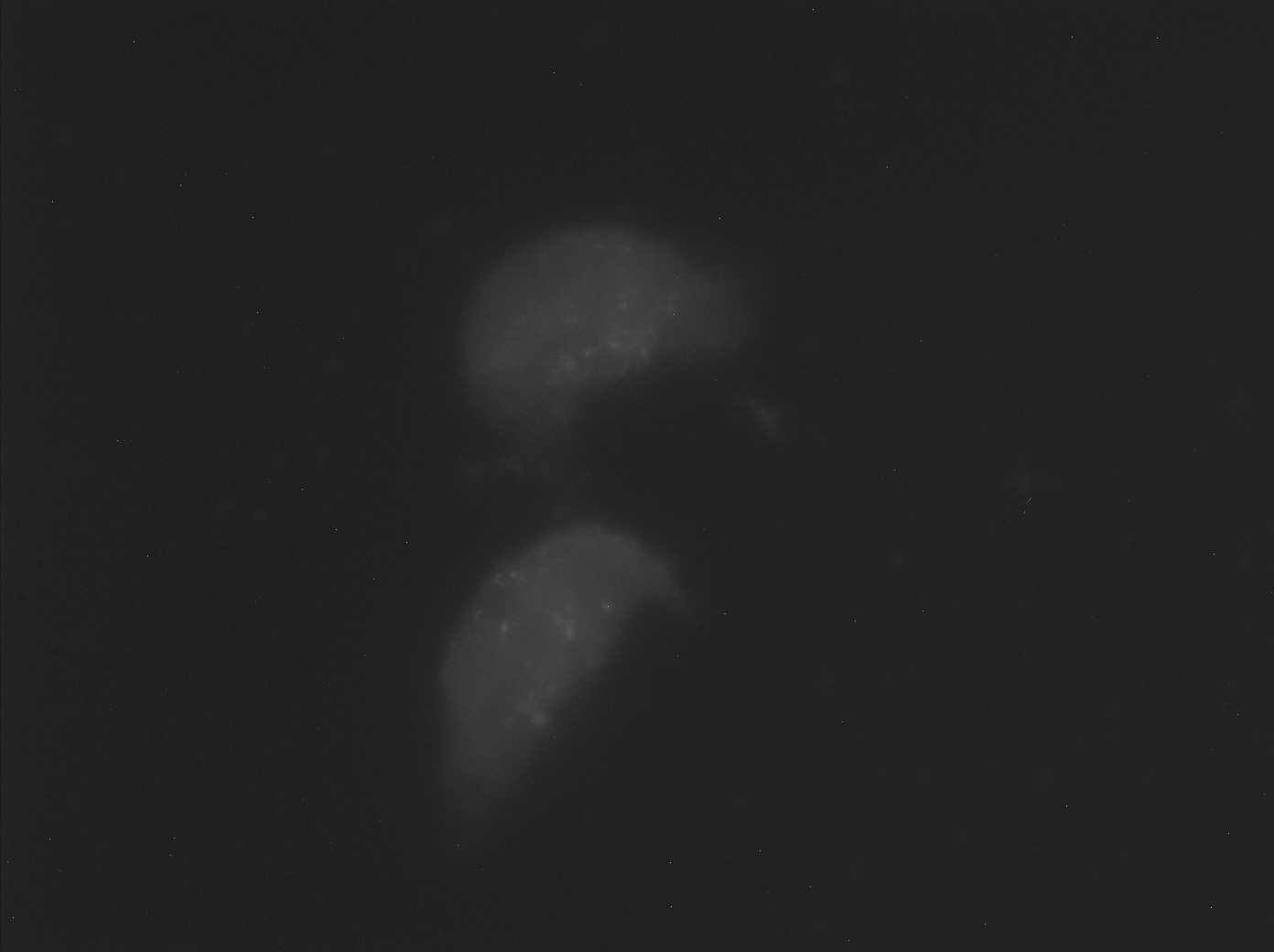

Supplement: Supplementary file 12 — Source data Fig. 3 part 2 [file 44319_2026_742_MOESM12_ESM.zip › Figure 3 Part 2/Fig 3b-e TTLL screen part 2/TTLL6-YFP R1 I1.Project Maximum Z_XY1663274991_Z0_T0_C2.tif]

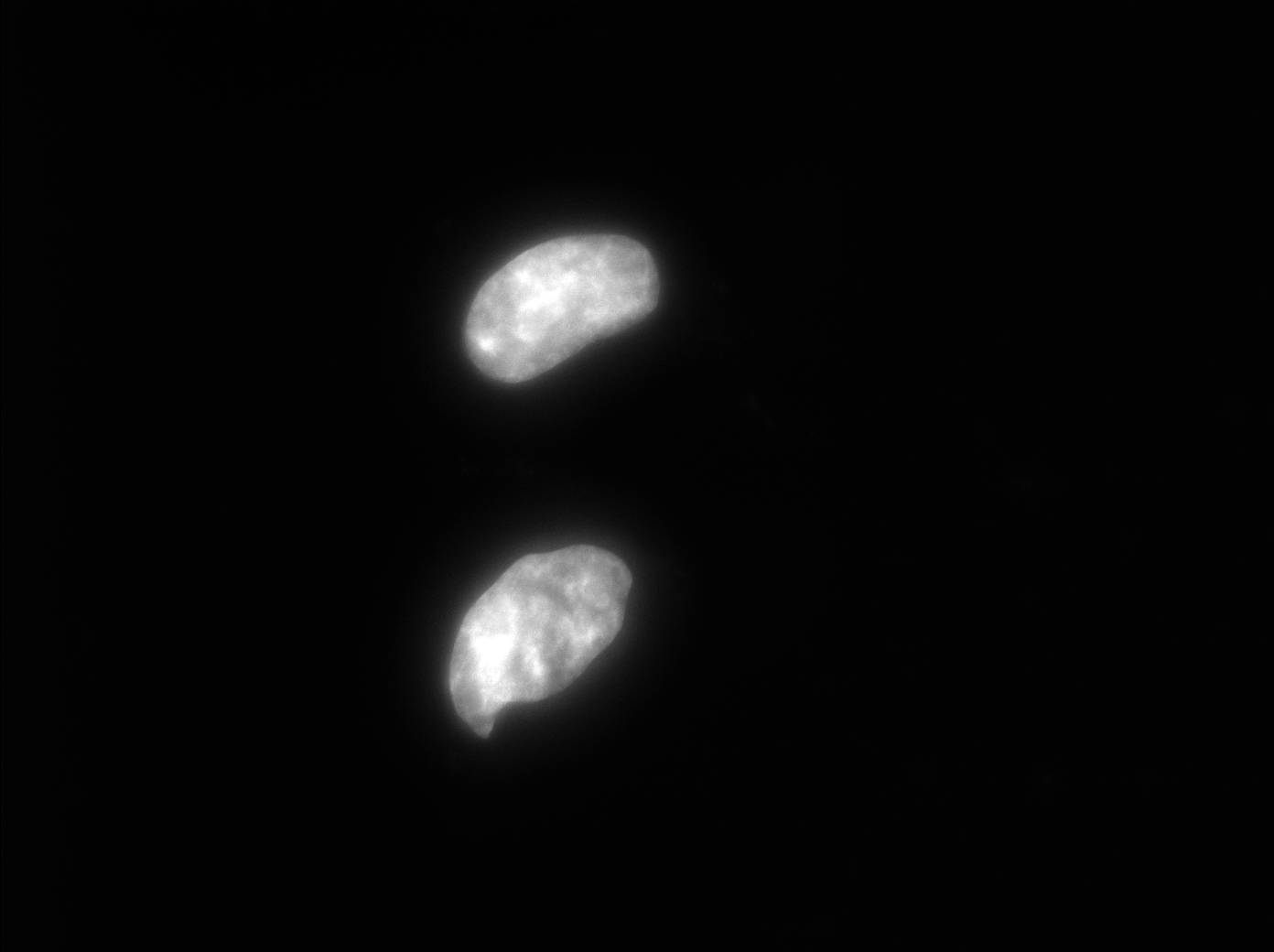

Supplement: Supplementary file 12 — Source data Fig. 3 part 2 [file 44319_2026_742_MOESM12_ESM.zip › Figure 3 Part 2/Fig 3b-e TTLL screen part 2/TTLL6-YFP R1 I1.Project Maximum Z_XY1663274991_Z0_T0_C0.tif]

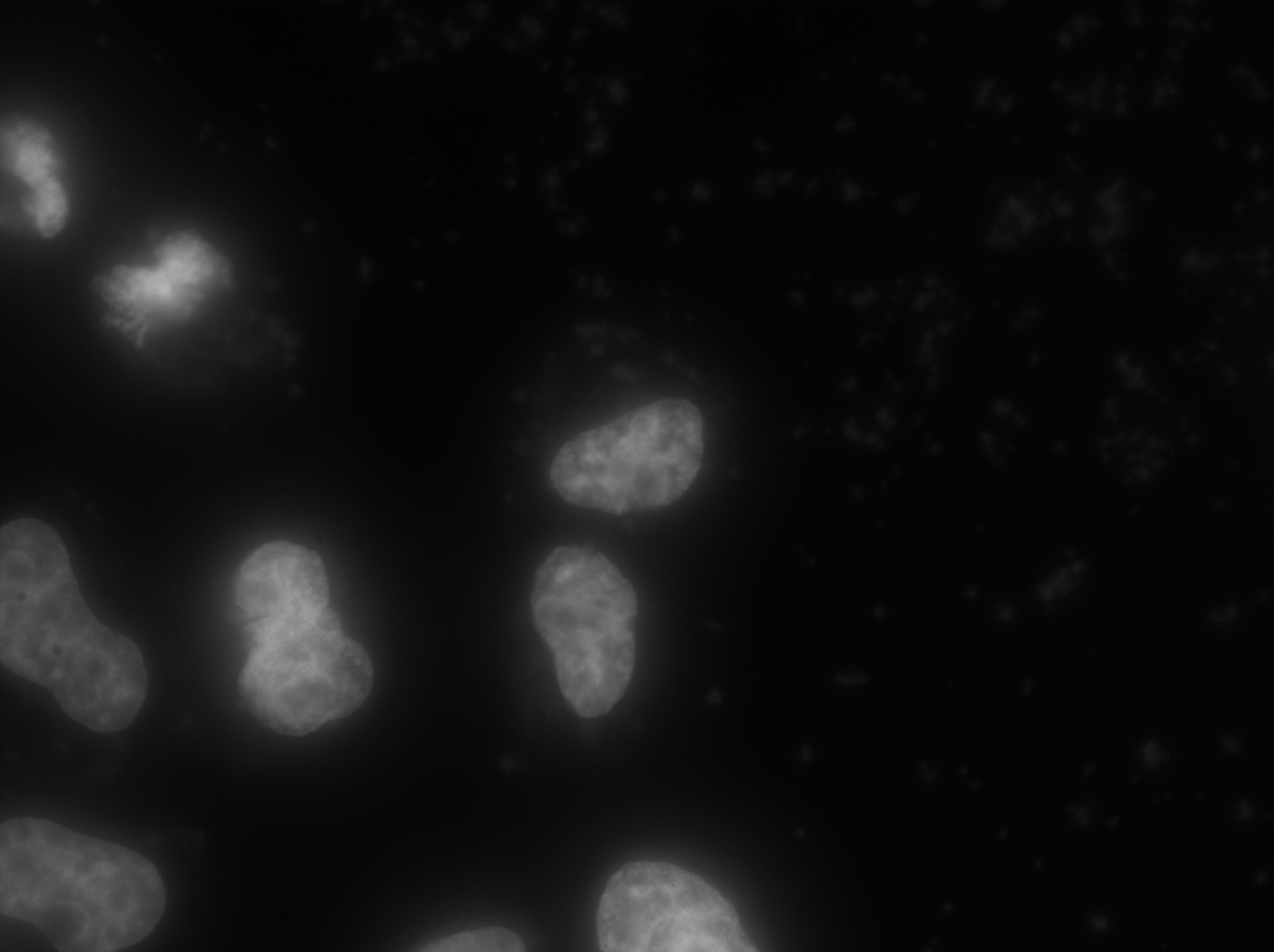

Supplement: Supplementary file 12 — Source data Fig. 3 part 2 [file 44319_2026_742_MOESM12_ESM.zip › Figure 3 Part 2/Fig 3b-e TTLL screen part 2/TTLL7-YFPy I16.Project Maximum Z_XY1679090685_Z0_T0_C0.tif]

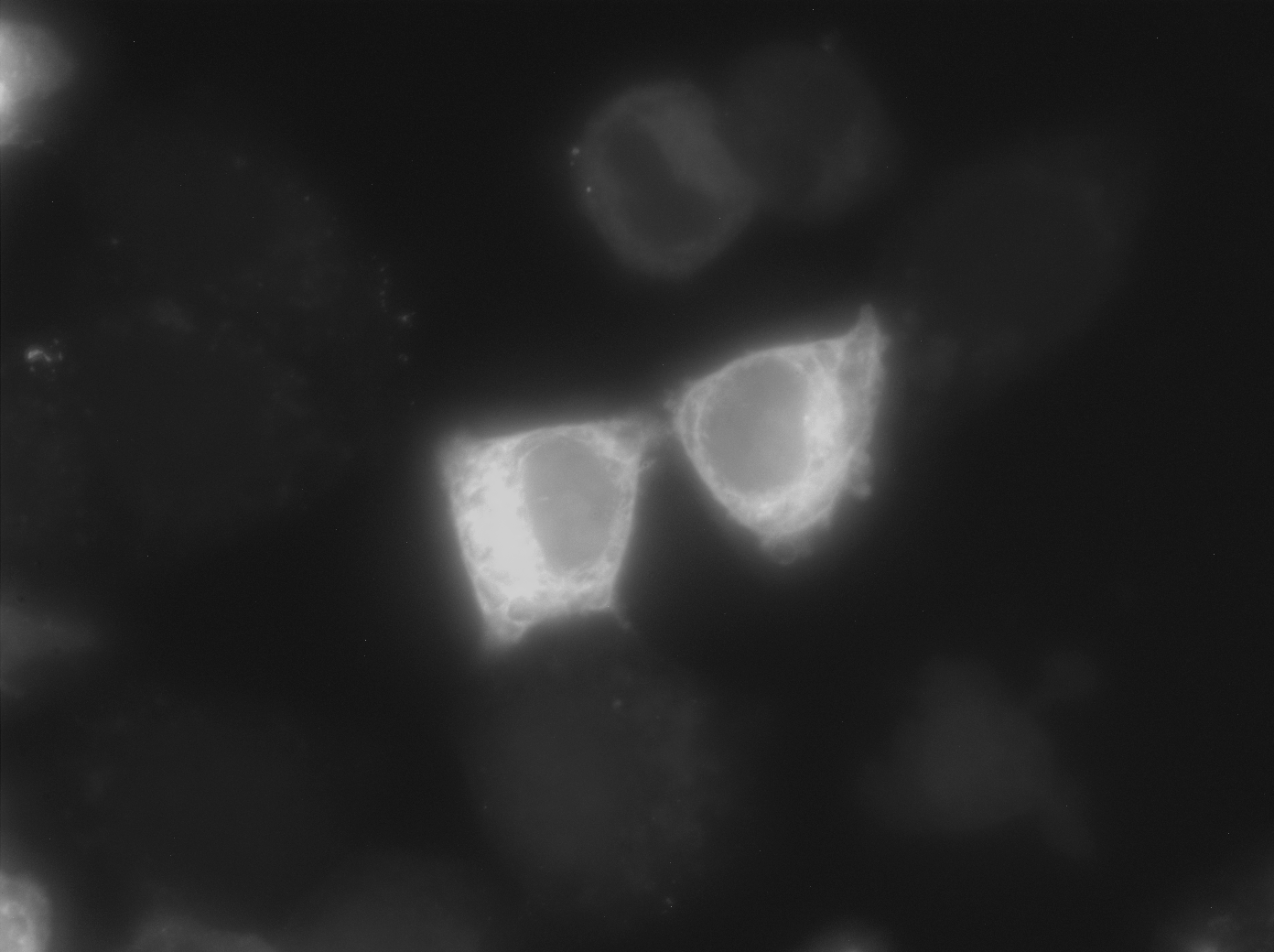

Supplement: Supplementary file 12 — Source data Fig. 3 part 2 [file 44319_2026_742_MOESM12_ESM.zip › Figure 3 Part 2/Fig 3b-e TTLL screen part 2/TTLL7-YFPy I14.Project Maximum Z_XY1679090342_Z0_T0_C2.tif]

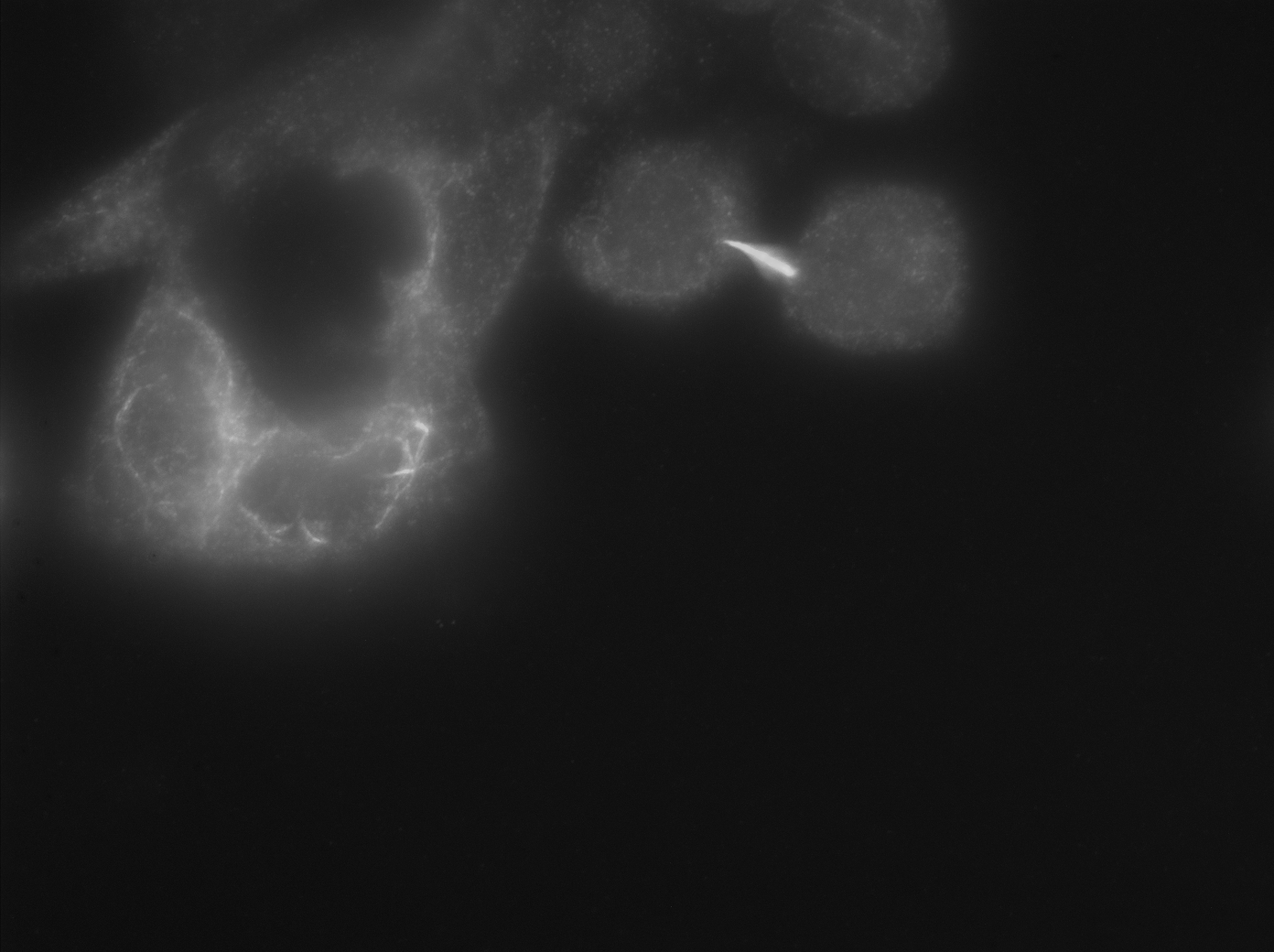

Supplement: Supplementary file 12 — Source data Fig. 3 part 2 [file 44319_2026_742_MOESM12_ESM.zip › Figure 3 Part 2/Fig 3b-e TTLL screen part 2/TTLL7-YFPy I11.Project Maximum Z_XY1679089317_Z0_T0_C1.tif]

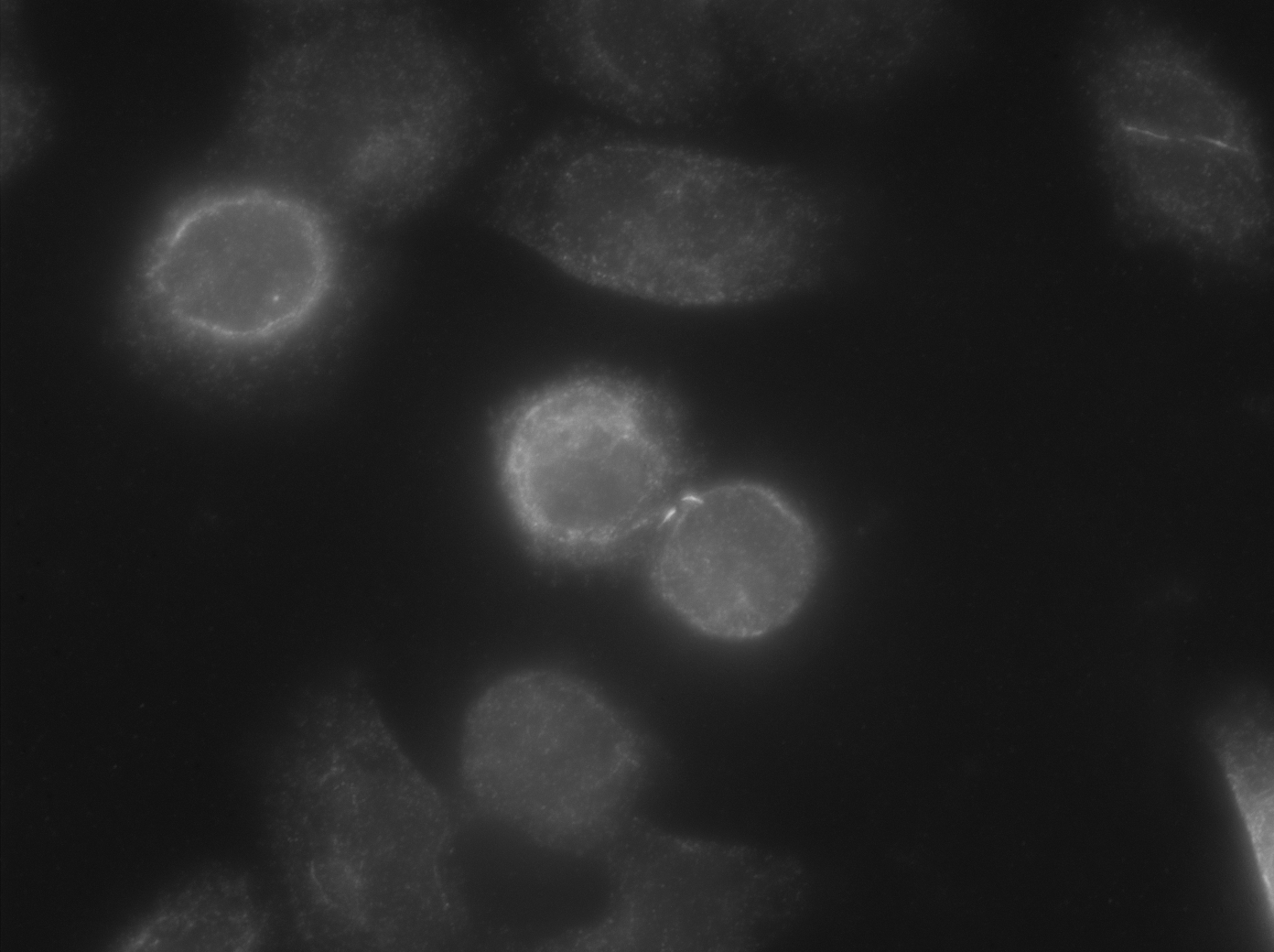

Supplement: Supplementary file 12 — Source data Fig. 3 part 2 [file 44319_2026_742_MOESM12_ESM.zip › Figure 3 Part 2/Fig 3b-e TTLL screen part 2/TTLL5-YFPy I11.Project Maximum Z_XY1679340264_Z0_T0_C1.tif]

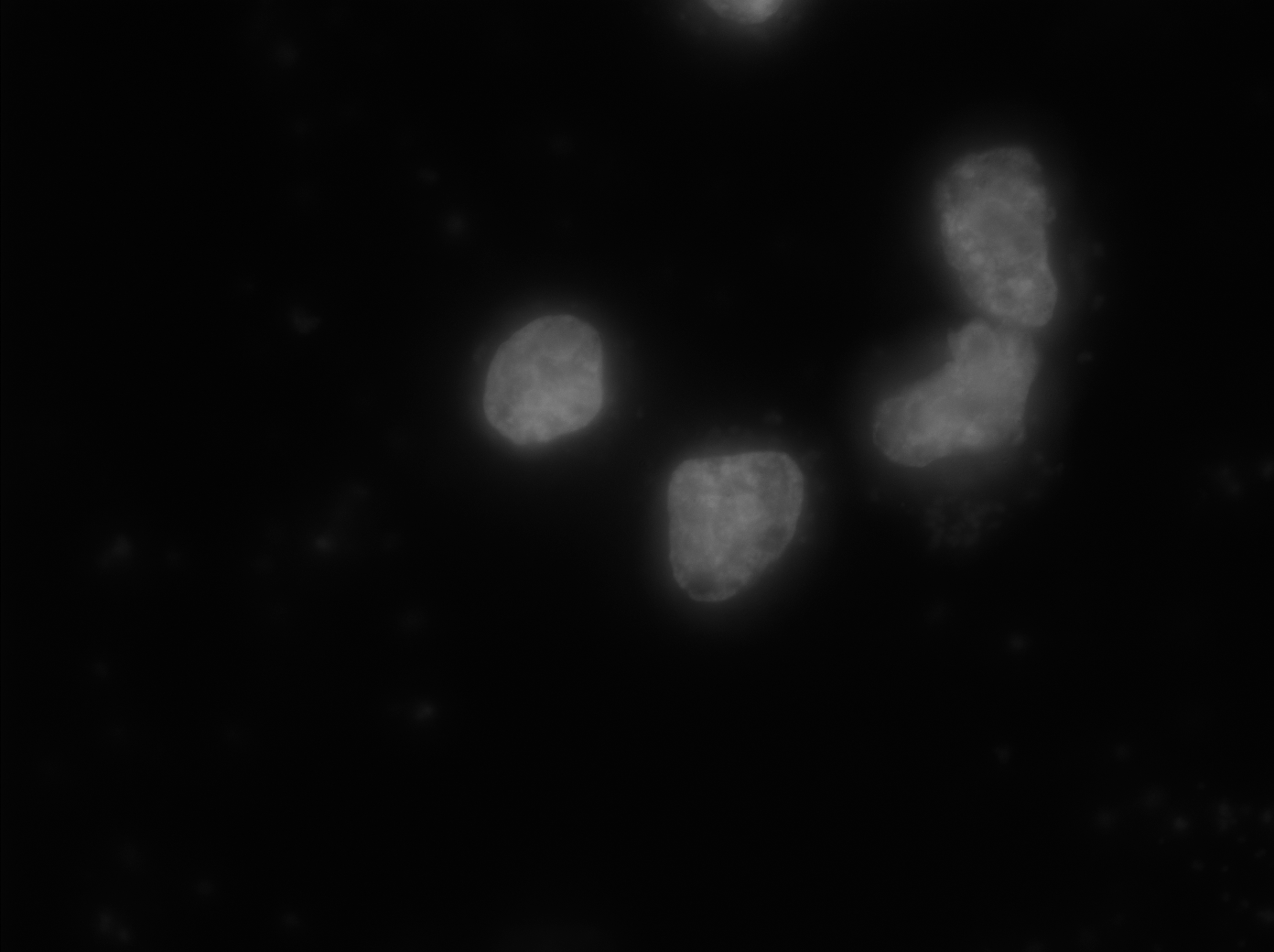

Supplement: Supplementary file 12 — Source data Fig. 3 part 2 [file 44319_2026_742_MOESM12_ESM.zip › Figure 3 Part 2/Fig 3b-e TTLL screen part 2/TTLL7-YFPy I13.Project Maximum Z_XY1679090168_Z0_T0_C0.tif]

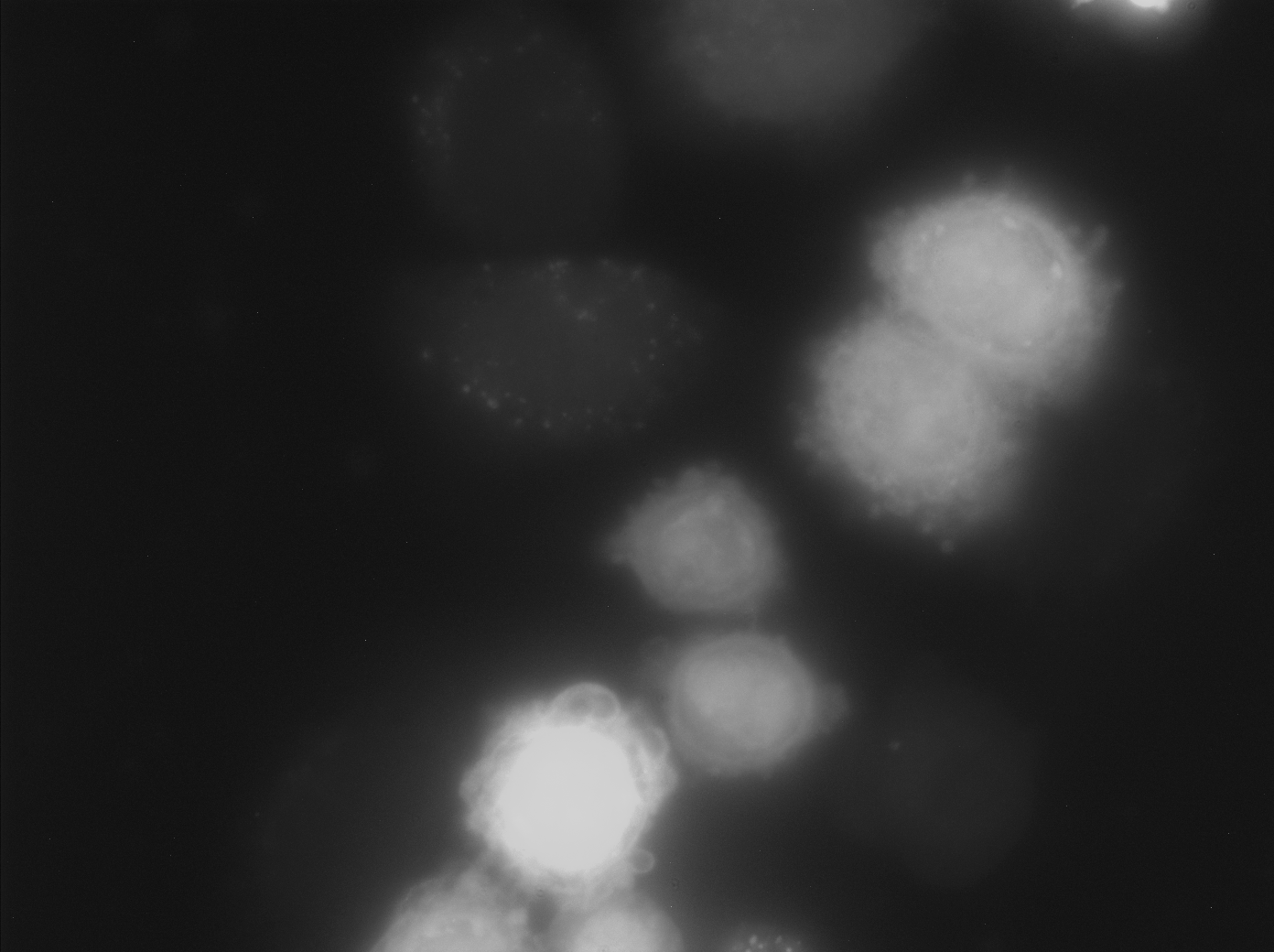

Supplement: Supplementary file 12 — Source data Fig. 3 part 2 [file 44319_2026_742_MOESM12_ESM.zip › Figure 3 Part 2/Fig 3b-e TTLL screen part 2/TTLL7-YFPy I19.Project Maximum Z_XY1679091282_Z0_T0_C2.tif]

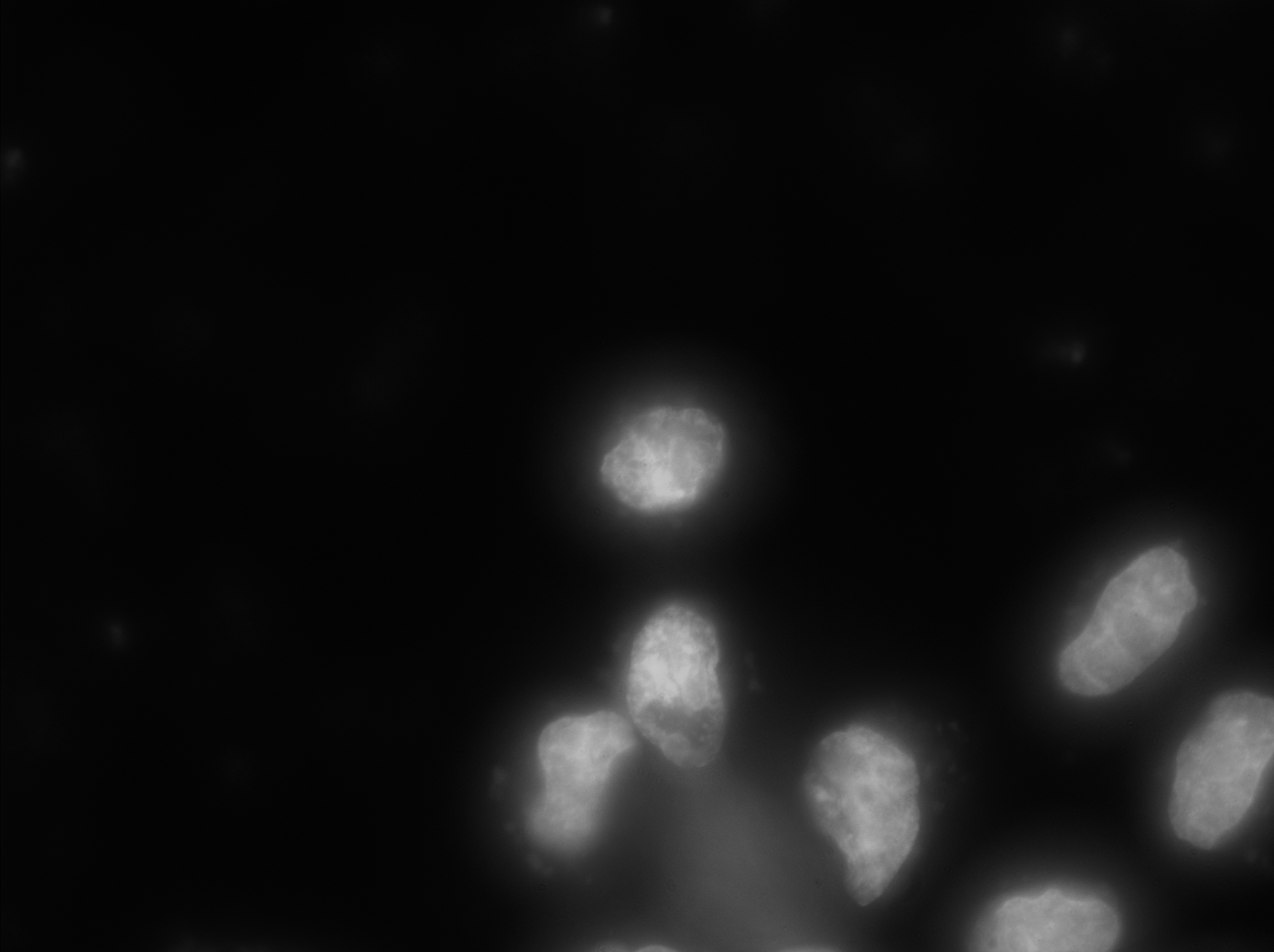

Supplement: Supplementary file 12 — Source data Fig. 3 part 2 [file 44319_2026_742_MOESM12_ESM.zip › Figure 3 Part 2/Fig 3b-e TTLL screen part 2/TTLL5-YFPy I18.Project Maximum Z_XY1679341389_Z0_T0_C0.tif]

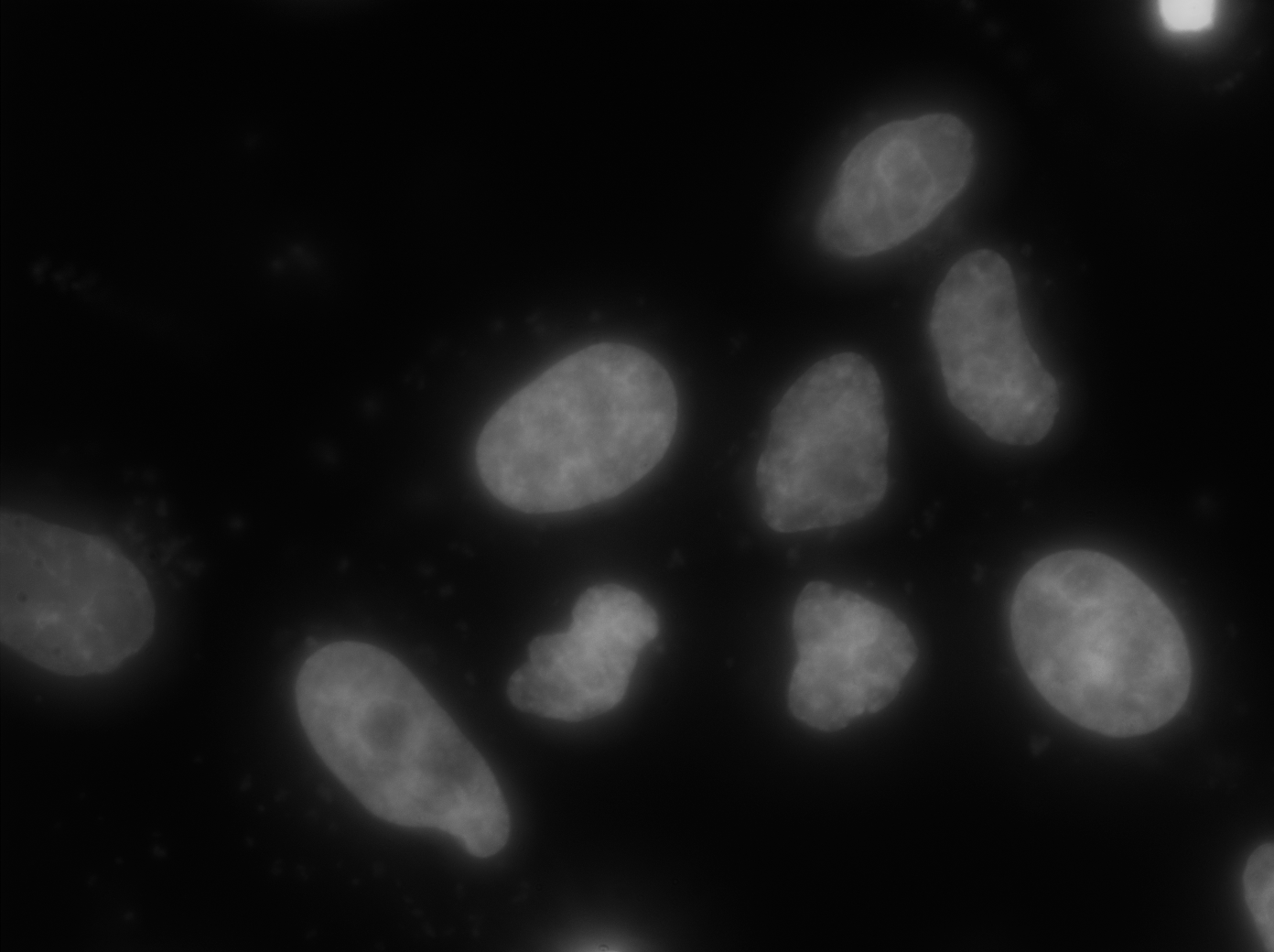

Supplement: Supplementary file 12 — Source data Fig. 3 part 2 [file 44319_2026_742_MOESM12_ESM.zip › Figure 3 Part 2/Fig 3b-e TTLL screen part 2/TTLL7-YFPy I7.Project Maximum Z_XY1679088493_Z0_T0_C0.tif]

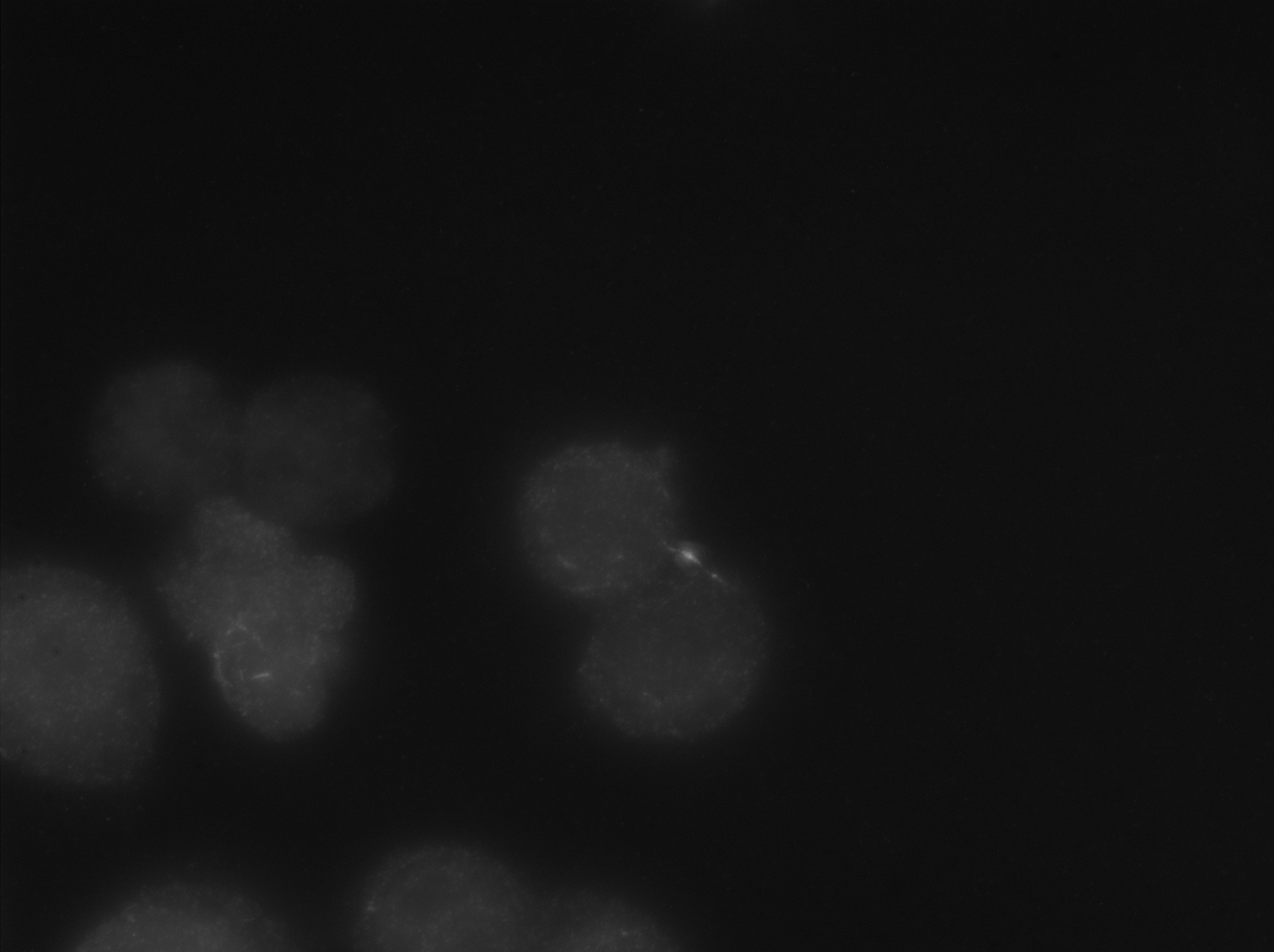

Supplement: Supplementary file 12 — Source data Fig. 3 part 2 [file 44319_2026_742_MOESM12_ESM.zip › Figure 3 Part 2/Fig 3b-e TTLL screen part 2/TTLL5-YFPy I1.Project Maximum Z_XY1679082481_Z0_T0_C1.tif]

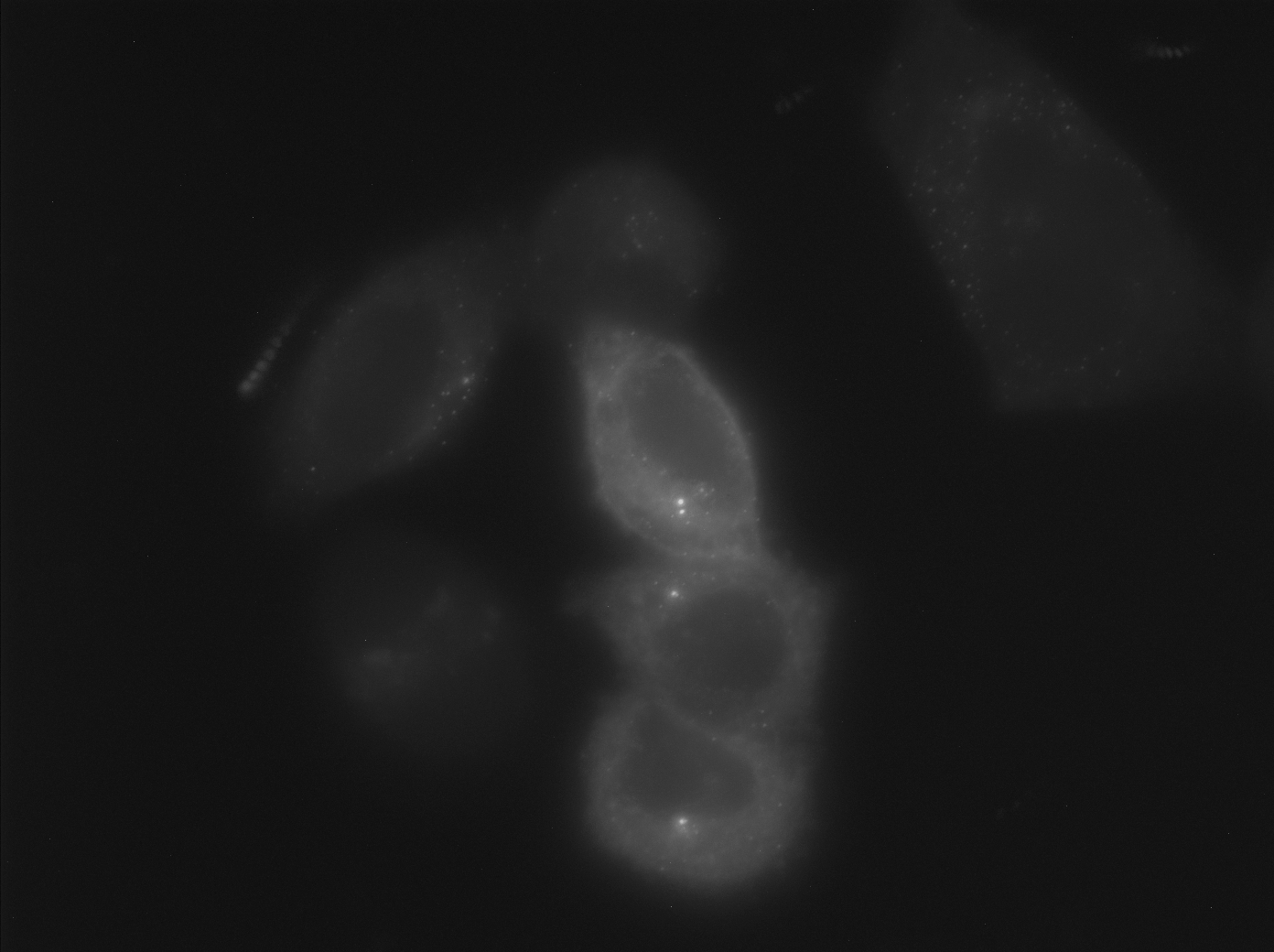

Supplement: Supplementary file 12 — Source data Fig. 3 part 2 [file 44319_2026_742_MOESM12_ESM.zip › Figure 3 Part 2/Fig 3b-e TTLL screen part 2/TTLL5-YFPy I2.Project Maximum Z_XY1679082686_Z0_T0_C2.tif]

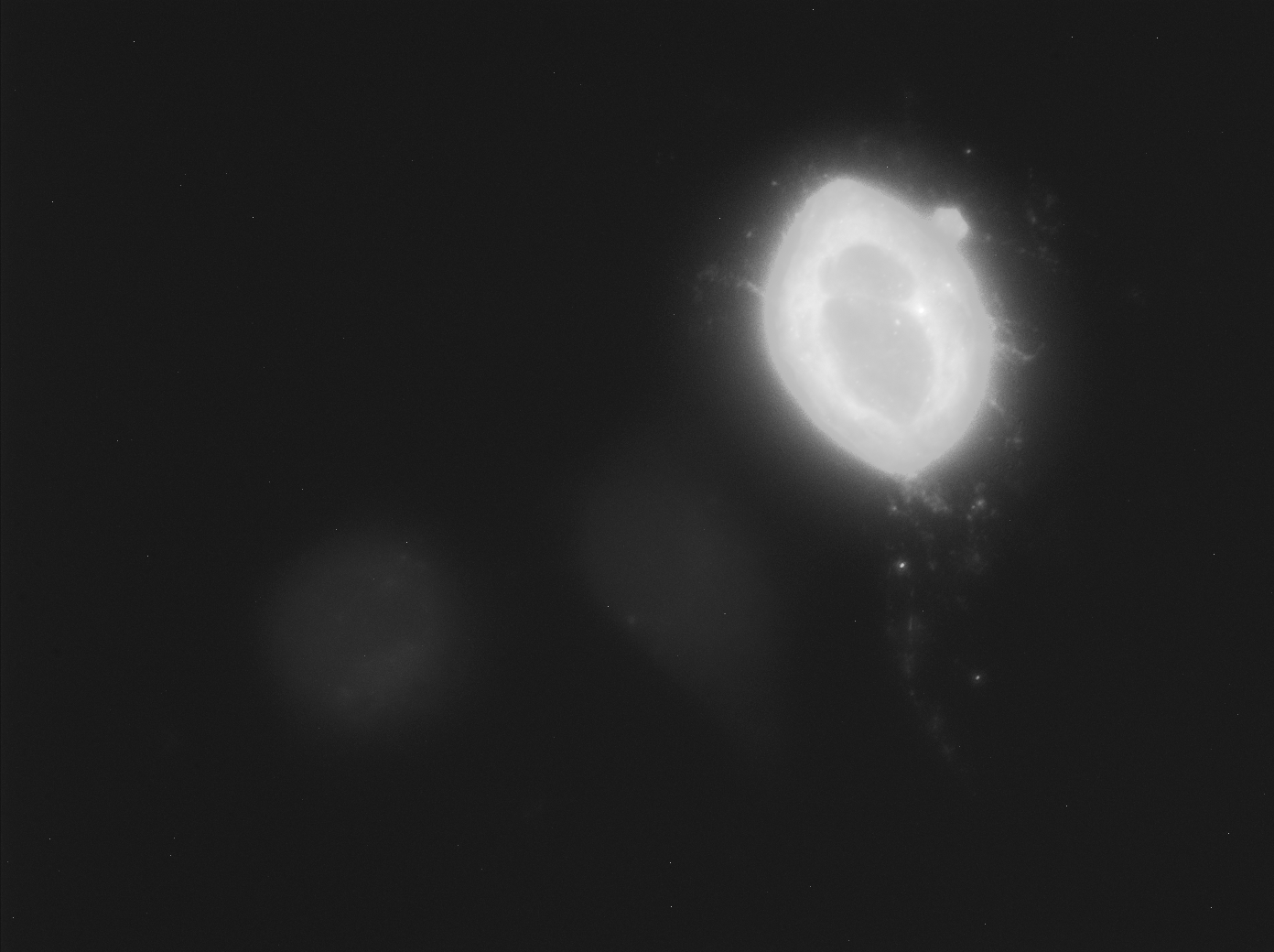

Supplement: Supplementary file 12 — Source data Fig. 3 part 2 [file 44319_2026_742_MOESM12_ESM.zip › Figure 3 Part 2/Fig 3b-e TTLL screen part 2/TTLL6-YFP R1 I5 w T high.Project Maximum Z_XY1661550001_Z0_T0_C2.tif]

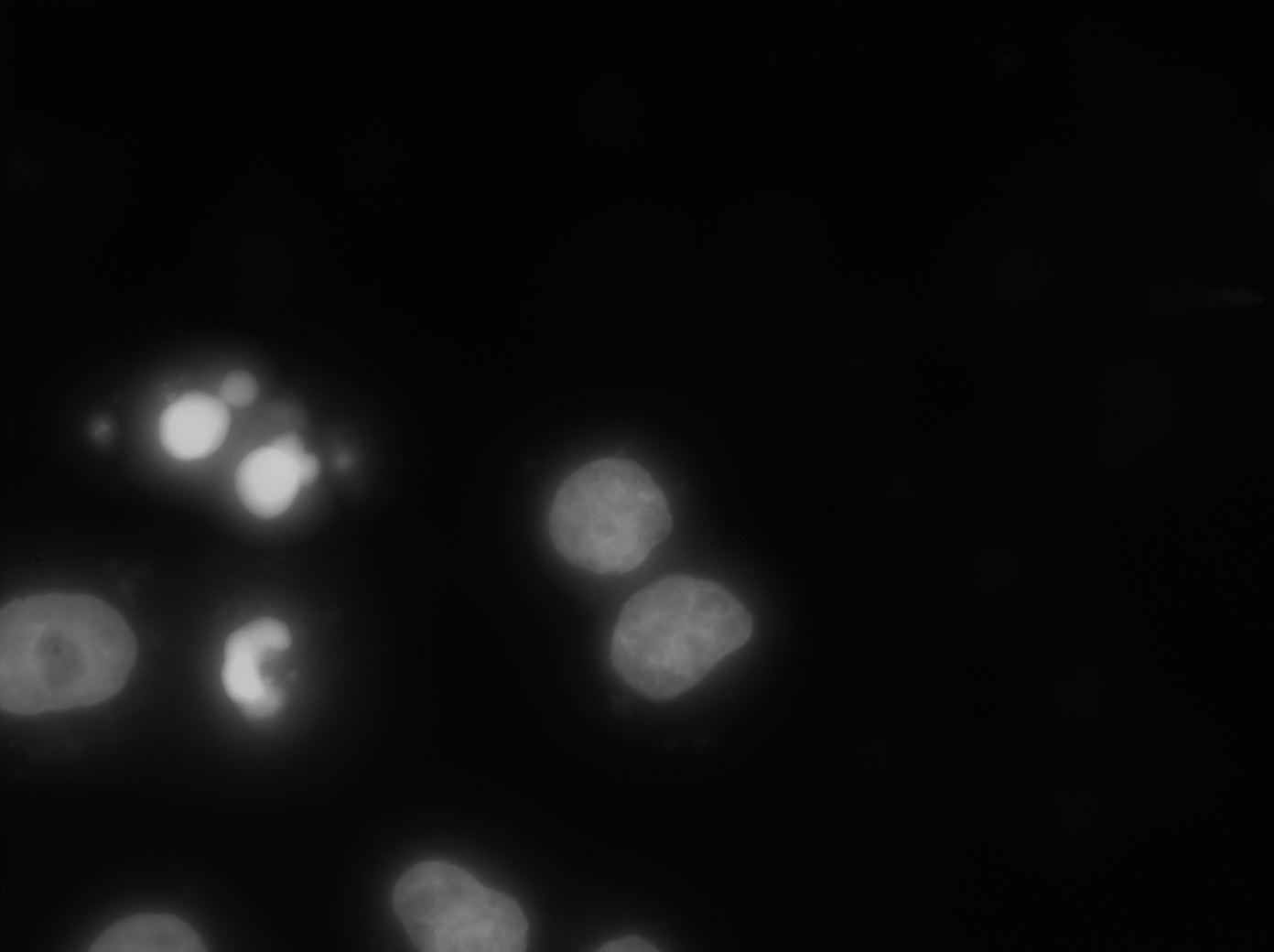

Supplement: Supplementary file 12 — Source data Fig. 3 part 2 [file 44319_2026_742_MOESM12_ESM.zip › Figure 3 Part 2/Fig 3b-e TTLL screen part 2/TTLL5-YFPy I1.Project Maximum Z_XY1679082481_Z0_T0_C0.tif]

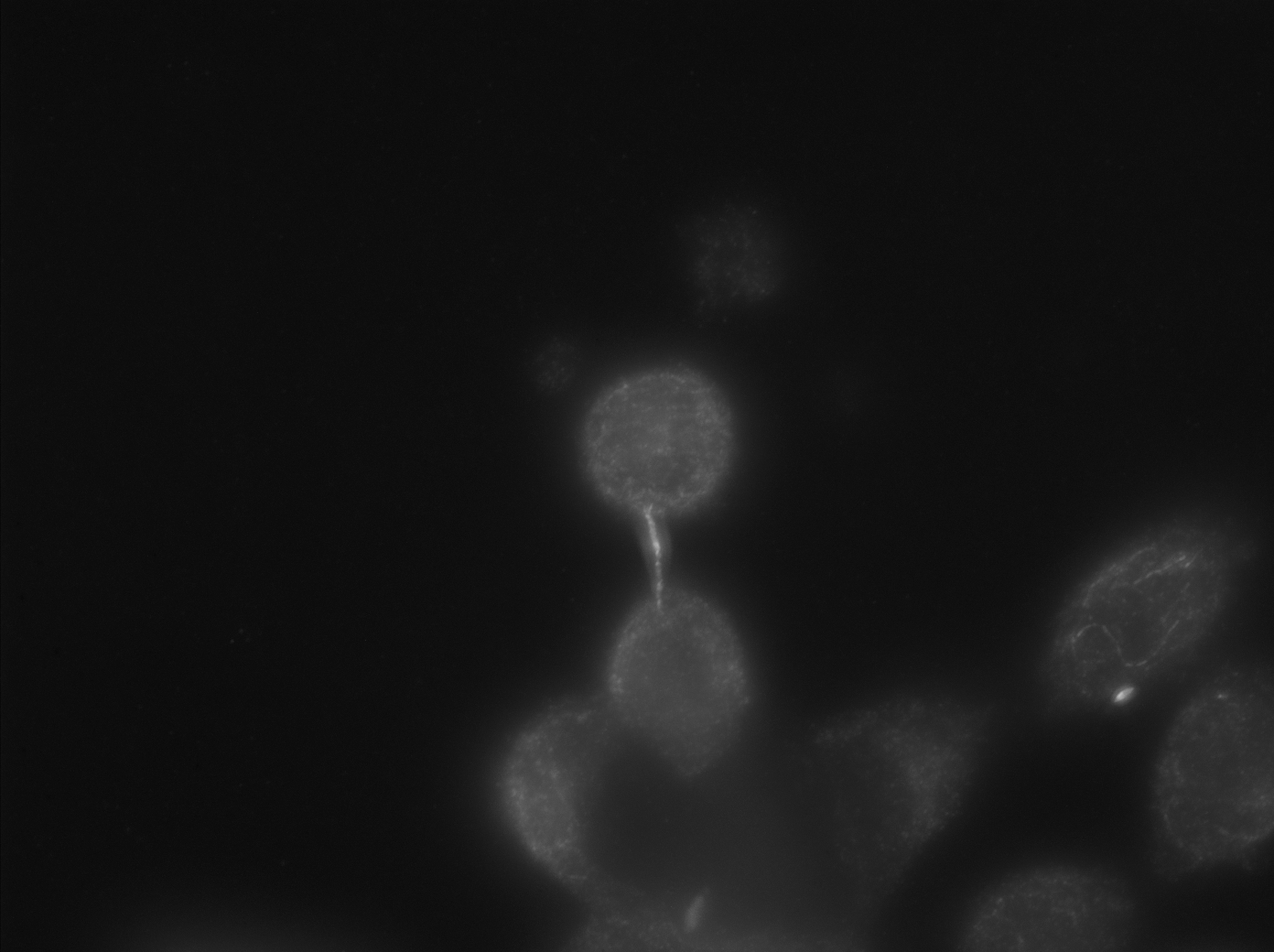

Supplement: Supplementary file 12 — Source data Fig. 3 part 2 [file 44319_2026_742_MOESM12_ESM.zip › Figure 3 Part 2/Fig 3b-e TTLL screen part 2/TTLL5-YFPy I18.Project Maximum Z_XY1679341389_Z0_T0_C1.tif]

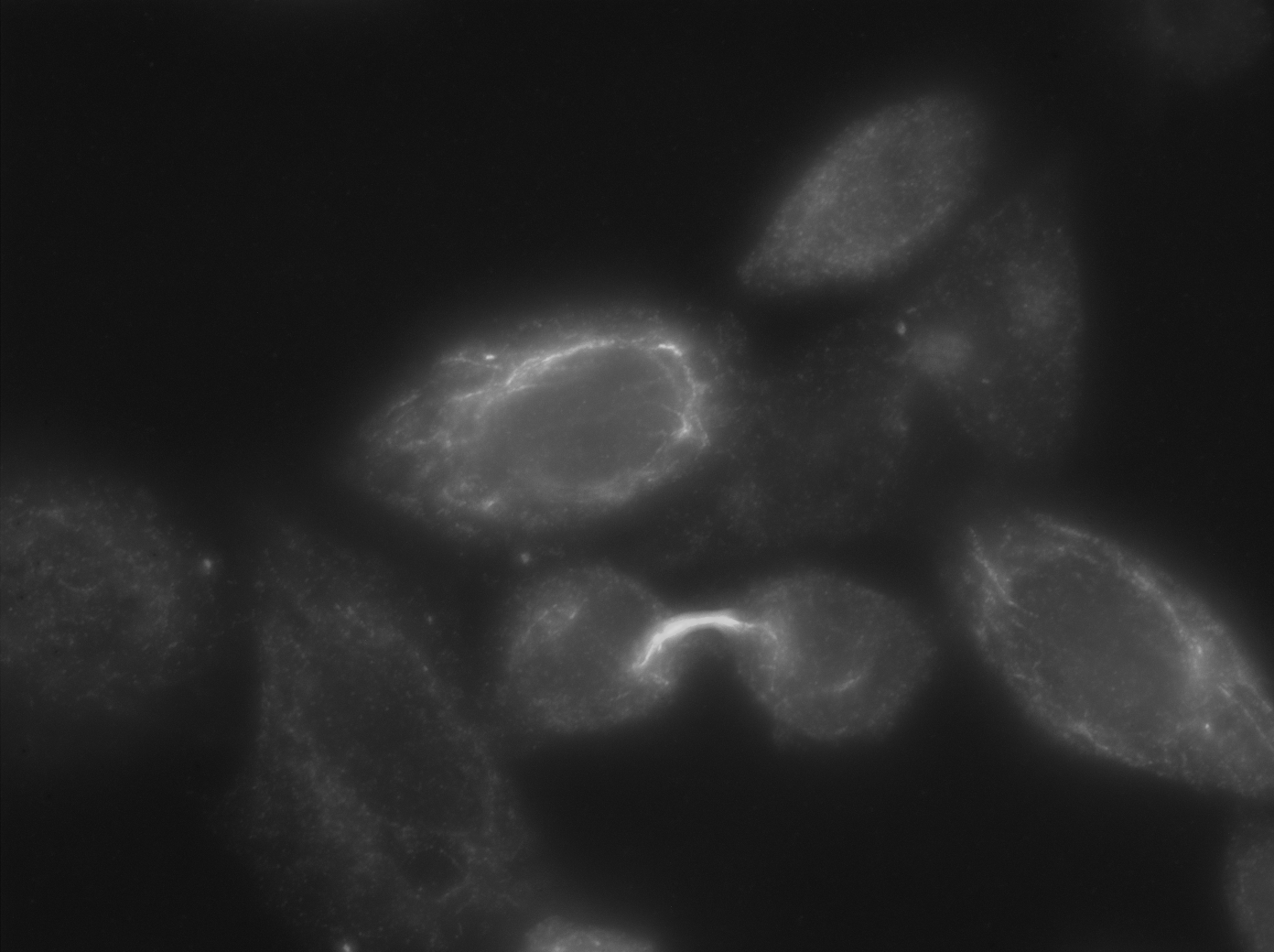

Supplement: Supplementary file 12 — Source data Fig. 3 part 2 [file 44319_2026_742_MOESM12_ESM.zip › Figure 3 Part 2/Fig 3b-e TTLL screen part 2/TTLL7-YFPy I7.Project Maximum Z_XY1679088493_Z0_T0_C1.tif]

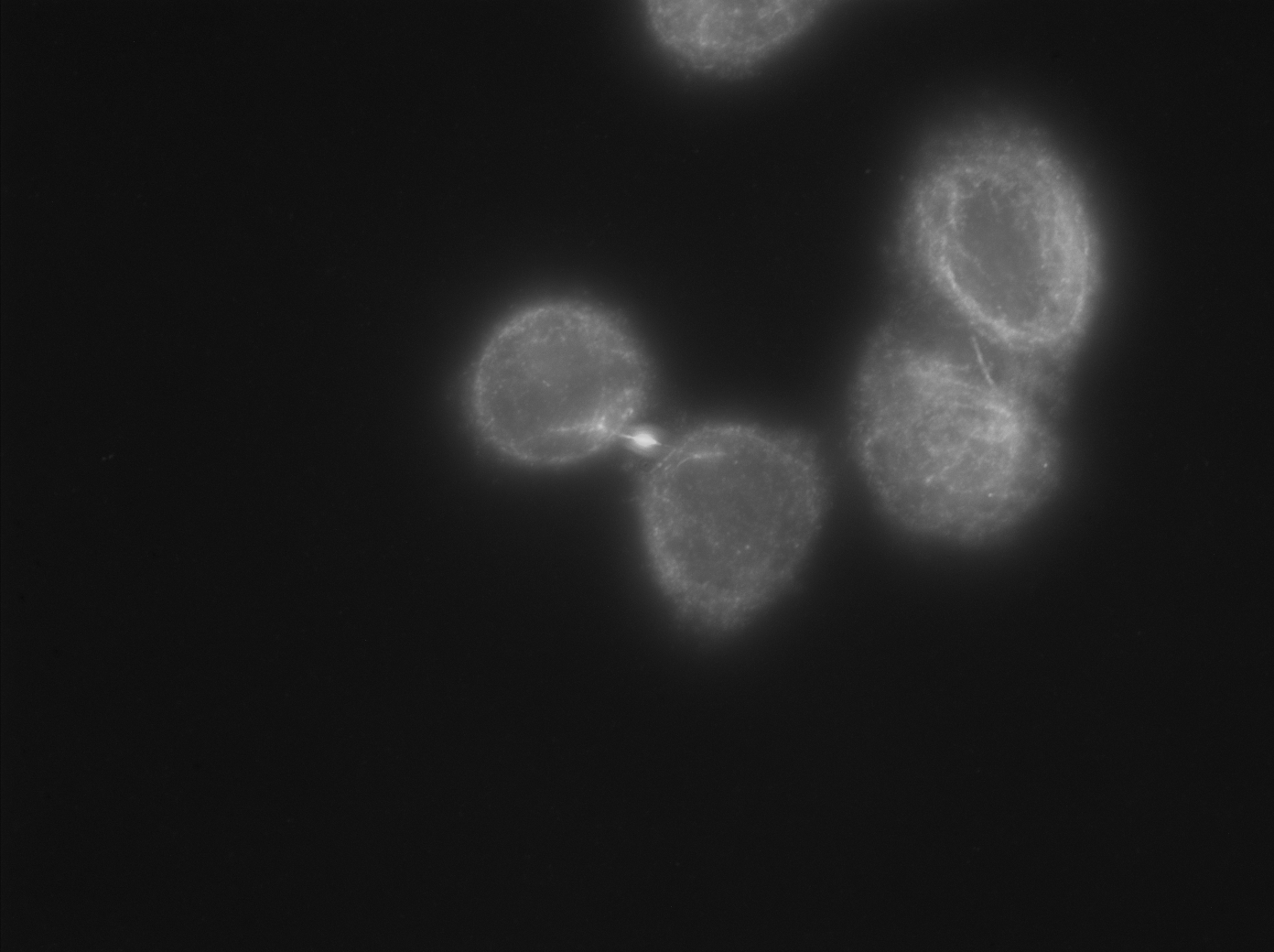

Supplement: Supplementary file 12 — Source data Fig. 3 part 2 [file 44319_2026_742_MOESM12_ESM.zip › Figure 3 Part 2/Fig 3b-e TTLL screen part 2/TTLL7-YFPy I13.Project Maximum Z_XY1679090168_Z0_T0_C1.tif]

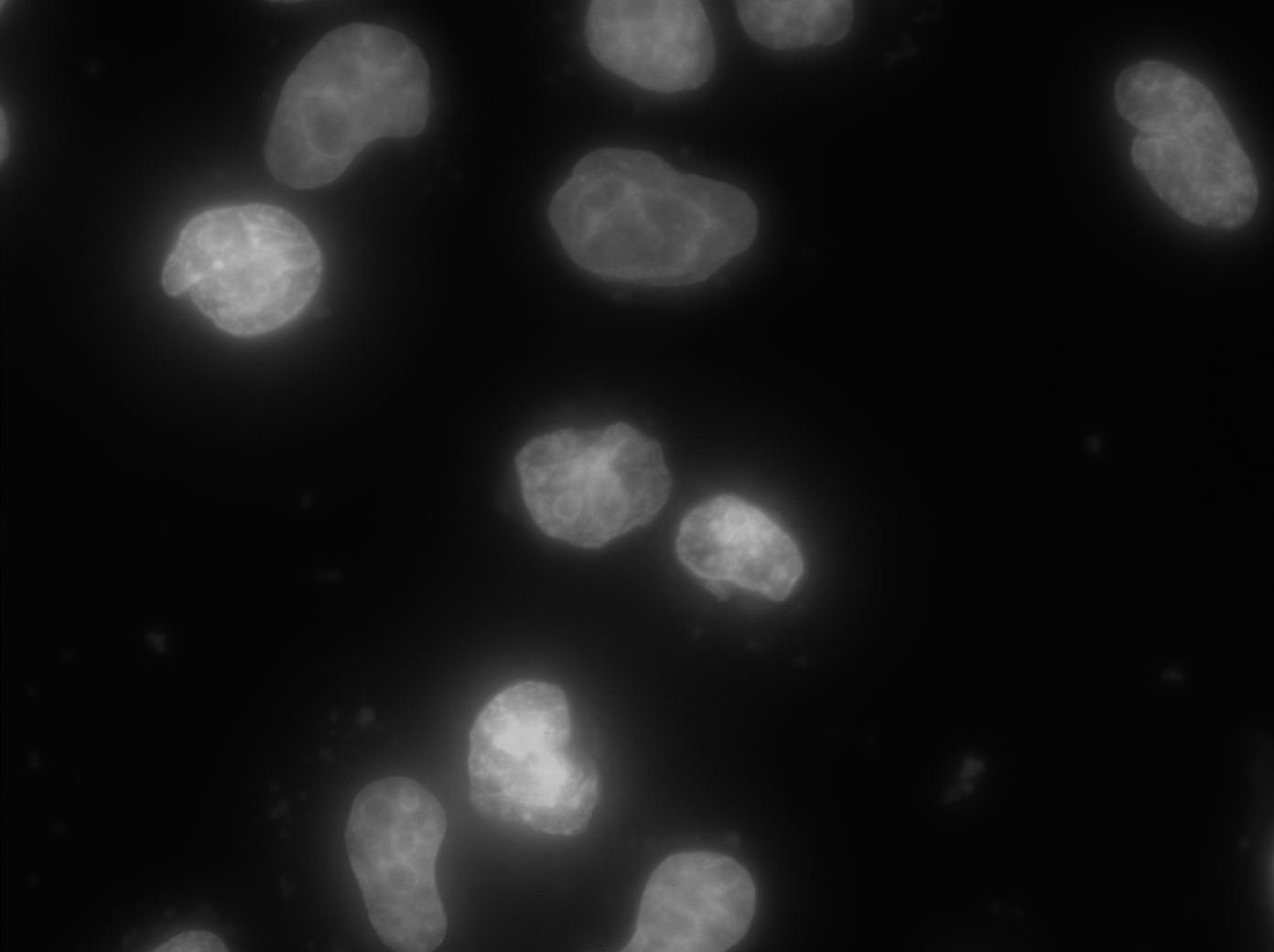

Supplement: Supplementary file 12 — Source data Fig. 3 part 2 [file 44319_2026_742_MOESM12_ESM.zip › Figure 3 Part 2/Fig 3b-e TTLL screen part 2/TTLL5-YFPy I11.Project Maximum Z_XY1679340264_Z0_T0_C0.tif]

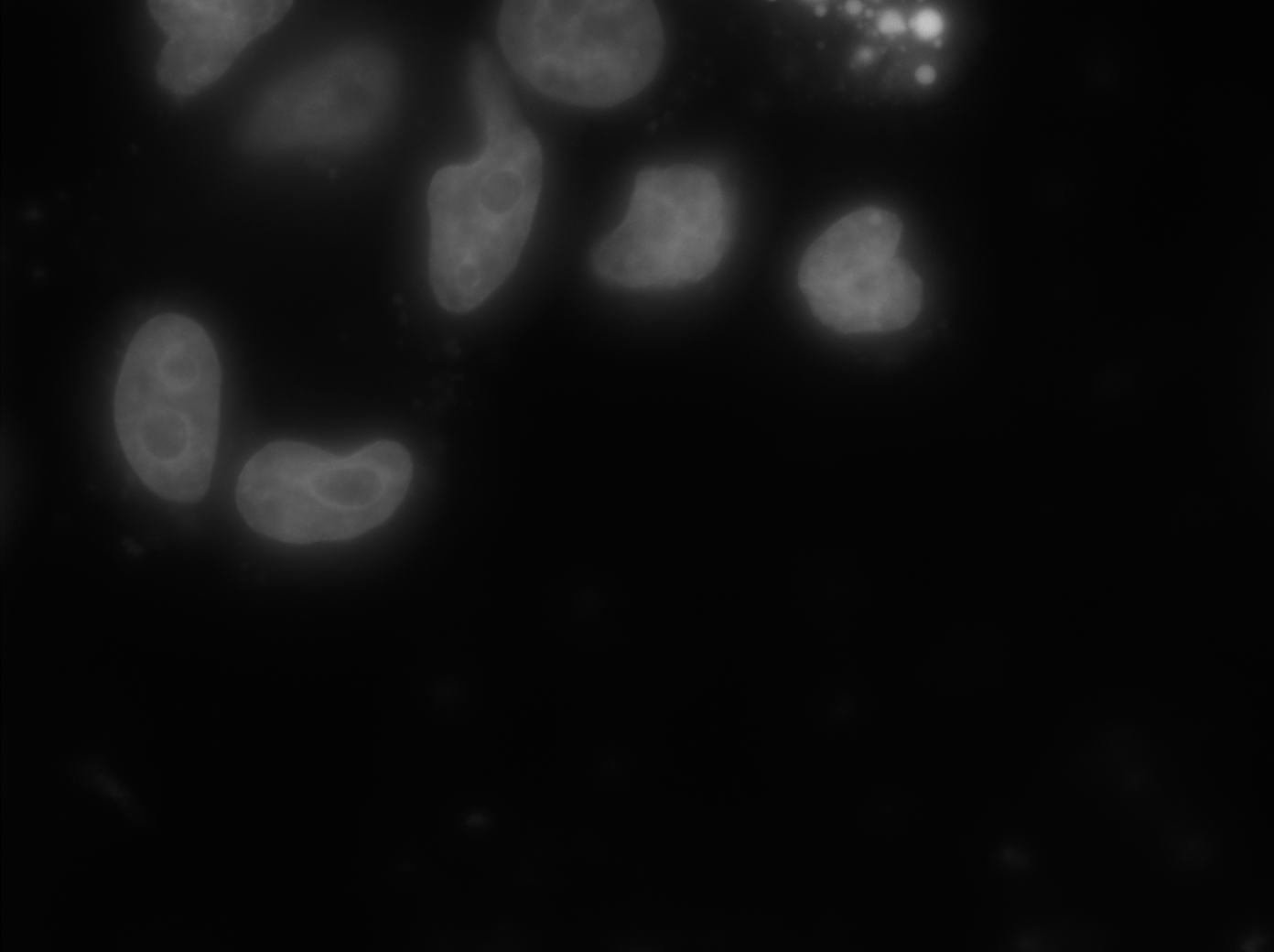

Supplement: Supplementary file 12 — Source data Fig. 3 part 2 [file 44319_2026_742_MOESM12_ESM.zip › Figure 3 Part 2/Fig 3b-e TTLL screen part 2/TTLL7-YFPy I11.Project Maximum Z_XY1679089317_Z0_T0_C0.tif]
