# Supplementary figures and images for: TPGS1 regulates central spindle microtubule glutamylation and remodeling during telophase and abscission (part 12 of 36)
Source: EMBO Rep. 2026 Mar 23;27(8):1944–63. doi: 10.1038/s44319-026-00742-3 (PMC13121839; doi:10.1038/s44319-026-00742-3)

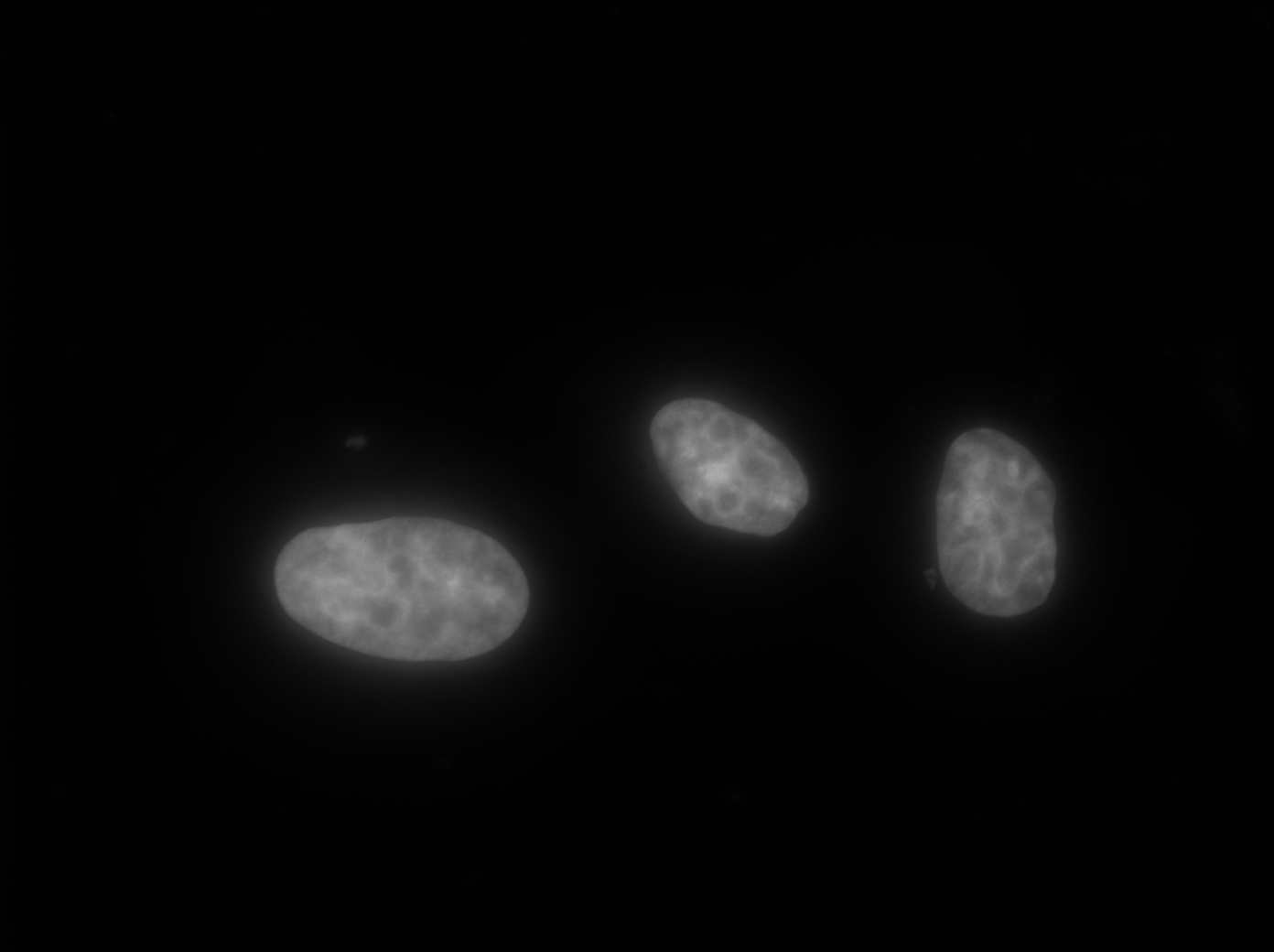

Supplement: Supplementary file 11 — Source data Fig. 3 part 1 [file 44319_2026_742_MOESM11_ESM.zip › Figure 3 Part 1/Fig 3b-e TTLL screen/EYFP MB I1.Project Maximum Z_XY1663874451_Z0_T0_C0.tif]

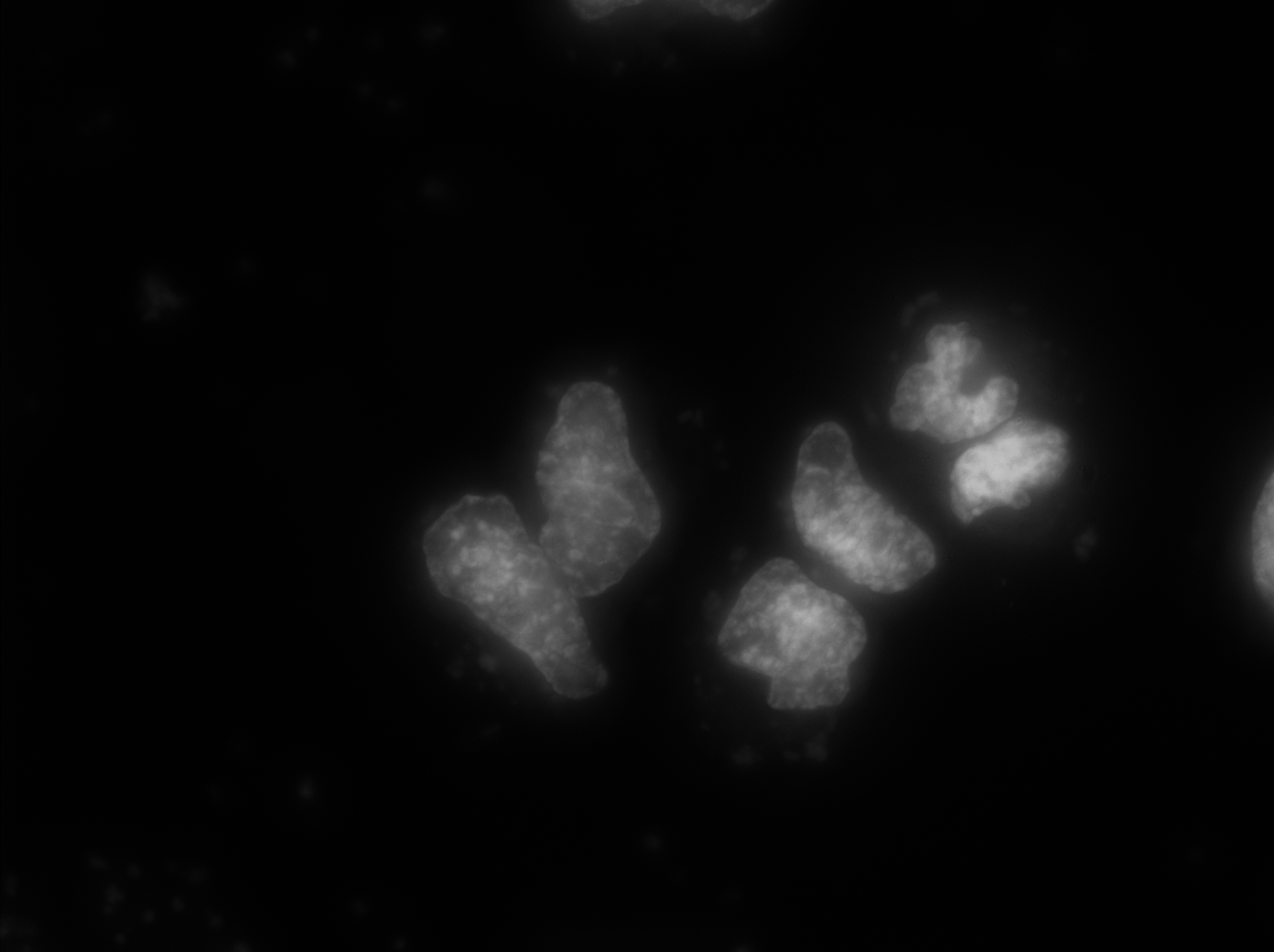

Supplement: Supplementary file 11 — Source data Fig. 3 part 1 [file 44319_2026_742_MOESM11_ESM.zip › Figure 3 Part 1/Fig 3b-e TTLL screen/TTLL1-GFP A4 I8.Project Maximum Z_XY1675962918_Z0_T0_C0.tif]

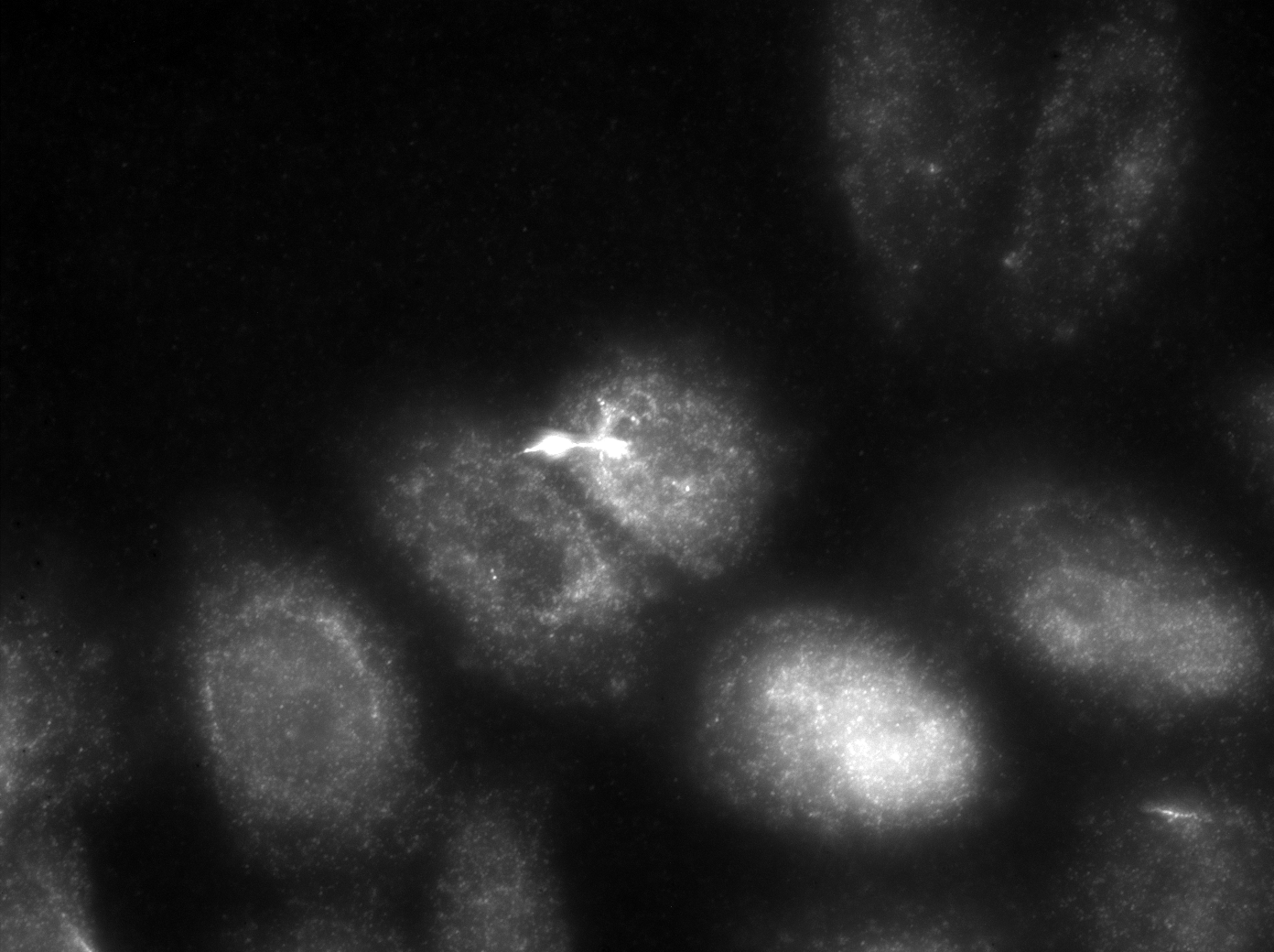

Supplement: Supplementary file 11 — Source data Fig. 3 part 1 [file 44319_2026_742_MOESM11_ESM.zip › Figure 3 Part 1/Fig 3b-e TTLL screen/TTLL1-GFP A3 I13.Project Maximum Z_XY1679696128_Z0_T0_C2.tif]

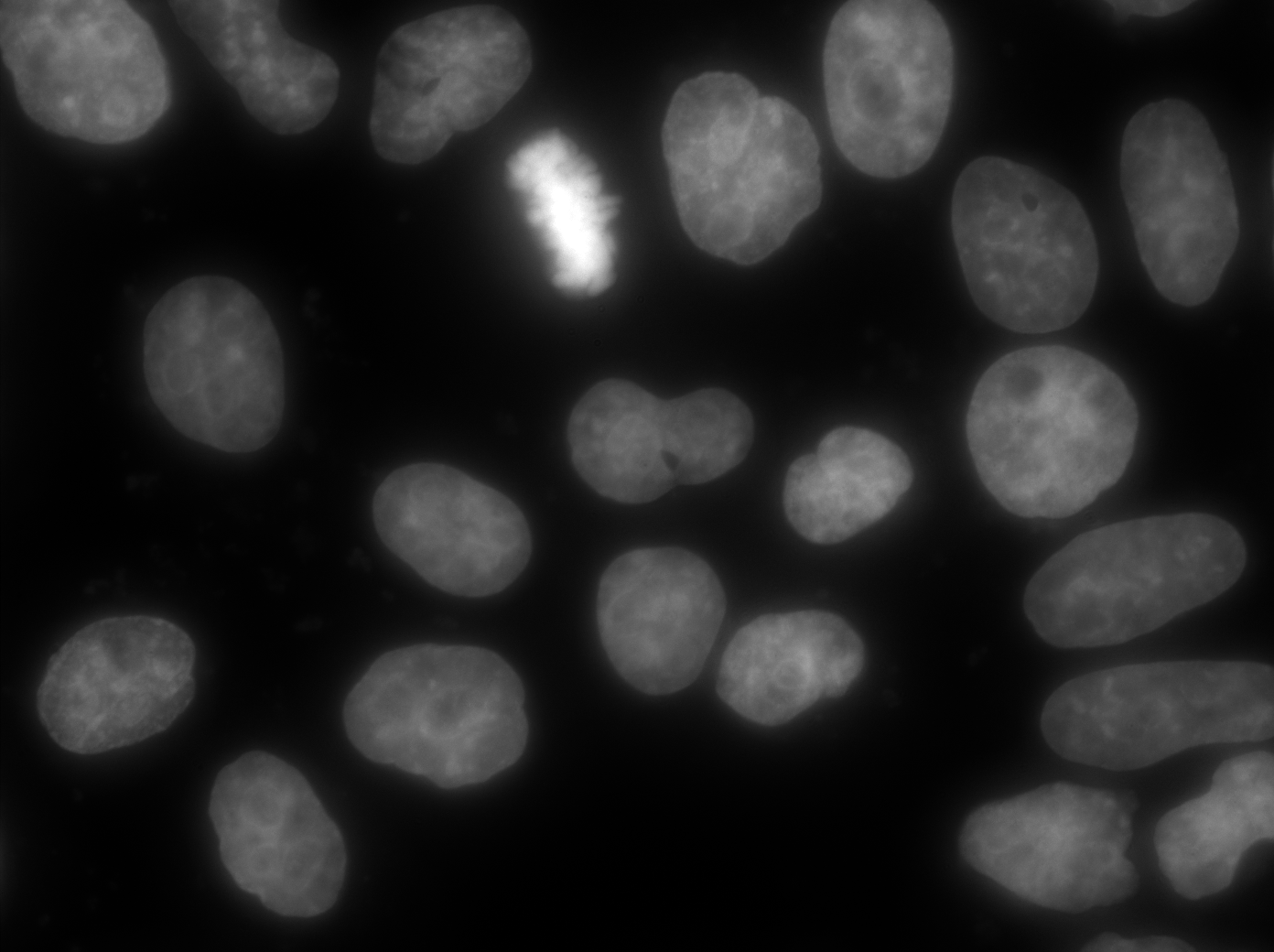

Supplement: Supplementary file 11 — Source data Fig. 3 part 1 [file 44319_2026_742_MOESM11_ESM.zip › Figure 3 Part 1/Fig 3b-e TTLL screen/TTLL1-GFP A3 I2 - 1.Project Maximum Z_XY1679694378_Z0_T0_C0.tif]

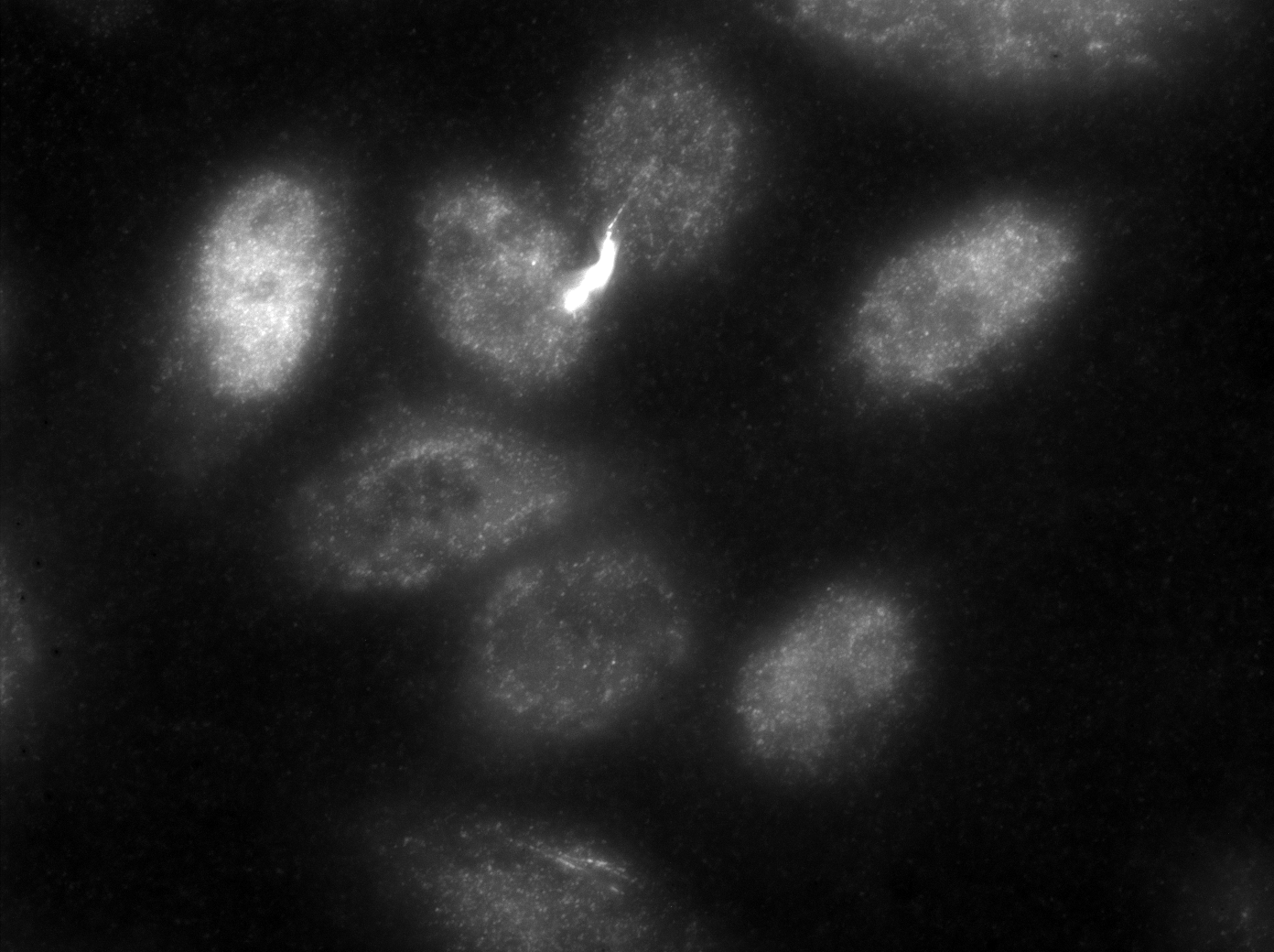

Supplement: Supplementary file 11 — Source data Fig. 3 part 1 [file 44319_2026_742_MOESM11_ESM.zip › Figure 3 Part 1/Fig 3b-e TTLL screen/TTLL1-GFP A3 I14.Project Maximum Z_XY1679696939_Z0_T0_C2.tif]

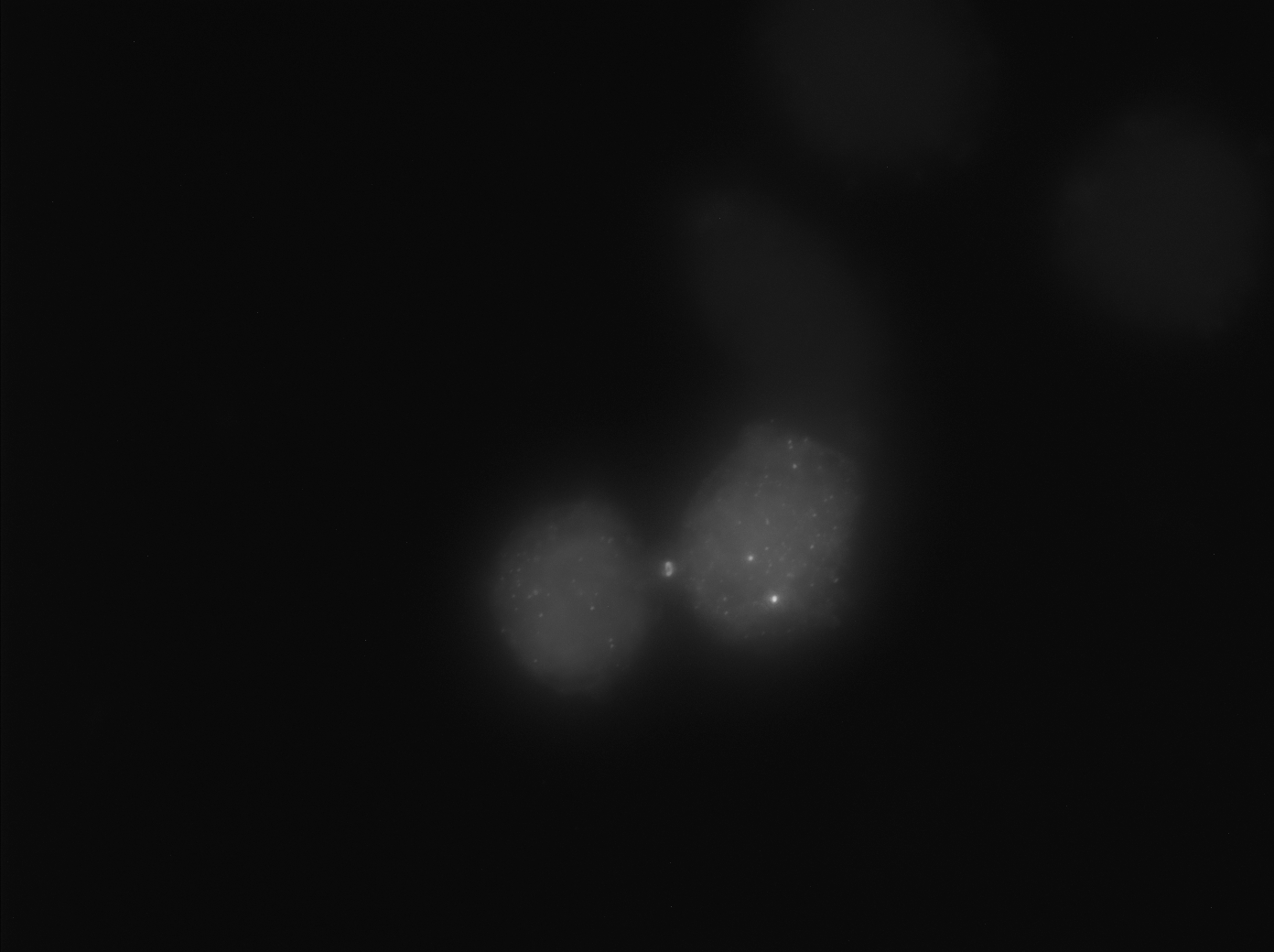

Supplement: Supplementary file 11 — Source data Fig. 3 part 1 [file 44319_2026_742_MOESM11_ESM.zip › Figure 3 Part 1/Fig 3b-e TTLL screen/TTLL1-GFP A4 I4.Project Maximum Z_XY1675962054_Z0_T0_C3.tif]

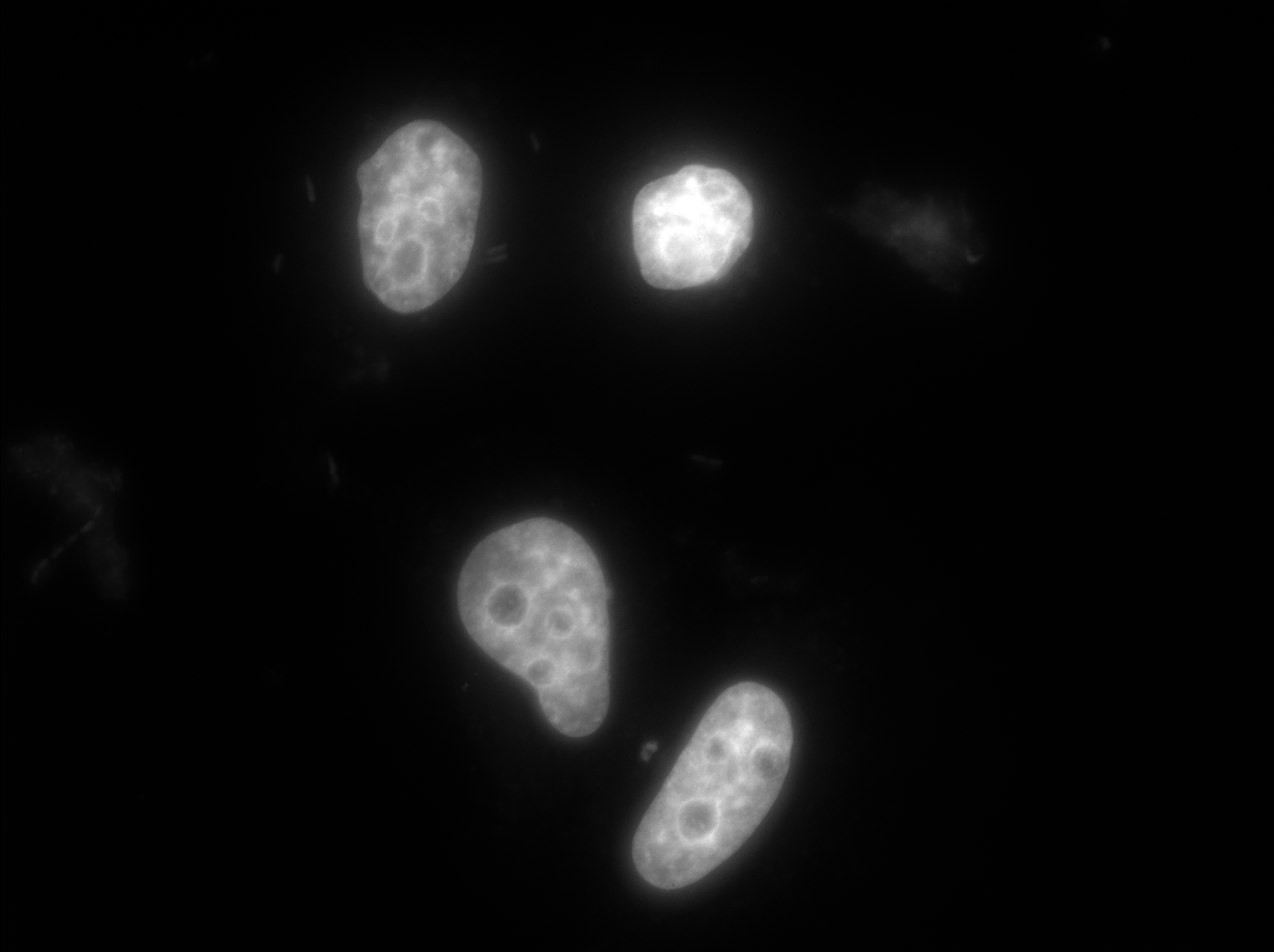

Supplement: Supplementary file 11 — Source data Fig. 3 part 1 [file 44319_2026_742_MOESM11_ESM.zip › Figure 3 Part 1/Fig 3b-e TTLL screen/TTLL1-GFP R1 I4.Project Maximum Z_XY1674164021_Z0_T0_C0.tif]

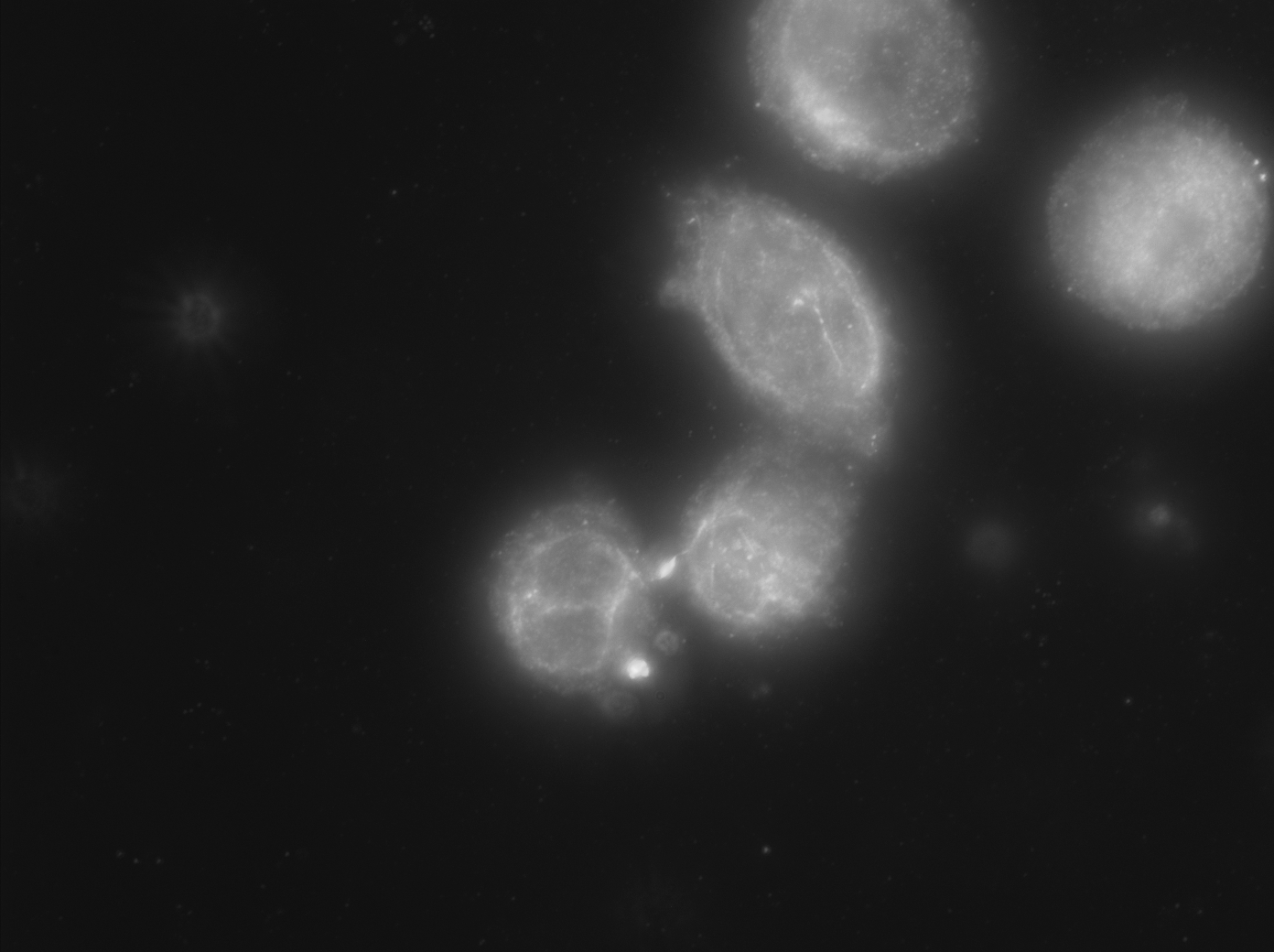

Supplement: Supplementary file 11 — Source data Fig. 3 part 1 [file 44319_2026_742_MOESM11_ESM.zip › Figure 3 Part 1/Fig 3b-e TTLL screen/TTLL1-GFP A4 I4.Project Maximum Z_XY1675962054_Z0_T0_C2.tif]

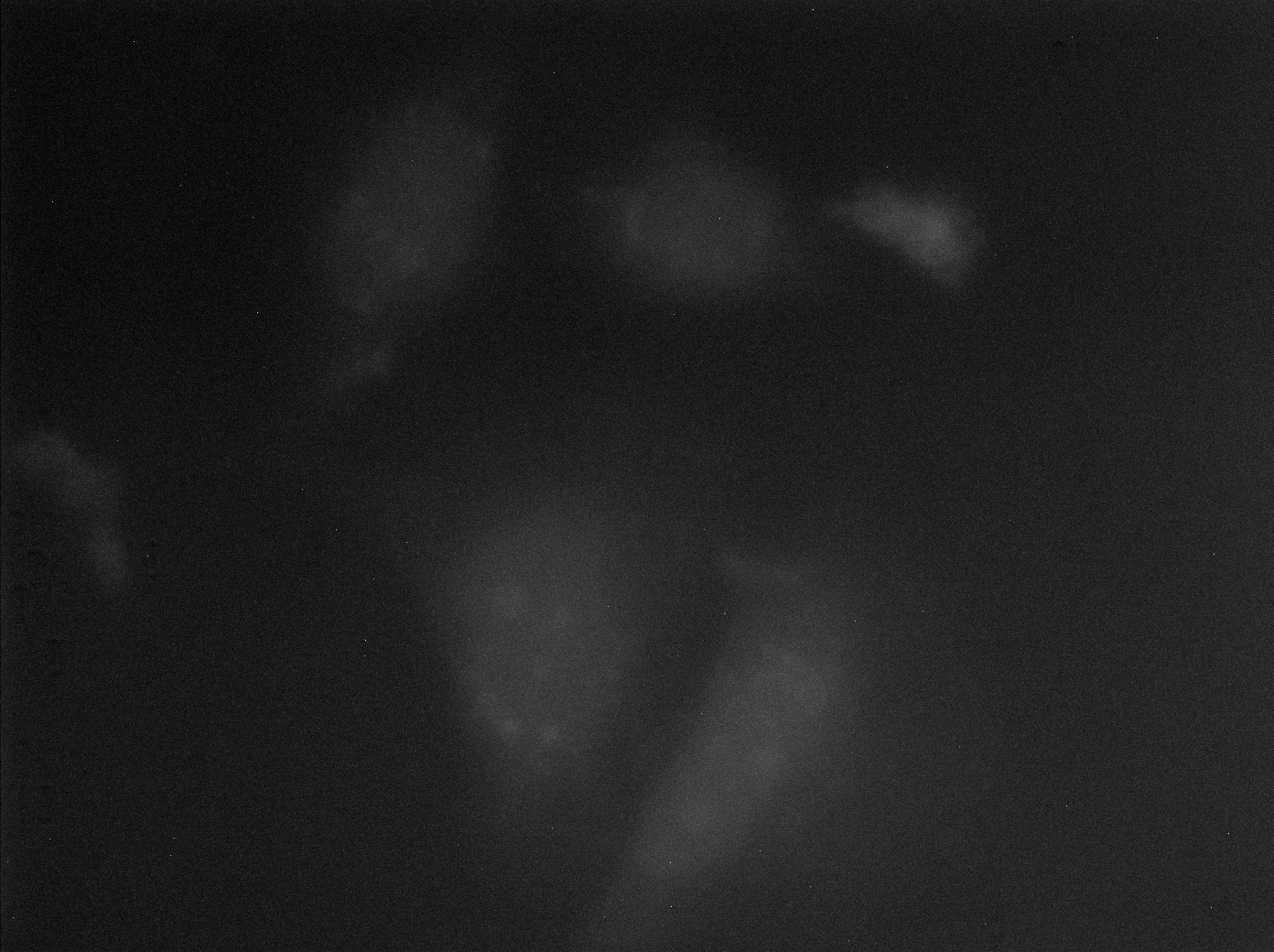

Supplement: Supplementary file 11 — Source data Fig. 3 part 1 [file 44319_2026_742_MOESM11_ESM.zip › Figure 3 Part 1/Fig 3b-e TTLL screen/TTLL1-GFP R1 I4.Project Maximum Z_XY1674164021_Z0_T0_C1.tif]

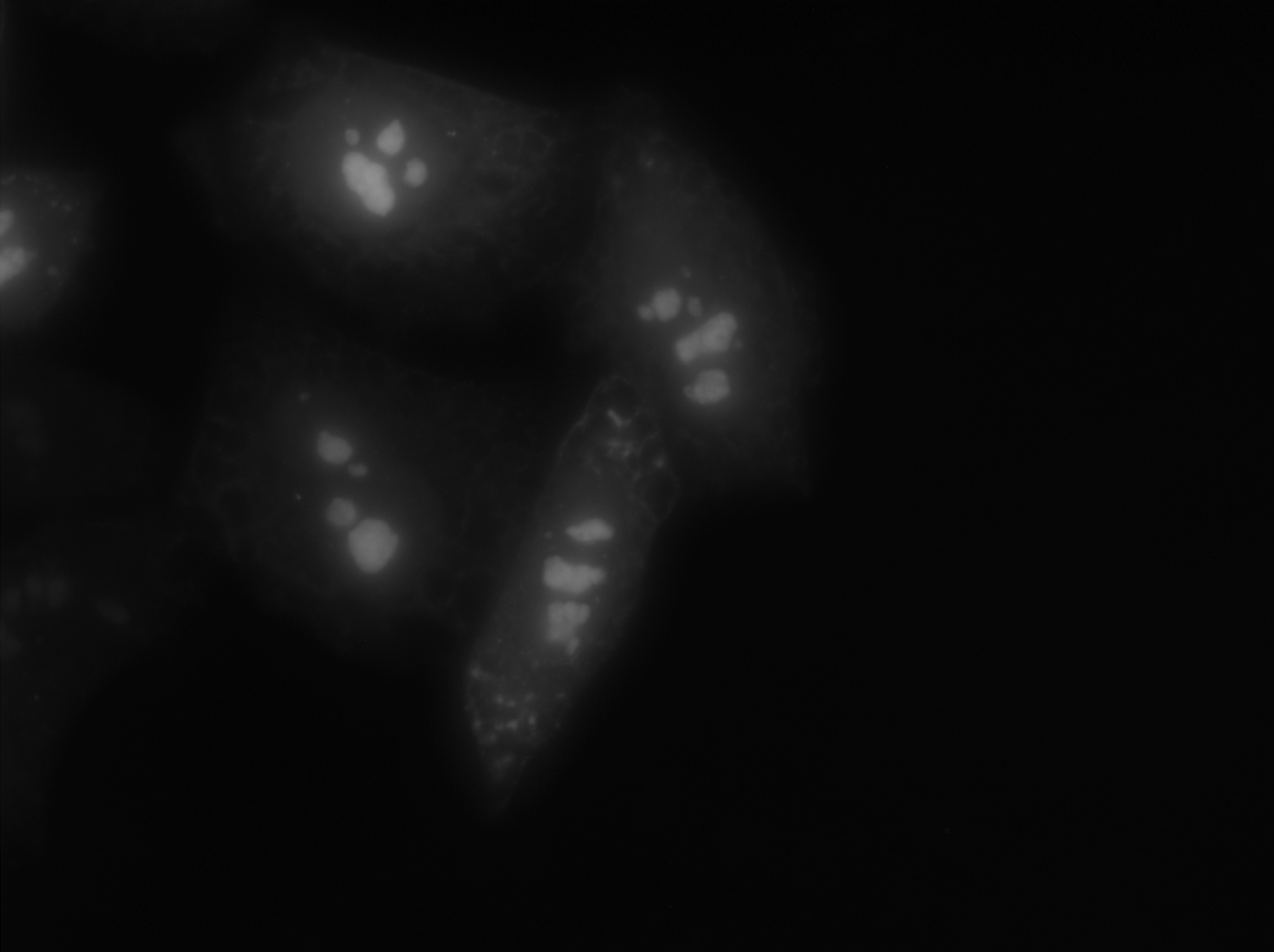

Supplement: Supplementary file 11 — Source data Fig. 3 part 1 [file 44319_2026_742_MOESM11_ESM.zip › Figure 3 Part 1/Fig 3b-e TTLL screen/TTLL4-YFPy I11.Project Maximum Z_XY1679336789_Z0_T0_C2.tif]

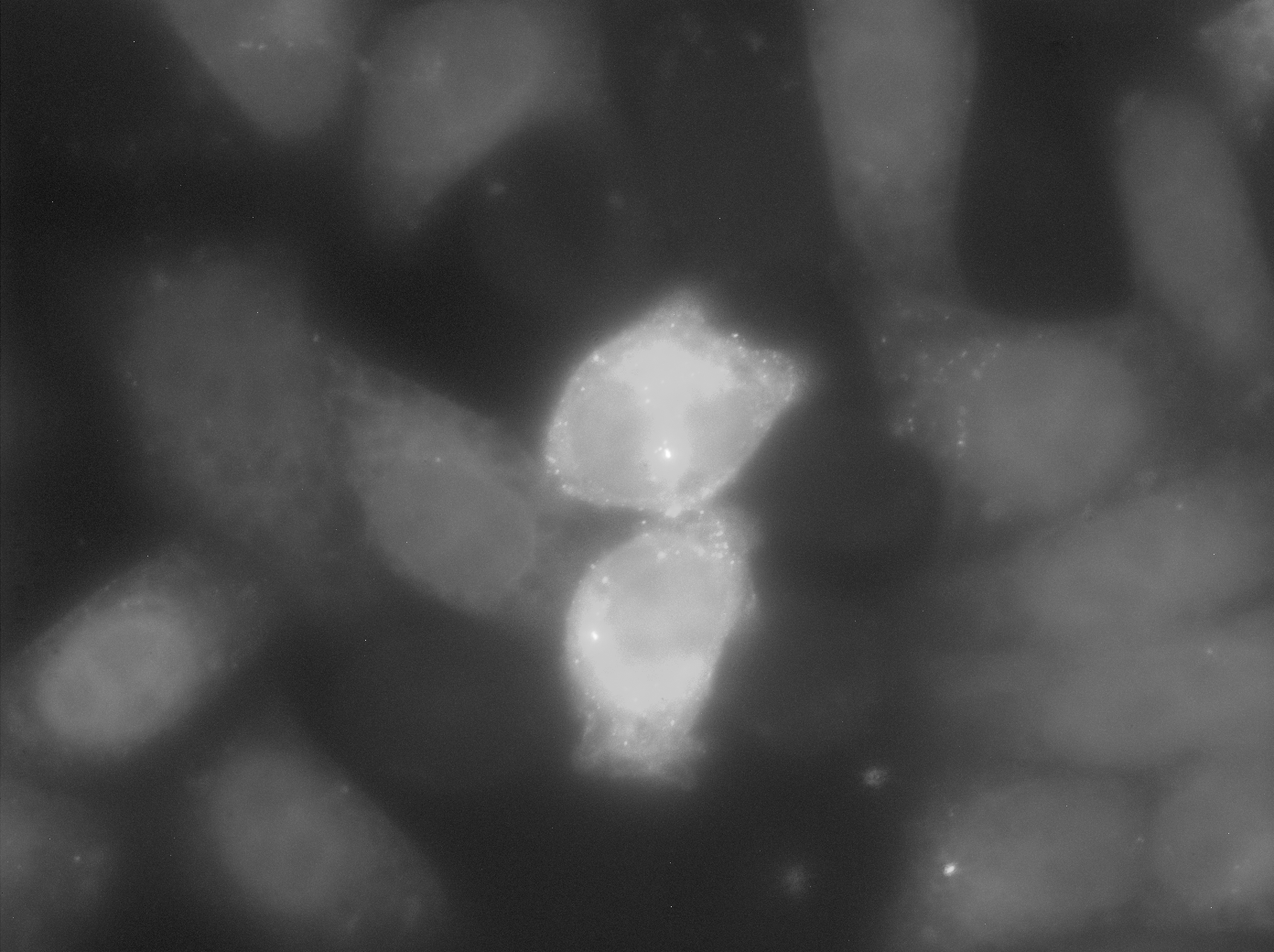

Supplement: Supplementary file 11 — Source data Fig. 3 part 1 [file 44319_2026_742_MOESM11_ESM.zip › Figure 3 Part 1/Fig 3b-e TTLL screen/TTLL1-GFP A3 I2 - 1.Project Maximum Z_XY1679694378_Z0_T0_C1.tif]

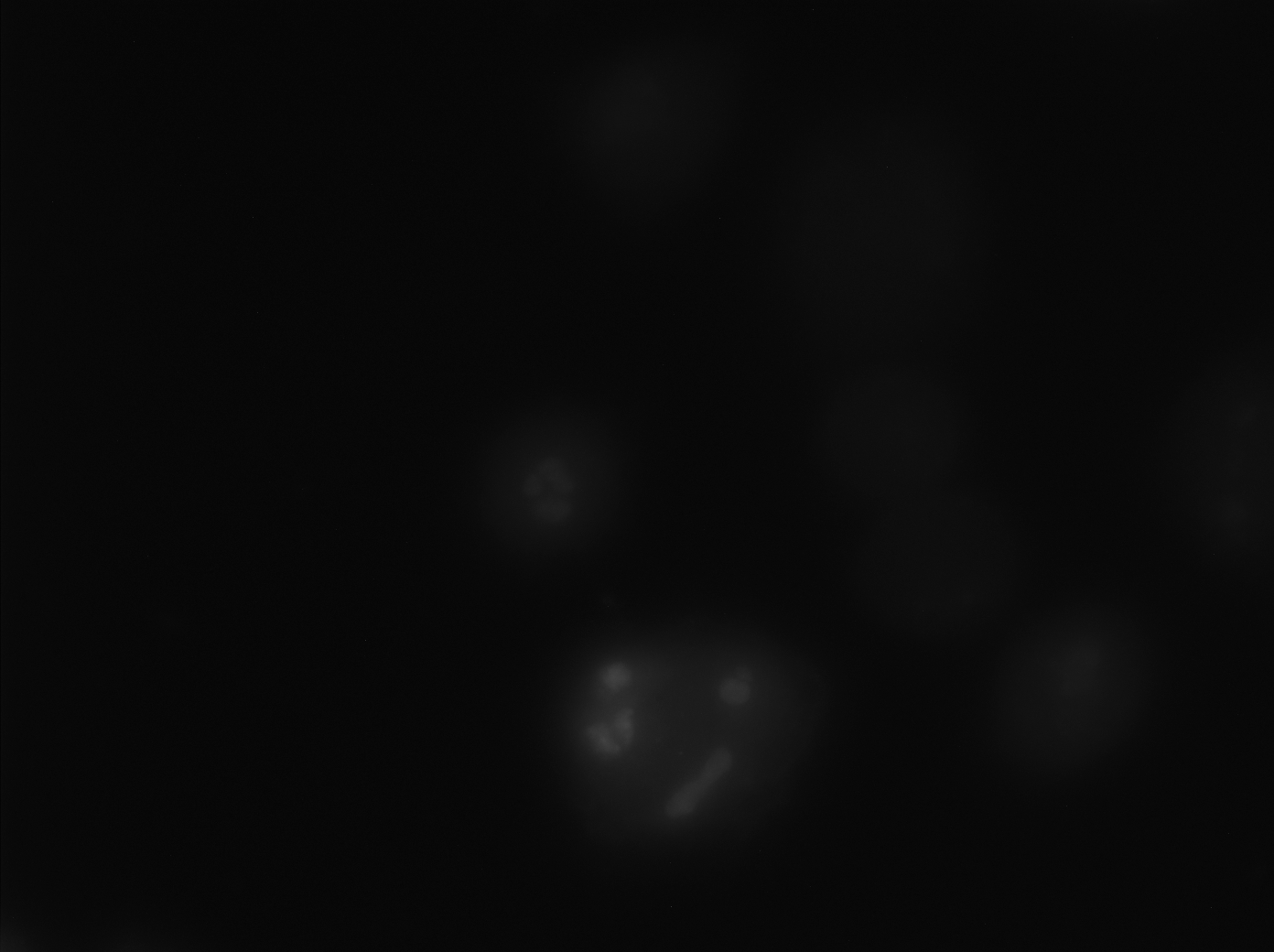

Supplement: Supplementary file 11 — Source data Fig. 3 part 1 [file 44319_2026_742_MOESM11_ESM.zip › Figure 3 Part 1/Fig 3b-e TTLL screen/TTLL4-YFPy I15.Project Maximum Z_XY1679337393_Z0_T0_C2.tif]

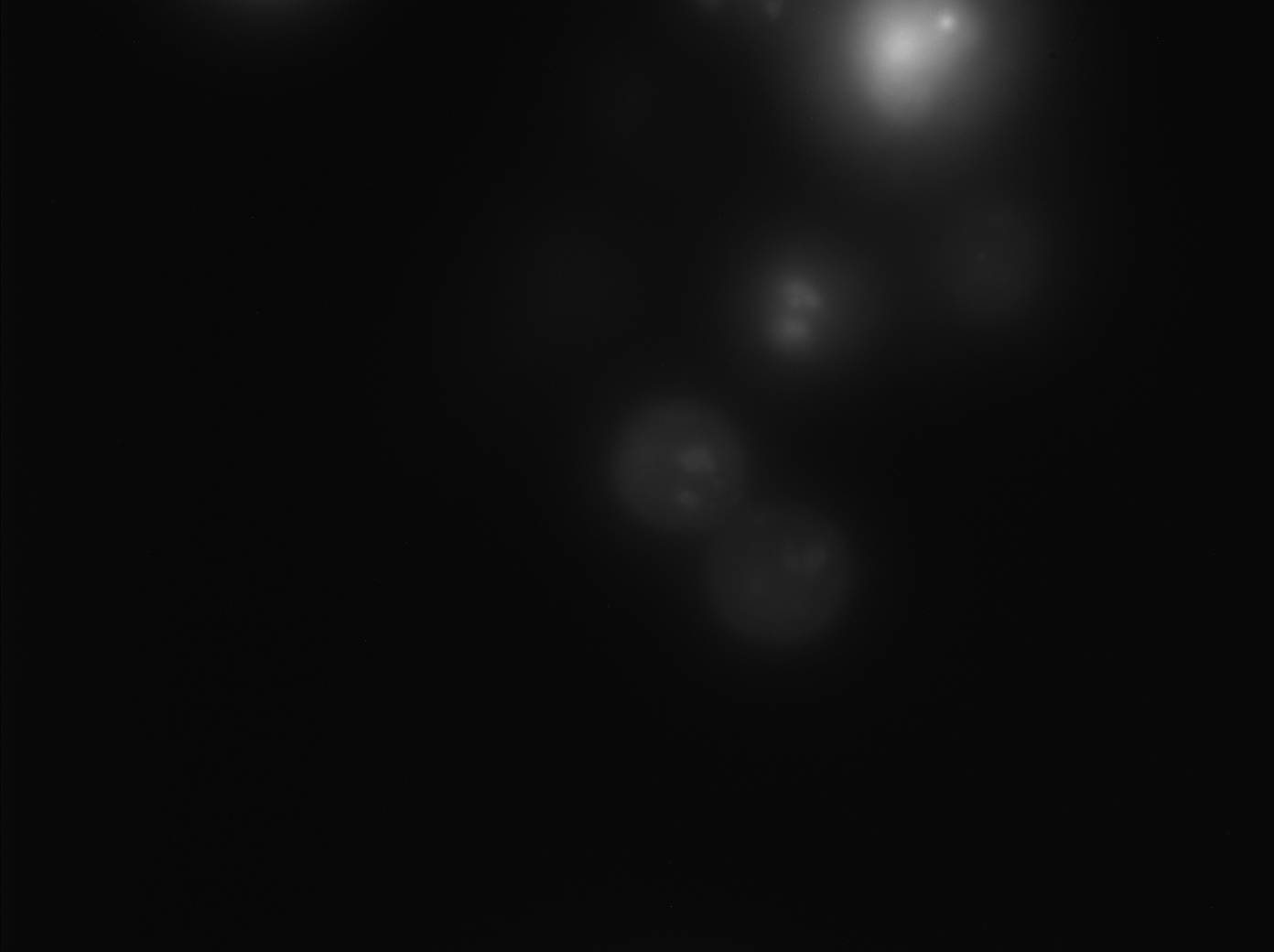

Supplement: Supplementary file 11 — Source data Fig. 3 part 1 [file 44319_2026_742_MOESM11_ESM.zip › Figure 3 Part 1/Fig 3b-e TTLL screen/TTLL4-YFPy I5.Project Maximum Z_XY1679076150_Z0_T0_C2.tif]

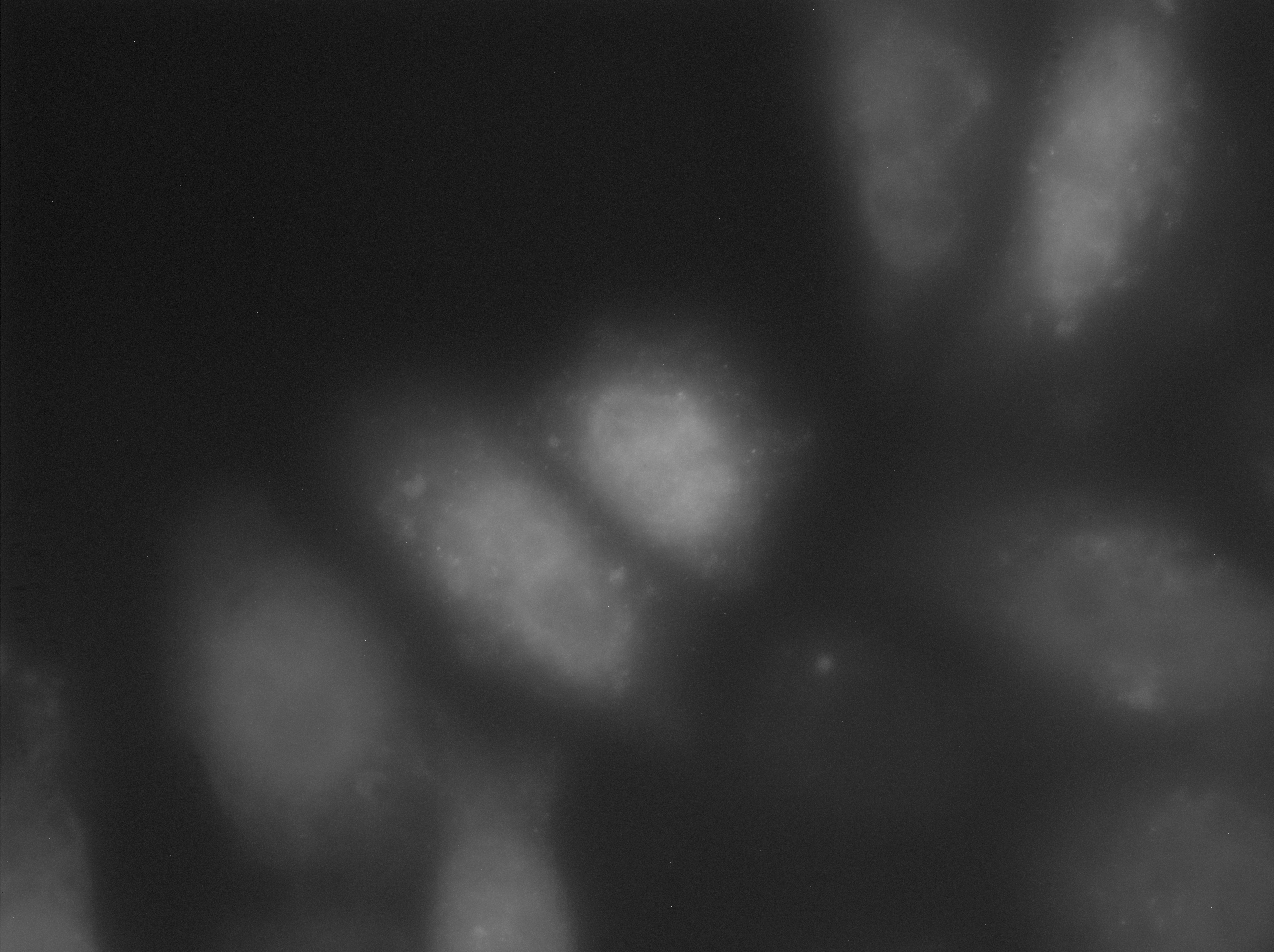

Supplement: Supplementary file 11 — Source data Fig. 3 part 1 [file 44319_2026_742_MOESM11_ESM.zip › Figure 3 Part 1/Fig 3b-e TTLL screen/TTLL1-GFP A3 I13.Project Maximum Z_XY1679696128_Z0_T0_C1.tif]

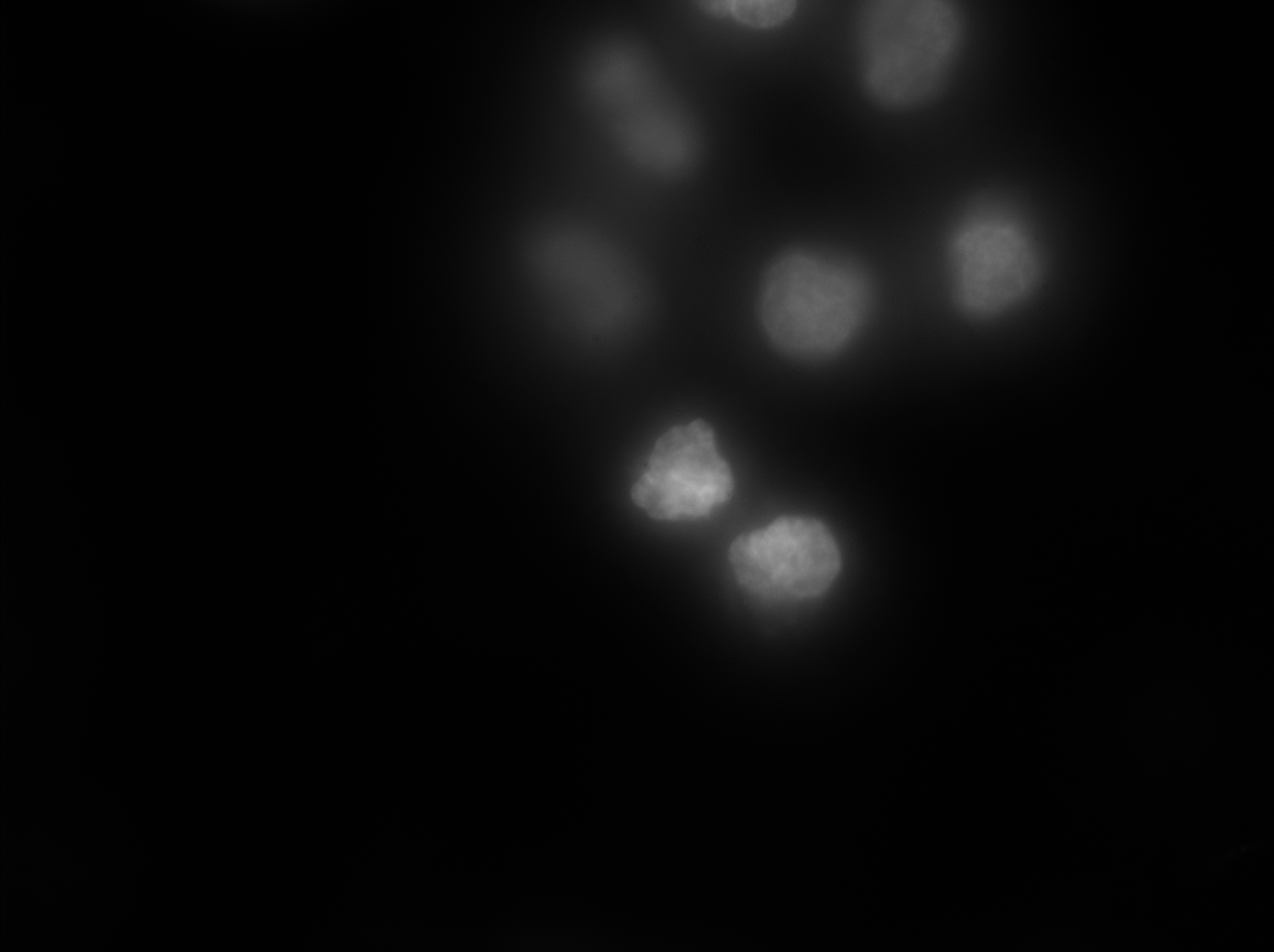

Supplement: Supplementary file 11 — Source data Fig. 3 part 1 [file 44319_2026_742_MOESM11_ESM.zip › Figure 3 Part 1/Fig 3b-e TTLL screen/TTLL4-YFPy I5.Project Maximum Z_XY1679076150_Z0_T0_C0.tif]

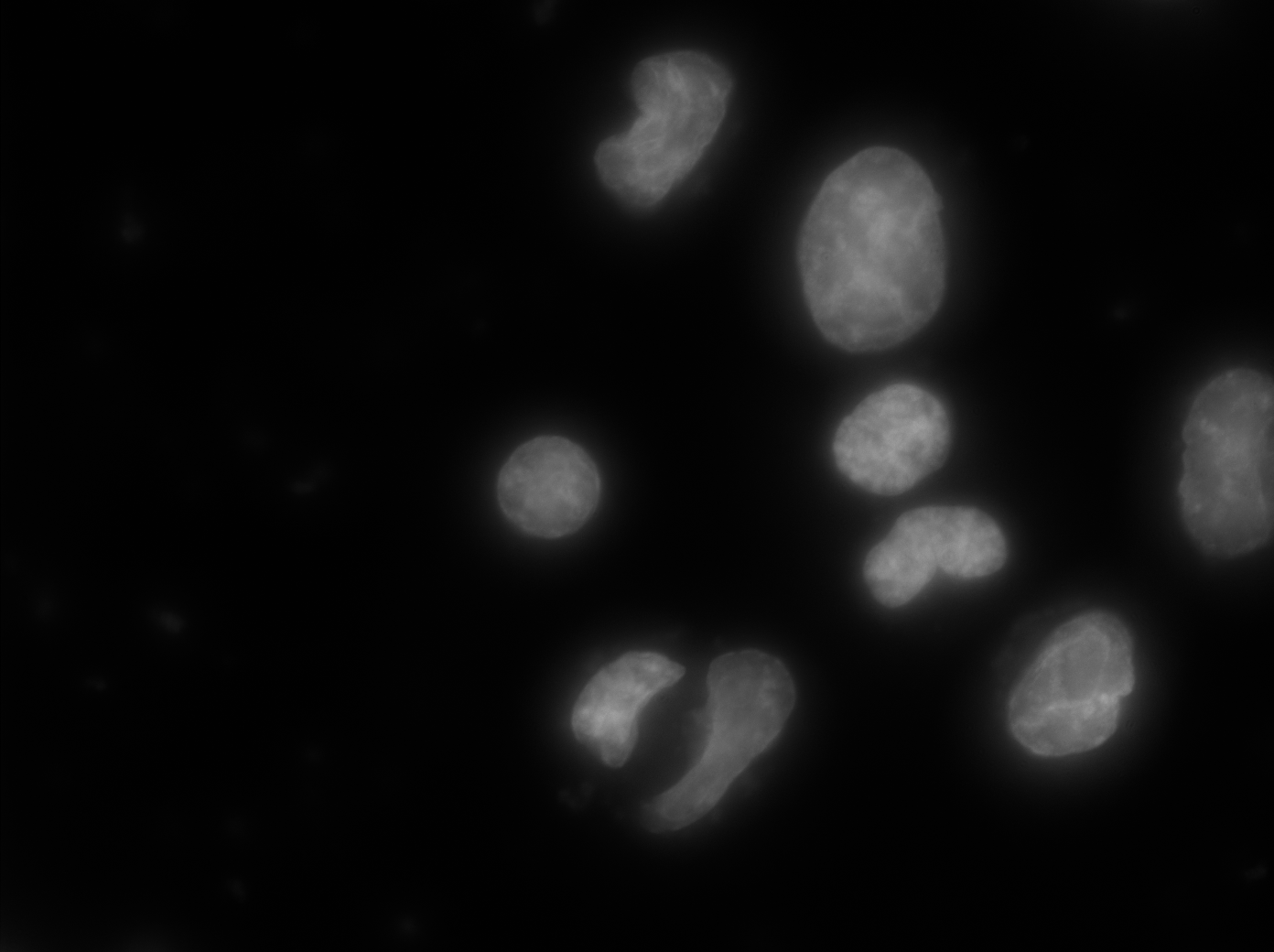

Supplement: Supplementary file 11 — Source data Fig. 3 part 1 [file 44319_2026_742_MOESM11_ESM.zip › Figure 3 Part 1/Fig 3b-e TTLL screen/TTLL4-YFPy I15.Project Maximum Z_XY1679337393_Z0_T0_C0.tif]

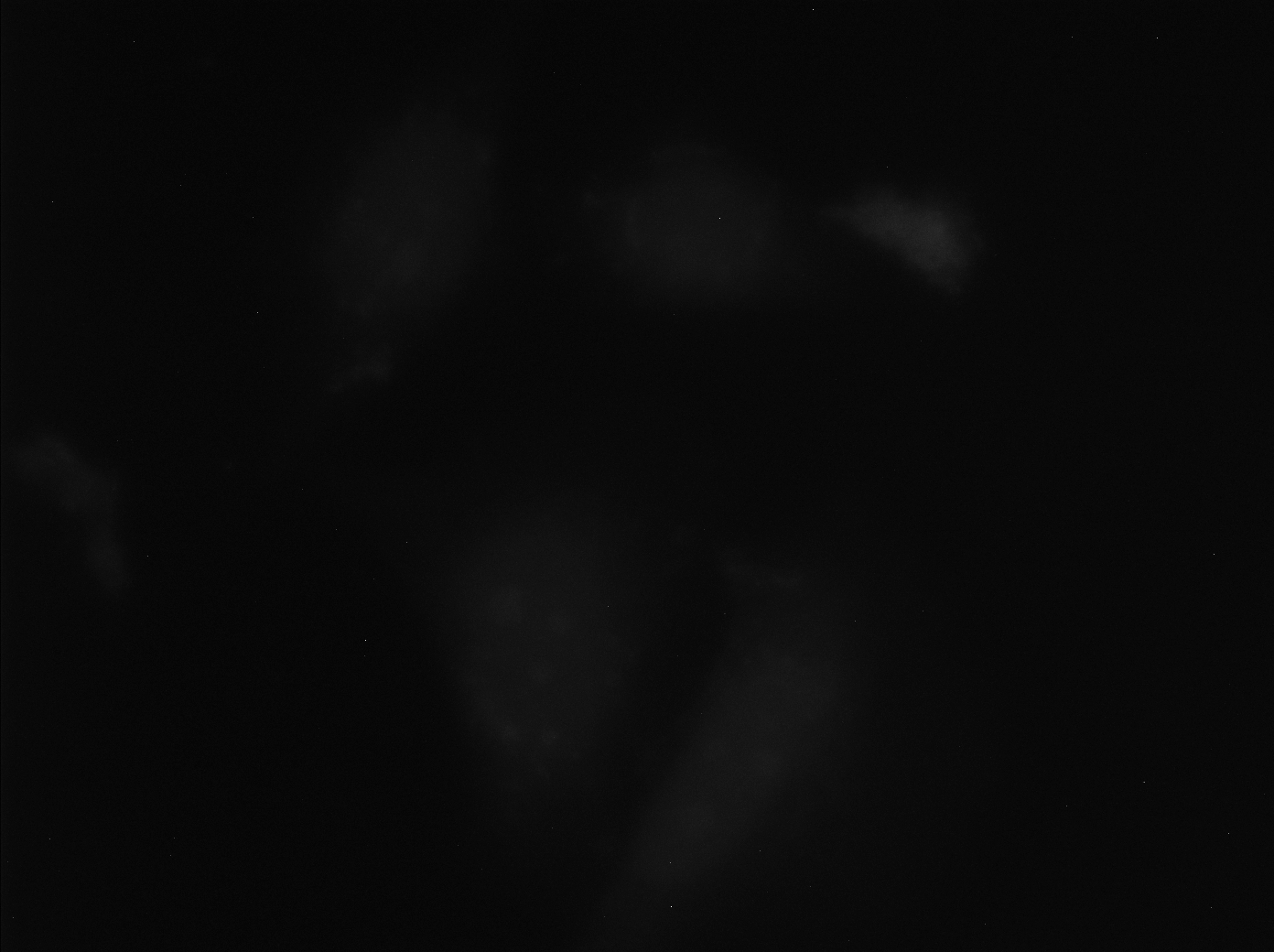

Supplement: Supplementary file 11 — Source data Fig. 3 part 1 [file 44319_2026_742_MOESM11_ESM.zip › Figure 3 Part 1/Fig 3b-e TTLL screen/TTLL1-GFP R1 I4.Project Maximum Z_XY1674164021_Z0_T0_C3.tif]

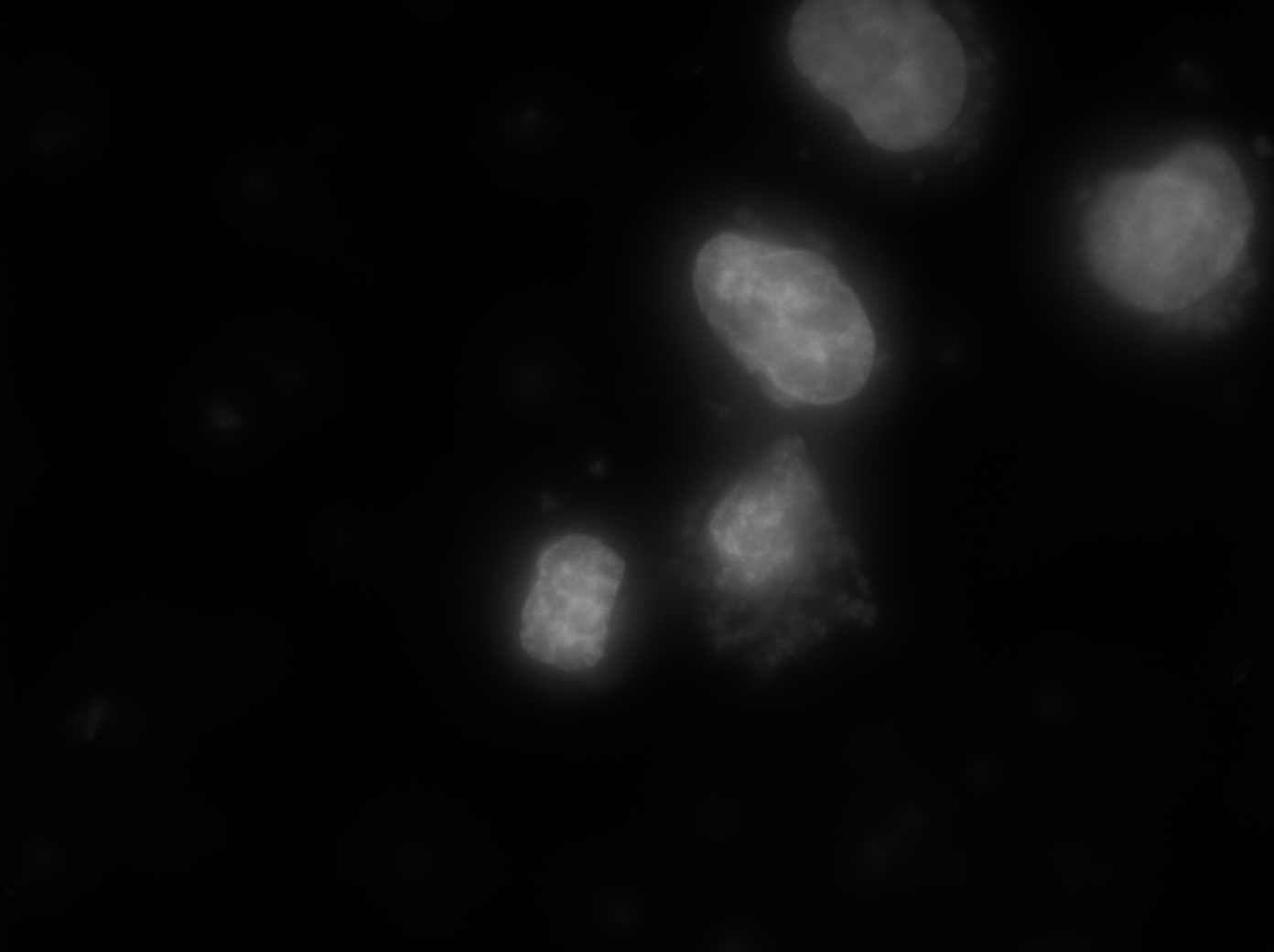

Supplement: Supplementary file 11 — Source data Fig. 3 part 1 [file 44319_2026_742_MOESM11_ESM.zip › Figure 3 Part 1/Fig 3b-e TTLL screen/TTLL1-GFP A4 I4.Project Maximum Z_XY1675962054_Z0_T0_C0.tif]

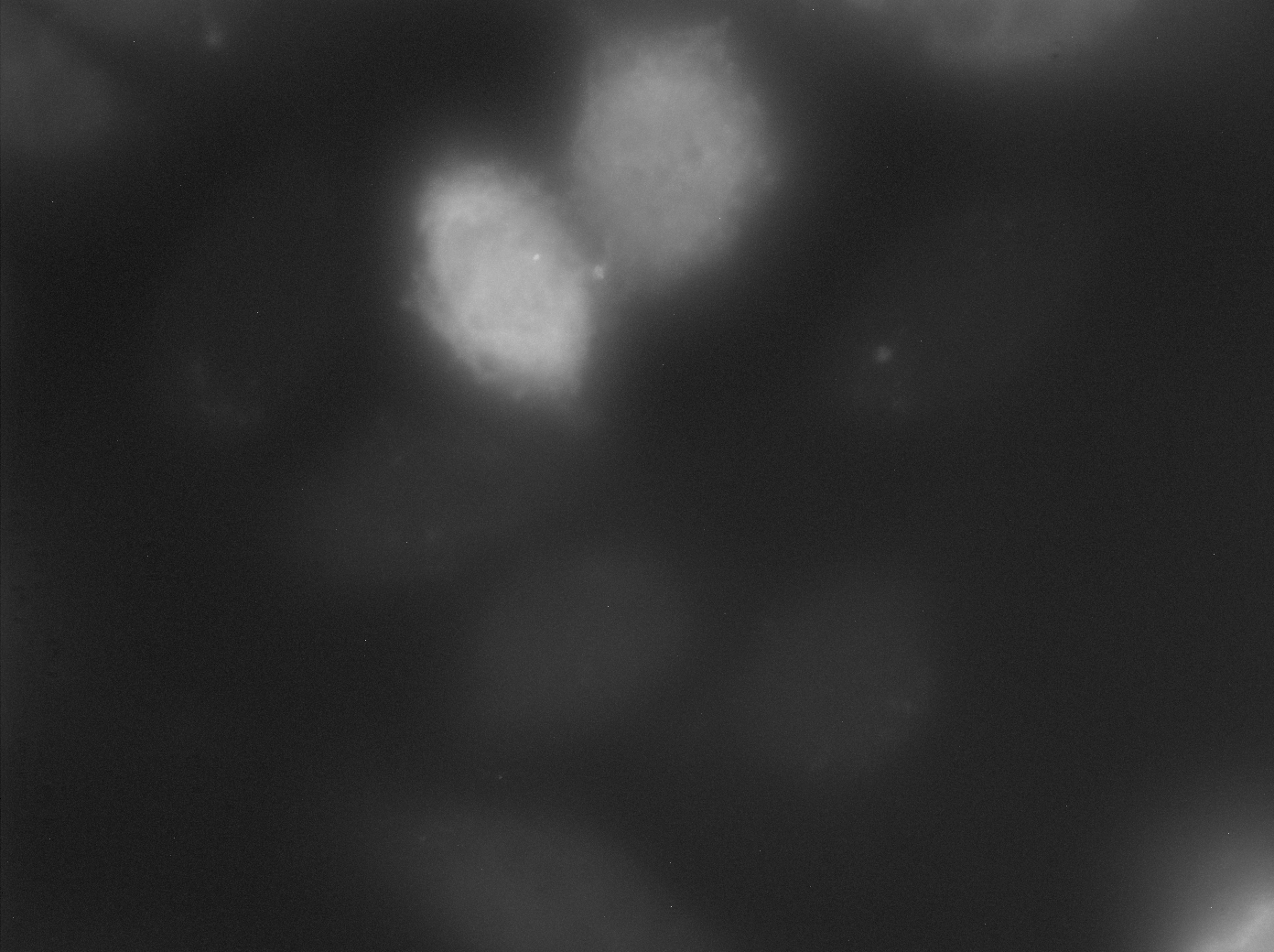

Supplement: Supplementary file 11 — Source data Fig. 3 part 1 [file 44319_2026_742_MOESM11_ESM.zip › Figure 3 Part 1/Fig 3b-e TTLL screen/TTLL1-GFP A3 I14.Project Maximum Z_XY1679696939_Z0_T0_C1.tif]

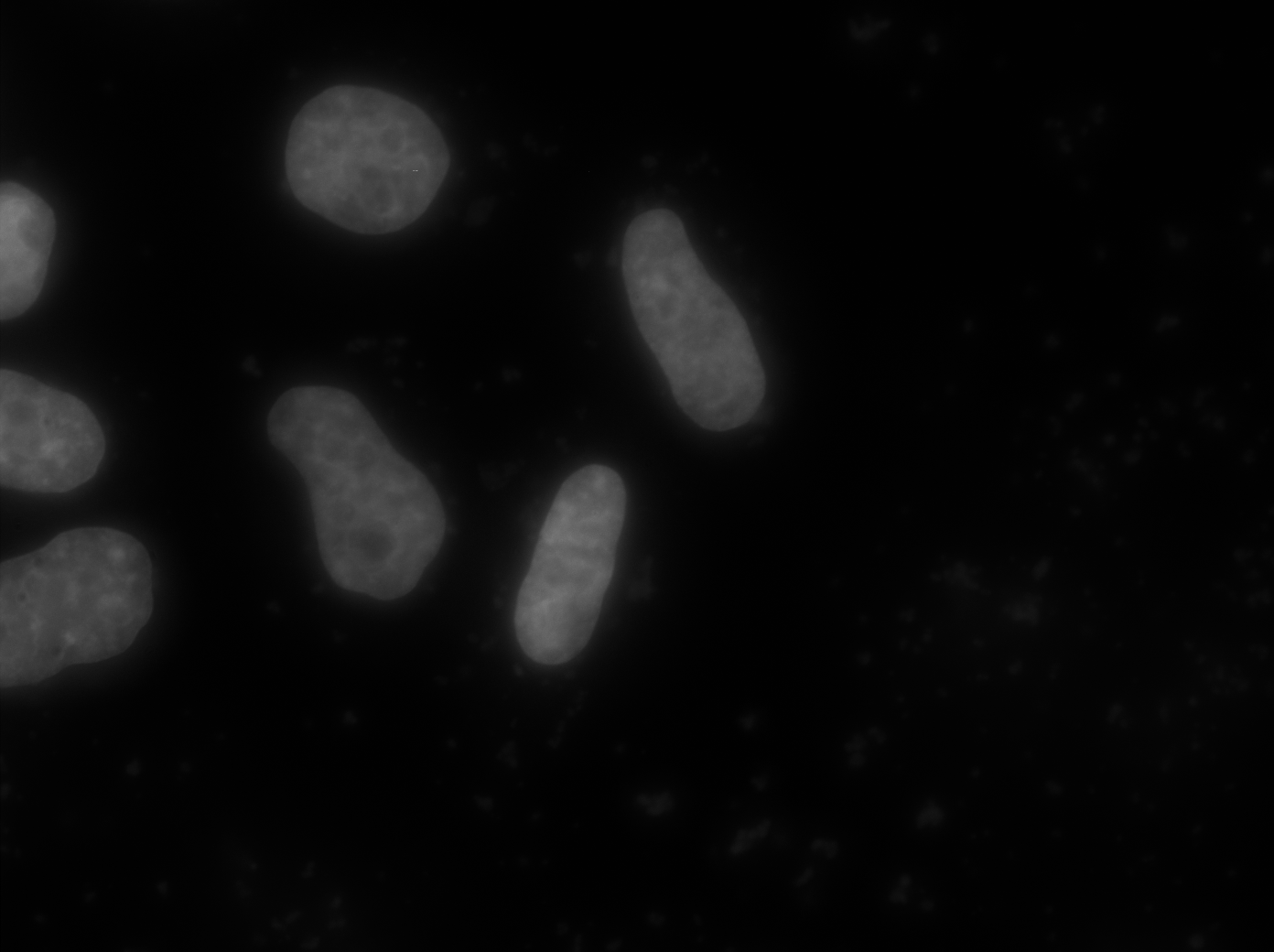

Supplement: Supplementary file 11 — Source data Fig. 3 part 1 [file 44319_2026_742_MOESM11_ESM.zip › Figure 3 Part 1/Fig 3b-e TTLL screen/TTLL4-YFPy I11.Project Maximum Z_XY1679336789_Z0_T0_C0.tif]

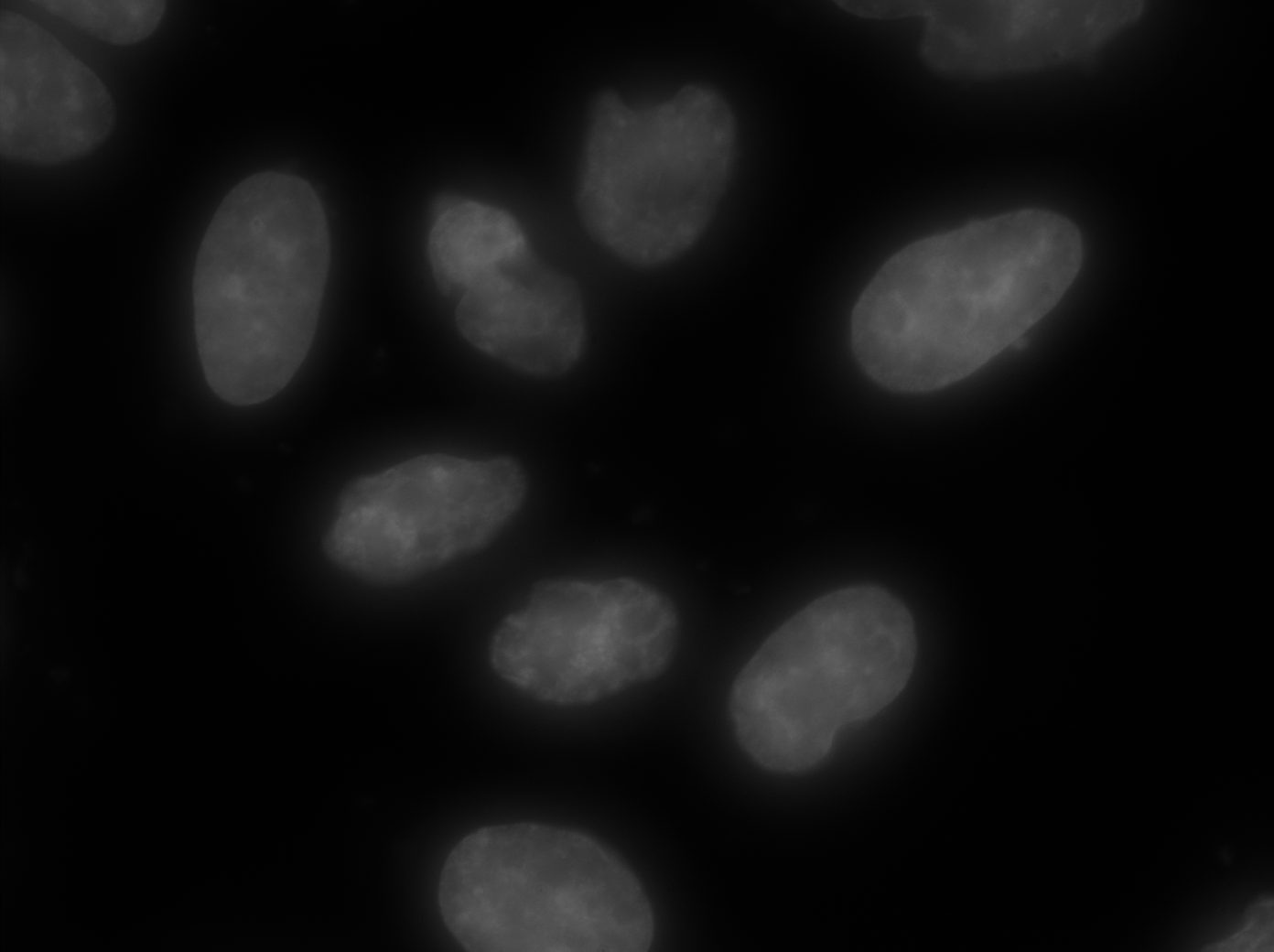

Supplement: Supplementary file 11 — Source data Fig. 3 part 1 [file 44319_2026_742_MOESM11_ESM.zip › Figure 3 Part 1/Fig 3b-e TTLL screen/TTLL1-GFP A3 I14.Project Maximum Z_XY1679696939_Z0_T0_C0.tif]

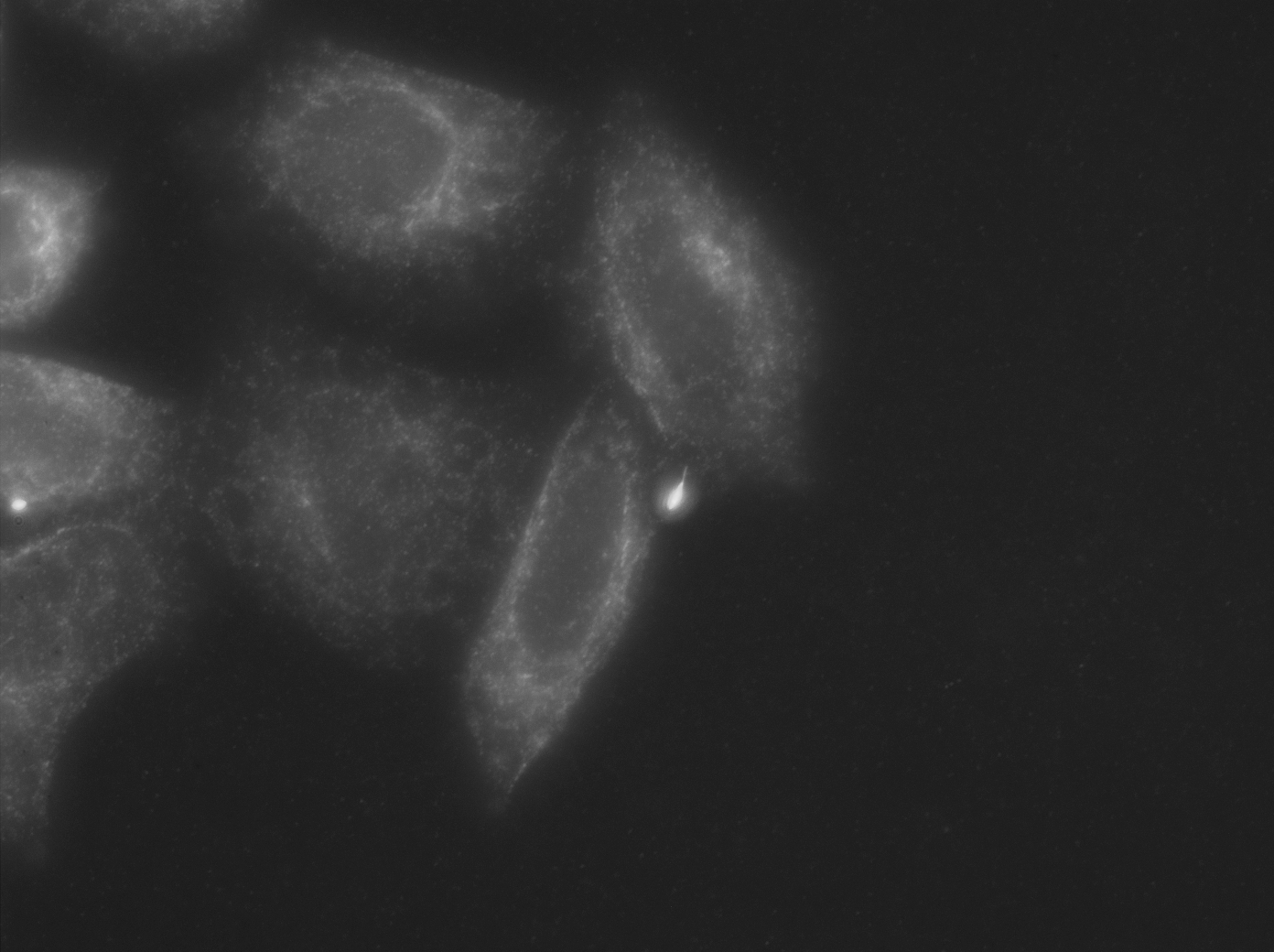

Supplement: Supplementary file 11 — Source data Fig. 3 part 1 [file 44319_2026_742_MOESM11_ESM.zip › Figure 3 Part 1/Fig 3b-e TTLL screen/TTLL4-YFPy I11.Project Maximum Z_XY1679336789_Z0_T0_C1.tif]

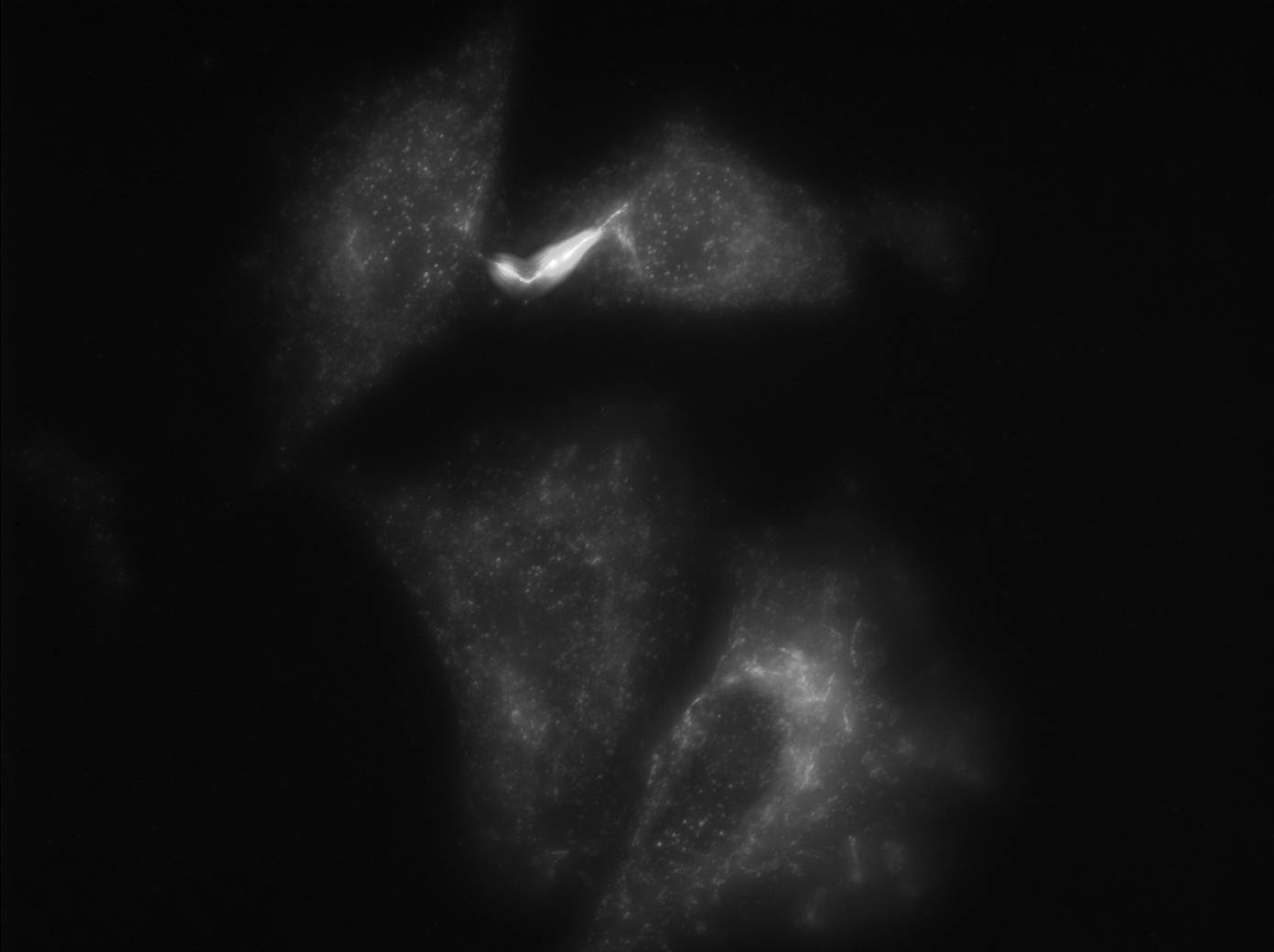

Supplement: Supplementary file 11 — Source data Fig. 3 part 1 [file 44319_2026_742_MOESM11_ESM.zip › Figure 3 Part 1/Fig 3b-e TTLL screen/TTLL1-GFP R1 I4.Project Maximum Z_XY1674164021_Z0_T0_C2.tif]

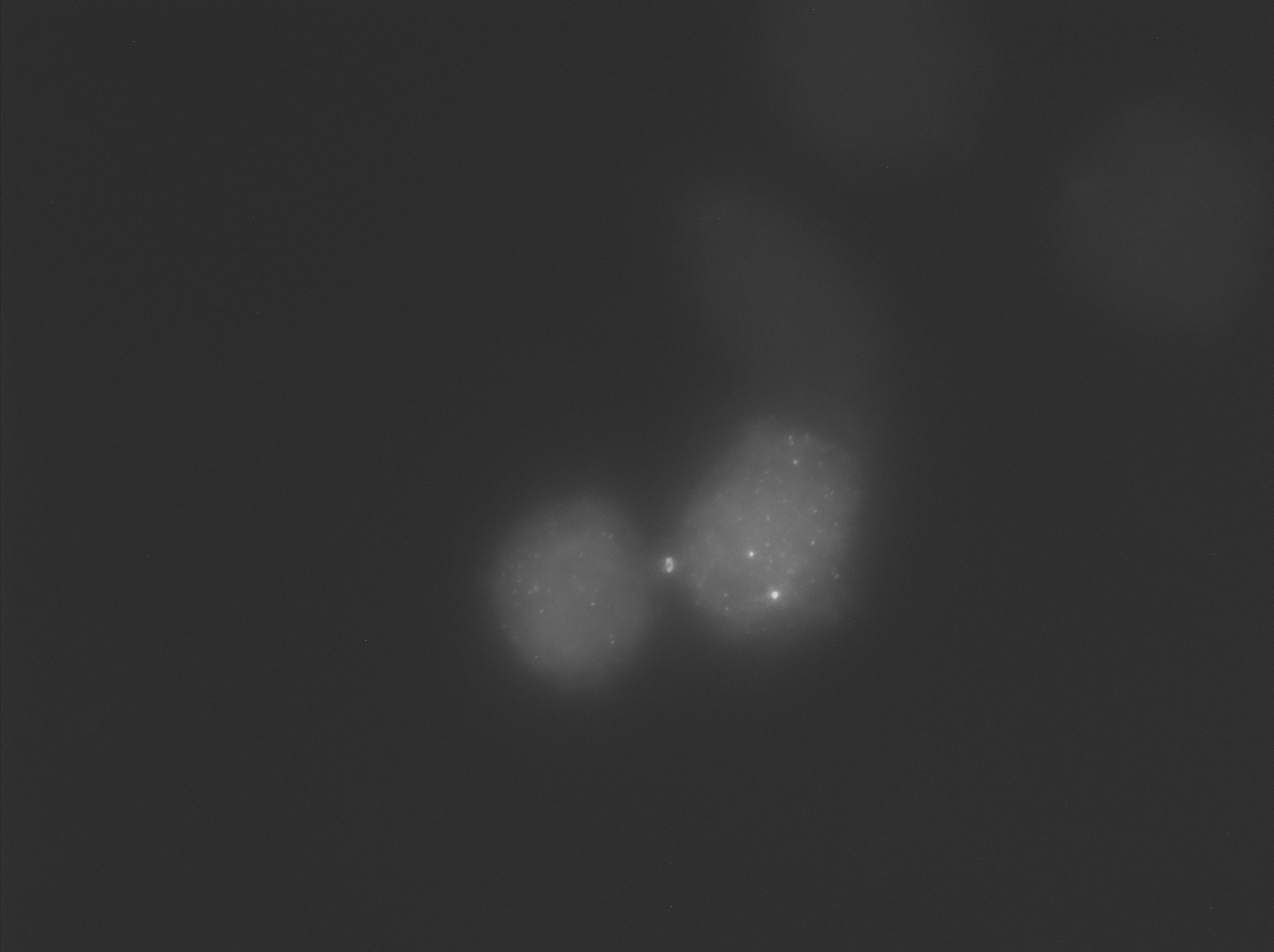

Supplement: Supplementary file 11 — Source data Fig. 3 part 1 [file 44319_2026_742_MOESM11_ESM.zip › Figure 3 Part 1/Fig 3b-e TTLL screen/TTLL1-GFP A4 I4.Project Maximum Z_XY1675962054_Z0_T0_C1.tif]

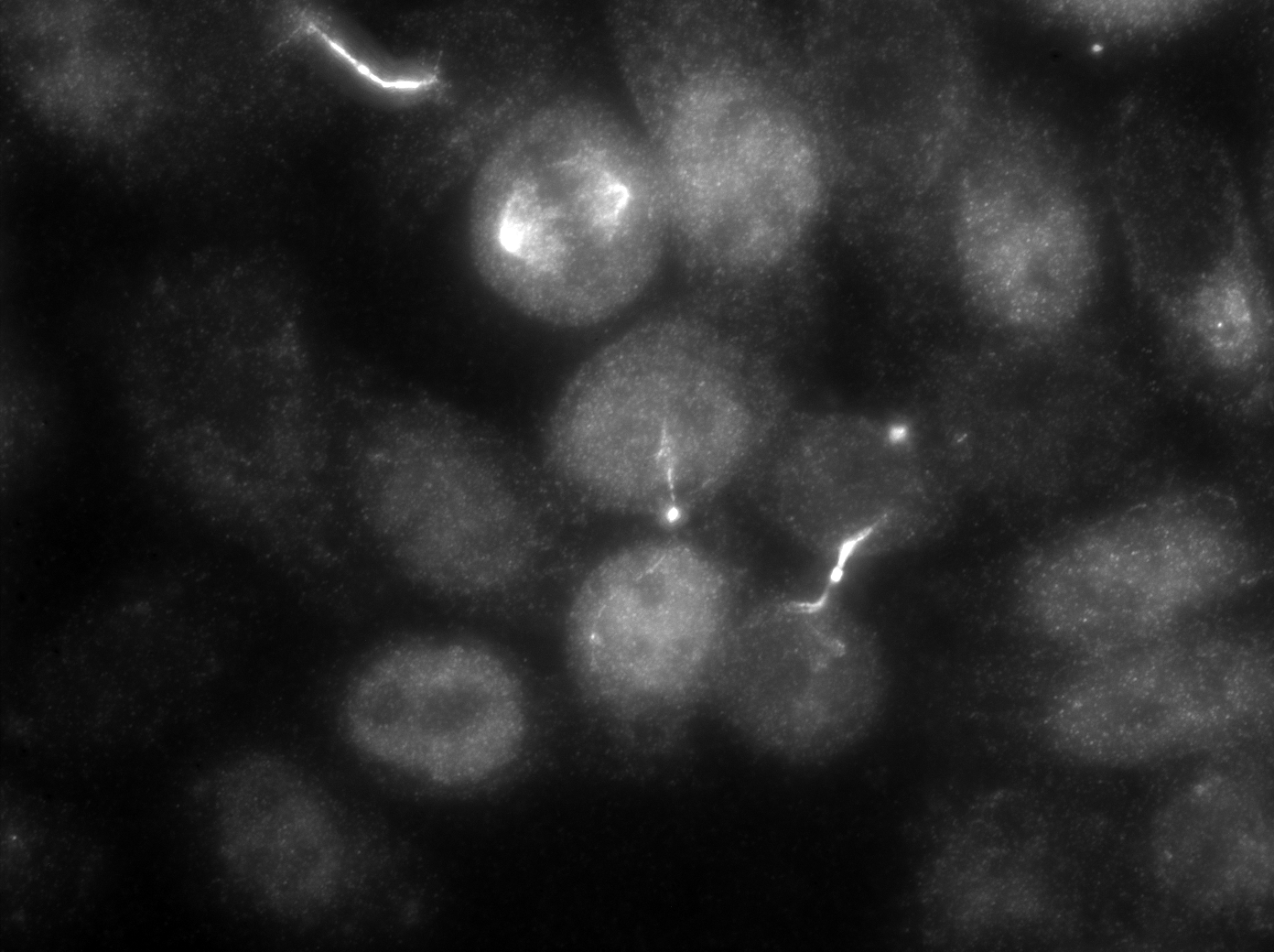

Supplement: Supplementary file 11 — Source data Fig. 3 part 1 [file 44319_2026_742_MOESM11_ESM.zip › Figure 3 Part 1/Fig 3b-e TTLL screen/TTLL1-GFP A3 I2 - 1.Project Maximum Z_XY1679694378_Z0_T0_C2.tif]

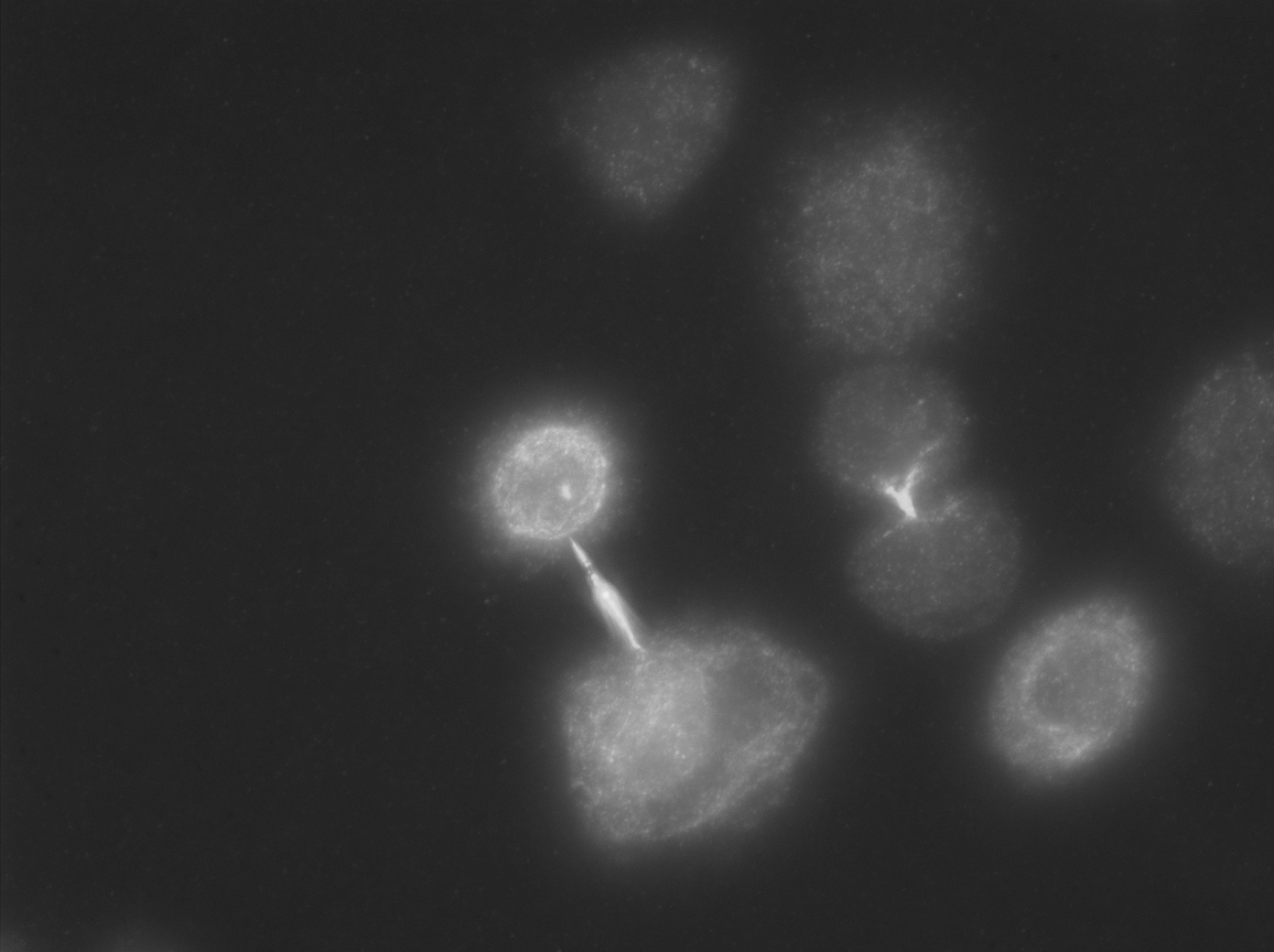

Supplement: Supplementary file 11 — Source data Fig. 3 part 1 [file 44319_2026_742_MOESM11_ESM.zip › Figure 3 Part 1/Fig 3b-e TTLL screen/TTLL4-YFPy I15.Project Maximum Z_XY1679337393_Z0_T0_C1.tif]

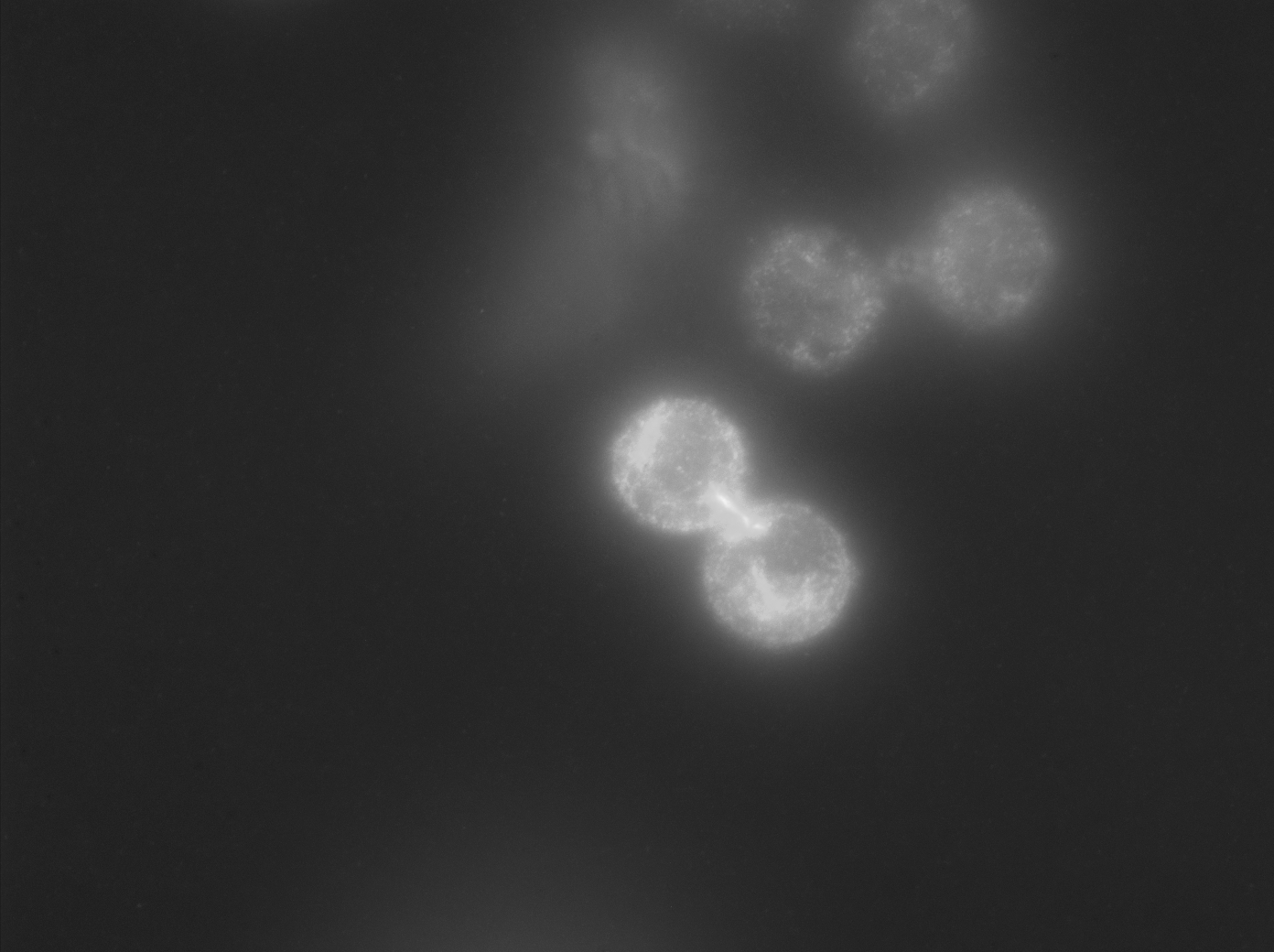

Supplement: Supplementary file 11 — Source data Fig. 3 part 1 [file 44319_2026_742_MOESM11_ESM.zip › Figure 3 Part 1/Fig 3b-e TTLL screen/TTLL4-YFPy I5.Project Maximum Z_XY1679076150_Z0_T0_C1.tif]

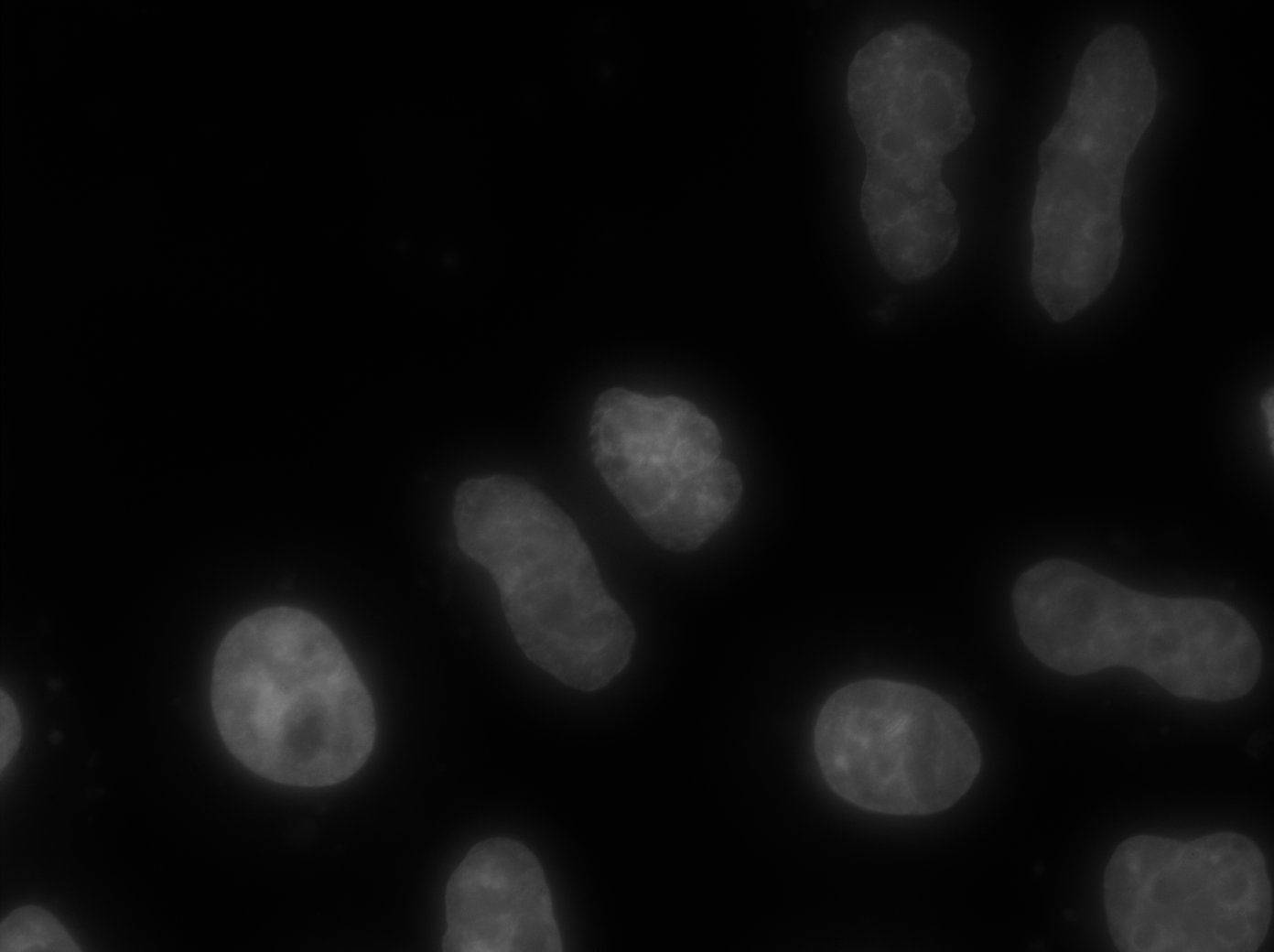

Supplement: Supplementary file 11 — Source data Fig. 3 part 1 [file 44319_2026_742_MOESM11_ESM.zip › Figure 3 Part 1/Fig 3b-e TTLL screen/TTLL1-GFP A3 I13.Project Maximum Z_XY1679696128_Z0_T0_C0.tif]

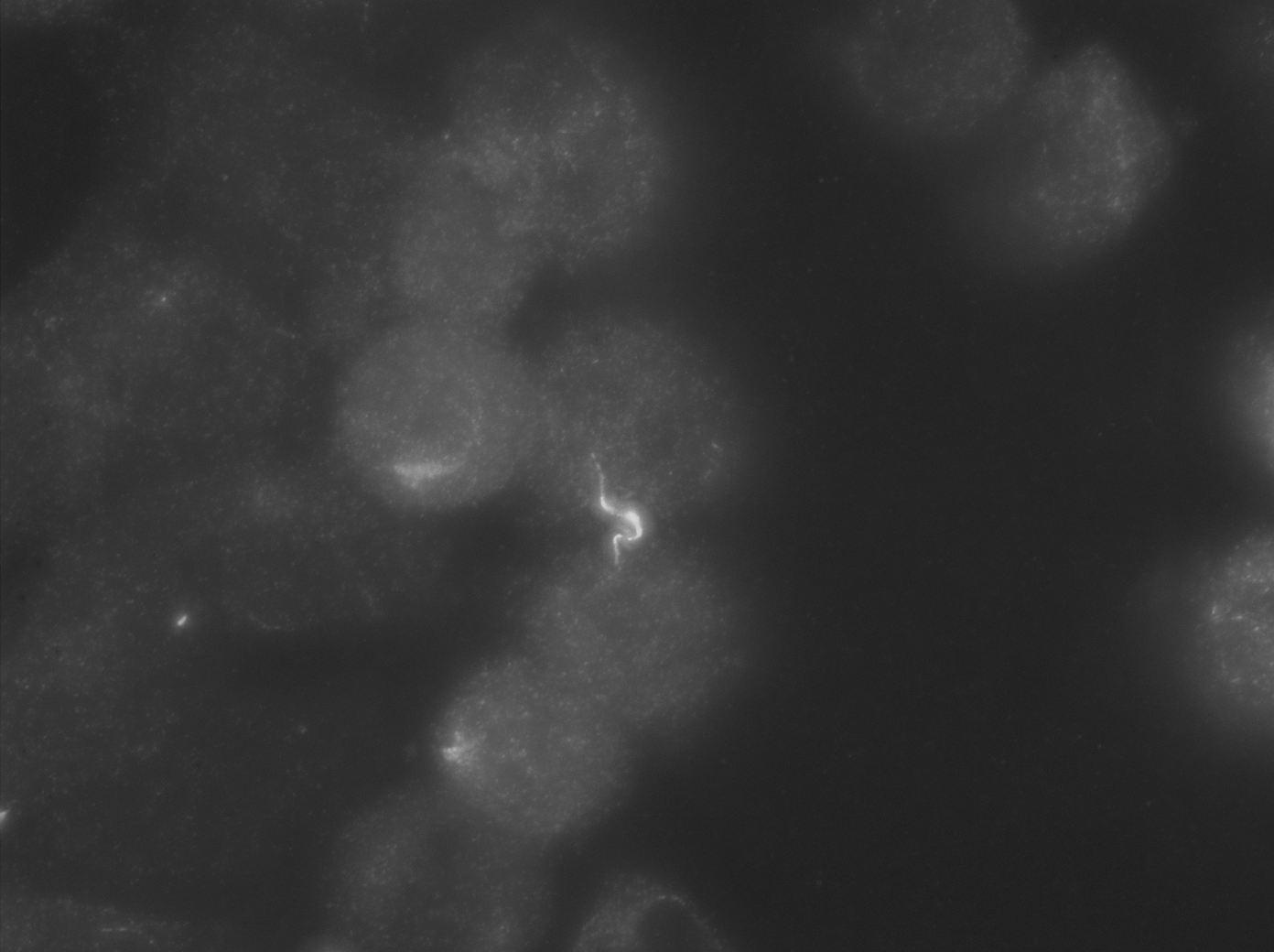

Supplement: Supplementary file 11 — Source data Fig. 3 part 1 [file 44319_2026_742_MOESM11_ESM.zip › Figure 3 Part 1/Fig 3b-e TTLL screen/TTLL4-YFPy I6 - 1.Project Maximum Z_XY1679076411_Z0_T0_C1.tif]

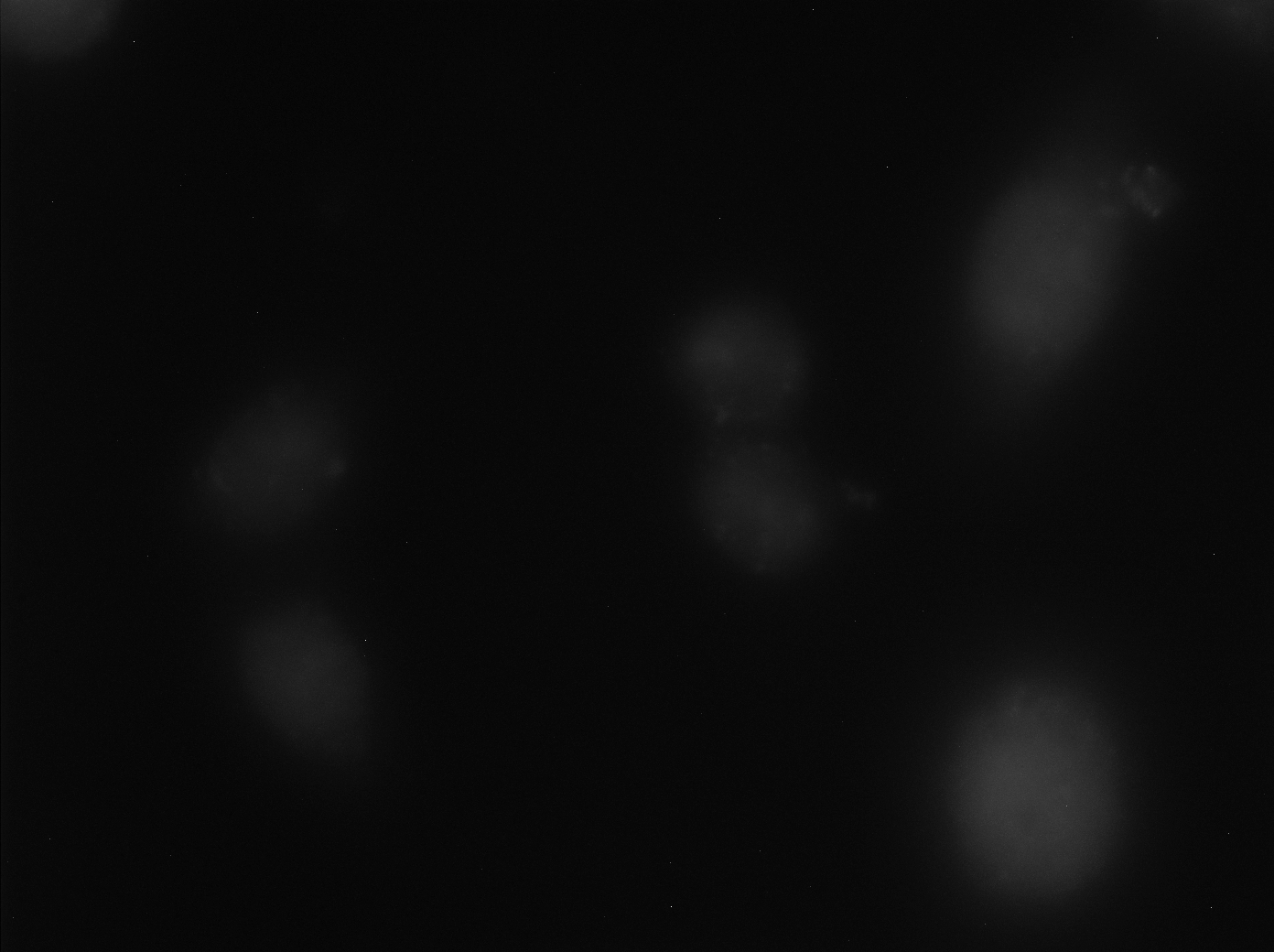

Supplement: Supplementary file 11 — Source data Fig. 3 part 1 [file 44319_2026_742_MOESM11_ESM.zip › Figure 3 Part 1/Fig 3b-e TTLL screen/TTLL1-GFP R1 I1.Project Maximum Z_XY1674162464_Z0_T0_C3.tif]

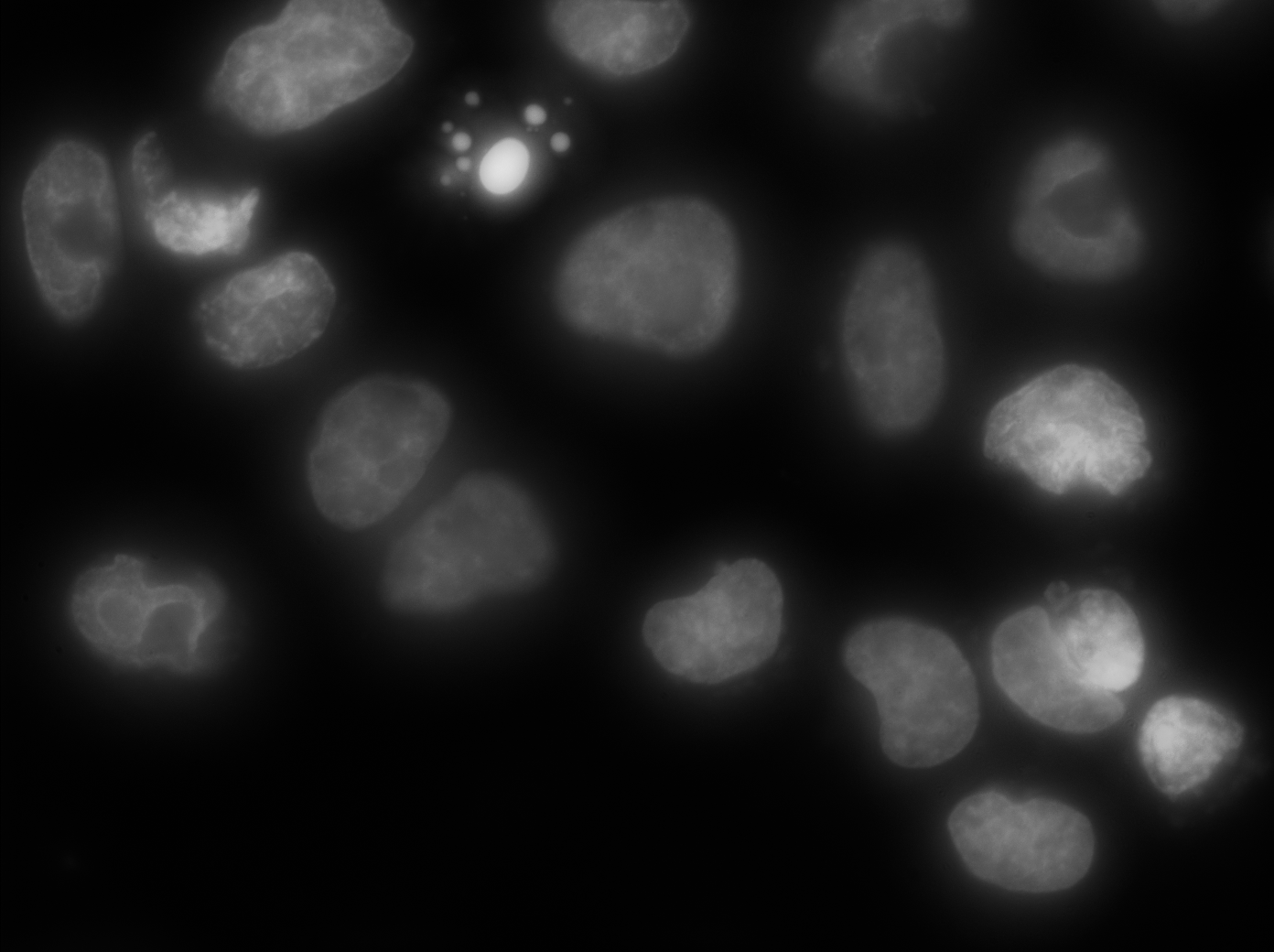

Supplement: Supplementary file 11 — Source data Fig. 3 part 1 [file 44319_2026_742_MOESM11_ESM.zip › Figure 3 Part 1/Fig 3b-e TTLL screen/TTLL4-YFPy I2.Project Maximum Z_XY1679075355_Z0_T0_C0.tif]

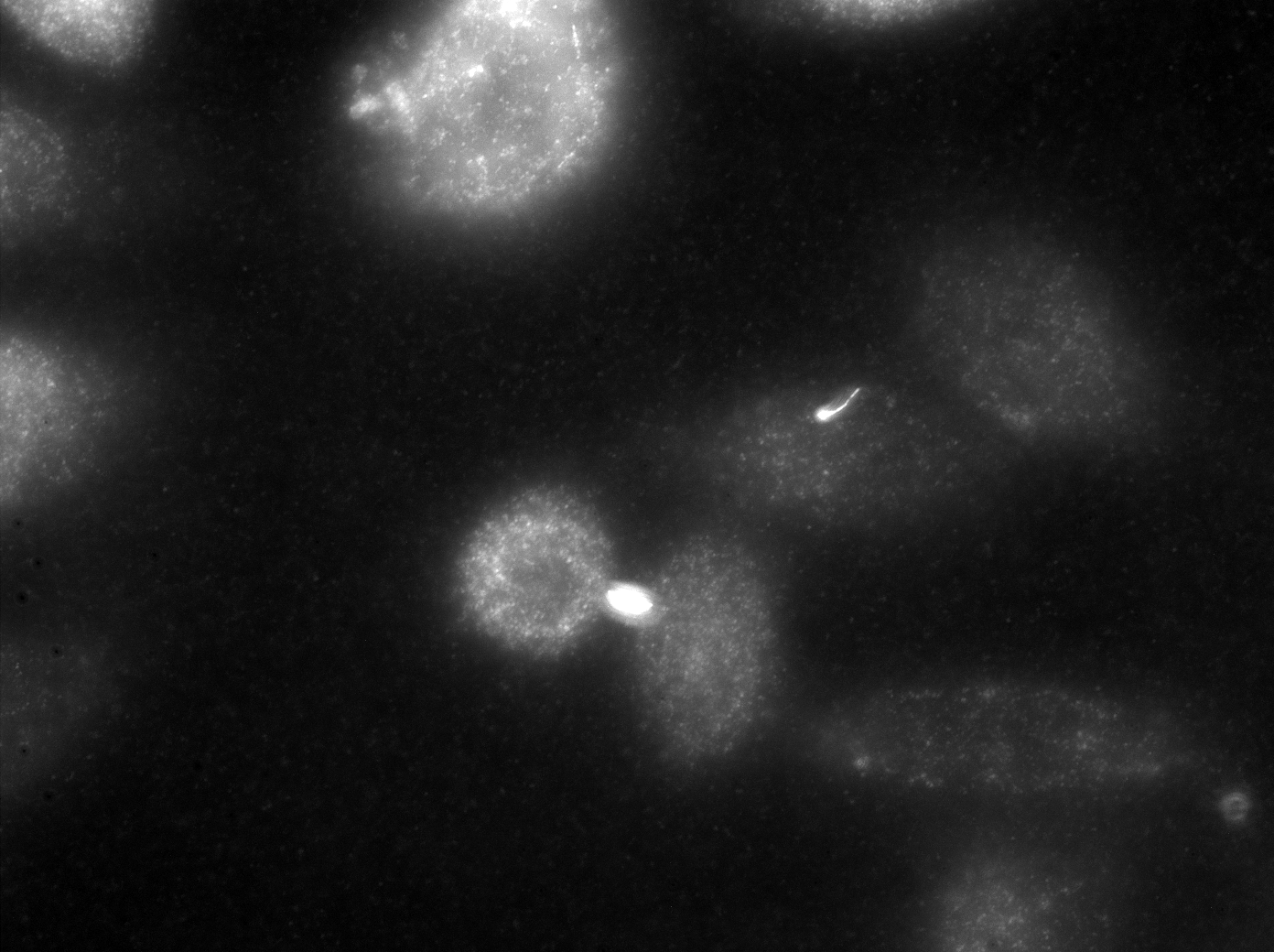

Supplement: Supplementary file 11 — Source data Fig. 3 part 1 [file 44319_2026_742_MOESM11_ESM.zip › Figure 3 Part 1/Fig 3b-e TTLL screen/TTLL1-GFP A3 I8.Project Maximum Z_XY1679695373_Z0_T0_C2.tif]

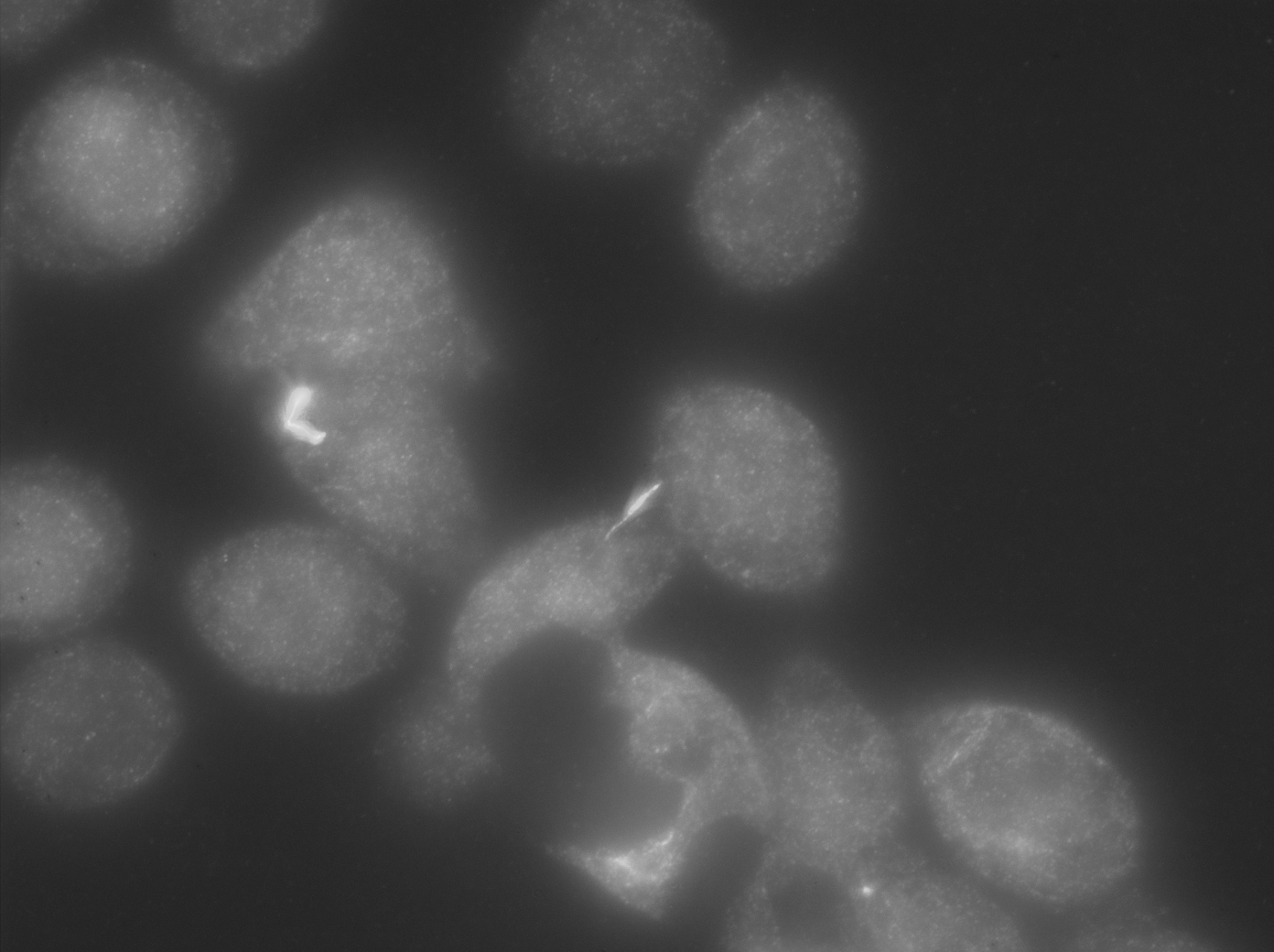

Supplement: Supplementary file 11 — Source data Fig. 3 part 1 [file 44319_2026_742_MOESM11_ESM.zip › Figure 3 Part 1/Fig 3b-e TTLL screen/TTLL4-YFPy I8.Project Maximum Z_XY1679081735_Z0_T0_C1.tif]

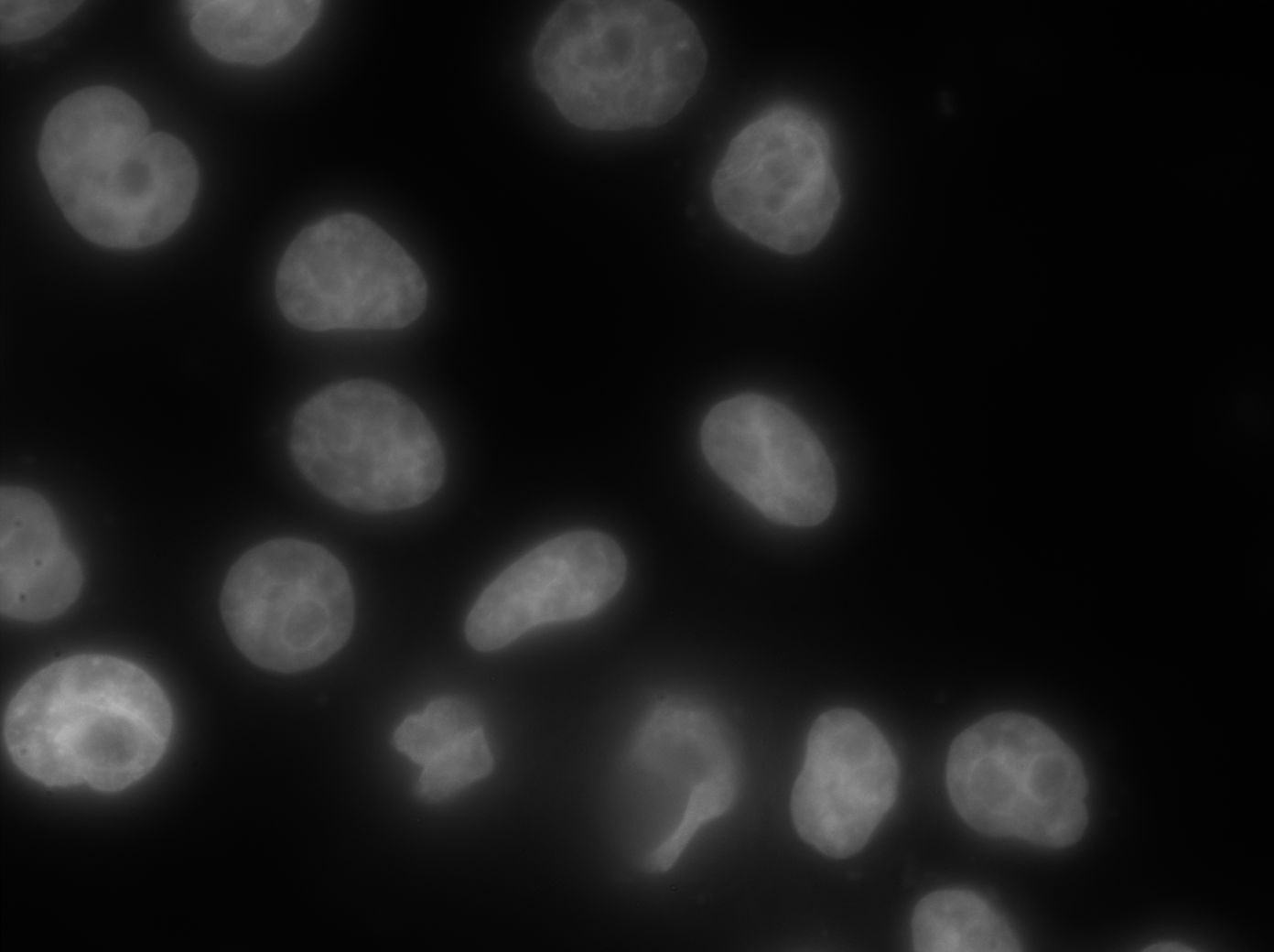

Supplement: Supplementary file 11 — Source data Fig. 3 part 1 [file 44319_2026_742_MOESM11_ESM.zip › Figure 3 Part 1/Fig 3b-e TTLL screen/TTLL4-YFPy I8.Project Maximum Z_XY1679081735_Z0_T0_C0.tif]

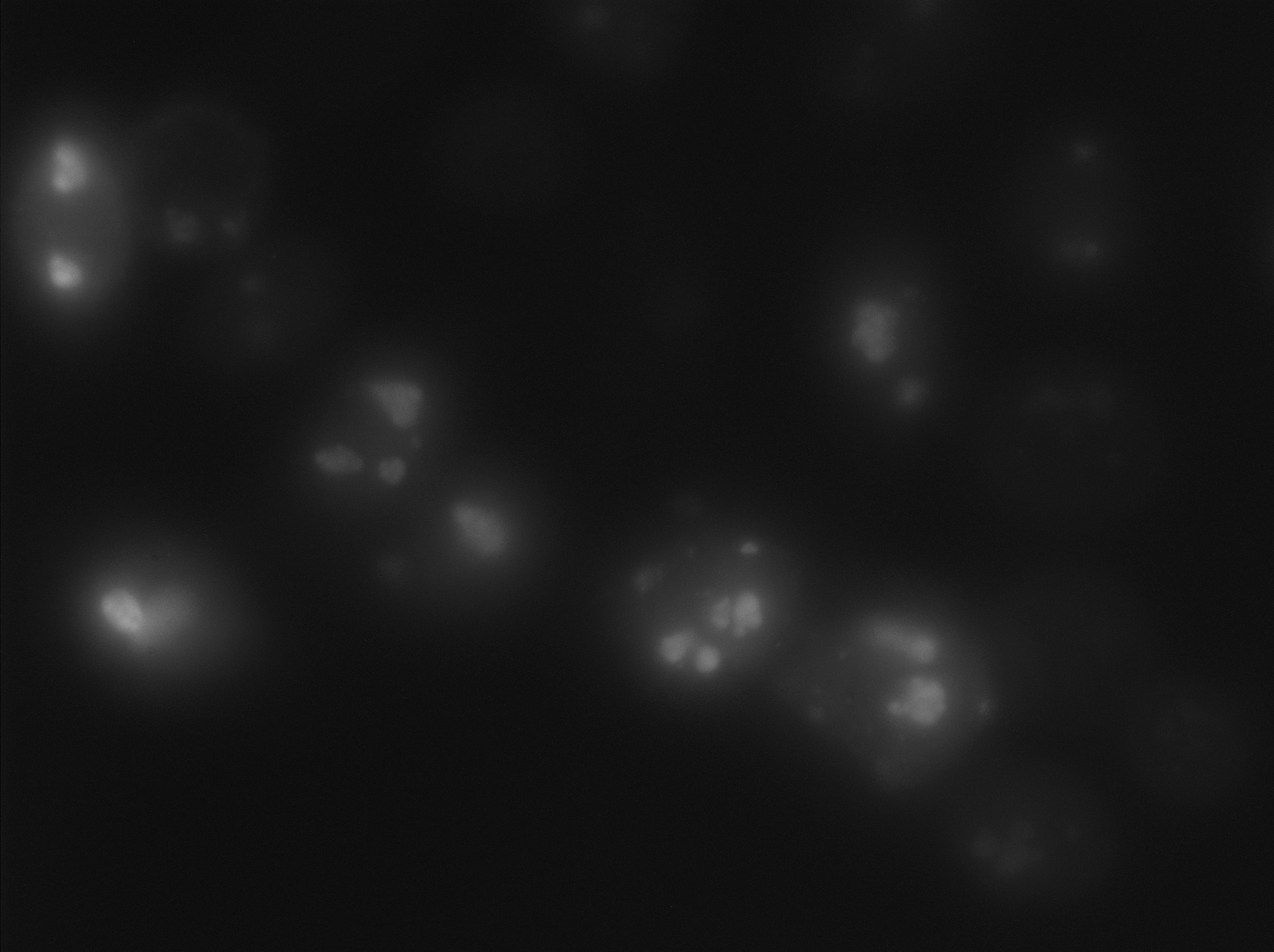

Supplement: Supplementary file 11 — Source data Fig. 3 part 1 [file 44319_2026_742_MOESM11_ESM.zip › Figure 3 Part 1/Fig 3b-e TTLL screen/TTLL4-YFPy I2.Project Maximum Z_XY1679075355_Z0_T0_C1.tif]

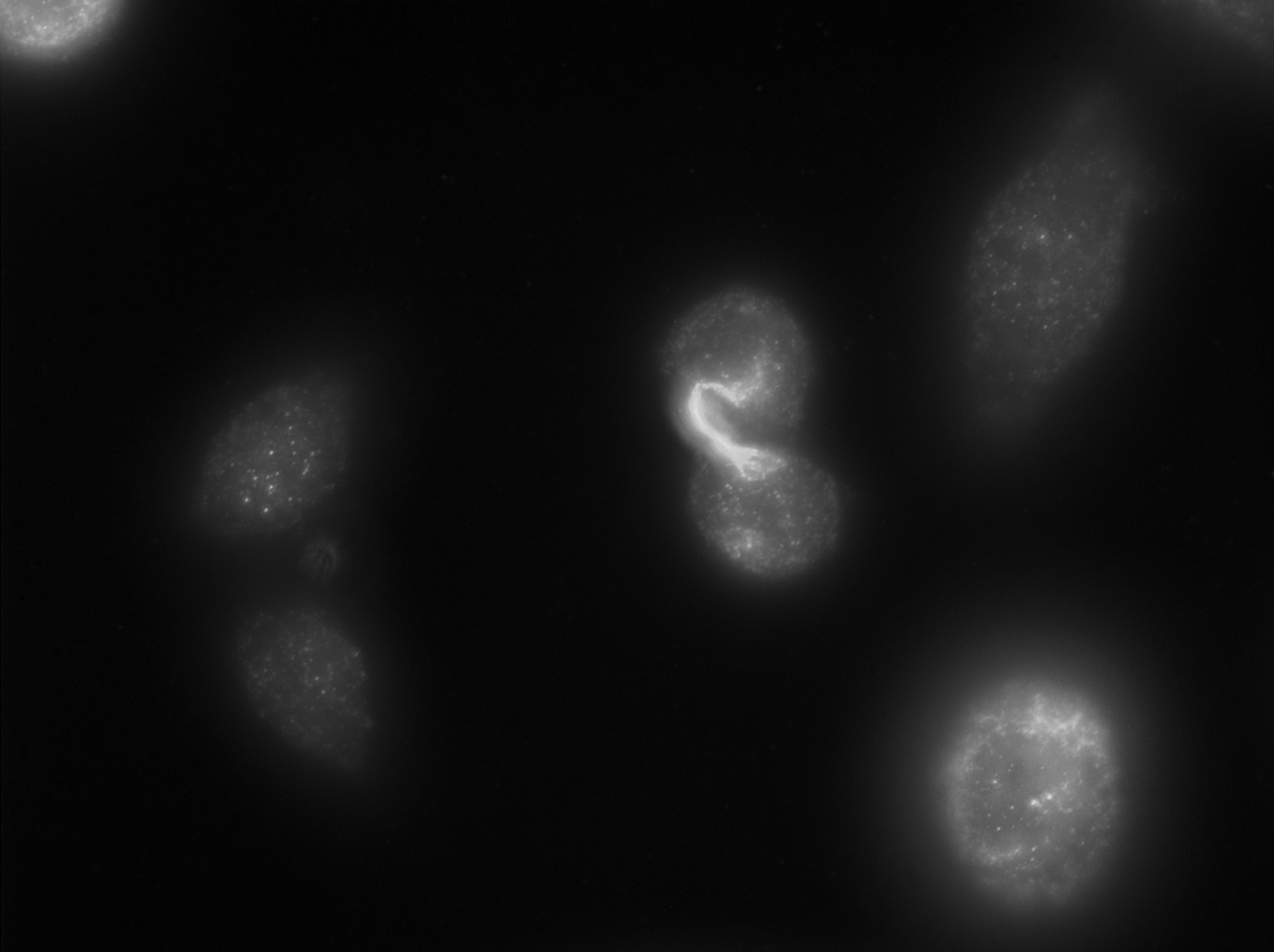

Supplement: Supplementary file 11 — Source data Fig. 3 part 1 [file 44319_2026_742_MOESM11_ESM.zip › Figure 3 Part 1/Fig 3b-e TTLL screen/TTLL1-GFP R1 I1.Project Maximum Z_XY1674162464_Z0_T0_C2.tif]

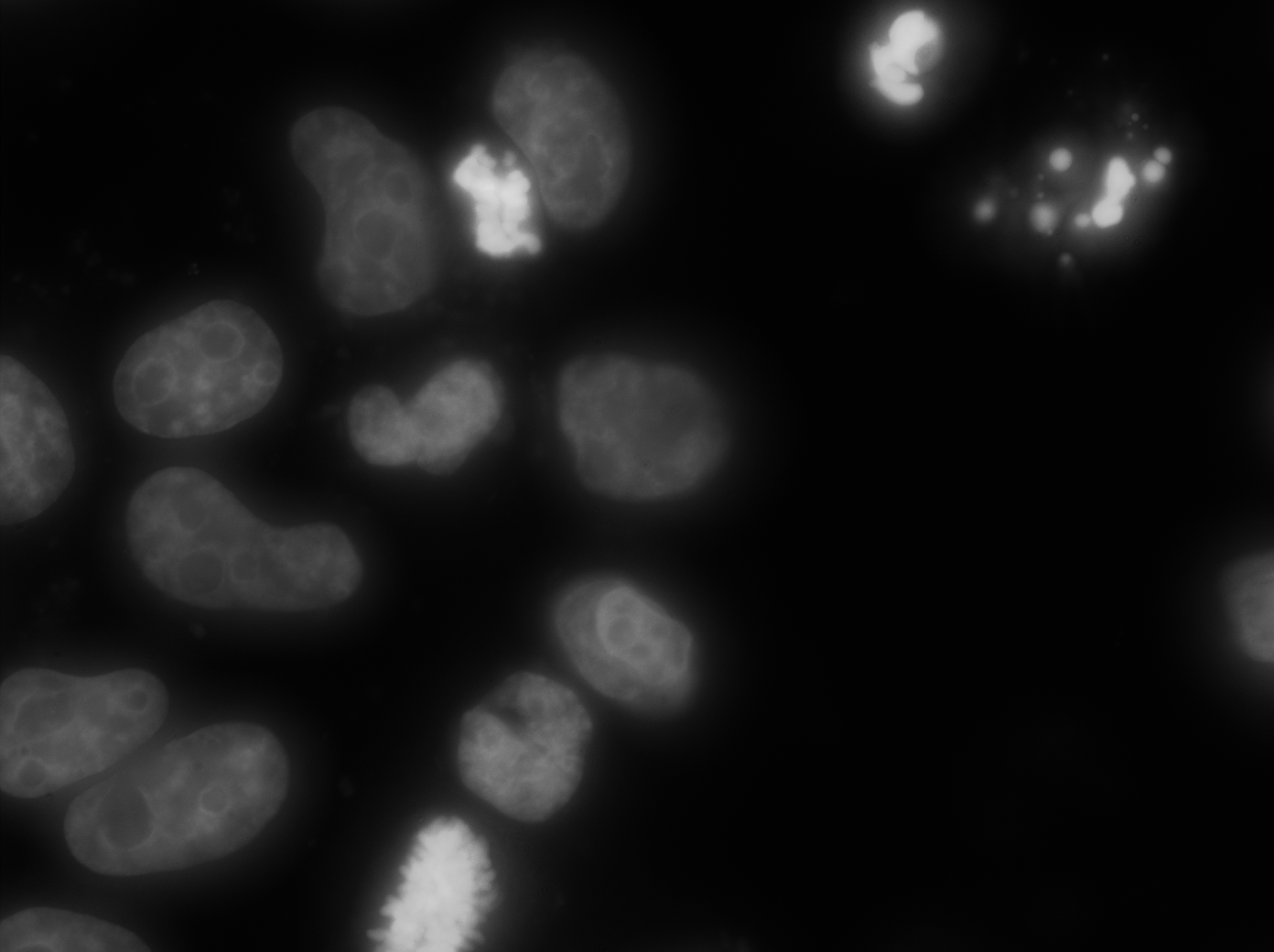

Supplement: Supplementary file 11 — Source data Fig. 3 part 1 [file 44319_2026_742_MOESM11_ESM.zip › Figure 3 Part 1/Fig 3b-e TTLL screen/TTLL4-YFPy I6 - 1.Project Maximum Z_XY1679076411_Z0_T0_C0.tif]

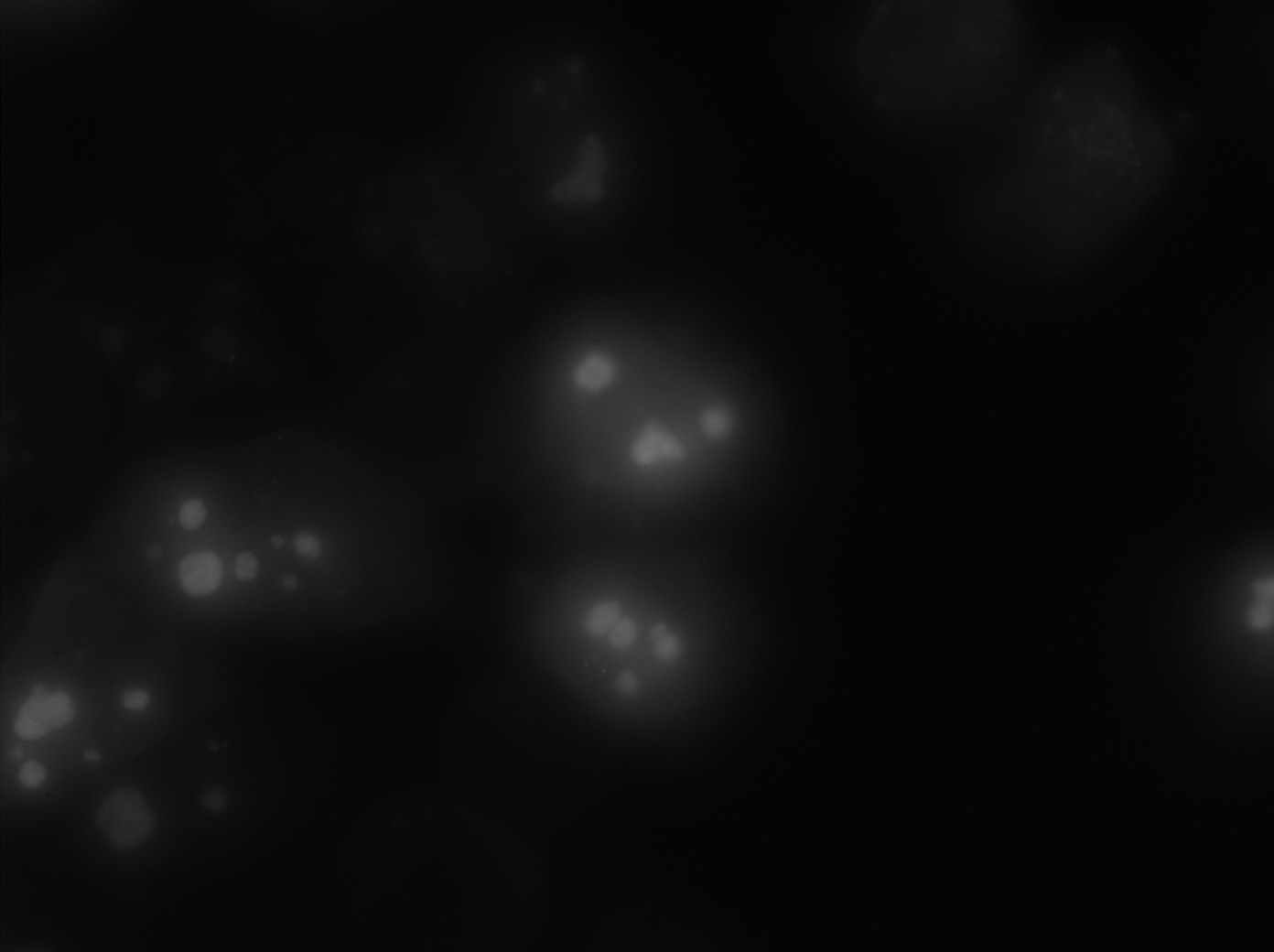

Supplement: Supplementary file 11 — Source data Fig. 3 part 1 [file 44319_2026_742_MOESM11_ESM.zip › Figure 3 Part 1/Fig 3b-e TTLL screen/TTLL4-YFPy I6 - 1.Project Maximum Z_XY1679076411_Z0_T0_C2.tif]

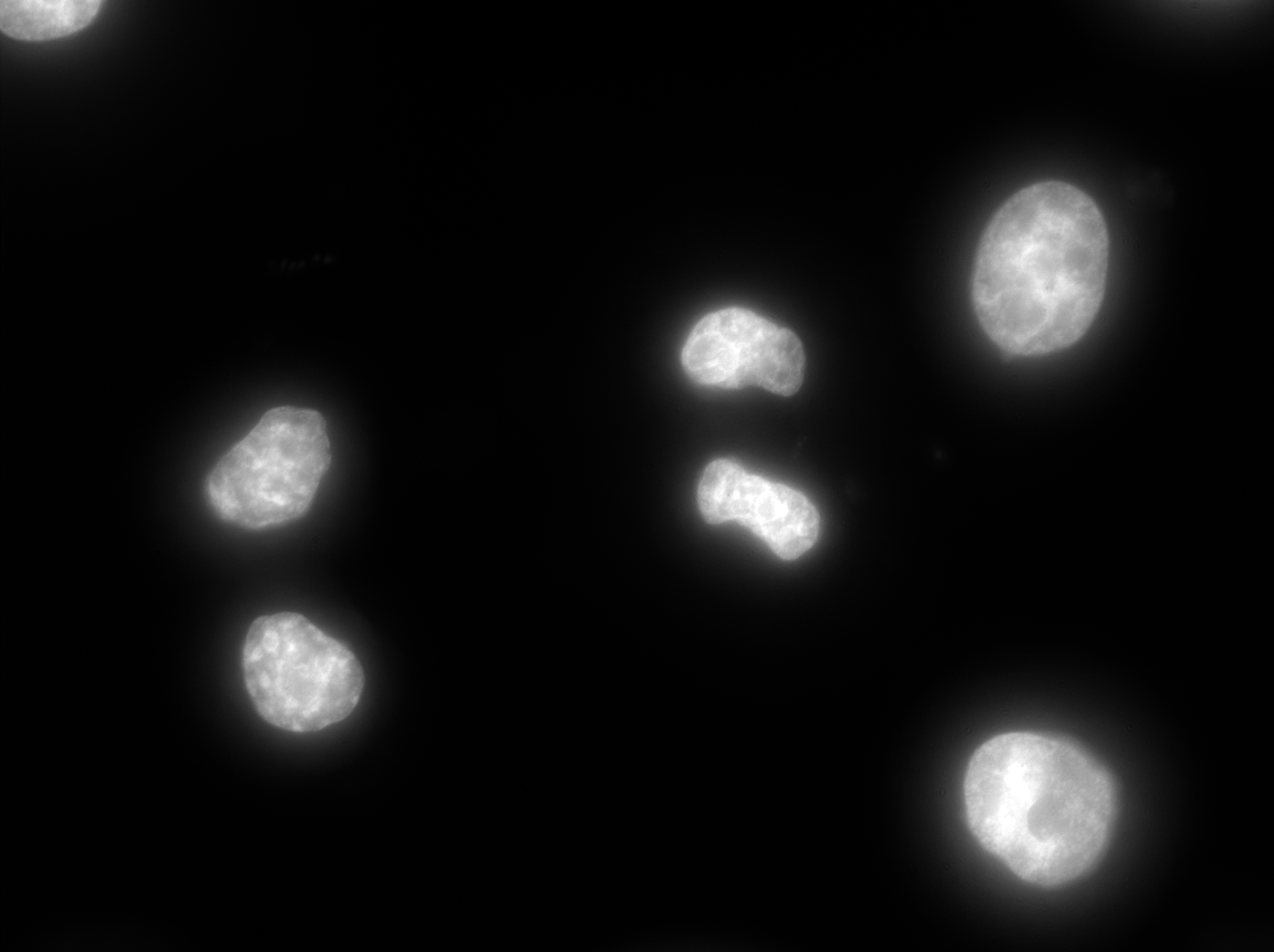

Supplement: Supplementary file 11 — Source data Fig. 3 part 1 [file 44319_2026_742_MOESM11_ESM.zip › Figure 3 Part 1/Fig 3b-e TTLL screen/TTLL1-GFP R1 I1.Project Maximum Z_XY1674162464_Z0_T0_C0.tif]

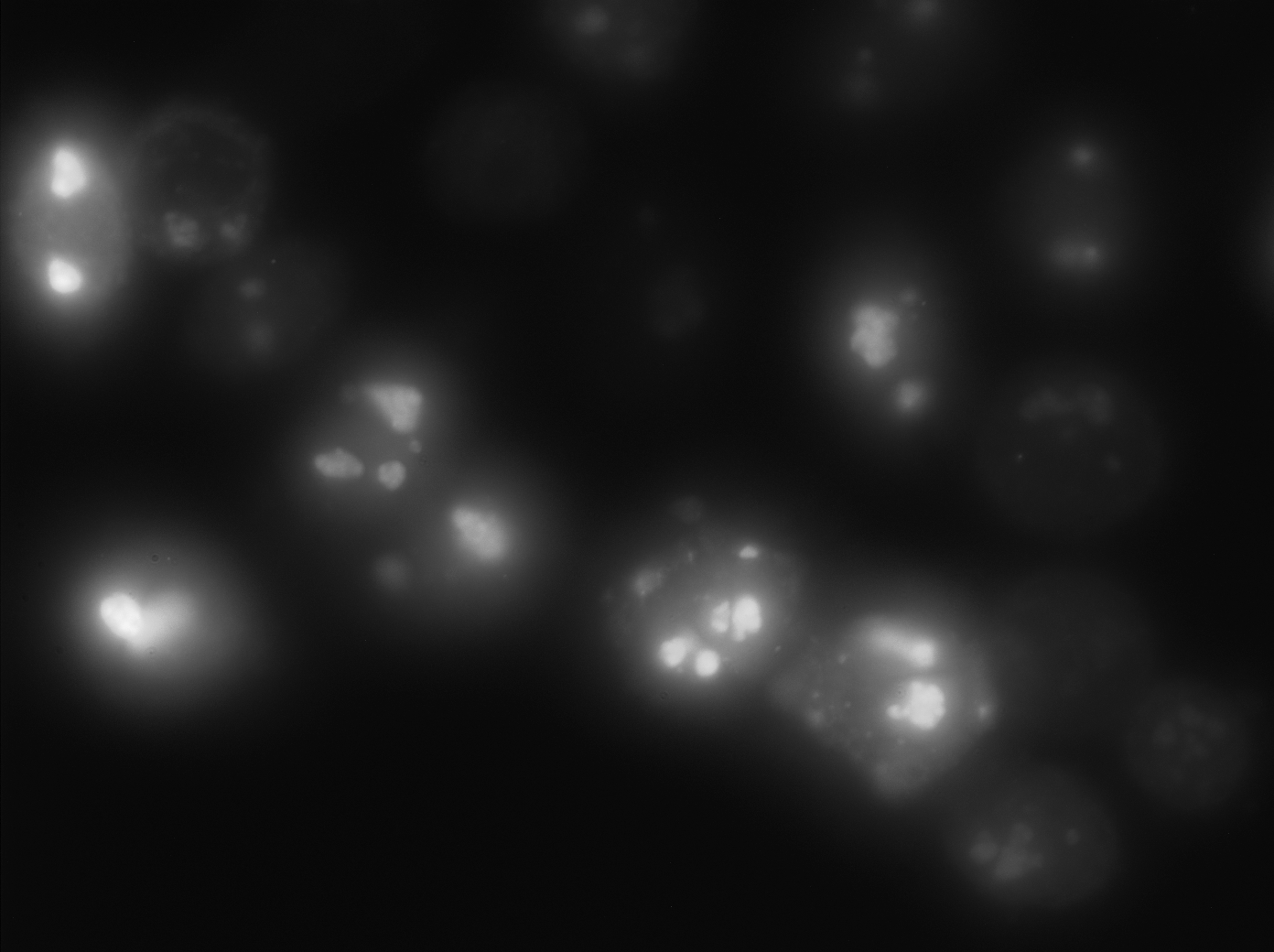

Supplement: Supplementary file 11 — Source data Fig. 3 part 1 [file 44319_2026_742_MOESM11_ESM.zip › Figure 3 Part 1/Fig 3b-e TTLL screen/TTLL4-YFPy I2.Project Maximum Z_XY1679075355_Z0_T0_C3.tif]

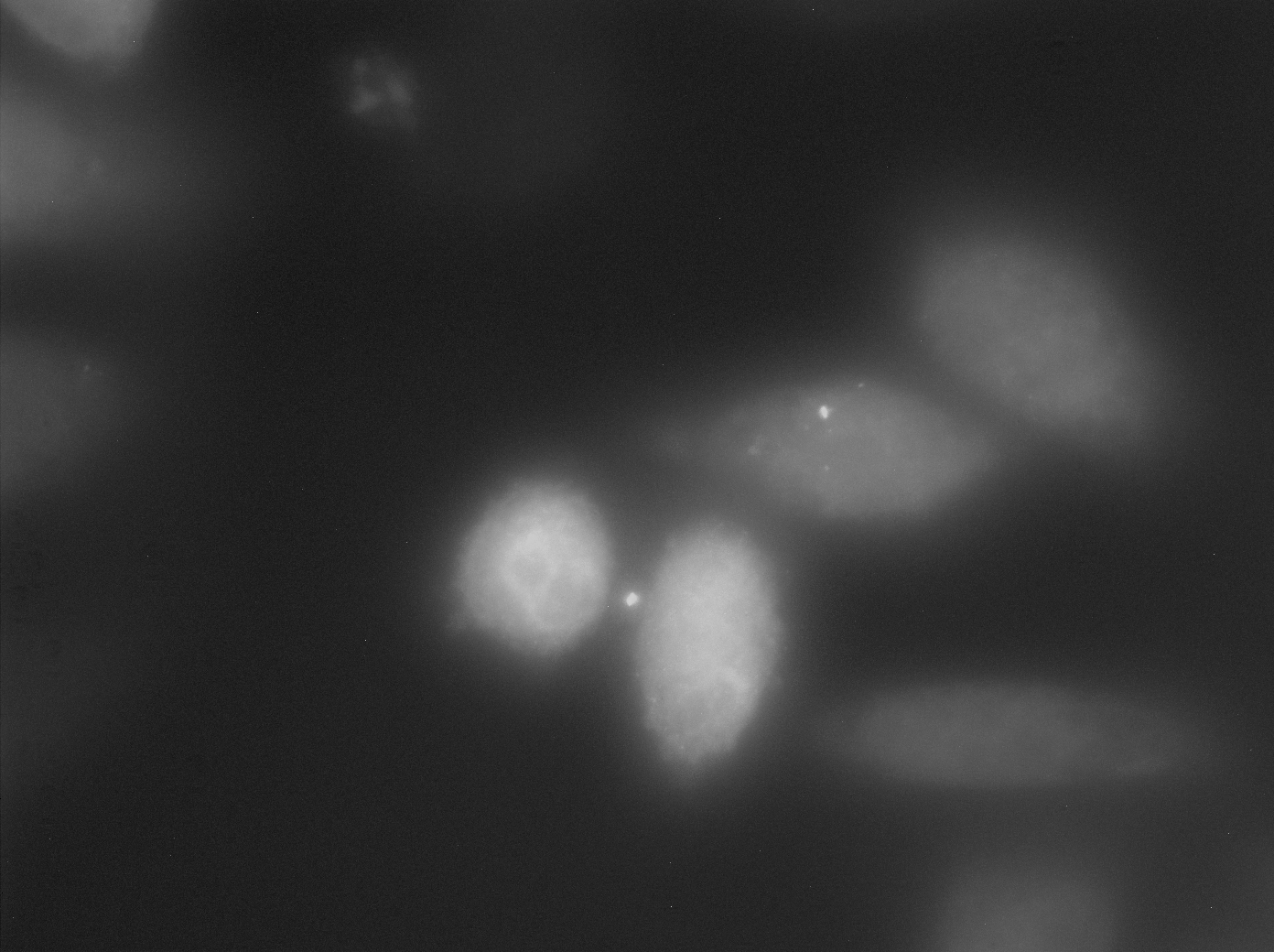

Supplement: Supplementary file 11 — Source data Fig. 3 part 1 [file 44319_2026_742_MOESM11_ESM.zip › Figure 3 Part 1/Fig 3b-e TTLL screen/TTLL1-GFP A3 I8.Project Maximum Z_XY1679695373_Z0_T0_C1.tif]

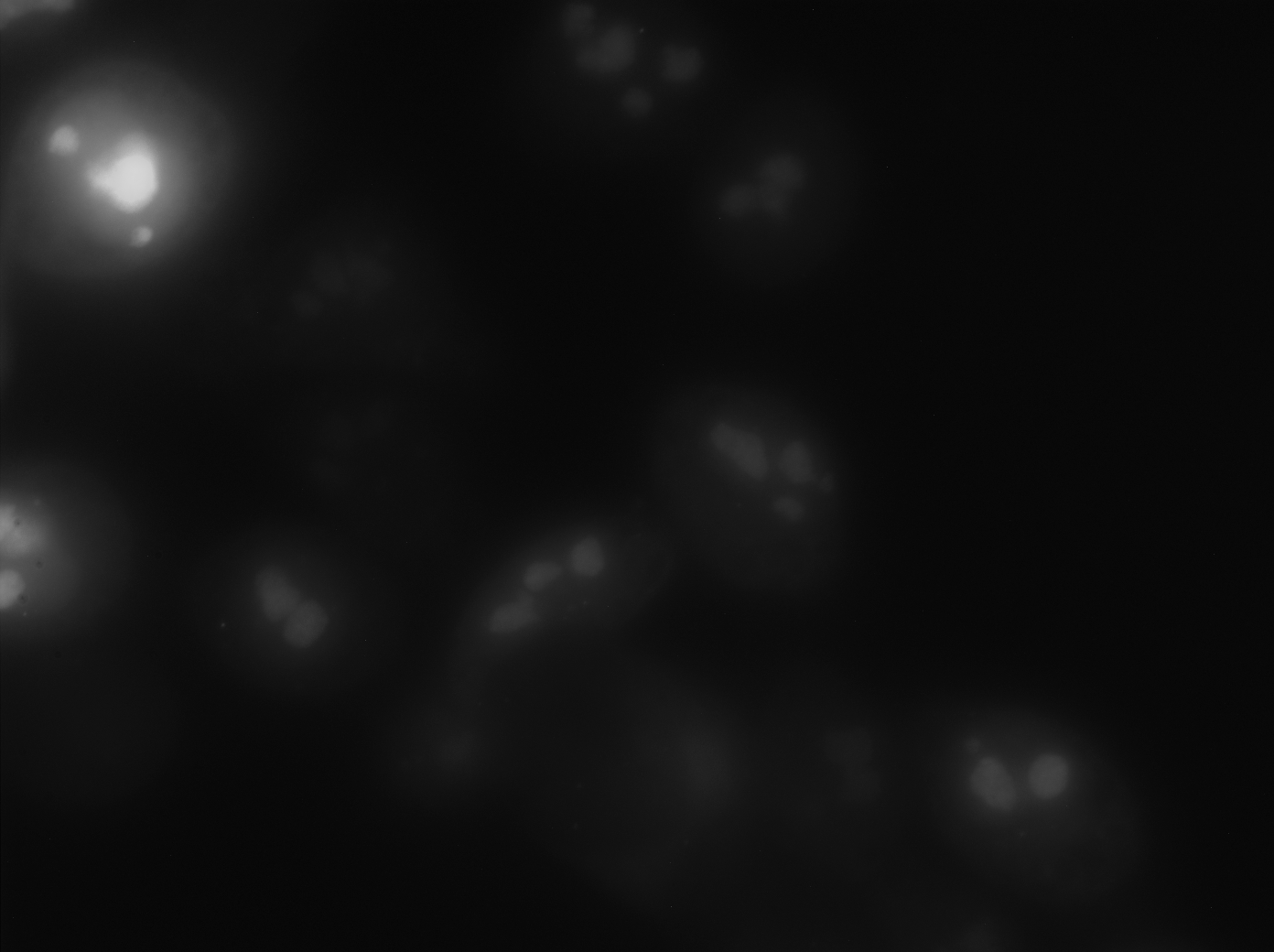

Supplement: Supplementary file 11 — Source data Fig. 3 part 1 [file 44319_2026_742_MOESM11_ESM.zip › Figure 3 Part 1/Fig 3b-e TTLL screen/TTLL4-YFPy I8.Project Maximum Z_XY1679081735_Z0_T0_C2.tif]

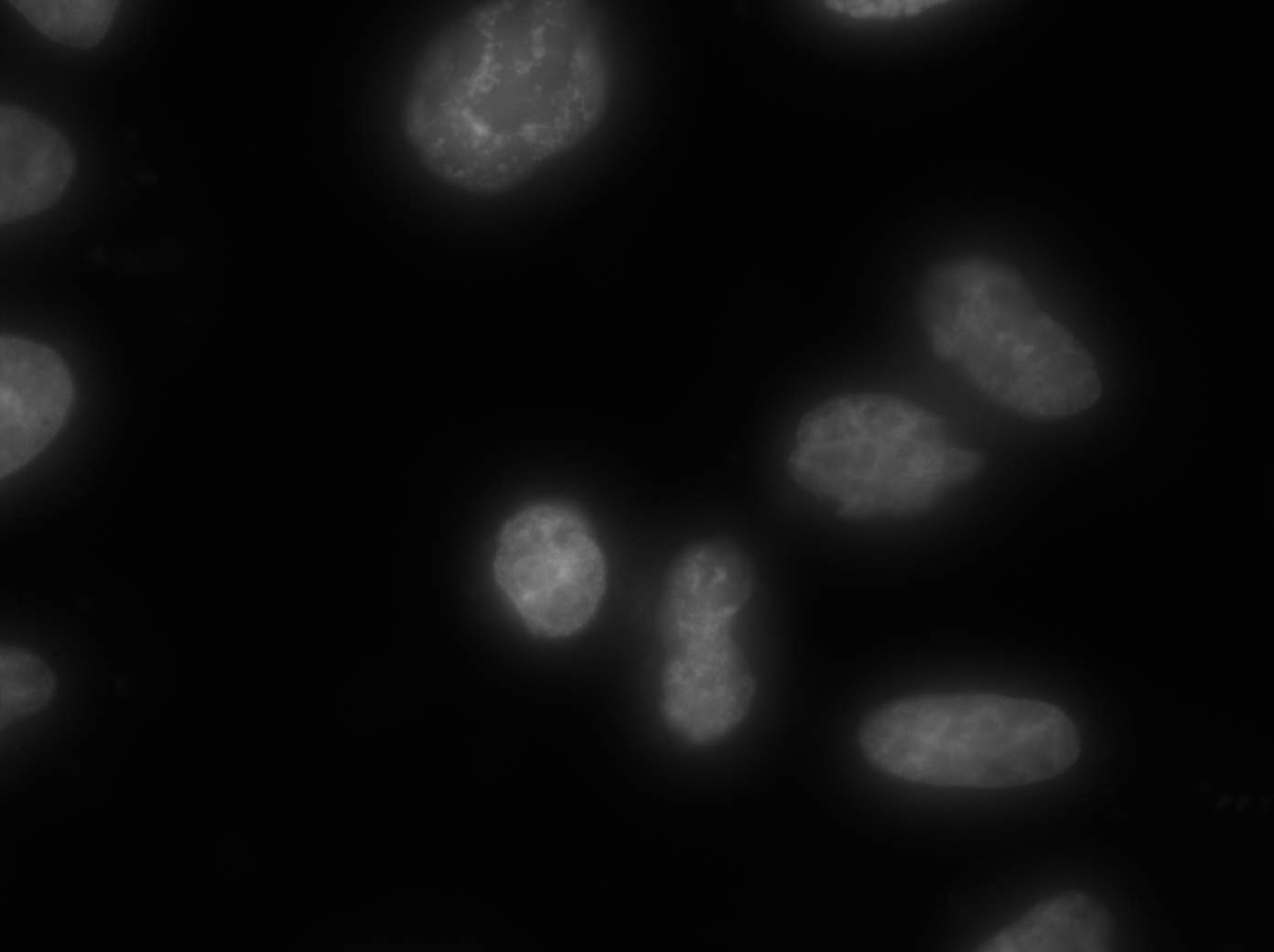

Supplement: Supplementary file 11 — Source data Fig. 3 part 1 [file 44319_2026_742_MOESM11_ESM.zip › Figure 3 Part 1/Fig 3b-e TTLL screen/TTLL1-GFP A3 I8.Project Maximum Z_XY1679695373_Z0_T0_C0.tif]

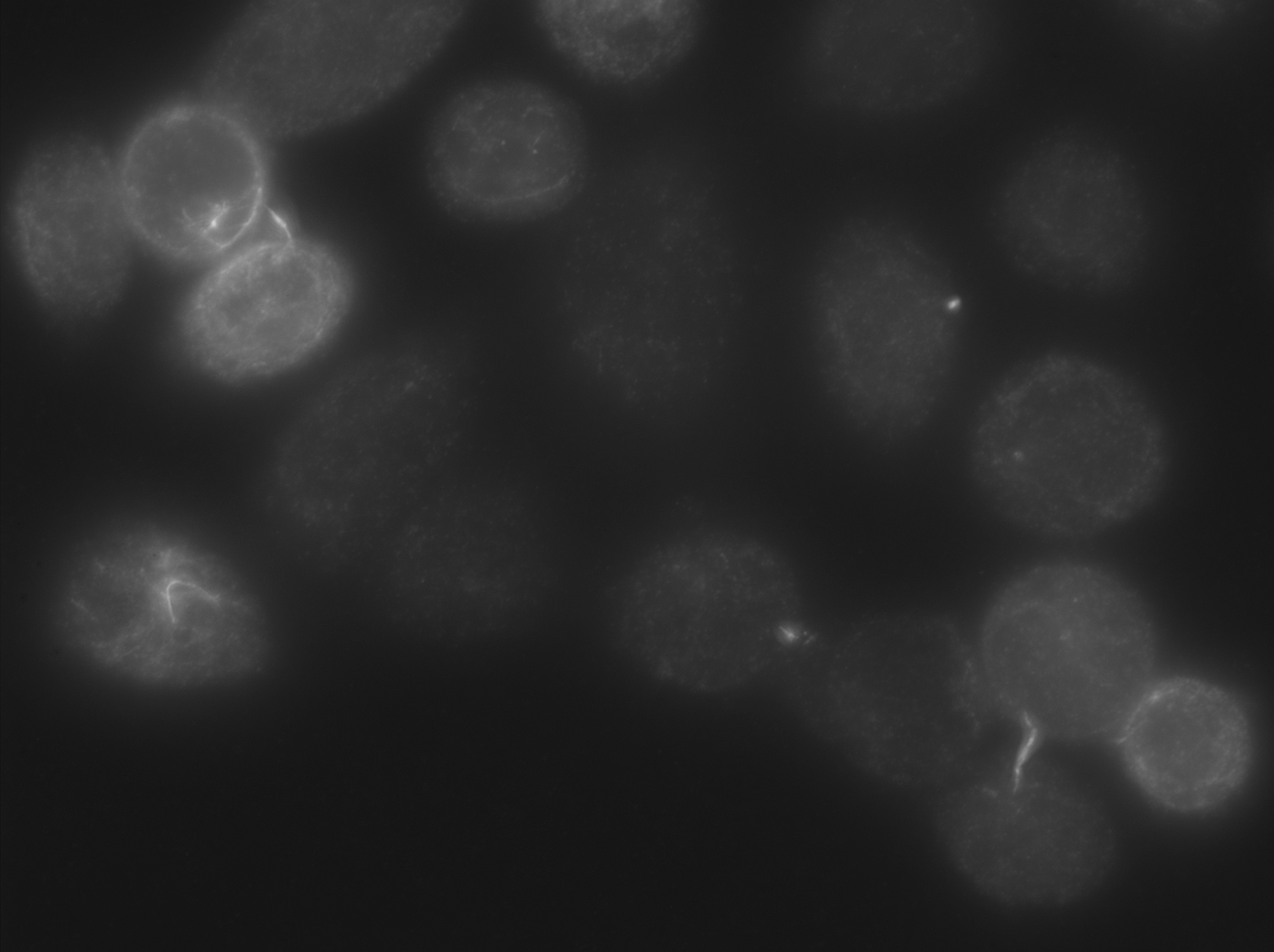

Supplement: Supplementary file 11 — Source data Fig. 3 part 1 [file 44319_2026_742_MOESM11_ESM.zip › Figure 3 Part 1/Fig 3b-e TTLL screen/TTLL4-YFPy I2.Project Maximum Z_XY1679075355_Z0_T0_C2.tif]

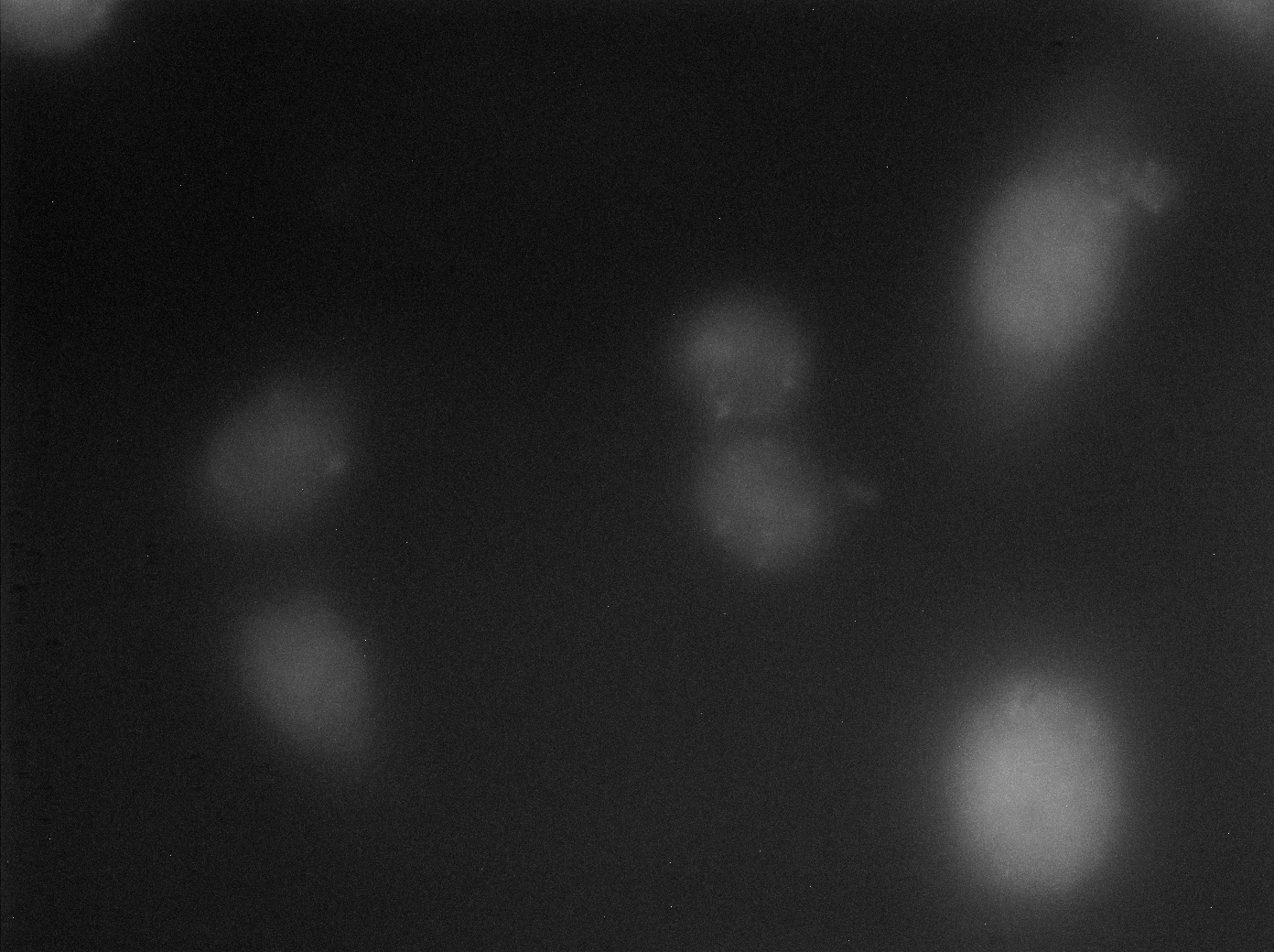

Supplement: Supplementary file 11 — Source data Fig. 3 part 1 [file 44319_2026_742_MOESM11_ESM.zip › Figure 3 Part 1/Fig 3b-e TTLL screen/TTLL1-GFP R1 I1.Project Maximum Z_XY1674162464_Z0_T0_C1.tif]

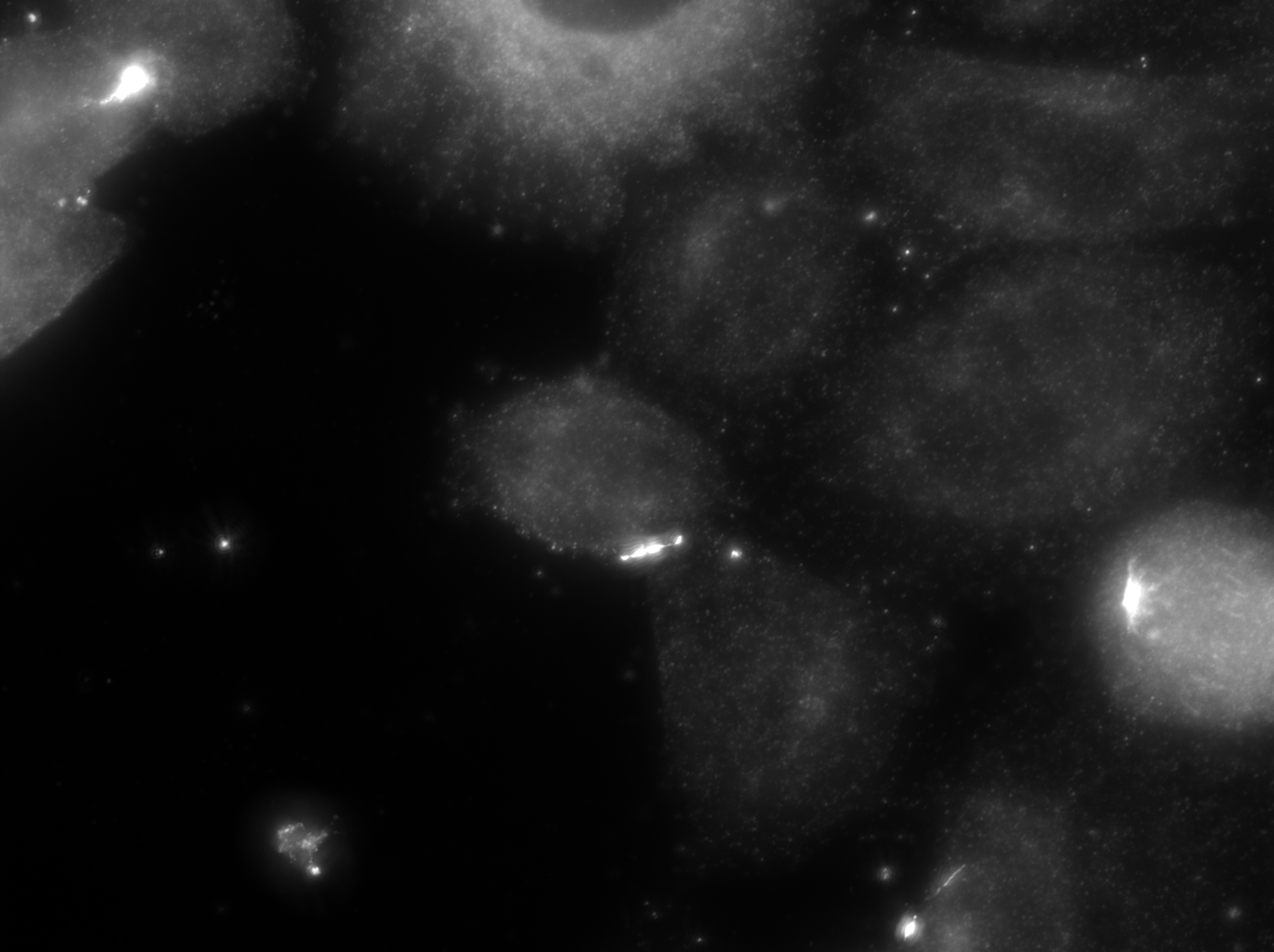

Supplement: Supplementary file 11 — Source data Fig. 3 part 1 [file 44319_2026_742_MOESM11_ESM.zip › Figure 3 Part 1/Fig 3b-e TTLL screen/TTLL1-GFP A4 I10.Project Maximum Z_XY1675963296_Z0_T0_C2.tif]

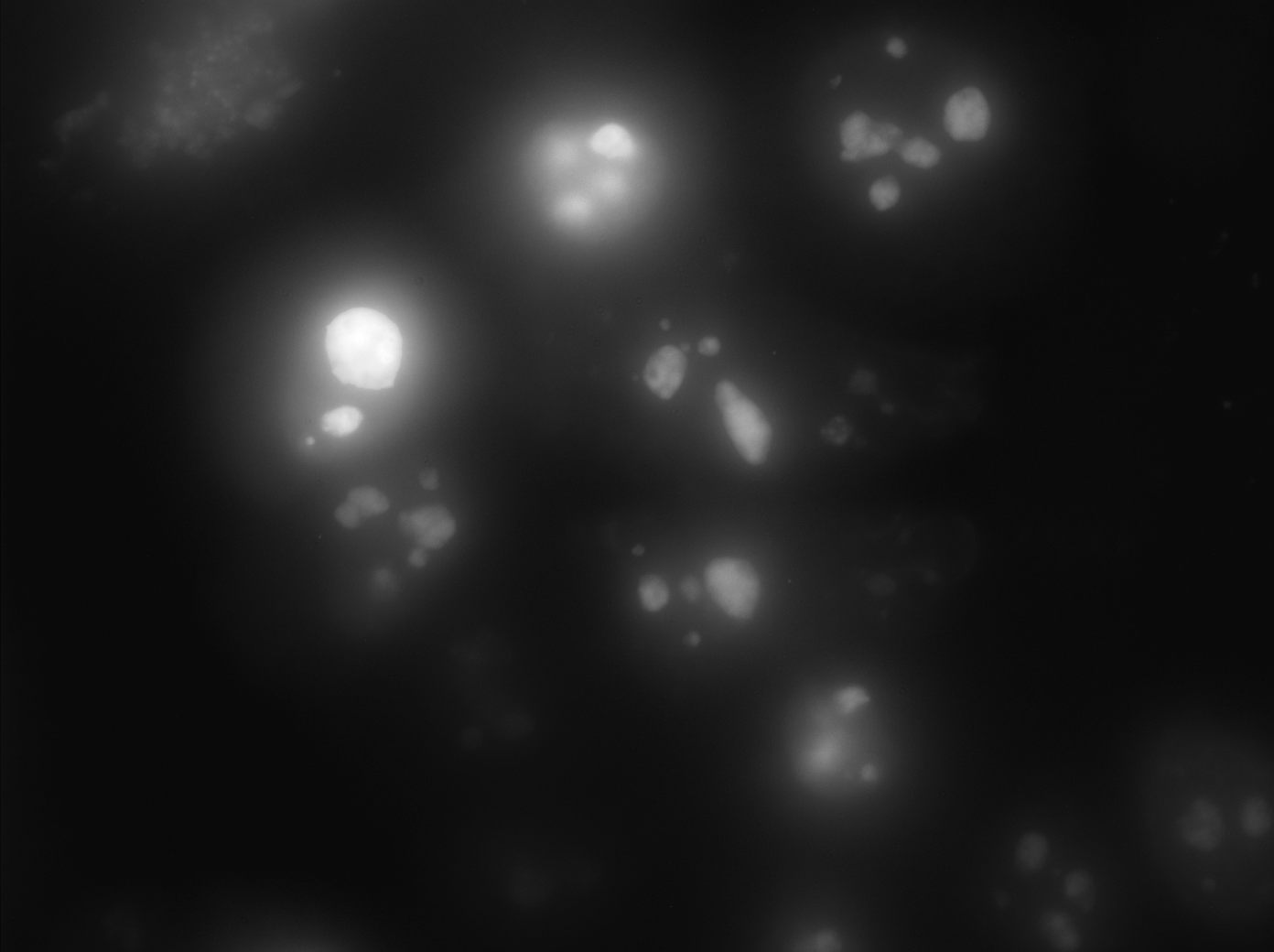

Supplement: Supplementary file 11 — Source data Fig. 3 part 1 [file 44319_2026_742_MOESM11_ESM.zip › Figure 3 Part 1/Fig 3b-e TTLL screen/TTLL4-YFPy I4.Project Maximum Z_XY1679075937_Z0_T0_C3.tif]

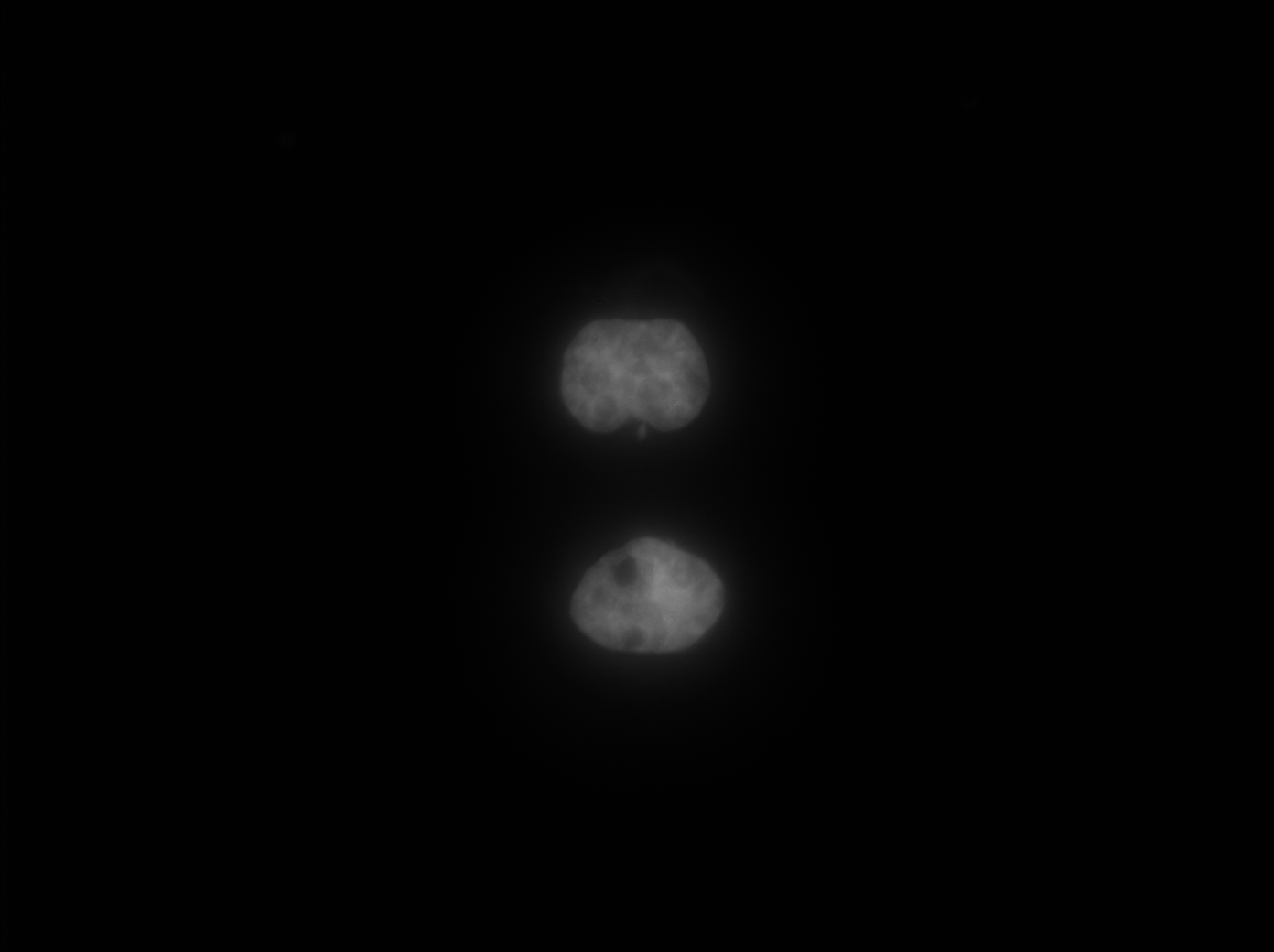

Supplement: Supplementary file 11 — Source data Fig. 3 part 1 [file 44319_2026_742_MOESM11_ESM.zip › Figure 3 Part 1/Fig 3b-e TTLL screen/TTLL1-GFP A3 I1.Project Maximum Z_XY1674673539_Z0_T0_C0.tif]

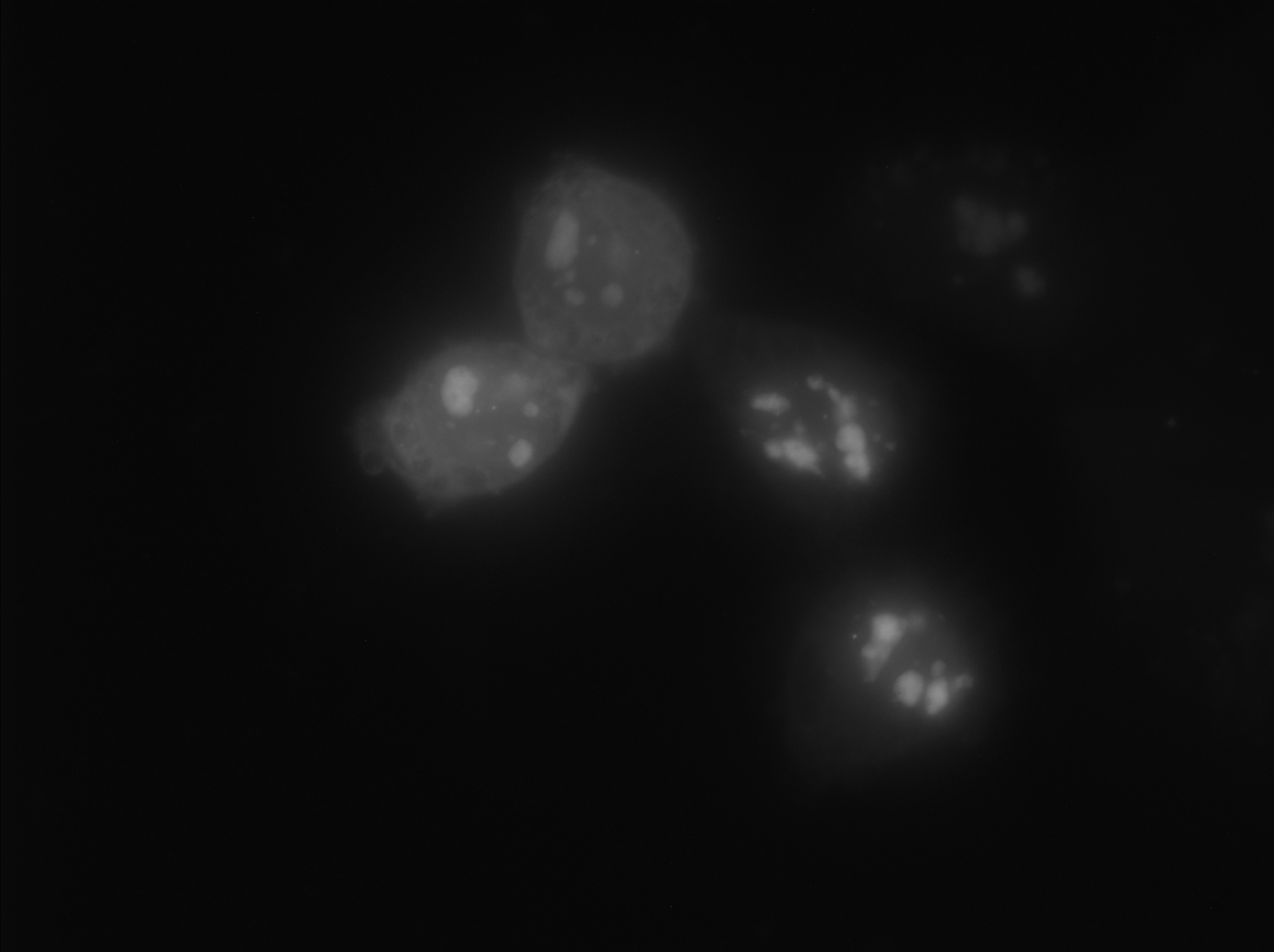

Supplement: Supplementary file 11 — Source data Fig. 3 part 1 [file 44319_2026_742_MOESM11_ESM.zip › Figure 3 Part 1/Fig 3b-e TTLL screen/TTLL4-YFPy I17.Project Maximum Z_XY1679337716_Z0_T0_C2.tif]

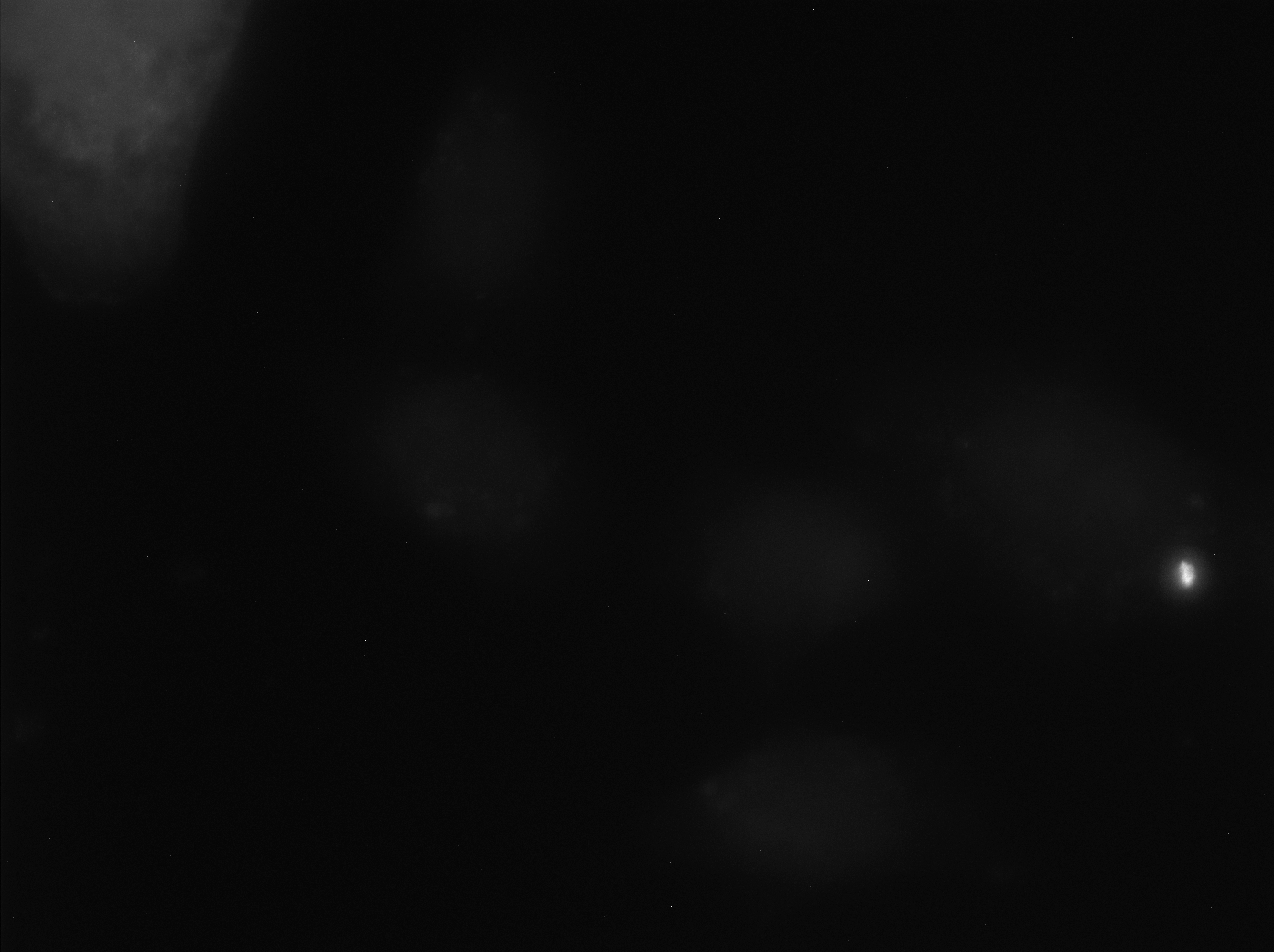

Supplement: Supplementary file 11 — Source data Fig. 3 part 1 [file 44319_2026_742_MOESM11_ESM.zip › Figure 3 Part 1/Fig 3b-e TTLL screen/TTLL1-GFP R1 I3.Project Maximum Z_XY1674163711_Z0_T0_C3.tif]

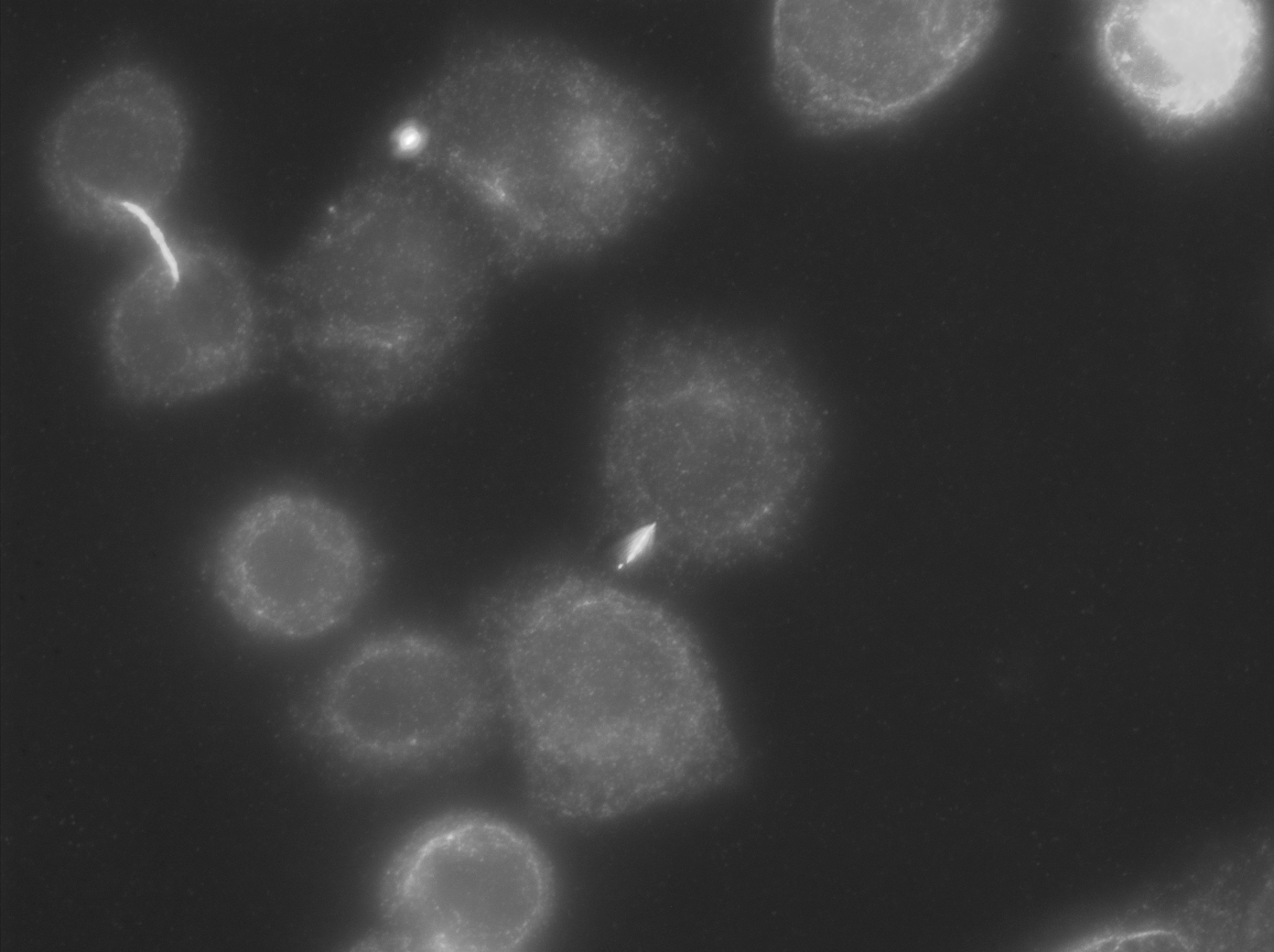

Supplement: Supplementary file 11 — Source data Fig. 3 part 1 [file 44319_2026_742_MOESM11_ESM.zip › Figure 3 Part 1/Fig 3b-e TTLL screen/TTLL4-YFPy I14.Project Maximum Z_XY1679337232_Z0_T0_C1.tif]

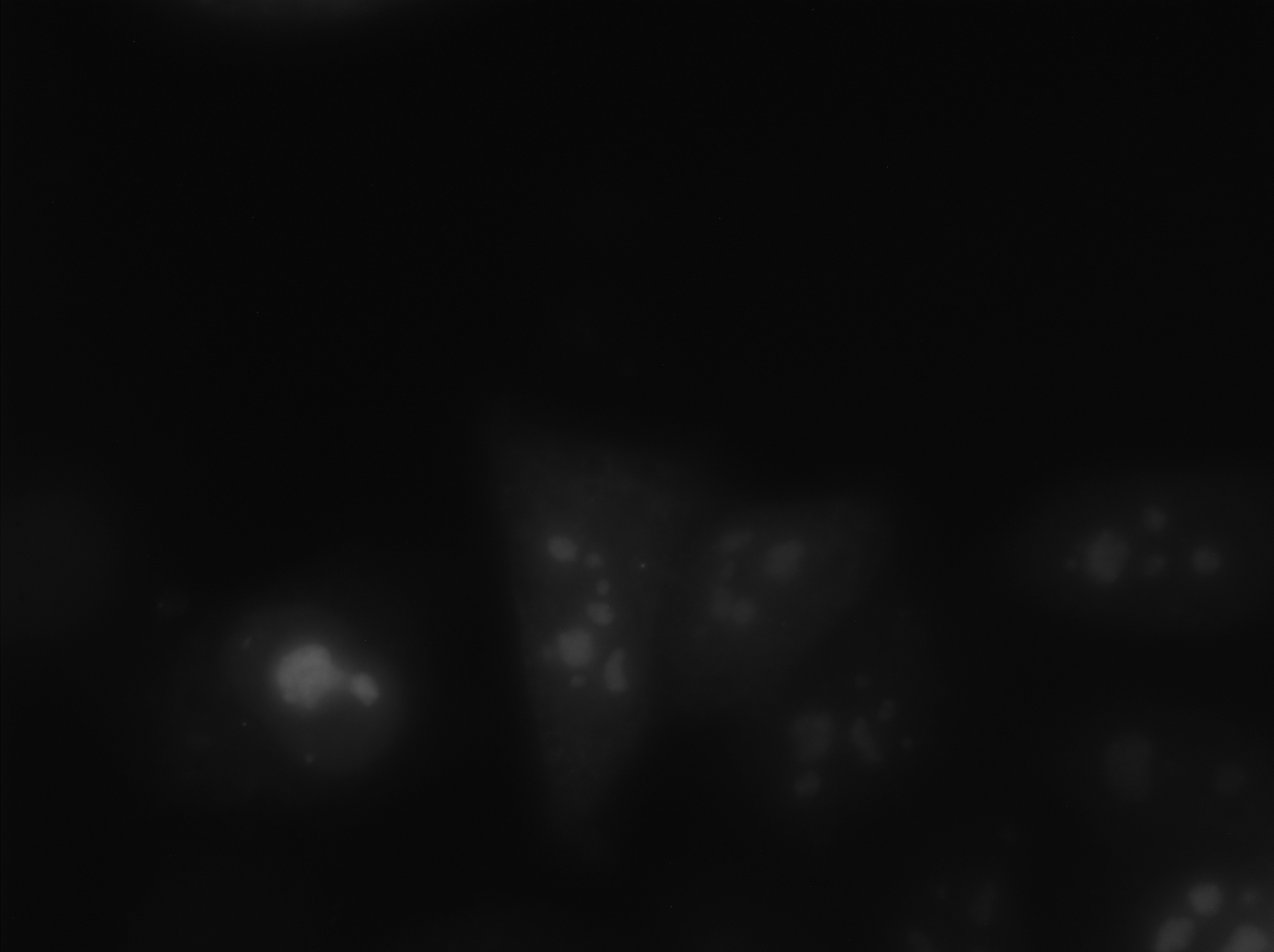

Supplement: Supplementary file 11 — Source data Fig. 3 part 1 [file 44319_2026_742_MOESM11_ESM.zip › Figure 3 Part 1/Fig 3b-e TTLL screen/TTLL4-YFPy I10.Project Maximum Z_XY1679082008_Z0_T0_C2.tif]

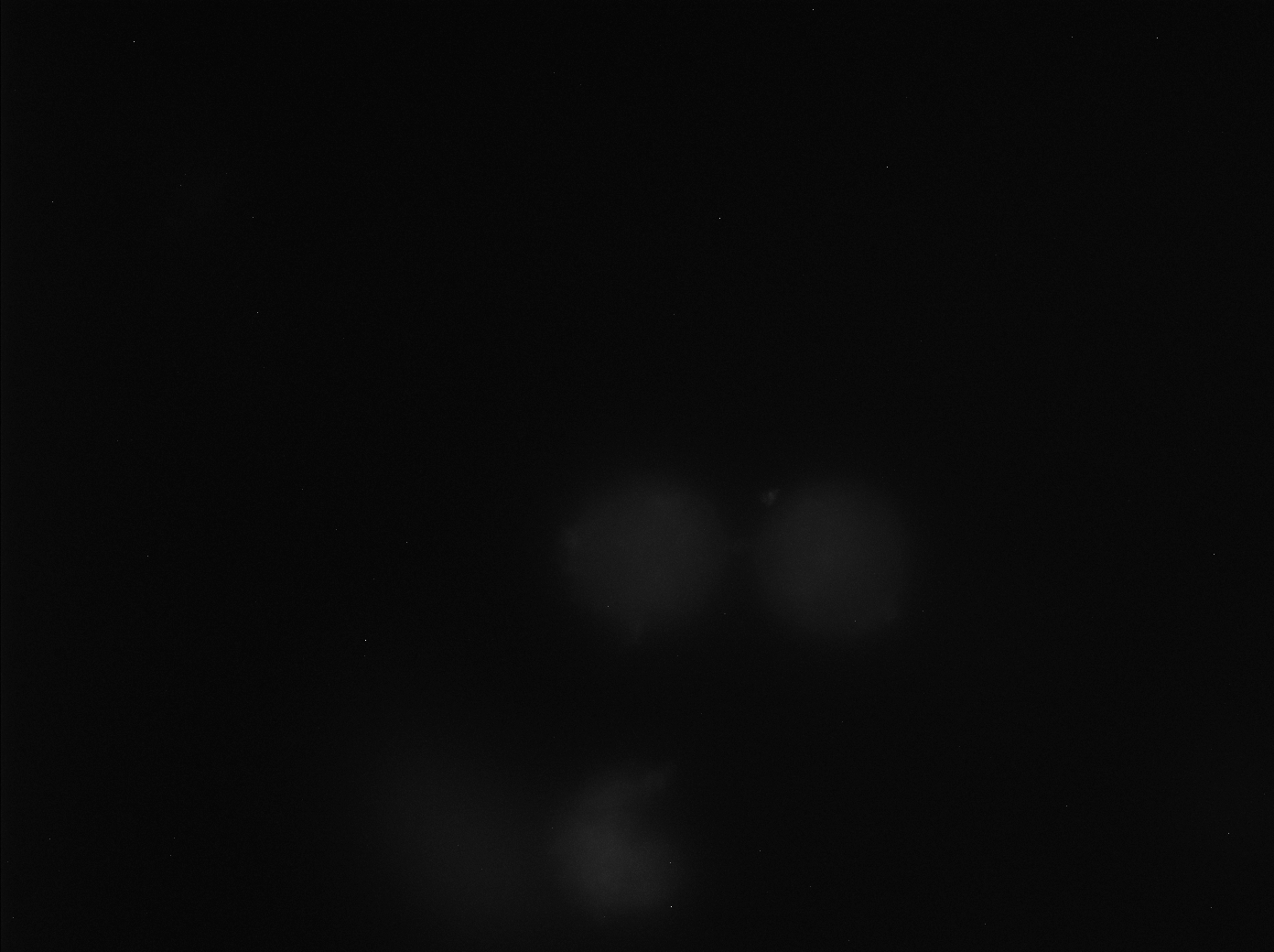

Supplement: Supplementary file 11 — Source data Fig. 3 part 1 [file 44319_2026_742_MOESM11_ESM.zip › Figure 3 Part 1/Fig 3b-e TTLL screen/TTLL1-GFP R1 I6 - 1.Project Maximum Z_XY1674164315_Z0_T0_C3.tif]

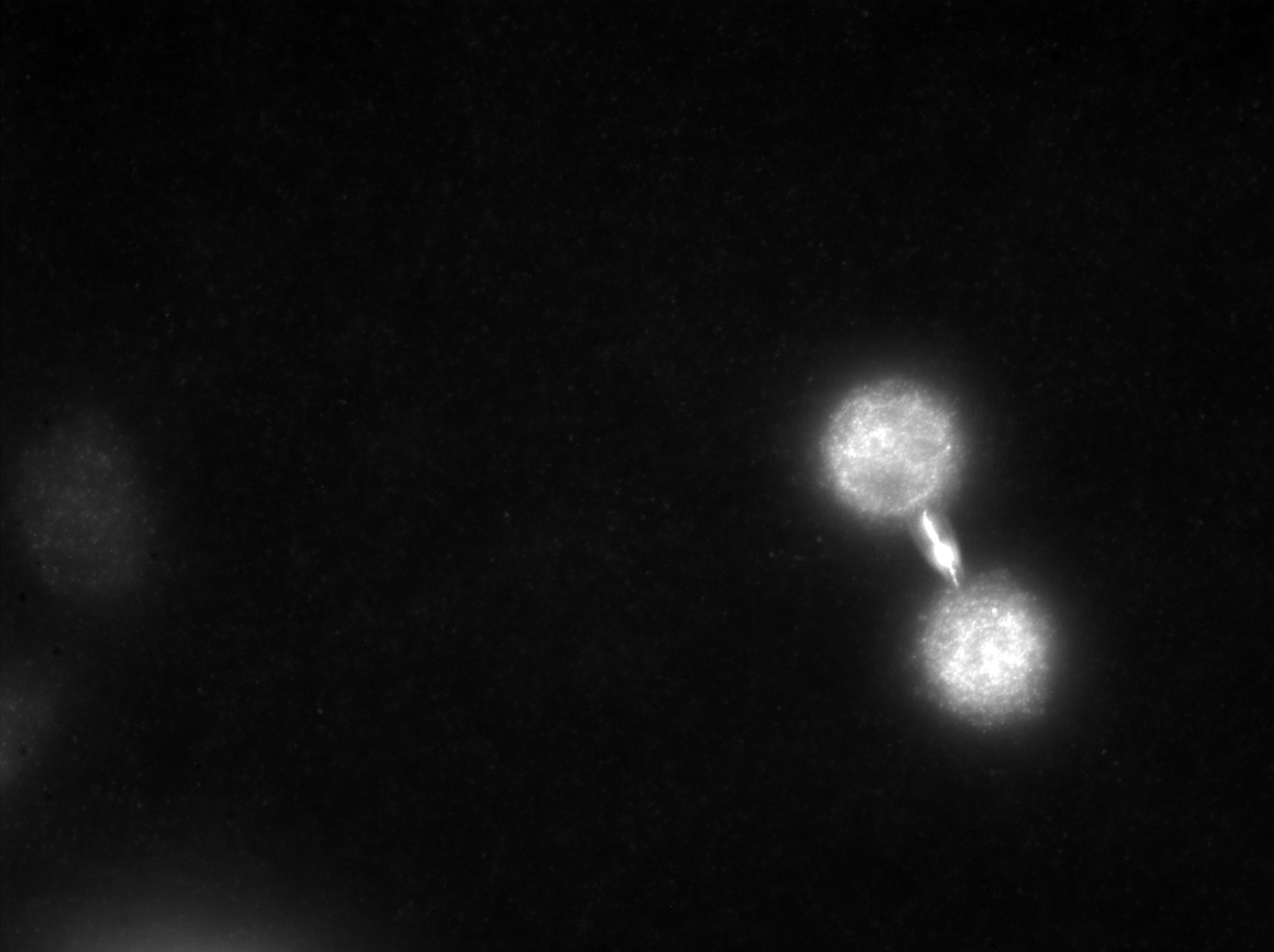

Supplement: Supplementary file 11 — Source data Fig. 3 part 1 [file 44319_2026_742_MOESM11_ESM.zip › Figure 3 Part 1/Fig 3b-e TTLL screen/TTLL1-GFP A3 I20.Project Maximum Z_XY1679698418_Z0_T0_C2.tif]

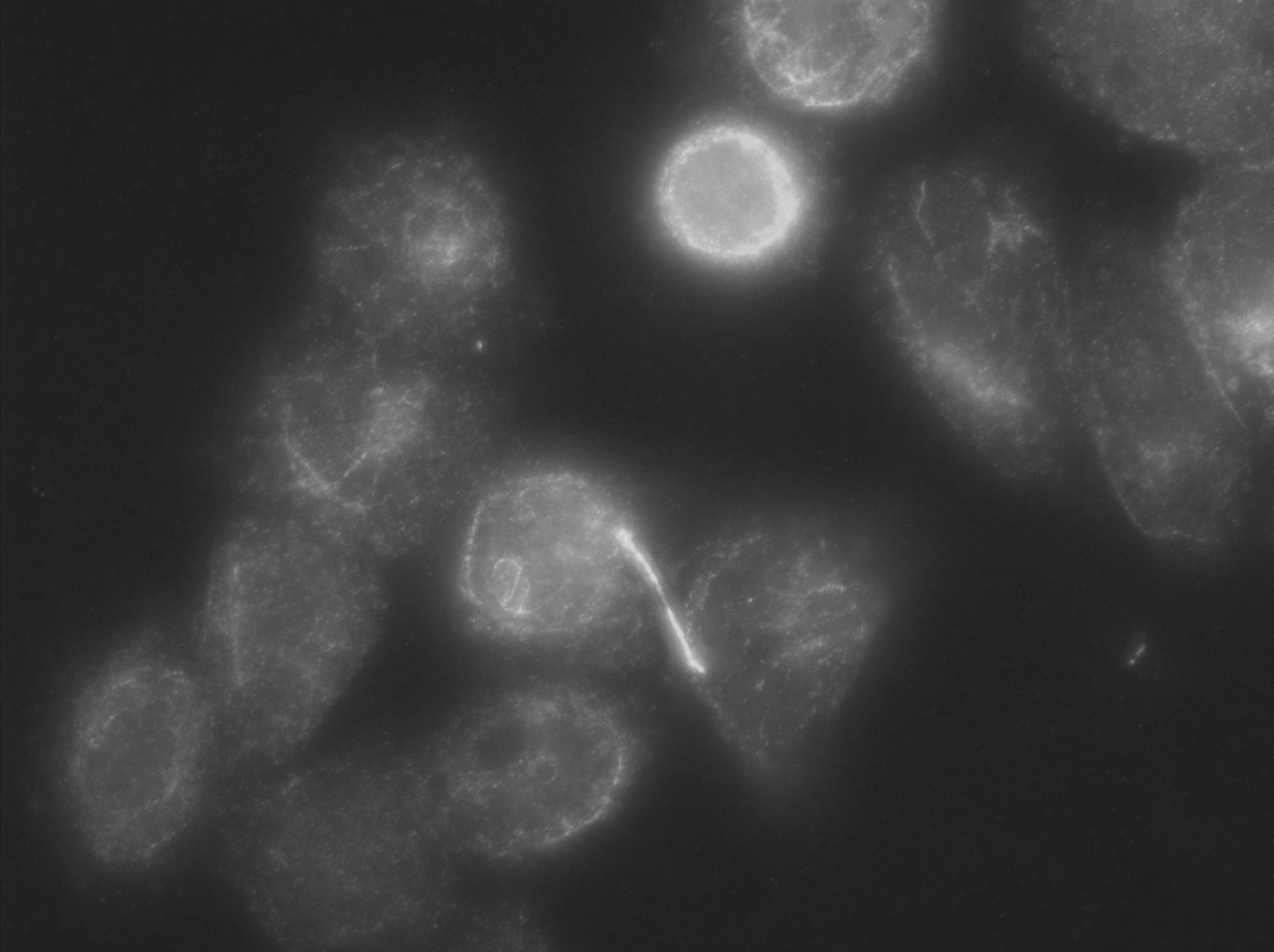

Supplement: Supplementary file 11 — Source data Fig. 3 part 1 [file 44319_2026_742_MOESM11_ESM.zip › Figure 3 Part 1/Fig 3b-e TTLL screen/TTLL4-YFPy I12.Project Maximum Z_XY1679336986_Z0_T0_C1.tif]

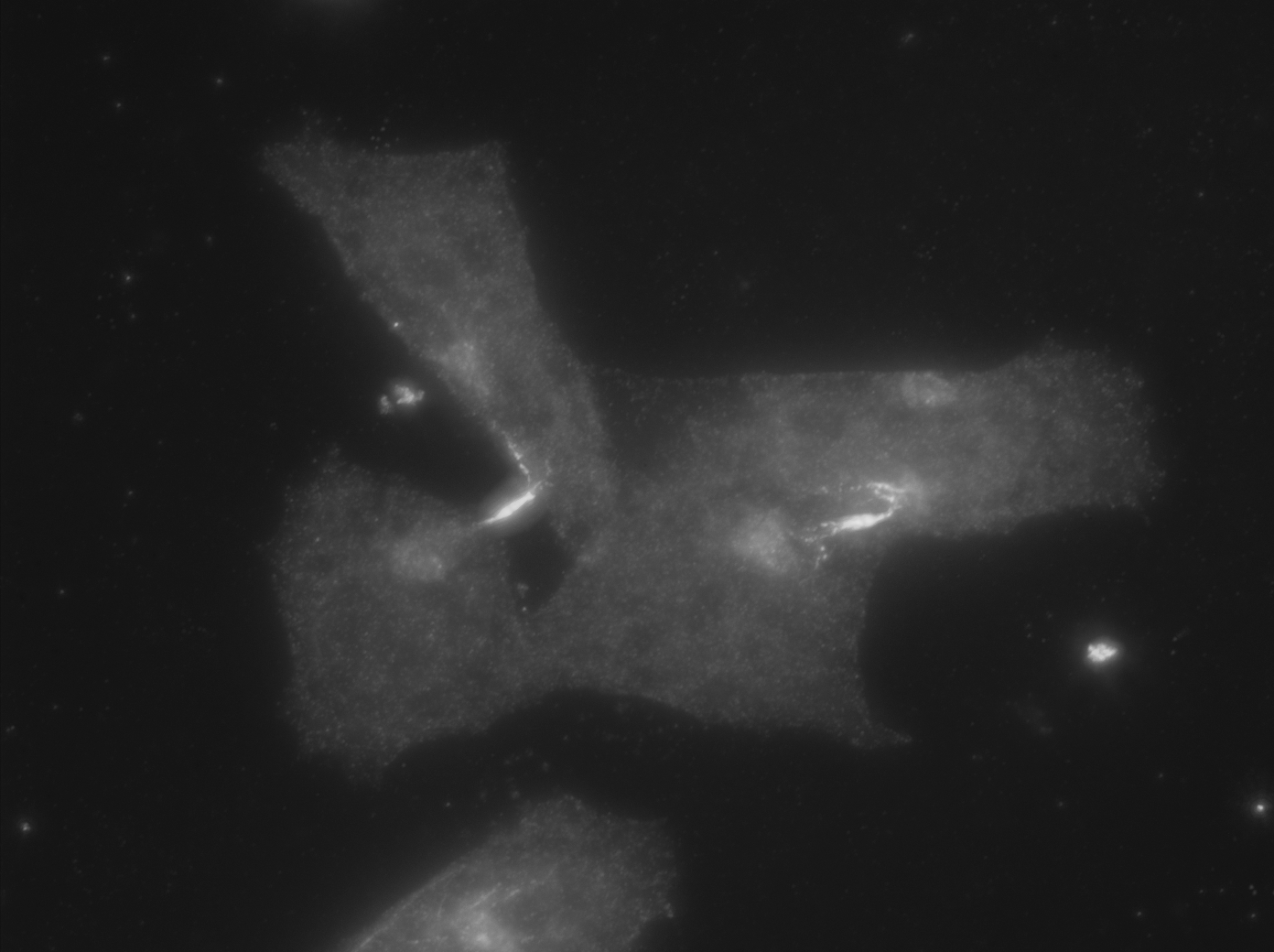

Supplement: Supplementary file 11 — Source data Fig. 3 part 1 [file 44319_2026_742_MOESM11_ESM.zip › Figure 3 Part 1/Fig 3b-e TTLL screen/TTLL1-GFP A4 I5.Project Maximum Z_XY1675962213_Z0_T0_C2.tif]

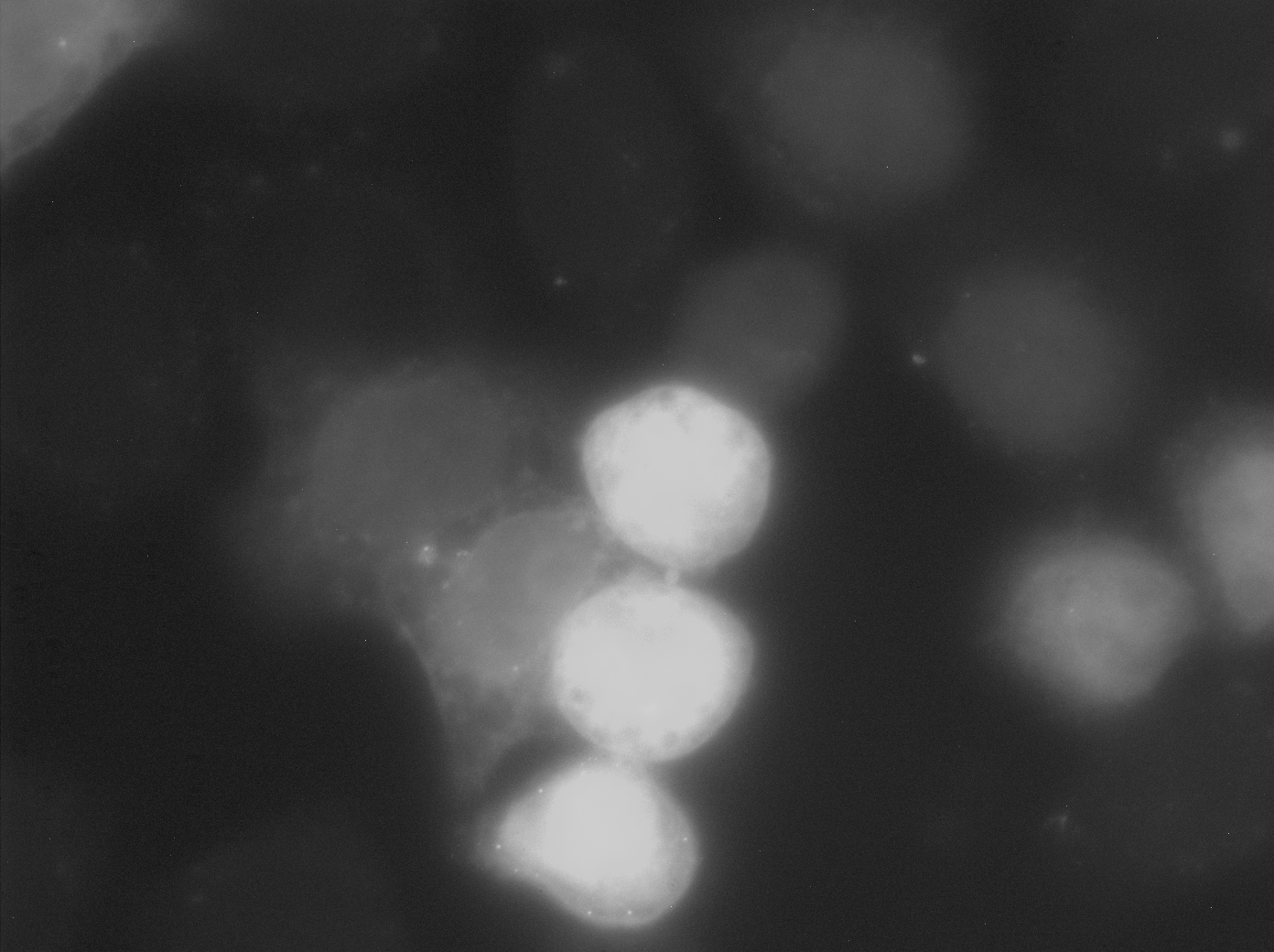

Supplement: Supplementary file 11 — Source data Fig. 3 part 1 [file 44319_2026_742_MOESM11_ESM.zip › Figure 3 Part 1/Fig 3b-e TTLL screen/TTLL1-GFP A3 I16.Project Maximum Z_XY1679697481_Z0_T0_C1.tif]

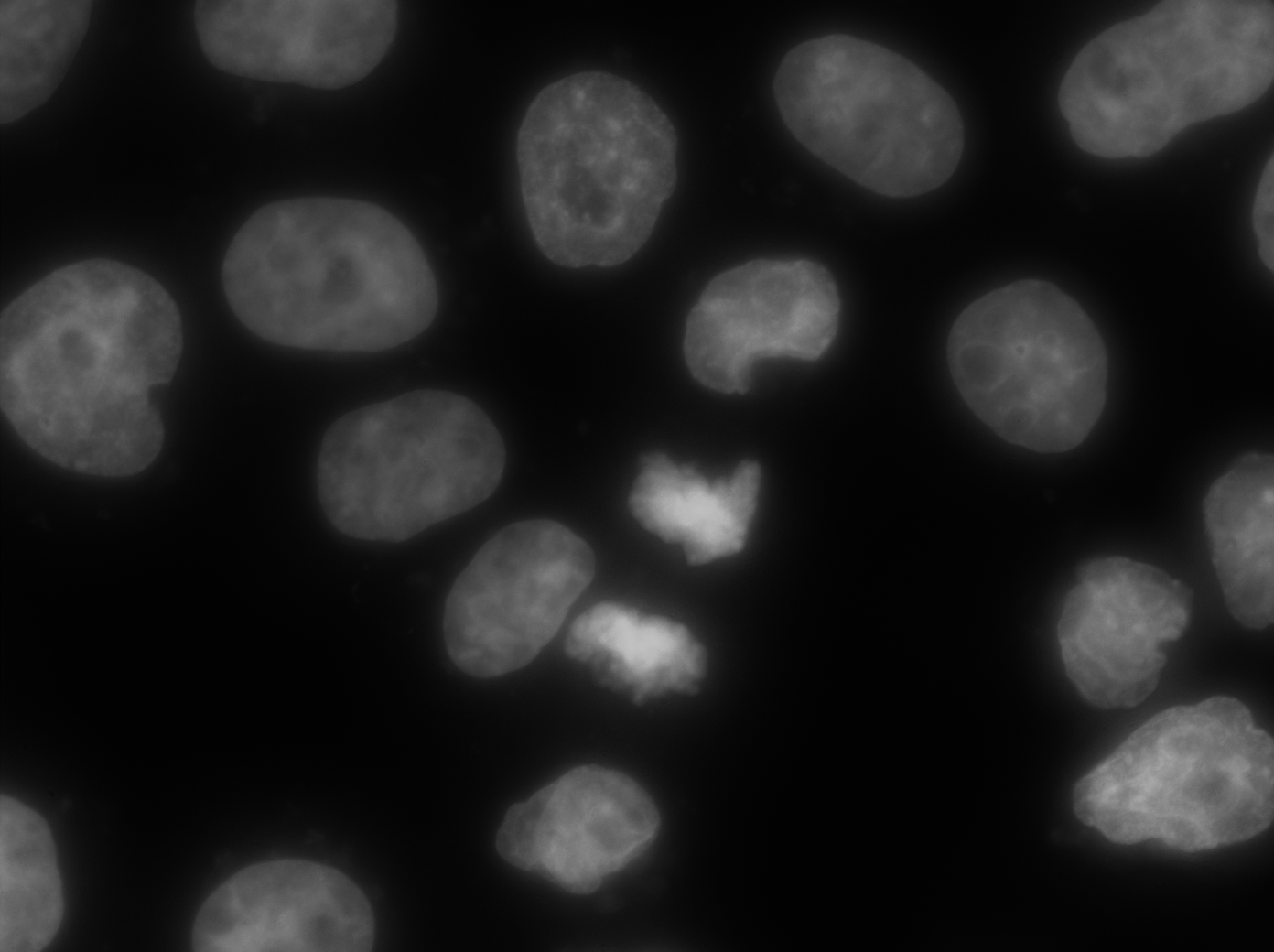

Supplement: Supplementary file 11 — Source data Fig. 3 part 1 [file 44319_2026_742_MOESM11_ESM.zip › Figure 3 Part 1/Fig 3b-e TTLL screen/TTLL1-GFP A3 I16.Project Maximum Z_XY1679697481_Z0_T0_C0.tif]

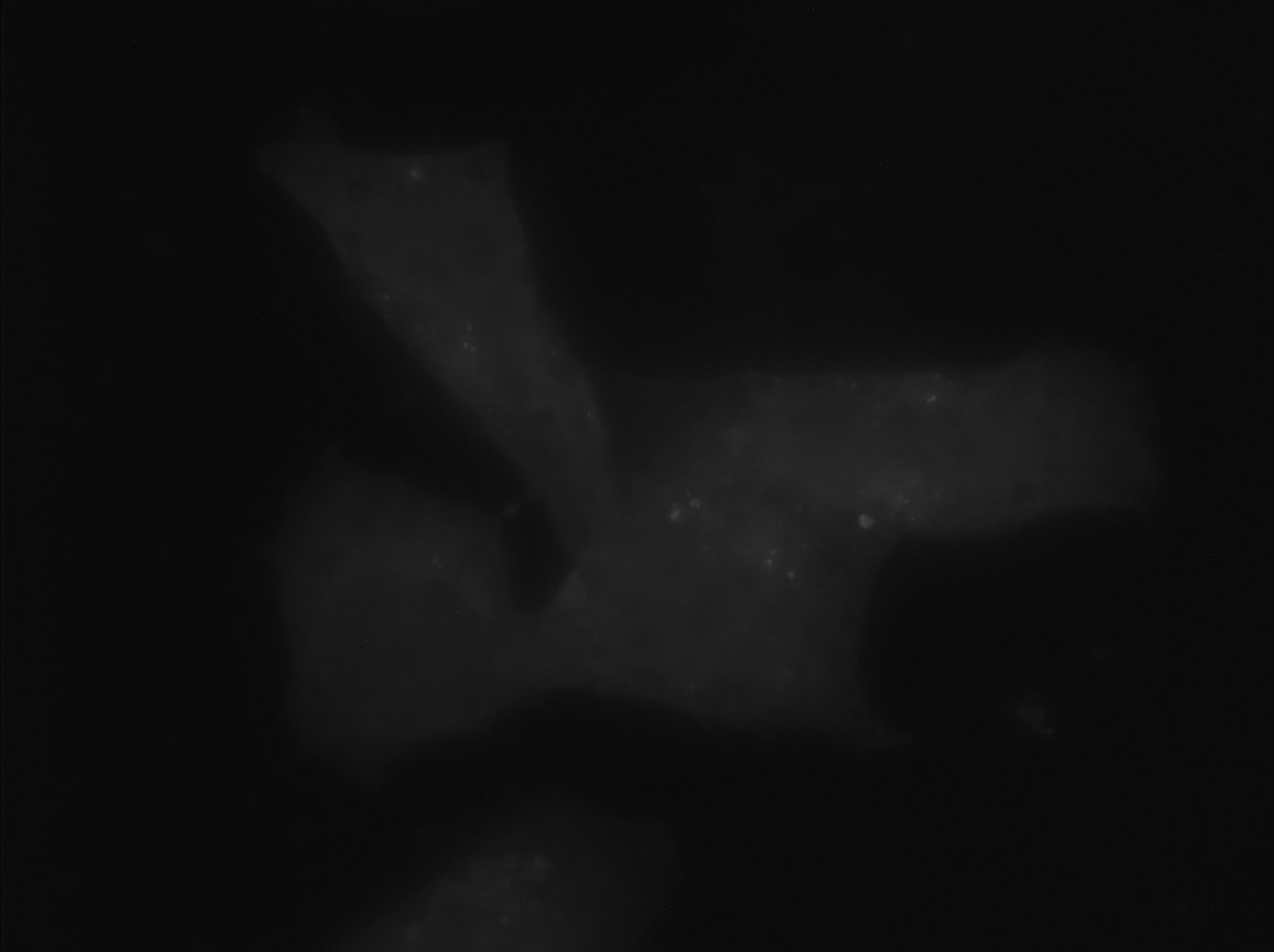

Supplement: Supplementary file 11 — Source data Fig. 3 part 1 [file 44319_2026_742_MOESM11_ESM.zip › Figure 3 Part 1/Fig 3b-e TTLL screen/TTLL1-GFP A4 I5.Project Maximum Z_XY1675962213_Z0_T0_C3.tif]

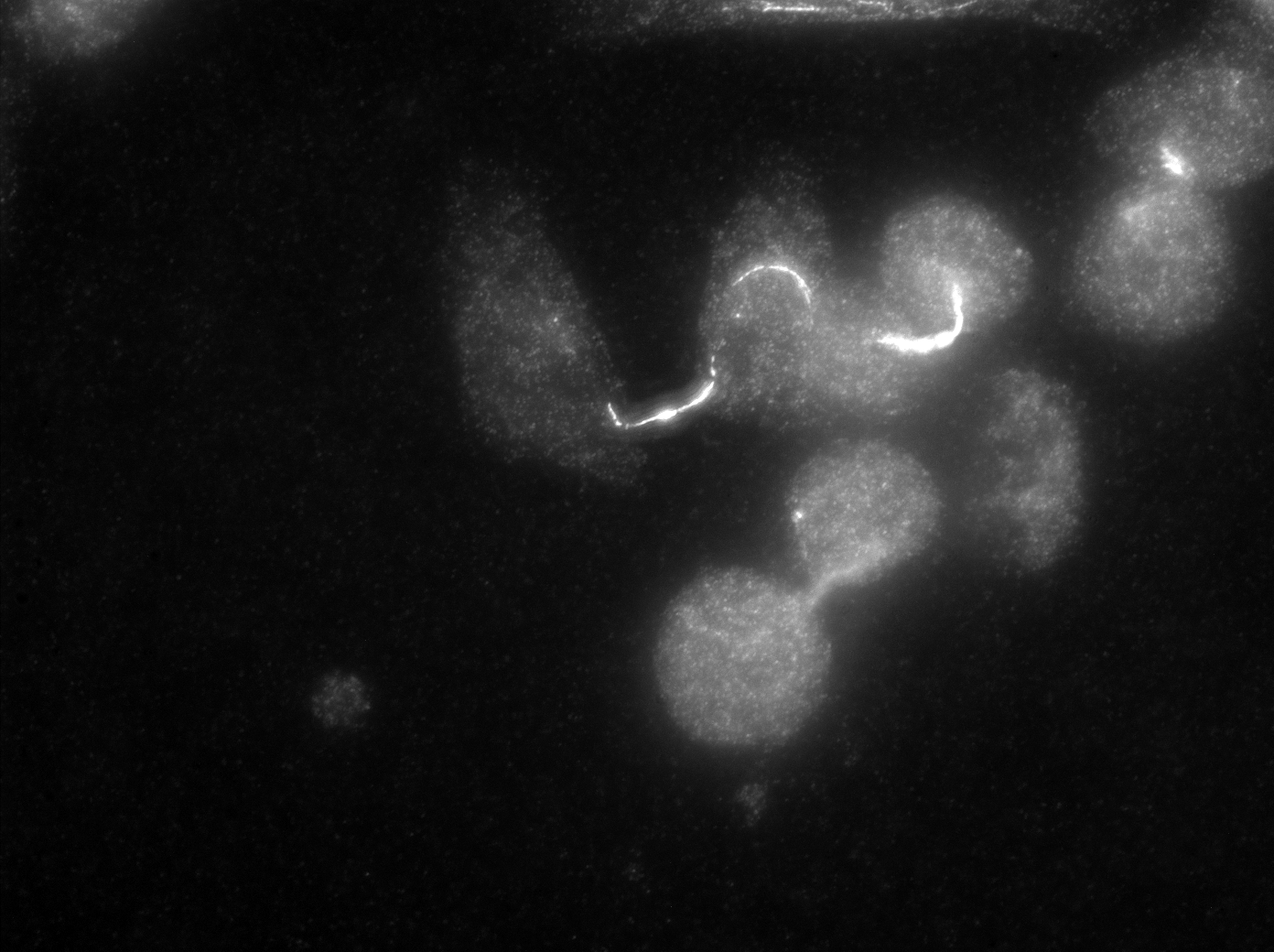

Supplement: Supplementary file 11 — Source data Fig. 3 part 1 [file 44319_2026_742_MOESM11_ESM.zip › Figure 3 Part 1/Fig 3b-e TTLL screen/TTLL1-GFP A3 I1 - 1.Project Maximum Z_XY1679694090_Z0_T0_C2.tif]

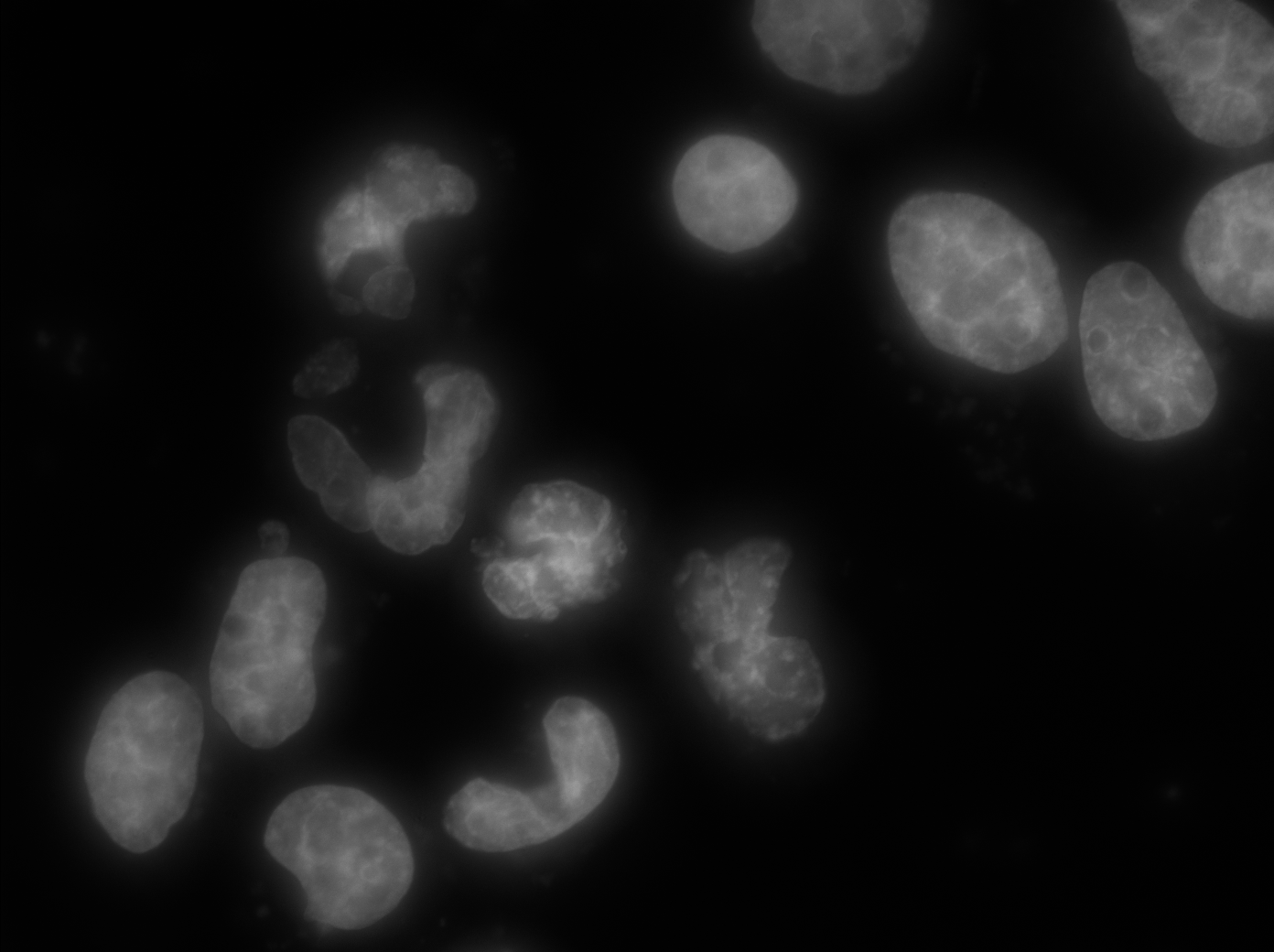

Supplement: Supplementary file 11 — Source data Fig. 3 part 1 [file 44319_2026_742_MOESM11_ESM.zip › Figure 3 Part 1/Fig 3b-e TTLL screen/TTLL4-YFPy I12.Project Maximum Z_XY1679336986_Z0_T0_C0.tif]

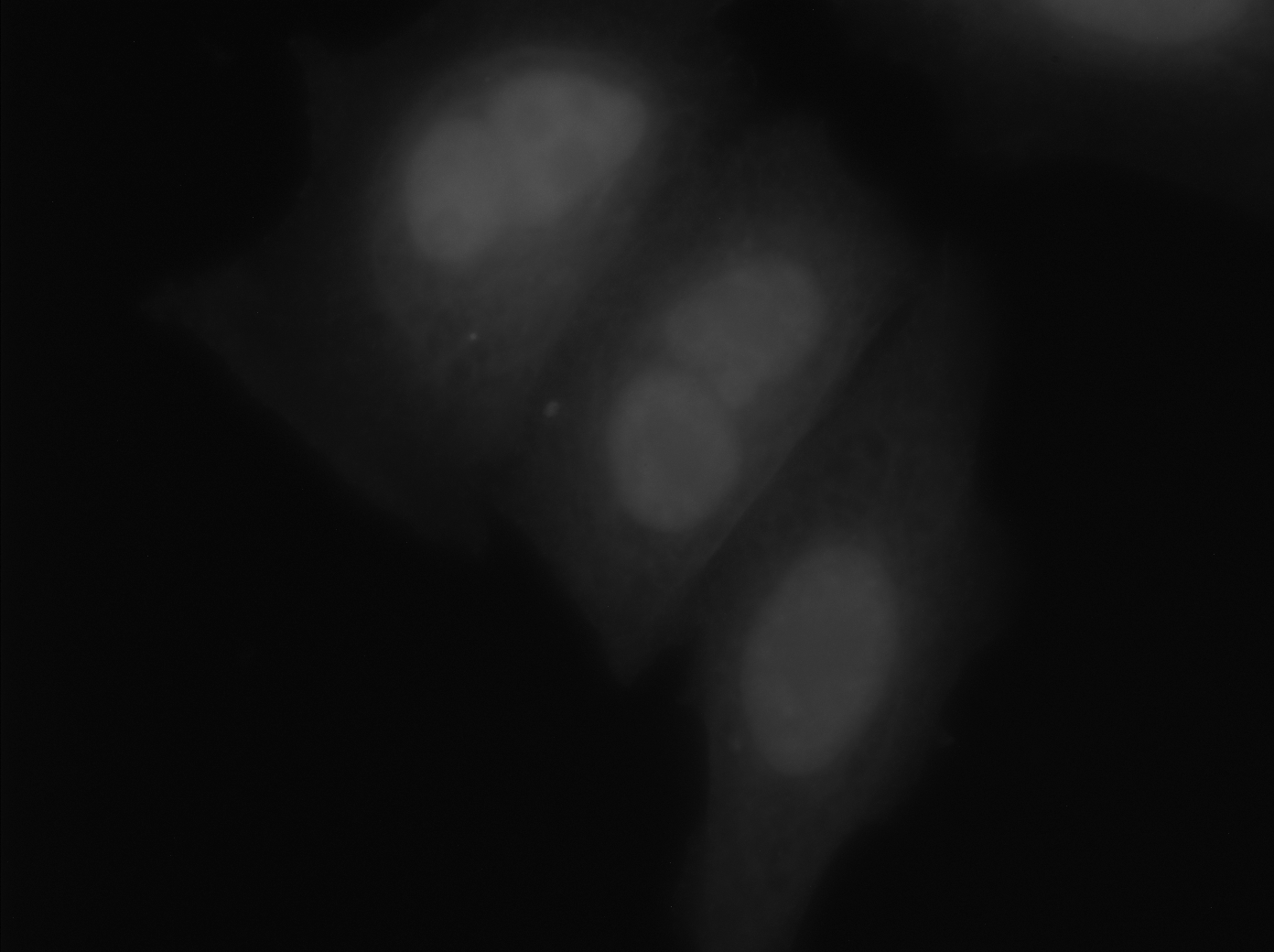

Supplement: Supplementary file 11 — Source data Fig. 3 part 1 [file 44319_2026_742_MOESM11_ESM.zip › Figure 3 Part 1/Fig 3b-e TTLL screen/EYFP MB multi I6.Project Maximum Z_XY1663875973_Z0_T0_C2.tif]

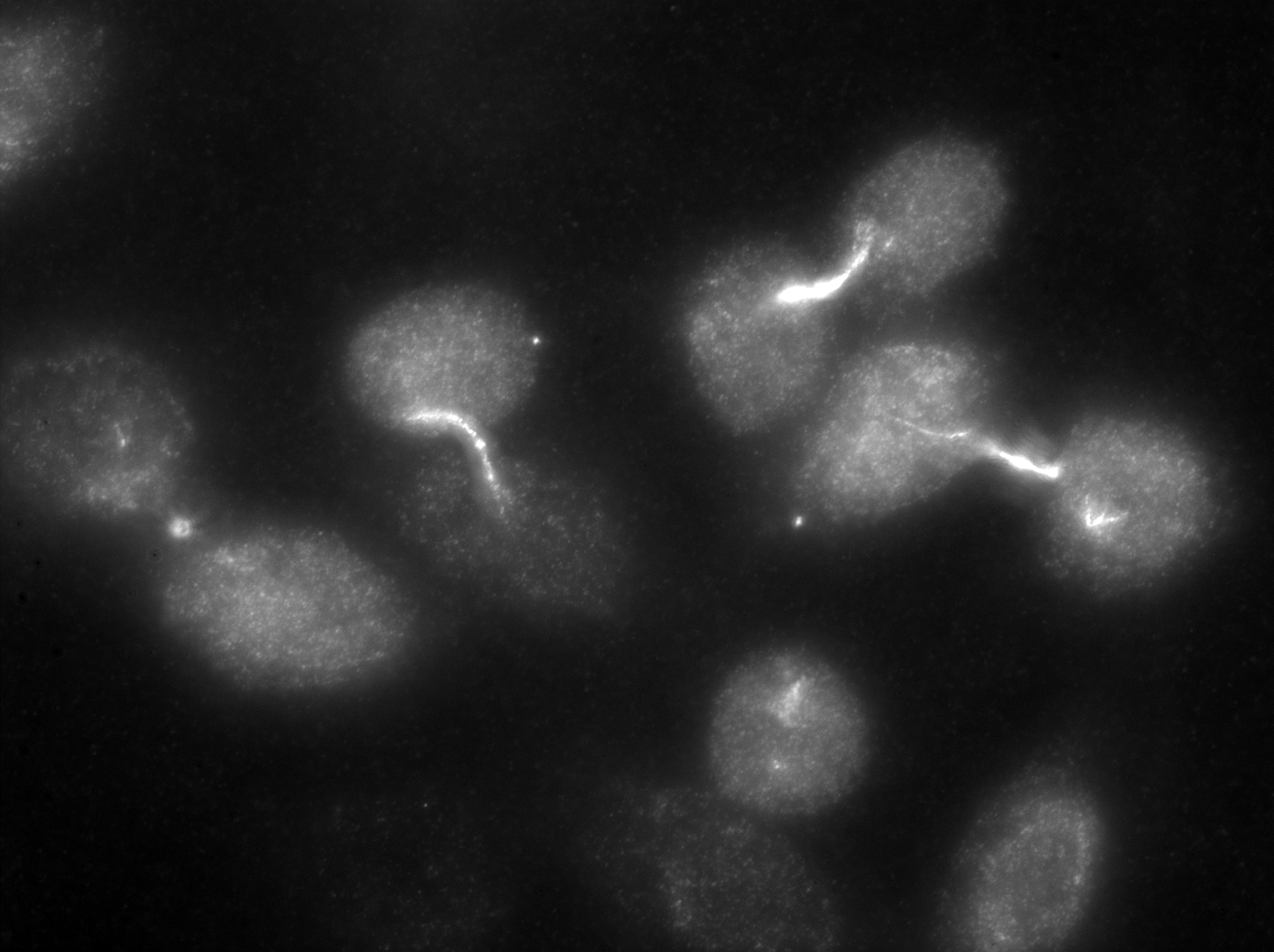

Supplement: Supplementary file 11 — Source data Fig. 3 part 1 [file 44319_2026_742_MOESM11_ESM.zip › Figure 3 Part 1/Fig 3b-e TTLL screen/TTLL1-GFP A3 I6.Project Maximum Z_XY1679695052_Z0_T0_C2.tif]

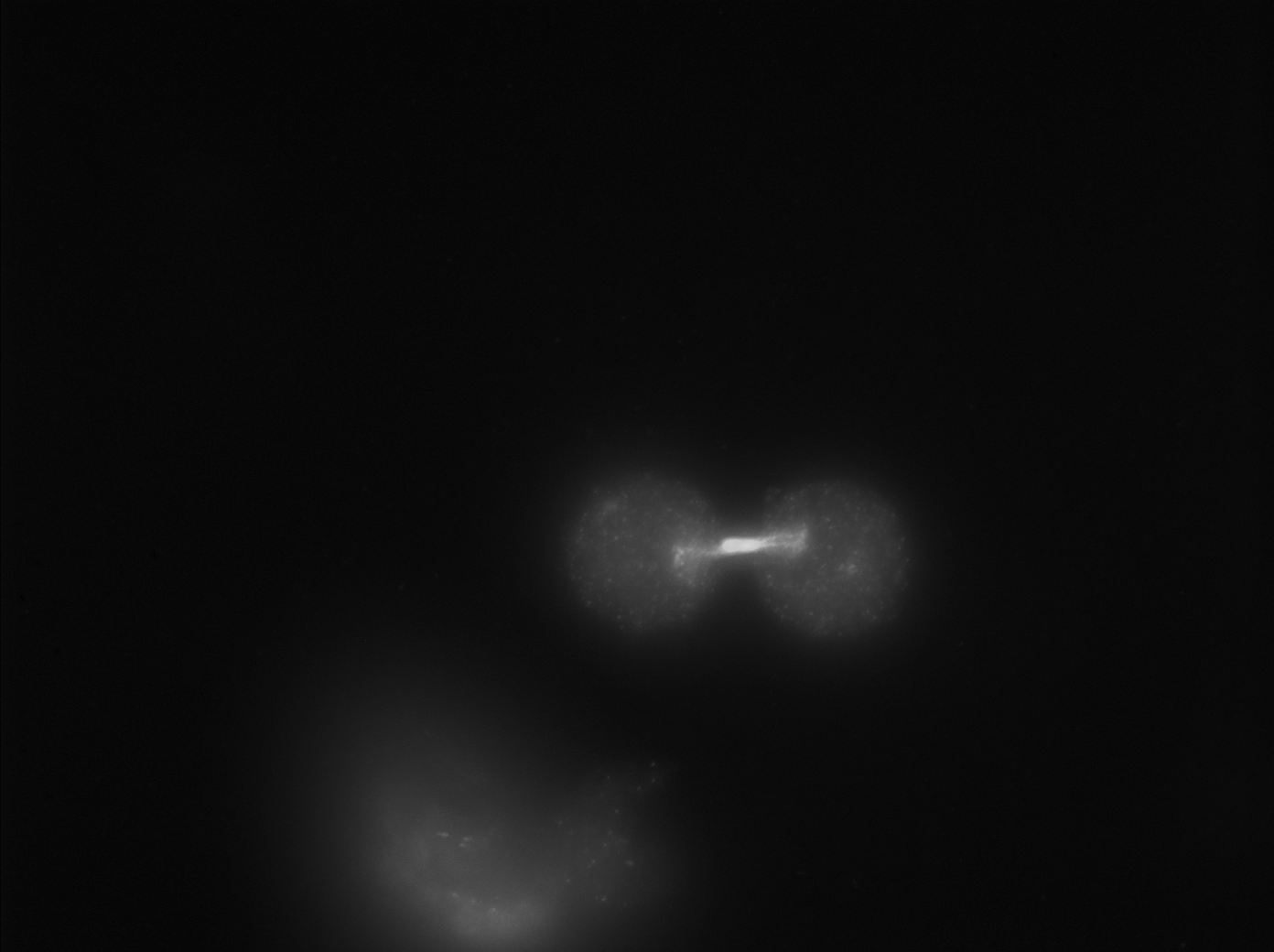

Supplement: Supplementary file 11 — Source data Fig. 3 part 1 [file 44319_2026_742_MOESM11_ESM.zip › Figure 3 Part 1/Fig 3b-e TTLL screen/TTLL1-GFP R1 I6 - 1.Project Maximum Z_XY1674164315_Z0_T0_C2.tif]

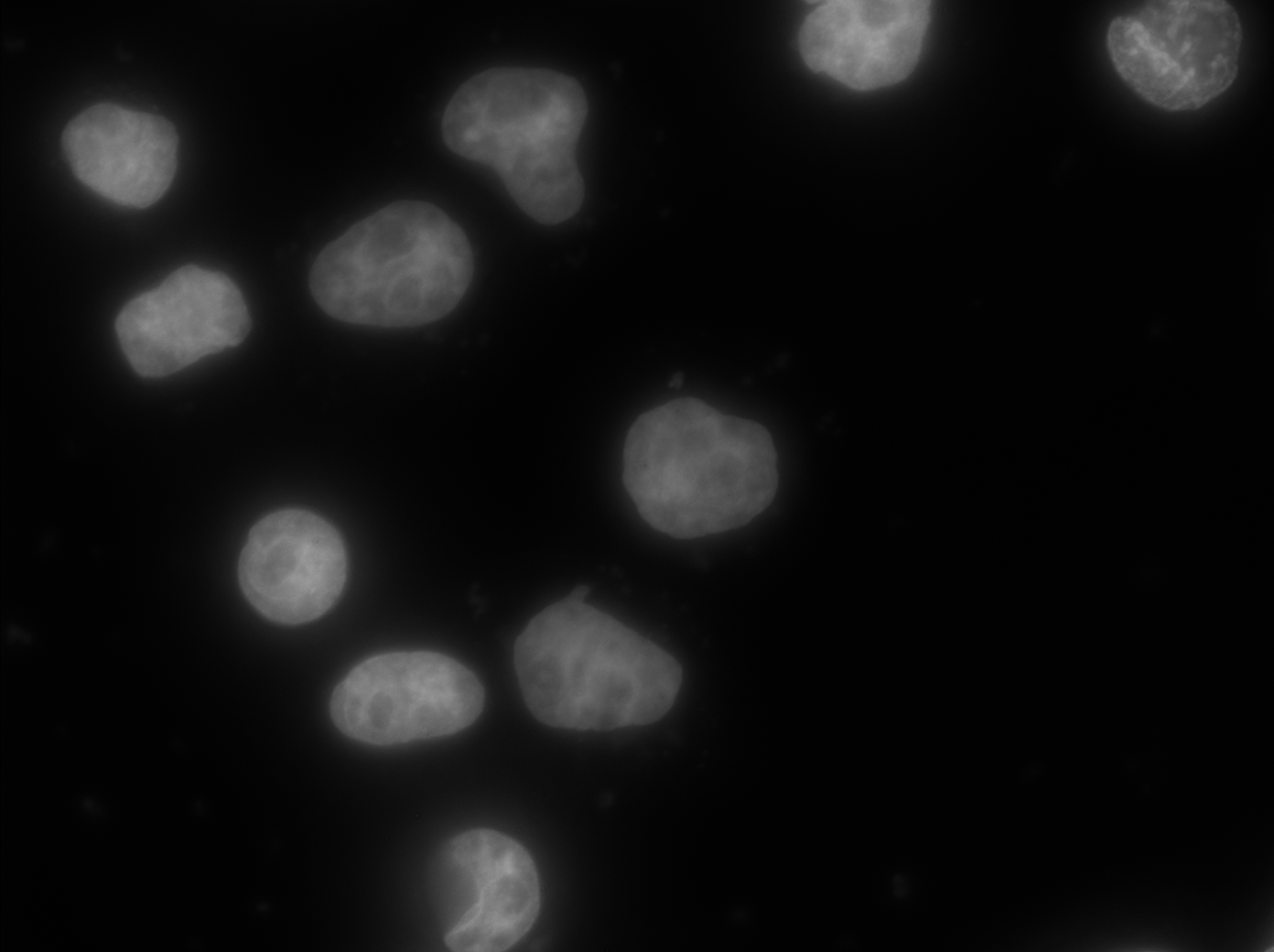

Supplement: Supplementary file 11 — Source data Fig. 3 part 1 [file 44319_2026_742_MOESM11_ESM.zip › Figure 3 Part 1/Fig 3b-e TTLL screen/TTLL4-YFPy I14.Project Maximum Z_XY1679337232_Z0_T0_C0.tif]

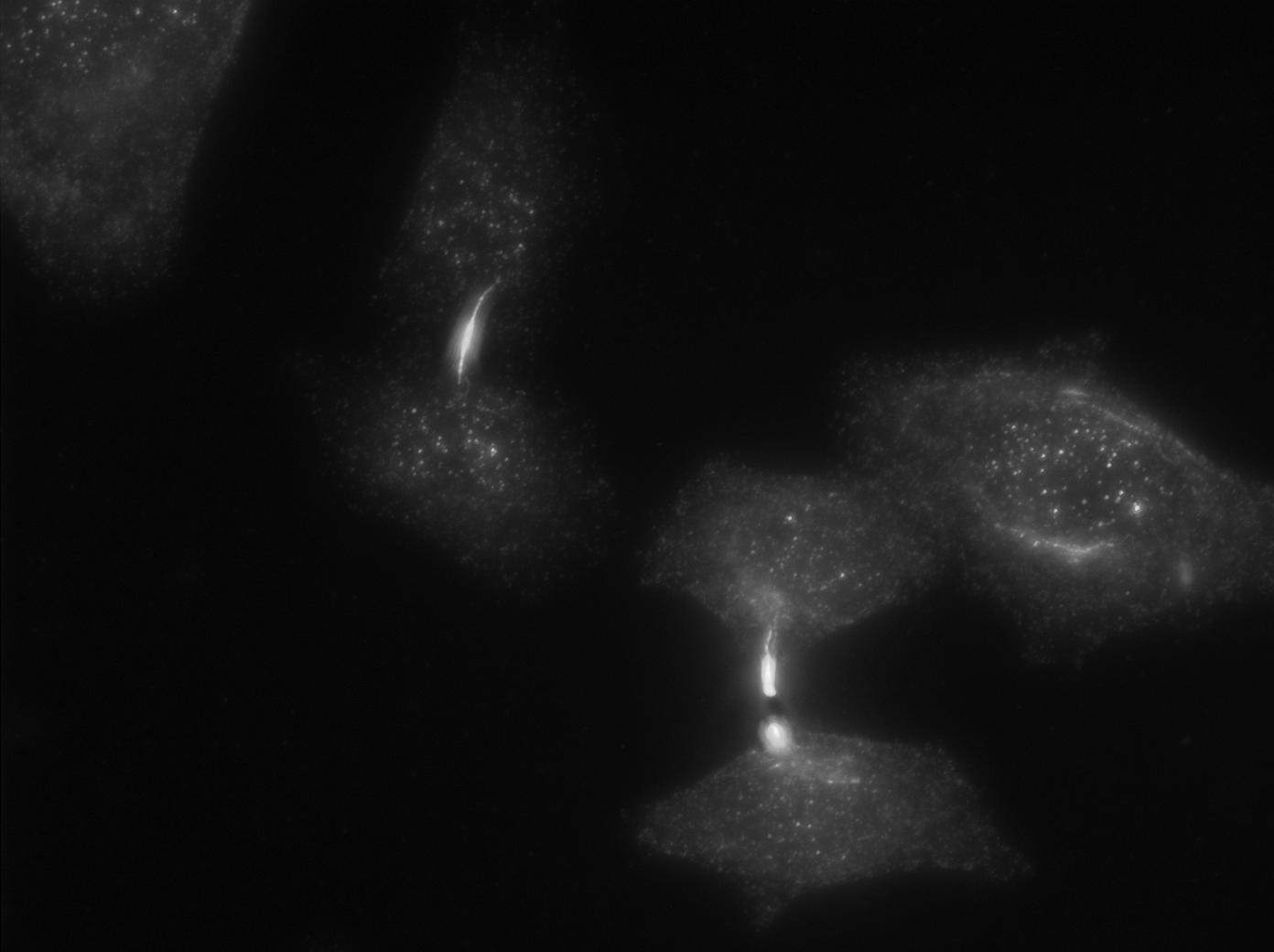

Supplement: Supplementary file 11 — Source data Fig. 3 part 1 [file 44319_2026_742_MOESM11_ESM.zip › Figure 3 Part 1/Fig 3b-e TTLL screen/TTLL1-GFP R1 I3.Project Maximum Z_XY1674163711_Z0_T0_C2.tif]

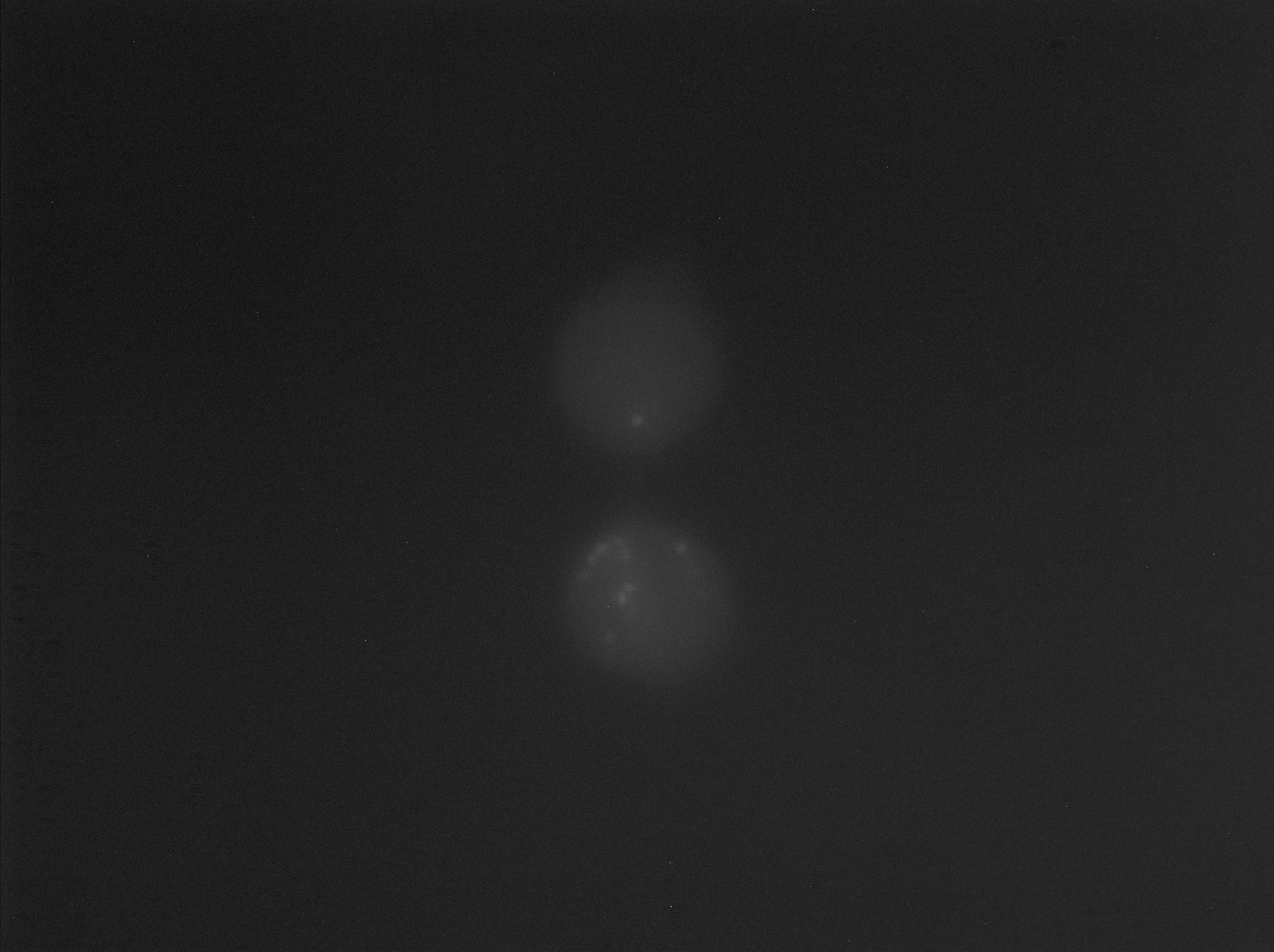

Supplement: Supplementary file 11 — Source data Fig. 3 part 1 [file 44319_2026_742_MOESM11_ESM.zip › Figure 3 Part 1/Fig 3b-e TTLL screen/TTLL1-GFP A3 I1.Project Maximum Z_XY1674673539_Z0_T0_C1.tif]

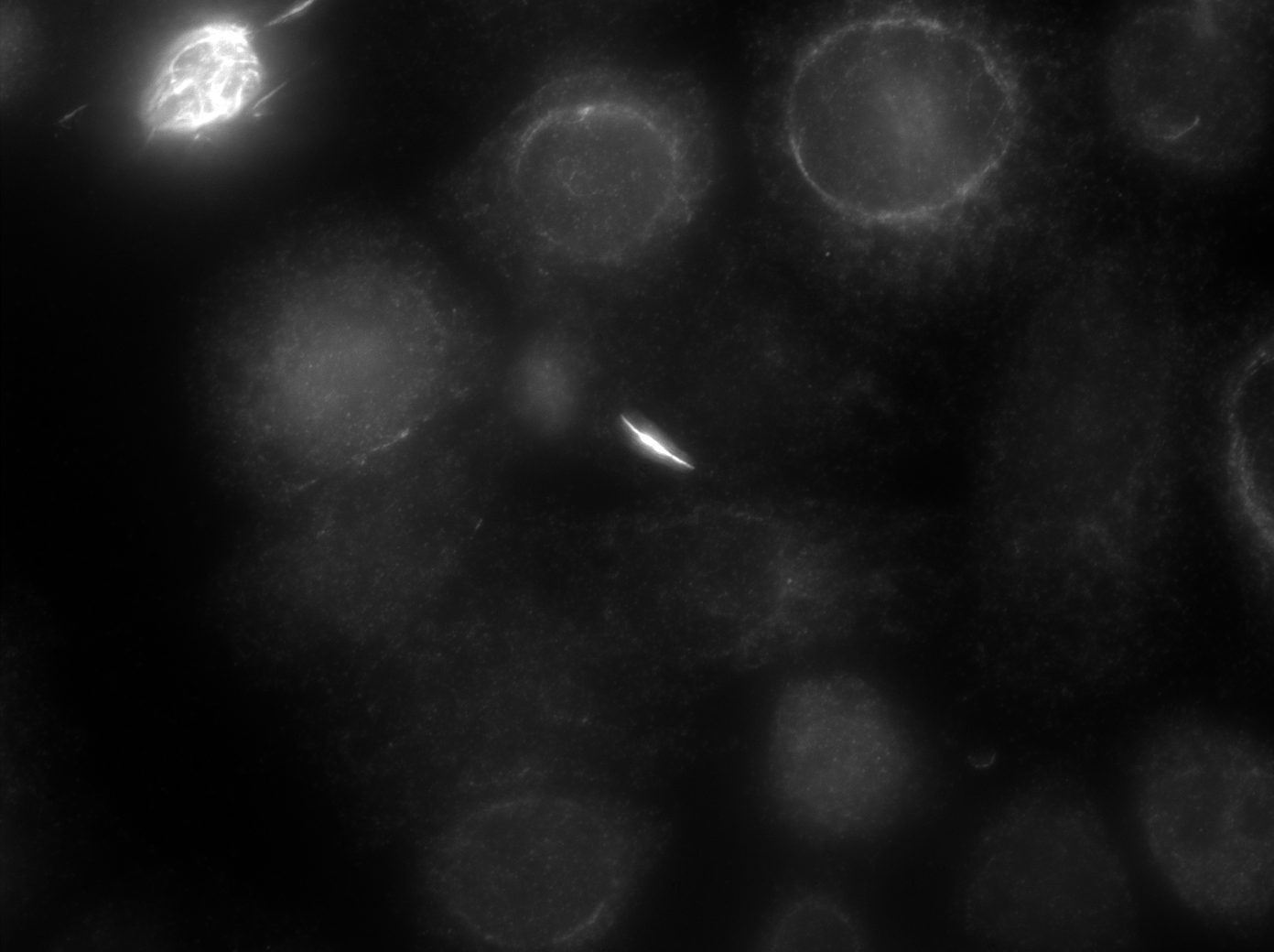

Supplement: Supplementary file 11 — Source data Fig. 3 part 1 [file 44319_2026_742_MOESM11_ESM.zip › Figure 3 Part 1/Fig 3b-e TTLL screen/TTLL4-YFPy I4.Project Maximum Z_XY1679075937_Z0_T0_C2.tif]

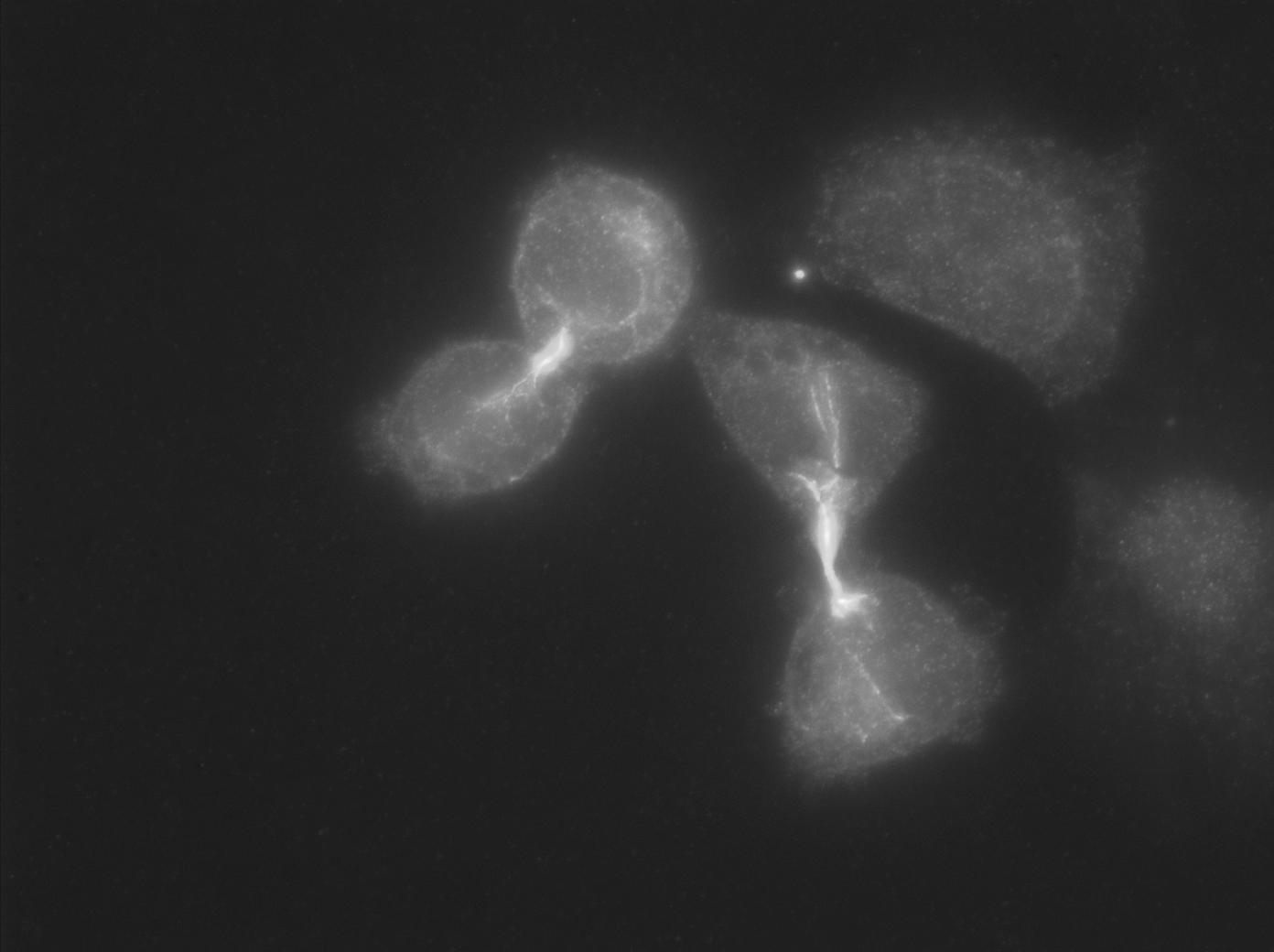

Supplement: Supplementary file 11 — Source data Fig. 3 part 1 [file 44319_2026_742_MOESM11_ESM.zip › Figure 3 Part 1/Fig 3b-e TTLL screen/TTLL4-YFPy I17.Project Maximum Z_XY1679337716_Z0_T0_C1.tif]

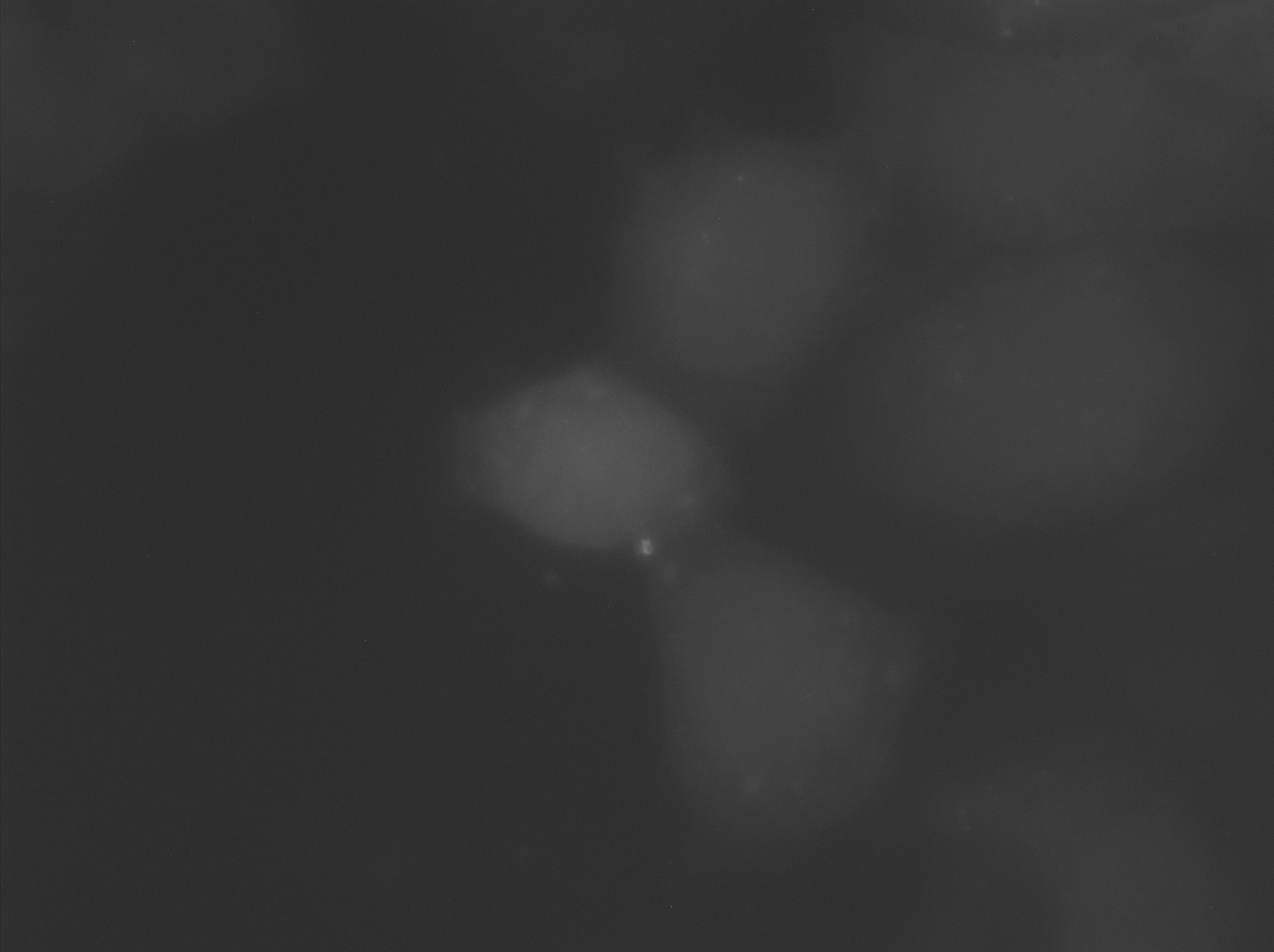

Supplement: Supplementary file 11 — Source data Fig. 3 part 1 [file 44319_2026_742_MOESM11_ESM.zip › Figure 3 Part 1/Fig 3b-e TTLL screen/TTLL1-GFP A4 I10.Project Maximum Z_XY1675963296_Z0_T0_C1.tif]

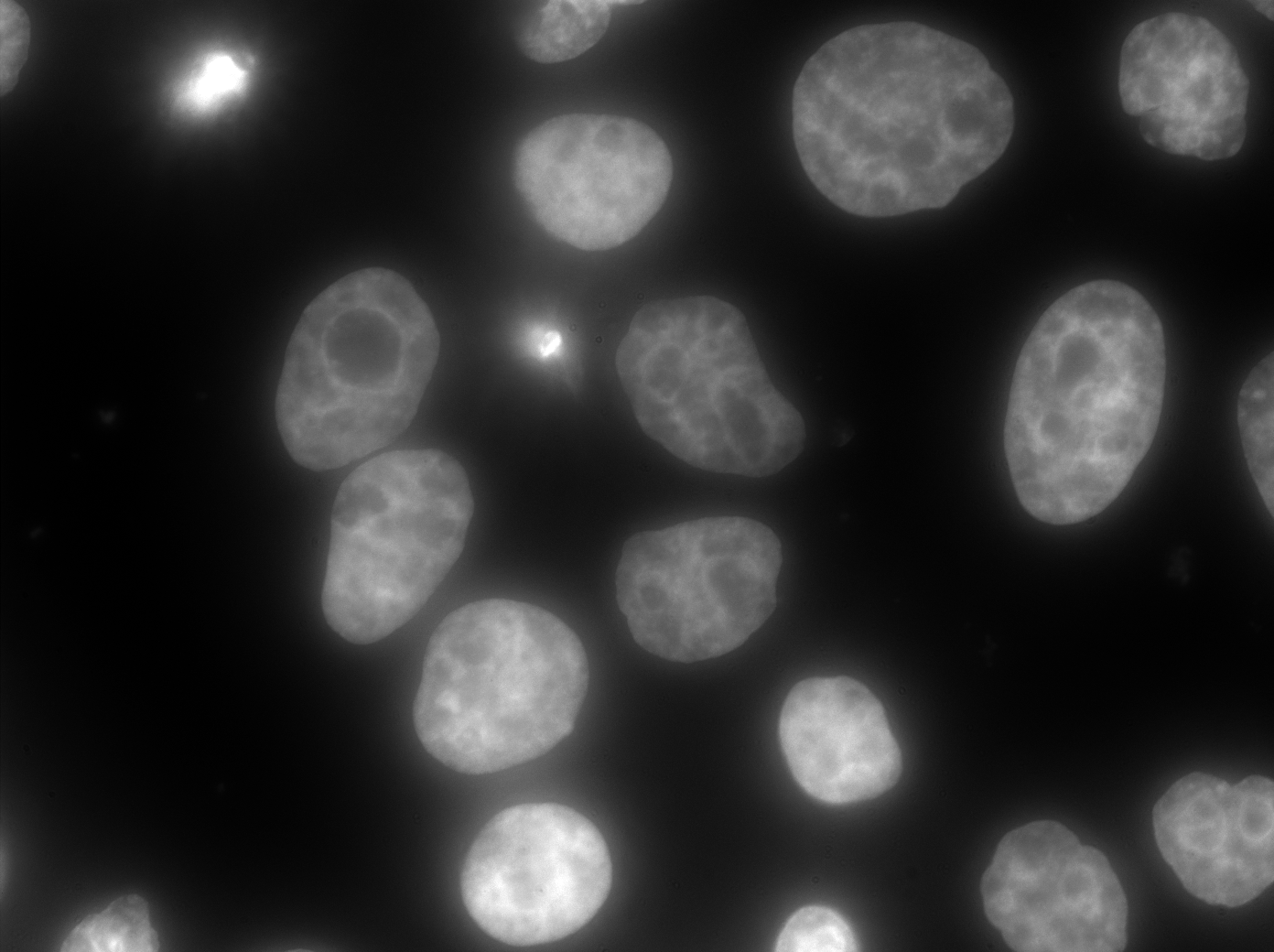

Supplement: Supplementary file 11 — Source data Fig. 3 part 1 [file 44319_2026_742_MOESM11_ESM.zip › Figure 3 Part 1/Fig 3b-e TTLL screen/TTLL4-YFPy I4.Project Maximum Z_XY1679075937_Z0_T0_C0.tif]

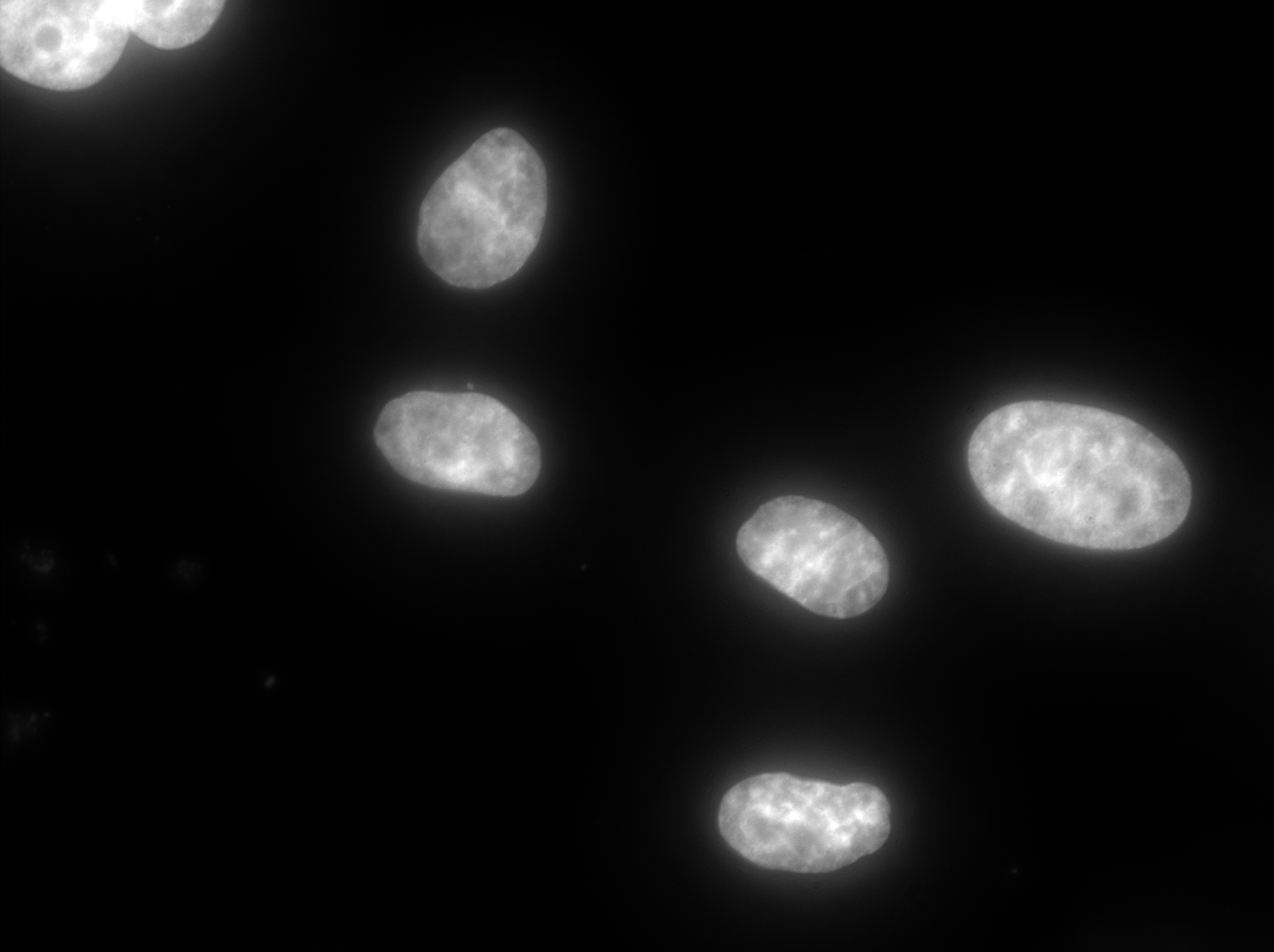

Supplement: Supplementary file 11 — Source data Fig. 3 part 1 [file 44319_2026_742_MOESM11_ESM.zip › Figure 3 Part 1/Fig 3b-e TTLL screen/TTLL1-GFP R1 I3.Project Maximum Z_XY1674163711_Z0_T0_C0.tif]

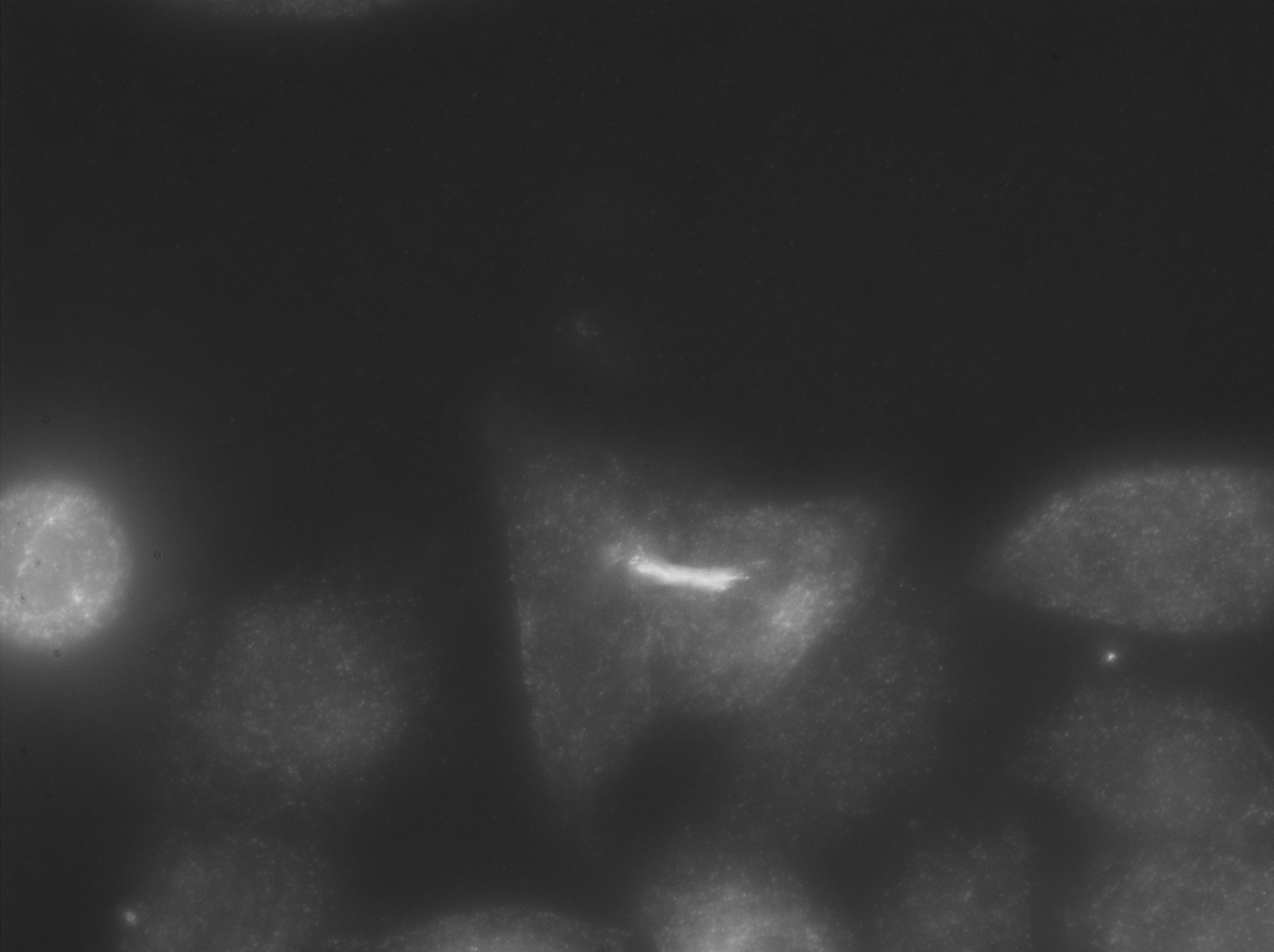

Supplement: Supplementary file 11 — Source data Fig. 3 part 1 [file 44319_2026_742_MOESM11_ESM.zip › Figure 3 Part 1/Fig 3b-e TTLL screen/TTLL4-YFPy I10.Project Maximum Z_XY1679082008_Z0_T0_C1.tif]

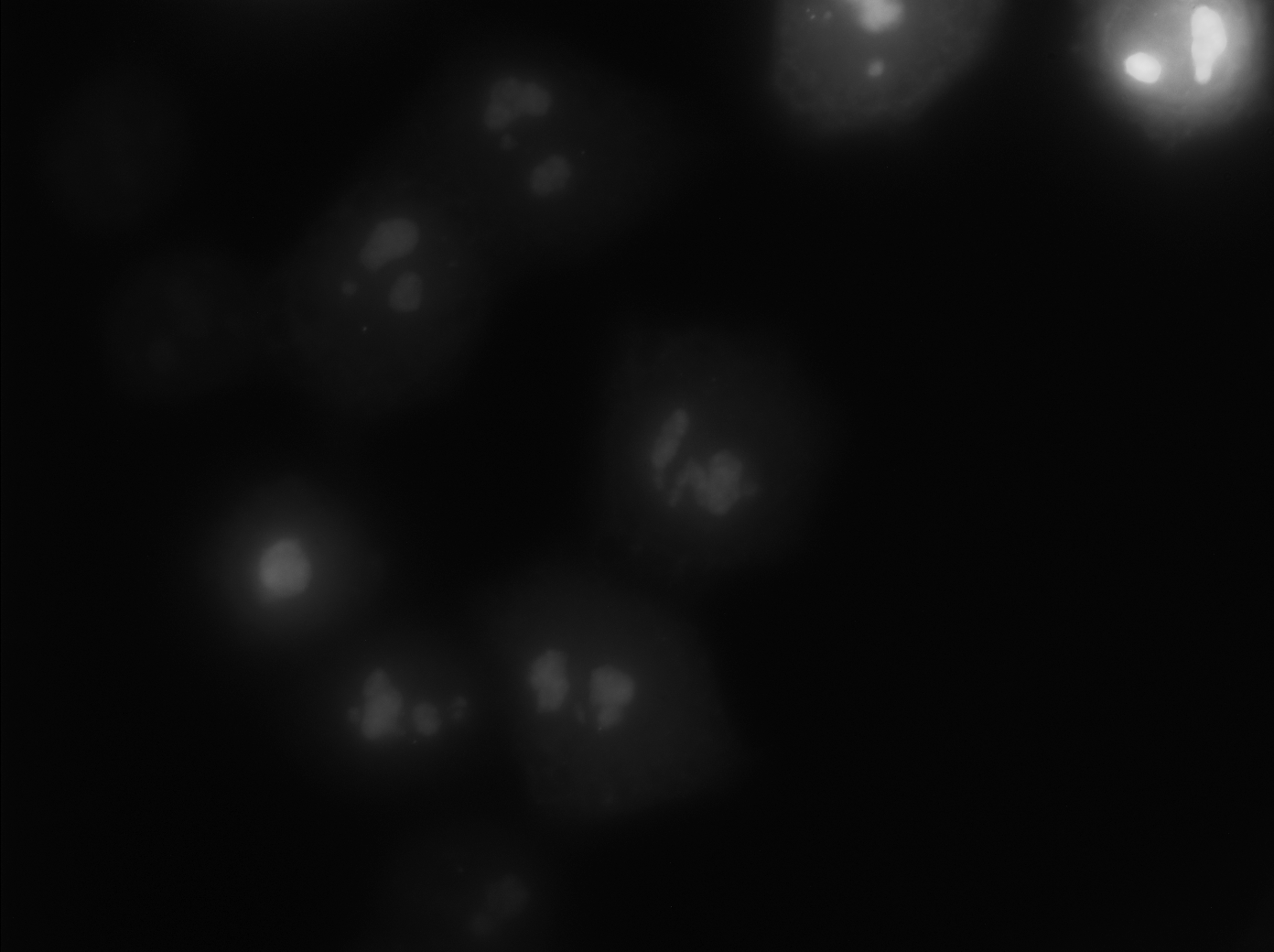

Supplement: Supplementary file 11 — Source data Fig. 3 part 1 [file 44319_2026_742_MOESM11_ESM.zip › Figure 3 Part 1/Fig 3b-e TTLL screen/TTLL4-YFPy I14.Project Maximum Z_XY1679337232_Z0_T0_C2.tif]

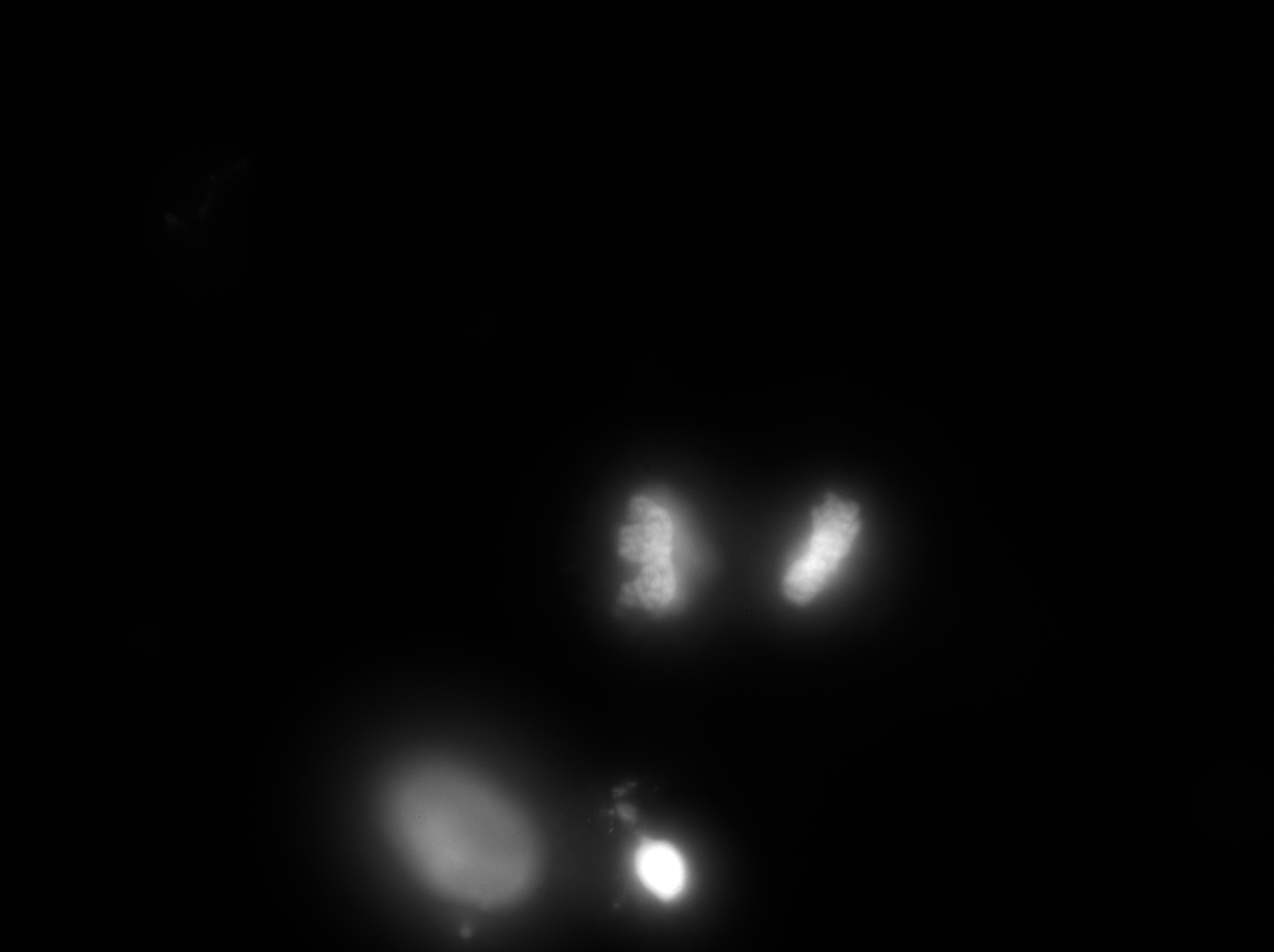

Supplement: Supplementary file 11 — Source data Fig. 3 part 1 [file 44319_2026_742_MOESM11_ESM.zip › Figure 3 Part 1/Fig 3b-e TTLL screen/TTLL1-GFP R1 I6 - 1.Project Maximum Z_XY1674164315_Z0_T0_C0.tif]

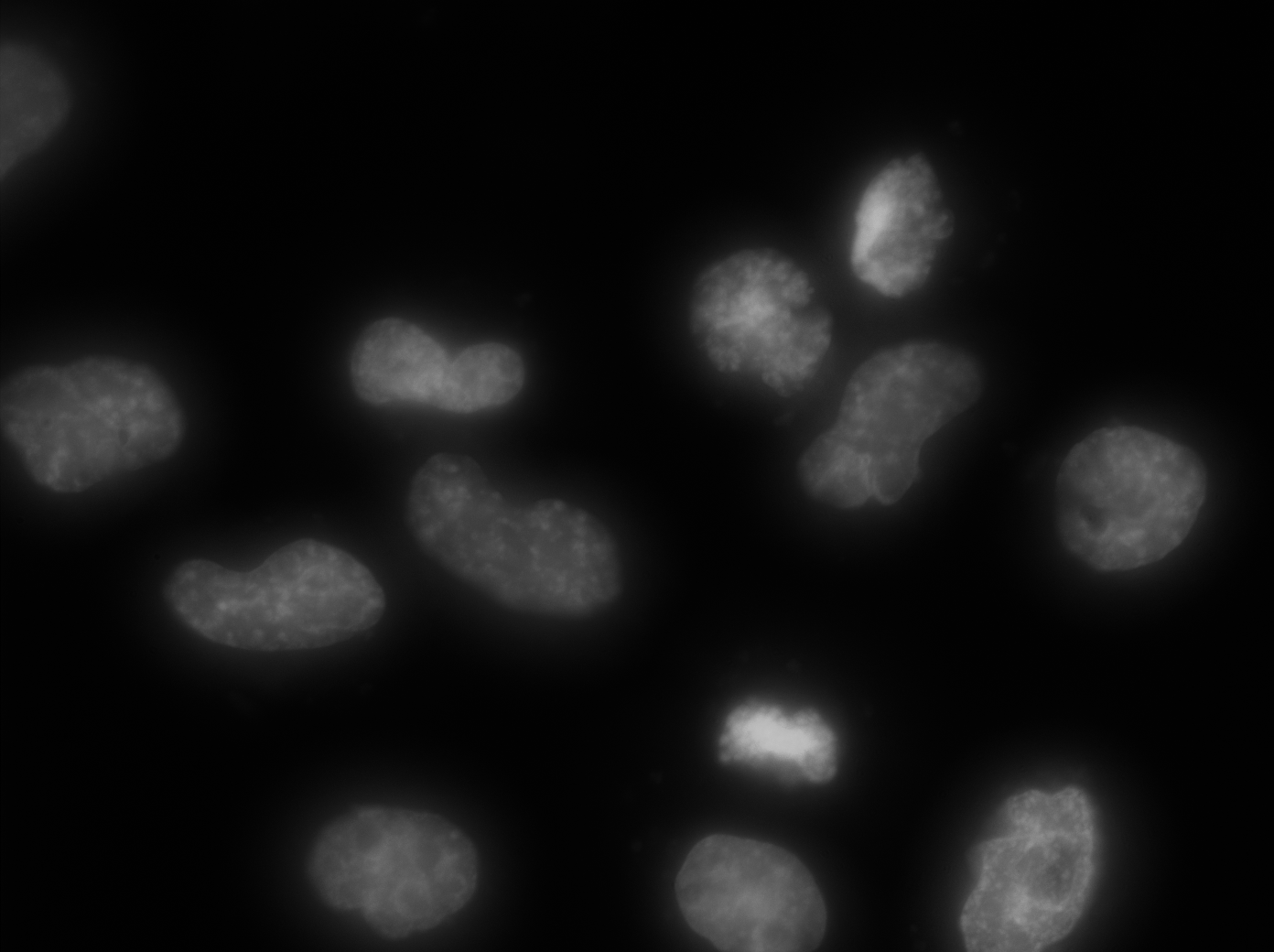

Supplement: Supplementary file 11 — Source data Fig. 3 part 1 [file 44319_2026_742_MOESM11_ESM.zip › Figure 3 Part 1/Fig 3b-e TTLL screen/TTLL1-GFP A3 I6.Project Maximum Z_XY1679695052_Z0_T0_C0.tif]

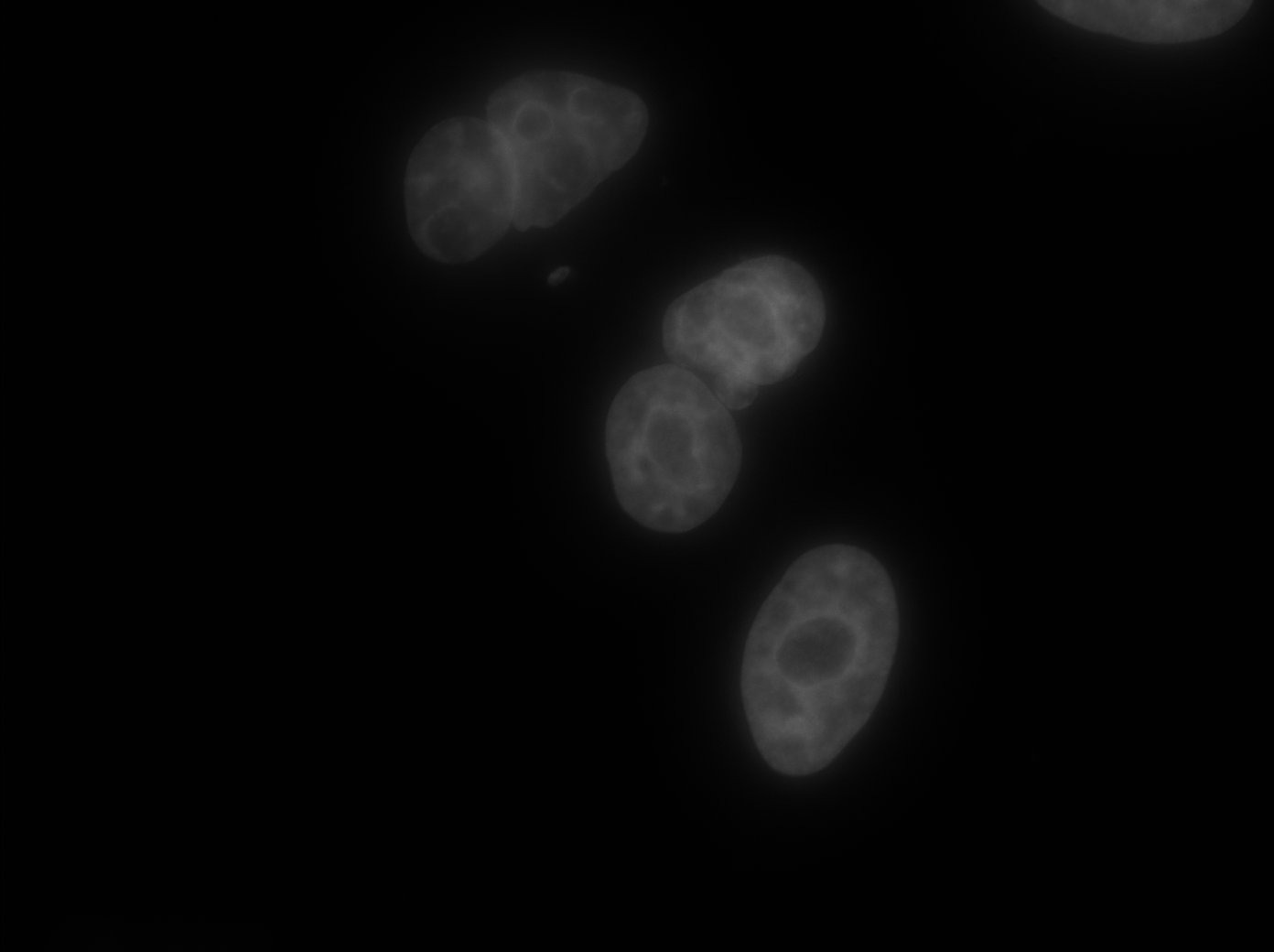

Supplement: Supplementary file 11 — Source data Fig. 3 part 1 [file 44319_2026_742_MOESM11_ESM.zip › Figure 3 Part 1/Fig 3b-e TTLL screen/EYFP MB multi I6.Project Maximum Z_XY1663875973_Z0_T0_C0.tif]

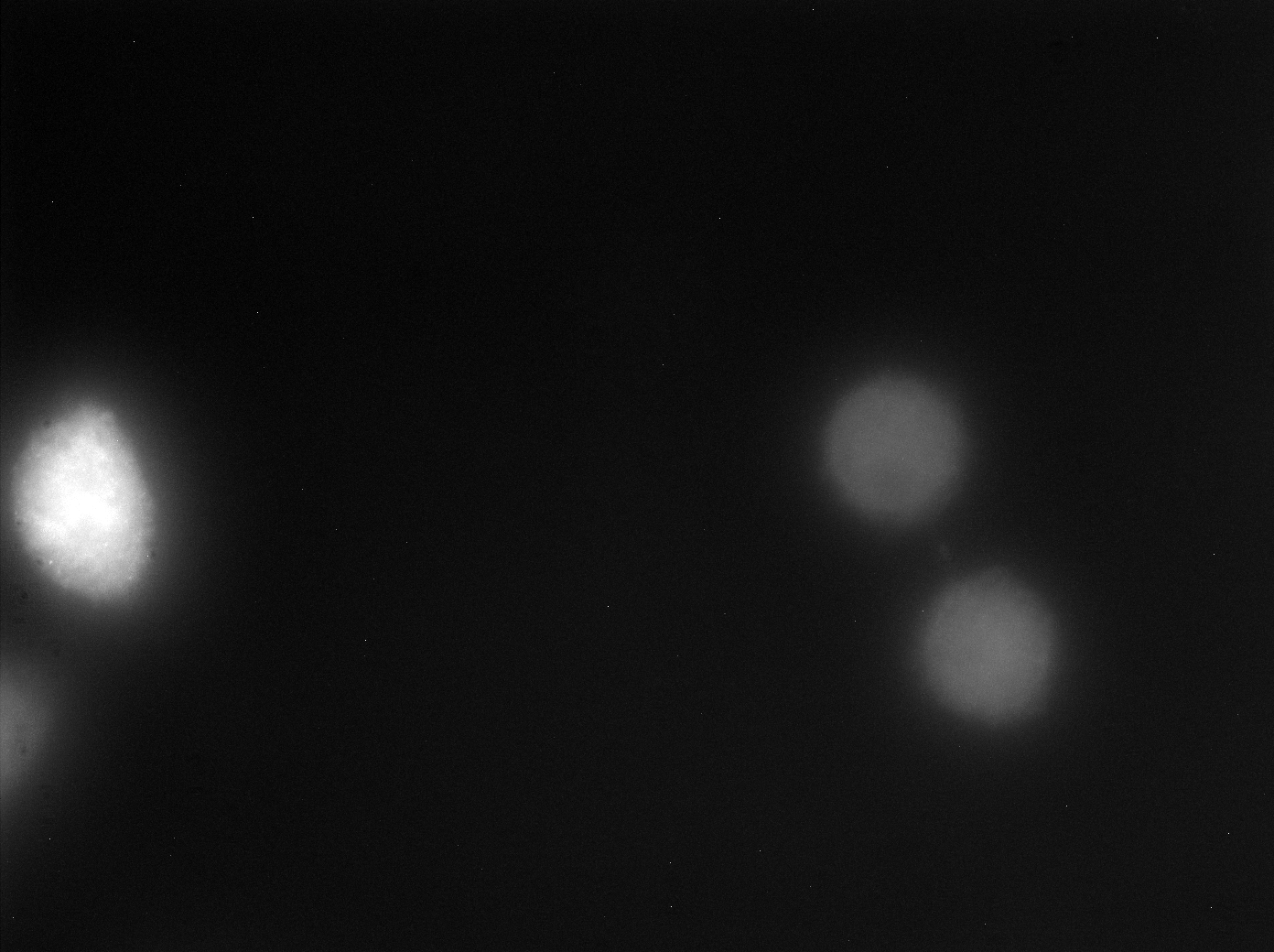

Supplement: Supplementary file 11 — Source data Fig. 3 part 1 [file 44319_2026_742_MOESM11_ESM.zip › Figure 3 Part 1/Fig 3b-e TTLL screen/TTLL1-GFP A3 I20.Project Maximum Z_XY1679698418_Z0_T0_C1.tif]

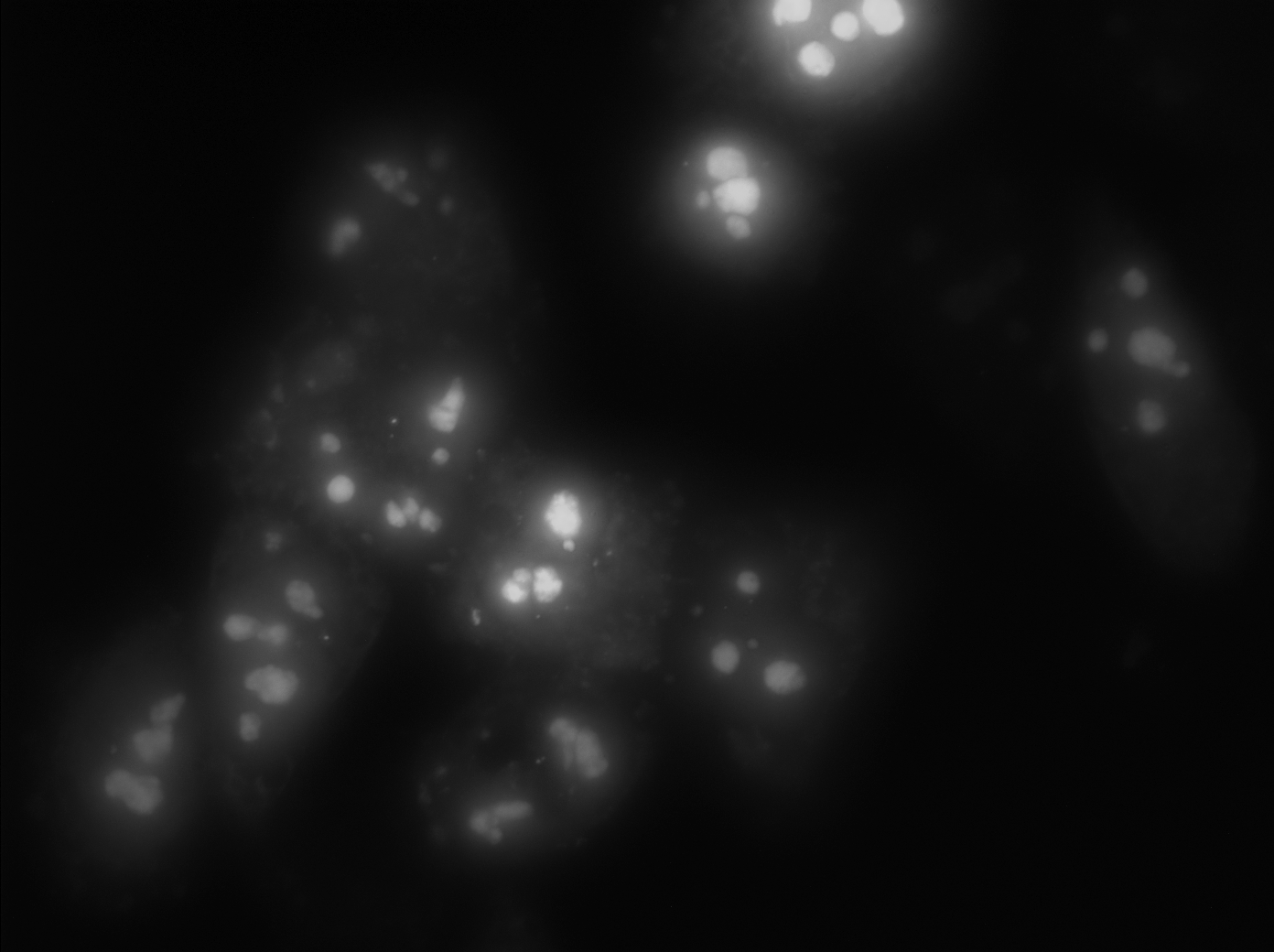

Supplement: Supplementary file 11 — Source data Fig. 3 part 1 [file 44319_2026_742_MOESM11_ESM.zip › Figure 3 Part 1/Fig 3b-e TTLL screen/TTLL4-YFPy I12.Project Maximum Z_XY1679336986_Z0_T0_C2.tif]

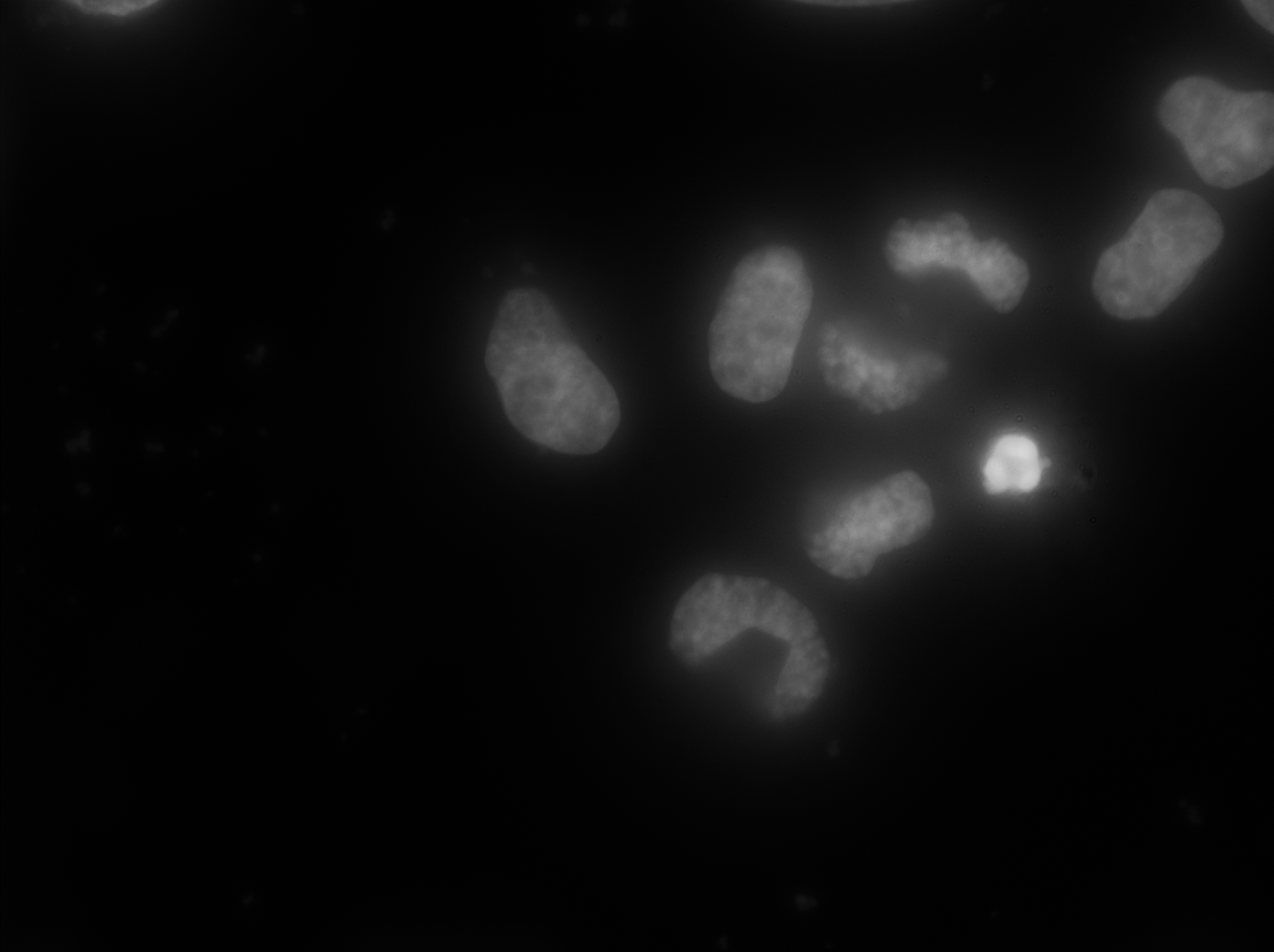

Supplement: Supplementary file 11 — Source data Fig. 3 part 1 [file 44319_2026_742_MOESM11_ESM.zip › Figure 3 Part 1/Fig 3b-e TTLL screen/TTLL1-GFP A3 I1 - 1.Project Maximum Z_XY1679694090_Z0_T0_C0.tif]

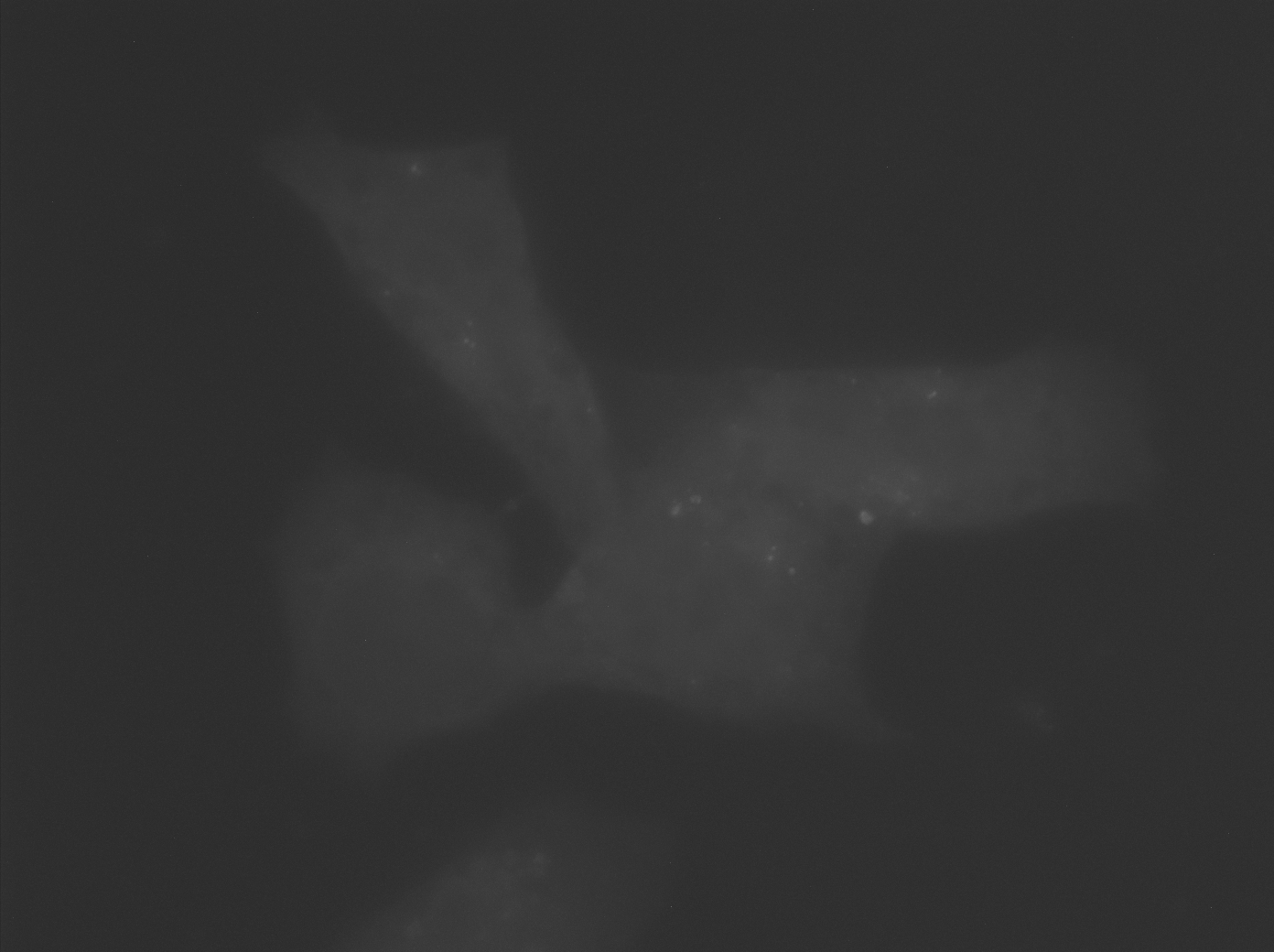

Supplement: Supplementary file 11 — Source data Fig. 3 part 1 [file 44319_2026_742_MOESM11_ESM.zip › Figure 3 Part 1/Fig 3b-e TTLL screen/TTLL1-GFP A4 I5.Project Maximum Z_XY1675962213_Z0_T0_C1.tif]

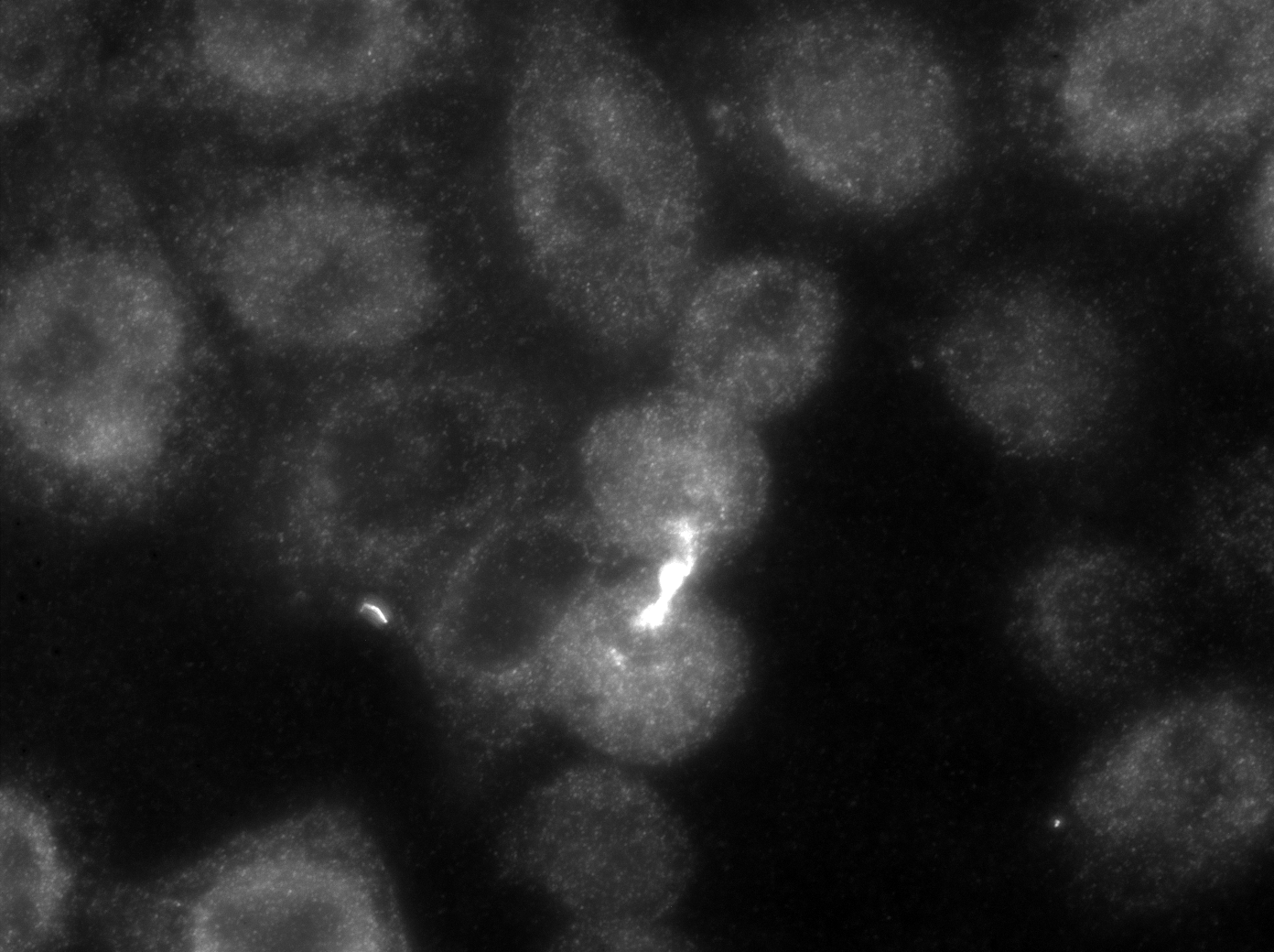

Supplement: Supplementary file 11 — Source data Fig. 3 part 1 [file 44319_2026_742_MOESM11_ESM.zip › Figure 3 Part 1/Fig 3b-e TTLL screen/TTLL1-GFP A3 I16.Project Maximum Z_XY1679697481_Z0_T0_C2.tif]

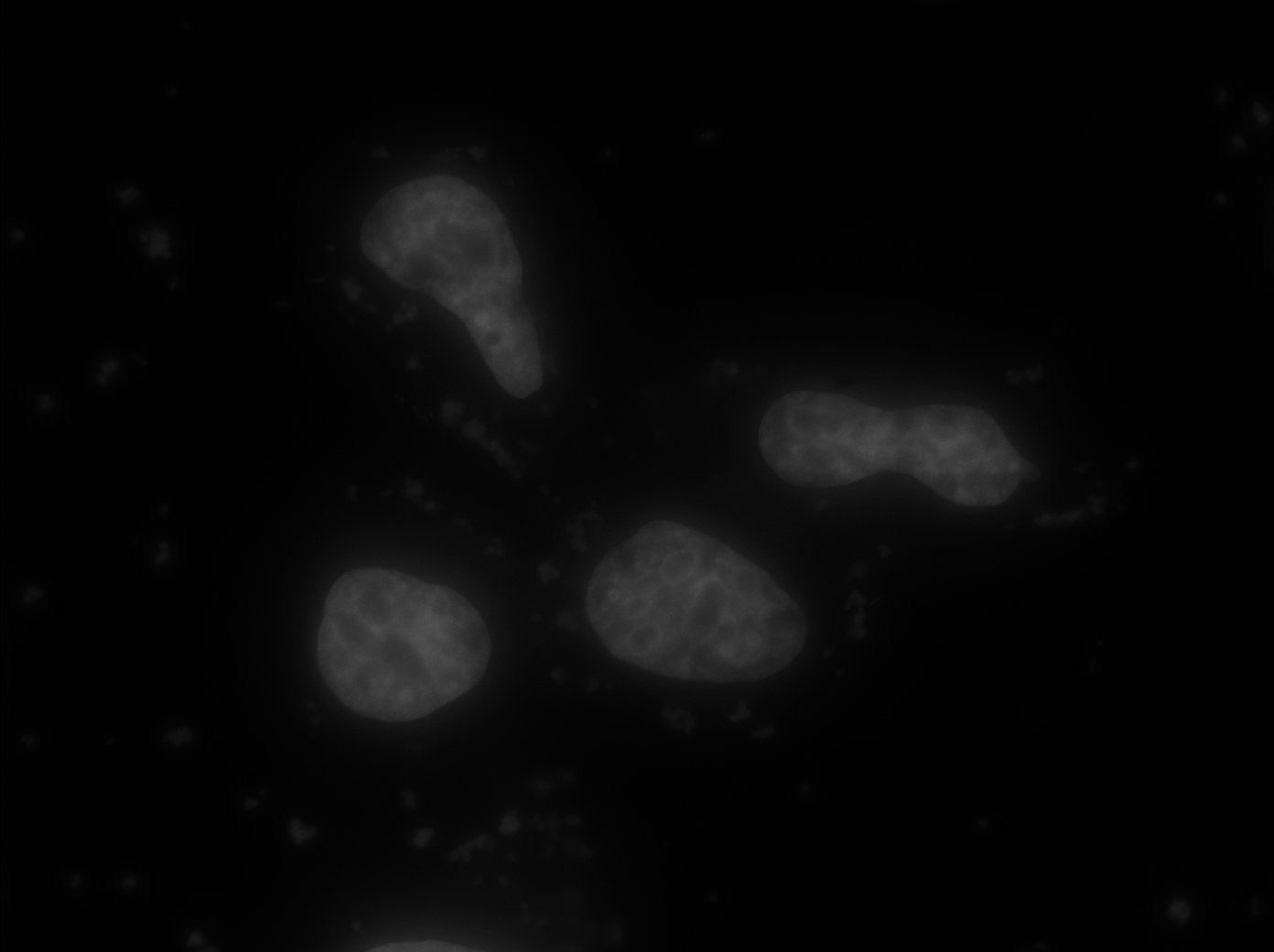

Supplement: Supplementary file 11 — Source data Fig. 3 part 1 [file 44319_2026_742_MOESM11_ESM.zip › Figure 3 Part 1/Fig 3b-e TTLL screen/TTLL1-GFP A4 I5.Project Maximum Z_XY1675962213_Z0_T0_C0.tif]

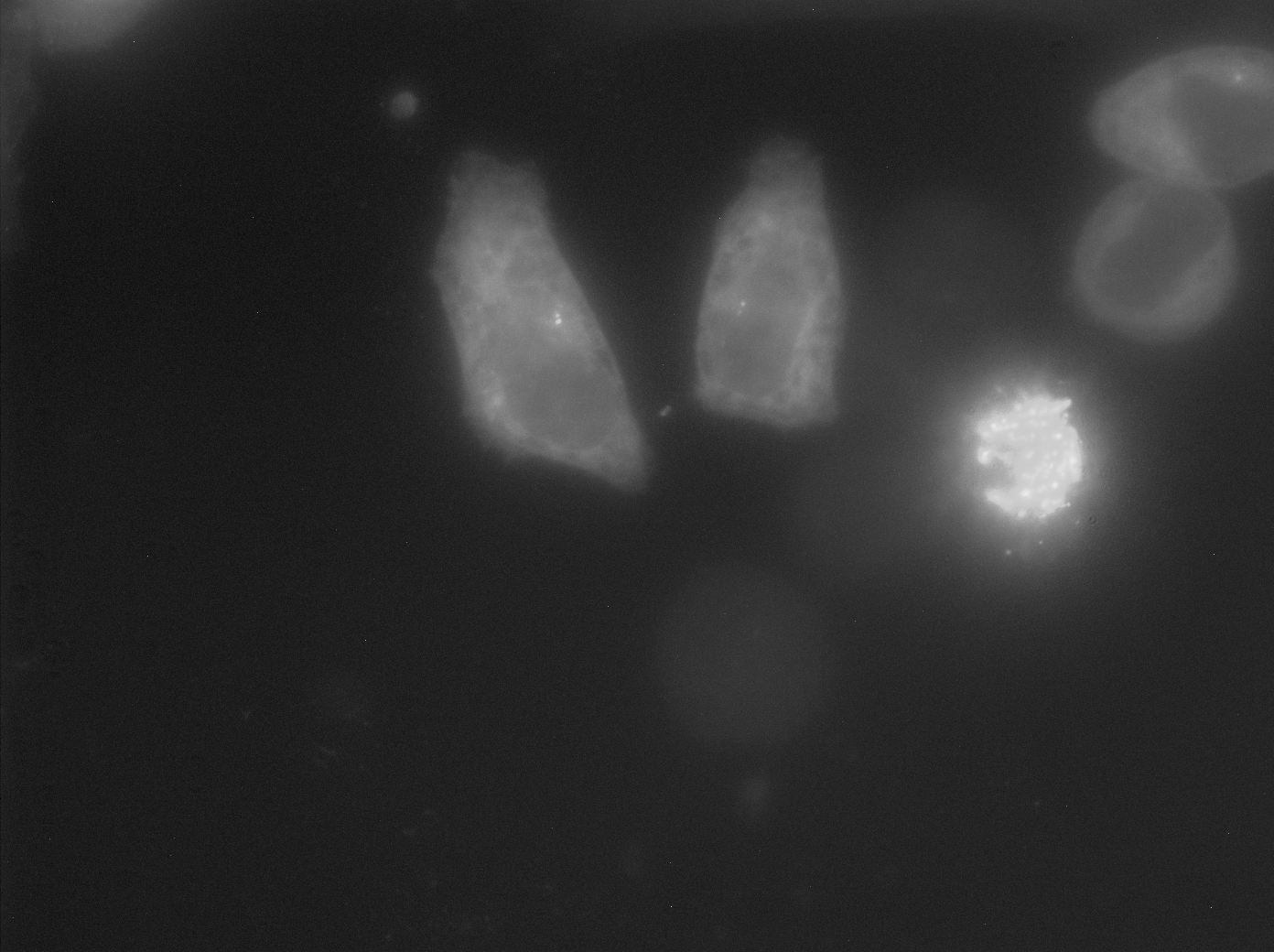

Supplement: Supplementary file 11 — Source data Fig. 3 part 1 [file 44319_2026_742_MOESM11_ESM.zip › Figure 3 Part 1/Fig 3b-e TTLL screen/TTLL1-GFP A3 I1 - 1.Project Maximum Z_XY1679694090_Z0_T0_C1.tif]

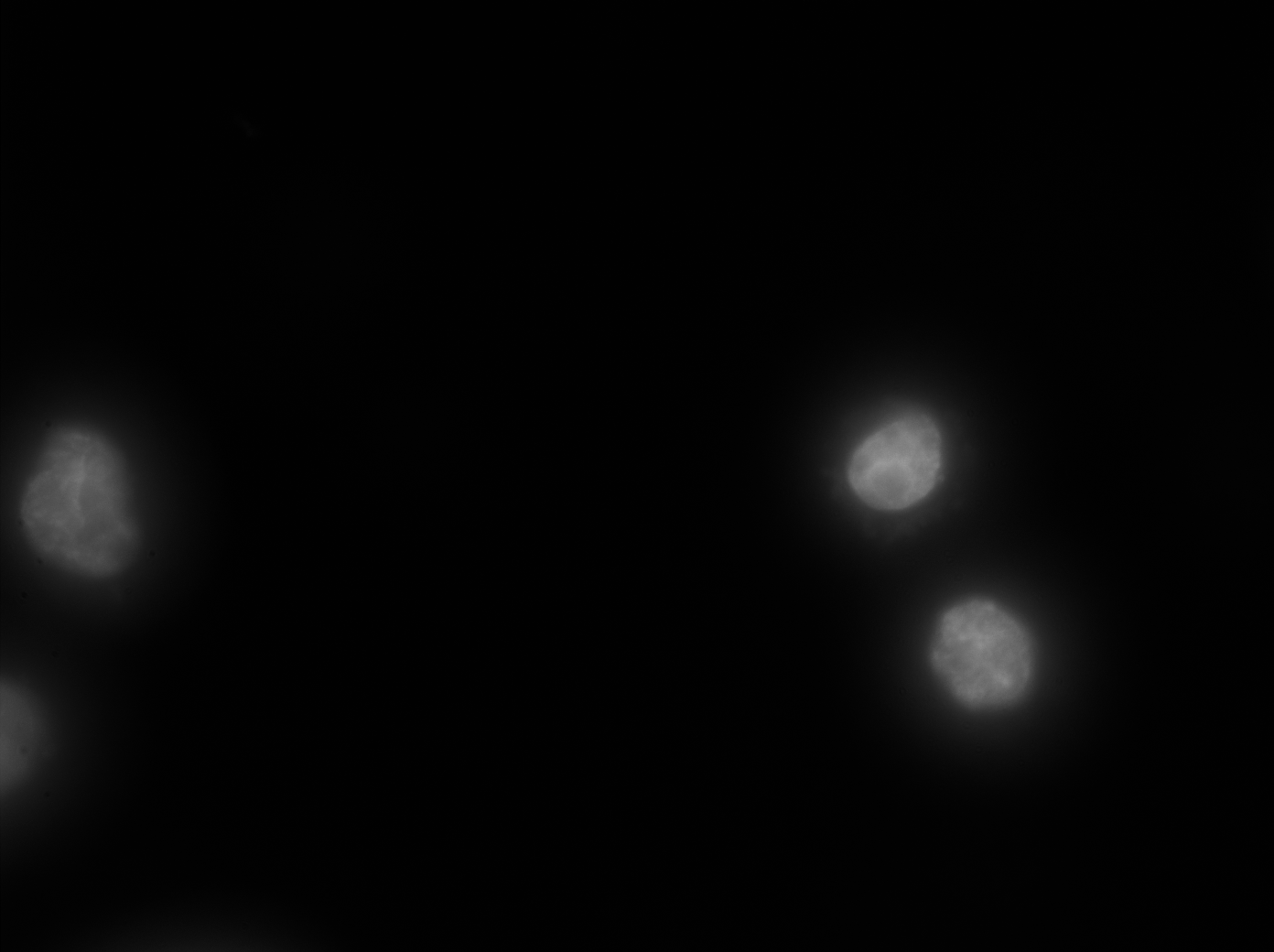

Supplement: Supplementary file 11 — Source data Fig. 3 part 1 [file 44319_2026_742_MOESM11_ESM.zip › Figure 3 Part 1/Fig 3b-e TTLL screen/TTLL1-GFP A3 I20.Project Maximum Z_XY1679698418_Z0_T0_C0.tif]

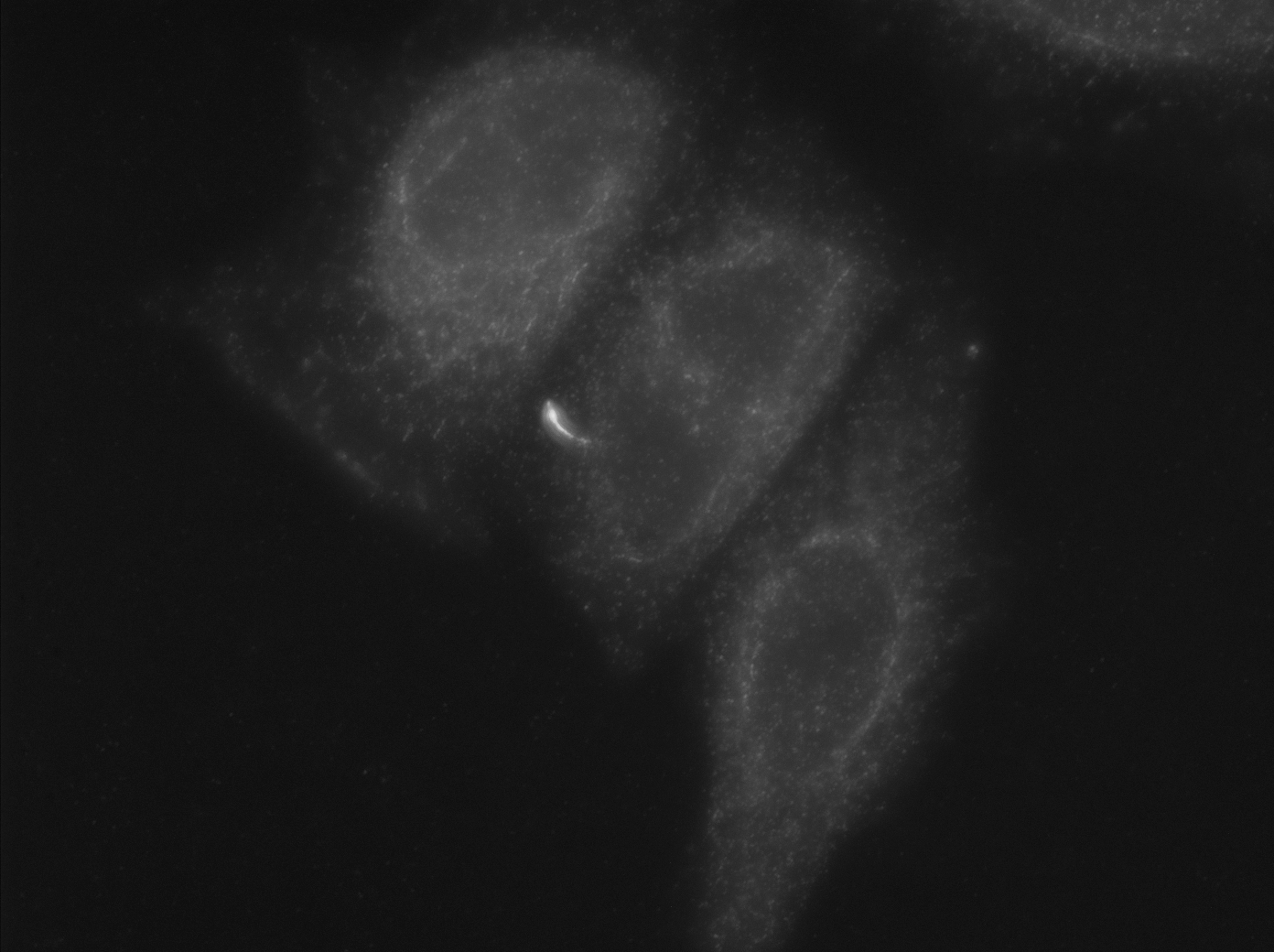

Supplement: Supplementary file 11 — Source data Fig. 3 part 1 [file 44319_2026_742_MOESM11_ESM.zip › Figure 3 Part 1/Fig 3b-e TTLL screen/EYFP MB multi I6.Project Maximum Z_XY1663875973_Z0_T0_C1.tif]

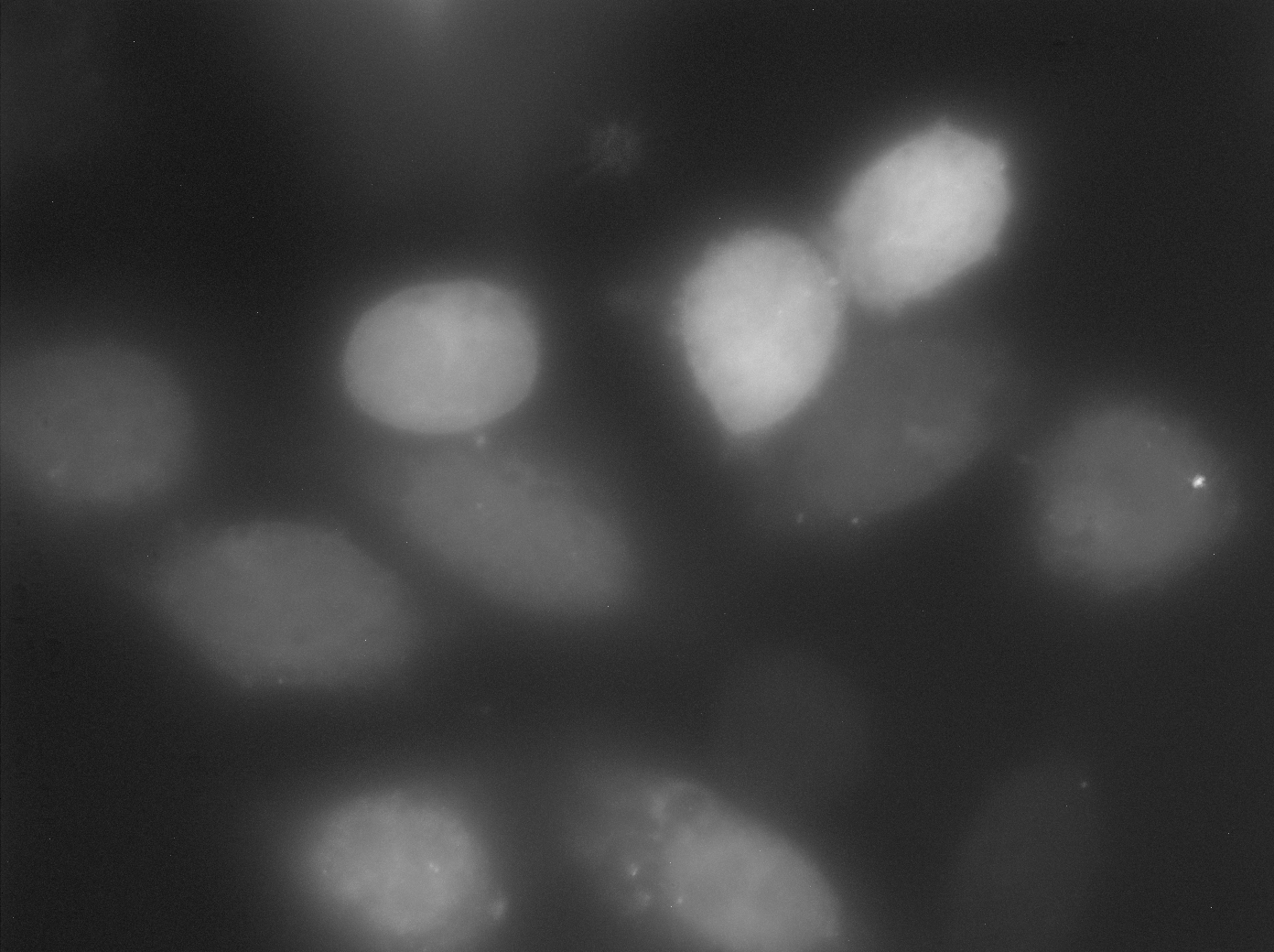

Supplement: Supplementary file 11 — Source data Fig. 3 part 1 [file 44319_2026_742_MOESM11_ESM.zip › Figure 3 Part 1/Fig 3b-e TTLL screen/TTLL1-GFP A3 I6.Project Maximum Z_XY1679695052_Z0_T0_C1.tif]

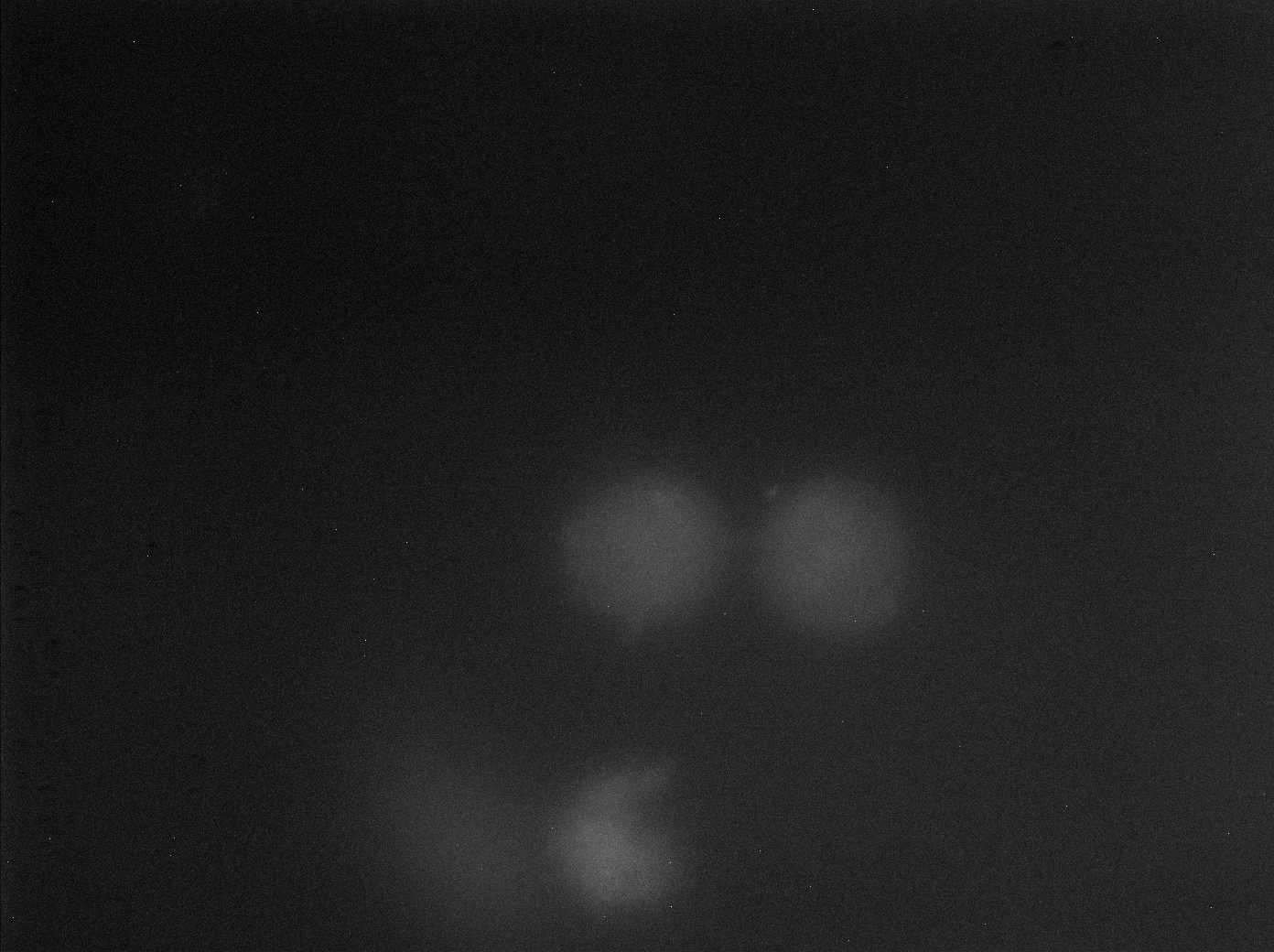

Supplement: Supplementary file 11 — Source data Fig. 3 part 1 [file 44319_2026_742_MOESM11_ESM.zip › Figure 3 Part 1/Fig 3b-e TTLL screen/TTLL1-GFP R1 I6 - 1.Project Maximum Z_XY1674164315_Z0_T0_C1.tif]

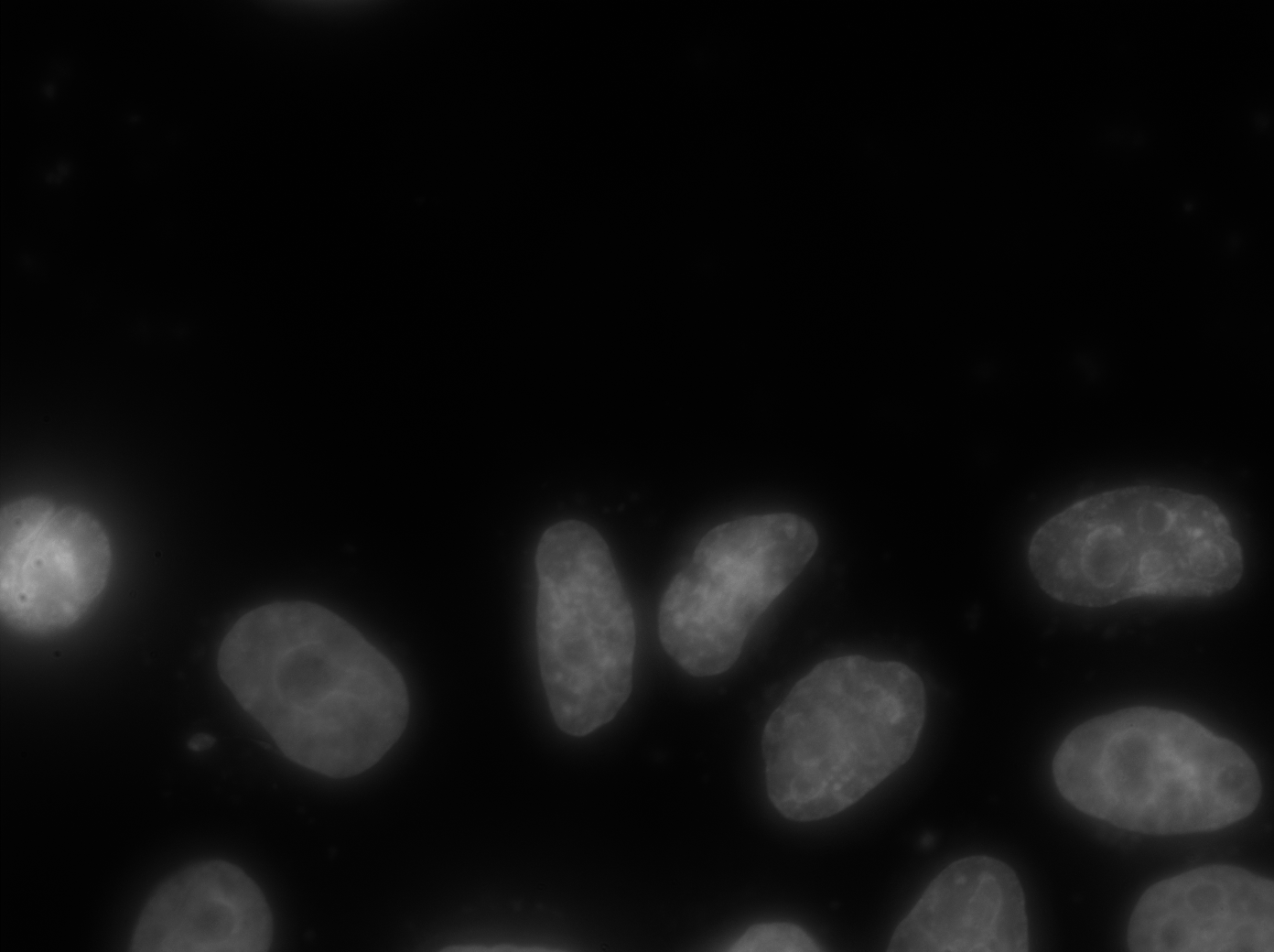

Supplement: Supplementary file 11 — Source data Fig. 3 part 1 [file 44319_2026_742_MOESM11_ESM.zip › Figure 3 Part 1/Fig 3b-e TTLL screen/TTLL4-YFPy I10.Project Maximum Z_XY1679082008_Z0_T0_C0.tif]

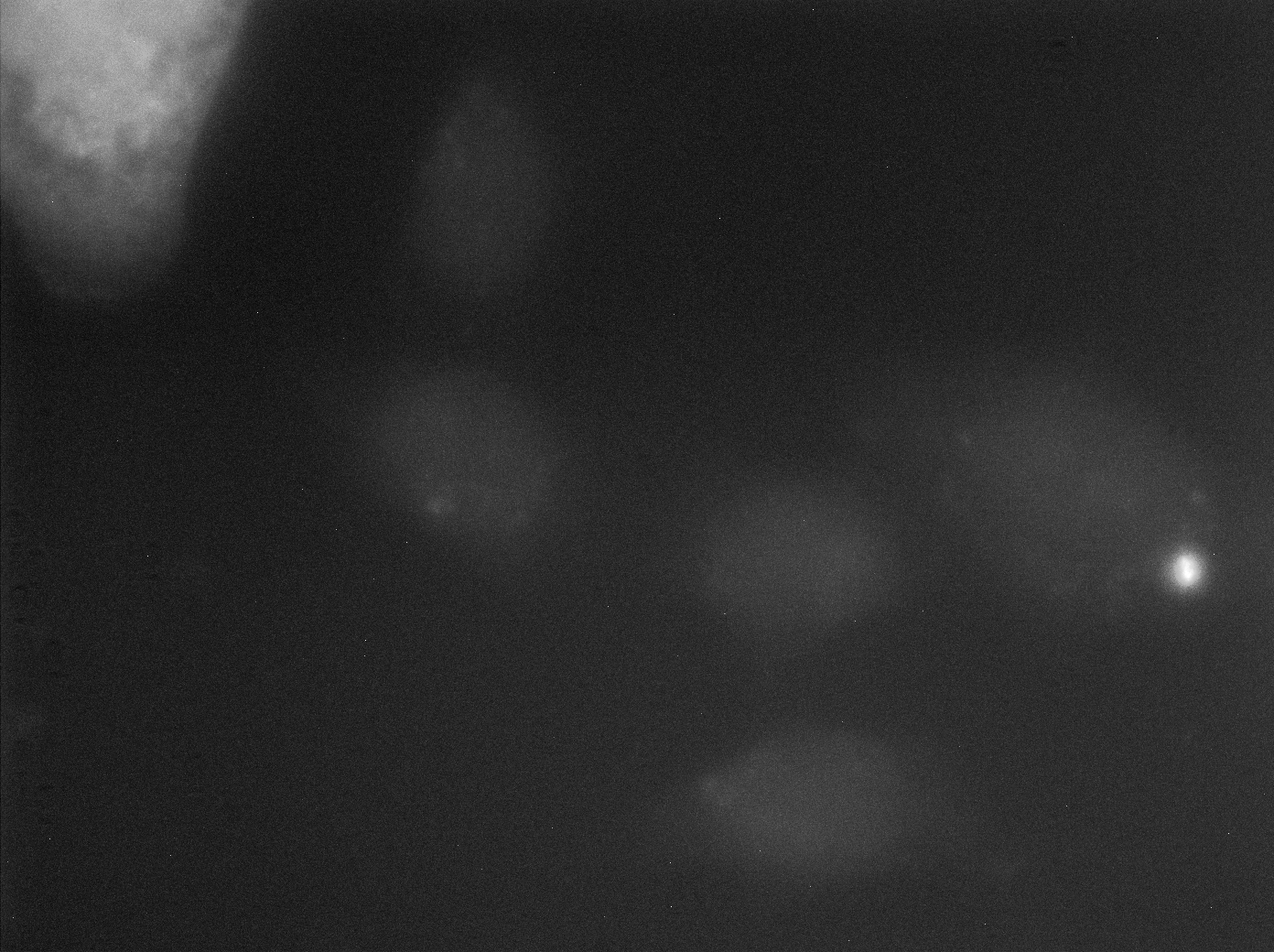

Supplement: Supplementary file 11 — Source data Fig. 3 part 1 [file 44319_2026_742_MOESM11_ESM.zip › Figure 3 Part 1/Fig 3b-e TTLL screen/TTLL1-GFP R1 I3.Project Maximum Z_XY1674163711_Z0_T0_C1.tif]

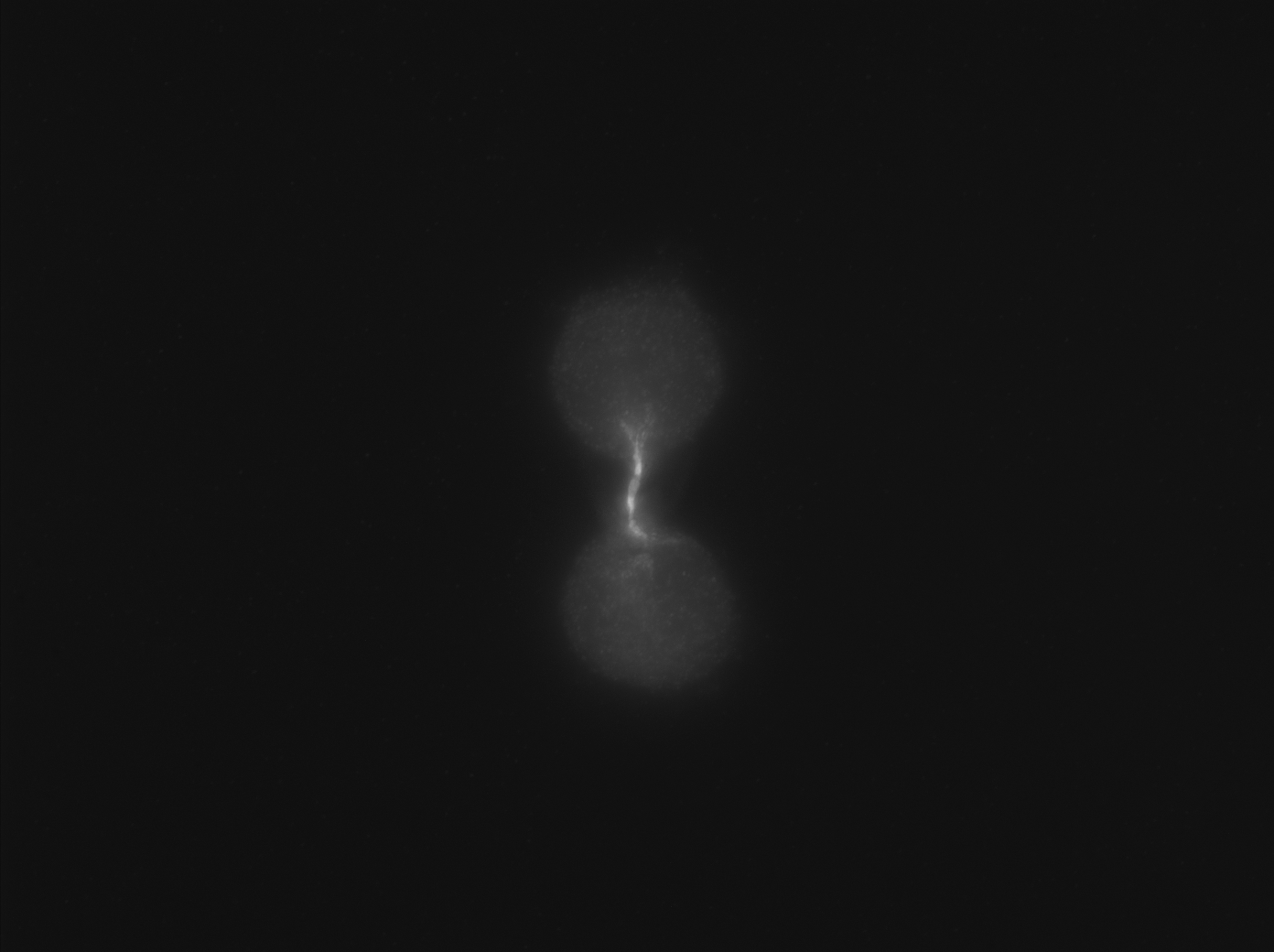

Supplement: Supplementary file 11 — Source data Fig. 3 part 1 [file 44319_2026_742_MOESM11_ESM.zip › Figure 3 Part 1/Fig 3b-e TTLL screen/TTLL1-GFP A3 I1.Project Maximum Z_XY1674673539_Z0_T0_C2.tif]

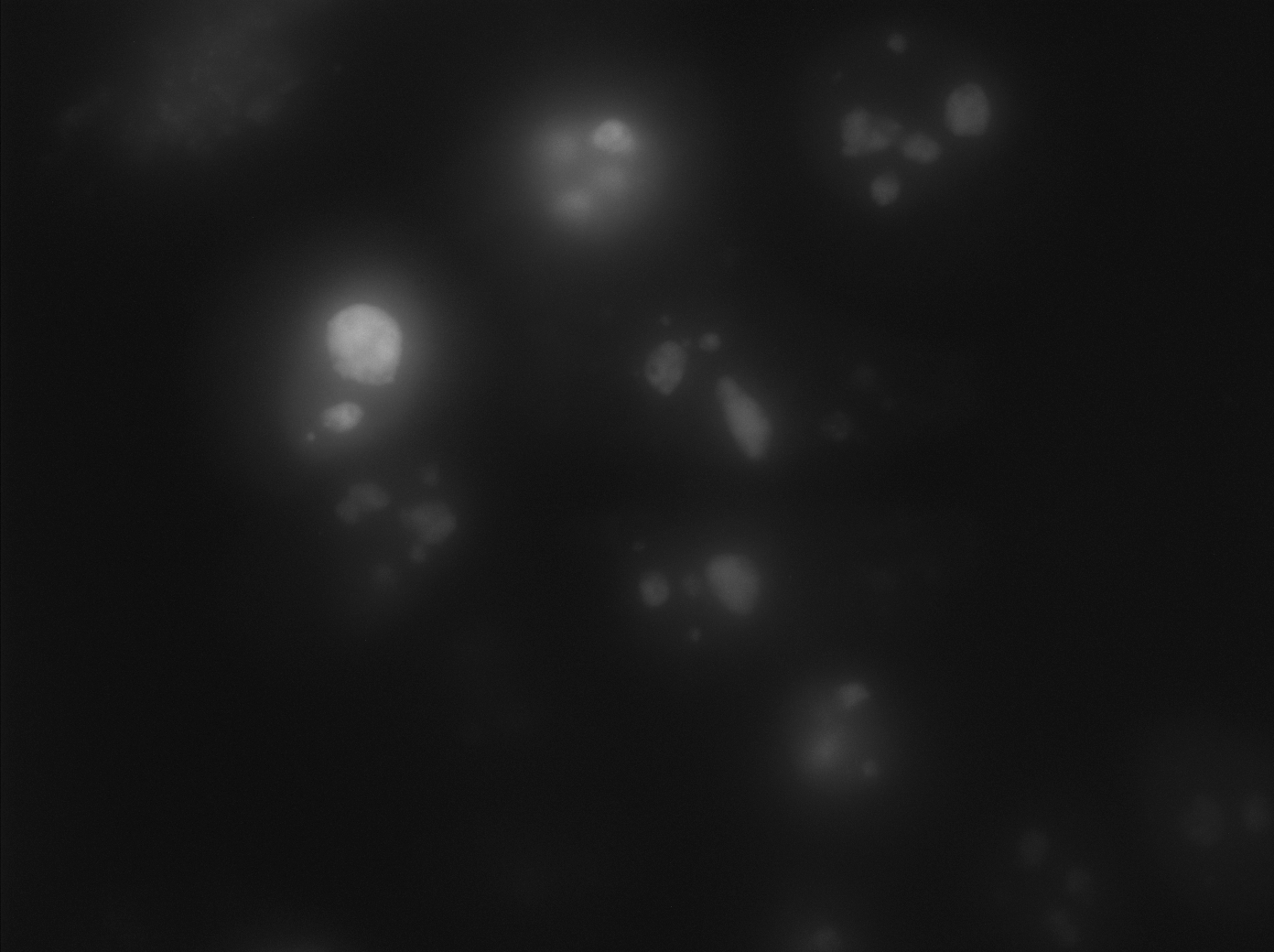

Supplement: Supplementary file 11 — Source data Fig. 3 part 1 [file 44319_2026_742_MOESM11_ESM.zip › Figure 3 Part 1/Fig 3b-e TTLL screen/TTLL4-YFPy I4.Project Maximum Z_XY1679075937_Z0_T0_C1.tif]

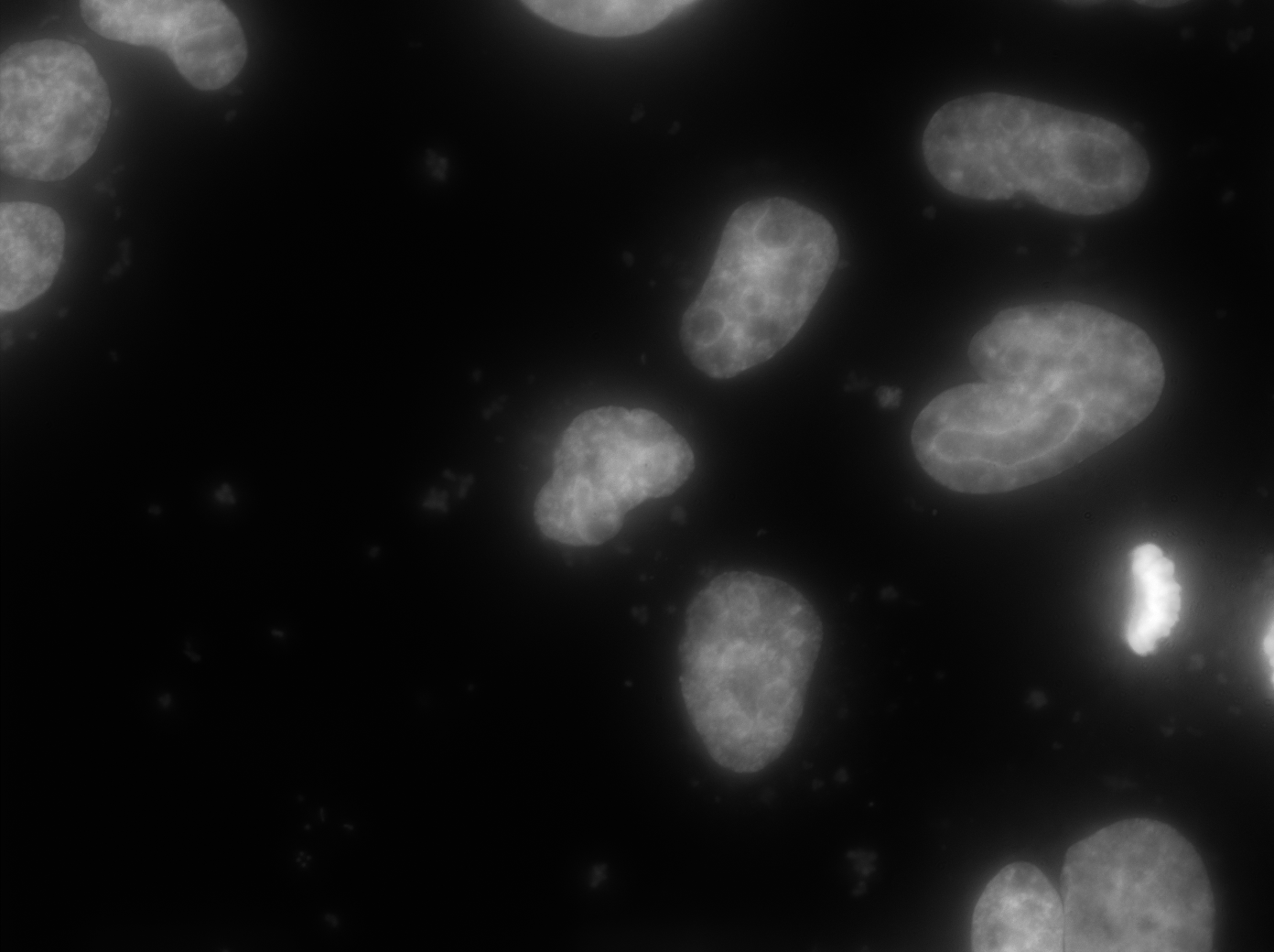

Supplement: Supplementary file 11 — Source data Fig. 3 part 1 [file 44319_2026_742_MOESM11_ESM.zip › Figure 3 Part 1/Fig 3b-e TTLL screen/TTLL1-GFP A4 I10.Project Maximum Z_XY1675963296_Z0_T0_C0.tif]

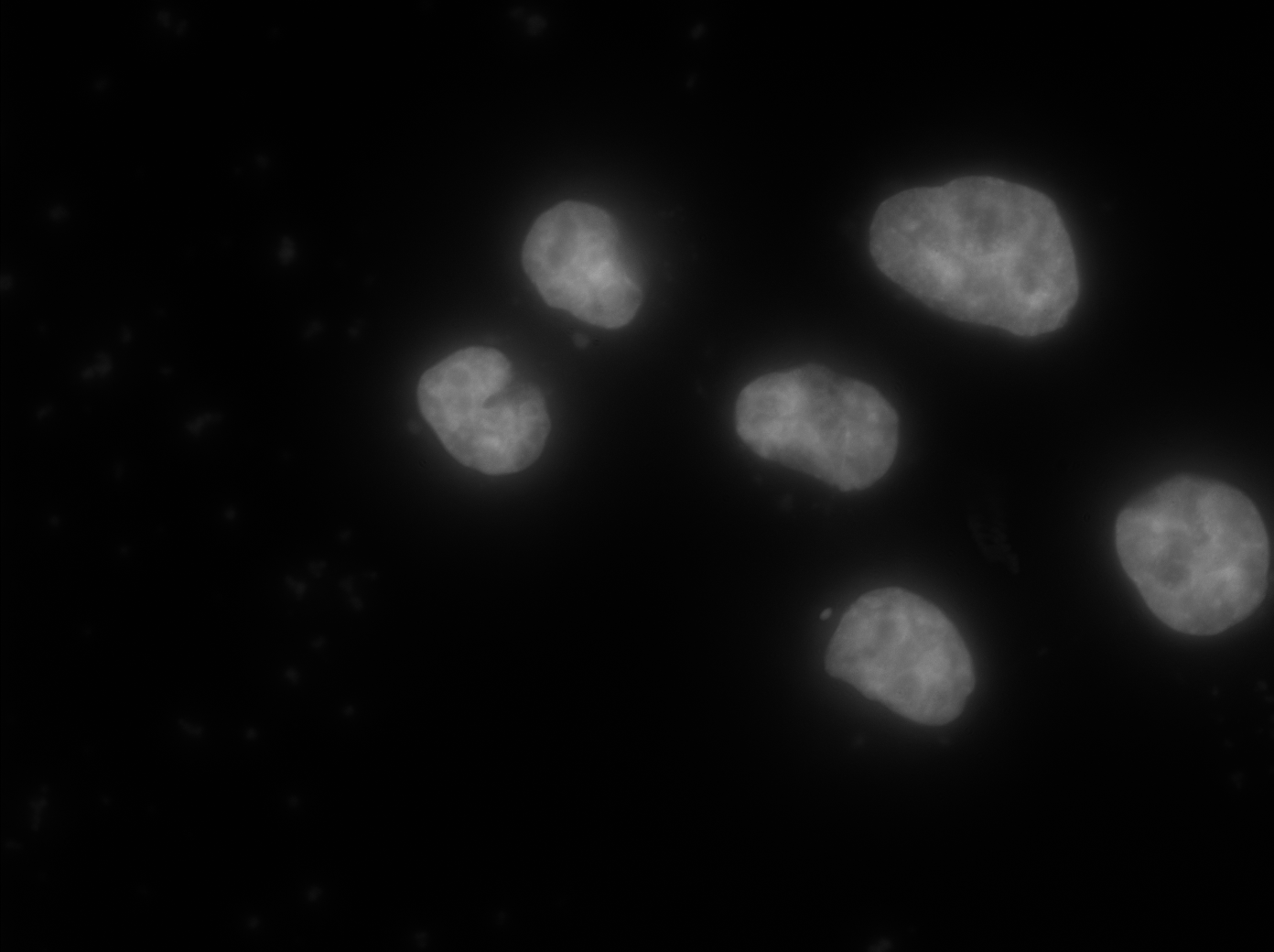

Supplement: Supplementary file 11 — Source data Fig. 3 part 1 [file 44319_2026_742_MOESM11_ESM.zip › Figure 3 Part 1/Fig 3b-e TTLL screen/TTLL4-YFPy I17.Project Maximum Z_XY1679337716_Z0_T0_C0.tif]

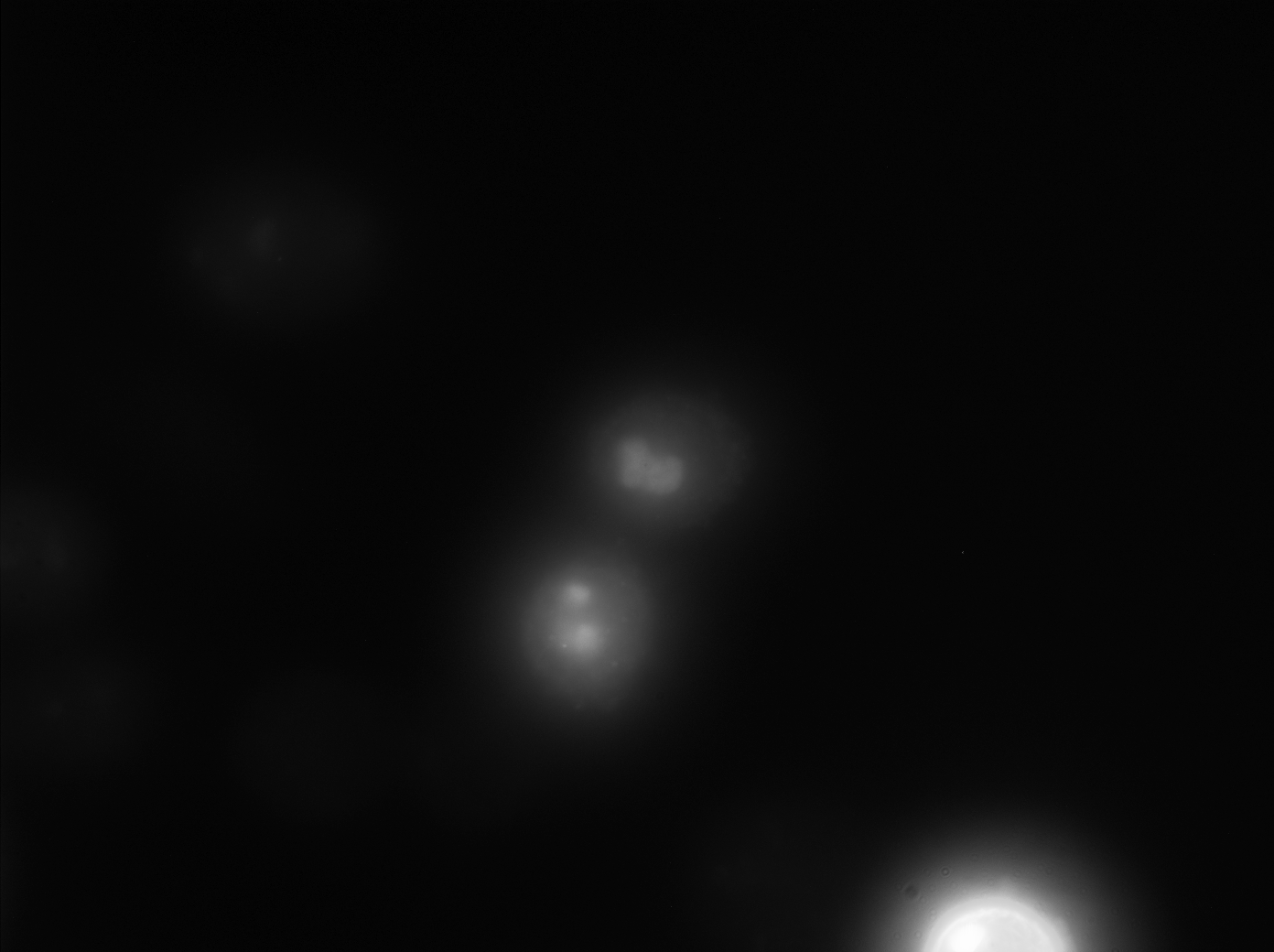

Supplement: Supplementary file 11 — Source data Fig. 3 part 1 [file 44319_2026_742_MOESM11_ESM.zip › Figure 3 Part 1/Fig 3b-e TTLL screen/TTLL4-YFPy I13.Project Maximum Z_XY1679337113_Z0_T0_C2.tif]

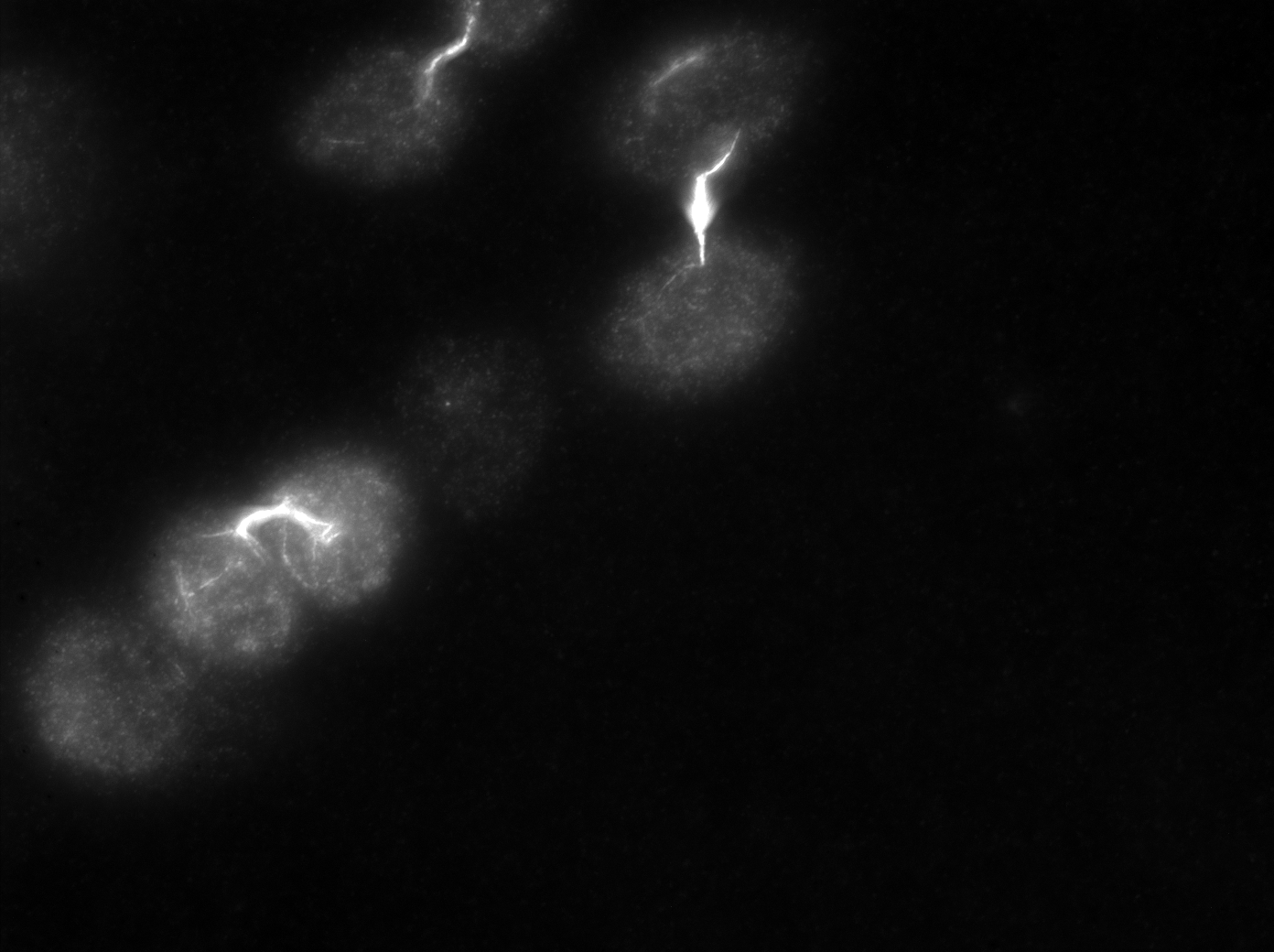

Supplement: Supplementary file 11 — Source data Fig. 3 part 1 [file 44319_2026_742_MOESM11_ESM.zip › Figure 3 Part 1/Fig 3b-e TTLL screen/TTLL1-GFP A3 I17.Project Maximum Z_XY1679697836_Z0_T0_C2.tif]

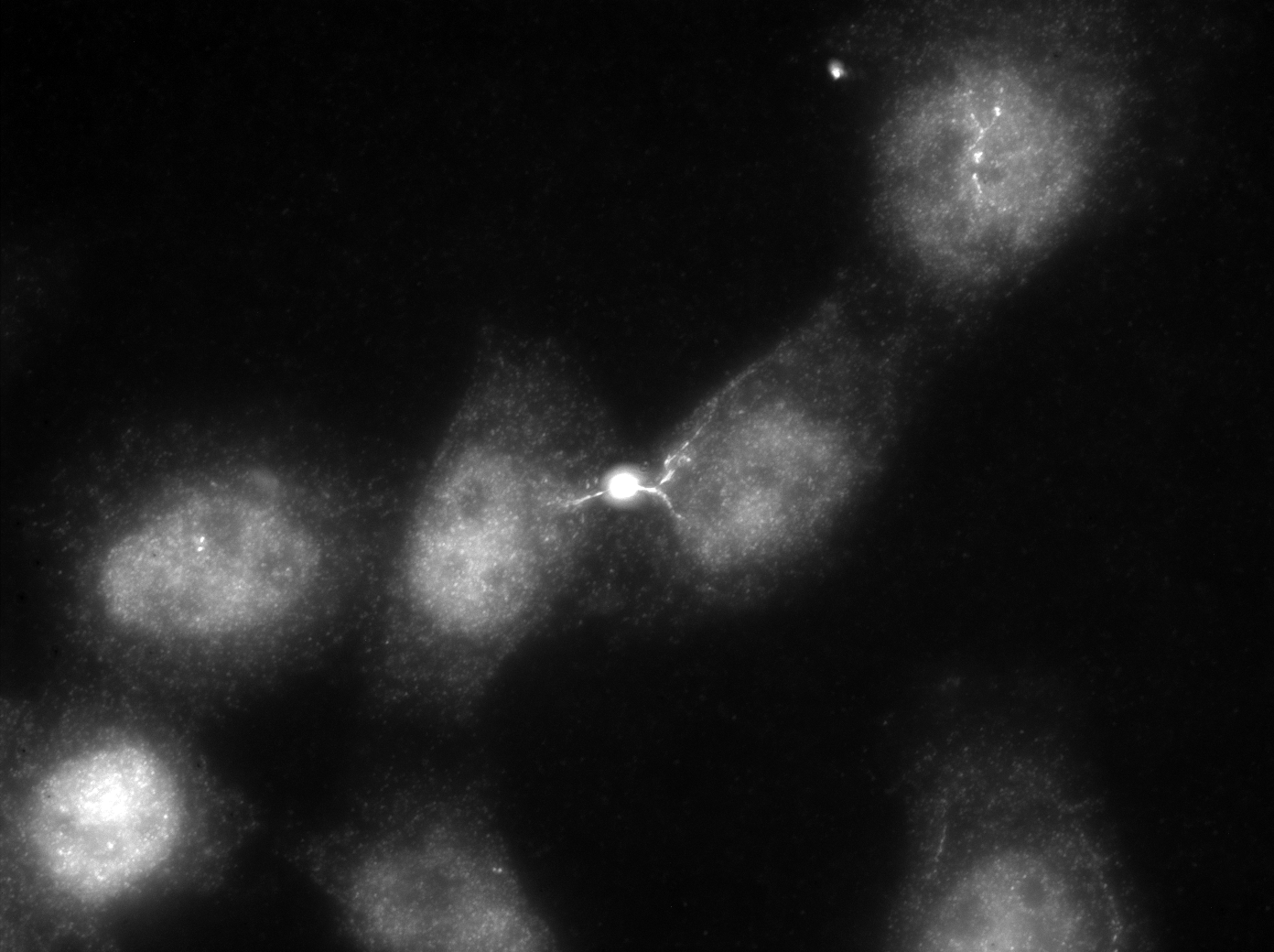

Supplement: Supplementary file 11 — Source data Fig. 3 part 1 [file 44319_2026_742_MOESM11_ESM.zip › Figure 3 Part 1/Fig 3b-e TTLL screen/TTLL1-GFP A3 I11.Project Maximum Z_XY1679695838_Z0_T0_C2.tif]

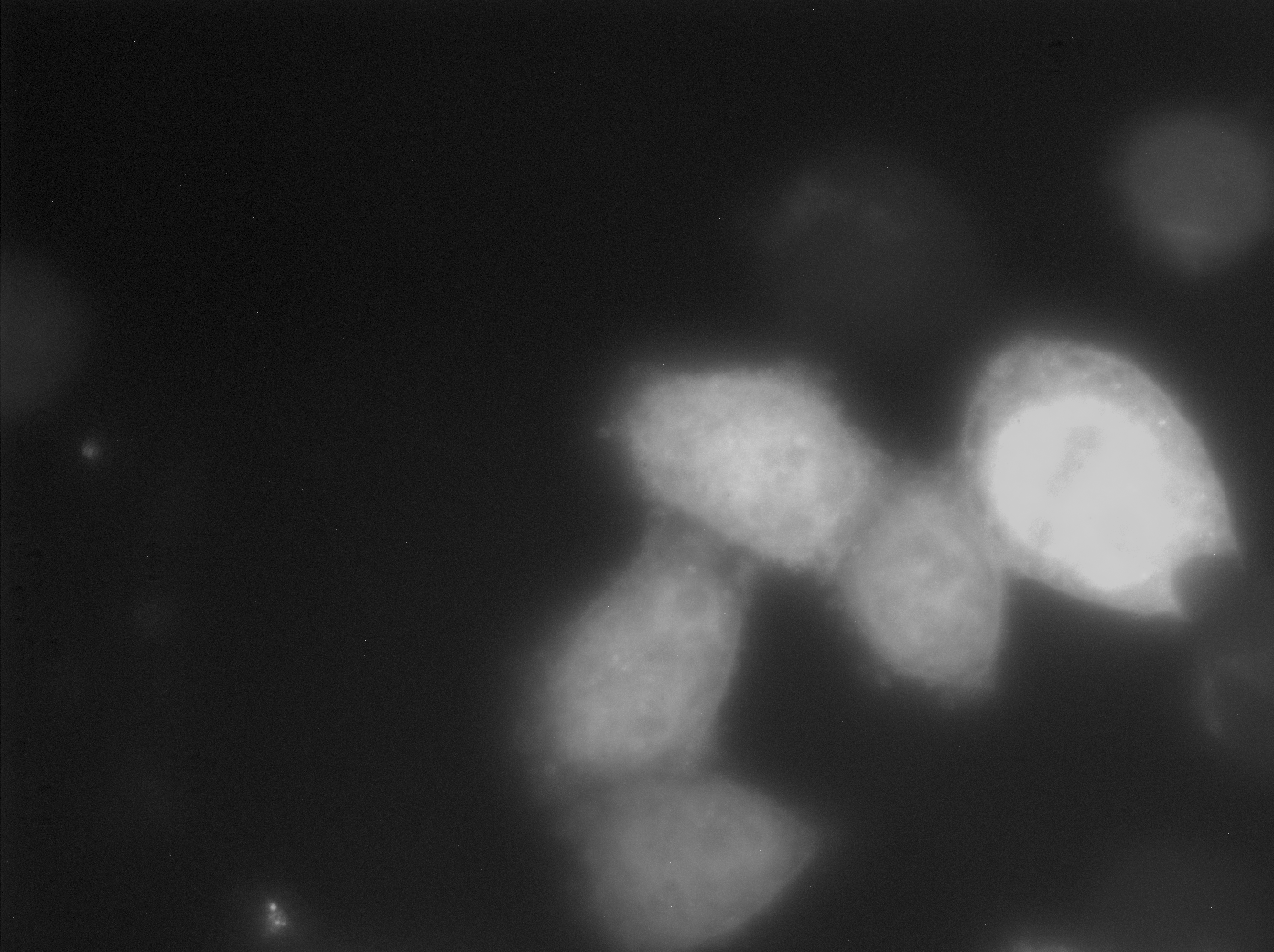

Supplement: Supplementary file 11 — Source data Fig. 3 part 1 [file 44319_2026_742_MOESM11_ESM.zip › Figure 3 Part 1/Fig 3b-e TTLL screen/TTLL1-GFPy I2.Project Maximum Z_XY1679086410_Z0_T0_C1.tif]

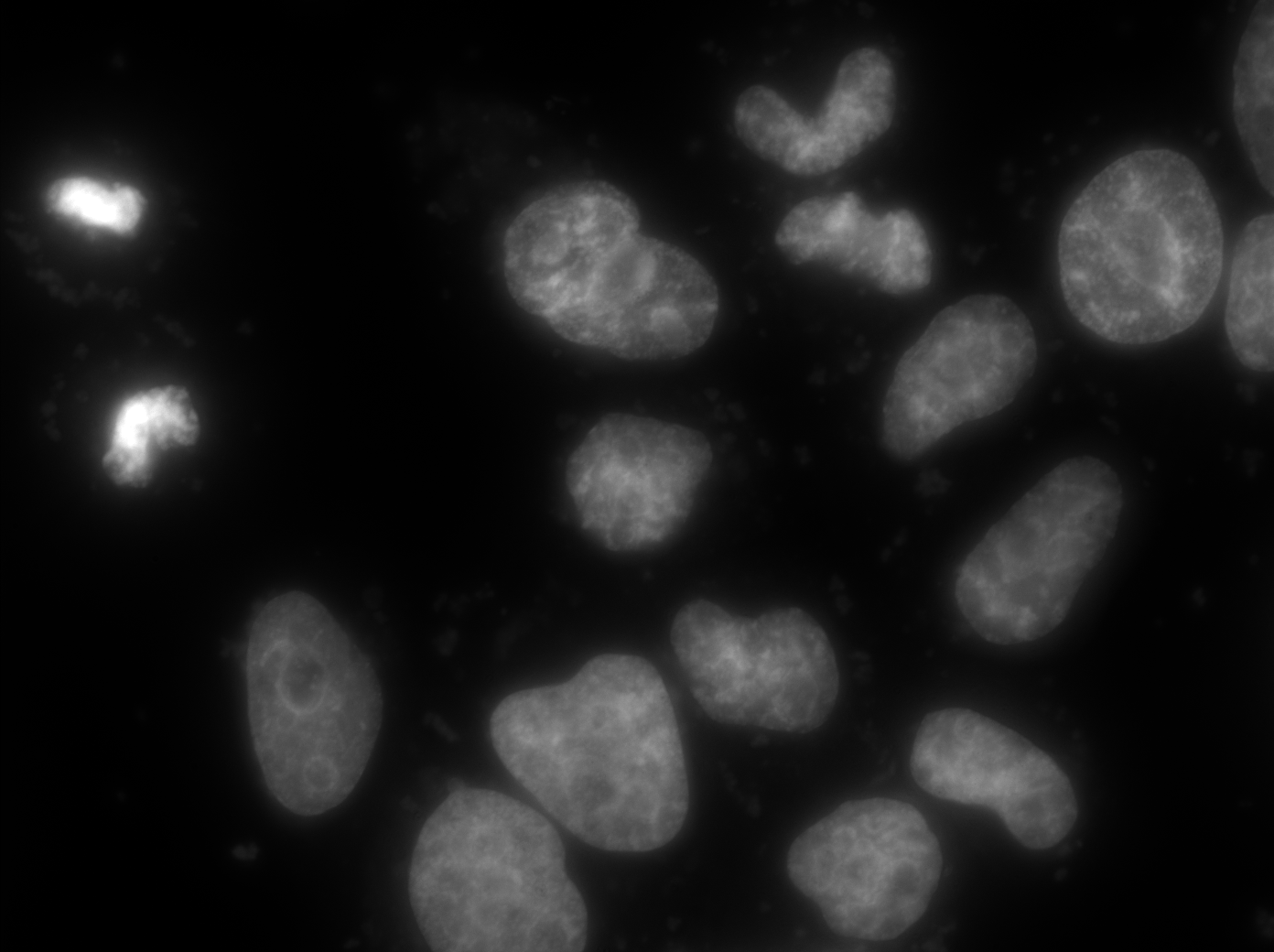

Supplement: Supplementary file 11 — Source data Fig. 3 part 1 [file 44319_2026_742_MOESM11_ESM.zip › Figure 3 Part 1/Fig 3b-e TTLL screen/TTLL1-GFP A4 I9.Project Maximum Z_XY1675963165_Z0_T0_C0.tif]

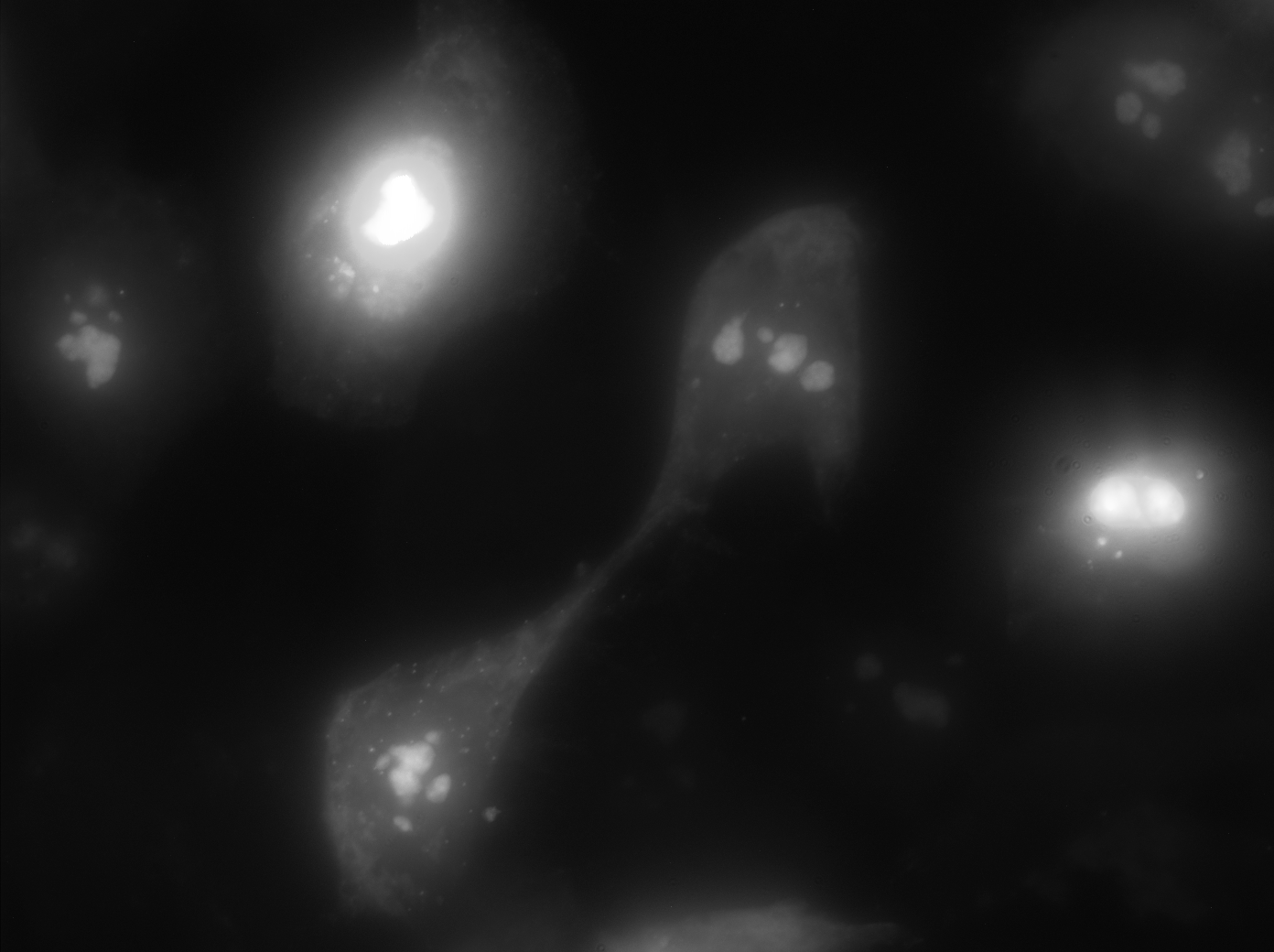

Supplement: Supplementary file 11 — Source data Fig. 3 part 1 [file 44319_2026_742_MOESM11_ESM.zip › Figure 3 Part 1/Fig 3b-e TTLL screen/TTLL4-YFPy I20.Project Maximum Z_XY1679338160_Z0_T0_C2.tif]
