# Supplementary figures and images for: TPGS1 regulates central spindle microtubule glutamylation and remodeling during telophase and abscission (part 14 of 36)
Source: EMBO Rep. 2026 Mar 23;27(8):1944–63. doi: 10.1038/s44319-026-00742-3 (PMC13121839; doi:10.1038/s44319-026-00742-3)

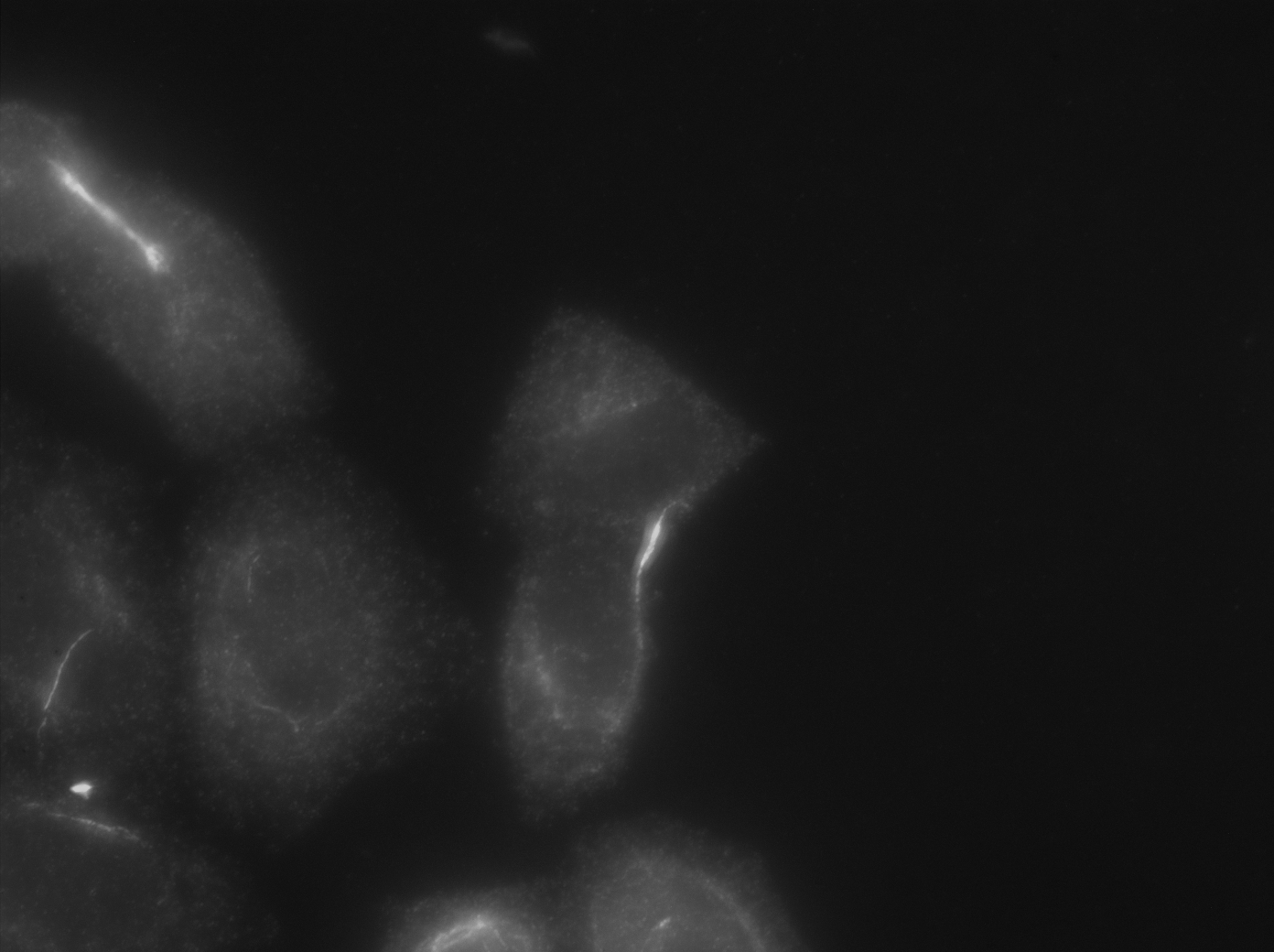

Supplement: Supplementary file 12 — Source data Fig. 3 part 2 [file 44319_2026_742_MOESM12_ESM.zip › Figure 3 Part 2/Fig 3b-e TTLL screen part 2/TTLL7-YFPy I16.Project Maximum Z_XY1679090685_Z0_T0_C1.tif]

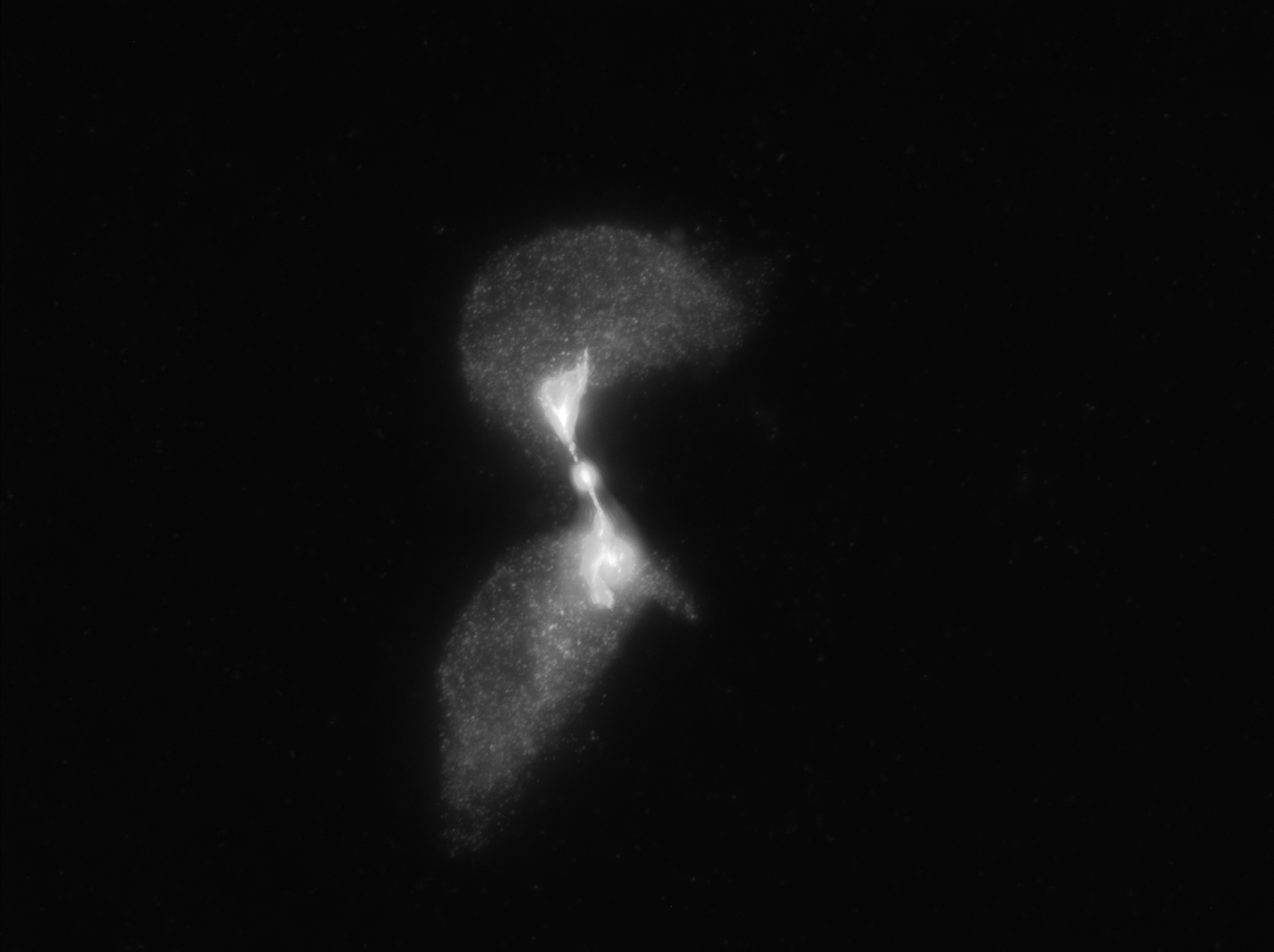

Supplement: Supplementary file 12 — Source data Fig. 3 part 2 [file 44319_2026_742_MOESM12_ESM.zip › Figure 3 Part 2/Fig 3b-e TTLL screen part 2/TTLL6-YFP R1 I1.Project Maximum Z_XY1663274991_Z0_T0_C1.tif]

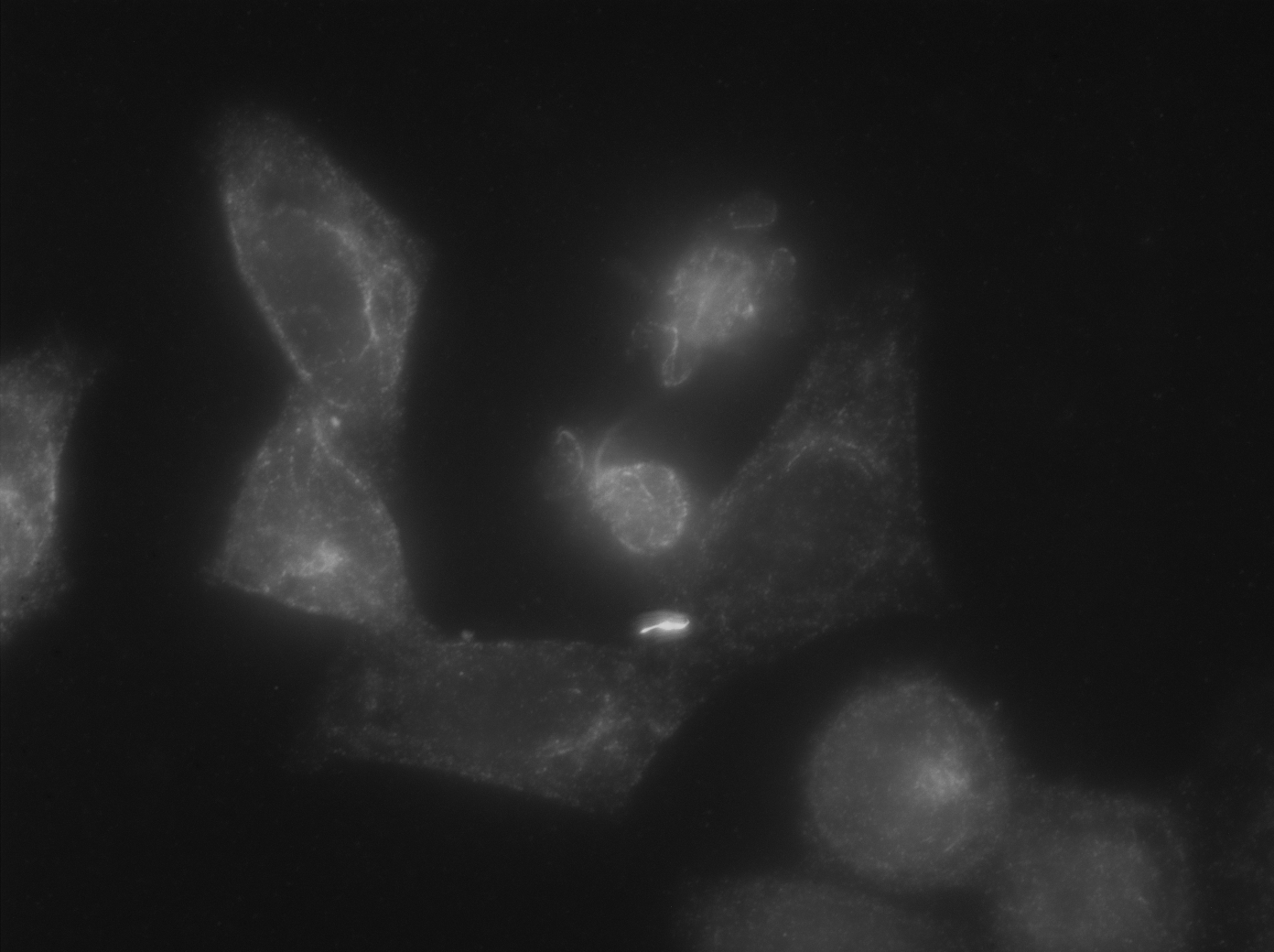

Supplement: Supplementary file 12 — Source data Fig. 3 part 2 [file 44319_2026_742_MOESM12_ESM.zip › Figure 3 Part 2/Fig 3b-e TTLL screen part 2/TTLL7-YFPy I1.Project Maximum Z_XY1679086971_Z0_T0_C1.tif]

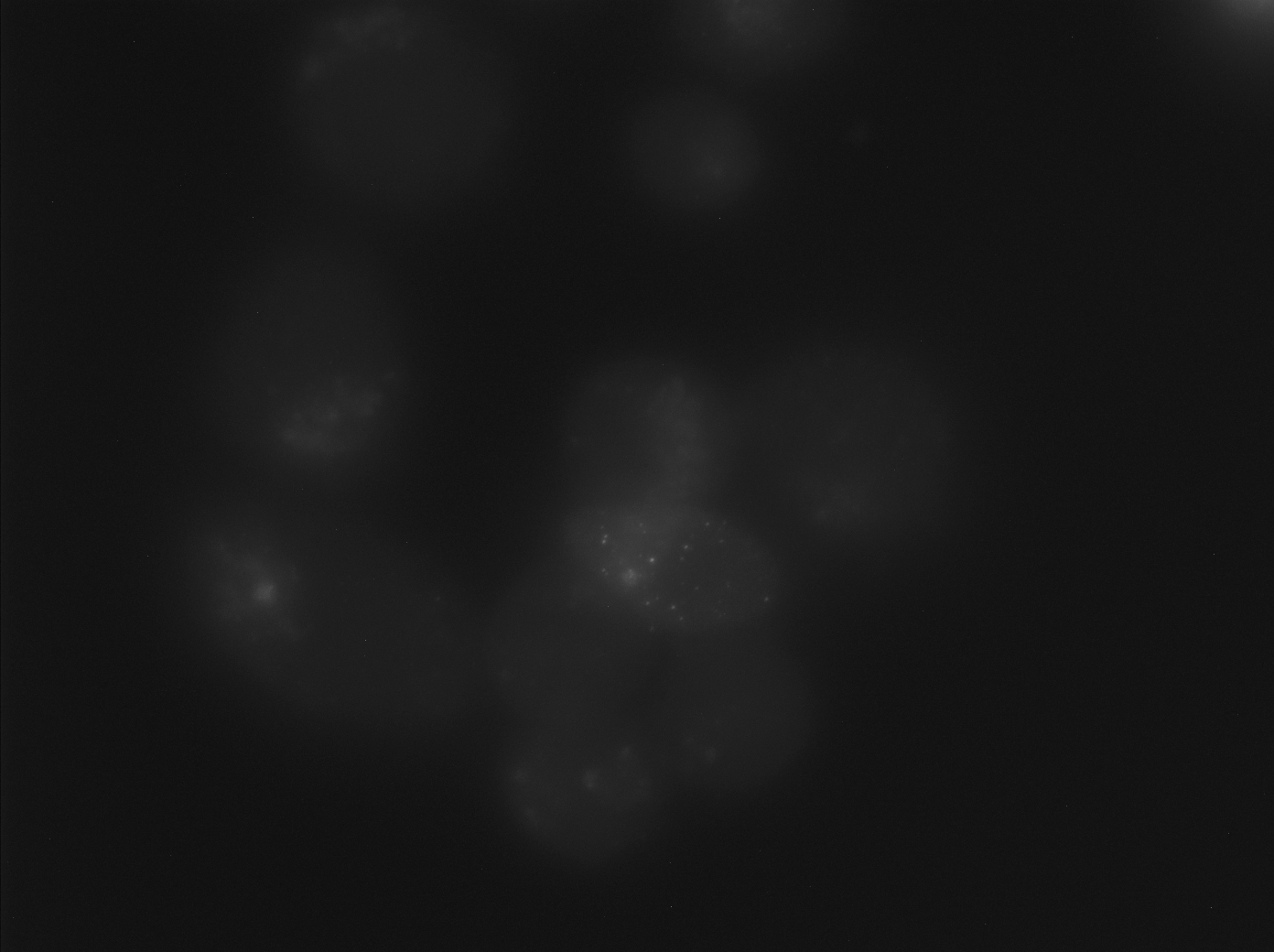

Supplement: Supplementary file 12 — Source data Fig. 3 part 2 [file 44319_2026_742_MOESM12_ESM.zip › Figure 3 Part 2/Fig 3b-e TTLL screen part 2/TTLL5-YFPy I13.Project Maximum Z_XY1679340570_Z0_T0_C2.tif]

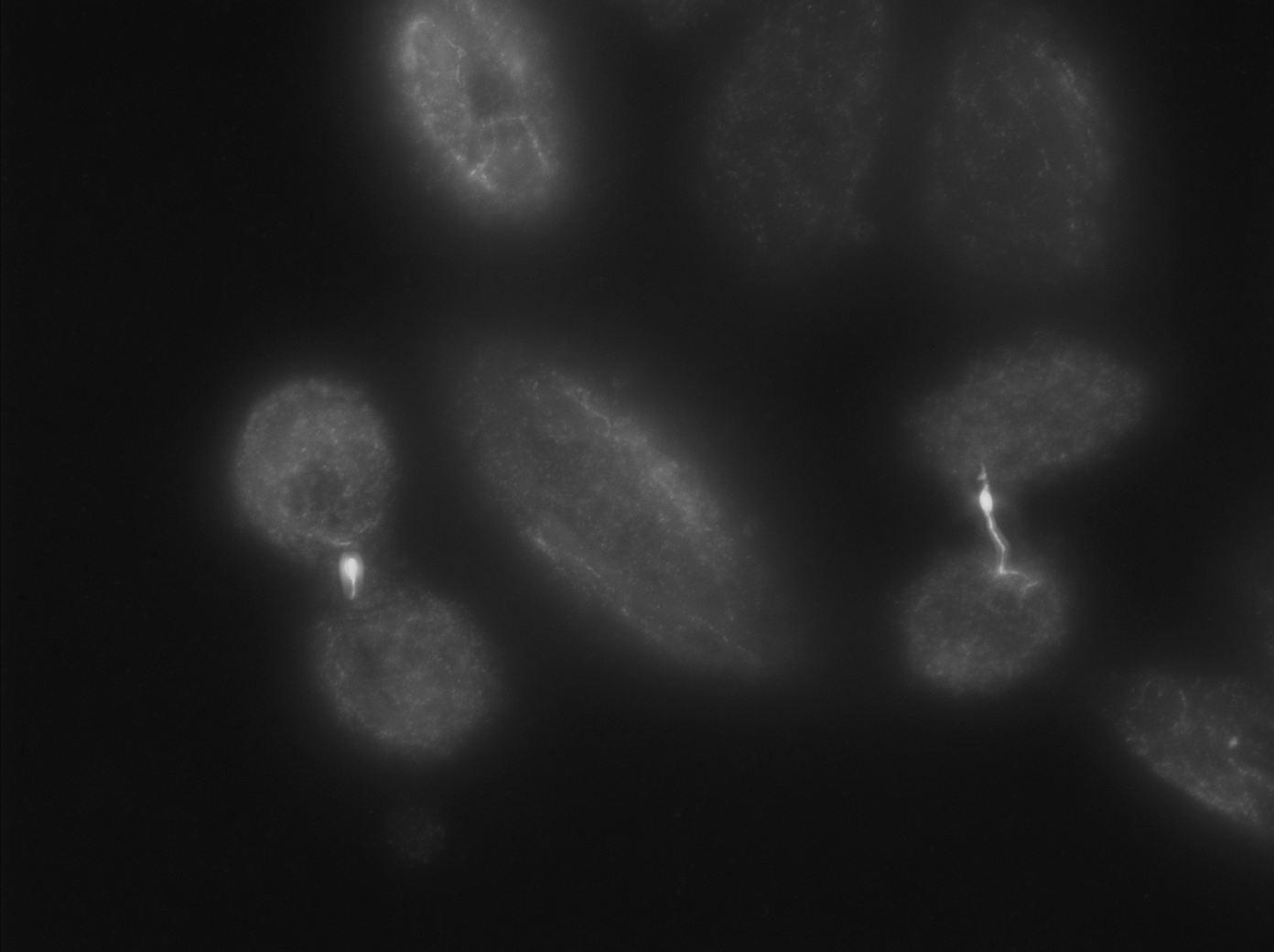

Supplement: Supplementary file 12 — Source data Fig. 3 part 2 [file 44319_2026_742_MOESM12_ESM.zip › Figure 3 Part 2/Fig 3b-e TTLL screen part 2/TTLL5-YFPy I15.Project Maximum Z_XY1679340888_Z0_T0_C1.tif]

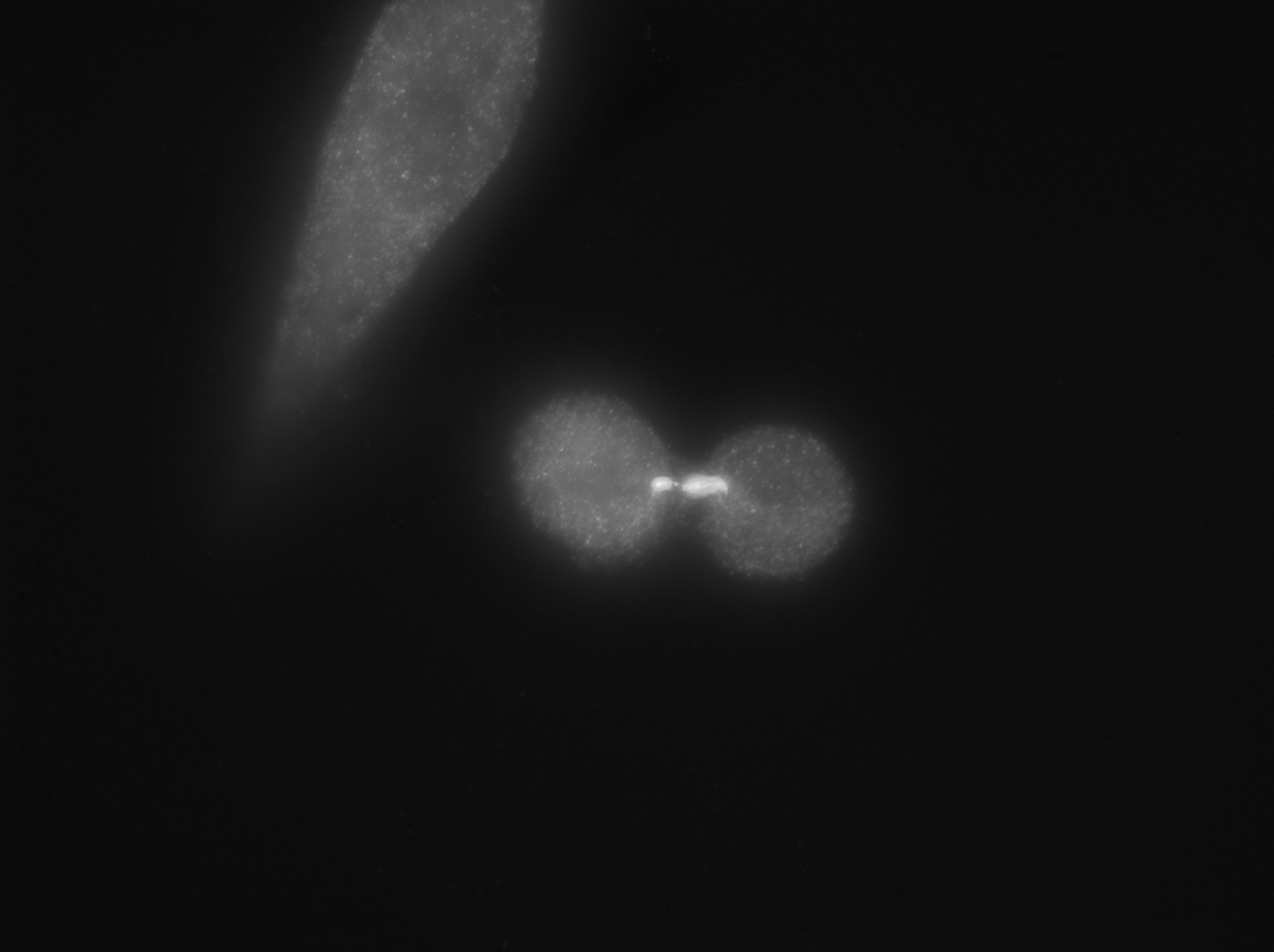

Supplement: Supplementary file 12 — Source data Fig. 3 part 2 [file 44319_2026_742_MOESM12_ESM.zip › Figure 3 Part 2/Fig 3b-e TTLL screen part 2/TTLL6-YFP R1 I2 low int.Project Maximum Z_XY1661546372_Z0_T0_C1.tif]

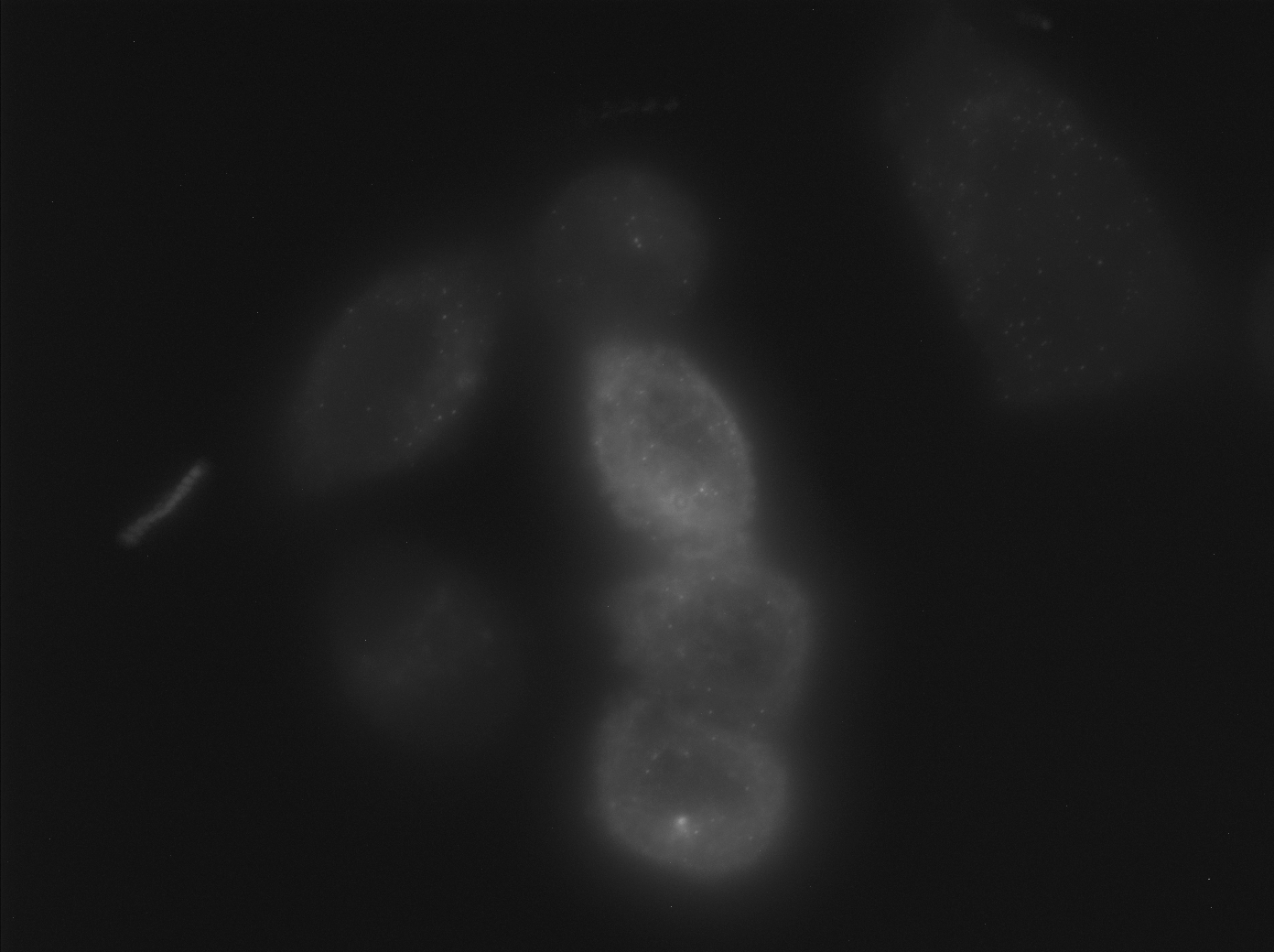

Supplement: Supplementary file 12 — Source data Fig. 3 part 2 [file 44319_2026_742_MOESM12_ESM.zip › Figure 3 Part 2/Fig 3b-e TTLL screen part 2/TTLL5-YFPy I3.Project Maximum Z_XY1679082773_Z0_T0_C2.tif]

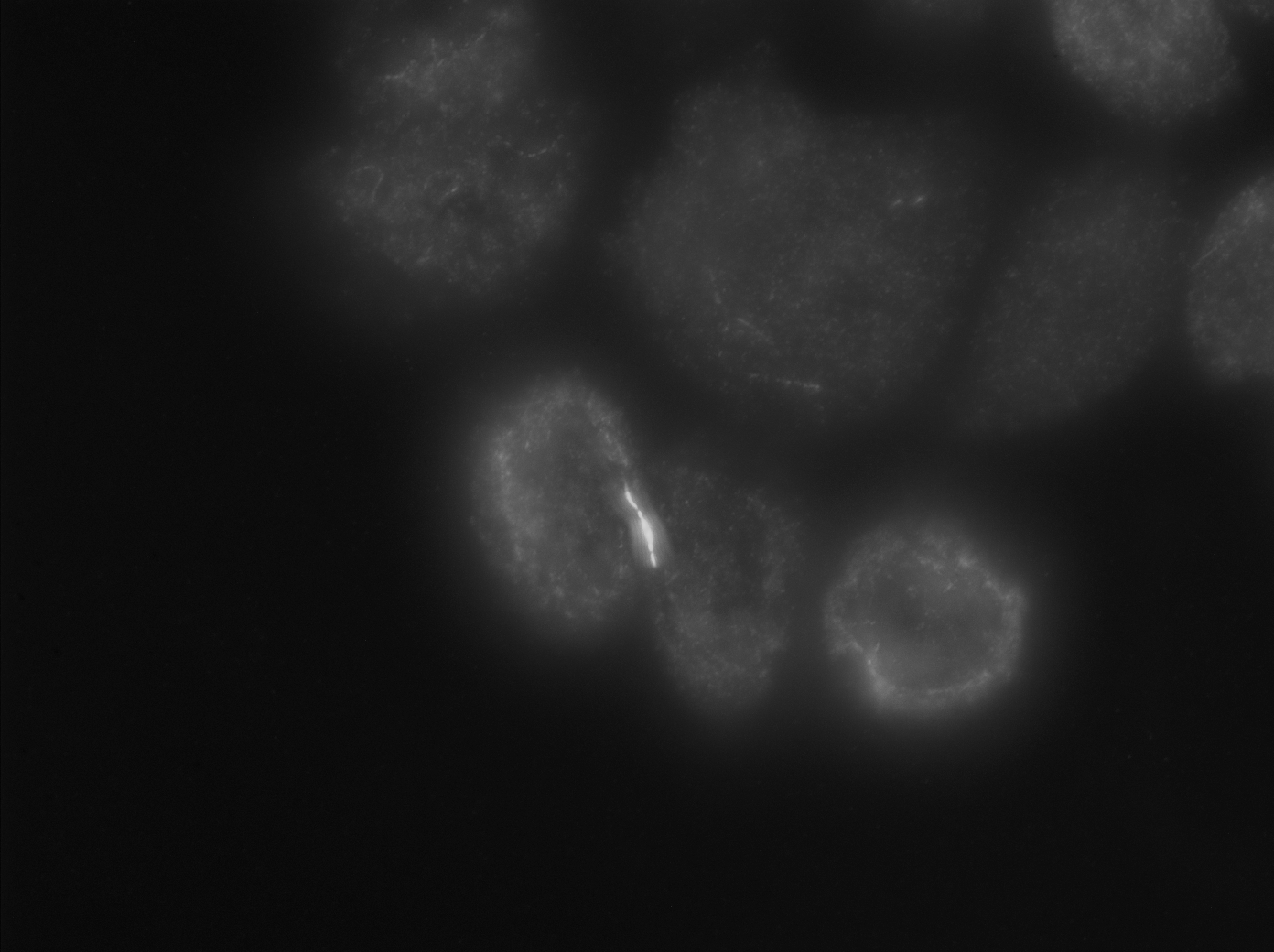

Supplement: Supplementary file 12 — Source data Fig. 3 part 2 [file 44319_2026_742_MOESM12_ESM.zip › Figure 3 Part 2/Fig 3b-e TTLL screen part 2/TTLL5-YFPy I4.Project Maximum Z_XY1679083019_Z0_T0_C1.tif]

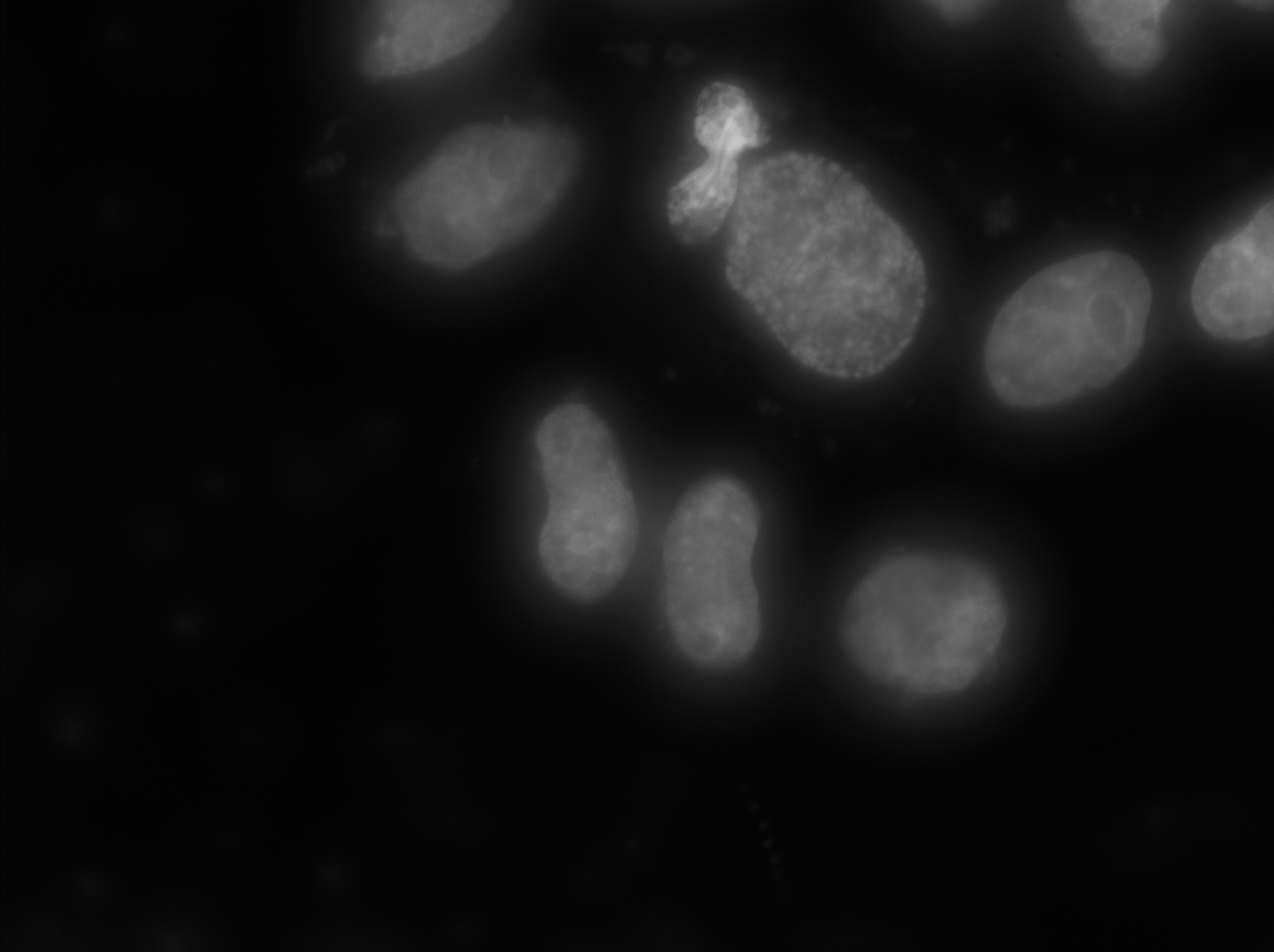

Supplement: Supplementary file 12 — Source data Fig. 3 part 2 [file 44319_2026_742_MOESM12_ESM.zip › Figure 3 Part 2/Fig 3b-e TTLL screen part 2/TTLL5-YFPy I4.Project Maximum Z_XY1679083019_Z0_T0_C0.tif]

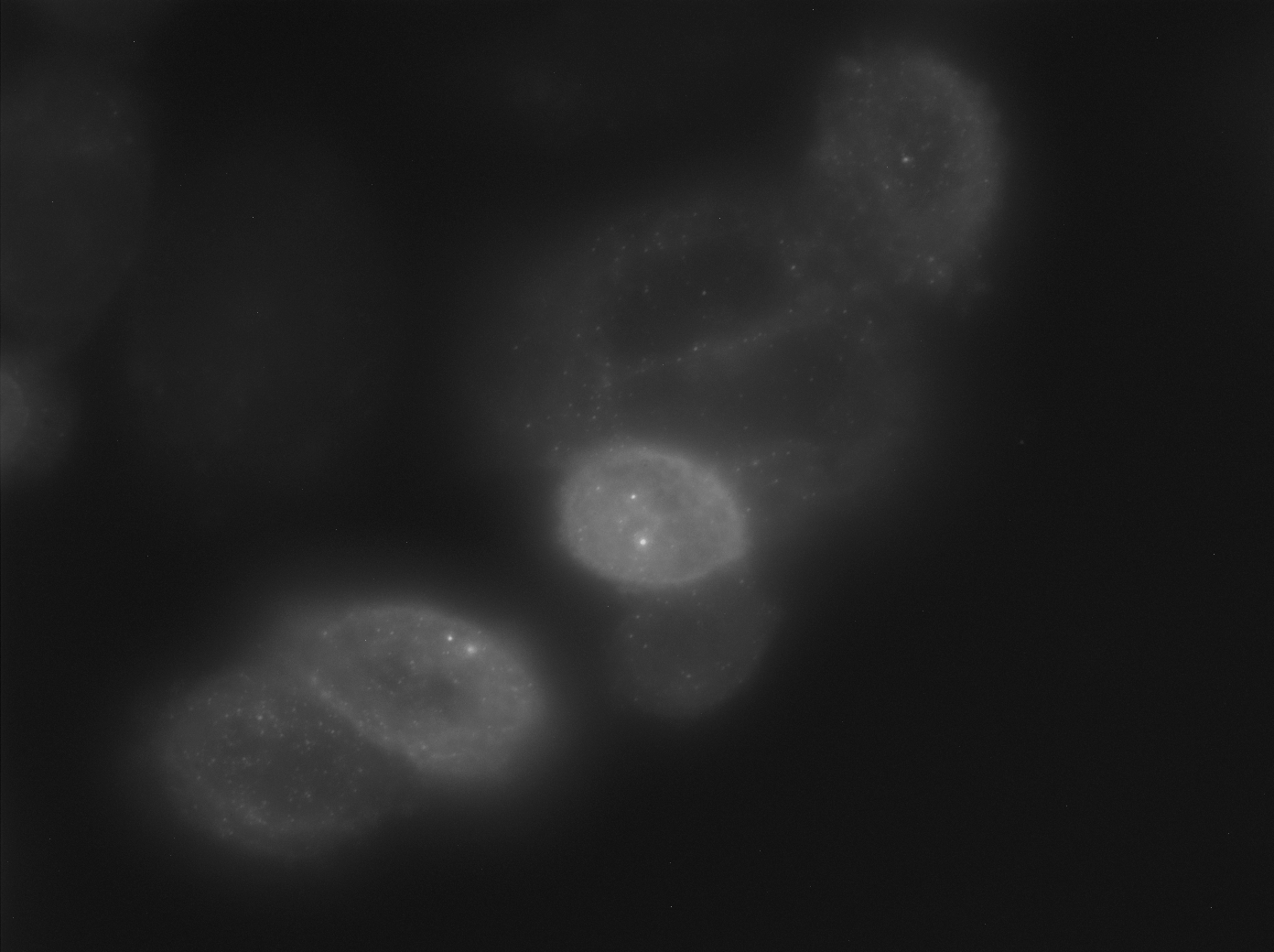

Supplement: Supplementary file 12 — Source data Fig. 3 part 2 [file 44319_2026_742_MOESM12_ESM.zip › Figure 3 Part 2/Fig 3b-e TTLL screen part 2/TTLL5-YFPy I9.Project Maximum Z_XY1679085208_Z0_T0_C2.tif]

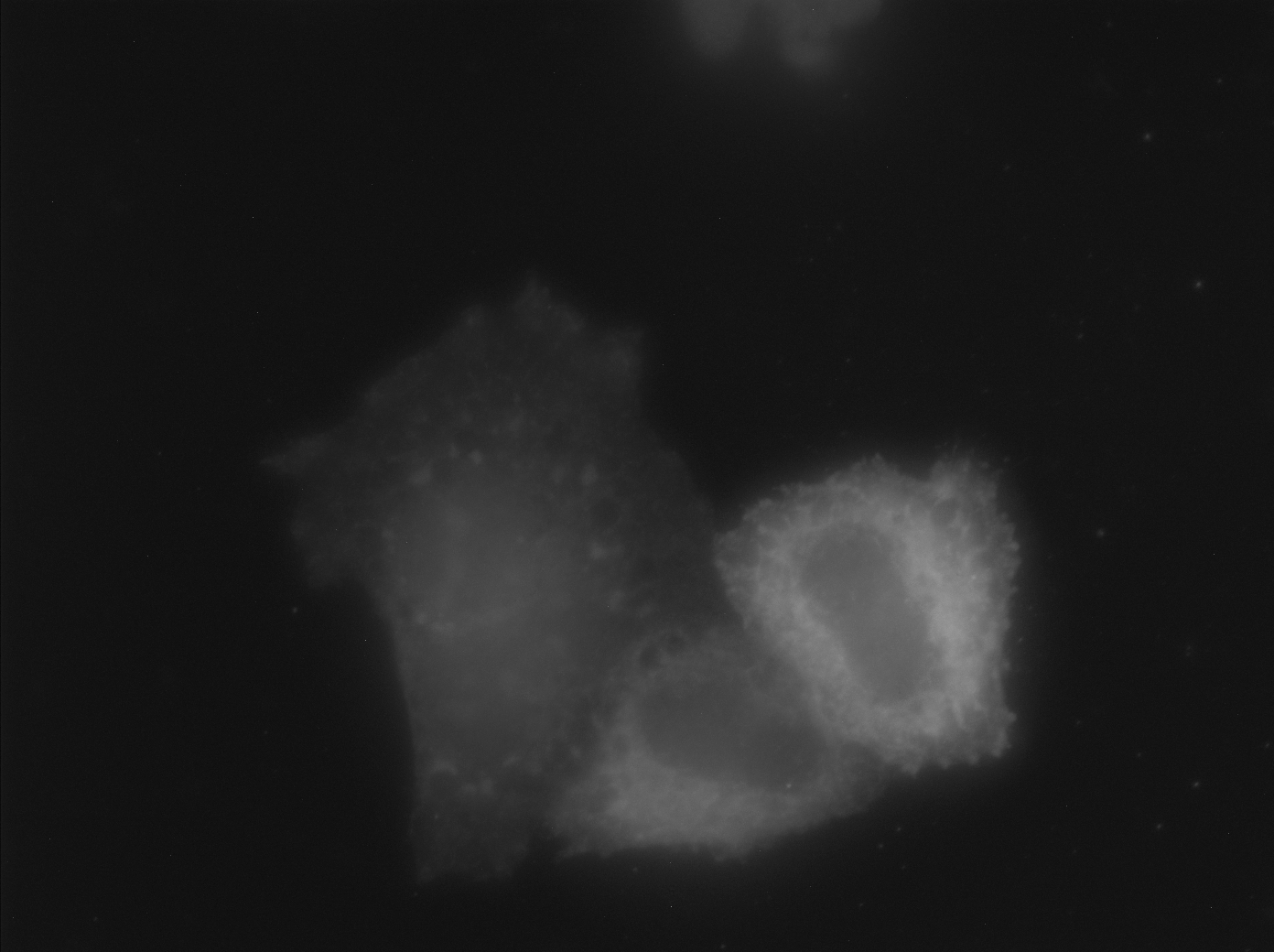

Supplement: Supplementary file 12 — Source data Fig. 3 part 2 [file 44319_2026_742_MOESM12_ESM.zip › Figure 3 Part 2/Fig 3b-e TTLL screen part 2/TTLL7-YFPy I4.Project Maximum Z_XY1679087897_Z0_T0_C2.tif]

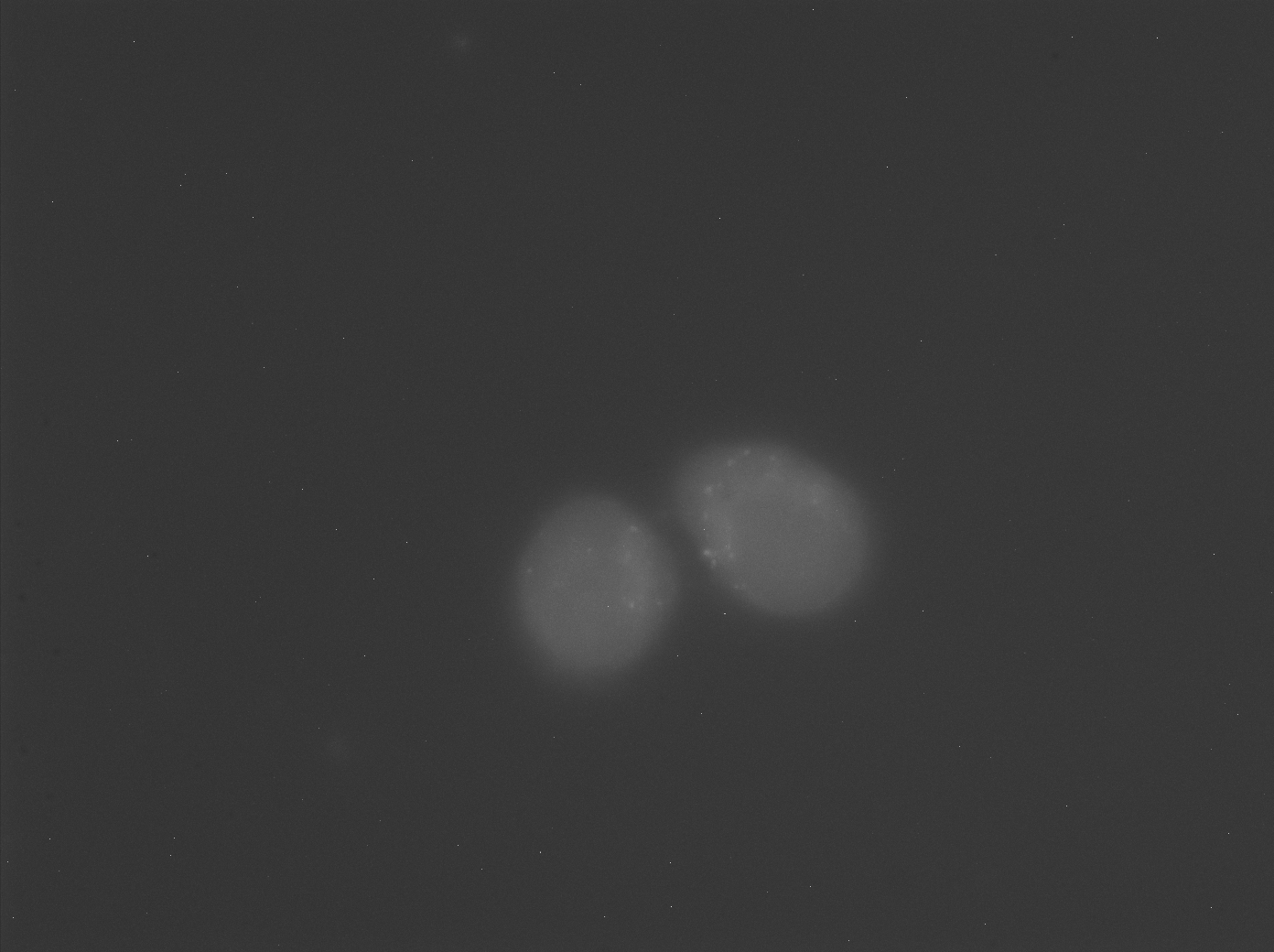

Supplement: Supplementary file 12 — Source data Fig. 3 part 2 [file 44319_2026_742_MOESM12_ESM.zip › Figure 3 Part 2/Fig 3b-e TTLL screen part 2/TTLL6-YFP R1 I3 low int.Project Maximum Z_XY1661547099_Z0_T0_C2.tif]

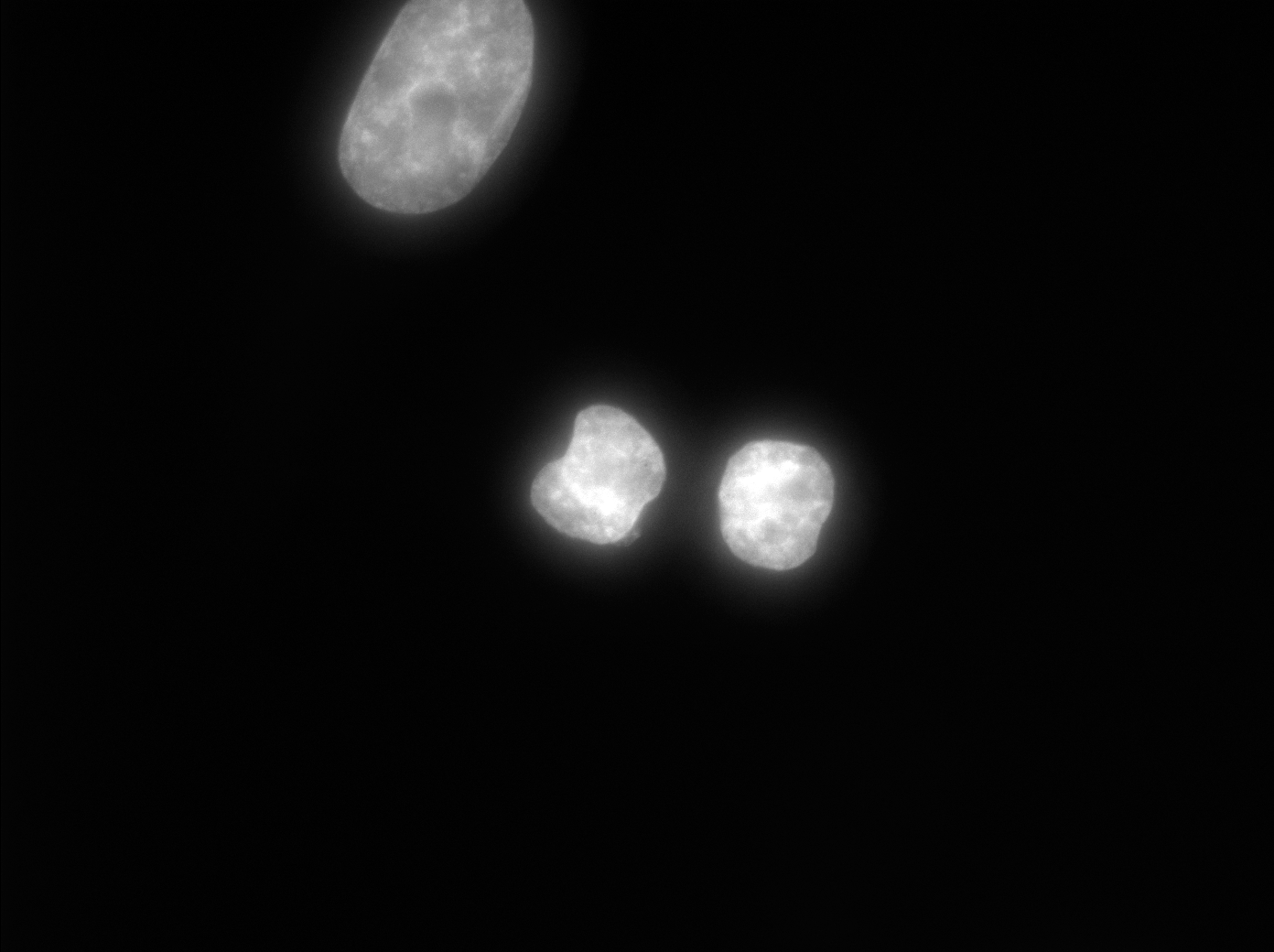

Supplement: Supplementary file 12 — Source data Fig. 3 part 2 [file 44319_2026_742_MOESM12_ESM.zip › Figure 3 Part 2/Fig 3b-e TTLL screen part 2/TTLL6-YFP R1 I2 low int.Project Maximum Z_XY1661546372_Z0_T0_C0.tif]

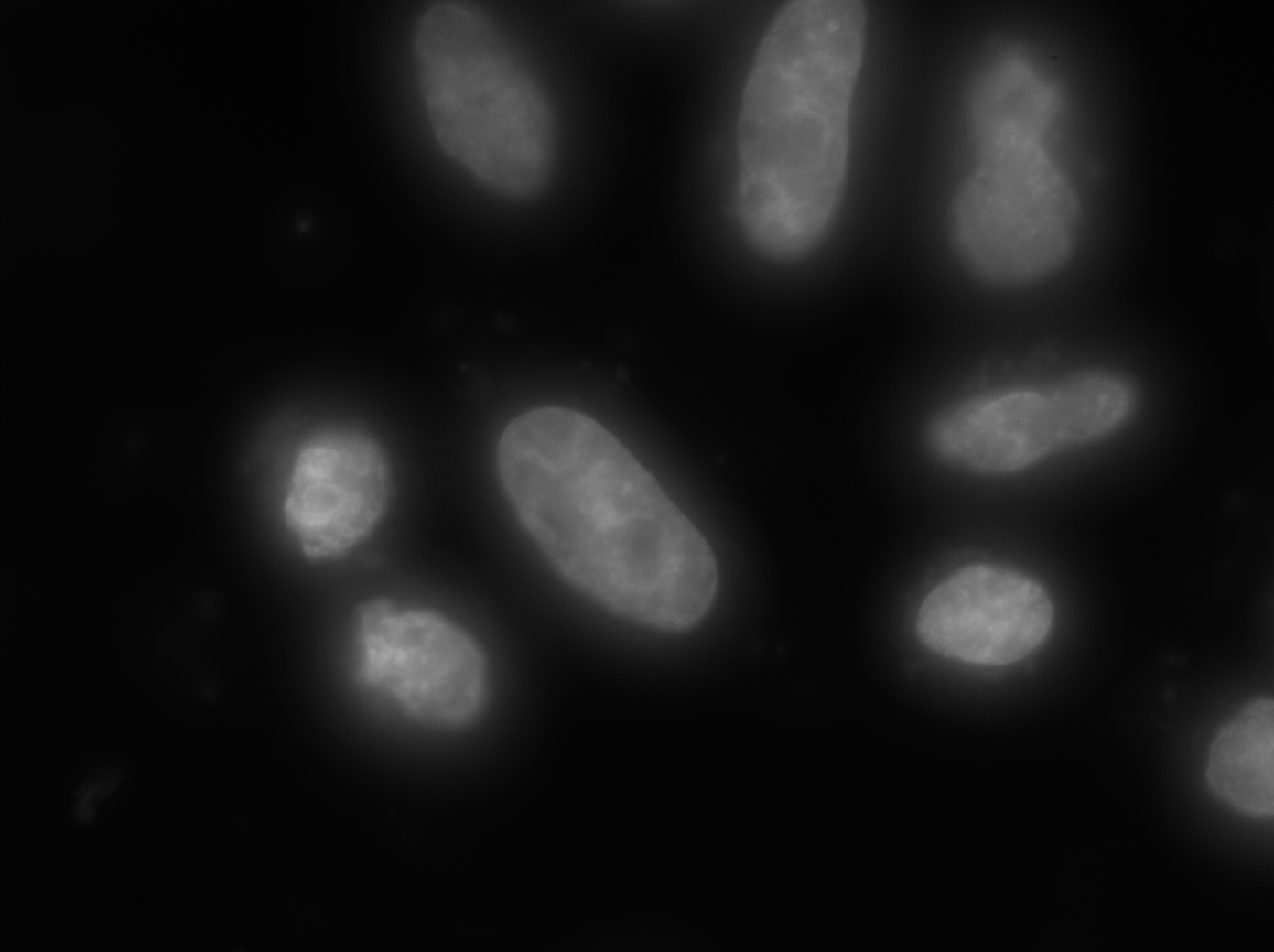

Supplement: Supplementary file 12 — Source data Fig. 3 part 2 [file 44319_2026_742_MOESM12_ESM.zip › Figure 3 Part 2/Fig 3b-e TTLL screen part 2/TTLL5-YFPy I15.Project Maximum Z_XY1679340888_Z0_T0_C0.tif]

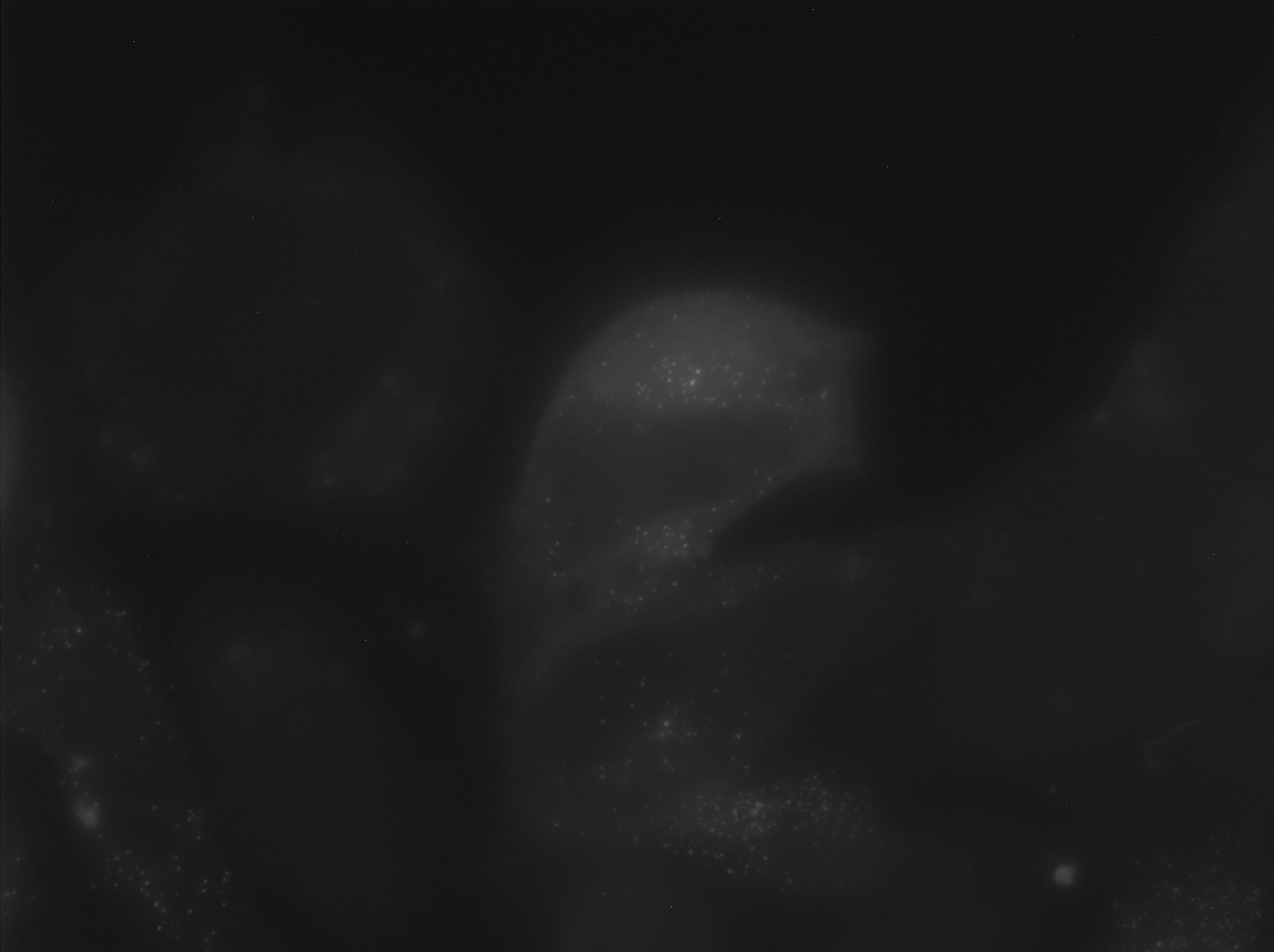

Supplement: Supplementary file 12 — Source data Fig. 3 part 2 [file 44319_2026_742_MOESM12_ESM.zip › Figure 3 Part 2/Fig 3b-e TTLL screen part 2/TTLL5-YFPy I10.Project Maximum Z_XY1679085407_Z0_T0_C2.tif]

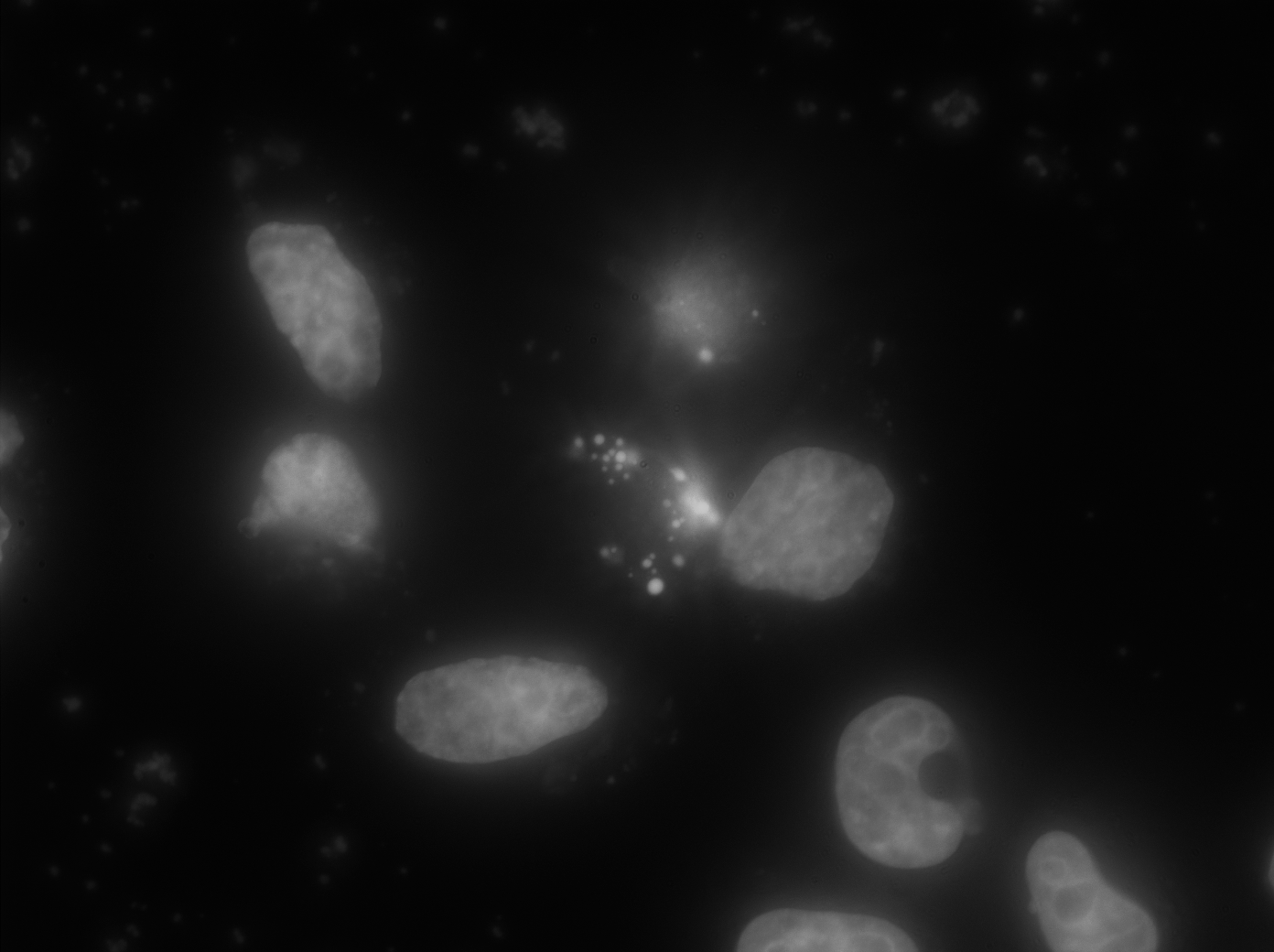

Supplement: Supplementary file 12 — Source data Fig. 3 part 2 [file 44319_2026_742_MOESM12_ESM.zip › Figure 3 Part 2/Fig 3b-e TTLL screen part 2/TTLL7-YFPy I1.Project Maximum Z_XY1679086971_Z0_T0_C0.tif]

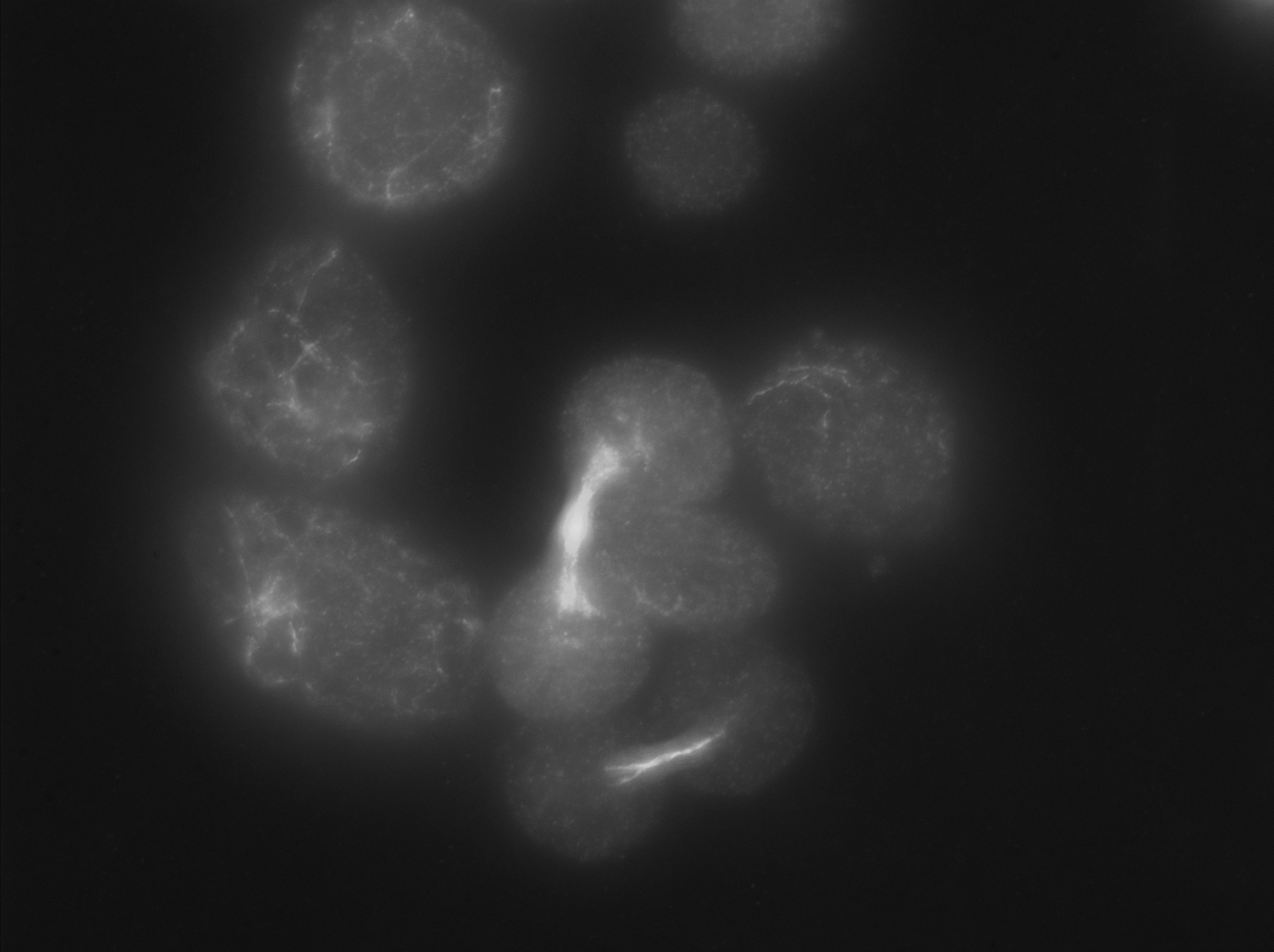

Supplement: Supplementary file 12 — Source data Fig. 3 part 2 [file 44319_2026_742_MOESM12_ESM.zip › Figure 3 Part 2/Fig 3b-e TTLL screen part 2/TTLL5-YFPy I13.Project Maximum Z_XY1679340570_Z0_T0_C1.tif]

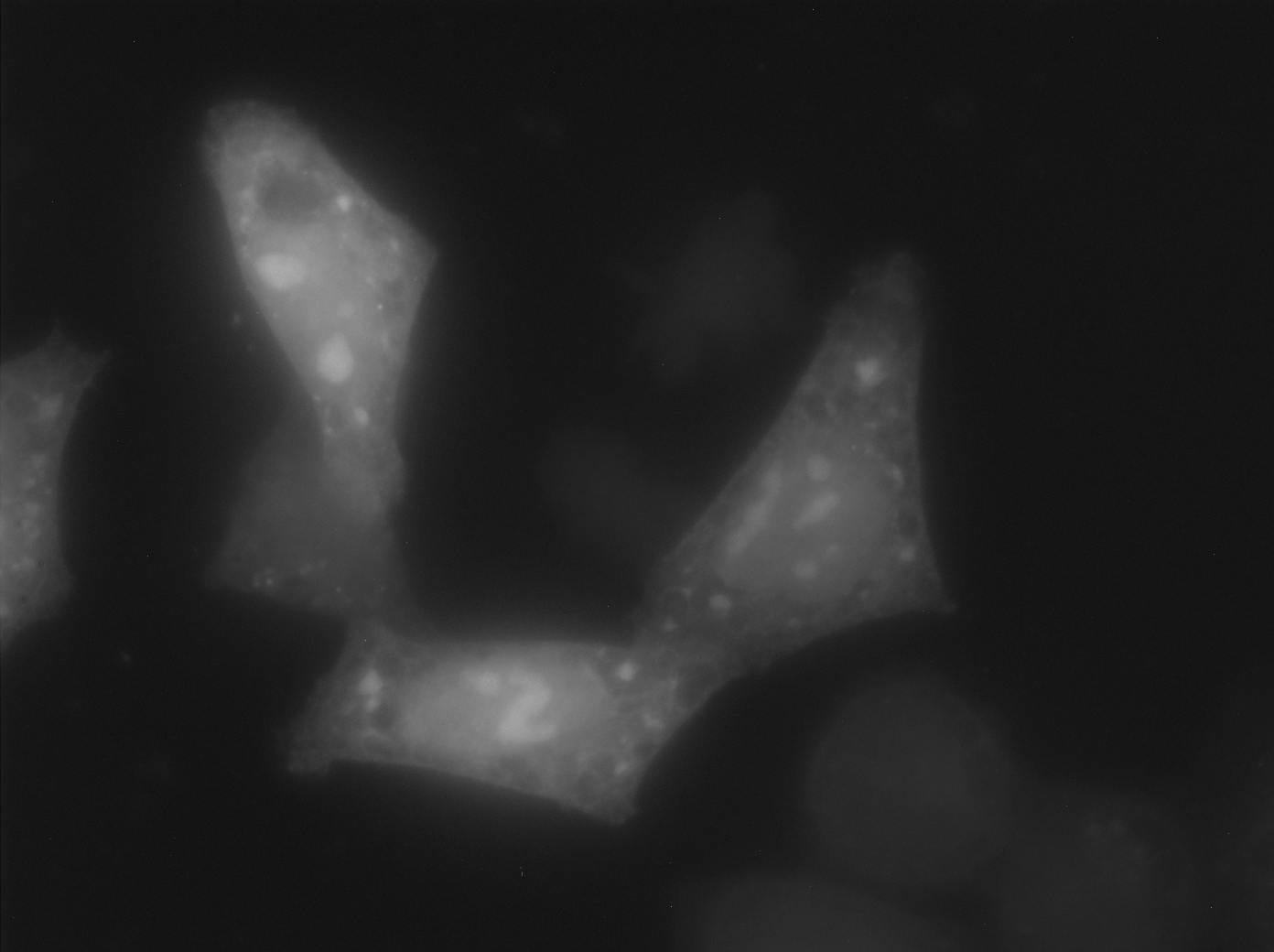

Supplement: Supplementary file 12 — Source data Fig. 3 part 2 [file 44319_2026_742_MOESM12_ESM.zip › Figure 3 Part 2/Fig 3b-e TTLL screen part 2/TTLL7-YFPy I1.Project Maximum Z_XY1679086971_Z0_T0_C2.tif]

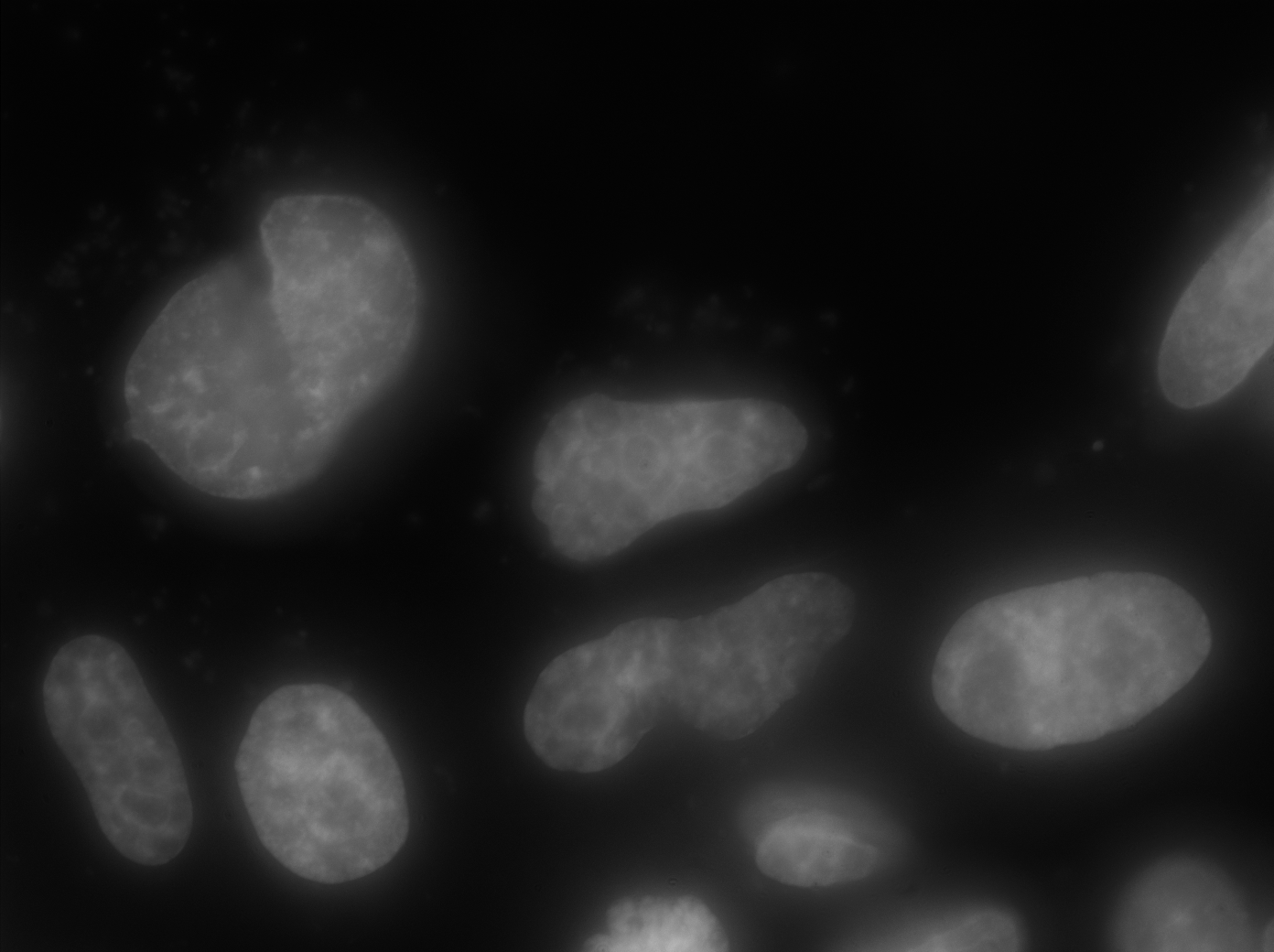

Supplement: Supplementary file 12 — Source data Fig. 3 part 2 [file 44319_2026_742_MOESM12_ESM.zip › Figure 3 Part 2/Fig 3b-e TTLL screen part 2/TTLL5-YFPy I10.Project Maximum Z_XY1679085407_Z0_T0_C0.tif]

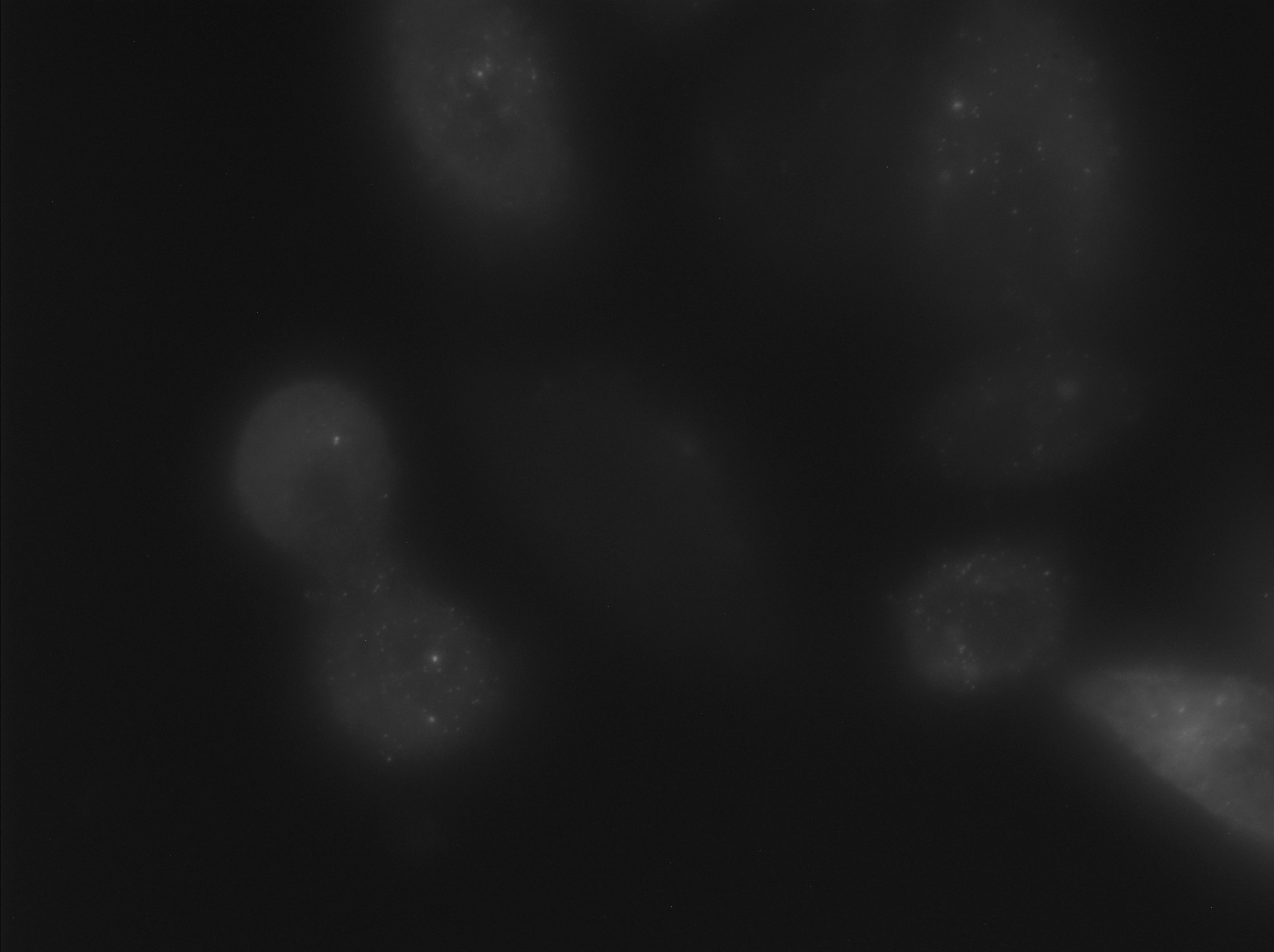

Supplement: Supplementary file 12 — Source data Fig. 3 part 2 [file 44319_2026_742_MOESM12_ESM.zip › Figure 3 Part 2/Fig 3b-e TTLL screen part 2/TTLL5-YFPy I15.Project Maximum Z_XY1679340888_Z0_T0_C2.tif]

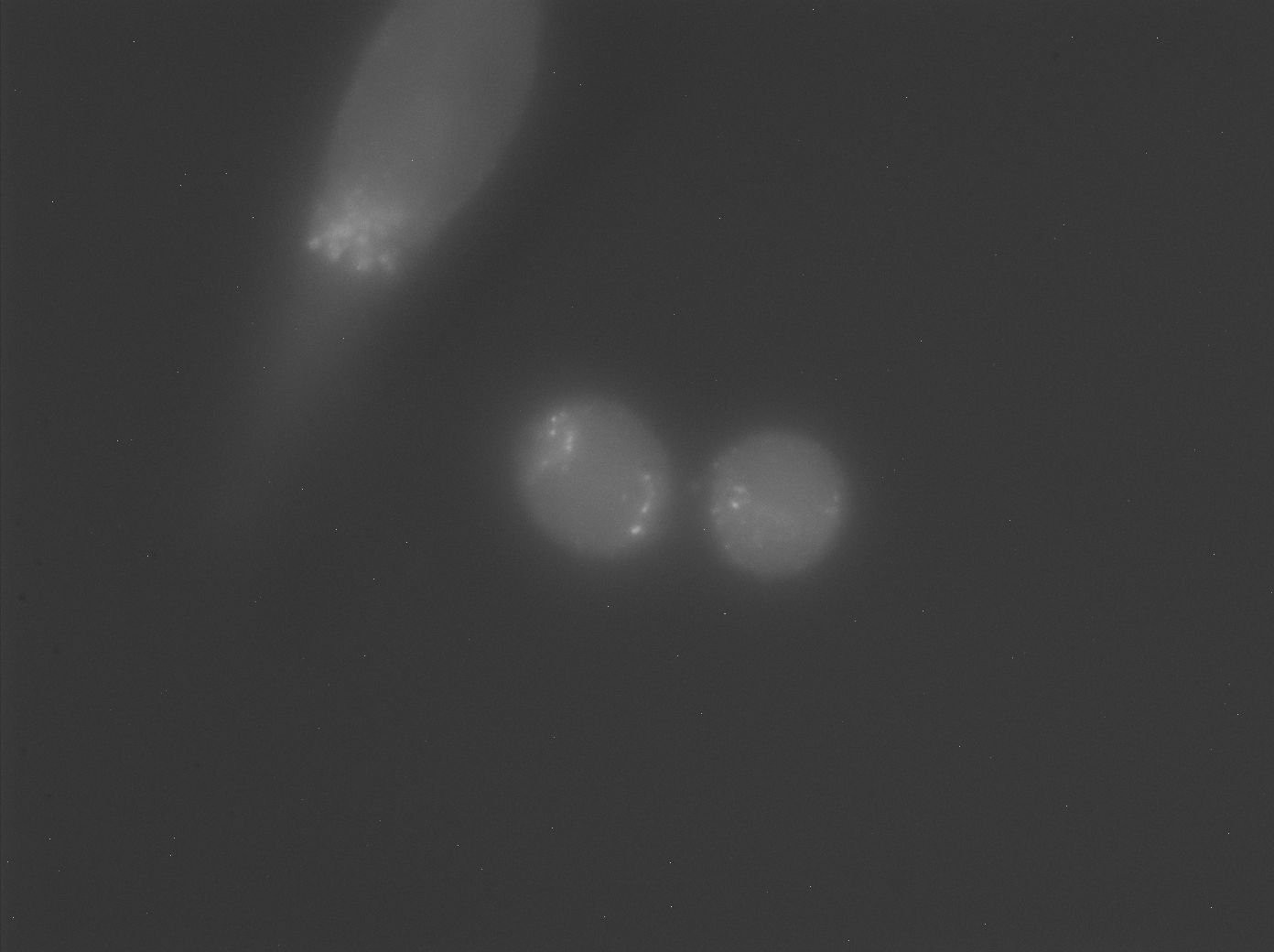

Supplement: Supplementary file 12 — Source data Fig. 3 part 2 [file 44319_2026_742_MOESM12_ESM.zip › Figure 3 Part 2/Fig 3b-e TTLL screen part 2/TTLL6-YFP R1 I2 low int.Project Maximum Z_XY1661546372_Z0_T0_C2.tif]

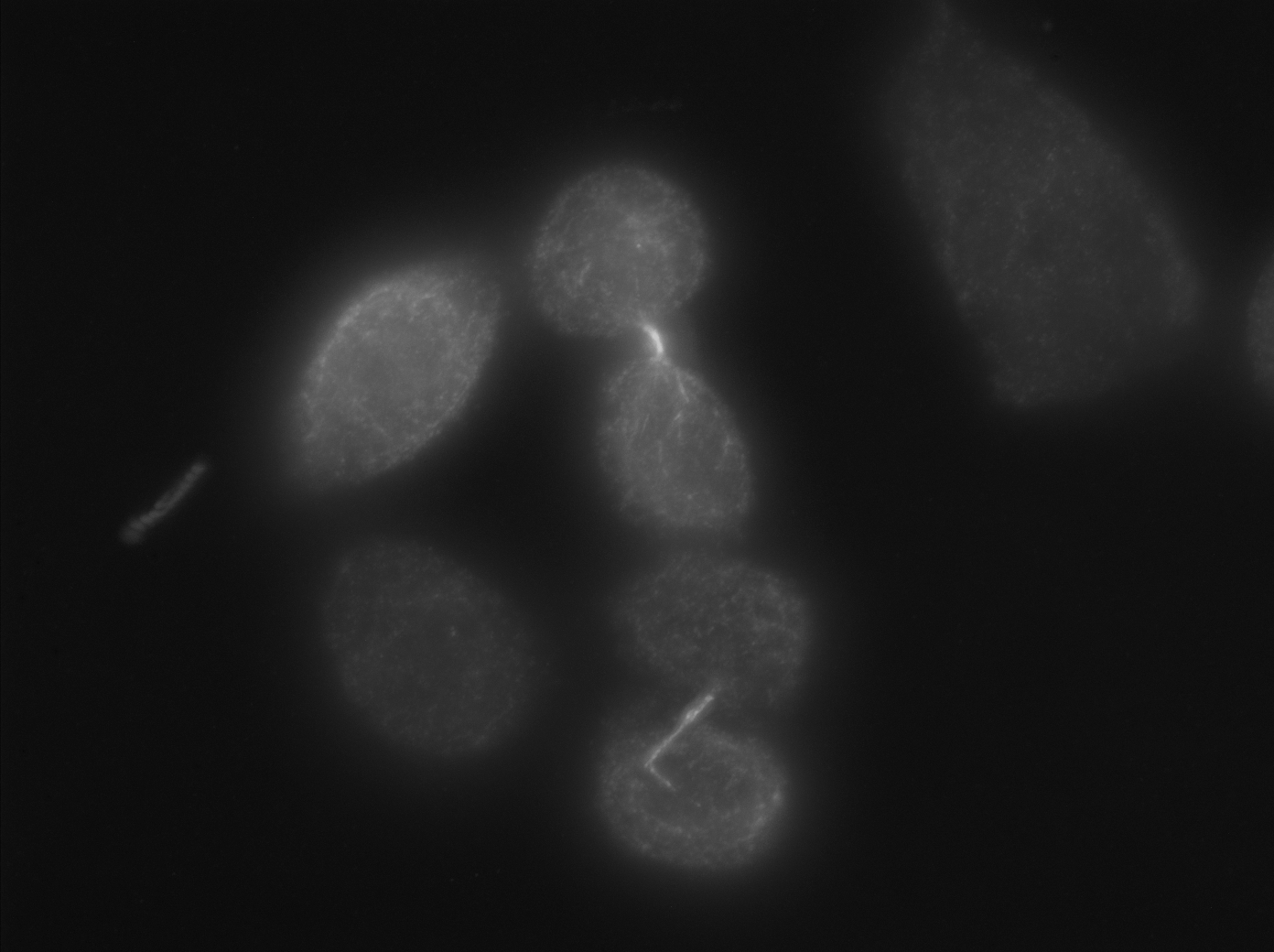

Supplement: Supplementary file 12 — Source data Fig. 3 part 2 [file 44319_2026_742_MOESM12_ESM.zip › Figure 3 Part 2/Fig 3b-e TTLL screen part 2/TTLL5-YFPy I3.Project Maximum Z_XY1679082773_Z0_T0_C1.tif]

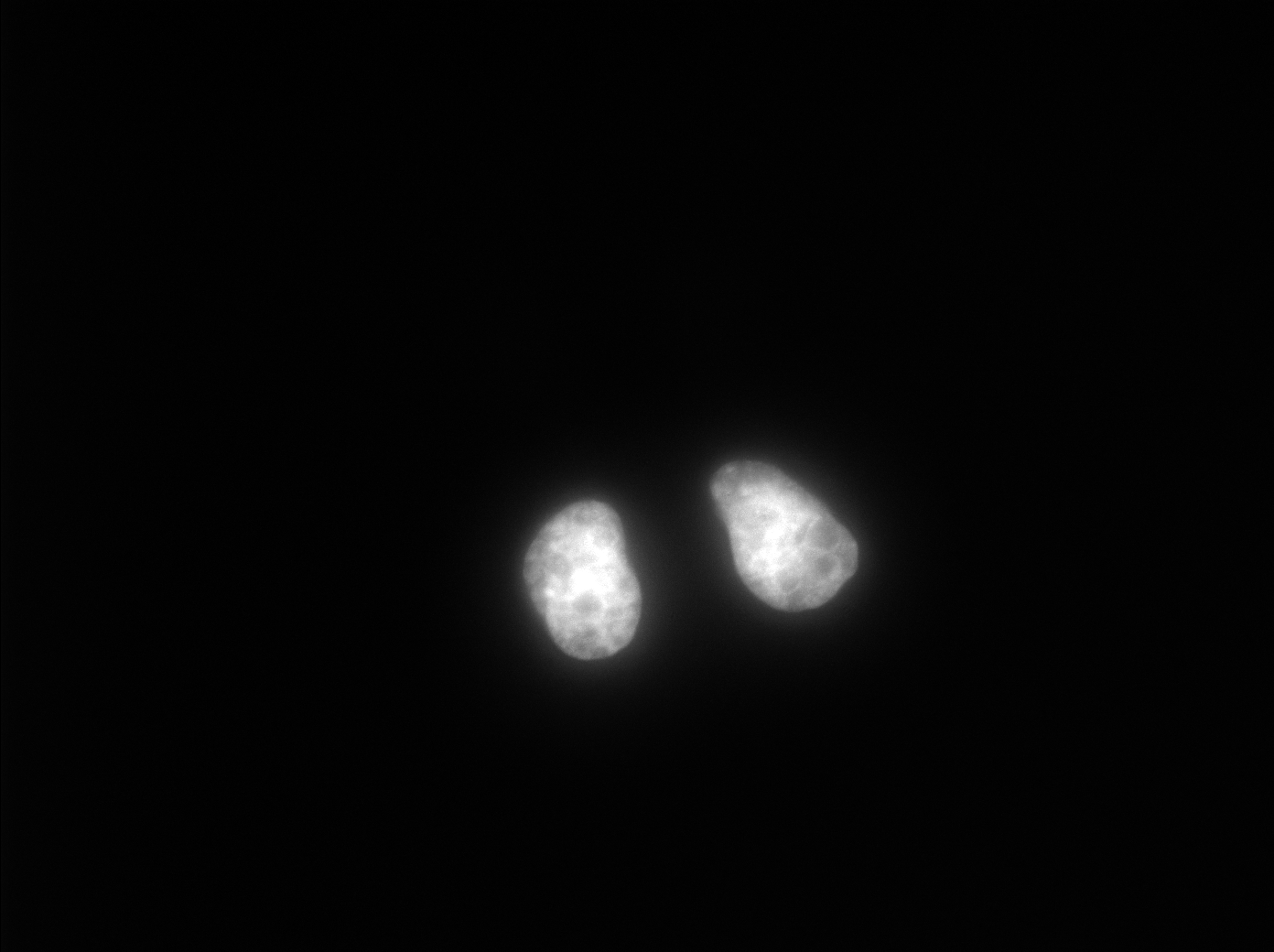

Supplement: Supplementary file 12 — Source data Fig. 3 part 2 [file 44319_2026_742_MOESM12_ESM.zip › Figure 3 Part 2/Fig 3b-e TTLL screen part 2/TTLL6-YFP R1 I3 low int.Project Maximum Z_XY1661547099_Z0_T0_C0.tif]

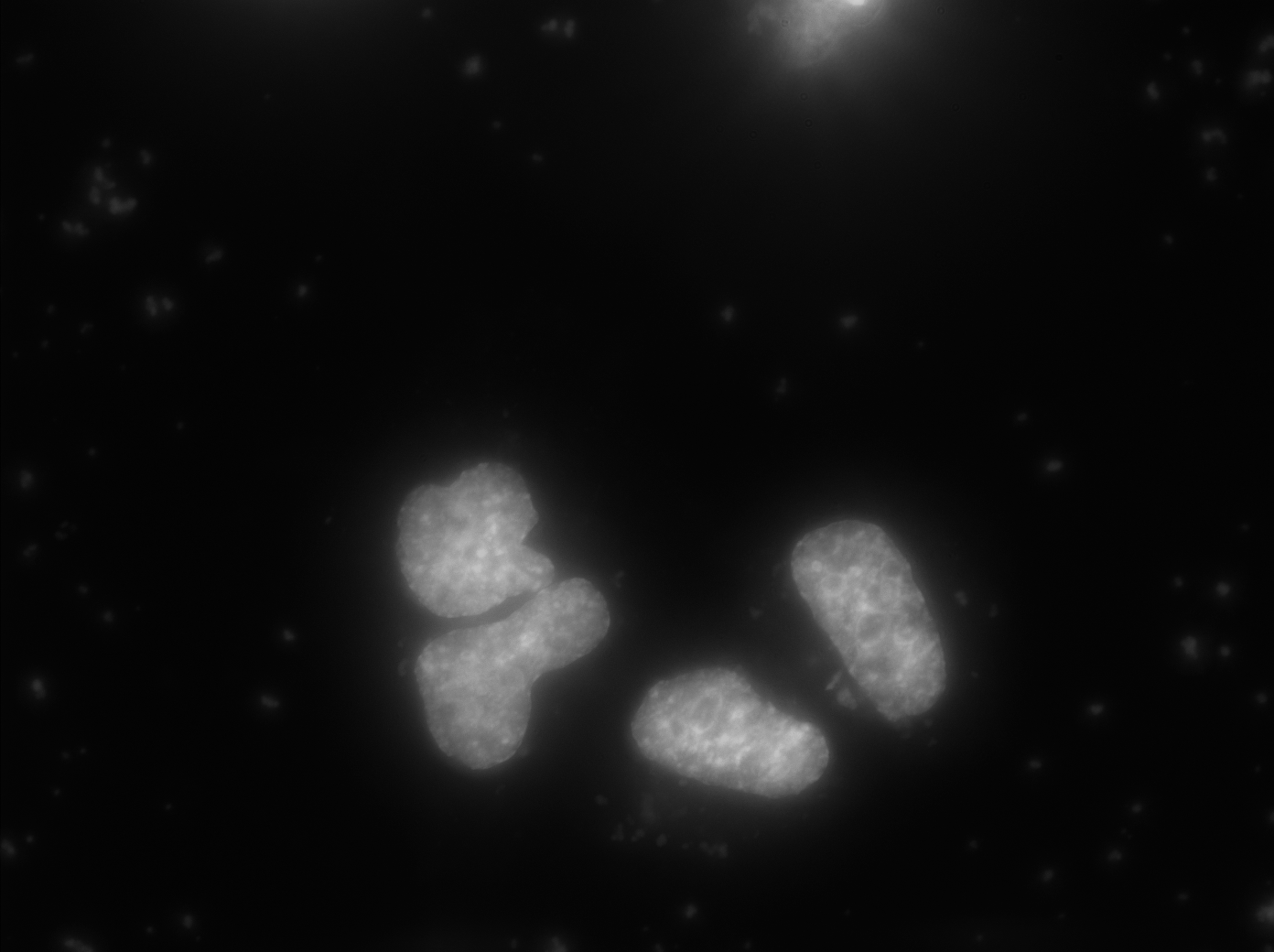

Supplement: Supplementary file 12 — Source data Fig. 3 part 2 [file 44319_2026_742_MOESM12_ESM.zip › Figure 3 Part 2/Fig 3b-e TTLL screen part 2/TTLL7-YFPy I4.Project Maximum Z_XY1679087897_Z0_T0_C0.tif]

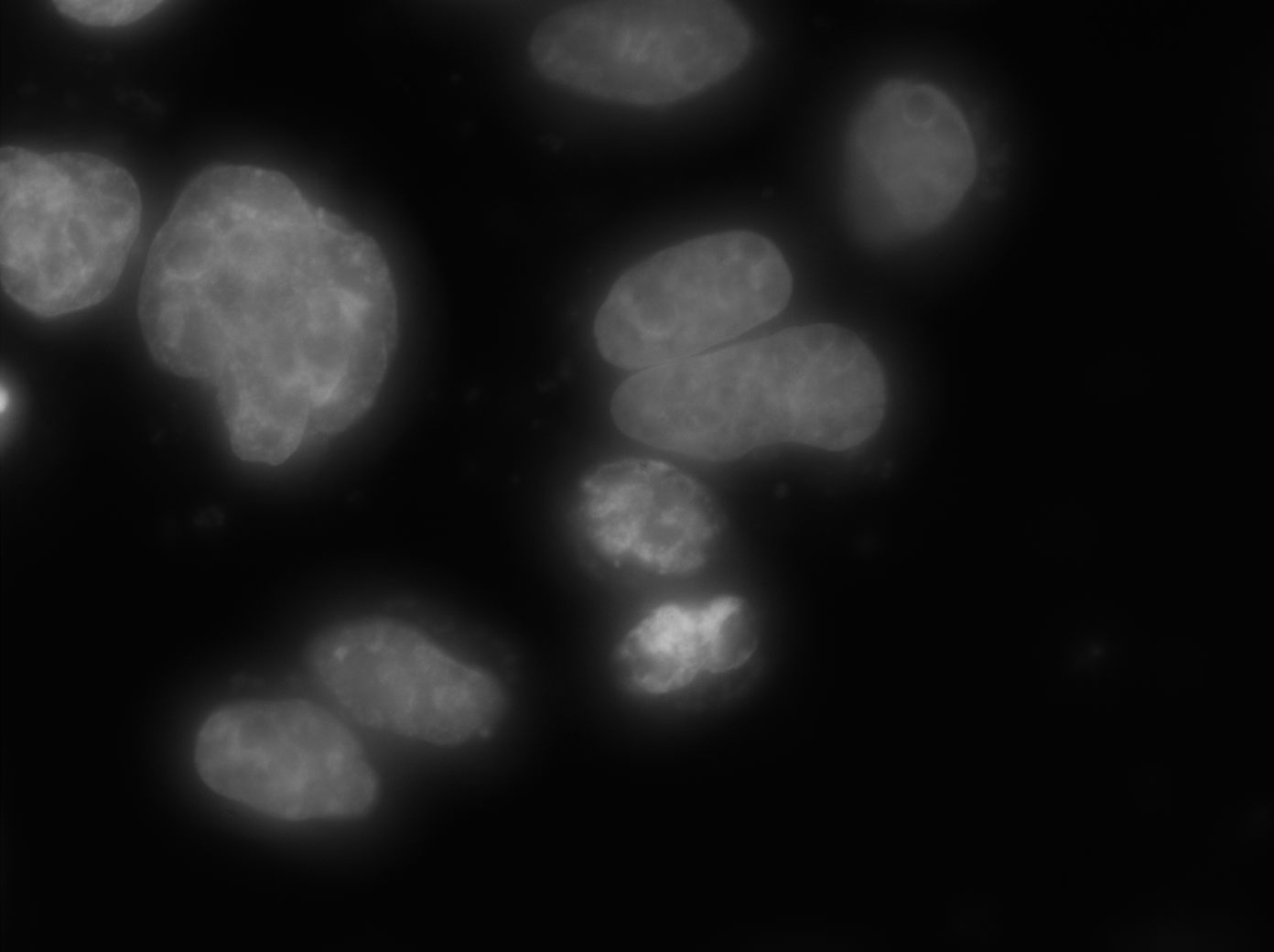

Supplement: Supplementary file 12 — Source data Fig. 3 part 2 [file 44319_2026_742_MOESM12_ESM.zip › Figure 3 Part 2/Fig 3b-e TTLL screen part 2/TTLL5-YFPy I9.Project Maximum Z_XY1679085208_Z0_T0_C0.tif]

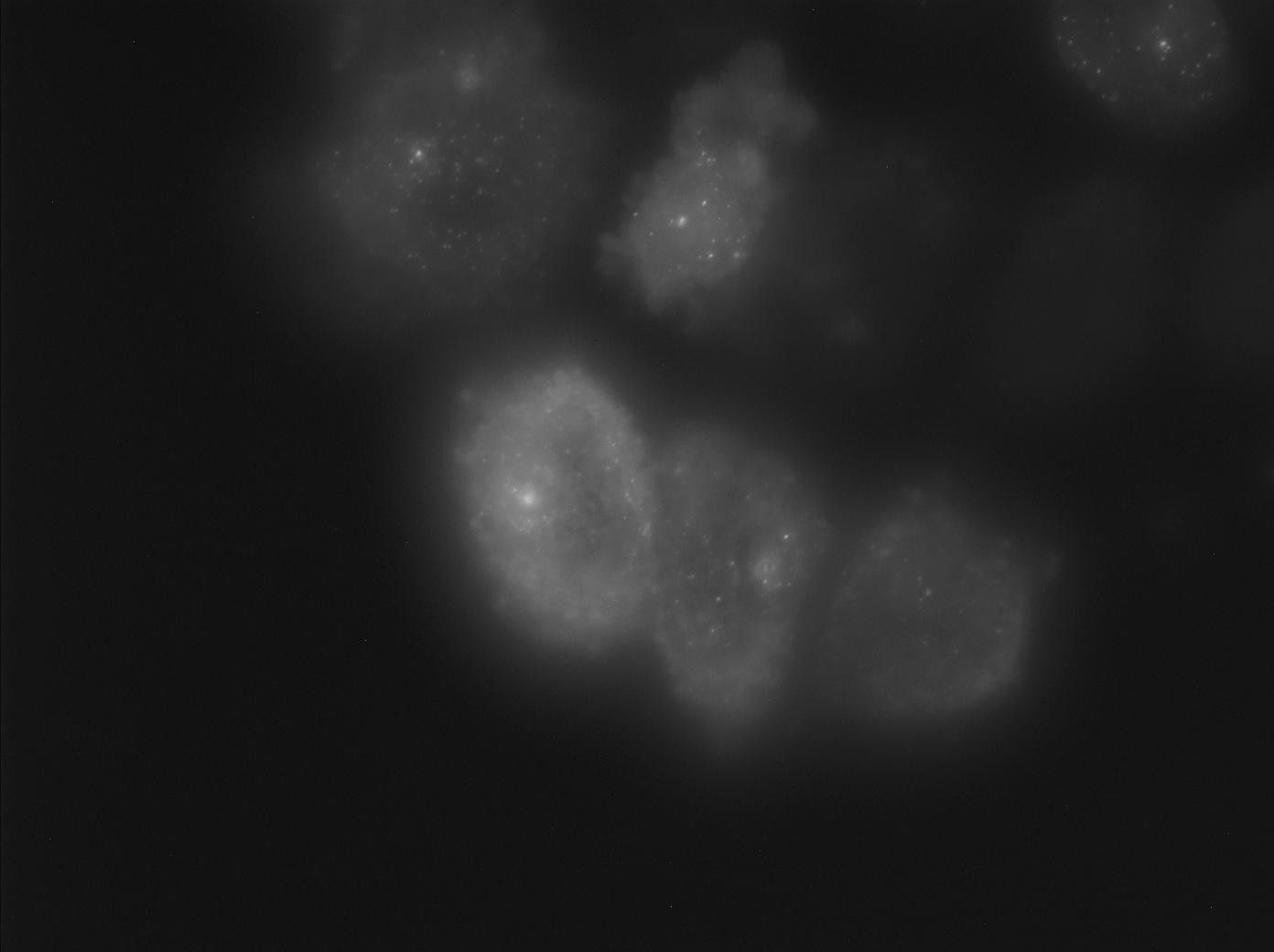

Supplement: Supplementary file 12 — Source data Fig. 3 part 2 [file 44319_2026_742_MOESM12_ESM.zip › Figure 3 Part 2/Fig 3b-e TTLL screen part 2/TTLL5-YFPy I4.Project Maximum Z_XY1679083019_Z0_T0_C2.tif]

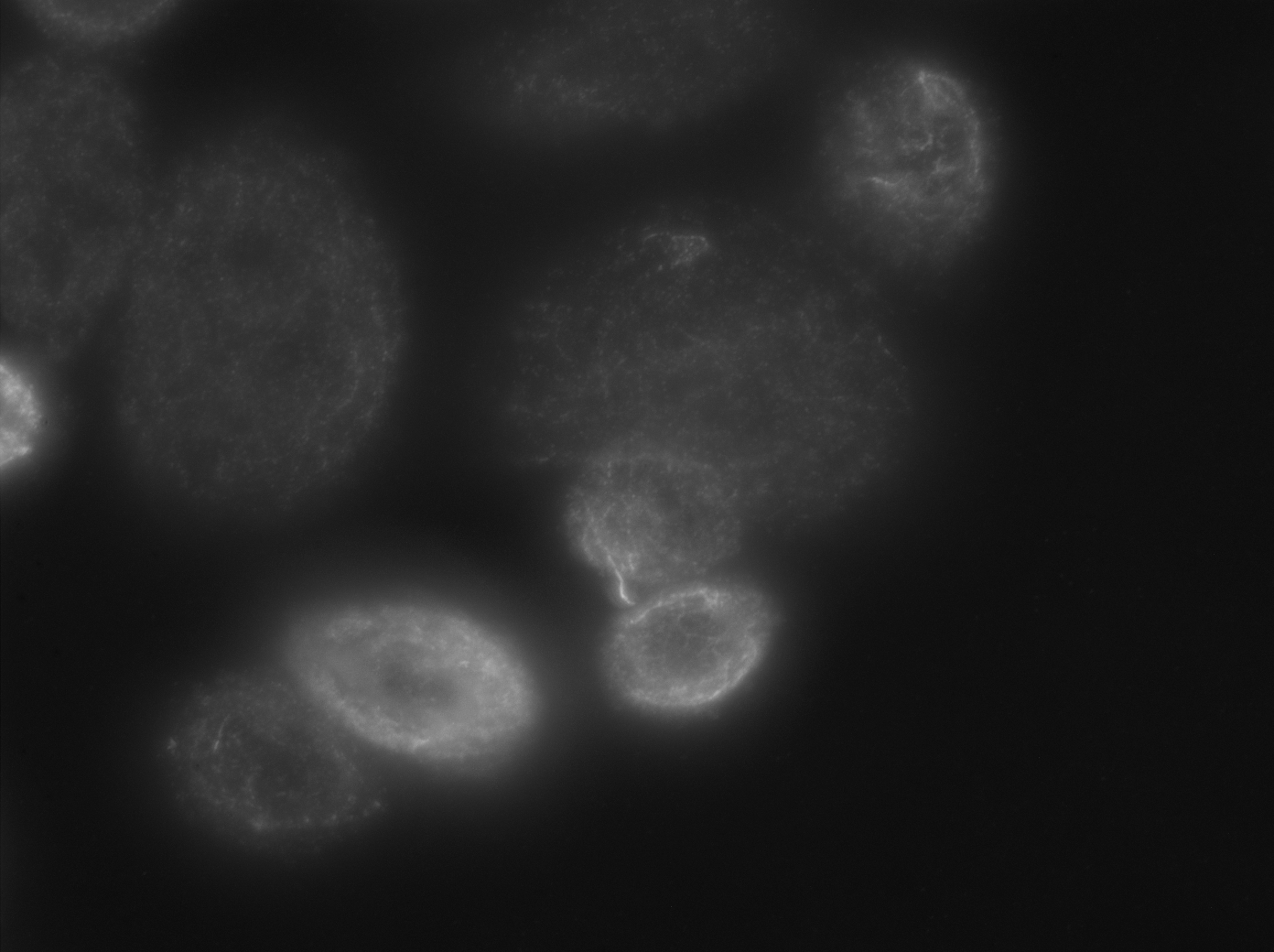

Supplement: Supplementary file 12 — Source data Fig. 3 part 2 [file 44319_2026_742_MOESM12_ESM.zip › Figure 3 Part 2/Fig 3b-e TTLL screen part 2/TTLL5-YFPy I9.Project Maximum Z_XY1679085208_Z0_T0_C1.tif]

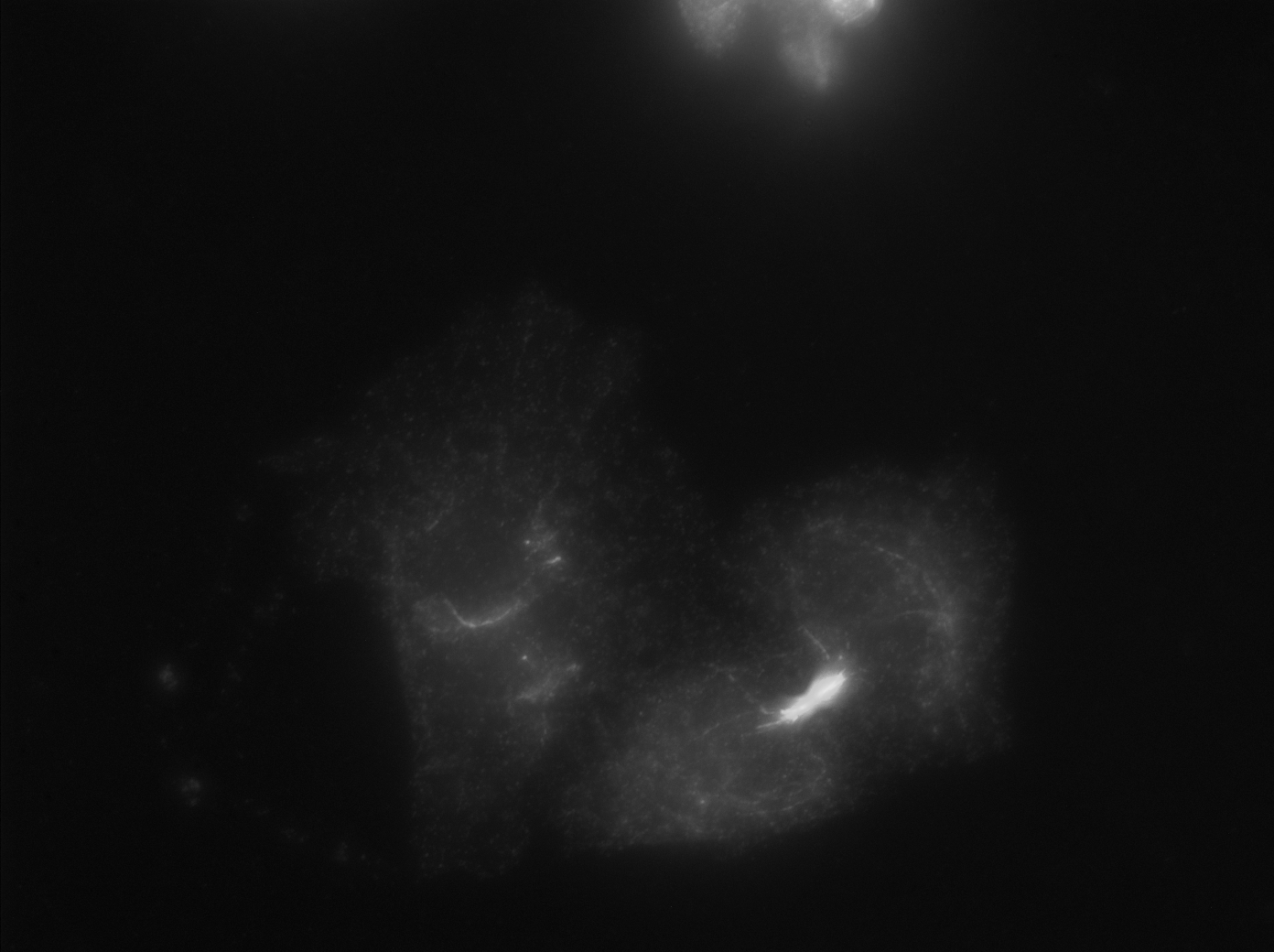

Supplement: Supplementary file 12 — Source data Fig. 3 part 2 [file 44319_2026_742_MOESM12_ESM.zip › Figure 3 Part 2/Fig 3b-e TTLL screen part 2/TTLL7-YFPy I4.Project Maximum Z_XY1679087897_Z0_T0_C1.tif]

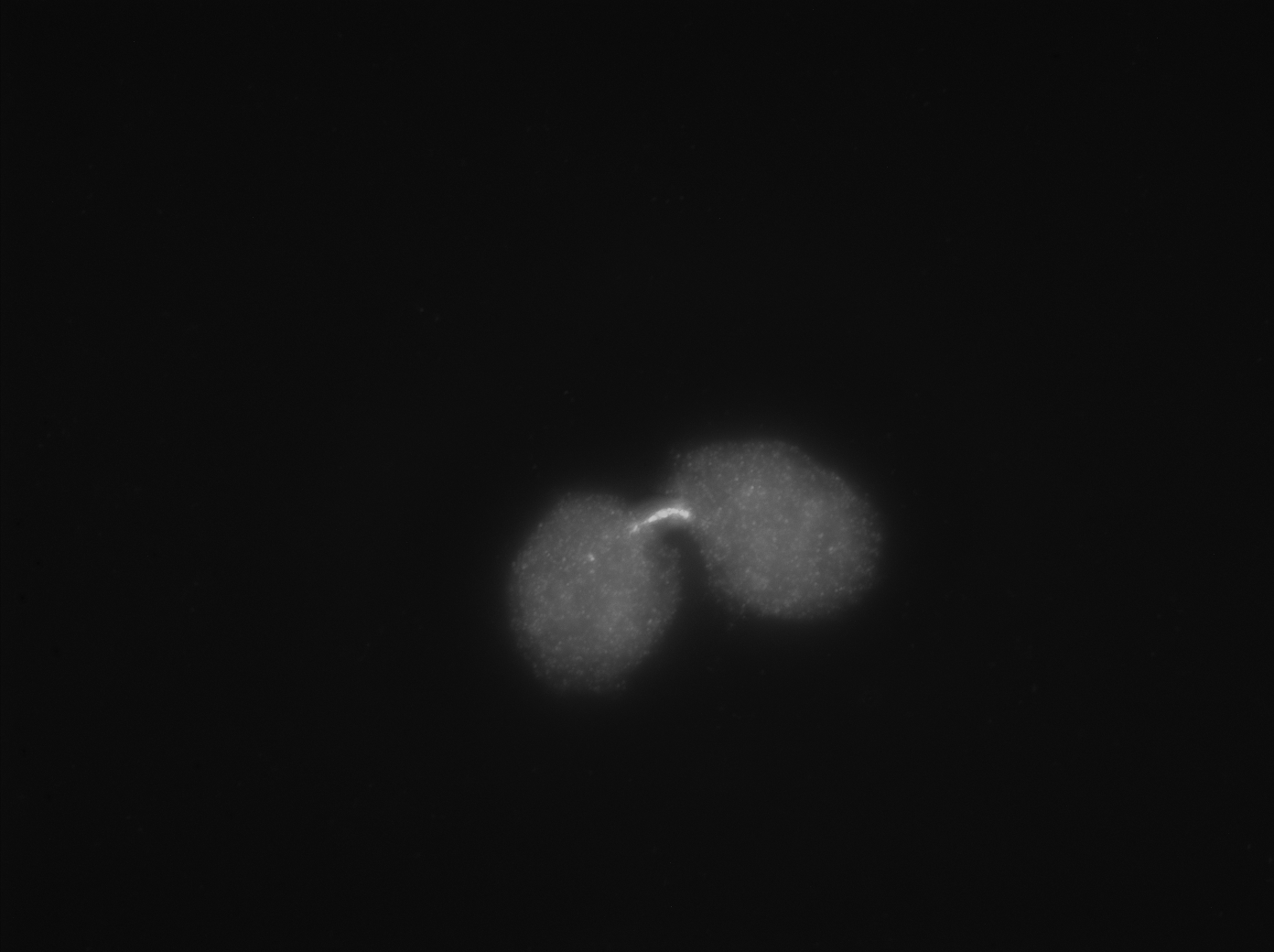

Supplement: Supplementary file 12 — Source data Fig. 3 part 2 [file 44319_2026_742_MOESM12_ESM.zip › Figure 3 Part 2/Fig 3b-e TTLL screen part 2/TTLL6-YFP R1 I3 low int.Project Maximum Z_XY1661547099_Z0_T0_C1.tif]

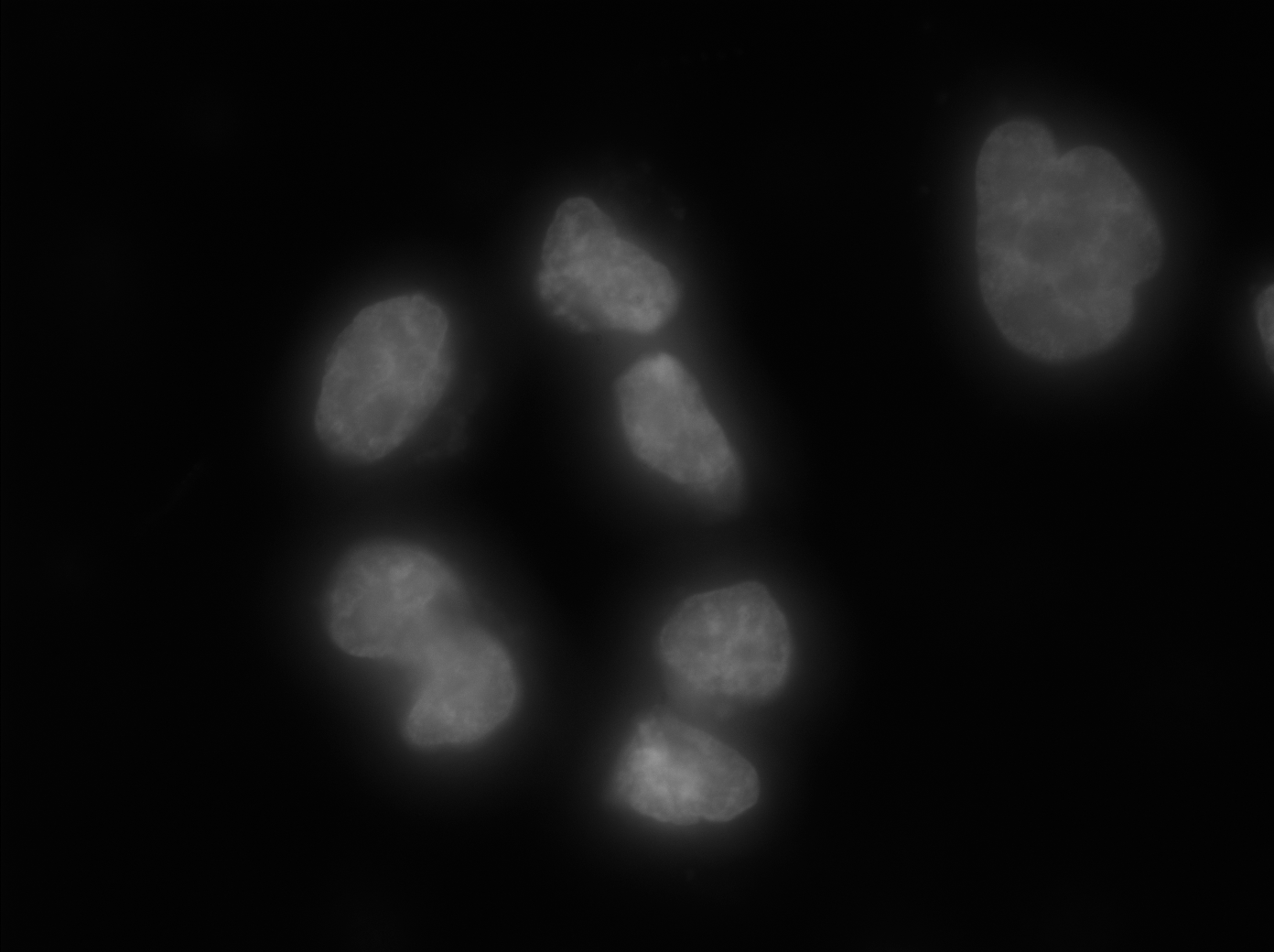

Supplement: Supplementary file 12 — Source data Fig. 3 part 2 [file 44319_2026_742_MOESM12_ESM.zip › Figure 3 Part 2/Fig 3b-e TTLL screen part 2/TTLL5-YFPy I3.Project Maximum Z_XY1679082773_Z0_T0_C0.tif]

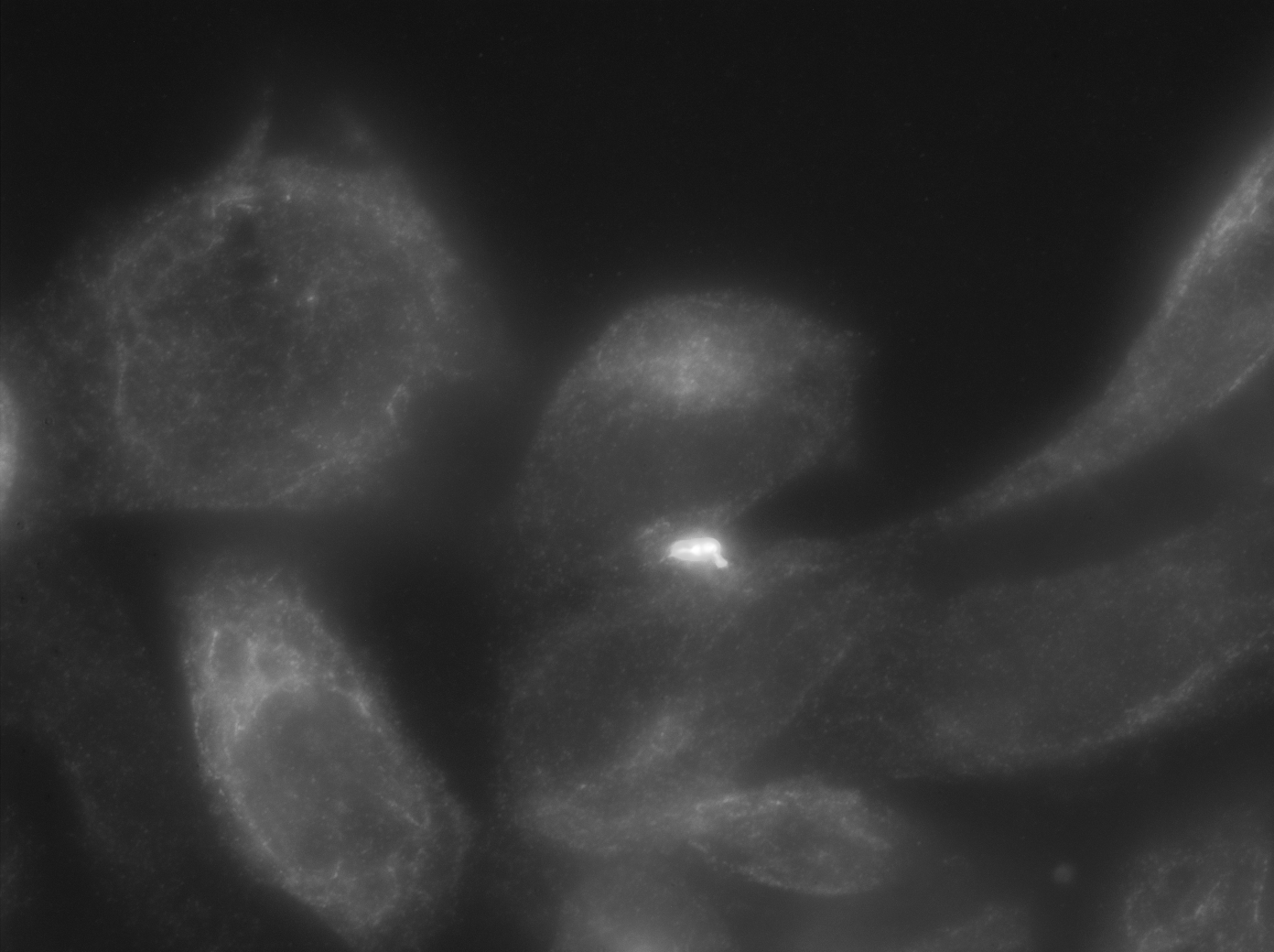

Supplement: Supplementary file 12 — Source data Fig. 3 part 2 [file 44319_2026_742_MOESM12_ESM.zip › Figure 3 Part 2/Fig 3b-e TTLL screen part 2/TTLL5-YFPy I10.Project Maximum Z_XY1679085407_Z0_T0_C1.tif]

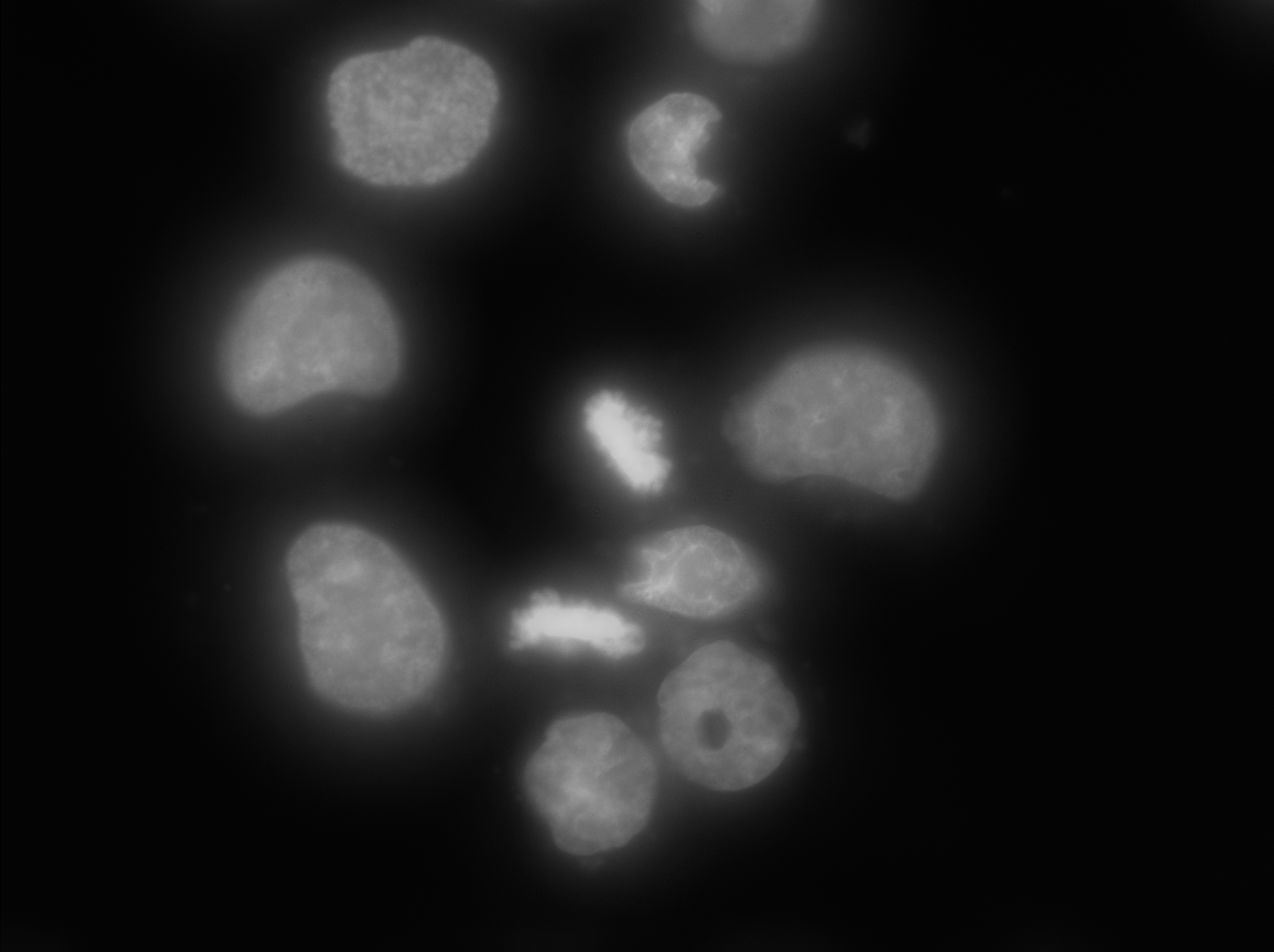

Supplement: Supplementary file 12 — Source data Fig. 3 part 2 [file 44319_2026_742_MOESM12_ESM.zip › Figure 3 Part 2/Fig 3b-e TTLL screen part 2/TTLL5-YFPy I13.Project Maximum Z_XY1679340570_Z0_T0_C0.tif]

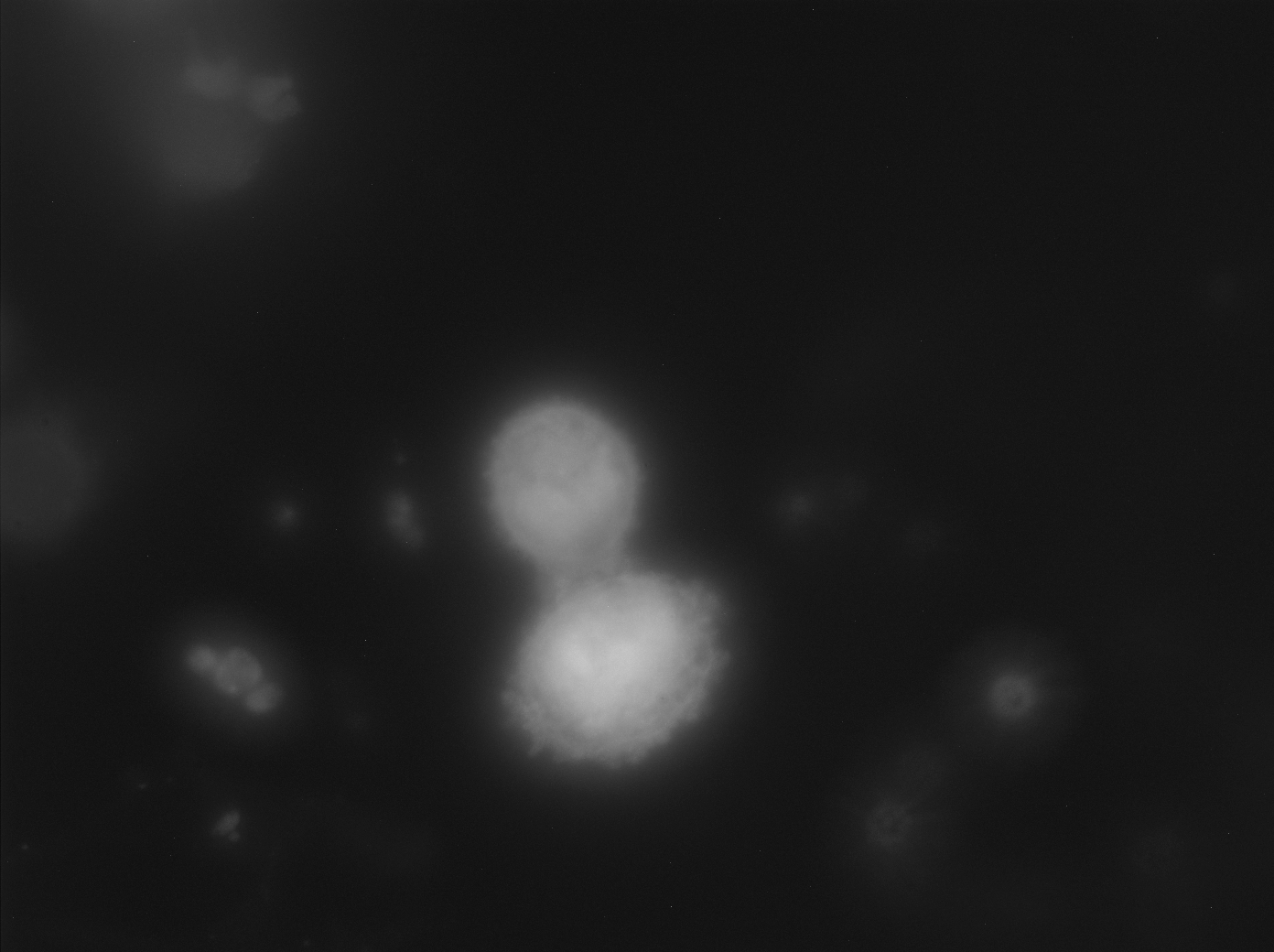

Supplement: Supplementary file 12 — Source data Fig. 3 part 2 [file 44319_2026_742_MOESM12_ESM.zip › Figure 3 Part 2/Fig 3b-e TTLL screen part 2/TTLL7-YFPy I17.Project Maximum Z_XY1679090864_Z0_T0_C2.tif]

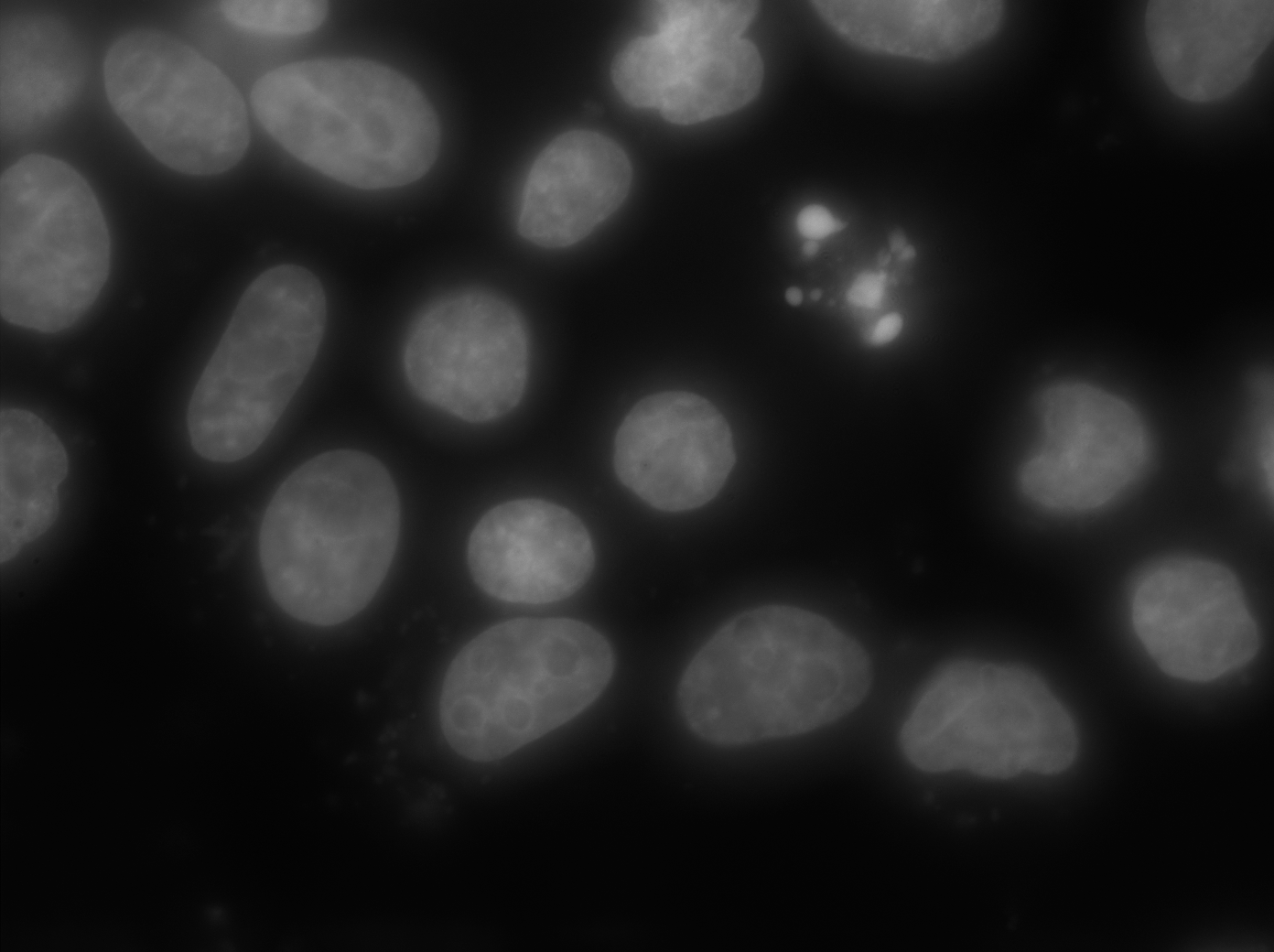

Supplement: Supplementary file 12 — Source data Fig. 3 part 2 [file 44319_2026_742_MOESM12_ESM.zip › Figure 3 Part 2/Fig 3b-e TTLL screen part 2/TTLL7-YFPy I10.Project Maximum Z_XY1679089174_Z0_T0_C0.tif]

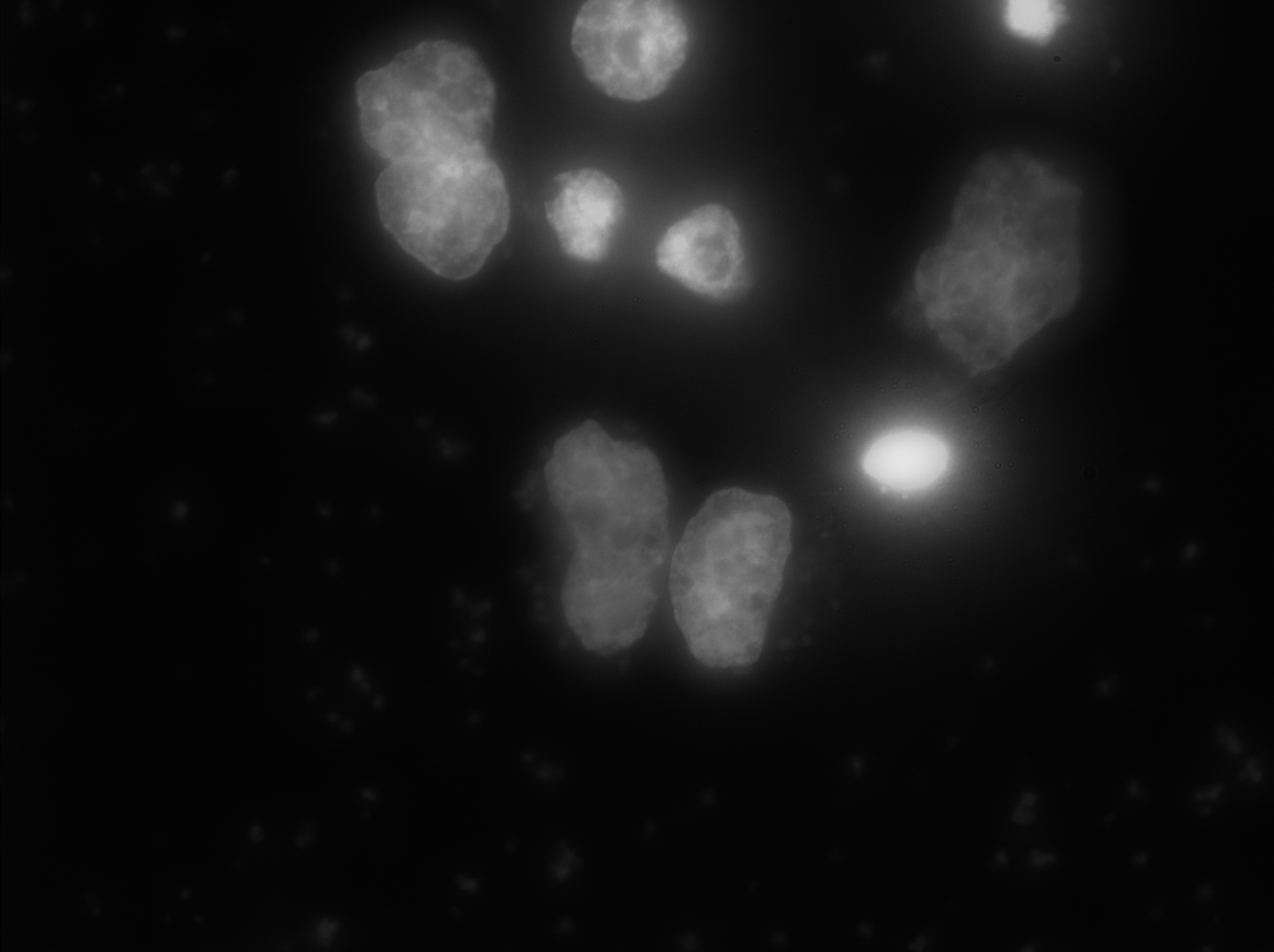

Supplement: Supplementary file 12 — Source data Fig. 3 part 2 [file 44319_2026_742_MOESM12_ESM.zip › Figure 3 Part 2/Fig 3b-e TTLL screen part 2/TTLL5-YFPy I16.Project Maximum Z_XY1679341063_Z0_T0_C0.tif]

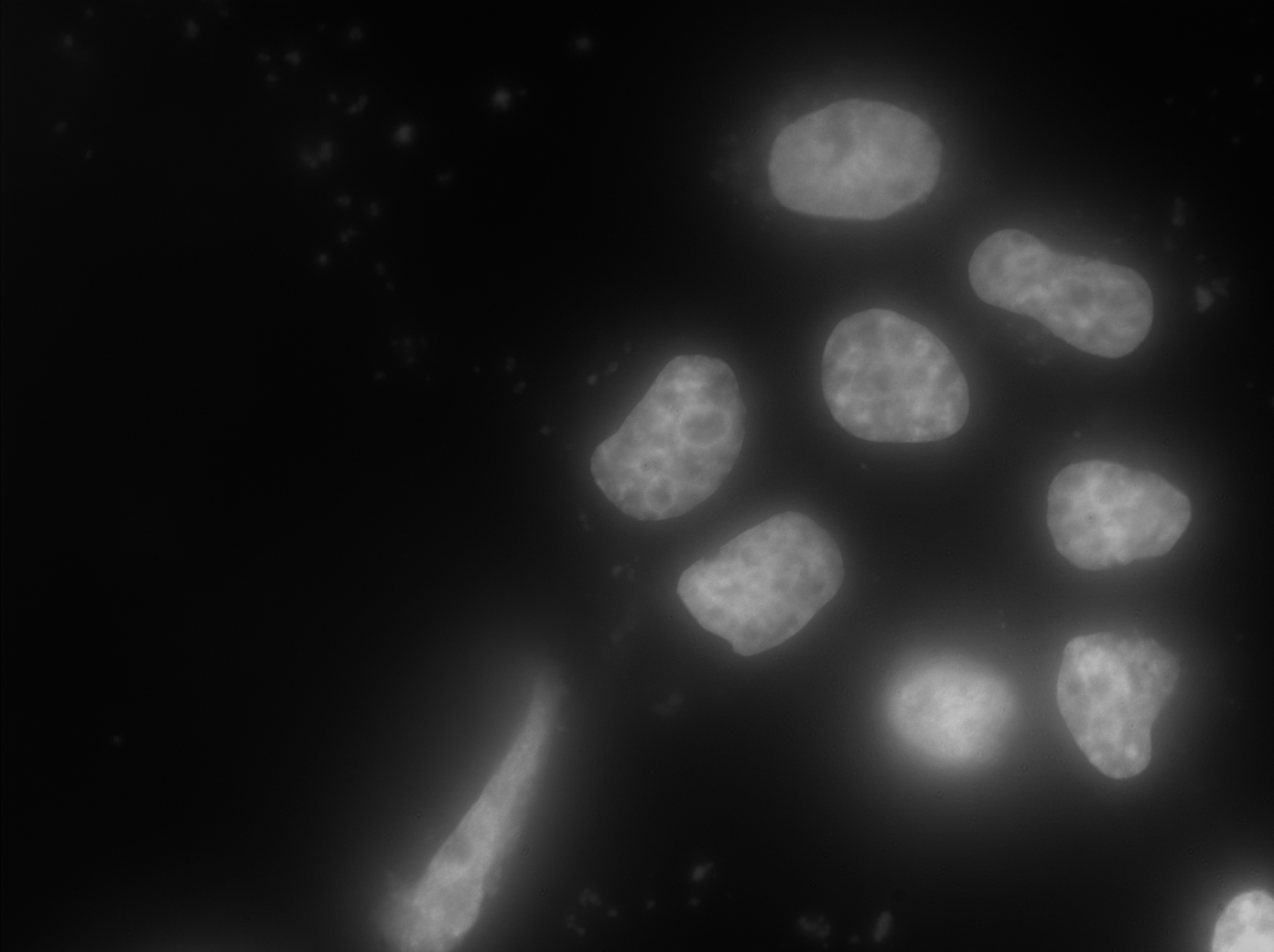

Supplement: Supplementary file 12 — Source data Fig. 3 part 2 [file 44319_2026_742_MOESM12_ESM.zip › Figure 3 Part 2/Fig 3b-e TTLL screen part 2/TTLL7-YFPy I6.Project Maximum Z_XY1679088203_Z0_T0_C0.tif]

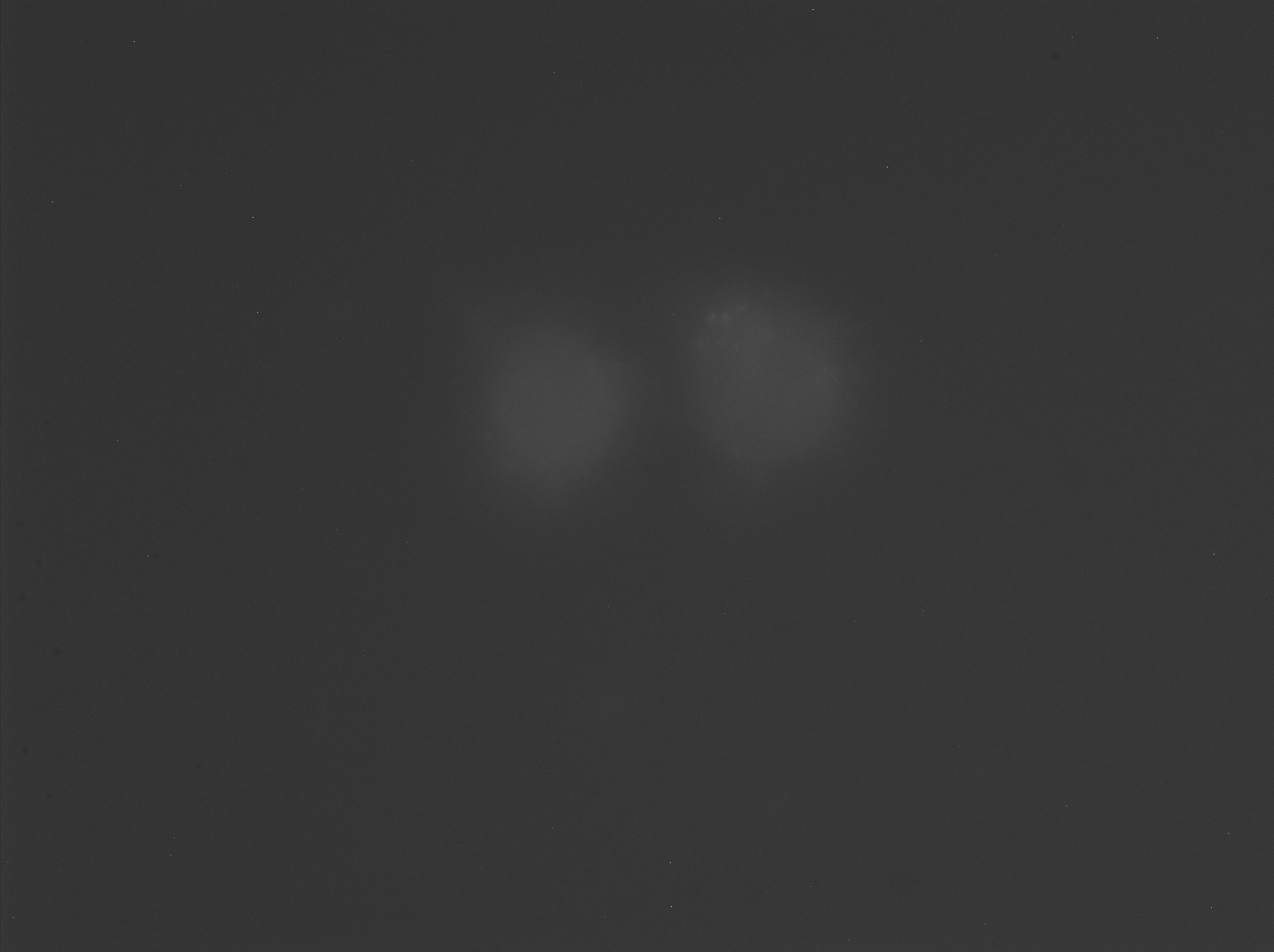

Supplement: Supplementary file 12 — Source data Fig. 3 part 2 [file 44319_2026_742_MOESM12_ESM.zip › Figure 3 Part 2/Fig 3b-e TTLL screen part 2/TTLL6-YFP MB light I2.Project Maximum Z_XY1663880938_Z0_T0_C2.tif]

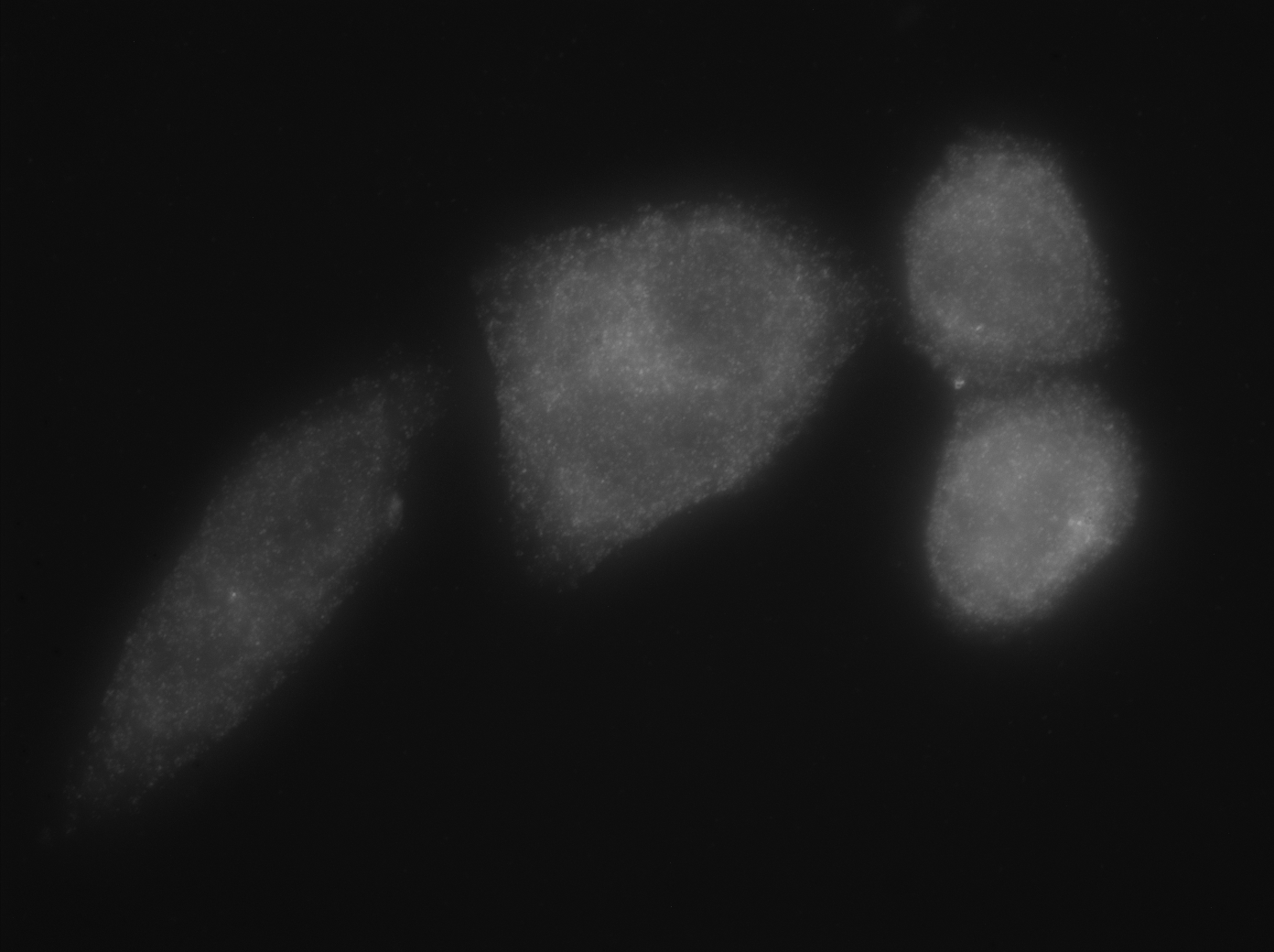

Supplement: Supplementary file 12 — Source data Fig. 3 part 2 [file 44319_2026_742_MOESM12_ESM.zip › Figure 3 Part 2/Fig 3b-e TTLL screen part 2/TTLL6-YFP R1 T2.Project Maximum Z_XY1661549228_Z0_T0_C1.tif]

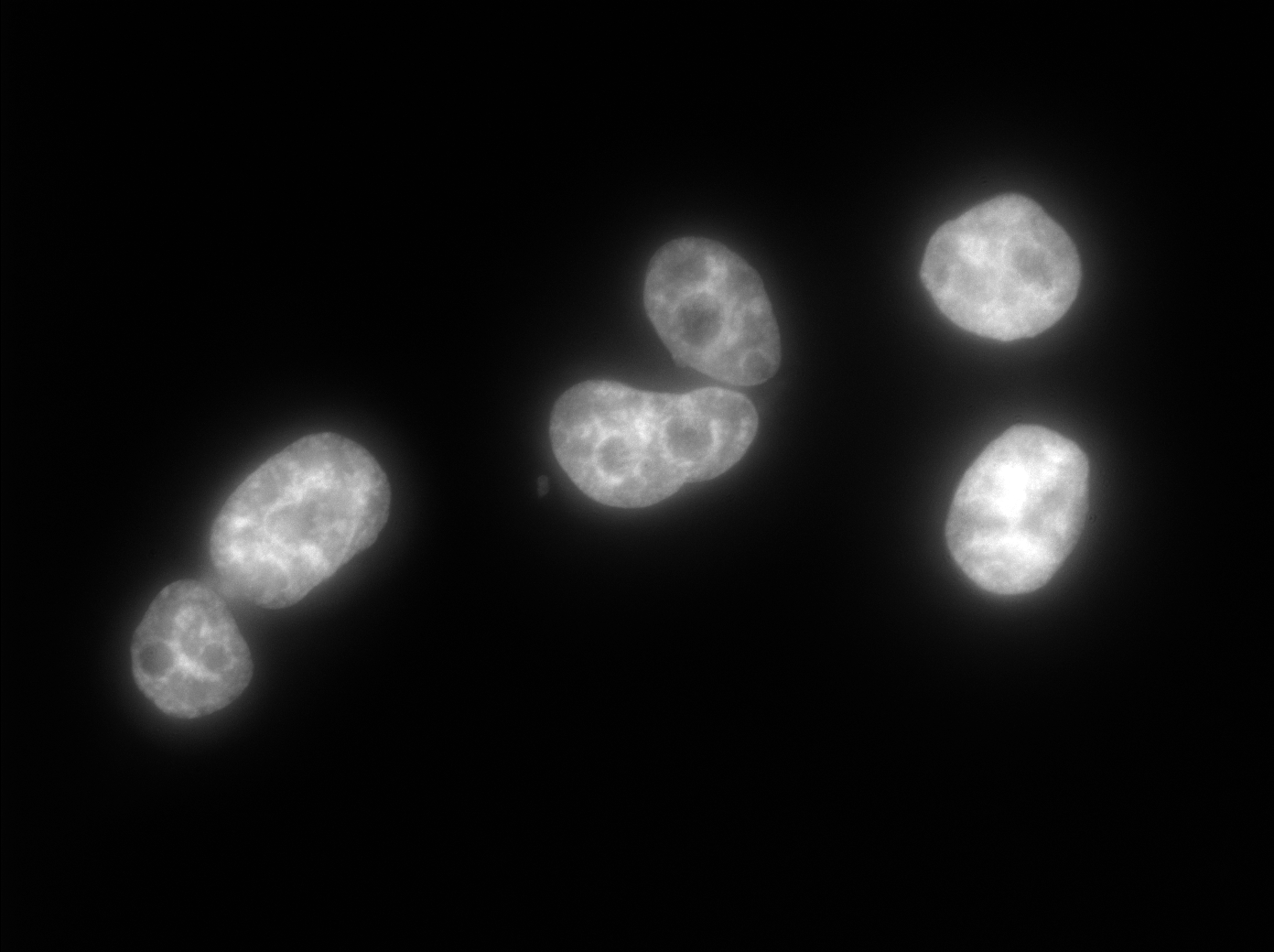

Supplement: Supplementary file 12 — Source data Fig. 3 part 2 [file 44319_2026_742_MOESM12_ESM.zip › Figure 3 Part 2/Fig 3b-e TTLL screen part 2/TTLL6-YFP R1 T2.Project Maximum Z_XY1661549228_Z0_T0_C0.tif]

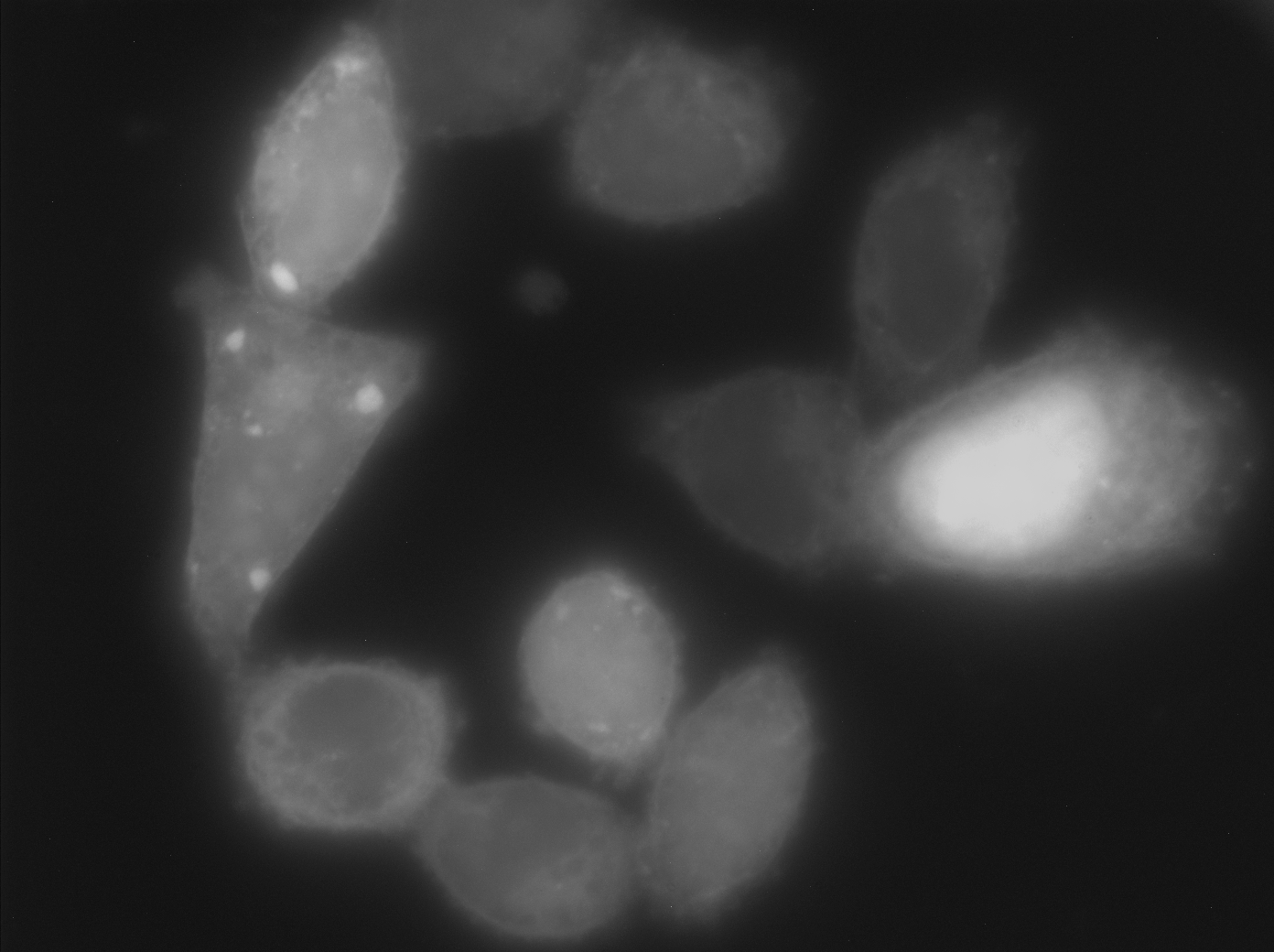

Supplement: Supplementary file 12 — Source data Fig. 3 part 2 [file 44319_2026_742_MOESM12_ESM.zip › Figure 3 Part 2/Fig 3b-e TTLL screen part 2/TTLL7-YFPy I8.Project Maximum Z_XY1679088689_Z0_T0_C2.tif]

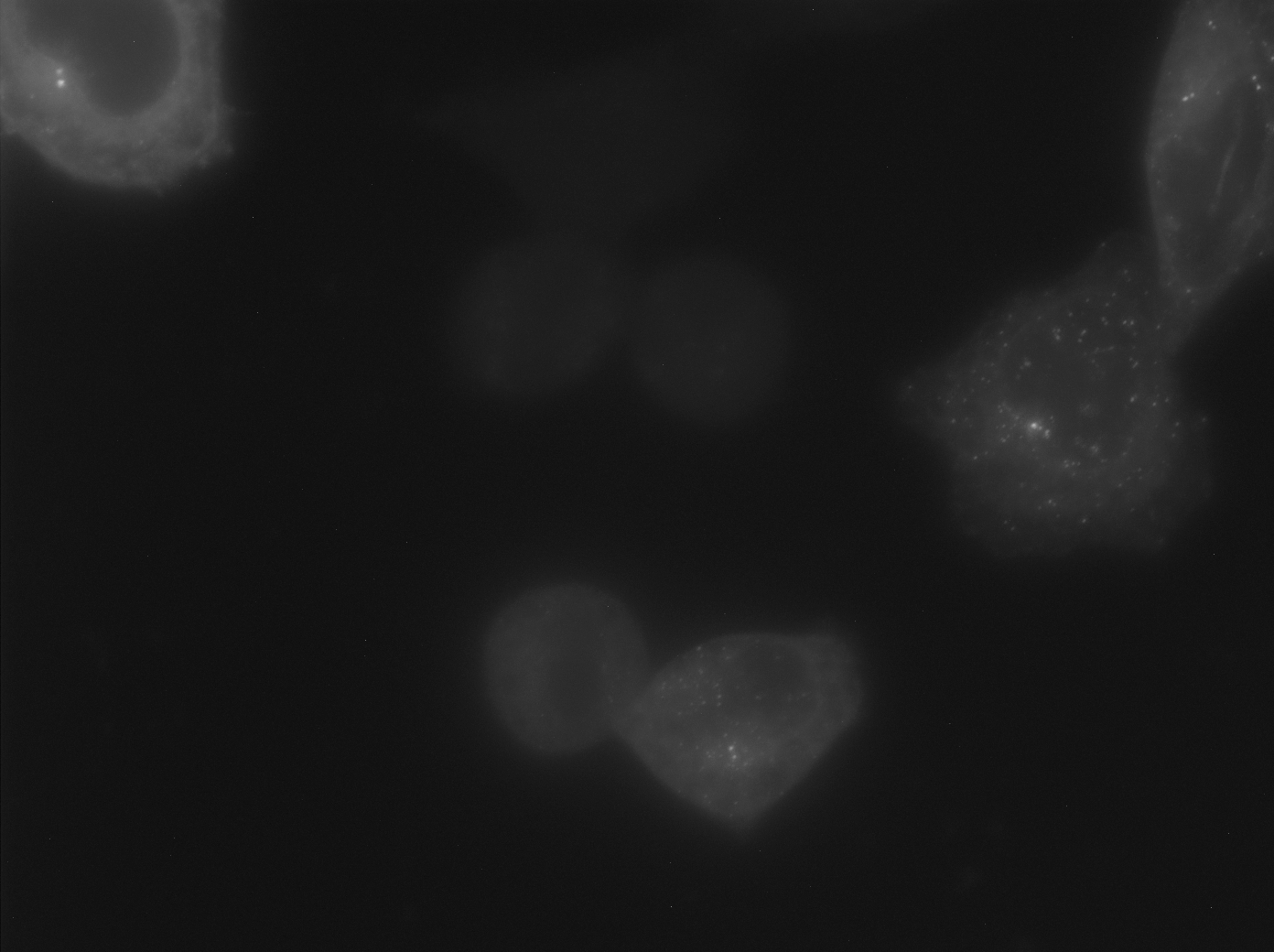

Supplement: Supplementary file 12 — Source data Fig. 3 part 2 [file 44319_2026_742_MOESM12_ESM.zip › Figure 3 Part 2/Fig 3b-e TTLL screen part 2/TTLL5-YFPy I7.Project Maximum Z_XY1679084862_Z0_T0_C2.tif]

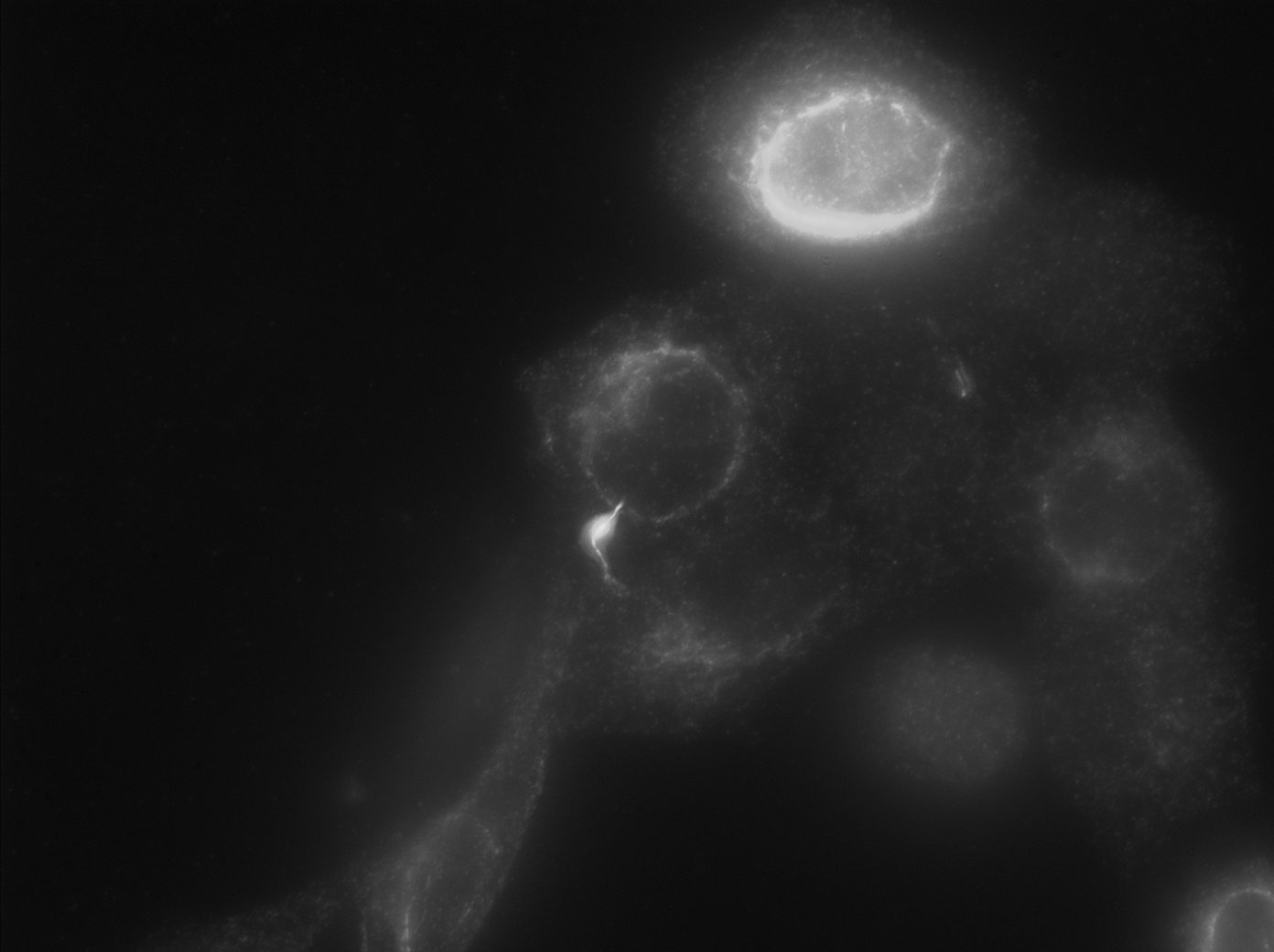

Supplement: Supplementary file 12 — Source data Fig. 3 part 2 [file 44319_2026_742_MOESM12_ESM.zip › Figure 3 Part 2/Fig 3b-e TTLL screen part 2/TTLL7-YFPy I6.Project Maximum Z_XY1679088203_Z0_T0_C1.tif]

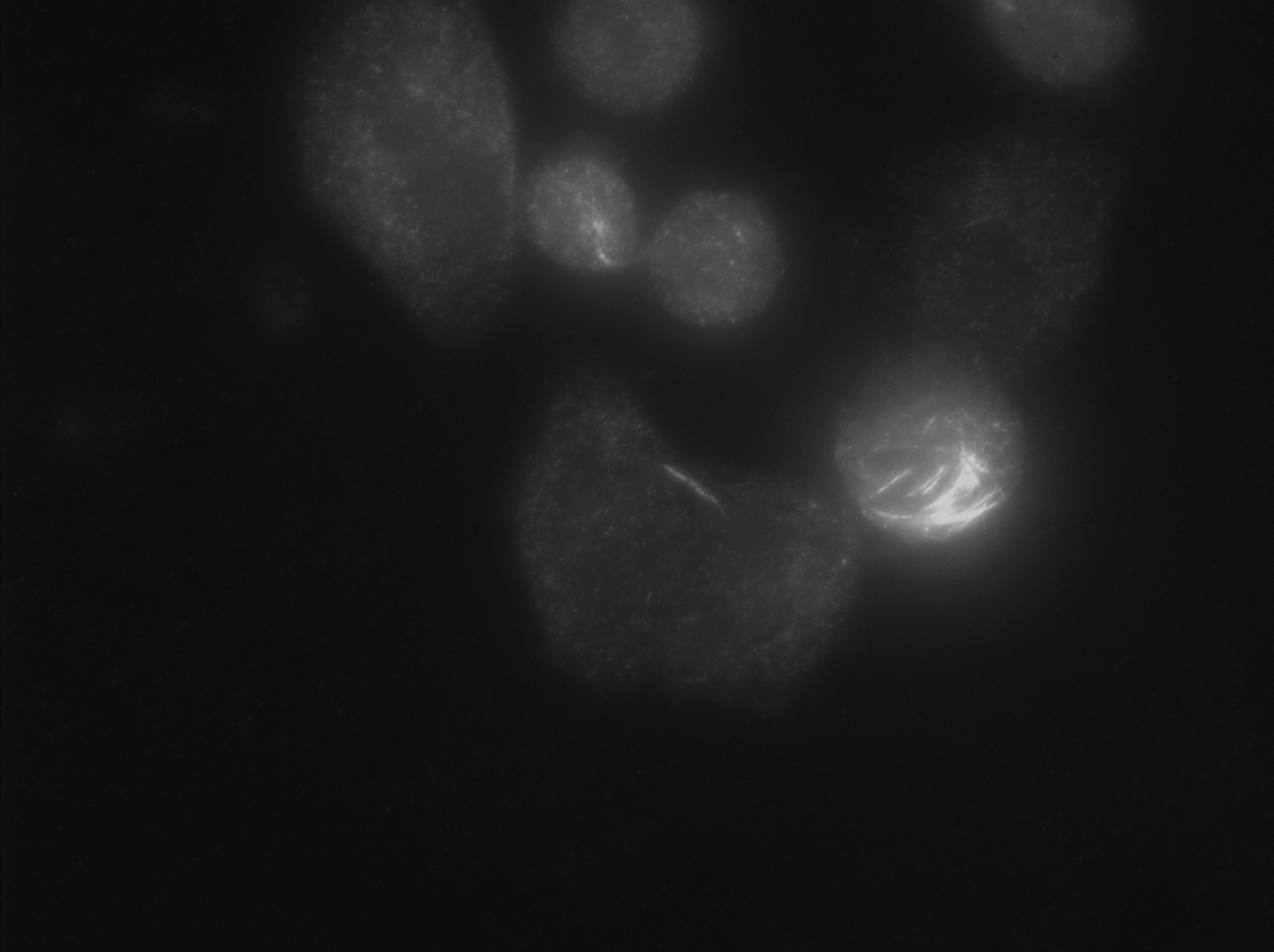

Supplement: Supplementary file 12 — Source data Fig. 3 part 2 [file 44319_2026_742_MOESM12_ESM.zip › Figure 3 Part 2/Fig 3b-e TTLL screen part 2/TTLL5-YFPy I16.Project Maximum Z_XY1679341063_Z0_T0_C1.tif]

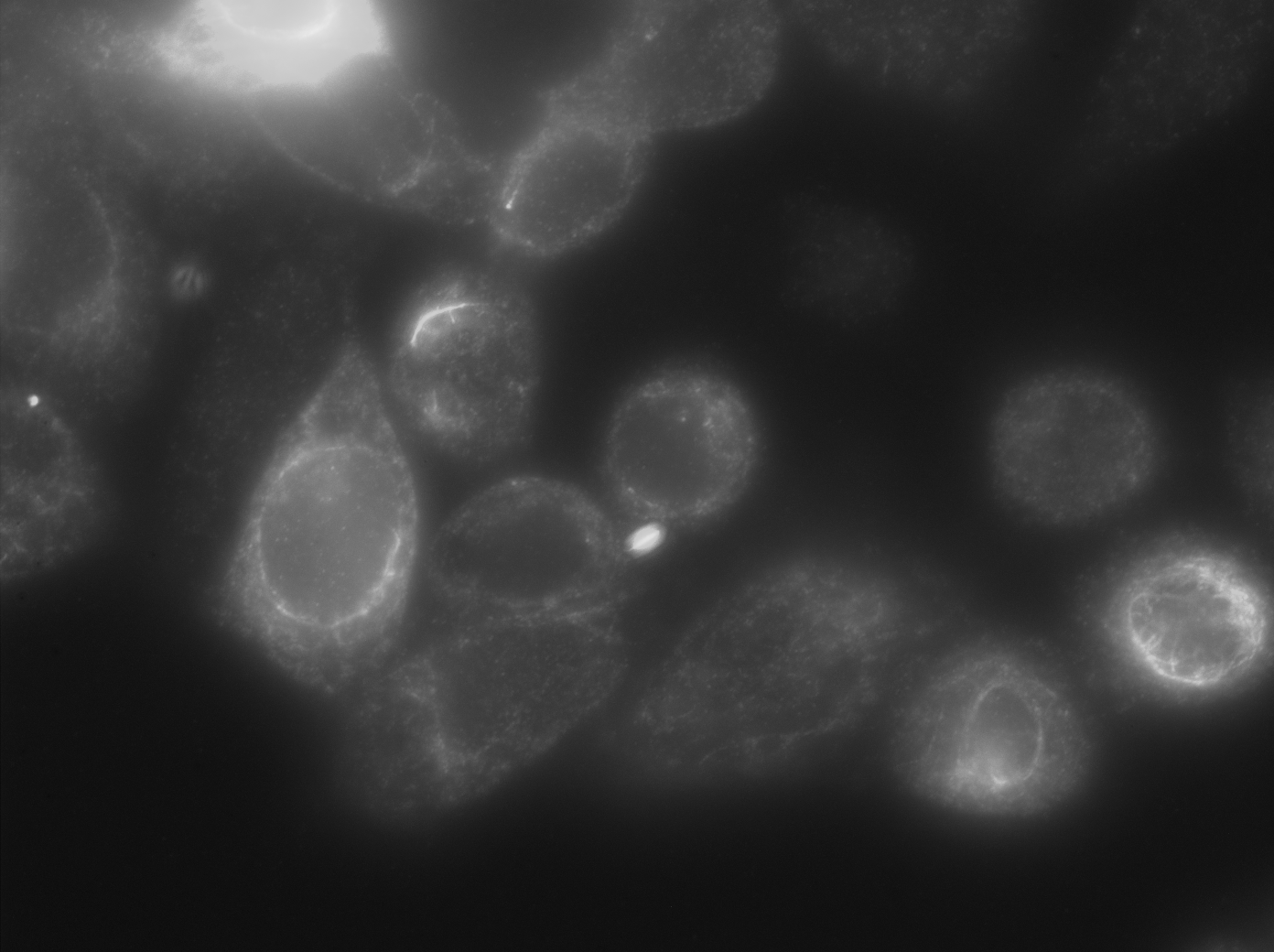

Supplement: Supplementary file 12 — Source data Fig. 3 part 2 [file 44319_2026_742_MOESM12_ESM.zip › Figure 3 Part 2/Fig 3b-e TTLL screen part 2/TTLL7-YFPy I10.Project Maximum Z_XY1679089174_Z0_T0_C1.tif]

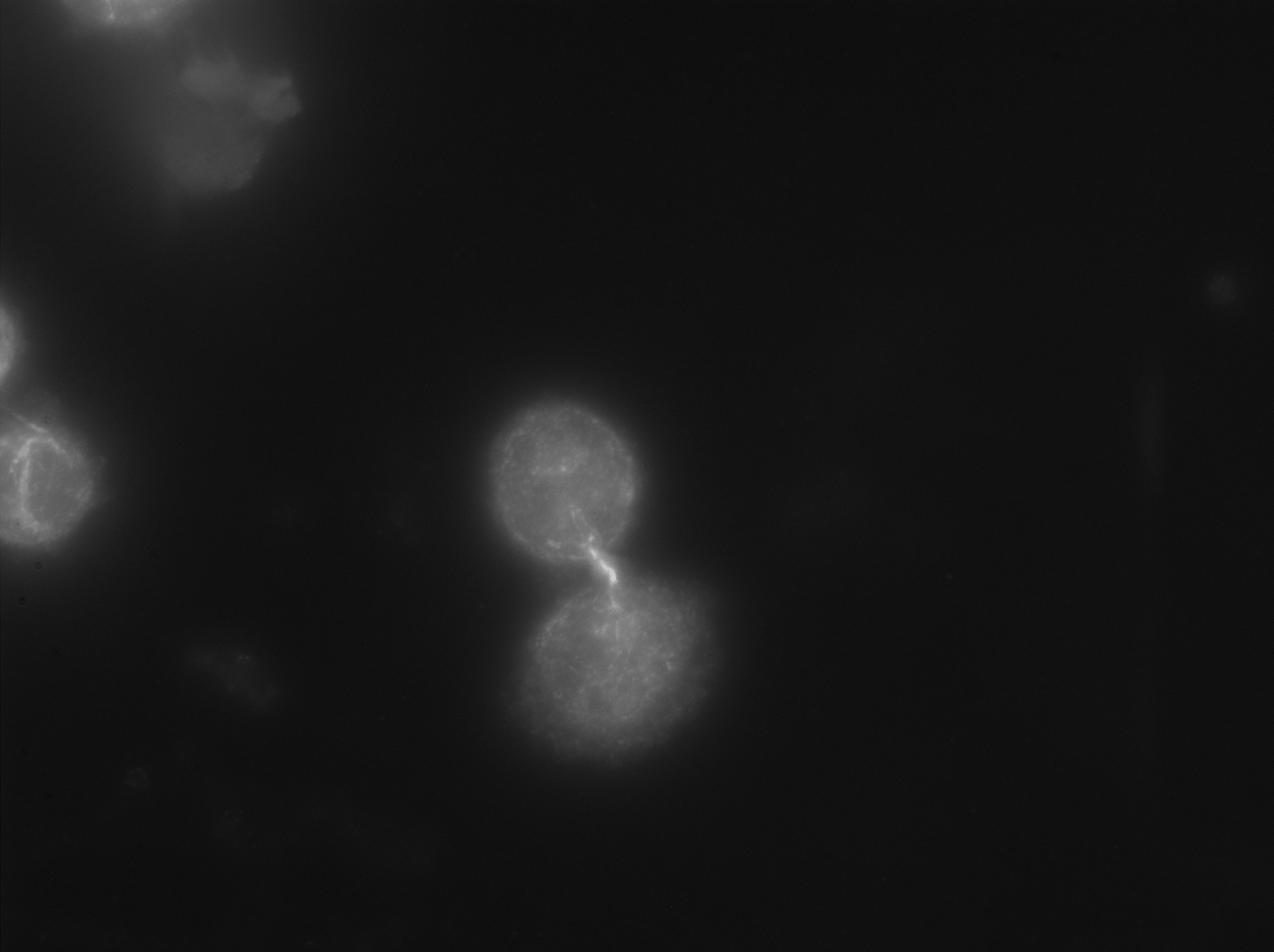

Supplement: Supplementary file 12 — Source data Fig. 3 part 2 [file 44319_2026_742_MOESM12_ESM.zip › Figure 3 Part 2/Fig 3b-e TTLL screen part 2/TTLL7-YFPy I17.Project Maximum Z_XY1679090864_Z0_T0_C1.tif]

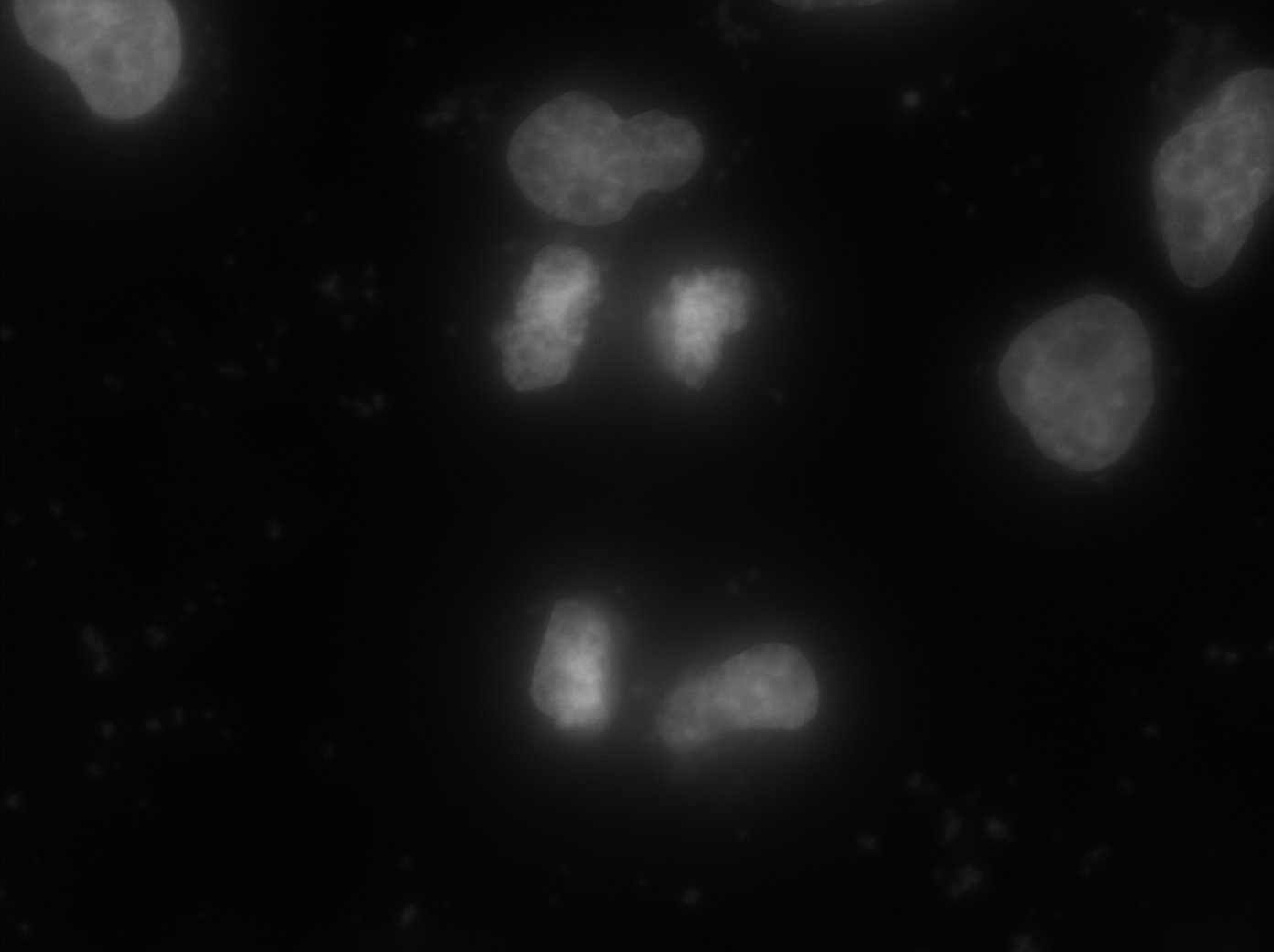

Supplement: Supplementary file 12 — Source data Fig. 3 part 2 [file 44319_2026_742_MOESM12_ESM.zip › Figure 3 Part 2/Fig 3b-e TTLL screen part 2/TTLL5-YFPy I7.Project Maximum Z_XY1679084862_Z0_T0_C0.tif]

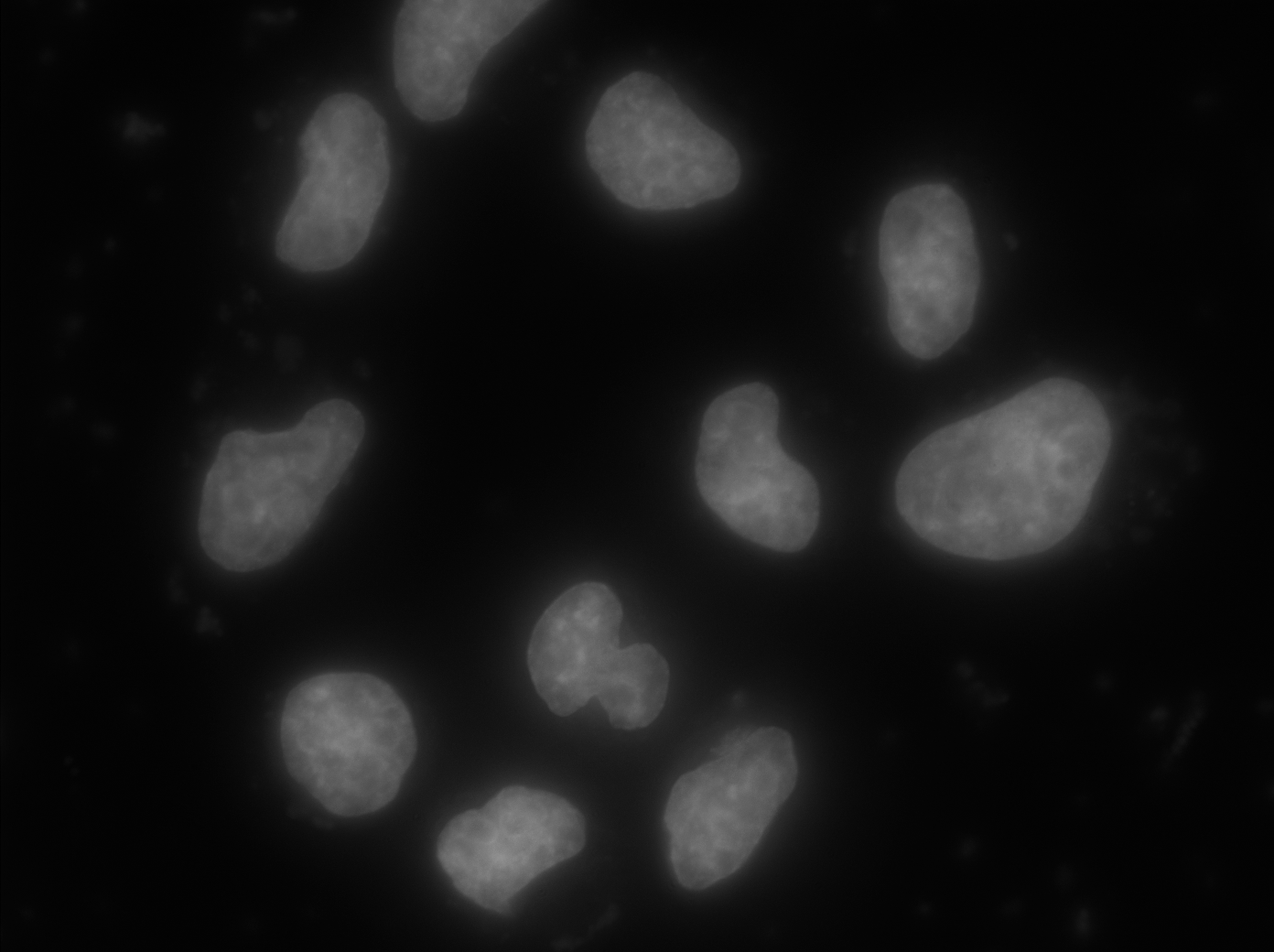

Supplement: Supplementary file 12 — Source data Fig. 3 part 2 [file 44319_2026_742_MOESM12_ESM.zip › Figure 3 Part 2/Fig 3b-e TTLL screen part 2/TTLL7-YFPy I8.Project Maximum Z_XY1679088689_Z0_T0_C0.tif]

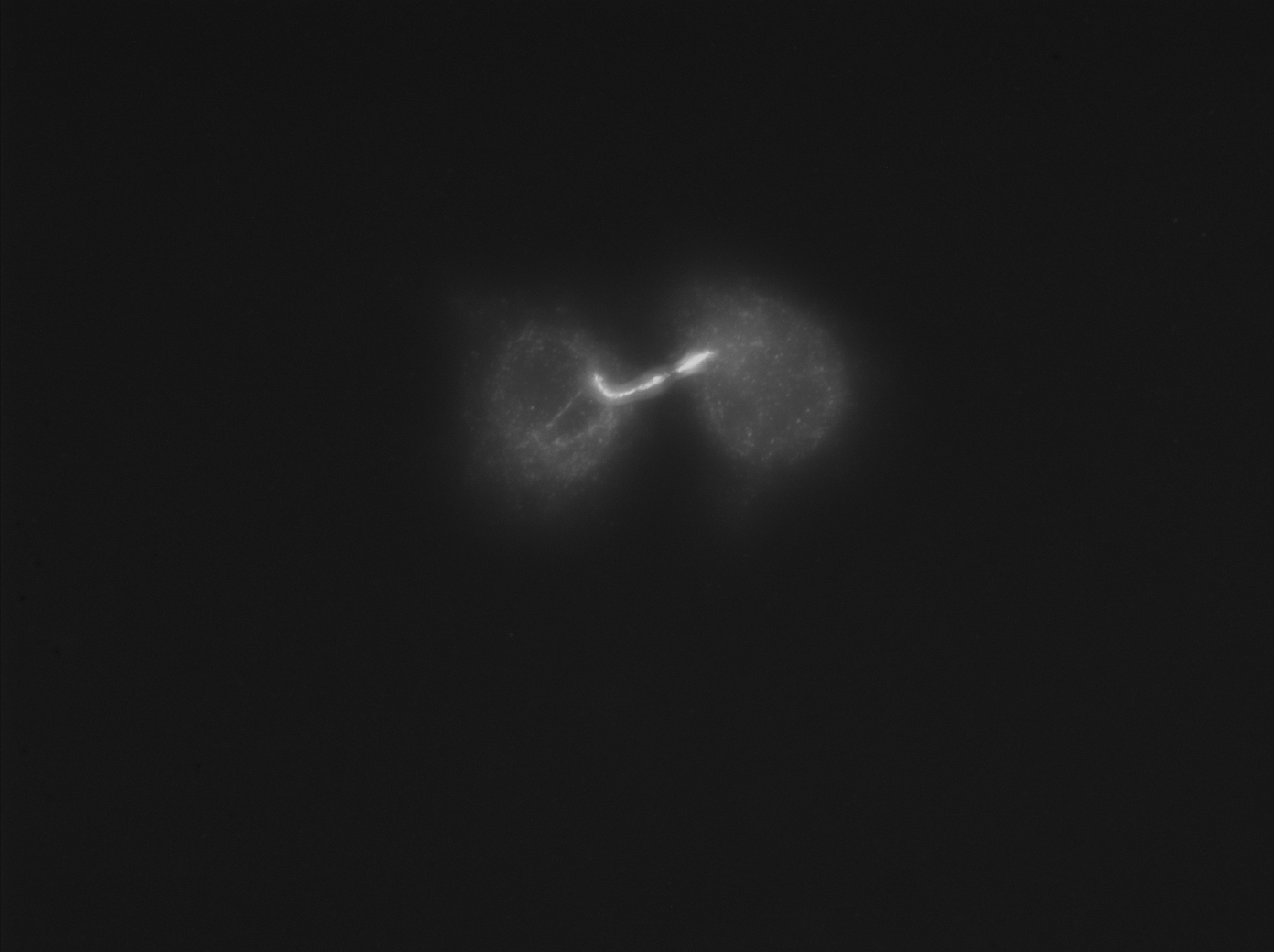

Supplement: Supplementary file 12 — Source data Fig. 3 part 2 [file 44319_2026_742_MOESM12_ESM.zip › Figure 3 Part 2/Fig 3b-e TTLL screen part 2/TTLL6-YFP MB light I2.Project Maximum Z_XY1663880938_Z0_T0_C1.tif]

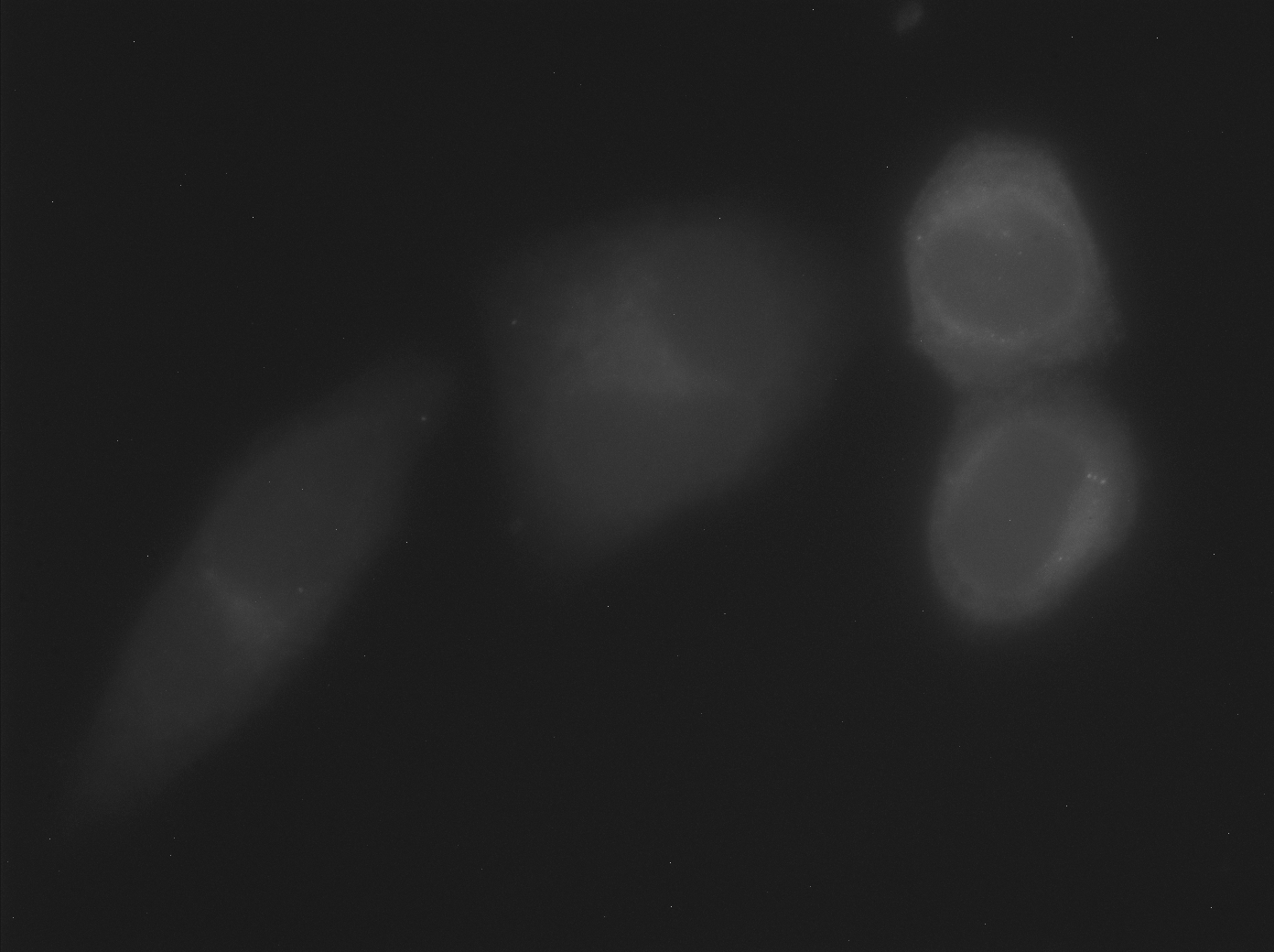

Supplement: Supplementary file 12 — Source data Fig. 3 part 2 [file 44319_2026_742_MOESM12_ESM.zip › Figure 3 Part 2/Fig 3b-e TTLL screen part 2/TTLL6-YFP R1 T2.Project Maximum Z_XY1661549228_Z0_T0_C2.tif]

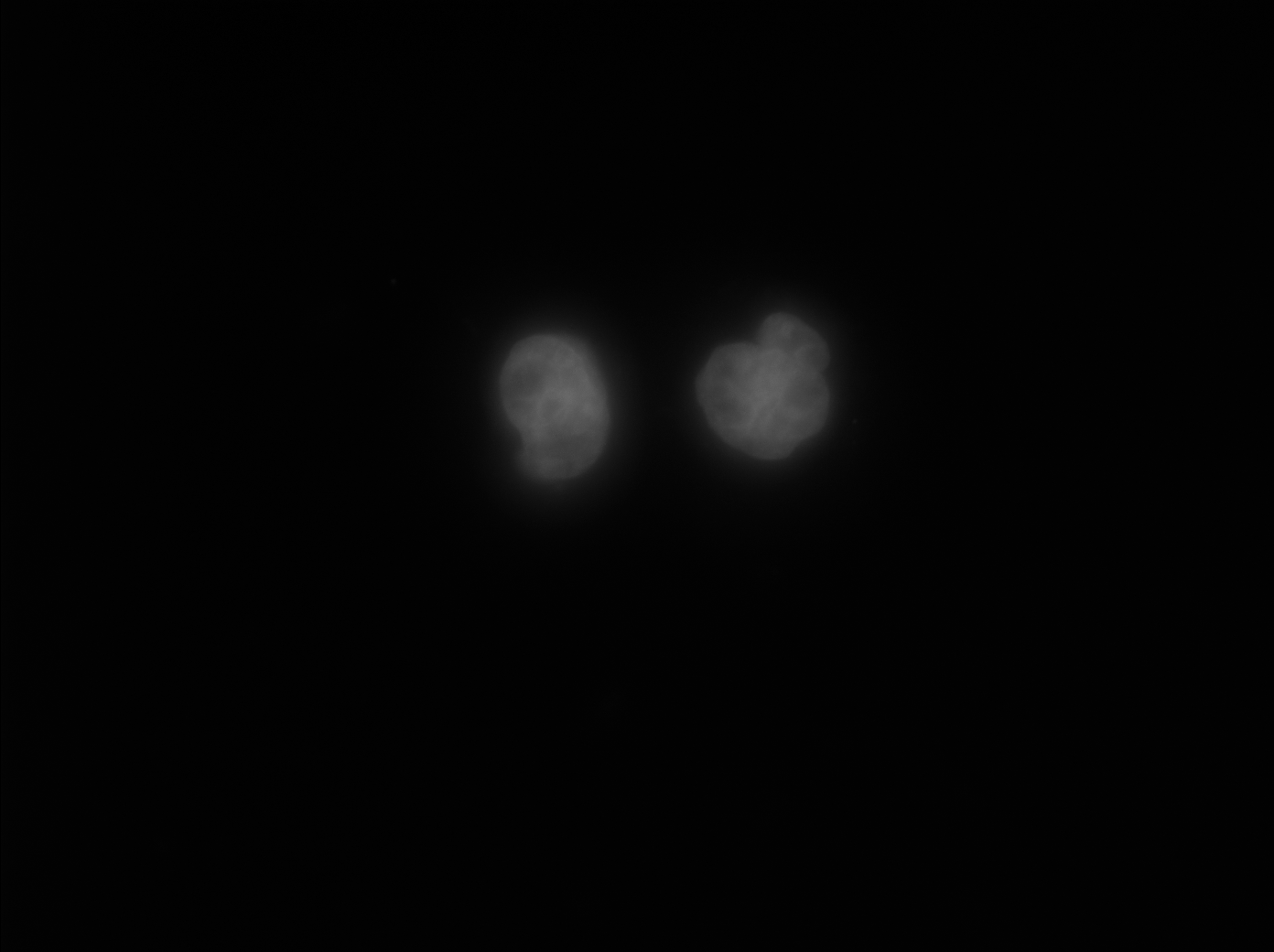

Supplement: Supplementary file 12 — Source data Fig. 3 part 2 [file 44319_2026_742_MOESM12_ESM.zip › Figure 3 Part 2/Fig 3b-e TTLL screen part 2/TTLL6-YFP MB light I2.Project Maximum Z_XY1663880938_Z0_T0_C0.tif]

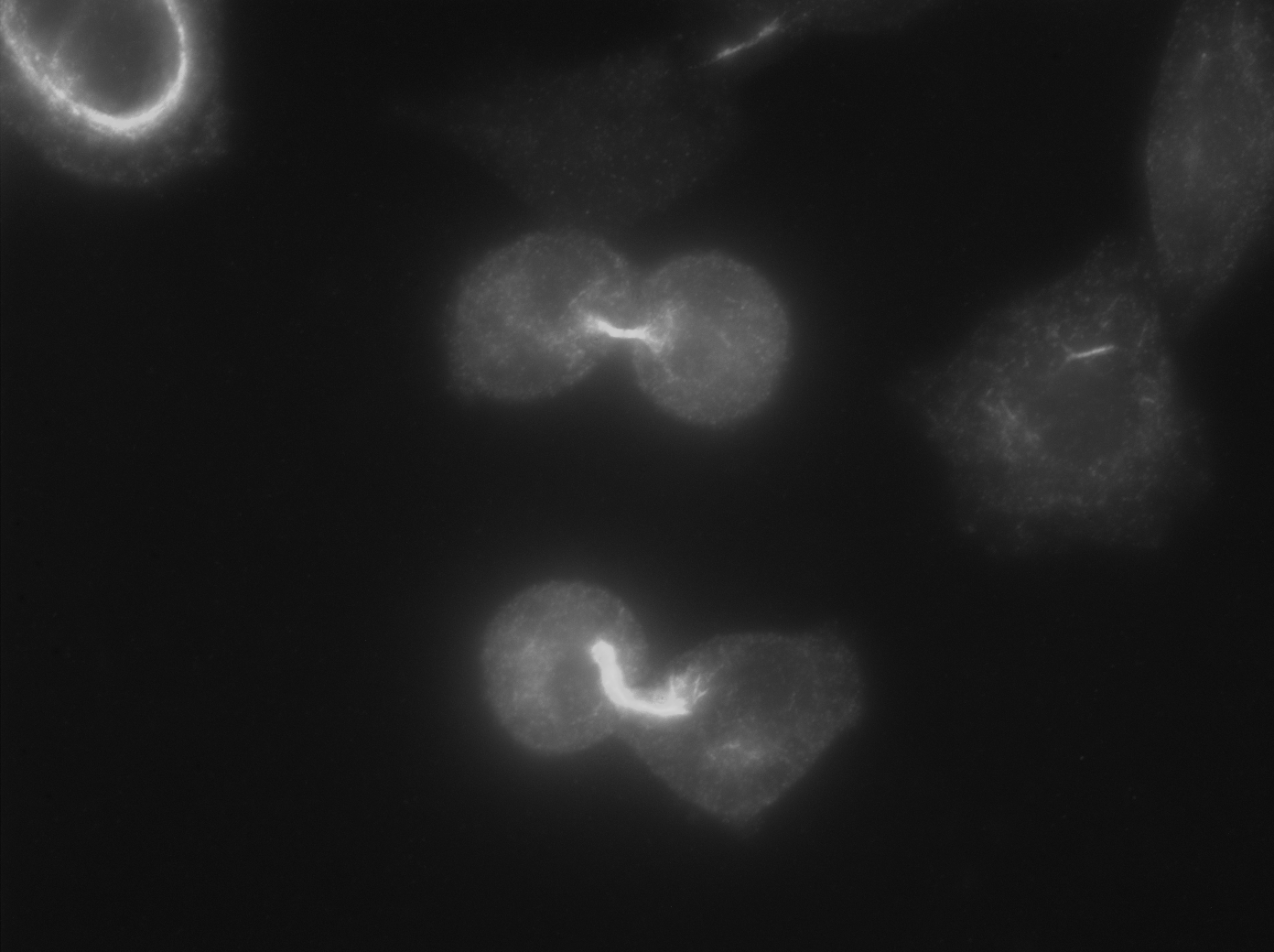

Supplement: Supplementary file 12 — Source data Fig. 3 part 2 [file 44319_2026_742_MOESM12_ESM.zip › Figure 3 Part 2/Fig 3b-e TTLL screen part 2/TTLL5-YFPy I7.Project Maximum Z_XY1679084862_Z0_T0_C1.tif]

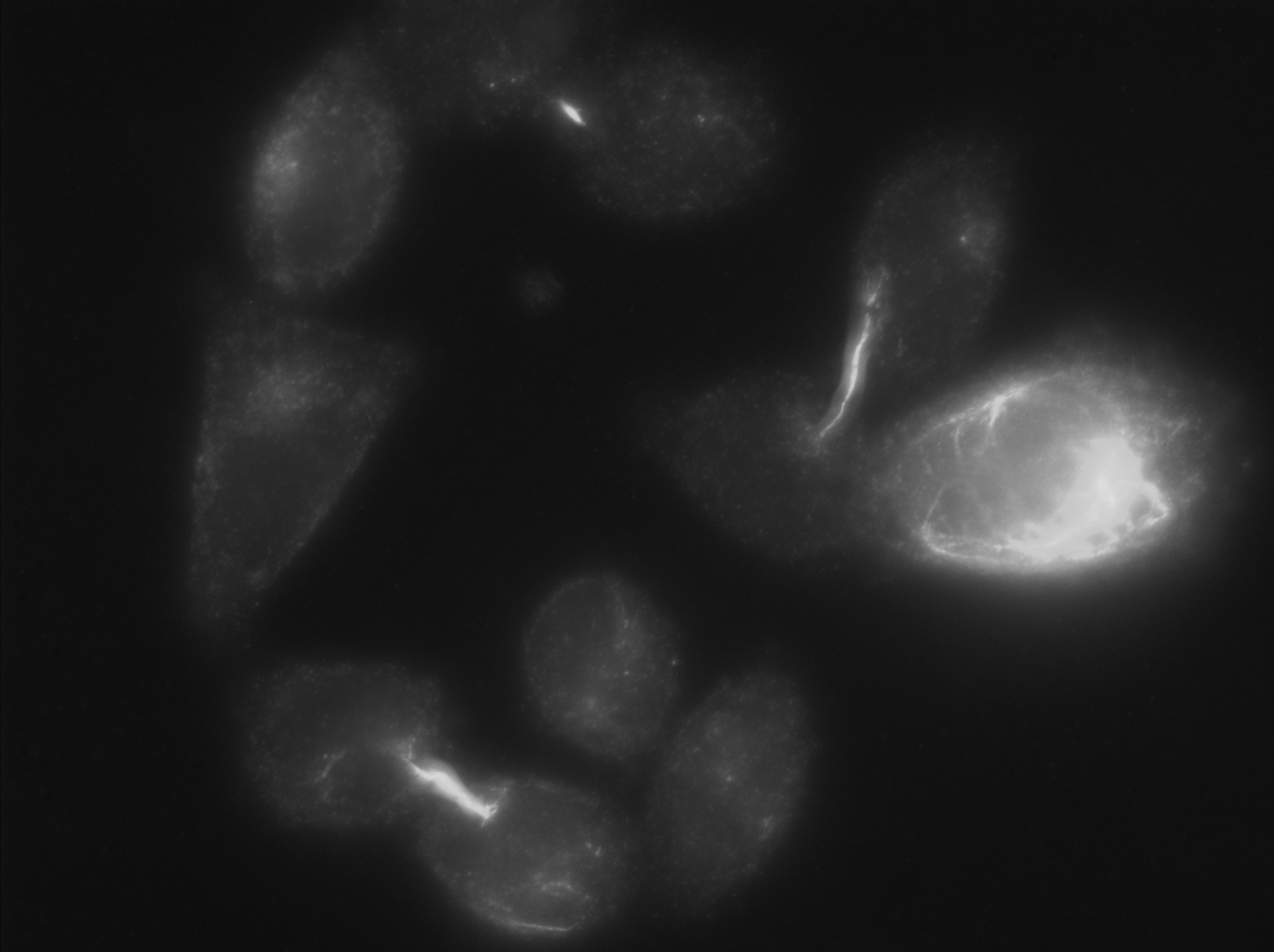

Supplement: Supplementary file 12 — Source data Fig. 3 part 2 [file 44319_2026_742_MOESM12_ESM.zip › Figure 3 Part 2/Fig 3b-e TTLL screen part 2/TTLL7-YFPy I8.Project Maximum Z_XY1679088689_Z0_T0_C1.tif]

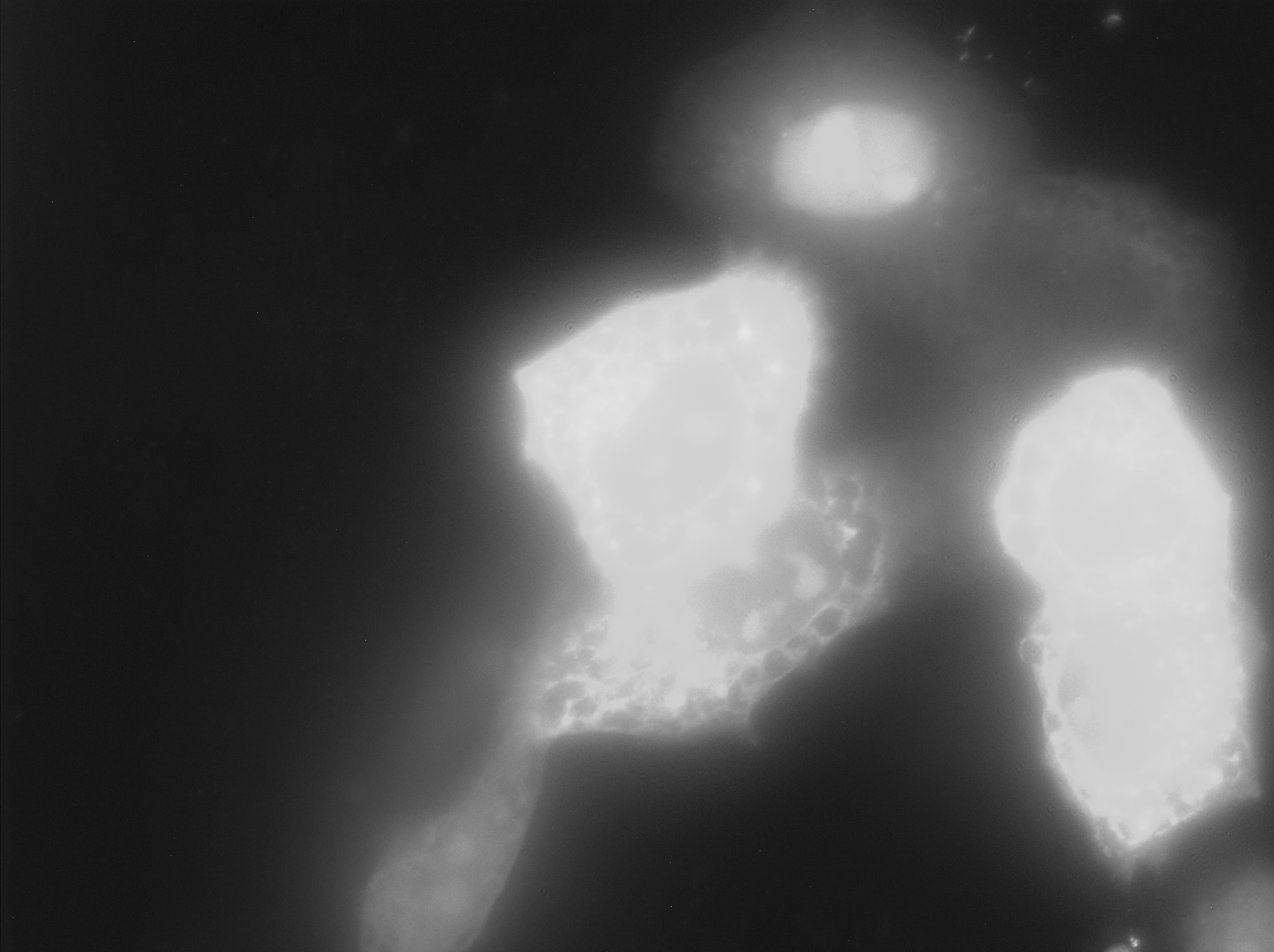

Supplement: Supplementary file 12 — Source data Fig. 3 part 2 [file 44319_2026_742_MOESM12_ESM.zip › Figure 3 Part 2/Fig 3b-e TTLL screen part 2/TTLL7-YFPy I6.Project Maximum Z_XY1679088203_Z0_T0_C2.tif]

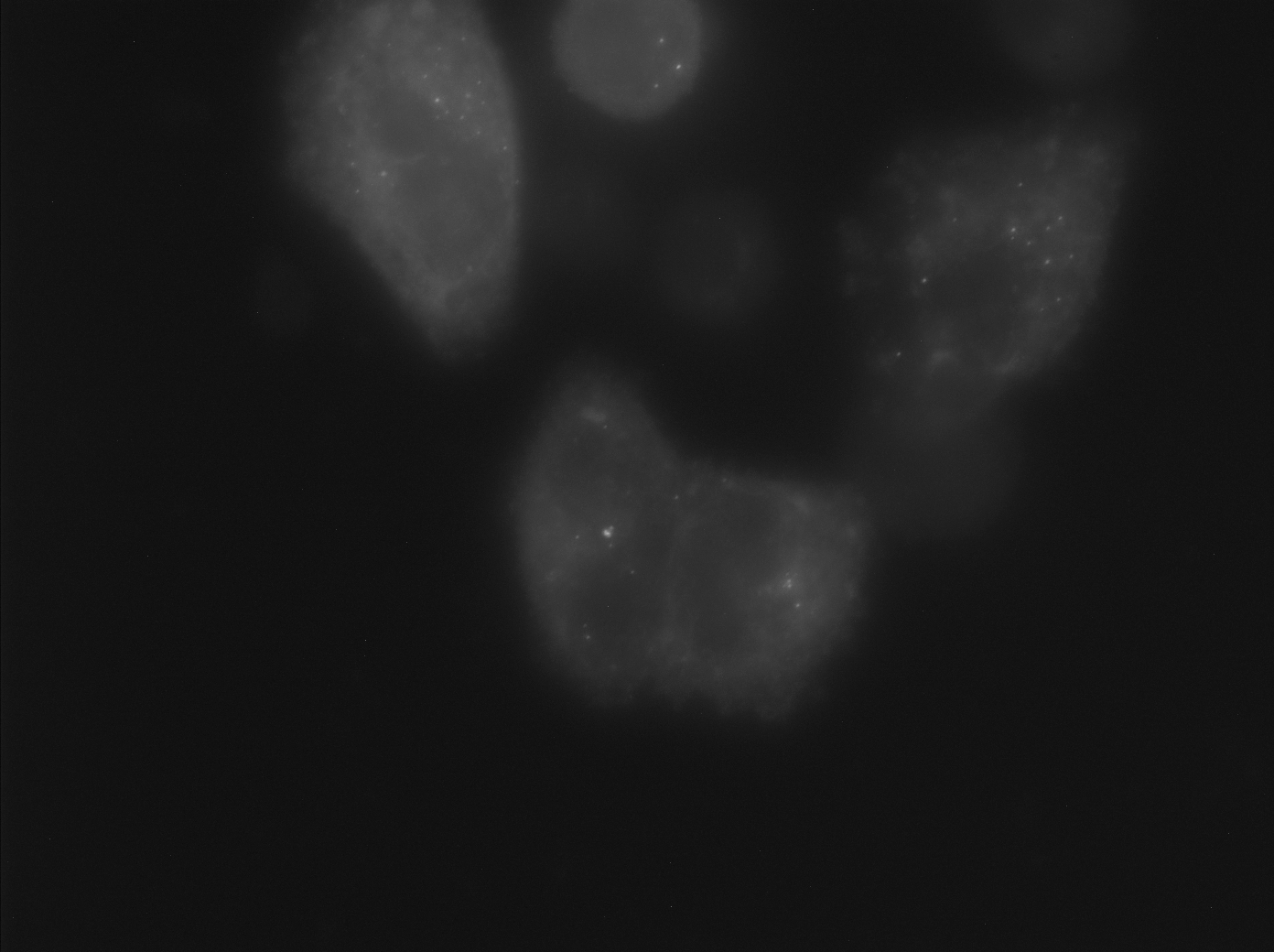

Supplement: Supplementary file 12 — Source data Fig. 3 part 2 [file 44319_2026_742_MOESM12_ESM.zip › Figure 3 Part 2/Fig 3b-e TTLL screen part 2/TTLL5-YFPy I16.Project Maximum Z_XY1679341063_Z0_T0_C2.tif]

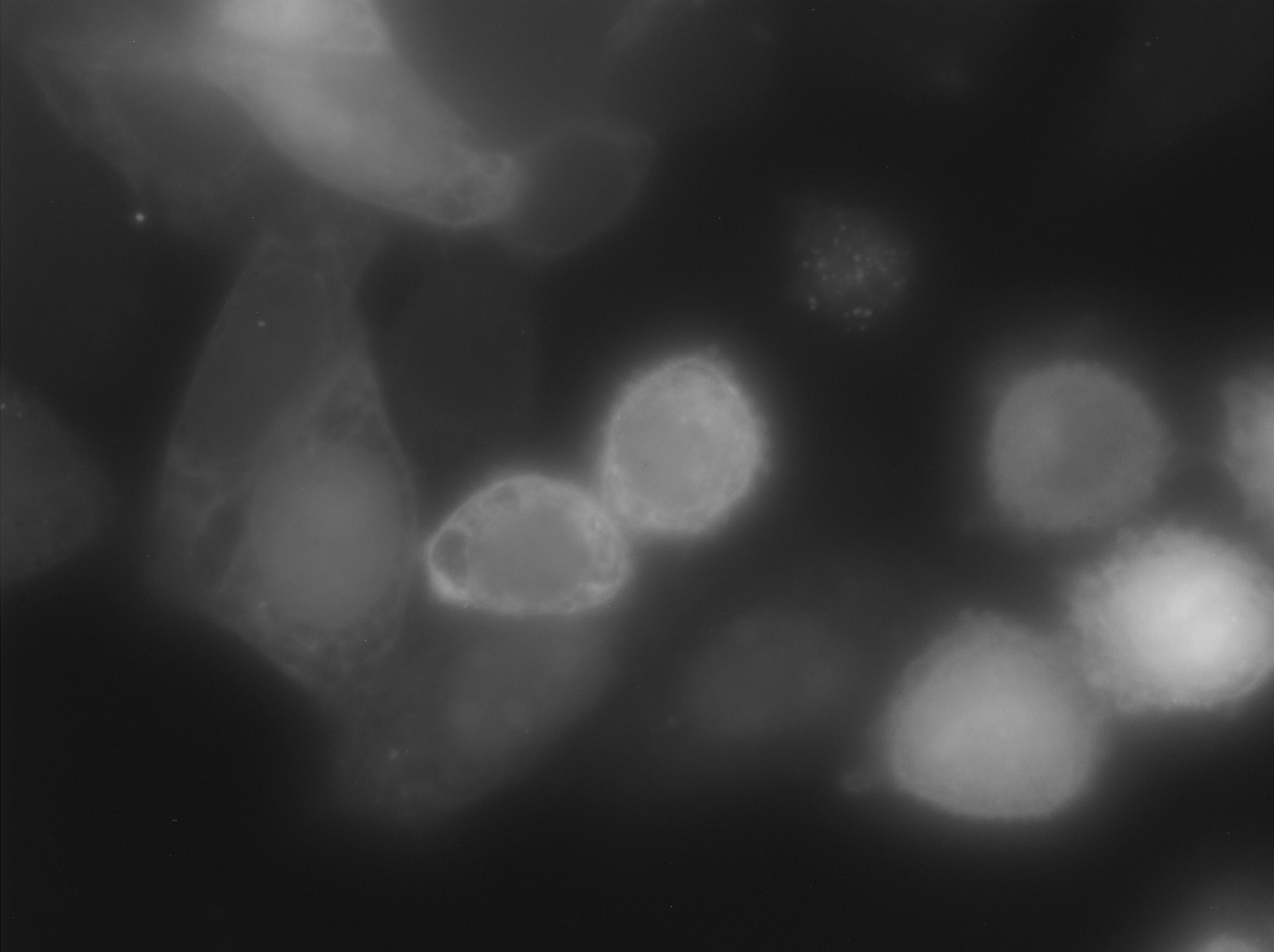

Supplement: Supplementary file 12 — Source data Fig. 3 part 2 [file 44319_2026_742_MOESM12_ESM.zip › Figure 3 Part 2/Fig 3b-e TTLL screen part 2/TTLL7-YFPy I10.Project Maximum Z_XY1679089174_Z0_T0_C2.tif]

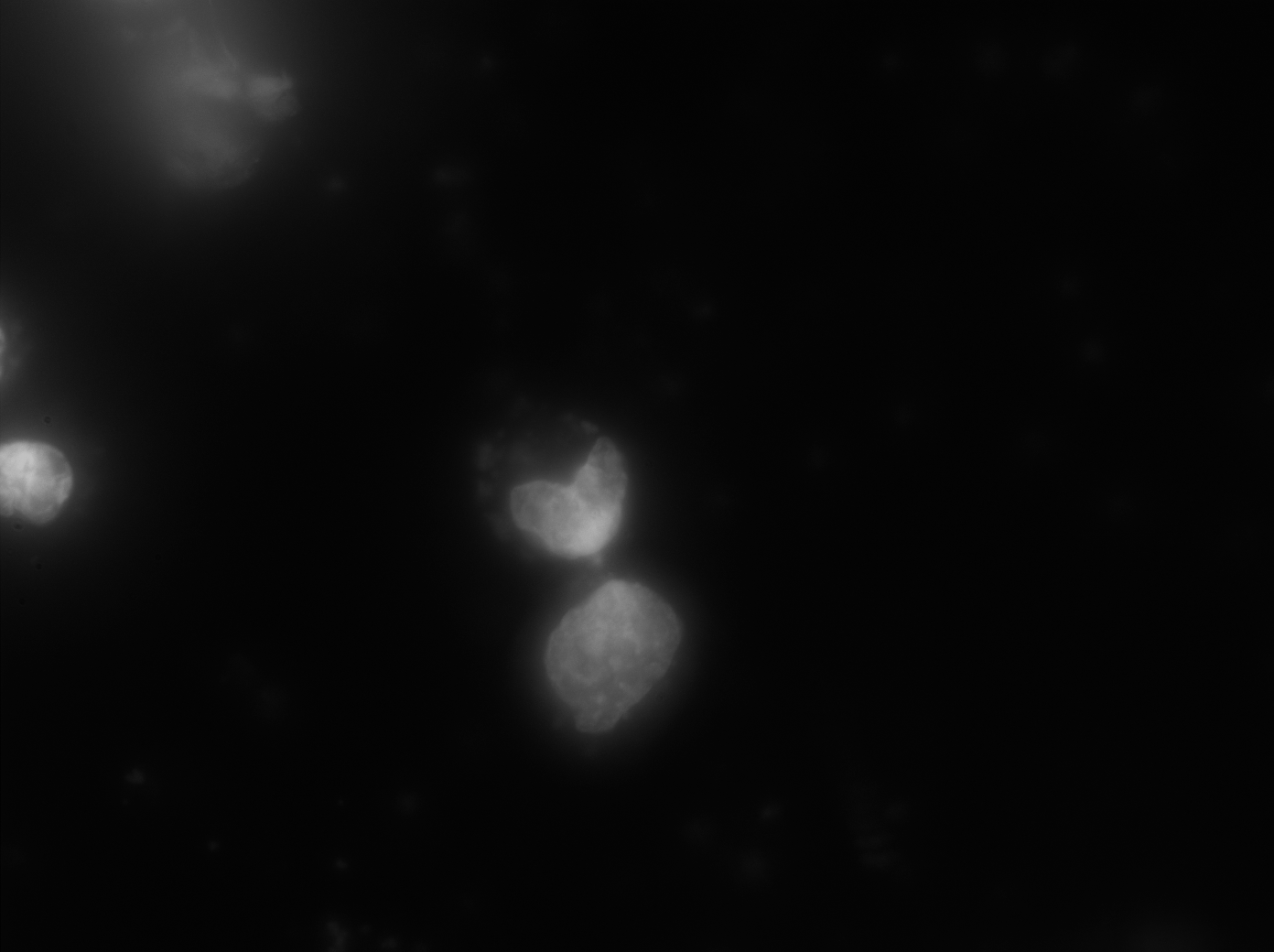

Supplement: Supplementary file 12 — Source data Fig. 3 part 2 [file 44319_2026_742_MOESM12_ESM.zip › Figure 3 Part 2/Fig 3b-e TTLL screen part 2/TTLL7-YFPy I17.Project Maximum Z_XY1679090864_Z0_T0_C0.tif]

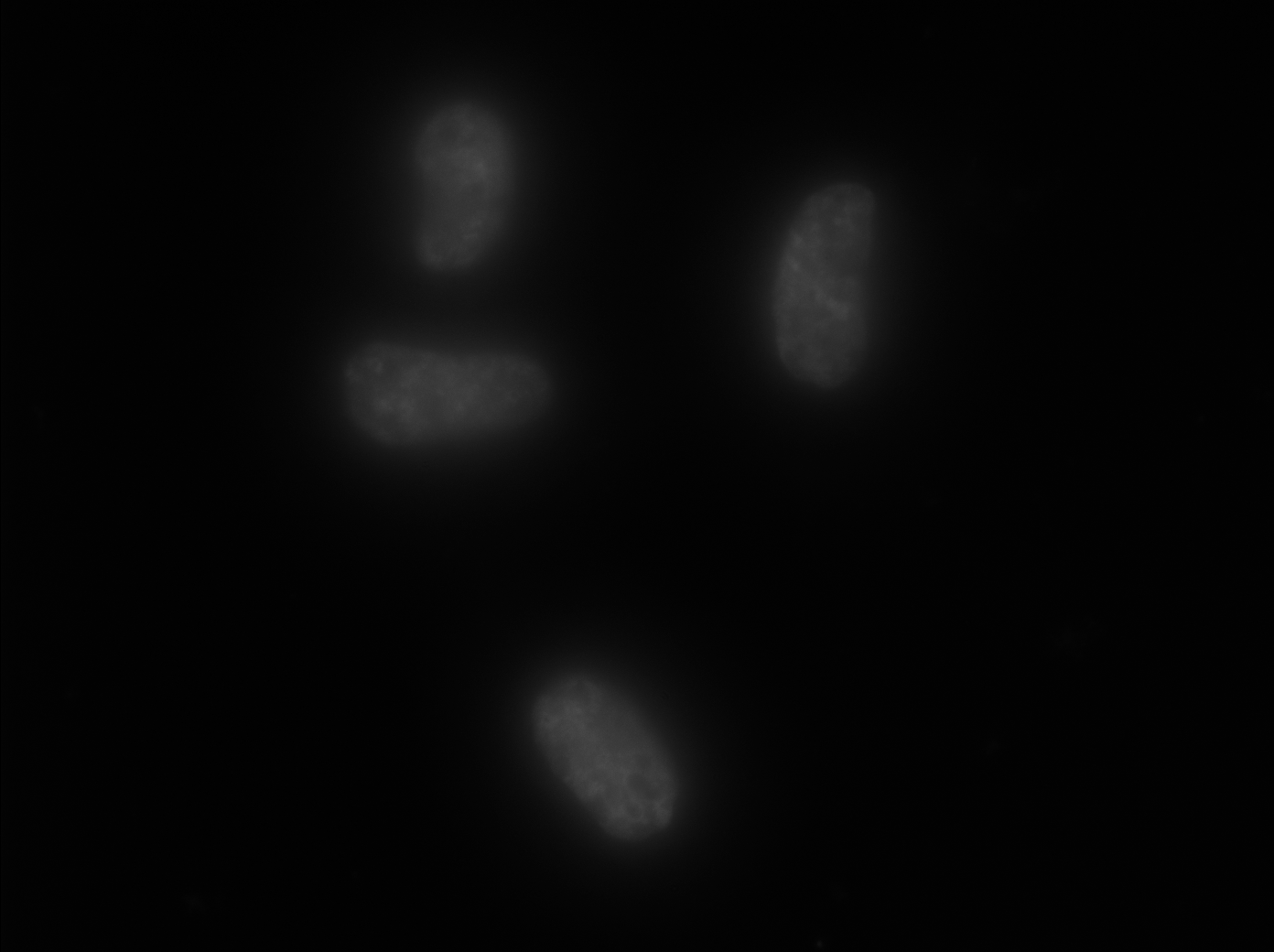

Supplement: Supplementary file 12 — Source data Fig. 3 part 2 [file 44319_2026_742_MOESM12_ESM.zip › Figure 3 Part 2/Fig 3b-e TTLL screen part 2/TTLL6-YFP MB light I3.Project Maximum Z_XY1663881282_Z0_T0_C0.tif]

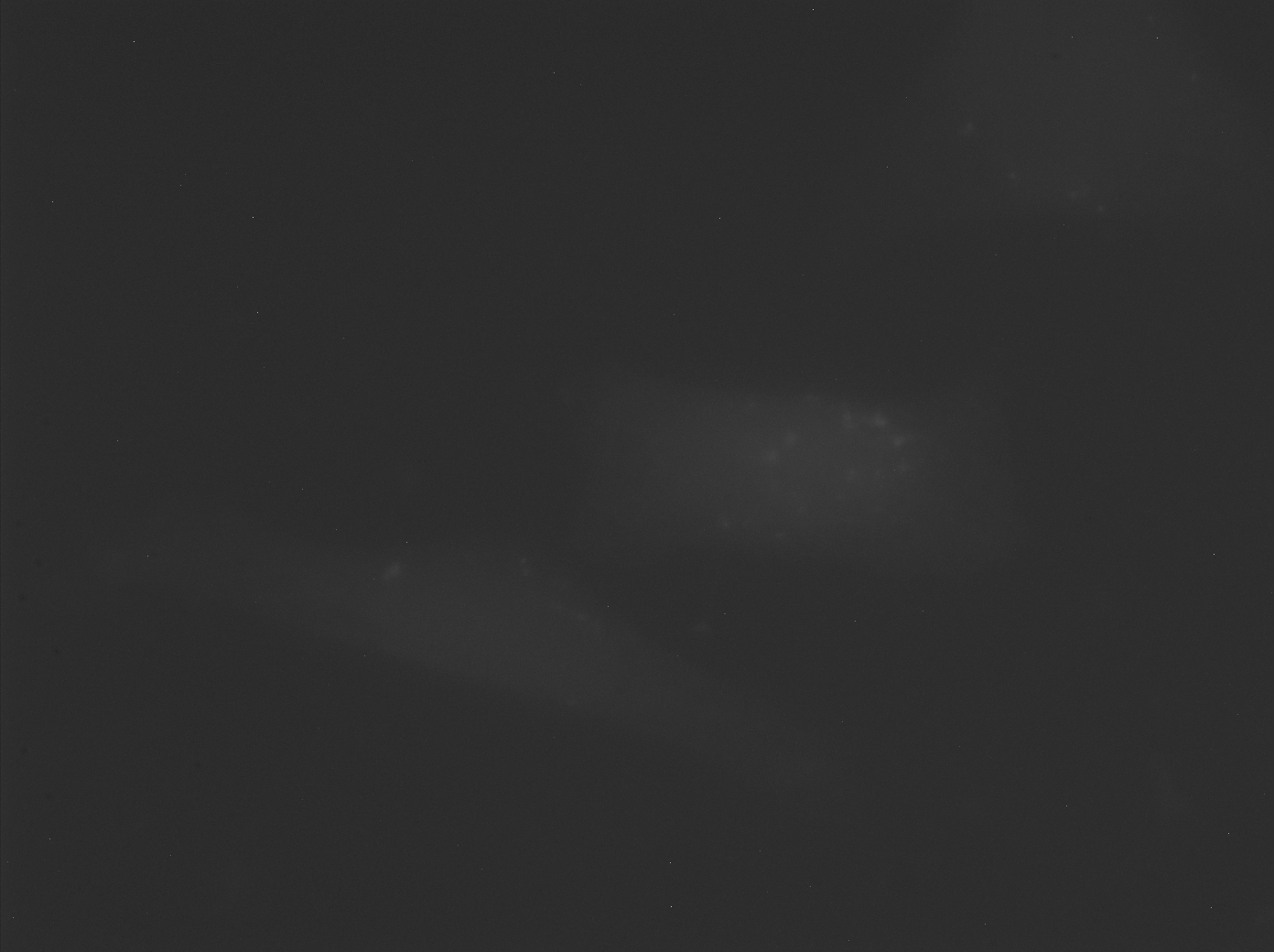

Supplement: Supplementary file 12 — Source data Fig. 3 part 2 [file 44319_2026_742_MOESM12_ESM.zip › Figure 3 Part 2/Fig 3b-e TTLL screen part 2/TTLL6-YFP MB light I4.Project Maximum Z_XY1663882222_Z0_T0_C2.tif]

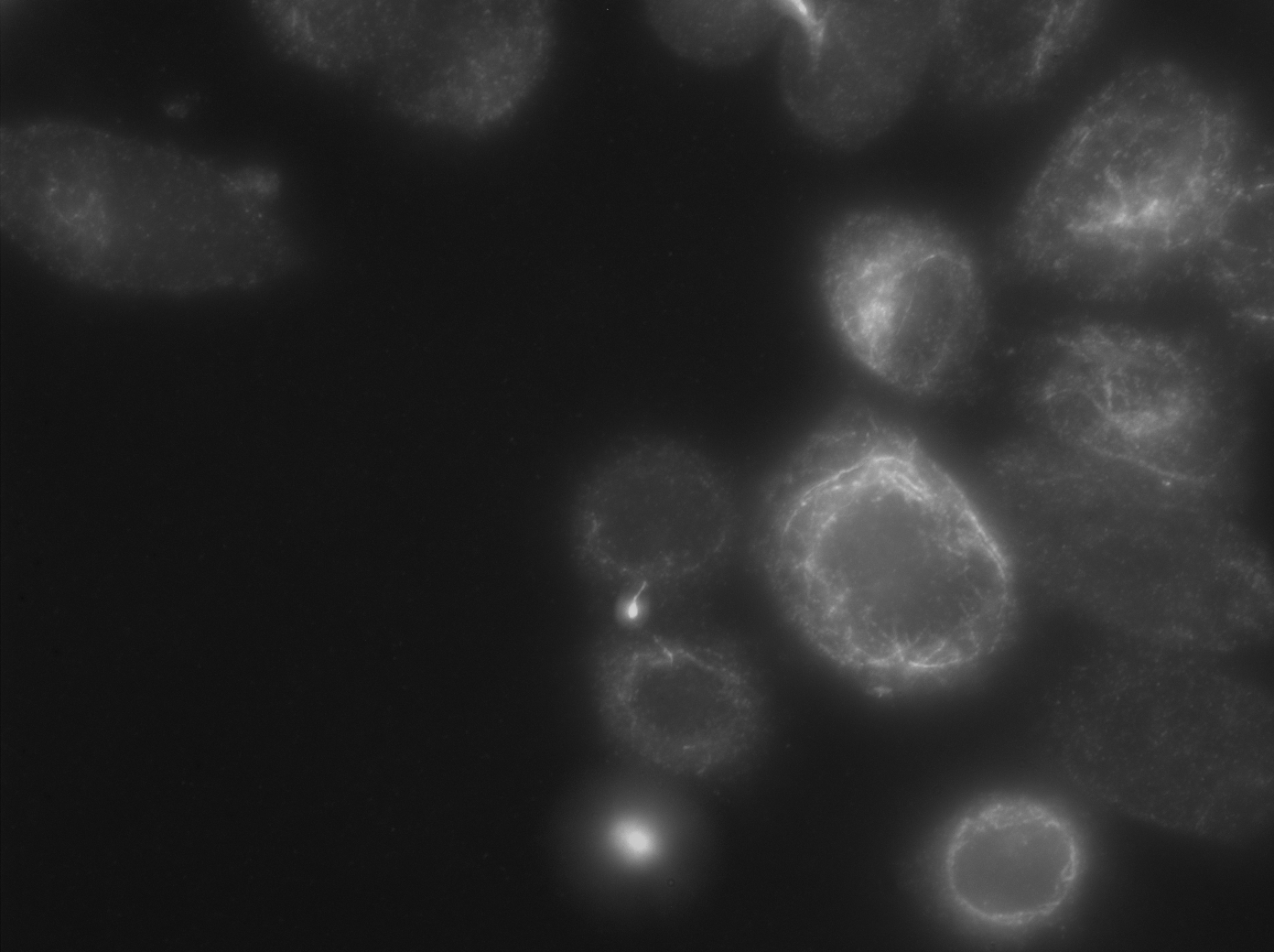

Supplement: Supplementary file 12 — Source data Fig. 3 part 2 [file 44319_2026_742_MOESM12_ESM.zip › Figure 3 Part 2/Fig 3b-e TTLL screen part 2/TTLL5-YFPy I12.Project Maximum Z_XY1679340402_Z0_T0_C1.tif]

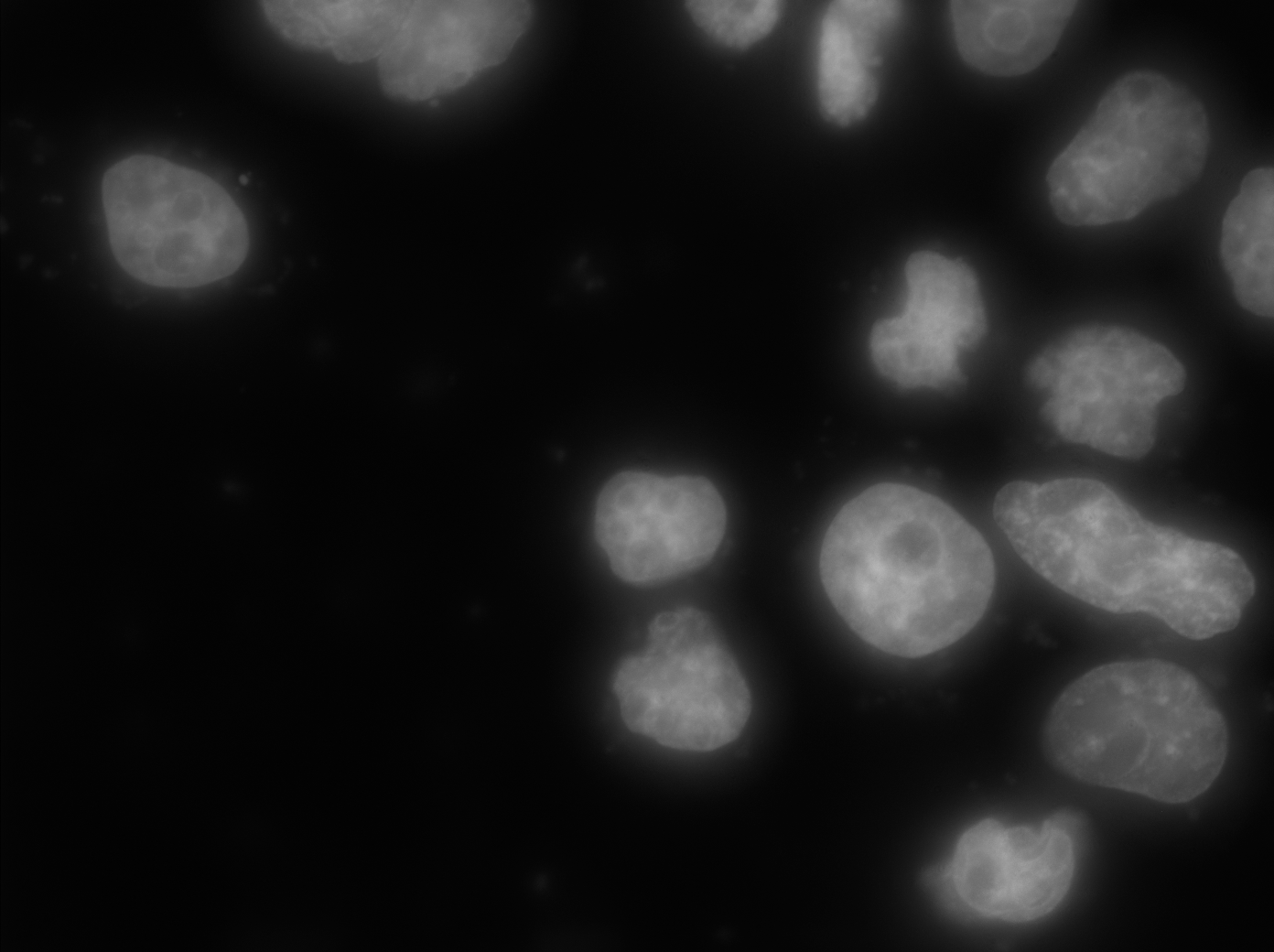

Supplement: Supplementary file 12 — Source data Fig. 3 part 2 [file 44319_2026_742_MOESM12_ESM.zip › Figure 3 Part 2/Fig 3b-e TTLL screen part 2/TTLL5-YFPy I12.Project Maximum Z_XY1679340402_Z0_T0_C0.tif]

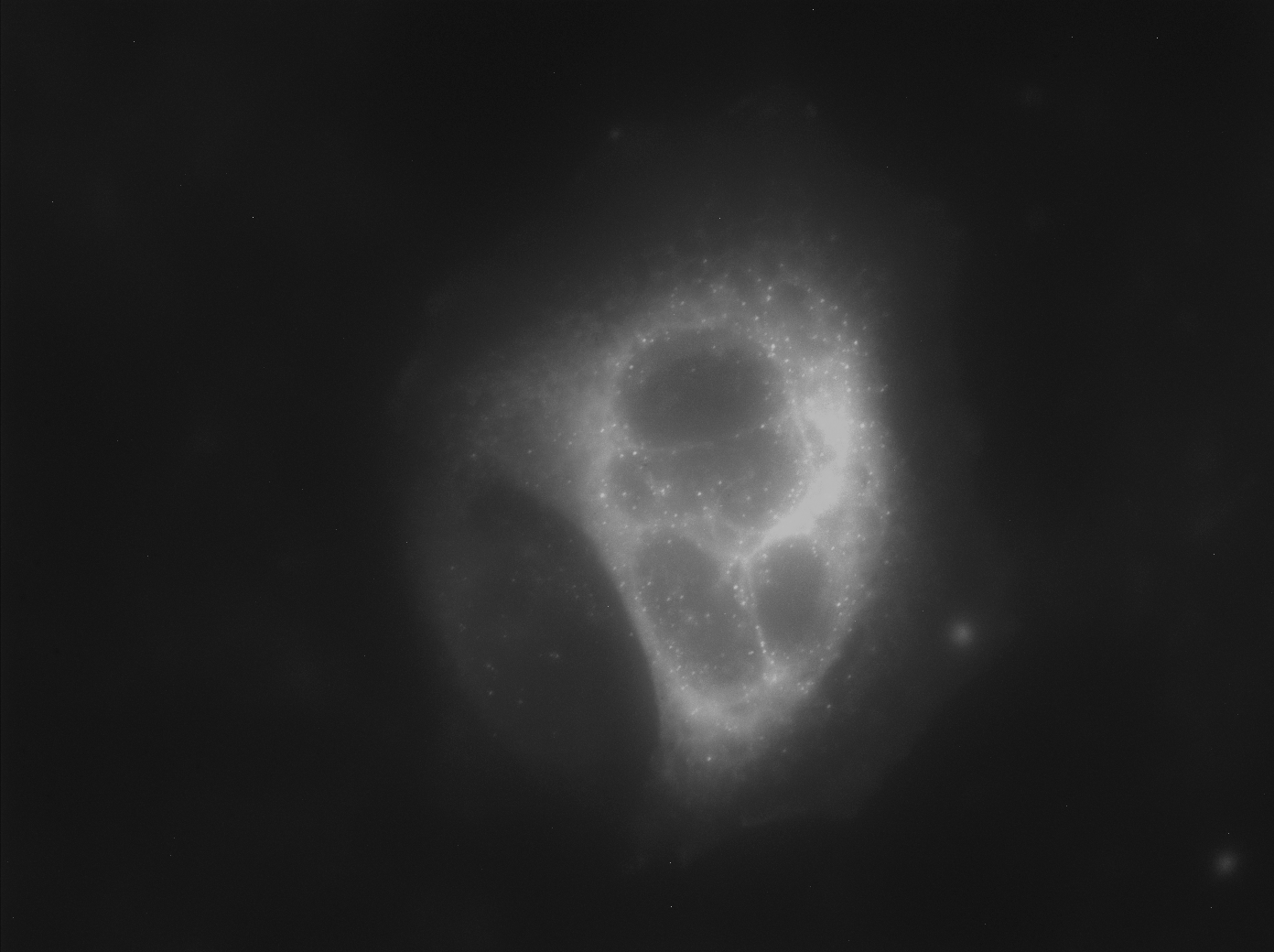

Supplement: Supplementary file 12 — Source data Fig. 3 part 2 [file 44319_2026_742_MOESM12_ESM.zip › Figure 3 Part 2/Fig 3b-e TTLL screen part 2/TTLL6-YFP R1 T4.Project Maximum Z_XY1661550469_Z0_T0_C2.tif]

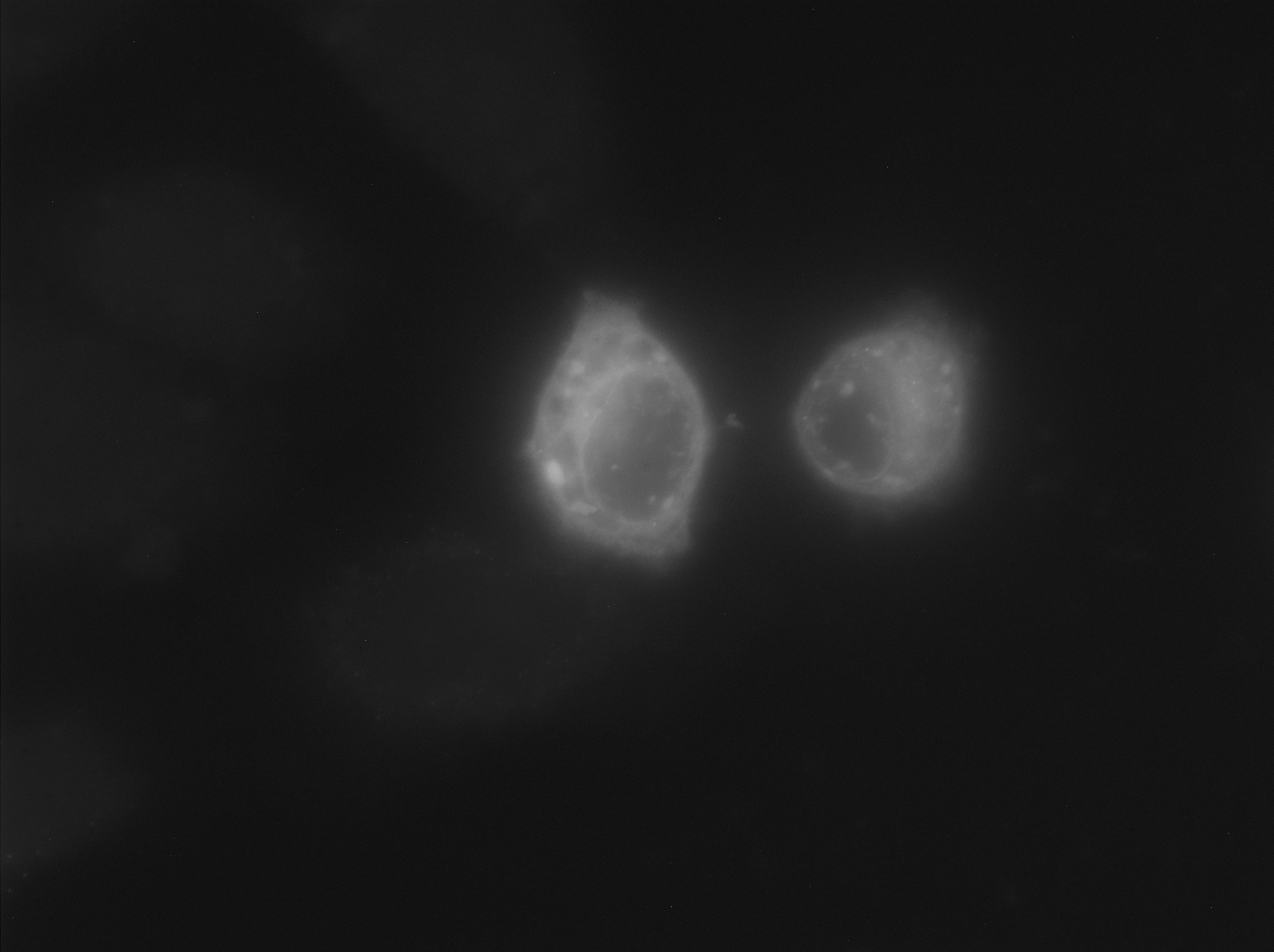

Supplement: Supplementary file 12 — Source data Fig. 3 part 2 [file 44319_2026_742_MOESM12_ESM.zip › Figure 3 Part 2/Fig 3b-e TTLL screen part 2/TTLL5-YFPy I17.Project Maximum Z_XY1679341238_Z0_T0_C2.tif]

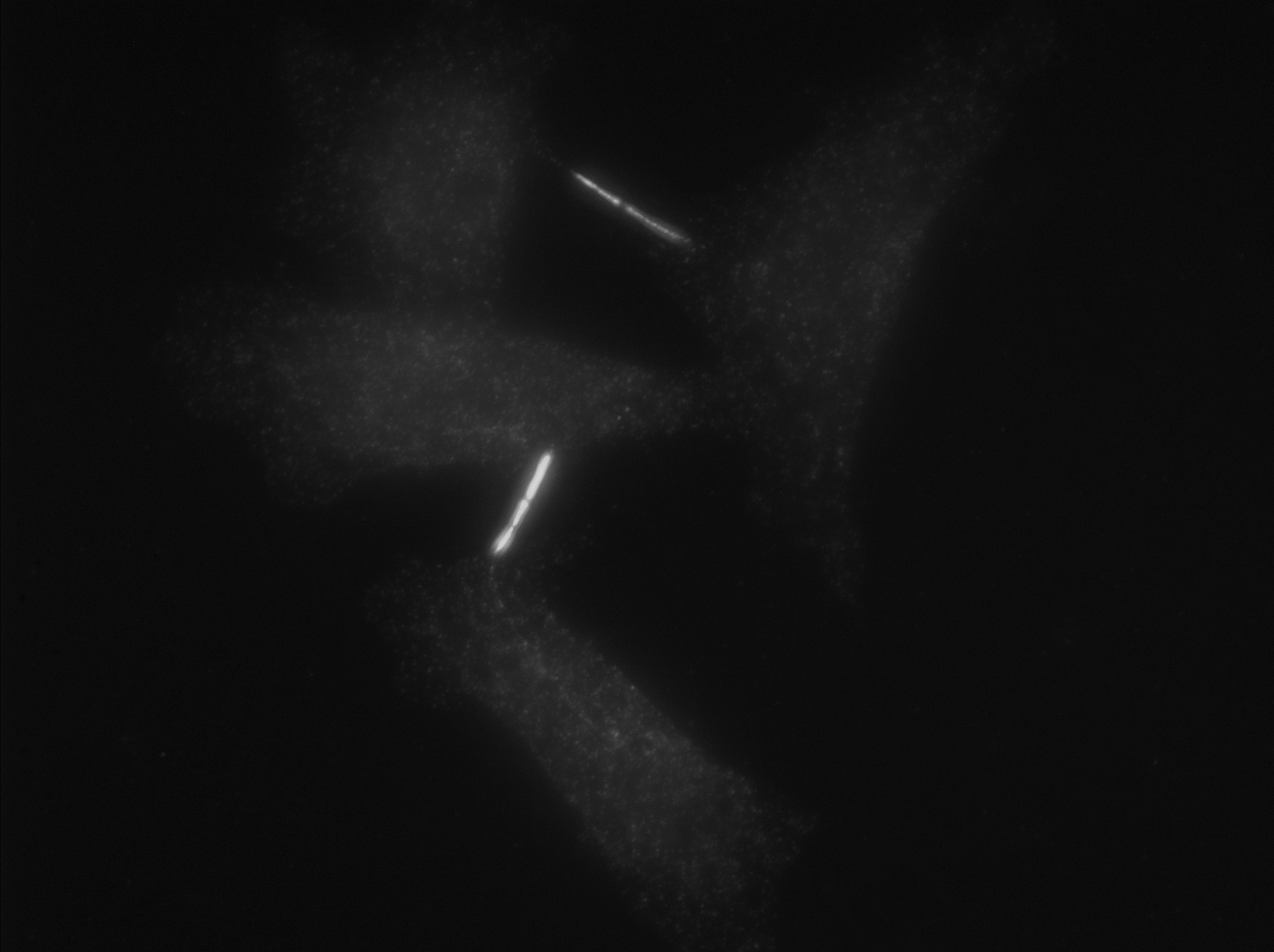

Supplement: Supplementary file 12 — Source data Fig. 3 part 2 [file 44319_2026_742_MOESM12_ESM.zip › Figure 3 Part 2/Fig 3b-e TTLL screen part 2/TTLL6-YFP MB light I3.Project Maximum Z_XY1663881282_Z0_T0_C1.tif]

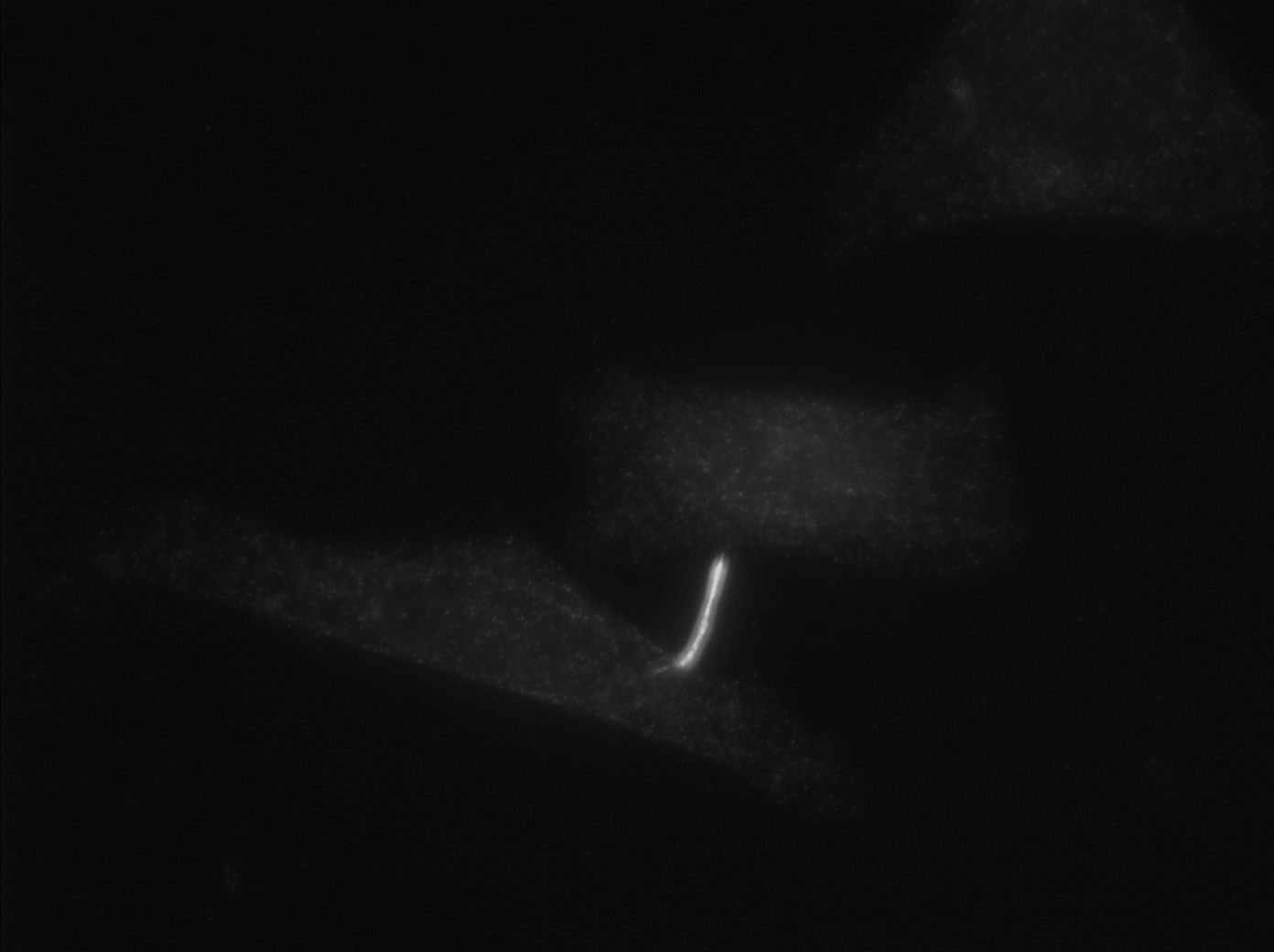

Supplement: Supplementary file 12 — Source data Fig. 3 part 2 [file 44319_2026_742_MOESM12_ESM.zip › Figure 3 Part 2/Fig 3b-e TTLL screen part 2/TTLL6-YFP MB light I4.Project Maximum Z_XY1663882222_Z0_T0_C1.tif]

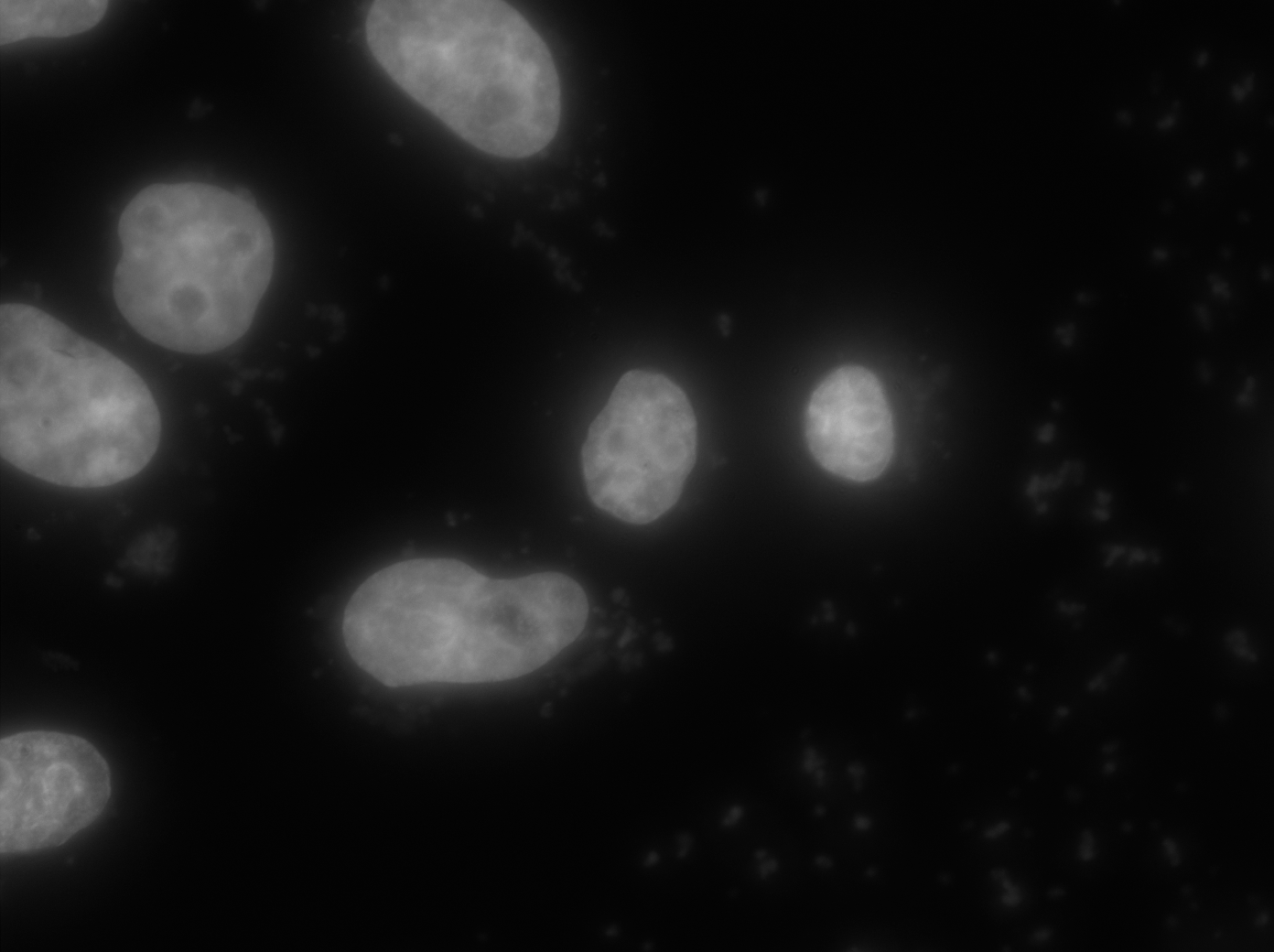

Supplement: Supplementary file 12 — Source data Fig. 3 part 2 [file 44319_2026_742_MOESM12_ESM.zip › Figure 3 Part 2/Fig 3b-e TTLL screen part 2/TTLL5-YFPy I17.Project Maximum Z_XY1679341238_Z0_T0_C0.tif]

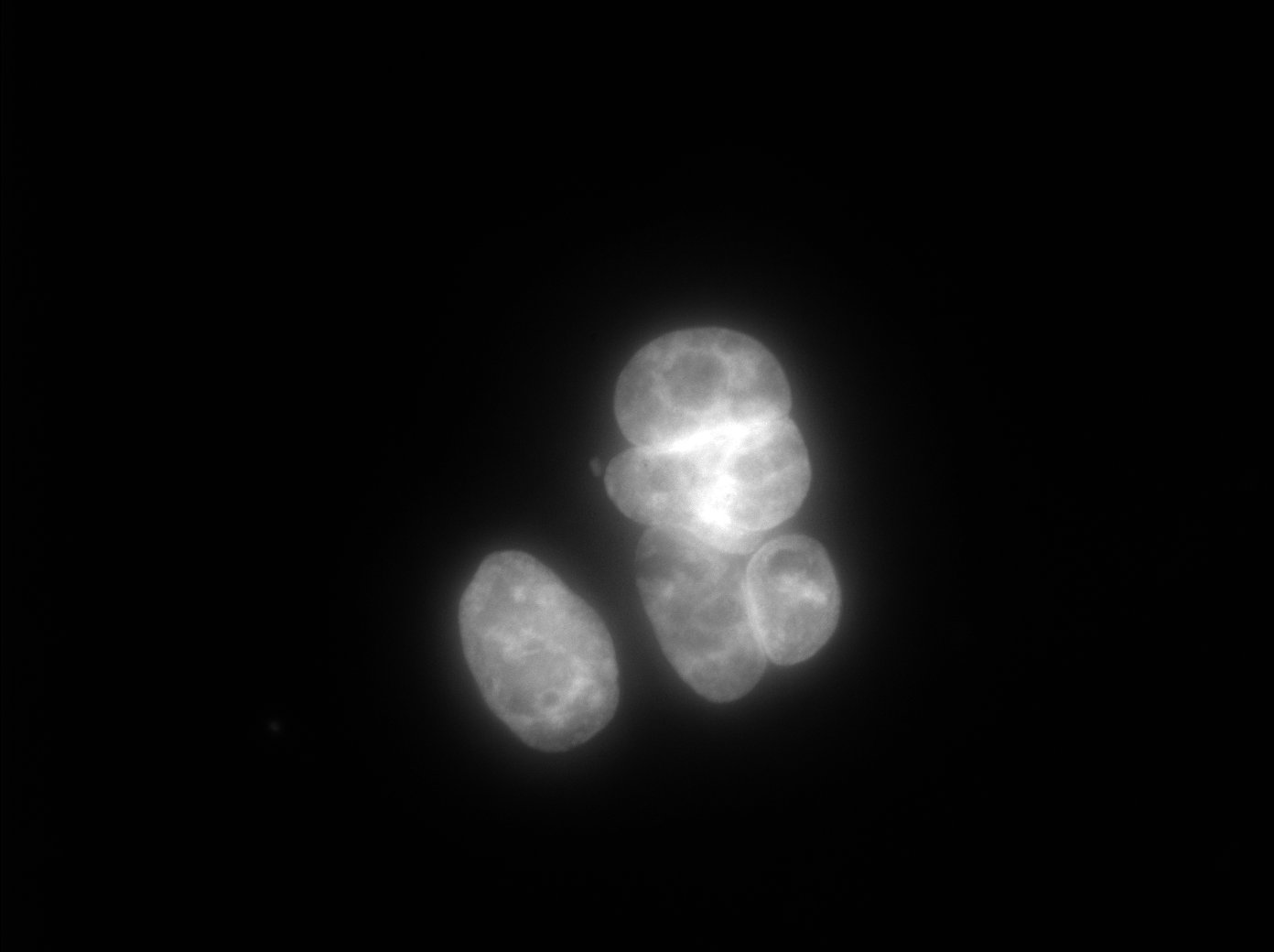

Supplement: Supplementary file 12 — Source data Fig. 3 part 2 [file 44319_2026_742_MOESM12_ESM.zip › Figure 3 Part 2/Fig 3b-e TTLL screen part 2/TTLL6-YFP R1 T4.Project Maximum Z_XY1661550469_Z0_T0_C0.tif]

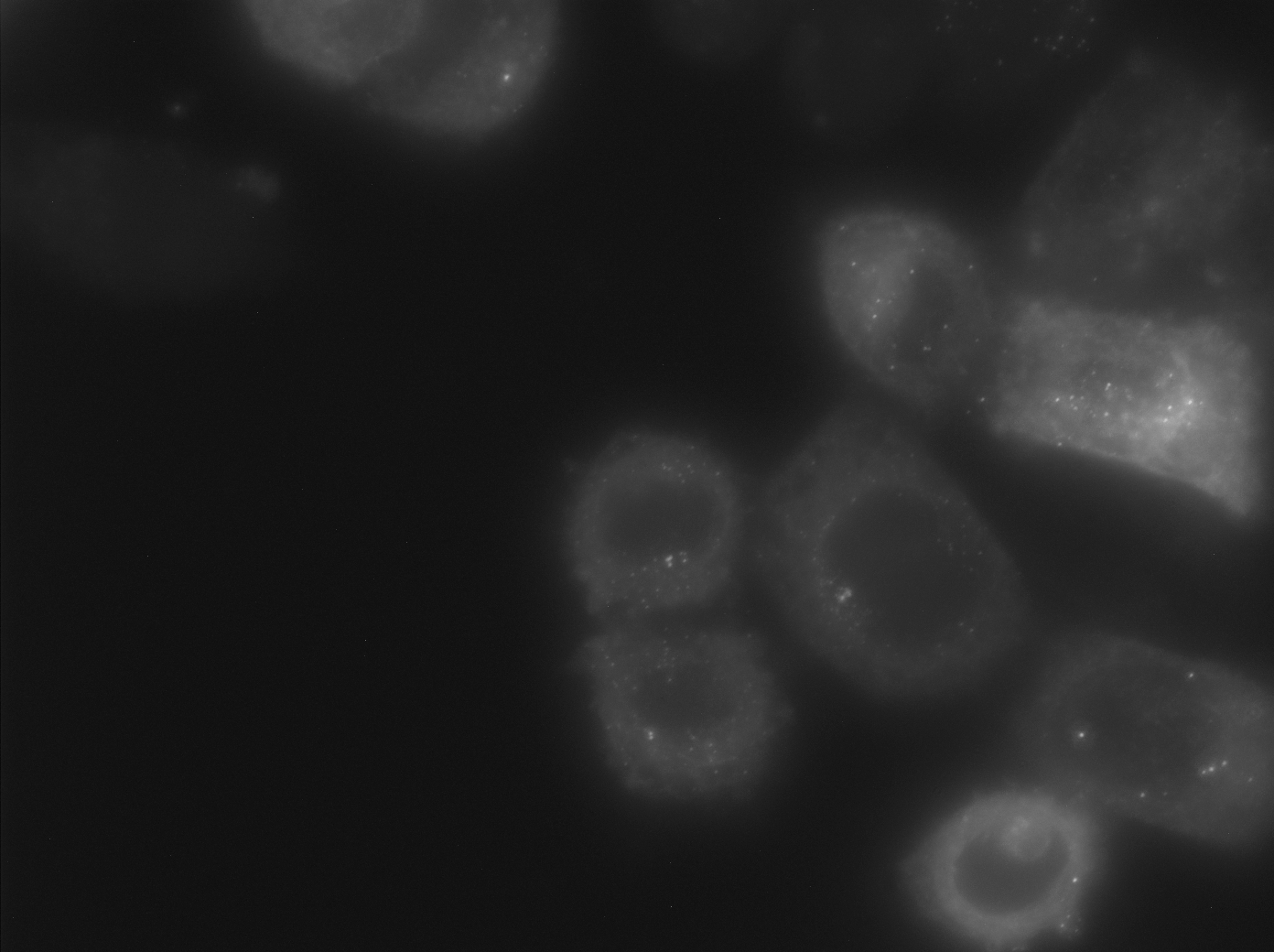

Supplement: Supplementary file 12 — Source data Fig. 3 part 2 [file 44319_2026_742_MOESM12_ESM.zip › Figure 3 Part 2/Fig 3b-e TTLL screen part 2/TTLL5-YFPy I12.Project Maximum Z_XY1679340402_Z0_T0_C2.tif]

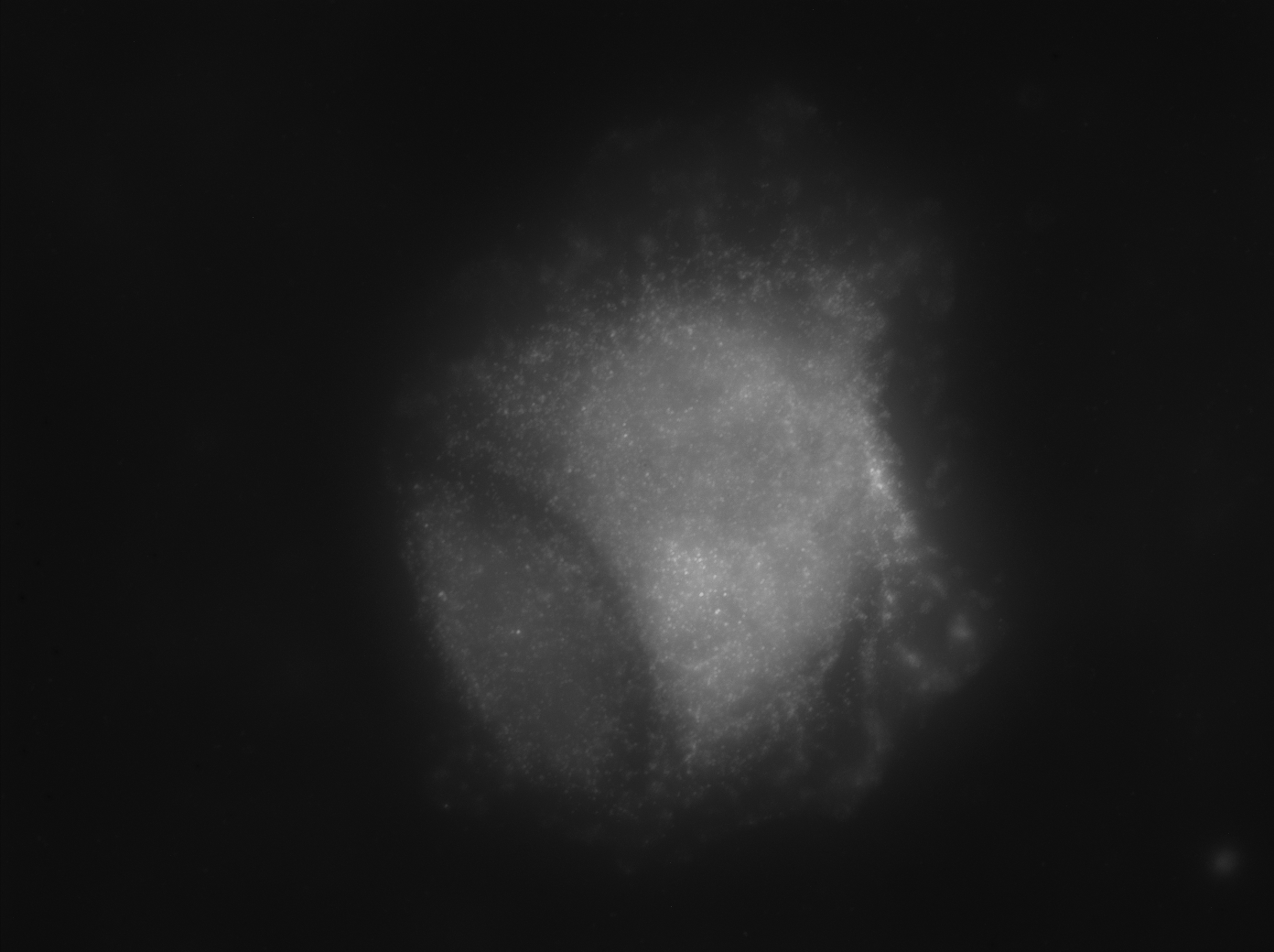

Supplement: Supplementary file 12 — Source data Fig. 3 part 2 [file 44319_2026_742_MOESM12_ESM.zip › Figure 3 Part 2/Fig 3b-e TTLL screen part 2/TTLL6-YFP R1 T4.Project Maximum Z_XY1661550469_Z0_T0_C1.tif]

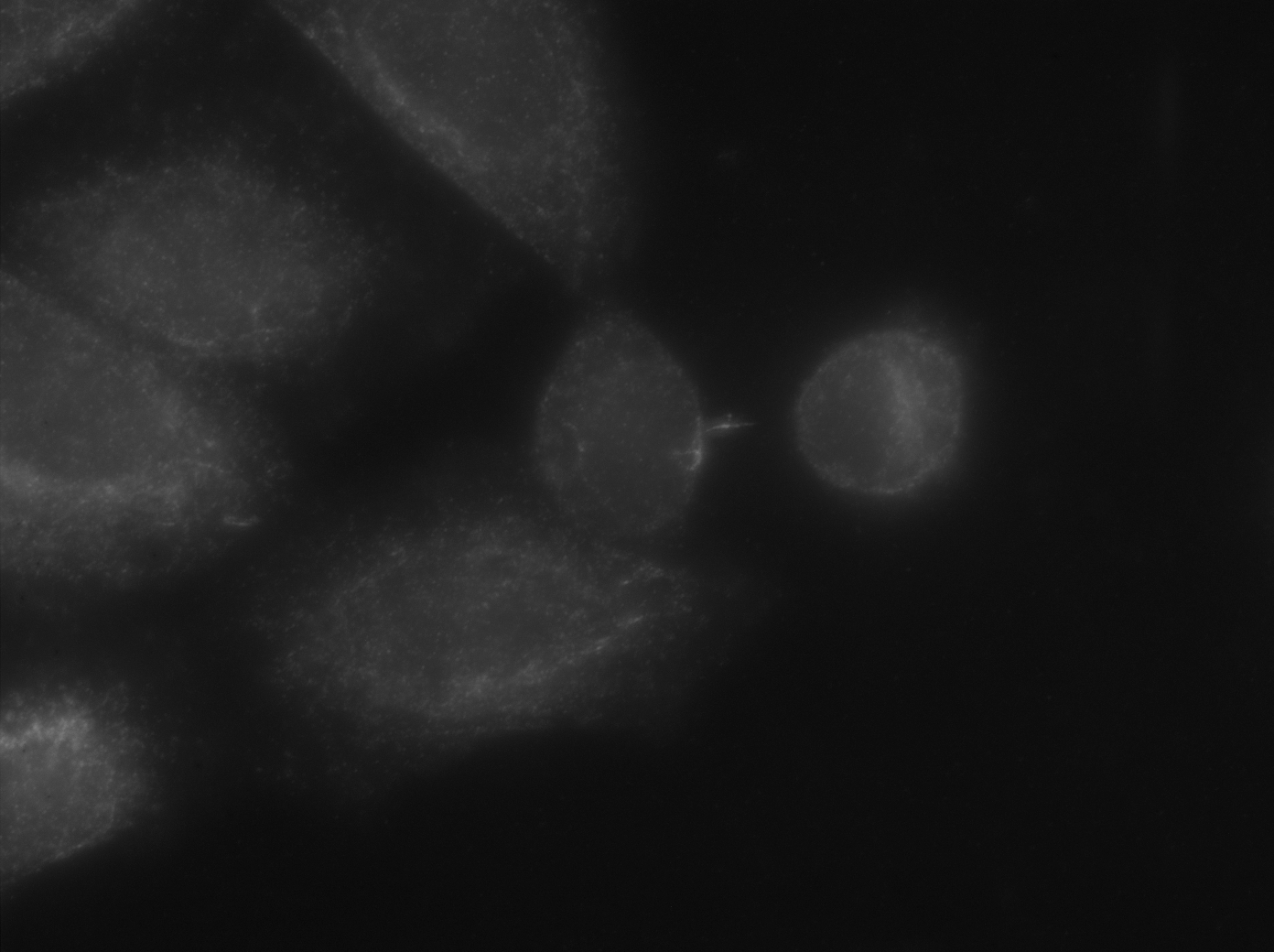

Supplement: Supplementary file 12 — Source data Fig. 3 part 2 [file 44319_2026_742_MOESM12_ESM.zip › Figure 3 Part 2/Fig 3b-e TTLL screen part 2/TTLL5-YFPy I17.Project Maximum Z_XY1679341238_Z0_T0_C1.tif]

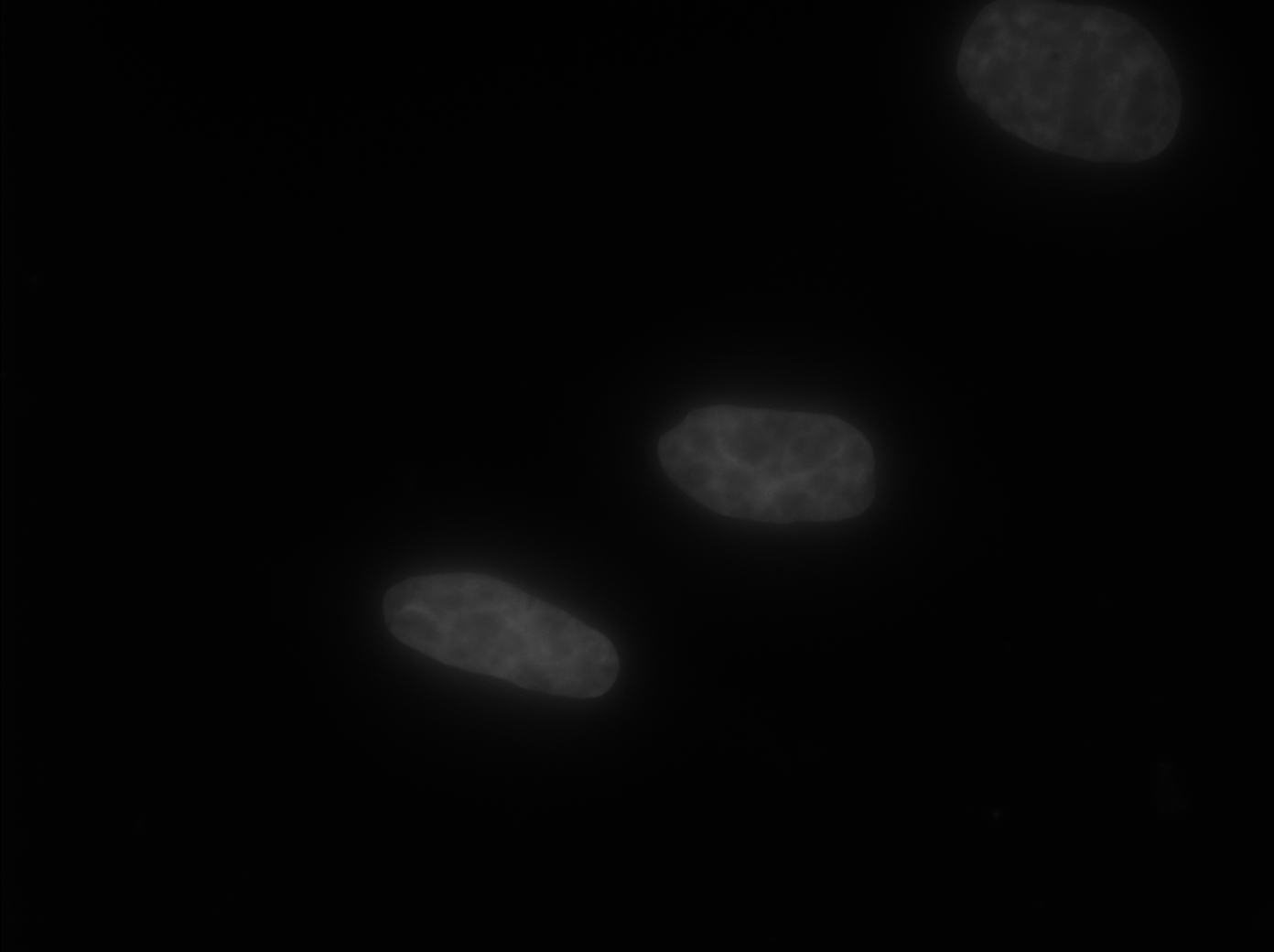

Supplement: Supplementary file 12 — Source data Fig. 3 part 2 [file 44319_2026_742_MOESM12_ESM.zip › Figure 3 Part 2/Fig 3b-e TTLL screen part 2/TTLL6-YFP MB light I4.Project Maximum Z_XY1663882222_Z0_T0_C0.tif]

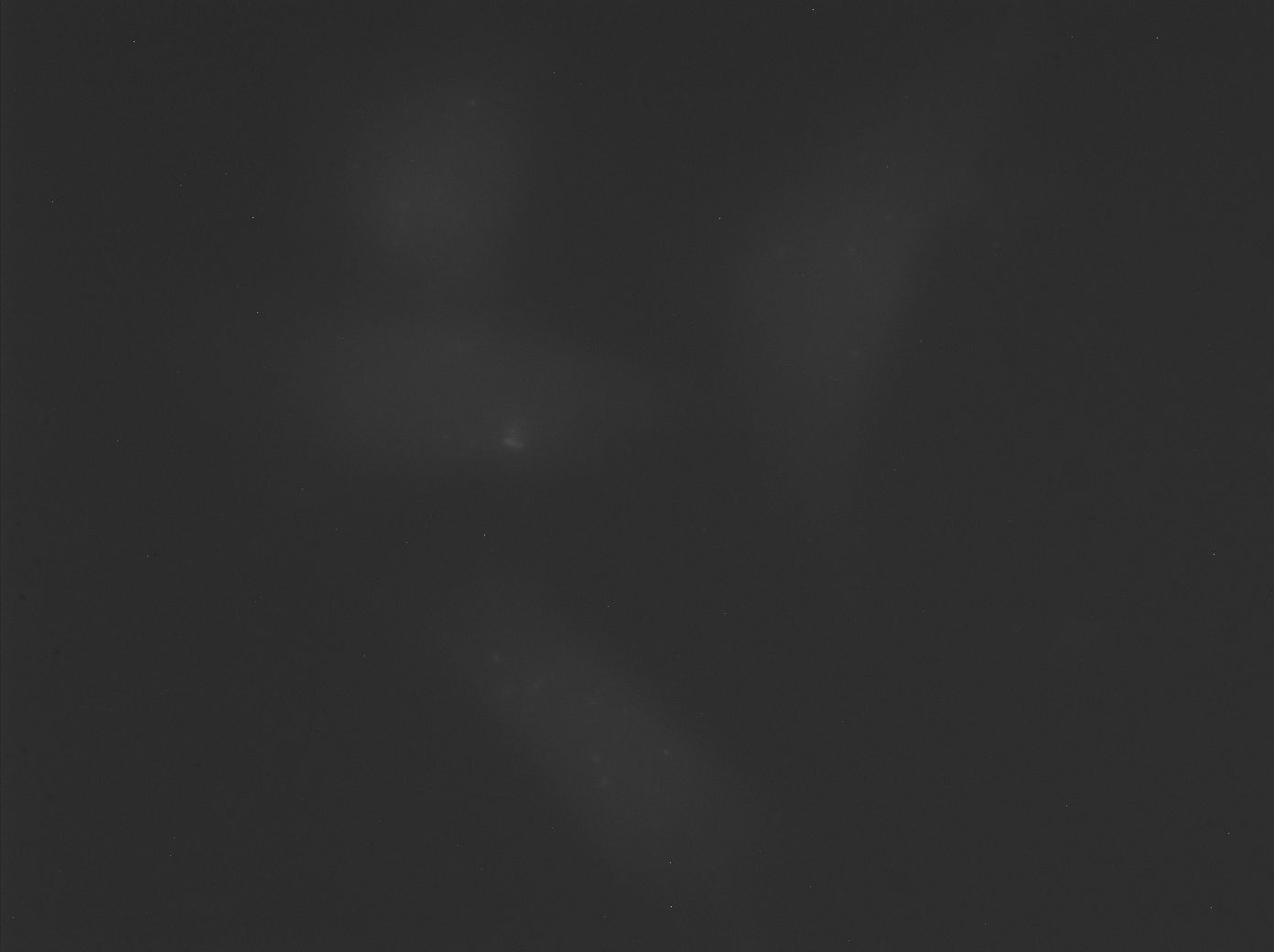

Supplement: Supplementary file 12 — Source data Fig. 3 part 2 [file 44319_2026_742_MOESM12_ESM.zip › Figure 3 Part 2/Fig 3b-e TTLL screen part 2/TTLL6-YFP MB light I3.Project Maximum Z_XY1663881282_Z0_T0_C2.tif]

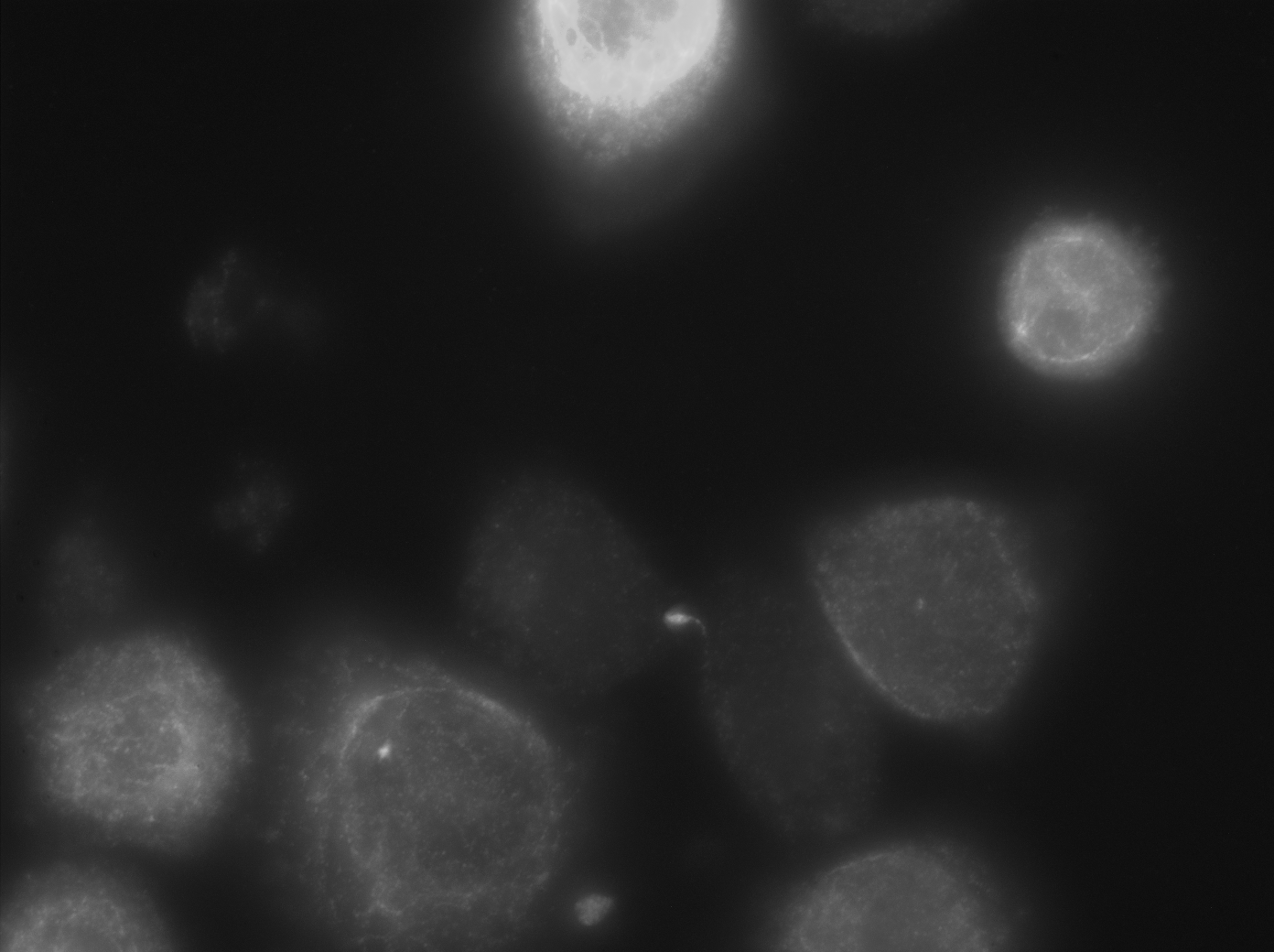

Supplement: Supplementary file 12 — Source data Fig. 3 part 2 [file 44319_2026_742_MOESM12_ESM.zip › Figure 3 Part 2/Fig 3b-e TTLL screen part 2/TTLL7-YFPy I20.Project Maximum Z_XY1679091425_Z0_T0_C1.tif]

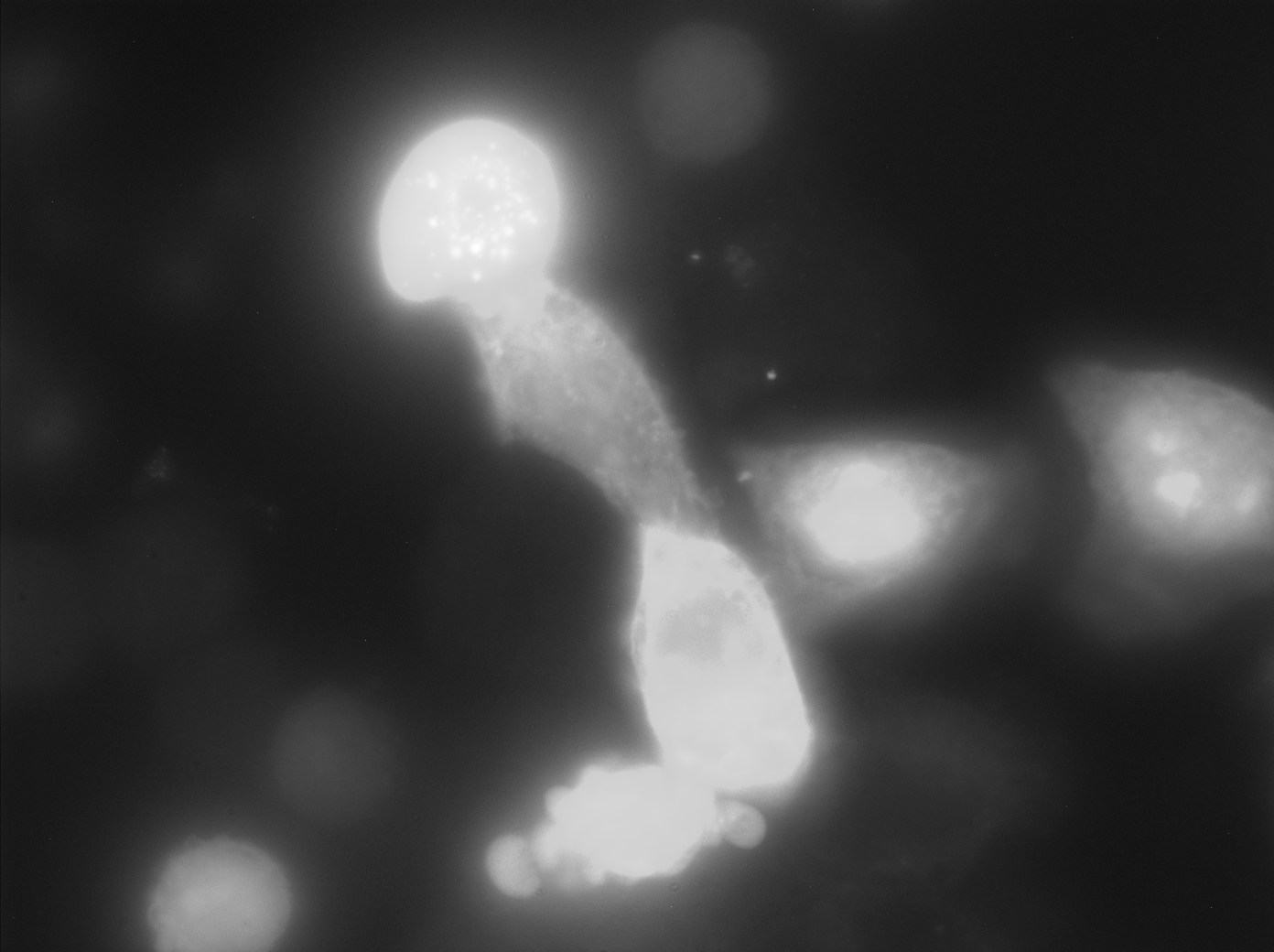

Supplement: Supplementary file 12 — Source data Fig. 3 part 2 [file 44319_2026_742_MOESM12_ESM.zip › Figure 3 Part 2/Fig 3b-e TTLL screen part 2/TTLL7-YFPy I5.Project Maximum Z_XY1679088039_Z0_T0_C2.tif]

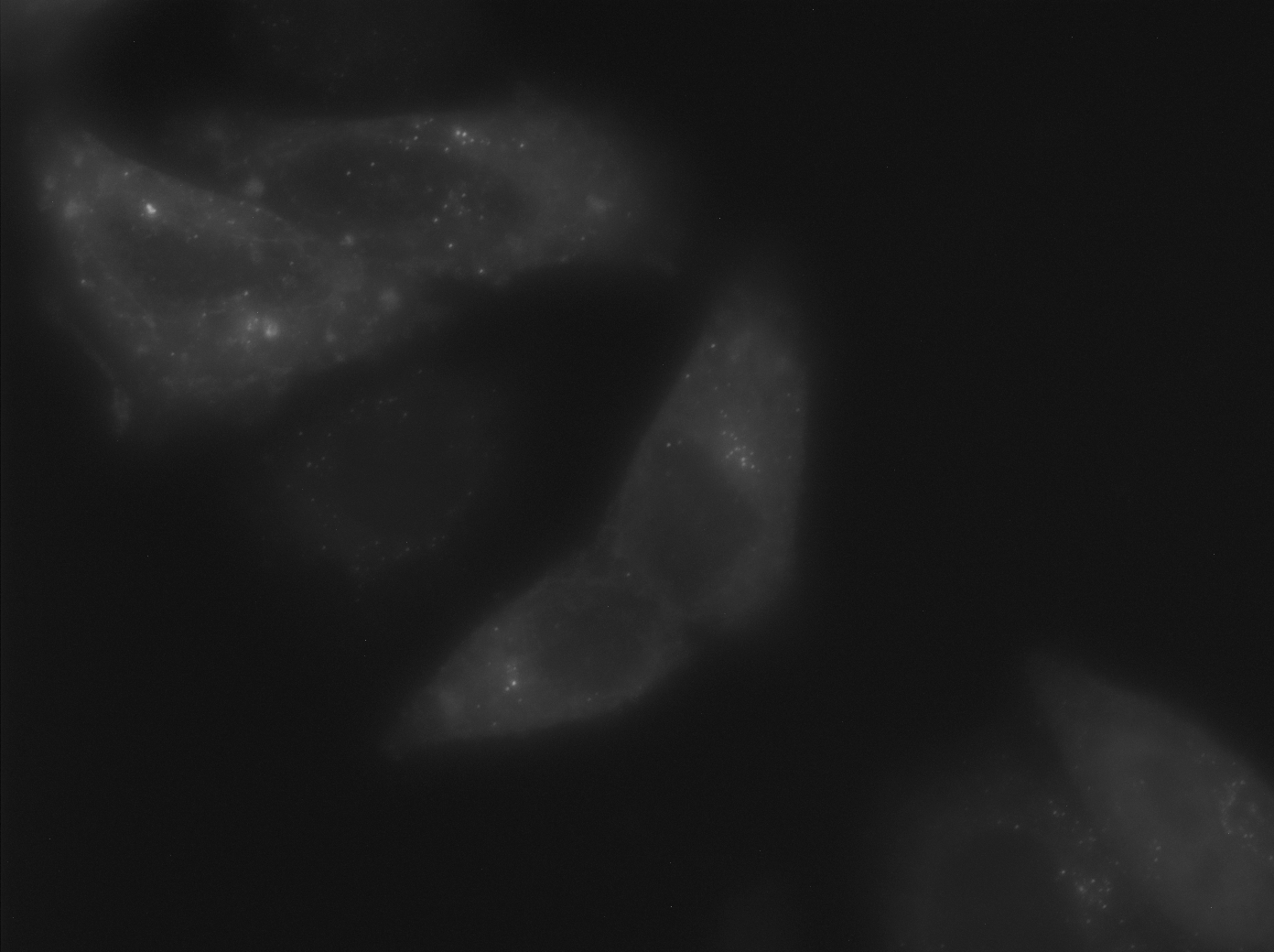

Supplement: Supplementary file 12 — Source data Fig. 3 part 2 [file 44319_2026_742_MOESM12_ESM.zip › Figure 3 Part 2/Fig 3b-e TTLL screen part 2/TTLL5-YFPy I14.Project Maximum Z_XY1679340750_Z0_T0_C2.tif]

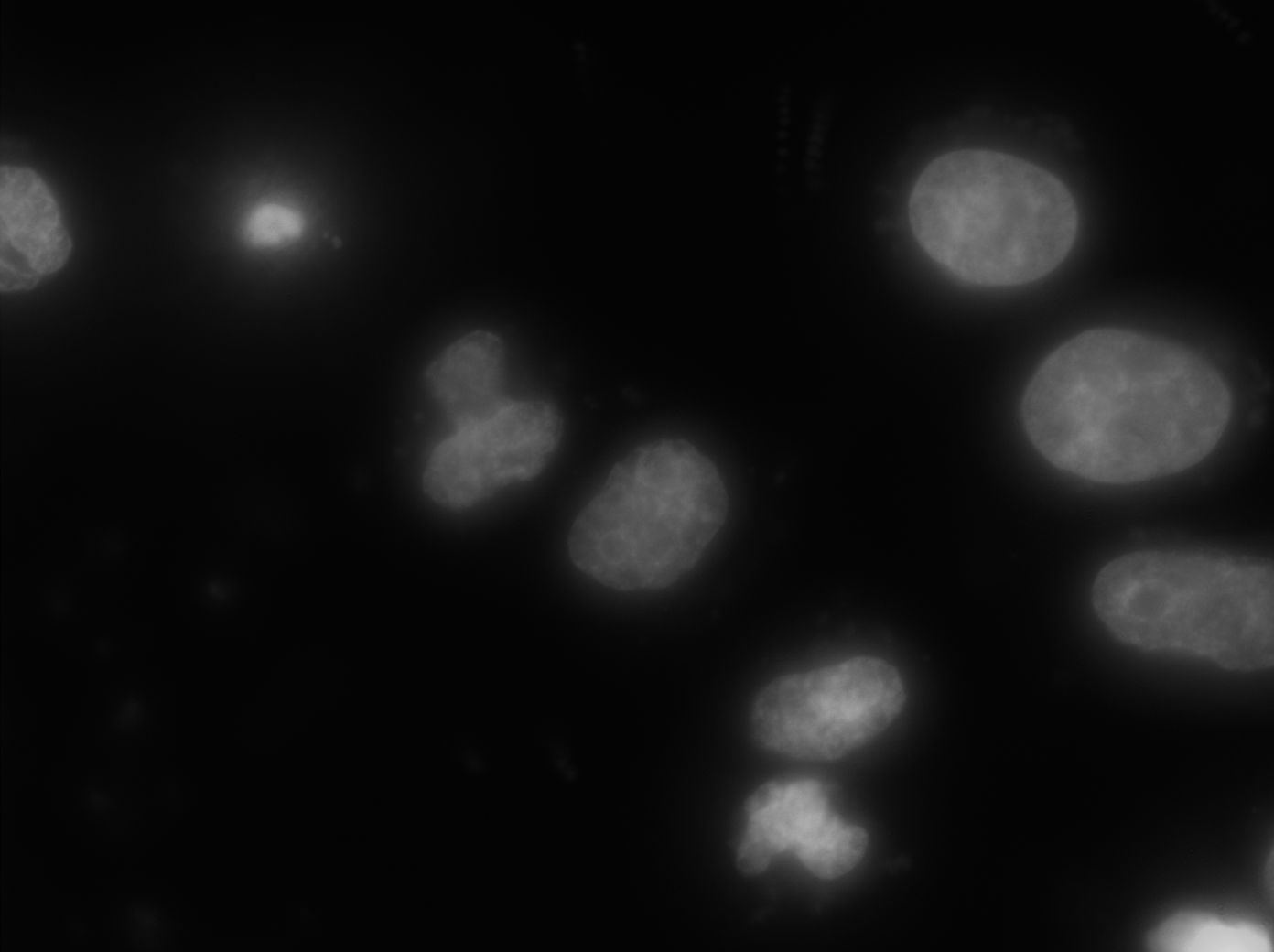

Supplement: Supplementary file 12 — Source data Fig. 3 part 2 [file 44319_2026_742_MOESM12_ESM.zip › Figure 3 Part 2/Fig 3b-e TTLL screen part 2/TTLL7-YFPy I18.Project Maximum Z_XY1679091040_Z0_T0_C0.tif]

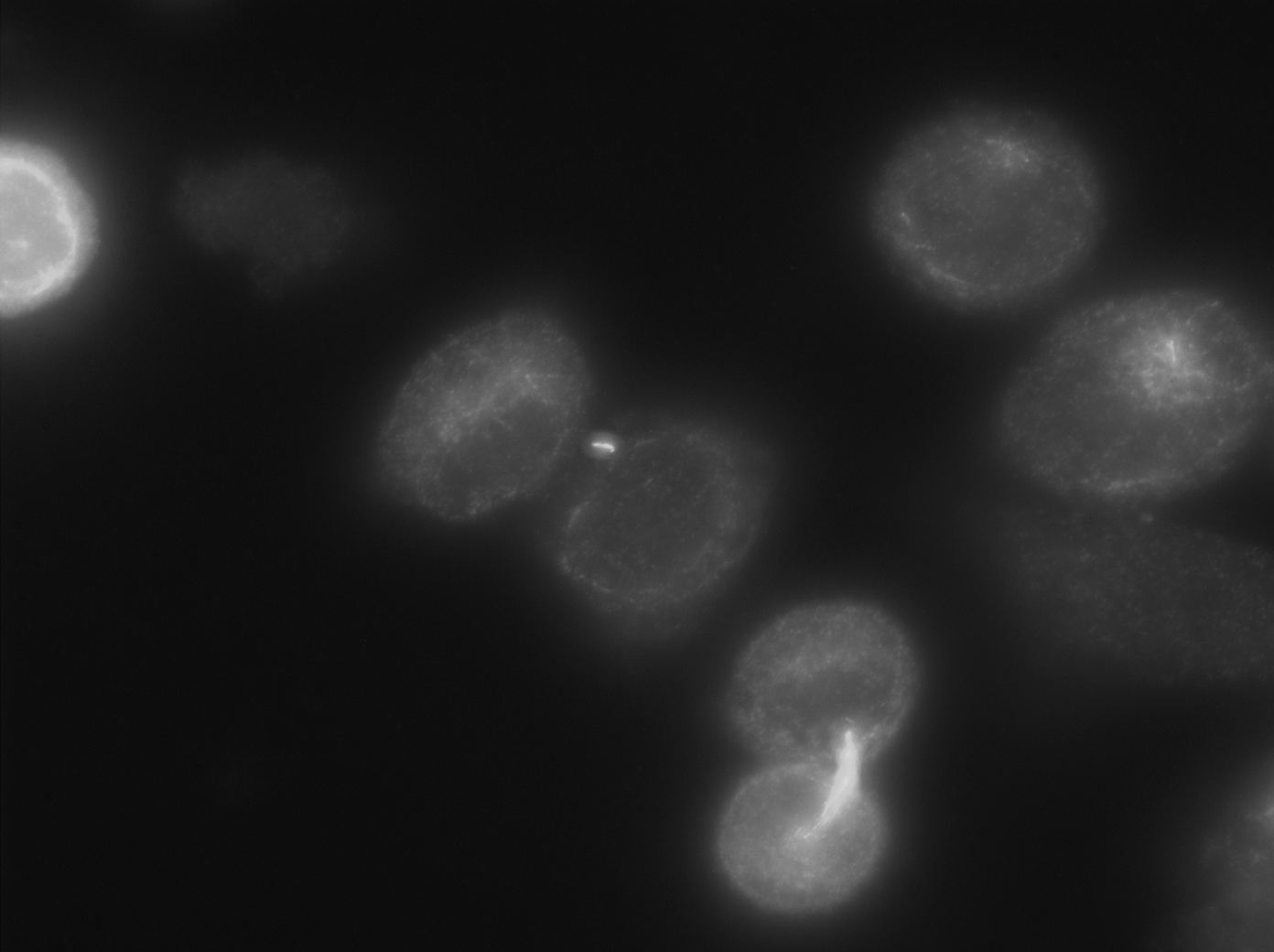

Supplement: Supplementary file 12 — Source data Fig. 3 part 2 [file 44319_2026_742_MOESM12_ESM.zip › Figure 3 Part 2/Fig 3b-e TTLL screen part 2/TTLL7-YFPy I18.Project Maximum Z_XY1679091040_Z0_T0_C1.tif]

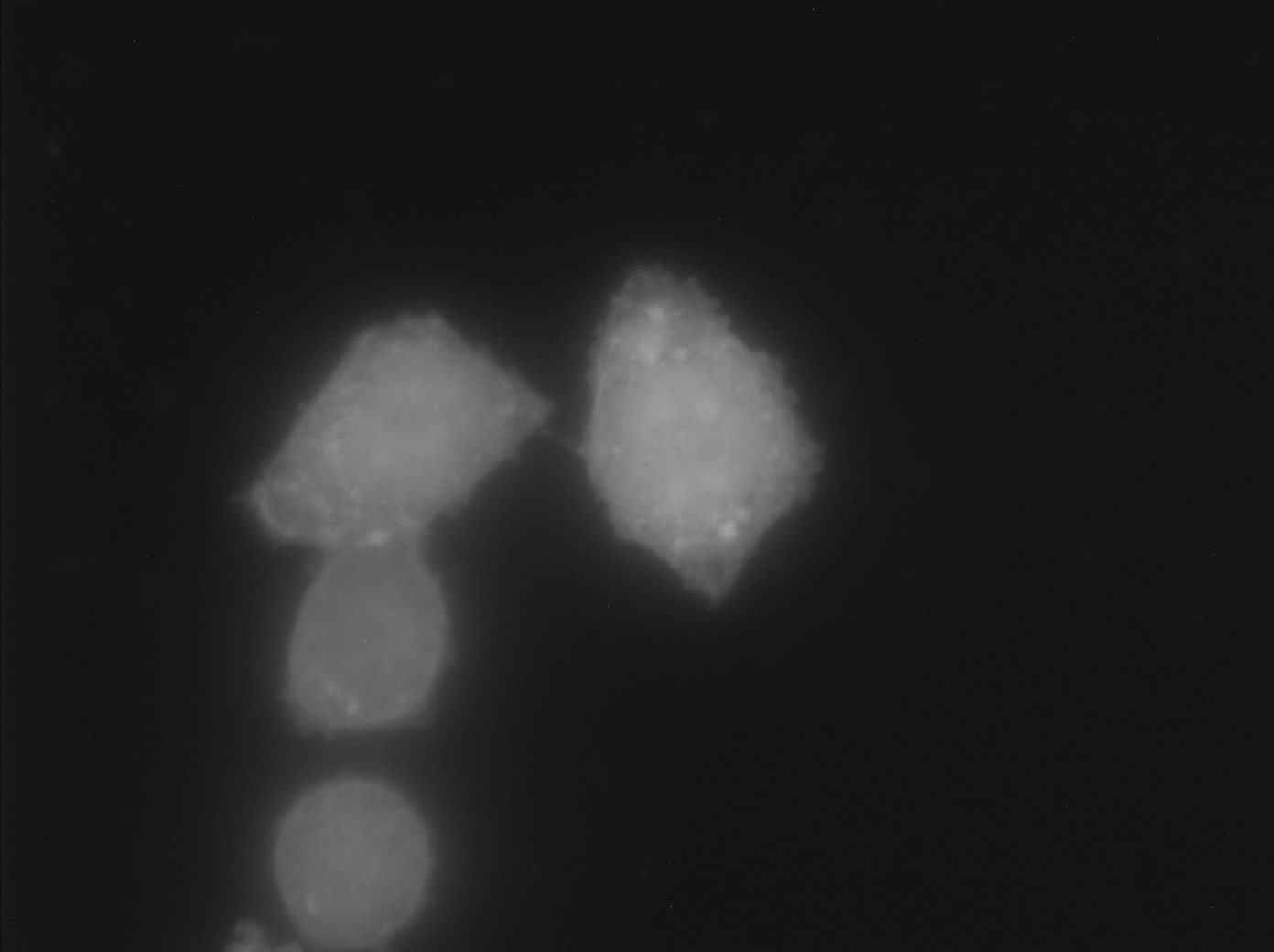

Supplement: Supplementary file 12 — Source data Fig. 3 part 2 [file 44319_2026_742_MOESM12_ESM.zip › Figure 3 Part 2/Fig 3b-e TTLL screen part 2/TTLL7-YFPy I15.Project Maximum Z_XY1679090498_Z0_T0_C2.tif]

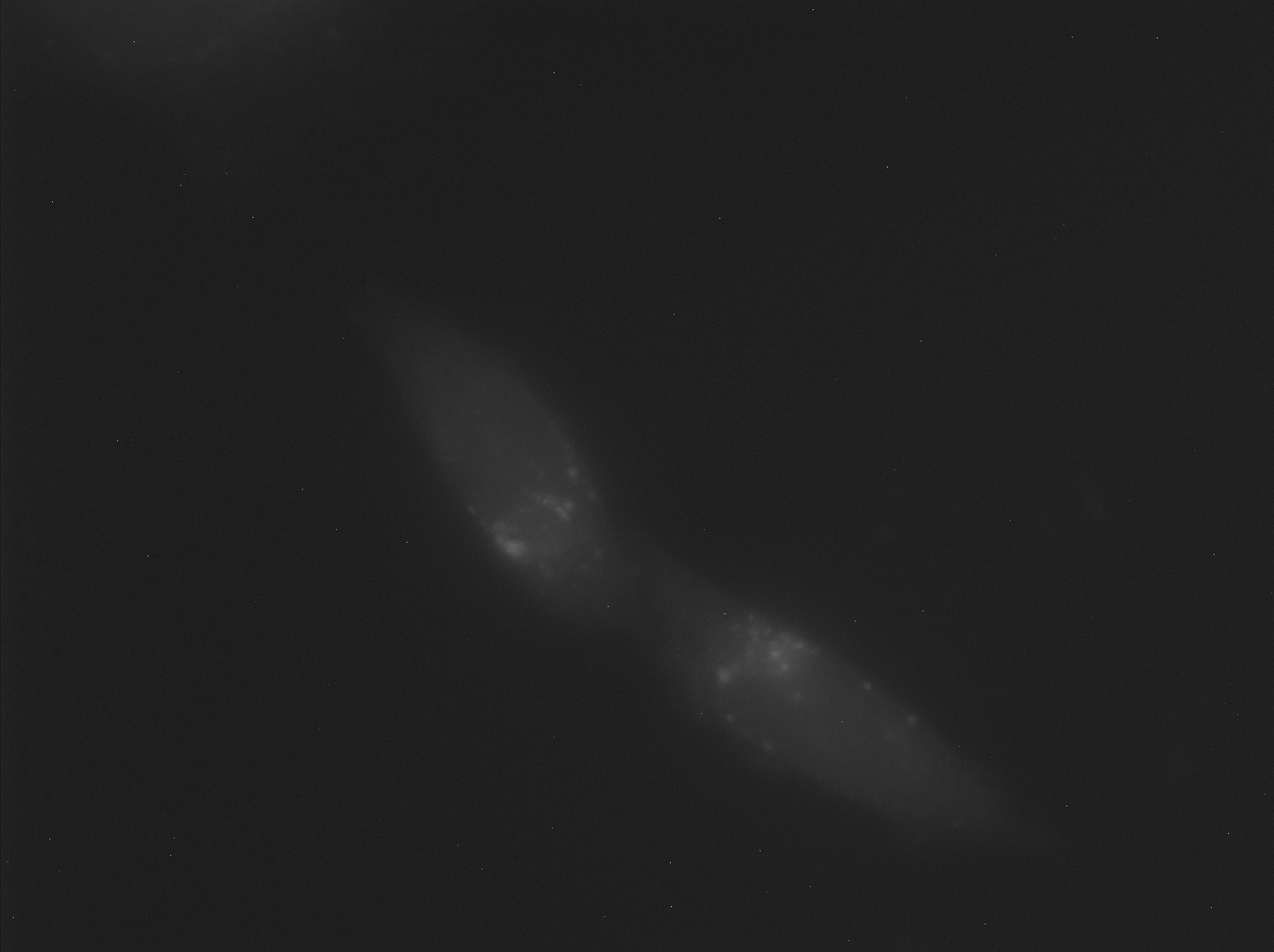

Supplement: Supplementary file 12 — Source data Fig. 3 part 2 [file 44319_2026_742_MOESM12_ESM.zip › Figure 3 Part 2/Fig 3b-e TTLL screen part 2/TTLL6-YFP R1 I3 C2 low.Project Maximum Z_XY1661793346_Z0_T0_C2.tif]

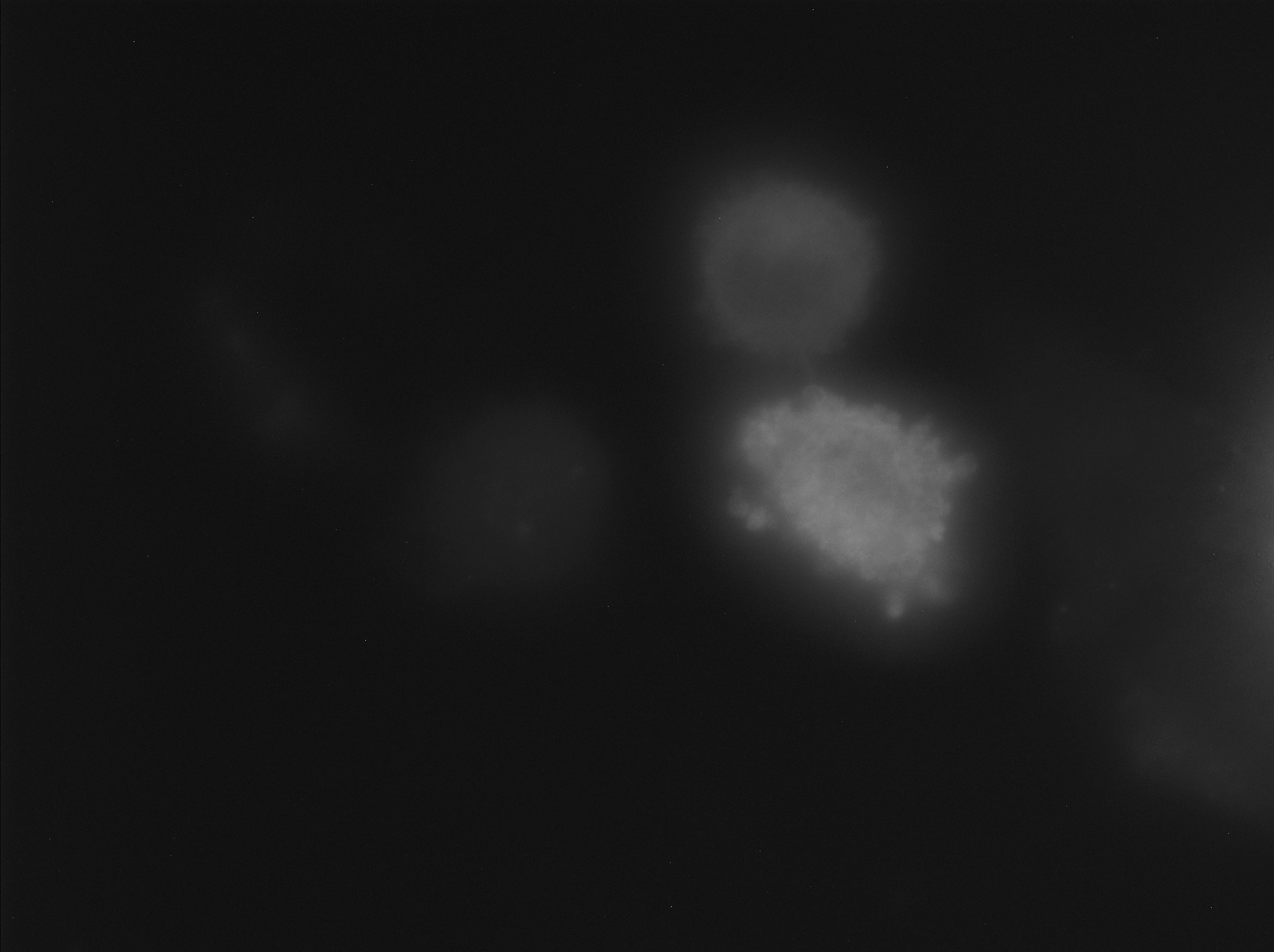

Supplement: Supplementary file 12 — Source data Fig. 3 part 2 [file 44319_2026_742_MOESM12_ESM.zip › Figure 3 Part 2/Fig 3b-e TTLL screen part 2/TTLL7-YFPy I3.Project Maximum Z_XY1679087380_Z0_T0_C2.tif]

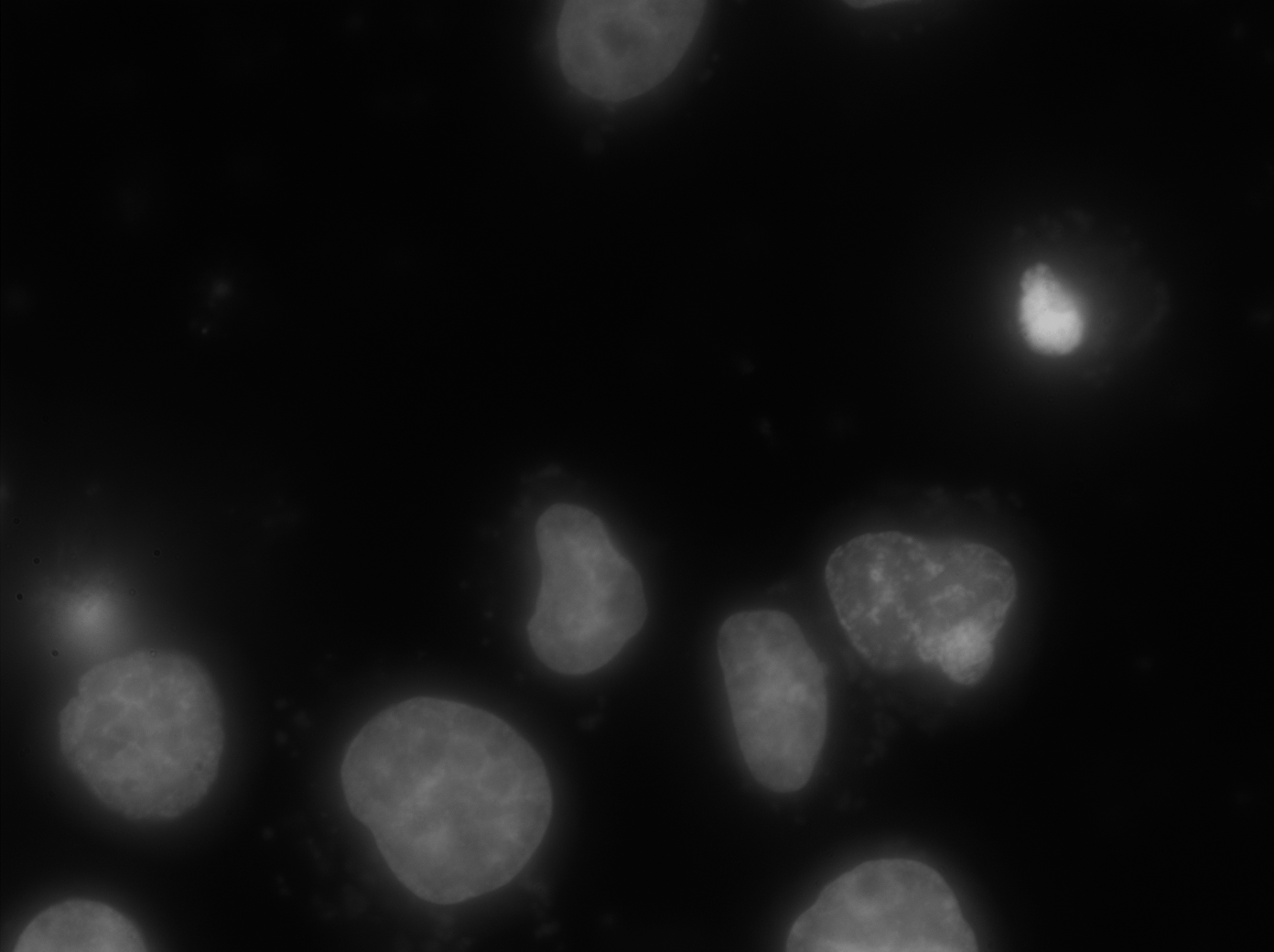

Supplement: Supplementary file 12 — Source data Fig. 3 part 2 [file 44319_2026_742_MOESM12_ESM.zip › Figure 3 Part 2/Fig 3b-e TTLL screen part 2/TTLL7-YFPy I20.Project Maximum Z_XY1679091425_Z0_T0_C0.tif]

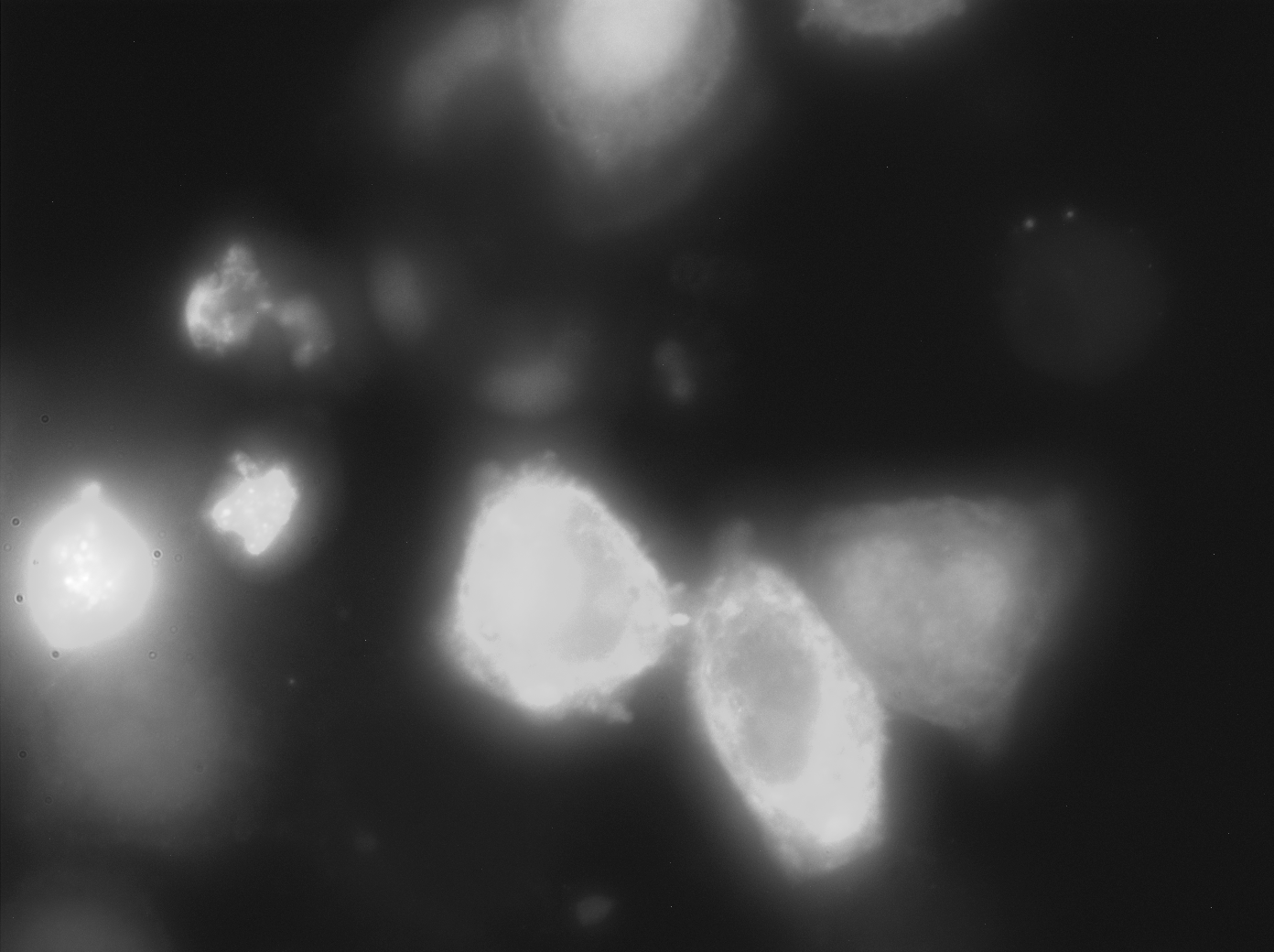

Supplement: Supplementary file 12 — Source data Fig. 3 part 2 [file 44319_2026_742_MOESM12_ESM.zip › Figure 3 Part 2/Fig 3b-e TTLL screen part 2/TTLL7-YFPy I20.Project Maximum Z_XY1679091425_Z0_T0_C2.tif]

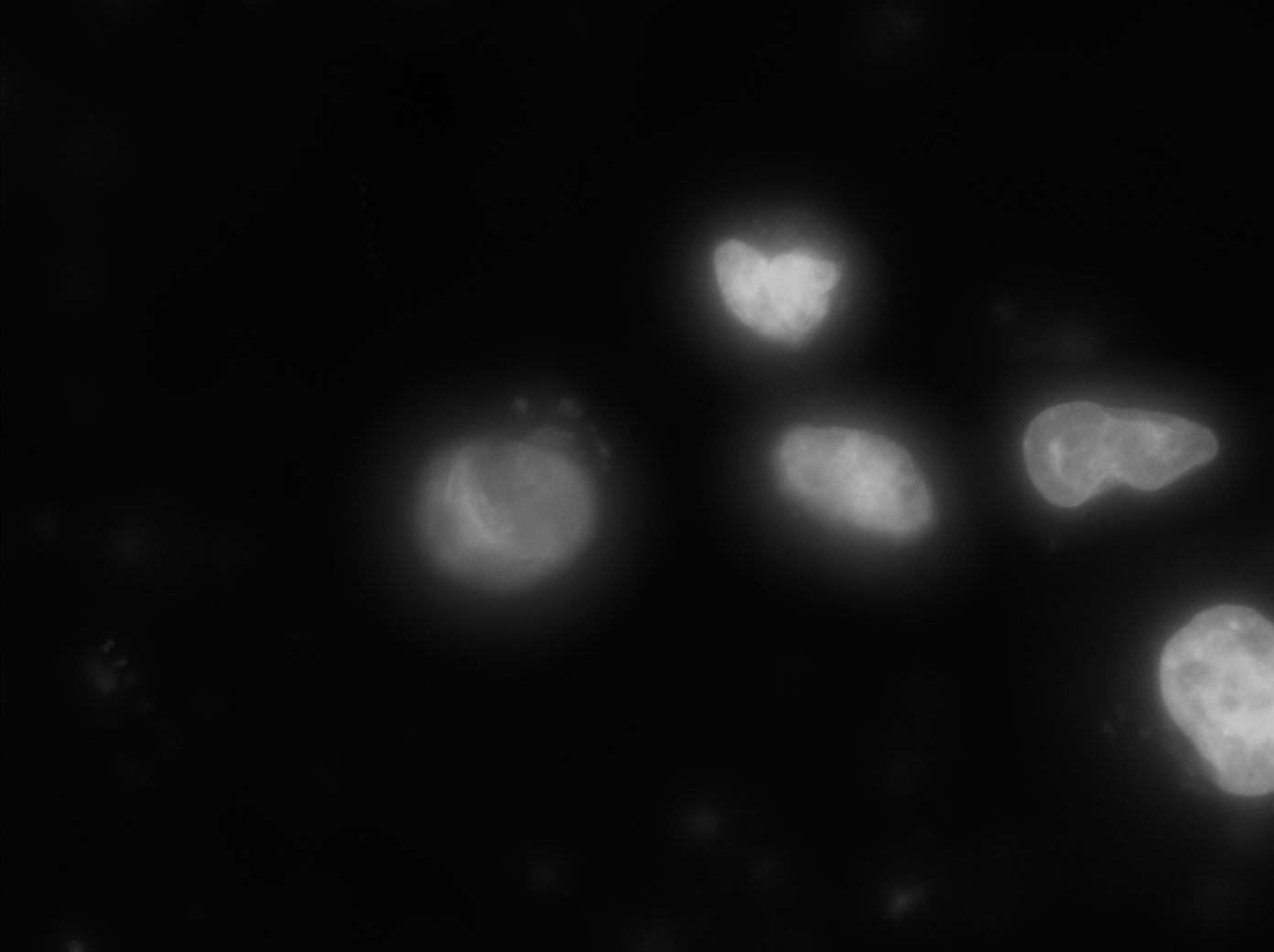

Supplement: Supplementary file 12 — Source data Fig. 3 part 2 [file 44319_2026_742_MOESM12_ESM.zip › Figure 3 Part 2/Fig 3b-e TTLL screen part 2/TTLL7-YFPy I3.Project Maximum Z_XY1679087380_Z0_T0_C0.tif]

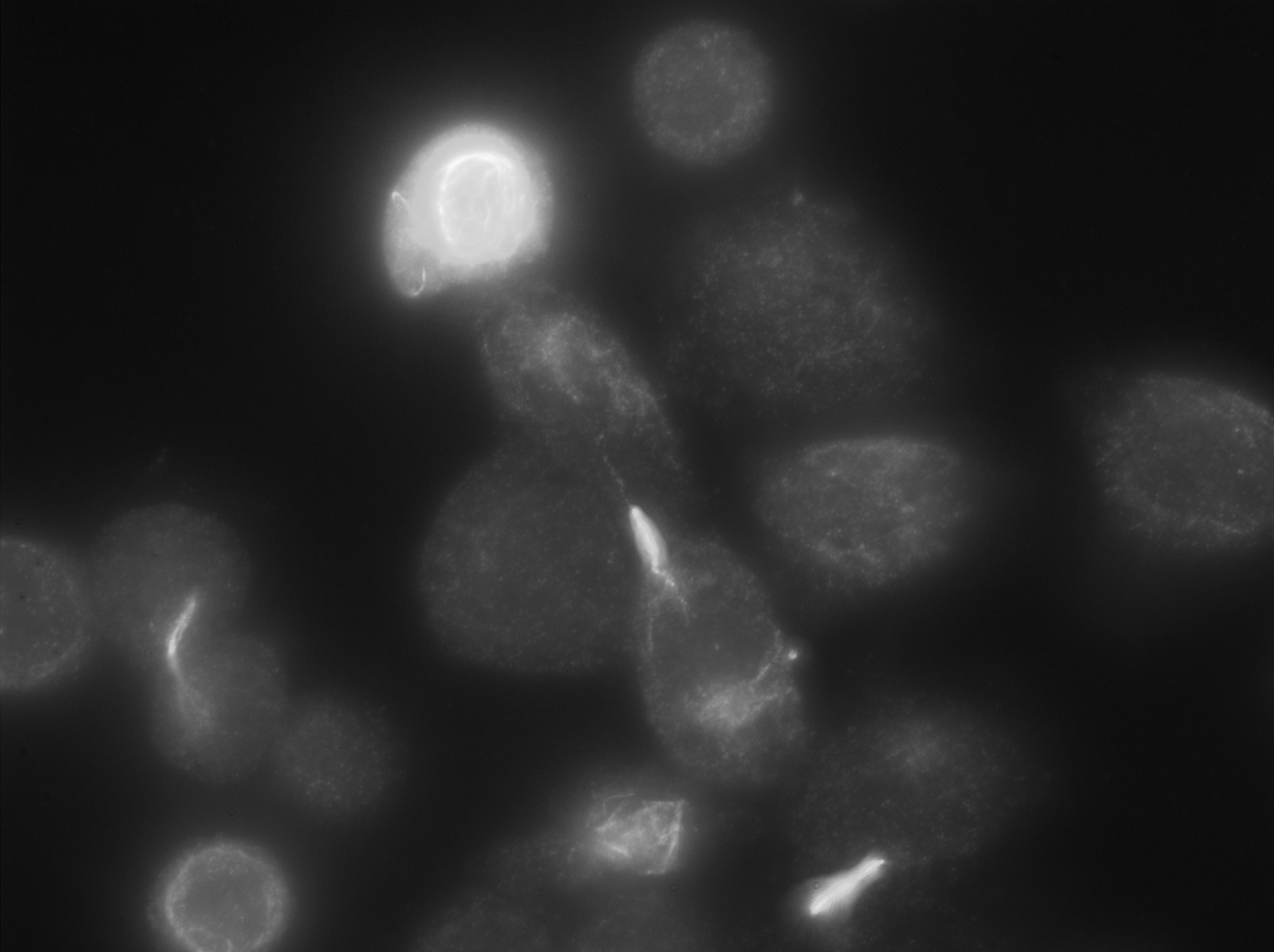

Supplement: Supplementary file 12 — Source data Fig. 3 part 2 [file 44319_2026_742_MOESM12_ESM.zip › Figure 3 Part 2/Fig 3b-e TTLL screen part 2/TTLL7-YFPy I5.Project Maximum Z_XY1679088039_Z0_T0_C1.tif]

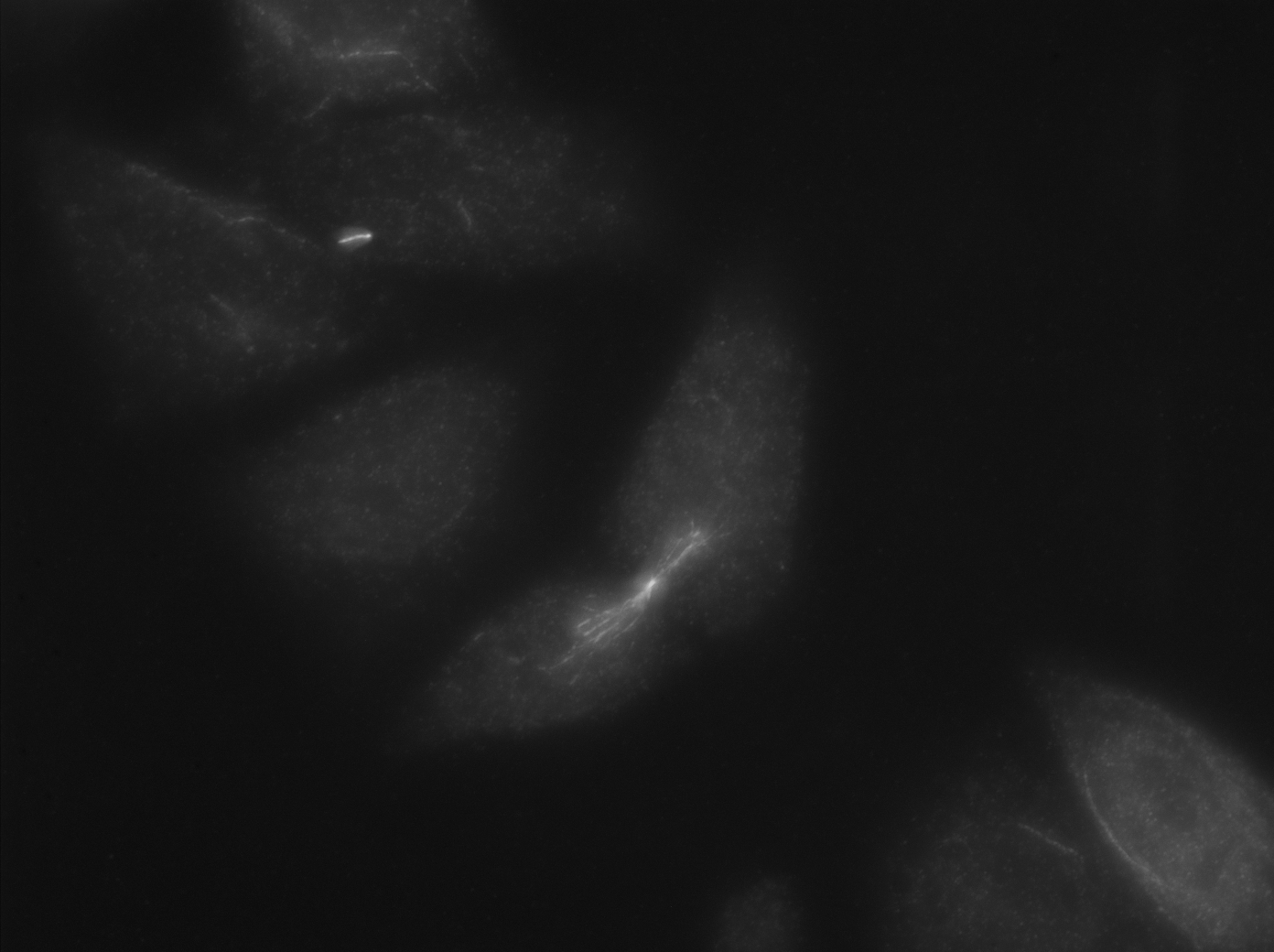

Supplement: Supplementary file 12 — Source data Fig. 3 part 2 [file 44319_2026_742_MOESM12_ESM.zip › Figure 3 Part 2/Fig 3b-e TTLL screen part 2/TTLL5-YFPy I14.Project Maximum Z_XY1679340750_Z0_T0_C1.tif]

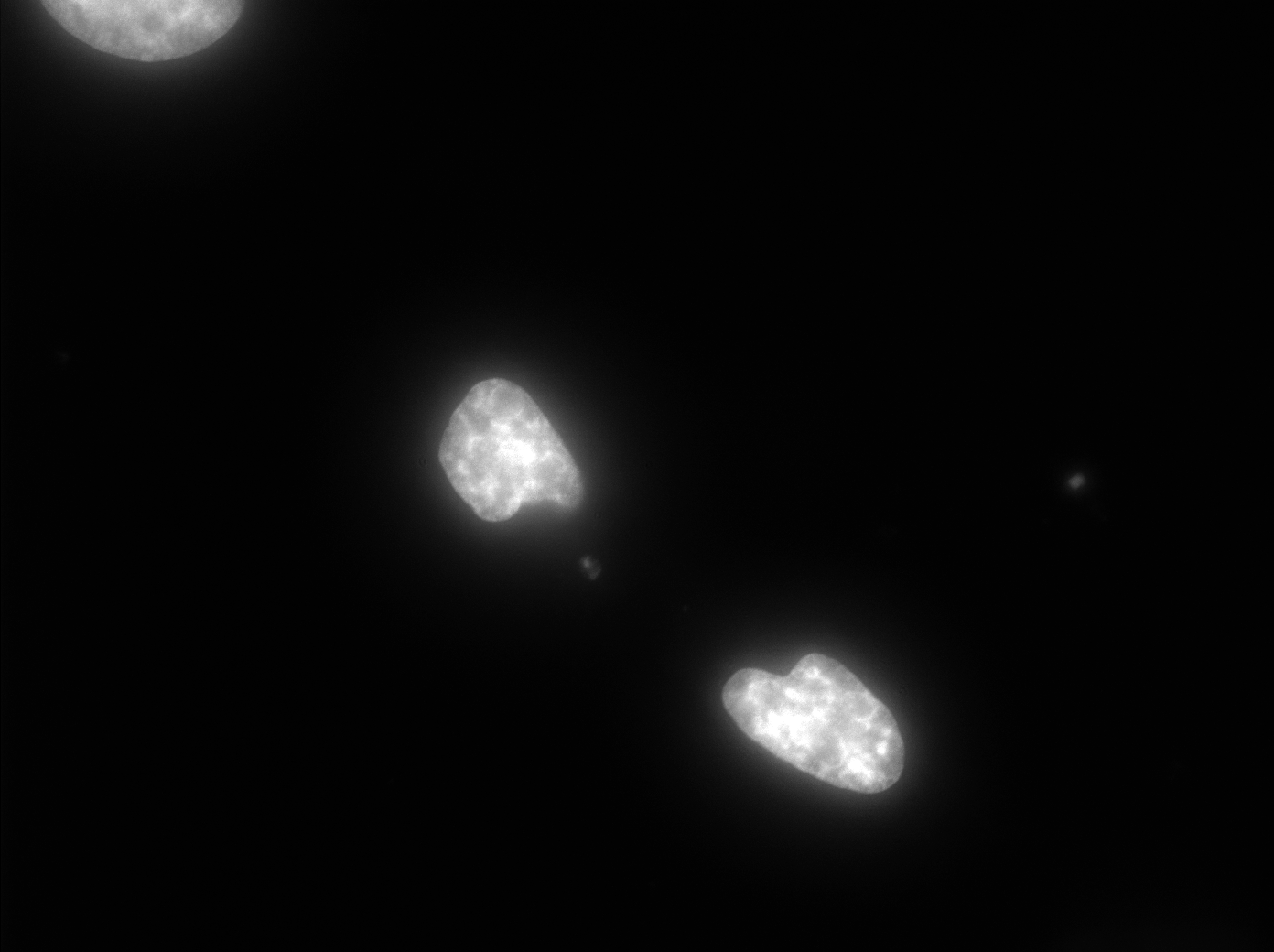

Supplement: Supplementary file 12 — Source data Fig. 3 part 2 [file 44319_2026_742_MOESM12_ESM.zip › Figure 3 Part 2/Fig 3b-e TTLL screen part 2/TTLL6-YFP R1 I3 C2 low.Project Maximum Z_XY1661793346_Z0_T0_C0.tif]

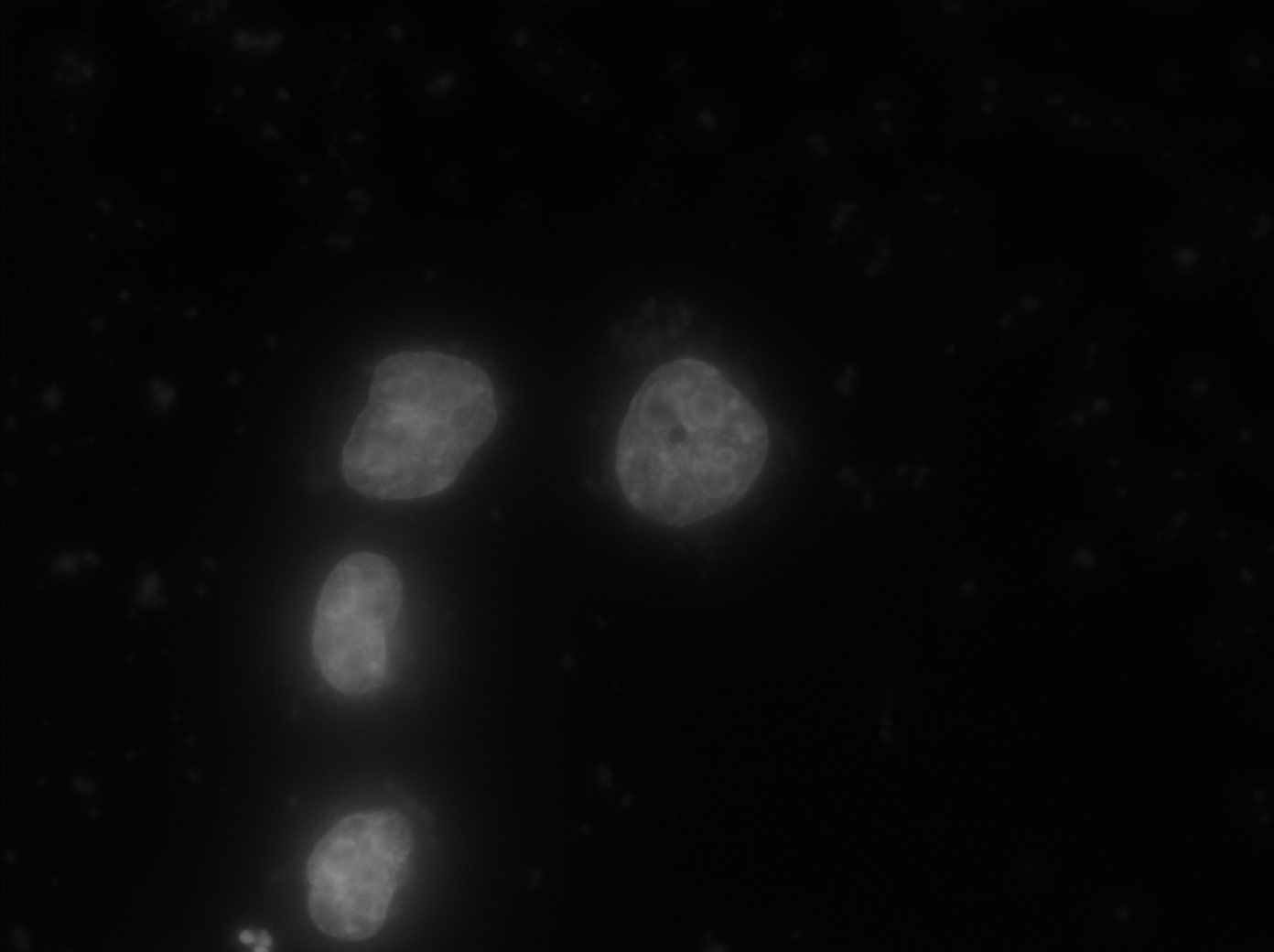

Supplement: Supplementary file 12 — Source data Fig. 3 part 2 [file 44319_2026_742_MOESM12_ESM.zip › Figure 3 Part 2/Fig 3b-e TTLL screen part 2/TTLL7-YFPy I15.Project Maximum Z_XY1679090498_Z0_T0_C0.tif]

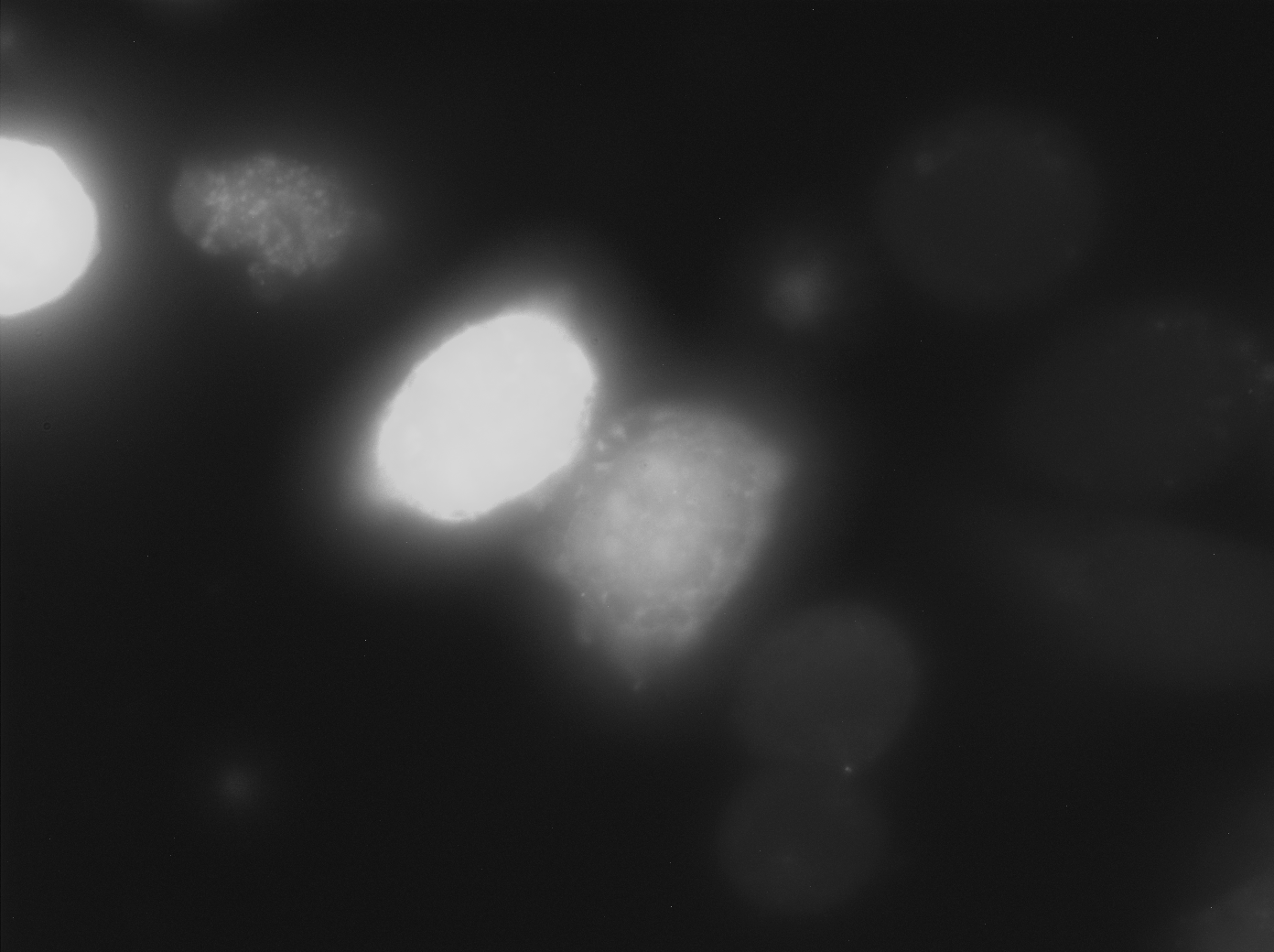

Supplement: Supplementary file 12 — Source data Fig. 3 part 2 [file 44319_2026_742_MOESM12_ESM.zip › Figure 3 Part 2/Fig 3b-e TTLL screen part 2/TTLL7-YFPy I18.Project Maximum Z_XY1679091040_Z0_T0_C2.tif]

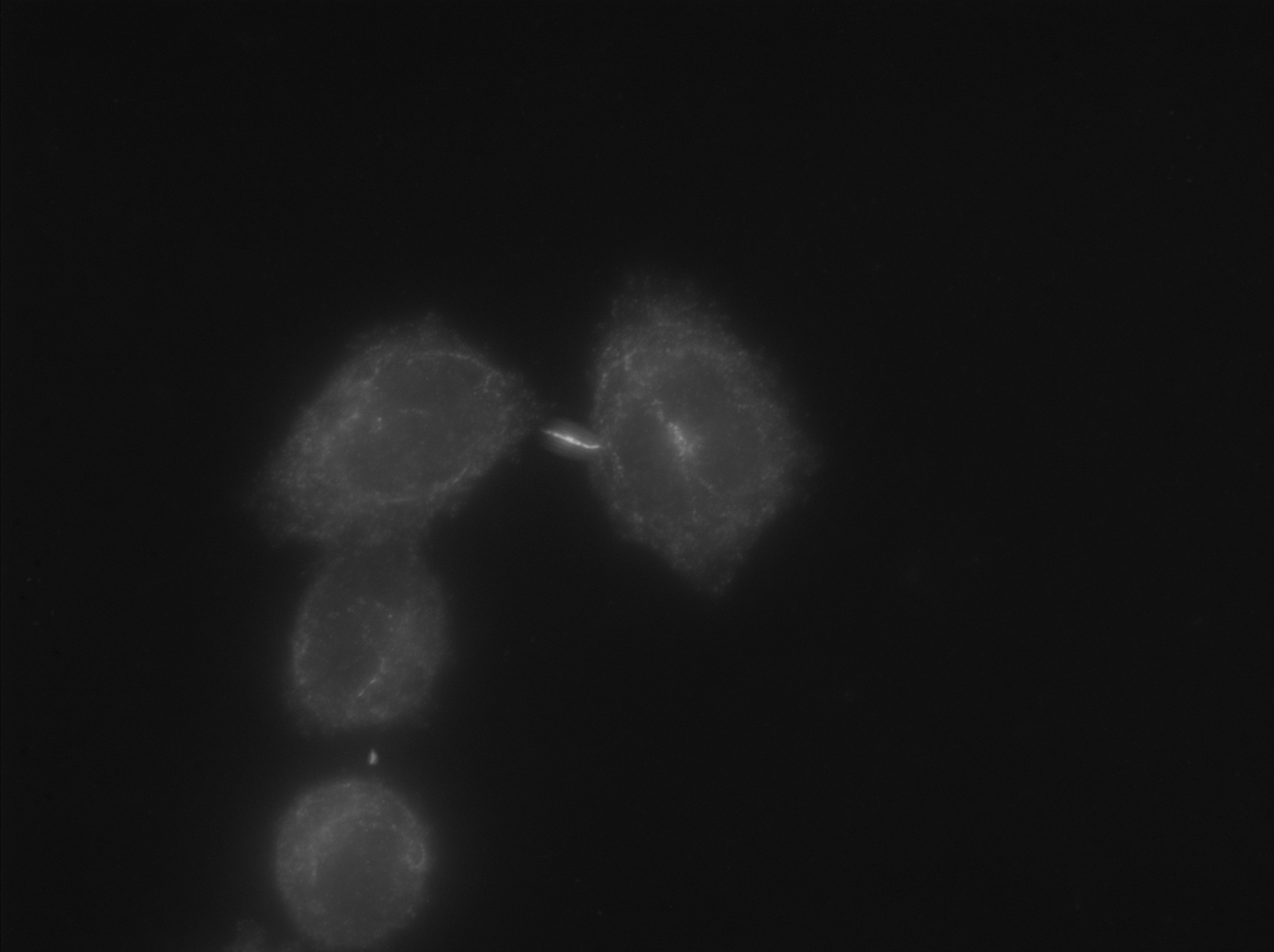

Supplement: Supplementary file 12 — Source data Fig. 3 part 2 [file 44319_2026_742_MOESM12_ESM.zip › Figure 3 Part 2/Fig 3b-e TTLL screen part 2/TTLL7-YFPy I15.Project Maximum Z_XY1679090498_Z0_T0_C1.tif]

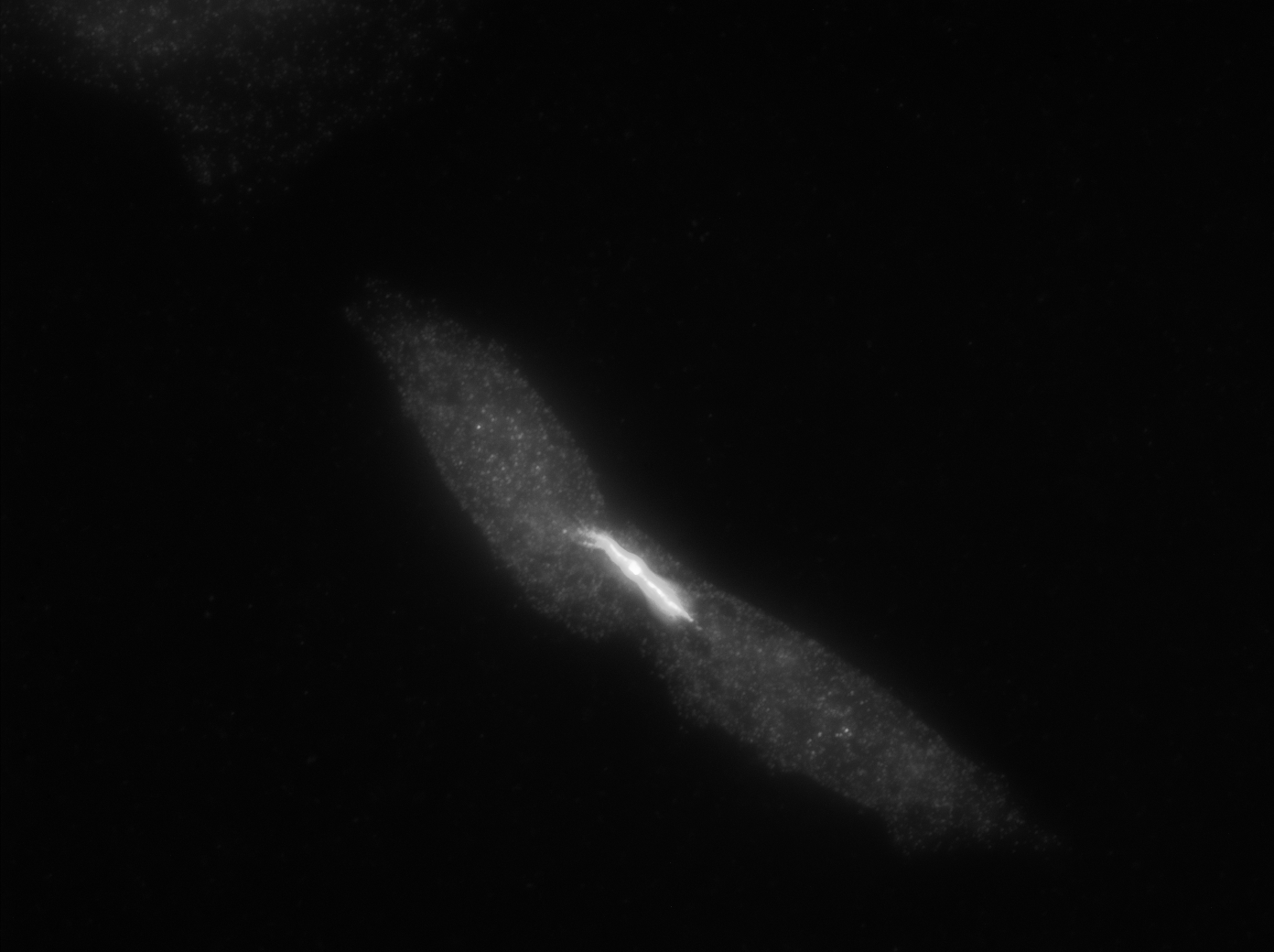

Supplement: Supplementary file 12 — Source data Fig. 3 part 2 [file 44319_2026_742_MOESM12_ESM.zip › Figure 3 Part 2/Fig 3b-e TTLL screen part 2/TTLL6-YFP R1 I3 C2 low.Project Maximum Z_XY1661793346_Z0_T0_C1.tif]

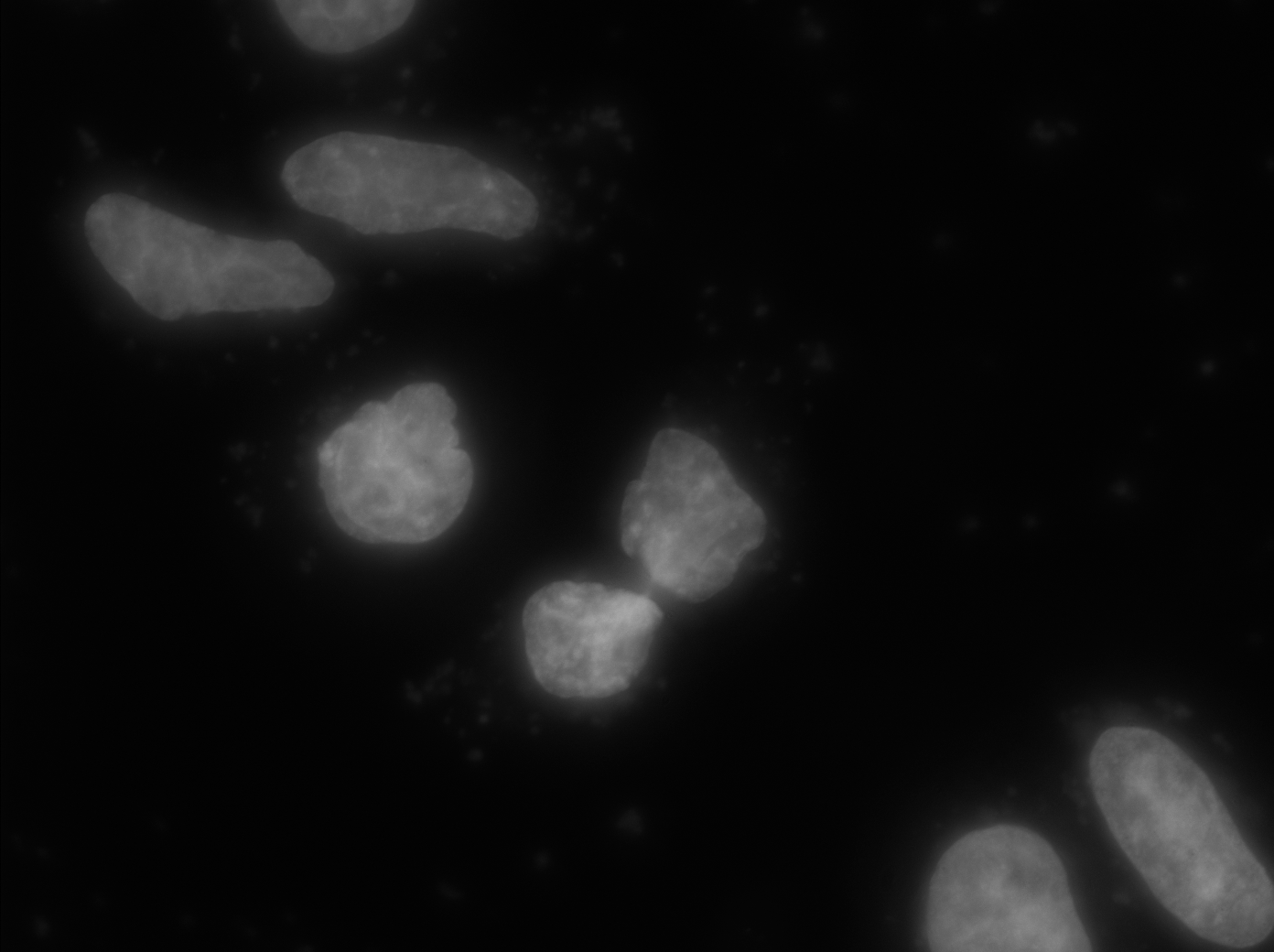

Supplement: Supplementary file 12 — Source data Fig. 3 part 2 [file 44319_2026_742_MOESM12_ESM.zip › Figure 3 Part 2/Fig 3b-e TTLL screen part 2/TTLL5-YFPy I14.Project Maximum Z_XY1679340750_Z0_T0_C0.tif]

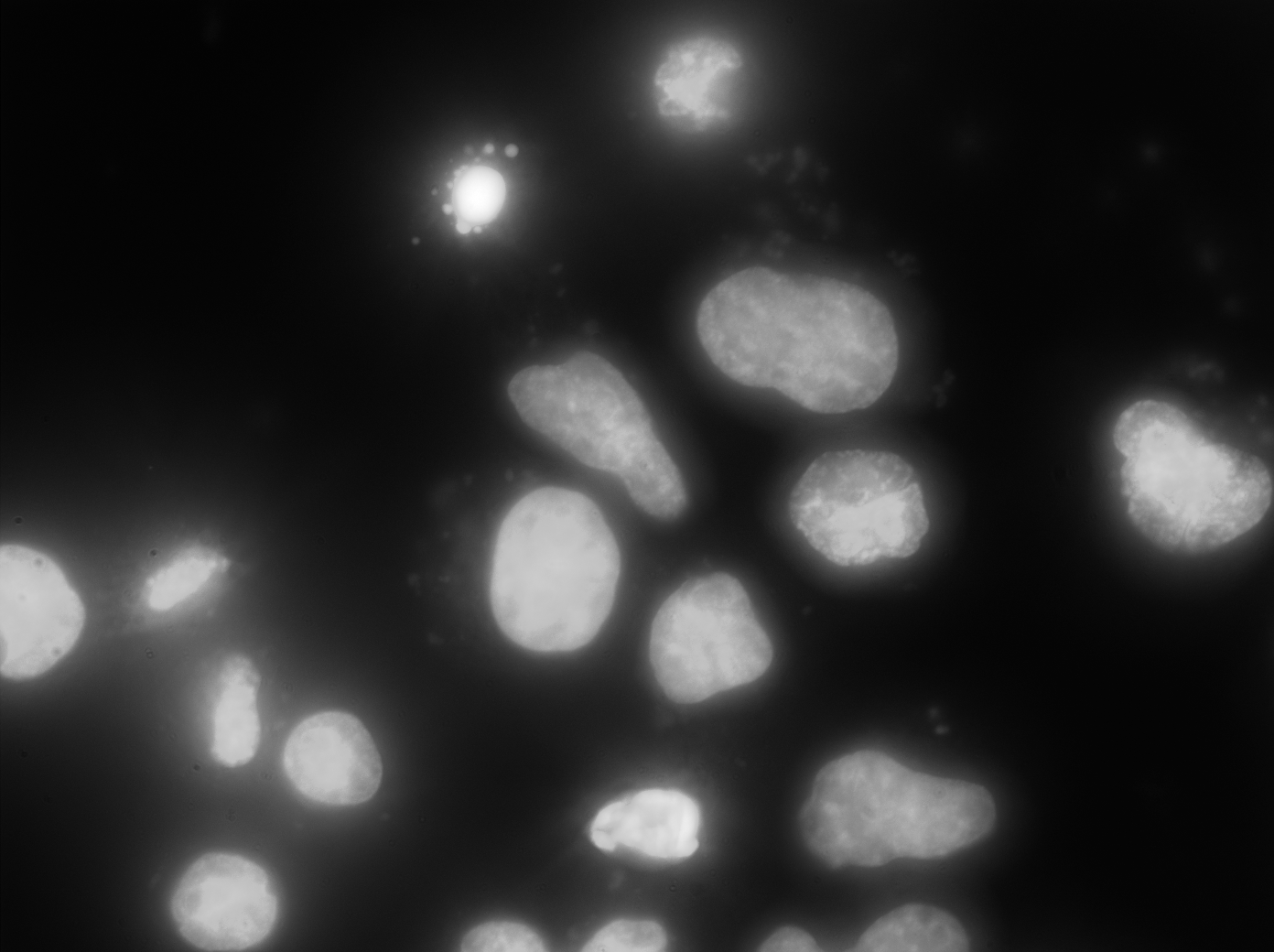

Supplement: Supplementary file 12 — Source data Fig. 3 part 2 [file 44319_2026_742_MOESM12_ESM.zip › Figure 3 Part 2/Fig 3b-e TTLL screen part 2/TTLL7-YFPy I5.Project Maximum Z_XY1679088039_Z0_T0_C0.tif]

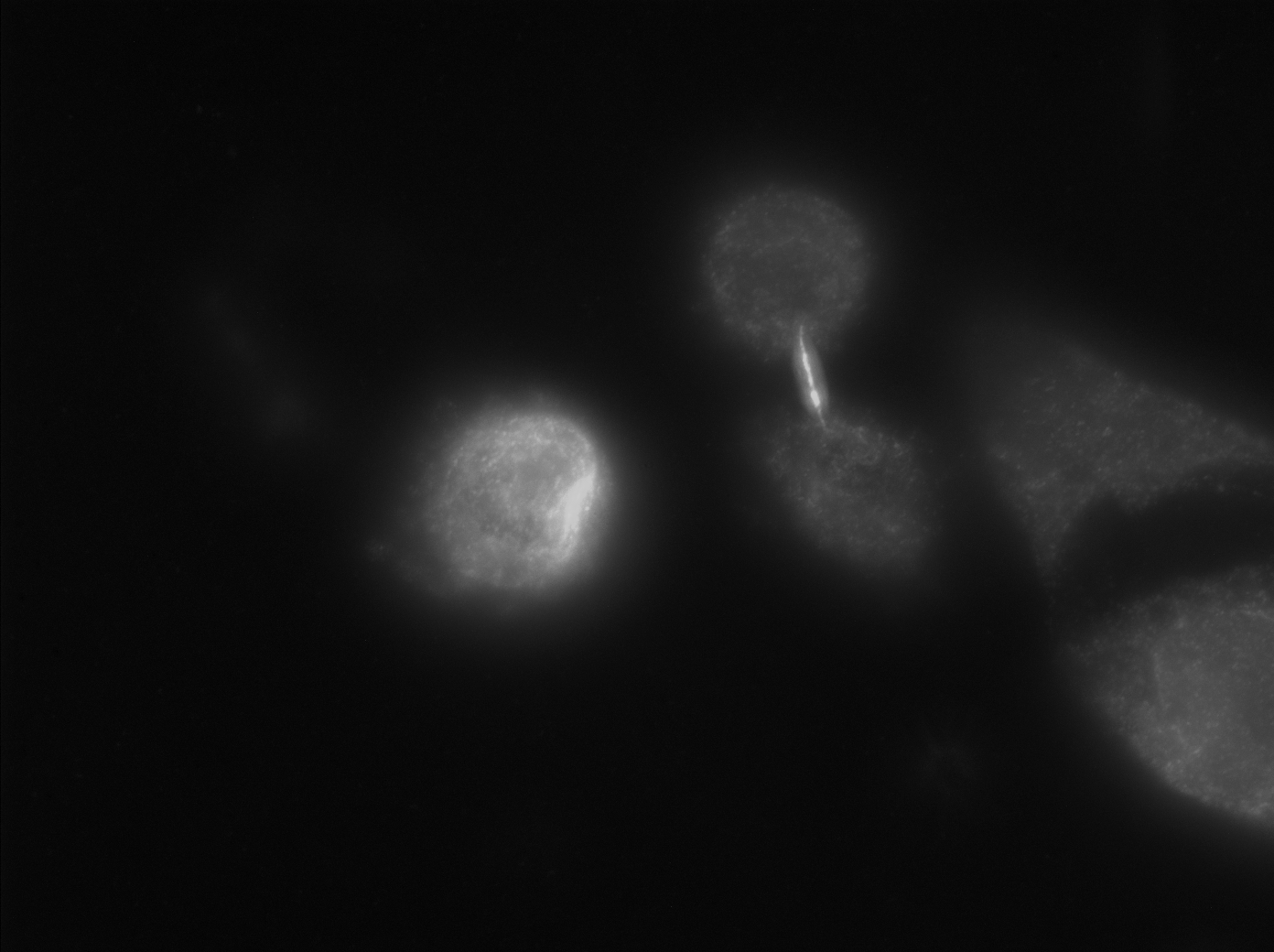

Supplement: Supplementary file 12 — Source data Fig. 3 part 2 [file 44319_2026_742_MOESM12_ESM.zip › Figure 3 Part 2/Fig 3b-e TTLL screen part 2/TTLL7-YFPy I3.Project Maximum Z_XY1679087380_Z0_T0_C1.tif]

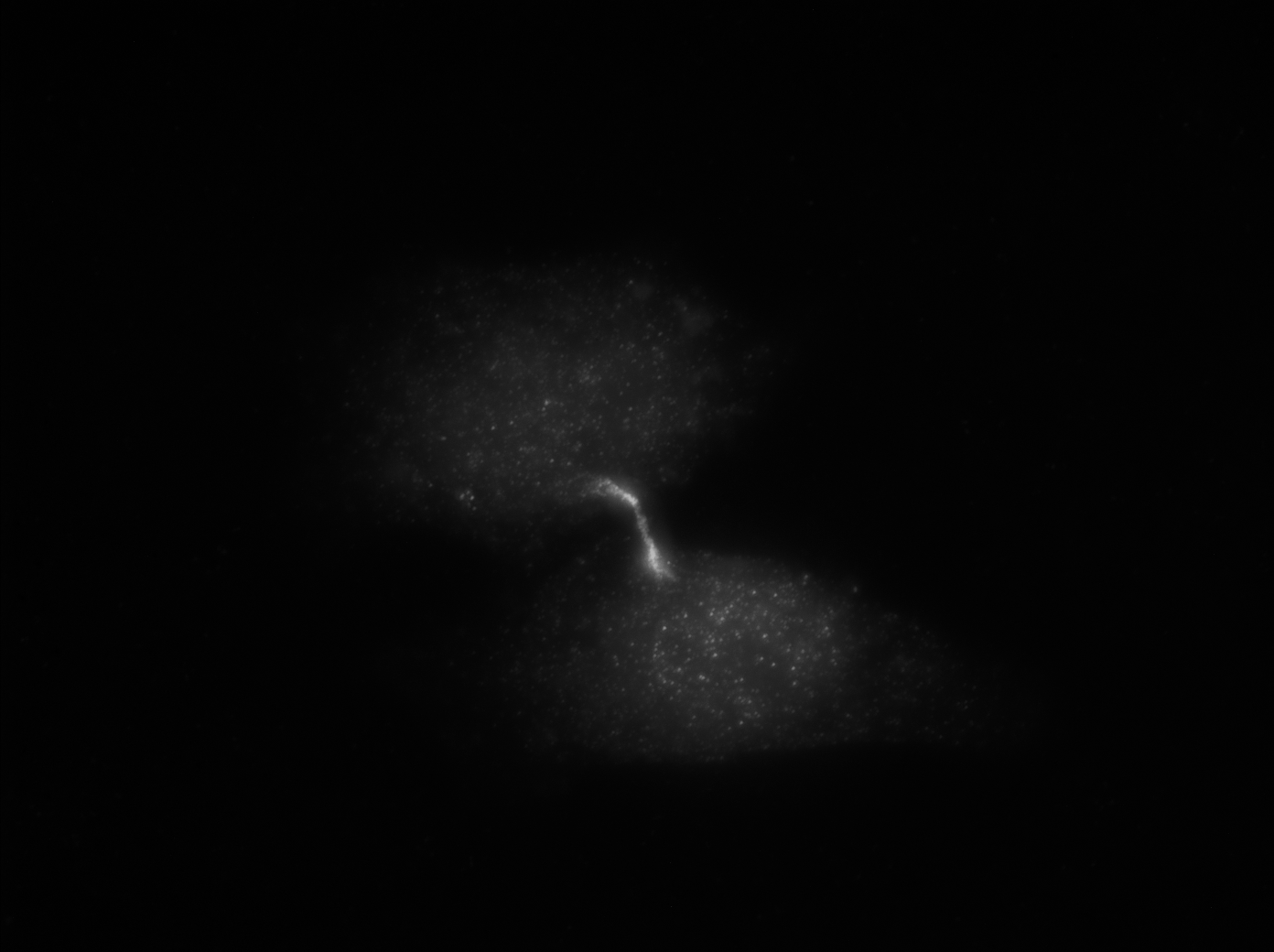

Supplement: Supplementary file 13 — Source data Fig. 3 part 3 [file 44319_2026_742_MOESM13_ESM.zip › Figure 3 Part 3/Fig 3b-e TTLL screen part 3/YFP Only R1 I2.Project Maximum Z_XY1663271353_Z0_T0_C1.tif]

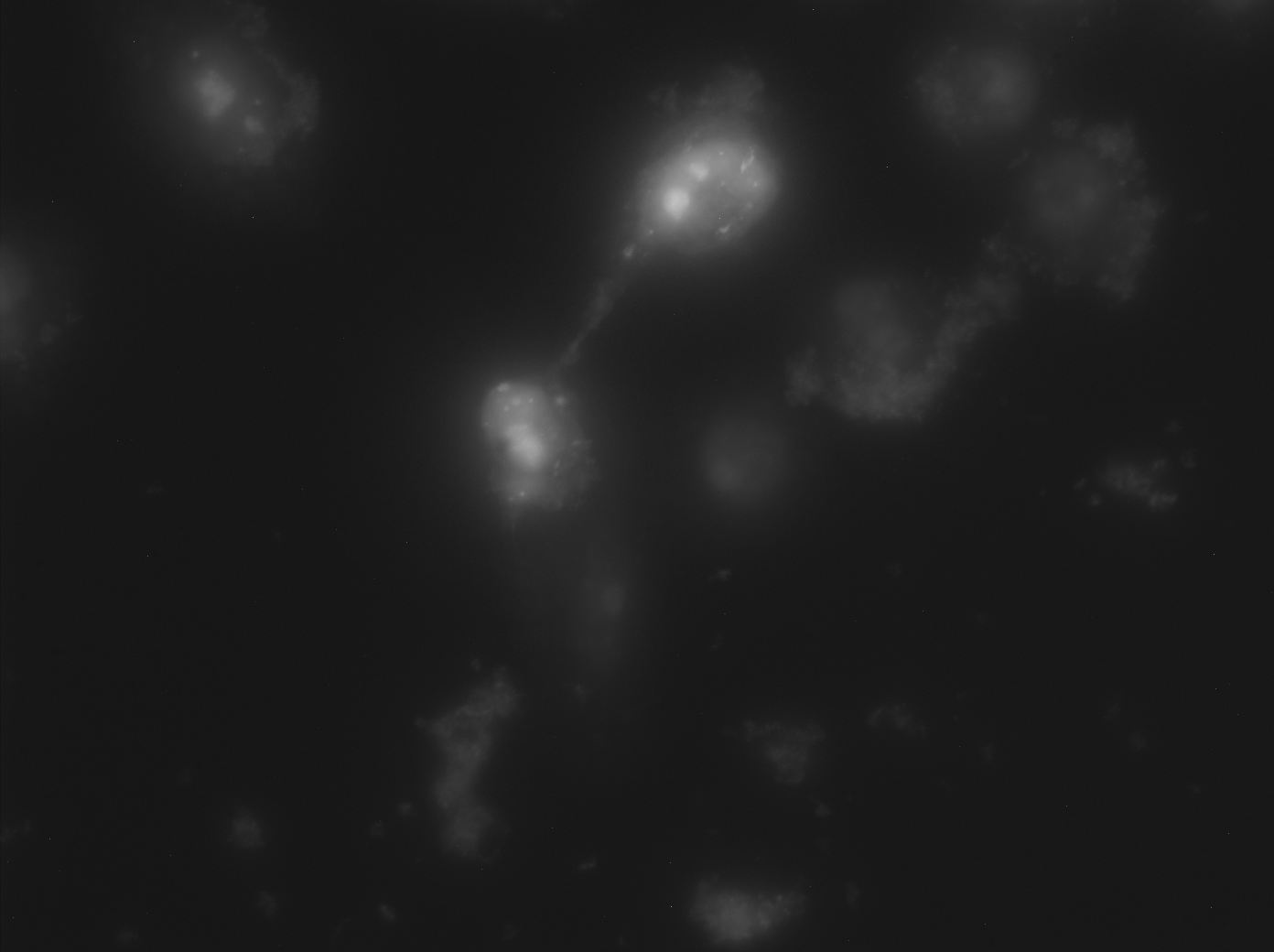

Supplement: Supplementary file 13 — Source data Fig. 3 part 3 [file 44319_2026_742_MOESM13_ESM.zip › Figure 3 Part 3/Fig 3b-e TTLL screen part 3/TTLL11-YFP Img 8 yfp2000.Project Maximum Z_XY1648578082_Z0_T0_C2.tif]

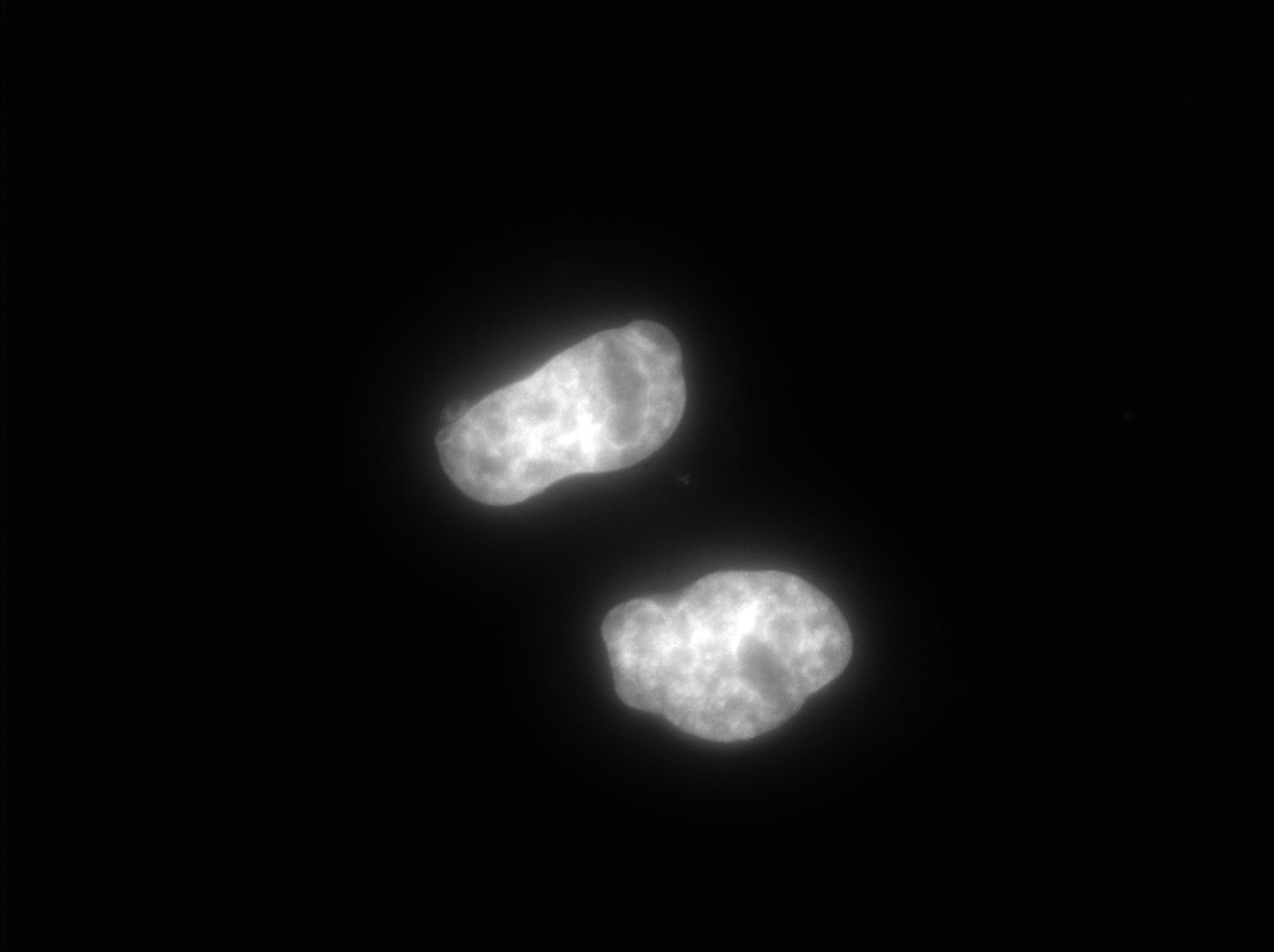

Supplement: Supplementary file 13 — Source data Fig. 3 part 3 [file 44319_2026_742_MOESM13_ESM.zip › Figure 3 Part 3/Fig 3b-e TTLL screen part 3/YFP Only R1 I2.Project Maximum Z_XY1663271353_Z0_T0_C0.tif]

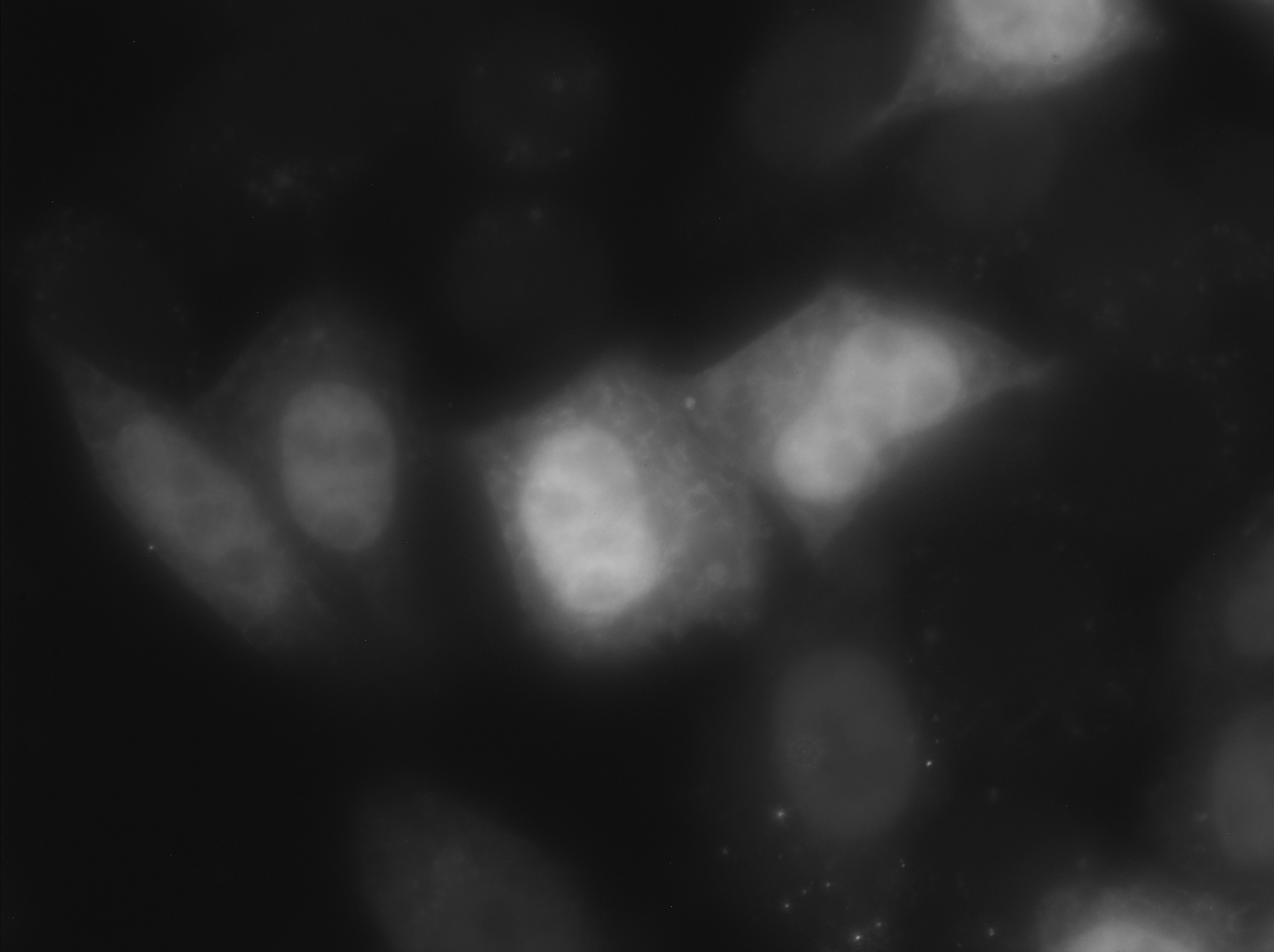

Supplement: Supplementary file 13 — Source data Fig. 3 part 3 [file 44319_2026_742_MOESM13_ESM.zip › Figure 3 Part 3/Fig 3b-e TTLL screen part 3/TTLL9-YFP A3 I8.Project Maximum Z_XY1679700378_Z0_T0_C2.tif]

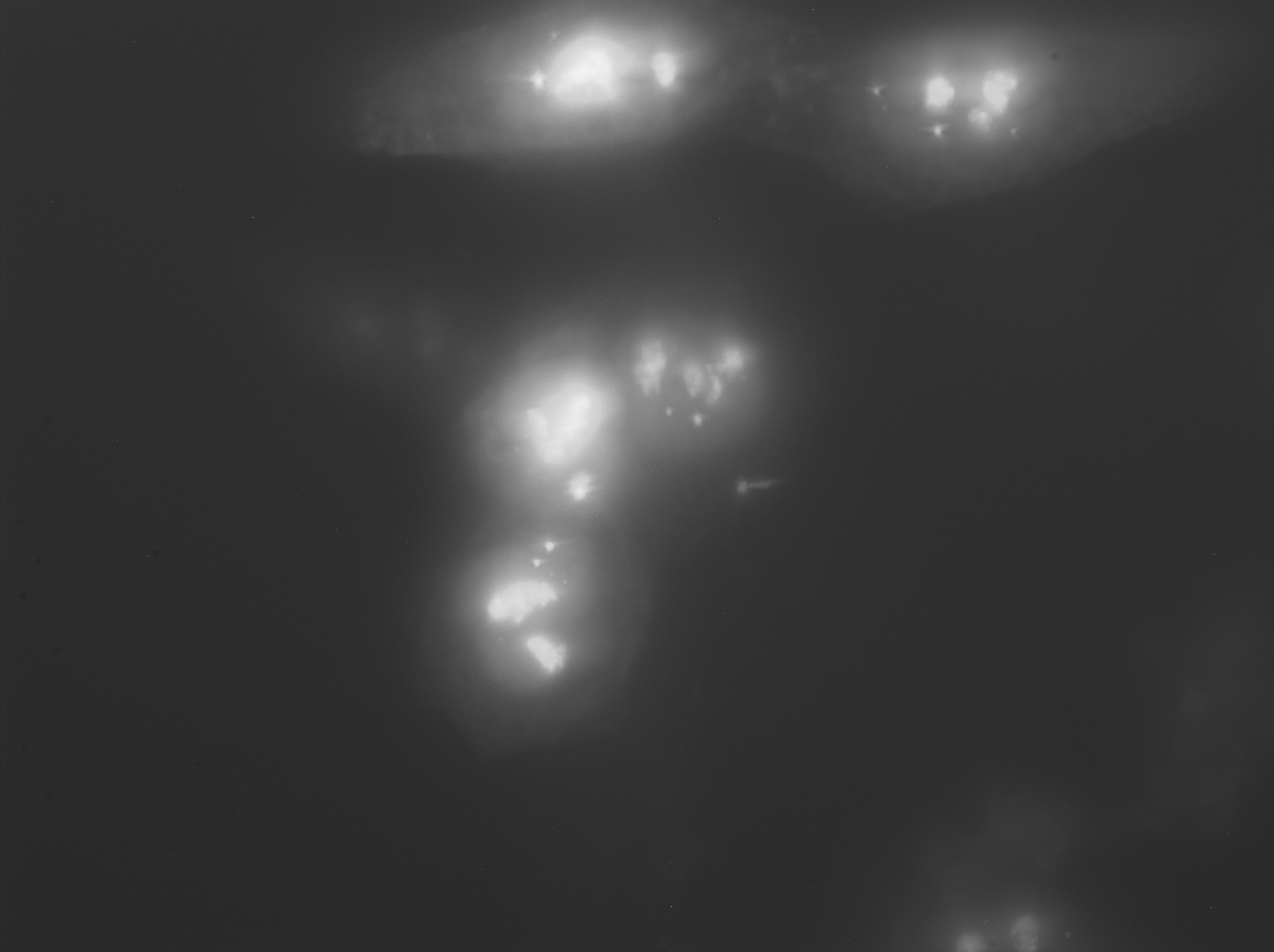

Supplement: Supplementary file 13 — Source data Fig. 3 part 3 [file 44319_2026_742_MOESM13_ESM.zip › Figure 3 Part 3/Fig 3b-e TTLL screen part 3/TTLL11-YFP A2 Img2.Project Maximum Z_XY1648574683_Z0_T0_C2.tif]

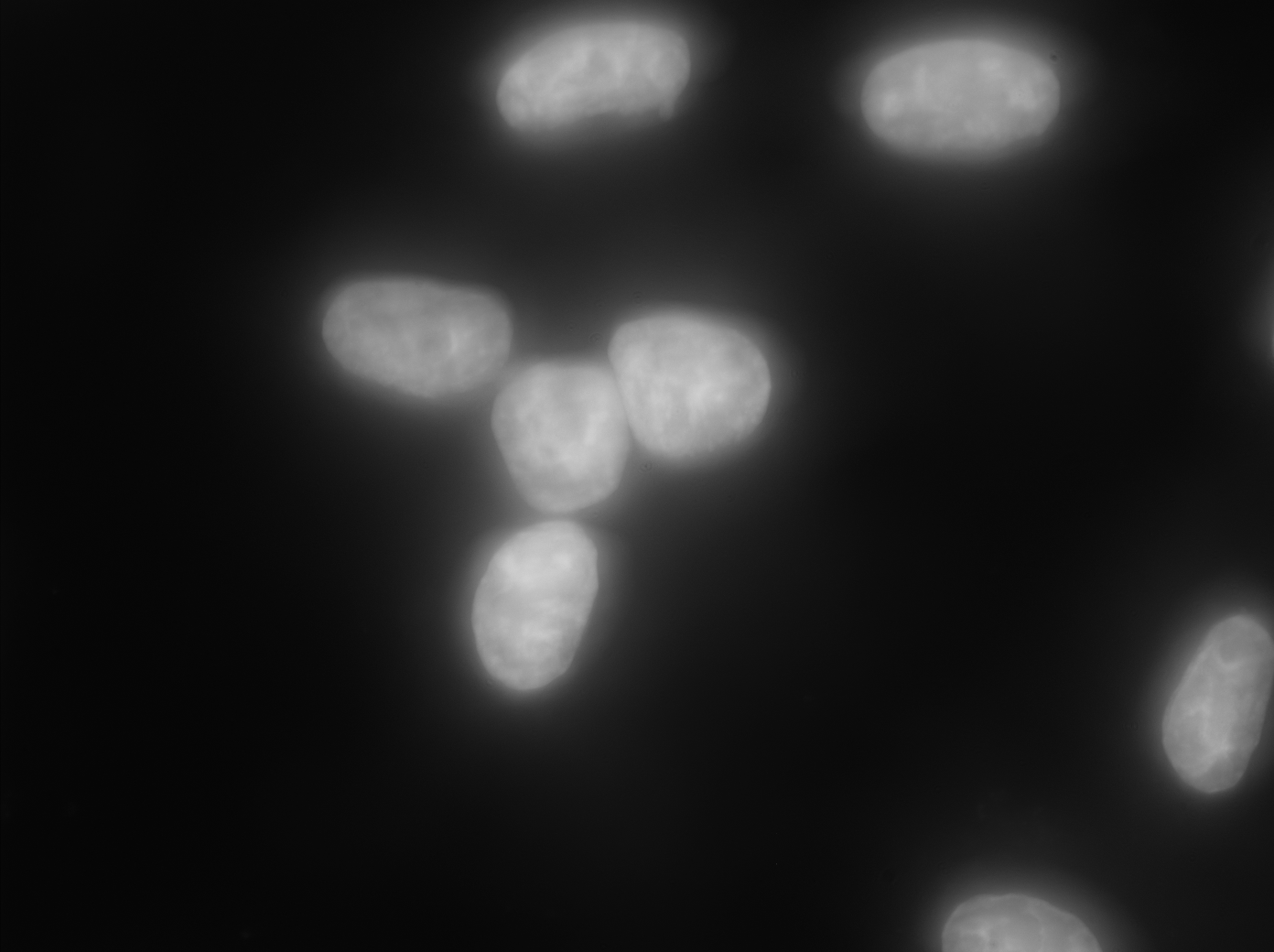

Supplement: Supplementary file 13 — Source data Fig. 3 part 3 [file 44319_2026_742_MOESM13_ESM.zip › Figure 3 Part 3/Fig 3b-e TTLL screen part 3/TTLL11-YFP A2 Img2.Project Maximum Z_XY1648574683_Z0_T0_C0.tif]

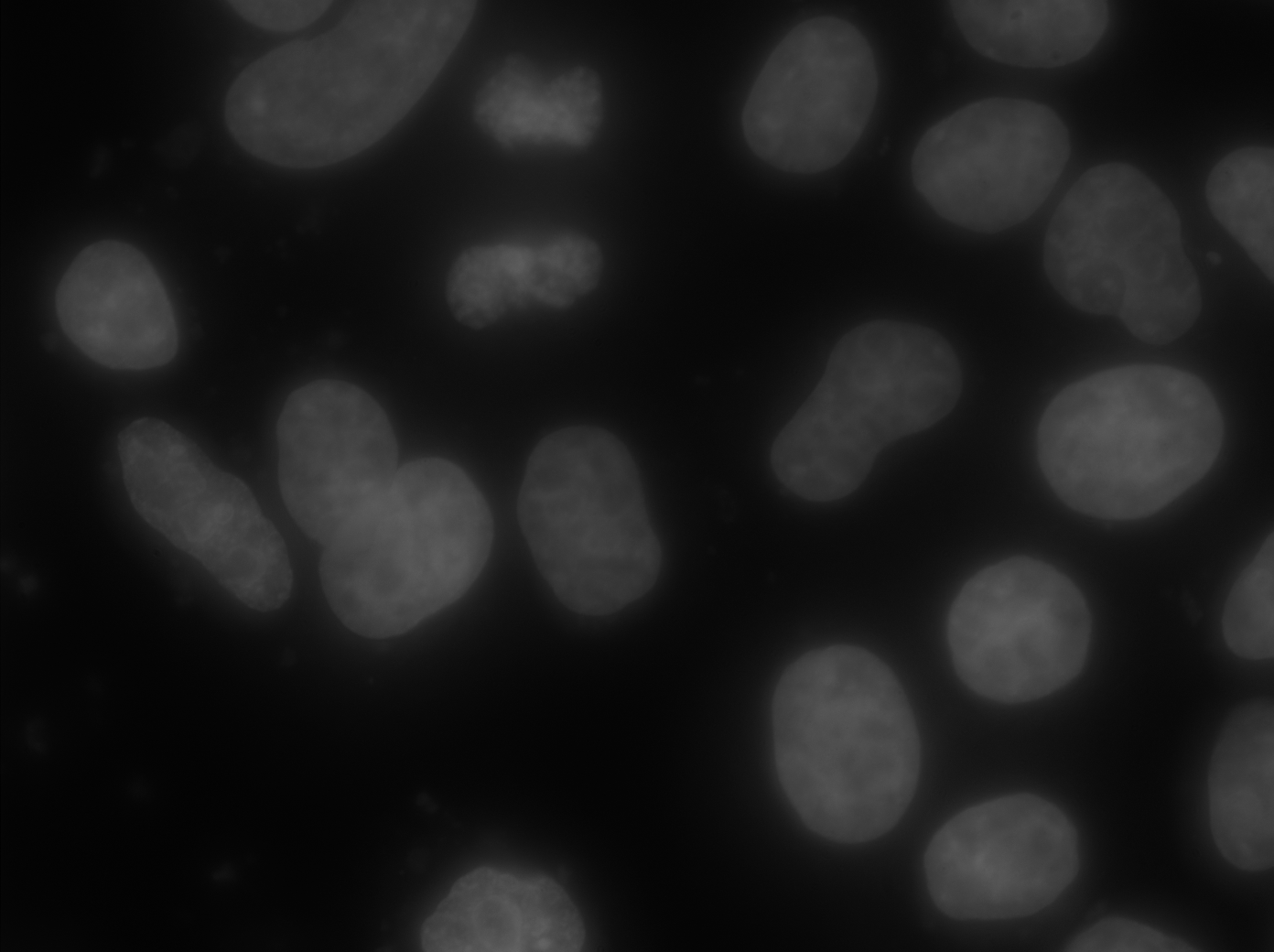

Supplement: Supplementary file 13 — Source data Fig. 3 part 3 [file 44319_2026_742_MOESM13_ESM.zip › Figure 3 Part 3/Fig 3b-e TTLL screen part 3/TTLL9-YFP A3 I8.Project Maximum Z_XY1679700378_Z0_T0_C0.tif]

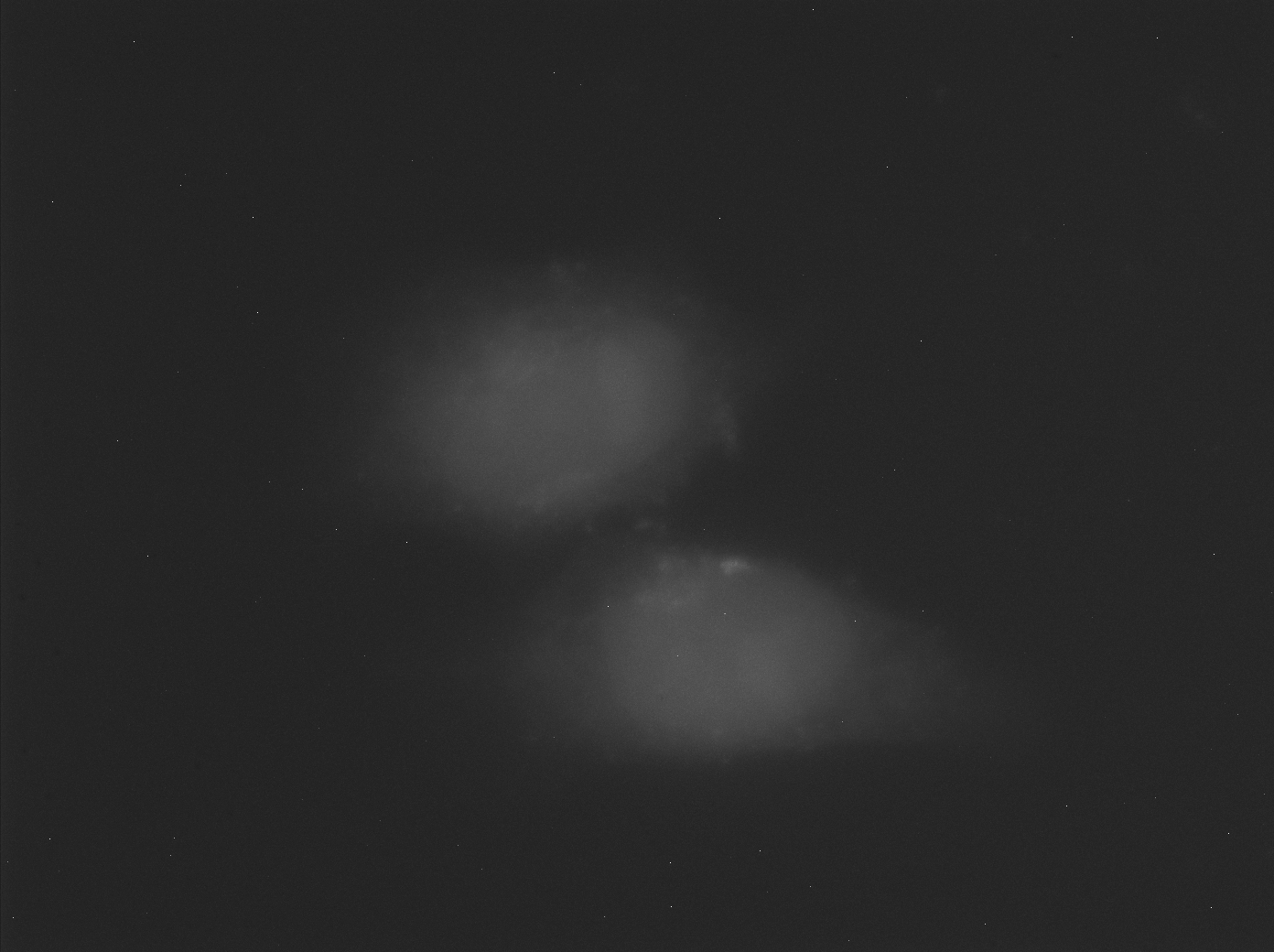

Supplement: Supplementary file 13 — Source data Fig. 3 part 3 [file 44319_2026_742_MOESM13_ESM.zip › Figure 3 Part 3/Fig 3b-e TTLL screen part 3/YFP Only R1 I2.Project Maximum Z_XY1663271353_Z0_T0_C2.tif]
