# Supplementary figures and images for: TPGS1 regulates central spindle microtubule glutamylation and remodeling during telophase and abscission (part 16 of 36)
Source: EMBO Rep. 2026 Mar 23;27(8):1944–63. doi: 10.1038/s44319-026-00742-3 (PMC13121839; doi:10.1038/s44319-026-00742-3)

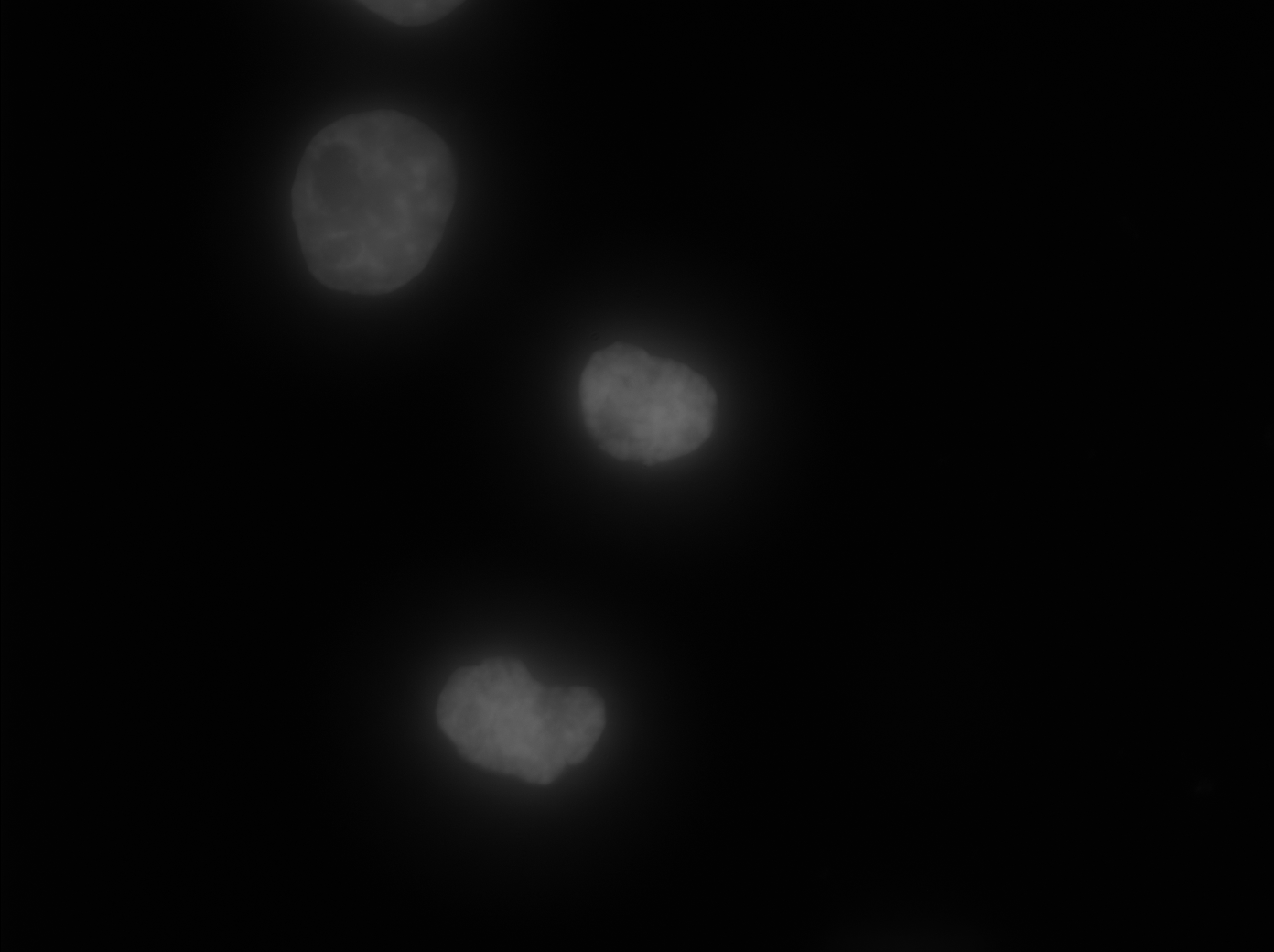

Supplement: Supplementary file 13 — Source data Fig. 3 part 3 [file 44319_2026_742_MOESM13_ESM.zip › Figure 3 Part 3/Fig 3b-e TTLL screen part 3/TTLL11-YFP A2 Img5.Project Maximum Z_XY1648754590_Z0_T0_C0.tif]

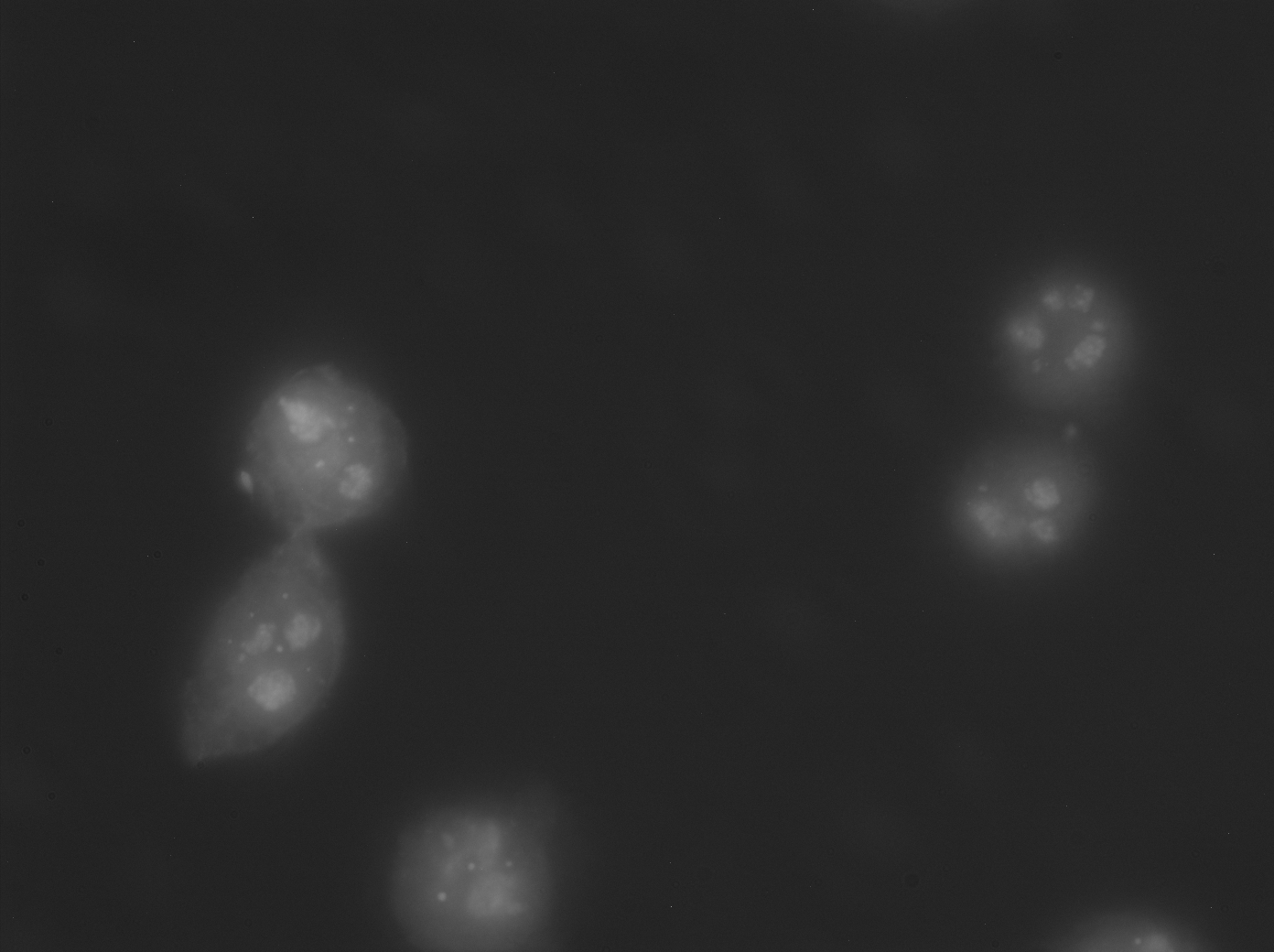

Supplement: Supplementary file 13 — Source data Fig. 3 part 3 [file 44319_2026_742_MOESM13_ESM.zip › Figure 3 Part 3/Fig 3b-e TTLL screen part 3/TTLL11-YFP Img 2 yfp2000.Project Maximum Z_XY1648156745_Z0_T0_C2.tif]

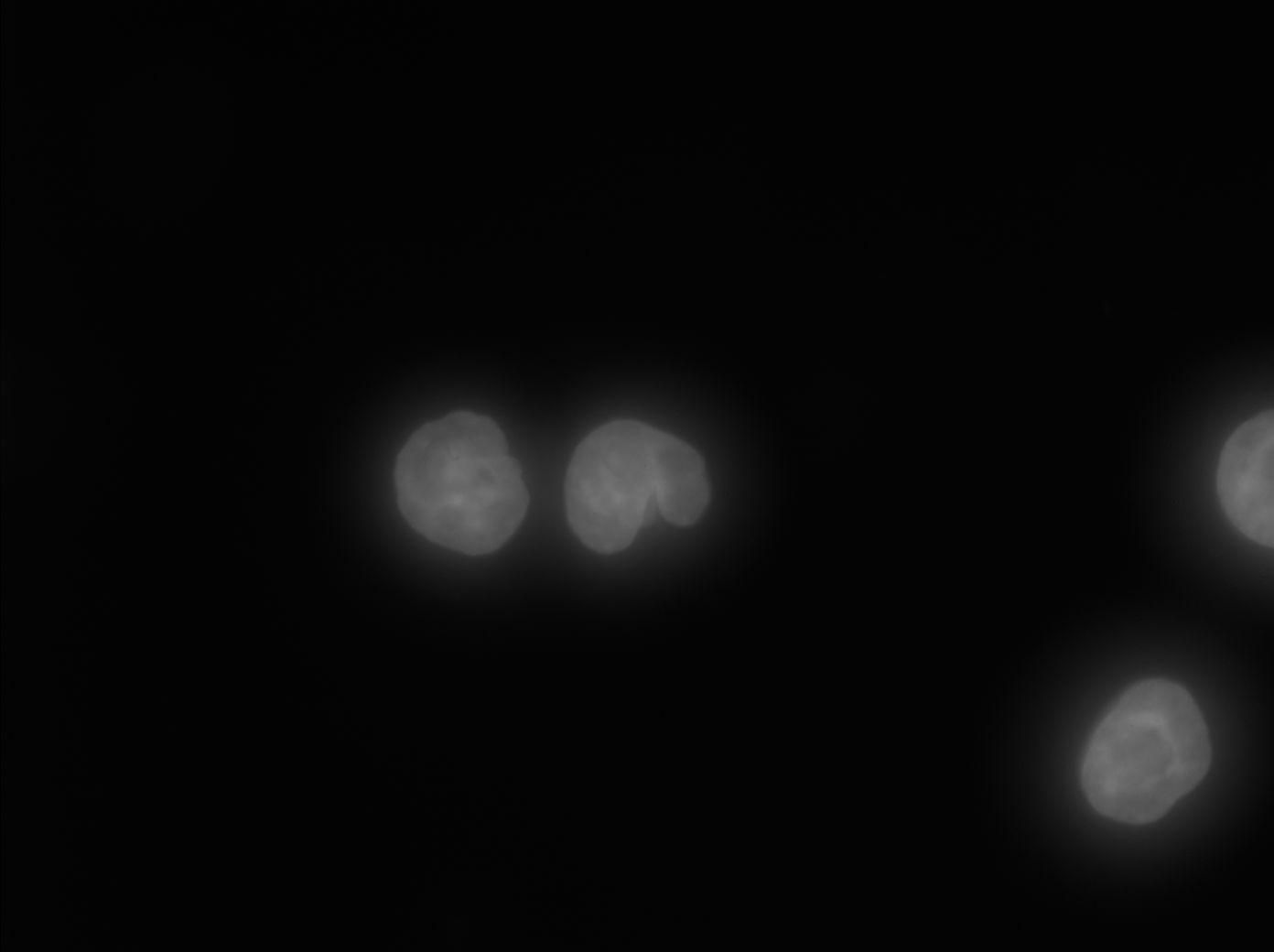

Supplement: Supplementary file 13 — Source data Fig. 3 part 3 [file 44319_2026_742_MOESM13_ESM.zip › Figure 3 Part 3/Fig 3b-e TTLL screen part 3/TTLL11-YFP Img 4 yfp2000 - 1.Project Maximum Z_XY1648157838_Z0_T0_C0.tif]

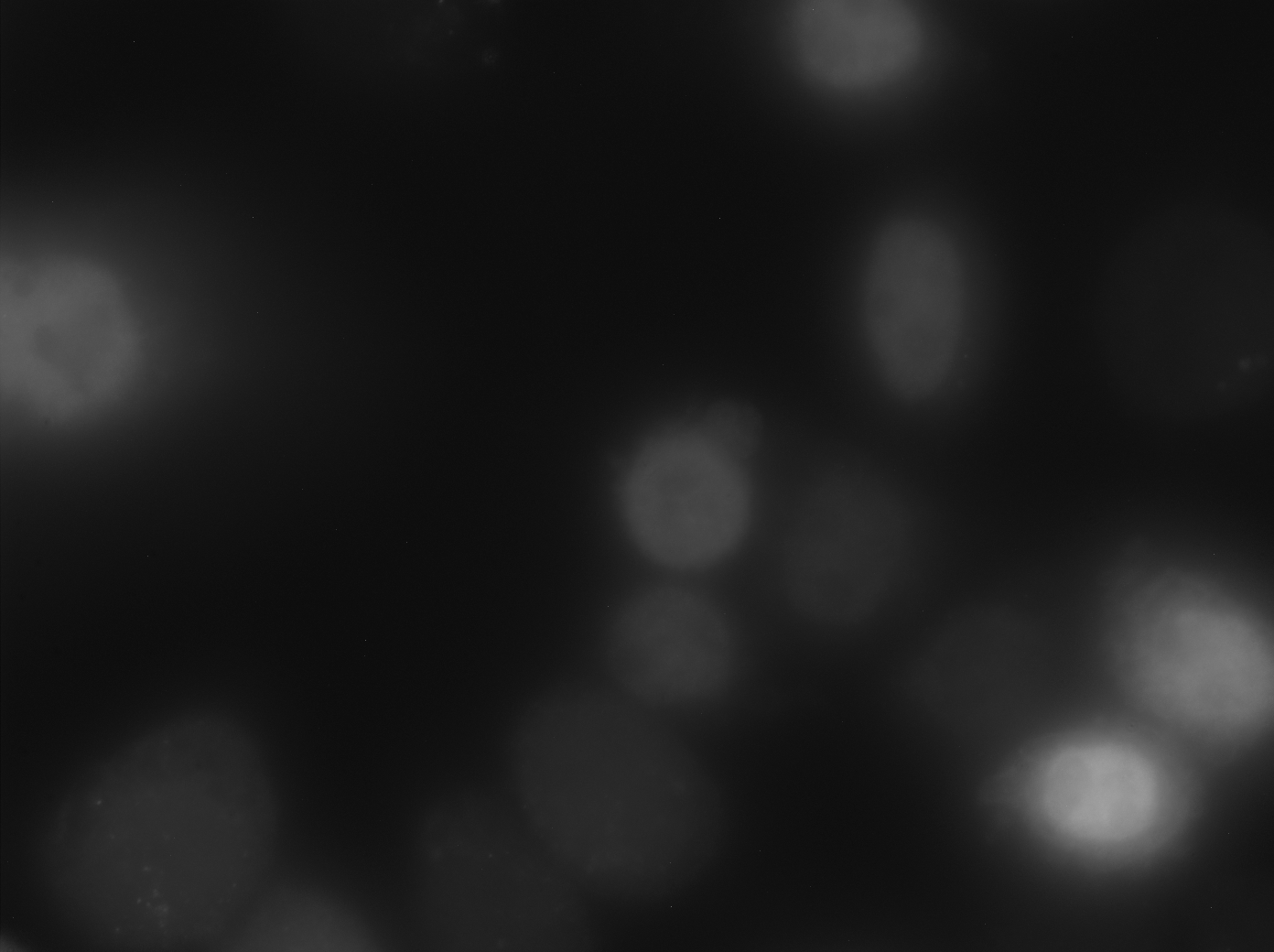

Supplement: Supplementary file 13 — Source data Fig. 3 part 3 [file 44319_2026_742_MOESM13_ESM.zip › Figure 3 Part 3/Fig 3b-e TTLL screen part 3/TTLL9-YFP A3 I20.Project Maximum Z_XY1679702138_Z0_T0_C2.tif]

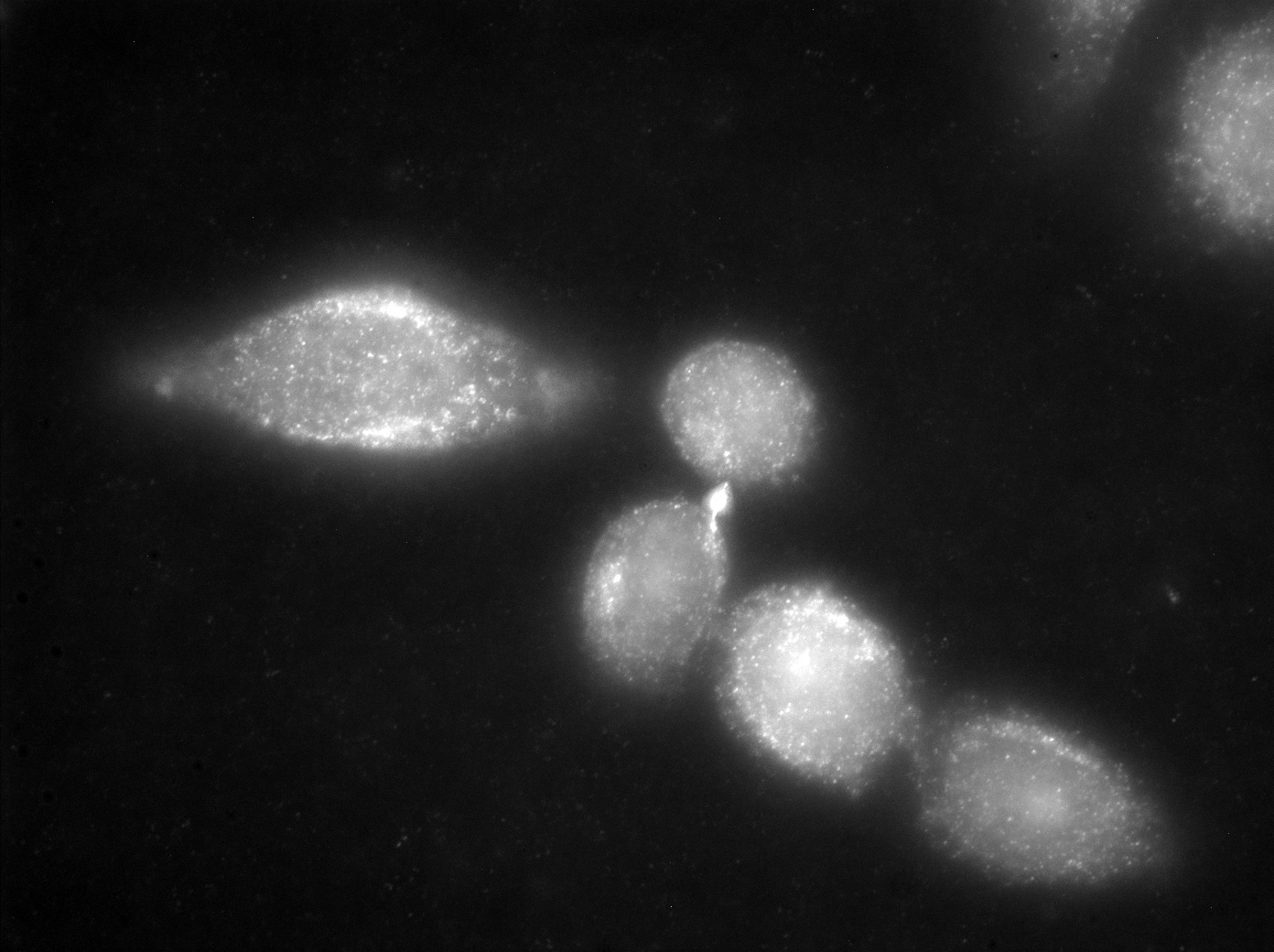

Supplement: Supplementary file 13 — Source data Fig. 3 part 3 [file 44319_2026_742_MOESM13_ESM.zip › Figure 3 Part 3/Fig 3b-e TTLL screen part 3/TTLL11-YFP A1 Img7.Project Maximum Z_XY1650055906_Z0_T0_C1.tif]

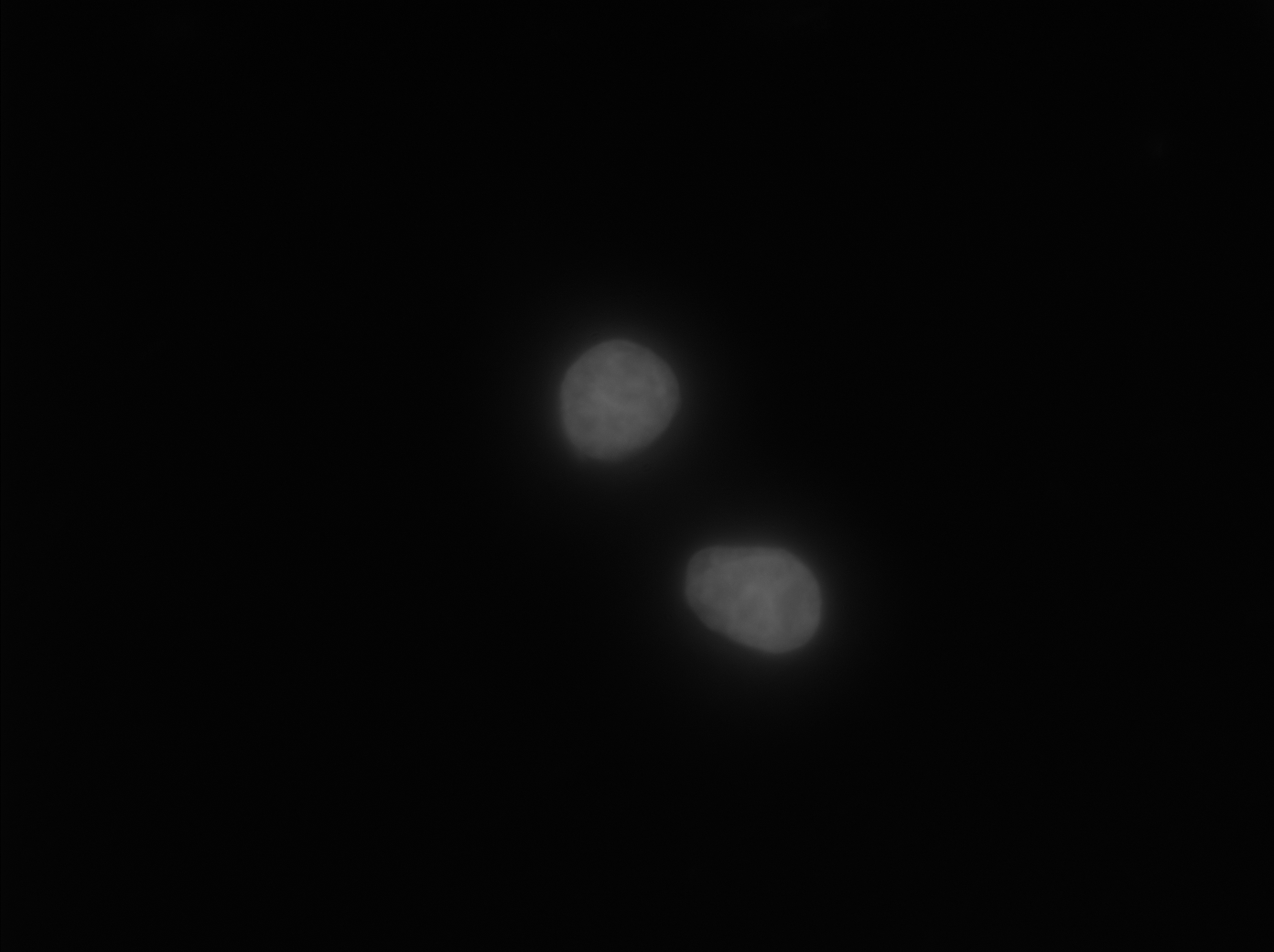

Supplement: Supplementary file 13 — Source data Fig. 3 part 3 [file 44319_2026_742_MOESM13_ESM.zip › Figure 3 Part 3/Fig 3b-e TTLL screen part 3/TTLL11-YFP A1 Img6.Project Maximum Z_XY1650055434_Z0_T0_C0.tif]

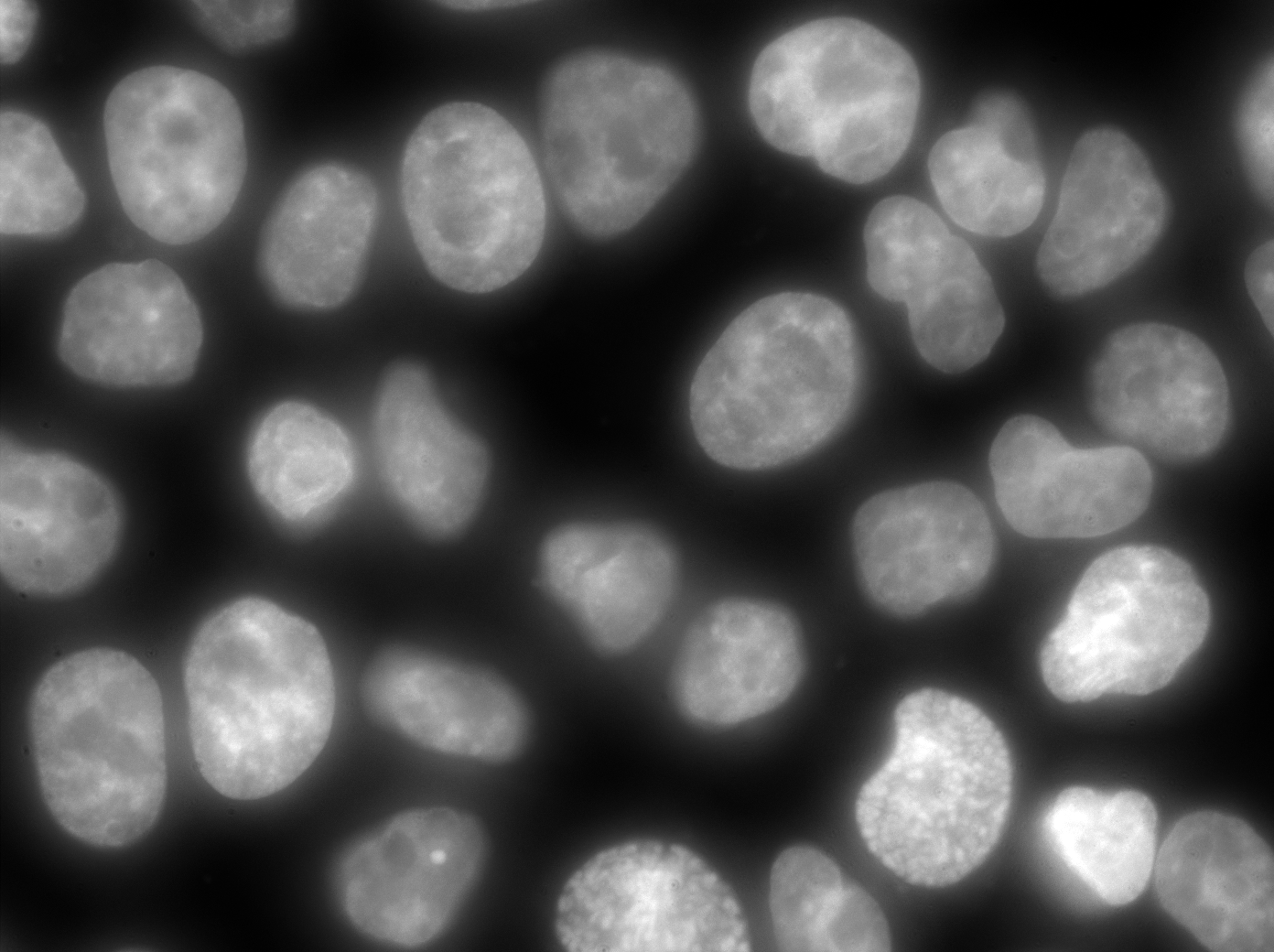

Supplement: Supplementary file 13 — Source data Fig. 3 part 3 [file 44319_2026_742_MOESM13_ESM.zip › Figure 3 Part 3/Fig 3b-e TTLL screen part 3/TTLL9-YFP A3 I2 - 1.Project Maximum Z_XY1679699371_Z0_T0_C0.tif]

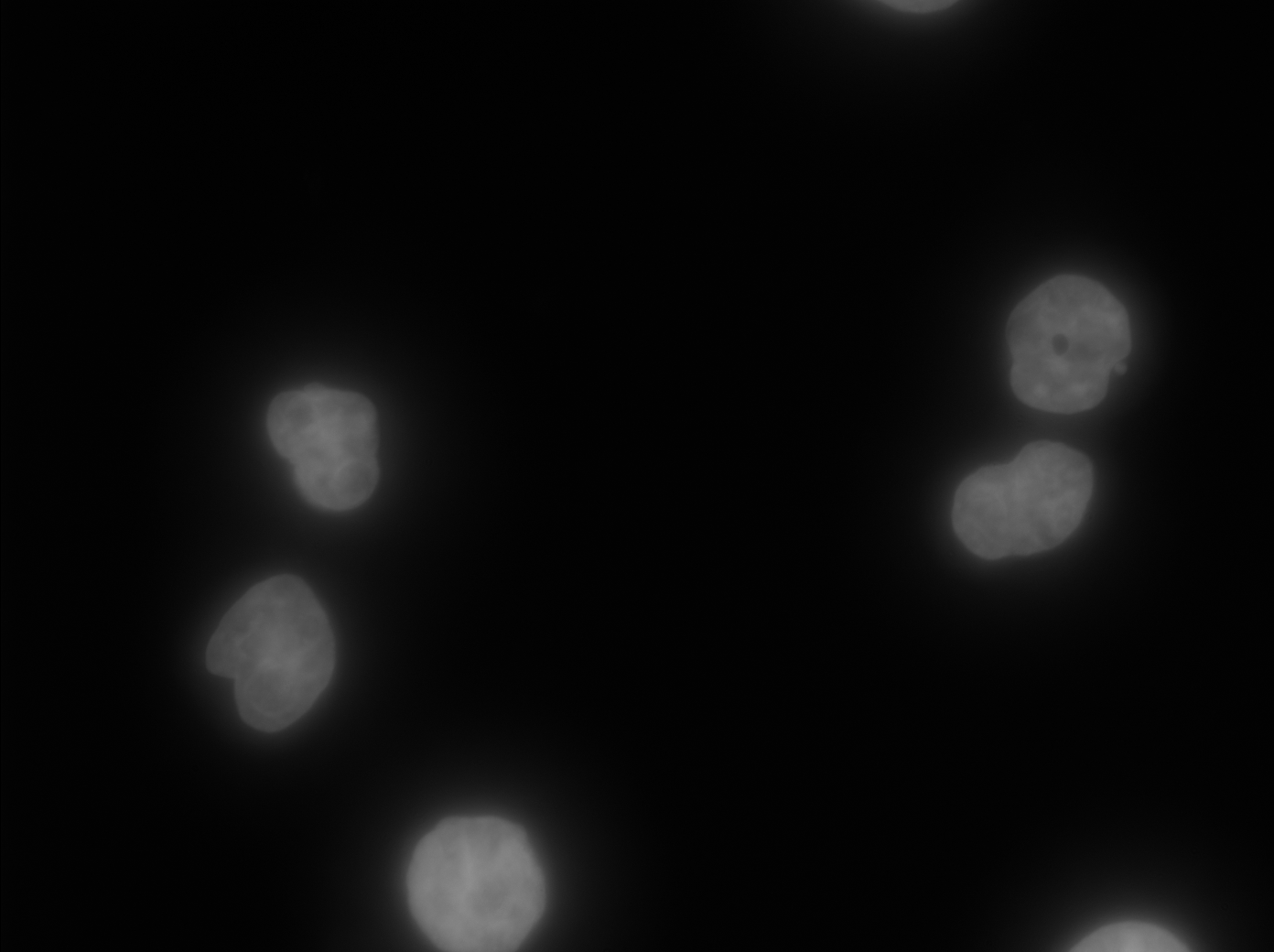

Supplement: Supplementary file 13 — Source data Fig. 3 part 3 [file 44319_2026_742_MOESM13_ESM.zip › Figure 3 Part 3/Fig 3b-e TTLL screen part 3/TTLL11-YFP Img 2 yfp2000 - 1.Project Maximum Z_XY1648156745_Z0_T0_C0.tif]

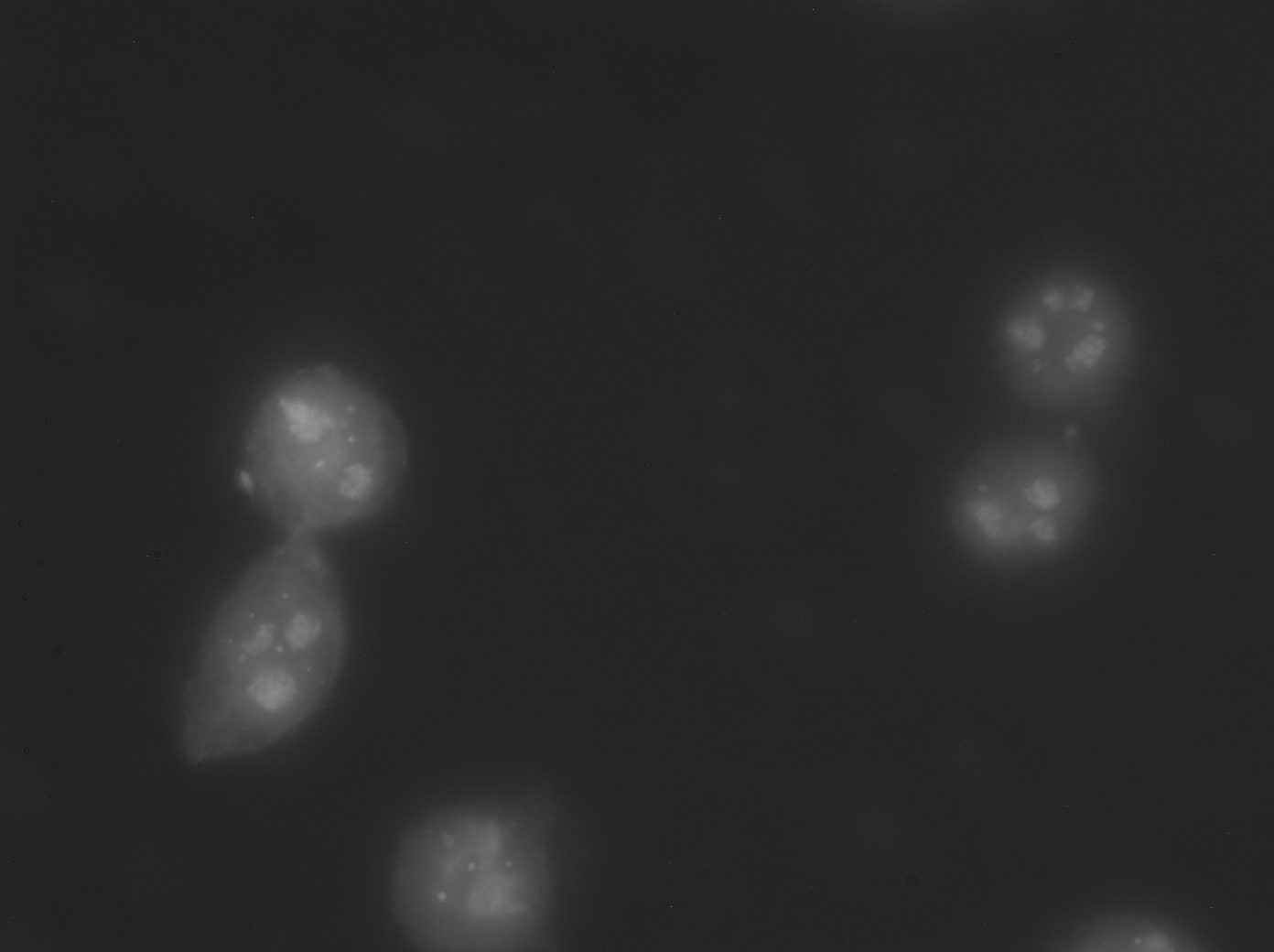

Supplement: Supplementary file 13 — Source data Fig. 3 part 3 [file 44319_2026_742_MOESM13_ESM.zip › Figure 3 Part 3/Fig 3b-e TTLL screen part 3/TTLL11-YFP Img 2 yfp2000 - 1.Project Maximum Z_XY1648156745_Z0_T0_C2.tif]

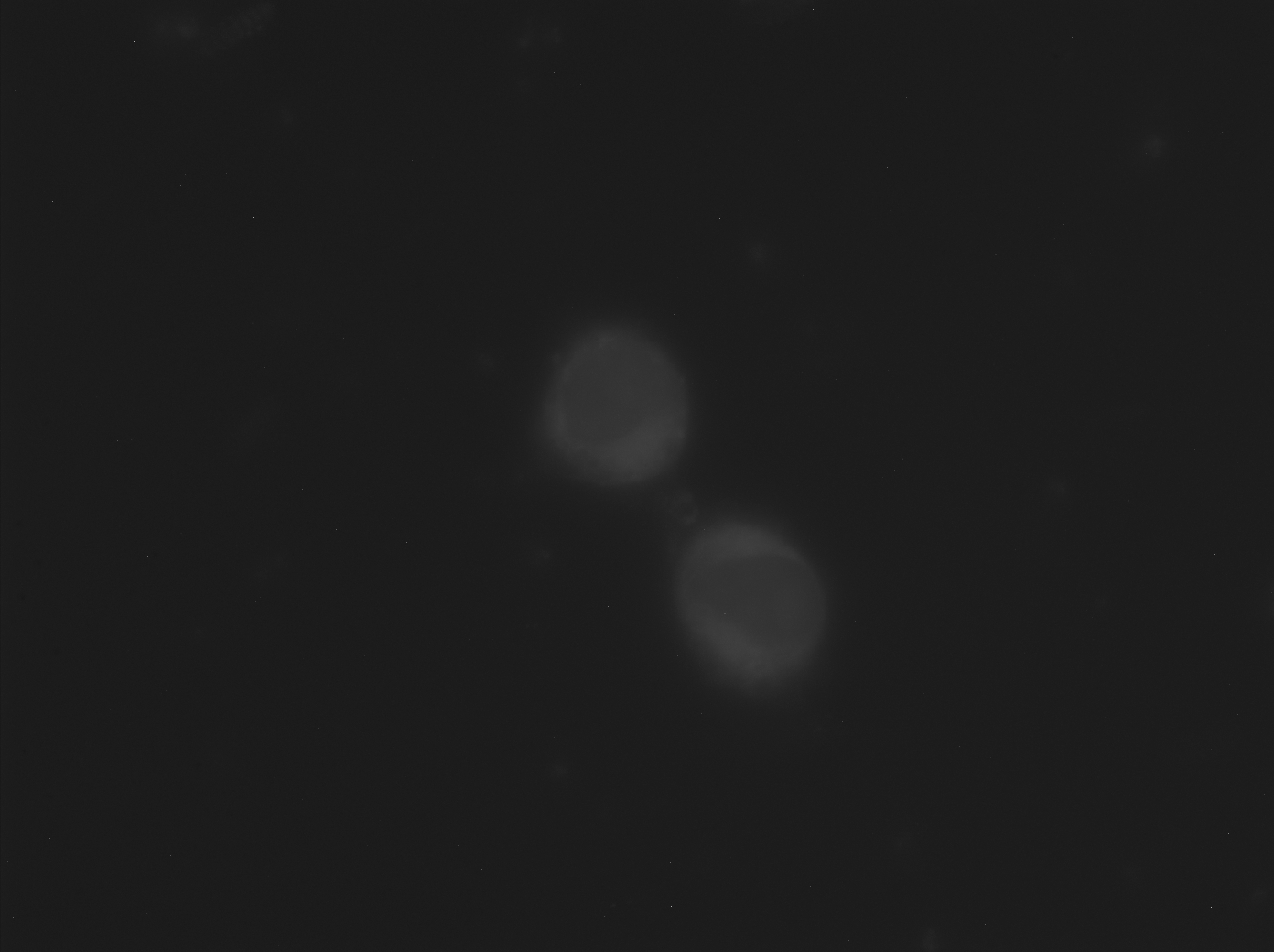

Supplement: Supplementary file 13 — Source data Fig. 3 part 3 [file 44319_2026_742_MOESM13_ESM.zip › Figure 3 Part 3/Fig 3b-e TTLL screen part 3/TTLL11-YFP A1 Img6.Project Maximum Z_XY1650055434_Z0_T0_C2.tif]

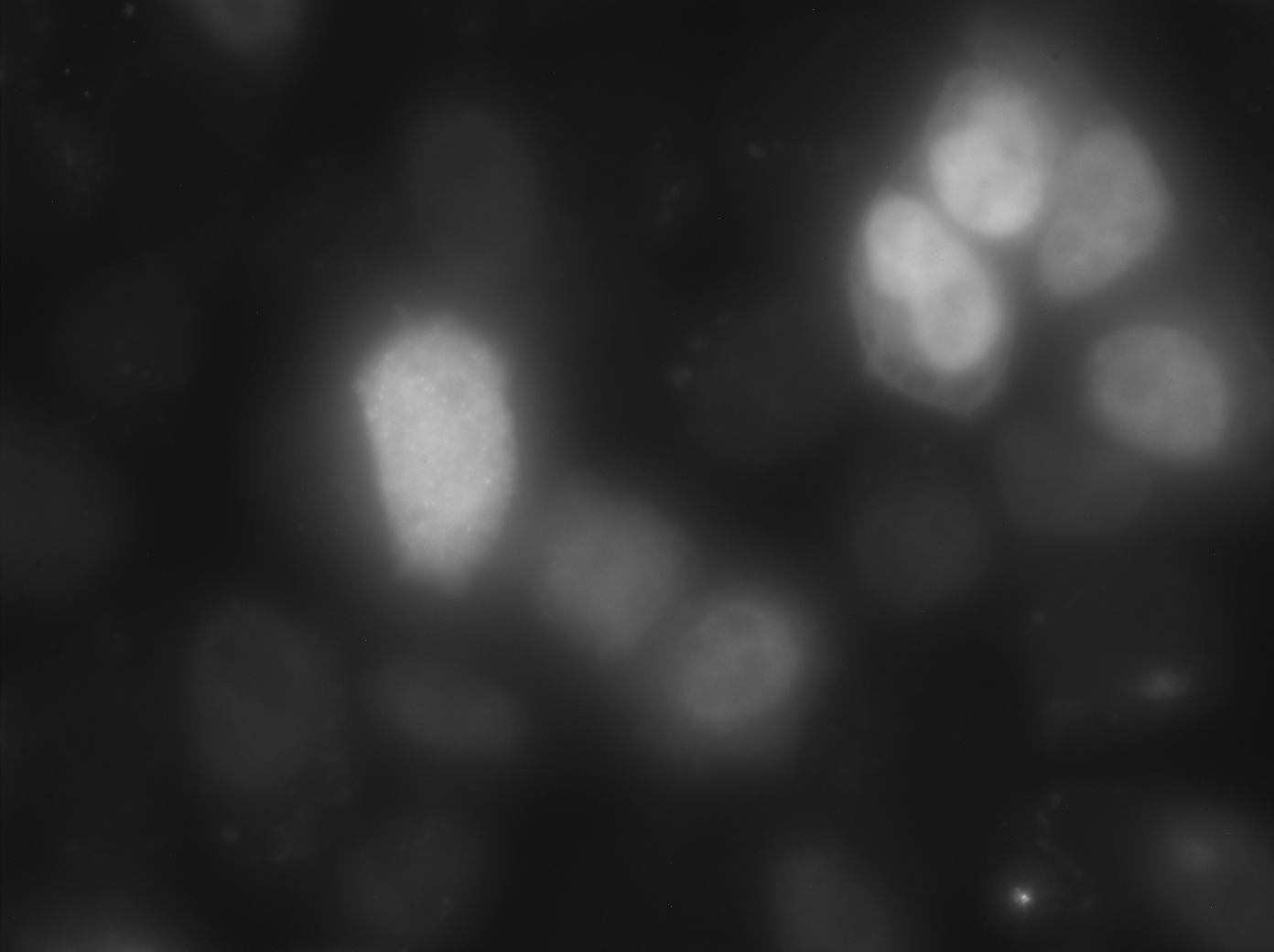

Supplement: Supplementary file 13 — Source data Fig. 3 part 3 [file 44319_2026_742_MOESM13_ESM.zip › Figure 3 Part 3/Fig 3b-e TTLL screen part 3/TTLL9-YFP A3 I2 - 1.Project Maximum Z_XY1679699371_Z0_T0_C2.tif]

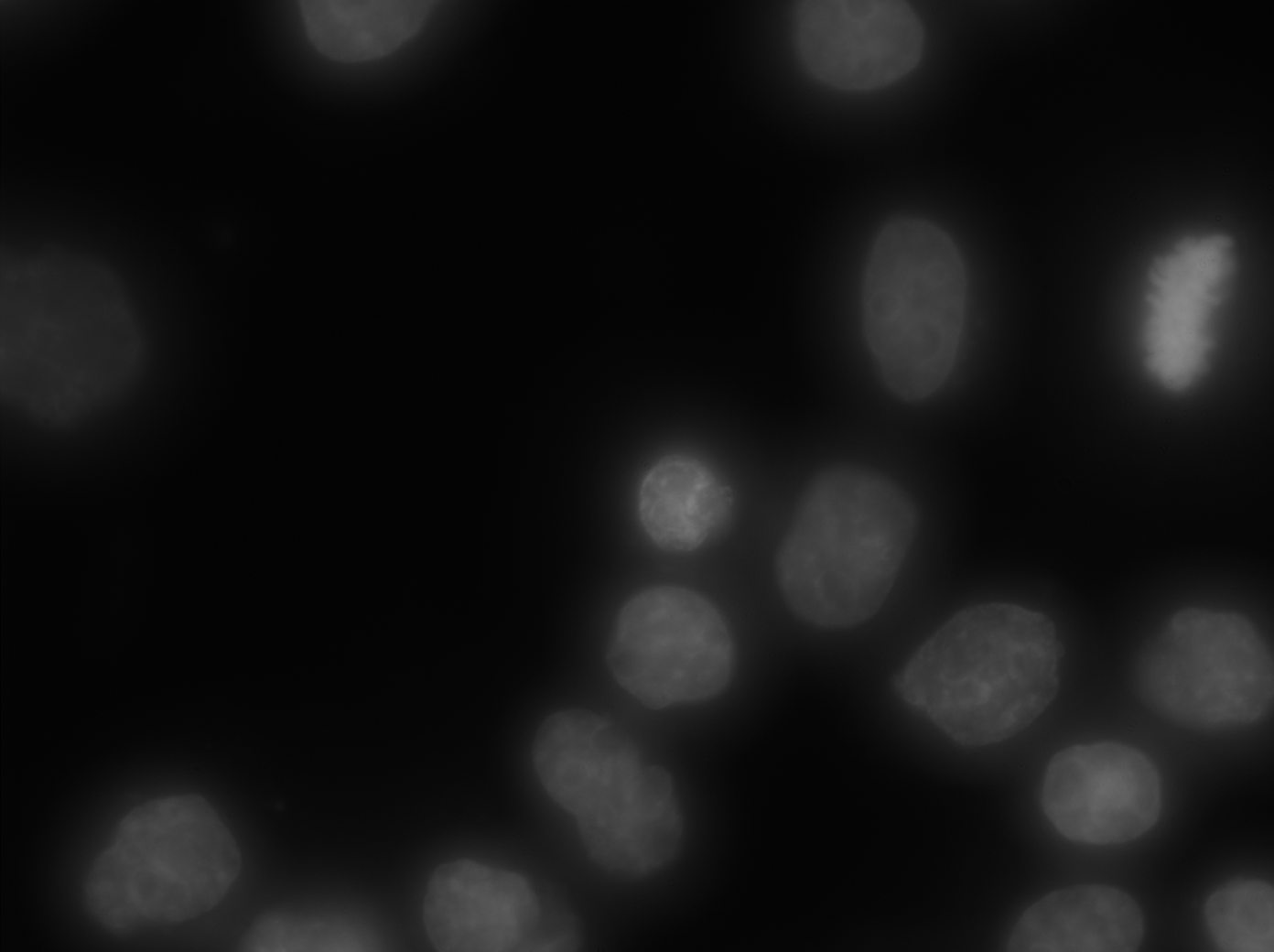

Supplement: Supplementary file 13 — Source data Fig. 3 part 3 [file 44319_2026_742_MOESM13_ESM.zip › Figure 3 Part 3/Fig 3b-e TTLL screen part 3/TTLL9-YFP A3 I20.Project Maximum Z_XY1679702138_Z0_T0_C0.tif]

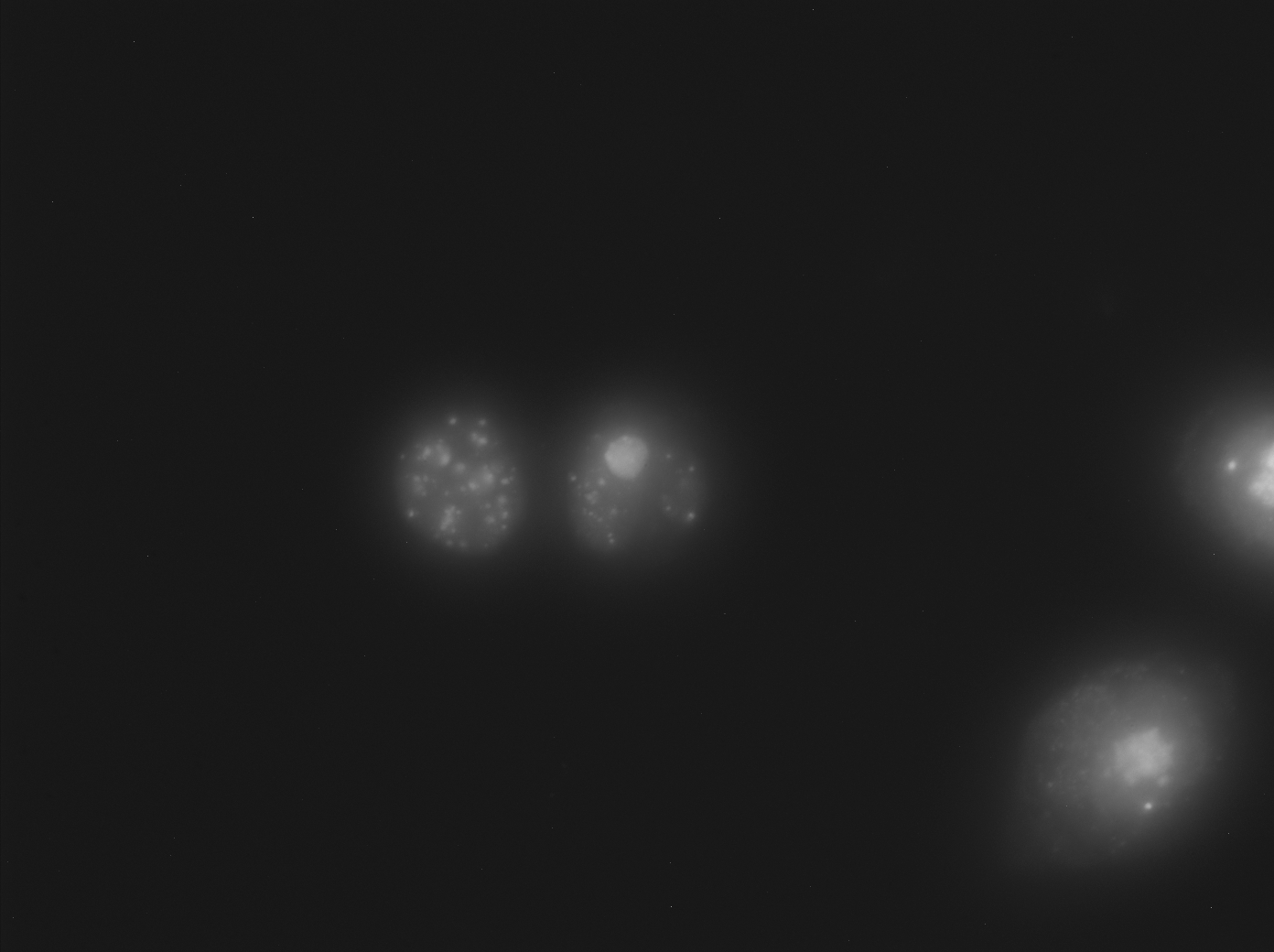

Supplement: Supplementary file 13 — Source data Fig. 3 part 3 [file 44319_2026_742_MOESM13_ESM.zip › Figure 3 Part 3/Fig 3b-e TTLL screen part 3/TTLL11-YFP Img 4 yfp2000 - 1.Project Maximum Z_XY1648157838_Z0_T0_C2.tif]

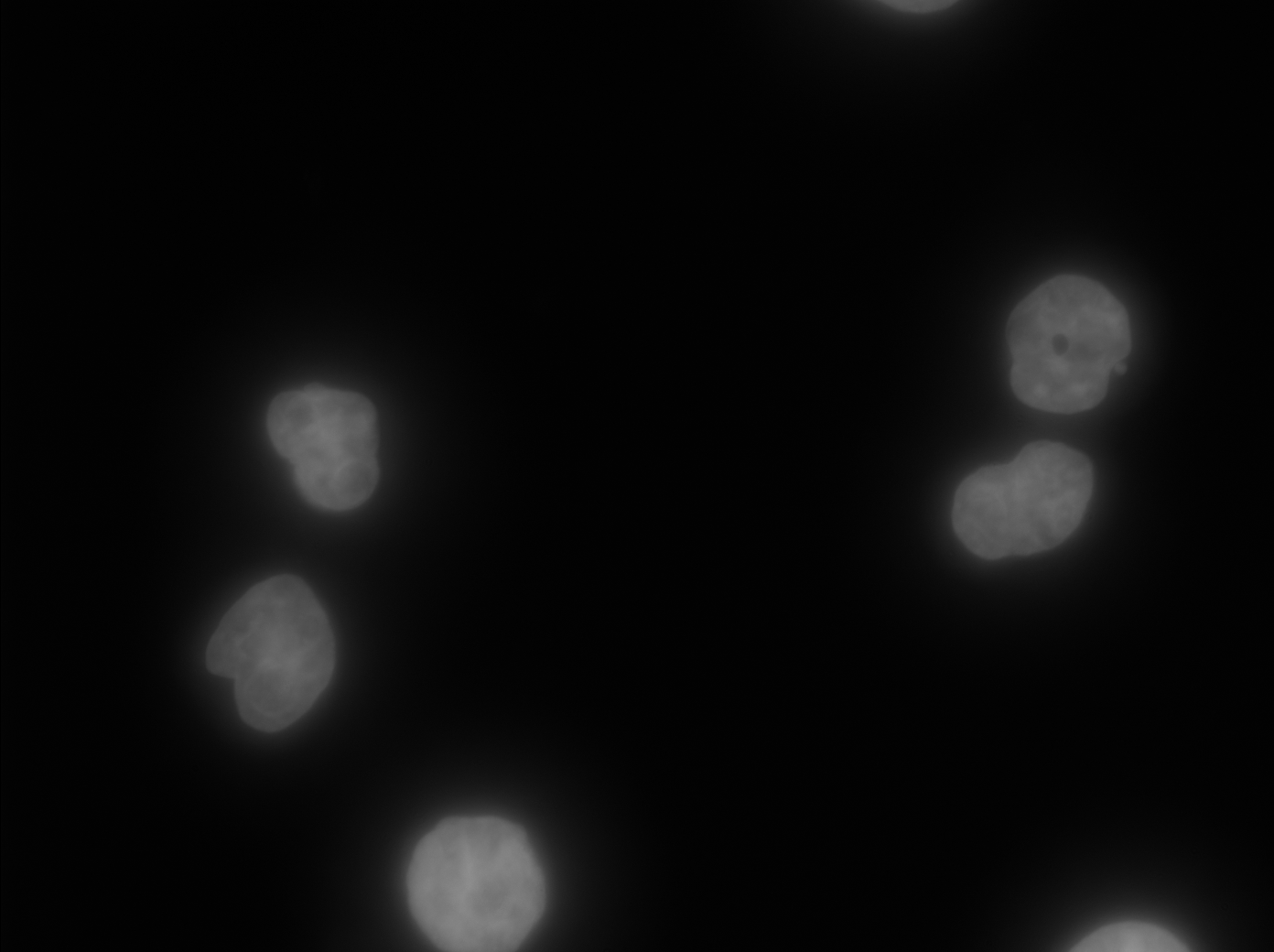

Supplement: Supplementary file 13 — Source data Fig. 3 part 3 [file 44319_2026_742_MOESM13_ESM.zip › Figure 3 Part 3/Fig 3b-e TTLL screen part 3/TTLL11-YFP Img 2 yfp2000.Project Maximum Z_XY1648156745_Z0_T0_C0.tif]

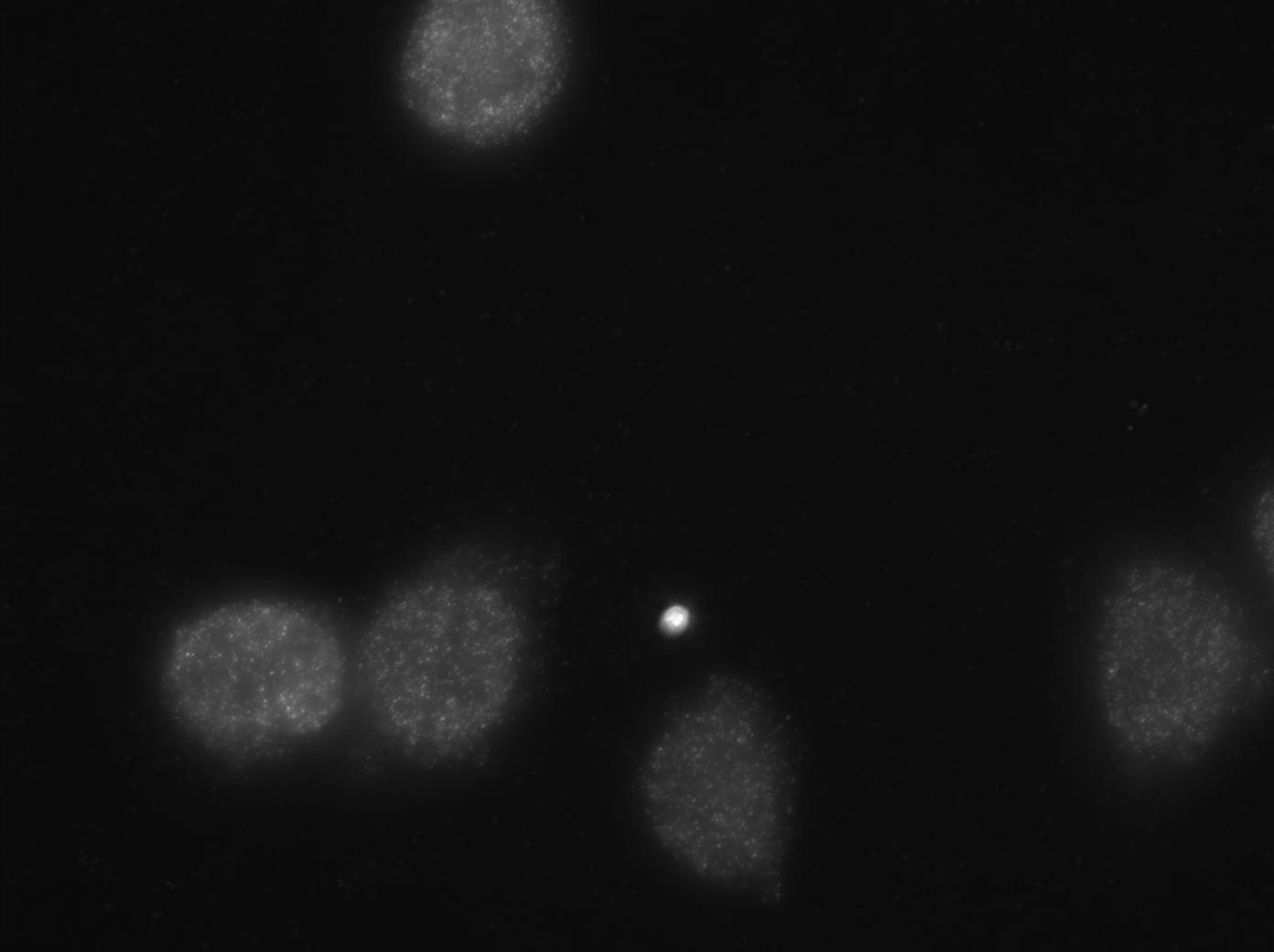

Supplement: Supplementary file 13 — Source data Fig. 3 part 3 [file 44319_2026_742_MOESM13_ESM.zip › Figure 3 Part 3/Fig 3b-e TTLL screen part 3/TTLL11-YFP Img 3 yfp2000.Project Maximum Z_XY1648157505_Z0_T0_C1.tif]

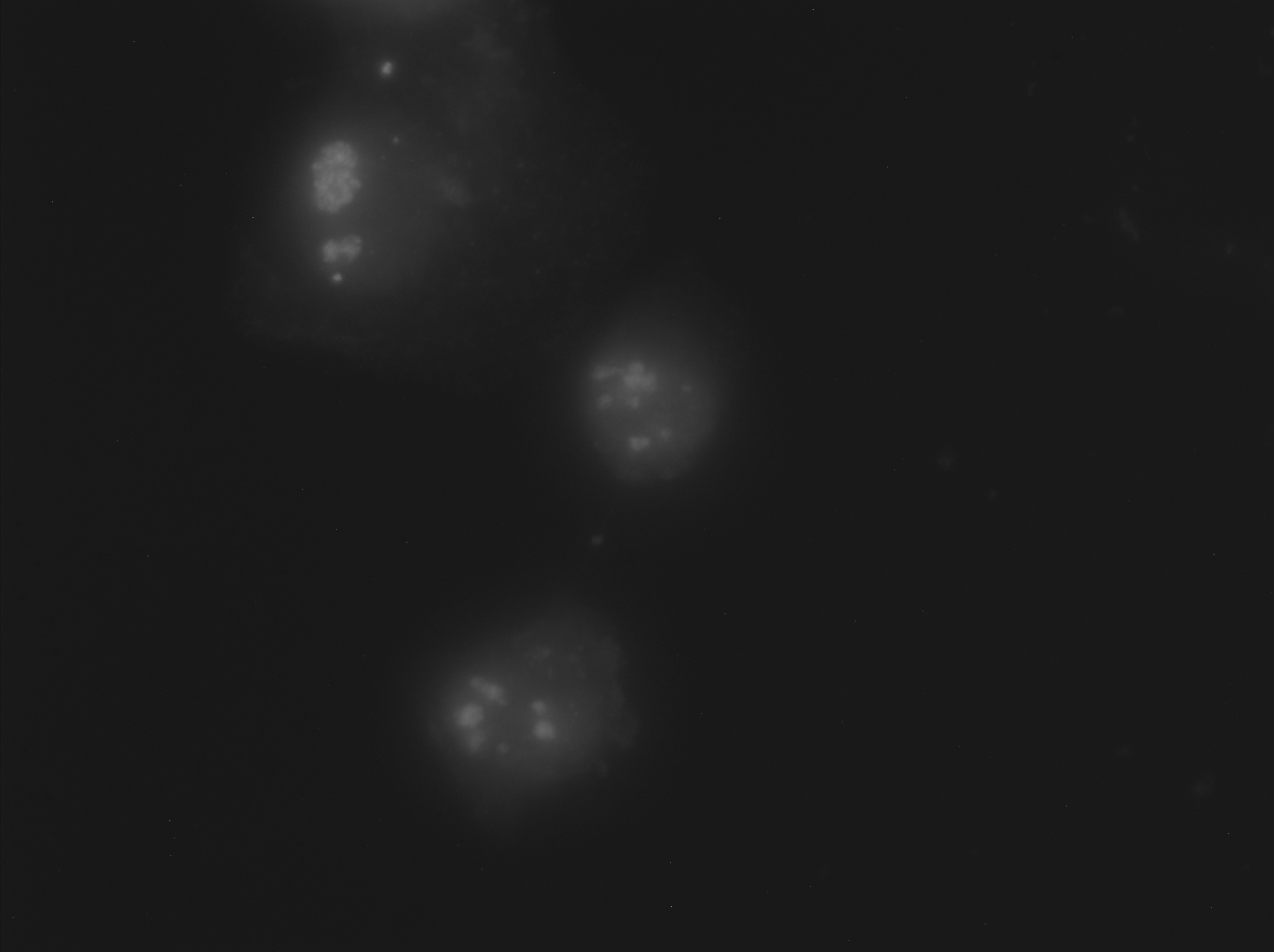

Supplement: Supplementary file 13 — Source data Fig. 3 part 3 [file 44319_2026_742_MOESM13_ESM.zip › Figure 3 Part 3/Fig 3b-e TTLL screen part 3/TTLL11-YFP A2 Img5.Project Maximum Z_XY1648754590_Z0_T0_C2.tif]

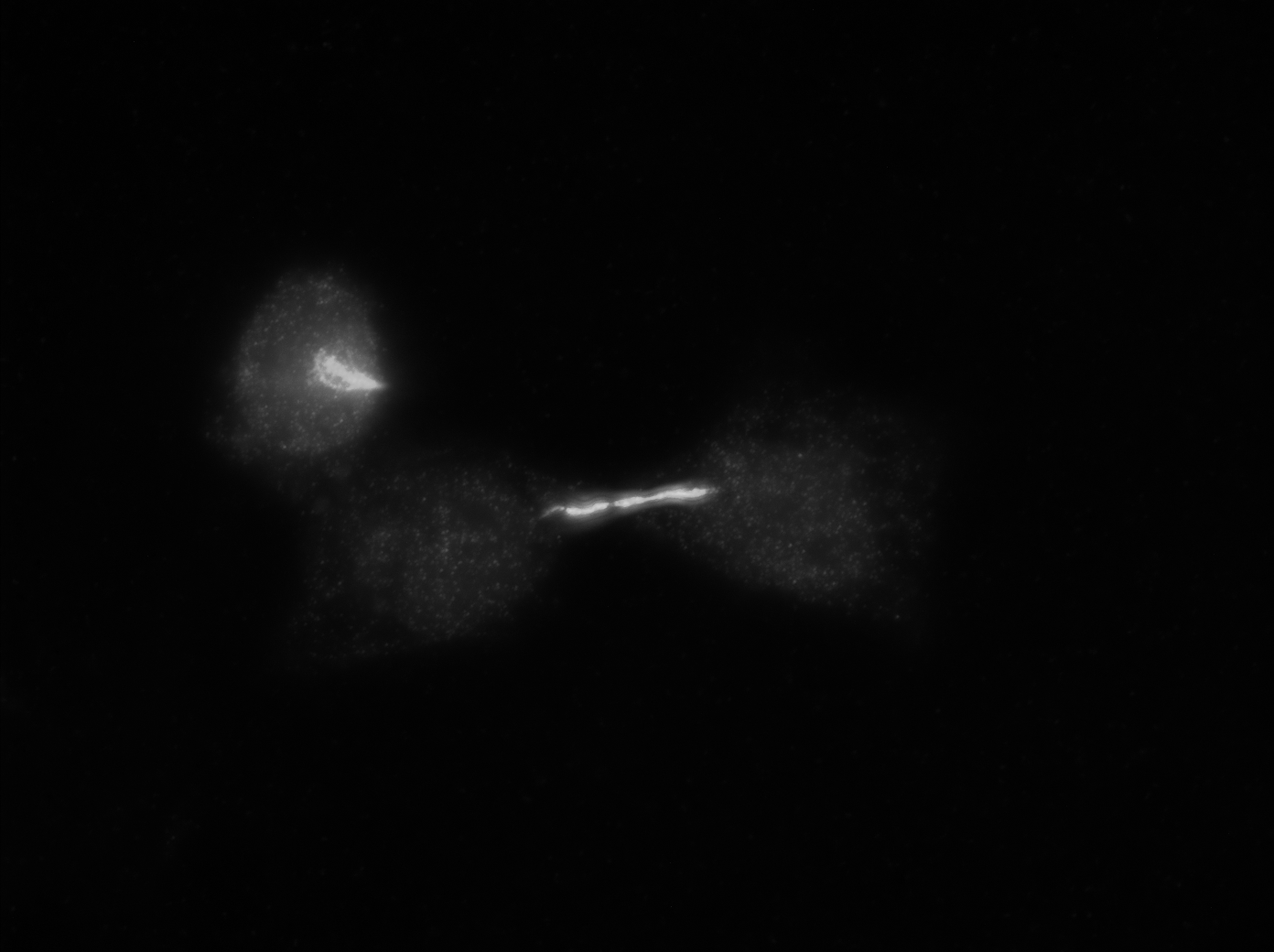

Supplement: Supplementary file 13 — Source data Fig. 3 part 3 [file 44319_2026_742_MOESM13_ESM.zip › Figure 3 Part 3/Fig 3b-e TTLL screen part 3/YFP Only R1 I1 - 1.Project Maximum Z_XY1663181796_Z0_T0_C1.tif]

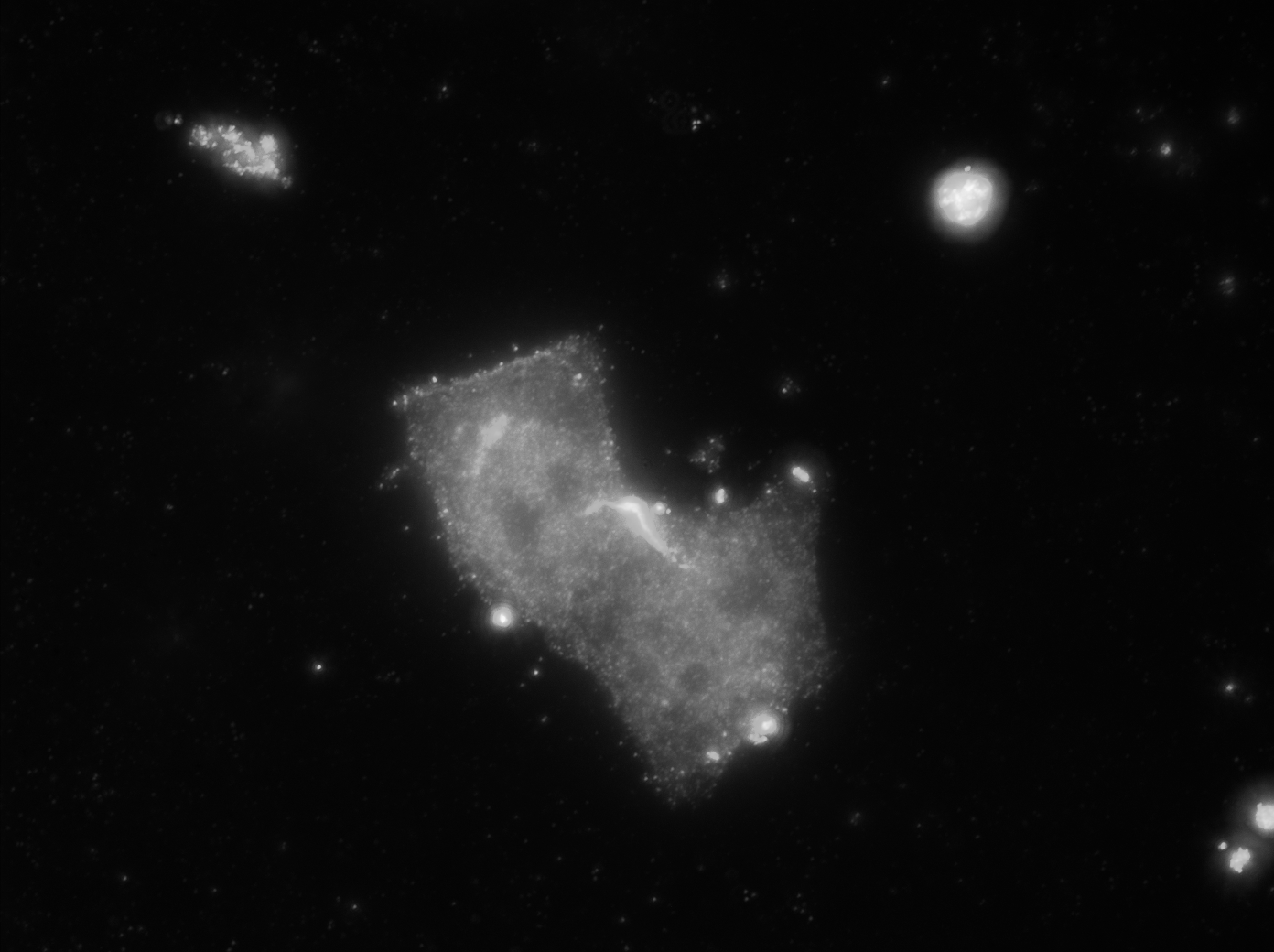

Supplement: Supplementary file 13 — Source data Fig. 3 part 3 [file 44319_2026_742_MOESM13_ESM.zip › Figure 3 Part 3/Fig 3b-e TTLL screen part 3/TTLL9-GFP A4 I5.Project Maximum Z_XY1675967797_Z0_T0_C1.tif]

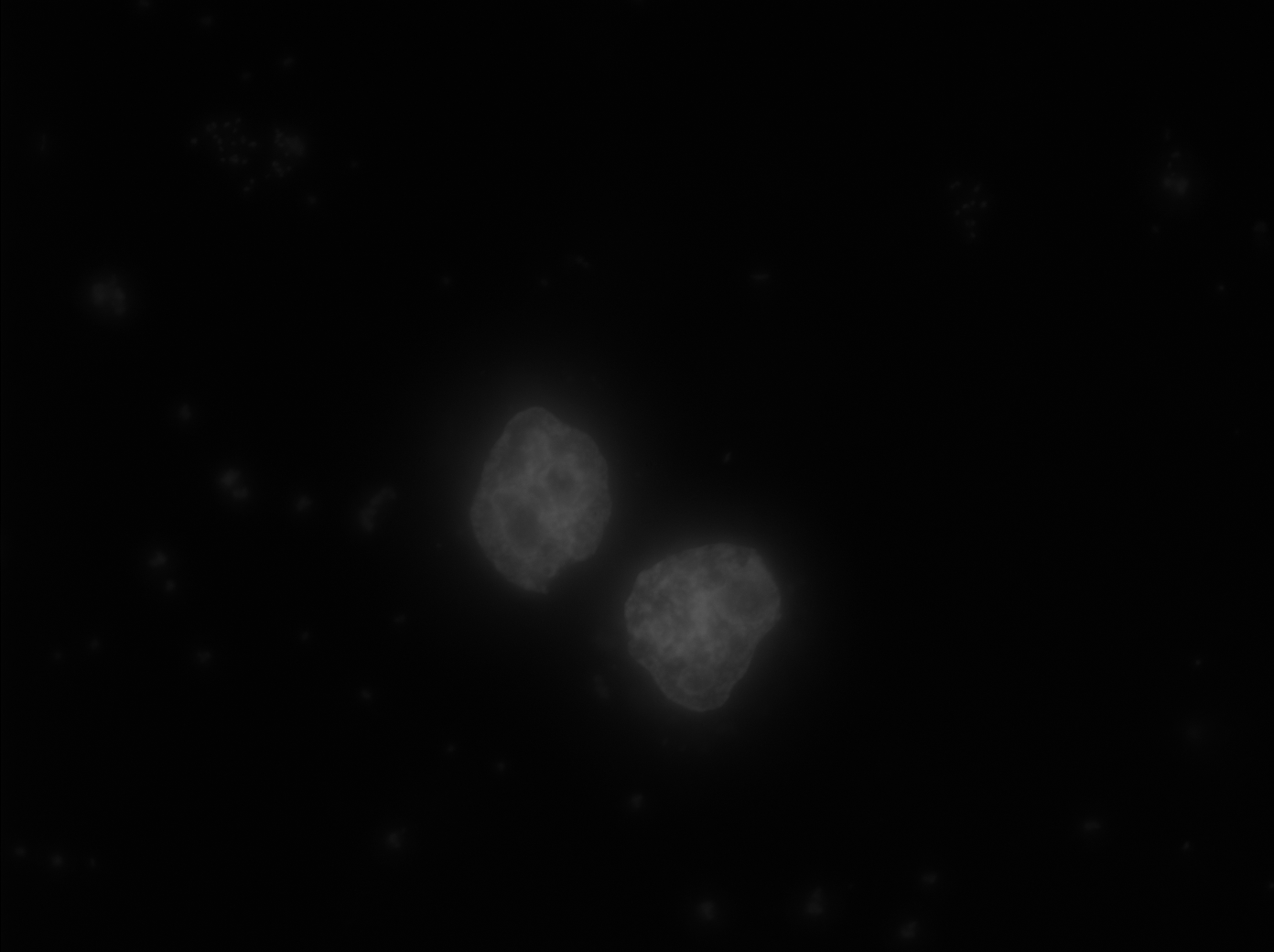

Supplement: Supplementary file 13 — Source data Fig. 3 part 3 [file 44319_2026_742_MOESM13_ESM.zip › Figure 3 Part 3/Fig 3b-e TTLL screen part 3/TTLL9-GFP A4 I5.Project Maximum Z_XY1675967797_Z0_T0_C0.tif]

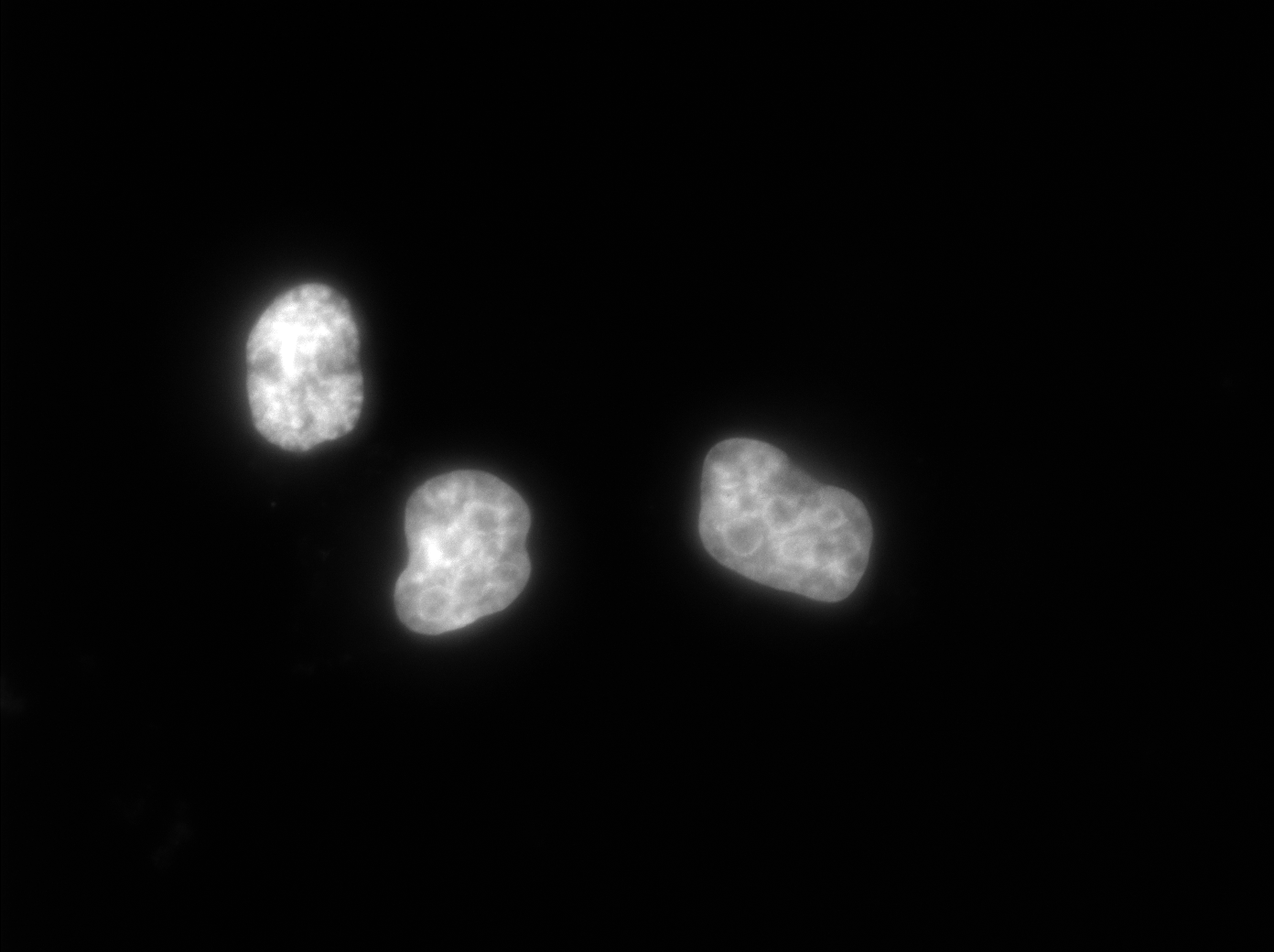

Supplement: Supplementary file 13 — Source data Fig. 3 part 3 [file 44319_2026_742_MOESM13_ESM.zip › Figure 3 Part 3/Fig 3b-e TTLL screen part 3/YFP Only R1 I1 - 1.Project Maximum Z_XY1663181796_Z0_T0_C0.tif]

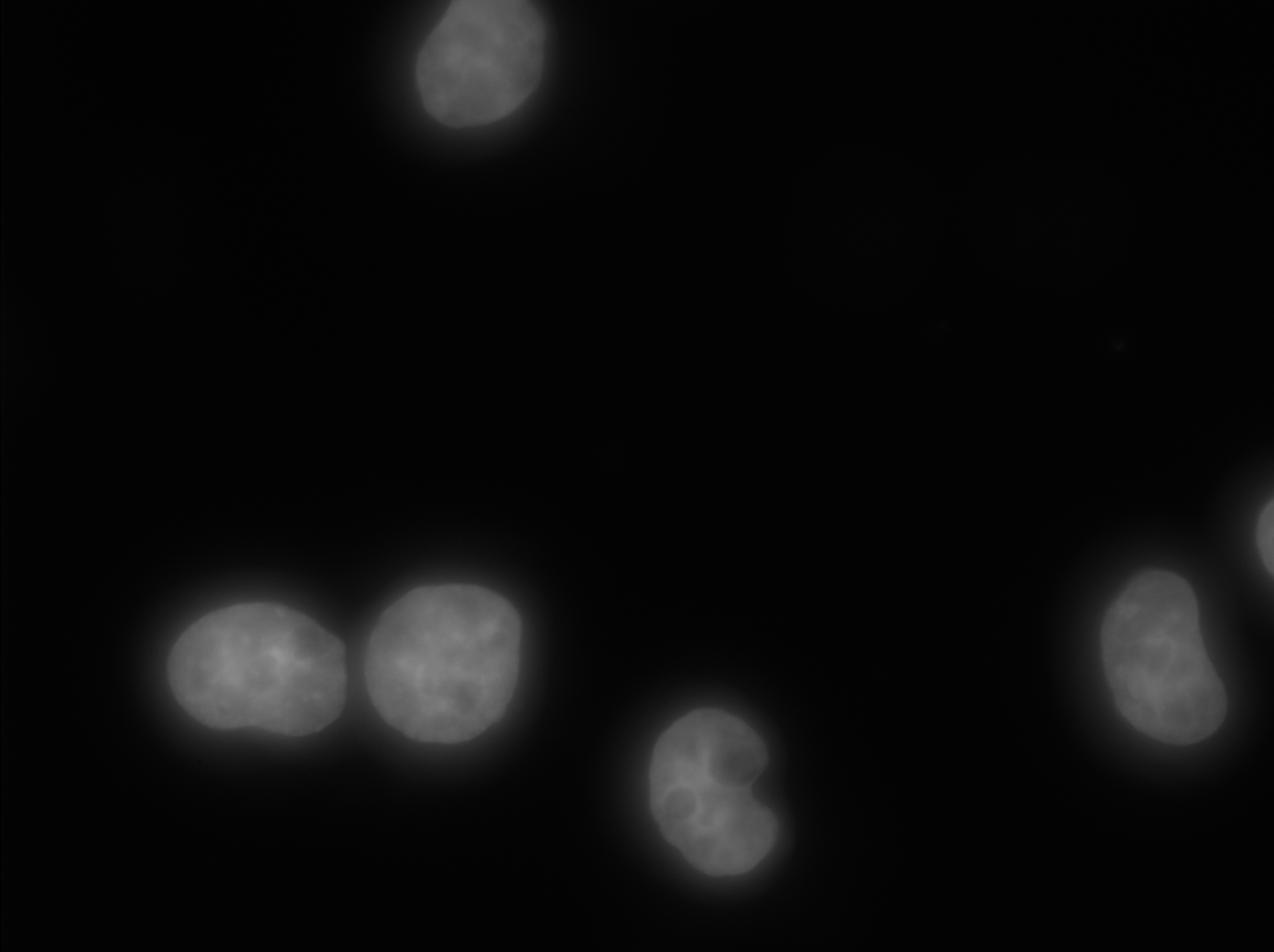

Supplement: Supplementary file 13 — Source data Fig. 3 part 3 [file 44319_2026_742_MOESM13_ESM.zip › Figure 3 Part 3/Fig 3b-e TTLL screen part 3/TTLL11-YFP Img 3 yfp2000.Project Maximum Z_XY1648157505_Z0_T0_C0.tif]

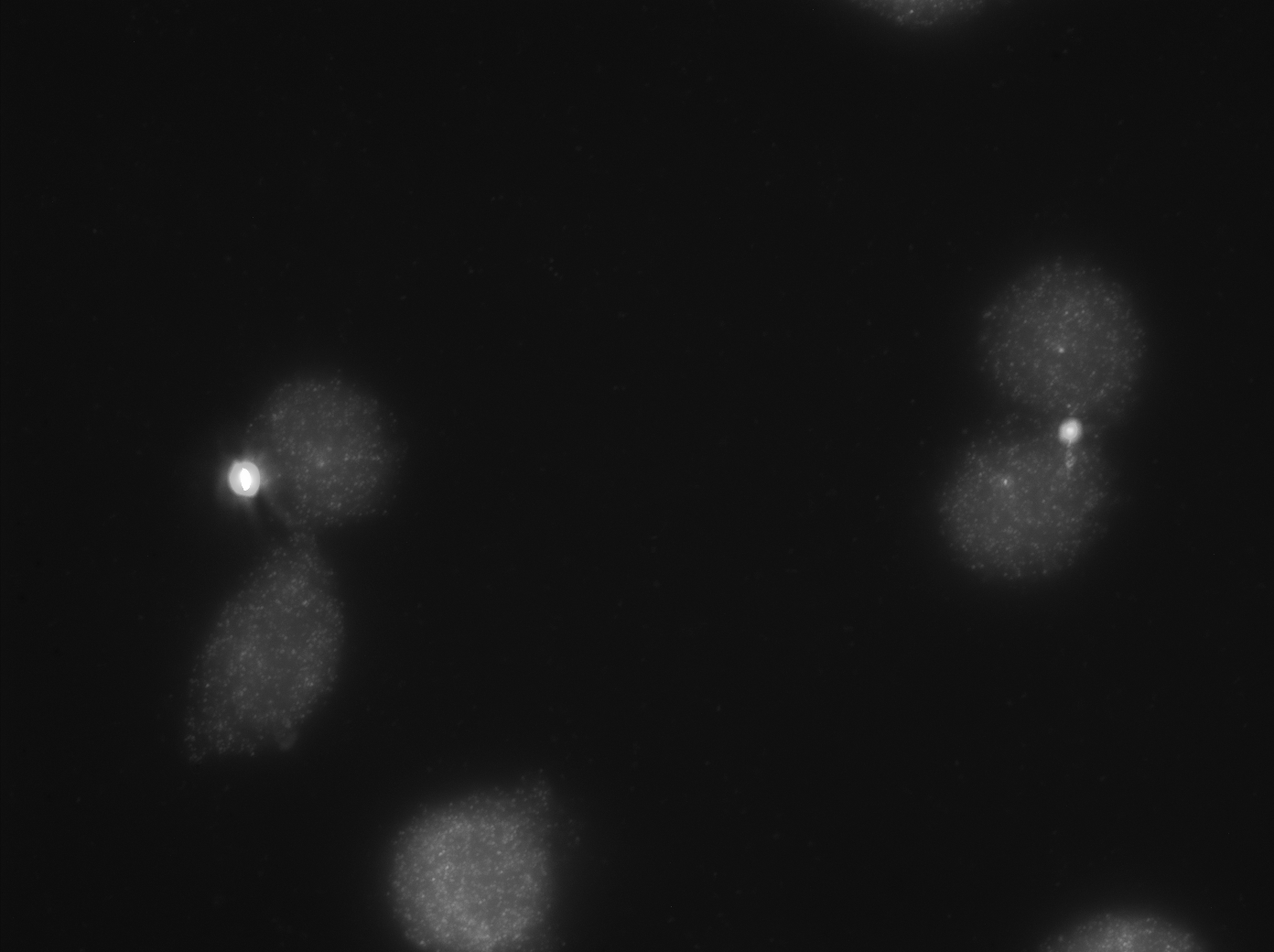

Supplement: Supplementary file 13 — Source data Fig. 3 part 3 [file 44319_2026_742_MOESM13_ESM.zip › Figure 3 Part 3/Fig 3b-e TTLL screen part 3/TTLL11-YFP Img 2 yfp2000.Project Maximum Z_XY1648156745_Z0_T0_C1.tif]

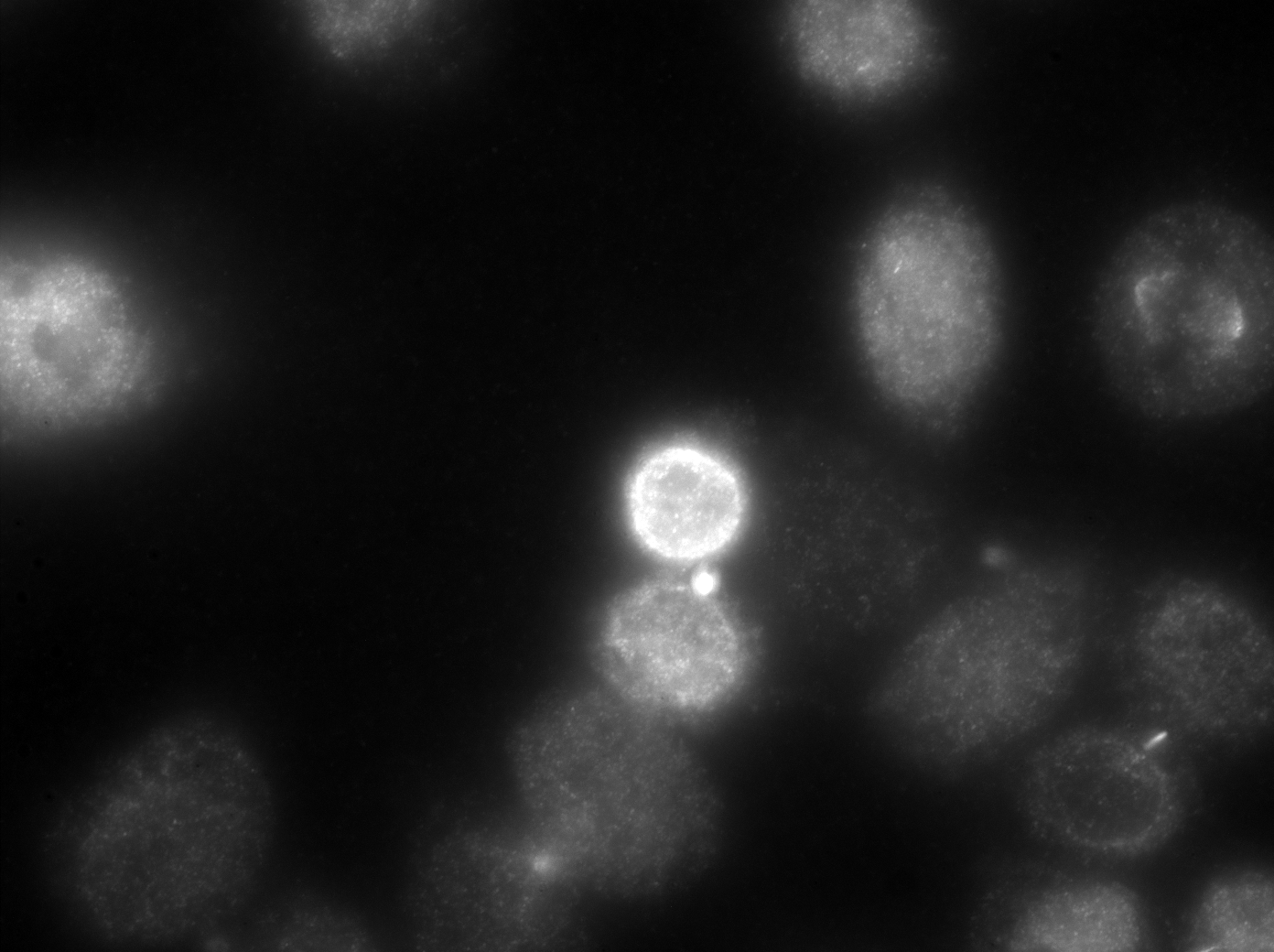

Supplement: Supplementary file 13 — Source data Fig. 3 part 3 [file 44319_2026_742_MOESM13_ESM.zip › Figure 3 Part 3/Fig 3b-e TTLL screen part 3/TTLL9-YFP A3 I20.Project Maximum Z_XY1679702138_Z0_T0_C1.tif]

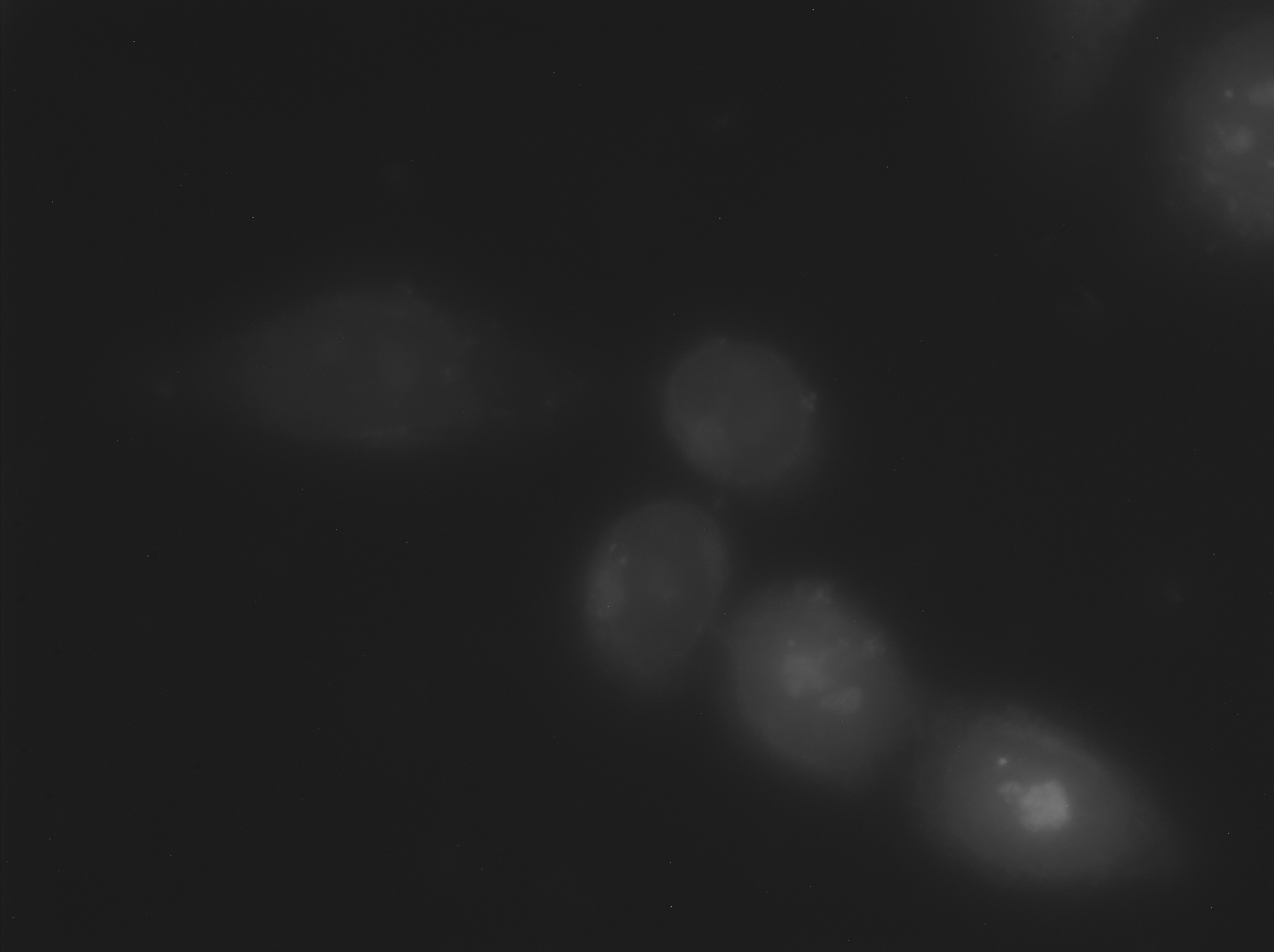

Supplement: Supplementary file 13 — Source data Fig. 3 part 3 [file 44319_2026_742_MOESM13_ESM.zip › Figure 3 Part 3/Fig 3b-e TTLL screen part 3/TTLL11-YFP A1 Img7.Project Maximum Z_XY1650055906_Z0_T0_C2.tif]

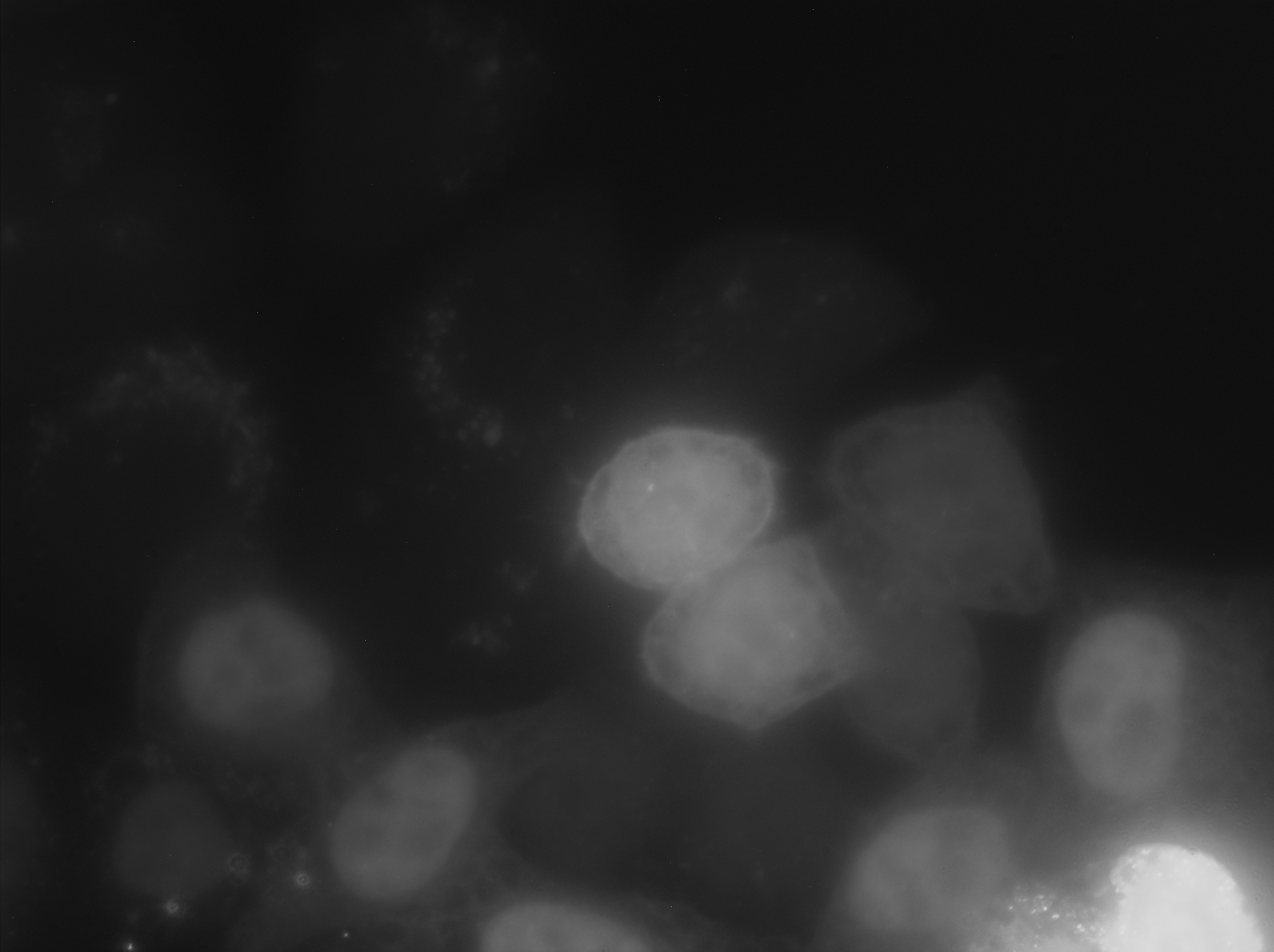

Supplement: Supplementary file 13 — Source data Fig. 3 part 3 [file 44319_2026_742_MOESM13_ESM.zip › Figure 3 Part 3/Fig 3b-e TTLL screen part 3/TTLL9-YFP A3 I6.Project Maximum Z_XY1679700103_Z0_T0_C2.tif]

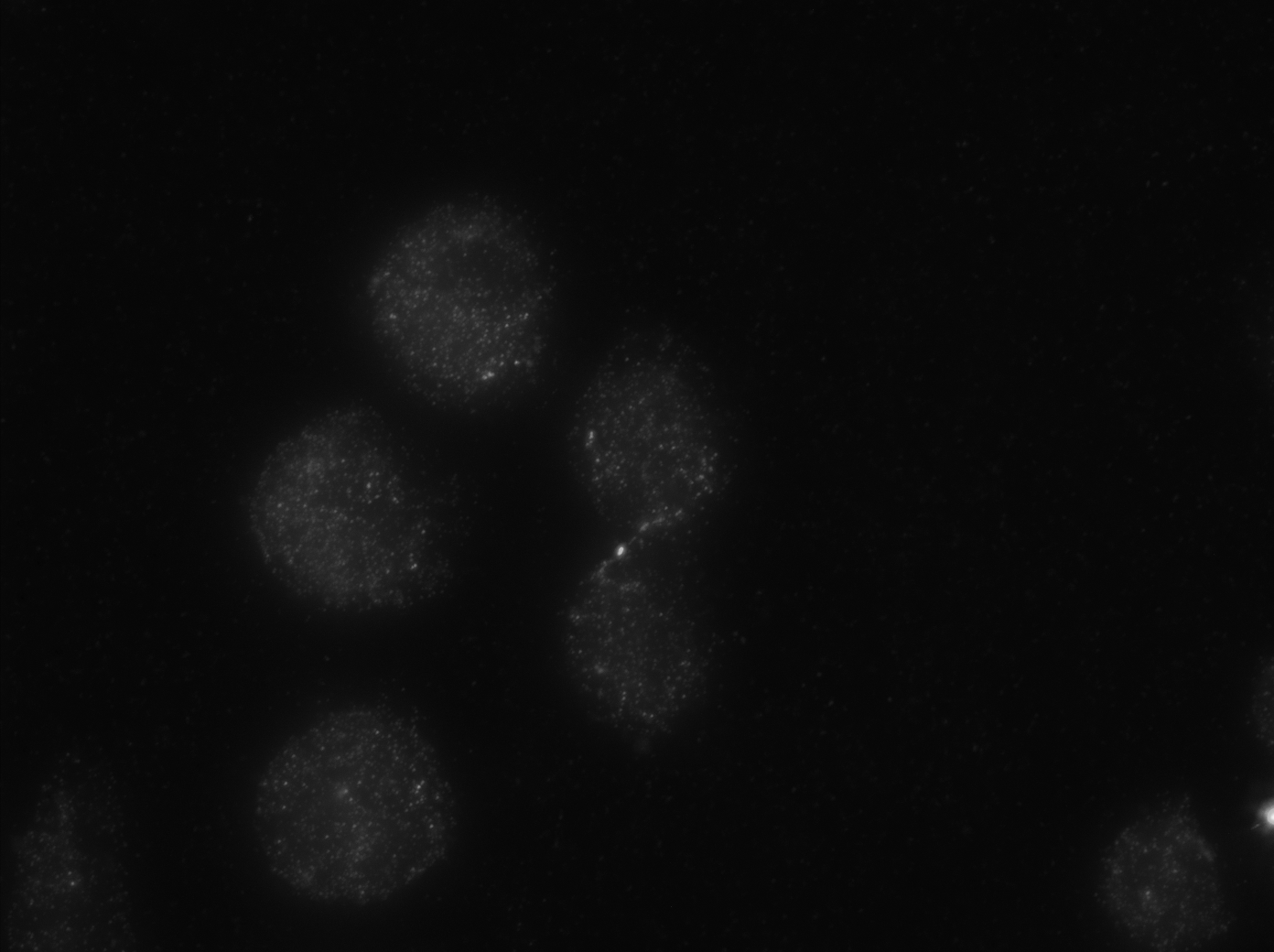

Supplement: Supplementary file 13 — Source data Fig. 3 part 3 [file 44319_2026_742_MOESM13_ESM.zip › Figure 3 Part 3/Fig 3b-e TTLL screen part 3/TTLL11-YFP A2 Img3 -Trey.Project Maximum Z_XY1648751169_Z0_T0_C1.tif]

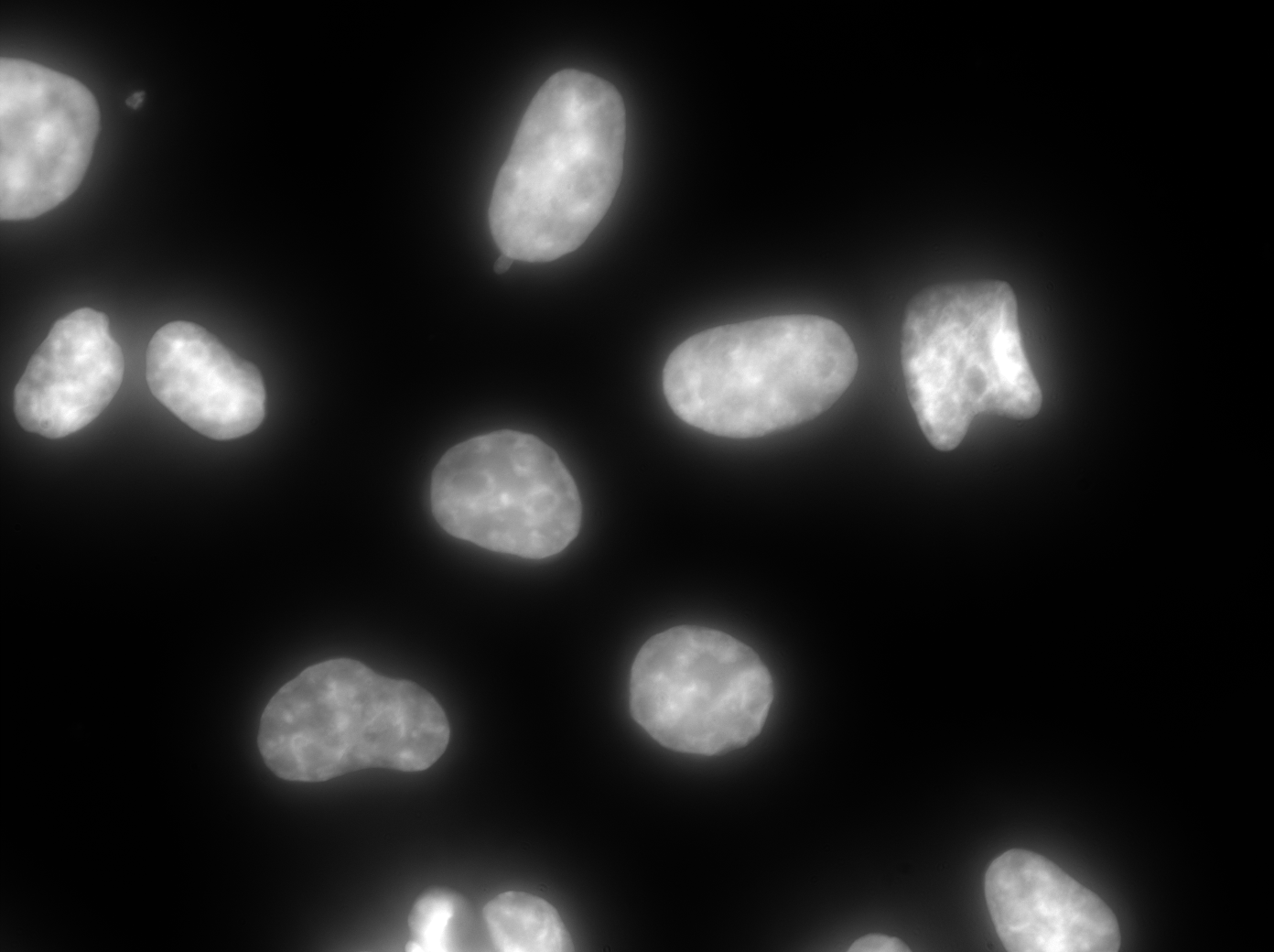

Supplement: Supplementary file 13 — Source data Fig. 3 part 3 [file 44319_2026_742_MOESM13_ESM.zip › Figure 3 Part 3/Fig 3b-e TTLL screen part 3/TTLL11-YFP Img 11 yfp2000.Project Maximum Z_XY1648579897_Z0_T0_C0.tif]

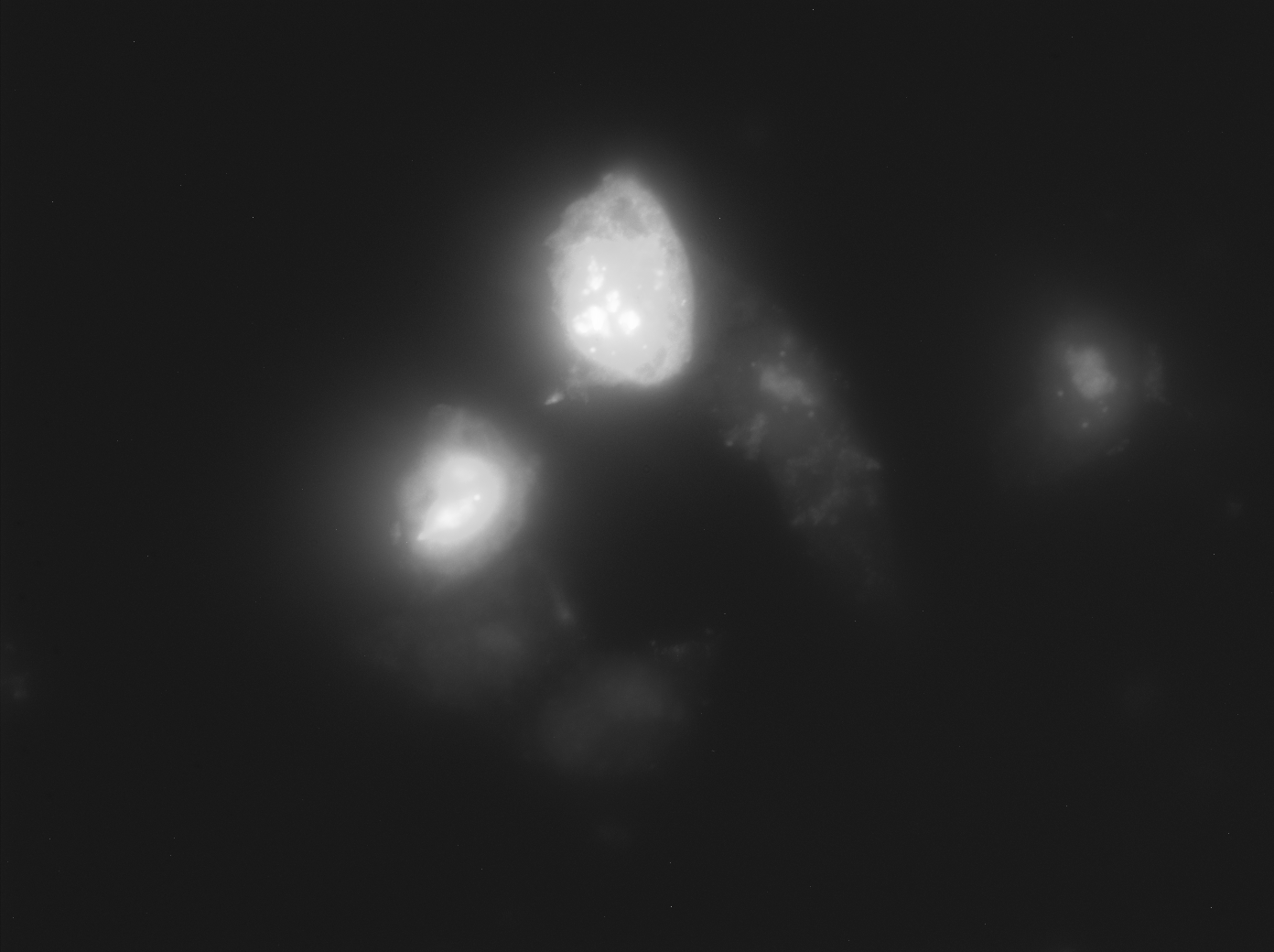

Supplement: Supplementary file 13 — Source data Fig. 3 part 3 [file 44319_2026_742_MOESM13_ESM.zip › Figure 3 Part 3/Fig 3b-e TTLL screen part 3/TTLL11-YFP Img 5 yfp2000.Project Maximum Z_XY1648573797_Z0_T0_C2.tif]

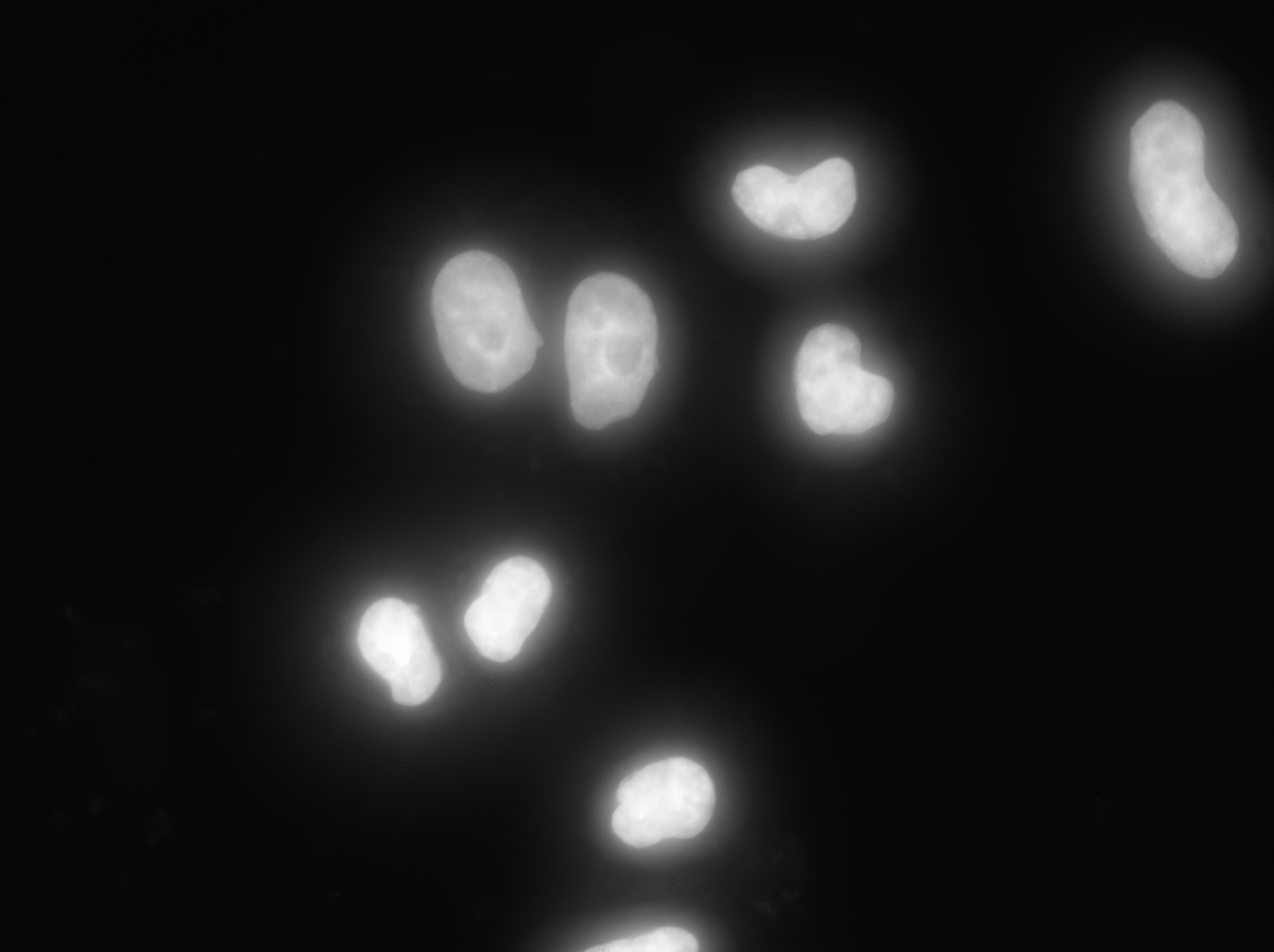

Supplement: Supplementary file 13 — Source data Fig. 3 part 3 [file 44319_2026_742_MOESM13_ESM.zip › Figure 3 Part 3/Fig 3b-e TTLL screen part 3/TTLL11-YFP Img 12 yfp2500 - 1.Project Maximum Z_XY1648580285_Z0_T0_C0.tif]

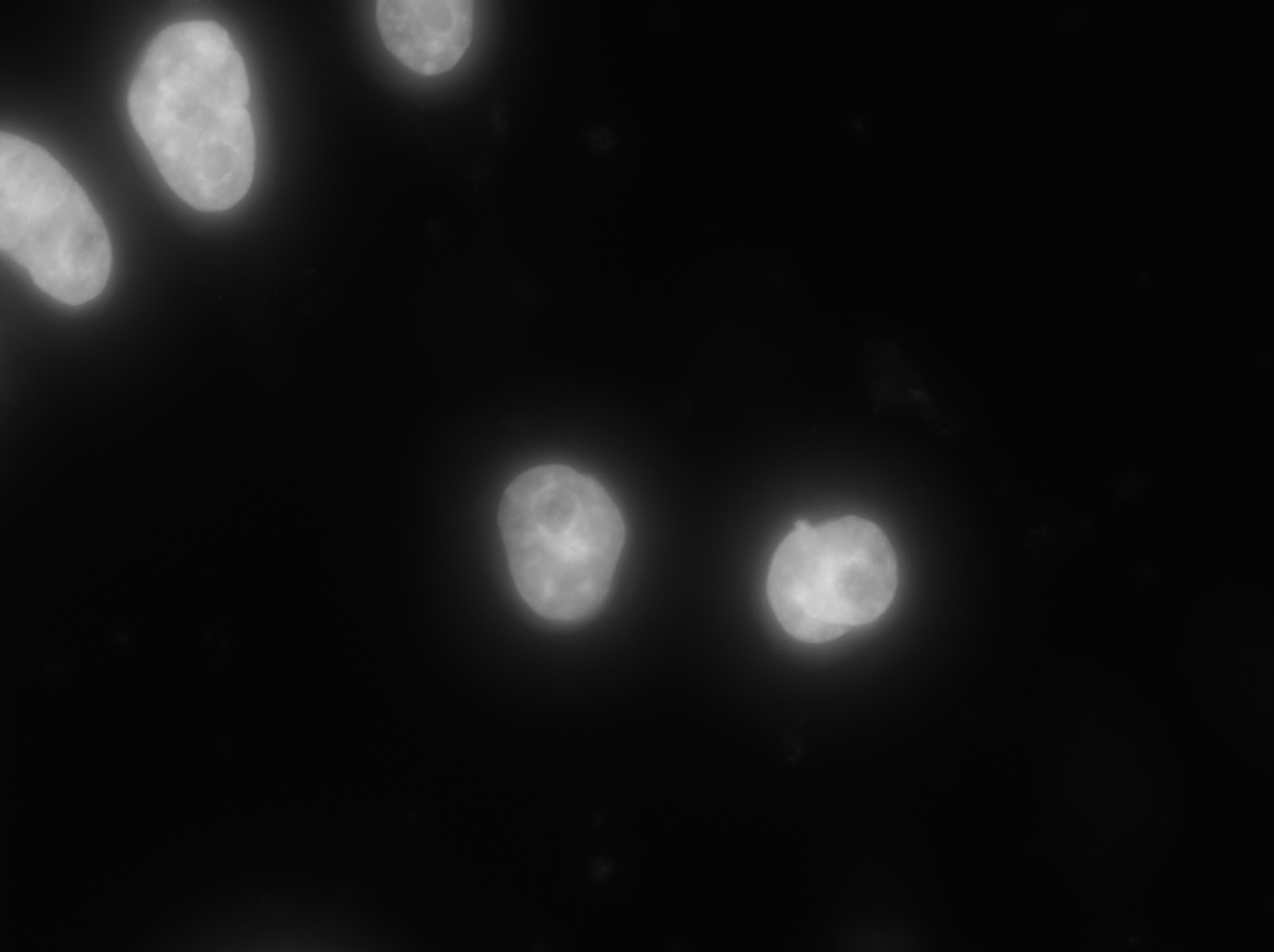

Supplement: Supplementary file 13 — Source data Fig. 3 part 3 [file 44319_2026_742_MOESM13_ESM.zip › Figure 3 Part 3/Fig 3b-e TTLL screen part 3/TTLL11-YFP Img 9 yfp2000 - 1.Project Maximum Z_XY1648578368_Z0_T0_C0.tif]

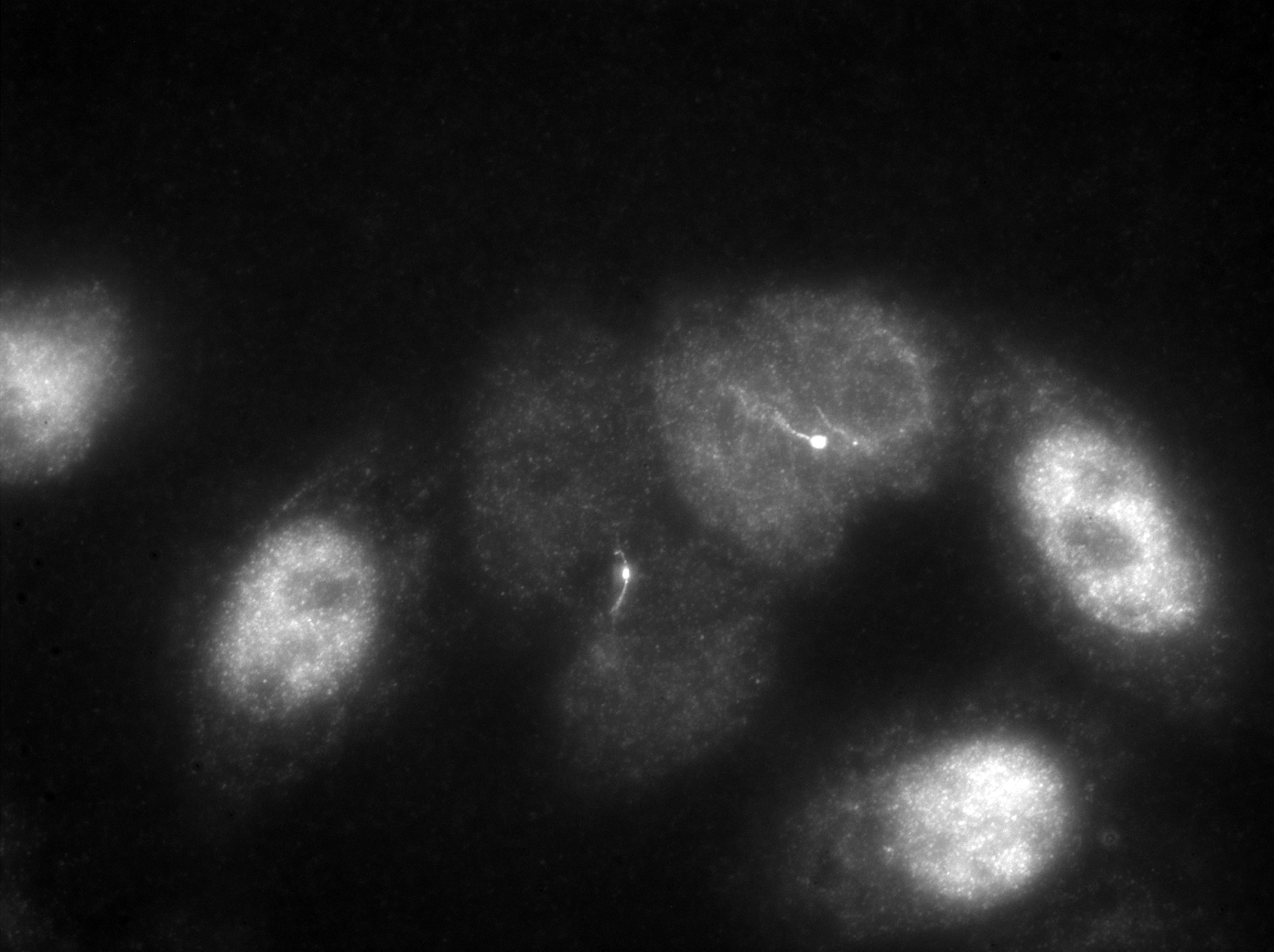

Supplement: Supplementary file 13 — Source data Fig. 3 part 3 [file 44319_2026_742_MOESM13_ESM.zip › Figure 3 Part 3/Fig 3b-e TTLL screen part 3/TTLL9-YFP A3 I7.Project Maximum Z_XY1679700241_Z0_T0_C1.tif]

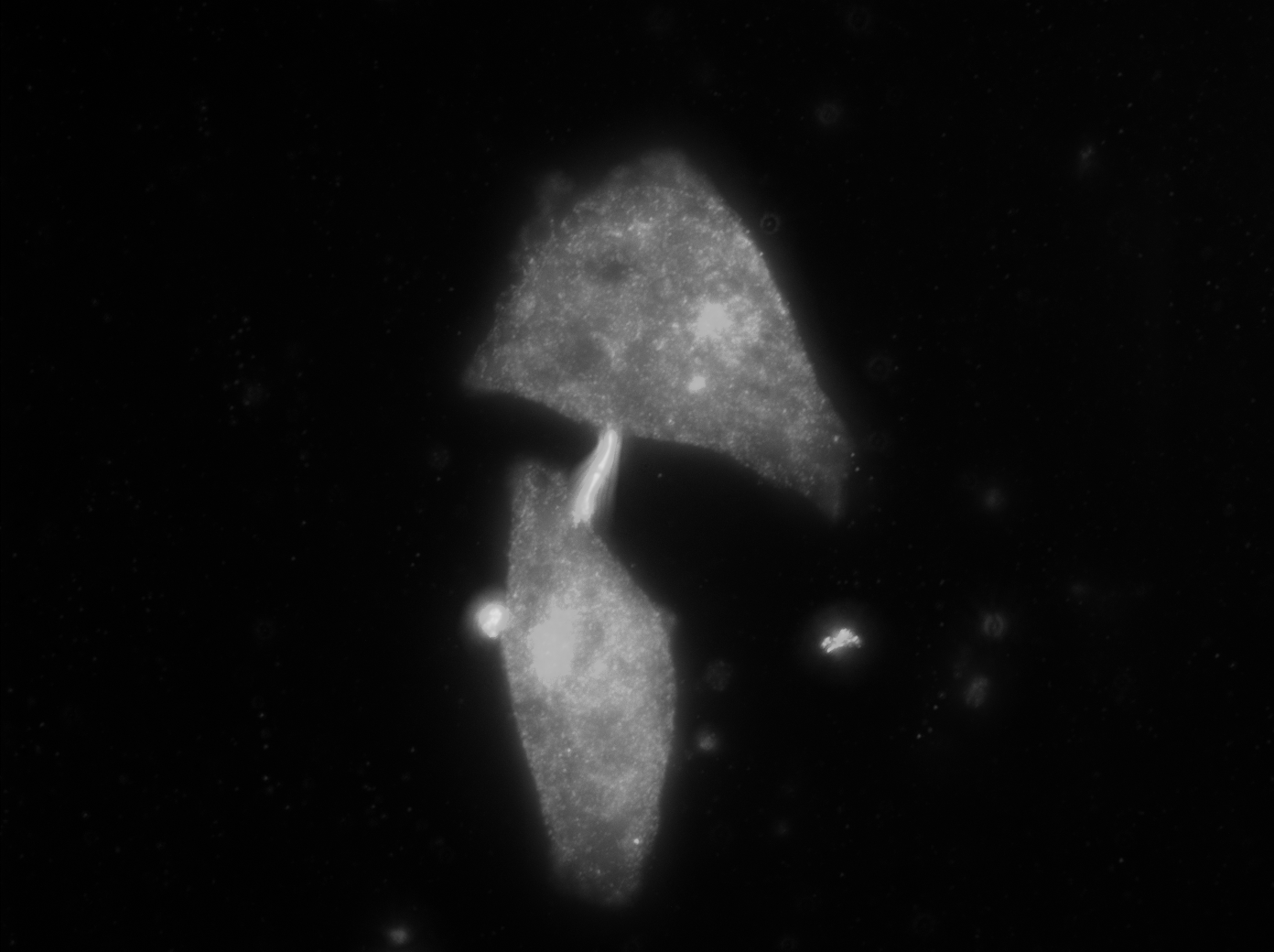

Supplement: Supplementary file 13 — Source data Fig. 3 part 3 [file 44319_2026_742_MOESM13_ESM.zip › Figure 3 Part 3/Fig 3b-e TTLL screen part 3/TTLL9-GFP A4 I1.Project Maximum Z_XY1675965339_Z0_T0_C1.tif]

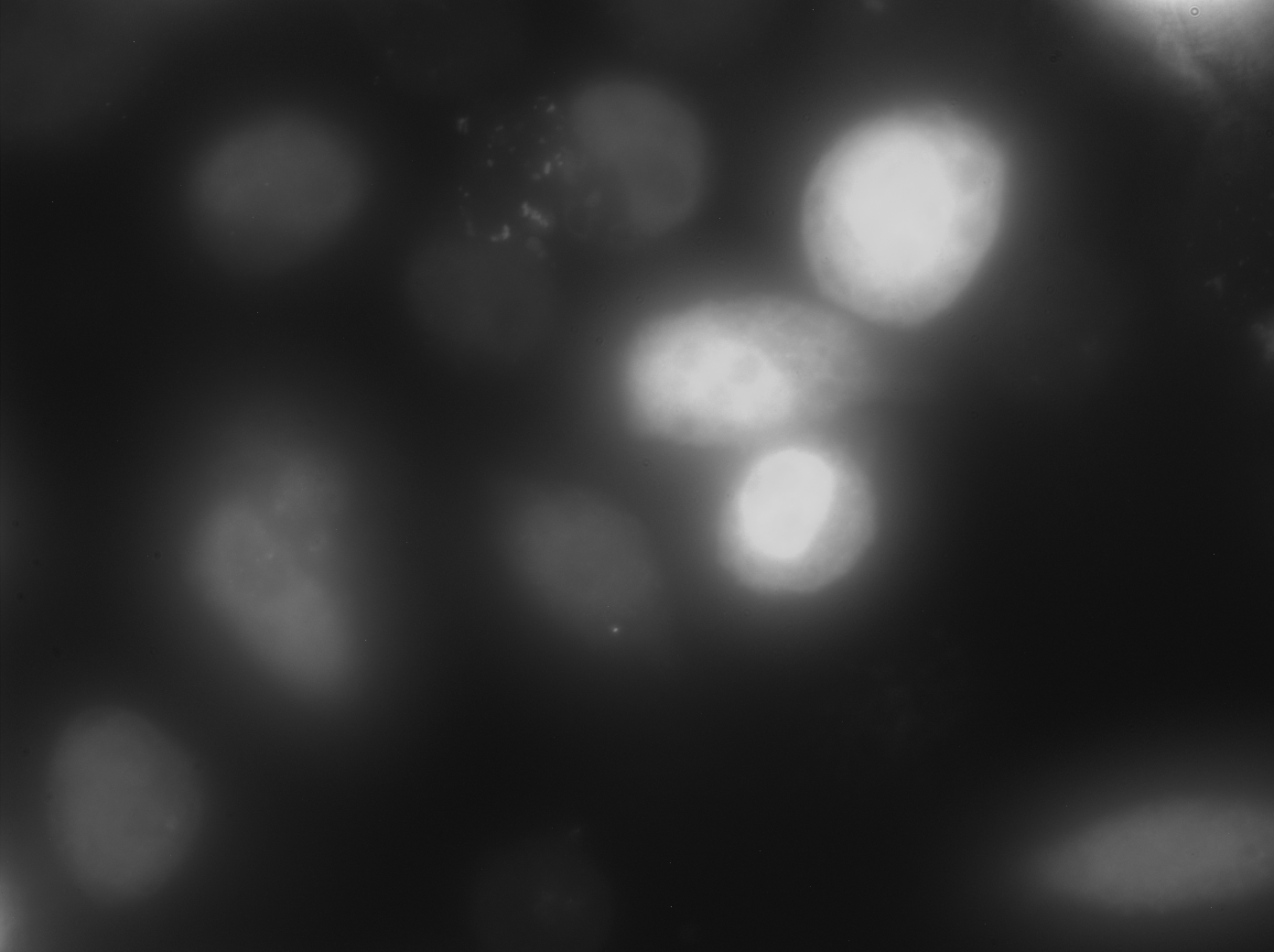

Supplement: Supplementary file 13 — Source data Fig. 3 part 3 [file 44319_2026_742_MOESM13_ESM.zip › Figure 3 Part 3/Fig 3b-e TTLL screen part 3/TTLL9-YFP A3 I13.Project Maximum Z_XY1679701070_Z0_T0_C2.tif]

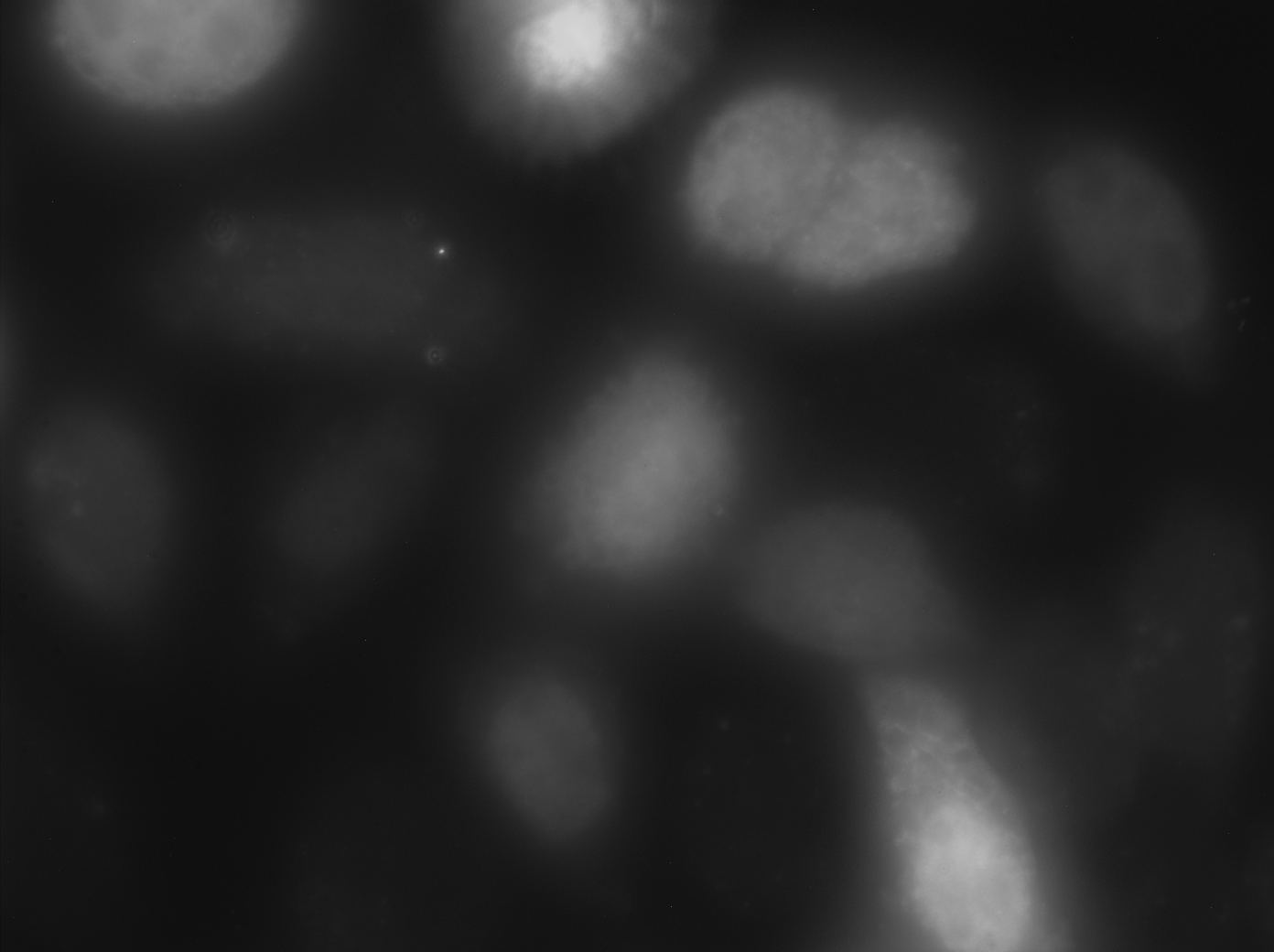

Supplement: Supplementary file 13 — Source data Fig. 3 part 3 [file 44319_2026_742_MOESM13_ESM.zip › Figure 3 Part 3/Fig 3b-e TTLL screen part 3/TTLL9-YFP A3 I4.Project Maximum Z_XY1679699772_Z0_T0_C2.tif]

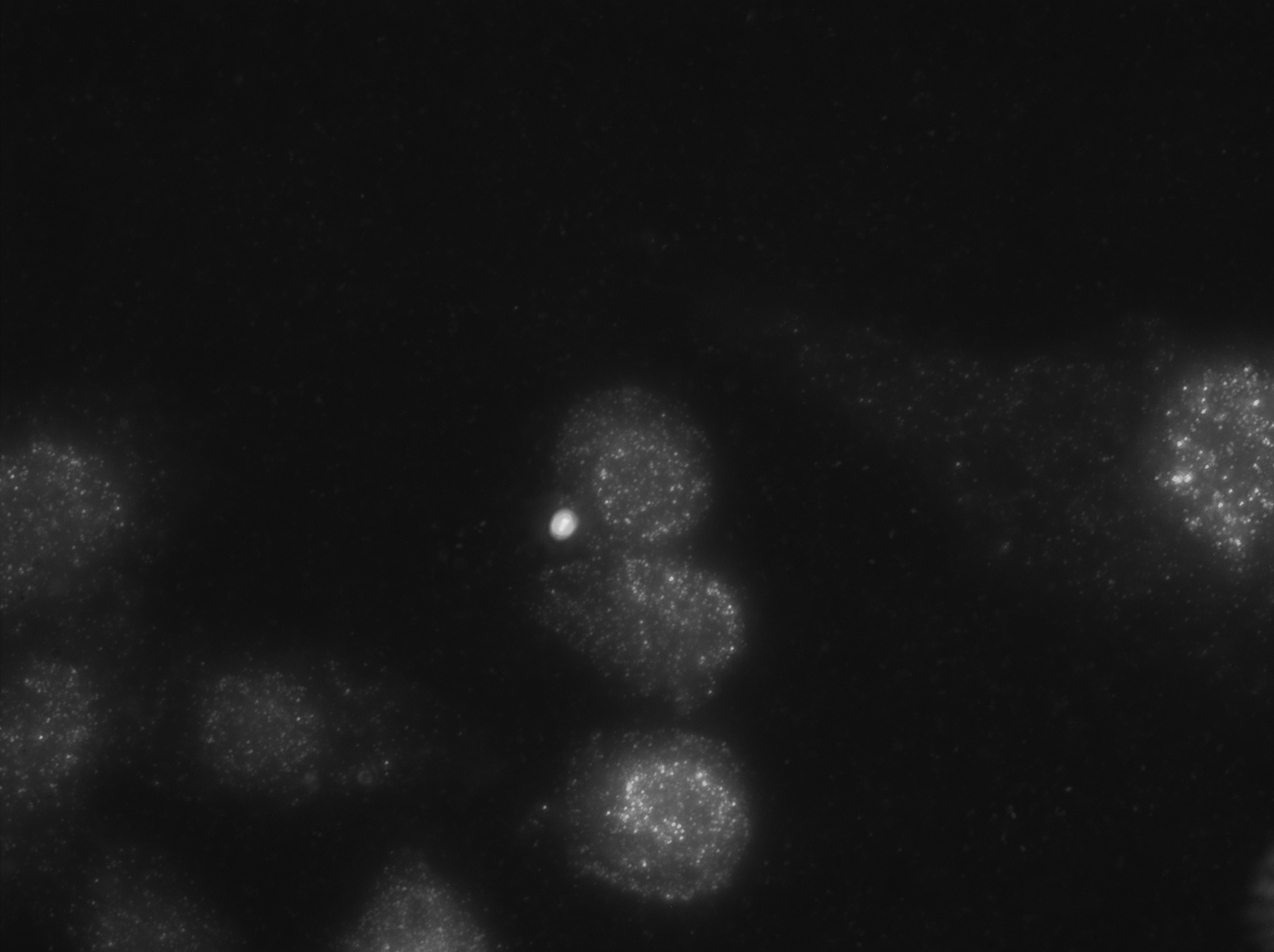

Supplement: Supplementary file 13 — Source data Fig. 3 part 3 [file 44319_2026_742_MOESM13_ESM.zip › Figure 3 Part 3/Fig 3b-e TTLL screen part 3/TTLL11-YFP Img 10 yfp2000.Project Maximum Z_XY1648579577_Z0_T0_C1.tif]

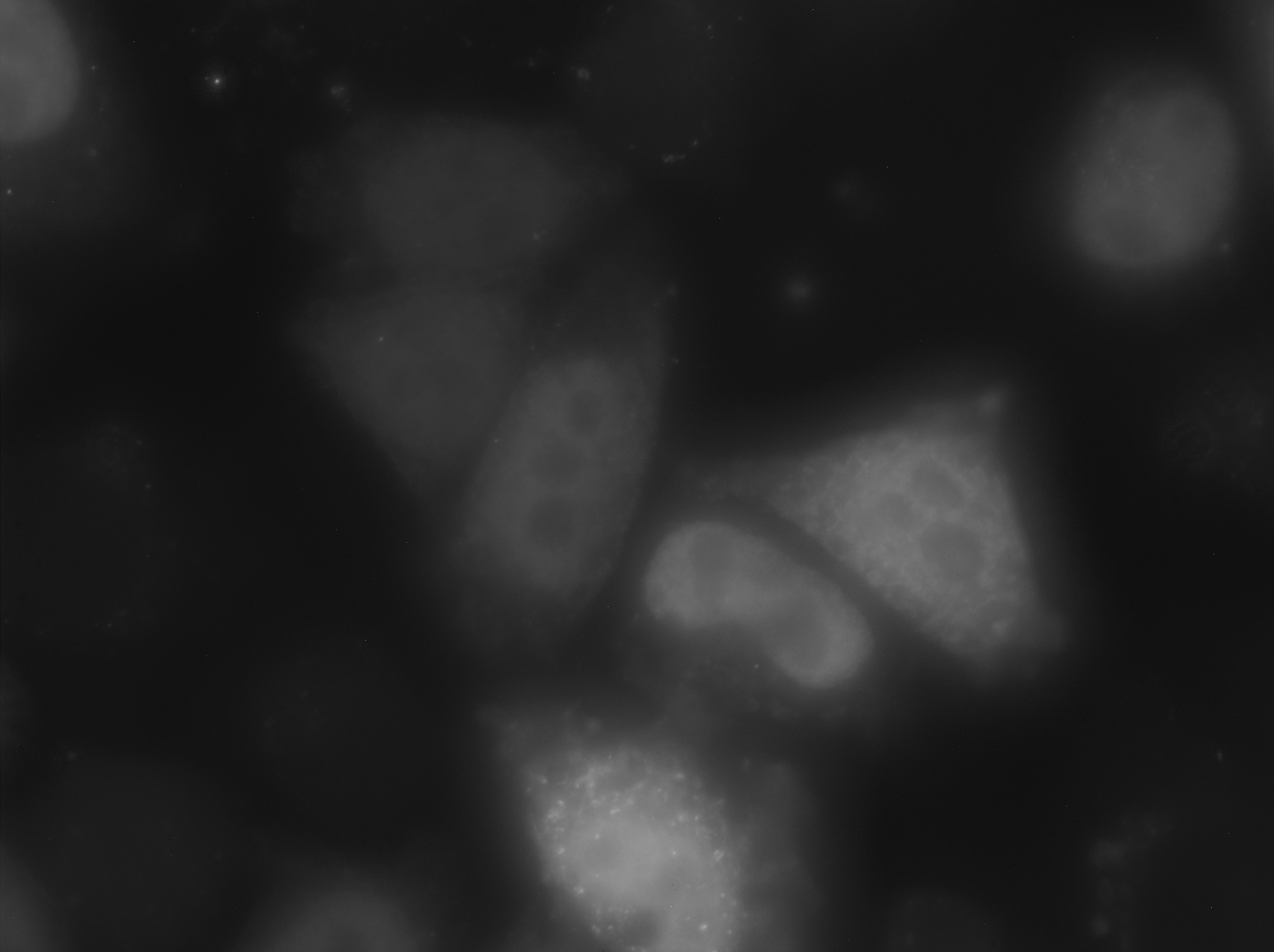

Supplement: Supplementary file 13 — Source data Fig. 3 part 3 [file 44319_2026_742_MOESM13_ESM.zip › Figure 3 Part 3/Fig 3b-e TTLL screen part 3/TTLL9-YFP A3 I18.Project Maximum Z_XY1679701787_Z0_T0_C2.tif]

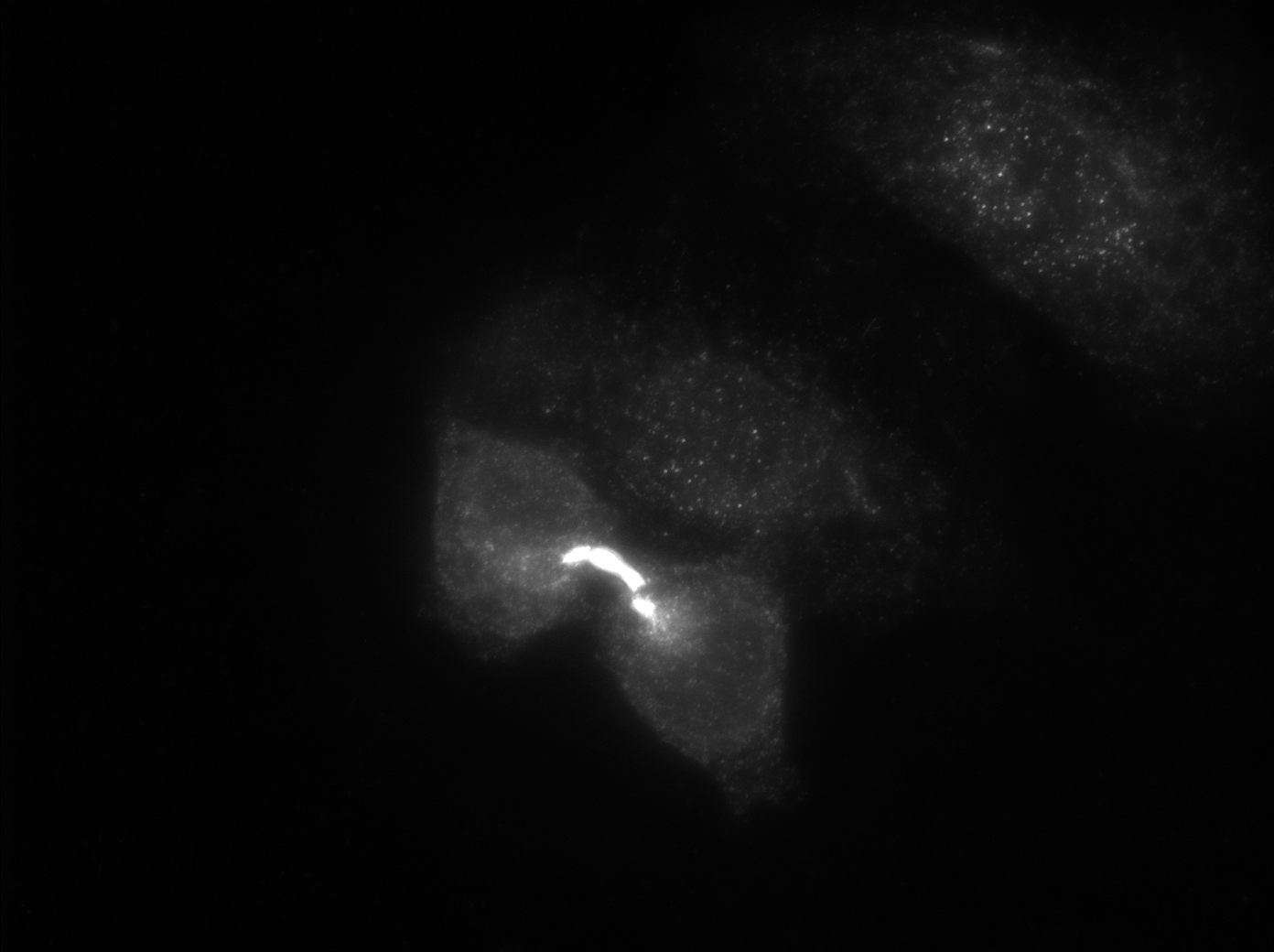

Supplement: Supplementary file 13 — Source data Fig. 3 part 3 [file 44319_2026_742_MOESM13_ESM.zip › Figure 3 Part 3/Fig 3b-e TTLL screen part 3/TTLL9-YFP R1 I4.Project Maximum Z_XY1674166819_Z0_T0_C1.tif]

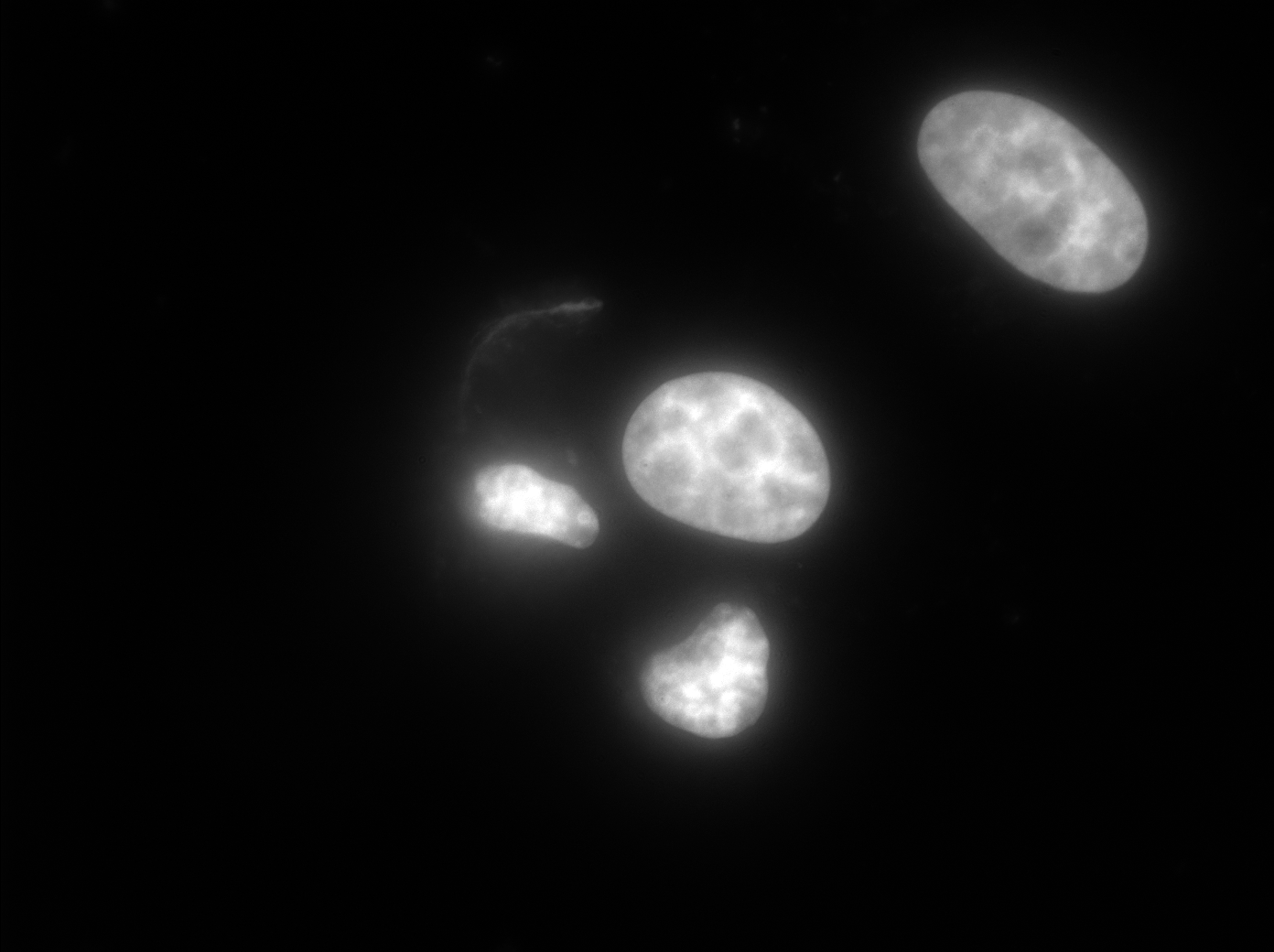

Supplement: Supplementary file 13 — Source data Fig. 3 part 3 [file 44319_2026_742_MOESM13_ESM.zip › Figure 3 Part 3/Fig 3b-e TTLL screen part 3/TTLL9-YFP R1 I4.Project Maximum Z_XY1674166819_Z0_T0_C0.tif]

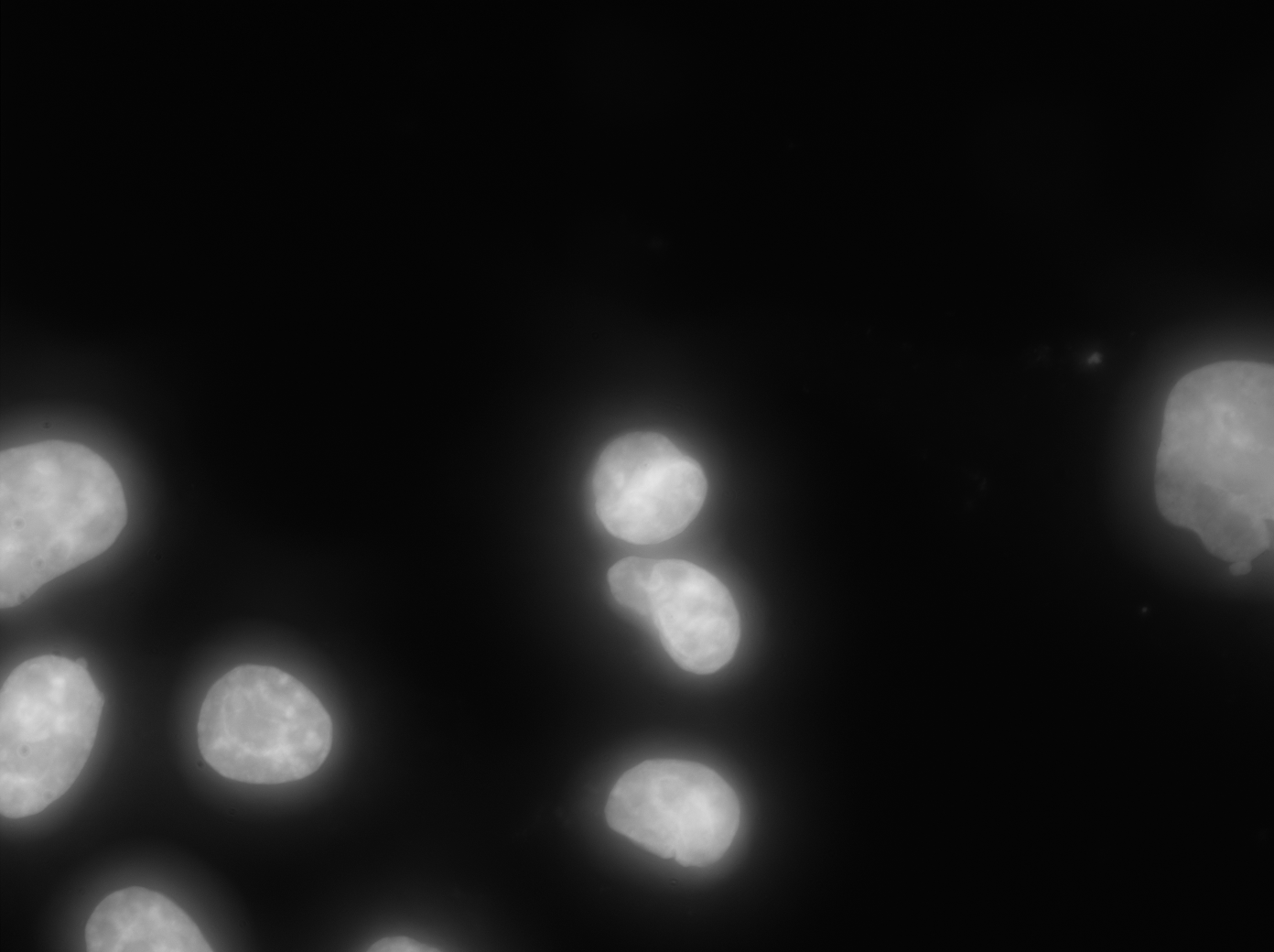

Supplement: Supplementary file 13 — Source data Fig. 3 part 3 [file 44319_2026_742_MOESM13_ESM.zip › Figure 3 Part 3/Fig 3b-e TTLL screen part 3/TTLL11-YFP Img 10 yfp2000.Project Maximum Z_XY1648579577_Z0_T0_C0.tif]

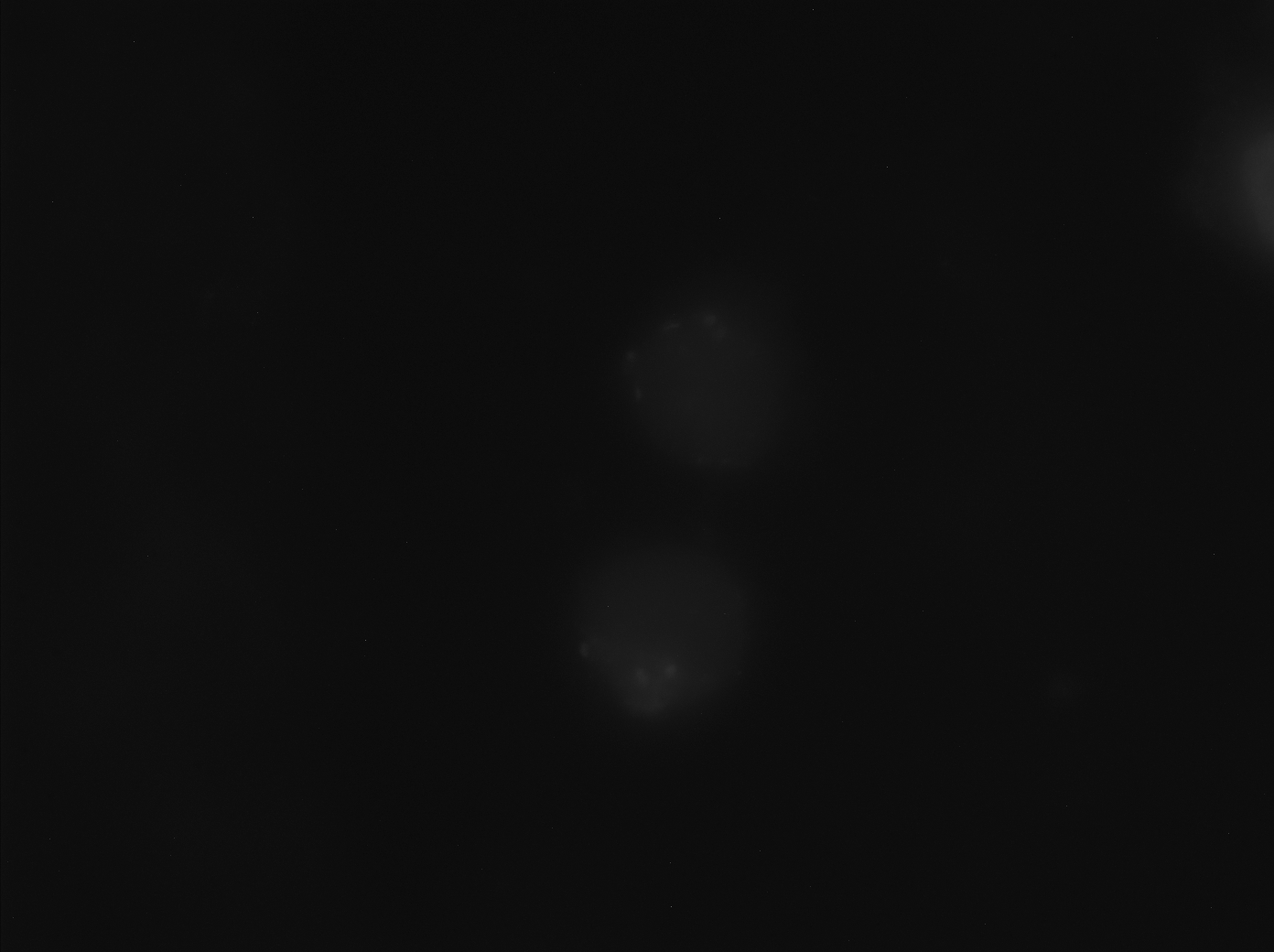

Supplement: Supplementary file 13 — Source data Fig. 3 part 3 [file 44319_2026_742_MOESM13_ESM.zip › Figure 3 Part 3/Fig 3b-e TTLL screen part 3/TTLL9-YFP A3 I3.Project Maximum Z_XY1674674979_Z0_T0_C2.tif]

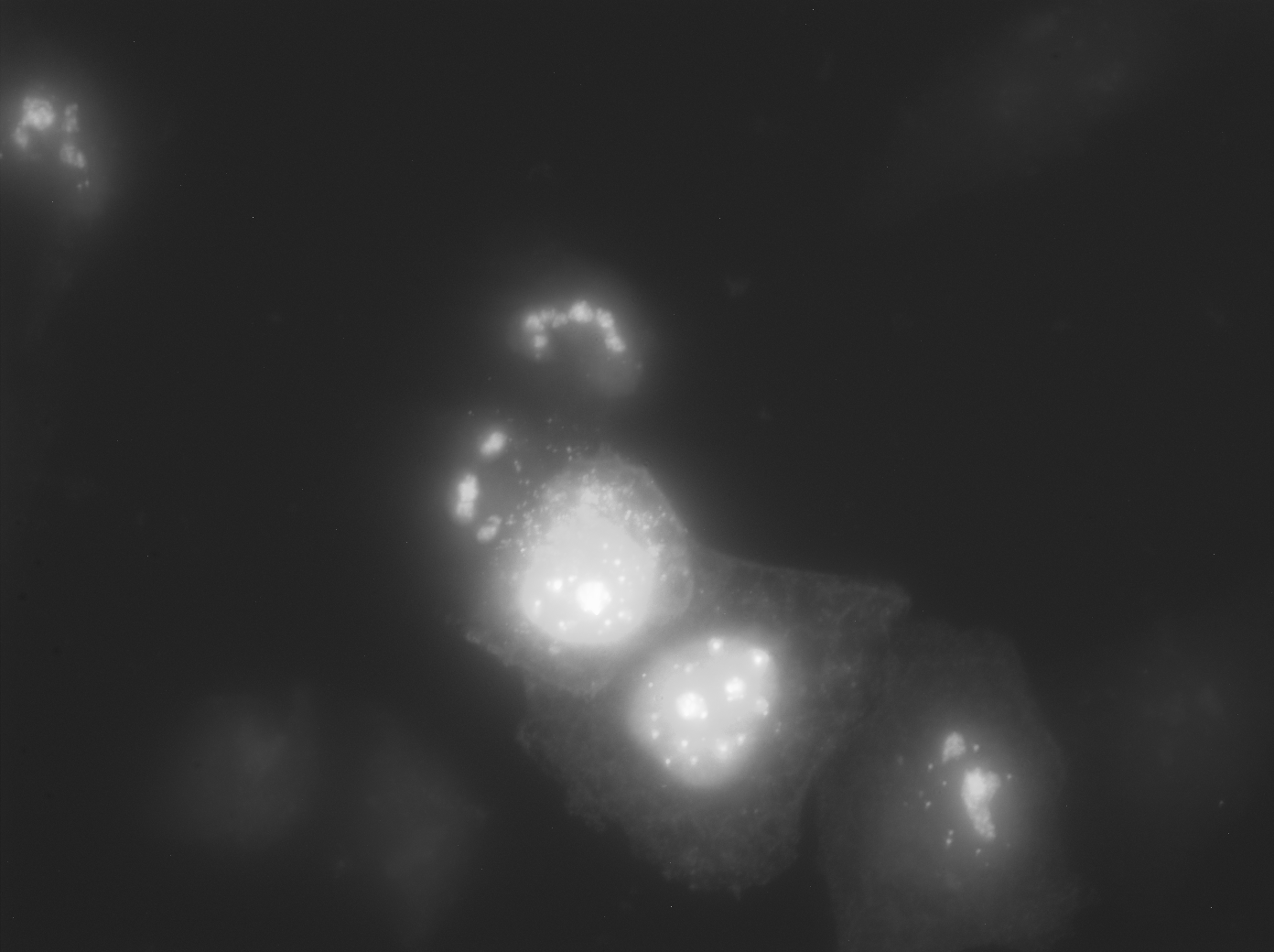

Supplement: Supplementary file 13 — Source data Fig. 3 part 3 [file 44319_2026_742_MOESM13_ESM.zip › Figure 3 Part 3/Fig 3b-e TTLL screen part 3/TTLL11-YFP Img 6 yfp2000 - 1.Project Maximum Z_XY1648574277_Z0_T0_C2.tif]

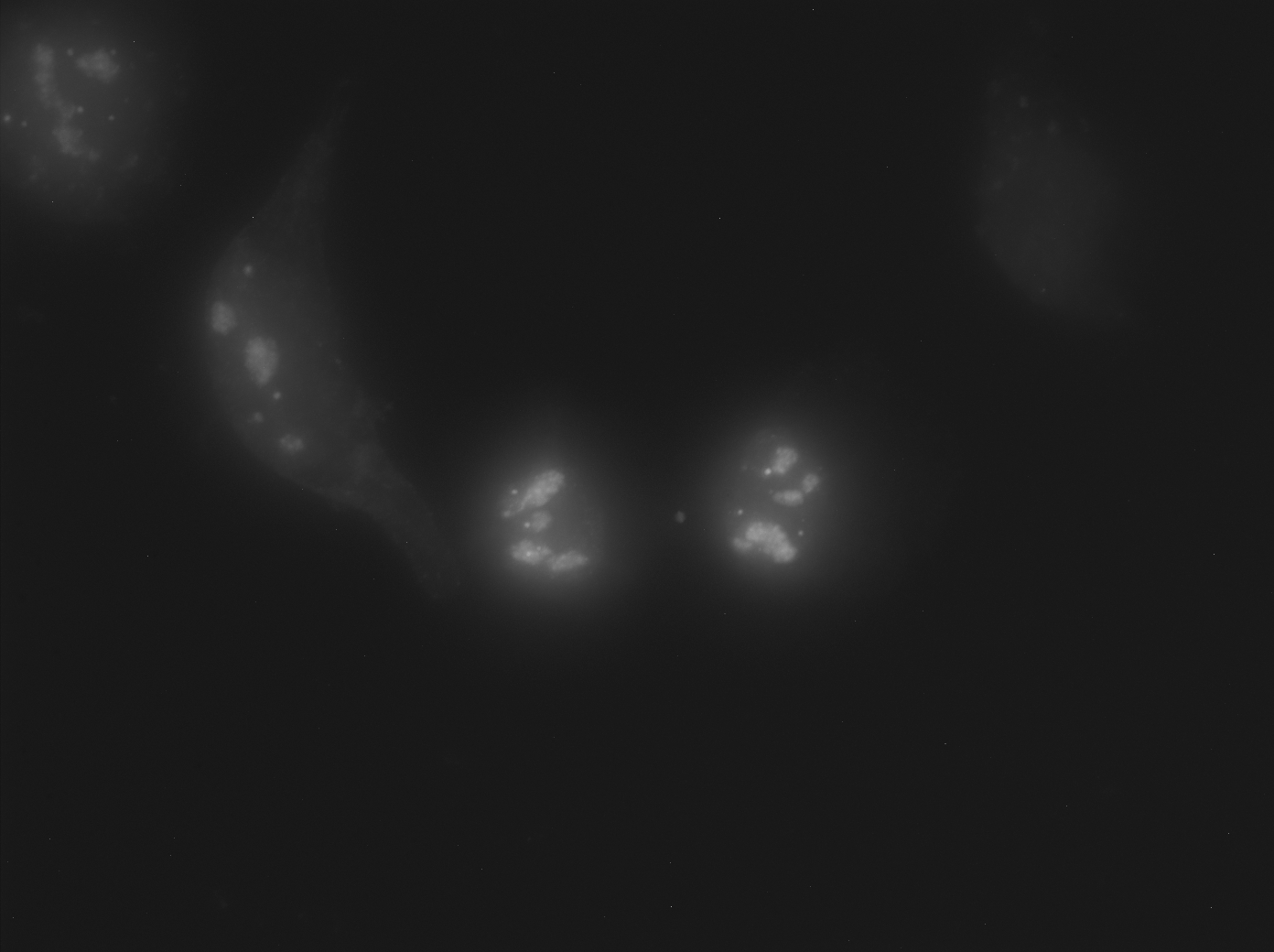

Supplement: Supplementary file 13 — Source data Fig. 3 part 3 [file 44319_2026_742_MOESM13_ESM.zip › Figure 3 Part 3/Fig 3b-e TTLL screen part 3/TTLL11-YFP A2 Img4.Project Maximum Z_XY1648752676_Z0_T0_C2.tif]

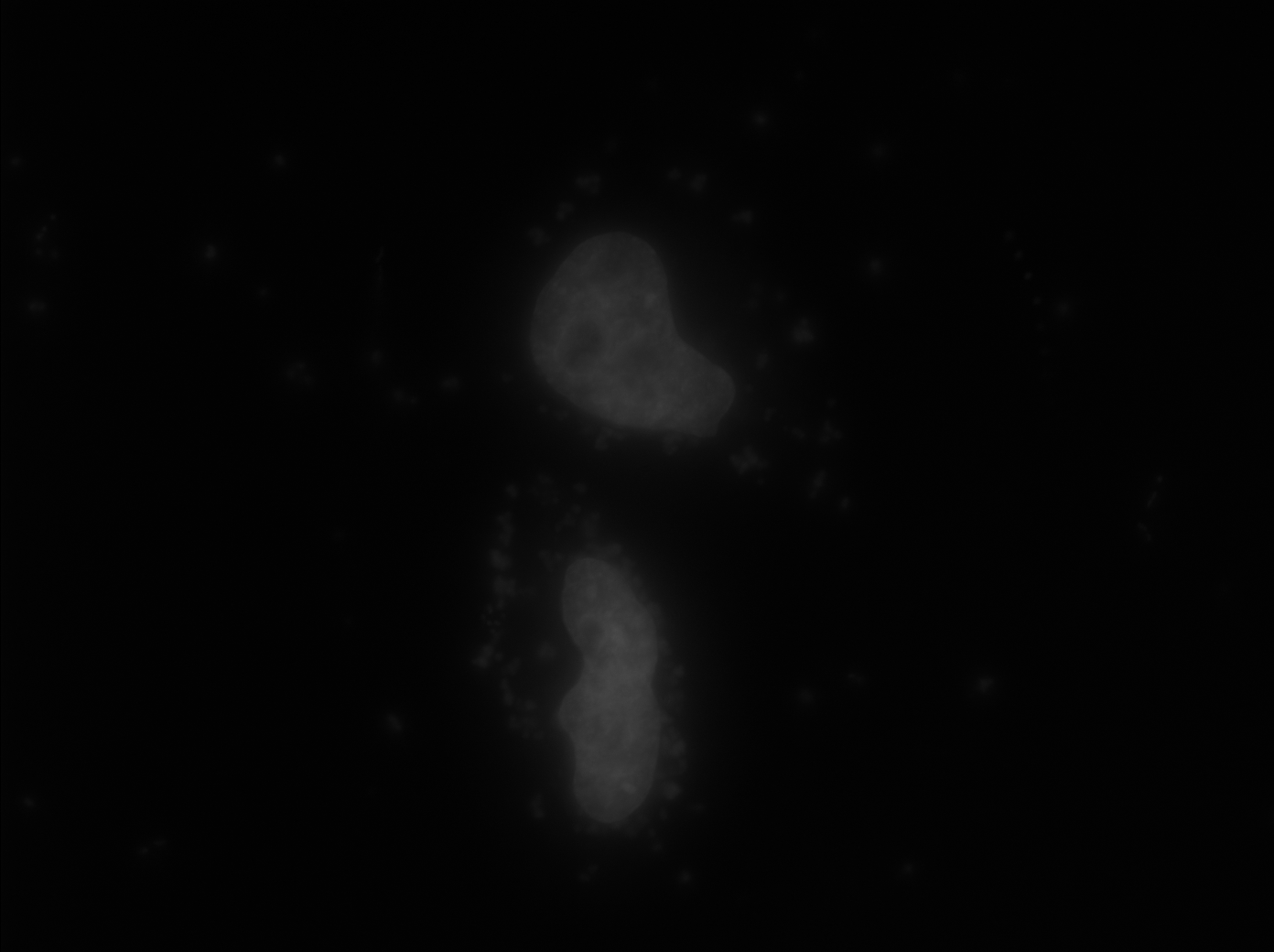

Supplement: Supplementary file 13 — Source data Fig. 3 part 3 [file 44319_2026_742_MOESM13_ESM.zip › Figure 3 Part 3/Fig 3b-e TTLL screen part 3/TTLL9-GFP A4 I1.Project Maximum Z_XY1675965339_Z0_T0_C0.tif]

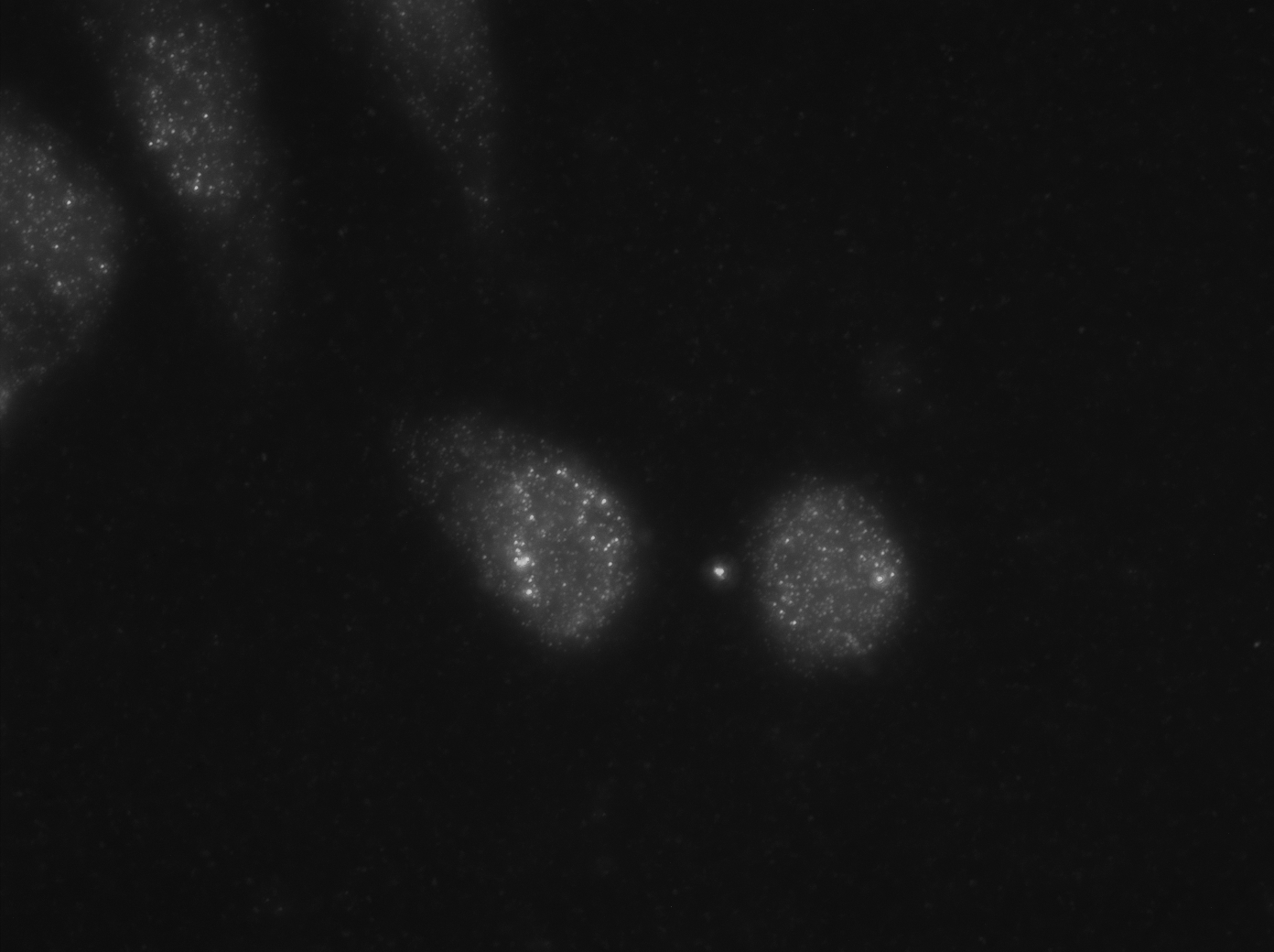

Supplement: Supplementary file 13 — Source data Fig. 3 part 3 [file 44319_2026_742_MOESM13_ESM.zip › Figure 3 Part 3/Fig 3b-e TTLL screen part 3/TTLL11-YFP Img 9 yfp2000 - 1.Project Maximum Z_XY1648578368_Z0_T0_C1.tif]

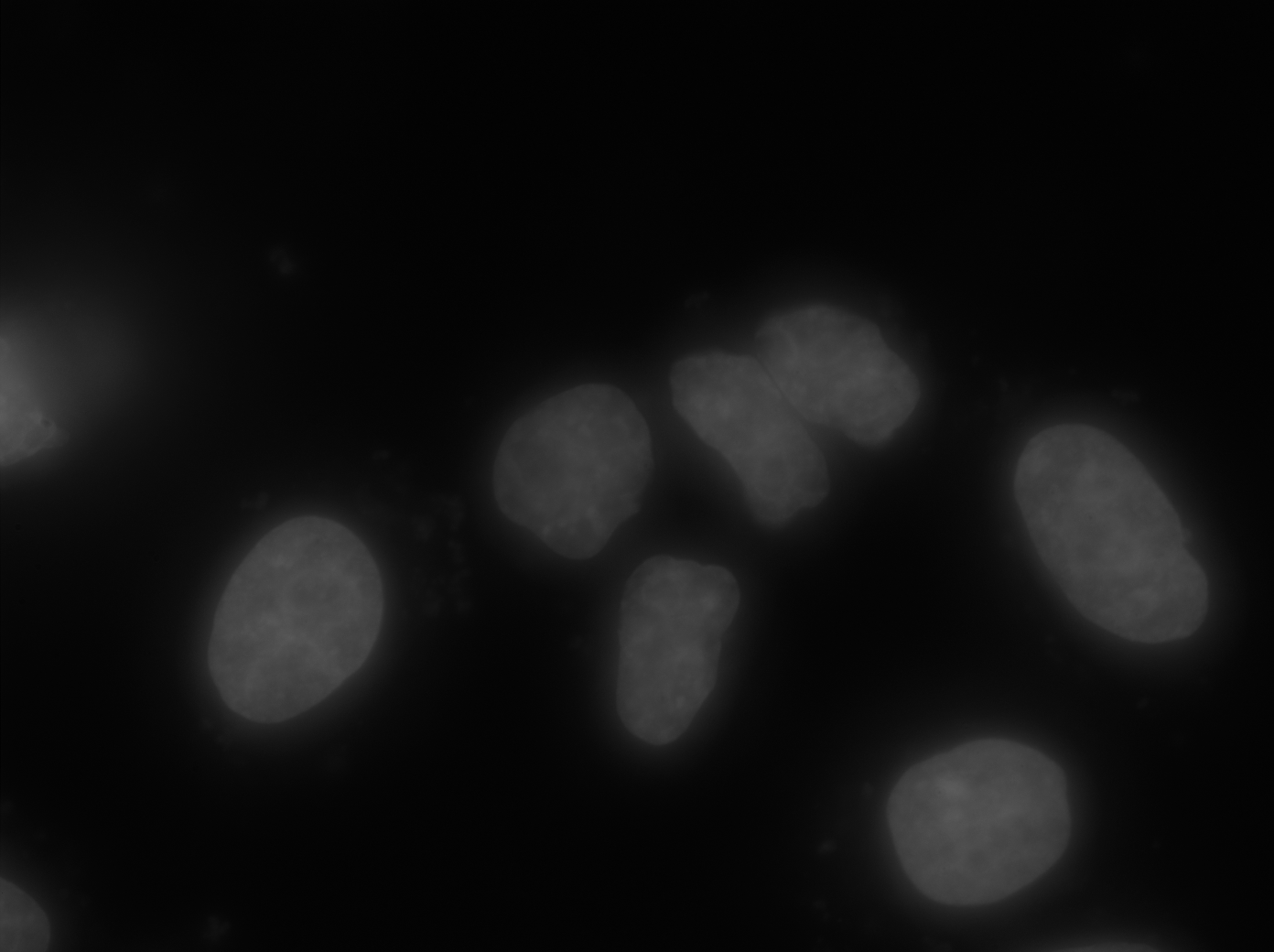

Supplement: Supplementary file 13 — Source data Fig. 3 part 3 [file 44319_2026_742_MOESM13_ESM.zip › Figure 3 Part 3/Fig 3b-e TTLL screen part 3/TTLL9-YFP A3 I7.Project Maximum Z_XY1679700241_Z0_T0_C0.tif]

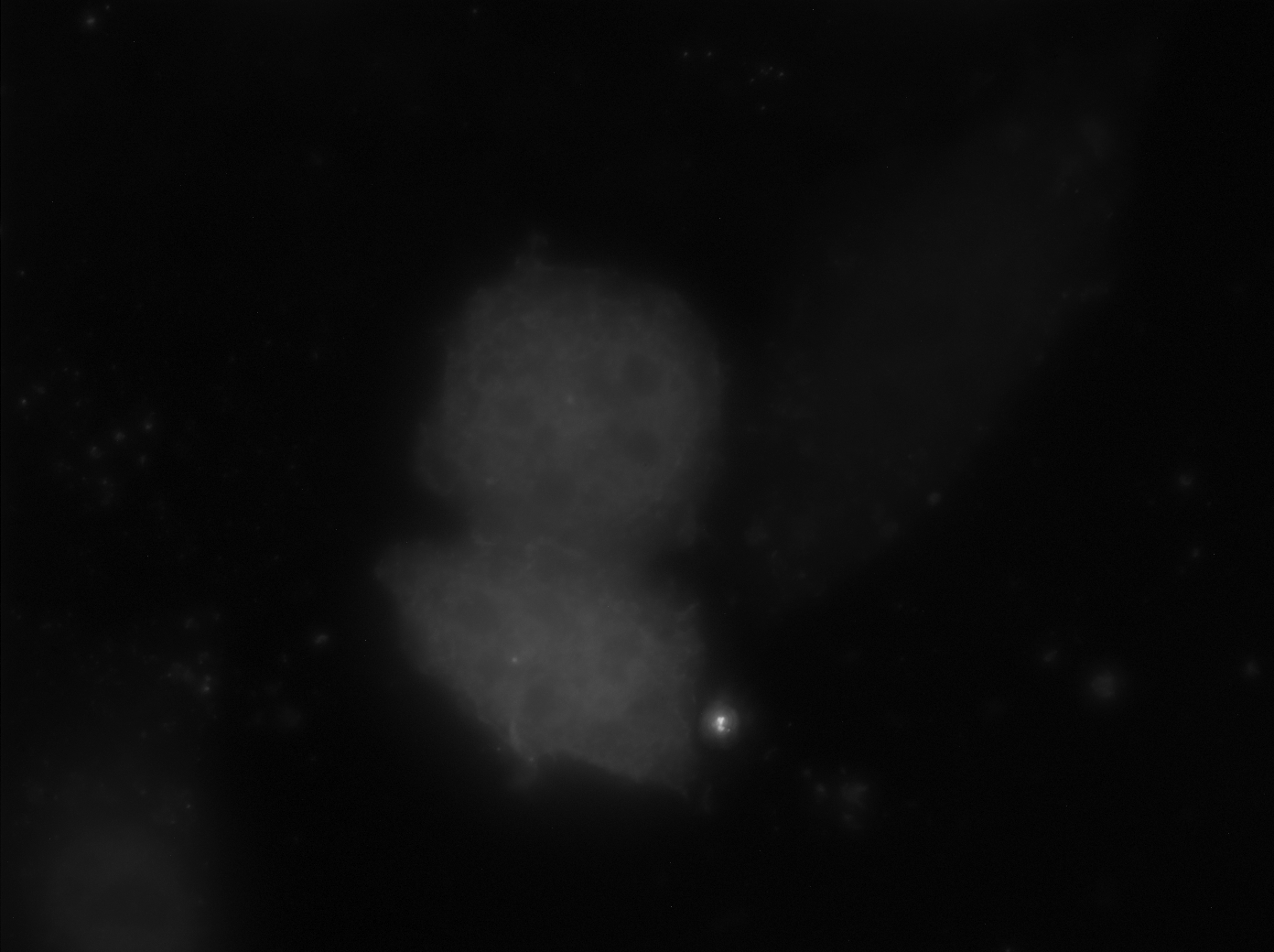

Supplement: Supplementary file 13 — Source data Fig. 3 part 3 [file 44319_2026_742_MOESM13_ESM.zip › Figure 3 Part 3/Fig 3b-e TTLL screen part 3/TTLL9-GFP A4 I6.Project Maximum Z_XY1675968056_Z0_T0_C2.tif]

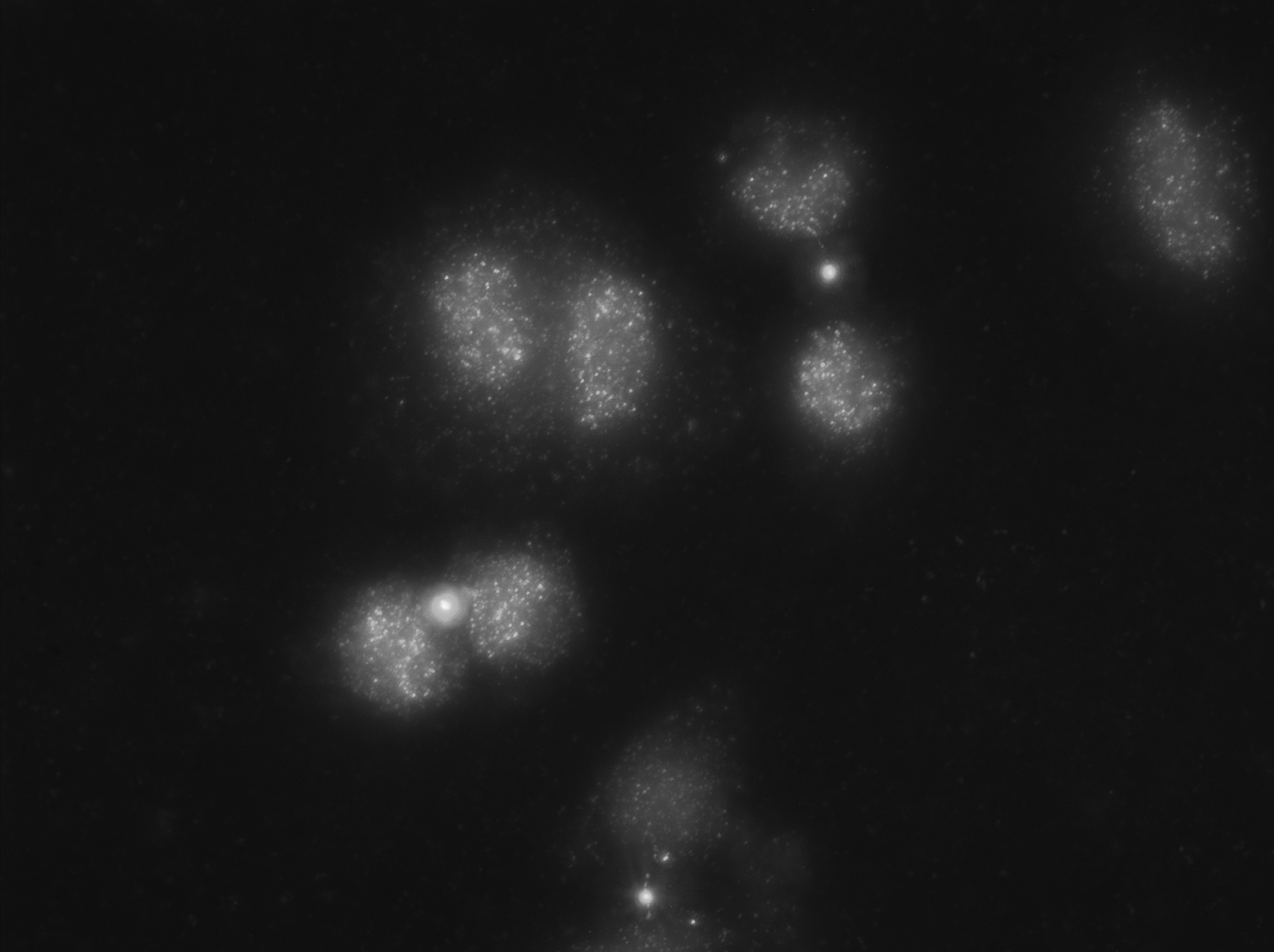

Supplement: Supplementary file 13 — Source data Fig. 3 part 3 [file 44319_2026_742_MOESM13_ESM.zip › Figure 3 Part 3/Fig 3b-e TTLL screen part 3/TTLL11-YFP Img 12 yfp2500 - 1.Project Maximum Z_XY1648580285_Z0_T0_C1.tif]

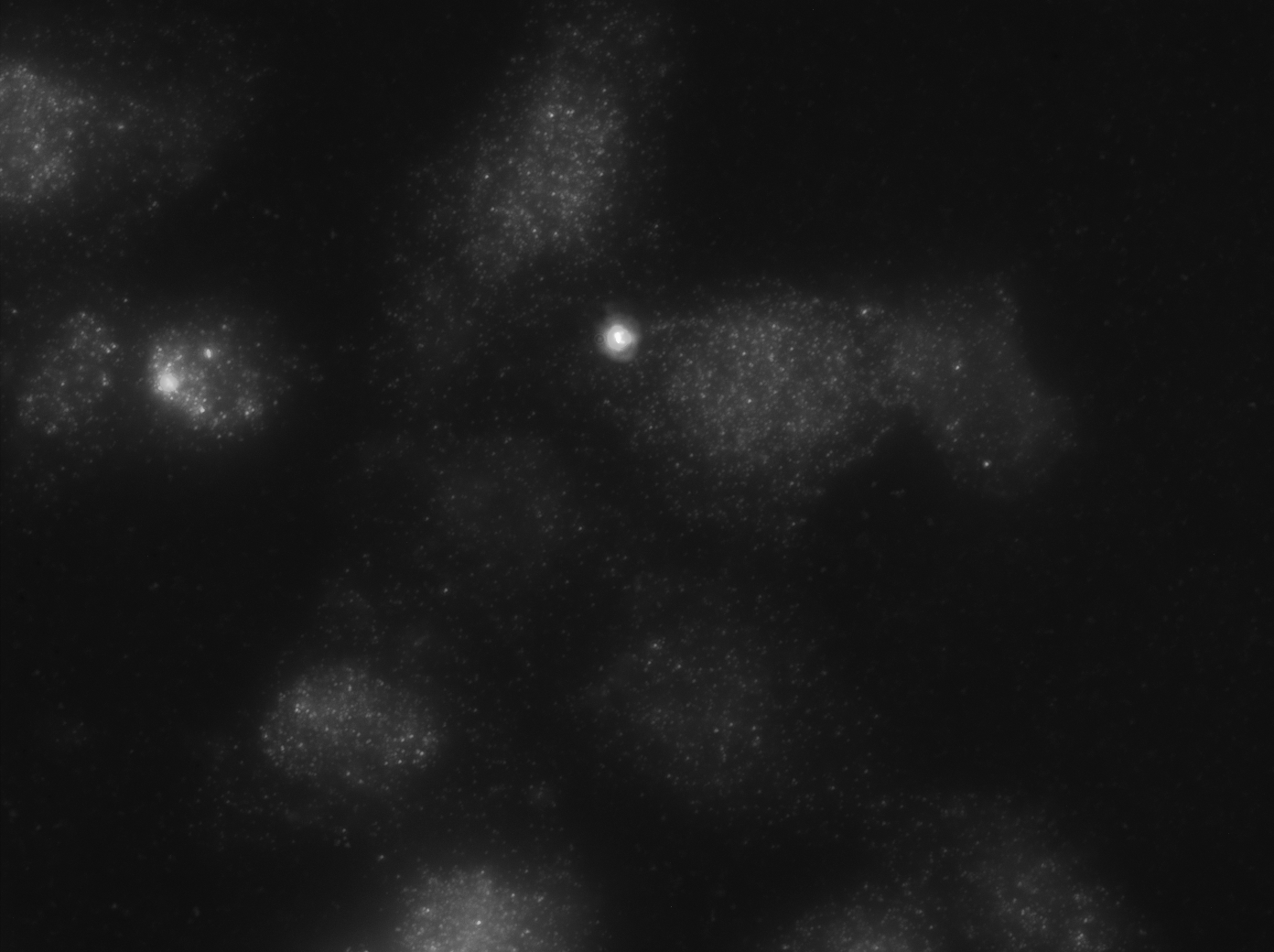

Supplement: Supplementary file 13 — Source data Fig. 3 part 3 [file 44319_2026_742_MOESM13_ESM.zip › Figure 3 Part 3/Fig 3b-e TTLL screen part 3/TTLL11-YFP Img 11 yfp2000.Project Maximum Z_XY1648579897_Z0_T0_C1.tif]

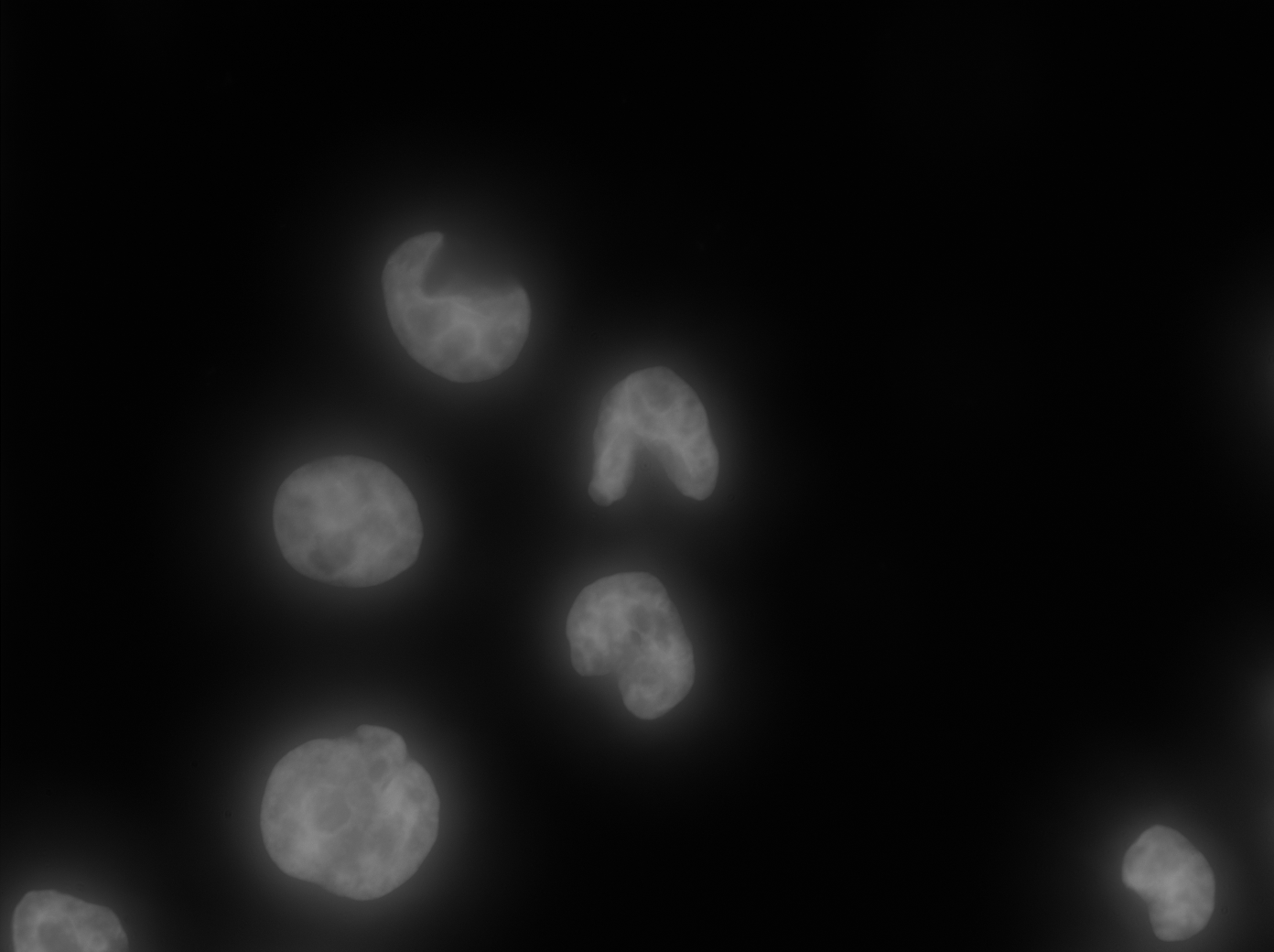

Supplement: Supplementary file 13 — Source data Fig. 3 part 3 [file 44319_2026_742_MOESM13_ESM.zip › Figure 3 Part 3/Fig 3b-e TTLL screen part 3/TTLL11-YFP A2 Img3 -Trey.Project Maximum Z_XY1648751169_Z0_T0_C0.tif]

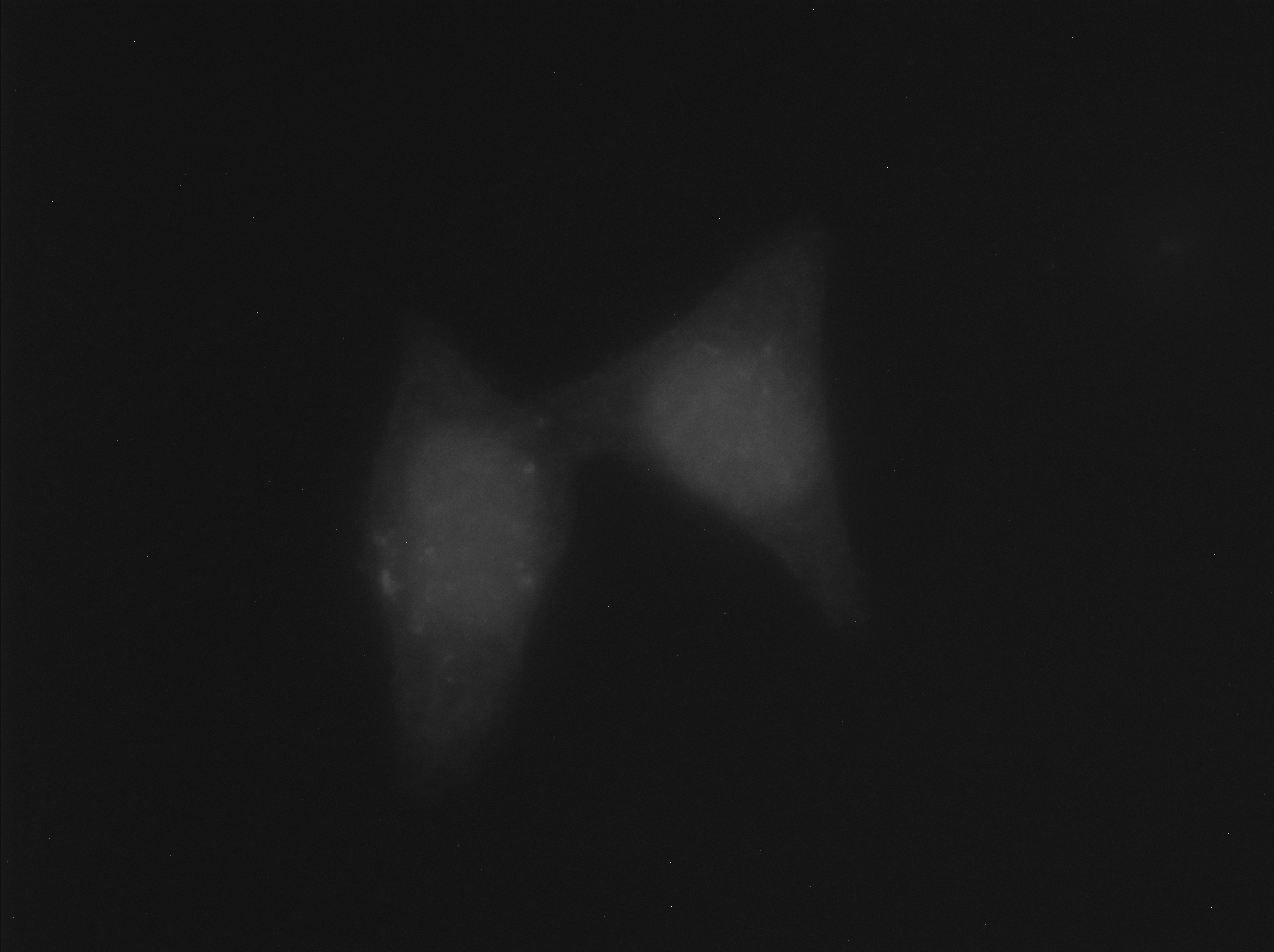

Supplement: Supplementary file 13 — Source data Fig. 3 part 3 [file 44319_2026_742_MOESM13_ESM.zip › Figure 3 Part 3/Fig 3b-e TTLL screen part 3/YFP Only R1 I7.Project Maximum Z_XY1663272885_Z0_T0_C2.tif]

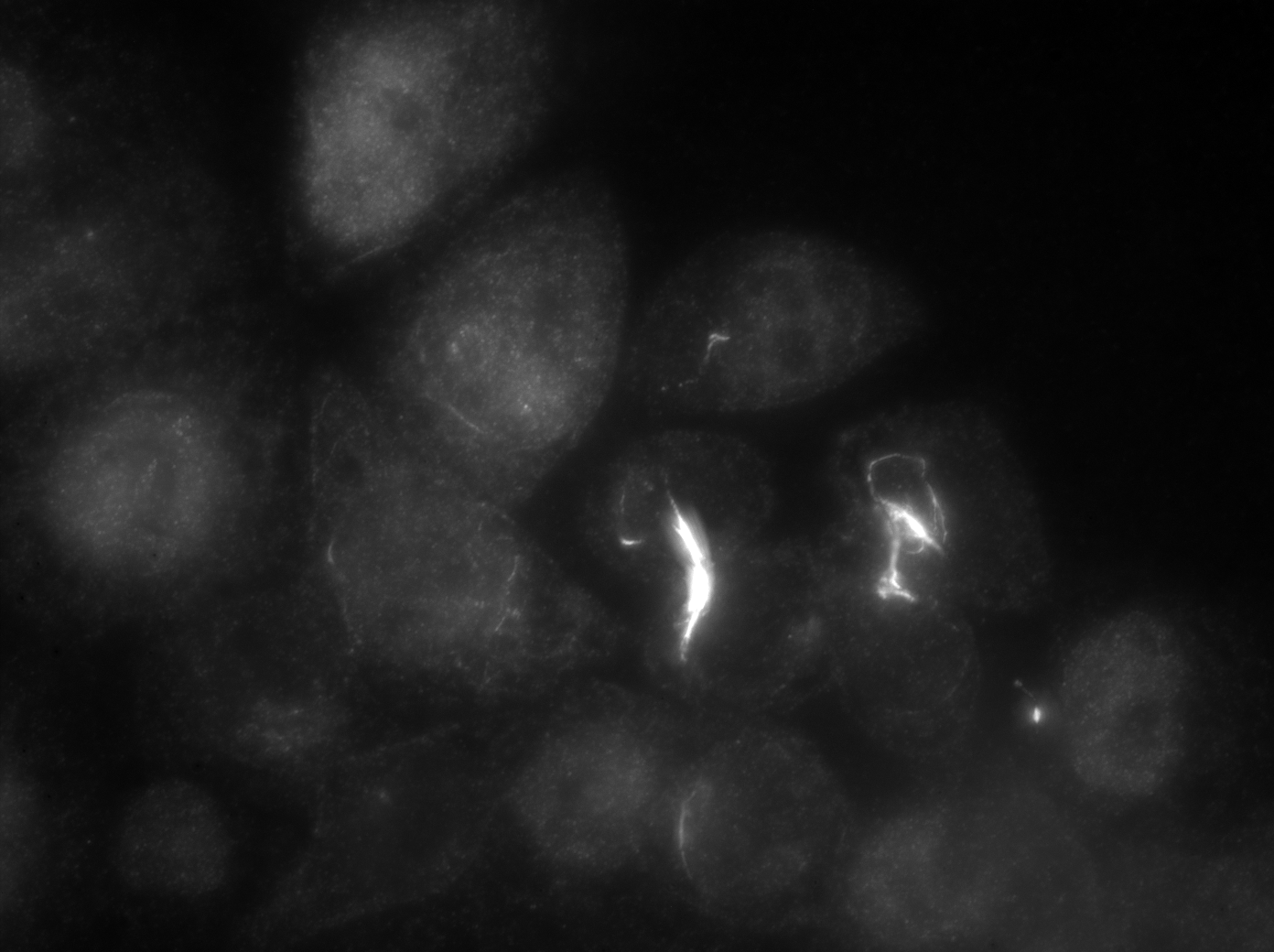

Supplement: Supplementary file 13 — Source data Fig. 3 part 3 [file 44319_2026_742_MOESM13_ESM.zip › Figure 3 Part 3/Fig 3b-e TTLL screen part 3/TTLL9-YFP A3 I6.Project Maximum Z_XY1679700103_Z0_T0_C1.tif]

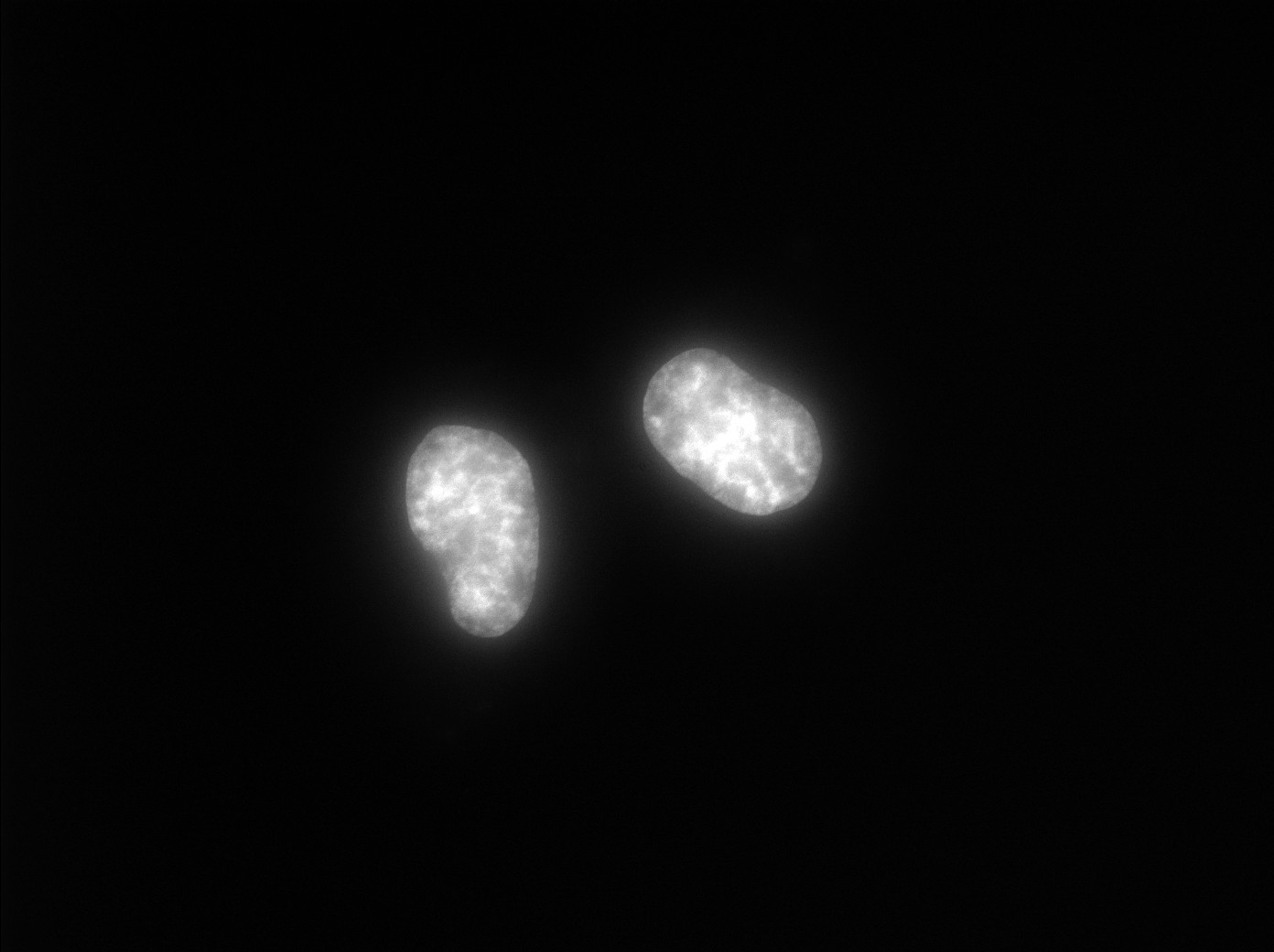

Supplement: Supplementary file 13 — Source data Fig. 3 part 3 [file 44319_2026_742_MOESM13_ESM.zip › Figure 3 Part 3/Fig 3b-e TTLL screen part 3/YFP Only R1 I7.Project Maximum Z_XY1663272885_Z0_T0_C0.tif]

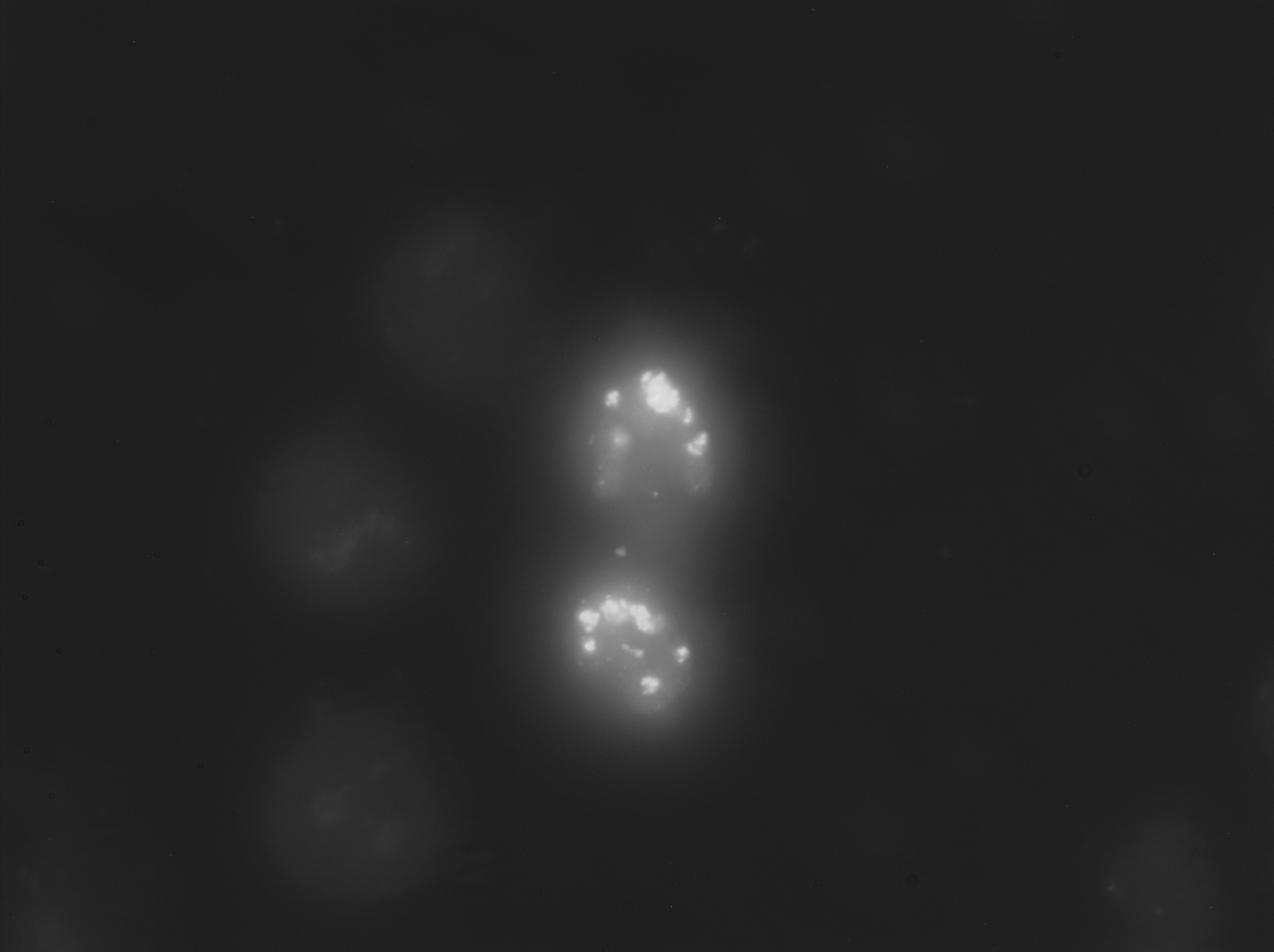

Supplement: Supplementary file 13 — Source data Fig. 3 part 3 [file 44319_2026_742_MOESM13_ESM.zip › Figure 3 Part 3/Fig 3b-e TTLL screen part 3/TTLL11-YFP A2 Img3 -Trey.Project Maximum Z_XY1648751169_Z0_T0_C2.tif]

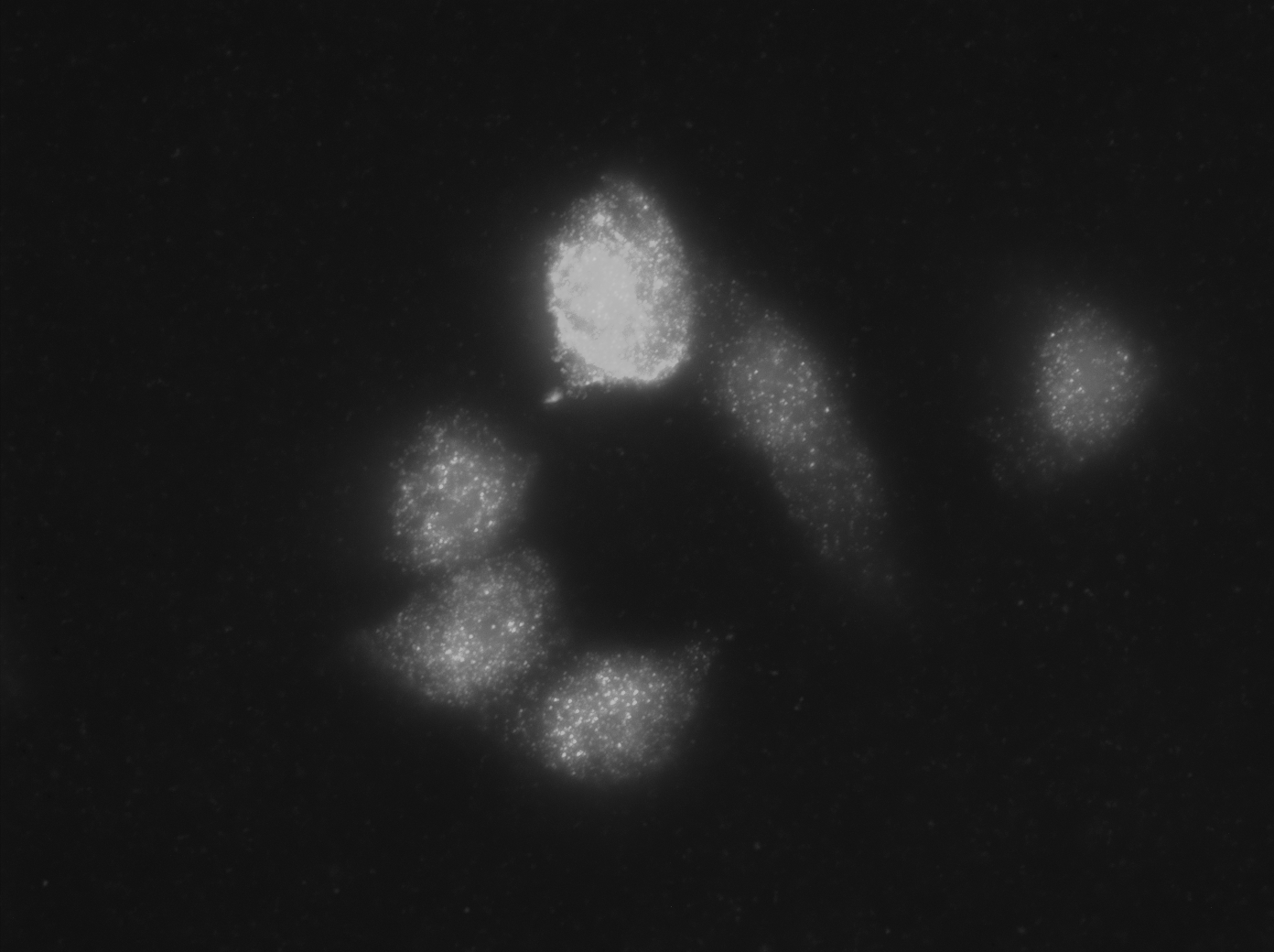

Supplement: Supplementary file 13 — Source data Fig. 3 part 3 [file 44319_2026_742_MOESM13_ESM.zip › Figure 3 Part 3/Fig 3b-e TTLL screen part 3/TTLL11-YFP Img 5 yfp2000.Project Maximum Z_XY1648573797_Z0_T0_C1.tif]

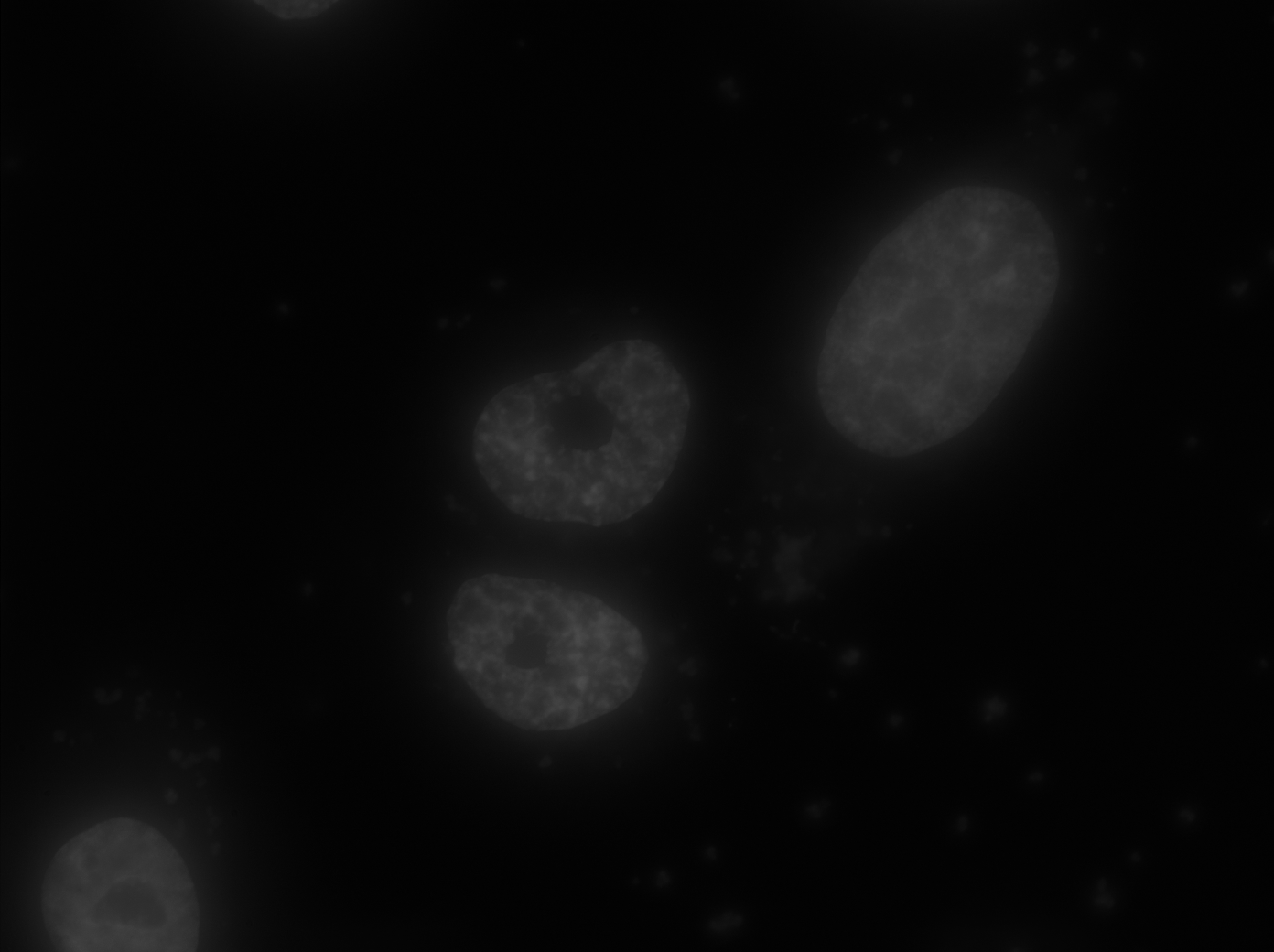

Supplement: Supplementary file 13 — Source data Fig. 3 part 3 [file 44319_2026_742_MOESM13_ESM.zip › Figure 3 Part 3/Fig 3b-e TTLL screen part 3/TTLL9-GFP A4 I6.Project Maximum Z_XY1675968056_Z0_T0_C0.tif]

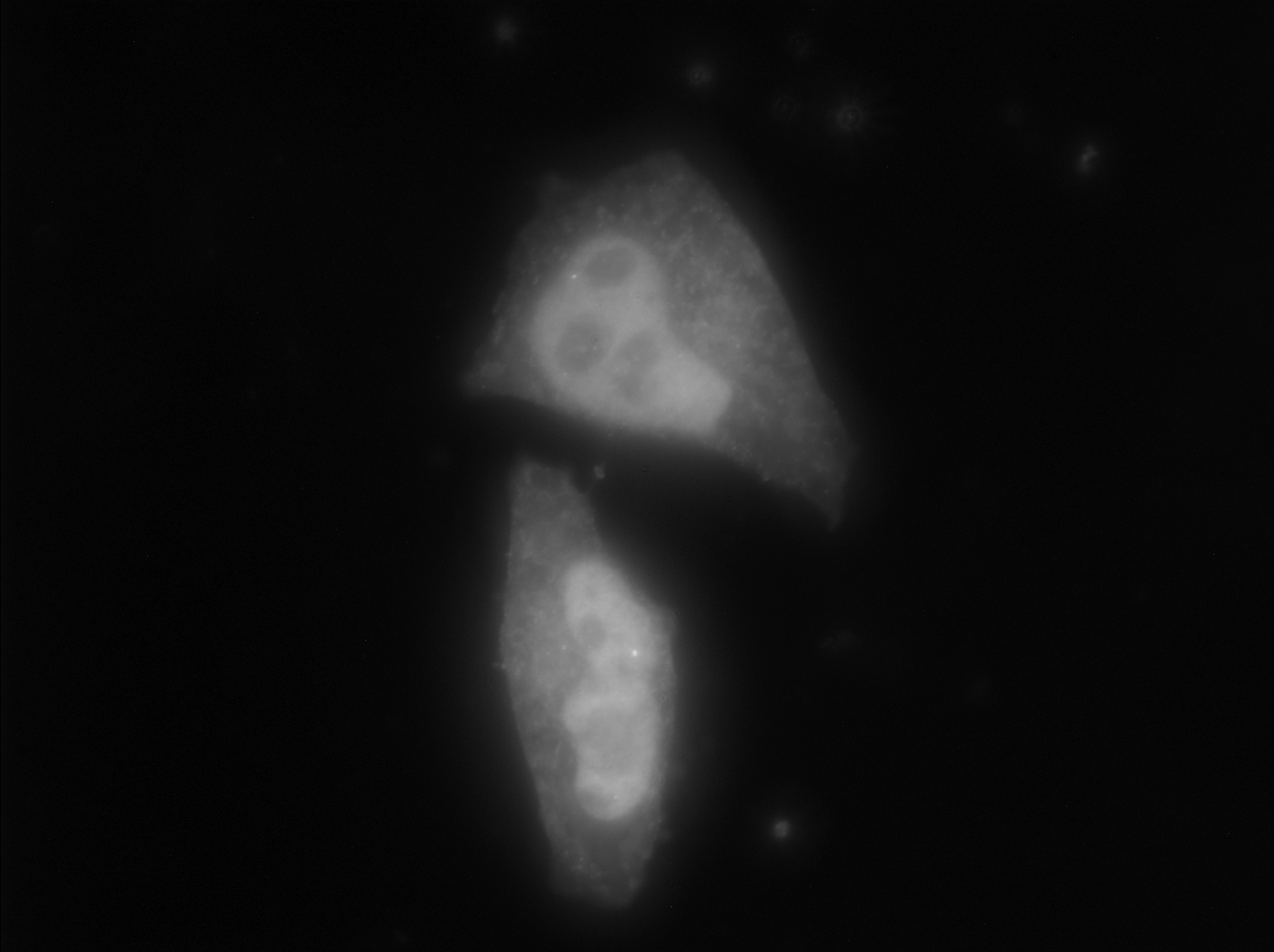

Supplement: Supplementary file 13 — Source data Fig. 3 part 3 [file 44319_2026_742_MOESM13_ESM.zip › Figure 3 Part 3/Fig 3b-e TTLL screen part 3/TTLL9-GFP A4 I1.Project Maximum Z_XY1675965339_Z0_T0_C2.tif]

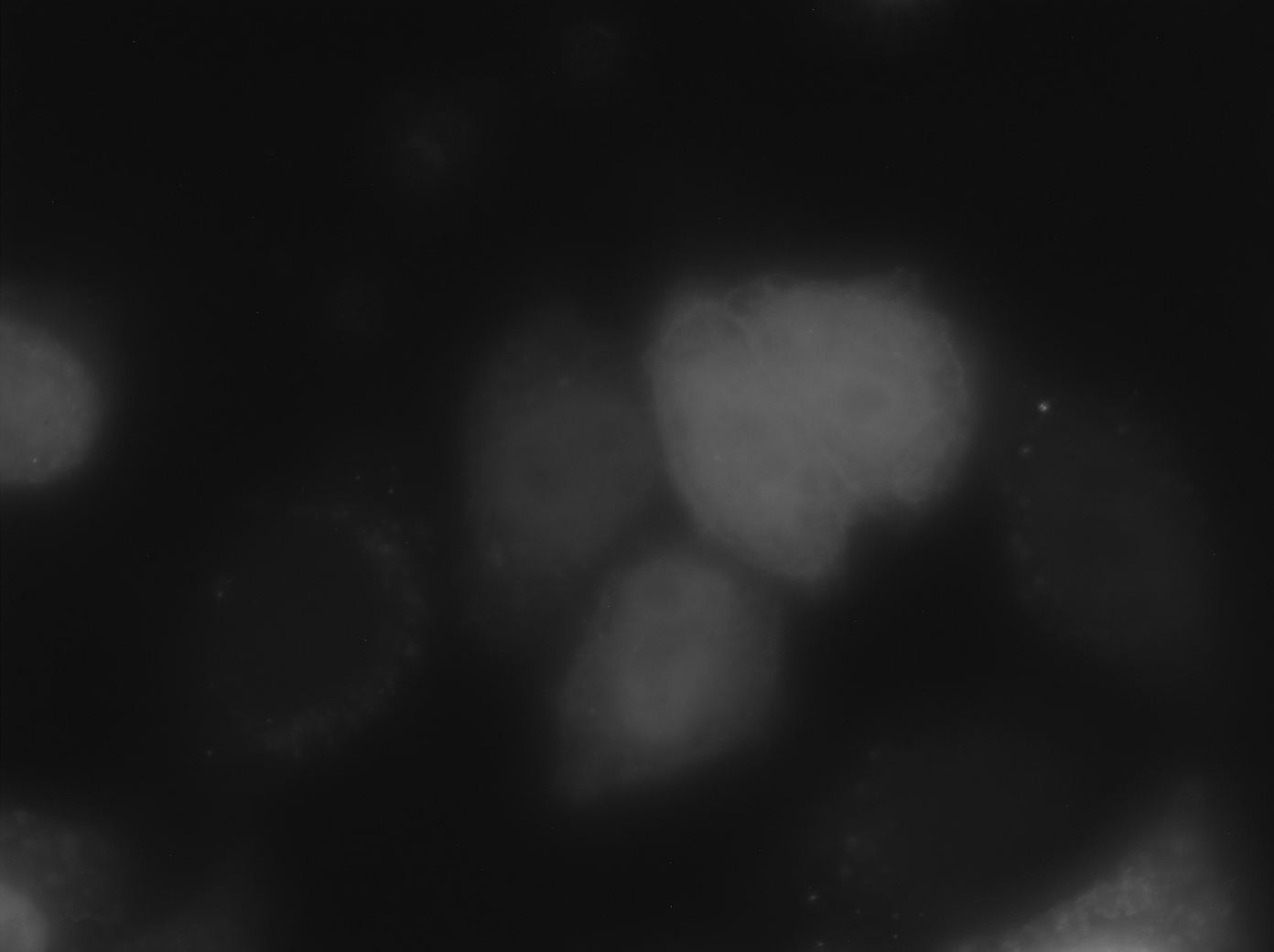

Supplement: Supplementary file 13 — Source data Fig. 3 part 3 [file 44319_2026_742_MOESM13_ESM.zip › Figure 3 Part 3/Fig 3b-e TTLL screen part 3/TTLL9-YFP A3 I7.Project Maximum Z_XY1679700241_Z0_T0_C2.tif]

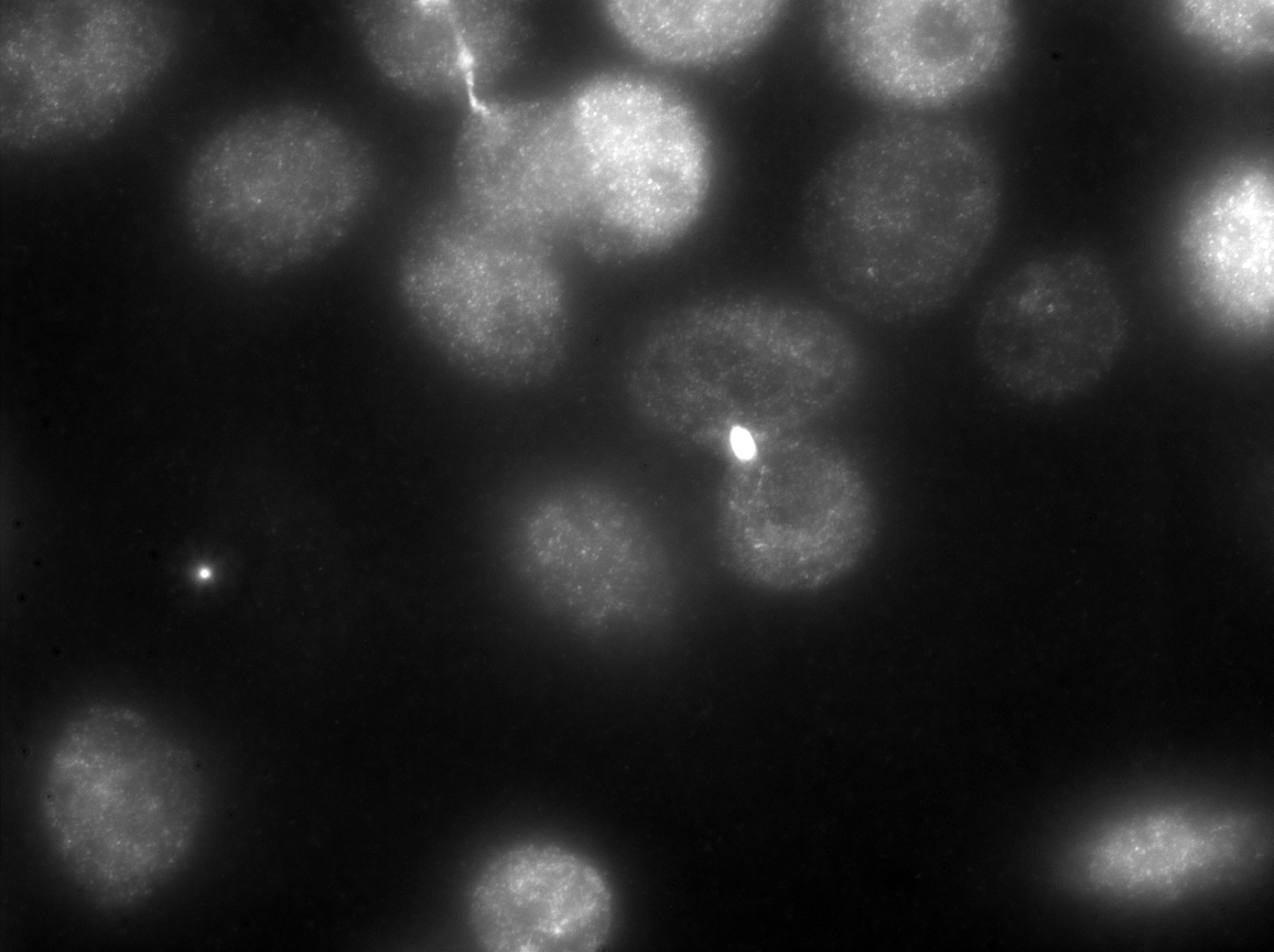

Supplement: Supplementary file 13 — Source data Fig. 3 part 3 [file 44319_2026_742_MOESM13_ESM.zip › Figure 3 Part 3/Fig 3b-e TTLL screen part 3/TTLL9-YFP A3 I13.Project Maximum Z_XY1679701070_Z0_T0_C1.tif]

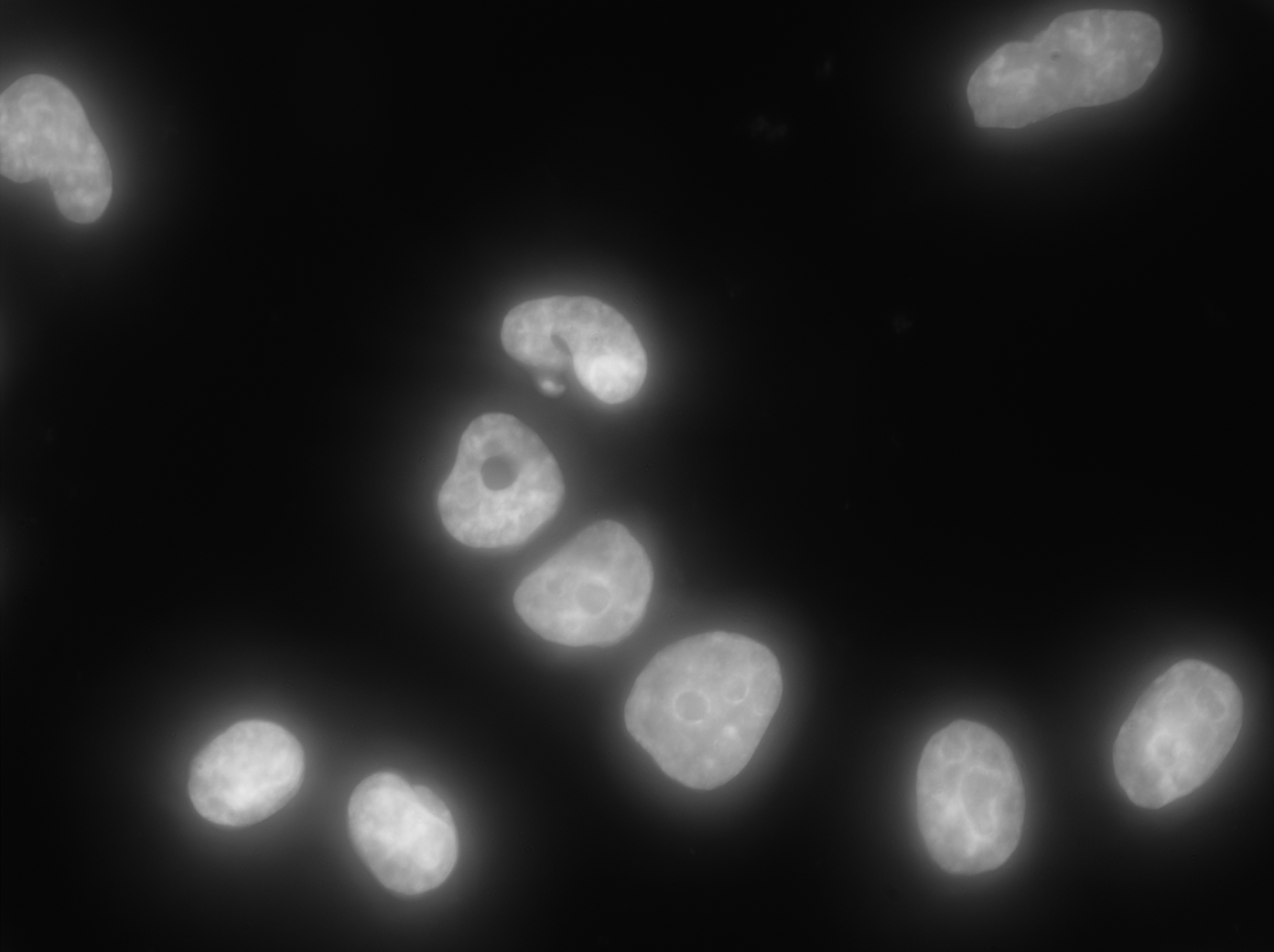

Supplement: Supplementary file 13 — Source data Fig. 3 part 3 [file 44319_2026_742_MOESM13_ESM.zip › Figure 3 Part 3/Fig 3b-e TTLL screen part 3/TTLL11-YFP Img 6 yfp2000 - 1.Project Maximum Z_XY1648574277_Z0_T0_C0.tif]

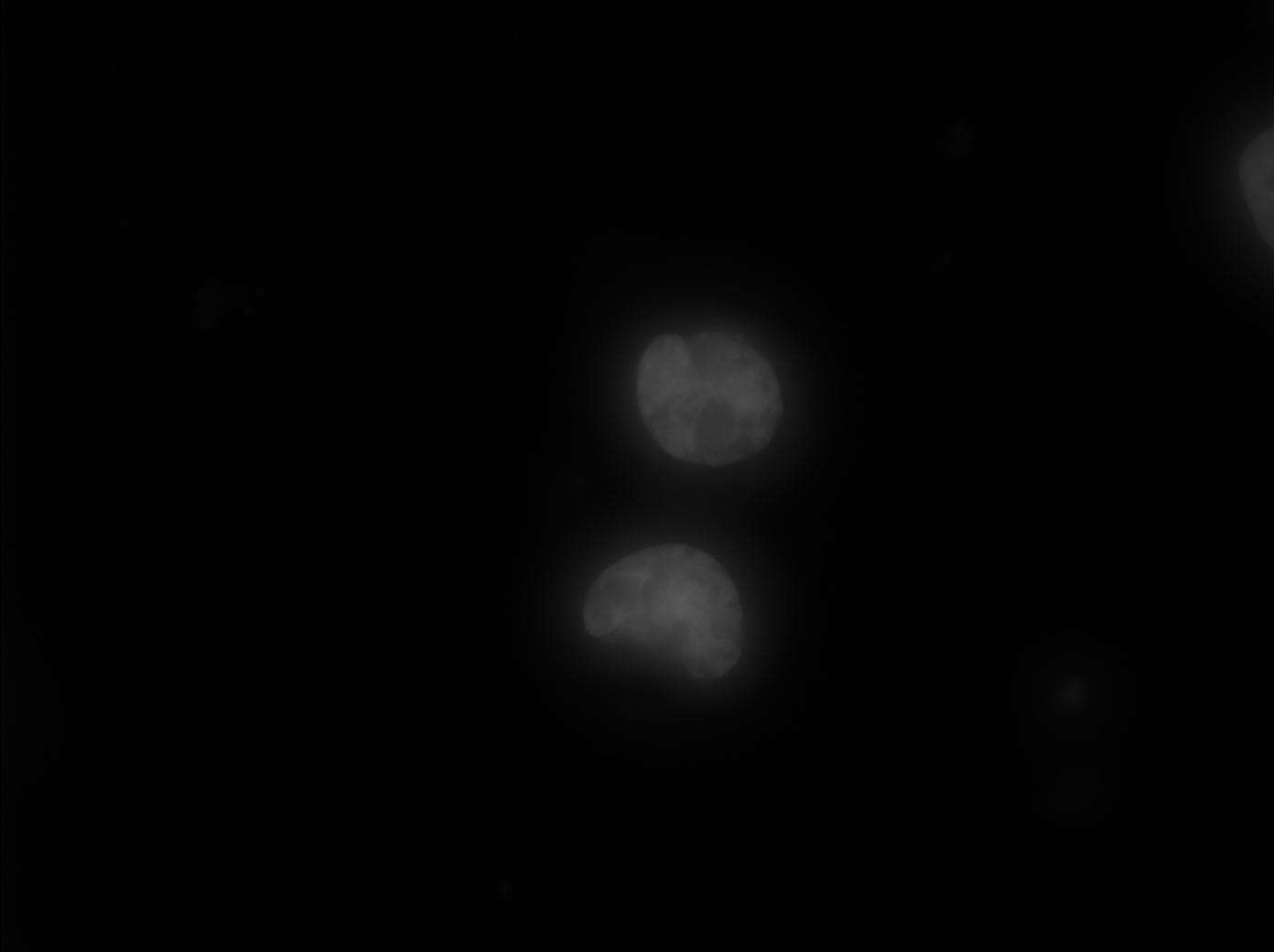

Supplement: Supplementary file 13 — Source data Fig. 3 part 3 [file 44319_2026_742_MOESM13_ESM.zip › Figure 3 Part 3/Fig 3b-e TTLL screen part 3/TTLL9-YFP A3 I3.Project Maximum Z_XY1674674979_Z0_T0_C0.tif]

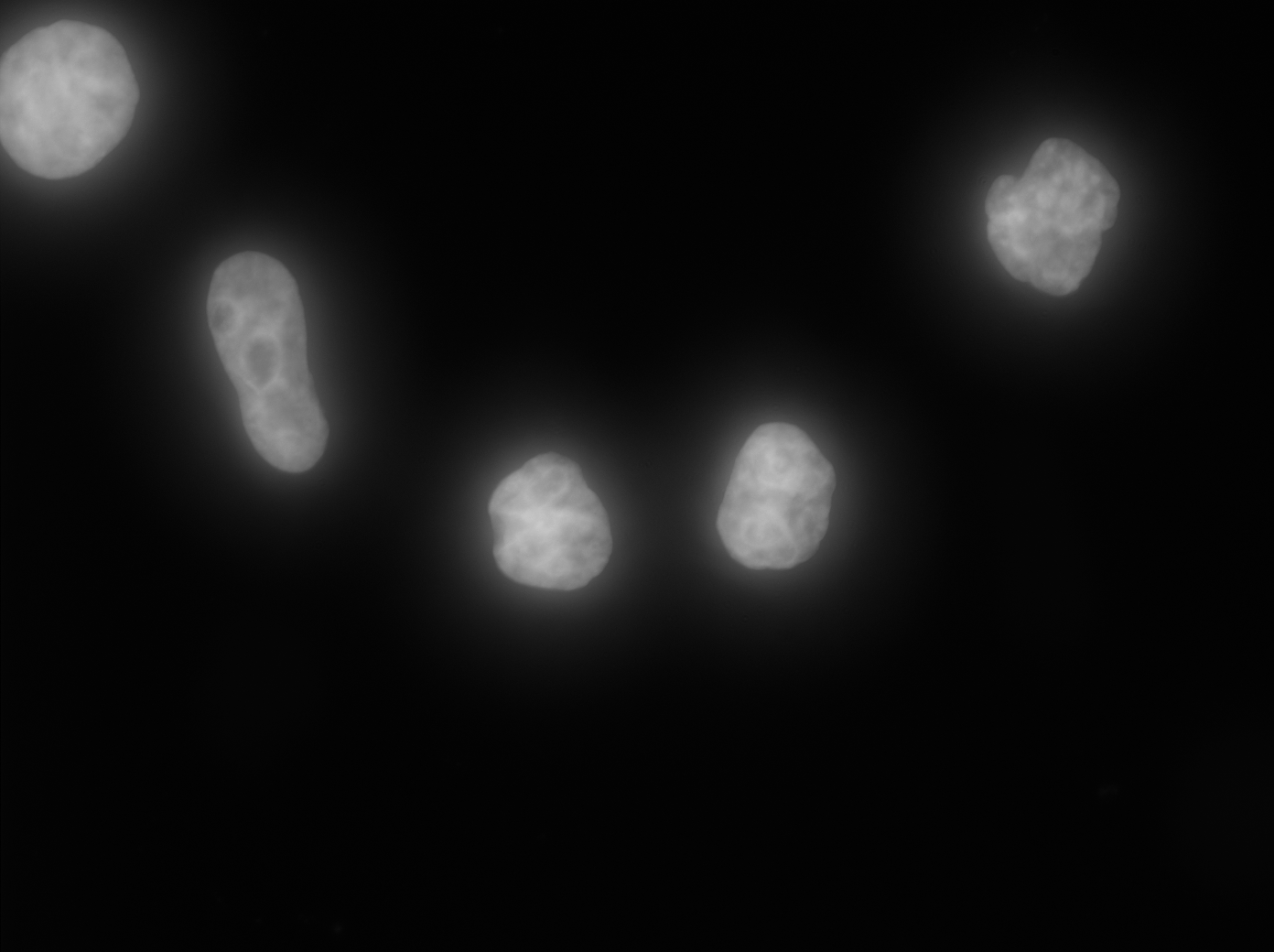

Supplement: Supplementary file 13 — Source data Fig. 3 part 3 [file 44319_2026_742_MOESM13_ESM.zip › Figure 3 Part 3/Fig 3b-e TTLL screen part 3/TTLL11-YFP A2 Img4.Project Maximum Z_XY1648752676_Z0_T0_C0.tif]

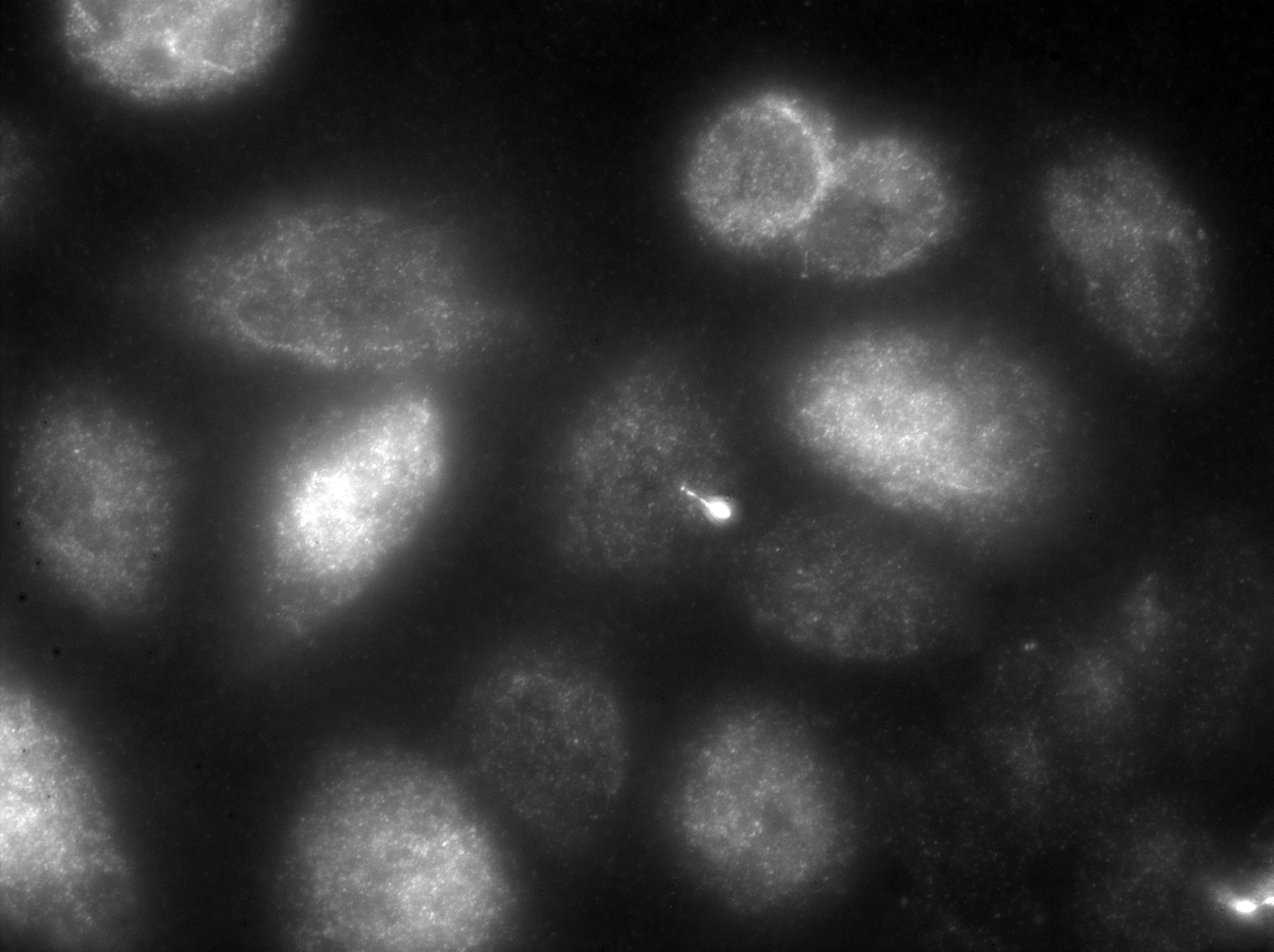

Supplement: Supplementary file 13 — Source data Fig. 3 part 3 [file 44319_2026_742_MOESM13_ESM.zip › Figure 3 Part 3/Fig 3b-e TTLL screen part 3/TTLL9-YFP A3 I4.Project Maximum Z_XY1679699772_Z0_T0_C1.tif]

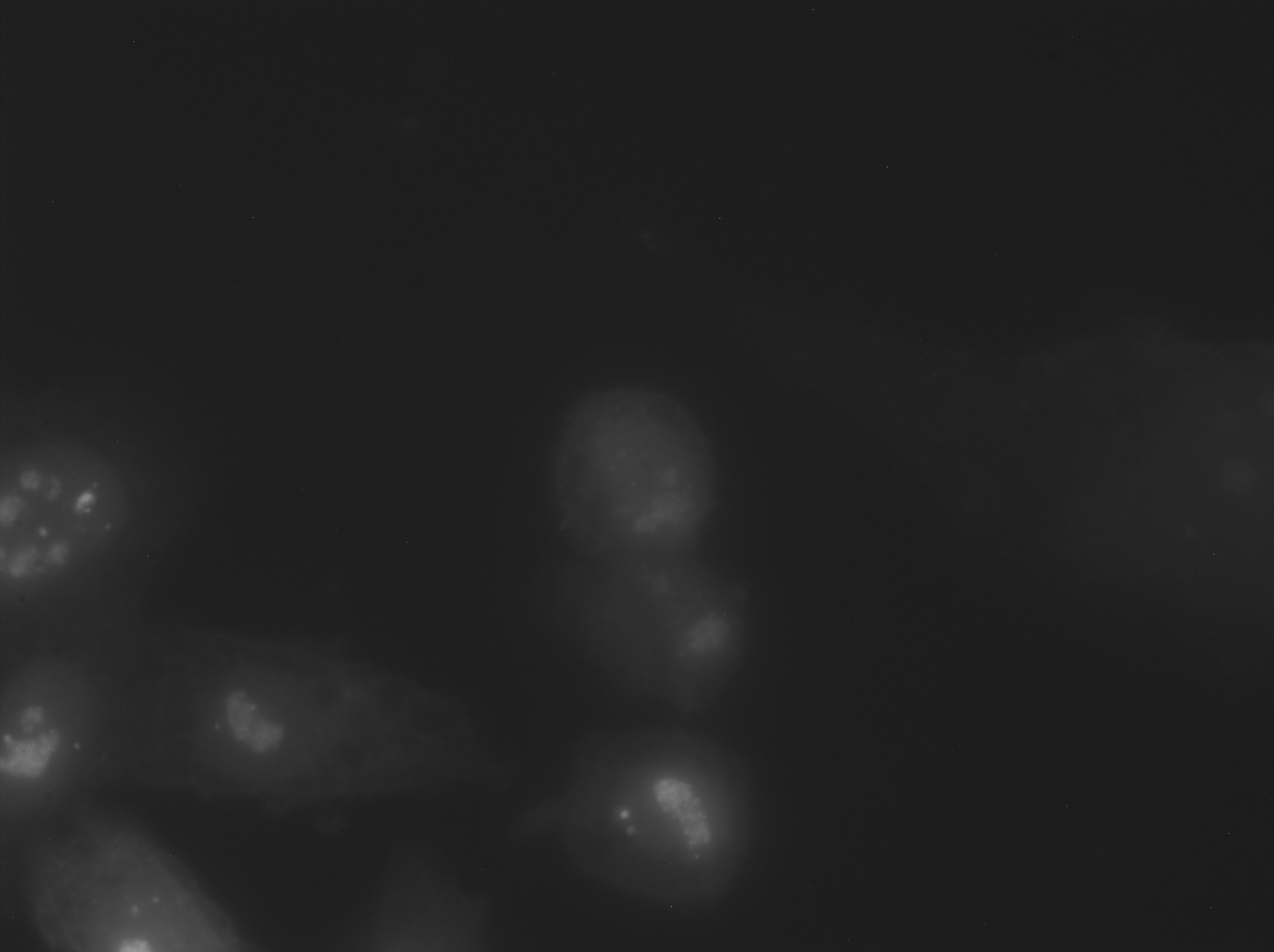

Supplement: Supplementary file 13 — Source data Fig. 3 part 3 [file 44319_2026_742_MOESM13_ESM.zip › Figure 3 Part 3/Fig 3b-e TTLL screen part 3/TTLL11-YFP Img 10 yfp2000.Project Maximum Z_XY1648579577_Z0_T0_C2.tif]

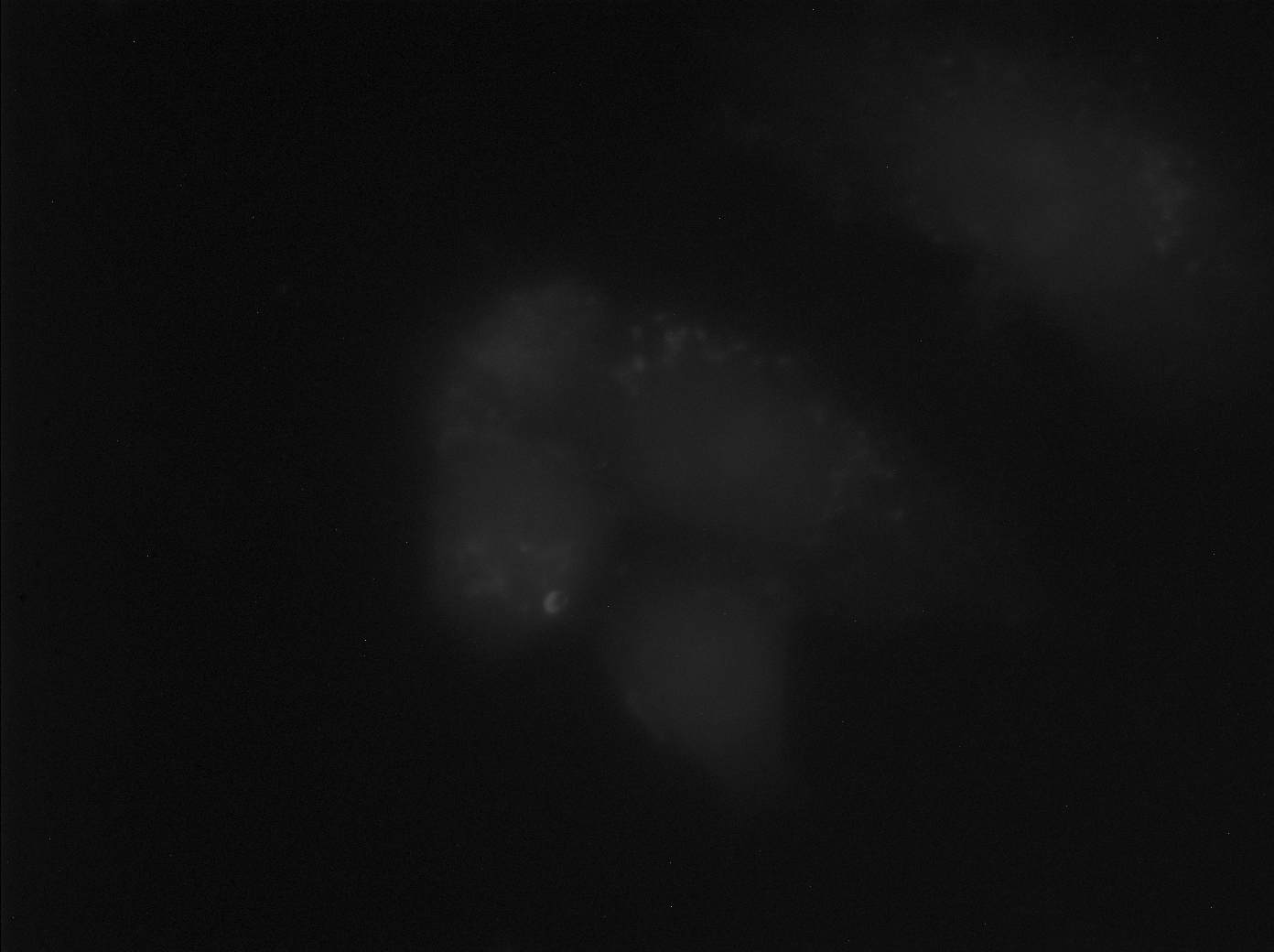

Supplement: Supplementary file 13 — Source data Fig. 3 part 3 [file 44319_2026_742_MOESM13_ESM.zip › Figure 3 Part 3/Fig 3b-e TTLL screen part 3/TTLL9-YFP R1 I4.Project Maximum Z_XY1674166819_Z0_T0_C2.tif]

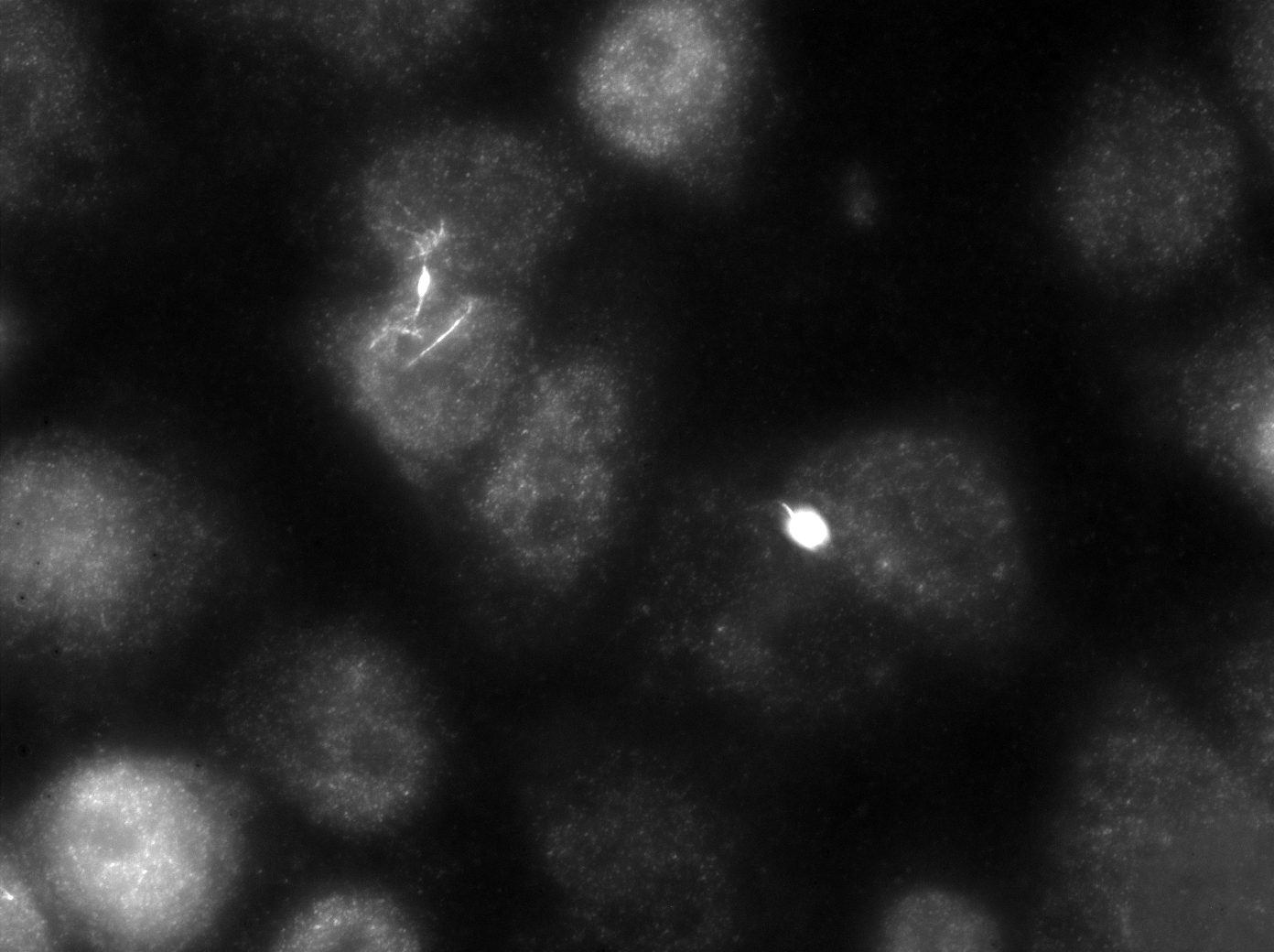

Supplement: Supplementary file 13 — Source data Fig. 3 part 3 [file 44319_2026_742_MOESM13_ESM.zip › Figure 3 Part 3/Fig 3b-e TTLL screen part 3/TTLL9-YFP A3 I18.Project Maximum Z_XY1679701787_Z0_T0_C1.tif]

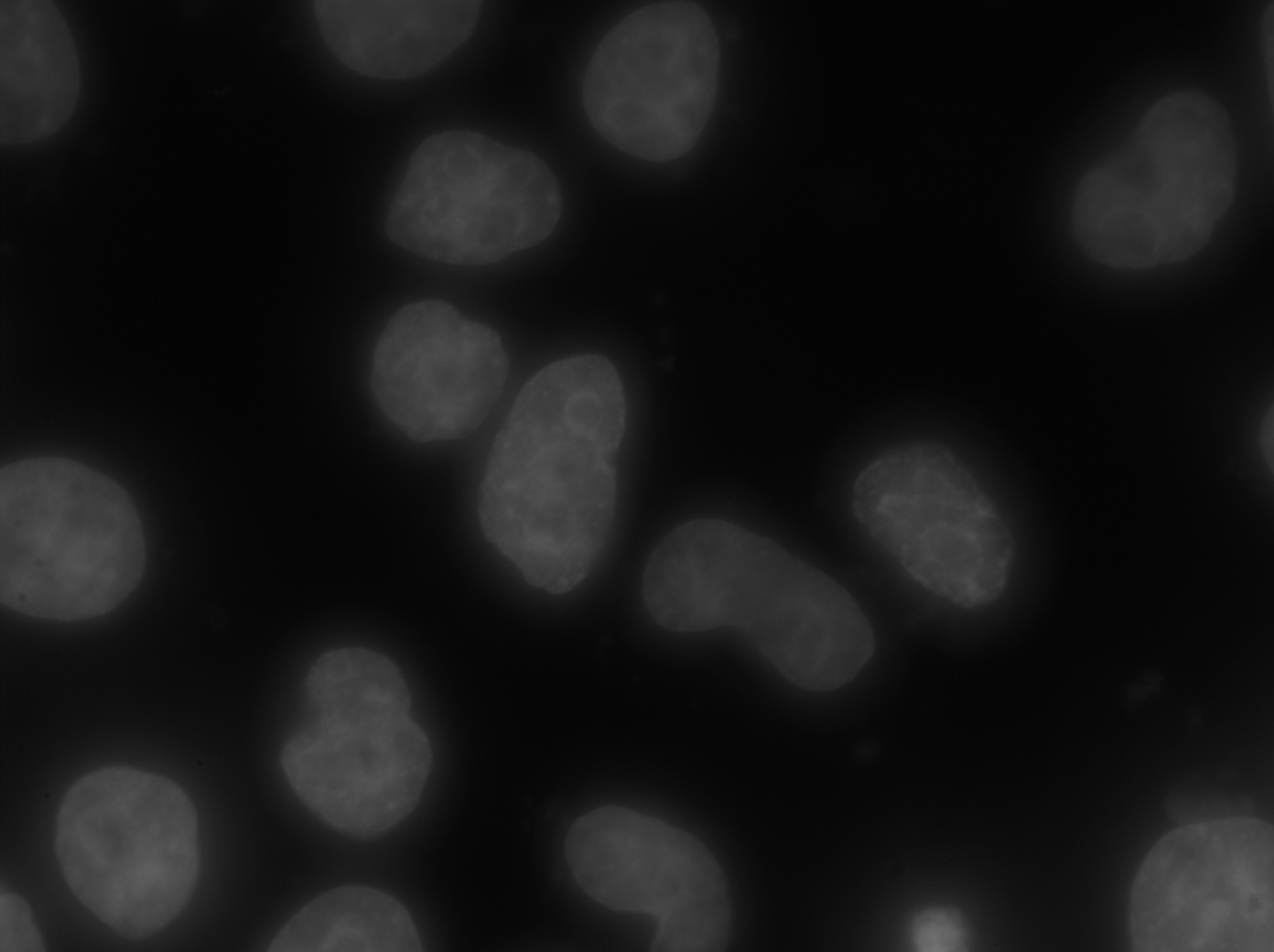

Supplement: Supplementary file 13 — Source data Fig. 3 part 3 [file 44319_2026_742_MOESM13_ESM.zip › Figure 3 Part 3/Fig 3b-e TTLL screen part 3/TTLL9-YFP A3 I18.Project Maximum Z_XY1679701787_Z0_T0_C0.tif]

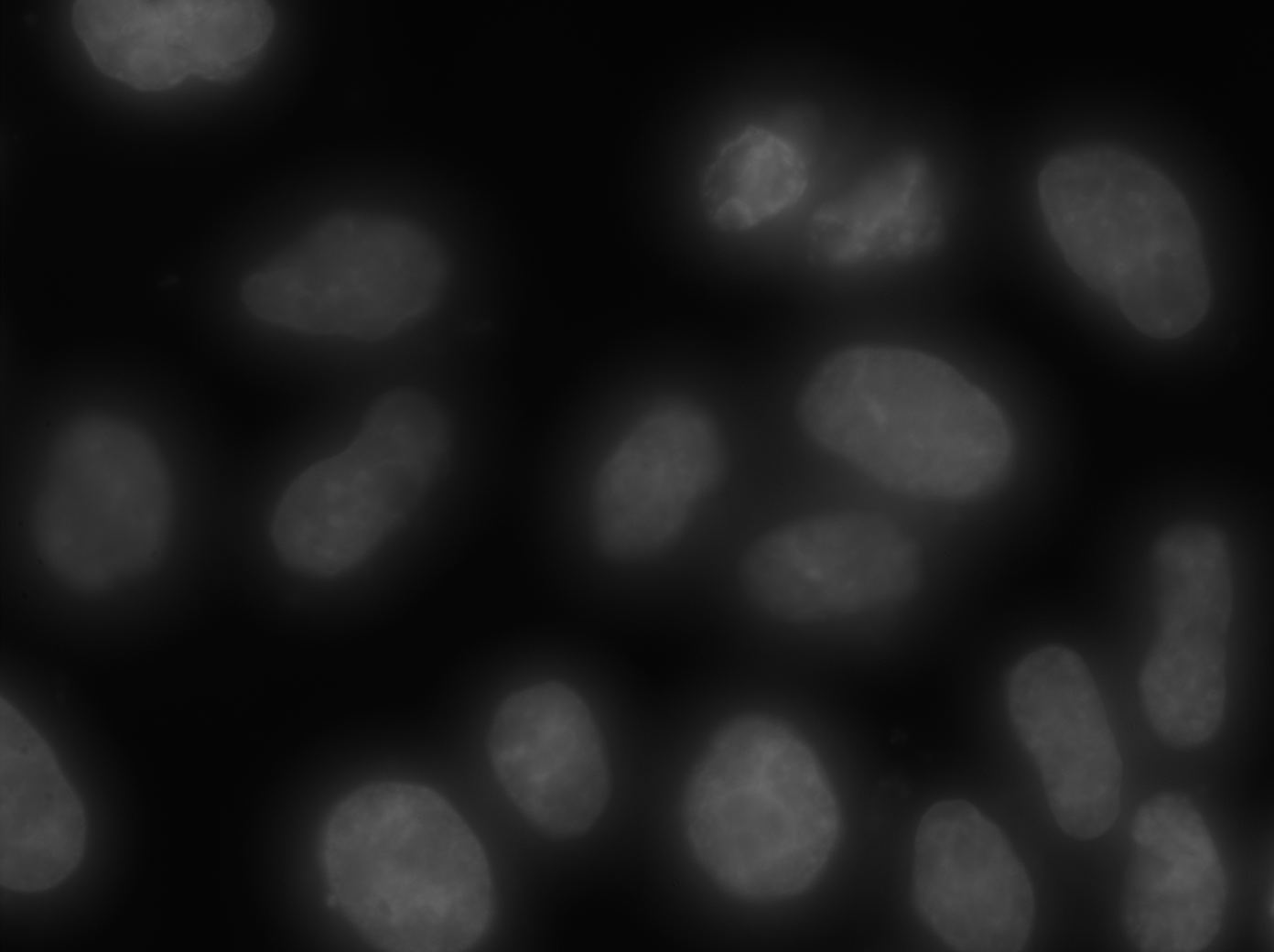

Supplement: Supplementary file 13 — Source data Fig. 3 part 3 [file 44319_2026_742_MOESM13_ESM.zip › Figure 3 Part 3/Fig 3b-e TTLL screen part 3/TTLL9-YFP A3 I4.Project Maximum Z_XY1679699772_Z0_T0_C0.tif]

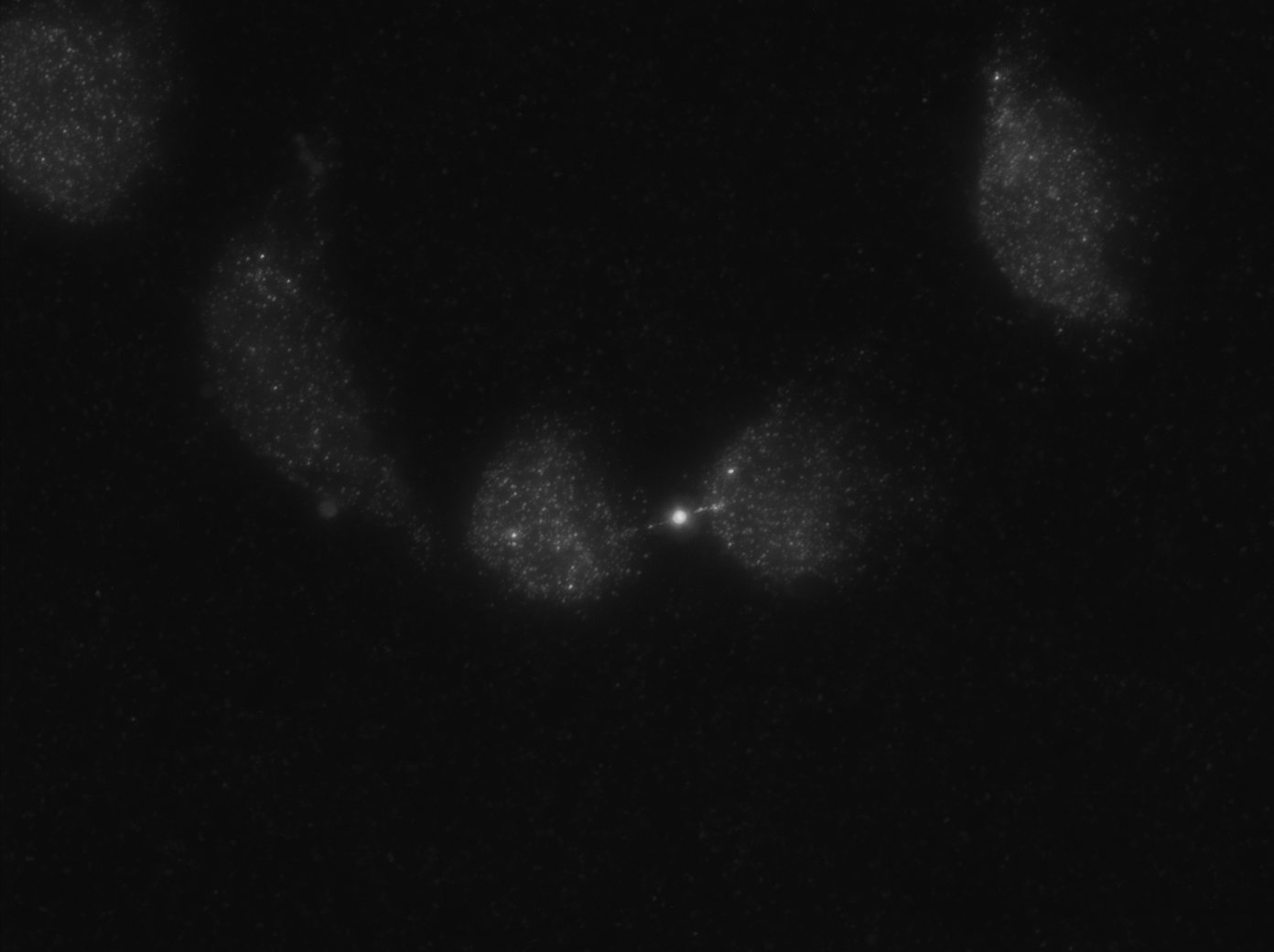

Supplement: Supplementary file 13 — Source data Fig. 3 part 3 [file 44319_2026_742_MOESM13_ESM.zip › Figure 3 Part 3/Fig 3b-e TTLL screen part 3/TTLL11-YFP A2 Img4.Project Maximum Z_XY1648752676_Z0_T0_C1.tif]

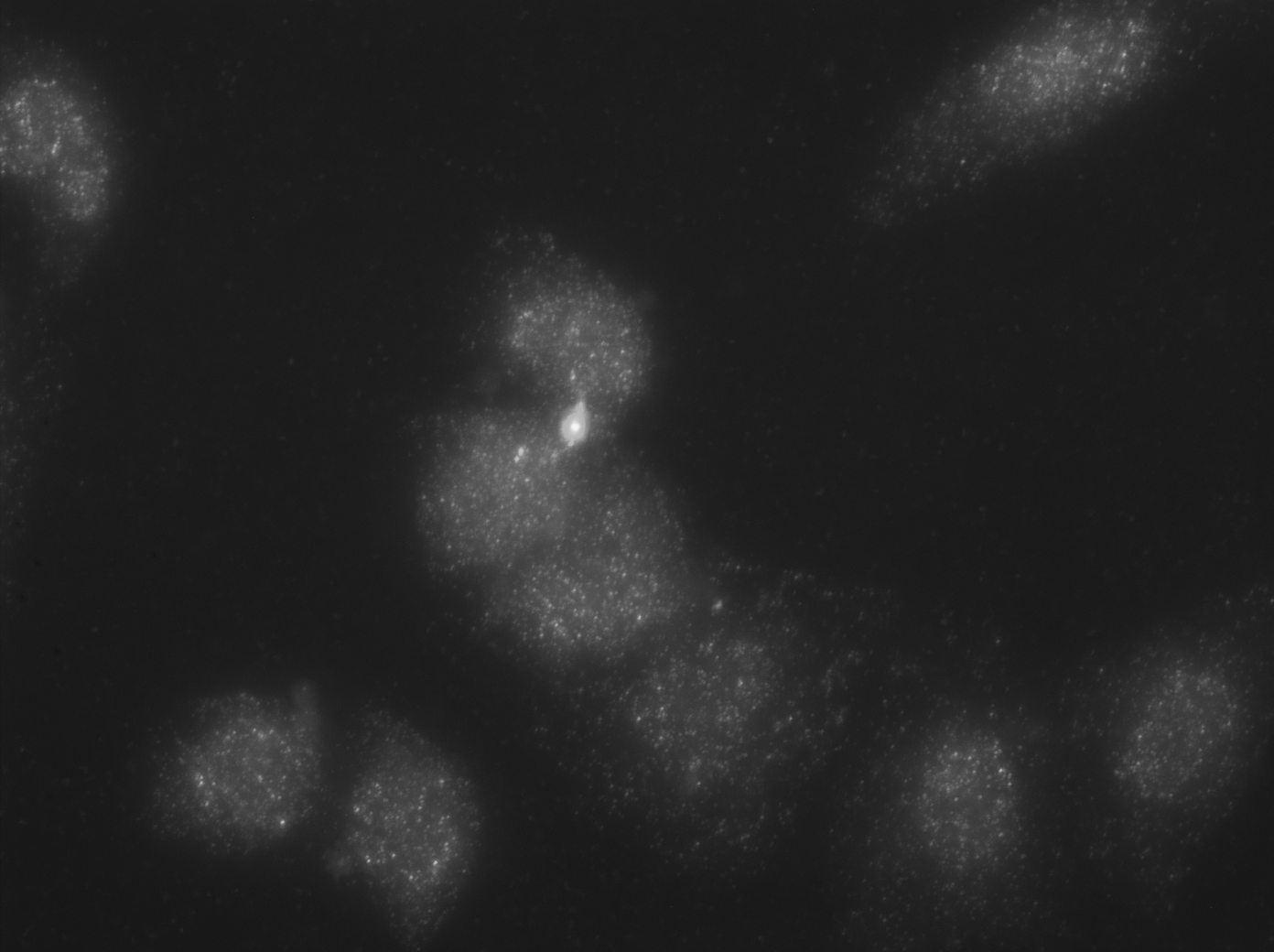

Supplement: Supplementary file 13 — Source data Fig. 3 part 3 [file 44319_2026_742_MOESM13_ESM.zip › Figure 3 Part 3/Fig 3b-e TTLL screen part 3/TTLL11-YFP Img 6 yfp2000 - 1.Project Maximum Z_XY1648574277_Z0_T0_C1.tif]

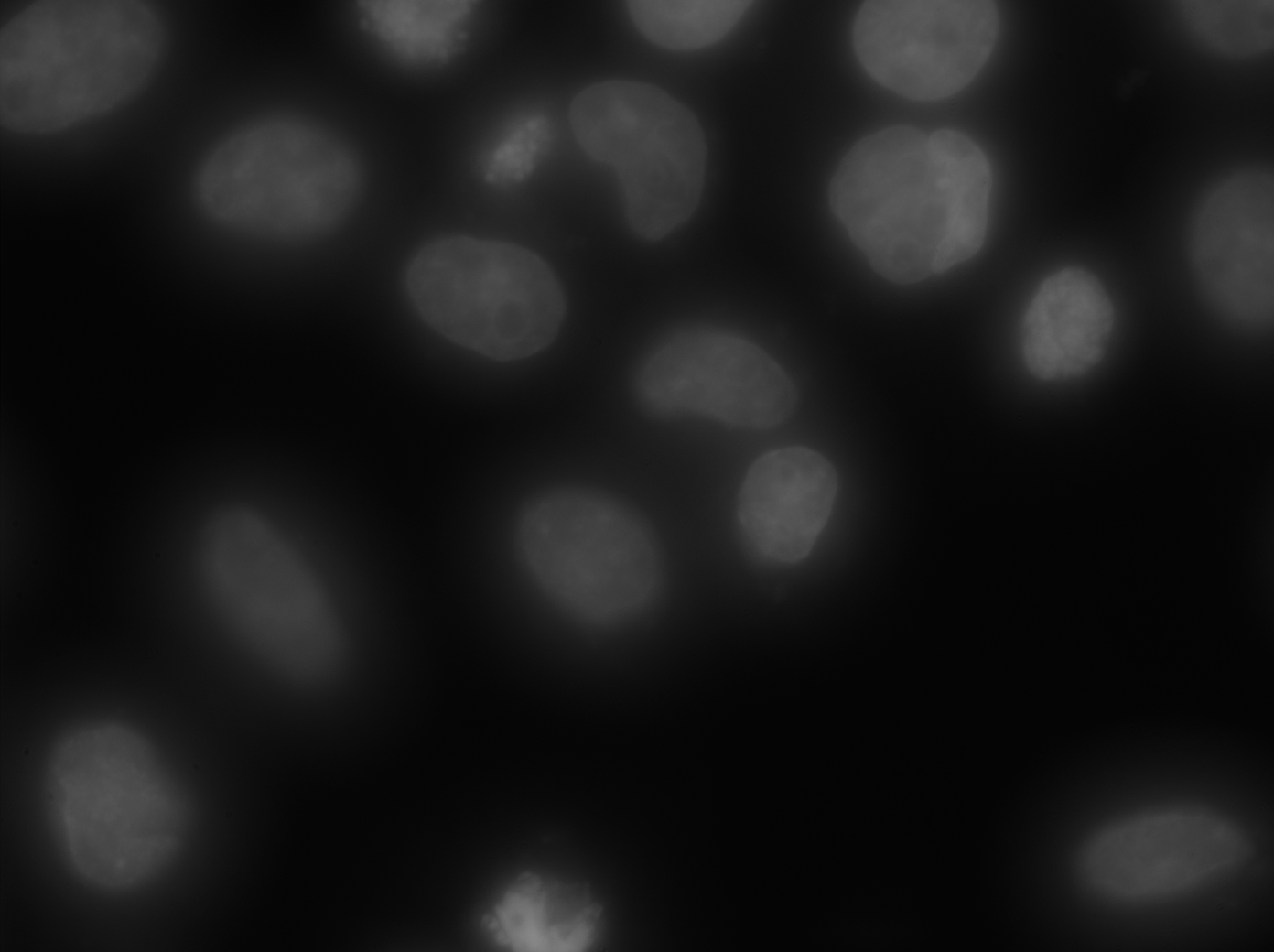

Supplement: Supplementary file 13 — Source data Fig. 3 part 3 [file 44319_2026_742_MOESM13_ESM.zip › Figure 3 Part 3/Fig 3b-e TTLL screen part 3/TTLL9-YFP A3 I13.Project Maximum Z_XY1679701070_Z0_T0_C0.tif]

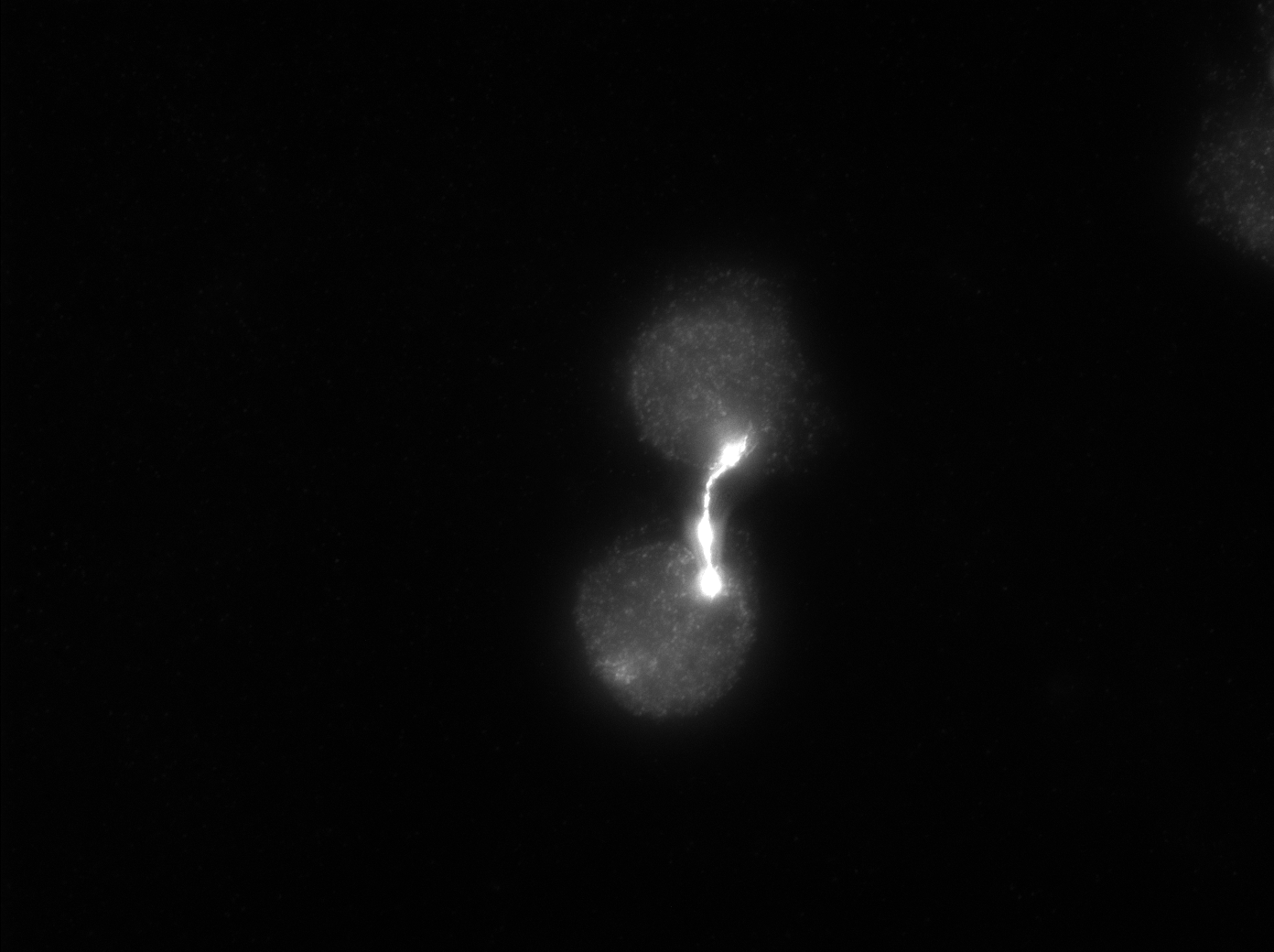

Supplement: Supplementary file 13 — Source data Fig. 3 part 3 [file 44319_2026_742_MOESM13_ESM.zip › Figure 3 Part 3/Fig 3b-e TTLL screen part 3/TTLL9-YFP A3 I3.Project Maximum Z_XY1674674979_Z0_T0_C1.tif]

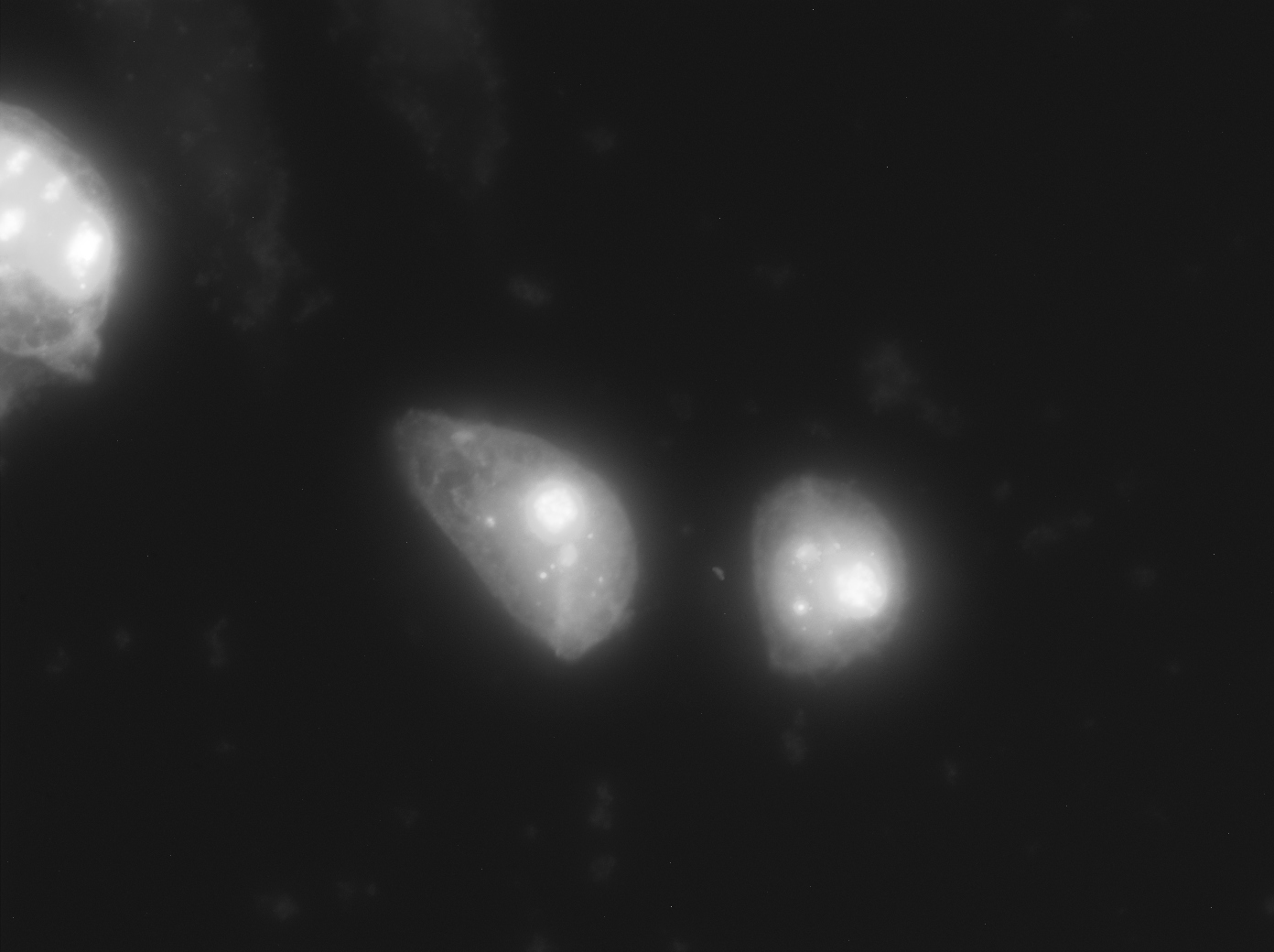

Supplement: Supplementary file 13 — Source data Fig. 3 part 3 [file 44319_2026_742_MOESM13_ESM.zip › Figure 3 Part 3/Fig 3b-e TTLL screen part 3/TTLL11-YFP Img 9 yfp2000 - 1.Project Maximum Z_XY1648578368_Z0_T0_C2.tif]

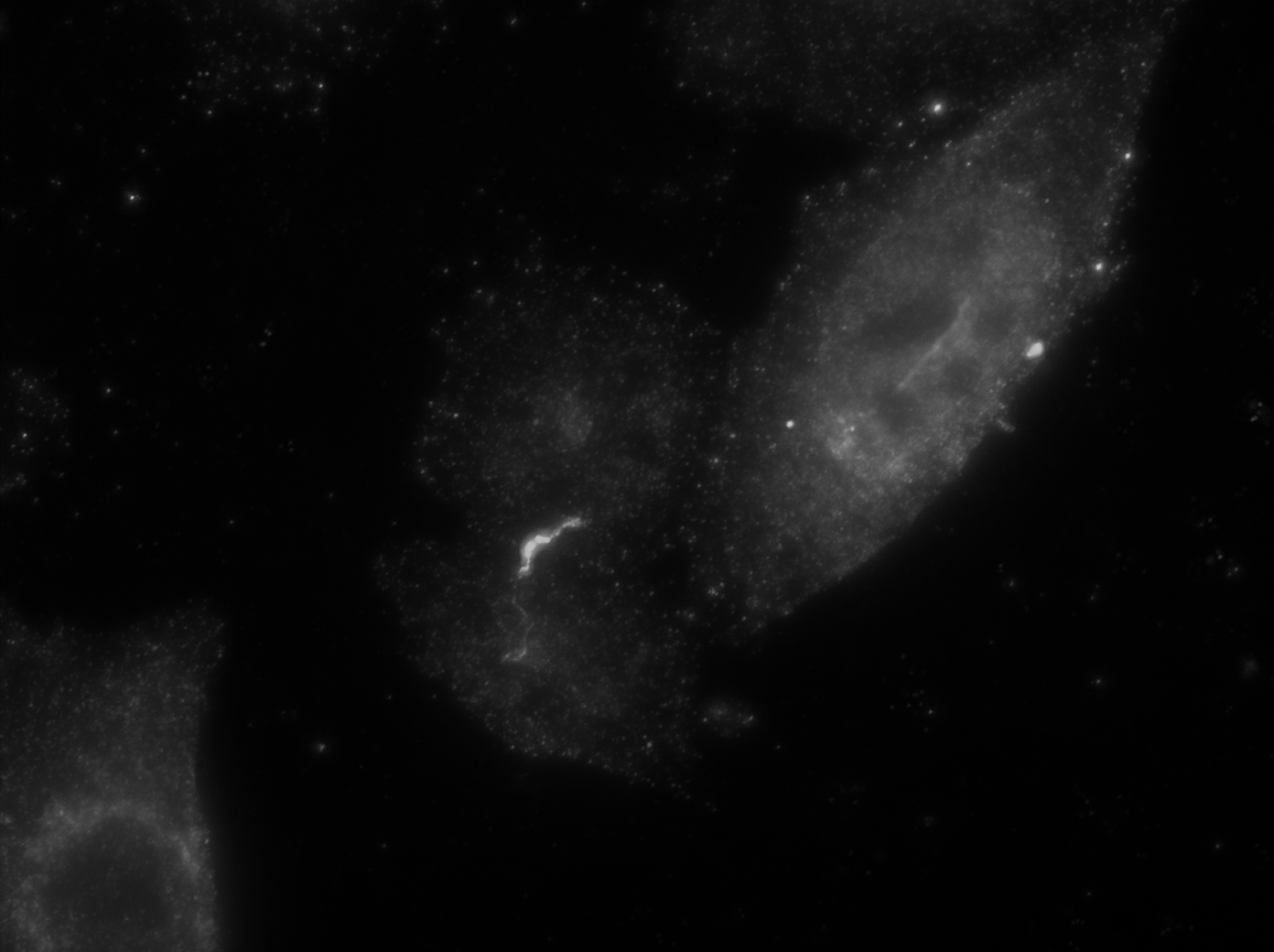

Supplement: Supplementary file 13 — Source data Fig. 3 part 3 [file 44319_2026_742_MOESM13_ESM.zip › Figure 3 Part 3/Fig 3b-e TTLL screen part 3/TTLL9-GFP A4 I6.Project Maximum Z_XY1675968056_Z0_T0_C1.tif]

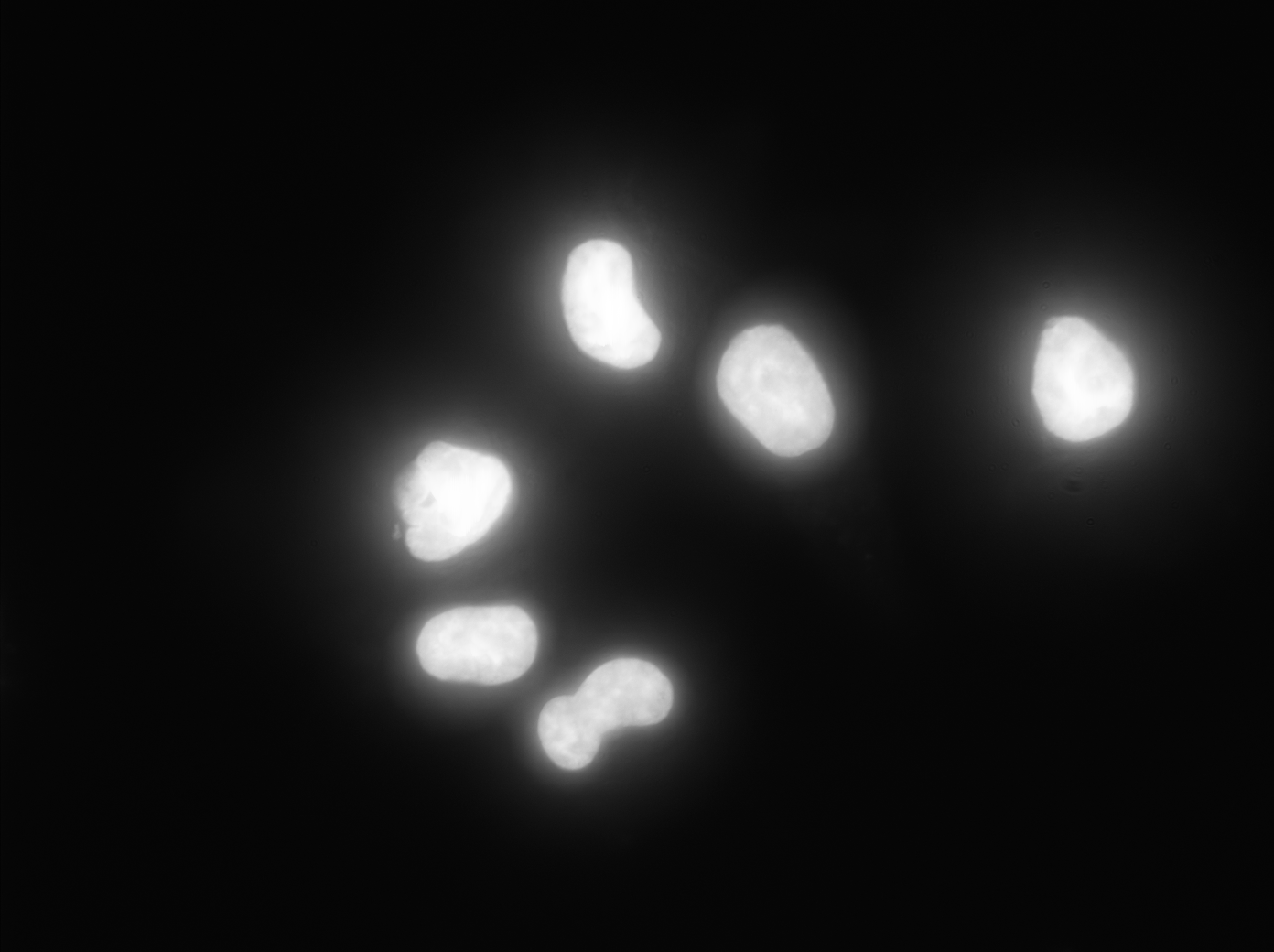

Supplement: Supplementary file 13 — Source data Fig. 3 part 3 [file 44319_2026_742_MOESM13_ESM.zip › Figure 3 Part 3/Fig 3b-e TTLL screen part 3/TTLL11-YFP Img 5 yfp2000.Project Maximum Z_XY1648573797_Z0_T0_C0.tif]

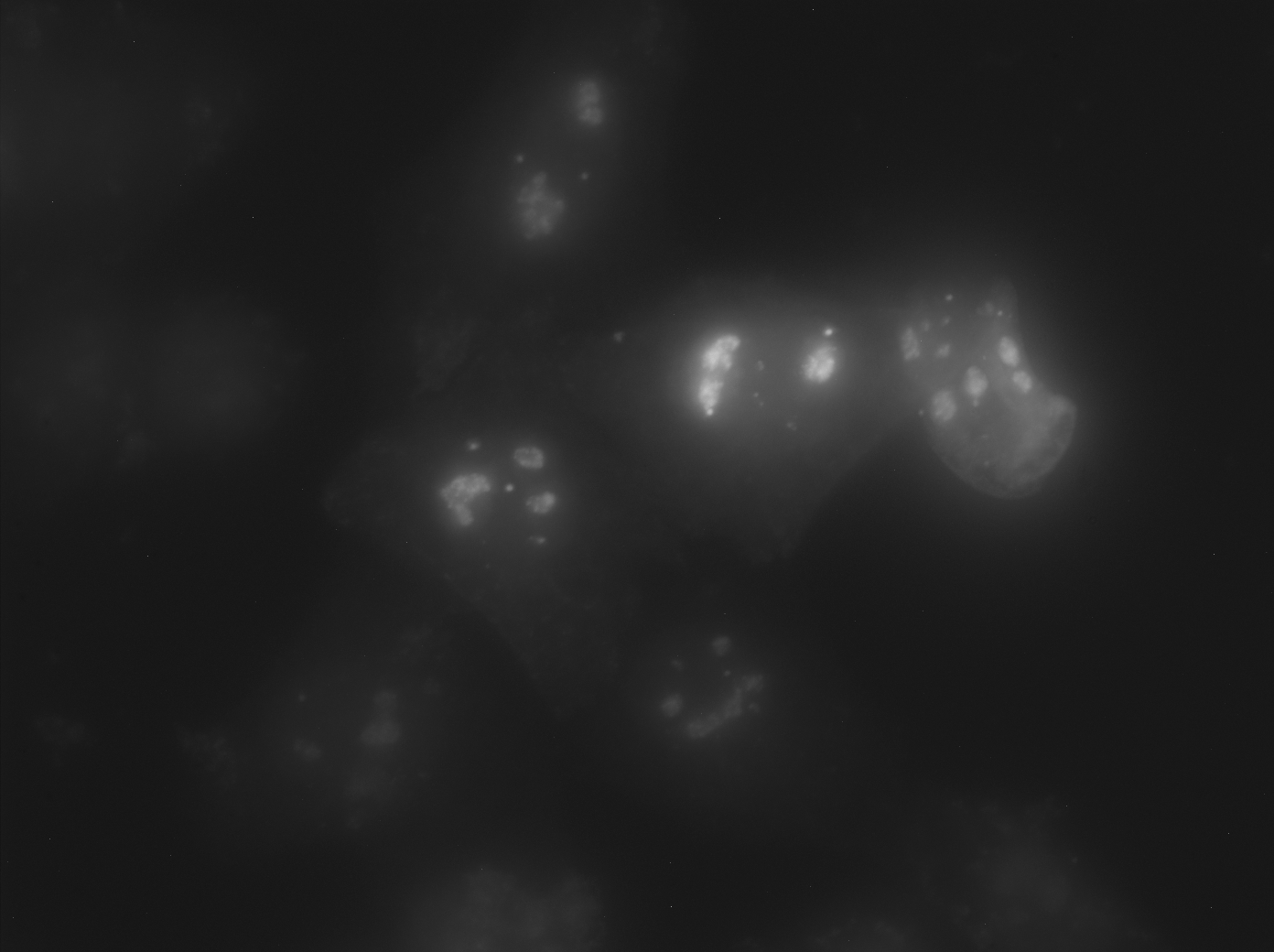

Supplement: Supplementary file 13 — Source data Fig. 3 part 3 [file 44319_2026_742_MOESM13_ESM.zip › Figure 3 Part 3/Fig 3b-e TTLL screen part 3/TTLL11-YFP Img 11 yfp2000.Project Maximum Z_XY1648579897_Z0_T0_C2.tif]

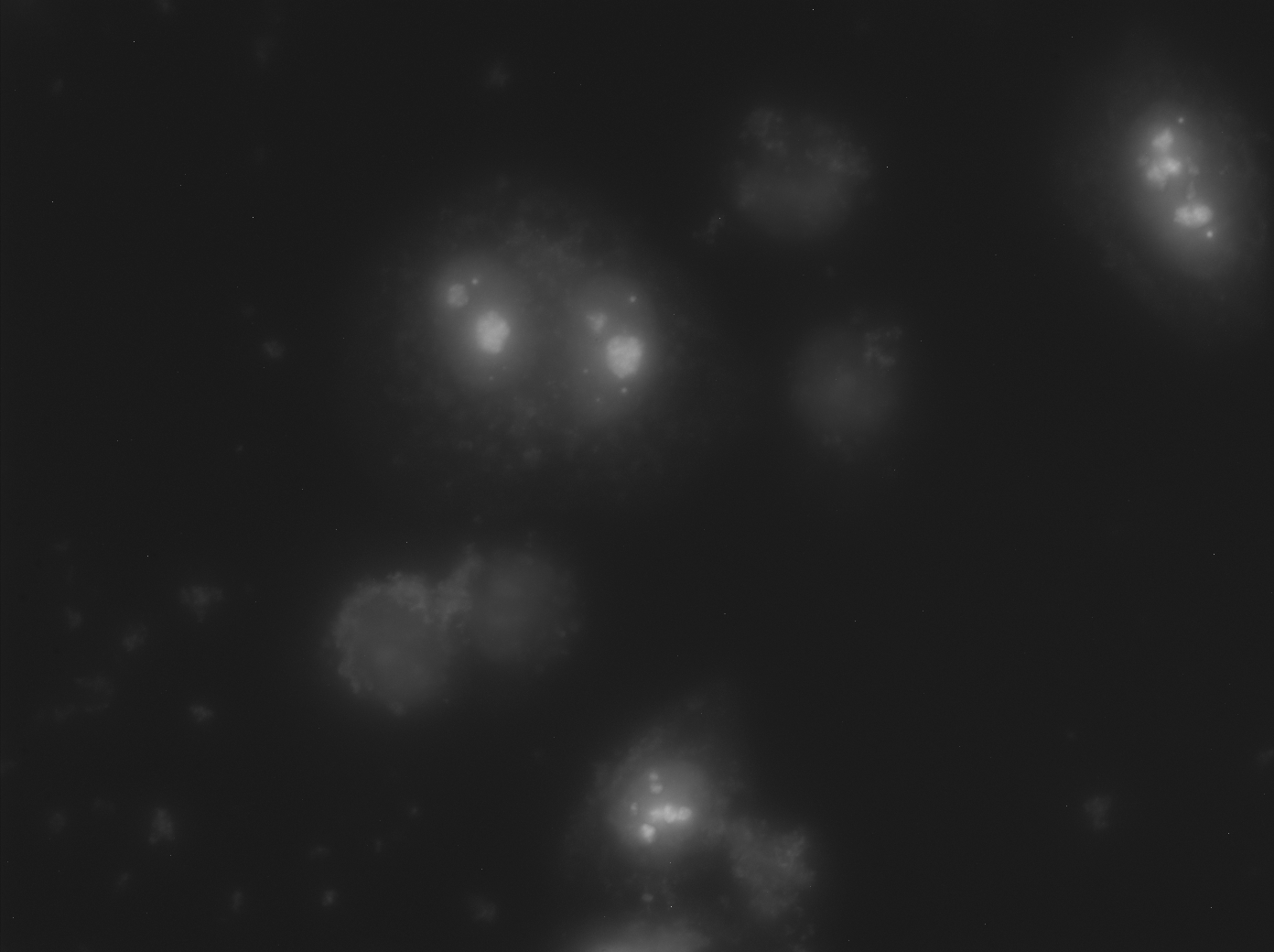

Supplement: Supplementary file 13 — Source data Fig. 3 part 3 [file 44319_2026_742_MOESM13_ESM.zip › Figure 3 Part 3/Fig 3b-e TTLL screen part 3/TTLL11-YFP Img 12 yfp2500 - 1.Project Maximum Z_XY1648580285_Z0_T0_C2.tif]

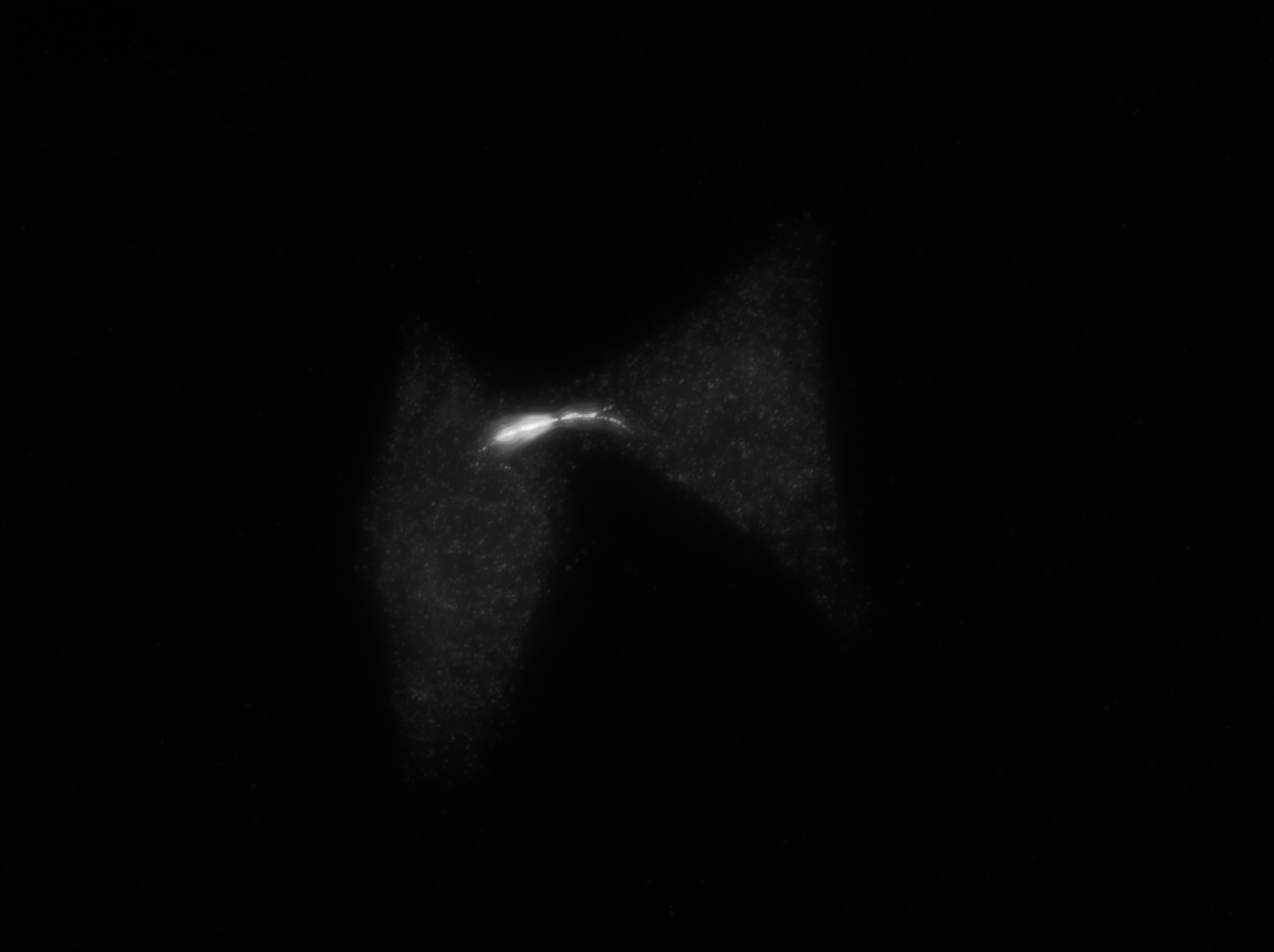

Supplement: Supplementary file 13 — Source data Fig. 3 part 3 [file 44319_2026_742_MOESM13_ESM.zip › Figure 3 Part 3/Fig 3b-e TTLL screen part 3/YFP Only R1 I7.Project Maximum Z_XY1663272885_Z0_T0_C1.tif]

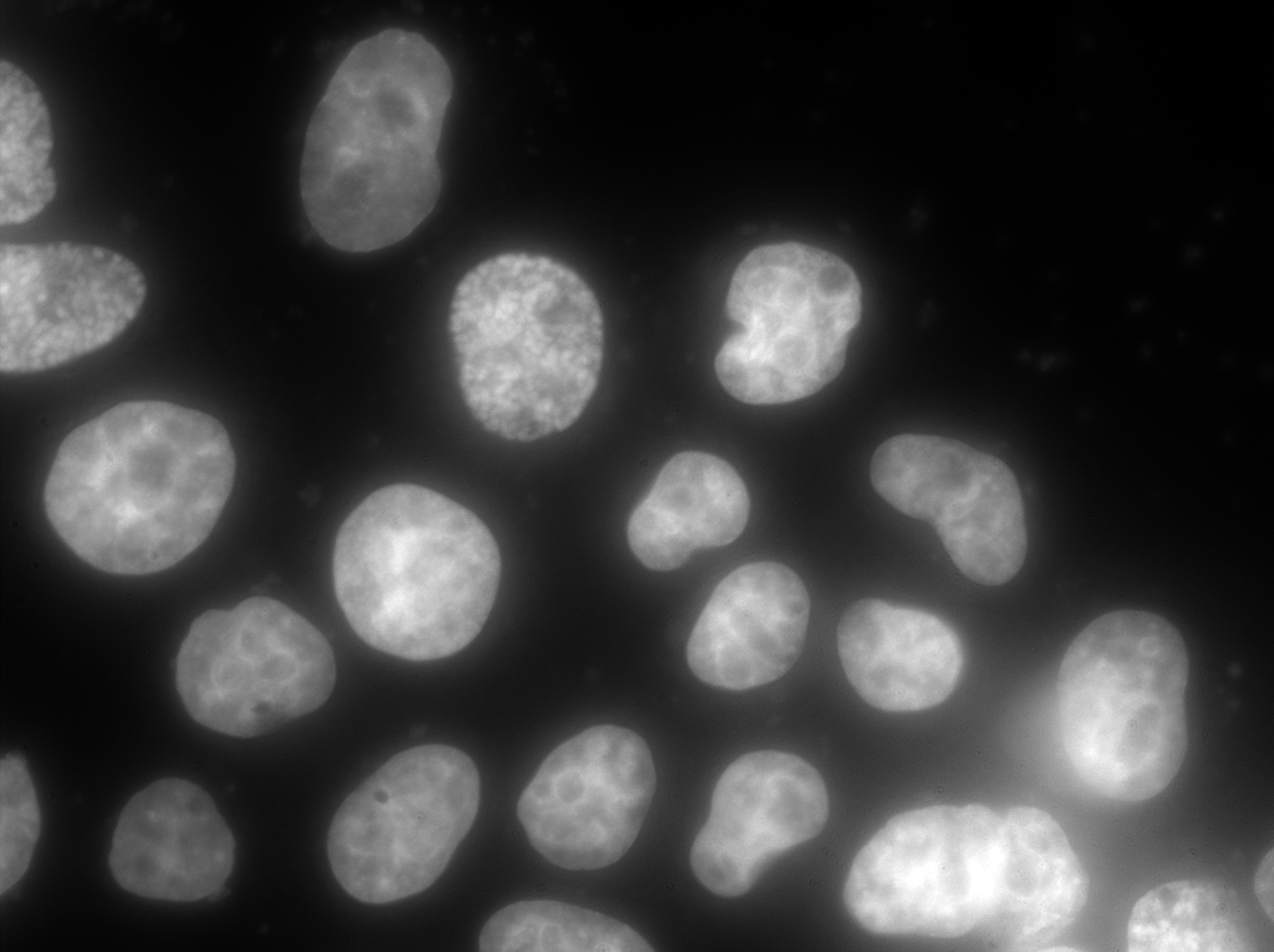

Supplement: Supplementary file 13 — Source data Fig. 3 part 3 [file 44319_2026_742_MOESM13_ESM.zip › Figure 3 Part 3/Fig 3b-e TTLL screen part 3/TTLL9-YFP A3 I6.Project Maximum Z_XY1679700103_Z0_T0_C0.tif]

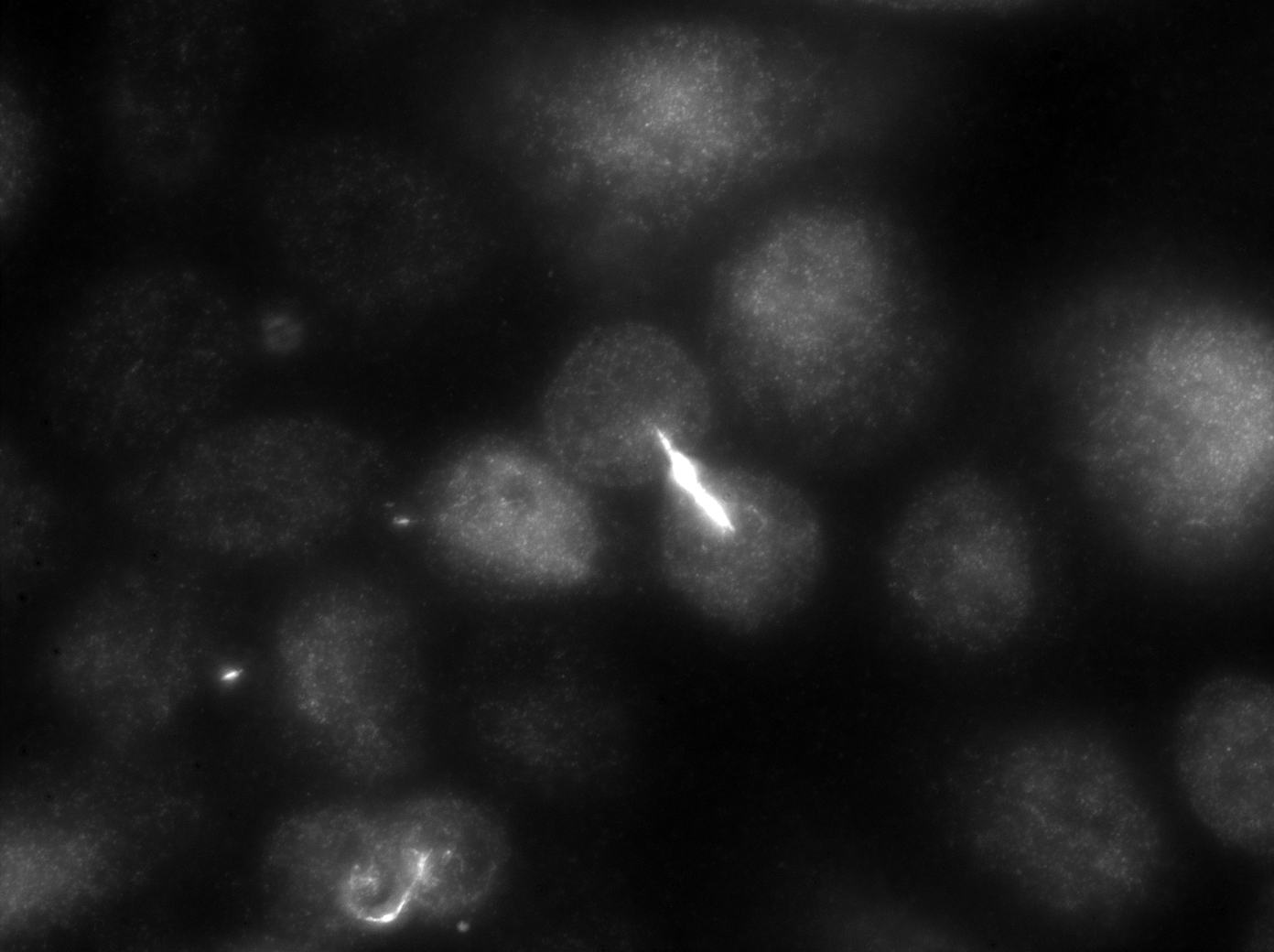

Supplement: Supplementary file 13 — Source data Fig. 3 part 3 [file 44319_2026_742_MOESM13_ESM.zip › Figure 3 Part 3/Fig 3b-e TTLL screen part 3/TTLL9-YFP A3 I1 - 1.Project Maximum Z_XY1679699160_Z0_T0_C1.tif]

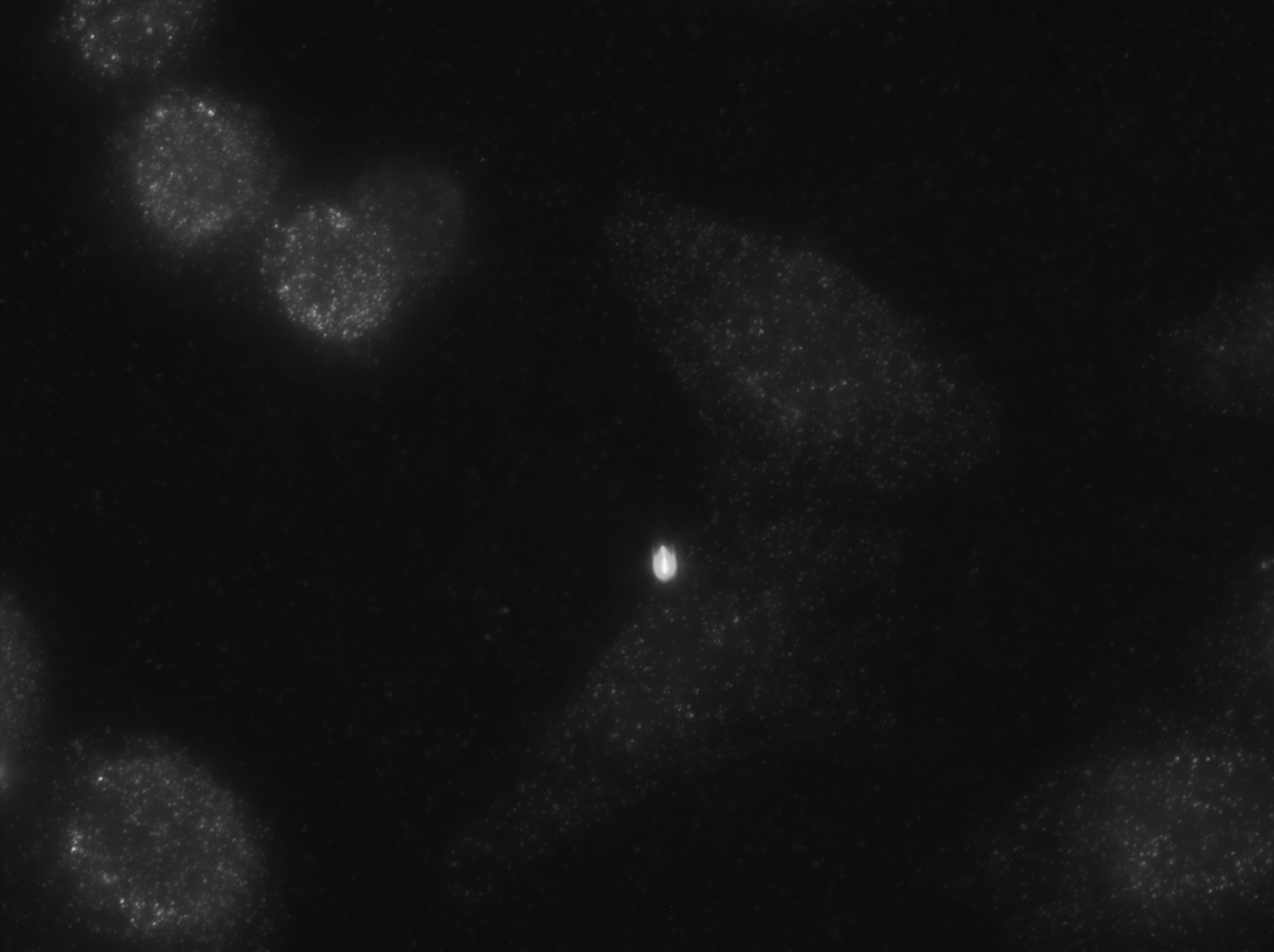

Supplement: Supplementary file 13 — Source data Fig. 3 part 3 [file 44319_2026_742_MOESM13_ESM.zip › Figure 3 Part 3/Fig 3b-e TTLL screen part 3/TTLL11-YFP Img 16 yfp 2000 - 1.Project Maximum Z_XY1648586920_Z0_T0_C1.tif]

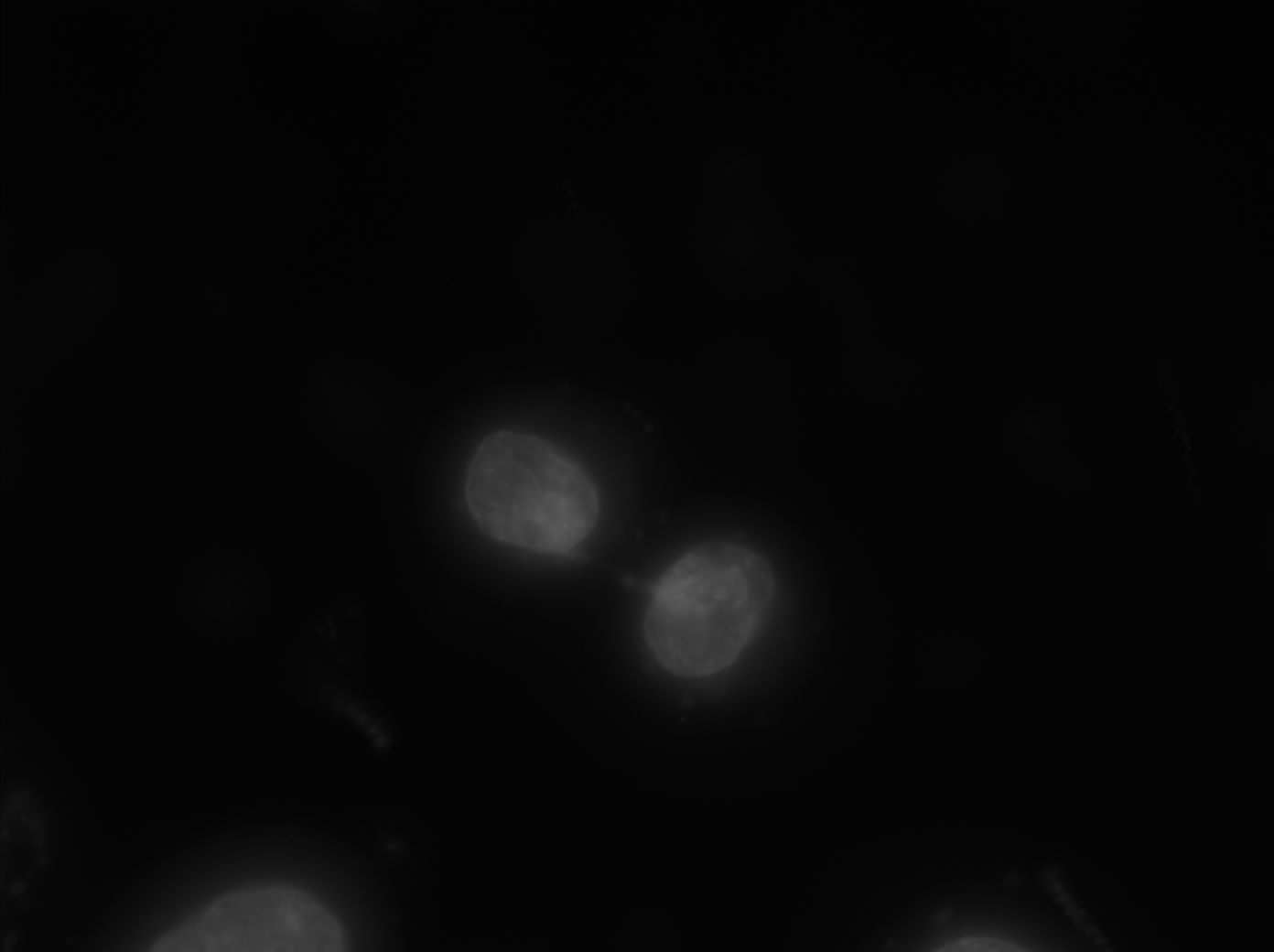

Supplement: Supplementary file 13 — Source data Fig. 3 part 3 [file 44319_2026_742_MOESM13_ESM.zip › Figure 3 Part 3/Fig 3b-e TTLL screen part 3/TTLL9-GFP A4 I2.Project Maximum Z_XY1675965557_Z0_T0_C0.tif]

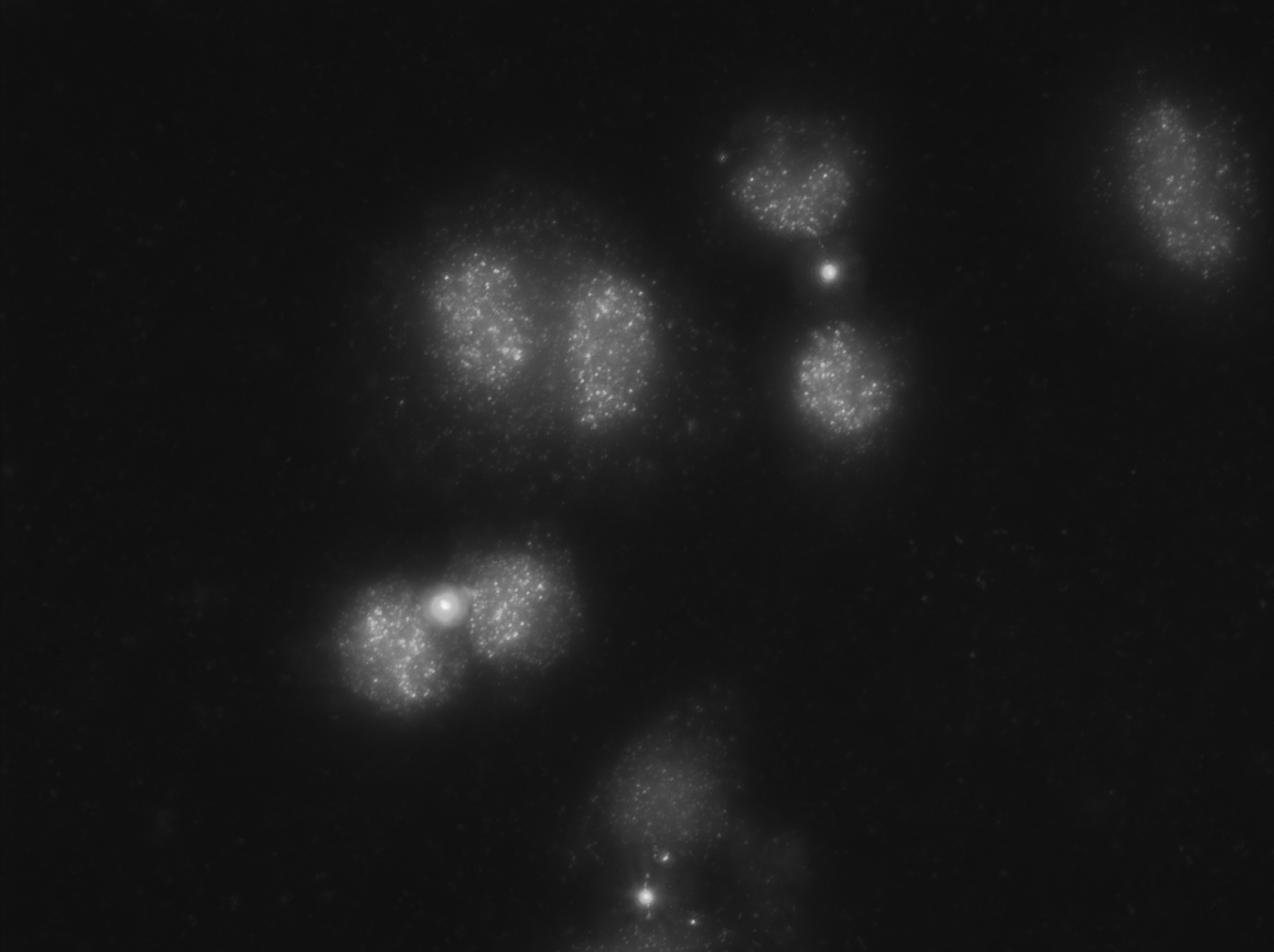

Supplement: Supplementary file 13 — Source data Fig. 3 part 3 [file 44319_2026_742_MOESM13_ESM.zip › Figure 3 Part 3/Fig 3b-e TTLL screen part 3/TTLL11-YFP Img 12 yfp2500.Project Maximum Z_XY1648580285_Z0_T0_C1.tif]

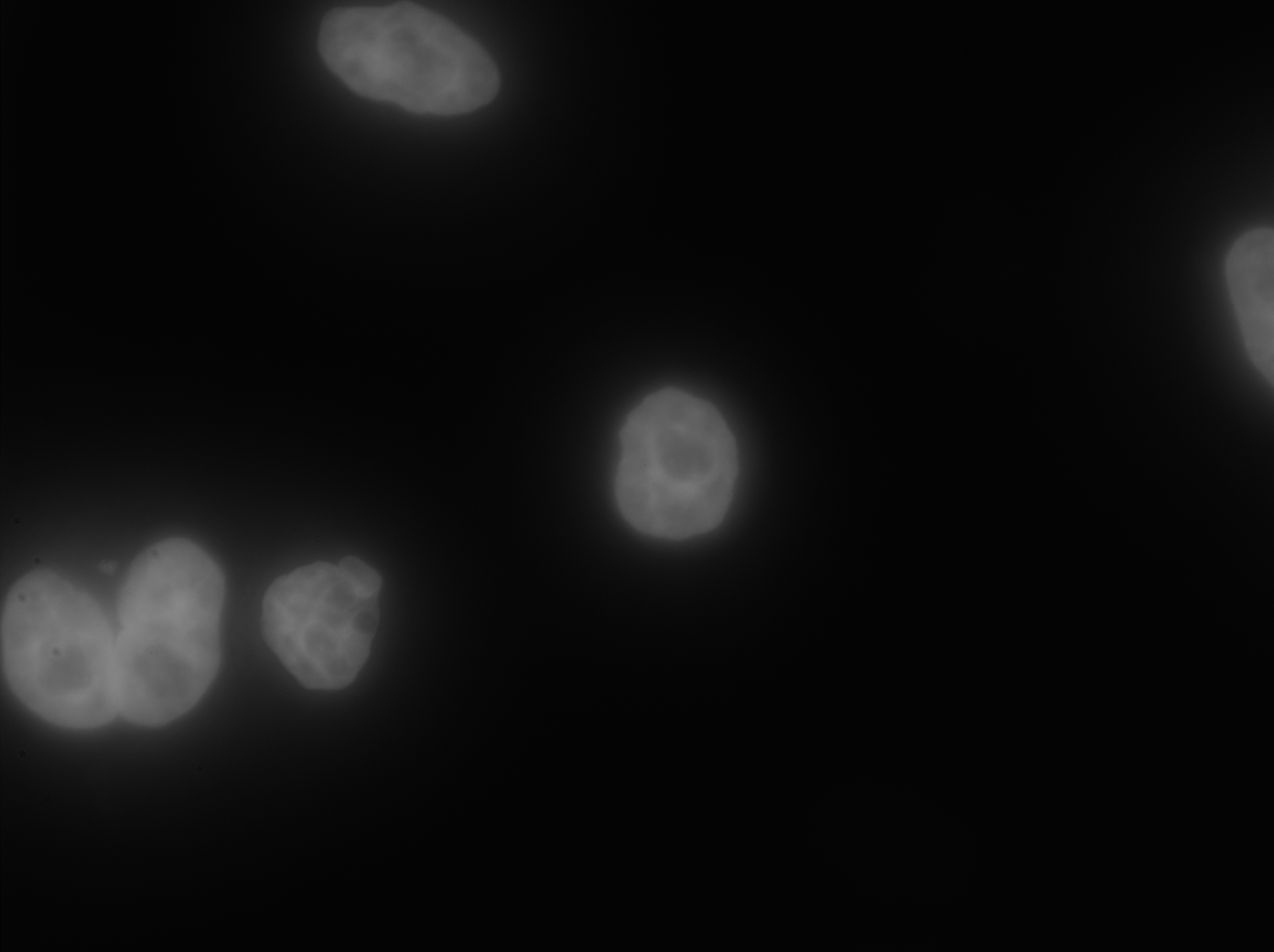

Supplement: Supplementary file 13 — Source data Fig. 3 part 3 [file 44319_2026_742_MOESM13_ESM.zip › Figure 3 Part 3/Fig 3b-e TTLL screen part 3/TTLL11-YFP Img 14 yfp 1200.Project Maximum Z_XY1648581343_Z0_T0_C0.tif]

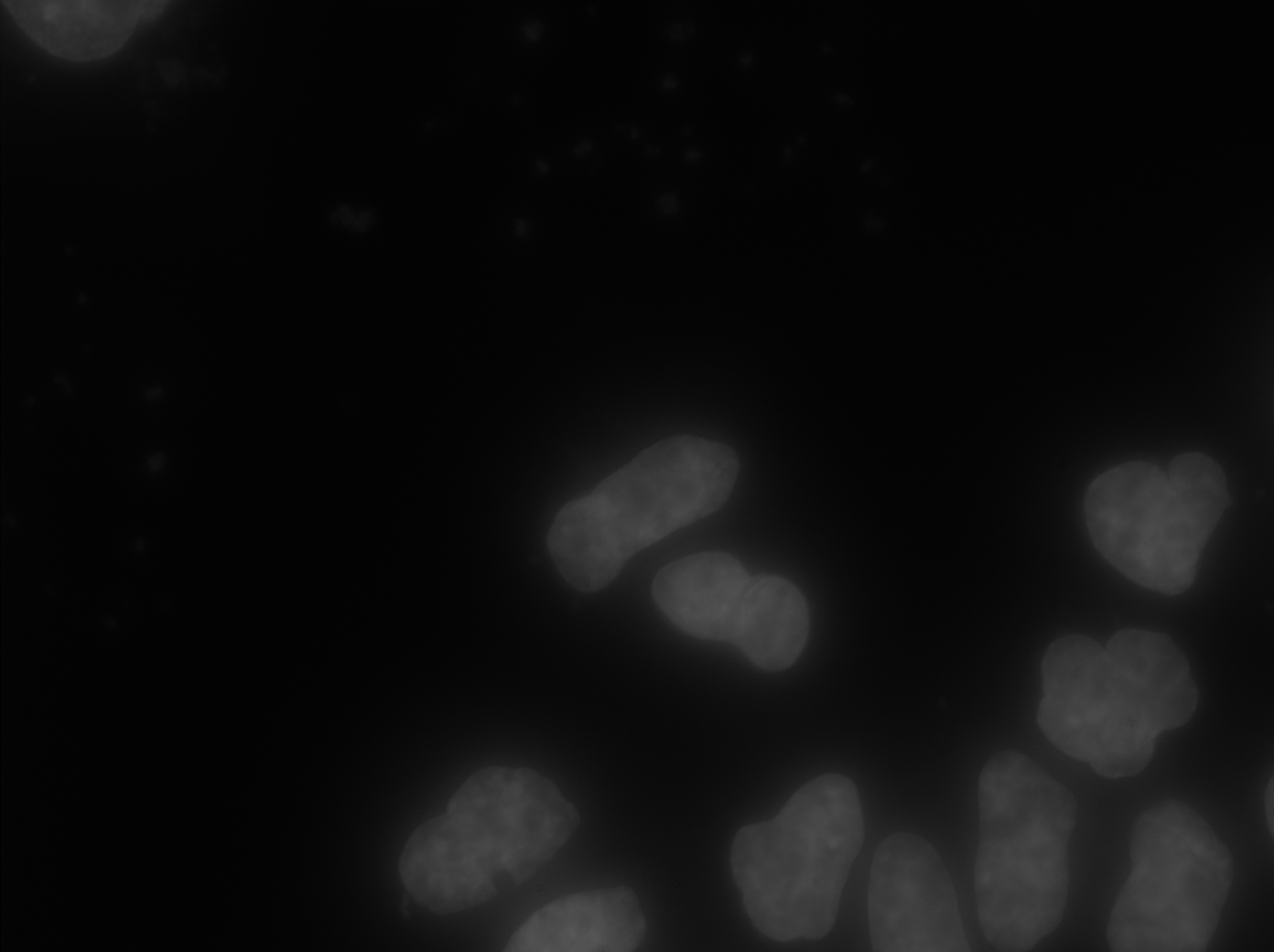

Supplement: Supplementary file 13 — Source data Fig. 3 part 3 [file 44319_2026_742_MOESM13_ESM.zip › Figure 3 Part 3/Fig 3b-e TTLL screen part 3/TTLL9-YFP A3 I17.Project Maximum Z_XY1679701639_Z0_T0_C0.tif]

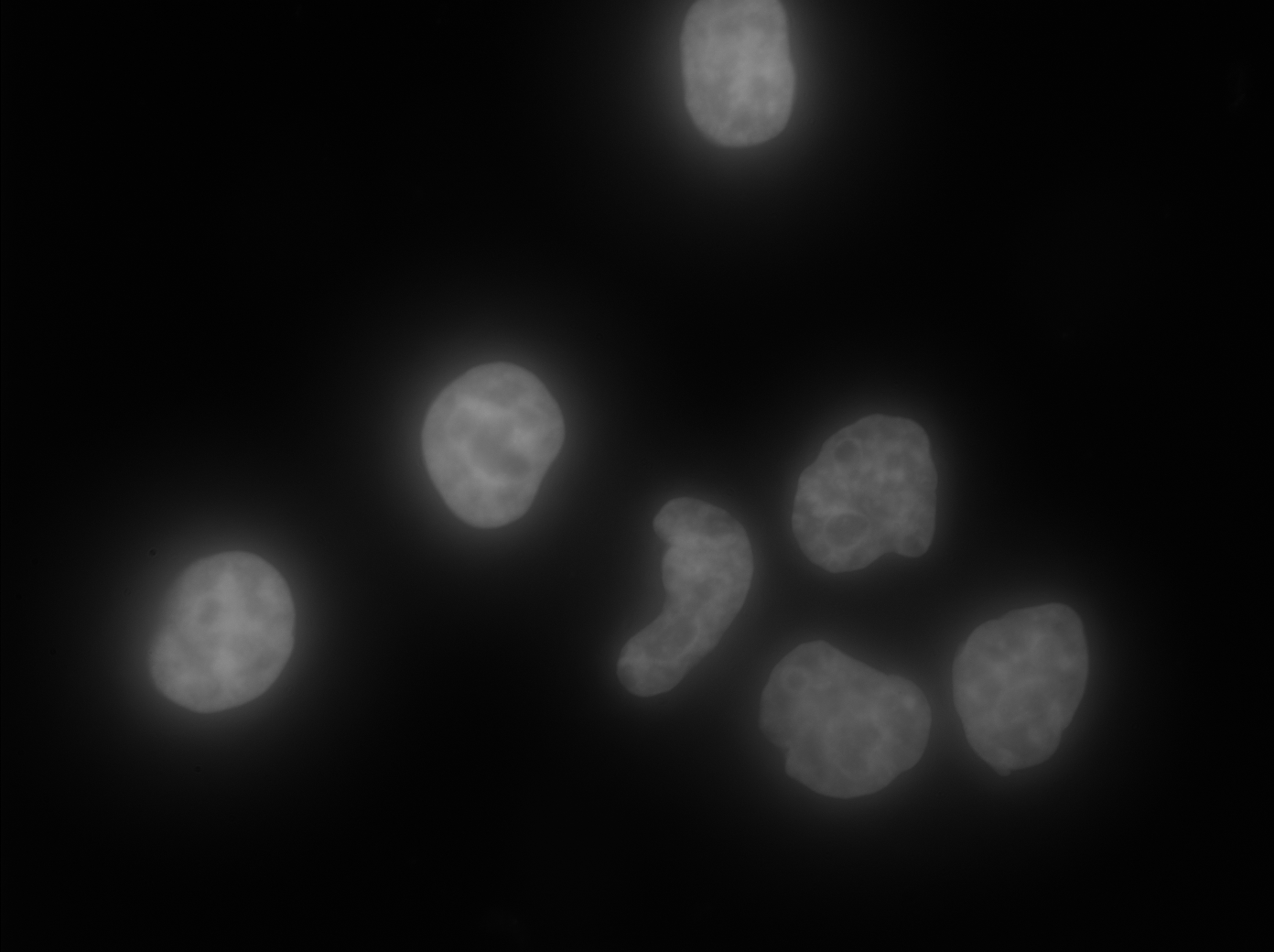

Supplement: Supplementary file 13 — Source data Fig. 3 part 3 [file 44319_2026_742_MOESM13_ESM.zip › Figure 3 Part 3/Fig 3b-e TTLL screen part 3/TTLL11-YFP Img 1 yfp1100 - 1.Project Maximum Z_XY1648156180_Z0_T0_C0.tif]

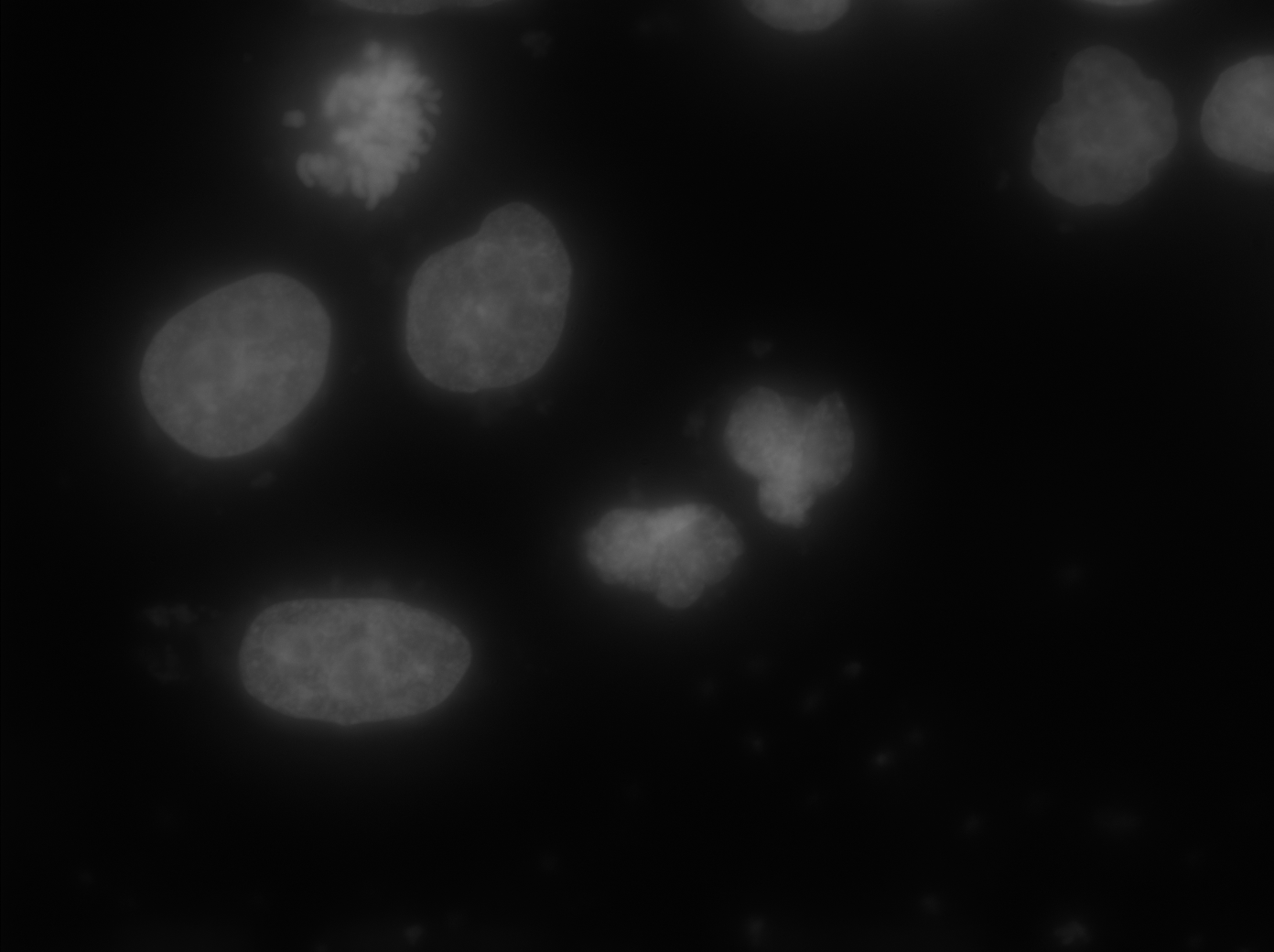

Supplement: Supplementary file 13 — Source data Fig. 3 part 3 [file 44319_2026_742_MOESM13_ESM.zip › Figure 3 Part 3/Fig 3b-e TTLL screen part 3/TTLL9-YFP A3 I11.Project Maximum Z_XY1679700787_Z0_T0_C0.tif]

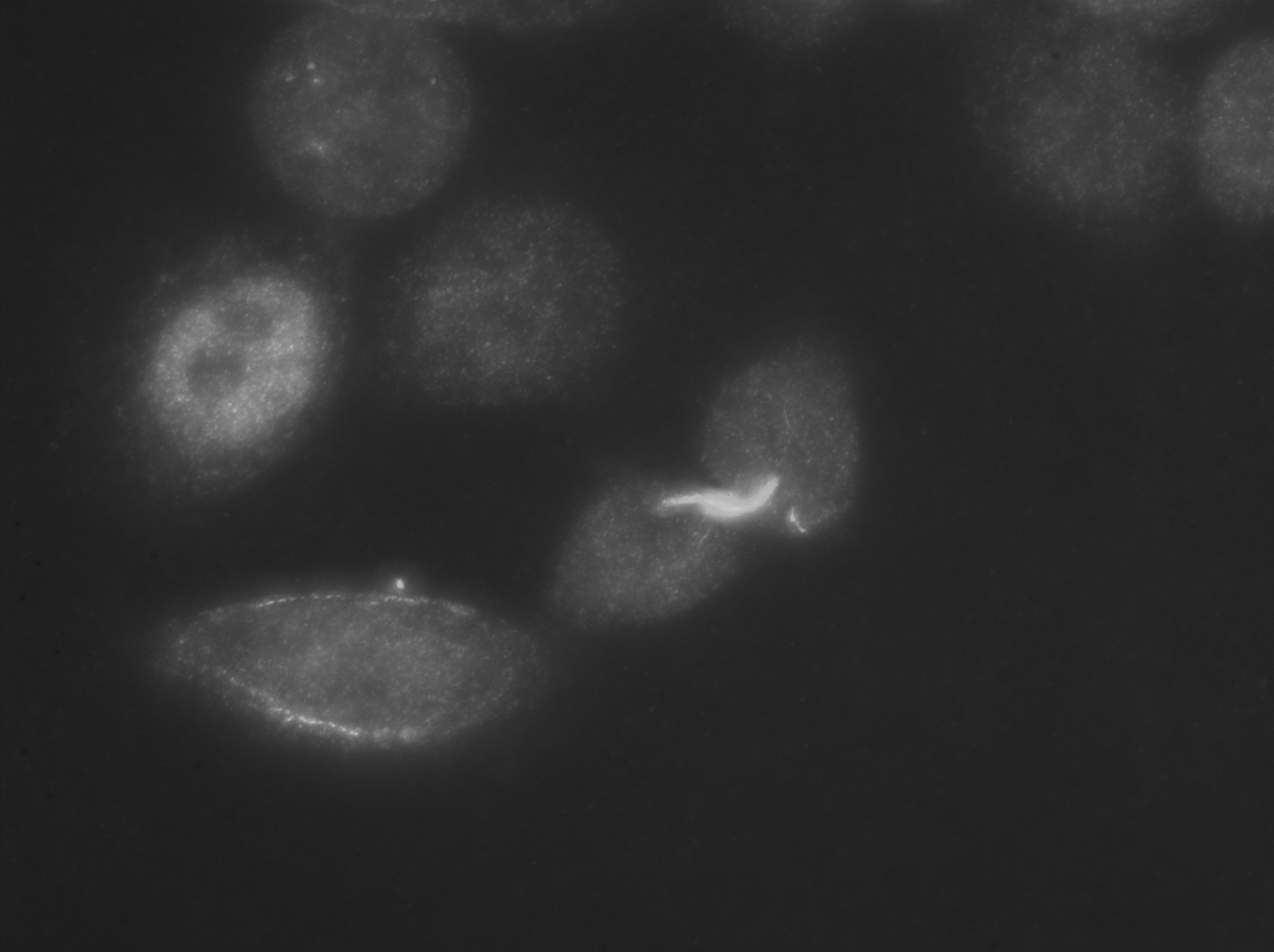

Supplement: Supplementary file 13 — Source data Fig. 3 part 3 [file 44319_2026_742_MOESM13_ESM.zip › Figure 3 Part 3/Fig 3b-e TTLL screen part 3/TTLL9-YFP A3 I11.Project Maximum Z_XY1679700787_Z0_T0_C1.tif]

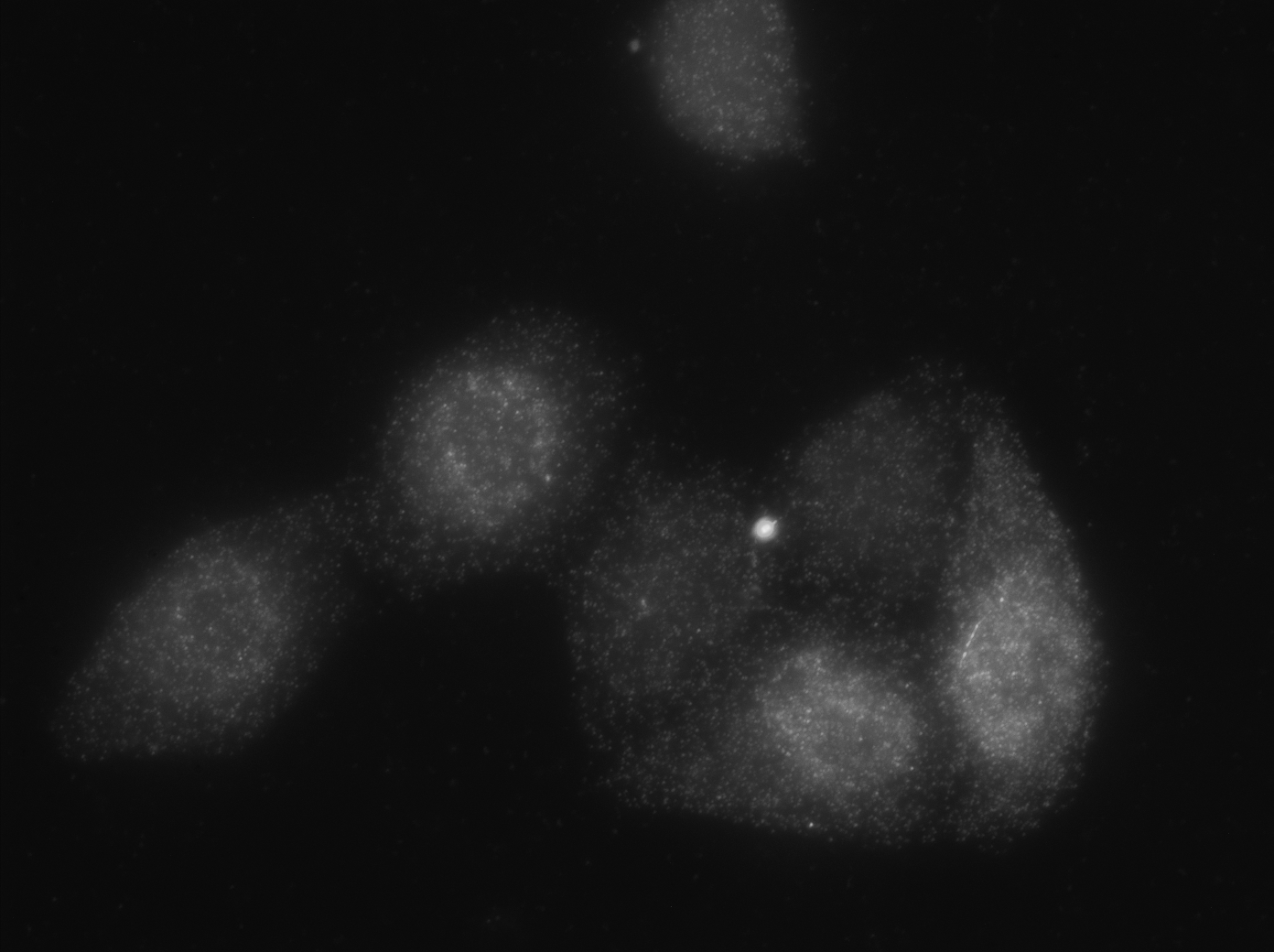

Supplement: Supplementary file 13 — Source data Fig. 3 part 3 [file 44319_2026_742_MOESM13_ESM.zip › Figure 3 Part 3/Fig 3b-e TTLL screen part 3/TTLL11-YFP Img 1 yfp1100 - 1.Project Maximum Z_XY1648156180_Z0_T0_C1.tif]

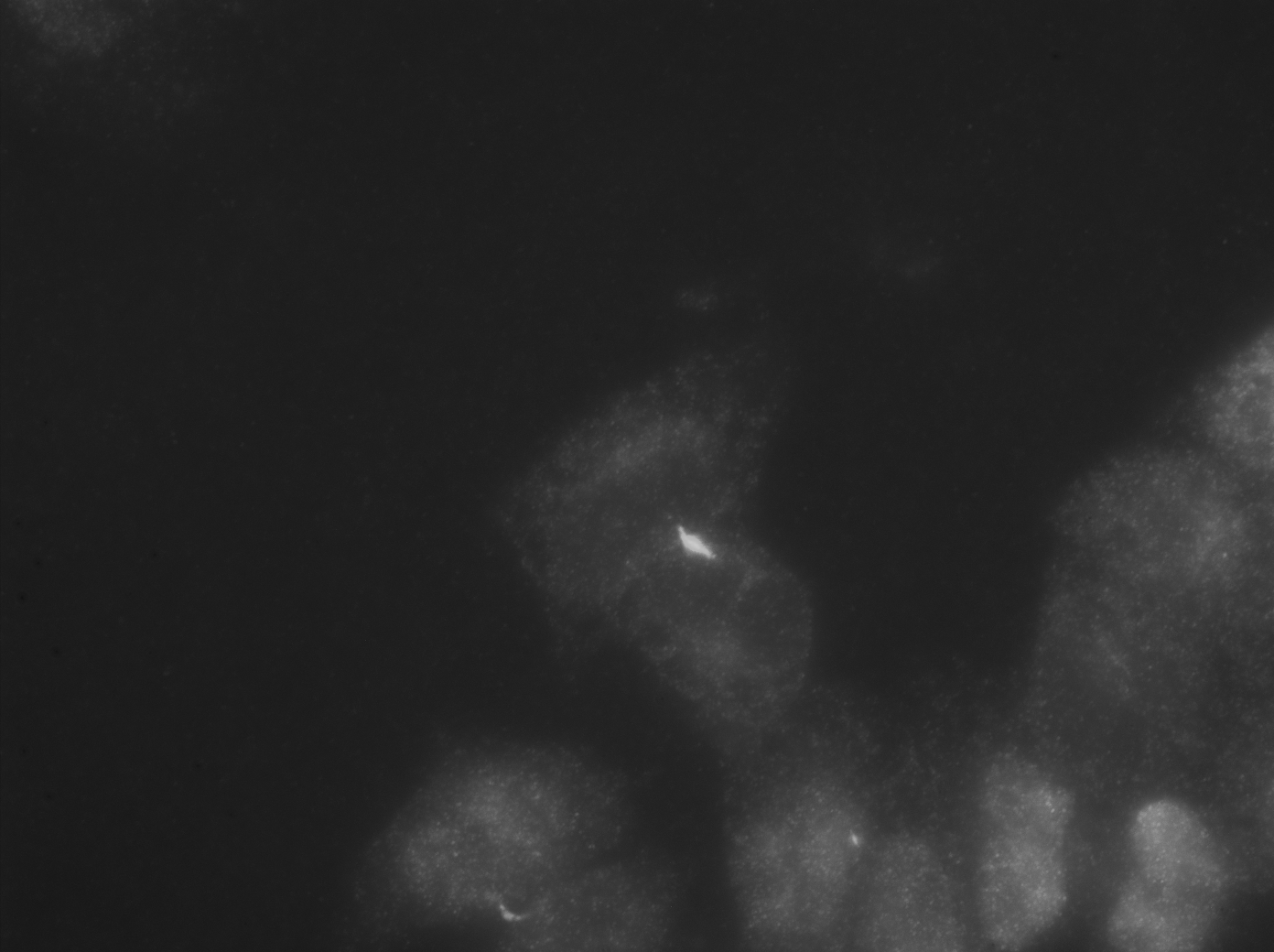

Supplement: Supplementary file 13 — Source data Fig. 3 part 3 [file 44319_2026_742_MOESM13_ESM.zip › Figure 3 Part 3/Fig 3b-e TTLL screen part 3/TTLL9-YFP A3 I17.Project Maximum Z_XY1679701639_Z0_T0_C1.tif]

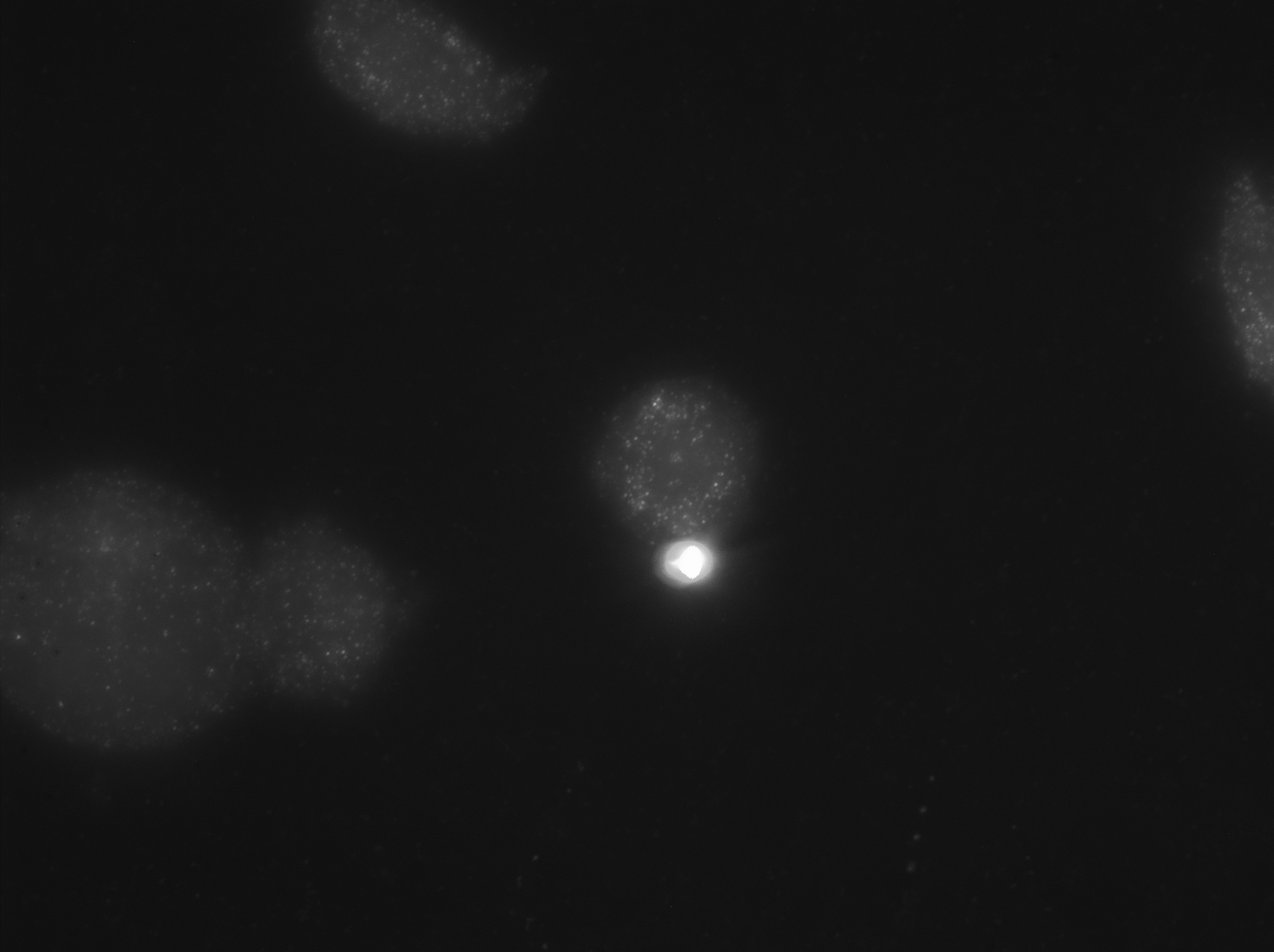

Supplement: Supplementary file 13 — Source data Fig. 3 part 3 [file 44319_2026_742_MOESM13_ESM.zip › Figure 3 Part 3/Fig 3b-e TTLL screen part 3/TTLL11-YFP Img 14 yfp 1200.Project Maximum Z_XY1648581343_Z0_T0_C1.tif]

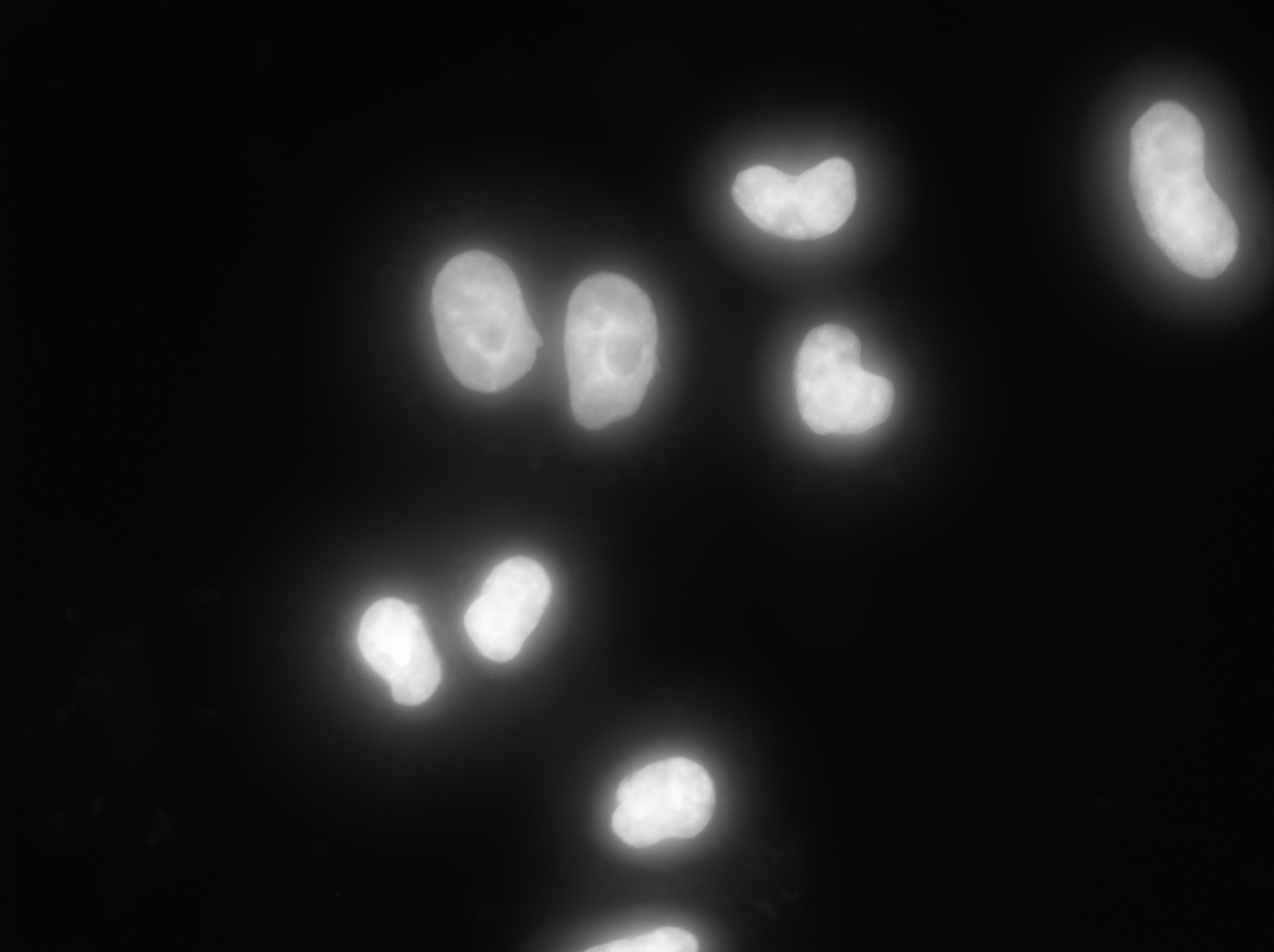

Supplement: Supplementary file 13 — Source data Fig. 3 part 3 [file 44319_2026_742_MOESM13_ESM.zip › Figure 3 Part 3/Fig 3b-e TTLL screen part 3/TTLL11-YFP Img 12 yfp2500.Project Maximum Z_XY1648580285_Z0_T0_C0.tif]

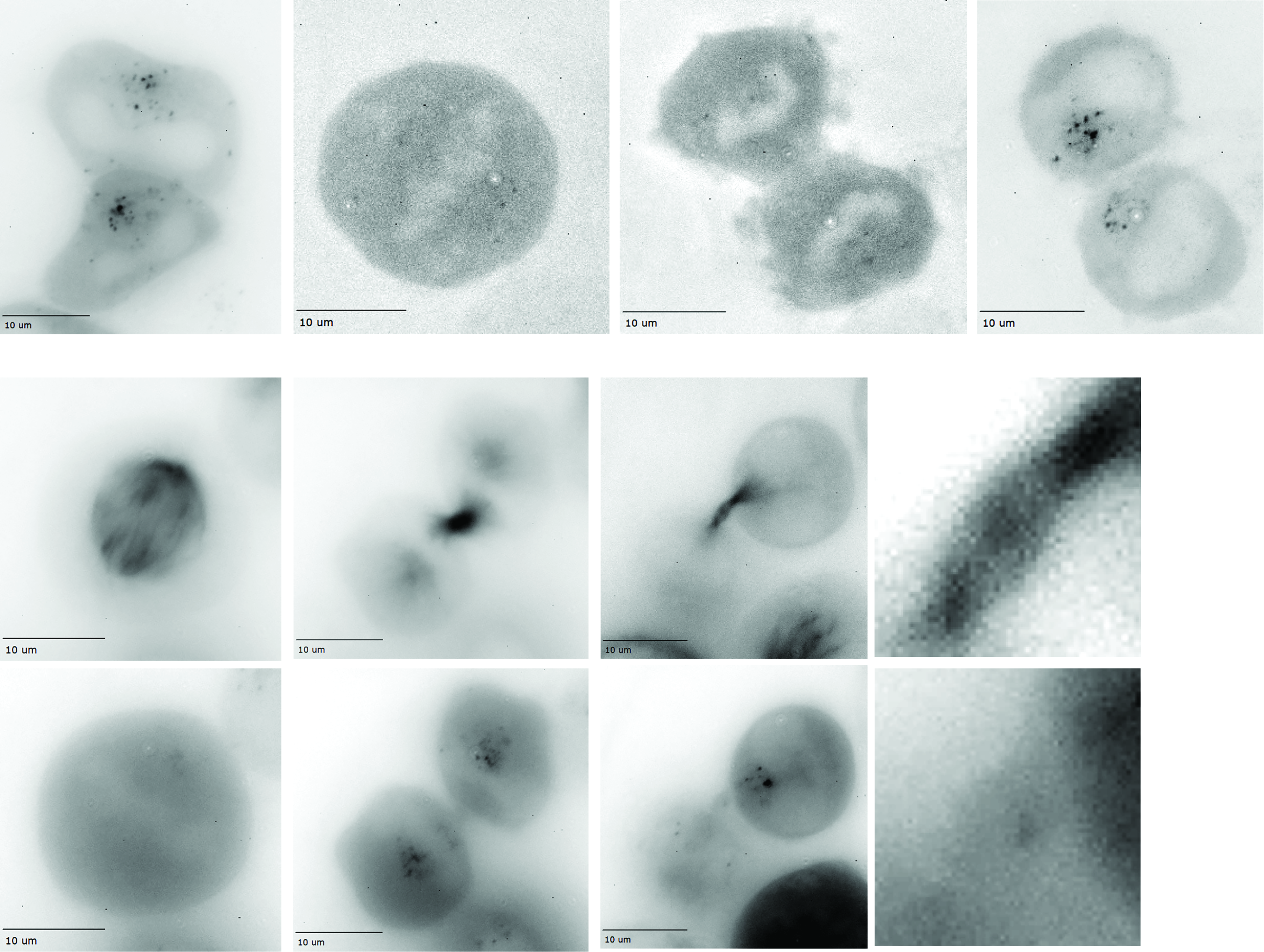

Supplement: Supplementary file 14 — Source data Fig. 4 [file 44319_2026_742_MOESM14_ESM.zip › Figure 4/4cd timelapse.tif]

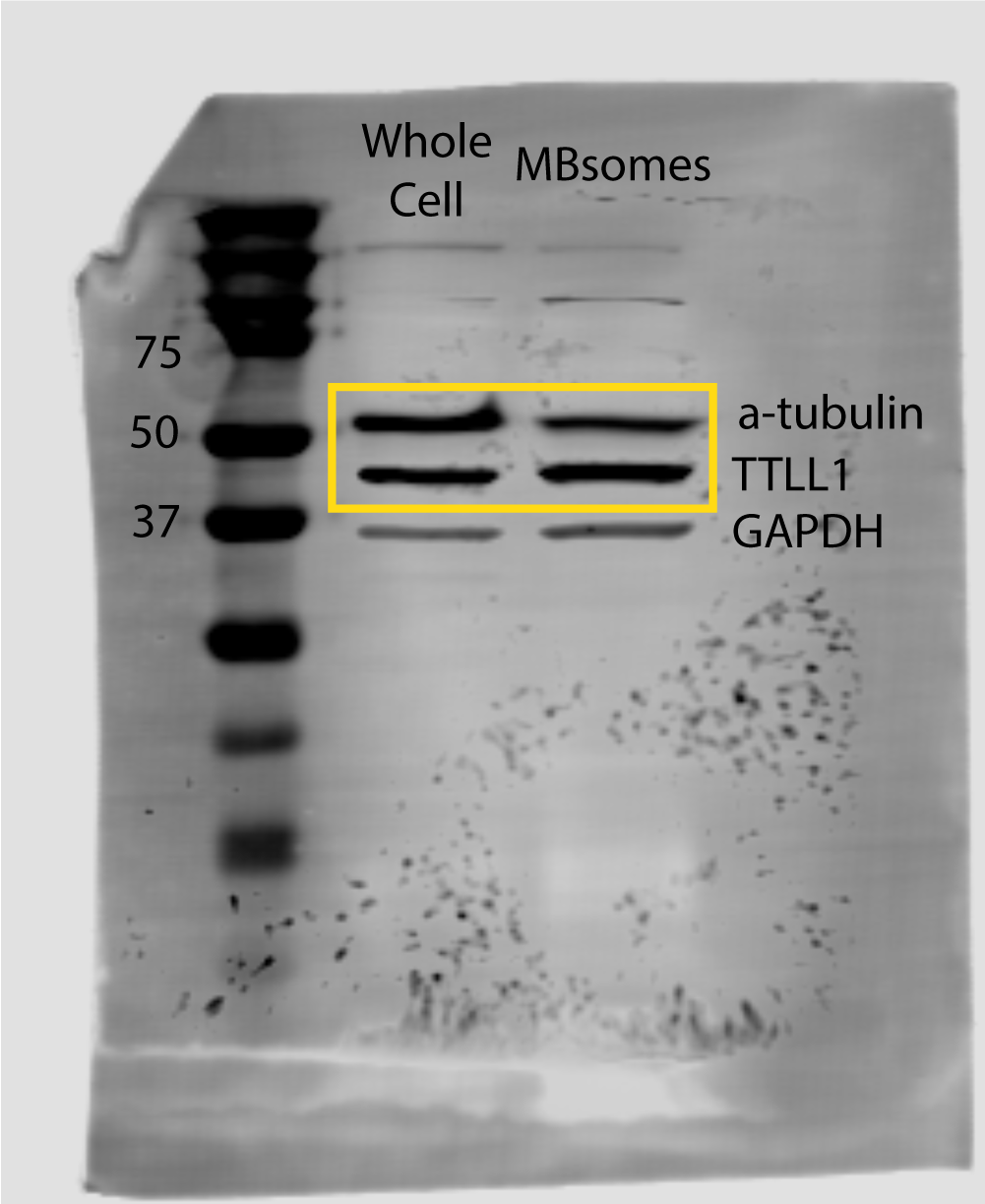

Supplement: Supplementary file 14 — Source data Fig. 4 [file 44319_2026_742_MOESM14_ESM.zip › Figure 4/Fig 4a TTLL1 wb/ttll1 mb WB annotated.tif]

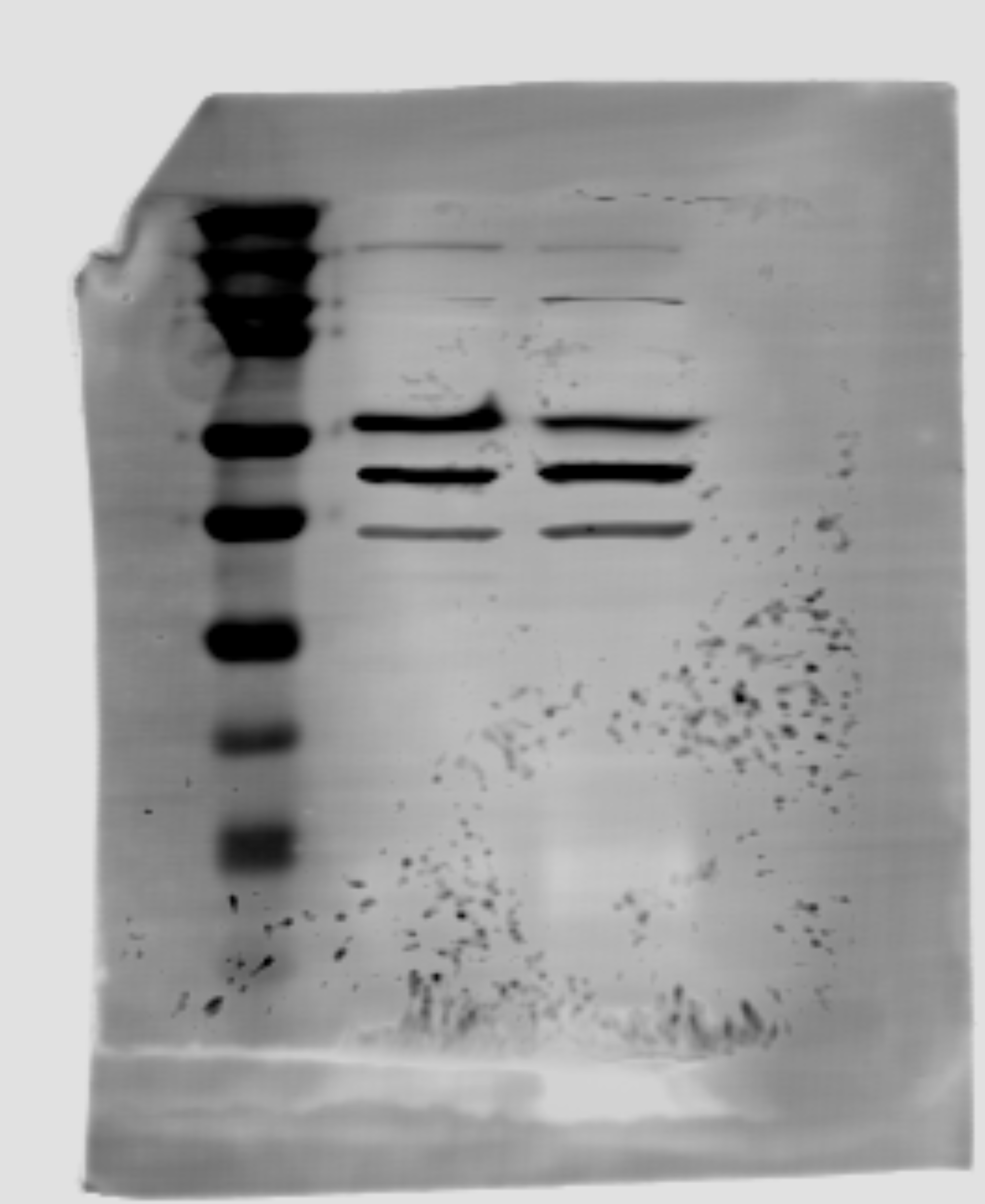

Supplement: Supplementary file 14 — Source data Fig. 4 [file 44319_2026_742_MOESM14_ESM.zip › Figure 4/Fig 4a TTLL1 wb/ttll1 mb WB.tif]

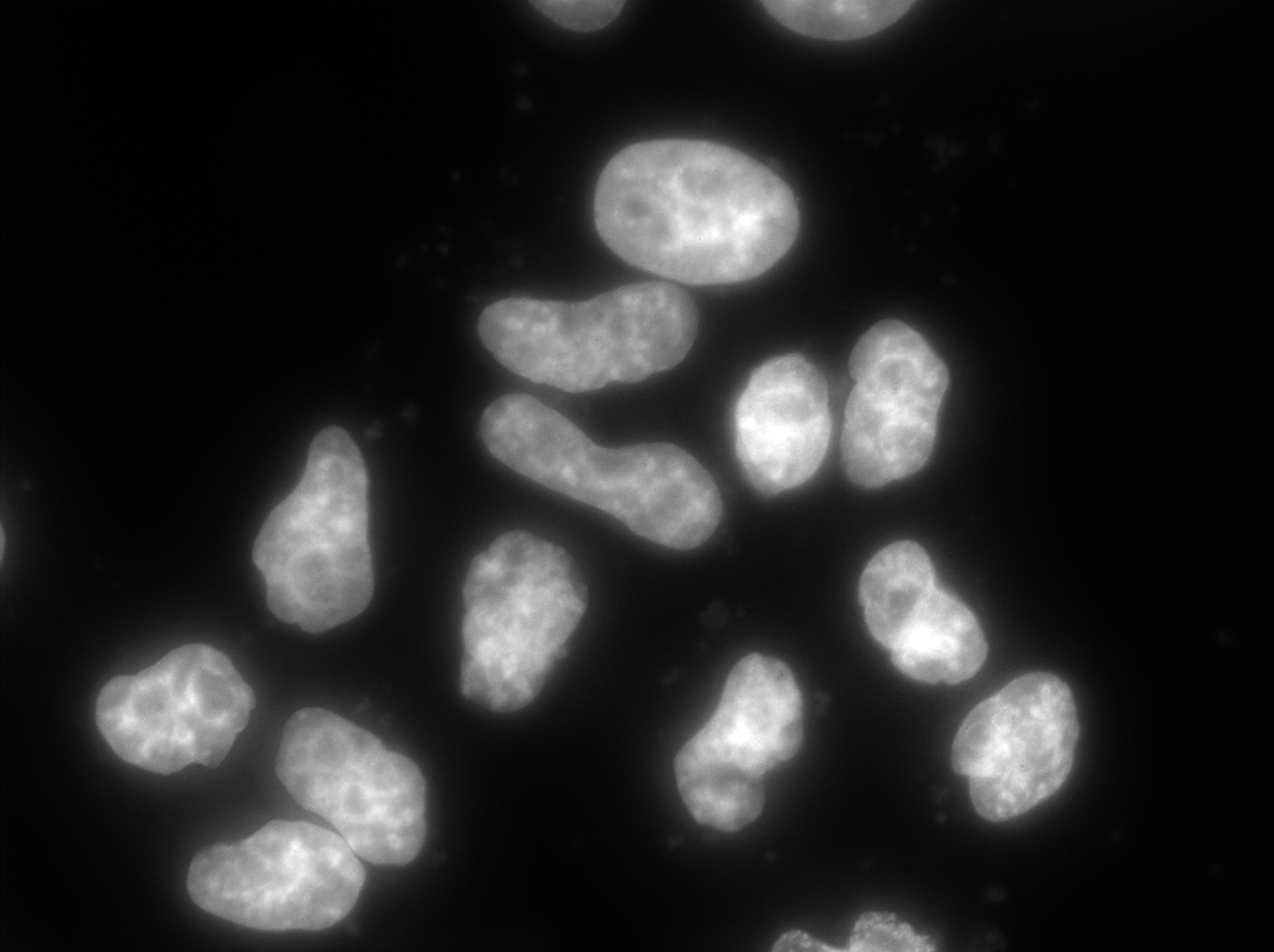

Supplement: Supplementary file 14 — Source data Fig. 4 [file 44319_2026_742_MOESM14_ESM.zip › Figure 4/Fig 4ef Cas9 TPGS1-EYFP-3'UTR acetylated tubulin/Cas9 TPGS1-3utr R3 2-5-25 LT10.Project Maximum Z_XY1738697780_Z0_T0_C0.tif]

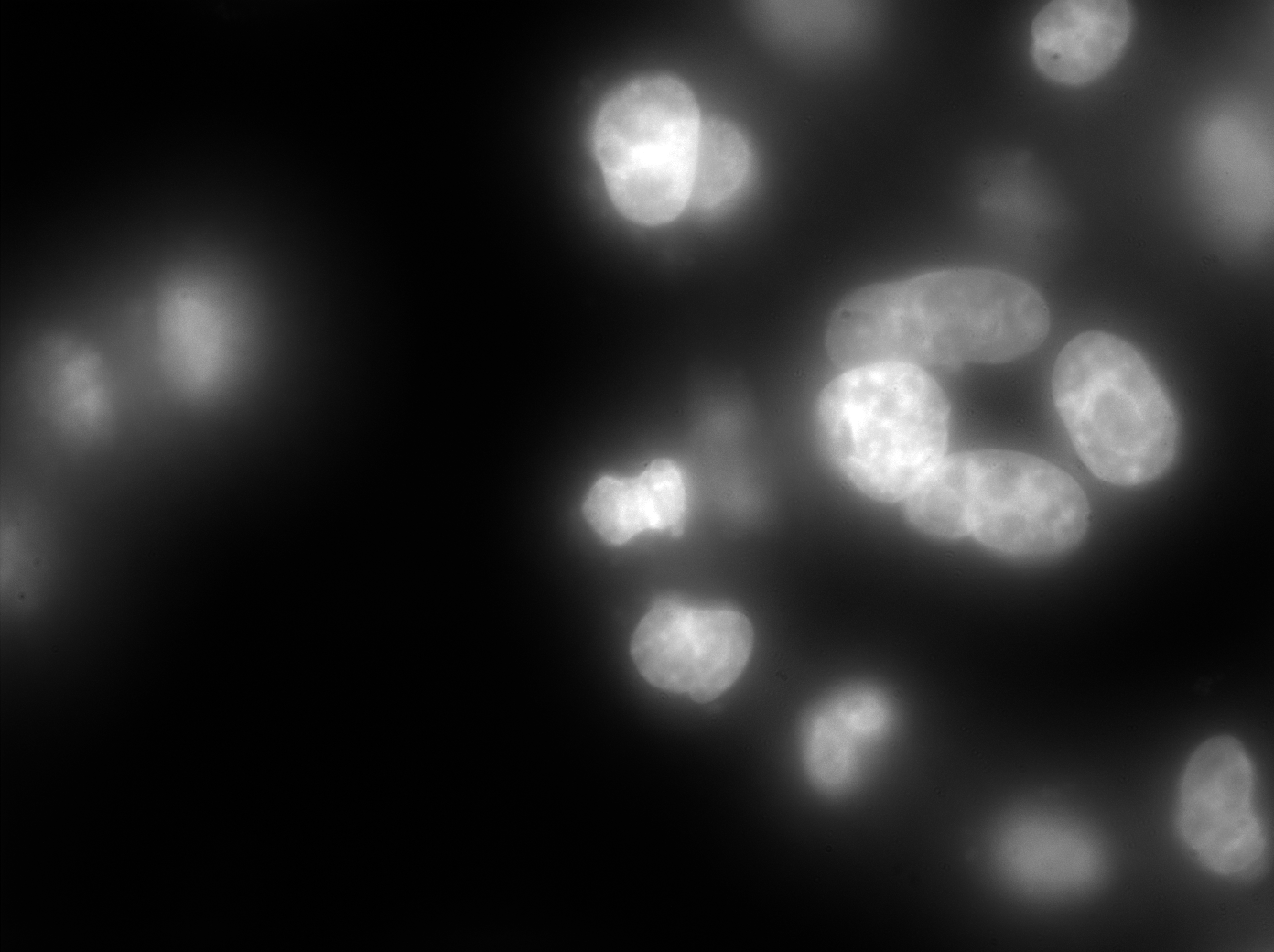

Supplement: Supplementary file 14 — Source data Fig. 4 [file 44319_2026_742_MOESM14_ESM.zip › Figure 4/Fig 4ef Cas9 TPGS1-EYFP-3'UTR acetylated tubulin/Cas9 TPGS1-3utr R1 1-28-24 ET8.Project Maximum Z_XY1738624870_Z0_T0_C0.tif]

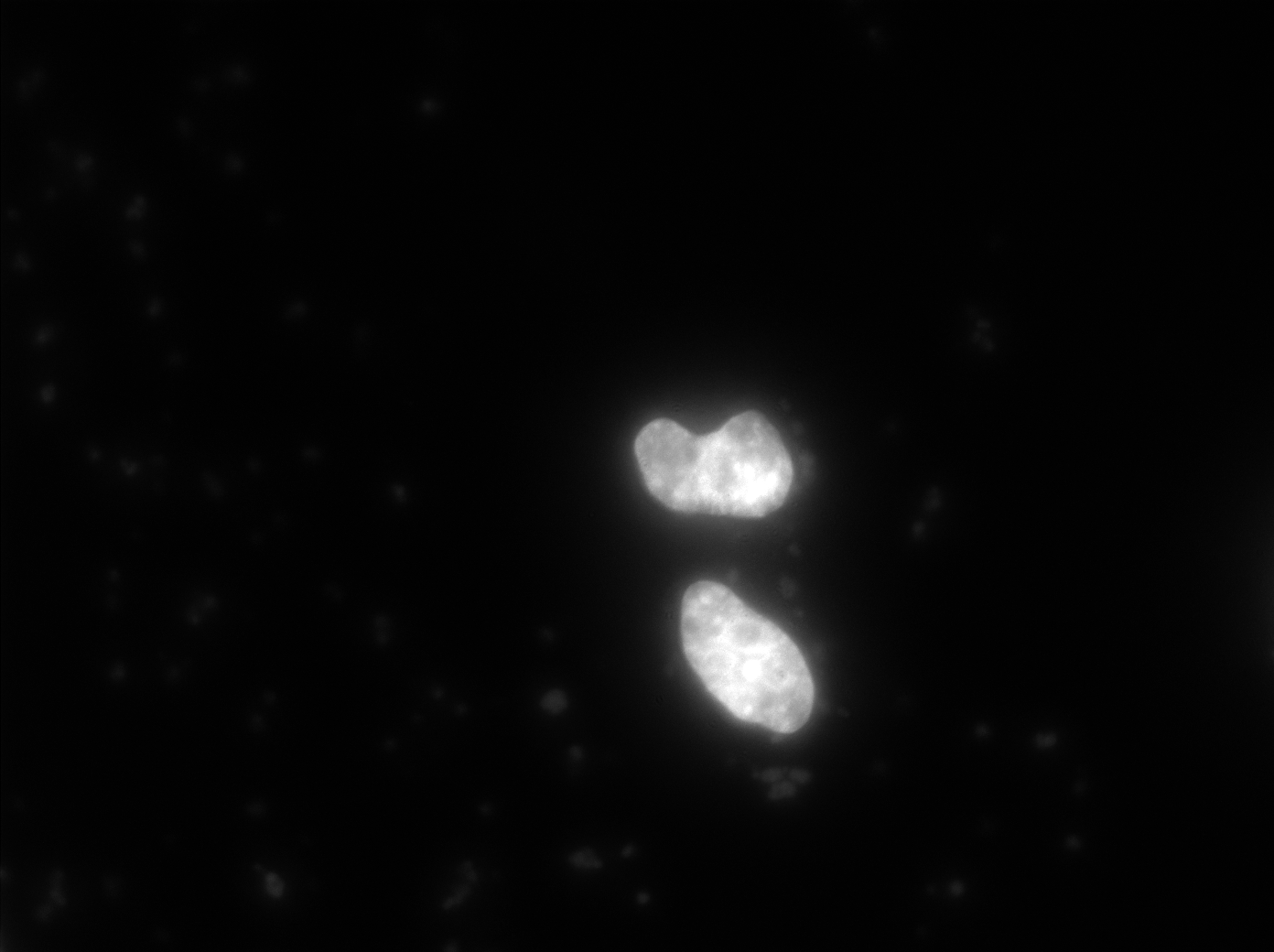

Supplement: Supplementary file 14 — Source data Fig. 4 [file 44319_2026_742_MOESM14_ESM.zip › Figure 4/Fig 4ef Cas9 TPGS1-EYFP-3'UTR acetylated tubulin/Cas9 TPGS1-3utr R3 2-5-25 LT8.Project Maximum Z_XY1738696612_Z0_T0_C0.tif]

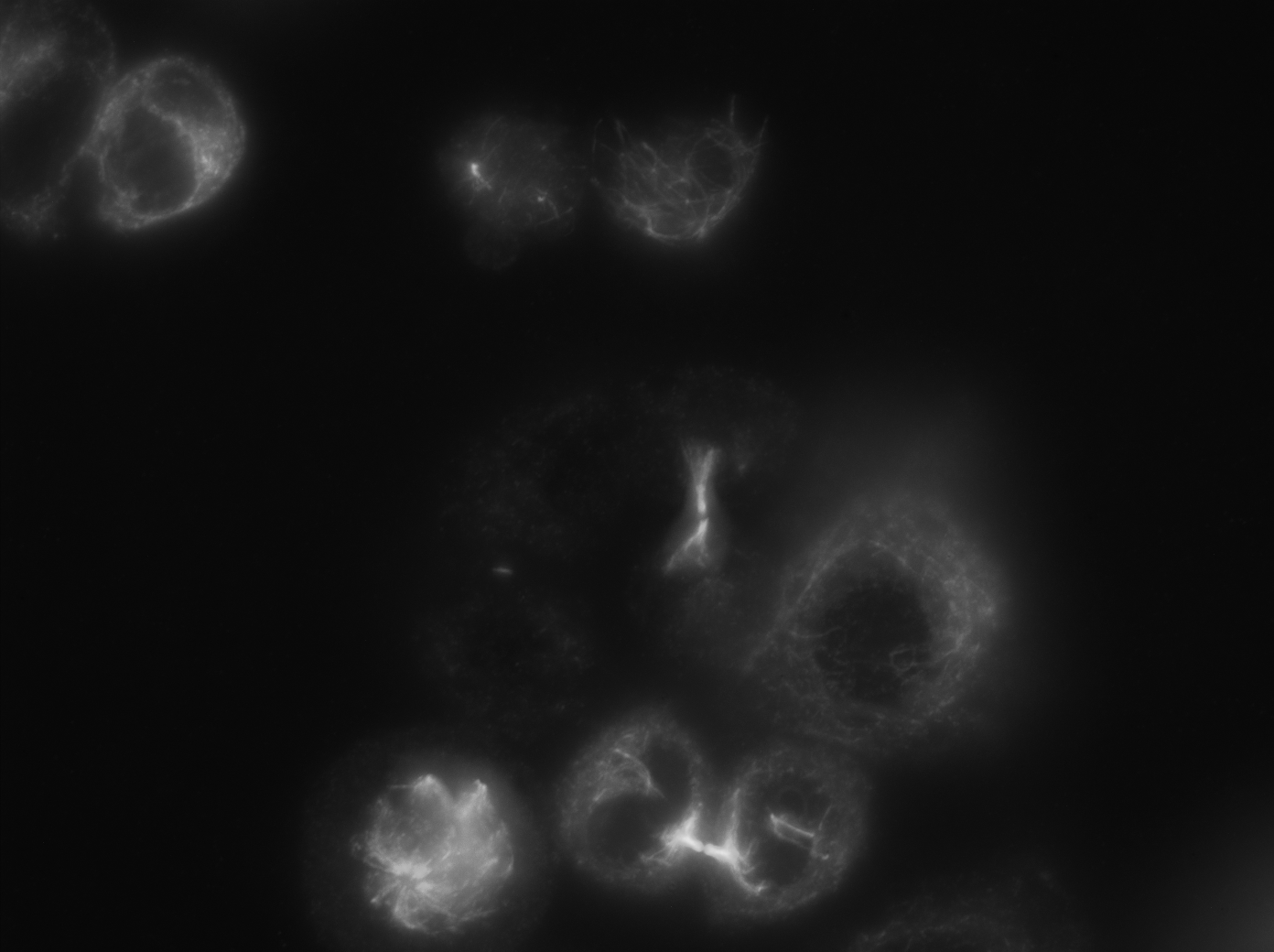

Supplement: Supplementary file 14 — Source data Fig. 4 [file 44319_2026_742_MOESM14_ESM.zip › Figure 4/Fig 4ef Cas9 TPGS1-EYFP-3'UTR acetylated tubulin/Cas9 TPGS1-3utr R2 2-5-25 ET2.Project Maximum Z_XY1738620516_Z0_T0_C1.tif]

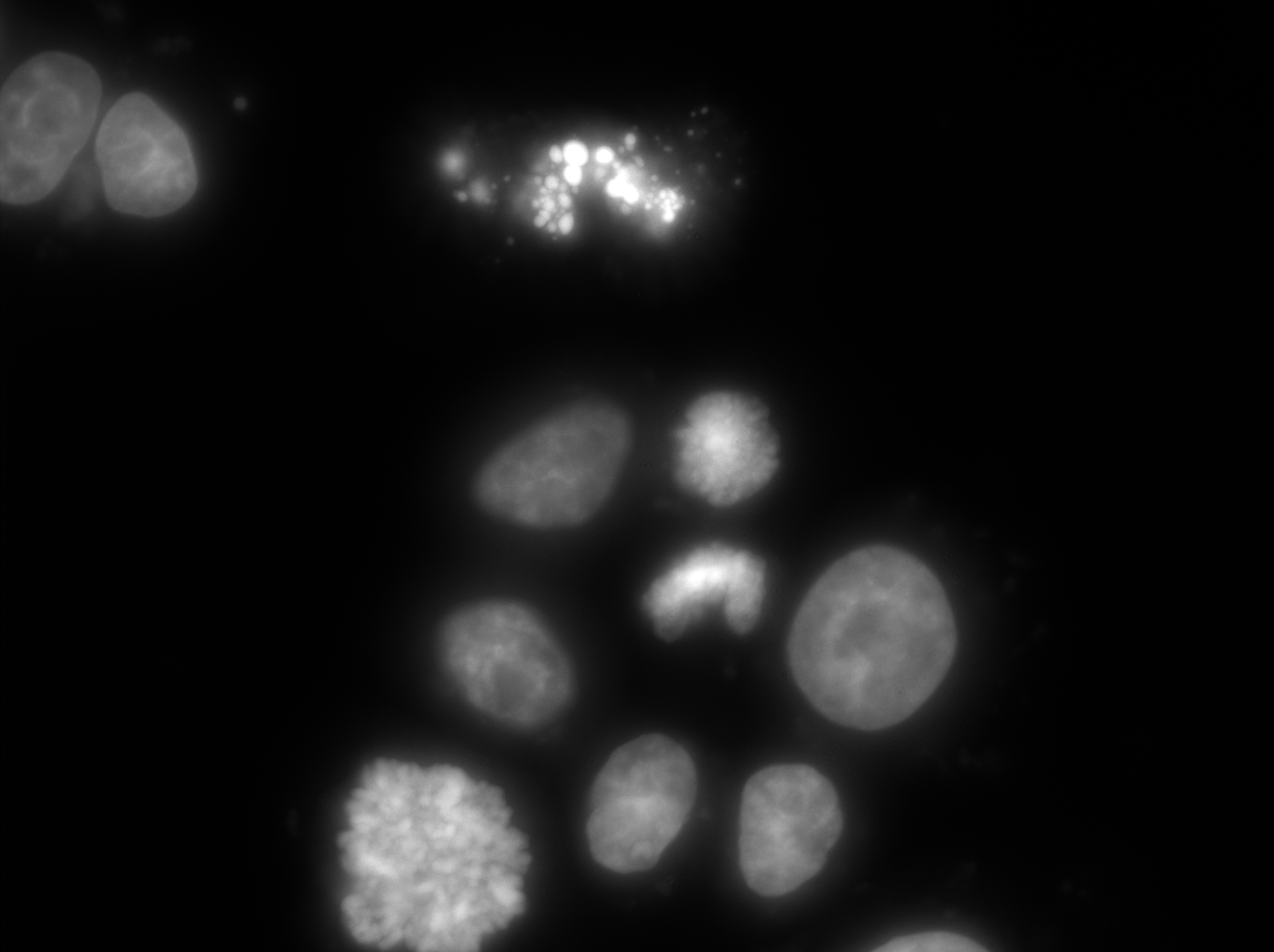

Supplement: Supplementary file 14 — Source data Fig. 4 [file 44319_2026_742_MOESM14_ESM.zip › Figure 4/Fig 4ef Cas9 TPGS1-EYFP-3'UTR acetylated tubulin/Cas9 TPGS1-3utr R2 2-5-25 ET2.Project Maximum Z_XY1738620516_Z0_T0_C0.tif]

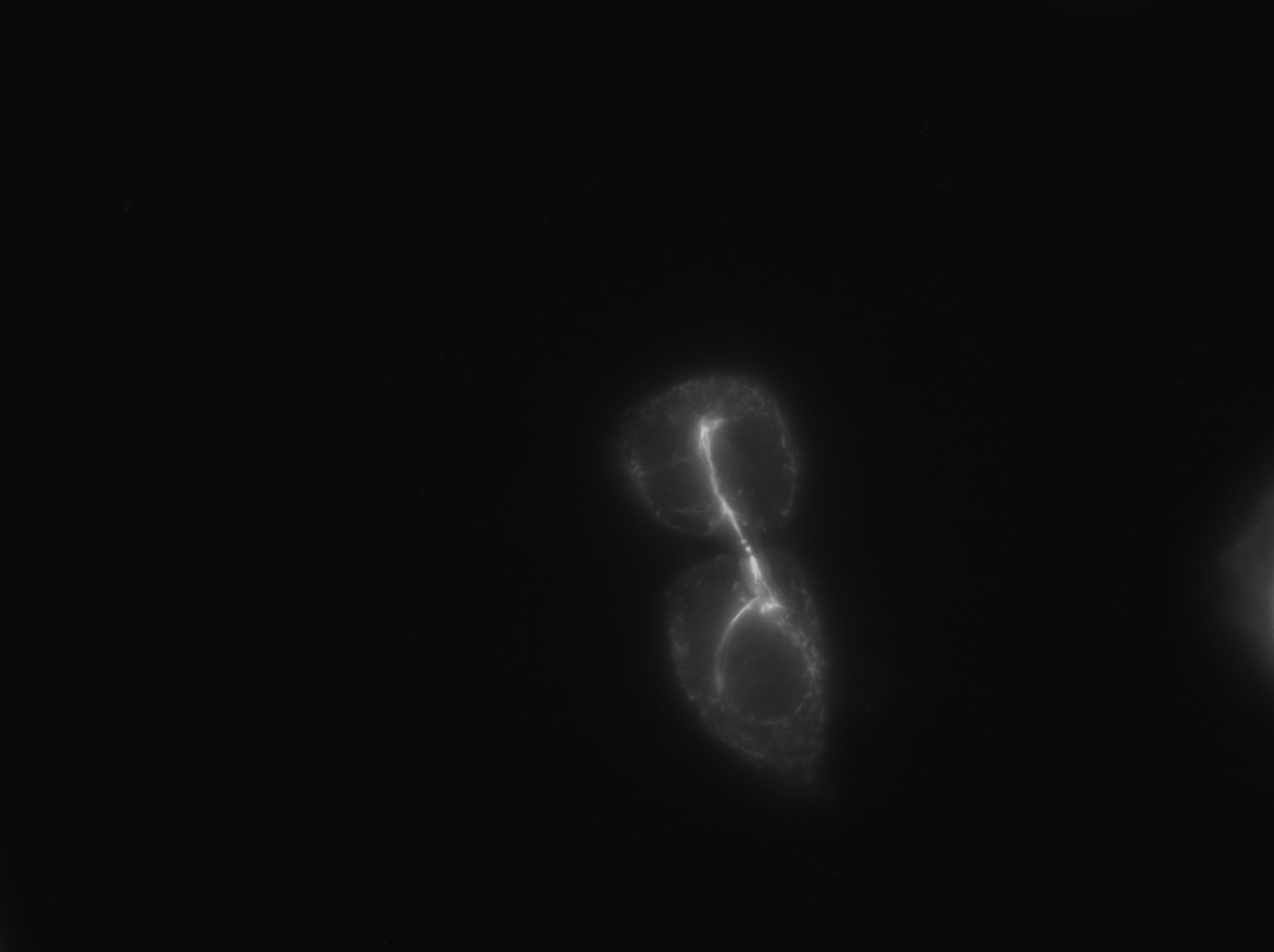

Supplement: Supplementary file 14 — Source data Fig. 4 [file 44319_2026_742_MOESM14_ESM.zip › Figure 4/Fig 4ef Cas9 TPGS1-EYFP-3'UTR acetylated tubulin/Cas9 TPGS1-3utr R3 2-5-25 LT8.Project Maximum Z_XY1738696612_Z0_T0_C1.tif]
