# Supplementary figures and images for: TPGS1 regulates central spindle microtubule glutamylation and remodeling during telophase and abscission (part 15 of 36)
Source: EMBO Rep. 2026 Mar 23;27(8):1944–63. doi: 10.1038/s44319-026-00742-3 (PMC13121839; doi:10.1038/s44319-026-00742-3)

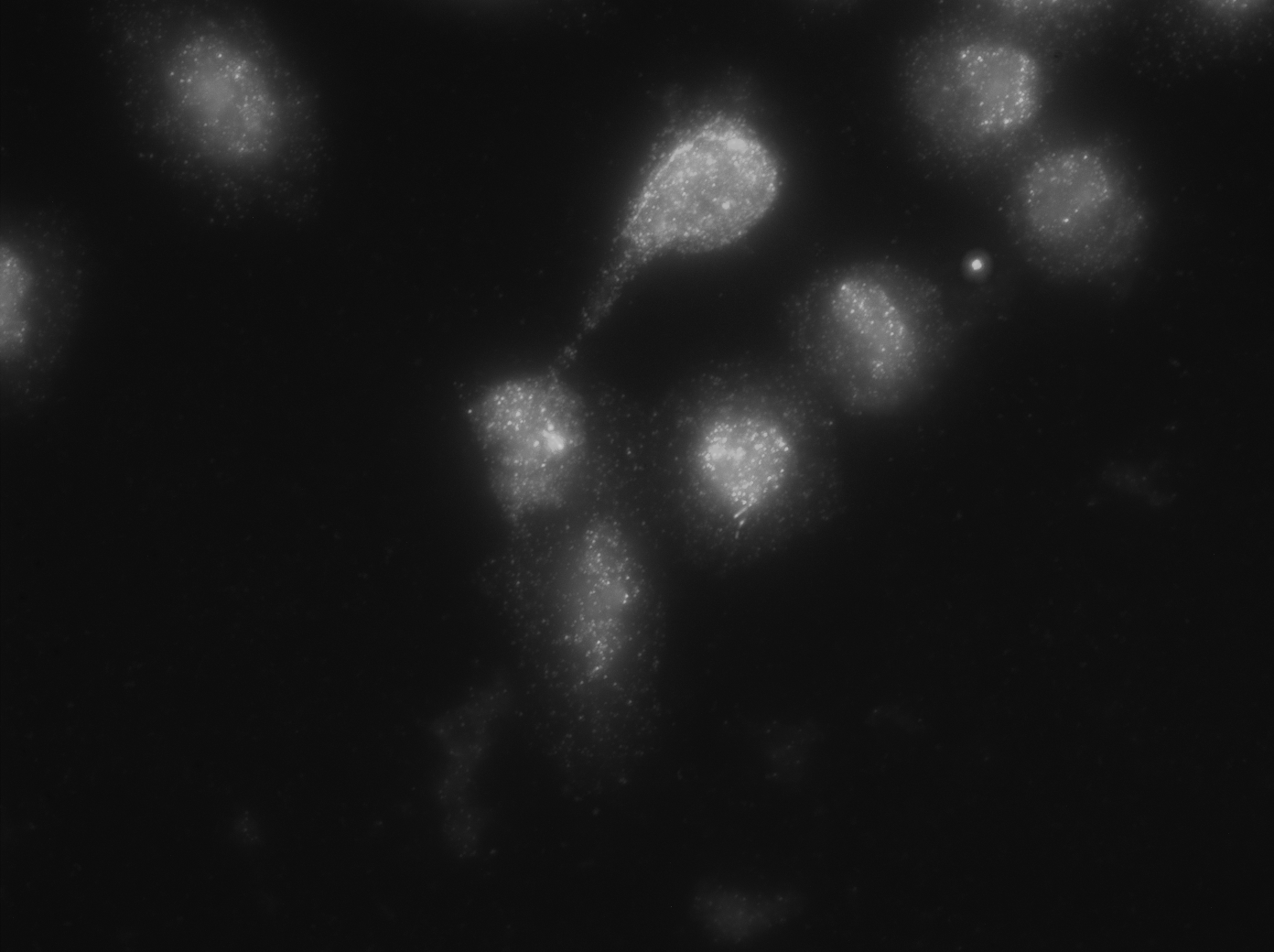

Supplement: Supplementary file 13 — Source data Fig. 3 part 3 [file 44319_2026_742_MOESM13_ESM.zip › Figure 3 Part 3/Fig 3b-e TTLL screen part 3/TTLL11-YFP Img 8 yfp2000.Project Maximum Z_XY1648578082_Z0_T0_C1.tif]

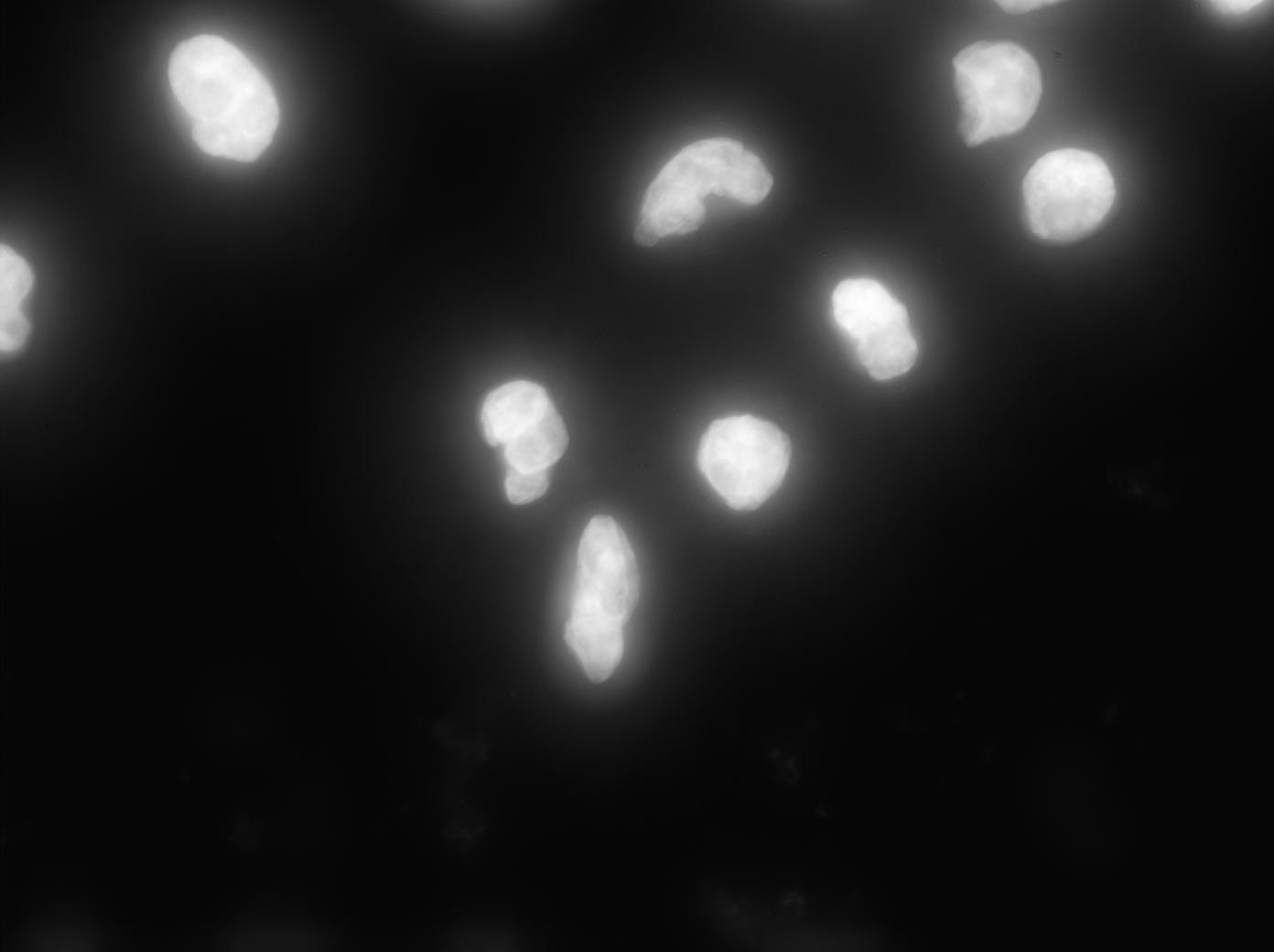

Supplement: Supplementary file 13 — Source data Fig. 3 part 3 [file 44319_2026_742_MOESM13_ESM.zip › Figure 3 Part 3/Fig 3b-e TTLL screen part 3/TTLL11-YFP Img 8 yfp2000.Project Maximum Z_XY1648578082_Z0_T0_C0.tif]

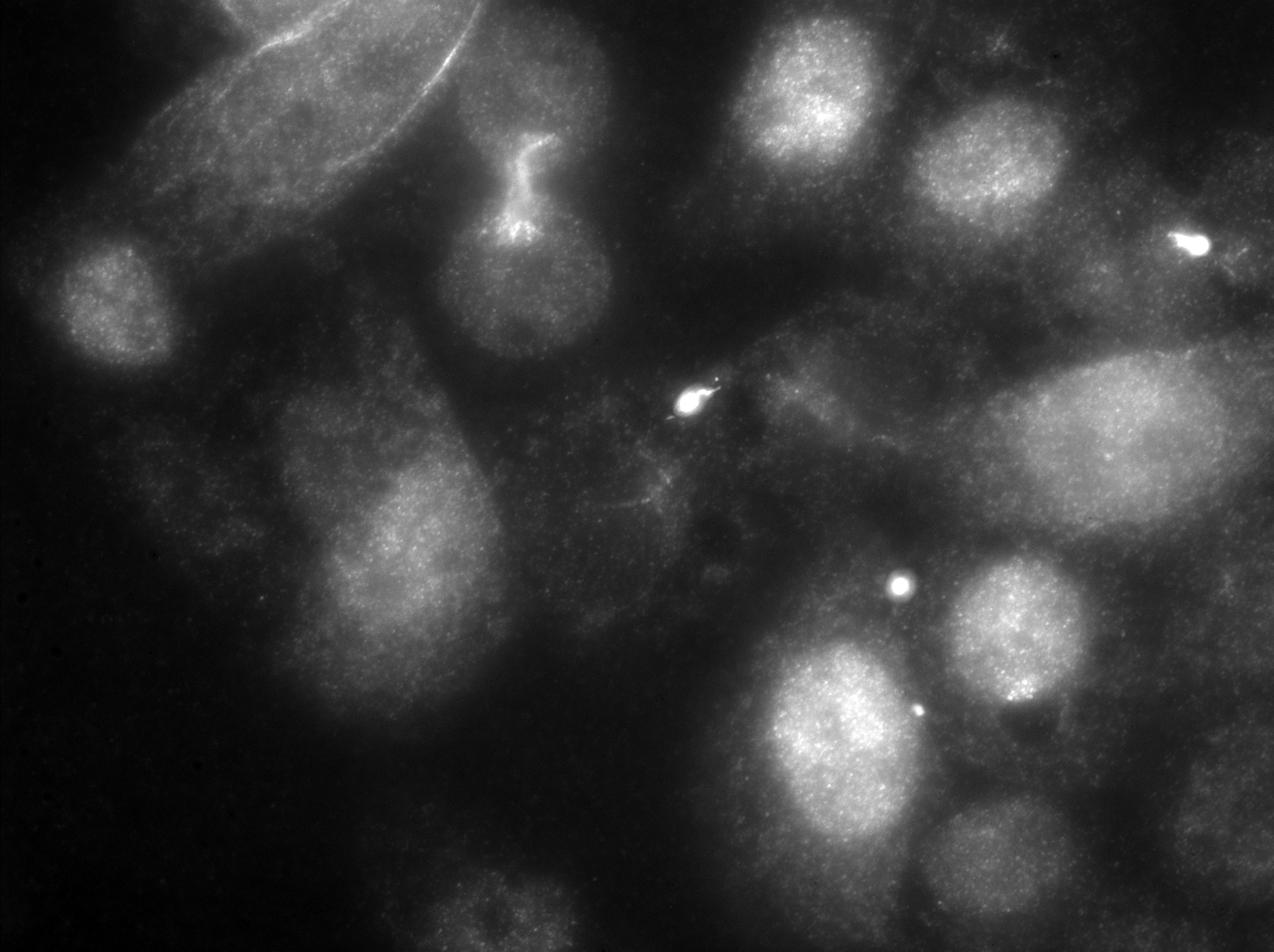

Supplement: Supplementary file 13 — Source data Fig. 3 part 3 [file 44319_2026_742_MOESM13_ESM.zip › Figure 3 Part 3/Fig 3b-e TTLL screen part 3/TTLL9-YFP A3 I8.Project Maximum Z_XY1679700378_Z0_T0_C1.tif]

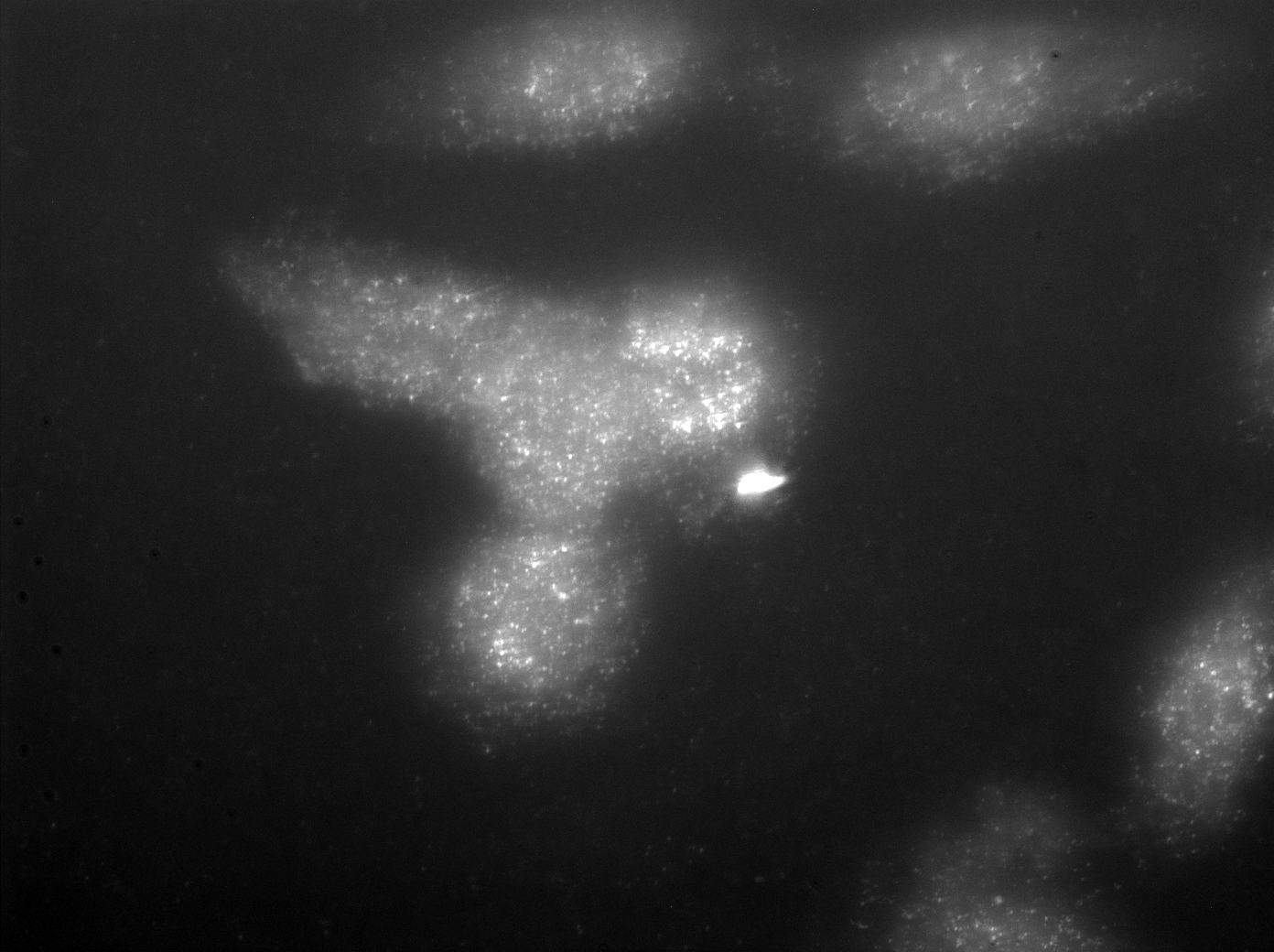

Supplement: Supplementary file 13 — Source data Fig. 3 part 3 [file 44319_2026_742_MOESM13_ESM.zip › Figure 3 Part 3/Fig 3b-e TTLL screen part 3/TTLL11-YFP A2 Img2.Project Maximum Z_XY1648574683_Z0_T0_C1.tif]

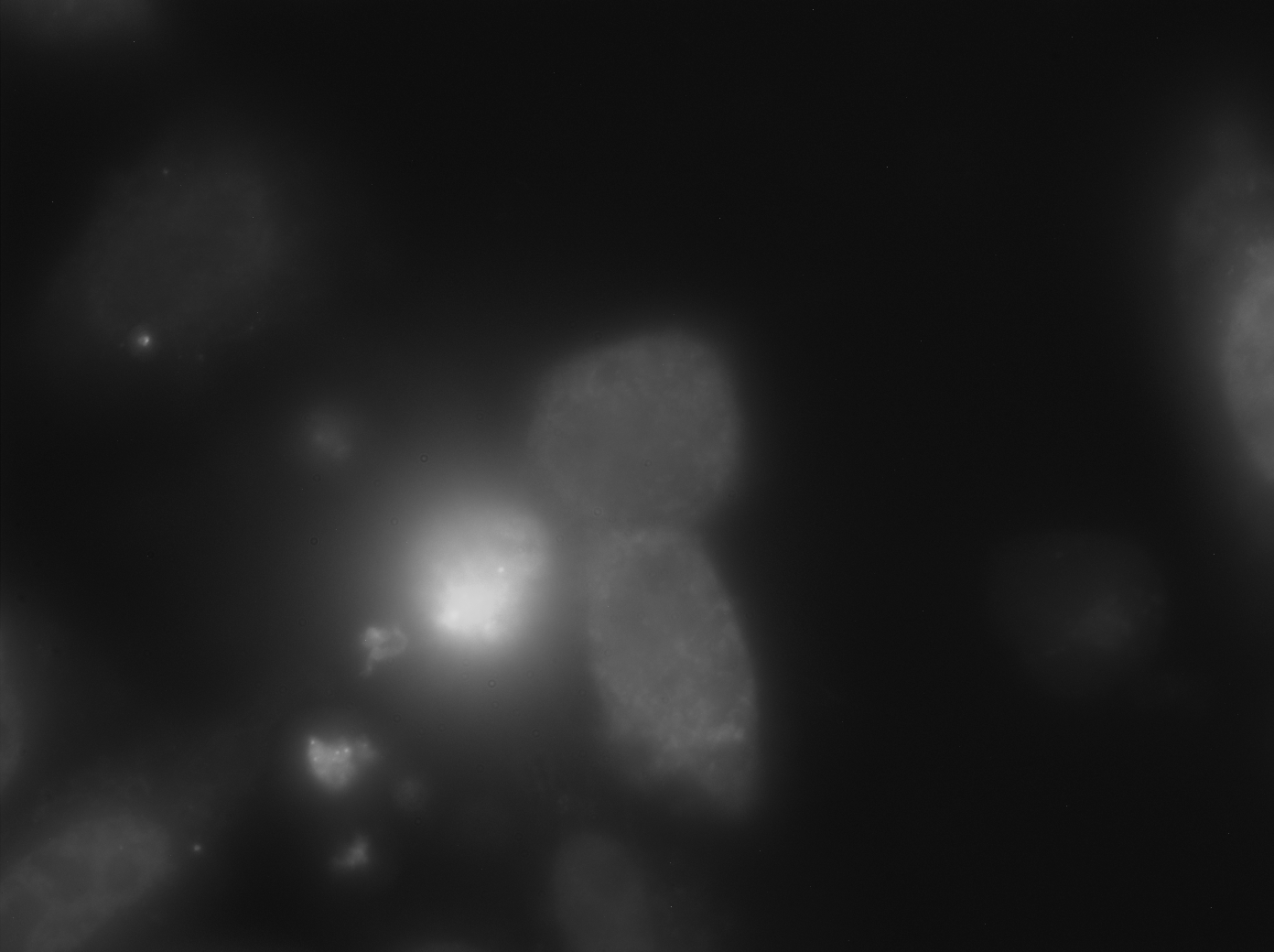

Supplement: Supplementary file 13 — Source data Fig. 3 part 3 [file 44319_2026_742_MOESM13_ESM.zip › Figure 3 Part 3/Fig 3b-e TTLL screen part 3/TTLL9-YFP A3 I16.Project Maximum Z_XY1679701502_Z0_T0_C2.tif]

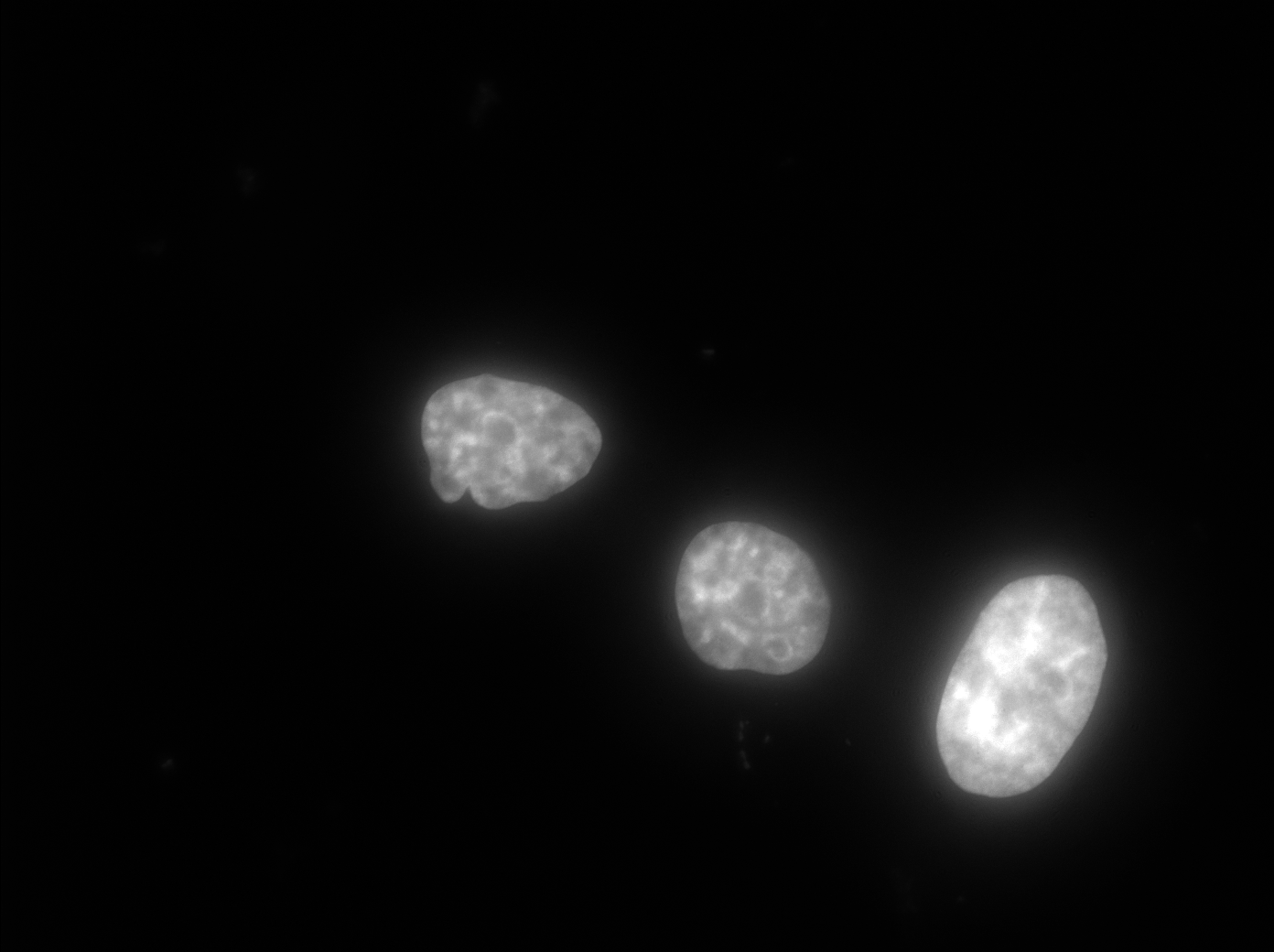

Supplement: Supplementary file 13 — Source data Fig. 3 part 3 [file 44319_2026_742_MOESM13_ESM.zip › Figure 3 Part 3/Fig 3b-e TTLL screen part 3/TTLL9-YFP R1 I5.Project Maximum Z_XY1674167006_Z0_T0_C0.tif]

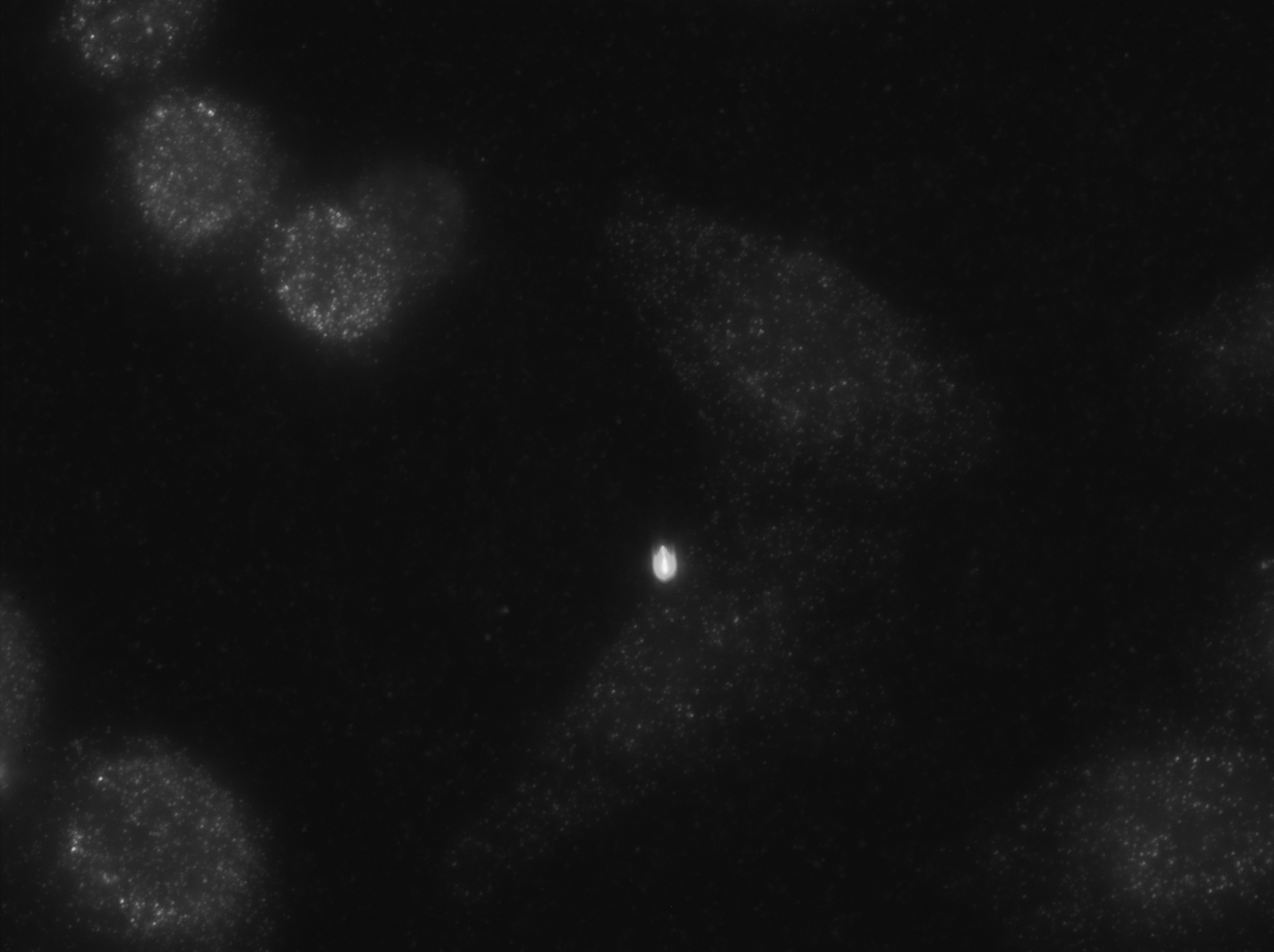

Supplement: Supplementary file 13 — Source data Fig. 3 part 3 [file 44319_2026_742_MOESM13_ESM.zip › Figure 3 Part 3/Fig 3b-e TTLL screen part 3/TTLL11-YFP Img 16 yfp 2000.Project Maximum Z_XY1648586920_Z0_T0_C1.tif]

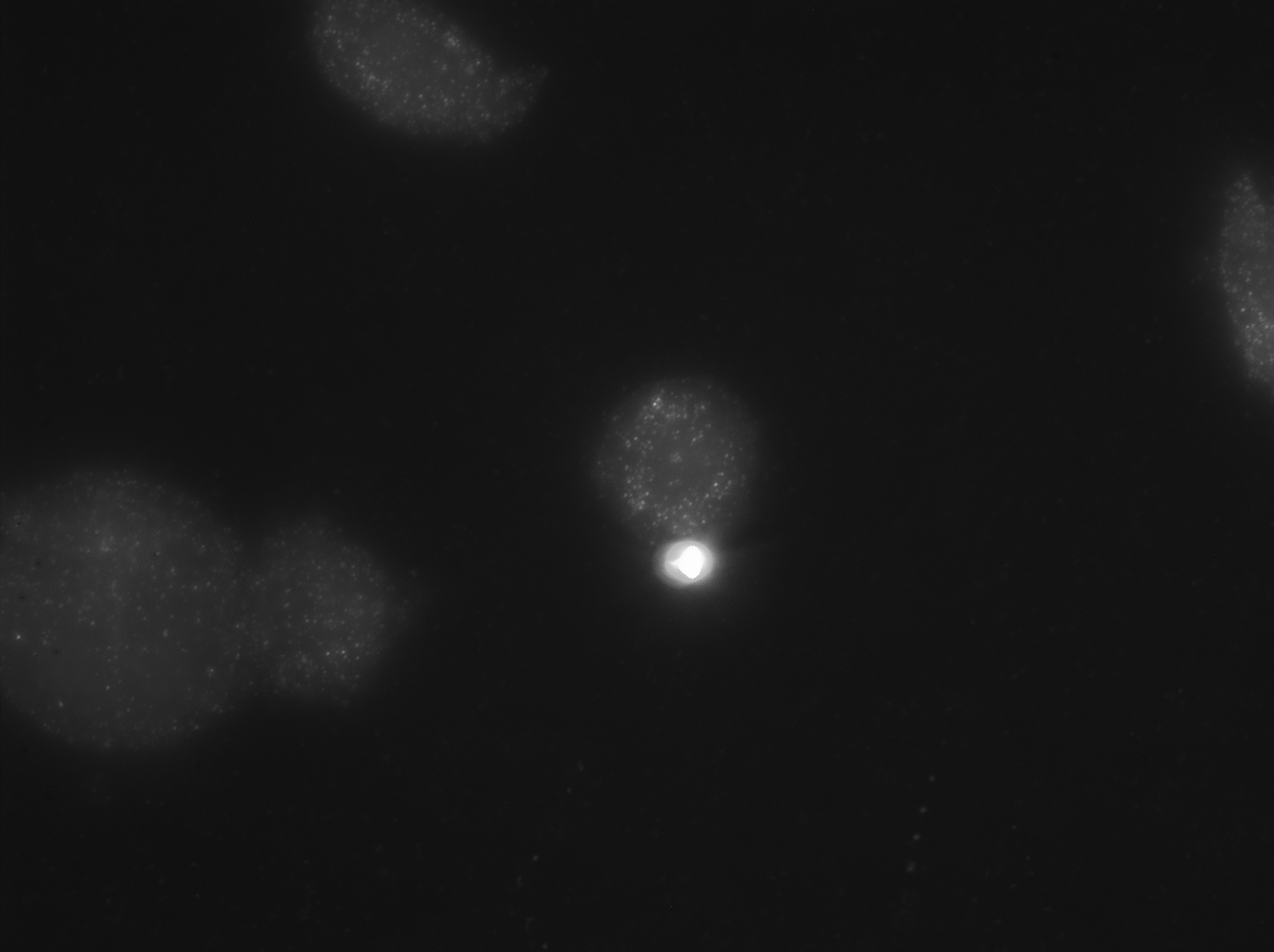

Supplement: Supplementary file 13 — Source data Fig. 3 part 3 [file 44319_2026_742_MOESM13_ESM.zip › Figure 3 Part 3/Fig 3b-e TTLL screen part 3/TTLL11-YFP Img 14 yfp 1200 - 1.Project Maximum Z_XY1648581343_Z0_T0_C1.tif]

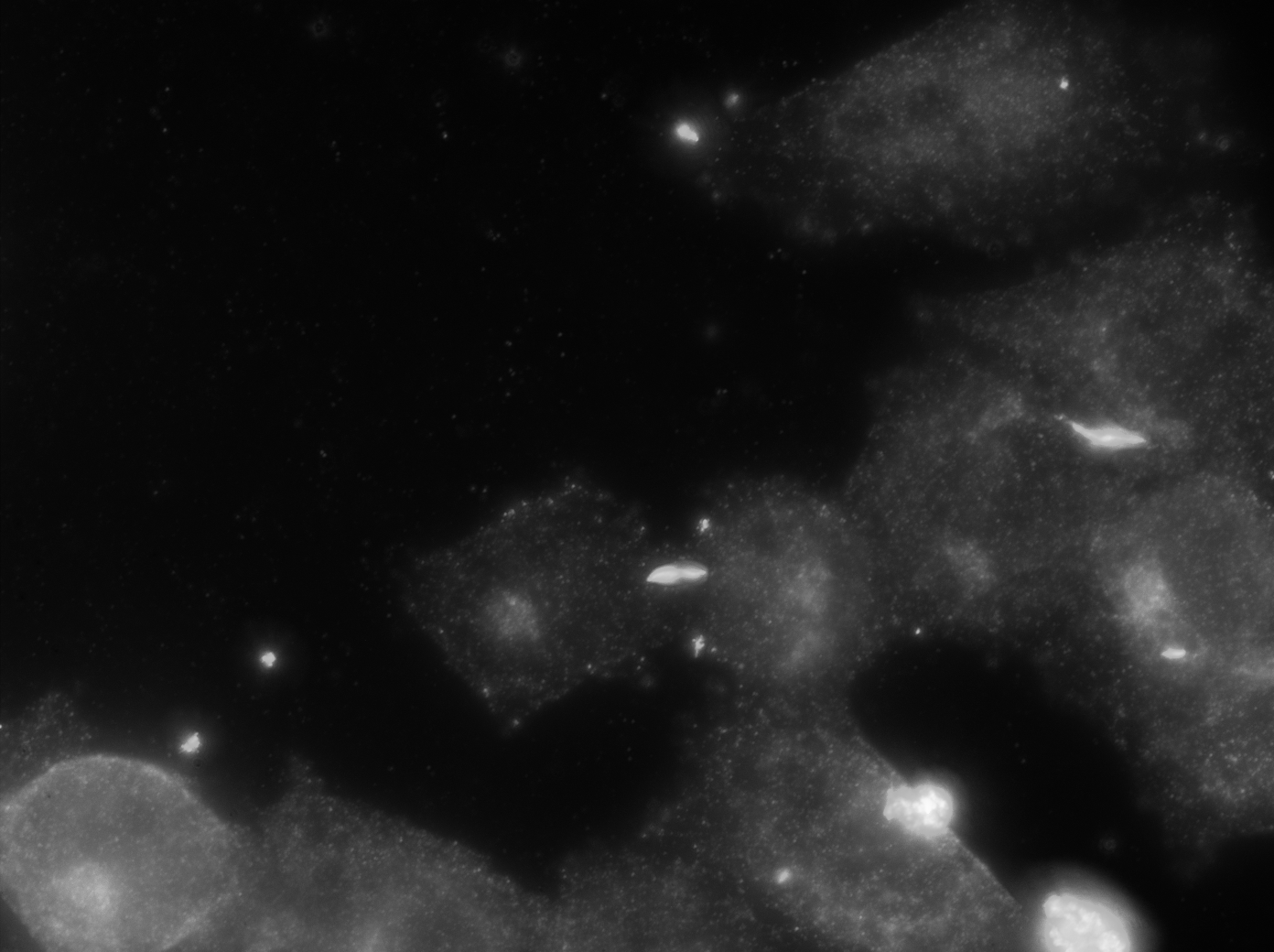

Supplement: Supplementary file 13 — Source data Fig. 3 part 3 [file 44319_2026_742_MOESM13_ESM.zip › Figure 3 Part 3/Fig 3b-e TTLL screen part 3/TTLL9-GFP A4 I3.Project Maximum Z_XY1675965809_Z0_T0_C1.tif]

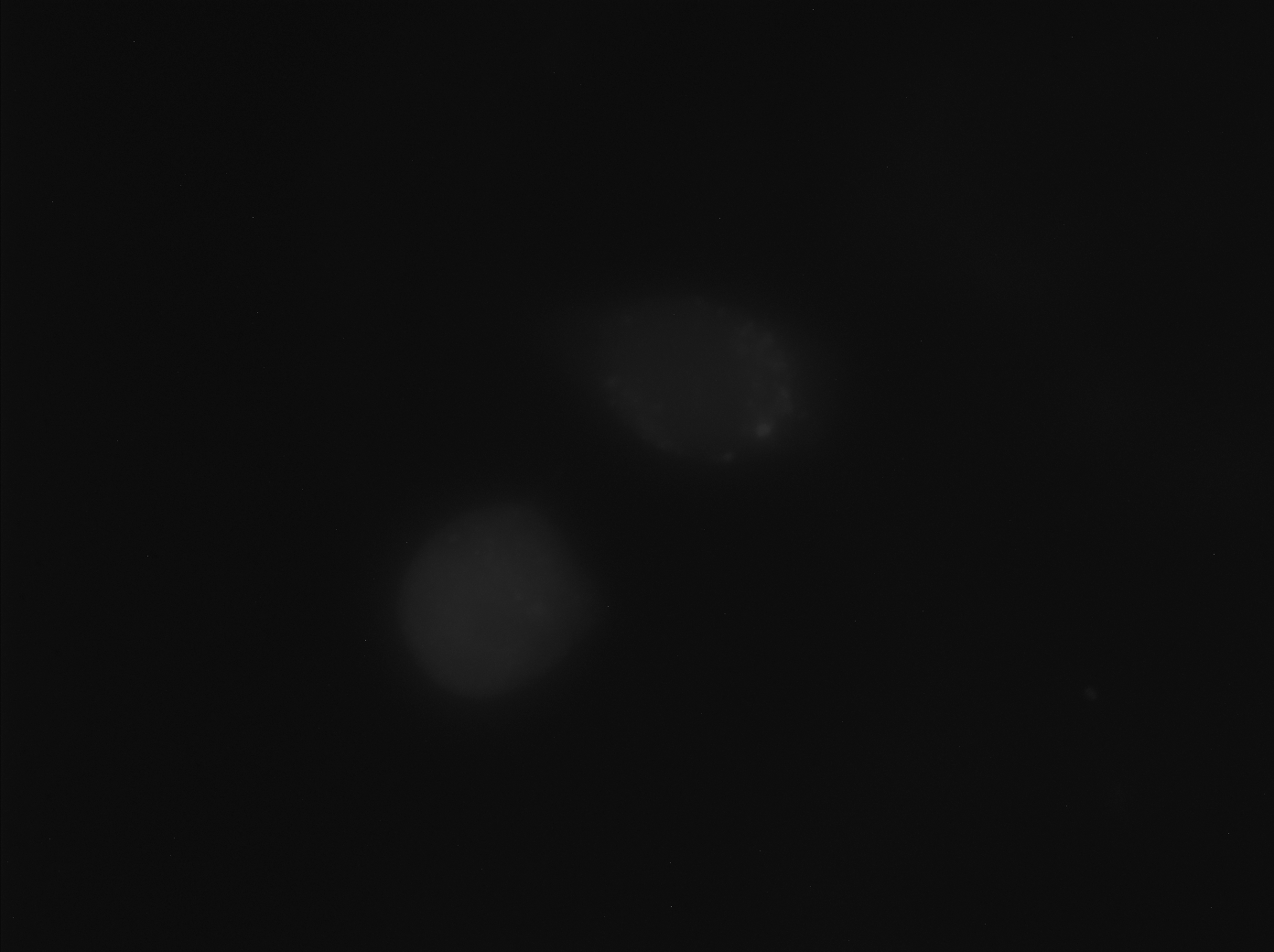

Supplement: Supplementary file 13 — Source data Fig. 3 part 3 [file 44319_2026_742_MOESM13_ESM.zip › Figure 3 Part 3/Fig 3b-e TTLL screen part 3/TTLL9-YFP A3 I1.Project Maximum Z_XY1674674408_Z0_T0_C2.tif]

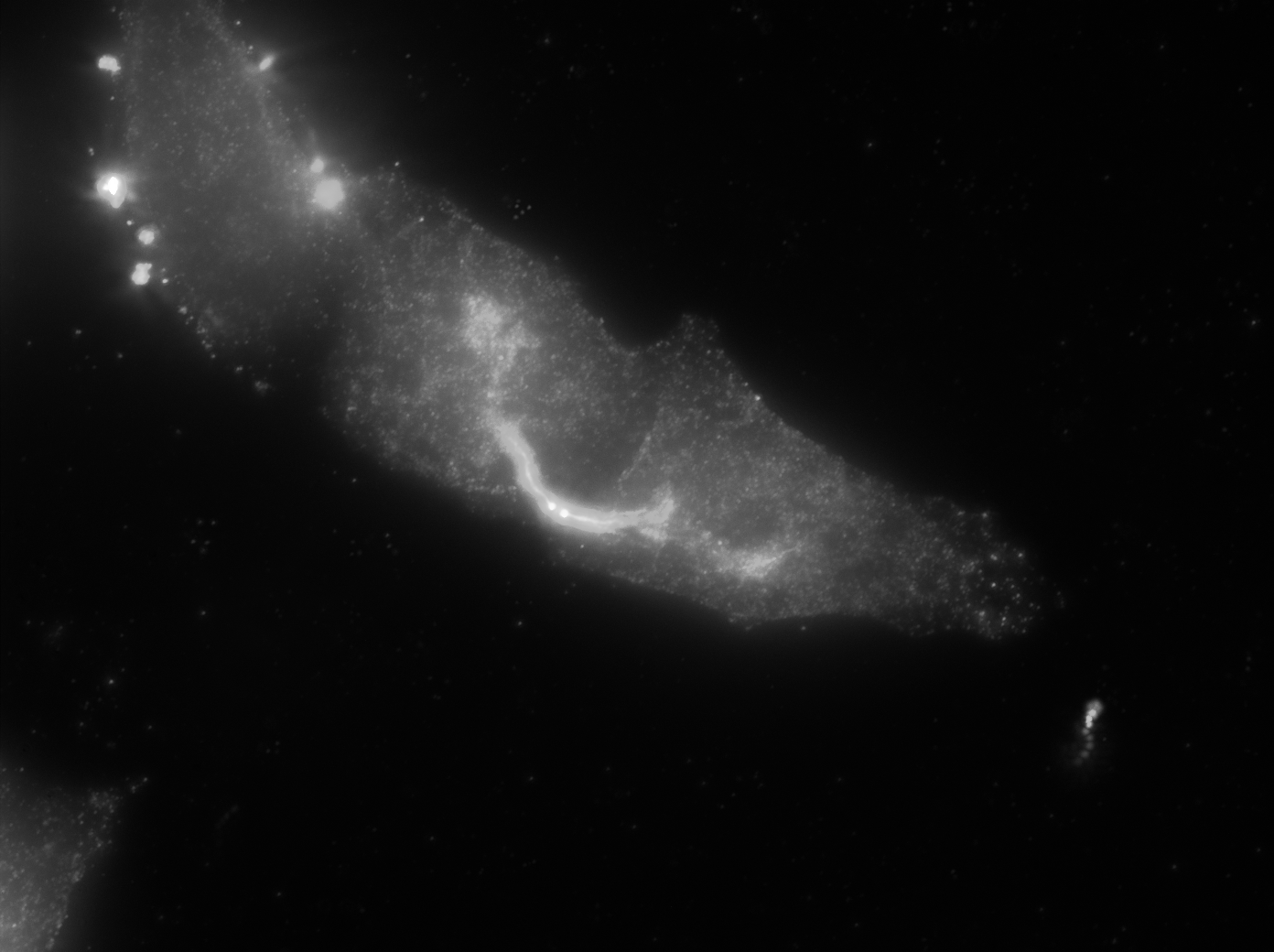

Supplement: Supplementary file 13 — Source data Fig. 3 part 3 [file 44319_2026_742_MOESM13_ESM.zip › Figure 3 Part 3/Fig 3b-e TTLL screen part 3/TTLL9-GFP A4 I4.Project Maximum Z_XY1675966027_Z0_T0_C1.tif]

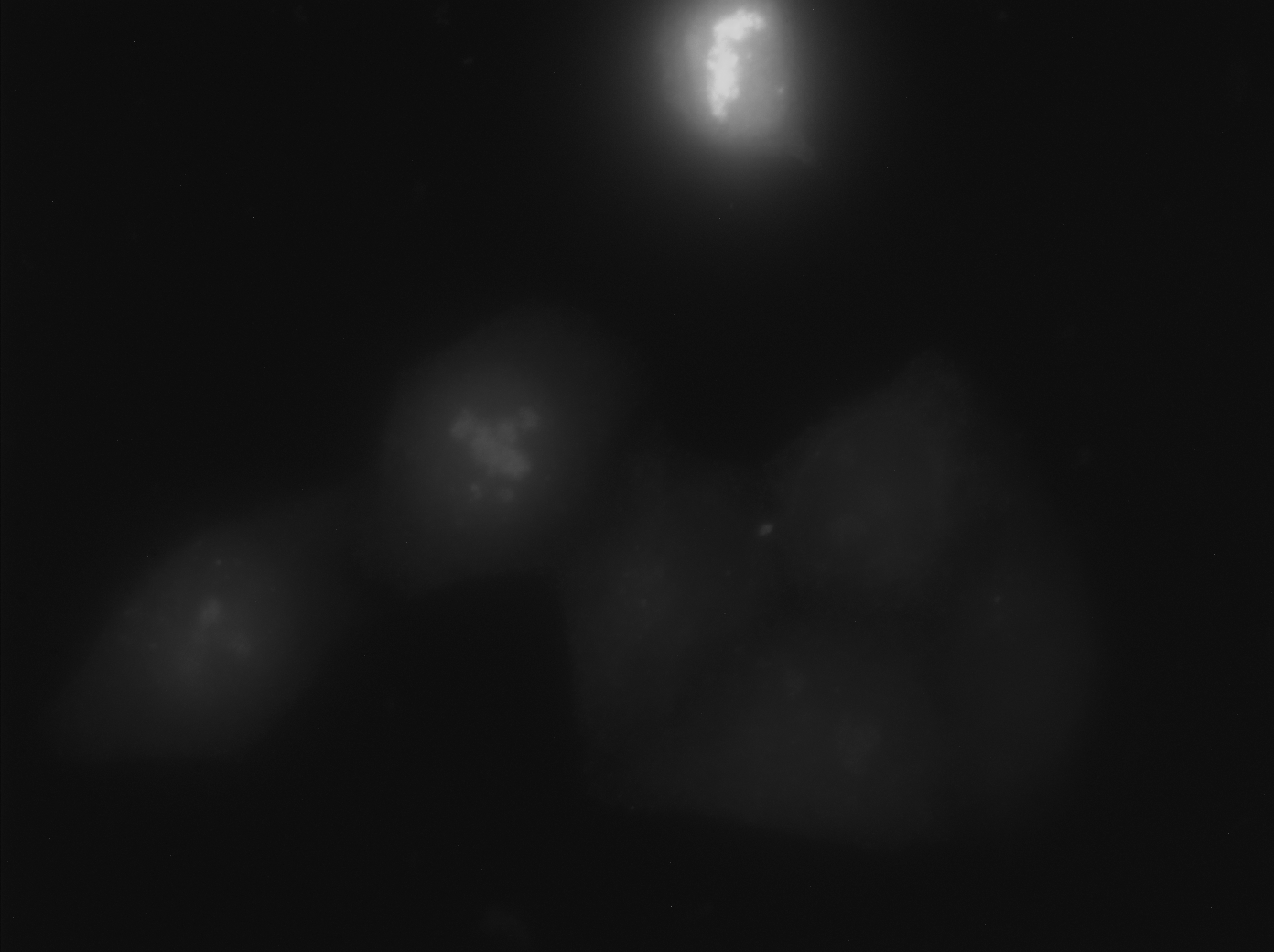

Supplement: Supplementary file 13 — Source data Fig. 3 part 3 [file 44319_2026_742_MOESM13_ESM.zip › Figure 3 Part 3/Fig 3b-e TTLL screen part 3/TTLL11-YFP Img 1 yfp1100.Project Maximum Z_XY1648156180_Z0_T0_C2.tif]

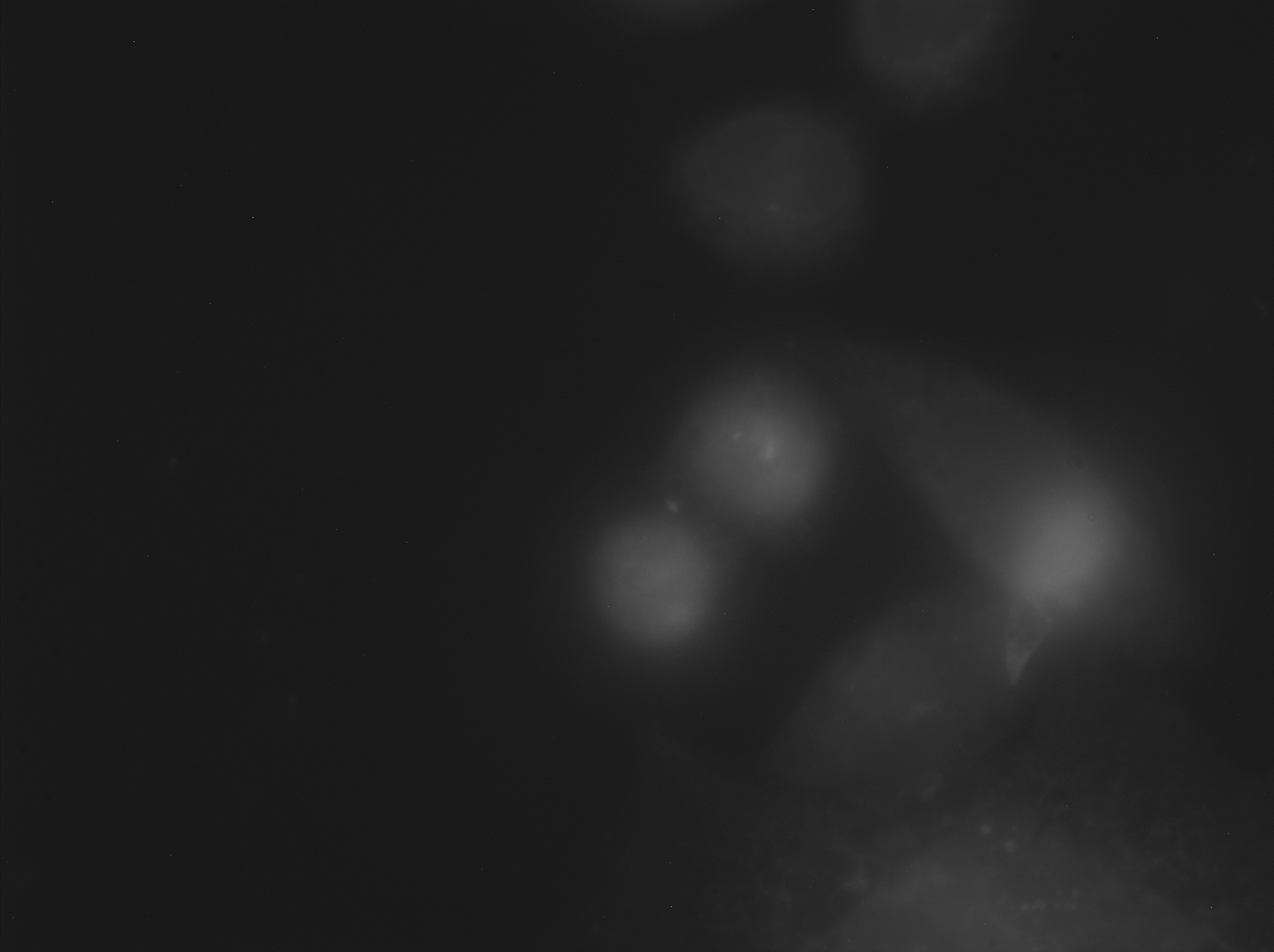

Supplement: Supplementary file 13 — Source data Fig. 3 part 3 [file 44319_2026_742_MOESM13_ESM.zip › Figure 3 Part 3/Fig 3b-e TTLL screen part 3/TTLL11-YFP A1 Img3.Project Maximum Z_XY1650054708_Z0_T0_C2.tif]

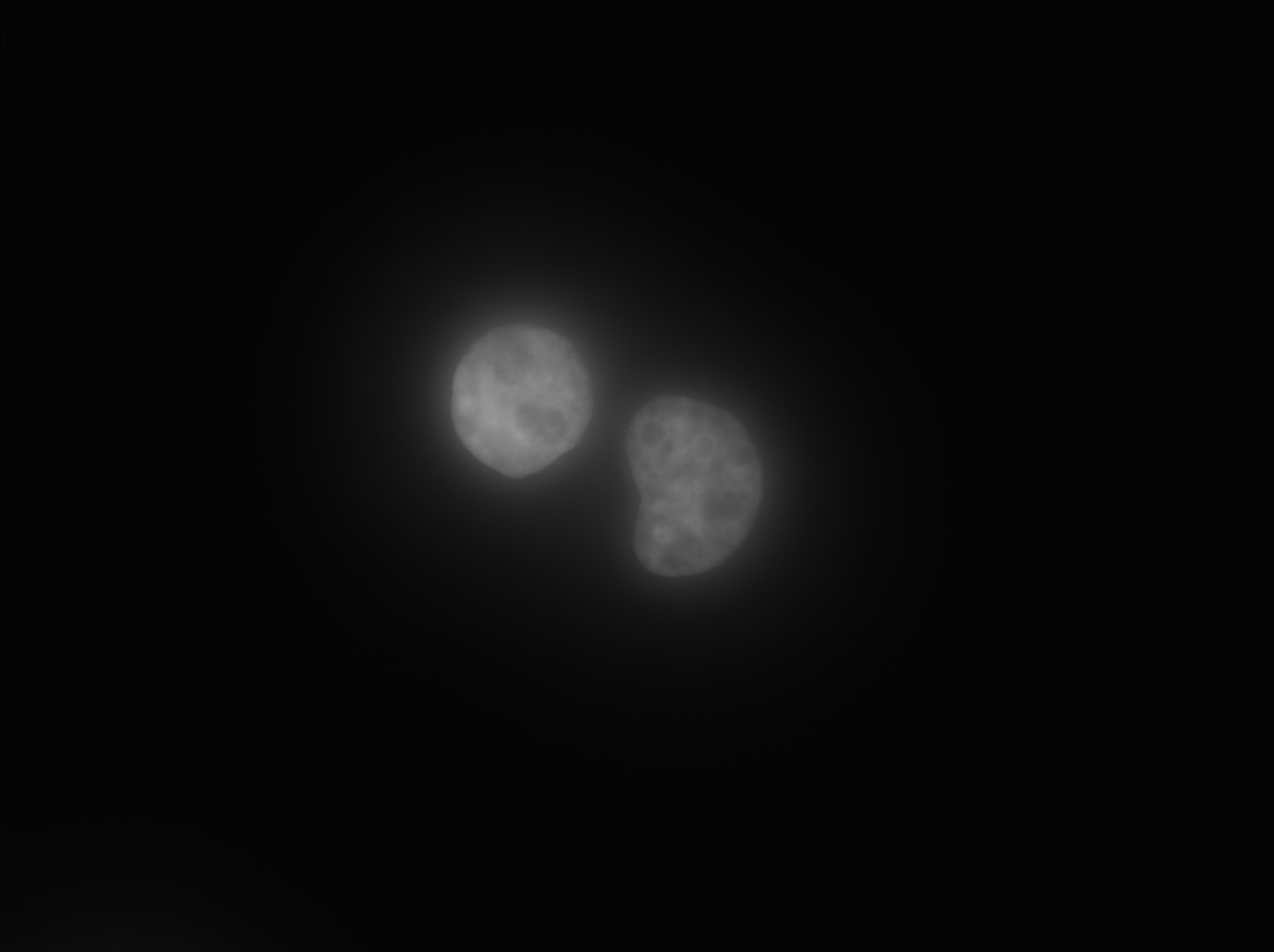

Supplement: Supplementary file 13 — Source data Fig. 3 part 3 [file 44319_2026_742_MOESM13_ESM.zip › Figure 3 Part 3/Fig 3b-e TTLL screen part 3/TTLL11-YFP Img 15 yfp 2000.Project Maximum Z_XY1648586329_Z0_T0_C0.tif]

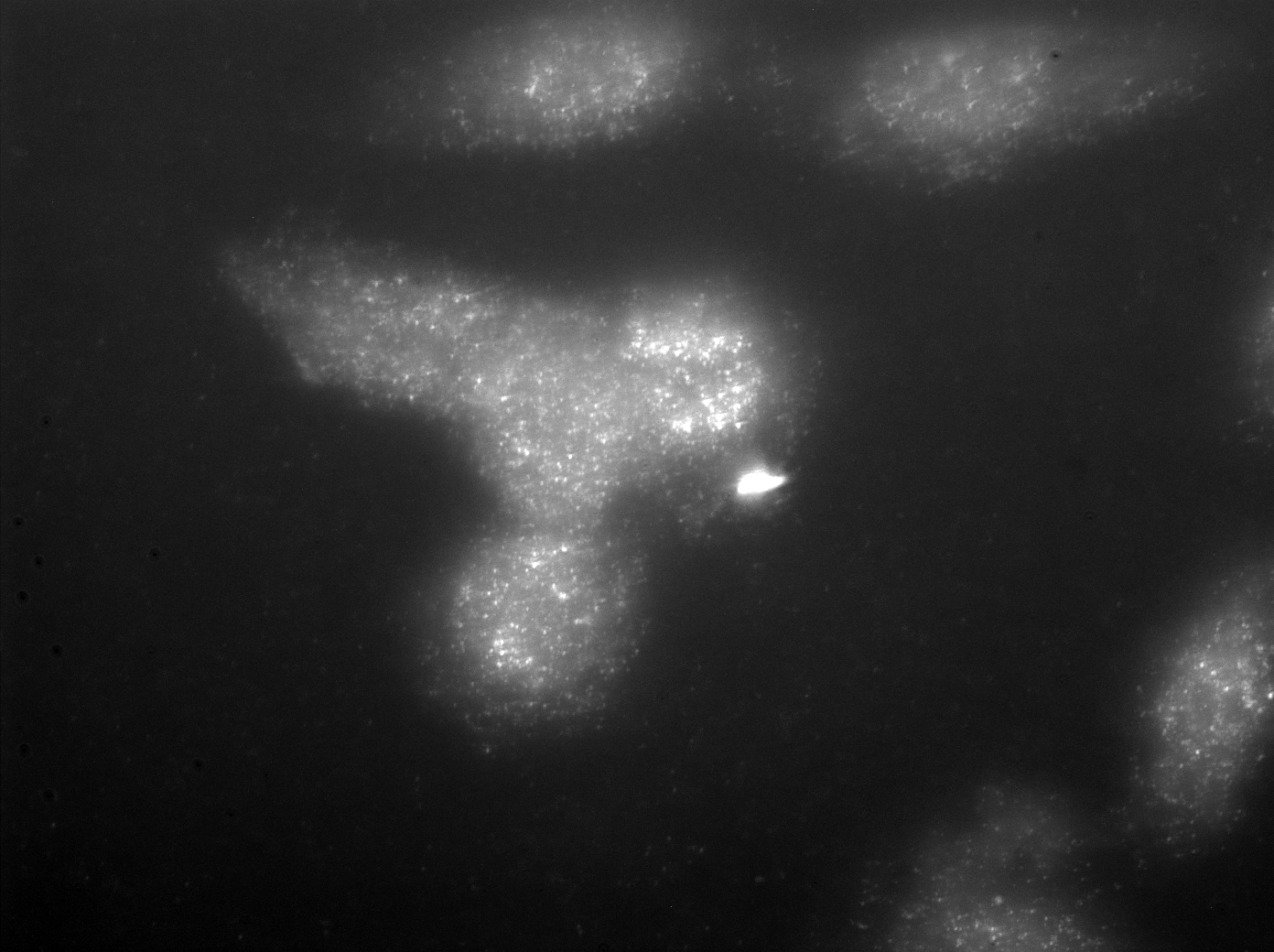

Supplement: Supplementary file 13 — Source data Fig. 3 part 3 [file 44319_2026_742_MOESM13_ESM.zip › Figure 3 Part 3/Fig 3b-e TTLL screen part 3/TTLL11-YFP Img 7 yfp2000.Project Maximum Z_XY1648574683_Z0_T0_C1.tif]

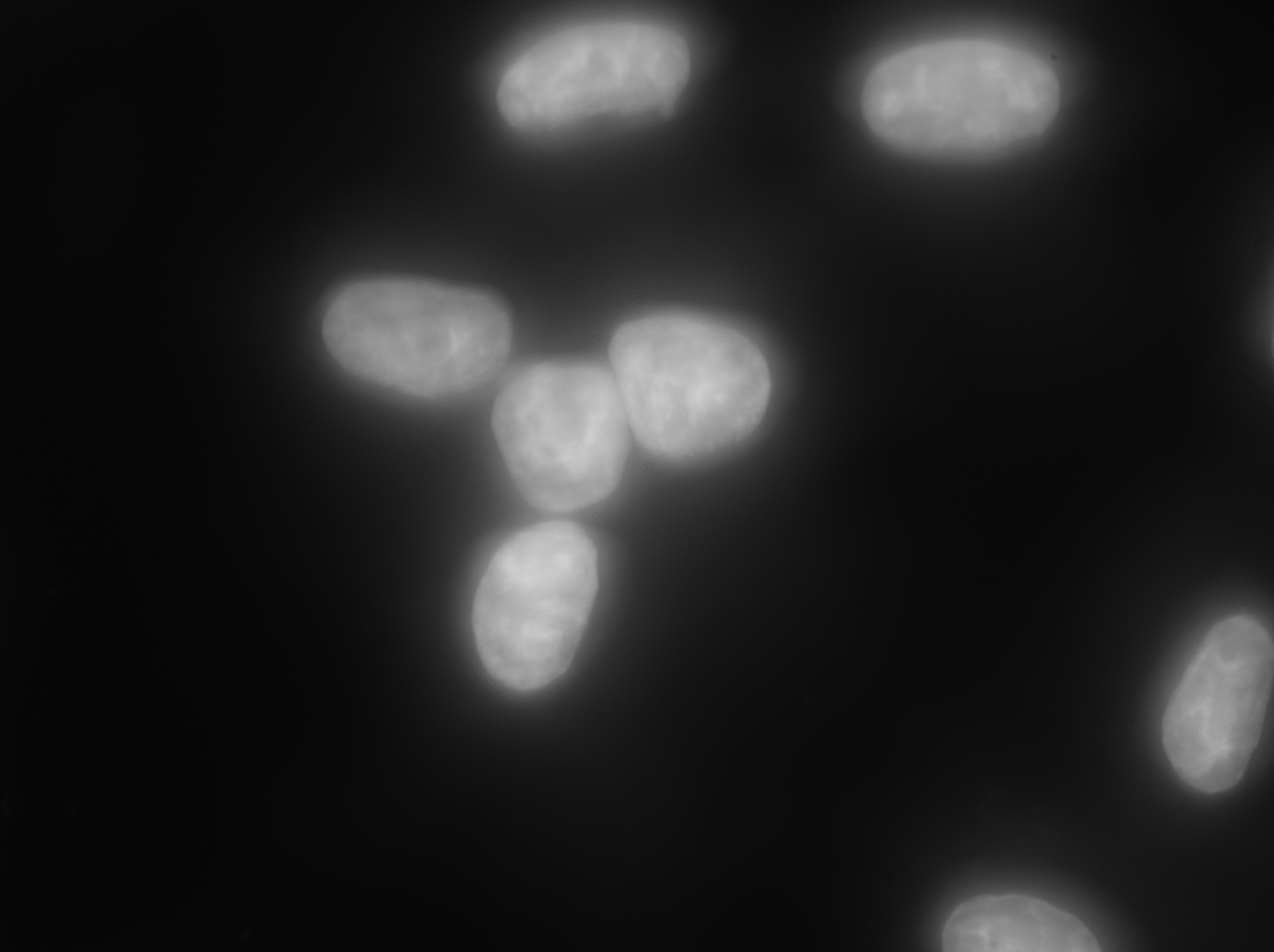

Supplement: Supplementary file 13 — Source data Fig. 3 part 3 [file 44319_2026_742_MOESM13_ESM.zip › Figure 3 Part 3/Fig 3b-e TTLL screen part 3/TTLL11-YFP Img 7 yfp2000.Project Maximum Z_XY1648574683_Z0_T0_C0.tif]

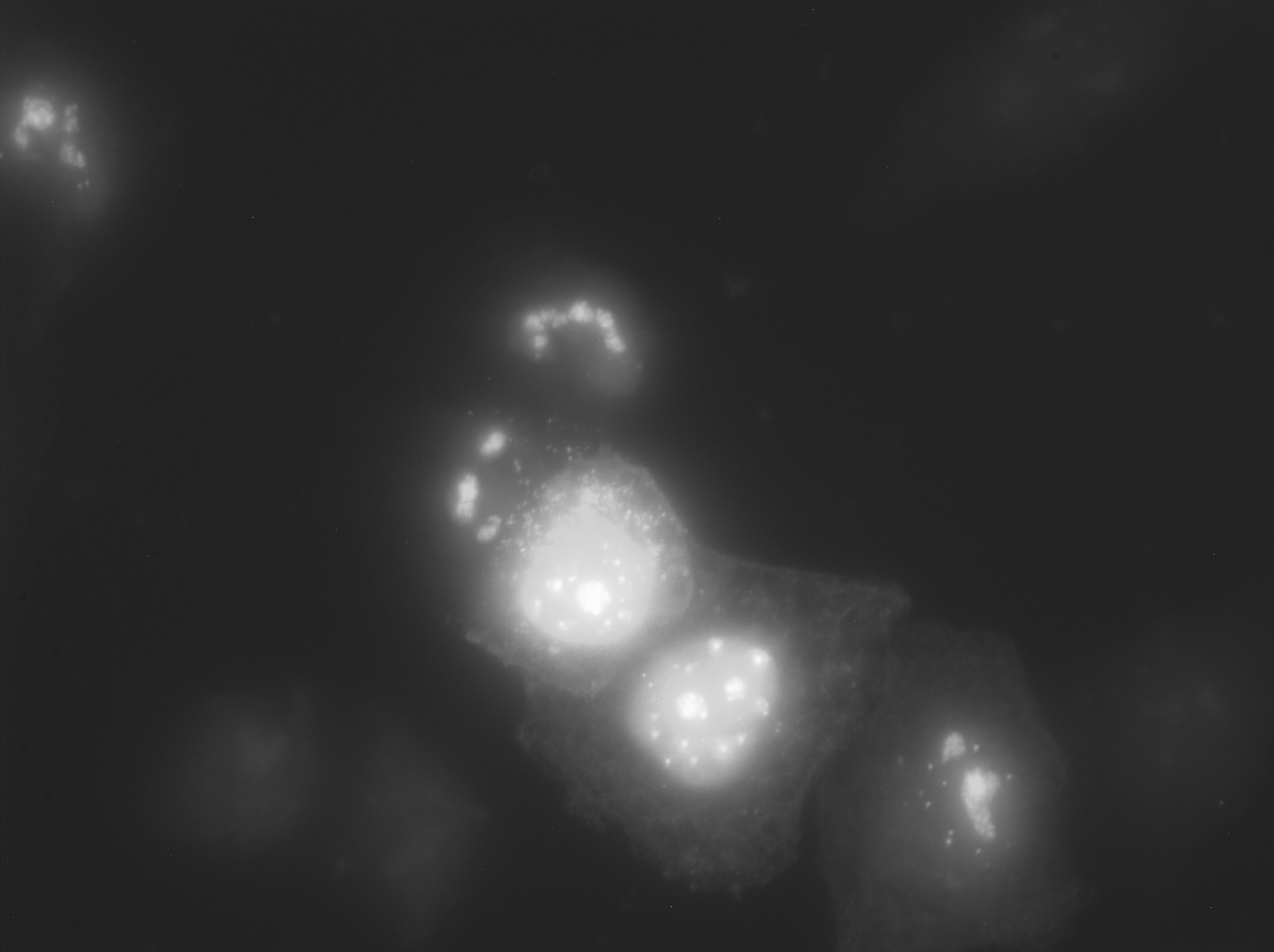

Supplement: Supplementary file 13 — Source data Fig. 3 part 3 [file 44319_2026_742_MOESM13_ESM.zip › Figure 3 Part 3/Fig 3b-e TTLL screen part 3/TTLL11-YFP Img 6 yfp2000.Project Maximum Z_XY1648574277_Z0_T0_C2.tif]

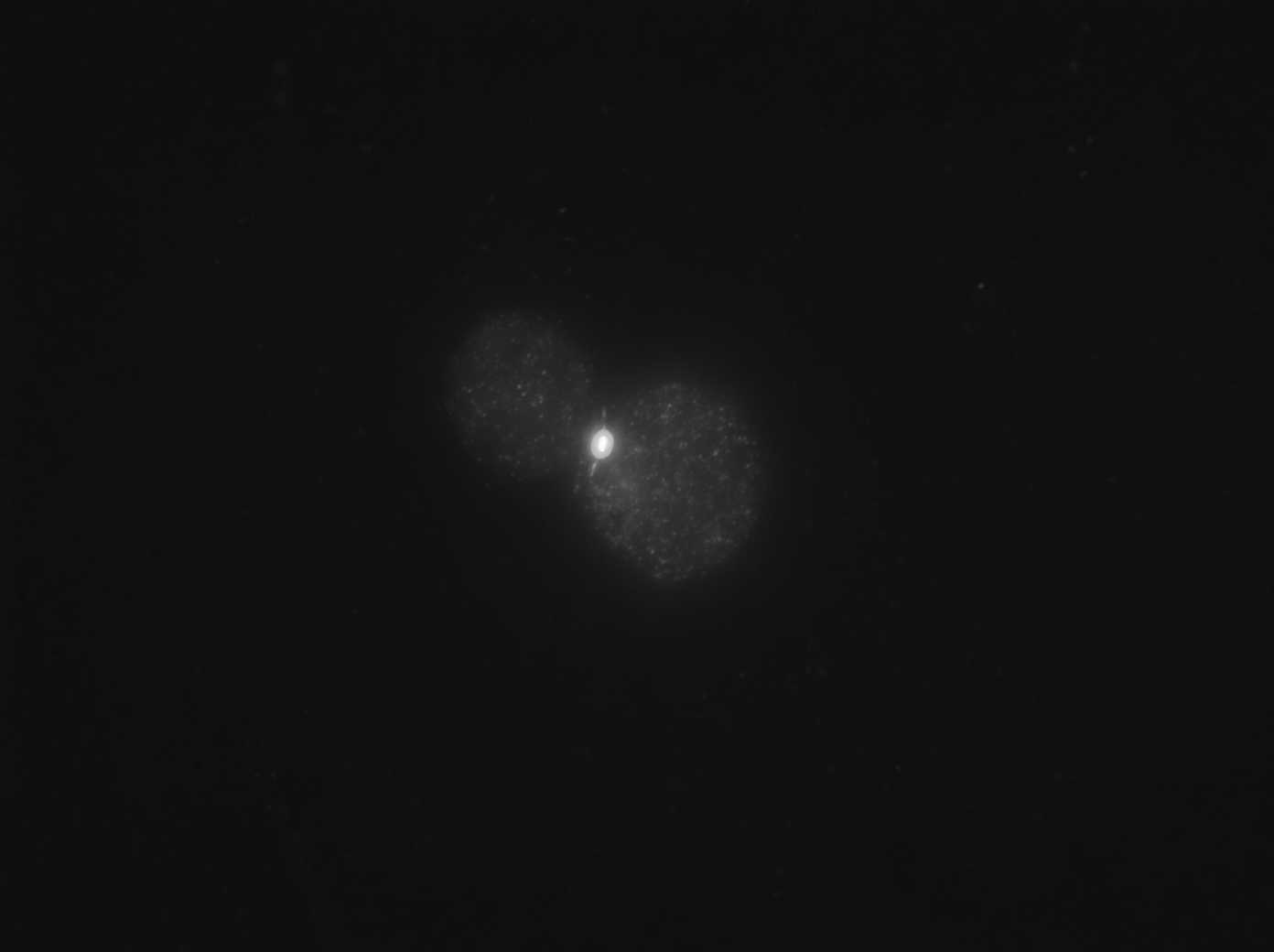

Supplement: Supplementary file 13 — Source data Fig. 3 part 3 [file 44319_2026_742_MOESM13_ESM.zip › Figure 3 Part 3/Fig 3b-e TTLL screen part 3/TTLL11-YFP Img 15 yfp 2000.Project Maximum Z_XY1648586329_Z0_T0_C1.tif]

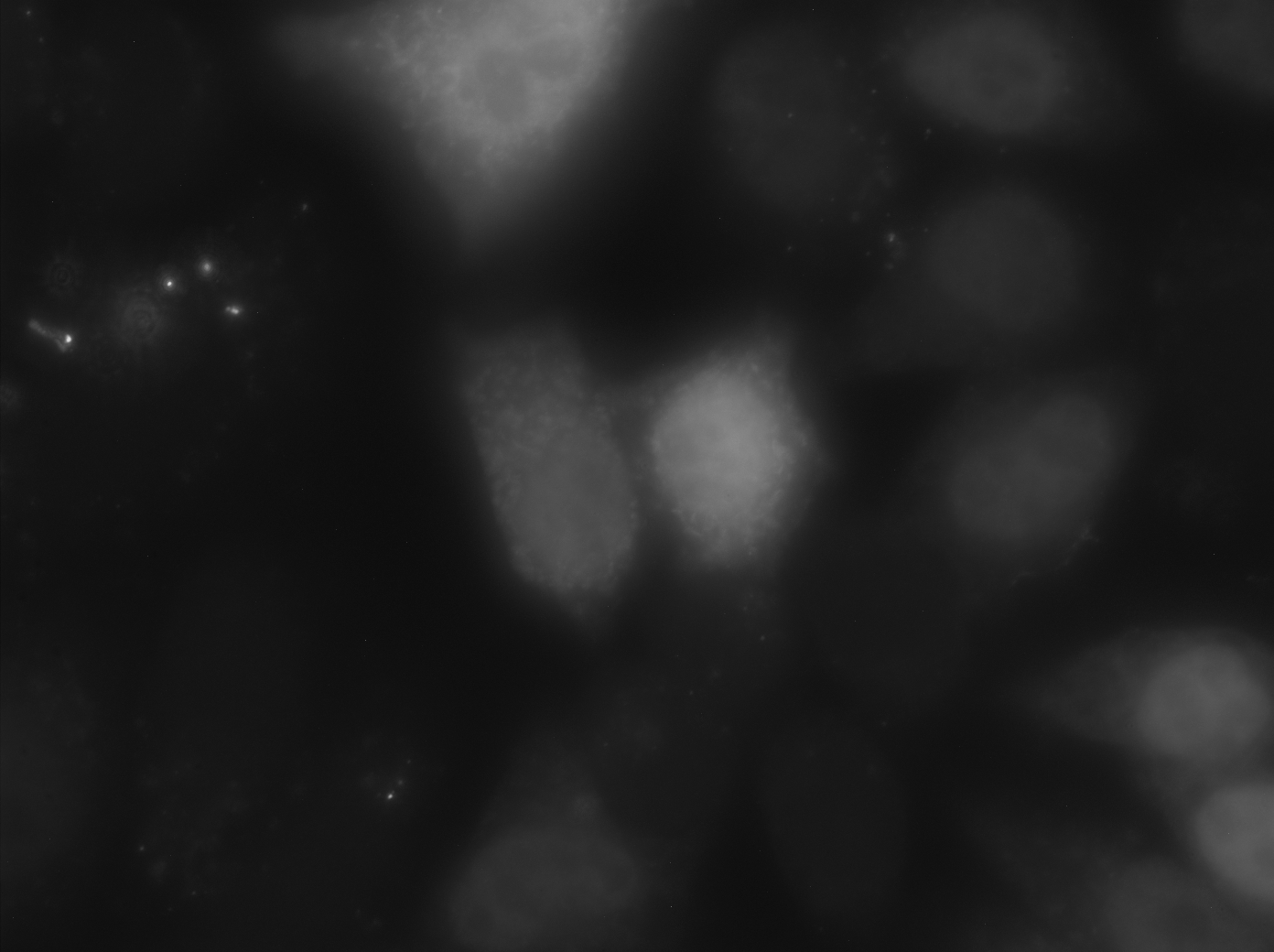

Supplement: Supplementary file 13 — Source data Fig. 3 part 3 [file 44319_2026_742_MOESM13_ESM.zip › Figure 3 Part 3/Fig 3b-e TTLL screen part 3/TTLL9-YFP A3 I3 - 1.Project Maximum Z_XY1679699600_Z0_T0_C2.tif]

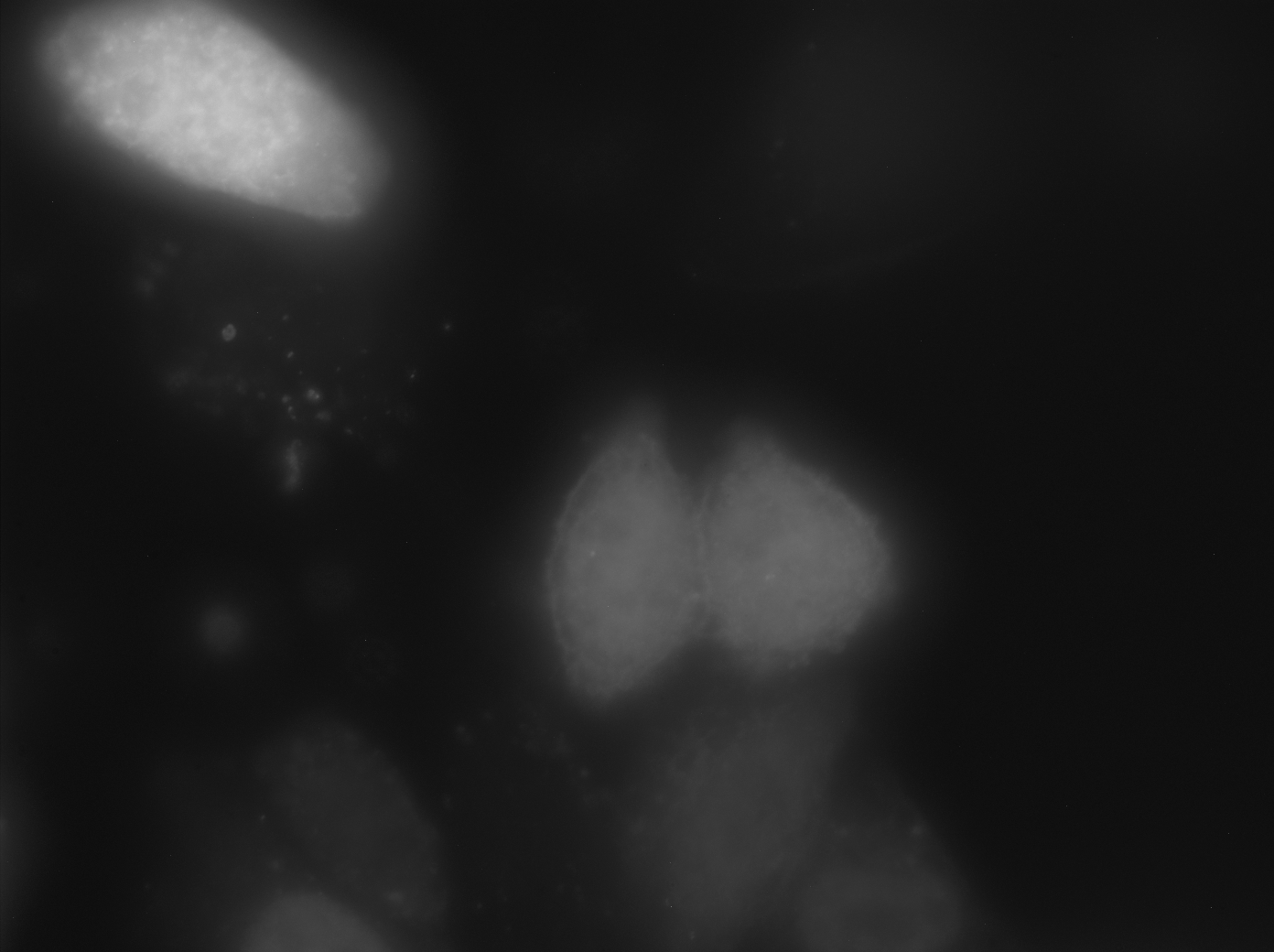

Supplement: Supplementary file 13 — Source data Fig. 3 part 3 [file 44319_2026_742_MOESM13_ESM.zip › Figure 3 Part 3/Fig 3b-e TTLL screen part 3/TTLL9-YFP A3 I9.Project Maximum Z_XY1679700536_Z0_T0_C2.tif]

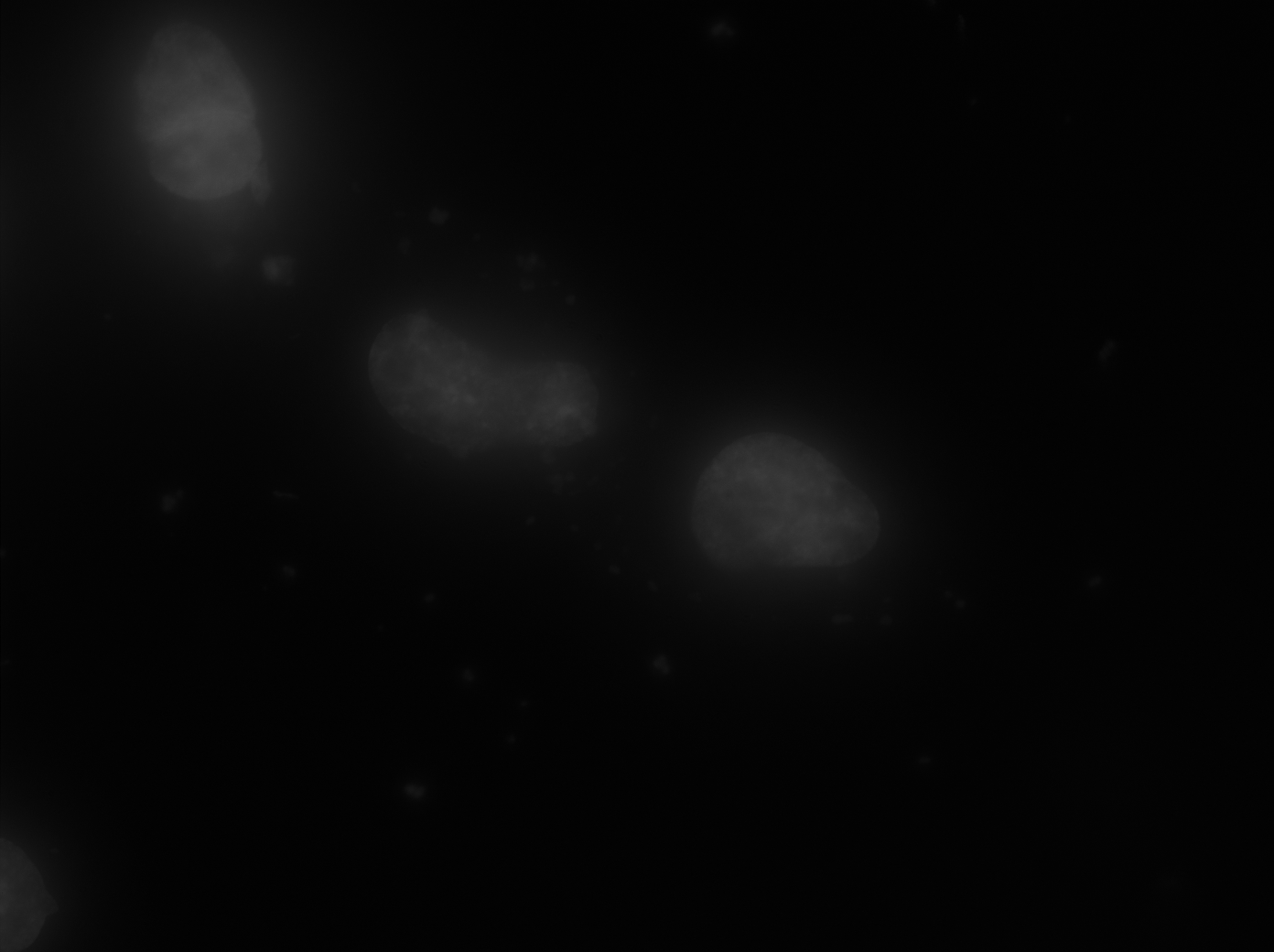

Supplement: Supplementary file 13 — Source data Fig. 3 part 3 [file 44319_2026_742_MOESM13_ESM.zip › Figure 3 Part 3/Fig 3b-e TTLL screen part 3/TTLL9-GFP A4 I4.Project Maximum Z_XY1675966027_Z0_T0_C0.tif]

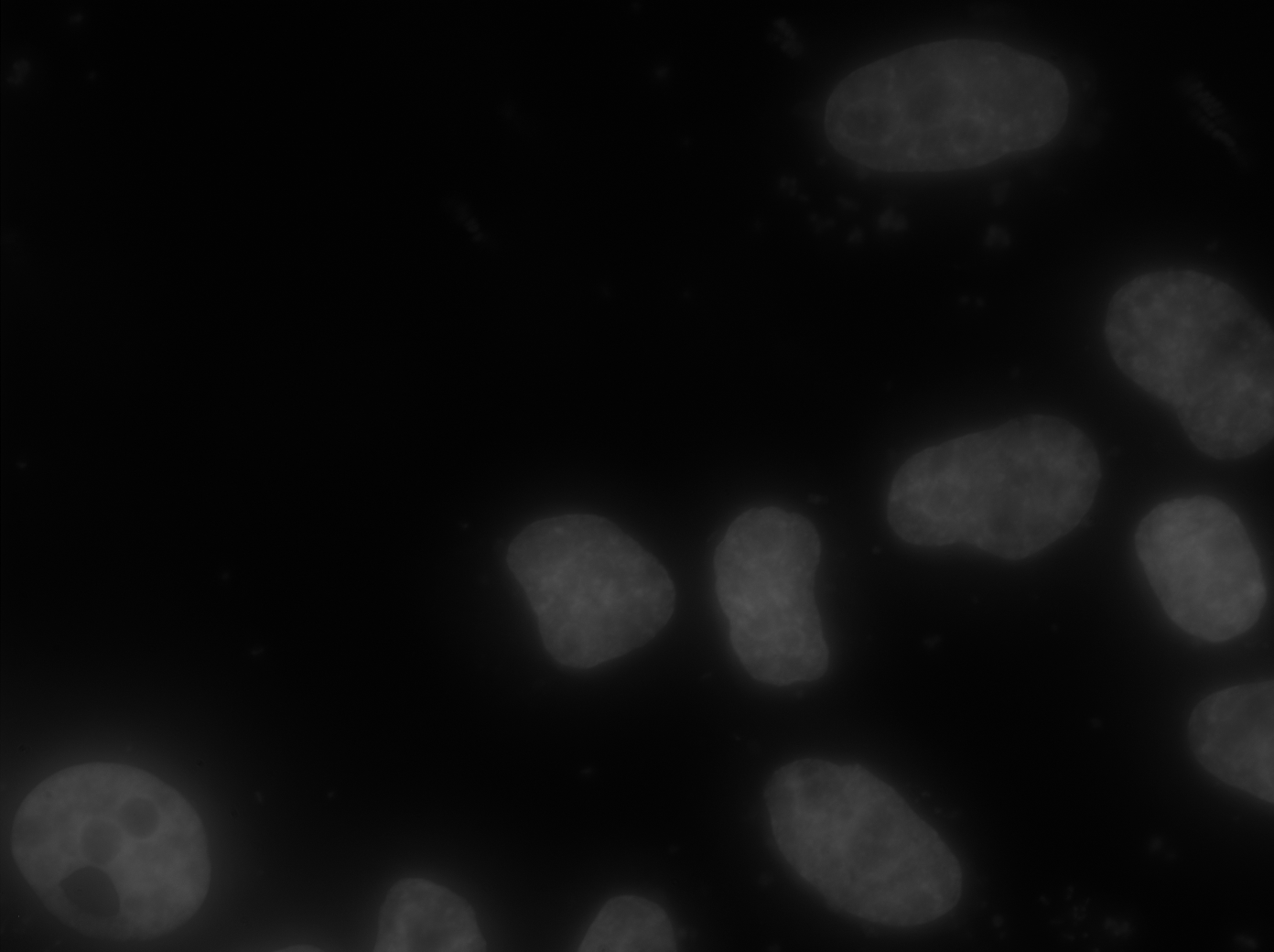

Supplement: Supplementary file 13 — Source data Fig. 3 part 3 [file 44319_2026_742_MOESM13_ESM.zip › Figure 3 Part 3/Fig 3b-e TTLL screen part 3/TTLL9-GFP A4 I3.Project Maximum Z_XY1675965809_Z0_T0_C0.tif]

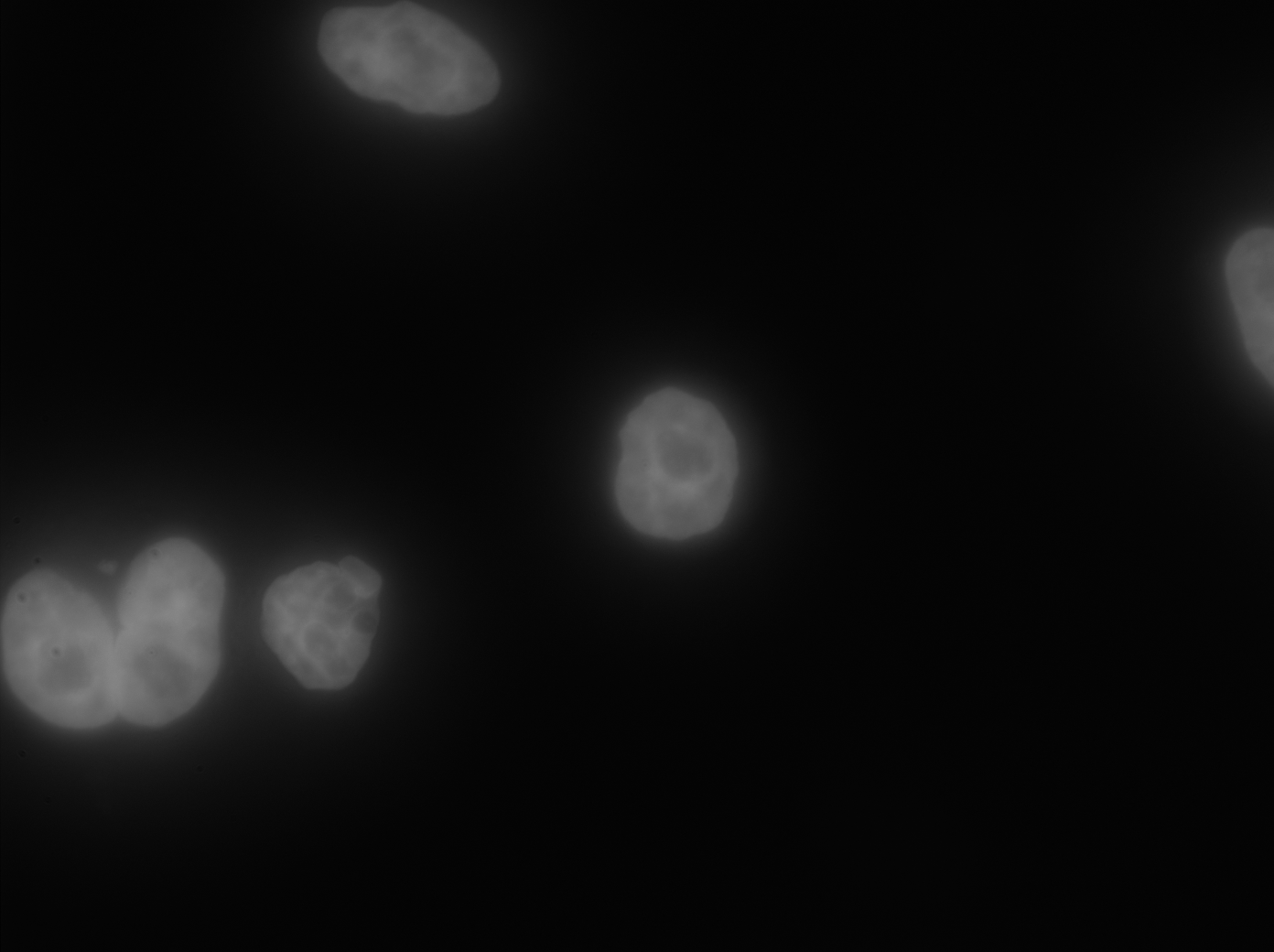

Supplement: Supplementary file 13 — Source data Fig. 3 part 3 [file 44319_2026_742_MOESM13_ESM.zip › Figure 3 Part 3/Fig 3b-e TTLL screen part 3/TTLL11-YFP Img 14 yfp 1200 - 1.Project Maximum Z_XY1648581343_Z0_T0_C0.tif]

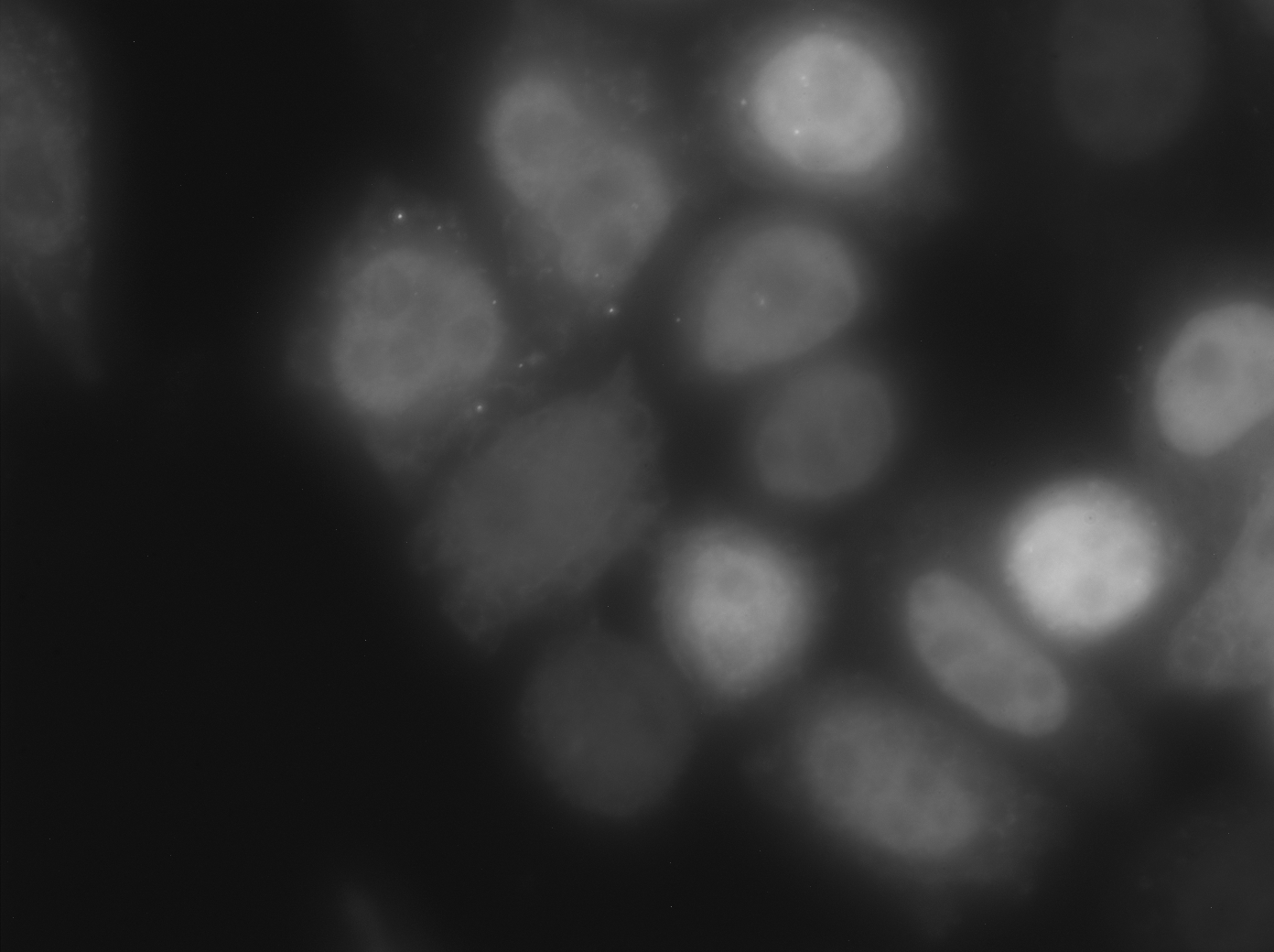

Supplement: Supplementary file 13 — Source data Fig. 3 part 3 [file 44319_2026_742_MOESM13_ESM.zip › Figure 3 Part 3/Fig 3b-e TTLL screen part 3/TTLL9-YFP A3 I14.Project Maximum Z_XY1679701191_Z0_T0_C2.tif]

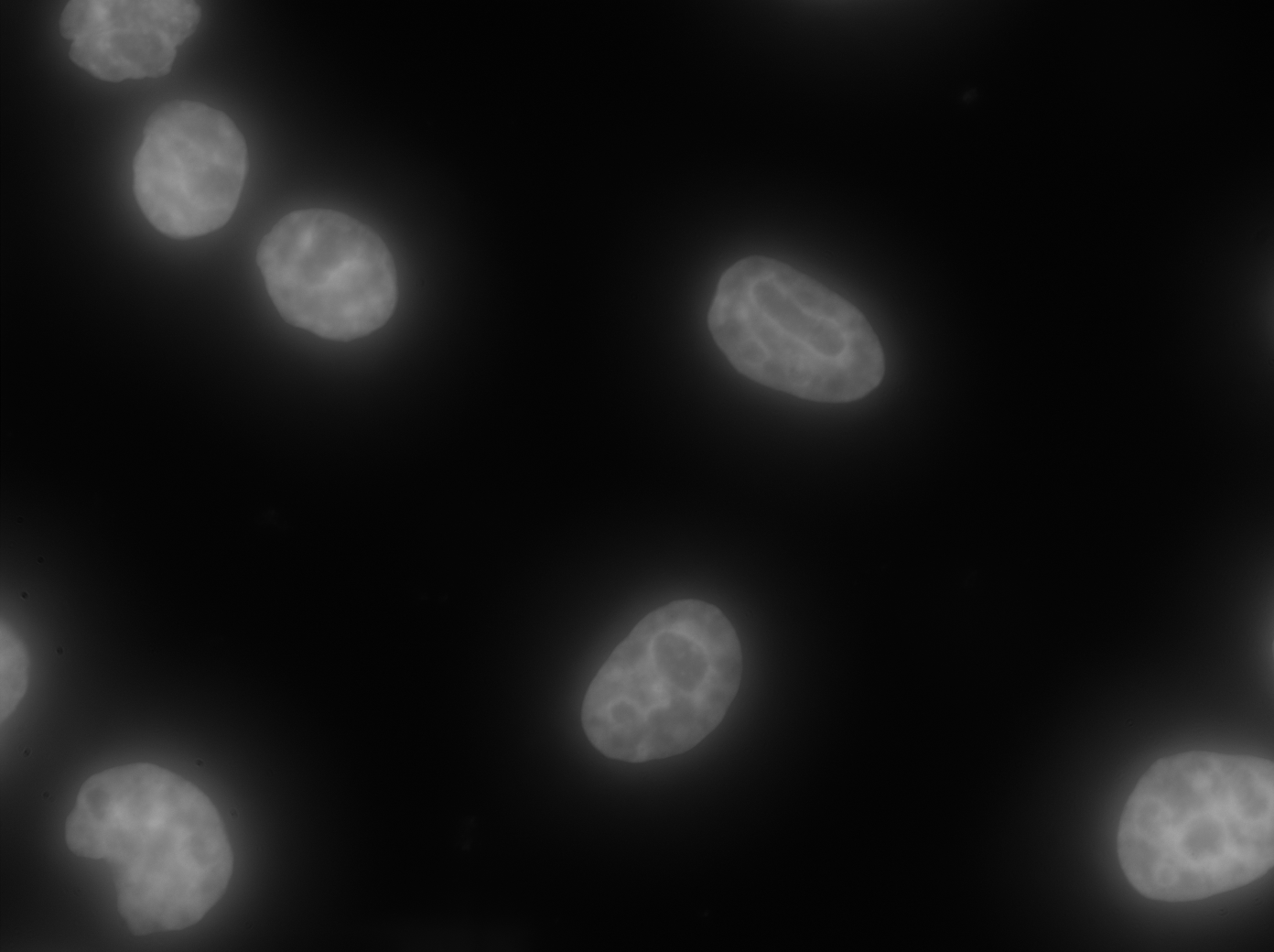

Supplement: Supplementary file 13 — Source data Fig. 3 part 3 [file 44319_2026_742_MOESM13_ESM.zip › Figure 3 Part 3/Fig 3b-e TTLL screen part 3/TTLL11-YFP Img 16 yfp 2000.Project Maximum Z_XY1648586920_Z0_T0_C0.tif]

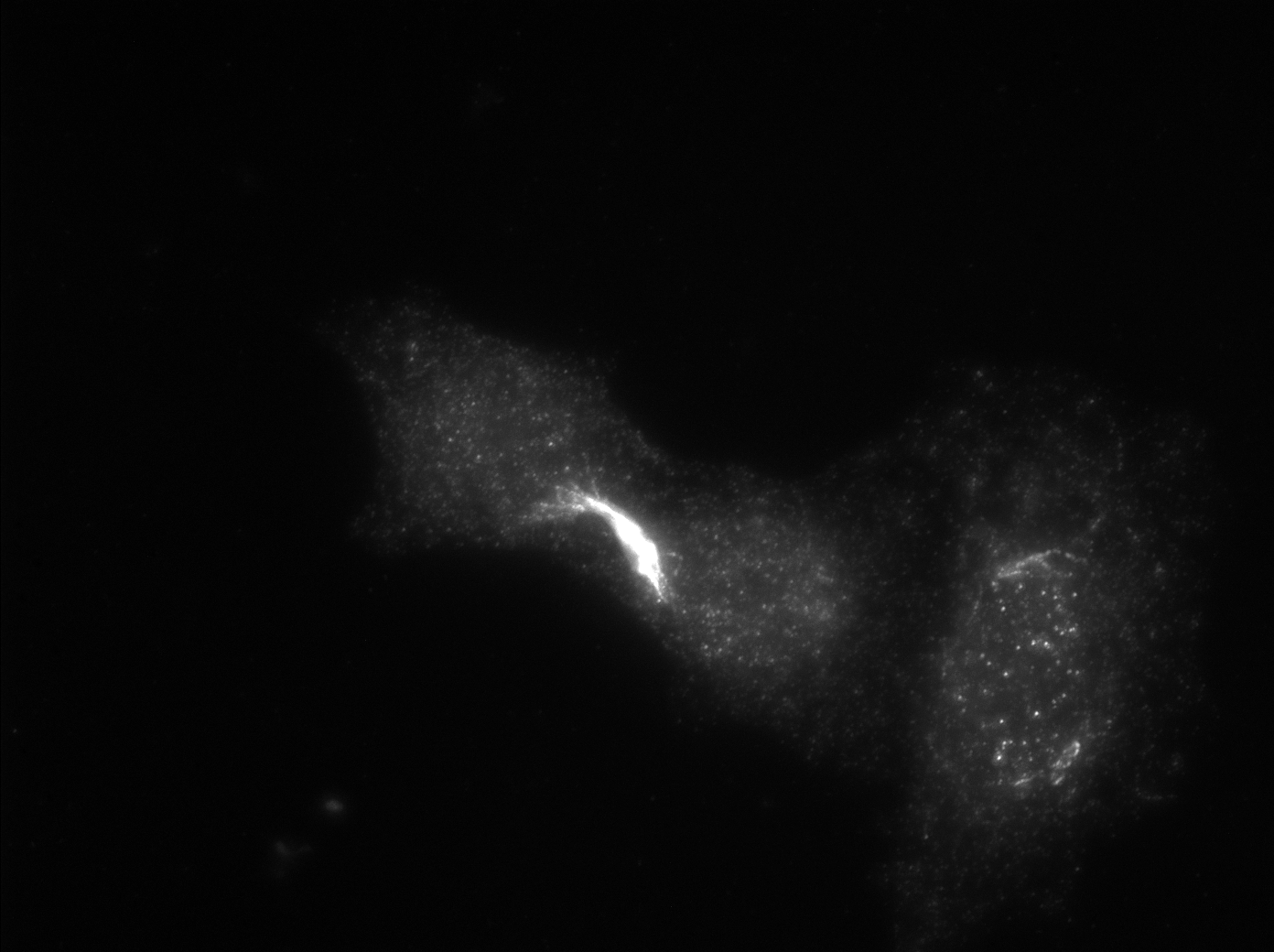

Supplement: Supplementary file 13 — Source data Fig. 3 part 3 [file 44319_2026_742_MOESM13_ESM.zip › Figure 3 Part 3/Fig 3b-e TTLL screen part 3/TTLL9-YFP R1 I5.Project Maximum Z_XY1674167006_Z0_T0_C1.tif]

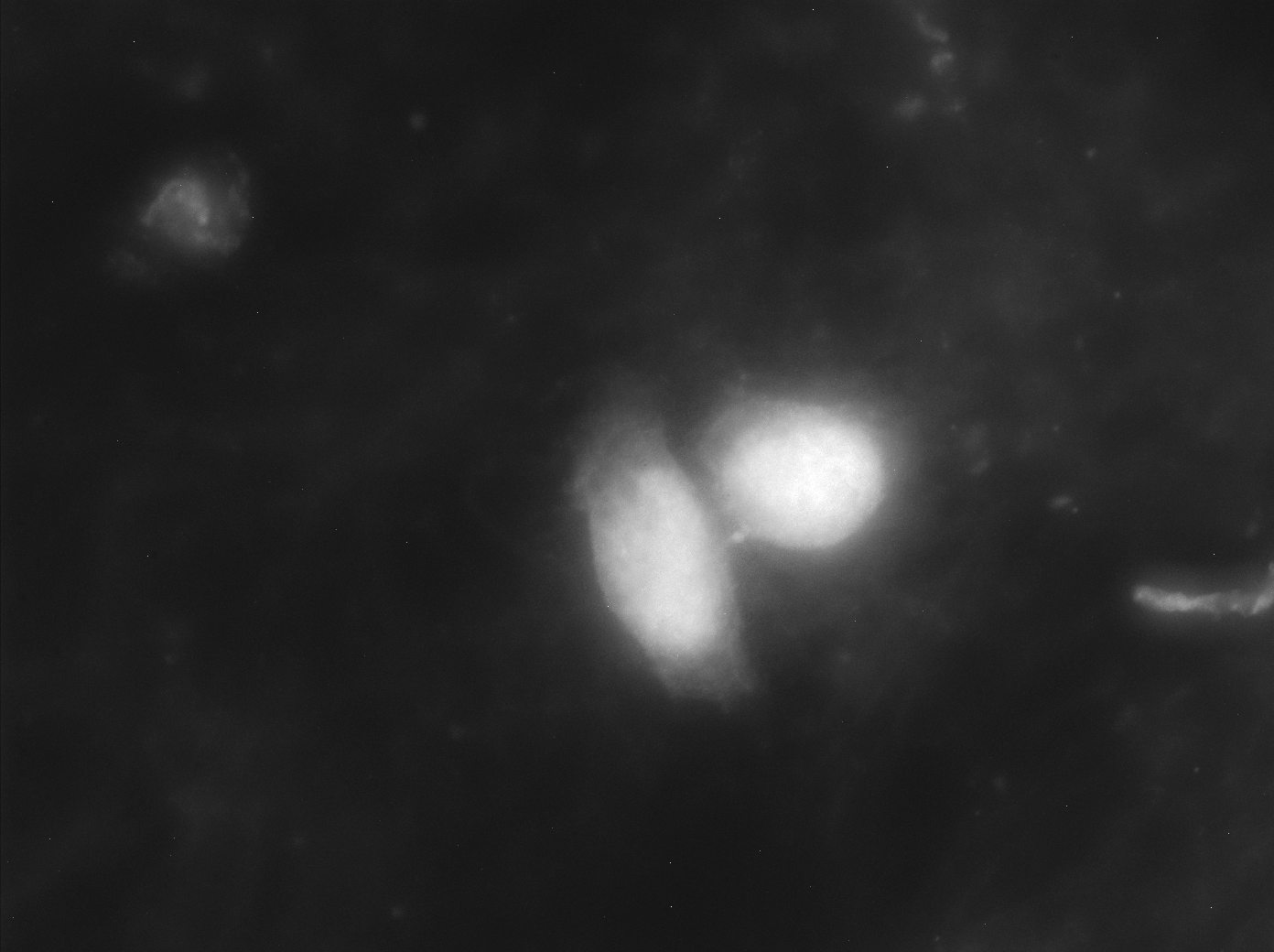

Supplement: Supplementary file 13 — Source data Fig. 3 part 3 [file 44319_2026_742_MOESM13_ESM.zip › Figure 3 Part 3/Fig 3b-e TTLL screen part 3/YFP Only R1 I4.Project Maximum Z_XY1663271976_Z0_T0_C2.tif]

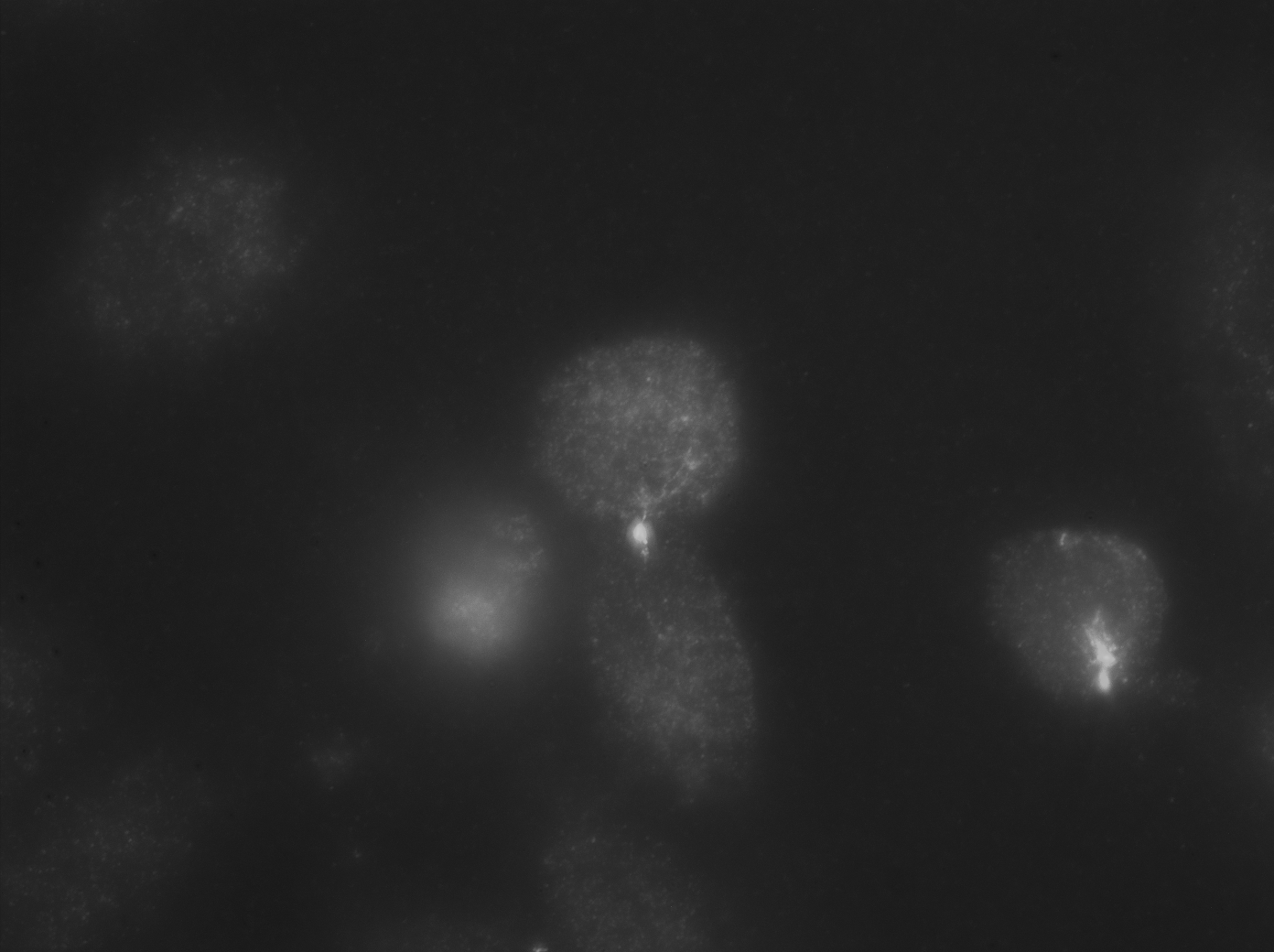

Supplement: Supplementary file 13 — Source data Fig. 3 part 3 [file 44319_2026_742_MOESM13_ESM.zip › Figure 3 Part 3/Fig 3b-e TTLL screen part 3/TTLL9-YFP A3 I16.Project Maximum Z_XY1679701502_Z0_T0_C1.tif]

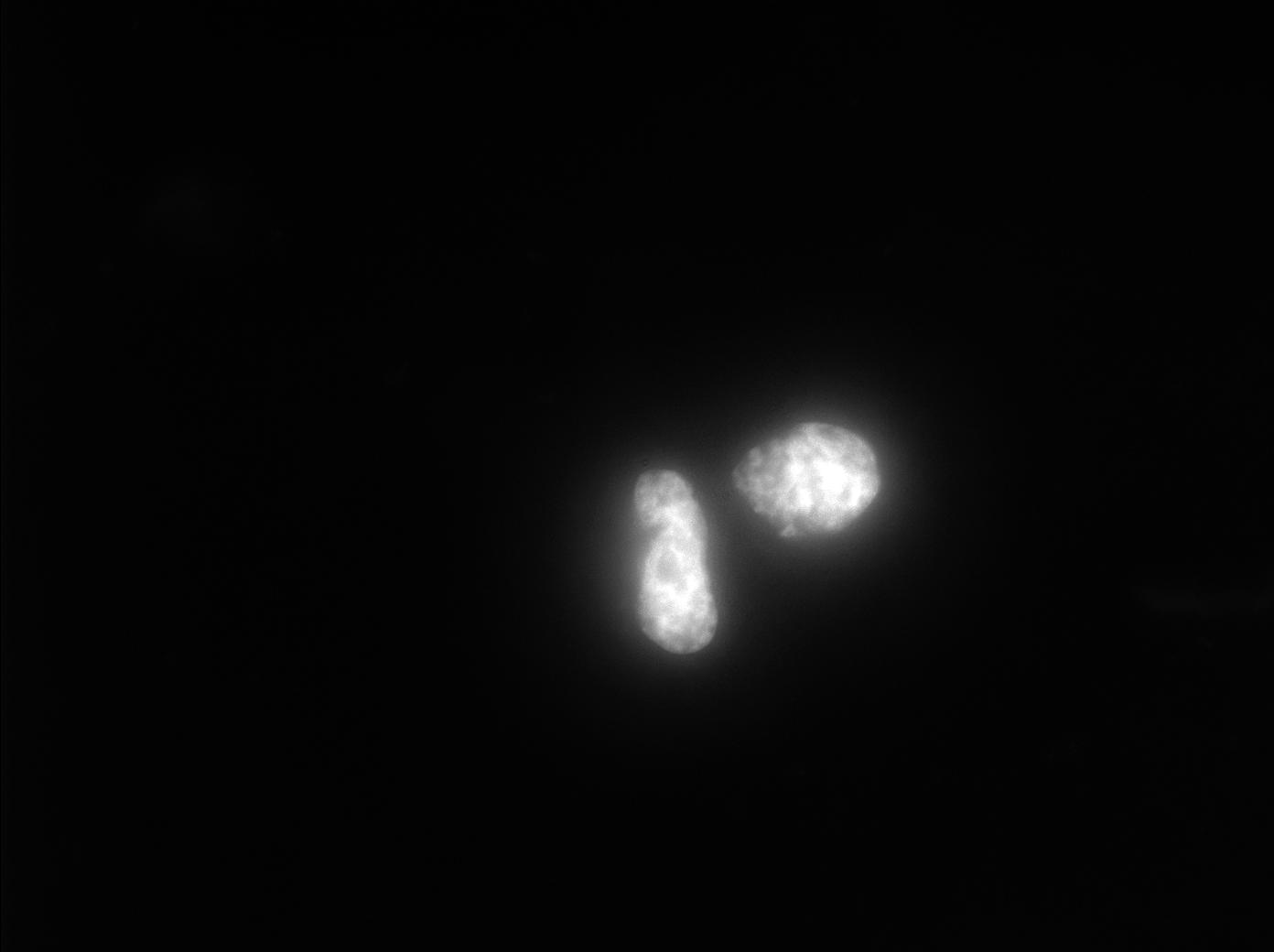

Supplement: Supplementary file 13 — Source data Fig. 3 part 3 [file 44319_2026_742_MOESM13_ESM.zip › Figure 3 Part 3/Fig 3b-e TTLL screen part 3/YFP Only R1 I4.Project Maximum Z_XY1663271976_Z0_T0_C0.tif]

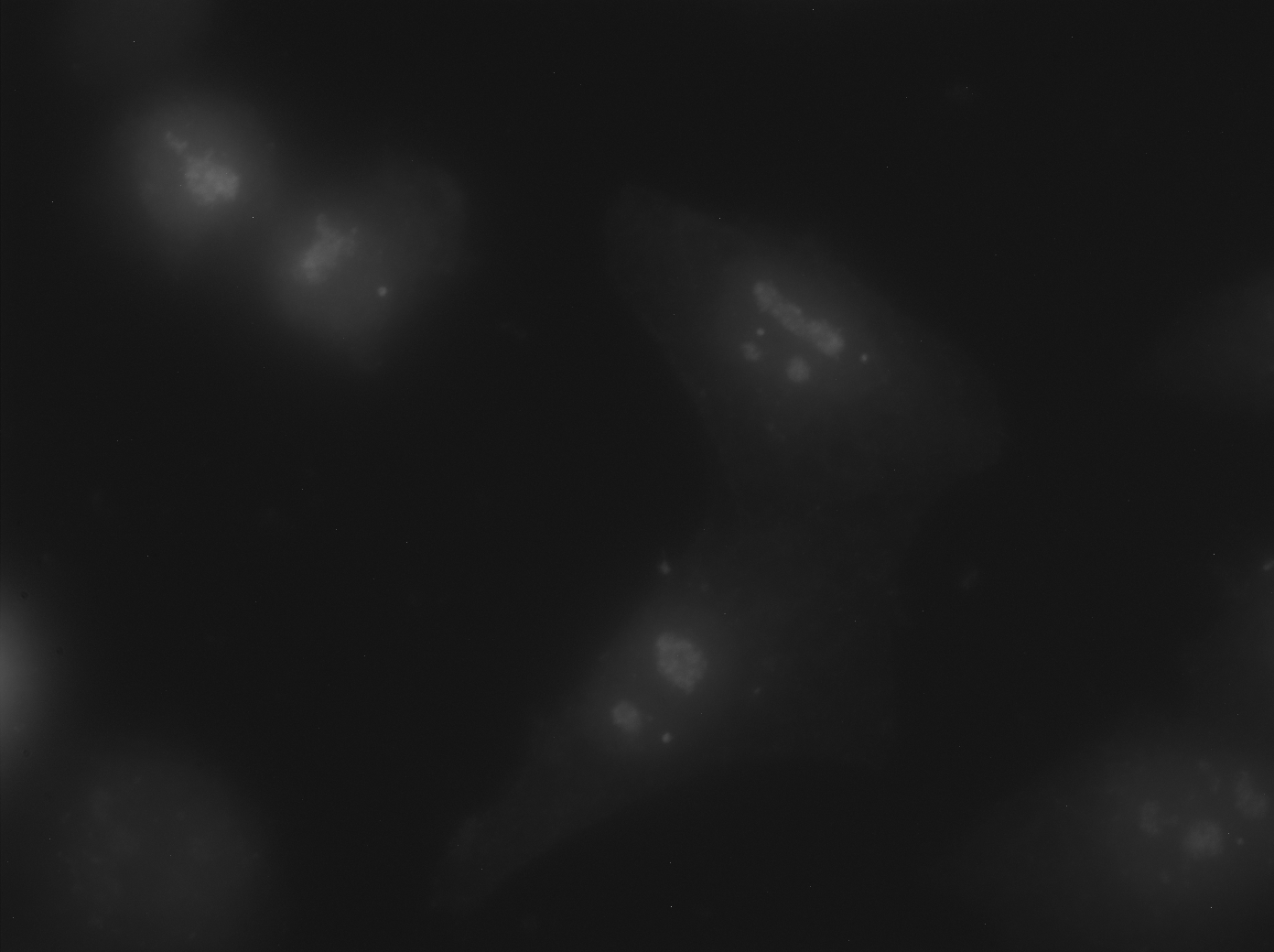

Supplement: Supplementary file 13 — Source data Fig. 3 part 3 [file 44319_2026_742_MOESM13_ESM.zip › Figure 3 Part 3/Fig 3b-e TTLL screen part 3/TTLL11-YFP Img 16 yfp 2000.Project Maximum Z_XY1648586920_Z0_T0_C2.tif]

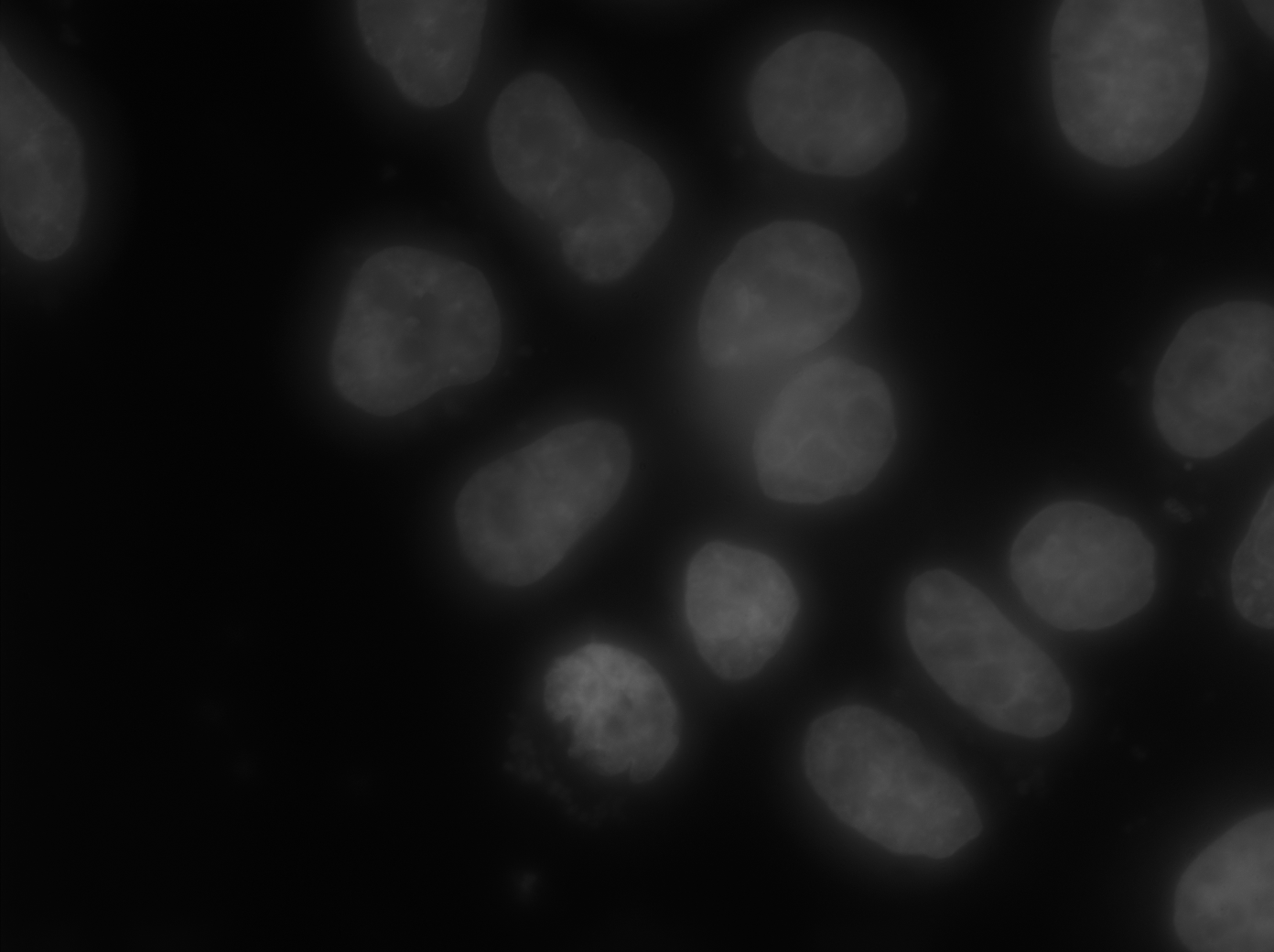

Supplement: Supplementary file 13 — Source data Fig. 3 part 3 [file 44319_2026_742_MOESM13_ESM.zip › Figure 3 Part 3/Fig 3b-e TTLL screen part 3/TTLL9-YFP A3 I14.Project Maximum Z_XY1679701191_Z0_T0_C0.tif]

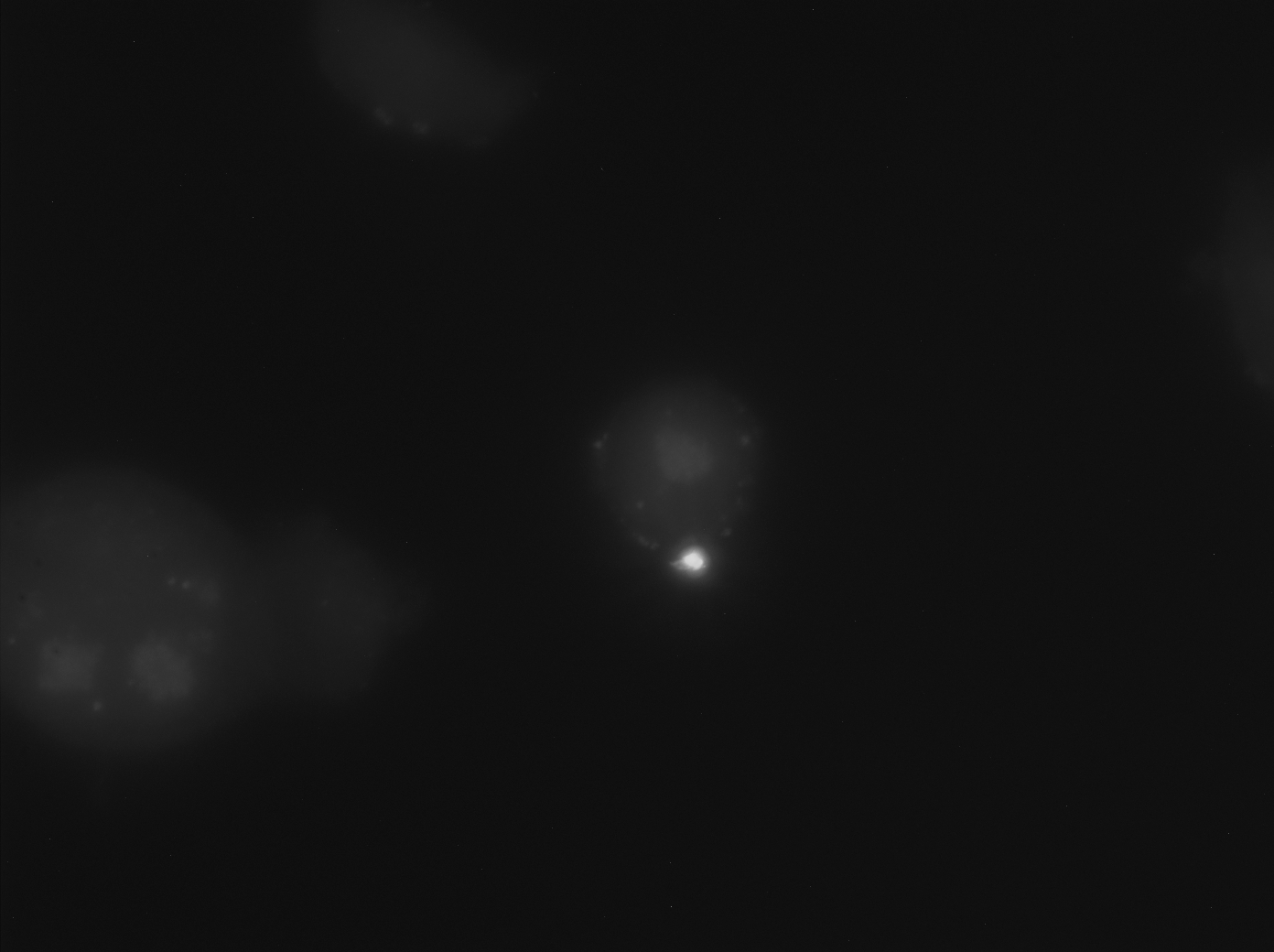

Supplement: Supplementary file 13 — Source data Fig. 3 part 3 [file 44319_2026_742_MOESM13_ESM.zip › Figure 3 Part 3/Fig 3b-e TTLL screen part 3/TTLL11-YFP Img 14 yfp 1200 - 1.Project Maximum Z_XY1648581343_Z0_T0_C2.tif]

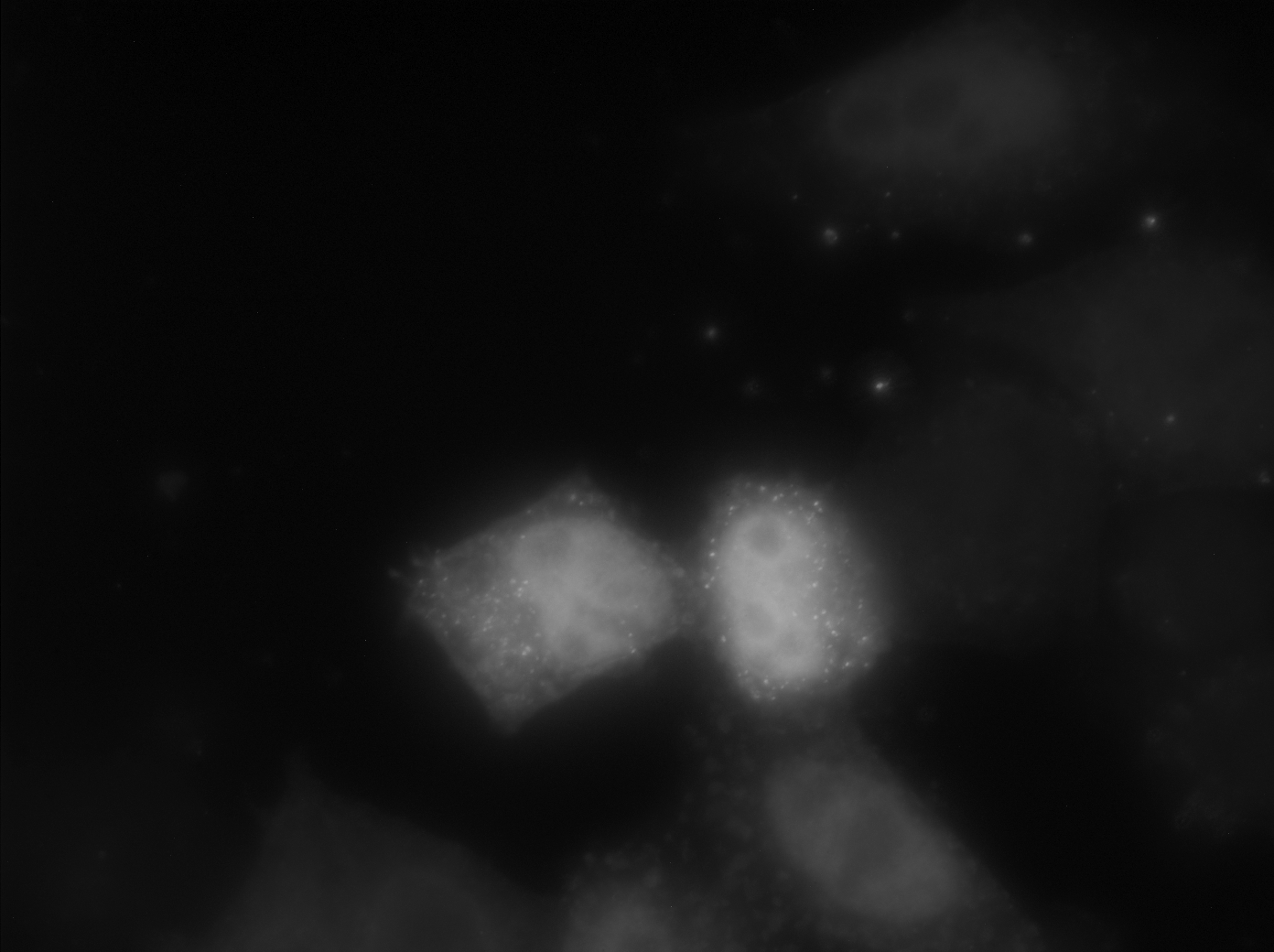

Supplement: Supplementary file 13 — Source data Fig. 3 part 3 [file 44319_2026_742_MOESM13_ESM.zip › Figure 3 Part 3/Fig 3b-e TTLL screen part 3/TTLL9-GFP A4 I3.Project Maximum Z_XY1675965809_Z0_T0_C2.tif]

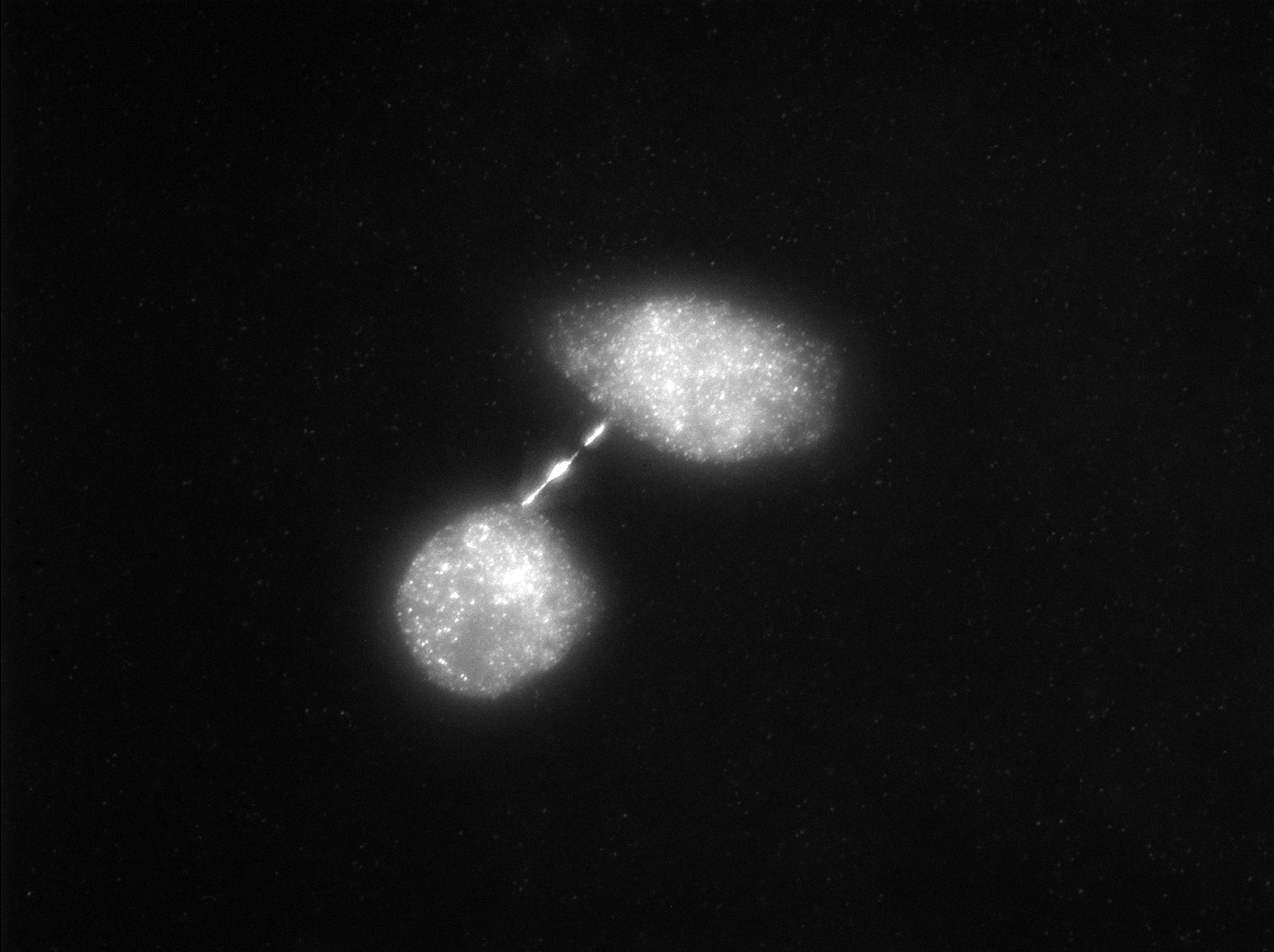

Supplement: Supplementary file 13 — Source data Fig. 3 part 3 [file 44319_2026_742_MOESM13_ESM.zip › Figure 3 Part 3/Fig 3b-e TTLL screen part 3/TTLL9-YFP A3 I1.Project Maximum Z_XY1674674408_Z0_T0_C1.tif]

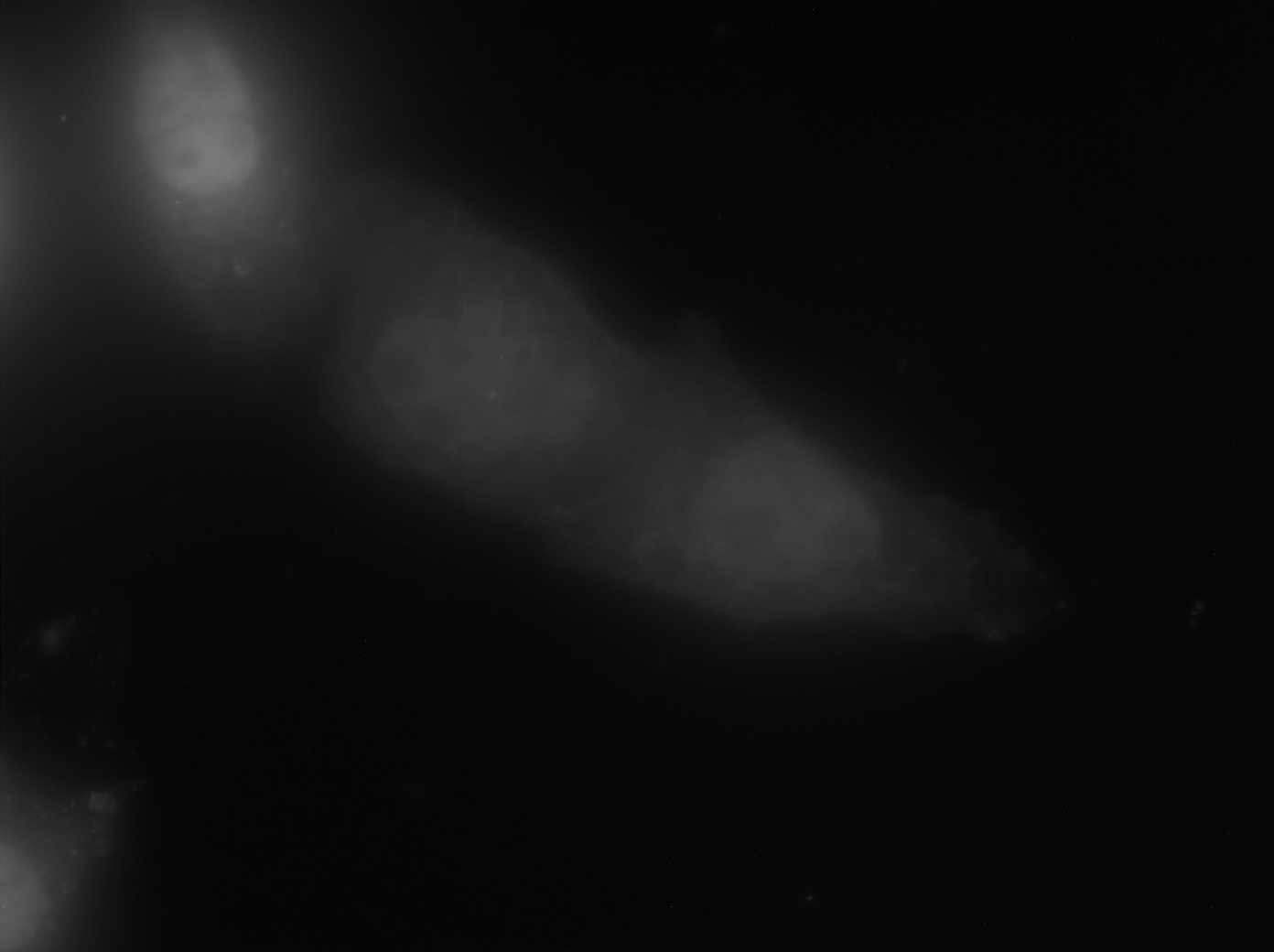

Supplement: Supplementary file 13 — Source data Fig. 3 part 3 [file 44319_2026_742_MOESM13_ESM.zip › Figure 3 Part 3/Fig 3b-e TTLL screen part 3/TTLL9-GFP A4 I4.Project Maximum Z_XY1675966027_Z0_T0_C2.tif]

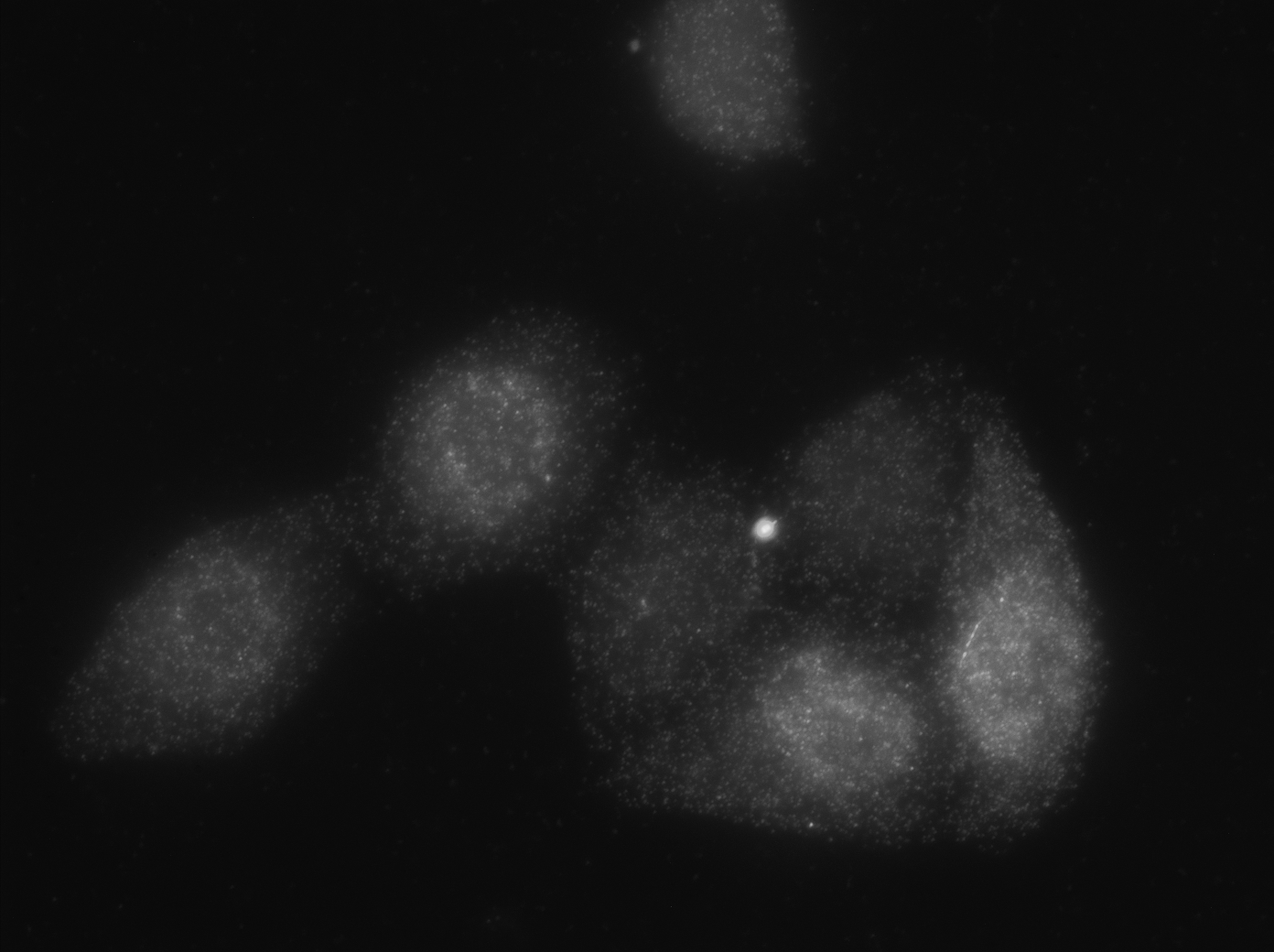

Supplement: Supplementary file 13 — Source data Fig. 3 part 3 [file 44319_2026_742_MOESM13_ESM.zip › Figure 3 Part 3/Fig 3b-e TTLL screen part 3/TTLL11-YFP Img 1 yfp1100.Project Maximum Z_XY1648156180_Z0_T0_C1.tif]

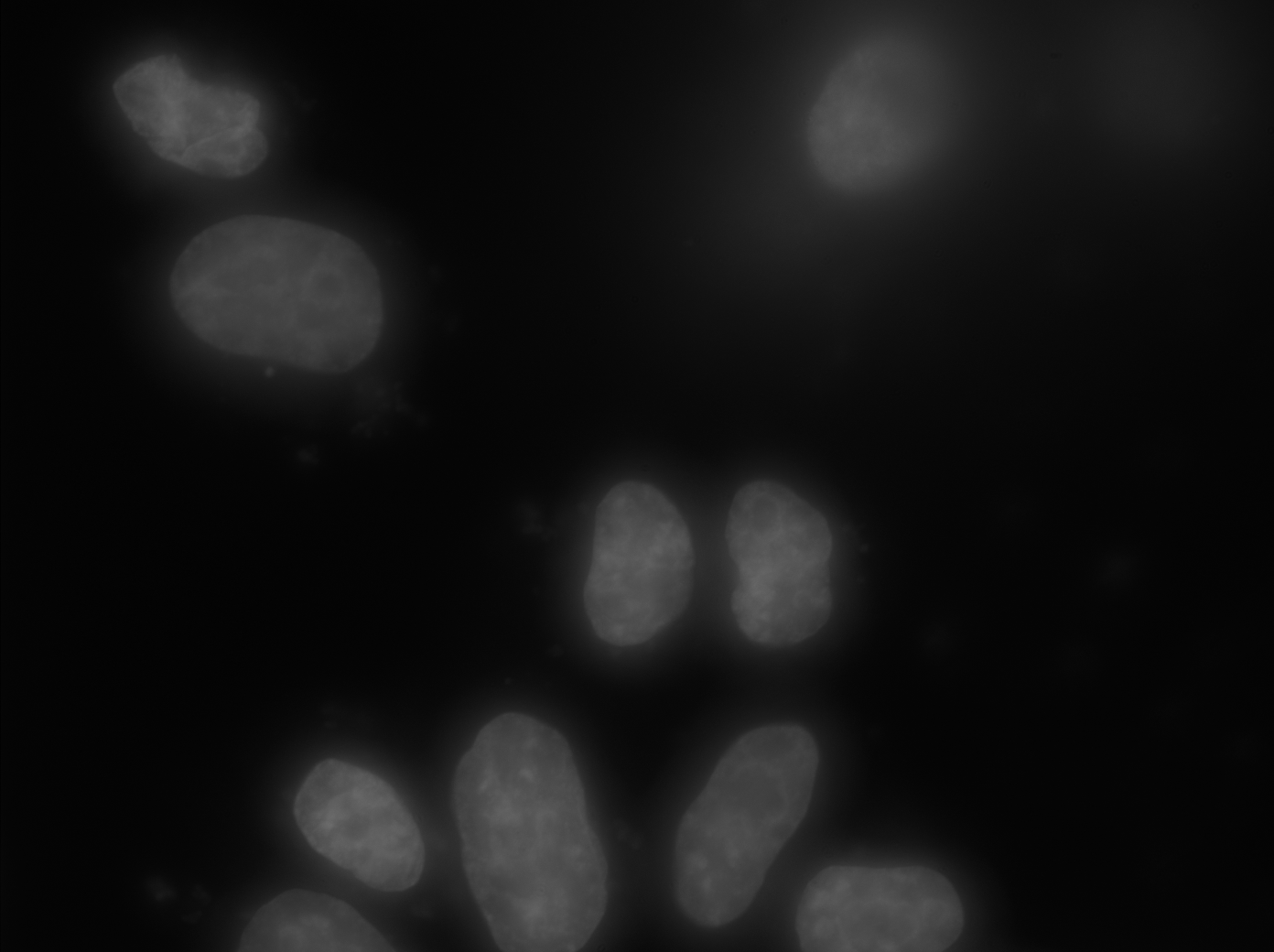

Supplement: Supplementary file 13 — Source data Fig. 3 part 3 [file 44319_2026_742_MOESM13_ESM.zip › Figure 3 Part 3/Fig 3b-e TTLL screen part 3/TTLL9-YFP A3 I9.Project Maximum Z_XY1679700536_Z0_T0_C0.tif]

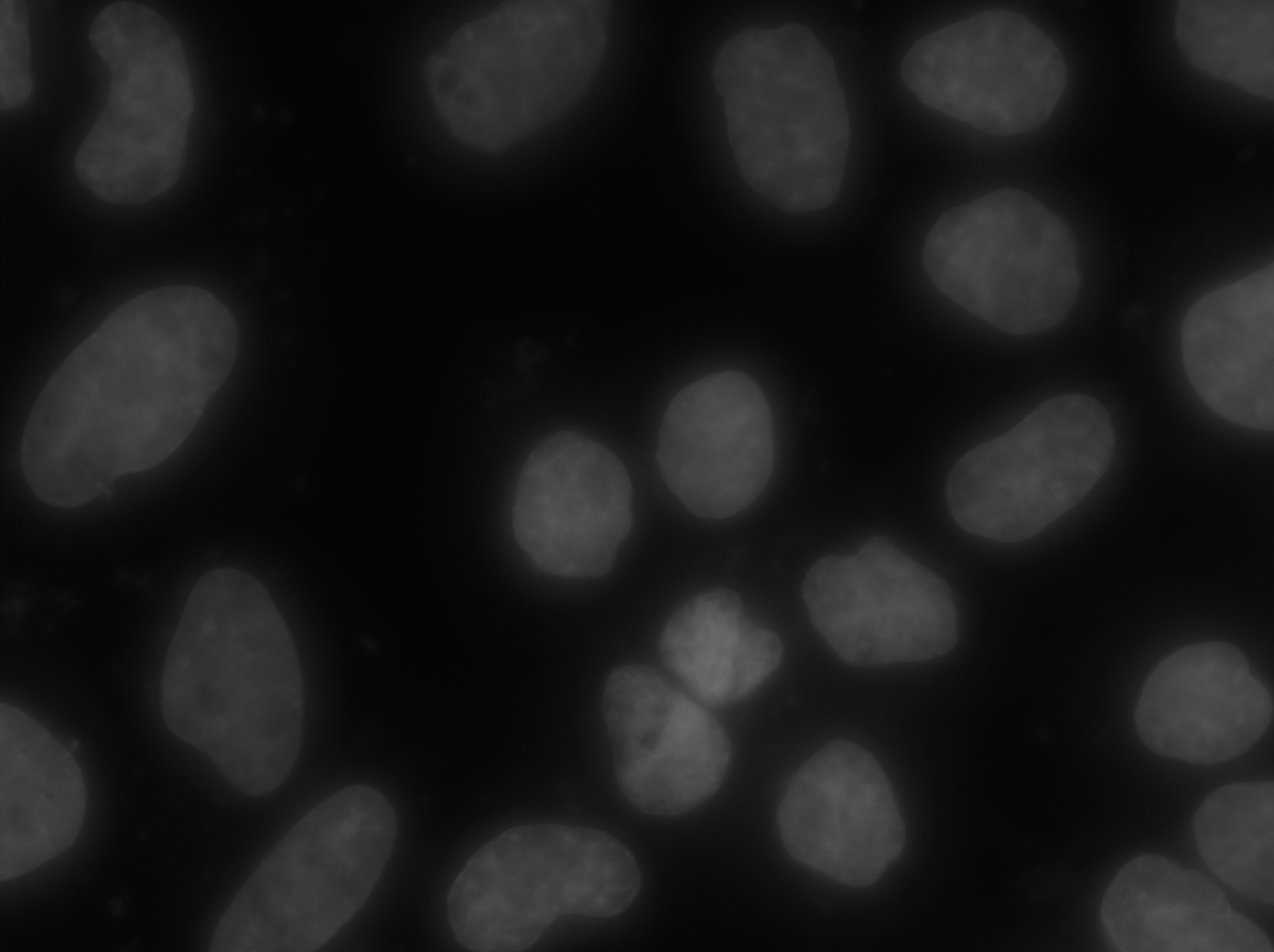

Supplement: Supplementary file 13 — Source data Fig. 3 part 3 [file 44319_2026_742_MOESM13_ESM.zip › Figure 3 Part 3/Fig 3b-e TTLL screen part 3/TTLL9-YFP A3 I3 - 1.Project Maximum Z_XY1679699600_Z0_T0_C0.tif]

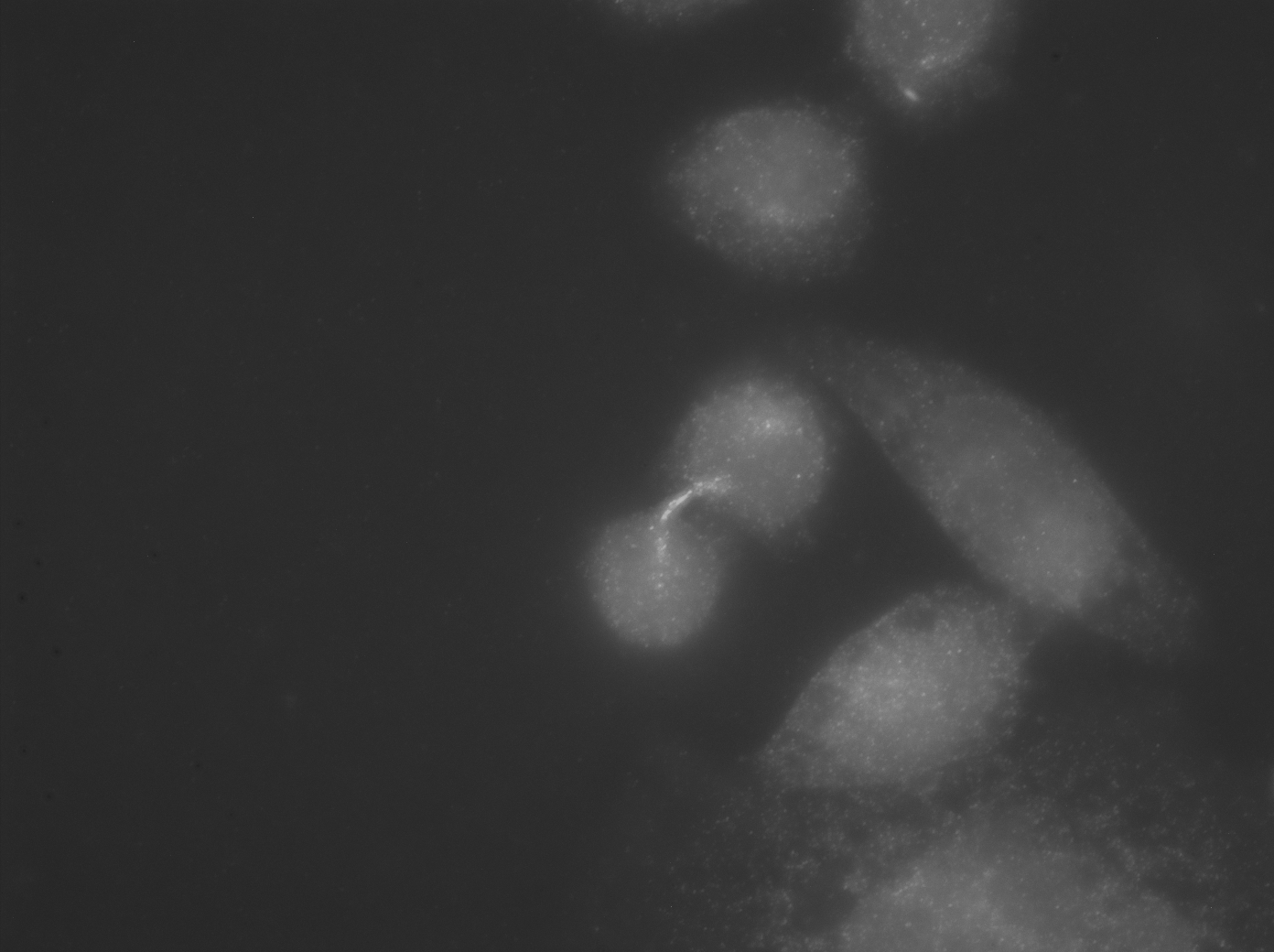

Supplement: Supplementary file 13 — Source data Fig. 3 part 3 [file 44319_2026_742_MOESM13_ESM.zip › Figure 3 Part 3/Fig 3b-e TTLL screen part 3/TTLL11-YFP A1 Img3.Project Maximum Z_XY1650054708_Z0_T0_C1.tif]

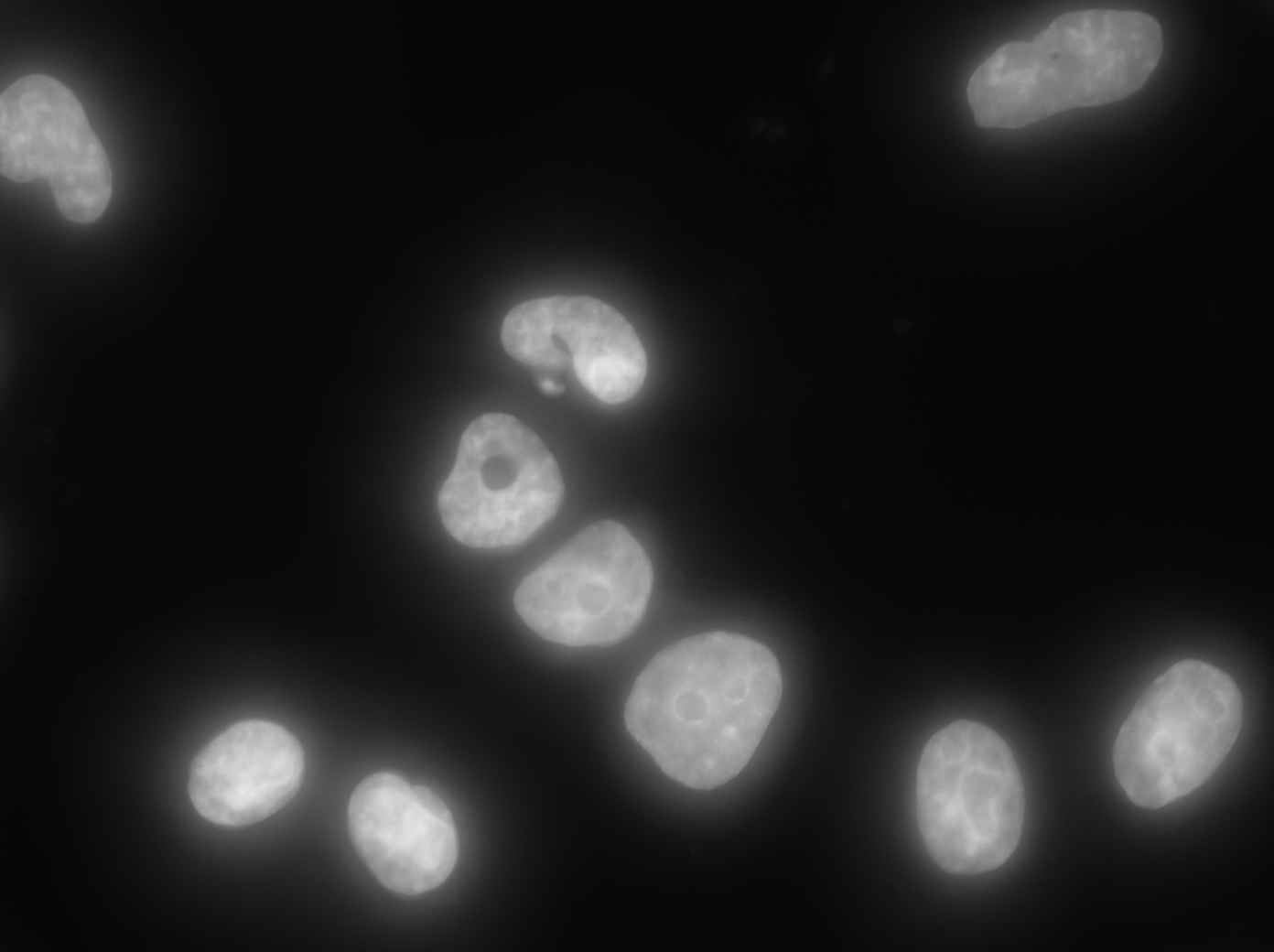

Supplement: Supplementary file 13 — Source data Fig. 3 part 3 [file 44319_2026_742_MOESM13_ESM.zip › Figure 3 Part 3/Fig 3b-e TTLL screen part 3/TTLL11-YFP Img 6 yfp2000.Project Maximum Z_XY1648574277_Z0_T0_C0.tif]

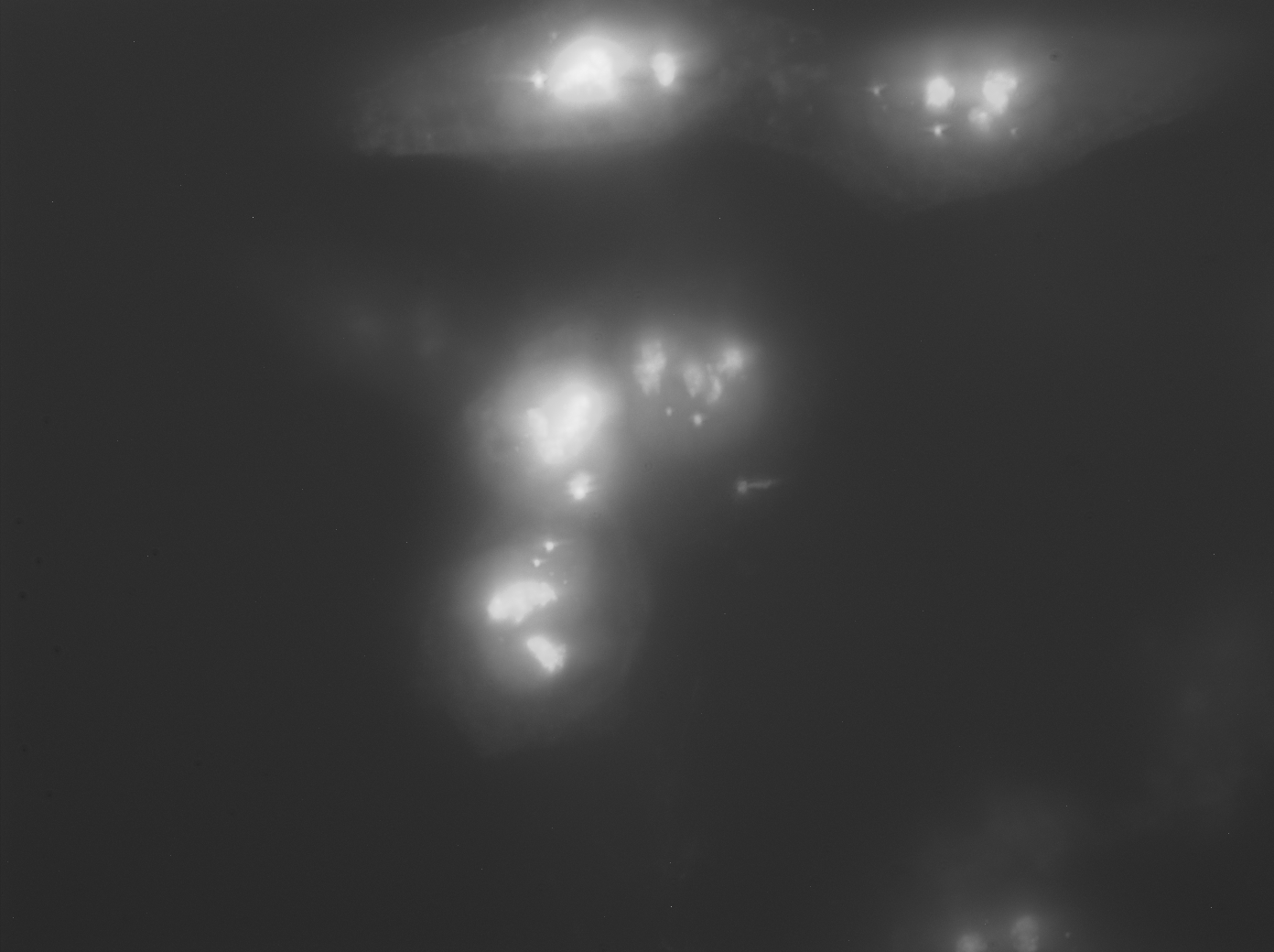

Supplement: Supplementary file 13 — Source data Fig. 3 part 3 [file 44319_2026_742_MOESM13_ESM.zip › Figure 3 Part 3/Fig 3b-e TTLL screen part 3/TTLL11-YFP Img 7 yfp2000.Project Maximum Z_XY1648574683_Z0_T0_C2.tif]

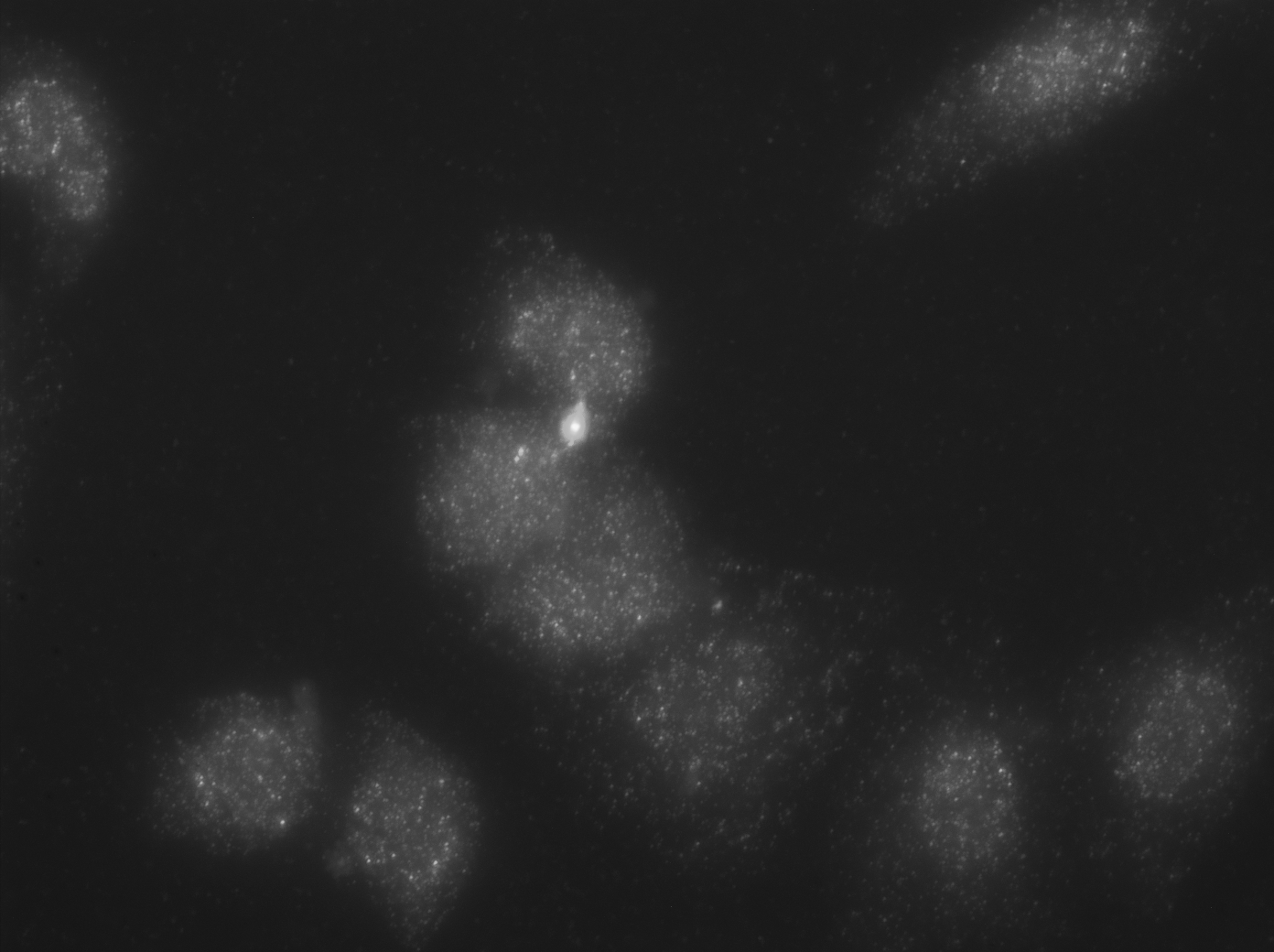

Supplement: Supplementary file 13 — Source data Fig. 3 part 3 [file 44319_2026_742_MOESM13_ESM.zip › Figure 3 Part 3/Fig 3b-e TTLL screen part 3/TTLL11-YFP Img 6 yfp2000.Project Maximum Z_XY1648574277_Z0_T0_C1.tif]

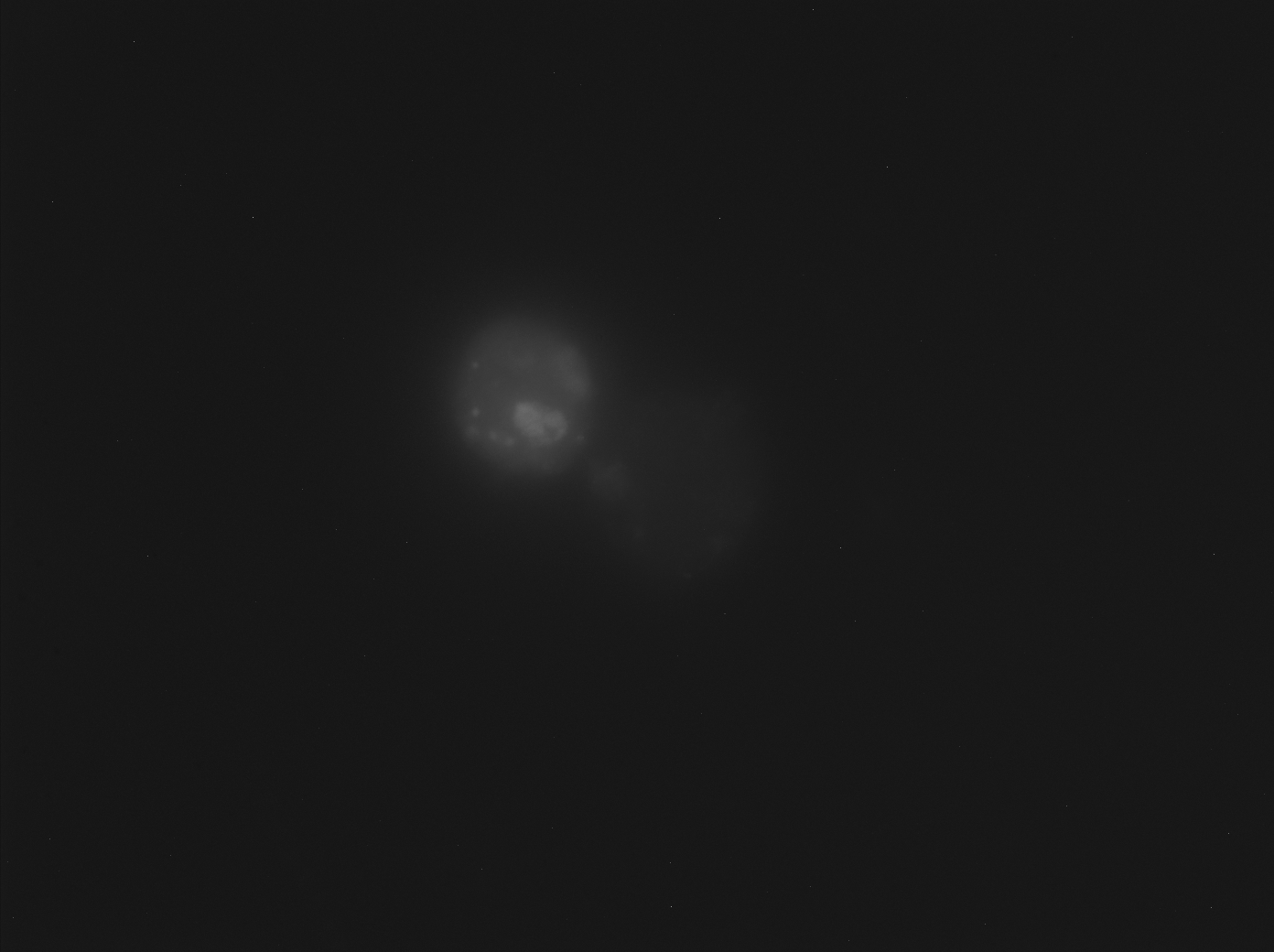

Supplement: Supplementary file 13 — Source data Fig. 3 part 3 [file 44319_2026_742_MOESM13_ESM.zip › Figure 3 Part 3/Fig 3b-e TTLL screen part 3/TTLL11-YFP Img 15 yfp 2000.Project Maximum Z_XY1648586329_Z0_T0_C2.tif]

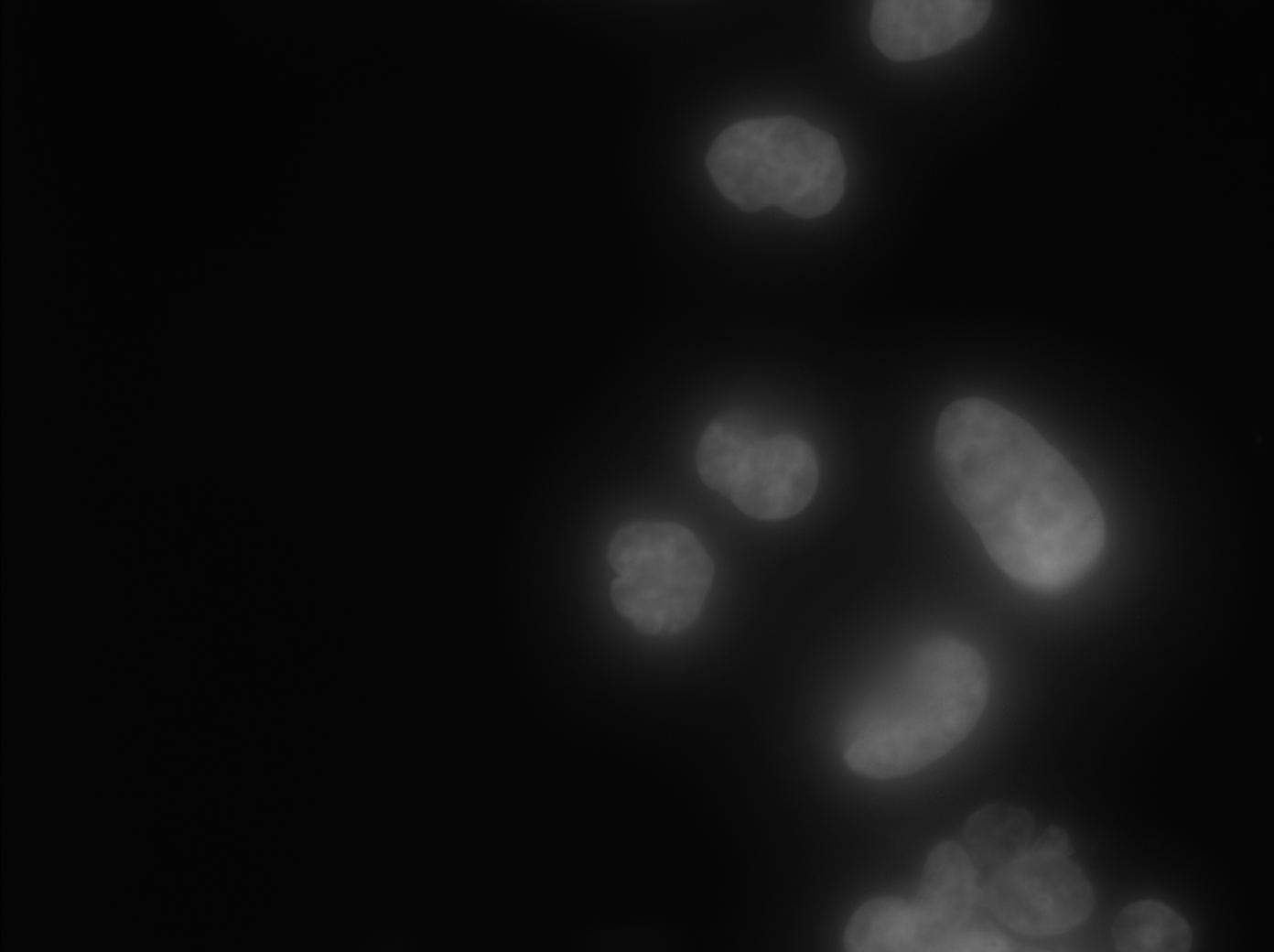

Supplement: Supplementary file 13 — Source data Fig. 3 part 3 [file 44319_2026_742_MOESM13_ESM.zip › Figure 3 Part 3/Fig 3b-e TTLL screen part 3/TTLL11-YFP A1 Img3.Project Maximum Z_XY1650054708_Z0_T0_C0.tif]

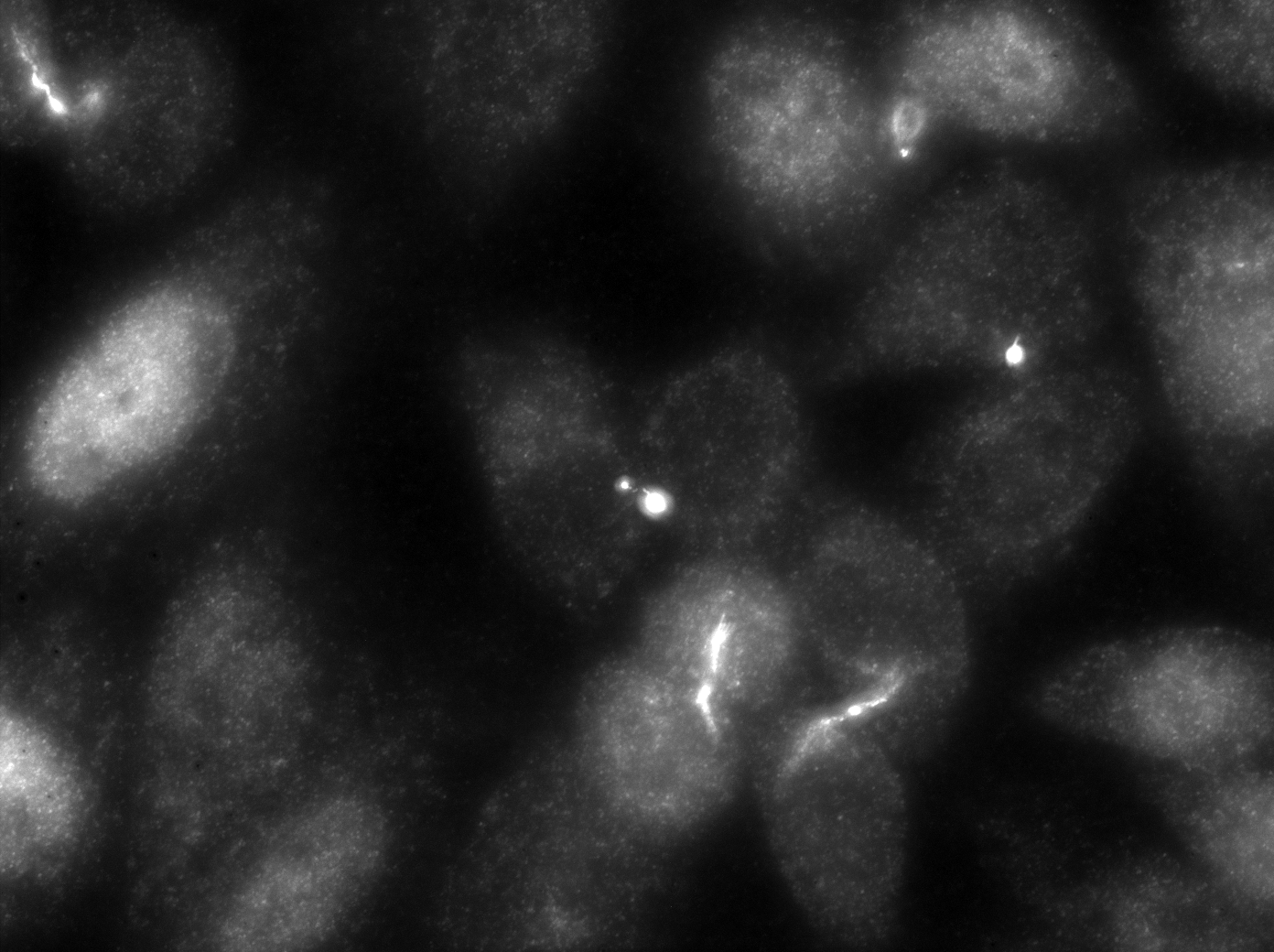

Supplement: Supplementary file 13 — Source data Fig. 3 part 3 [file 44319_2026_742_MOESM13_ESM.zip › Figure 3 Part 3/Fig 3b-e TTLL screen part 3/TTLL9-YFP A3 I3 - 1.Project Maximum Z_XY1679699600_Z0_T0_C1.tif]

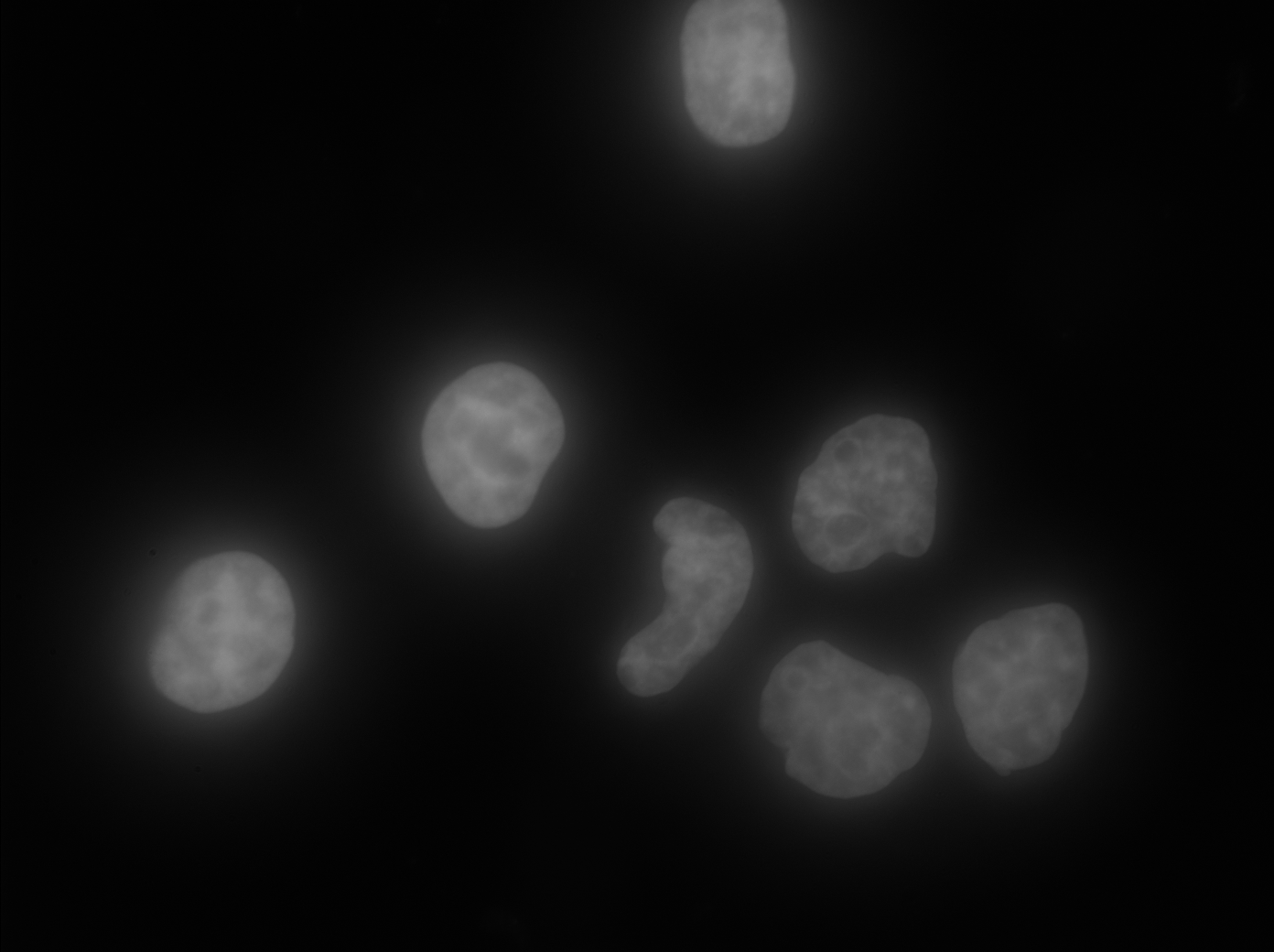

Supplement: Supplementary file 13 — Source data Fig. 3 part 3 [file 44319_2026_742_MOESM13_ESM.zip › Figure 3 Part 3/Fig 3b-e TTLL screen part 3/TTLL11-YFP Img 1 yfp1100.Project Maximum Z_XY1648156180_Z0_T0_C0.tif]

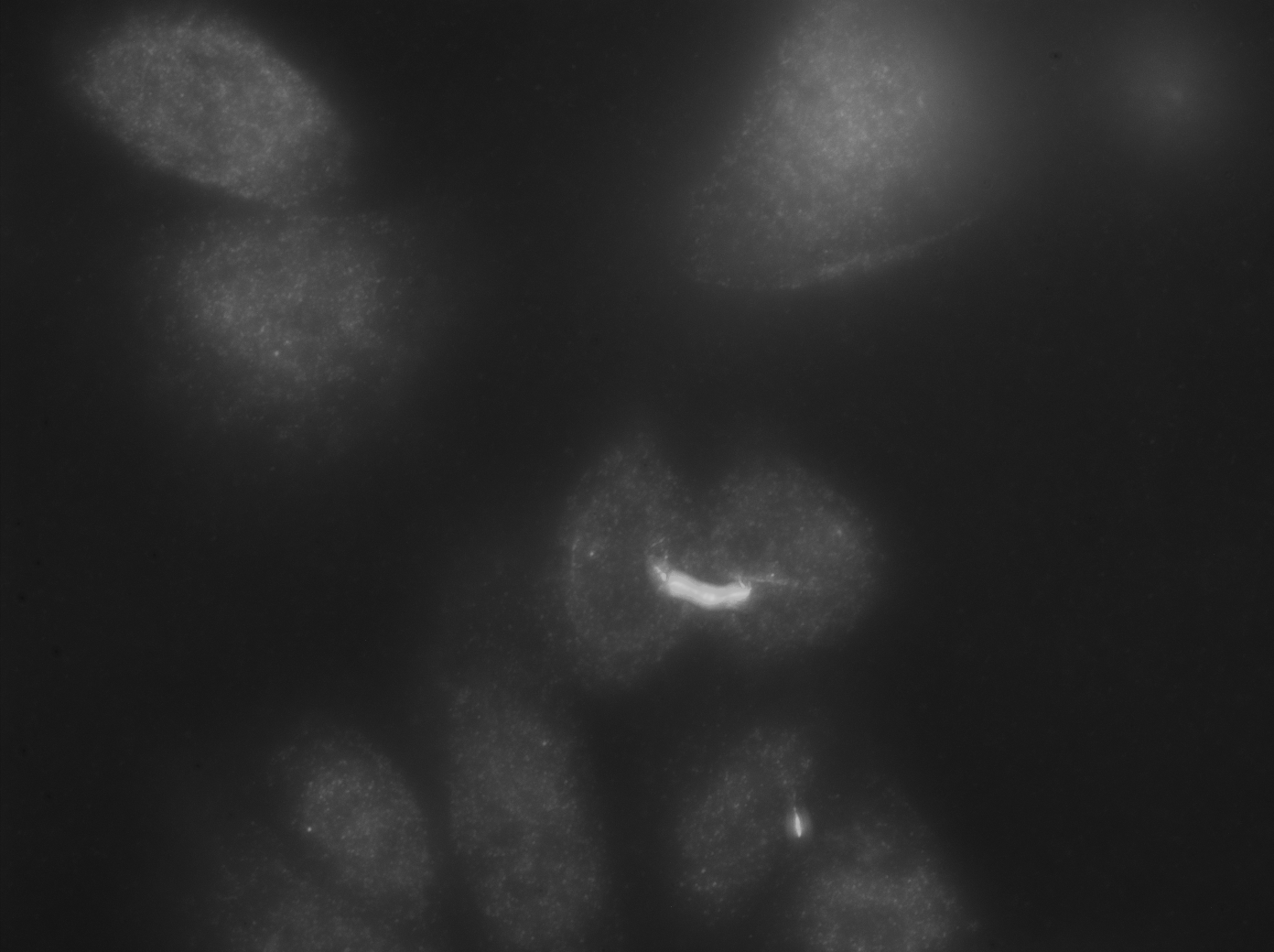

Supplement: Supplementary file 13 — Source data Fig. 3 part 3 [file 44319_2026_742_MOESM13_ESM.zip › Figure 3 Part 3/Fig 3b-e TTLL screen part 3/TTLL9-YFP A3 I9.Project Maximum Z_XY1679700536_Z0_T0_C1.tif]

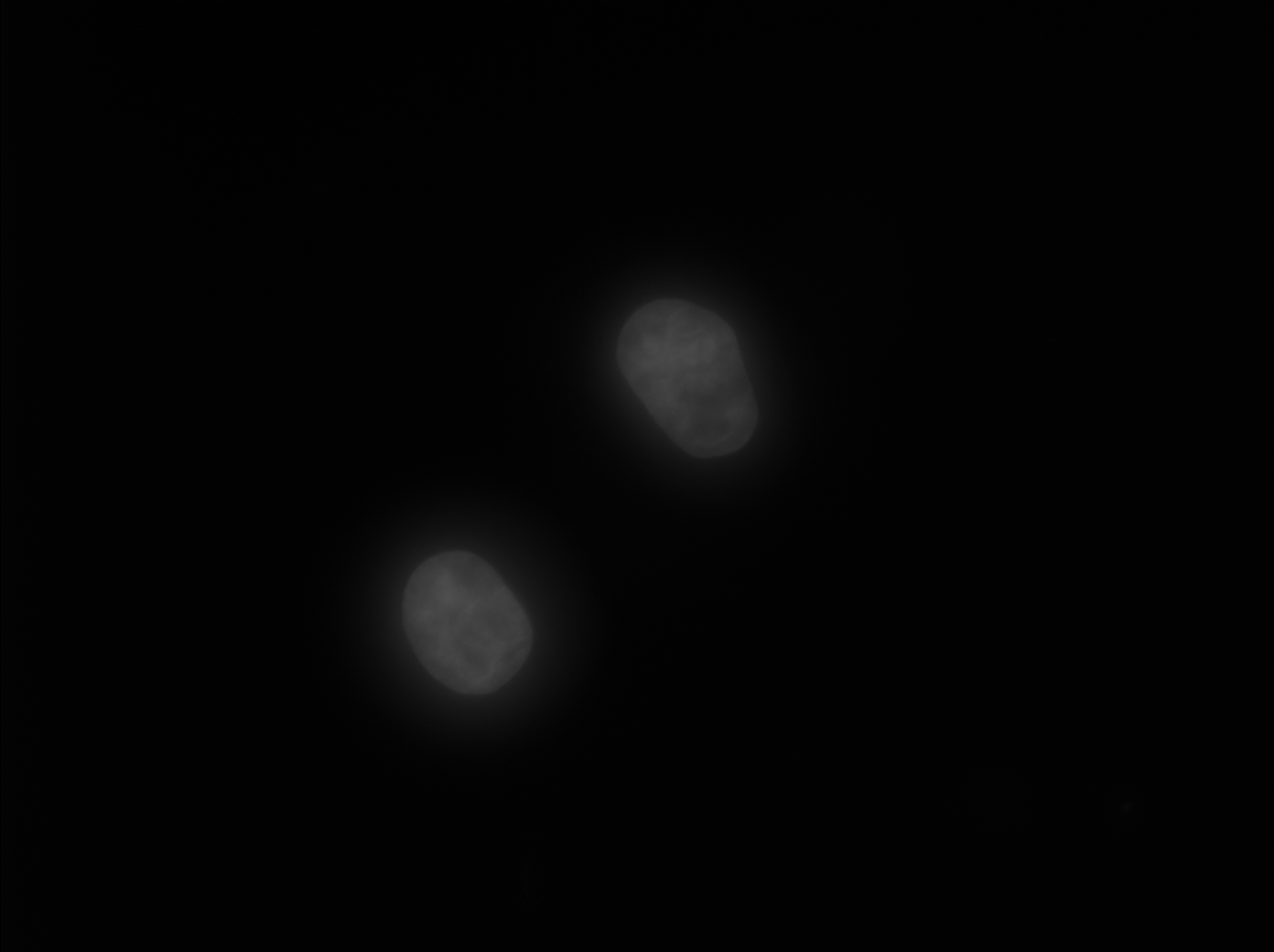

Supplement: Supplementary file 13 — Source data Fig. 3 part 3 [file 44319_2026_742_MOESM13_ESM.zip › Figure 3 Part 3/Fig 3b-e TTLL screen part 3/TTLL9-YFP A3 I1.Project Maximum Z_XY1674674408_Z0_T0_C0.tif]

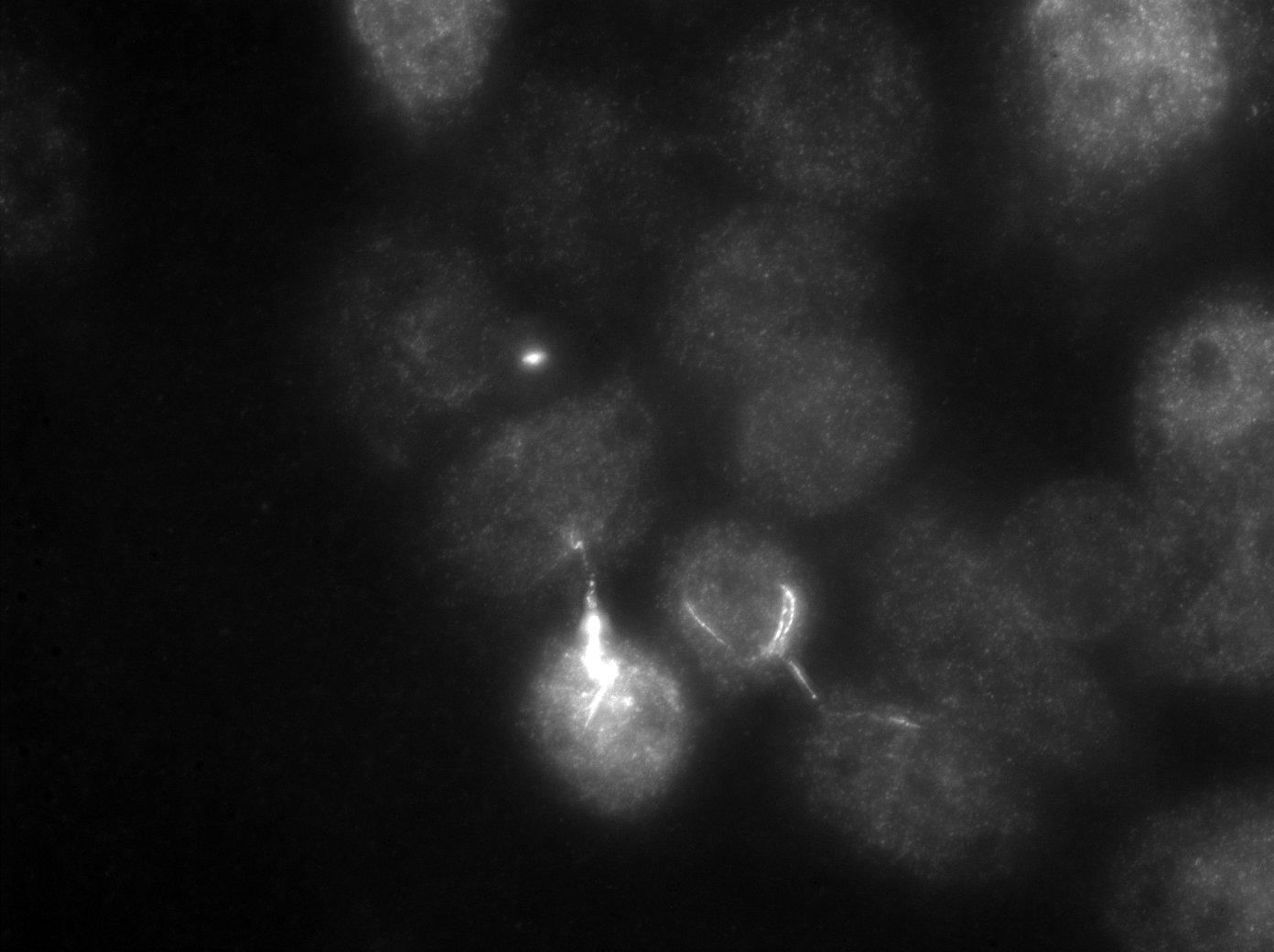

Supplement: Supplementary file 13 — Source data Fig. 3 part 3 [file 44319_2026_742_MOESM13_ESM.zip › Figure 3 Part 3/Fig 3b-e TTLL screen part 3/TTLL9-YFP A3 I14.Project Maximum Z_XY1679701191_Z0_T0_C1.tif]

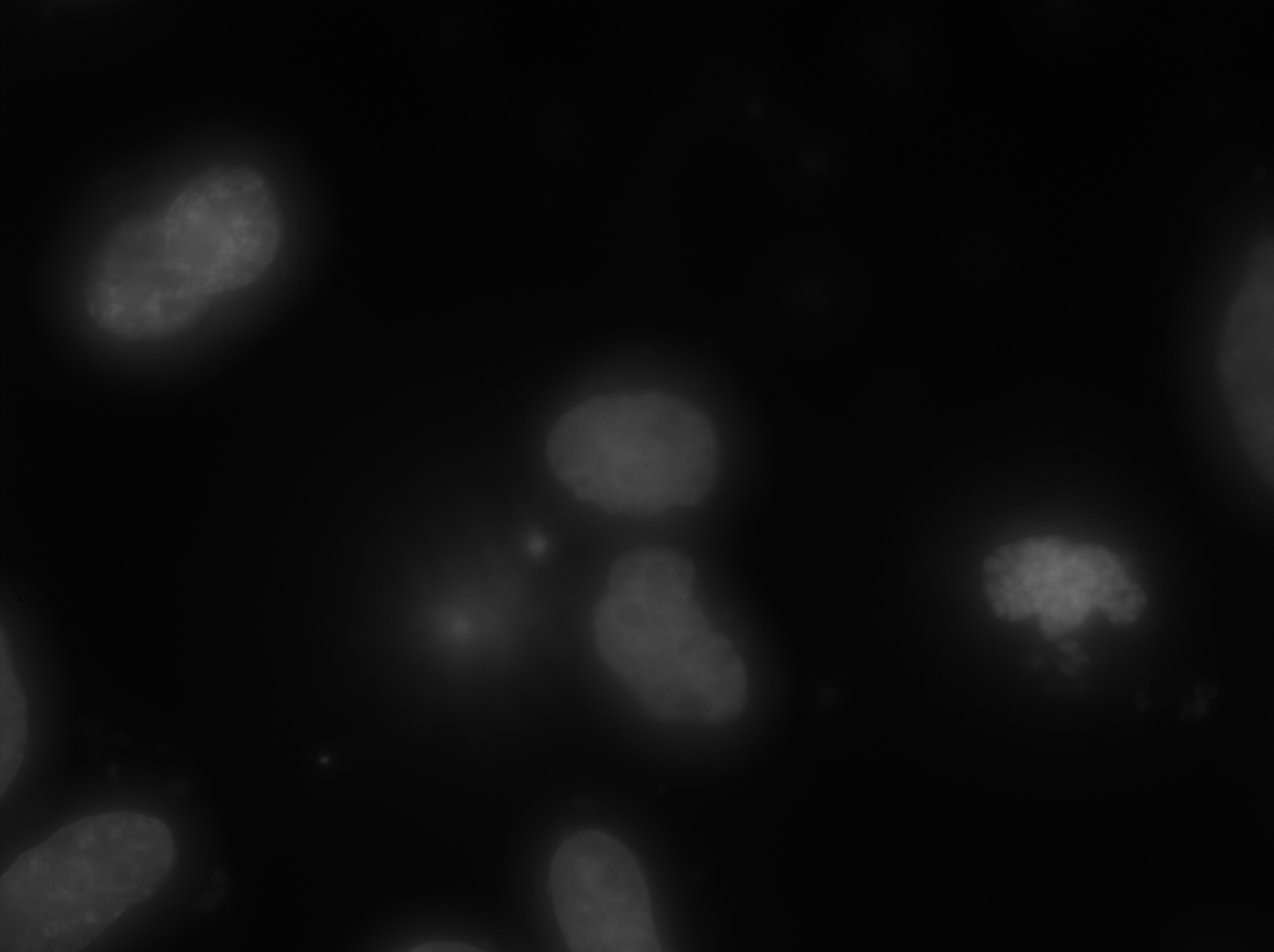

Supplement: Supplementary file 13 — Source data Fig. 3 part 3 [file 44319_2026_742_MOESM13_ESM.zip › Figure 3 Part 3/Fig 3b-e TTLL screen part 3/TTLL9-YFP A3 I16.Project Maximum Z_XY1679701502_Z0_T0_C0.tif]

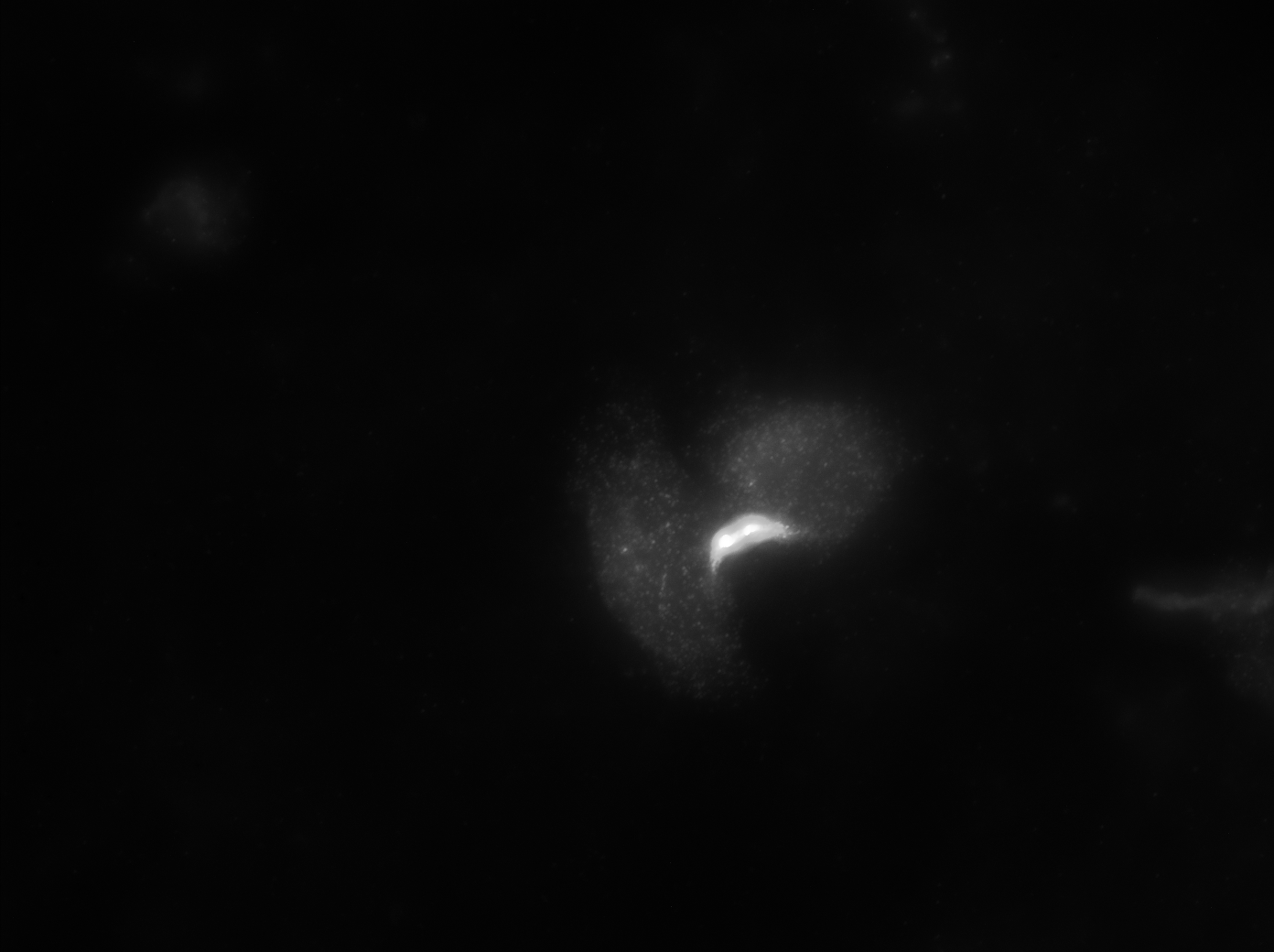

Supplement: Supplementary file 13 — Source data Fig. 3 part 3 [file 44319_2026_742_MOESM13_ESM.zip › Figure 3 Part 3/Fig 3b-e TTLL screen part 3/YFP Only R1 I4.Project Maximum Z_XY1663271976_Z0_T0_C1.tif]

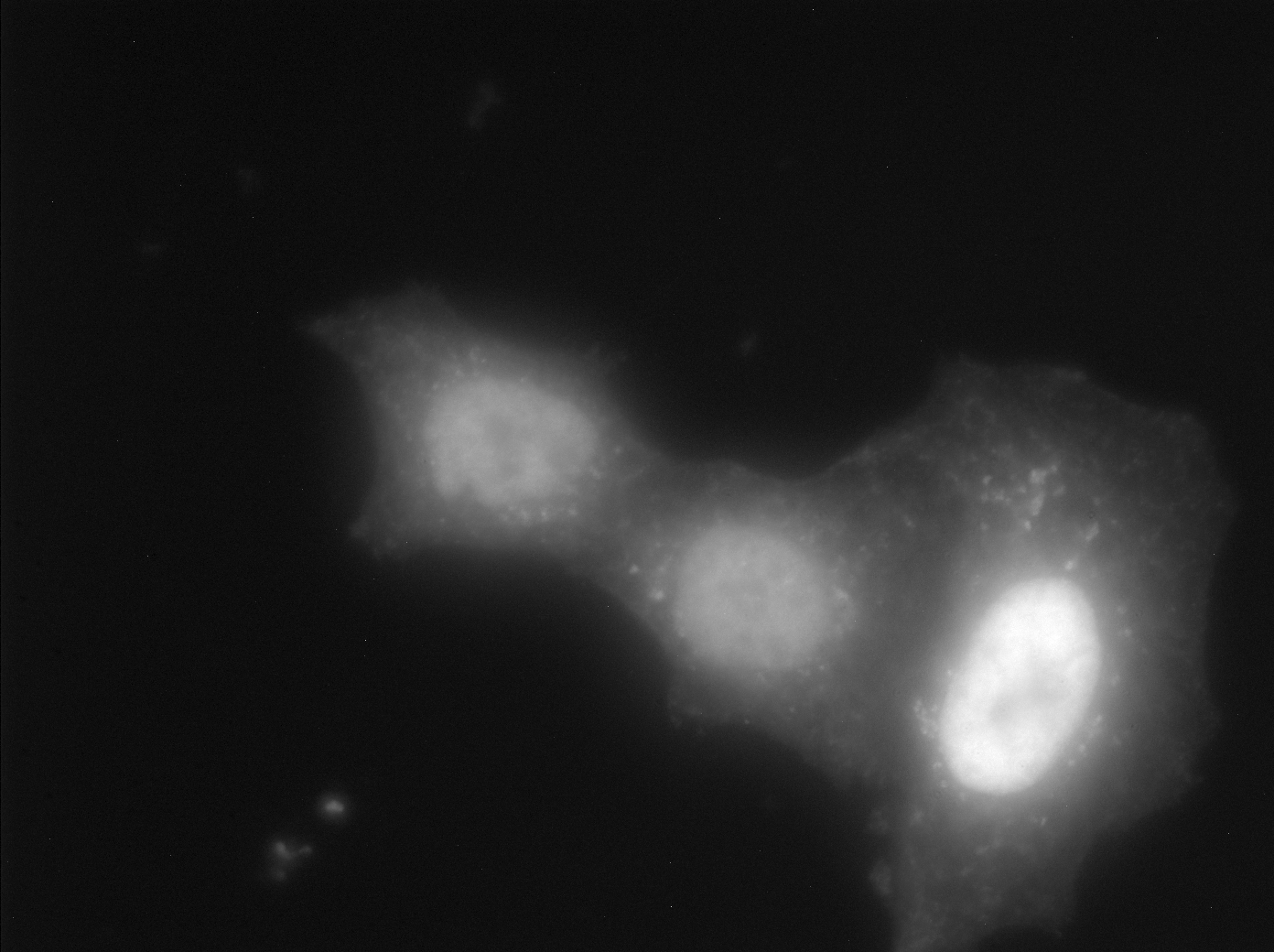

Supplement: Supplementary file 13 — Source data Fig. 3 part 3 [file 44319_2026_742_MOESM13_ESM.zip › Figure 3 Part 3/Fig 3b-e TTLL screen part 3/TTLL9-YFP R1 I5.Project Maximum Z_XY1674167006_Z0_T0_C2.tif]

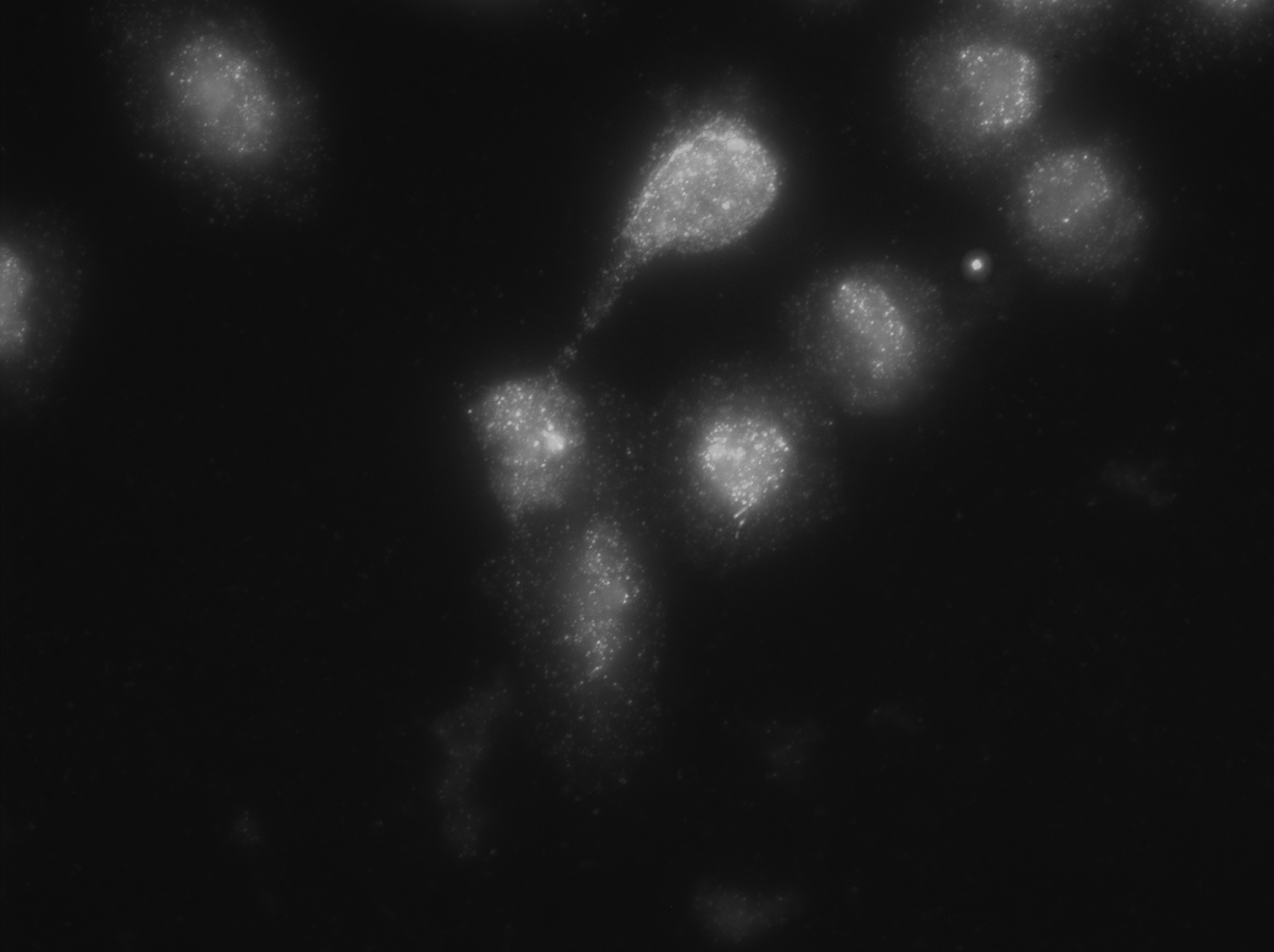

Supplement: Supplementary file 13 — Source data Fig. 3 part 3 [file 44319_2026_742_MOESM13_ESM.zip › Figure 3 Part 3/Fig 3b-e TTLL screen part 3/TTLL11-YFP Img 8 yfp2000 - 1.Project Maximum Z_XY1648578082_Z0_T0_C1.tif]

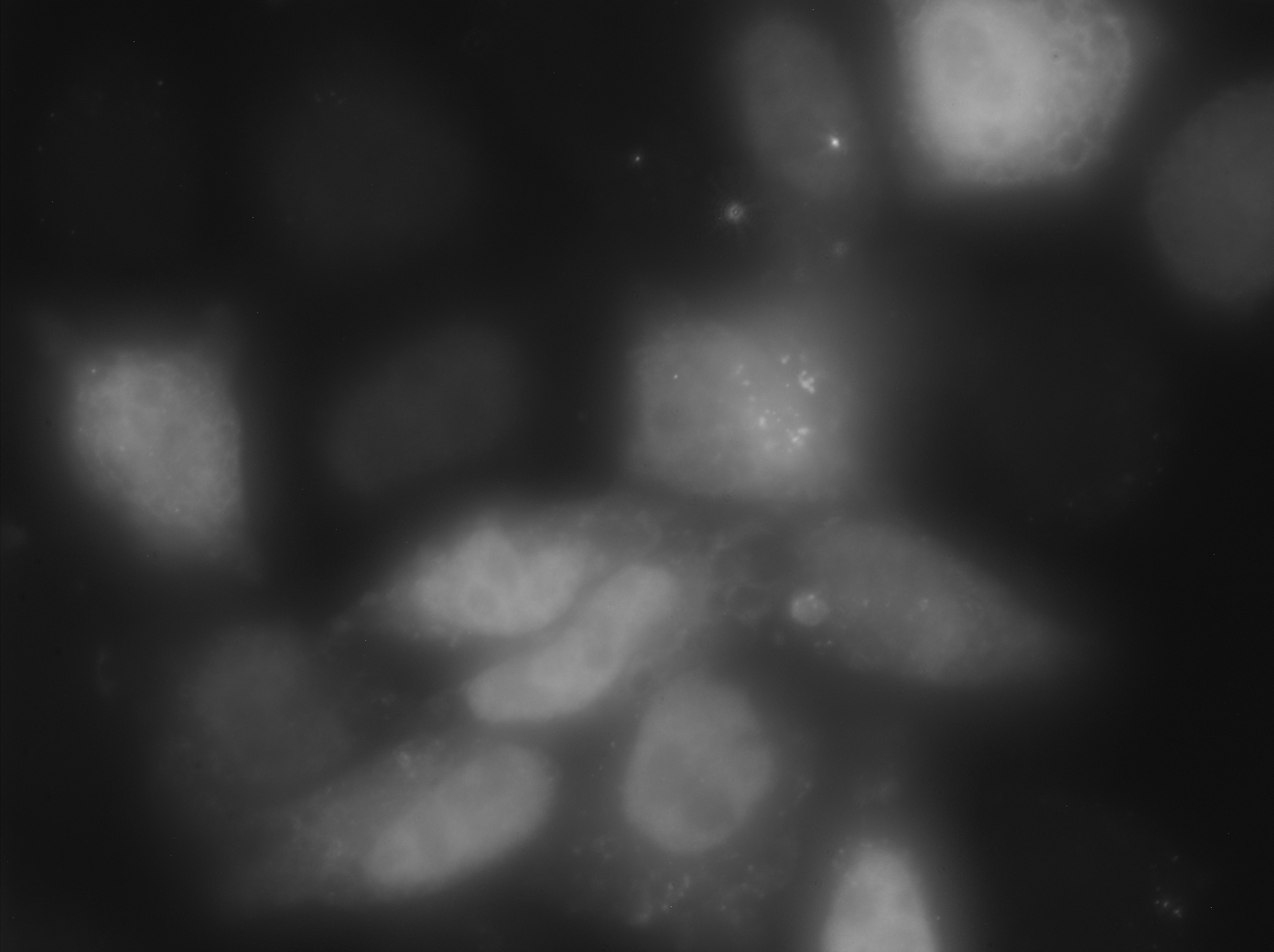

Supplement: Supplementary file 13 — Source data Fig. 3 part 3 [file 44319_2026_742_MOESM13_ESM.zip › Figure 3 Part 3/Fig 3b-e TTLL screen part 3/TTLL9-YFP A3 I19.Project Maximum Z_XY1679701974_Z0_T0_C2.tif]

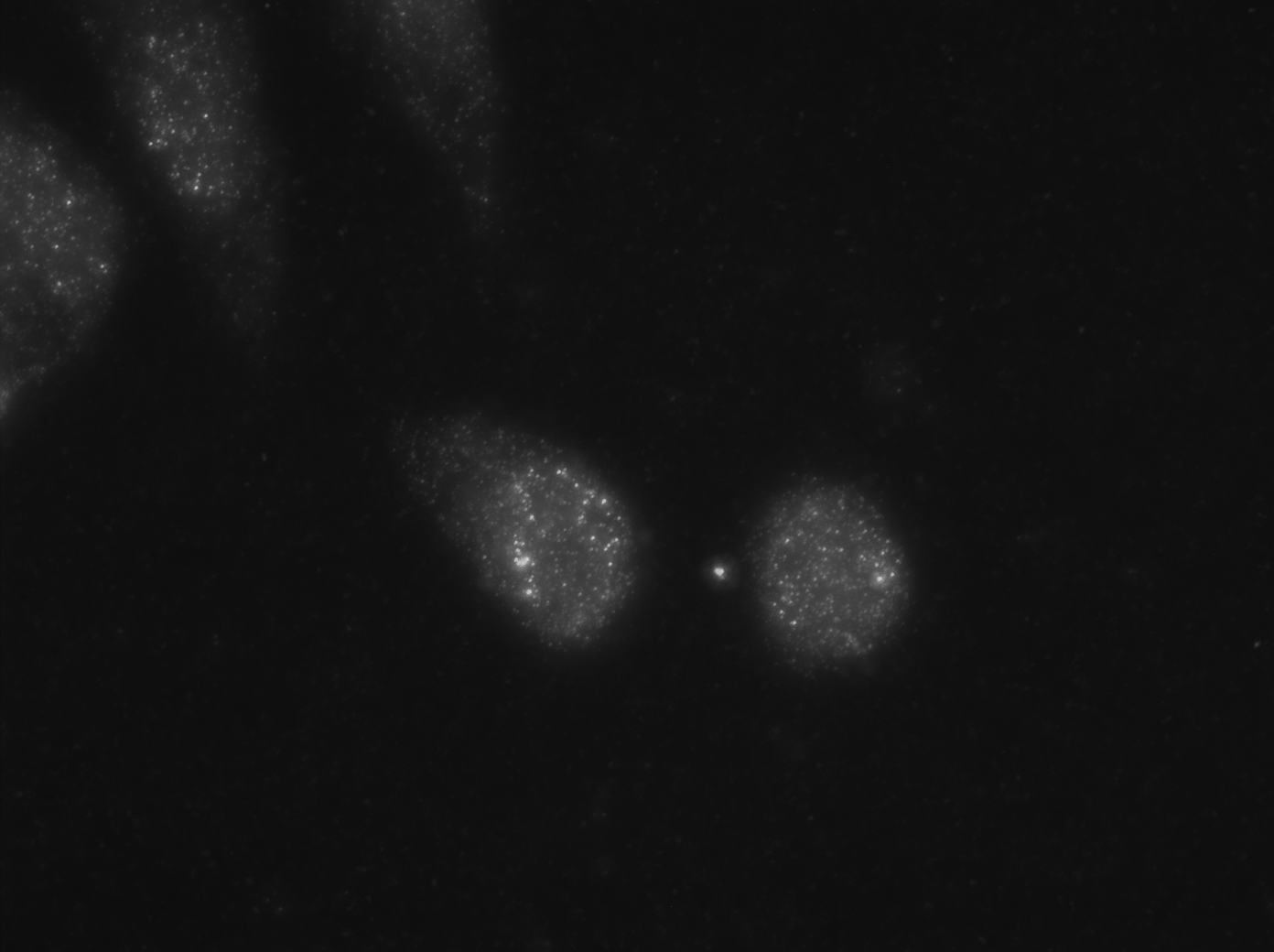

Supplement: Supplementary file 13 — Source data Fig. 3 part 3 [file 44319_2026_742_MOESM13_ESM.zip › Figure 3 Part 3/Fig 3b-e TTLL screen part 3/TTLL11-YFP Img 9 yfp2000.Project Maximum Z_XY1648578368_Z0_T0_C1.tif]

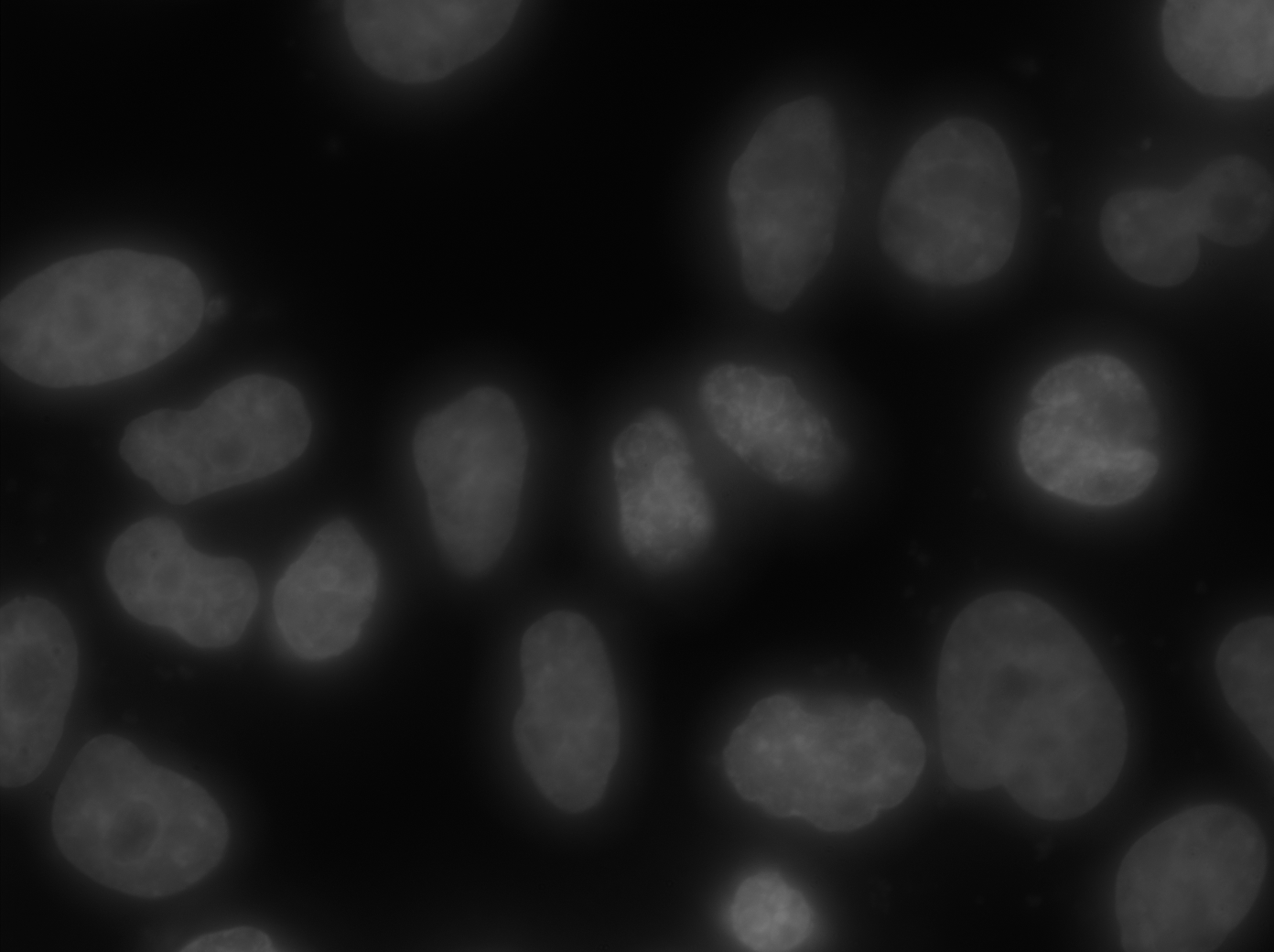

Supplement: Supplementary file 13 — Source data Fig. 3 part 3 [file 44319_2026_742_MOESM13_ESM.zip › Figure 3 Part 3/Fig 3b-e TTLL screen part 3/TTLL9-YFP A3 I15.Project Maximum Z_XY1679701360_Z0_T0_C0.tif]

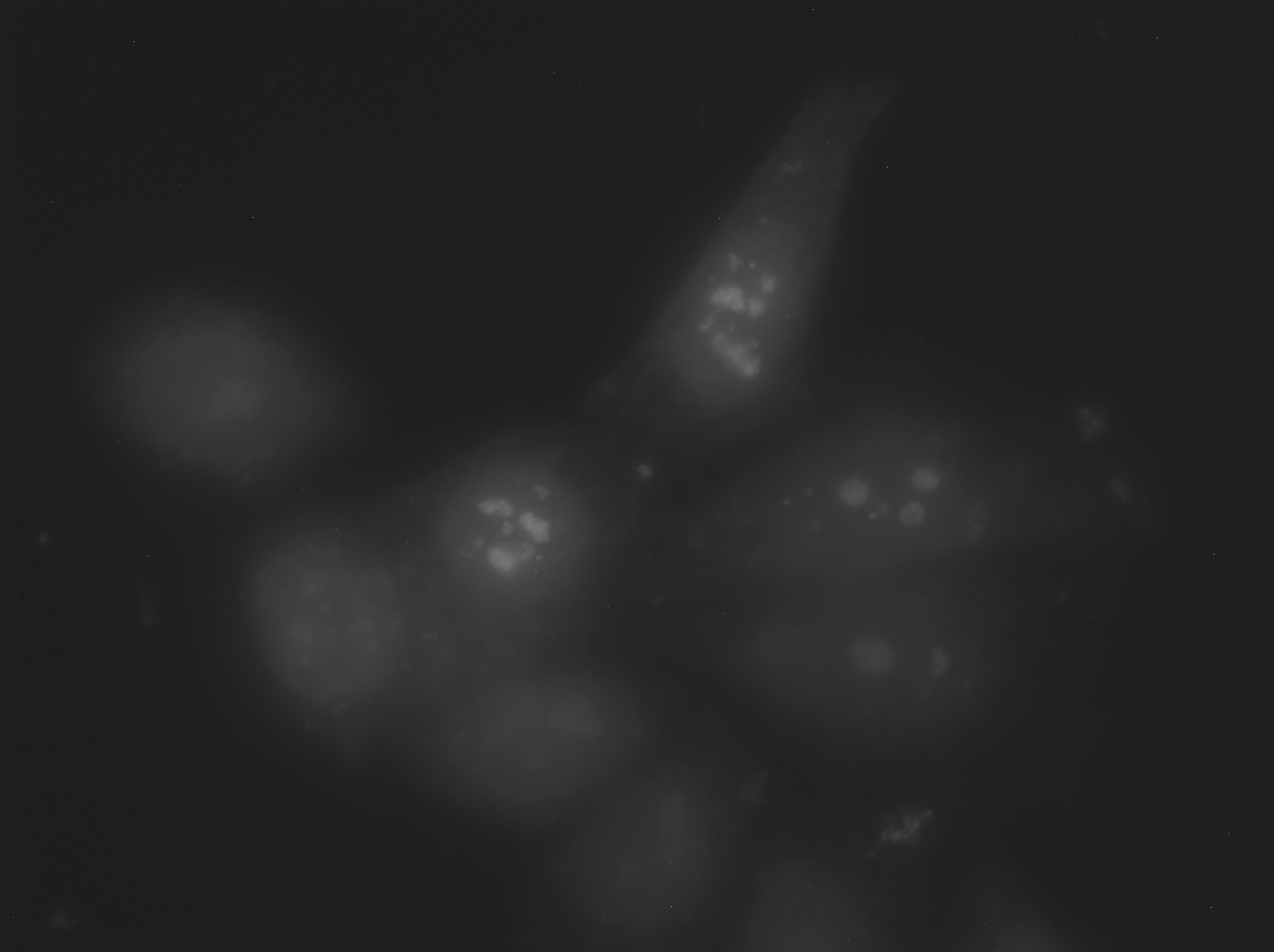

Supplement: Supplementary file 13 — Source data Fig. 3 part 3 [file 44319_2026_742_MOESM13_ESM.zip › Figure 3 Part 3/Fig 3b-e TTLL screen part 3/TTLL11-YFP A1 Img9.Project Maximum Z_XY1650056953_Z0_T0_C2.tif]

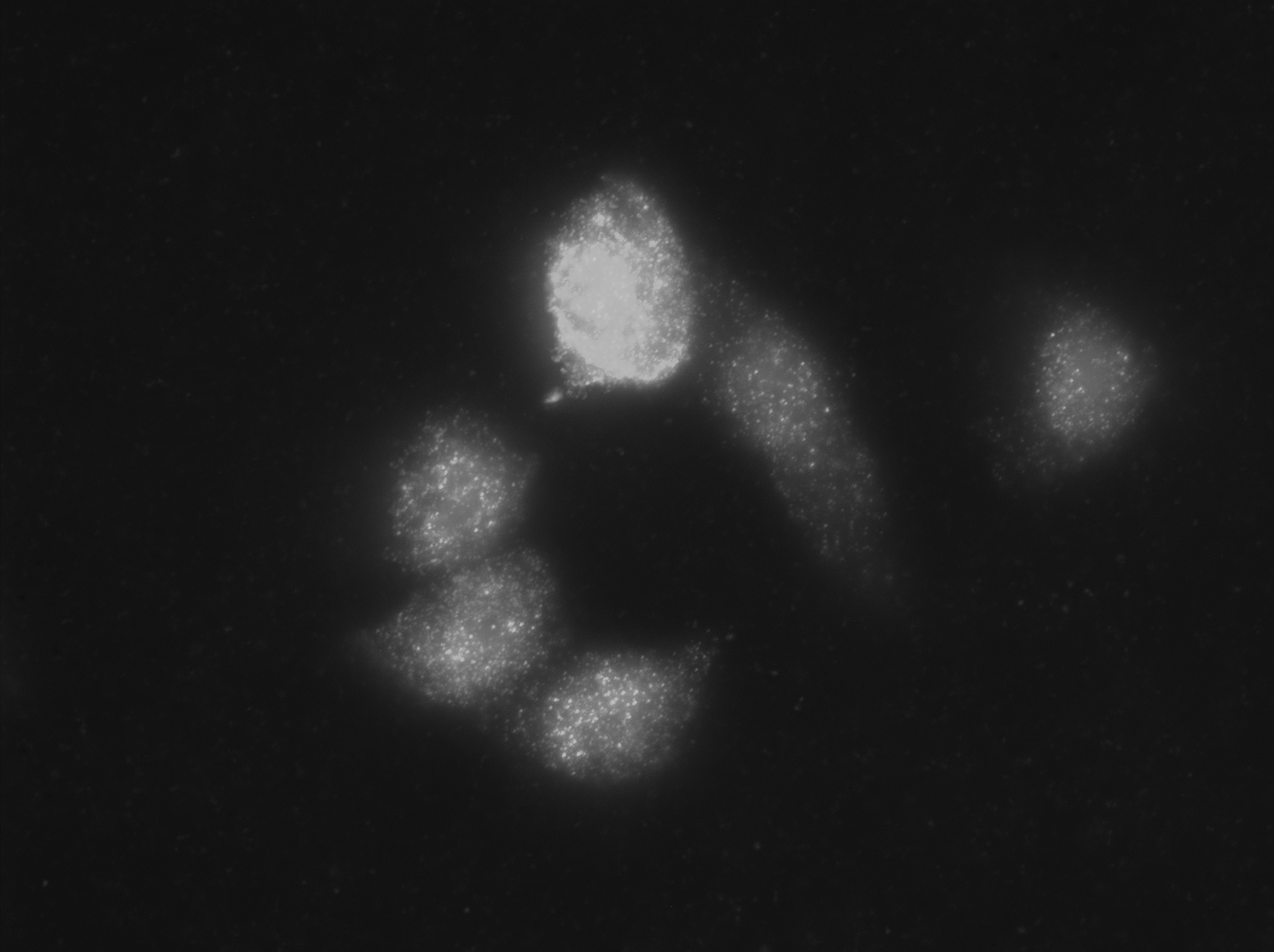

Supplement: Supplementary file 13 — Source data Fig. 3 part 3 [file 44319_2026_742_MOESM13_ESM.zip › Figure 3 Part 3/Fig 3b-e TTLL screen part 3/TTLL11-YFP A2 Img 1.Project Maximum Z_XY1648573797_Z0_T0_C1.tif]

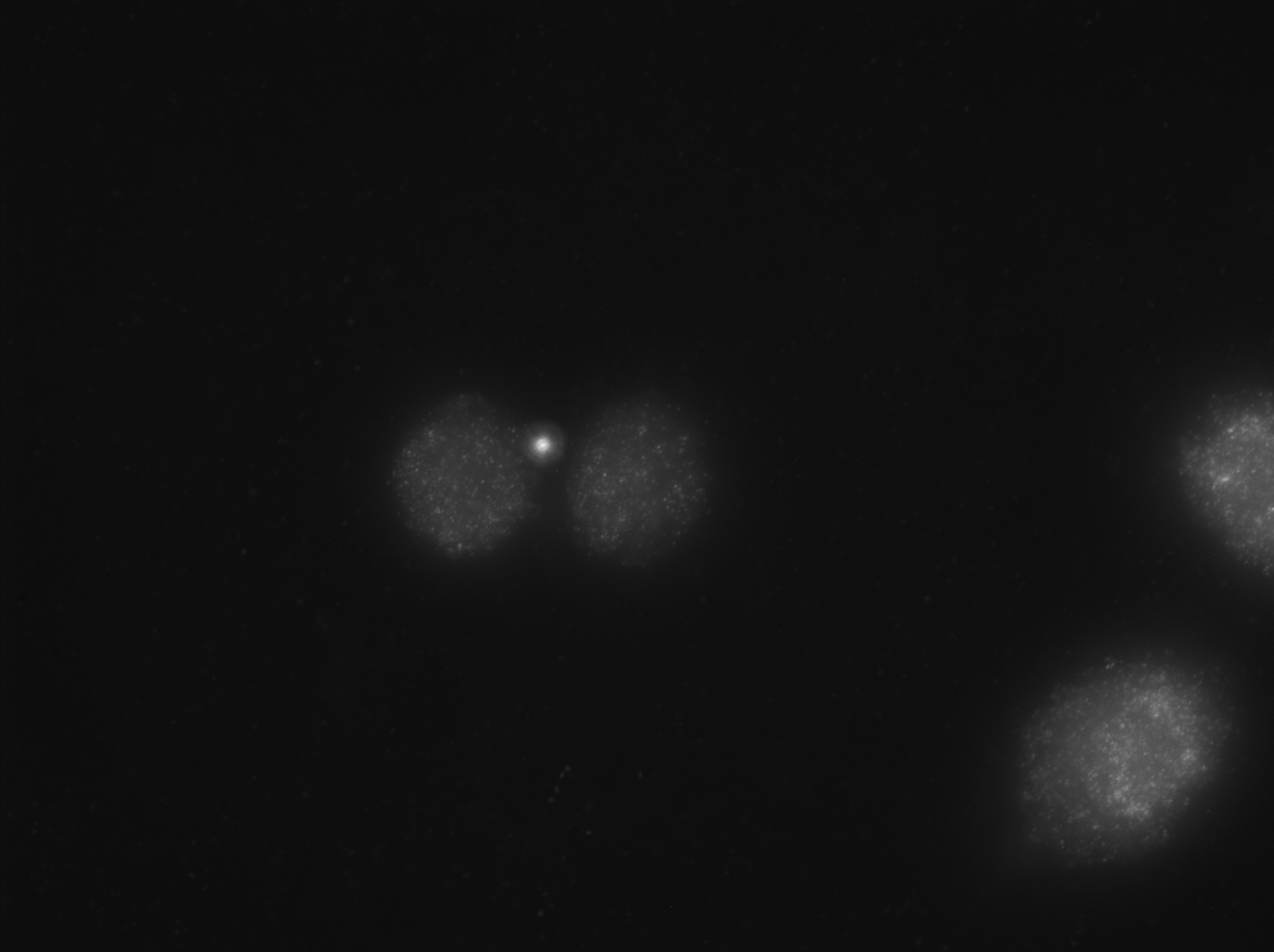

Supplement: Supplementary file 13 — Source data Fig. 3 part 3 [file 44319_2026_742_MOESM13_ESM.zip › Figure 3 Part 3/Fig 3b-e TTLL screen part 3/TTLL11-YFP Img 4 yfp2000.Project Maximum Z_XY1648157838_Z0_T0_C1.tif]

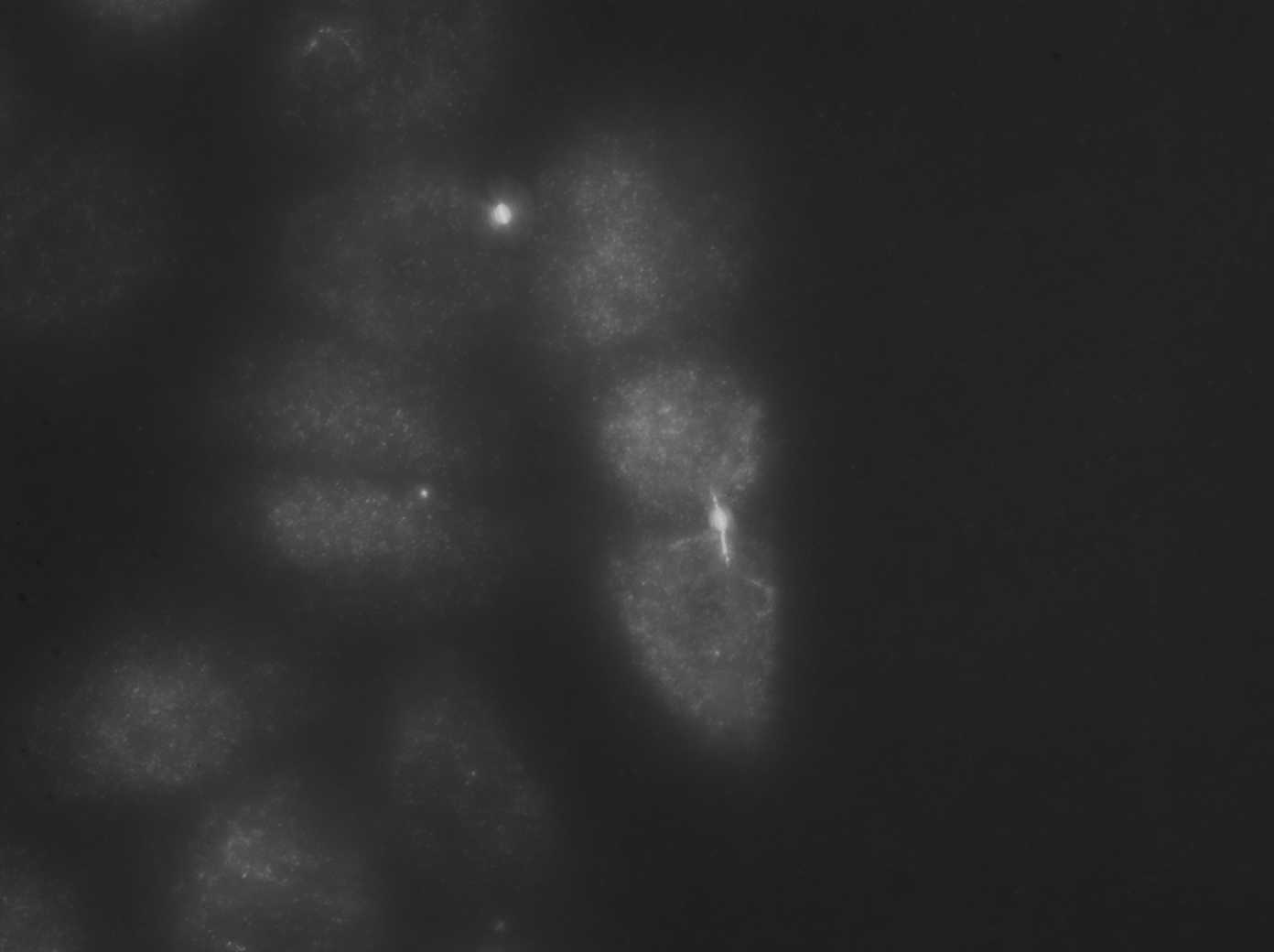

Supplement: Supplementary file 13 — Source data Fig. 3 part 3 [file 44319_2026_742_MOESM13_ESM.zip › Figure 3 Part 3/Fig 3b-e TTLL screen part 3/TTLL9-YFP A3 I12.Project Maximum Z_XY1679700912_Z0_T0_C1.tif]

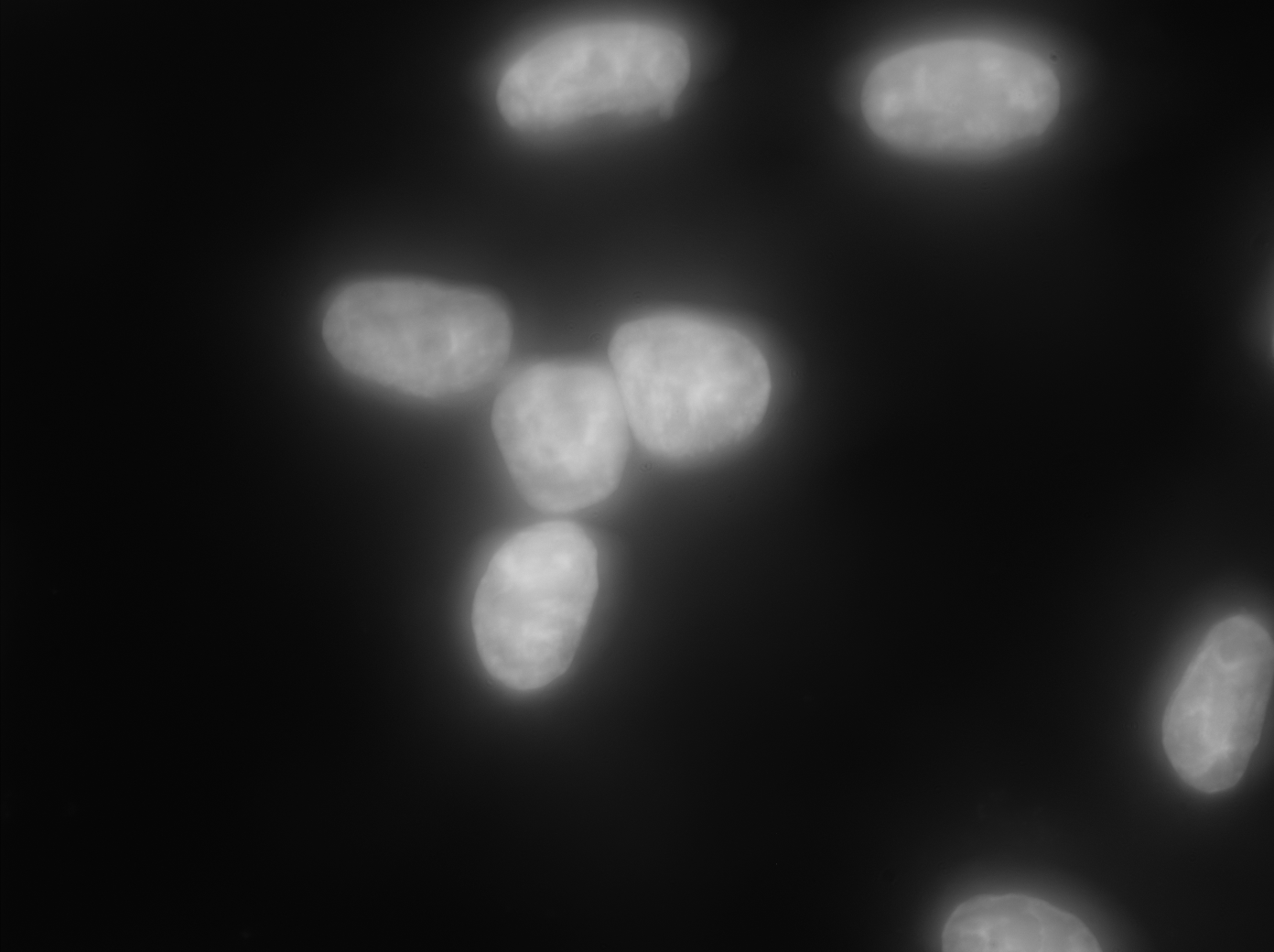

Supplement: Supplementary file 13 — Source data Fig. 3 part 3 [file 44319_2026_742_MOESM13_ESM.zip › Figure 3 Part 3/Fig 3b-e TTLL screen part 3/TTLL11-YFP A2 Img2 - 1.Project Maximum Z_XY1648574683_Z0_T0_C0.tif]

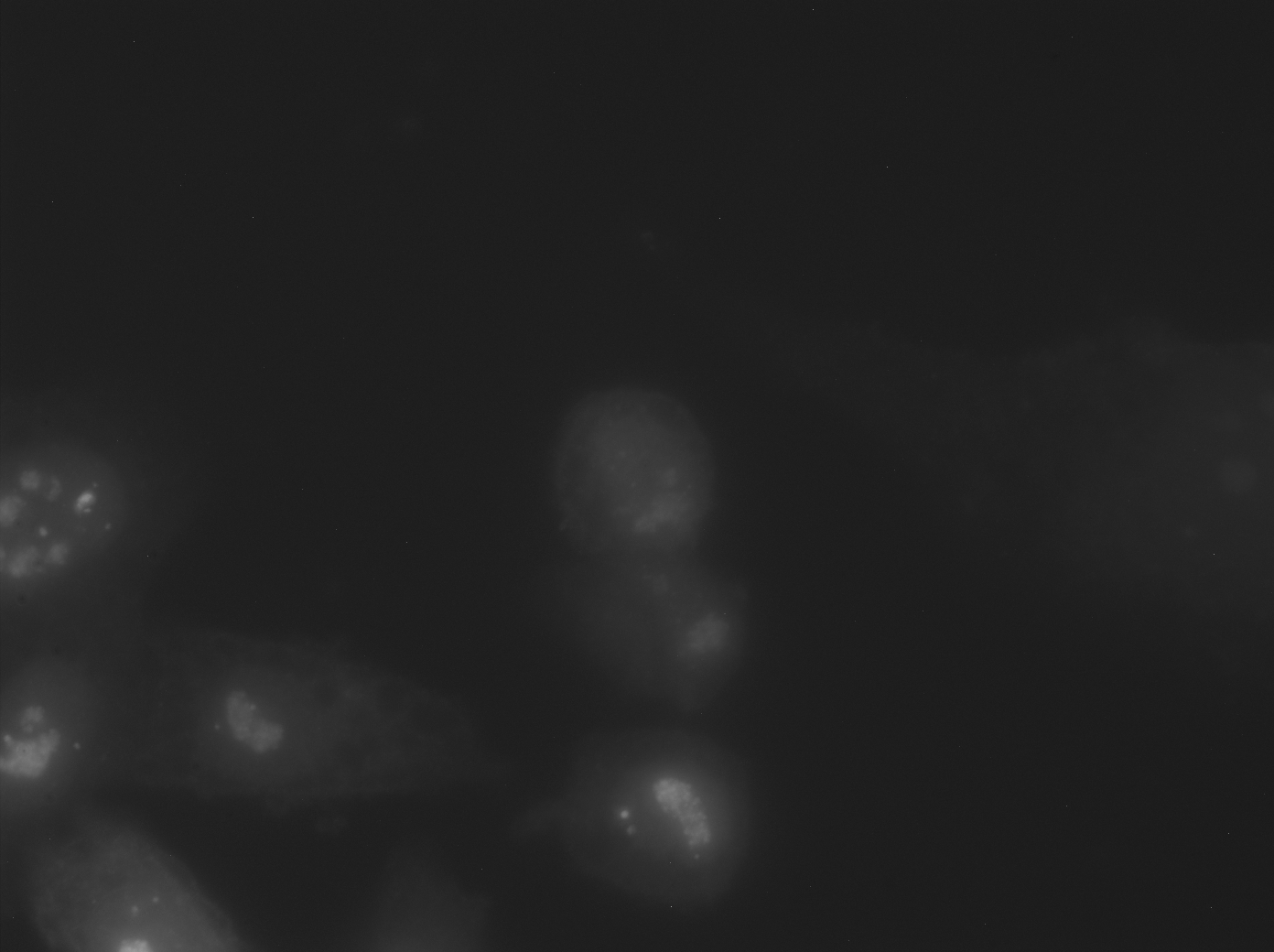

Supplement: Supplementary file 13 — Source data Fig. 3 part 3 [file 44319_2026_742_MOESM13_ESM.zip › Figure 3 Part 3/Fig 3b-e TTLL screen part 3/TTLL11-YFP Img 10 yfp2000 - 1.Project Maximum Z_XY1648579577_Z0_T0_C2.tif]

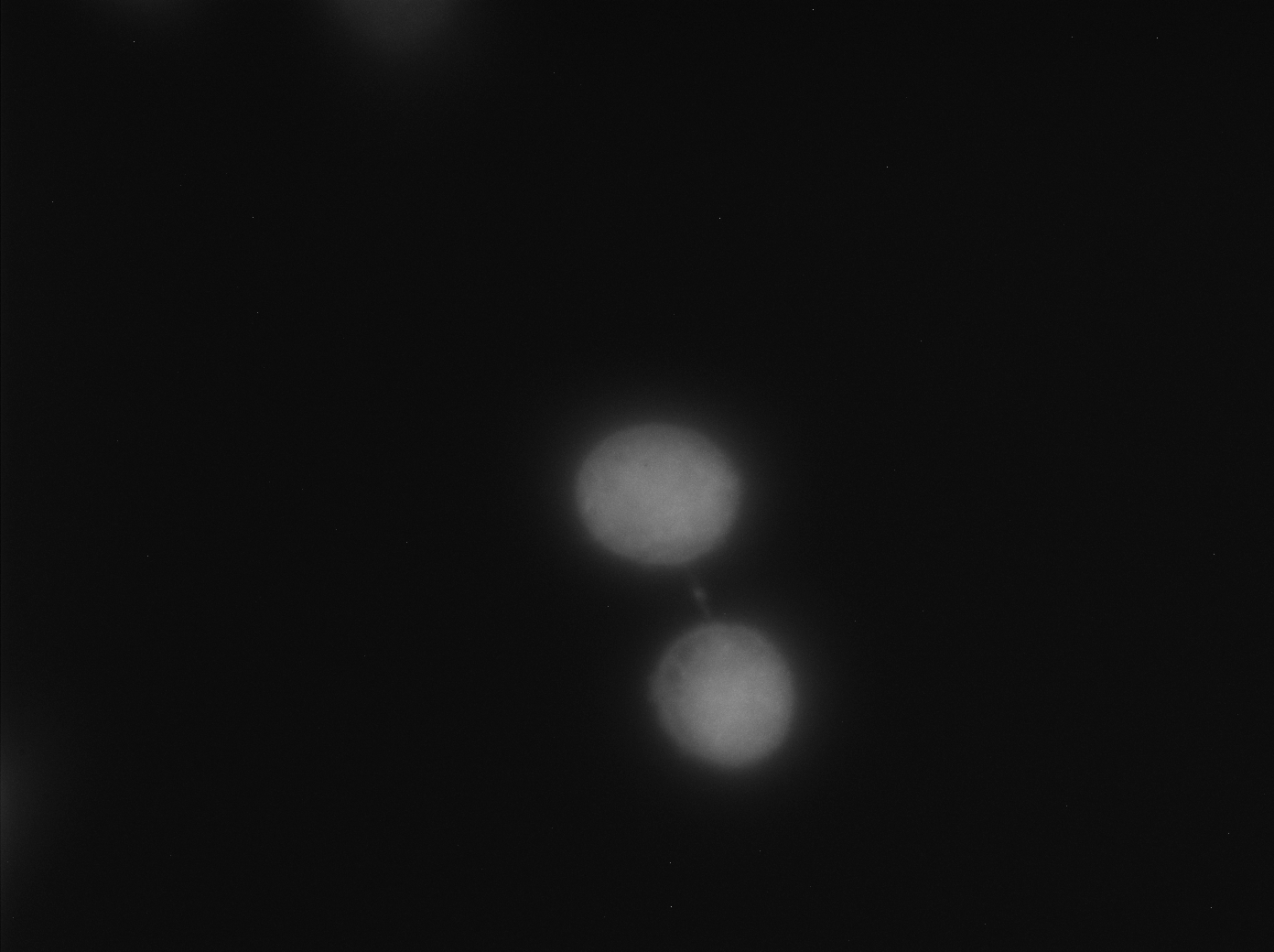

Supplement: Supplementary file 13 — Source data Fig. 3 part 3 [file 44319_2026_742_MOESM13_ESM.zip › Figure 3 Part 3/Fig 3b-e TTLL screen part 3/YFP Only R1 I5.Project Maximum Z_XY1663272254_Z0_T0_C2.tif]

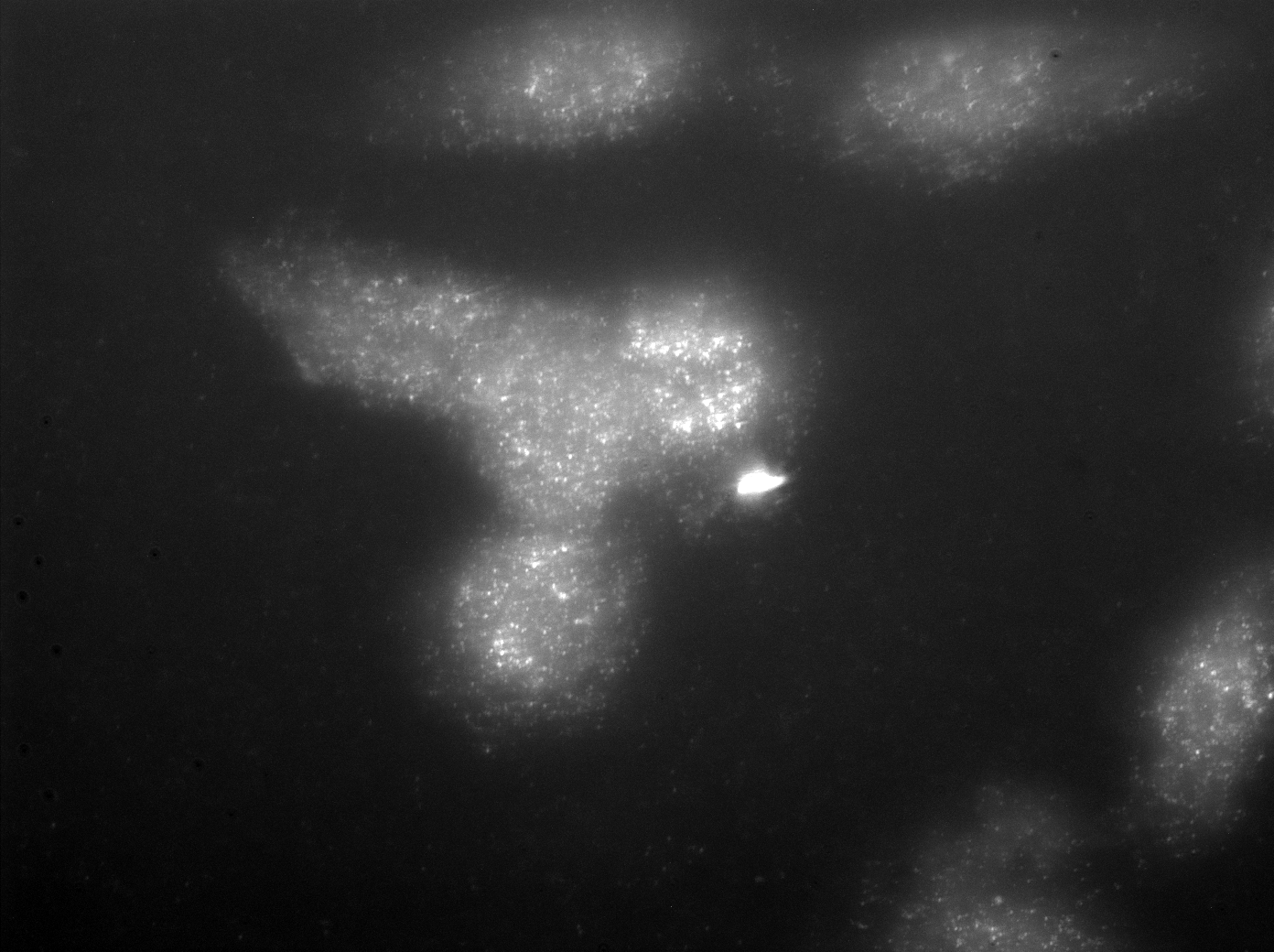

Supplement: Supplementary file 13 — Source data Fig. 3 part 3 [file 44319_2026_742_MOESM13_ESM.zip › Figure 3 Part 3/Fig 3b-e TTLL screen part 3/TTLL11-YFP A2 Img2 - 1.Project Maximum Z_XY1648574683_Z0_T0_C1.tif]

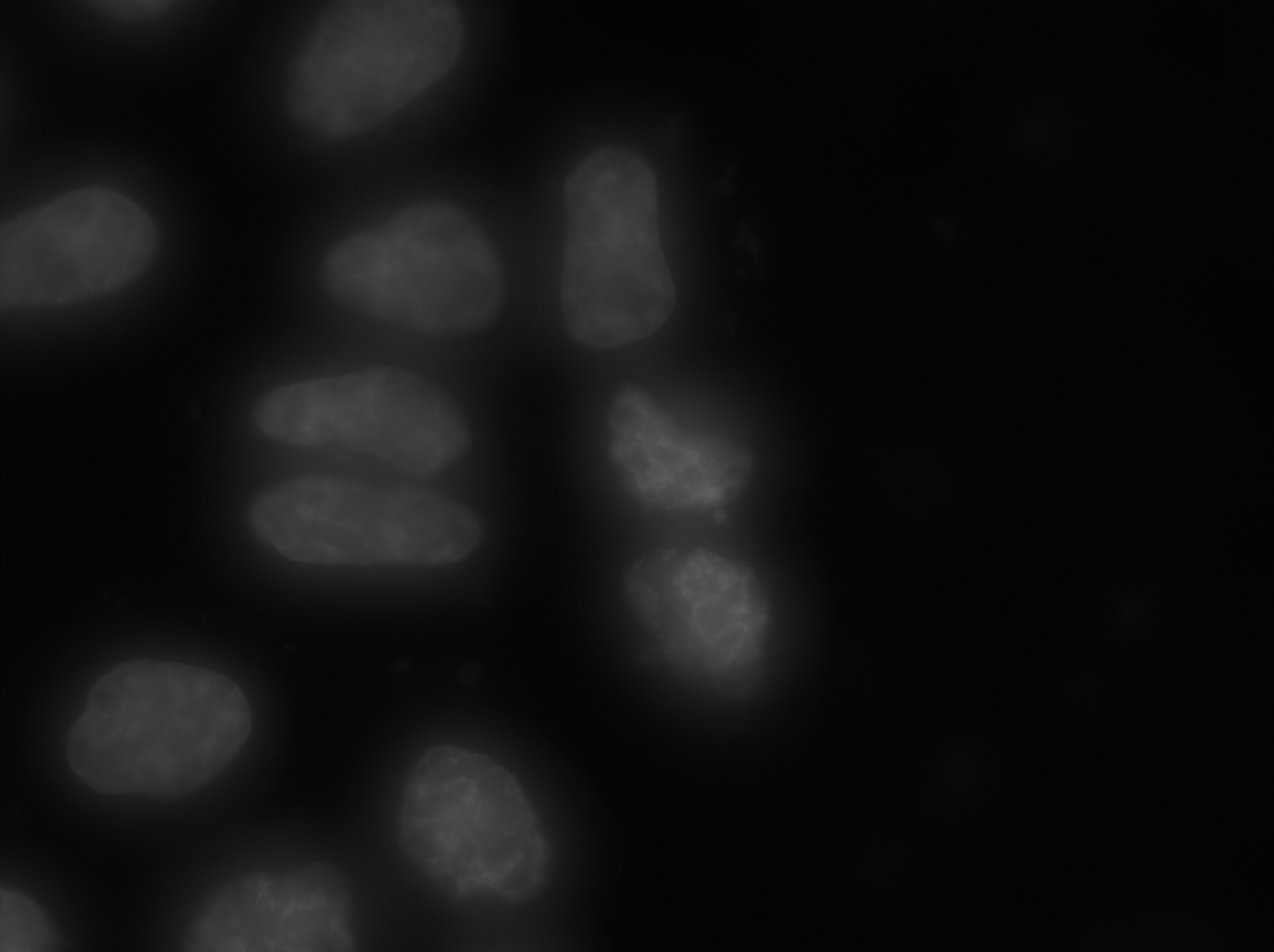

Supplement: Supplementary file 13 — Source data Fig. 3 part 3 [file 44319_2026_742_MOESM13_ESM.zip › Figure 3 Part 3/Fig 3b-e TTLL screen part 3/TTLL9-YFP A3 I12.Project Maximum Z_XY1679700912_Z0_T0_C0.tif]

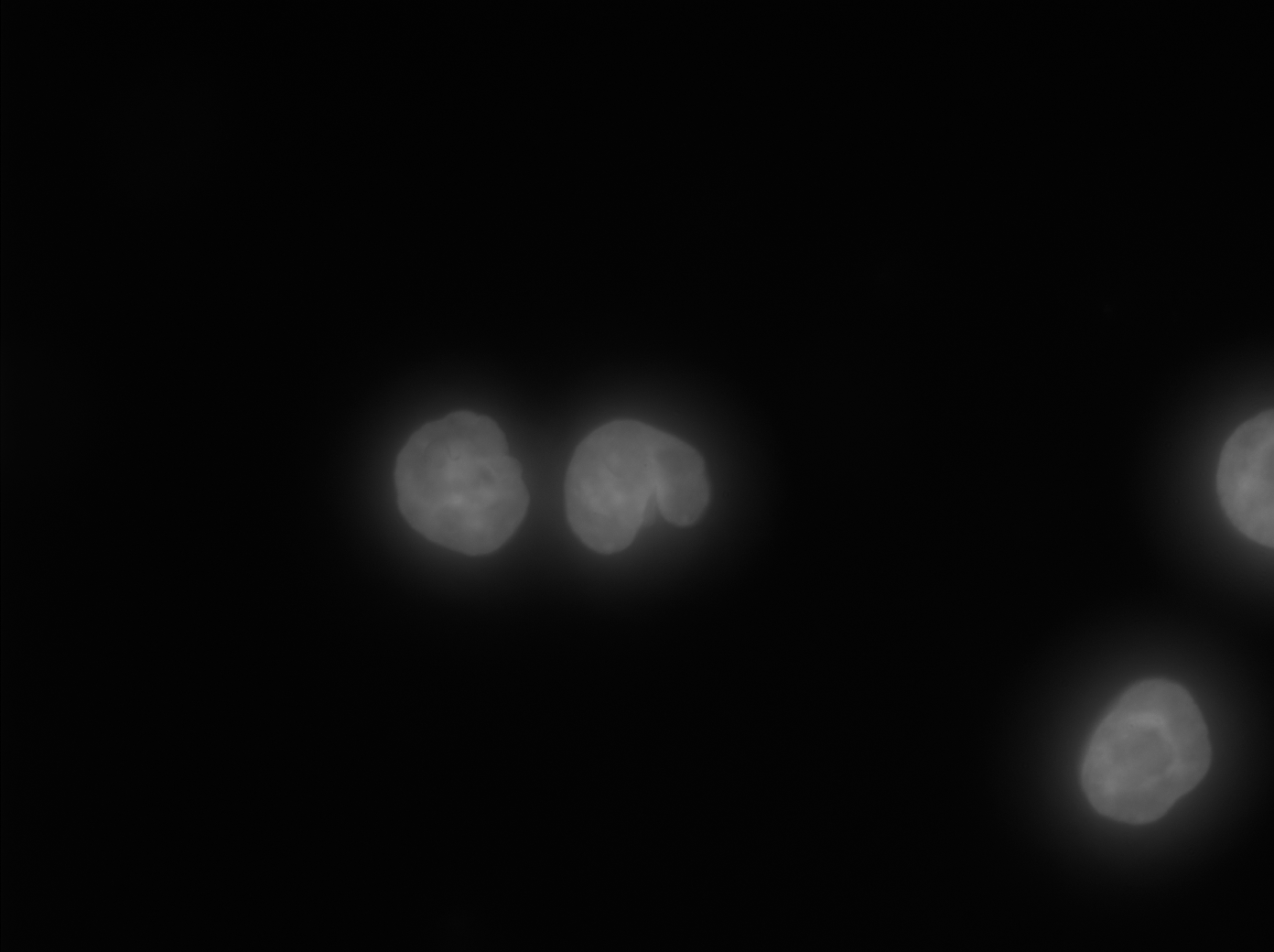

Supplement: Supplementary file 13 — Source data Fig. 3 part 3 [file 44319_2026_742_MOESM13_ESM.zip › Figure 3 Part 3/Fig 3b-e TTLL screen part 3/TTLL11-YFP Img 4 yfp2000.Project Maximum Z_XY1648157838_Z0_T0_C0.tif]

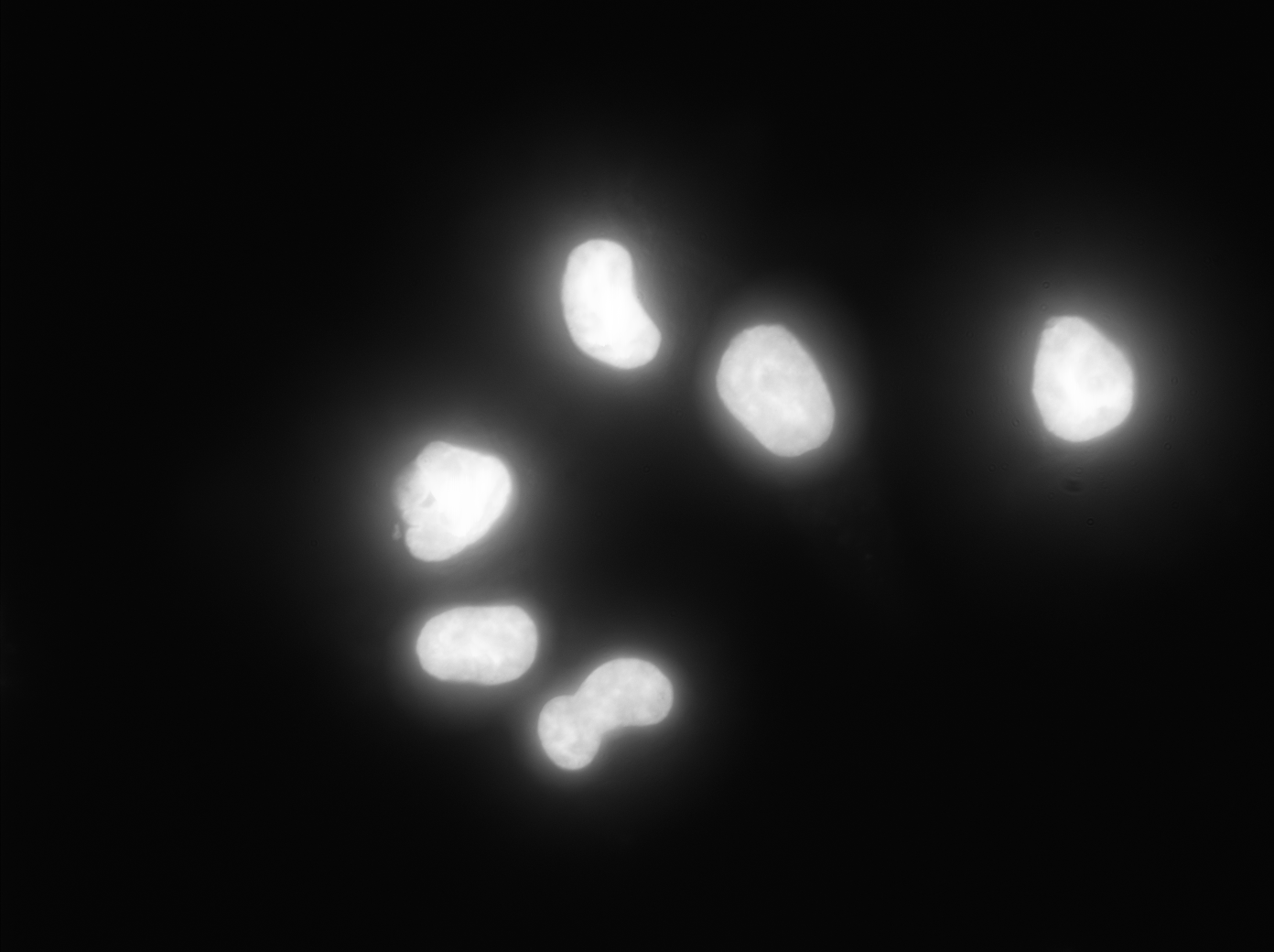

Supplement: Supplementary file 13 — Source data Fig. 3 part 3 [file 44319_2026_742_MOESM13_ESM.zip › Figure 3 Part 3/Fig 3b-e TTLL screen part 3/TTLL11-YFP A2 Img 1.Project Maximum Z_XY1648573797_Z0_T0_C0.tif]

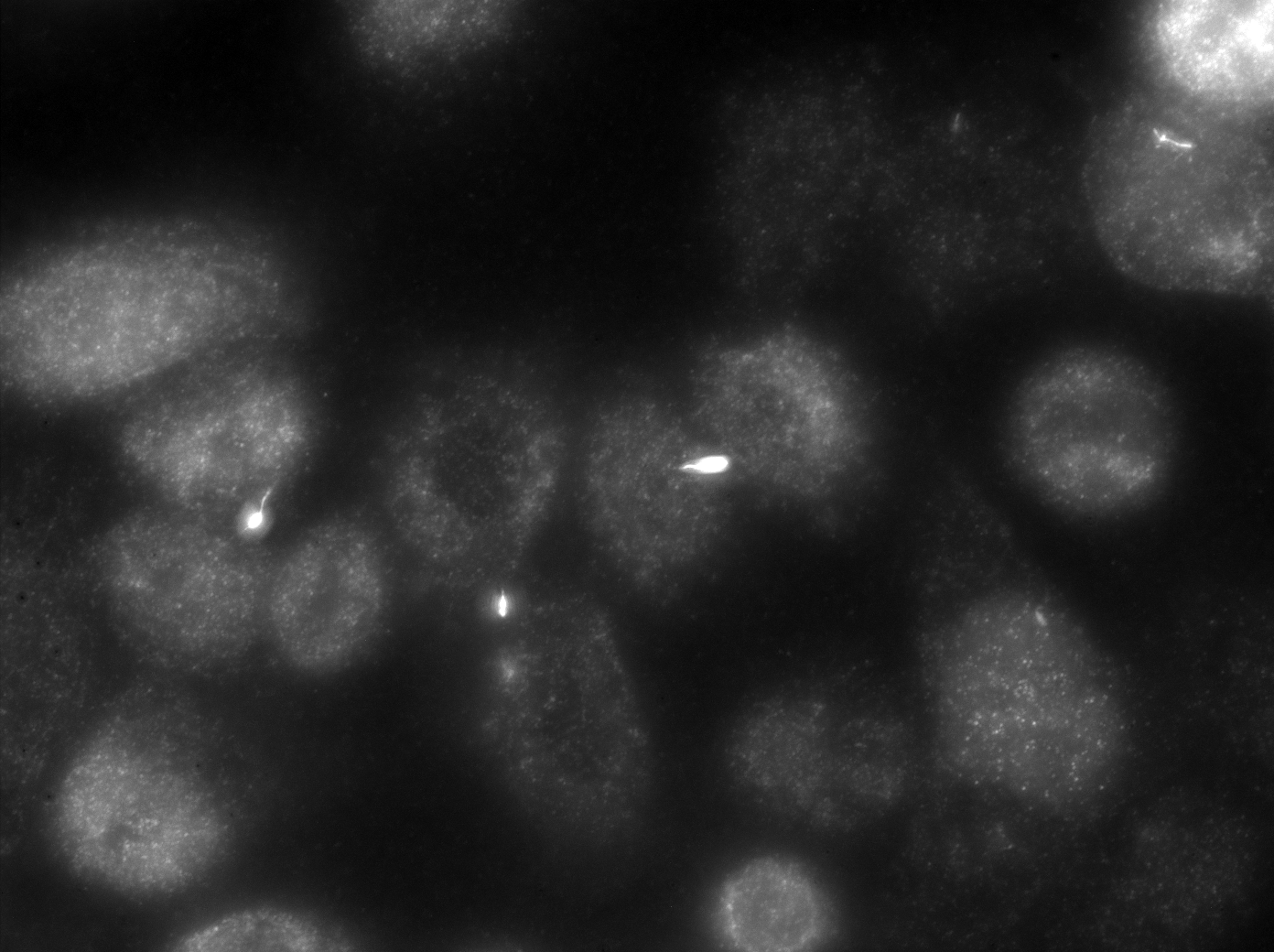

Supplement: Supplementary file 13 — Source data Fig. 3 part 3 [file 44319_2026_742_MOESM13_ESM.zip › Figure 3 Part 3/Fig 3b-e TTLL screen part 3/TTLL9-YFP A3 I15.Project Maximum Z_XY1679701360_Z0_T0_C1.tif]

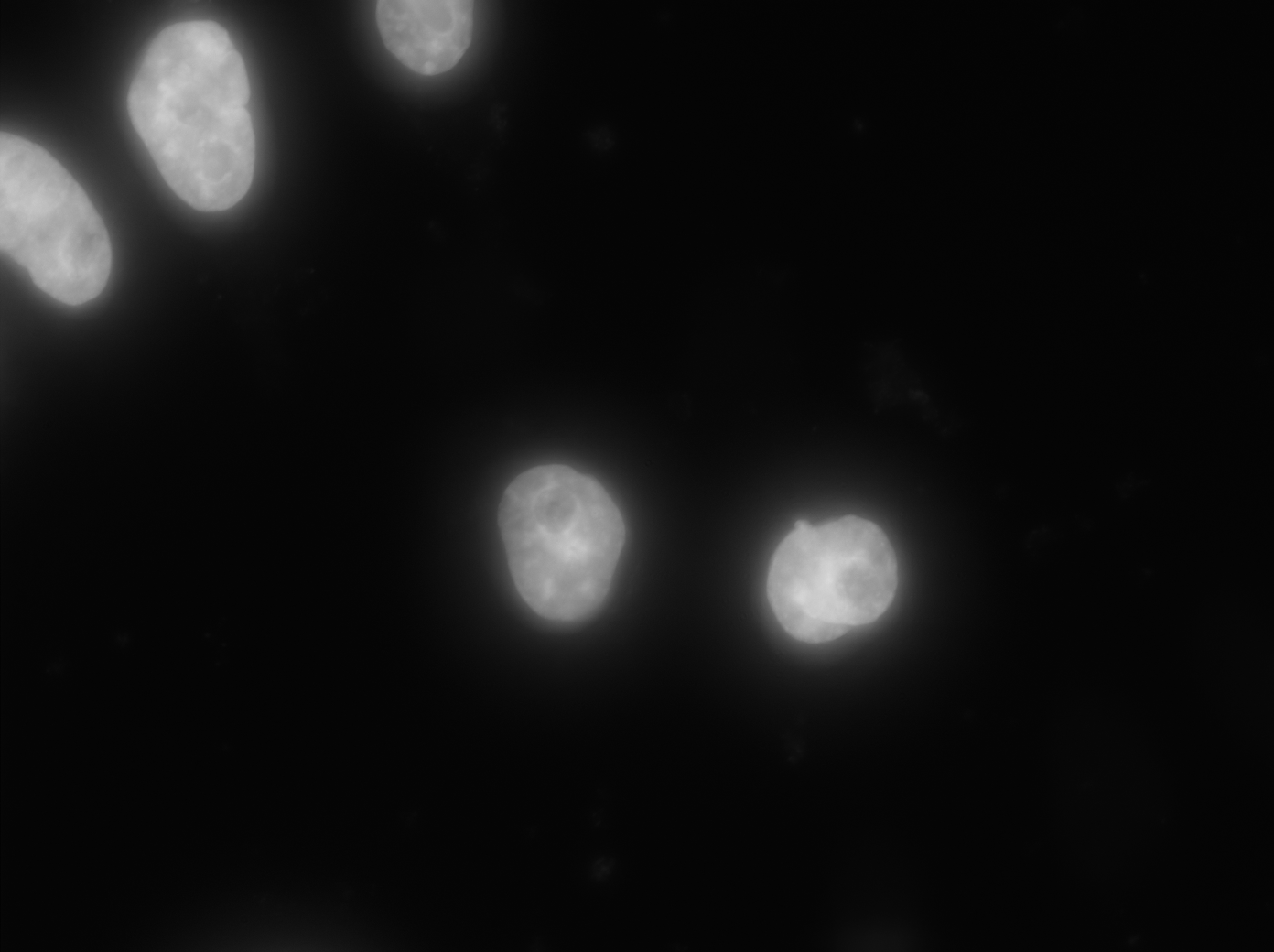

Supplement: Supplementary file 13 — Source data Fig. 3 part 3 [file 44319_2026_742_MOESM13_ESM.zip › Figure 3 Part 3/Fig 3b-e TTLL screen part 3/TTLL11-YFP Img 9 yfp2000.Project Maximum Z_XY1648578368_Z0_T0_C0.tif]

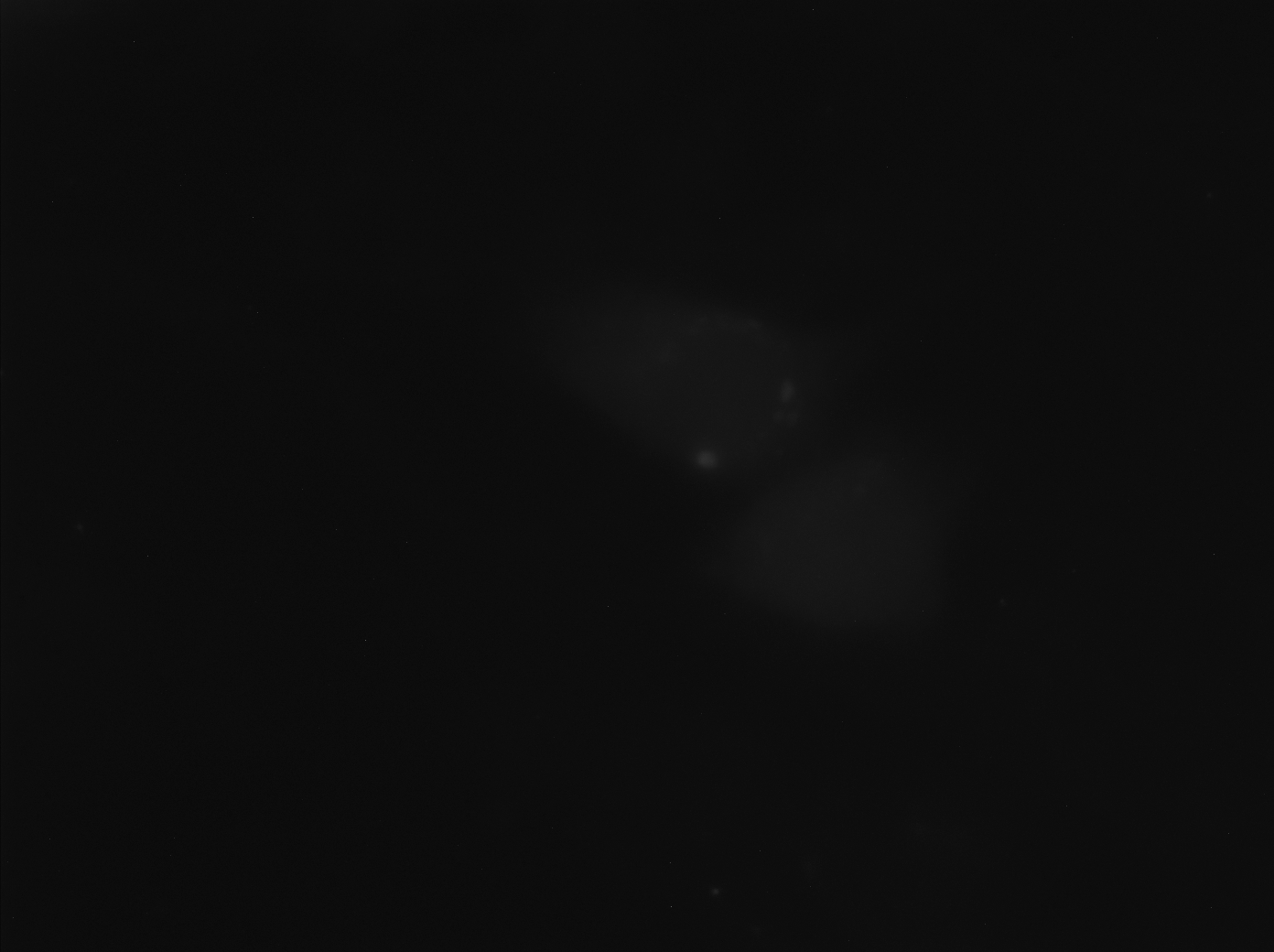

Supplement: Supplementary file 13 — Source data Fig. 3 part 3 [file 44319_2026_742_MOESM13_ESM.zip › Figure 3 Part 3/Fig 3b-e TTLL screen part 3/TTLL9-YFP A3 I2.Project Maximum Z_XY1674674772_Z0_T0_C2.tif]

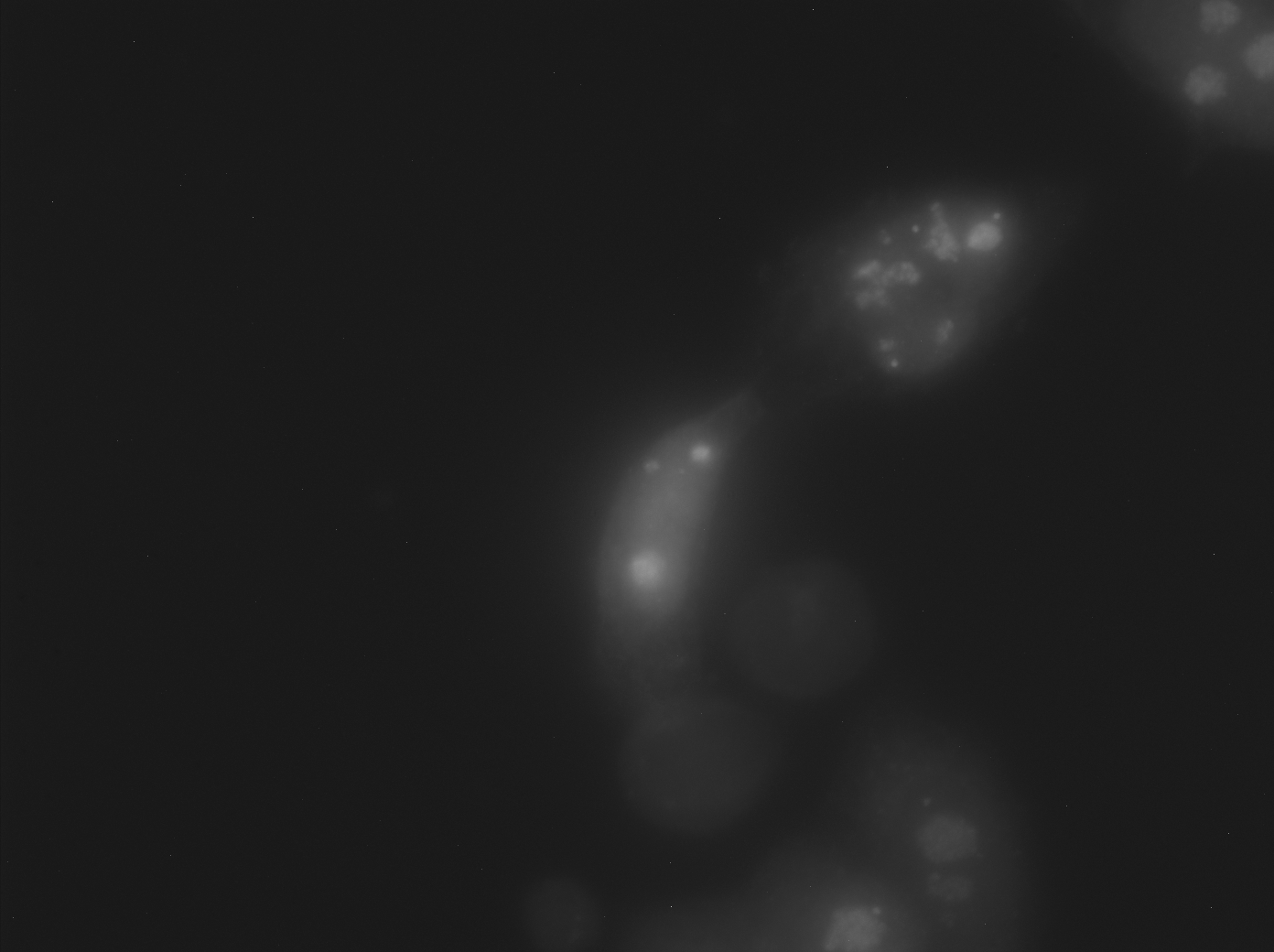

Supplement: Supplementary file 13 — Source data Fig. 3 part 3 [file 44319_2026_742_MOESM13_ESM.zip › Figure 3 Part 3/Fig 3b-e TTLL screen part 3/TTLL11-YFP Img 13 yfp2500.Project Maximum Z_XY1648580917_Z0_T0_C2.tif]

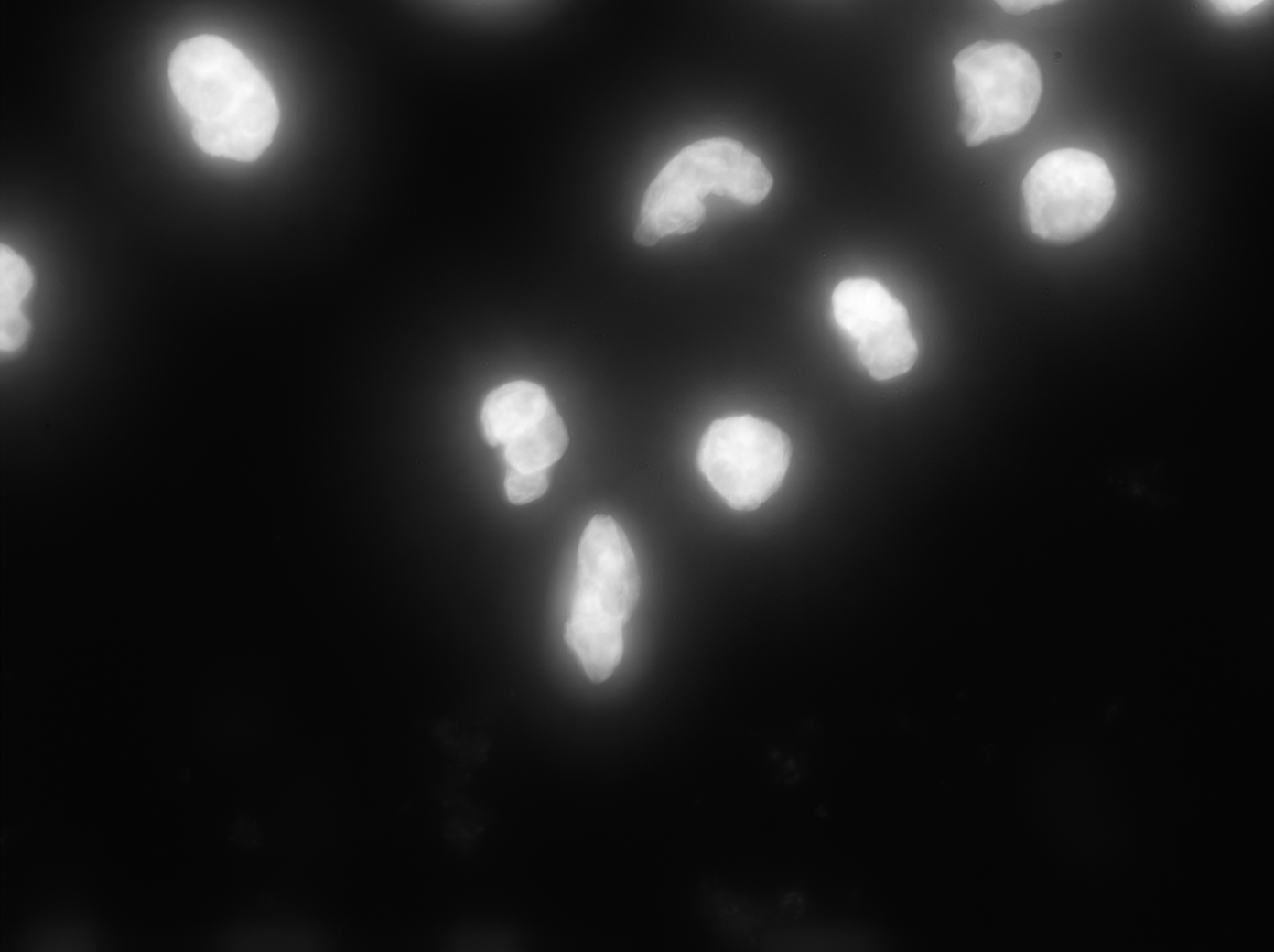

Supplement: Supplementary file 13 — Source data Fig. 3 part 3 [file 44319_2026_742_MOESM13_ESM.zip › Figure 3 Part 3/Fig 3b-e TTLL screen part 3/TTLL11-YFP Img 8 yfp2000 - 1.Project Maximum Z_XY1648578082_Z0_T0_C0.tif]

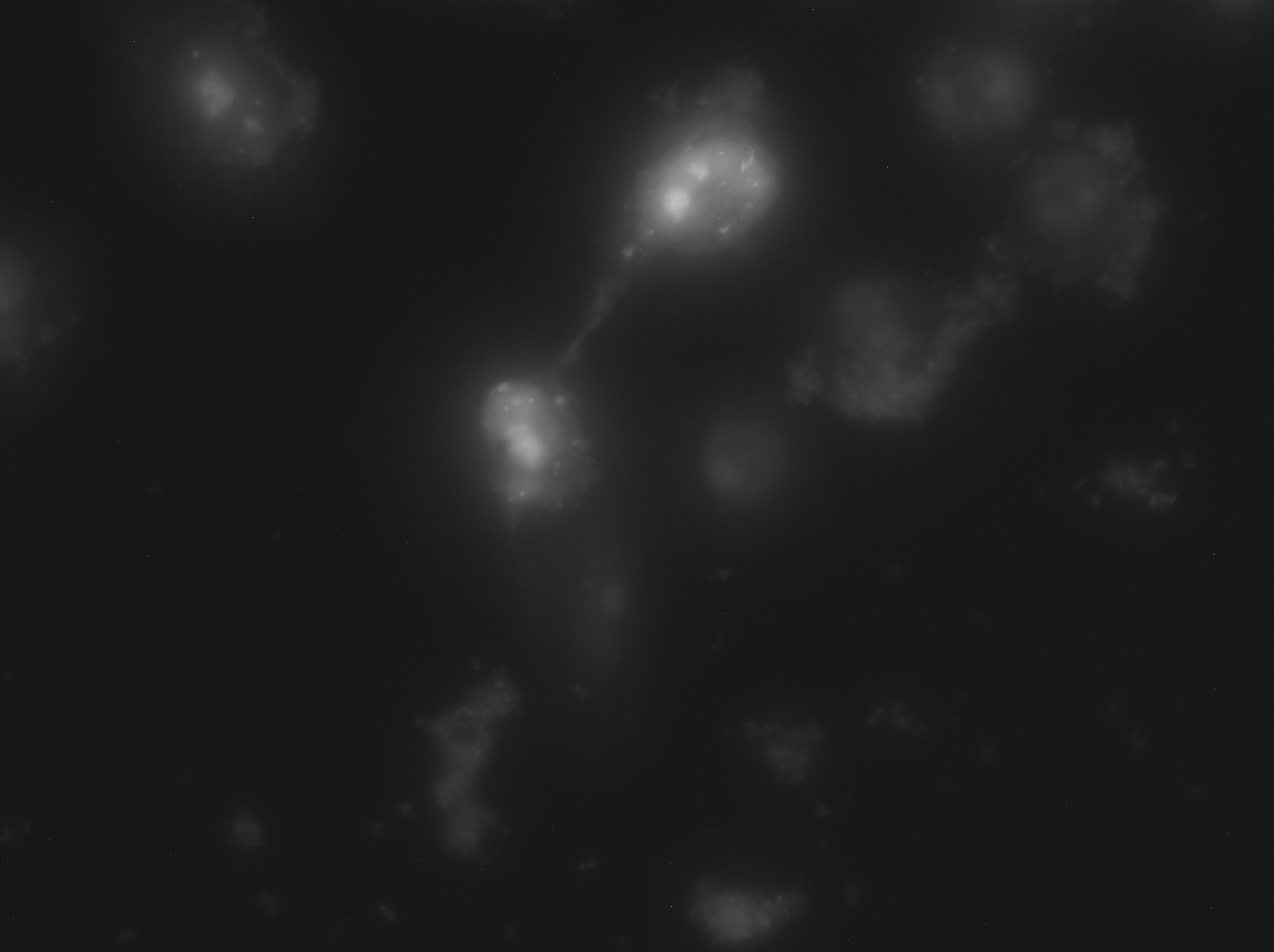

Supplement: Supplementary file 13 — Source data Fig. 3 part 3 [file 44319_2026_742_MOESM13_ESM.zip › Figure 3 Part 3/Fig 3b-e TTLL screen part 3/TTLL11-YFP Img 8 yfp2000 - 1.Project Maximum Z_XY1648578082_Z0_T0_C2.tif]

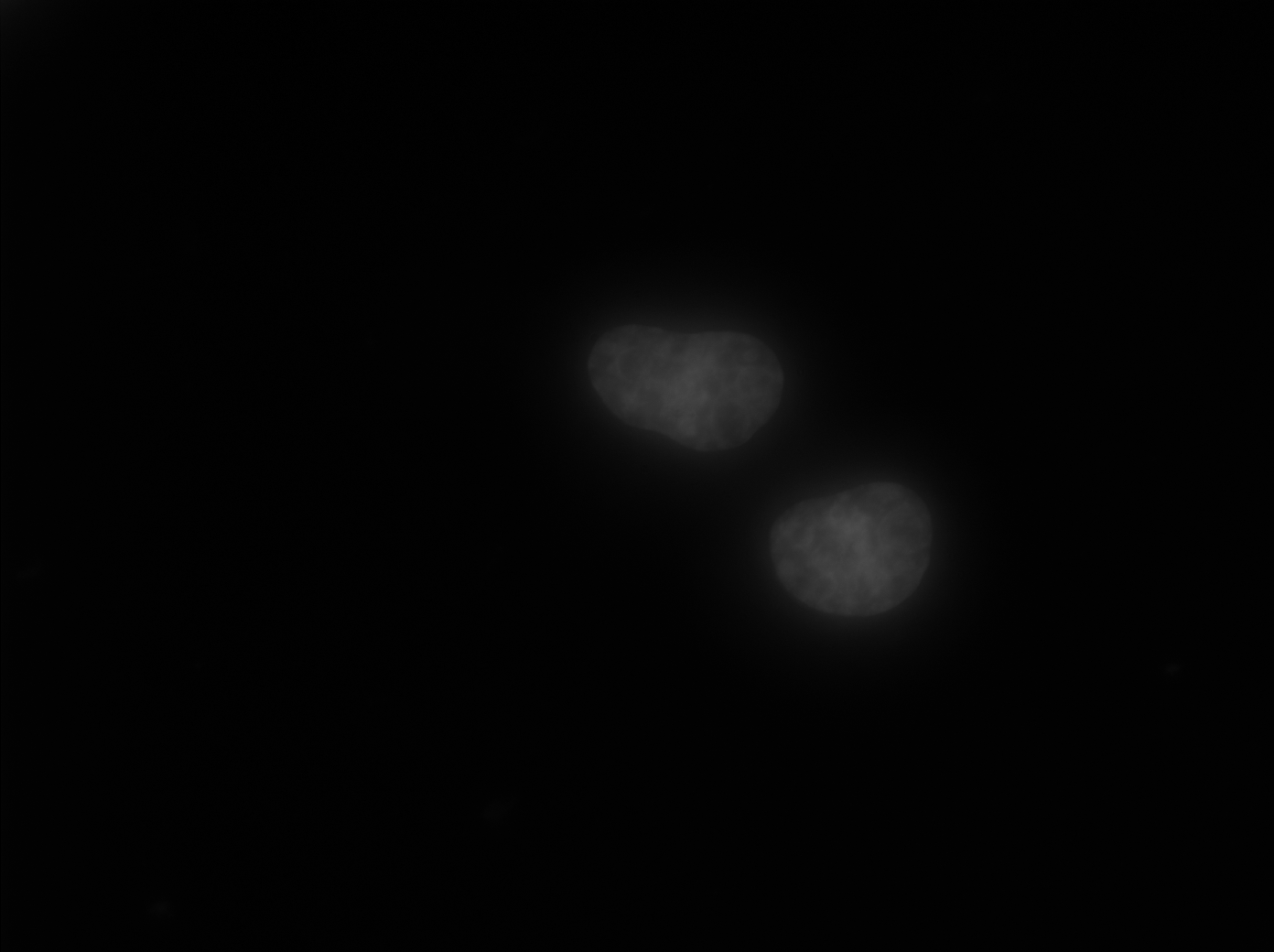

Supplement: Supplementary file 13 — Source data Fig. 3 part 3 [file 44319_2026_742_MOESM13_ESM.zip › Figure 3 Part 3/Fig 3b-e TTLL screen part 3/TTLL9-YFP A3 I2.Project Maximum Z_XY1674674772_Z0_T0_C0.tif]

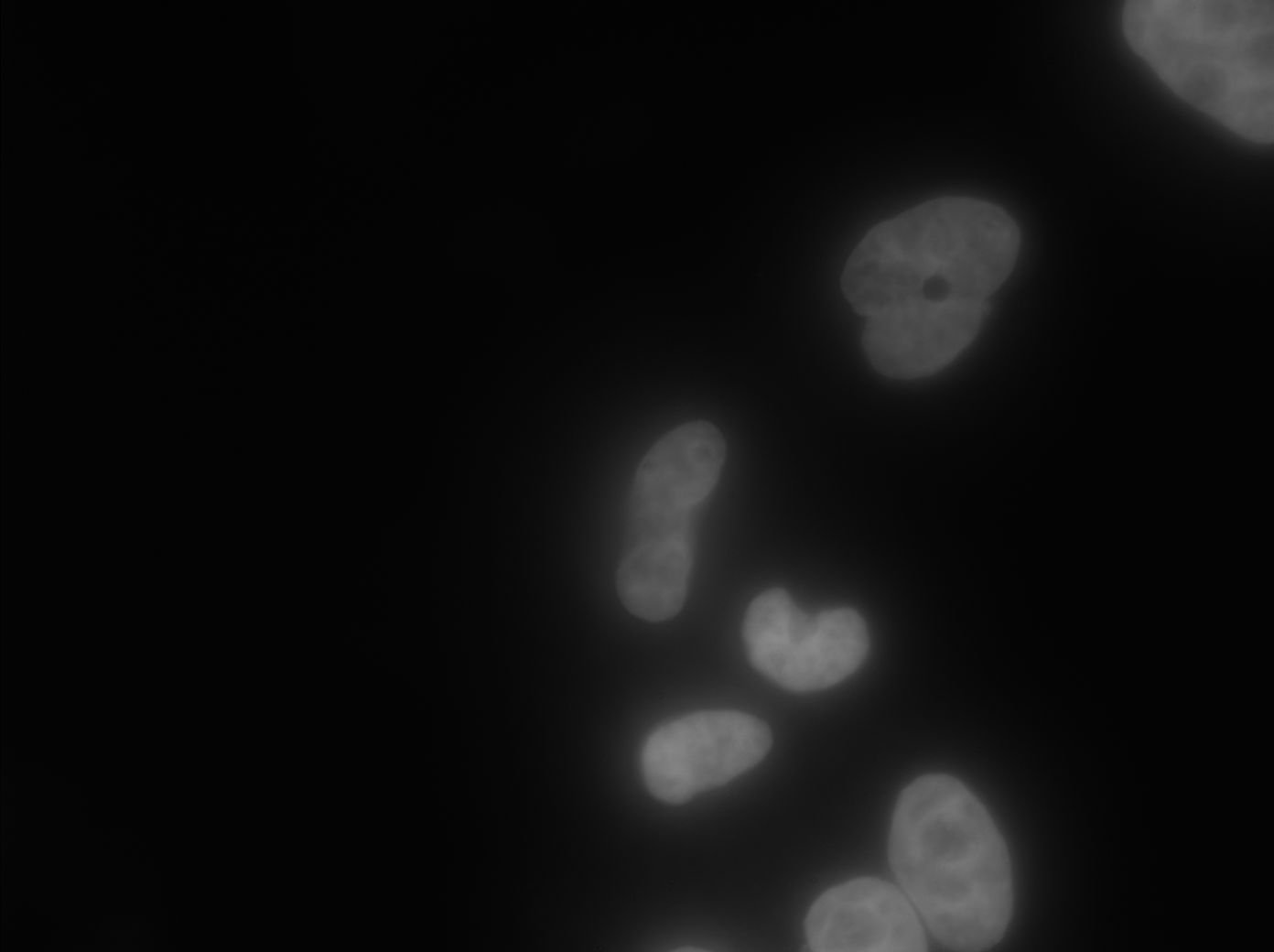

Supplement: Supplementary file 13 — Source data Fig. 3 part 3 [file 44319_2026_742_MOESM13_ESM.zip › Figure 3 Part 3/Fig 3b-e TTLL screen part 3/TTLL11-YFP Img 13 yfp2500.Project Maximum Z_XY1648580917_Z0_T0_C0.tif]

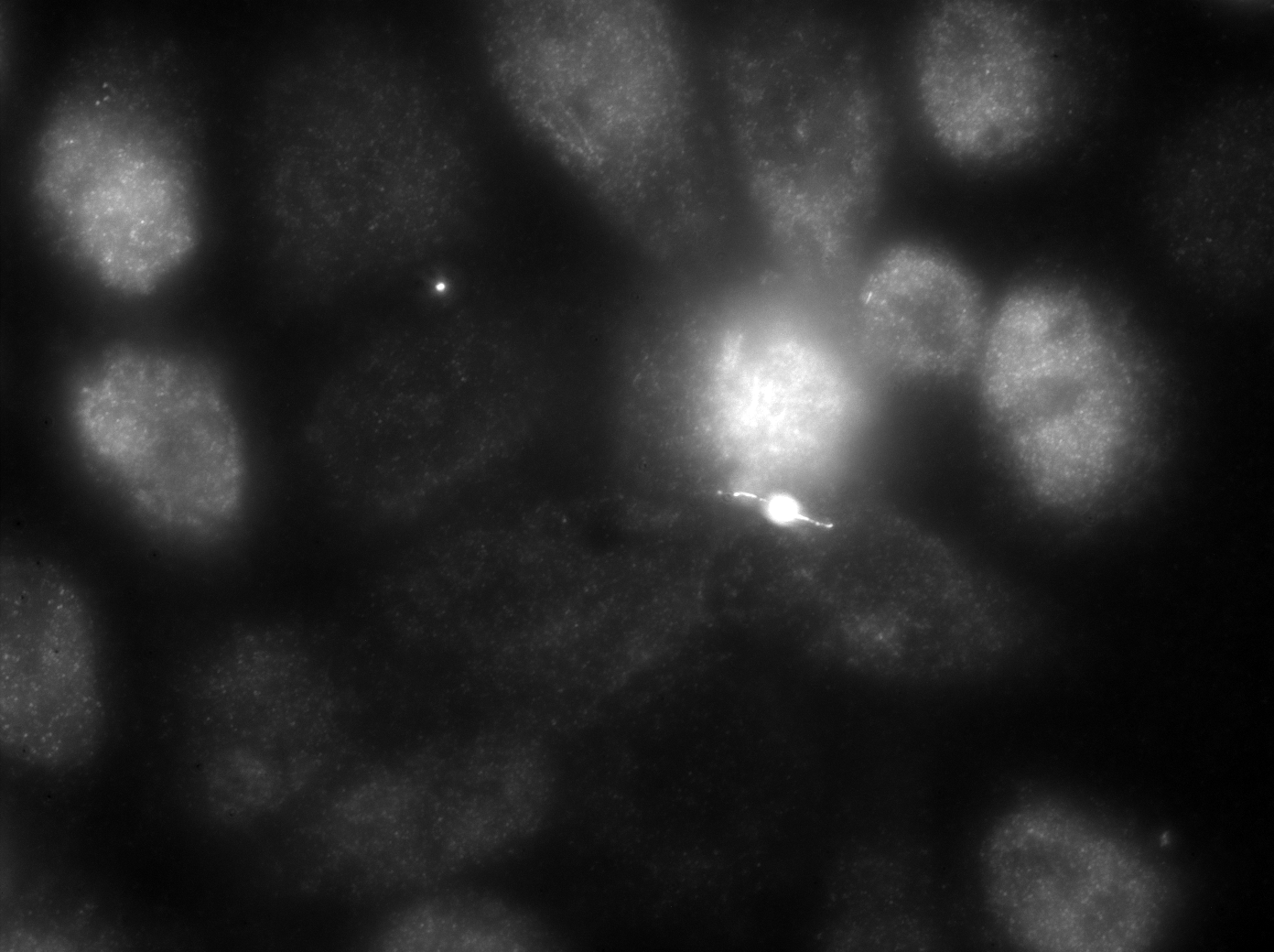

Supplement: Supplementary file 13 — Source data Fig. 3 part 3 [file 44319_2026_742_MOESM13_ESM.zip › Figure 3 Part 3/Fig 3b-e TTLL screen part 3/TTLL9-YFP A3 I19.Project Maximum Z_XY1679701974_Z0_T0_C1.tif]

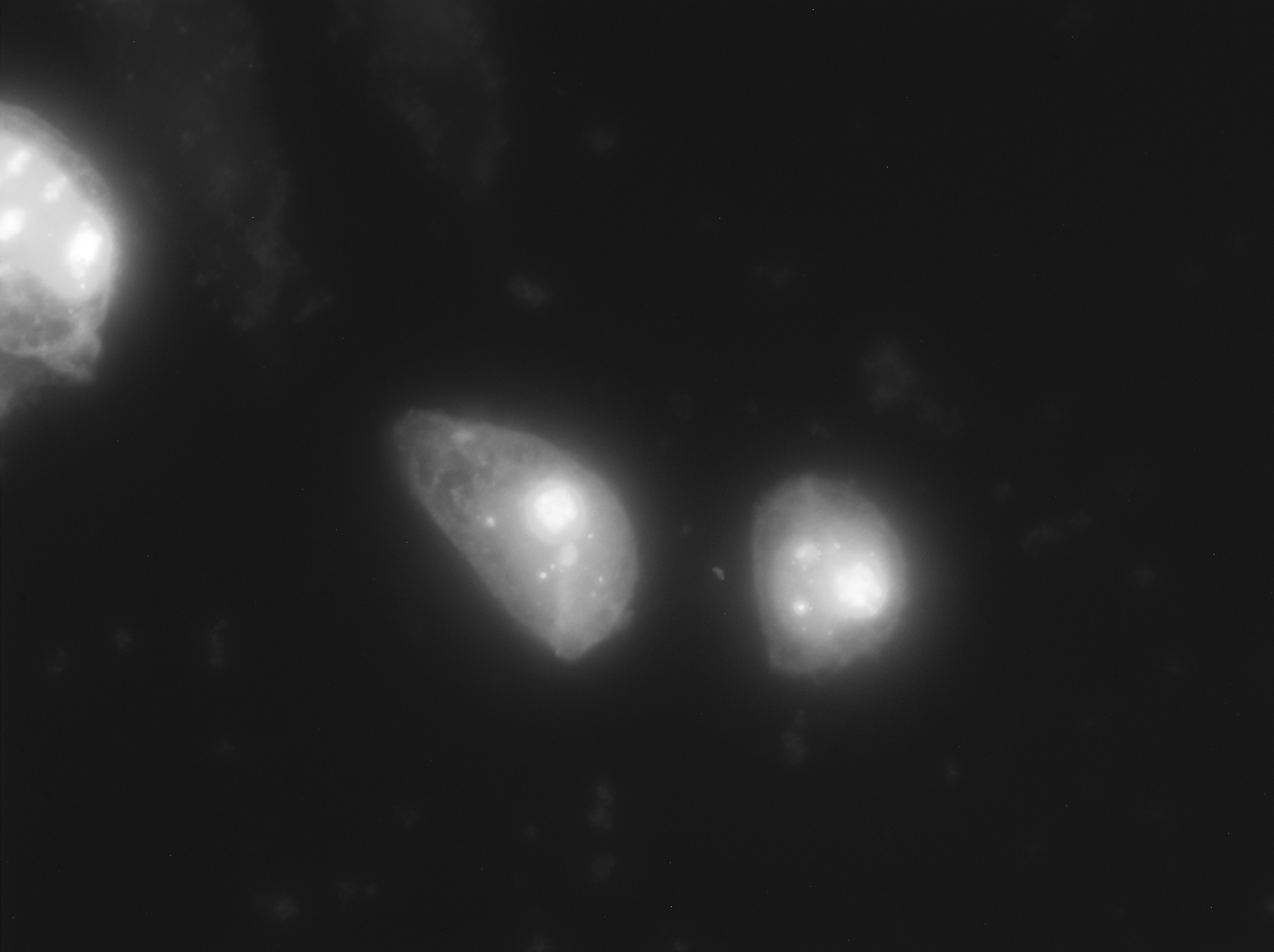

Supplement: Supplementary file 13 — Source data Fig. 3 part 3 [file 44319_2026_742_MOESM13_ESM.zip › Figure 3 Part 3/Fig 3b-e TTLL screen part 3/TTLL11-YFP Img 9 yfp2000.Project Maximum Z_XY1648578368_Z0_T0_C2.tif]

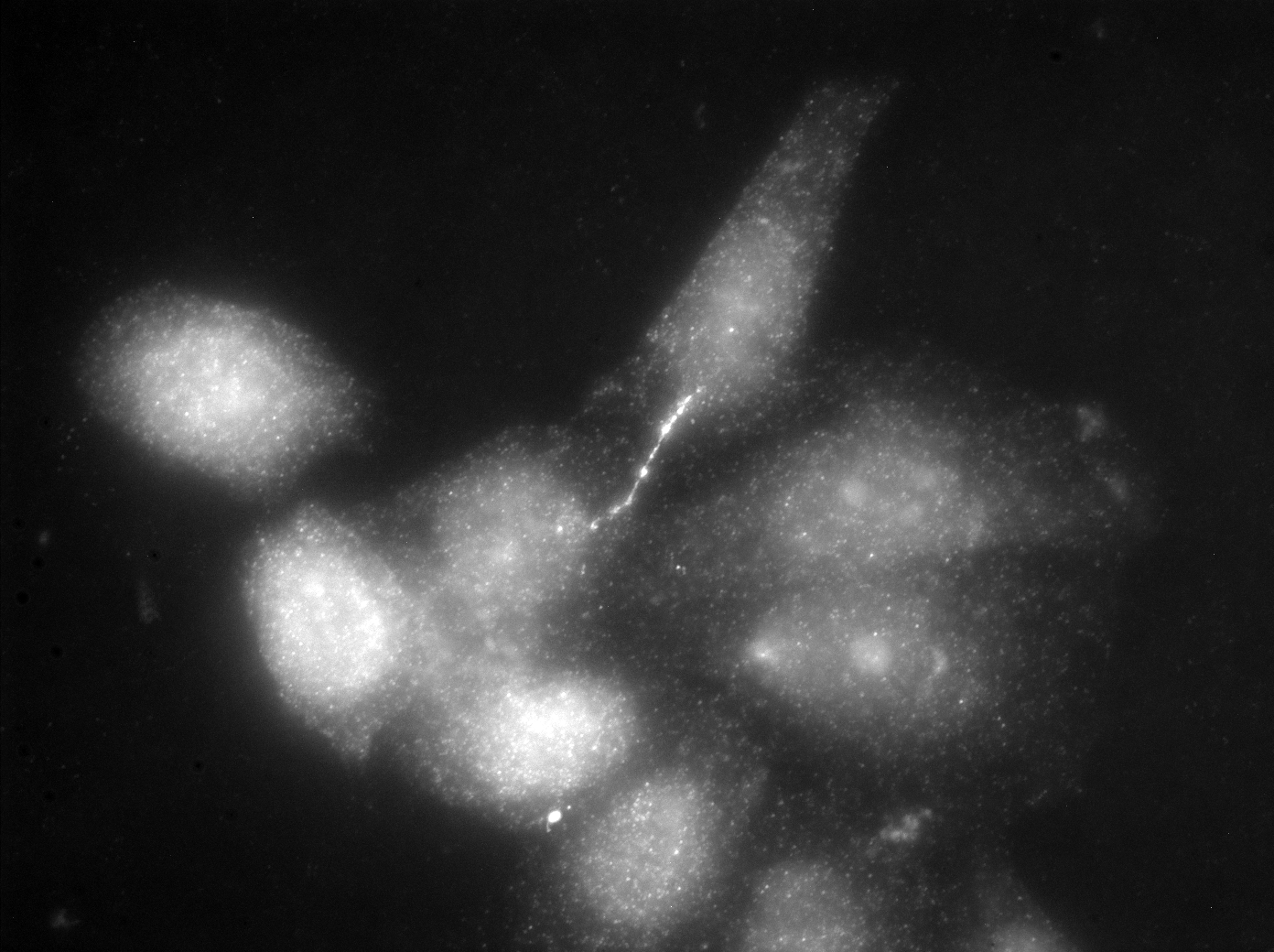

Supplement: Supplementary file 13 — Source data Fig. 3 part 3 [file 44319_2026_742_MOESM13_ESM.zip › Figure 3 Part 3/Fig 3b-e TTLL screen part 3/TTLL11-YFP A1 Img9.Project Maximum Z_XY1650056953_Z0_T0_C1.tif]

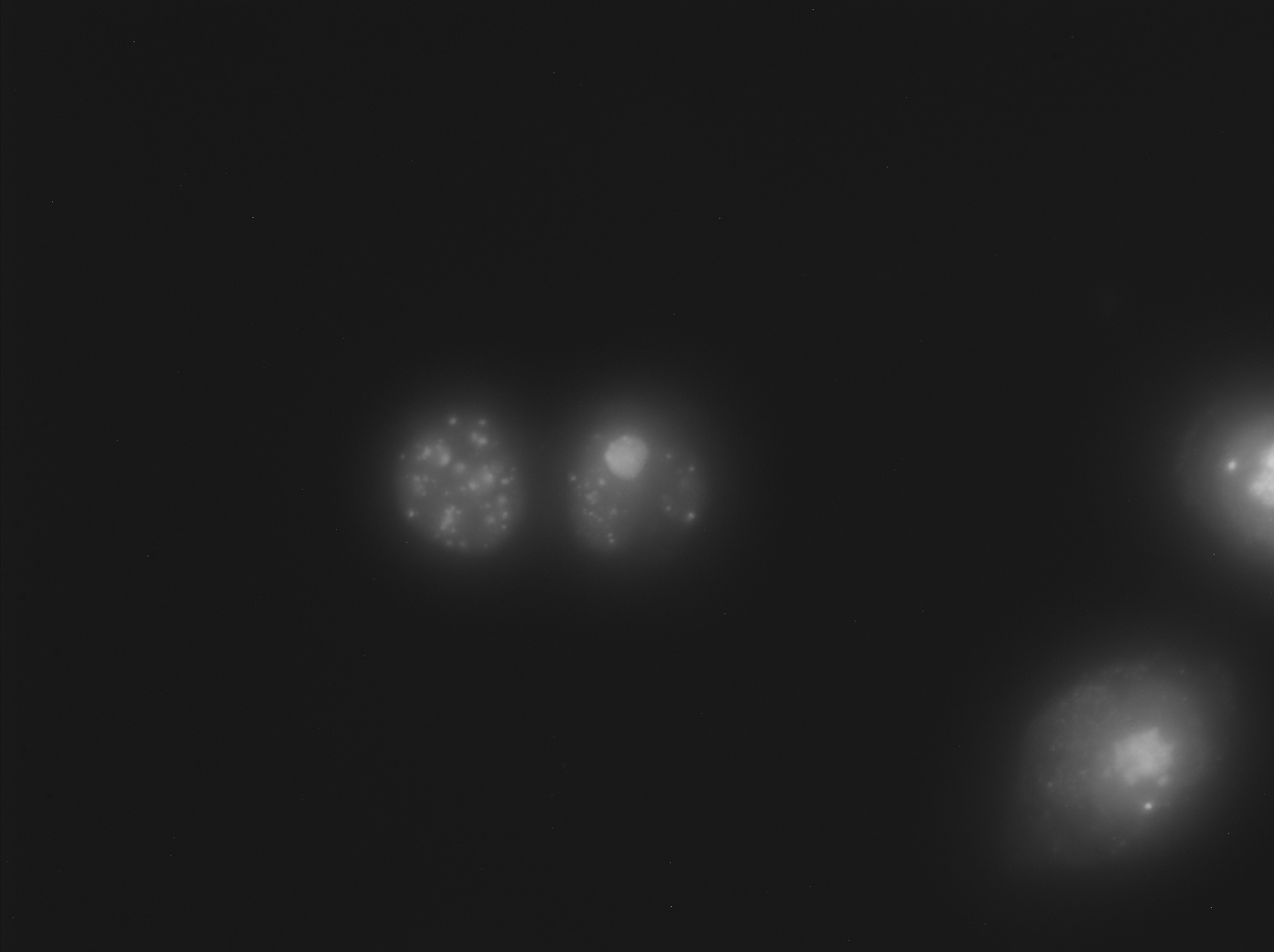

Supplement: Supplementary file 13 — Source data Fig. 3 part 3 [file 44319_2026_742_MOESM13_ESM.zip › Figure 3 Part 3/Fig 3b-e TTLL screen part 3/TTLL11-YFP Img 4 yfp2000.Project Maximum Z_XY1648157838_Z0_T0_C2.tif]

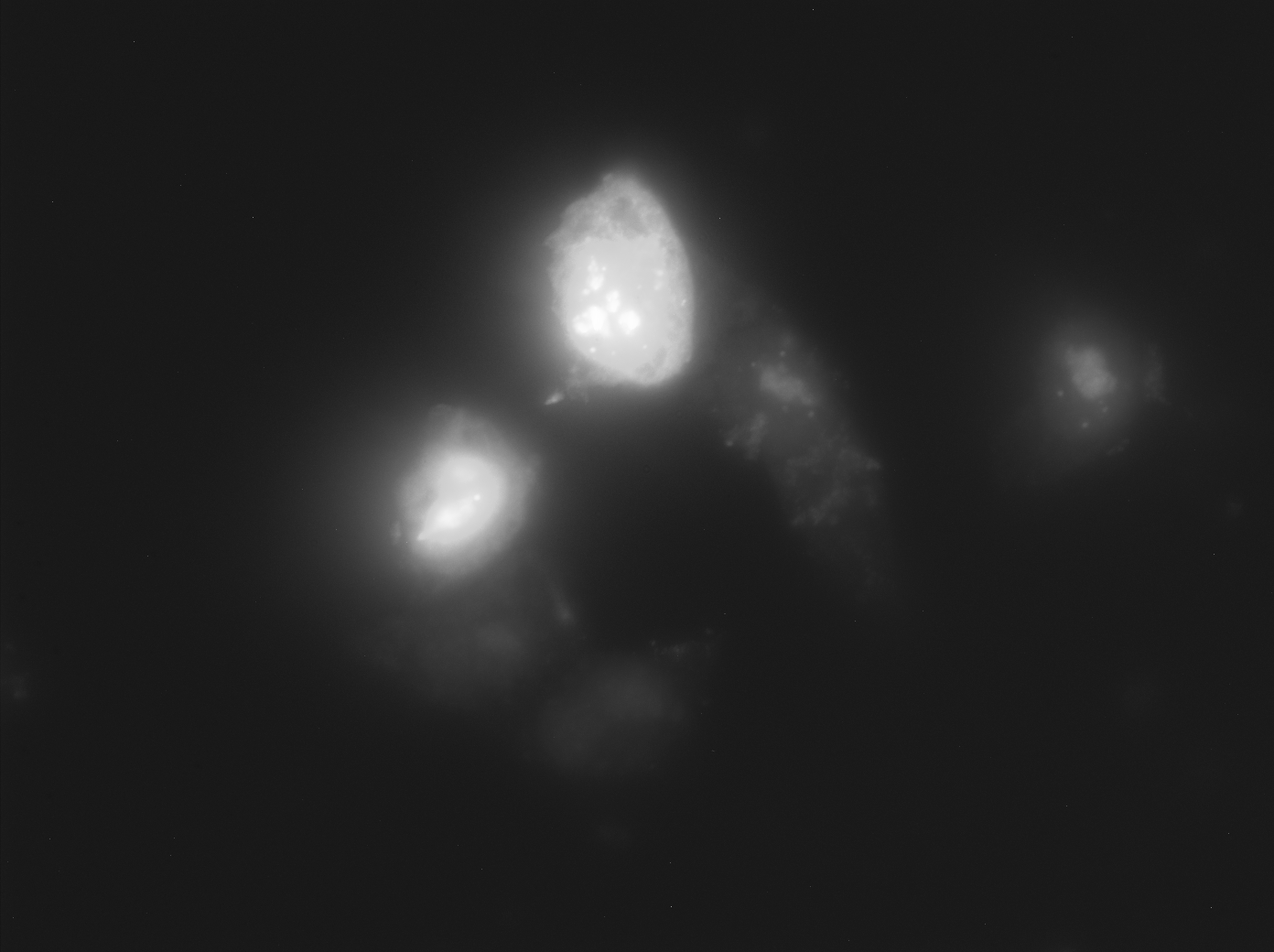

Supplement: Supplementary file 13 — Source data Fig. 3 part 3 [file 44319_2026_742_MOESM13_ESM.zip › Figure 3 Part 3/Fig 3b-e TTLL screen part 3/TTLL11-YFP A2 Img 1.Project Maximum Z_XY1648573797_Z0_T0_C2.tif]

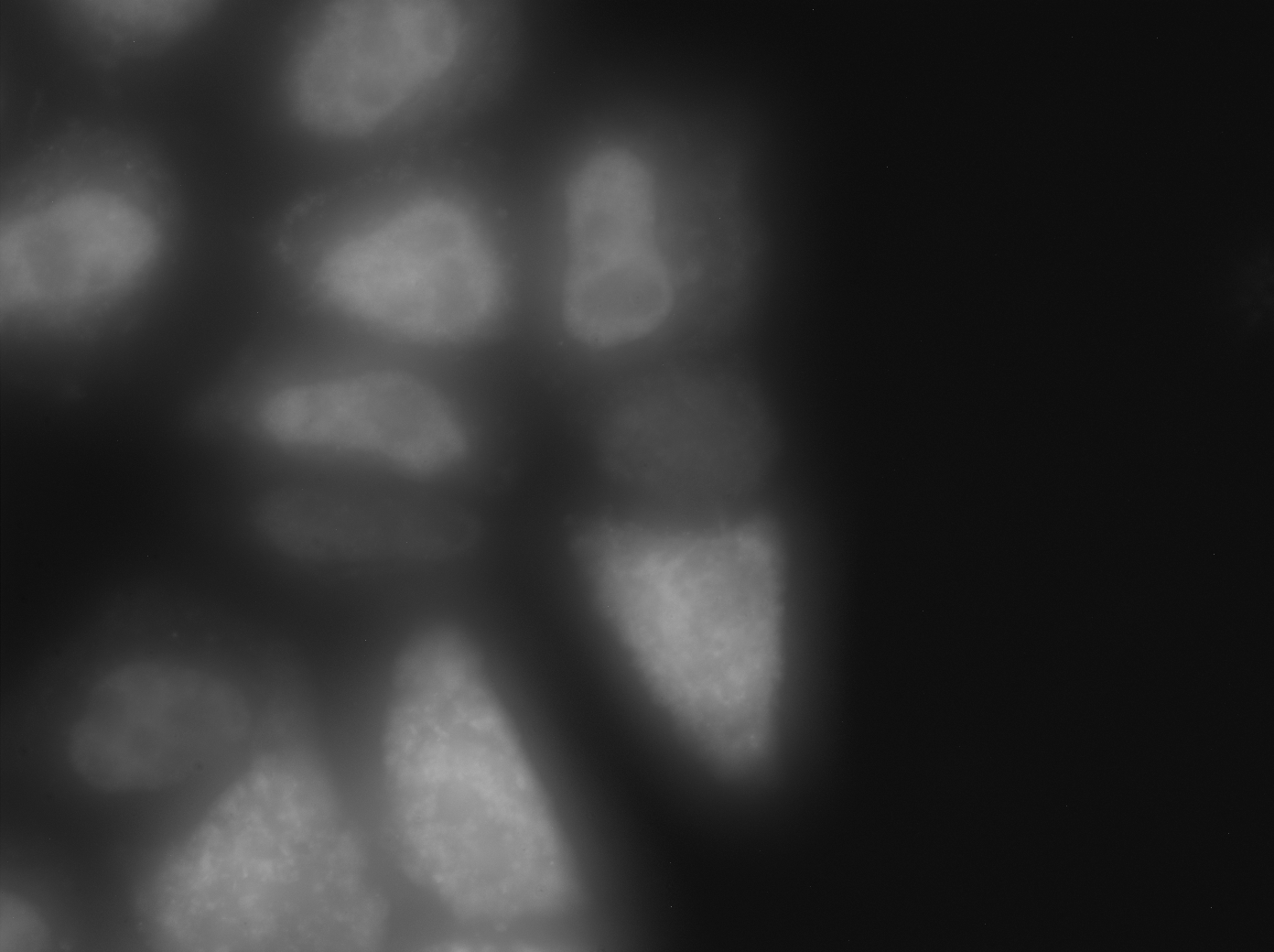

Supplement: Supplementary file 13 — Source data Fig. 3 part 3 [file 44319_2026_742_MOESM13_ESM.zip › Figure 3 Part 3/Fig 3b-e TTLL screen part 3/TTLL9-YFP A3 I12.Project Maximum Z_XY1679700912_Z0_T0_C2.tif]

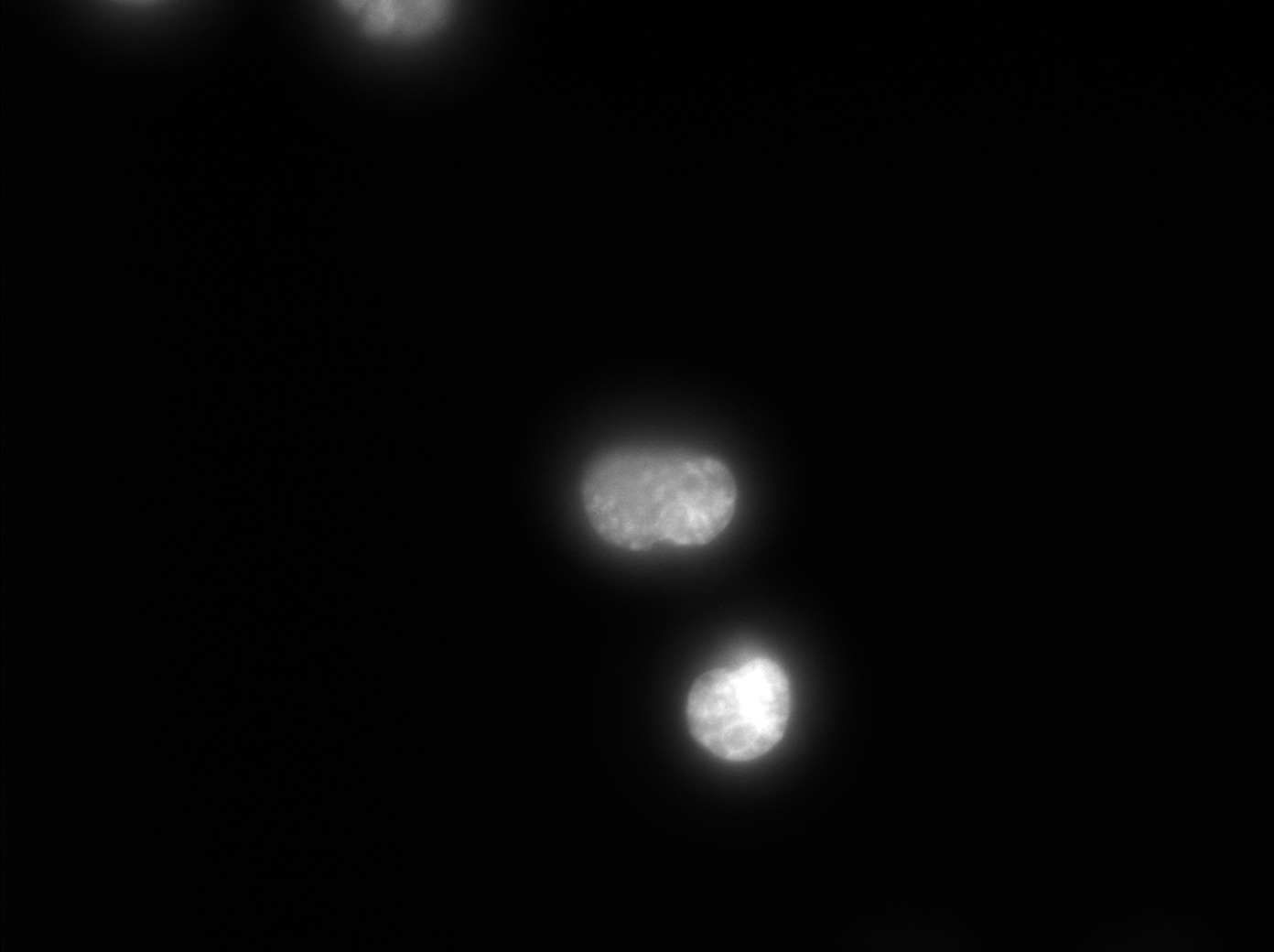

Supplement: Supplementary file 13 — Source data Fig. 3 part 3 [file 44319_2026_742_MOESM13_ESM.zip › Figure 3 Part 3/Fig 3b-e TTLL screen part 3/YFP Only R1 I5.Project Maximum Z_XY1663272254_Z0_T0_C0.tif]

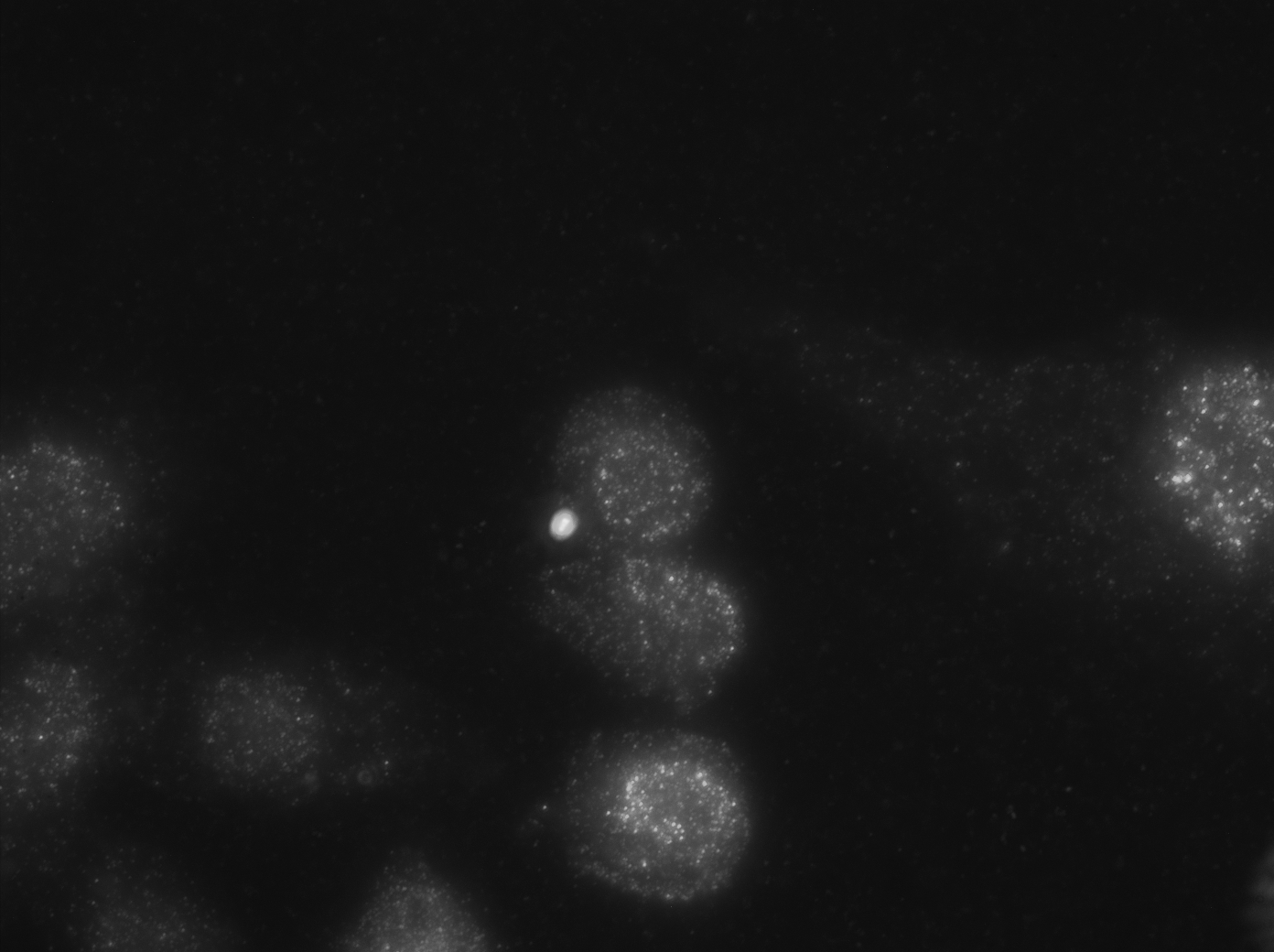

Supplement: Supplementary file 13 — Source data Fig. 3 part 3 [file 44319_2026_742_MOESM13_ESM.zip › Figure 3 Part 3/Fig 3b-e TTLL screen part 3/TTLL11-YFP Img 10 yfp2000 - 1.Project Maximum Z_XY1648579577_Z0_T0_C1.tif]

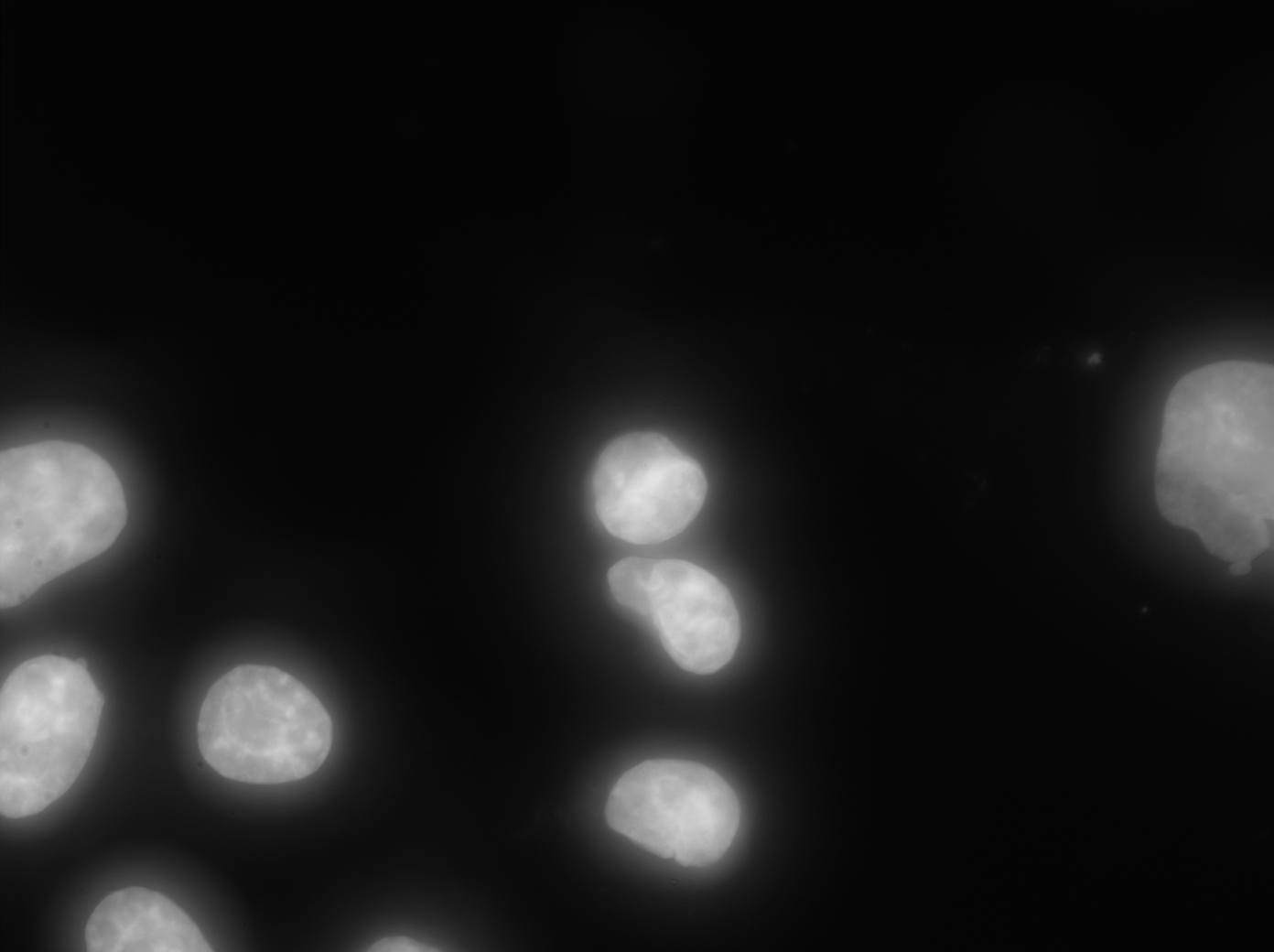

Supplement: Supplementary file 13 — Source data Fig. 3 part 3 [file 44319_2026_742_MOESM13_ESM.zip › Figure 3 Part 3/Fig 3b-e TTLL screen part 3/TTLL11-YFP Img 10 yfp2000 - 1.Project Maximum Z_XY1648579577_Z0_T0_C0.tif]

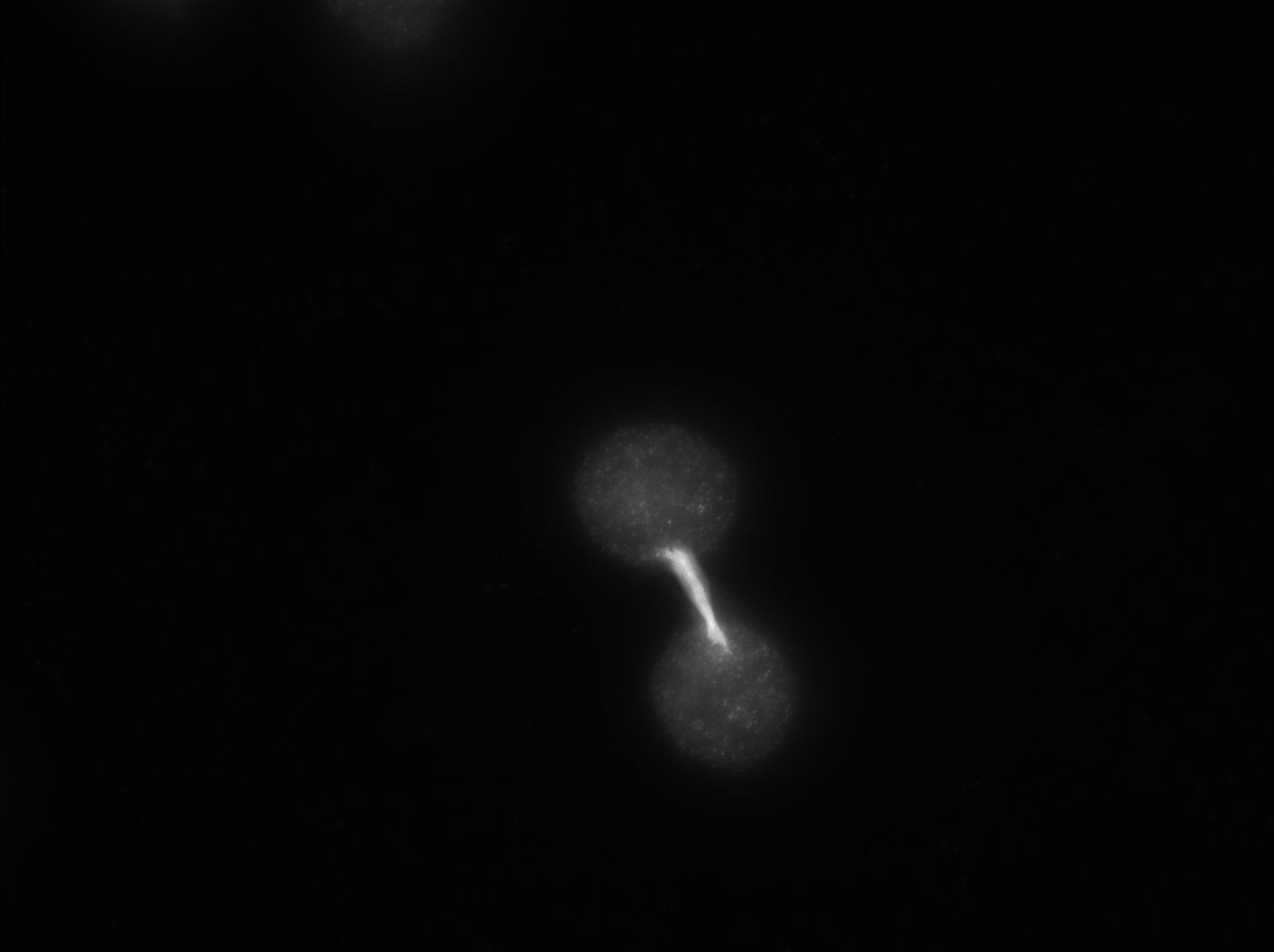

Supplement: Supplementary file 13 — Source data Fig. 3 part 3 [file 44319_2026_742_MOESM13_ESM.zip › Figure 3 Part 3/Fig 3b-e TTLL screen part 3/YFP Only R1 I5.Project Maximum Z_XY1663272254_Z0_T0_C1.tif]

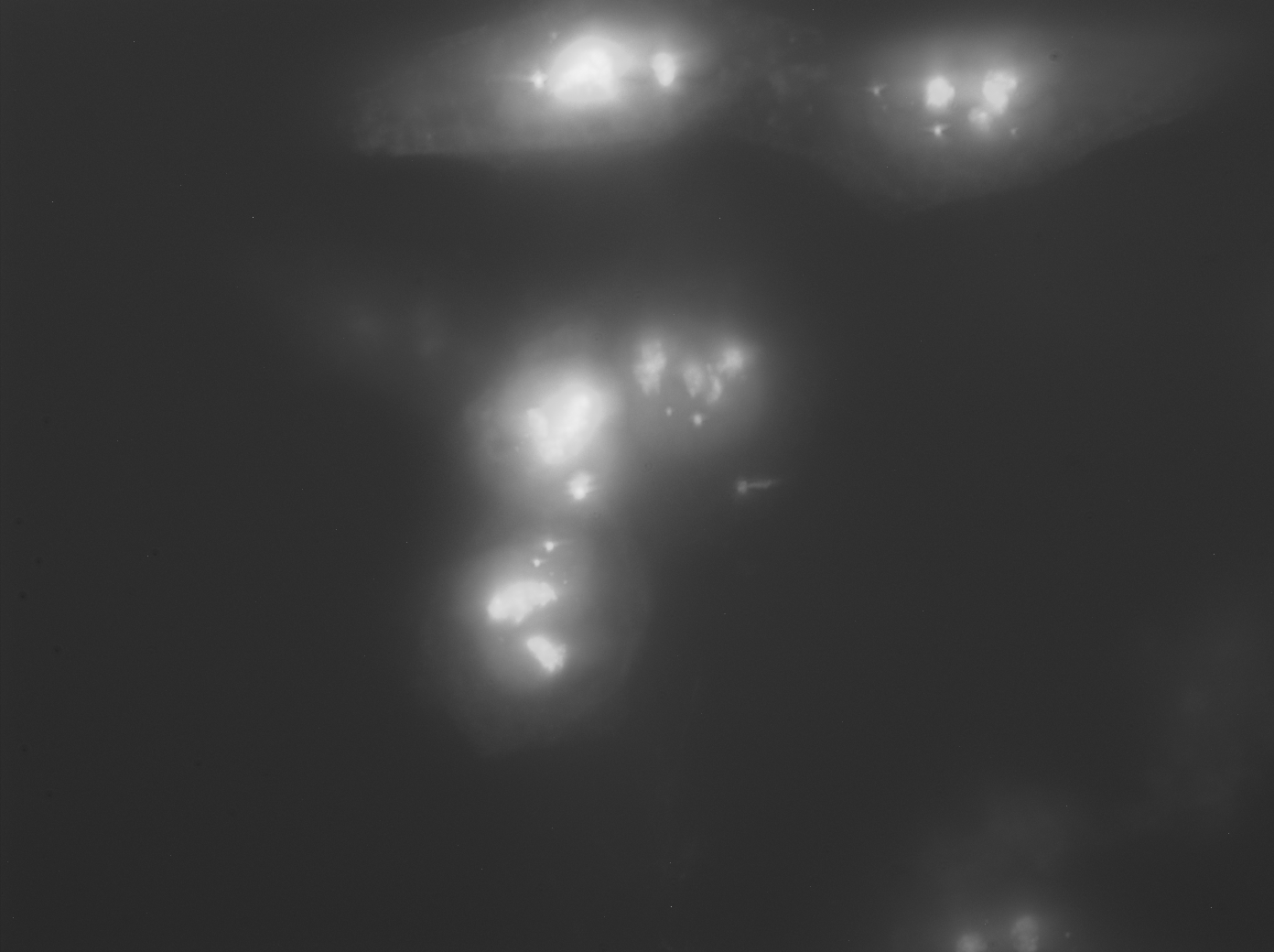

Supplement: Supplementary file 13 — Source data Fig. 3 part 3 [file 44319_2026_742_MOESM13_ESM.zip › Figure 3 Part 3/Fig 3b-e TTLL screen part 3/TTLL11-YFP A2 Img2 - 1.Project Maximum Z_XY1648574683_Z0_T0_C2.tif]

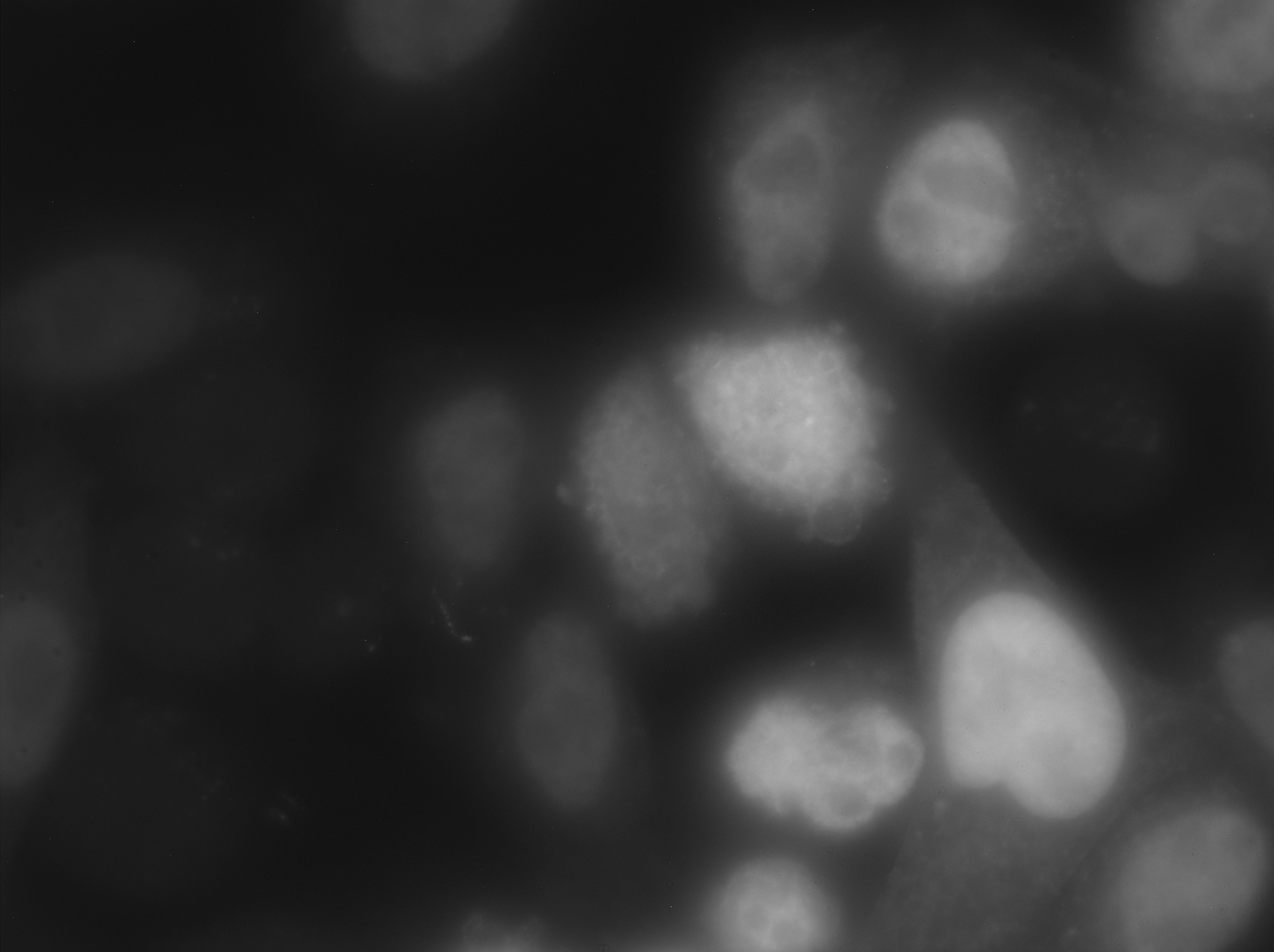

Supplement: Supplementary file 13 — Source data Fig. 3 part 3 [file 44319_2026_742_MOESM13_ESM.zip › Figure 3 Part 3/Fig 3b-e TTLL screen part 3/TTLL9-YFP A3 I15.Project Maximum Z_XY1679701360_Z0_T0_C2.tif]

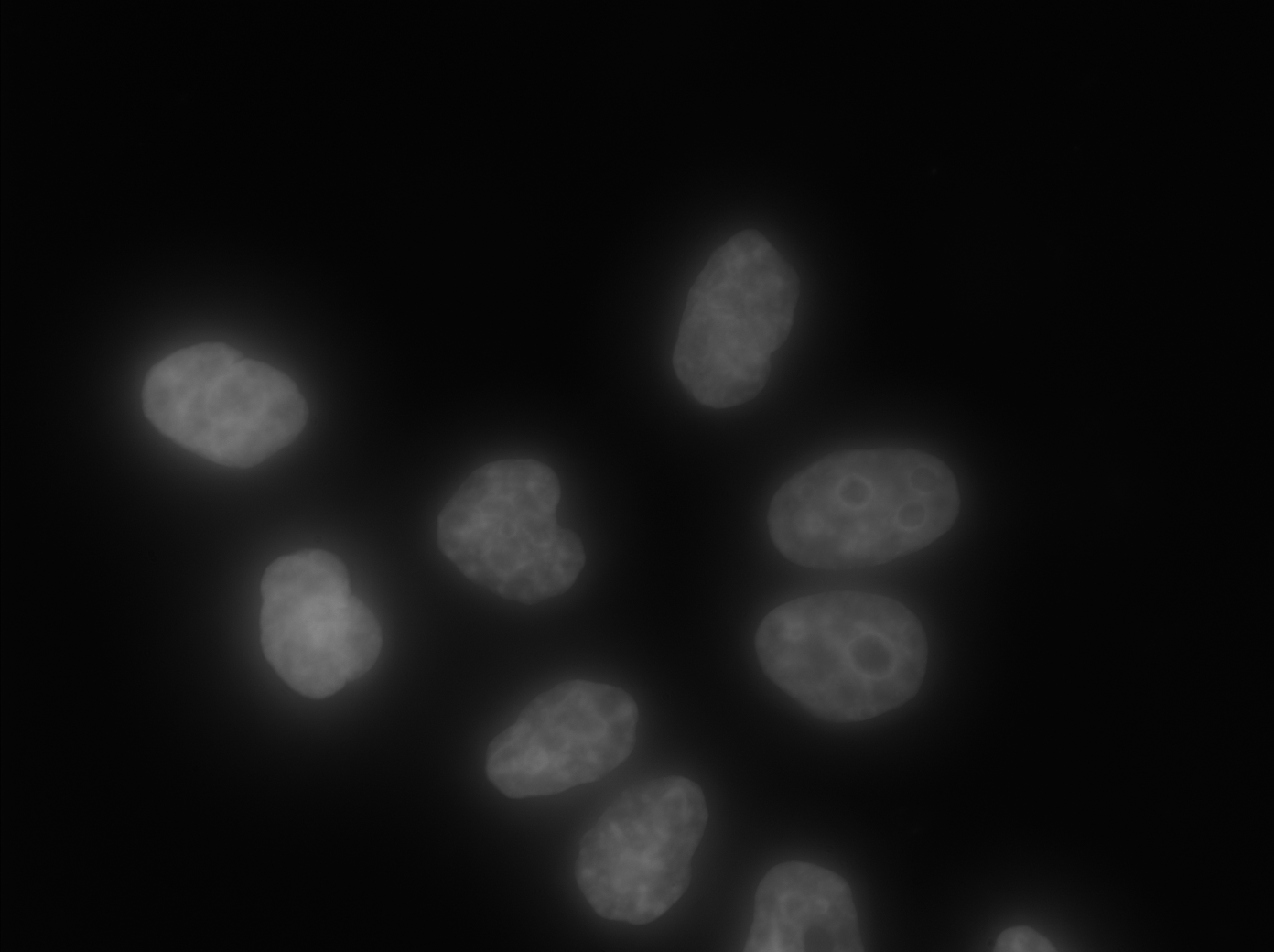

Supplement: Supplementary file 13 — Source data Fig. 3 part 3 [file 44319_2026_742_MOESM13_ESM.zip › Figure 3 Part 3/Fig 3b-e TTLL screen part 3/TTLL11-YFP A1 Img9.Project Maximum Z_XY1650056953_Z0_T0_C0.tif]

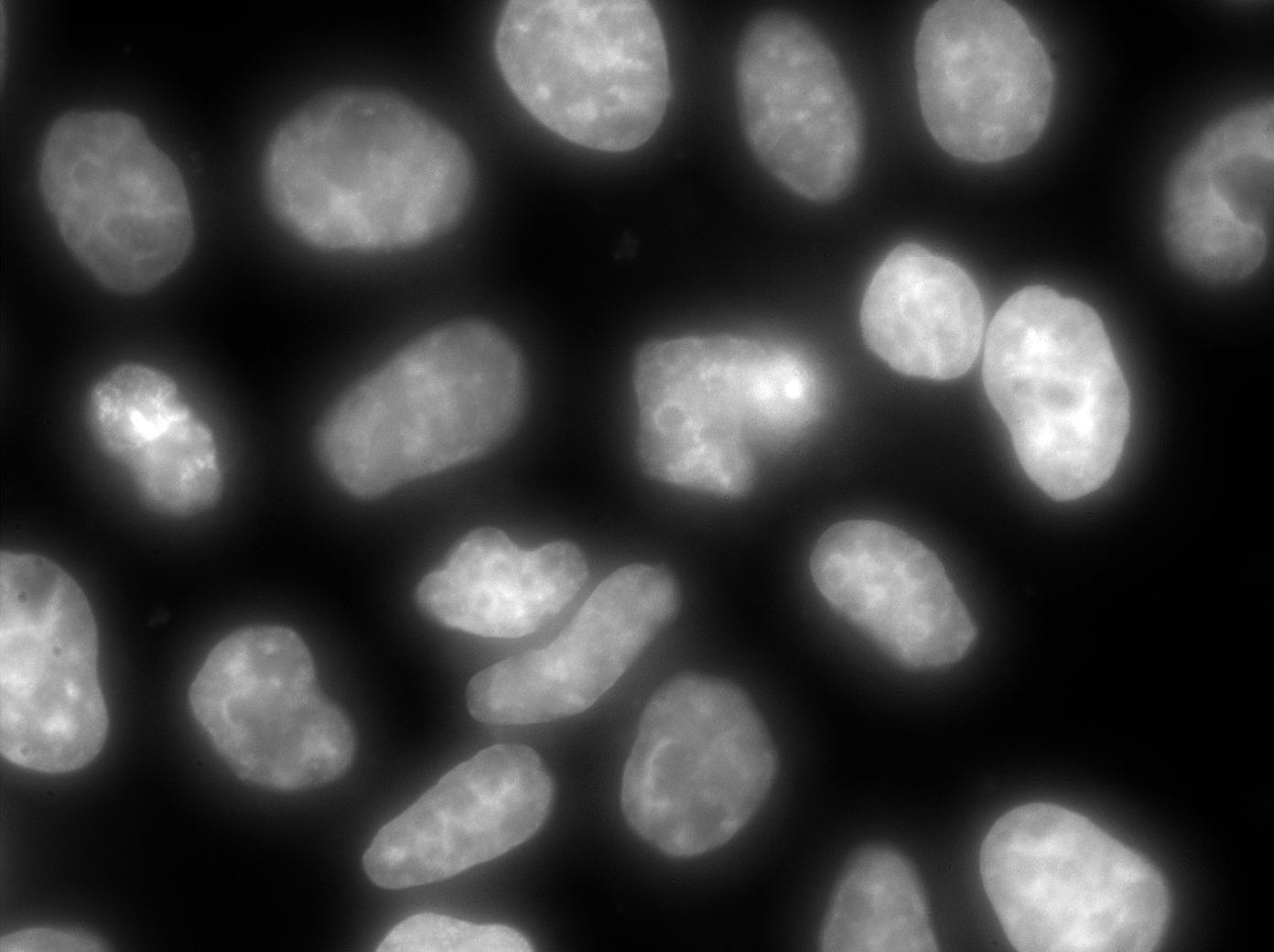

Supplement: Supplementary file 13 — Source data Fig. 3 part 3 [file 44319_2026_742_MOESM13_ESM.zip › Figure 3 Part 3/Fig 3b-e TTLL screen part 3/TTLL9-YFP A3 I19.Project Maximum Z_XY1679701974_Z0_T0_C0.tif]

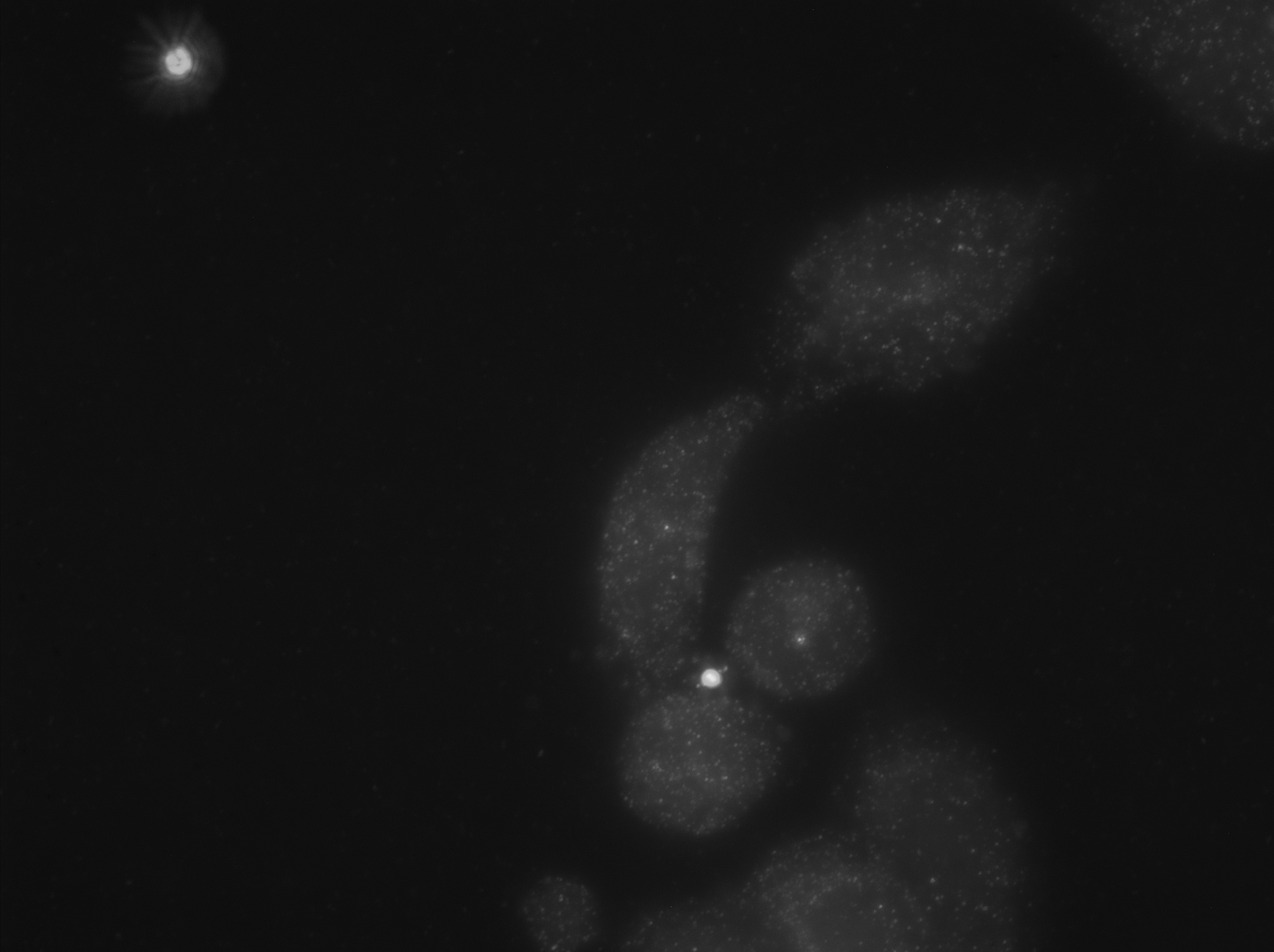

Supplement: Supplementary file 13 — Source data Fig. 3 part 3 [file 44319_2026_742_MOESM13_ESM.zip › Figure 3 Part 3/Fig 3b-e TTLL screen part 3/TTLL11-YFP Img 13 yfp2500.Project Maximum Z_XY1648580917_Z0_T0_C1.tif]

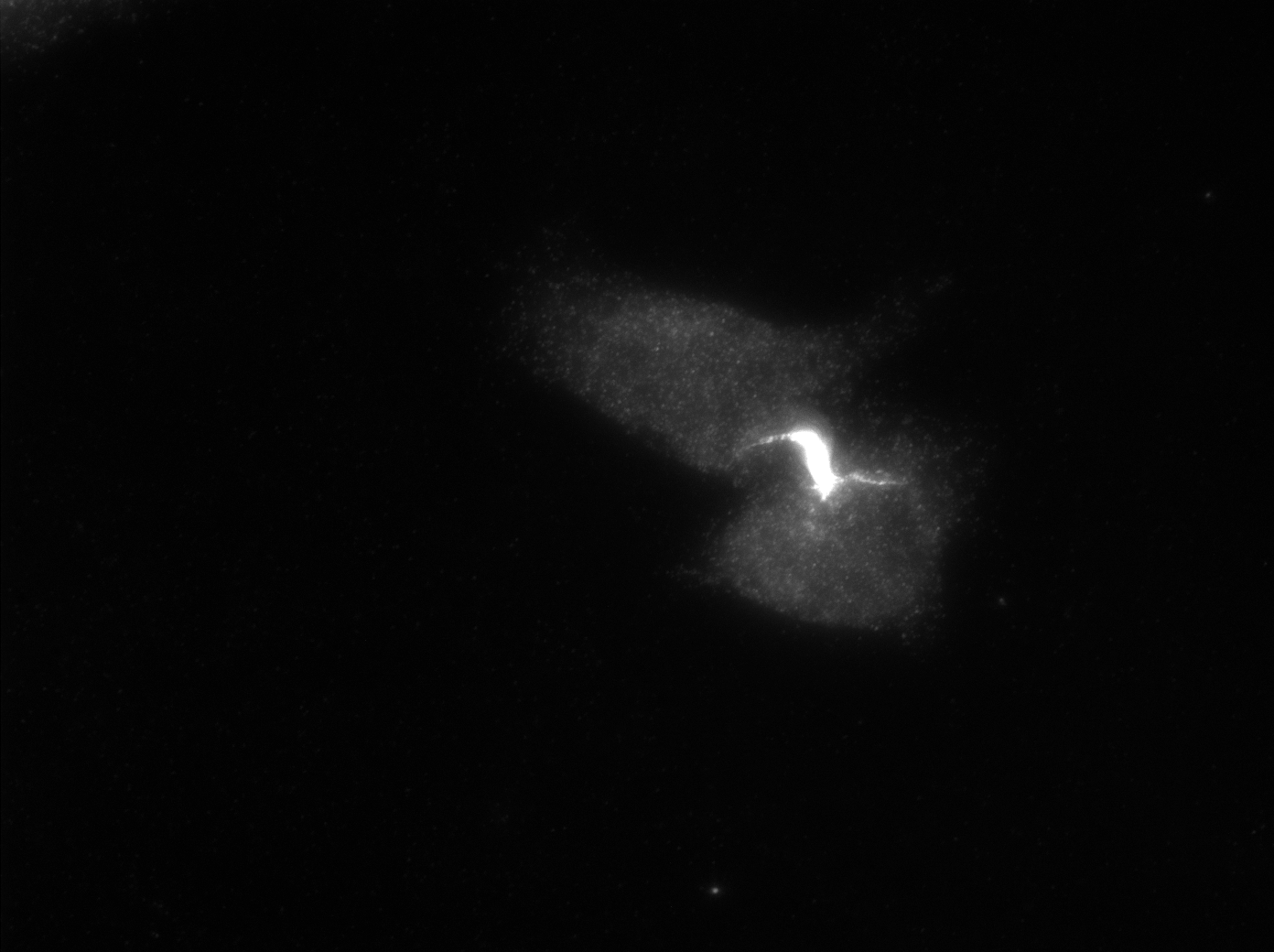

Supplement: Supplementary file 13 — Source data Fig. 3 part 3 [file 44319_2026_742_MOESM13_ESM.zip › Figure 3 Part 3/Fig 3b-e TTLL screen part 3/TTLL9-YFP A3 I2.Project Maximum Z_XY1674674772_Z0_T0_C1.tif]

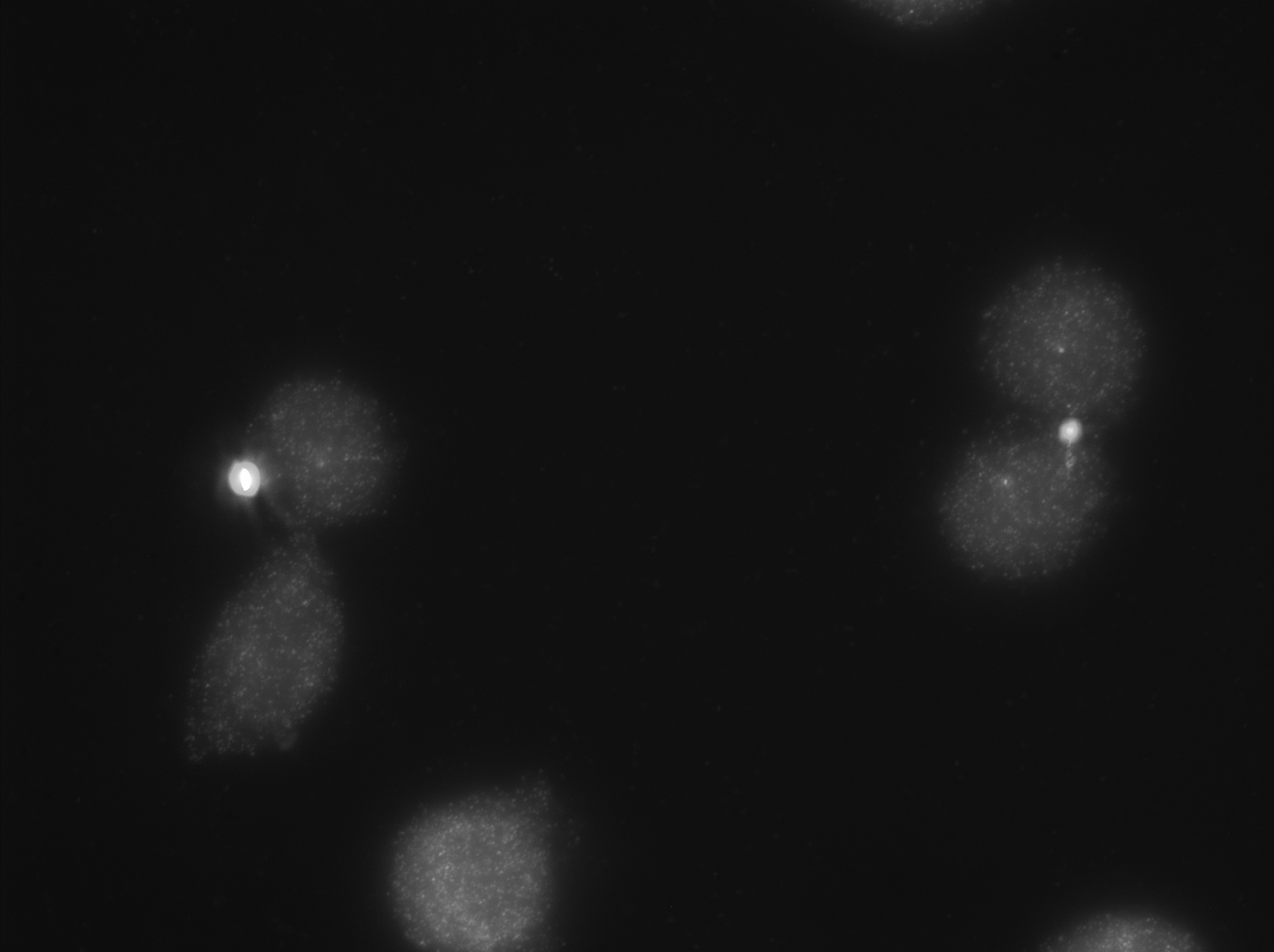

Supplement: Supplementary file 13 — Source data Fig. 3 part 3 [file 44319_2026_742_MOESM13_ESM.zip › Figure 3 Part 3/Fig 3b-e TTLL screen part 3/TTLL11-YFP Img 2 yfp2000 - 1.Project Maximum Z_XY1648156745_Z0_T0_C1.tif]

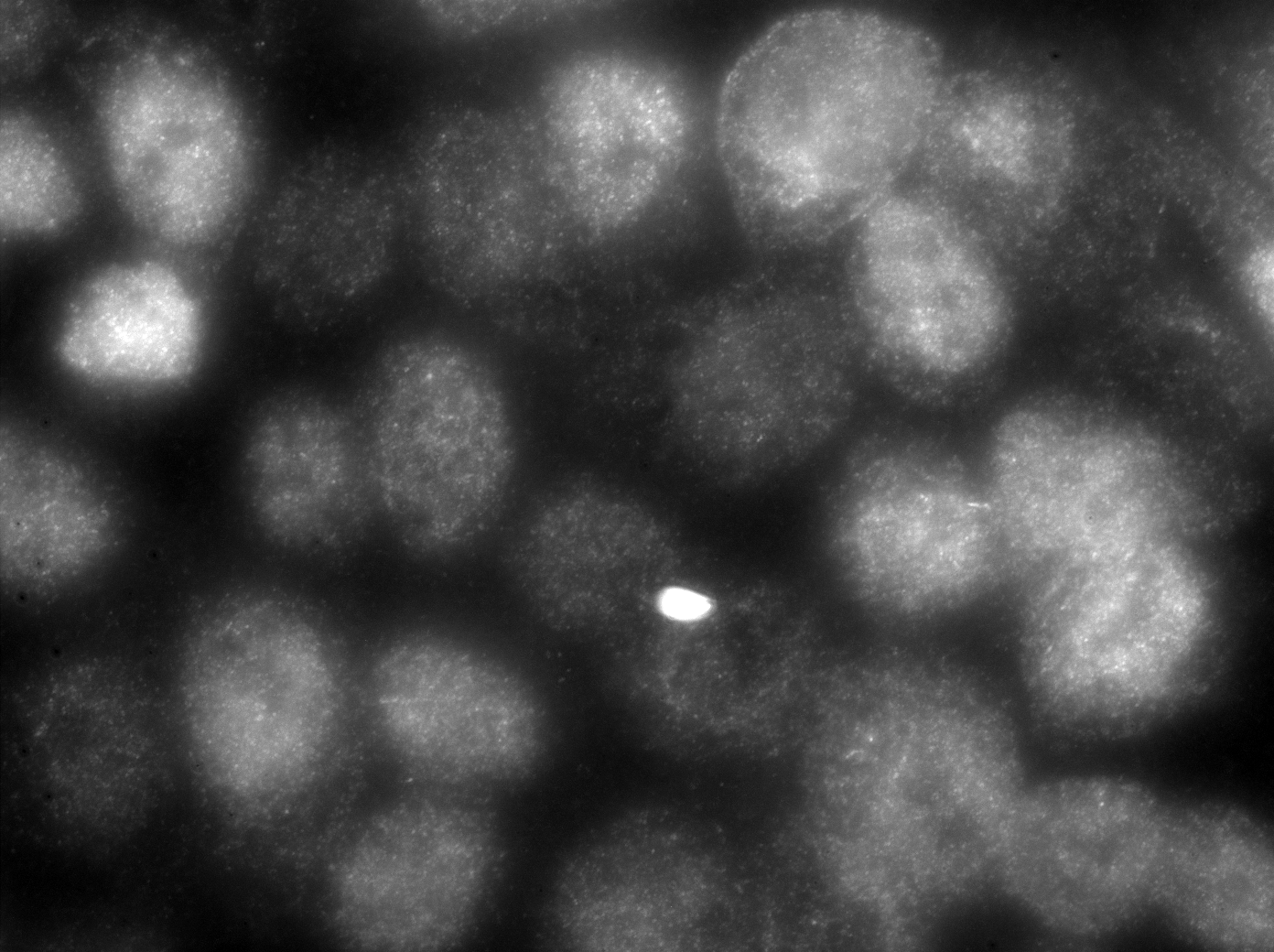

Supplement: Supplementary file 13 — Source data Fig. 3 part 3 [file 44319_2026_742_MOESM13_ESM.zip › Figure 3 Part 3/Fig 3b-e TTLL screen part 3/TTLL9-YFP A3 I2 - 1.Project Maximum Z_XY1679699371_Z0_T0_C1.tif]

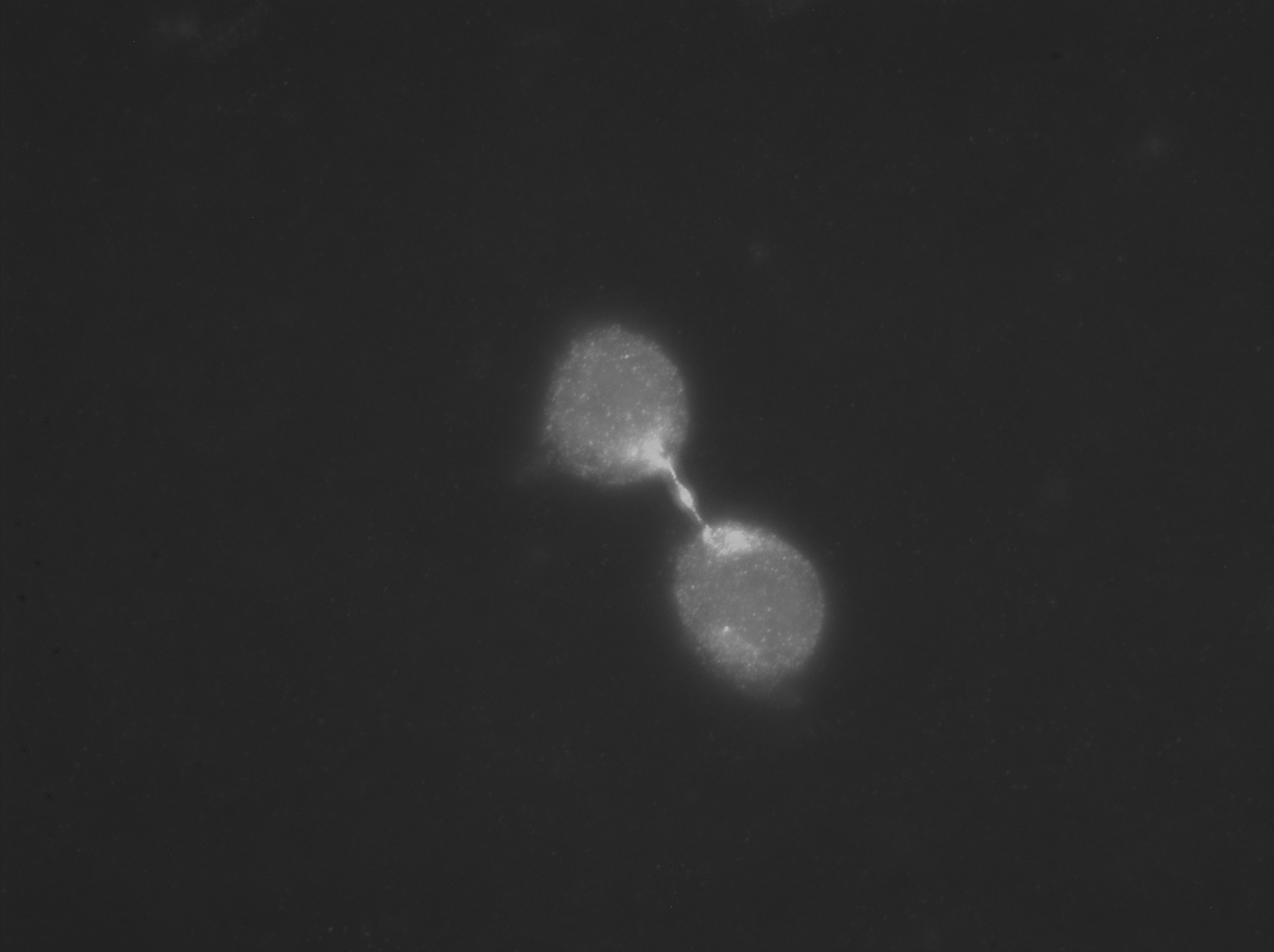

Supplement: Supplementary file 13 — Source data Fig. 3 part 3 [file 44319_2026_742_MOESM13_ESM.zip › Figure 3 Part 3/Fig 3b-e TTLL screen part 3/TTLL11-YFP A1 Img6.Project Maximum Z_XY1650055434_Z0_T0_C1.tif]

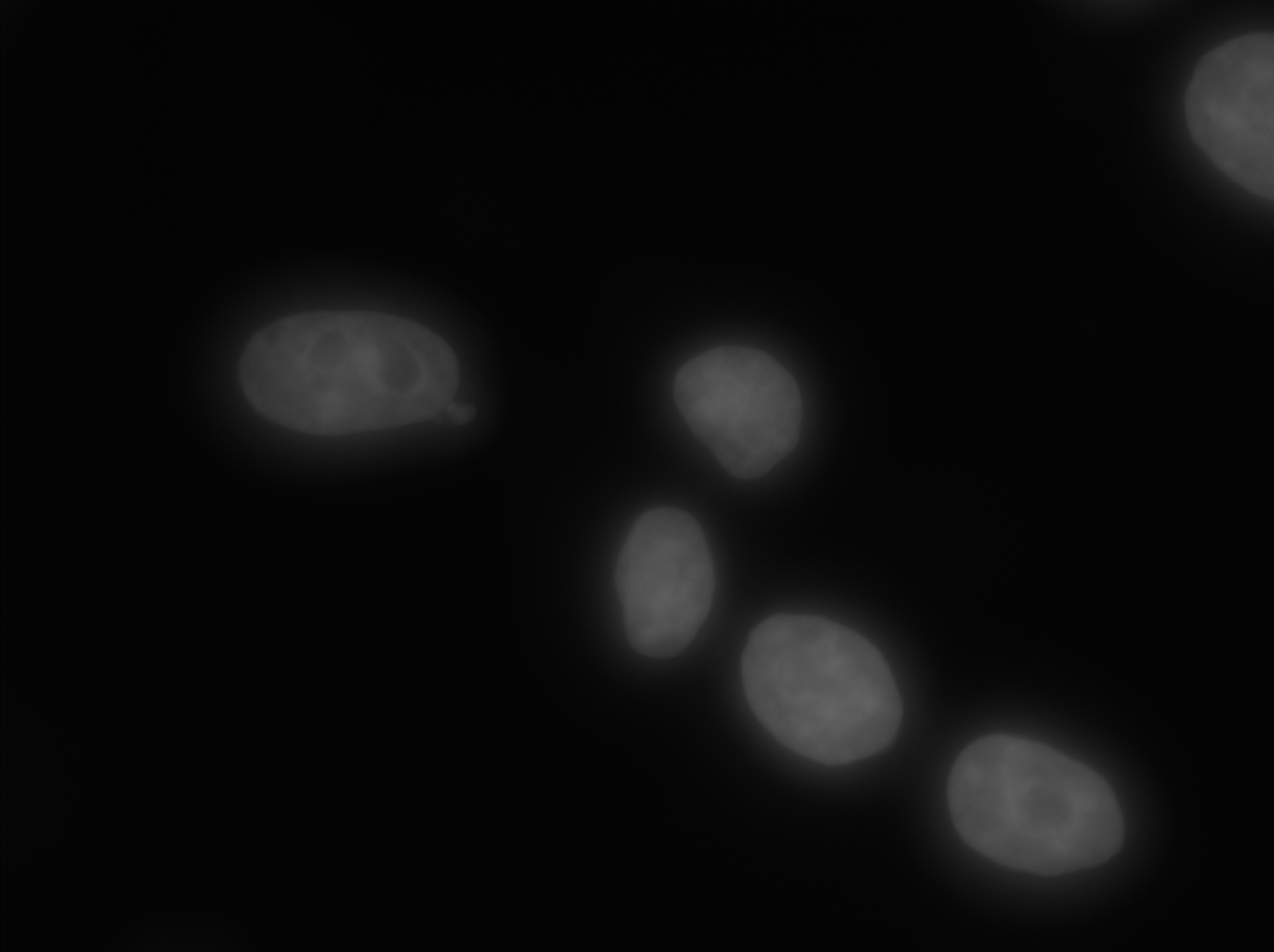

Supplement: Supplementary file 13 — Source data Fig. 3 part 3 [file 44319_2026_742_MOESM13_ESM.zip › Figure 3 Part 3/Fig 3b-e TTLL screen part 3/TTLL11-YFP A1 Img7.Project Maximum Z_XY1650055906_Z0_T0_C0.tif]

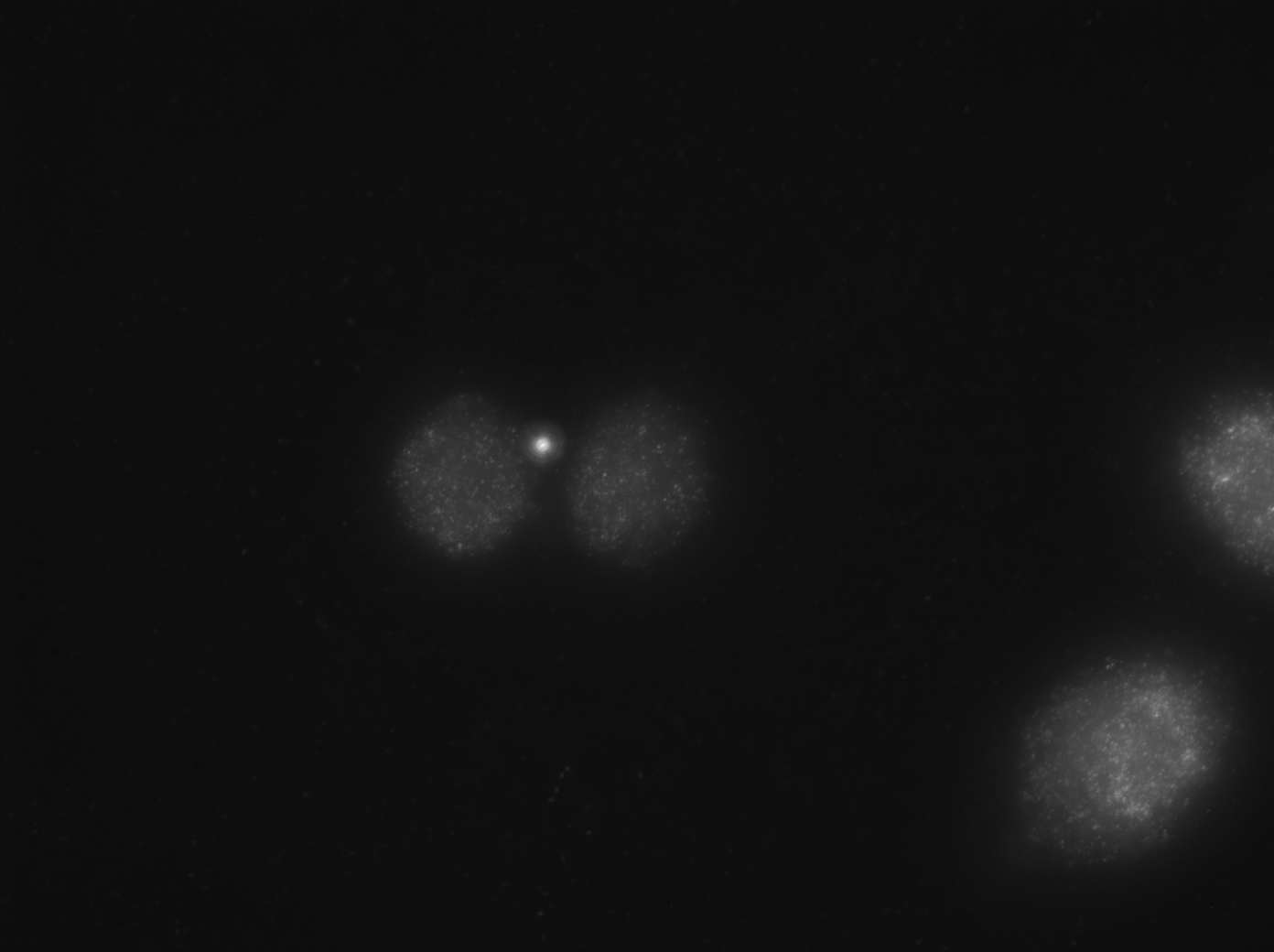

Supplement: Supplementary file 13 — Source data Fig. 3 part 3 [file 44319_2026_742_MOESM13_ESM.zip › Figure 3 Part 3/Fig 3b-e TTLL screen part 3/TTLL11-YFP Img 4 yfp2000 - 1.Project Maximum Z_XY1648157838_Z0_T0_C1.tif]

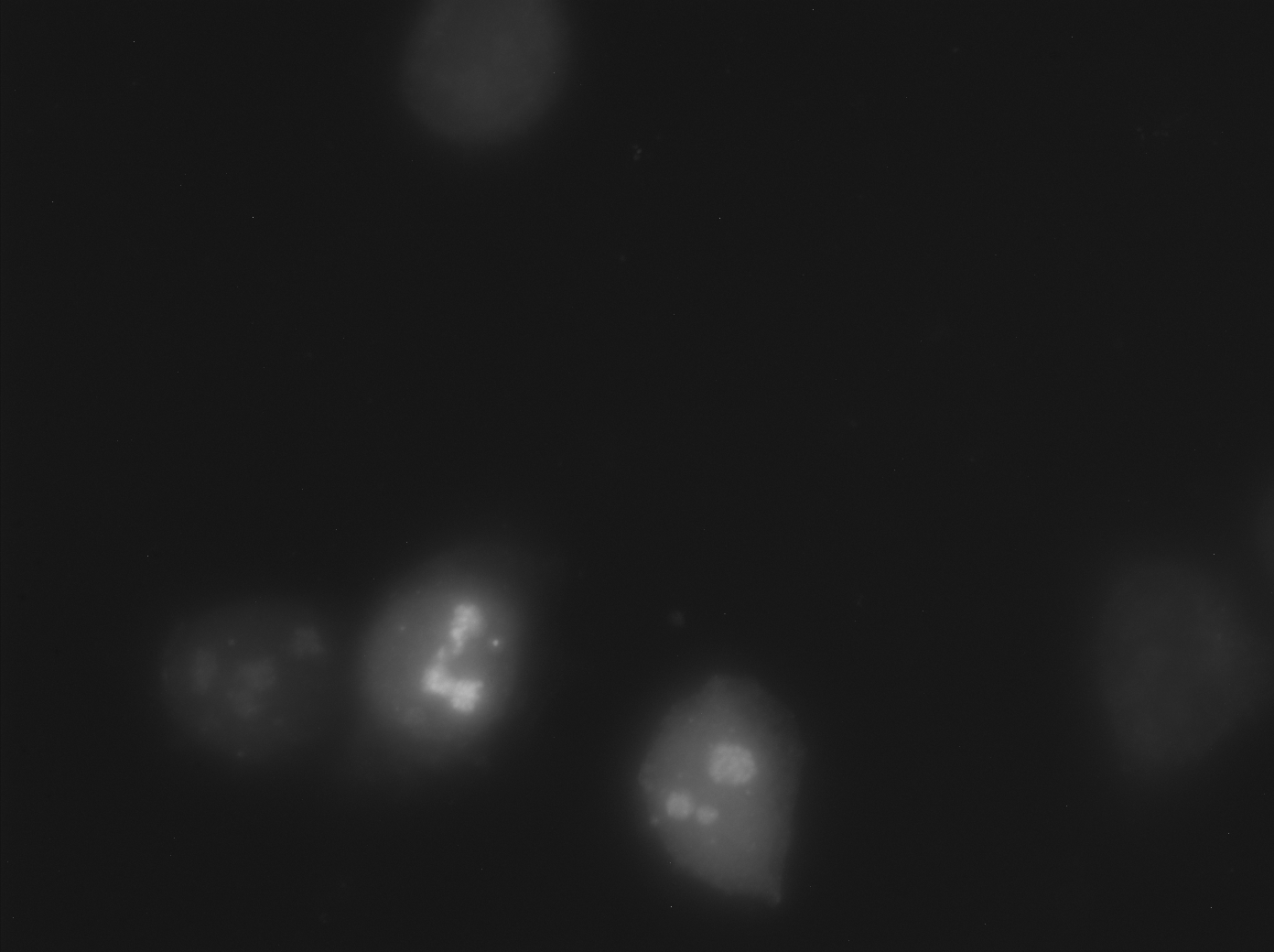

Supplement: Supplementary file 13 — Source data Fig. 3 part 3 [file 44319_2026_742_MOESM13_ESM.zip › Figure 3 Part 3/Fig 3b-e TTLL screen part 3/TTLL11-YFP Img 3 yfp2000.Project Maximum Z_XY1648157505_Z0_T0_C2.tif]

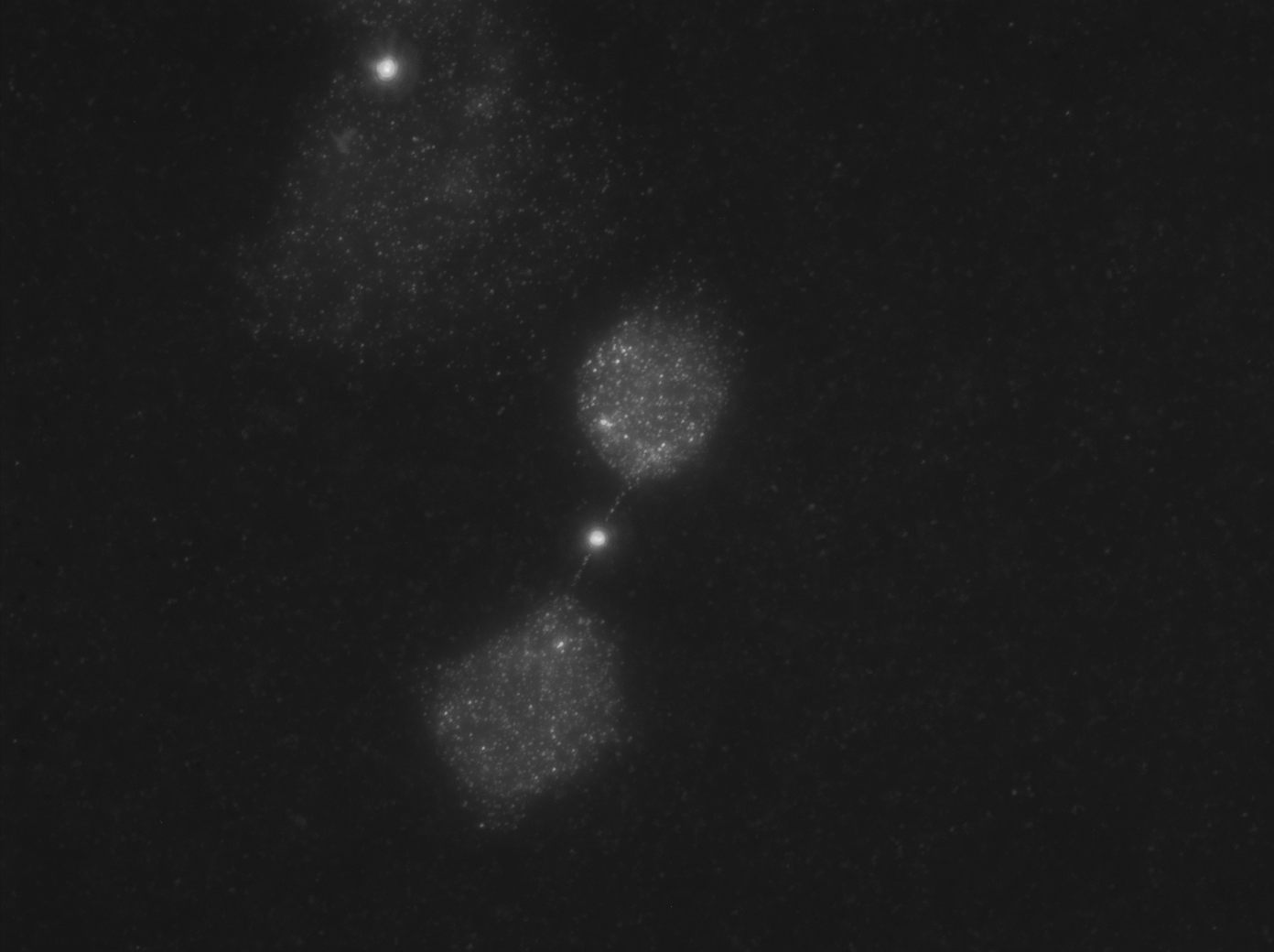

Supplement: Supplementary file 13 — Source data Fig. 3 part 3 [file 44319_2026_742_MOESM13_ESM.zip › Figure 3 Part 3/Fig 3b-e TTLL screen part 3/TTLL11-YFP A2 Img5.Project Maximum Z_XY1648754590_Z0_T0_C1.tif]

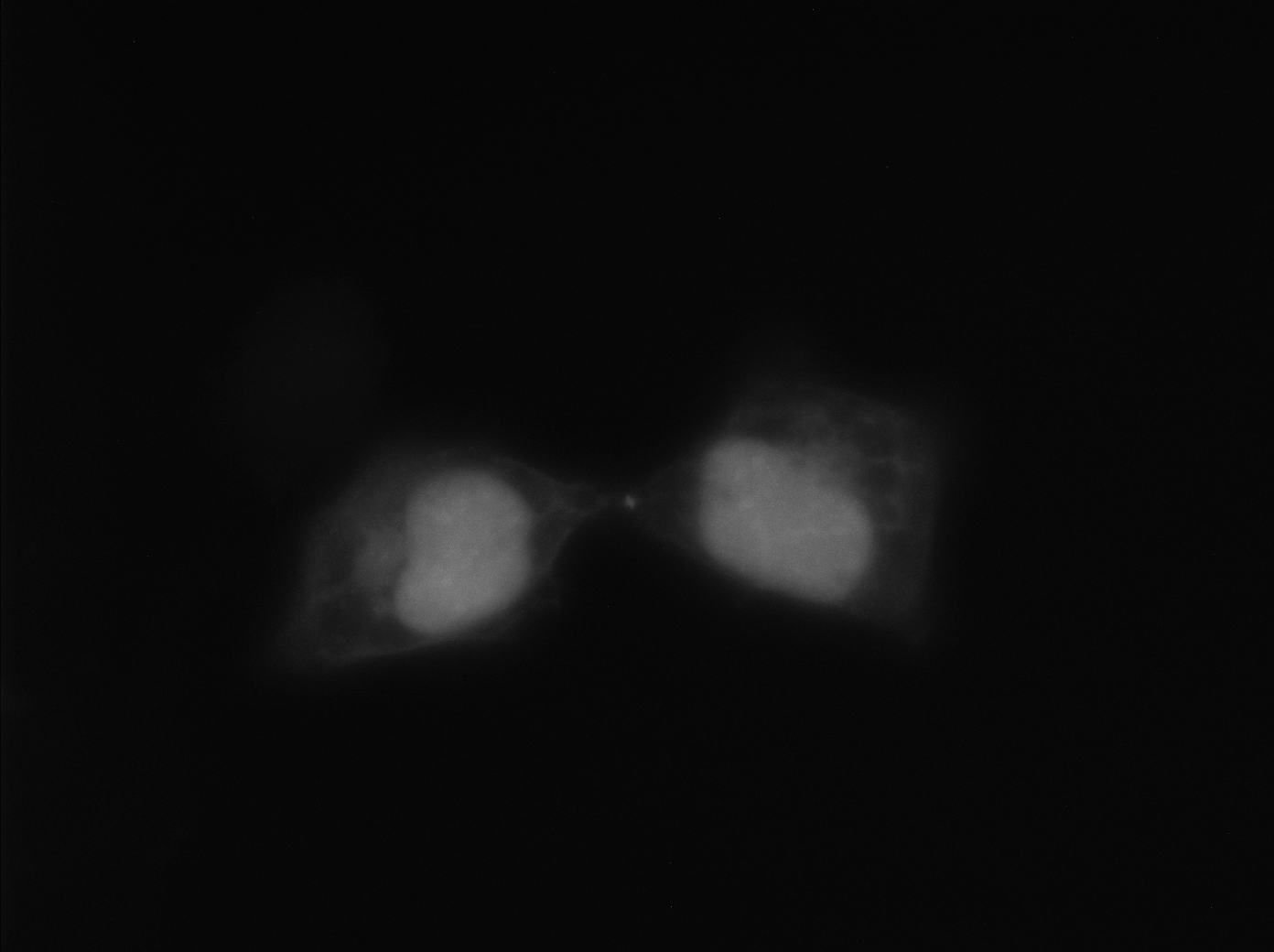

Supplement: Supplementary file 13 — Source data Fig. 3 part 3 [file 44319_2026_742_MOESM13_ESM.zip › Figure 3 Part 3/Fig 3b-e TTLL screen part 3/YFP Only R1 I1 - 1.Project Maximum Z_XY1663181796_Z0_T0_C2.tif]

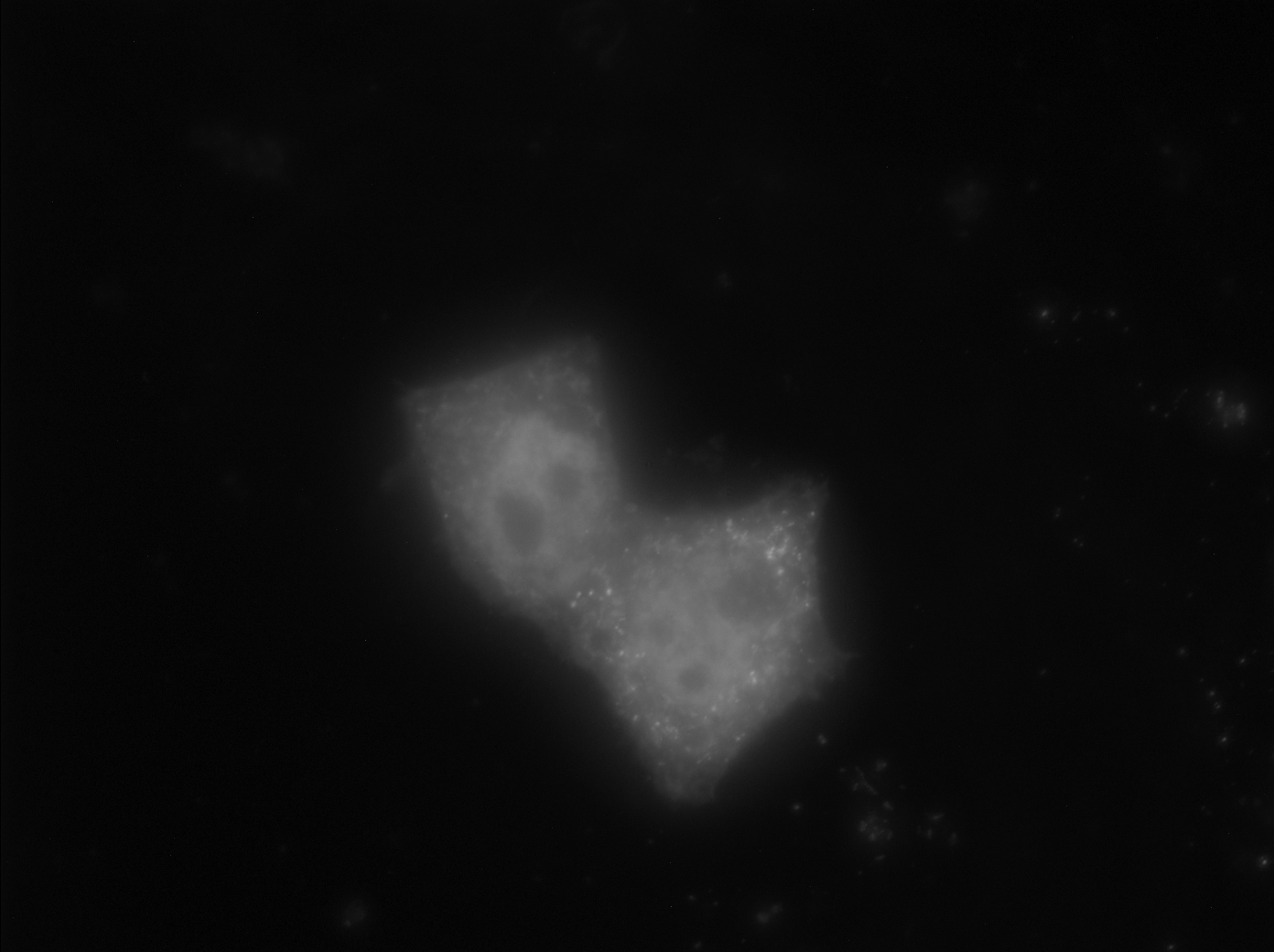

Supplement: Supplementary file 13 — Source data Fig. 3 part 3 [file 44319_2026_742_MOESM13_ESM.zip › Figure 3 Part 3/Fig 3b-e TTLL screen part 3/TTLL9-GFP A4 I5.Project Maximum Z_XY1675967797_Z0_T0_C2.tif]
