# Supplementary figures and images for: TPGS1 regulates central spindle microtubule glutamylation and remodeling during telophase and abscission (part 18 of 36)
Source: EMBO Rep. 2026 Mar 23;27(8):1944–63. doi: 10.1038/s44319-026-00742-3 (PMC13121839; doi:10.1038/s44319-026-00742-3)

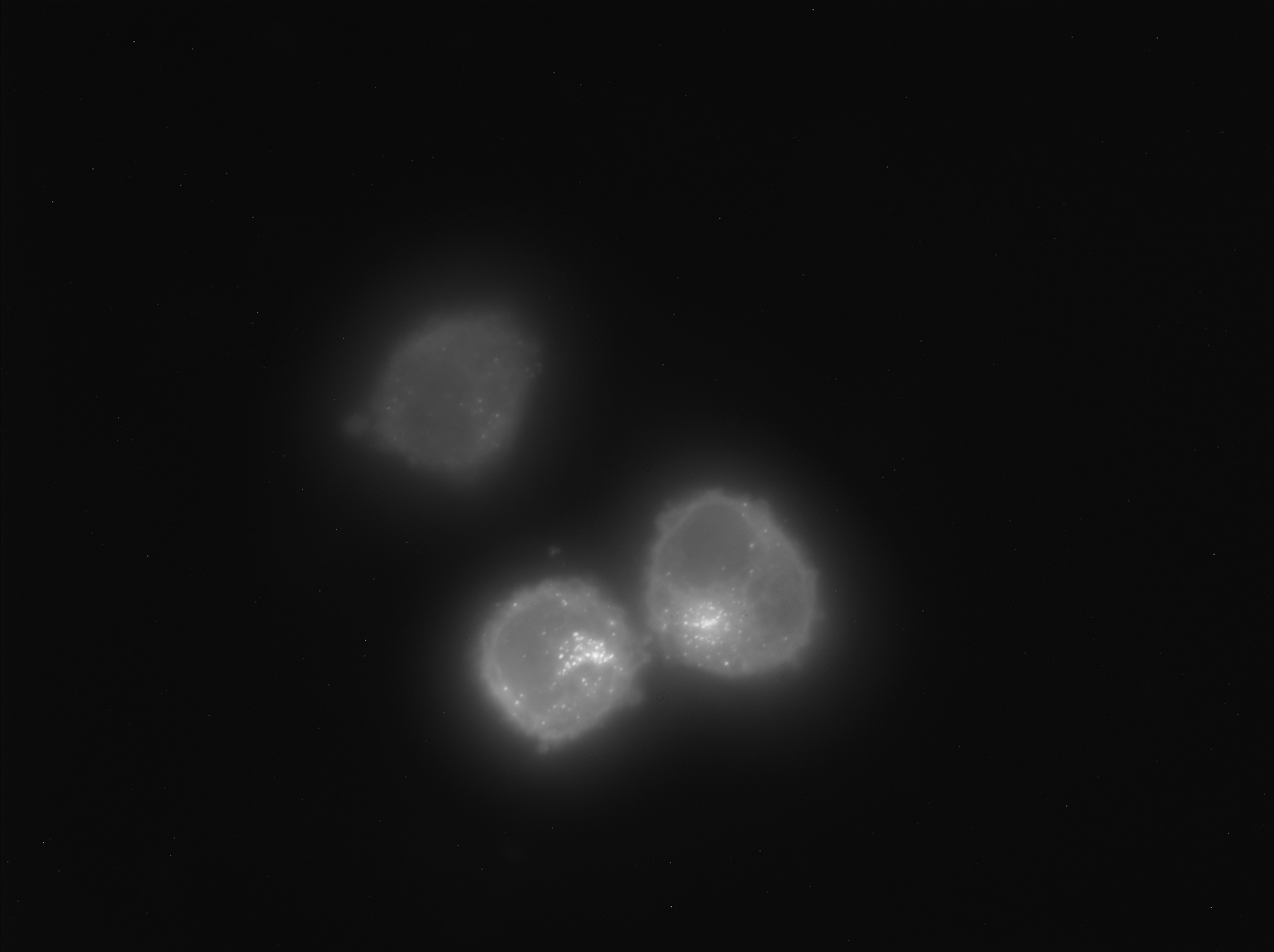

Supplement: Supplementary file 14 — Source data Fig. 4 [file 44319_2026_742_MOESM14_ESM.zip › Figure 4/Fig 4ef Cas9 TPGS1-EYFP-3'UTR acetylated tubulin/Cas9 TPGS1-3utr R1 1-28-24 LT5.Project Maximum Z_XY1738102322_Z0_T0_C2.tif]

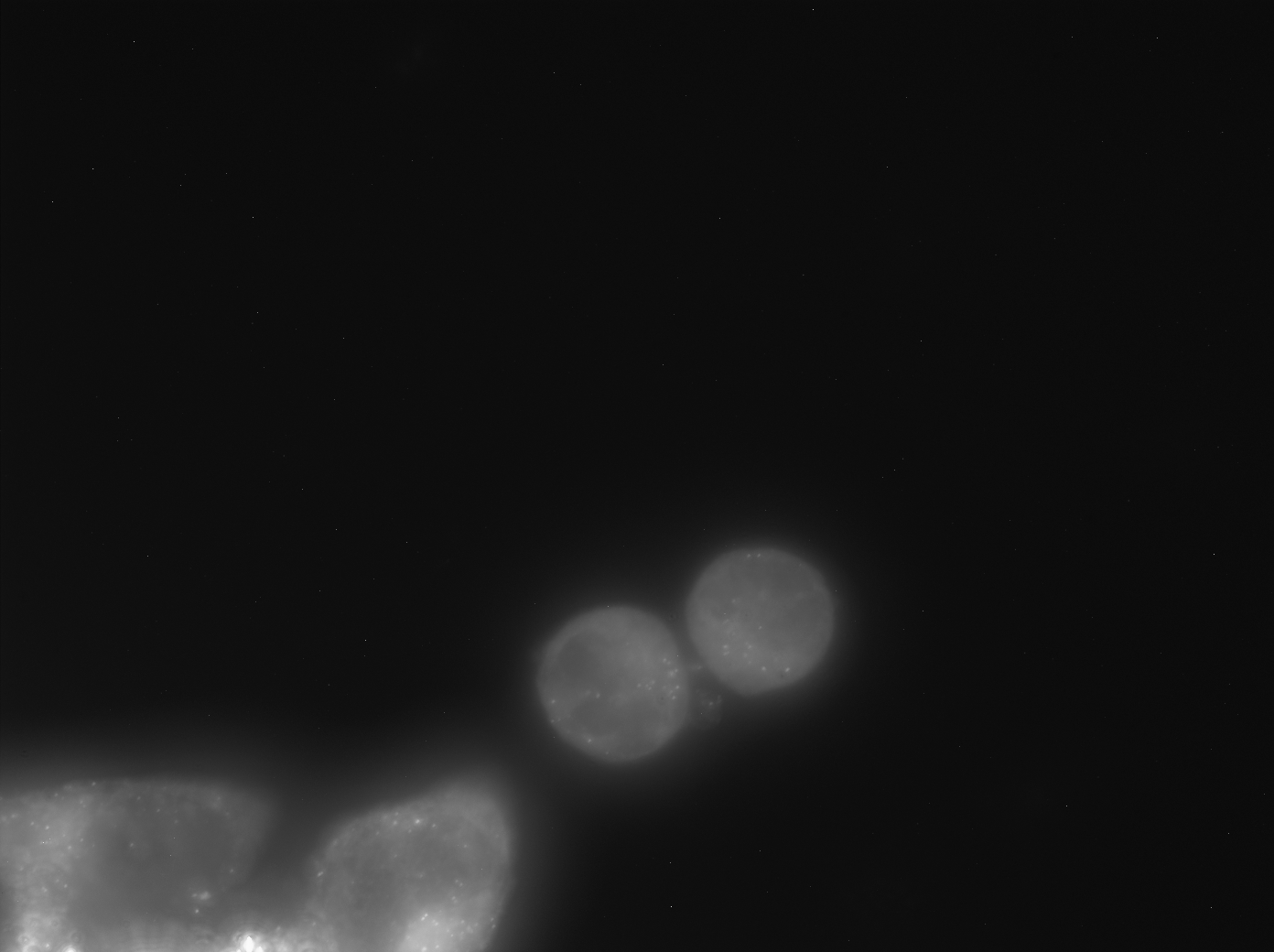

Supplement: Supplementary file 14 — Source data Fig. 4 [file 44319_2026_742_MOESM14_ESM.zip › Figure 4/Fig 4ef Cas9 TPGS1-EYFP-3'UTR acetylated tubulin/Cas9 TPGS1-3utr R1 1-28-24 ET5.Project Maximum Z_XY1738102003_Z0_T0_C2.tif]

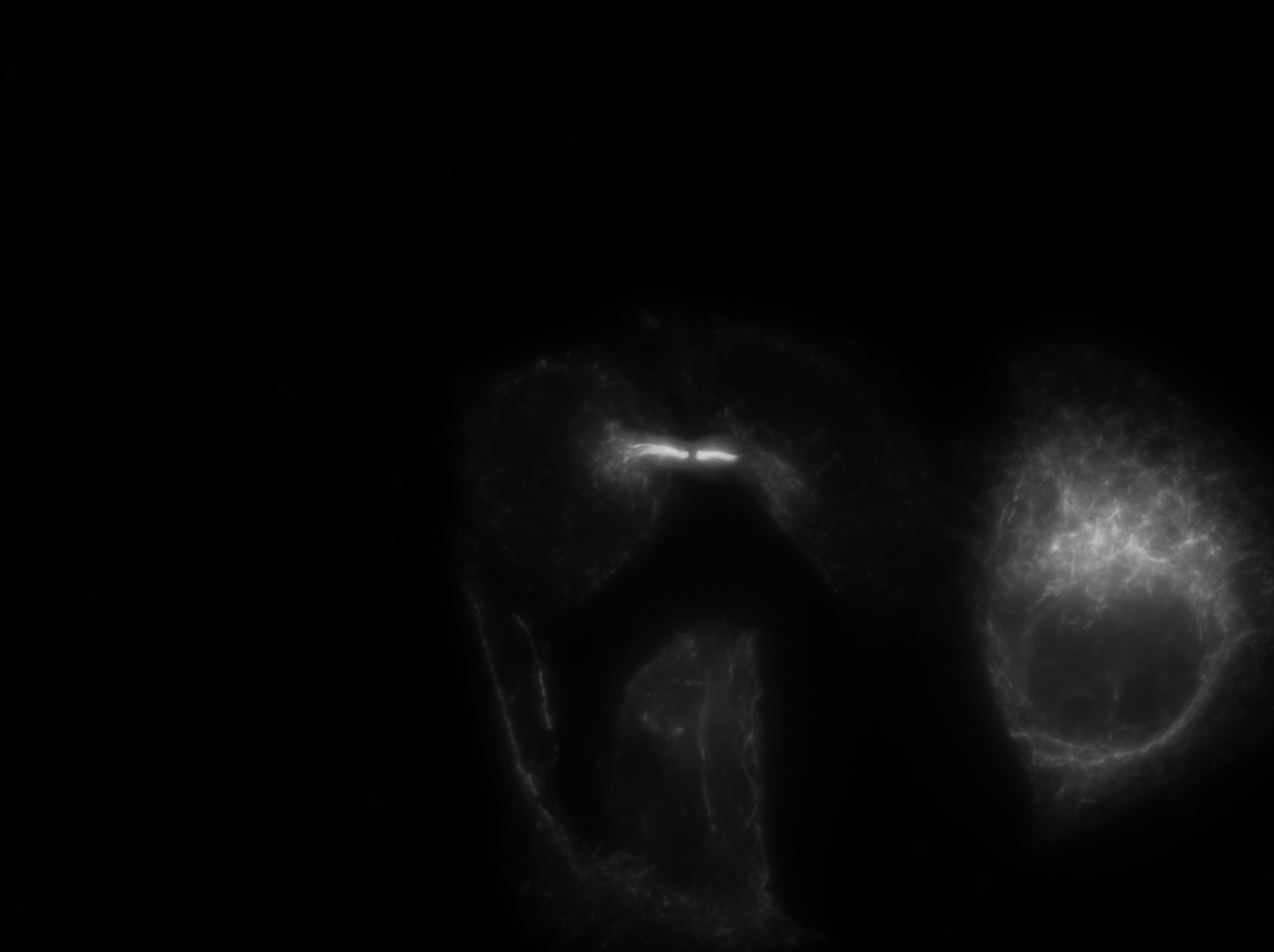

Supplement: Supplementary file 14 — Source data Fig. 4 [file 44319_2026_742_MOESM14_ESM.zip › Figure 4/Fig 4ef Cas9 TPGS1-EYFP-3'UTR acetylated tubulin/Cas9 TPGS1-3utr R1 1-28-24 LT2.Project Maximum Z_XY1738100690_Z0_T0_C1.tif]

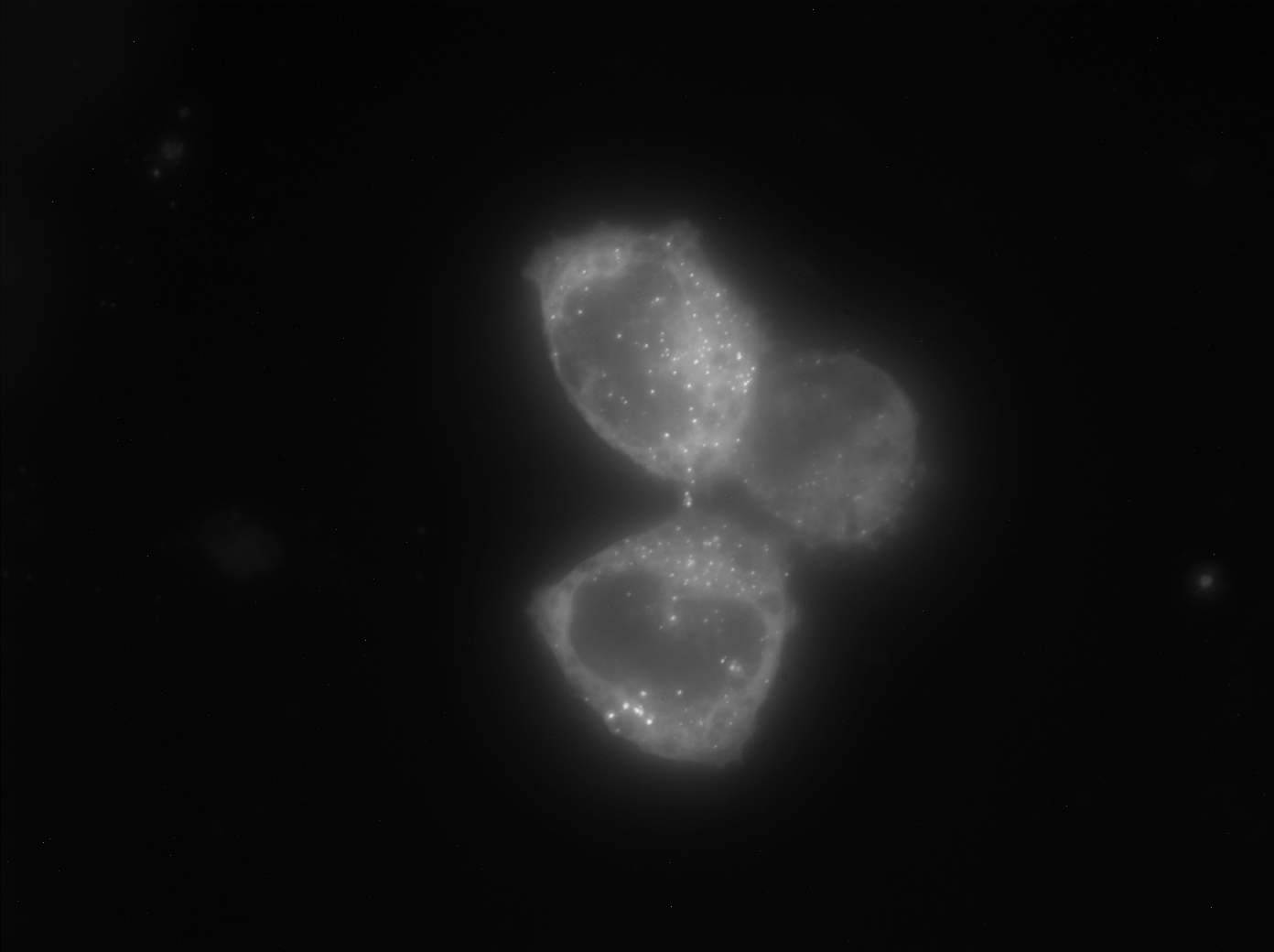

Supplement: Supplementary file 14 — Source data Fig. 4 [file 44319_2026_742_MOESM14_ESM.zip › Figure 4/Fig 4ef Cas9 TPGS1-EYFP-3'UTR acetylated tubulin/Cas9 TPGS1-3utr R1 1-28-24 LT4.Project Maximum Z_XY1738101264_Z0_T0_C2.tif]

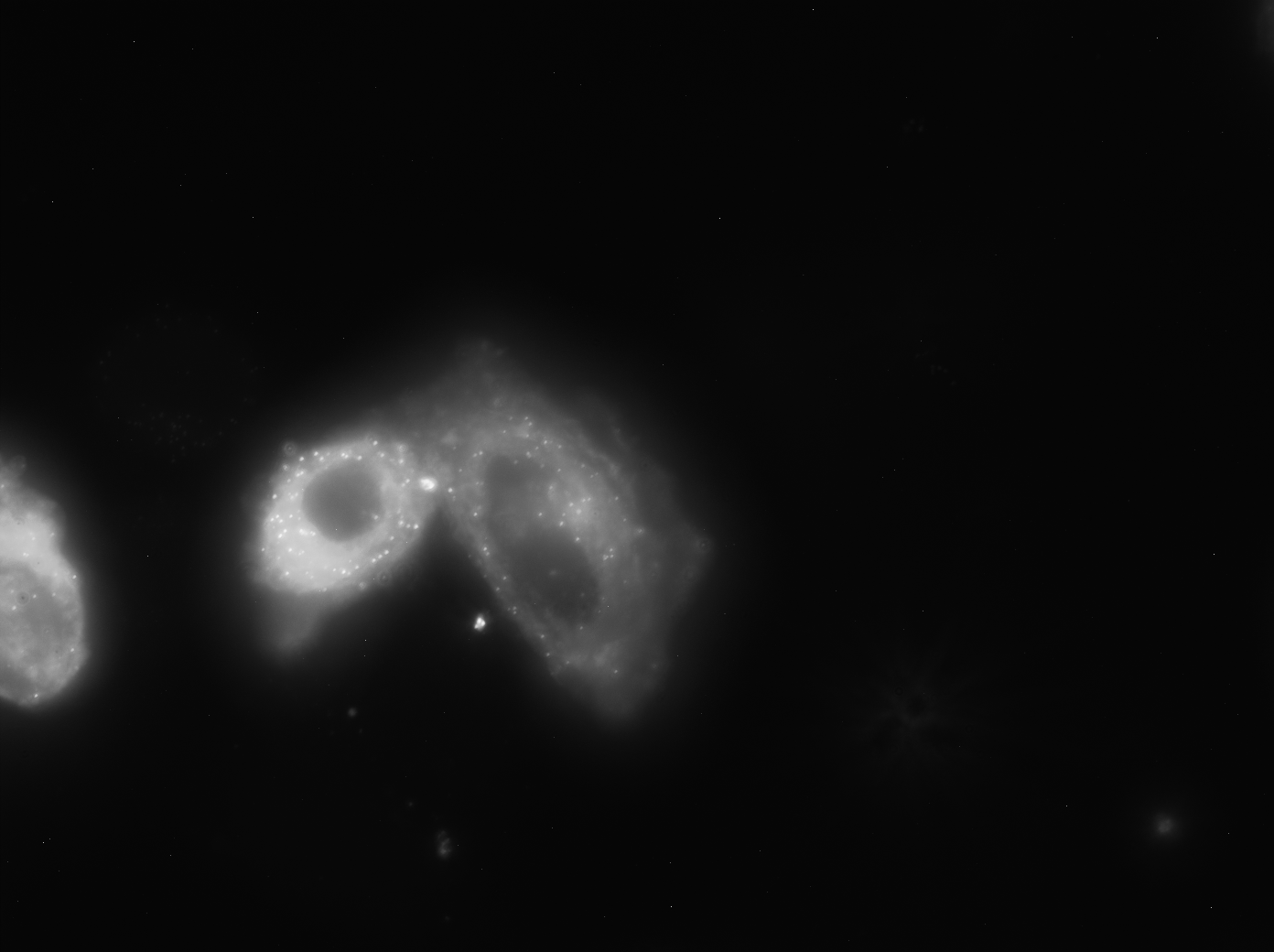

Supplement: Supplementary file 14 — Source data Fig. 4 [file 44319_2026_742_MOESM14_ESM.zip › Figure 4/Fig 4ef Cas9 TPGS1-EYFP-3'UTR acetylated tubulin/Cas9 TPGS1-3utr R2 2-5-25 LT8.Project Maximum Z_XY1738625347_Z0_T0_C2.tif]

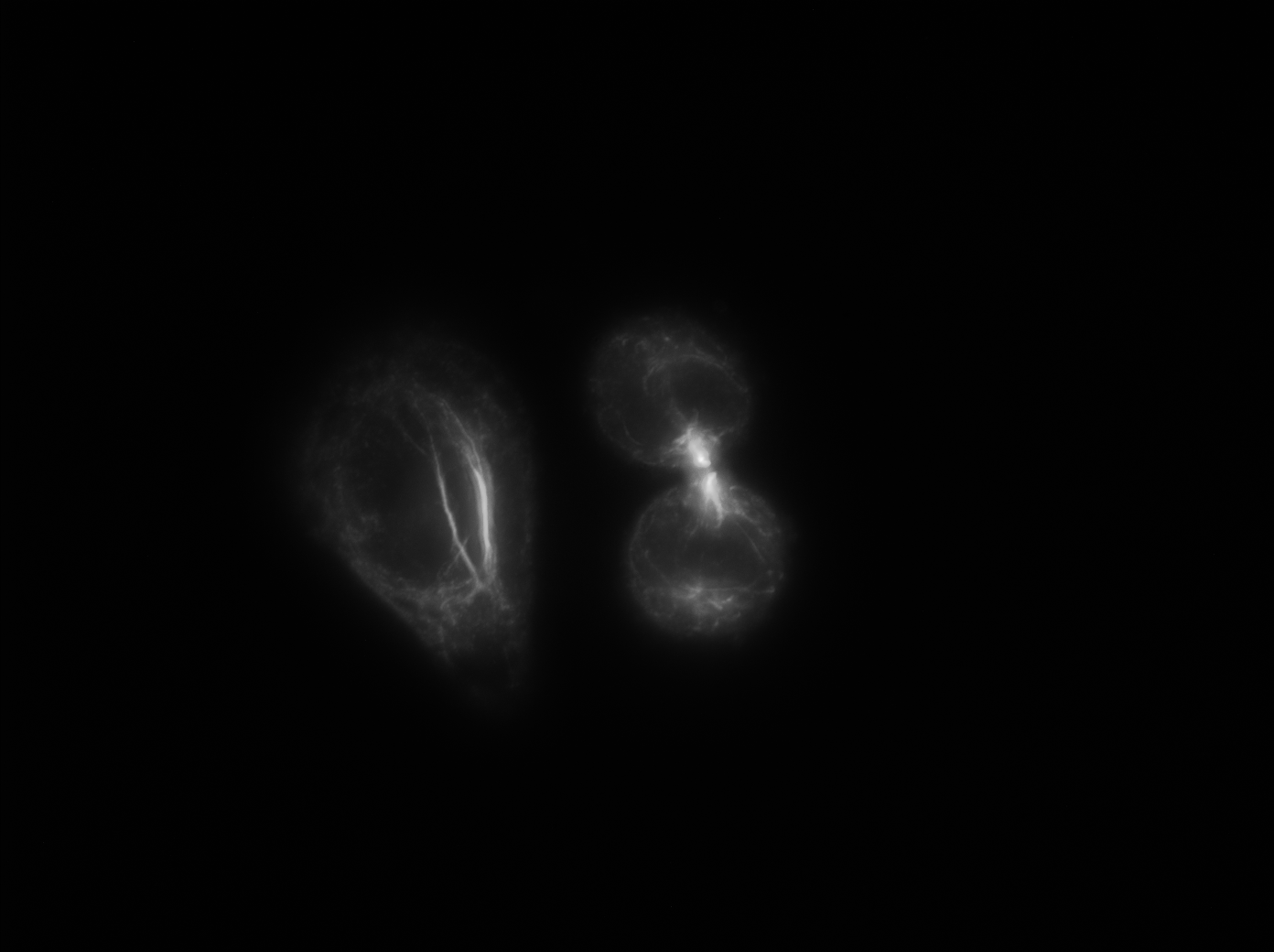

Supplement: Supplementary file 14 — Source data Fig. 4 [file 44319_2026_742_MOESM14_ESM.zip › Figure 4/Fig 4ef Cas9 TPGS1-EYFP-3'UTR acetylated tubulin/Cas9 TPGS1-3utr R1 1-28-24 ET6.Project Maximum Z_XY1738103674_Z0_T0_C1.tif]

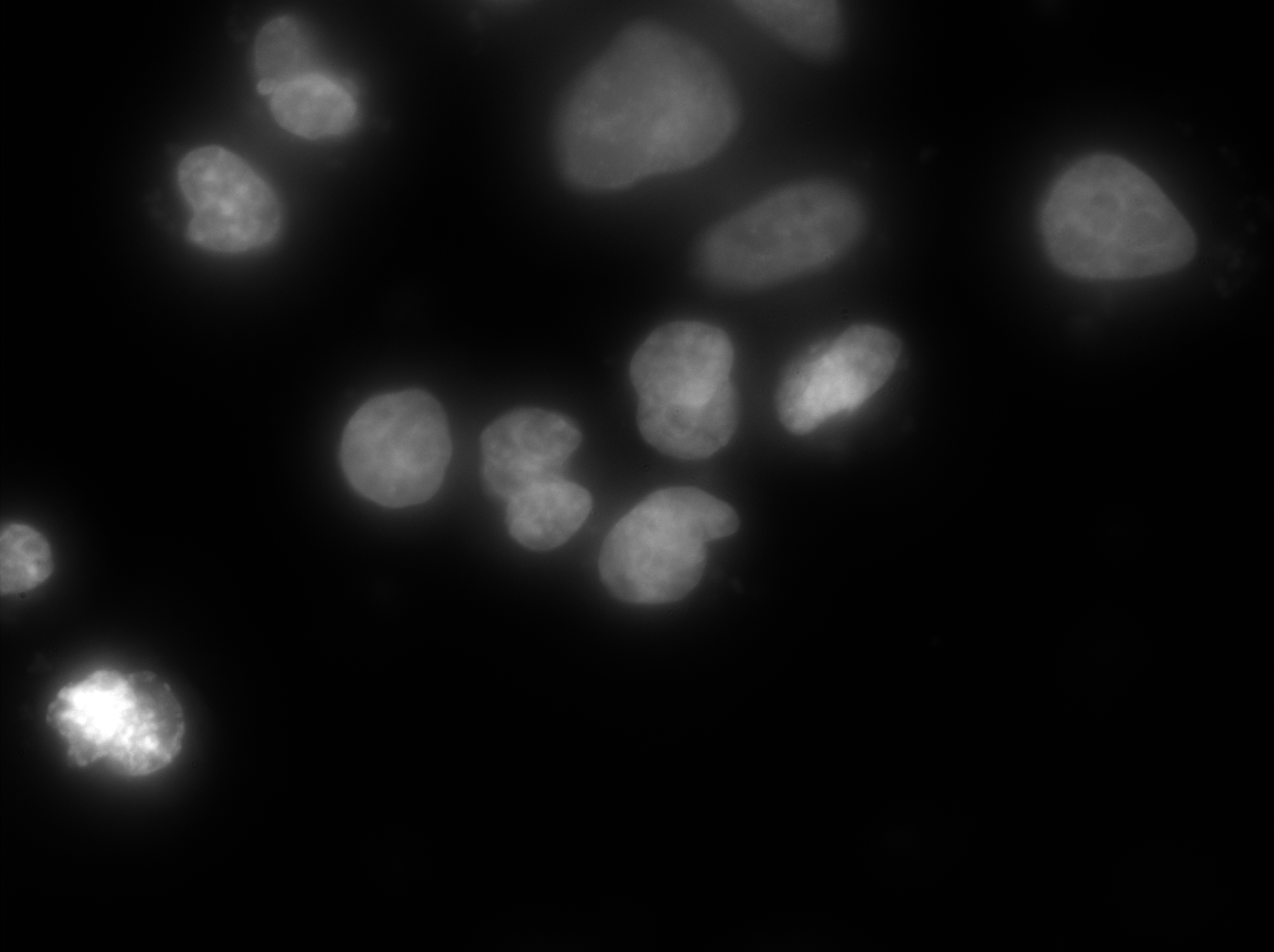

Supplement: Supplementary file 14 — Source data Fig. 4 [file 44319_2026_742_MOESM14_ESM.zip › Figure 4/Fig 4ef Cas9 TPGS1-EYFP-3'UTR acetylated tubulin/Cas9 TPGS1-3utr R3 2-5-25 ET5.Project Maximum Z_XY1738695158_Z0_T0_C0.tif]

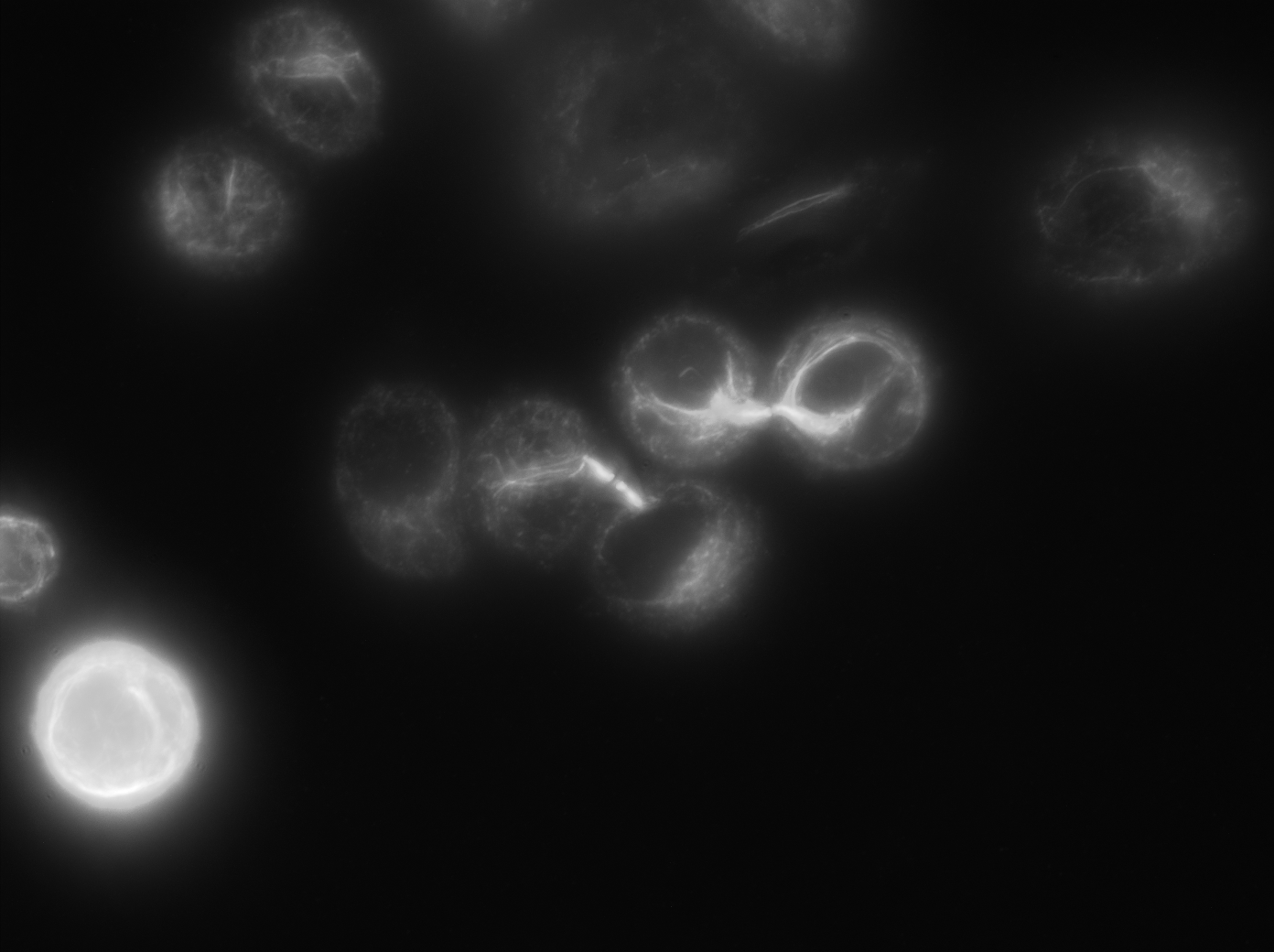

Supplement: Supplementary file 14 — Source data Fig. 4 [file 44319_2026_742_MOESM14_ESM.zip › Figure 4/Fig 4ef Cas9 TPGS1-EYFP-3'UTR acetylated tubulin/Cas9 TPGS1-3utr R3 2-5-25 ET5.Project Maximum Z_XY1738695158_Z0_T0_C1.tif]

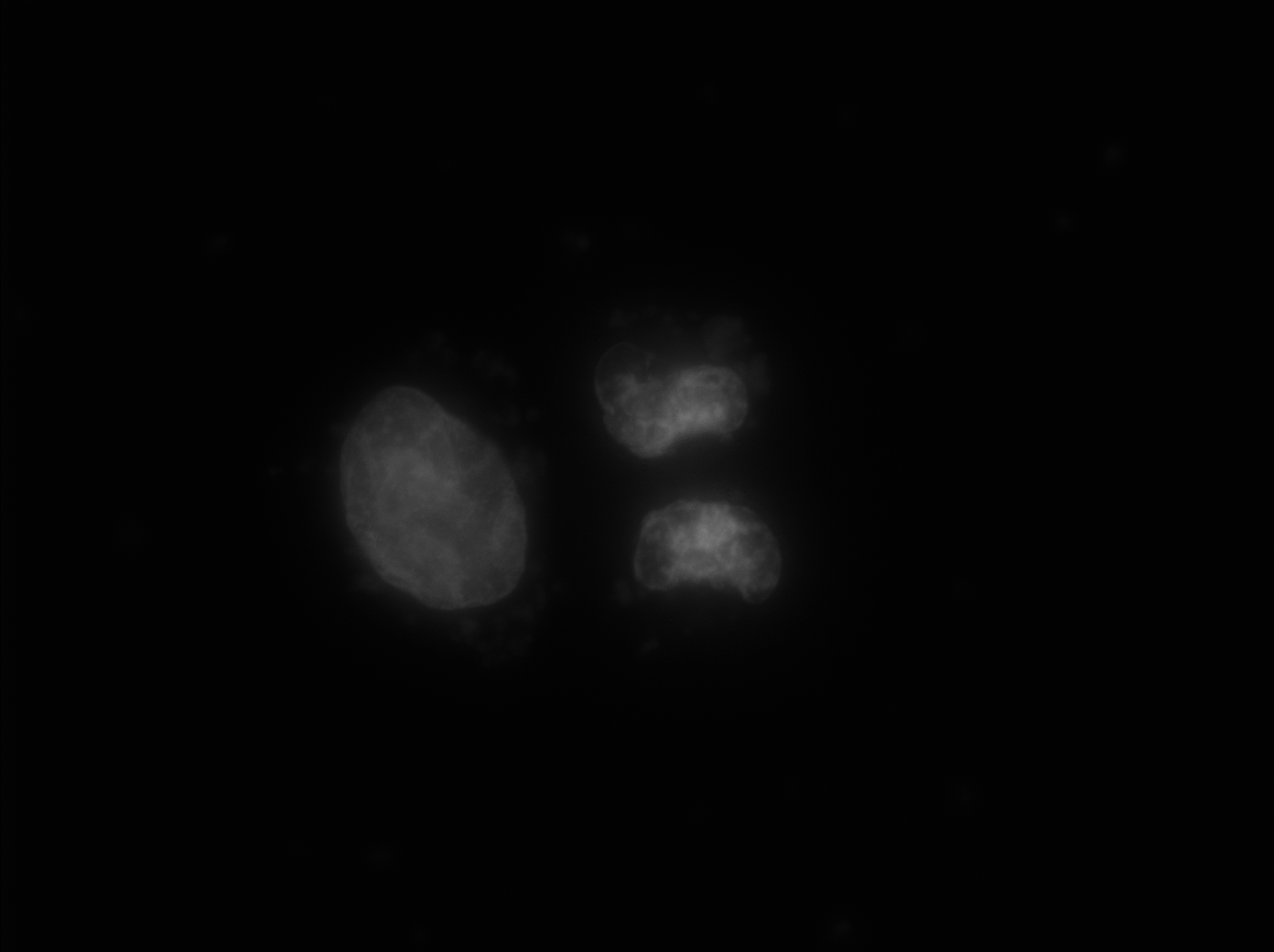

Supplement: Supplementary file 14 — Source data Fig. 4 [file 44319_2026_742_MOESM14_ESM.zip › Figure 4/Fig 4ef Cas9 TPGS1-EYFP-3'UTR acetylated tubulin/Cas9 TPGS1-3utr R1 1-28-24 ET6.Project Maximum Z_XY1738103674_Z0_T0_C0.tif]

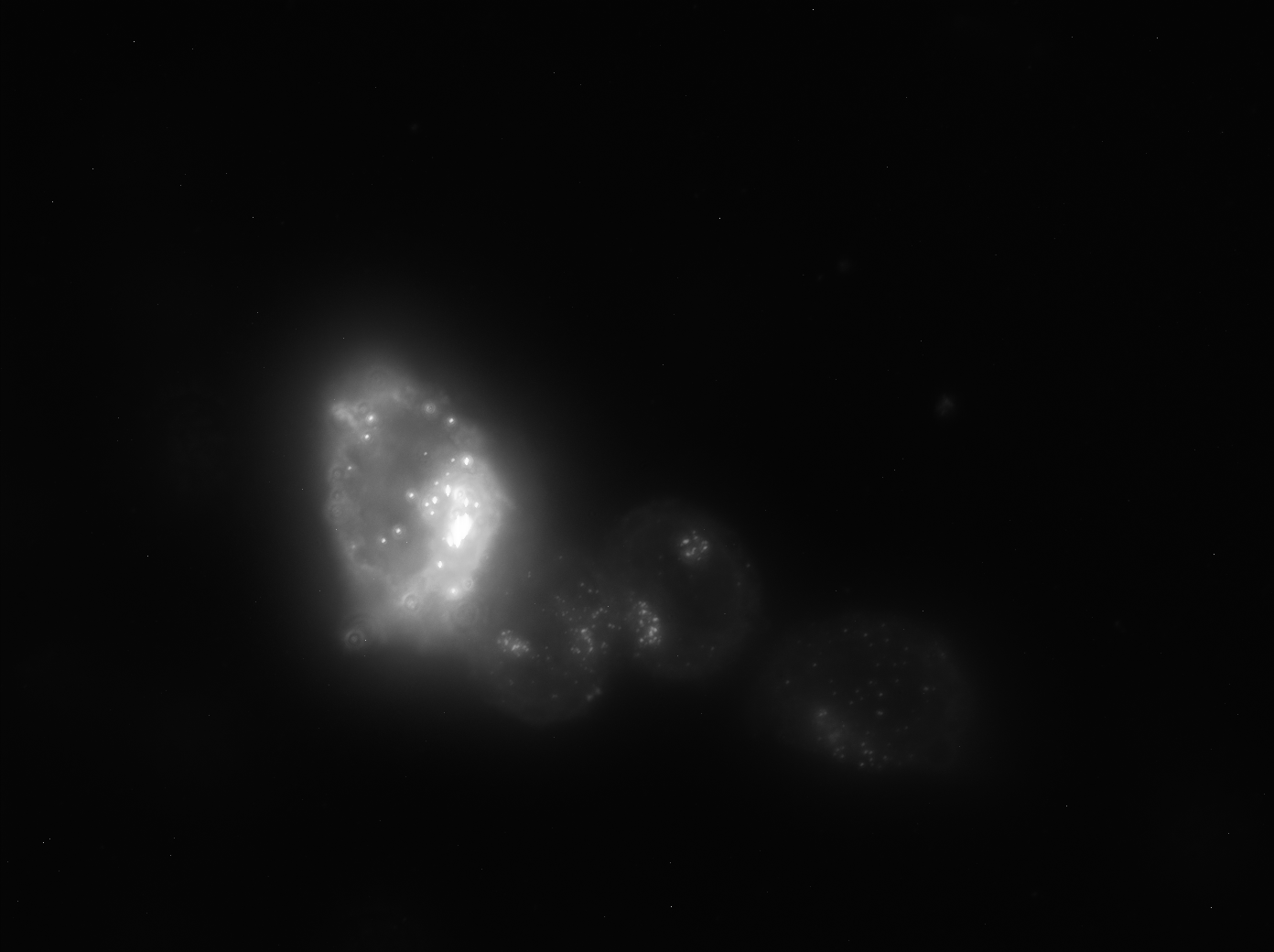

Supplement: Supplementary file 14 — Source data Fig. 4 [file 44319_2026_742_MOESM14_ESM.zip › Figure 4/Fig 4ef Cas9 TPGS1-EYFP-3'UTR acetylated tubulin/Cas9 TPGS1-3utr R2 2-5-25 ET4 EXIMG.Project Maximum Z_XY1738621115_Z0_T0_C2.tif]

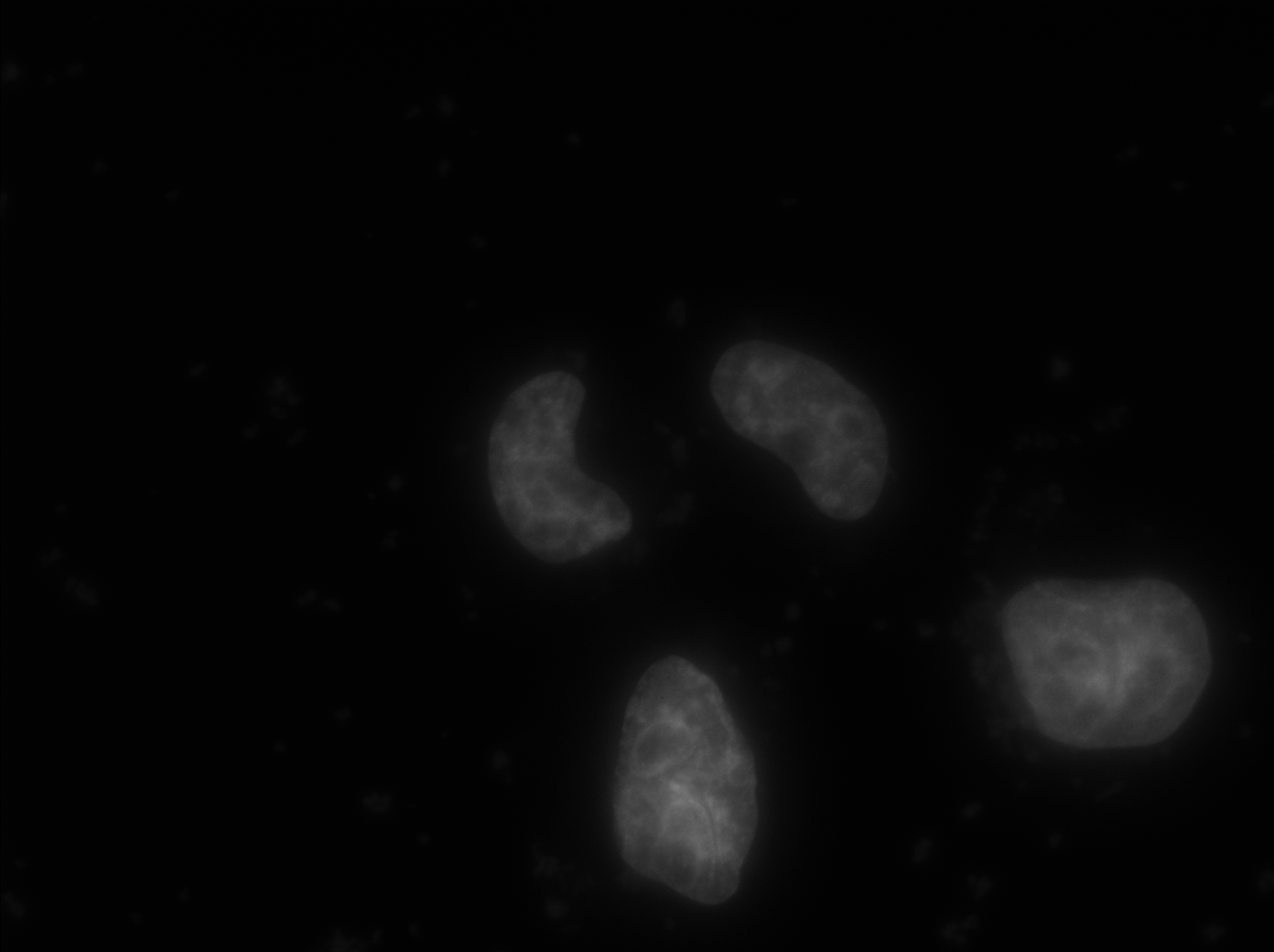

Supplement: Supplementary file 14 — Source data Fig. 4 [file 44319_2026_742_MOESM14_ESM.zip › Figure 4/Fig 4ef Cas9 TPGS1-EYFP-3'UTR acetylated tubulin/Cas9 TPGS1-3utr R1 1-28-24 LT2.Project Maximum Z_XY1738100690_Z0_T0_C0.tif]

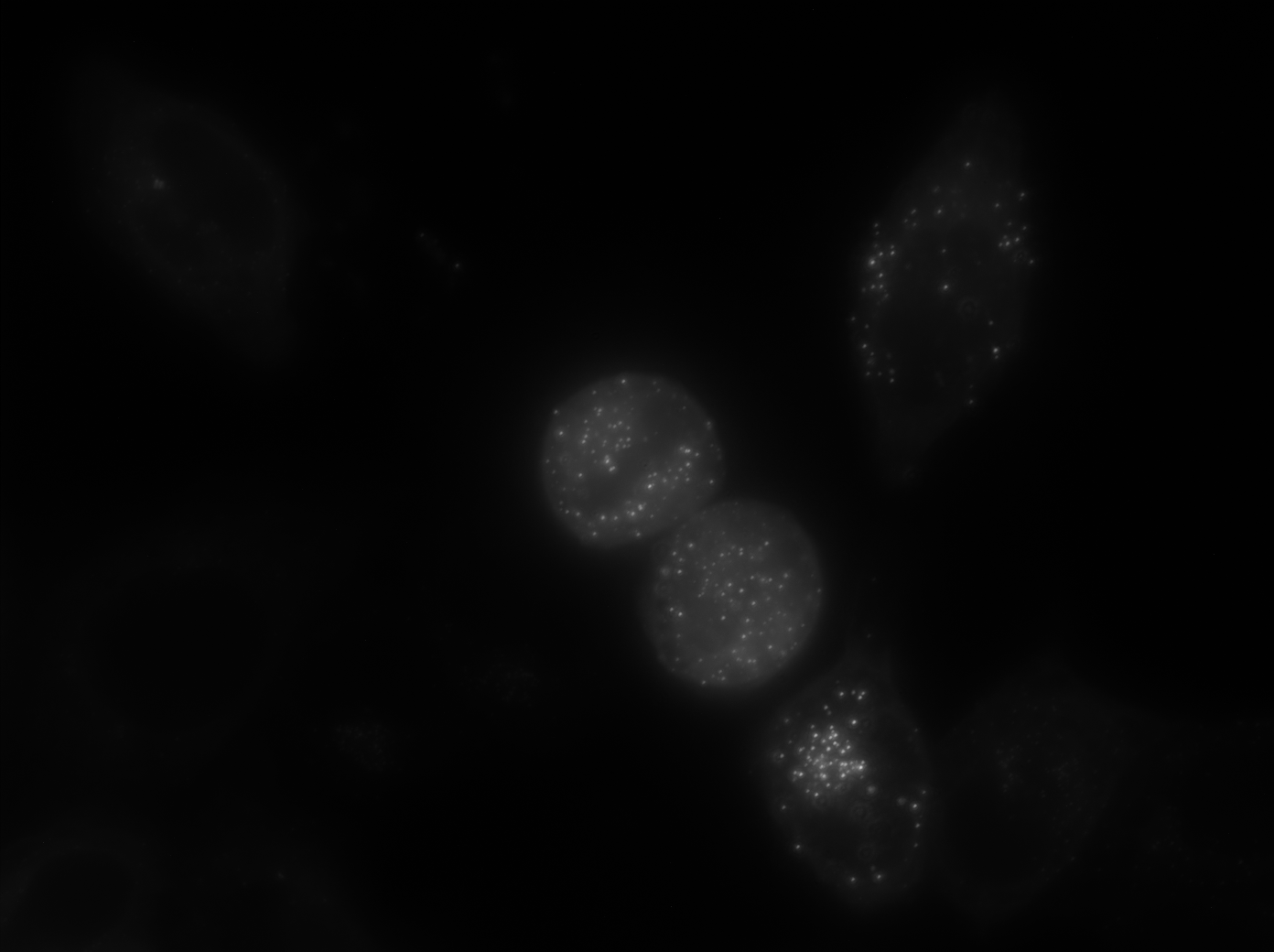

Supplement: Supplementary file 14 — Source data Fig. 4 [file 44319_2026_742_MOESM14_ESM.zip › Figure 4/Fig 4ef Cas9 TPGS1-EYFP-3'UTR acetylated tubulin/Cas9 TPGS1-3utr R3 2-5-25 ET9.Project Maximum Z_XY1738697075_Z0_T0_C2.tif]

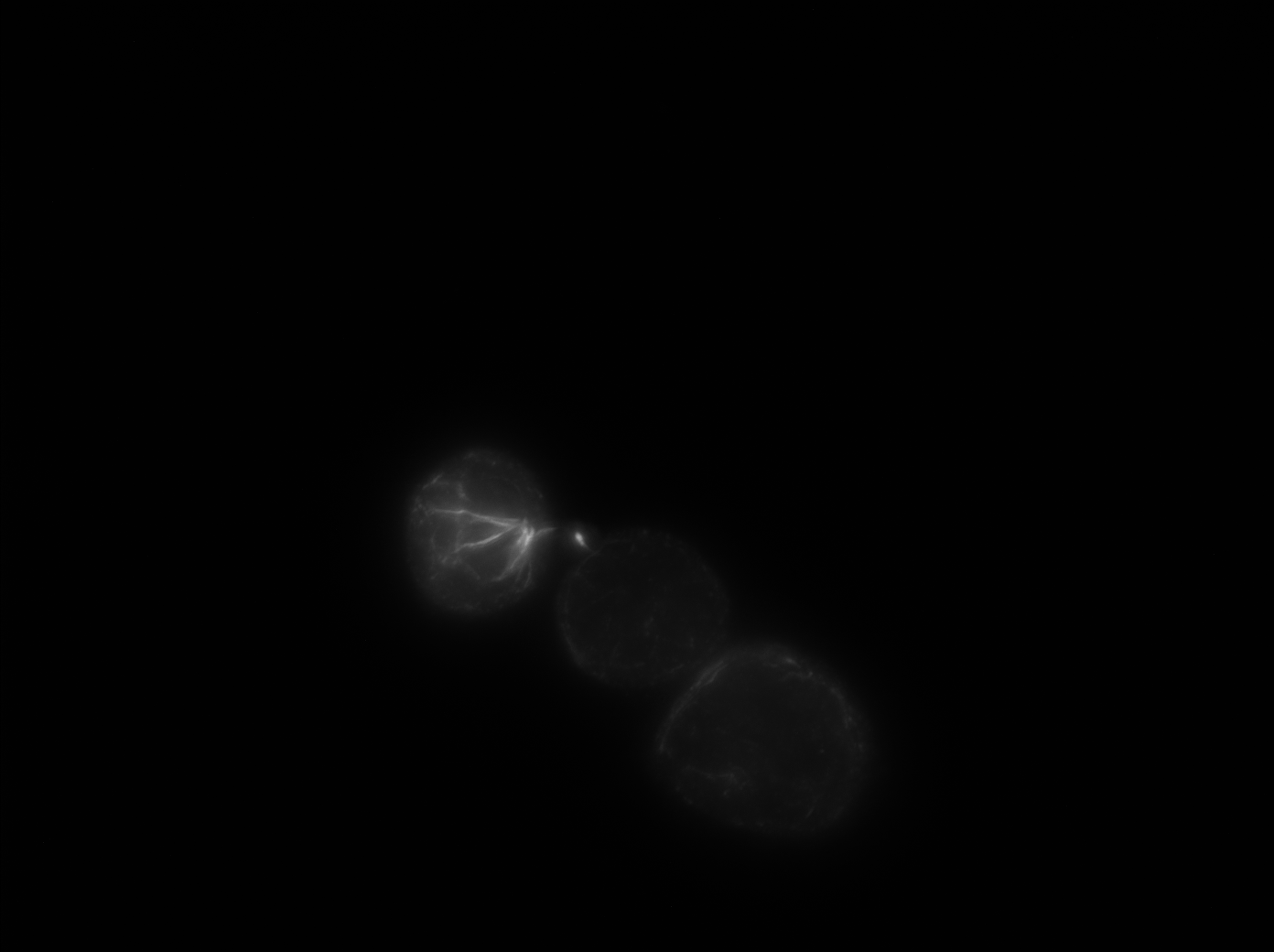

Supplement: Supplementary file 14 — Source data Fig. 4 [file 44319_2026_742_MOESM14_ESM.zip › Figure 4/Fig 4ef Cas9 TPGS1-EYFP-3'UTR acetylated tubulin/Cas9 TPGS1-3utr R1 1-28-24 ET7.Project Maximum Z_XY1738180583_Z0_T0_C1.tif]

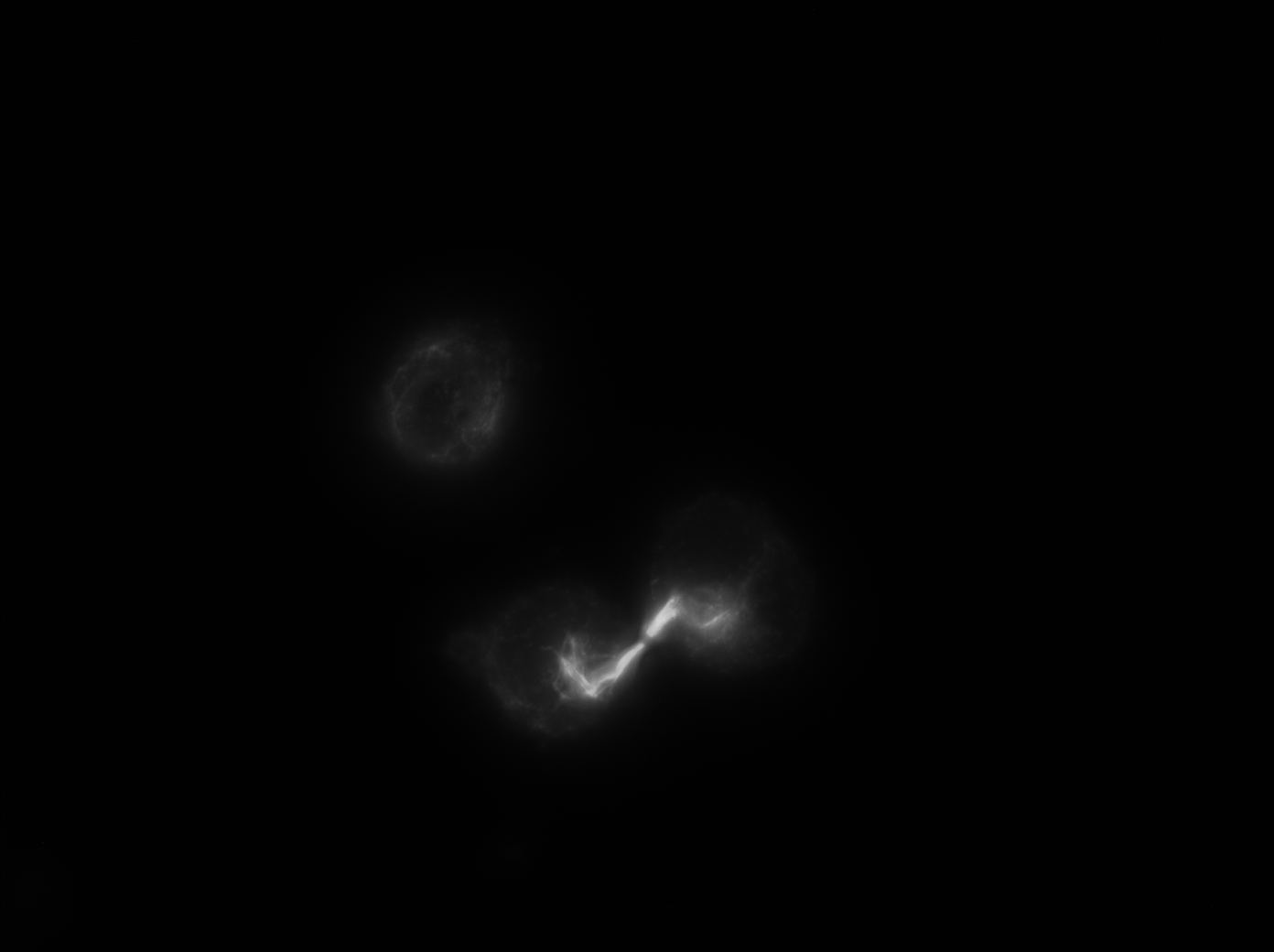

Supplement: Supplementary file 14 — Source data Fig. 4 [file 44319_2026_742_MOESM14_ESM.zip › Figure 4/Fig 4ef Cas9 TPGS1-EYFP-3'UTR acetylated tubulin/Cas9 TPGS1-3utr R1 1-28-24 LT5.Project Maximum Z_XY1738102322_Z0_T0_C1.tif]

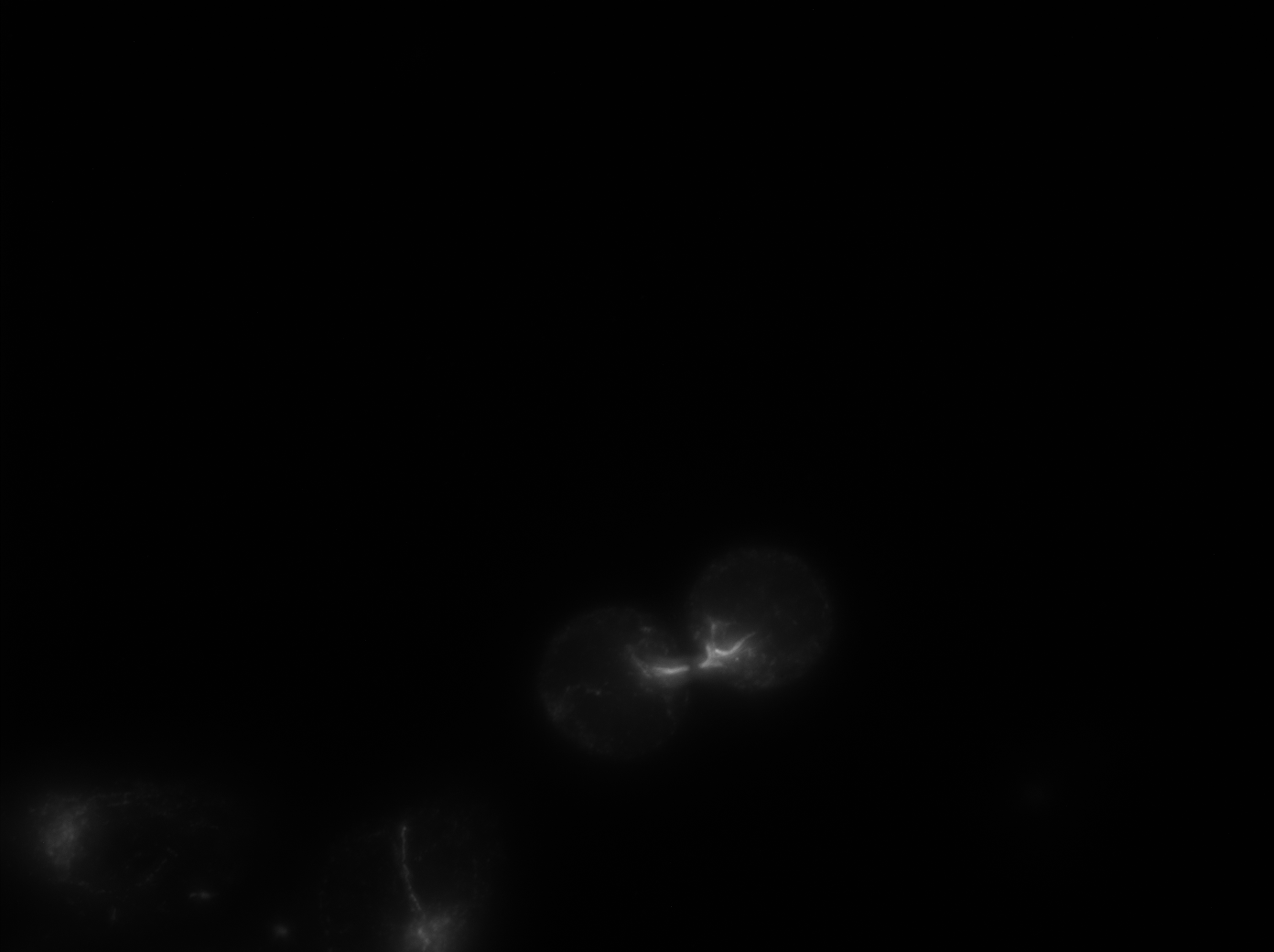

Supplement: Supplementary file 14 — Source data Fig. 4 [file 44319_2026_742_MOESM14_ESM.zip › Figure 4/Fig 4ef Cas9 TPGS1-EYFP-3'UTR acetylated tubulin/Cas9 TPGS1-3utr R1 1-28-24 ET5.Project Maximum Z_XY1738102003_Z0_T0_C1.tif]

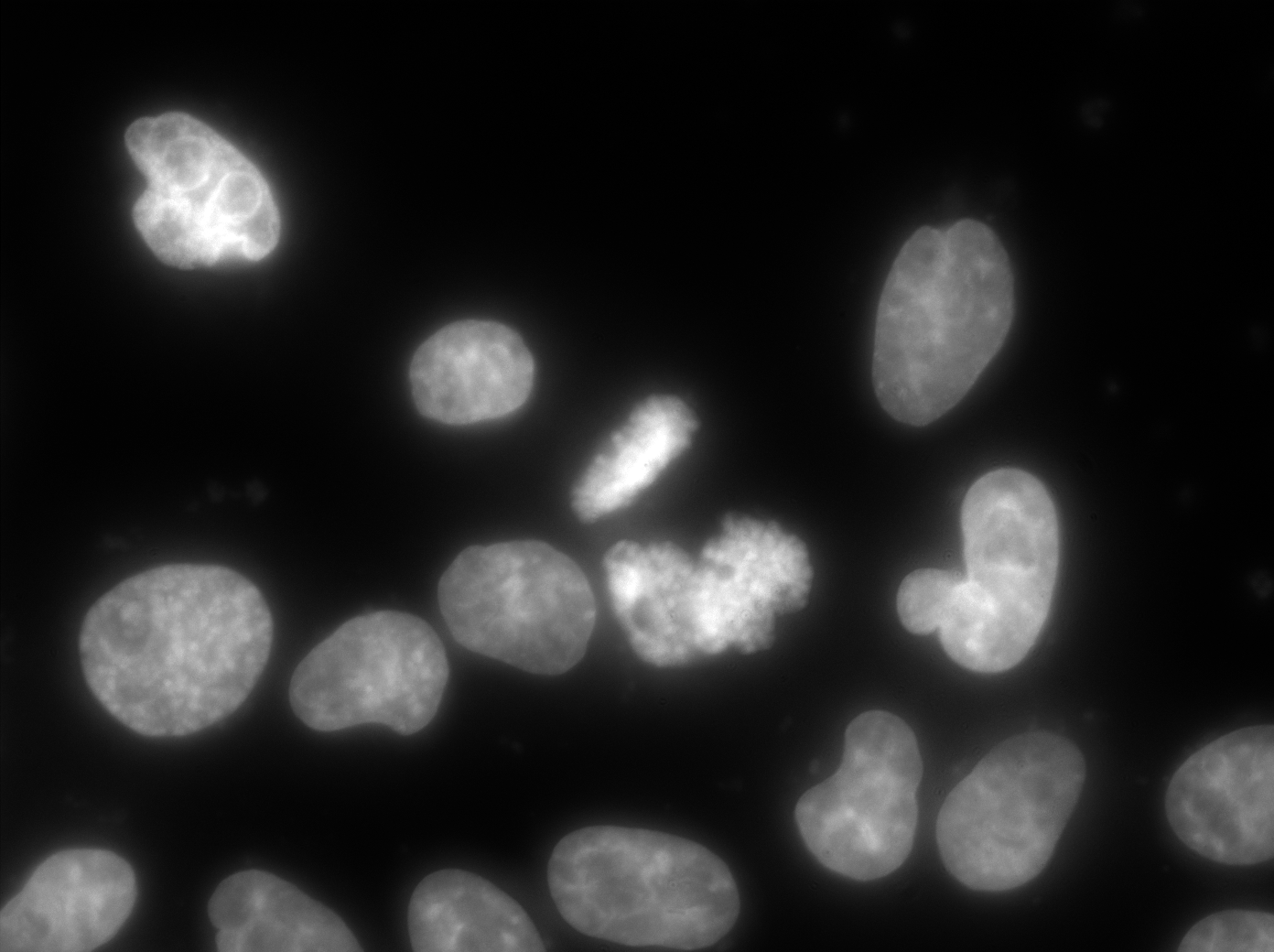

Supplement: Supplementary file 14 — Source data Fig. 4 [file 44319_2026_742_MOESM14_ESM.zip › Figure 4/Fig 4ef Cas9 TPGS1-EYFP-3'UTR acetylated tubulin/Cas9 TPGS1-3utr R3 2-5-25 ET9.Project Maximum Z_XY1738697075_Z0_T0_C0.tif]

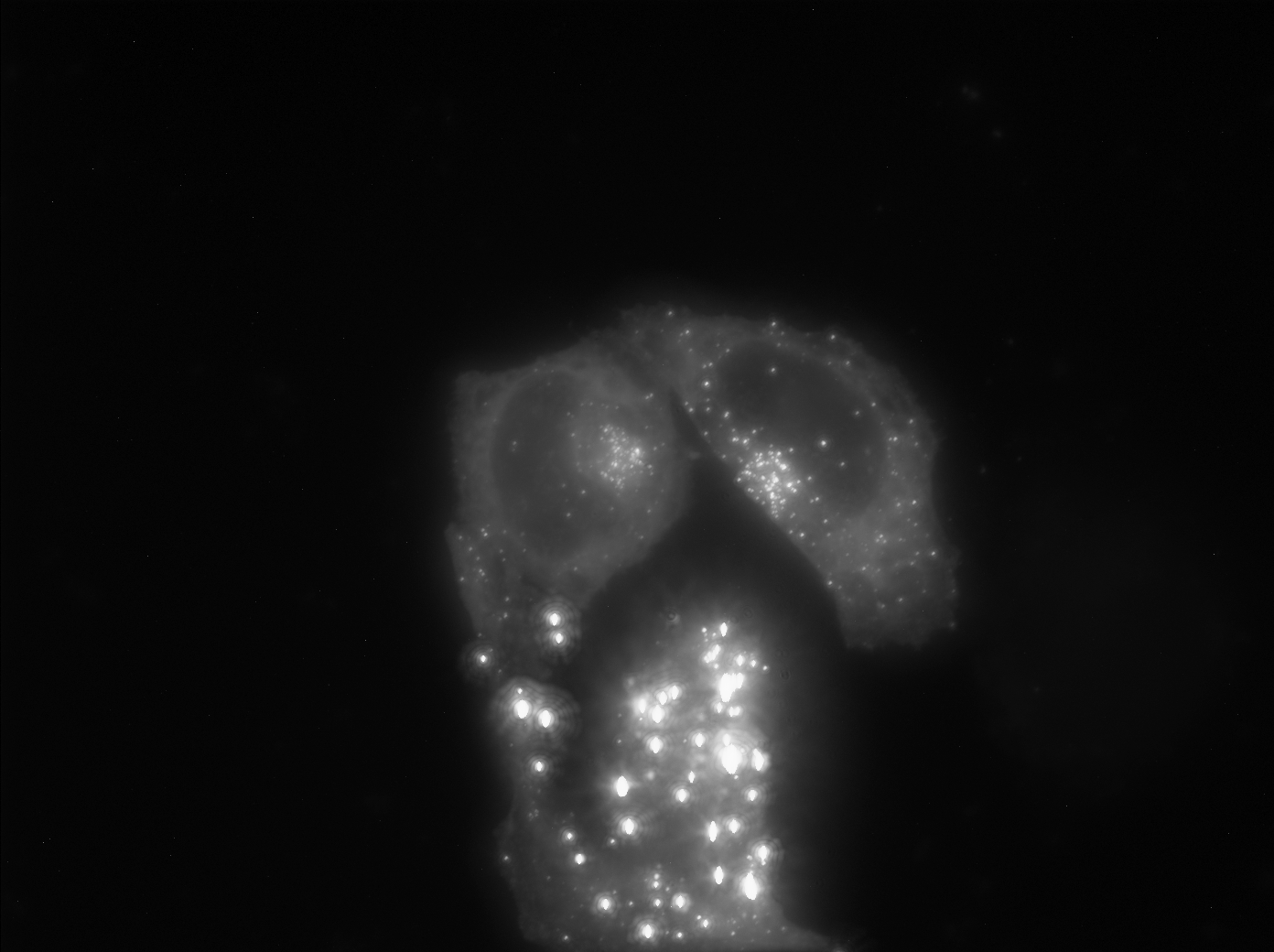

Supplement: Supplementary file 14 — Source data Fig. 4 [file 44319_2026_742_MOESM14_ESM.zip › Figure 4/Fig 4ef Cas9 TPGS1-EYFP-3'UTR acetylated tubulin/Cas9 TPGS1-3utr R1 1-28-24 LT2.Project Maximum Z_XY1738100690_Z0_T0_C2.tif]

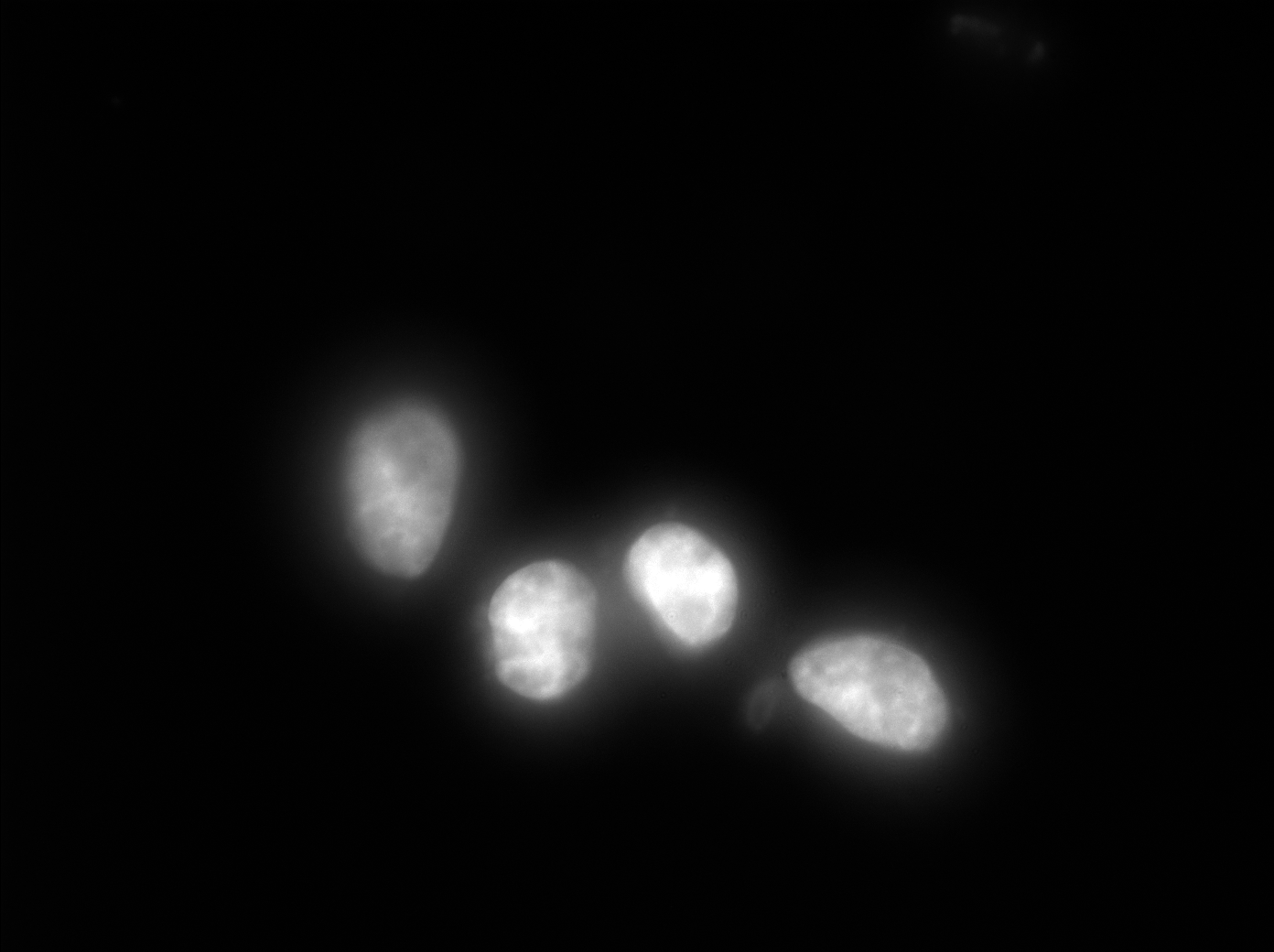

Supplement: Supplementary file 14 — Source data Fig. 4 [file 44319_2026_742_MOESM14_ESM.zip › Figure 4/Fig 4ef Cas9 TPGS1-EYFP-3'UTR acetylated tubulin/Cas9 TPGS1-3utr R2 2-5-25 ET4 EXIMG.Project Maximum Z_XY1738621115_Z0_T0_C0.tif]

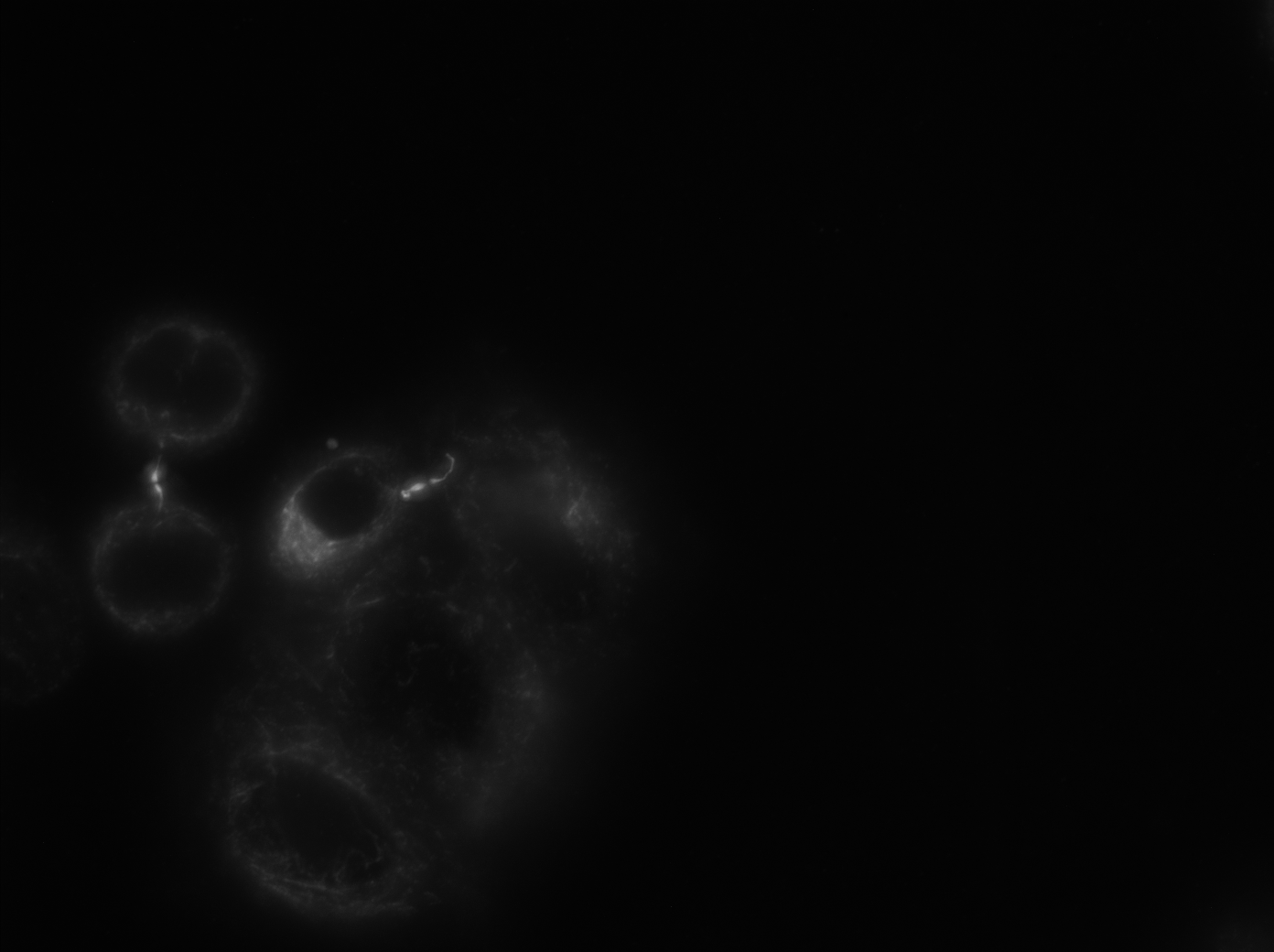

Supplement: Supplementary file 14 — Source data Fig. 4 [file 44319_2026_742_MOESM14_ESM.zip › Figure 4/Fig 4ef Cas9 TPGS1-EYFP-3'UTR acetylated tubulin/Cas9 TPGS1-3utr R2 2-5-25 LT8.Project Maximum Z_XY1738625347_Z0_T0_C1.tif]

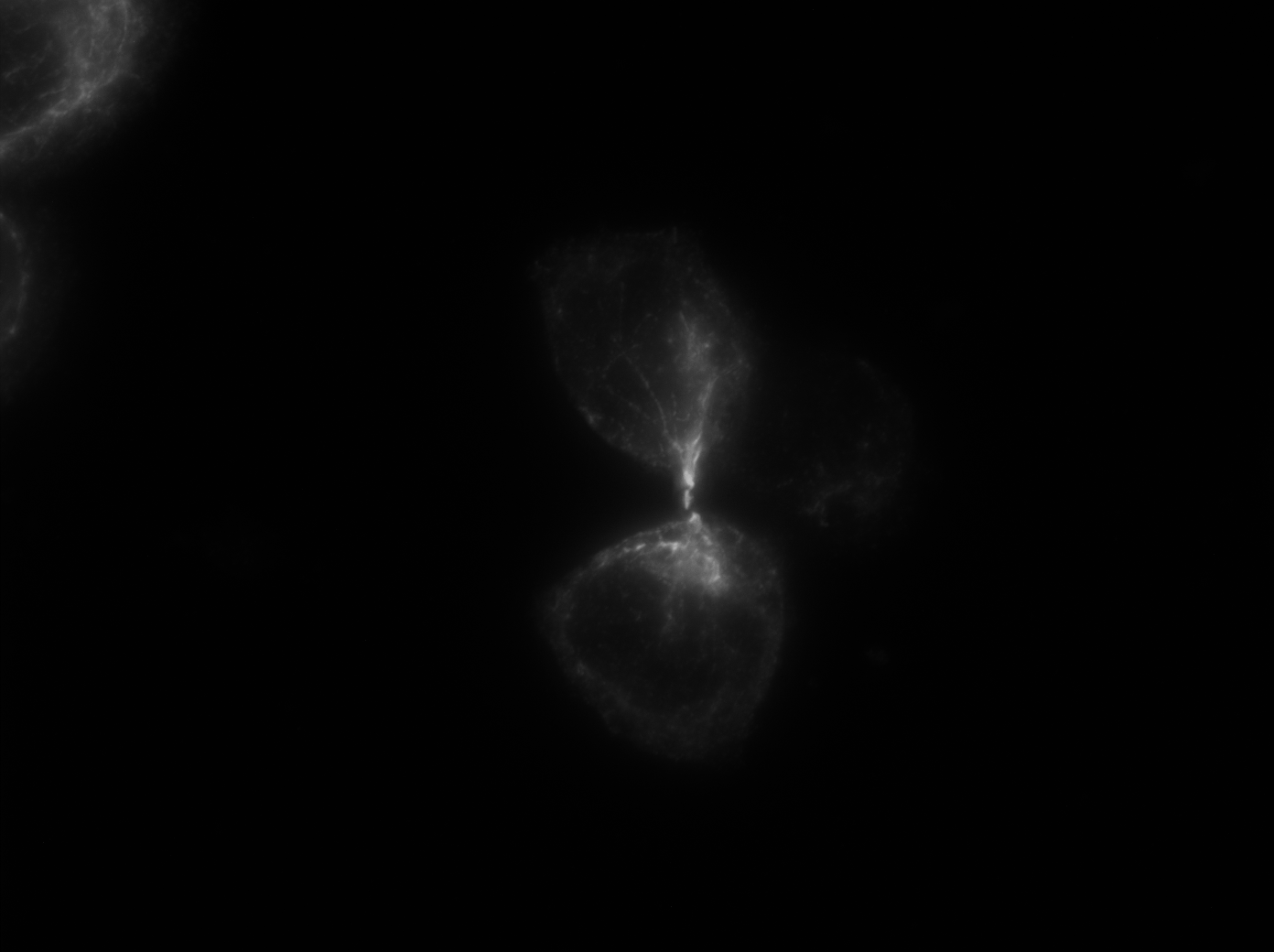

Supplement: Supplementary file 14 — Source data Fig. 4 [file 44319_2026_742_MOESM14_ESM.zip › Figure 4/Fig 4ef Cas9 TPGS1-EYFP-3'UTR acetylated tubulin/Cas9 TPGS1-3utr R1 1-28-24 LT4.Project Maximum Z_XY1738101264_Z0_T0_C1.tif]

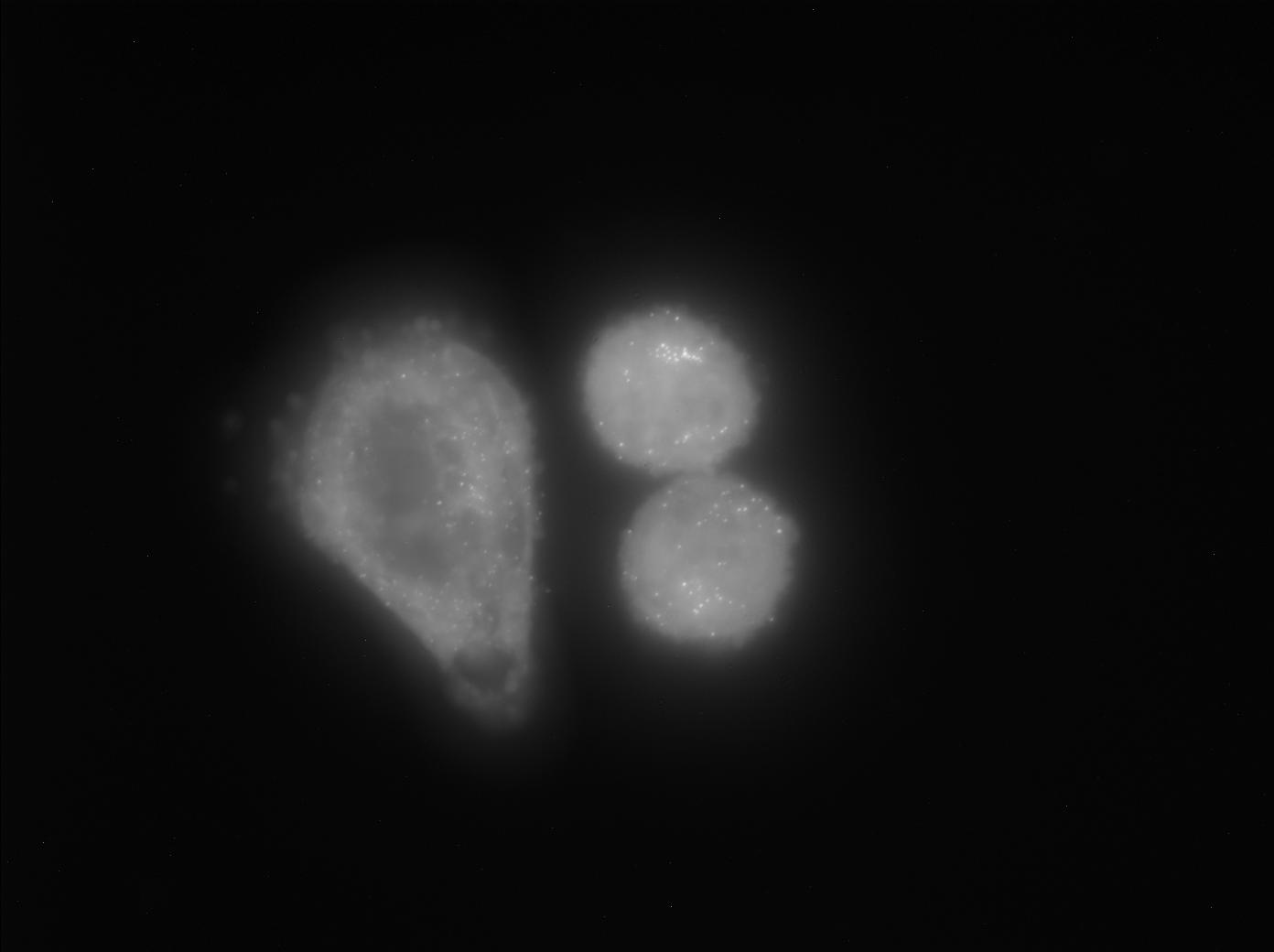

Supplement: Supplementary file 14 — Source data Fig. 4 [file 44319_2026_742_MOESM14_ESM.zip › Figure 4/Fig 4ef Cas9 TPGS1-EYFP-3'UTR acetylated tubulin/Cas9 TPGS1-3utr R1 1-28-24 ET6.Project Maximum Z_XY1738103674_Z0_T0_C2.tif]

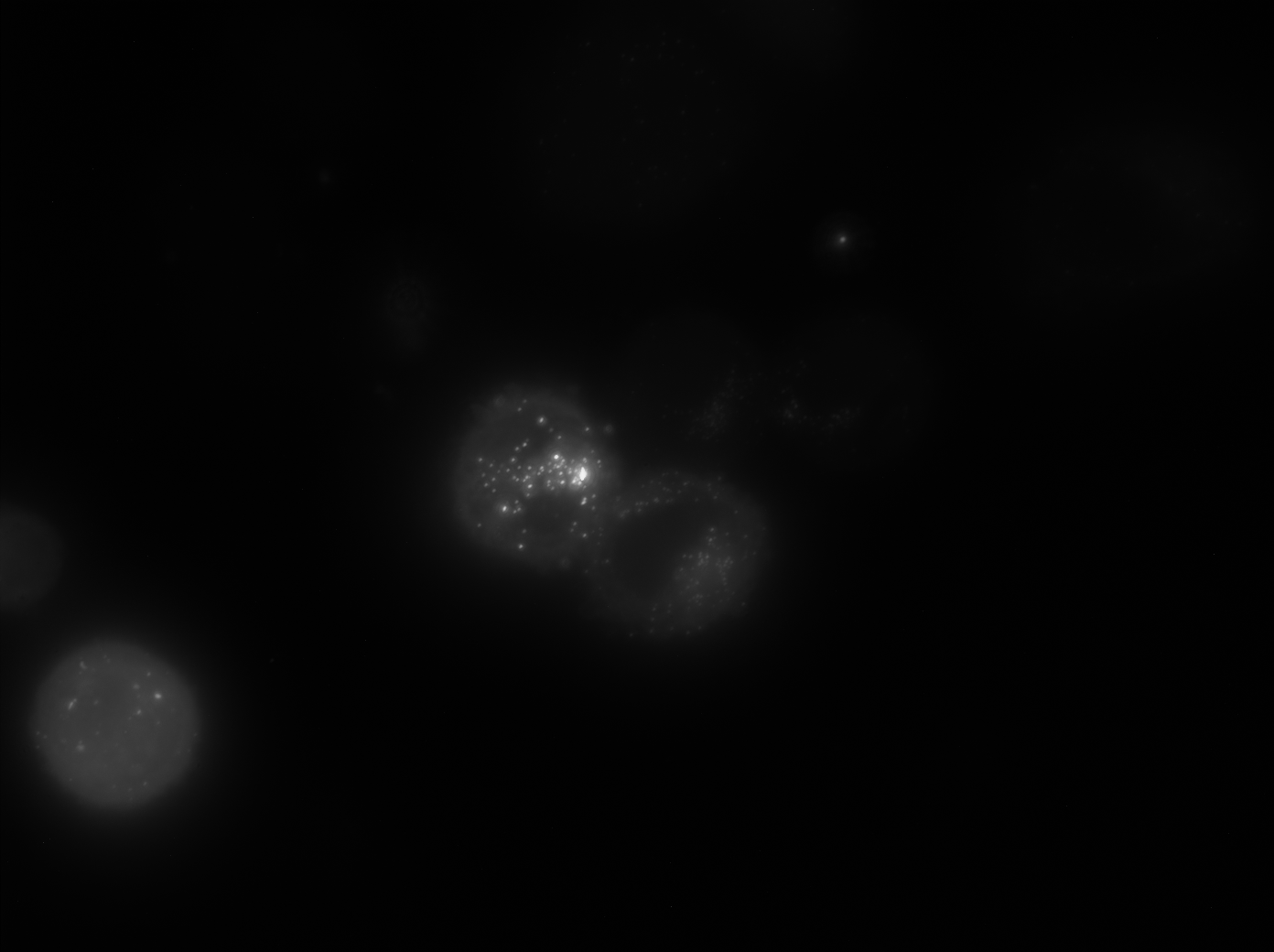

Supplement: Supplementary file 14 — Source data Fig. 4 [file 44319_2026_742_MOESM14_ESM.zip › Figure 4/Fig 4ef Cas9 TPGS1-EYFP-3'UTR acetylated tubulin/Cas9 TPGS1-3utr R3 2-5-25 ET5.Project Maximum Z_XY1738695158_Z0_T0_C2.tif]

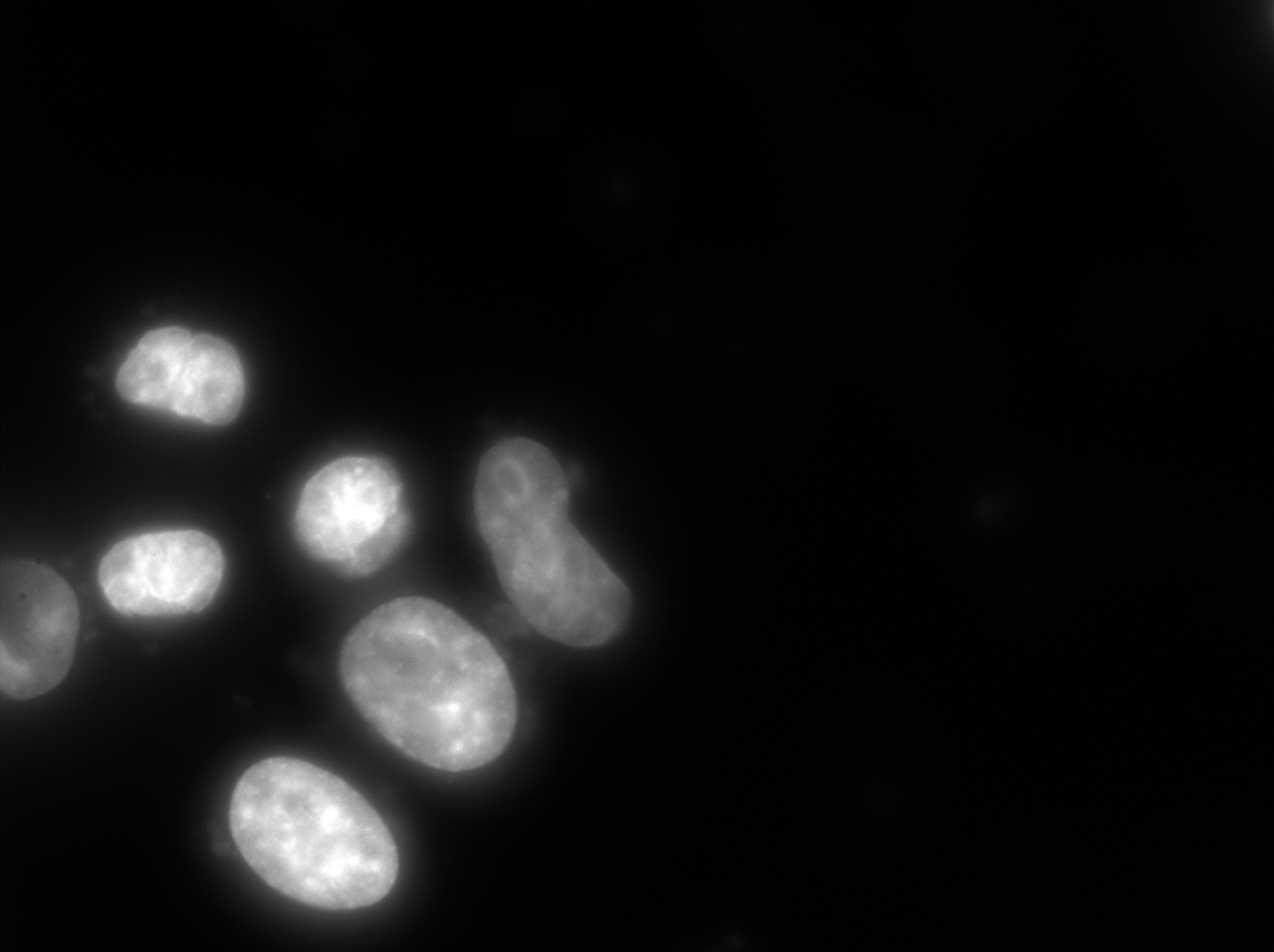

Supplement: Supplementary file 14 — Source data Fig. 4 [file 44319_2026_742_MOESM14_ESM.zip › Figure 4/Fig 4ef Cas9 TPGS1-EYFP-3'UTR acetylated tubulin/Cas9 TPGS1-3utr R2 2-5-25 LT8.Project Maximum Z_XY1738625347_Z0_T0_C0.tif]

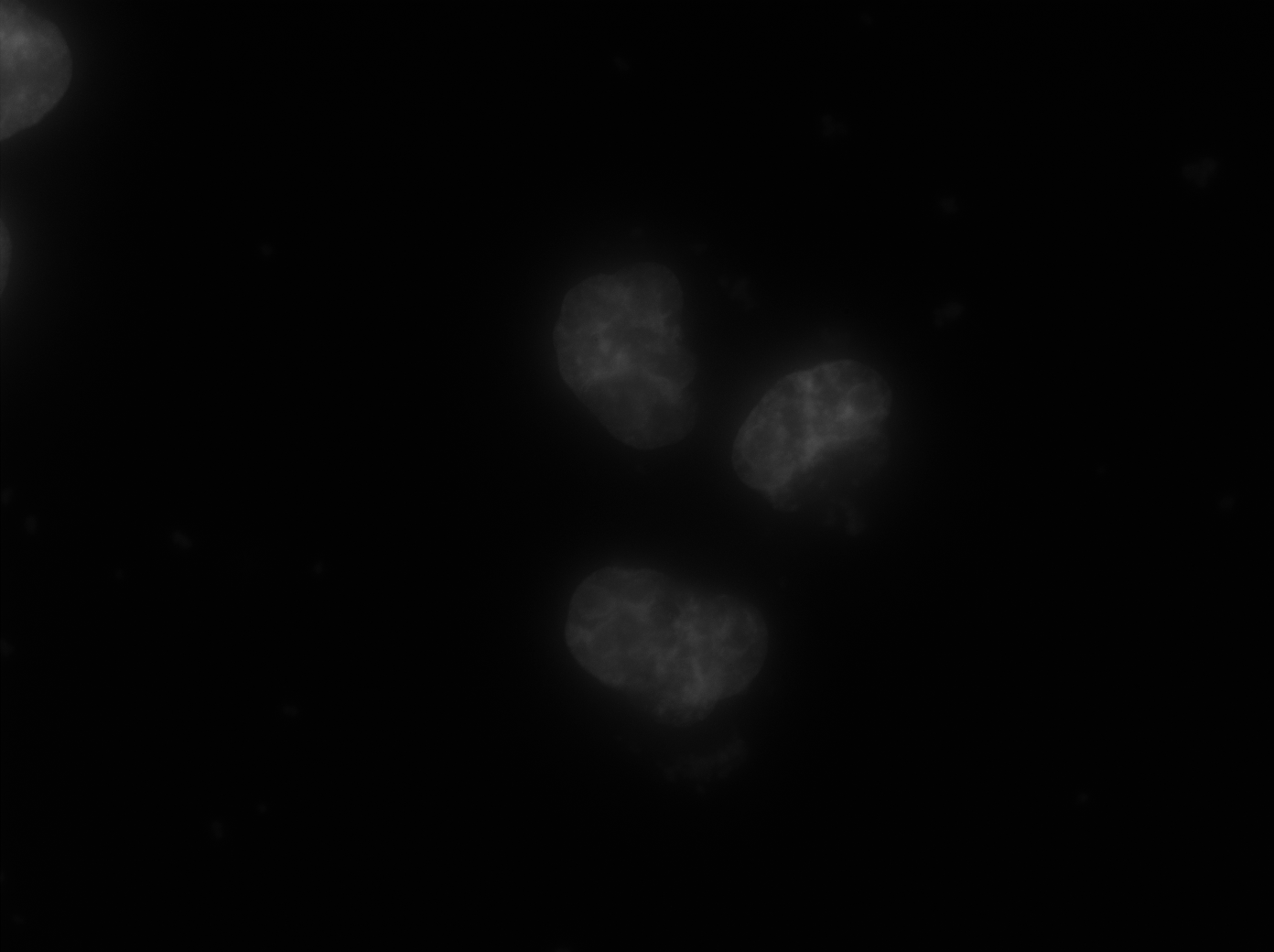

Supplement: Supplementary file 14 — Source data Fig. 4 [file 44319_2026_742_MOESM14_ESM.zip › Figure 4/Fig 4ef Cas9 TPGS1-EYFP-3'UTR acetylated tubulin/Cas9 TPGS1-3utr R1 1-28-24 LT4.Project Maximum Z_XY1738101264_Z0_T0_C0.tif]

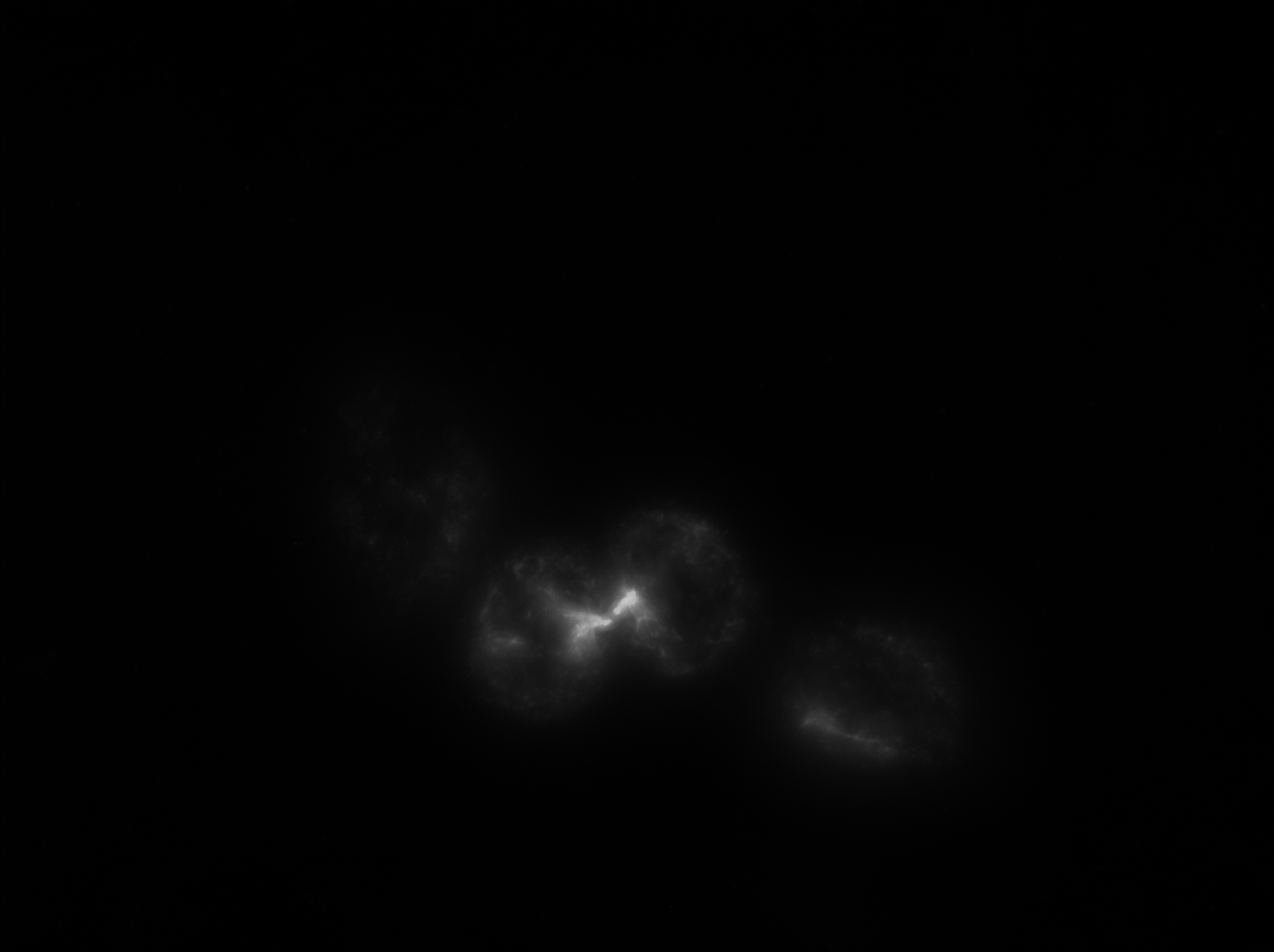

Supplement: Supplementary file 14 — Source data Fig. 4 [file 44319_2026_742_MOESM14_ESM.zip › Figure 4/Fig 4ef Cas9 TPGS1-EYFP-3'UTR acetylated tubulin/Cas9 TPGS1-3utr R2 2-5-25 ET4 EXIMG.Project Maximum Z_XY1738621115_Z0_T0_C1.tif]

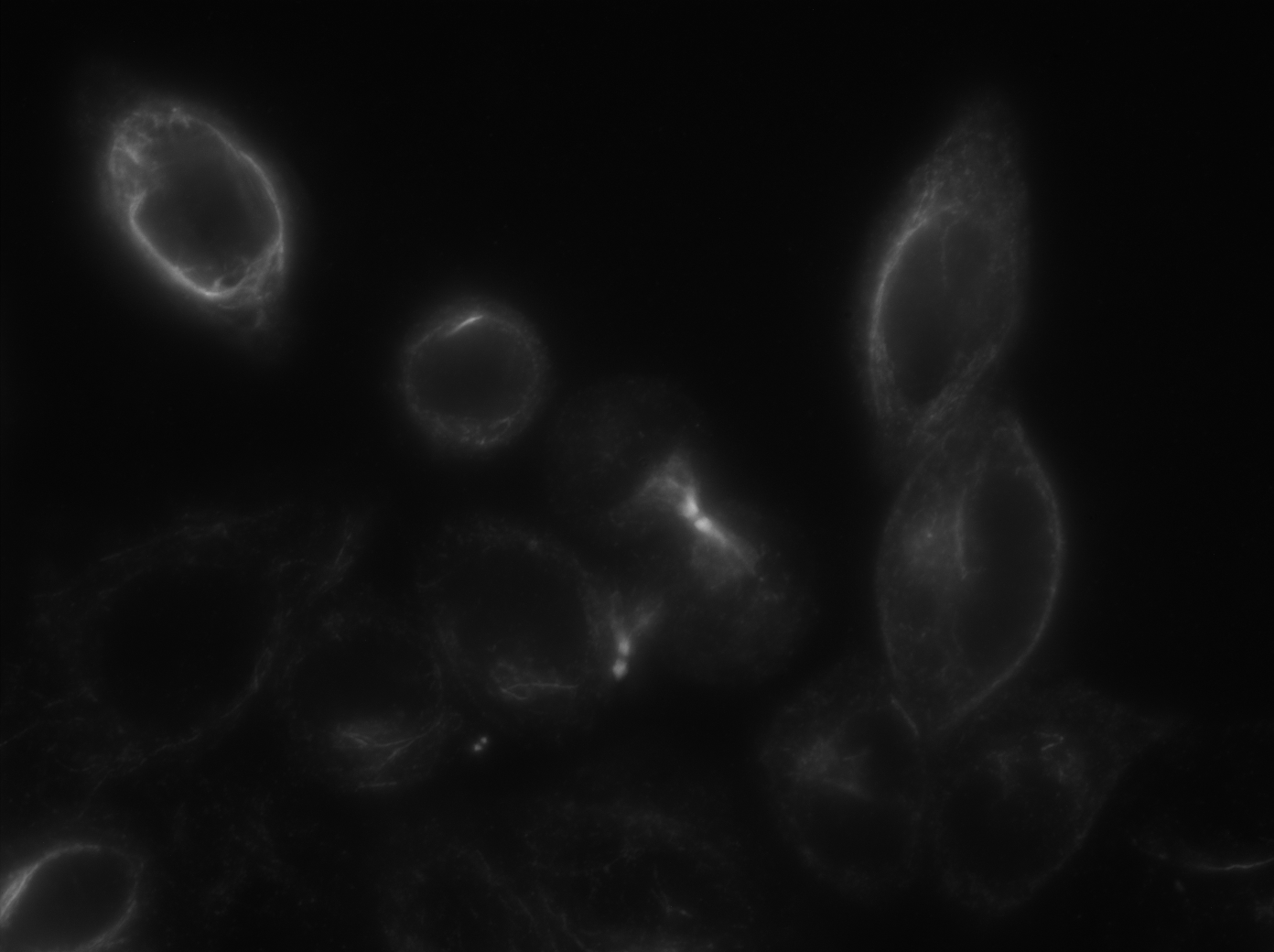

Supplement: Supplementary file 14 — Source data Fig. 4 [file 44319_2026_742_MOESM14_ESM.zip › Figure 4/Fig 4ef Cas9 TPGS1-EYFP-3'UTR acetylated tubulin/Cas9 TPGS1-3utr R3 2-5-25 ET9.Project Maximum Z_XY1738697075_Z0_T0_C1.tif]

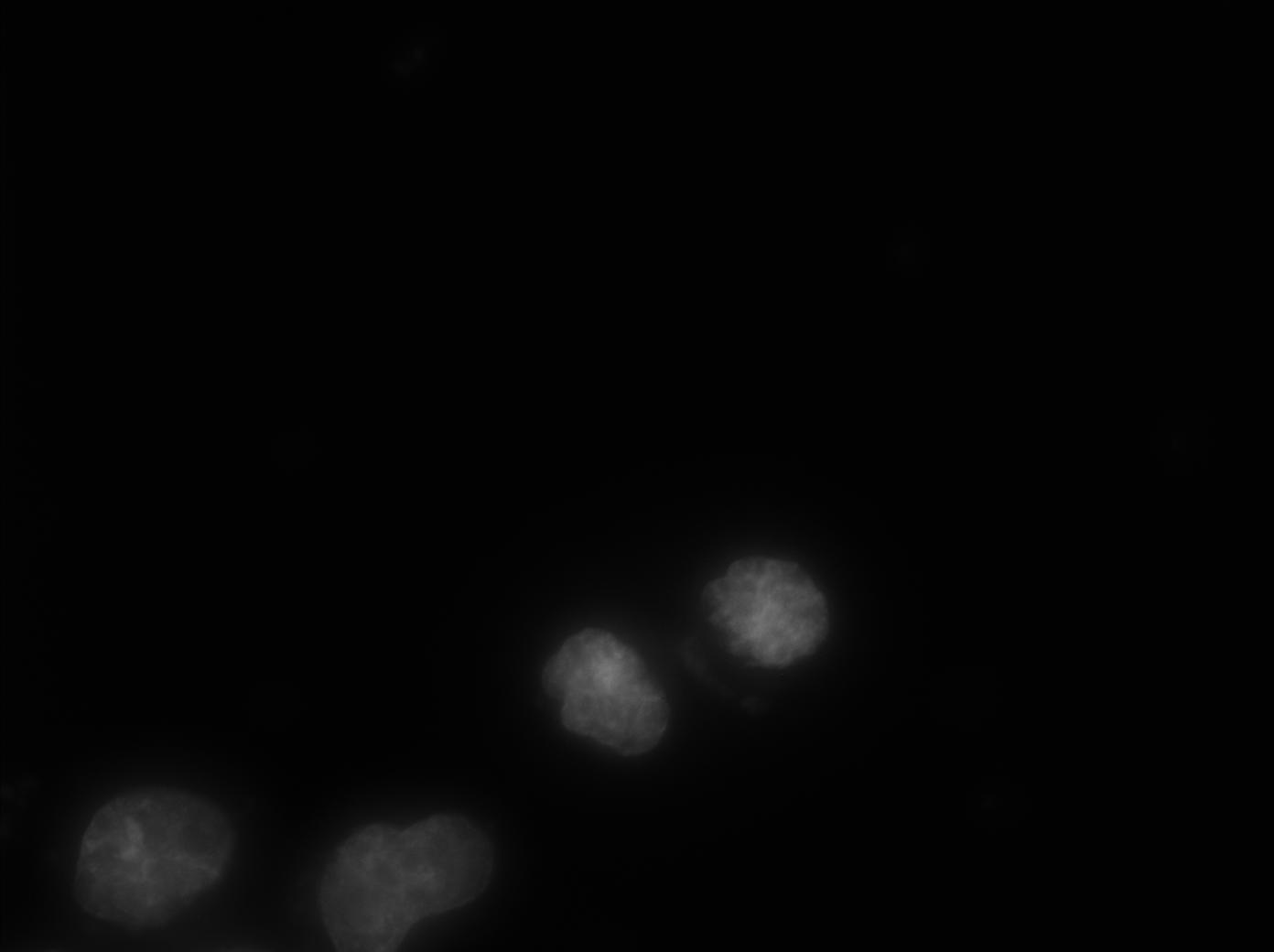

Supplement: Supplementary file 14 — Source data Fig. 4 [file 44319_2026_742_MOESM14_ESM.zip › Figure 4/Fig 4ef Cas9 TPGS1-EYFP-3'UTR acetylated tubulin/Cas9 TPGS1-3utr R1 1-28-24 ET5.Project Maximum Z_XY1738102003_Z0_T0_C0.tif]

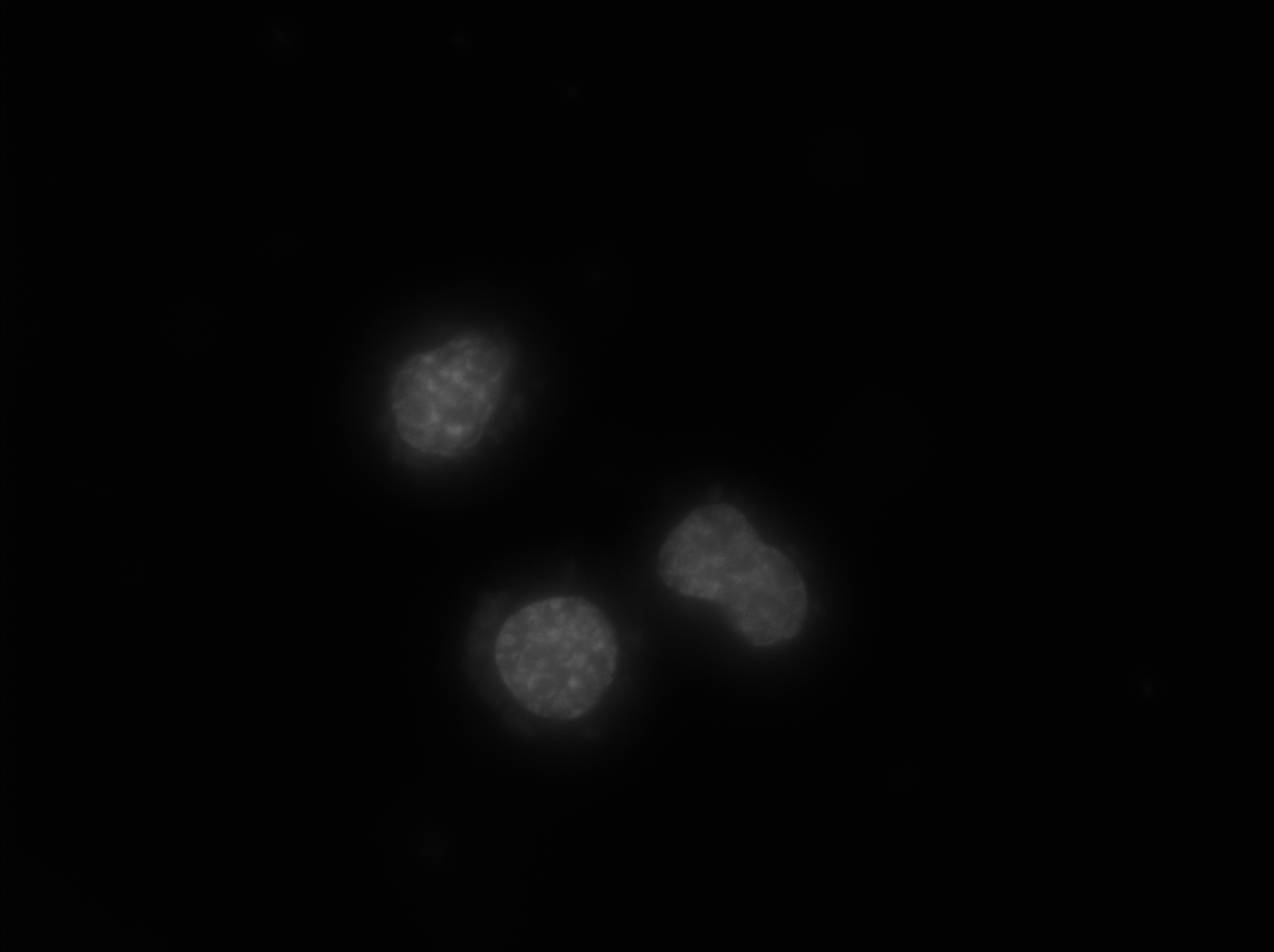

Supplement: Supplementary file 14 — Source data Fig. 4 [file 44319_2026_742_MOESM14_ESM.zip › Figure 4/Fig 4ef Cas9 TPGS1-EYFP-3'UTR acetylated tubulin/Cas9 TPGS1-3utr R1 1-28-24 LT5.Project Maximum Z_XY1738102322_Z0_T0_C0.tif]

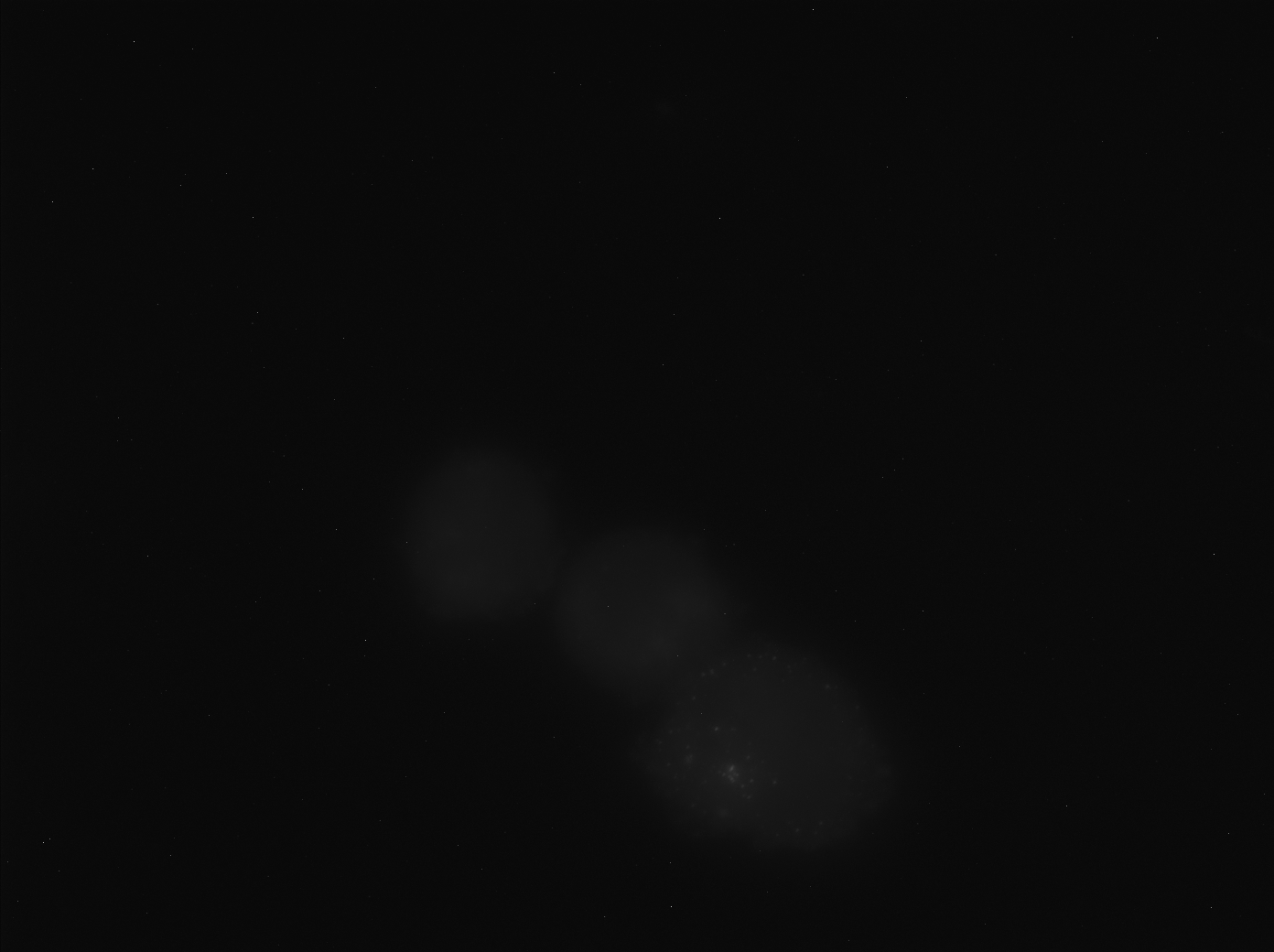

Supplement: Supplementary file 14 — Source data Fig. 4 [file 44319_2026_742_MOESM14_ESM.zip › Figure 4/Fig 4ef Cas9 TPGS1-EYFP-3'UTR acetylated tubulin/Cas9 TPGS1-3utr R1 1-28-24 ET7.Project Maximum Z_XY1738180583_Z0_T0_C2.tif]

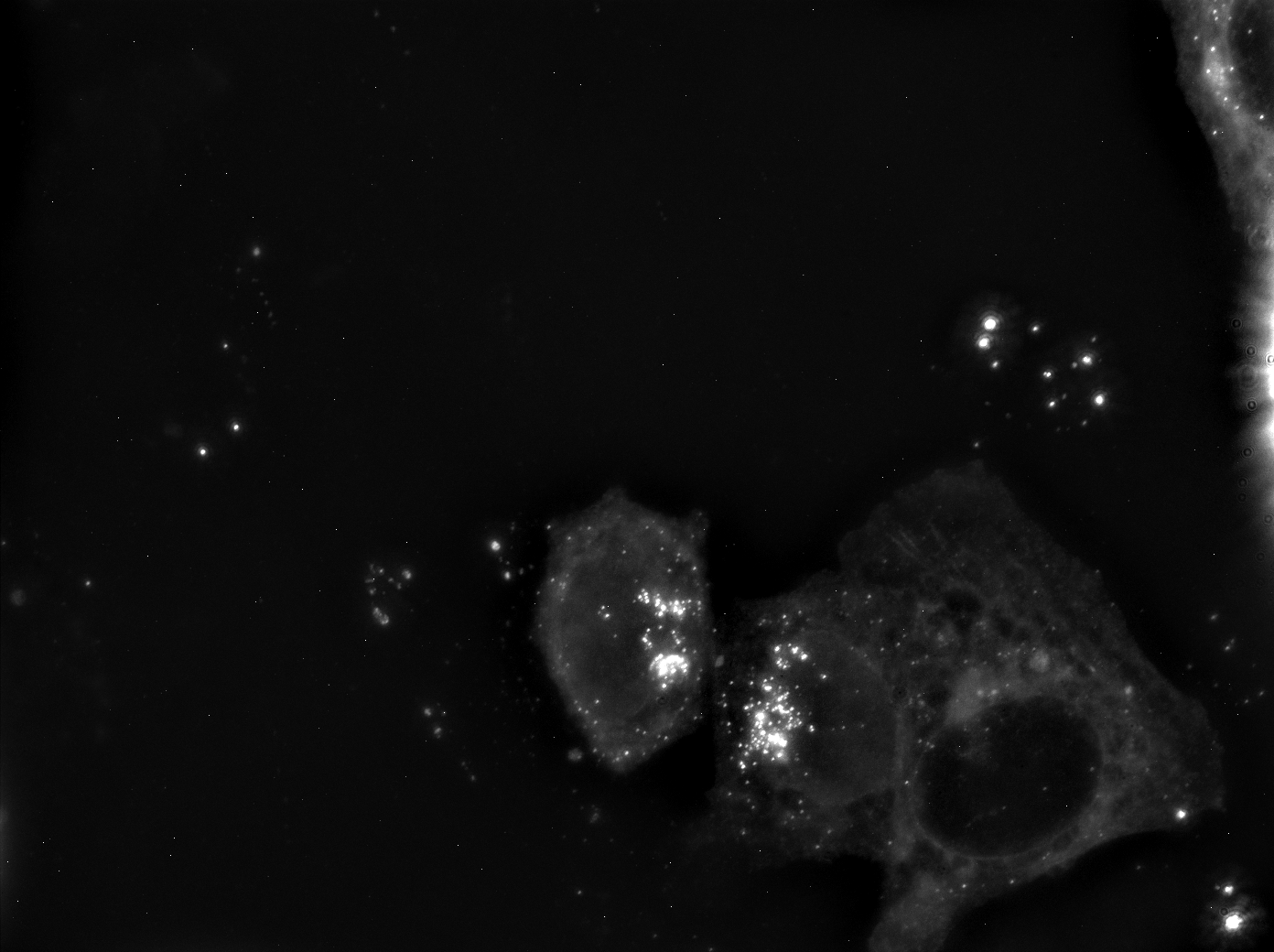

Supplement: Supplementary file 14 — Source data Fig. 4 [file 44319_2026_742_MOESM14_ESM.zip › Figure 4/Fig 4ef Cas9 TPGS1-EYFP-3'UTR acetylated tubulin/Cas9 TPGS1-3utr R3 2-5-25 LT1 exim.NearN.Project Maximum Z_XY1738692427_Z0_T0_C2.tif]

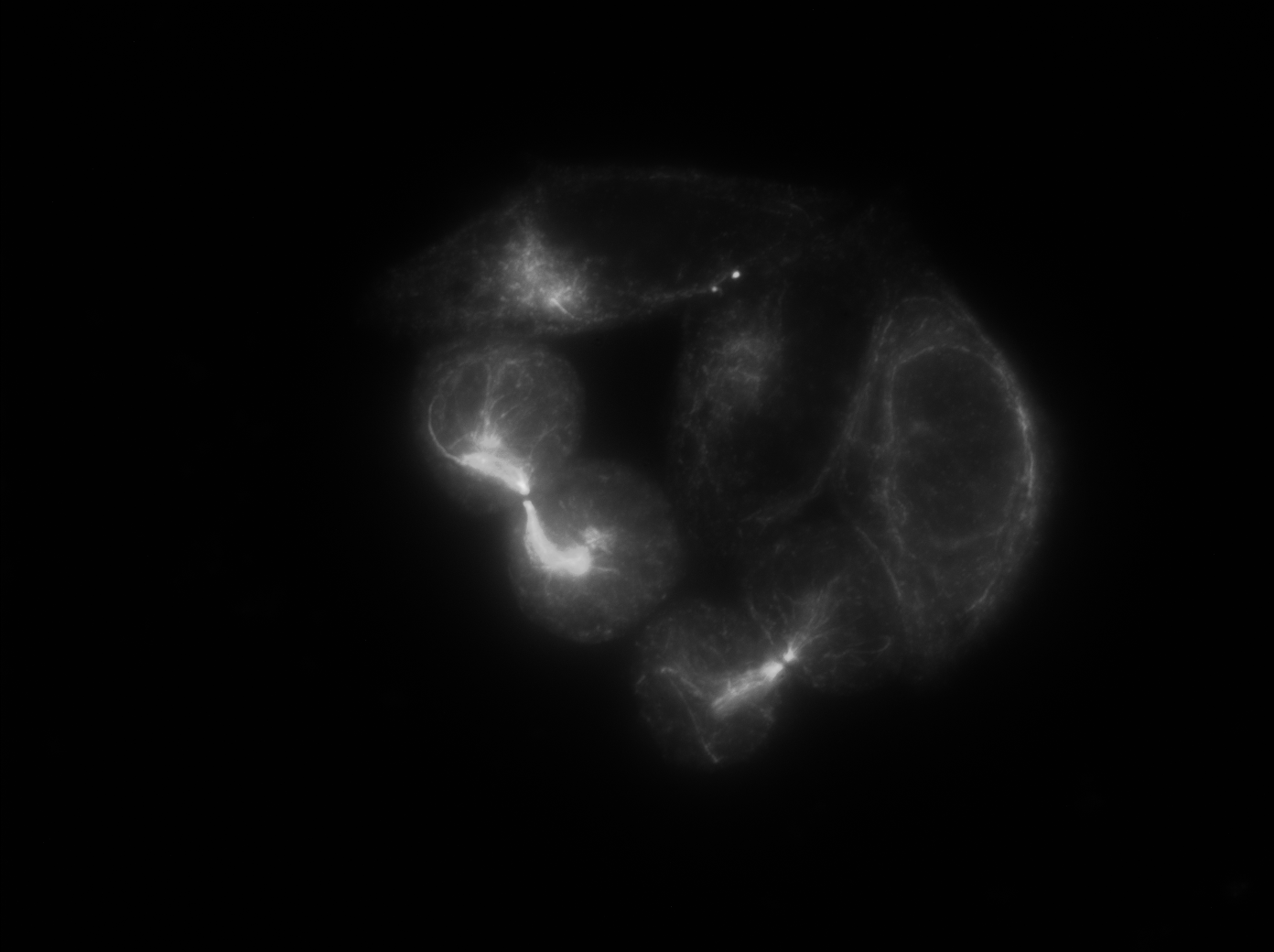

Supplement: Supplementary file 14 — Source data Fig. 4 [file 44319_2026_742_MOESM14_ESM.zip › Figure 4/Fig 4ef Cas9 TPGS1-EYFP-3'UTR acetylated tubulin/Cas9 TPGS1-3utr R1 1-28-24 ET2.Project Maximum Z_XY1738100903_Z0_T0_C1.tif]

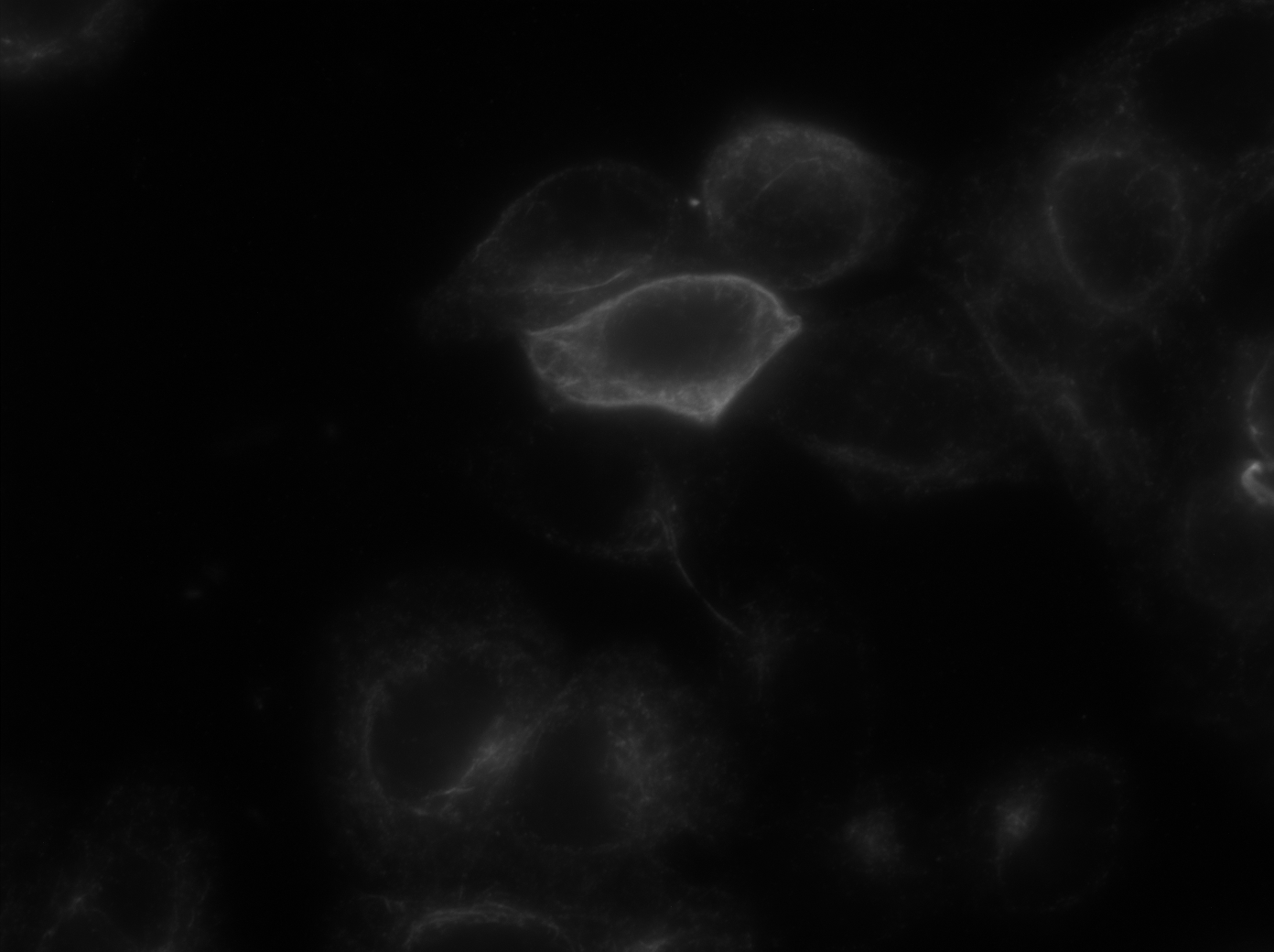

Supplement: Supplementary file 14 — Source data Fig. 4 [file 44319_2026_742_MOESM14_ESM.zip › Figure 4/Fig 4ef Cas9 TPGS1-EYFP-3'UTR acetylated tubulin/Cas9 TPGS1-3utr R3 2-5-25 LT2.Project Maximum Z_XY1738693347_Z0_T0_C1.tif]

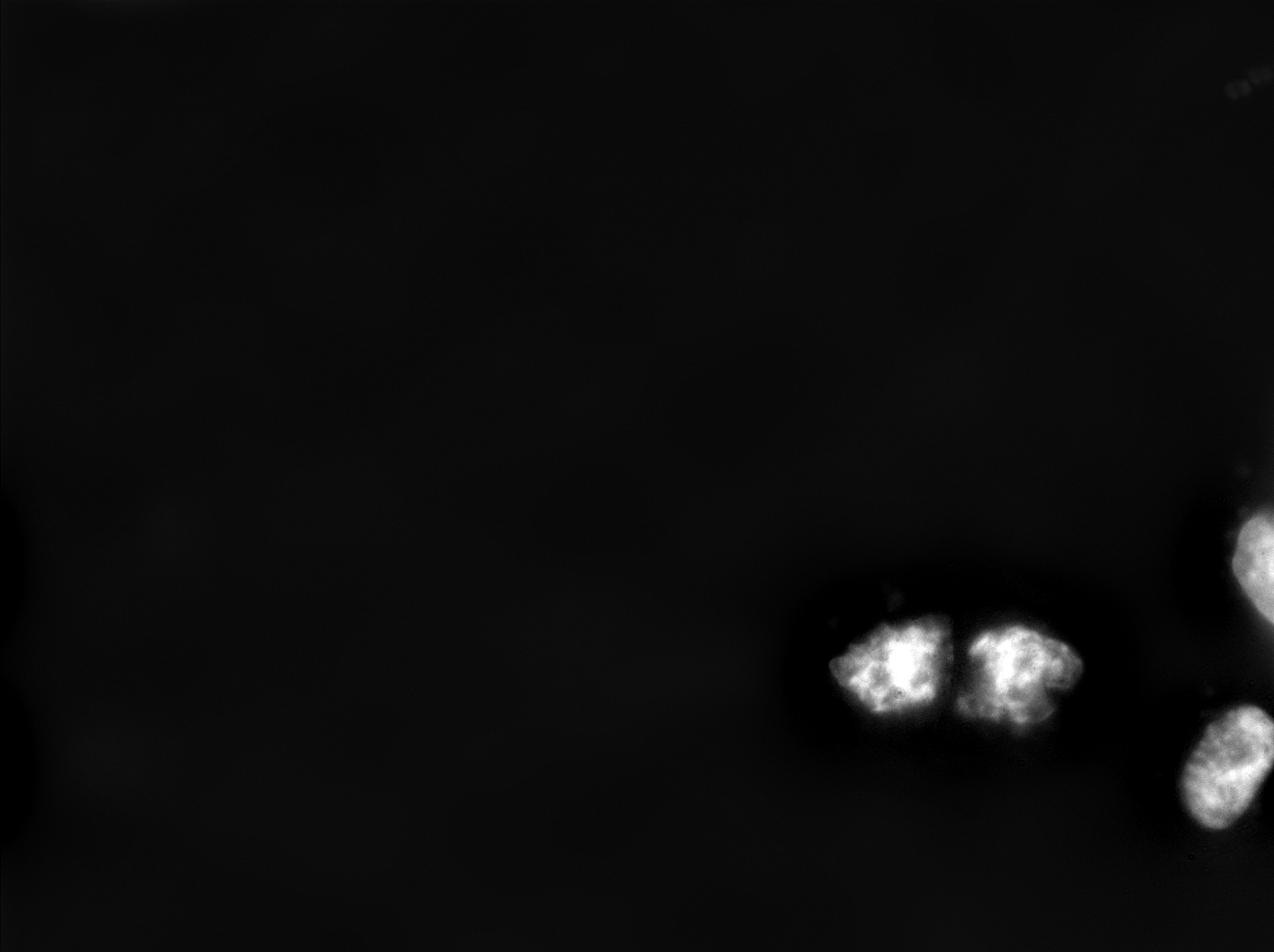

Supplement: Supplementary file 14 — Source data Fig. 4 [file 44319_2026_742_MOESM14_ESM.zip › Figure 4/Fig 4ef Cas9 TPGS1-EYFP-3'UTR acetylated tubulin/Cas9 TPGS1-3utr R2 2-5-25 ET10.Project Maximum Z - 1_XY1738624693_Z0_T0_C0.tif]

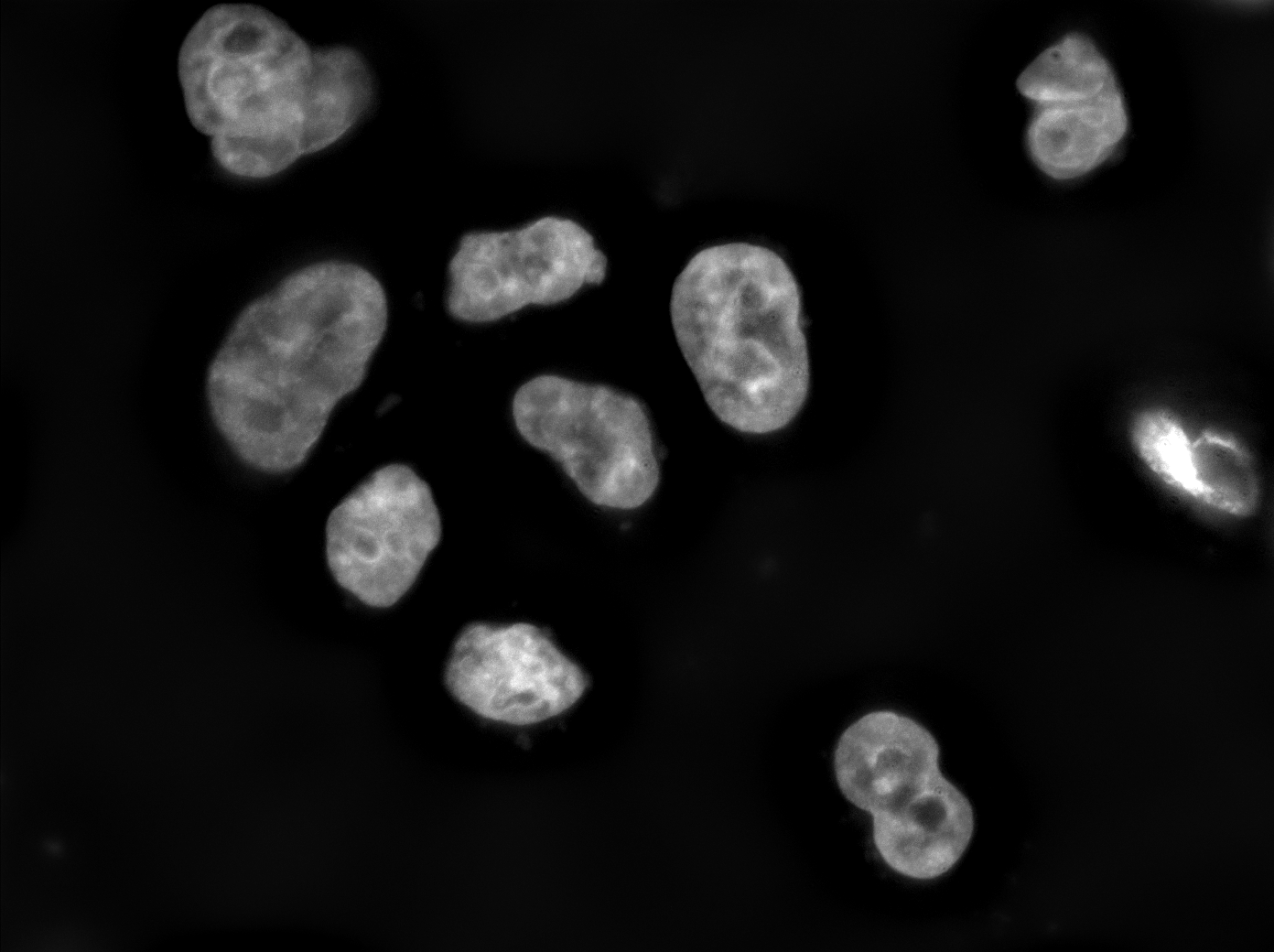

Supplement: Supplementary file 14 — Source data Fig. 4 [file 44319_2026_742_MOESM14_ESM.zip › Figure 4/Fig 4ef Cas9 TPGS1-EYFP-3'UTR acetylated tubulin/Cas9 TPGS1-3utr R1 1-28-24 LT10.Project Maximum Z - 1_XY1738626891_Z0_T0_C0.tif]

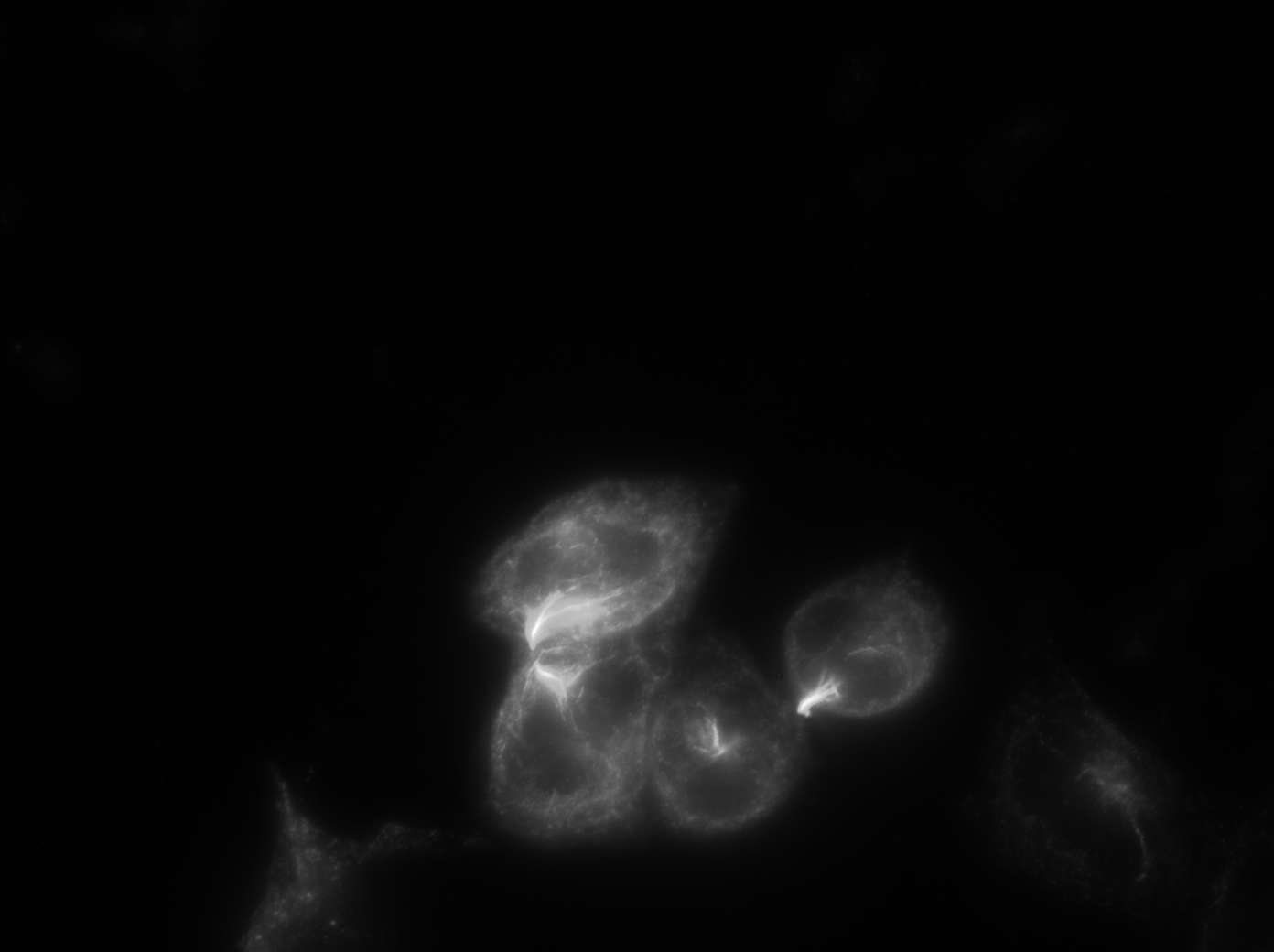

Supplement: Supplementary file 14 — Source data Fig. 4 [file 44319_2026_742_MOESM14_ESM.zip › Figure 4/Fig 4ef Cas9 TPGS1-EYFP-3'UTR acetylated tubulin/Cas9 TPGS1-3utr R2 2-5-25 L10.Project Maximum Z_XY1738626318_Z0_T0_C1.tif]

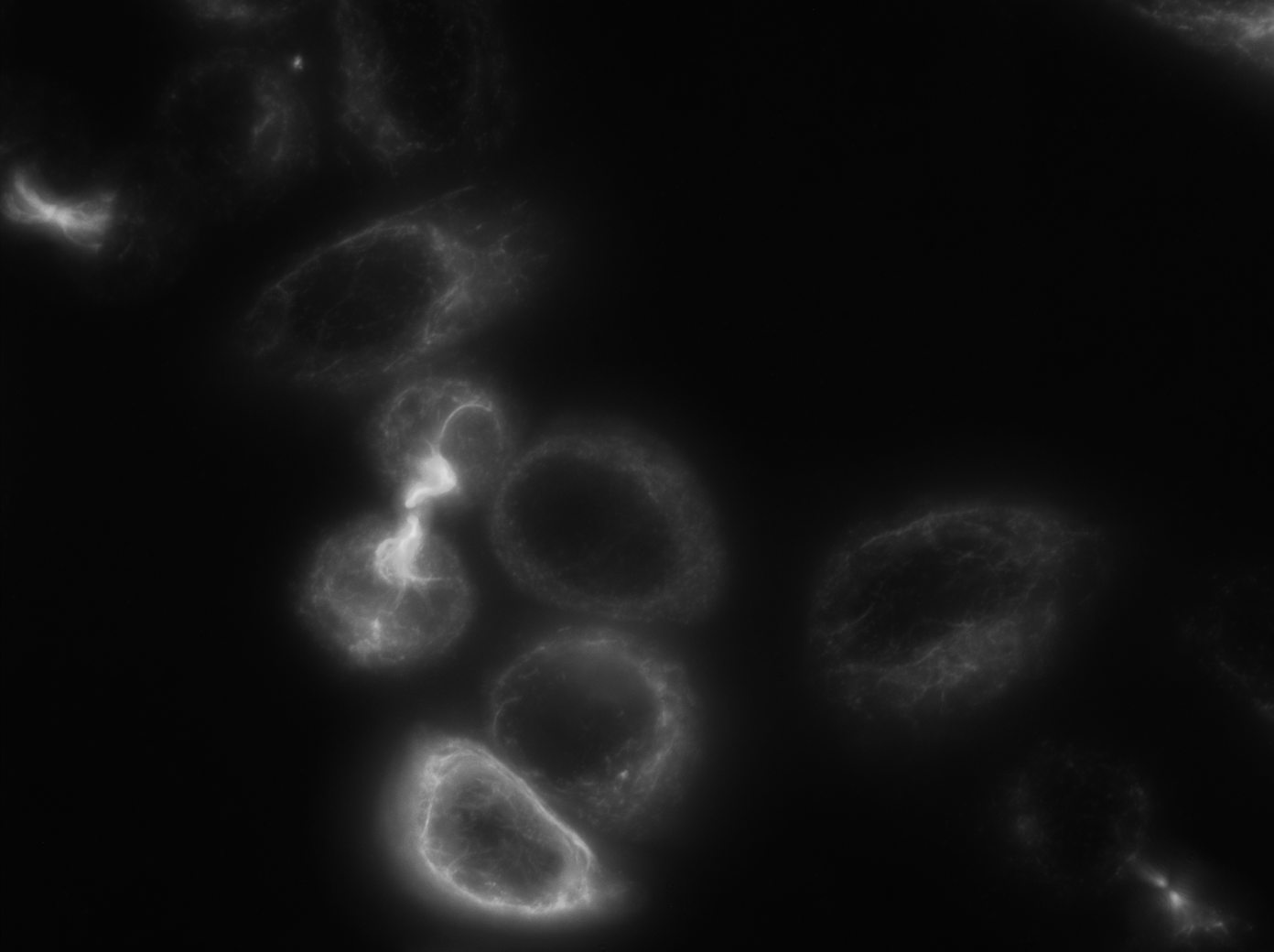

Supplement: Supplementary file 14 — Source data Fig. 4 [file 44319_2026_742_MOESM14_ESM.zip › Figure 4/Fig 4ef Cas9 TPGS1-EYFP-3'UTR acetylated tubulin/Cas9 TPGS1-3utr R2 2-5-25 ET3.Project Maximum Z_XY1738620849_Z0_T0_C1.tif]

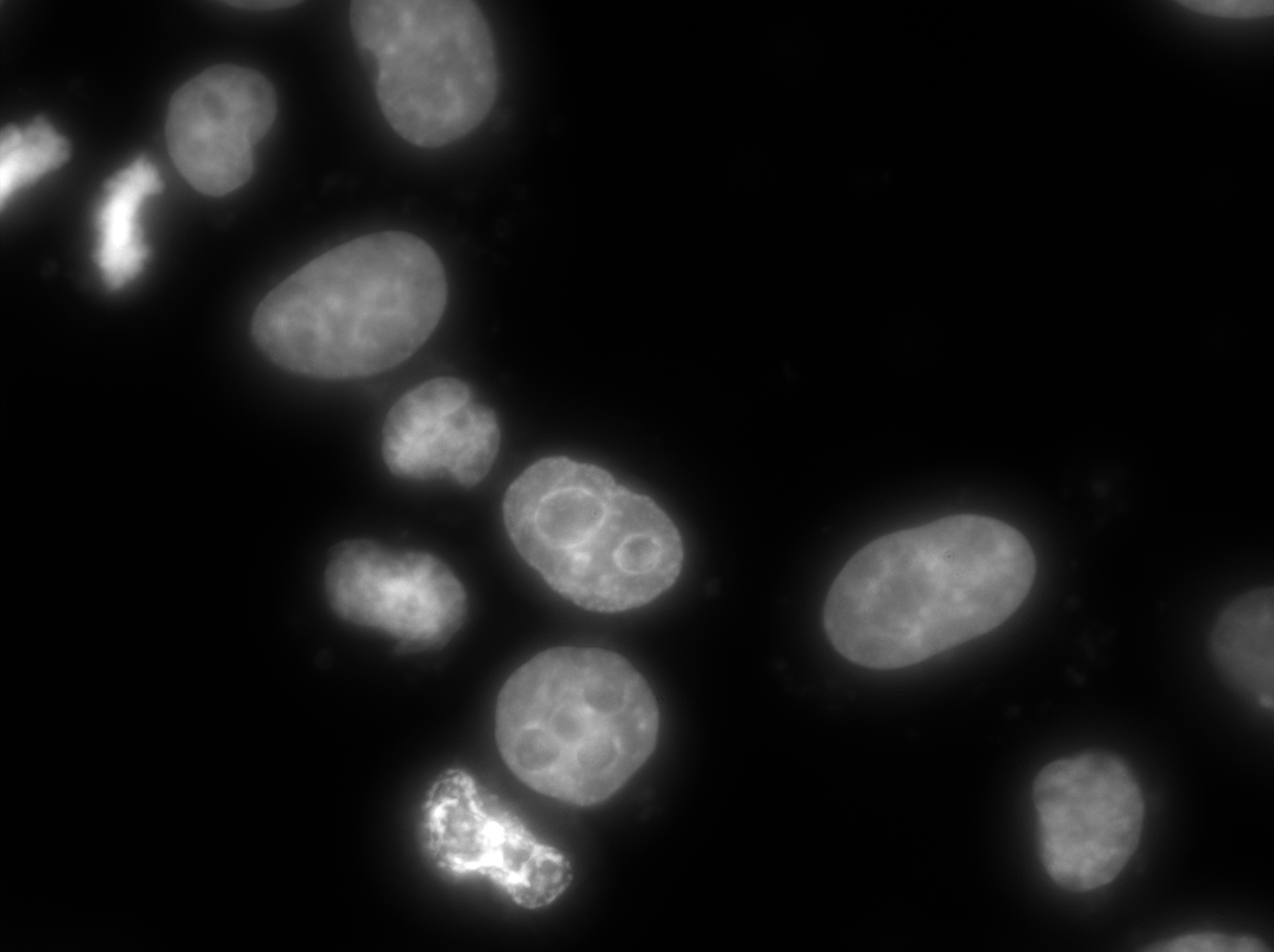

Supplement: Supplementary file 14 — Source data Fig. 4 [file 44319_2026_742_MOESM14_ESM.zip › Figure 4/Fig 4ef Cas9 TPGS1-EYFP-3'UTR acetylated tubulin/Cas9 TPGS1-3utr R2 2-5-25 ET3.Project Maximum Z_XY1738620849_Z0_T0_C0.tif]

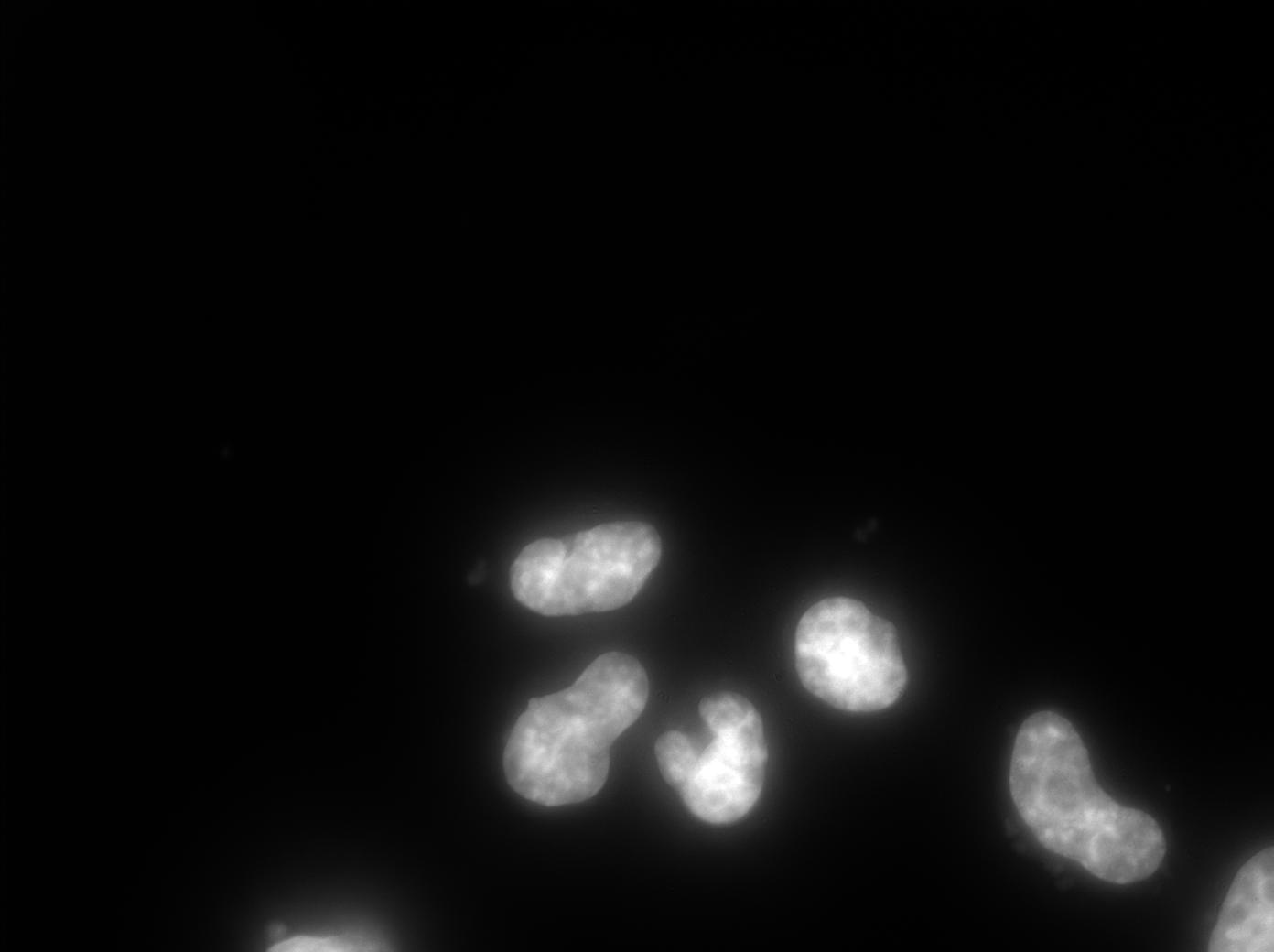

Supplement: Supplementary file 14 — Source data Fig. 4 [file 44319_2026_742_MOESM14_ESM.zip › Figure 4/Fig 4ef Cas9 TPGS1-EYFP-3'UTR acetylated tubulin/Cas9 TPGS1-3utr R2 2-5-25 L10.Project Maximum Z_XY1738626318_Z0_T0_C0.tif]

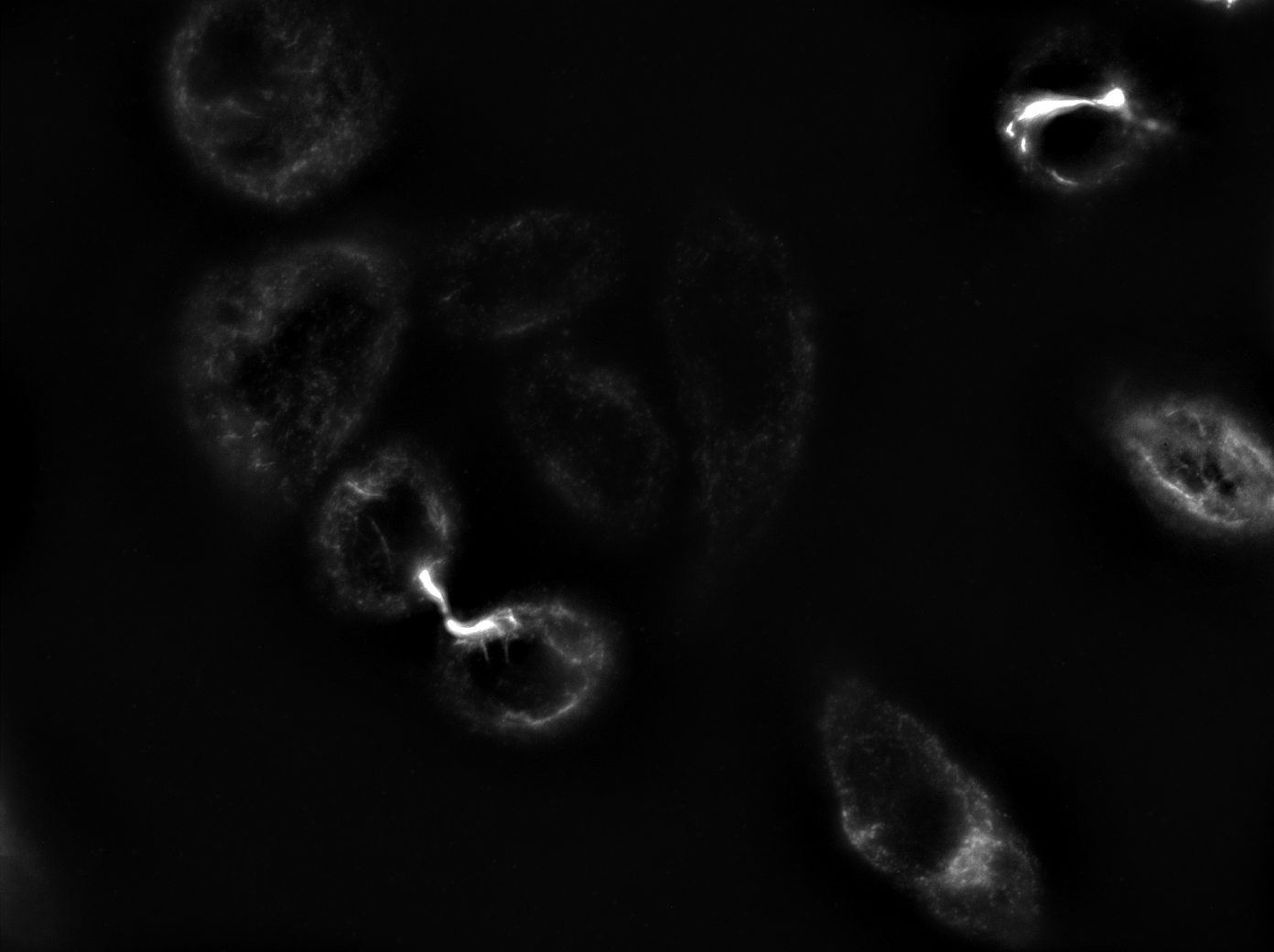

Supplement: Supplementary file 14 — Source data Fig. 4 [file 44319_2026_742_MOESM14_ESM.zip › Figure 4/Fig 4ef Cas9 TPGS1-EYFP-3'UTR acetylated tubulin/Cas9 TPGS1-3utr R1 1-28-24 LT10.Project Maximum Z - 1_XY1738626891_Z0_T0_C1.tif]

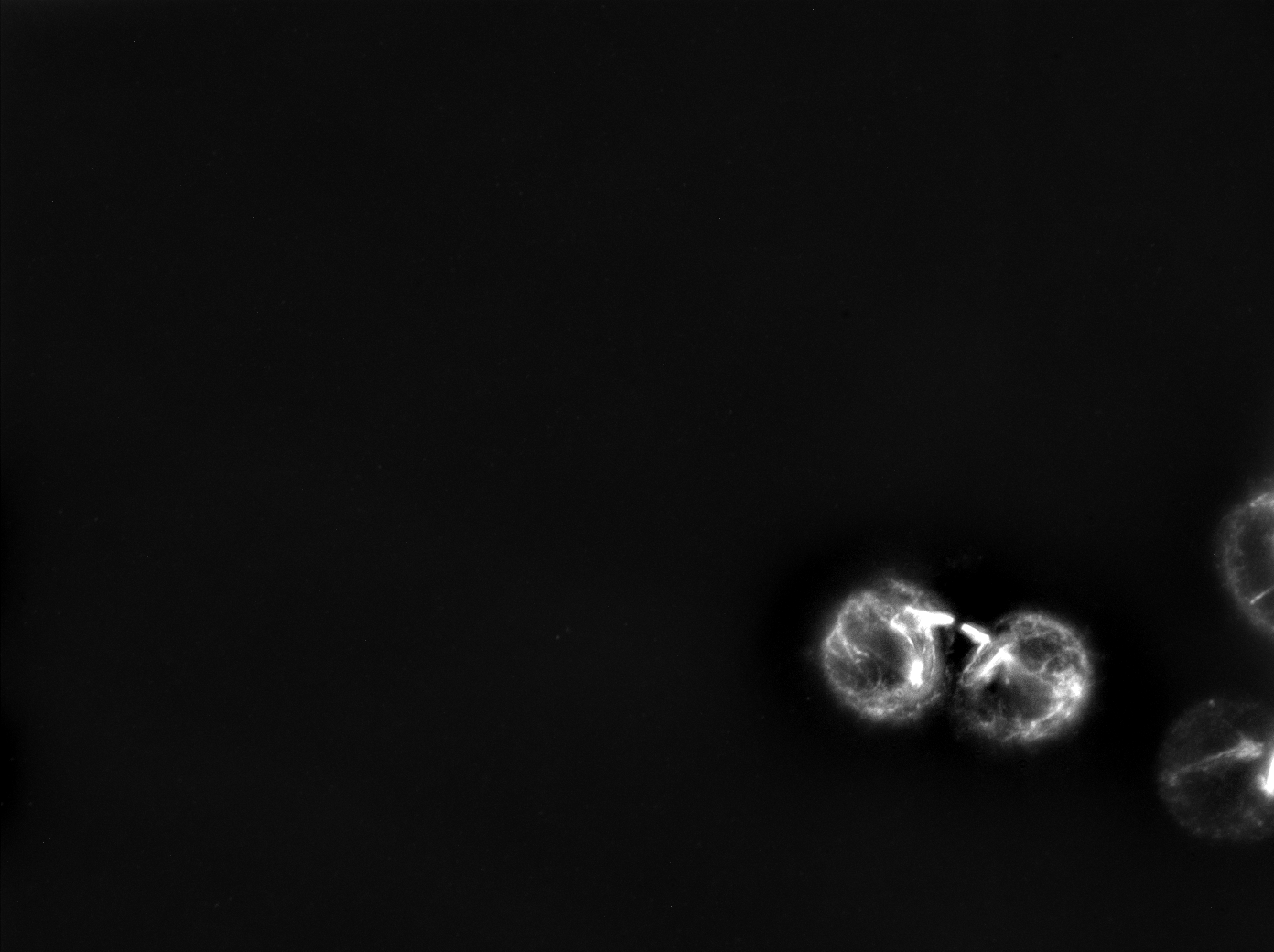

Supplement: Supplementary file 14 — Source data Fig. 4 [file 44319_2026_742_MOESM14_ESM.zip › Figure 4/Fig 4ef Cas9 TPGS1-EYFP-3'UTR acetylated tubulin/Cas9 TPGS1-3utr R2 2-5-25 ET10.Project Maximum Z - 1_XY1738624693_Z0_T0_C1.tif]

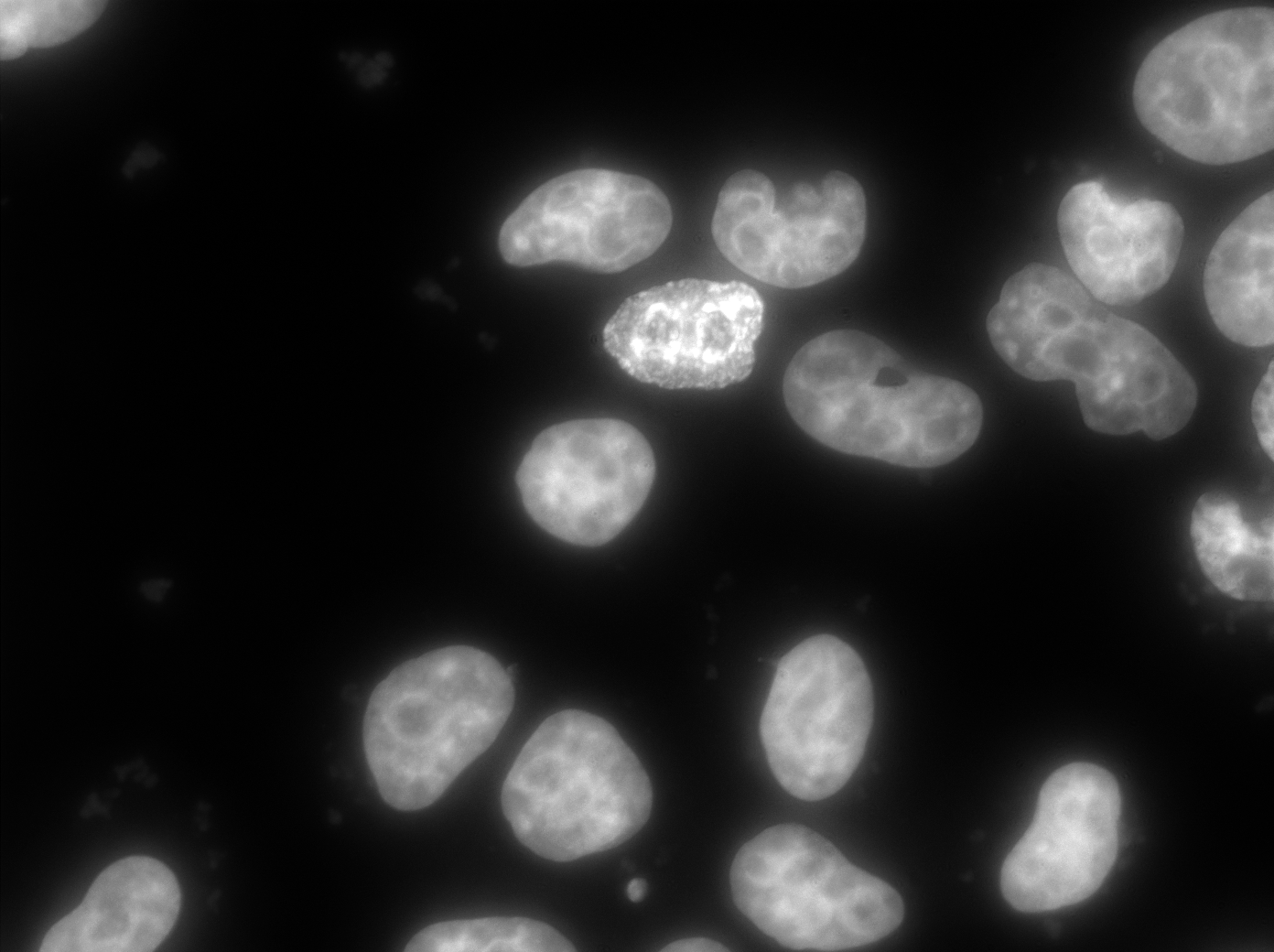

Supplement: Supplementary file 14 — Source data Fig. 4 [file 44319_2026_742_MOESM14_ESM.zip › Figure 4/Fig 4ef Cas9 TPGS1-EYFP-3'UTR acetylated tubulin/Cas9 TPGS1-3utr R3 2-5-25 LT2.Project Maximum Z_XY1738693347_Z0_T0_C0.tif]

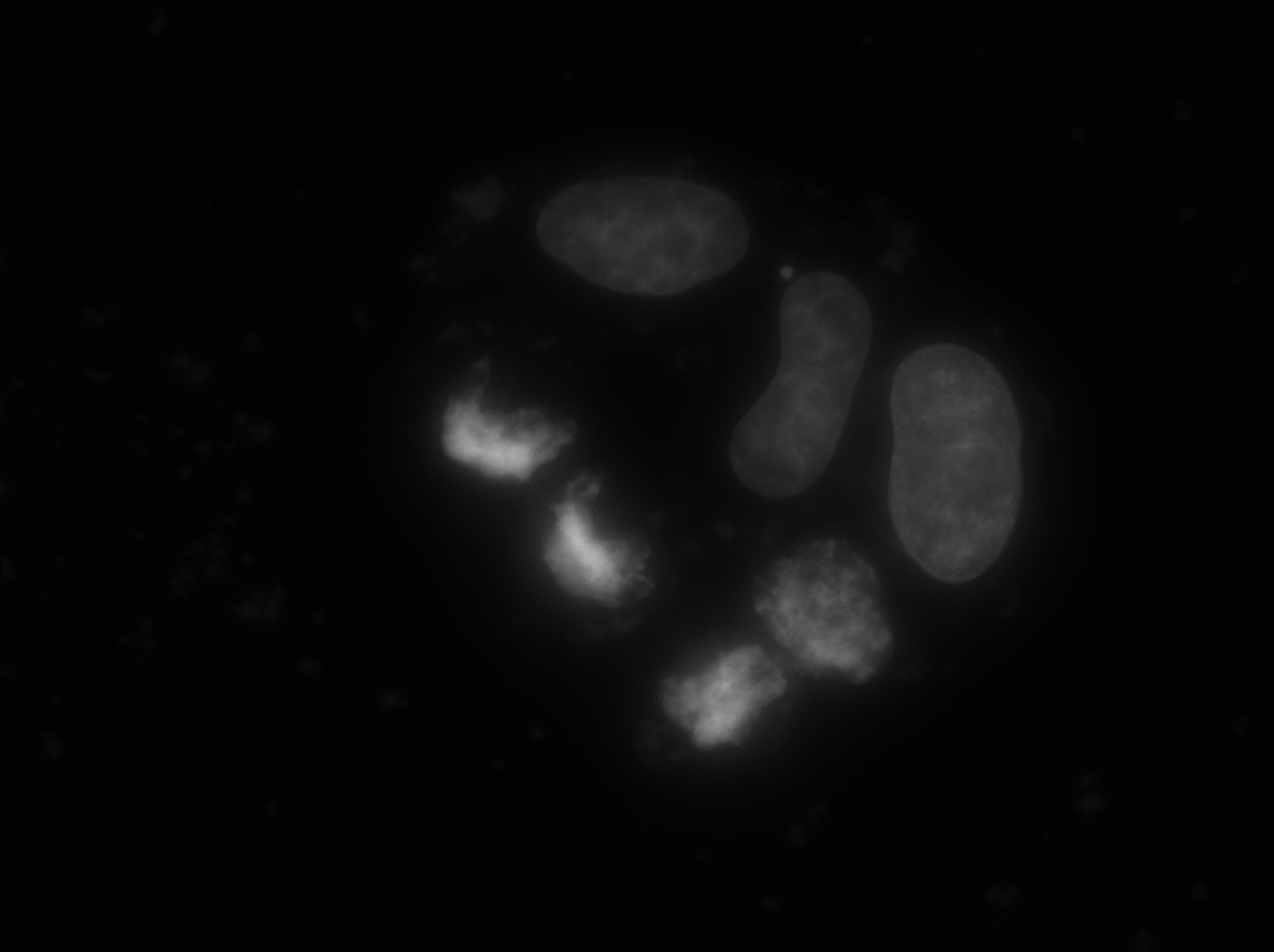

Supplement: Supplementary file 14 — Source data Fig. 4 [file 44319_2026_742_MOESM14_ESM.zip › Figure 4/Fig 4ef Cas9 TPGS1-EYFP-3'UTR acetylated tubulin/Cas9 TPGS1-3utr R1 1-28-24 ET2.Project Maximum Z_XY1738100903_Z0_T0_C0.tif]

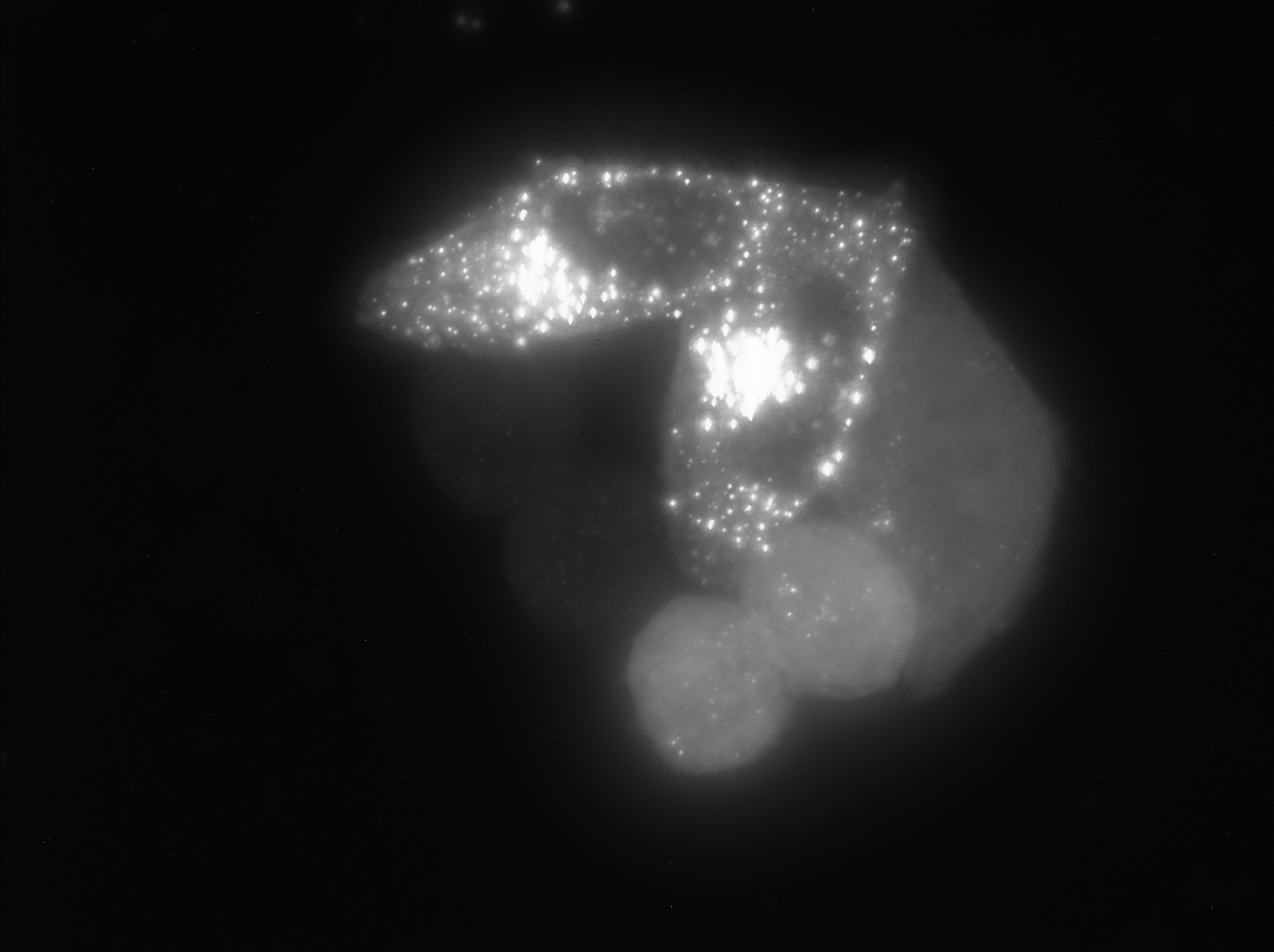

Supplement: Supplementary file 14 — Source data Fig. 4 [file 44319_2026_742_MOESM14_ESM.zip › Figure 4/Fig 4ef Cas9 TPGS1-EYFP-3'UTR acetylated tubulin/Cas9 TPGS1-3utr R1 1-28-24 ET2.Project Maximum Z_XY1738100903_Z0_T0_C2.tif]

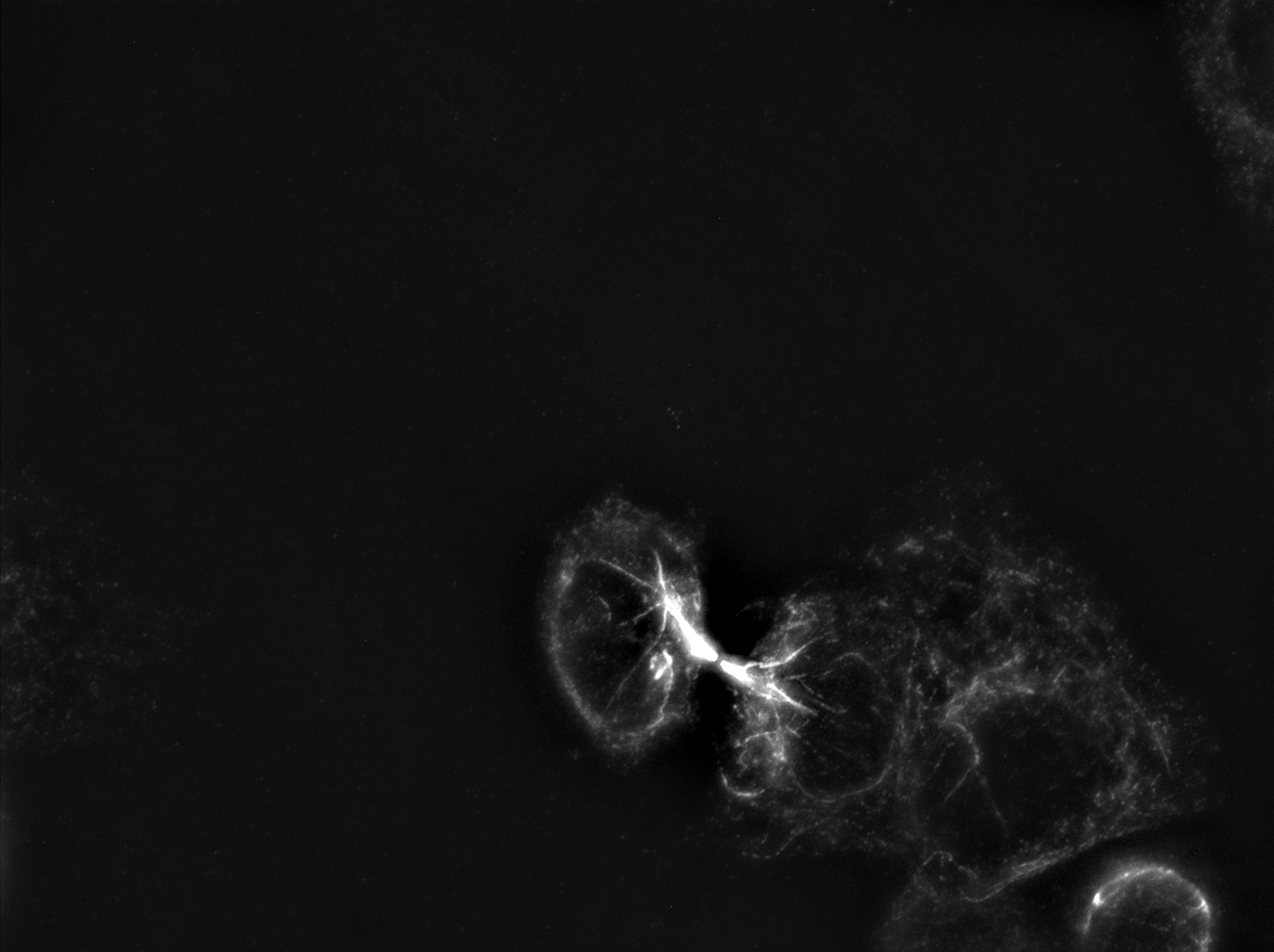

Supplement: Supplementary file 14 — Source data Fig. 4 [file 44319_2026_742_MOESM14_ESM.zip › Figure 4/Fig 4ef Cas9 TPGS1-EYFP-3'UTR acetylated tubulin/Cas9 TPGS1-3utr R3 2-5-25 LT1 exim.NearN.Project Maximum Z_XY1738692427_Z0_T0_C1.tif]

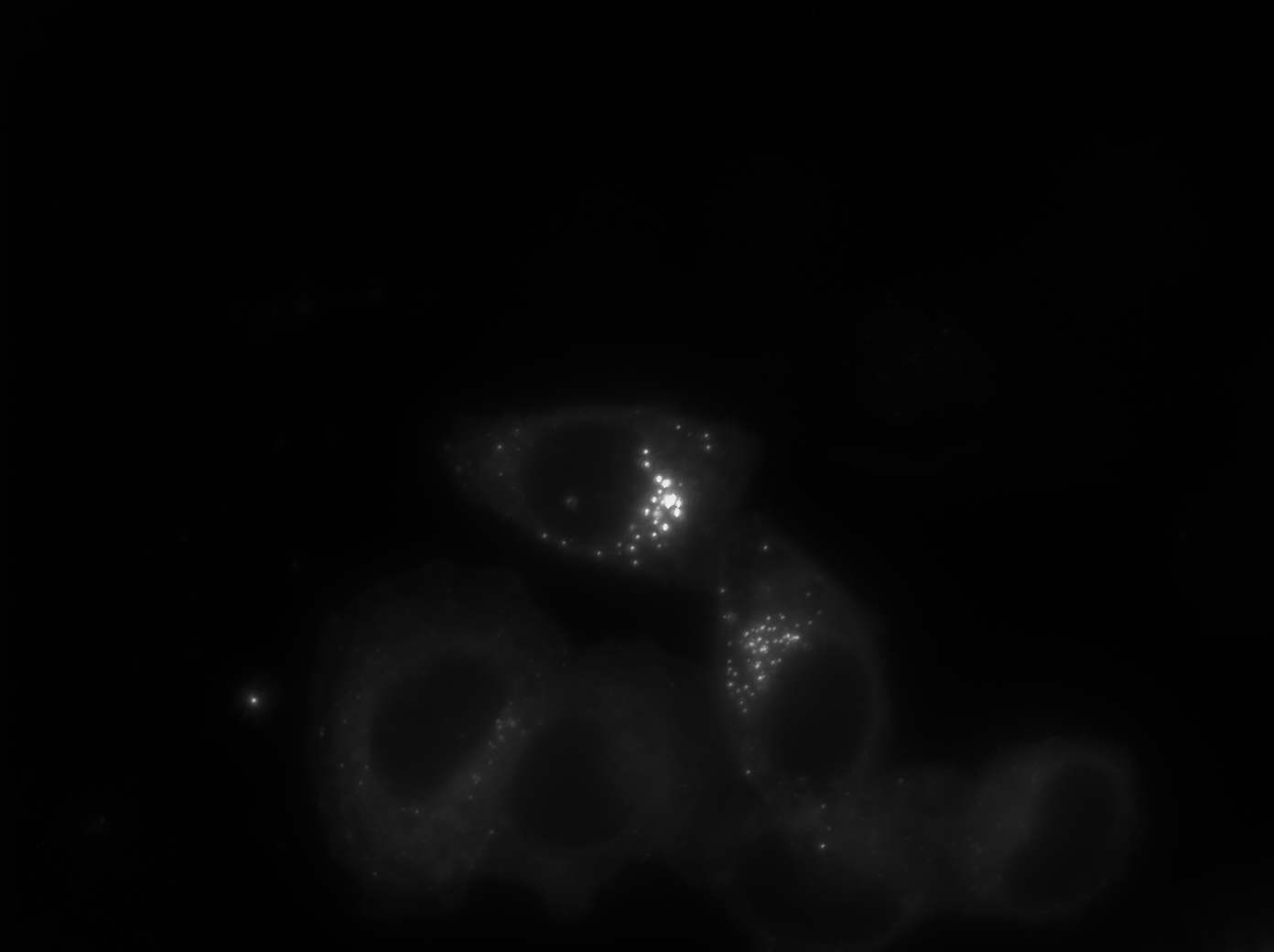

Supplement: Supplementary file 14 — Source data Fig. 4 [file 44319_2026_742_MOESM14_ESM.zip › Figure 4/Fig 4ef Cas9 TPGS1-EYFP-3'UTR acetylated tubulin/Cas9 TPGS1-3utr R3 2-5-25 LT2.Project Maximum Z_XY1738693347_Z0_T0_C2.tif]

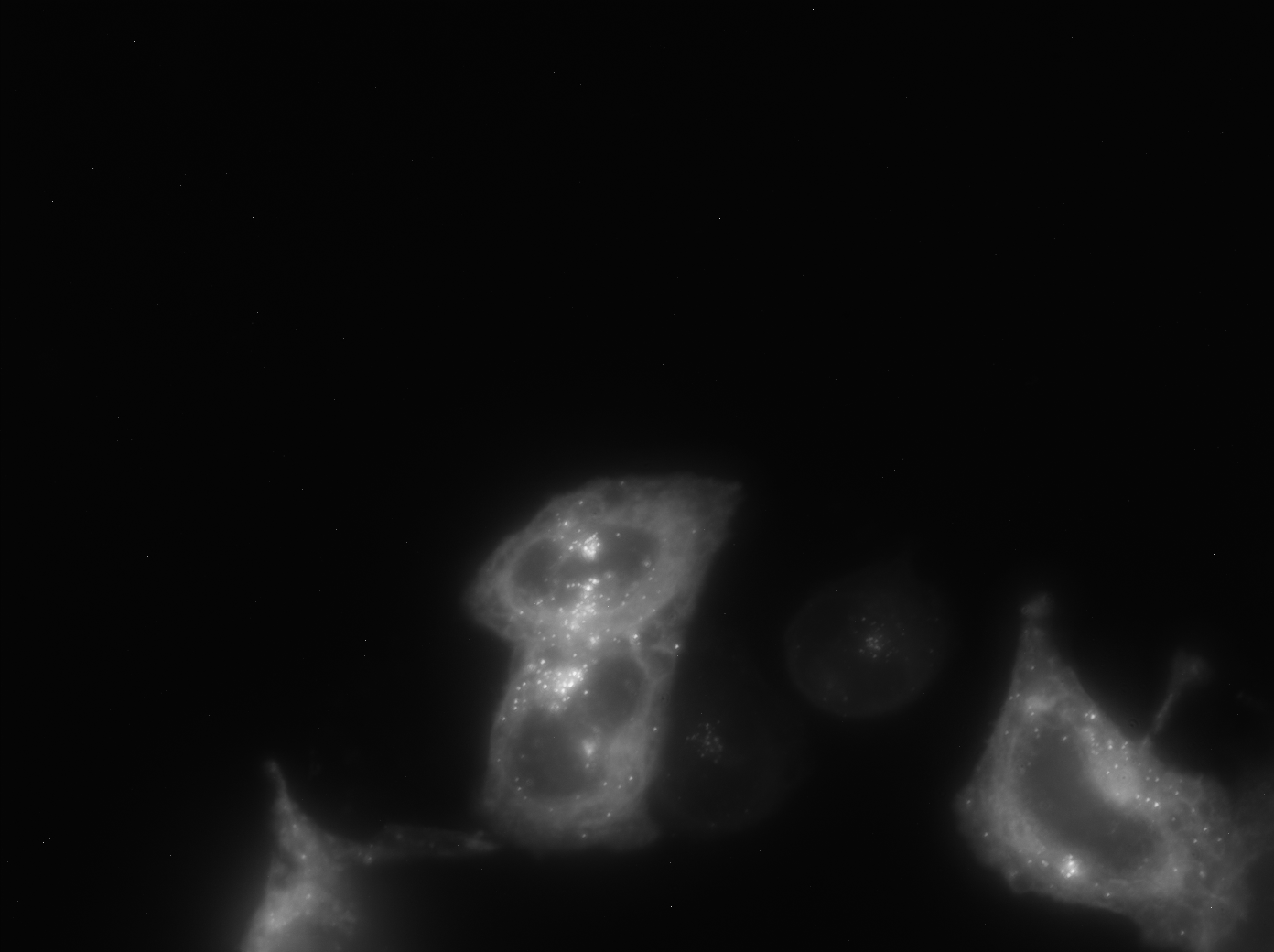

Supplement: Supplementary file 14 — Source data Fig. 4 [file 44319_2026_742_MOESM14_ESM.zip › Figure 4/Fig 4ef Cas9 TPGS1-EYFP-3'UTR acetylated tubulin/Cas9 TPGS1-3utr R2 2-5-25 L10.Project Maximum Z_XY1738626318_Z0_T0_C2.tif]

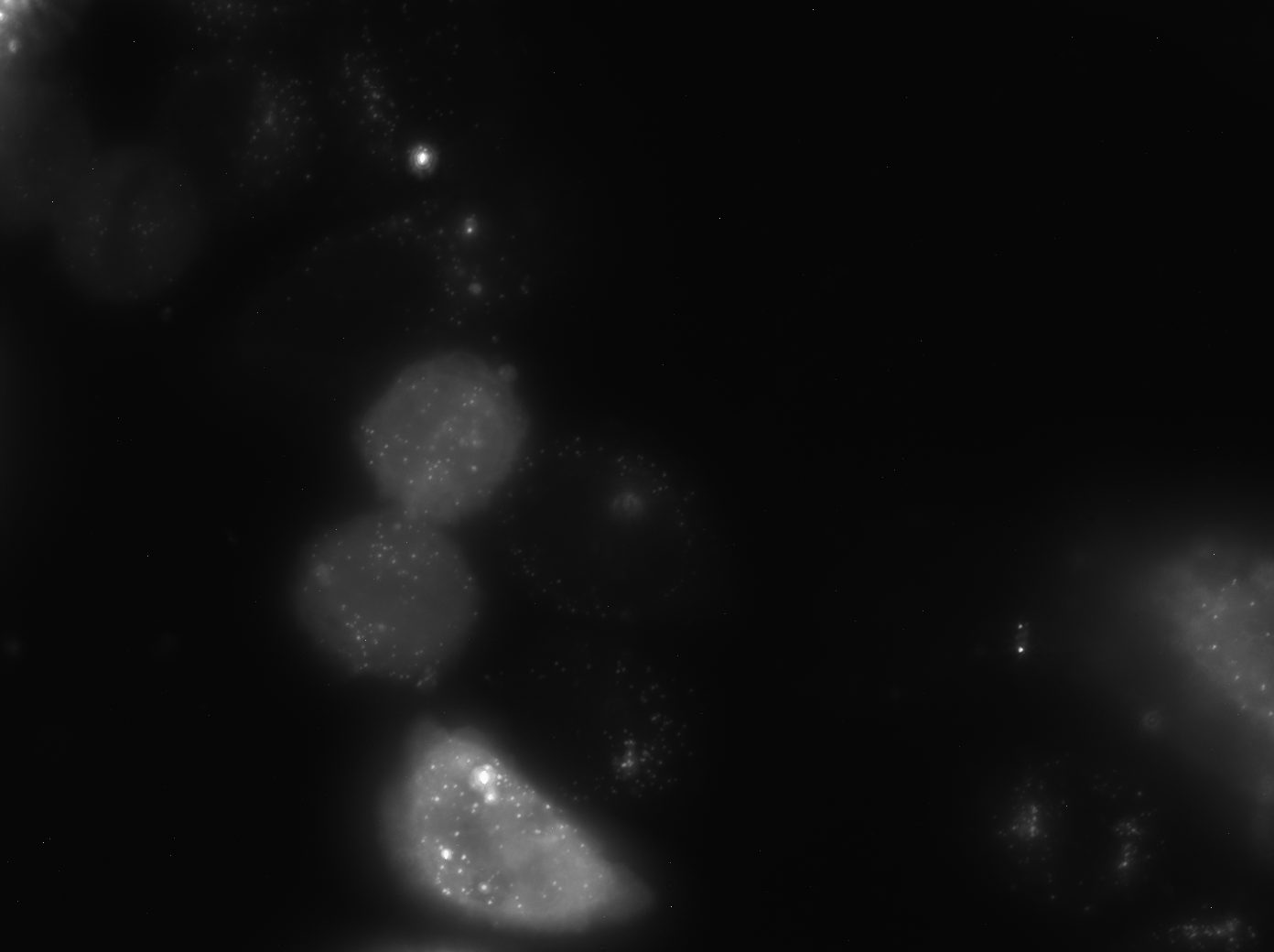

Supplement: Supplementary file 14 — Source data Fig. 4 [file 44319_2026_742_MOESM14_ESM.zip › Figure 4/Fig 4ef Cas9 TPGS1-EYFP-3'UTR acetylated tubulin/Cas9 TPGS1-3utr R2 2-5-25 ET3.Project Maximum Z_XY1738620849_Z0_T0_C2.tif]

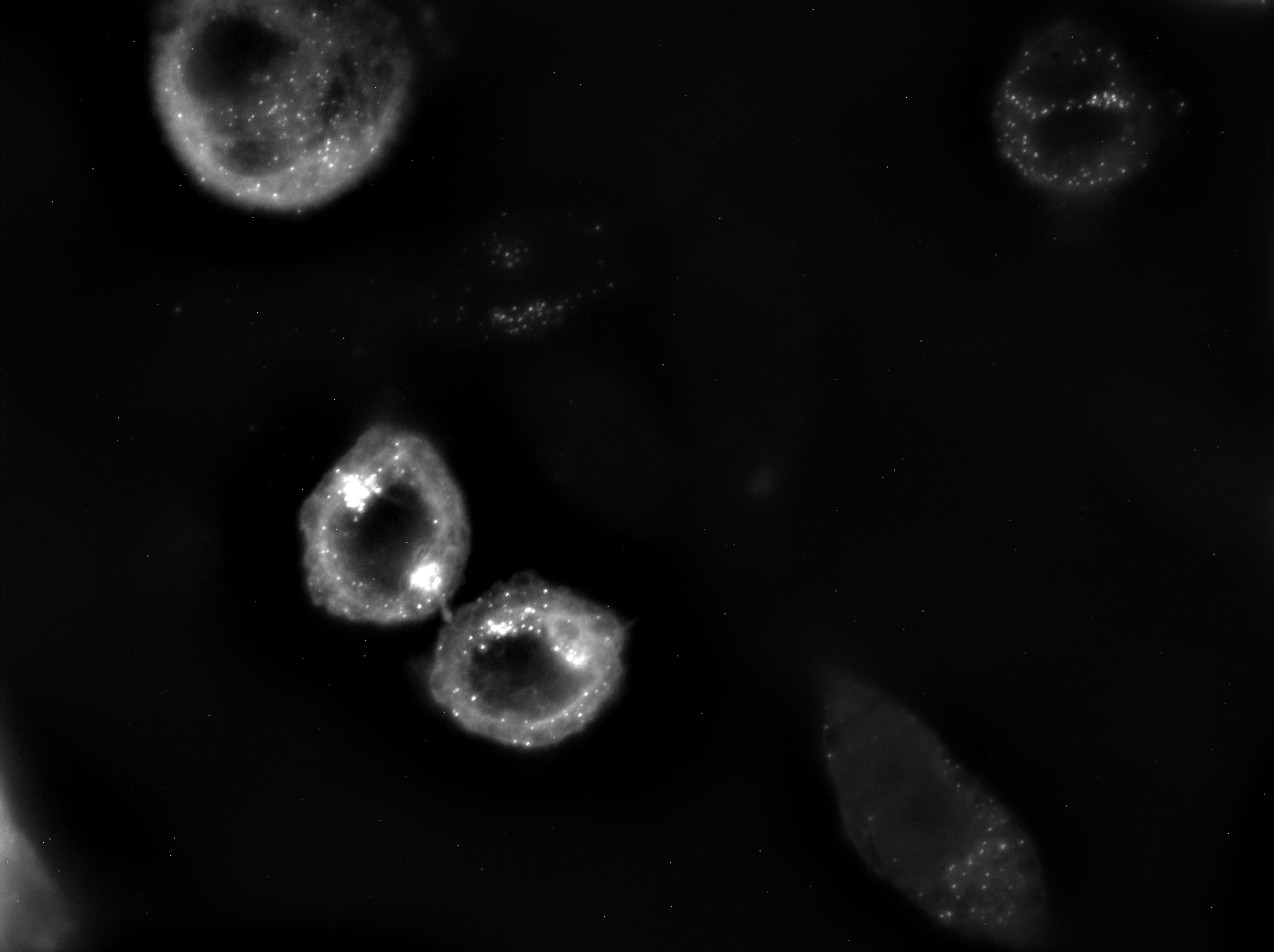

Supplement: Supplementary file 14 — Source data Fig. 4 [file 44319_2026_742_MOESM14_ESM.zip › Figure 4/Fig 4ef Cas9 TPGS1-EYFP-3'UTR acetylated tubulin/Cas9 TPGS1-3utr R1 1-28-24 LT10.Project Maximum Z - 1_XY1738626891_Z0_T0_C2.tif]

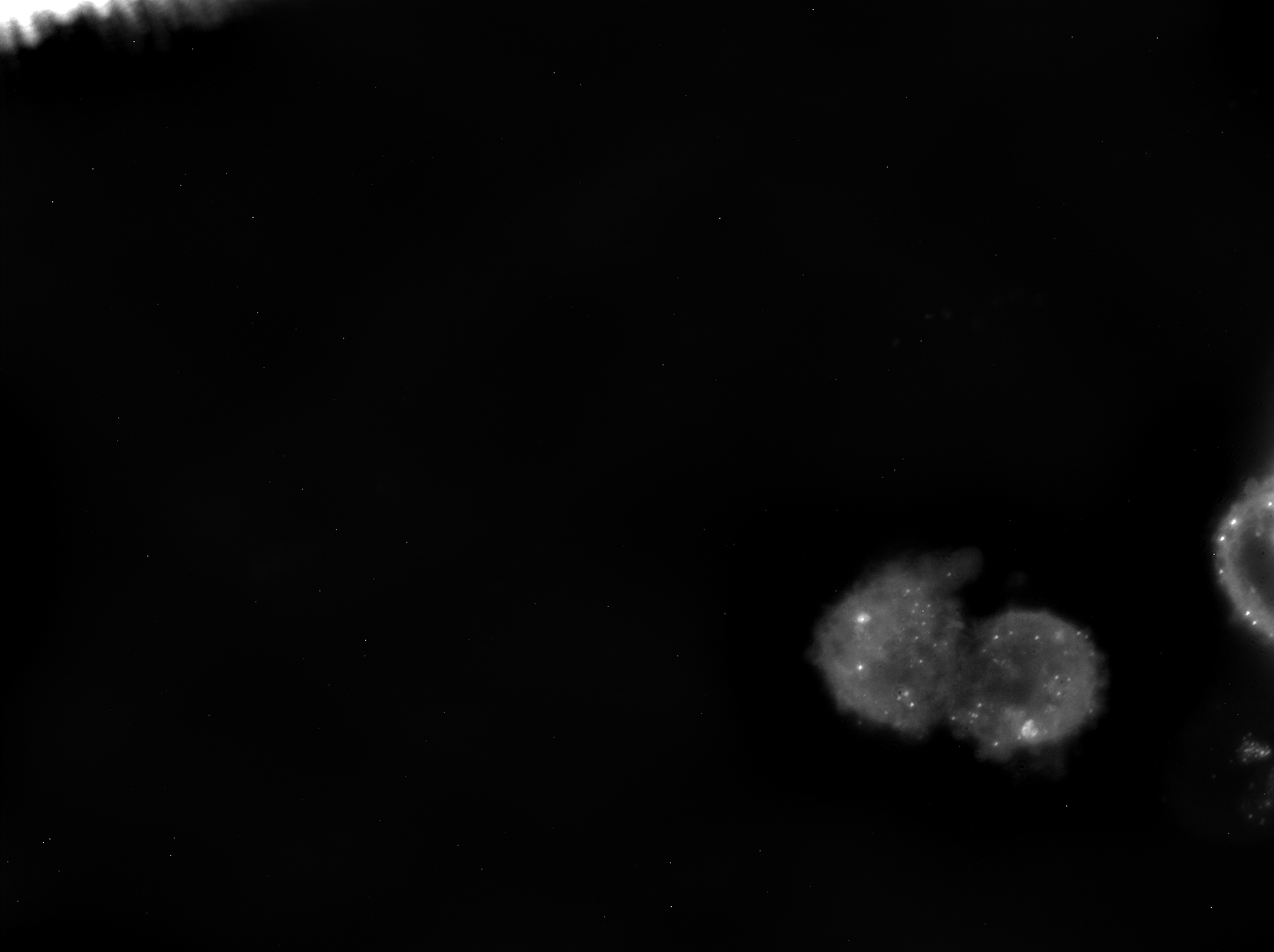

Supplement: Supplementary file 14 — Source data Fig. 4 [file 44319_2026_742_MOESM14_ESM.zip › Figure 4/Fig 4ef Cas9 TPGS1-EYFP-3'UTR acetylated tubulin/Cas9 TPGS1-3utr R2 2-5-25 ET10.Project Maximum Z - 1_XY1738624693_Z0_T0_C2.tif]

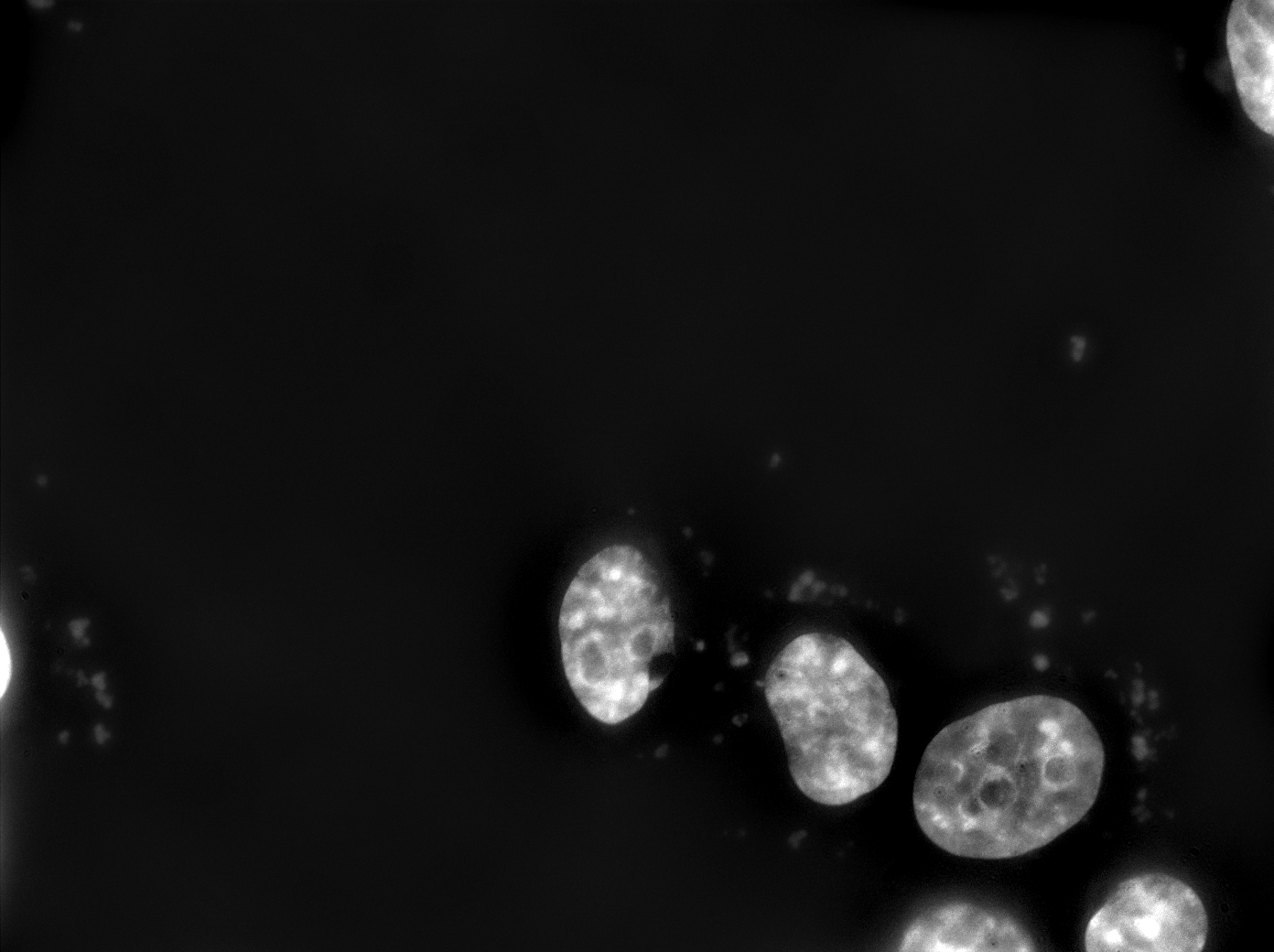

Supplement: Supplementary file 14 — Source data Fig. 4 [file 44319_2026_742_MOESM14_ESM.zip › Figure 4/Fig 4ef Cas9 TPGS1-EYFP-3'UTR acetylated tubulin/Cas9 TPGS1-3utr R3 2-5-25 LT1 exim.NearN.Project Maximum Z_XY1738692427_Z0_T0_C0.tif]

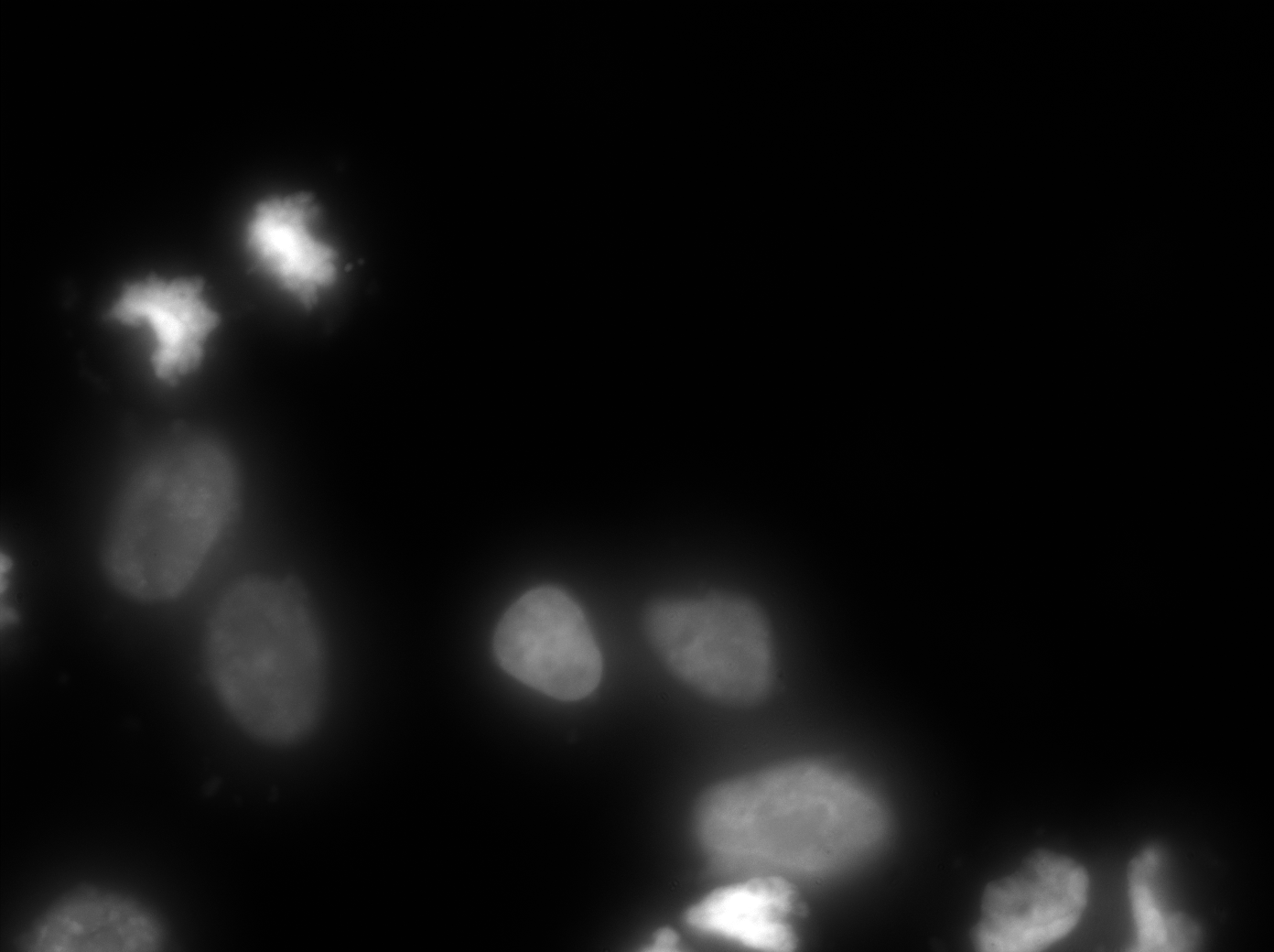

Supplement: Supplementary file 14 — Source data Fig. 4 [file 44319_2026_742_MOESM14_ESM.zip › Figure 4/Fig 4ef Cas9 TPGS1-EYFP-3'UTR acetylated tubulin/Cas9 TPGS1-3utr R3 2-5-25 ET4.Project Maximum Z_XY1738694957_Z0_T0_C0.tif]

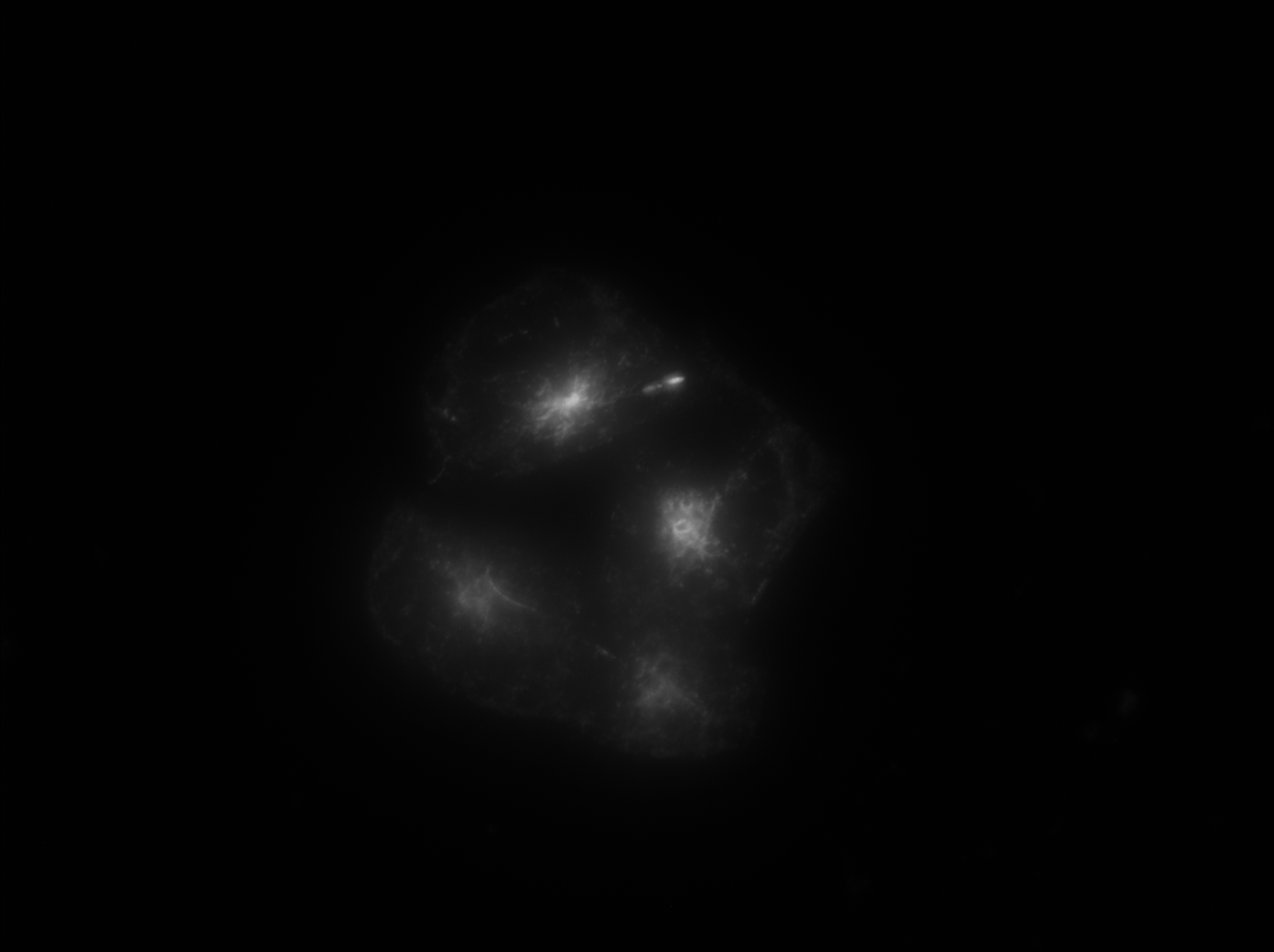

Supplement: Supplementary file 14 — Source data Fig. 4 [file 44319_2026_742_MOESM14_ESM.zip › Figure 4/Fig 4ef Cas9 TPGS1-EYFP-3'UTR acetylated tubulin/Cas9 TPGS1-3utr R1 1-28-24 LT3.Project Maximum Z_XY1738101074_Z0_T0_C1.tif]

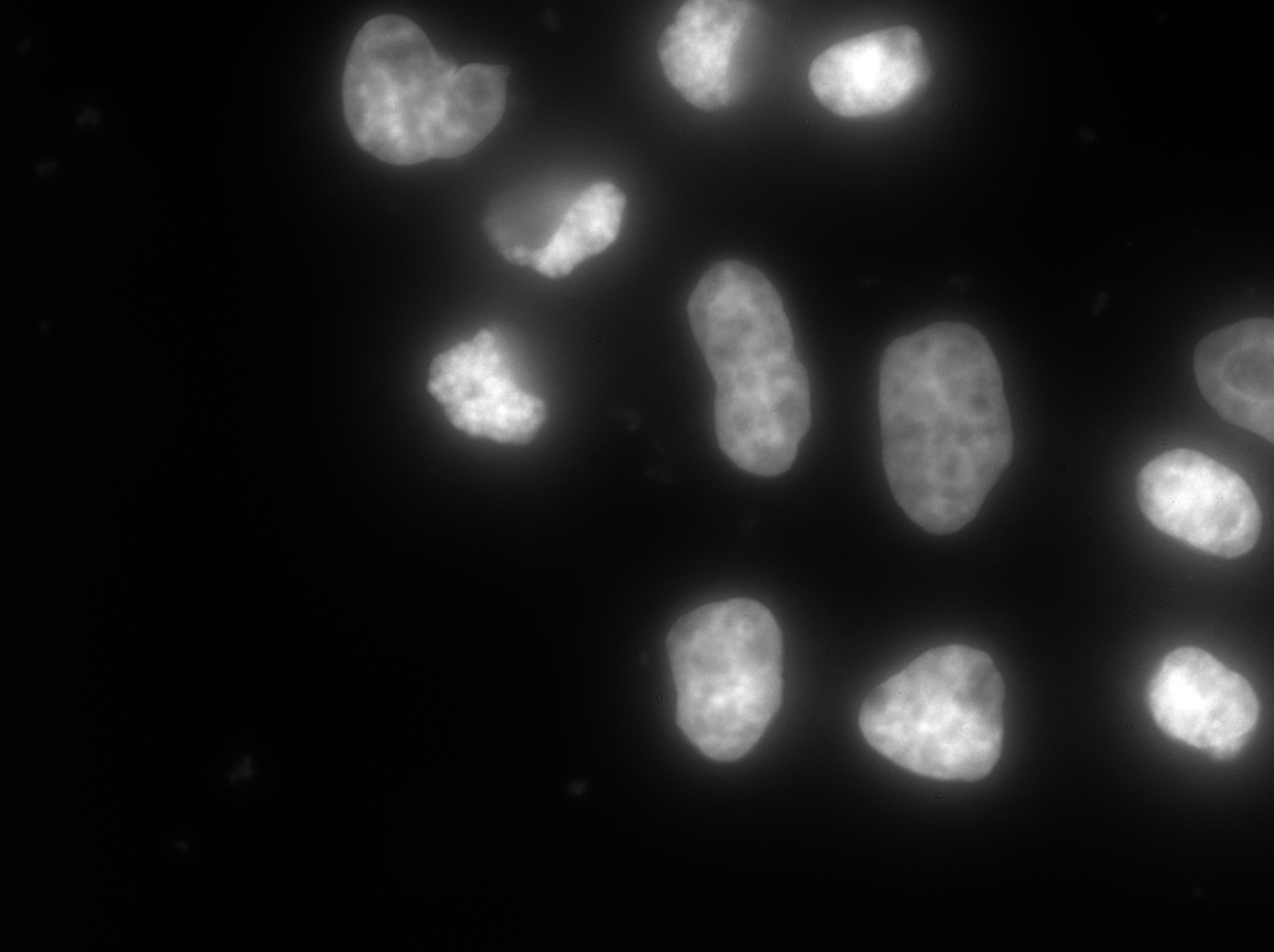

Supplement: Supplementary file 14 — Source data Fig. 4 [file 44319_2026_742_MOESM14_ESM.zip › Figure 4/Fig 4ef Cas9 TPGS1-EYFP-3'UTR acetylated tubulin/Cas9 TPGS1-3utr R3 2-5-25 LT9.Project Maximum Z_XY1738697664_Z0_T0_C0.tif]

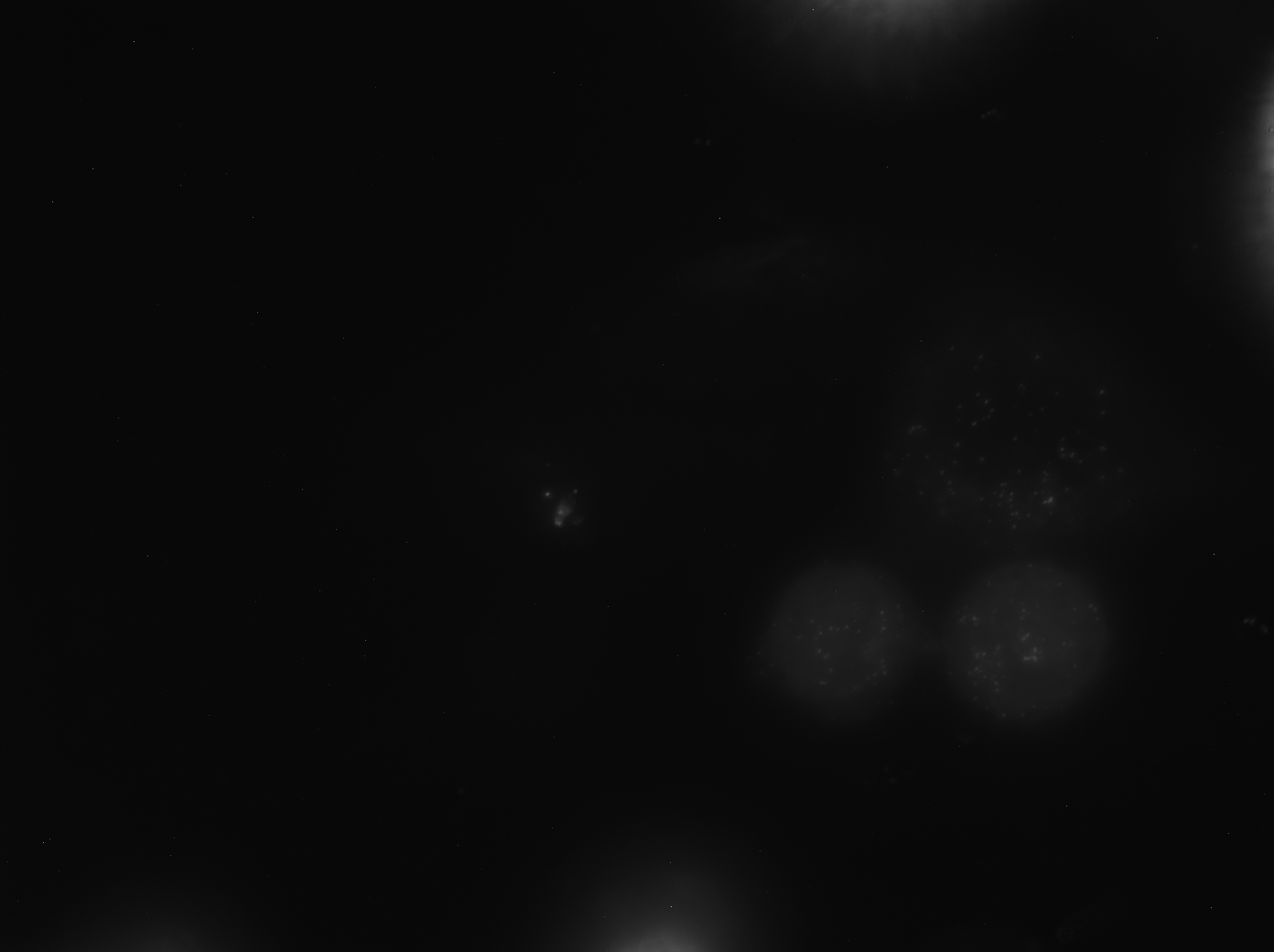

Supplement: Supplementary file 14 — Source data Fig. 4 [file 44319_2026_742_MOESM14_ESM.zip › Figure 4/Fig 4ef Cas9 TPGS1-EYFP-3'UTR acetylated tubulin/Cas9 TPGS1-3utr R3 2-5-25 ET8.Project Maximum Z_XY1738696890_Z0_T0_C2.tif]

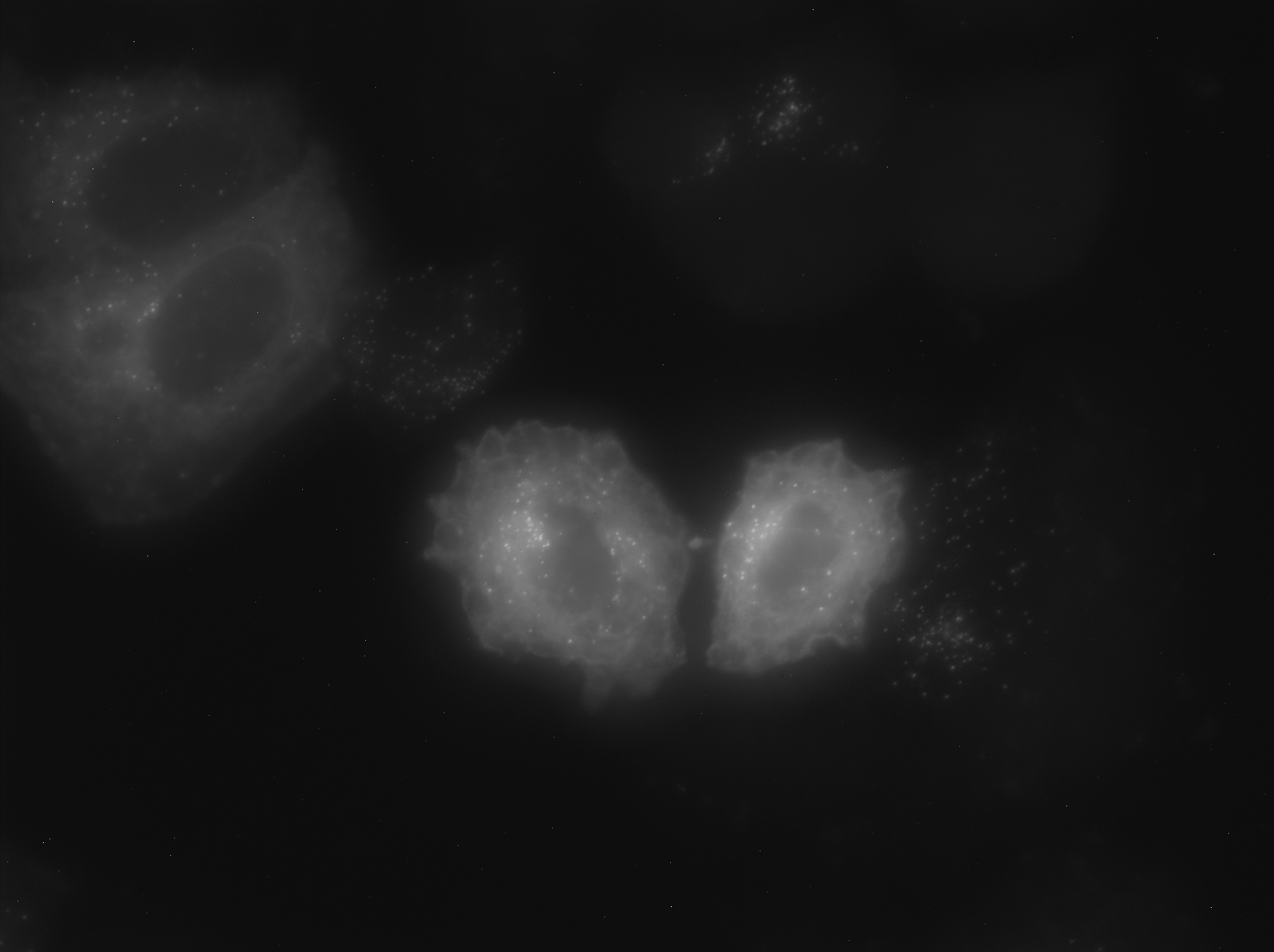

Supplement: Supplementary file 14 — Source data Fig. 4 [file 44319_2026_742_MOESM14_ESM.zip › Figure 4/Fig 4ef Cas9 TPGS1-EYFP-3'UTR acetylated tubulin/Cas9 TPGS1-3utr R1 1-28-24 LT9.Project Maximum Z_XY1738626725_Z0_T0_C2.tif]

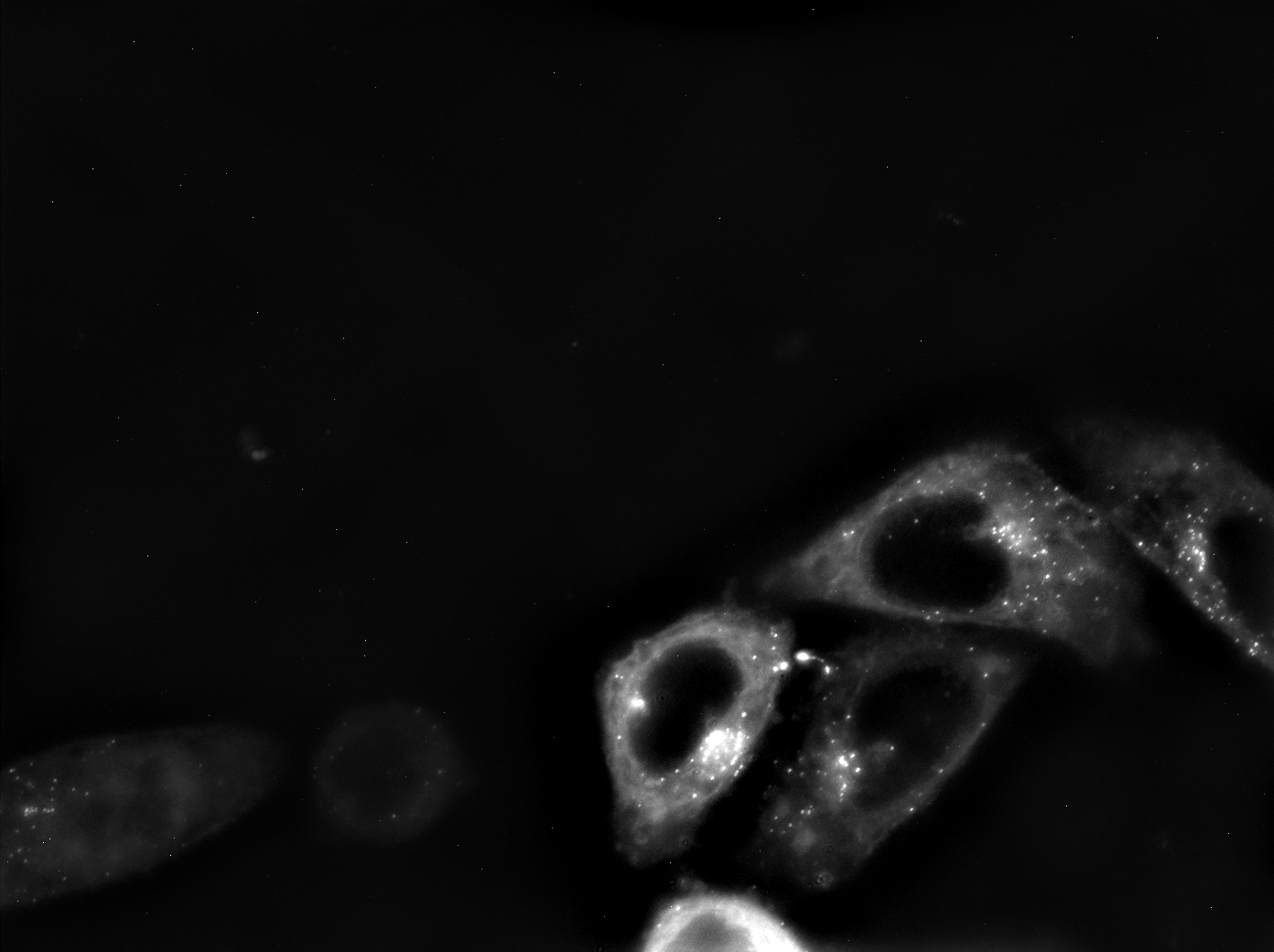

Supplement: Supplementary file 14 — Source data Fig. 4 [file 44319_2026_742_MOESM14_ESM.zip › Figure 4/Fig 4ef Cas9 TPGS1-EYFP-3'UTR acetylated tubulin/Cas9 TPGS1-3utr R2 2-5-25 LT4 figimg.NearN.Project Maximum Z_XY1738620138_Z0_T0_C2.tif]

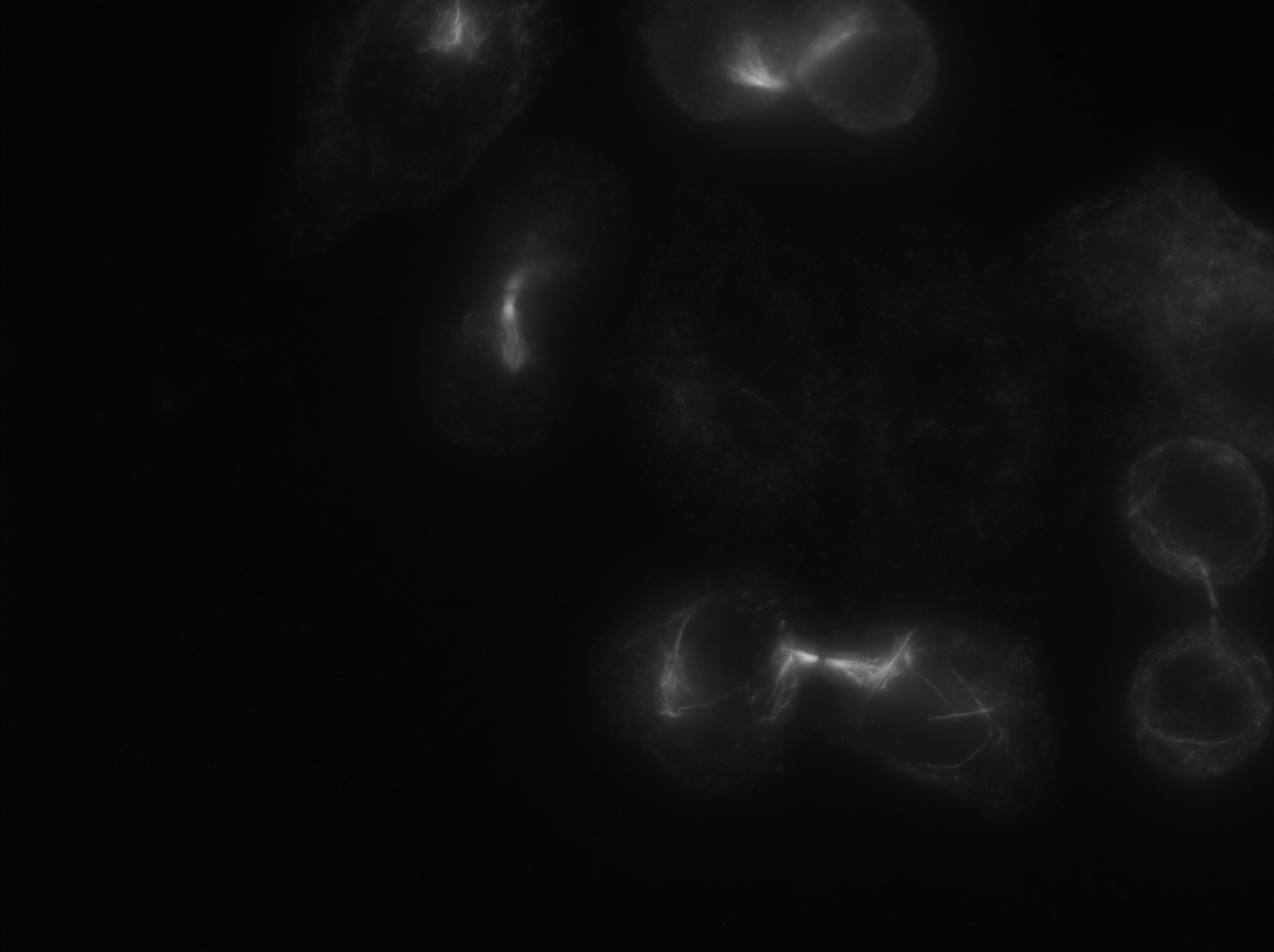

Supplement: Supplementary file 14 — Source data Fig. 4 [file 44319_2026_742_MOESM14_ESM.zip › Figure 4/Fig 4ef Cas9 TPGS1-EYFP-3'UTR acetylated tubulin/Cas9 TPGS1-3utr R3 2-5-25 LT9.Project Maximum Z_XY1738697664_Z0_T0_C1.tif]

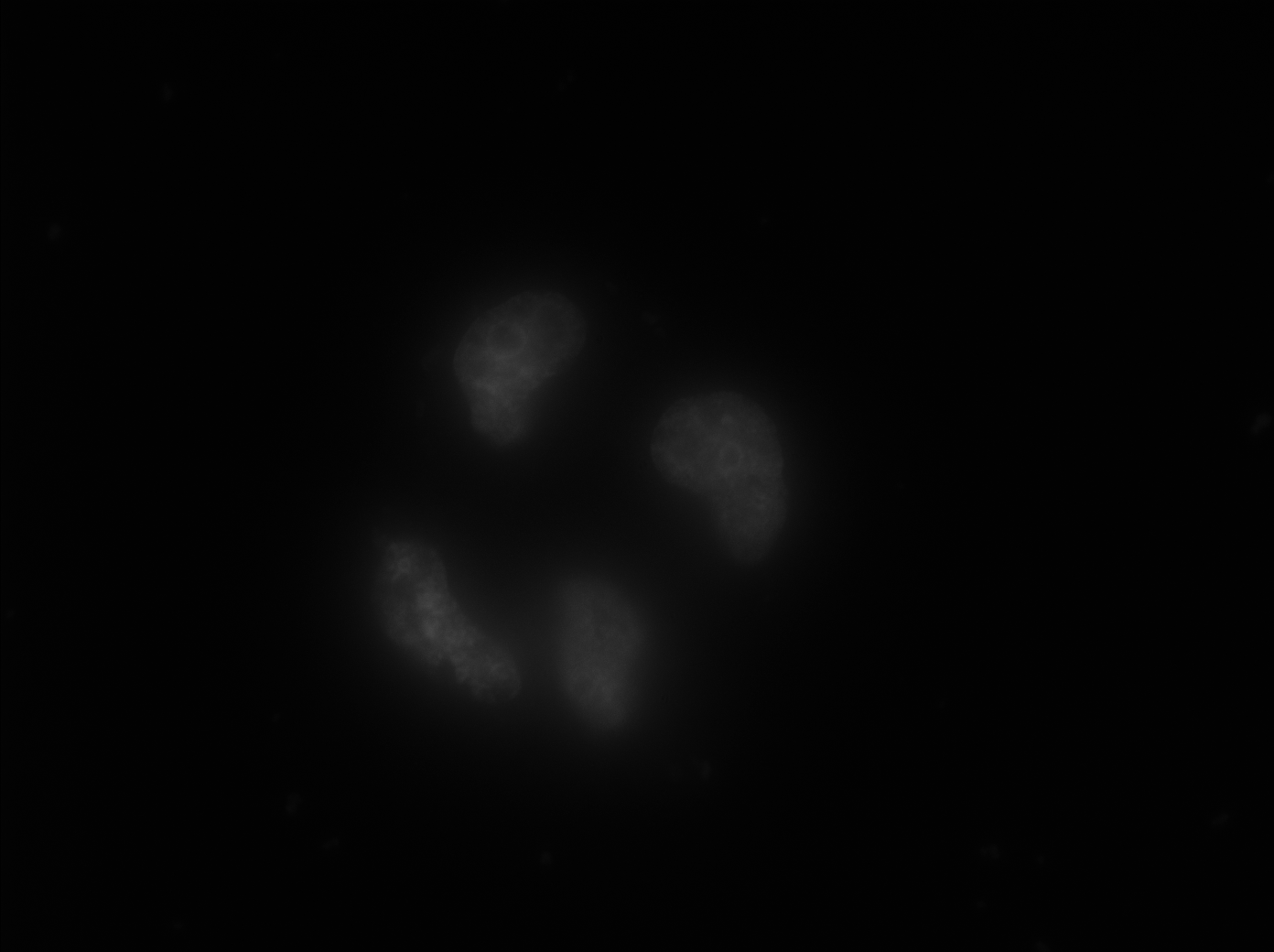

Supplement: Supplementary file 14 — Source data Fig. 4 [file 44319_2026_742_MOESM14_ESM.zip › Figure 4/Fig 4ef Cas9 TPGS1-EYFP-3'UTR acetylated tubulin/Cas9 TPGS1-3utr R1 1-28-24 LT3.Project Maximum Z_XY1738101074_Z0_T0_C0.tif]

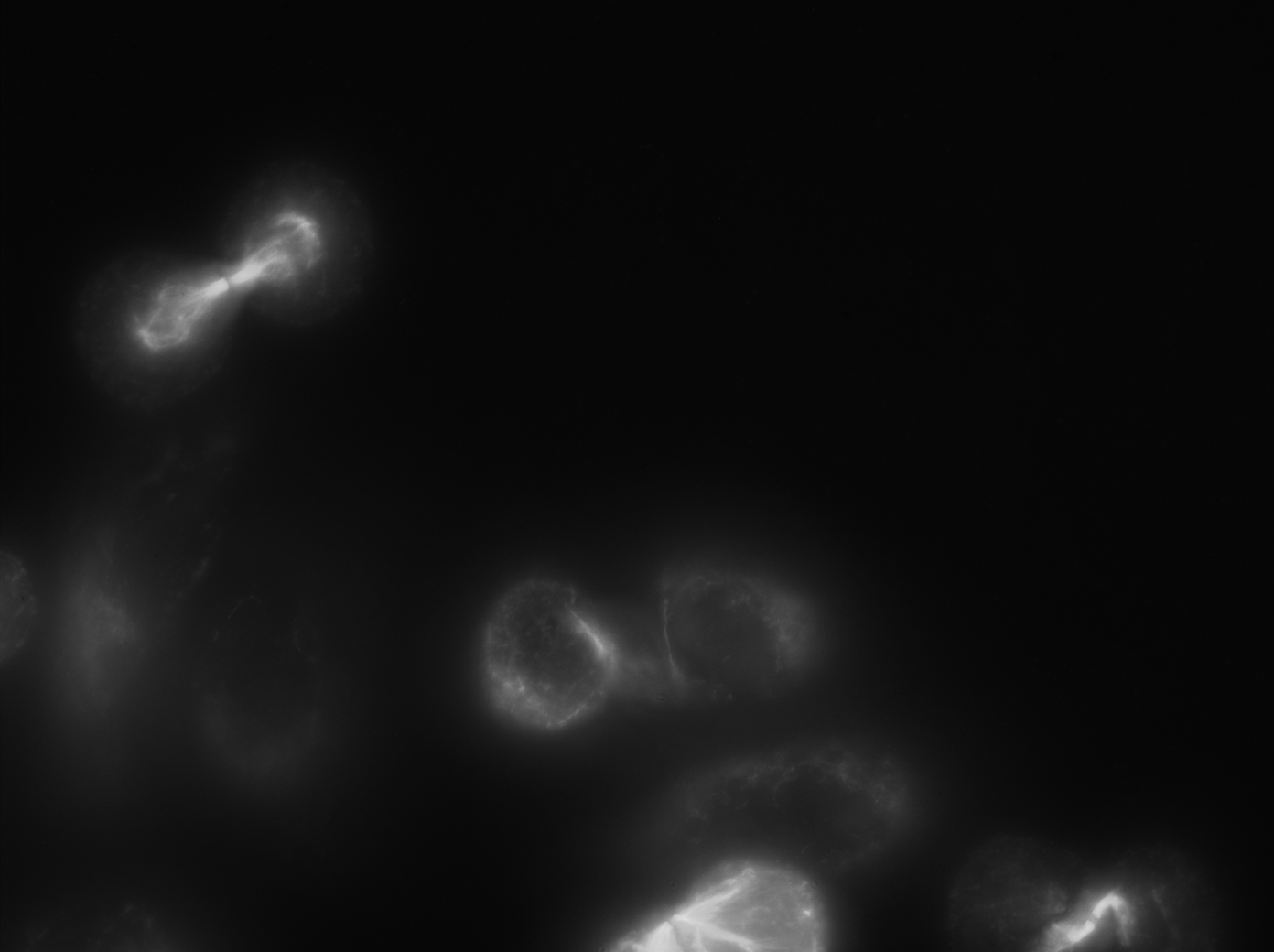

Supplement: Supplementary file 14 — Source data Fig. 4 [file 44319_2026_742_MOESM14_ESM.zip › Figure 4/Fig 4ef Cas9 TPGS1-EYFP-3'UTR acetylated tubulin/Cas9 TPGS1-3utr R3 2-5-25 ET4.Project Maximum Z_XY1738694957_Z0_T0_C1.tif]

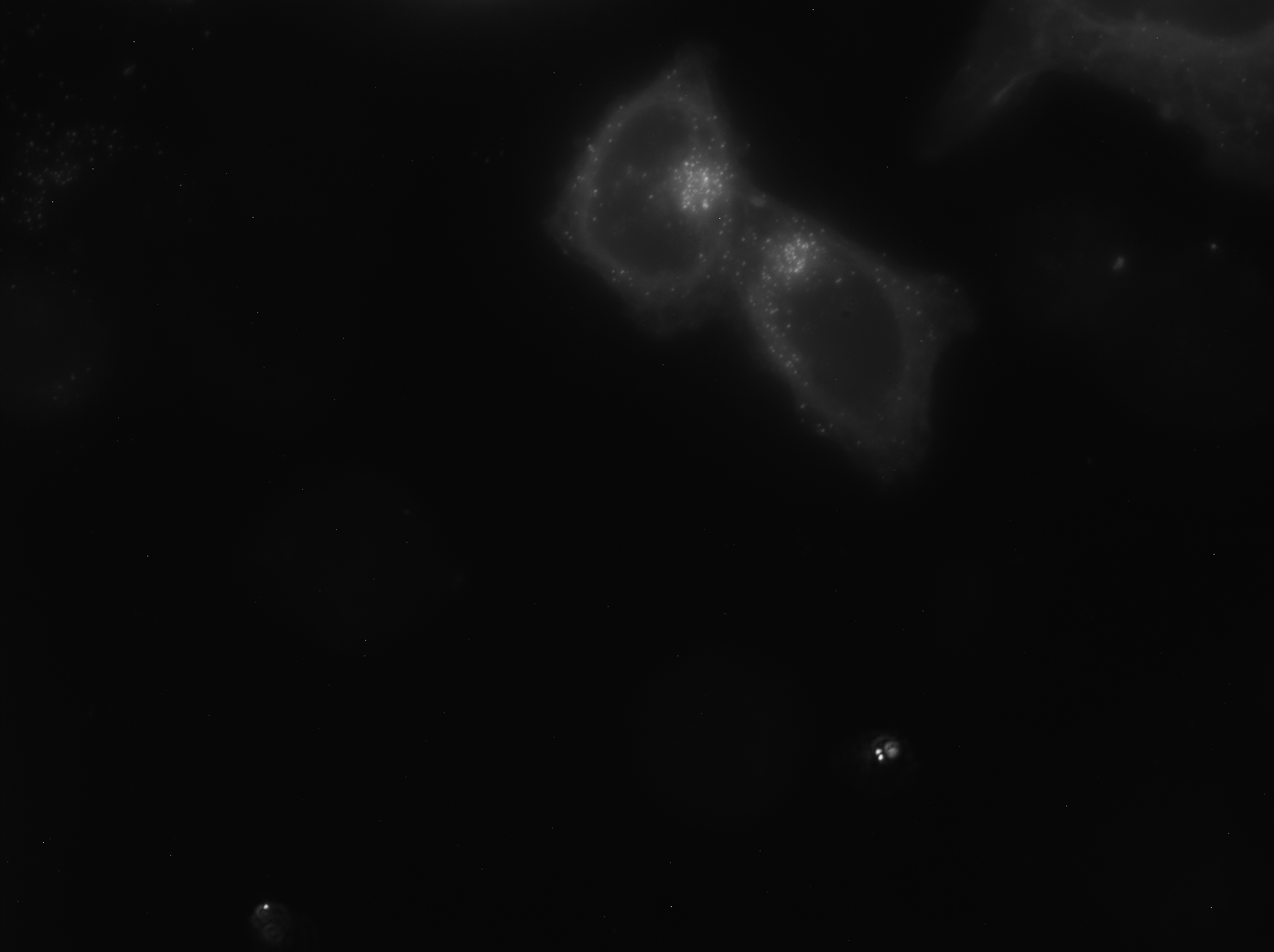

Supplement: Supplementary file 14 — Source data Fig. 4 [file 44319_2026_742_MOESM14_ESM.zip › Figure 4/Fig 4ef Cas9 TPGS1-EYFP-3'UTR acetylated tubulin/Cas9 TPGS1-3utr R2 2-5-25 LT5.Project Maximum Z_XY1738622161_Z0_T0_C2.tif]

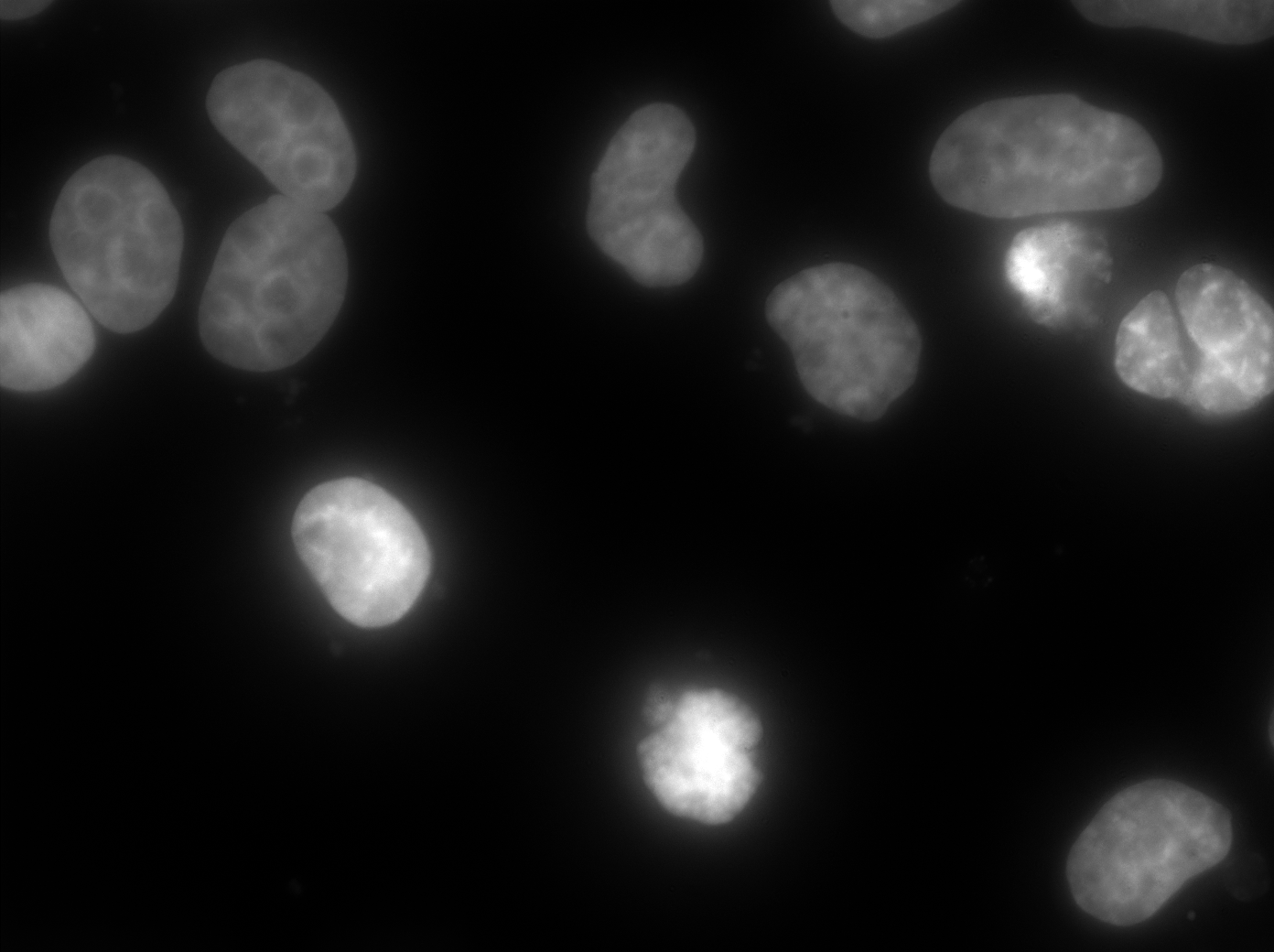

Supplement: Supplementary file 14 — Source data Fig. 4 [file 44319_2026_742_MOESM14_ESM.zip › Figure 4/Fig 4ef Cas9 TPGS1-EYFP-3'UTR acetylated tubulin/Cas9 TPGS1-3utr R2 2-5-25 LT5.Project Maximum Z_XY1738622161_Z0_T0_C0.tif]

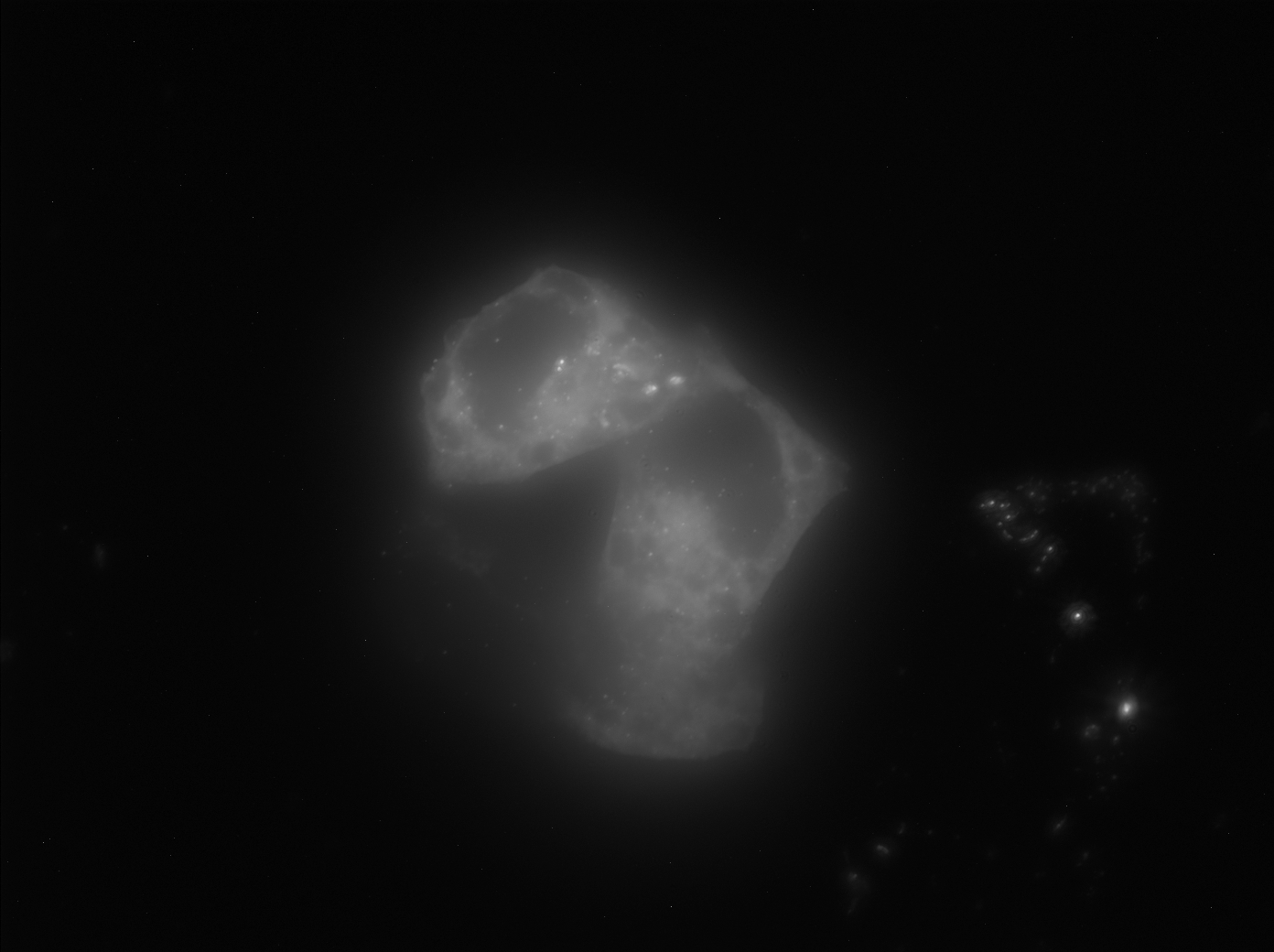

Supplement: Supplementary file 14 — Source data Fig. 4 [file 44319_2026_742_MOESM14_ESM.zip › Figure 4/Fig 4ef Cas9 TPGS1-EYFP-3'UTR acetylated tubulin/Cas9 TPGS1-3utr R1 1-28-24 LT3.Project Maximum Z_XY1738101074_Z0_T0_C2.tif]

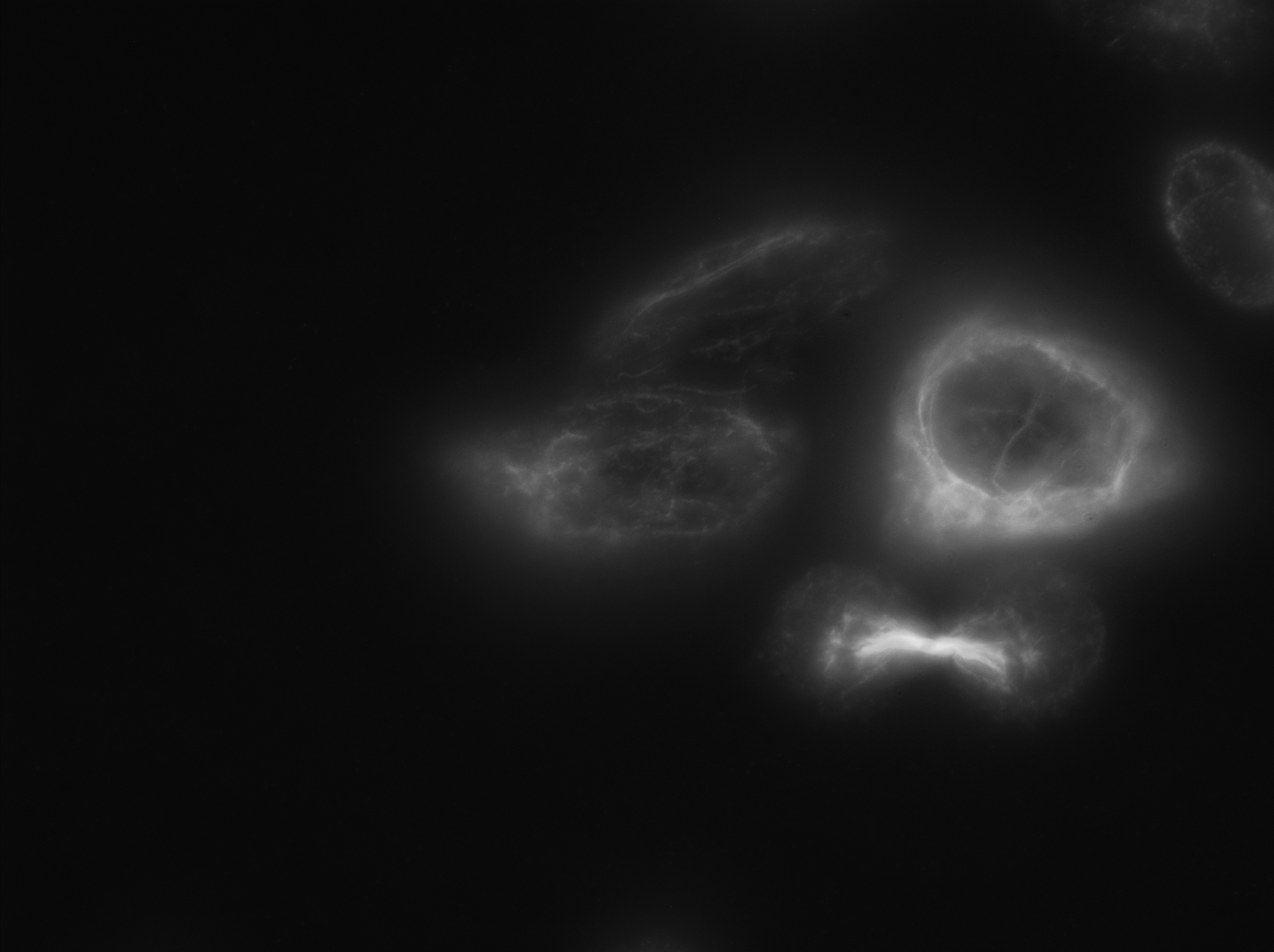

Supplement: Supplementary file 14 — Source data Fig. 4 [file 44319_2026_742_MOESM14_ESM.zip › Figure 4/Fig 4ef Cas9 TPGS1-EYFP-3'UTR acetylated tubulin/Cas9 TPGS1-3utr R3 2-5-25 ET8.Project Maximum Z_XY1738696890_Z0_T0_C1.tif]

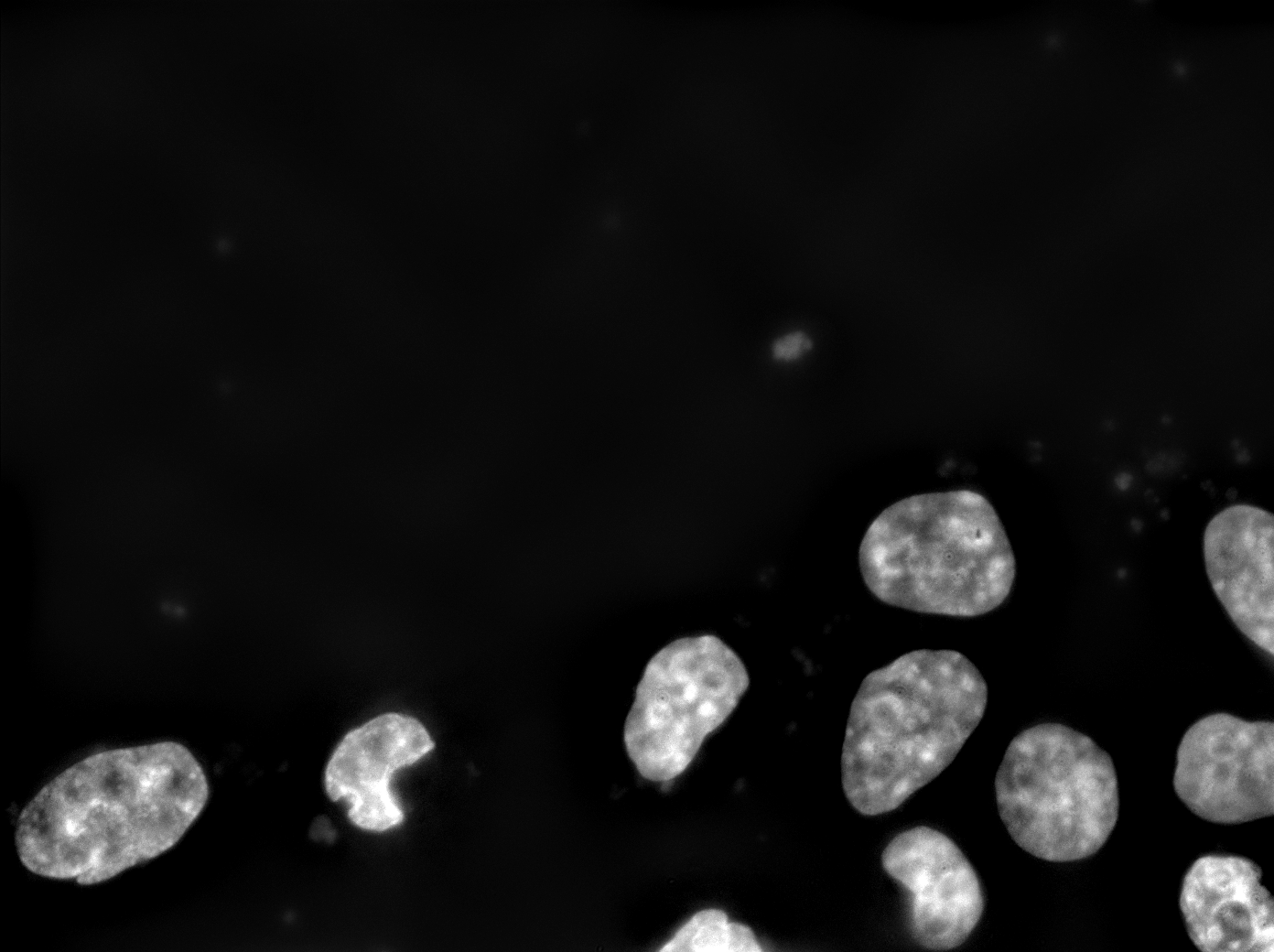

Supplement: Supplementary file 14 — Source data Fig. 4 [file 44319_2026_742_MOESM14_ESM.zip › Figure 4/Fig 4ef Cas9 TPGS1-EYFP-3'UTR acetylated tubulin/Cas9 TPGS1-3utr R2 2-5-25 LT4 figimg.NearN.Project Maximum Z_XY1738620138_Z0_T0_C0.tif]

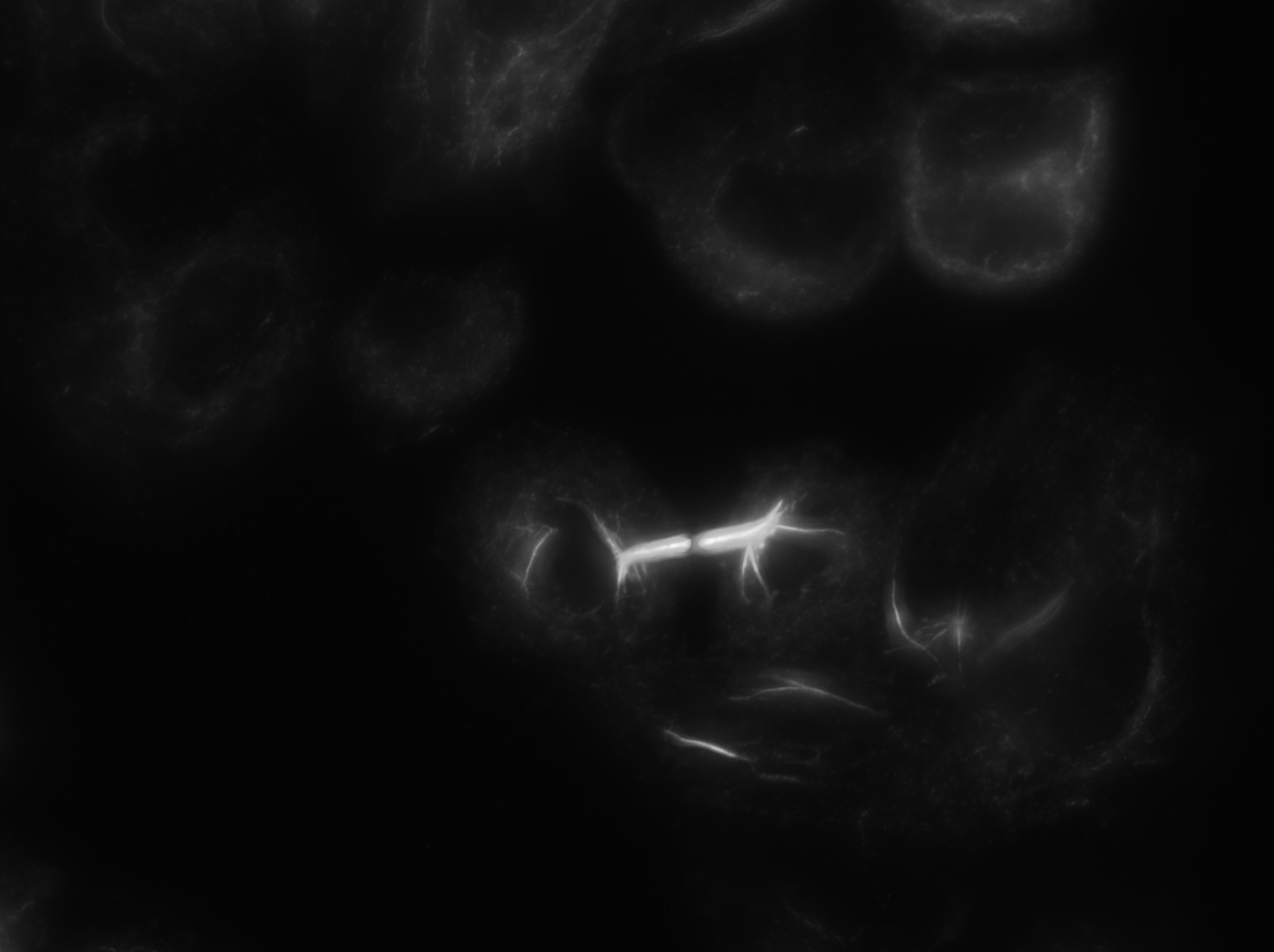

Supplement: Supplementary file 14 — Source data Fig. 4 [file 44319_2026_742_MOESM14_ESM.zip › Figure 4/Fig 4ef Cas9 TPGS1-EYFP-3'UTR acetylated tubulin/Cas9 TPGS1-3utr R1 1-28-24 LT9.Project Maximum Z_XY1738626725_Z0_T0_C1.tif]

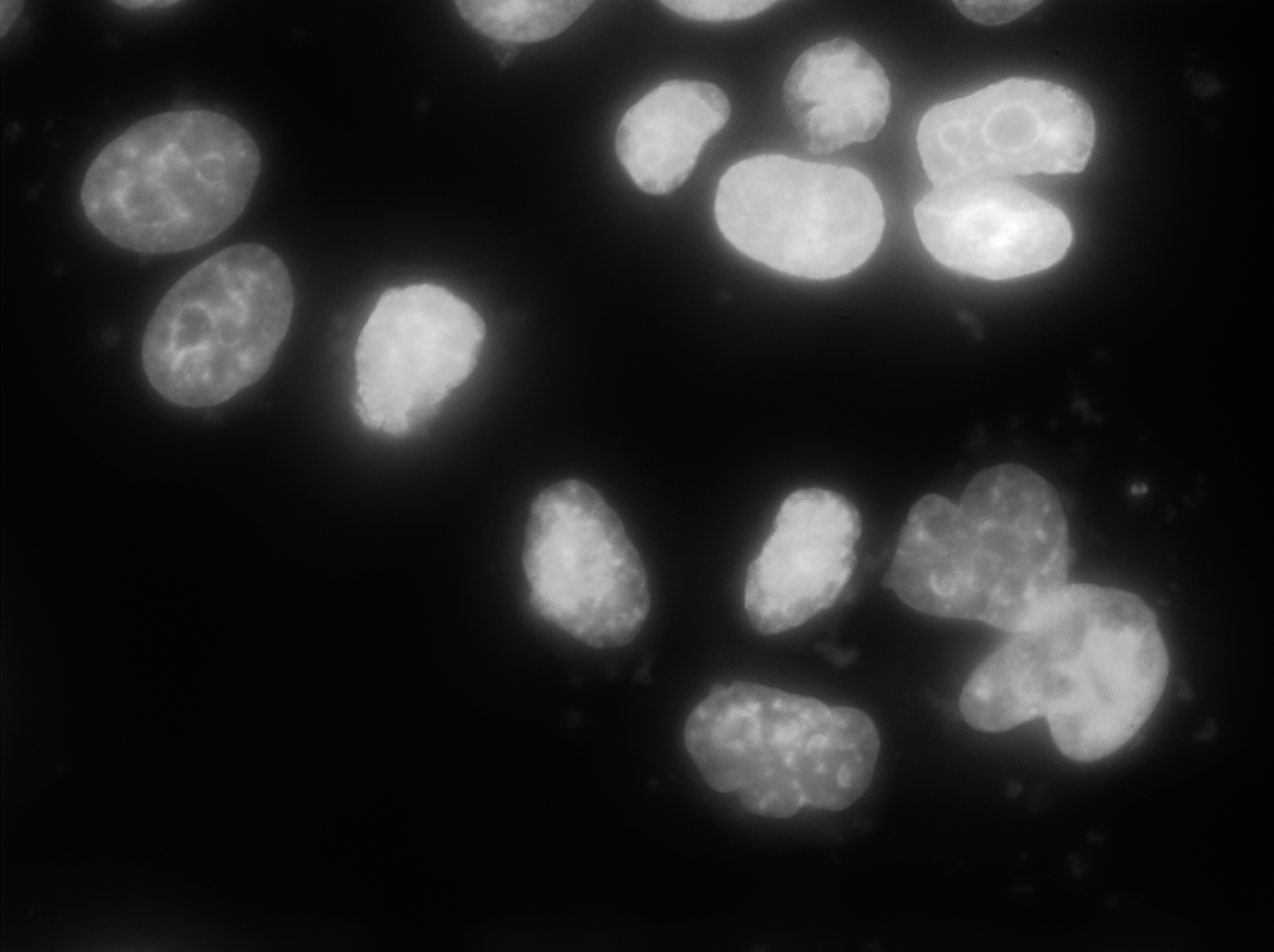

Supplement: Supplementary file 14 — Source data Fig. 4 [file 44319_2026_742_MOESM14_ESM.zip › Figure 4/Fig 4ef Cas9 TPGS1-EYFP-3'UTR acetylated tubulin/Cas9 TPGS1-3utr R1 1-28-24 LT9.Project Maximum Z_XY1738626725_Z0_T0_C0.tif]

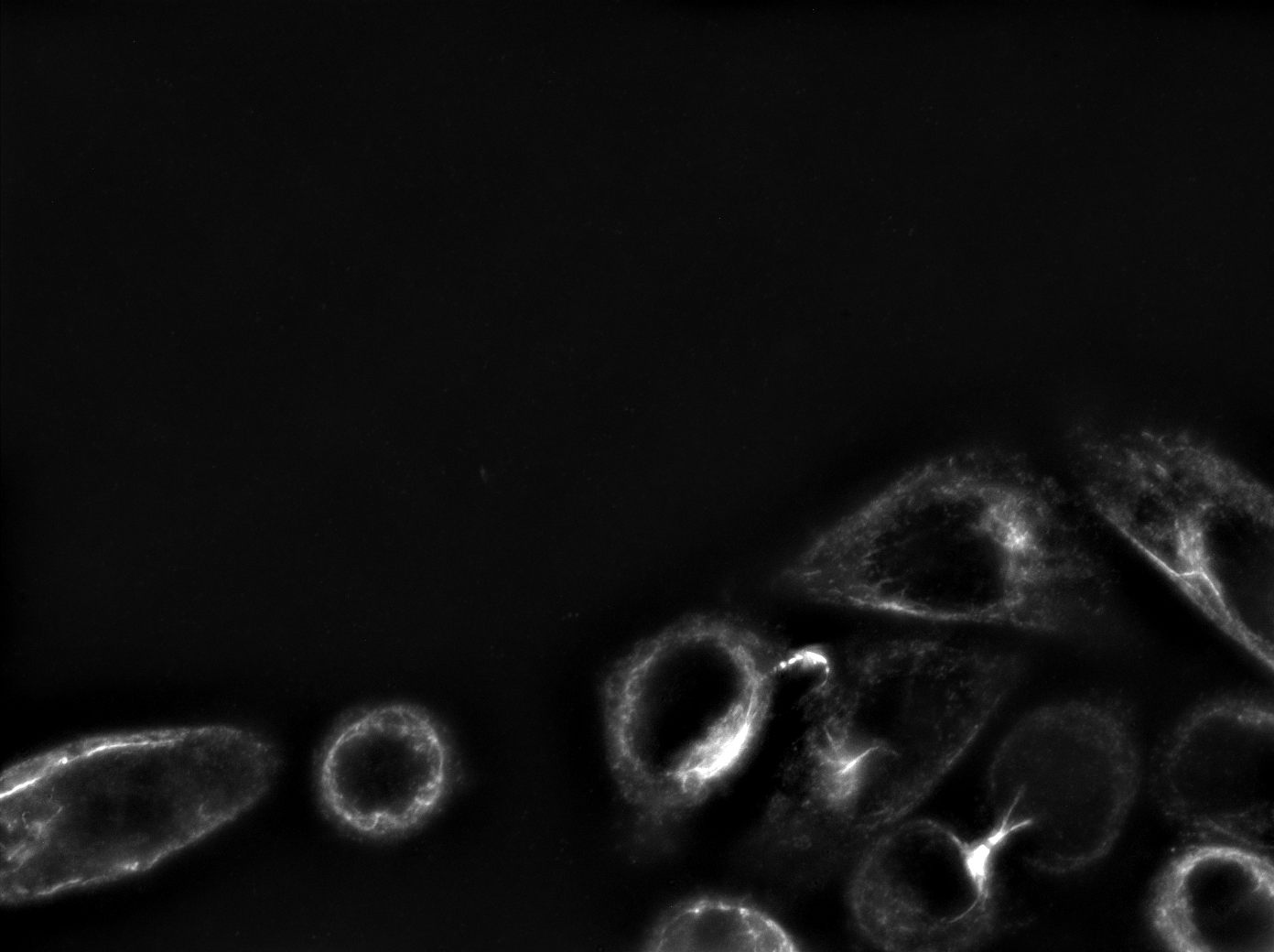

Supplement: Supplementary file 14 — Source data Fig. 4 [file 44319_2026_742_MOESM14_ESM.zip › Figure 4/Fig 4ef Cas9 TPGS1-EYFP-3'UTR acetylated tubulin/Cas9 TPGS1-3utr R2 2-5-25 LT4 figimg.NearN.Project Maximum Z_XY1738620138_Z0_T0_C1.tif]

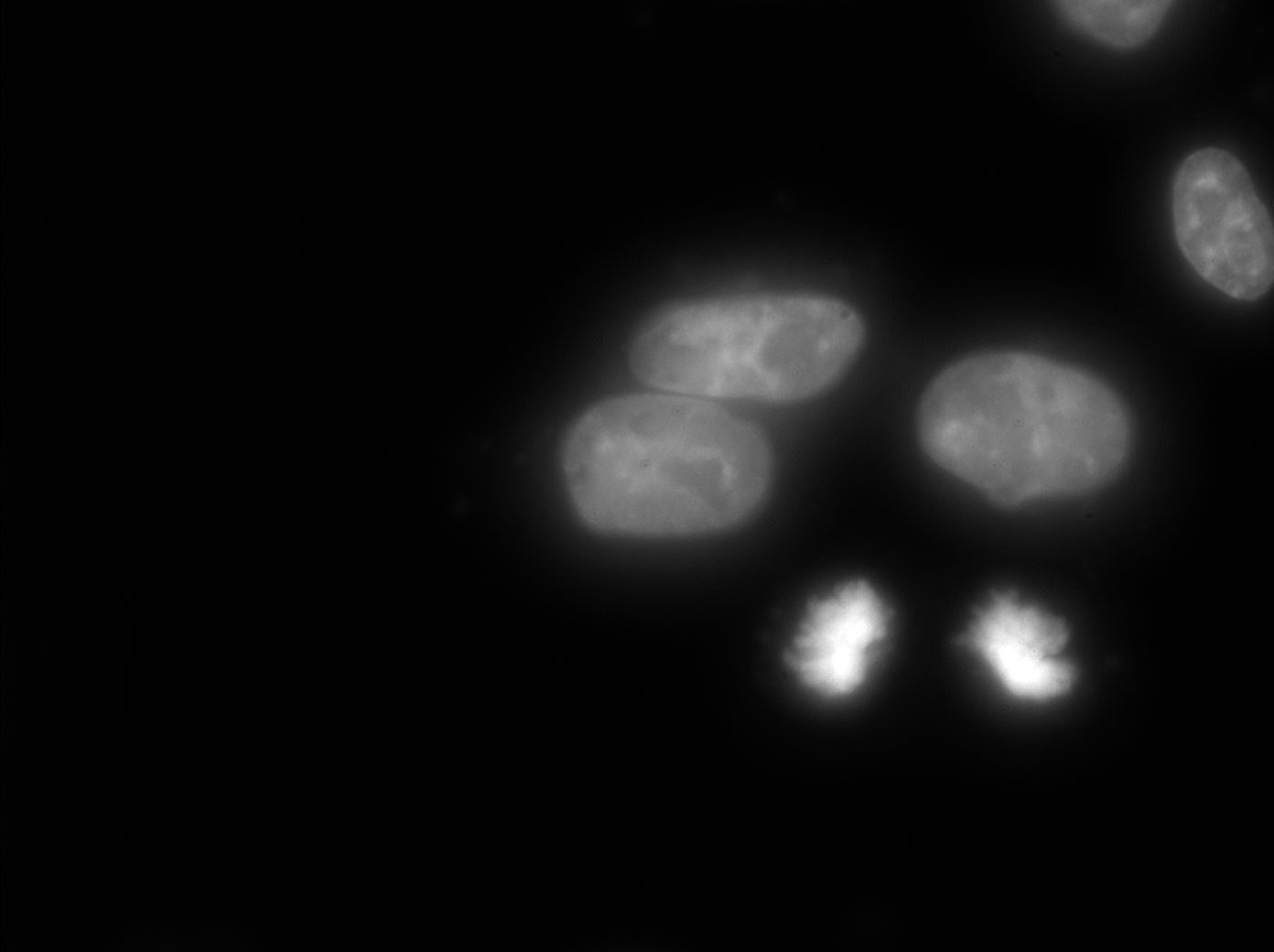

Supplement: Supplementary file 14 — Source data Fig. 4 [file 44319_2026_742_MOESM14_ESM.zip › Figure 4/Fig 4ef Cas9 TPGS1-EYFP-3'UTR acetylated tubulin/Cas9 TPGS1-3utr R3 2-5-25 ET8.Project Maximum Z_XY1738696890_Z0_T0_C0.tif]

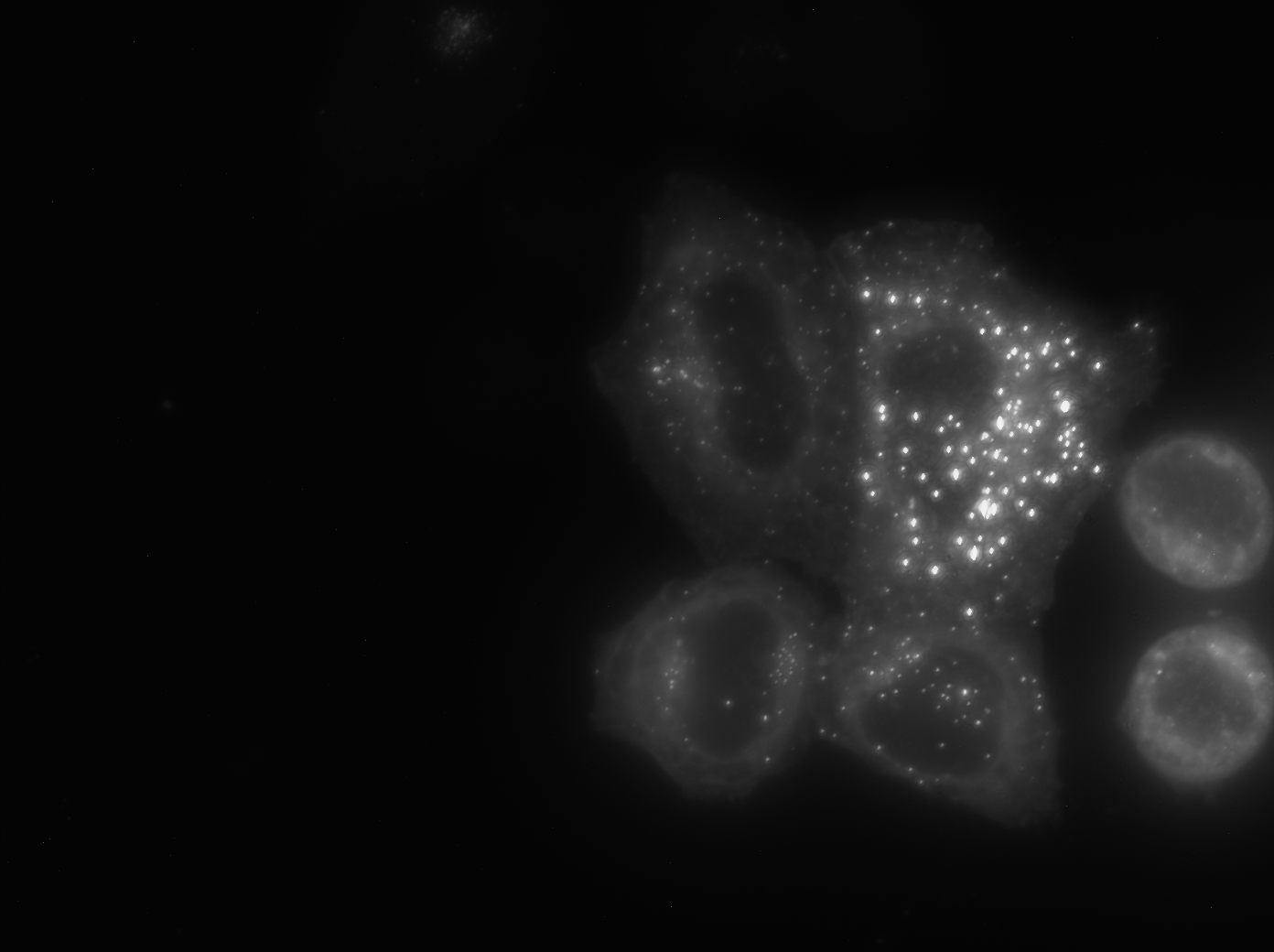

Supplement: Supplementary file 14 — Source data Fig. 4 [file 44319_2026_742_MOESM14_ESM.zip › Figure 4/Fig 4ef Cas9 TPGS1-EYFP-3'UTR acetylated tubulin/Cas9 TPGS1-3utr R3 2-5-25 LT9.Project Maximum Z_XY1738697664_Z0_T0_C2.tif]

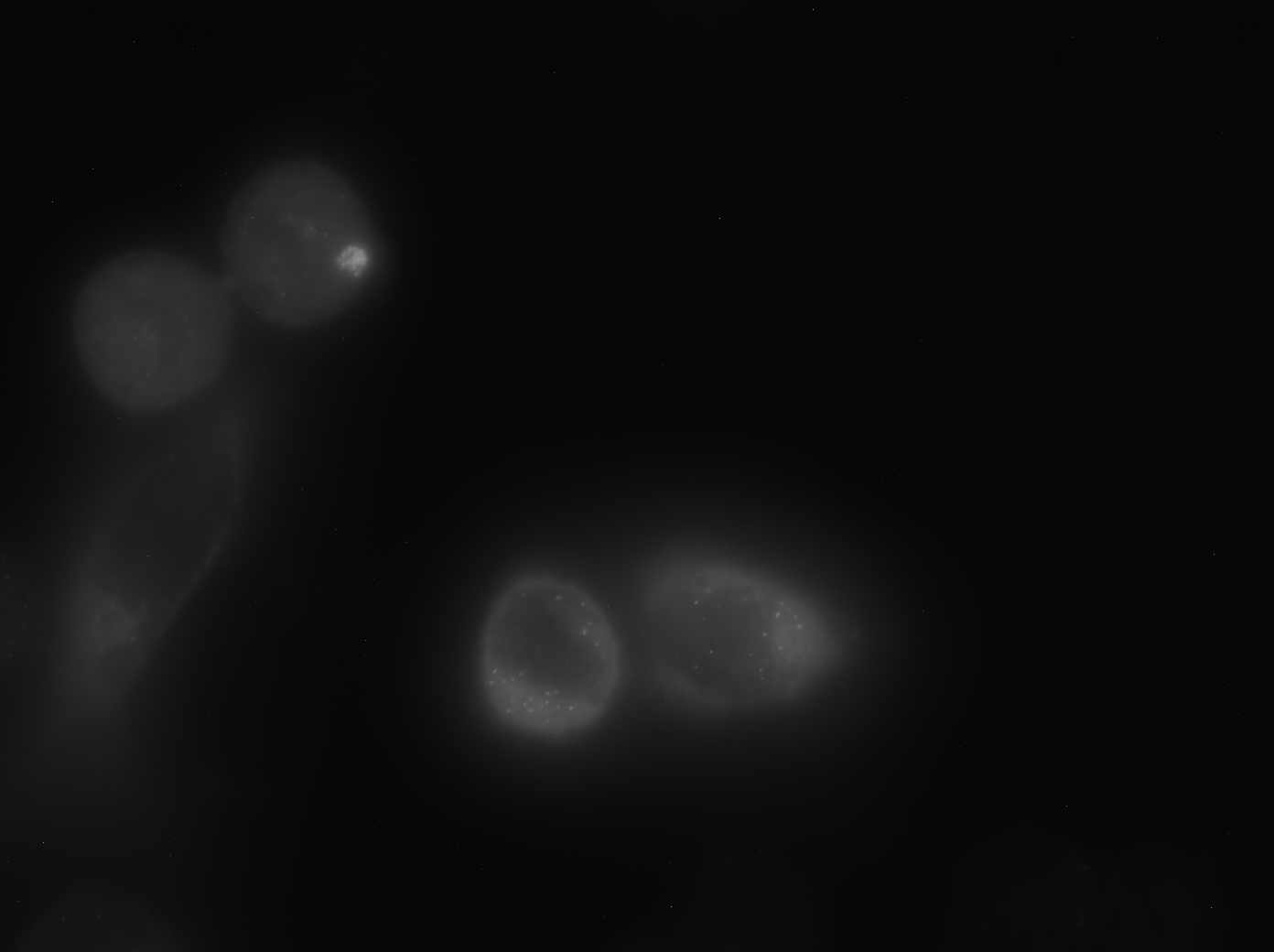

Supplement: Supplementary file 14 — Source data Fig. 4 [file 44319_2026_742_MOESM14_ESM.zip › Figure 4/Fig 4ef Cas9 TPGS1-EYFP-3'UTR acetylated tubulin/Cas9 TPGS1-3utr R3 2-5-25 ET4.Project Maximum Z_XY1738694957_Z0_T0_C2.tif]

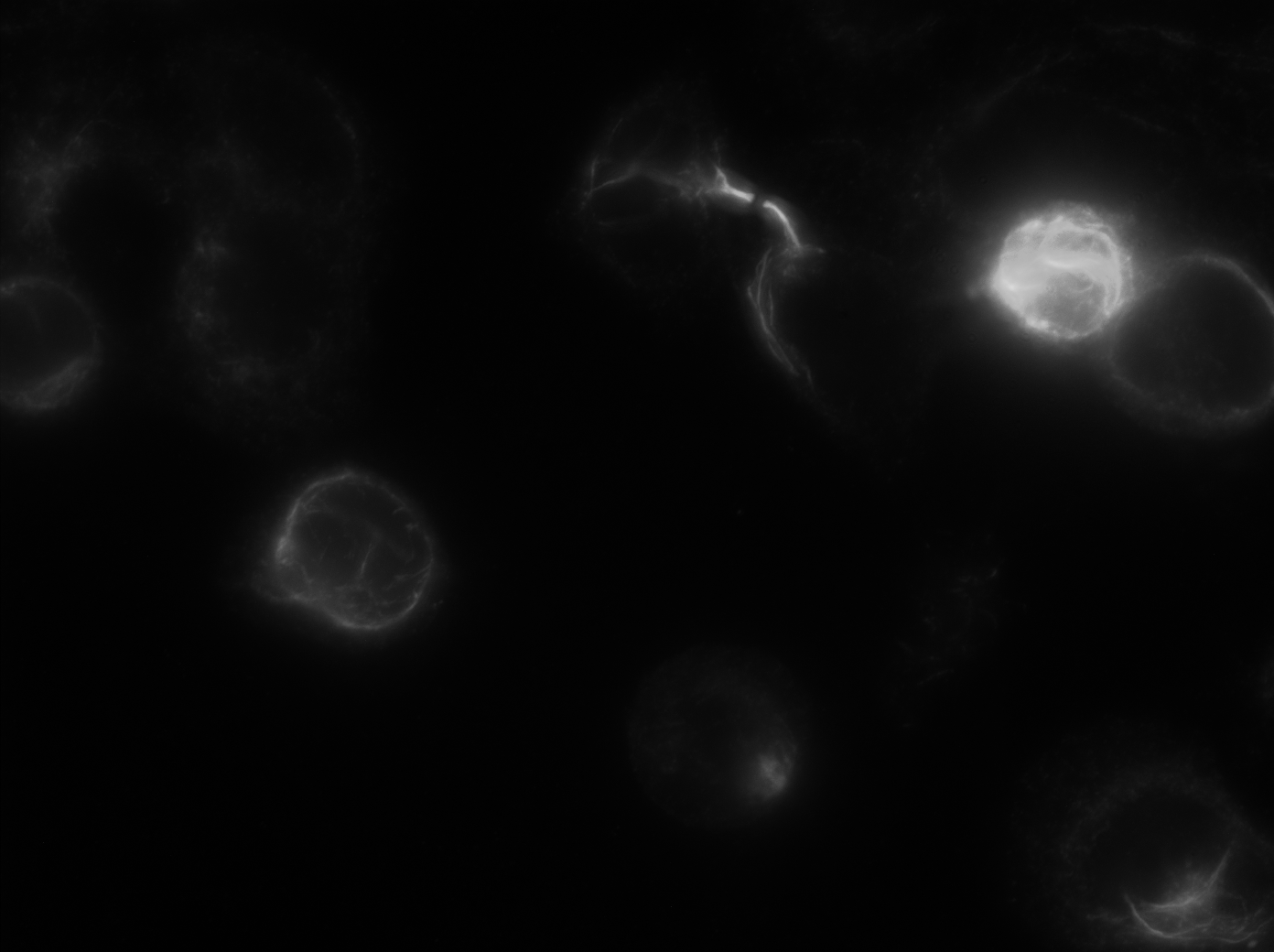

Supplement: Supplementary file 14 — Source data Fig. 4 [file 44319_2026_742_MOESM14_ESM.zip › Figure 4/Fig 4ef Cas9 TPGS1-EYFP-3'UTR acetylated tubulin/Cas9 TPGS1-3utr R2 2-5-25 LT5.Project Maximum Z_XY1738622161_Z0_T0_C1.tif]

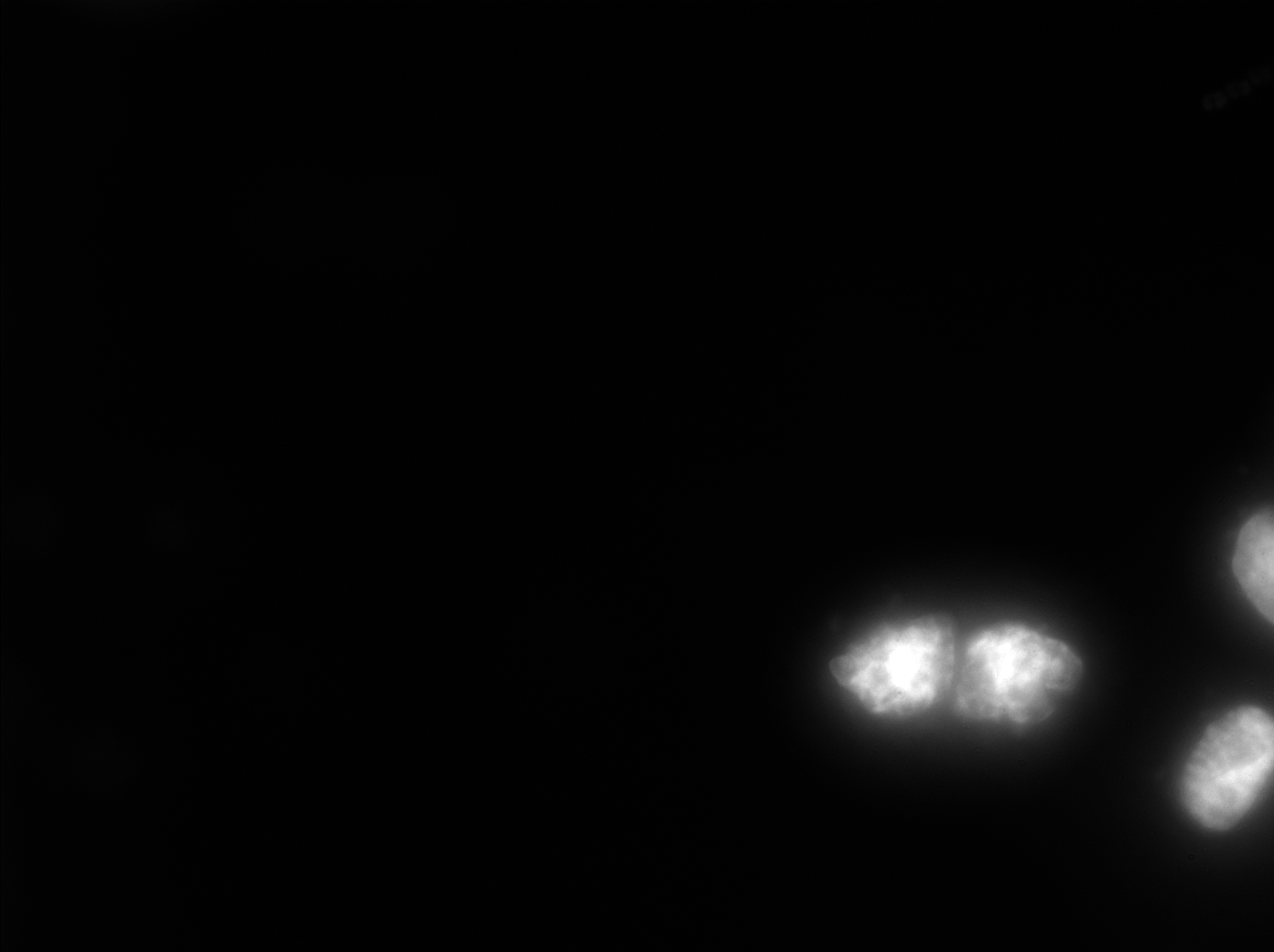

Supplement: Supplementary file 14 — Source data Fig. 4 [file 44319_2026_742_MOESM14_ESM.zip › Figure 4/Fig 4ef Cas9 TPGS1-EYFP-3'UTR acetylated tubulin/Cas9 TPGS1-3utr R2 2-5-25 ET10.Project Maximum Z_XY1738624693_Z0_T0_C0.tif]

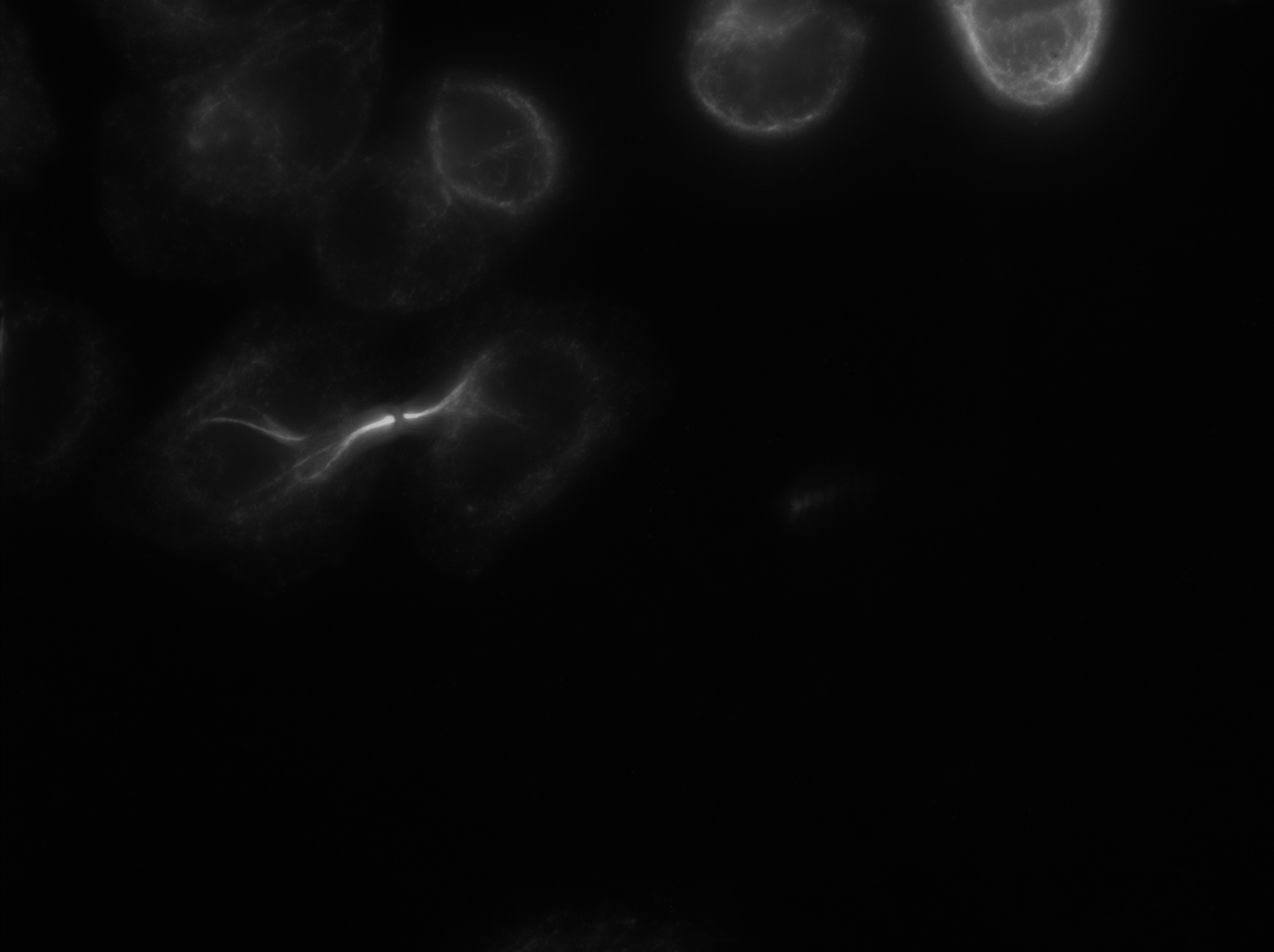

Supplement: Supplementary file 14 — Source data Fig. 4 [file 44319_2026_742_MOESM14_ESM.zip › Figure 4/Fig 4ef Cas9 TPGS1-EYFP-3'UTR acetylated tubulin/Cas9 TPGS1-3utr R2 2-5-25 LT9.Project Maximum Z_XY1738625881_Z0_T0_C1.tif]

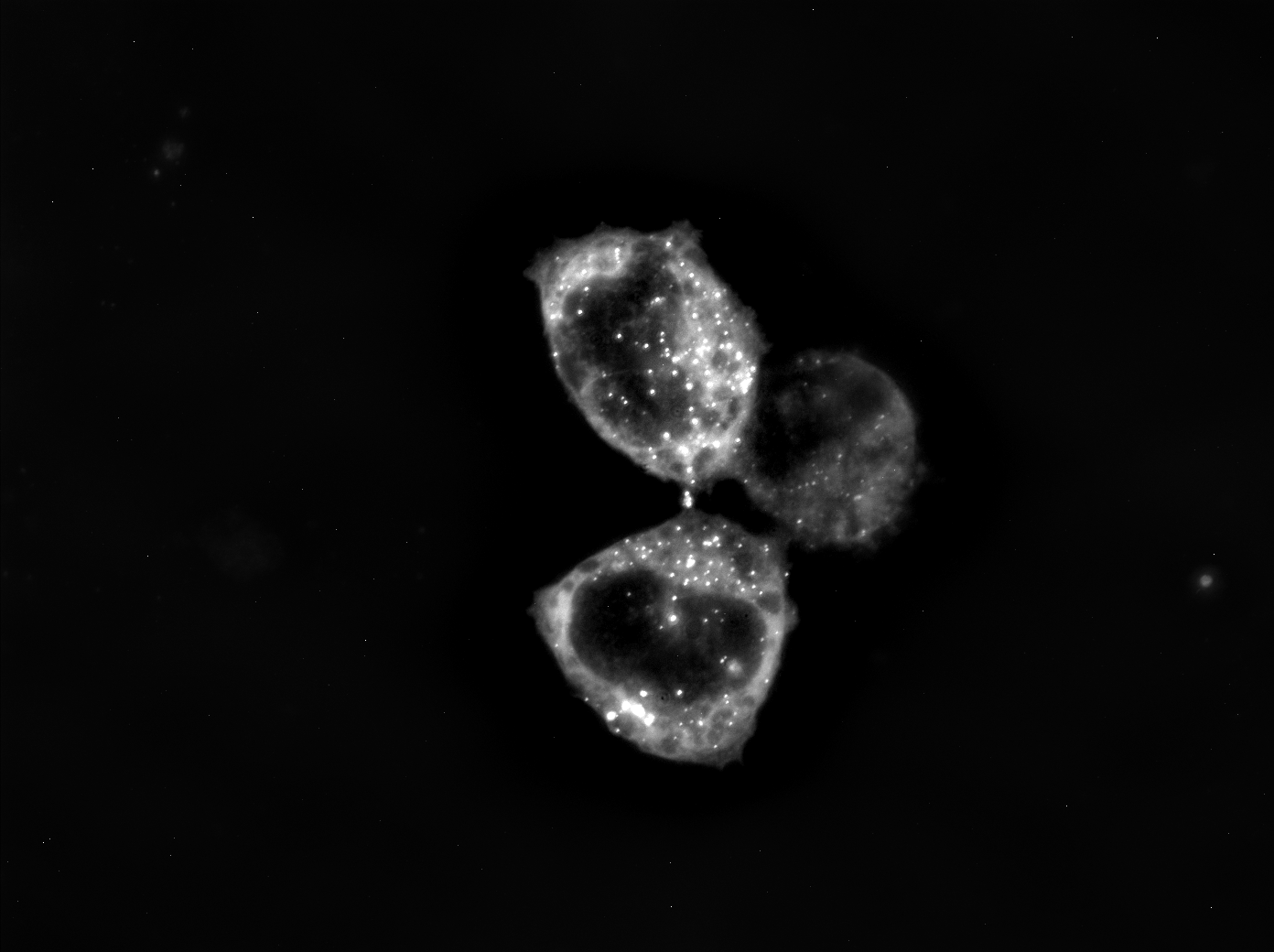

Supplement: Supplementary file 14 — Source data Fig. 4 [file 44319_2026_742_MOESM14_ESM.zip › Figure 4/Fig 4ef Cas9 TPGS1-EYFP-3'UTR acetylated tubulin/Cas9 TPGS1-3utr R1 1-28-24 LT4.NearN.Project Maximum Z_XY1738101264_Z0_T0_C2.tif]

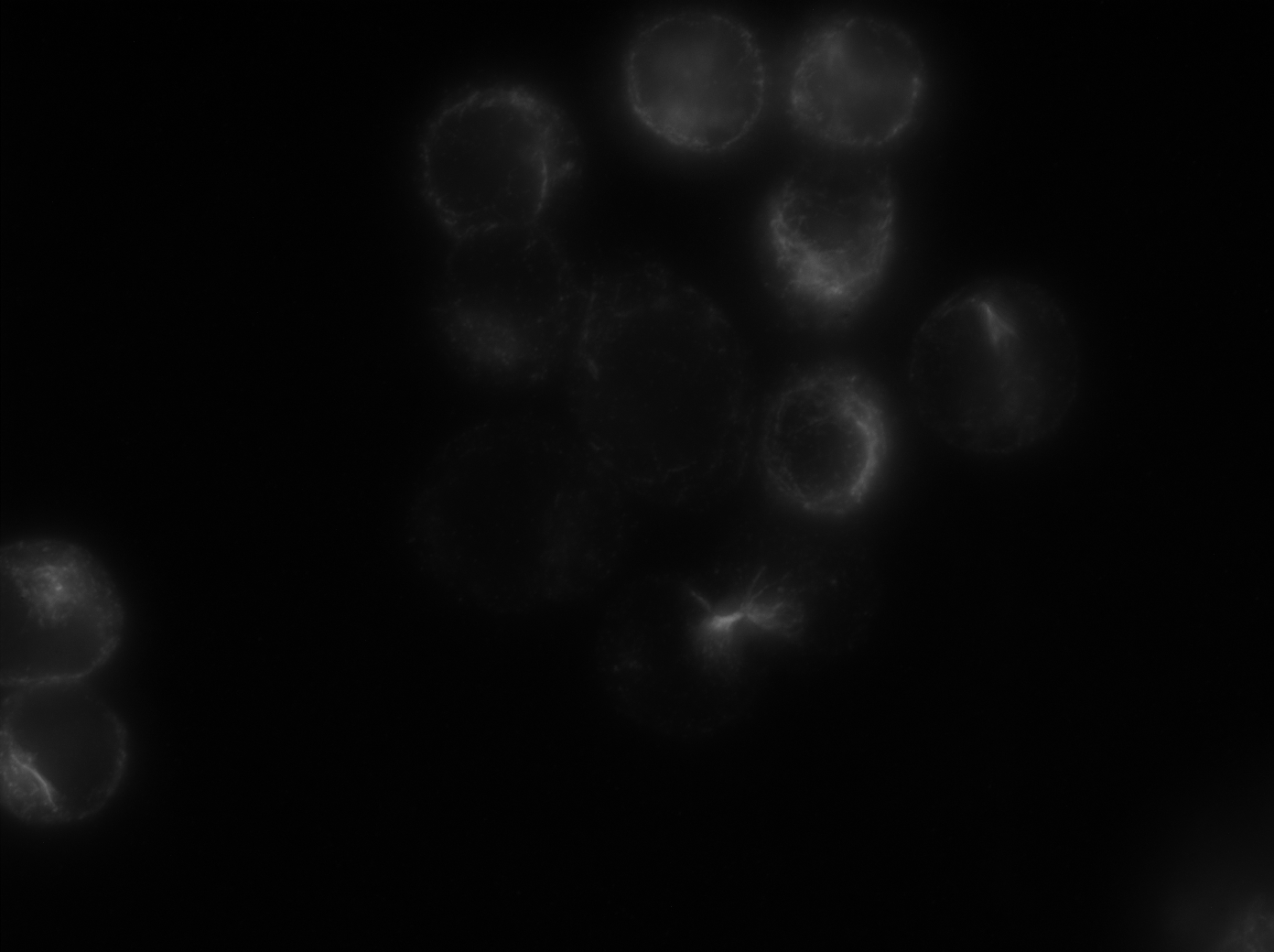

Supplement: Supplementary file 14 — Source data Fig. 4 [file 44319_2026_742_MOESM14_ESM.zip › Figure 4/Fig 4ef Cas9 TPGS1-EYFP-3'UTR acetylated tubulin/Cas9 TPGS1-3utr R3 2-5-25 ET3.Project Maximum Z_XY1738694282_Z0_T0_C1.tif]

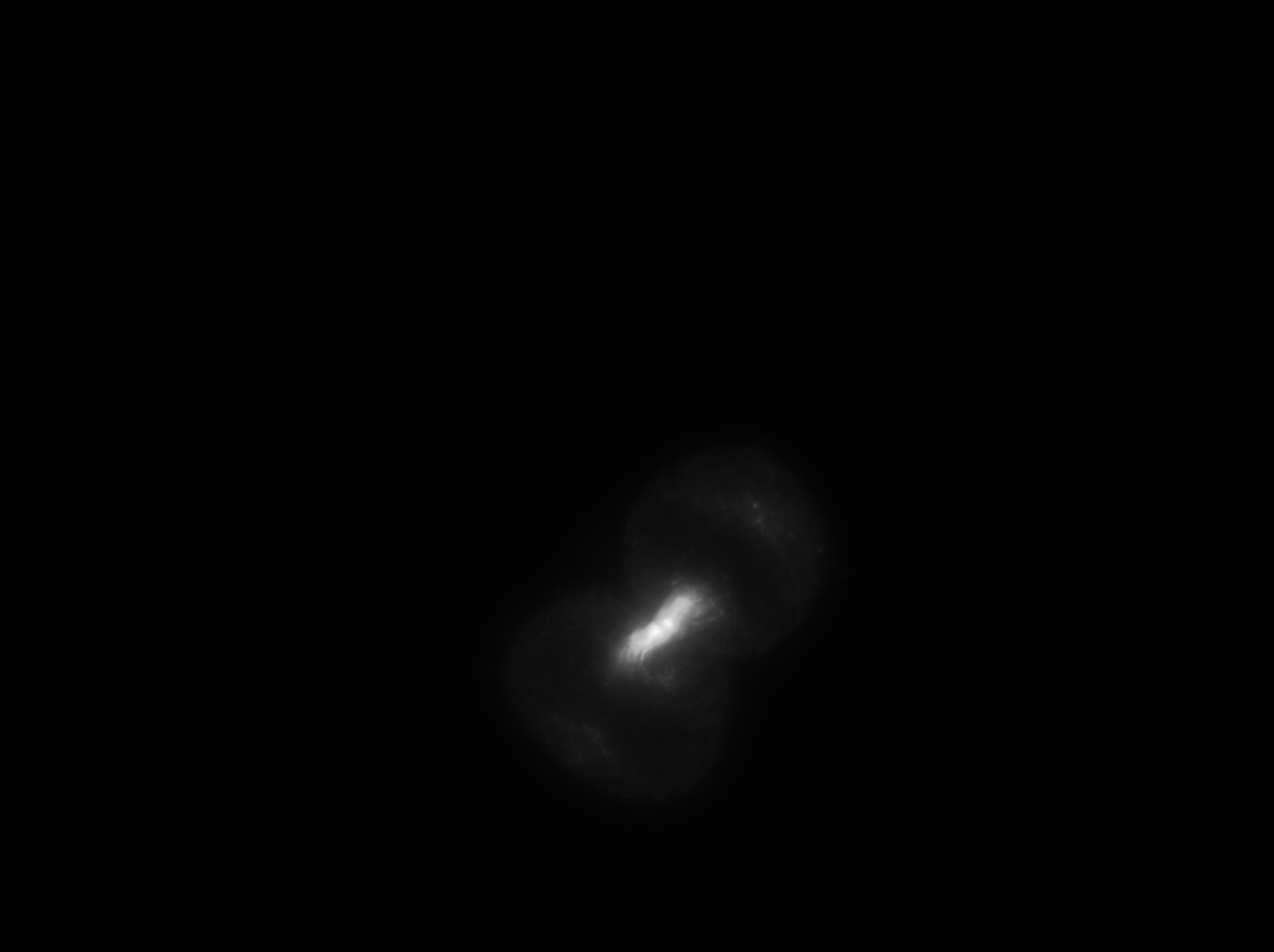

Supplement: Supplementary file 14 — Source data Fig. 4 [file 44319_2026_742_MOESM14_ESM.zip › Figure 4/Fig 4ef Cas9 TPGS1-EYFP-3'UTR acetylated tubulin/Cas9 TPGS1-3utr R1 1-28-24 ET3.Project Maximum Z_XY1738101547_Z0_T0_C1.tif]

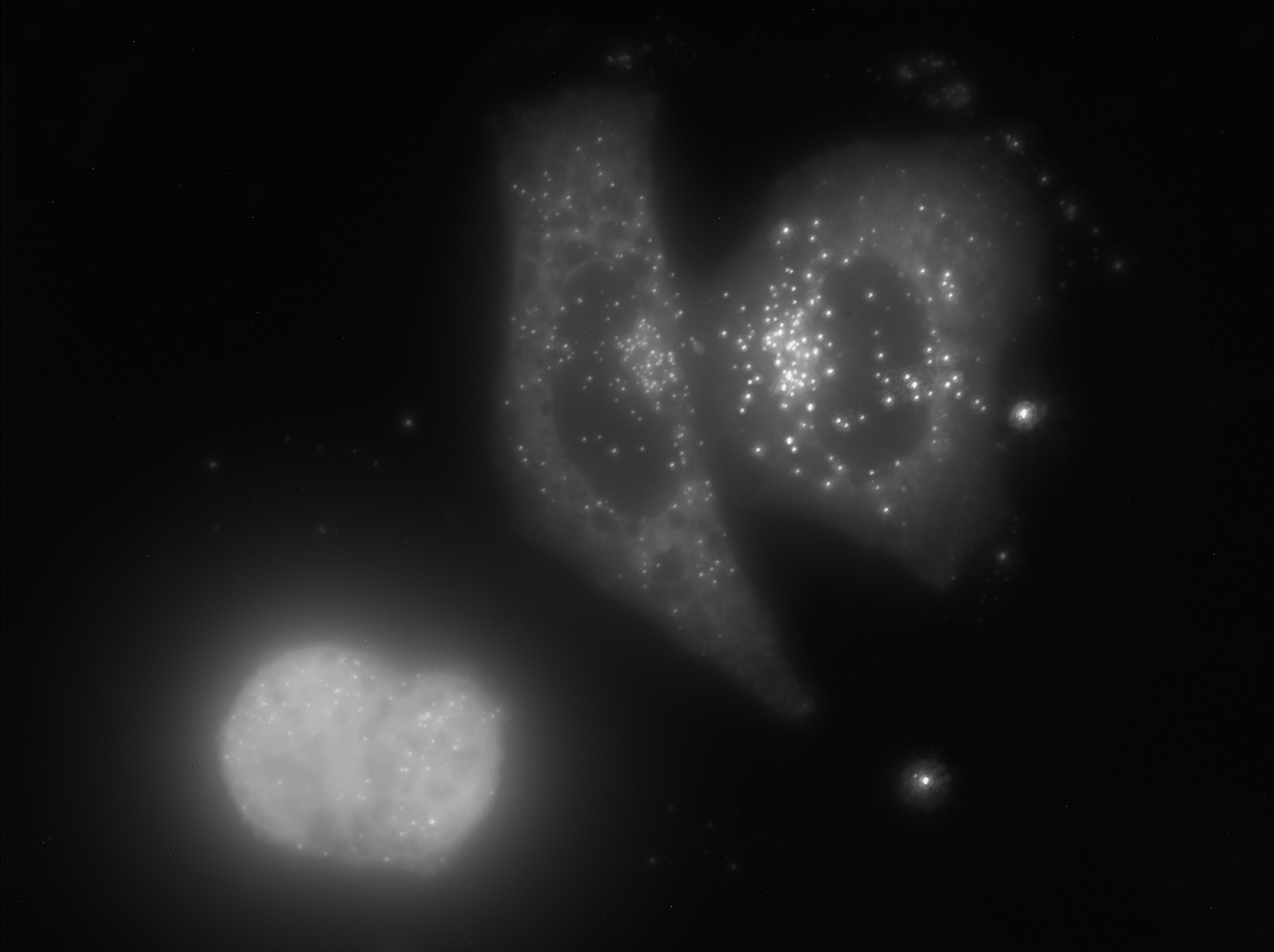

Supplement: Supplementary file 14 — Source data Fig. 4 [file 44319_2026_742_MOESM14_ESM.zip › Figure 4/Fig 4ef Cas9 TPGS1-EYFP-3'UTR acetylated tubulin/Cas9 TPGS1-3utr R1 1-28-24 LT7.Project Maximum Z_XY1738103345_Z0_T0_C2.tif]

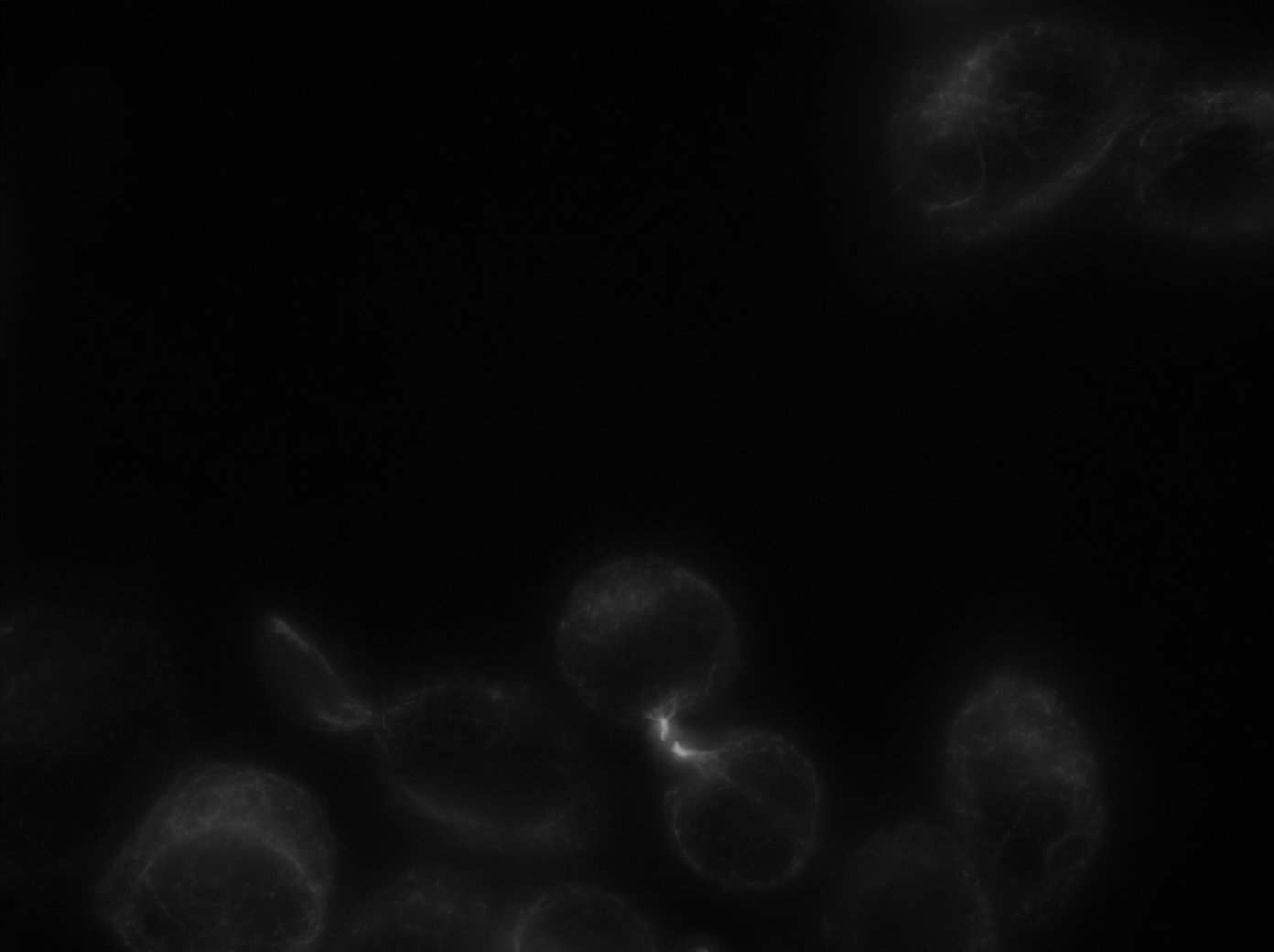

Supplement: Supplementary file 14 — Source data Fig. 4 [file 44319_2026_742_MOESM14_ESM.zip › Figure 4/Fig 4ef Cas9 TPGS1-EYFP-3'UTR acetylated tubulin/Cas9 TPGS1-3utr R3 2-5-25 ET2.Project Maximum Z_XY1738692835_Z0_T0_C1.tif]

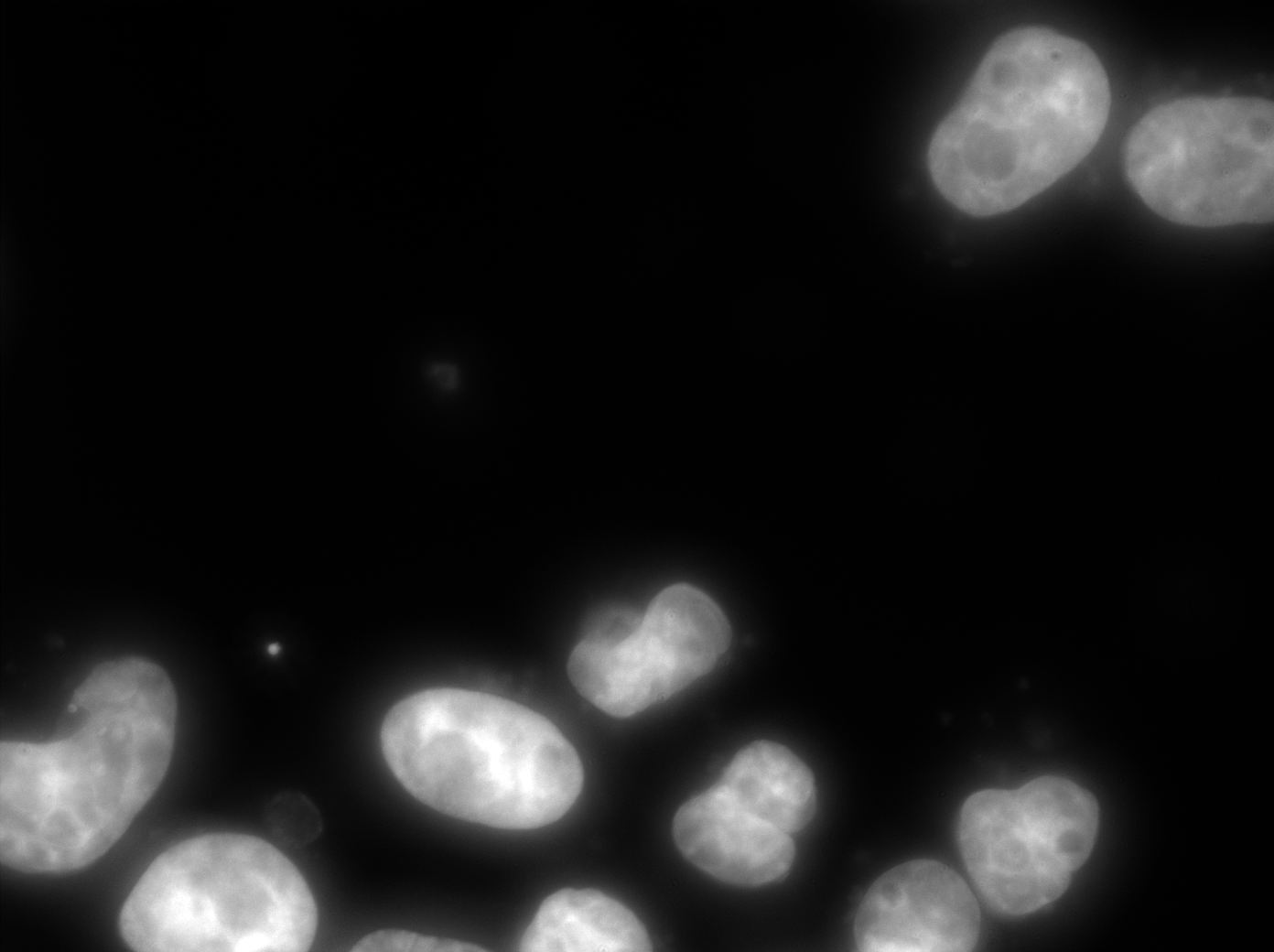

Supplement: Supplementary file 14 — Source data Fig. 4 [file 44319_2026_742_MOESM14_ESM.zip › Figure 4/Fig 4ef Cas9 TPGS1-EYFP-3'UTR acetylated tubulin/Cas9 TPGS1-3utr R3 2-5-25 ET2.Project Maximum Z_XY1738692835_Z0_T0_C0.tif]

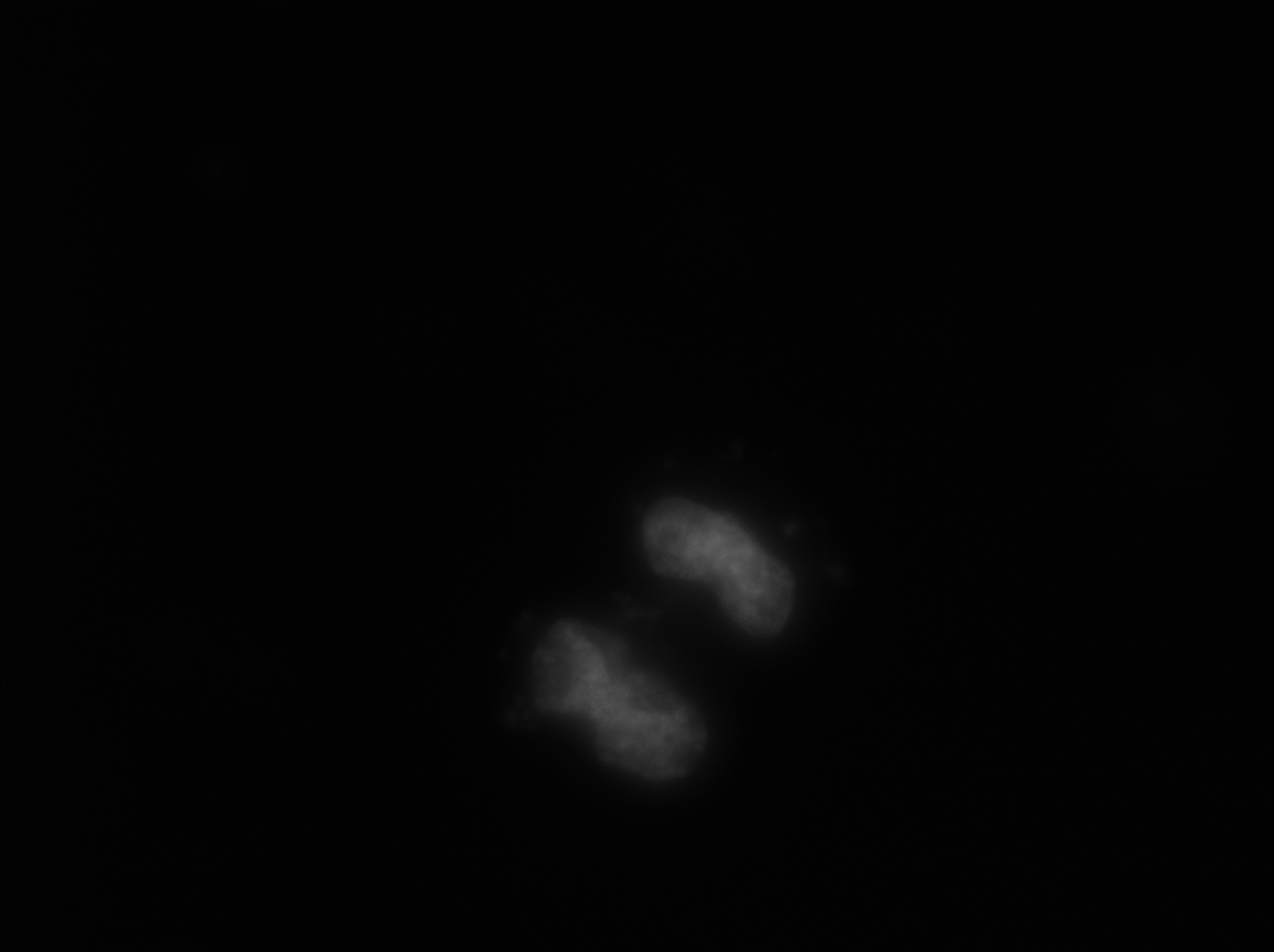

Supplement: Supplementary file 14 — Source data Fig. 4 [file 44319_2026_742_MOESM14_ESM.zip › Figure 4/Fig 4ef Cas9 TPGS1-EYFP-3'UTR acetylated tubulin/Cas9 TPGS1-3utr R1 1-28-24 ET3.Project Maximum Z_XY1738101547_Z0_T0_C0.tif]

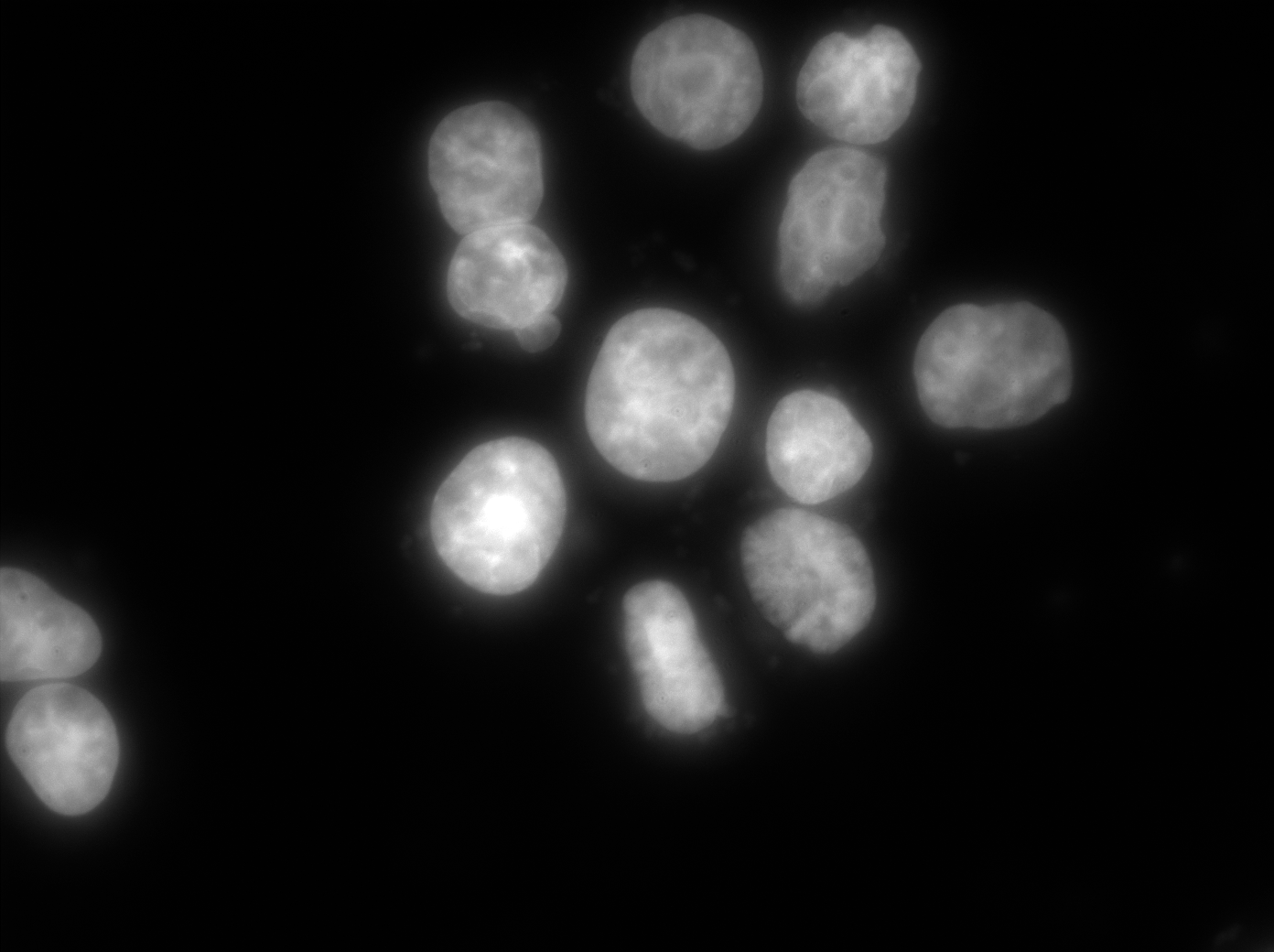

Supplement: Supplementary file 14 — Source data Fig. 4 [file 44319_2026_742_MOESM14_ESM.zip › Figure 4/Fig 4ef Cas9 TPGS1-EYFP-3'UTR acetylated tubulin/Cas9 TPGS1-3utr R3 2-5-25 ET3.Project Maximum Z_XY1738694282_Z0_T0_C0.tif]

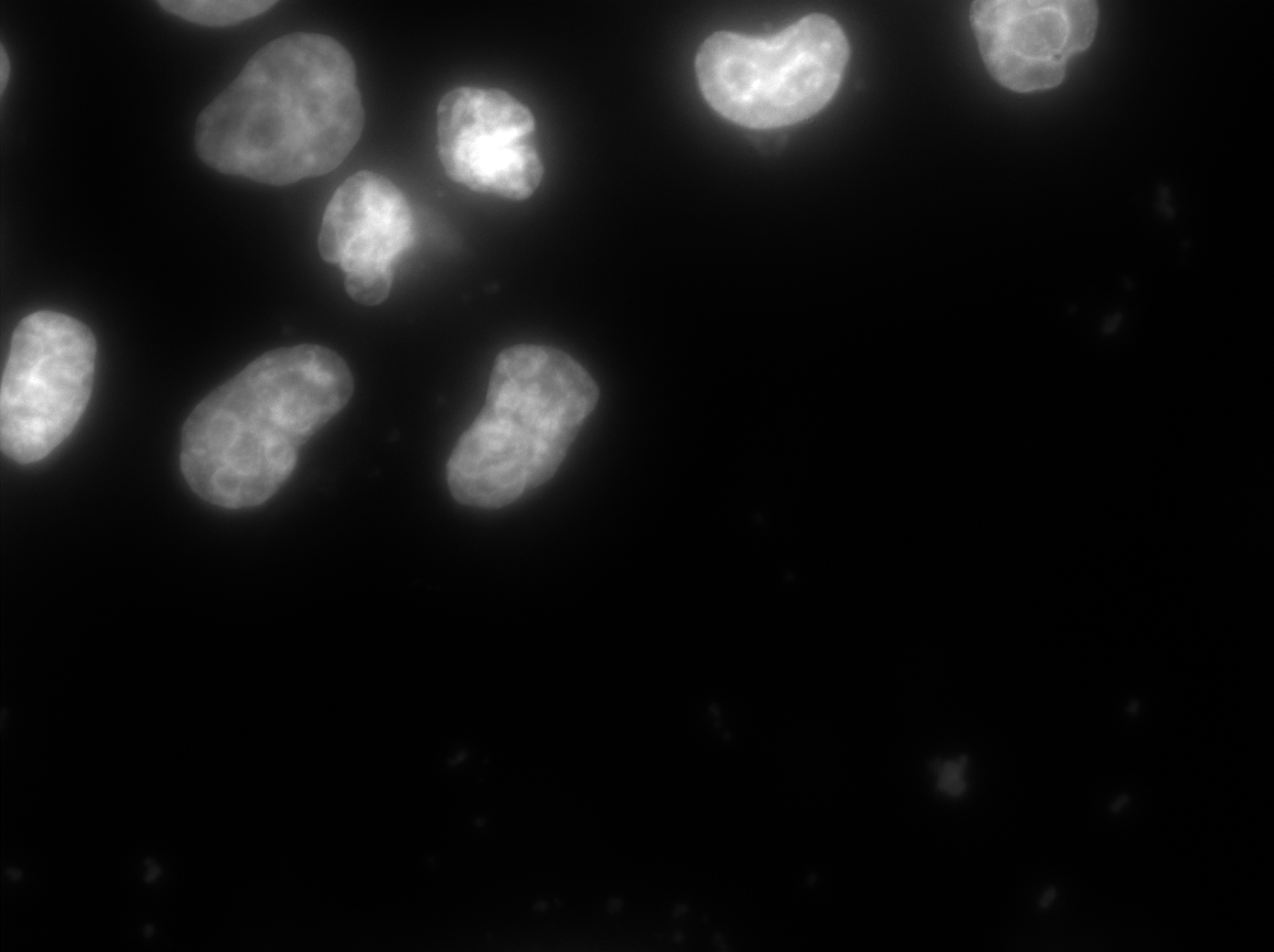

Supplement: Supplementary file 14 — Source data Fig. 4 [file 44319_2026_742_MOESM14_ESM.zip › Figure 4/Fig 4ef Cas9 TPGS1-EYFP-3'UTR acetylated tubulin/Cas9 TPGS1-3utr R2 2-5-25 LT9.Project Maximum Z_XY1738625881_Z0_T0_C0.tif]

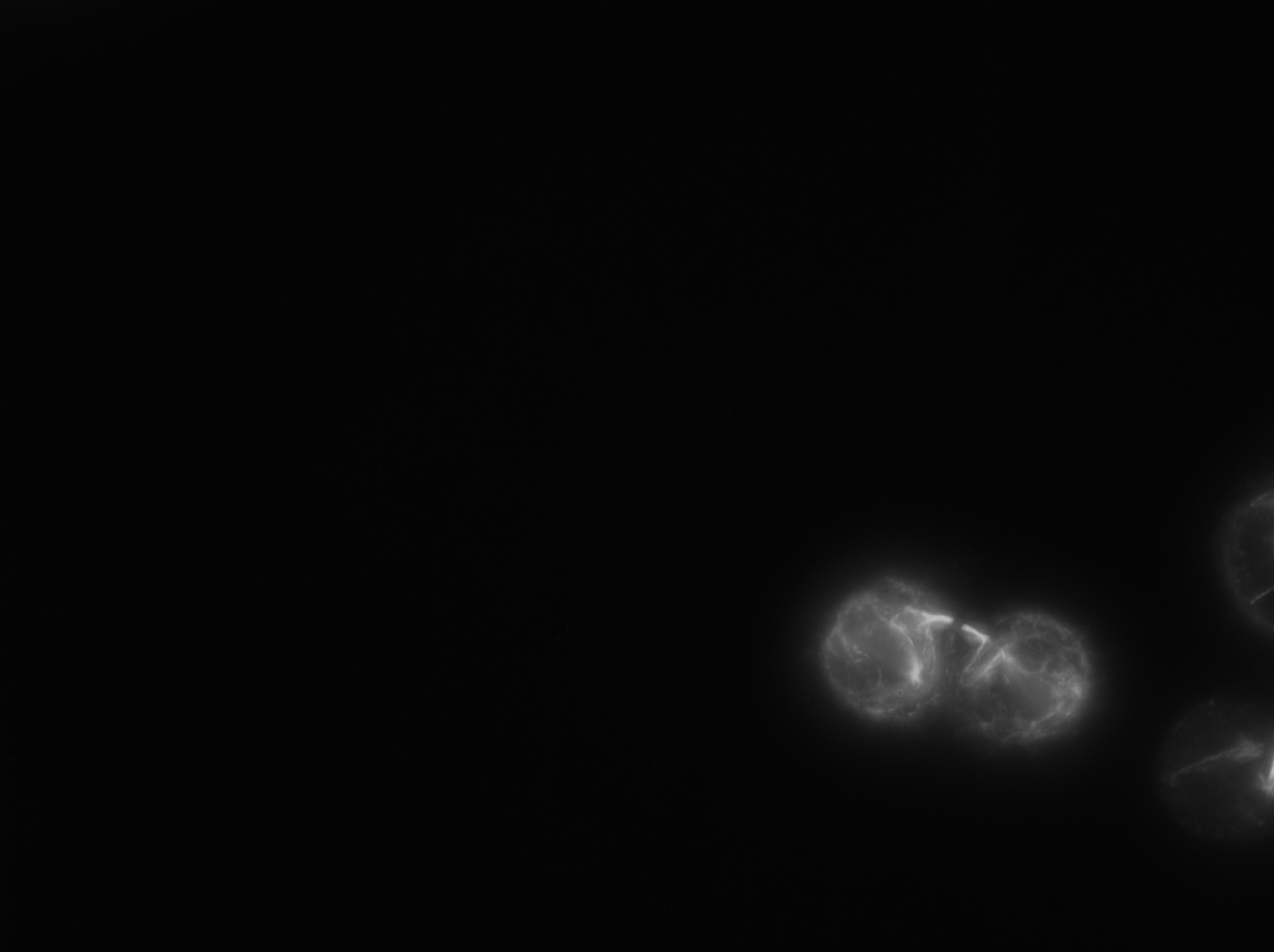

Supplement: Supplementary file 14 — Source data Fig. 4 [file 44319_2026_742_MOESM14_ESM.zip › Figure 4/Fig 4ef Cas9 TPGS1-EYFP-3'UTR acetylated tubulin/Cas9 TPGS1-3utr R2 2-5-25 ET10.Project Maximum Z_XY1738624693_Z0_T0_C1.tif]

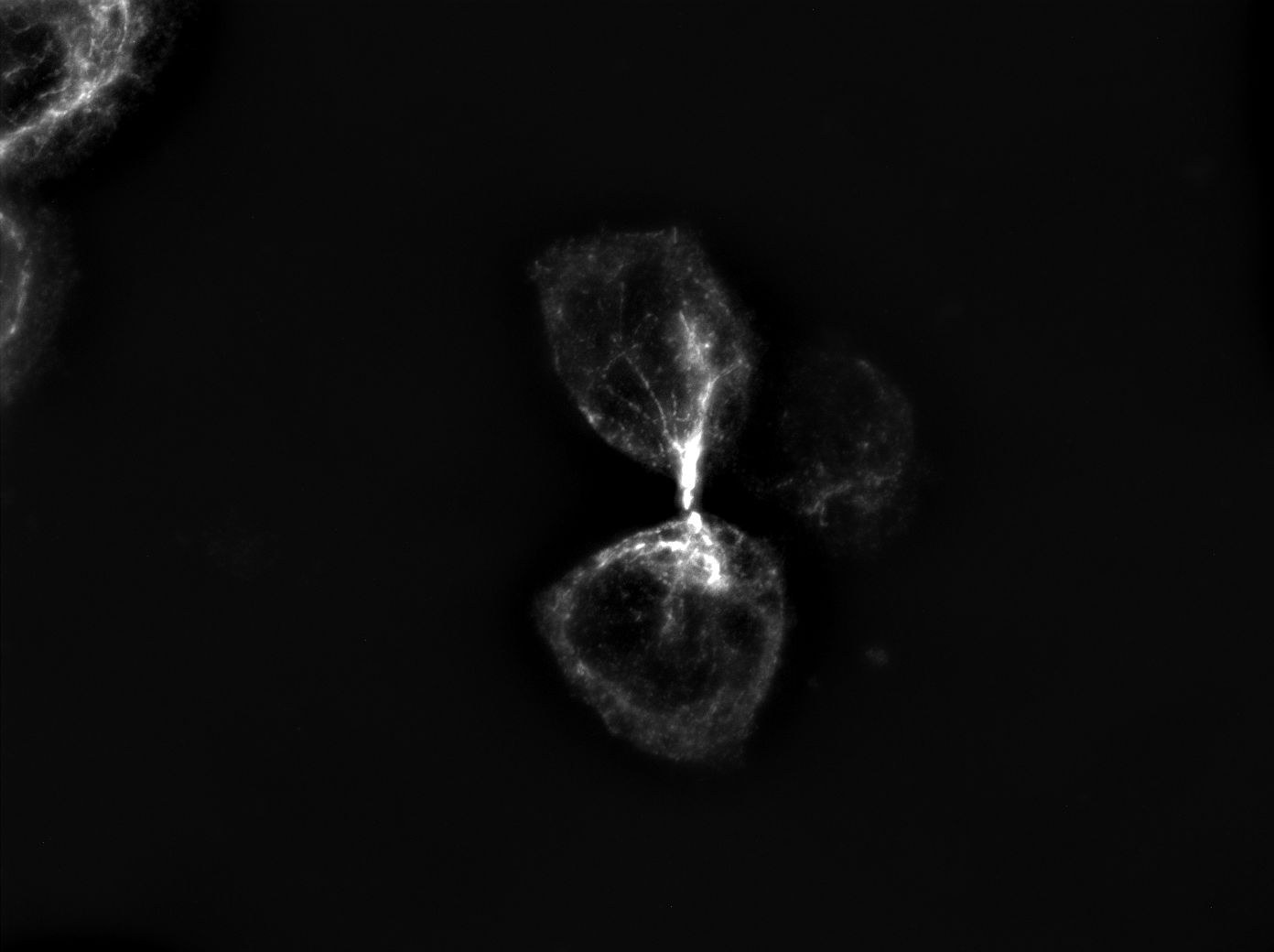

Supplement: Supplementary file 14 — Source data Fig. 4 [file 44319_2026_742_MOESM14_ESM.zip › Figure 4/Fig 4ef Cas9 TPGS1-EYFP-3'UTR acetylated tubulin/Cas9 TPGS1-3utr R1 1-28-24 LT4.NearN.Project Maximum Z_XY1738101264_Z0_T0_C1.tif]

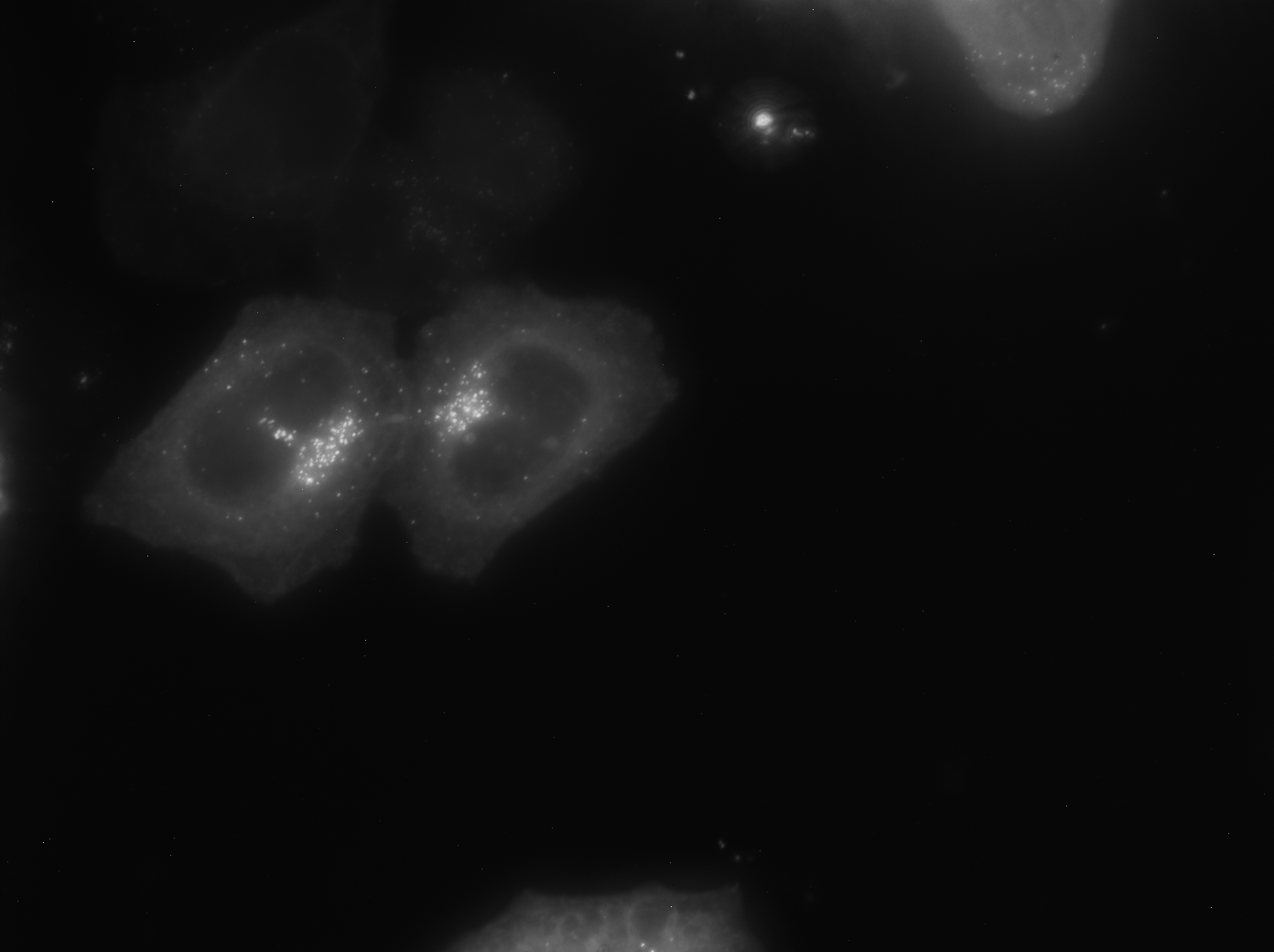

Supplement: Supplementary file 14 — Source data Fig. 4 [file 44319_2026_742_MOESM14_ESM.zip › Figure 4/Fig 4ef Cas9 TPGS1-EYFP-3'UTR acetylated tubulin/Cas9 TPGS1-3utr R2 2-5-25 LT9.Project Maximum Z_XY1738625881_Z0_T0_C2.tif]

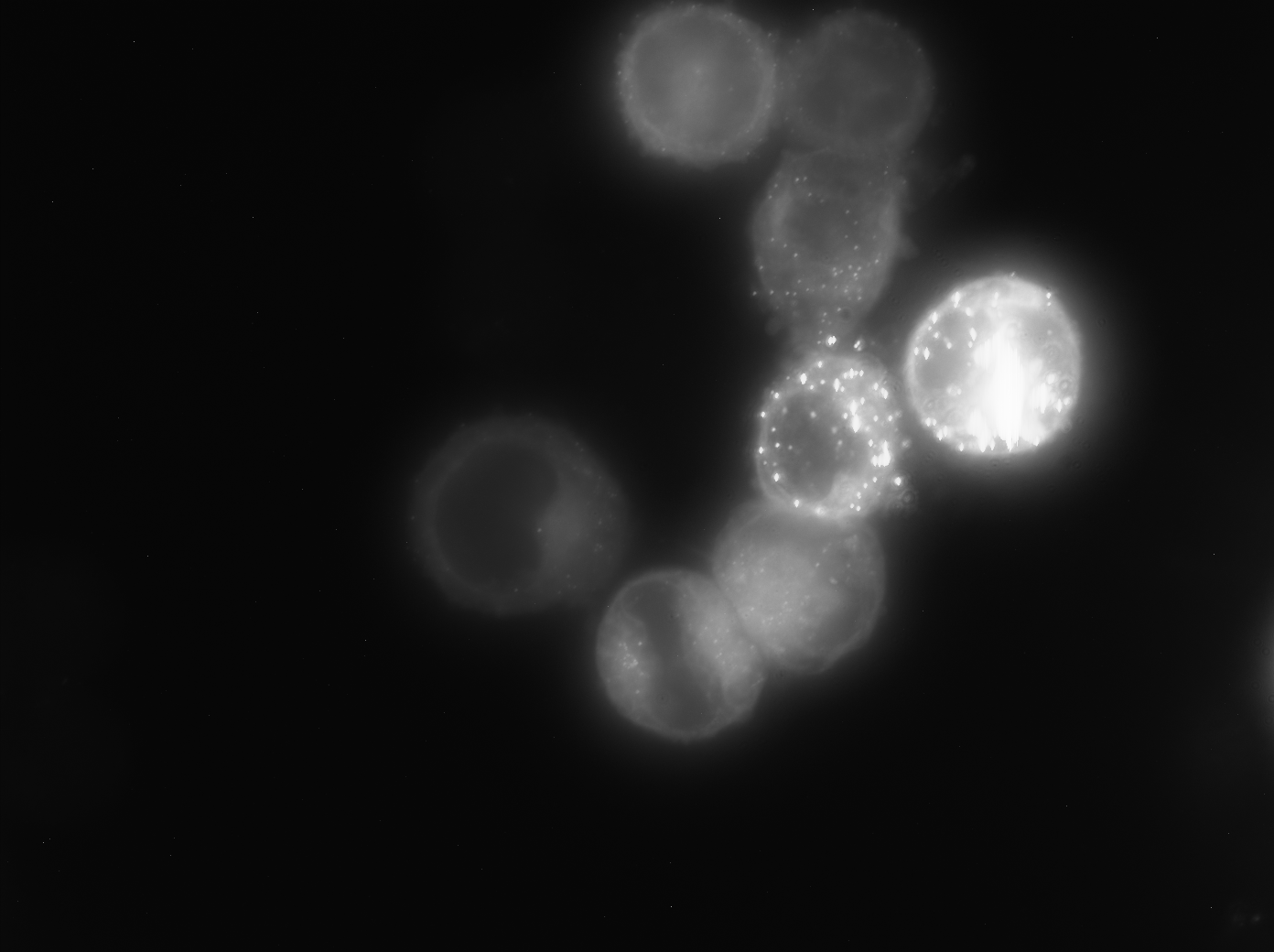

Supplement: Supplementary file 14 — Source data Fig. 4 [file 44319_2026_742_MOESM14_ESM.zip › Figure 4/Fig 4ef Cas9 TPGS1-EYFP-3'UTR acetylated tubulin/Cas9 TPGS1-3utr R3 2-5-25 ET3.Project Maximum Z_XY1738694282_Z0_T0_C2.tif]

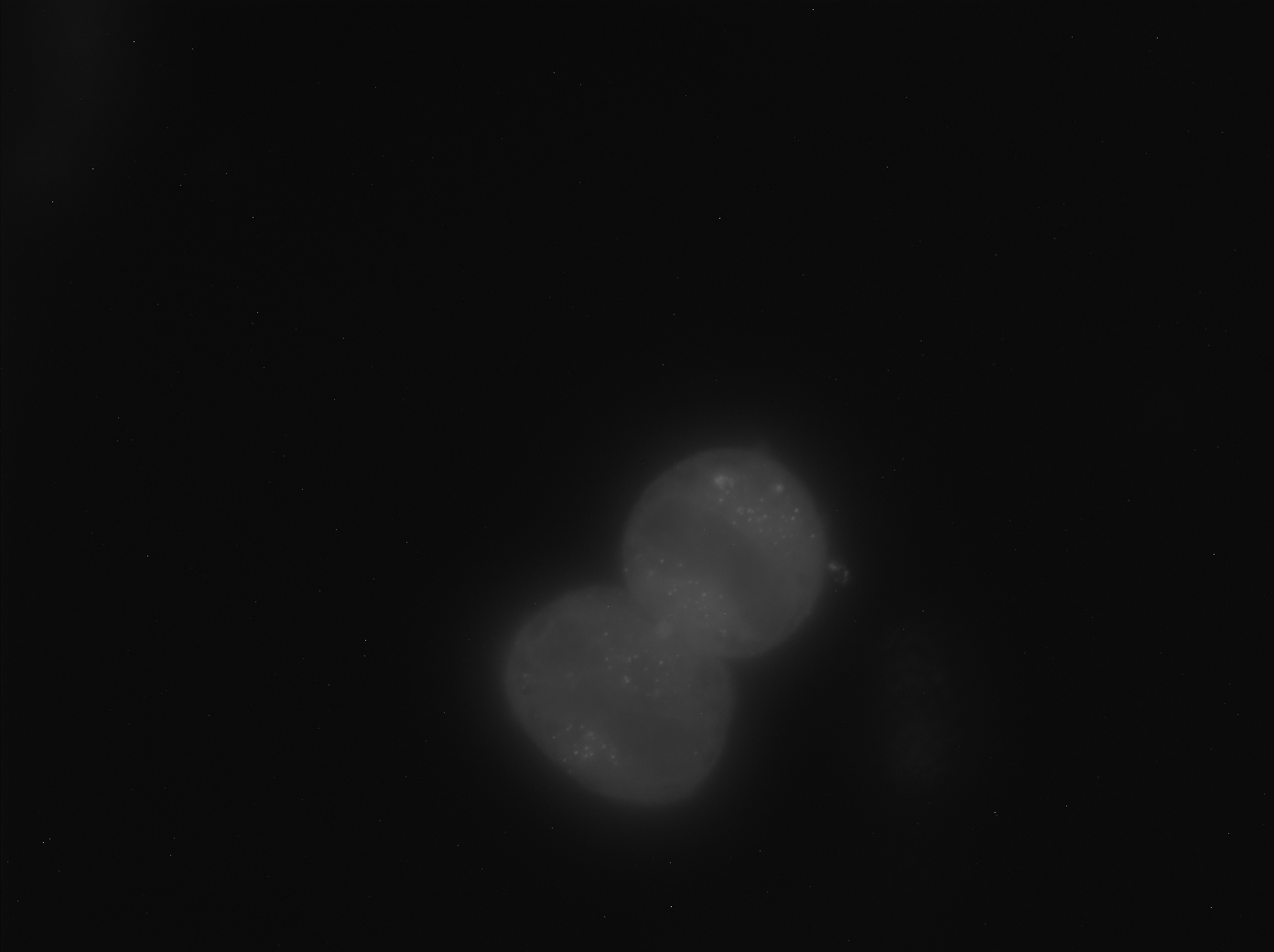

Supplement: Supplementary file 14 — Source data Fig. 4 [file 44319_2026_742_MOESM14_ESM.zip › Figure 4/Fig 4ef Cas9 TPGS1-EYFP-3'UTR acetylated tubulin/Cas9 TPGS1-3utr R1 1-28-24 ET3.Project Maximum Z_XY1738101547_Z0_T0_C2.tif]

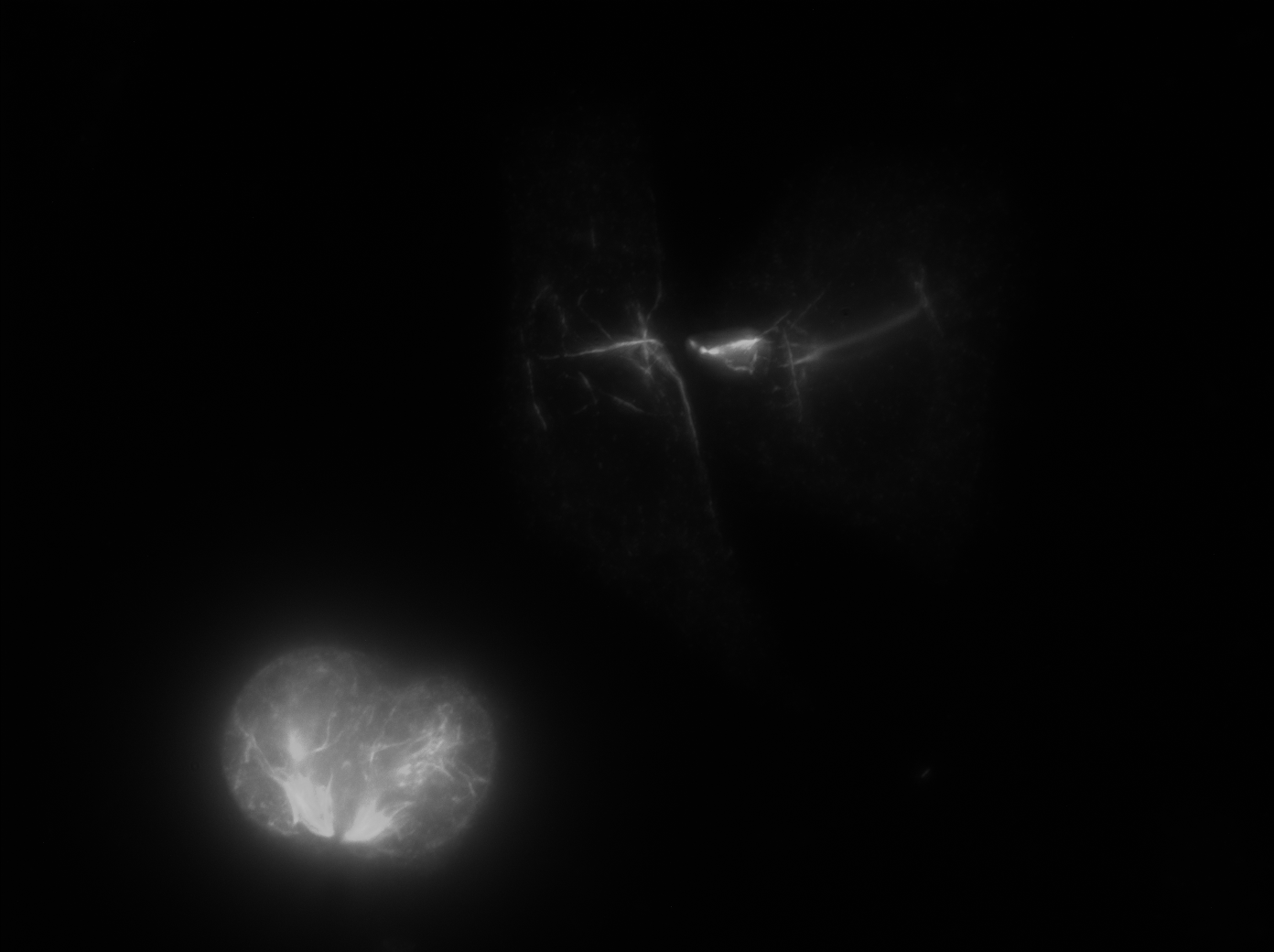

Supplement: Supplementary file 14 — Source data Fig. 4 [file 44319_2026_742_MOESM14_ESM.zip › Figure 4/Fig 4ef Cas9 TPGS1-EYFP-3'UTR acetylated tubulin/Cas9 TPGS1-3utr R1 1-28-24 LT7.Project Maximum Z_XY1738103345_Z0_T0_C1.tif]

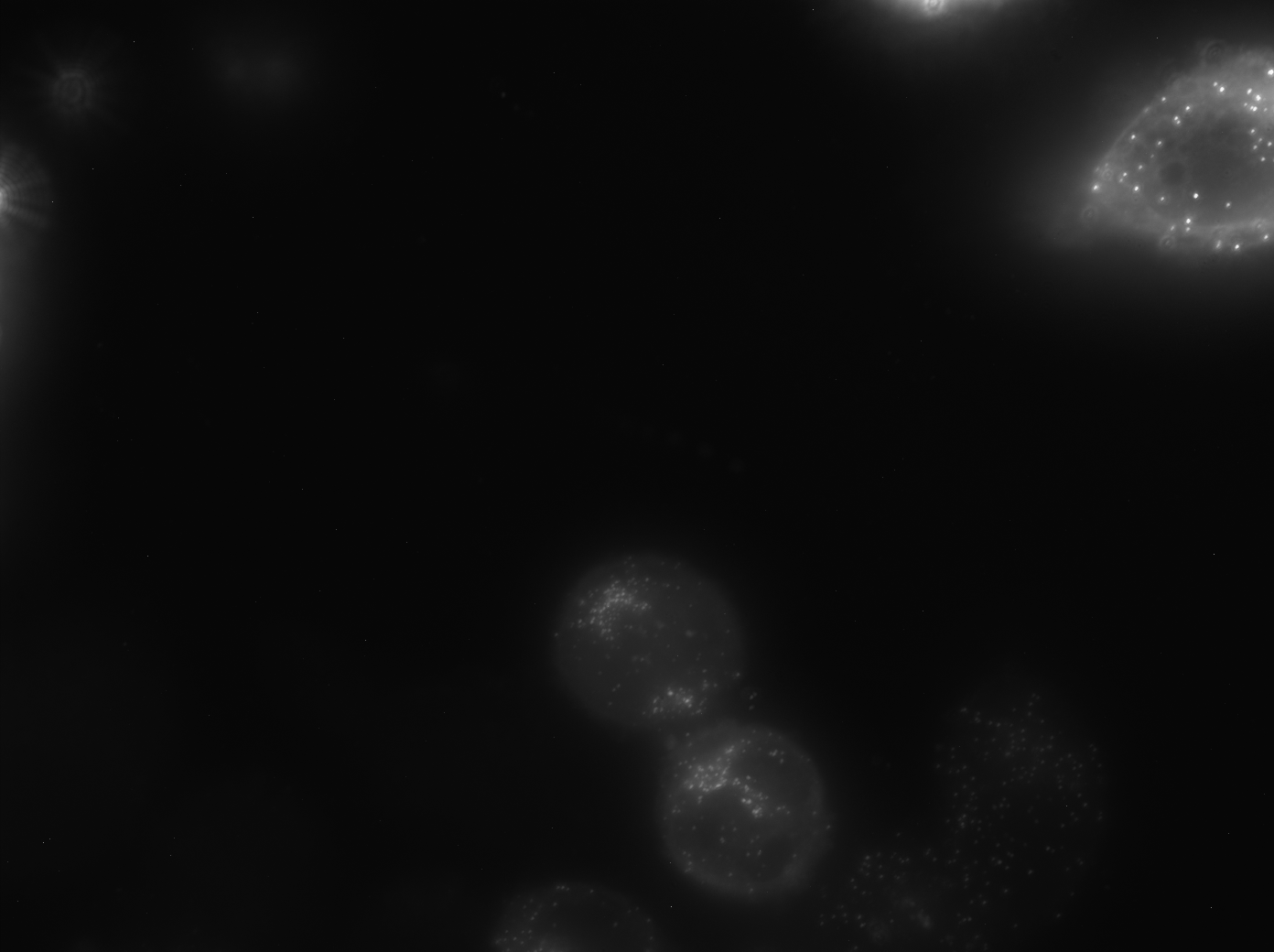

Supplement: Supplementary file 14 — Source data Fig. 4 [file 44319_2026_742_MOESM14_ESM.zip › Figure 4/Fig 4ef Cas9 TPGS1-EYFP-3'UTR acetylated tubulin/Cas9 TPGS1-3utr R3 2-5-25 ET2.Project Maximum Z_XY1738692835_Z0_T0_C2.tif]

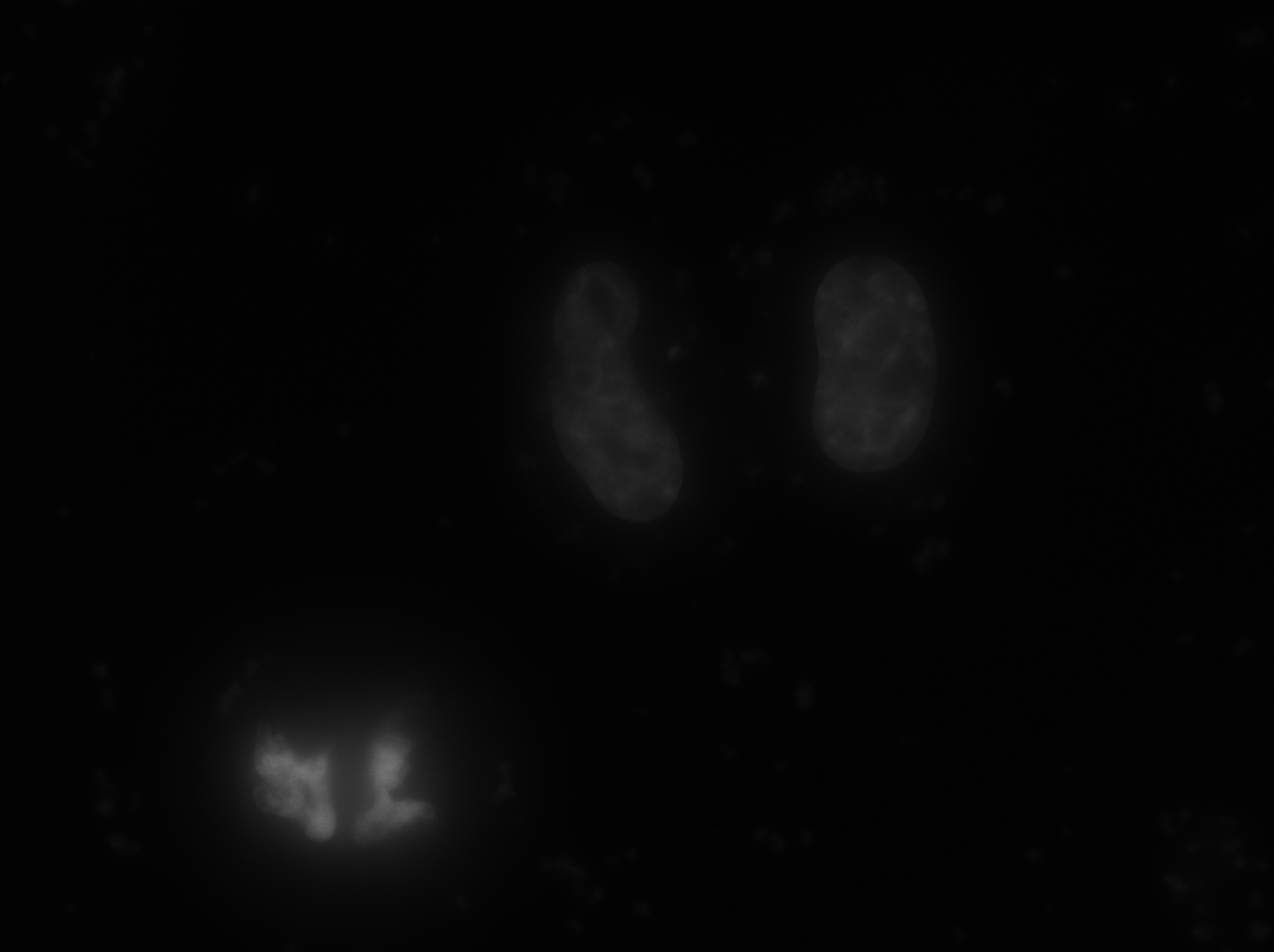

Supplement: Supplementary file 14 — Source data Fig. 4 [file 44319_2026_742_MOESM14_ESM.zip › Figure 4/Fig 4ef Cas9 TPGS1-EYFP-3'UTR acetylated tubulin/Cas9 TPGS1-3utr R1 1-28-24 LT7.Project Maximum Z_XY1738103345_Z0_T0_C0.tif]

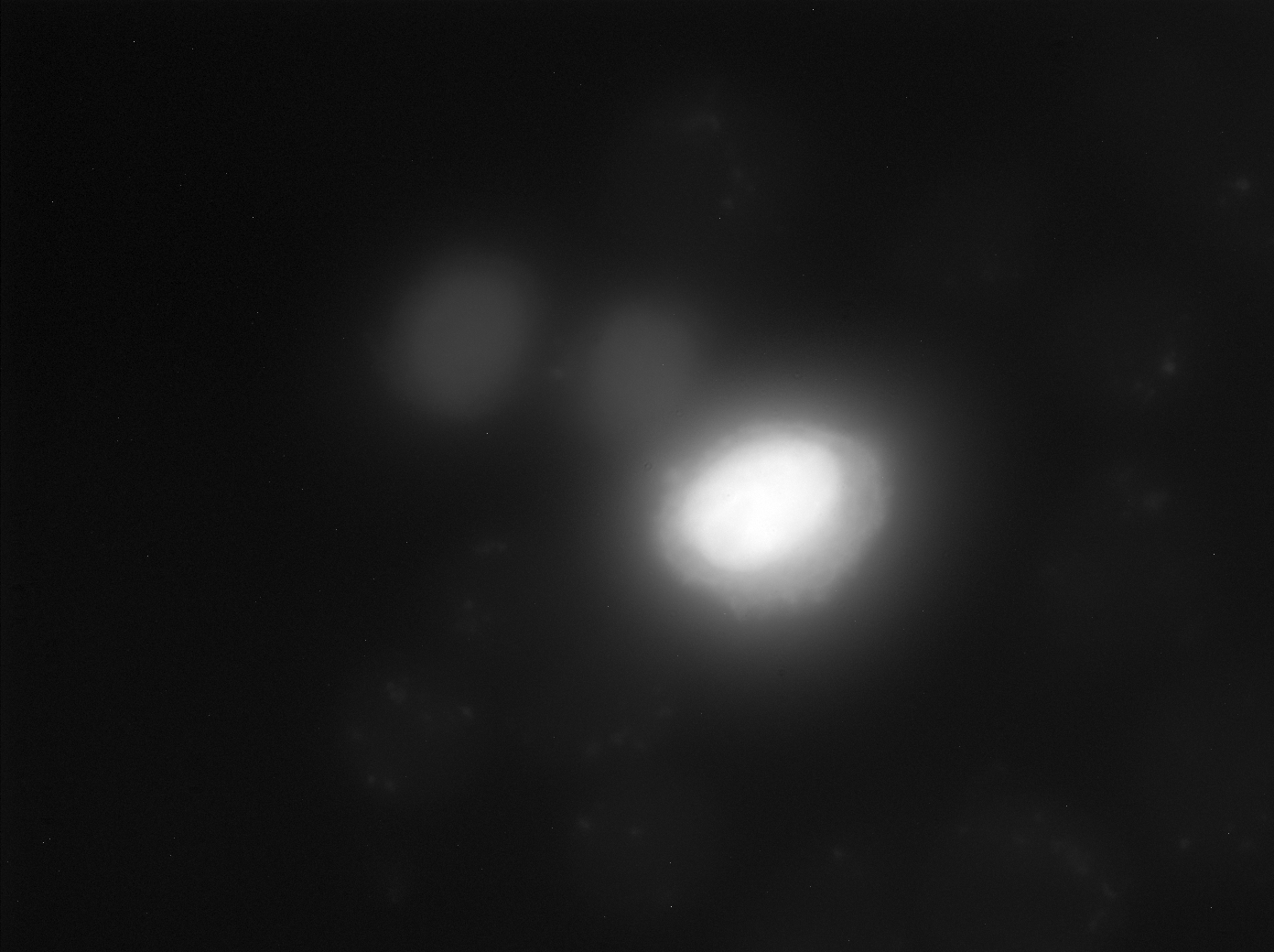

Supplement: Supplementary file 18 — Source data Fig. 5 part 4 [file 44319_2026_742_MOESM18_ESM.zip › Figure 5 Part 4/Fig 5ab WT and KO hela TTLL1-e326g atubulin/EGFP/EGFP-N3 atub R1 LT6.Project Maximum Z_XY1724714341_Z0_T0_C1.tif]

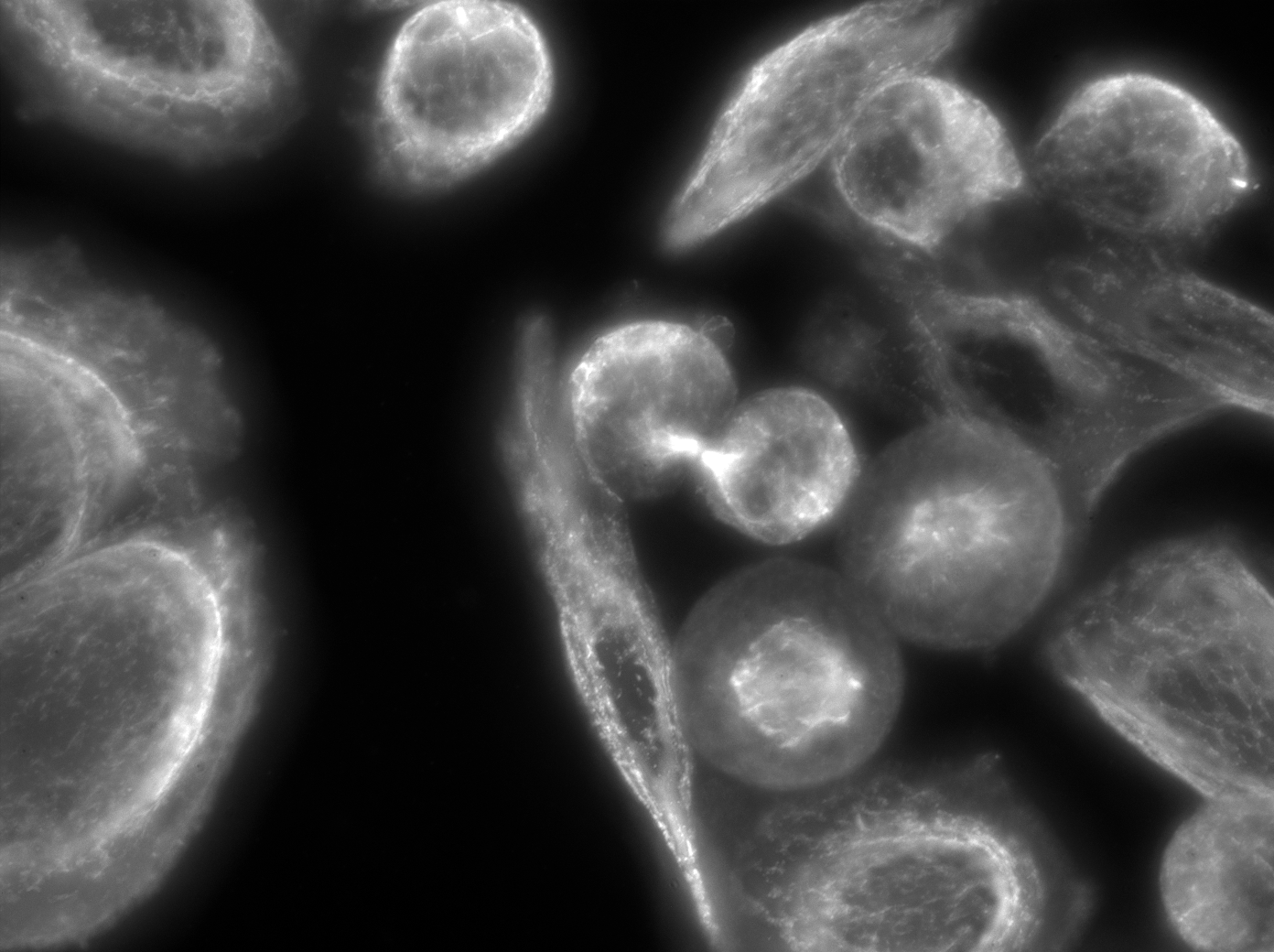

Supplement: Supplementary file 18 — Source data Fig. 5 part 4 [file 44319_2026_742_MOESM18_ESM.zip › Figure 5 Part 4/Fig 5ab WT and KO hela TTLL1-e326g atubulin/EGFP/EGFP-N2 8-23-24 atub R2 ET3 M1M2.Project Maximum Z_XY1725569489_Z0_T0_C2.tif]

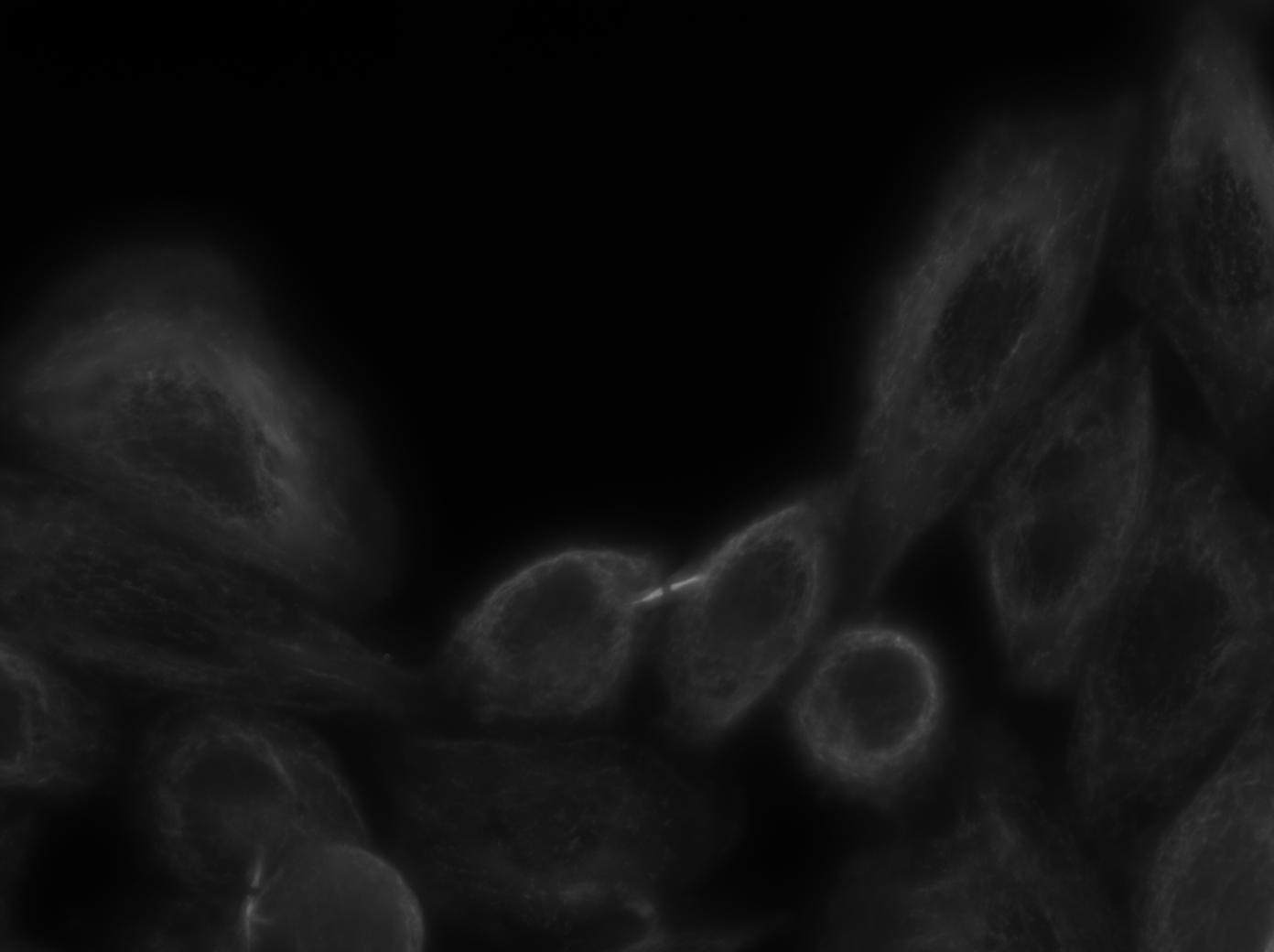

Supplement: Supplementary file 18 — Source data Fig. 5 part 4 [file 44319_2026_742_MOESM18_ESM.zip › Figure 5 Part 4/Fig 5ab WT and KO hela TTLL1-e326g atubulin/EGFP/EGFP-N2 8-23-24 atub R2 LT5.Project Maximum Z_XY1725568784_Z0_T0_C2.tif]

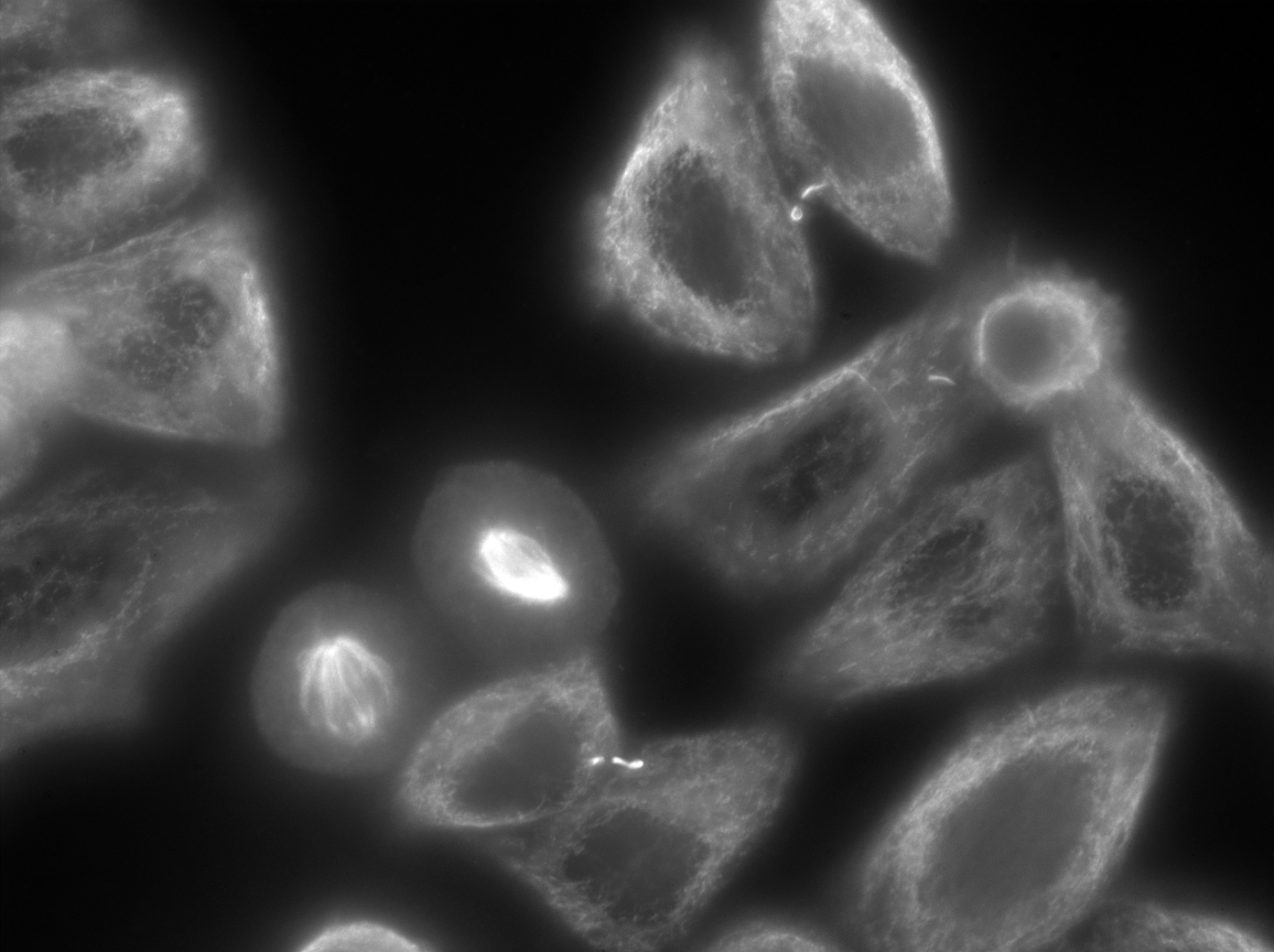

Supplement: Supplementary file 18 — Source data Fig. 5 part 4 [file 44319_2026_742_MOESM18_ESM.zip › Figure 5 Part 4/Fig 5ab WT and KO hela TTLL1-e326g atubulin/EGFP/EGFP-N3 atub R1 LT7LT8LT9.Project Maximum Z_XY1724714518_Z0_T0_C2.tif]

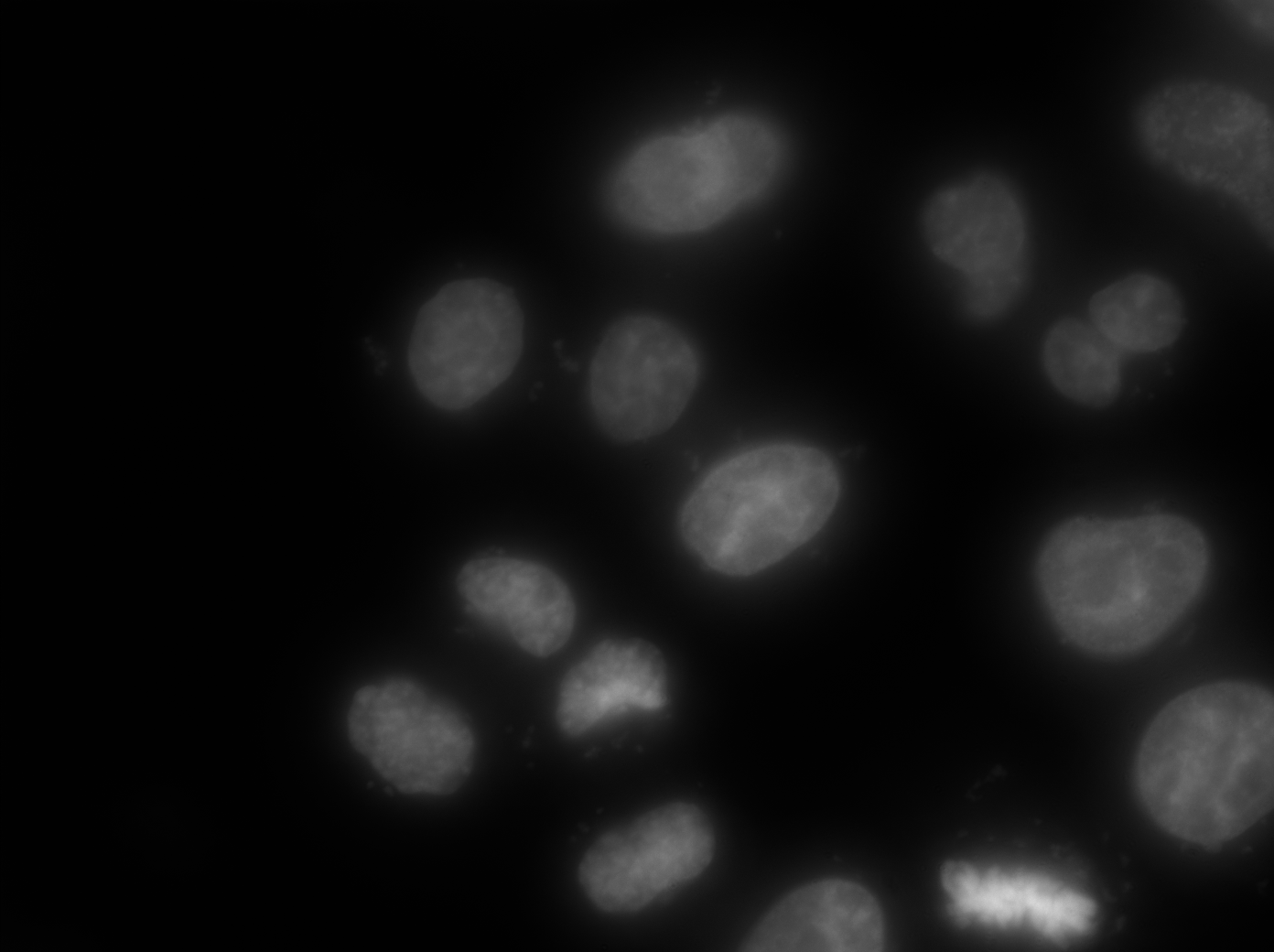

Supplement: Supplementary file 18 — Source data Fig. 5 part 4 [file 44319_2026_742_MOESM18_ESM.zip › Figure 5 Part 4/Fig 5ab WT and KO hela TTLL1-e326g atubulin/EGFP/EGFP-N3 atub R1 LT6.Project Maximum Z_XY1724714341_Z0_T0_C0.tif]

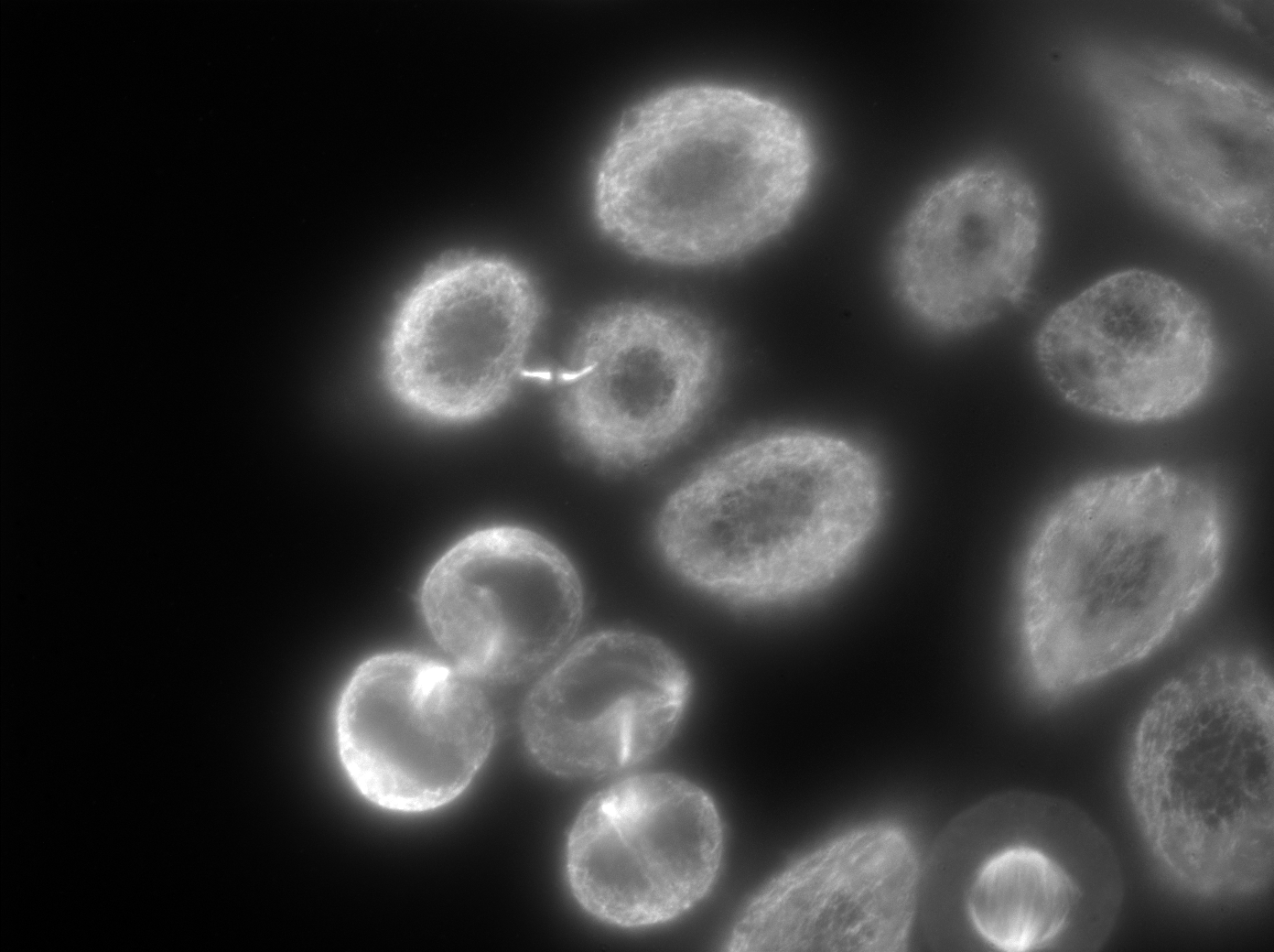

Supplement: Supplementary file 18 — Source data Fig. 5 part 4 [file 44319_2026_742_MOESM18_ESM.zip › Figure 5 Part 4/Fig 5ab WT and KO hela TTLL1-e326g atubulin/EGFP/EGFP-N3 atub R1 LT6.Project Maximum Z_XY1724714341_Z0_T0_C2.tif]

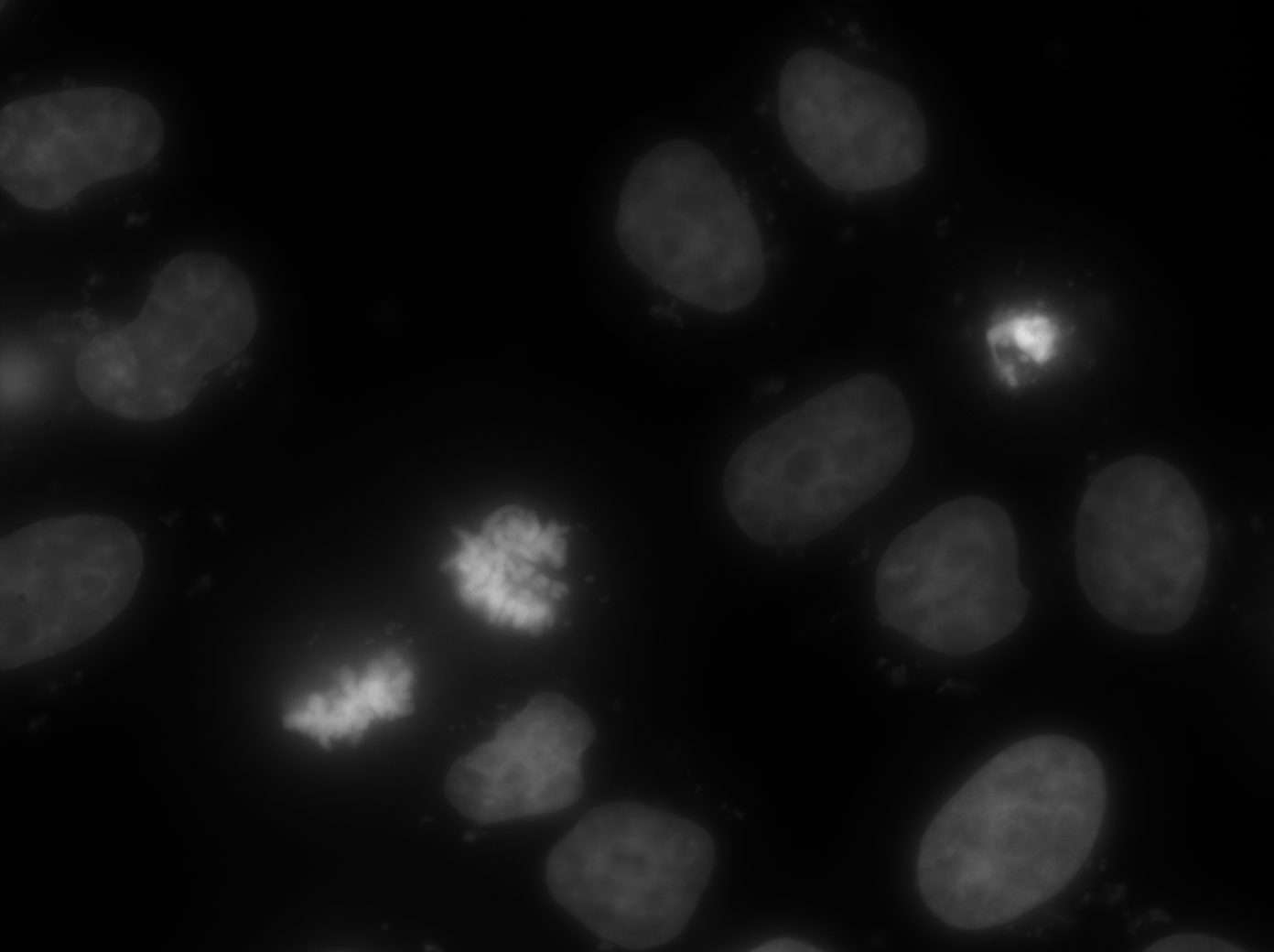

Supplement: Supplementary file 18 — Source data Fig. 5 part 4 [file 44319_2026_742_MOESM18_ESM.zip › Figure 5 Part 4/Fig 5ab WT and KO hela TTLL1-e326g atubulin/EGFP/EGFP-N3 atub R1 LT7LT8LT9.Project Maximum Z_XY1724714518_Z0_T0_C0.tif]

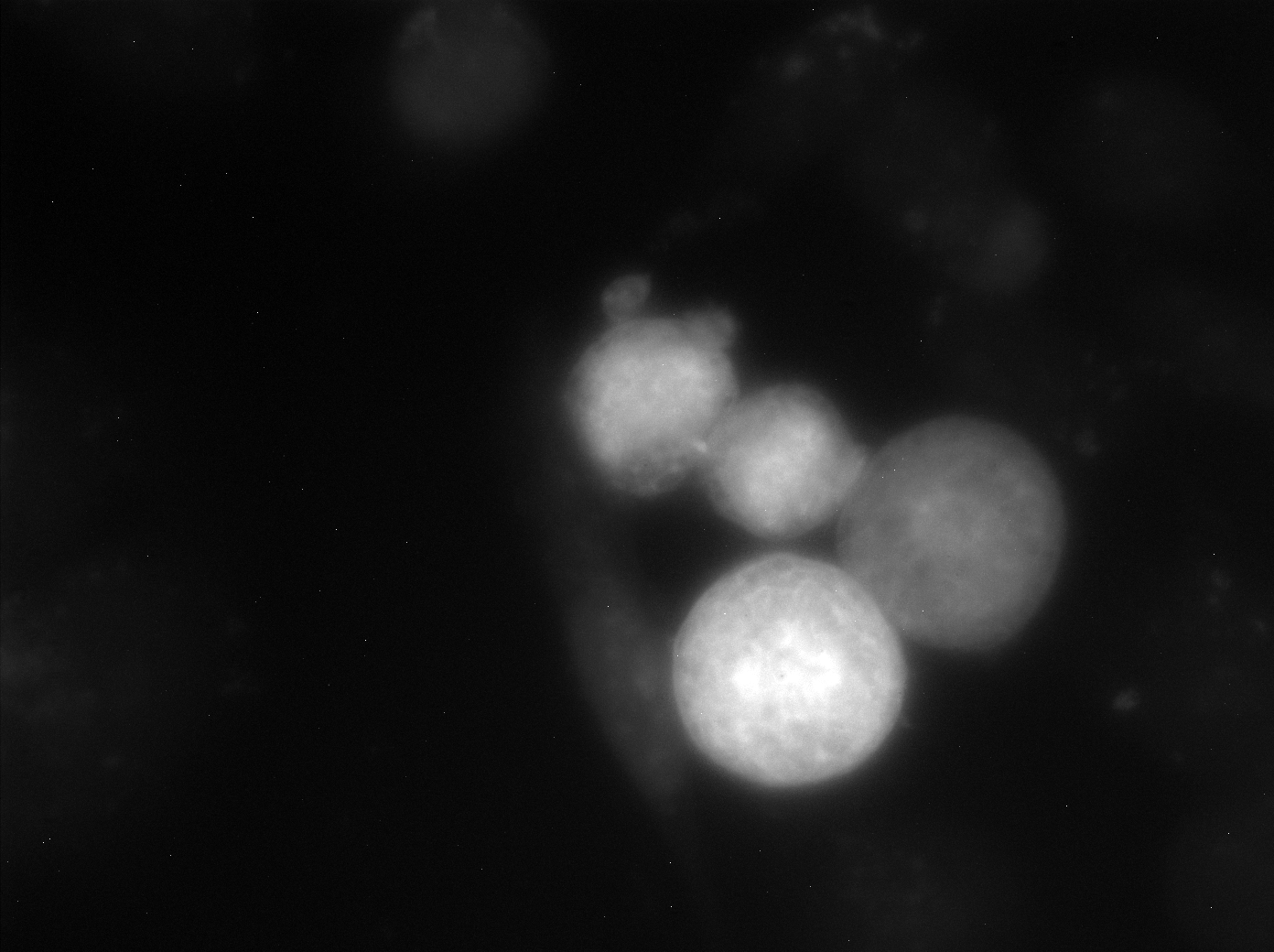

Supplement: Supplementary file 18 — Source data Fig. 5 part 4 [file 44319_2026_742_MOESM18_ESM.zip › Figure 5 Part 4/Fig 5ab WT and KO hela TTLL1-e326g atubulin/EGFP/EGFP-N2 8-23-24 atub R2 ET3 M1M2.Project Maximum Z_XY1725569489_Z0_T0_C1.tif]

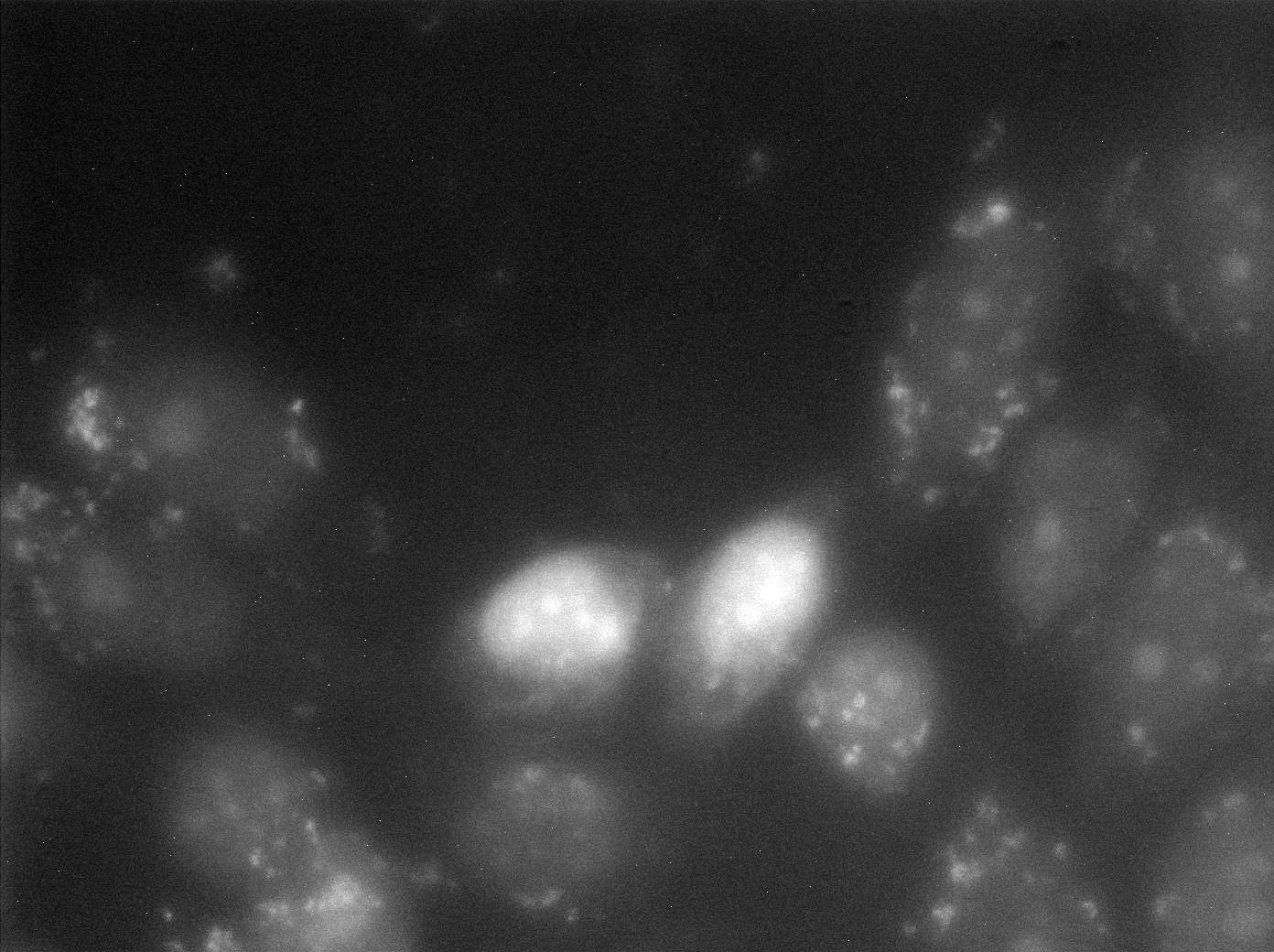

Supplement: Supplementary file 18 — Source data Fig. 5 part 4 [file 44319_2026_742_MOESM18_ESM.zip › Figure 5 Part 4/Fig 5ab WT and KO hela TTLL1-e326g atubulin/EGFP/EGFP-N2 8-23-24 atub R2 LT5.Project Maximum Z_XY1725568784_Z0_T0_C1.tif]

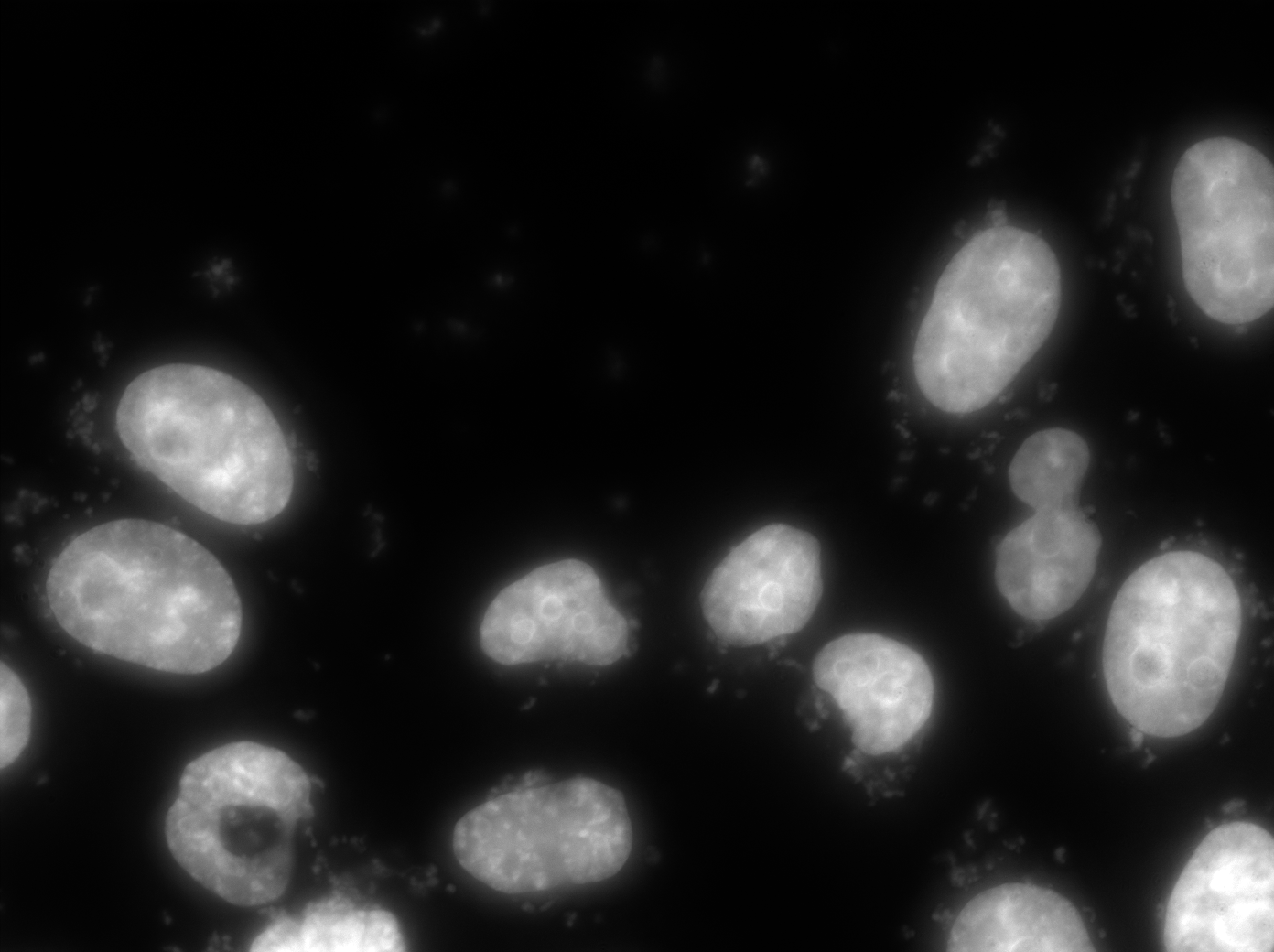

Supplement: Supplementary file 18 — Source data Fig. 5 part 4 [file 44319_2026_742_MOESM18_ESM.zip › Figure 5 Part 4/Fig 5ab WT and KO hela TTLL1-e326g atubulin/EGFP/EGFP-N2 8-23-24 atub R2 LT5.Project Maximum Z_XY1725568784_Z0_T0_C0.tif]
