# Supplementary figures and images for: TPGS1 regulates central spindle microtubule glutamylation and remodeling during telophase and abscission (part 20 of 36)
Source: EMBO Rep. 2026 Mar 23;27(8):1944–63. doi: 10.1038/s44319-026-00742-3 (PMC13121839; doi:10.1038/s44319-026-00742-3)

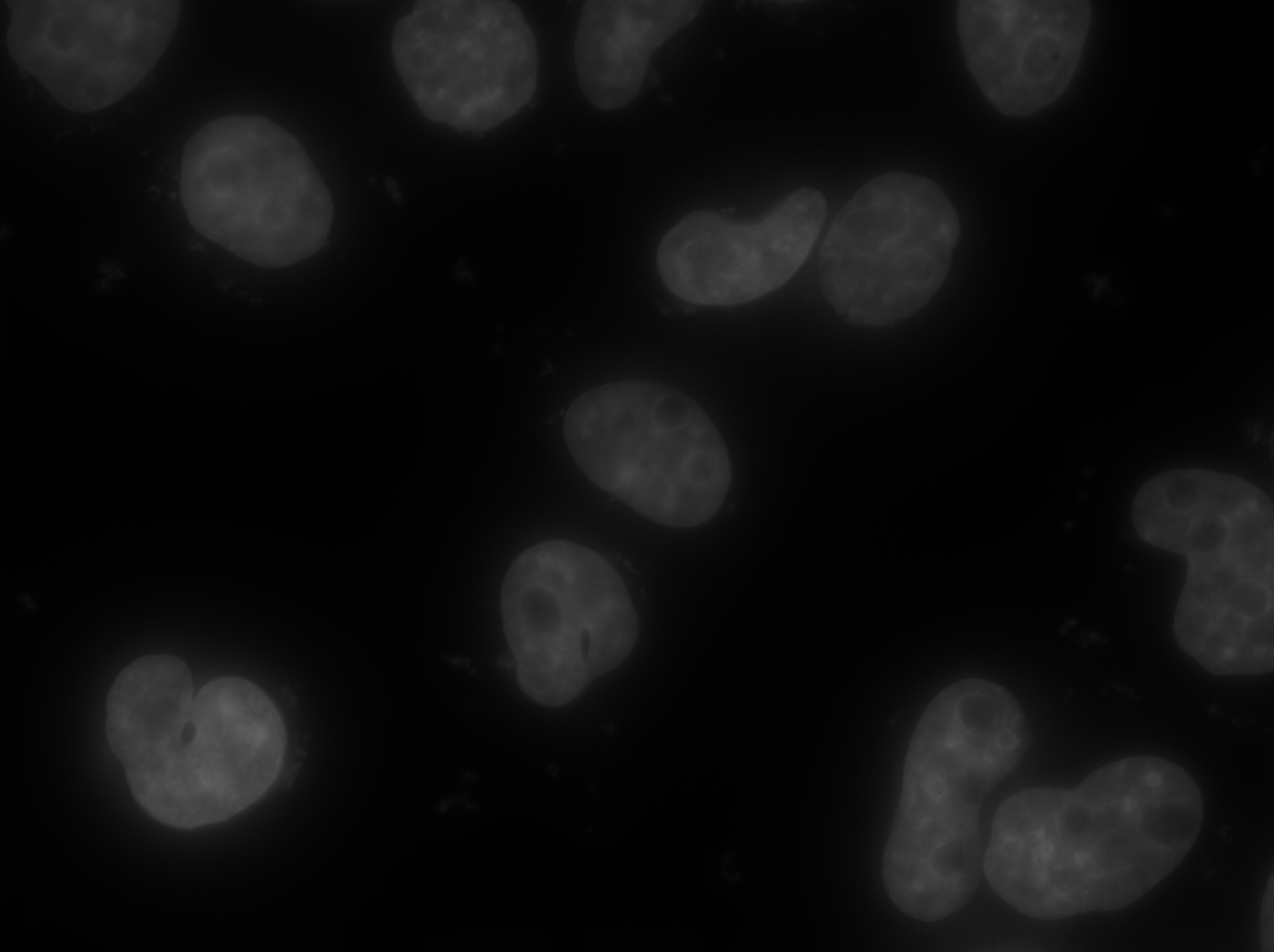

Supplement: Supplementary file 18 — Source data Fig. 5 part 4 [file 44319_2026_742_MOESM18_ESM.zip › Figure 5 Part 4/Fig 5ab WT and KO hela TTLL1-e326g atubulin/Control/TTLL1-mut atub R1 LT9.Project Maximum Z_XY1724441133_Z0_T0_C0.tif]

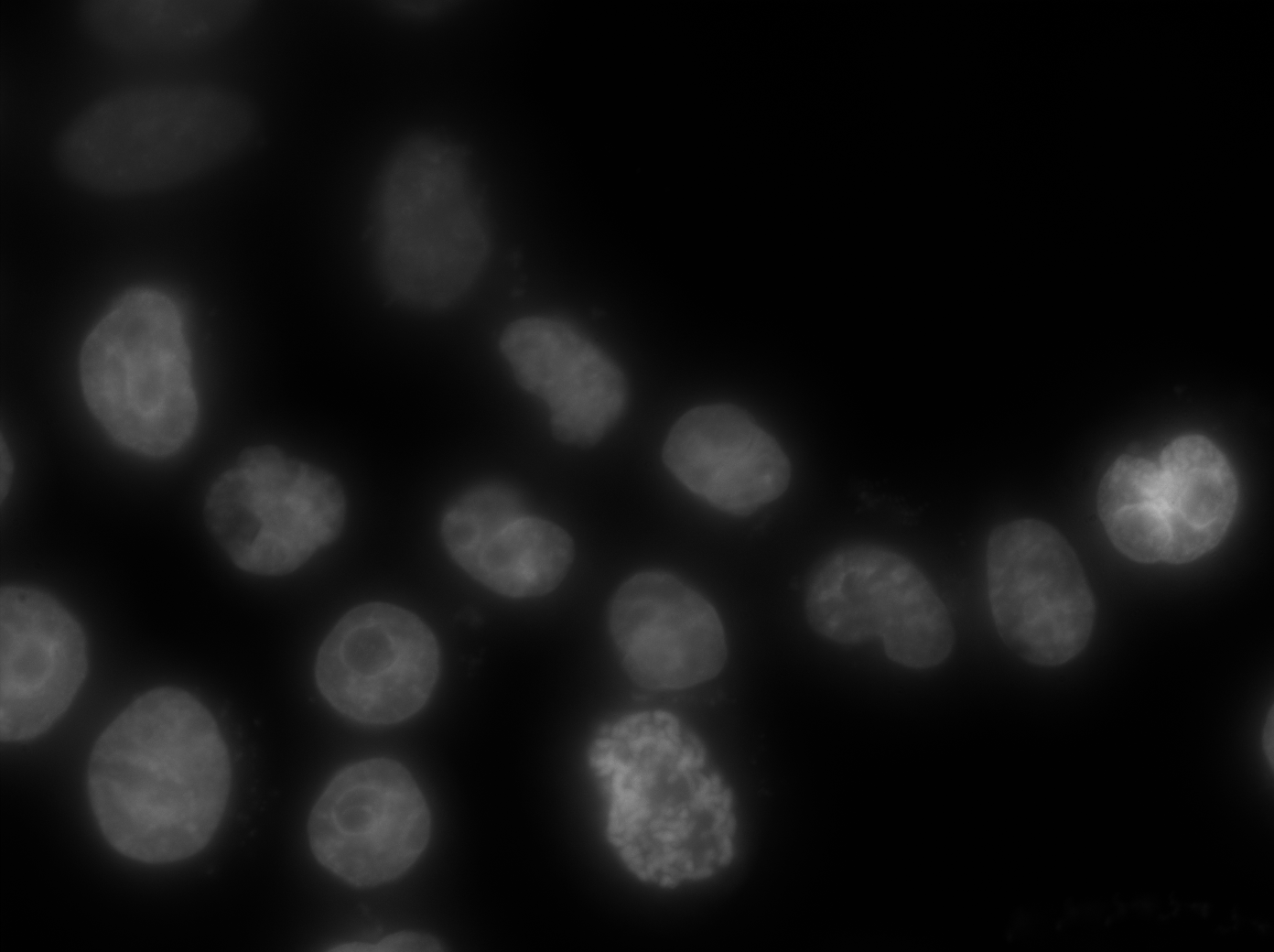

Supplement: Supplementary file 18 — Source data Fig. 5 part 4 [file 44319_2026_742_MOESM18_ESM.zip › Figure 5 Part 4/Fig 5ab WT and KO hela TTLL1-e326g atubulin/Control/WT Hela TTLL1-mut R2 LT5.Project Maximum Z_XY1731543093_Z0_T0_C0.tif]

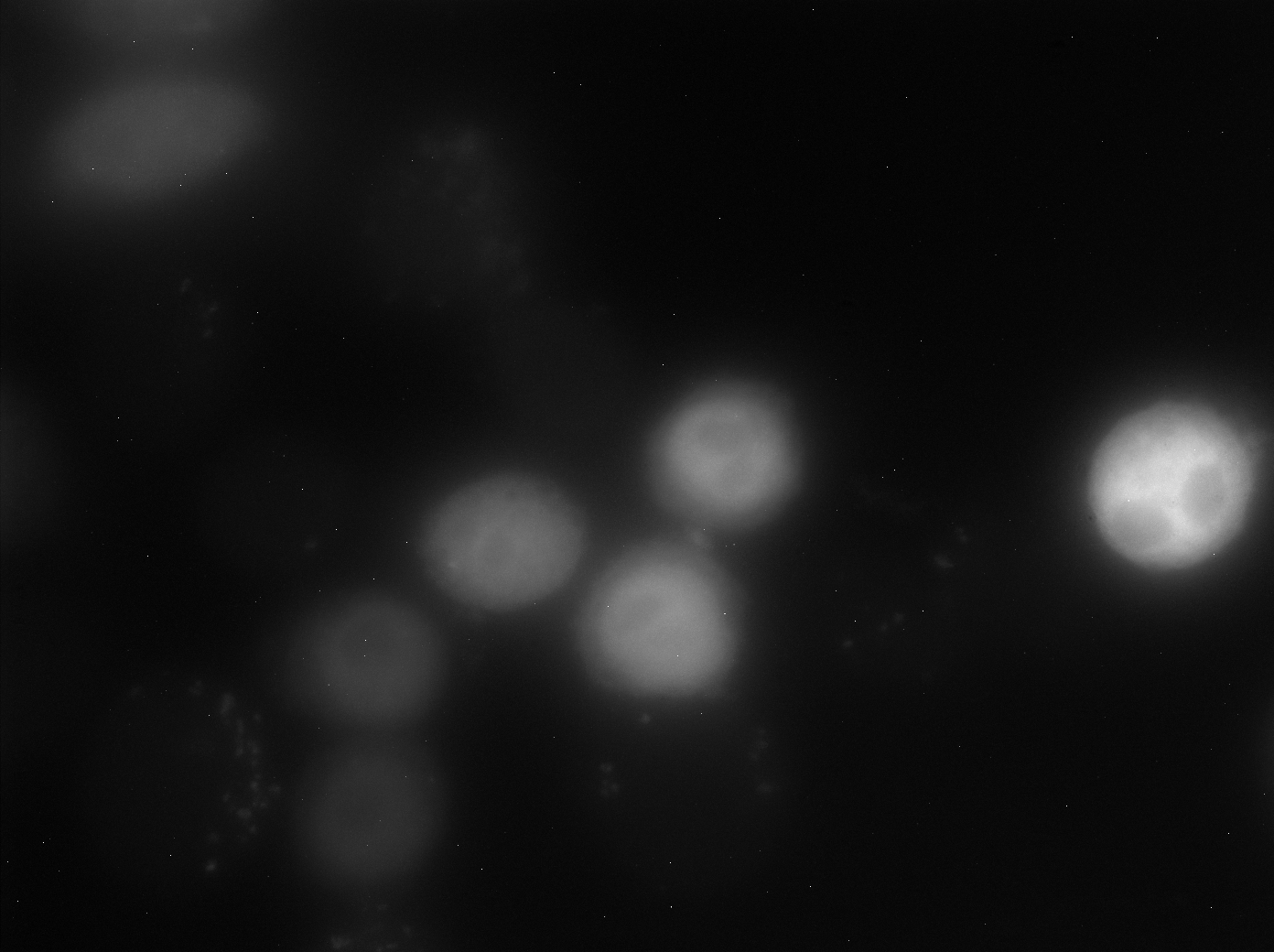

Supplement: Supplementary file 18 — Source data Fig. 5 part 4 [file 44319_2026_742_MOESM18_ESM.zip › Figure 5 Part 4/Fig 5ab WT and KO hela TTLL1-e326g atubulin/Control/WT Hela TTLL1-mut R2 LT5.Project Maximum Z_XY1731543093_Z0_T0_C1.tif]

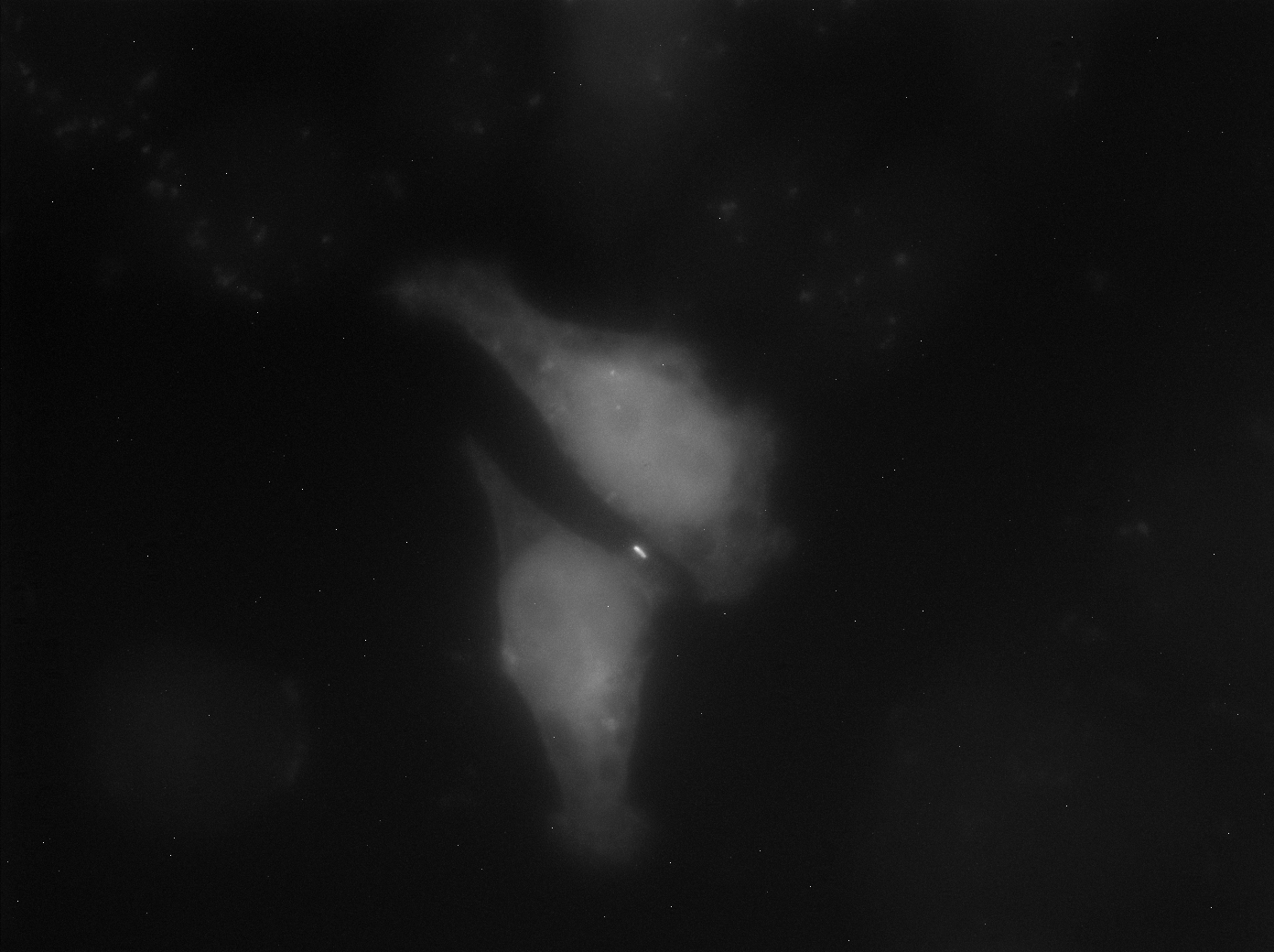

Supplement: Supplementary file 18 — Source data Fig. 5 part 4 [file 44319_2026_742_MOESM18_ESM.zip › Figure 5 Part 4/Fig 5ab WT and KO hela TTLL1-e326g atubulin/Control/TTLL1-mut atub R1 LT9.Project Maximum Z_XY1724441133_Z0_T0_C1.tif]

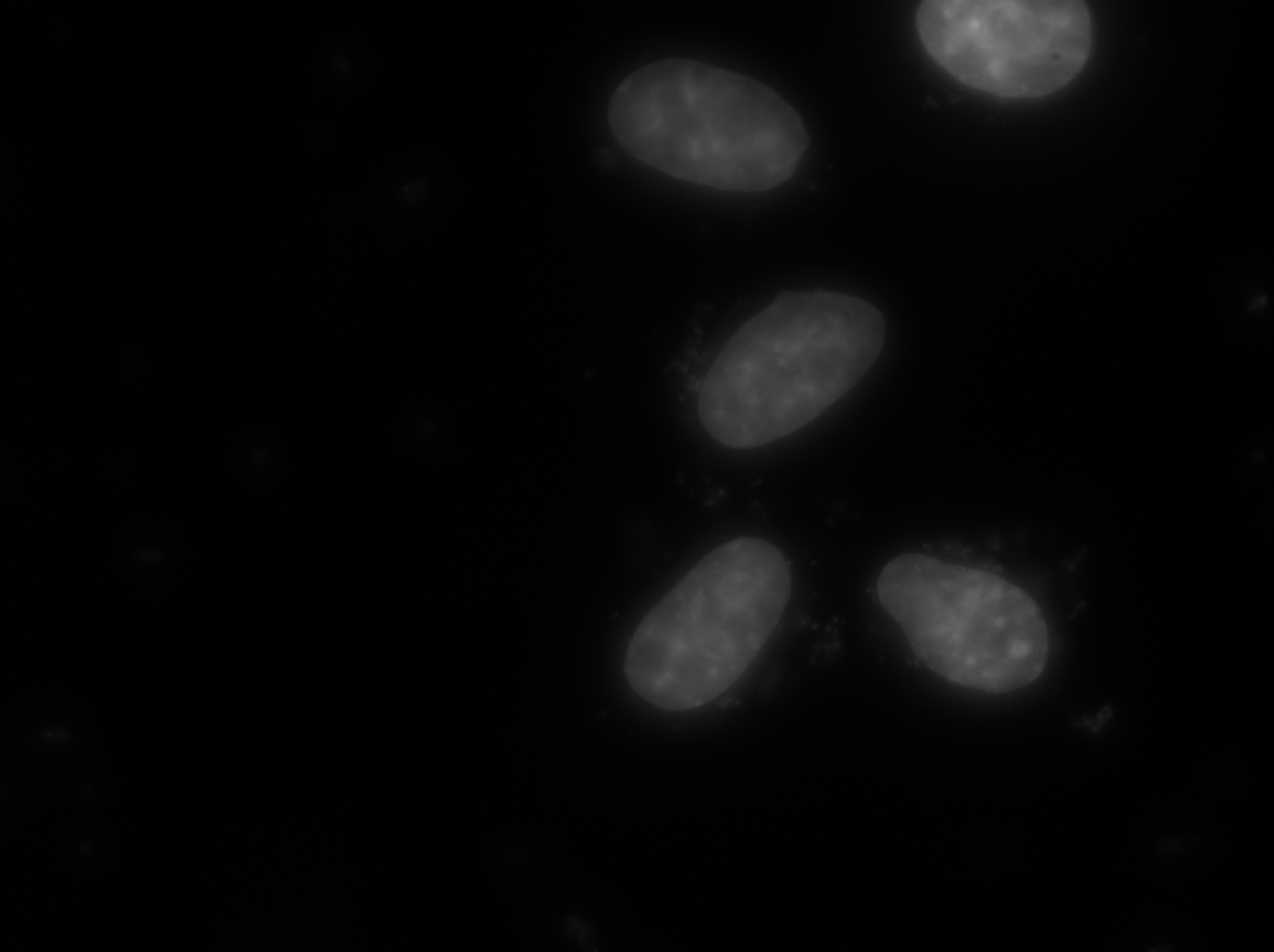

Supplement: Supplementary file 18 — Source data Fig. 5 part 4 [file 44319_2026_742_MOESM18_ESM.zip › Figure 5 Part 4/Fig 5ab WT and KO hela TTLL1-e326g atubulin/Control/TTLL1-mut atub R1 LT7.Project Maximum Z_XY1724440601_Z0_T0_C0.tif]

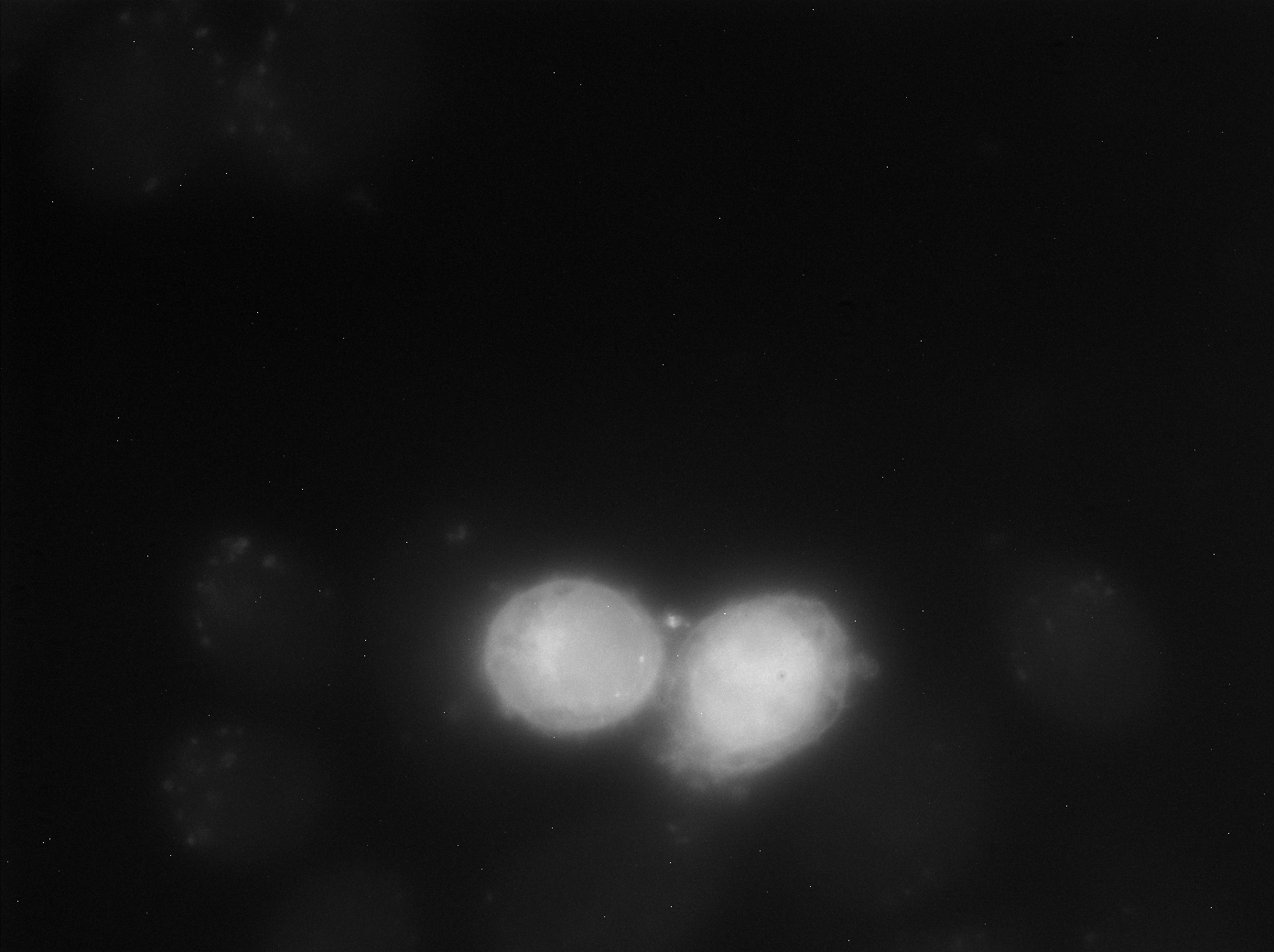

Supplement: Supplementary file 18 — Source data Fig. 5 part 4 [file 44319_2026_742_MOESM18_ESM.zip › Figure 5 Part 4/Fig 5ab WT and KO hela TTLL1-e326g atubulin/Control/TTLL1-mut atub R1 LT4.Project Maximum Z_XY1724439804_Z0_T0_C1.tif]

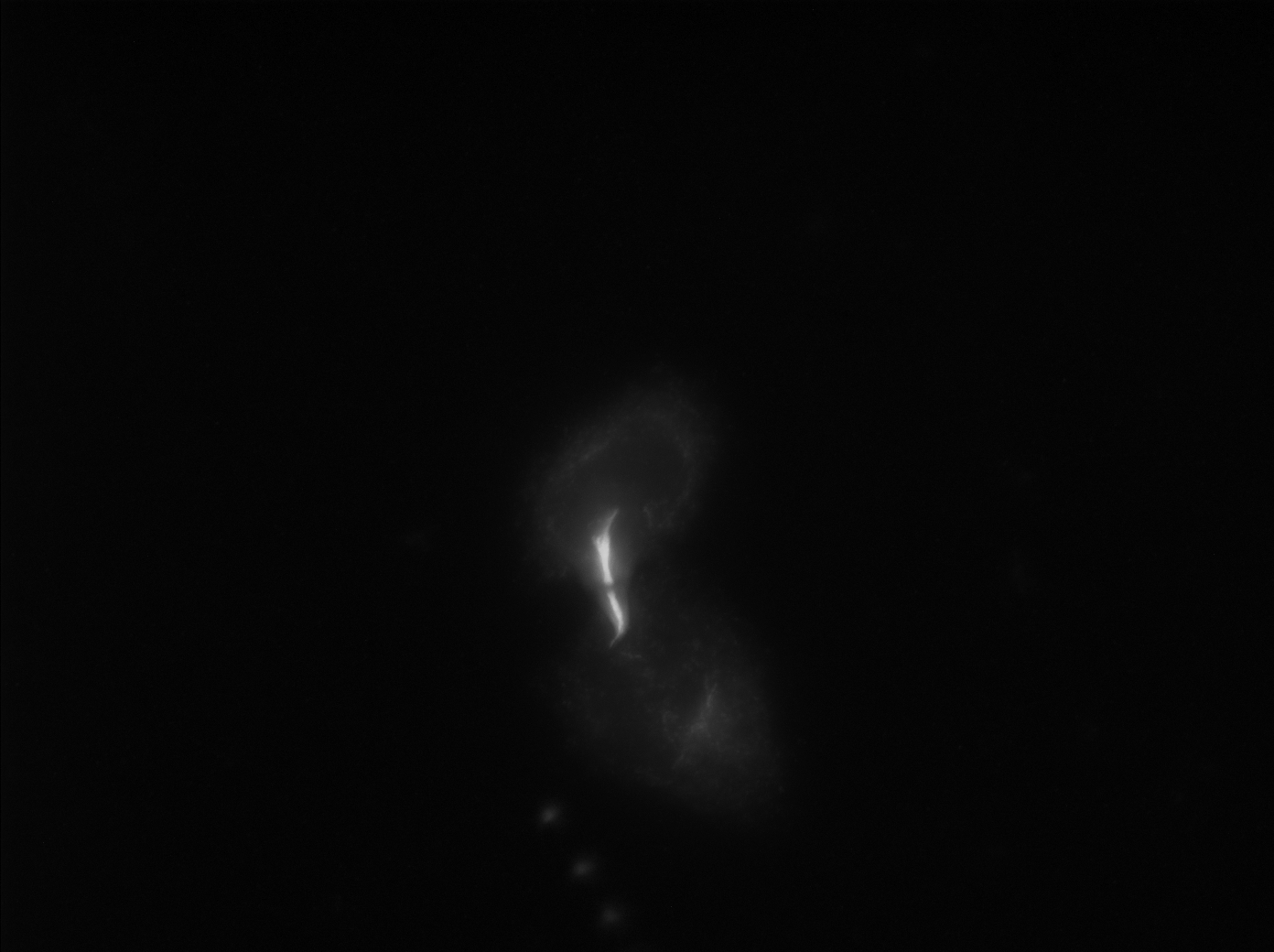

Supplement: Supplementary file 18 — Source data Fig. 5 part 4 [file 44319_2026_742_MOESM18_ESM.zip › Figure 5 Part 4/Fig 5ab WT and KO hela TTLL1-e326g atubulin/Control/WT Hela TTLL1-mut R2 LT2.Project Maximum Z_XY1731542188_Z0_T0_C2.tif]

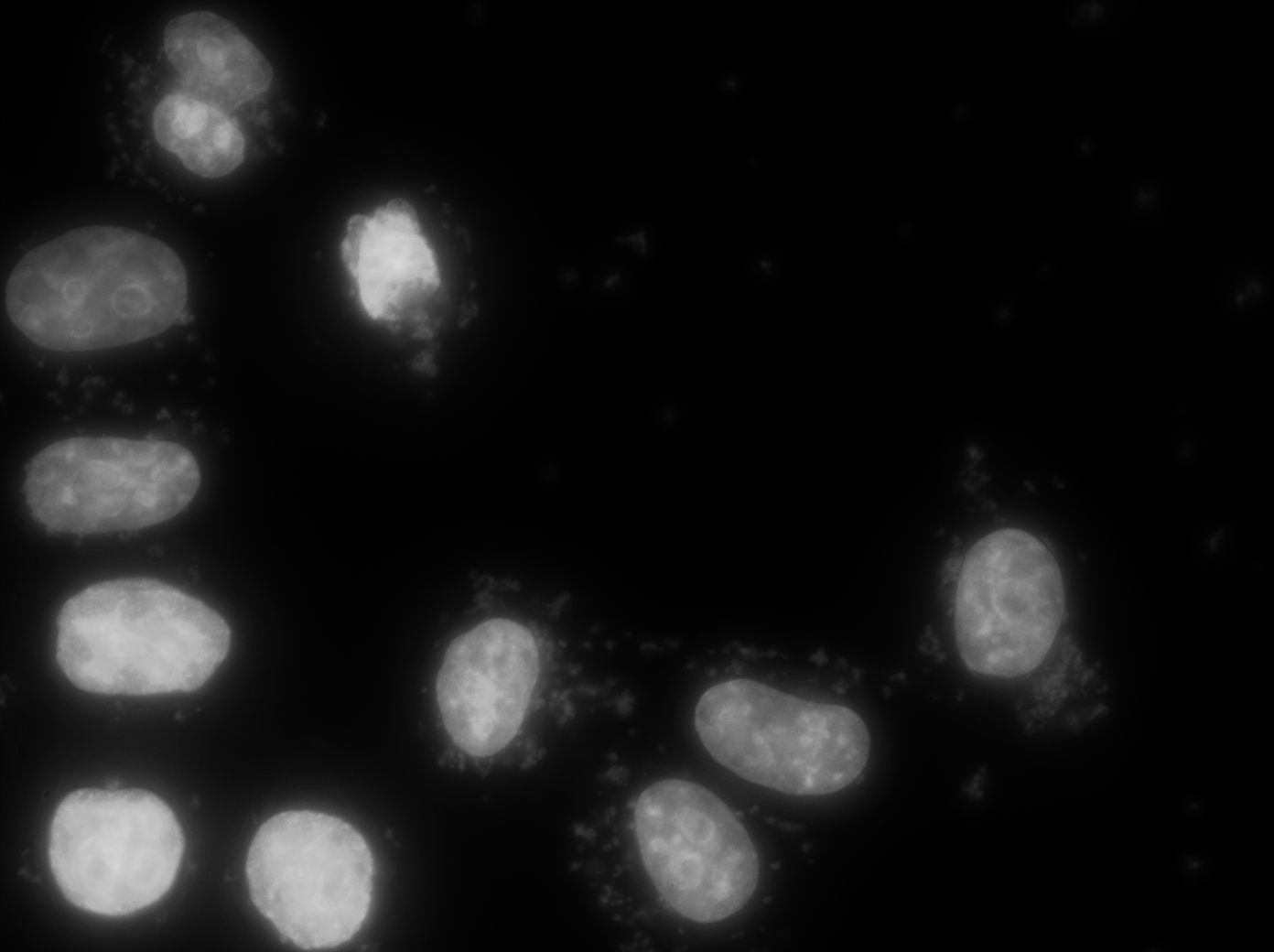

Supplement: Supplementary file 18 — Source data Fig. 5 part 4 [file 44319_2026_742_MOESM18_ESM.zip › Figure 5 Part 4/Fig 5ab WT and KO hela TTLL1-e326g atubulin/Control/TTLL1-mut atub R2 LT1.Project Maximum Z_XY1724950427_Z0_T0_C0.tif]

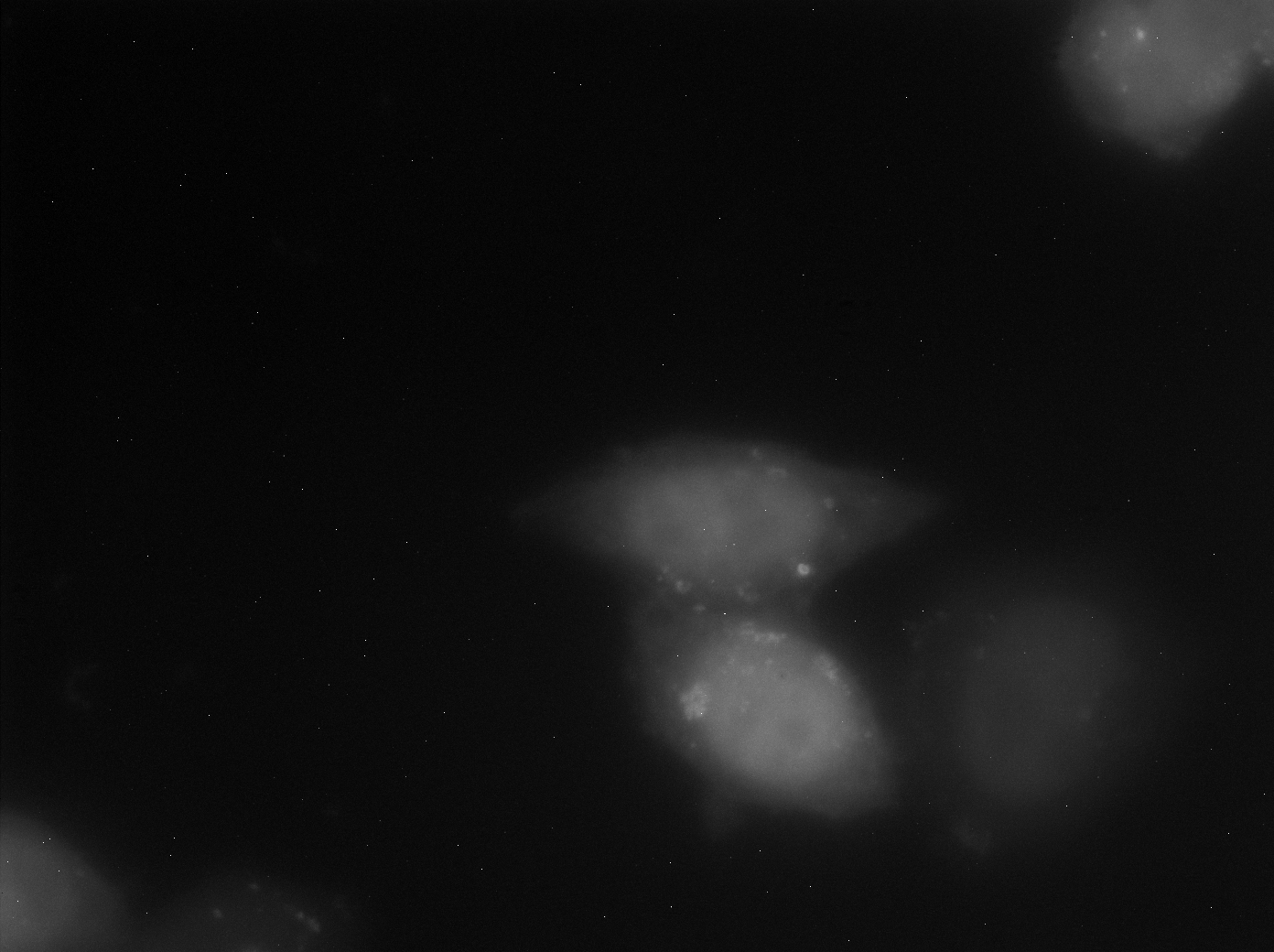

Supplement: Supplementary file 18 — Source data Fig. 5 part 4 [file 44319_2026_742_MOESM18_ESM.zip › Figure 5 Part 4/Fig 5ab WT and KO hela TTLL1-e326g atubulin/Control/WT Hela TTLL1-mut R3 11-13-24 LT6.Project Maximum Z_XY1731546721_Z0_T0_C1.tif]

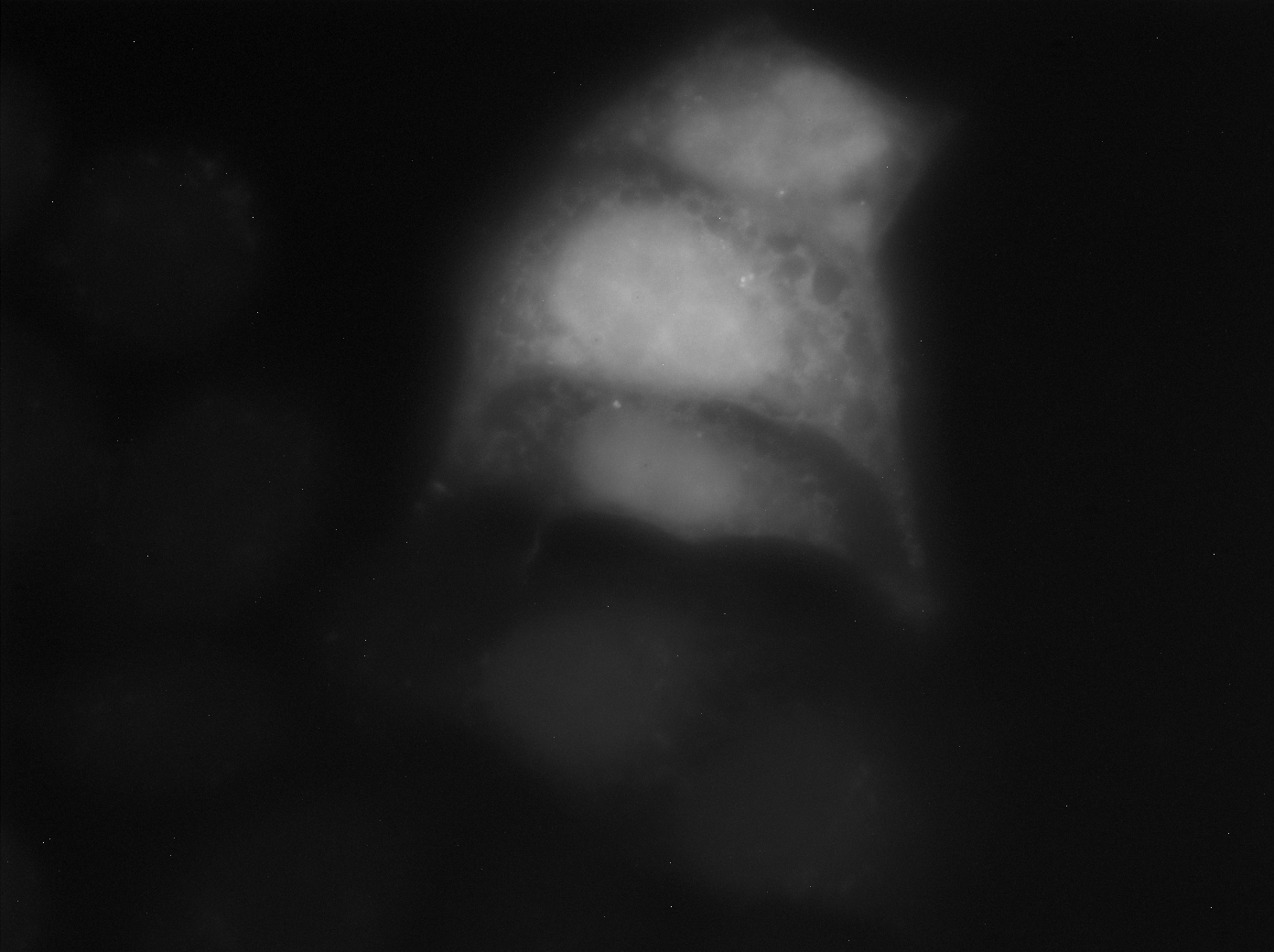

Supplement: Supplementary file 18 — Source data Fig. 5 part 4 [file 44319_2026_742_MOESM18_ESM.zip › Figure 5 Part 4/Fig 5ab WT and KO hela TTLL1-e326g atubulin/Control/TTLL1-mut atub R2 LT7.Project Maximum Z_XY1724952155_Z0_T0_C1.tif]

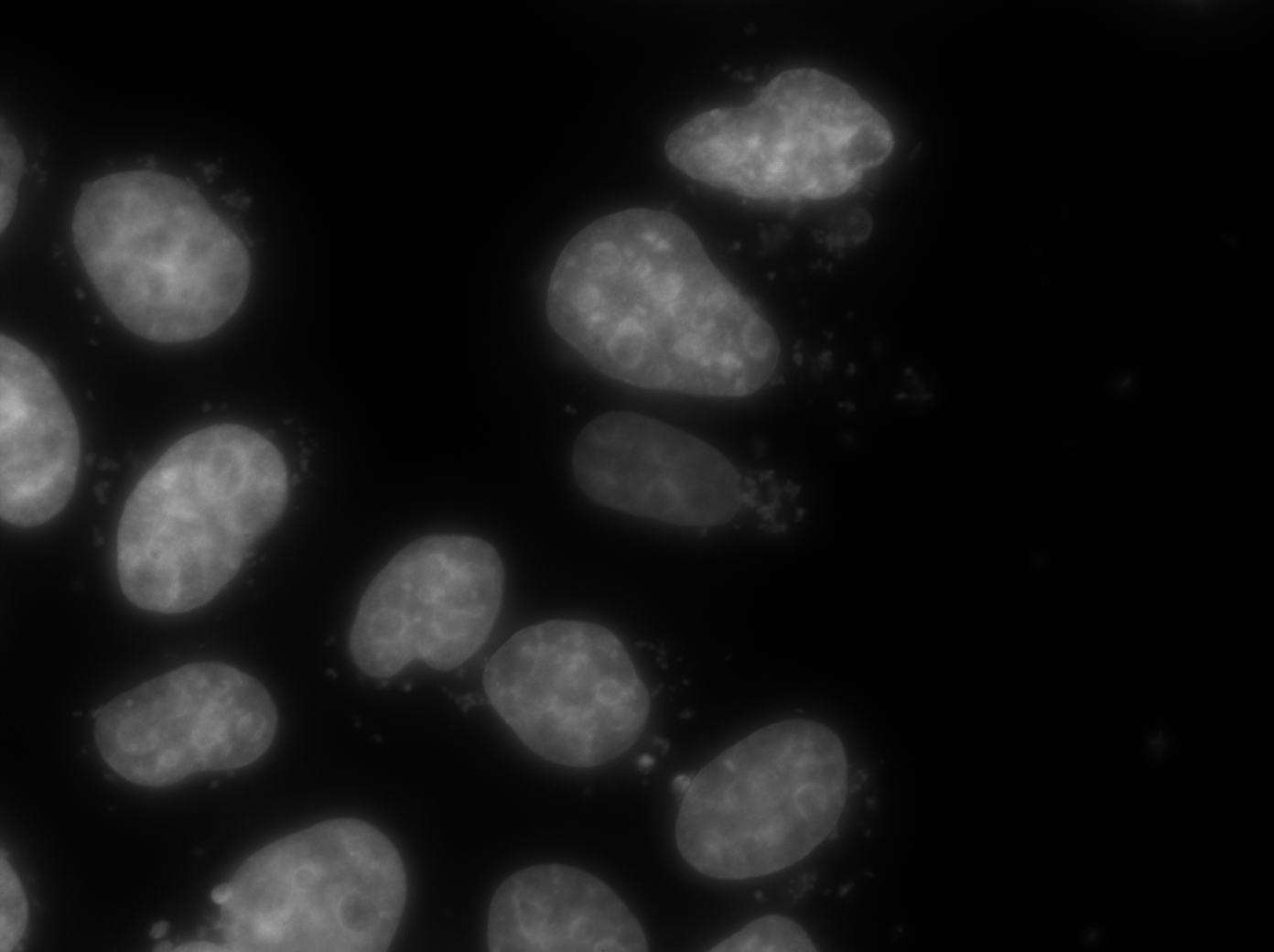

Supplement: Supplementary file 18 — Source data Fig. 5 part 4 [file 44319_2026_742_MOESM18_ESM.zip › Figure 5 Part 4/Fig 5ab WT and KO hela TTLL1-e326g atubulin/Control/TTLL1-mut atub R2 LT7.Project Maximum Z_XY1724952155_Z0_T0_C0.tif]

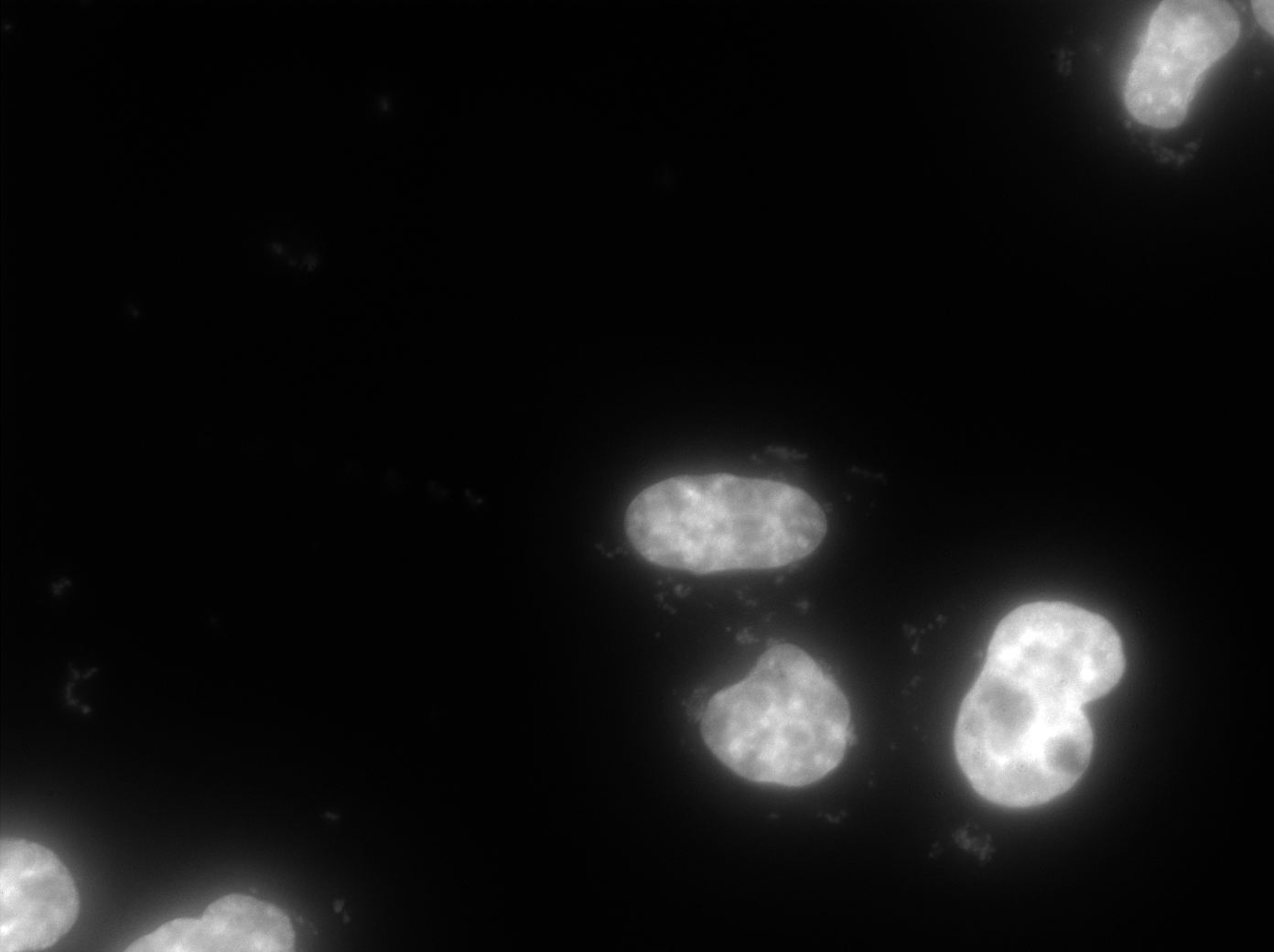

Supplement: Supplementary file 18 — Source data Fig. 5 part 4 [file 44319_2026_742_MOESM18_ESM.zip › Figure 5 Part 4/Fig 5ab WT and KO hela TTLL1-e326g atubulin/Control/WT Hela TTLL1-mut R3 11-13-24 LT6.Project Maximum Z_XY1731546721_Z0_T0_C0.tif]

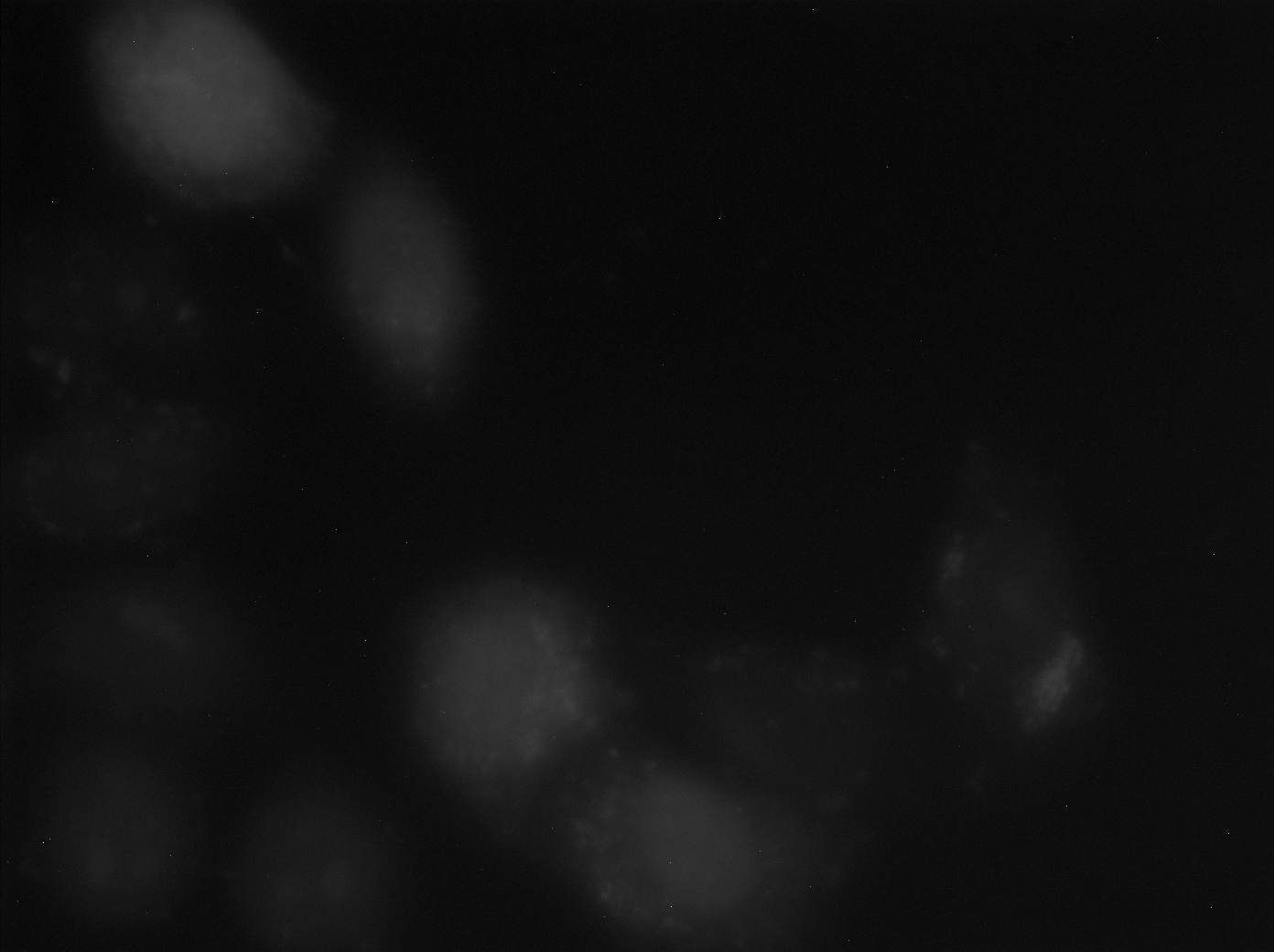

Supplement: Supplementary file 18 — Source data Fig. 5 part 4 [file 44319_2026_742_MOESM18_ESM.zip › Figure 5 Part 4/Fig 5ab WT and KO hela TTLL1-e326g atubulin/Control/TTLL1-mut atub R2 LT1.Project Maximum Z_XY1724950427_Z0_T0_C1.tif]

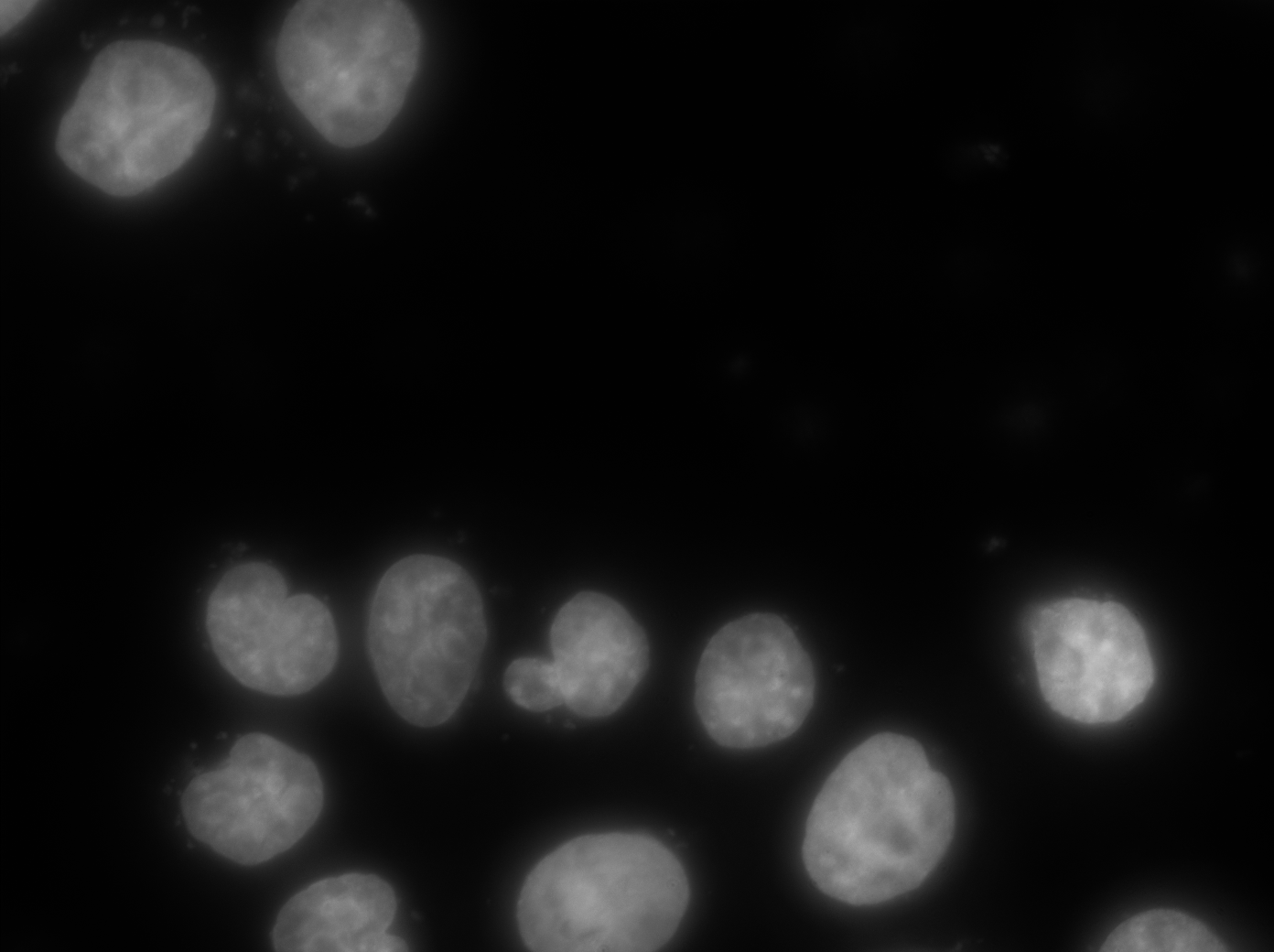

Supplement: Supplementary file 18 — Source data Fig. 5 part 4 [file 44319_2026_742_MOESM18_ESM.zip › Figure 5 Part 4/Fig 5ab WT and KO hela TTLL1-e326g atubulin/Control/TTLL1-mut atub R1 LT4.Project Maximum Z_XY1724439804_Z0_T0_C0.tif]

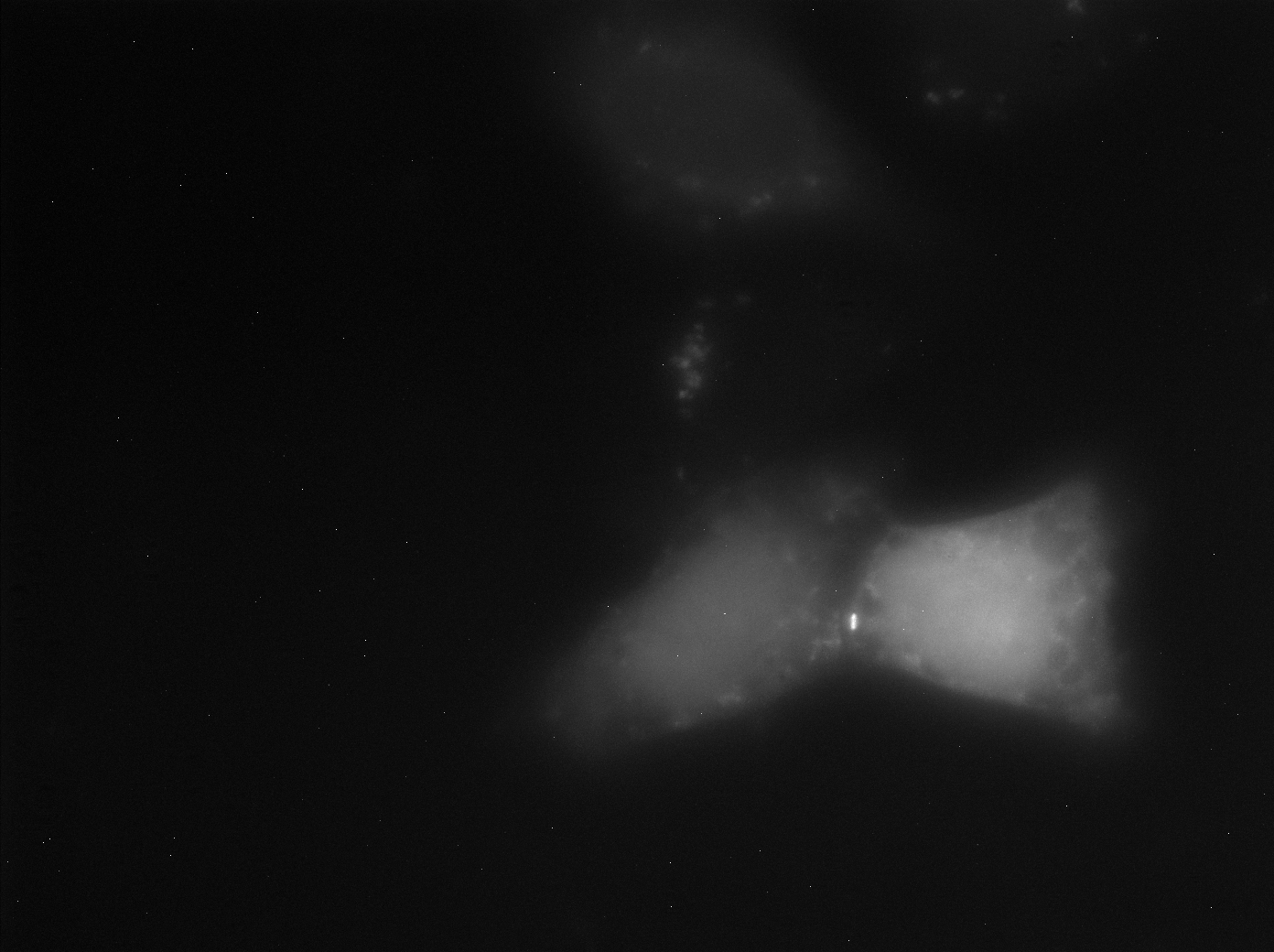

Supplement: Supplementary file 18 — Source data Fig. 5 part 4 [file 44319_2026_742_MOESM18_ESM.zip › Figure 5 Part 4/Fig 5ab WT and KO hela TTLL1-e326g atubulin/Control/TTLL1-mut atub R1 LT7.Project Maximum Z_XY1724440601_Z0_T0_C1.tif]

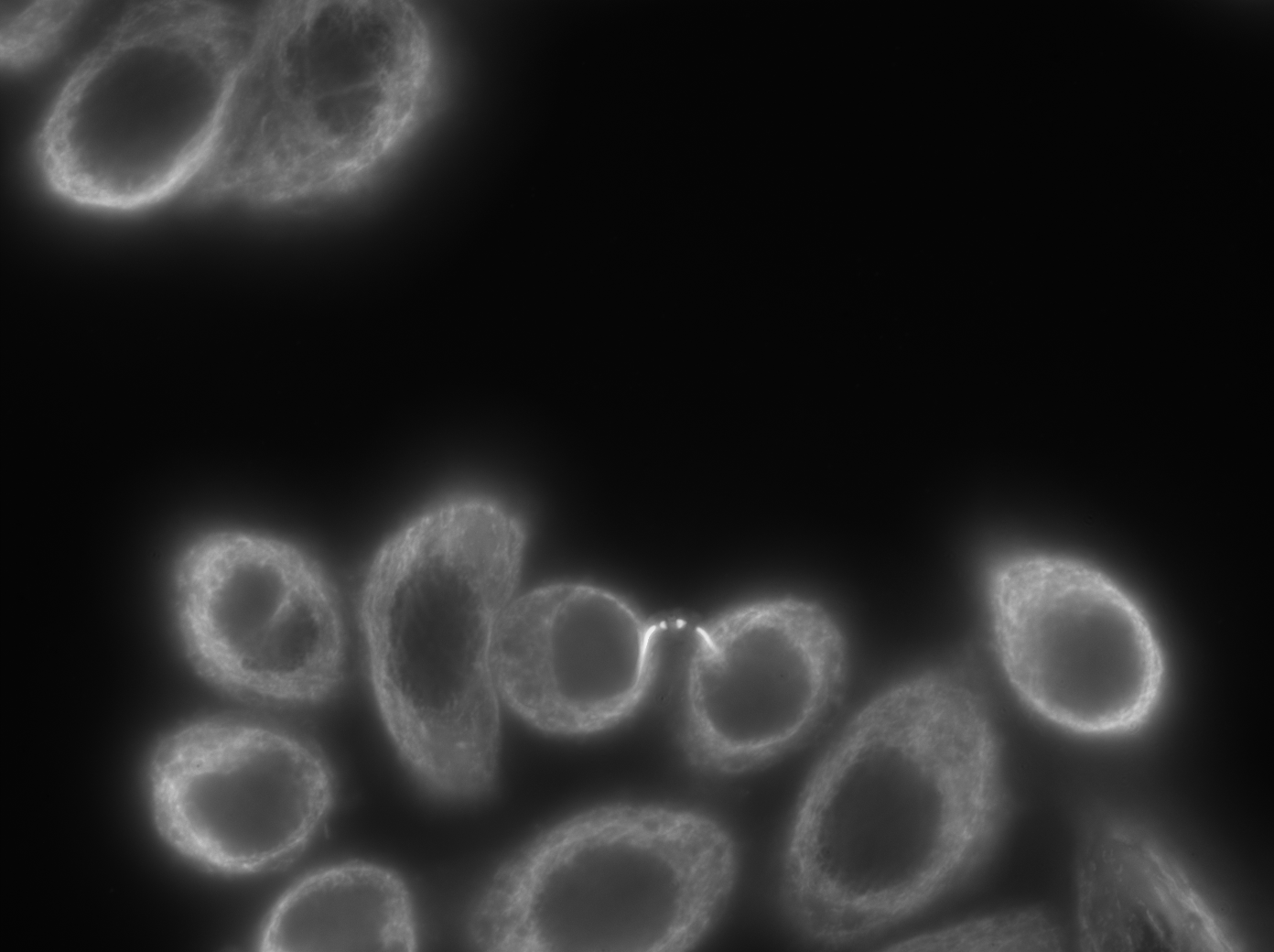

Supplement: Supplementary file 18 — Source data Fig. 5 part 4 [file 44319_2026_742_MOESM18_ESM.zip › Figure 5 Part 4/Fig 5ab WT and KO hela TTLL1-e326g atubulin/Control/TTLL1-mut atub R1 LT4.Project Maximum Z_XY1724439804_Z0_T0_C2.tif]

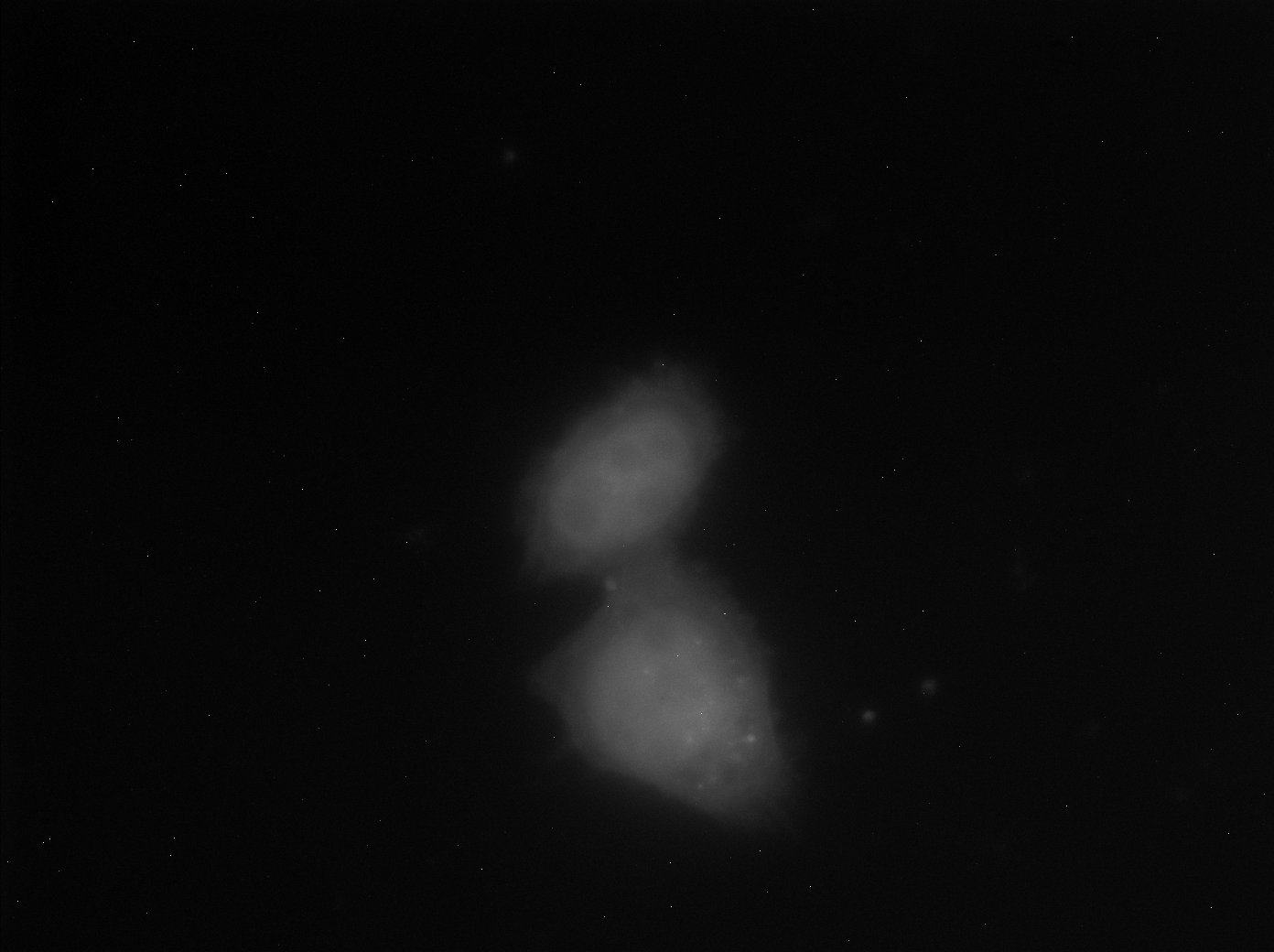

Supplement: Supplementary file 18 — Source data Fig. 5 part 4 [file 44319_2026_742_MOESM18_ESM.zip › Figure 5 Part 4/Fig 5ab WT and KO hela TTLL1-e326g atubulin/Control/WT Hela TTLL1-mut R2 LT2.Project Maximum Z_XY1731542188_Z0_T0_C1.tif]

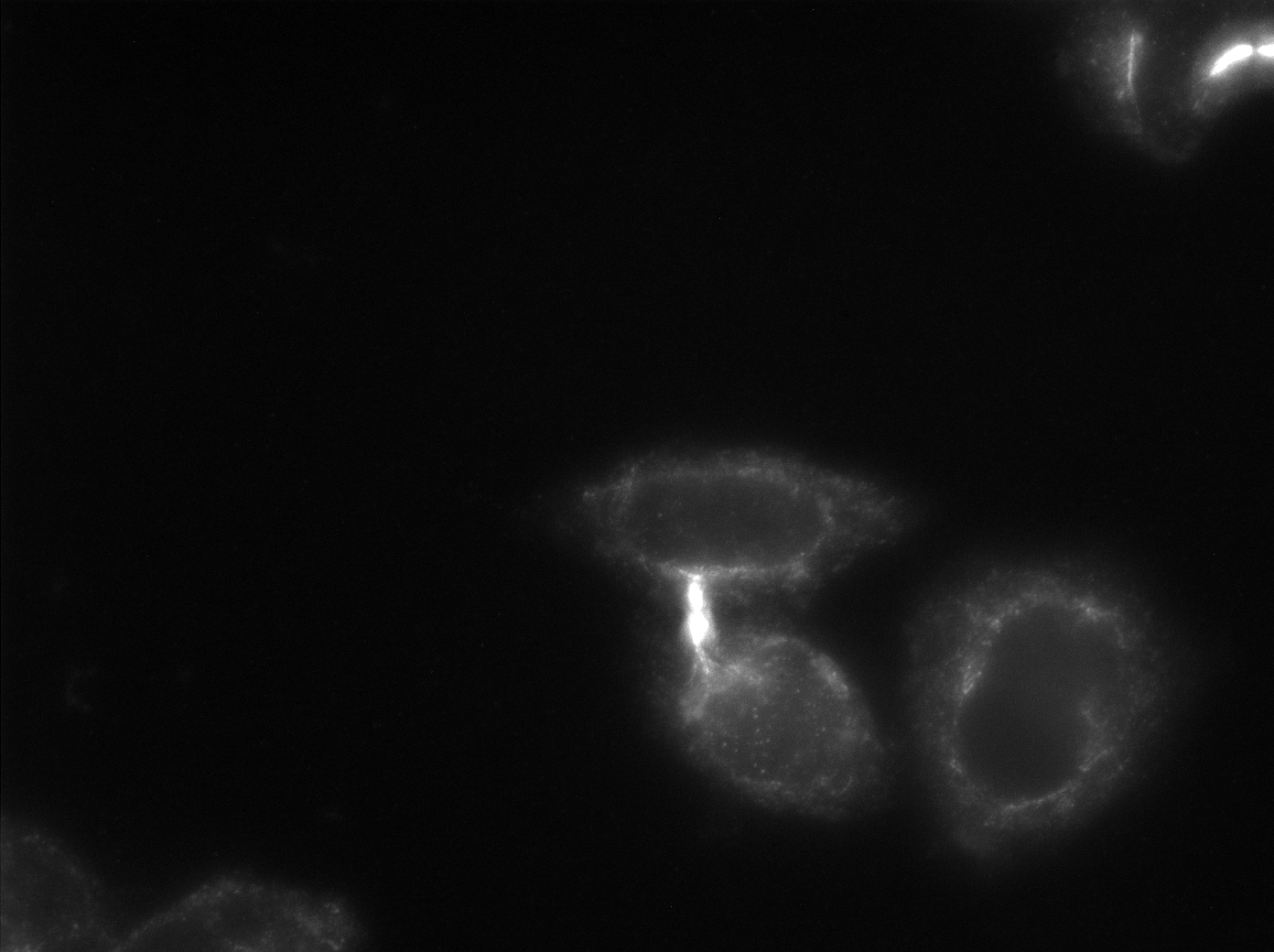

Supplement: Supplementary file 18 — Source data Fig. 5 part 4 [file 44319_2026_742_MOESM18_ESM.zip › Figure 5 Part 4/Fig 5ab WT and KO hela TTLL1-e326g atubulin/Control/WT Hela TTLL1-mut R3 11-13-24 LT6.Project Maximum Z_XY1731546721_Z0_T0_C2.tif]

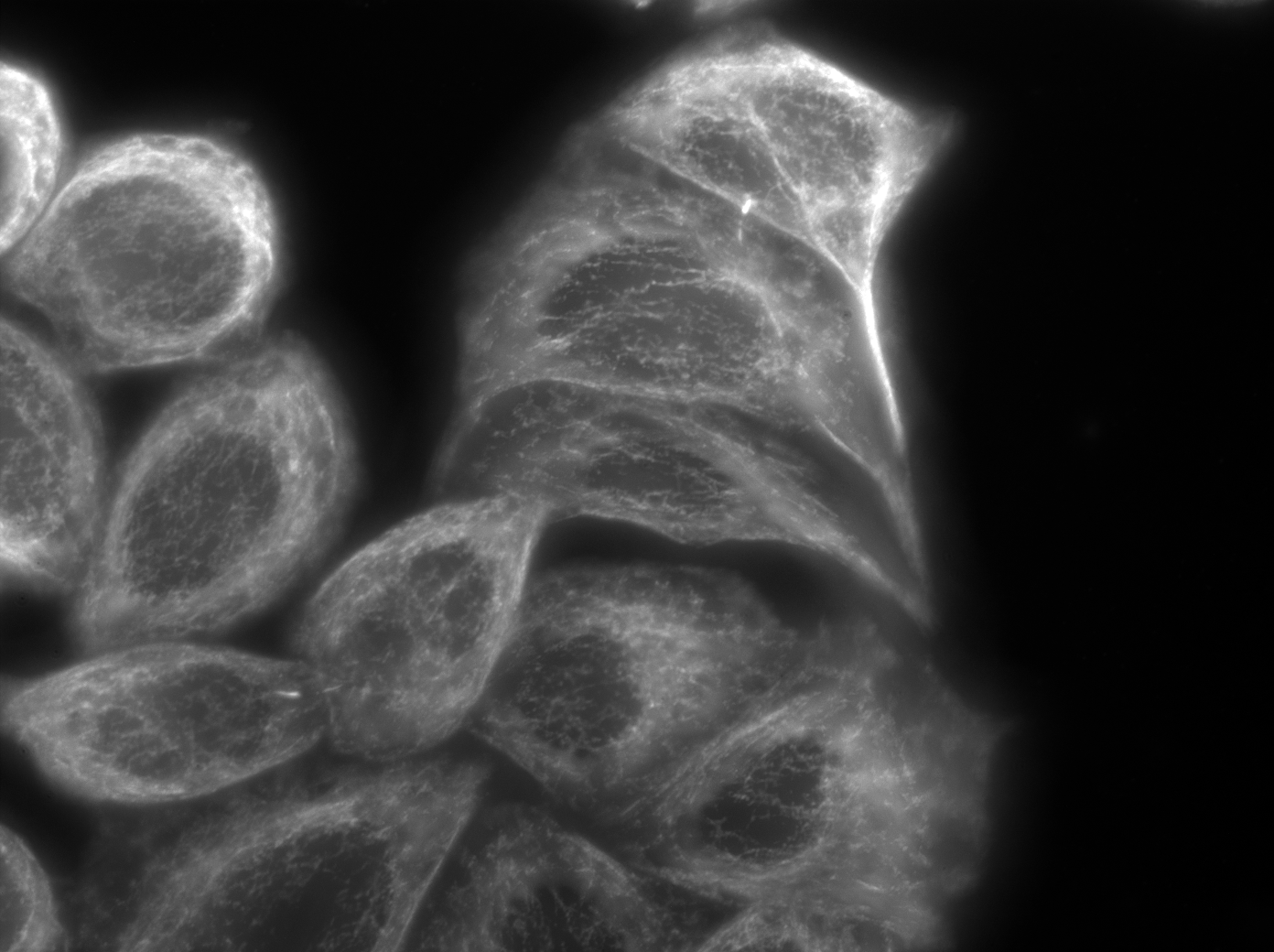

Supplement: Supplementary file 18 — Source data Fig. 5 part 4 [file 44319_2026_742_MOESM18_ESM.zip › Figure 5 Part 4/Fig 5ab WT and KO hela TTLL1-e326g atubulin/Control/TTLL1-mut atub R2 LT7.Project Maximum Z_XY1724952155_Z0_T0_C2.tif]

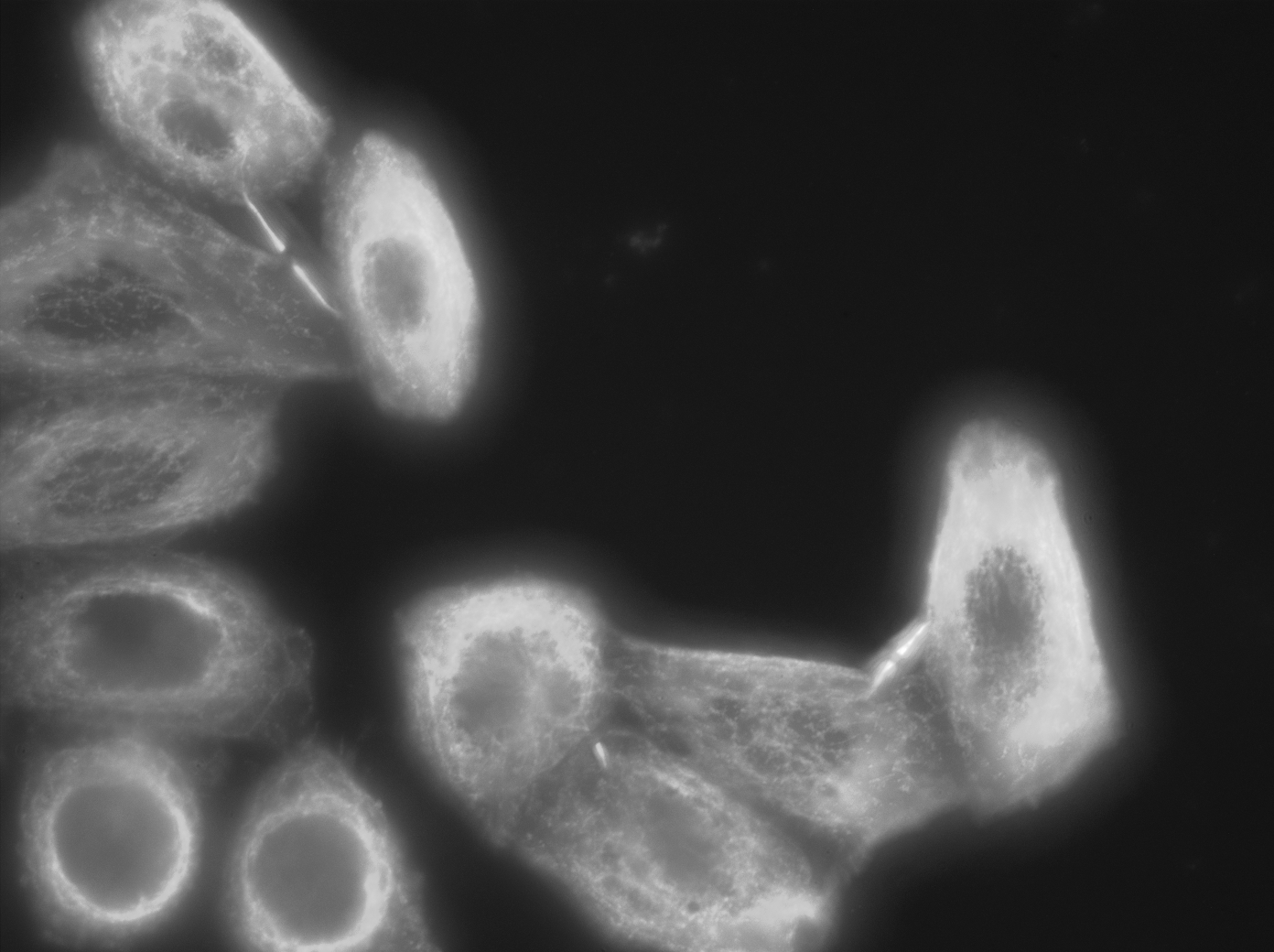

Supplement: Supplementary file 18 — Source data Fig. 5 part 4 [file 44319_2026_742_MOESM18_ESM.zip › Figure 5 Part 4/Fig 5ab WT and KO hela TTLL1-e326g atubulin/Control/TTLL1-mut atub R2 LT1.Project Maximum Z_XY1724950427_Z0_T0_C2.tif]

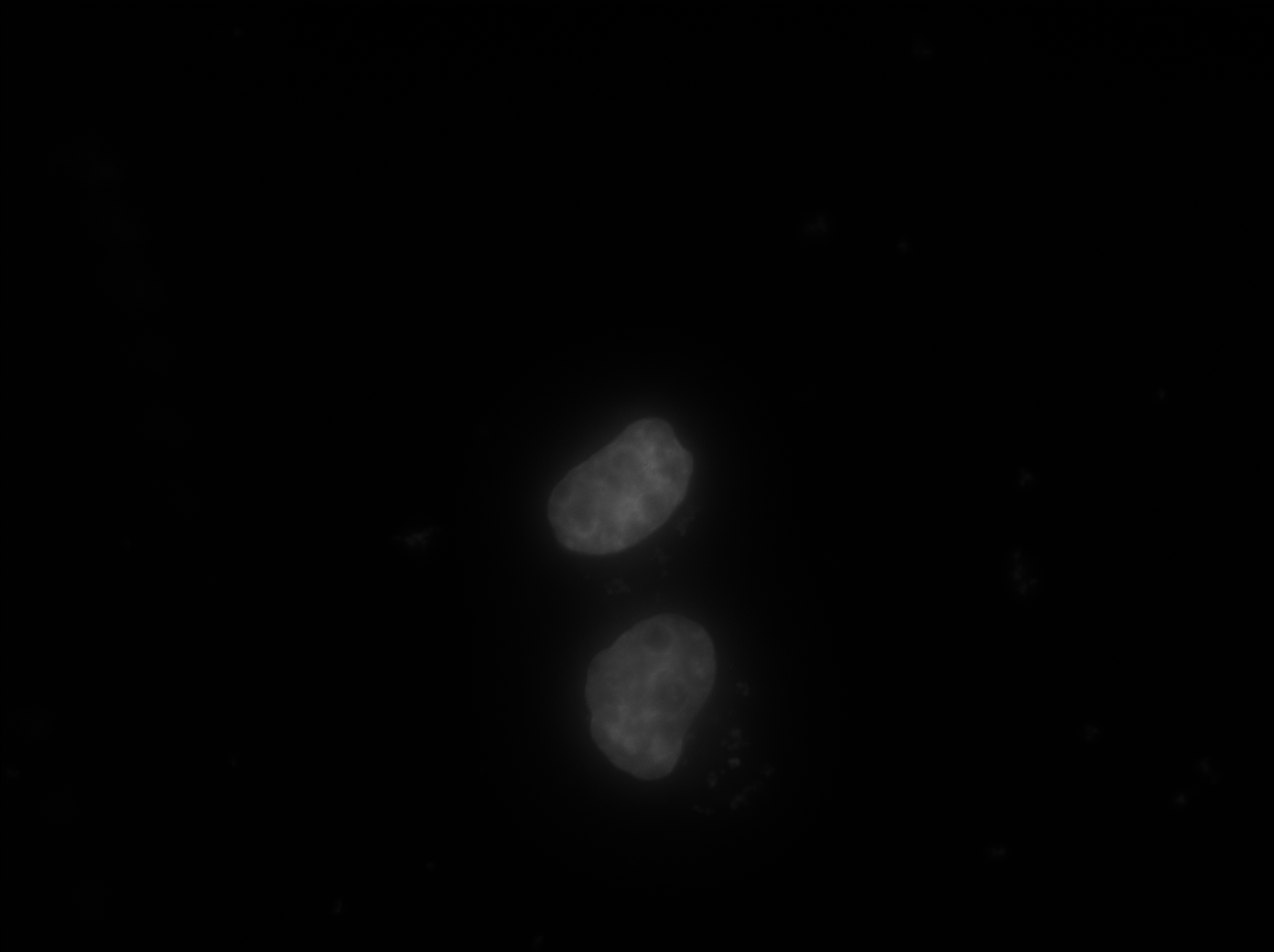

Supplement: Supplementary file 18 — Source data Fig. 5 part 4 [file 44319_2026_742_MOESM18_ESM.zip › Figure 5 Part 4/Fig 5ab WT and KO hela TTLL1-e326g atubulin/Control/WT Hela TTLL1-mut R2 LT2.Project Maximum Z_XY1731542188_Z0_T0_C0.tif]

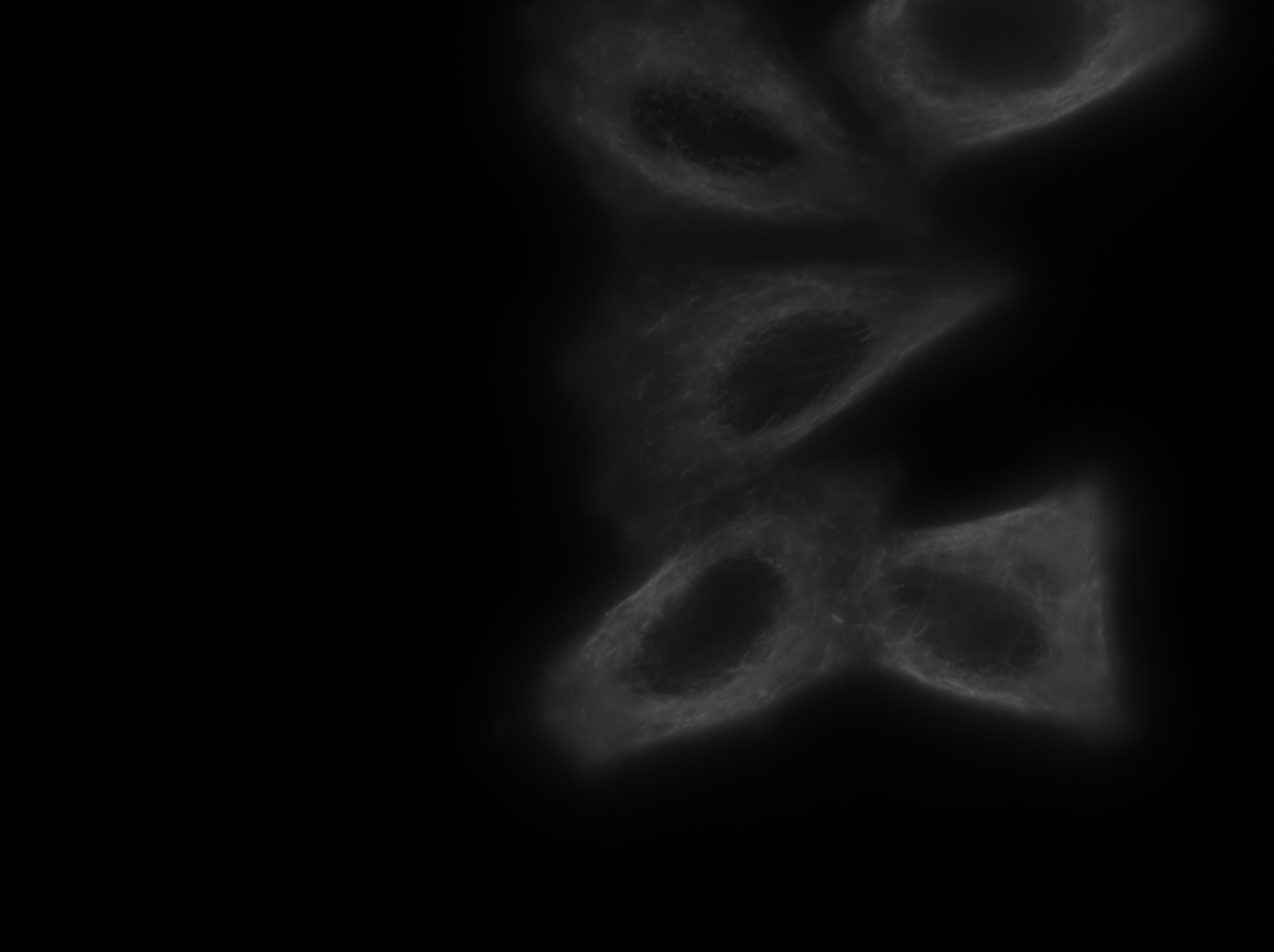

Supplement: Supplementary file 18 — Source data Fig. 5 part 4 [file 44319_2026_742_MOESM18_ESM.zip › Figure 5 Part 4/Fig 5ab WT and KO hela TTLL1-e326g atubulin/Control/TTLL1-mut atub R1 LT7.Project Maximum Z_XY1724440601_Z0_T0_C2.tif]

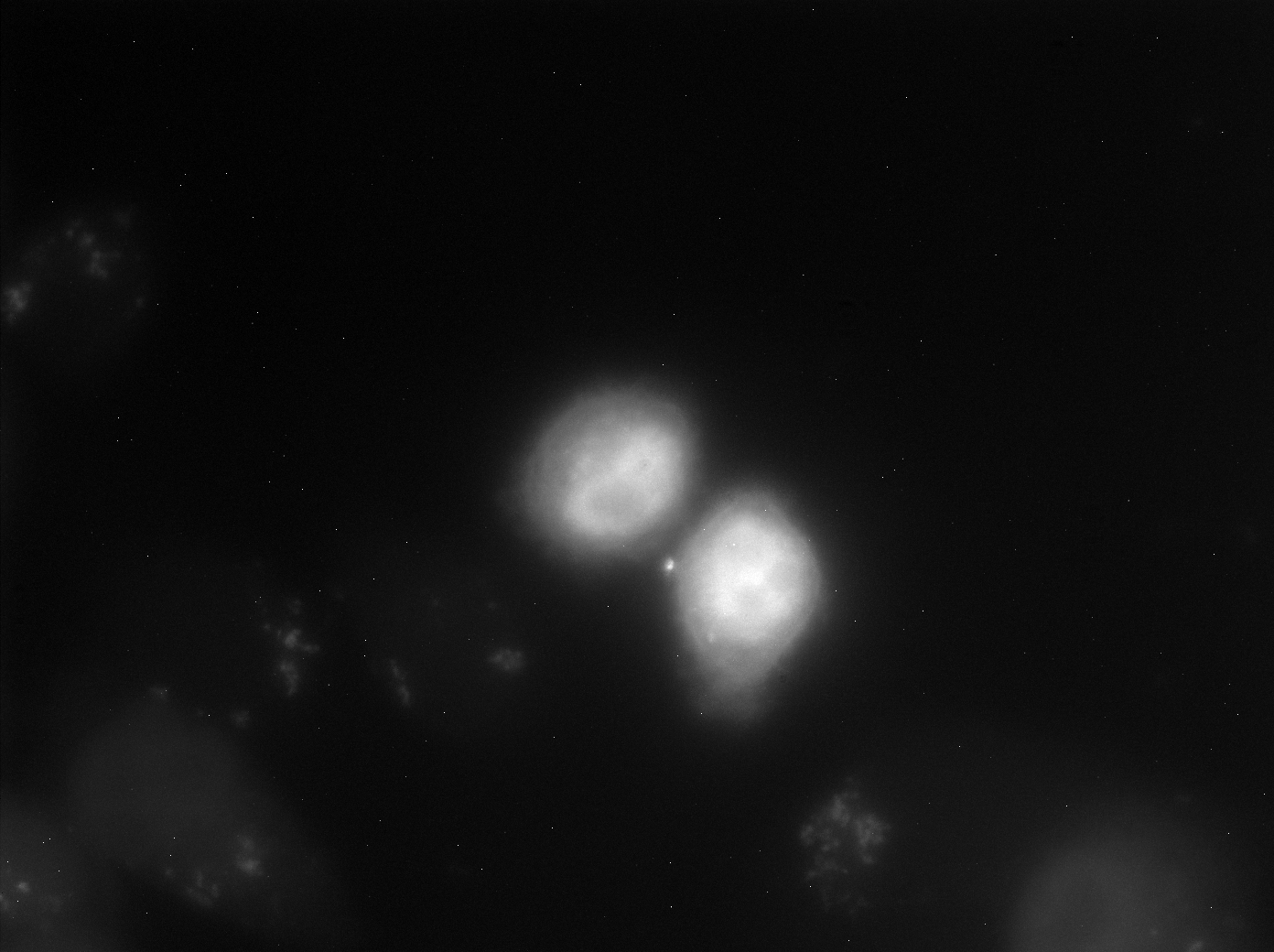

Supplement: Supplementary file 18 — Source data Fig. 5 part 4 [file 44319_2026_742_MOESM18_ESM.zip › Figure 5 Part 4/Fig 5ab WT and KO hela TTLL1-e326g atubulin/Control/WT Hela TTLL1-mut R3 11-13-24 LT10.Project Maximum Z_XY1731547834_Z0_T0_C1.tif]

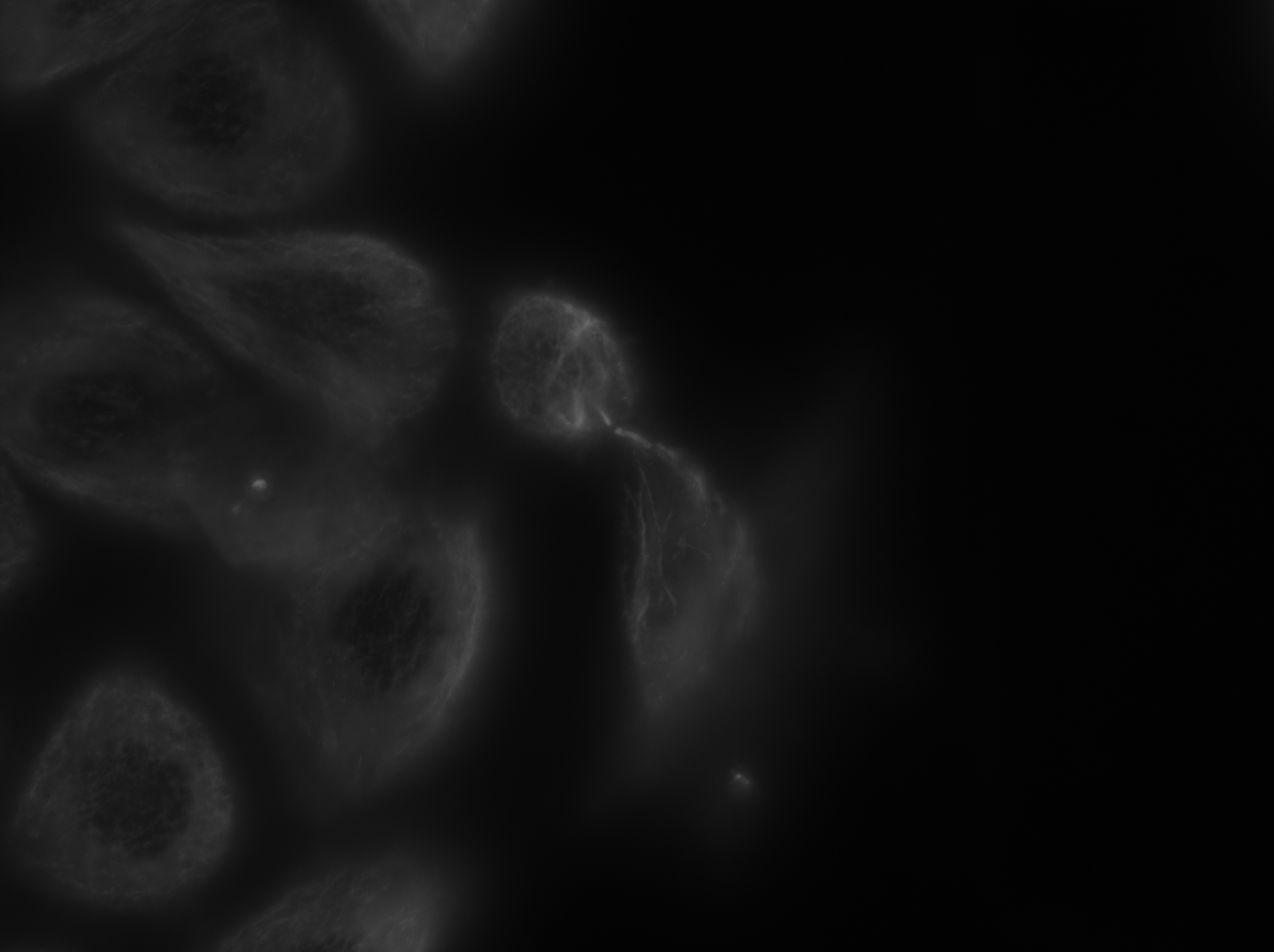

Supplement: Supplementary file 18 — Source data Fig. 5 part 4 [file 44319_2026_742_MOESM18_ESM.zip › Figure 5 Part 4/Fig 5ab WT and KO hela TTLL1-e326g atubulin/Control/TTLL1-mut atub R1 LT1.Project Maximum Z_XY1724435566_Z0_T0_C2.tif]

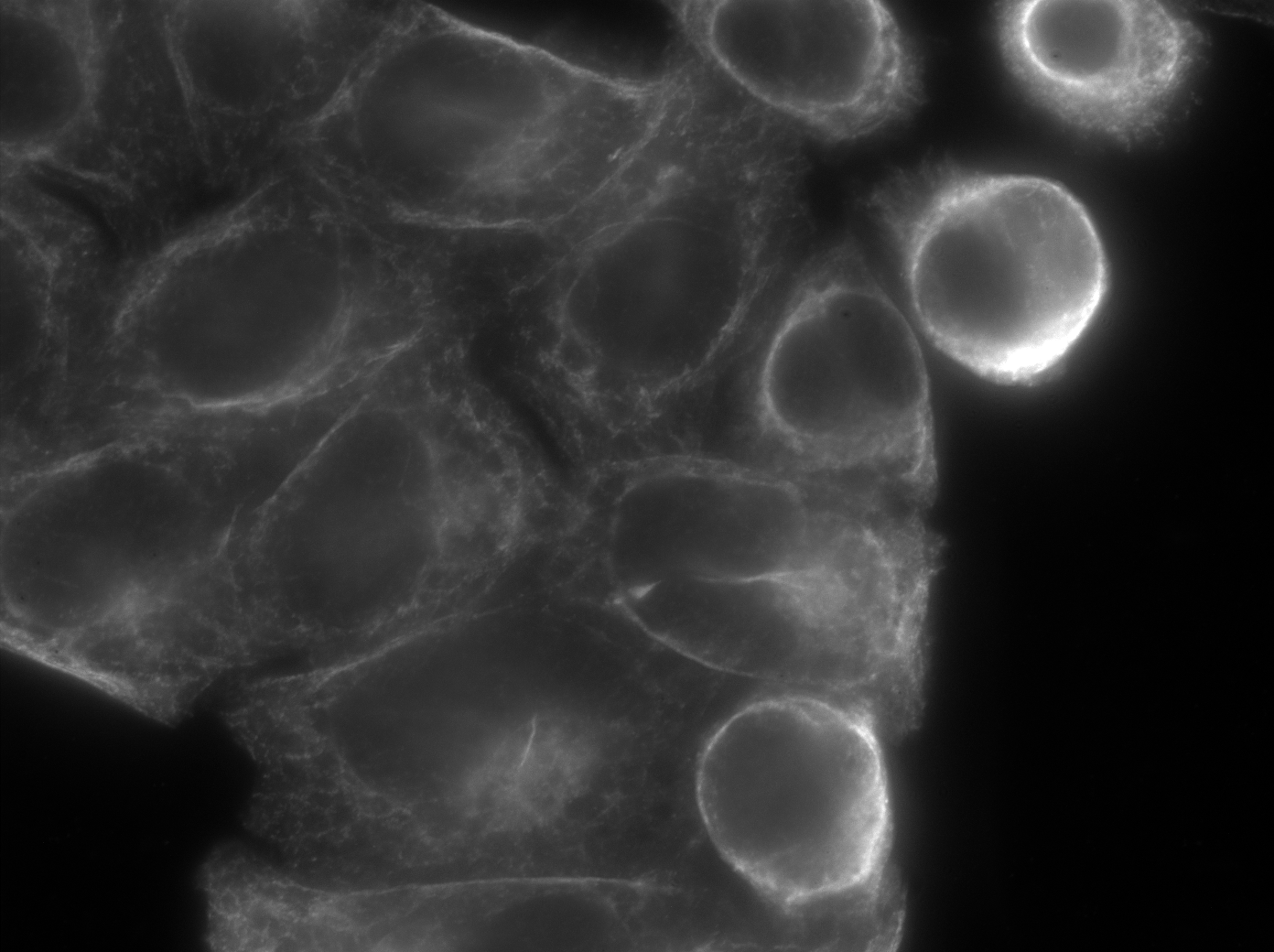

Supplement: Supplementary file 18 — Source data Fig. 5 part 4 [file 44319_2026_742_MOESM18_ESM.zip › Figure 5 Part 4/Fig 5ab WT and KO hela TTLL1-e326g atubulin/Control/TTLL1-mut atub R2 LT5.Project Maximum Z_XY1724951535_Z0_T0_C2.tif]

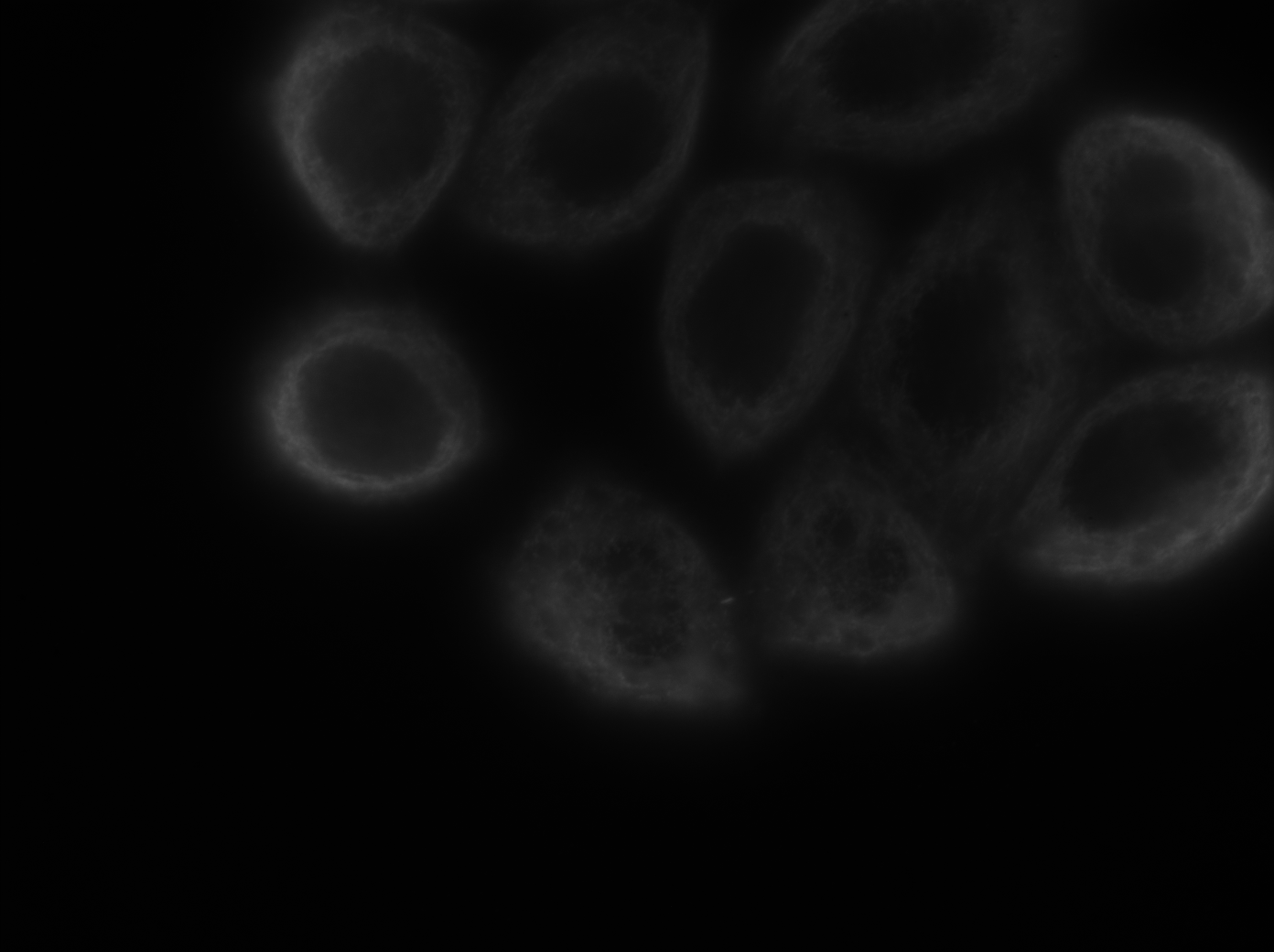

Supplement: Supplementary file 18 — Source data Fig. 5 part 4 [file 44319_2026_742_MOESM18_ESM.zip › Figure 5 Part 4/Fig 5ab WT and KO hela TTLL1-e326g atubulin/Control/TTLL1-mut atub R1 LT5.Project Maximum Z_XY1724440046_Z0_T0_C2.tif]

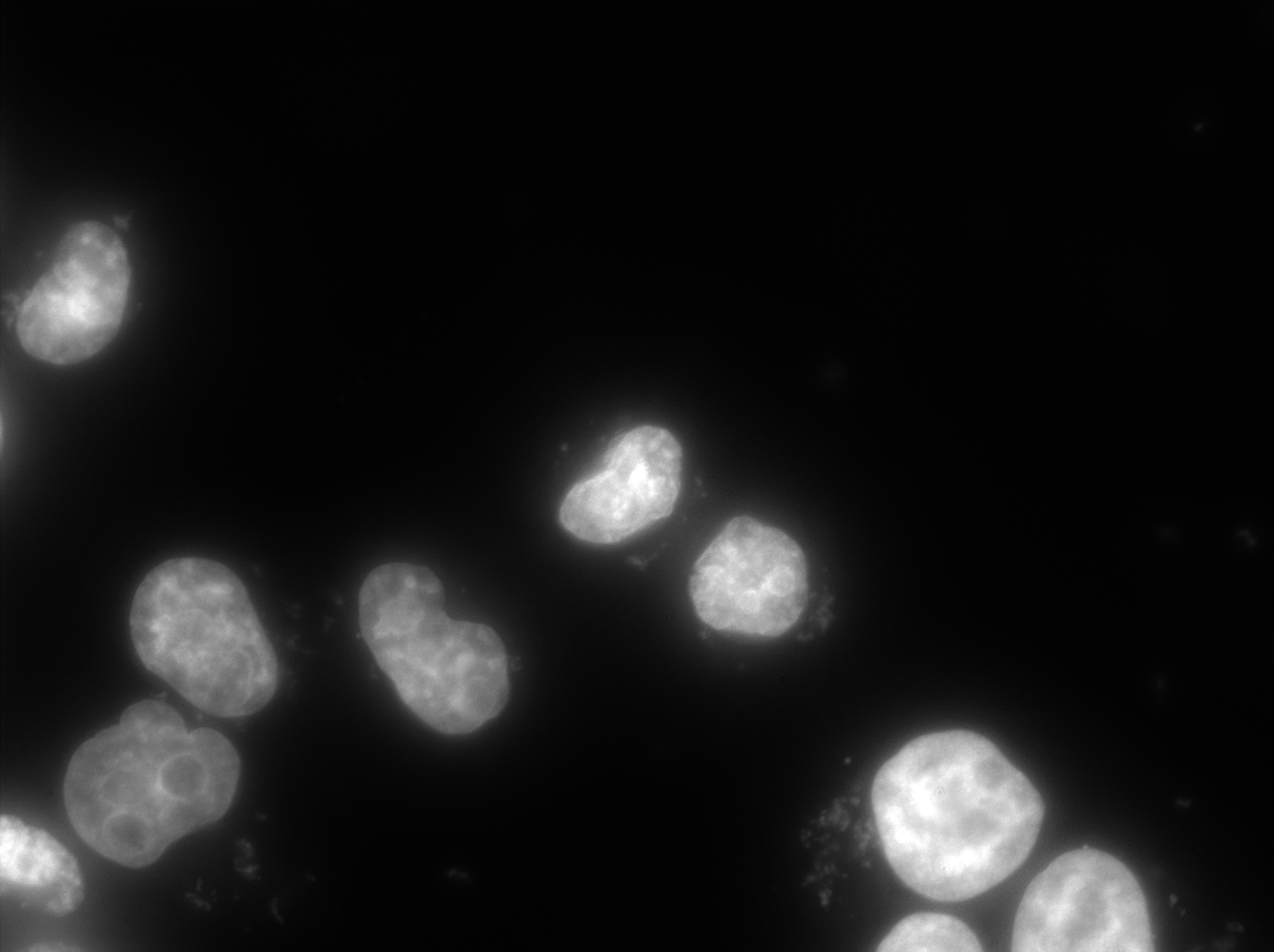

Supplement: Supplementary file 18 — Source data Fig. 5 part 4 [file 44319_2026_742_MOESM18_ESM.zip › Figure 5 Part 4/Fig 5ab WT and KO hela TTLL1-e326g atubulin/Control/WT Hela TTLL1-mut R3 11-13-24 LT10.Project Maximum Z_XY1731547834_Z0_T0_C0.tif]

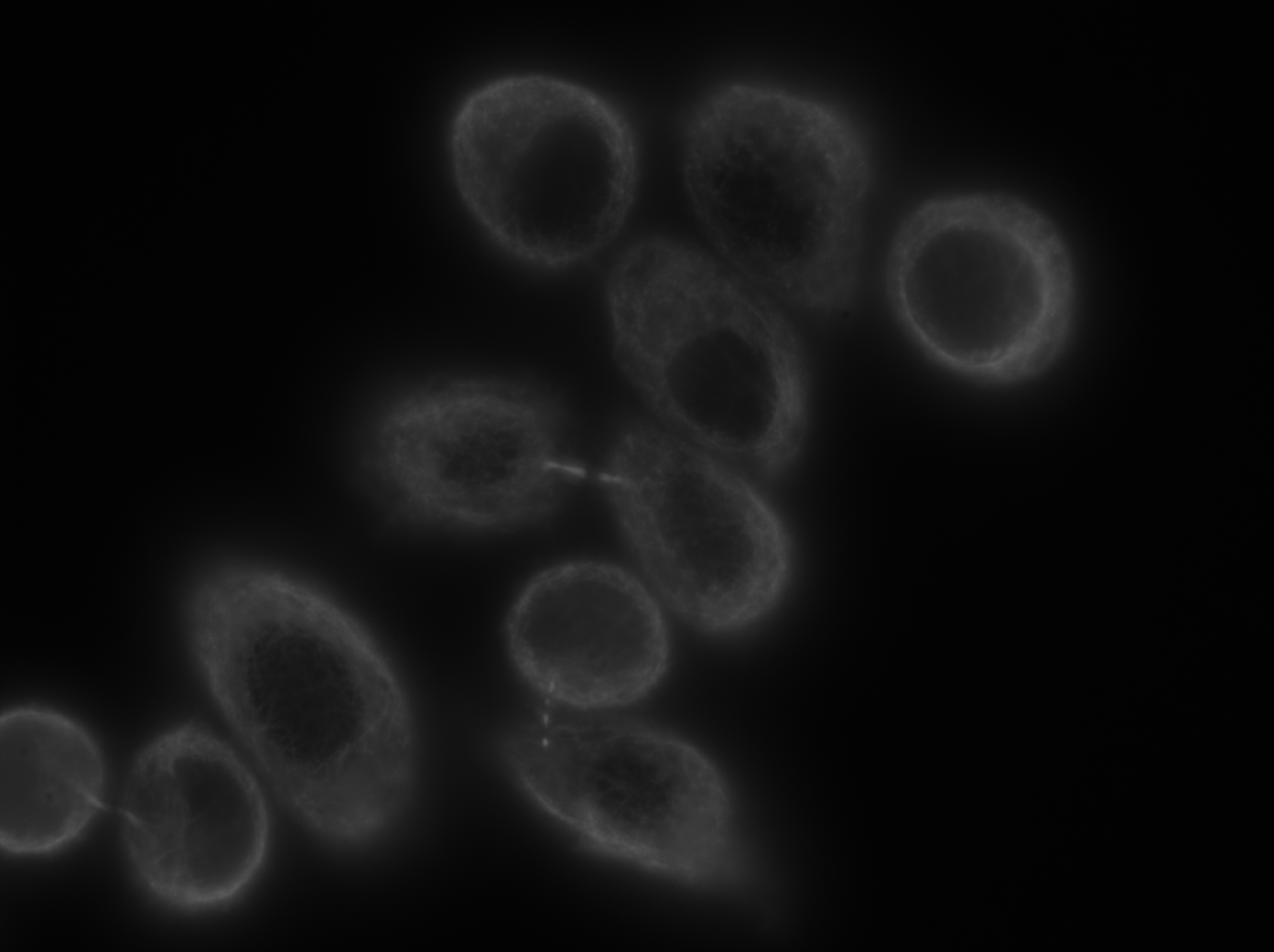

Supplement: Supplementary file 18 — Source data Fig. 5 part 4 [file 44319_2026_742_MOESM18_ESM.zip › Figure 5 Part 4/Fig 5ab WT and KO hela TTLL1-e326g atubulin/Control/TTLL1-mut atub R1 LT8.Project Maximum Z_XY1724441009_Z0_T0_C2.tif]

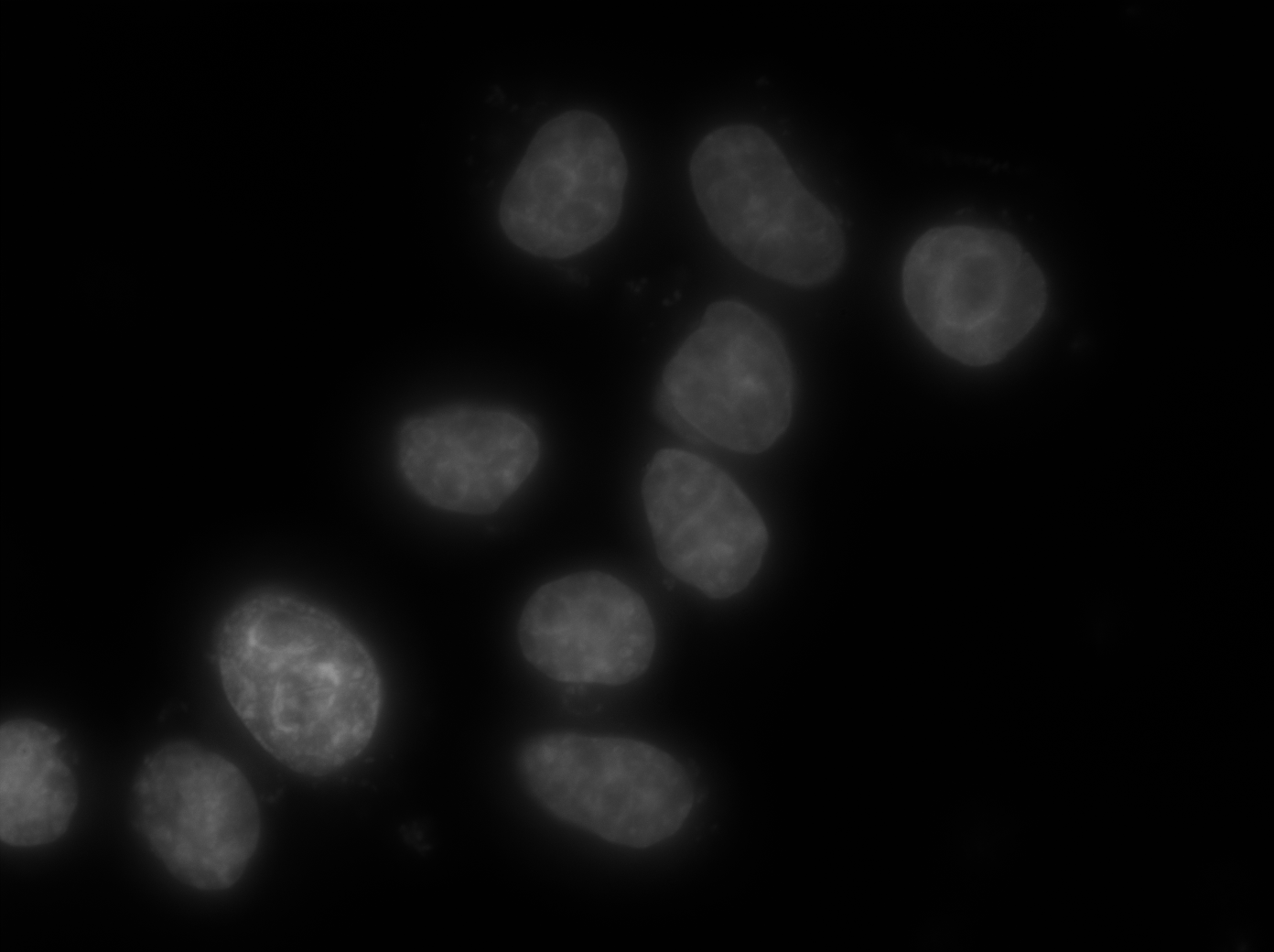

Supplement: Supplementary file 18 — Source data Fig. 5 part 4 [file 44319_2026_742_MOESM18_ESM.zip › Figure 5 Part 4/Fig 5ab WT and KO hela TTLL1-e326g atubulin/Control/TTLL1-mut atub R1 LT8.Project Maximum Z_XY1724441009_Z0_T0_C0.tif]

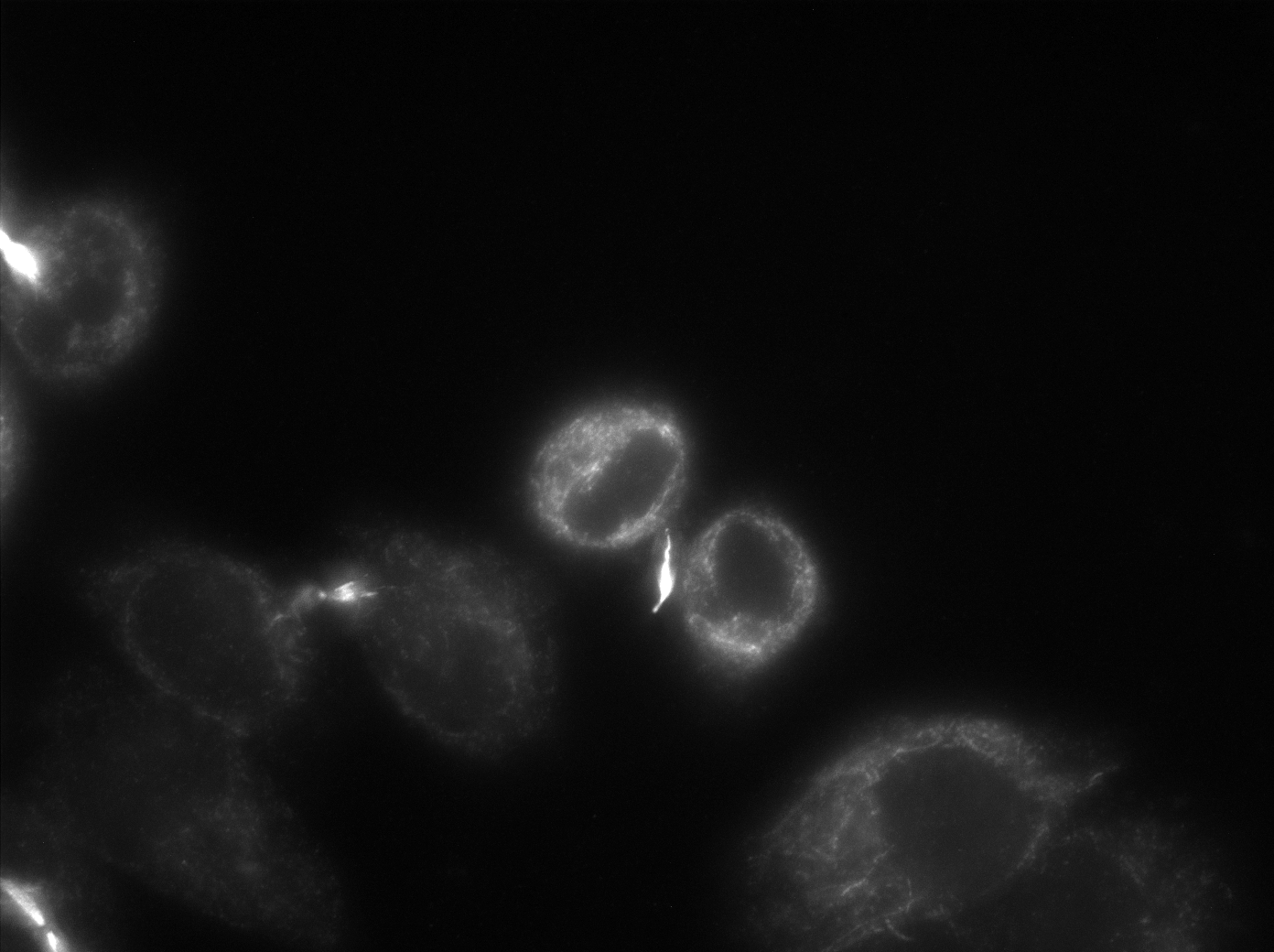

Supplement: Supplementary file 18 — Source data Fig. 5 part 4 [file 44319_2026_742_MOESM18_ESM.zip › Figure 5 Part 4/Fig 5ab WT and KO hela TTLL1-e326g atubulin/Control/WT Hela TTLL1-mut R3 11-13-24 LT10.Project Maximum Z_XY1731547834_Z0_T0_C2.tif]

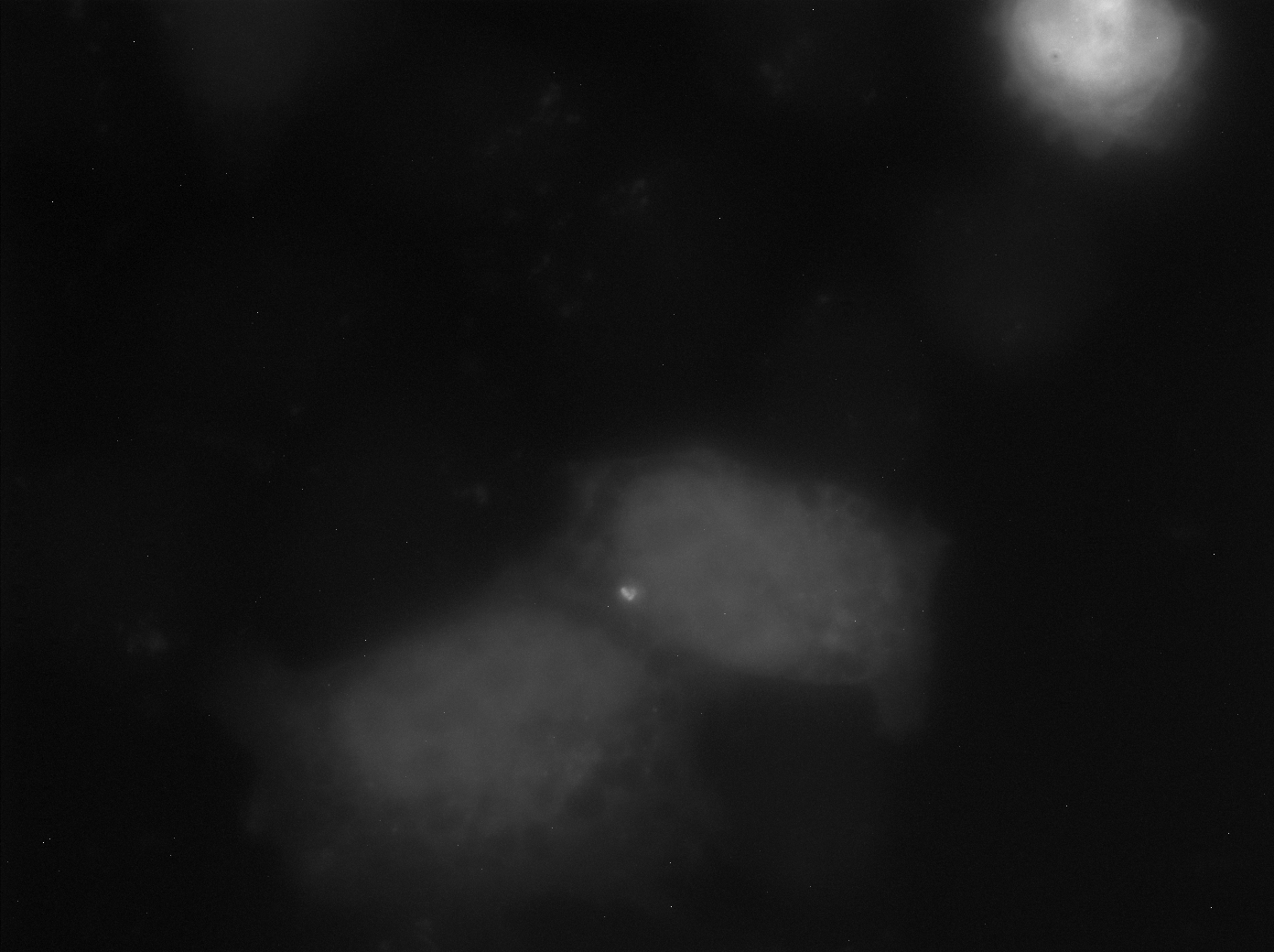

Supplement: Supplementary file 18 — Source data Fig. 5 part 4 [file 44319_2026_742_MOESM18_ESM.zip › Figure 5 Part 4/Fig 5ab WT and KO hela TTLL1-e326g atubulin/Control/TTLL1-mut atub R2 LT5.Project Maximum Z_XY1724951535_Z0_T0_C1.tif]

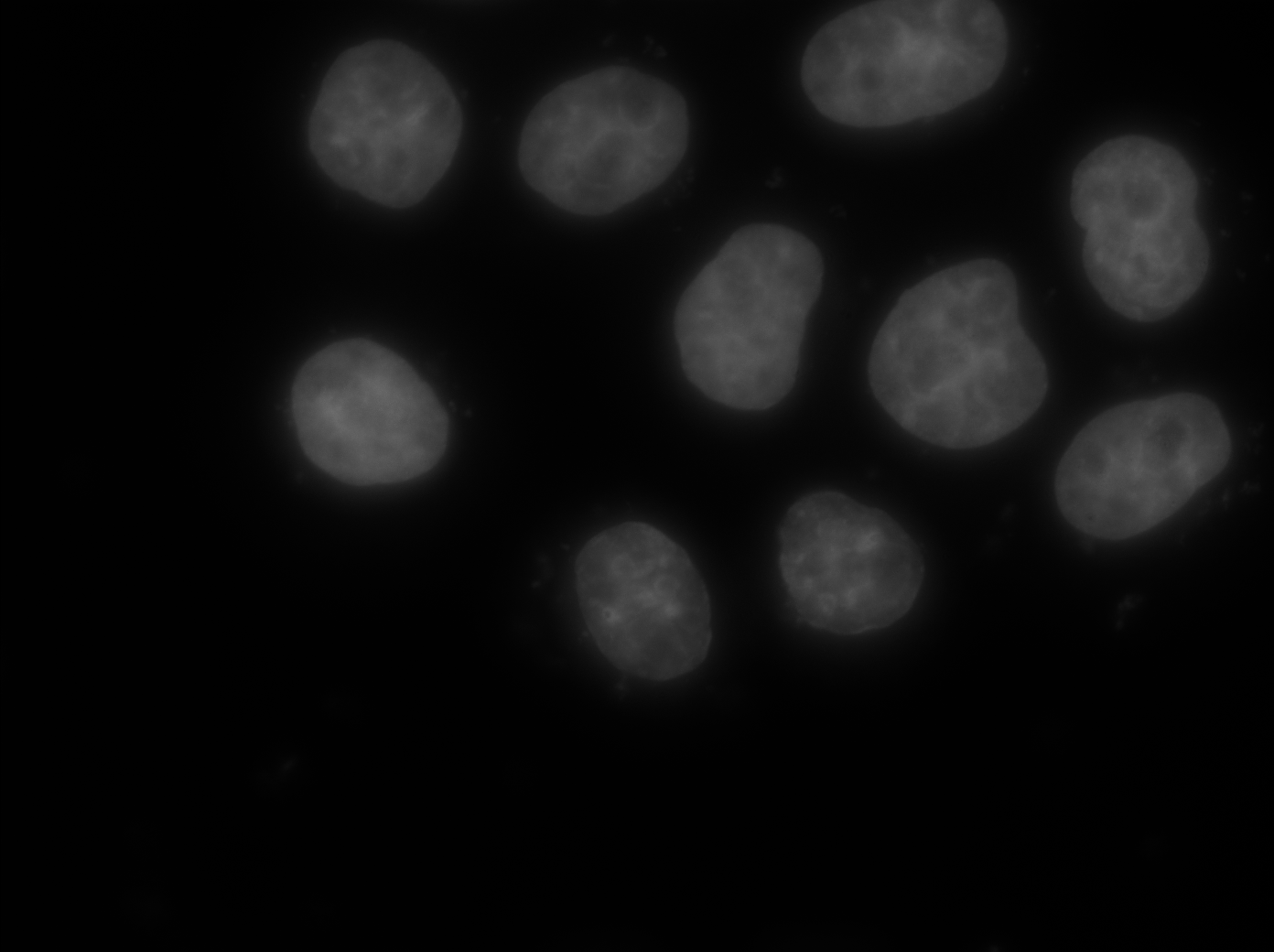

Supplement: Supplementary file 18 — Source data Fig. 5 part 4 [file 44319_2026_742_MOESM18_ESM.zip › Figure 5 Part 4/Fig 5ab WT and KO hela TTLL1-e326g atubulin/Control/TTLL1-mut atub R1 LT5.Project Maximum Z_XY1724440046_Z0_T0_C0.tif]

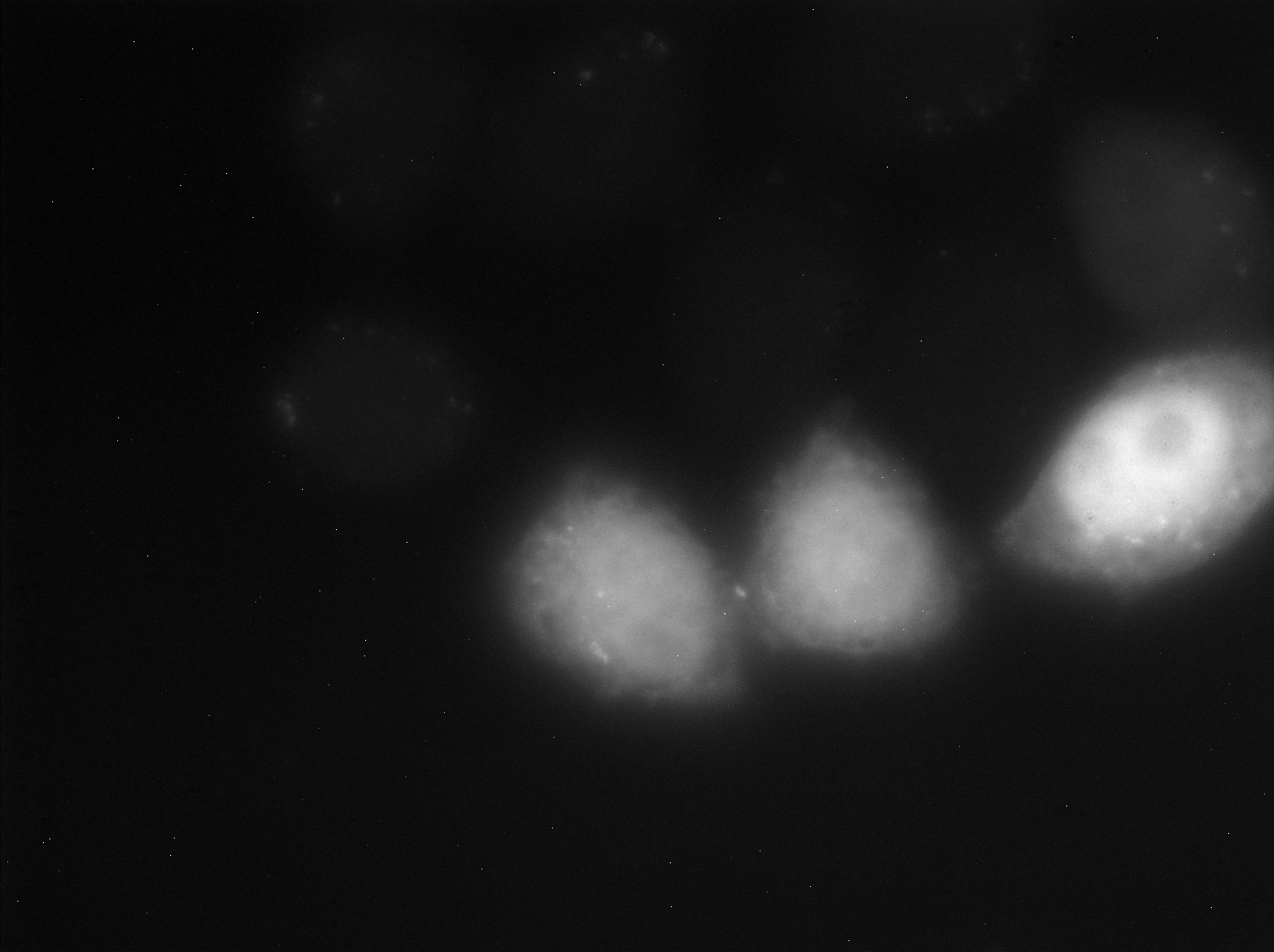

Supplement: Supplementary file 18 — Source data Fig. 5 part 4 [file 44319_2026_742_MOESM18_ESM.zip › Figure 5 Part 4/Fig 5ab WT and KO hela TTLL1-e326g atubulin/Control/TTLL1-mut atub R1 LT5.Project Maximum Z_XY1724440046_Z0_T0_C1.tif]

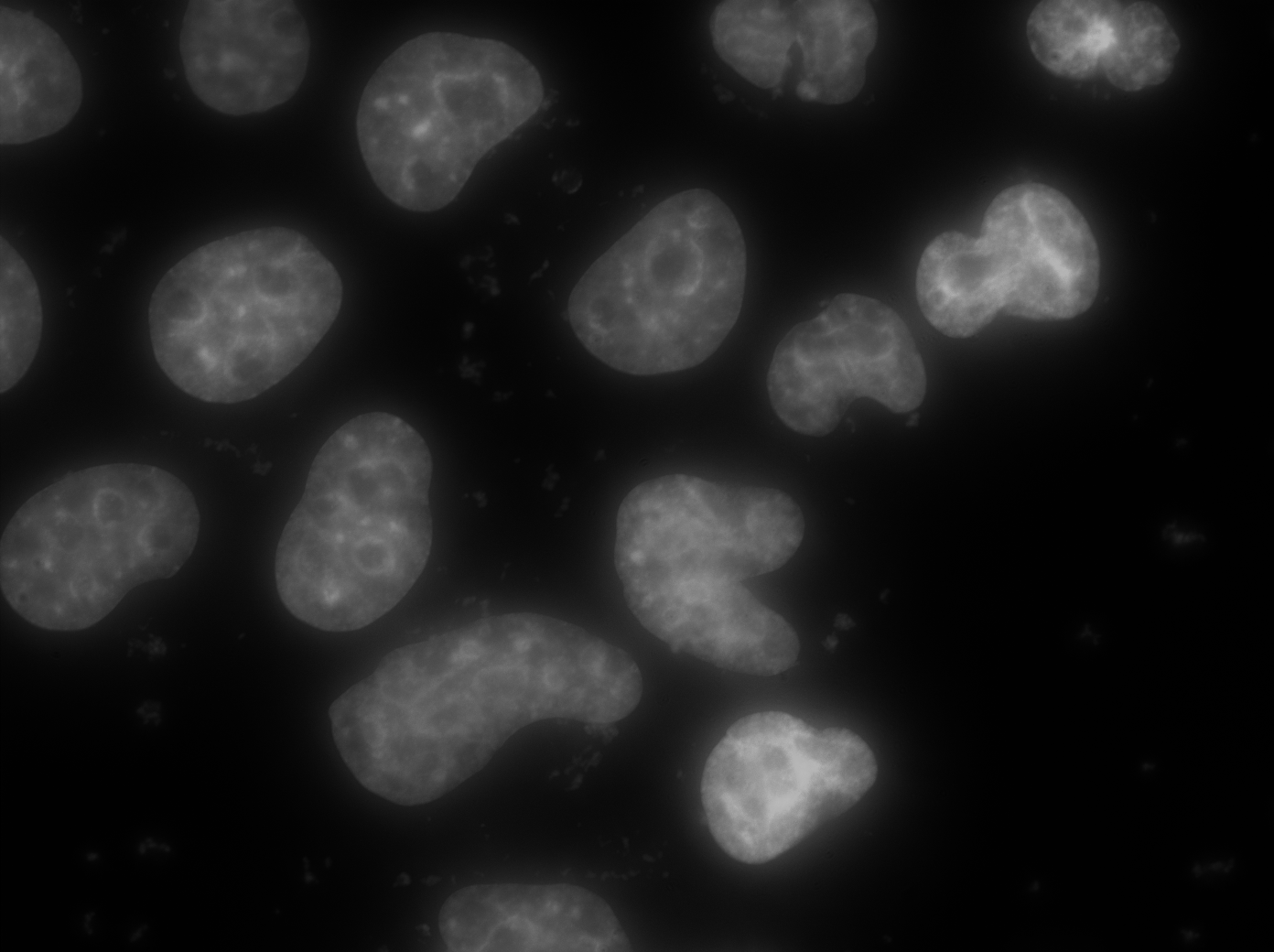

Supplement: Supplementary file 18 — Source data Fig. 5 part 4 [file 44319_2026_742_MOESM18_ESM.zip › Figure 5 Part 4/Fig 5ab WT and KO hela TTLL1-e326g atubulin/Control/TTLL1-mut atub R2 LT5.Project Maximum Z_XY1724951535_Z0_T0_C0.tif]

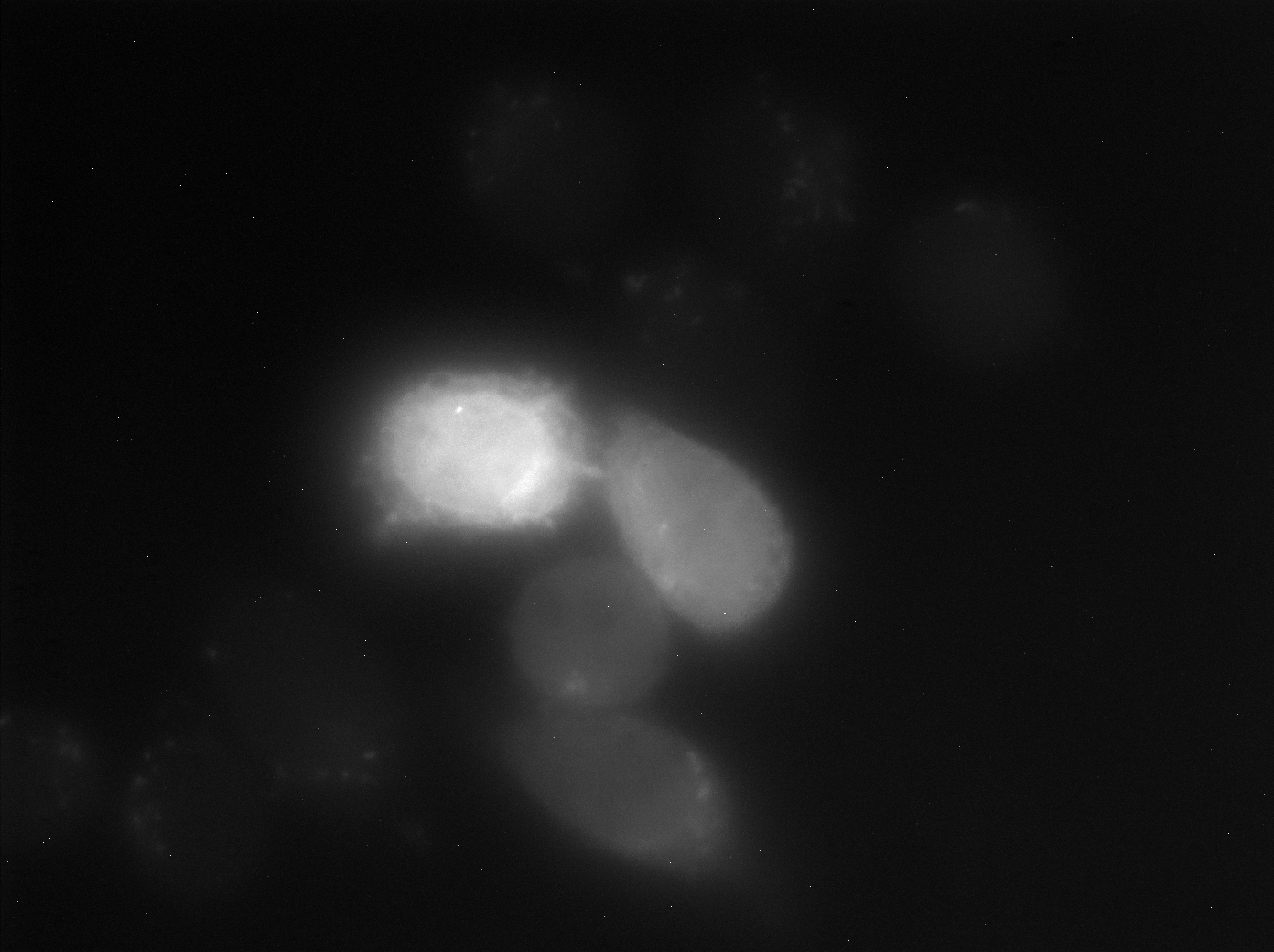

Supplement: Supplementary file 18 — Source data Fig. 5 part 4 [file 44319_2026_742_MOESM18_ESM.zip › Figure 5 Part 4/Fig 5ab WT and KO hela TTLL1-e326g atubulin/Control/TTLL1-mut atub R1 LT8.Project Maximum Z_XY1724441009_Z0_T0_C1.tif]

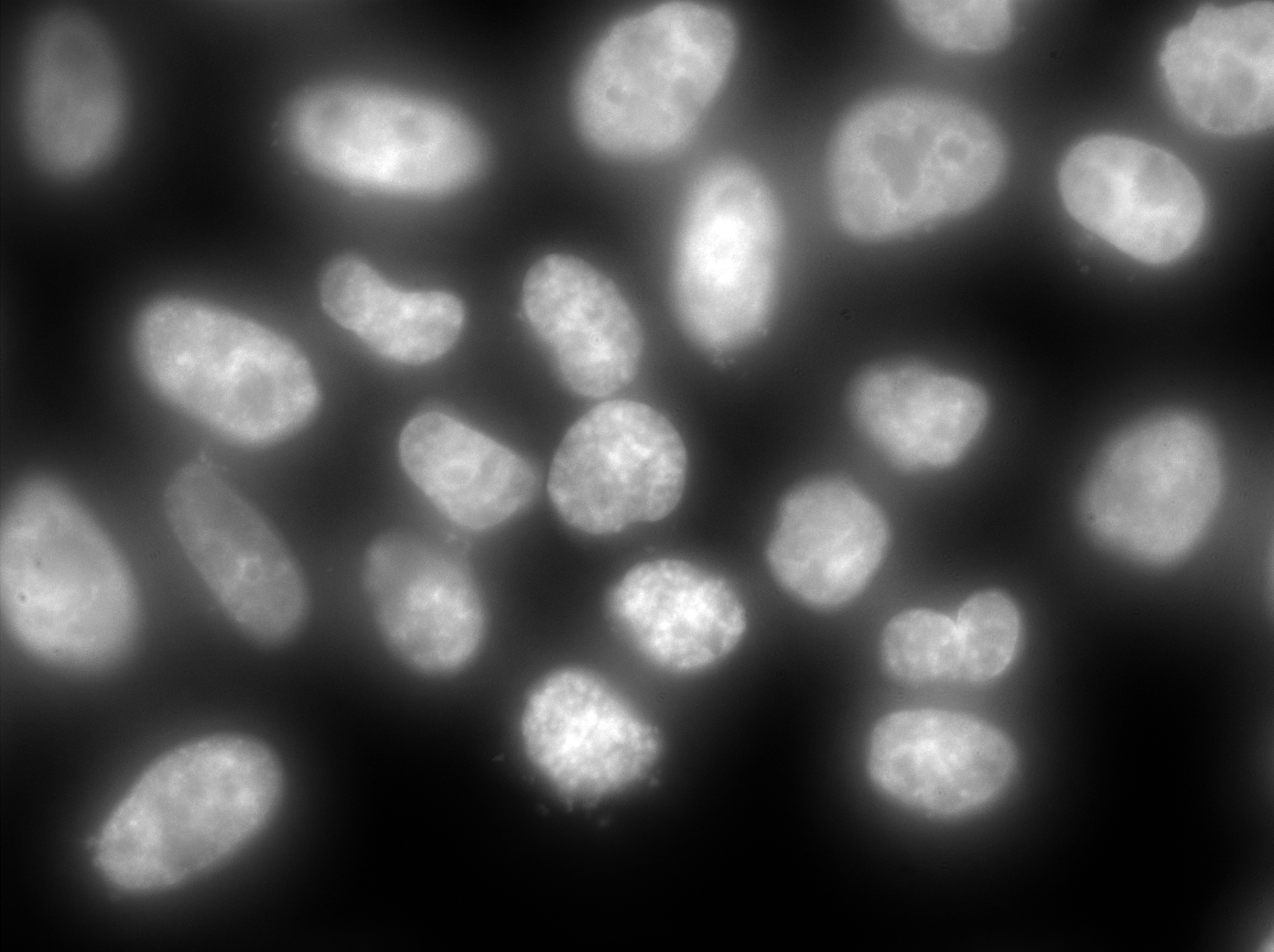

Supplement: Supplementary file 18 — Source data Fig. 5 part 4 [file 44319_2026_742_MOESM18_ESM.zip › Figure 5 Part 4/Fig 5ab WT and KO hela TTLL1-e326g atubulin/Control/WT Hela TTLL1-mut R3 11-13-24 LT7LT8.Project Maximum Z_XY1731547036_Z0_T0_C0.tif]

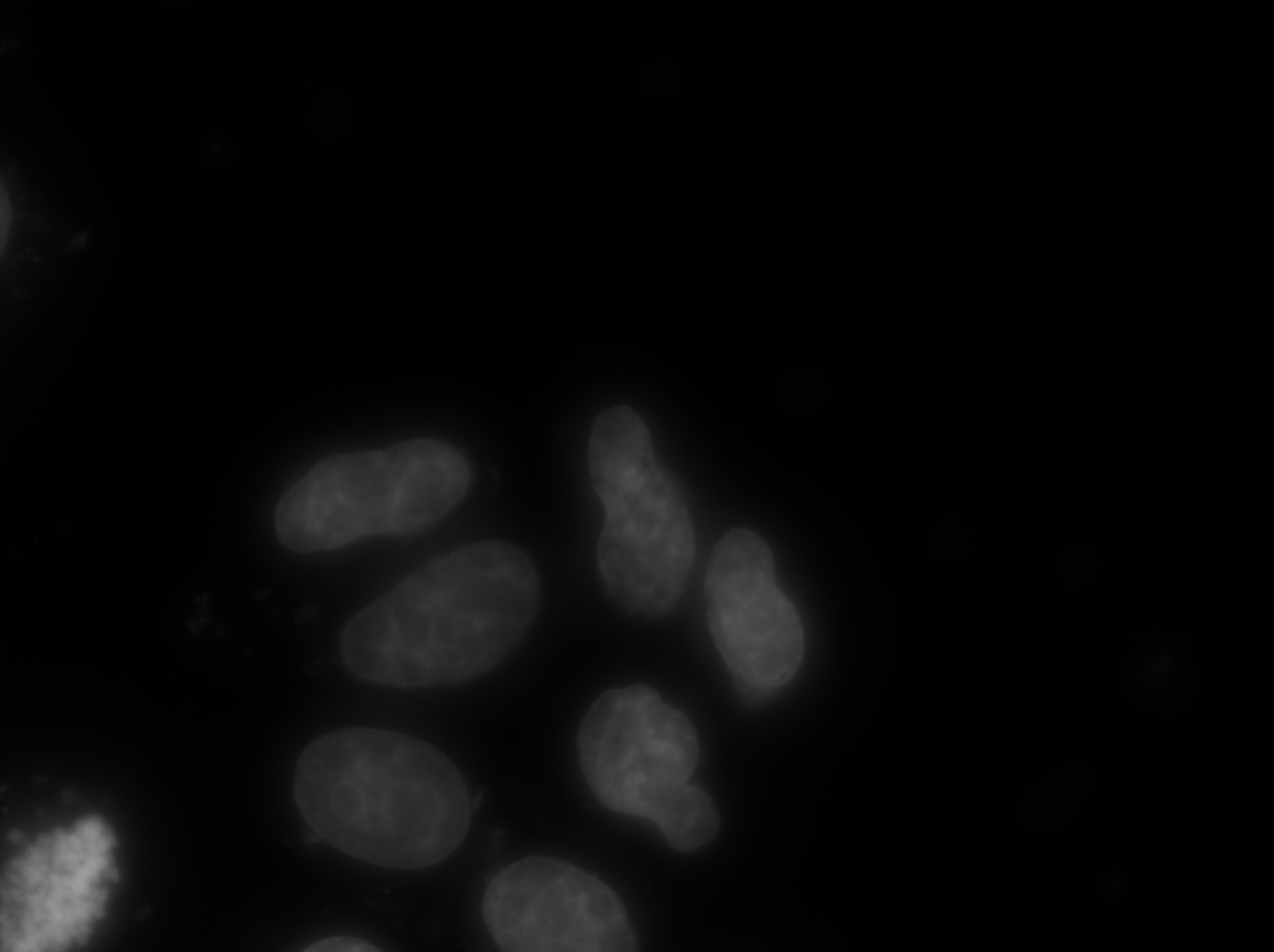

Supplement: Supplementary file 18 — Source data Fig. 5 part 4 [file 44319_2026_742_MOESM18_ESM.zip › Figure 5 Part 4/Fig 5ab WT and KO hela TTLL1-e326g atubulin/Control/TTLL1-mut atub R1 LT6.Project Maximum Z_XY1724440388_Z0_T0_C0.tif]

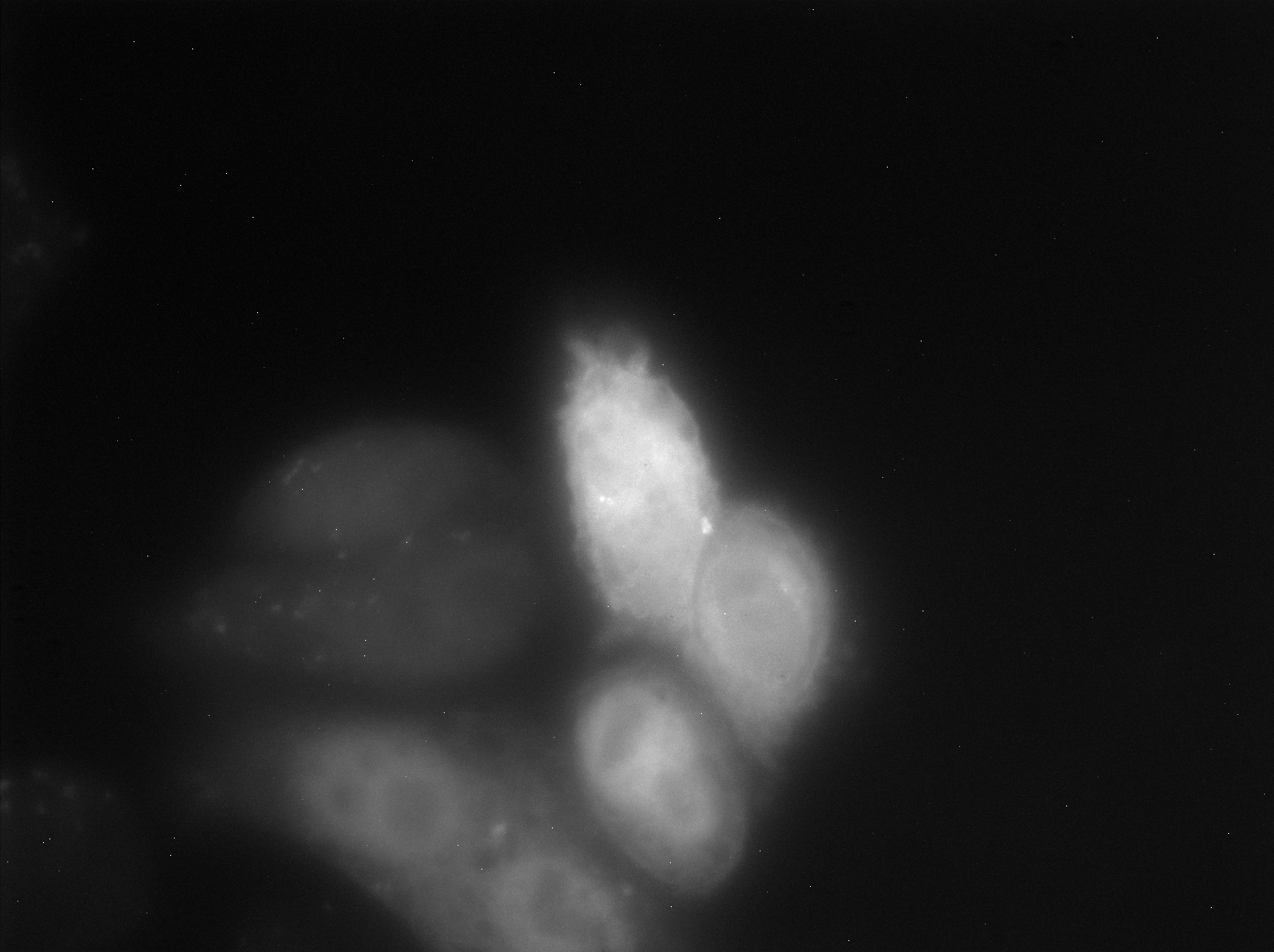

Supplement: Supplementary file 18 — Source data Fig. 5 part 4 [file 44319_2026_742_MOESM18_ESM.zip › Figure 5 Part 4/Fig 5ab WT and KO hela TTLL1-e326g atubulin/Control/TTLL1-mut atub R1 LT6.Project Maximum Z_XY1724440388_Z0_T0_C1.tif]

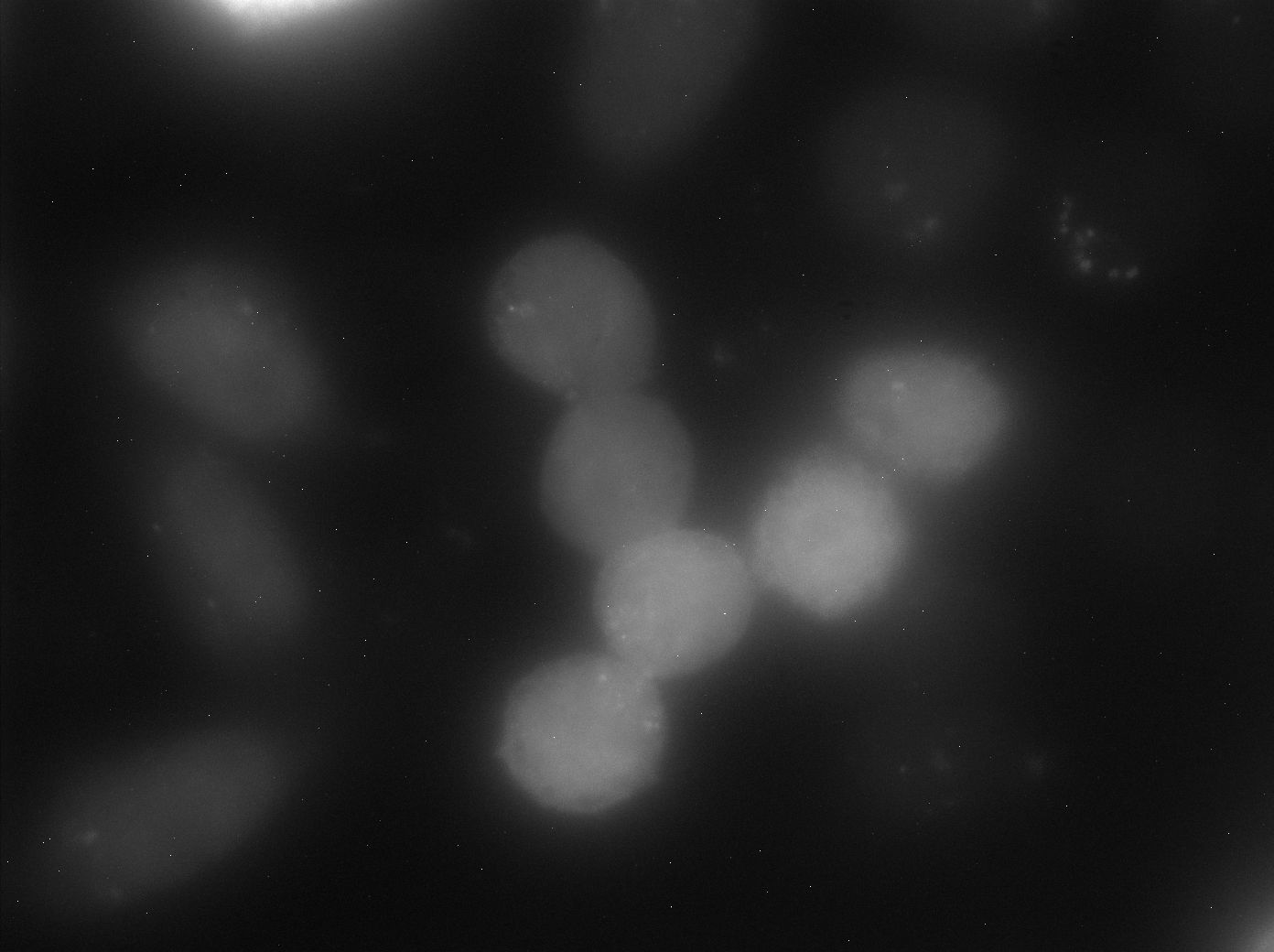

Supplement: Supplementary file 18 — Source data Fig. 5 part 4 [file 44319_2026_742_MOESM18_ESM.zip › Figure 5 Part 4/Fig 5ab WT and KO hela TTLL1-e326g atubulin/Control/WT Hela TTLL1-mut R3 11-13-24 LT7LT8.Project Maximum Z_XY1731547036_Z0_T0_C1.tif]

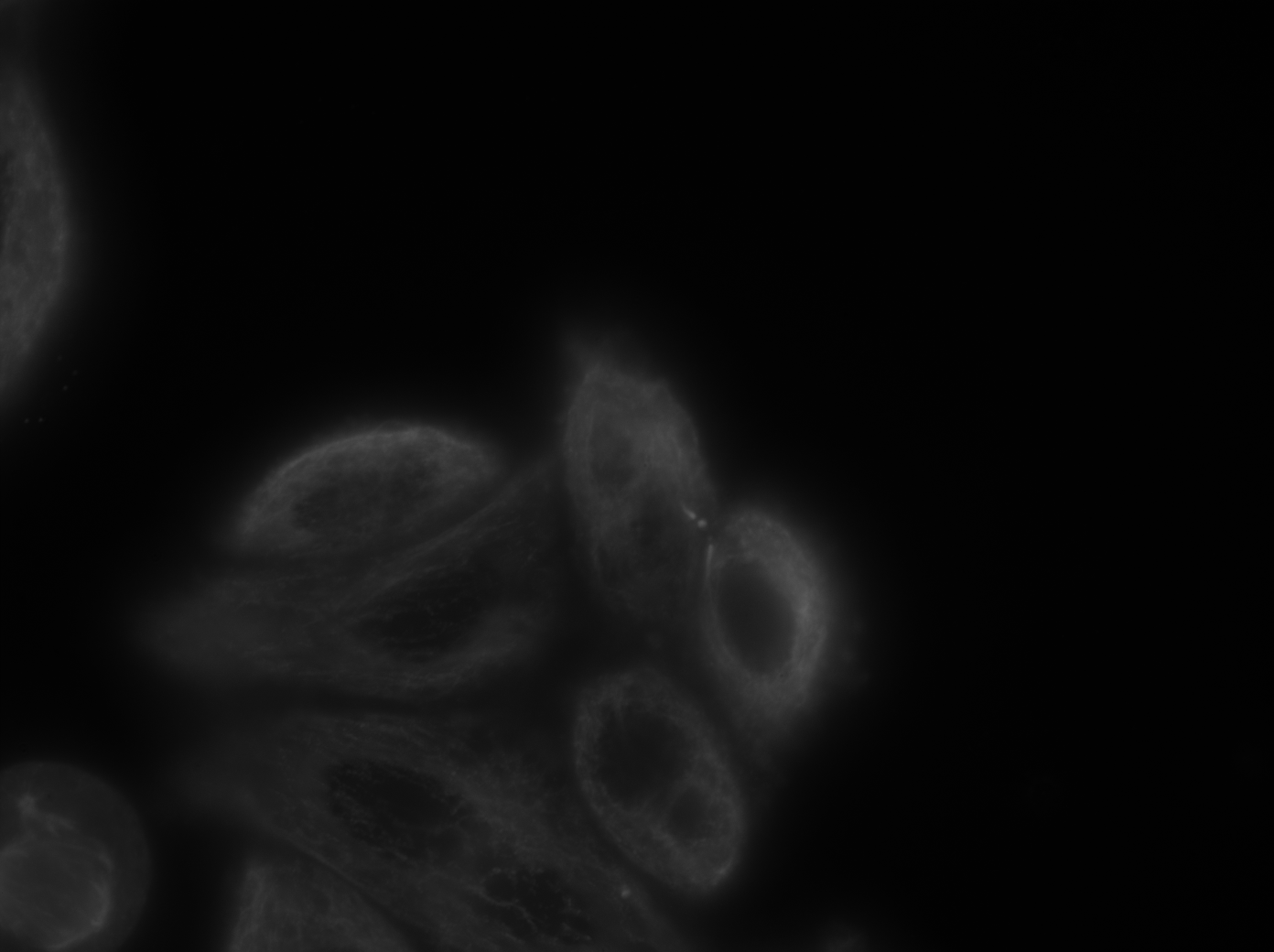

Supplement: Supplementary file 18 — Source data Fig. 5 part 4 [file 44319_2026_742_MOESM18_ESM.zip › Figure 5 Part 4/Fig 5ab WT and KO hela TTLL1-e326g atubulin/Control/TTLL1-mut atub R1 LT6.Project Maximum Z_XY1724440388_Z0_T0_C2.tif]

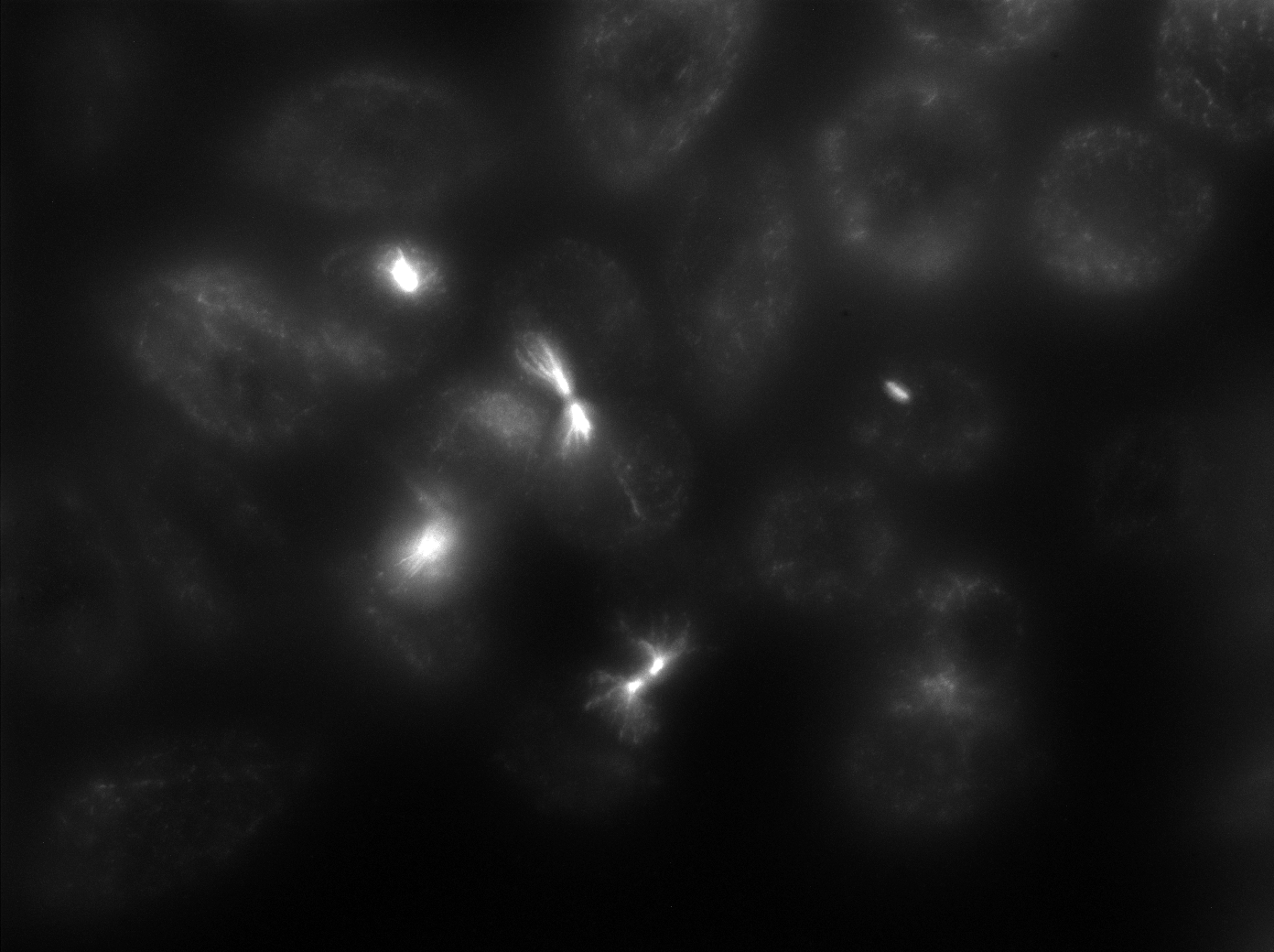

Supplement: Supplementary file 18 — Source data Fig. 5 part 4 [file 44319_2026_742_MOESM18_ESM.zip › Figure 5 Part 4/Fig 5ab WT and KO hela TTLL1-e326g atubulin/Control/WT Hela TTLL1-mut R3 11-13-24 LT7LT8.Project Maximum Z_XY1731547036_Z0_T0_C2.tif]

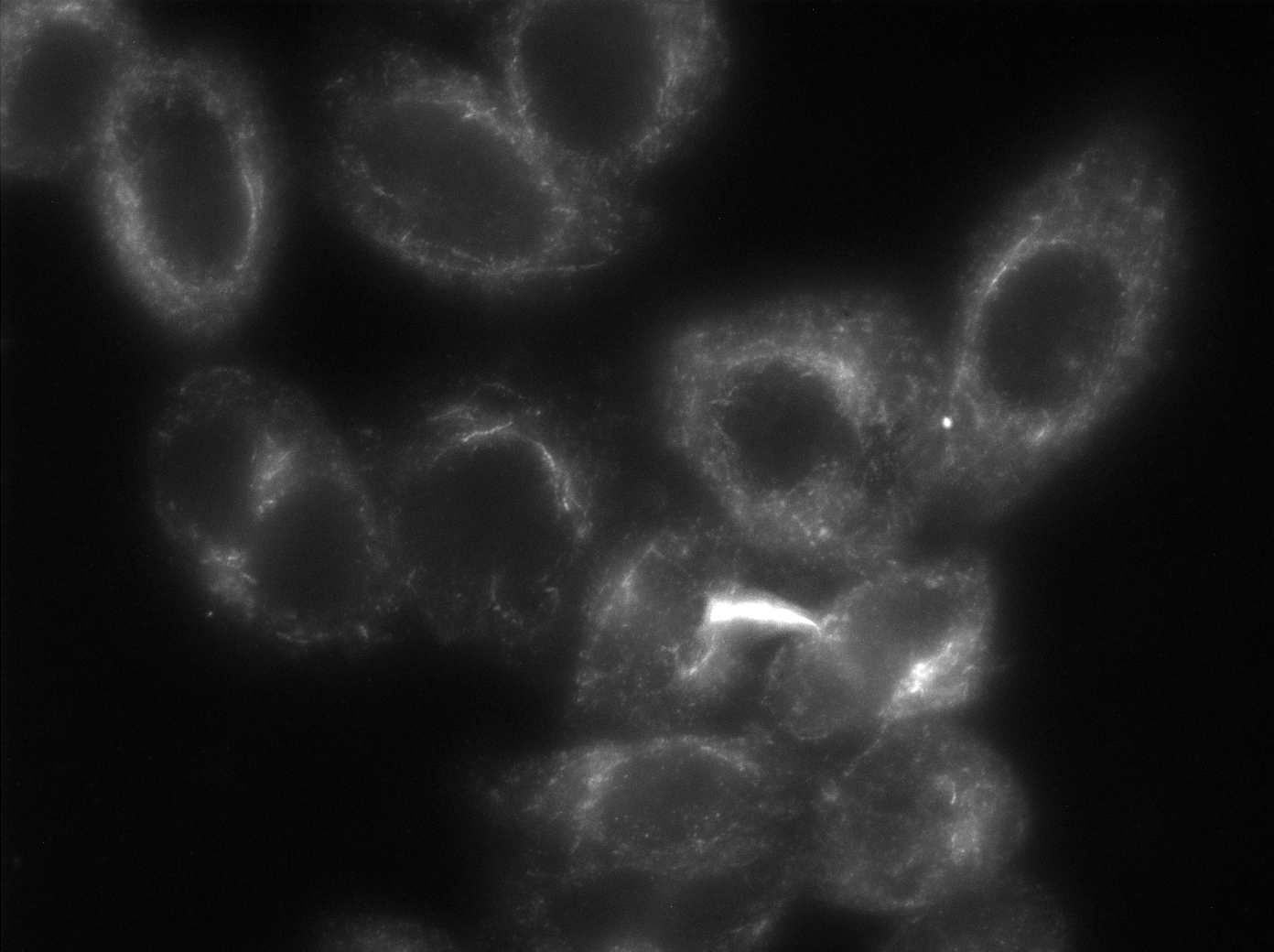

Supplement: Supplementary file 18 — Source data Fig. 5 part 4 [file 44319_2026_742_MOESM18_ESM.zip › Figure 5 Part 4/Fig 5ab WT and KO hela TTLL1-e326g atubulin/Control/WT Hela TTLL1-mut R3 11-13-24 LT3.Project Maximum Z_XY1731546288_Z0_T0_C2.tif]

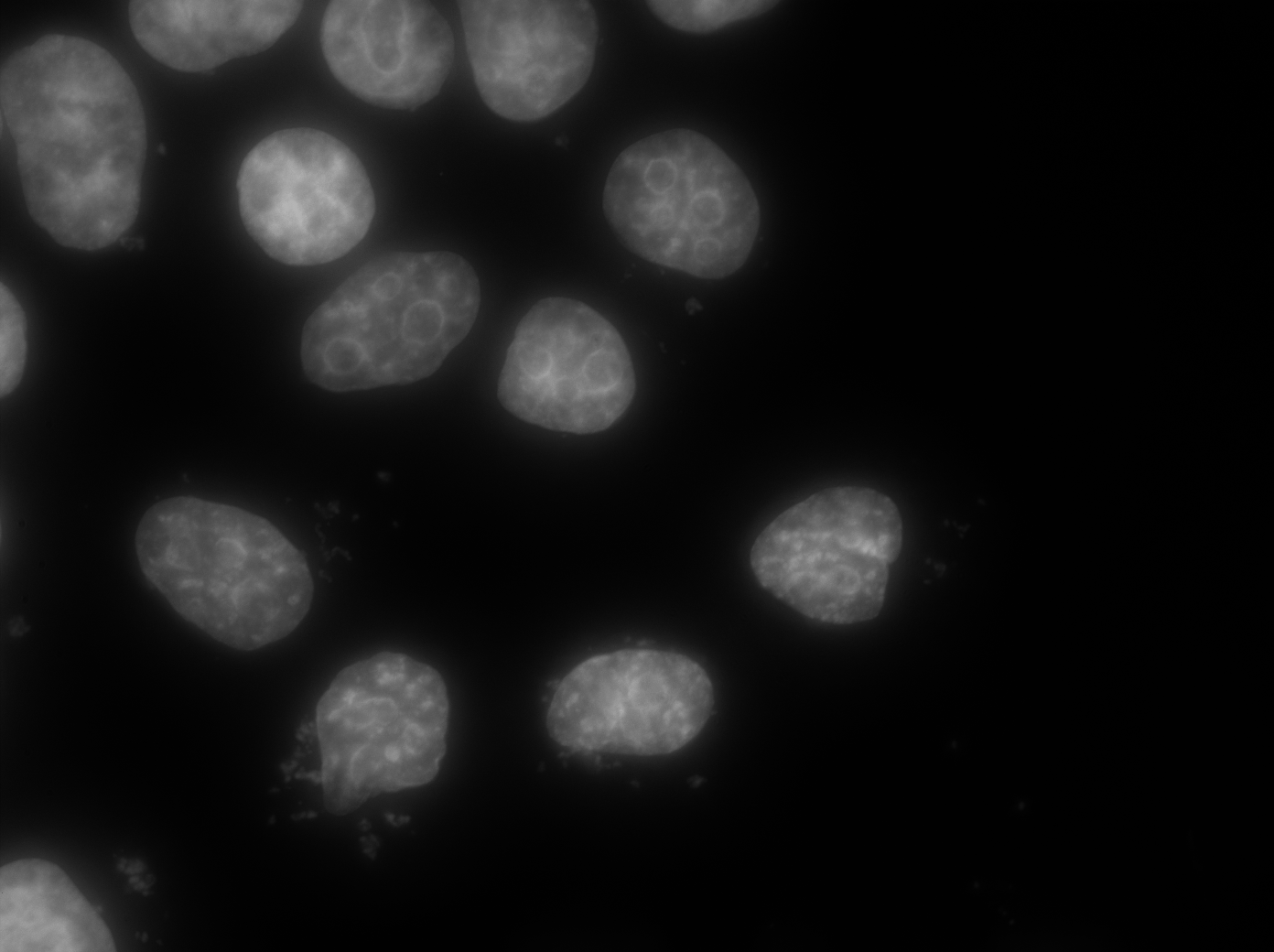

Supplement: Supplementary file 18 — Source data Fig. 5 part 4 [file 44319_2026_742_MOESM18_ESM.zip › Figure 5 Part 4/Fig 5ab WT and KO hela TTLL1-e326g atubulin/Control/TTLL1-mut atub R2 LT8.Project Maximum Z_XY1724952399_Z0_T0_C0.tif]

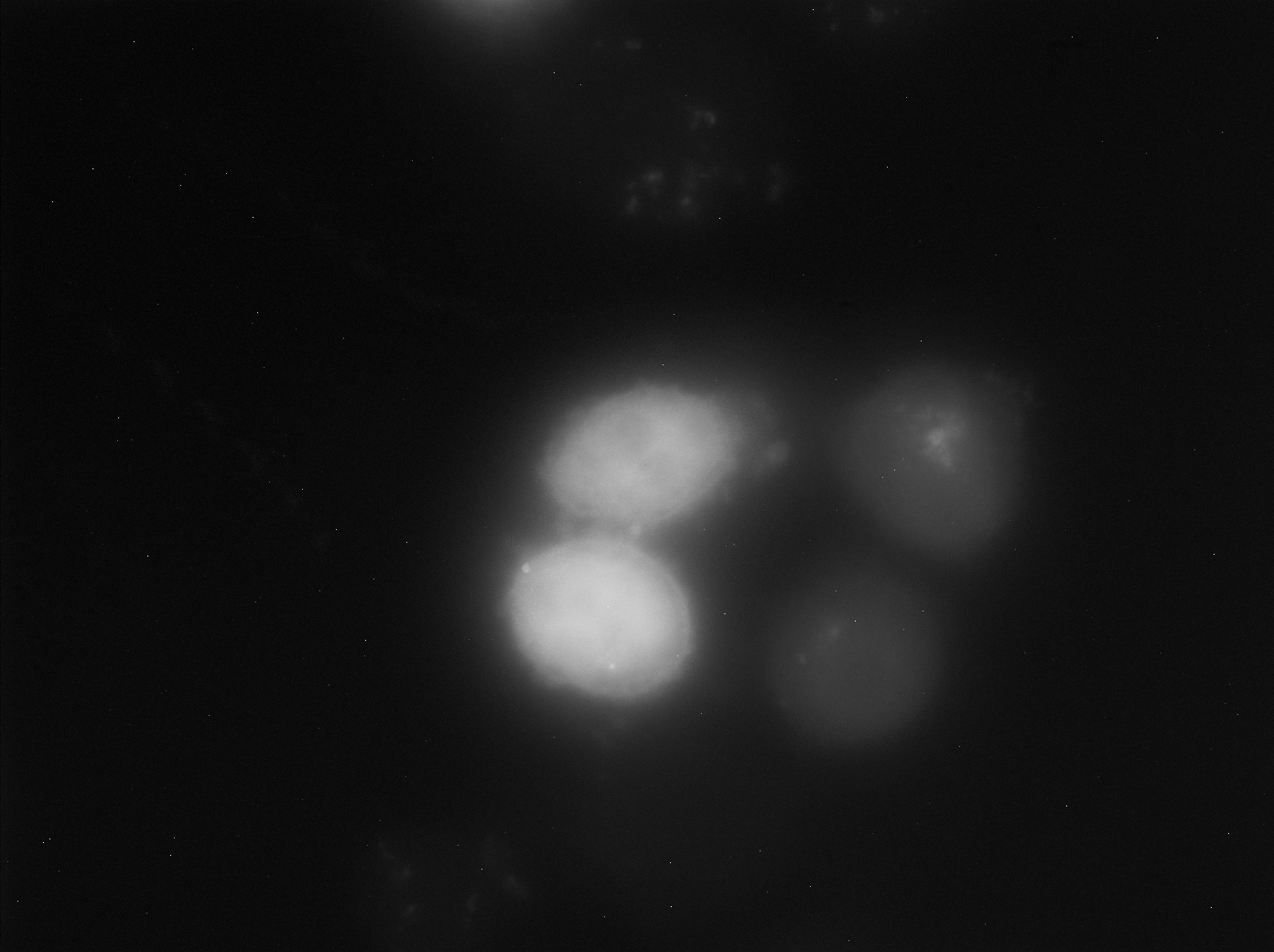

Supplement: Supplementary file 18 — Source data Fig. 5 part 4 [file 44319_2026_742_MOESM18_ESM.zip › Figure 5 Part 4/Fig 5ab WT and KO hela TTLL1-e326g atubulin/Control/WT Hela TTLL1-mut R2 LT1.Project Maximum Z_XY1731542067_Z0_T0_C1.tif]

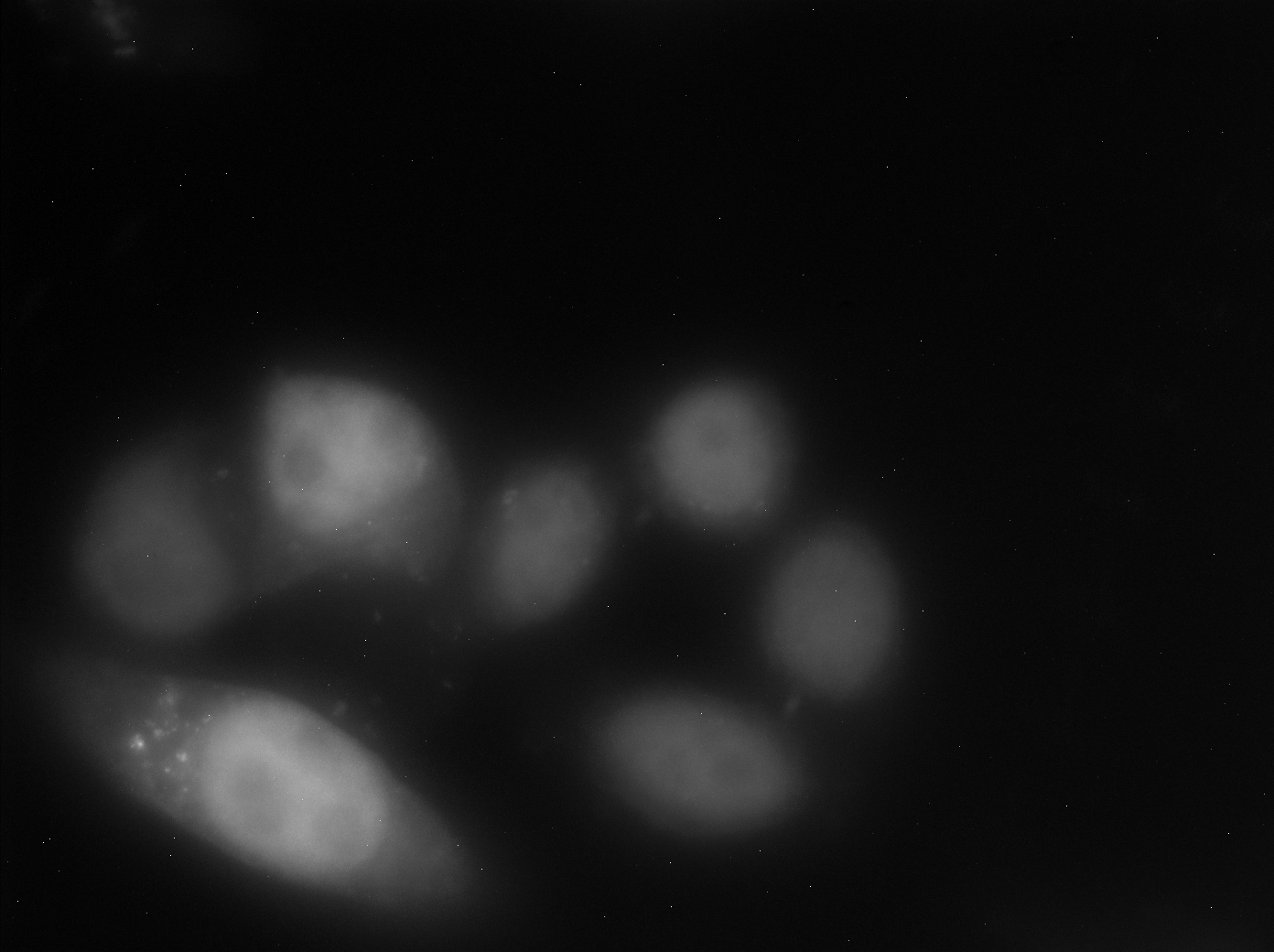

Supplement: Supplementary file 18 — Source data Fig. 5 part 4 [file 44319_2026_742_MOESM18_ESM.zip › Figure 5 Part 4/Fig 5ab WT and KO hela TTLL1-e326g atubulin/Control/WT Hela TTLL1-mut R2 LT3LT4.Project Maximum Z_XY1731542668_Z0_T0_C1.tif]

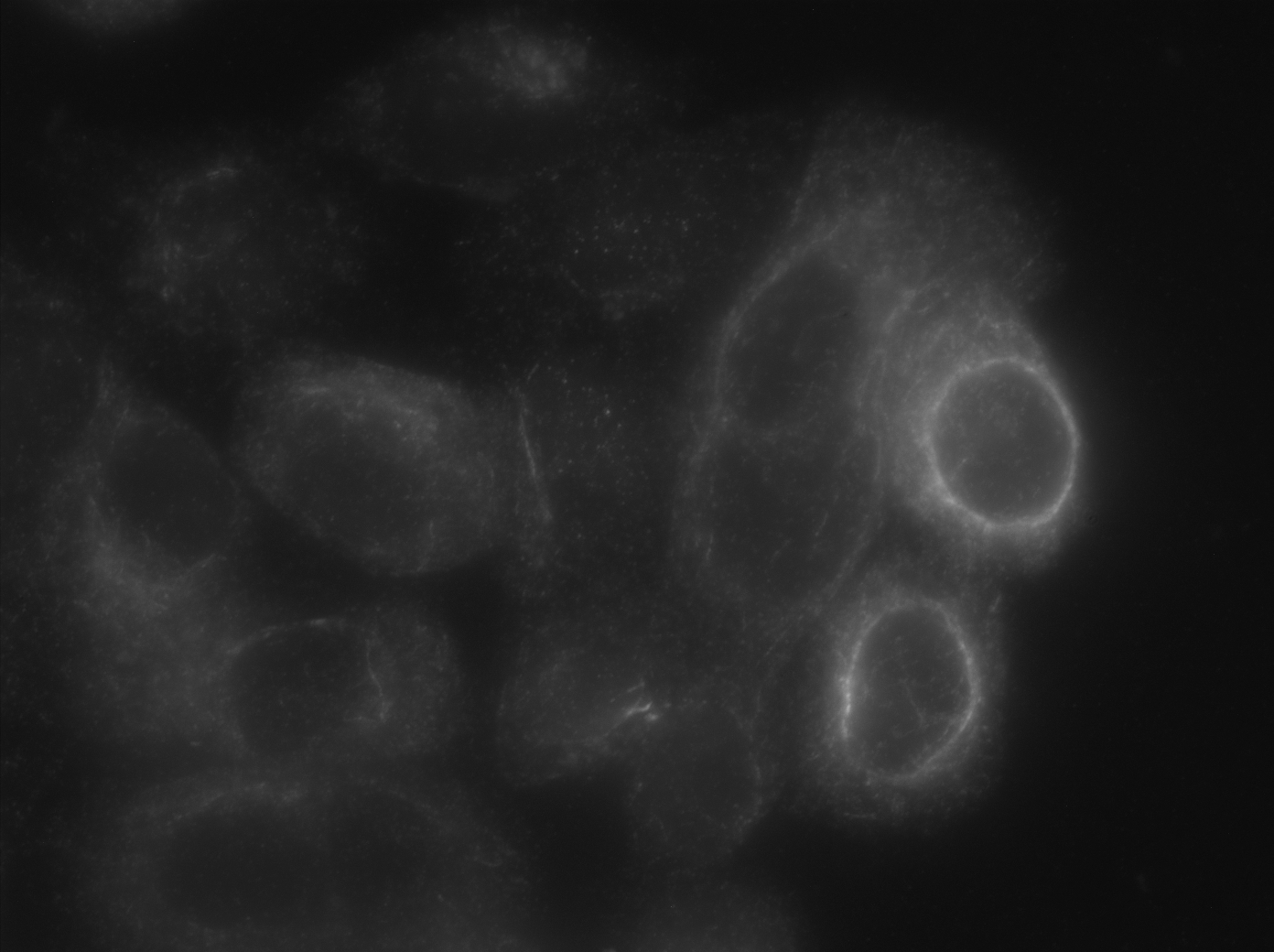

Supplement: Supplementary file 18 — Source data Fig. 5 part 4 [file 44319_2026_742_MOESM18_ESM.zip › Figure 5 Part 4/Fig 5ab WT and KO hela TTLL1-e326g atubulin/Control/WT Hela TTLL1-mut R2 LT7.Project Maximum Z_XY1731543950_Z0_T0_C2.tif]

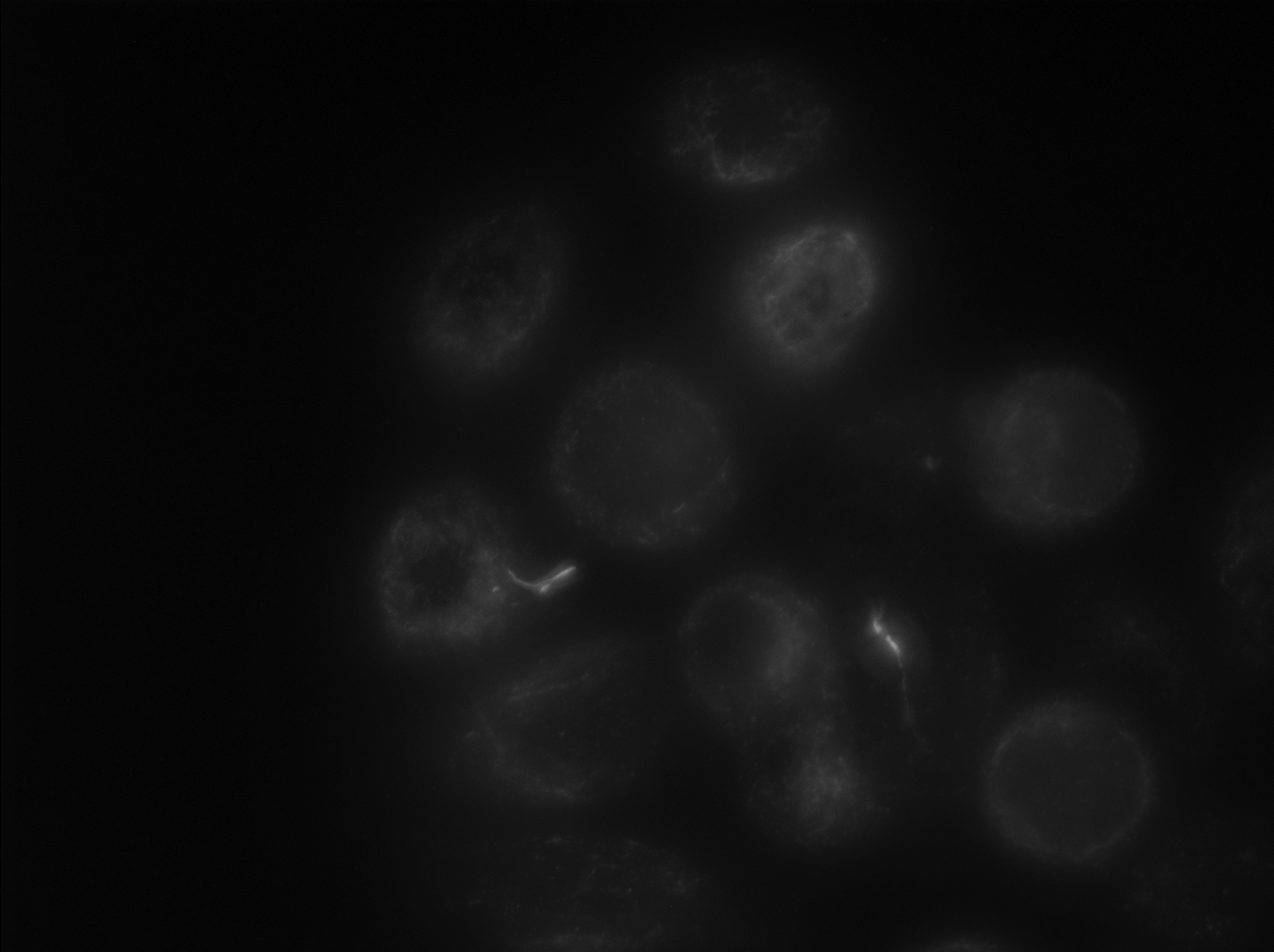

Supplement: Supplementary file 18 — Source data Fig. 5 part 4 [file 44319_2026_742_MOESM18_ESM.zip › Figure 5 Part 4/Fig 5ab WT and KO hela TTLL1-e326g atubulin/Control/WT Hela TTLL1-mut R2 LT10.Project Maximum Z_XY1731544792_Z0_T0_C2.tif]

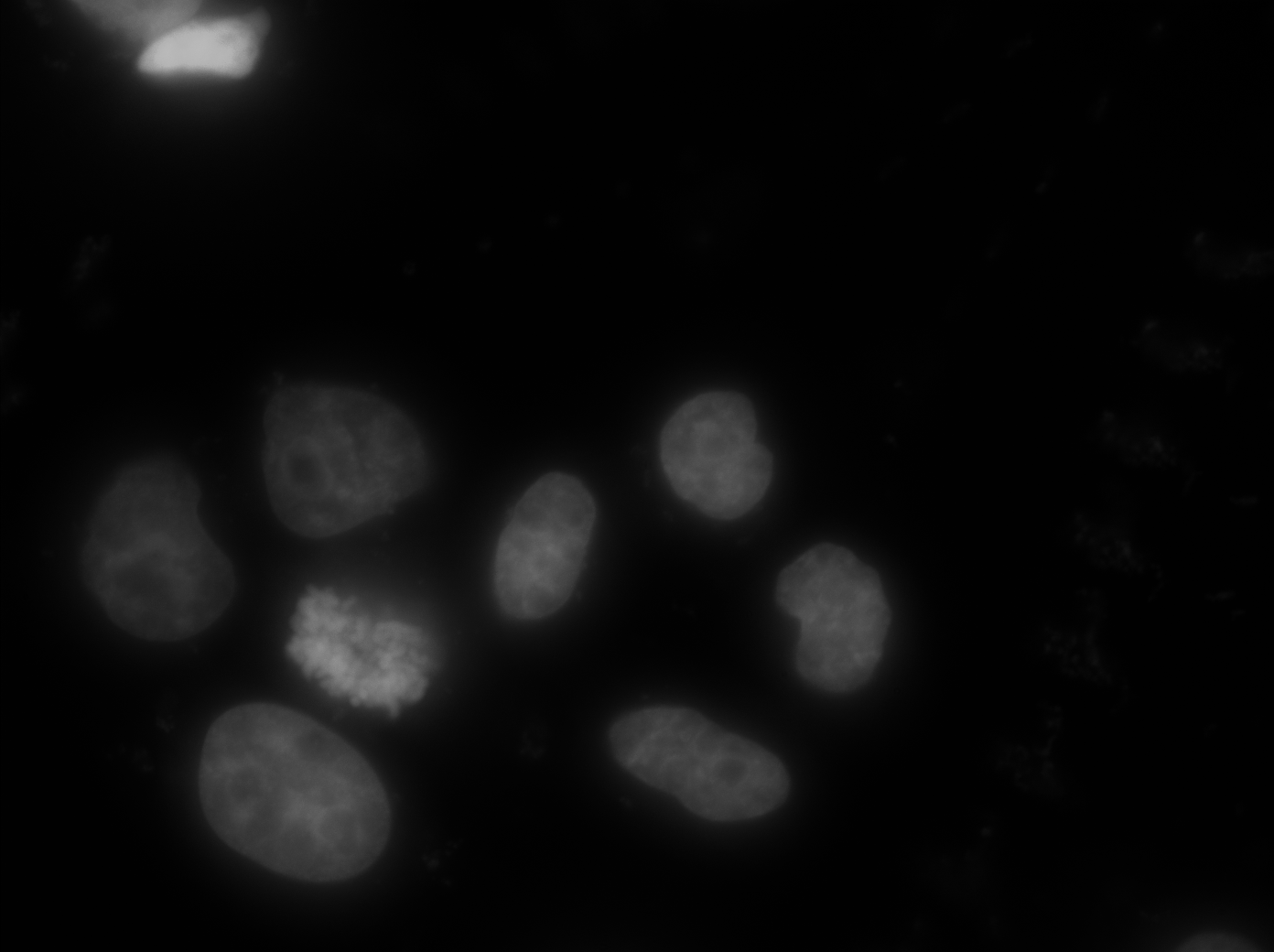

Supplement: Supplementary file 18 — Source data Fig. 5 part 4 [file 44319_2026_742_MOESM18_ESM.zip › Figure 5 Part 4/Fig 5ab WT and KO hela TTLL1-e326g atubulin/Control/WT Hela TTLL1-mut R2 LT3LT4.Project Maximum Z_XY1731542668_Z0_T0_C0.tif]

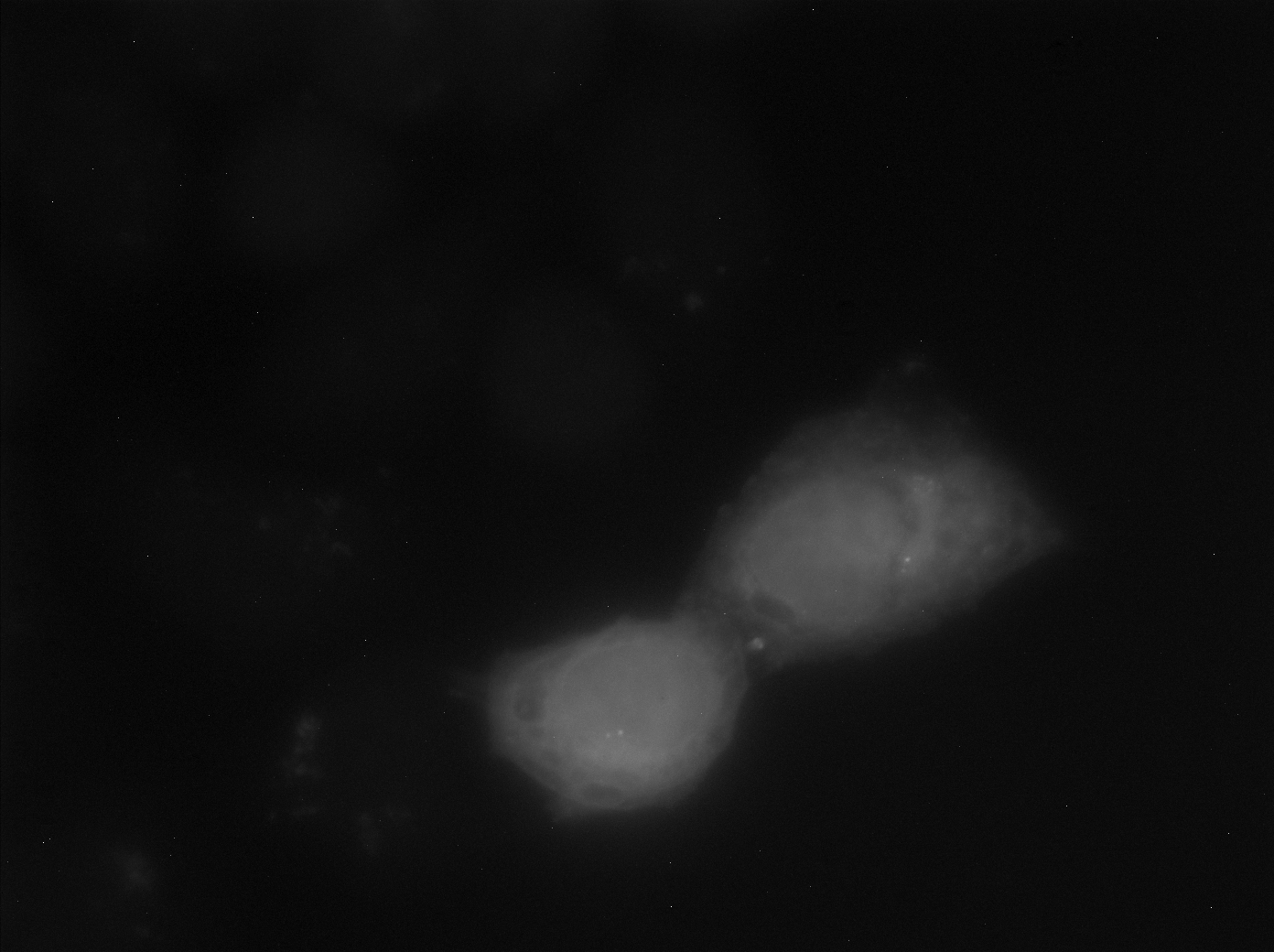

Supplement: Supplementary file 18 — Source data Fig. 5 part 4 [file 44319_2026_742_MOESM18_ESM.zip › Figure 5 Part 4/Fig 5ab WT and KO hela TTLL1-e326g atubulin/Control/TTLL1-mut atub R2 LT8.Project Maximum Z_XY1724952399_Z0_T0_C1.tif]

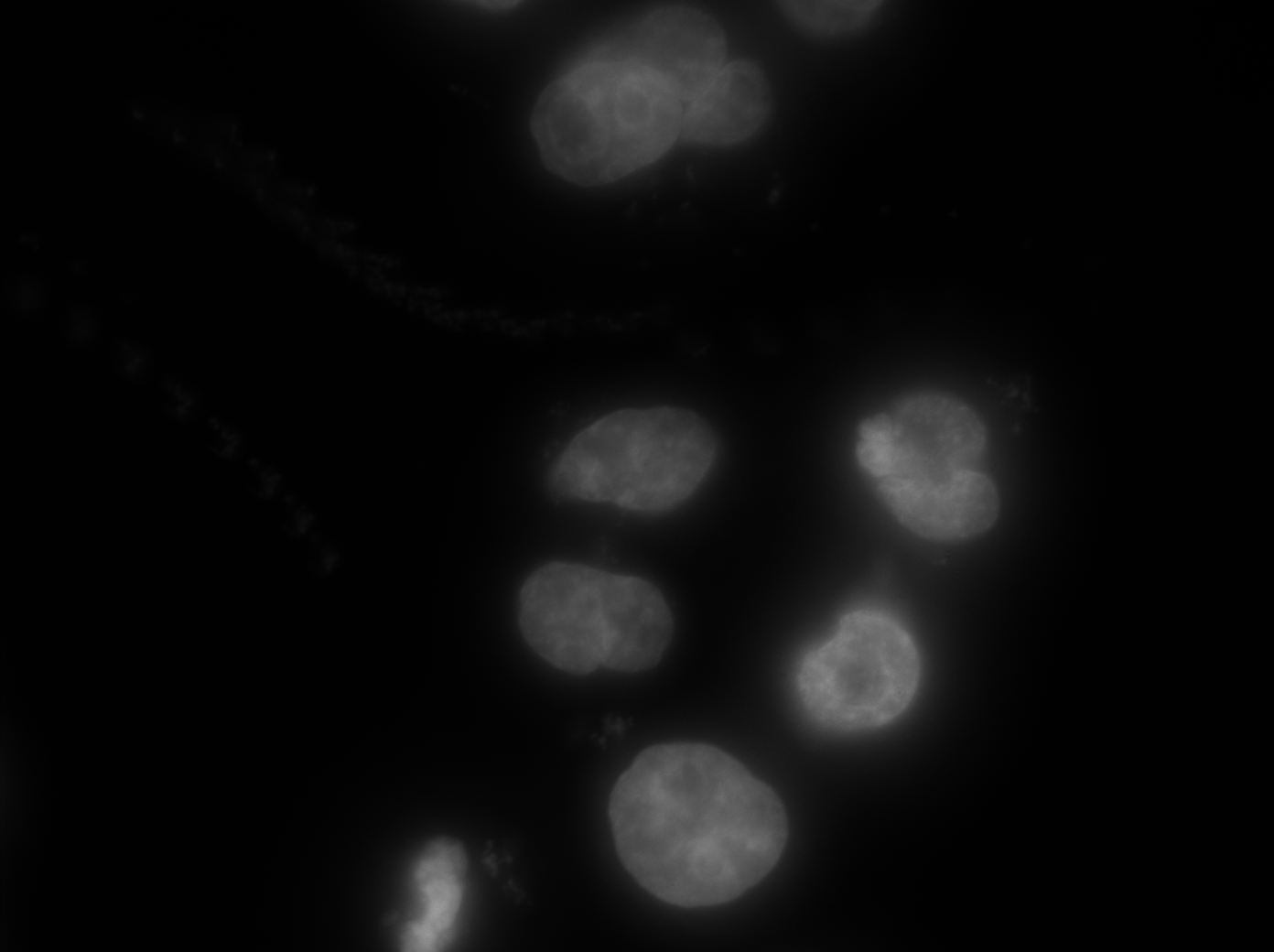

Supplement: Supplementary file 18 — Source data Fig. 5 part 4 [file 44319_2026_742_MOESM18_ESM.zip › Figure 5 Part 4/Fig 5ab WT and KO hela TTLL1-e326g atubulin/Control/WT Hela TTLL1-mut R2 LT1.Project Maximum Z_XY1731542067_Z0_T0_C0.tif]

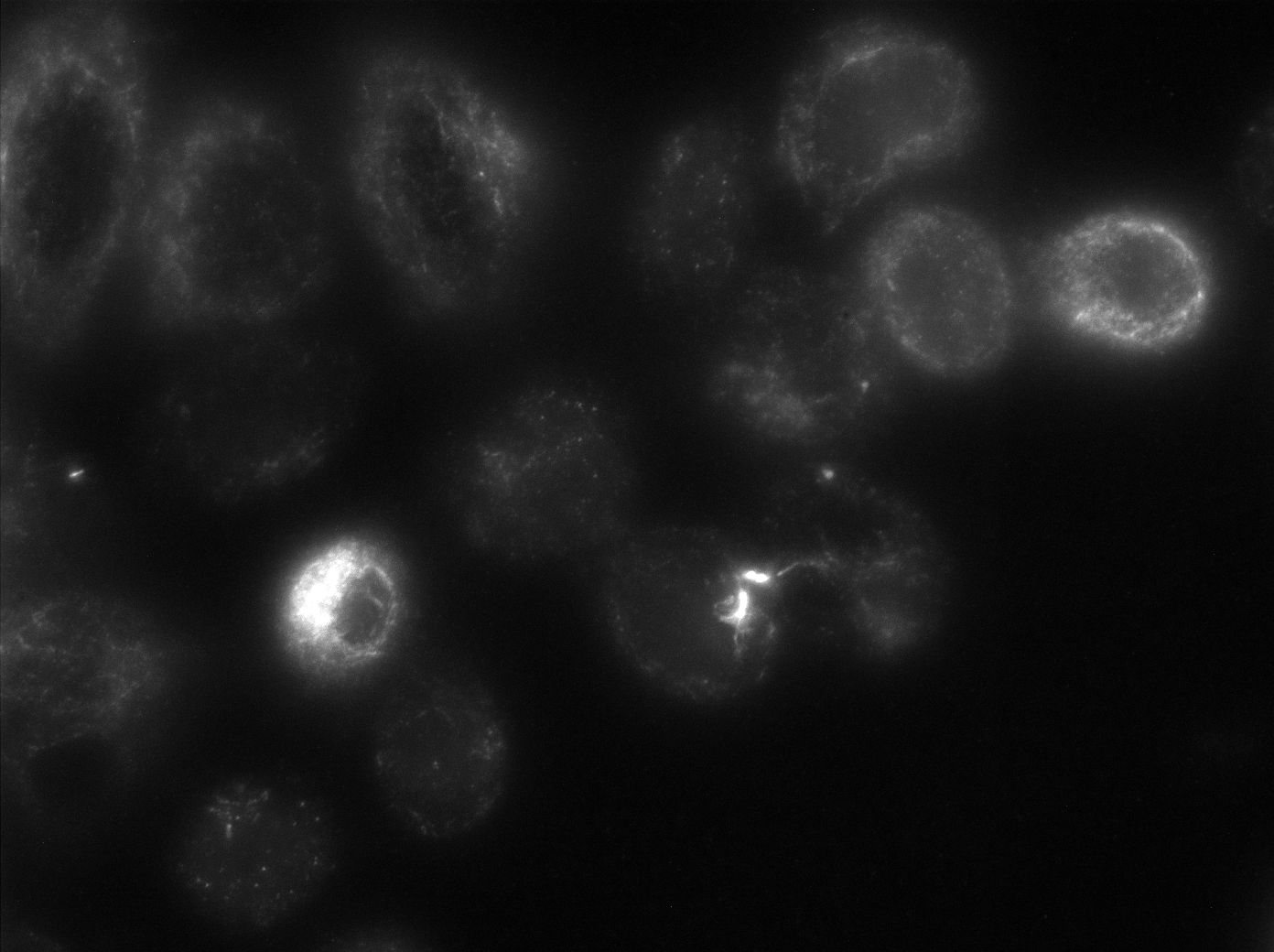

Supplement: Supplementary file 18 — Source data Fig. 5 part 4 [file 44319_2026_742_MOESM18_ESM.zip › Figure 5 Part 4/Fig 5ab WT and KO hela TTLL1-e326g atubulin/Control/WT Hela TTLL1-mut R3 11-13-24 LT2.Project Maximum Z_XY1731545983_Z0_T0_C2.tif]

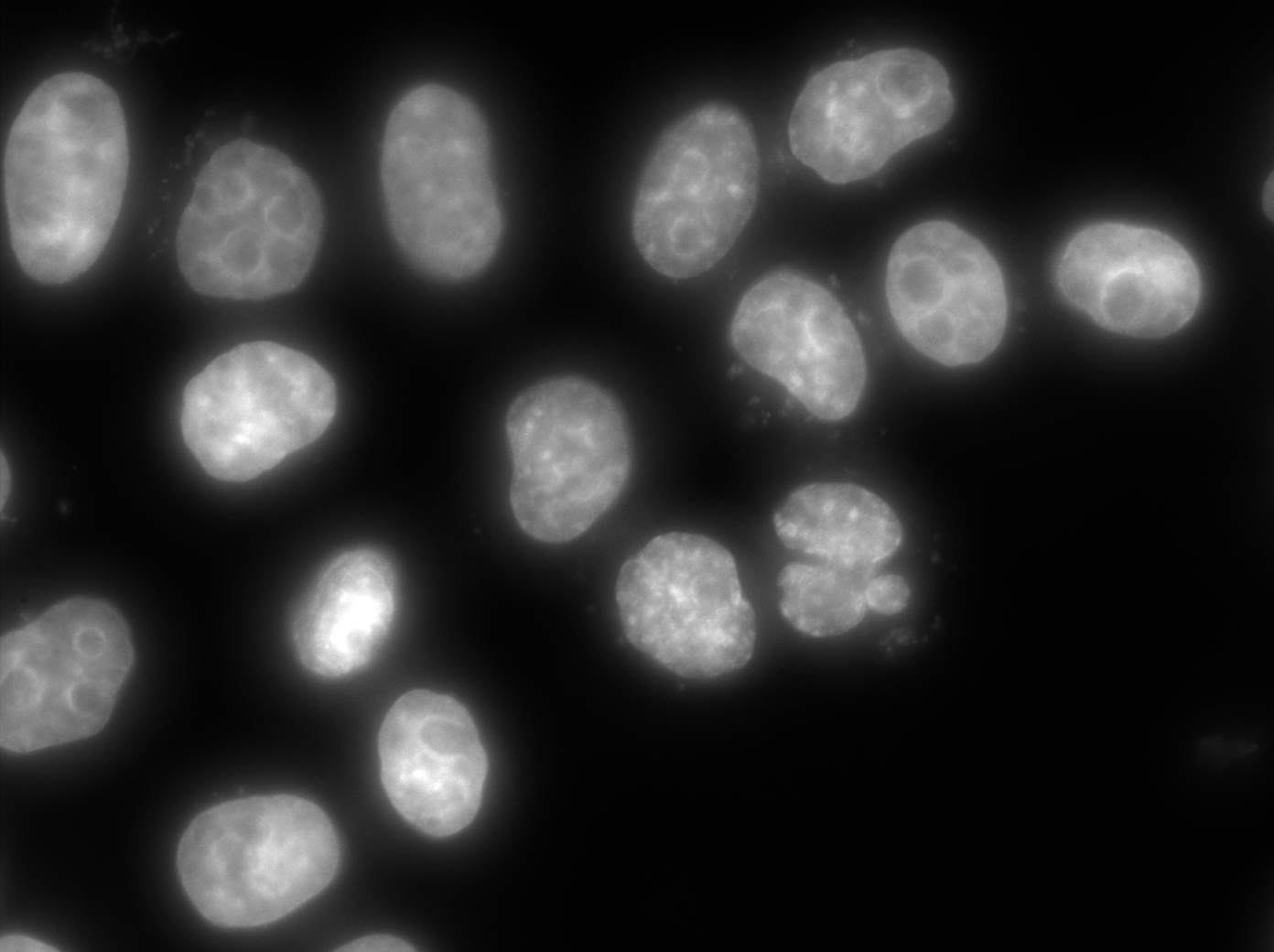

Supplement: Supplementary file 18 — Source data Fig. 5 part 4 [file 44319_2026_742_MOESM18_ESM.zip › Figure 5 Part 4/Fig 5ab WT and KO hela TTLL1-e326g atubulin/Control/WT Hela TTLL1-mut R3 11-13-24 LT2.Project Maximum Z_XY1731545983_Z0_T0_C0.tif]

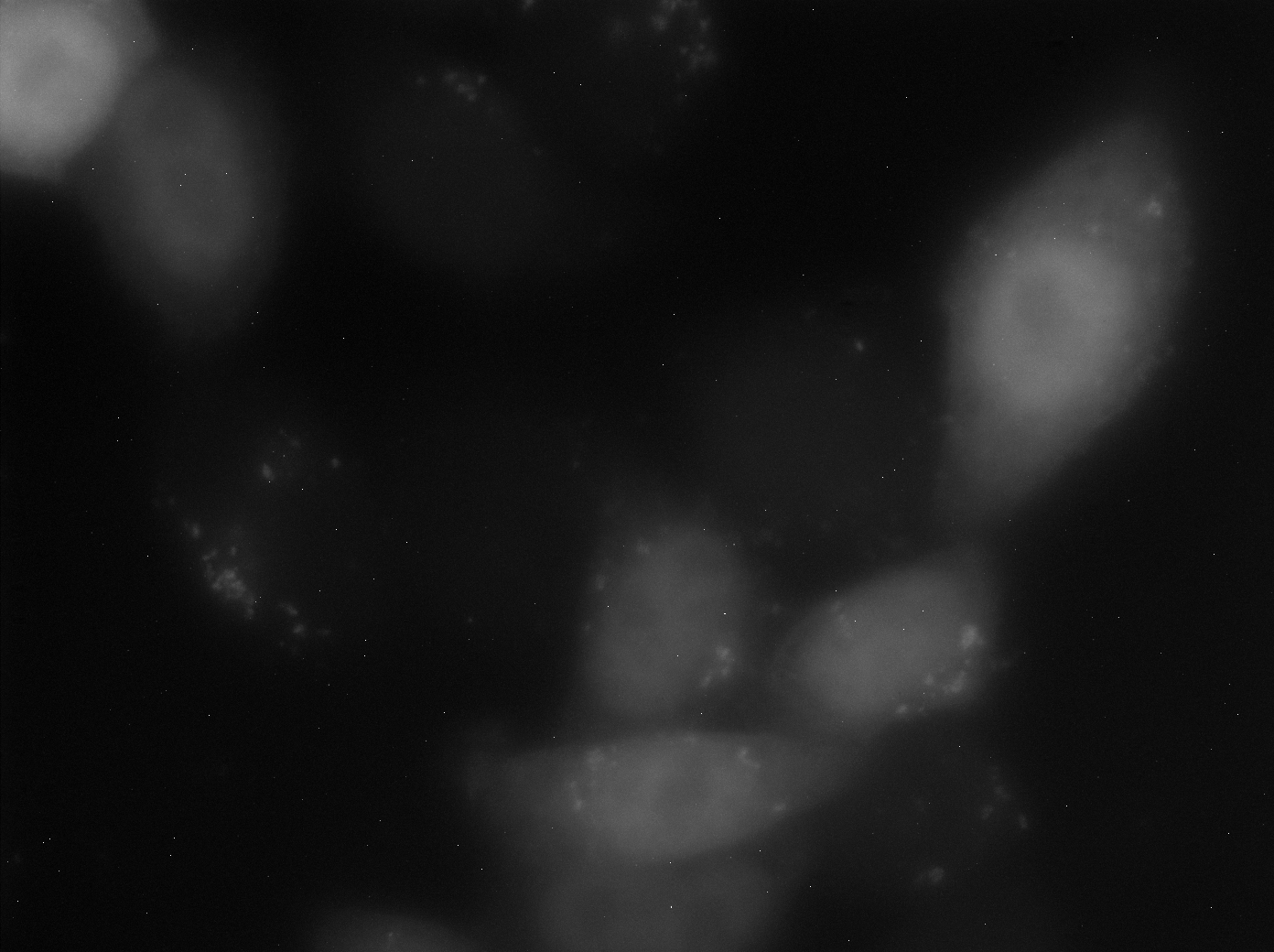

Supplement: Supplementary file 18 — Source data Fig. 5 part 4 [file 44319_2026_742_MOESM18_ESM.zip › Figure 5 Part 4/Fig 5ab WT and KO hela TTLL1-e326g atubulin/Control/WT Hela TTLL1-mut R3 11-13-24 LT3.Project Maximum Z_XY1731546288_Z0_T0_C1.tif]

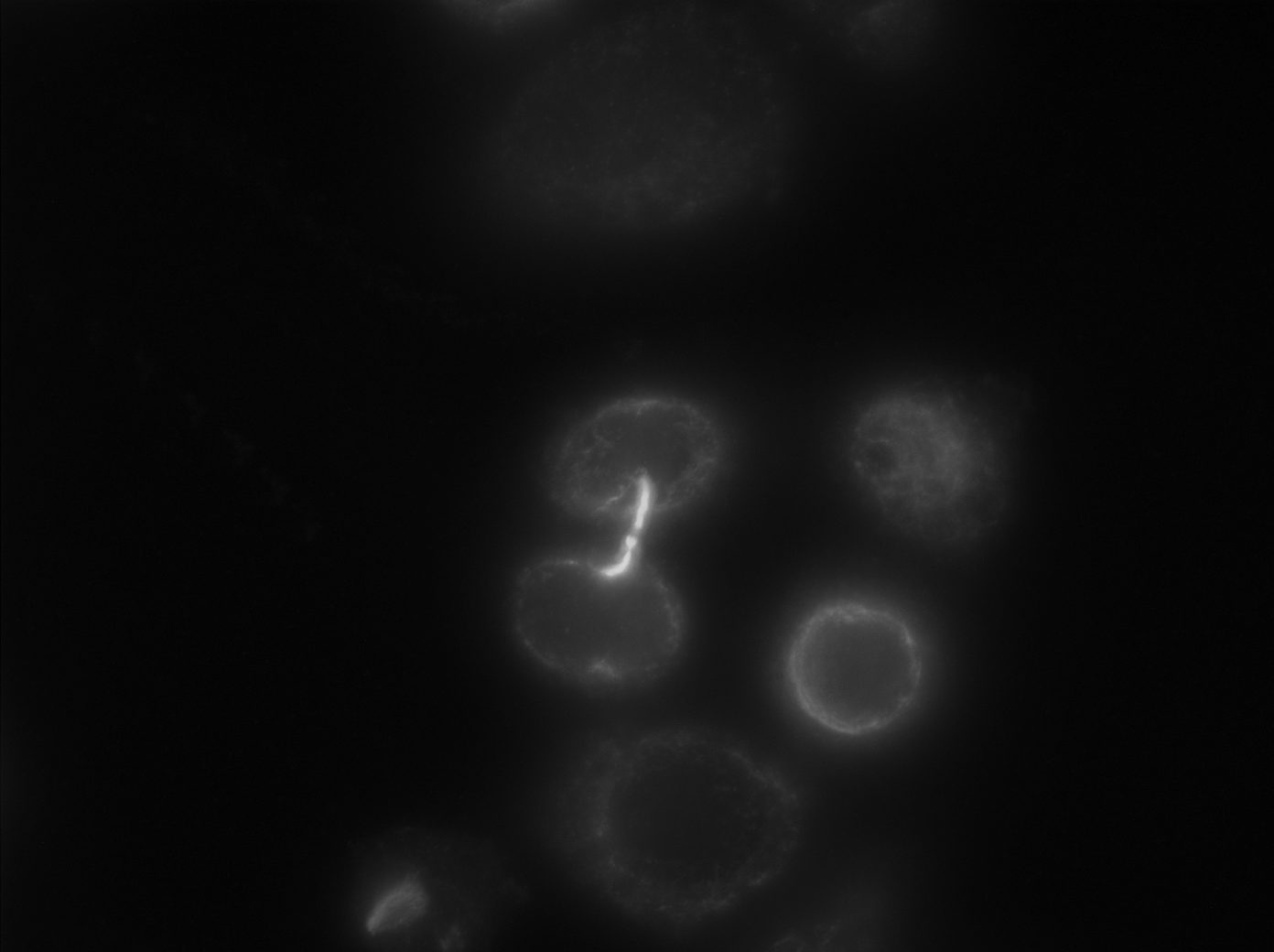

Supplement: Supplementary file 18 — Source data Fig. 5 part 4 [file 44319_2026_742_MOESM18_ESM.zip › Figure 5 Part 4/Fig 5ab WT and KO hela TTLL1-e326g atubulin/Control/WT Hela TTLL1-mut R2 LT1.Project Maximum Z_XY1731542067_Z0_T0_C2.tif]

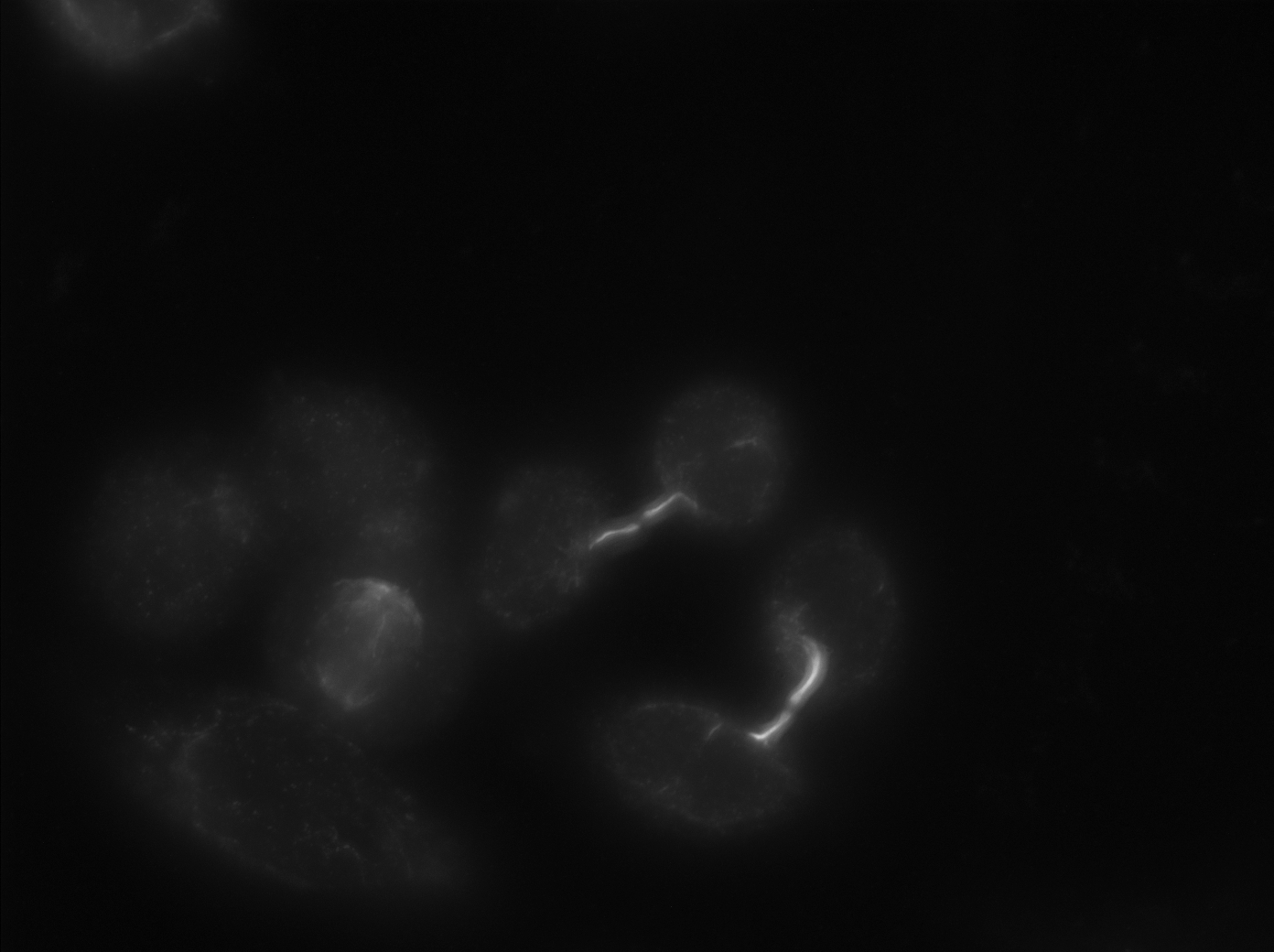

Supplement: Supplementary file 18 — Source data Fig. 5 part 4 [file 44319_2026_742_MOESM18_ESM.zip › Figure 5 Part 4/Fig 5ab WT and KO hela TTLL1-e326g atubulin/Control/WT Hela TTLL1-mut R2 LT3LT4.Project Maximum Z_XY1731542668_Z0_T0_C2.tif]

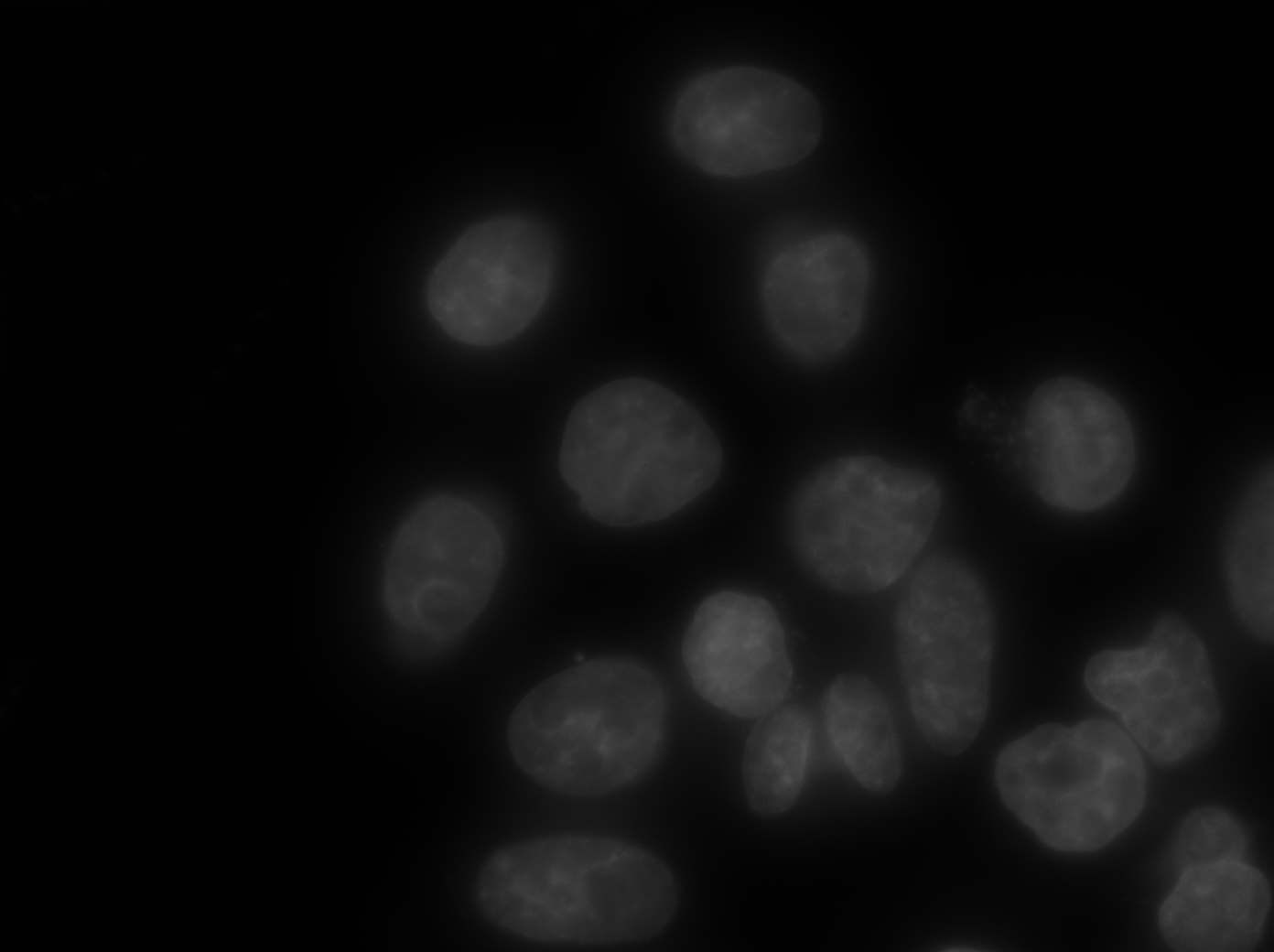

Supplement: Supplementary file 18 — Source data Fig. 5 part 4 [file 44319_2026_742_MOESM18_ESM.zip › Figure 5 Part 4/Fig 5ab WT and KO hela TTLL1-e326g atubulin/Control/WT Hela TTLL1-mut R2 LT10.Project Maximum Z_XY1731544792_Z0_T0_C0.tif]

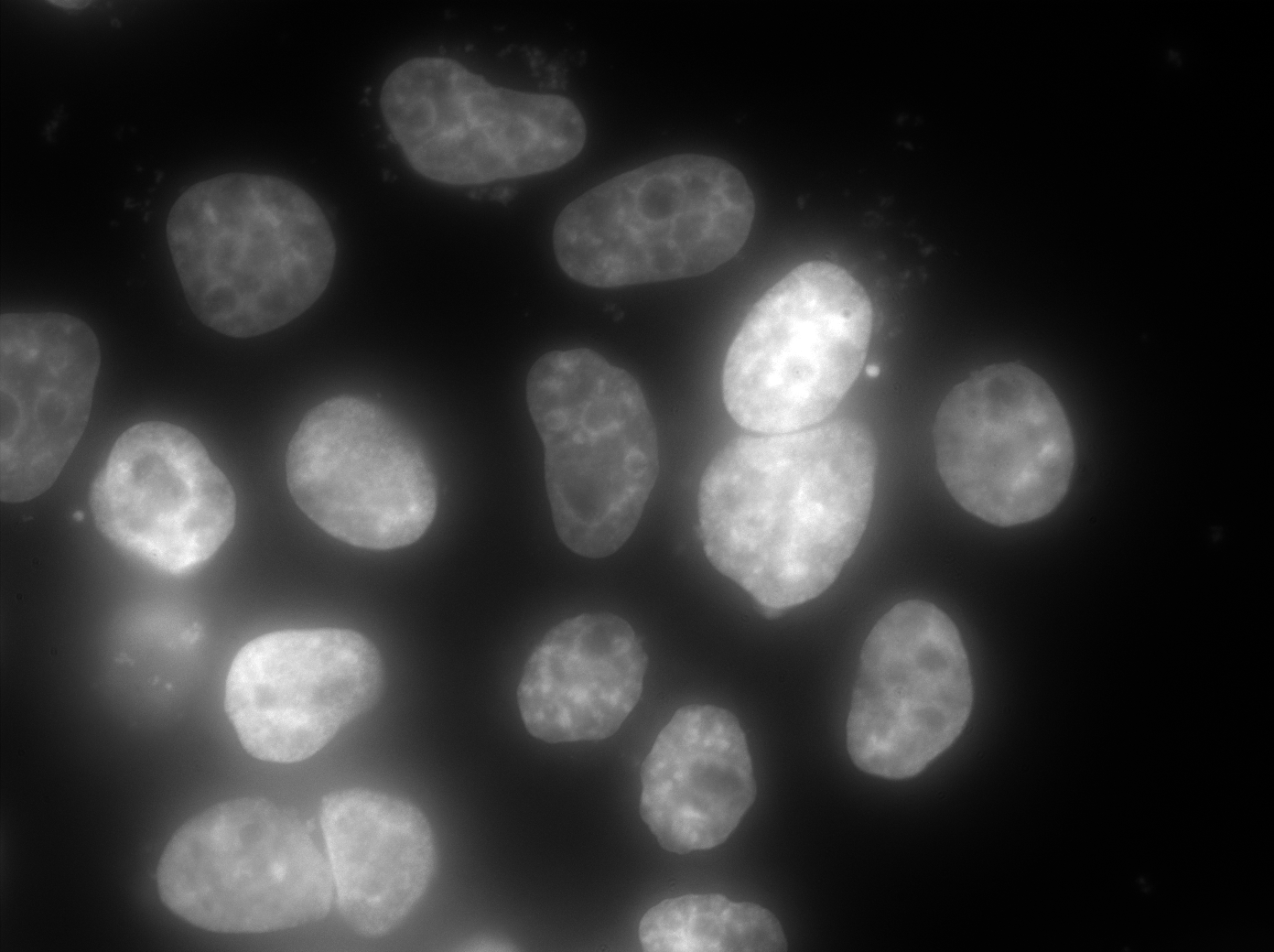

Supplement: Supplementary file 18 — Source data Fig. 5 part 4 [file 44319_2026_742_MOESM18_ESM.zip › Figure 5 Part 4/Fig 5ab WT and KO hela TTLL1-e326g atubulin/Control/WT Hela TTLL1-mut R2 LT7.Project Maximum Z_XY1731543950_Z0_T0_C0.tif]

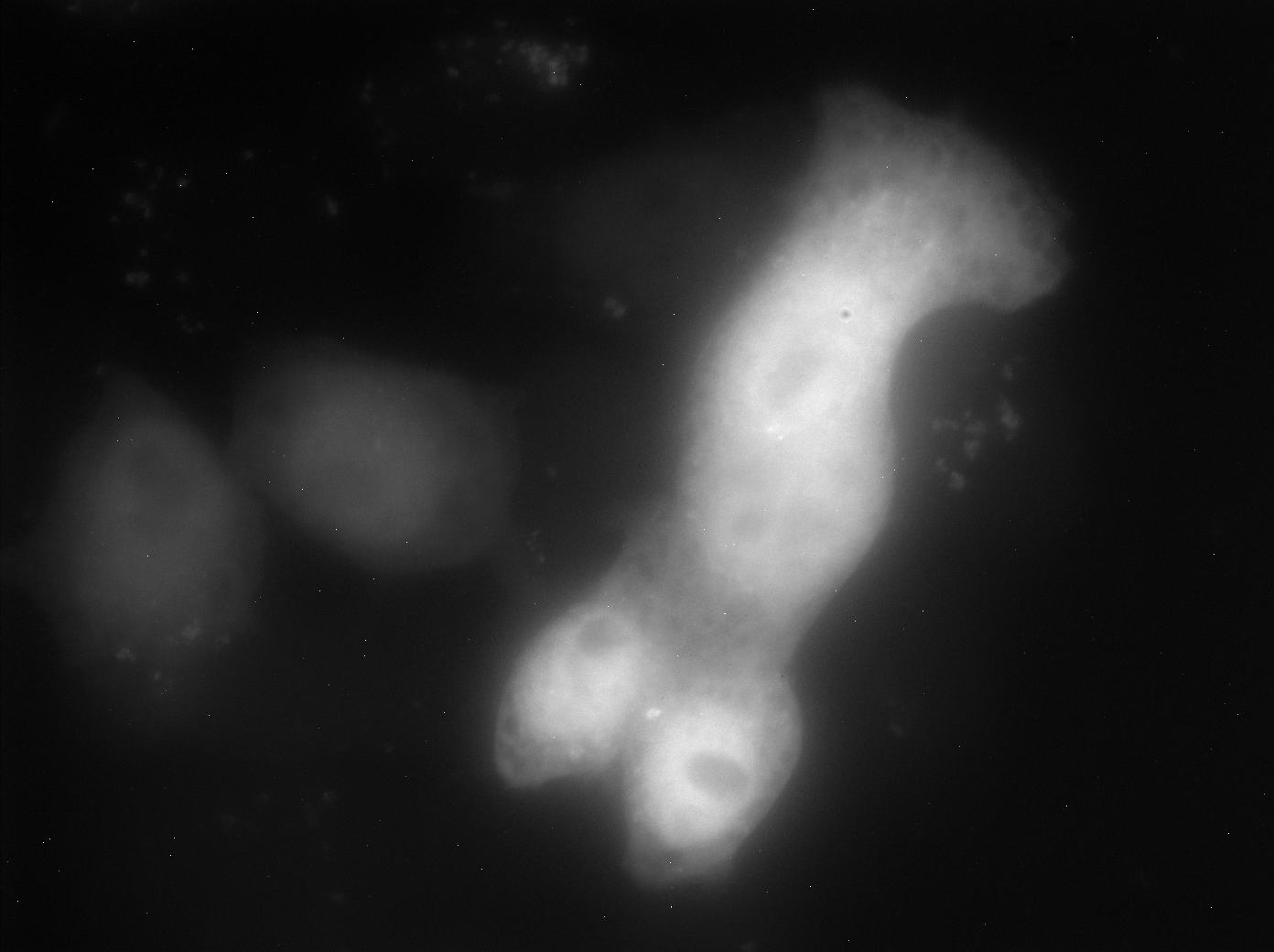

Supplement: Supplementary file 18 — Source data Fig. 5 part 4 [file 44319_2026_742_MOESM18_ESM.zip › Figure 5 Part 4/Fig 5ab WT and KO hela TTLL1-e326g atubulin/Control/WT Hela TTLL1-mut R2 LT7.Project Maximum Z_XY1731543950_Z0_T0_C1.tif]

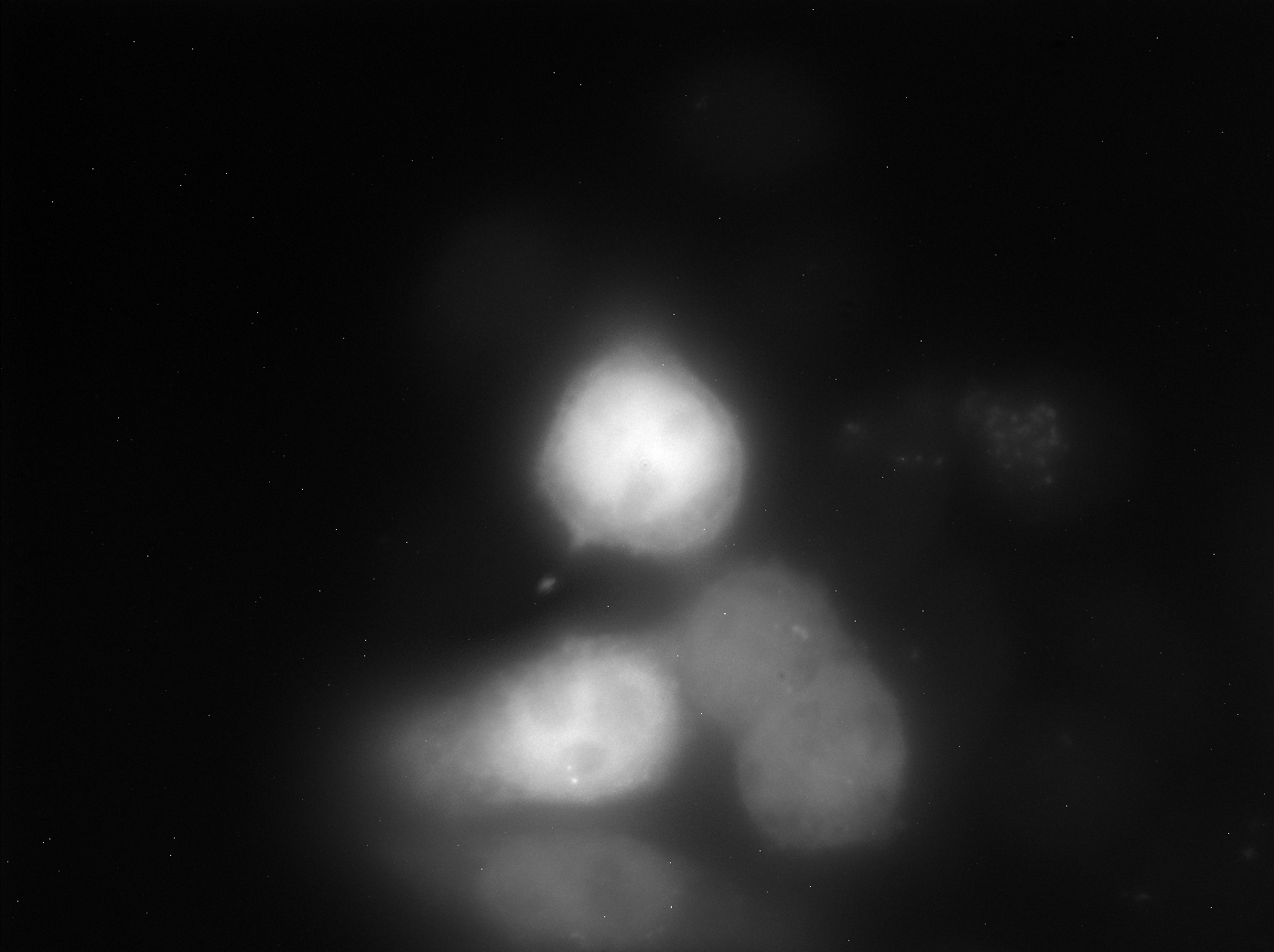

Supplement: Supplementary file 18 — Source data Fig. 5 part 4 [file 44319_2026_742_MOESM18_ESM.zip › Figure 5 Part 4/Fig 5ab WT and KO hela TTLL1-e326g atubulin/Control/WT Hela TTLL1-mut R2 LT10.Project Maximum Z_XY1731544792_Z0_T0_C1.tif]

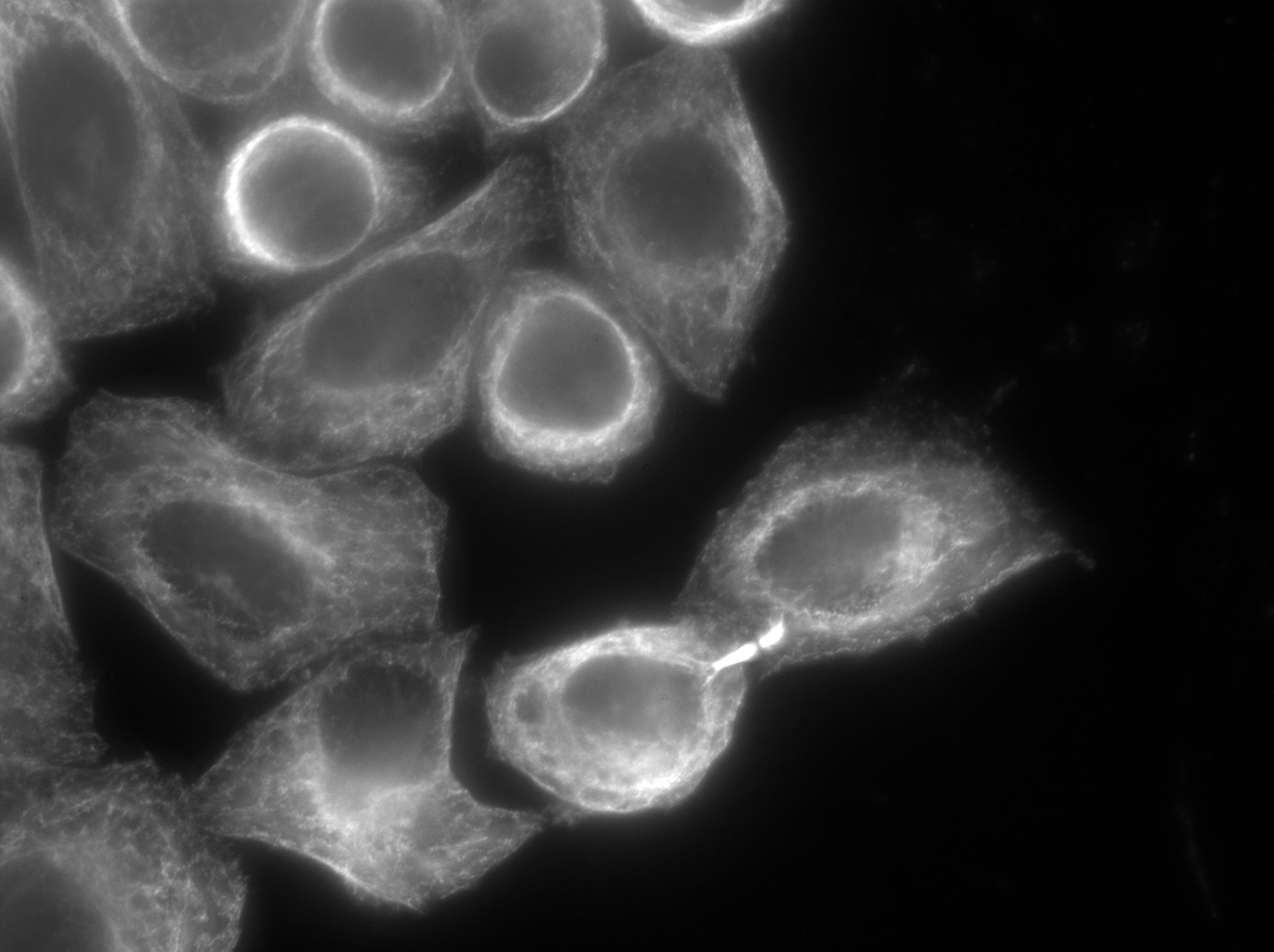

Supplement: Supplementary file 18 — Source data Fig. 5 part 4 [file 44319_2026_742_MOESM18_ESM.zip › Figure 5 Part 4/Fig 5ab WT and KO hela TTLL1-e326g atubulin/Control/TTLL1-mut atub R2 LT8.Project Maximum Z_XY1724952399_Z0_T0_C2.tif]

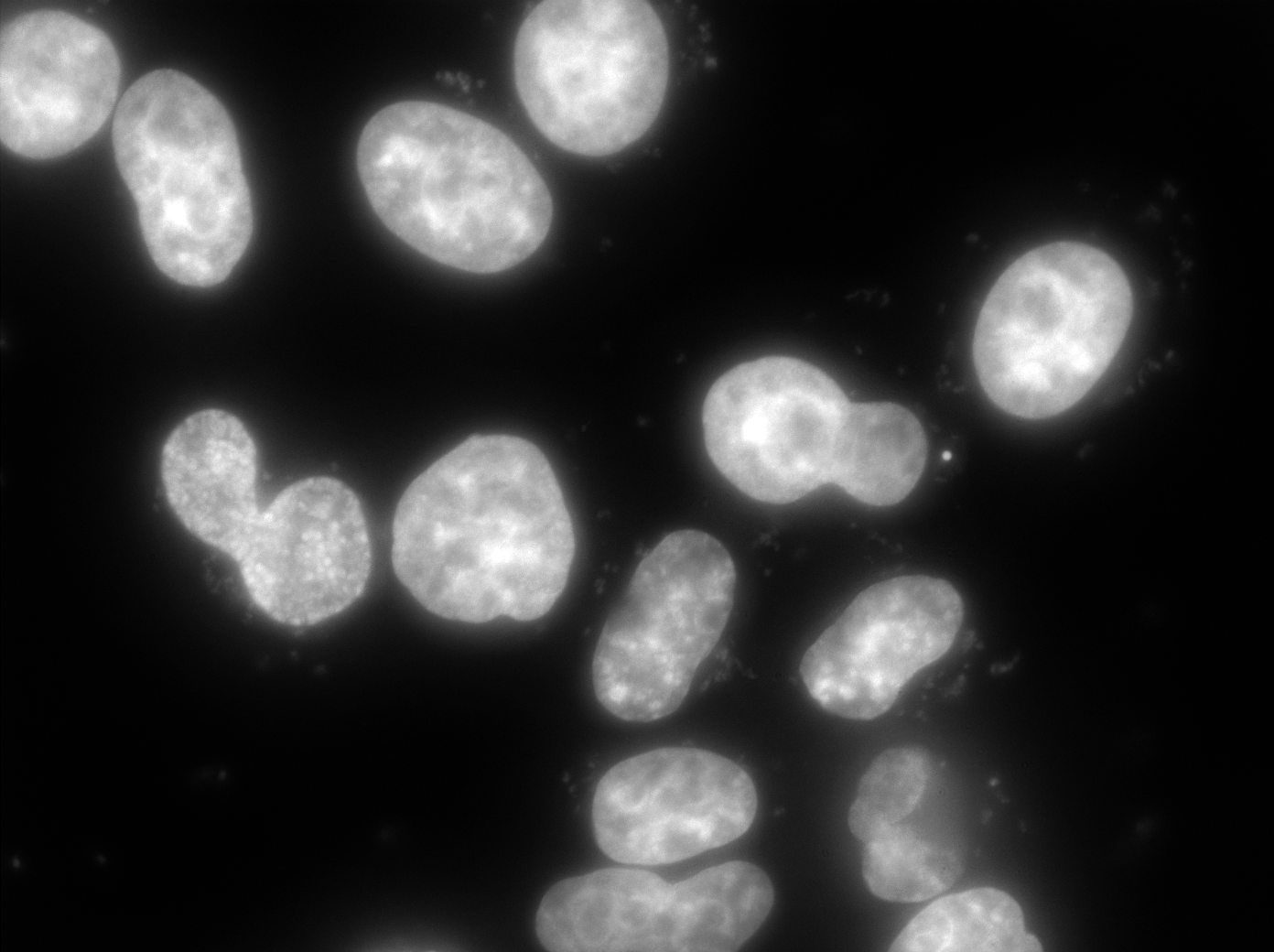

Supplement: Supplementary file 18 — Source data Fig. 5 part 4 [file 44319_2026_742_MOESM18_ESM.zip › Figure 5 Part 4/Fig 5ab WT and KO hela TTLL1-e326g atubulin/Control/WT Hela TTLL1-mut R3 11-13-24 LT3.Project Maximum Z_XY1731546288_Z0_T0_C0.tif]

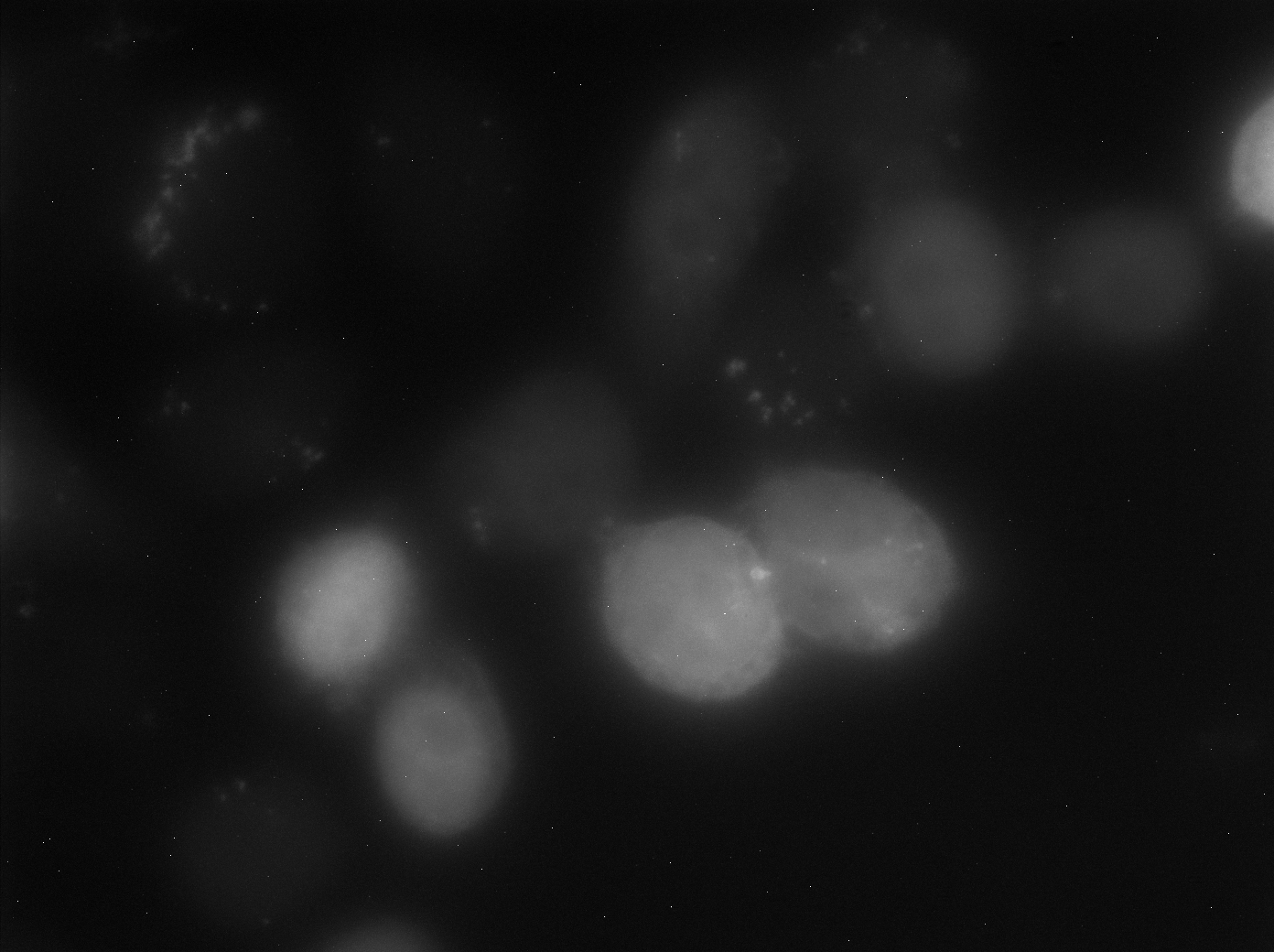

Supplement: Supplementary file 18 — Source data Fig. 5 part 4 [file 44319_2026_742_MOESM18_ESM.zip › Figure 5 Part 4/Fig 5ab WT and KO hela TTLL1-e326g atubulin/Control/WT Hela TTLL1-mut R3 11-13-24 LT2.Project Maximum Z_XY1731545983_Z0_T0_C1.tif]

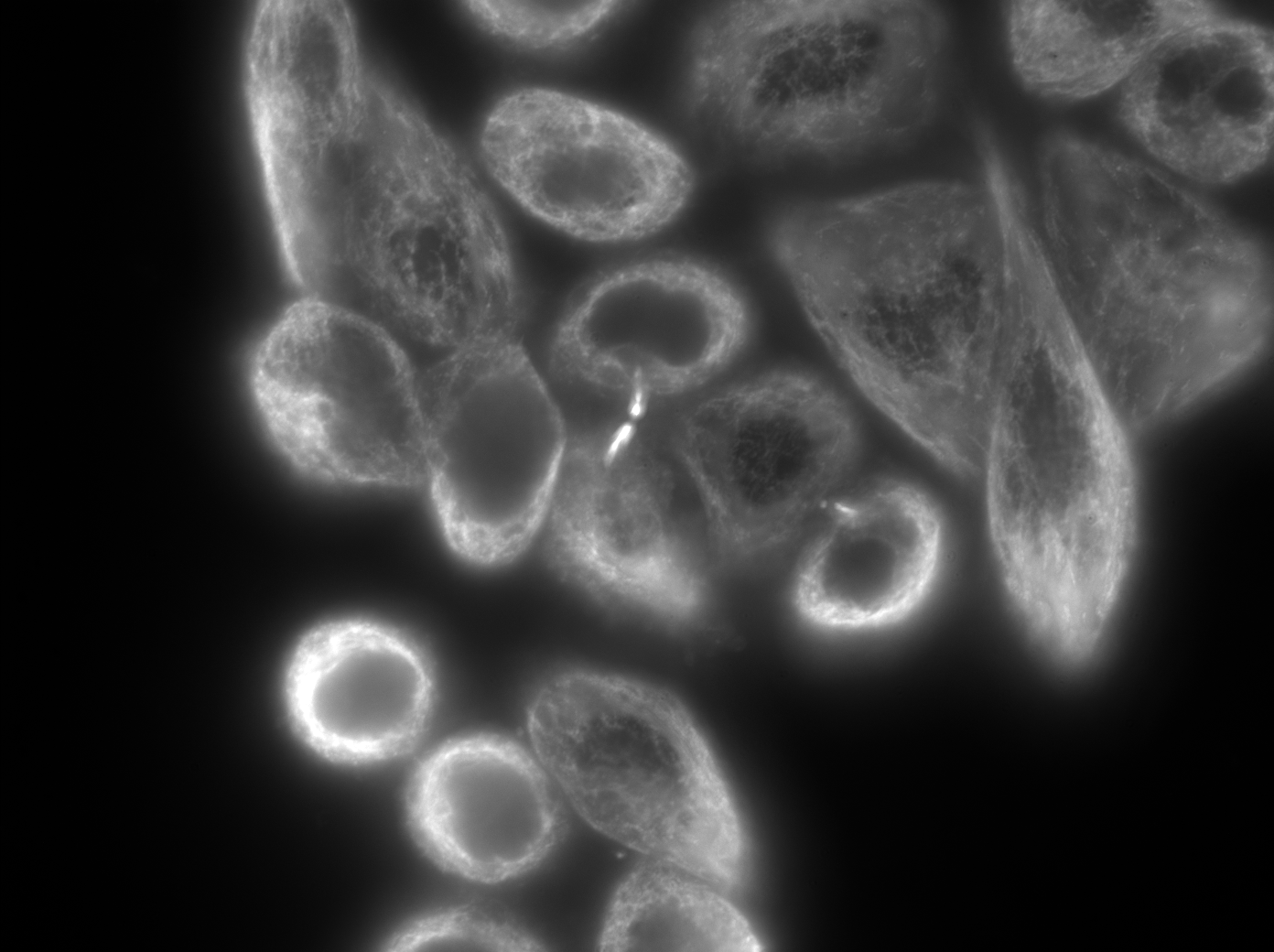

Supplement: Supplementary file 18 — Source data Fig. 5 part 4 [file 44319_2026_742_MOESM18_ESM.zip › Figure 5 Part 4/Fig 5ab WT and KO hela TTLL1-e326g atubulin/Control/TTLL1-mut atub R1 LT3.Project Maximum Z_XY1724439533_Z0_T0_C2.tif]

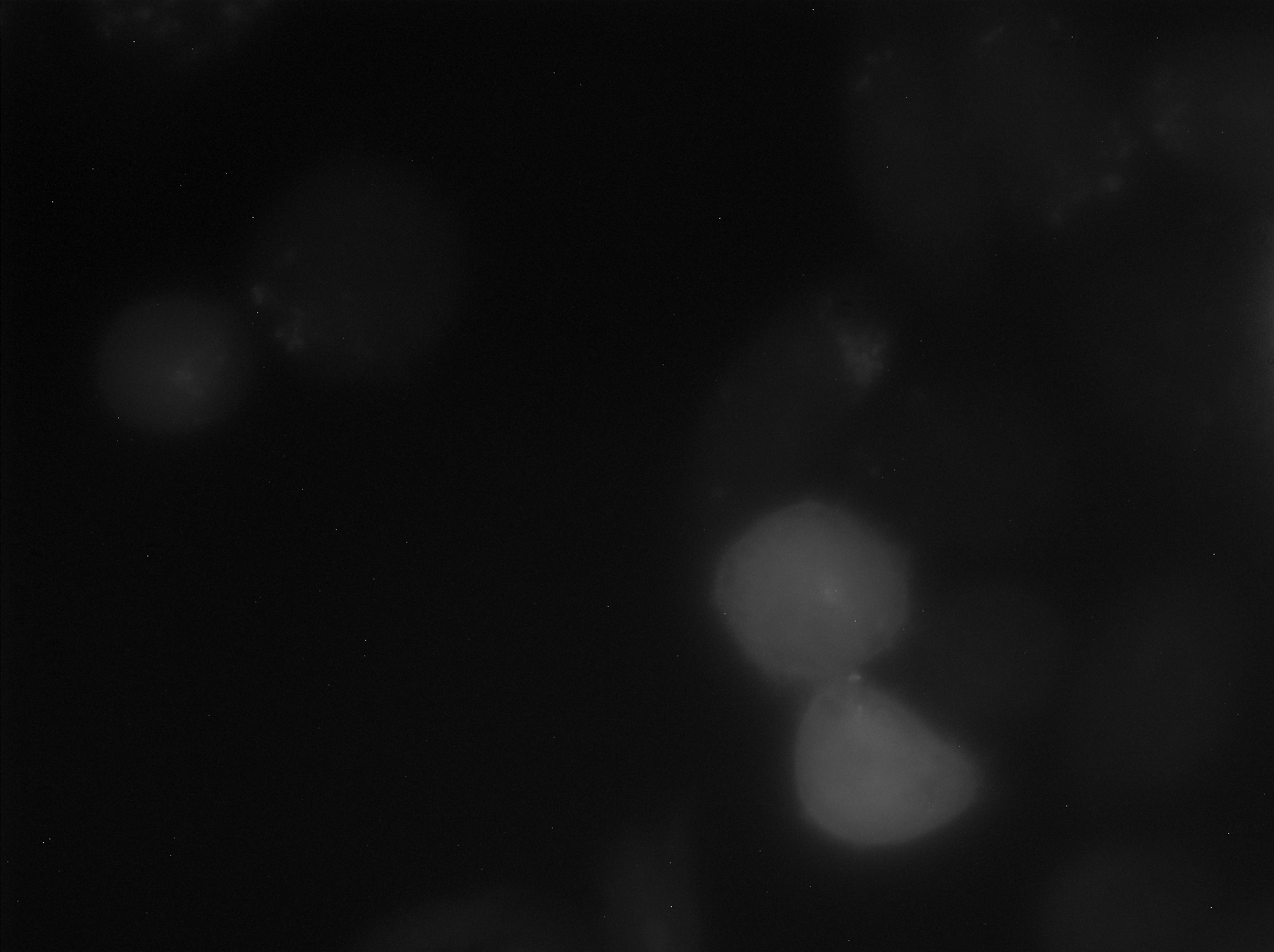

Supplement: Supplementary file 18 — Source data Fig. 5 part 4 [file 44319_2026_742_MOESM18_ESM.zip › Figure 5 Part 4/Fig 5ab WT and KO hela TTLL1-e326g atubulin/Control/TTLL1-mut atub R2 LT9.Project Maximum Z_XY1724952600_Z0_T0_C1.tif]

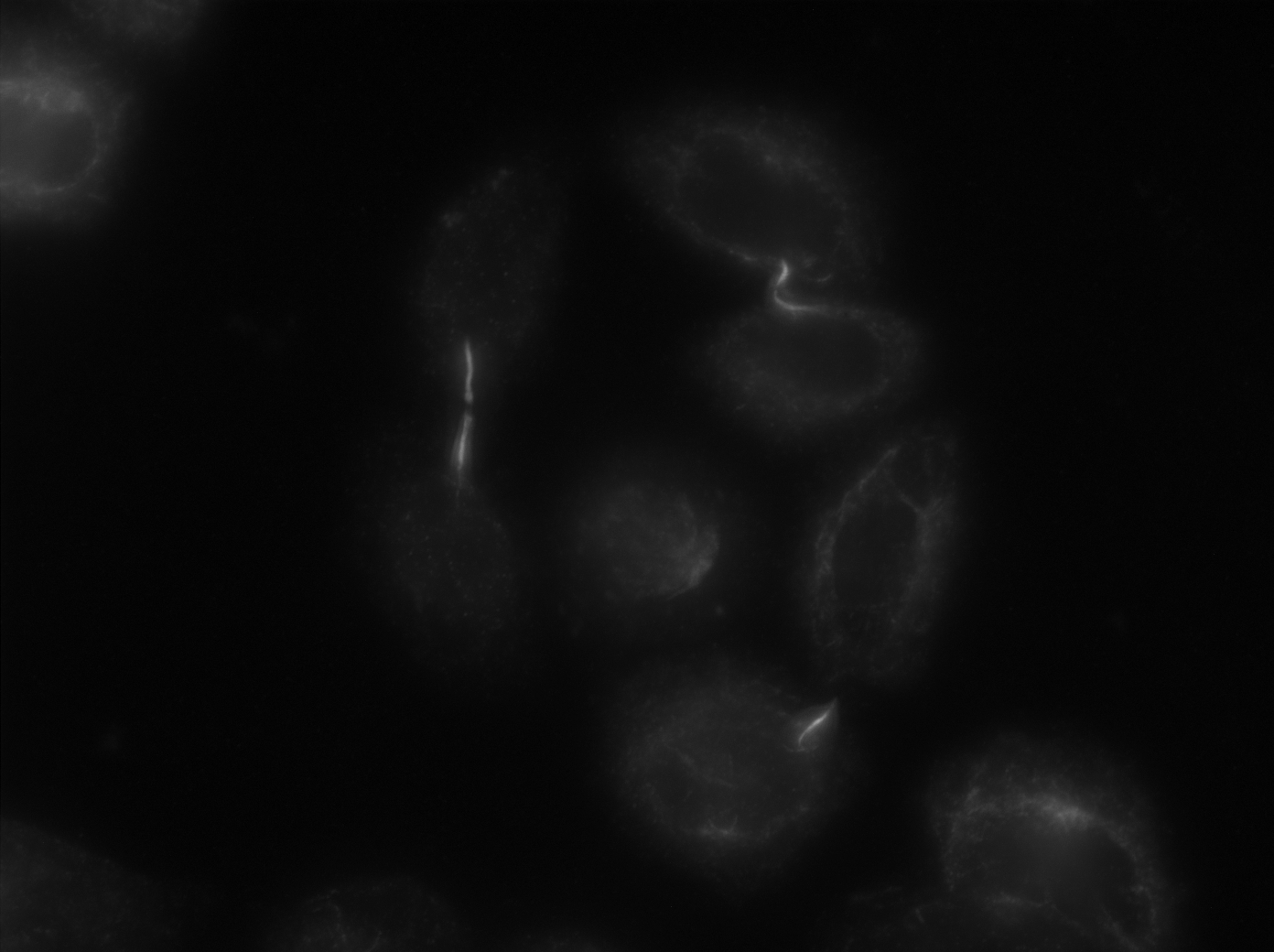

Supplement: Supplementary file 18 — Source data Fig. 5 part 4 [file 44319_2026_742_MOESM18_ESM.zip › Figure 5 Part 4/Fig 5ab WT and KO hela TTLL1-e326g atubulin/Control/WT Hela TTLL1-mut R2 LT6.Project Maximum Z_XY1731543506_Z0_T0_C2.tif]

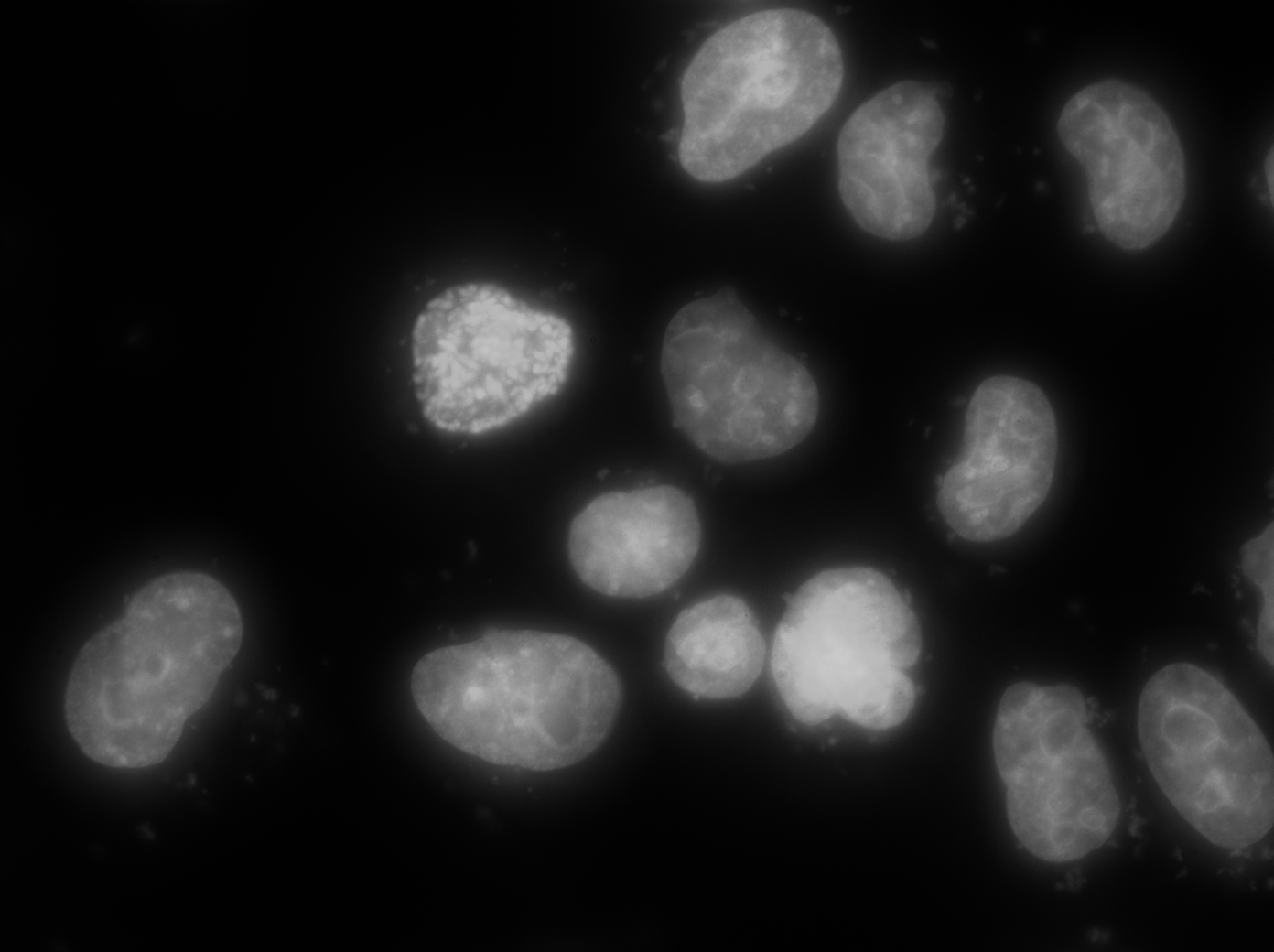

Supplement: Supplementary file 18 — Source data Fig. 5 part 4 [file 44319_2026_742_MOESM18_ESM.zip › Figure 5 Part 4/Fig 5ab WT and KO hela TTLL1-e326g atubulin/Control/TTLL1-mut atub R2 LT6.Project Maximum Z_XY1724951880_Z0_T0_C0.tif]

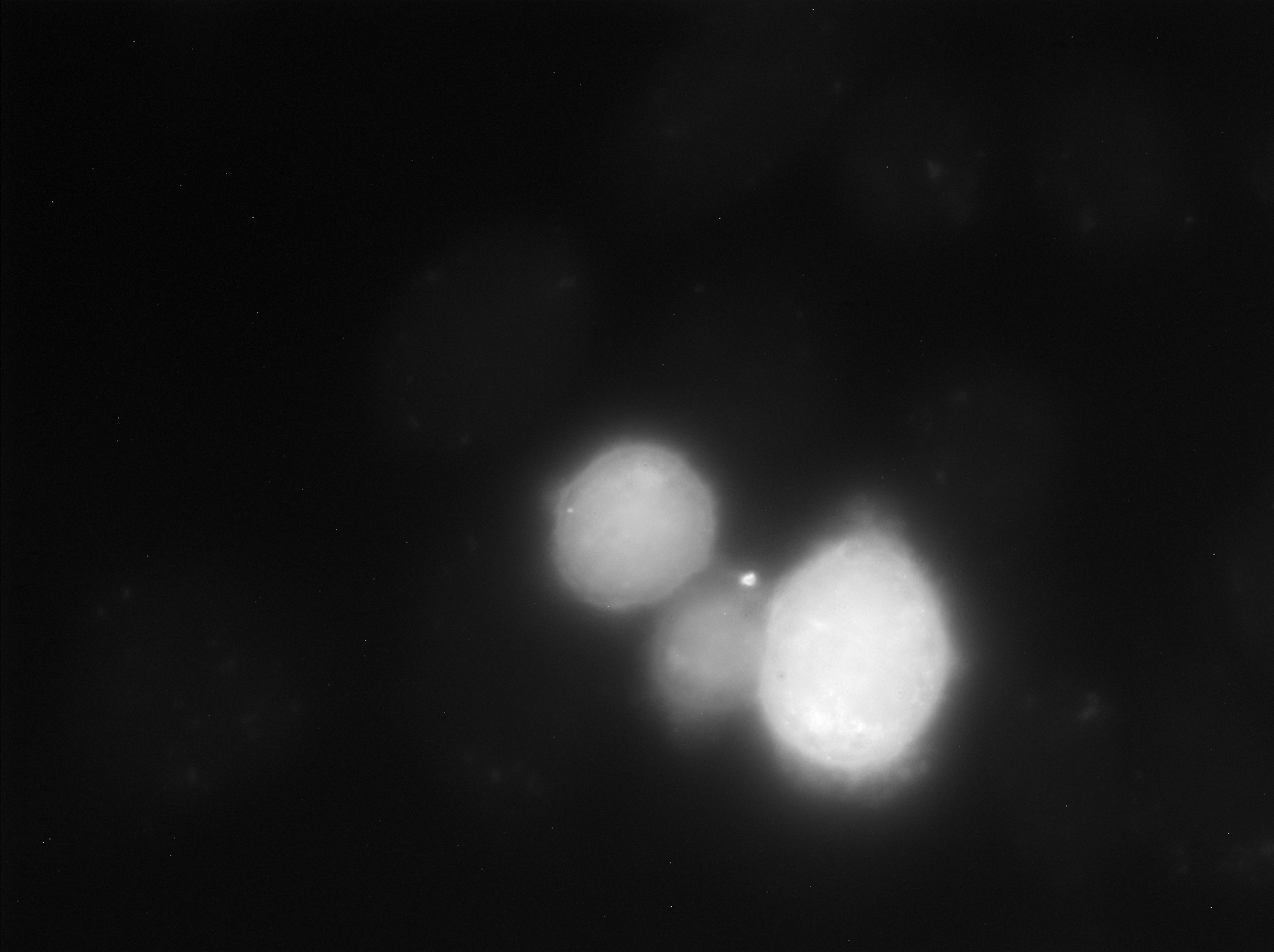

Supplement: Supplementary file 18 — Source data Fig. 5 part 4 [file 44319_2026_742_MOESM18_ESM.zip › Figure 5 Part 4/Fig 5ab WT and KO hela TTLL1-e326g atubulin/Control/TTLL1-mut atub R2 LT6.Project Maximum Z_XY1724951880_Z0_T0_C1.tif]

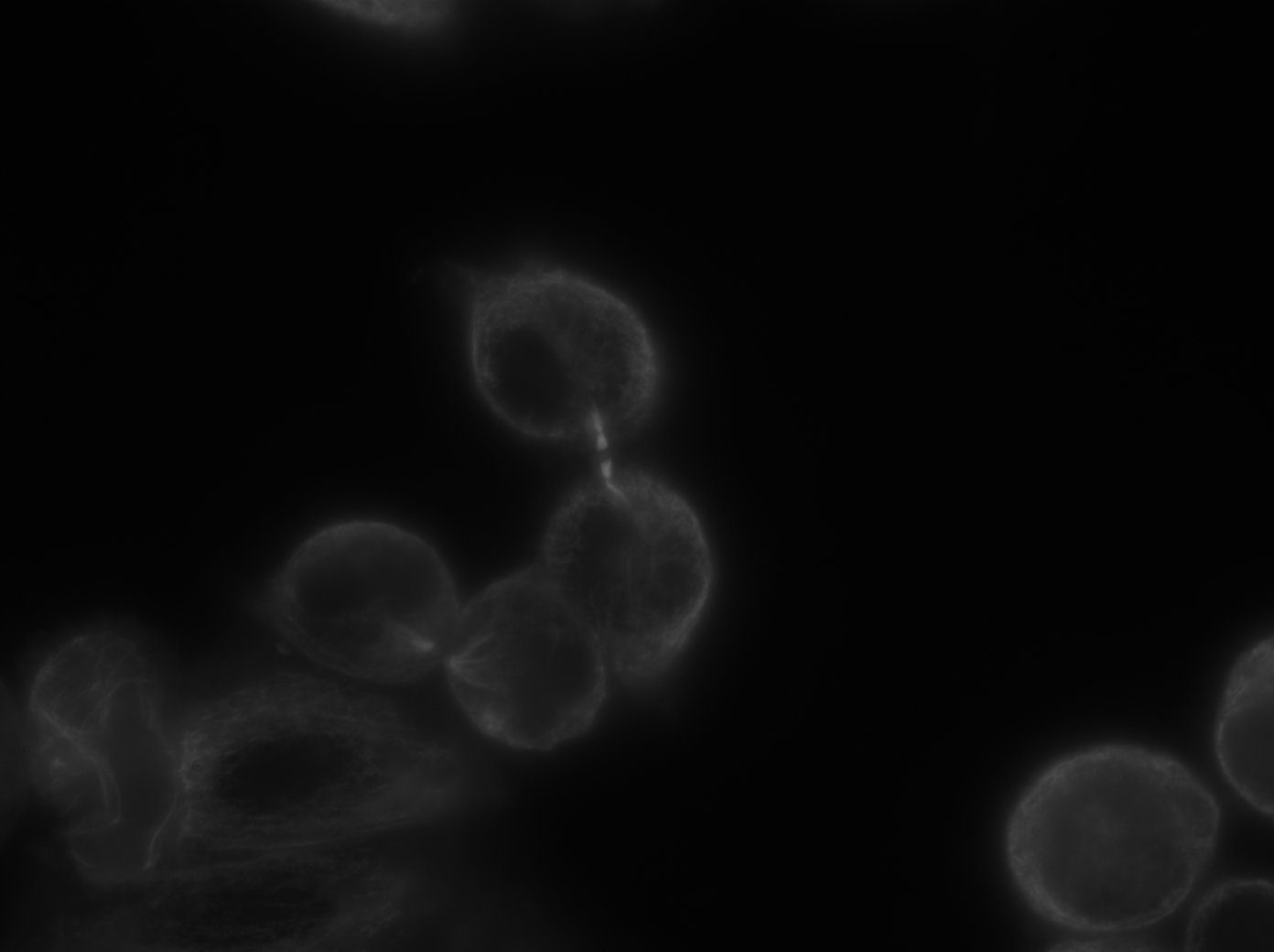

Supplement: Supplementary file 18 — Source data Fig. 5 part 4 [file 44319_2026_742_MOESM18_ESM.zip › Figure 5 Part 4/Fig 5ab WT and KO hela TTLL1-e326g atubulin/Control/TTLL1-mut atub R1 LT2 ET1.Project Maximum Z_XY1724438861_Z0_T0_C2.tif]

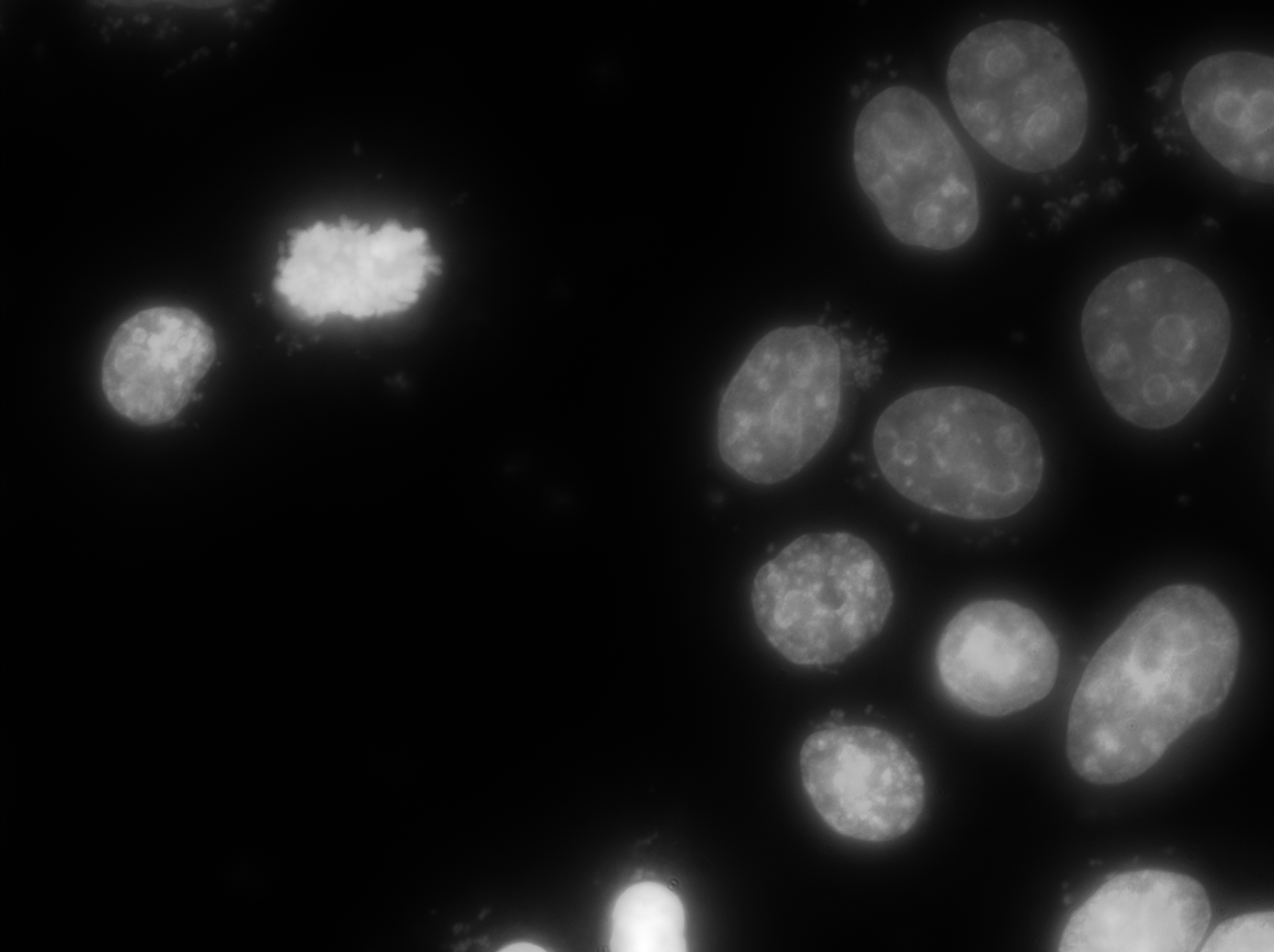

Supplement: Supplementary file 18 — Source data Fig. 5 part 4 [file 44319_2026_742_MOESM18_ESM.zip › Figure 5 Part 4/Fig 5ab WT and KO hela TTLL1-e326g atubulin/Control/TTLL1-mut atub R2 LT9.Project Maximum Z_XY1724952600_Z0_T0_C0.tif]

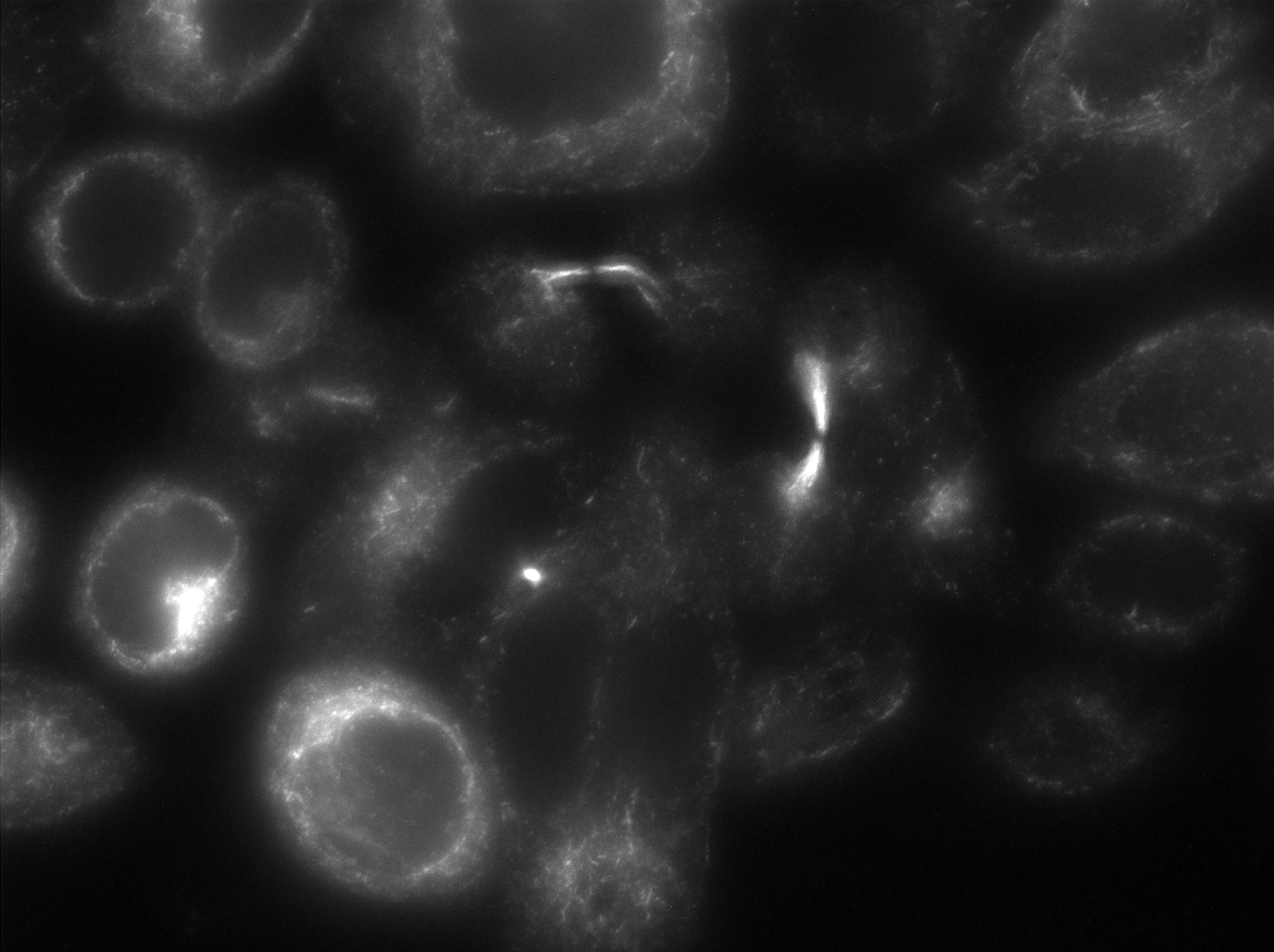

Supplement: Supplementary file 18 — Source data Fig. 5 part 4 [file 44319_2026_742_MOESM18_ESM.zip › Figure 5 Part 4/Fig 5ab WT and KO hela TTLL1-e326g atubulin/Control/WT Hela TTLL1-mut R3 11-13-24 LT4LT5.Project Maximum Z_XY1731546412_Z0_T0_C2.tif]

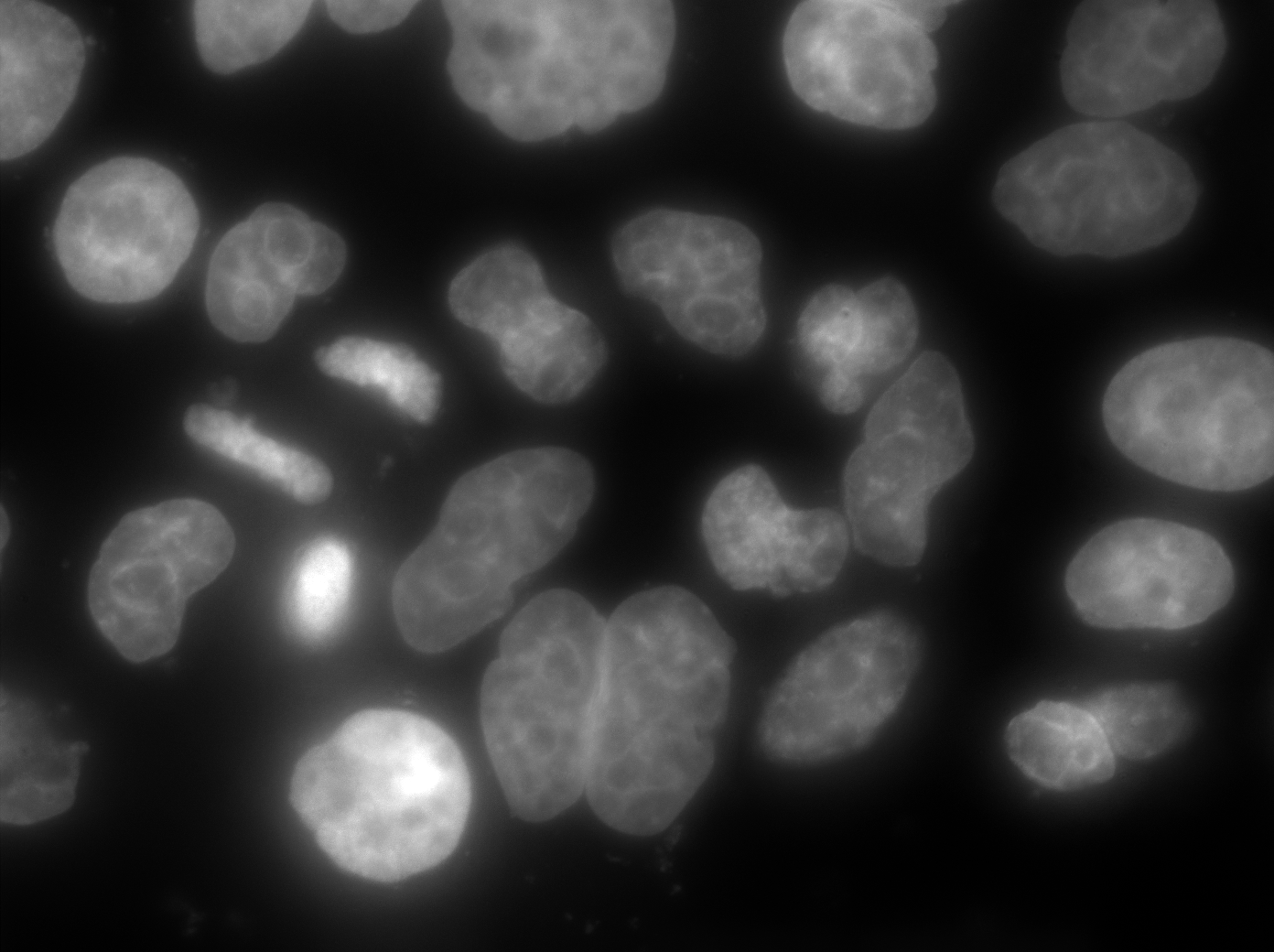

Supplement: Supplementary file 18 — Source data Fig. 5 part 4 [file 44319_2026_742_MOESM18_ESM.zip › Figure 5 Part 4/Fig 5ab WT and KO hela TTLL1-e326g atubulin/Control/WT Hela TTLL1-mut R3 11-13-24 LT4LT5.Project Maximum Z_XY1731546412_Z0_T0_C0.tif]

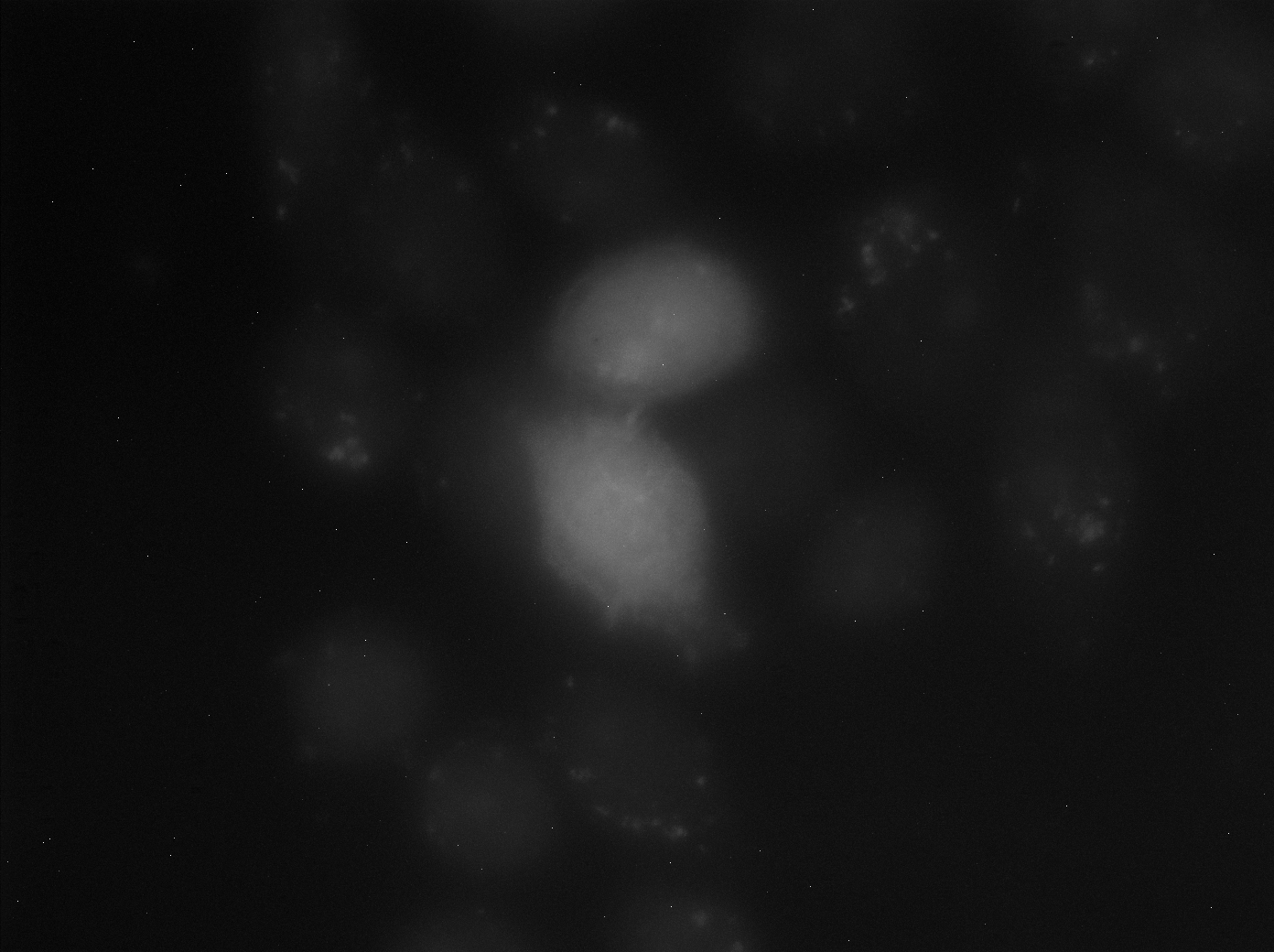

Supplement: Supplementary file 18 — Source data Fig. 5 part 4 [file 44319_2026_742_MOESM18_ESM.zip › Figure 5 Part 4/Fig 5ab WT and KO hela TTLL1-e326g atubulin/Control/TTLL1-mut atub R1 LT3.Project Maximum Z_XY1724439533_Z0_T0_C1.tif]

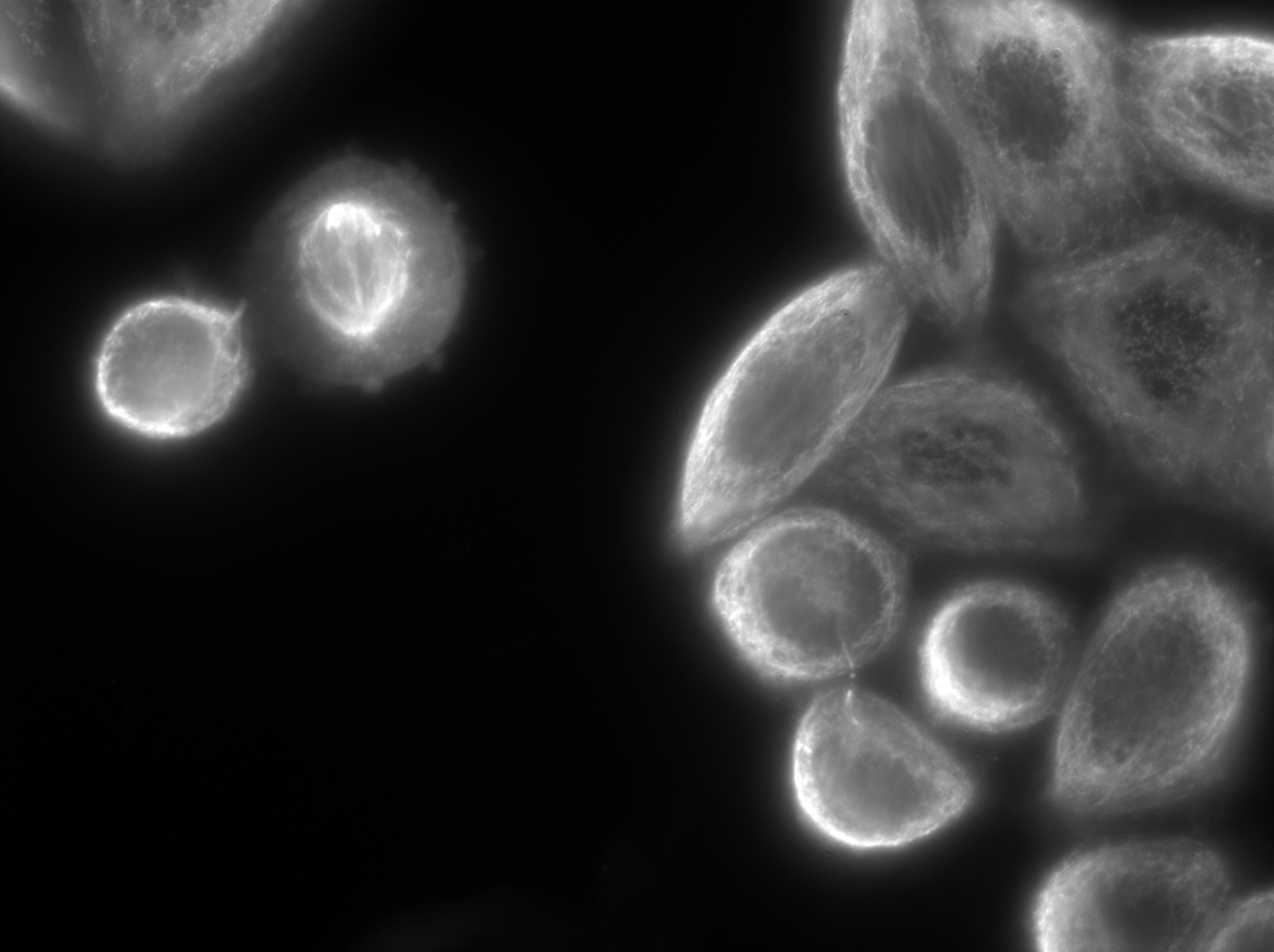

Supplement: Supplementary file 18 — Source data Fig. 5 part 4 [file 44319_2026_742_MOESM18_ESM.zip › Figure 5 Part 4/Fig 5ab WT and KO hela TTLL1-e326g atubulin/Control/TTLL1-mut atub R2 LT9.Project Maximum Z_XY1724952600_Z0_T0_C2.tif]

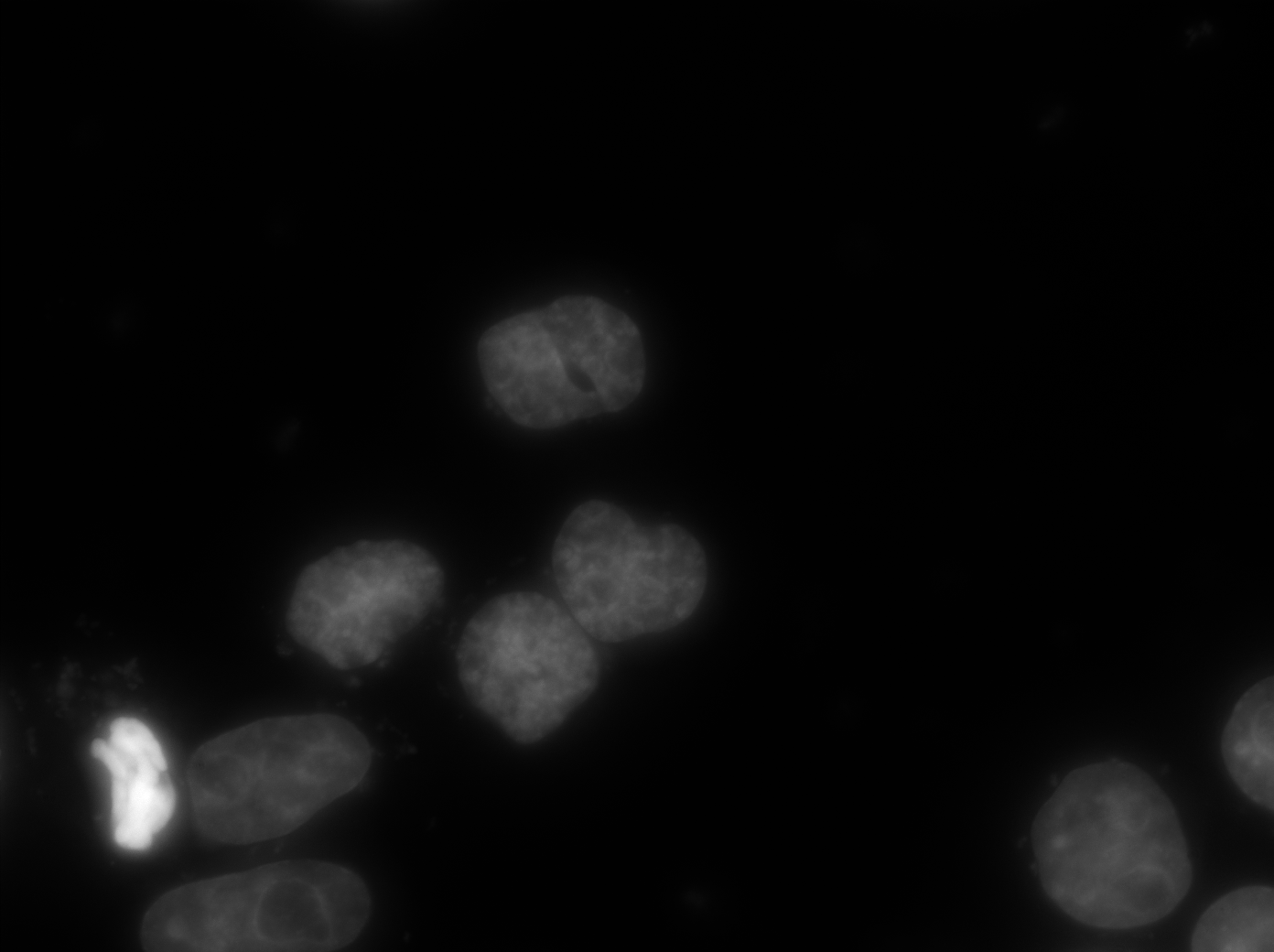

Supplement: Supplementary file 18 — Source data Fig. 5 part 4 [file 44319_2026_742_MOESM18_ESM.zip › Figure 5 Part 4/Fig 5ab WT and KO hela TTLL1-e326g atubulin/Control/TTLL1-mut atub R1 LT2 ET1.Project Maximum Z_XY1724438861_Z0_T0_C0.tif]

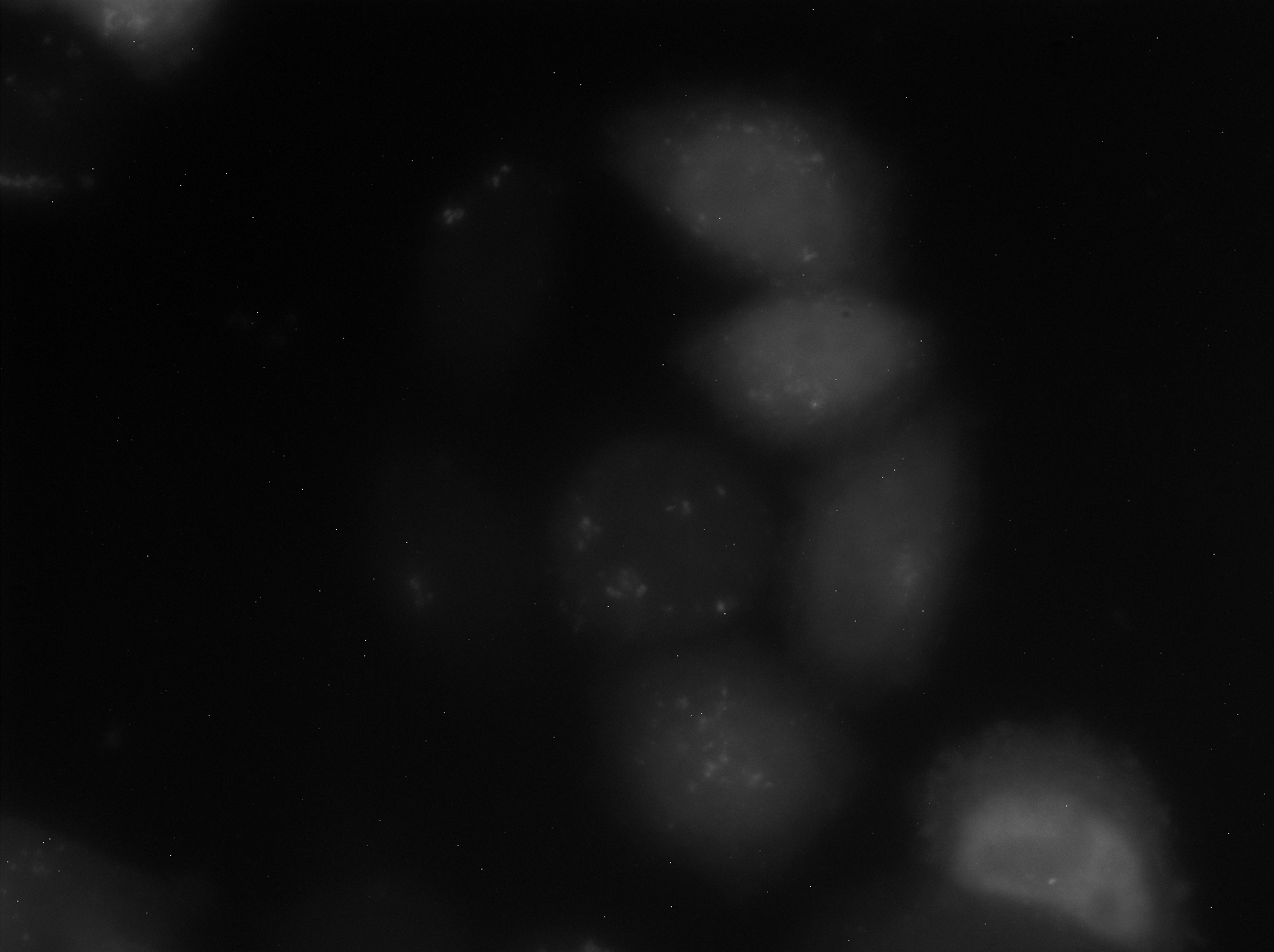

Supplement: Supplementary file 18 — Source data Fig. 5 part 4 [file 44319_2026_742_MOESM18_ESM.zip › Figure 5 Part 4/Fig 5ab WT and KO hela TTLL1-e326g atubulin/Control/WT Hela TTLL1-mut R2 LT6.Project Maximum Z_XY1731543506_Z0_T0_C1.tif]

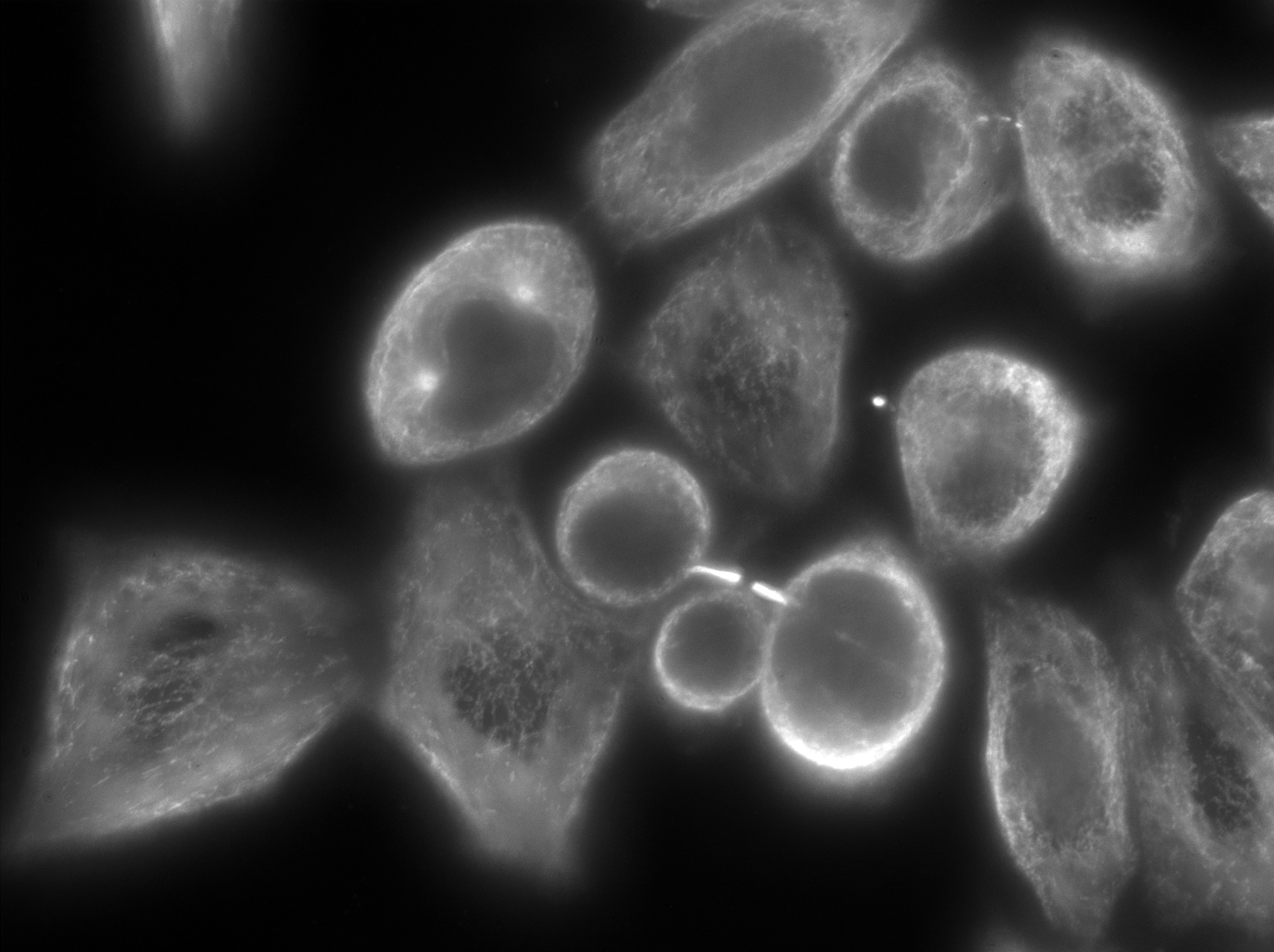

Supplement: Supplementary file 18 — Source data Fig. 5 part 4 [file 44319_2026_742_MOESM18_ESM.zip › Figure 5 Part 4/Fig 5ab WT and KO hela TTLL1-e326g atubulin/Control/TTLL1-mut atub R2 LT6.Project Maximum Z_XY1724951880_Z0_T0_C2.tif]

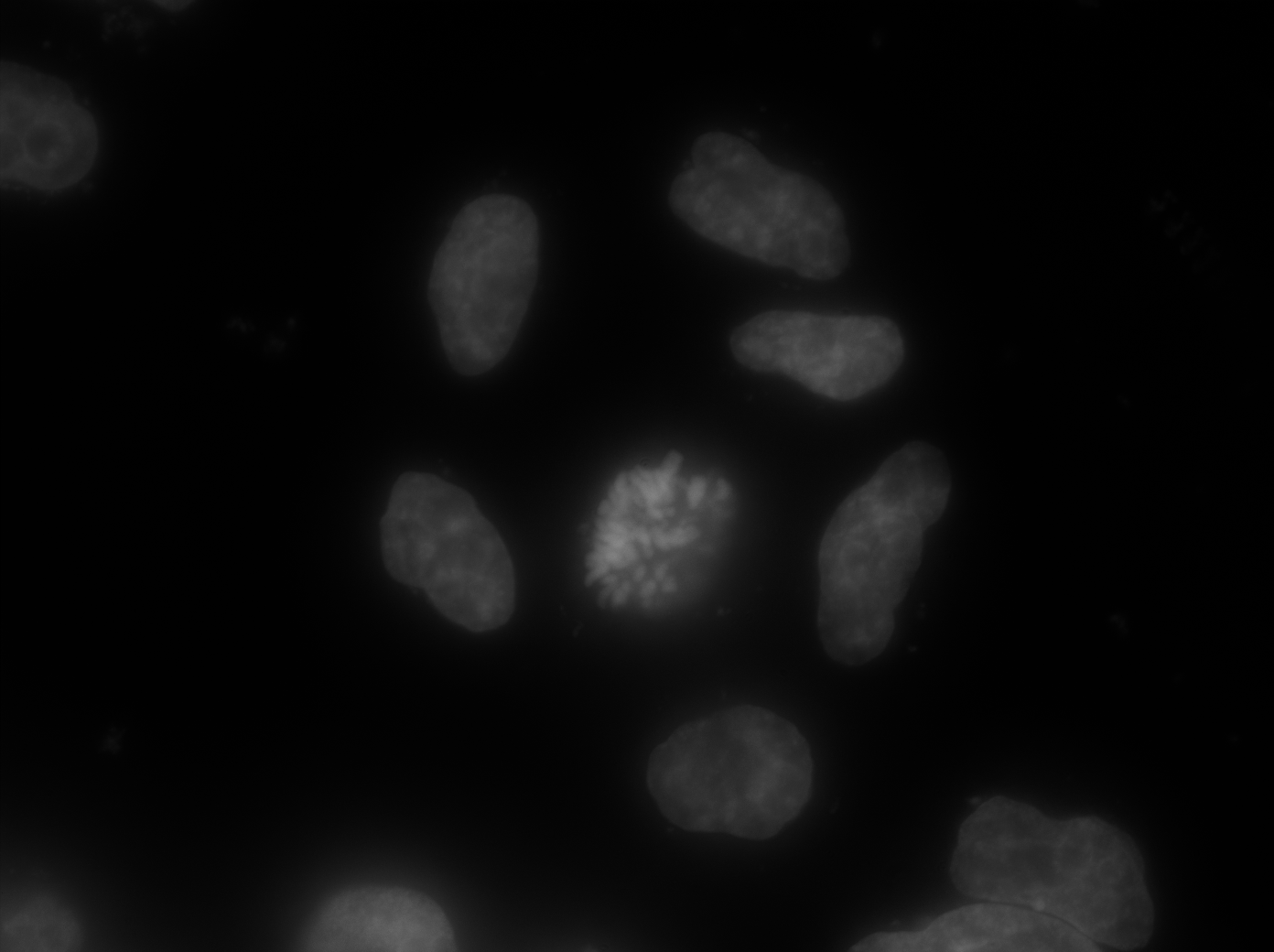

Supplement: Supplementary file 18 — Source data Fig. 5 part 4 [file 44319_2026_742_MOESM18_ESM.zip › Figure 5 Part 4/Fig 5ab WT and KO hela TTLL1-e326g atubulin/Control/WT Hela TTLL1-mut R2 LT6.Project Maximum Z_XY1731543506_Z0_T0_C0.tif]

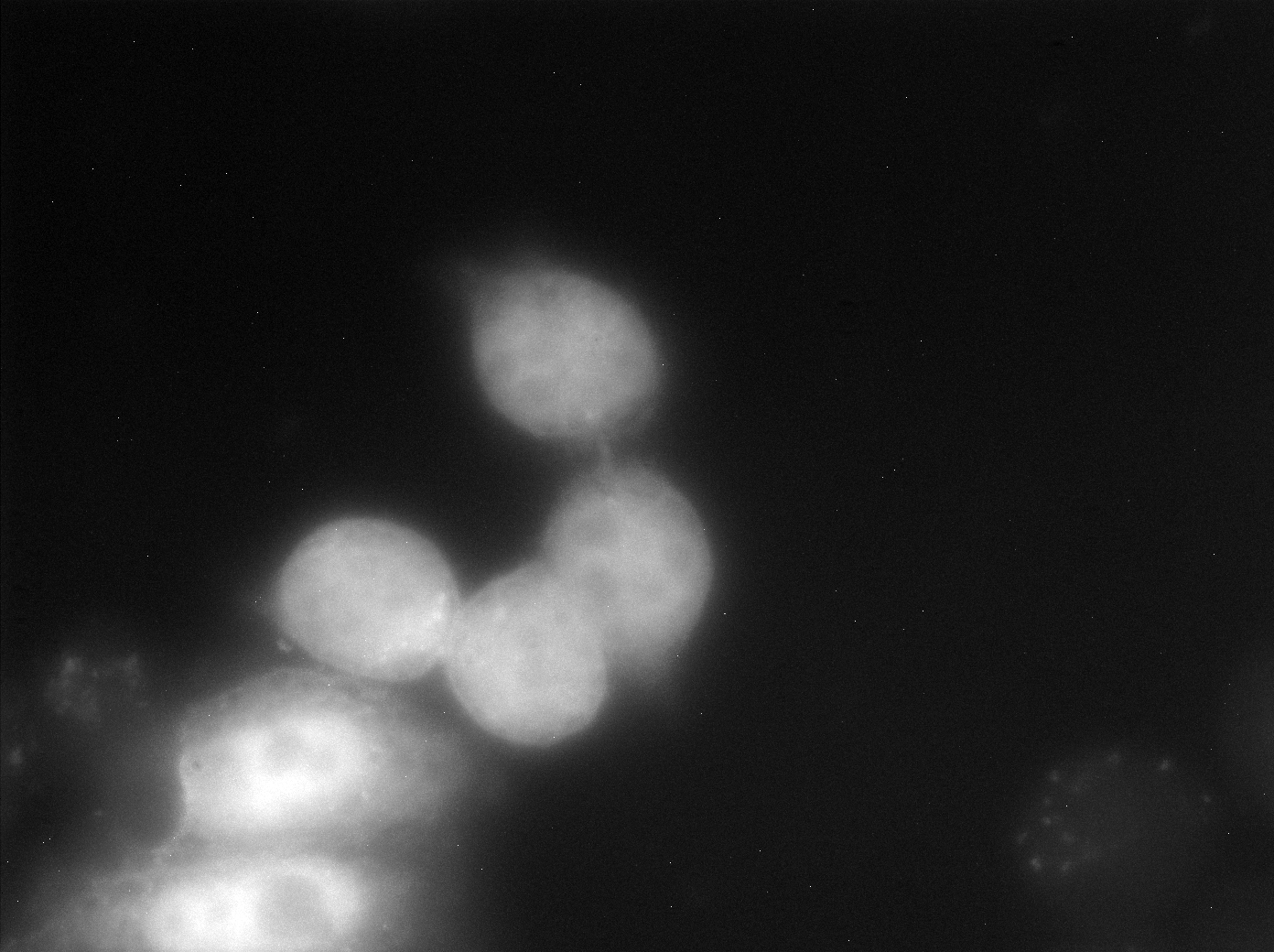

Supplement: Supplementary file 18 — Source data Fig. 5 part 4 [file 44319_2026_742_MOESM18_ESM.zip › Figure 5 Part 4/Fig 5ab WT and KO hela TTLL1-e326g atubulin/Control/TTLL1-mut atub R1 LT2 ET1.Project Maximum Z_XY1724438861_Z0_T0_C1.tif]

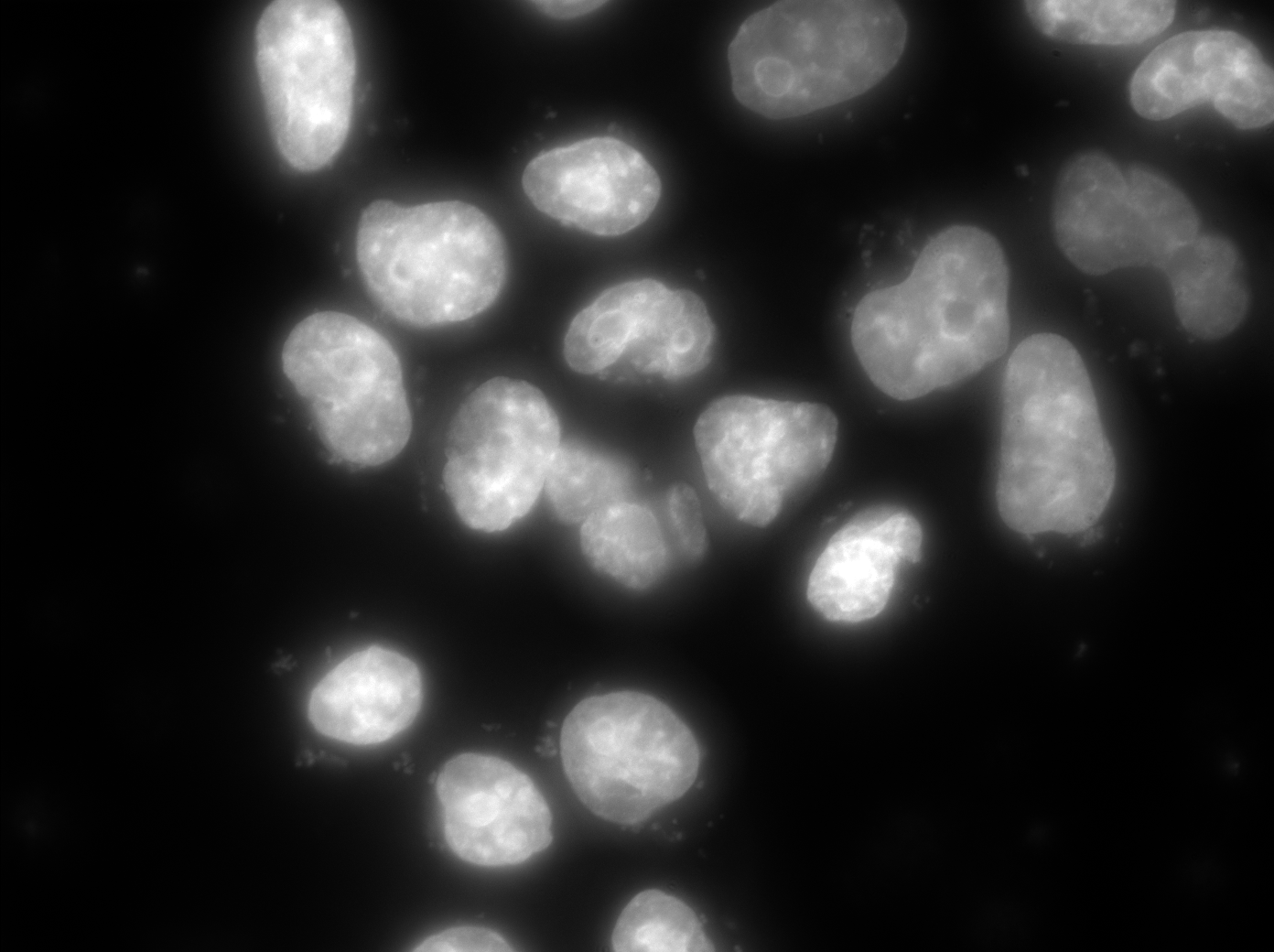

Supplement: Supplementary file 18 — Source data Fig. 5 part 4 [file 44319_2026_742_MOESM18_ESM.zip › Figure 5 Part 4/Fig 5ab WT and KO hela TTLL1-e326g atubulin/Control/TTLL1-mut atub R1 LT3.Project Maximum Z_XY1724439533_Z0_T0_C0.tif]

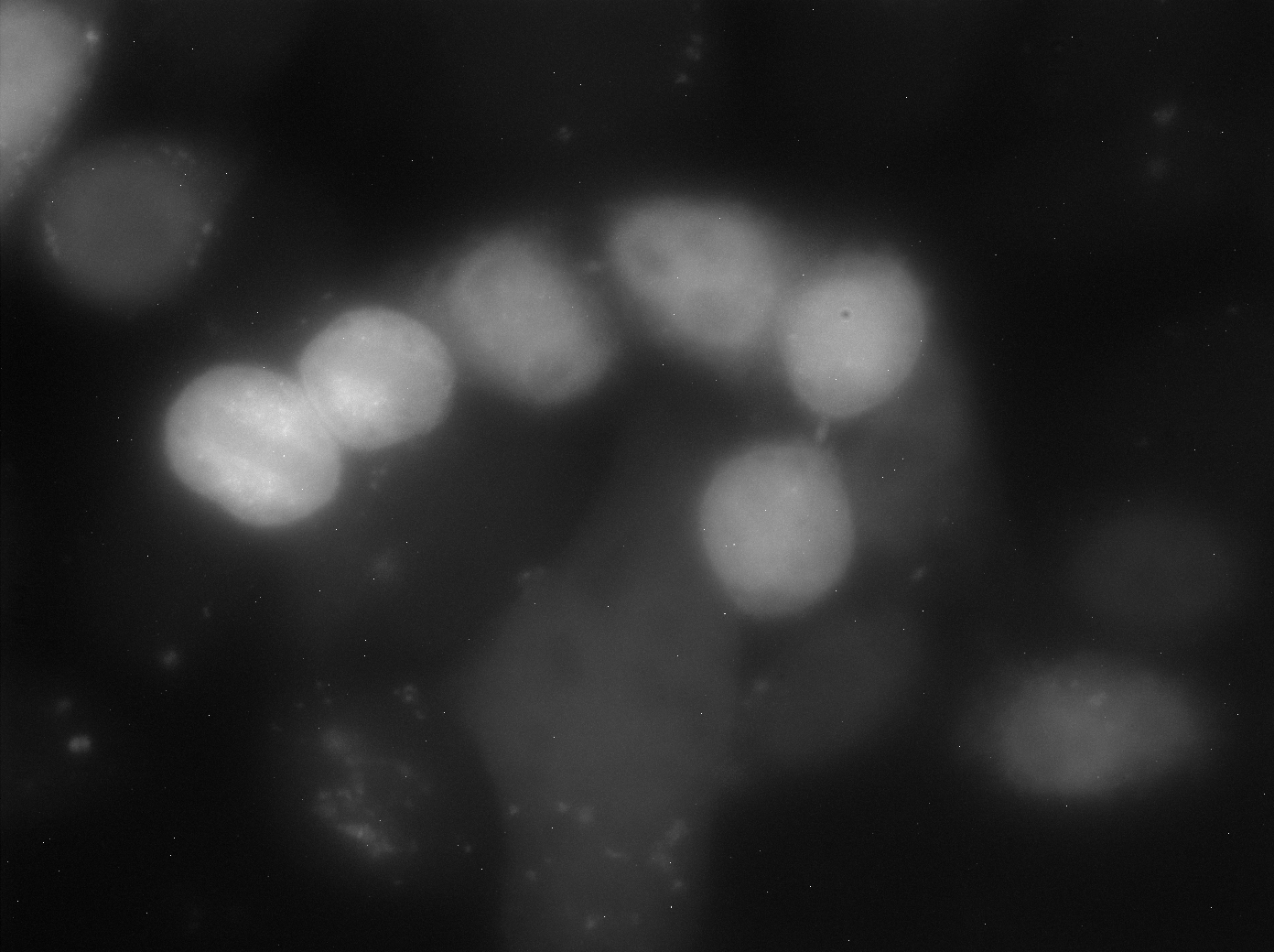

Supplement: Supplementary file 18 — Source data Fig. 5 part 4 [file 44319_2026_742_MOESM18_ESM.zip › Figure 5 Part 4/Fig 5ab WT and KO hela TTLL1-e326g atubulin/Control/WT Hela TTLL1-mut R3 11-13-24 LT4LT5.Project Maximum Z_XY1731546412_Z0_T0_C1.tif]

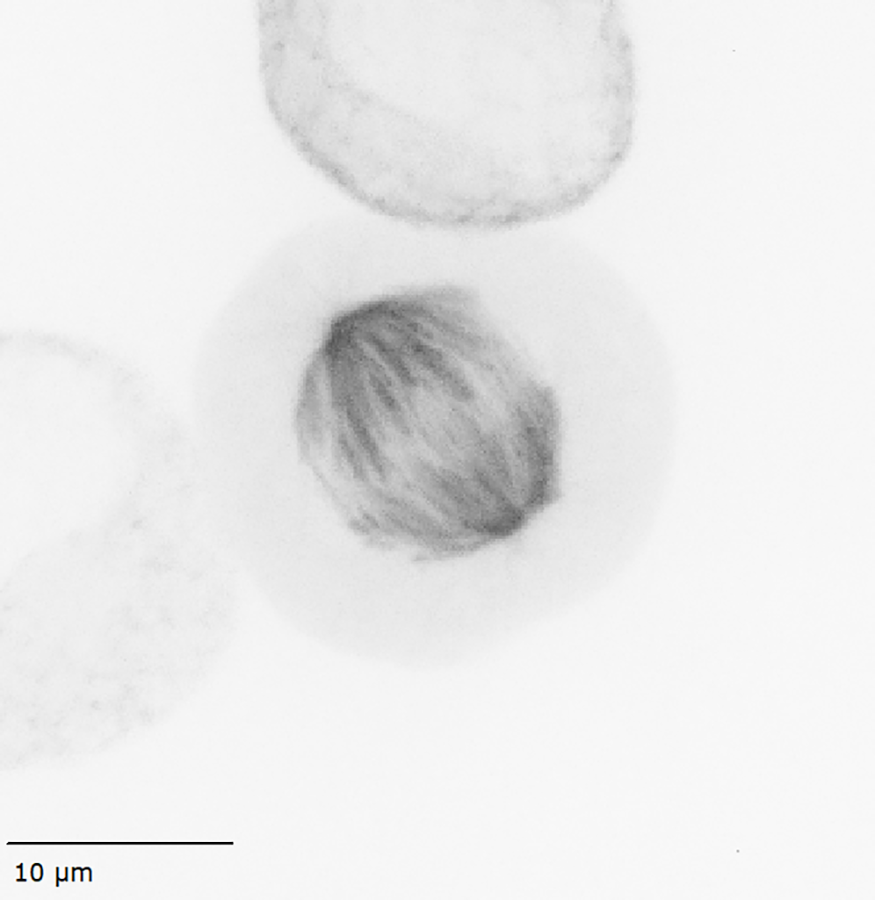

Supplement: Supplementary file 19 — Source data Fig. 5 part 5 [file 44319_2026_742_MOESM19_ESM.zip › Figure 5 Part 5/Fig 5e Time lapse control and tpgs1 gfp-tubulin/time lapse control/comtrol 1.tif]

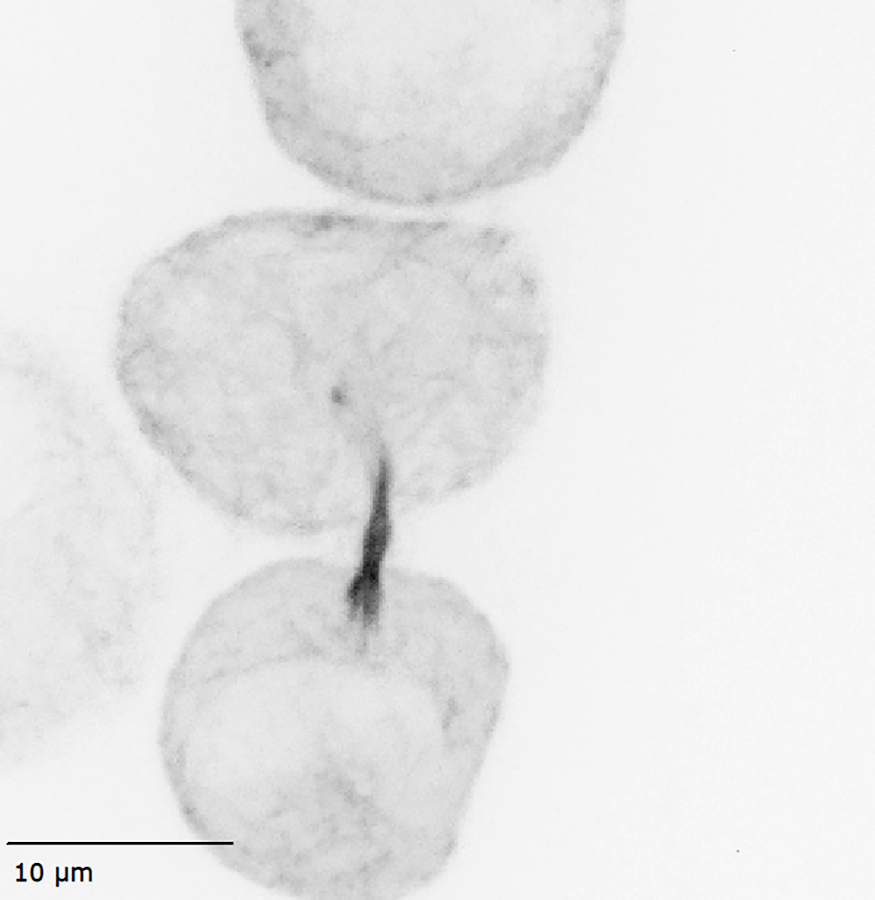

Supplement: Supplementary file 19 — Source data Fig. 5 part 5 [file 44319_2026_742_MOESM19_ESM.zip › Figure 5 Part 5/Fig 5e Time lapse control and tpgs1 gfp-tubulin/time lapse control/control 5.tif]

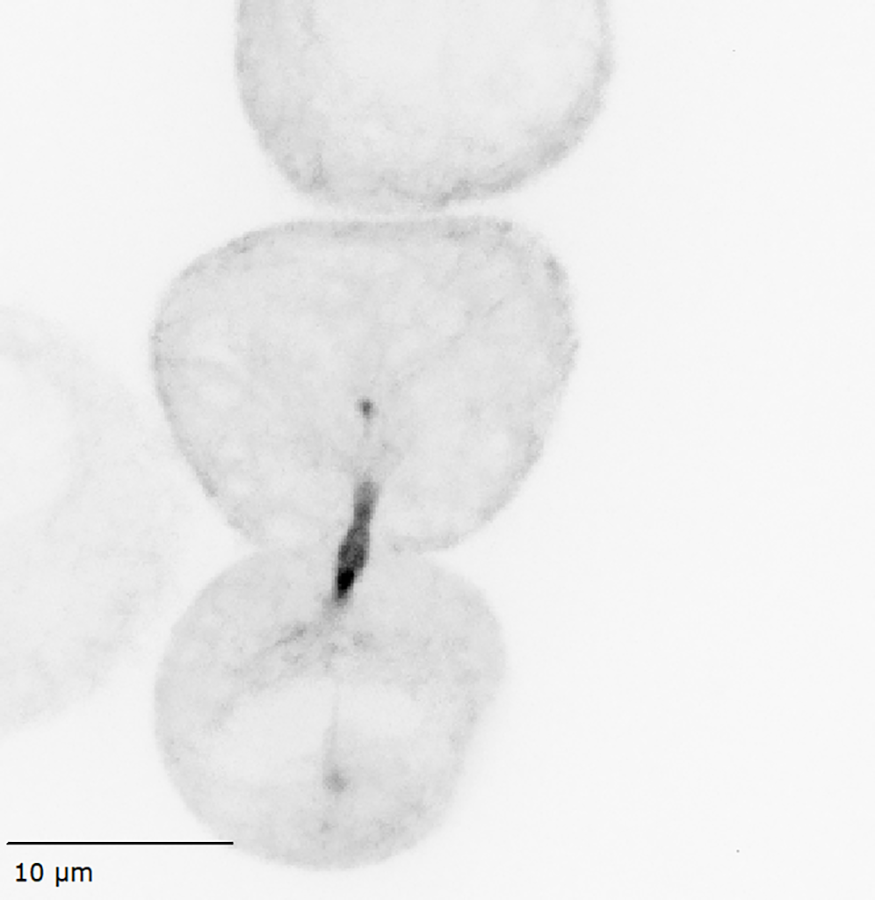

Supplement: Supplementary file 19 — Source data Fig. 5 part 5 [file 44319_2026_742_MOESM19_ESM.zip › Figure 5 Part 5/Fig 5e Time lapse control and tpgs1 gfp-tubulin/time lapse control/control 4.tif]

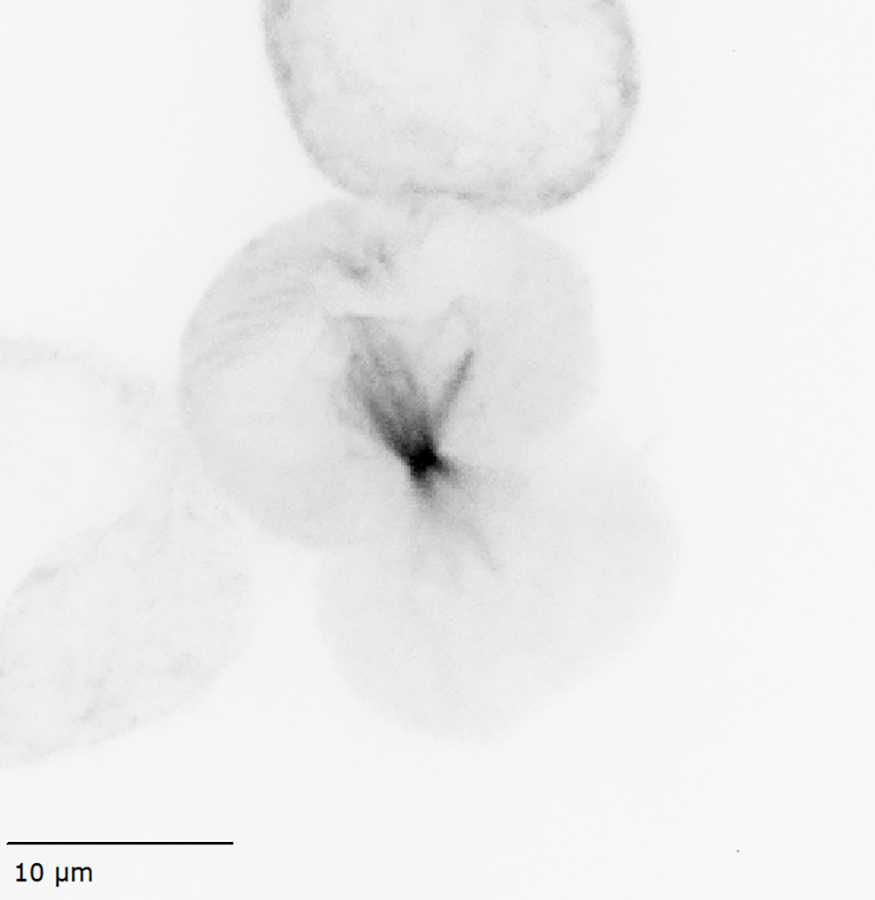

Supplement: Supplementary file 19 — Source data Fig. 5 part 5 [file 44319_2026_742_MOESM19_ESM.zip › Figure 5 Part 5/Fig 5e Time lapse control and tpgs1 gfp-tubulin/time lapse control/control 3.tif]

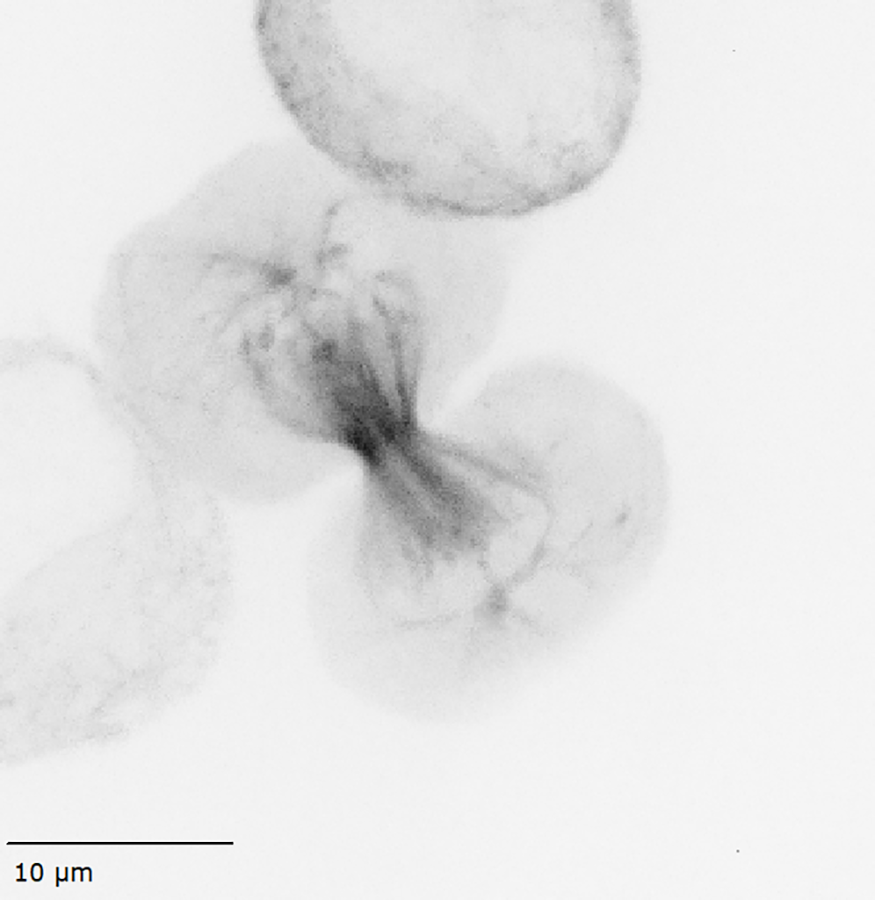

Supplement: Supplementary file 19 — Source data Fig. 5 part 5 [file 44319_2026_742_MOESM19_ESM.zip › Figure 5 Part 5/Fig 5e Time lapse control and tpgs1 gfp-tubulin/time lapse control/control 2.tif]

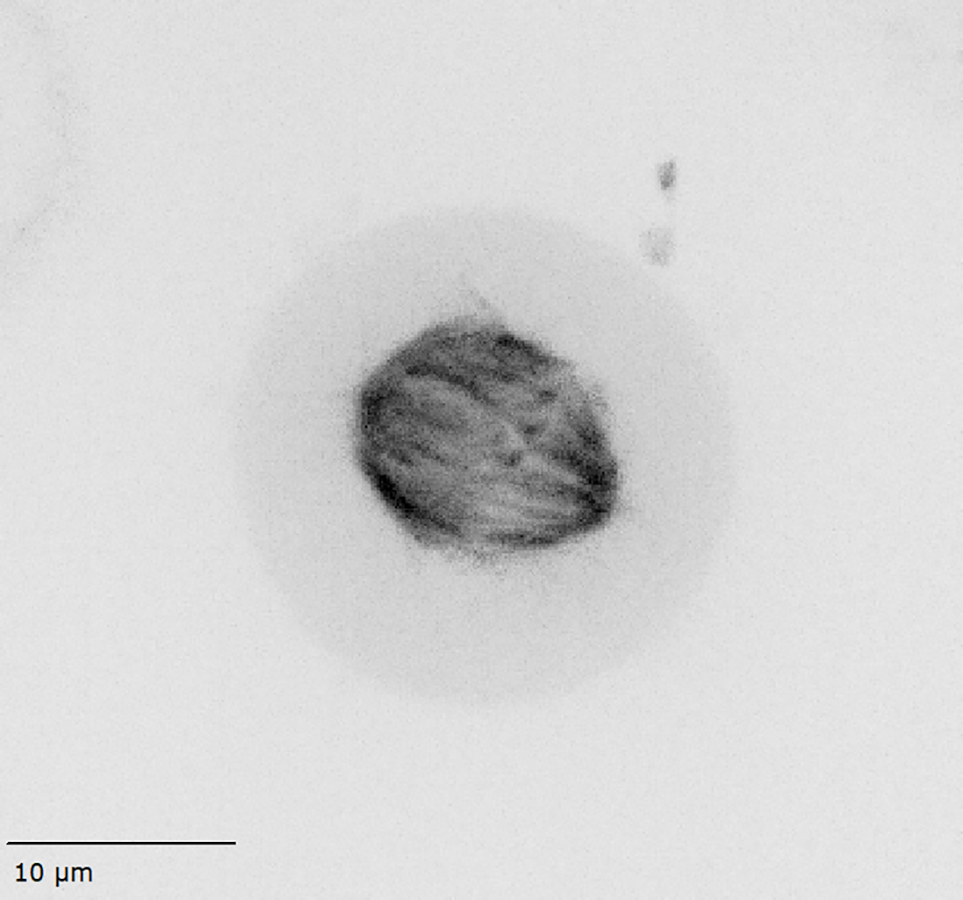

Supplement: Supplementary file 19 — Source data Fig. 5 part 5 [file 44319_2026_742_MOESM19_ESM.zip › Figure 5 Part 5/Fig 5e Time lapse control and tpgs1 gfp-tubulin/time lapse ko/KO 1.tif]

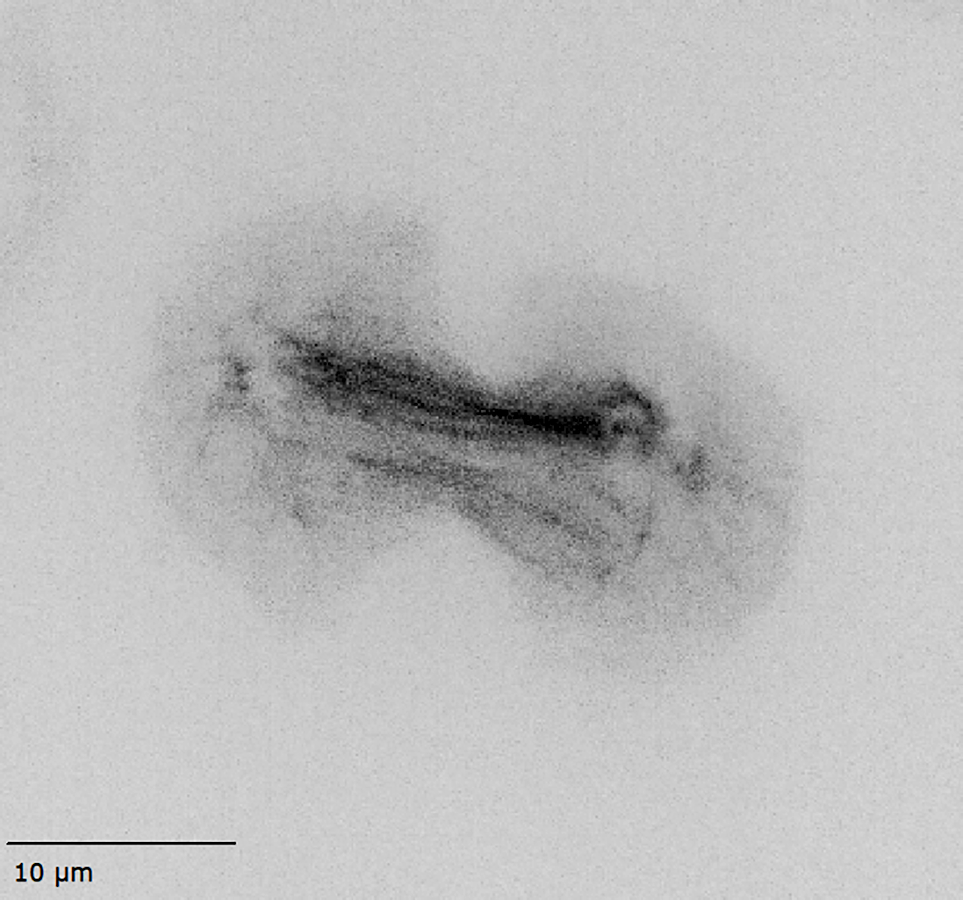

Supplement: Supplementary file 19 — Source data Fig. 5 part 5 [file 44319_2026_742_MOESM19_ESM.zip › Figure 5 Part 5/Fig 5e Time lapse control and tpgs1 gfp-tubulin/time lapse ko/KO 2.tif]

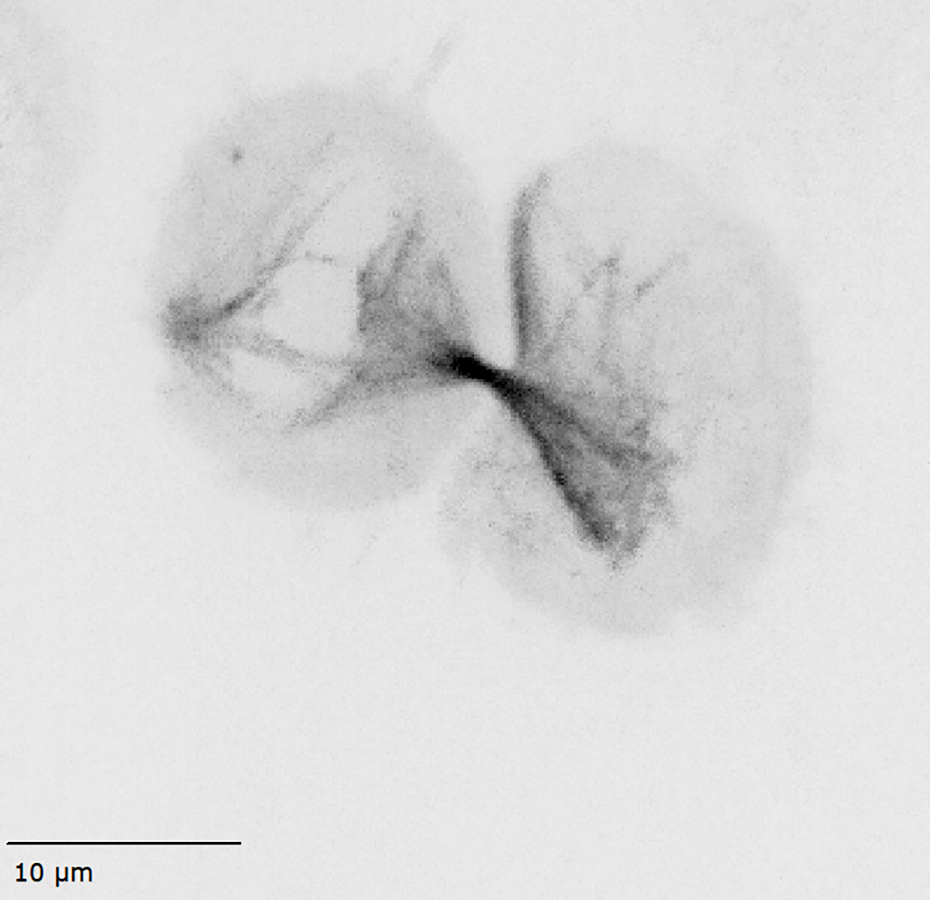

Supplement: Supplementary file 19 — Source data Fig. 5 part 5 [file 44319_2026_742_MOESM19_ESM.zip › Figure 5 Part 5/Fig 5e Time lapse control and tpgs1 gfp-tubulin/time lapse ko/KO 3.tif]

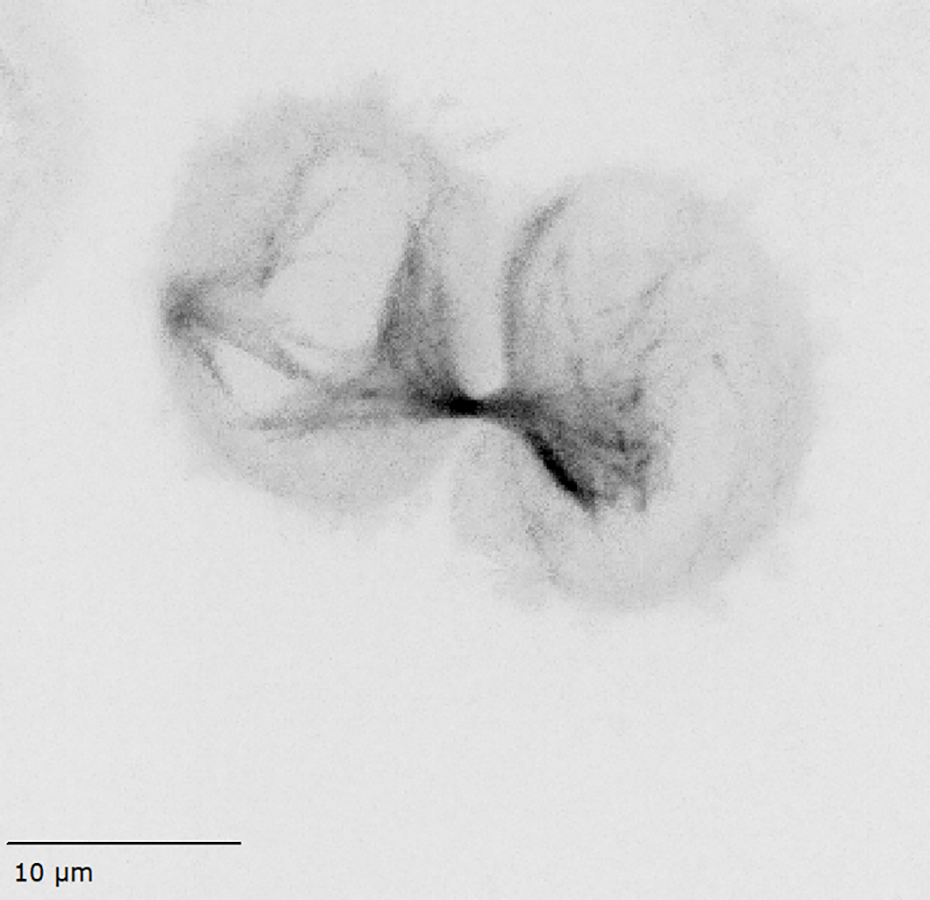

Supplement: Supplementary file 19 — Source data Fig. 5 part 5 [file 44319_2026_742_MOESM19_ESM.zip › Figure 5 Part 5/Fig 5e Time lapse control and tpgs1 gfp-tubulin/time lapse ko/KO 4.tif]

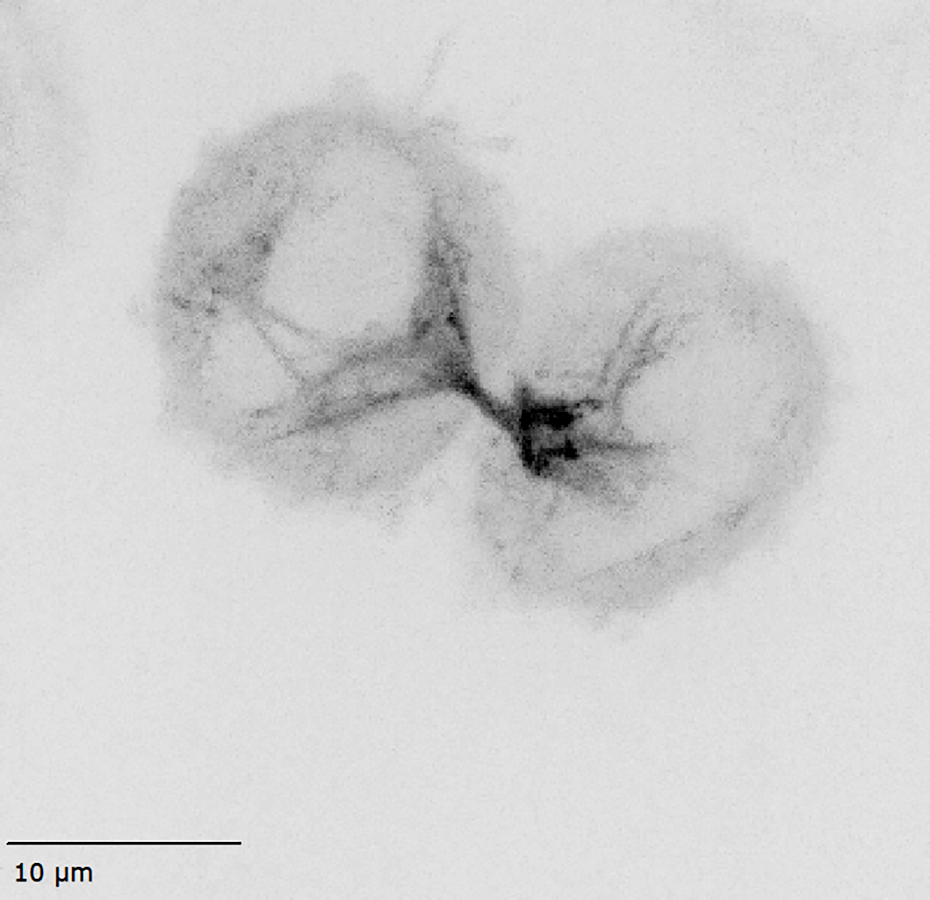

Supplement: Supplementary file 19 — Source data Fig. 5 part 5 [file 44319_2026_742_MOESM19_ESM.zip › Figure 5 Part 5/Fig 5e Time lapse control and tpgs1 gfp-tubulin/time lapse ko/KO 5.tif]

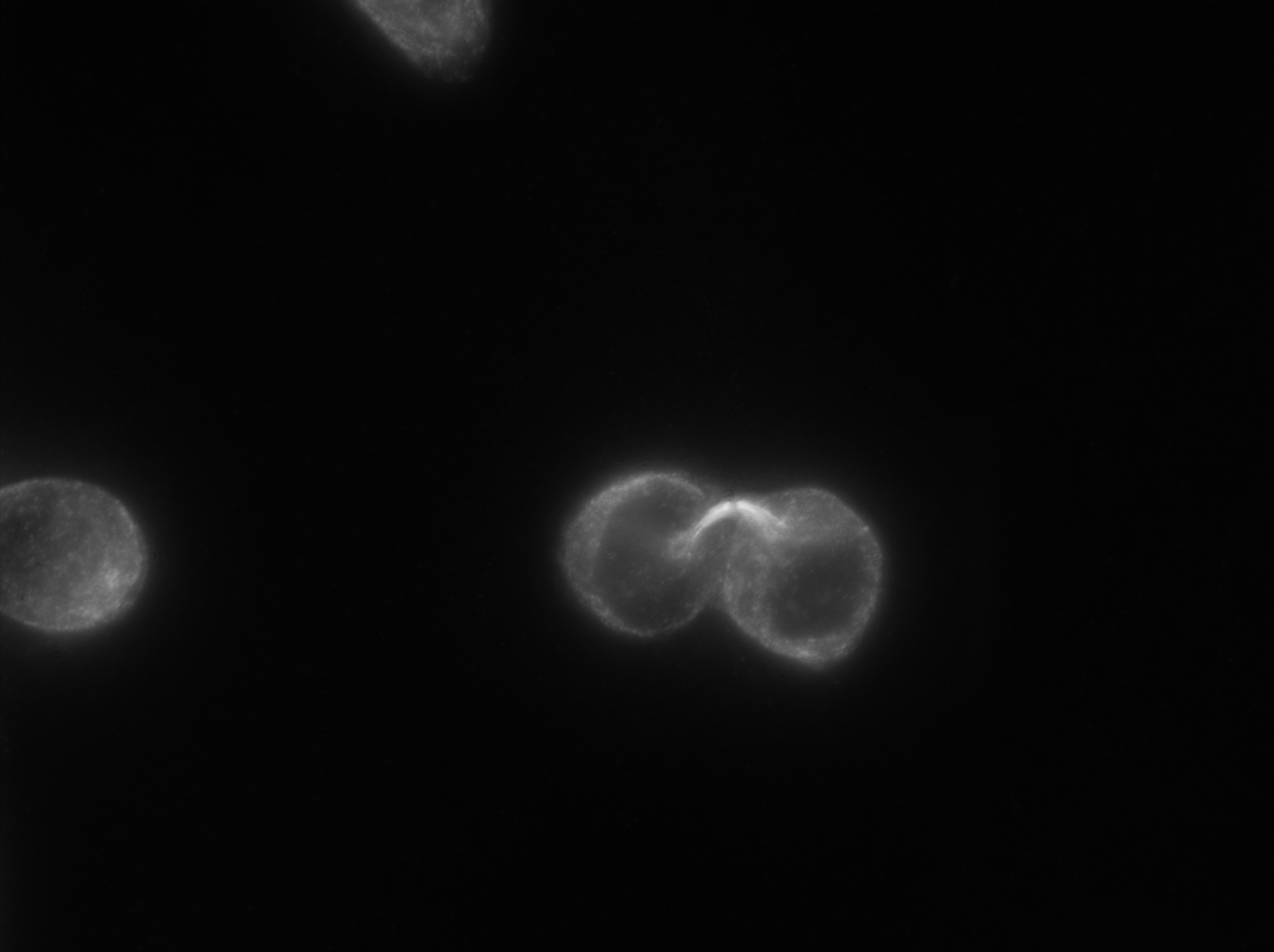

Supplement: Supplementary file 19 — Source data Fig. 5 part 5 [file 44319_2026_742_MOESM19_ESM.zip › Figure 5 Part 5/Fig 5ab WT and KO hela TTLL1-e326g atubulin part 2/TPGS1-KO/TPGS1-KO TTLL1-mut 10-15-24 R1 LT1.Project Maximum Z_XY1729022368_Z0_T0_C2.tif]

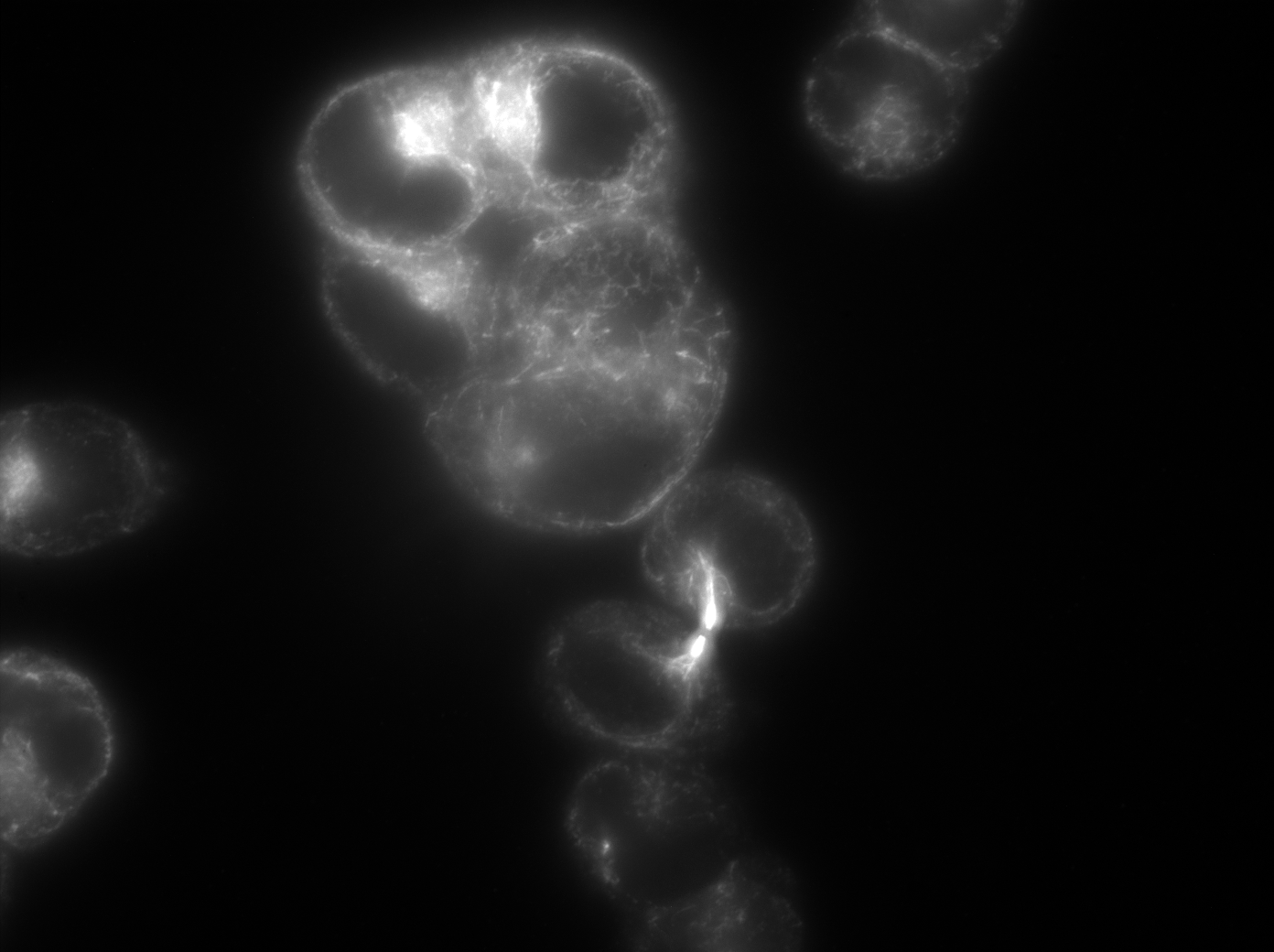

Supplement: Supplementary file 19 — Source data Fig. 5 part 5 [file 44319_2026_742_MOESM19_ESM.zip › Figure 5 Part 5/Fig 5ab WT and KO hela TTLL1-e326g atubulin part 2/TPGS1-KO/TPGS1-KO TTLL1-mut 10-22-24 R2 LT9.Project Maximum Z_XY1730227721_Z0_T0_C2.tif]

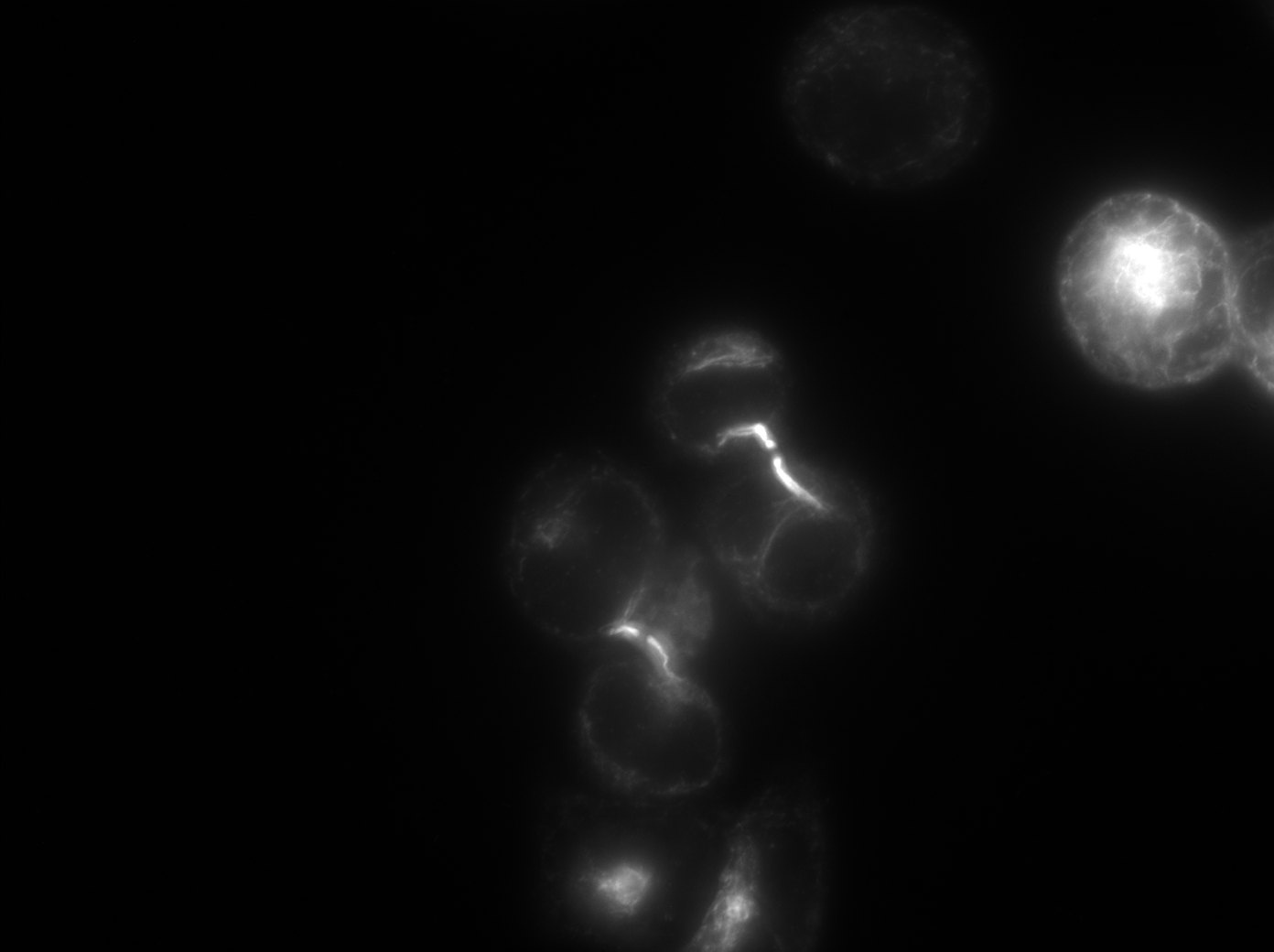

Supplement: Supplementary file 19 — Source data Fig. 5 part 5 [file 44319_2026_742_MOESM19_ESM.zip › Figure 5 Part 5/Fig 5ab WT and KO hela TTLL1-e326g atubulin part 2/TPGS1-KO/TPGS1-KO TTLL1-mut 10-22-24 R2 LT10LT11.Project Maximum Z_XY1730227999_Z0_T0_C2.tif]

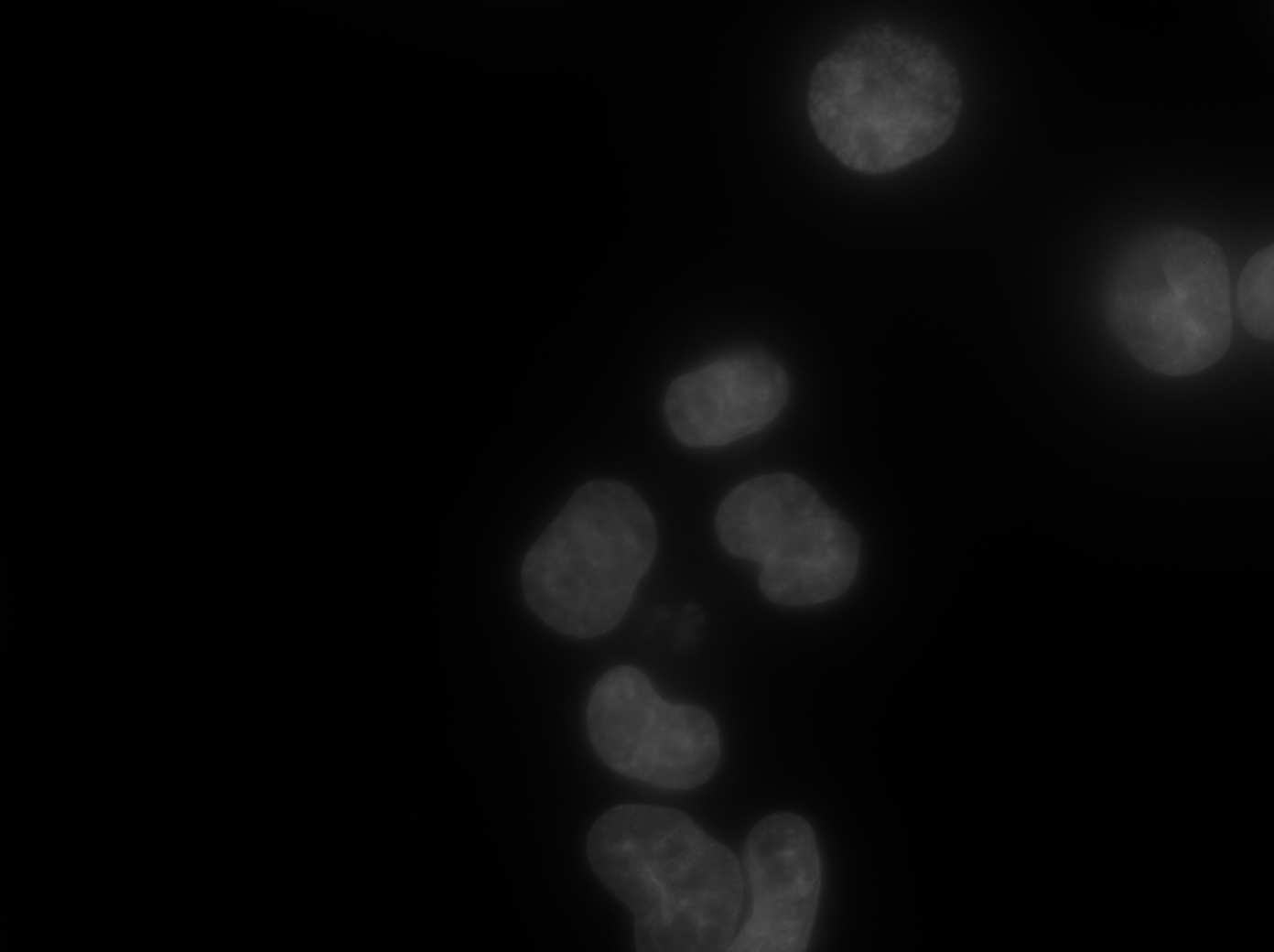

Supplement: Supplementary file 19 — Source data Fig. 5 part 5 [file 44319_2026_742_MOESM19_ESM.zip › Figure 5 Part 5/Fig 5ab WT and KO hela TTLL1-e326g atubulin part 2/TPGS1-KO/TPGS1-KO TTLL1-mut 10-22-24 R2 LT10LT11.Project Maximum Z_XY1730227999_Z0_T0_C0.tif]

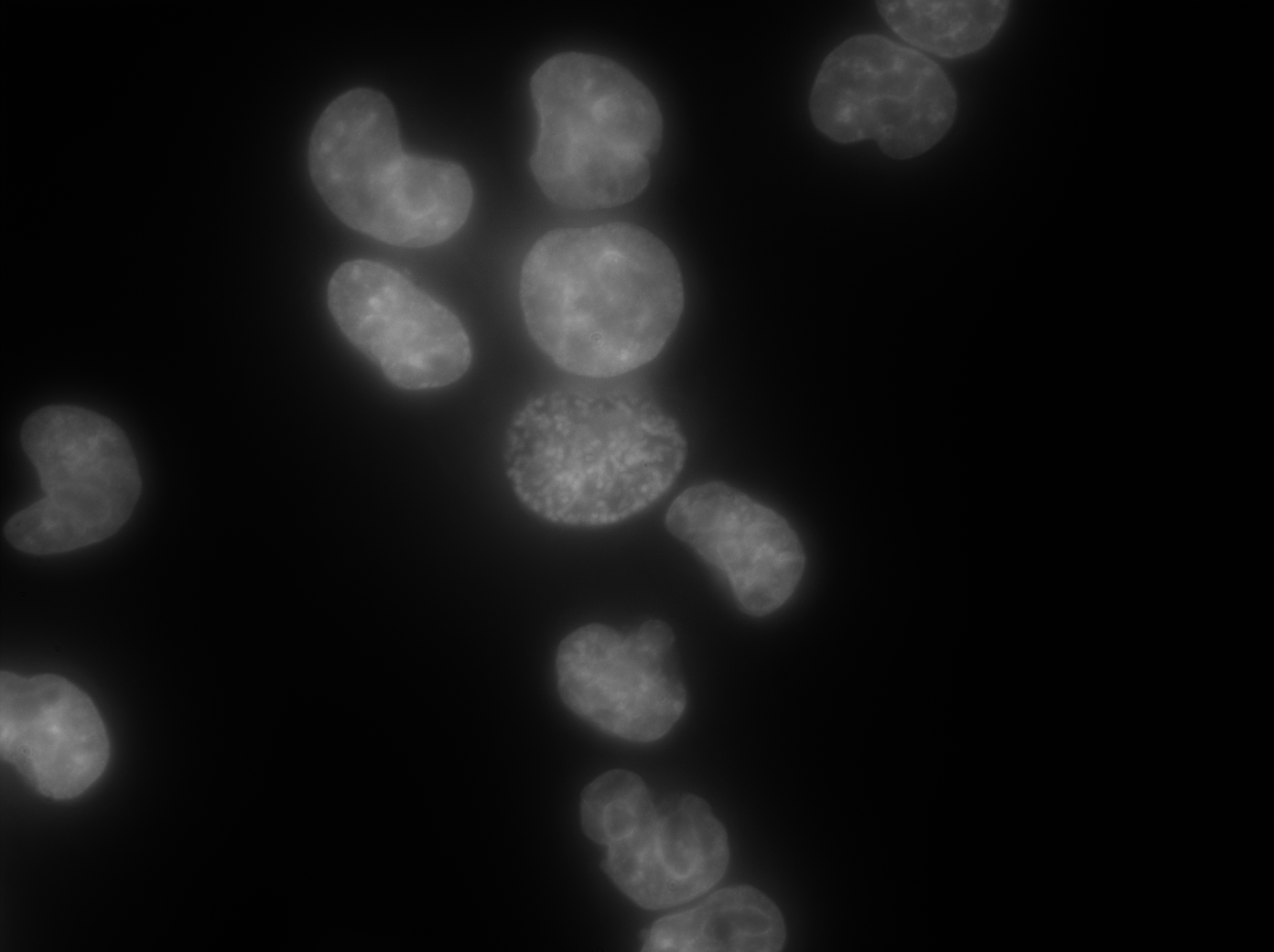

Supplement: Supplementary file 19 — Source data Fig. 5 part 5 [file 44319_2026_742_MOESM19_ESM.zip › Figure 5 Part 5/Fig 5ab WT and KO hela TTLL1-e326g atubulin part 2/TPGS1-KO/TPGS1-KO TTLL1-mut 10-22-24 R2 LT9.Project Maximum Z_XY1730227721_Z0_T0_C0.tif]

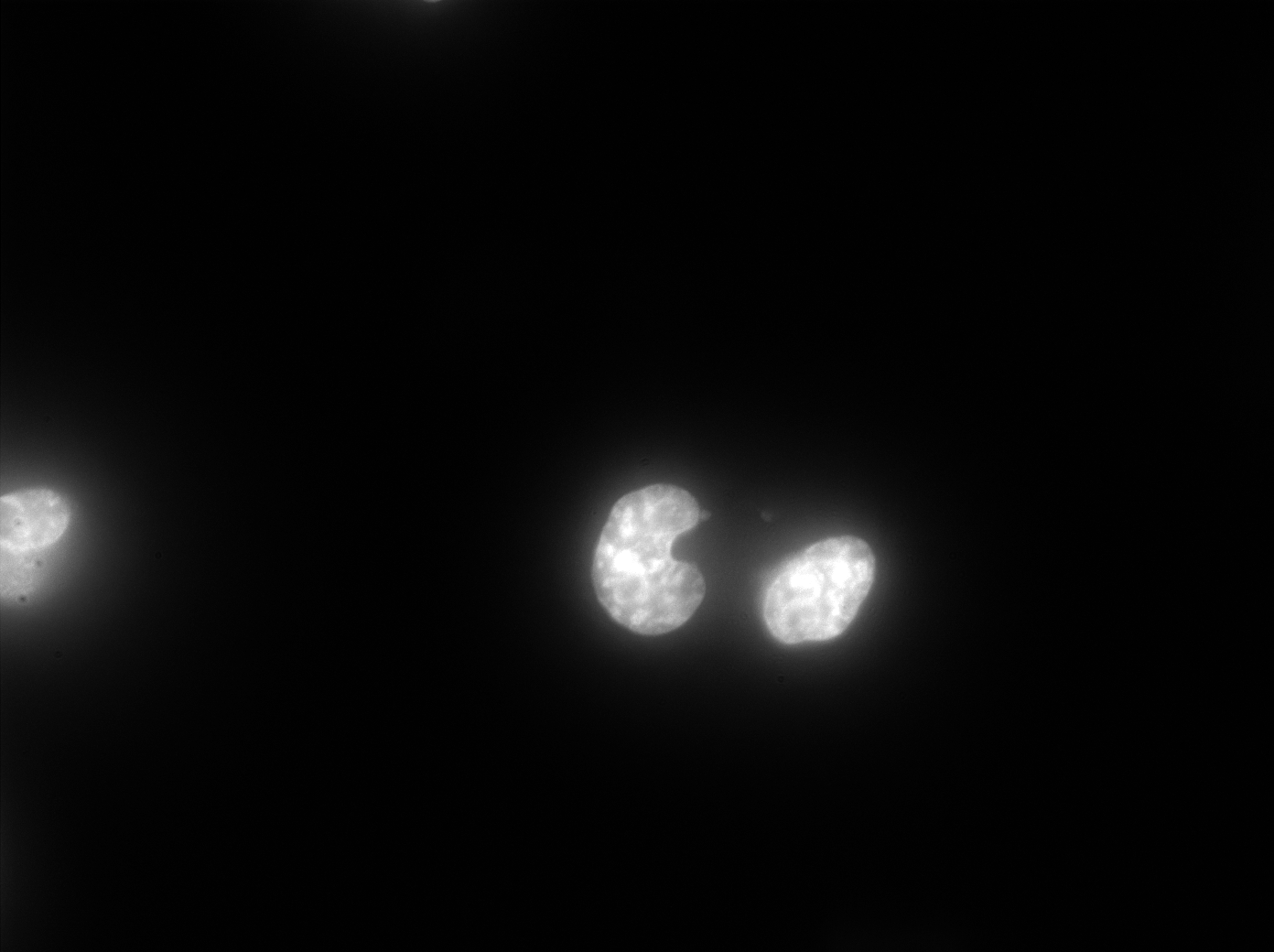

Supplement: Supplementary file 19 — Source data Fig. 5 part 5 [file 44319_2026_742_MOESM19_ESM.zip › Figure 5 Part 5/Fig 5ab WT and KO hela TTLL1-e326g atubulin part 2/TPGS1-KO/TPGS1-KO TTLL1-mut 10-15-24 R1 LT1.Project Maximum Z_XY1729022368_Z0_T0_C0.tif]

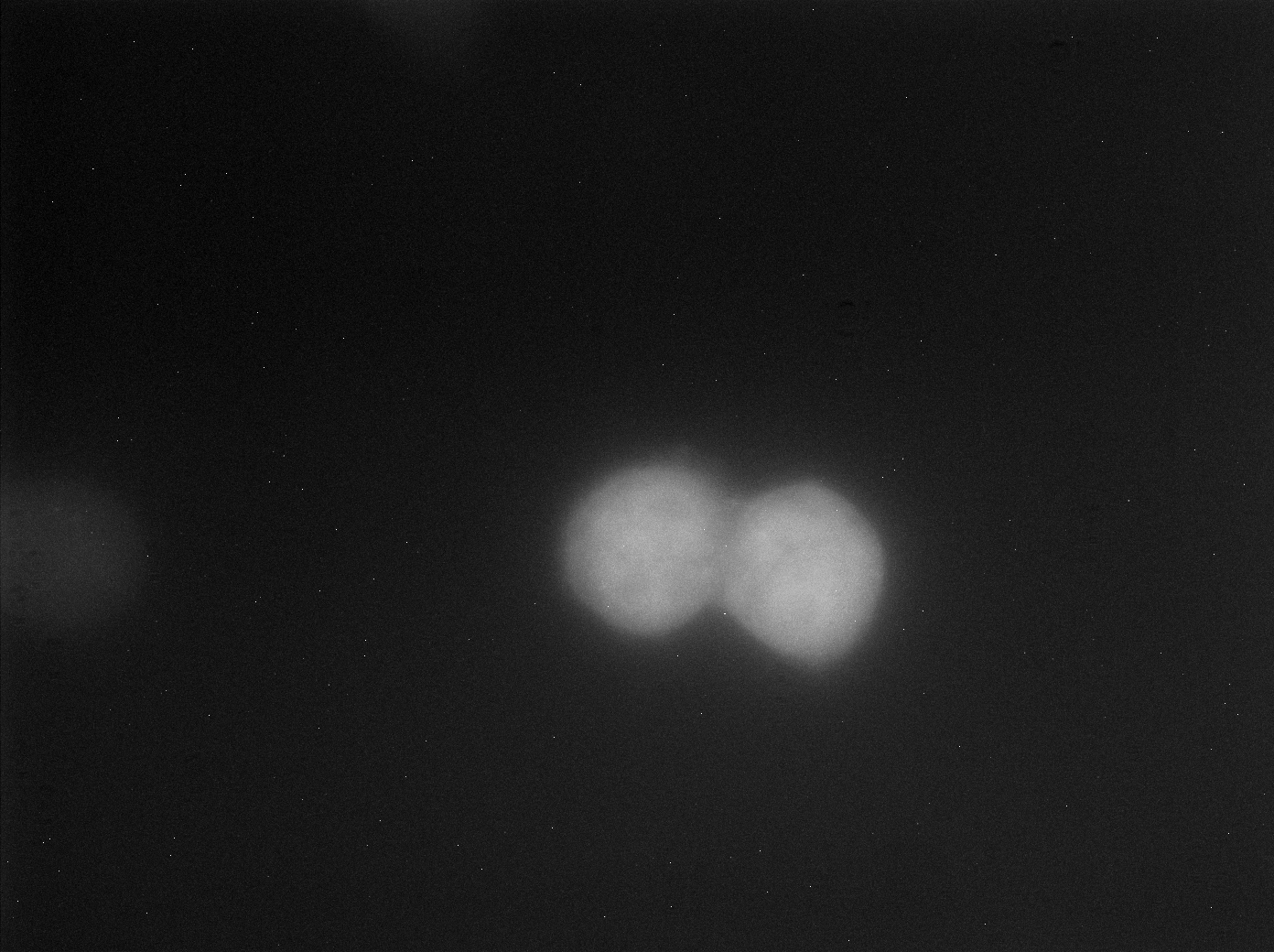

Supplement: Supplementary file 19 — Source data Fig. 5 part 5 [file 44319_2026_742_MOESM19_ESM.zip › Figure 5 Part 5/Fig 5ab WT and KO hela TTLL1-e326g atubulin part 2/TPGS1-KO/TPGS1-KO TTLL1-mut 10-15-24 R1 LT1.Project Maximum Z_XY1729022368_Z0_T0_C1.tif]

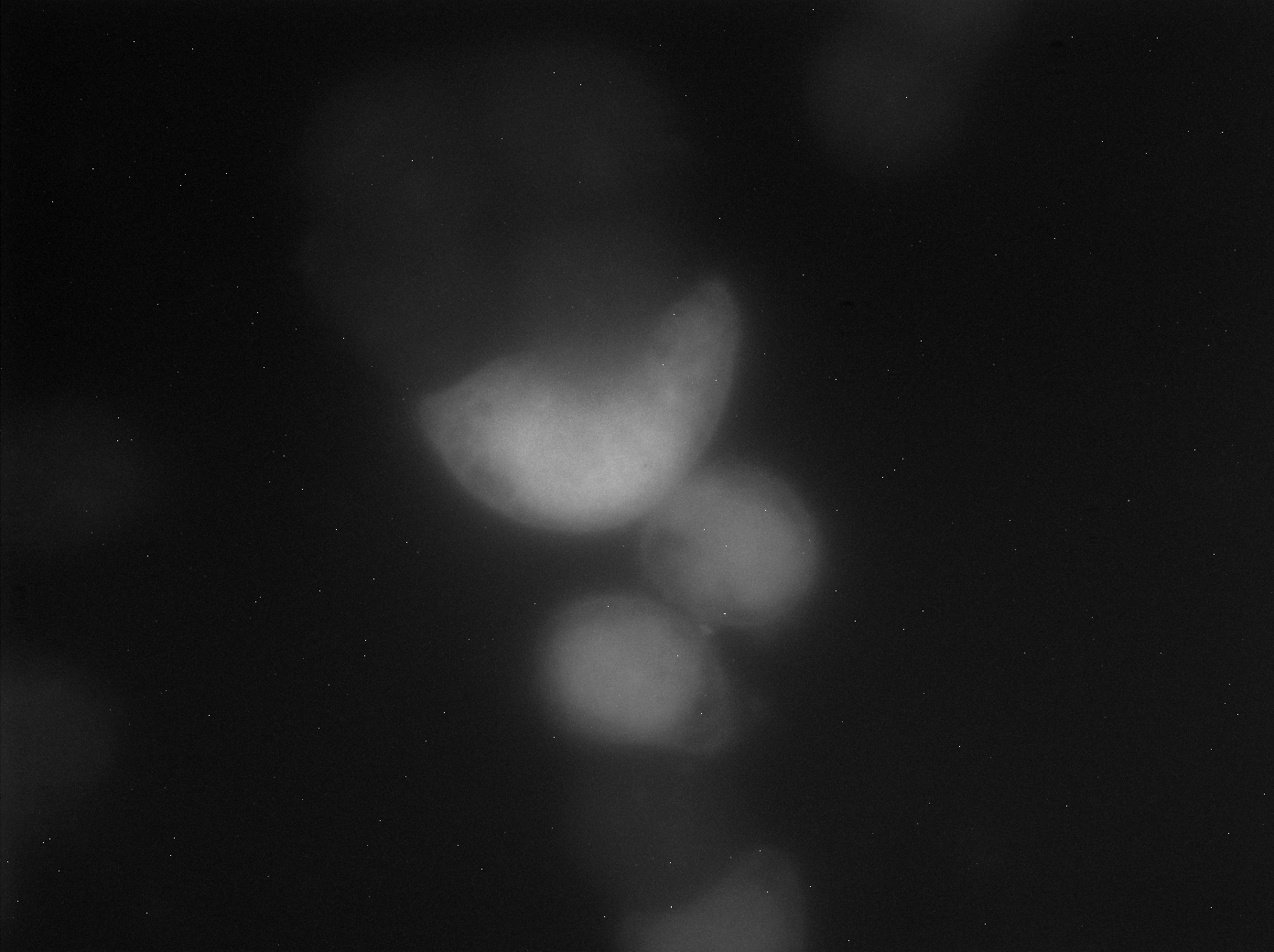

Supplement: Supplementary file 19 — Source data Fig. 5 part 5 [file 44319_2026_742_MOESM19_ESM.zip › Figure 5 Part 5/Fig 5ab WT and KO hela TTLL1-e326g atubulin part 2/TPGS1-KO/TPGS1-KO TTLL1-mut 10-22-24 R2 LT9.Project Maximum Z_XY1730227721_Z0_T0_C1.tif]

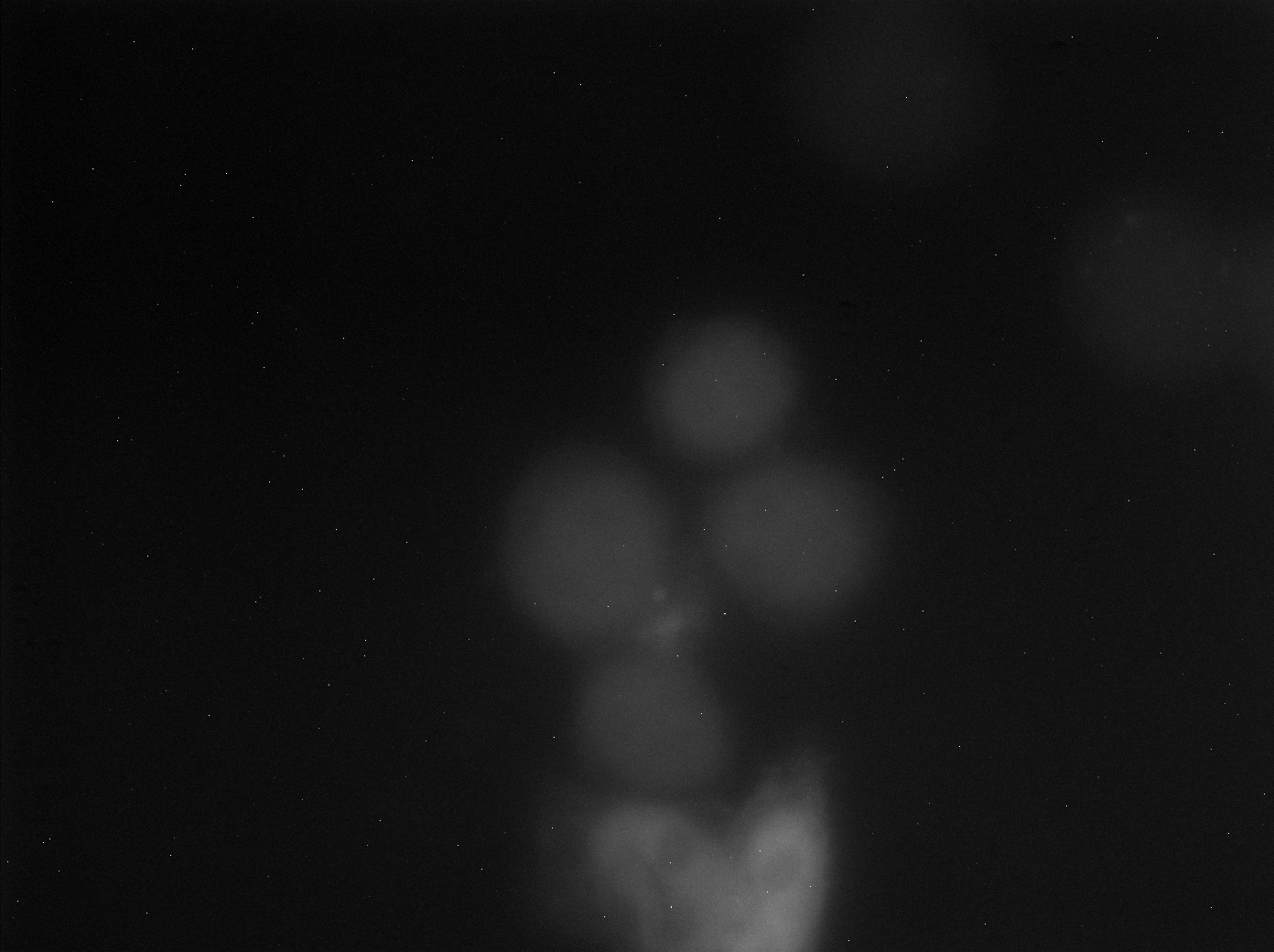

Supplement: Supplementary file 19 — Source data Fig. 5 part 5 [file 44319_2026_742_MOESM19_ESM.zip › Figure 5 Part 5/Fig 5ab WT and KO hela TTLL1-e326g atubulin part 2/TPGS1-KO/TPGS1-KO TTLL1-mut 10-22-24 R2 LT10LT11.Project Maximum Z_XY1730227999_Z0_T0_C1.tif]

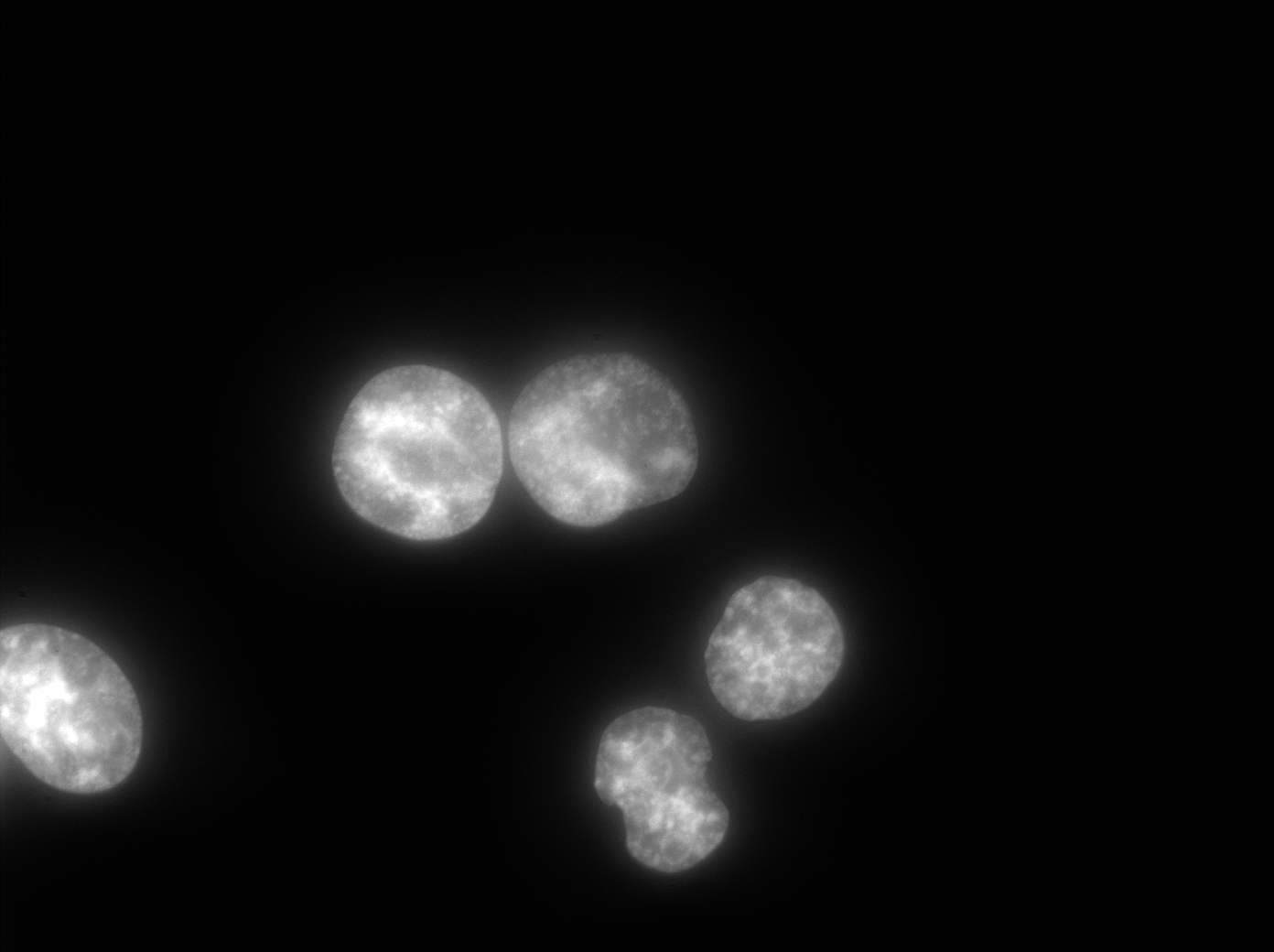

Supplement: Supplementary file 19 — Source data Fig. 5 part 5 [file 44319_2026_742_MOESM19_ESM.zip › Figure 5 Part 5/Fig 5ab WT and KO hela TTLL1-e326g atubulin part 2/TPGS1-KO/TPGS1-KO TTLL1-mut 10-15-24 R1 LT4.Project Maximum Z_XY1729023288_Z0_T0_C0.tif]
