# Supplementary figures and images for: TPGS1 regulates central spindle microtubule glutamylation and remodeling during telophase and abscission (part 19 of 36)
Source: EMBO Rep. 2026 Mar 23;27(8):1944–63. doi: 10.1038/s44319-026-00742-3 (PMC13121839; doi:10.1038/s44319-026-00742-3)

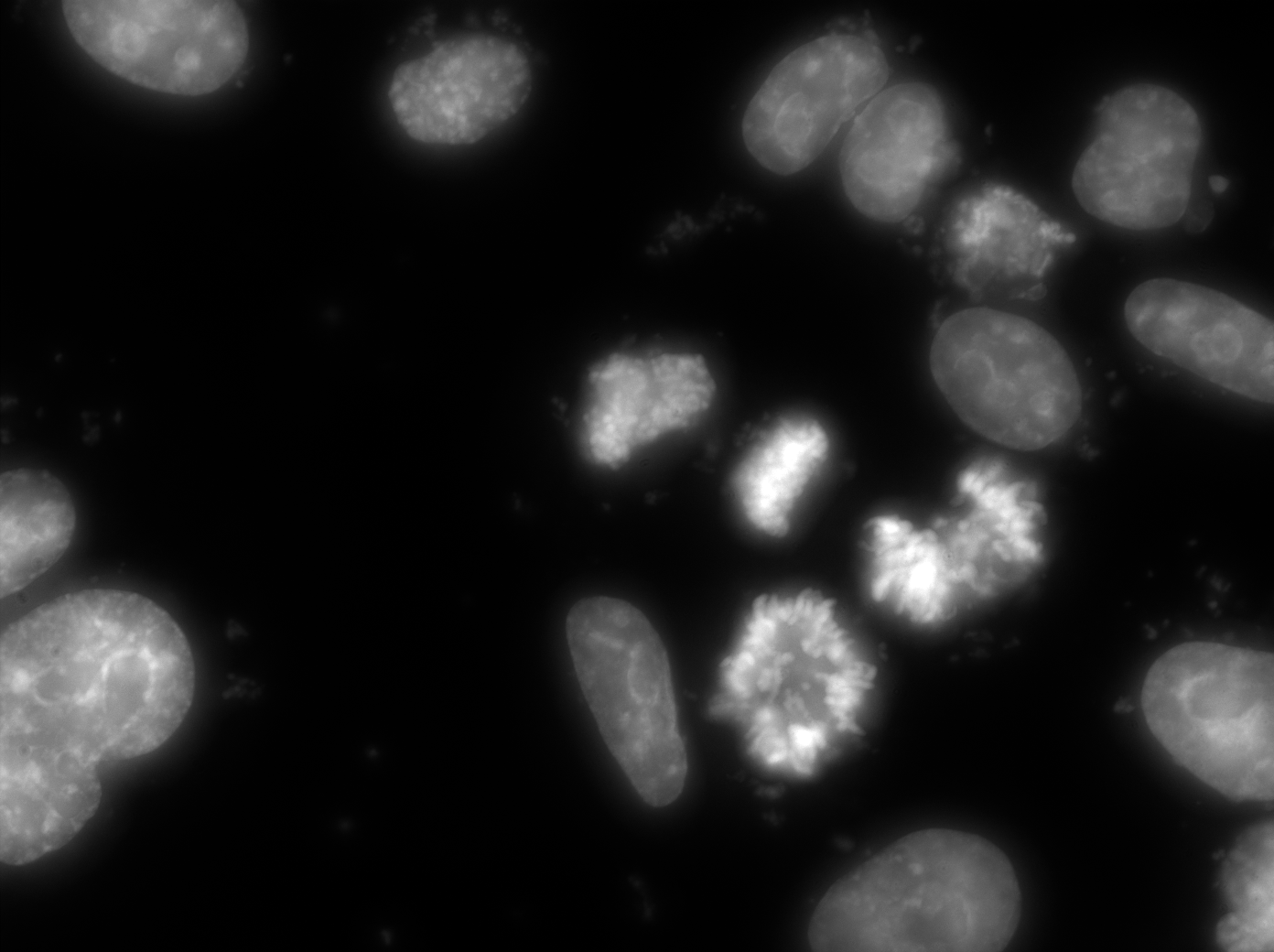

Supplement: Supplementary file 18 — Source data Fig. 5 part 4 [file 44319_2026_742_MOESM18_ESM.zip › Figure 5 Part 4/Fig 5ab WT and KO hela TTLL1-e326g atubulin/EGFP/EGFP-N2 8-23-24 atub R2 ET3 M1M2.Project Maximum Z_XY1725569489_Z0_T0_C0.tif]

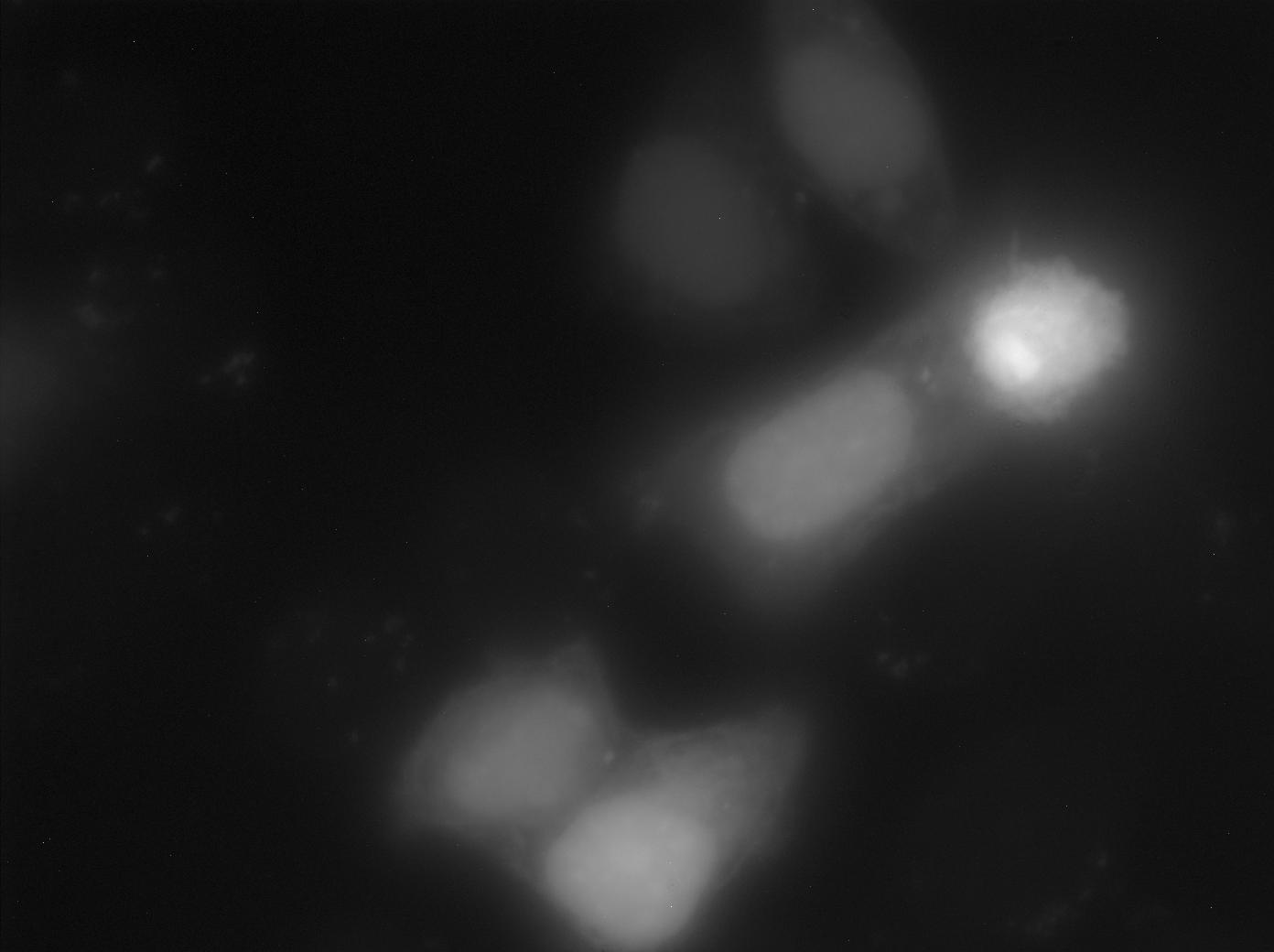

Supplement: Supplementary file 18 — Source data Fig. 5 part 4 [file 44319_2026_742_MOESM18_ESM.zip › Figure 5 Part 4/Fig 5ab WT and KO hela TTLL1-e326g atubulin/EGFP/EGFP-N3 atub R1 LT7LT8LT9.Project Maximum Z_XY1724714518_Z0_T0_C1.tif]

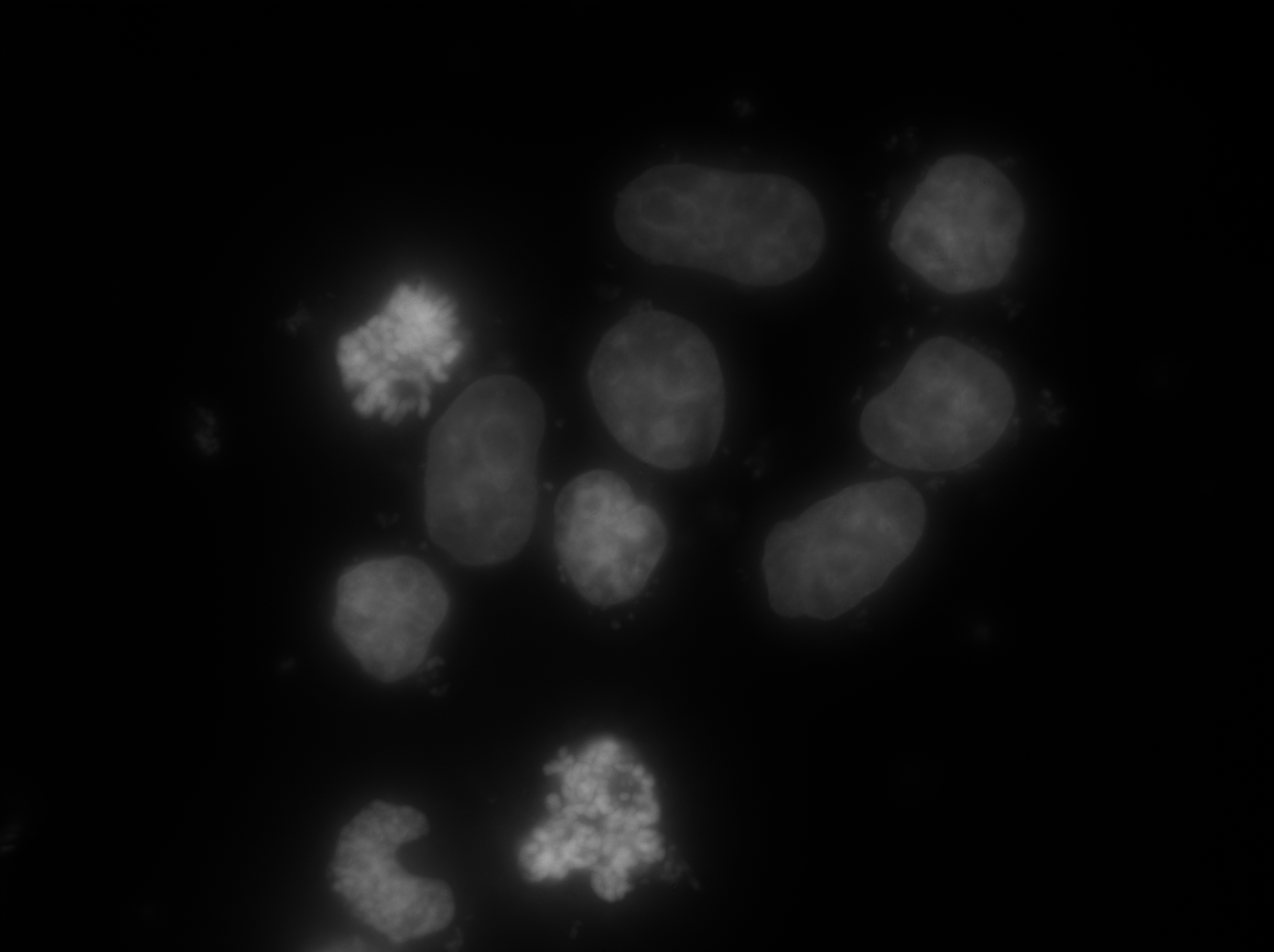

Supplement: Supplementary file 18 — Source data Fig. 5 part 4 [file 44319_2026_742_MOESM18_ESM.zip › Figure 5 Part 4/Fig 5ab WT and KO hela TTLL1-e326g atubulin/EGFP/EGFP-N3 atub R1 LT4.Project Maximum Z_XY1724713880_Z0_T0_C0.tif]

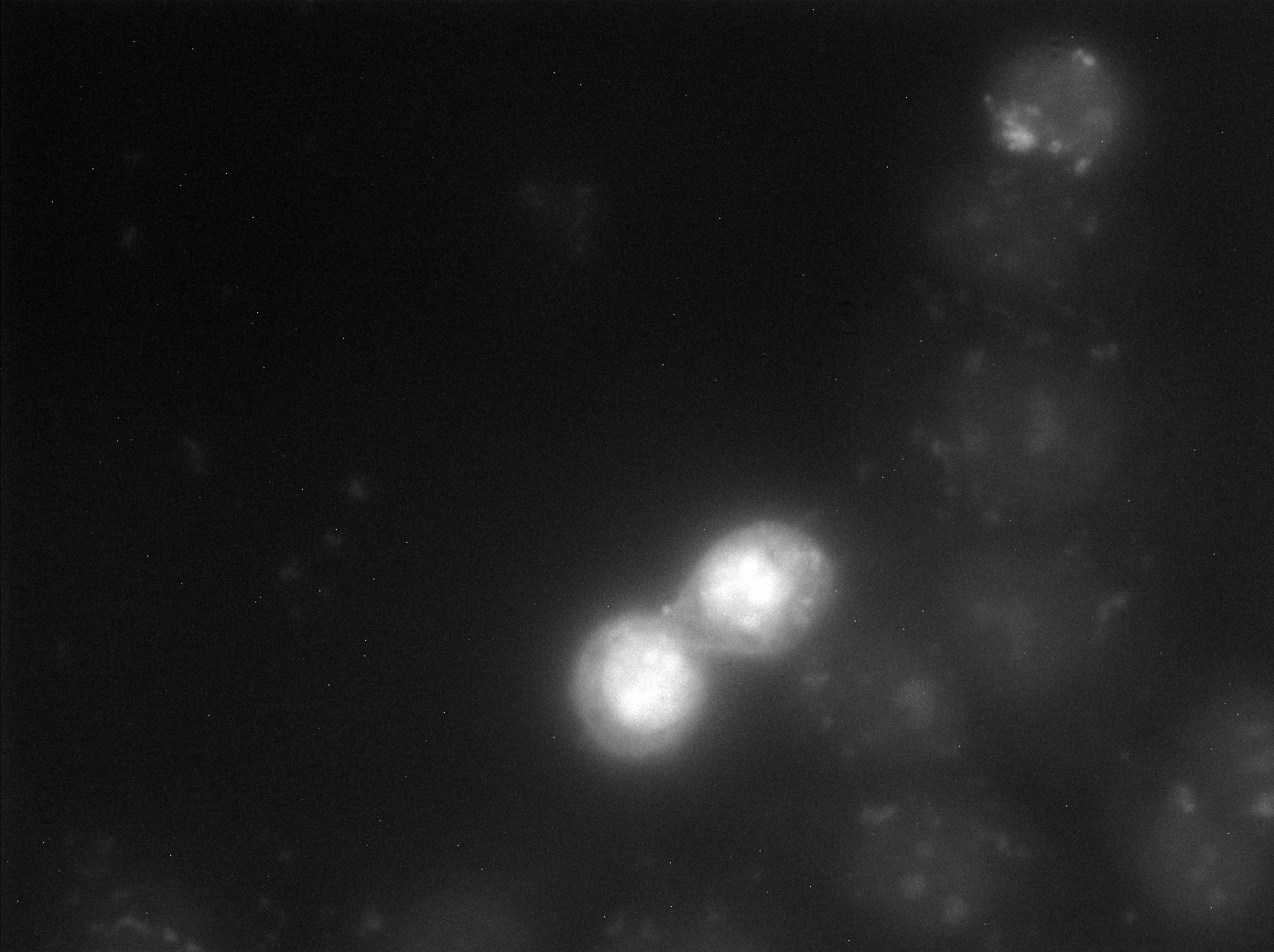

Supplement: Supplementary file 18 — Source data Fig. 5 part 4 [file 44319_2026_742_MOESM18_ESM.zip › Figure 5 Part 4/Fig 5ab WT and KO hela TTLL1-e326g atubulin/EGFP/EGFP-N2 8-23-24 atub R2 LT2.Project Maximum Z_XY1725568039_Z0_T0_C1.tif]

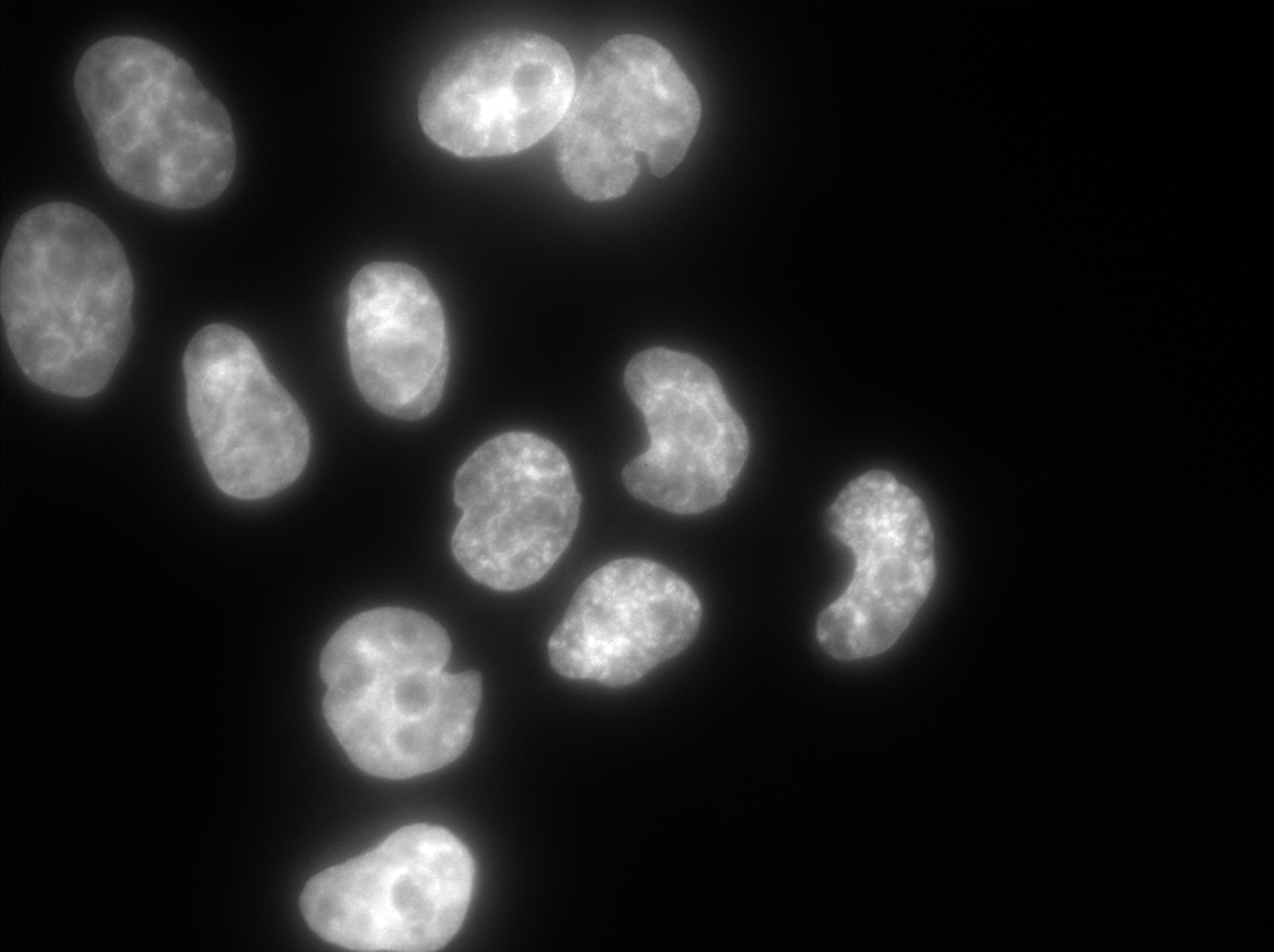

Supplement: Supplementary file 18 — Source data Fig. 5 part 4 [file 44319_2026_742_MOESM18_ESM.zip › Figure 5 Part 4/Fig 5ab WT and KO hela TTLL1-e326g atubulin/EGFP/Cas9 EGFP-N3 10-15-24 R3 LT10.Project Maximum Z_XY1729031131_Z0_T0_C0.tif]

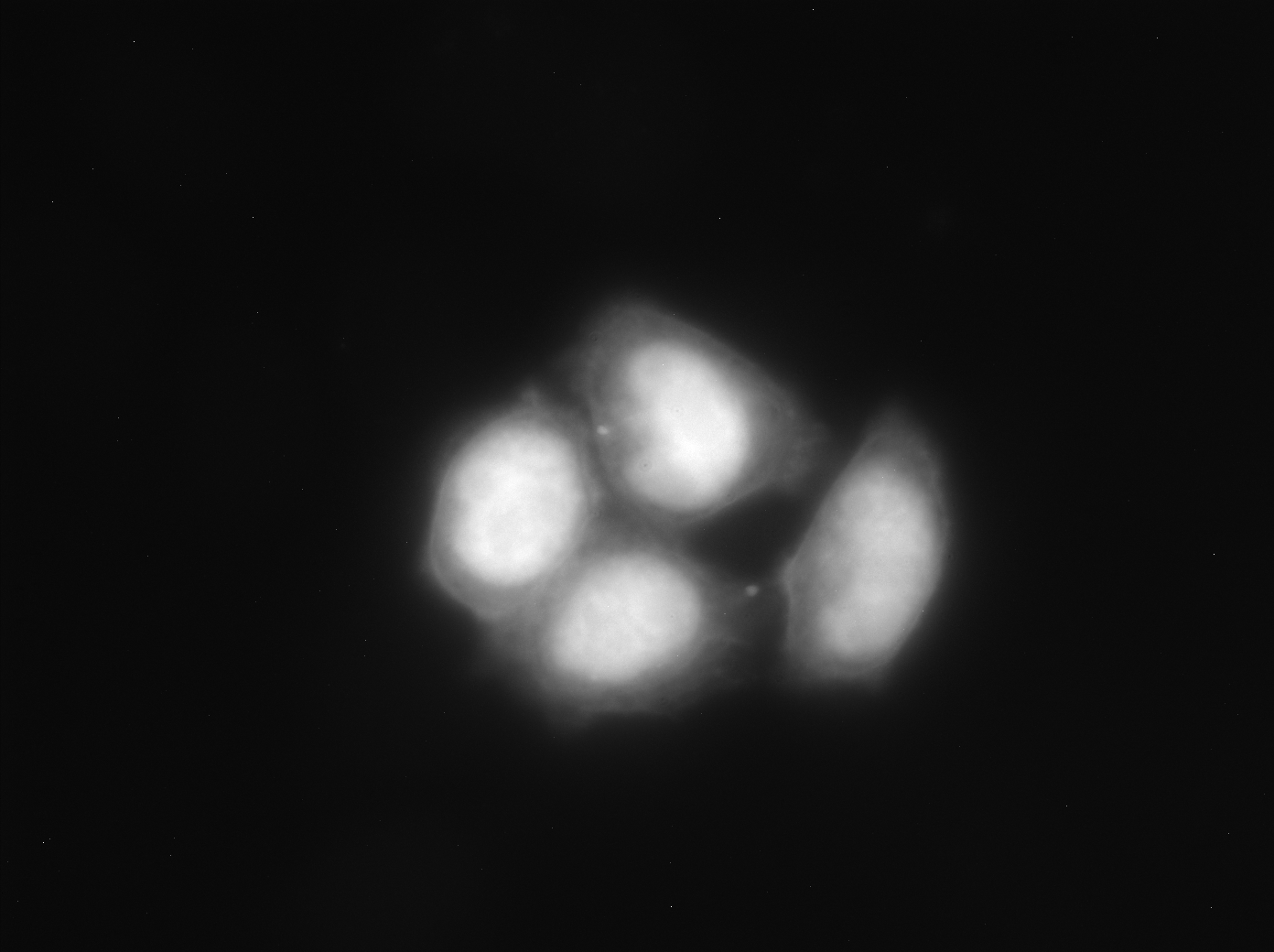

Supplement: Supplementary file 18 — Source data Fig. 5 part 4 [file 44319_2026_742_MOESM18_ESM.zip › Figure 5 Part 4/Fig 5ab WT and KO hela TTLL1-e326g atubulin/EGFP/Cas9 EGFP-N3 10-15-24 R3 LT10.Project Maximum Z_XY1729031131_Z0_T0_C1.tif]

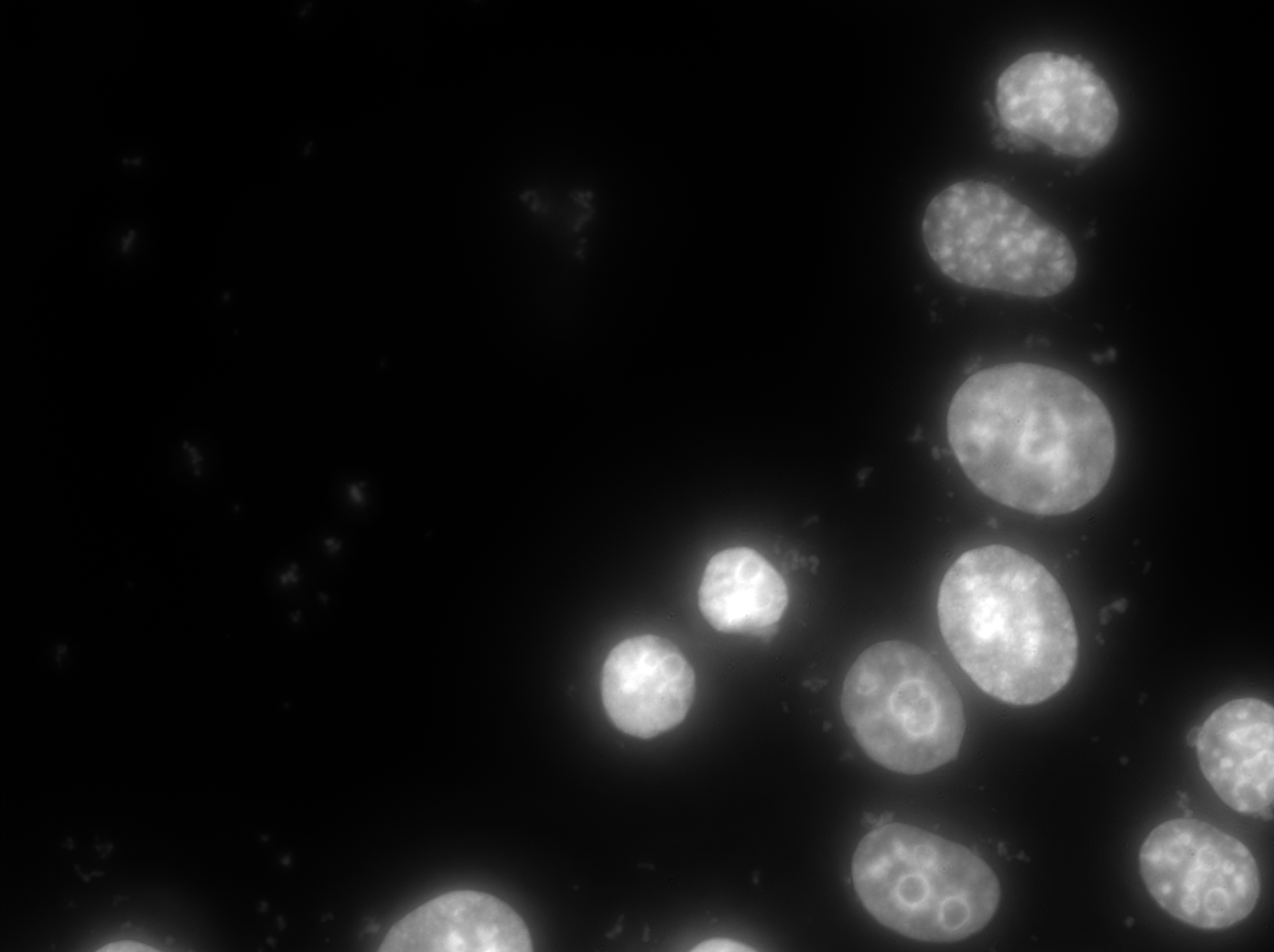

Supplement: Supplementary file 18 — Source data Fig. 5 part 4 [file 44319_2026_742_MOESM18_ESM.zip › Figure 5 Part 4/Fig 5ab WT and KO hela TTLL1-e326g atubulin/EGFP/EGFP-N2 8-23-24 atub R2 LT2.Project Maximum Z_XY1725568039_Z0_T0_C0.tif]

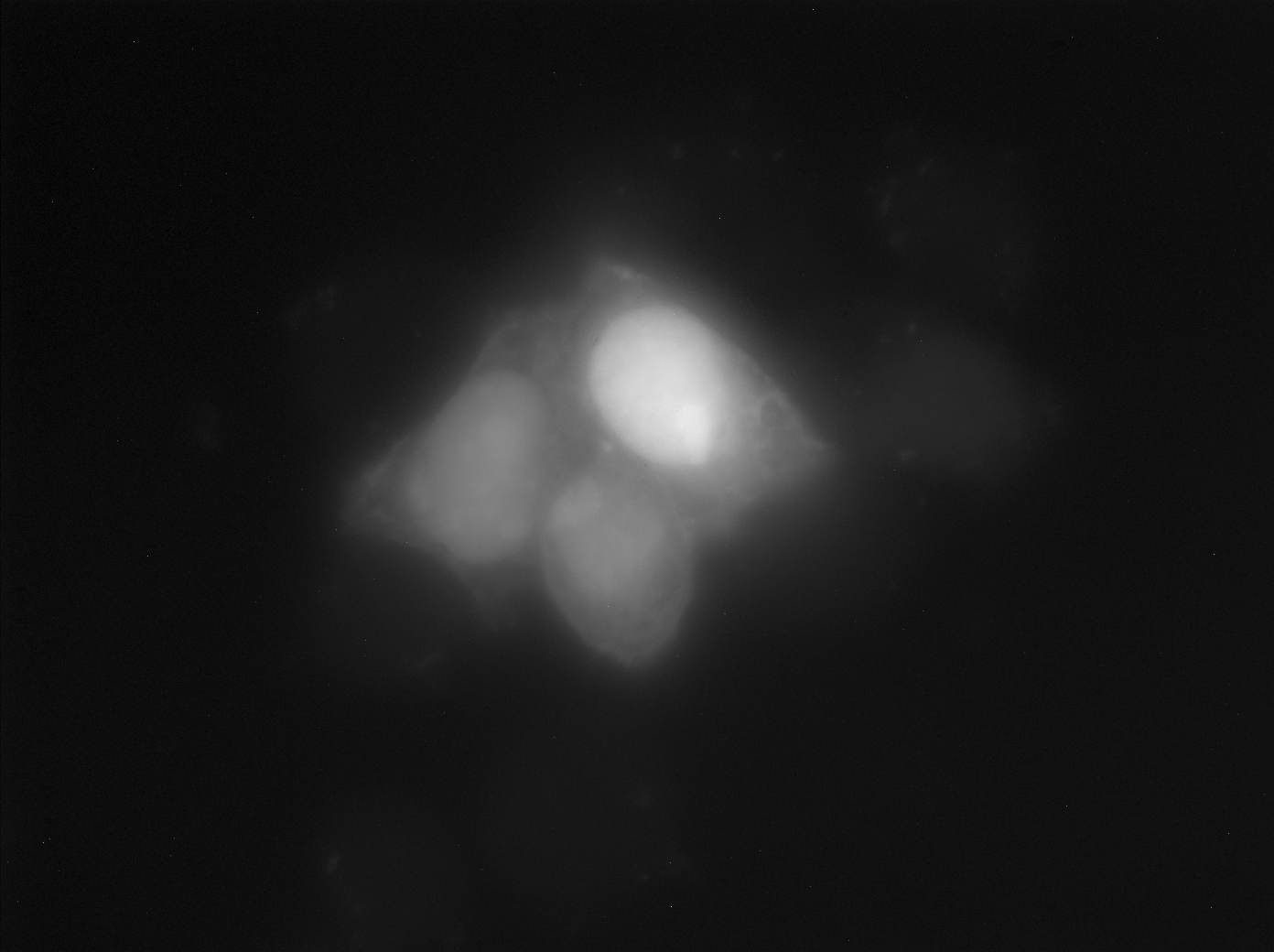

Supplement: Supplementary file 18 — Source data Fig. 5 part 4 [file 44319_2026_742_MOESM18_ESM.zip › Figure 5 Part 4/Fig 5ab WT and KO hela TTLL1-e326g atubulin/EGFP/EGFP-N3 atub R1 LT4.Project Maximum Z_XY1724713880_Z0_T0_C1.tif]

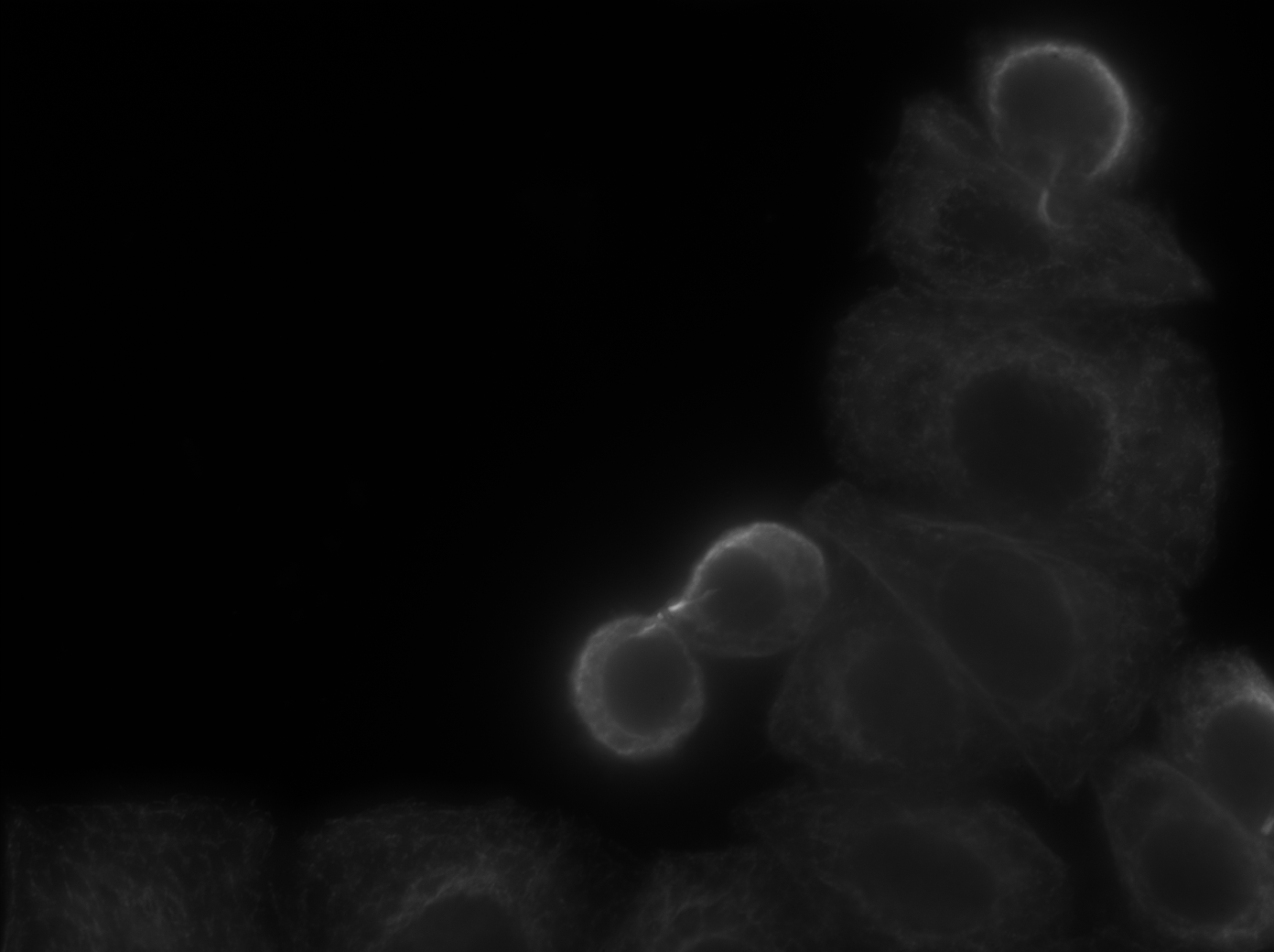

Supplement: Supplementary file 18 — Source data Fig. 5 part 4 [file 44319_2026_742_MOESM18_ESM.zip › Figure 5 Part 4/Fig 5ab WT and KO hela TTLL1-e326g atubulin/EGFP/EGFP-N2 8-23-24 atub R2 LT2.Project Maximum Z_XY1725568039_Z0_T0_C2.tif]

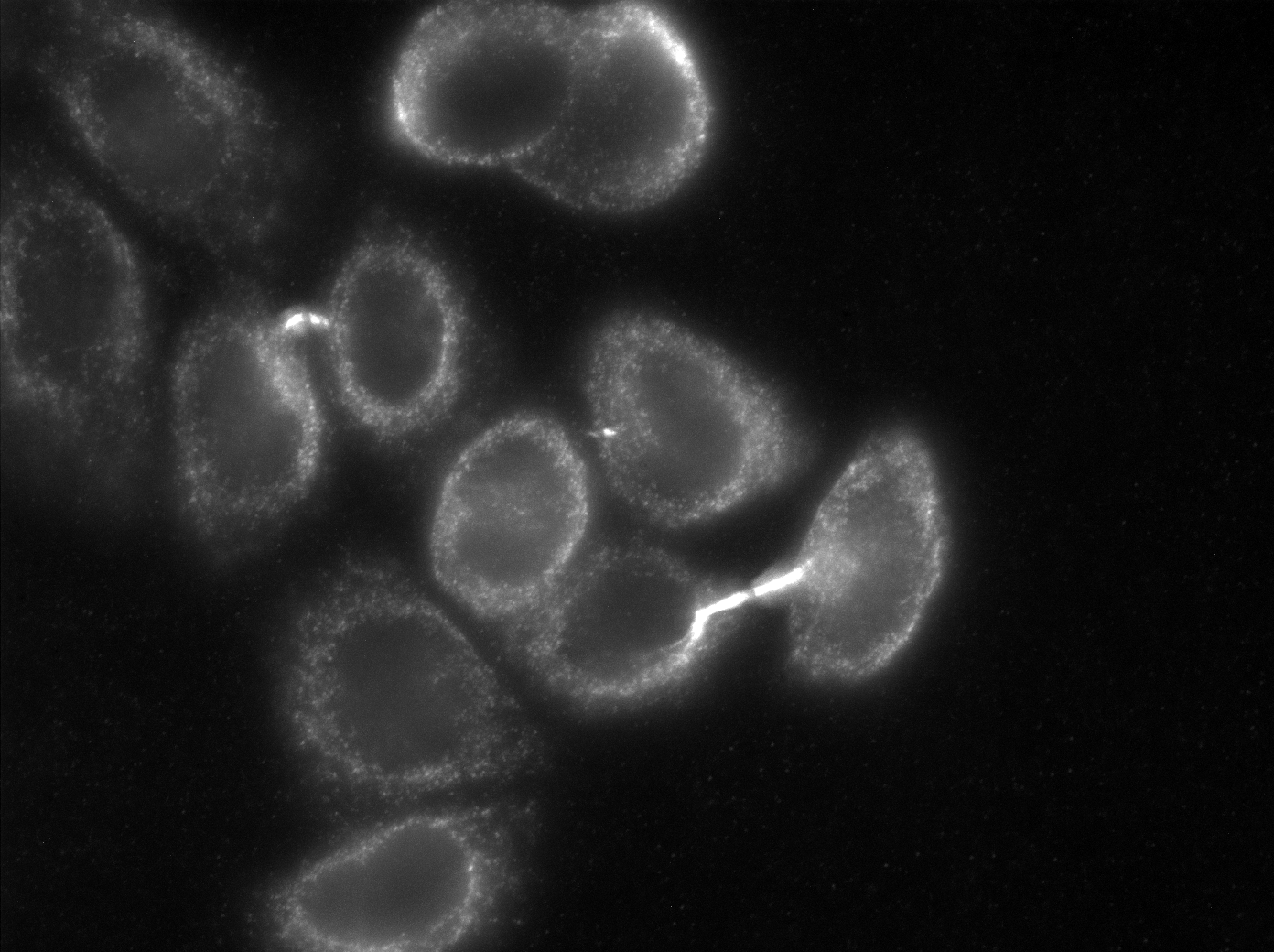

Supplement: Supplementary file 18 — Source data Fig. 5 part 4 [file 44319_2026_742_MOESM18_ESM.zip › Figure 5 Part 4/Fig 5ab WT and KO hela TTLL1-e326g atubulin/EGFP/Cas9 EGFP-N3 10-15-24 R3 LT10.Project Maximum Z_XY1729031131_Z0_T0_C2.tif]

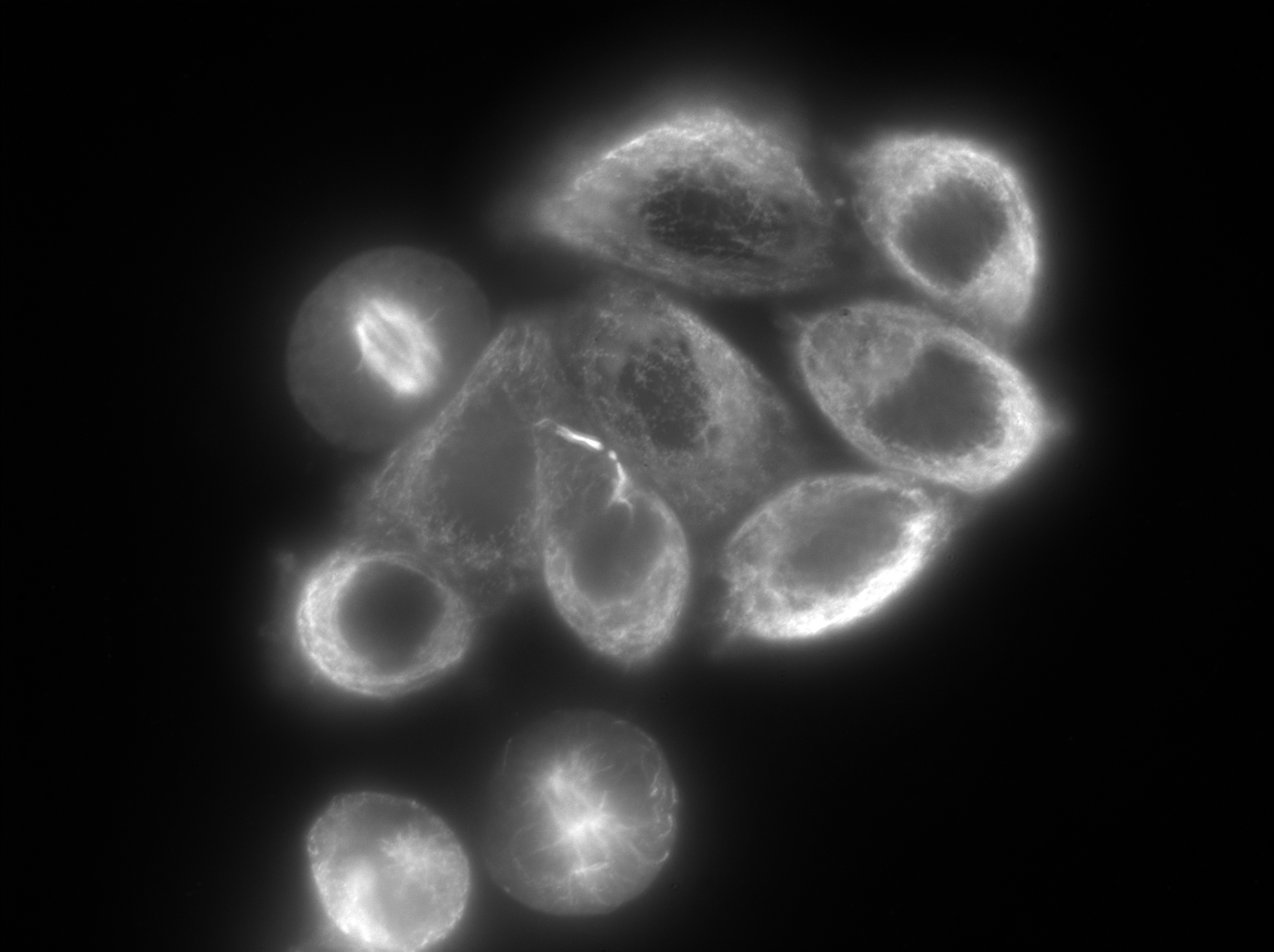

Supplement: Supplementary file 18 — Source data Fig. 5 part 4 [file 44319_2026_742_MOESM18_ESM.zip › Figure 5 Part 4/Fig 5ab WT and KO hela TTLL1-e326g atubulin/EGFP/EGFP-N3 atub R1 LT4.Project Maximum Z_XY1724713880_Z0_T0_C2.tif]

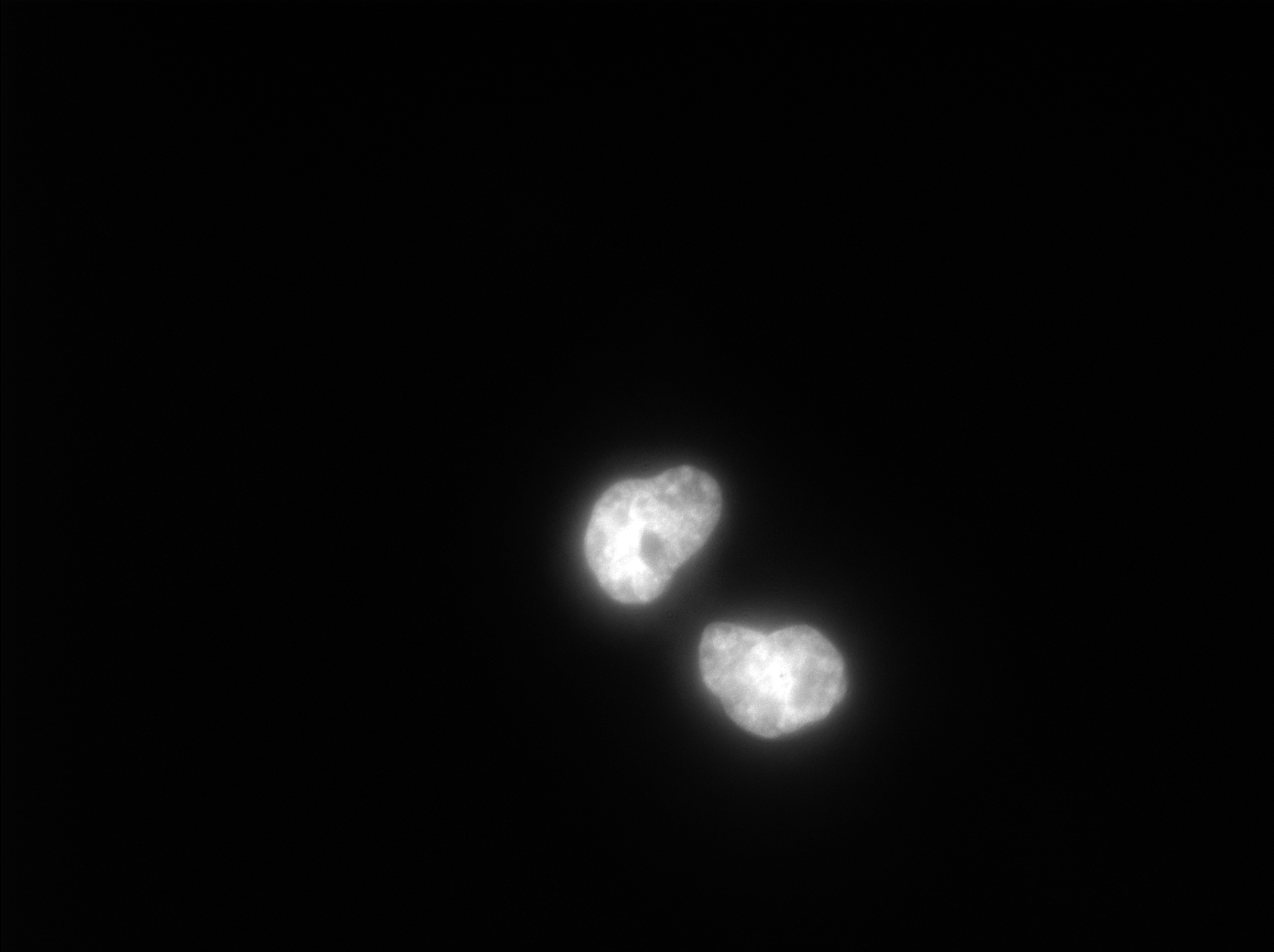

Supplement: Supplementary file 18 — Source data Fig. 5 part 4 [file 44319_2026_742_MOESM18_ESM.zip › Figure 5 Part 4/Fig 5ab WT and KO hela TTLL1-e326g atubulin/EGFP/Cas9 EGFP-N3 10-15-24 R3 LT3.Project Maximum Z_XY1729029741_Z0_T0_C0.tif]

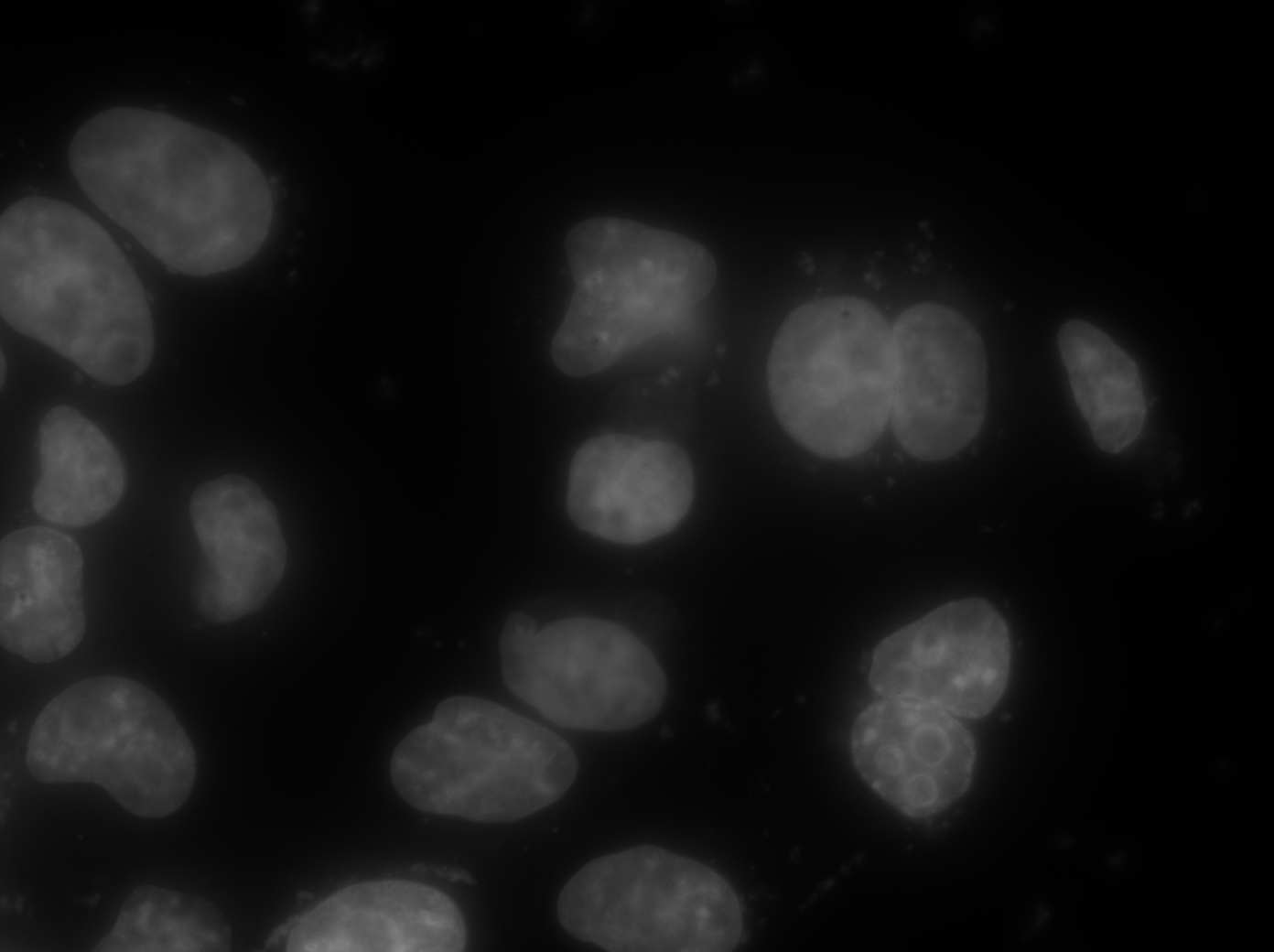

Supplement: Supplementary file 18 — Source data Fig. 5 part 4 [file 44319_2026_742_MOESM18_ESM.zip › Figure 5 Part 4/Fig 5ab WT and KO hela TTLL1-e326g atubulin/EGFP/EGFP-N3 atub R1 LT3.Project Maximum Z_XY1724713596_Z0_T0_C0.tif]

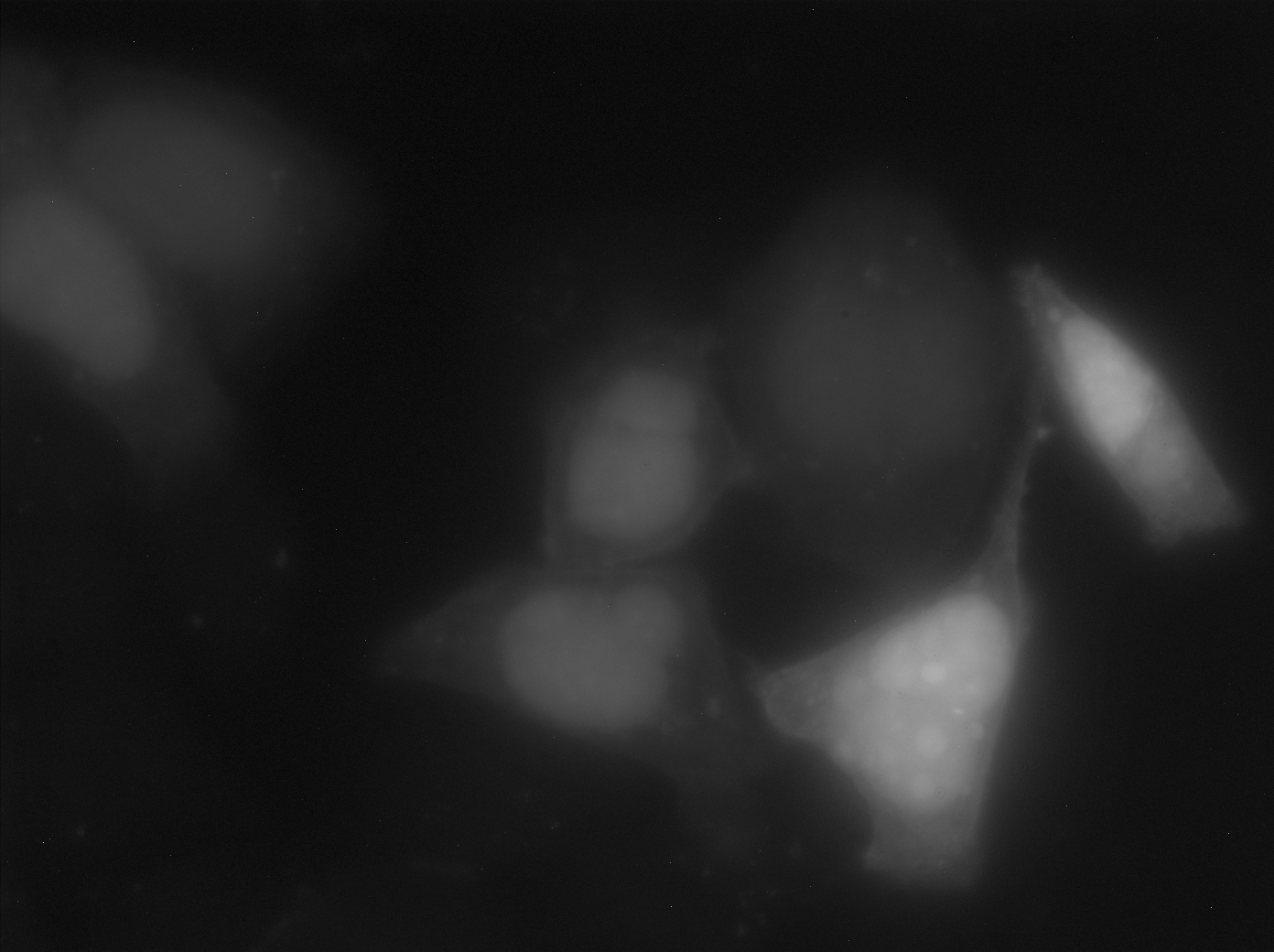

Supplement: Supplementary file 18 — Source data Fig. 5 part 4 [file 44319_2026_742_MOESM18_ESM.zip › Figure 5 Part 4/Fig 5ab WT and KO hela TTLL1-e326g atubulin/EGFP/EGFP-N3 atub R1 LT3.Project Maximum Z_XY1724713596_Z0_T0_C1.tif]

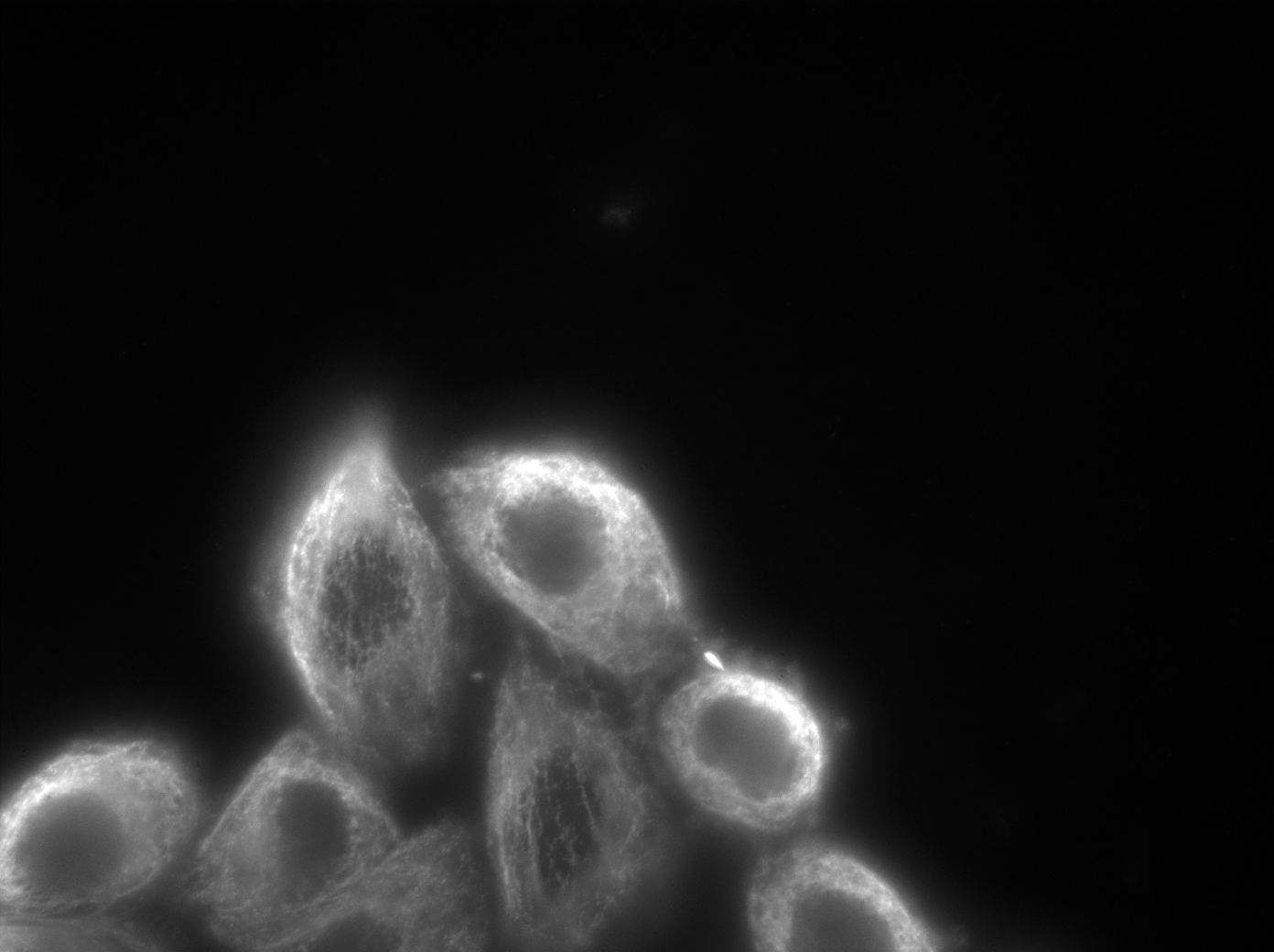

Supplement: Supplementary file 18 — Source data Fig. 5 part 4 [file 44319_2026_742_MOESM18_ESM.zip › Figure 5 Part 4/Fig 5ab WT and KO hela TTLL1-e326g atubulin/EGFP/EGFP-N3 atub R1 LT10.Project Maximum Z_XY1724714918_Z0_T0_C2.tif]

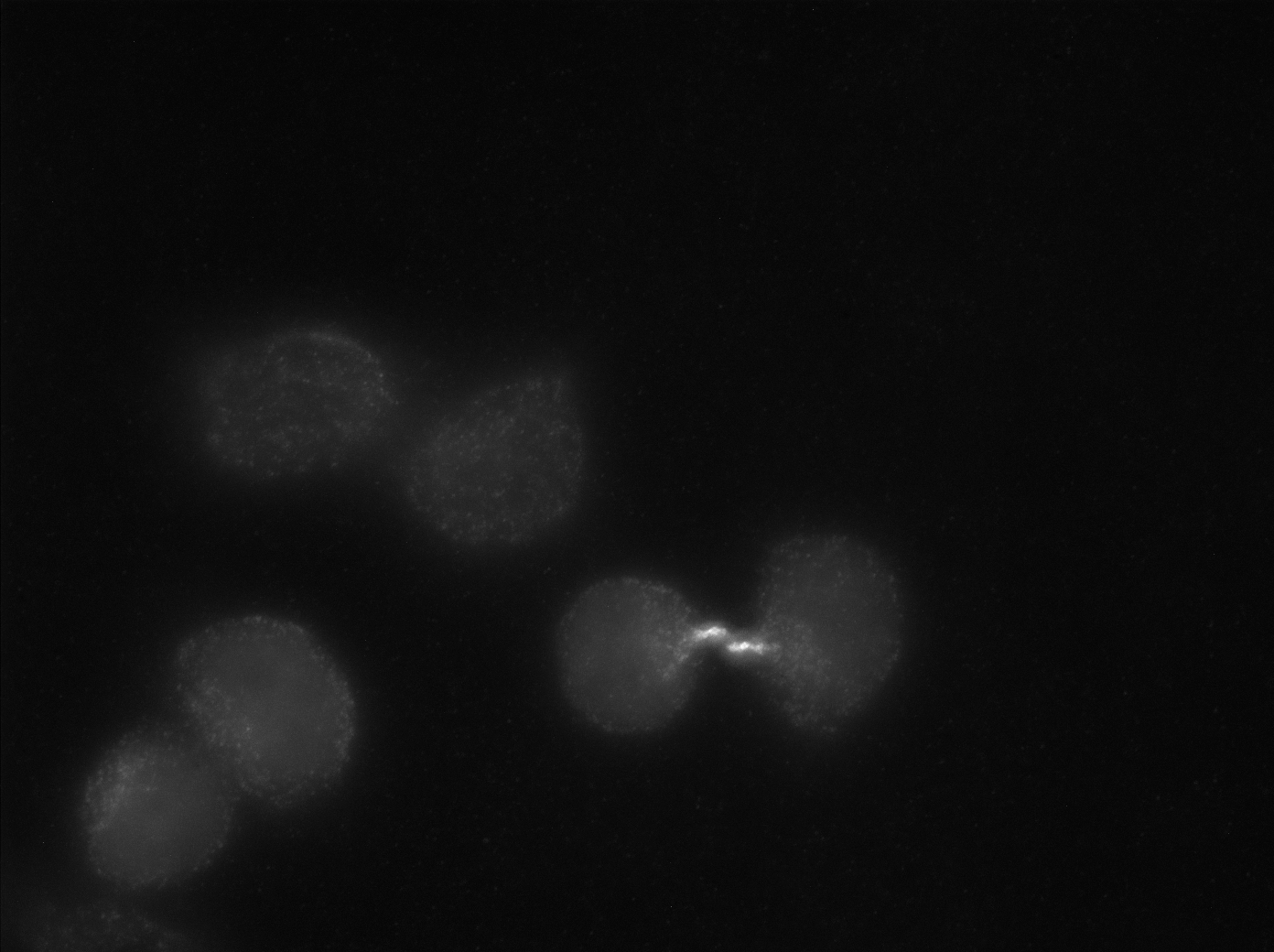

Supplement: Supplementary file 18 — Source data Fig. 5 part 4 [file 44319_2026_742_MOESM18_ESM.zip › Figure 5 Part 4/Fig 5ab WT and KO hela TTLL1-e326g atubulin/EGFP/Cas9 EGFP-N3 10-15-24 R3 LT9.Project Maximum Z_XY1729030913_Z0_T0_C2.tif]

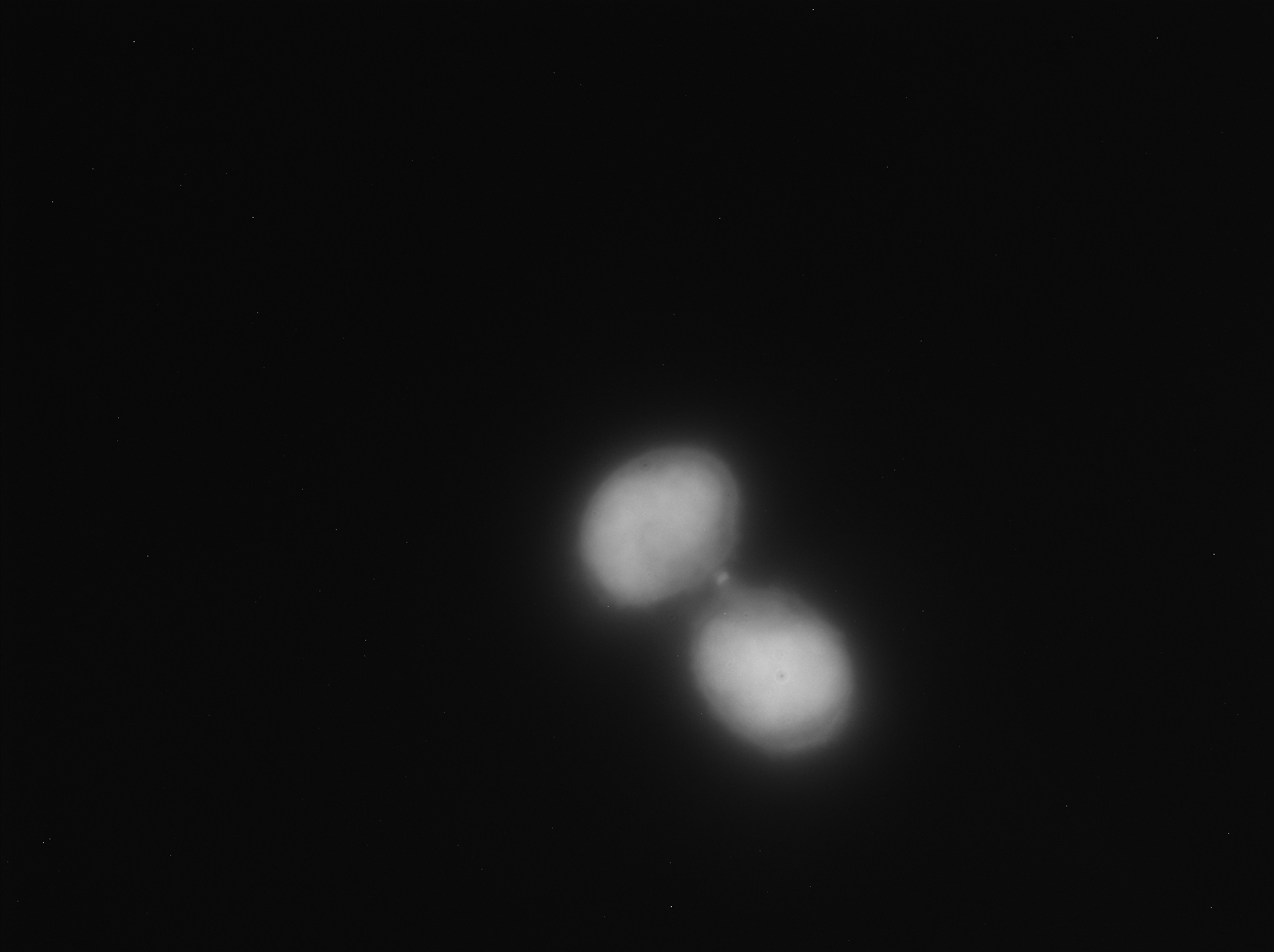

Supplement: Supplementary file 18 — Source data Fig. 5 part 4 [file 44319_2026_742_MOESM18_ESM.zip › Figure 5 Part 4/Fig 5ab WT and KO hela TTLL1-e326g atubulin/EGFP/Cas9 EGFP-N3 10-15-24 R3 LT3.Project Maximum Z_XY1729029741_Z0_T0_C1.tif]

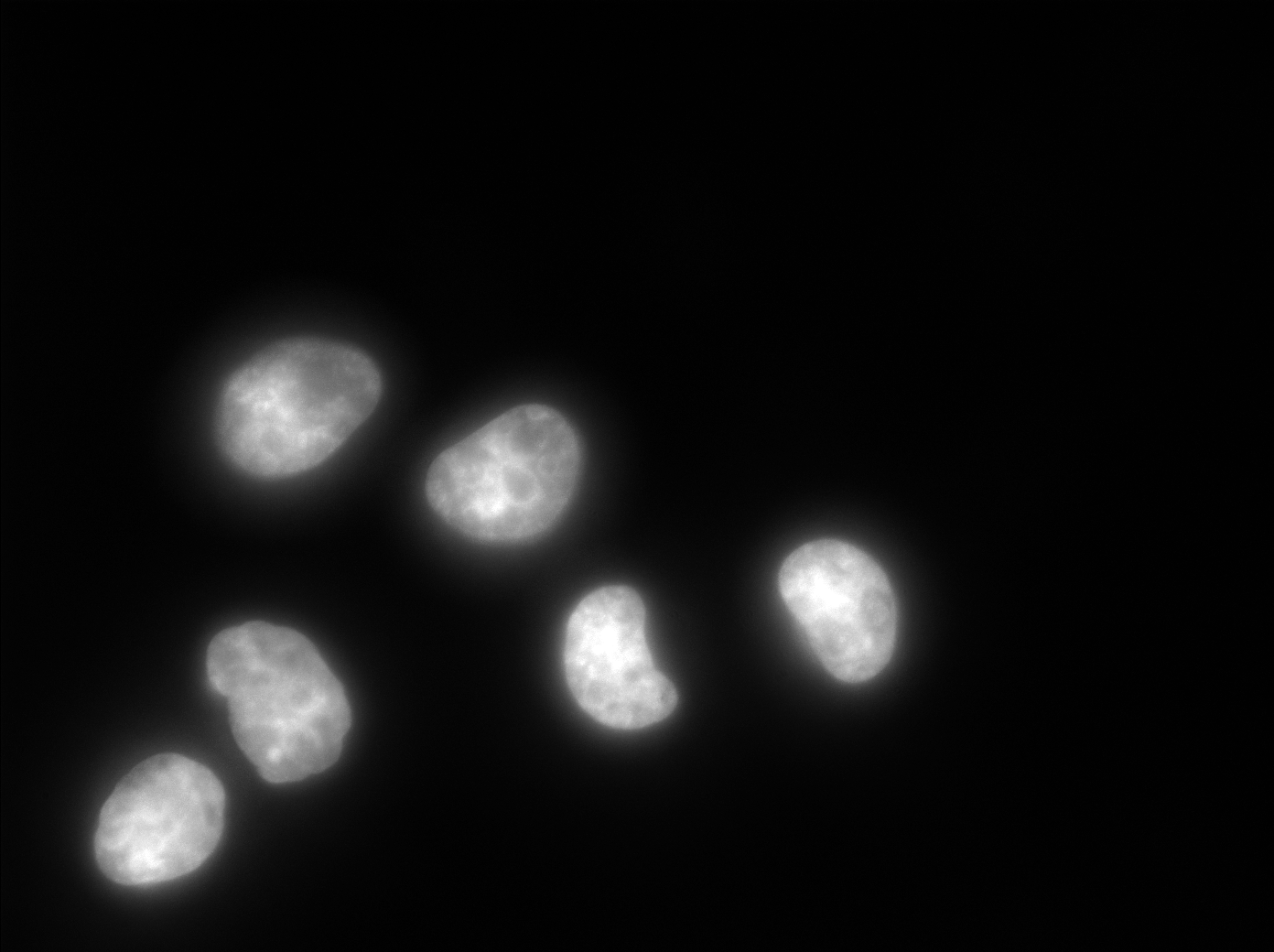

Supplement: Supplementary file 18 — Source data Fig. 5 part 4 [file 44319_2026_742_MOESM18_ESM.zip › Figure 5 Part 4/Fig 5ab WT and KO hela TTLL1-e326g atubulin/EGFP/Cas9 EGFP-N3 10-15-24 R3 LT9.Project Maximum Z_XY1729030913_Z0_T0_C0.tif]

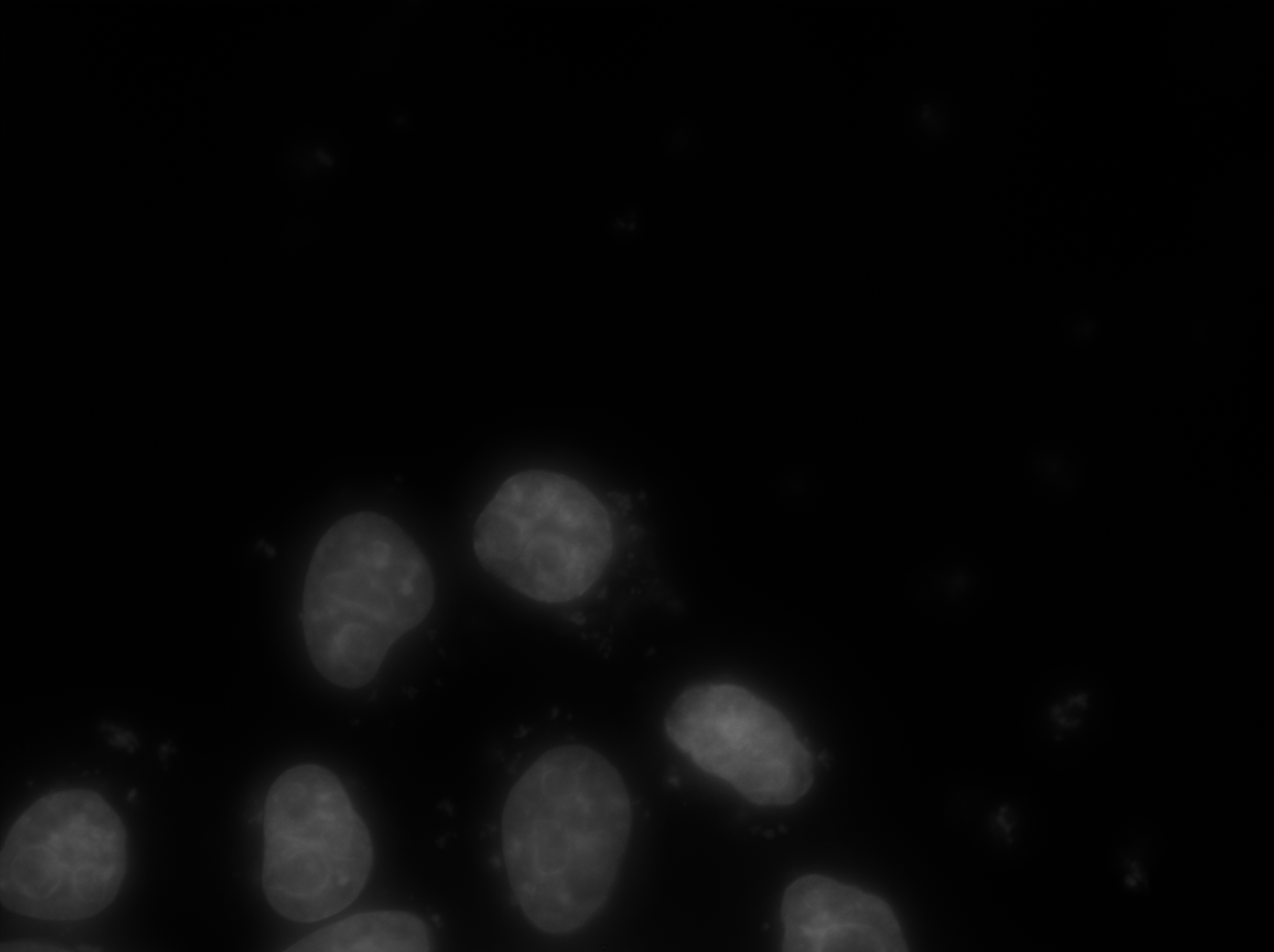

Supplement: Supplementary file 18 — Source data Fig. 5 part 4 [file 44319_2026_742_MOESM18_ESM.zip › Figure 5 Part 4/Fig 5ab WT and KO hela TTLL1-e326g atubulin/EGFP/EGFP-N3 atub R1 LT10.Project Maximum Z_XY1724714918_Z0_T0_C0.tif]

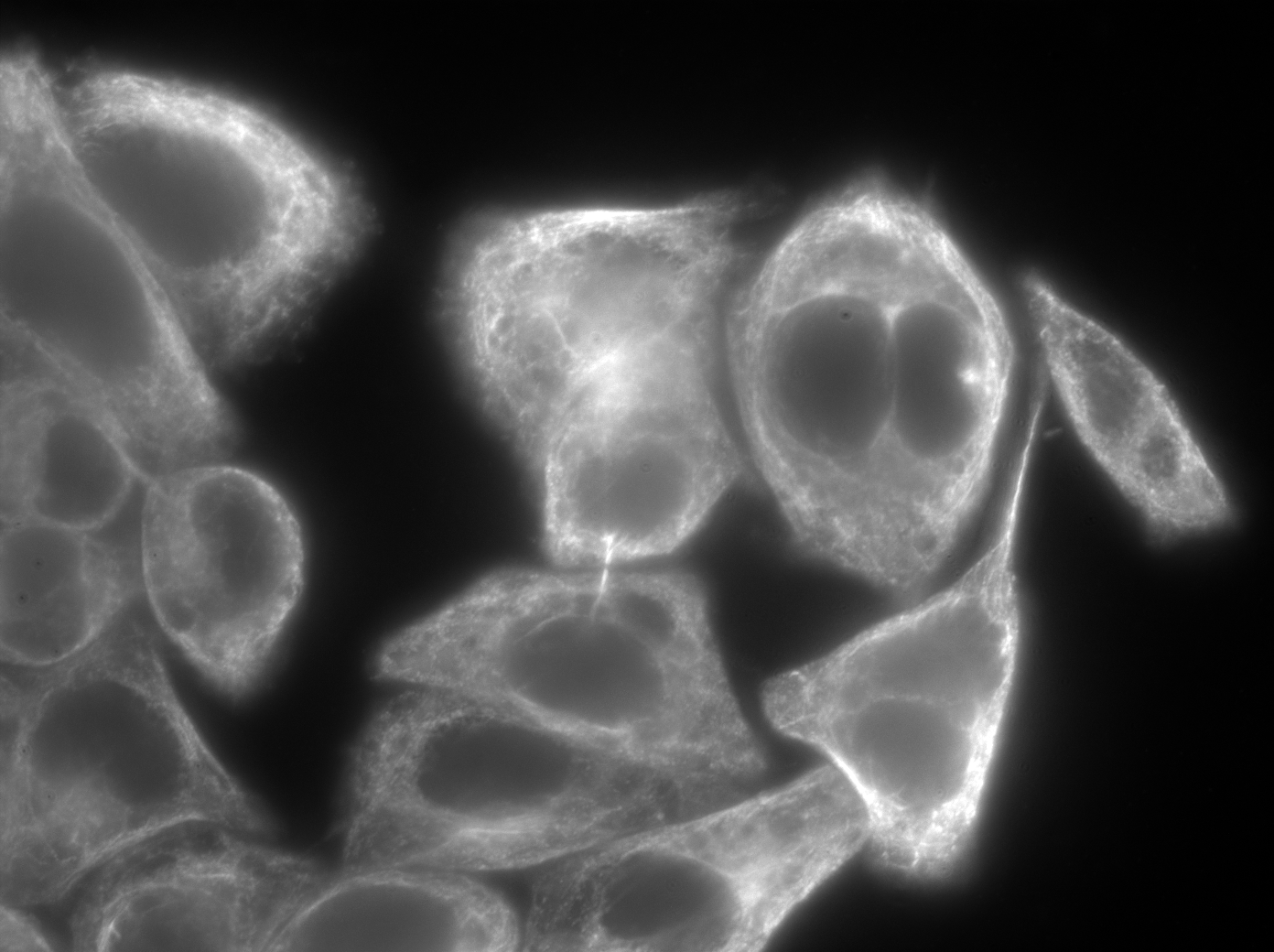

Supplement: Supplementary file 18 — Source data Fig. 5 part 4 [file 44319_2026_742_MOESM18_ESM.zip › Figure 5 Part 4/Fig 5ab WT and KO hela TTLL1-e326g atubulin/EGFP/EGFP-N3 atub R1 LT3.Project Maximum Z_XY1724713596_Z0_T0_C2.tif]

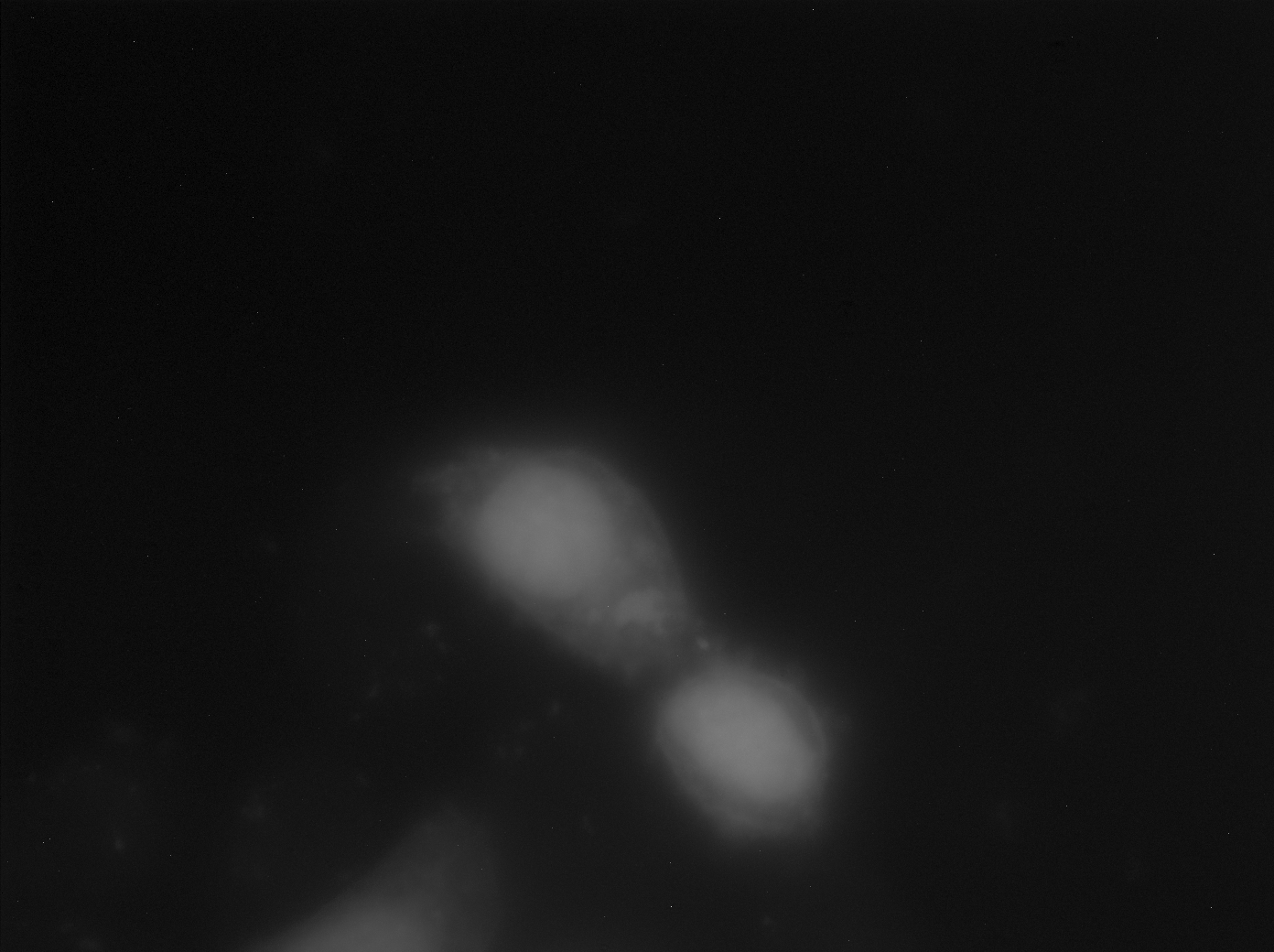

Supplement: Supplementary file 18 — Source data Fig. 5 part 4 [file 44319_2026_742_MOESM18_ESM.zip › Figure 5 Part 4/Fig 5ab WT and KO hela TTLL1-e326g atubulin/EGFP/EGFP-N3 atub R1 LT10.Project Maximum Z_XY1724714918_Z0_T0_C1.tif]

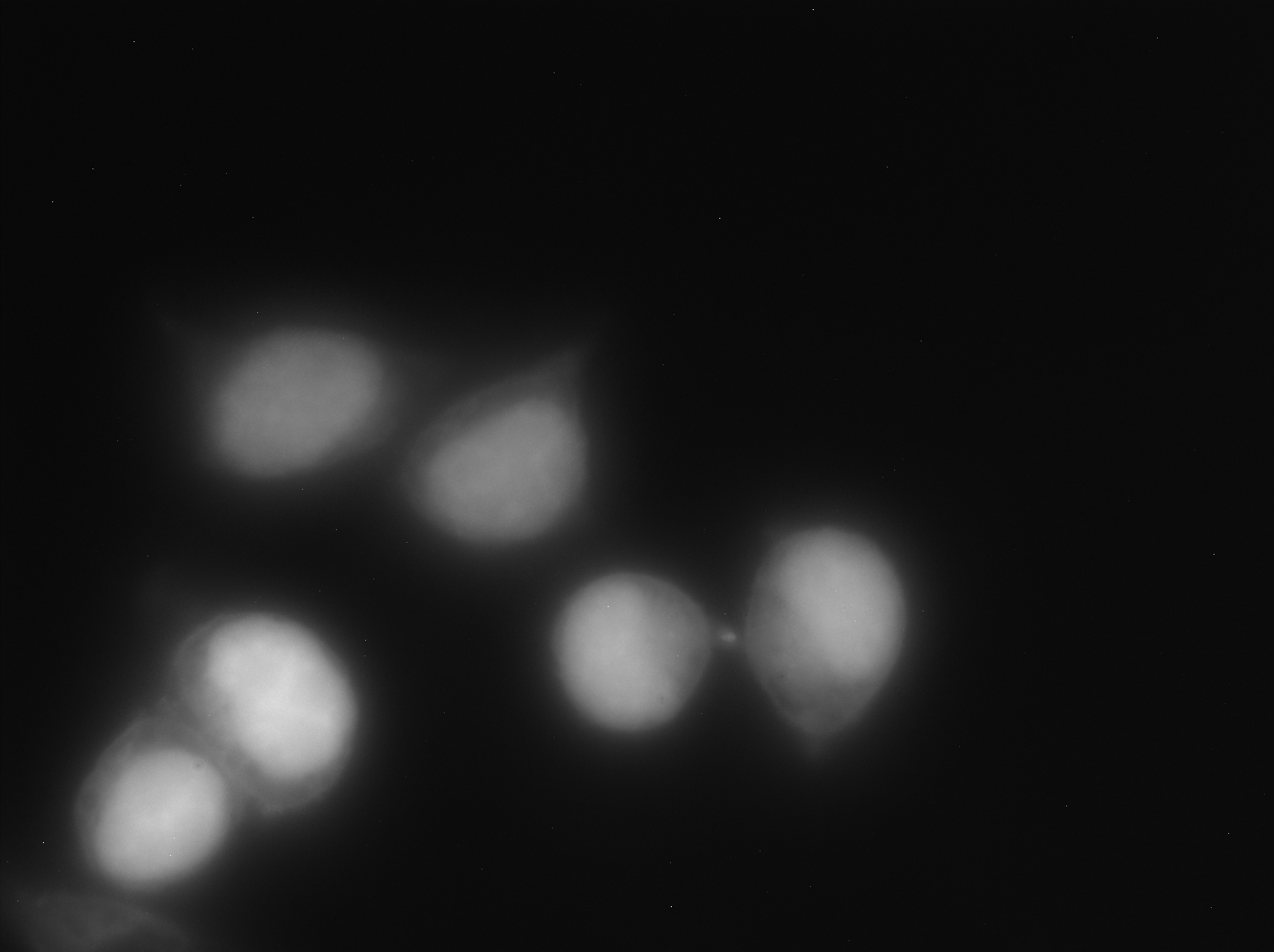

Supplement: Supplementary file 18 — Source data Fig. 5 part 4 [file 44319_2026_742_MOESM18_ESM.zip › Figure 5 Part 4/Fig 5ab WT and KO hela TTLL1-e326g atubulin/EGFP/Cas9 EGFP-N3 10-15-24 R3 LT9.Project Maximum Z_XY1729030913_Z0_T0_C1.tif]

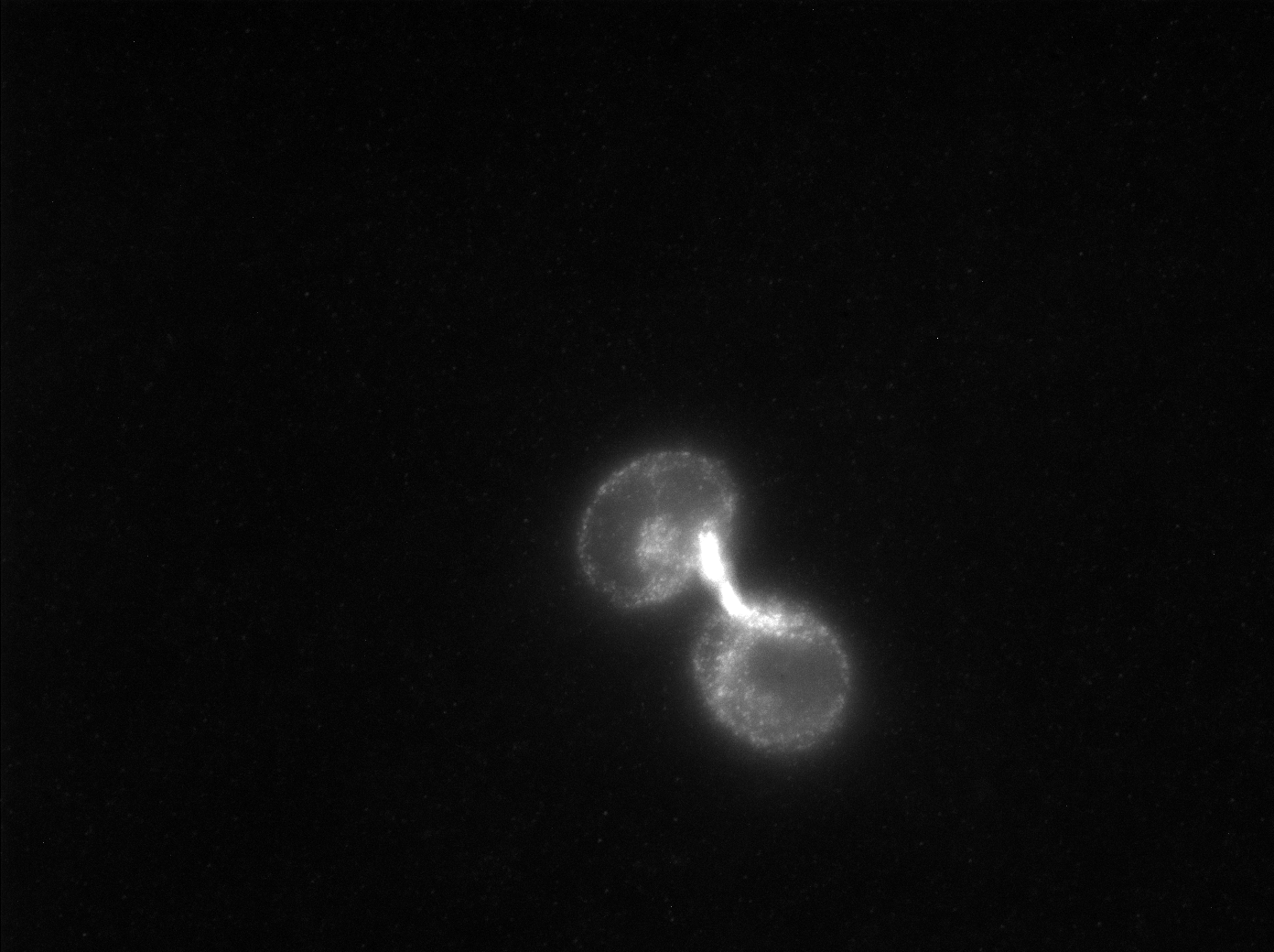

Supplement: Supplementary file 18 — Source data Fig. 5 part 4 [file 44319_2026_742_MOESM18_ESM.zip › Figure 5 Part 4/Fig 5ab WT and KO hela TTLL1-e326g atubulin/EGFP/Cas9 EGFP-N3 10-15-24 R3 LT3.Project Maximum Z_XY1729029741_Z0_T0_C2.tif]

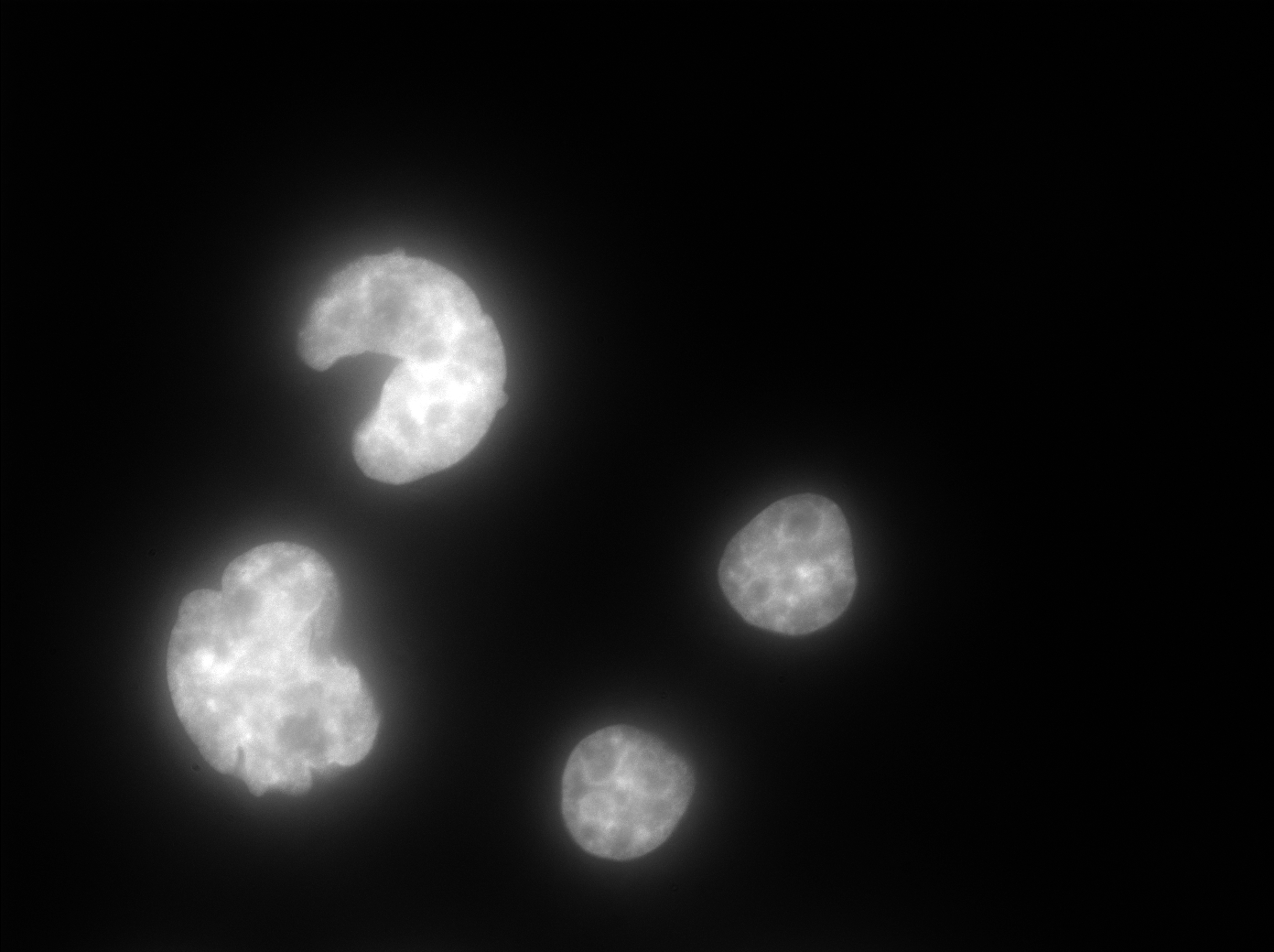

Supplement: Supplementary file 18 — Source data Fig. 5 part 4 [file 44319_2026_742_MOESM18_ESM.zip › Figure 5 Part 4/Fig 5ab WT and KO hela TTLL1-e326g atubulin/EGFP/Cas9 EGFP-N3 10-15-24 R3 LT4.Project Maximum Z_XY1729029898_Z0_T0_C0.tif]

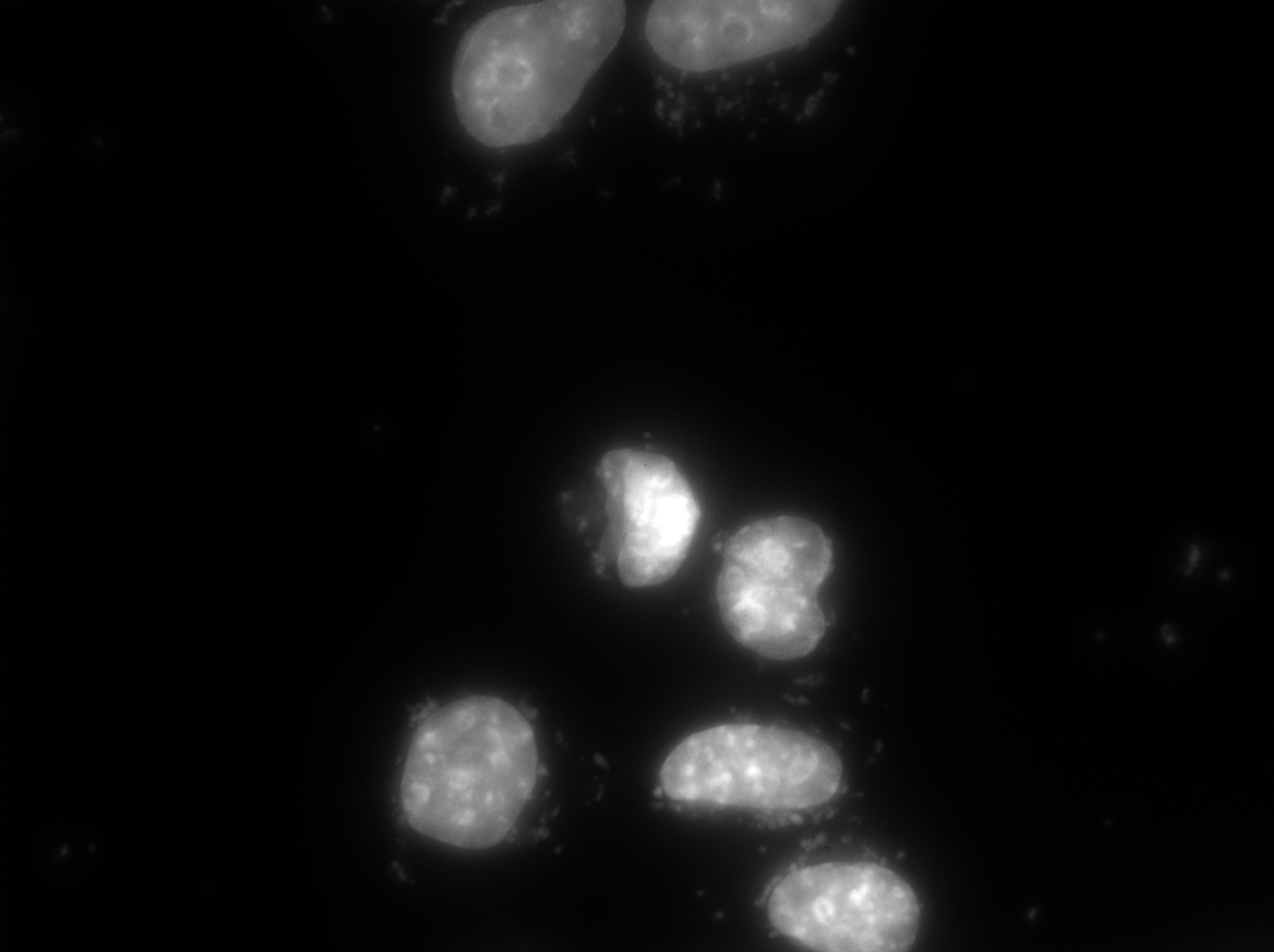

Supplement: Supplementary file 18 — Source data Fig. 5 part 4 [file 44319_2026_742_MOESM18_ESM.zip › Figure 5 Part 4/Fig 5ab WT and KO hela TTLL1-e326g atubulin/EGFP/EGFP-N2 8-23-24 atub R2 LT4.Project Maximum Z_XY1725568598_Z0_T0_C0.tif]

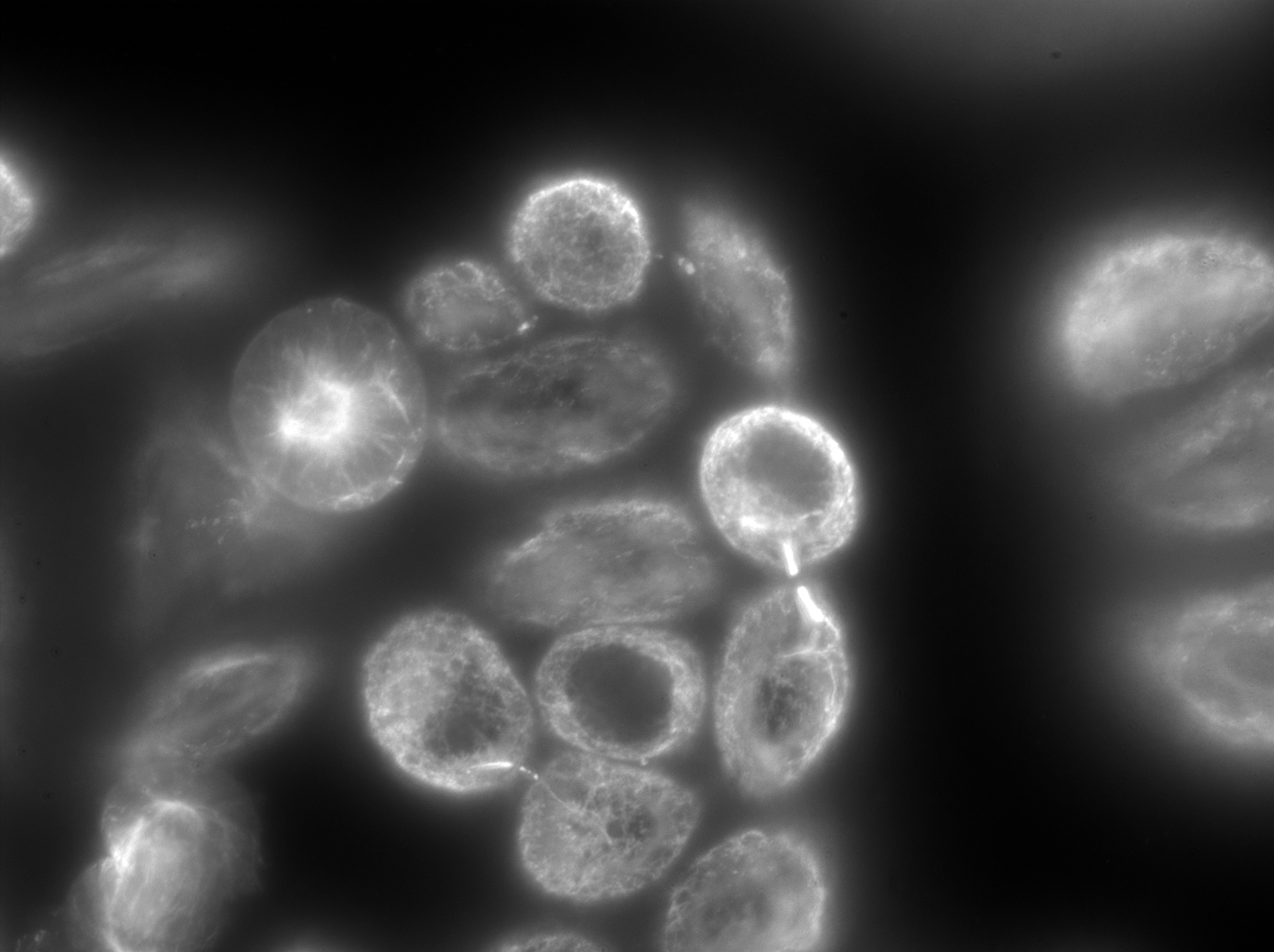

Supplement: Supplementary file 18 — Source data Fig. 5 part 4 [file 44319_2026_742_MOESM18_ESM.zip › Figure 5 Part 4/Fig 5ab WT and KO hela TTLL1-e326g atubulin/EGFP/EGFP-N3 atub R1 LT2.Project Maximum Z_XY1724713435_Z0_T0_C2.tif]

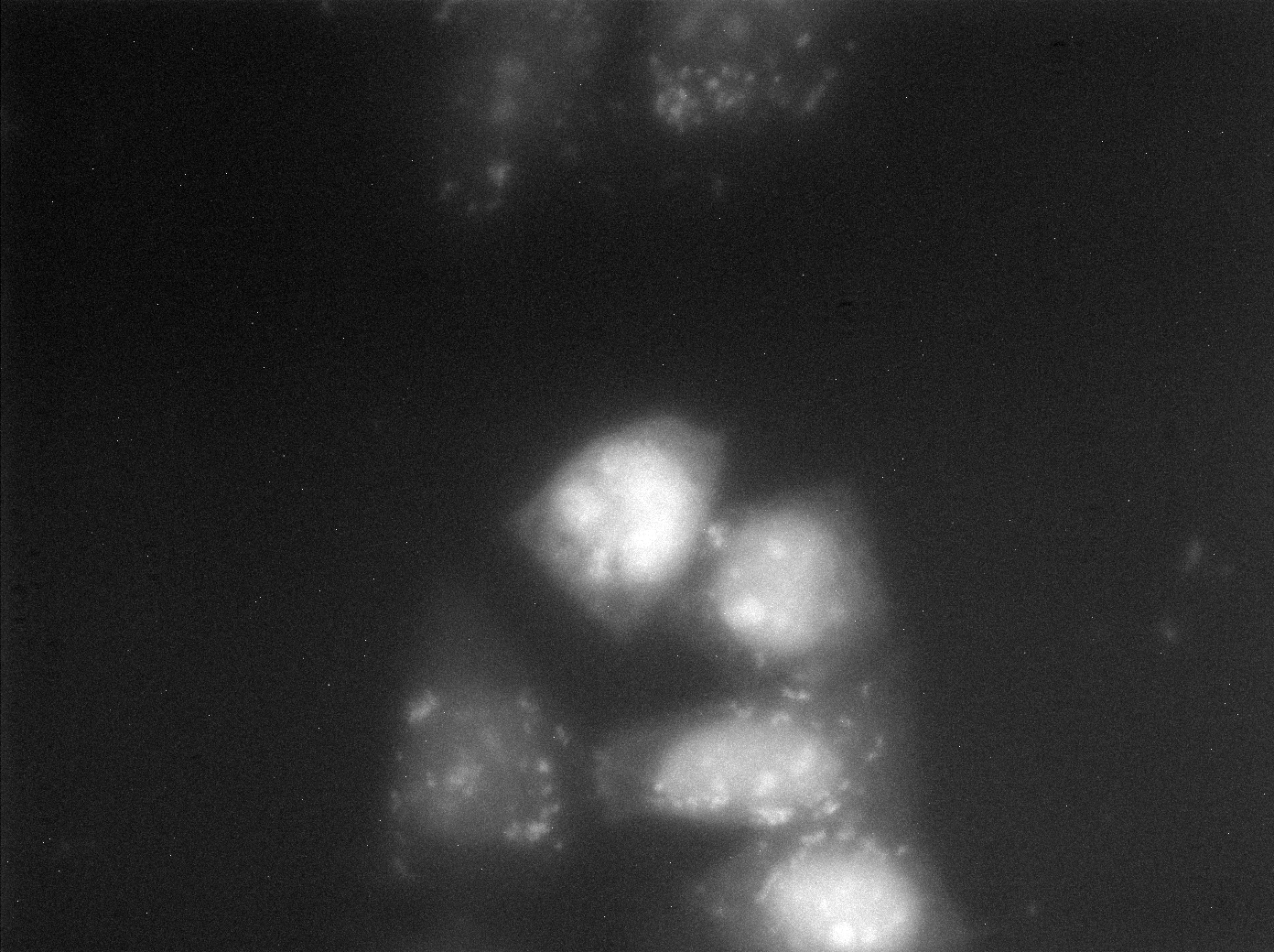

Supplement: Supplementary file 18 — Source data Fig. 5 part 4 [file 44319_2026_742_MOESM18_ESM.zip › Figure 5 Part 4/Fig 5ab WT and KO hela TTLL1-e326g atubulin/EGFP/EGFP-N2 8-23-24 atub R2 LT4.Project Maximum Z_XY1725568598_Z0_T0_C1.tif]

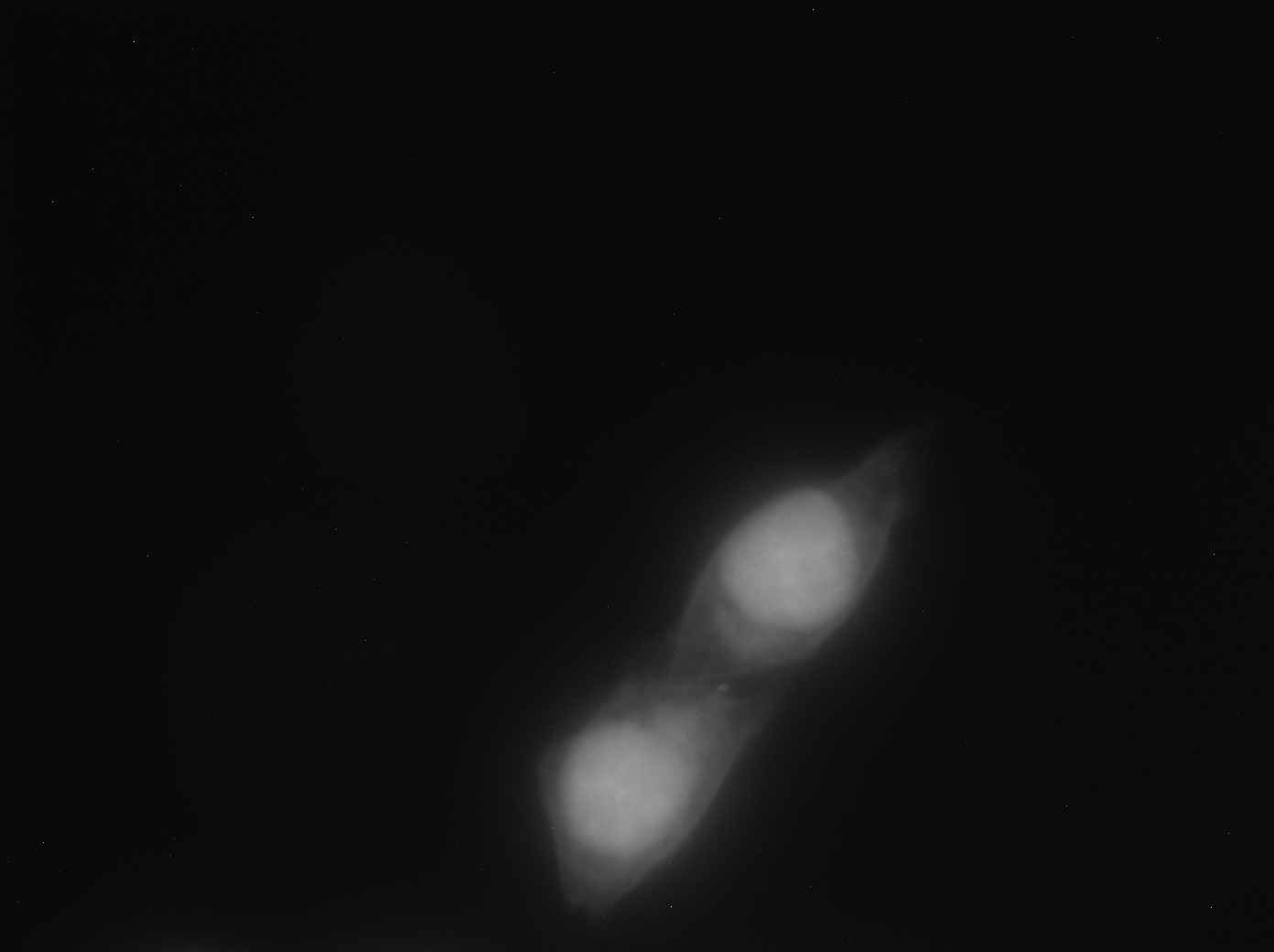

Supplement: Supplementary file 18 — Source data Fig. 5 part 4 [file 44319_2026_742_MOESM18_ESM.zip › Figure 5 Part 4/Fig 5ab WT and KO hela TTLL1-e326g atubulin/EGFP/Cas9 EGFP-N3 10-15-24 R3 LT4.Project Maximum Z_XY1729029898_Z0_T0_C1.tif]

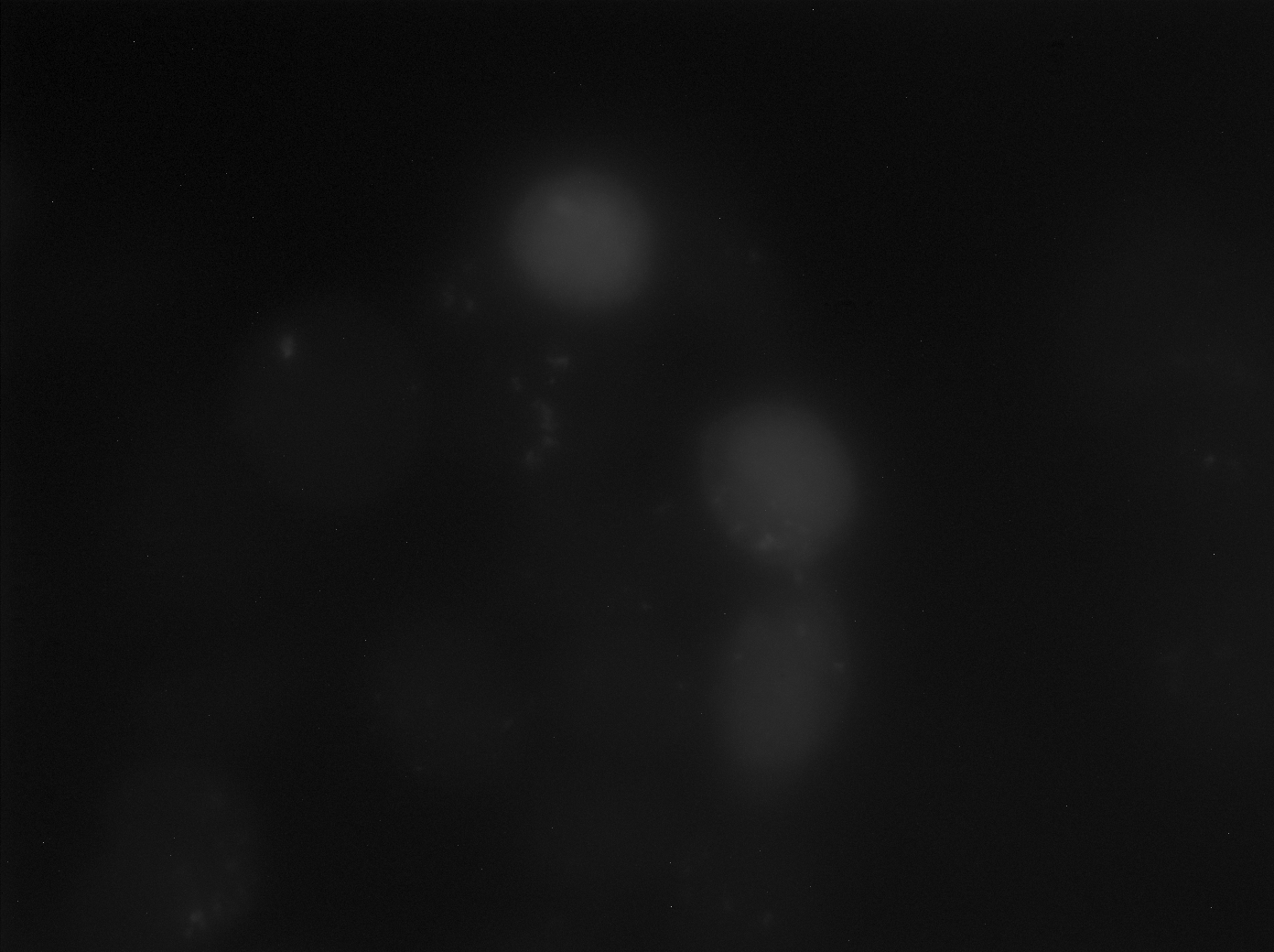

Supplement: Supplementary file 18 — Source data Fig. 5 part 4 [file 44319_2026_742_MOESM18_ESM.zip › Figure 5 Part 4/Fig 5ab WT and KO hela TTLL1-e326g atubulin/EGFP/EGFP-N3 atub R1 LT2.Project Maximum Z_XY1724713435_Z0_T0_C1.tif]

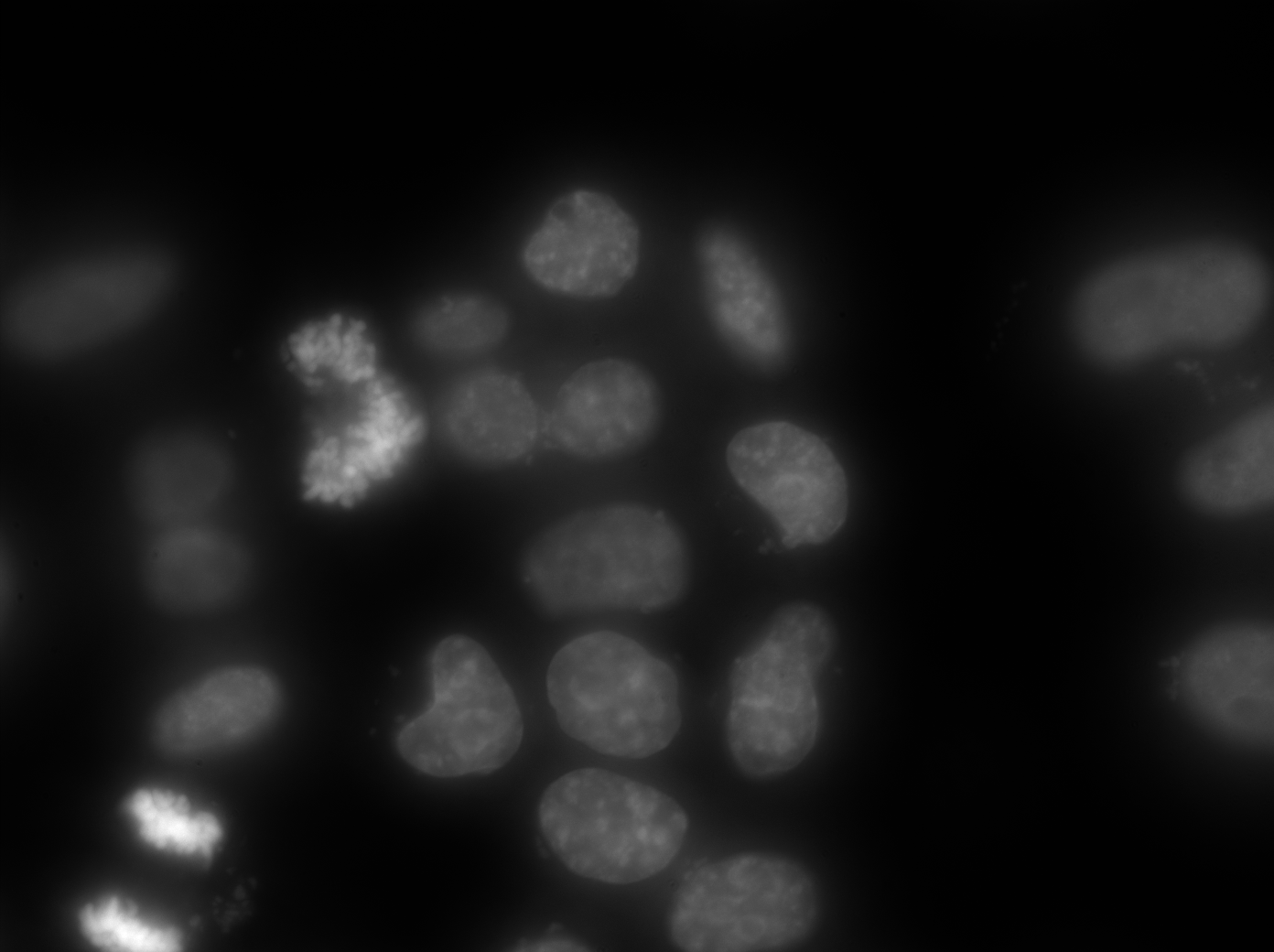

Supplement: Supplementary file 18 — Source data Fig. 5 part 4 [file 44319_2026_742_MOESM18_ESM.zip › Figure 5 Part 4/Fig 5ab WT and KO hela TTLL1-e326g atubulin/EGFP/EGFP-N3 atub R1 LT2.Project Maximum Z_XY1724713435_Z0_T0_C0.tif]

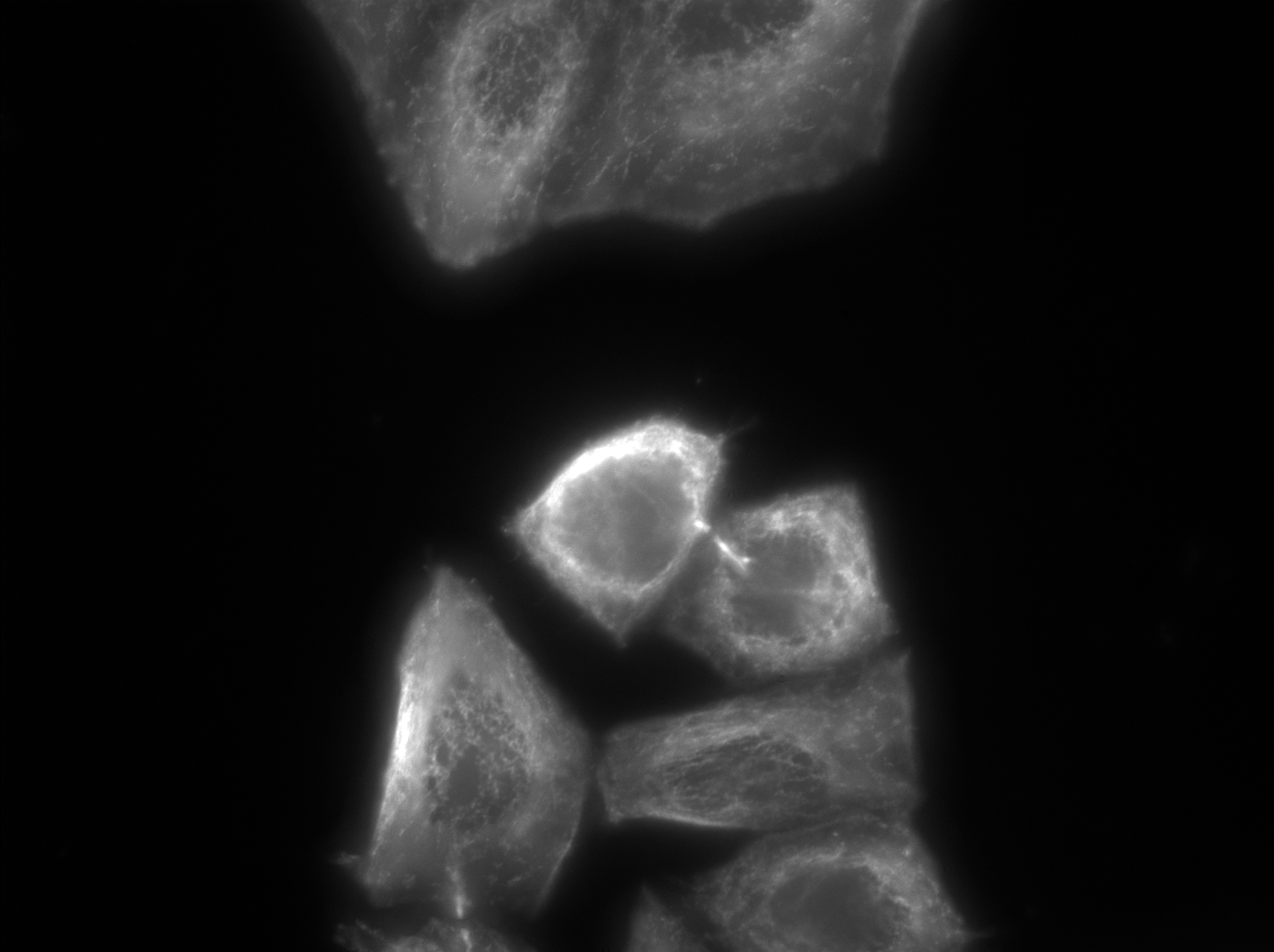

Supplement: Supplementary file 18 — Source data Fig. 5 part 4 [file 44319_2026_742_MOESM18_ESM.zip › Figure 5 Part 4/Fig 5ab WT and KO hela TTLL1-e326g atubulin/EGFP/EGFP-N2 8-23-24 atub R2 LT4.Project Maximum Z_XY1725568598_Z0_T0_C2.tif]

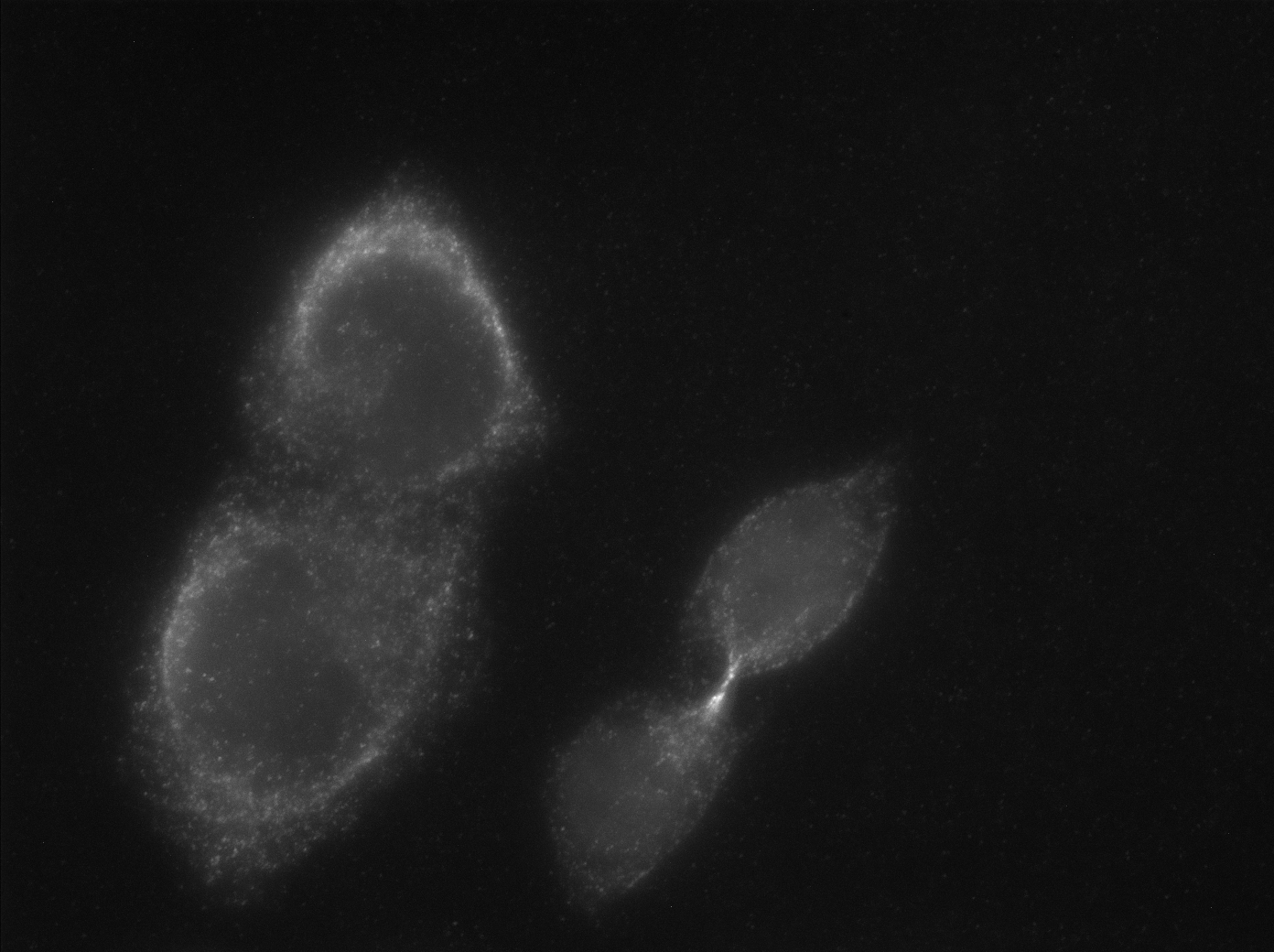

Supplement: Supplementary file 18 — Source data Fig. 5 part 4 [file 44319_2026_742_MOESM18_ESM.zip › Figure 5 Part 4/Fig 5ab WT and KO hela TTLL1-e326g atubulin/EGFP/Cas9 EGFP-N3 10-15-24 R3 LT4.Project Maximum Z_XY1729029898_Z0_T0_C2.tif]

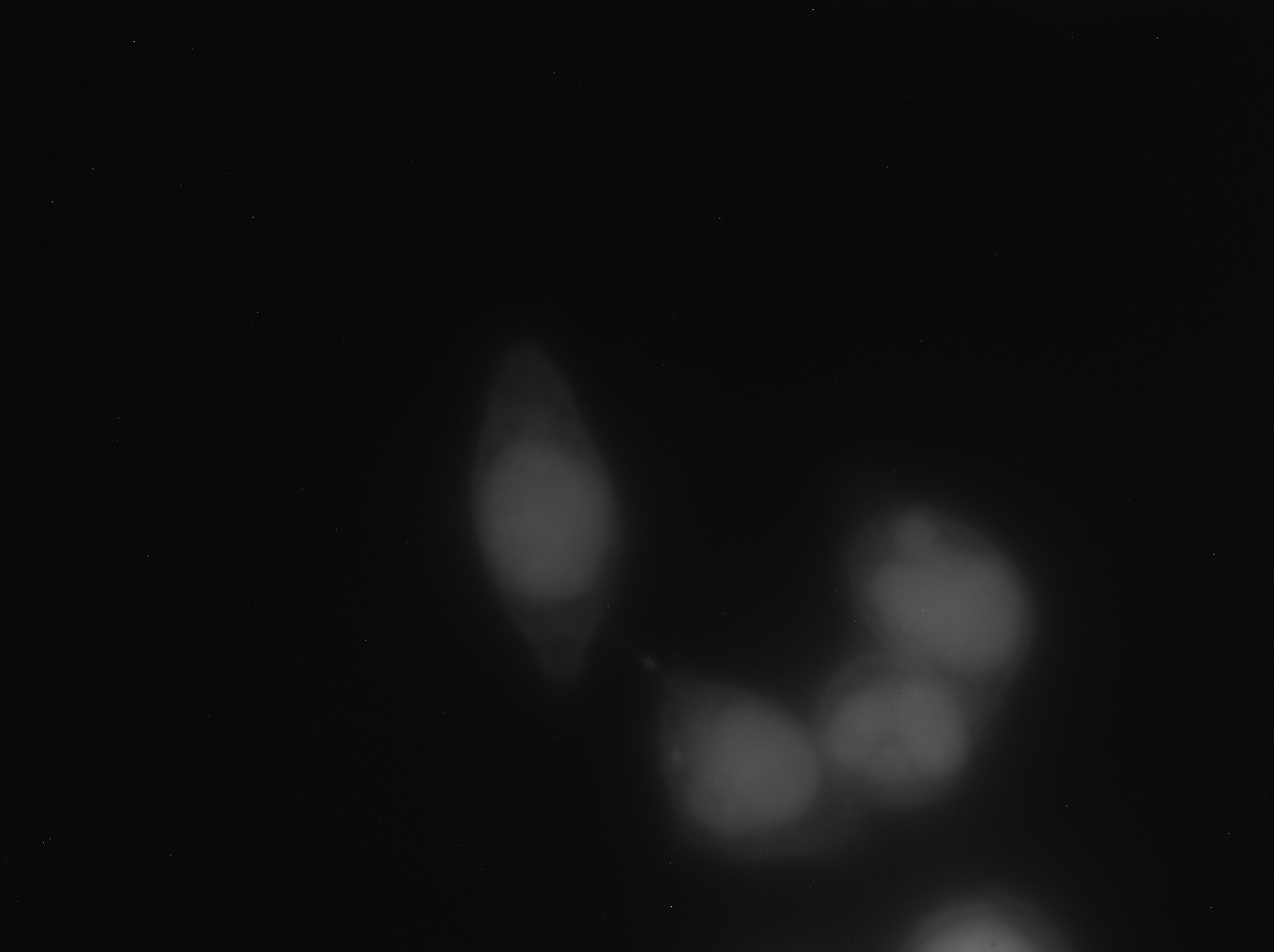

Supplement: Supplementary file 18 — Source data Fig. 5 part 4 [file 44319_2026_742_MOESM18_ESM.zip › Figure 5 Part 4/Fig 5ab WT and KO hela TTLL1-e326g atubulin/EGFP/Cas9 EGFP-N3 10-15-24 R3 LT7.Project Maximum Z_XY1729030371_Z0_T0_C1.tif]

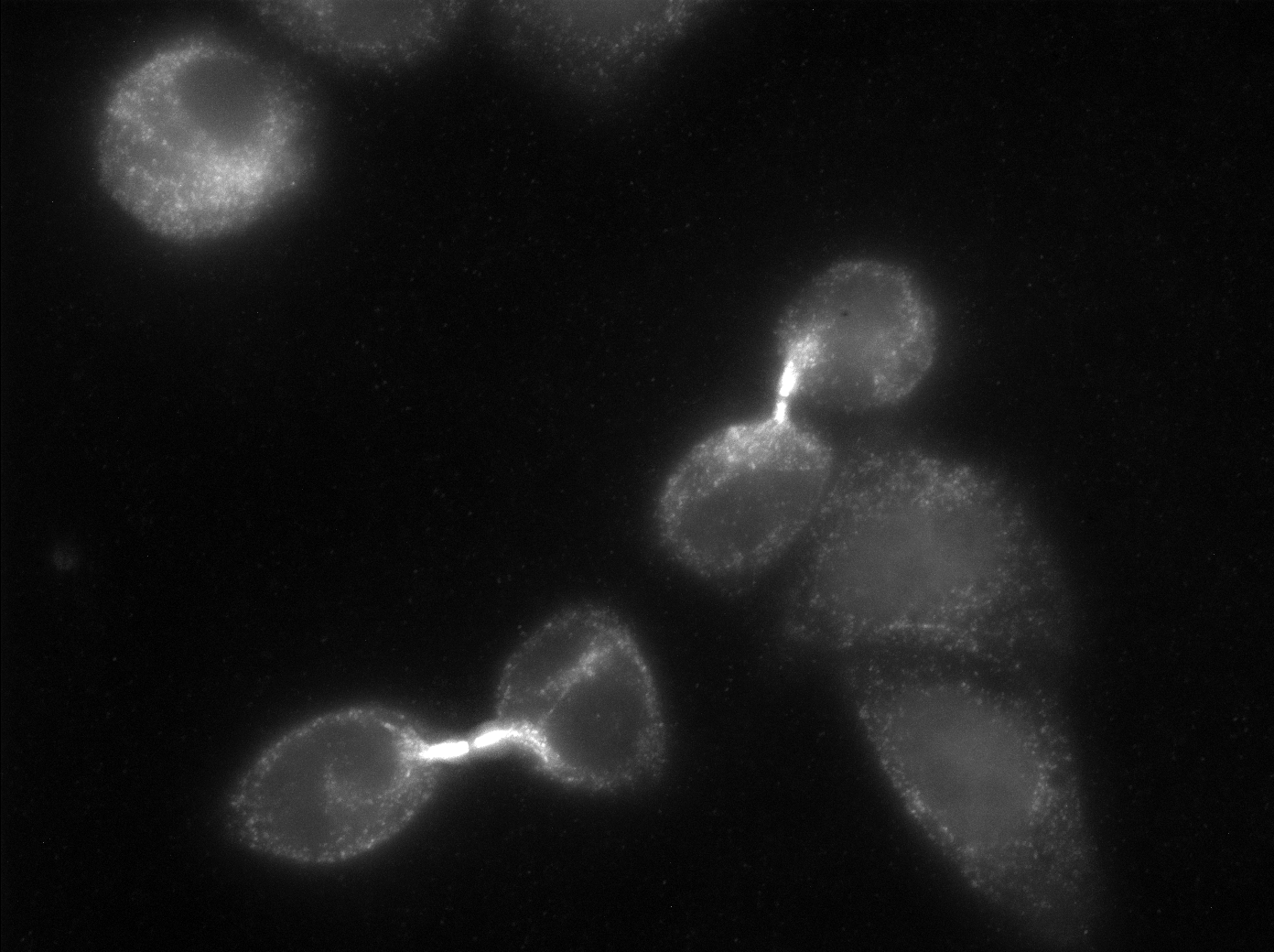

Supplement: Supplementary file 18 — Source data Fig. 5 part 4 [file 44319_2026_742_MOESM18_ESM.zip › Figure 5 Part 4/Fig 5ab WT and KO hela TTLL1-e326g atubulin/EGFP/Cas9 EGFP-N3 10-15-24 R3 LT5LT6.Project Maximum Z_XY1729030238_Z0_T0_C2.tif]

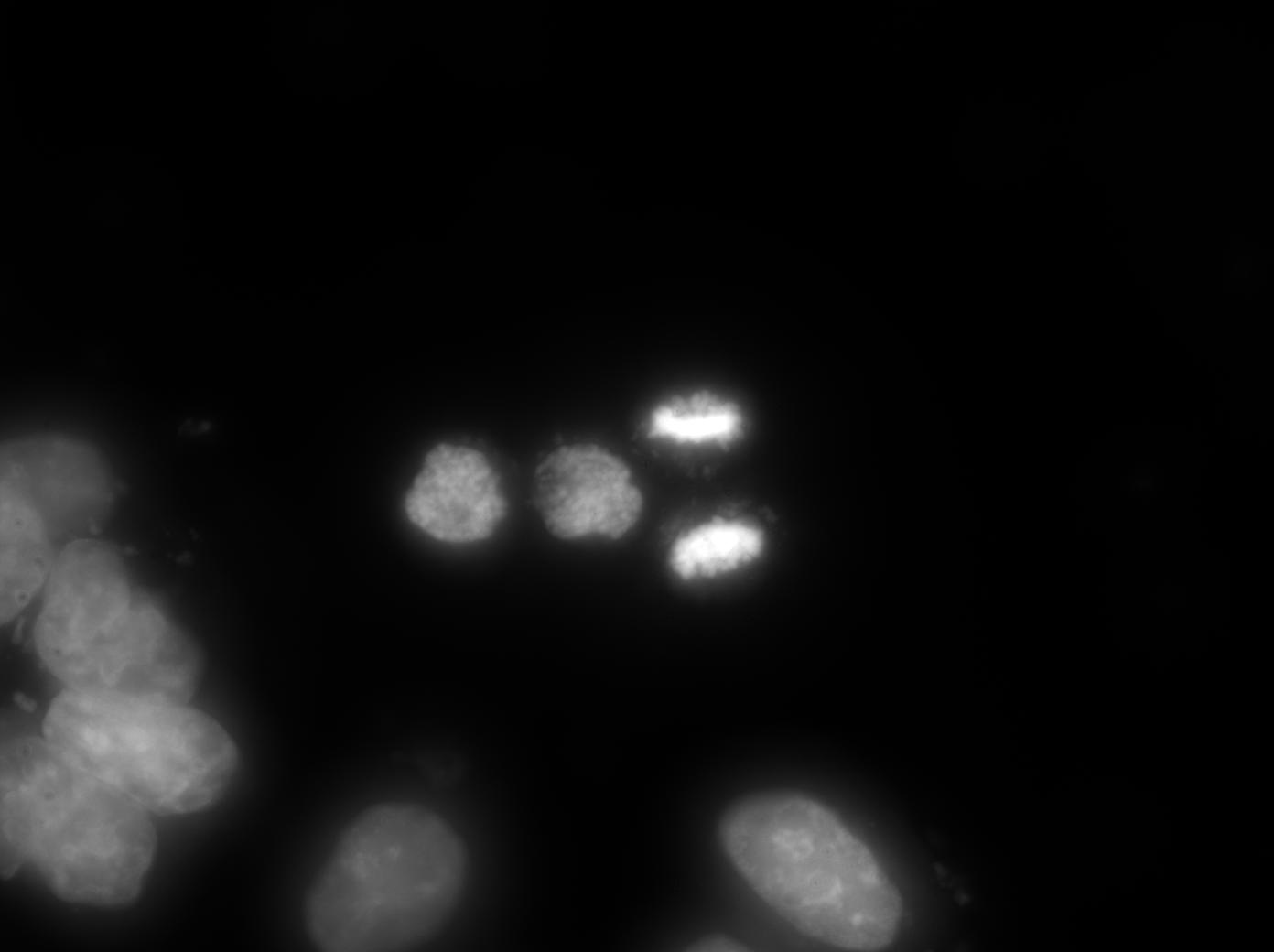

Supplement: Supplementary file 18 — Source data Fig. 5 part 4 [file 44319_2026_742_MOESM18_ESM.zip › Figure 5 Part 4/Fig 5ab WT and KO hela TTLL1-e326g atubulin/EGFP/EGFP-N2 8-23-24 atub R2 ET1ET2.Project Maximum Z_XY1725568286_Z0_T0_C0.tif]

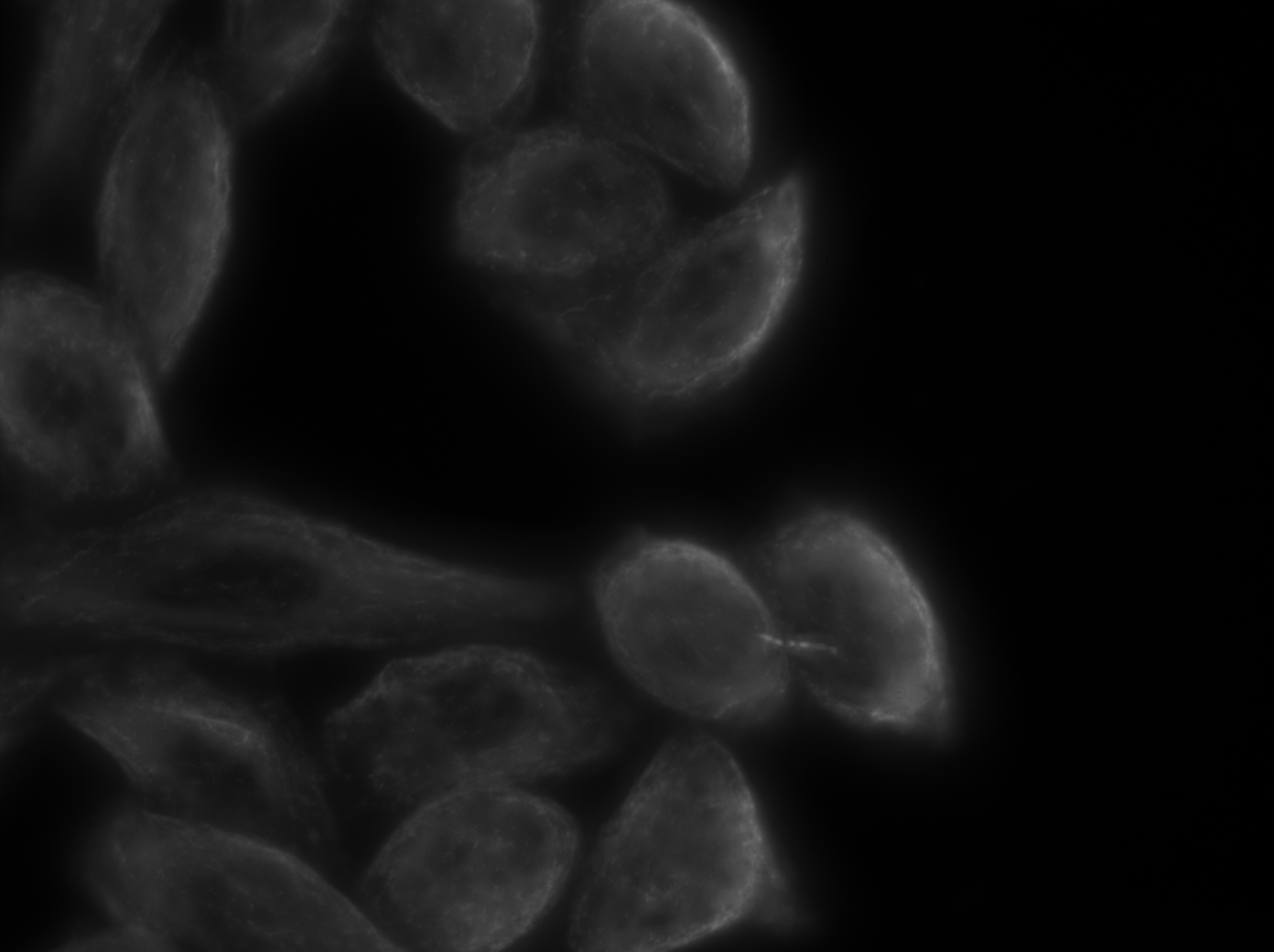

Supplement: Supplementary file 18 — Source data Fig. 5 part 4 [file 44319_2026_742_MOESM18_ESM.zip › Figure 5 Part 4/Fig 5ab WT and KO hela TTLL1-e326g atubulin/EGFP/EGFP-N2 8-23-24 atub R2 LT10.Project Maximum Z_XY1725569595_Z0_T0_C2.tif]

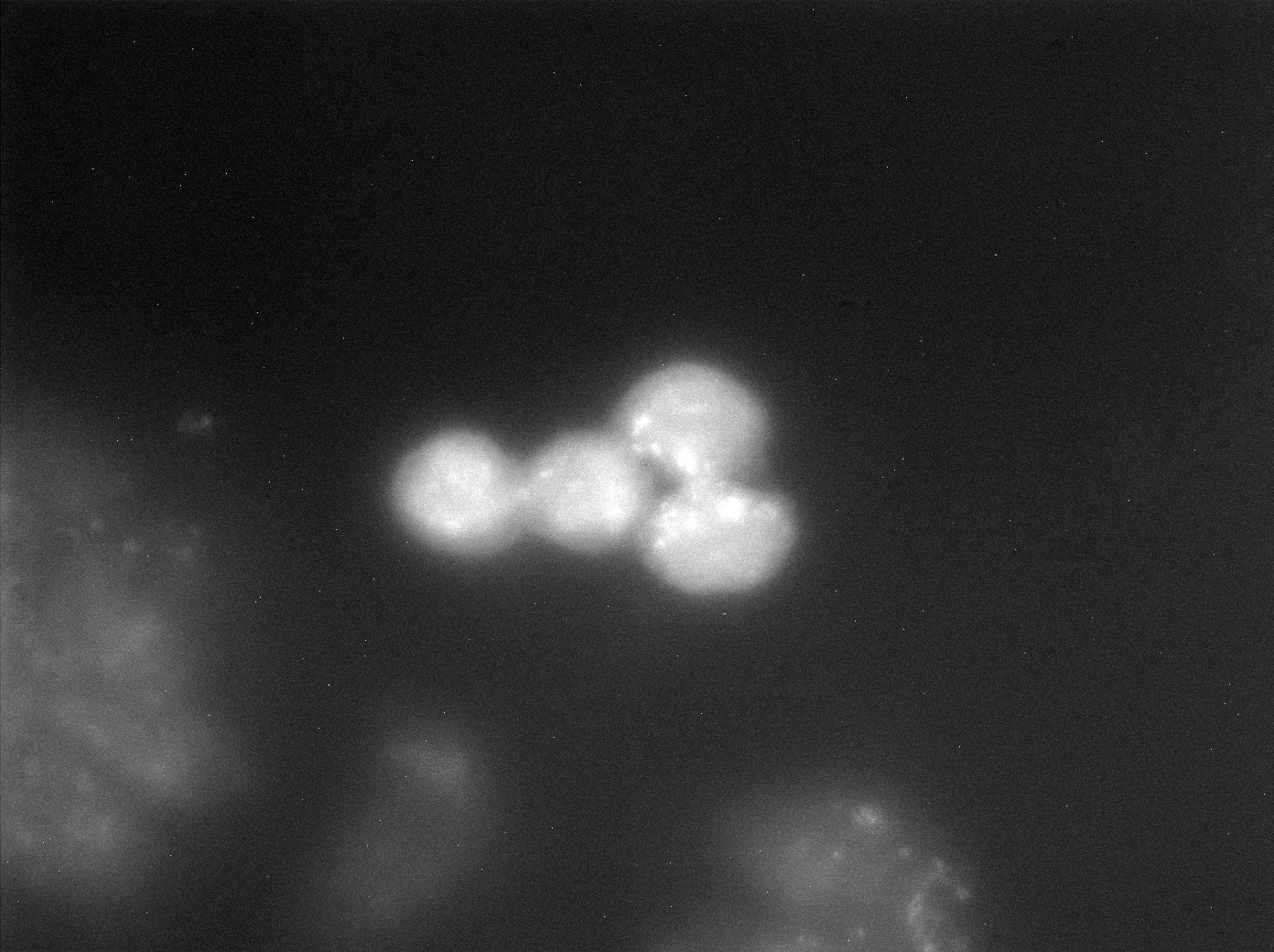

Supplement: Supplementary file 18 — Source data Fig. 5 part 4 [file 44319_2026_742_MOESM18_ESM.zip › Figure 5 Part 4/Fig 5ab WT and KO hela TTLL1-e326g atubulin/EGFP/EGFP-N2 8-23-24 atub R2 ET1ET2.Project Maximum Z_XY1725568286_Z0_T0_C1.tif]

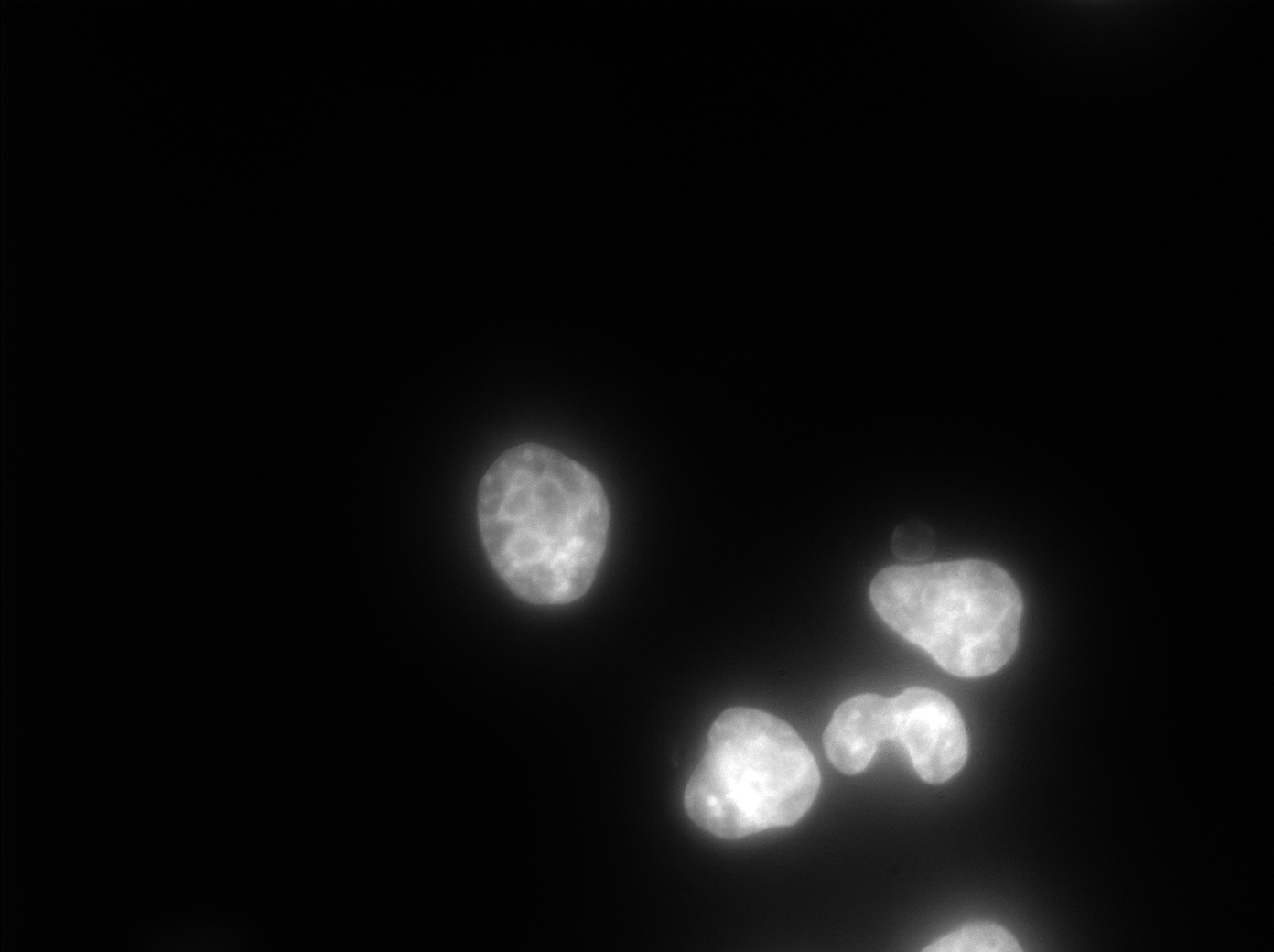

Supplement: Supplementary file 18 — Source data Fig. 5 part 4 [file 44319_2026_742_MOESM18_ESM.zip › Figure 5 Part 4/Fig 5ab WT and KO hela TTLL1-e326g atubulin/EGFP/Cas9 EGFP-N3 10-15-24 R3 LT7.Project Maximum Z_XY1729030371_Z0_T0_C0.tif]

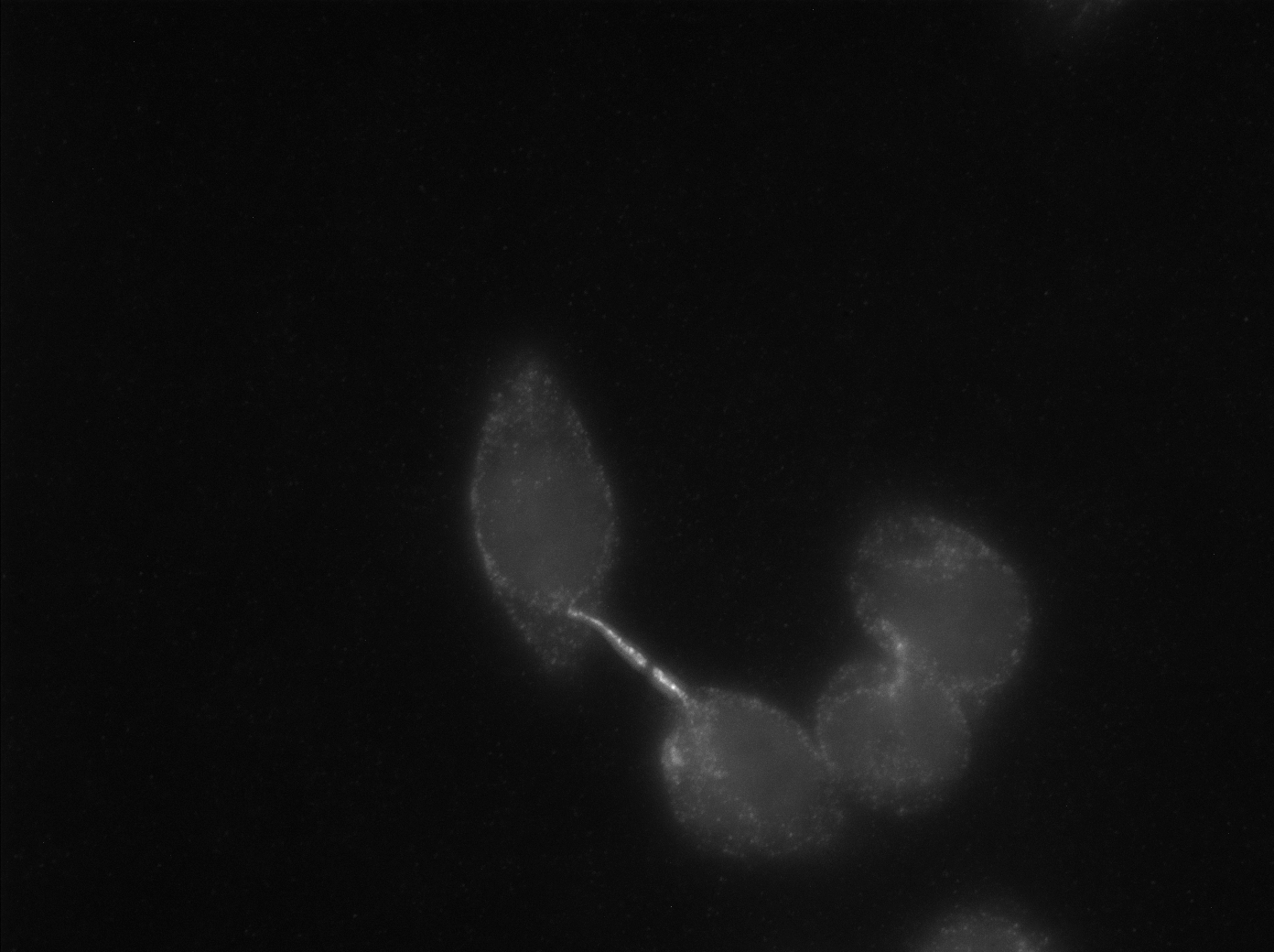

Supplement: Supplementary file 18 — Source data Fig. 5 part 4 [file 44319_2026_742_MOESM18_ESM.zip › Figure 5 Part 4/Fig 5ab WT and KO hela TTLL1-e326g atubulin/EGFP/Cas9 EGFP-N3 10-15-24 R3 LT7.Project Maximum Z_XY1729030371_Z0_T0_C2.tif]

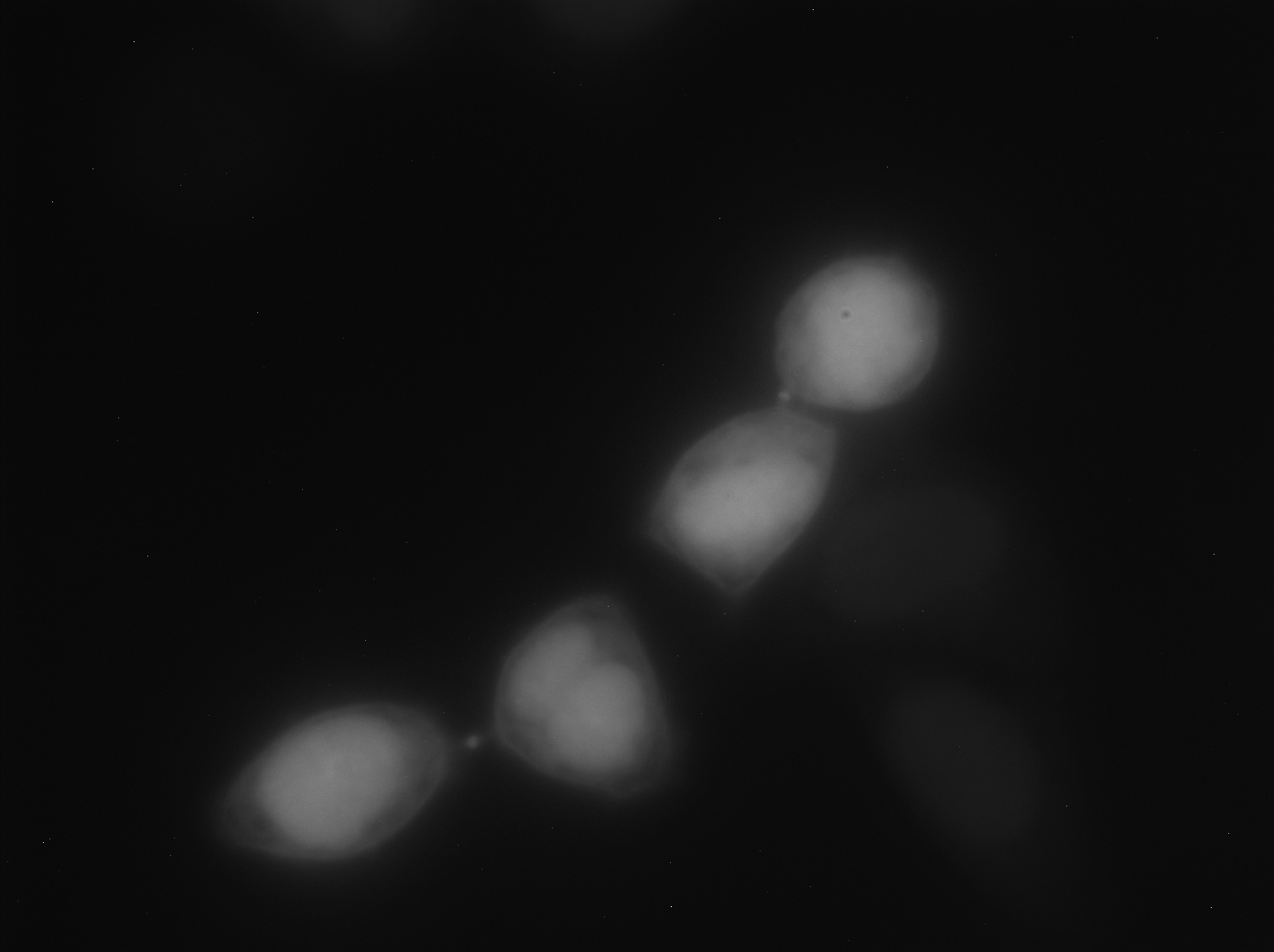

Supplement: Supplementary file 18 — Source data Fig. 5 part 4 [file 44319_2026_742_MOESM18_ESM.zip › Figure 5 Part 4/Fig 5ab WT and KO hela TTLL1-e326g atubulin/EGFP/Cas9 EGFP-N3 10-15-24 R3 LT5LT6.Project Maximum Z_XY1729030238_Z0_T0_C1.tif]

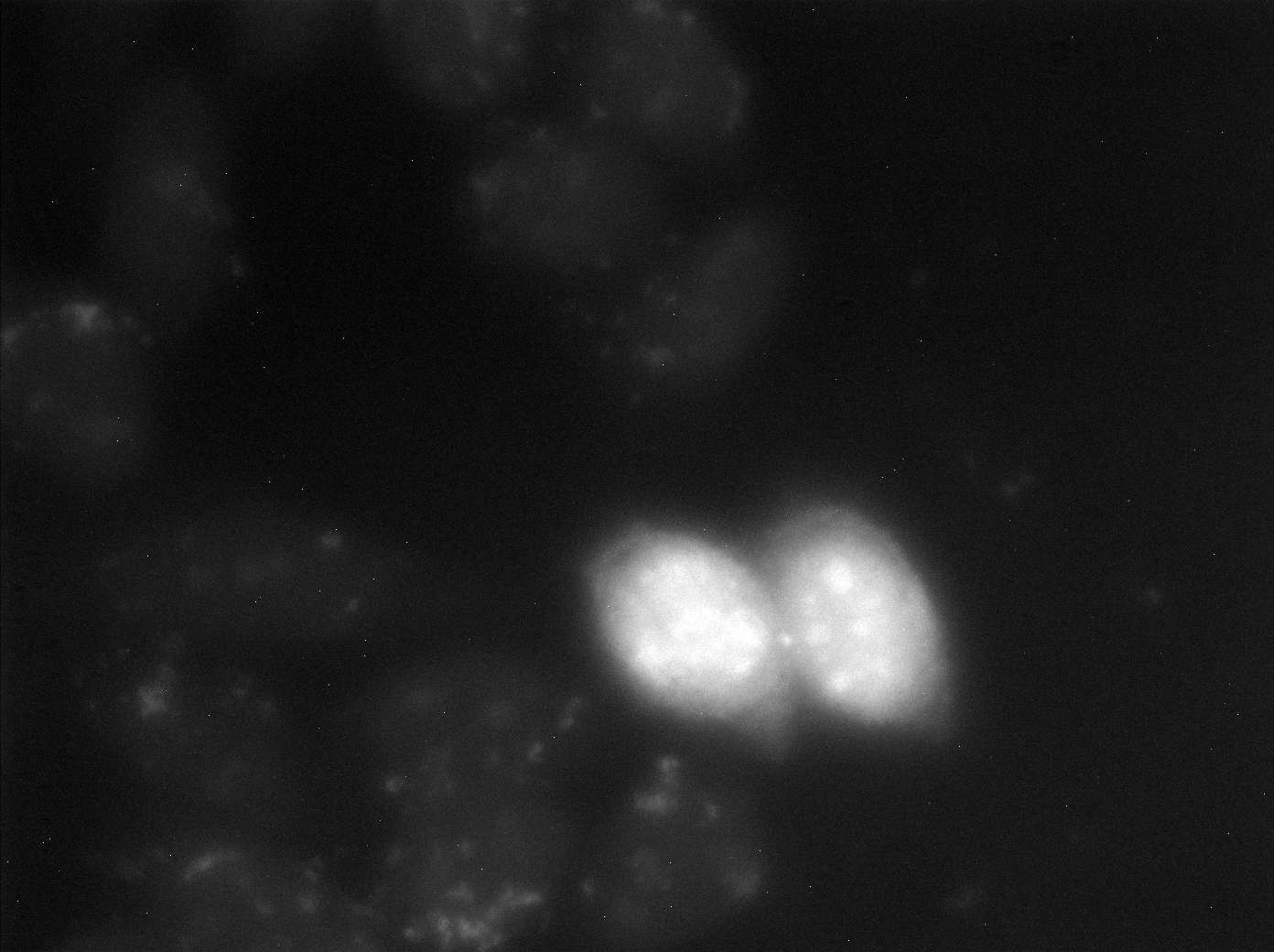

Supplement: Supplementary file 18 — Source data Fig. 5 part 4 [file 44319_2026_742_MOESM18_ESM.zip › Figure 5 Part 4/Fig 5ab WT and KO hela TTLL1-e326g atubulin/EGFP/EGFP-N2 8-23-24 atub R2 LT10.Project Maximum Z_XY1725569595_Z0_T0_C1.tif]

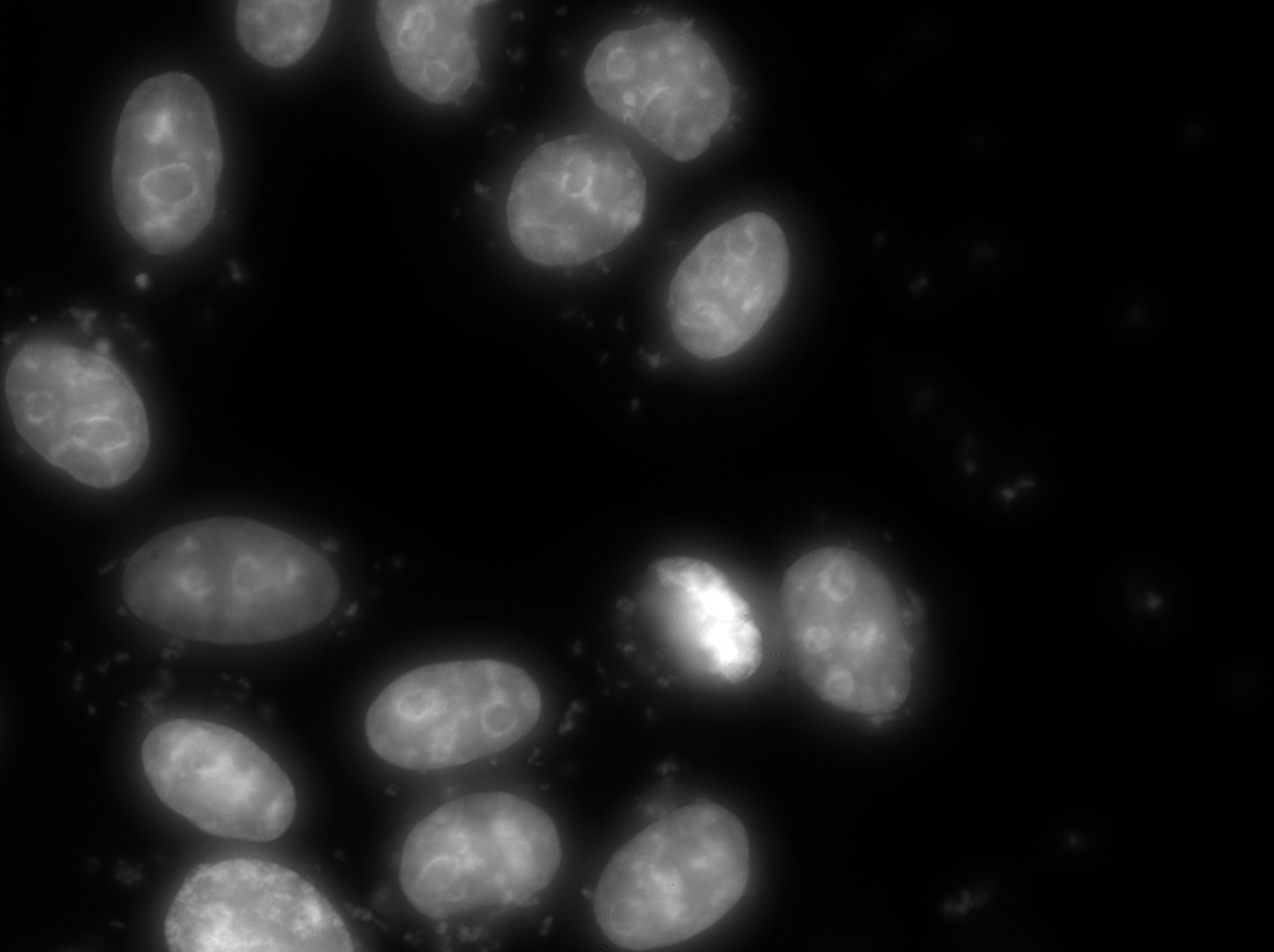

Supplement: Supplementary file 18 — Source data Fig. 5 part 4 [file 44319_2026_742_MOESM18_ESM.zip › Figure 5 Part 4/Fig 5ab WT and KO hela TTLL1-e326g atubulin/EGFP/EGFP-N2 8-23-24 atub R2 LT10.Project Maximum Z_XY1725569595_Z0_T0_C0.tif]

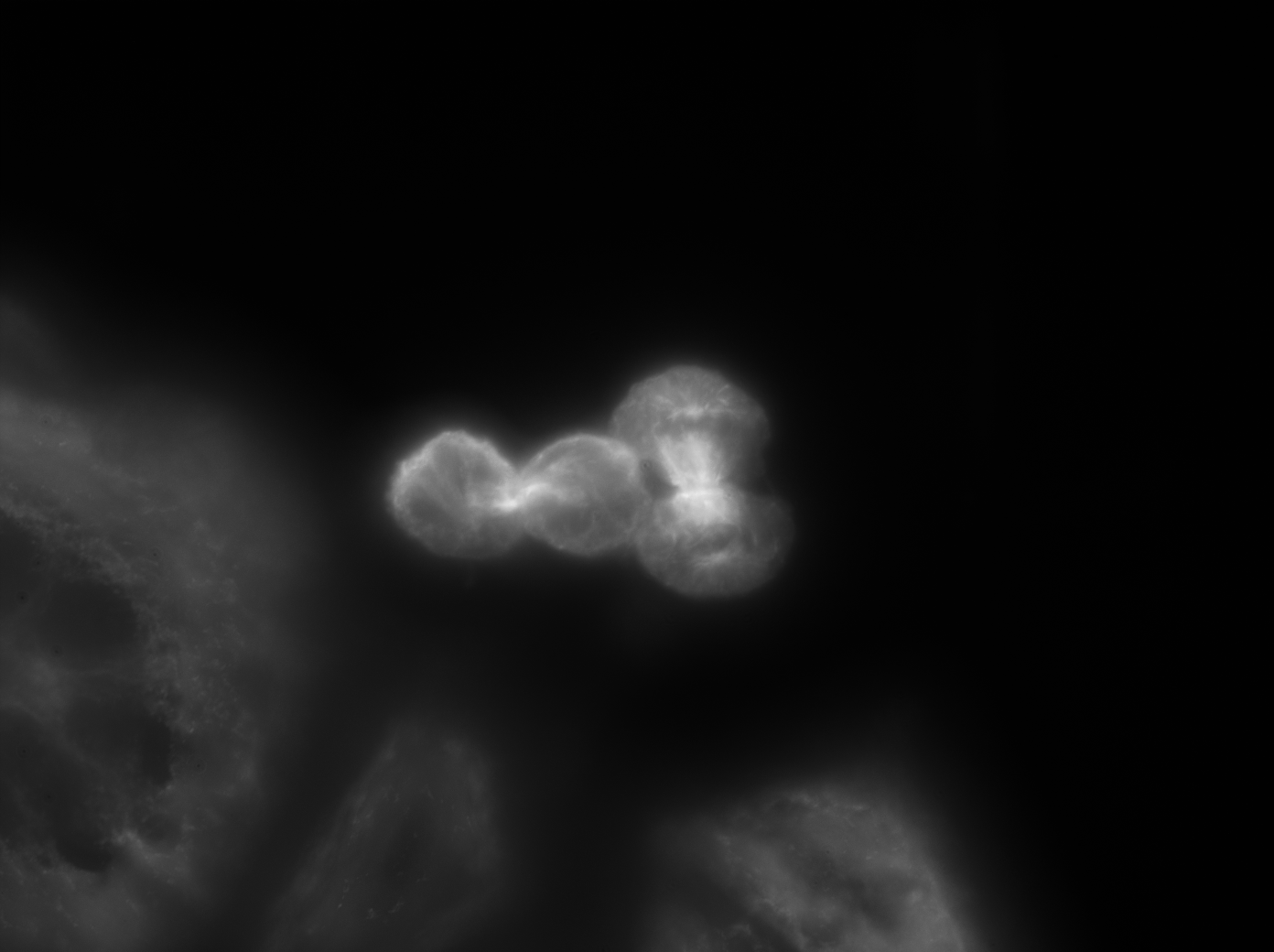

Supplement: Supplementary file 18 — Source data Fig. 5 part 4 [file 44319_2026_742_MOESM18_ESM.zip › Figure 5 Part 4/Fig 5ab WT and KO hela TTLL1-e326g atubulin/EGFP/EGFP-N2 8-23-24 atub R2 ET1ET2.Project Maximum Z_XY1725568286_Z0_T0_C2.tif]

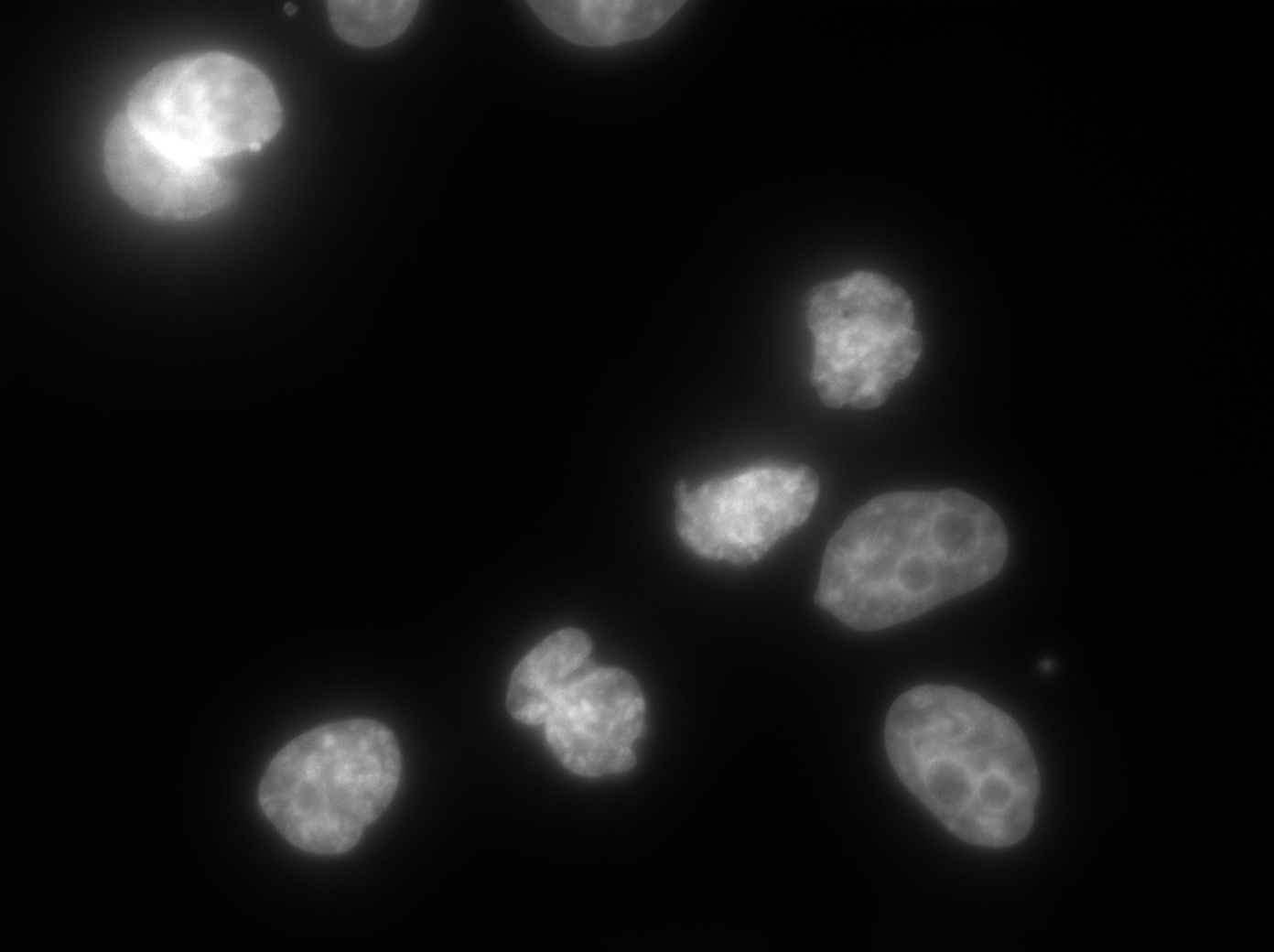

Supplement: Supplementary file 18 — Source data Fig. 5 part 4 [file 44319_2026_742_MOESM18_ESM.zip › Figure 5 Part 4/Fig 5ab WT and KO hela TTLL1-e326g atubulin/EGFP/Cas9 EGFP-N3 10-15-24 R3 LT5LT6.Project Maximum Z_XY1729030238_Z0_T0_C0.tif]

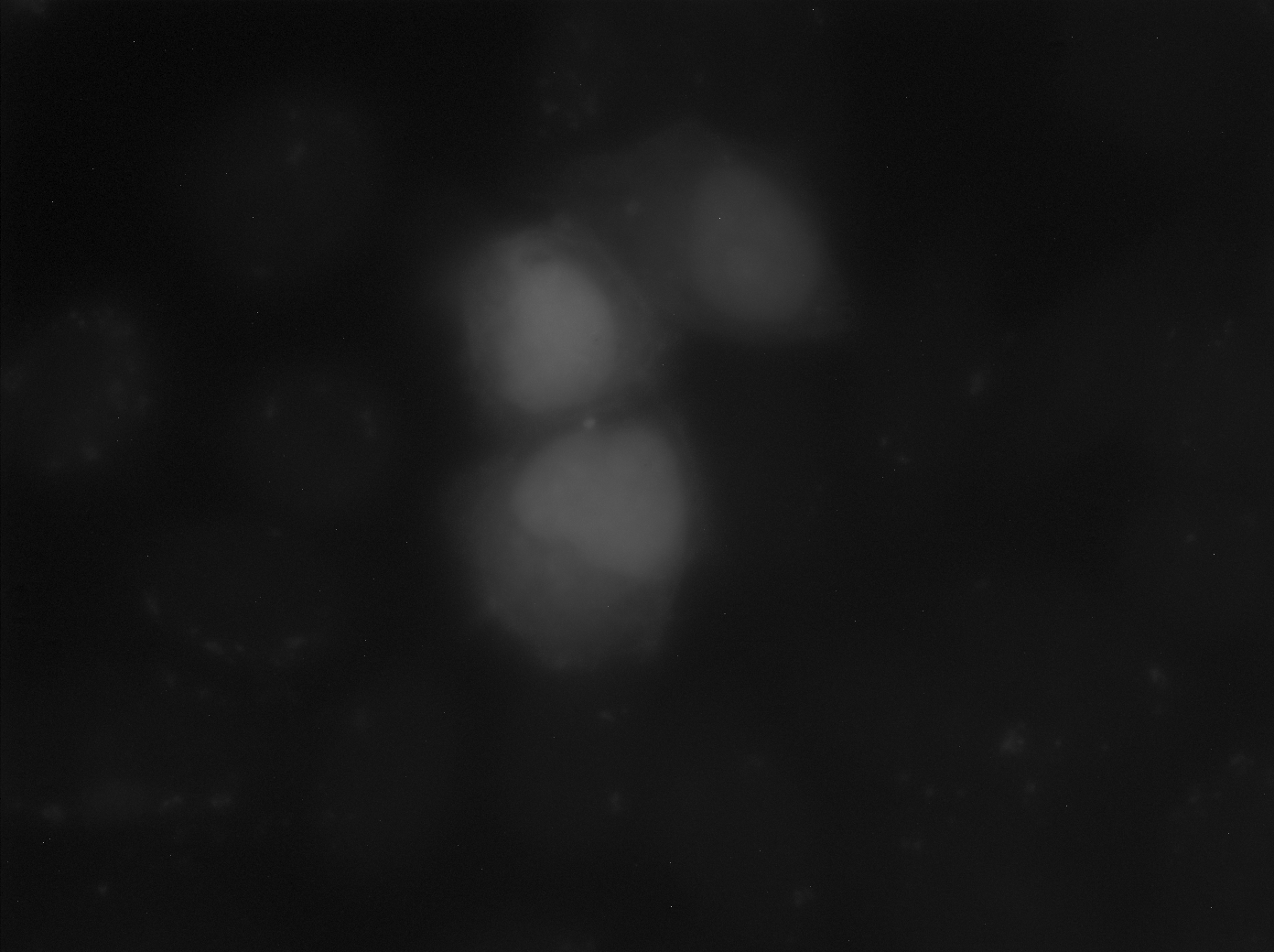

Supplement: Supplementary file 18 — Source data Fig. 5 part 4 [file 44319_2026_742_MOESM18_ESM.zip › Figure 5 Part 4/Fig 5ab WT and KO hela TTLL1-e326g atubulin/EGFP/EGFP-N3 atub R1 LT1.Project Maximum Z_XY1724713100_Z0_T0_C1.tif]

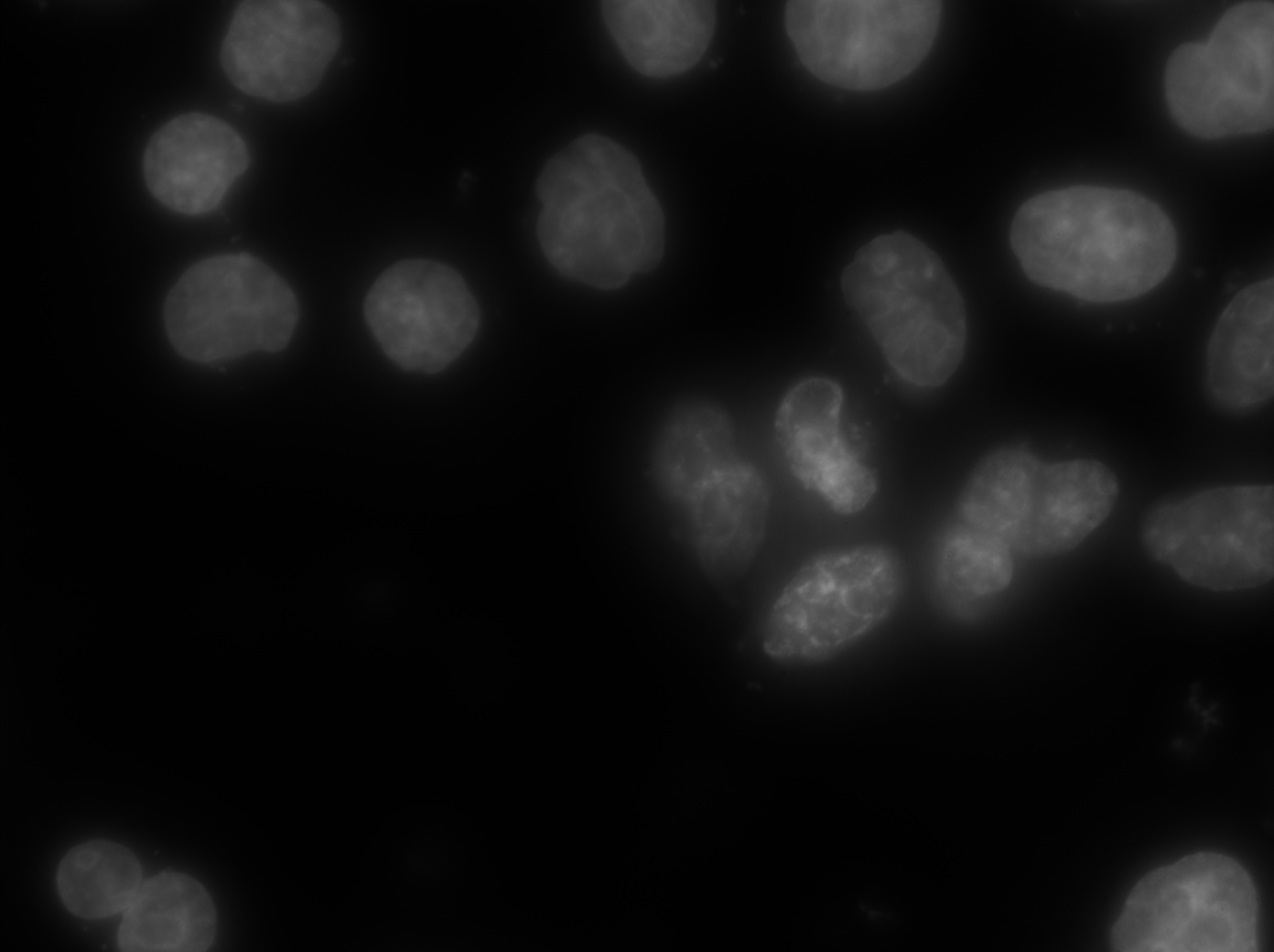

Supplement: Supplementary file 18 — Source data Fig. 5 part 4 [file 44319_2026_742_MOESM18_ESM.zip › Figure 5 Part 4/Fig 5ab WT and KO hela TTLL1-e326g atubulin/EGFP/EGFP-N3 atub R1 LT5.Project Maximum Z_XY1724714080_Z0_T0_C0.tif]

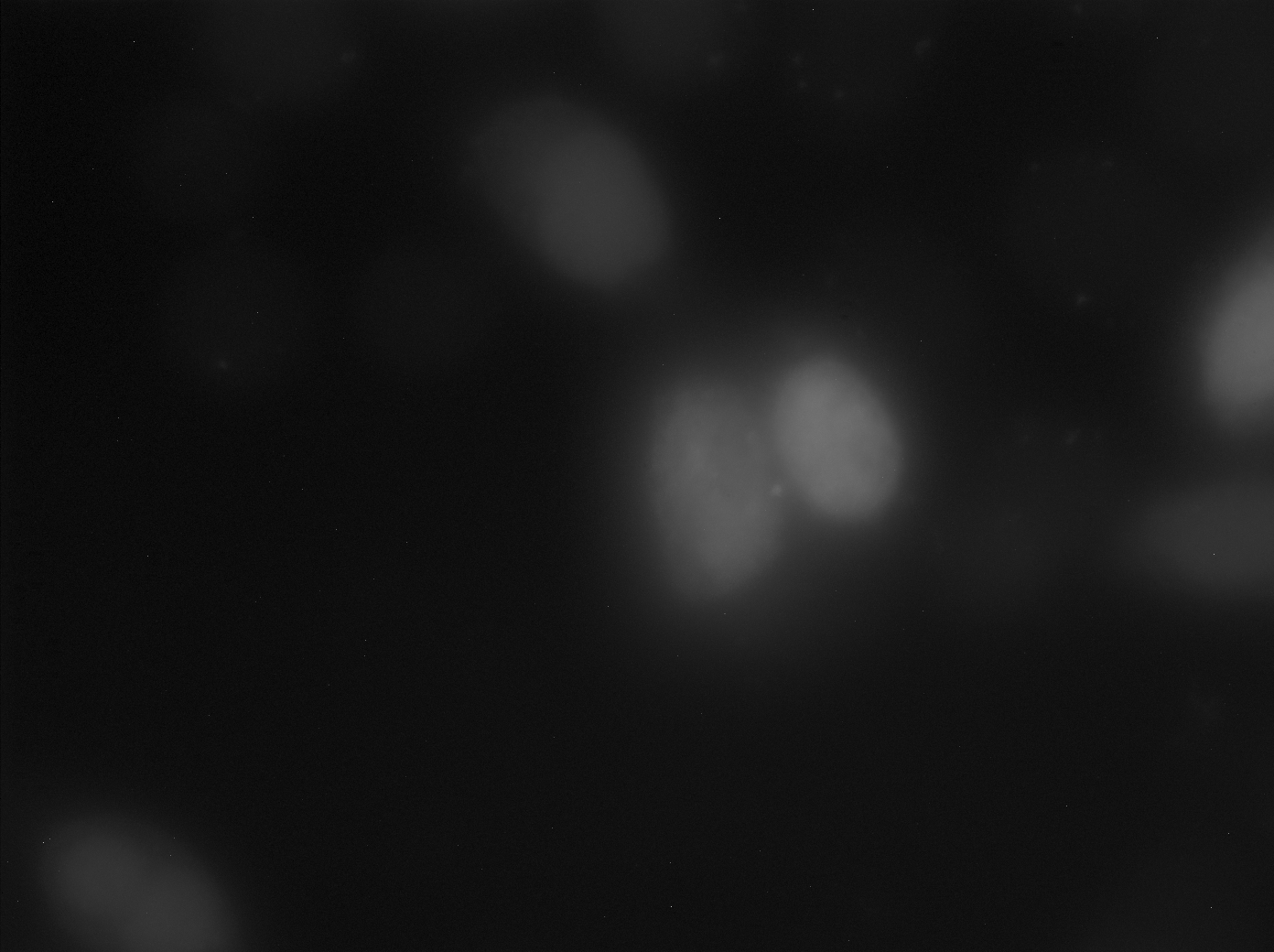

Supplement: Supplementary file 18 — Source data Fig. 5 part 4 [file 44319_2026_742_MOESM18_ESM.zip › Figure 5 Part 4/Fig 5ab WT and KO hela TTLL1-e326g atubulin/EGFP/EGFP-N3 atub R1 LT5.Project Maximum Z_XY1724714080_Z0_T0_C1.tif]

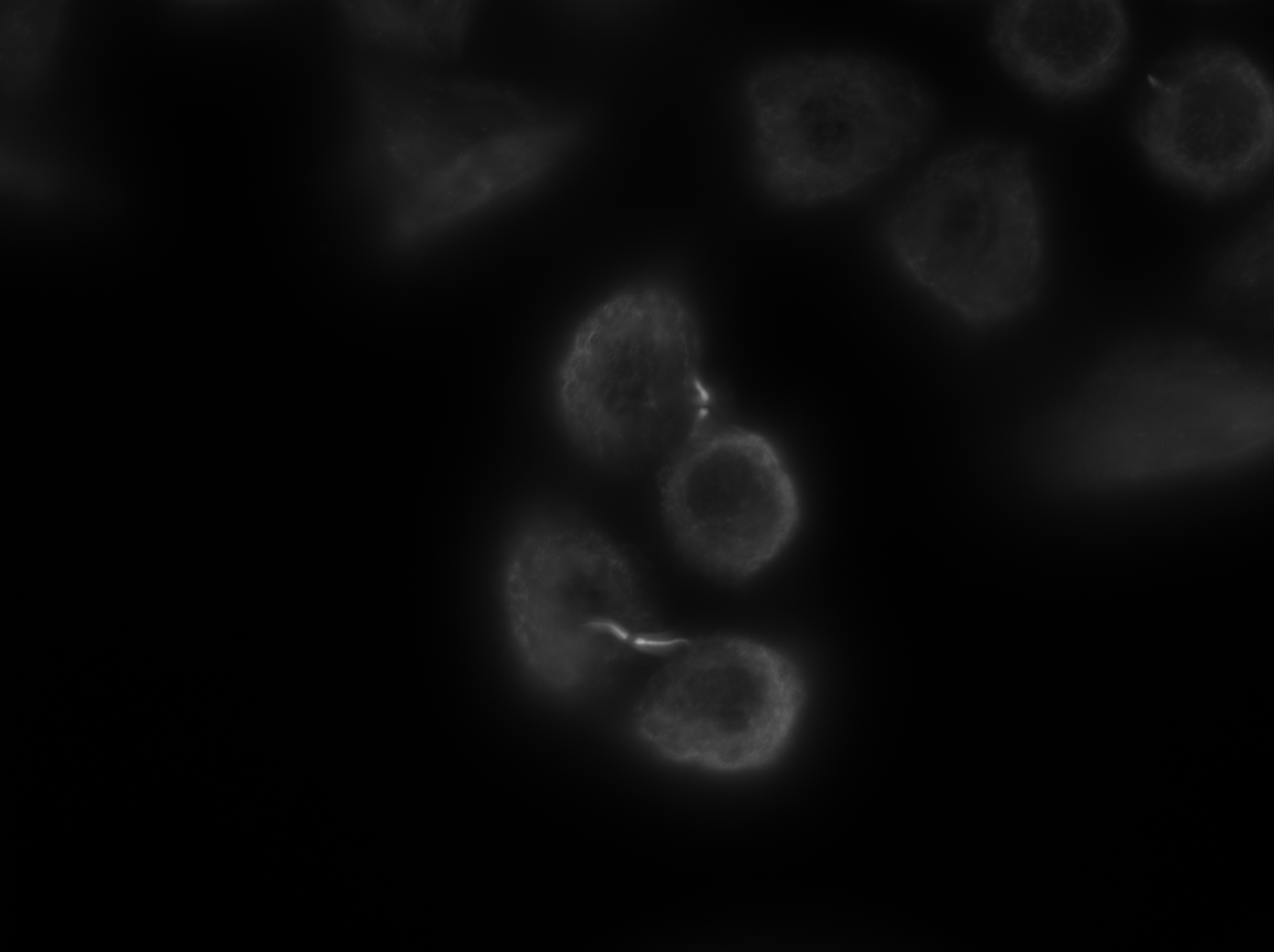

Supplement: Supplementary file 18 — Source data Fig. 5 part 4 [file 44319_2026_742_MOESM18_ESM.zip › Figure 5 Part 4/Fig 5ab WT and KO hela TTLL1-e326g atubulin/EGFP/EGFP-N2 8-23-24 atub R2 LT3.Project Maximum Z_XY1725568473_Z0_T0_C2.tif]

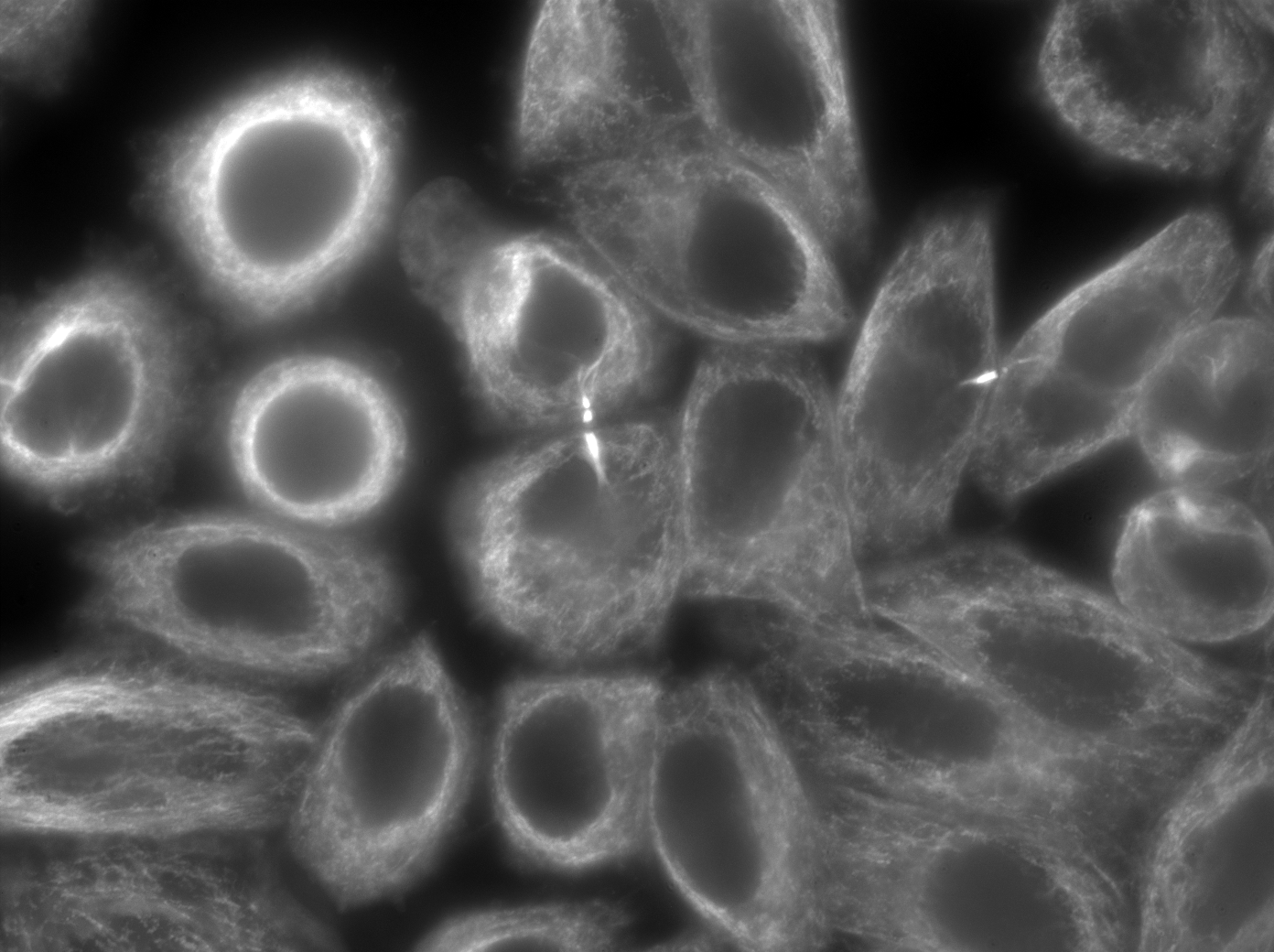

Supplement: Supplementary file 18 — Source data Fig. 5 part 4 [file 44319_2026_742_MOESM18_ESM.zip › Figure 5 Part 4/Fig 5ab WT and KO hela TTLL1-e326g atubulin/EGFP/EGFP-N3 atub R1 LT1.Project Maximum Z_XY1724713100_Z0_T0_C2.tif]

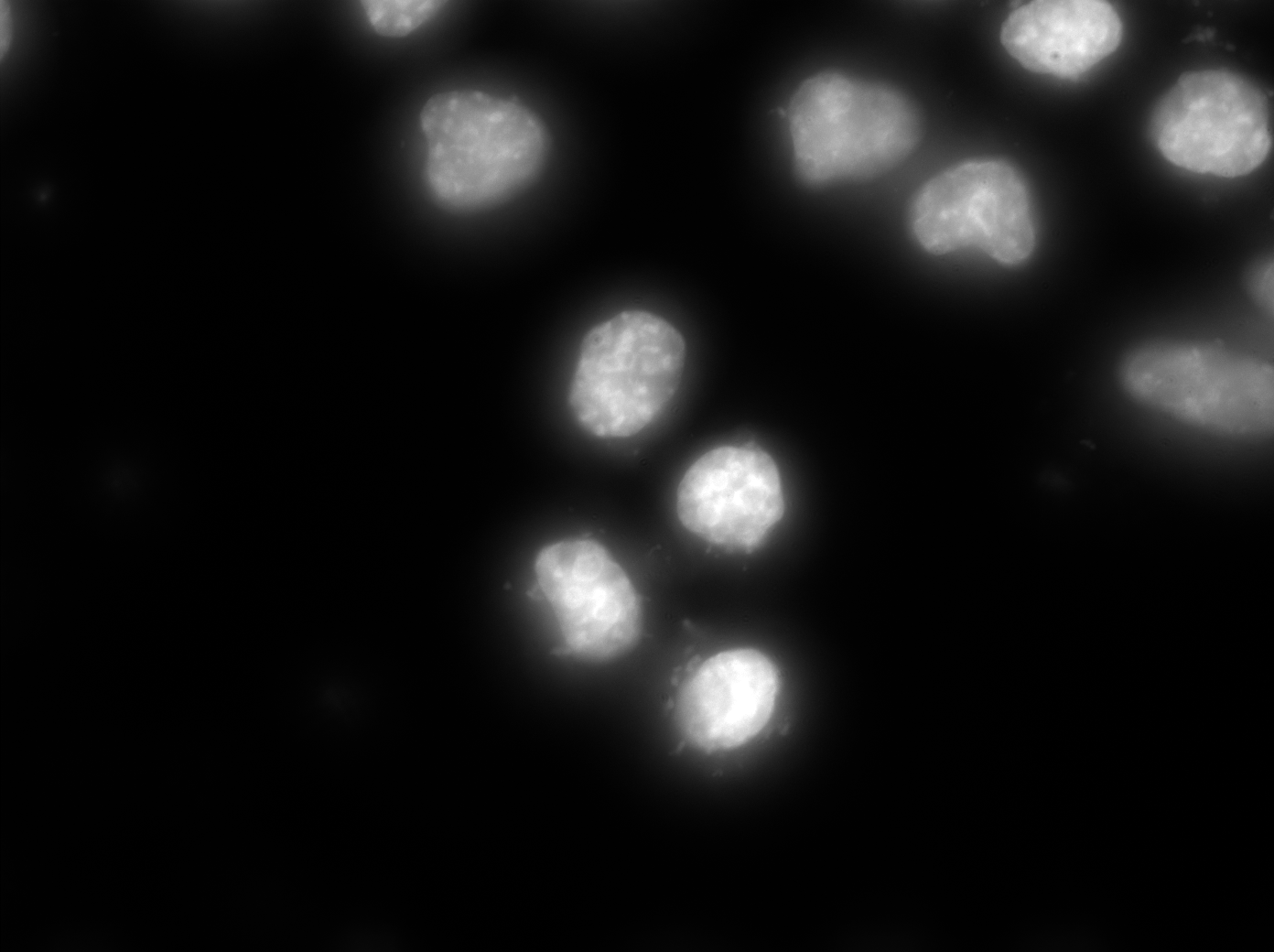

Supplement: Supplementary file 18 — Source data Fig. 5 part 4 [file 44319_2026_742_MOESM18_ESM.zip › Figure 5 Part 4/Fig 5ab WT and KO hela TTLL1-e326g atubulin/EGFP/EGFP-N2 8-23-24 atub R2 LT3.Project Maximum Z_XY1725568473_Z0_T0_C0.tif]

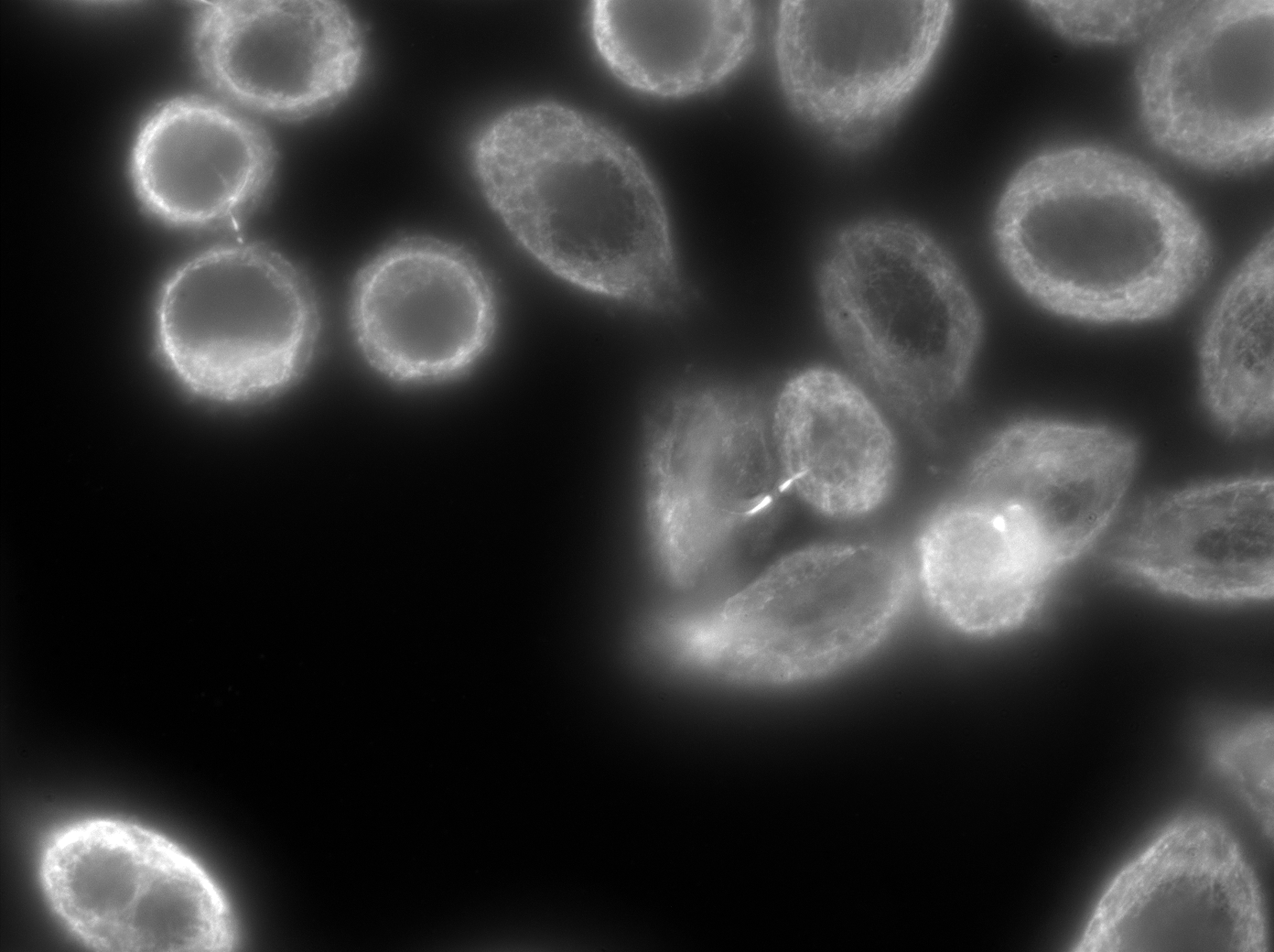

Supplement: Supplementary file 18 — Source data Fig. 5 part 4 [file 44319_2026_742_MOESM18_ESM.zip › Figure 5 Part 4/Fig 5ab WT and KO hela TTLL1-e326g atubulin/EGFP/EGFP-N3 atub R1 LT5.Project Maximum Z_XY1724714080_Z0_T0_C2.tif]

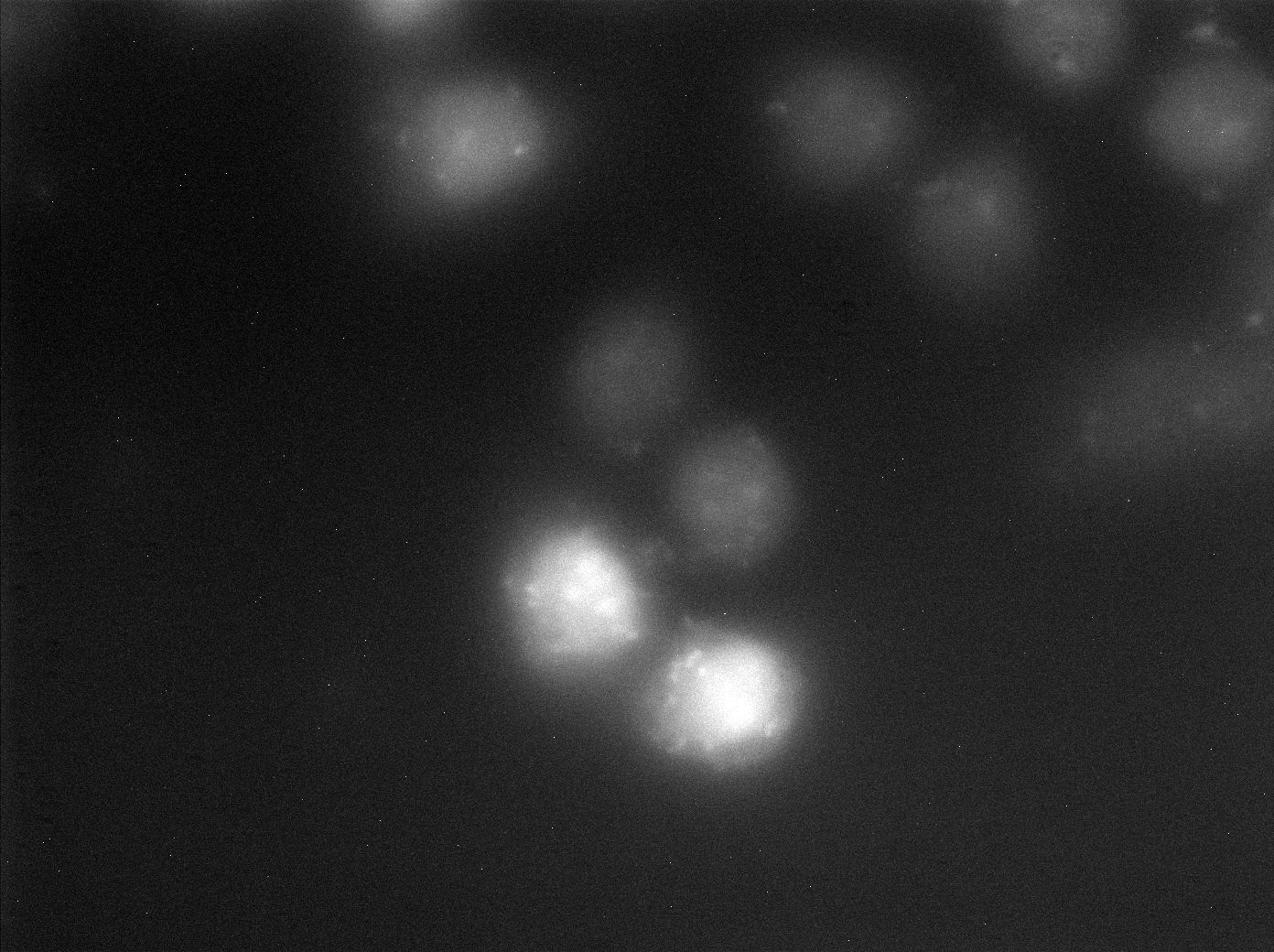

Supplement: Supplementary file 18 — Source data Fig. 5 part 4 [file 44319_2026_742_MOESM18_ESM.zip › Figure 5 Part 4/Fig 5ab WT and KO hela TTLL1-e326g atubulin/EGFP/EGFP-N2 8-23-24 atub R2 LT3.Project Maximum Z_XY1725568473_Z0_T0_C1.tif]

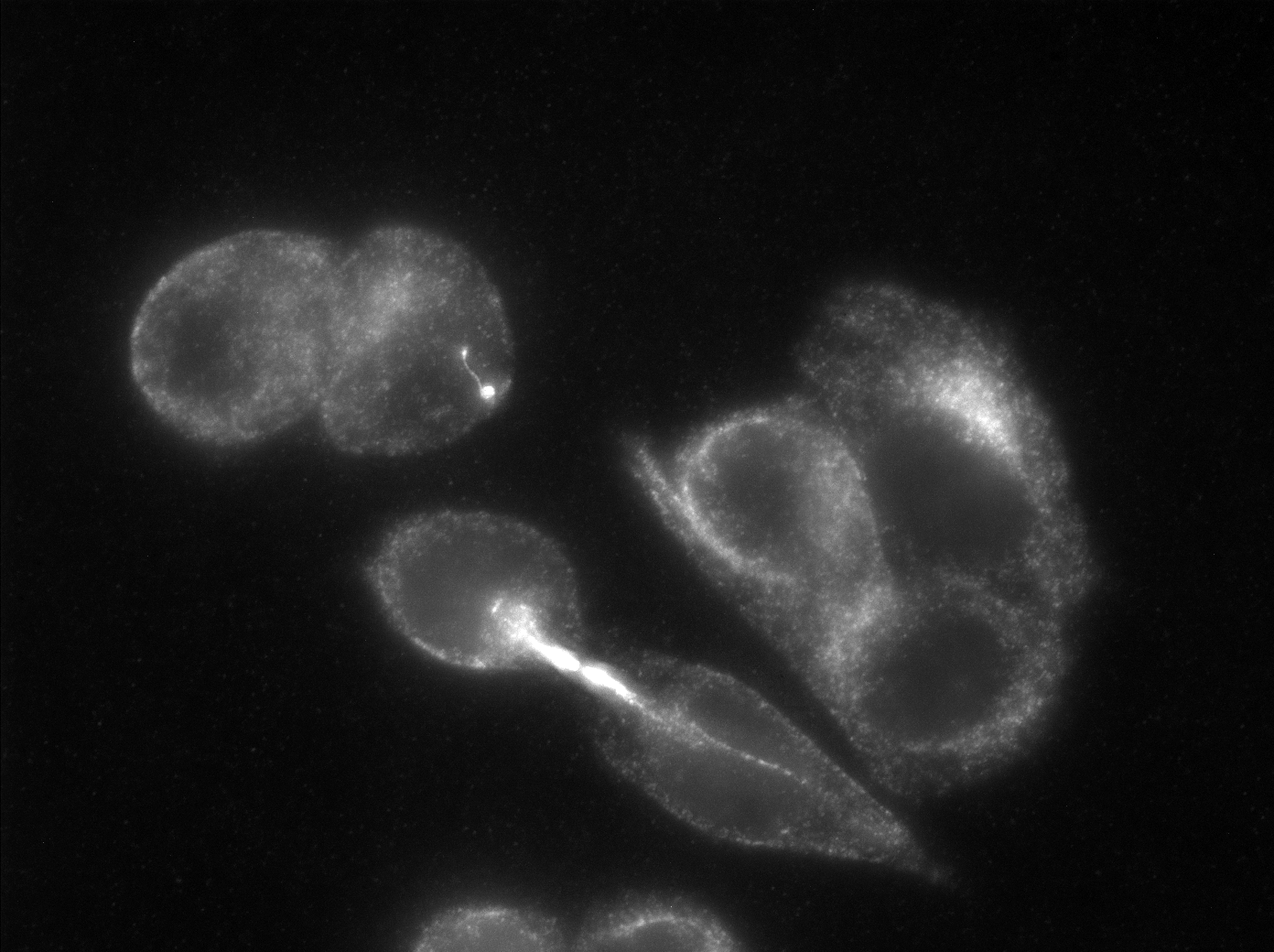

Supplement: Supplementary file 18 — Source data Fig. 5 part 4 [file 44319_2026_742_MOESM18_ESM.zip › Figure 5 Part 4/Fig 5ab WT and KO hela TTLL1-e326g atubulin/EGFP/Cas9 EGFP-N3 10-15-24 R3 LT1.Project Maximum Z_XY1729029365_Z0_T0_C2.tif]

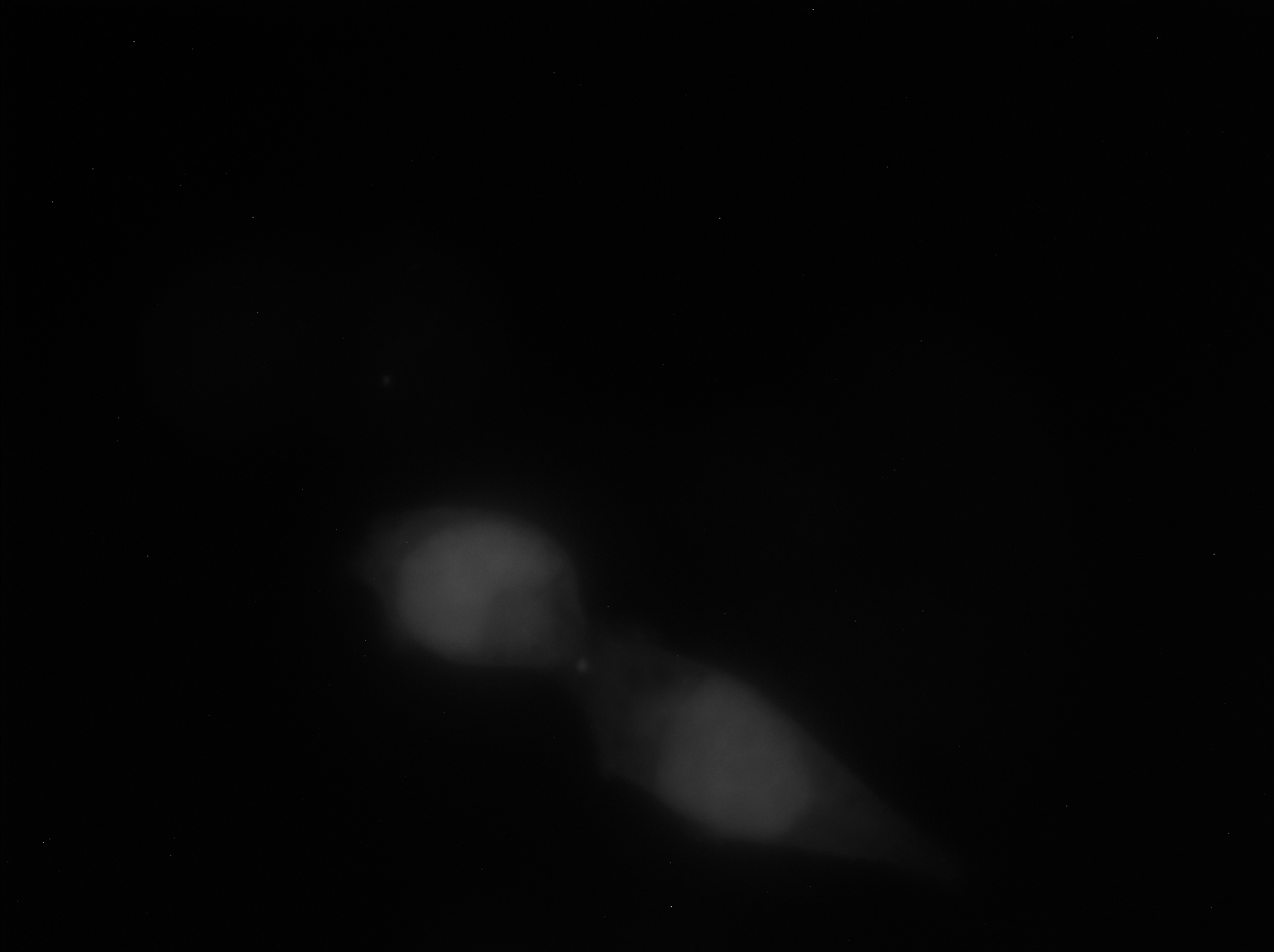

Supplement: Supplementary file 18 — Source data Fig. 5 part 4 [file 44319_2026_742_MOESM18_ESM.zip › Figure 5 Part 4/Fig 5ab WT and KO hela TTLL1-e326g atubulin/EGFP/Cas9 EGFP-N3 10-15-24 R3 LT1.Project Maximum Z_XY1729029365_Z0_T0_C1.tif]

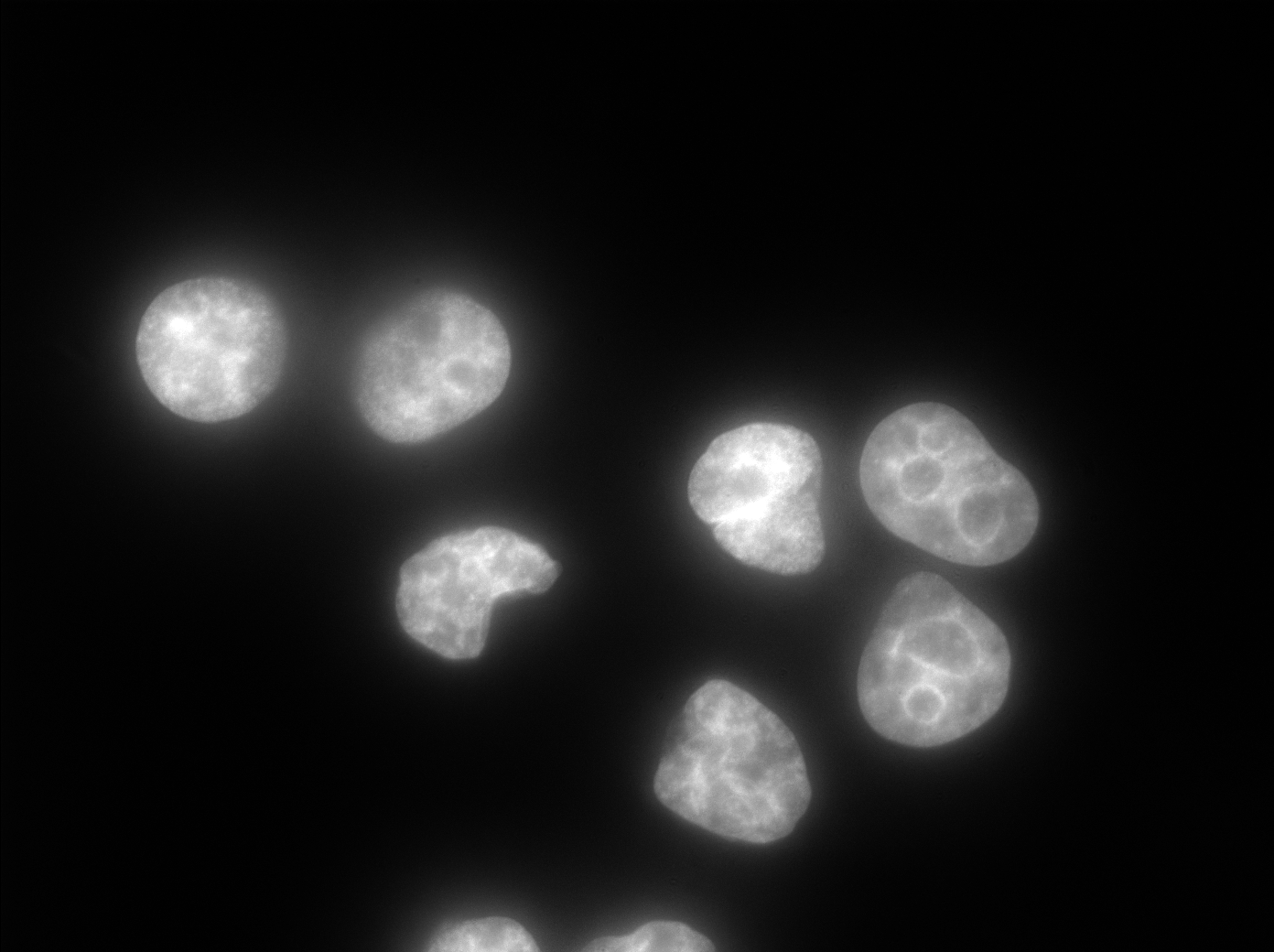

Supplement: Supplementary file 18 — Source data Fig. 5 part 4 [file 44319_2026_742_MOESM18_ESM.zip › Figure 5 Part 4/Fig 5ab WT and KO hela TTLL1-e326g atubulin/EGFP/Cas9 EGFP-N3 10-15-24 R3 LT1.Project Maximum Z_XY1729029365_Z0_T0_C0.tif]

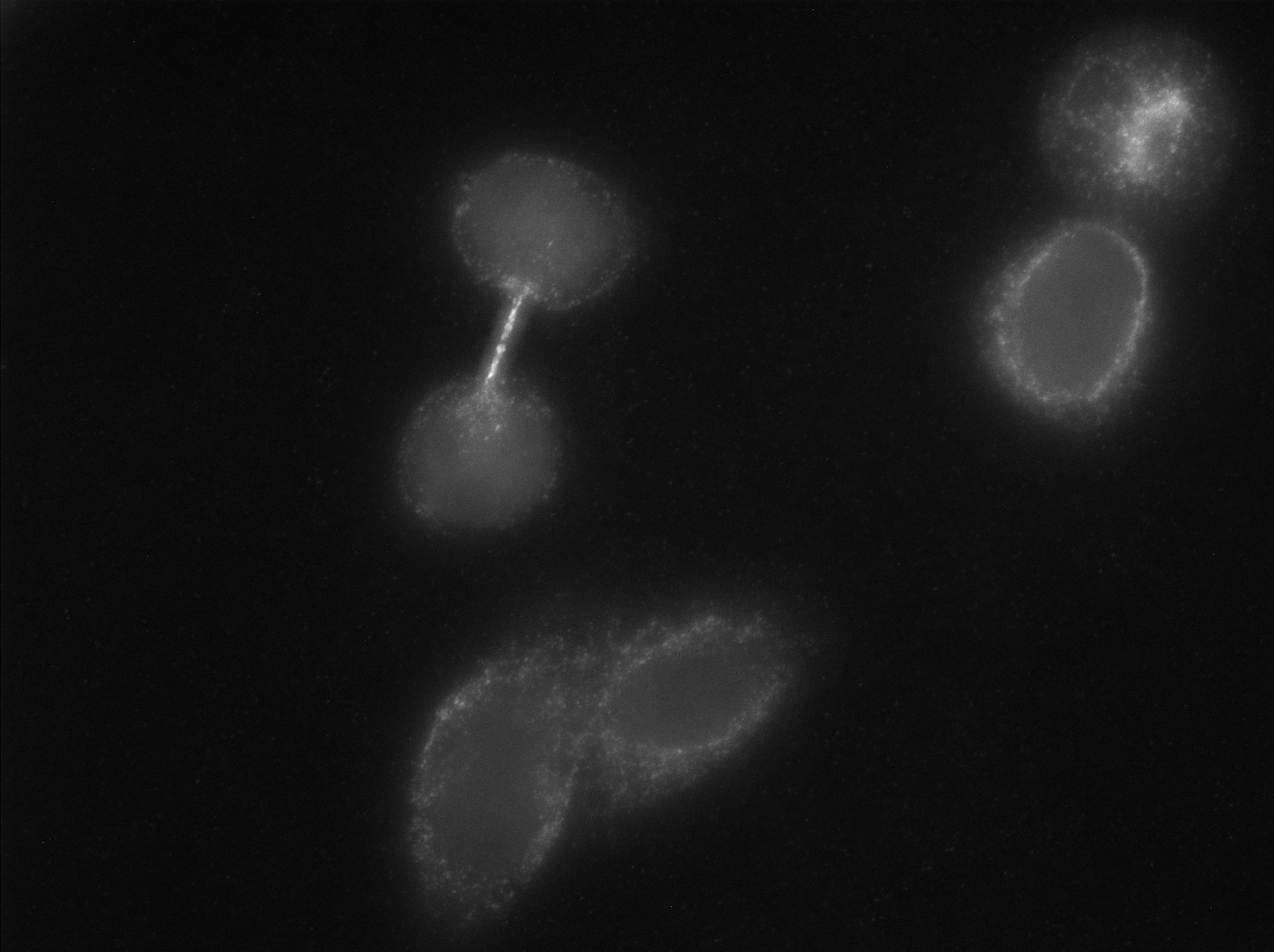

Supplement: Supplementary file 18 — Source data Fig. 5 part 4 [file 44319_2026_742_MOESM18_ESM.zip › Figure 5 Part 4/Fig 5ab WT and KO hela TTLL1-e326g atubulin/EGFP/Cas9 EGFP-N3 10-15-24 R3 LT8.Project Maximum Z_XY1729030584_Z0_T0_C2.tif]

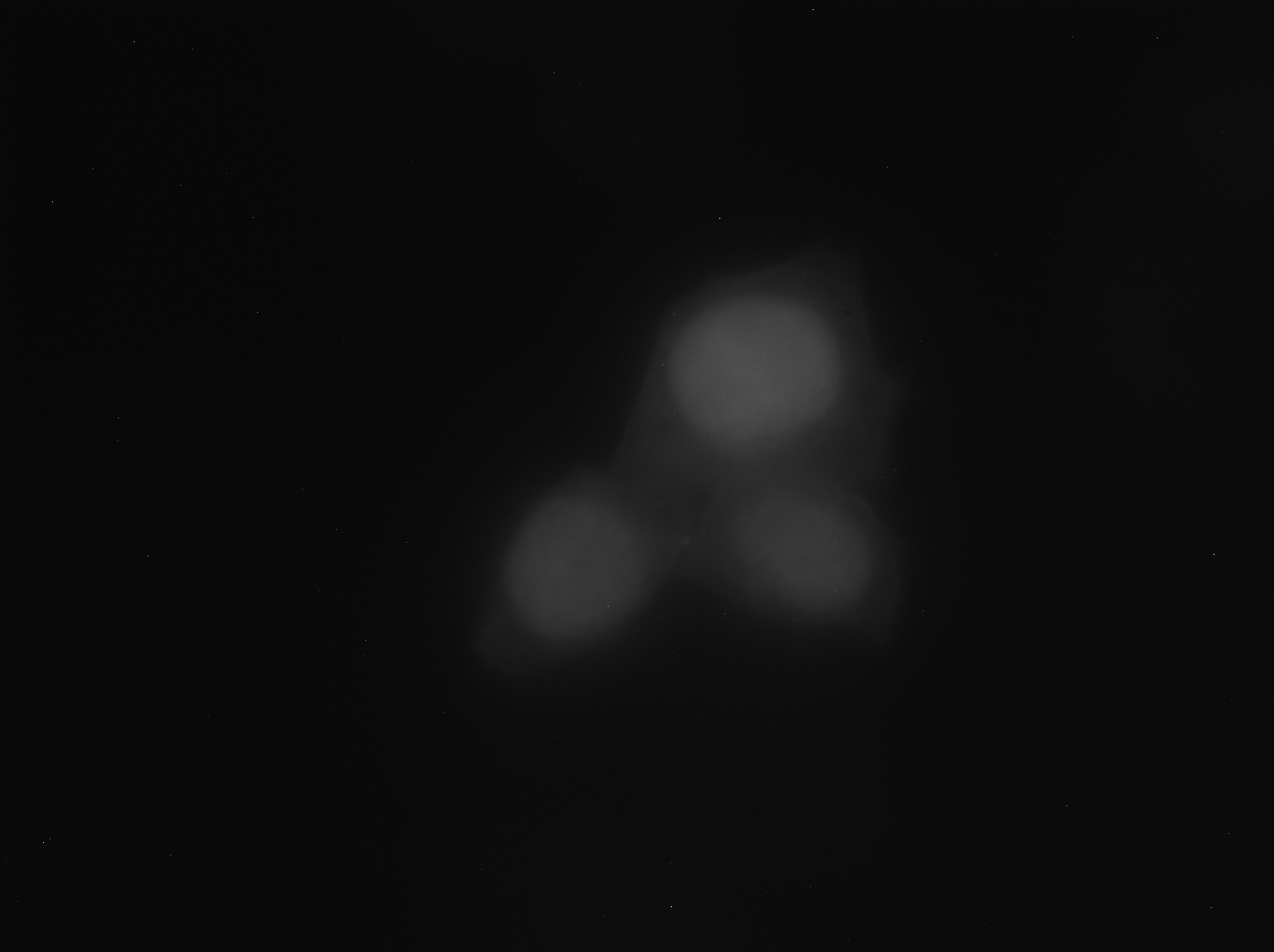

Supplement: Supplementary file 18 — Source data Fig. 5 part 4 [file 44319_2026_742_MOESM18_ESM.zip › Figure 5 Part 4/Fig 5ab WT and KO hela TTLL1-e326g atubulin/EGFP/Cas9 EGFP-N3 10-15-24 R3 LT2.Project Maximum Z_XY1729029607_Z0_T0_C1.tif]

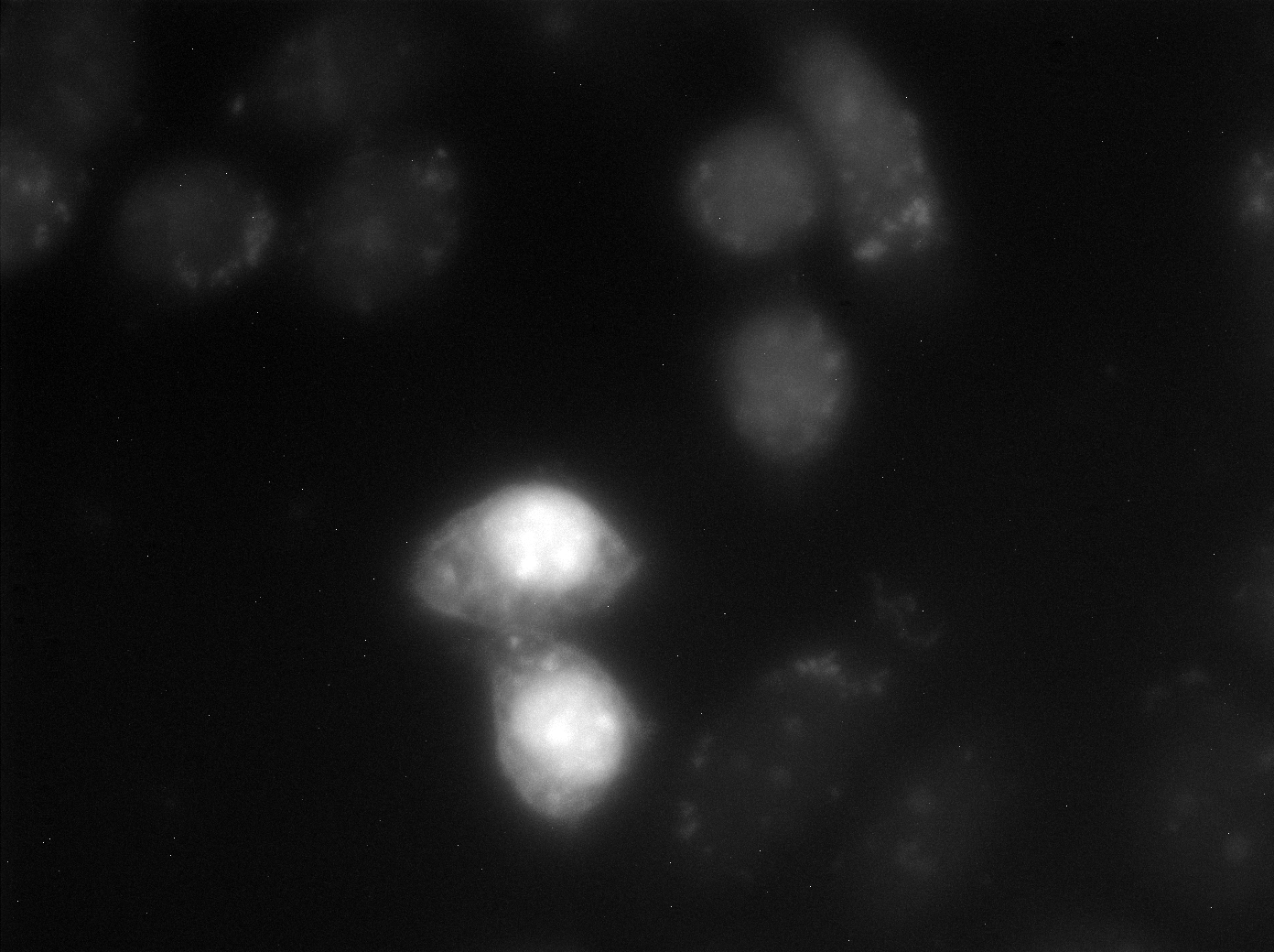

Supplement: Supplementary file 18 — Source data Fig. 5 part 4 [file 44319_2026_742_MOESM18_ESM.zip › Figure 5 Part 4/Fig 5ab WT and KO hela TTLL1-e326g atubulin/EGFP/EGFP-N2 8-23-24 atub R2 LT6LT7.Project Maximum Z_XY1725568941_Z0_T0_C1.tif]

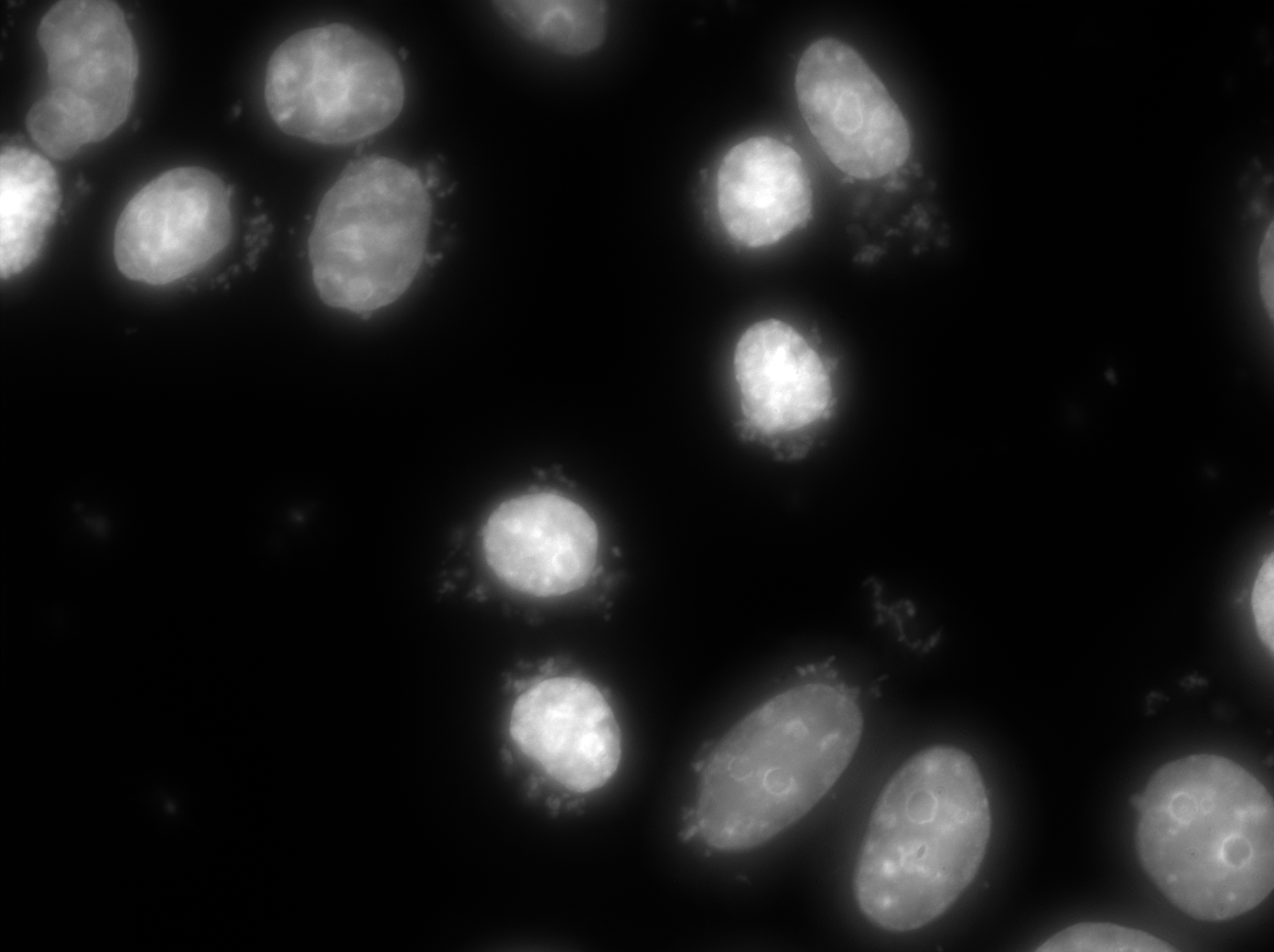

Supplement: Supplementary file 18 — Source data Fig. 5 part 4 [file 44319_2026_742_MOESM18_ESM.zip › Figure 5 Part 4/Fig 5ab WT and KO hela TTLL1-e326g atubulin/EGFP/EGFP-N2 8-23-24 atub R2 LT6LT7.Project Maximum Z_XY1725568941_Z0_T0_C0.tif]

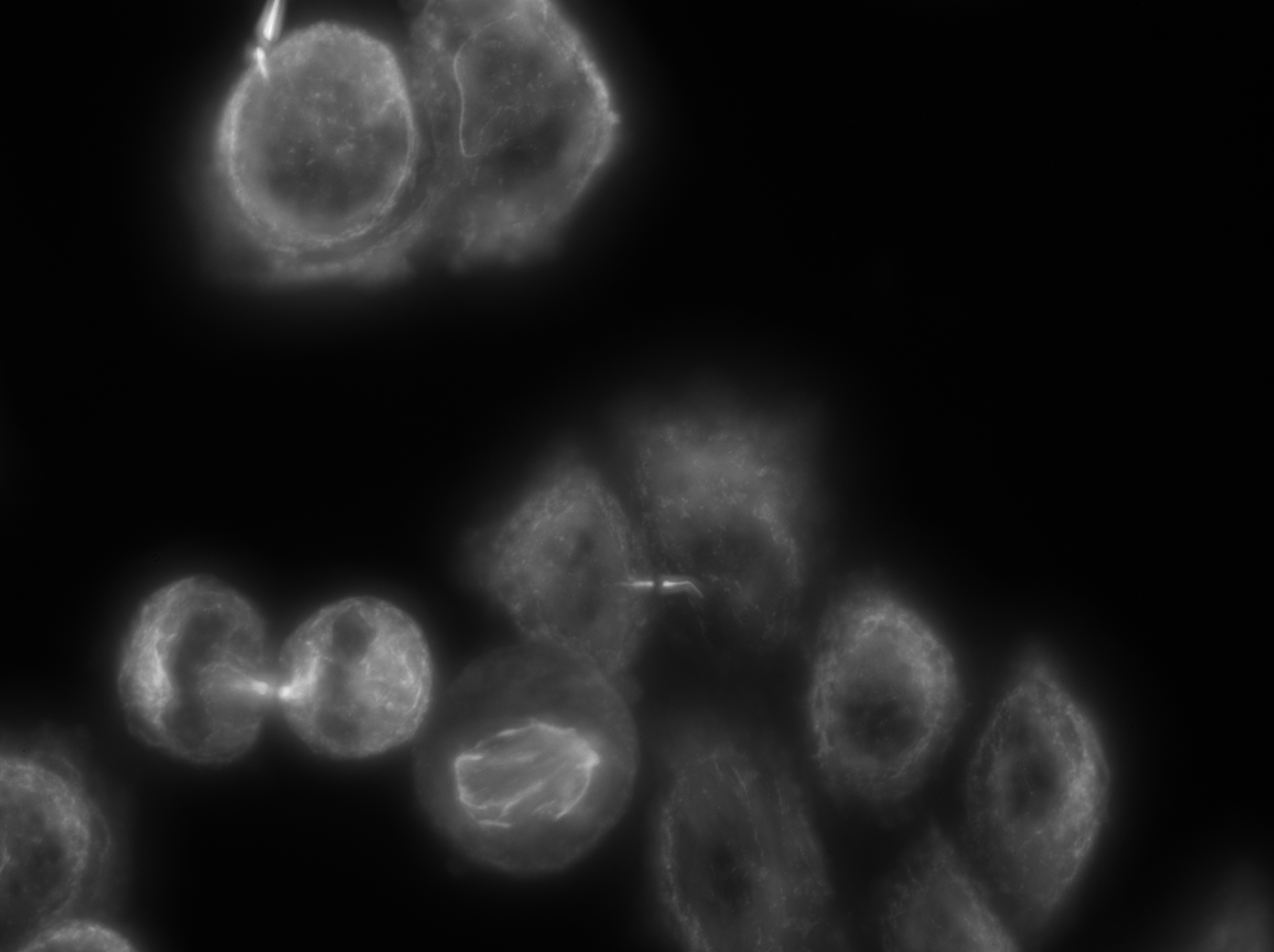

Supplement: Supplementary file 18 — Source data Fig. 5 part 4 [file 44319_2026_742_MOESM18_ESM.zip › Figure 5 Part 4/Fig 5ab WT and KO hela TTLL1-e326g atubulin/EGFP/EGFP-N2 8-23-24 atub R2 LT8.Project Maximum Z_XY1725569218_Z0_T0_C2.tif]

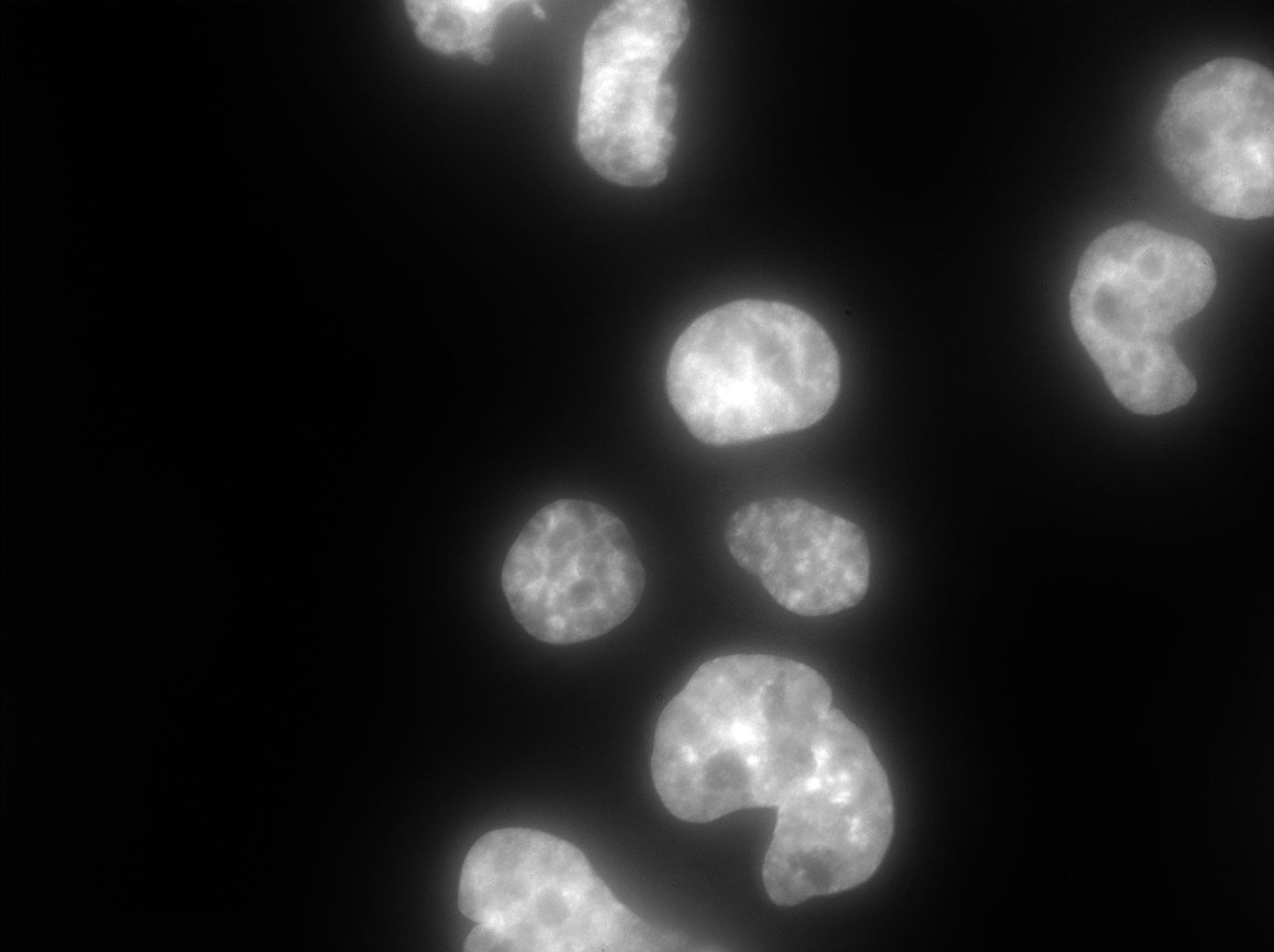

Supplement: Supplementary file 18 — Source data Fig. 5 part 4 [file 44319_2026_742_MOESM18_ESM.zip › Figure 5 Part 4/Fig 5ab WT and KO hela TTLL1-e326g atubulin/EGFP/Cas9 EGFP-N3 10-15-24 R3 LT2.Project Maximum Z_XY1729029607_Z0_T0_C0.tif]

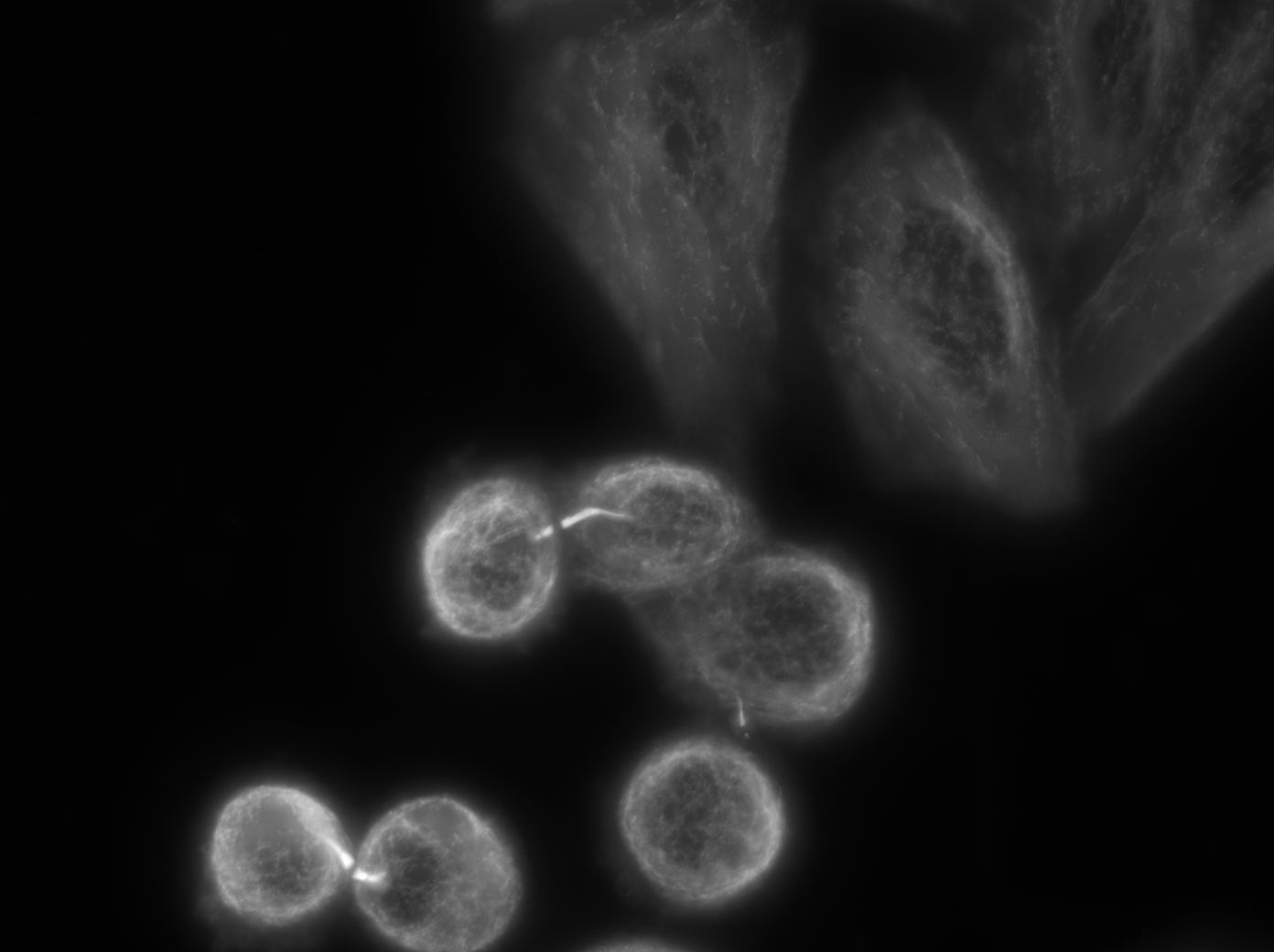

Supplement: Supplementary file 18 — Source data Fig. 5 part 4 [file 44319_2026_742_MOESM18_ESM.zip › Figure 5 Part 4/Fig 5ab WT and KO hela TTLL1-e326g atubulin/EGFP/EGFP-N2 8-23-24 atub R2 LT9.Project Maximum Z_XY1725569333_Z0_T0_C2.tif]

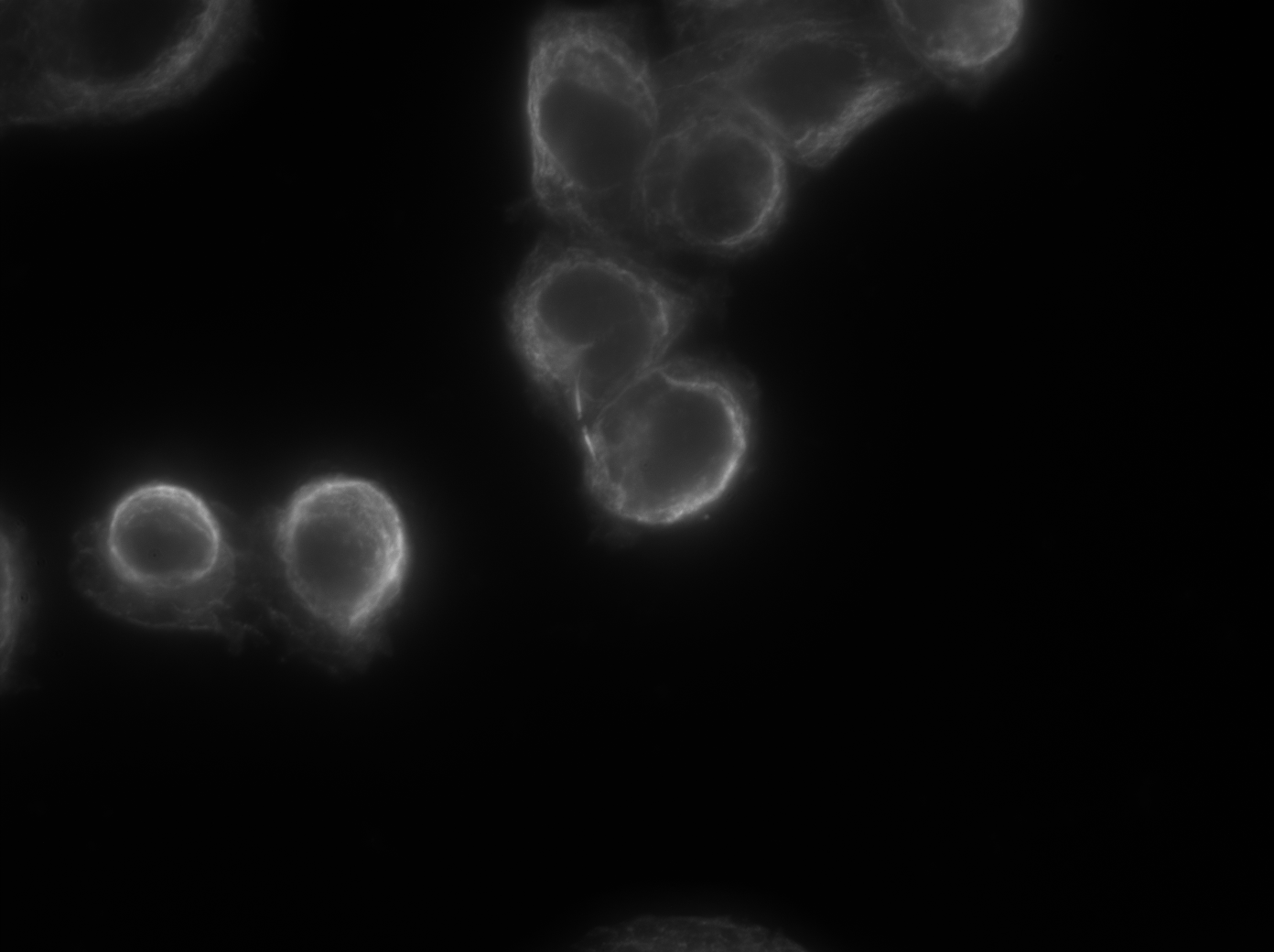

Supplement: Supplementary file 18 — Source data Fig. 5 part 4 [file 44319_2026_742_MOESM18_ESM.zip › Figure 5 Part 4/Fig 5ab WT and KO hela TTLL1-e326g atubulin/EGFP/EGFP-N2 8-23-24 atub R2 LT1.Project Maximum Z_XY1725567854_Z0_T0_C2.tif]

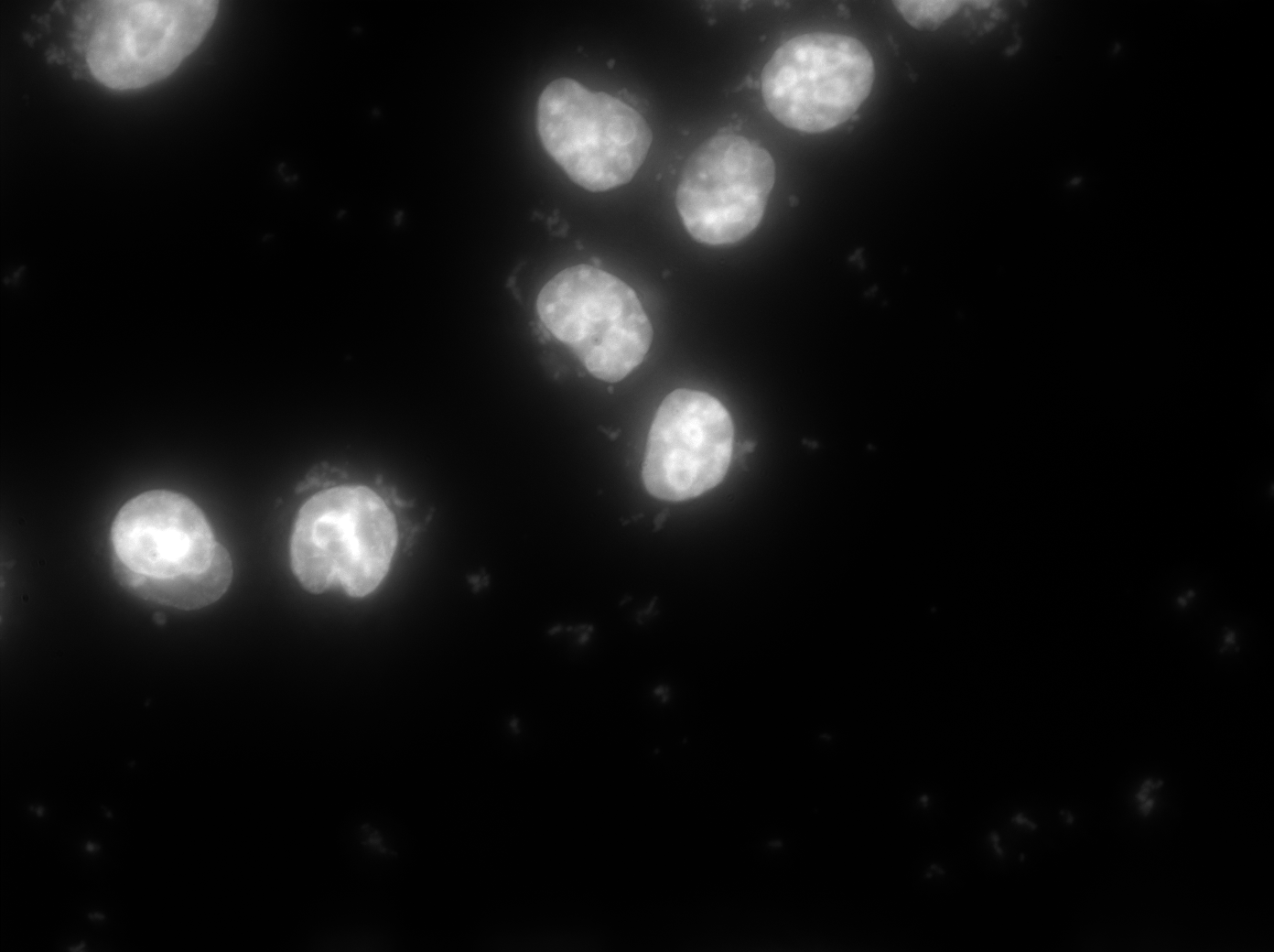

Supplement: Supplementary file 18 — Source data Fig. 5 part 4 [file 44319_2026_742_MOESM18_ESM.zip › Figure 5 Part 4/Fig 5ab WT and KO hela TTLL1-e326g atubulin/EGFP/EGFP-N2 8-23-24 atub R2 LT1.Project Maximum Z_XY1725567854_Z0_T0_C0.tif]

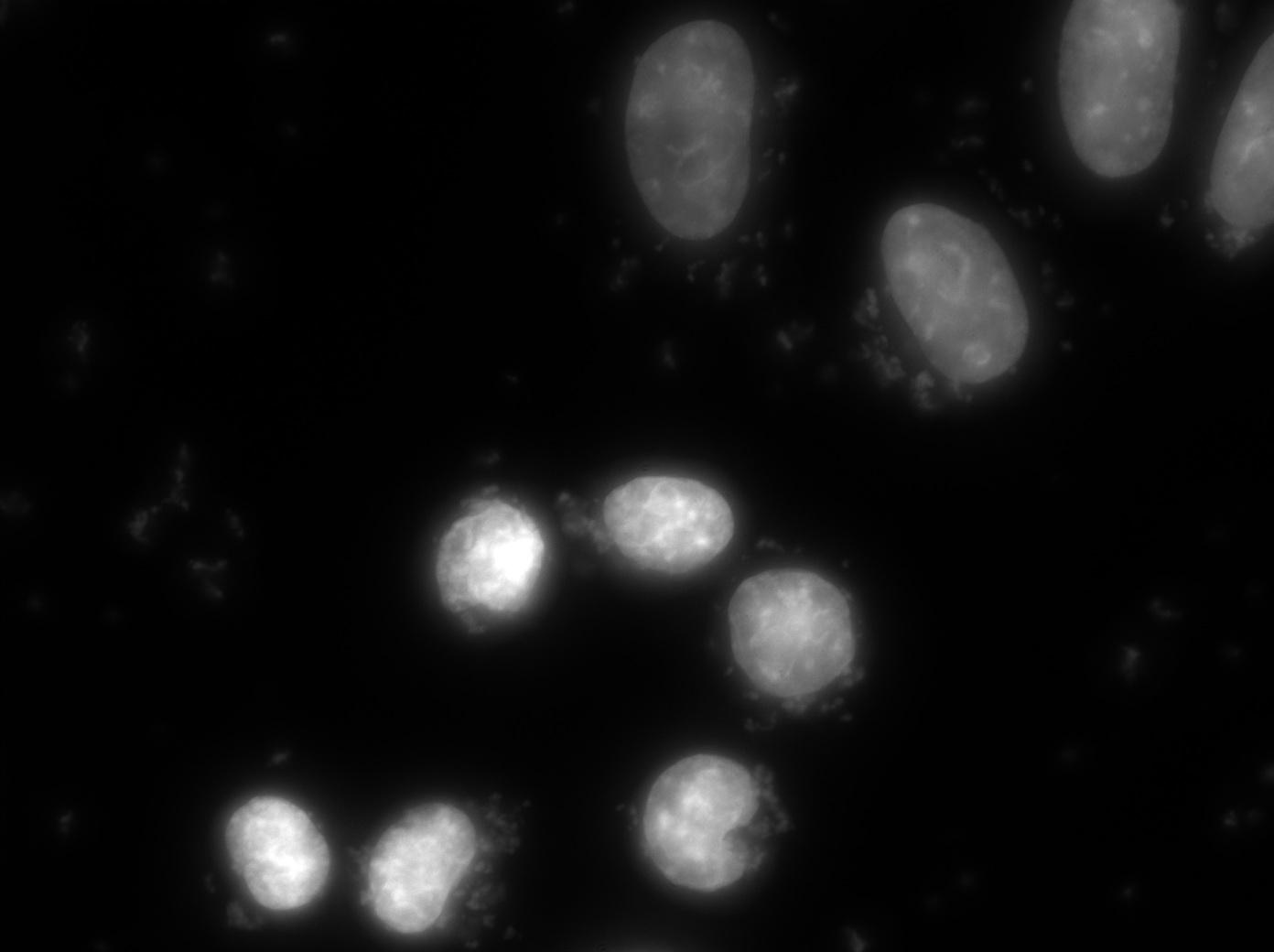

Supplement: Supplementary file 18 — Source data Fig. 5 part 4 [file 44319_2026_742_MOESM18_ESM.zip › Figure 5 Part 4/Fig 5ab WT and KO hela TTLL1-e326g atubulin/EGFP/EGFP-N2 8-23-24 atub R2 LT9.Project Maximum Z_XY1725569333_Z0_T0_C0.tif]

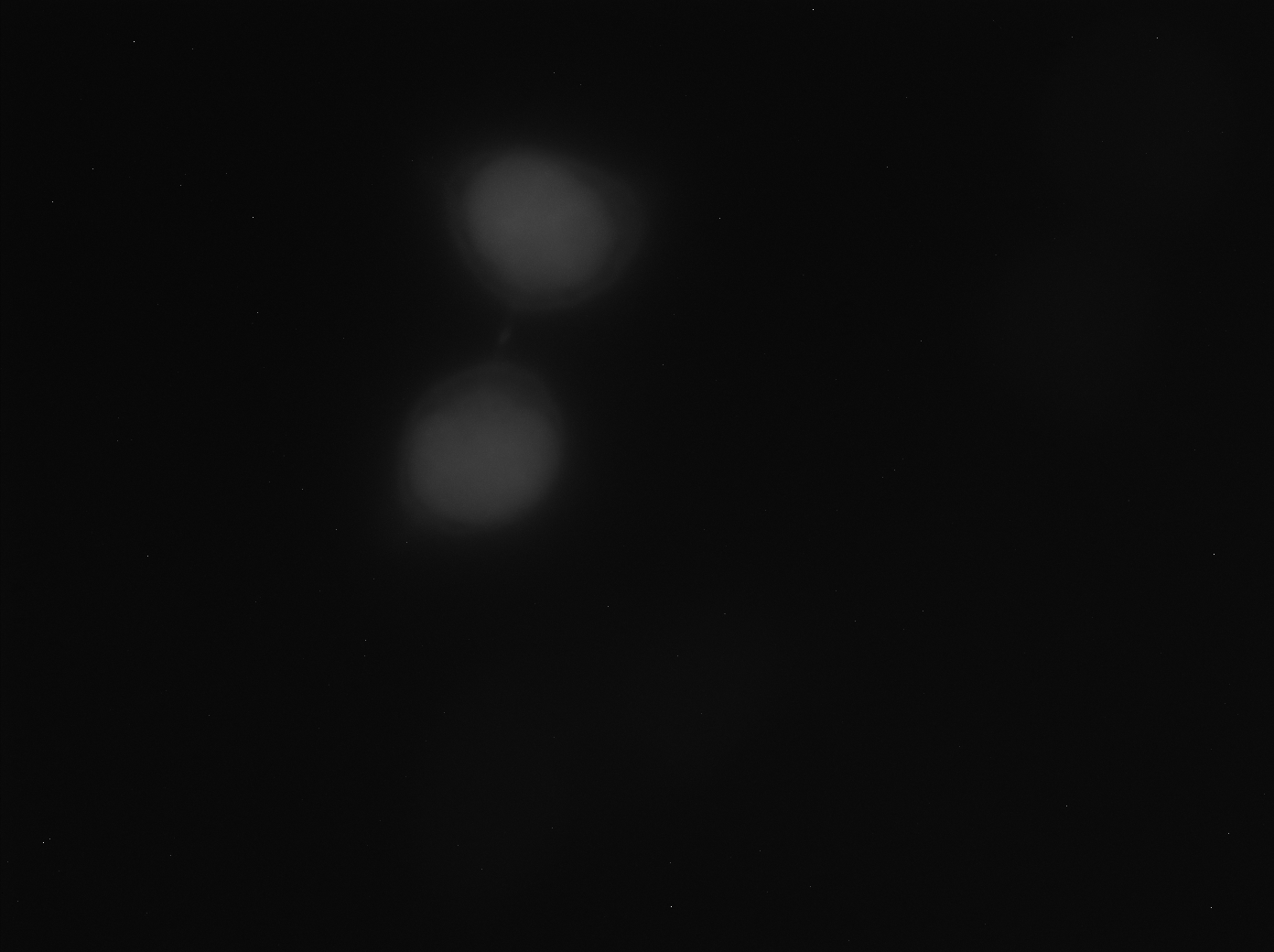

Supplement: Supplementary file 18 — Source data Fig. 5 part 4 [file 44319_2026_742_MOESM18_ESM.zip › Figure 5 Part 4/Fig 5ab WT and KO hela TTLL1-e326g atubulin/EGFP/Cas9 EGFP-N3 10-15-24 R3 LT8.Project Maximum Z_XY1729030584_Z0_T0_C1.tif]

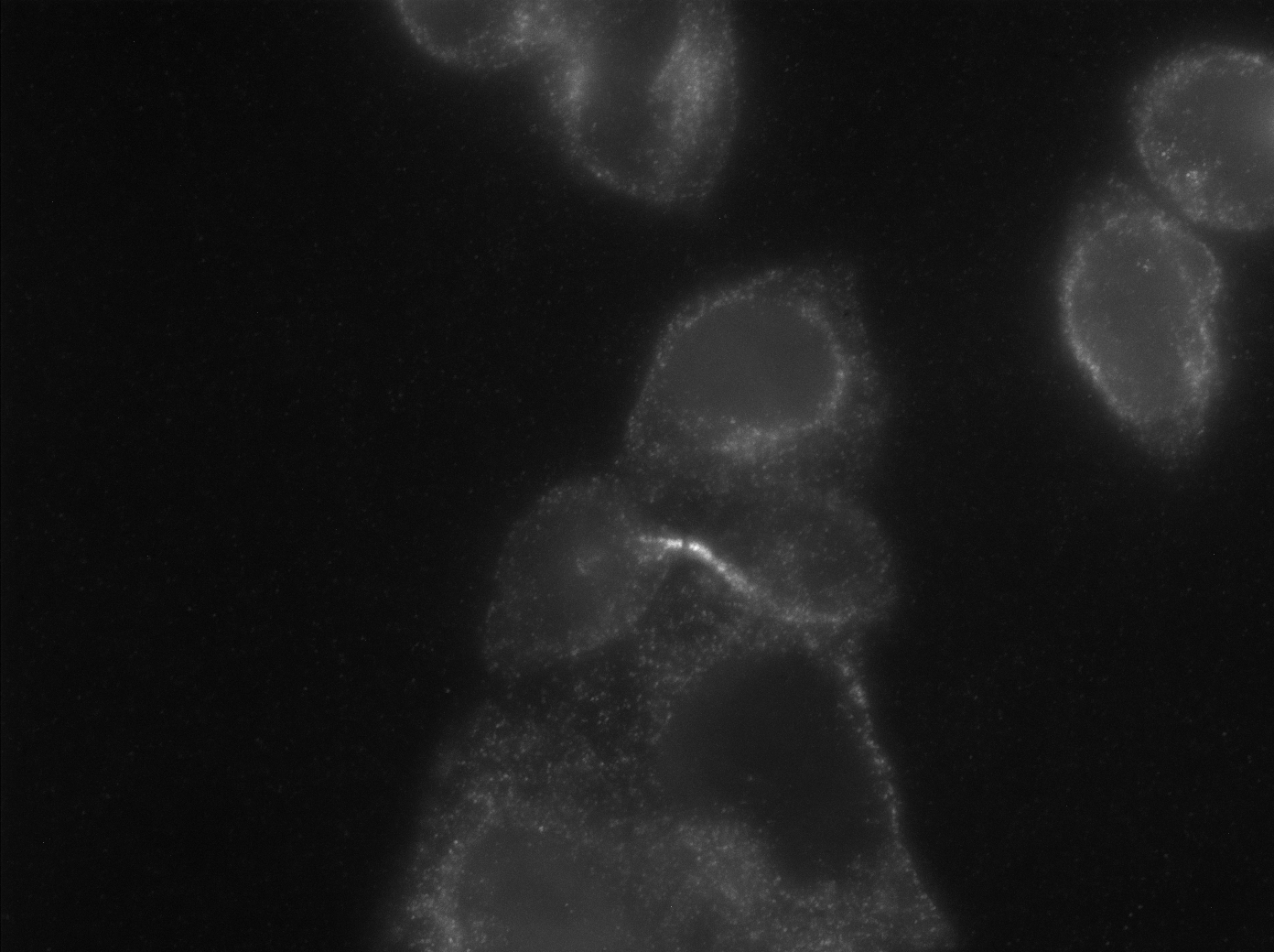

Supplement: Supplementary file 18 — Source data Fig. 5 part 4 [file 44319_2026_742_MOESM18_ESM.zip › Figure 5 Part 4/Fig 5ab WT and KO hela TTLL1-e326g atubulin/EGFP/Cas9 EGFP-N3 10-15-24 R3 LT2.Project Maximum Z_XY1729029607_Z0_T0_C2.tif]

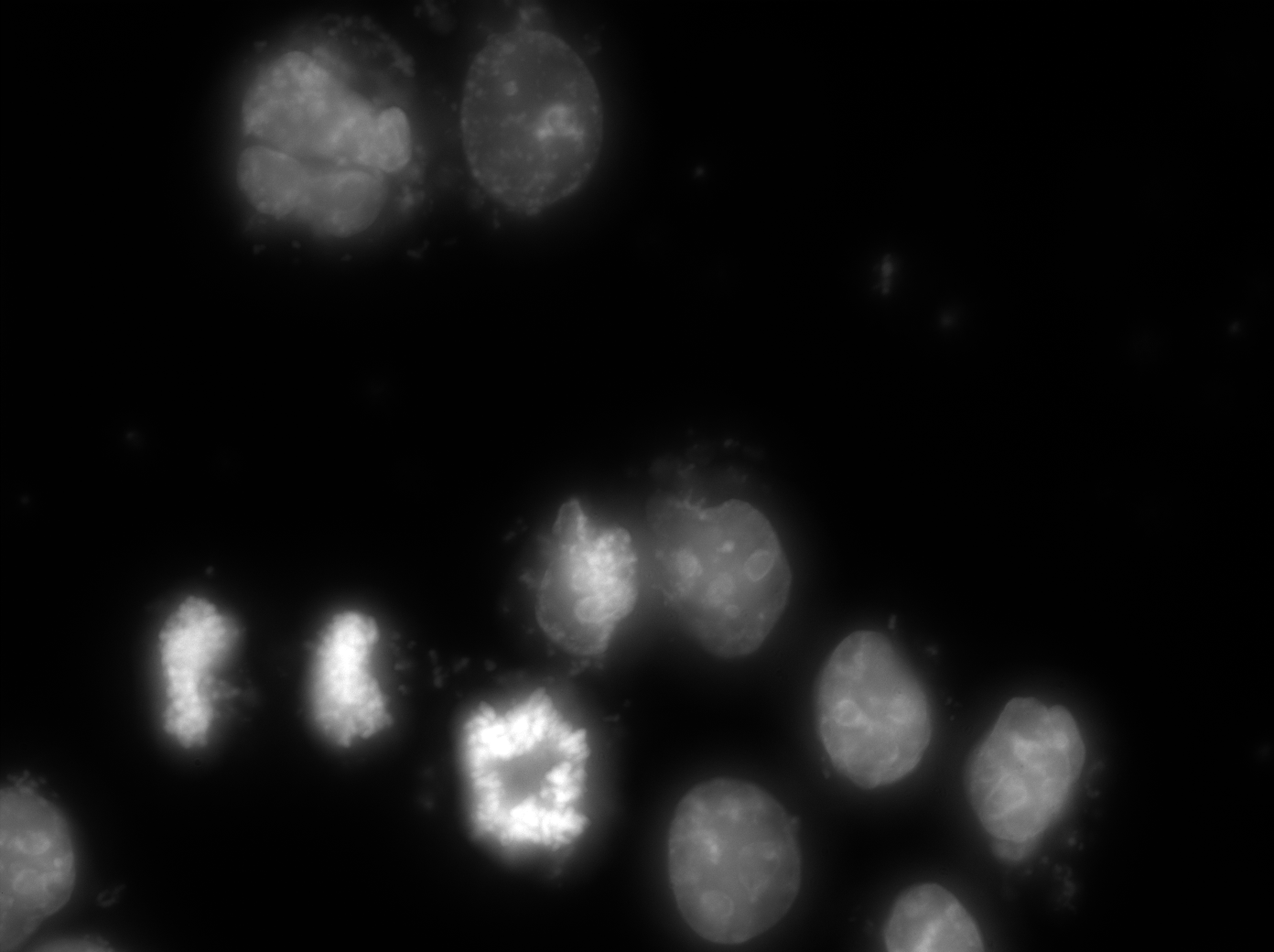

Supplement: Supplementary file 18 — Source data Fig. 5 part 4 [file 44319_2026_742_MOESM18_ESM.zip › Figure 5 Part 4/Fig 5ab WT and KO hela TTLL1-e326g atubulin/EGFP/EGFP-N2 8-23-24 atub R2 LT8.Project Maximum Z_XY1725569218_Z0_T0_C0.tif]

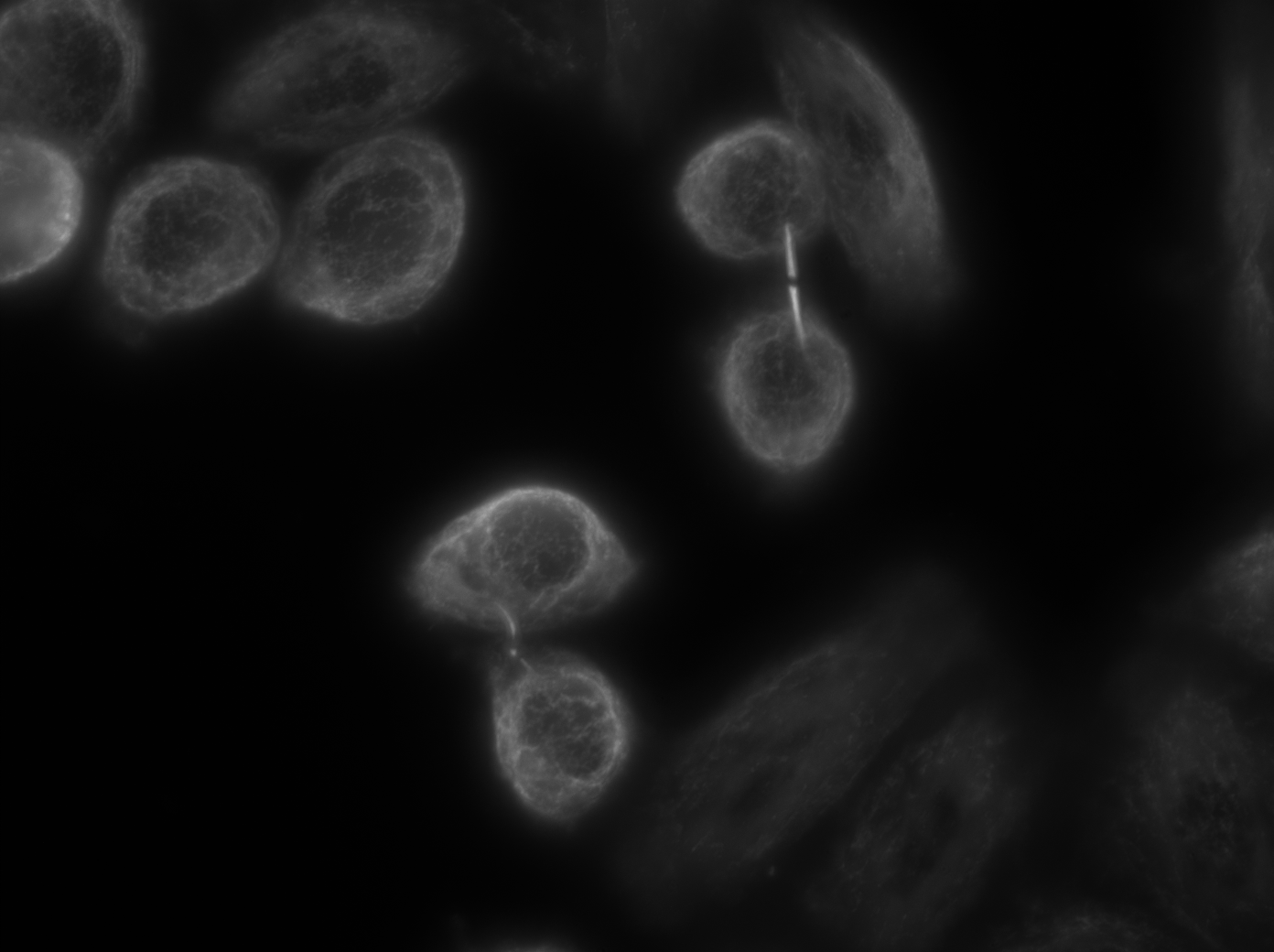

Supplement: Supplementary file 18 — Source data Fig. 5 part 4 [file 44319_2026_742_MOESM18_ESM.zip › Figure 5 Part 4/Fig 5ab WT and KO hela TTLL1-e326g atubulin/EGFP/EGFP-N2 8-23-24 atub R2 LT6LT7.Project Maximum Z_XY1725568941_Z0_T0_C2.tif]

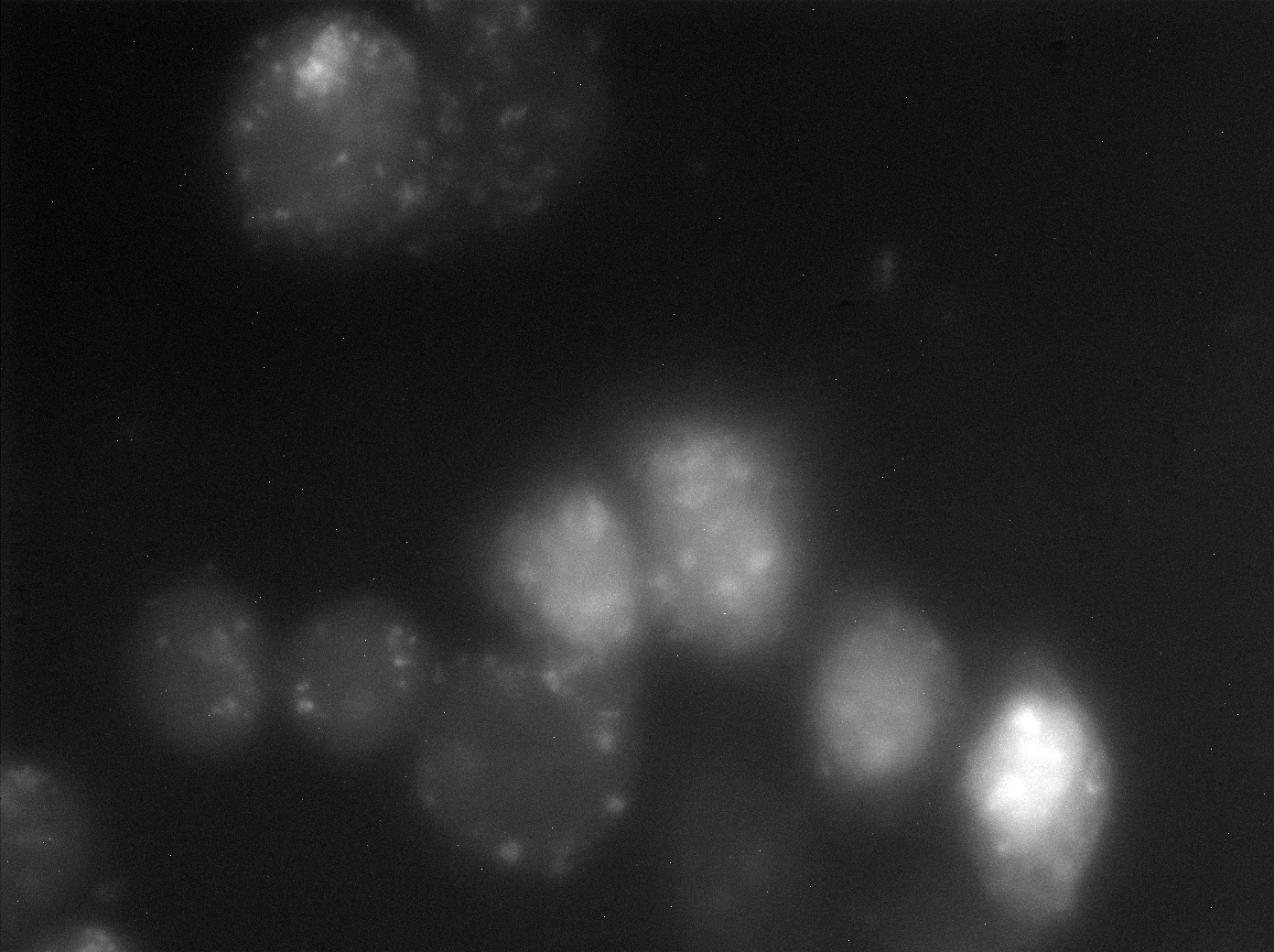

Supplement: Supplementary file 18 — Source data Fig. 5 part 4 [file 44319_2026_742_MOESM18_ESM.zip › Figure 5 Part 4/Fig 5ab WT and KO hela TTLL1-e326g atubulin/EGFP/EGFP-N2 8-23-24 atub R2 LT8.Project Maximum Z_XY1725569218_Z0_T0_C1.tif]

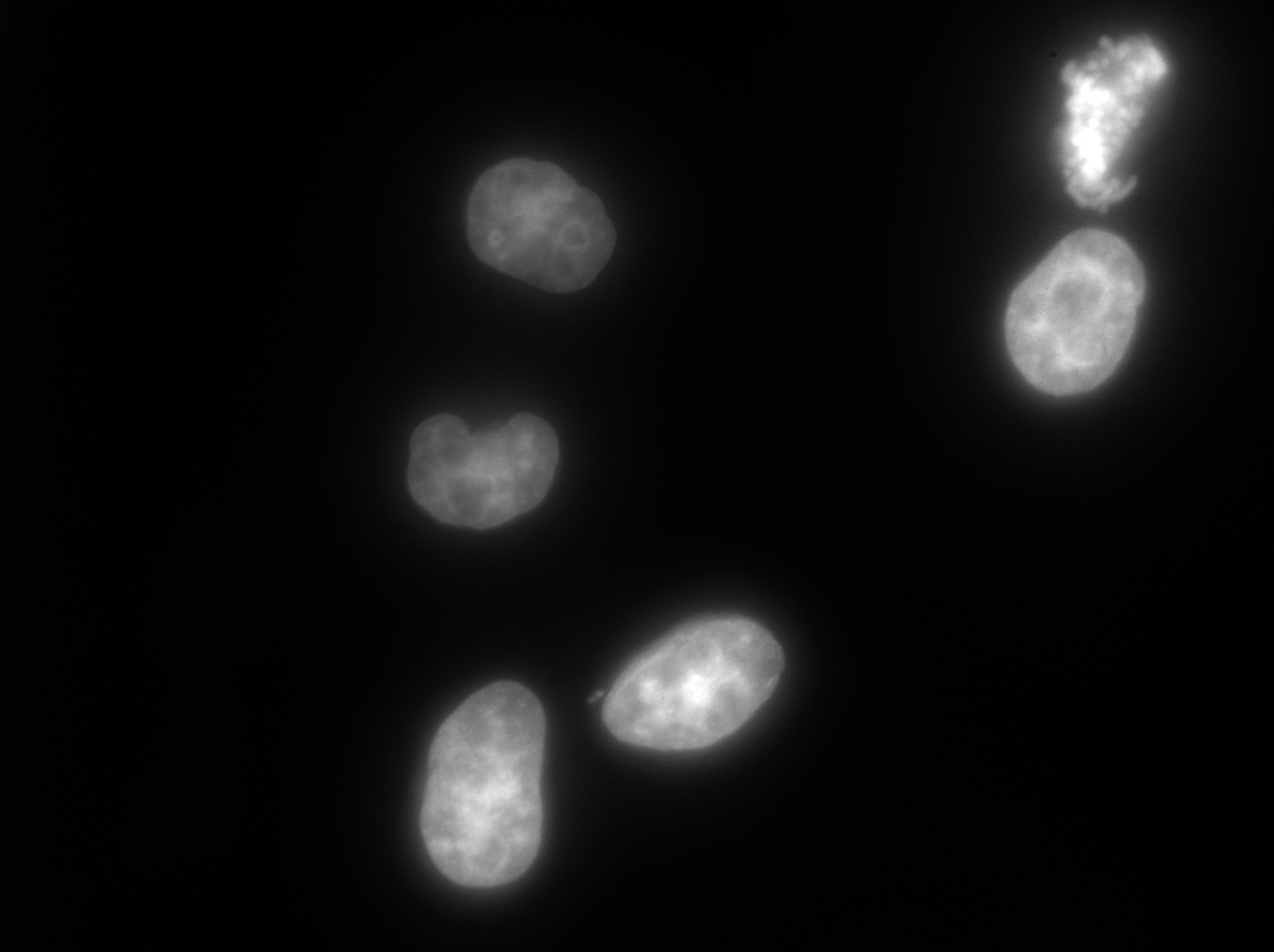

Supplement: Supplementary file 18 — Source data Fig. 5 part 4 [file 44319_2026_742_MOESM18_ESM.zip › Figure 5 Part 4/Fig 5ab WT and KO hela TTLL1-e326g atubulin/EGFP/Cas9 EGFP-N3 10-15-24 R3 LT8.Project Maximum Z_XY1729030584_Z0_T0_C0.tif]

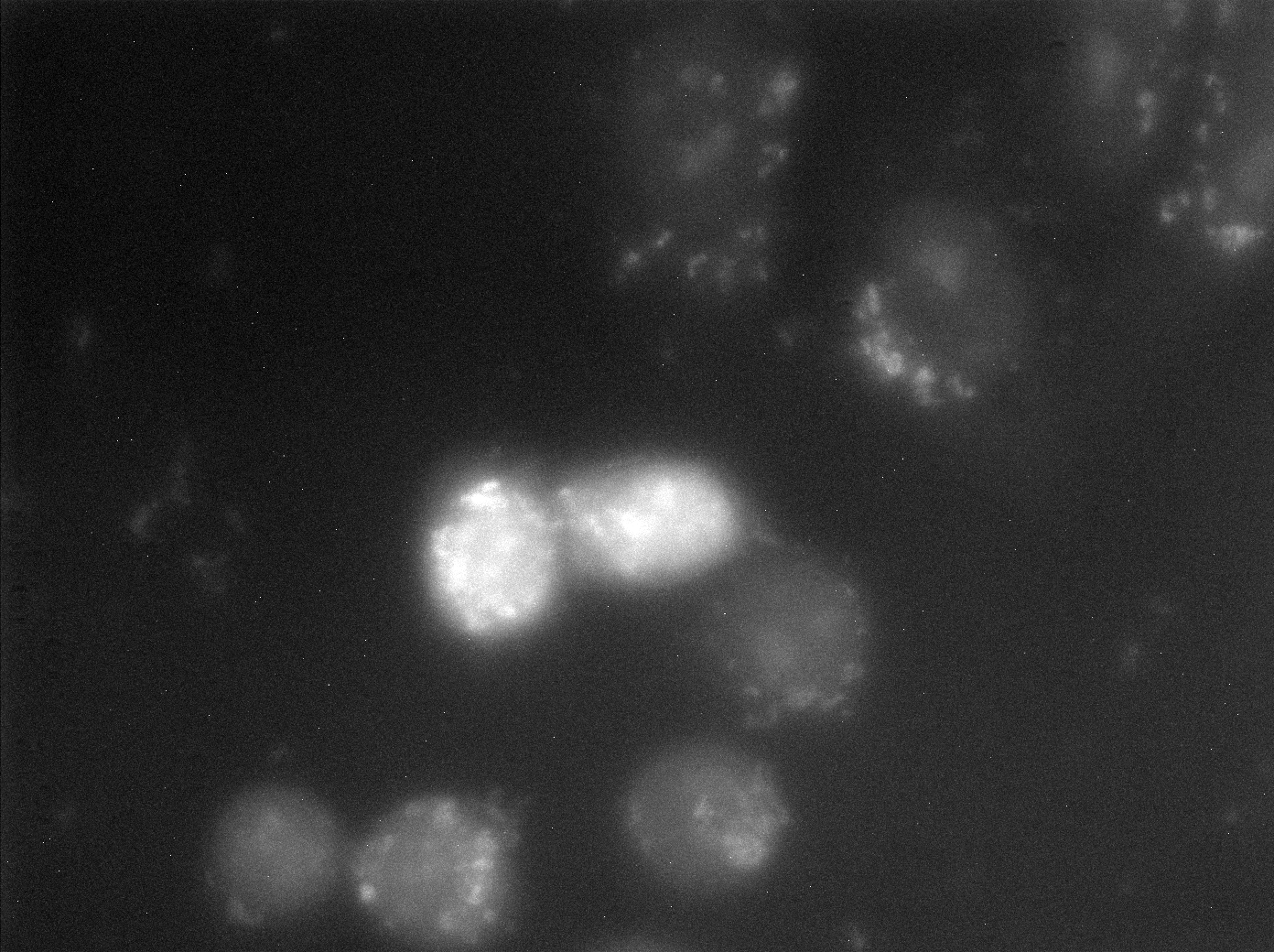

Supplement: Supplementary file 18 — Source data Fig. 5 part 4 [file 44319_2026_742_MOESM18_ESM.zip › Figure 5 Part 4/Fig 5ab WT and KO hela TTLL1-e326g atubulin/EGFP/EGFP-N2 8-23-24 atub R2 LT9.Project Maximum Z_XY1725569333_Z0_T0_C1.tif]

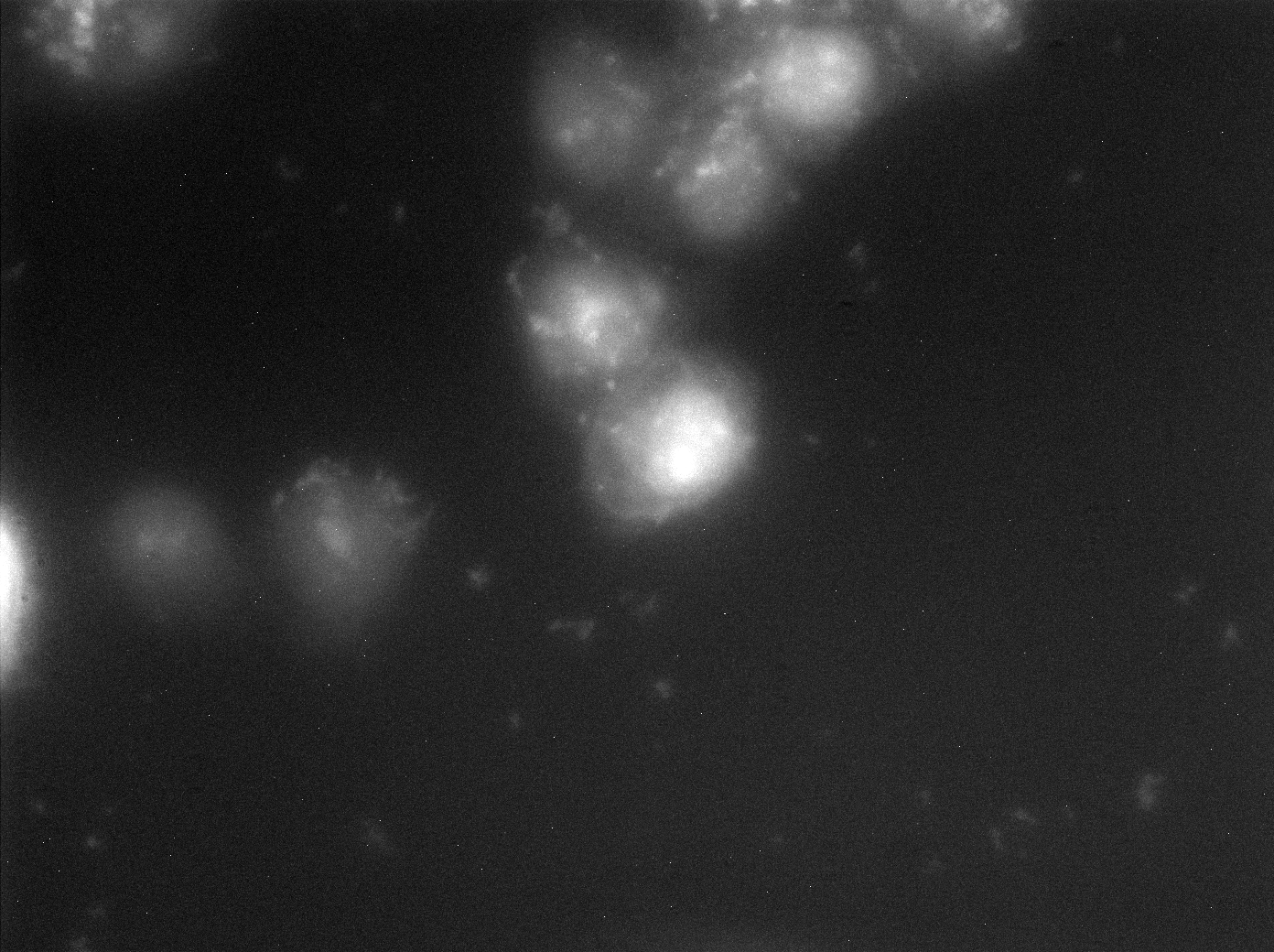

Supplement: Supplementary file 18 — Source data Fig. 5 part 4 [file 44319_2026_742_MOESM18_ESM.zip › Figure 5 Part 4/Fig 5ab WT and KO hela TTLL1-e326g atubulin/EGFP/EGFP-N2 8-23-24 atub R2 LT1.Project Maximum Z_XY1725567854_Z0_T0_C1.tif]

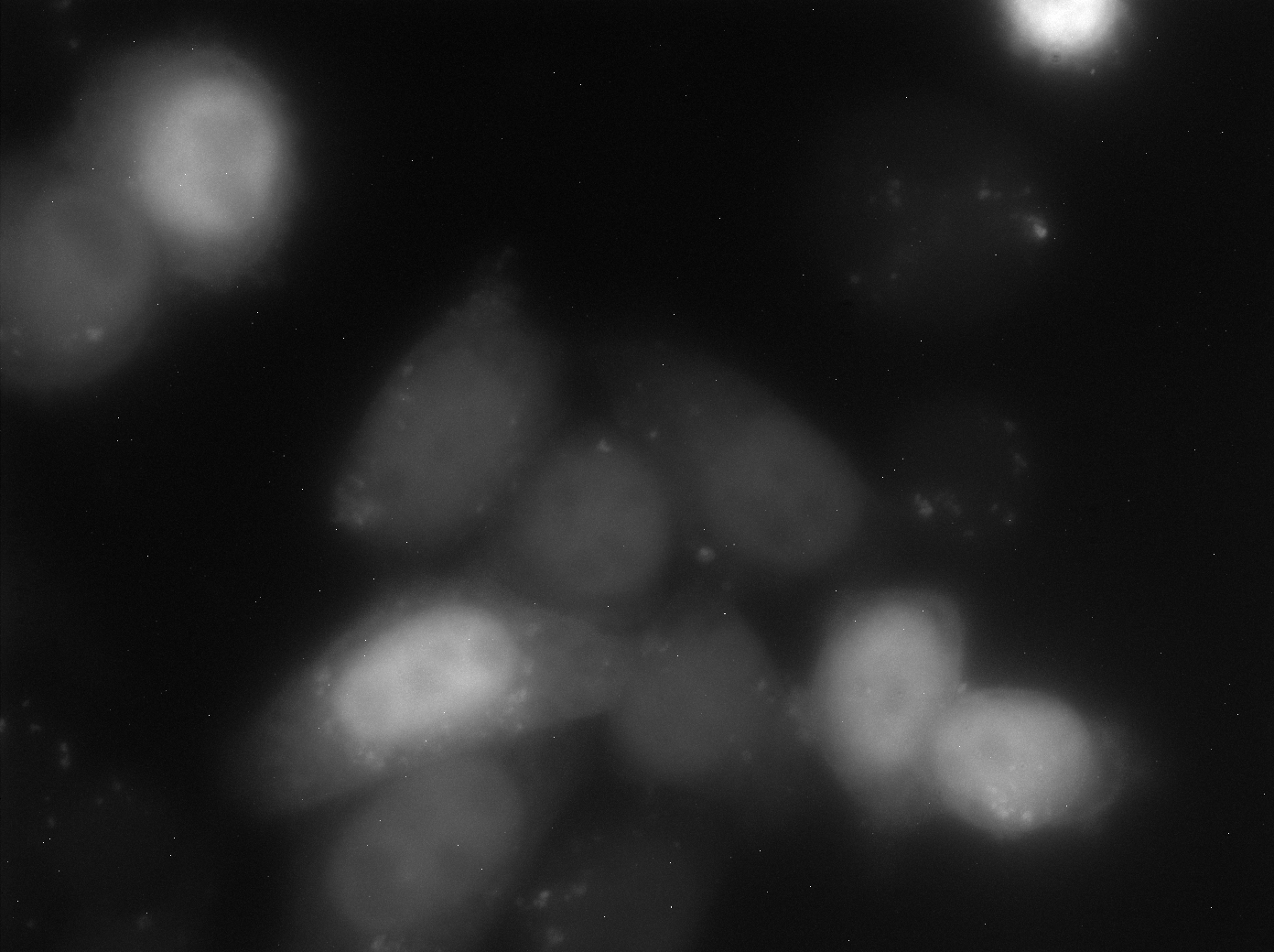

Supplement: Supplementary file 18 — Source data Fig. 5 part 4 [file 44319_2026_742_MOESM18_ESM.zip › Figure 5 Part 4/Fig 5ab WT and KO hela TTLL1-e326g atubulin/Control/WT Hela TTLL1-mut R3 11-13-24 LT1.Project Maximum Z_XY1731545726_Z0_T0_C1.tif]

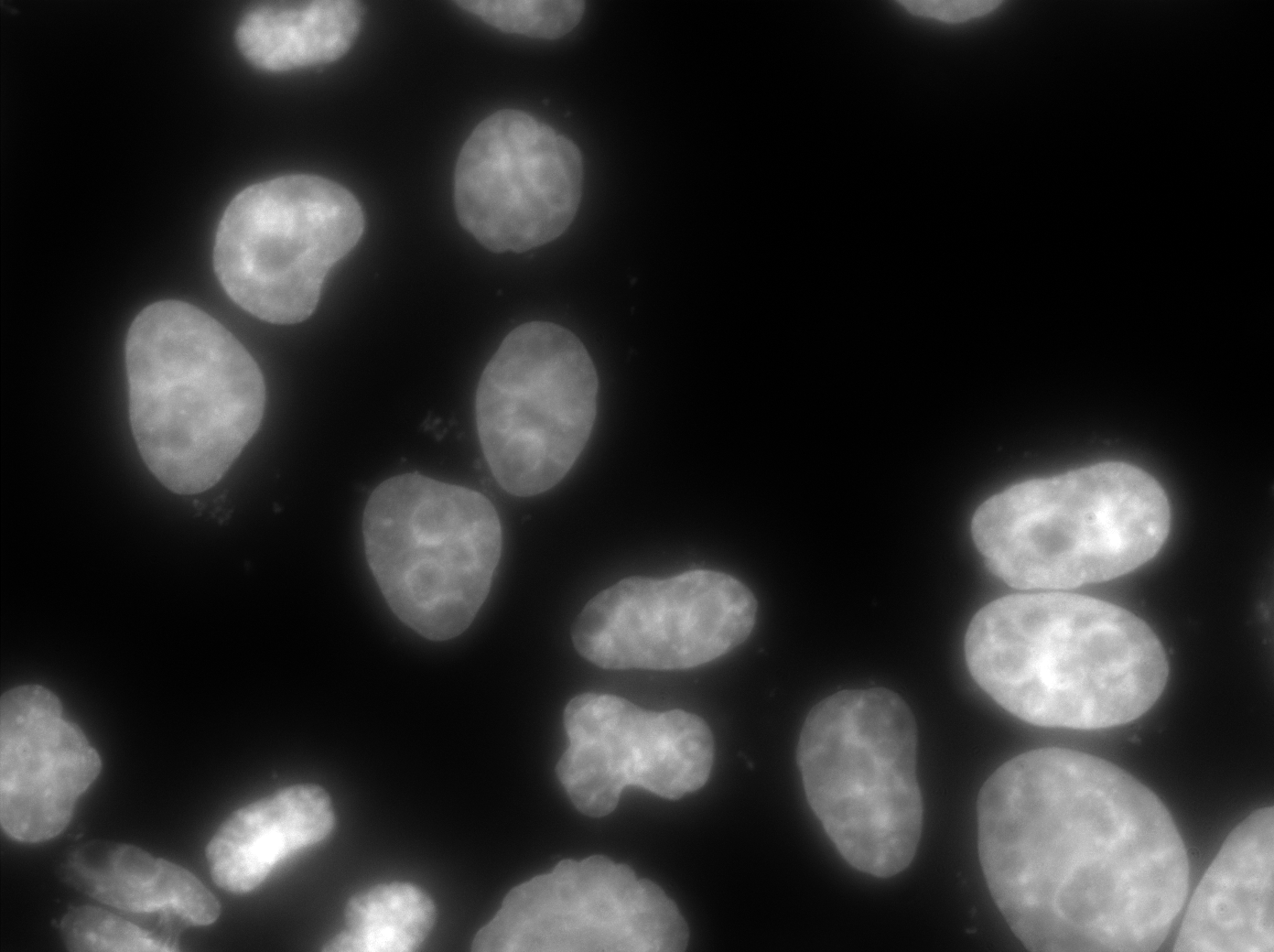

Supplement: Supplementary file 18 — Source data Fig. 5 part 4 [file 44319_2026_742_MOESM18_ESM.zip › Figure 5 Part 4/Fig 5ab WT and KO hela TTLL1-e326g atubulin/Control/WT Hela TTLL1-mut R3 11-13-24 LT9.Project Maximum Z_XY1731547173_Z0_T0_C0.tif]

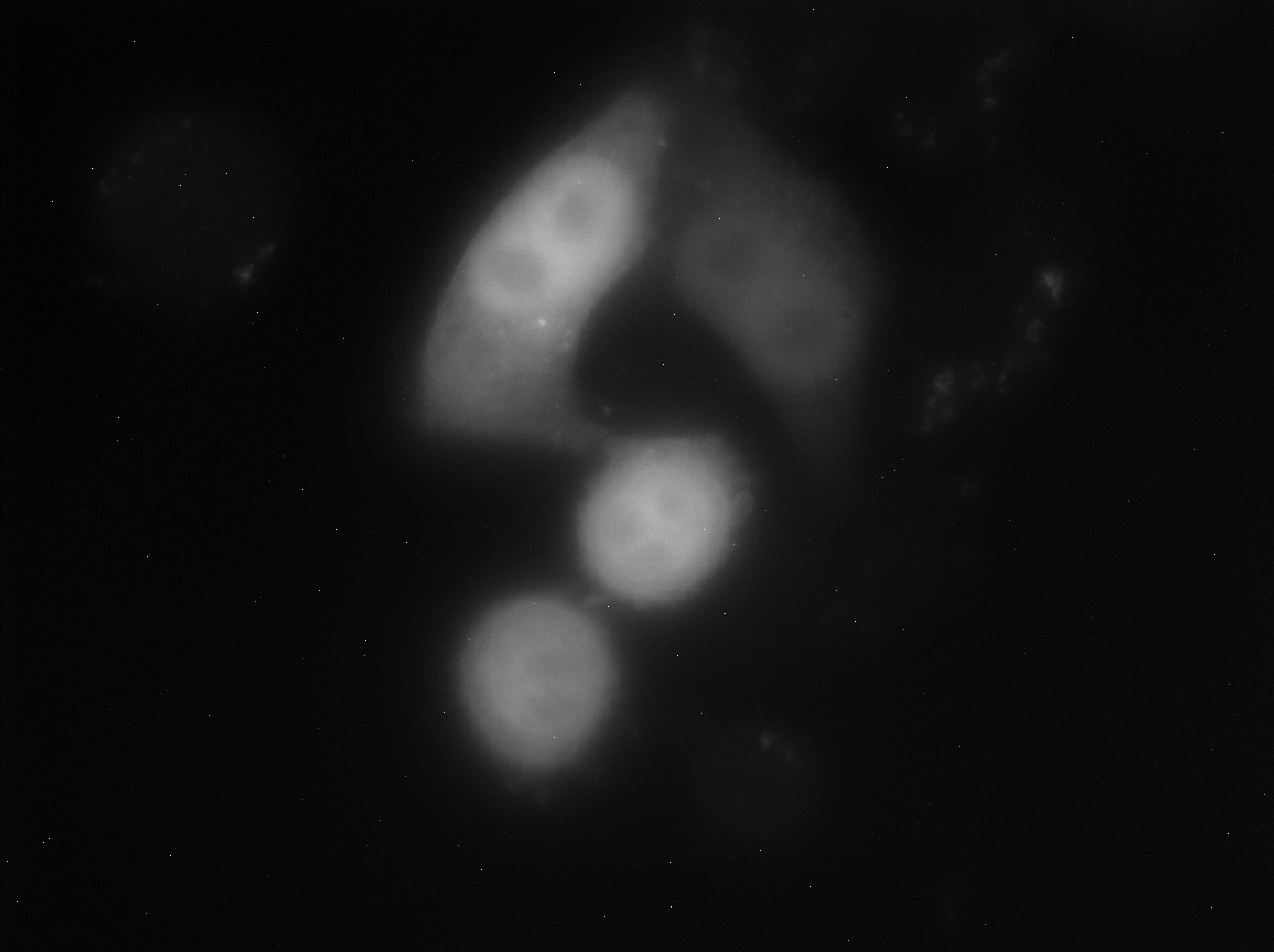

Supplement: Supplementary file 18 — Source data Fig. 5 part 4 [file 44319_2026_742_MOESM18_ESM.zip › Figure 5 Part 4/Fig 5ab WT and KO hela TTLL1-e326g atubulin/Control/WT Hela TTLL1-mut R2 LT9.Project Maximum Z_XY1731544345_Z0_T0_C1.tif]

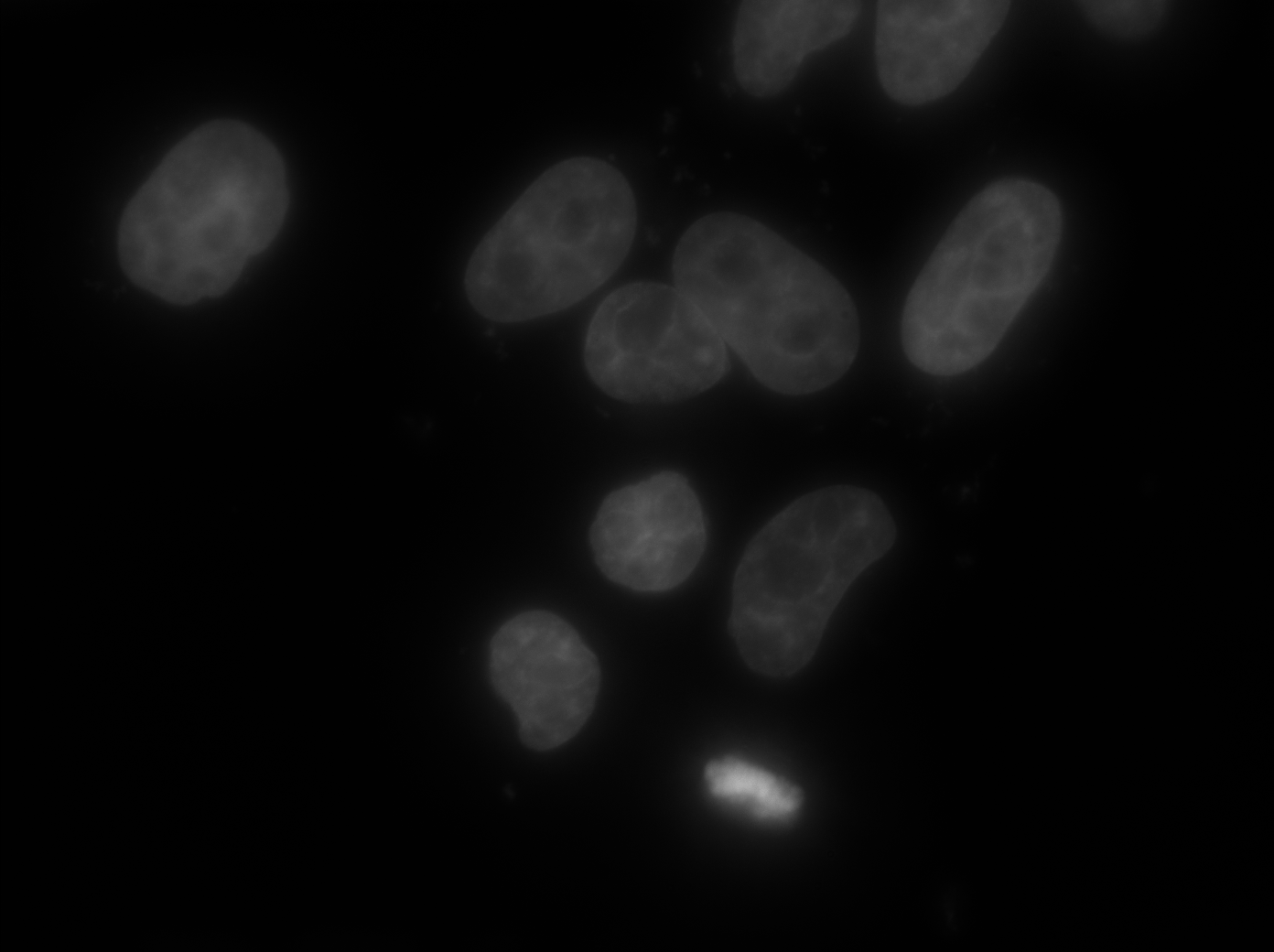

Supplement: Supplementary file 18 — Source data Fig. 5 part 4 [file 44319_2026_742_MOESM18_ESM.zip › Figure 5 Part 4/Fig 5ab WT and KO hela TTLL1-e326g atubulin/Control/WT Hela TTLL1-mut R2 LT9.Project Maximum Z_XY1731544345_Z0_T0_C0.tif]

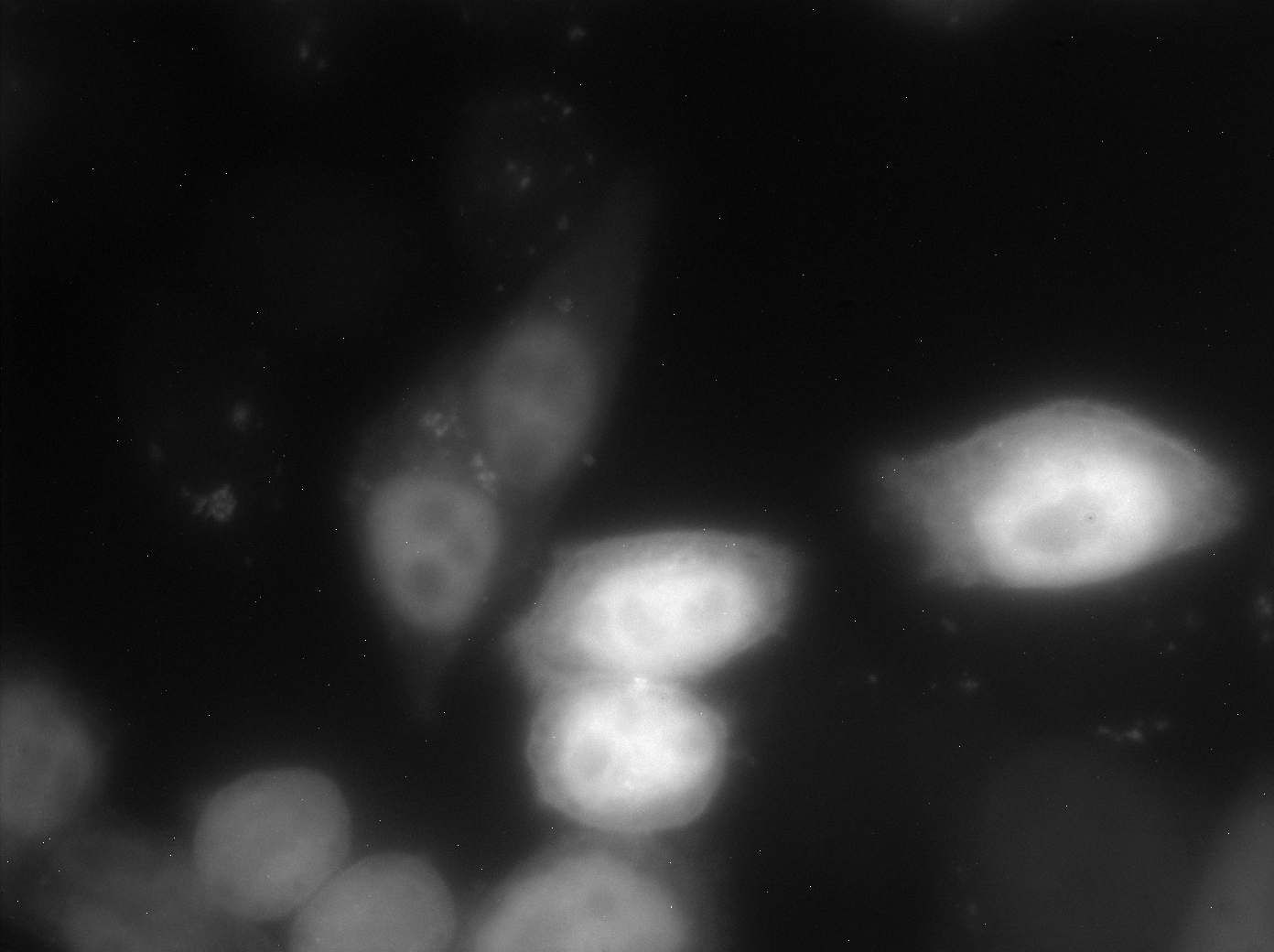

Supplement: Supplementary file 18 — Source data Fig. 5 part 4 [file 44319_2026_742_MOESM18_ESM.zip › Figure 5 Part 4/Fig 5ab WT and KO hela TTLL1-e326g atubulin/Control/WT Hela TTLL1-mut R3 11-13-24 LT9.Project Maximum Z_XY1731547173_Z0_T0_C1.tif]

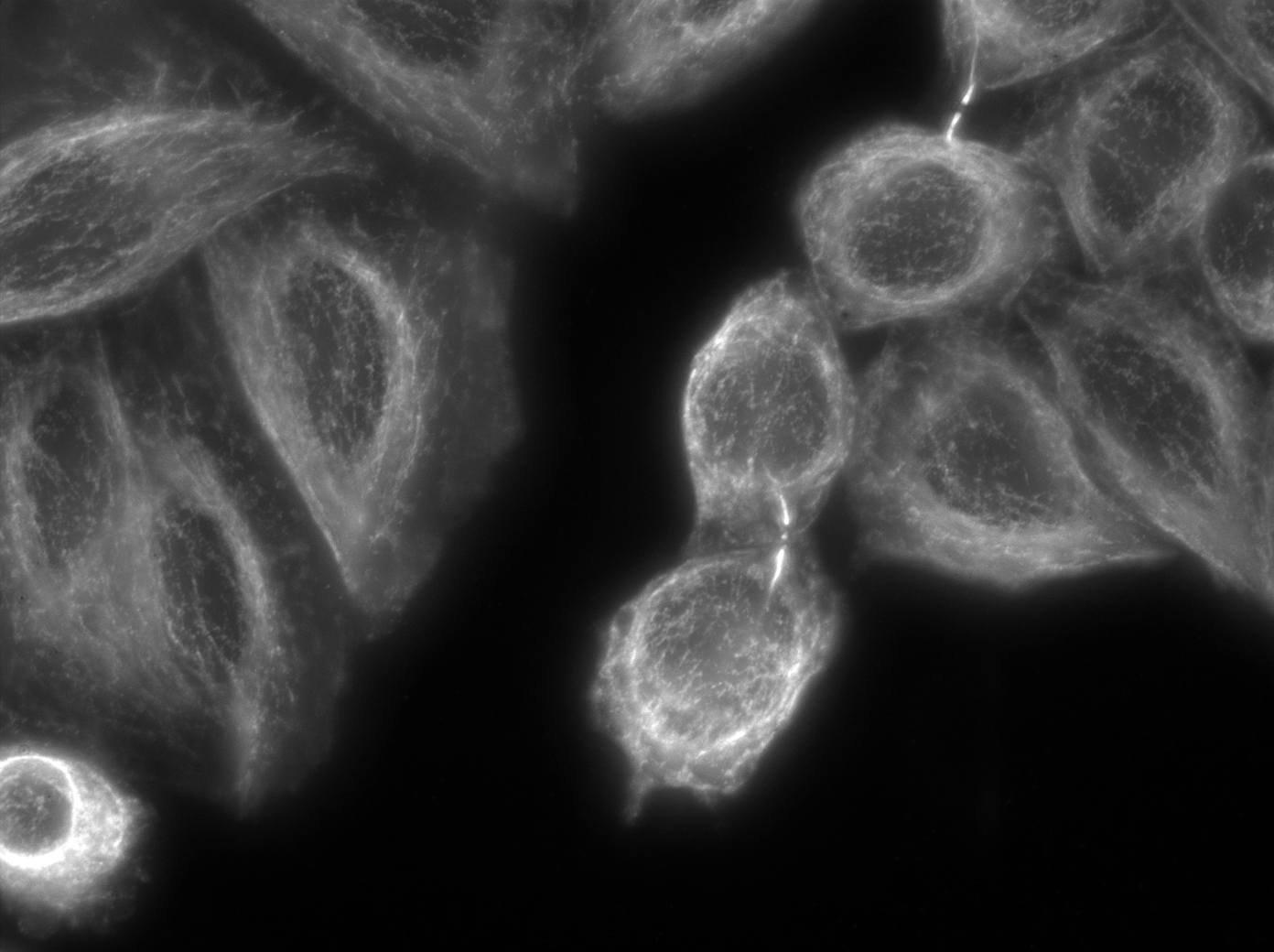

Supplement: Supplementary file 18 — Source data Fig. 5 part 4 [file 44319_2026_742_MOESM18_ESM.zip › Figure 5 Part 4/Fig 5ab WT and KO hela TTLL1-e326g atubulin/Control/TTLL1-mut atub R2 LT4.Project Maximum Z_XY1724951081_Z0_T0_C2.tif]

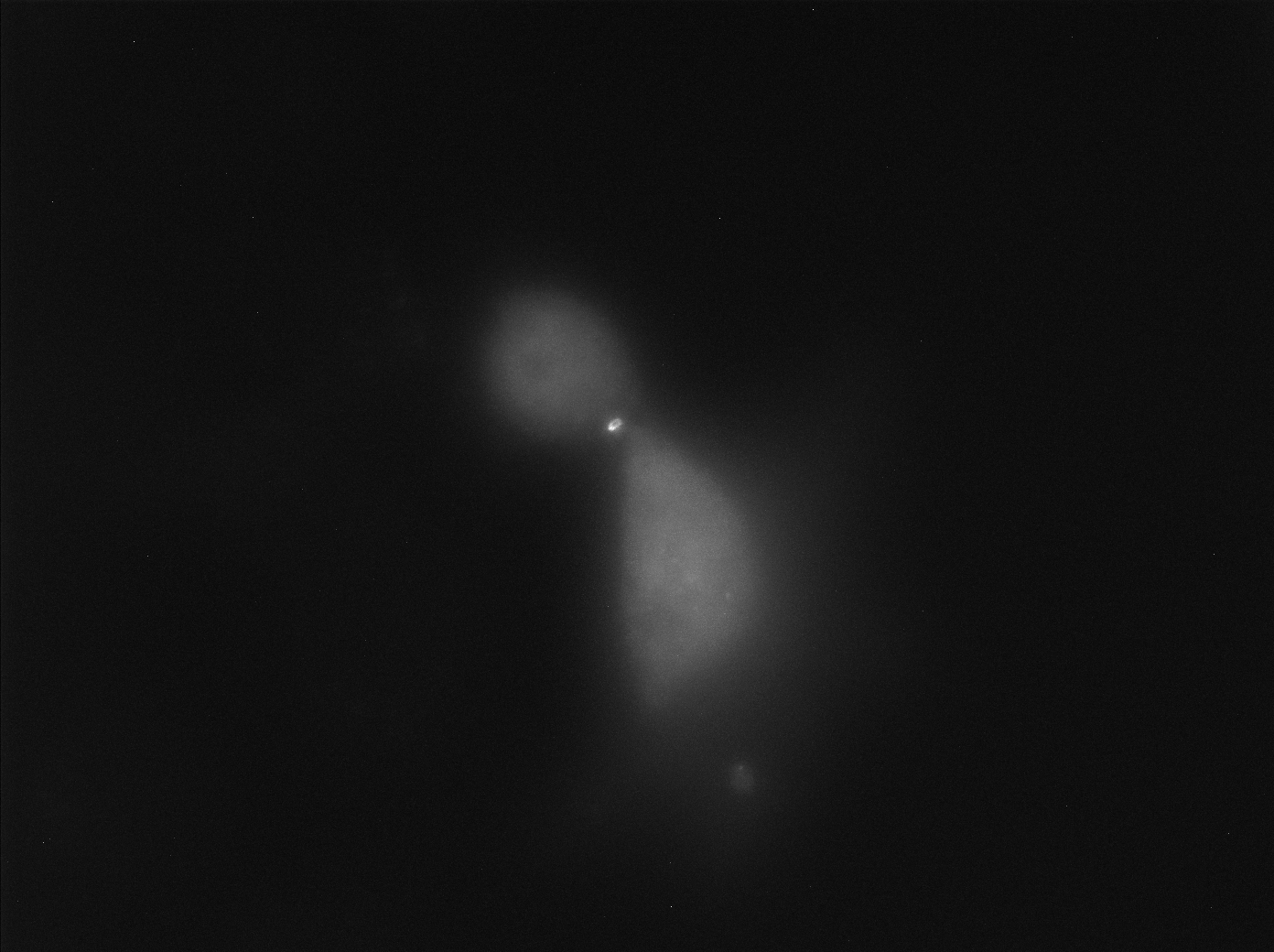

Supplement: Supplementary file 18 — Source data Fig. 5 part 4 [file 44319_2026_742_MOESM18_ESM.zip › Figure 5 Part 4/Fig 5ab WT and KO hela TTLL1-e326g atubulin/Control/TTLL1-mut atub R1 LT1.Project Maximum Z_XY1724435566_Z0_T0_C1-01.tif]

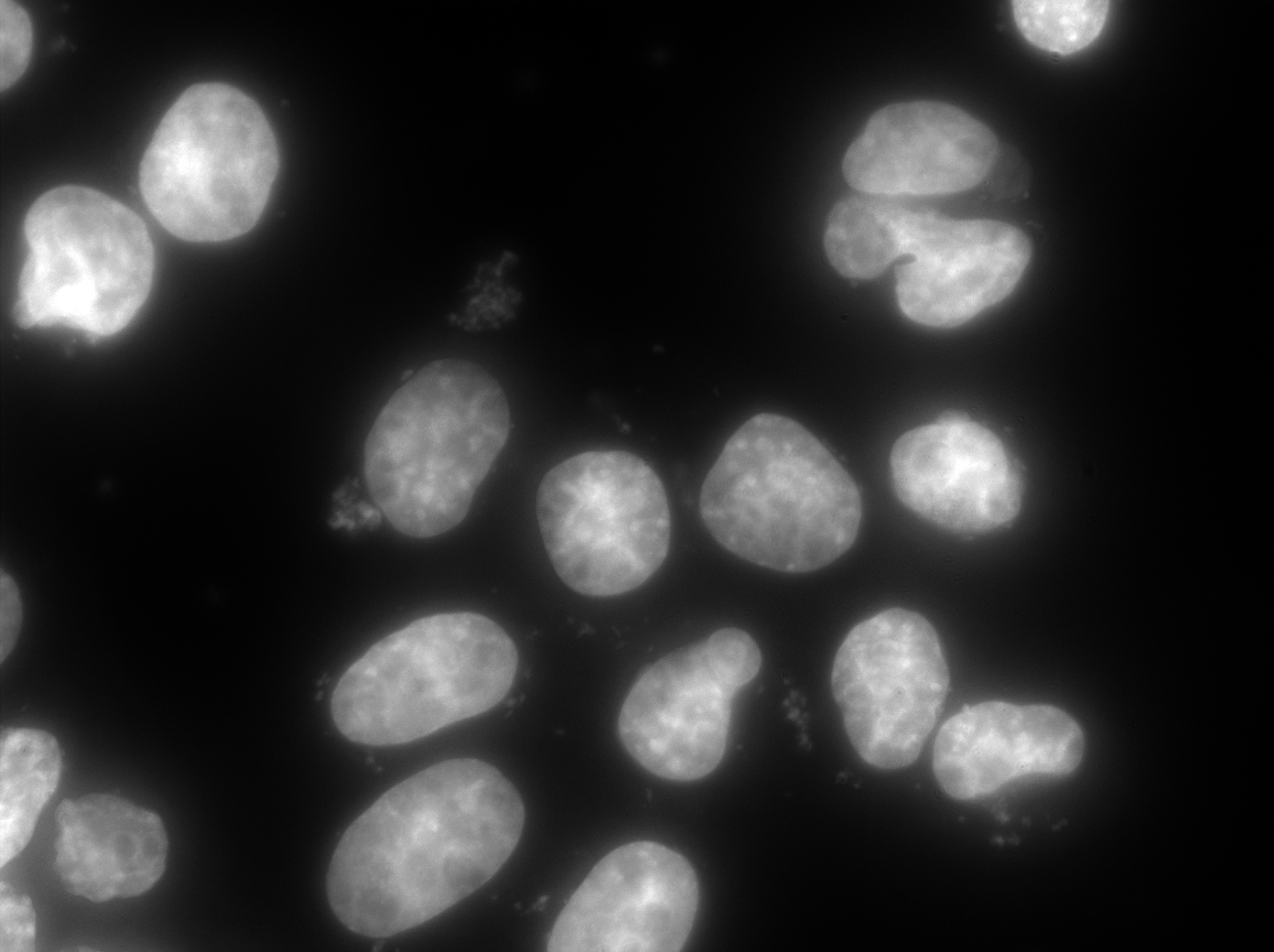

Supplement: Supplementary file 18 — Source data Fig. 5 part 4 [file 44319_2026_742_MOESM18_ESM.zip › Figure 5 Part 4/Fig 5ab WT and KO hela TTLL1-e326g atubulin/Control/WT Hela TTLL1-mut R3 11-13-24 LT1.Project Maximum Z_XY1731545726_Z0_T0_C0.tif]

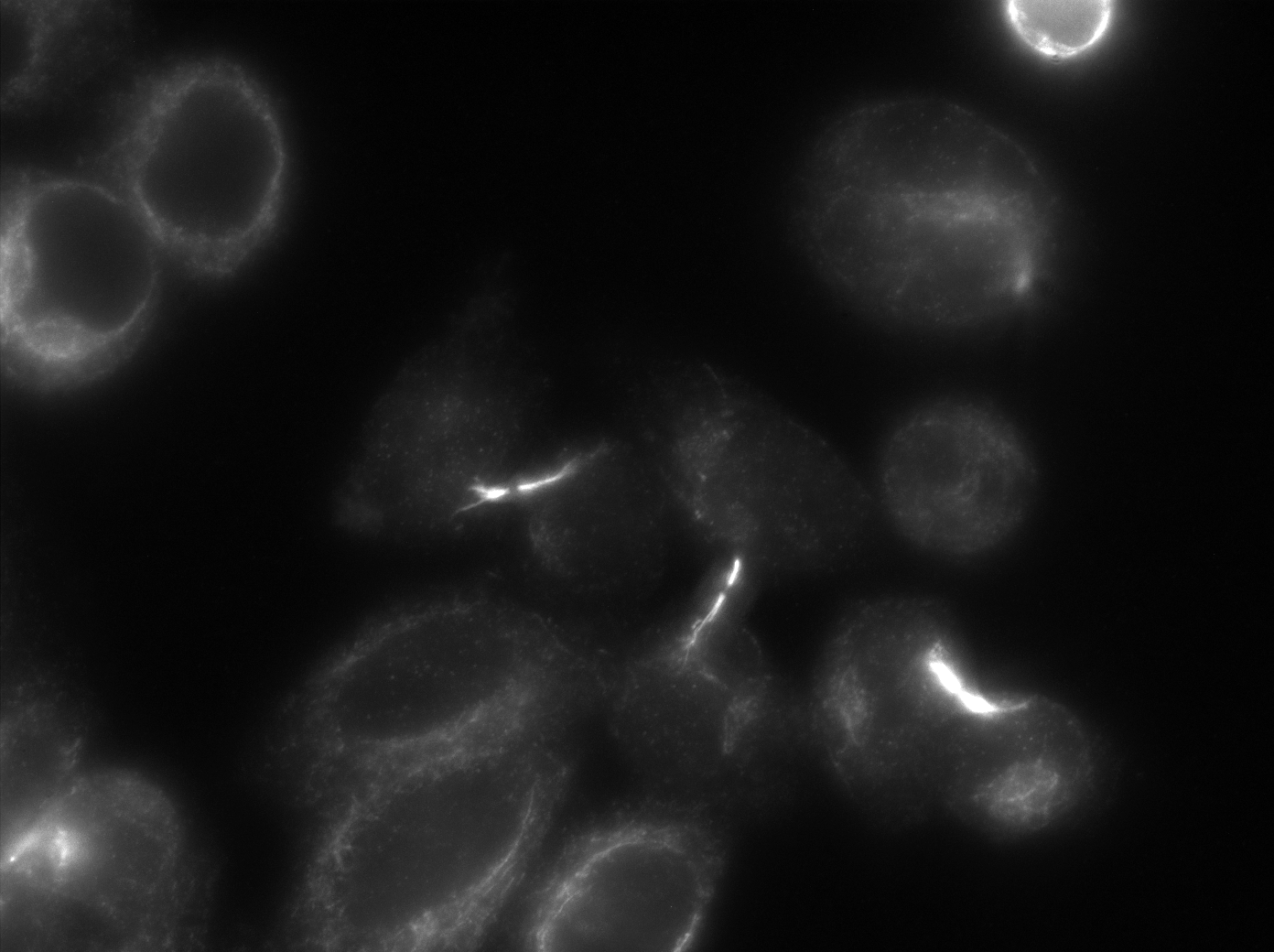

Supplement: Supplementary file 18 — Source data Fig. 5 part 4 [file 44319_2026_742_MOESM18_ESM.zip › Figure 5 Part 4/Fig 5ab WT and KO hela TTLL1-e326g atubulin/Control/WT Hela TTLL1-mut R3 11-13-24 LT1.Project Maximum Z_XY1731545726_Z0_T0_C2.tif]

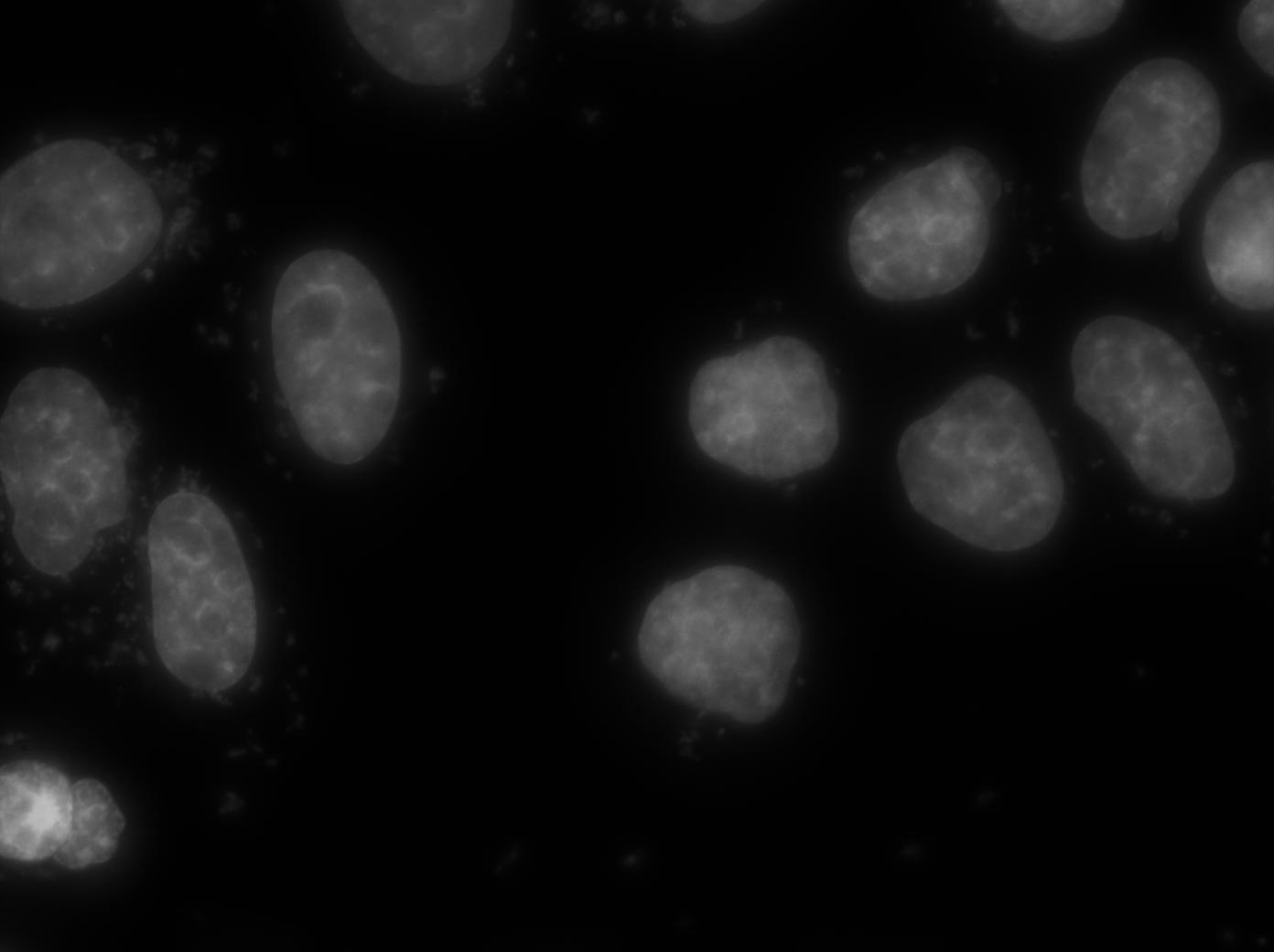

Supplement: Supplementary file 18 — Source data Fig. 5 part 4 [file 44319_2026_742_MOESM18_ESM.zip › Figure 5 Part 4/Fig 5ab WT and KO hela TTLL1-e326g atubulin/Control/TTLL1-mut atub R2 LT4.Project Maximum Z_XY1724951081_Z0_T0_C0.tif]

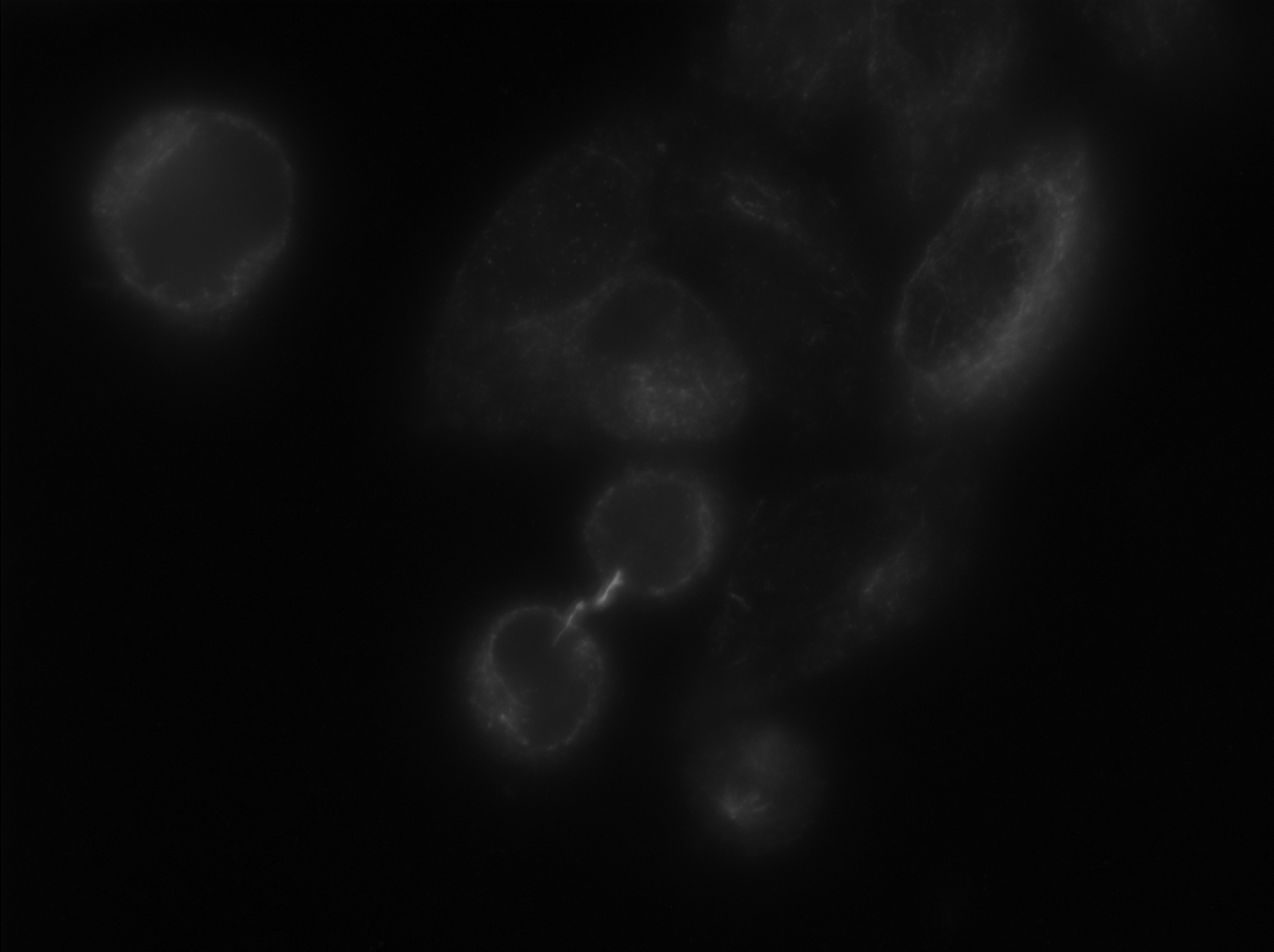

Supplement: Supplementary file 18 — Source data Fig. 5 part 4 [file 44319_2026_742_MOESM18_ESM.zip › Figure 5 Part 4/Fig 5ab WT and KO hela TTLL1-e326g atubulin/Control/WT Hela TTLL1-mut R2 LT9.Project Maximum Z_XY1731544345_Z0_T0_C2.tif]

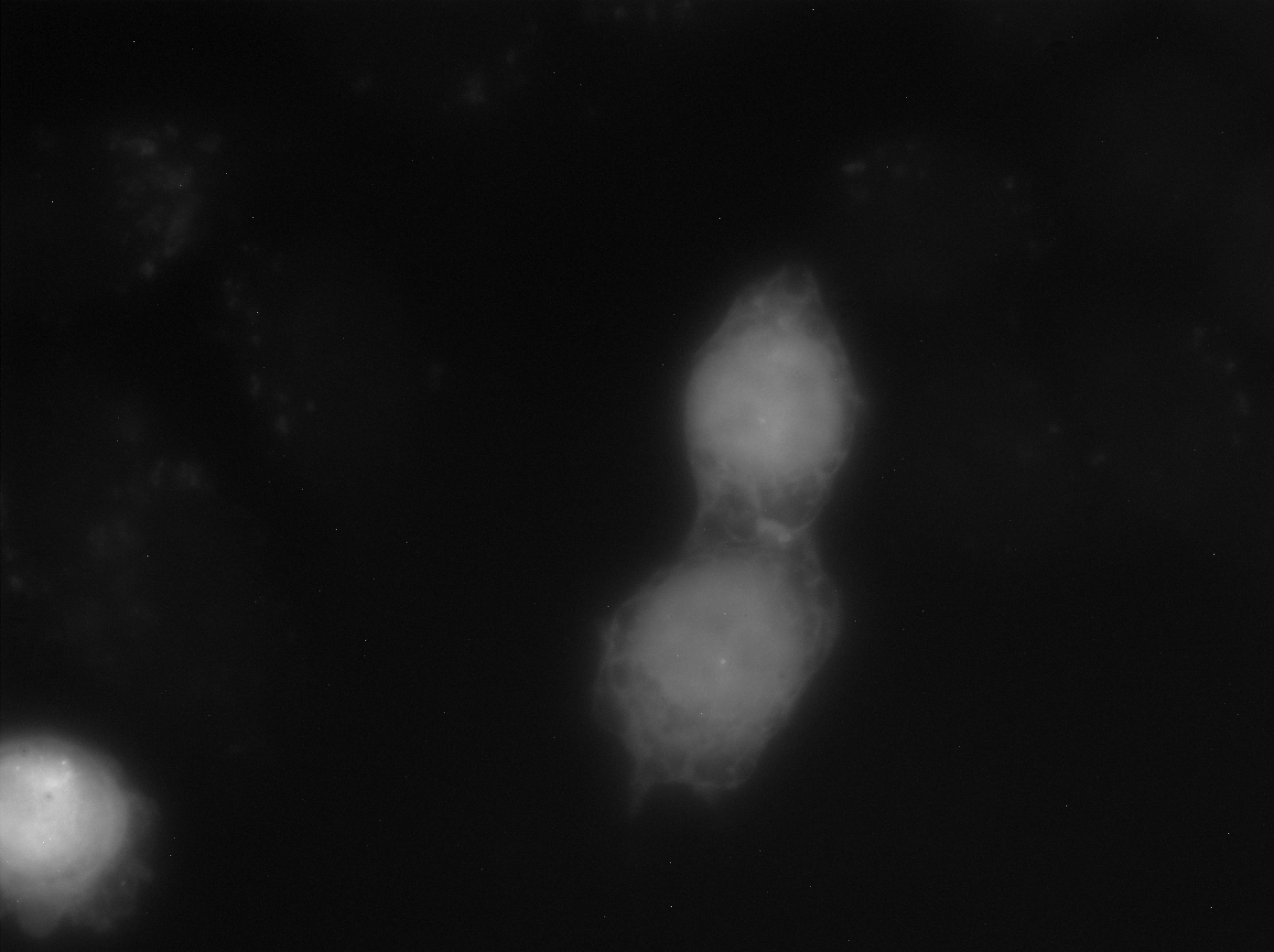

Supplement: Supplementary file 18 — Source data Fig. 5 part 4 [file 44319_2026_742_MOESM18_ESM.zip › Figure 5 Part 4/Fig 5ab WT and KO hela TTLL1-e326g atubulin/Control/TTLL1-mut atub R2 LT4.Project Maximum Z_XY1724951081_Z0_T0_C1.tif]

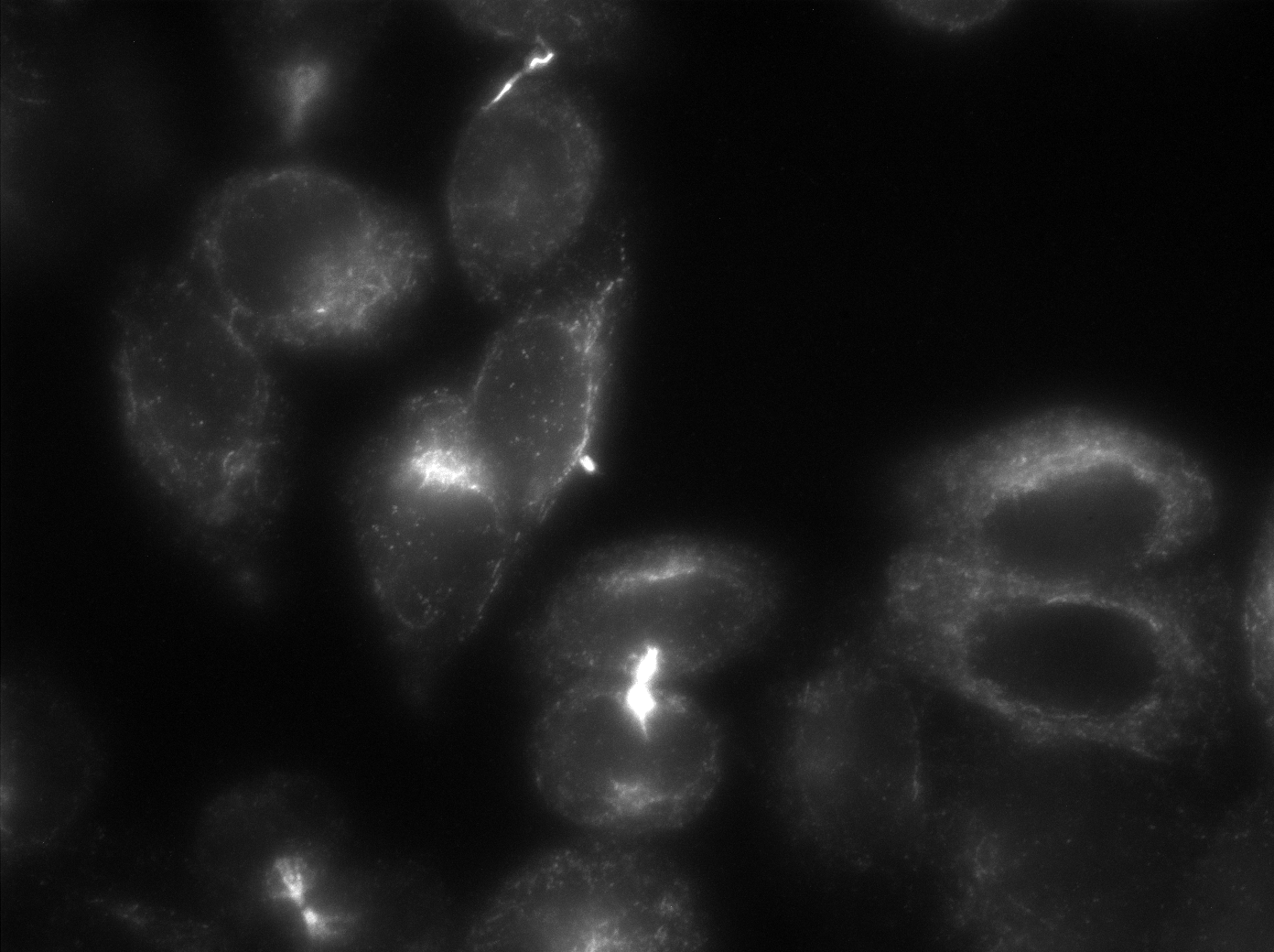

Supplement: Supplementary file 18 — Source data Fig. 5 part 4 [file 44319_2026_742_MOESM18_ESM.zip › Figure 5 Part 4/Fig 5ab WT and KO hela TTLL1-e326g atubulin/Control/WT Hela TTLL1-mut R3 11-13-24 LT9.Project Maximum Z_XY1731547173_Z0_T0_C2.tif]

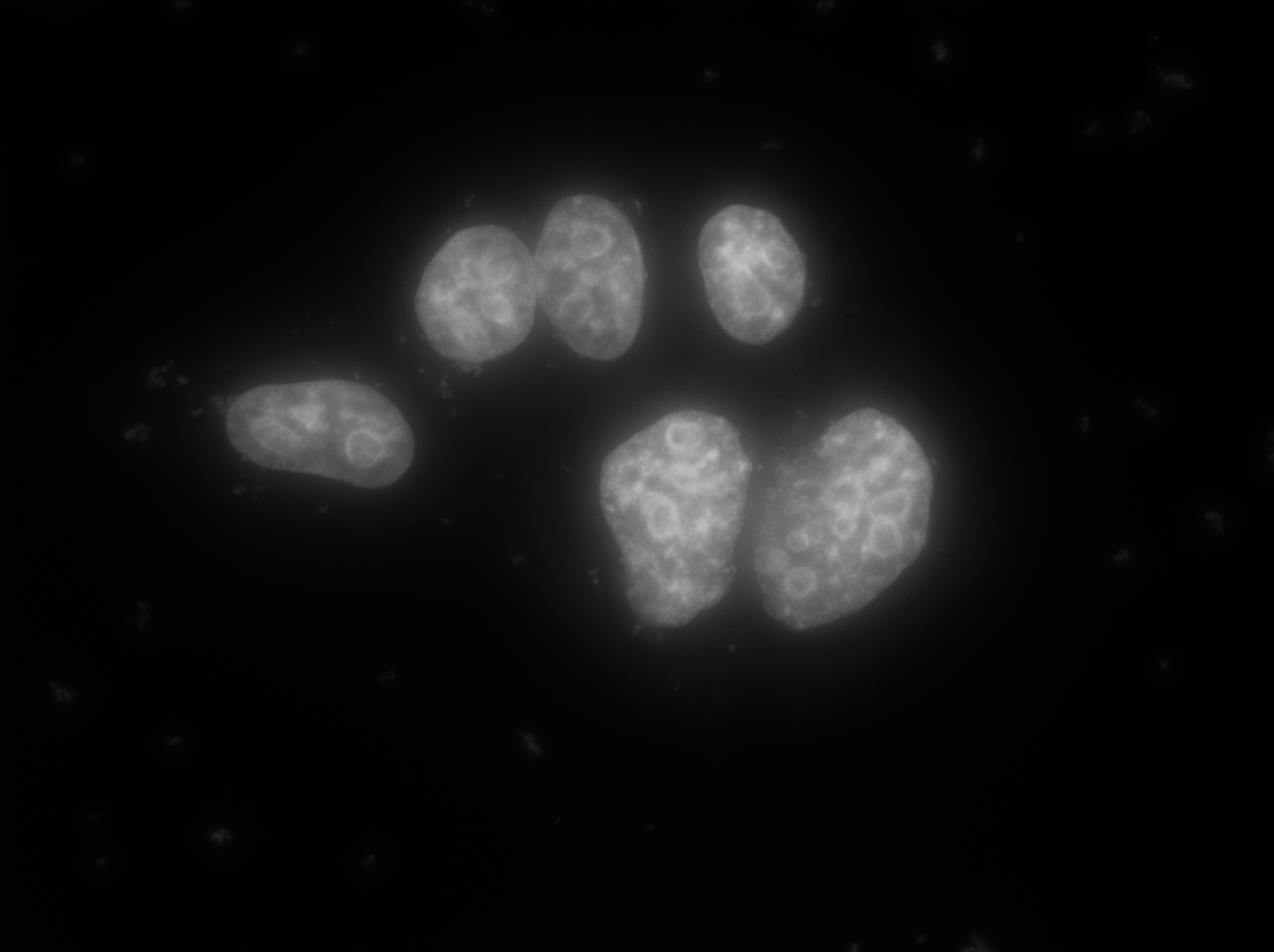

Supplement: Supplementary file 18 — Source data Fig. 5 part 4 [file 44319_2026_742_MOESM18_ESM.zip › Figure 5 Part 4/Fig 5ab WT and KO hela TTLL1-e326g atubulin/Control/TTLL1-mut atub R1 LT10.Project Maximum Z_XY1724442323_Z0_T0_C0.tif]

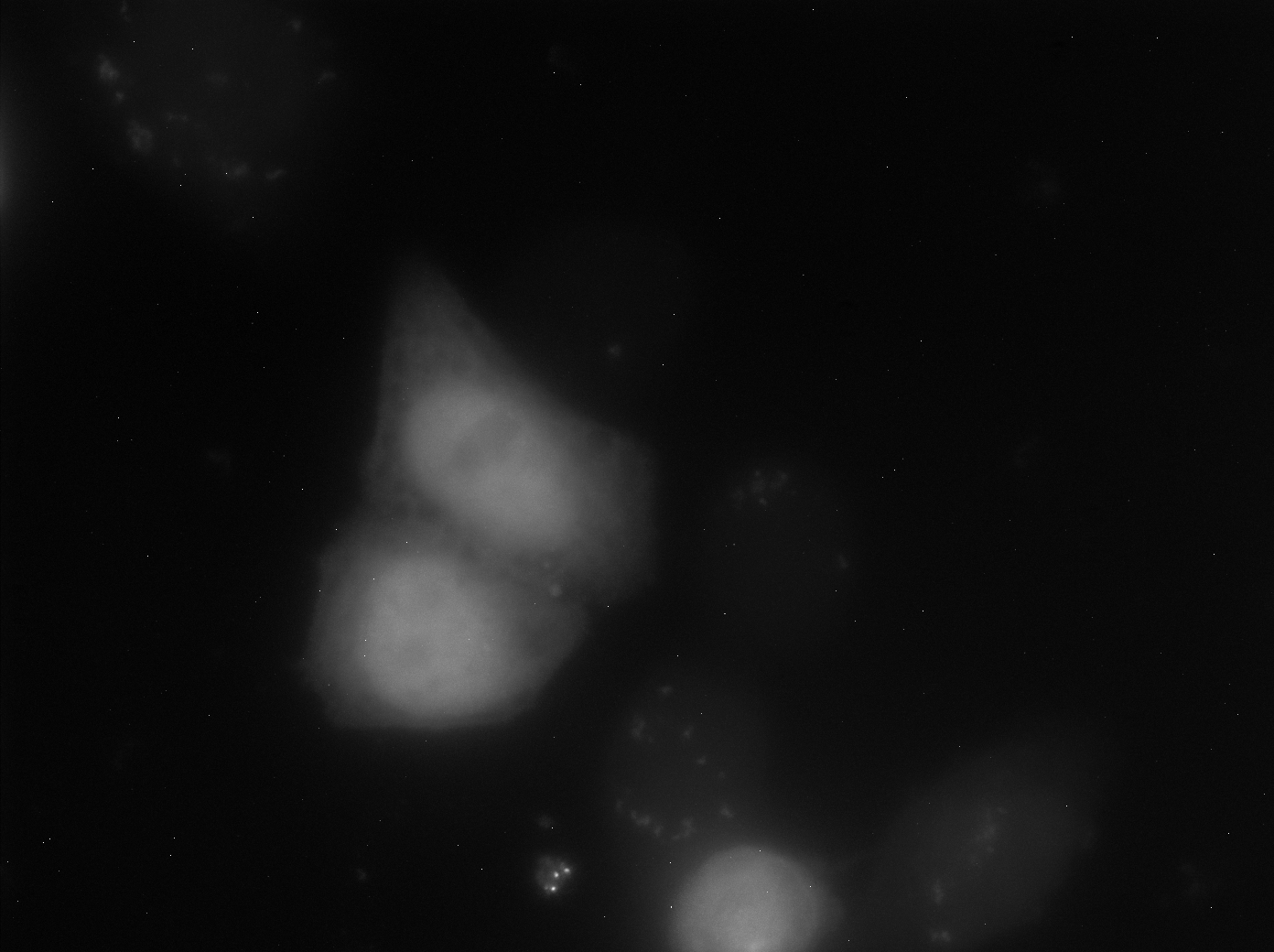

Supplement: Supplementary file 18 — Source data Fig. 5 part 4 [file 44319_2026_742_MOESM18_ESM.zip › Figure 5 Part 4/Fig 5ab WT and KO hela TTLL1-e326g atubulin/Control/WT Hela TTLL1-mut R2 LT8.Project Maximum Z_XY1731544140_Z0_T0_C1.tif]

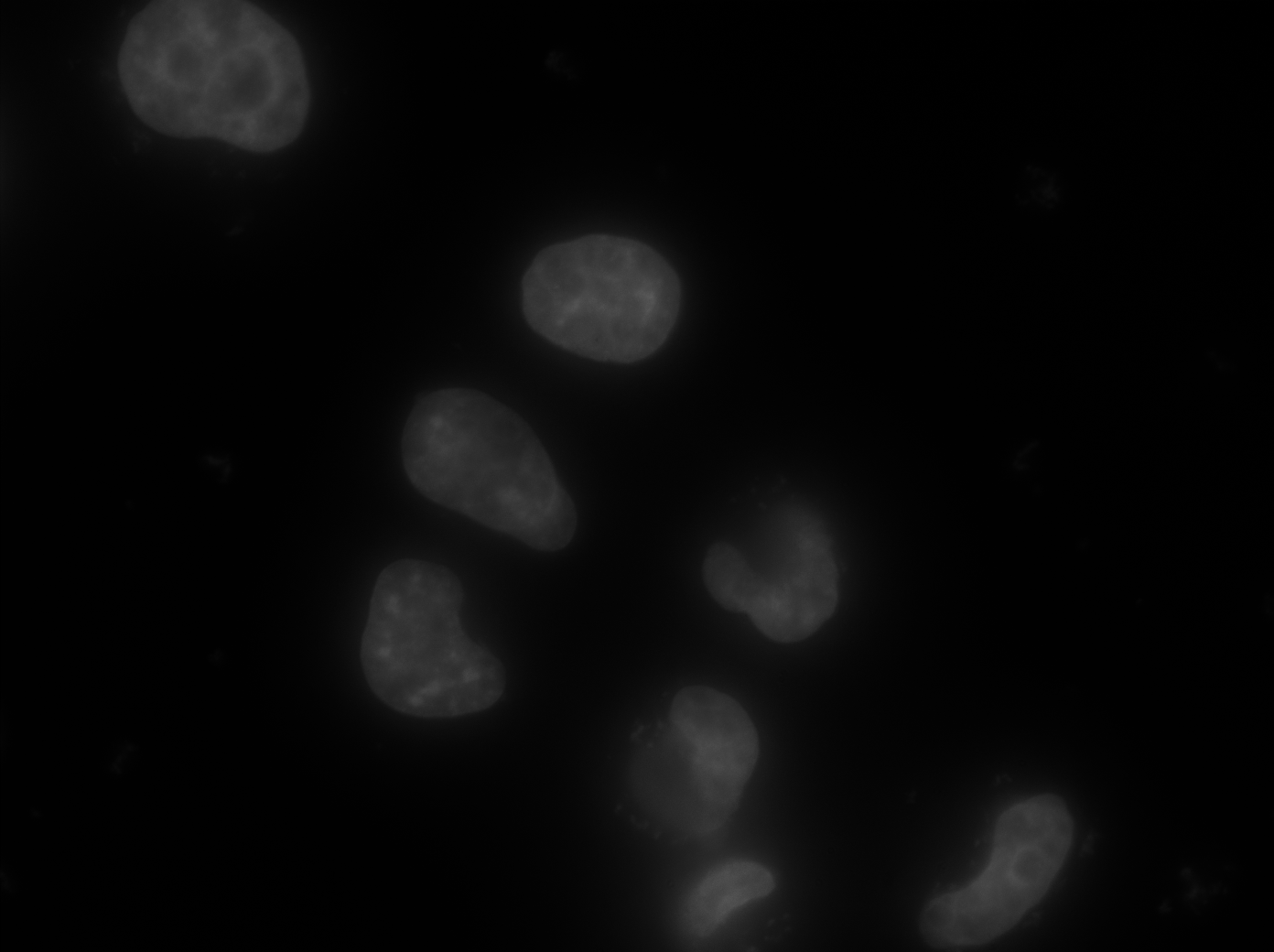

Supplement: Supplementary file 18 — Source data Fig. 5 part 4 [file 44319_2026_742_MOESM18_ESM.zip › Figure 5 Part 4/Fig 5ab WT and KO hela TTLL1-e326g atubulin/Control/WT Hela TTLL1-mut R2 LT8.Project Maximum Z_XY1731544140_Z0_T0_C0.tif]

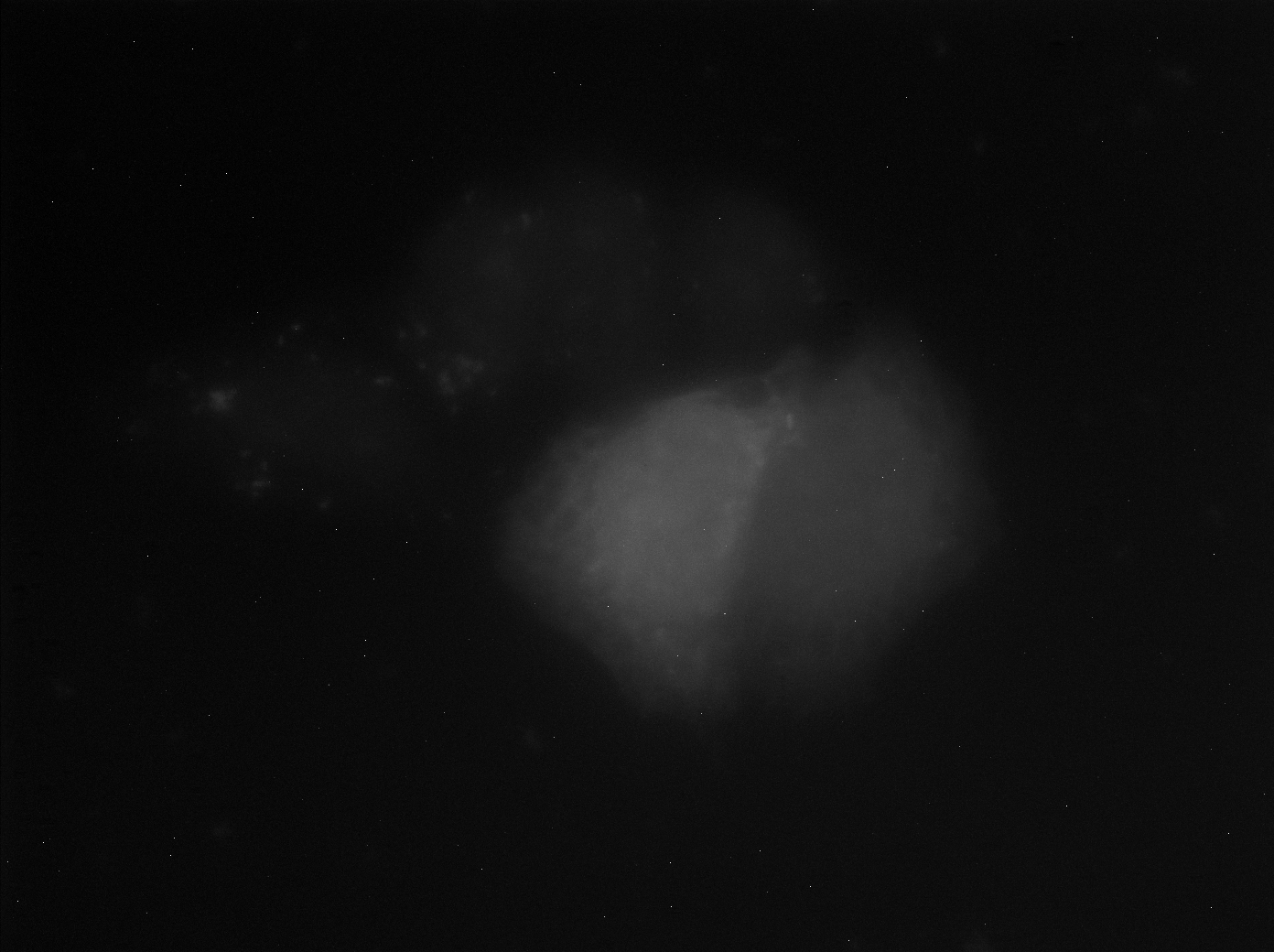

Supplement: Supplementary file 18 — Source data Fig. 5 part 4 [file 44319_2026_742_MOESM18_ESM.zip › Figure 5 Part 4/Fig 5ab WT and KO hela TTLL1-e326g atubulin/Control/TTLL1-mut atub R1 LT10.Project Maximum Z_XY1724442323_Z0_T0_C1.tif]

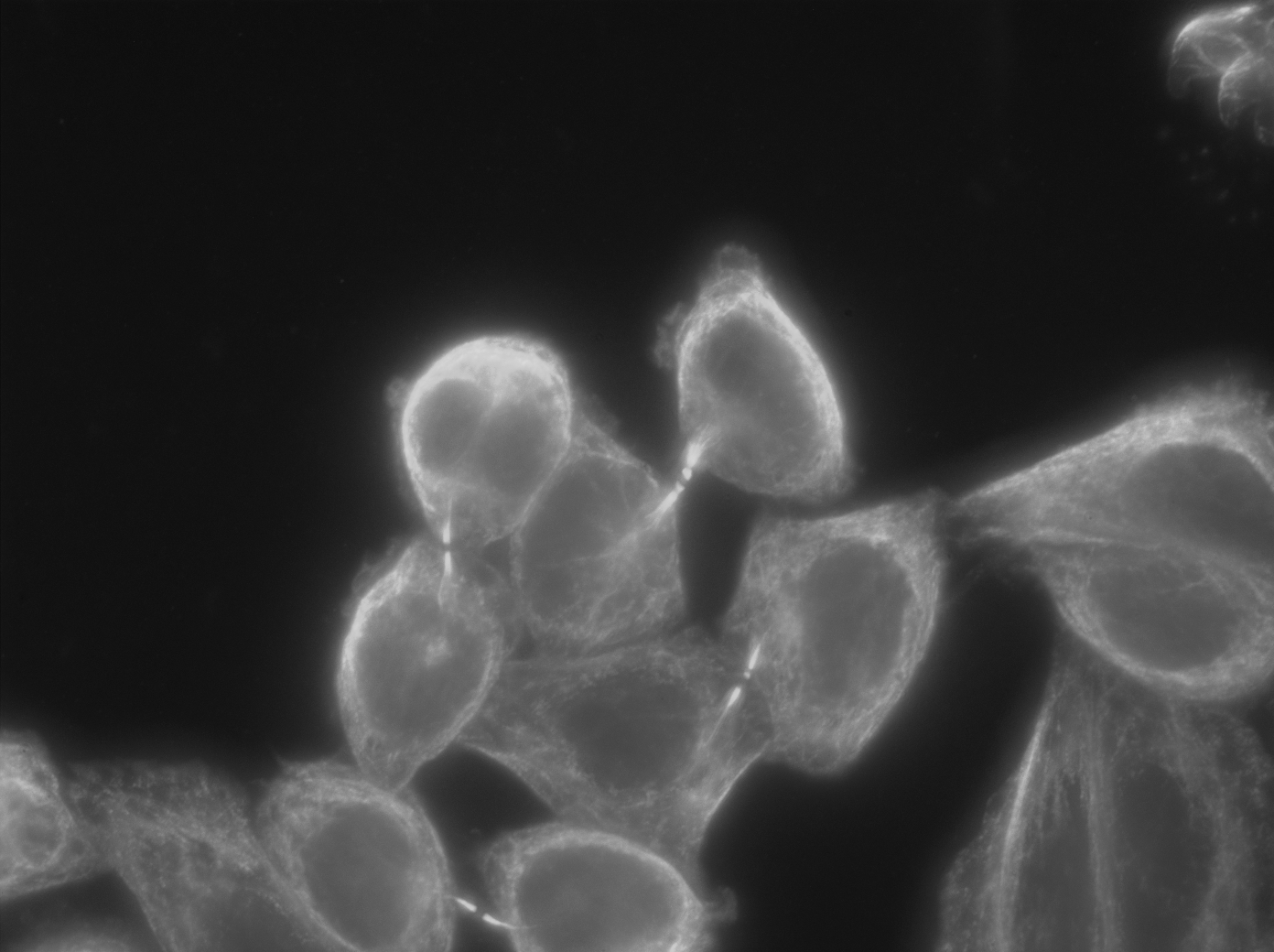

Supplement: Supplementary file 18 — Source data Fig. 5 part 4 [file 44319_2026_742_MOESM18_ESM.zip › Figure 5 Part 4/Fig 5ab WT and KO hela TTLL1-e326g atubulin/Control/TTLL1-mut atub R2 LT2.Project Maximum Z_XY1724950608_Z0_T0_C2.tif]

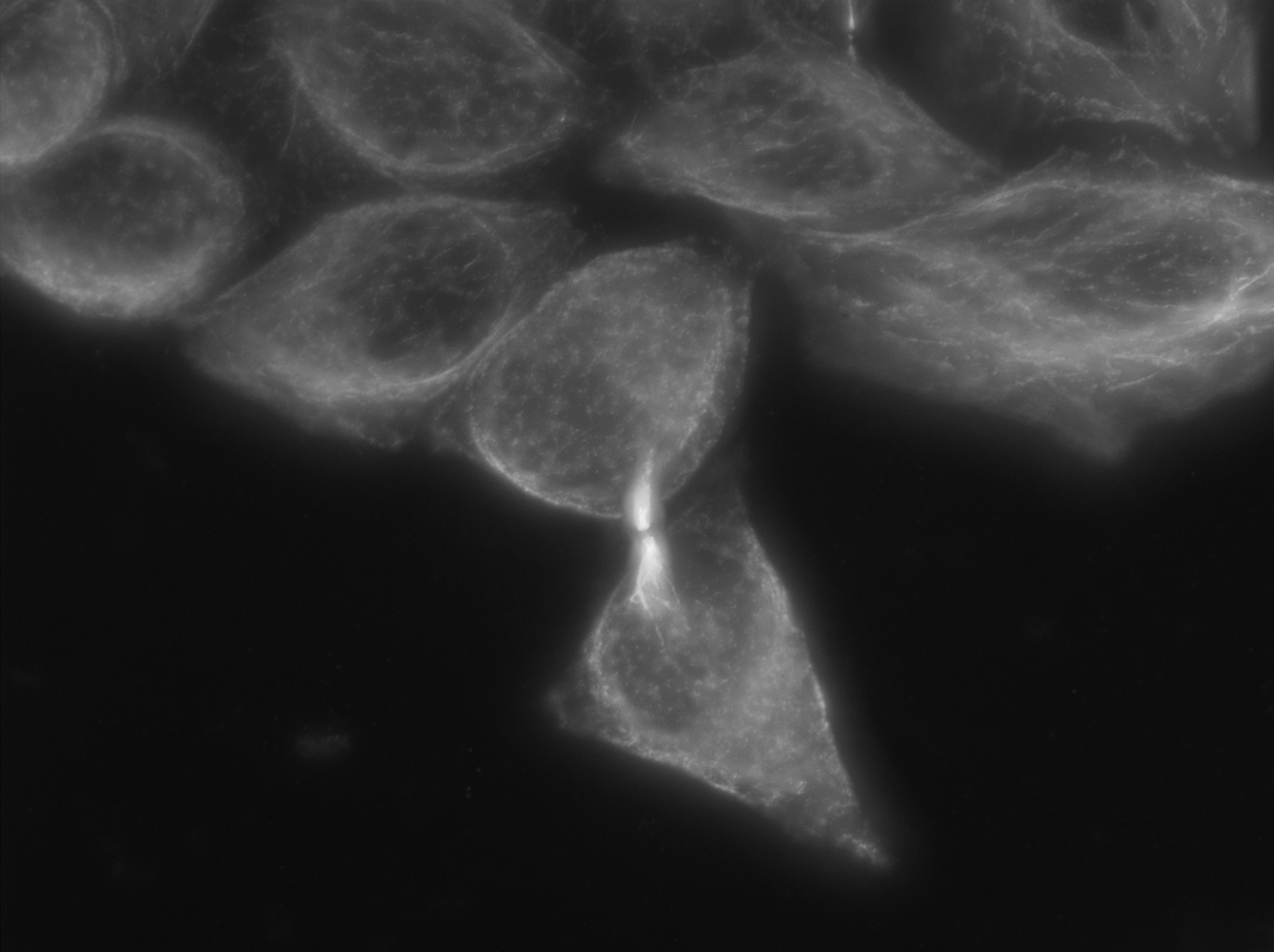

Supplement: Supplementary file 18 — Source data Fig. 5 part 4 [file 44319_2026_742_MOESM18_ESM.zip › Figure 5 Part 4/Fig 5ab WT and KO hela TTLL1-e326g atubulin/Control/TTLL1-mut atub R2 LT3.Project Maximum Z_XY1724950815_Z0_T0_C2.tif]

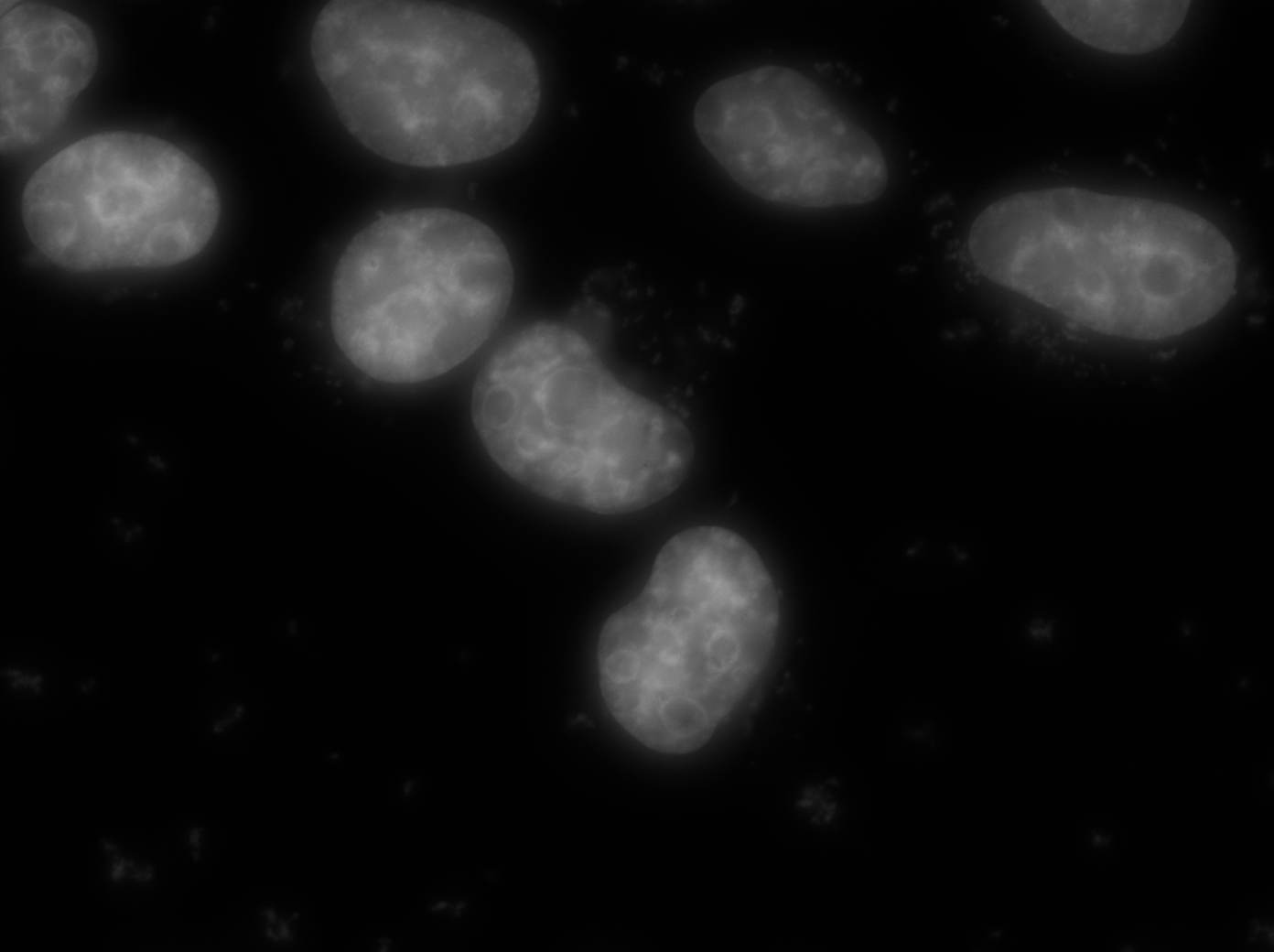

Supplement: Supplementary file 18 — Source data Fig. 5 part 4 [file 44319_2026_742_MOESM18_ESM.zip › Figure 5 Part 4/Fig 5ab WT and KO hela TTLL1-e326g atubulin/Control/TTLL1-mut atub R2 LT3.Project Maximum Z_XY1724950815_Z0_T0_C0.tif]

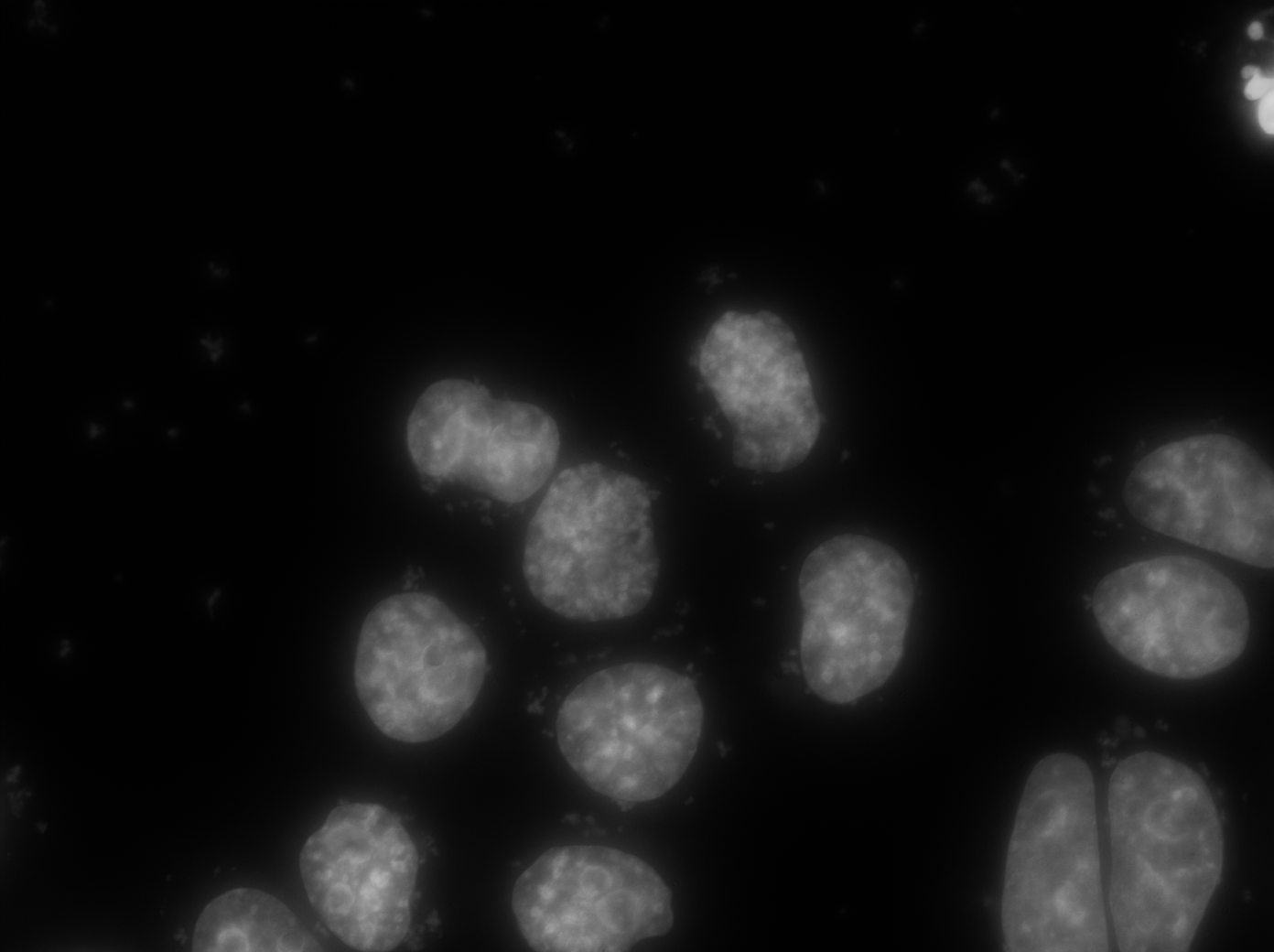

Supplement: Supplementary file 18 — Source data Fig. 5 part 4 [file 44319_2026_742_MOESM18_ESM.zip › Figure 5 Part 4/Fig 5ab WT and KO hela TTLL1-e326g atubulin/Control/TTLL1-mut atub R2 LT2.Project Maximum Z_XY1724950608_Z0_T0_C0.tif]

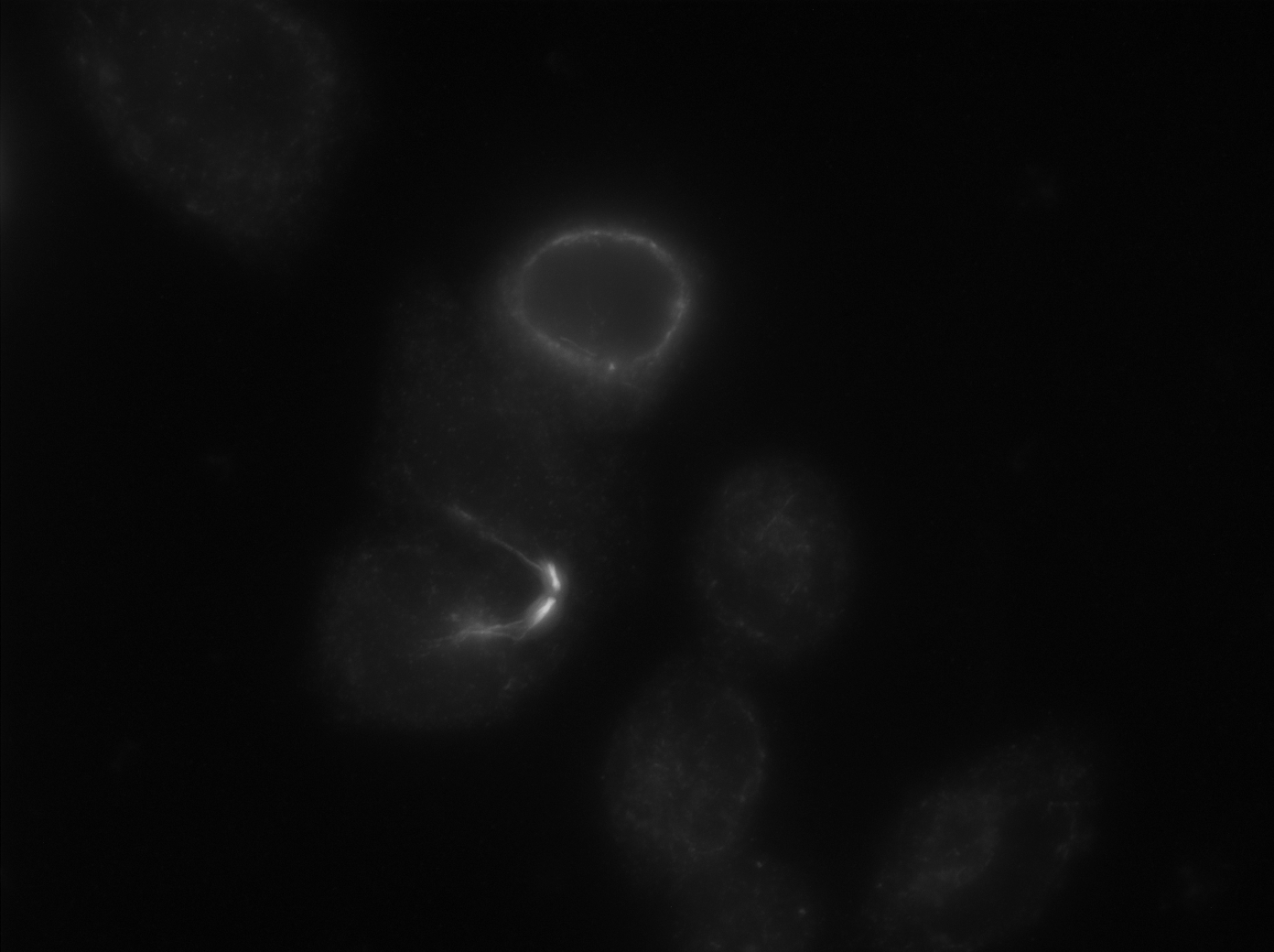

Supplement: Supplementary file 18 — Source data Fig. 5 part 4 [file 44319_2026_742_MOESM18_ESM.zip › Figure 5 Part 4/Fig 5ab WT and KO hela TTLL1-e326g atubulin/Control/WT Hela TTLL1-mut R2 LT8.Project Maximum Z_XY1731544140_Z0_T0_C2.tif]

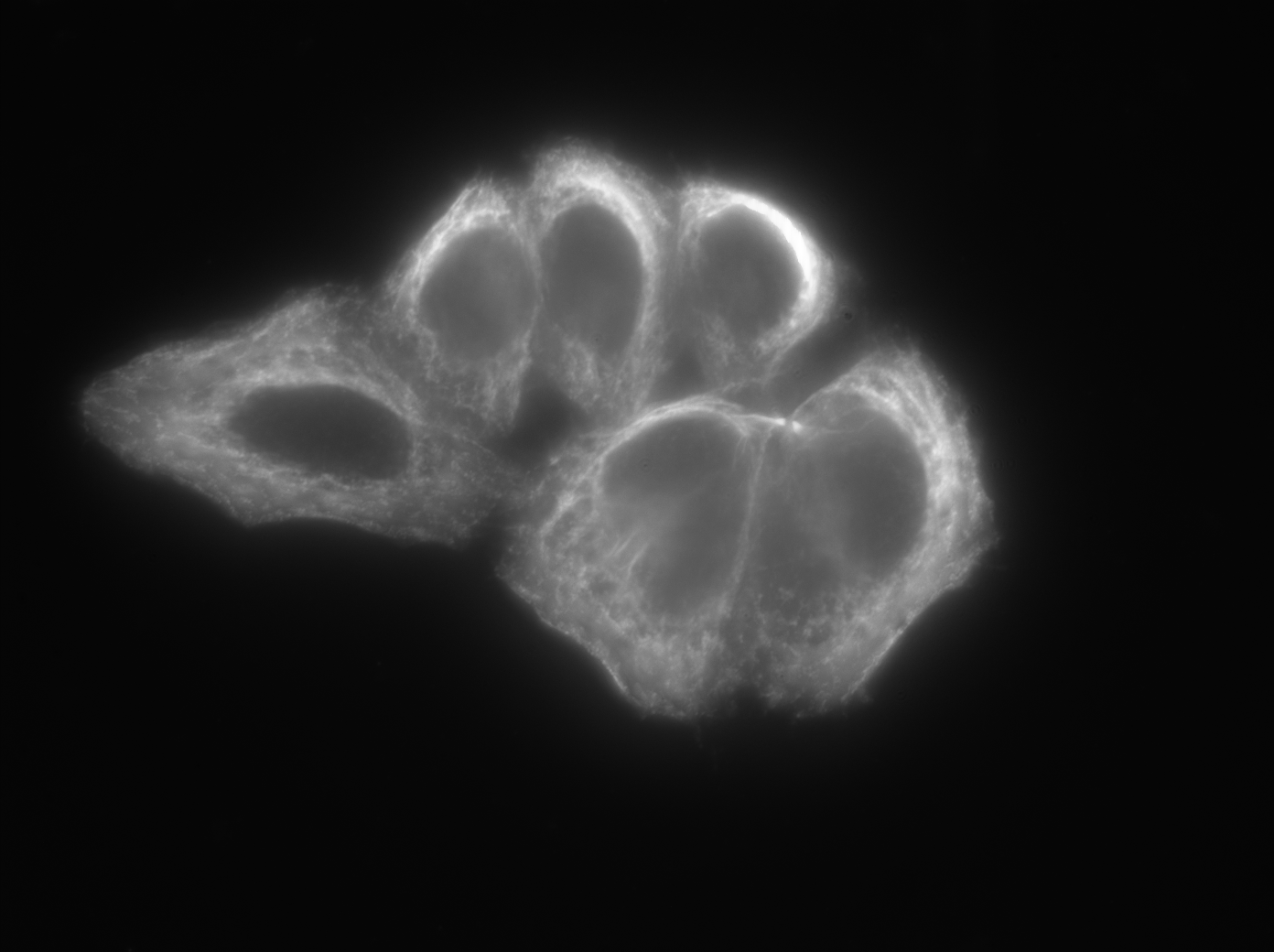

Supplement: Supplementary file 18 — Source data Fig. 5 part 4 [file 44319_2026_742_MOESM18_ESM.zip › Figure 5 Part 4/Fig 5ab WT and KO hela TTLL1-e326g atubulin/Control/TTLL1-mut atub R1 LT10.Project Maximum Z_XY1724442323_Z0_T0_C2.tif]

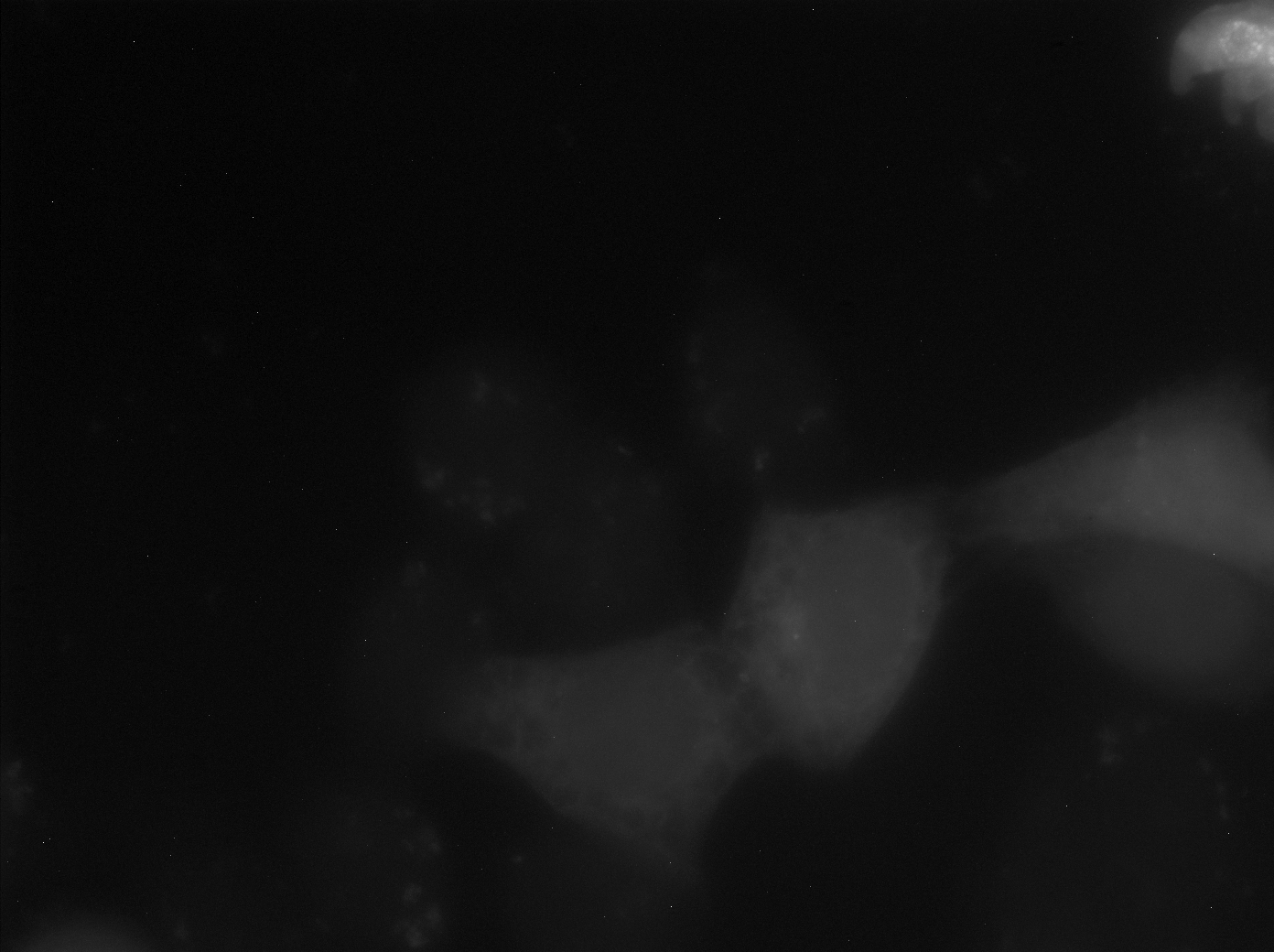

Supplement: Supplementary file 18 — Source data Fig. 5 part 4 [file 44319_2026_742_MOESM18_ESM.zip › Figure 5 Part 4/Fig 5ab WT and KO hela TTLL1-e326g atubulin/Control/TTLL1-mut atub R2 LT2.Project Maximum Z_XY1724950608_Z0_T0_C1.tif]

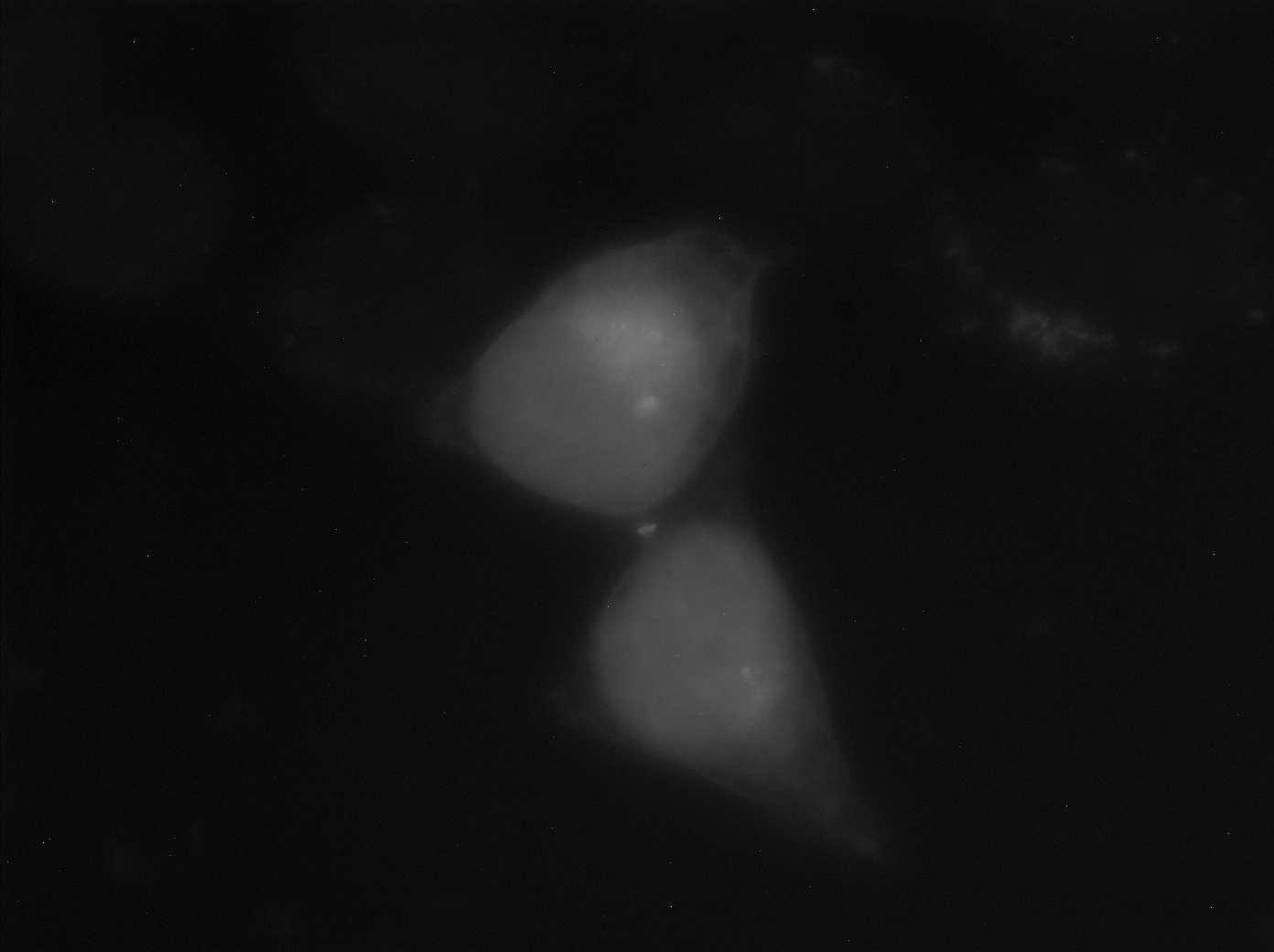

Supplement: Supplementary file 18 — Source data Fig. 5 part 4 [file 44319_2026_742_MOESM18_ESM.zip › Figure 5 Part 4/Fig 5ab WT and KO hela TTLL1-e326g atubulin/Control/TTLL1-mut atub R2 LT3.Project Maximum Z_XY1724950815_Z0_T0_C1.tif]

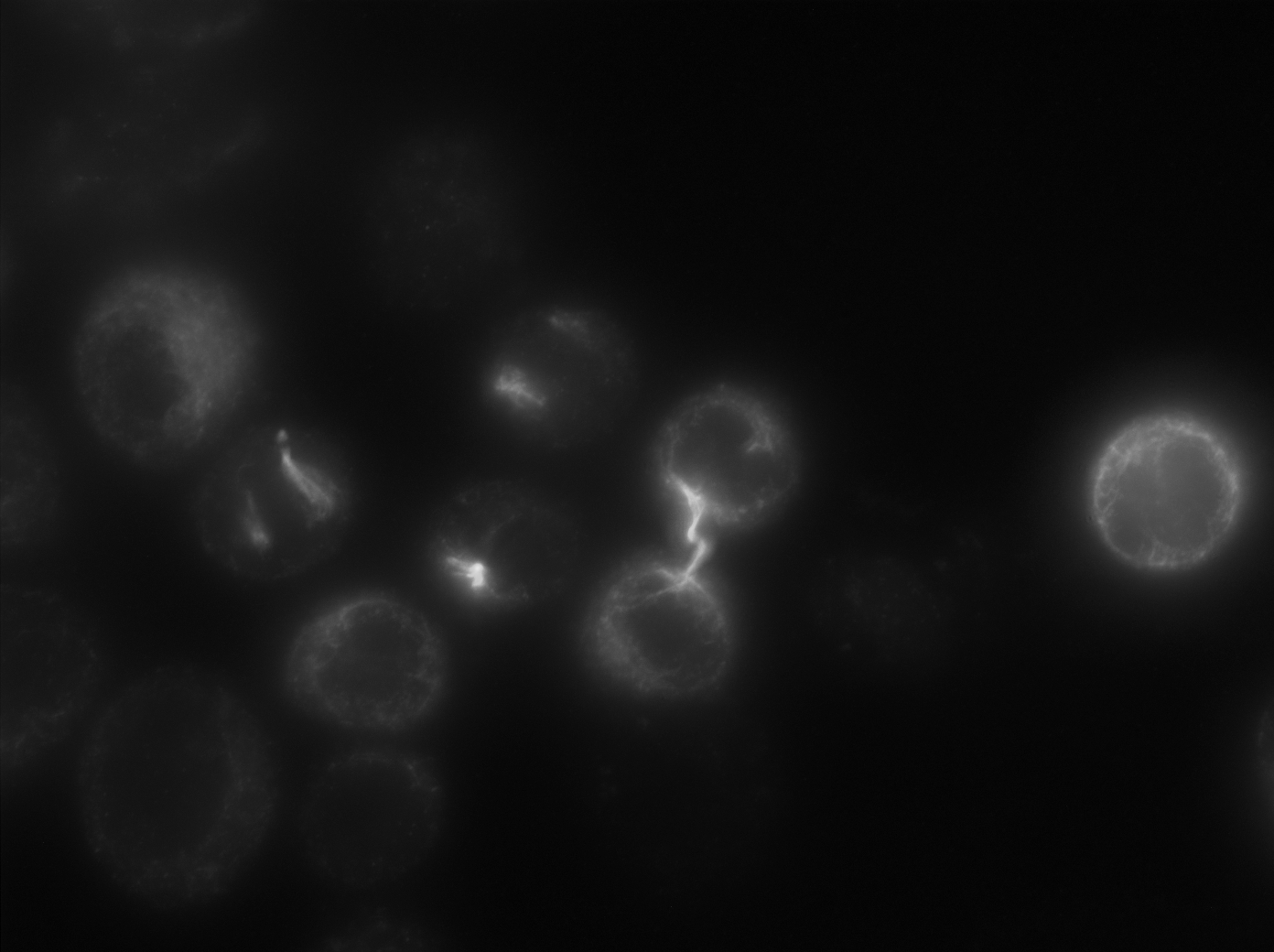

Supplement: Supplementary file 18 — Source data Fig. 5 part 4 [file 44319_2026_742_MOESM18_ESM.zip › Figure 5 Part 4/Fig 5ab WT and KO hela TTLL1-e326g atubulin/Control/WT Hela TTLL1-mut R2 LT5.Project Maximum Z_XY1731543093_Z0_T0_C2.tif]

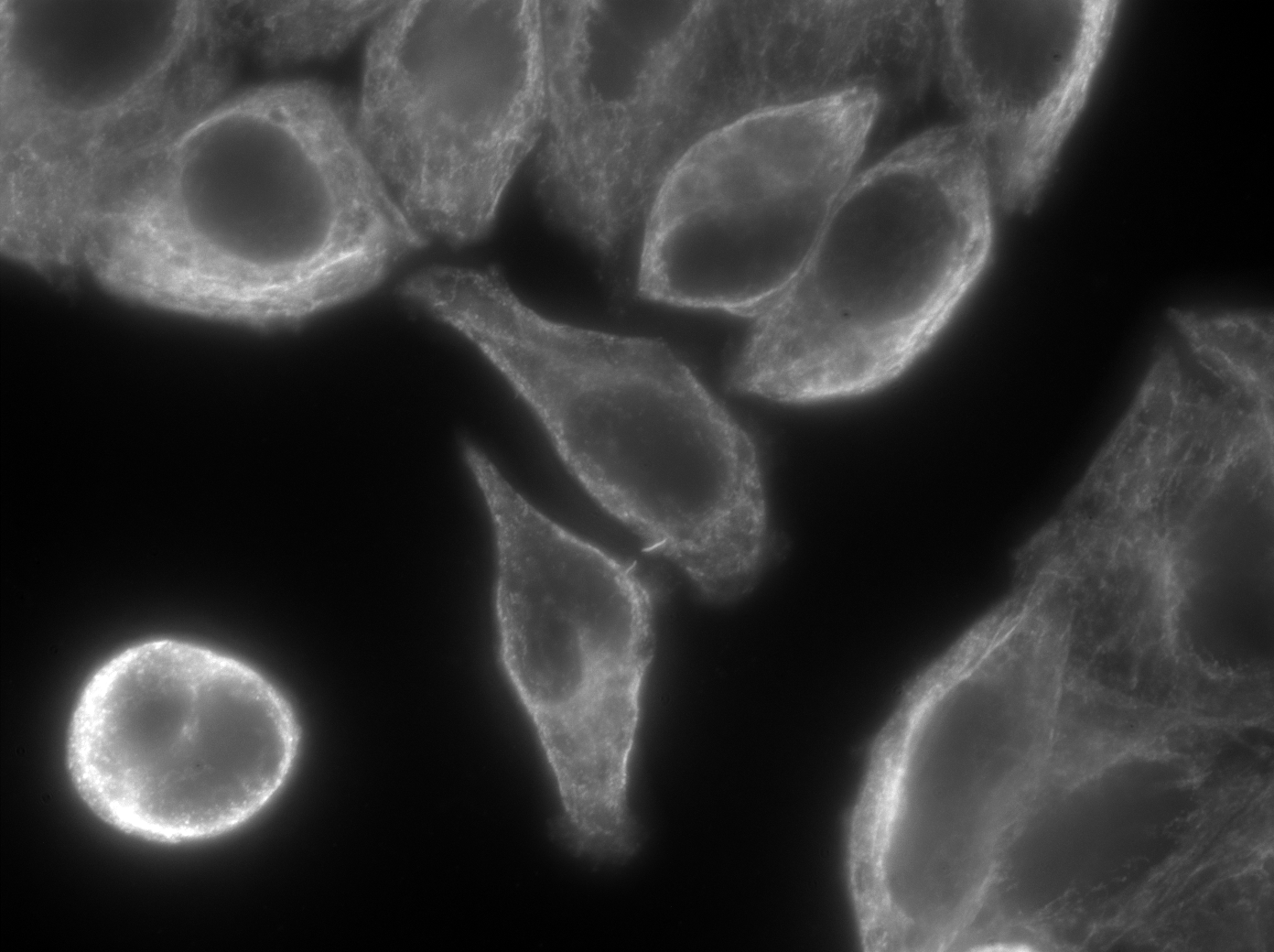

Supplement: Supplementary file 18 — Source data Fig. 5 part 4 [file 44319_2026_742_MOESM18_ESM.zip › Figure 5 Part 4/Fig 5ab WT and KO hela TTLL1-e326g atubulin/Control/TTLL1-mut atub R1 LT9.Project Maximum Z_XY1724441133_Z0_T0_C2.tif]
