# Supplementary figures and images for: TPGS1 regulates central spindle microtubule glutamylation and remodeling during telophase and abscission (part 21 of 36)
Source: EMBO Rep. 2026 Mar 23;27(8):1944–63. doi: 10.1038/s44319-026-00742-3 (PMC13121839; doi:10.1038/s44319-026-00742-3)

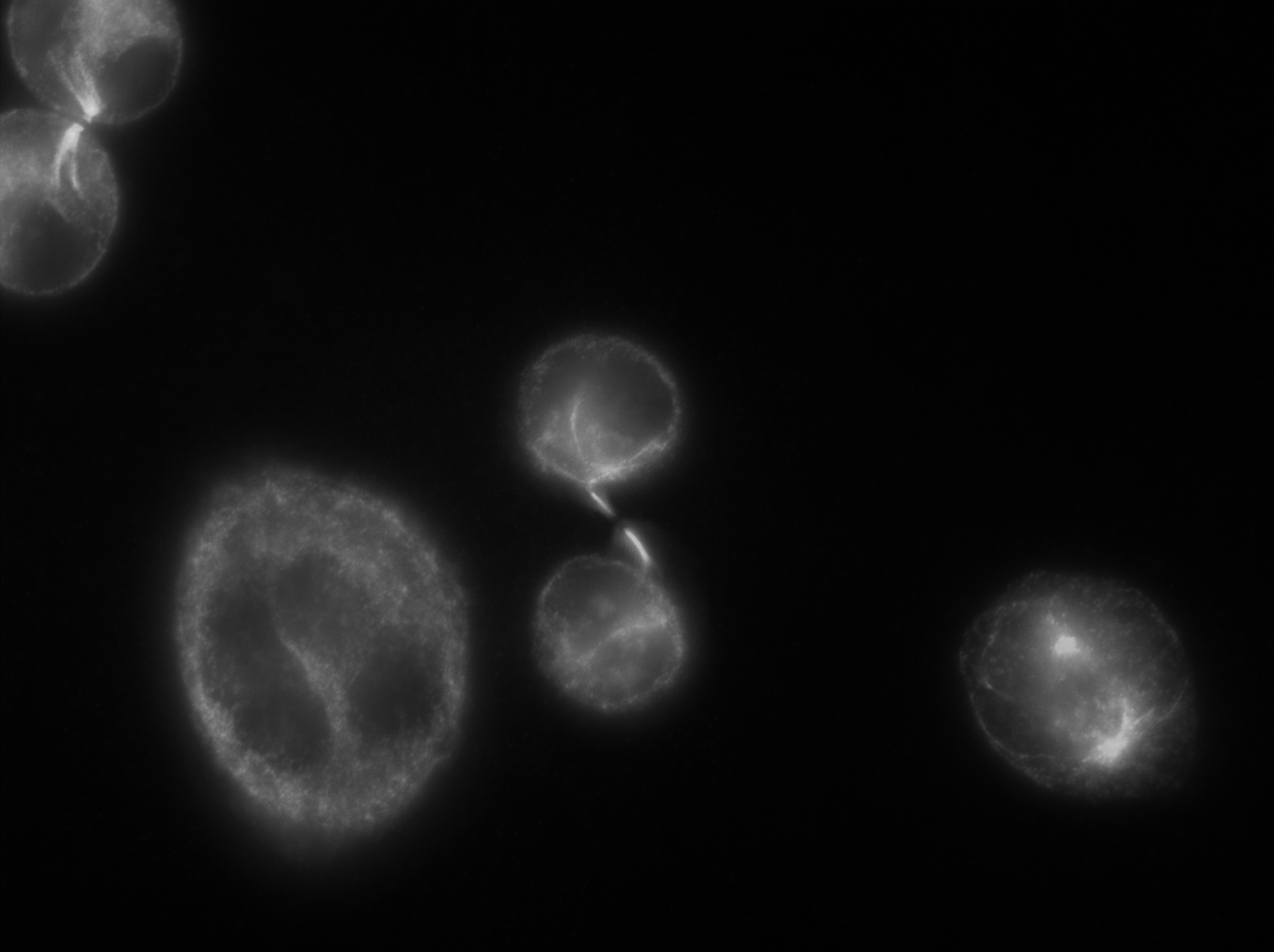

Supplement: Supplementary file 19 — Source data Fig. 5 part 5 [file 44319_2026_742_MOESM19_ESM.zip › Figure 5 Part 5/Fig 5ab WT and KO hela TTLL1-e326g atubulin part 2/TPGS1-KO/TPGS1-KO TTLL1-mut 10-15-24 R1 LT6 P2.Project Maximum Z_XY1729023653_Z0_T0_C2.tif]

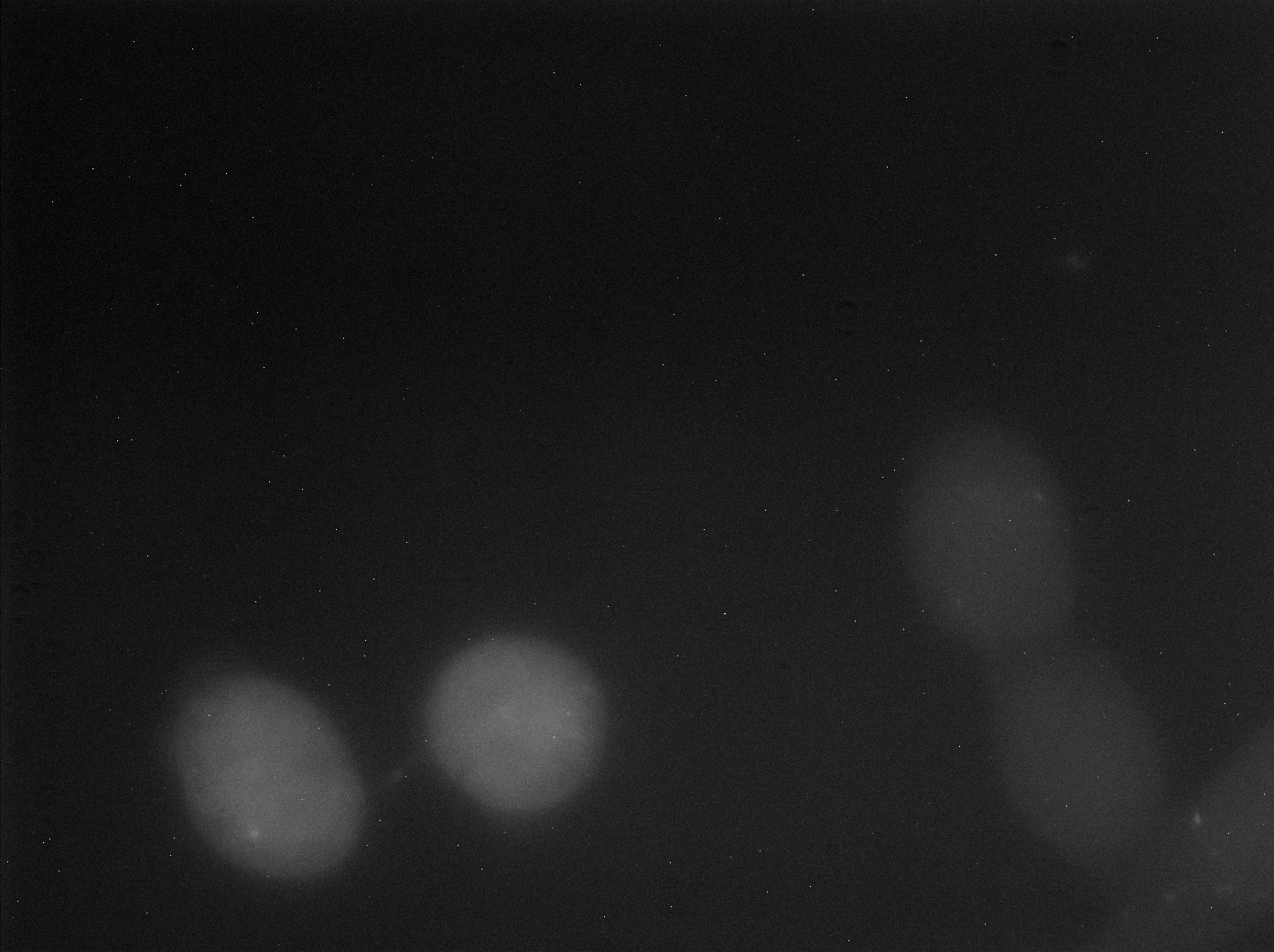

Supplement: Supplementary file 19 — Source data Fig. 5 part 5 [file 44319_2026_742_MOESM19_ESM.zip › Figure 5 Part 5/Fig 5ab WT and KO hela TTLL1-e326g atubulin part 2/TPGS1-KO/TPGS1-KO TTLL1-mut 10-15-24 R1 LT11.Project Maximum Z_XY1729025322_Z0_T0_C1.tif]

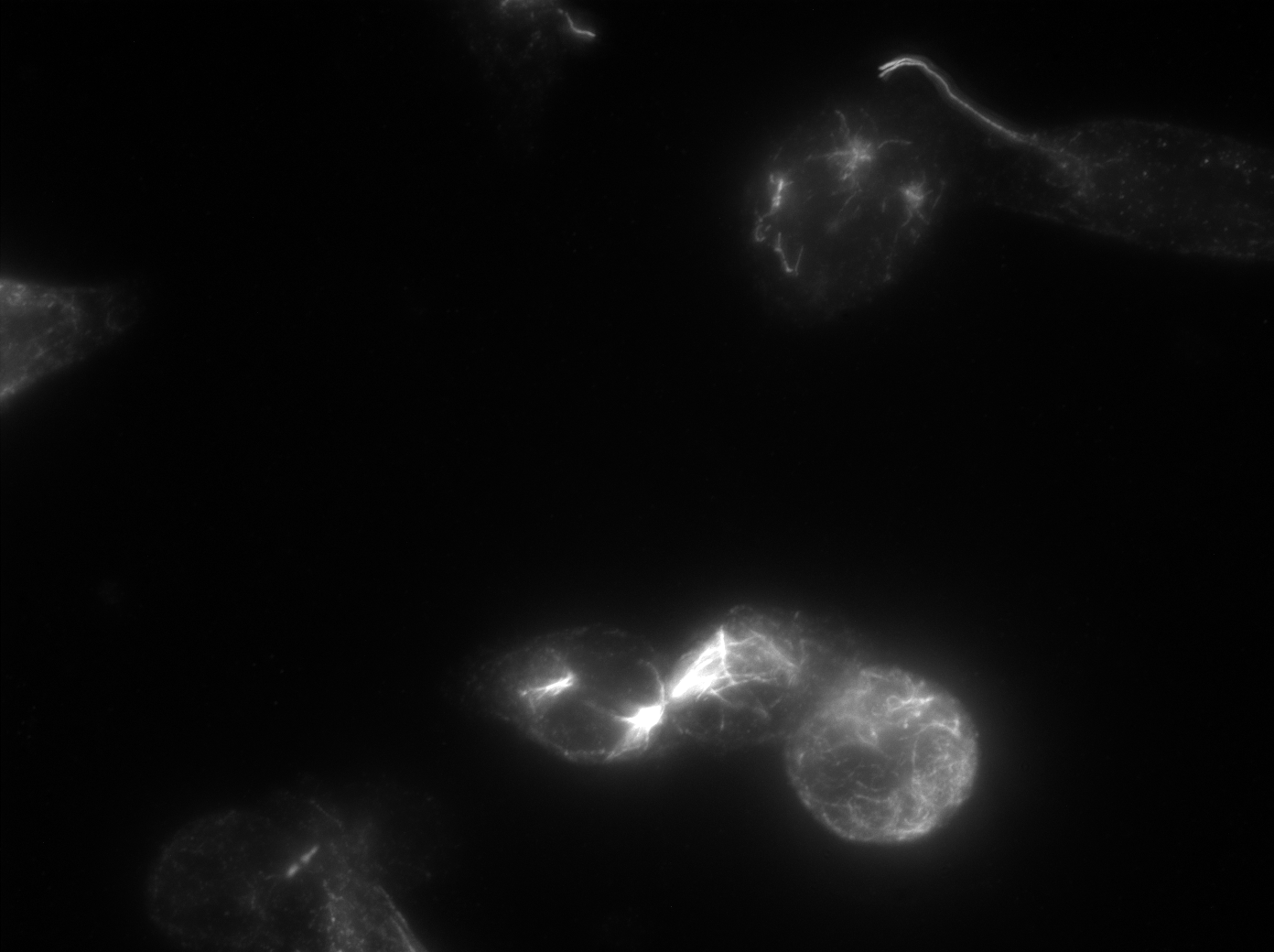

Supplement: Supplementary file 19 — Source data Fig. 5 part 5 [file 44319_2026_742_MOESM19_ESM.zip › Figure 5 Part 5/Fig 5ab WT and KO hela TTLL1-e326g atubulin part 2/TPGS1-KO/TPGS1-KO TTLL1-mut 10-22-24 R2 LT1.Project Maximum Z_XY1730225539_Z0_T0_C2.tif]

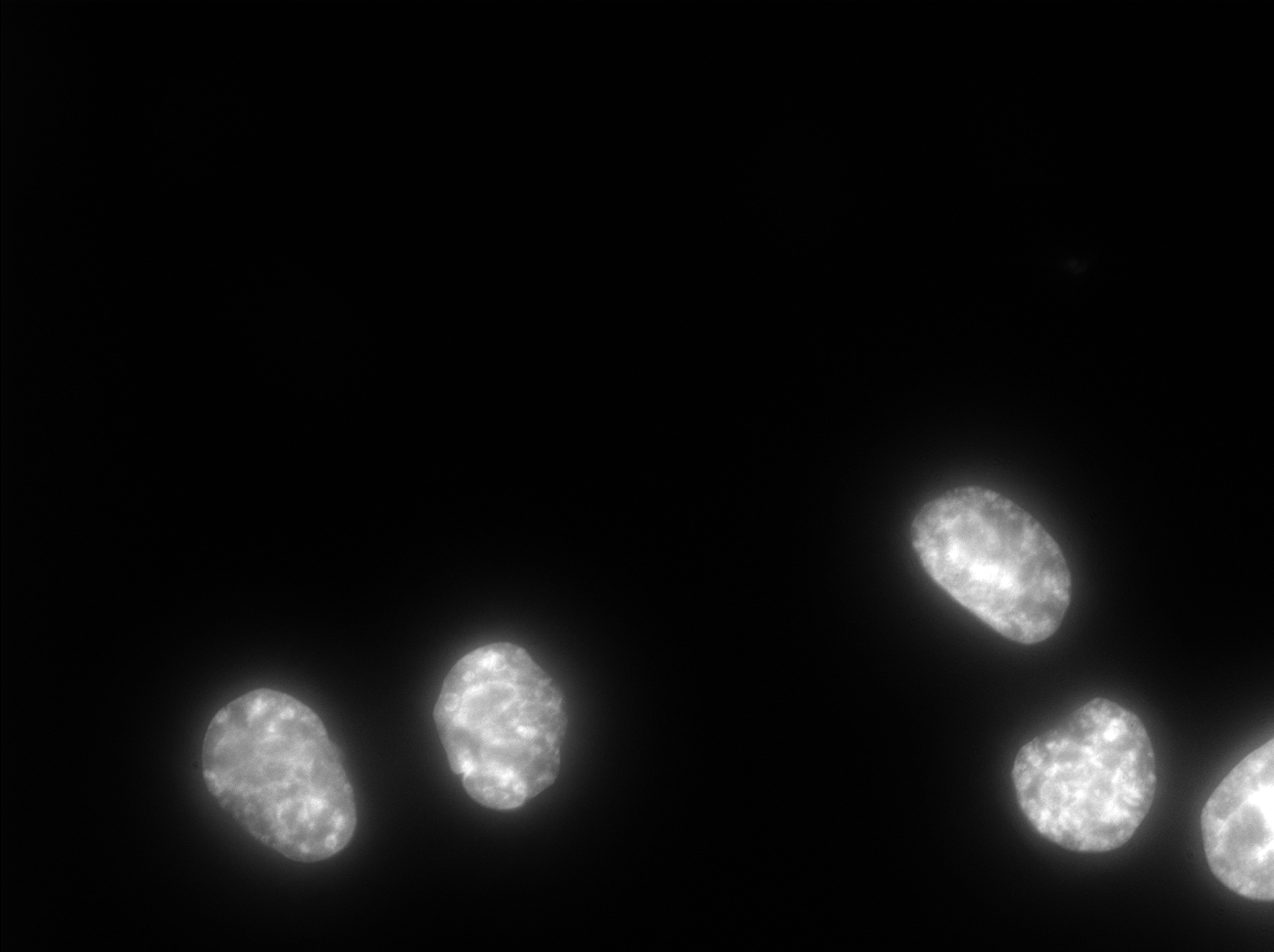

Supplement: Supplementary file 19 — Source data Fig. 5 part 5 [file 44319_2026_742_MOESM19_ESM.zip › Figure 5 Part 5/Fig 5ab WT and KO hela TTLL1-e326g atubulin part 2/TPGS1-KO/TPGS1-KO TTLL1-mut 10-15-24 R1 LT11.Project Maximum Z_XY1729025322_Z0_T0_C0.tif]

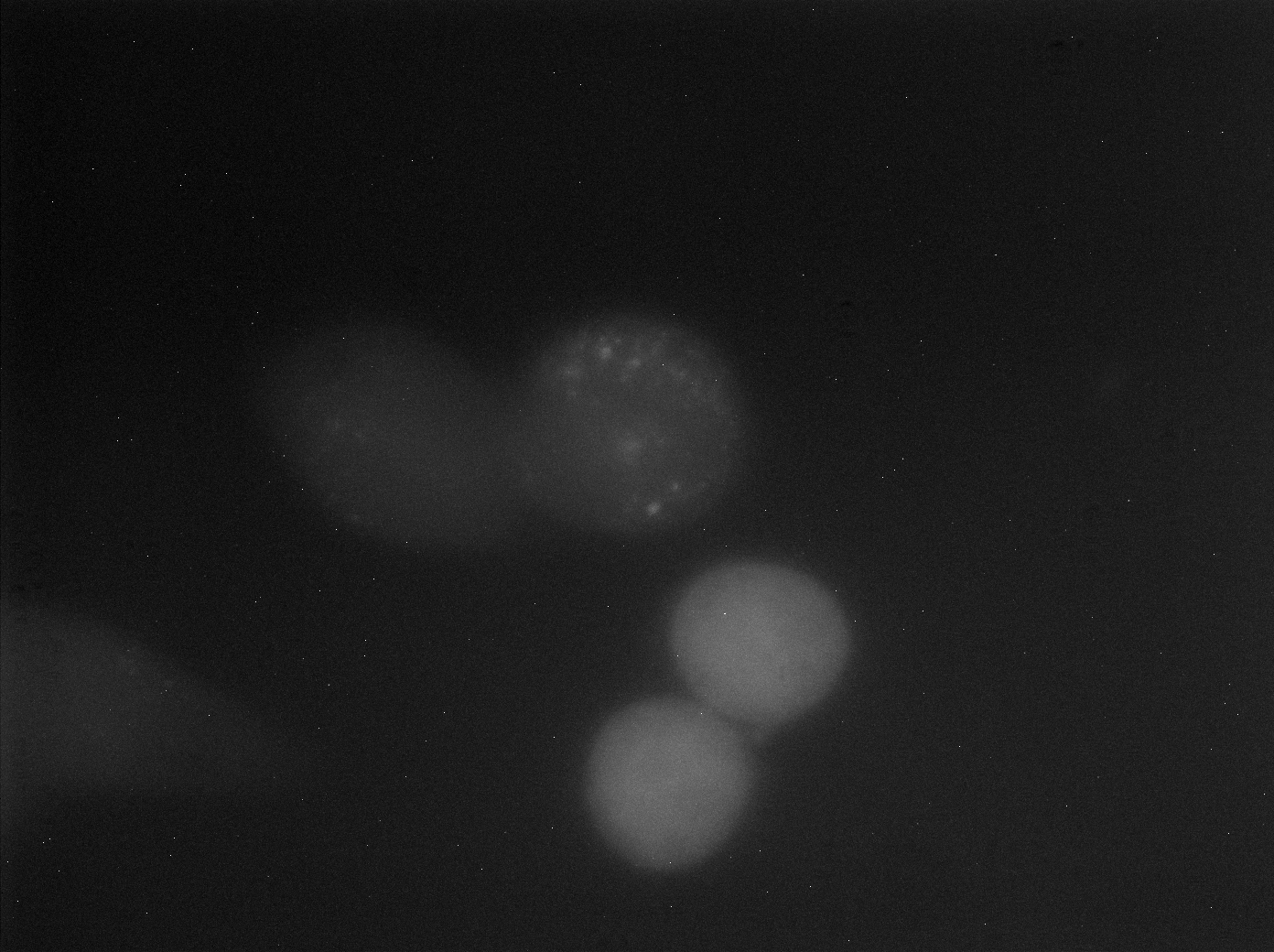

Supplement: Supplementary file 19 — Source data Fig. 5 part 5 [file 44319_2026_742_MOESM19_ESM.zip › Figure 5 Part 5/Fig 5ab WT and KO hela TTLL1-e326g atubulin part 2/TPGS1-KO/TPGS1-KO TTLL1-mut 10-15-24 R1 LT4.Project Maximum Z_XY1729023288_Z0_T0_C1.tif]

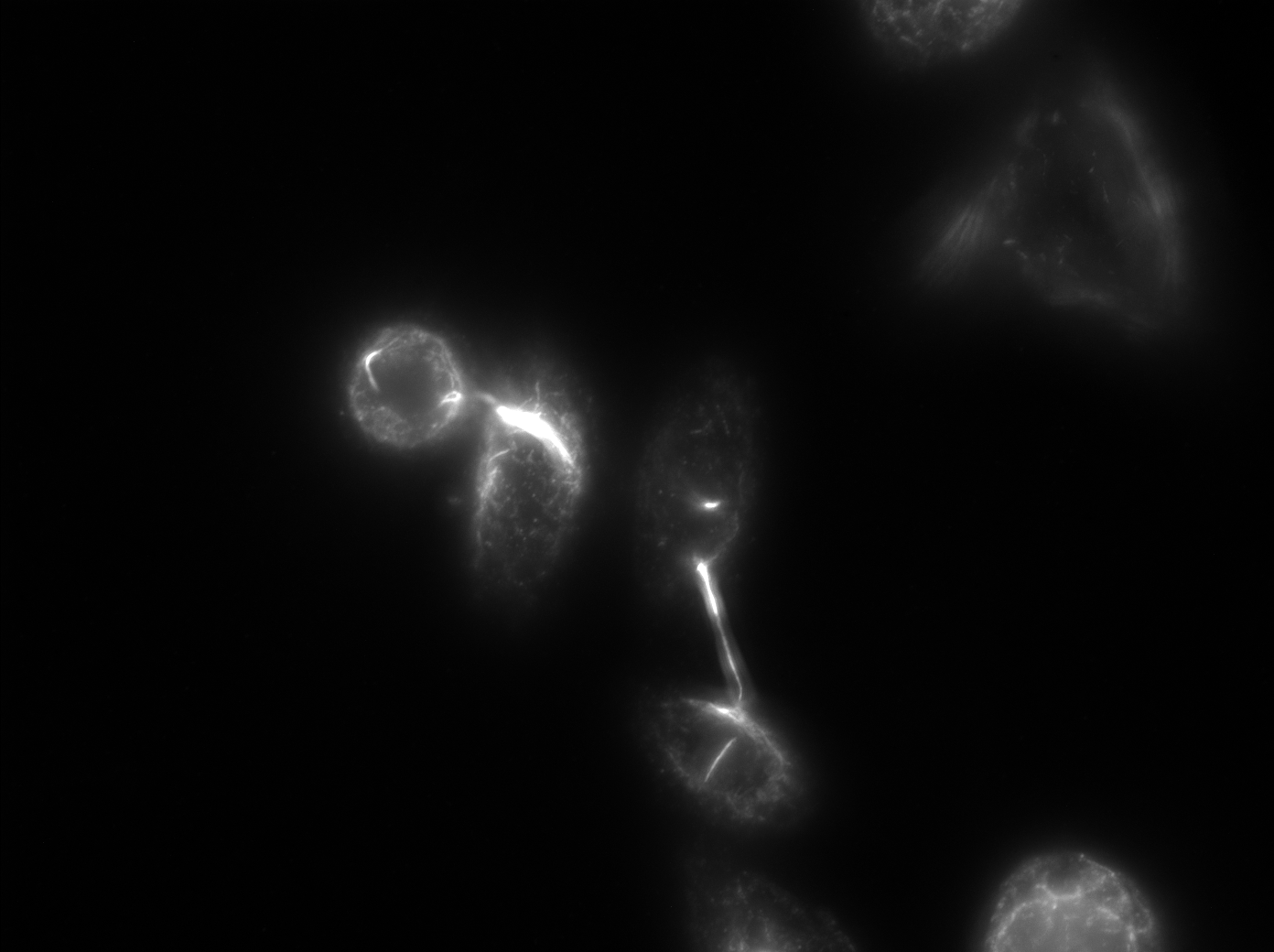

Supplement: Supplementary file 19 — Source data Fig. 5 part 5 [file 44319_2026_742_MOESM19_ESM.zip › Figure 5 Part 5/Fig 5ab WT and KO hela TTLL1-e326g atubulin part 2/TPGS1-KO/TPGS1-KO TTLL1-mut 10-22-24 R3 LT3.Project Maximum Z_XY1730228605_Z0_T0_C2.tif]

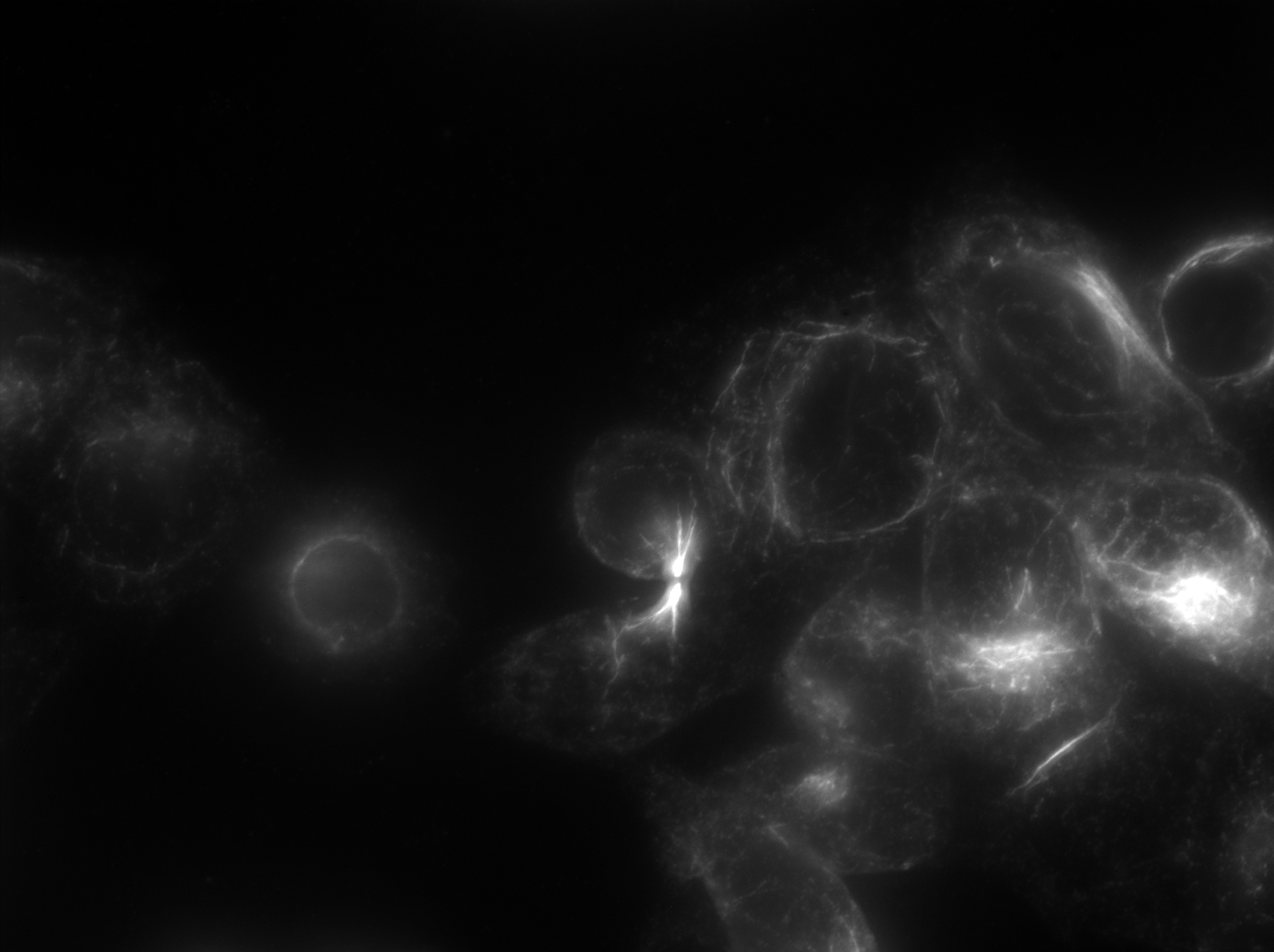

Supplement: Supplementary file 19 — Source data Fig. 5 part 5 [file 44319_2026_742_MOESM19_ESM.zip › Figure 5 Part 5/Fig 5ab WT and KO hela TTLL1-e326g atubulin part 2/TPGS1-KO/TPGS1-KO TTLL1-mut 10-22-24 R3 LT9.Project Maximum Z_XY1730230416_Z0_T0_C2.tif]

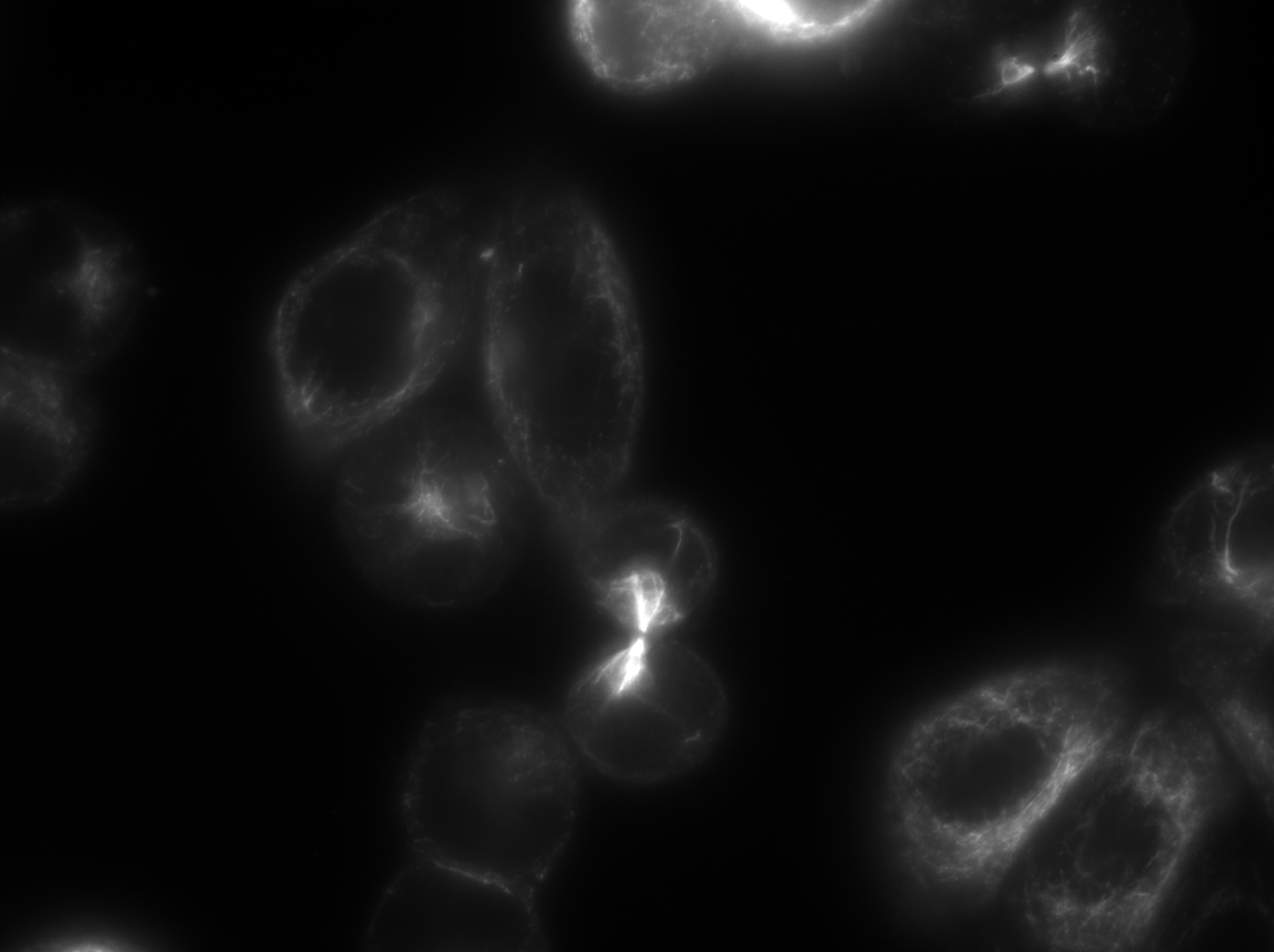

Supplement: Supplementary file 19 — Source data Fig. 5 part 5 [file 44319_2026_742_MOESM19_ESM.zip › Figure 5 Part 5/Fig 5ab WT and KO hela TTLL1-e326g atubulin part 2/TPGS1-KO/TPGS1-KO TTLL1-mut 10-22-24 R2 LT7.Project Maximum Z_XY1730227336_Z0_T0_C2.tif]

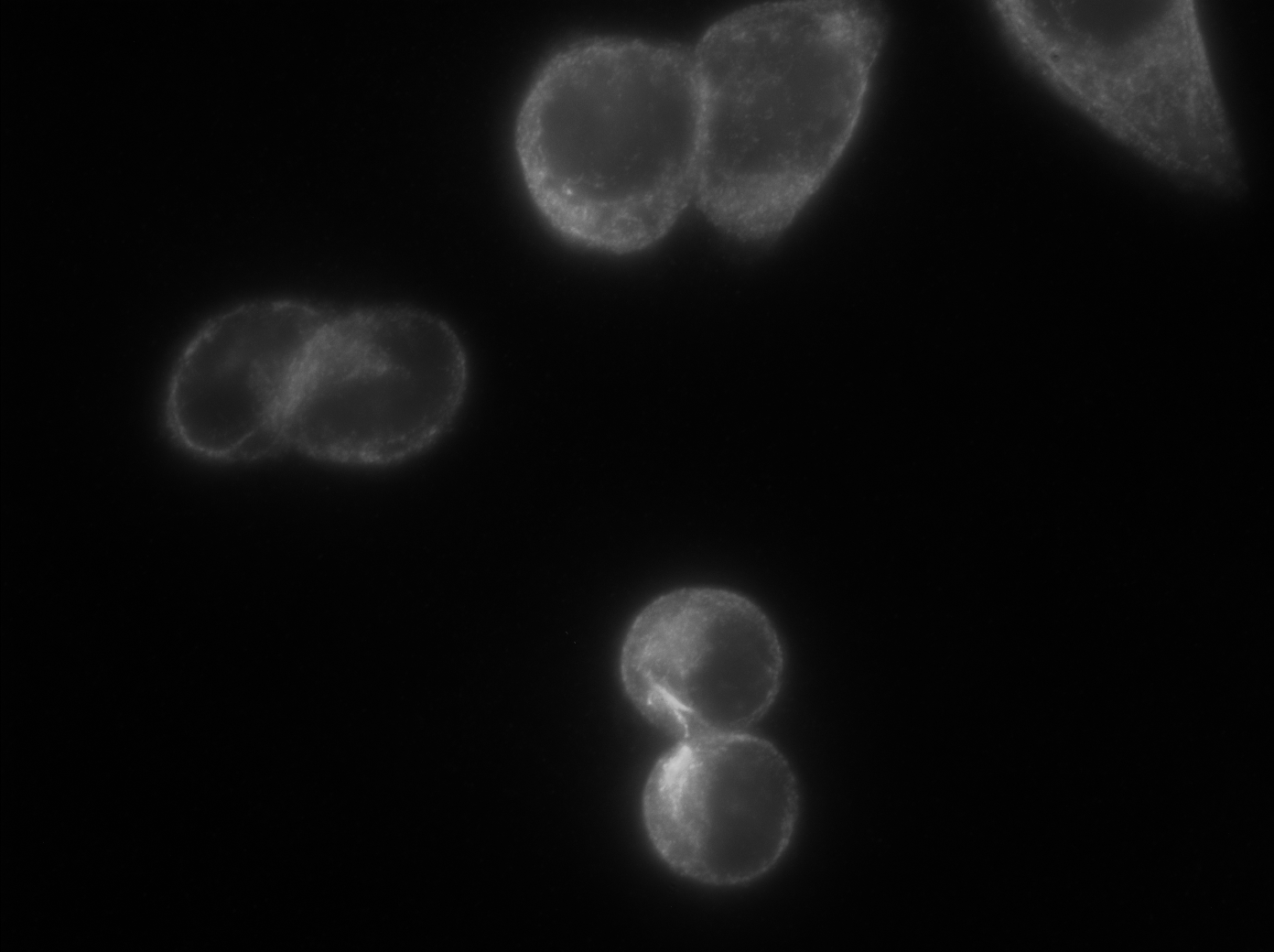

Supplement: Supplementary file 19 — Source data Fig. 5 part 5 [file 44319_2026_742_MOESM19_ESM.zip › Figure 5 Part 5/Fig 5ab WT and KO hela TTLL1-e326g atubulin part 2/TPGS1-KO/TPGS1-KO TTLL1-mut 10-15-24 R1 LT3.Project Maximum Z_XY1729022834_Z0_T0_C2.tif]

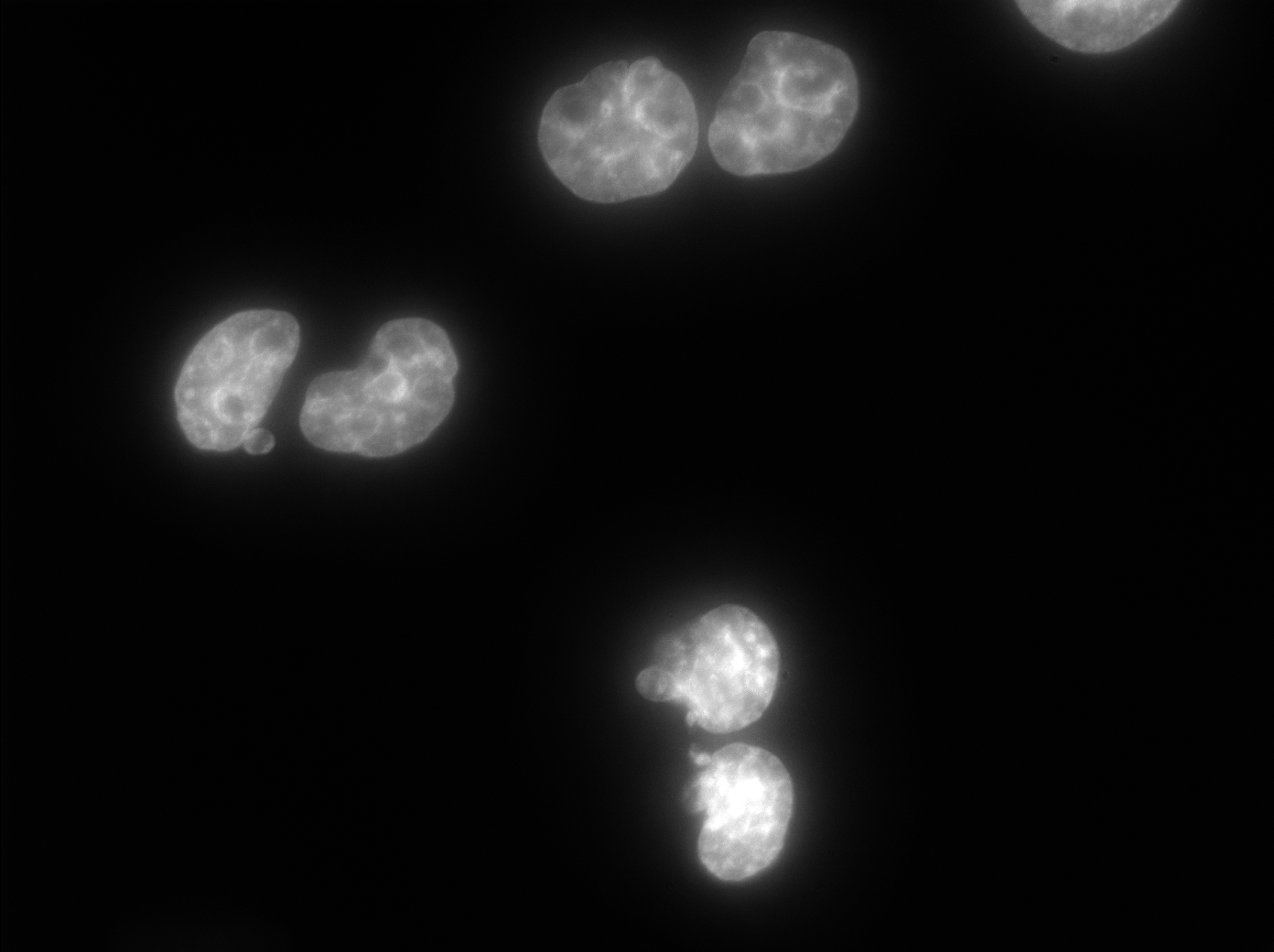

Supplement: Supplementary file 19 — Source data Fig. 5 part 5 [file 44319_2026_742_MOESM19_ESM.zip › Figure 5 Part 5/Fig 5ab WT and KO hela TTLL1-e326g atubulin part 2/TPGS1-KO/TPGS1-KO TTLL1-mut 10-15-24 R1 LT3.Project Maximum Z_XY1729022834_Z0_T0_C0.tif]

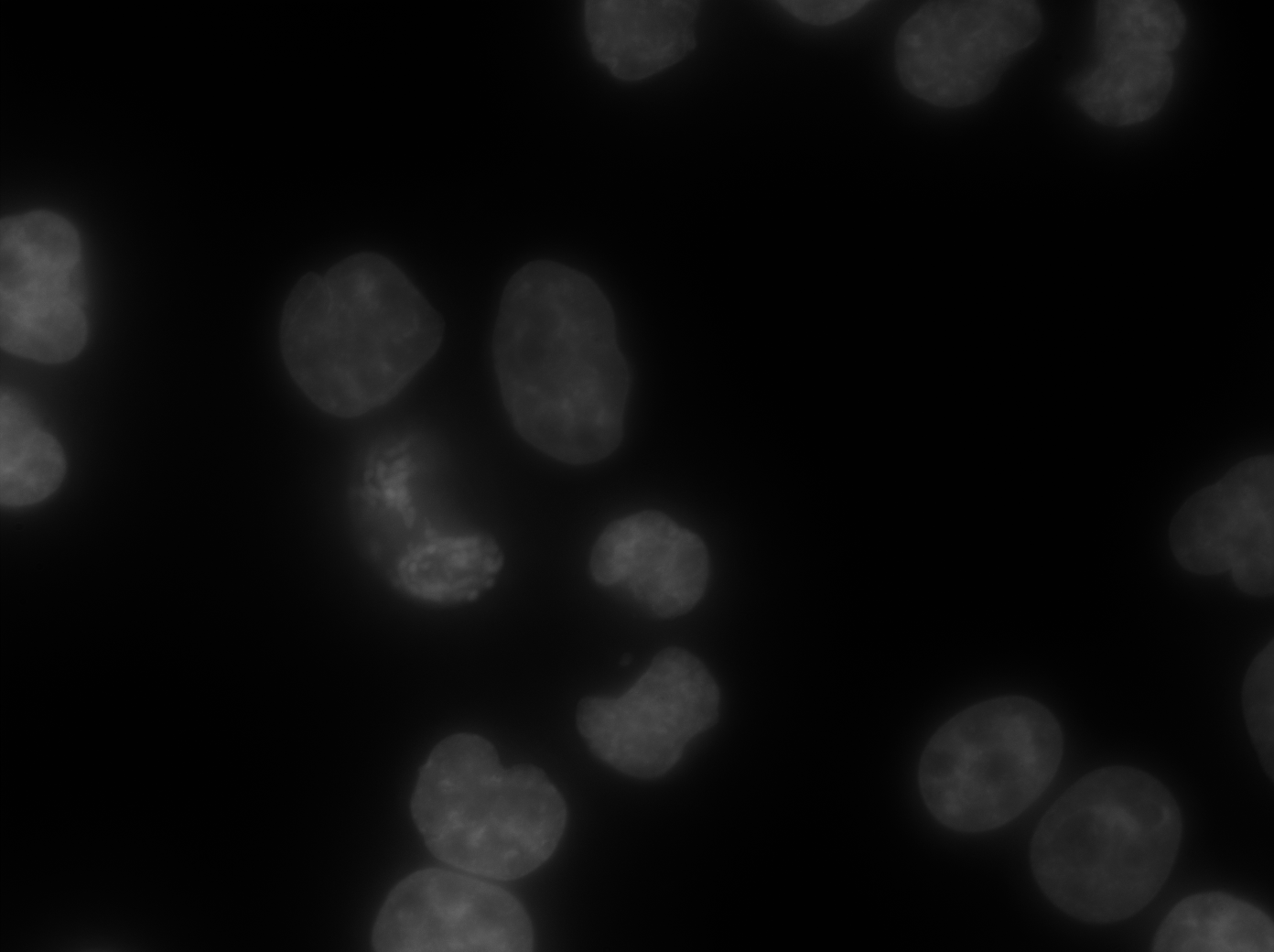

Supplement: Supplementary file 19 — Source data Fig. 5 part 5 [file 44319_2026_742_MOESM19_ESM.zip › Figure 5 Part 5/Fig 5ab WT and KO hela TTLL1-e326g atubulin part 2/TPGS1-KO/TPGS1-KO TTLL1-mut 10-22-24 R2 LT7.Project Maximum Z_XY1730227336_Z0_T0_C0.tif]

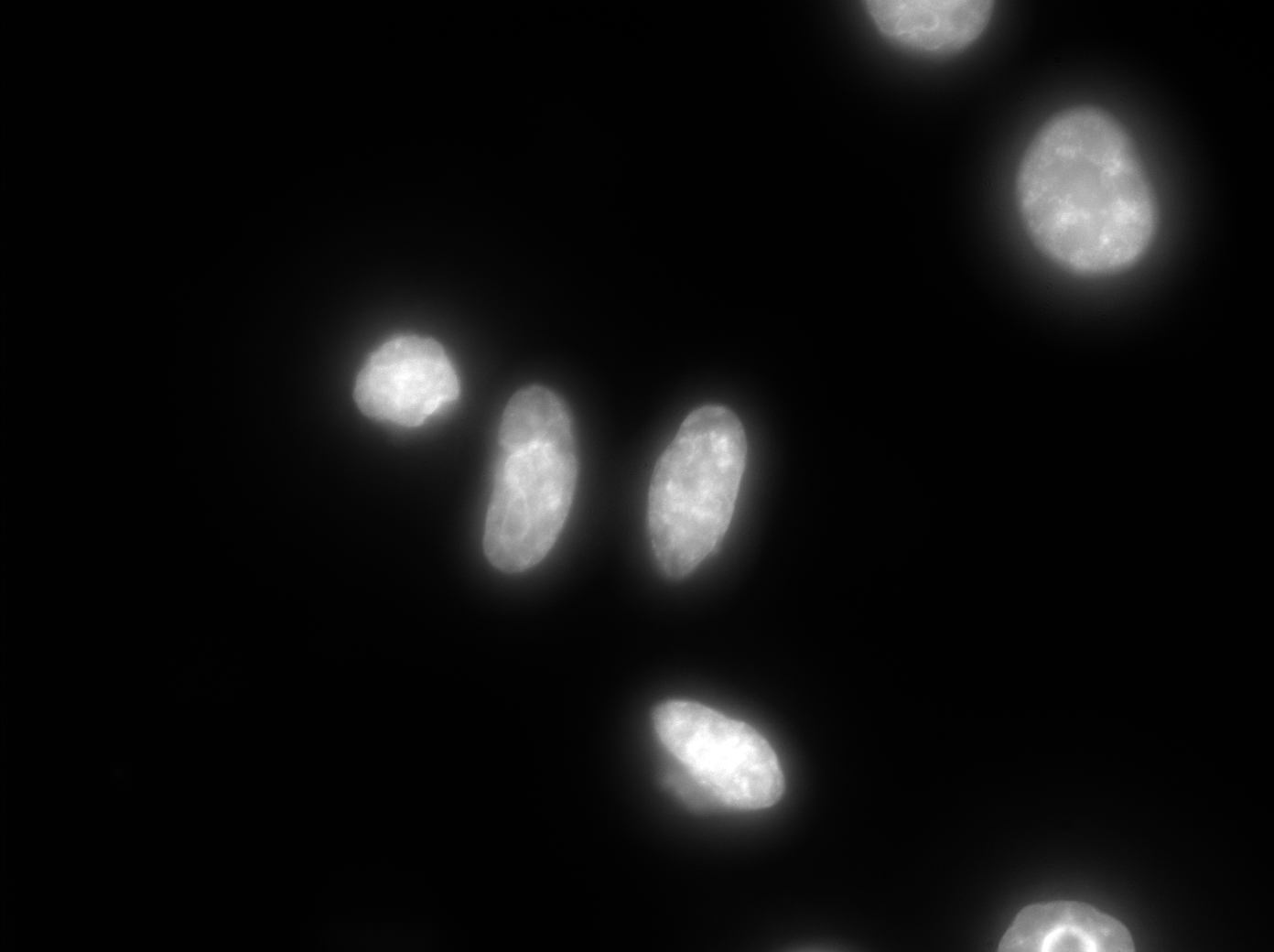

Supplement: Supplementary file 19 — Source data Fig. 5 part 5 [file 44319_2026_742_MOESM19_ESM.zip › Figure 5 Part 5/Fig 5ab WT and KO hela TTLL1-e326g atubulin part 2/TPGS1-KO/TPGS1-KO TTLL1-mut 10-22-24 R3 LT3.Project Maximum Z_XY1730228605_Z0_T0_C0.tif]

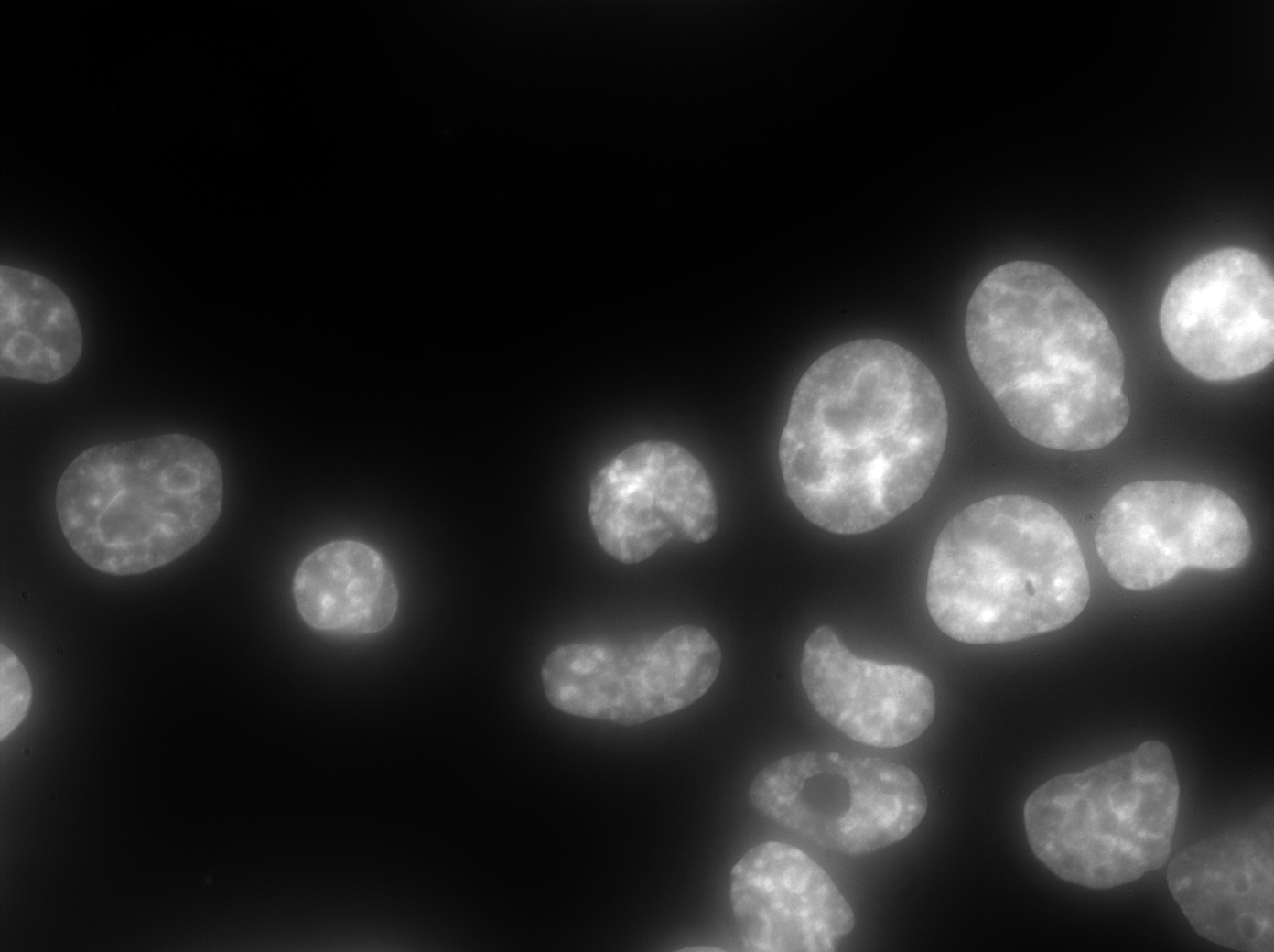

Supplement: Supplementary file 19 — Source data Fig. 5 part 5 [file 44319_2026_742_MOESM19_ESM.zip › Figure 5 Part 5/Fig 5ab WT and KO hela TTLL1-e326g atubulin part 2/TPGS1-KO/TPGS1-KO TTLL1-mut 10-22-24 R3 LT9.Project Maximum Z_XY1730230416_Z0_T0_C0.tif]

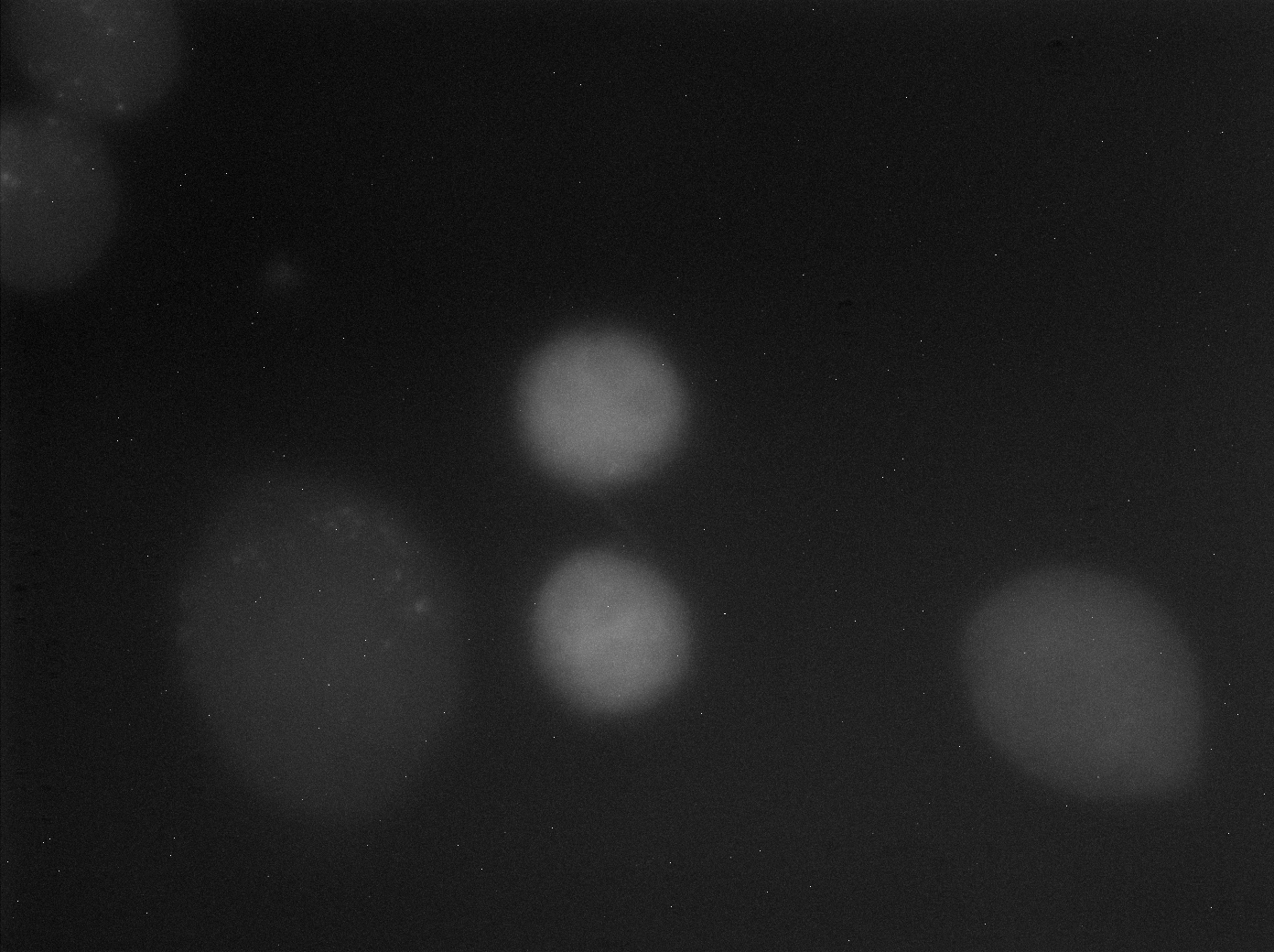

Supplement: Supplementary file 19 — Source data Fig. 5 part 5 [file 44319_2026_742_MOESM19_ESM.zip › Figure 5 Part 5/Fig 5ab WT and KO hela TTLL1-e326g atubulin part 2/TPGS1-KO/TPGS1-KO TTLL1-mut 10-15-24 R1 LT6 P2.Project Maximum Z_XY1729023653_Z0_T0_C1.tif]

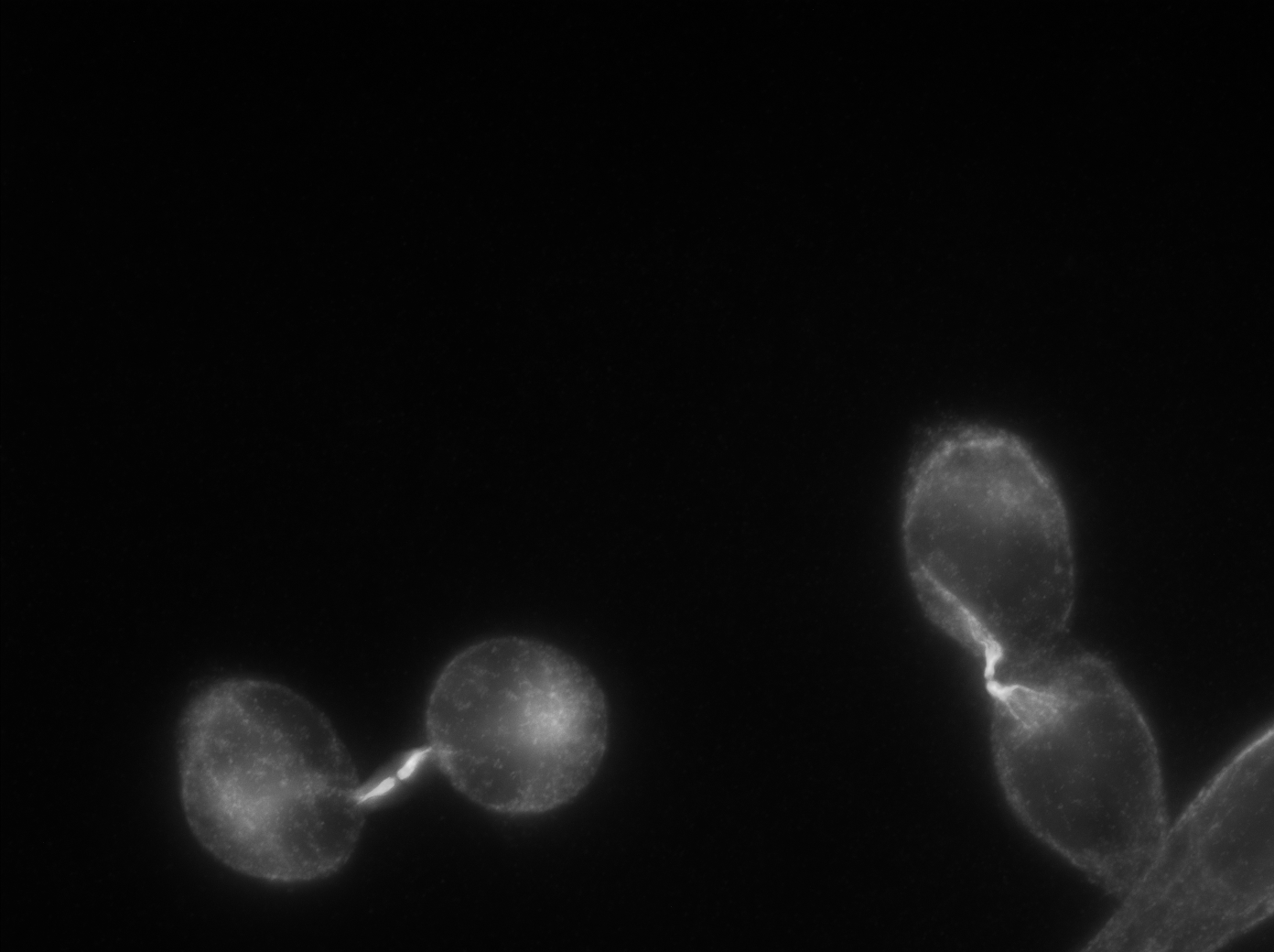

Supplement: Supplementary file 19 — Source data Fig. 5 part 5 [file 44319_2026_742_MOESM19_ESM.zip › Figure 5 Part 5/Fig 5ab WT and KO hela TTLL1-e326g atubulin part 2/TPGS1-KO/TPGS1-KO TTLL1-mut 10-15-24 R1 LT11.Project Maximum Z_XY1729025322_Z0_T0_C2.tif]

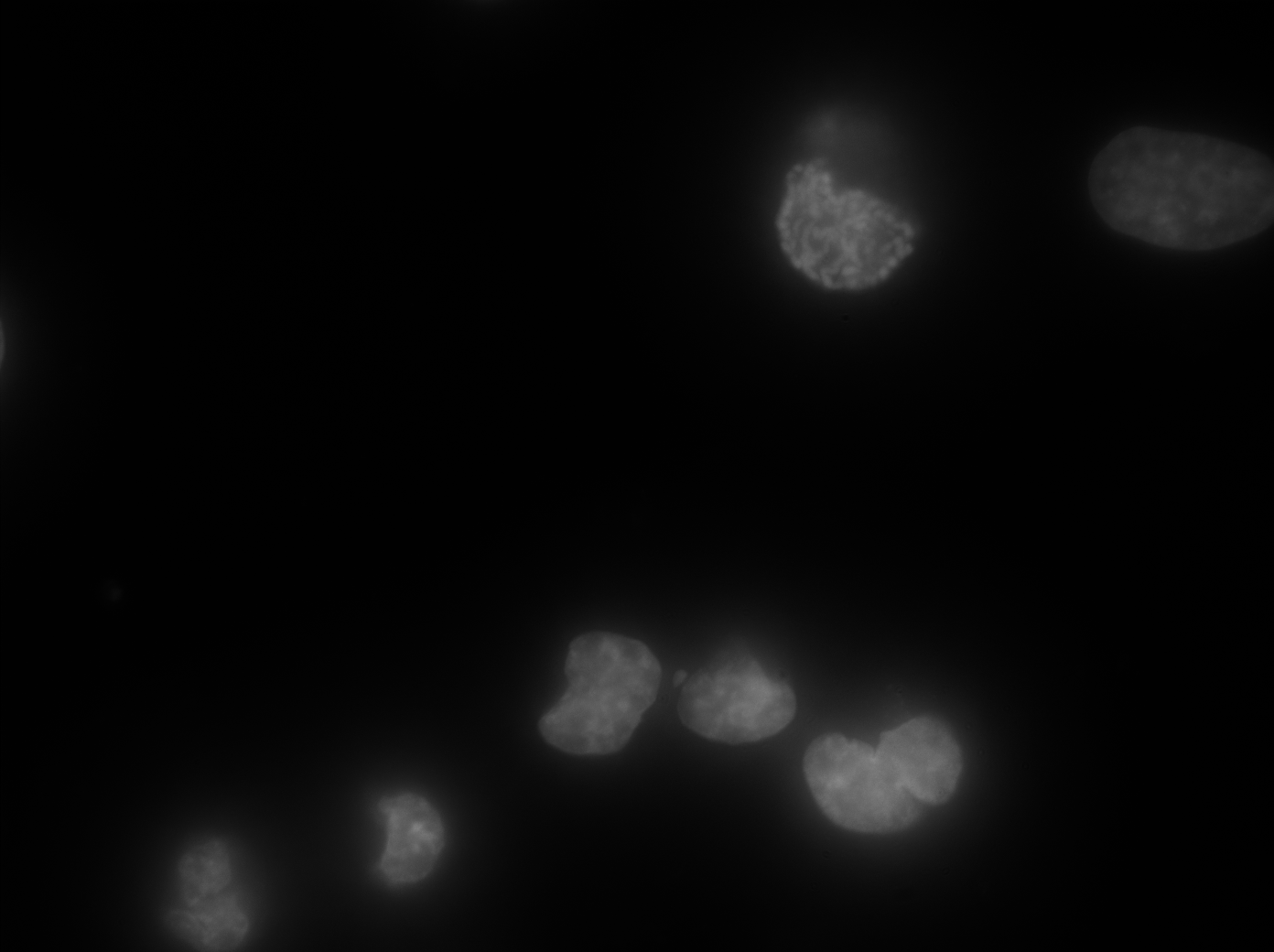

Supplement: Supplementary file 19 — Source data Fig. 5 part 5 [file 44319_2026_742_MOESM19_ESM.zip › Figure 5 Part 5/Fig 5ab WT and KO hela TTLL1-e326g atubulin part 2/TPGS1-KO/TPGS1-KO TTLL1-mut 10-22-24 R2 LT1.Project Maximum Z_XY1730225539_Z0_T0_C0.tif]

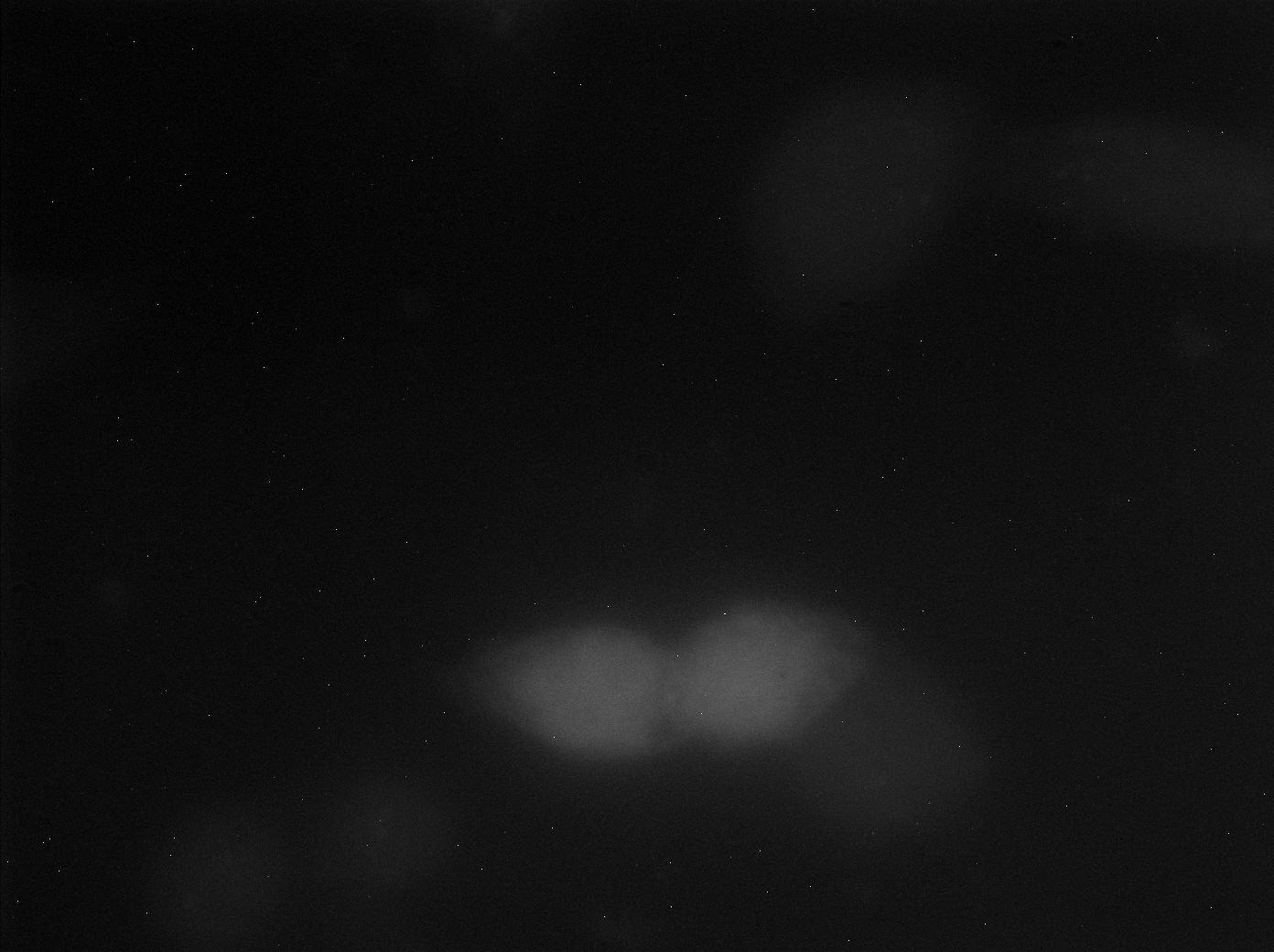

Supplement: Supplementary file 19 — Source data Fig. 5 part 5 [file 44319_2026_742_MOESM19_ESM.zip › Figure 5 Part 5/Fig 5ab WT and KO hela TTLL1-e326g atubulin part 2/TPGS1-KO/TPGS1-KO TTLL1-mut 10-22-24 R2 LT1.Project Maximum Z_XY1730225539_Z0_T0_C1.tif]

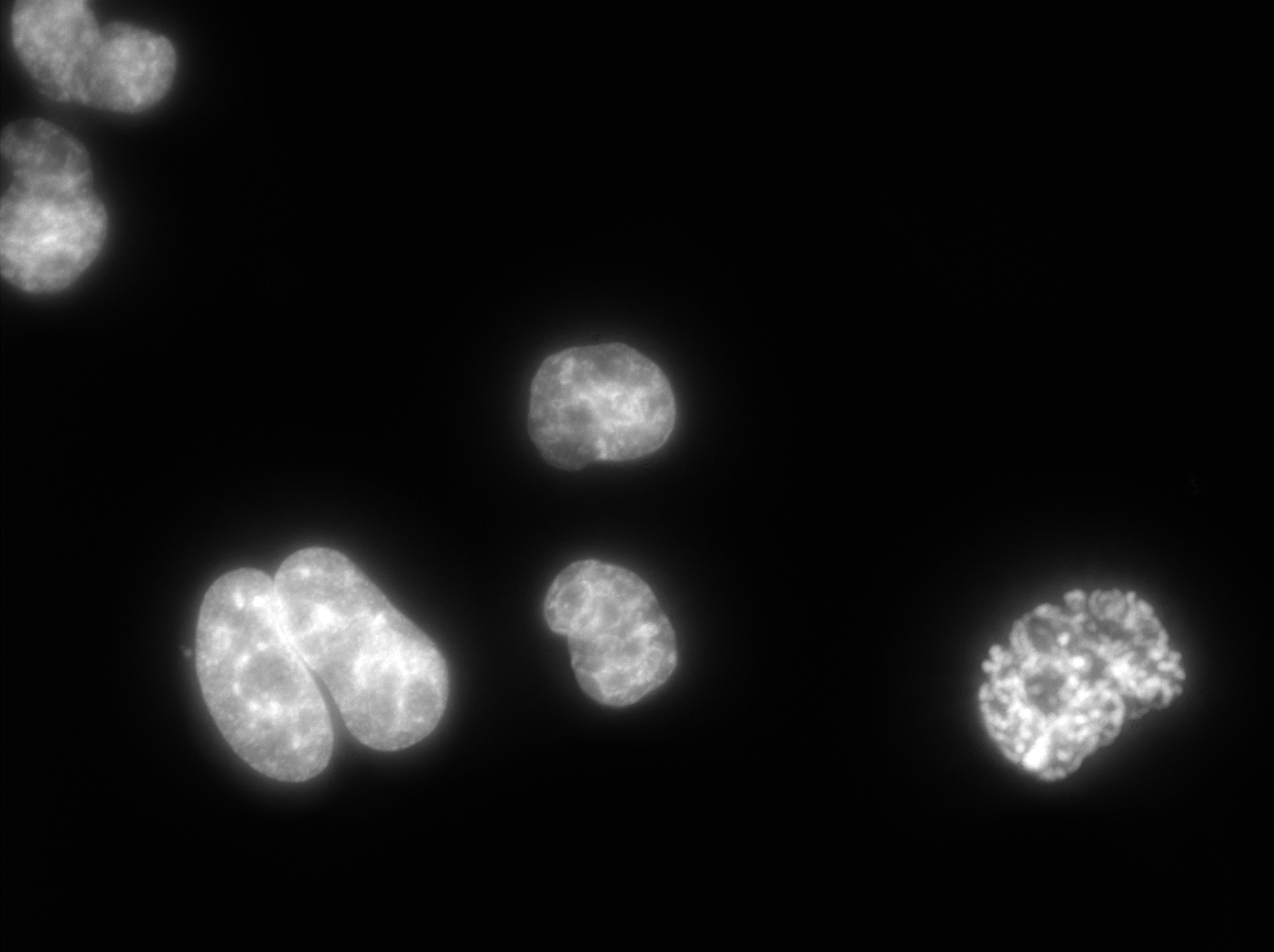

Supplement: Supplementary file 19 — Source data Fig. 5 part 5 [file 44319_2026_742_MOESM19_ESM.zip › Figure 5 Part 5/Fig 5ab WT and KO hela TTLL1-e326g atubulin part 2/TPGS1-KO/TPGS1-KO TTLL1-mut 10-15-24 R1 LT6 P2.Project Maximum Z_XY1729023653_Z0_T0_C0.tif]

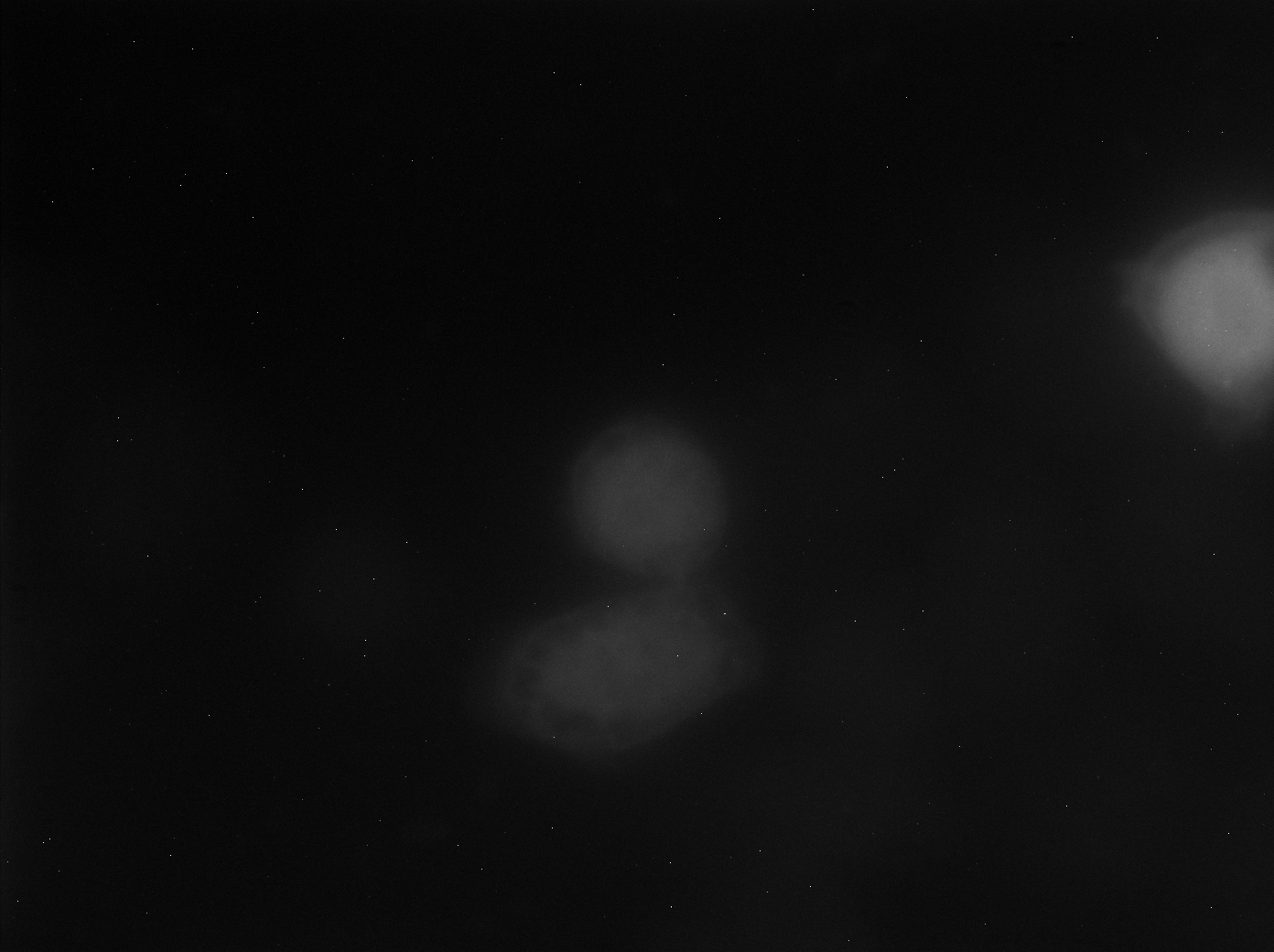

Supplement: Supplementary file 19 — Source data Fig. 5 part 5 [file 44319_2026_742_MOESM19_ESM.zip › Figure 5 Part 5/Fig 5ab WT and KO hela TTLL1-e326g atubulin part 2/TPGS1-KO/TPGS1-KO TTLL1-mut 10-22-24 R3 LT9.Project Maximum Z_XY1730230416_Z0_T0_C1.tif]

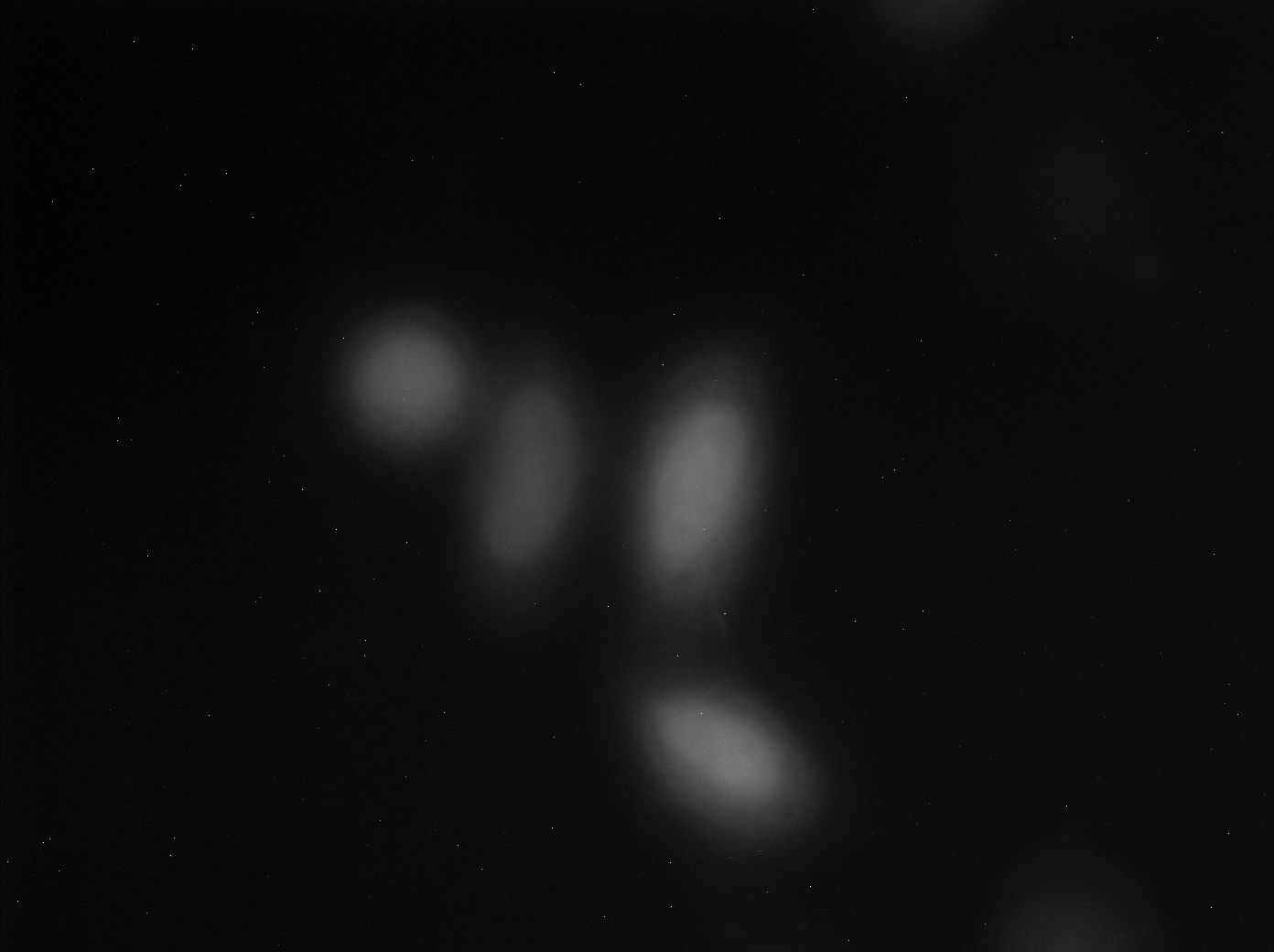

Supplement: Supplementary file 19 — Source data Fig. 5 part 5 [file 44319_2026_742_MOESM19_ESM.zip › Figure 5 Part 5/Fig 5ab WT and KO hela TTLL1-e326g atubulin part 2/TPGS1-KO/TPGS1-KO TTLL1-mut 10-22-24 R3 LT3.Project Maximum Z_XY1730228605_Z0_T0_C1.tif]

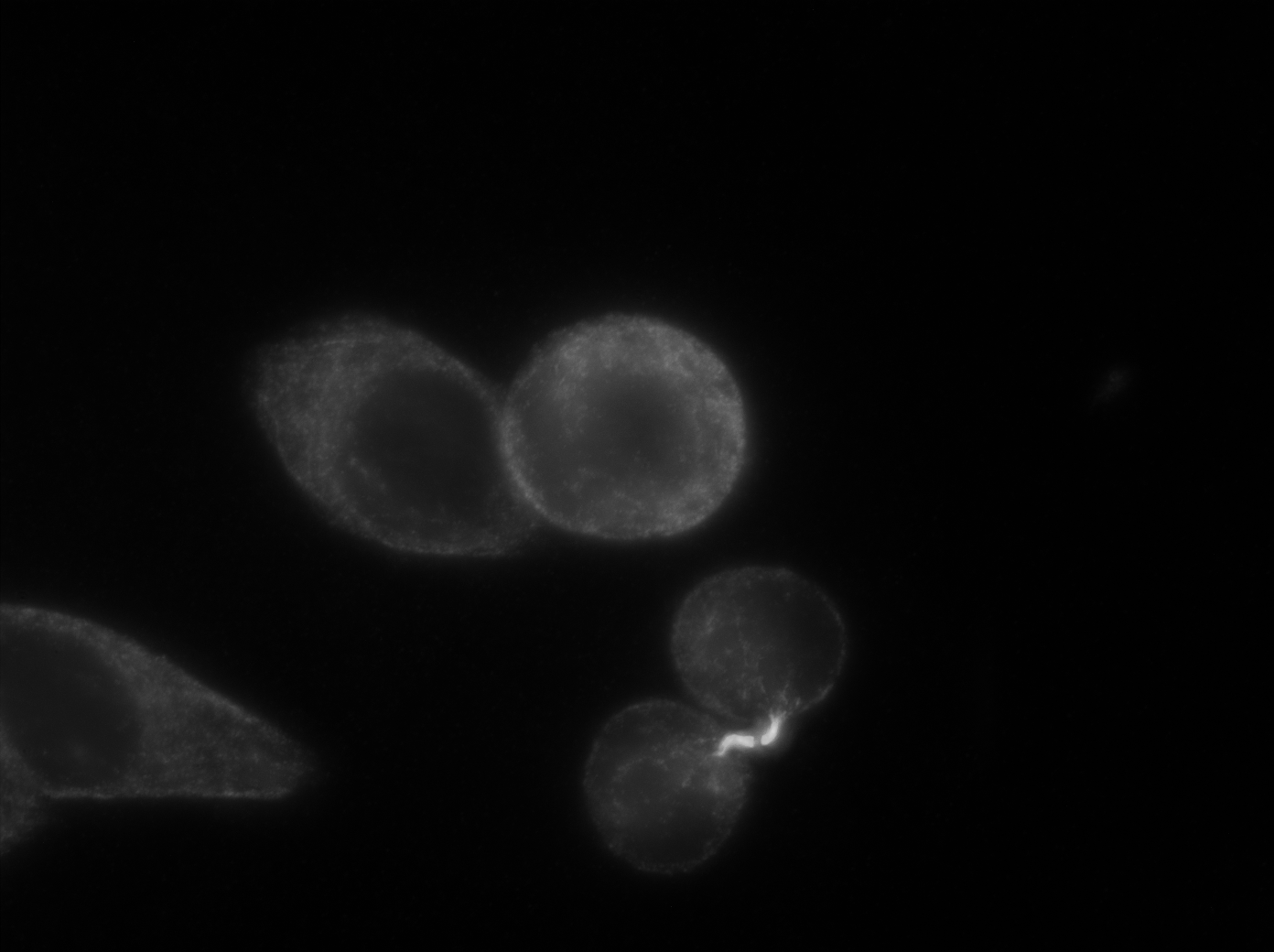

Supplement: Supplementary file 19 — Source data Fig. 5 part 5 [file 44319_2026_742_MOESM19_ESM.zip › Figure 5 Part 5/Fig 5ab WT and KO hela TTLL1-e326g atubulin part 2/TPGS1-KO/TPGS1-KO TTLL1-mut 10-15-24 R1 LT4.Project Maximum Z_XY1729023288_Z0_T0_C2.tif]

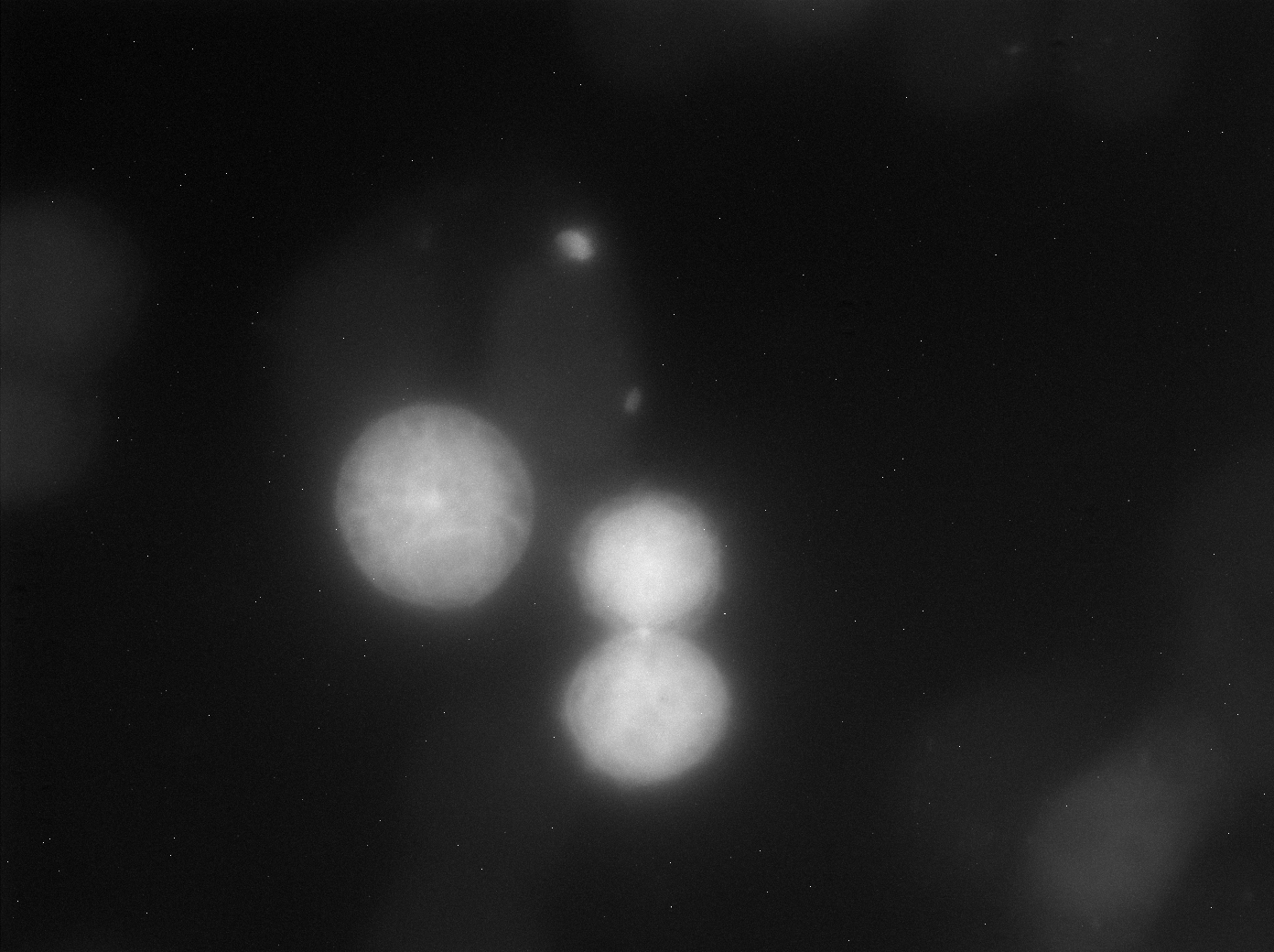

Supplement: Supplementary file 19 — Source data Fig. 5 part 5 [file 44319_2026_742_MOESM19_ESM.zip › Figure 5 Part 5/Fig 5ab WT and KO hela TTLL1-e326g atubulin part 2/TPGS1-KO/TPGS1-KO TTLL1-mut 10-22-24 R2 LT7.Project Maximum Z_XY1730227336_Z0_T0_C1.tif]

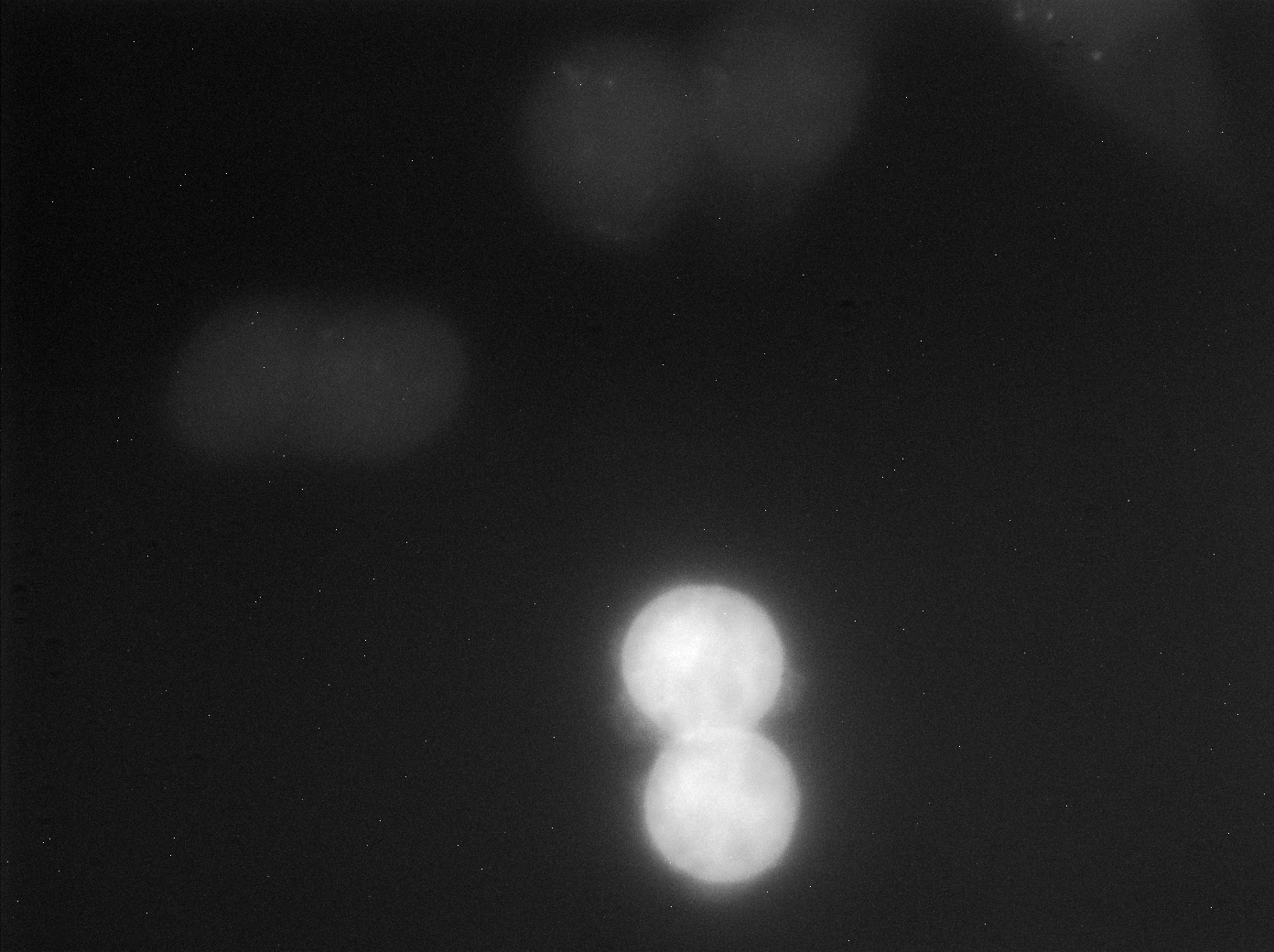

Supplement: Supplementary file 19 — Source data Fig. 5 part 5 [file 44319_2026_742_MOESM19_ESM.zip › Figure 5 Part 5/Fig 5ab WT and KO hela TTLL1-e326g atubulin part 2/TPGS1-KO/TPGS1-KO TTLL1-mut 10-15-24 R1 LT3.Project Maximum Z_XY1729022834_Z0_T0_C1.tif]

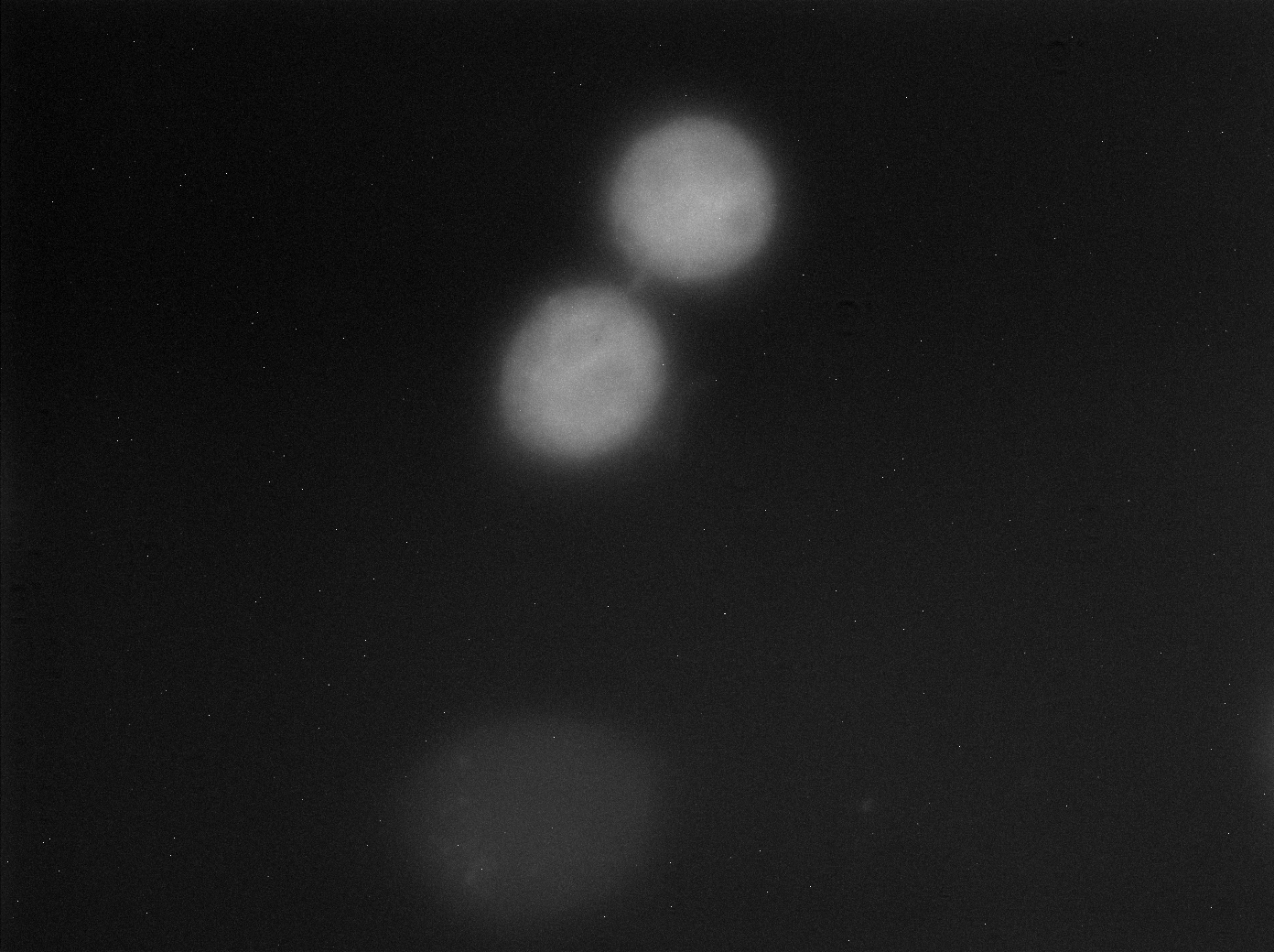

Supplement: Supplementary file 19 — Source data Fig. 5 part 5 [file 44319_2026_742_MOESM19_ESM.zip › Figure 5 Part 5/Fig 5ab WT and KO hela TTLL1-e326g atubulin part 2/TPGS1-KO/TPGS1-KO TTLL1-mut 10-15-24 R1 LT10.Project Maximum Z_XY1729025235_Z0_T0_C1.tif]

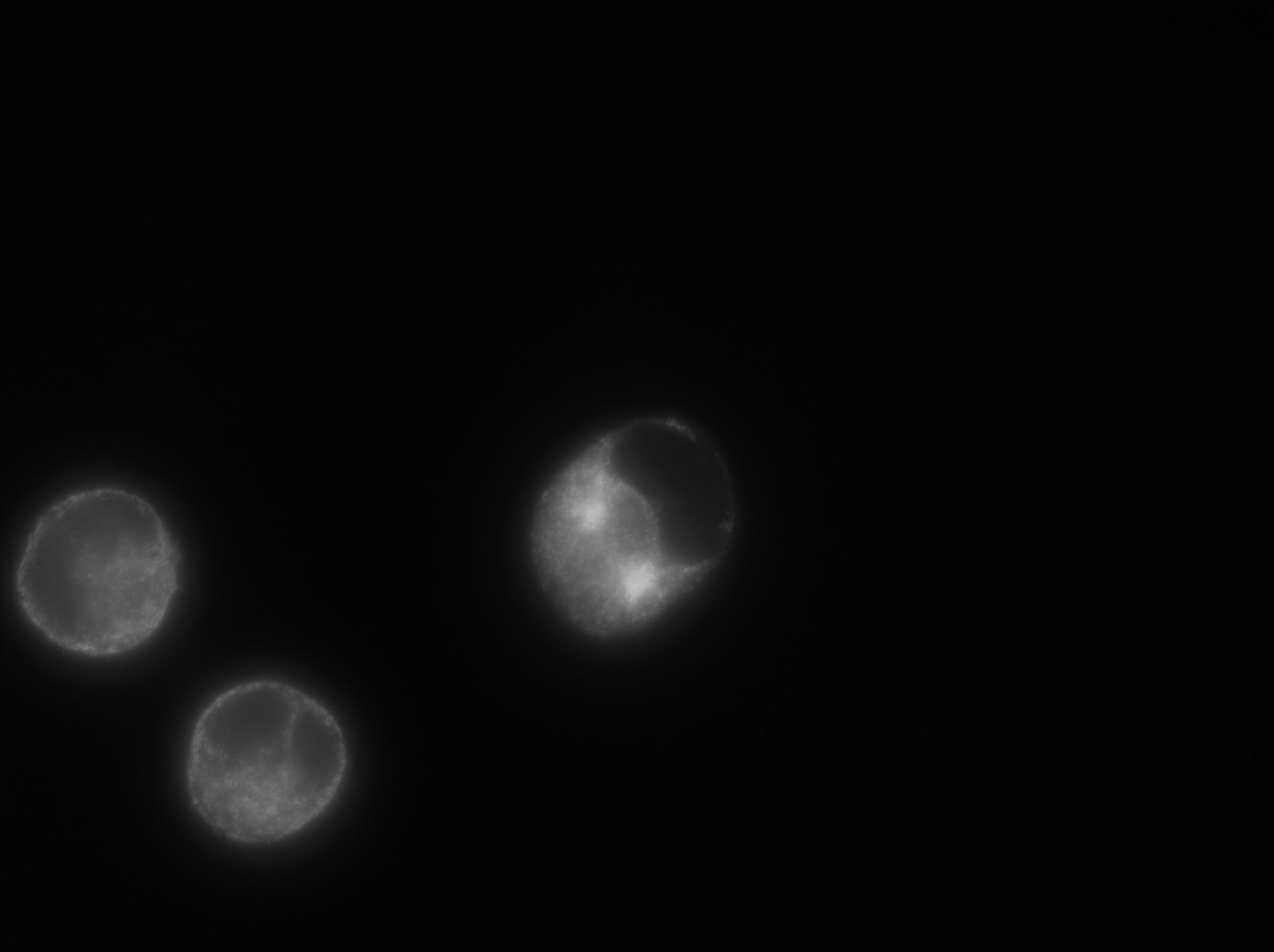

Supplement: Supplementary file 19 — Source data Fig. 5 part 5 [file 44319_2026_742_MOESM19_ESM.zip › Figure 5 Part 5/Fig 5ab WT and KO hela TTLL1-e326g atubulin part 2/TPGS1-KO/TPGS1-KO TTLL1-mut 10-15-24 R1 P1.Project Maximum Z_XY1729022038_Z0_T0_C2.tif]

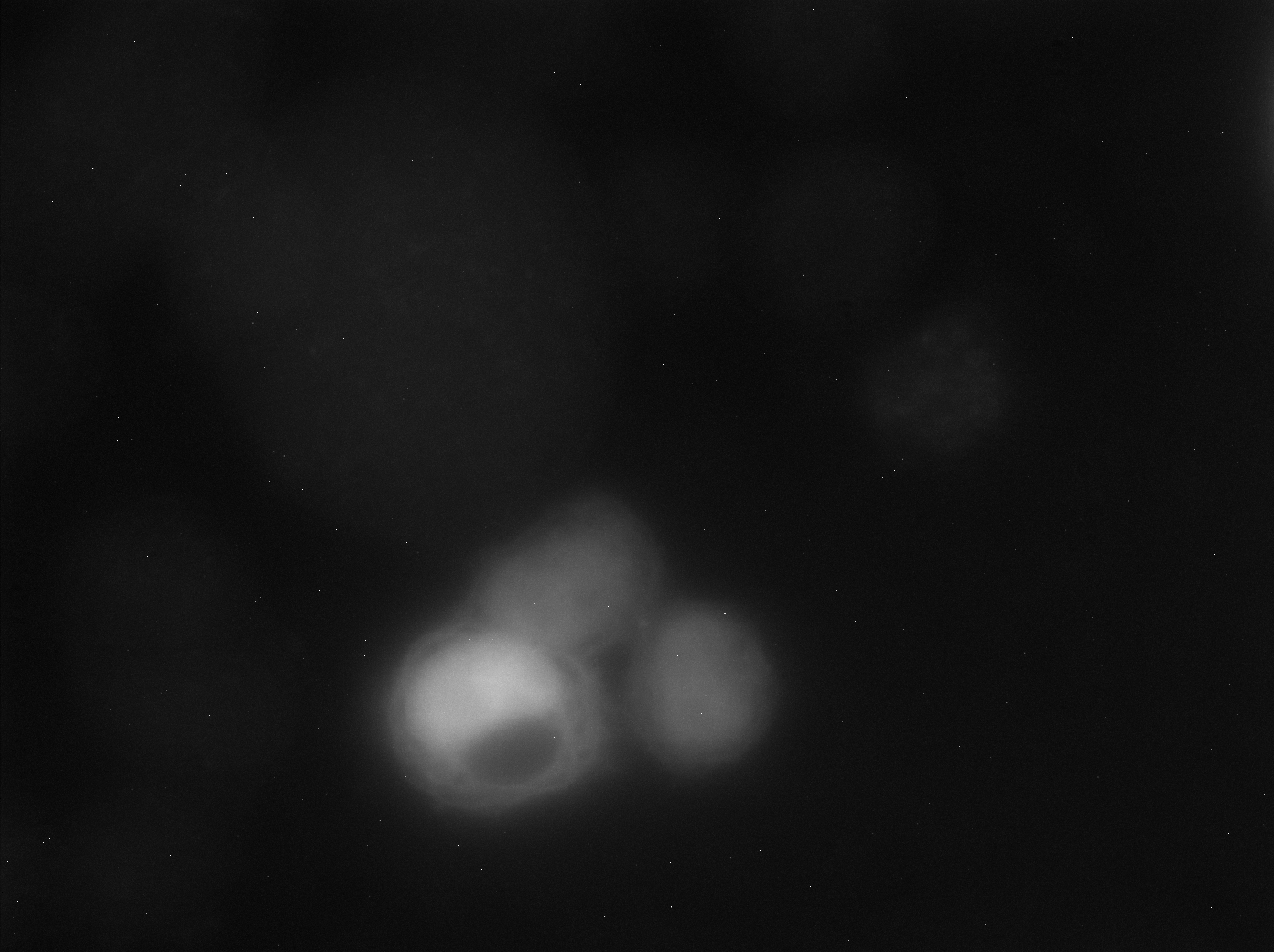

Supplement: Supplementary file 19 — Source data Fig. 5 part 5 [file 44319_2026_742_MOESM19_ESM.zip › Figure 5 Part 5/Fig 5ab WT and KO hela TTLL1-e326g atubulin part 2/TPGS1-KO/TPGS1-KO TTLL1-mut 10-22-24 R3 LT5.Project Maximum Z_XY1730229357_Z0_T0_C1.tif]

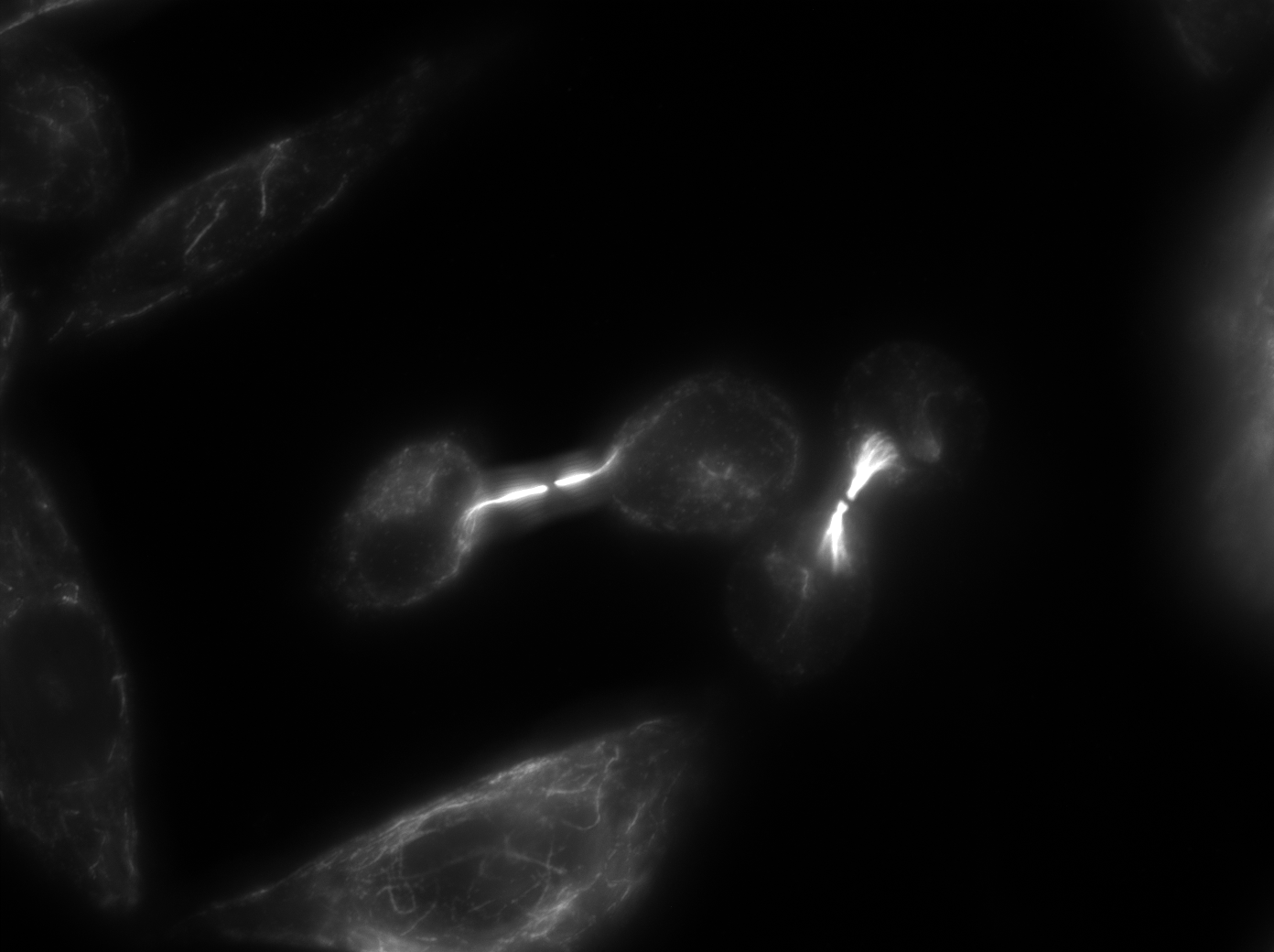

Supplement: Supplementary file 19 — Source data Fig. 5 part 5 [file 44319_2026_742_MOESM19_ESM.zip › Figure 5 Part 5/Fig 5ab WT and KO hela TTLL1-e326g atubulin part 2/TPGS1-KO/TPGS1-KO TTLL1-mut 10-22-24 R2 LT3LT4.Project Maximum Z_XY1730226457_Z0_T0_C2.tif]

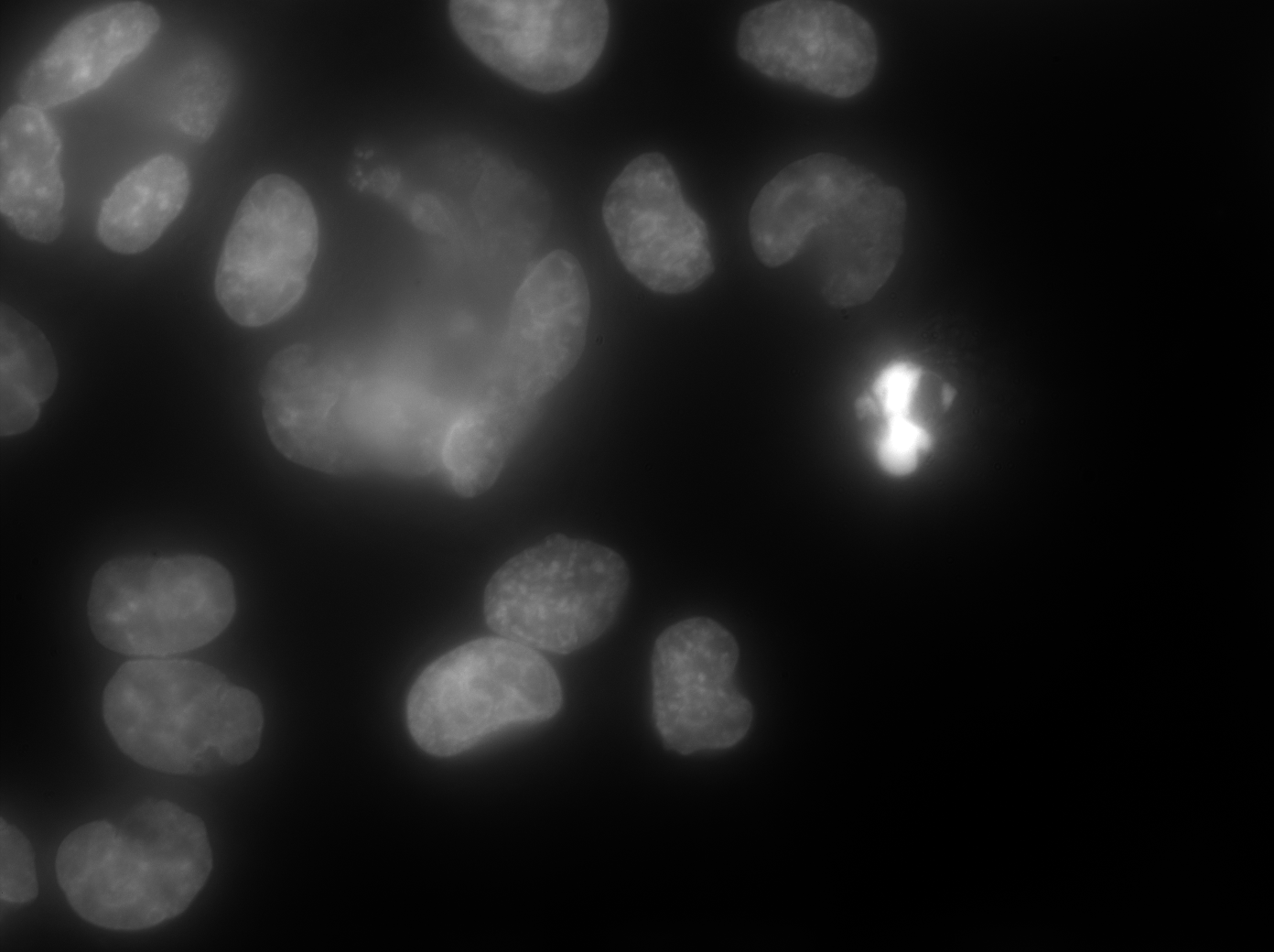

Supplement: Supplementary file 19 — Source data Fig. 5 part 5 [file 44319_2026_742_MOESM19_ESM.zip › Figure 5 Part 5/Fig 5ab WT and KO hela TTLL1-e326g atubulin part 2/TPGS1-KO/TPGS1-KO TTLL1-mut 10-22-24 R3 LT5.Project Maximum Z_XY1730229357_Z0_T0_C0.tif]

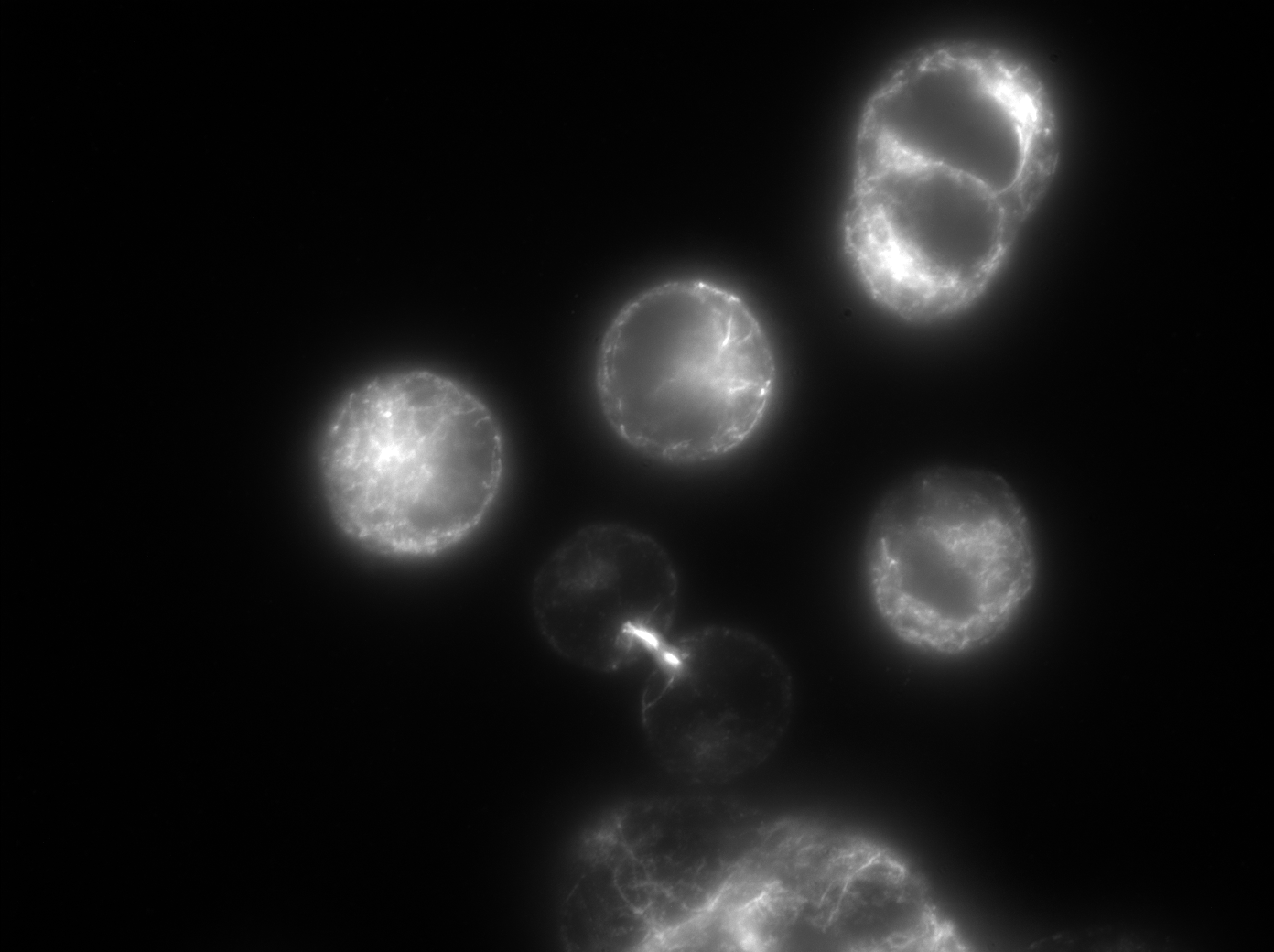

Supplement: Supplementary file 19 — Source data Fig. 5 part 5 [file 44319_2026_742_MOESM19_ESM.zip › Figure 5 Part 5/Fig 5ab WT and KO hela TTLL1-e326g atubulin part 2/TPGS1-KO/TPGS1-KO TTLL1-mut 10-22-24 R3 LT8.Project Maximum Z_XY1730229897_Z0_T0_C2.tif]

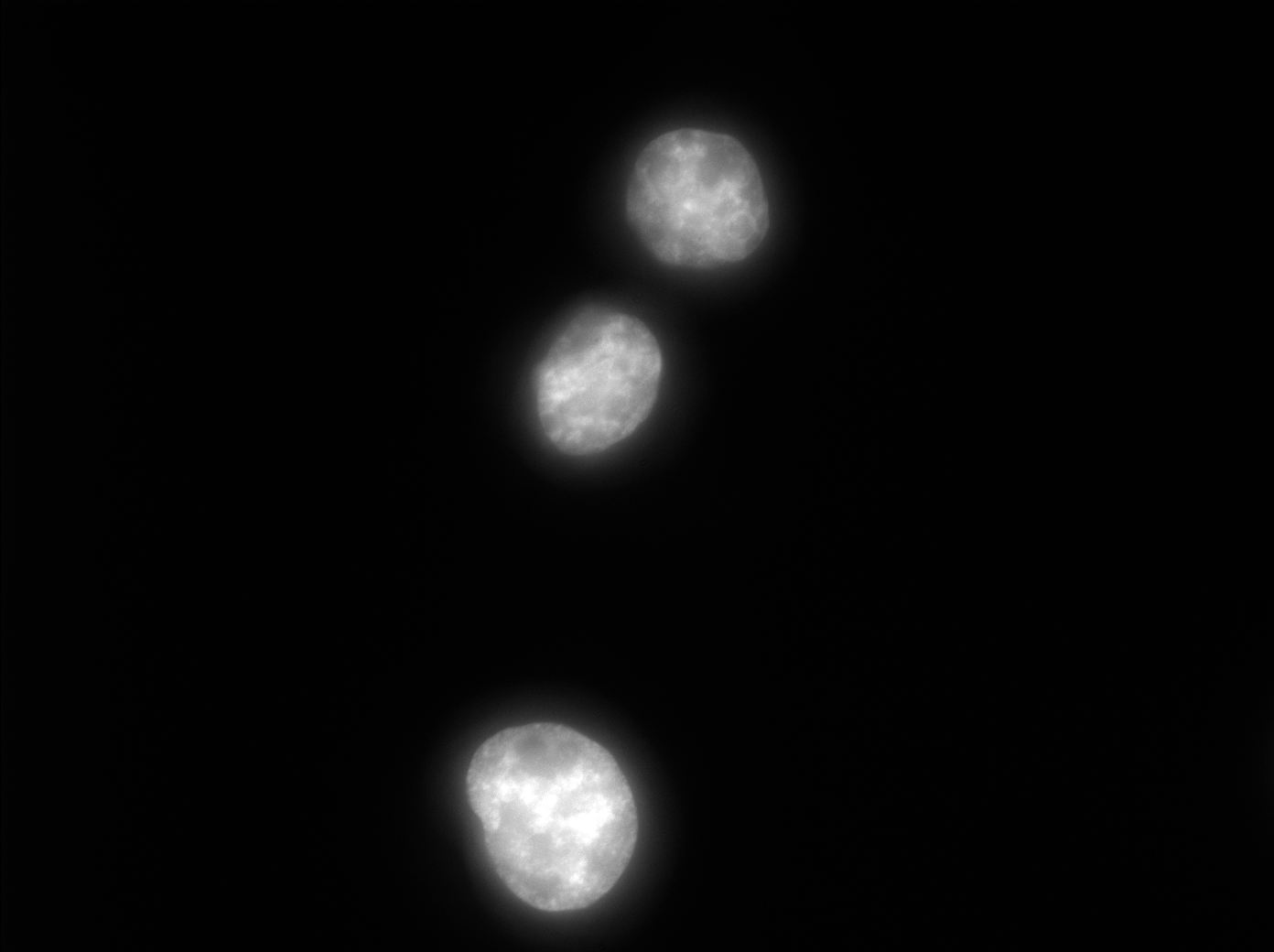

Supplement: Supplementary file 19 — Source data Fig. 5 part 5 [file 44319_2026_742_MOESM19_ESM.zip › Figure 5 Part 5/Fig 5ab WT and KO hela TTLL1-e326g atubulin part 2/TPGS1-KO/TPGS1-KO TTLL1-mut 10-15-24 R1 LT10.Project Maximum Z_XY1729025235_Z0_T0_C0.tif]

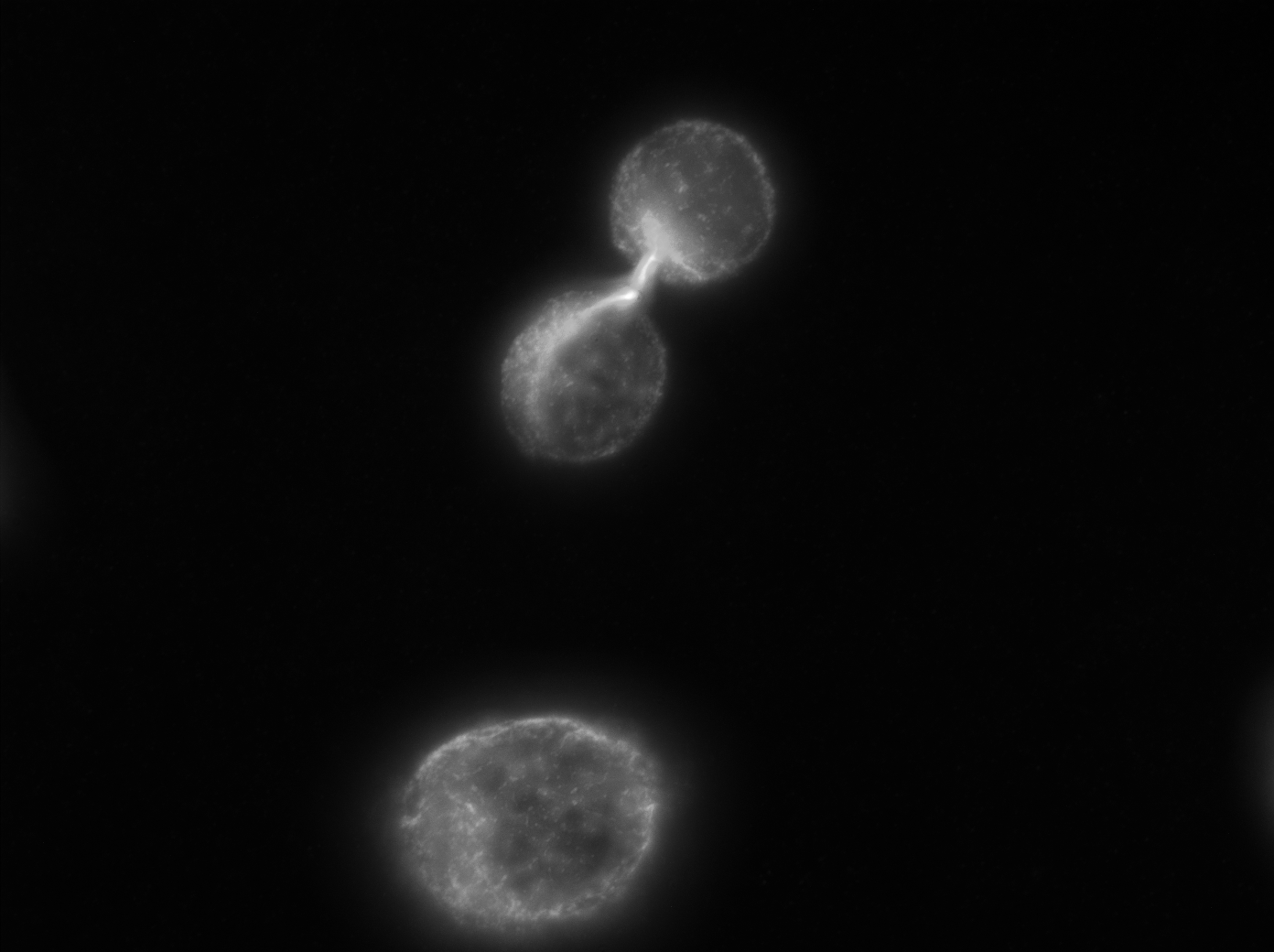

Supplement: Supplementary file 19 — Source data Fig. 5 part 5 [file 44319_2026_742_MOESM19_ESM.zip › Figure 5 Part 5/Fig 5ab WT and KO hela TTLL1-e326g atubulin part 2/TPGS1-KO/TPGS1-KO TTLL1-mut 10-15-24 R1 LT10.Project Maximum Z_XY1729025235_Z0_T0_C2.tif]

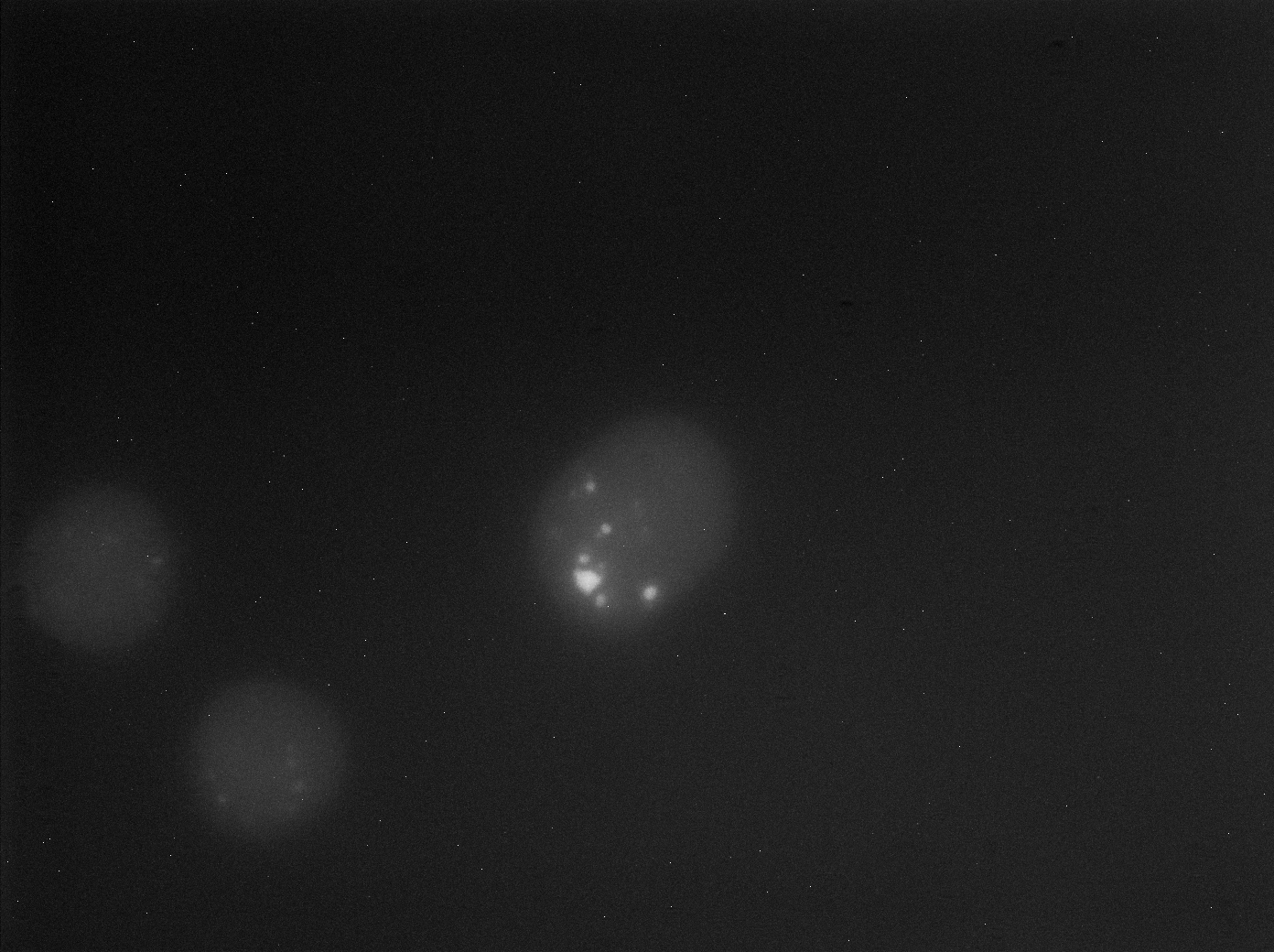

Supplement: Supplementary file 19 — Source data Fig. 5 part 5 [file 44319_2026_742_MOESM19_ESM.zip › Figure 5 Part 5/Fig 5ab WT and KO hela TTLL1-e326g atubulin part 2/TPGS1-KO/TPGS1-KO TTLL1-mut 10-15-24 R1 P1.Project Maximum Z_XY1729022038_Z0_T0_C1.tif]

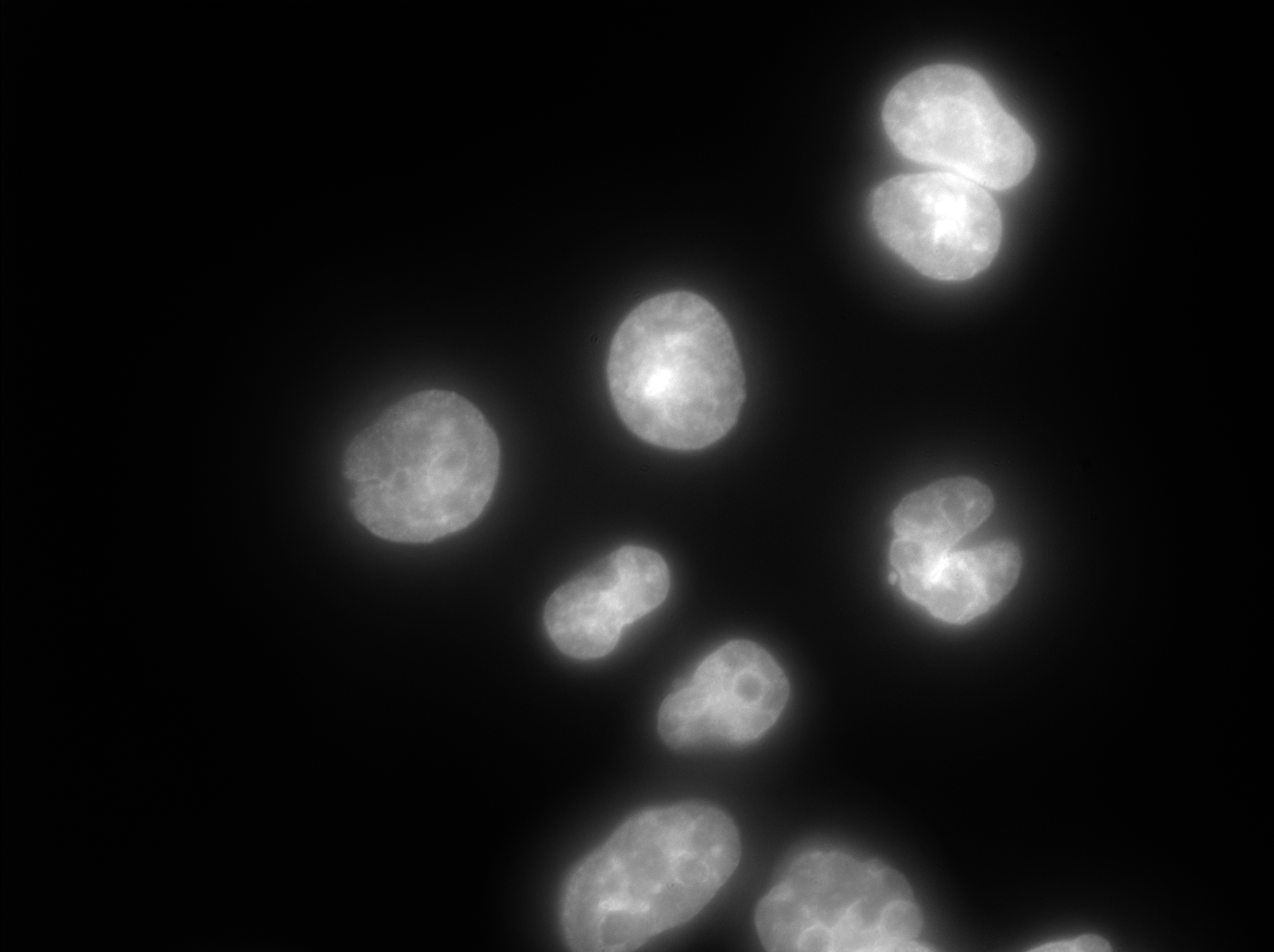

Supplement: Supplementary file 19 — Source data Fig. 5 part 5 [file 44319_2026_742_MOESM19_ESM.zip › Figure 5 Part 5/Fig 5ab WT and KO hela TTLL1-e326g atubulin part 2/TPGS1-KO/TPGS1-KO TTLL1-mut 10-22-24 R3 LT8.Project Maximum Z_XY1730229897_Z0_T0_C0.tif]

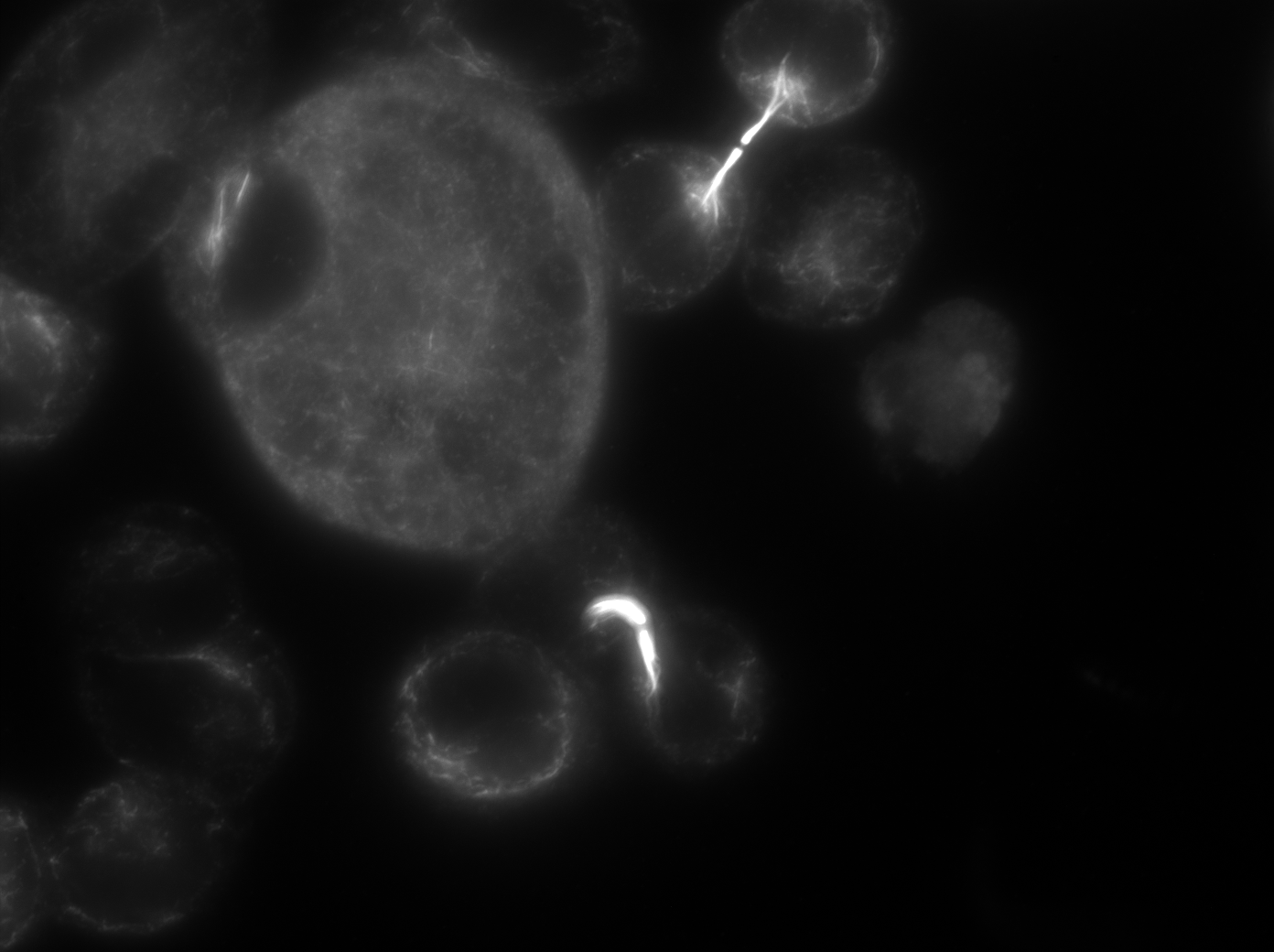

Supplement: Supplementary file 19 — Source data Fig. 5 part 5 [file 44319_2026_742_MOESM19_ESM.zip › Figure 5 Part 5/Fig 5ab WT and KO hela TTLL1-e326g atubulin part 2/TPGS1-KO/TPGS1-KO TTLL1-mut 10-22-24 R3 LT5.Project Maximum Z_XY1730229357_Z0_T0_C2.tif]

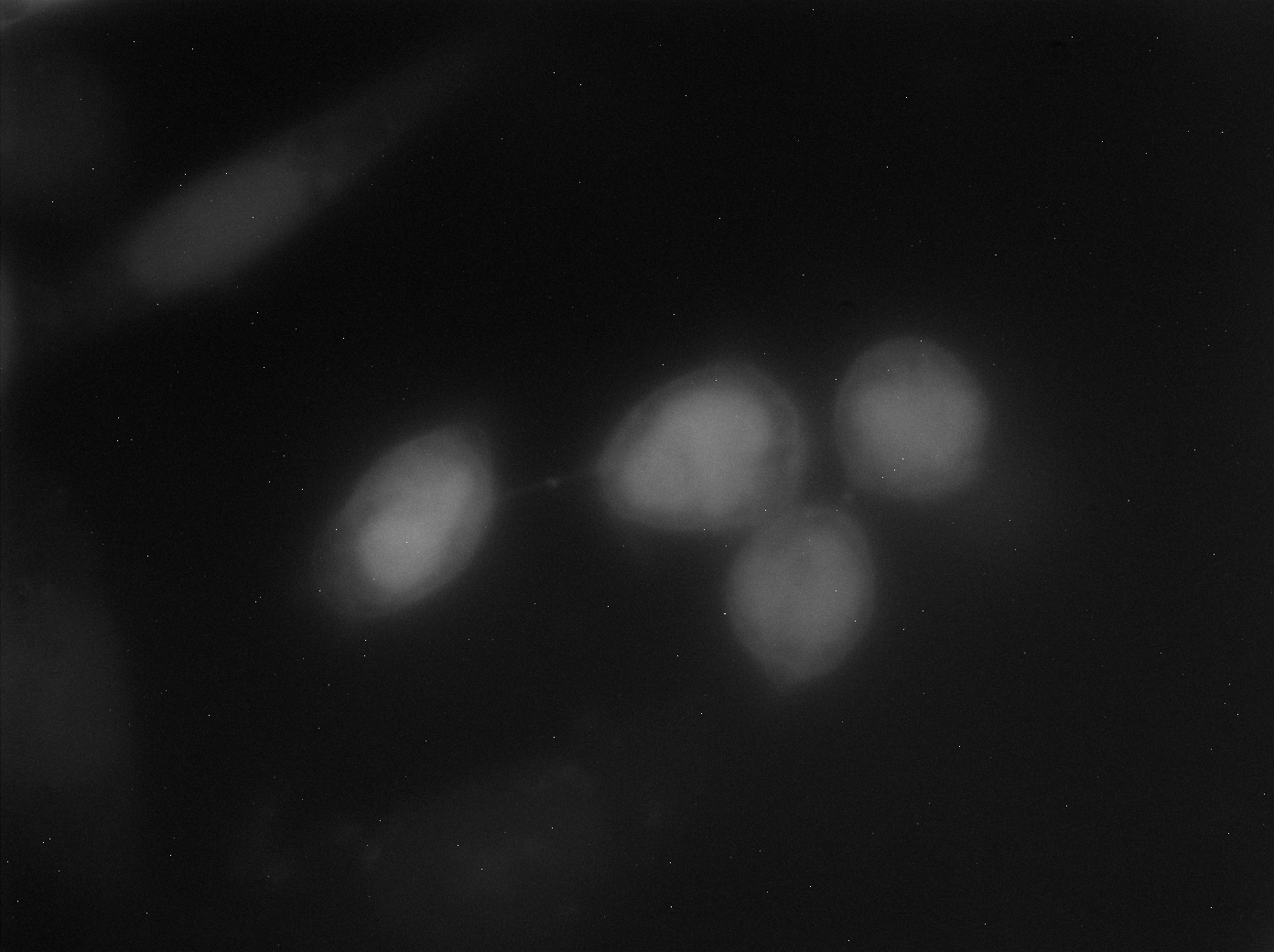

Supplement: Supplementary file 19 — Source data Fig. 5 part 5 [file 44319_2026_742_MOESM19_ESM.zip › Figure 5 Part 5/Fig 5ab WT and KO hela TTLL1-e326g atubulin part 2/TPGS1-KO/TPGS1-KO TTLL1-mut 10-22-24 R2 LT3LT4.Project Maximum Z_XY1730226457_Z0_T0_C1.tif]

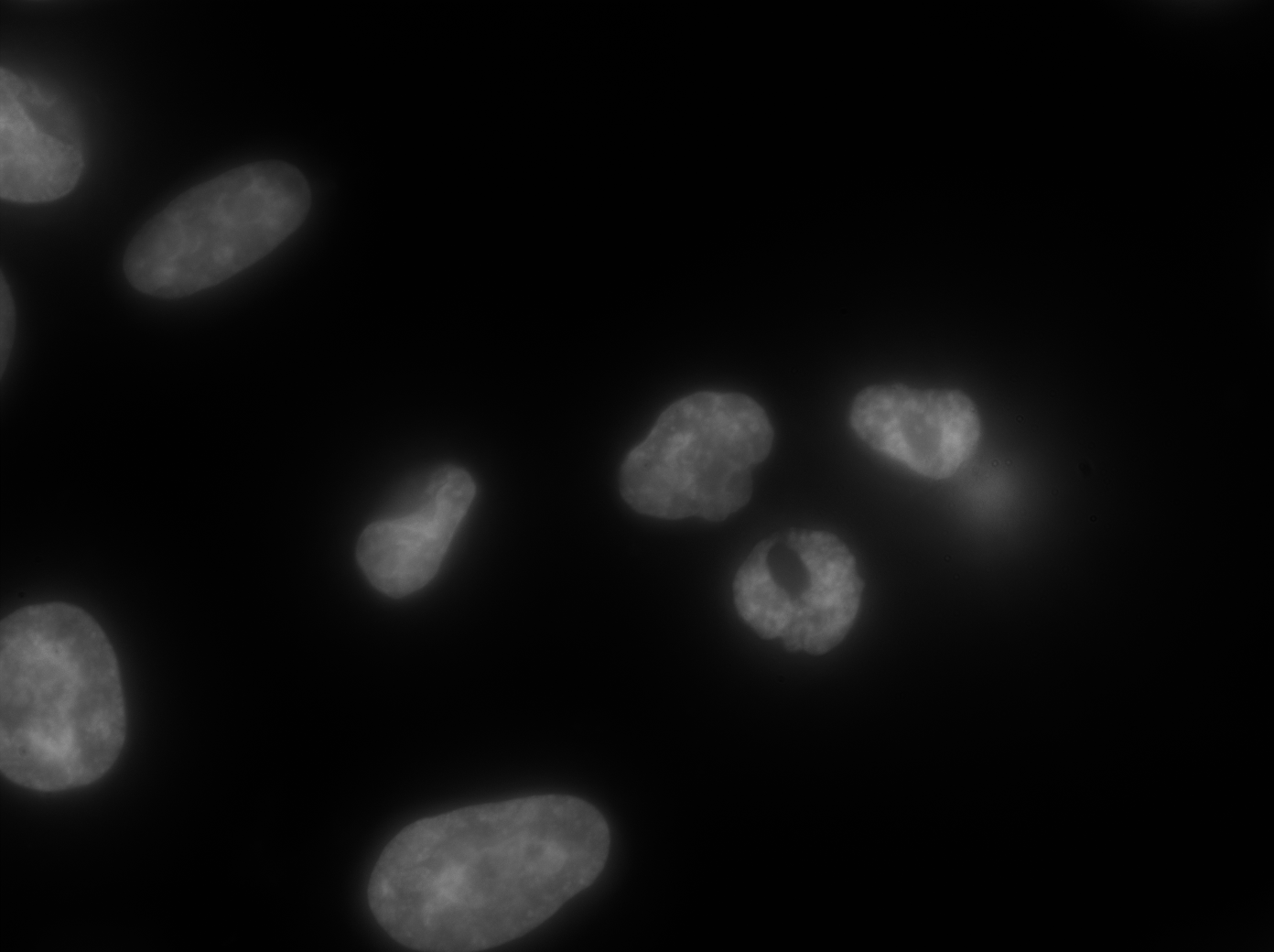

Supplement: Supplementary file 19 — Source data Fig. 5 part 5 [file 44319_2026_742_MOESM19_ESM.zip › Figure 5 Part 5/Fig 5ab WT and KO hela TTLL1-e326g atubulin part 2/TPGS1-KO/TPGS1-KO TTLL1-mut 10-22-24 R2 LT3LT4.Project Maximum Z_XY1730226457_Z0_T0_C0.tif]

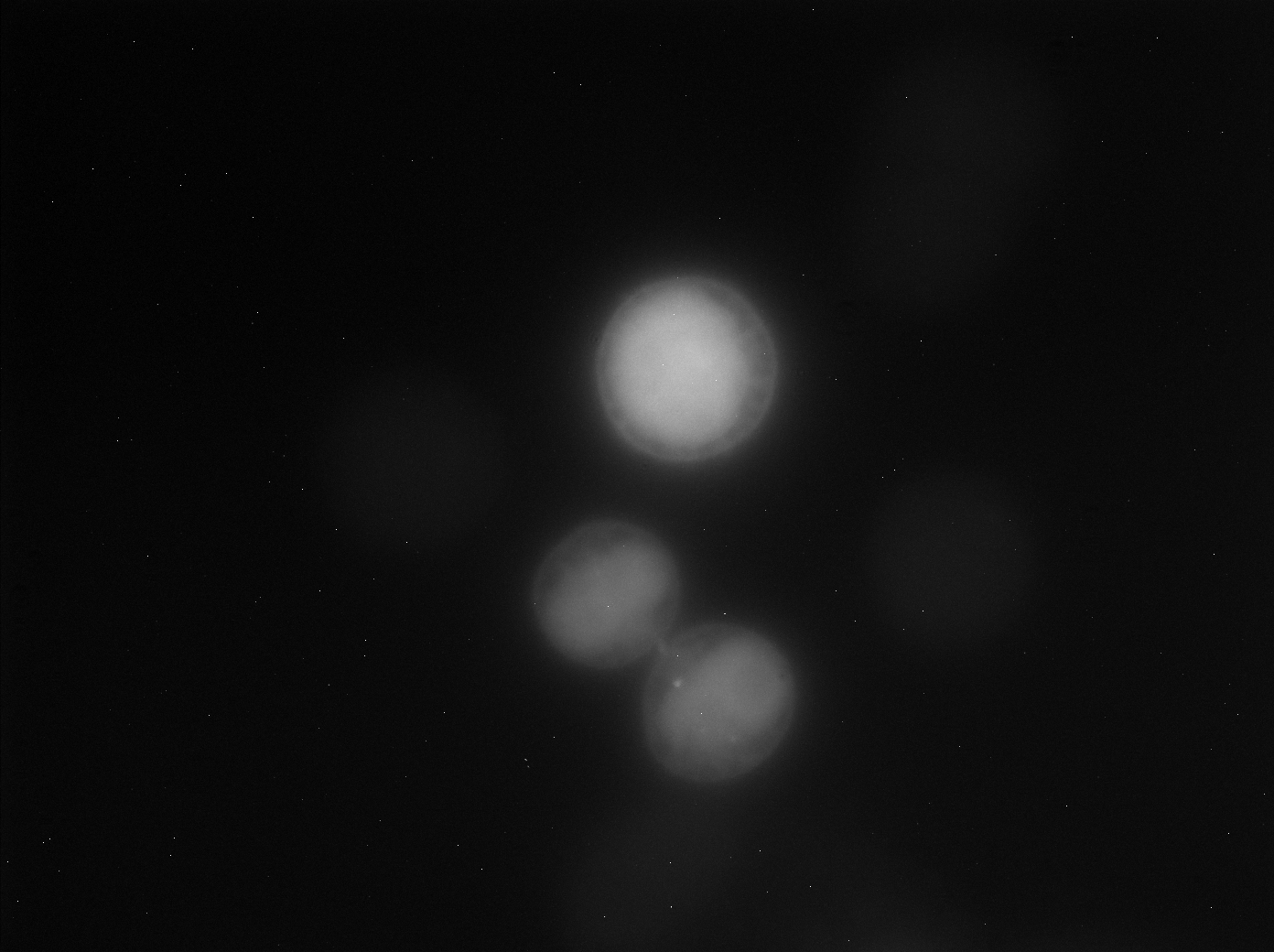

Supplement: Supplementary file 19 — Source data Fig. 5 part 5 [file 44319_2026_742_MOESM19_ESM.zip › Figure 5 Part 5/Fig 5ab WT and KO hela TTLL1-e326g atubulin part 2/TPGS1-KO/TPGS1-KO TTLL1-mut 10-22-24 R3 LT8.Project Maximum Z_XY1730229897_Z0_T0_C1.tif]

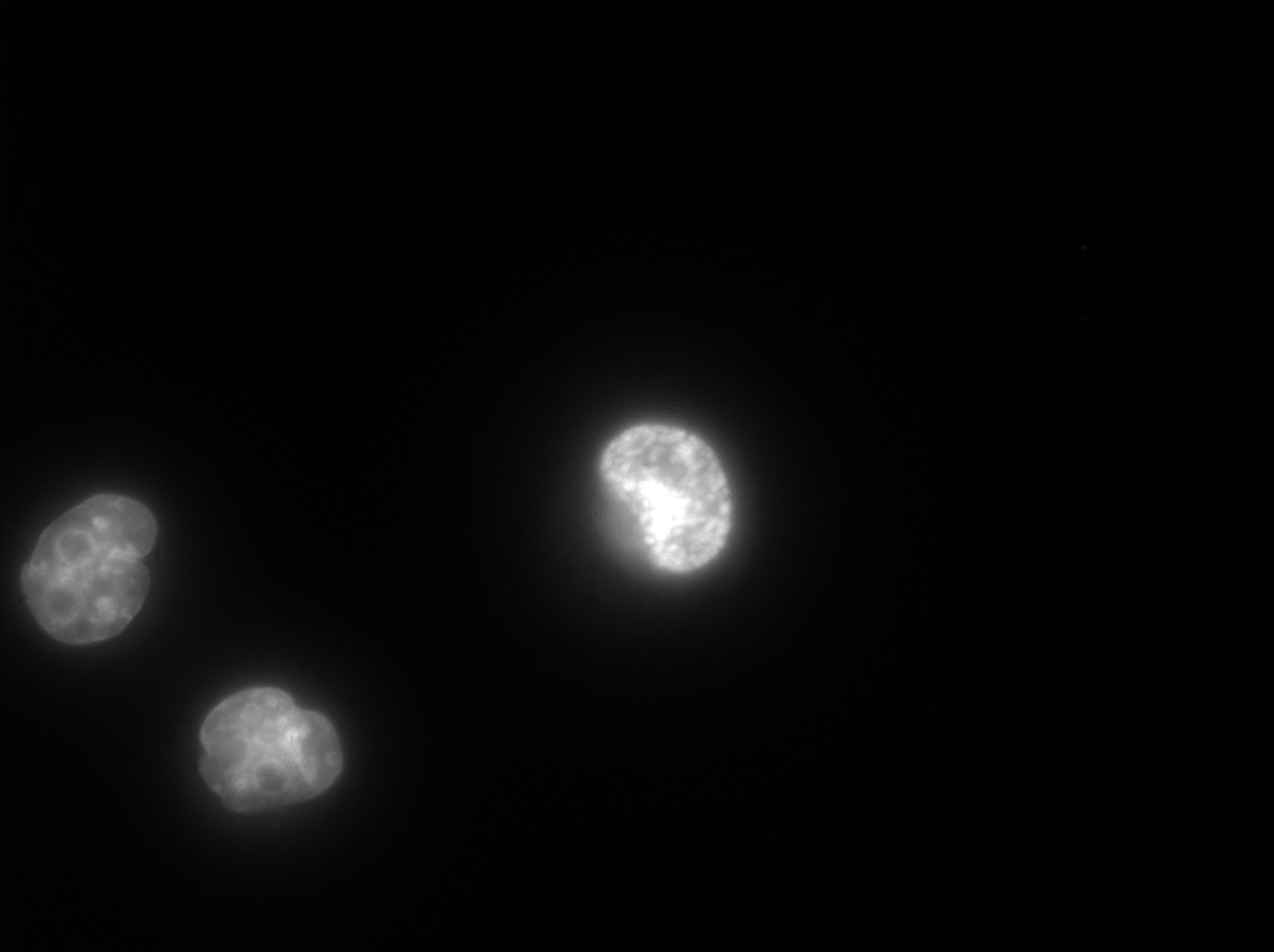

Supplement: Supplementary file 19 — Source data Fig. 5 part 5 [file 44319_2026_742_MOESM19_ESM.zip › Figure 5 Part 5/Fig 5ab WT and KO hela TTLL1-e326g atubulin part 2/TPGS1-KO/TPGS1-KO TTLL1-mut 10-15-24 R1 P1.Project Maximum Z_XY1729022038_Z0_T0_C0.tif]

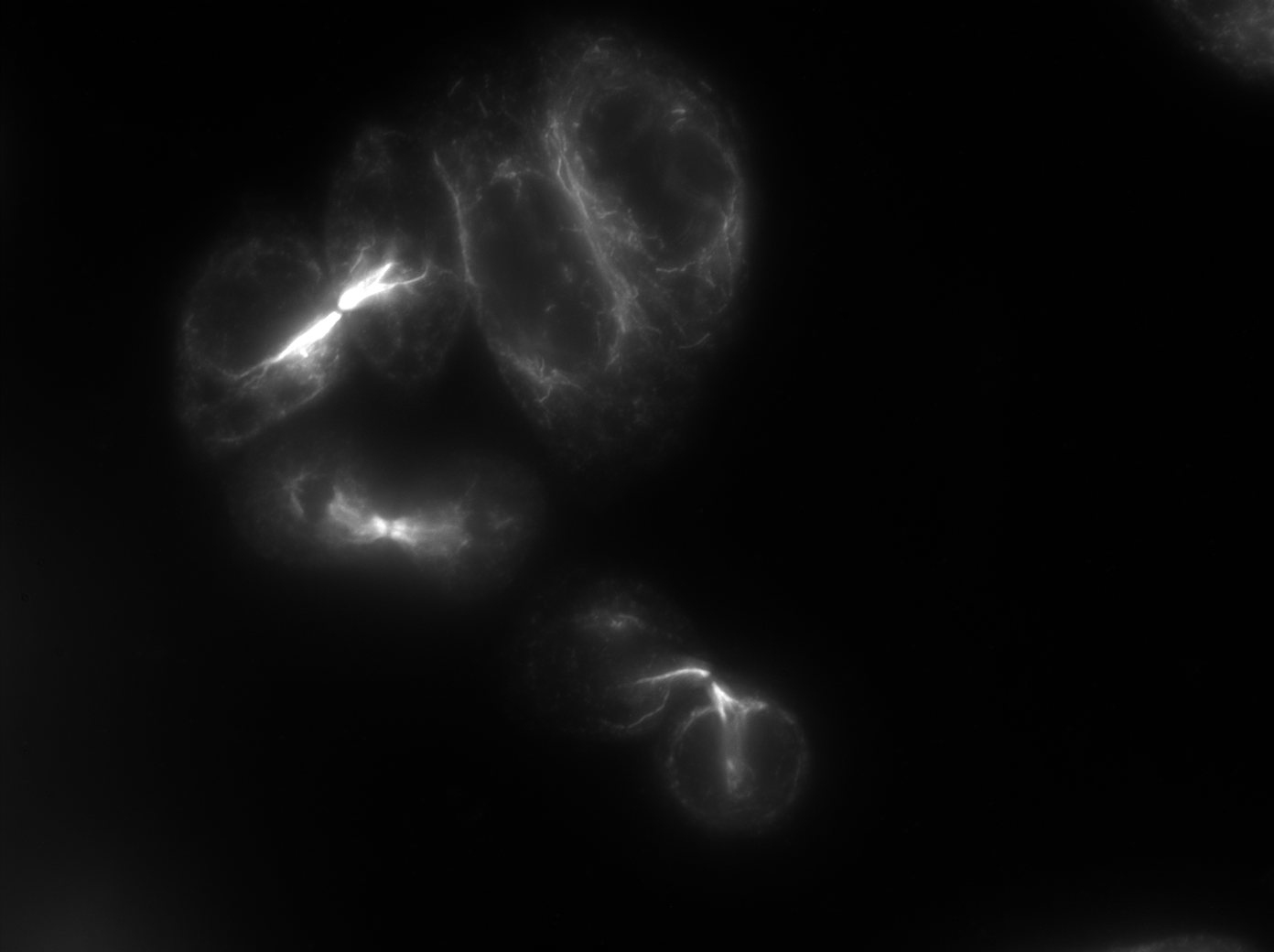

Supplement: Supplementary file 19 — Source data Fig. 5 part 5 [file 44319_2026_742_MOESM19_ESM.zip › Figure 5 Part 5/Fig 5ab WT and KO hela TTLL1-e326g atubulin part 2/TPGS1-KO/TPGS1-KO TTLL1-mut 10-22-24 R2 LT8.Project Maximum Z_XY1730227623_Z0_T0_C2.tif]

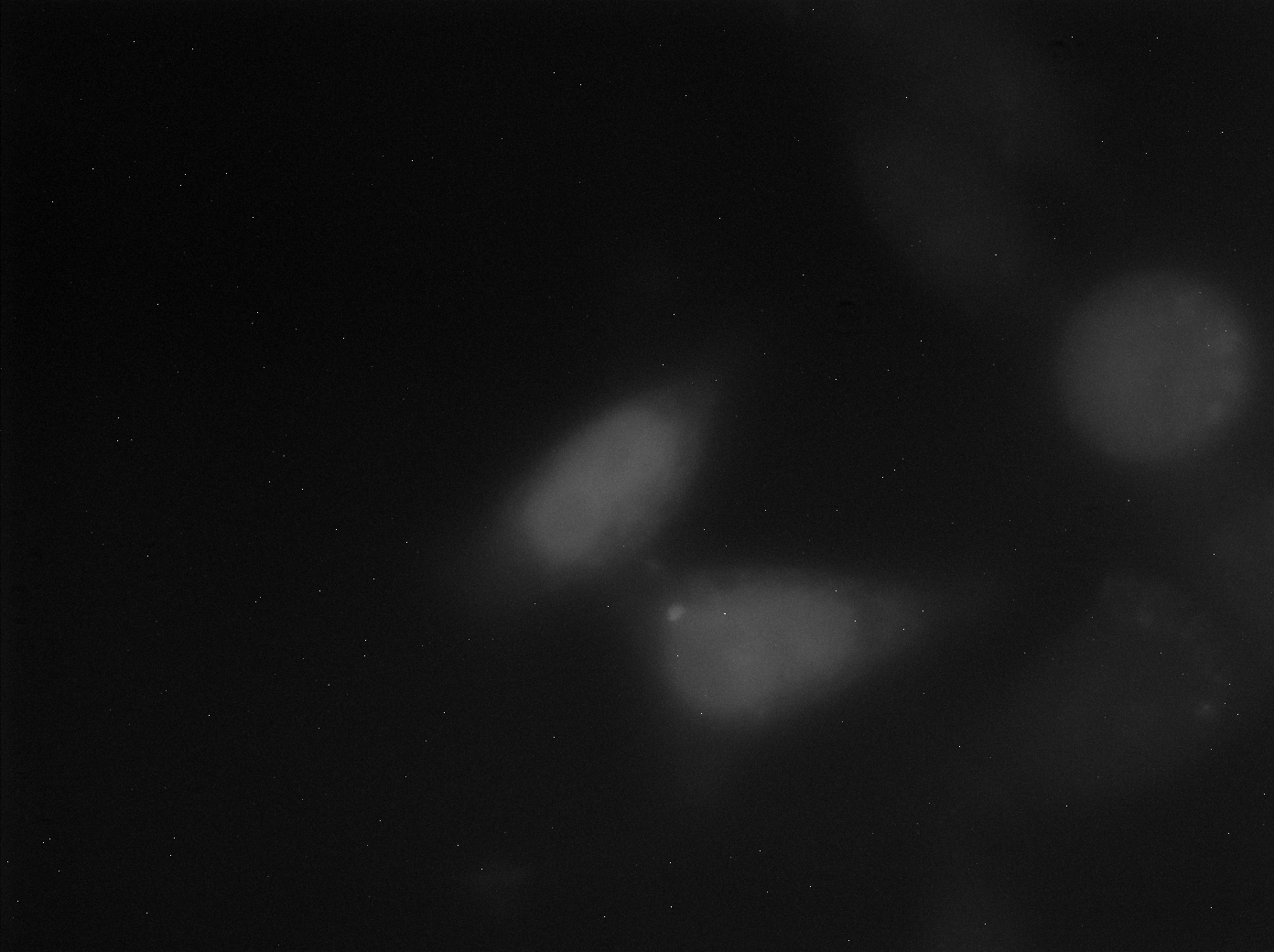

Supplement: Supplementary file 19 — Source data Fig. 5 part 5 [file 44319_2026_742_MOESM19_ESM.zip › Figure 5 Part 5/Fig 5ab WT and KO hela TTLL1-e326g atubulin part 2/TPGS1-KO/TPGS1-KO TTLL1-mut 10-22-24 R2 LT2.Project Maximum Z_XY1730225703_Z0_T0_C1.tif]

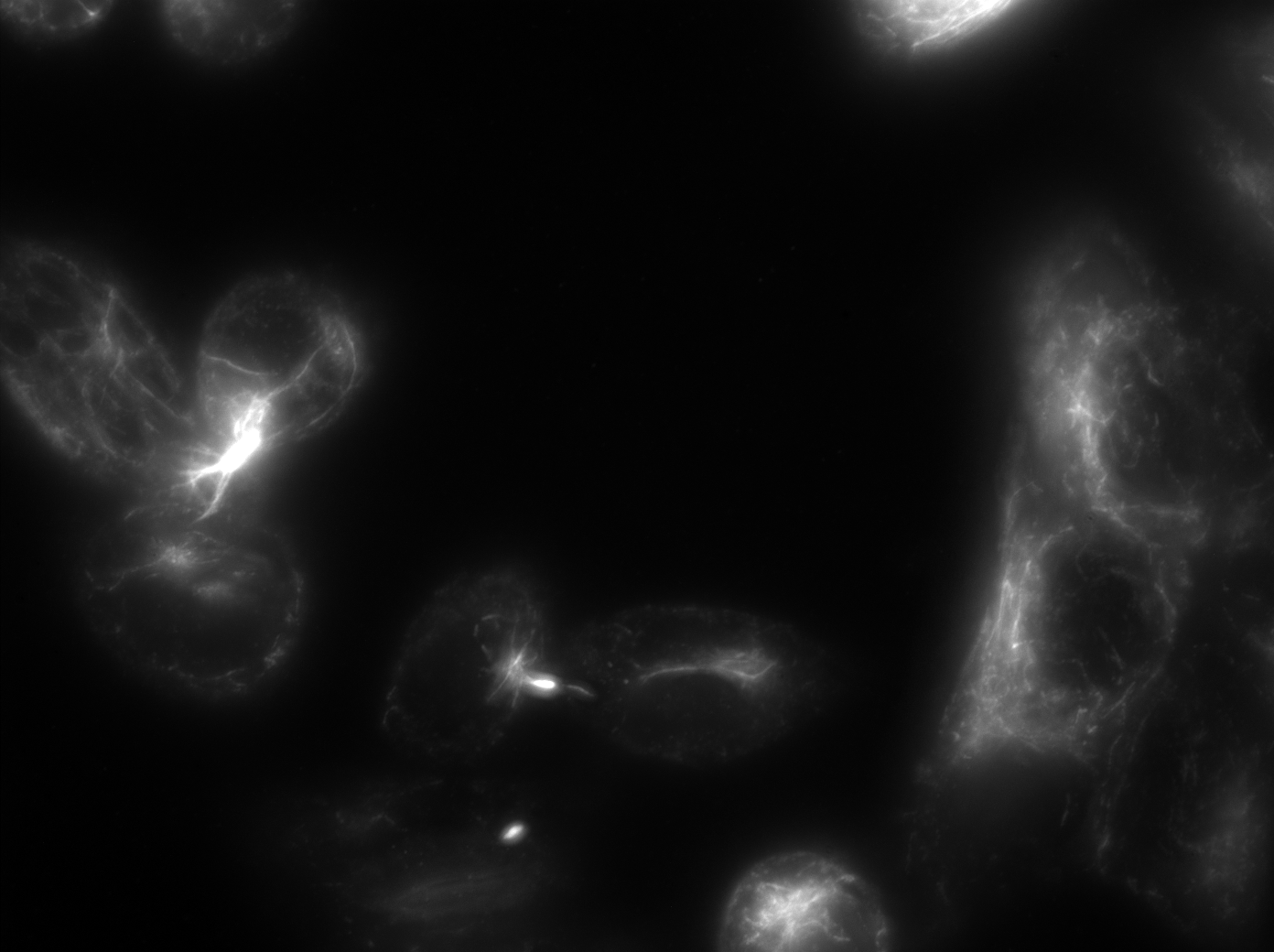

Supplement: Supplementary file 19 — Source data Fig. 5 part 5 [file 44319_2026_742_MOESM19_ESM.zip › Figure 5 Part 5/Fig 5ab WT and KO hela TTLL1-e326g atubulin part 2/TPGS1-KO/TPGS1-KO TTLL1-mut 10-22-24 R3 LT10.Project Maximum Z_XY1730230612_Z0_T0_C2.tif]

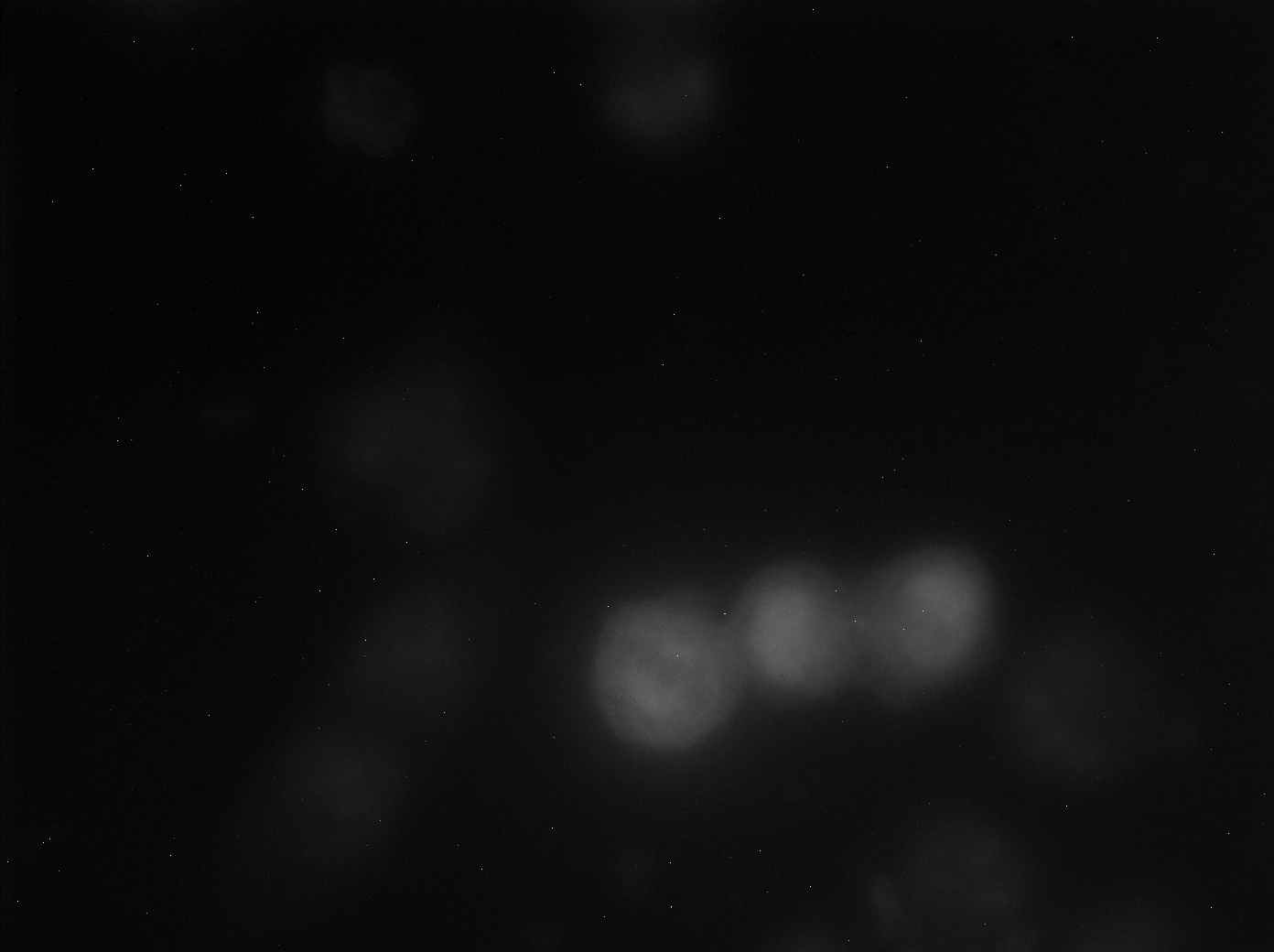

Supplement: Supplementary file 19 — Source data Fig. 5 part 5 [file 44319_2026_742_MOESM19_ESM.zip › Figure 5 Part 5/Fig 5ab WT and KO hela TTLL1-e326g atubulin part 2/TPGS1-KO/TPGS1-KO TTLL1-mut 10-22-24 R3 LT4.Project Maximum Z_XY1730228790_Z0_T0_C1.tif]

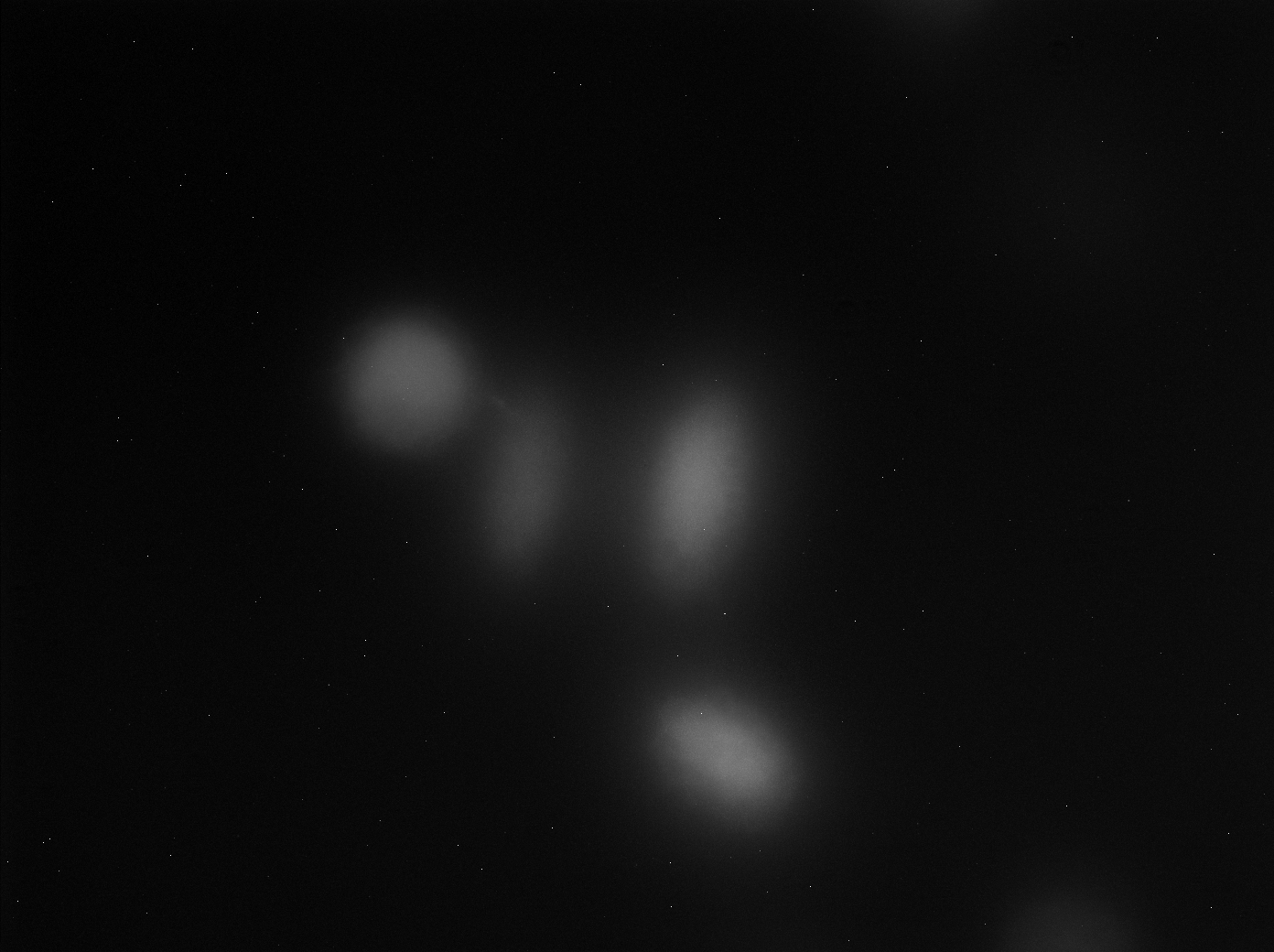

Supplement: Supplementary file 19 — Source data Fig. 5 part 5 [file 44319_2026_742_MOESM19_ESM.zip › Figure 5 Part 5/Fig 5ab WT and KO hela TTLL1-e326g atubulin part 2/TPGS1-KO/TPGS1-KO TTLL1-mut 10-22-24 R3 LT2.Project Maximum Z_XY1730228528_Z0_T0_C1.tif]

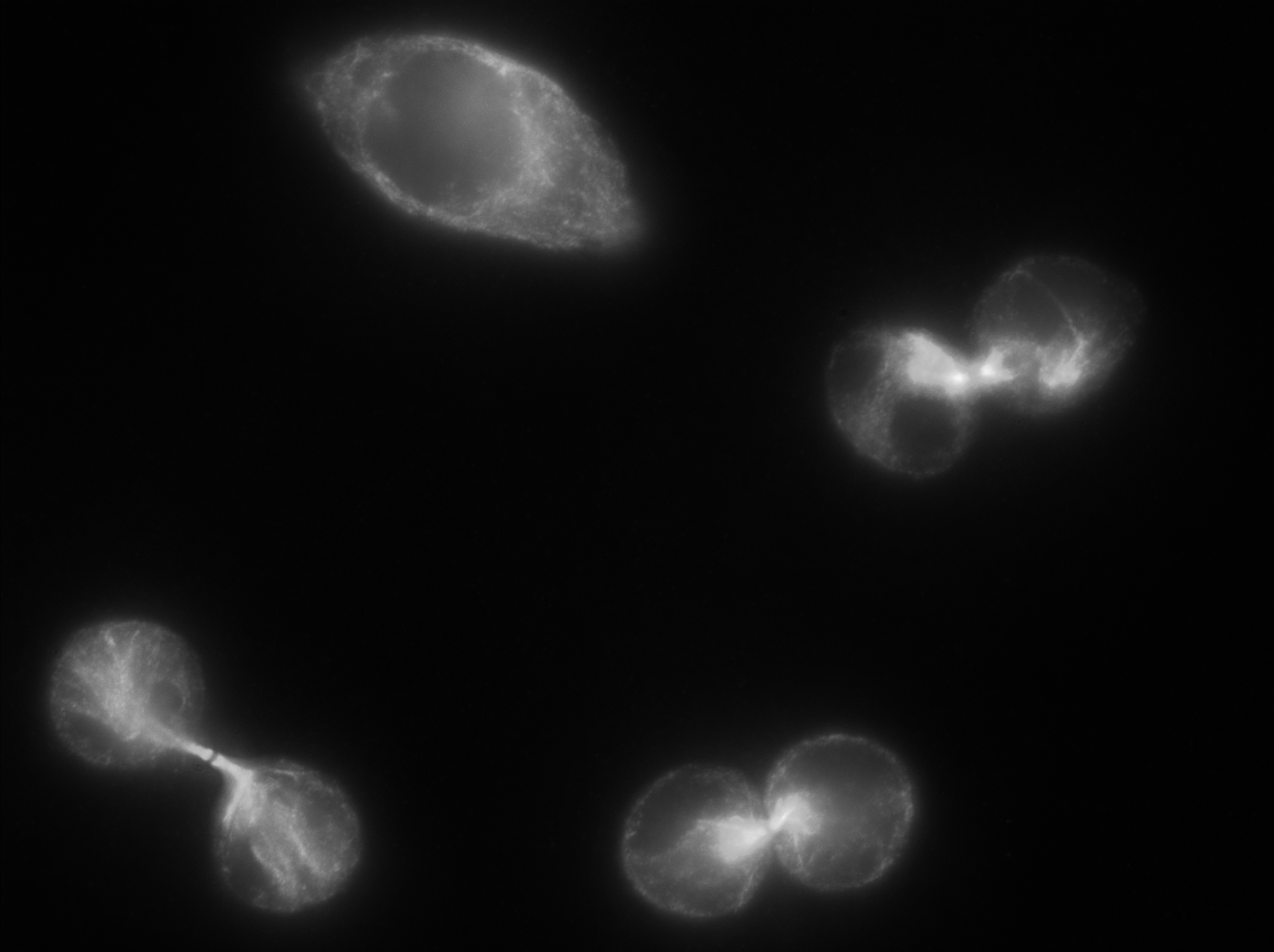

Supplement: Supplementary file 19 — Source data Fig. 5 part 5 [file 44319_2026_742_MOESM19_ESM.zip › Figure 5 Part 5/Fig 5ab WT and KO hela TTLL1-e326g atubulin part 2/TPGS1-KO/TPGS1-KO TTLL1-mut 10-15-24 R1 LT2ET1ET2.Project Maximum Z_XY1729022587_Z0_T0_C2.tif]

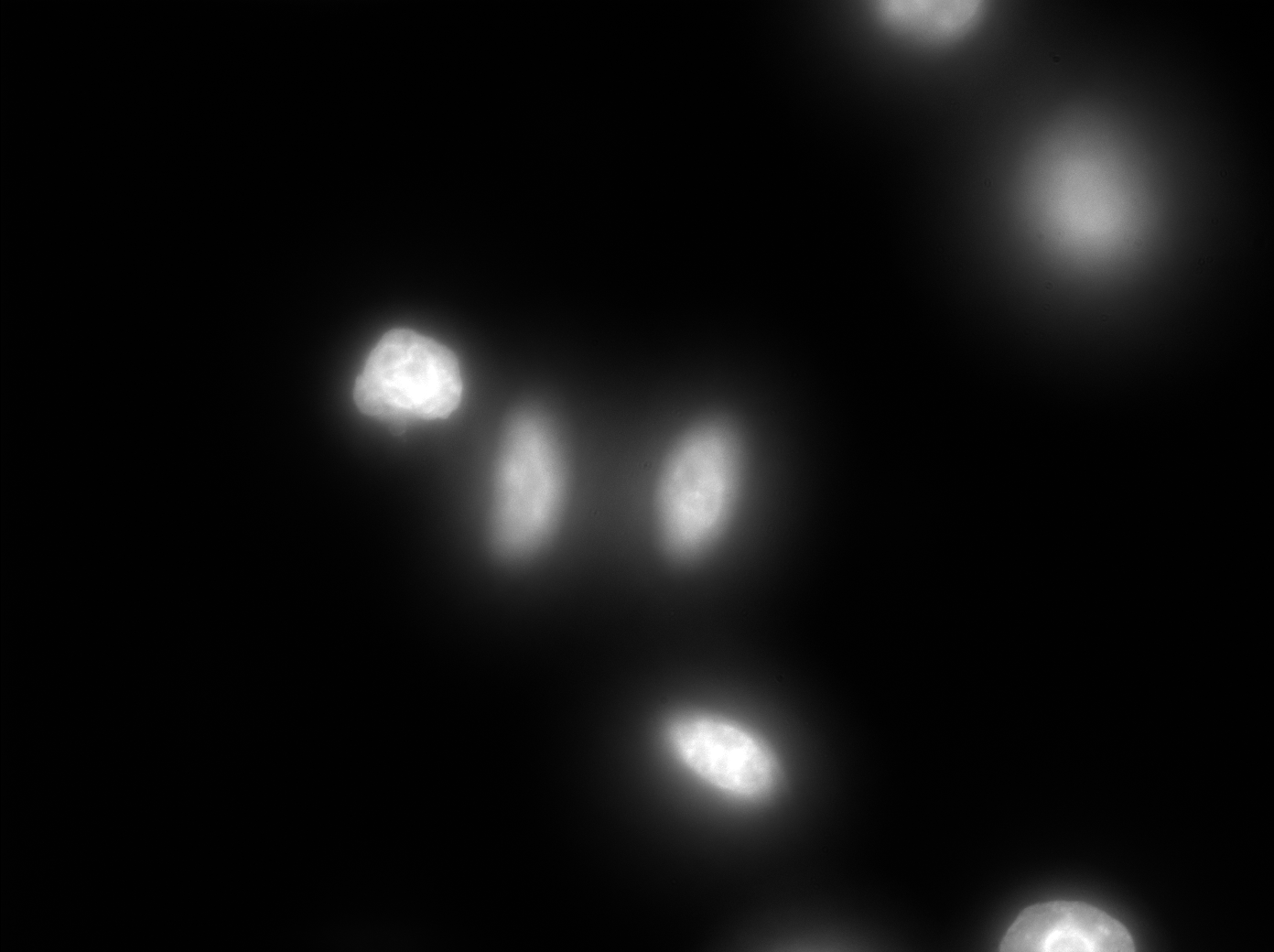

Supplement: Supplementary file 19 — Source data Fig. 5 part 5 [file 44319_2026_742_MOESM19_ESM.zip › Figure 5 Part 5/Fig 5ab WT and KO hela TTLL1-e326g atubulin part 2/TPGS1-KO/TPGS1-KO TTLL1-mut 10-22-24 R3 LT2.Project Maximum Z_XY1730228528_Z0_T0_C0.tif]

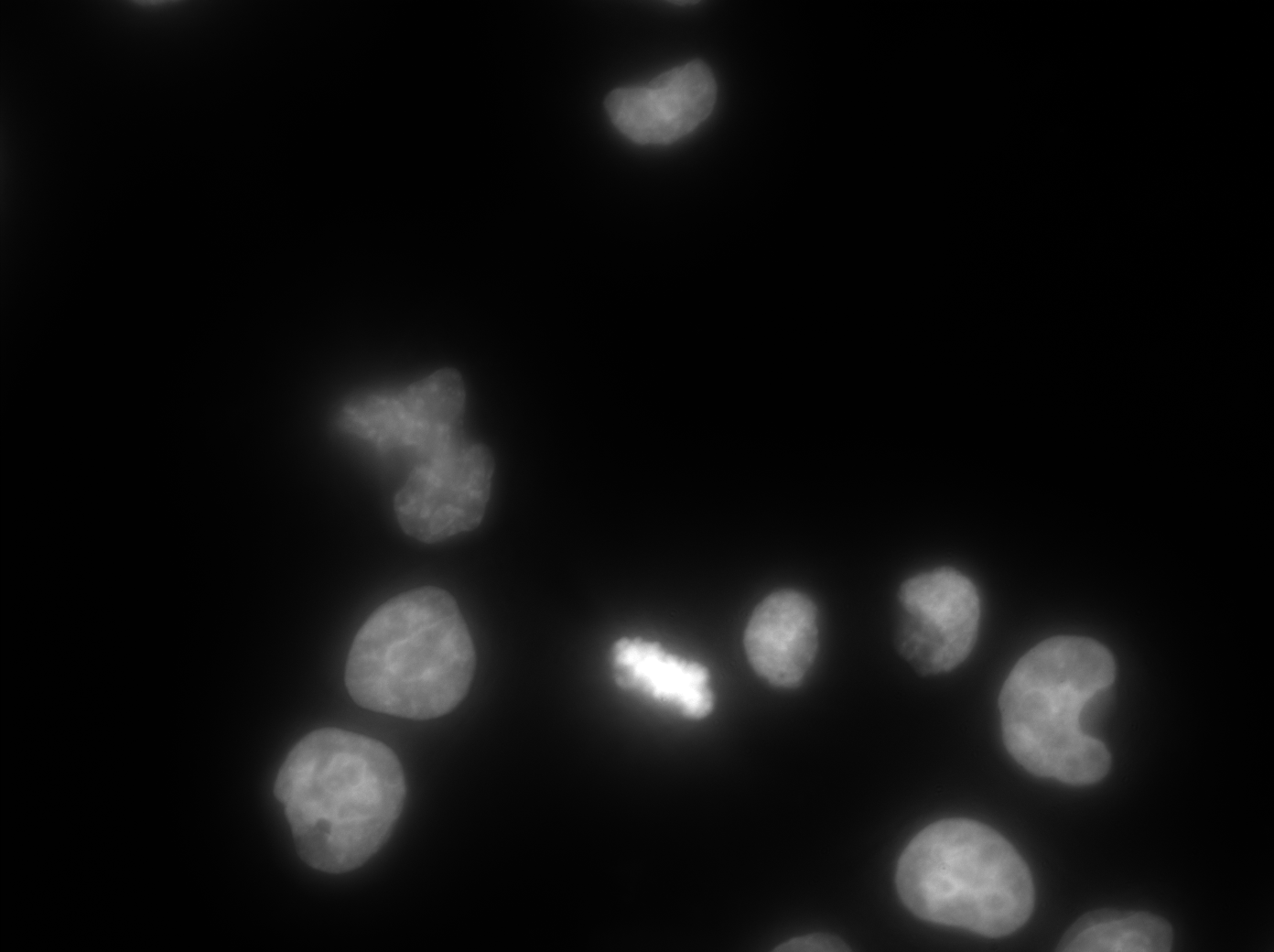

Supplement: Supplementary file 19 — Source data Fig. 5 part 5 [file 44319_2026_742_MOESM19_ESM.zip › Figure 5 Part 5/Fig 5ab WT and KO hela TTLL1-e326g atubulin part 2/TPGS1-KO/TPGS1-KO TTLL1-mut 10-22-24 R3 LT4.Project Maximum Z_XY1730228790_Z0_T0_C0.tif]

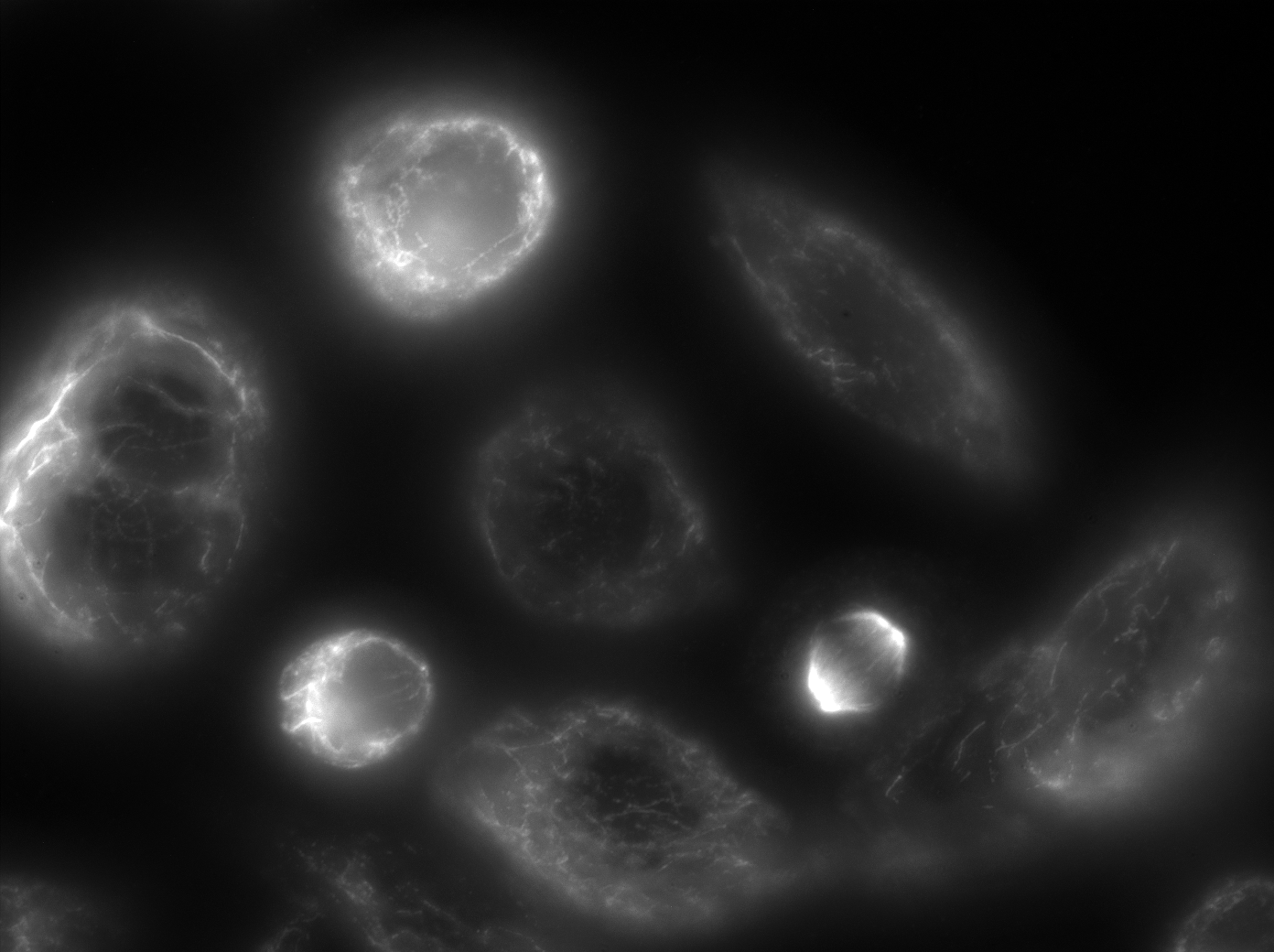

Supplement: Supplementary file 19 — Source data Fig. 5 part 5 [file 44319_2026_742_MOESM19_ESM.zip › Figure 5 Part 5/Fig 5ab WT and KO hela TTLL1-e326g atubulin part 2/TPGS1-KO/TPGS1-KO TTLL1-mut 10-22-24 R2 M1.Project Maximum Z_XY1730226171_Z0_T0_C2.tif]

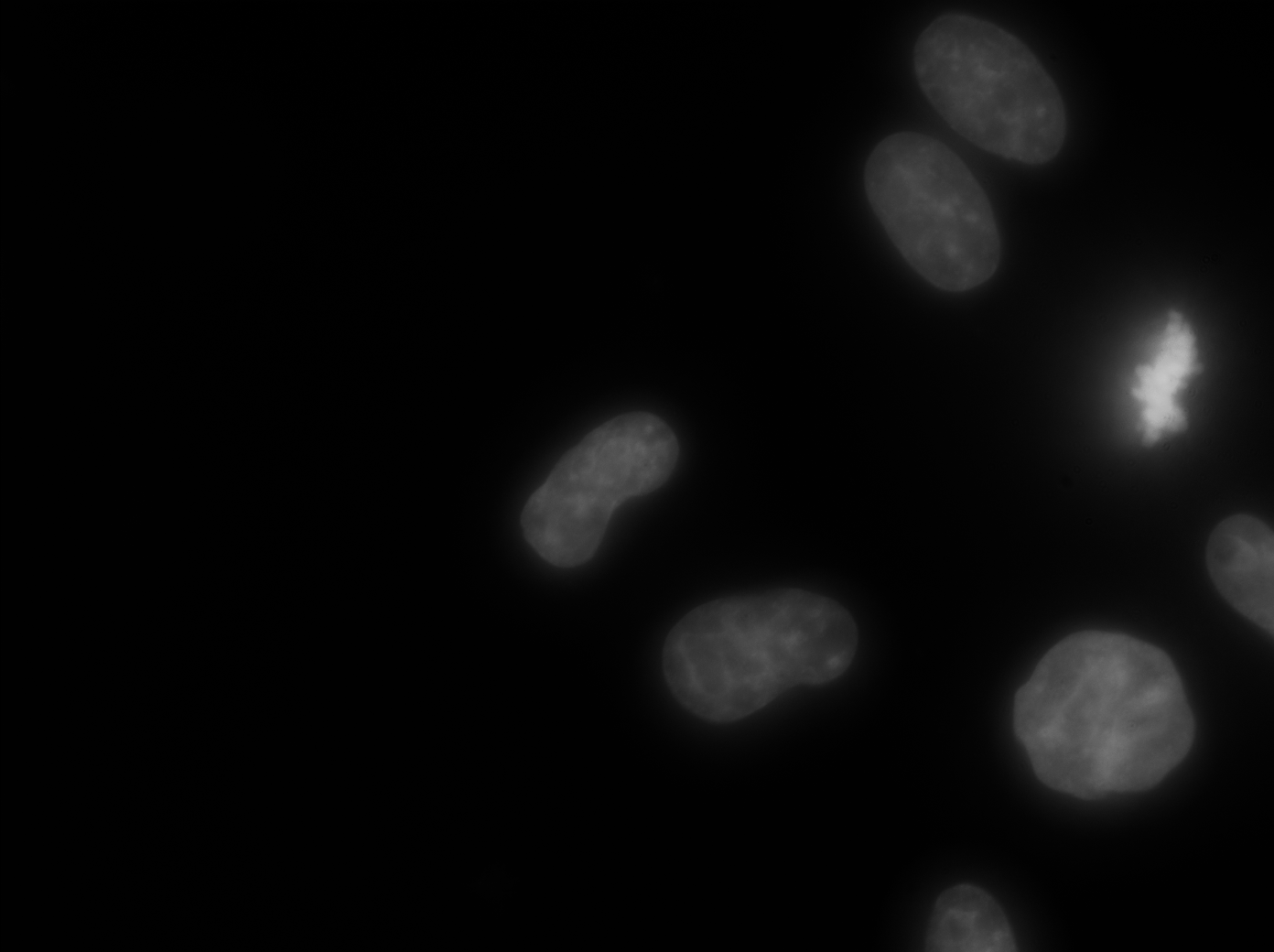

Supplement: Supplementary file 19 — Source data Fig. 5 part 5 [file 44319_2026_742_MOESM19_ESM.zip › Figure 5 Part 5/Fig 5ab WT and KO hela TTLL1-e326g atubulin part 2/TPGS1-KO/TPGS1-KO TTLL1-mut 10-22-24 R2 LT2.Project Maximum Z_XY1730225703_Z0_T0_C0.tif]

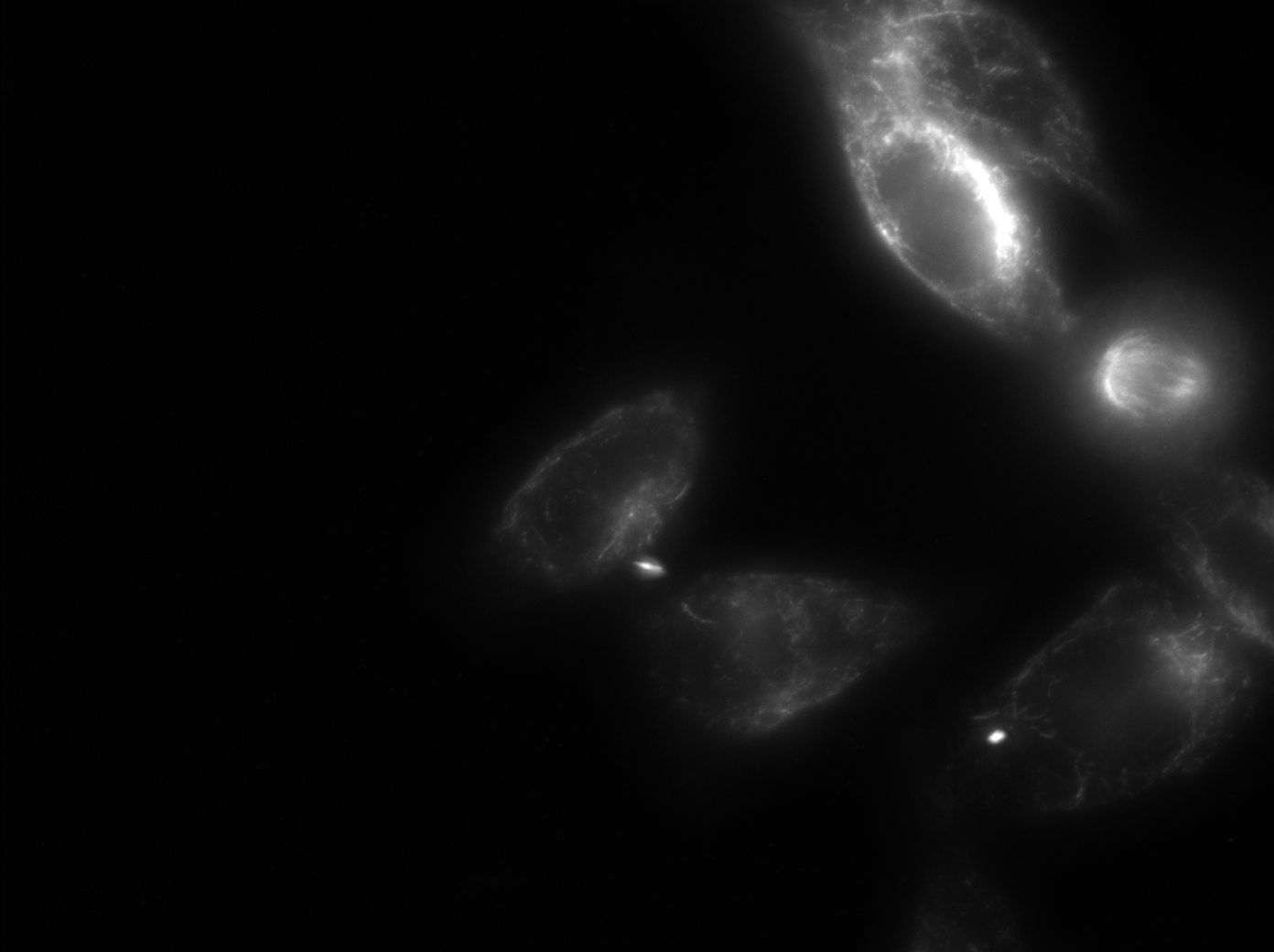

Supplement: Supplementary file 19 — Source data Fig. 5 part 5 [file 44319_2026_742_MOESM19_ESM.zip › Figure 5 Part 5/Fig 5ab WT and KO hela TTLL1-e326g atubulin part 2/TPGS1-KO/TPGS1-KO TTLL1-mut 10-22-24 R2 LT2.Project Maximum Z_XY1730225703_Z0_T0_C2.tif]

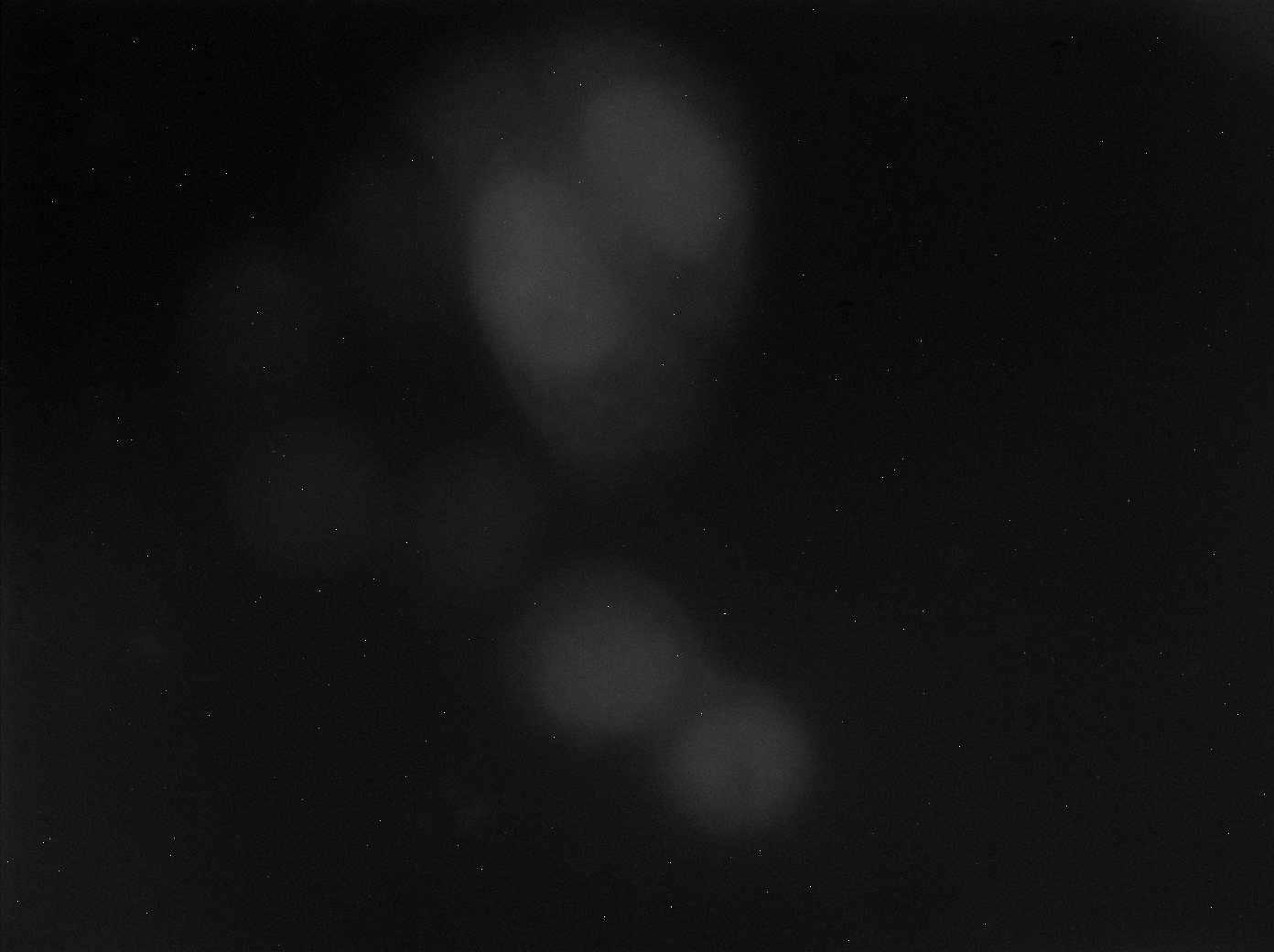

Supplement: Supplementary file 19 — Source data Fig. 5 part 5 [file 44319_2026_742_MOESM19_ESM.zip › Figure 5 Part 5/Fig 5ab WT and KO hela TTLL1-e326g atubulin part 2/TPGS1-KO/TPGS1-KO TTLL1-mut 10-22-24 R2 LT8.Project Maximum Z_XY1730227623_Z0_T0_C1.tif]

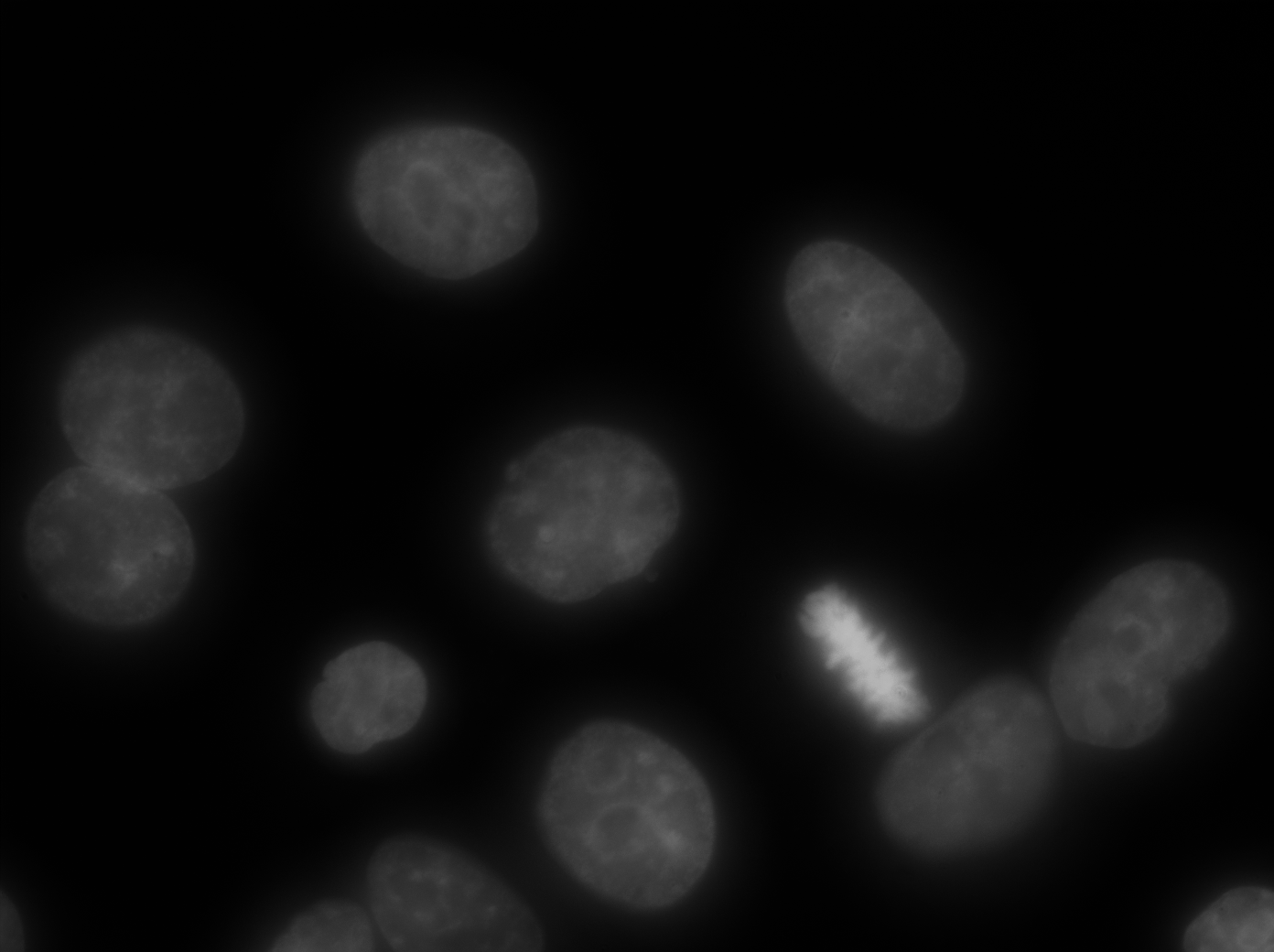

Supplement: Supplementary file 19 — Source data Fig. 5 part 5 [file 44319_2026_742_MOESM19_ESM.zip › Figure 5 Part 5/Fig 5ab WT and KO hela TTLL1-e326g atubulin part 2/TPGS1-KO/TPGS1-KO TTLL1-mut 10-22-24 R2 M1.Project Maximum Z_XY1730226171_Z0_T0_C0.tif]

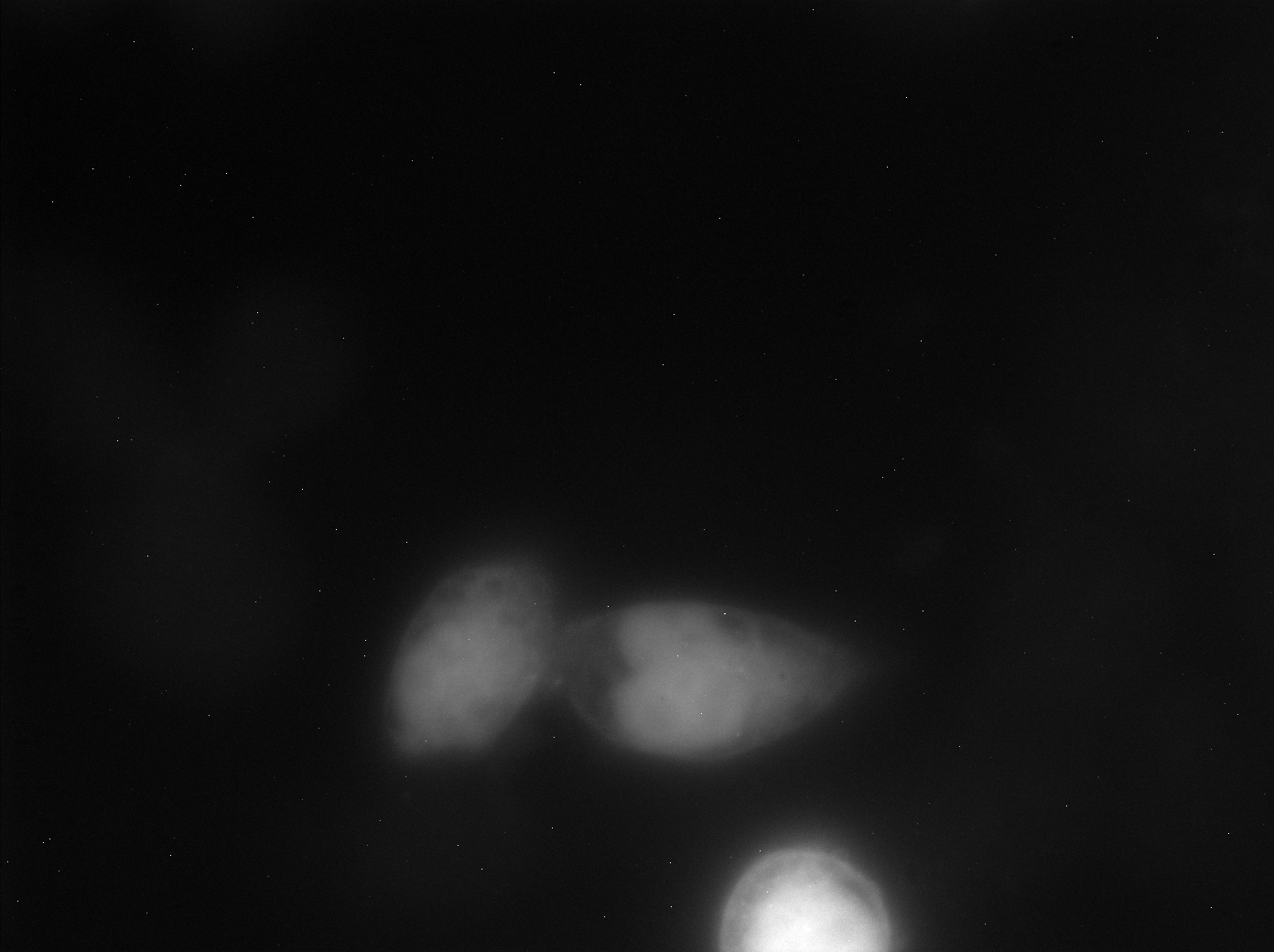

Supplement: Supplementary file 19 — Source data Fig. 5 part 5 [file 44319_2026_742_MOESM19_ESM.zip › Figure 5 Part 5/Fig 5ab WT and KO hela TTLL1-e326g atubulin part 2/TPGS1-KO/TPGS1-KO TTLL1-mut 10-22-24 R3 LT10.Project Maximum Z_XY1730230612_Z0_T0_C1.tif]

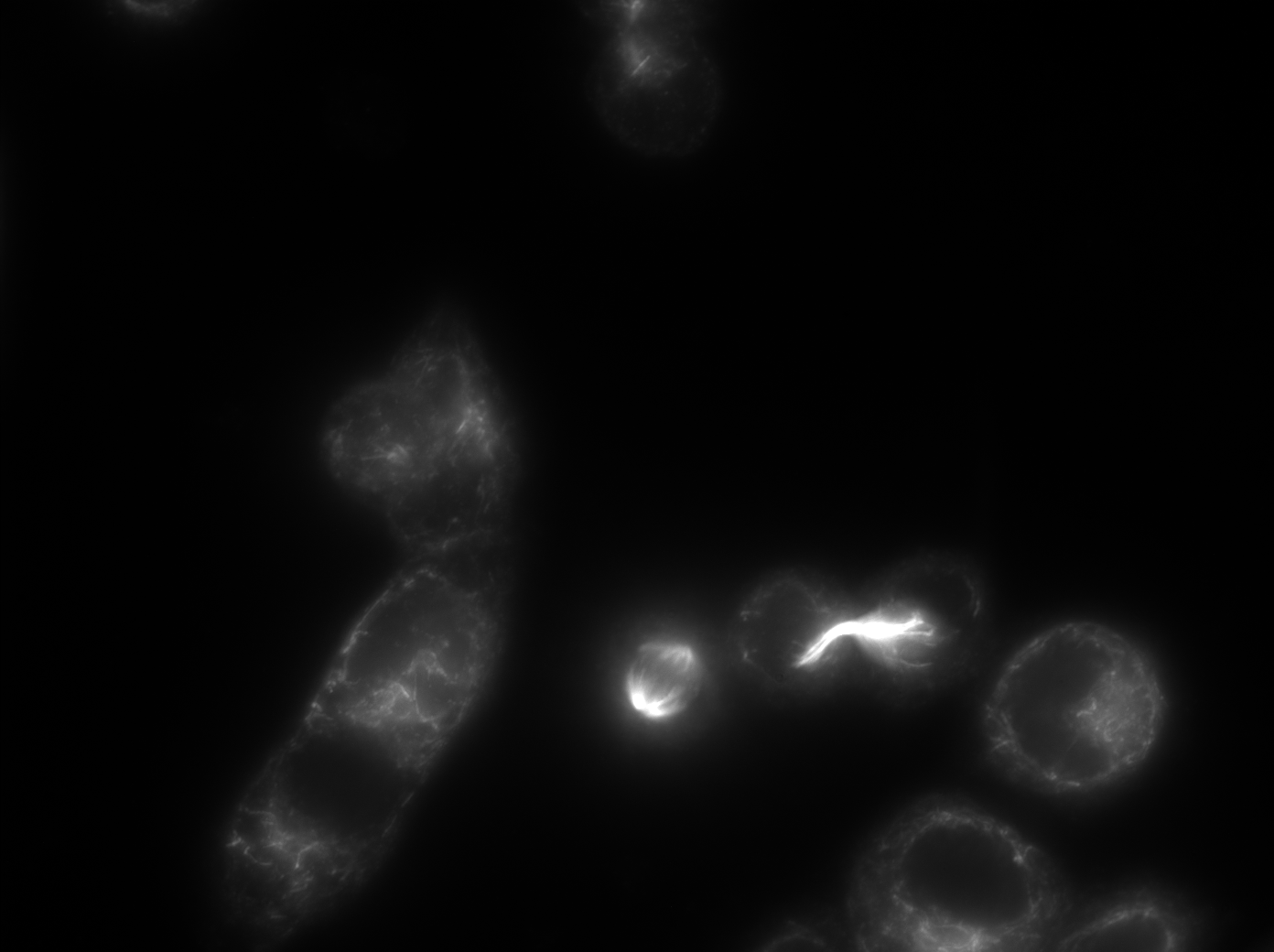

Supplement: Supplementary file 19 — Source data Fig. 5 part 5 [file 44319_2026_742_MOESM19_ESM.zip › Figure 5 Part 5/Fig 5ab WT and KO hela TTLL1-e326g atubulin part 2/TPGS1-KO/TPGS1-KO TTLL1-mut 10-22-24 R3 LT4.Project Maximum Z_XY1730228790_Z0_T0_C2.tif]

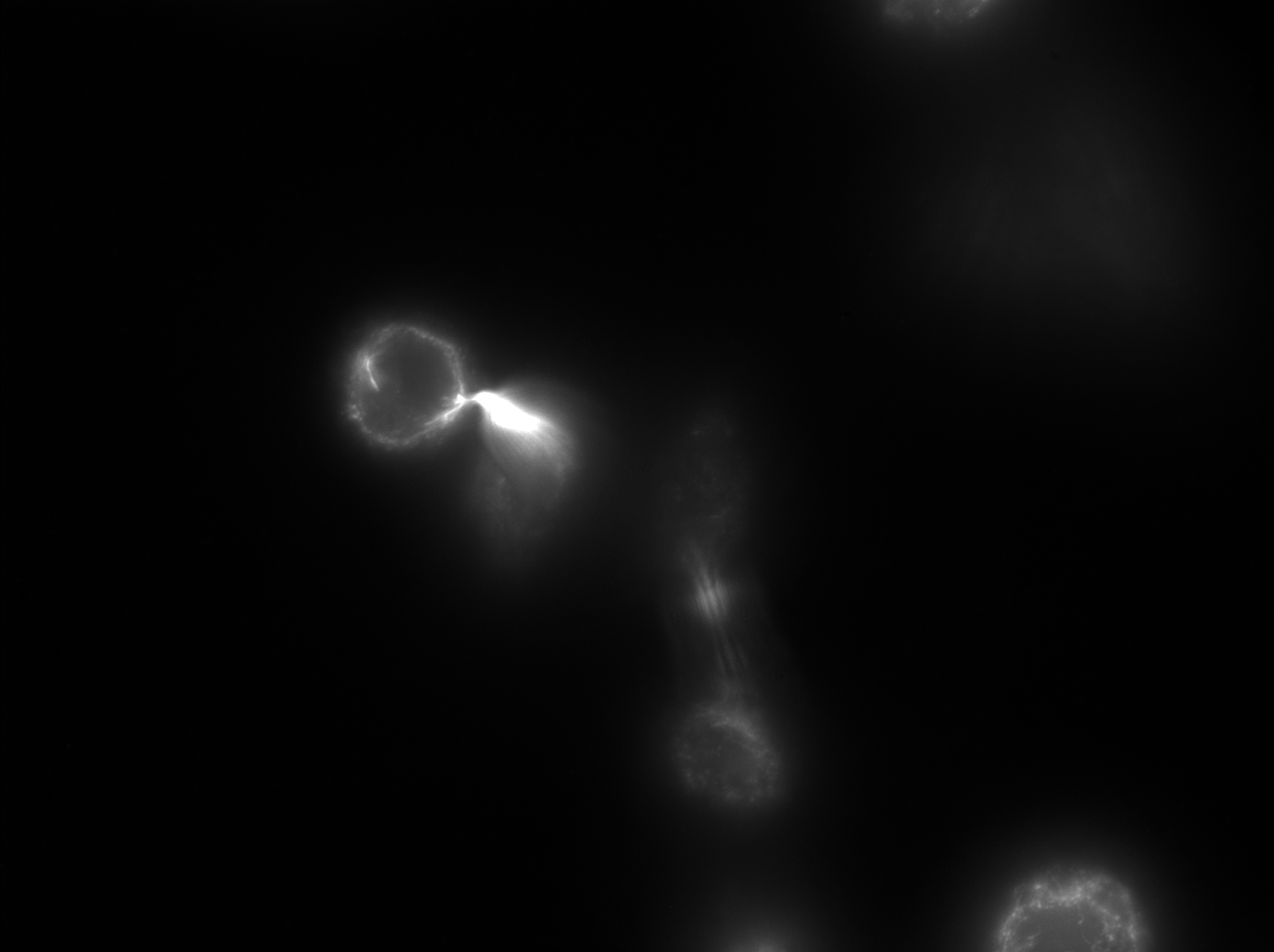

Supplement: Supplementary file 19 — Source data Fig. 5 part 5 [file 44319_2026_742_MOESM19_ESM.zip › Figure 5 Part 5/Fig 5ab WT and KO hela TTLL1-e326g atubulin part 2/TPGS1-KO/TPGS1-KO TTLL1-mut 10-22-24 R3 LT2.Project Maximum Z_XY1730228528_Z0_T0_C2.tif]

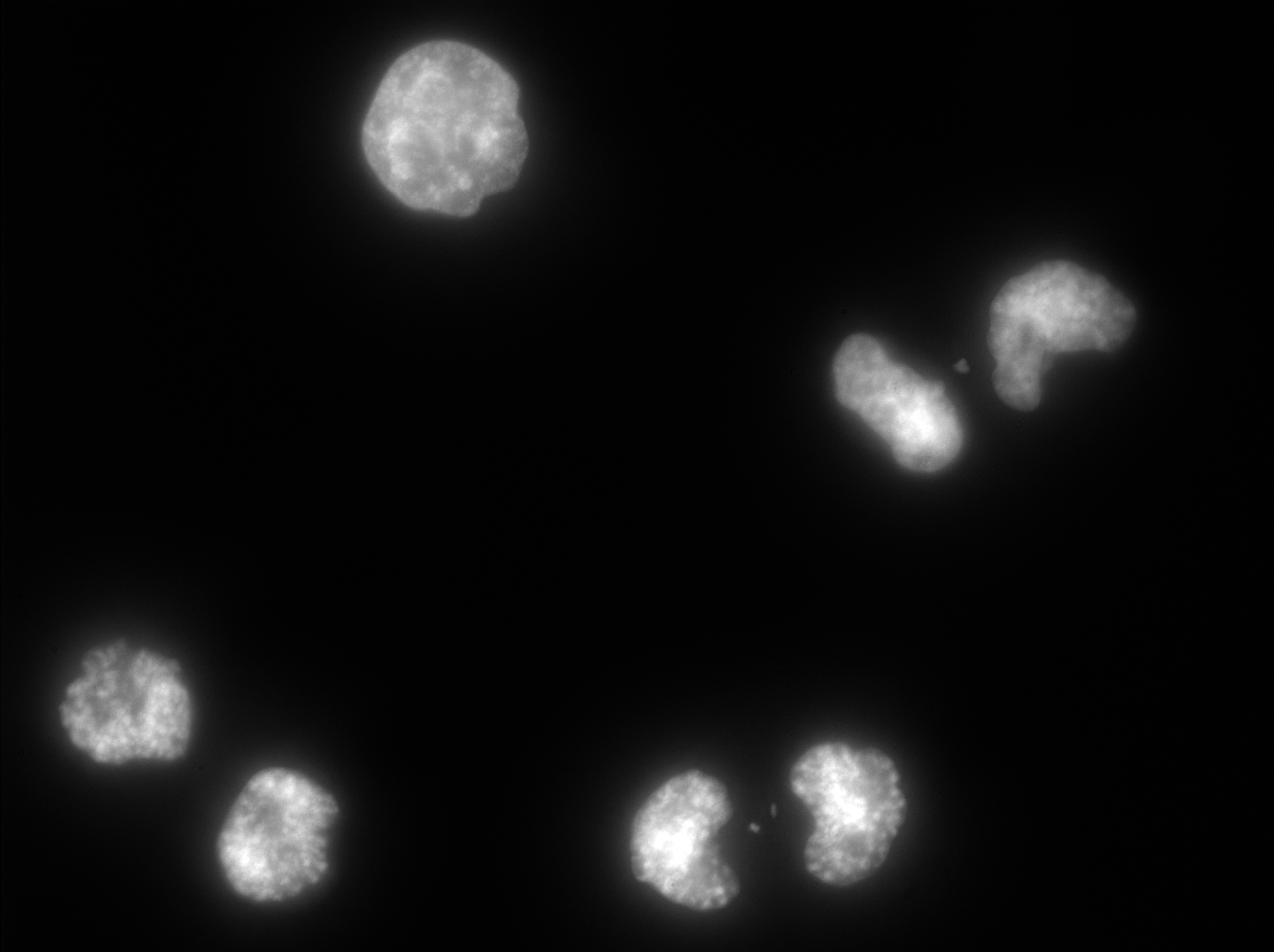

Supplement: Supplementary file 19 — Source data Fig. 5 part 5 [file 44319_2026_742_MOESM19_ESM.zip › Figure 5 Part 5/Fig 5ab WT and KO hela TTLL1-e326g atubulin part 2/TPGS1-KO/TPGS1-KO TTLL1-mut 10-15-24 R1 LT2ET1ET2.Project Maximum Z_XY1729022587_Z0_T0_C0.tif]

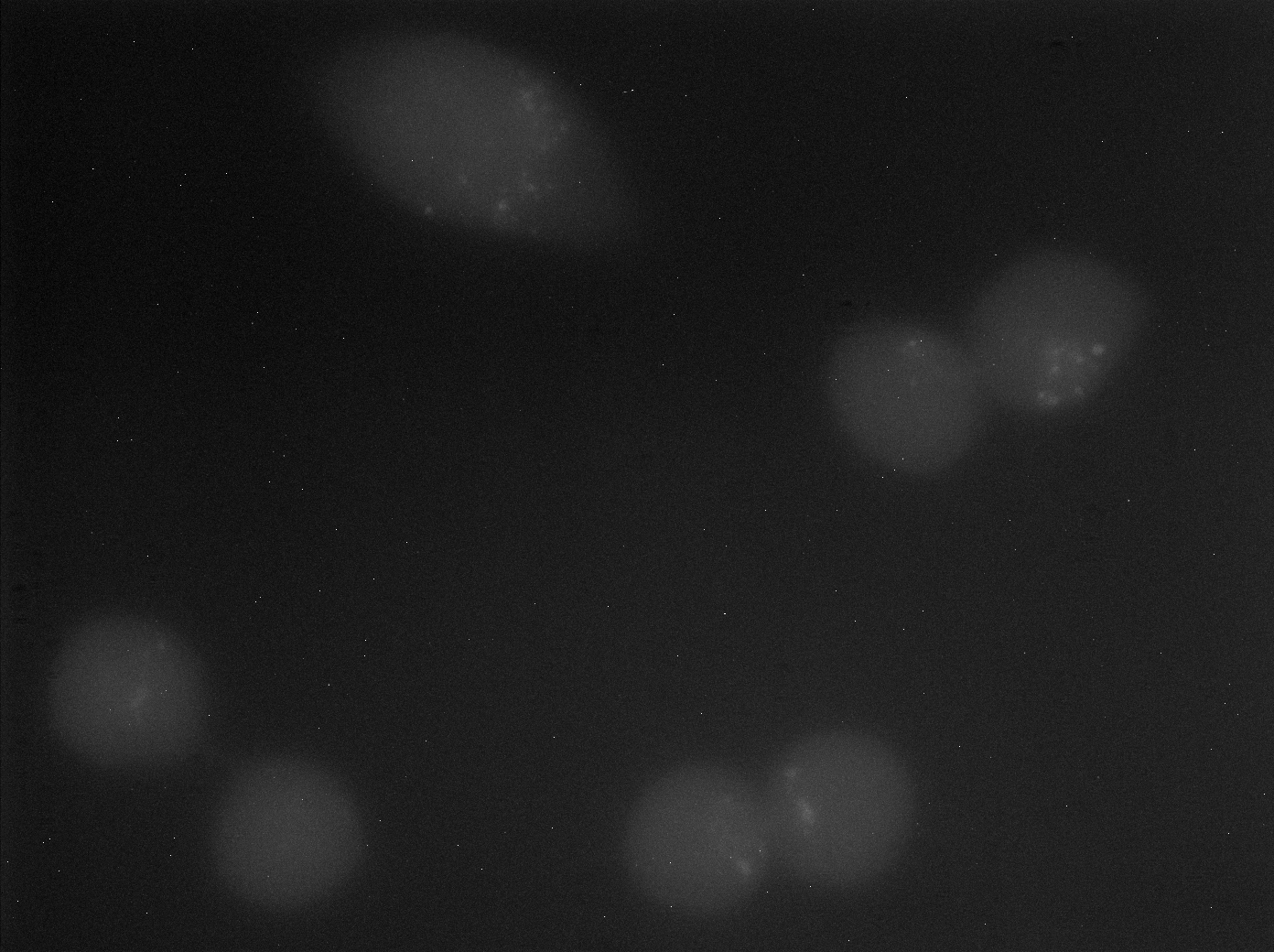

Supplement: Supplementary file 19 — Source data Fig. 5 part 5 [file 44319_2026_742_MOESM19_ESM.zip › Figure 5 Part 5/Fig 5ab WT and KO hela TTLL1-e326g atubulin part 2/TPGS1-KO/TPGS1-KO TTLL1-mut 10-15-24 R1 LT2ET1ET2.Project Maximum Z_XY1729022587_Z0_T0_C1.tif]

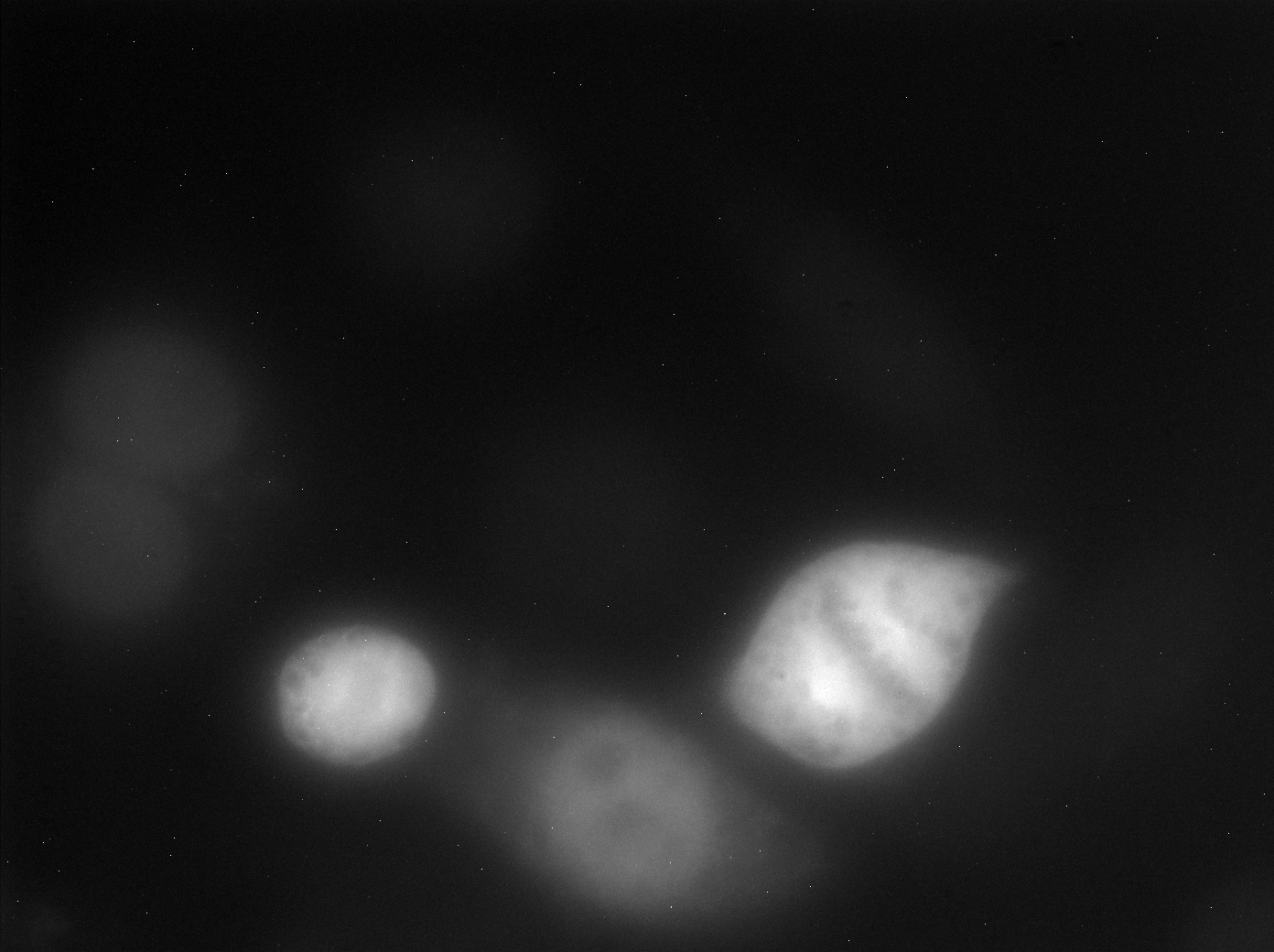

Supplement: Supplementary file 19 — Source data Fig. 5 part 5 [file 44319_2026_742_MOESM19_ESM.zip › Figure 5 Part 5/Fig 5ab WT and KO hela TTLL1-e326g atubulin part 2/TPGS1-KO/TPGS1-KO TTLL1-mut 10-22-24 R2 M1.Project Maximum Z_XY1730226171_Z0_T0_C1.tif]

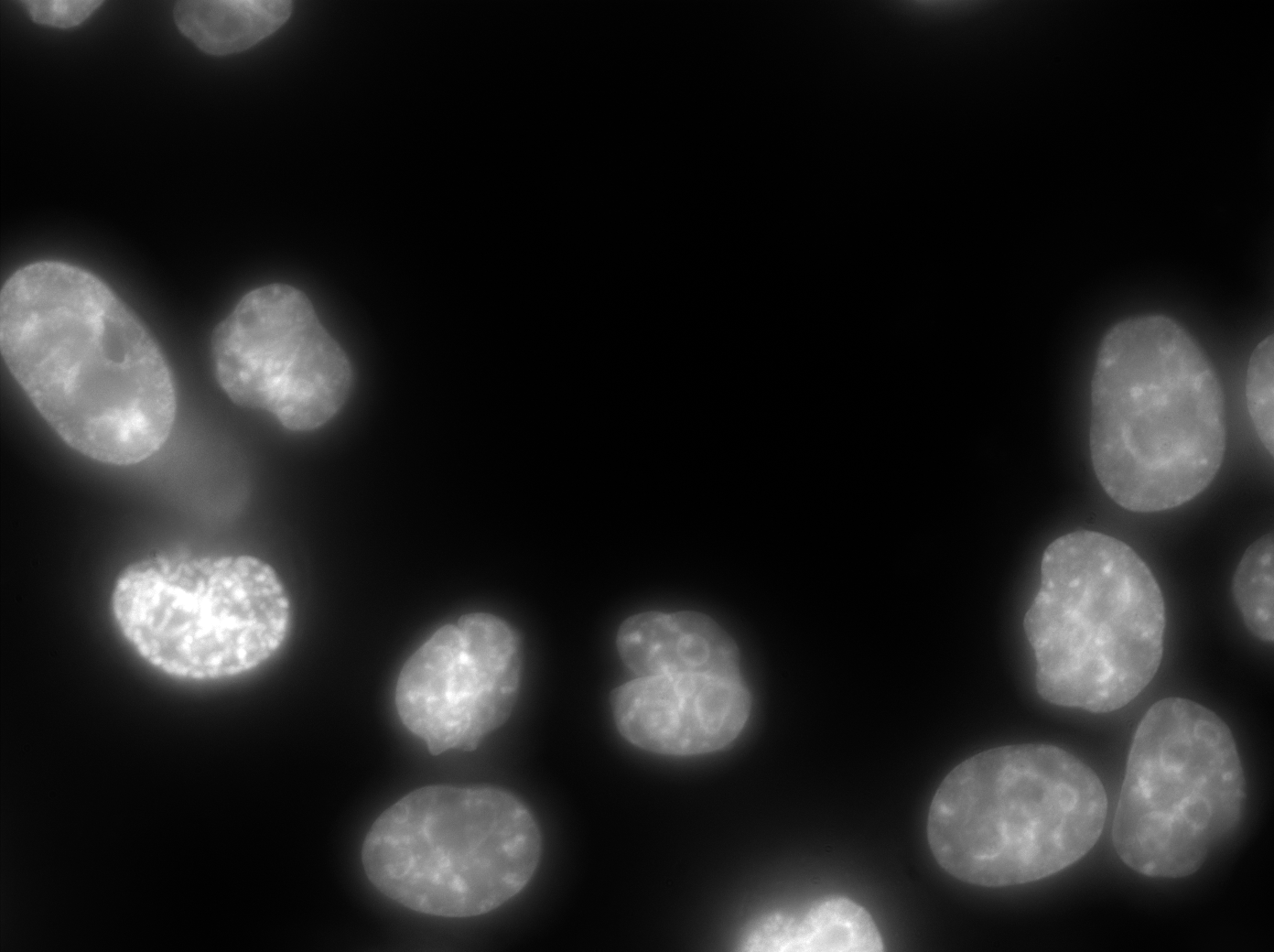

Supplement: Supplementary file 19 — Source data Fig. 5 part 5 [file 44319_2026_742_MOESM19_ESM.zip › Figure 5 Part 5/Fig 5ab WT and KO hela TTLL1-e326g atubulin part 2/TPGS1-KO/TPGS1-KO TTLL1-mut 10-22-24 R3 LT10.Project Maximum Z_XY1730230612_Z0_T0_C0.tif]

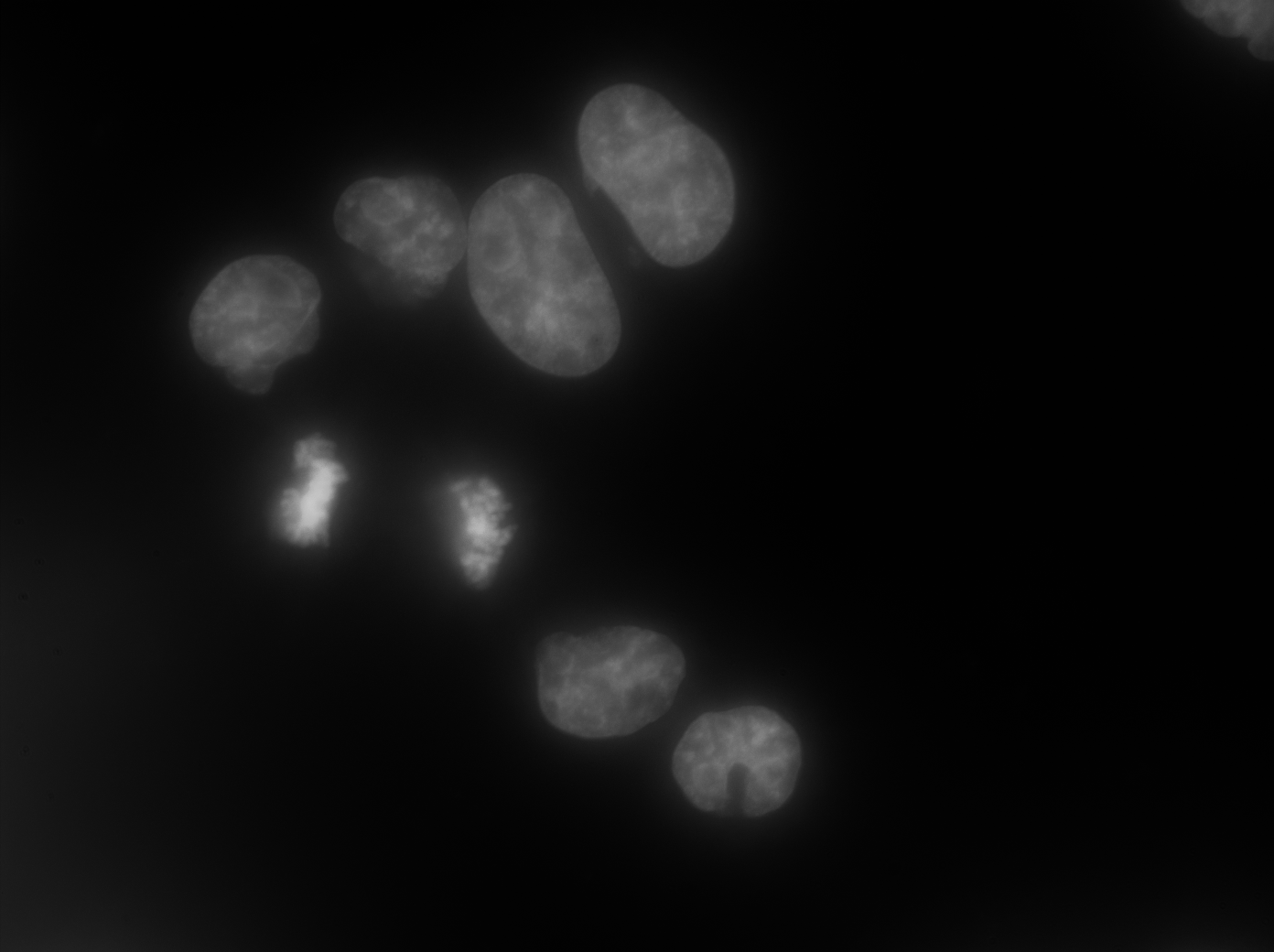

Supplement: Supplementary file 19 — Source data Fig. 5 part 5 [file 44319_2026_742_MOESM19_ESM.zip › Figure 5 Part 5/Fig 5ab WT and KO hela TTLL1-e326g atubulin part 2/TPGS1-KO/TPGS1-KO TTLL1-mut 10-22-24 R2 LT8.Project Maximum Z_XY1730227623_Z0_T0_C0.tif]

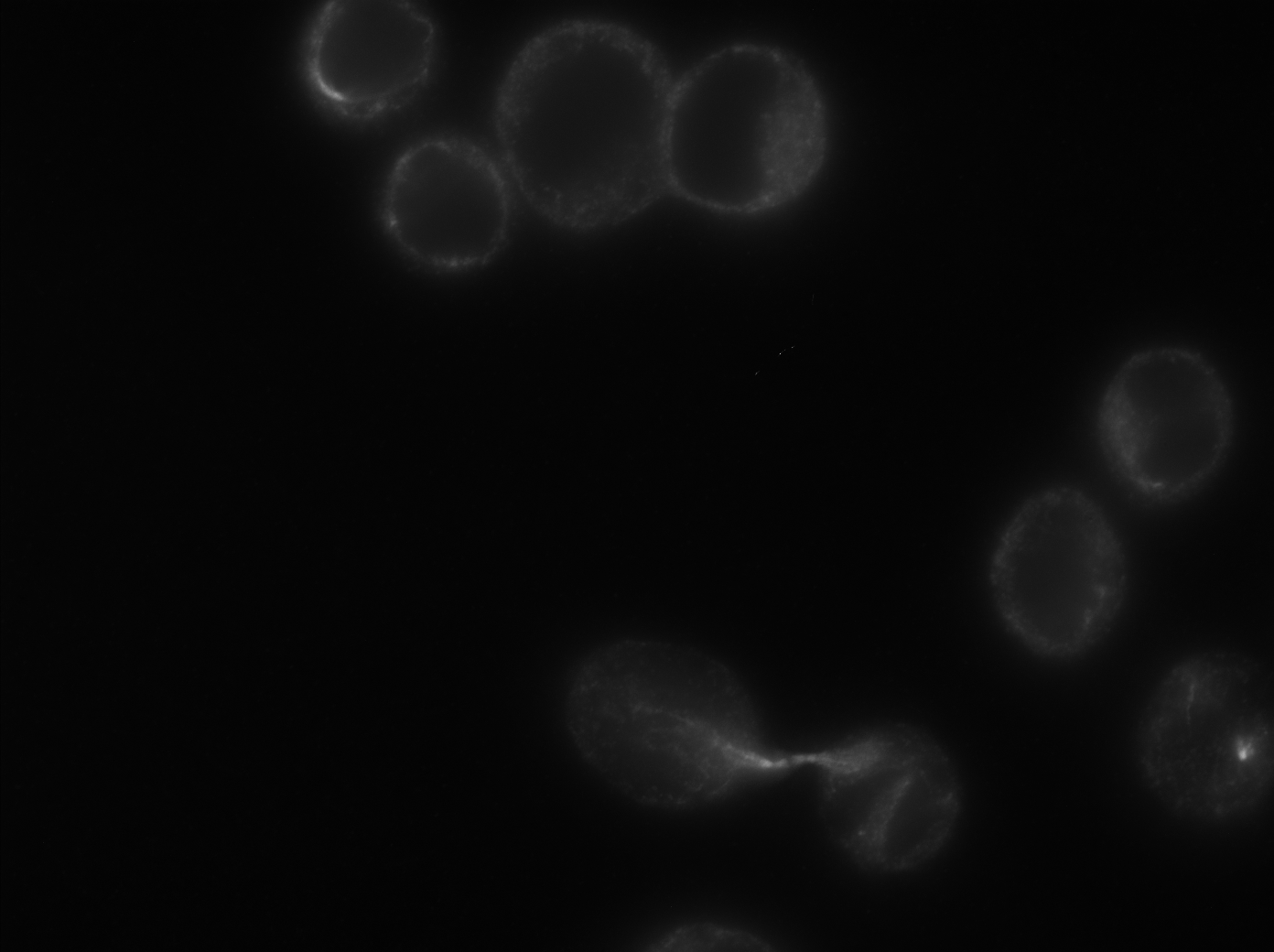

Supplement: Supplementary file 19 — Source data Fig. 5 part 5 [file 44319_2026_742_MOESM19_ESM.zip › Figure 5 Part 5/Fig 5ab WT and KO hela TTLL1-e326g atubulin part 2/TPGS1-KO/TPGS1-KO TTLL1-mut 10-15-24 R1 LT8.Project Maximum Z_XY1729024233_Z0_T0_C2.tif]

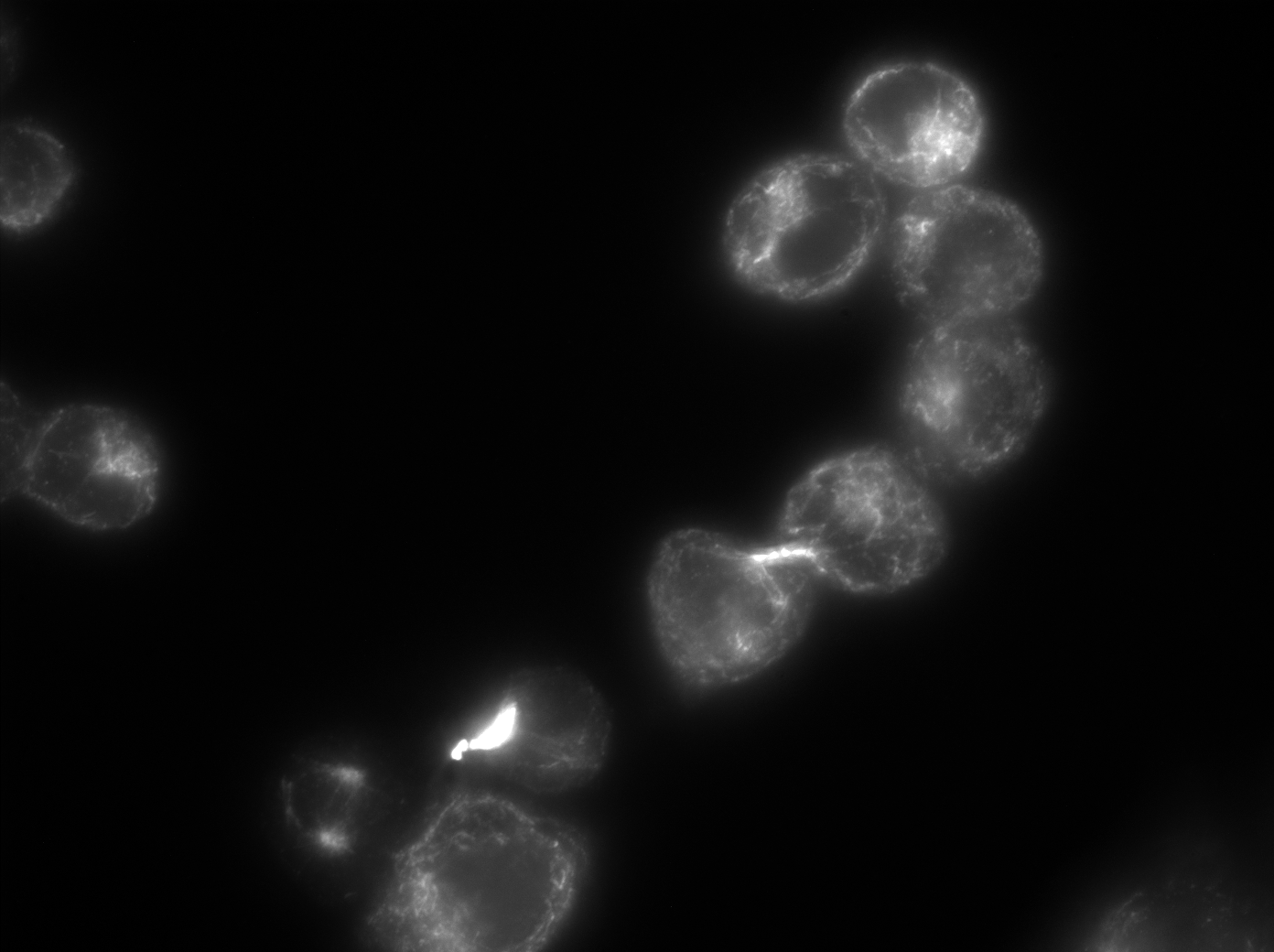

Supplement: Supplementary file 19 — Source data Fig. 5 part 5 [file 44319_2026_742_MOESM19_ESM.zip › Figure 5 Part 5/Fig 5ab WT and KO hela TTLL1-e326g atubulin part 2/TPGS1-KO/TPGS1-KO TTLL1-mut 10-22-24 R2 LT6.Project Maximum Z_XY1730226788_Z0_T0_C2.tif]

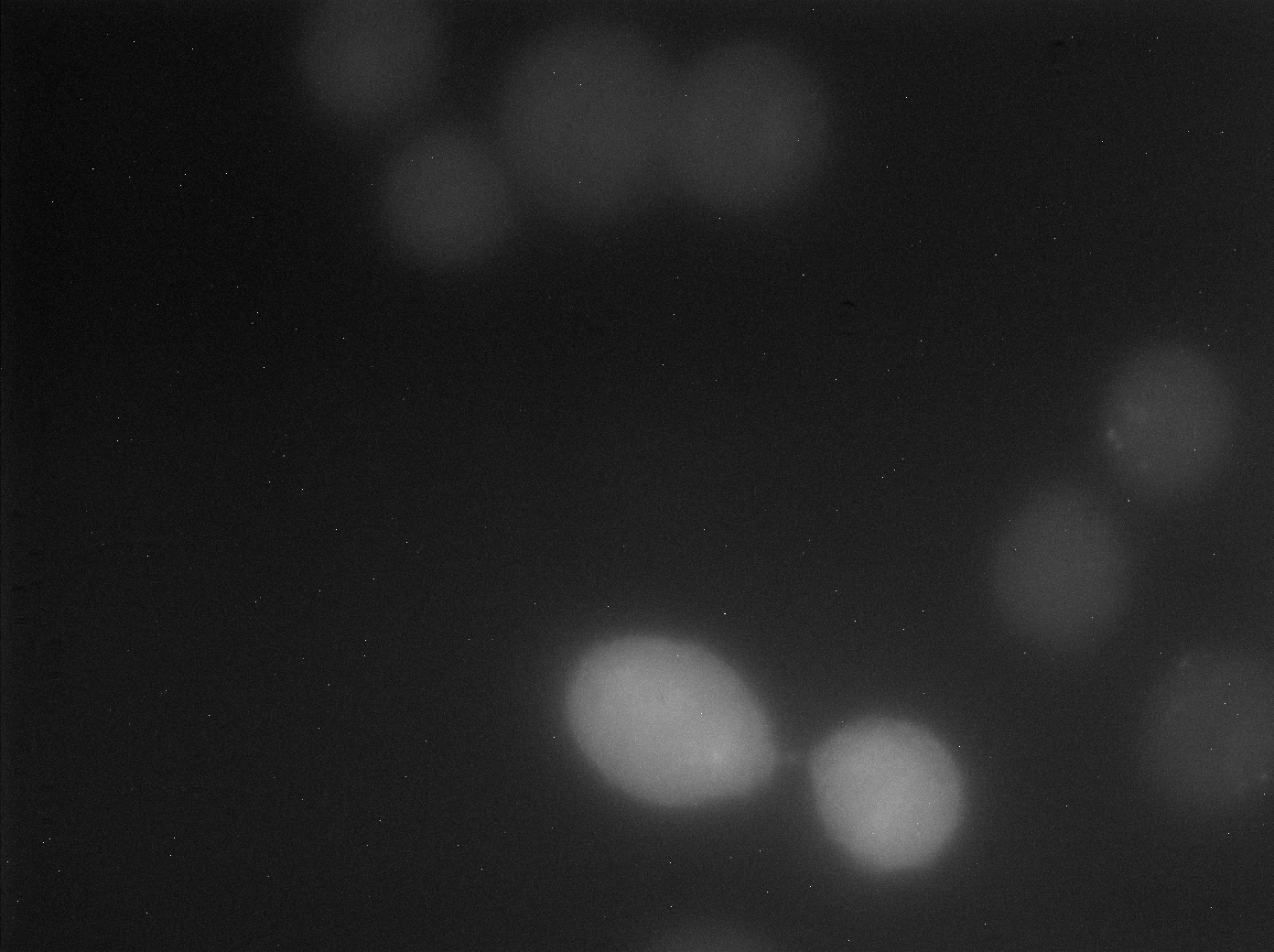

Supplement: Supplementary file 19 — Source data Fig. 5 part 5 [file 44319_2026_742_MOESM19_ESM.zip › Figure 5 Part 5/Fig 5ab WT and KO hela TTLL1-e326g atubulin part 2/TPGS1-KO/TPGS1-KO TTLL1-mut 10-15-24 R1 LT8.Project Maximum Z_XY1729024233_Z0_T0_C1.tif]

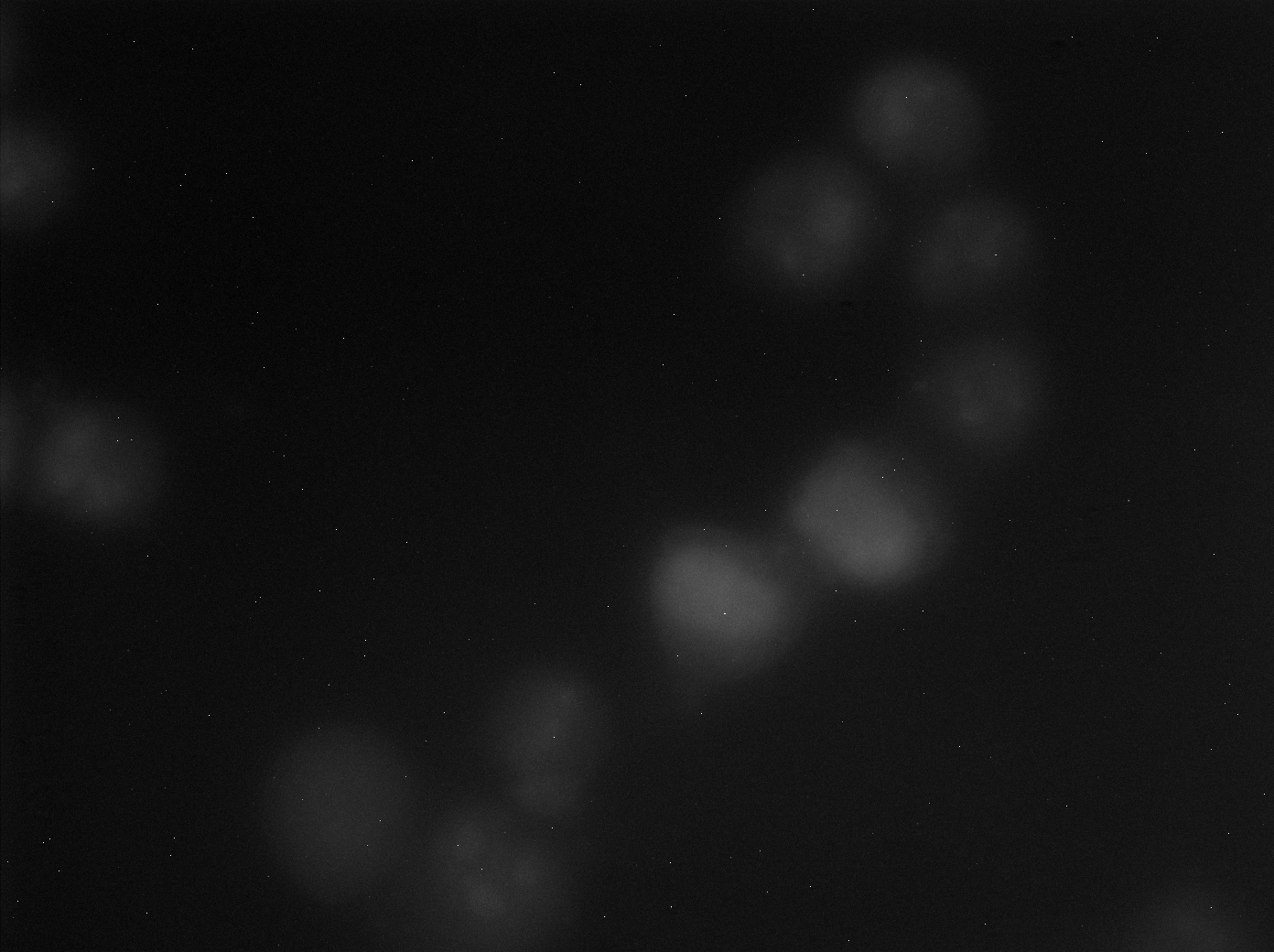

Supplement: Supplementary file 19 — Source data Fig. 5 part 5 [file 44319_2026_742_MOESM19_ESM.zip › Figure 5 Part 5/Fig 5ab WT and KO hela TTLL1-e326g atubulin part 2/TPGS1-KO/TPGS1-KO TTLL1-mut 10-22-24 R2 LT6.Project Maximum Z_XY1730226788_Z0_T0_C1.tif]

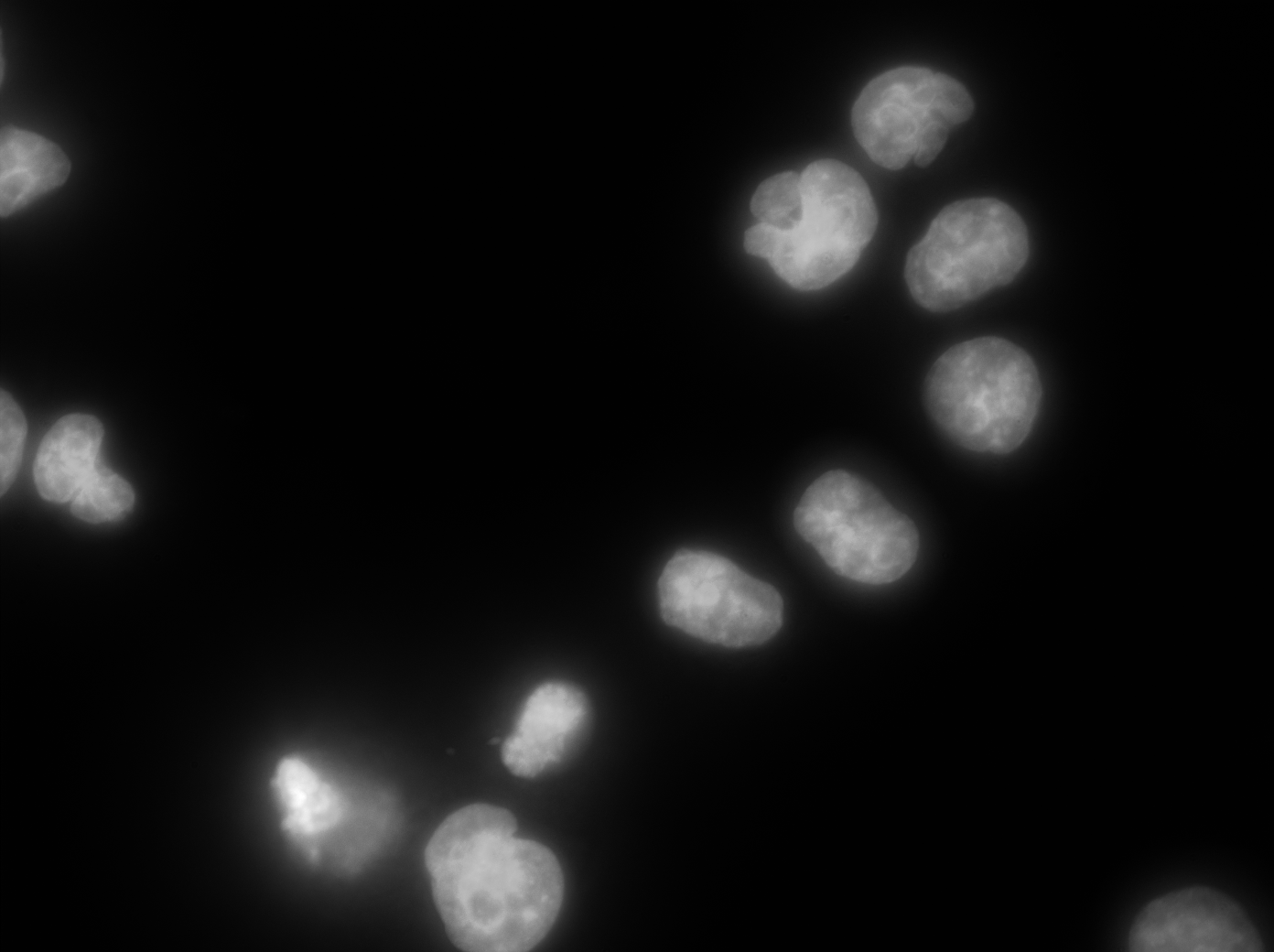

Supplement: Supplementary file 19 — Source data Fig. 5 part 5 [file 44319_2026_742_MOESM19_ESM.zip › Figure 5 Part 5/Fig 5ab WT and KO hela TTLL1-e326g atubulin part 2/TPGS1-KO/TPGS1-KO TTLL1-mut 10-22-24 R2 LT6.Project Maximum Z_XY1730226788_Z0_T0_C0.tif]

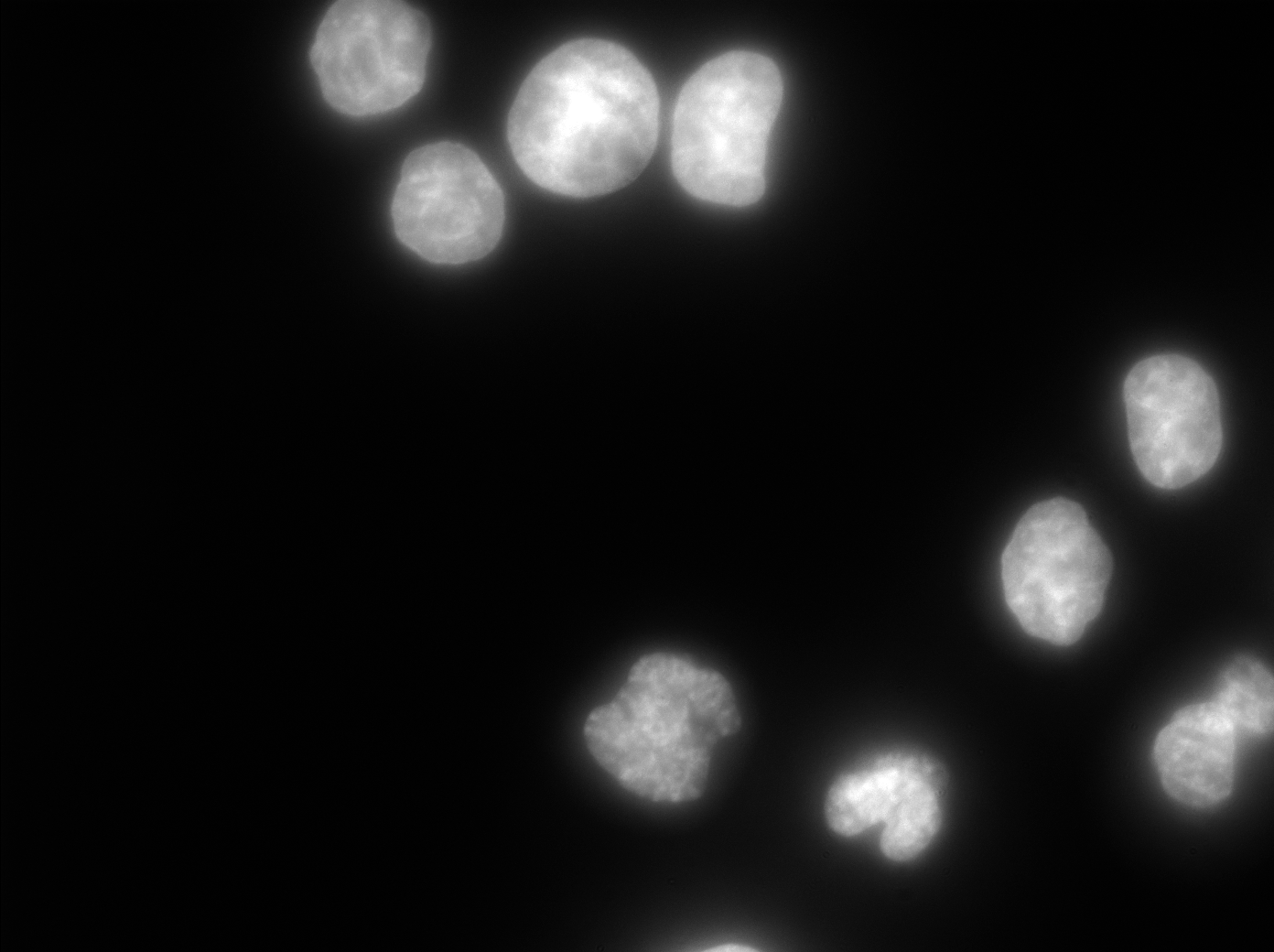

Supplement: Supplementary file 19 — Source data Fig. 5 part 5 [file 44319_2026_742_MOESM19_ESM.zip › Figure 5 Part 5/Fig 5ab WT and KO hela TTLL1-e326g atubulin part 2/TPGS1-KO/TPGS1-KO TTLL1-mut 10-15-24 R1 LT8.Project Maximum Z_XY1729024233_Z0_T0_C0.tif]

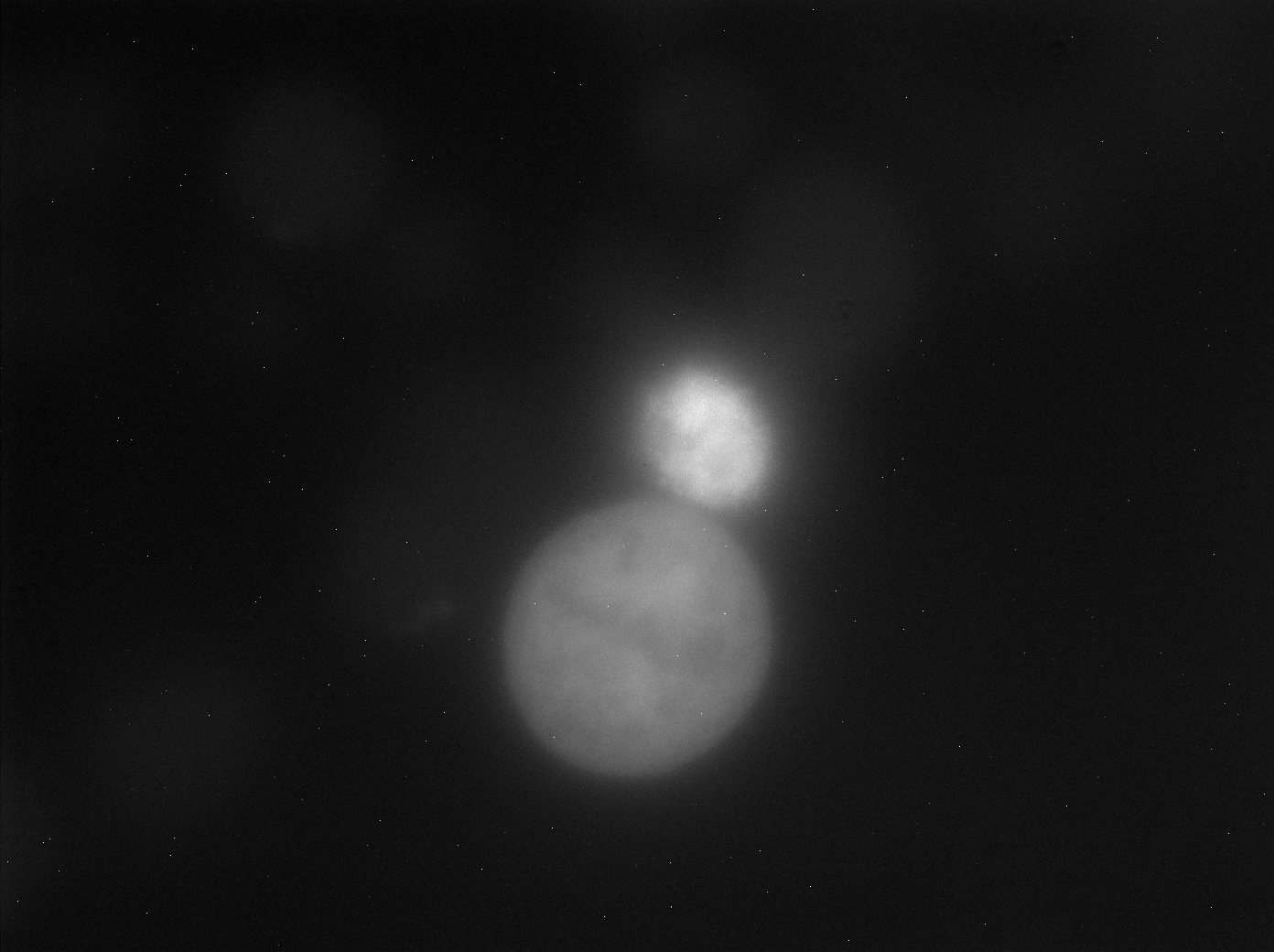

Supplement: Supplementary file 19 — Source data Fig. 5 part 5 [file 44319_2026_742_MOESM19_ESM.zip › Figure 5 Part 5/Fig 5ab WT and KO hela TTLL1-e326g atubulin part 2/TPGS1-KO/TPGS1-KO TTLL1-mut 10-22-24 R2 M2.Project Maximum Z_XY1730227192_Z0_T0_C1.tif]

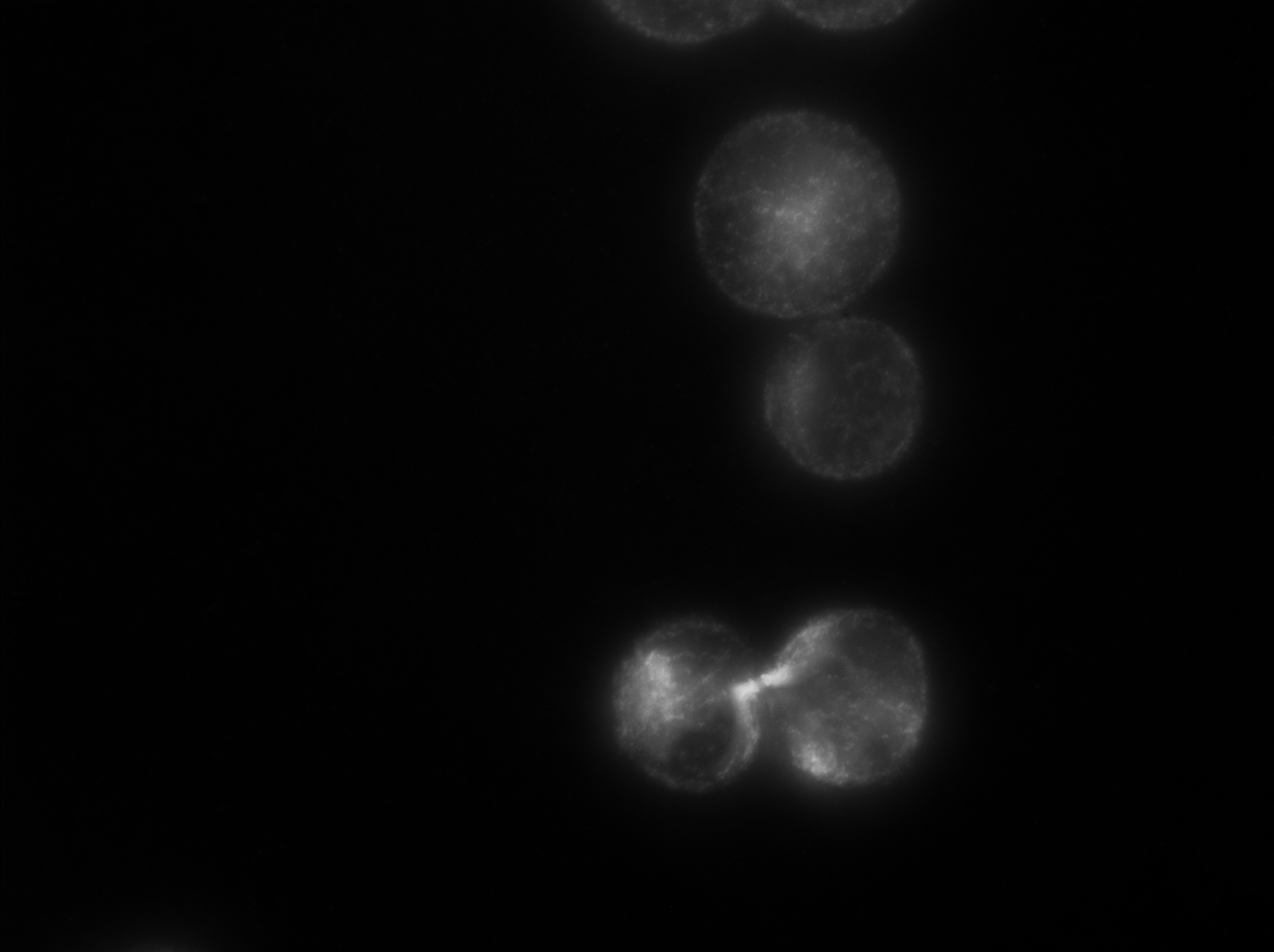

Supplement: Supplementary file 19 — Source data Fig. 5 part 5 [file 44319_2026_742_MOESM19_ESM.zip › Figure 5 Part 5/Fig 5ab WT and KO hela TTLL1-e326g atubulin part 2/TPGS1-KO/TPGS1-KO TTLL1-mut 10-15-24 R1 LT5.Project Maximum Z_XY1729023419_Z0_T0_C2.tif]

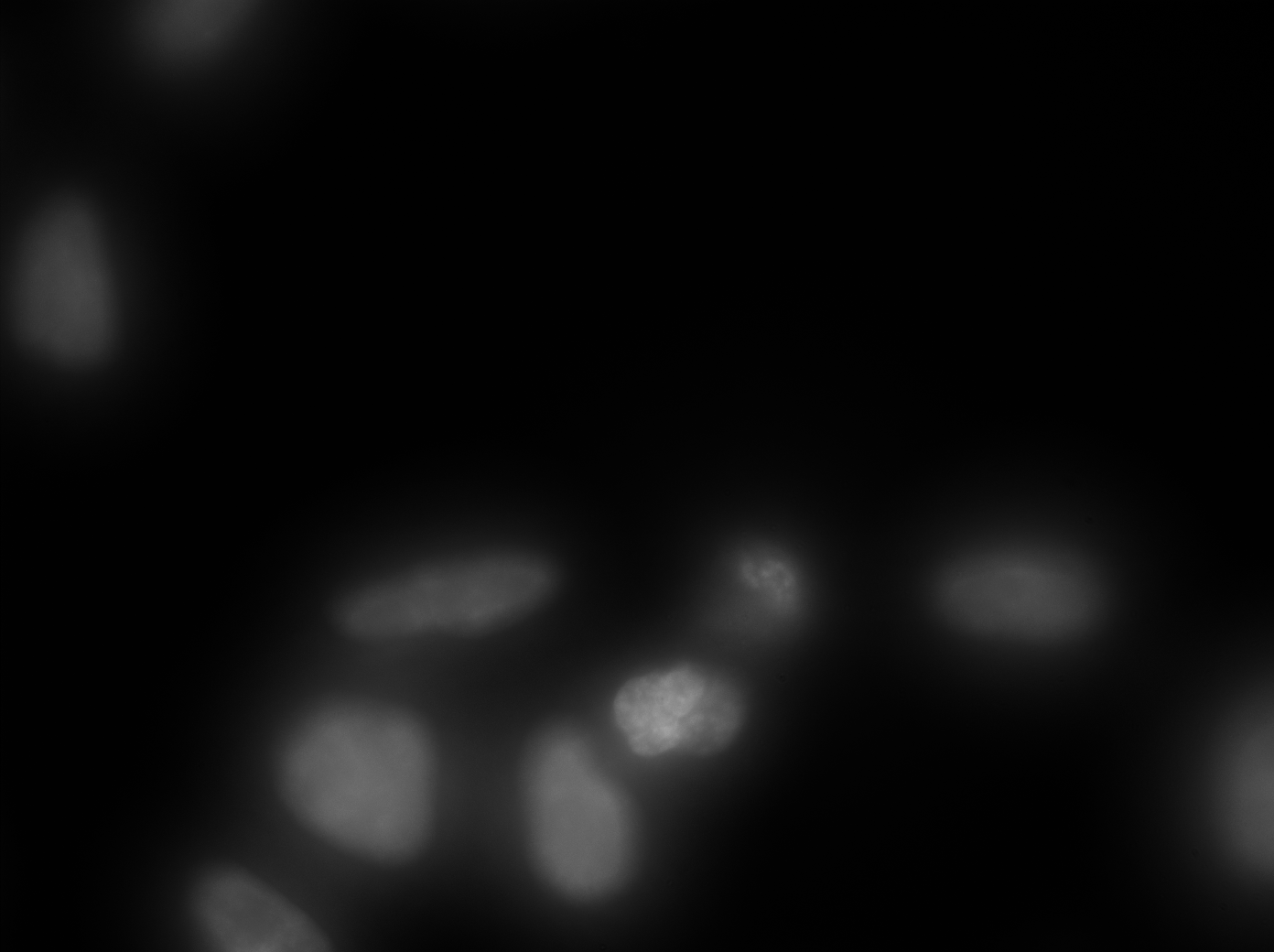

Supplement: Supplementary file 19 — Source data Fig. 5 part 5 [file 44319_2026_742_MOESM19_ESM.zip › Figure 5 Part 5/Fig 5ab WT and KO hela TTLL1-e326g atubulin part 2/TPGS1-KO/TPGS1-KO TTLL1-mut 10-22-24 R2 LT5.Project Maximum Z_XY1730226622_Z0_T0_C0.tif]

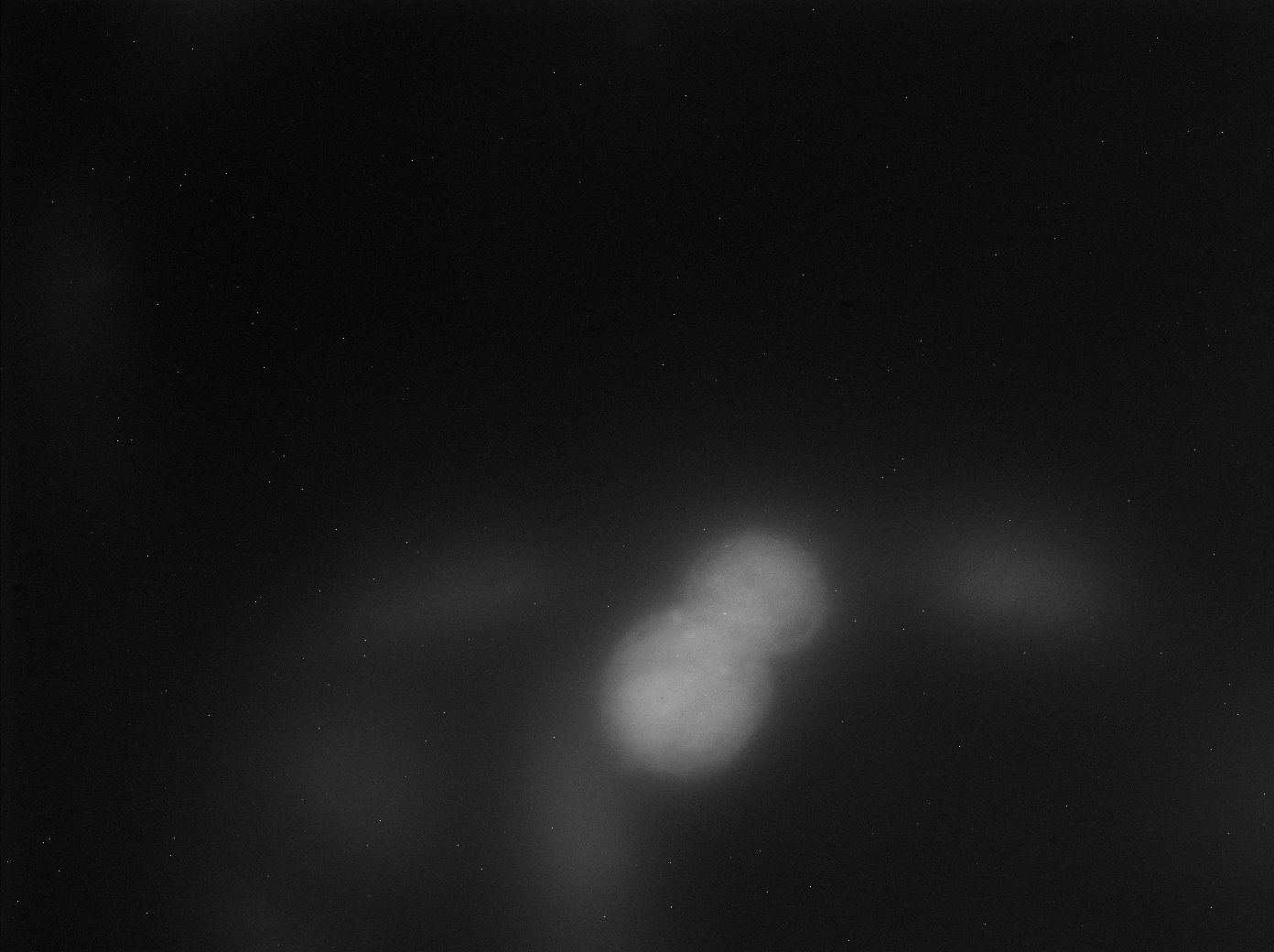

Supplement: Supplementary file 19 — Source data Fig. 5 part 5 [file 44319_2026_742_MOESM19_ESM.zip › Figure 5 Part 5/Fig 5ab WT and KO hela TTLL1-e326g atubulin part 2/TPGS1-KO/TPGS1-KO TTLL1-mut 10-22-24 R2 LT5.Project Maximum Z_XY1730226622_Z0_T0_C1.tif]

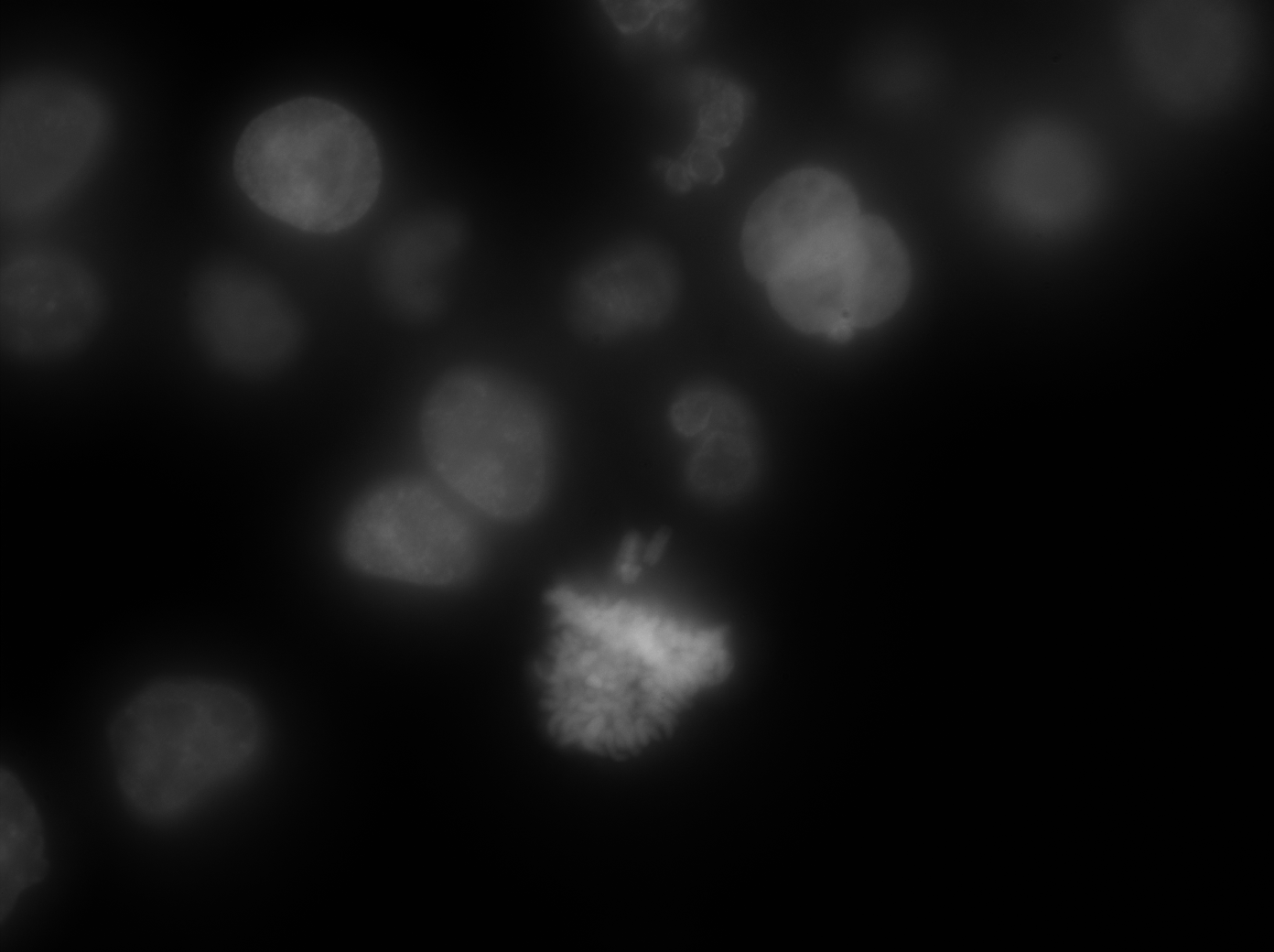

Supplement: Supplementary file 19 — Source data Fig. 5 part 5 [file 44319_2026_742_MOESM19_ESM.zip › Figure 5 Part 5/Fig 5ab WT and KO hela TTLL1-e326g atubulin part 2/TPGS1-KO/TPGS1-KO TTLL1-mut 10-22-24 R2 M2.Project Maximum Z_XY1730227192_Z0_T0_C0.tif]

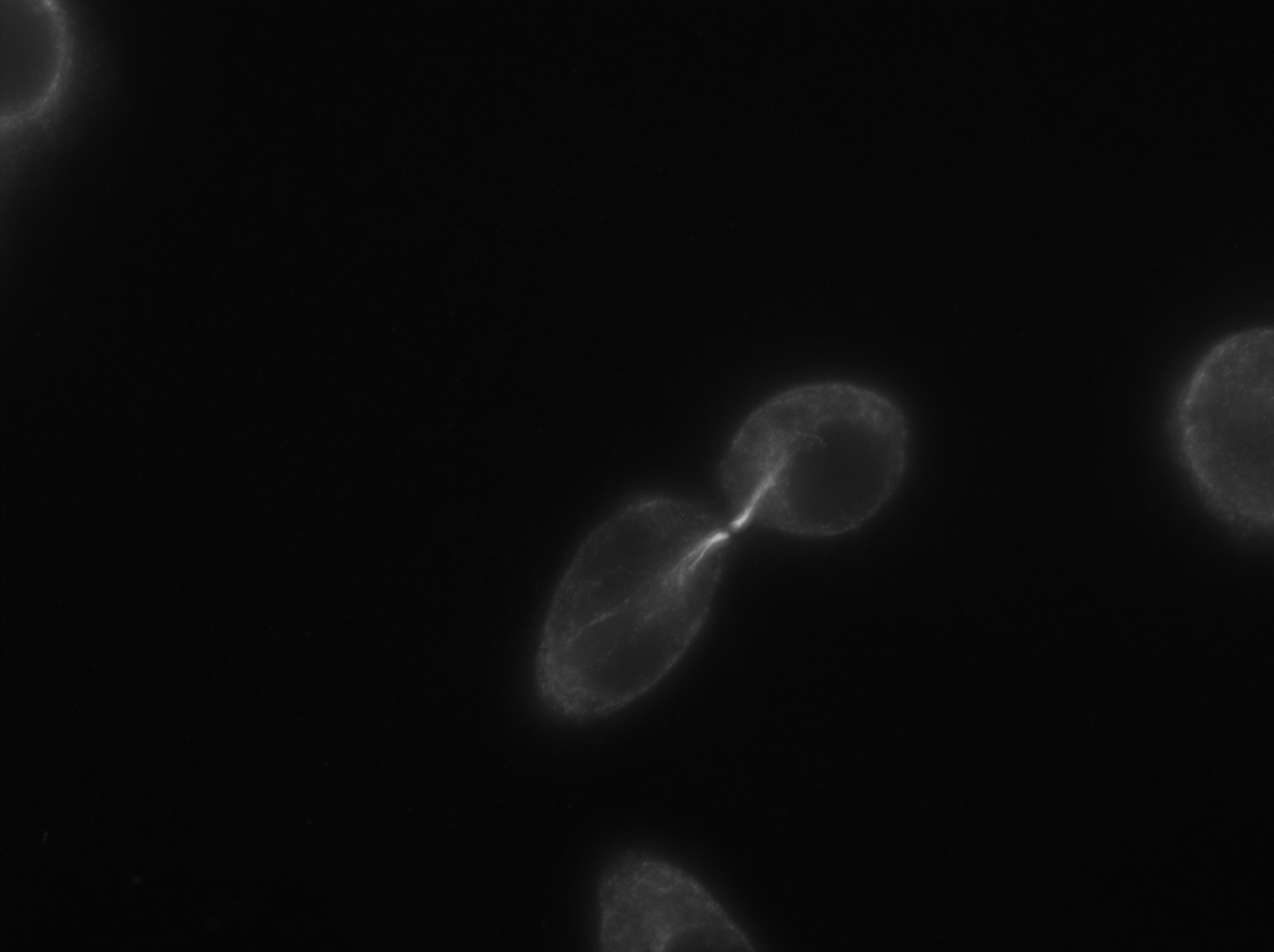

Supplement: Supplementary file 19 — Source data Fig. 5 part 5 [file 44319_2026_742_MOESM19_ESM.zip › Figure 5 Part 5/Fig 5ab WT and KO hela TTLL1-e326g atubulin part 2/TPGS1-KO/TPGS1-KO TTLL1-mut 10-15-24 R1 LT9.Project Maximum Z_XY1729024576_Z0_T0_C2.tif]

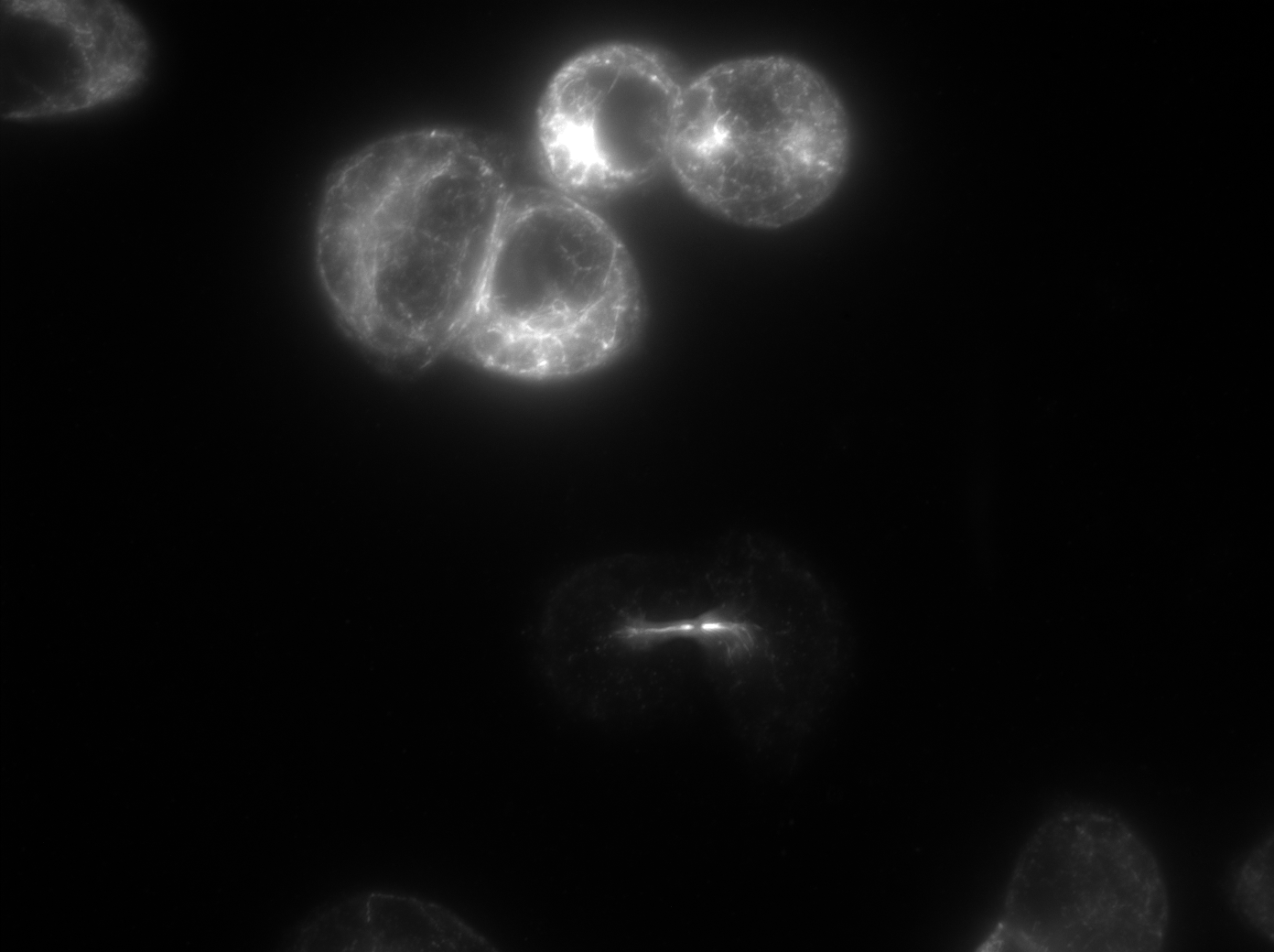

Supplement: Supplementary file 19 — Source data Fig. 5 part 5 [file 44319_2026_742_MOESM19_ESM.zip › Figure 5 Part 5/Fig 5ab WT and KO hela TTLL1-e326g atubulin part 2/TPGS1-KO/TPGS1-KO TTLL1-mut 10-22-24 R3 LT1.Project Maximum Z_XY1730228286_Z0_T0_C2.tif]

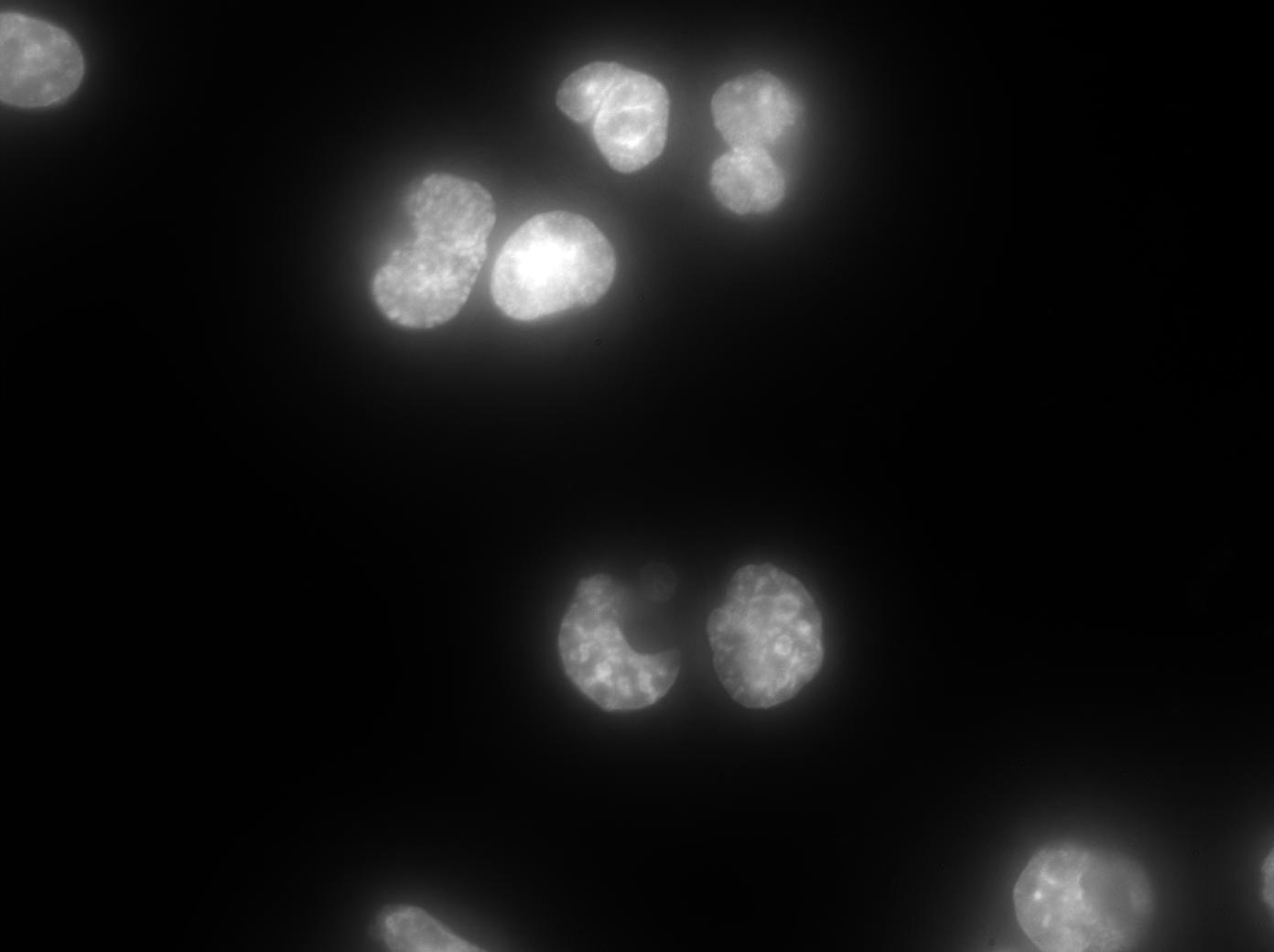

Supplement: Supplementary file 19 — Source data Fig. 5 part 5 [file 44319_2026_742_MOESM19_ESM.zip › Figure 5 Part 5/Fig 5ab WT and KO hela TTLL1-e326g atubulin part 2/TPGS1-KO/TPGS1-KO TTLL1-mut 10-22-24 R3 LT1.Project Maximum Z_XY1730228286_Z0_T0_C0.tif]

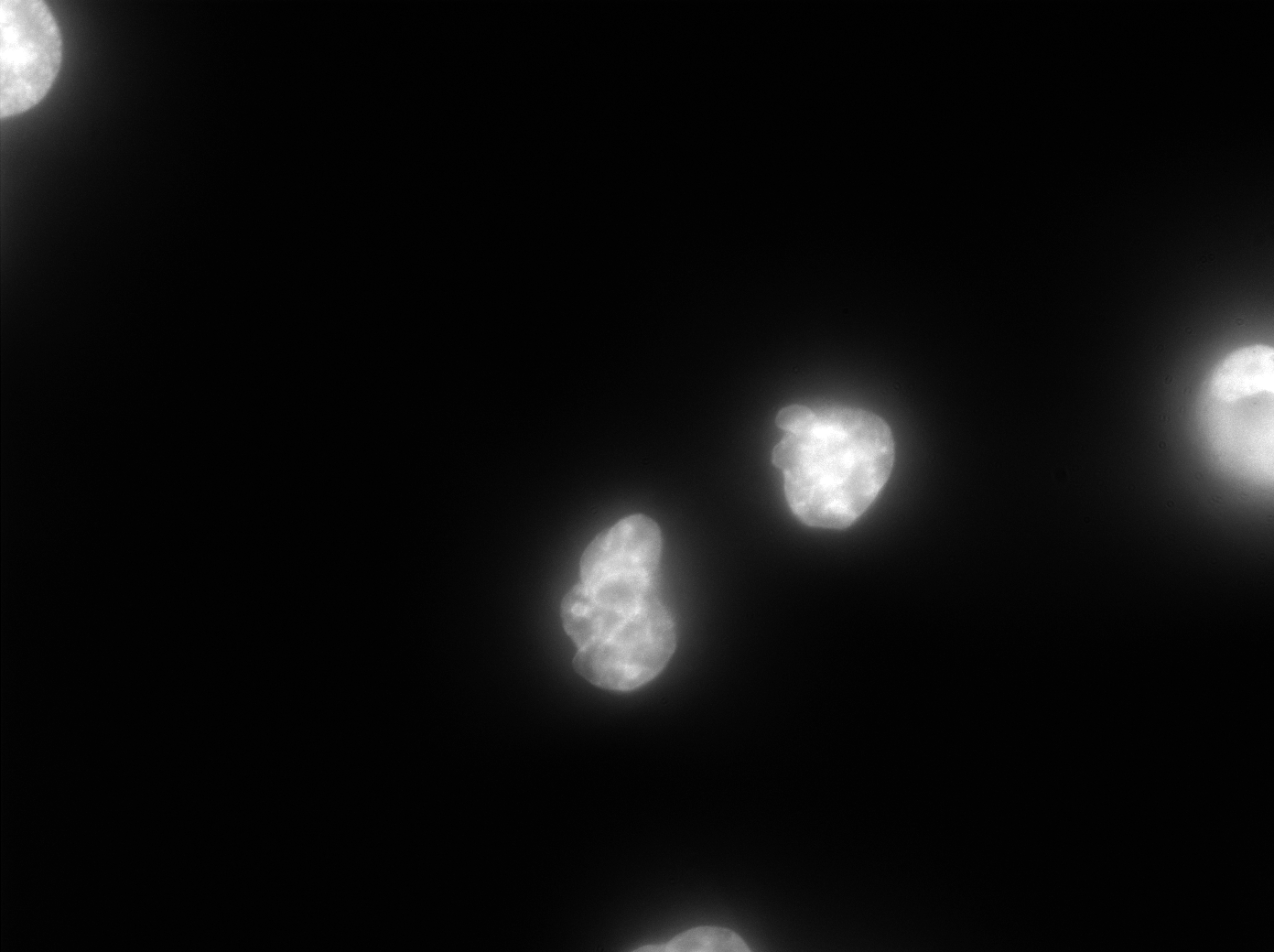

Supplement: Supplementary file 19 — Source data Fig. 5 part 5 [file 44319_2026_742_MOESM19_ESM.zip › Figure 5 Part 5/Fig 5ab WT and KO hela TTLL1-e326g atubulin part 2/TPGS1-KO/TPGS1-KO TTLL1-mut 10-15-24 R1 LT9.Project Maximum Z_XY1729024576_Z0_T0_C0.tif]

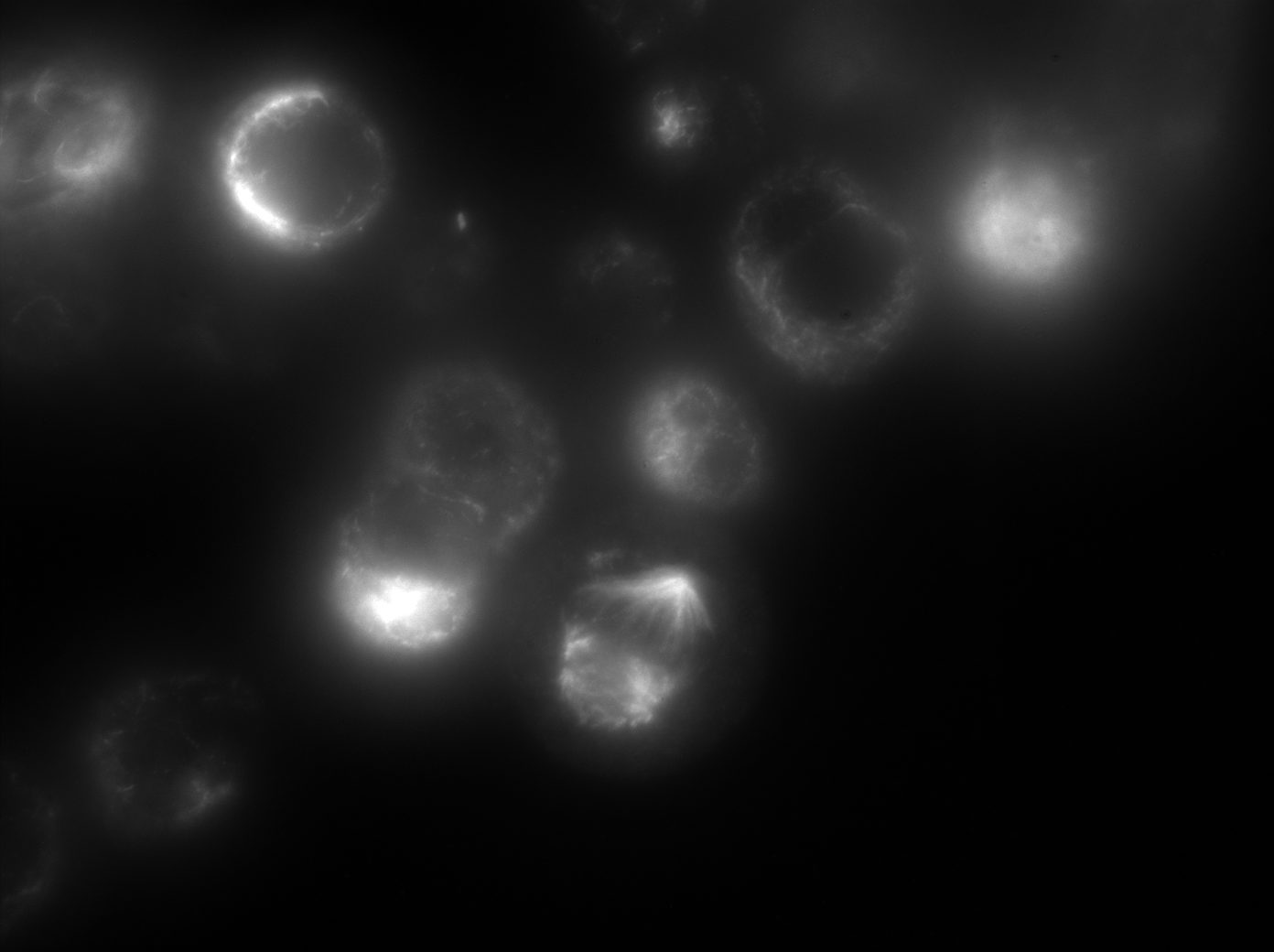

Supplement: Supplementary file 19 — Source data Fig. 5 part 5 [file 44319_2026_742_MOESM19_ESM.zip › Figure 5 Part 5/Fig 5ab WT and KO hela TTLL1-e326g atubulin part 2/TPGS1-KO/TPGS1-KO TTLL1-mut 10-22-24 R2 M2.Project Maximum Z_XY1730227192_Z0_T0_C2.tif]

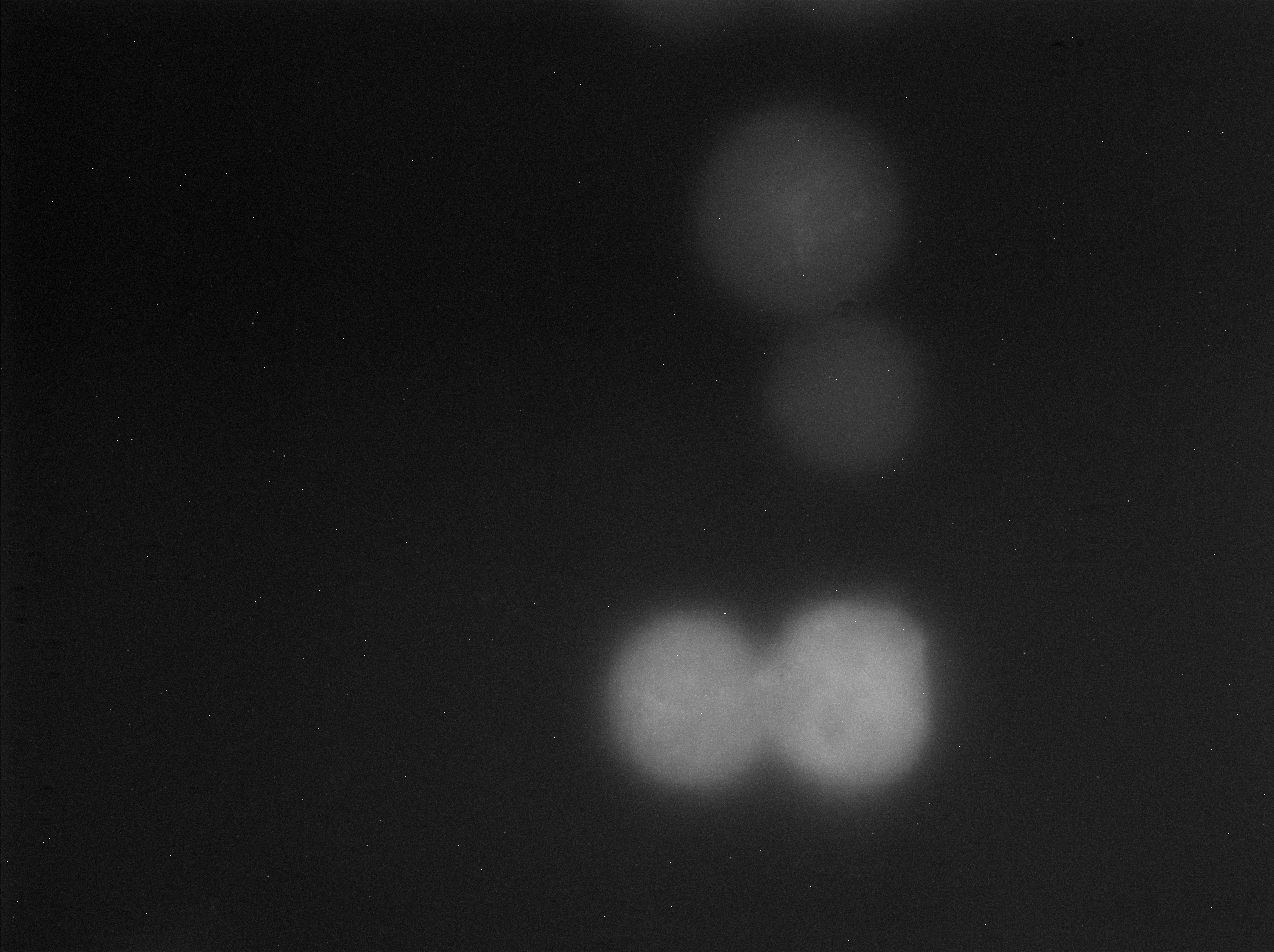

Supplement: Supplementary file 19 — Source data Fig. 5 part 5 [file 44319_2026_742_MOESM19_ESM.zip › Figure 5 Part 5/Fig 5ab WT and KO hela TTLL1-e326g atubulin part 2/TPGS1-KO/TPGS1-KO TTLL1-mut 10-15-24 R1 LT5.Project Maximum Z_XY1729023419_Z0_T0_C1.tif]

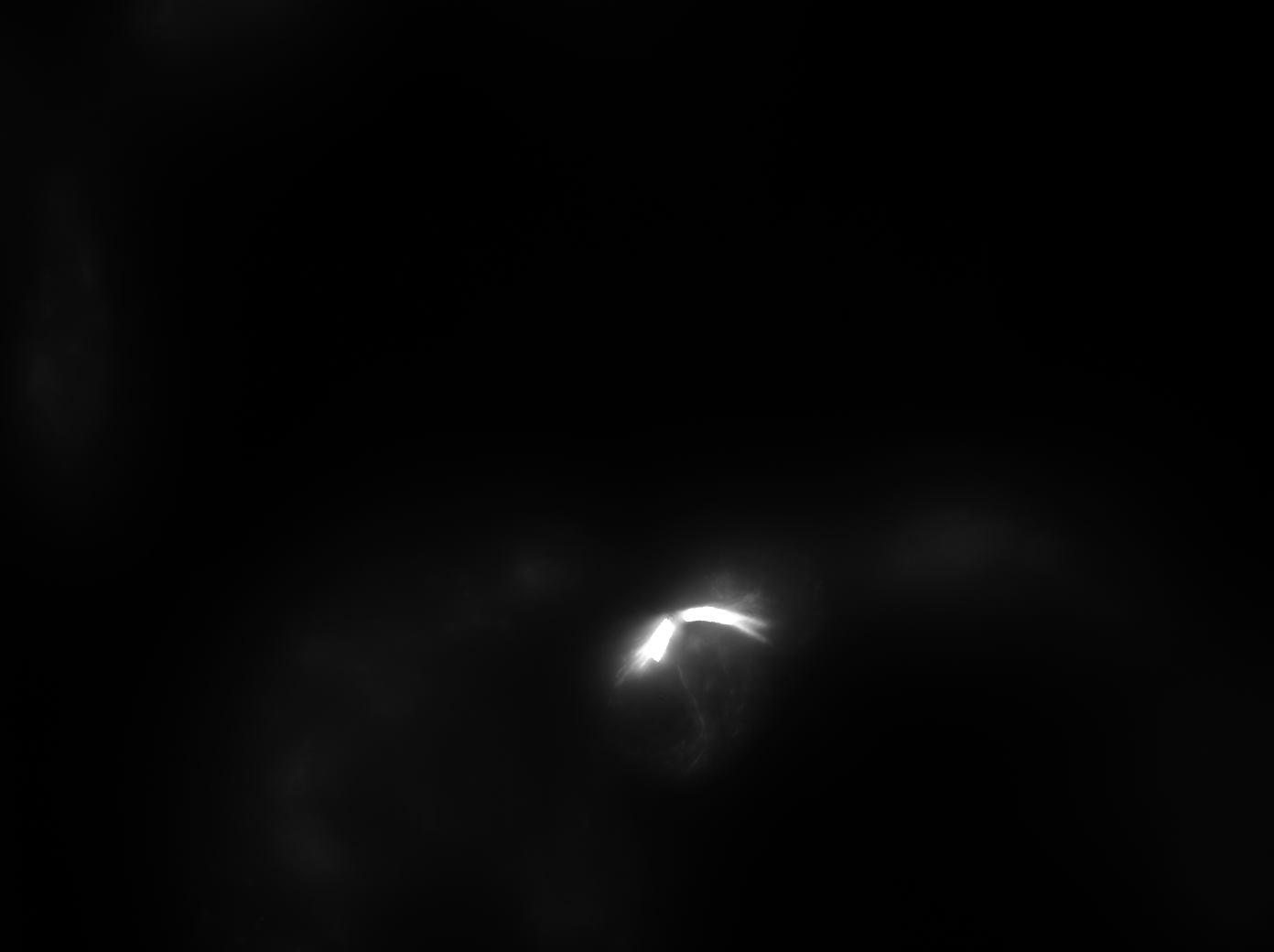

Supplement: Supplementary file 19 — Source data Fig. 5 part 5 [file 44319_2026_742_MOESM19_ESM.zip › Figure 5 Part 5/Fig 5ab WT and KO hela TTLL1-e326g atubulin part 2/TPGS1-KO/TPGS1-KO TTLL1-mut 10-22-24 R2 LT5.Project Maximum Z_XY1730226622_Z0_T0_C2.tif]

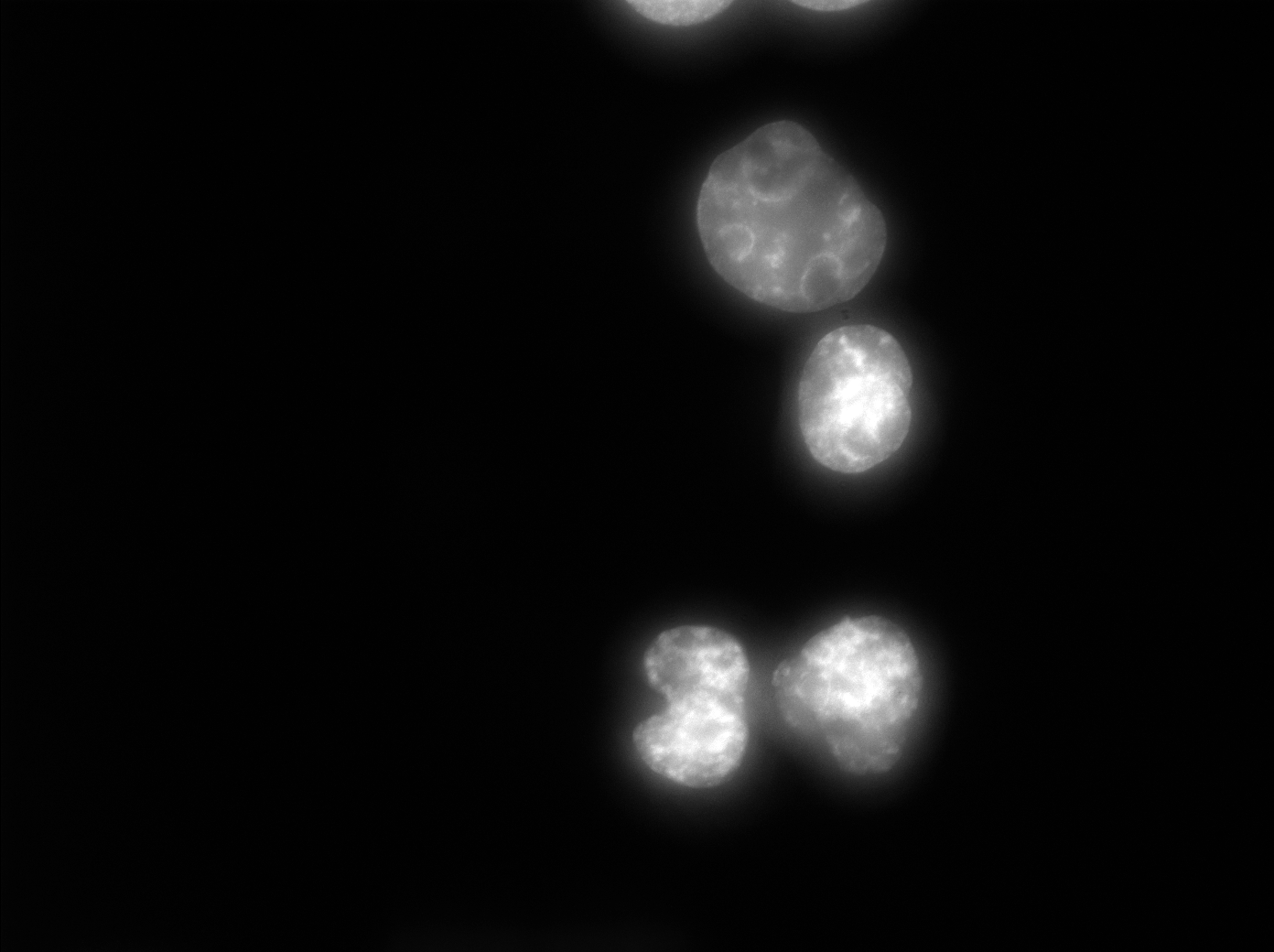

Supplement: Supplementary file 19 — Source data Fig. 5 part 5 [file 44319_2026_742_MOESM19_ESM.zip › Figure 5 Part 5/Fig 5ab WT and KO hela TTLL1-e326g atubulin part 2/TPGS1-KO/TPGS1-KO TTLL1-mut 10-15-24 R1 LT5.Project Maximum Z_XY1729023419_Z0_T0_C0.tif]

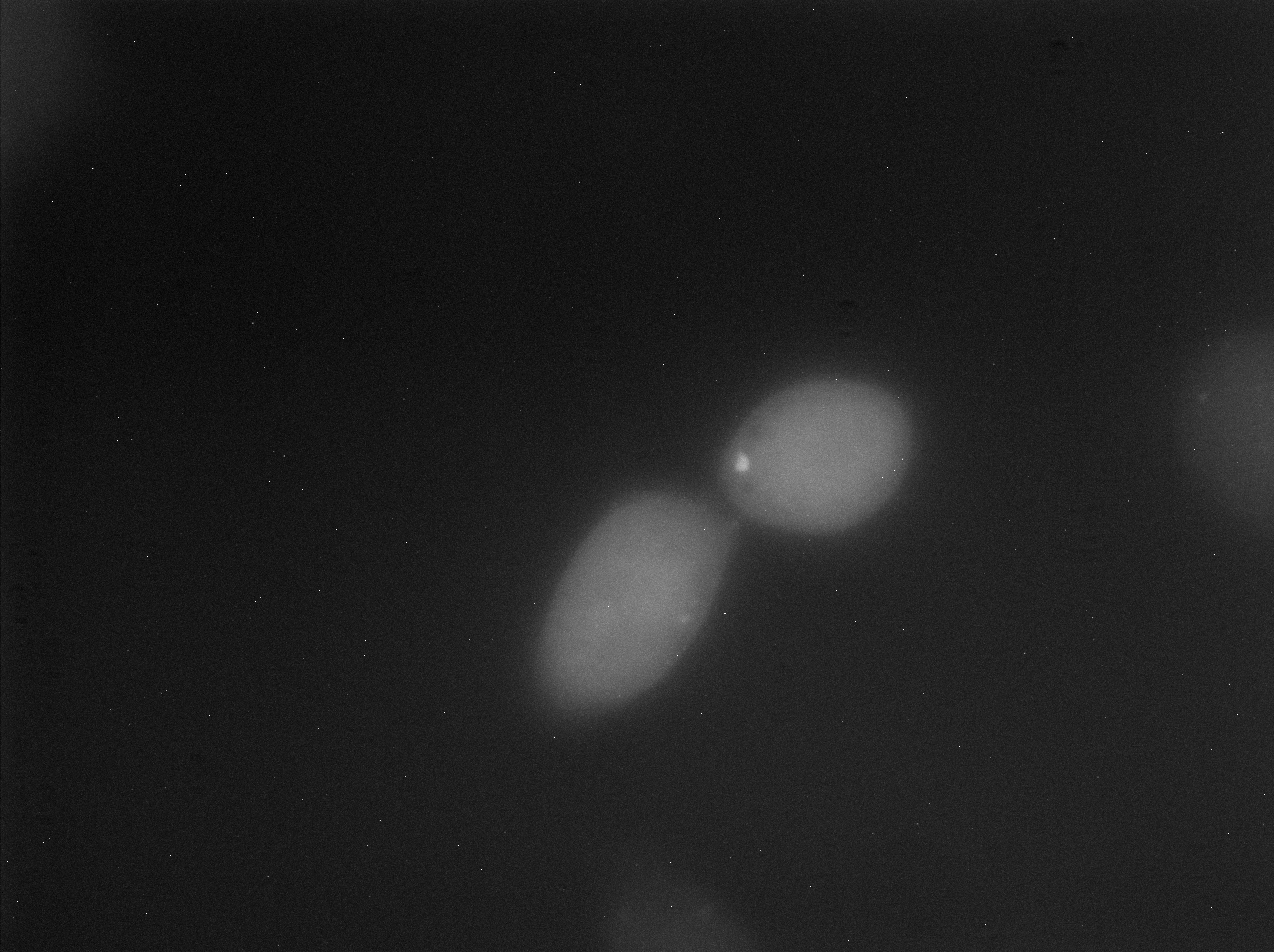

Supplement: Supplementary file 19 — Source data Fig. 5 part 5 [file 44319_2026_742_MOESM19_ESM.zip › Figure 5 Part 5/Fig 5ab WT and KO hela TTLL1-e326g atubulin part 2/TPGS1-KO/TPGS1-KO TTLL1-mut 10-15-24 R1 LT9.Project Maximum Z_XY1729024576_Z0_T0_C1.tif]

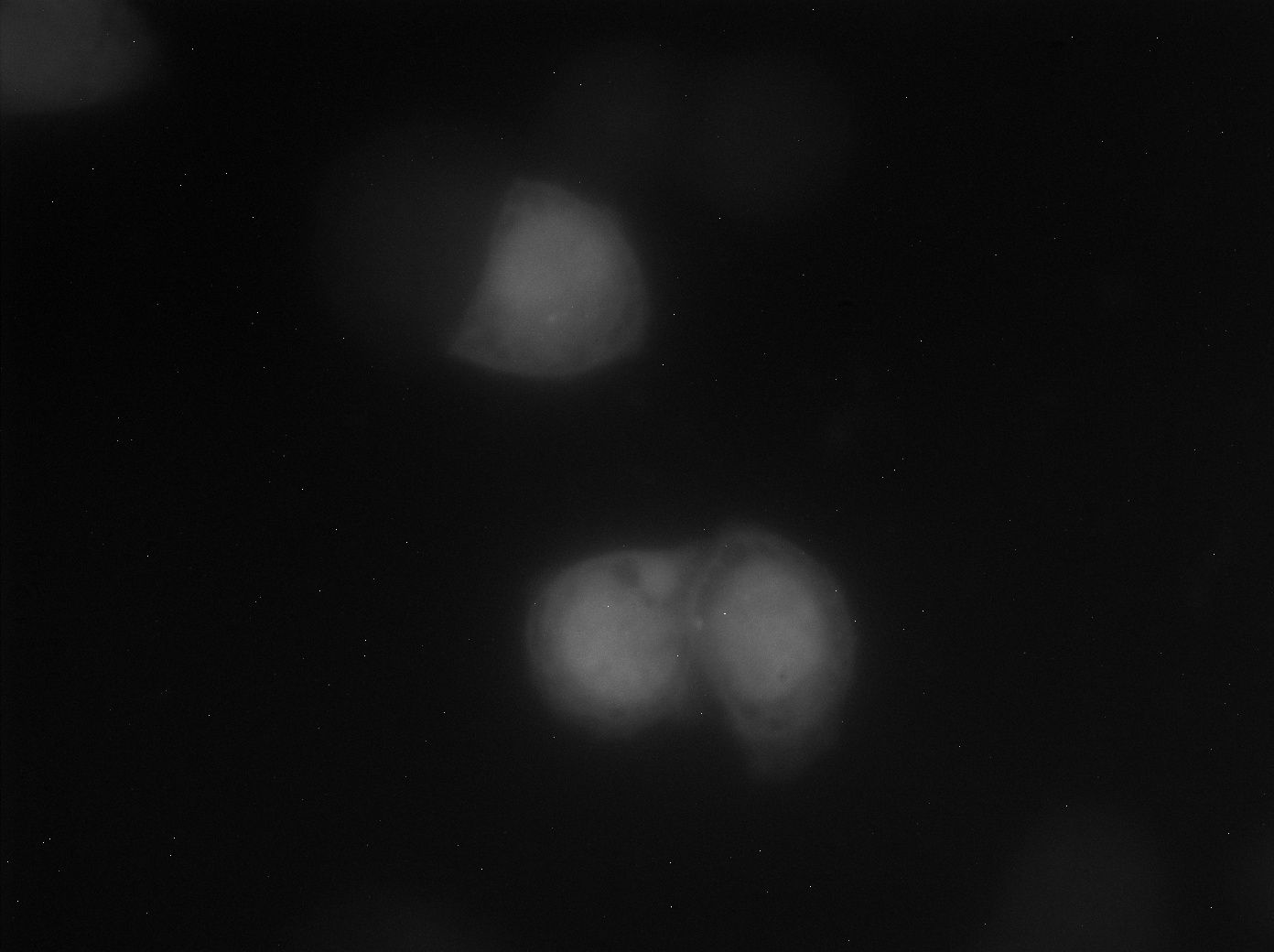

Supplement: Supplementary file 19 — Source data Fig. 5 part 5 [file 44319_2026_742_MOESM19_ESM.zip › Figure 5 Part 5/Fig 5ab WT and KO hela TTLL1-e326g atubulin part 2/TPGS1-KO/TPGS1-KO TTLL1-mut 10-22-24 R3 LT1.Project Maximum Z_XY1730228286_Z0_T0_C1.tif]

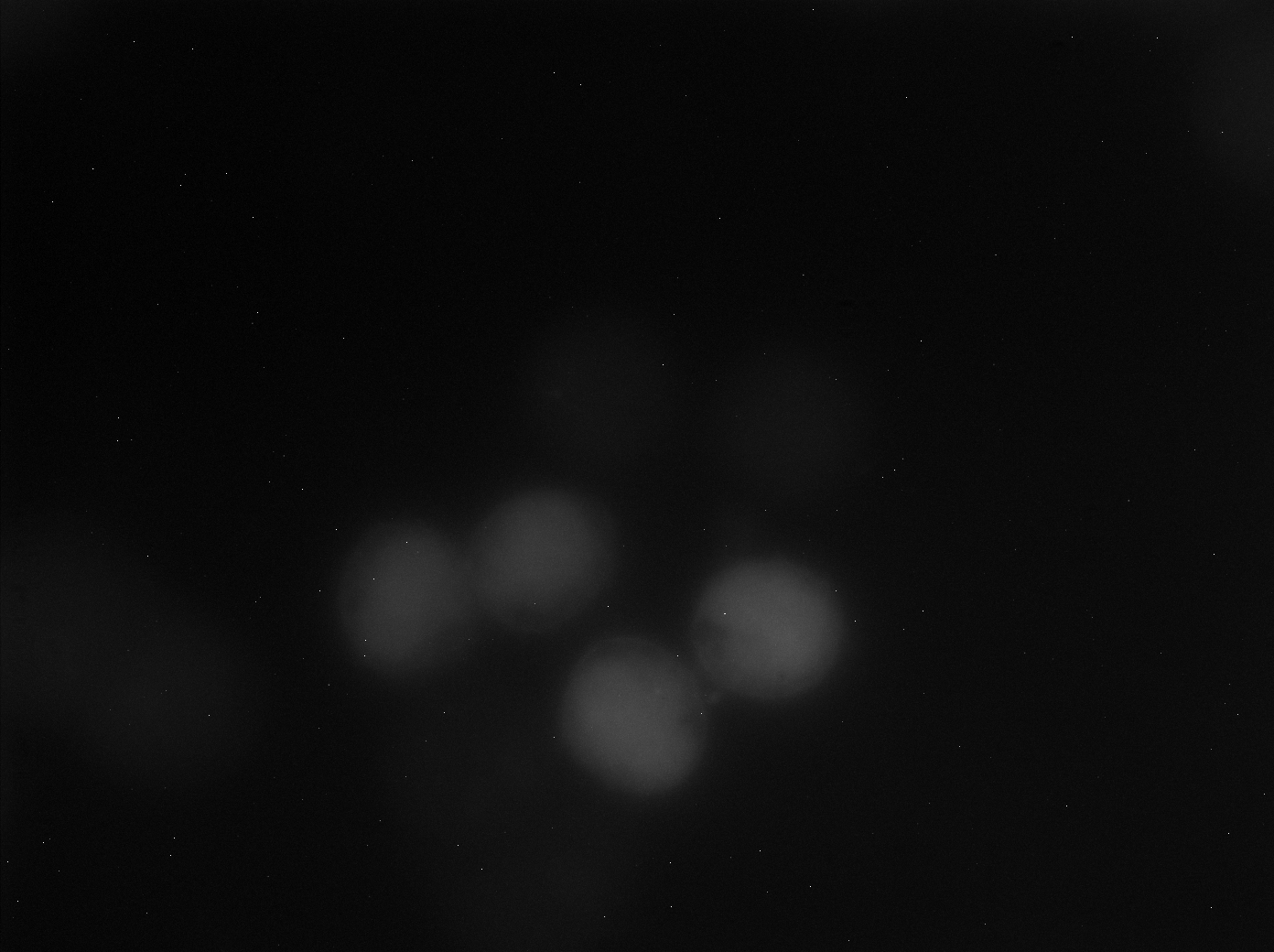

Supplement: Supplementary file 19 — Source data Fig. 5 part 5 [file 44319_2026_742_MOESM19_ESM.zip › Figure 5 Part 5/Fig 5ab WT and KO hela TTLL1-e326g atubulin part 2/TPGS1-KO/TPGS1-KO TTLL1-mut 10-22-24 R3 LT6LT7.Project Maximum Z_XY1730229549_Z0_T0_C1.tif]

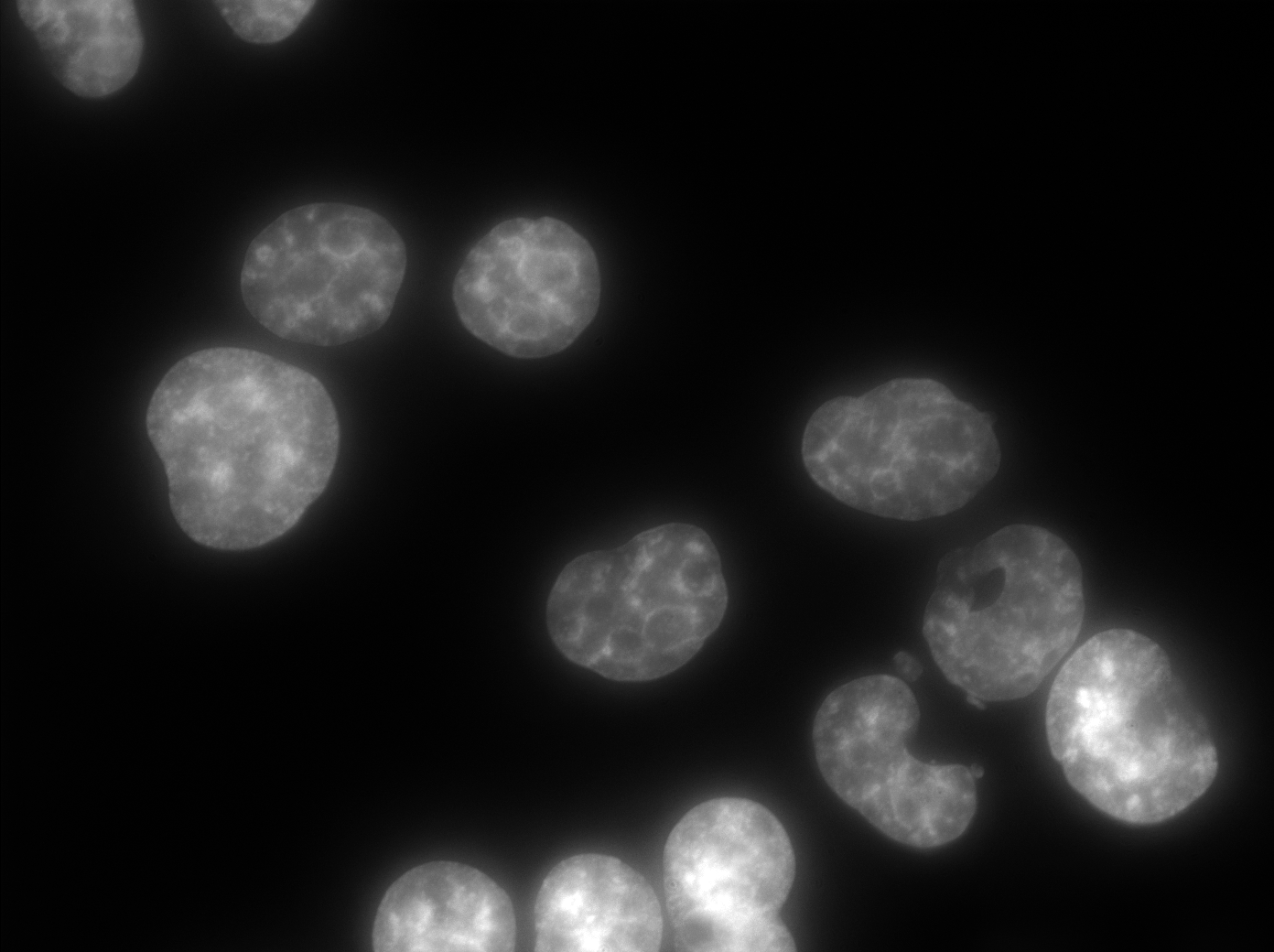

Supplement: Supplementary file 19 — Source data Fig. 5 part 5 [file 44319_2026_742_MOESM19_ESM.zip › Figure 5 Part 5/Fig 5ab WT and KO hela TTLL1-e326g atubulin part 2/TPGS1-KO/TPGS1-KO TTLL1-mut 10-15-24 R1 LT7.Project Maximum Z_XY1729023927_Z0_T0_C0.tif]

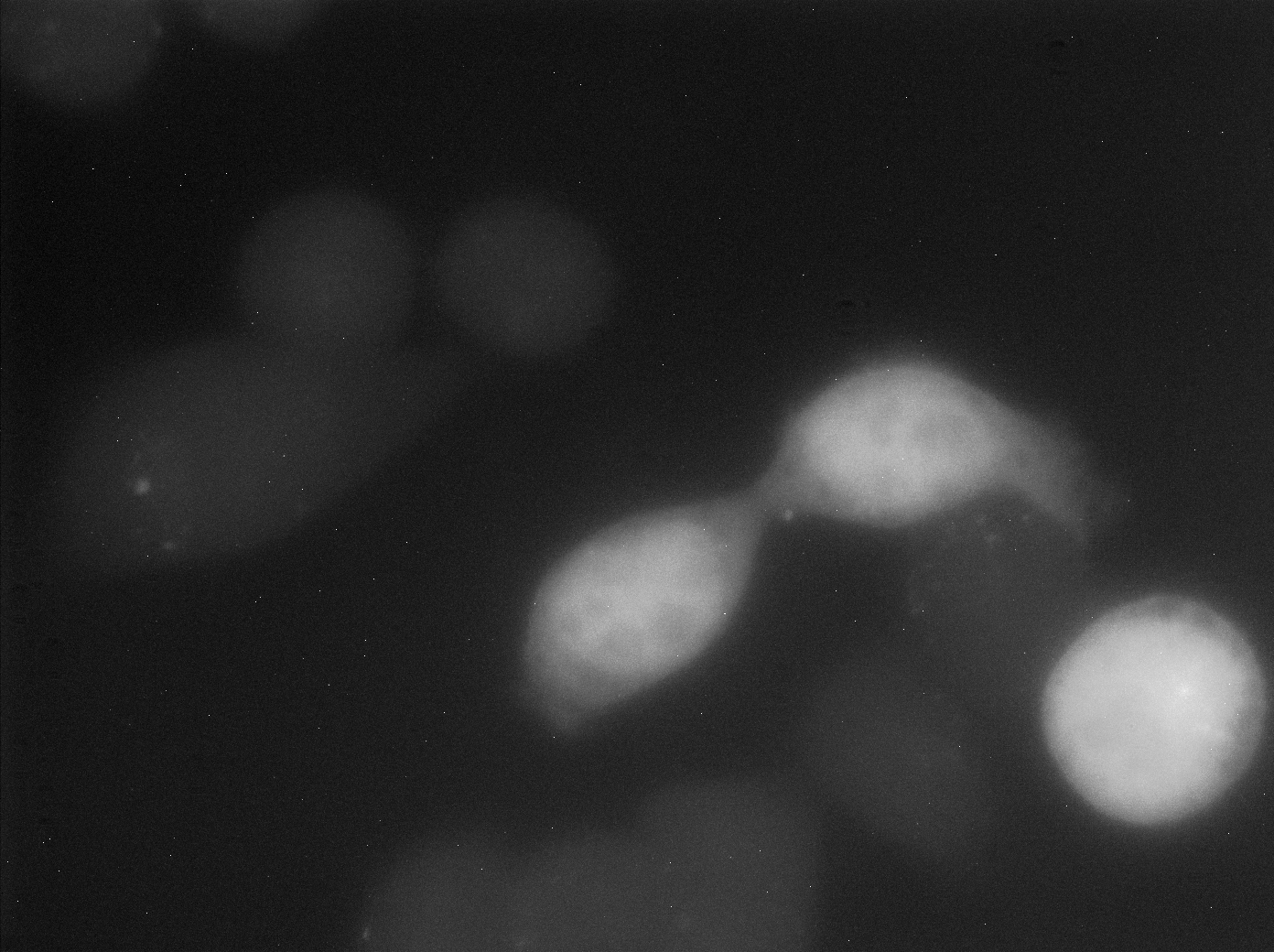

Supplement: Supplementary file 19 — Source data Fig. 5 part 5 [file 44319_2026_742_MOESM19_ESM.zip › Figure 5 Part 5/Fig 5ab WT and KO hela TTLL1-e326g atubulin part 2/TPGS1-KO/TPGS1-KO TTLL1-mut 10-15-24 R1 LT7.Project Maximum Z_XY1729023927_Z0_T0_C1.tif]

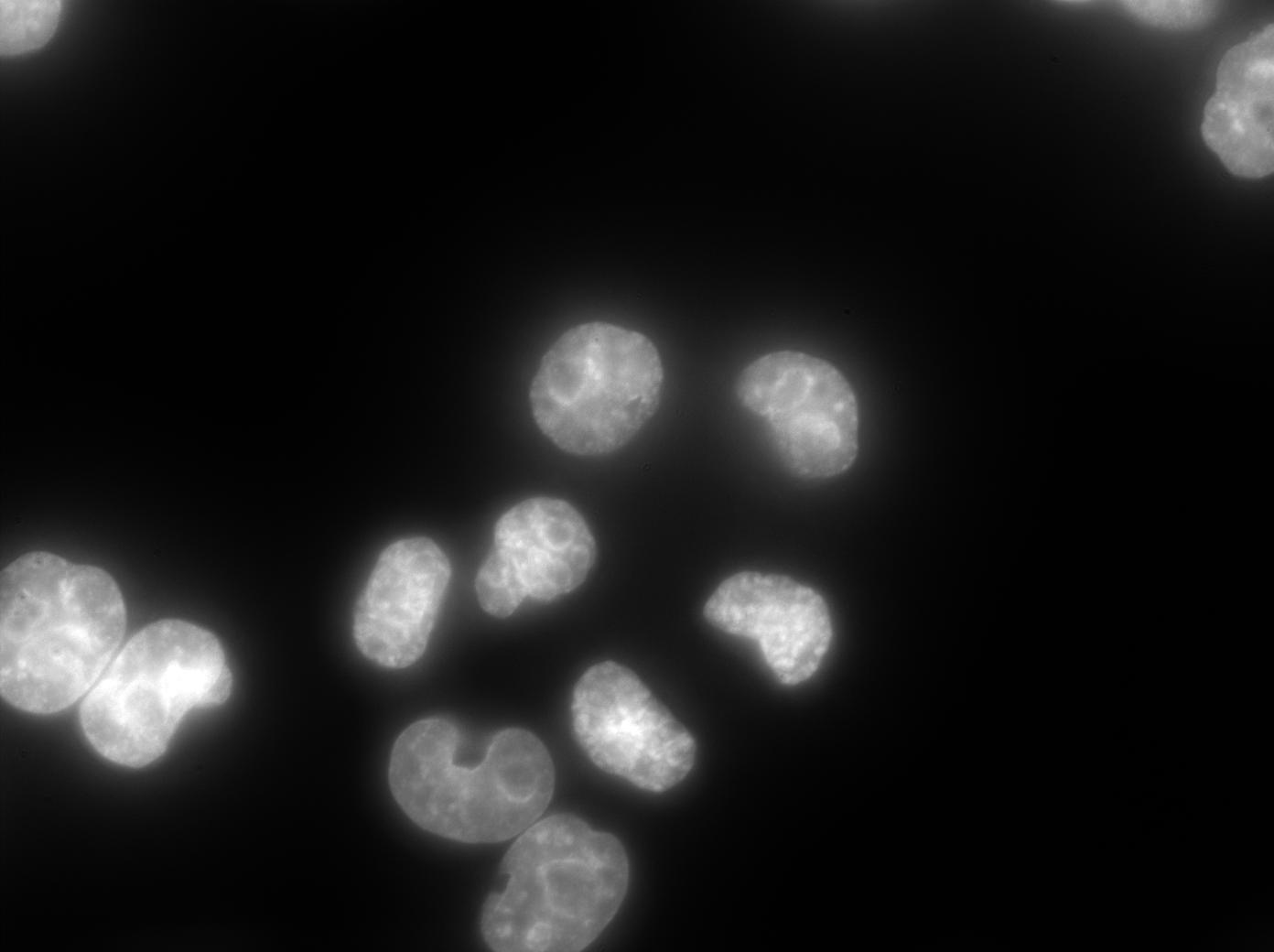

Supplement: Supplementary file 19 — Source data Fig. 5 part 5 [file 44319_2026_742_MOESM19_ESM.zip › Figure 5 Part 5/Fig 5ab WT and KO hela TTLL1-e326g atubulin part 2/TPGS1-KO/TPGS1-KO TTLL1-mut 10-22-24 R3 LT6LT7.Project Maximum Z_XY1730229549_Z0_T0_C0.tif]

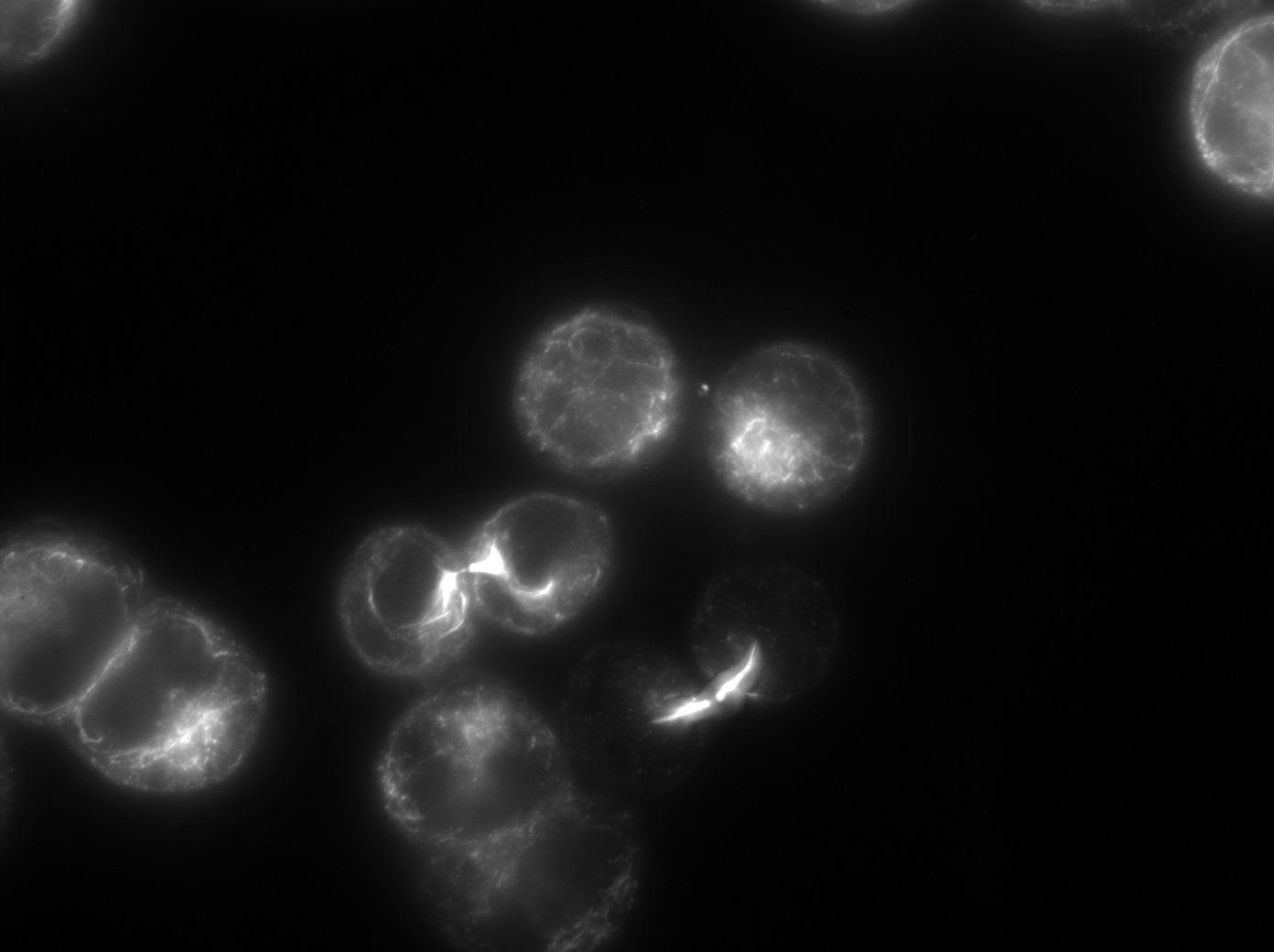

Supplement: Supplementary file 19 — Source data Fig. 5 part 5 [file 44319_2026_742_MOESM19_ESM.zip › Figure 5 Part 5/Fig 5ab WT and KO hela TTLL1-e326g atubulin part 2/TPGS1-KO/TPGS1-KO TTLL1-mut 10-22-24 R3 LT6LT7.Project Maximum Z_XY1730229549_Z0_T0_C2.tif]

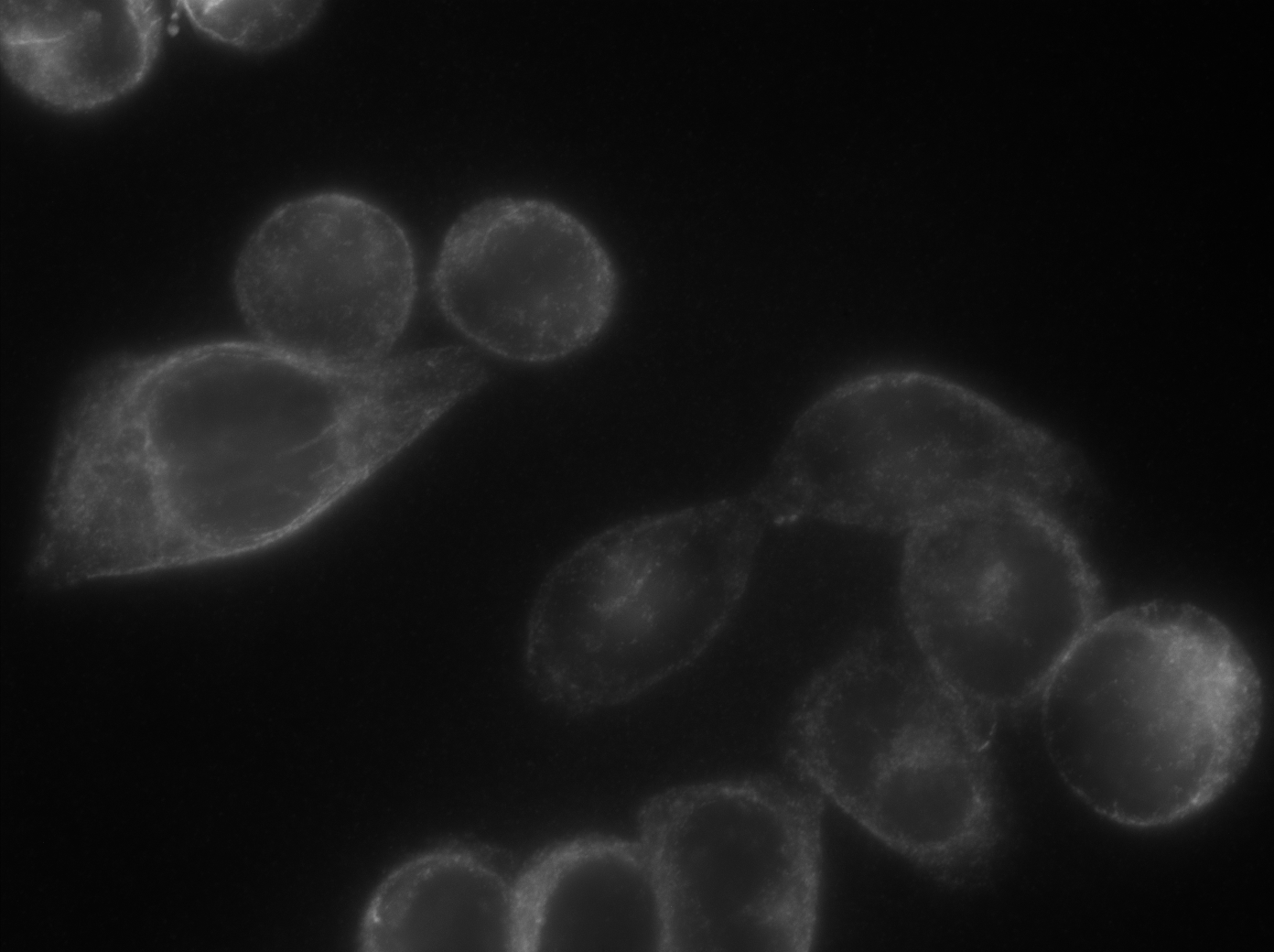

Supplement: Supplementary file 19 — Source data Fig. 5 part 5 [file 44319_2026_742_MOESM19_ESM.zip › Figure 5 Part 5/Fig 5ab WT and KO hela TTLL1-e326g atubulin part 2/TPGS1-KO/TPGS1-KO TTLL1-mut 10-15-24 R1 LT7.Project Maximum Z_XY1729023927_Z0_T0_C2.tif]

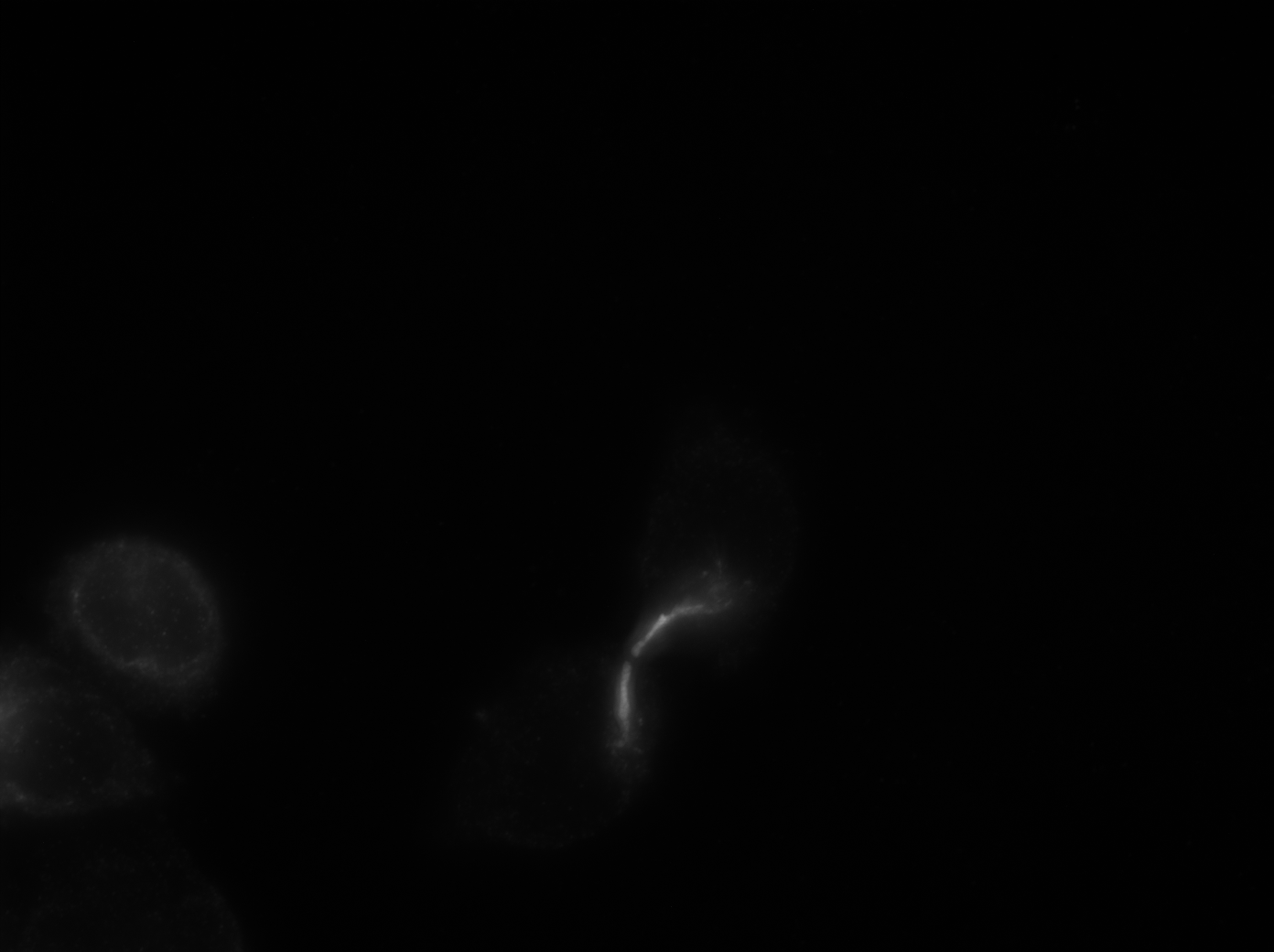

Supplement: Supplementary file 20 — Source data Fig. 6 part 1 [file 44319_2026_742_MOESM20_ESM.zip › Figure 6 Part 1/Fig 6abcd Cas9 TPGS1-KO acetylated tubulin atubulin/Cas9 R3 9-13-24 LT26.Project Maximum Z_XY1726767497_Z0_T0_C2.tif]

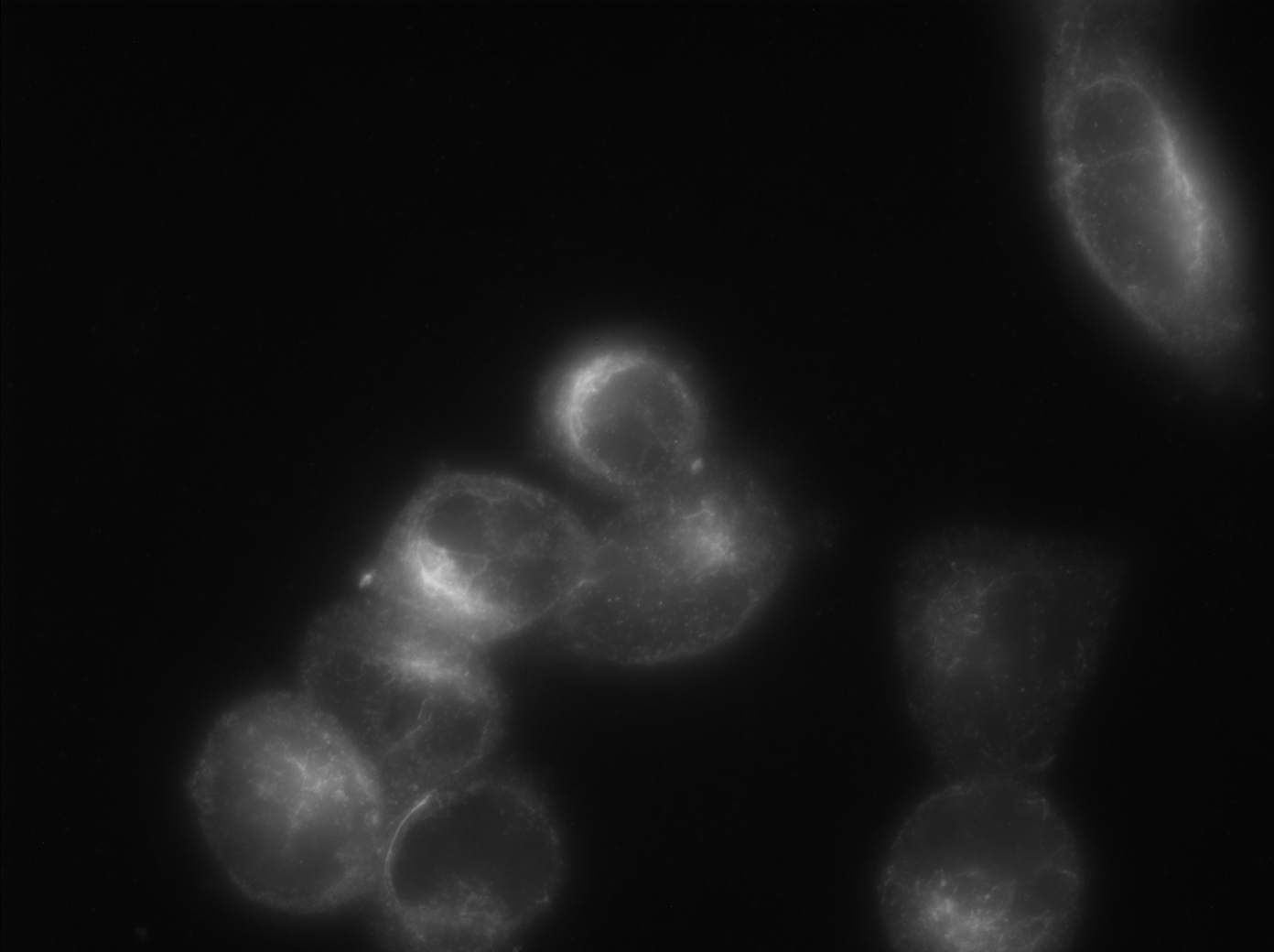

Supplement: Supplementary file 20 — Source data Fig. 6 part 1 [file 44319_2026_742_MOESM20_ESM.zip › Figure 6 Part 1/Fig 6abcd Cas9 TPGS1-KO acetylated tubulin atubulin/Cas9 R2 9-11-24 PA6PA7.Project Maximum Z_XY1726173793_Z0_T0_C2.tif]

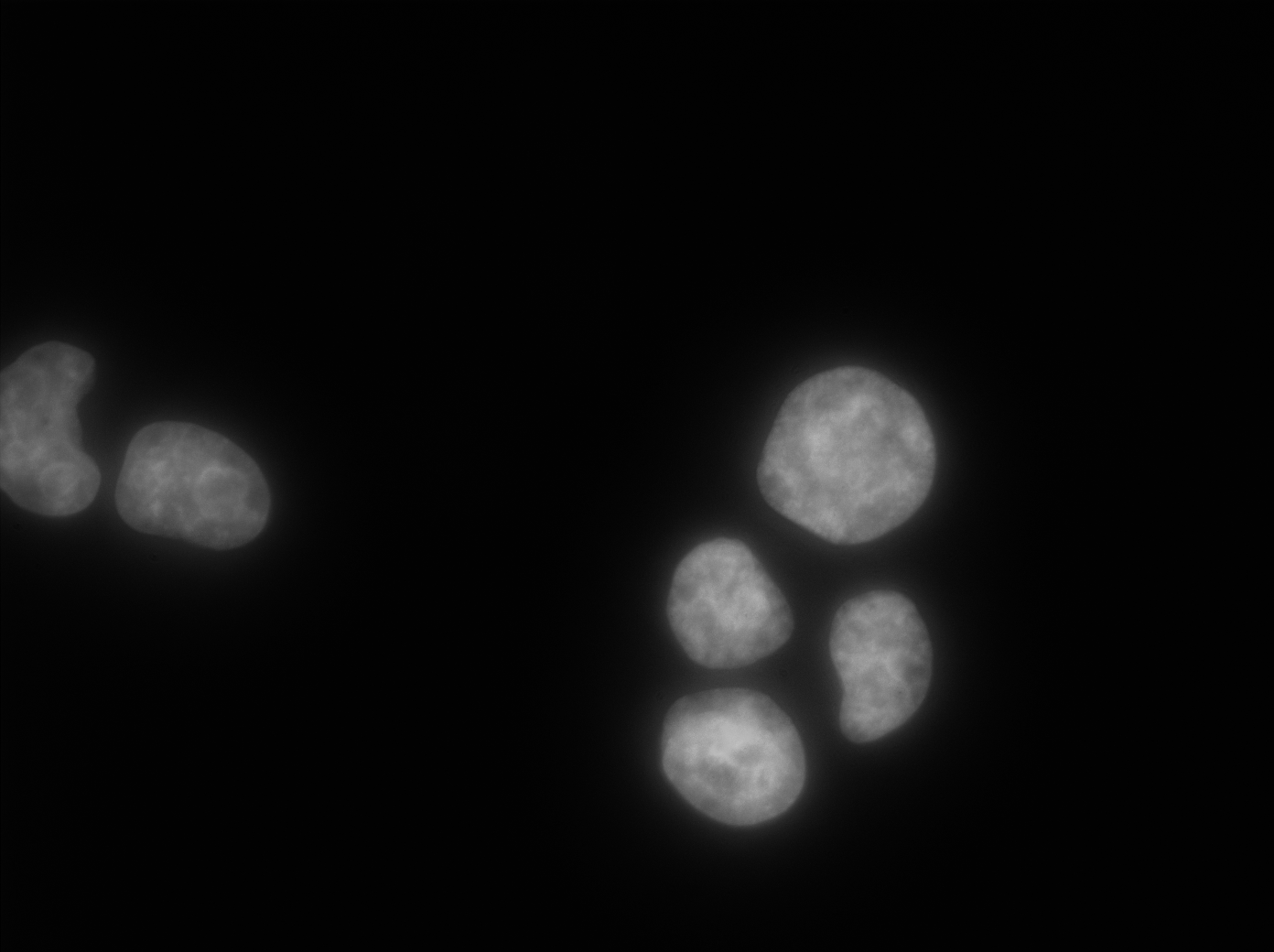

Supplement: Supplementary file 20 — Source data Fig. 6 part 1 [file 44319_2026_742_MOESM20_ESM.zip › Figure 6 Part 1/Fig 6abcd Cas9 TPGS1-KO acetylated tubulin atubulin/Cas9 R2 9-11-24 LT15.Project Maximum Z_XY1726174550_Z0_T0_C0.tif]

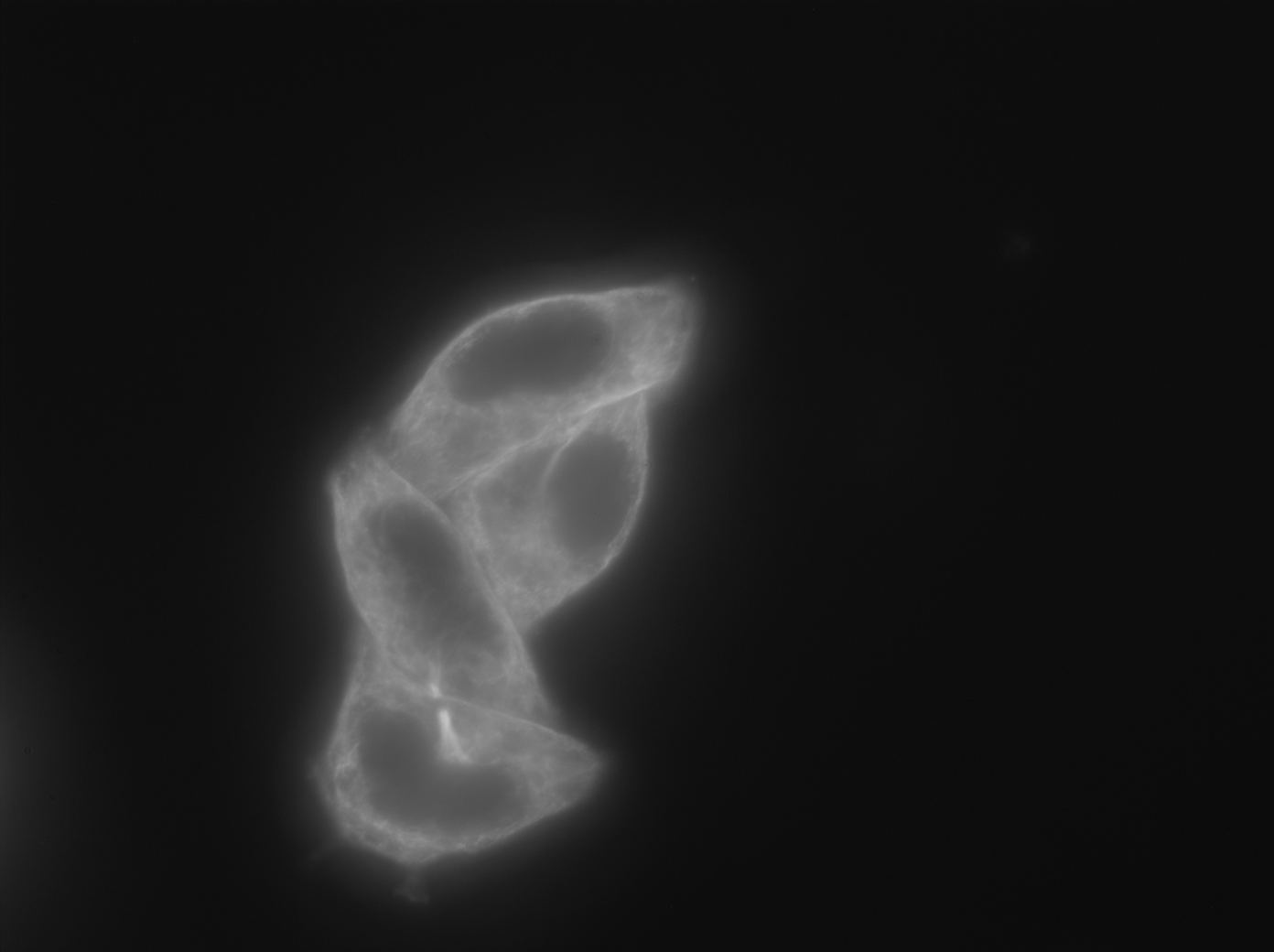

Supplement: Supplementary file 20 — Source data Fig. 6 part 1 [file 44319_2026_742_MOESM20_ESM.zip › Figure 6 Part 1/Fig 6abcd Cas9 TPGS1-KO acetylated tubulin atubulin/Cas9 R2 9-11-24 LT19 PA9.Project Maximum Z_XY1726178474_Z0_T0_C1.tif]

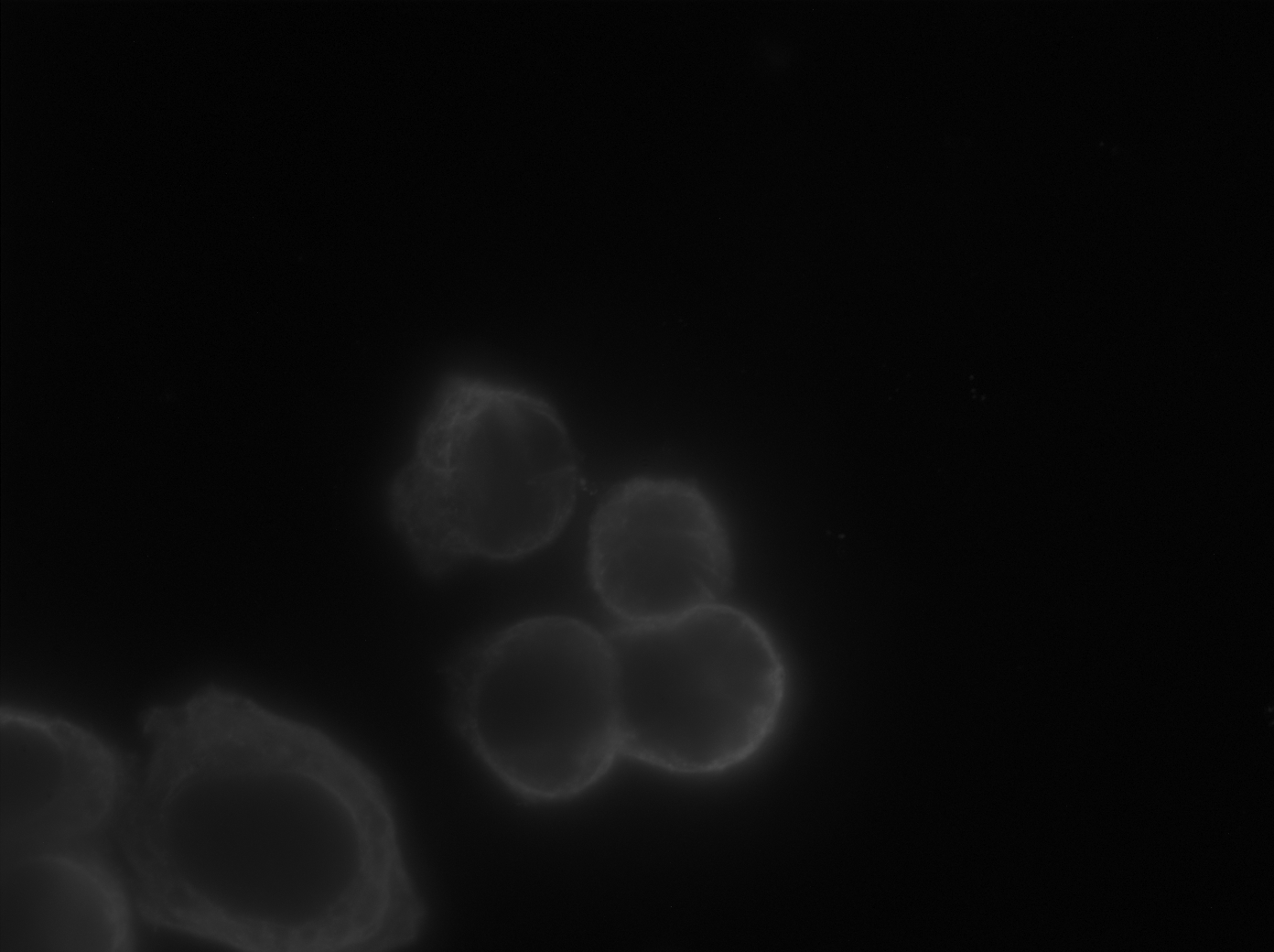

Supplement: Supplementary file 20 — Source data Fig. 6 part 1 [file 44319_2026_742_MOESM20_ESM.zip › Figure 6 Part 1/Fig 6abcd Cas9 TPGS1-KO acetylated tubulin atubulin/Cas9 R2 9-11-24 PA29.Project Maximum Z_XY1726181930_Z0_T0_C1.tif]

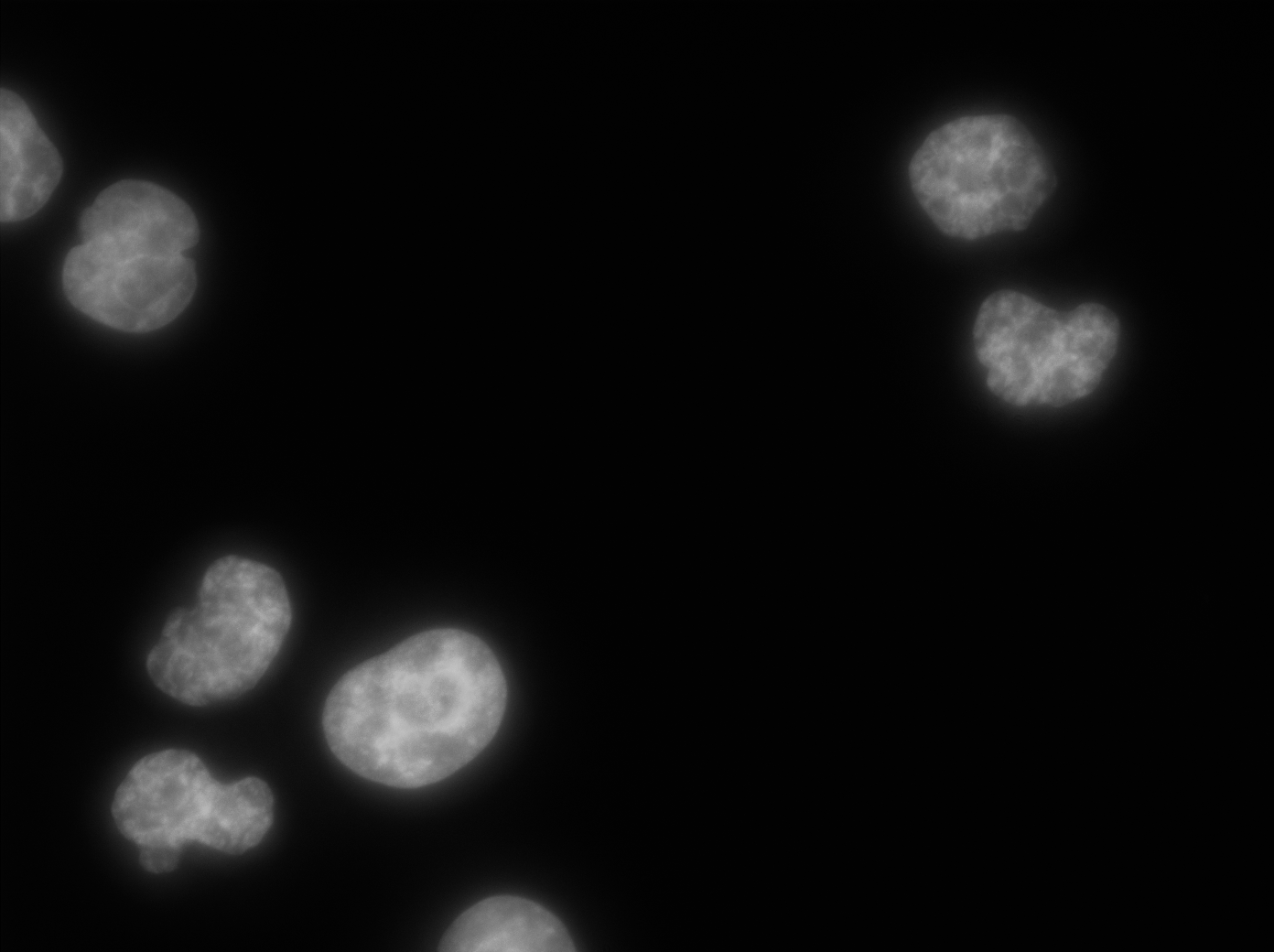

Supplement: Supplementary file 20 — Source data Fig. 6 part 1 [file 44319_2026_742_MOESM20_ESM.zip › Figure 6 Part 1/Fig 6abcd Cas9 TPGS1-KO acetylated tubulin atubulin/Cas9 R2 9-11-24 LT7LT8.Project Maximum Z_XY1726173377_Z0_T0_C0.tif]

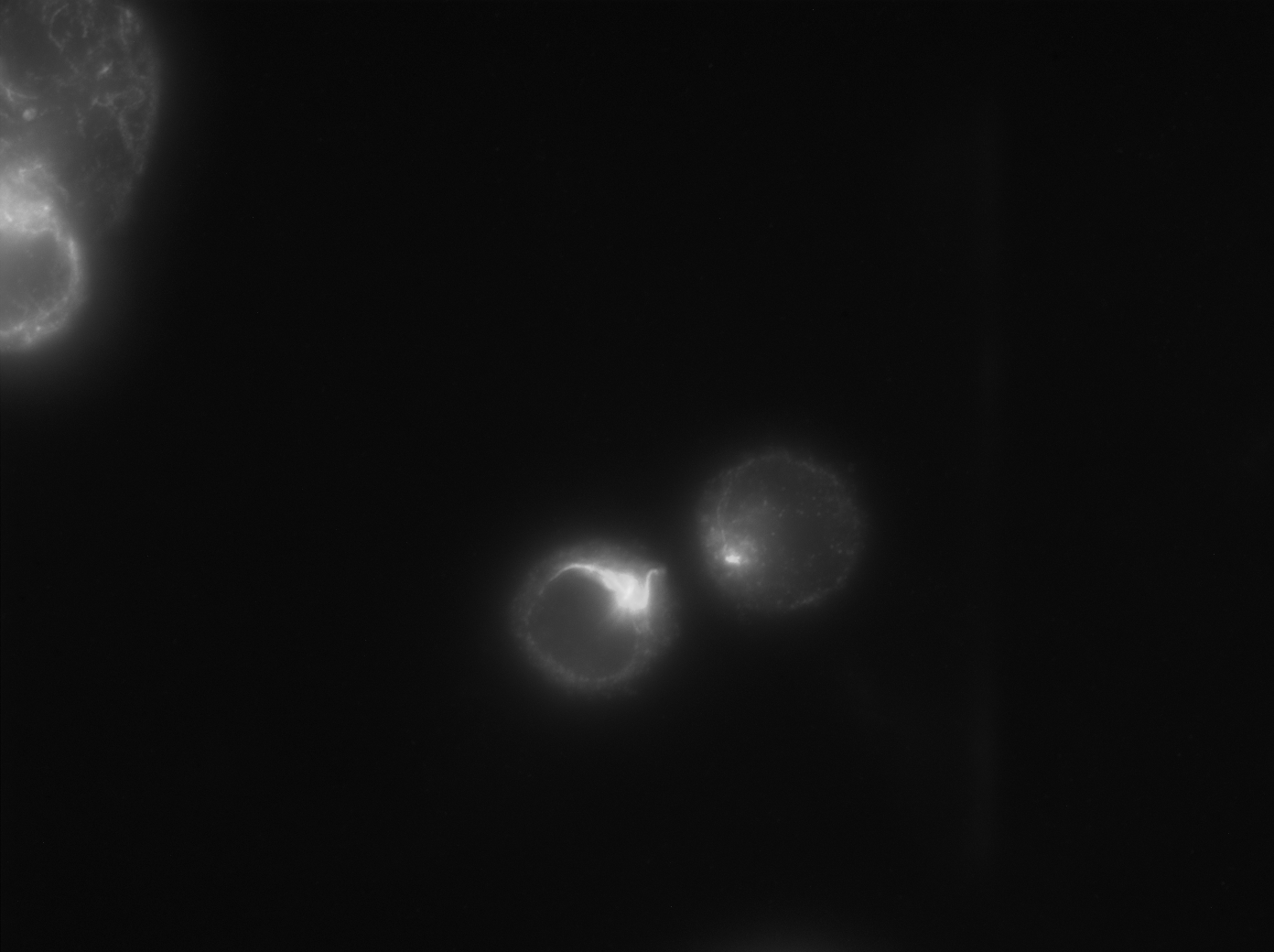

Supplement: Supplementary file 20 — Source data Fig. 6 part 1 [file 44319_2026_742_MOESM20_ESM.zip › Figure 6 Part 1/Fig 6abcd Cas9 TPGS1-KO acetylated tubulin atubulin/Cas9 R2 9-11-24 PA8.Project Maximum Z_XY1726178188_Z0_T0_C2.tif]

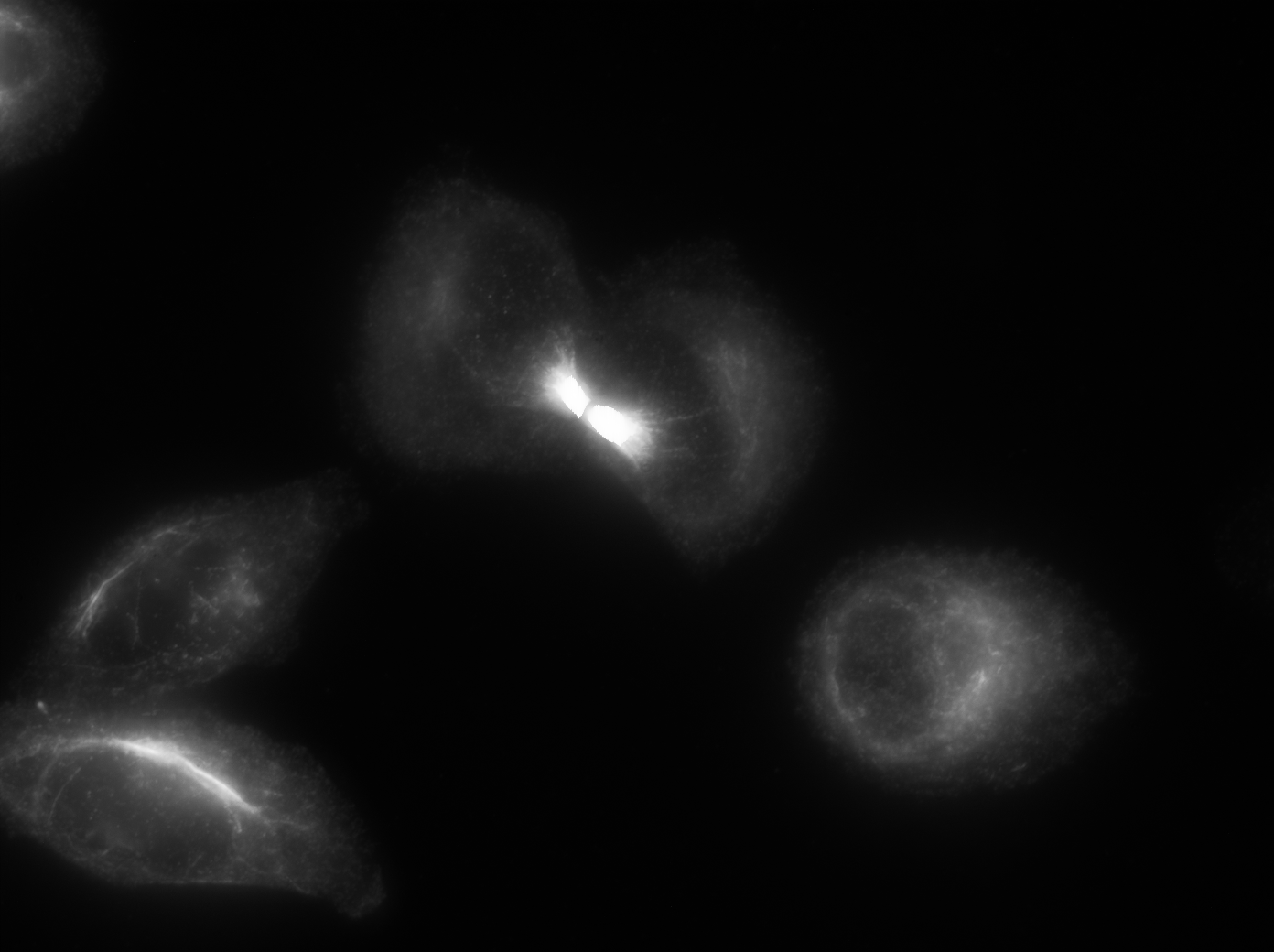

Supplement: Supplementary file 20 — Source data Fig. 6 part 1 [file 44319_2026_742_MOESM20_ESM.zip › Figure 6 Part 1/Fig 6abcd Cas9 TPGS1-KO acetylated tubulin atubulin/Cas9 R3 9-13-24 LT1.Project Maximum Z_XY1726765271_Z0_T0_C2.tif]

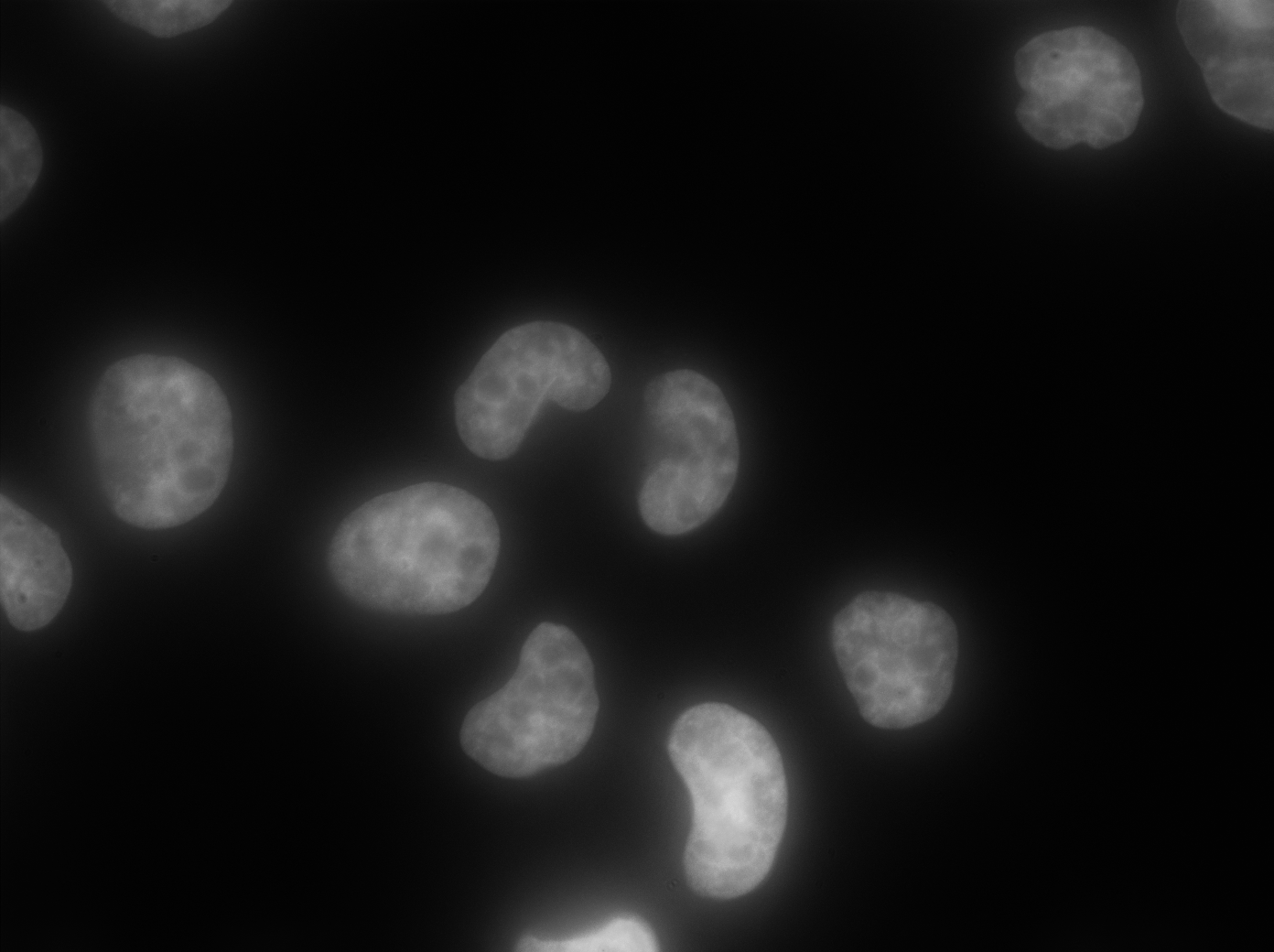

Supplement: Supplementary file 20 — Source data Fig. 6 part 1 [file 44319_2026_742_MOESM20_ESM.zip › Figure 6 Part 1/Fig 6abcd Cas9 TPGS1-KO acetylated tubulin atubulin/Cas9 R2 9-11-24 PA30.Project Maximum Z_XY1726182071_Z0_T0_C0.tif]

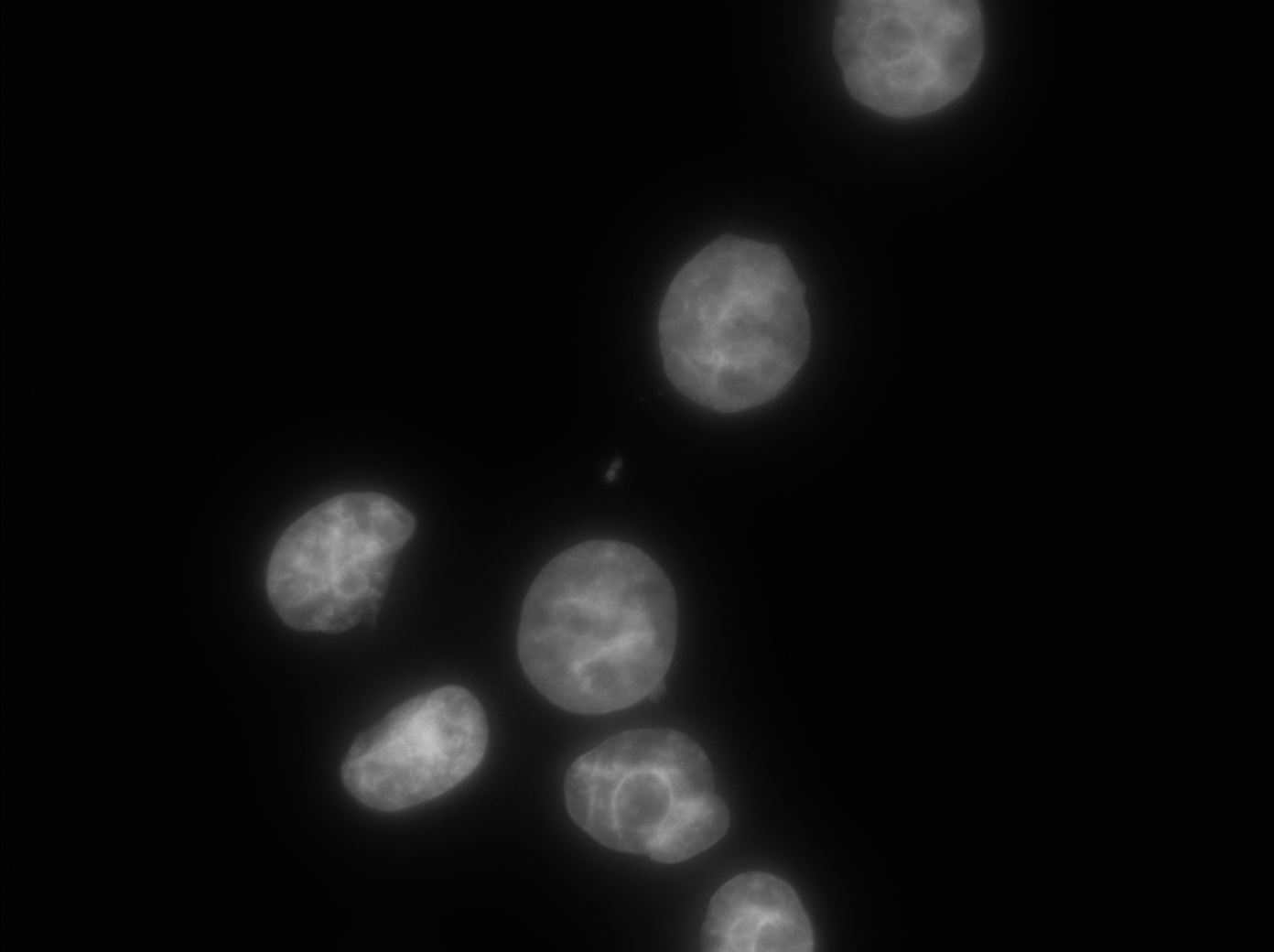

Supplement: Supplementary file 20 — Source data Fig. 6 part 1 [file 44319_2026_742_MOESM20_ESM.zip › Figure 6 Part 1/Fig 6abcd Cas9 TPGS1-KO acetylated tubulin atubulin/Cas9 R2 9-11-24 LT6.Project Maximum Z_XY1726173281_Z0_T0_C0.tif]

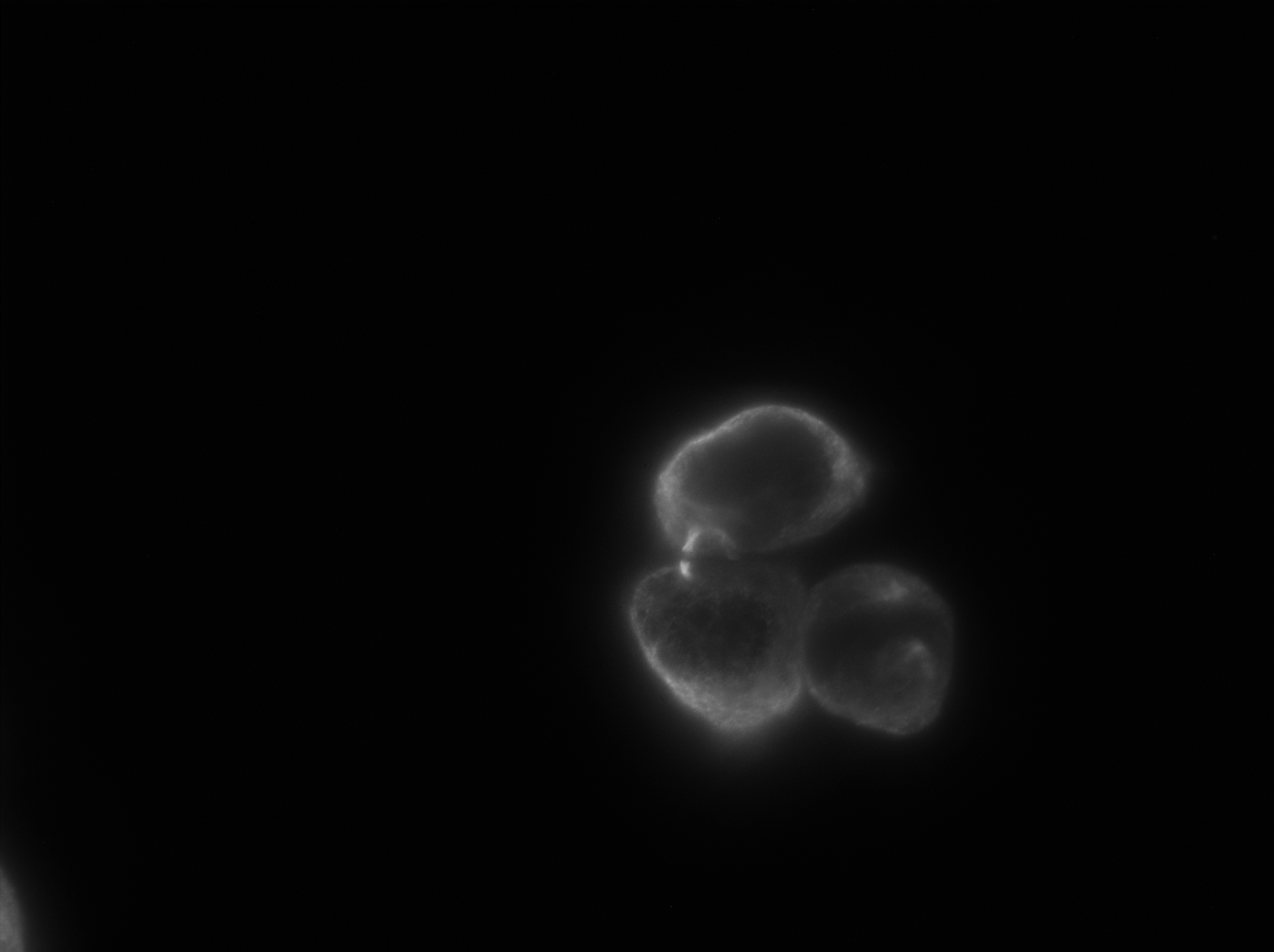

Supplement: Supplementary file 20 — Source data Fig. 6 part 1 [file 44319_2026_742_MOESM20_ESM.zip › Figure 6 Part 1/Fig 6abcd Cas9 TPGS1-KO acetylated tubulin atubulin/Cas9 R3 9-13-24 LT20.Project Maximum Z_XY1726766906_Z0_T0_C1.tif]

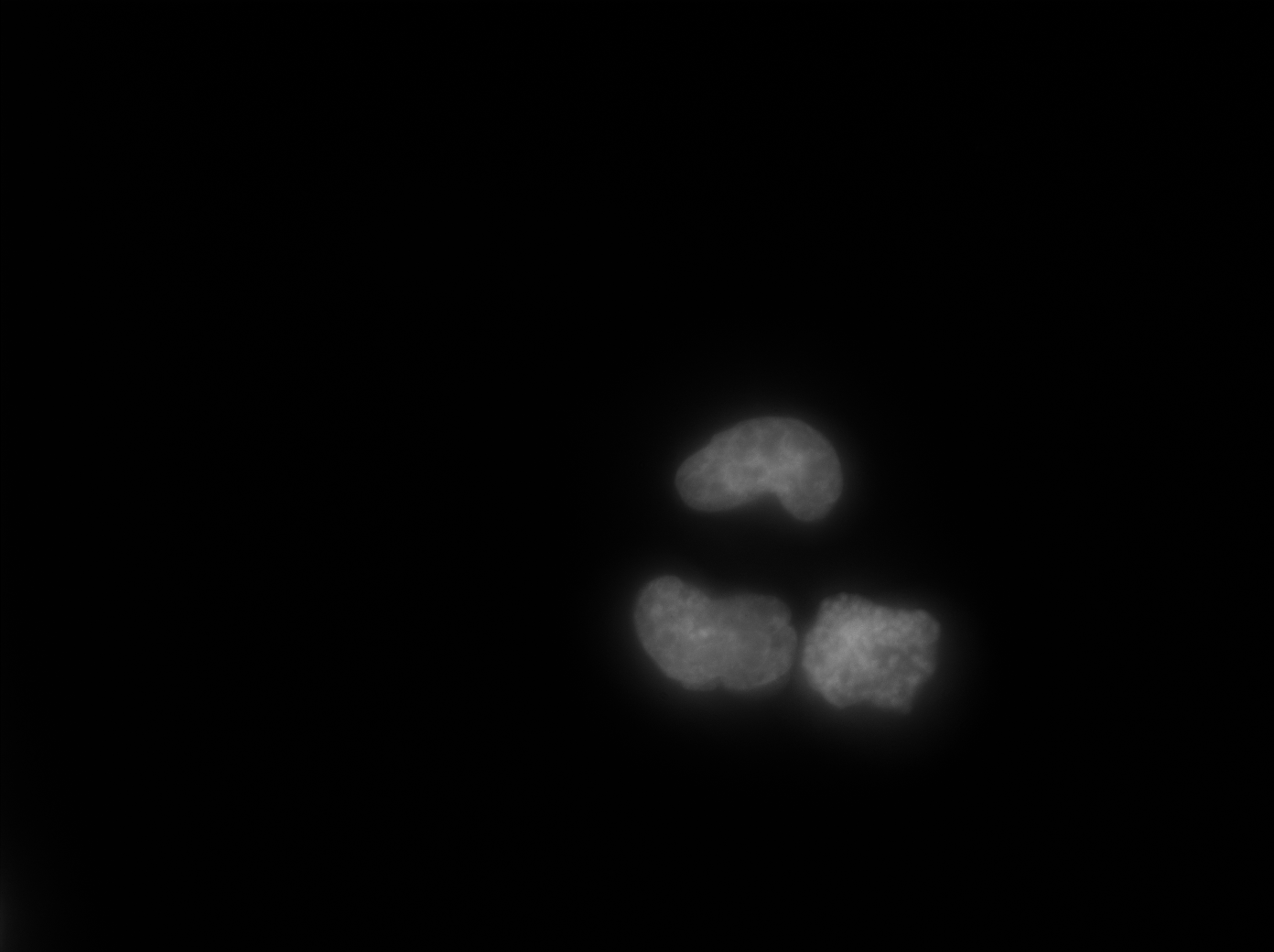

Supplement: Supplementary file 20 — Source data Fig. 6 part 1 [file 44319_2026_742_MOESM20_ESM.zip › Figure 6 Part 1/Fig 6abcd Cas9 TPGS1-KO acetylated tubulin atubulin/Cas9 R3 9-13-24 LT20.Project Maximum Z_XY1726766906_Z0_T0_C0.tif]

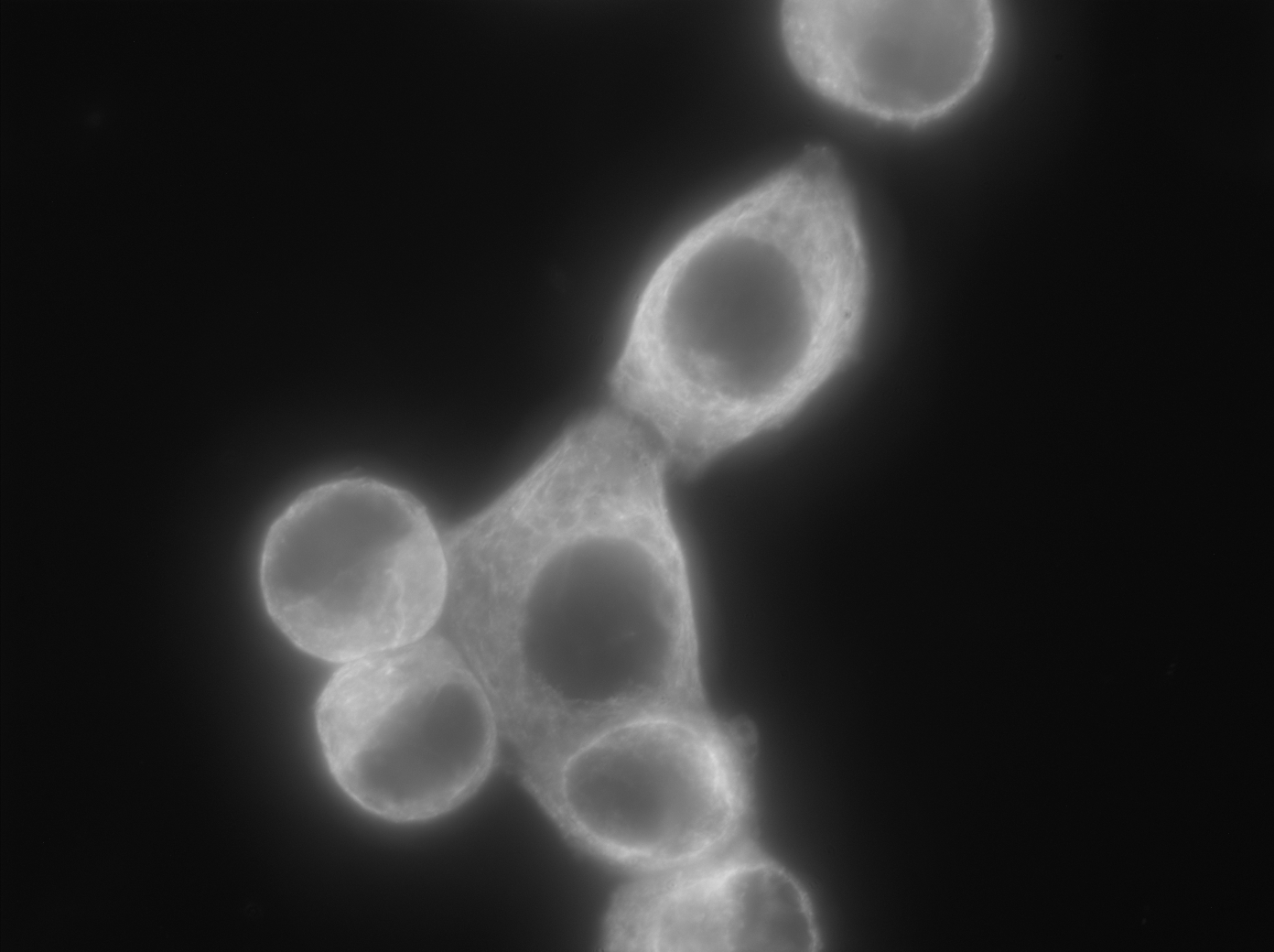

Supplement: Supplementary file 20 — Source data Fig. 6 part 1 [file 44319_2026_742_MOESM20_ESM.zip › Figure 6 Part 1/Fig 6abcd Cas9 TPGS1-KO acetylated tubulin atubulin/Cas9 R2 9-11-24 LT6.Project Maximum Z_XY1726173281_Z0_T0_C1.tif]

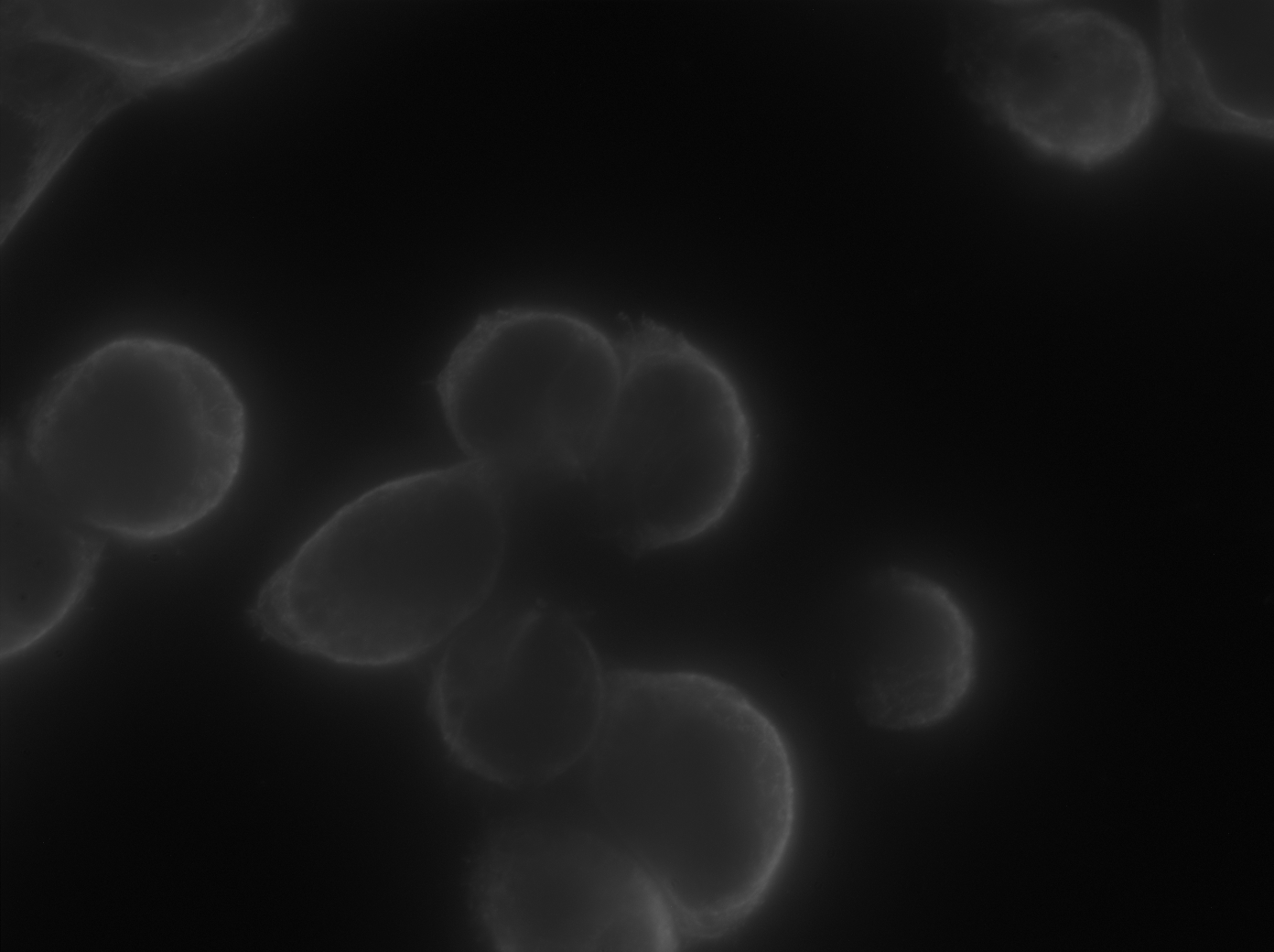

Supplement: Supplementary file 20 — Source data Fig. 6 part 1 [file 44319_2026_742_MOESM20_ESM.zip › Figure 6 Part 1/Fig 6abcd Cas9 TPGS1-KO acetylated tubulin atubulin/Cas9 R2 9-11-24 PA30.Project Maximum Z_XY1726182071_Z0_T0_C1.tif]
