# Supplementary figures and images for: TPGS1 regulates central spindle microtubule glutamylation and remodeling during telophase and abscission (part 23 of 36)
Source: EMBO Rep. 2026 Mar 23;27(8):1944–63. doi: 10.1038/s44319-026-00742-3 (PMC13121839; doi:10.1038/s44319-026-00742-3)

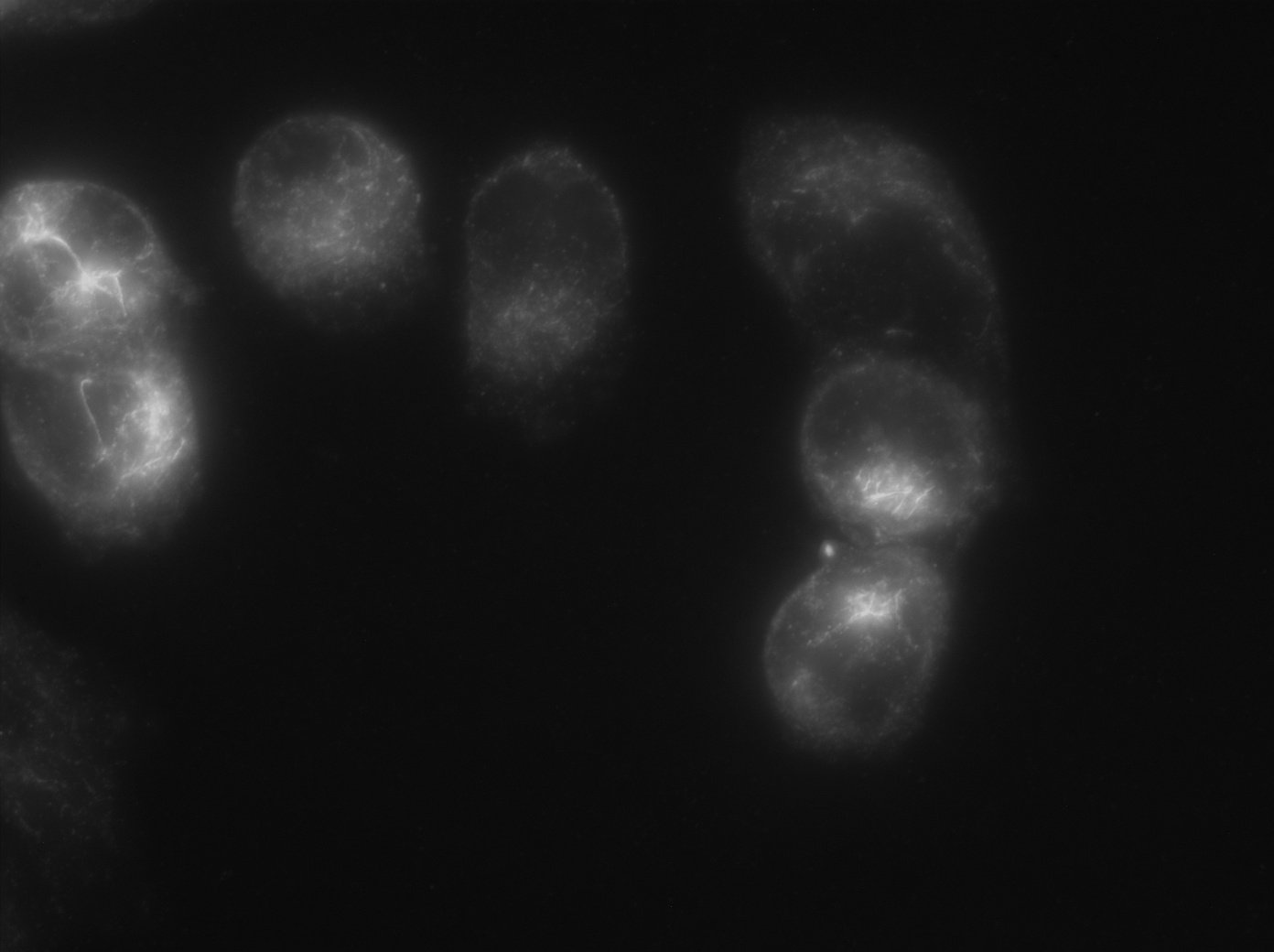

Supplement: Supplementary file 20 — Source data Fig. 6 part 1 [file 44319_2026_742_MOESM20_ESM.zip › Figure 6 Part 1/Fig 6abcd Cas9 TPGS1-KO acetylated tubulin atubulin/Cas9 R2 9-11-24 PA19.Project Maximum Z_XY1726180240_Z0_T0_C2.tif]

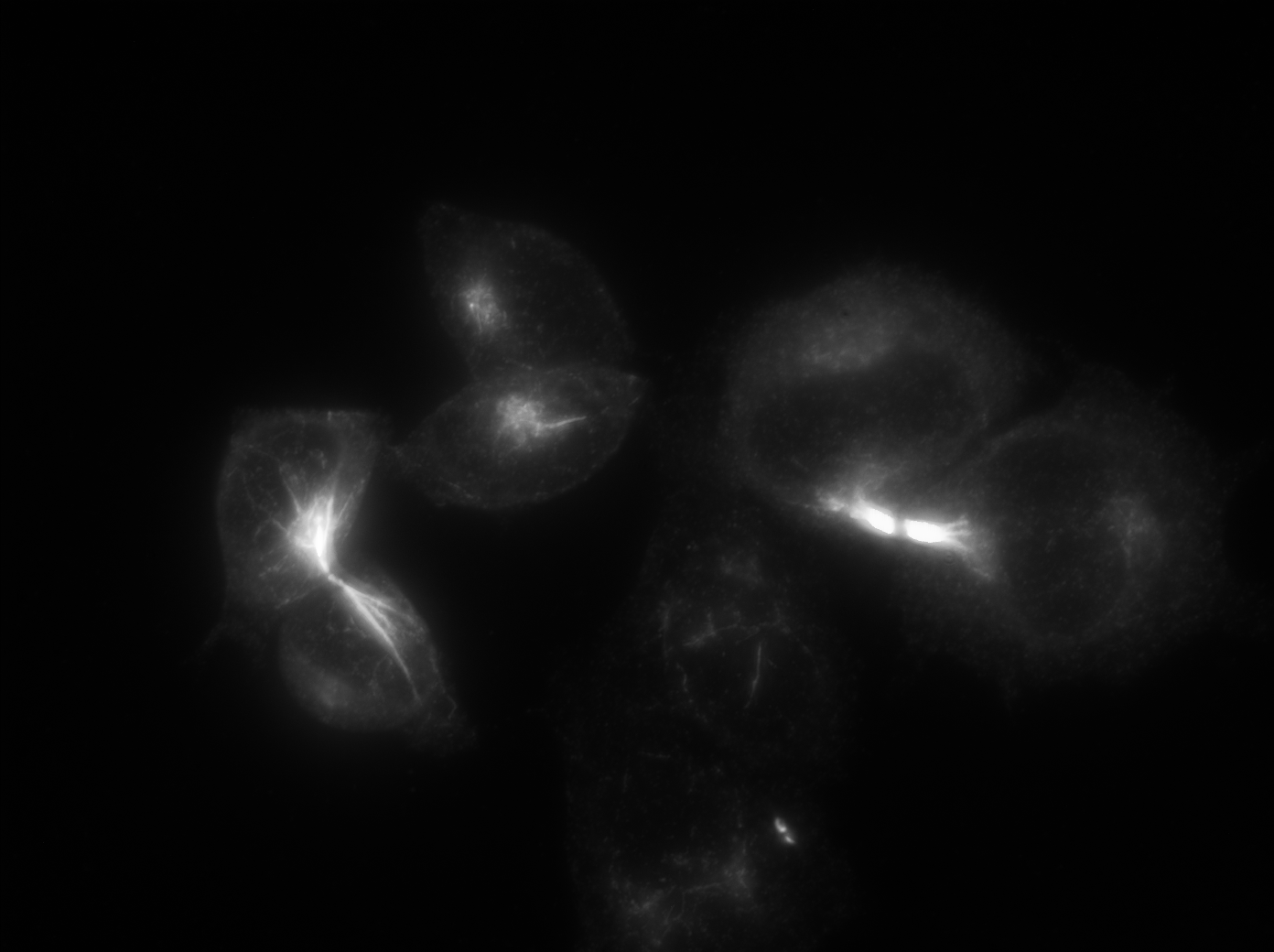

Supplement: Supplementary file 20 — Source data Fig. 6 part 1 [file 44319_2026_742_MOESM20_ESM.zip › Figure 6 Part 1/Fig 6abcd Cas9 TPGS1-KO acetylated tubulin atubulin/Cas9 R3 9-13-24 LT8LT9.Project Maximum Z_XY1726765747_Z0_T0_C2.tif]

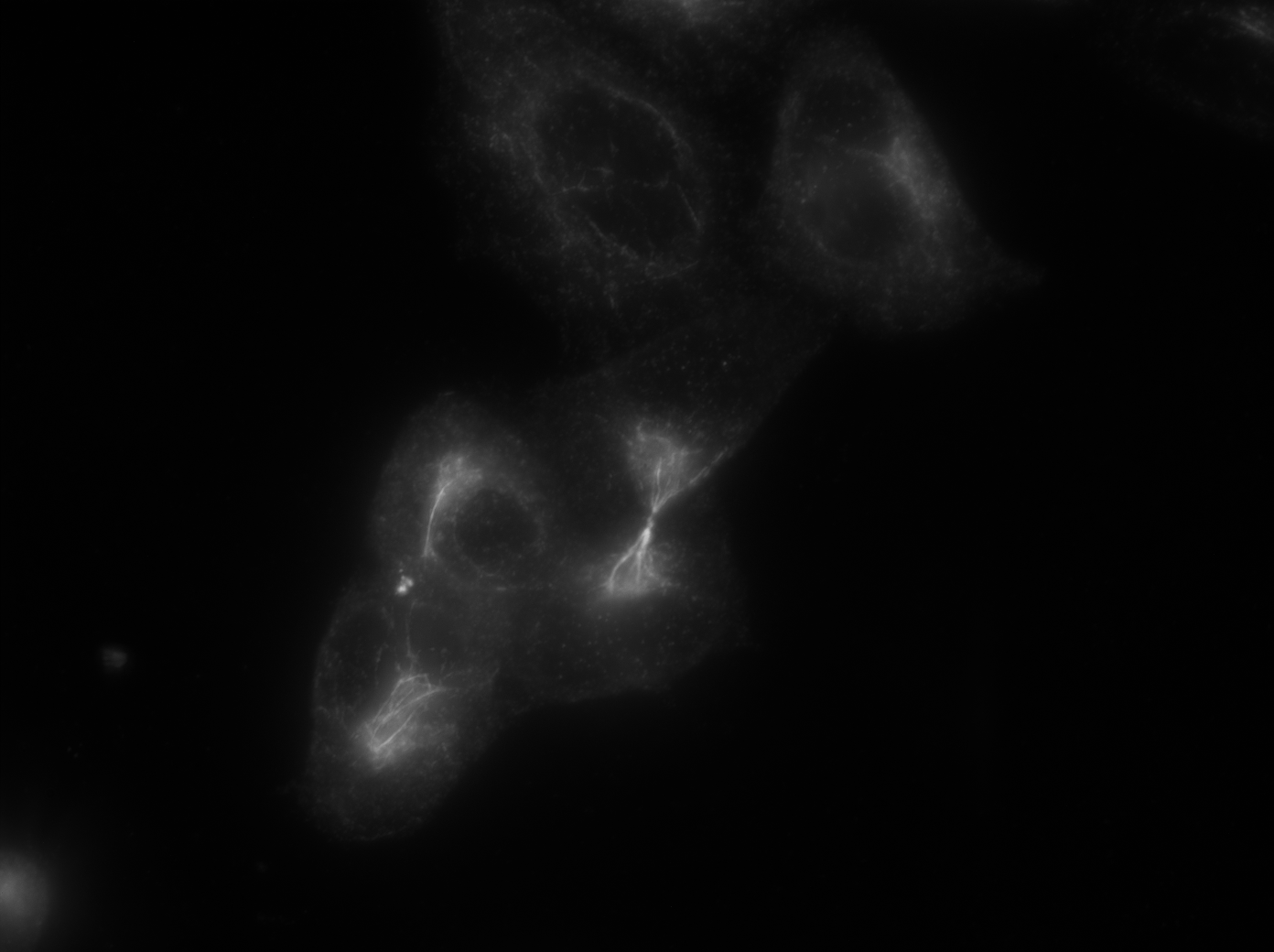

Supplement: Supplementary file 20 — Source data Fig. 6 part 1 [file 44319_2026_742_MOESM20_ESM.zip › Figure 6 Part 1/Fig 6abcd Cas9 TPGS1-KO acetylated tubulin atubulin/Cas9 R3 9-13-24 LT13.Project Maximum Z_XY1726766091_Z0_T0_C2.tif]

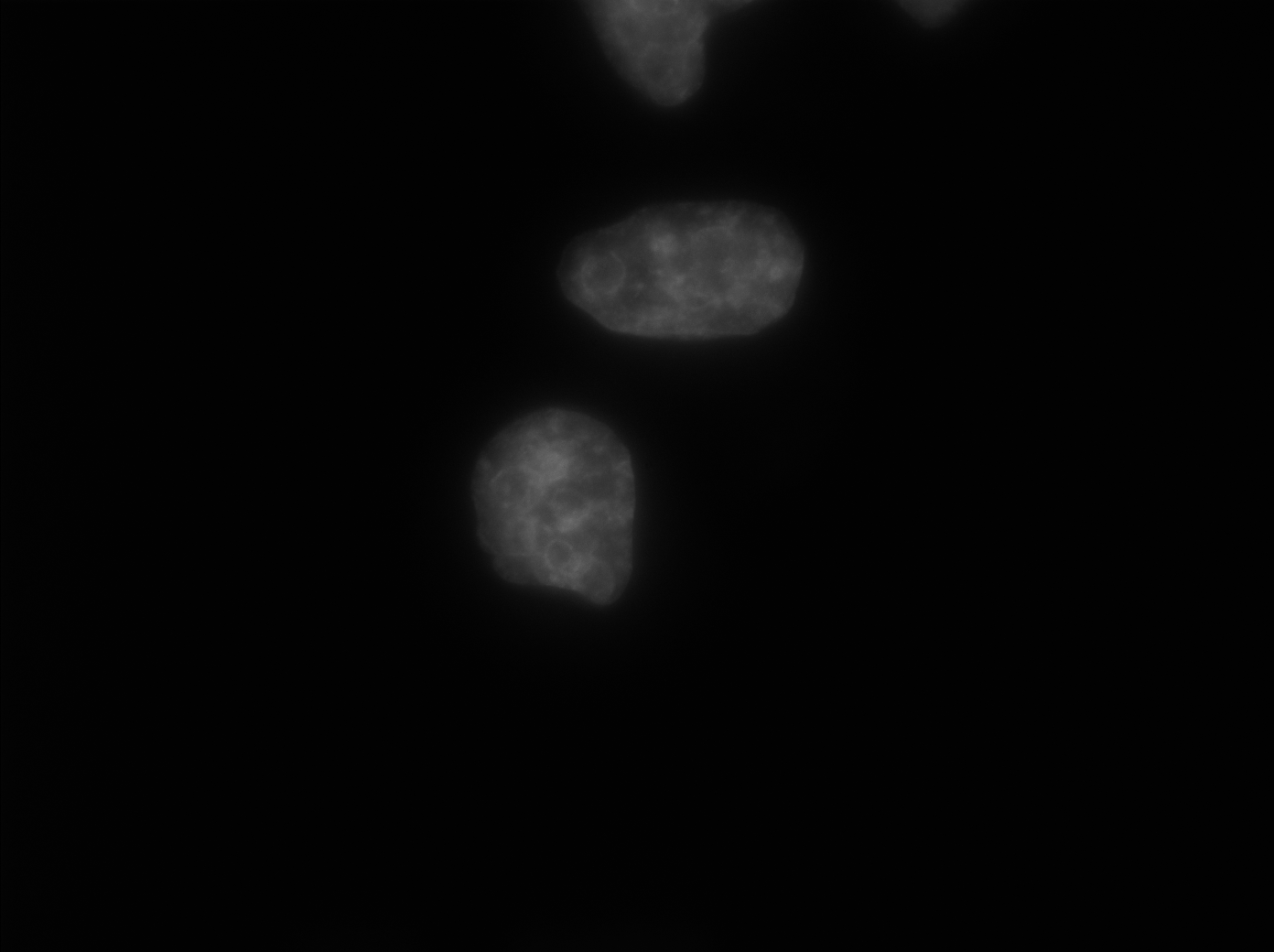

Supplement: Supplementary file 20 — Source data Fig. 6 part 1 [file 44319_2026_742_MOESM20_ESM.zip › Figure 6 Part 1/Fig 6abcd Cas9 TPGS1-KO acetylated tubulin atubulin/Cas9 R2 9-11-24 LT26.Project Maximum Z_XY1726180996_Z0_T0_C0.tif]

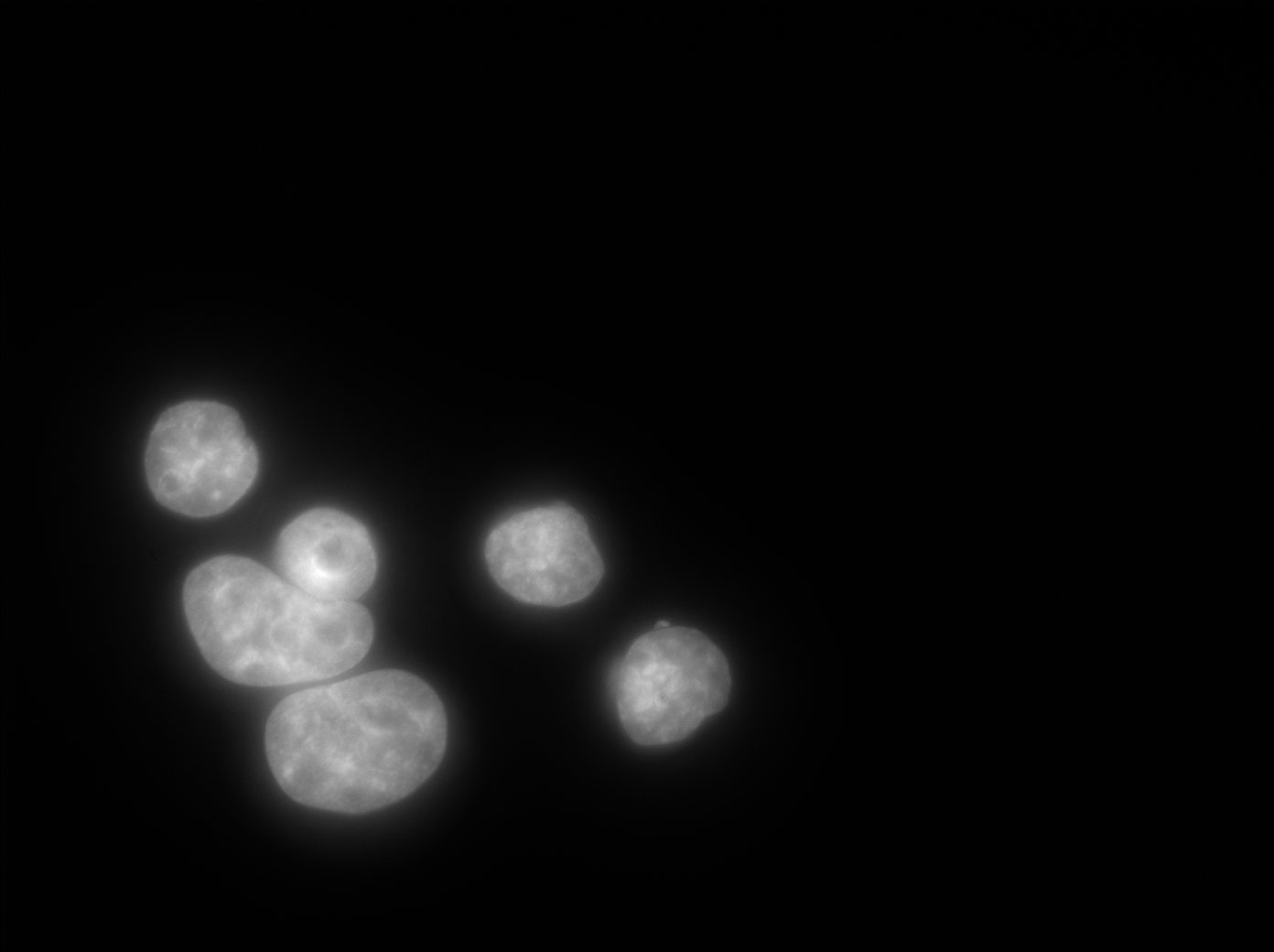

Supplement: Supplementary file 20 — Source data Fig. 6 part 1 [file 44319_2026_742_MOESM20_ESM.zip › Figure 6 Part 1/Fig 6abcd Cas9 TPGS1-KO acetylated tubulin atubulin/Cas9 R3 9-13-24 LT18.Project Maximum Z_XY1726766620_Z0_T0_C0.tif]

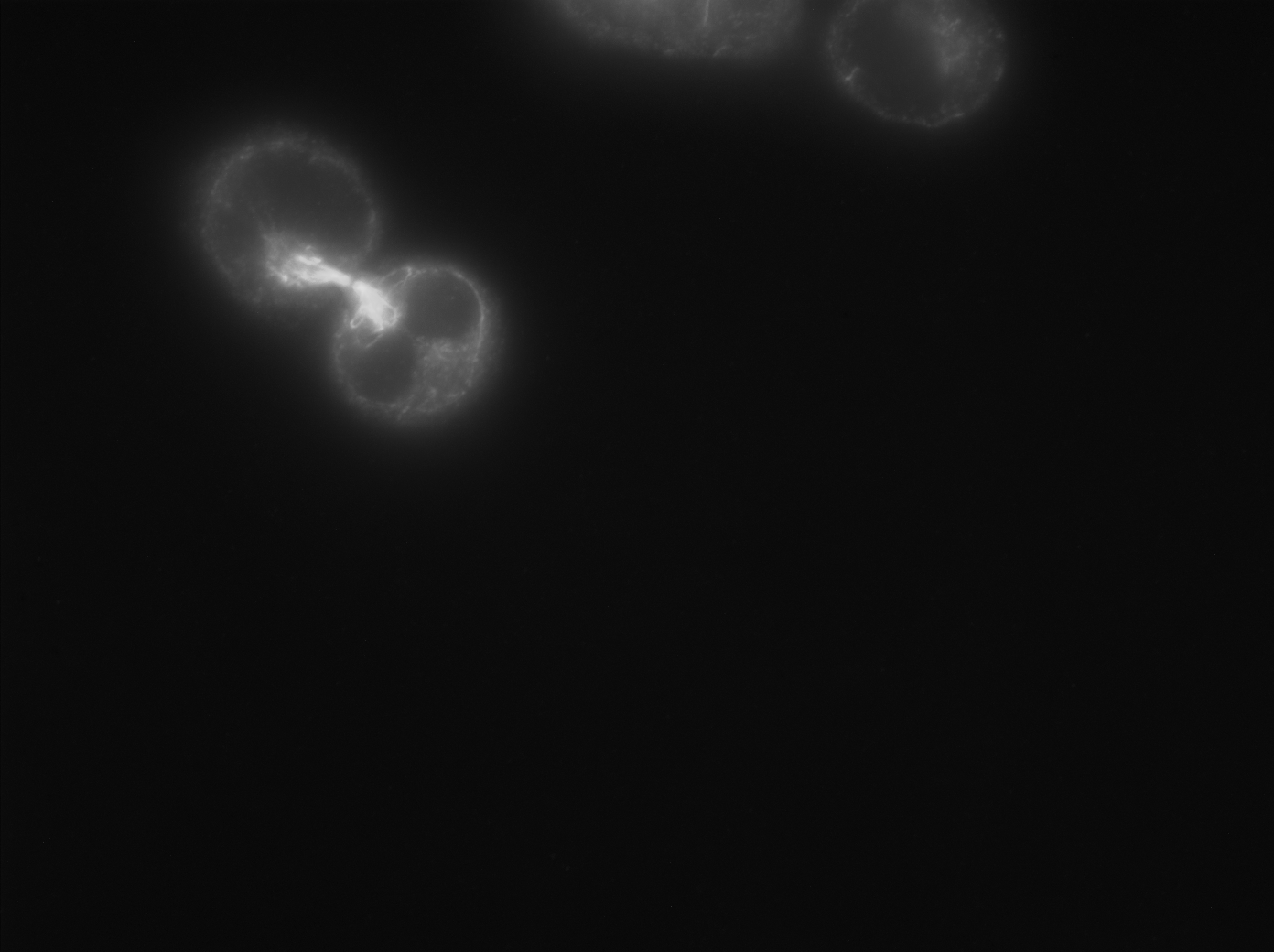

Supplement: Supplementary file 20 — Source data Fig. 6 part 1 [file 44319_2026_742_MOESM20_ESM.zip › Figure 6 Part 1/Fig 6abcd Cas9 TPGS1-KO acetylated tubulin atubulin/Cas9 R2 9-11-24 LT20.Project Maximum Z_XY1726178865_Z0_T0_C2.tif]

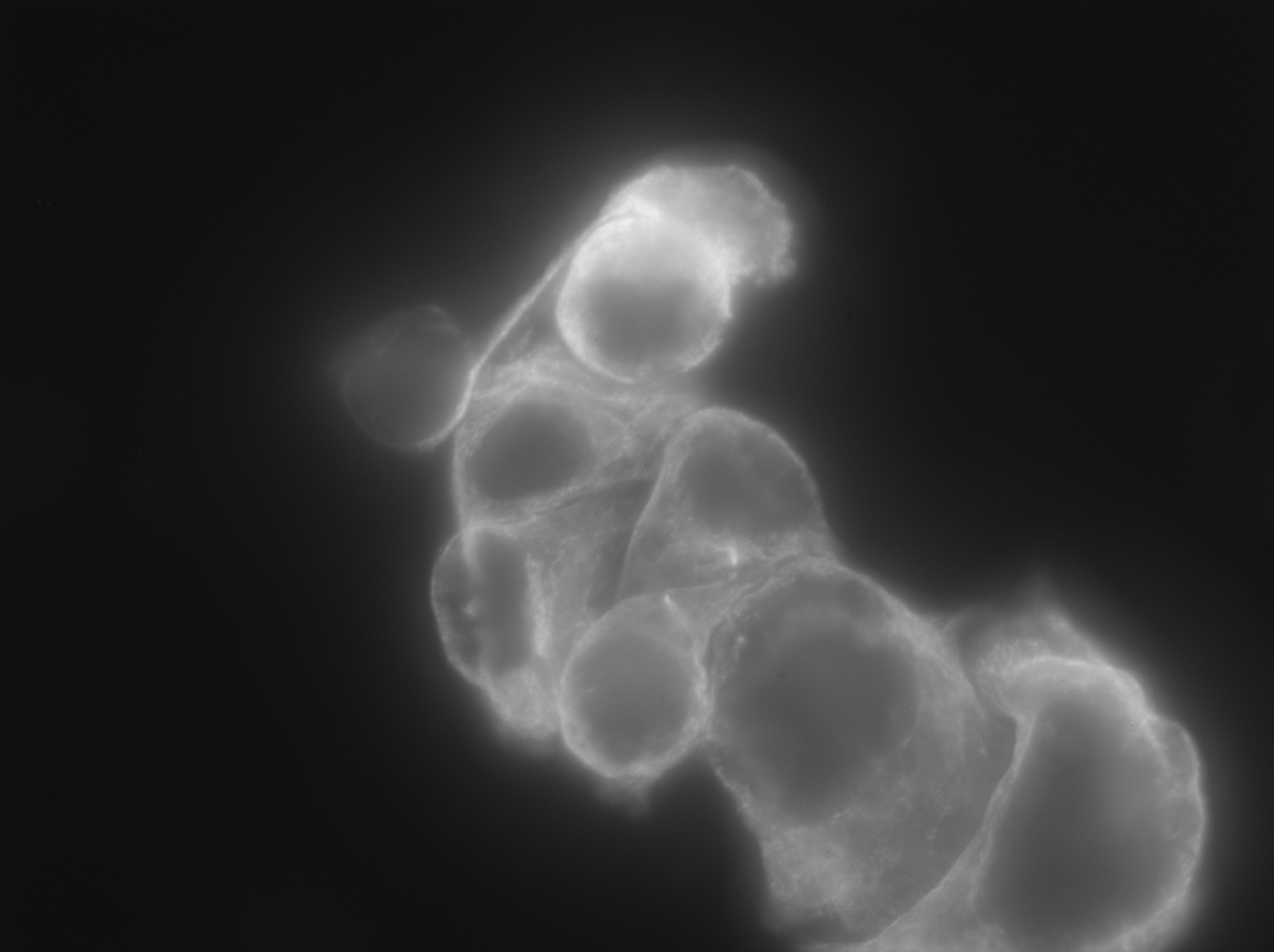

Supplement: Supplementary file 20 — Source data Fig. 6 part 1 [file 44319_2026_742_MOESM20_ESM.zip › Figure 6 Part 1/Fig 6abcd Cas9 TPGS1-KO acetylated tubulin atubulin/Cas9 R2 9-11-24 PA11.Project Maximum Z_XY1726179132_Z0_T0_C1.tif]

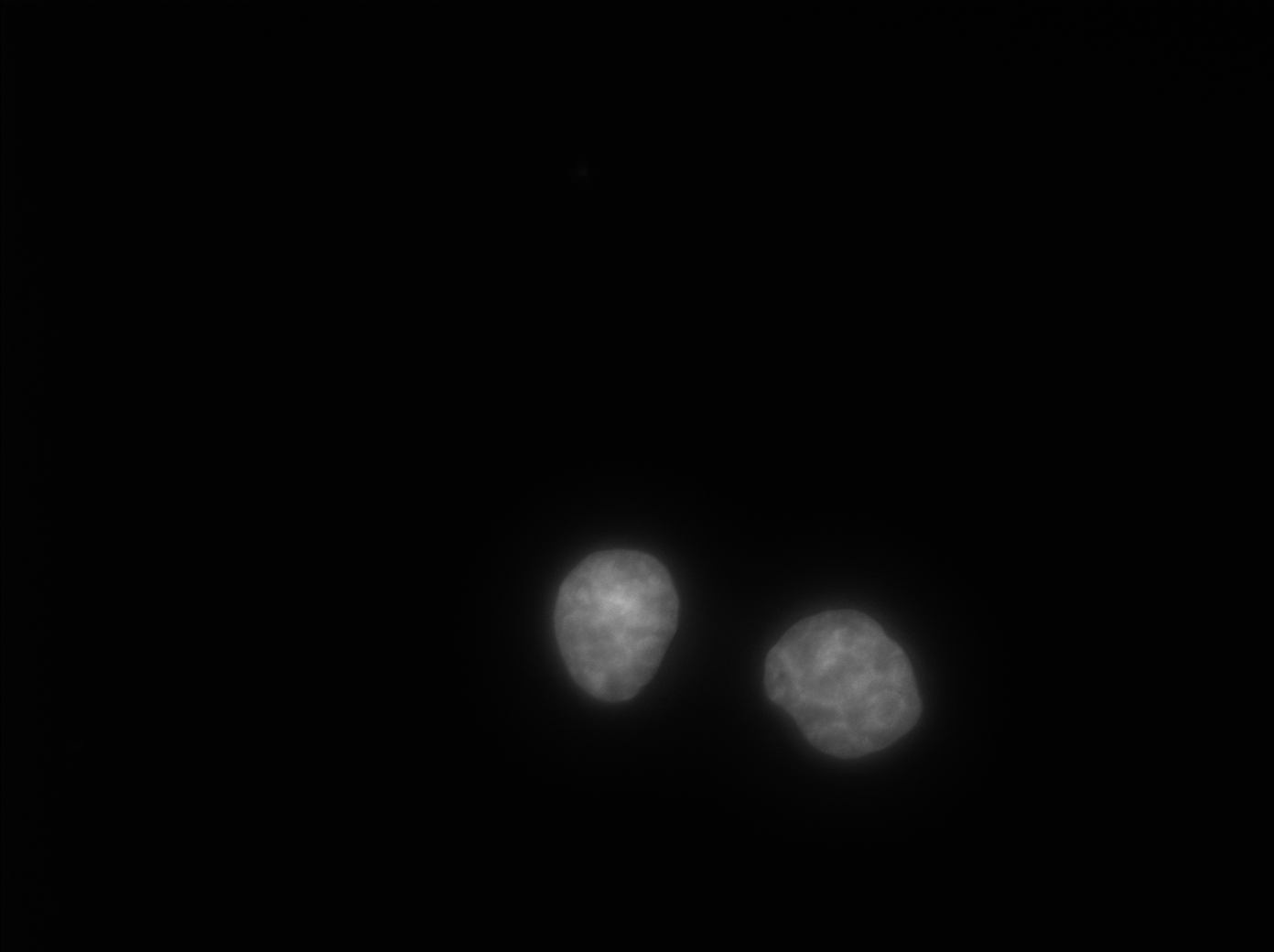

Supplement: Supplementary file 20 — Source data Fig. 6 part 1 [file 44319_2026_742_MOESM20_ESM.zip › Figure 6 Part 1/Fig 6abcd Cas9 TPGS1-KO acetylated tubulin atubulin/Cas9 R2 9-11-24 PA5.Project Maximum Z_XY1726173656_Z0_T0_C0.tif]

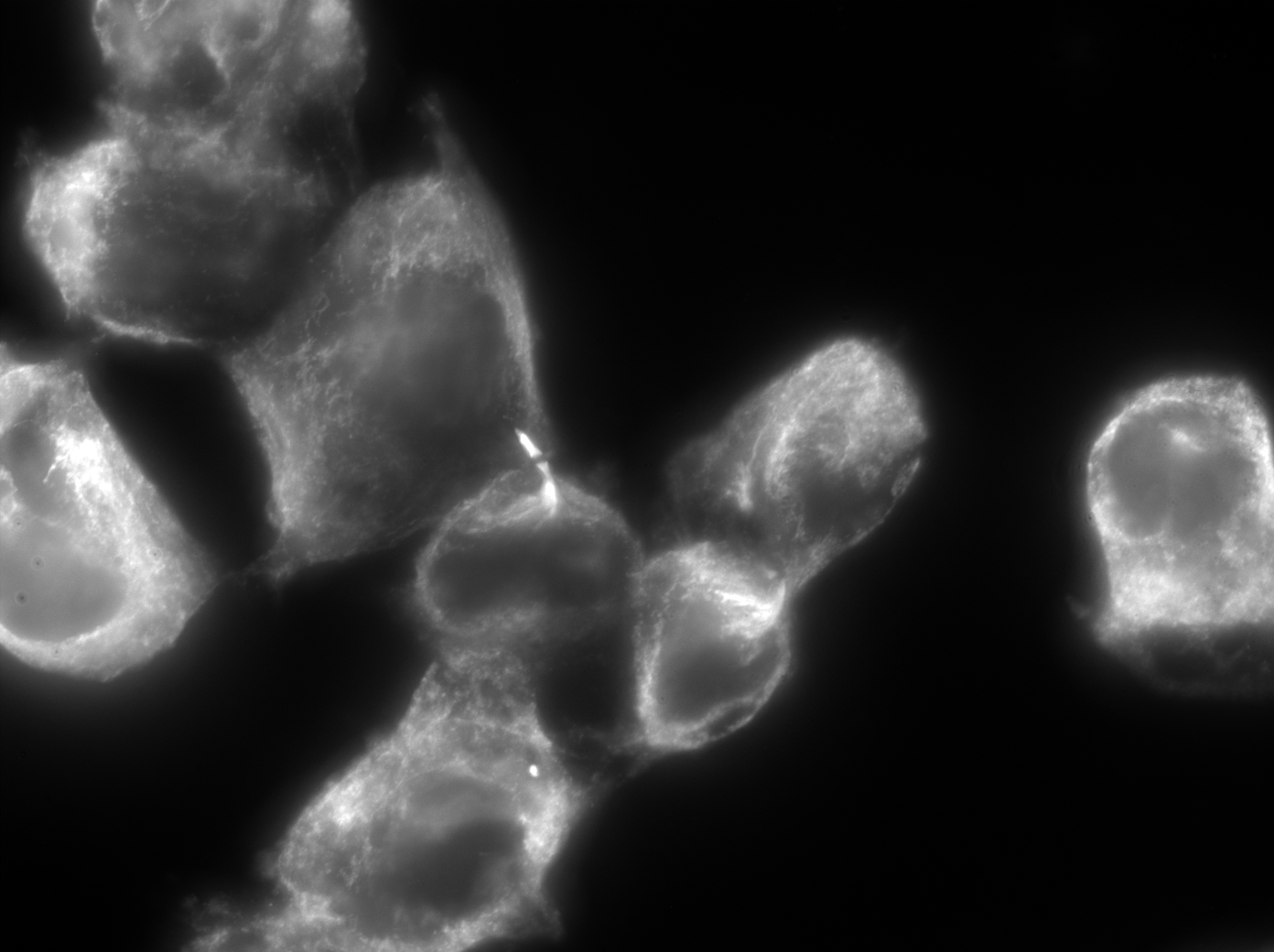

Supplement: Supplementary file 20 — Source data Fig. 6 part 1 [file 44319_2026_742_MOESM20_ESM.zip › Figure 6 Part 1/Fig 6abcd Cas9 TPGS1-KO acetylated tubulin atubulin/Cas9 R2 9-11-24 LT24 PA21.Project Maximum Z_XY1726180572_Z0_T0_C1.tif]

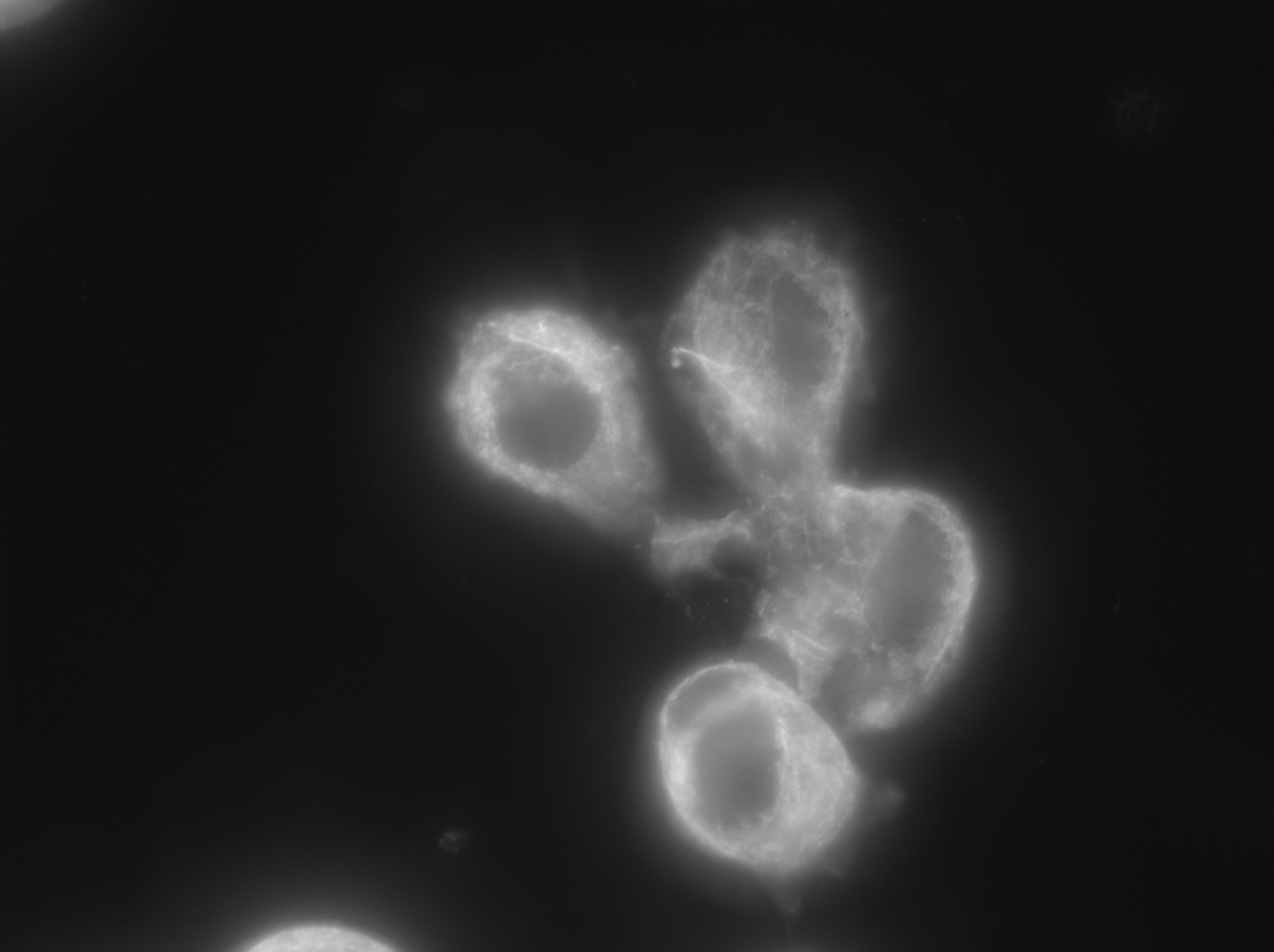

Supplement: Supplementary file 20 — Source data Fig. 6 part 1 [file 44319_2026_742_MOESM20_ESM.zip › Figure 6 Part 1/Fig 6abcd Cas9 TPGS1-KO acetylated tubulin atubulin/Cas9 R2 9-11-24 PA25PA26.Project Maximum Z_XY1726181279_Z0_T0_C1.tif]

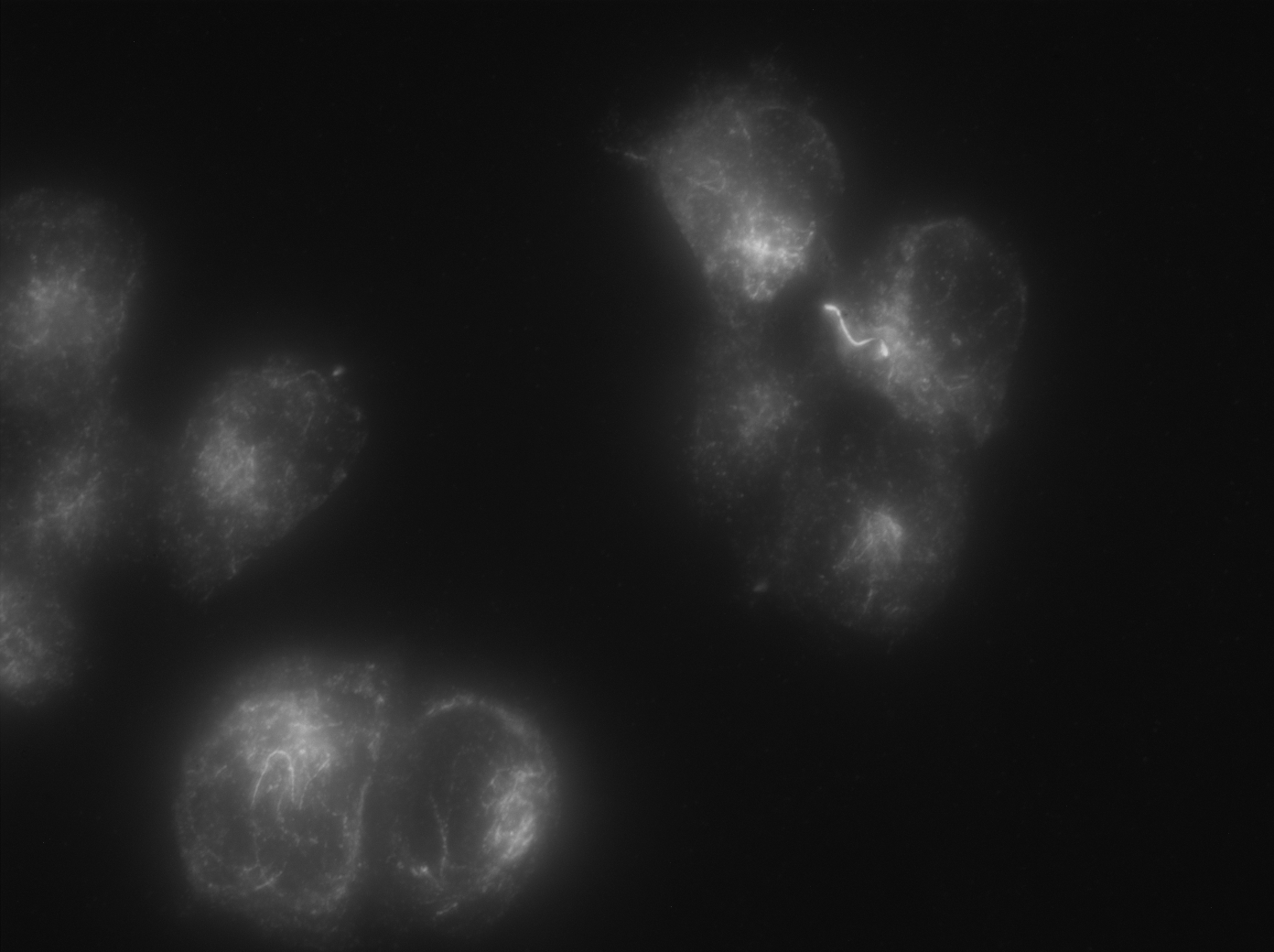

Supplement: Supplementary file 20 — Source data Fig. 6 part 1 [file 44319_2026_742_MOESM20_ESM.zip › Figure 6 Part 1/Fig 6abcd Cas9 TPGS1-KO acetylated tubulin atubulin/Cas9 R2 9-11-24 PA15.Project Maximum Z_XY1726179658_Z0_T0_C2.tif]

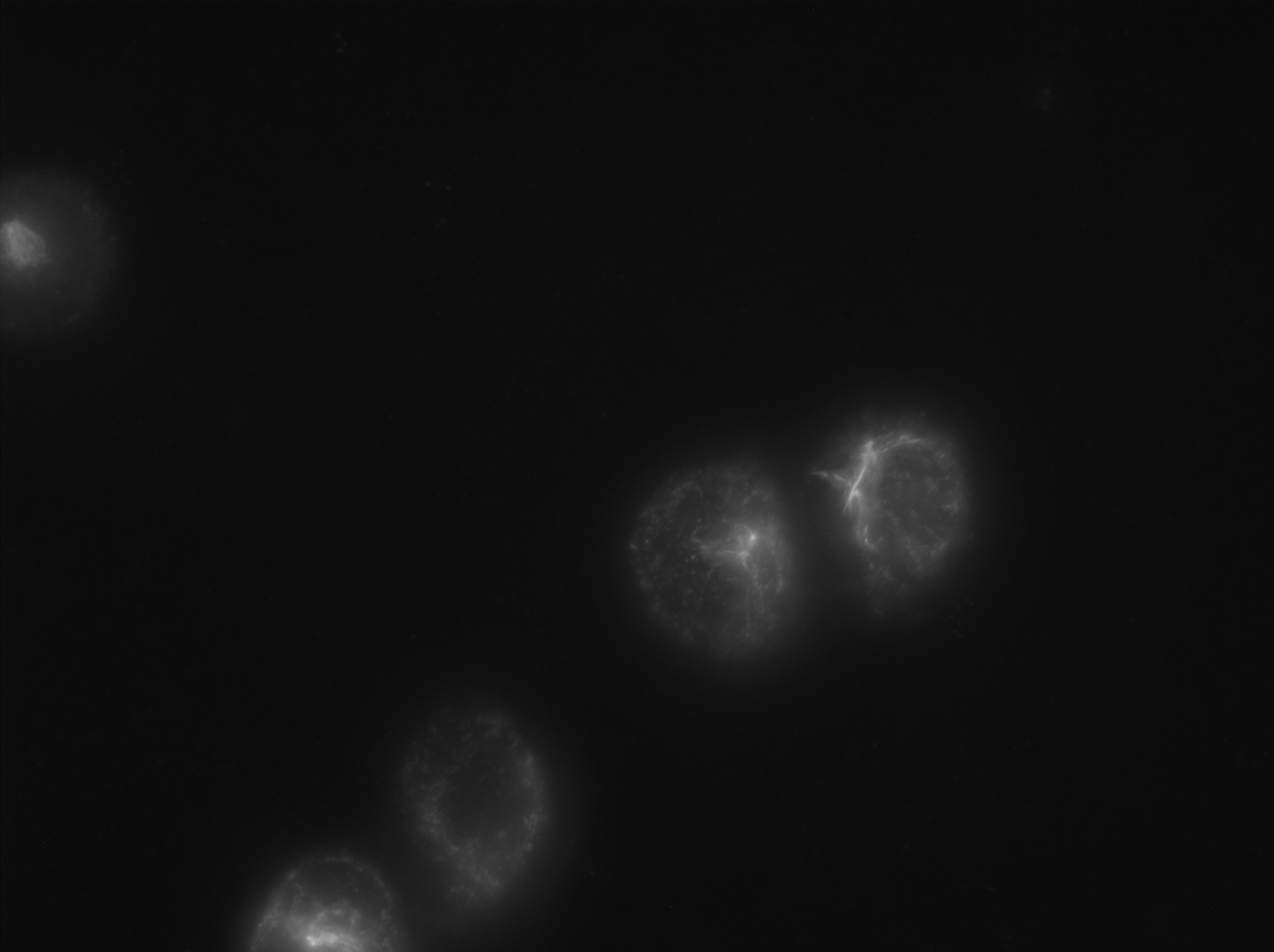

Supplement: Supplementary file 20 — Source data Fig. 6 part 1 [file 44319_2026_742_MOESM20_ESM.zip › Figure 6 Part 1/Fig 6abcd Cas9 TPGS1-KO acetylated tubulin atubulin/Cas9 R2 9-11-24 PA16.Project Maximum Z_XY1726179753_Z0_T0_C2.tif]

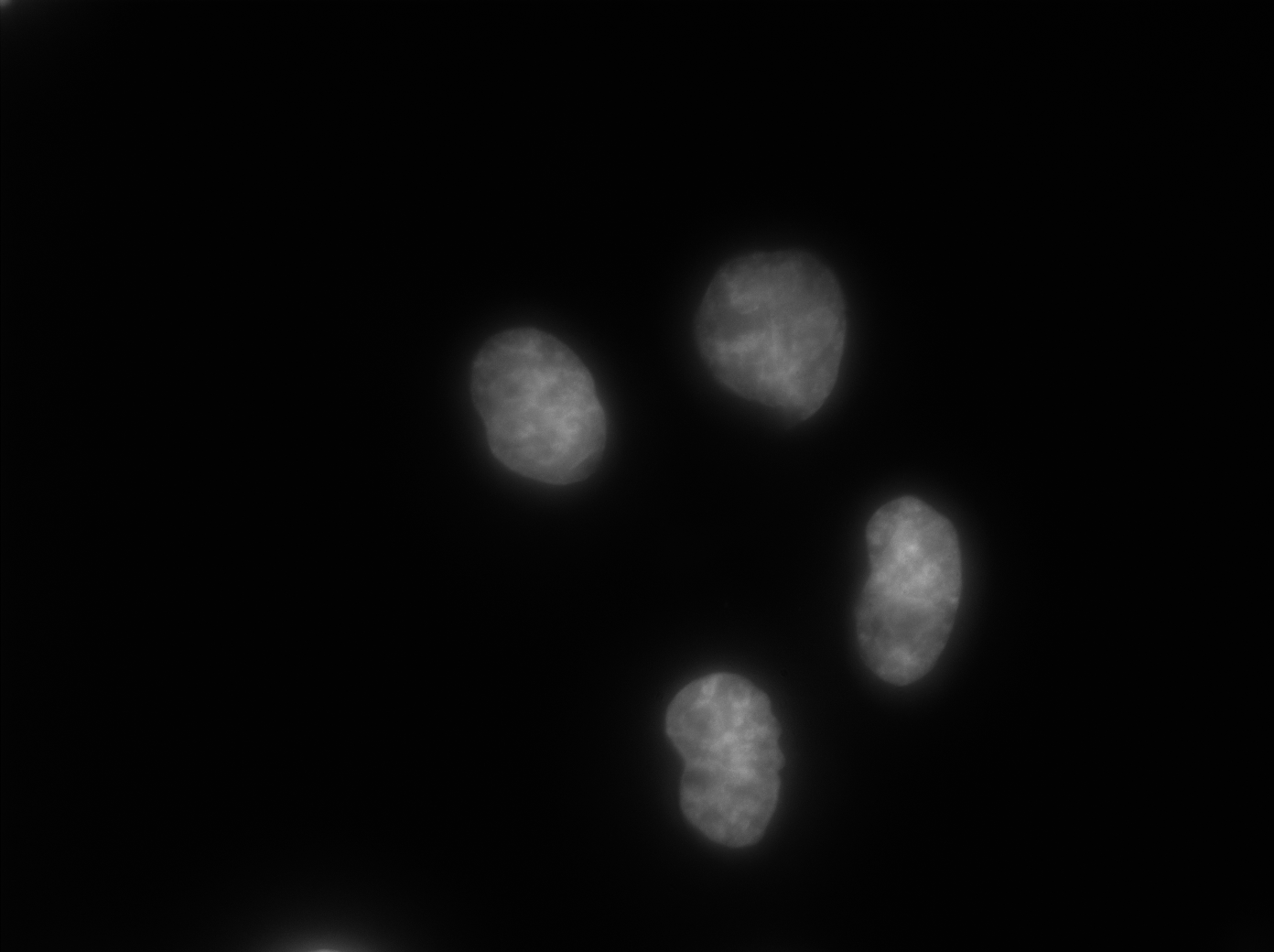

Supplement: Supplementary file 20 — Source data Fig. 6 part 1 [file 44319_2026_742_MOESM20_ESM.zip › Figure 6 Part 1/Fig 6abcd Cas9 TPGS1-KO acetylated tubulin atubulin/Cas9 R2 9-11-24 PA25PA26.Project Maximum Z_XY1726181279_Z0_T0_C0.tif]

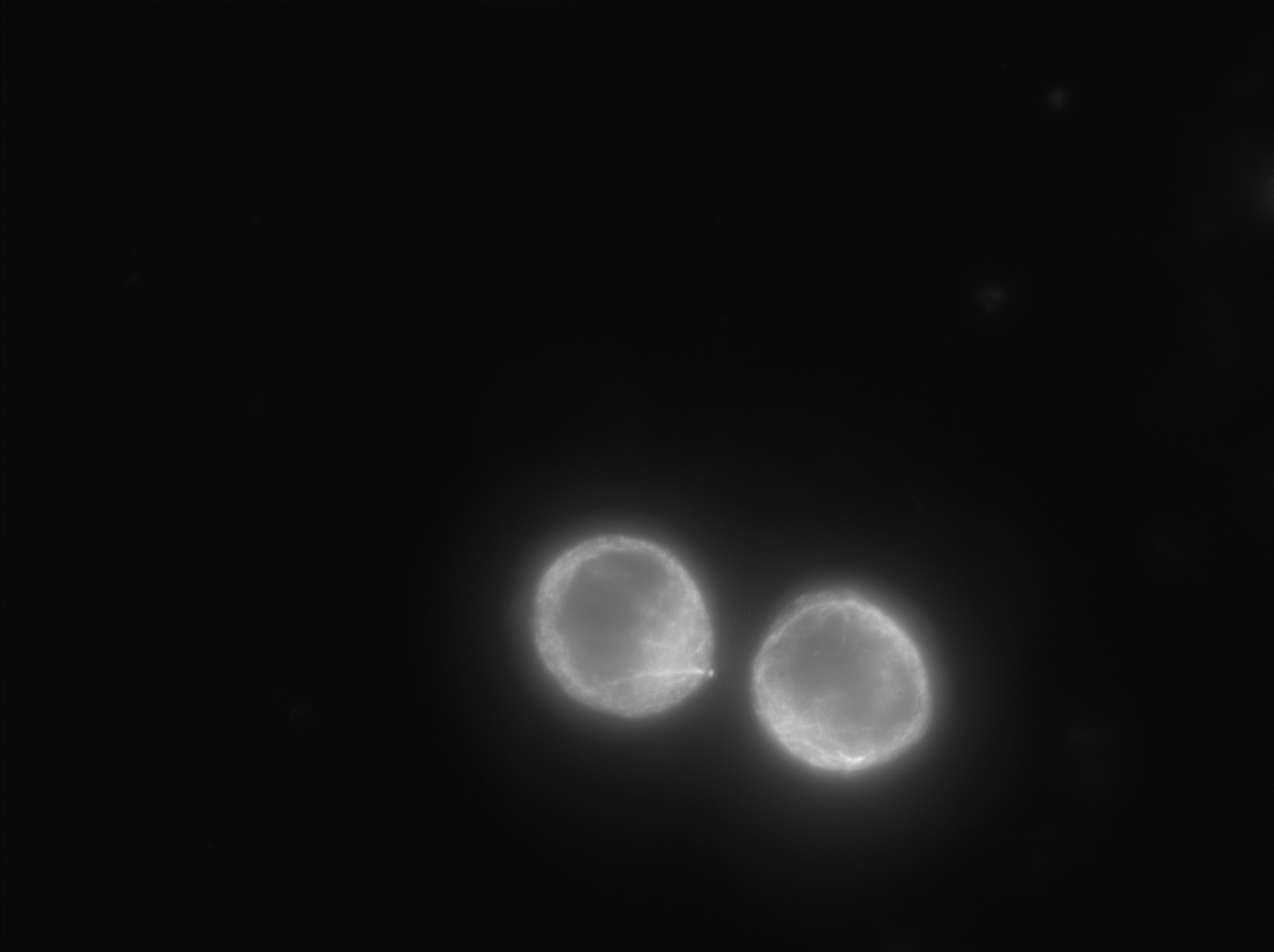

Supplement: Supplementary file 20 — Source data Fig. 6 part 1 [file 44319_2026_742_MOESM20_ESM.zip › Figure 6 Part 1/Fig 6abcd Cas9 TPGS1-KO acetylated tubulin atubulin/Cas9 R2 9-11-24 PA5.Project Maximum Z_XY1726173656_Z0_T0_C1.tif]

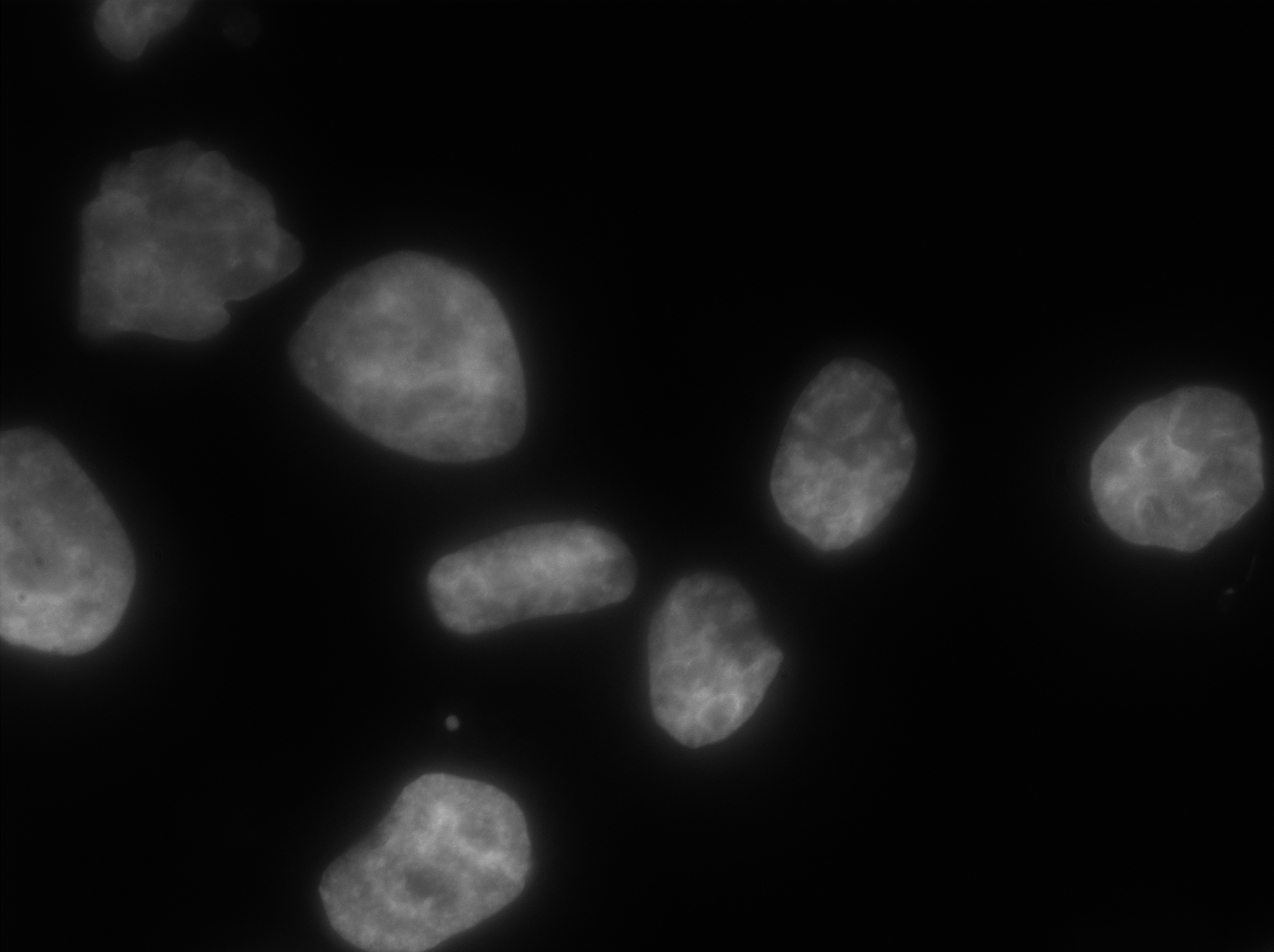

Supplement: Supplementary file 20 — Source data Fig. 6 part 1 [file 44319_2026_742_MOESM20_ESM.zip › Figure 6 Part 1/Fig 6abcd Cas9 TPGS1-KO acetylated tubulin atubulin/Cas9 R2 9-11-24 LT24 PA21.Project Maximum Z_XY1726180572_Z0_T0_C0.tif]

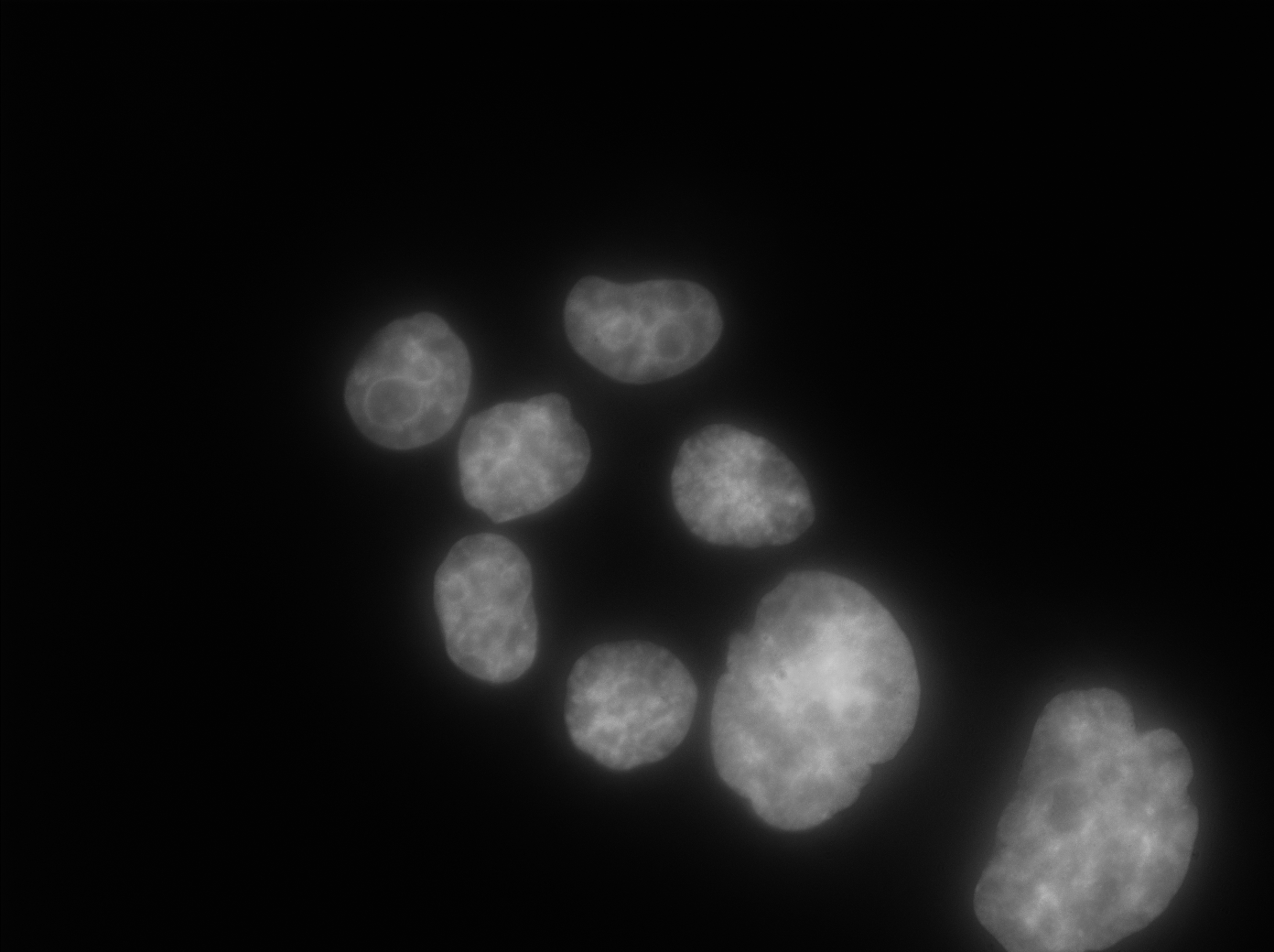

Supplement: Supplementary file 20 — Source data Fig. 6 part 1 [file 44319_2026_742_MOESM20_ESM.zip › Figure 6 Part 1/Fig 6abcd Cas9 TPGS1-KO acetylated tubulin atubulin/Cas9 R2 9-11-24 PA11.Project Maximum Z_XY1726179132_Z0_T0_C0.tif]

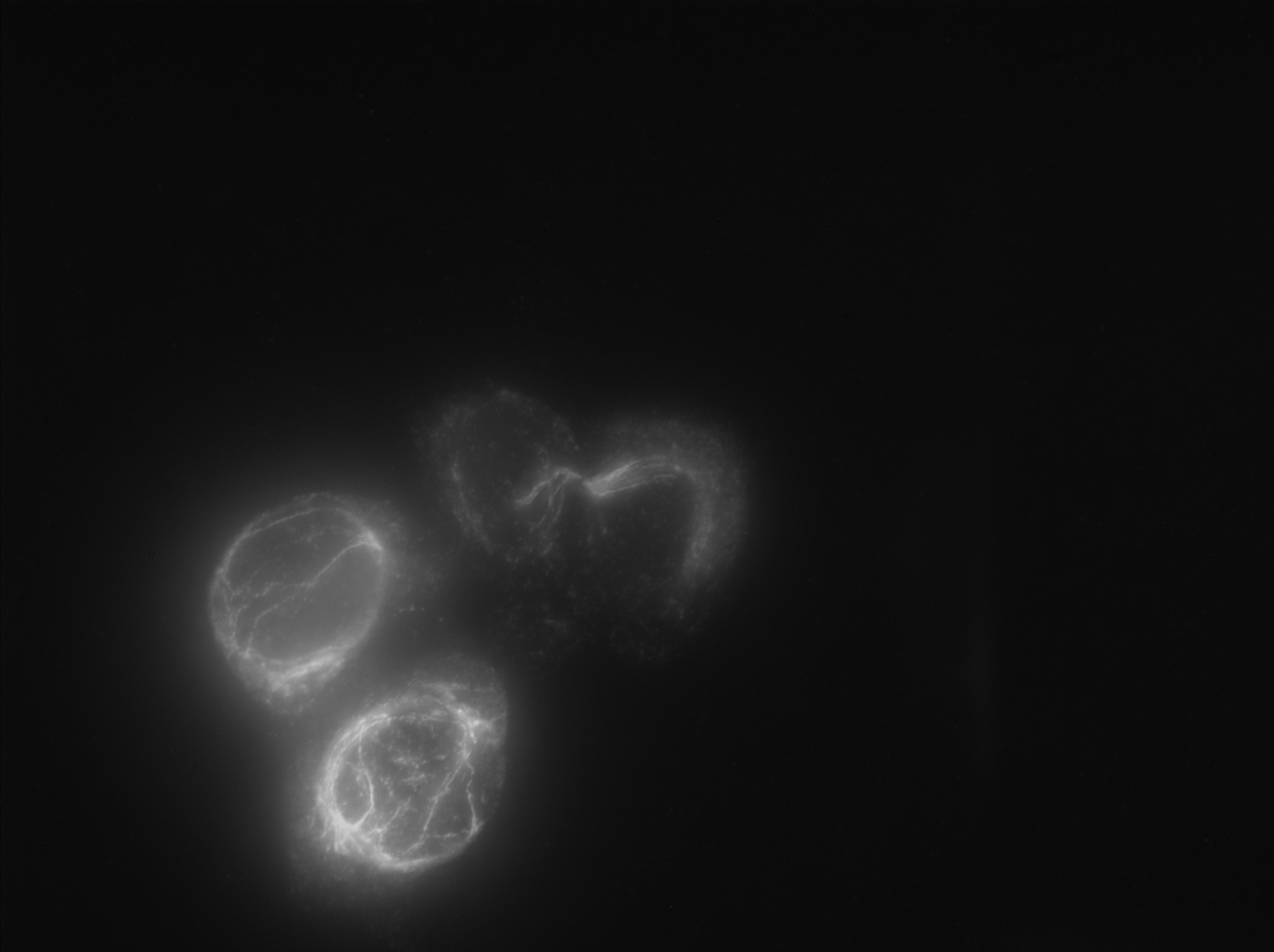

Supplement: Supplementary file 20 — Source data Fig. 6 part 1 [file 44319_2026_742_MOESM20_ESM.zip › Figure 6 Part 1/Fig 6abcd Cas9 TPGS1-KO acetylated tubulin atubulin/Cas9 R2 9-11-24 LT23.Project Maximum Z_XY1726180452_Z0_T0_C2.tif]

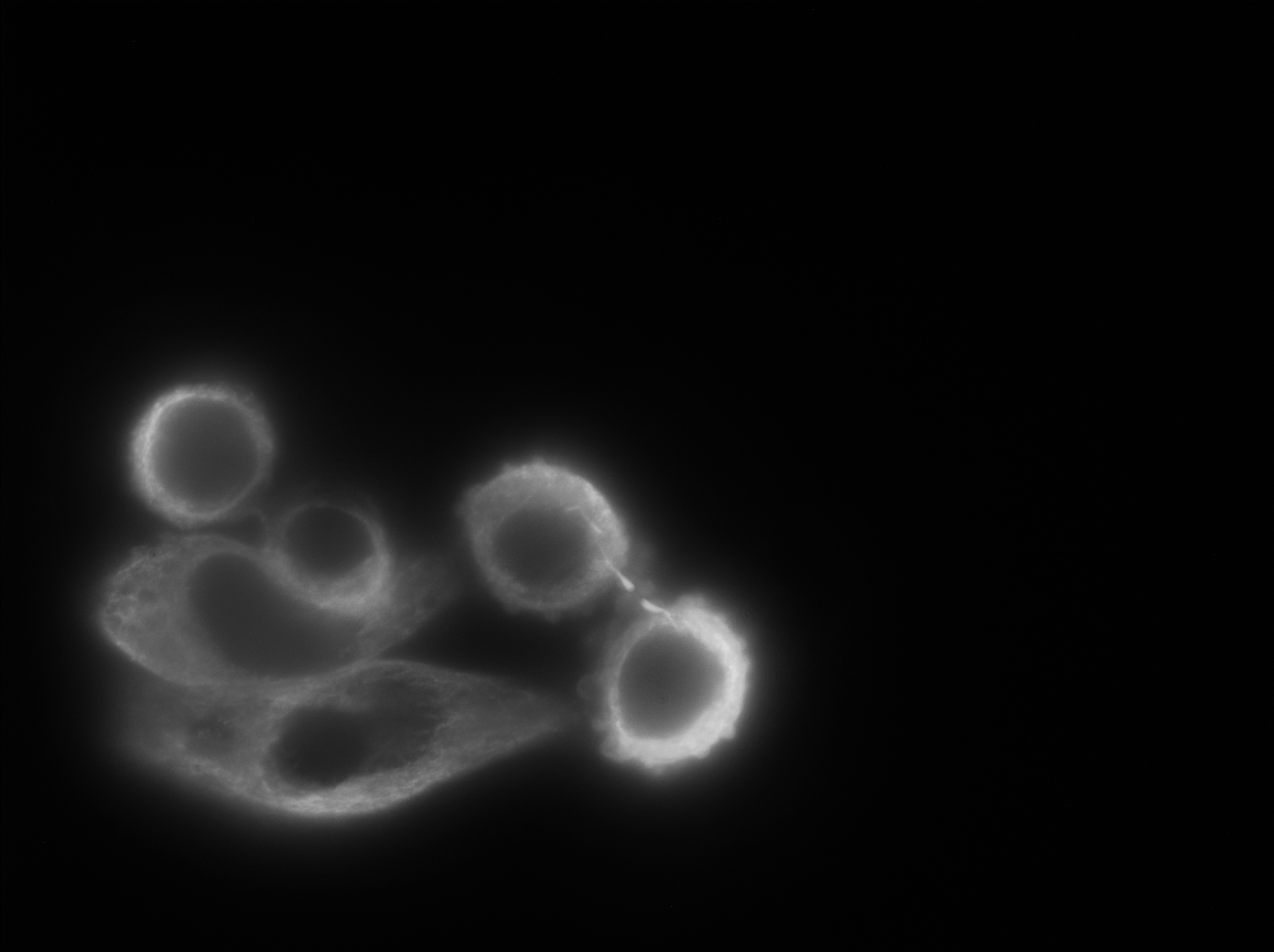

Supplement: Supplementary file 20 — Source data Fig. 6 part 1 [file 44319_2026_742_MOESM20_ESM.zip › Figure 6 Part 1/Fig 6abcd Cas9 TPGS1-KO acetylated tubulin atubulin/Cas9 R3 9-13-24 LT18.Project Maximum Z_XY1726766620_Z0_T0_C1.tif]

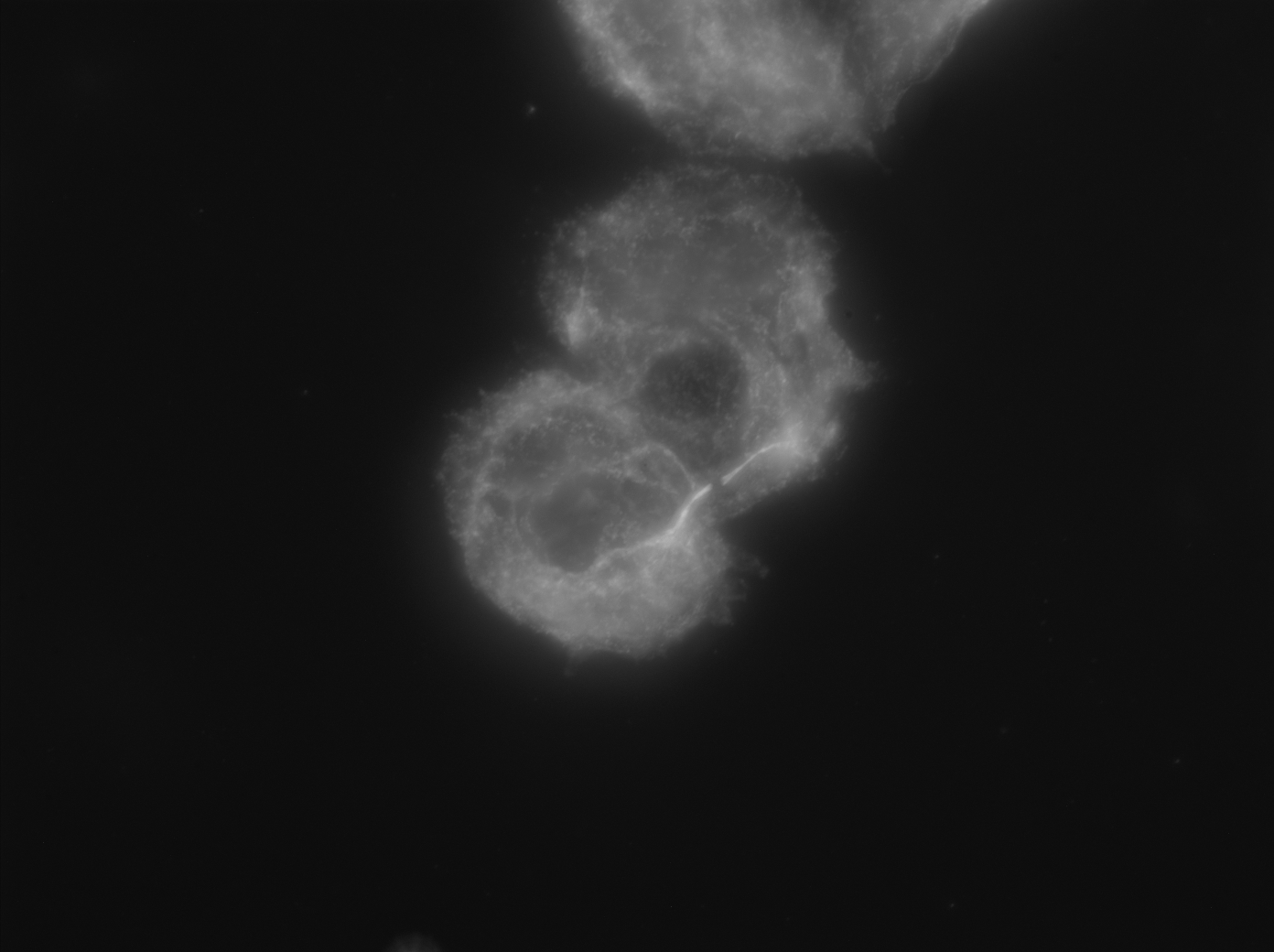

Supplement: Supplementary file 20 — Source data Fig. 6 part 1 [file 44319_2026_742_MOESM20_ESM.zip › Figure 6 Part 1/Fig 6abcd Cas9 TPGS1-KO acetylated tubulin atubulin/Cas9 R2 9-11-24 LT26.Project Maximum Z_XY1726180996_Z0_T0_C1.tif]

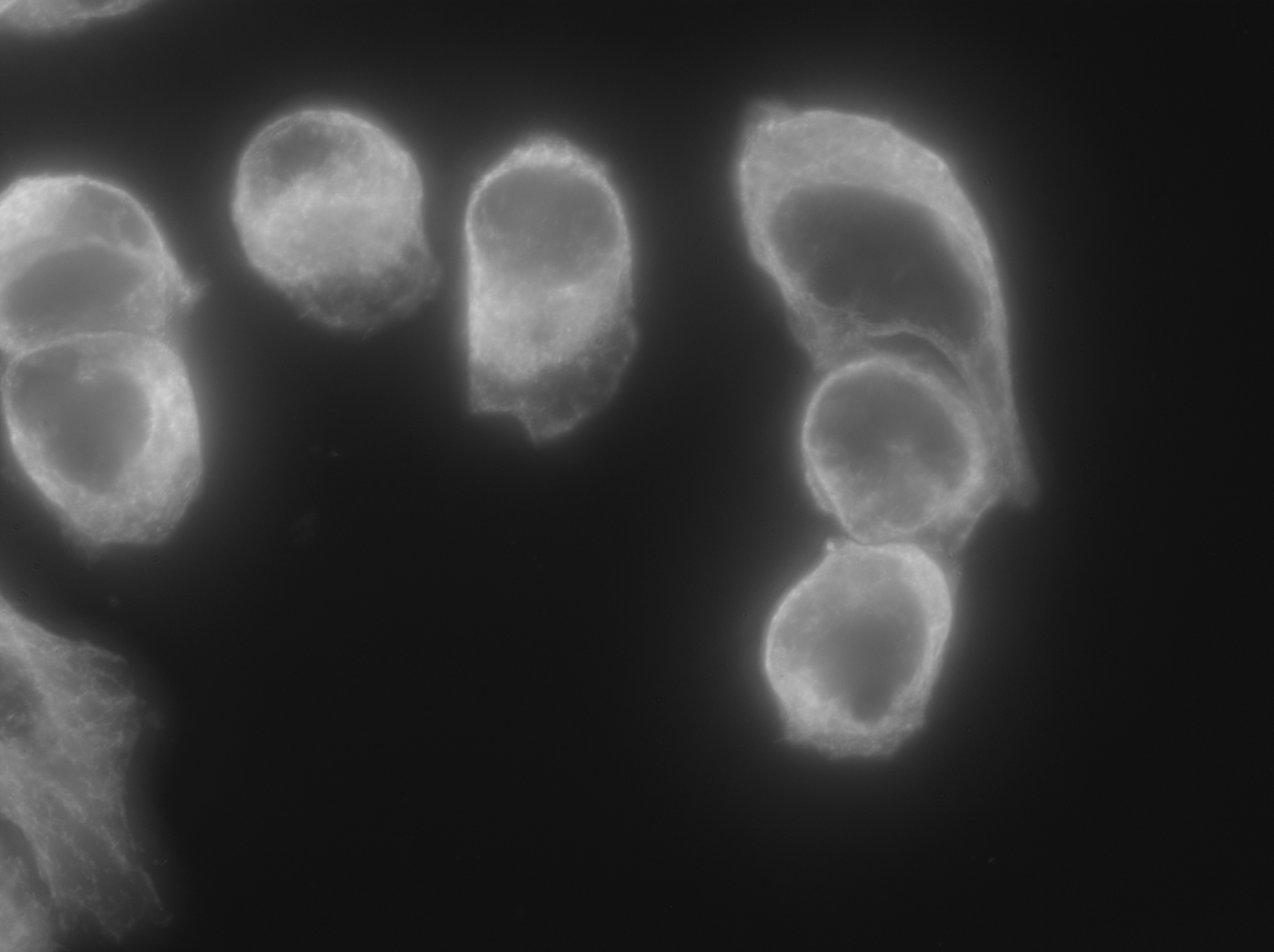

Supplement: Supplementary file 20 — Source data Fig. 6 part 1 [file 44319_2026_742_MOESM20_ESM.zip › Figure 6 Part 1/Fig 6abcd Cas9 TPGS1-KO acetylated tubulin atubulin/Cas9 R2 9-11-24 PA19.Project Maximum Z_XY1726180240_Z0_T0_C1.tif]

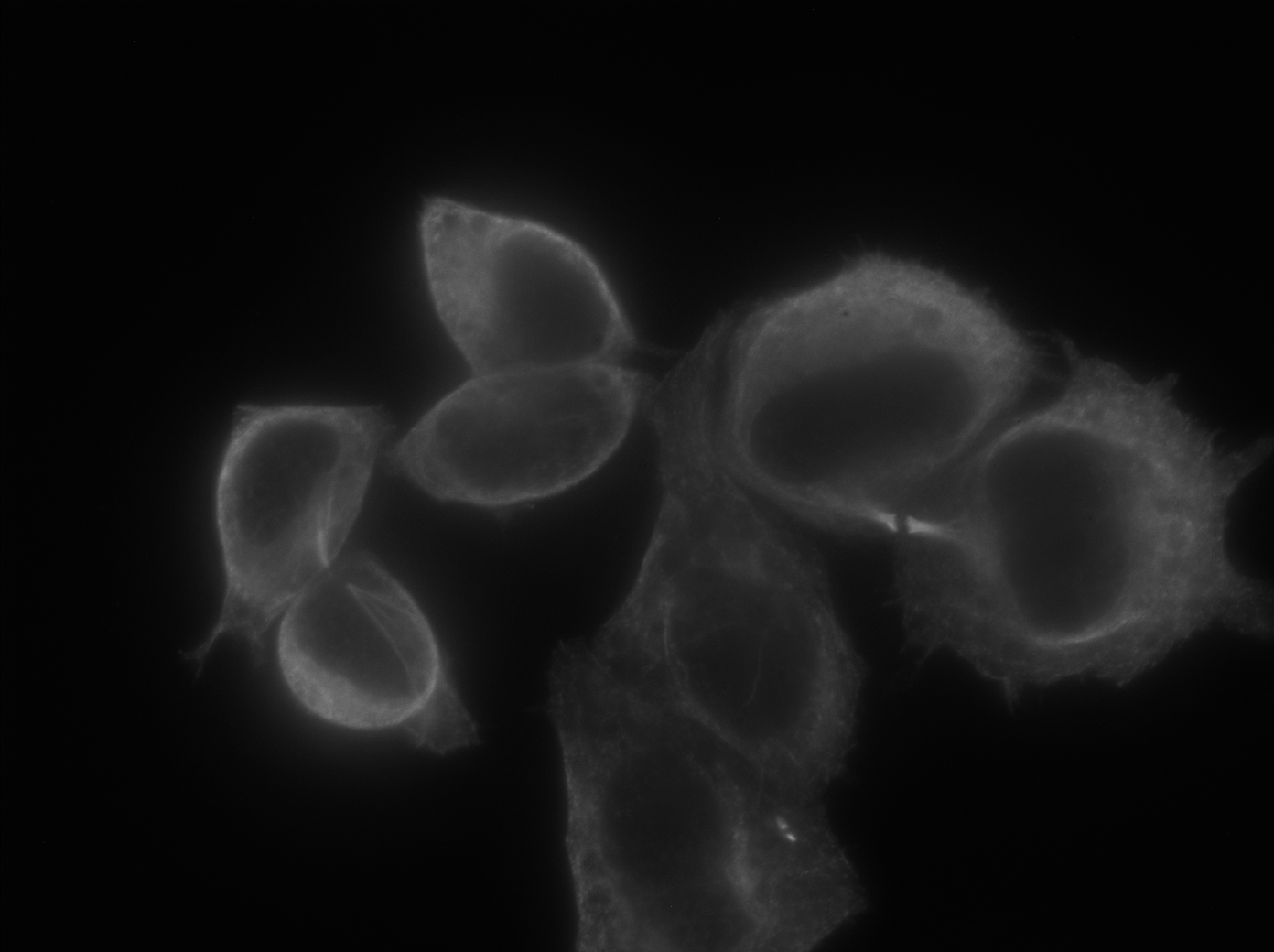

Supplement: Supplementary file 20 — Source data Fig. 6 part 1 [file 44319_2026_742_MOESM20_ESM.zip › Figure 6 Part 1/Fig 6abcd Cas9 TPGS1-KO acetylated tubulin atubulin/Cas9 R3 9-13-24 LT8LT9.Project Maximum Z_XY1726765747_Z0_T0_C1.tif]

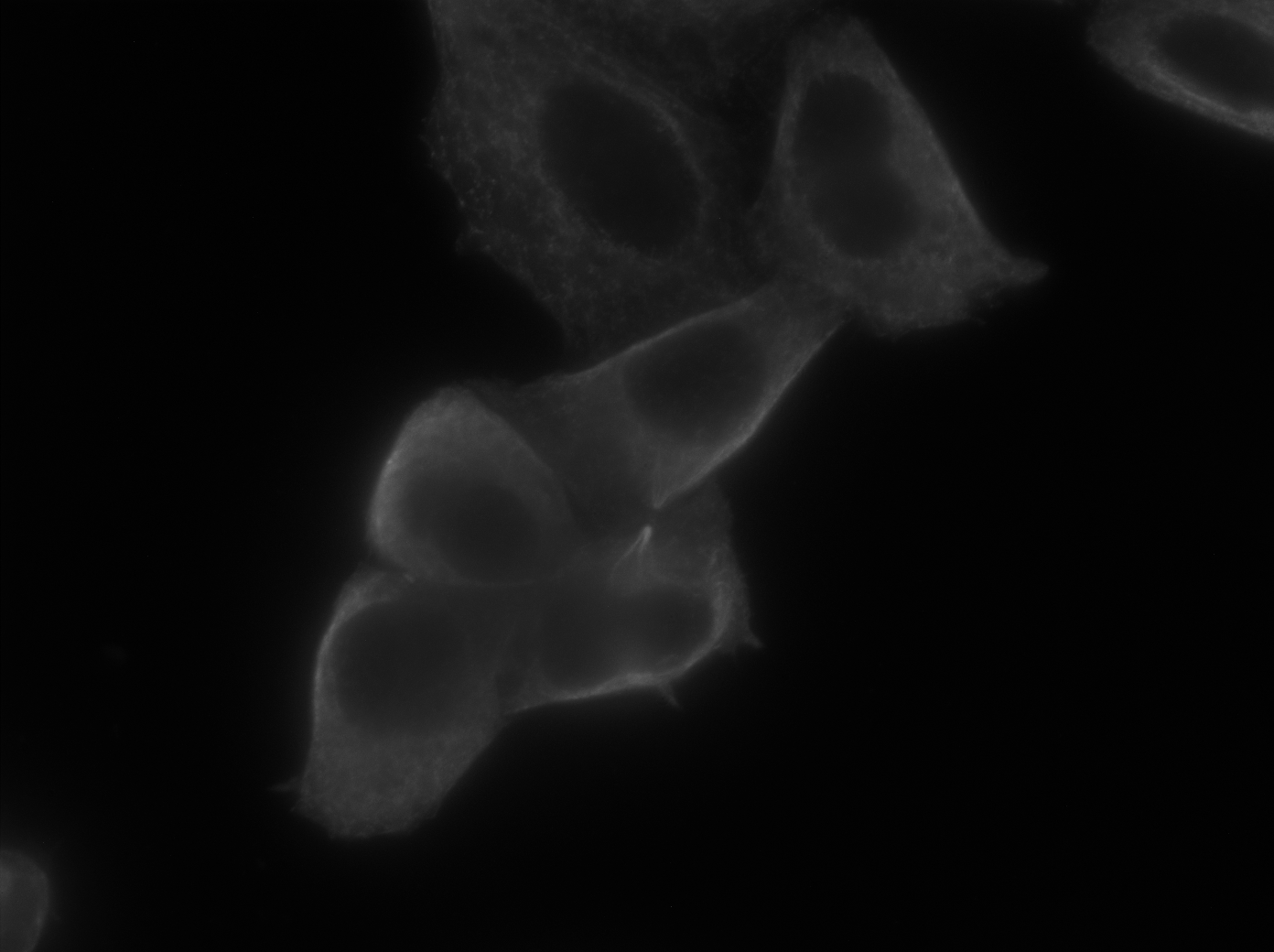

Supplement: Supplementary file 20 — Source data Fig. 6 part 1 [file 44319_2026_742_MOESM20_ESM.zip › Figure 6 Part 1/Fig 6abcd Cas9 TPGS1-KO acetylated tubulin atubulin/Cas9 R3 9-13-24 LT13.Project Maximum Z_XY1726766091_Z0_T0_C1.tif]

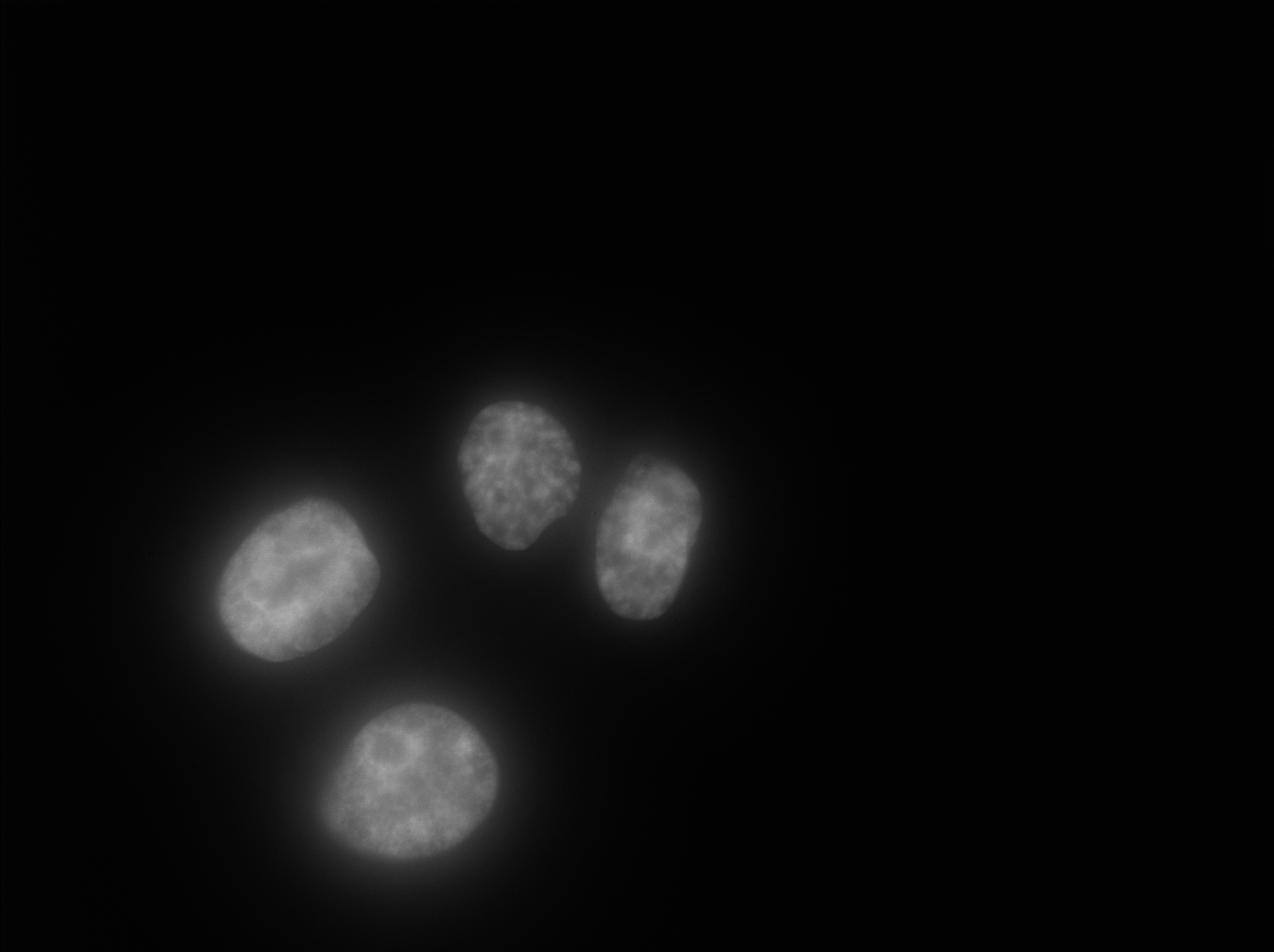

Supplement: Supplementary file 20 — Source data Fig. 6 part 1 [file 44319_2026_742_MOESM20_ESM.zip › Figure 6 Part 1/Fig 6abcd Cas9 TPGS1-KO acetylated tubulin atubulin/Cas9 R2 9-11-24 LT23.Project Maximum Z_XY1726180452_Z0_T0_C0.tif]

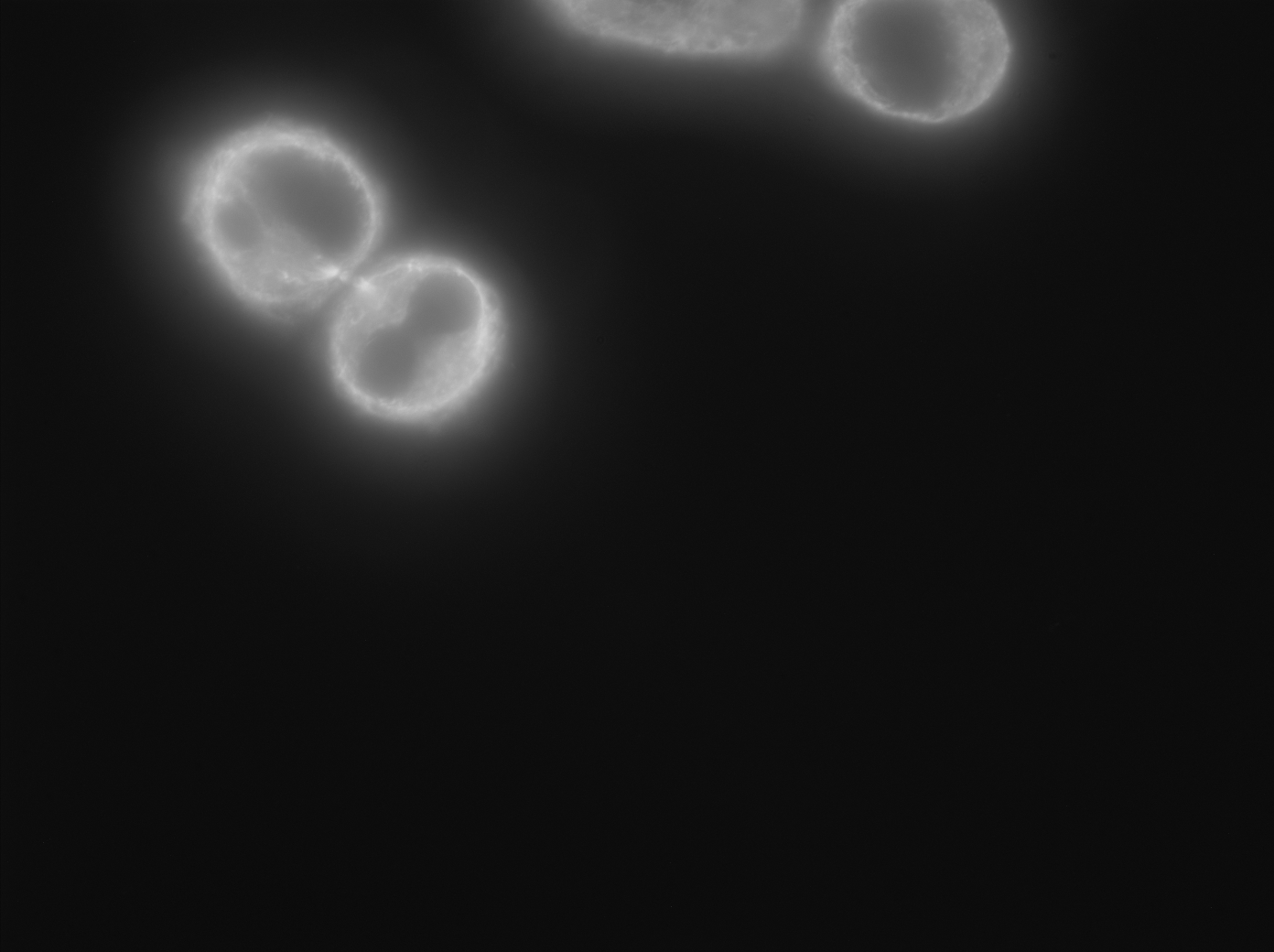

Supplement: Supplementary file 20 — Source data Fig. 6 part 1 [file 44319_2026_742_MOESM20_ESM.zip › Figure 6 Part 1/Fig 6abcd Cas9 TPGS1-KO acetylated tubulin atubulin/Cas9 R2 9-11-24 LT20.Project Maximum Z_XY1726178865_Z0_T0_C1.tif]

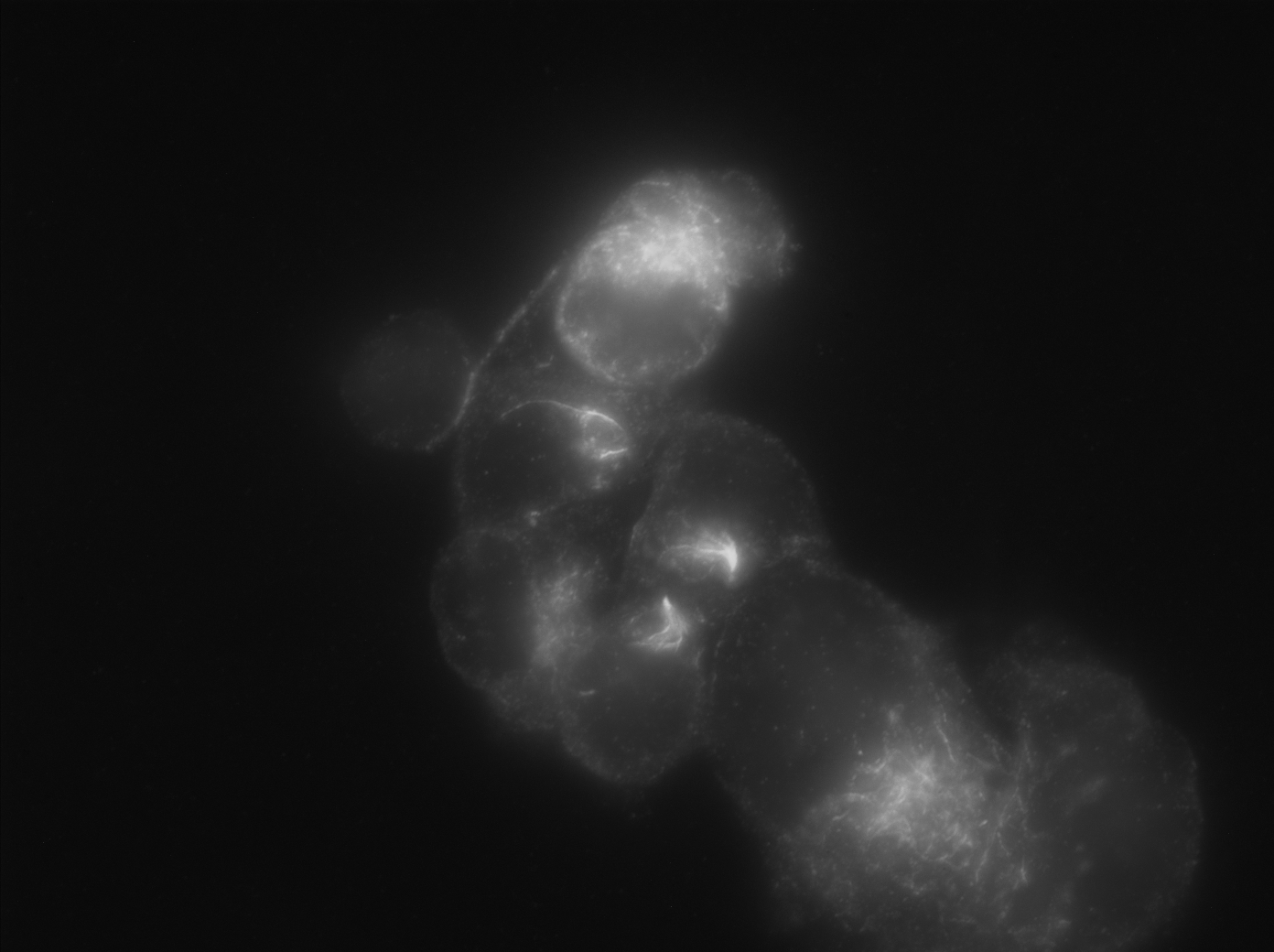

Supplement: Supplementary file 20 — Source data Fig. 6 part 1 [file 44319_2026_742_MOESM20_ESM.zip › Figure 6 Part 1/Fig 6abcd Cas9 TPGS1-KO acetylated tubulin atubulin/Cas9 R2 9-11-24 PA11.Project Maximum Z_XY1726179132_Z0_T0_C2.tif]

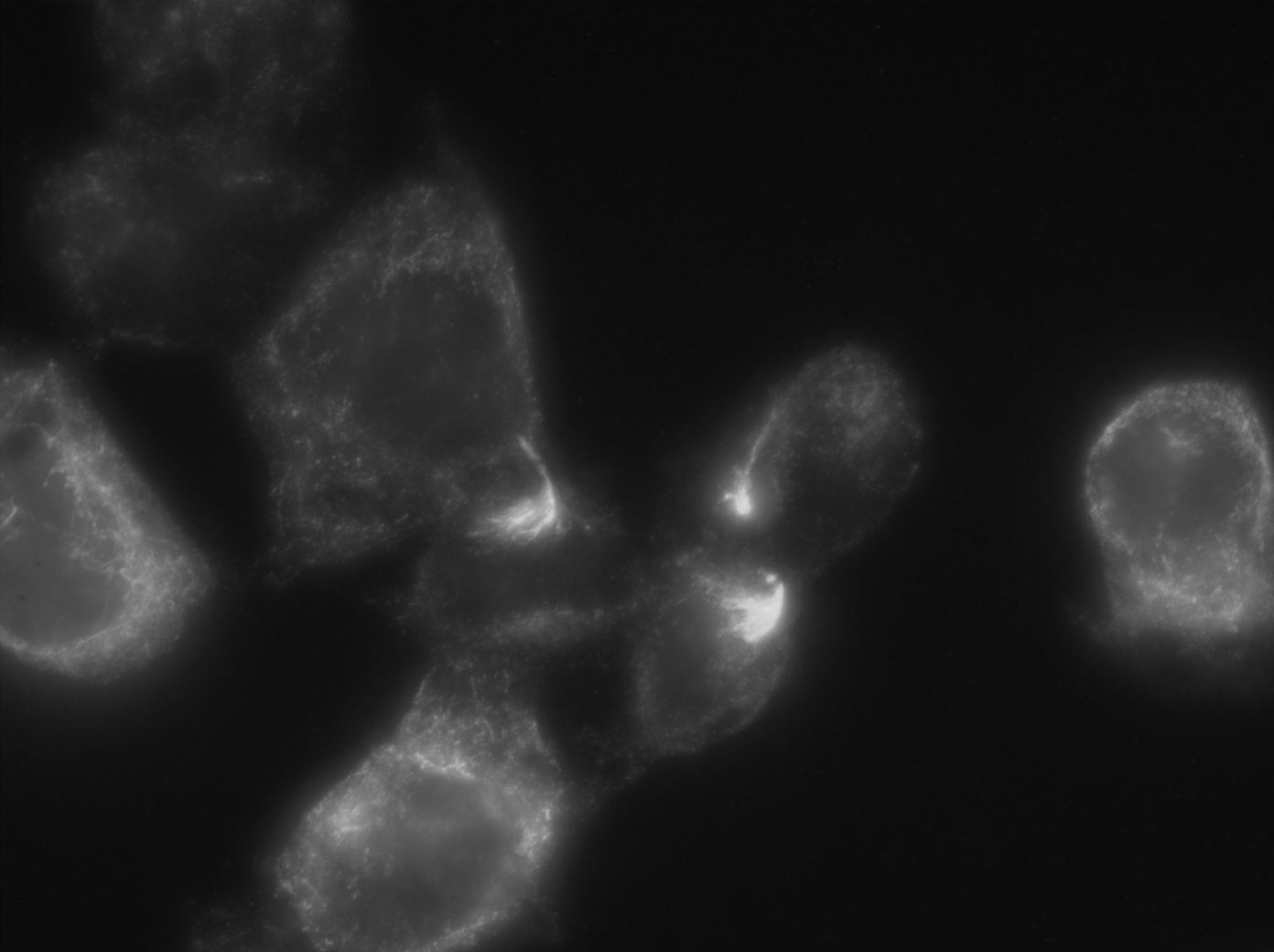

Supplement: Supplementary file 20 — Source data Fig. 6 part 1 [file 44319_2026_742_MOESM20_ESM.zip › Figure 6 Part 1/Fig 6abcd Cas9 TPGS1-KO acetylated tubulin atubulin/Cas9 R2 9-11-24 LT24 PA21.Project Maximum Z_XY1726180572_Z0_T0_C2.tif]

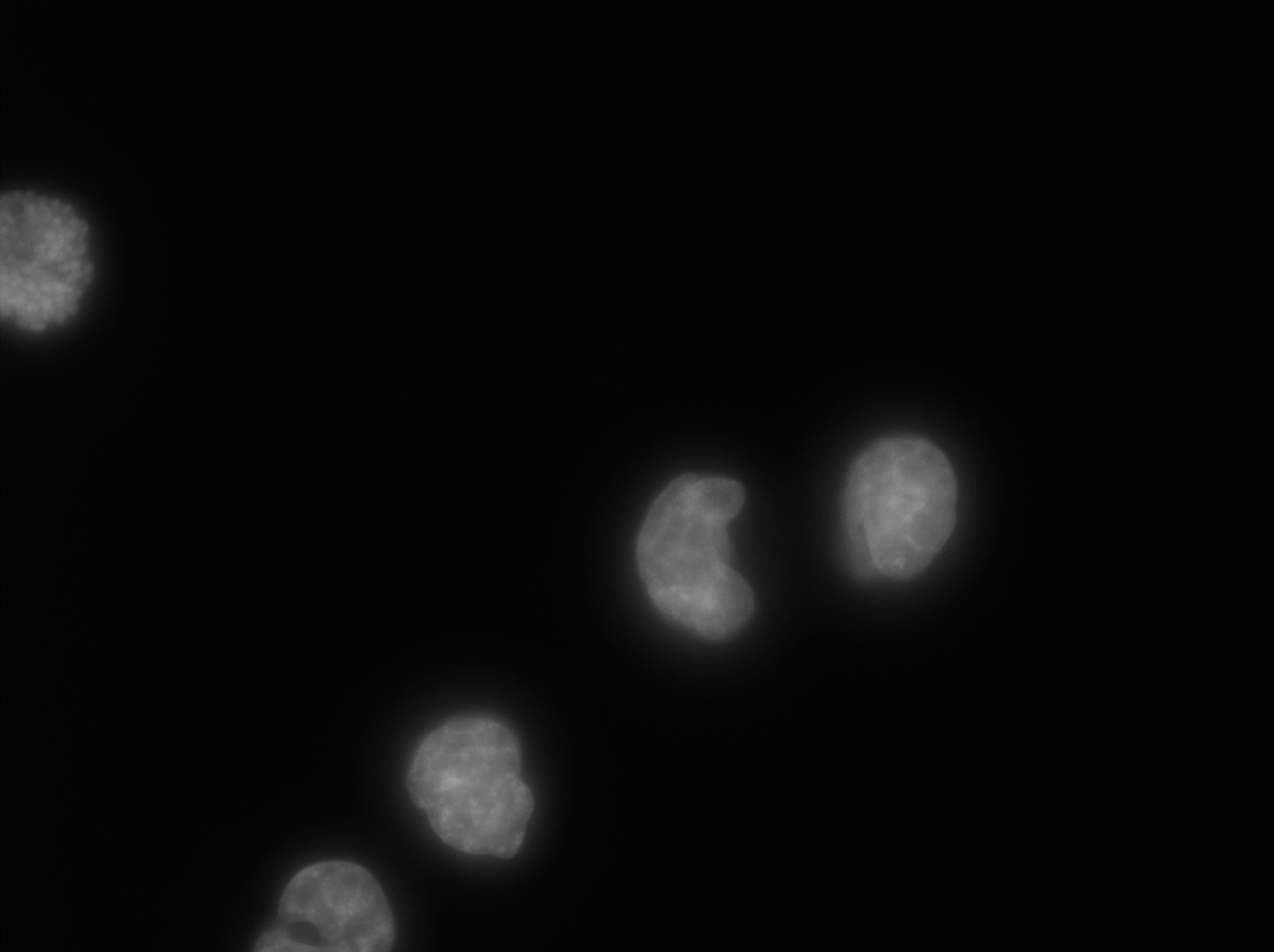

Supplement: Supplementary file 20 — Source data Fig. 6 part 1 [file 44319_2026_742_MOESM20_ESM.zip › Figure 6 Part 1/Fig 6abcd Cas9 TPGS1-KO acetylated tubulin atubulin/Cas9 R2 9-11-24 PA16.Project Maximum Z_XY1726179753_Z0_T0_C0.tif]

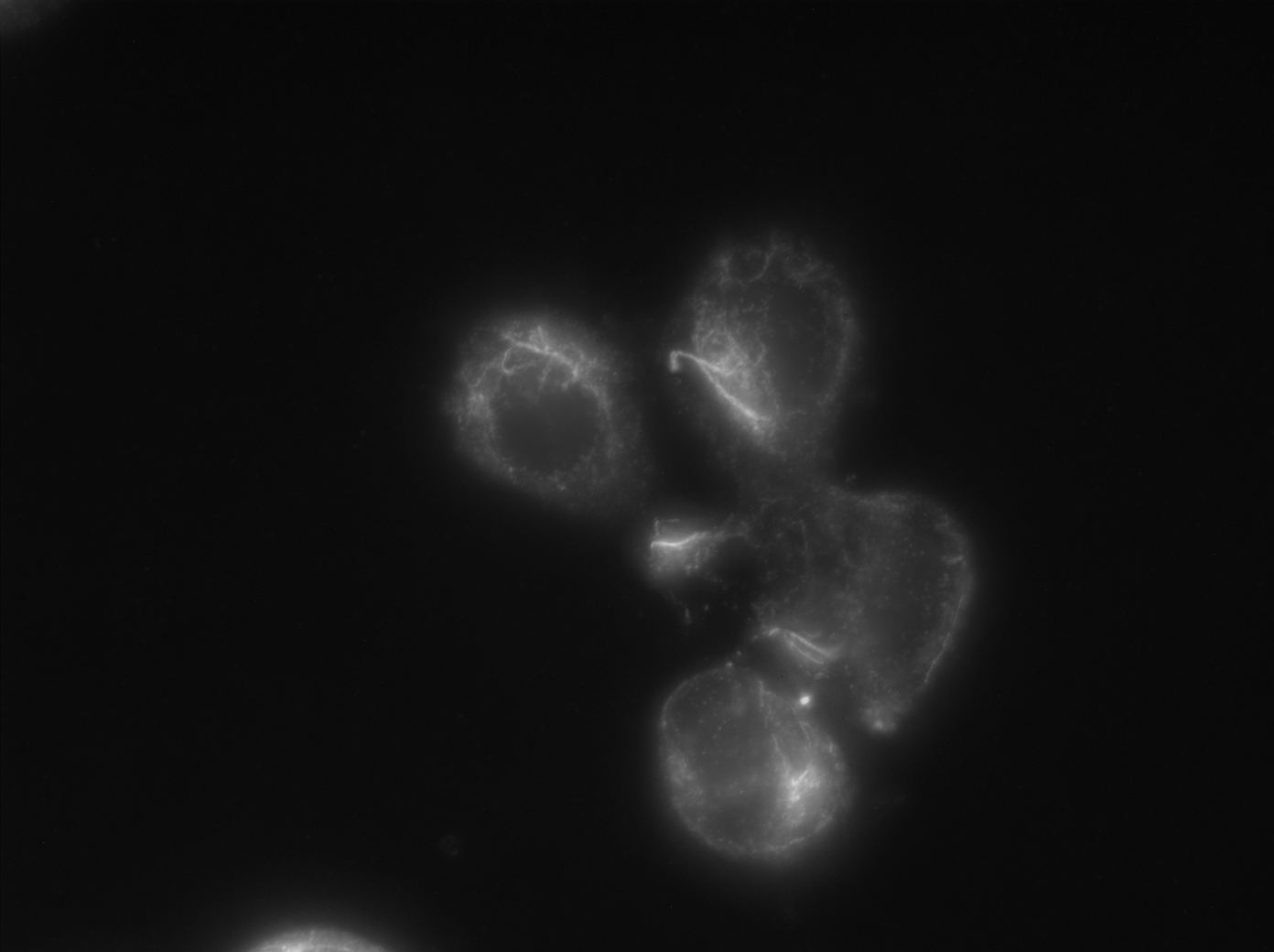

Supplement: Supplementary file 20 — Source data Fig. 6 part 1 [file 44319_2026_742_MOESM20_ESM.zip › Figure 6 Part 1/Fig 6abcd Cas9 TPGS1-KO acetylated tubulin atubulin/Cas9 R2 9-11-24 PA25PA26.Project Maximum Z_XY1726181279_Z0_T0_C2.tif]

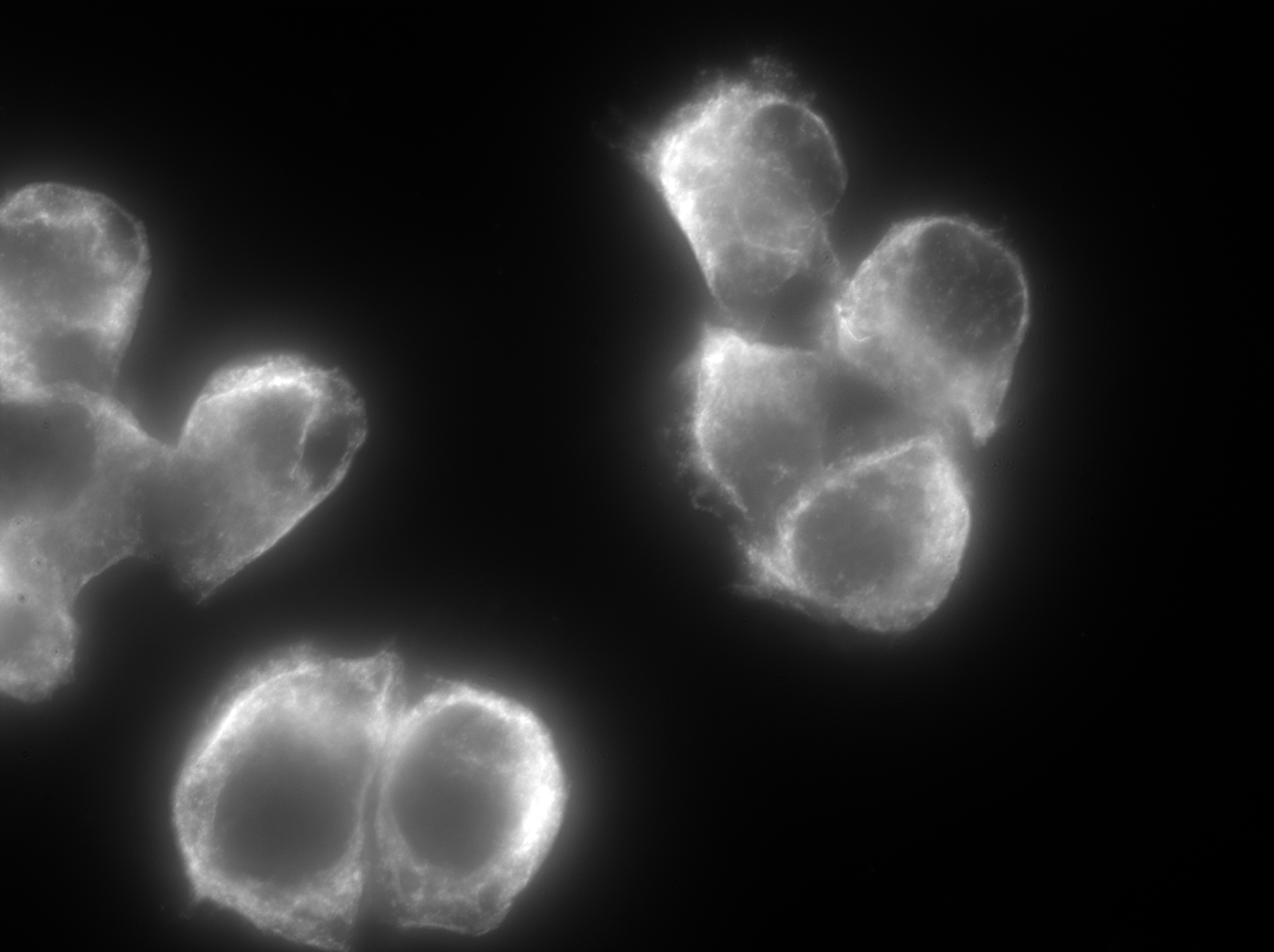

Supplement: Supplementary file 20 — Source data Fig. 6 part 1 [file 44319_2026_742_MOESM20_ESM.zip › Figure 6 Part 1/Fig 6abcd Cas9 TPGS1-KO acetylated tubulin atubulin/Cas9 R2 9-11-24 PA15.Project Maximum Z_XY1726179658_Z0_T0_C1.tif]

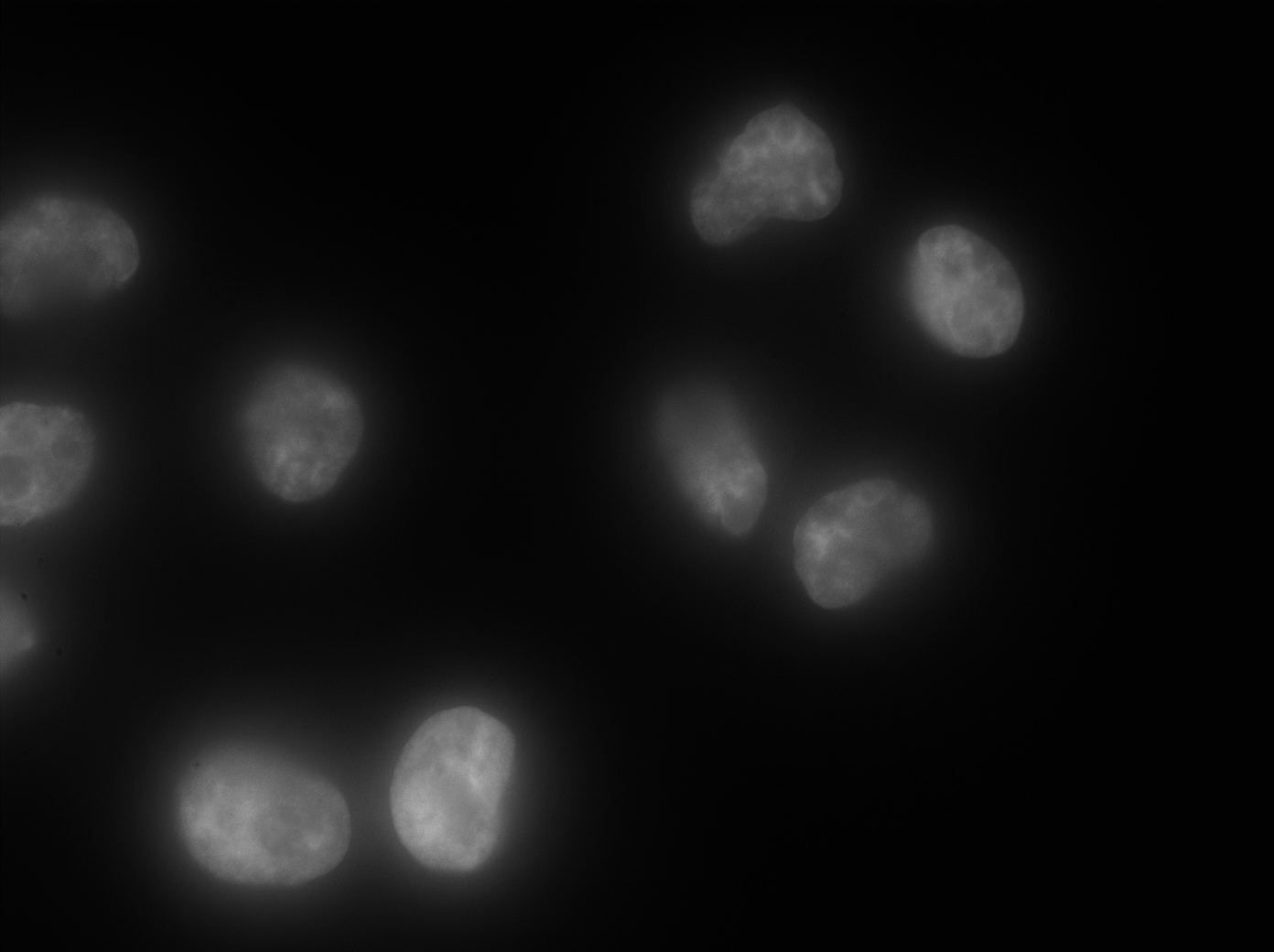

Supplement: Supplementary file 20 — Source data Fig. 6 part 1 [file 44319_2026_742_MOESM20_ESM.zip › Figure 6 Part 1/Fig 6abcd Cas9 TPGS1-KO acetylated tubulin atubulin/Cas9 R2 9-11-24 PA15.Project Maximum Z_XY1726179658_Z0_T0_C0.tif]

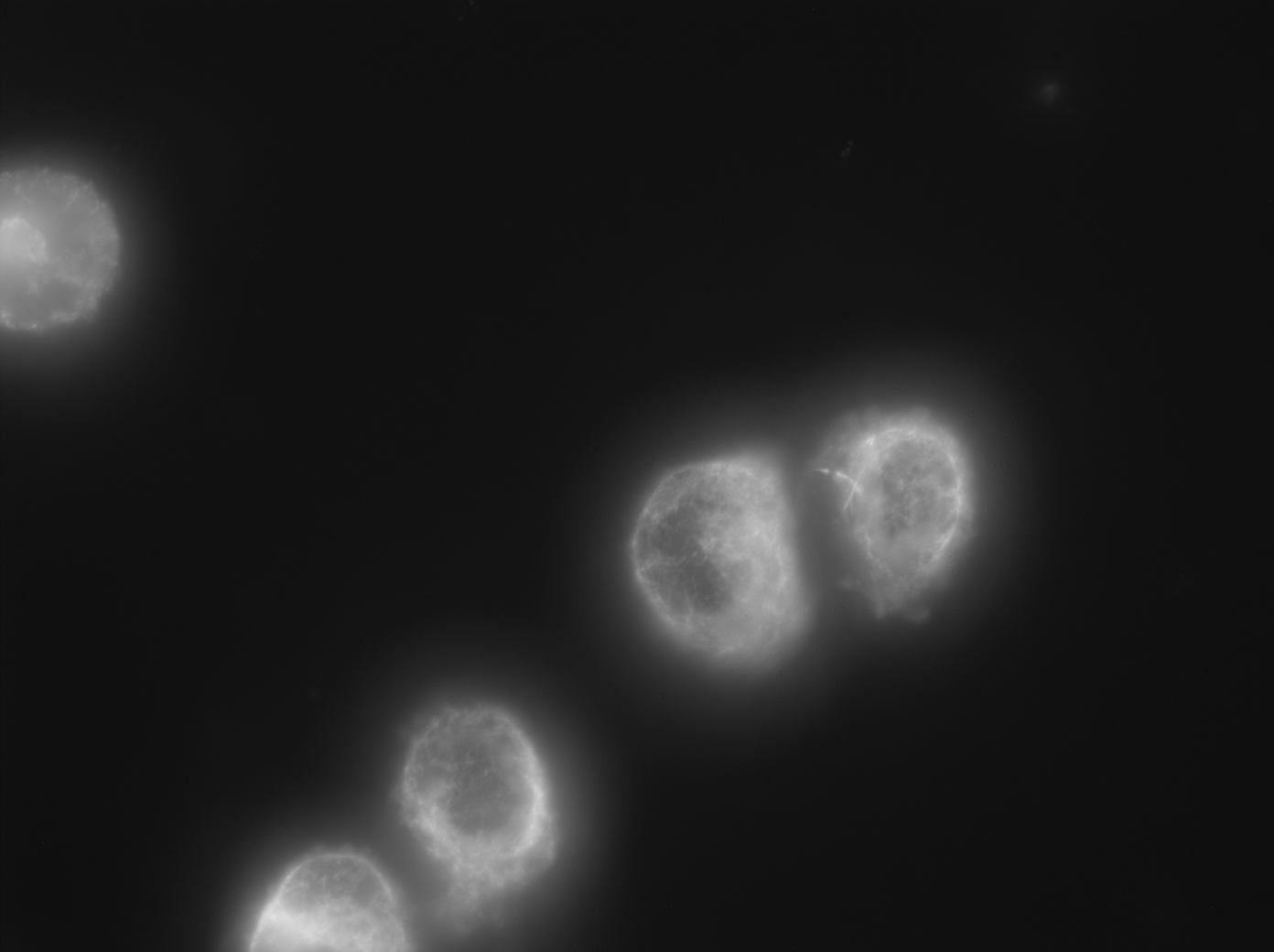

Supplement: Supplementary file 20 — Source data Fig. 6 part 1 [file 44319_2026_742_MOESM20_ESM.zip › Figure 6 Part 1/Fig 6abcd Cas9 TPGS1-KO acetylated tubulin atubulin/Cas9 R2 9-11-24 PA16.Project Maximum Z_XY1726179753_Z0_T0_C1.tif]

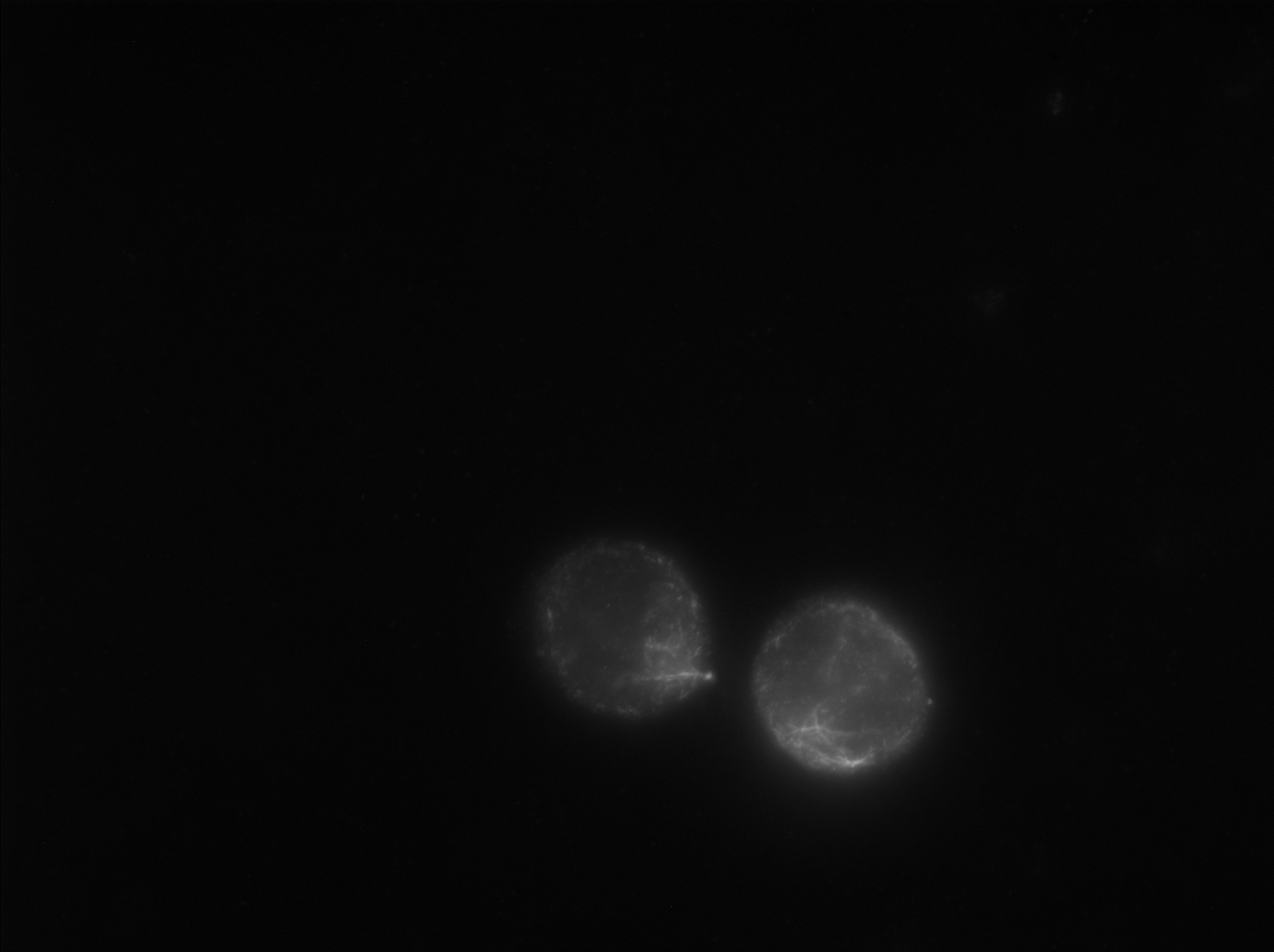

Supplement: Supplementary file 20 — Source data Fig. 6 part 1 [file 44319_2026_742_MOESM20_ESM.zip › Figure 6 Part 1/Fig 6abcd Cas9 TPGS1-KO acetylated tubulin atubulin/Cas9 R2 9-11-24 PA5.Project Maximum Z_XY1726173656_Z0_T0_C2.tif]

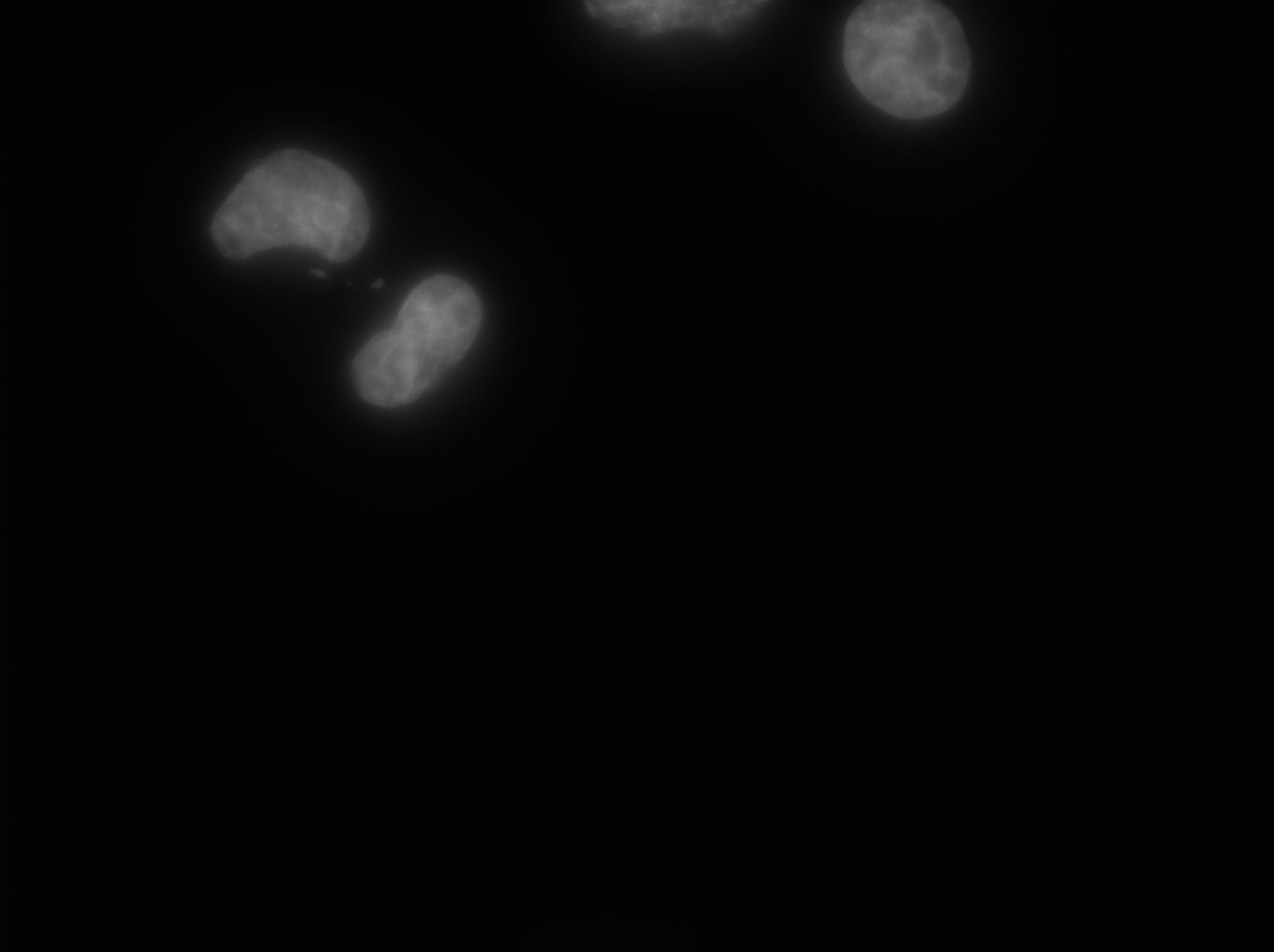

Supplement: Supplementary file 20 — Source data Fig. 6 part 1 [file 44319_2026_742_MOESM20_ESM.zip › Figure 6 Part 1/Fig 6abcd Cas9 TPGS1-KO acetylated tubulin atubulin/Cas9 R2 9-11-24 LT20.Project Maximum Z_XY1726178865_Z0_T0_C0.tif]

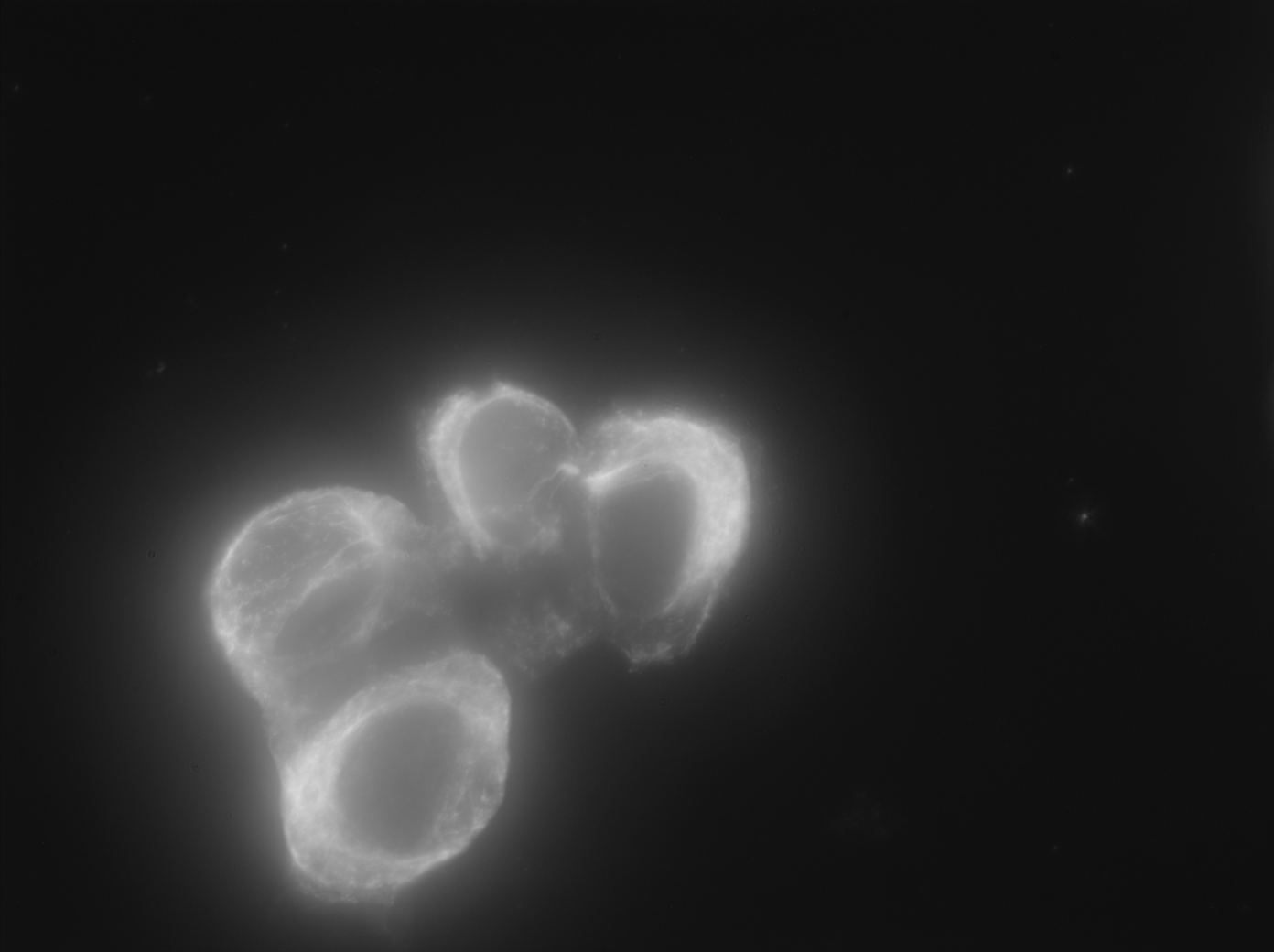

Supplement: Supplementary file 20 — Source data Fig. 6 part 1 [file 44319_2026_742_MOESM20_ESM.zip › Figure 6 Part 1/Fig 6abcd Cas9 TPGS1-KO acetylated tubulin atubulin/Cas9 R2 9-11-24 LT23.Project Maximum Z_XY1726180452_Z0_T0_C1.tif]

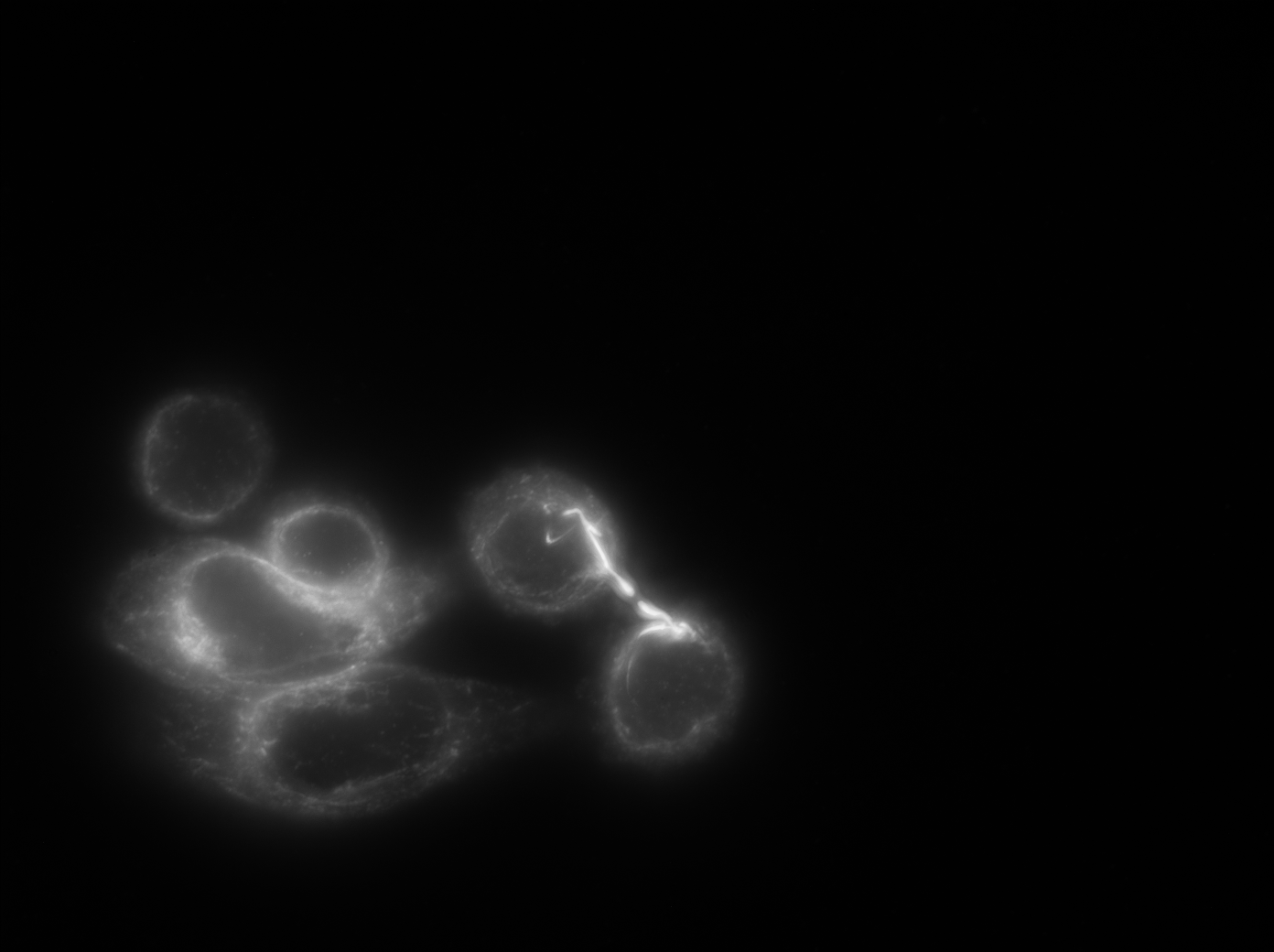

Supplement: Supplementary file 20 — Source data Fig. 6 part 1 [file 44319_2026_742_MOESM20_ESM.zip › Figure 6 Part 1/Fig 6abcd Cas9 TPGS1-KO acetylated tubulin atubulin/Cas9 R3 9-13-24 LT18.Project Maximum Z_XY1726766620_Z0_T0_C2.tif]

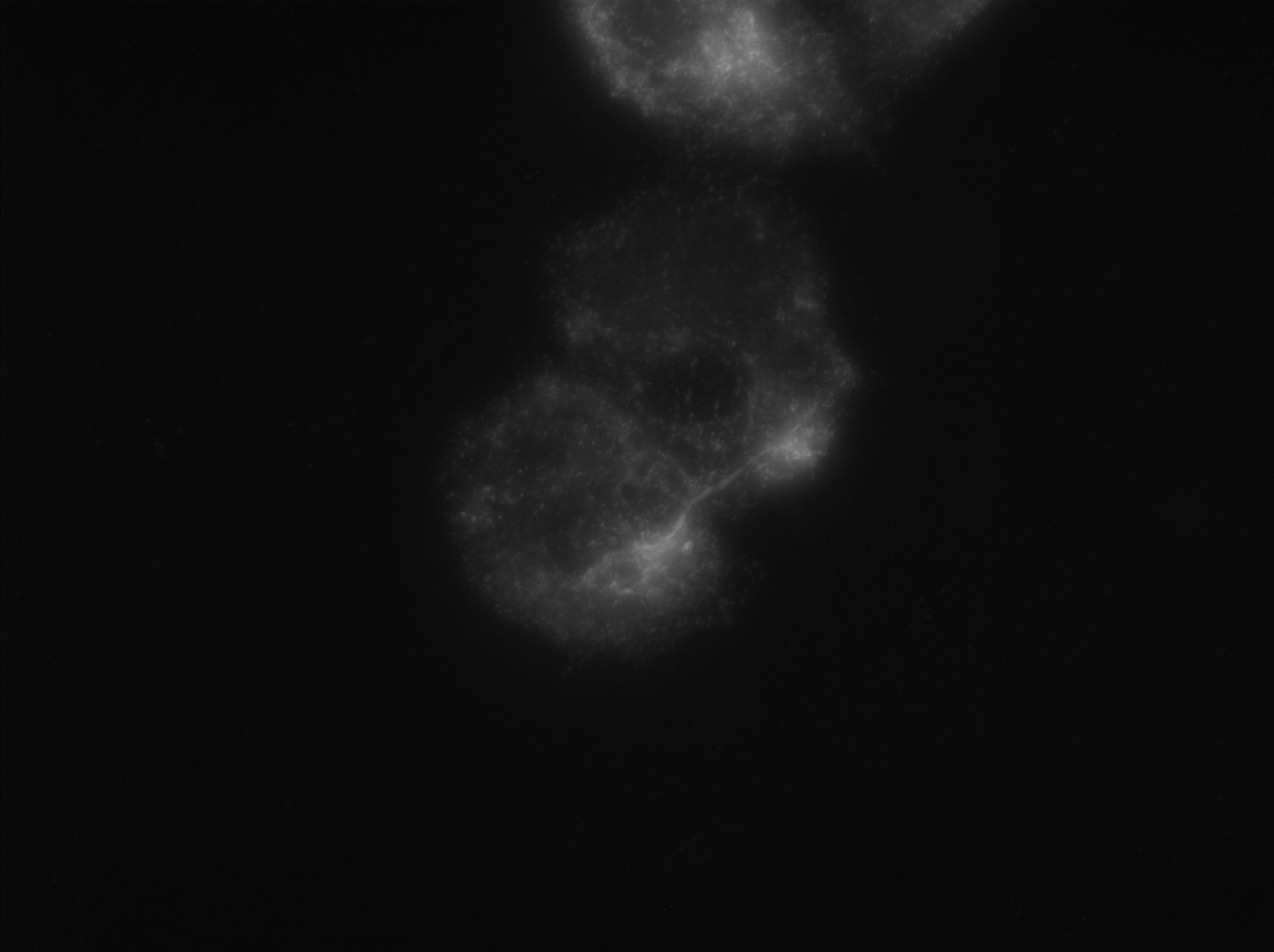

Supplement: Supplementary file 20 — Source data Fig. 6 part 1 [file 44319_2026_742_MOESM20_ESM.zip › Figure 6 Part 1/Fig 6abcd Cas9 TPGS1-KO acetylated tubulin atubulin/Cas9 R2 9-11-24 LT26.Project Maximum Z_XY1726180996_Z0_T0_C2.tif]

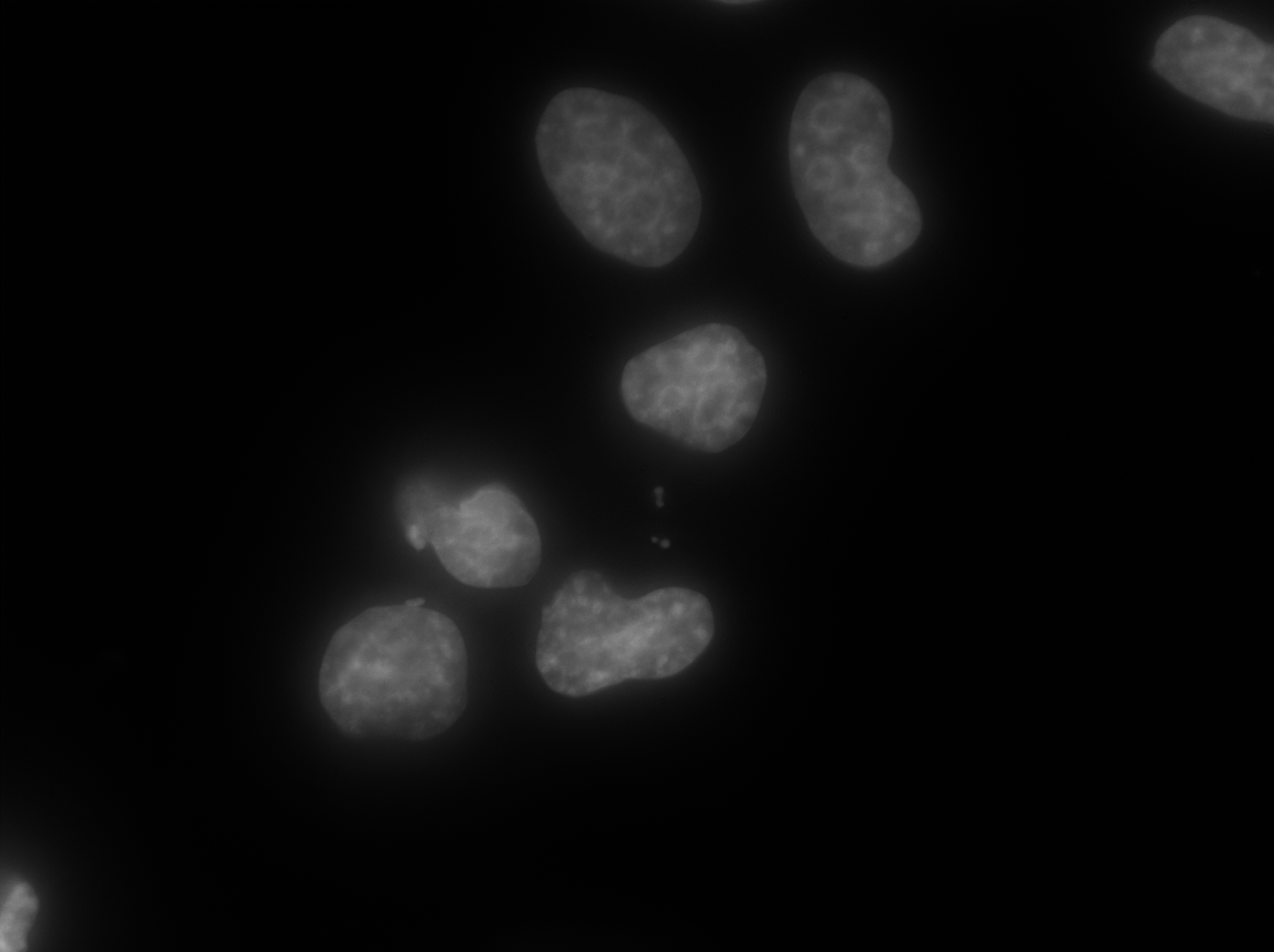

Supplement: Supplementary file 20 — Source data Fig. 6 part 1 [file 44319_2026_742_MOESM20_ESM.zip › Figure 6 Part 1/Fig 6abcd Cas9 TPGS1-KO acetylated tubulin atubulin/Cas9 R3 9-13-24 LT13.Project Maximum Z_XY1726766091_Z0_T0_C0.tif]

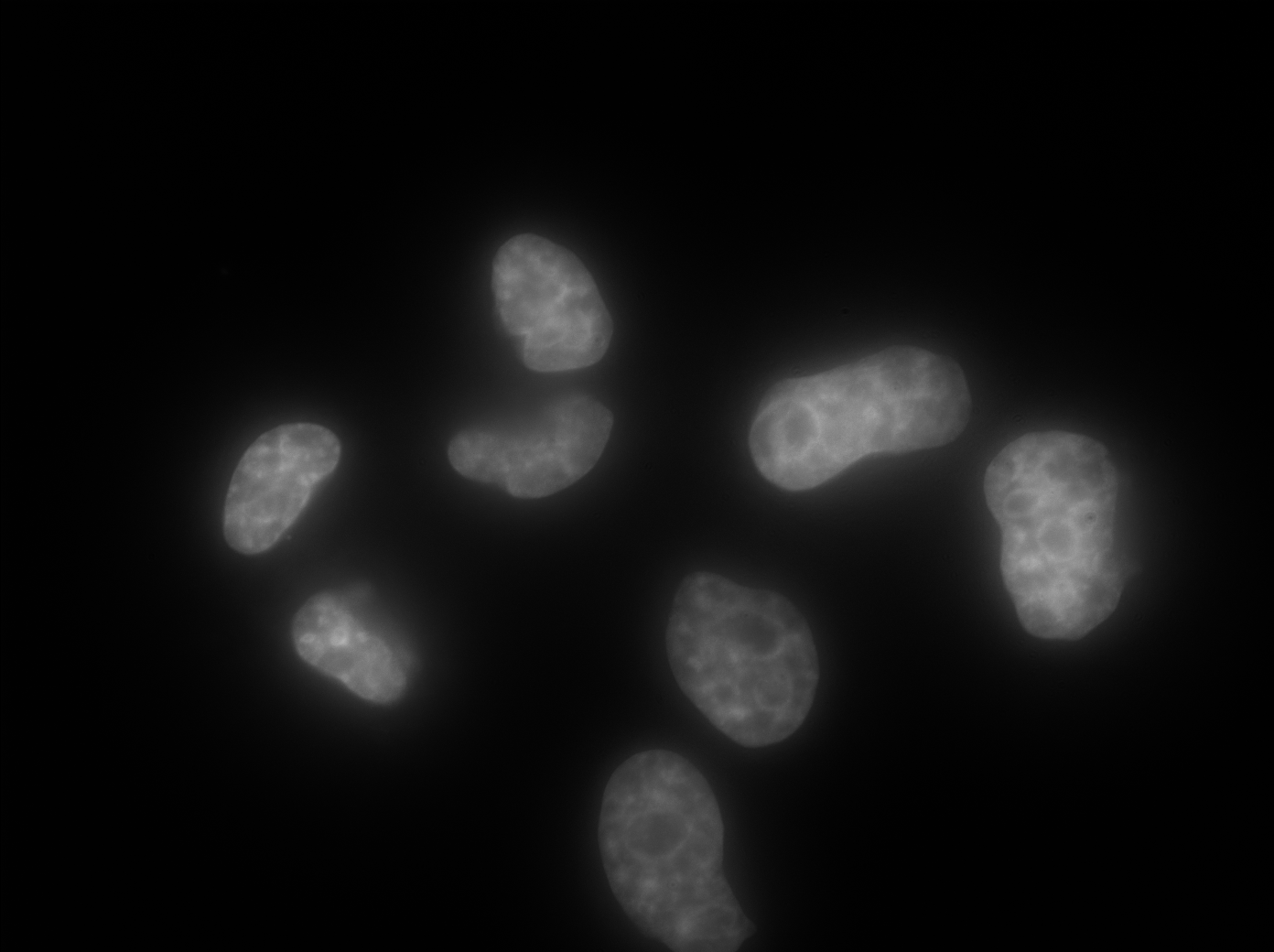

Supplement: Supplementary file 20 — Source data Fig. 6 part 1 [file 44319_2026_742_MOESM20_ESM.zip › Figure 6 Part 1/Fig 6abcd Cas9 TPGS1-KO acetylated tubulin atubulin/Cas9 R3 9-13-24 LT8LT9.Project Maximum Z_XY1726765747_Z0_T0_C0.tif]

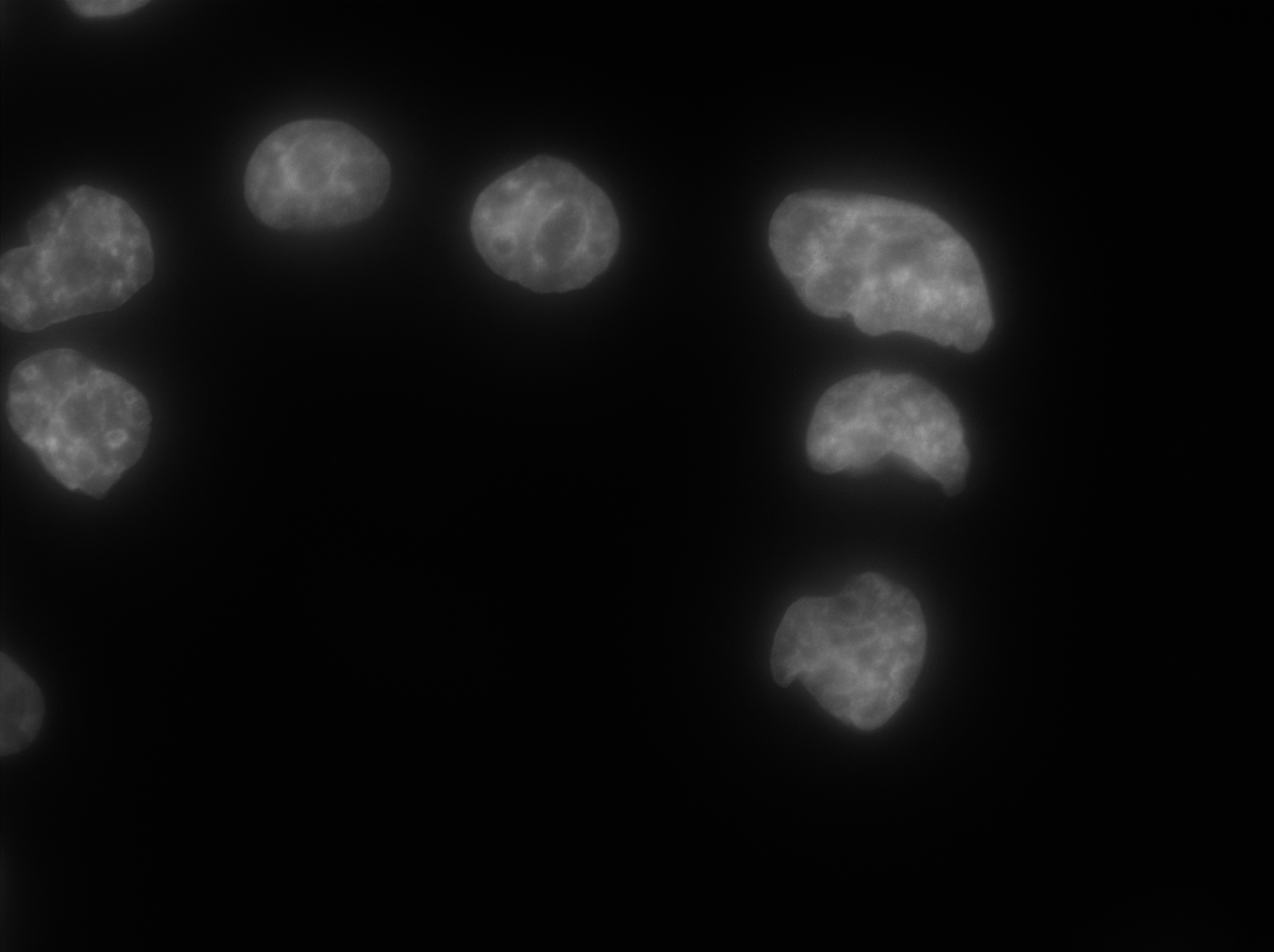

Supplement: Supplementary file 20 — Source data Fig. 6 part 1 [file 44319_2026_742_MOESM20_ESM.zip › Figure 6 Part 1/Fig 6abcd Cas9 TPGS1-KO acetylated tubulin atubulin/Cas9 R2 9-11-24 PA19.Project Maximum Z_XY1726180240_Z0_T0_C0.tif]

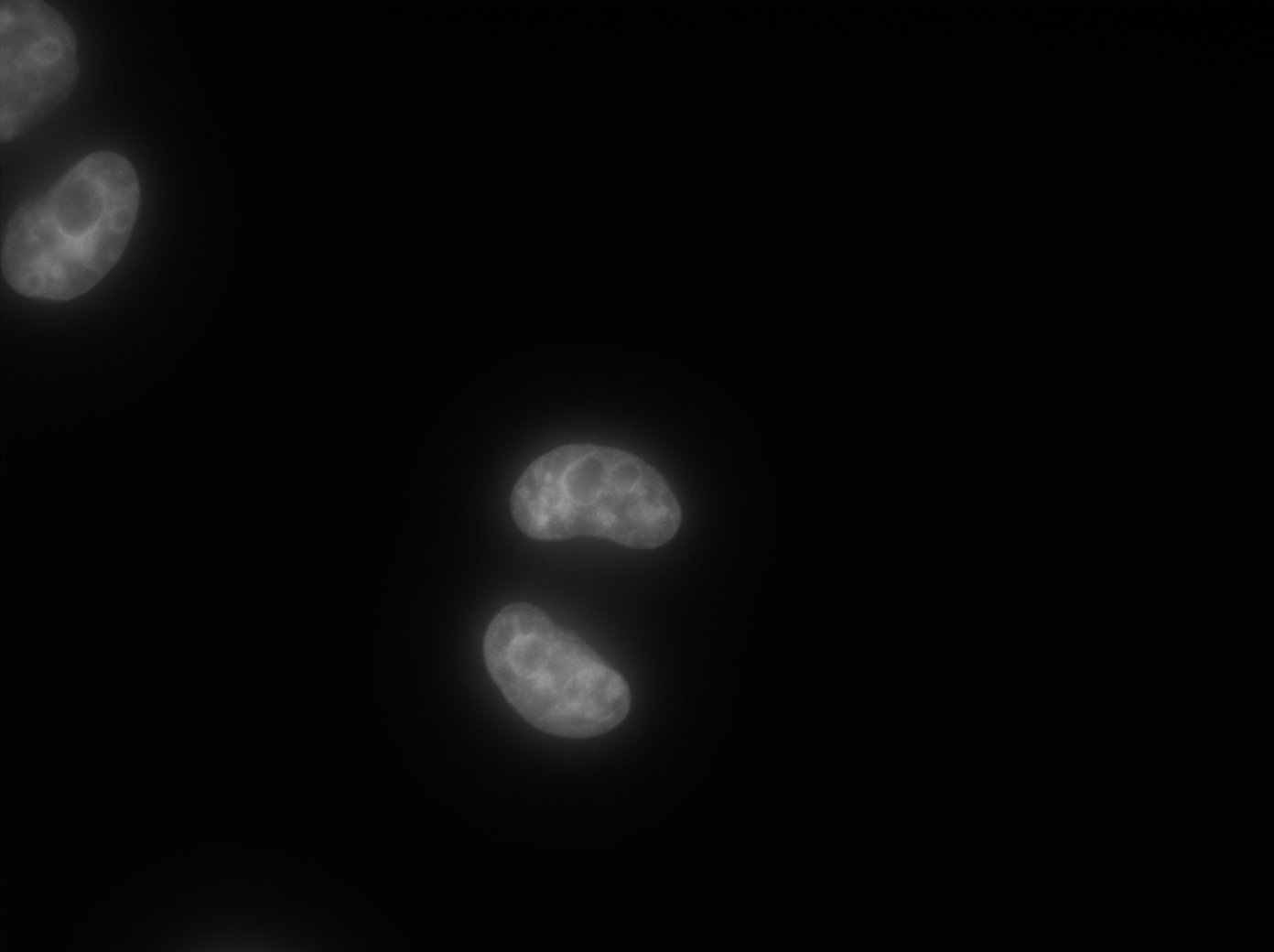

Supplement: Supplementary file 20 — Source data Fig. 6 part 1 [file 44319_2026_742_MOESM20_ESM.zip › Figure 6 Part 1/Fig 6abcd Cas9 TPGS1-KO acetylated tubulin atubulin/Cas9 R2 9-11-24 LT9.Project Maximum Z_XY1726173531_Z0_T0_C0.tif]

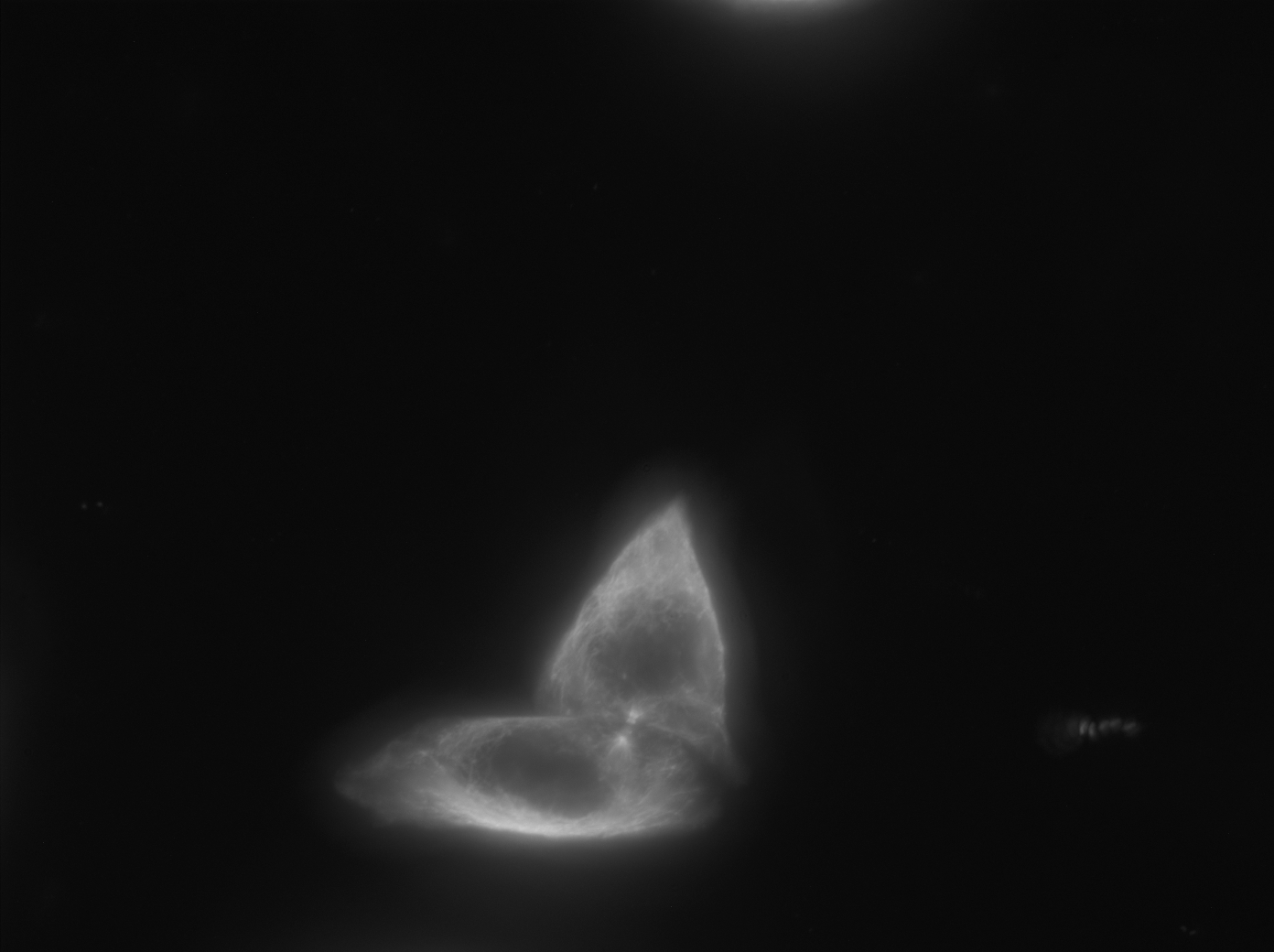

Supplement: Supplementary file 20 — Source data Fig. 6 part 1 [file 44319_2026_742_MOESM20_ESM.zip › Figure 6 Part 1/Fig 6abcd Cas9 TPGS1-KO acetylated tubulin atubulin/Cas9 R2 9-11-24 LT30.Project Maximum Z_XY1726181687_Z0_T0_C1.tif]

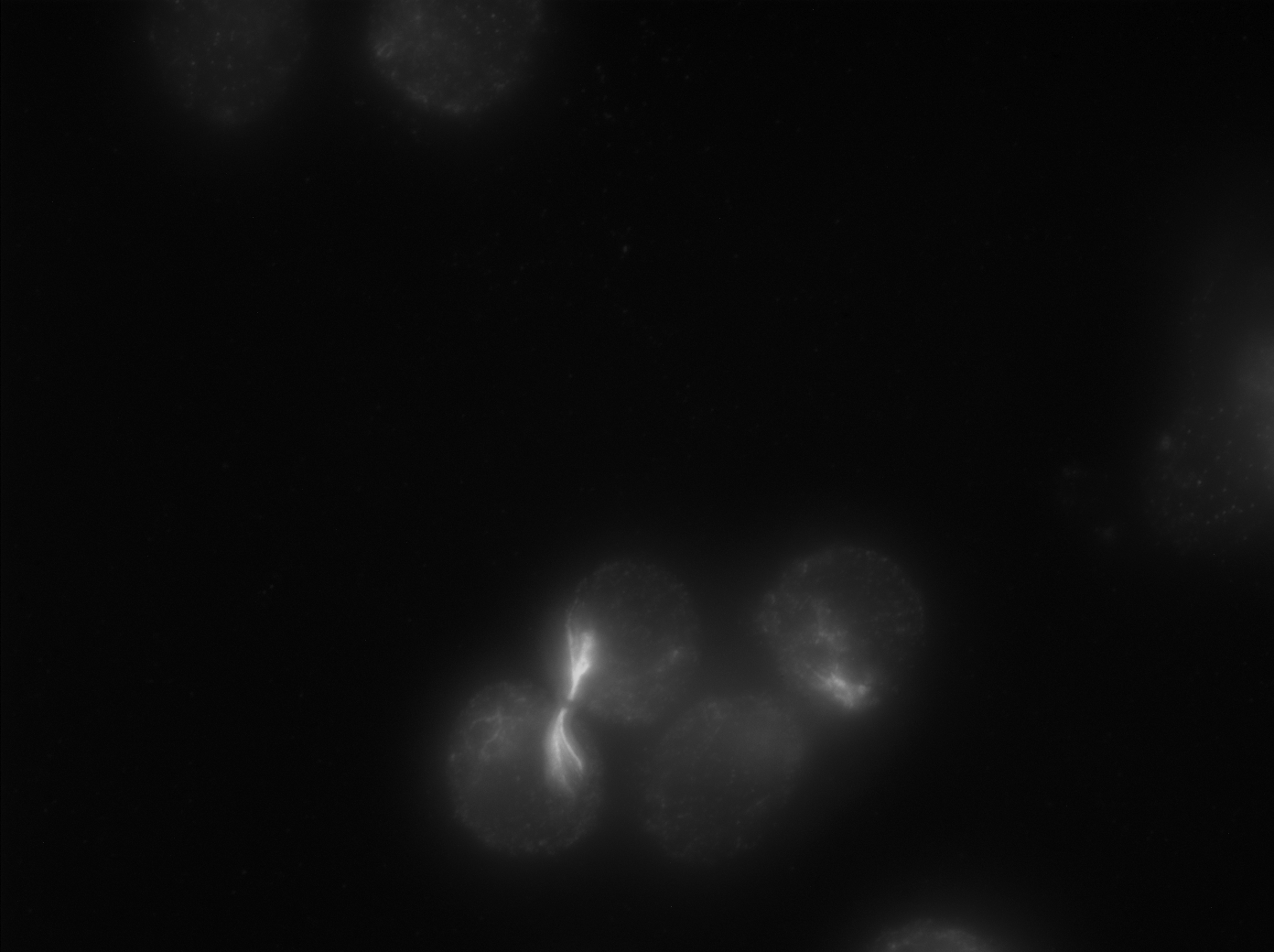

Supplement: Supplementary file 20 — Source data Fig. 6 part 1 [file 44319_2026_742_MOESM20_ESM.zip › Figure 6 Part 1/Fig 6abcd Cas9 TPGS1-KO acetylated tubulin atubulin/Cas9 R2 9-11-24 LT1.Project Maximum Z_XY1726172198_Z0_T0_C2.tif]

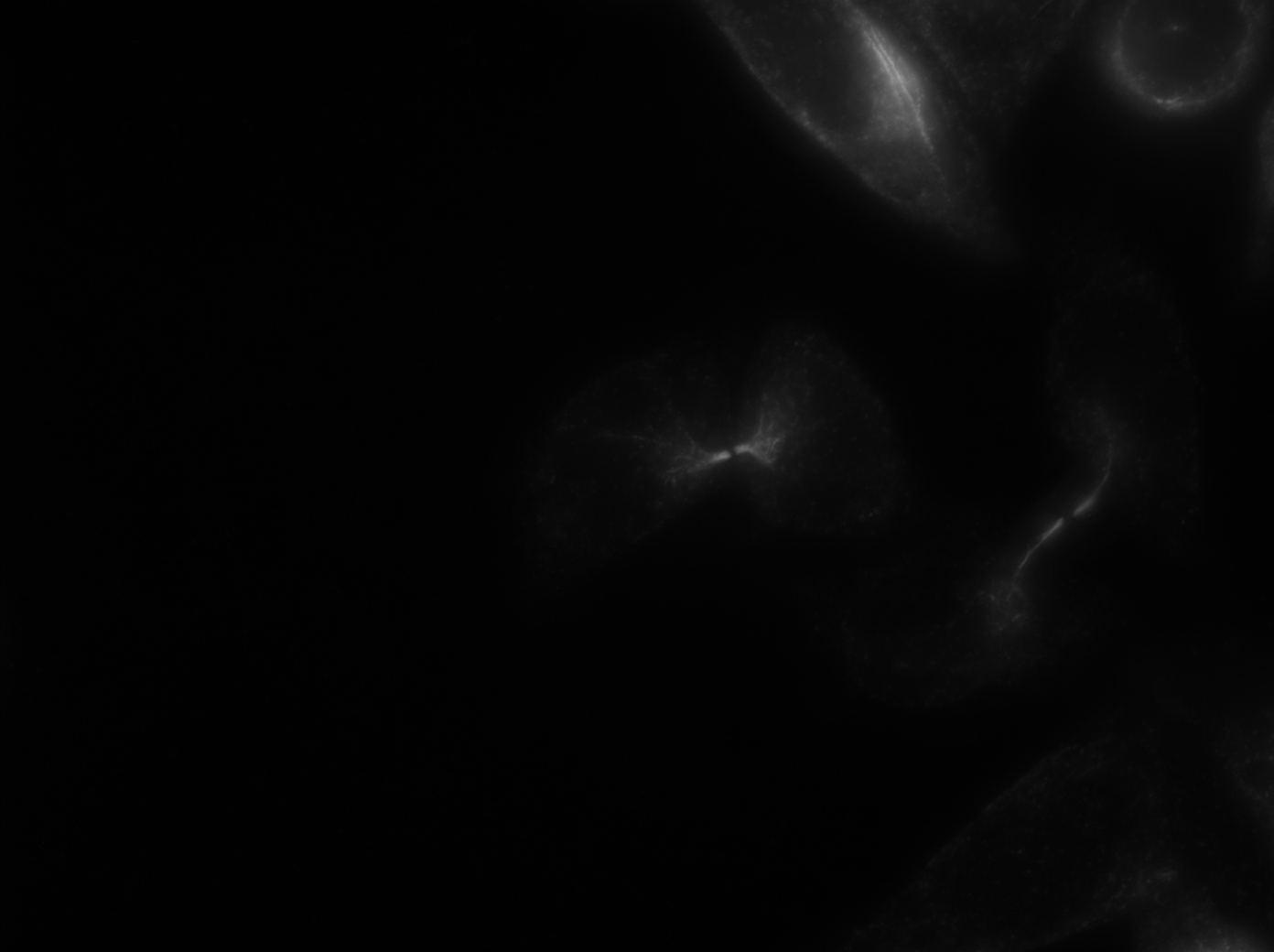

Supplement: Supplementary file 20 — Source data Fig. 6 part 1 [file 44319_2026_742_MOESM20_ESM.zip › Figure 6 Part 1/Fig 6abcd Cas9 TPGS1-KO acetylated tubulin atubulin/Cas9 R3 9-13-24 LT10LT11.Project Maximum Z_XY1726765869_Z0_T0_C2.tif]

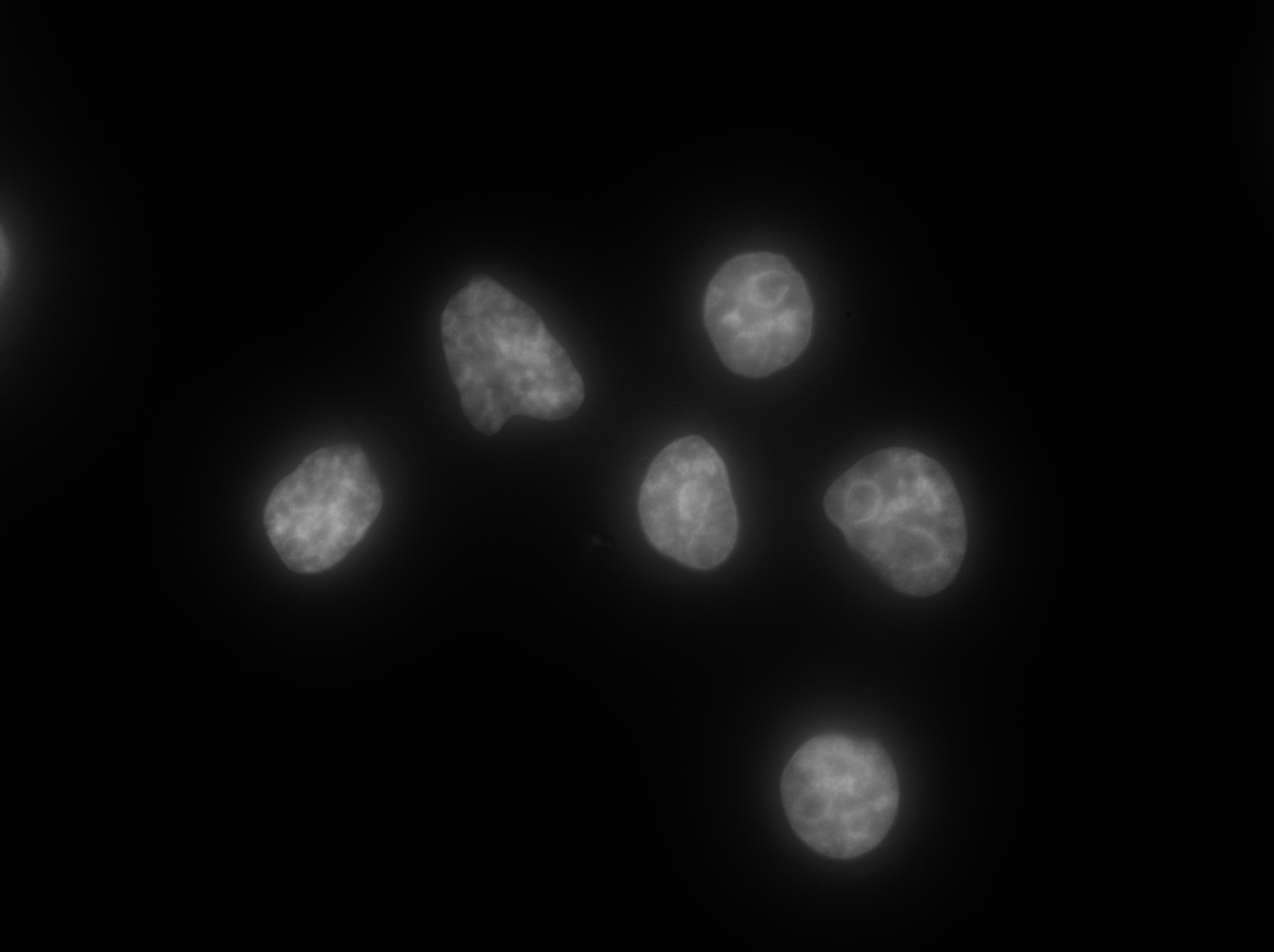

Supplement: Supplementary file 20 — Source data Fig. 6 part 1 [file 44319_2026_742_MOESM20_ESM.zip › Figure 6 Part 1/Fig 6abcd Cas9 TPGS1-KO acetylated tubulin atubulin/Cas9 R3 9-13-24 LT4 PA1.Project Maximum Z_XY1726765489_Z0_T0_C0.tif]

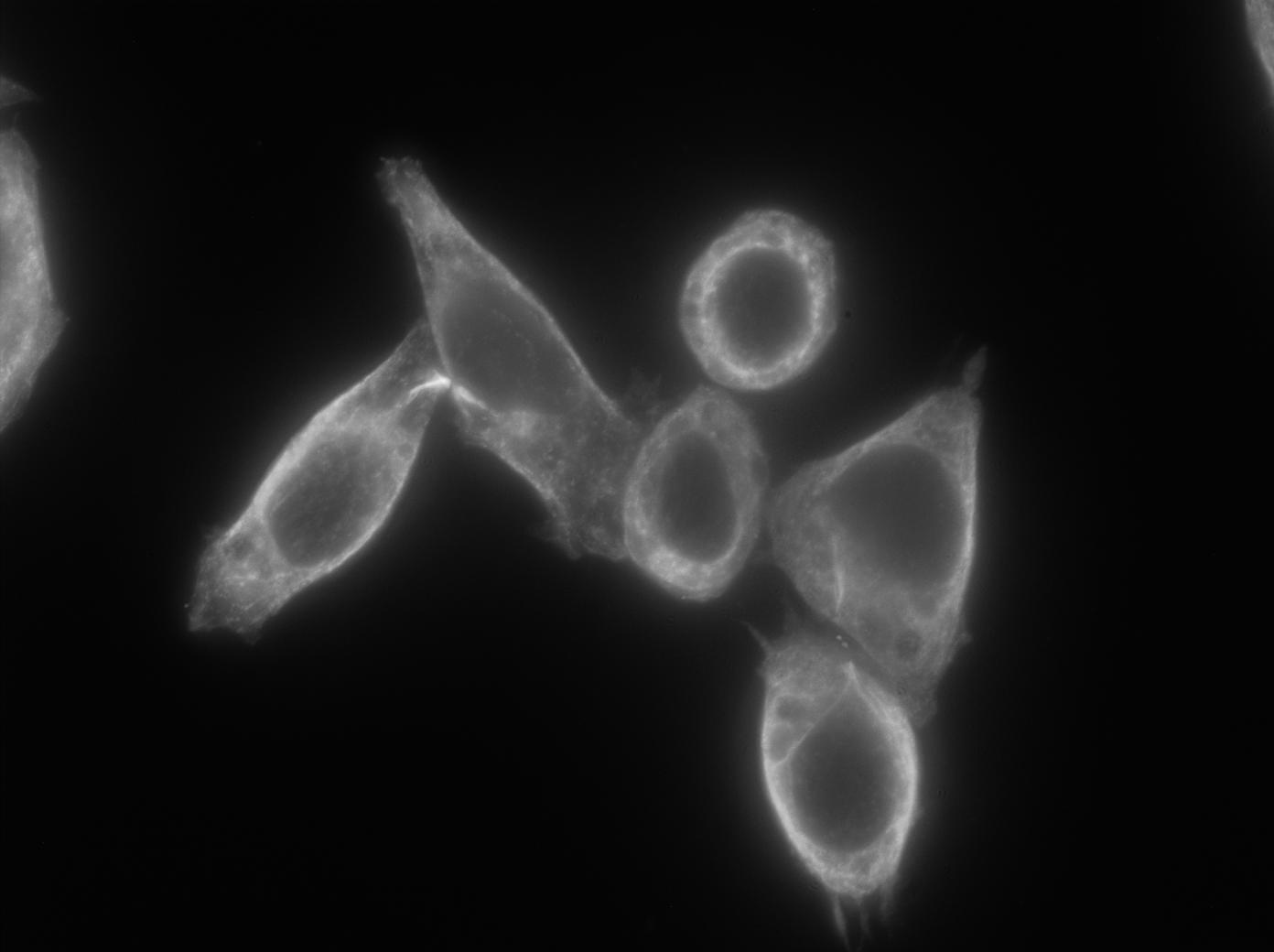

Supplement: Supplementary file 20 — Source data Fig. 6 part 1 [file 44319_2026_742_MOESM20_ESM.zip › Figure 6 Part 1/Fig 6abcd Cas9 TPGS1-KO acetylated tubulin atubulin/Cas9 R3 9-13-24 LT4 PA1.Project Maximum Z_XY1726765489_Z0_T0_C1.tif]

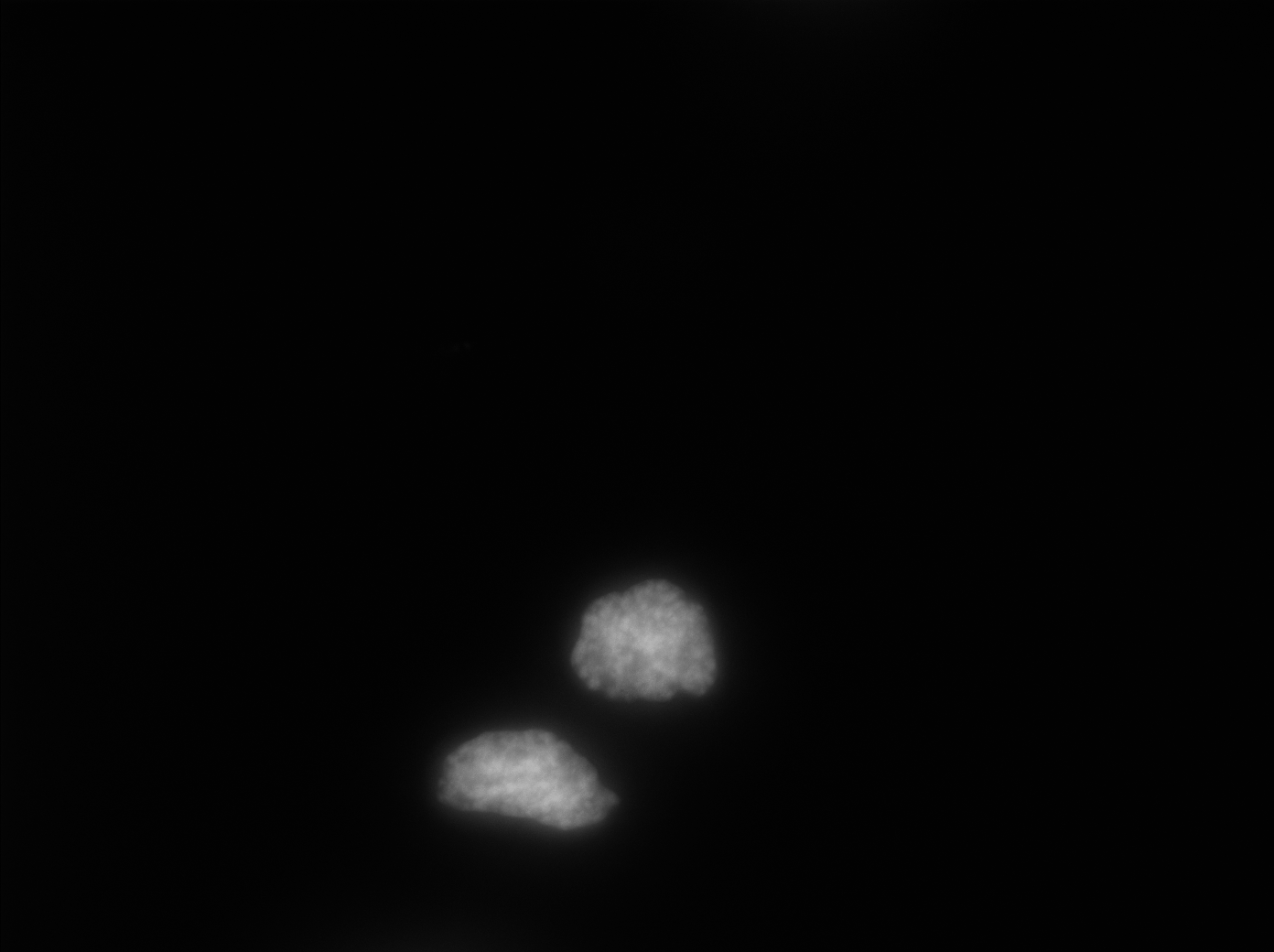

Supplement: Supplementary file 20 — Source data Fig. 6 part 1 [file 44319_2026_742_MOESM20_ESM.zip › Figure 6 Part 1/Fig 6abcd Cas9 TPGS1-KO acetylated tubulin atubulin/Cas9 R2 9-11-24 LT30.Project Maximum Z_XY1726181687_Z0_T0_C0.tif]

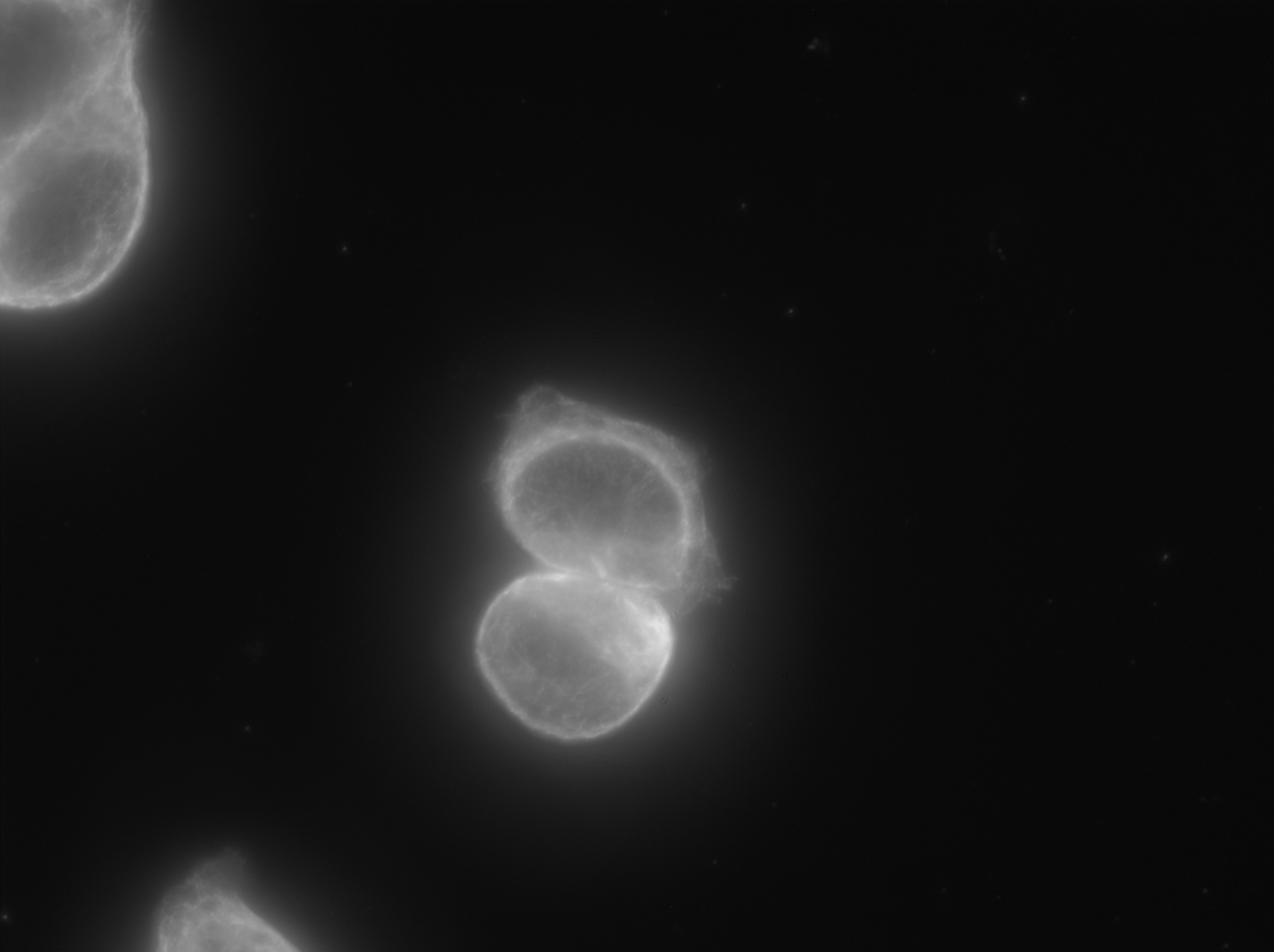

Supplement: Supplementary file 20 — Source data Fig. 6 part 1 [file 44319_2026_742_MOESM20_ESM.zip › Figure 6 Part 1/Fig 6abcd Cas9 TPGS1-KO acetylated tubulin atubulin/Cas9 R2 9-11-24 LT9.Project Maximum Z_XY1726173531_Z0_T0_C1.tif]

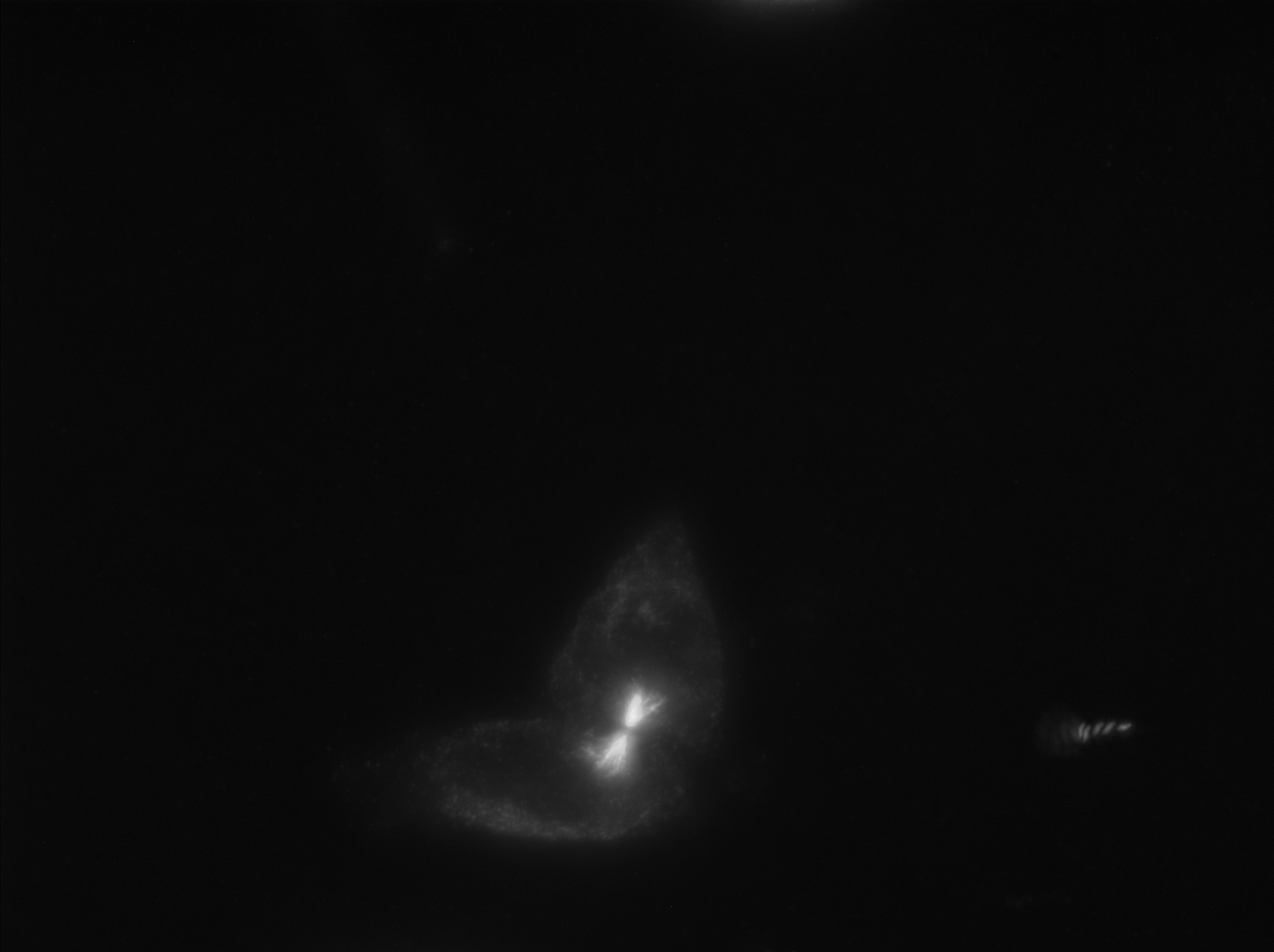

Supplement: Supplementary file 20 — Source data Fig. 6 part 1 [file 44319_2026_742_MOESM20_ESM.zip › Figure 6 Part 1/Fig 6abcd Cas9 TPGS1-KO acetylated tubulin atubulin/Cas9 R2 9-11-24 LT30.Project Maximum Z_XY1726181687_Z0_T0_C2.tif]

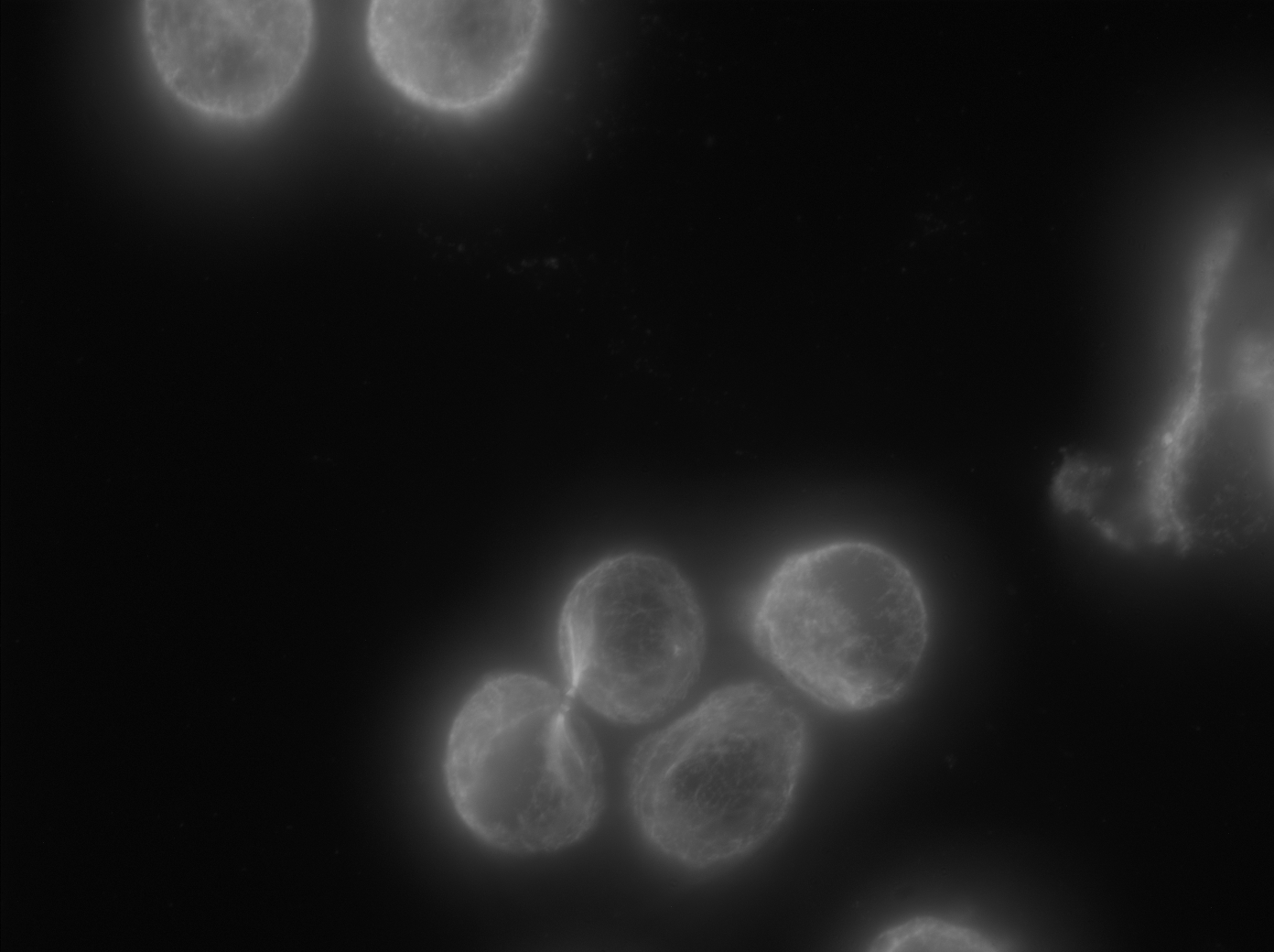

Supplement: Supplementary file 20 — Source data Fig. 6 part 1 [file 44319_2026_742_MOESM20_ESM.zip › Figure 6 Part 1/Fig 6abcd Cas9 TPGS1-KO acetylated tubulin atubulin/Cas9 R2 9-11-24 LT1.Project Maximum Z_XY1726172198_Z0_T0_C1.tif]

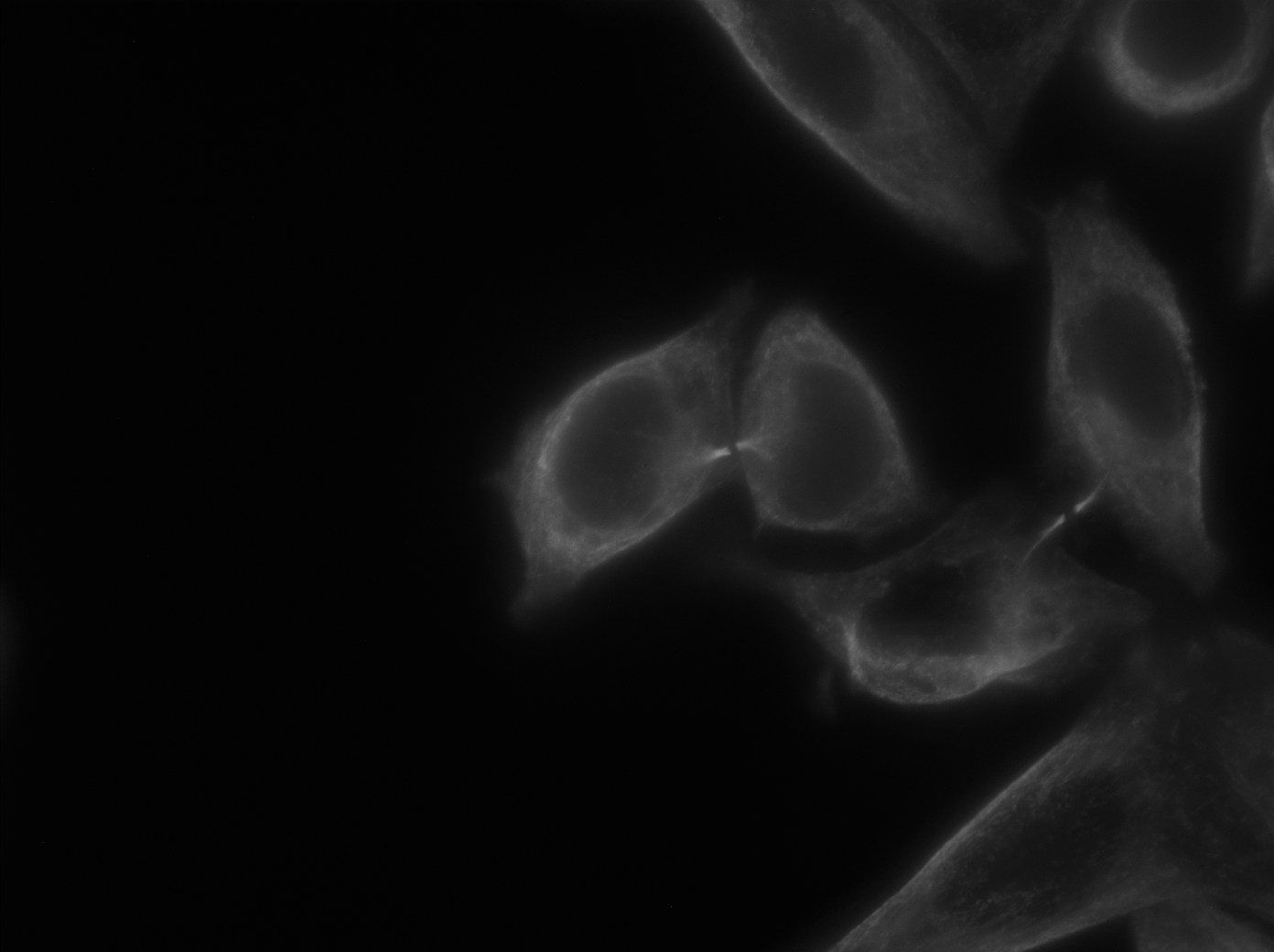

Supplement: Supplementary file 20 — Source data Fig. 6 part 1 [file 44319_2026_742_MOESM20_ESM.zip › Figure 6 Part 1/Fig 6abcd Cas9 TPGS1-KO acetylated tubulin atubulin/Cas9 R3 9-13-24 LT10LT11.Project Maximum Z_XY1726765869_Z0_T0_C1.tif]

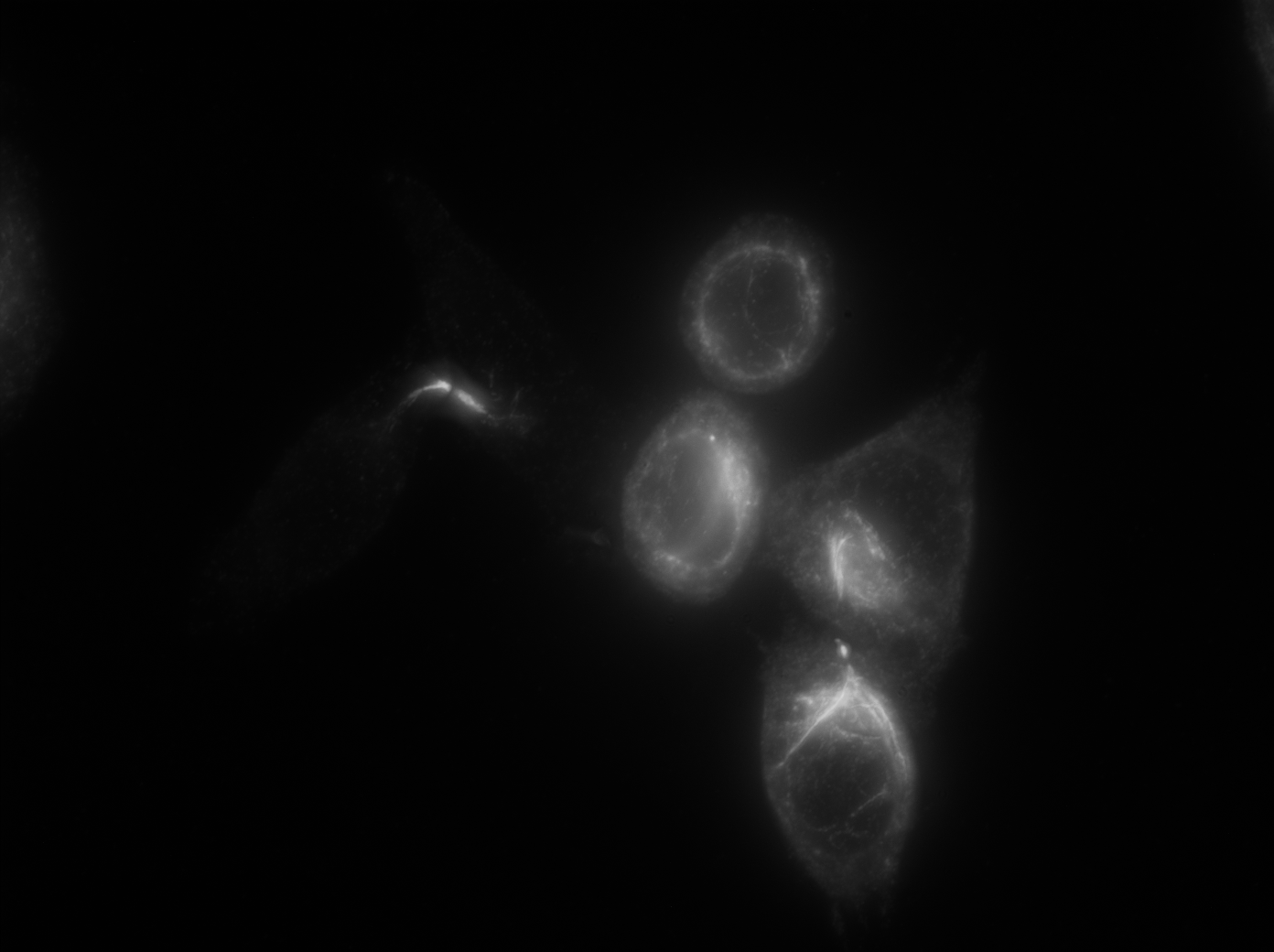

Supplement: Supplementary file 20 — Source data Fig. 6 part 1 [file 44319_2026_742_MOESM20_ESM.zip › Figure 6 Part 1/Fig 6abcd Cas9 TPGS1-KO acetylated tubulin atubulin/Cas9 R3 9-13-24 LT4 PA1.Project Maximum Z_XY1726765489_Z0_T0_C2.tif]

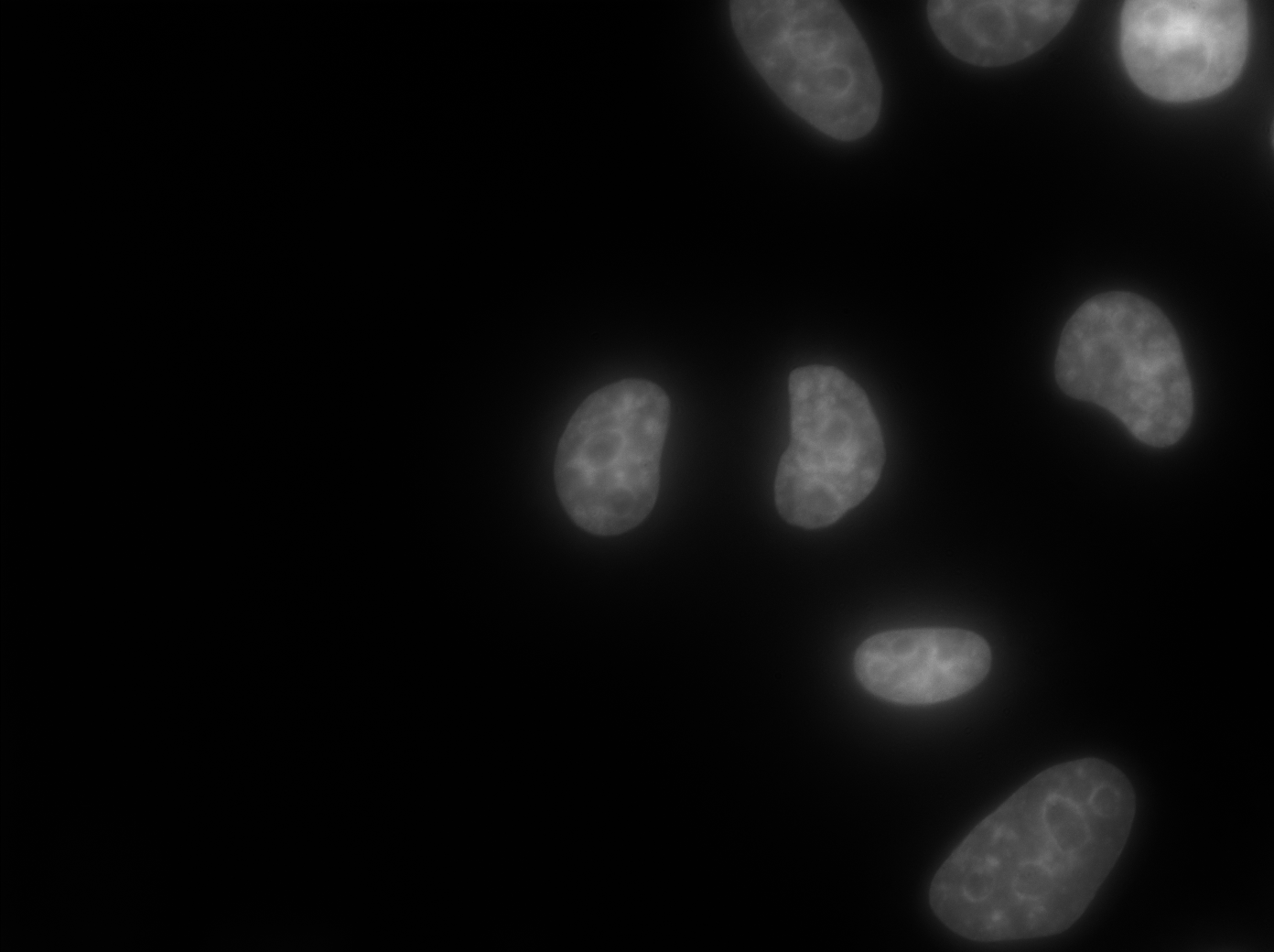

Supplement: Supplementary file 20 — Source data Fig. 6 part 1 [file 44319_2026_742_MOESM20_ESM.zip › Figure 6 Part 1/Fig 6abcd Cas9 TPGS1-KO acetylated tubulin atubulin/Cas9 R3 9-13-24 LT10LT11.Project Maximum Z_XY1726765869_Z0_T0_C0.tif]

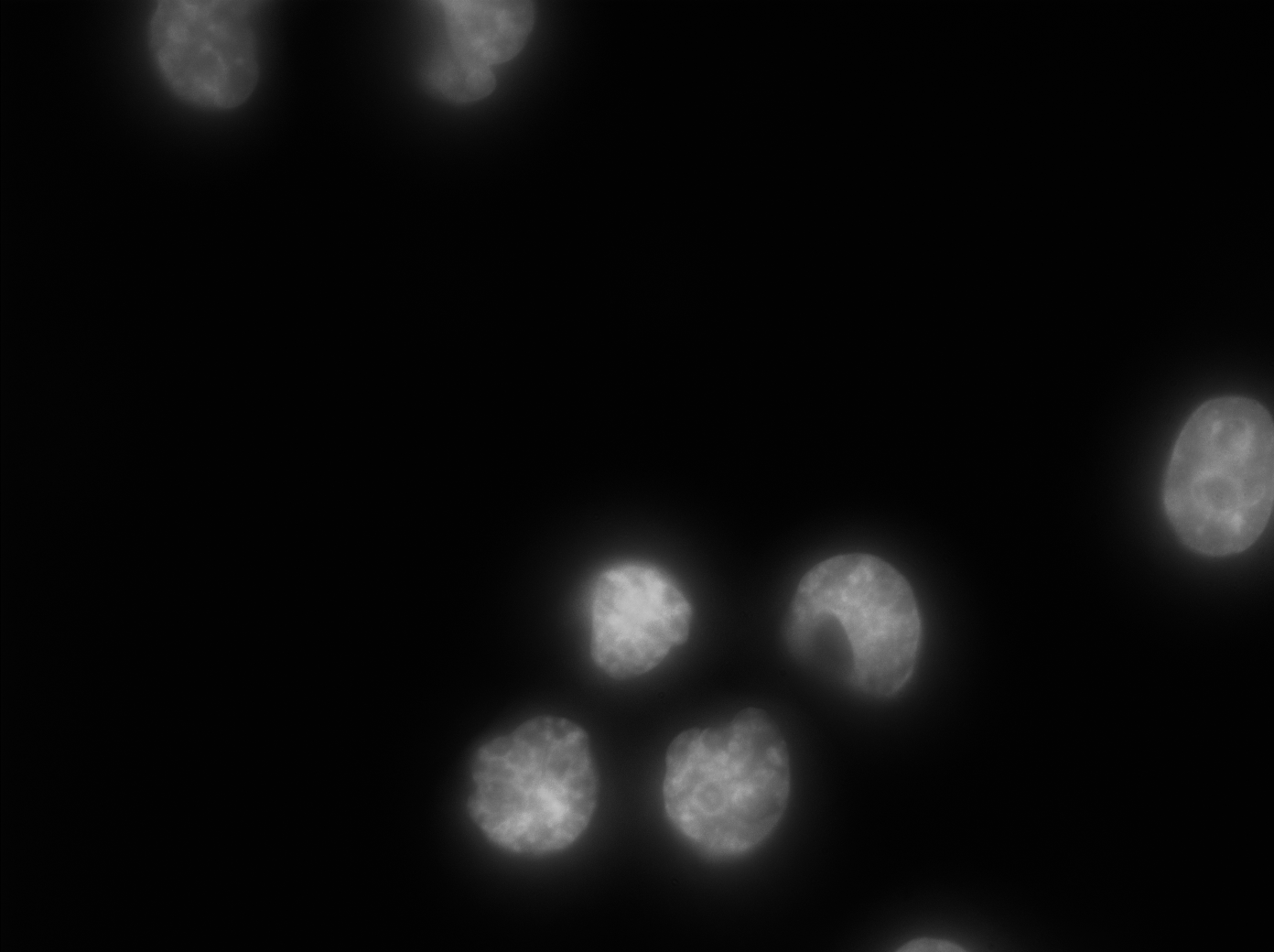

Supplement: Supplementary file 20 — Source data Fig. 6 part 1 [file 44319_2026_742_MOESM20_ESM.zip › Figure 6 Part 1/Fig 6abcd Cas9 TPGS1-KO acetylated tubulin atubulin/Cas9 R2 9-11-24 LT1.Project Maximum Z_XY1726172198_Z0_T0_C0.tif]

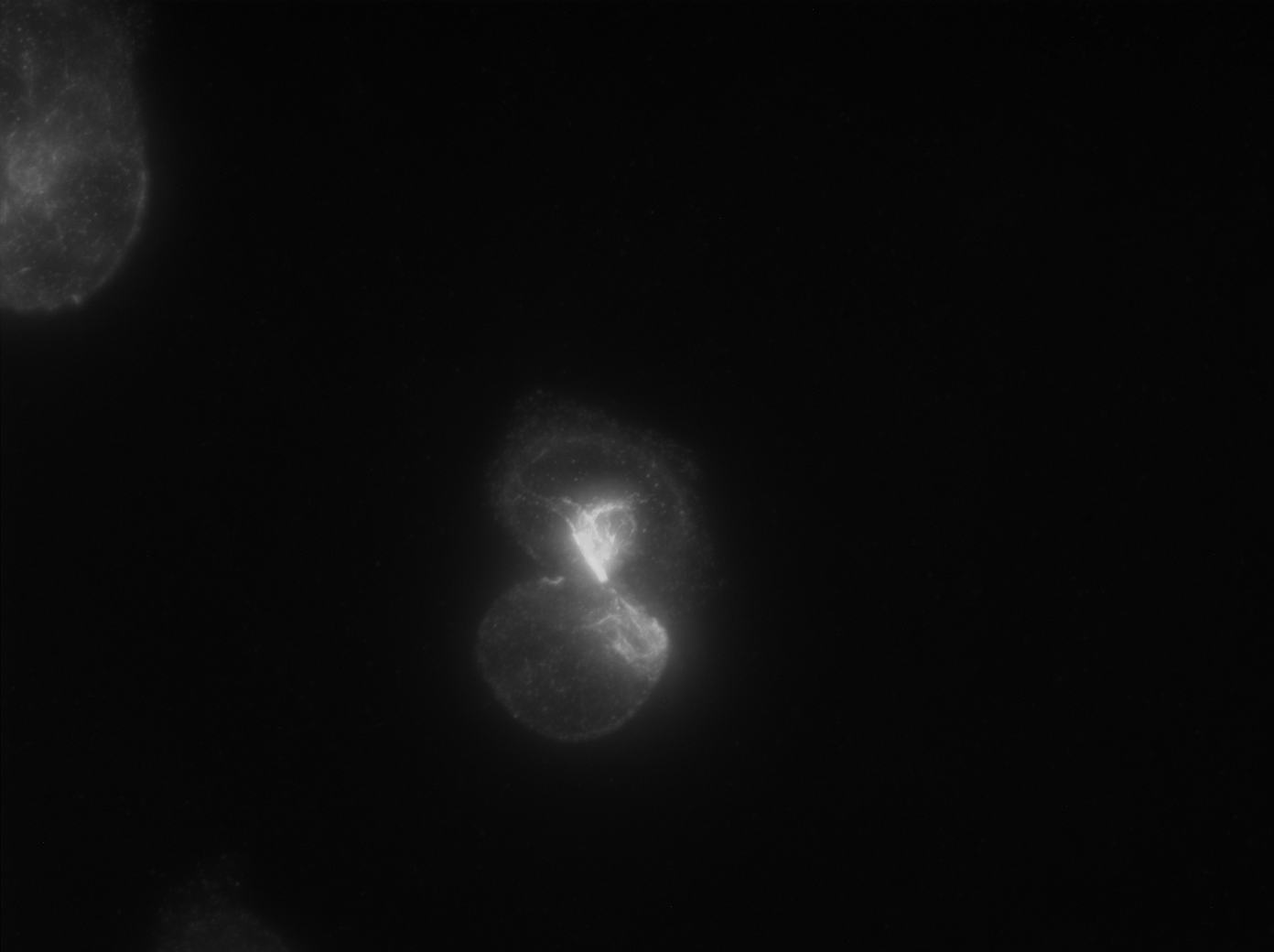

Supplement: Supplementary file 20 — Source data Fig. 6 part 1 [file 44319_2026_742_MOESM20_ESM.zip › Figure 6 Part 1/Fig 6abcd Cas9 TPGS1-KO acetylated tubulin atubulin/Cas9 R2 9-11-24 LT9.Project Maximum Z_XY1726173531_Z0_T0_C2.tif]

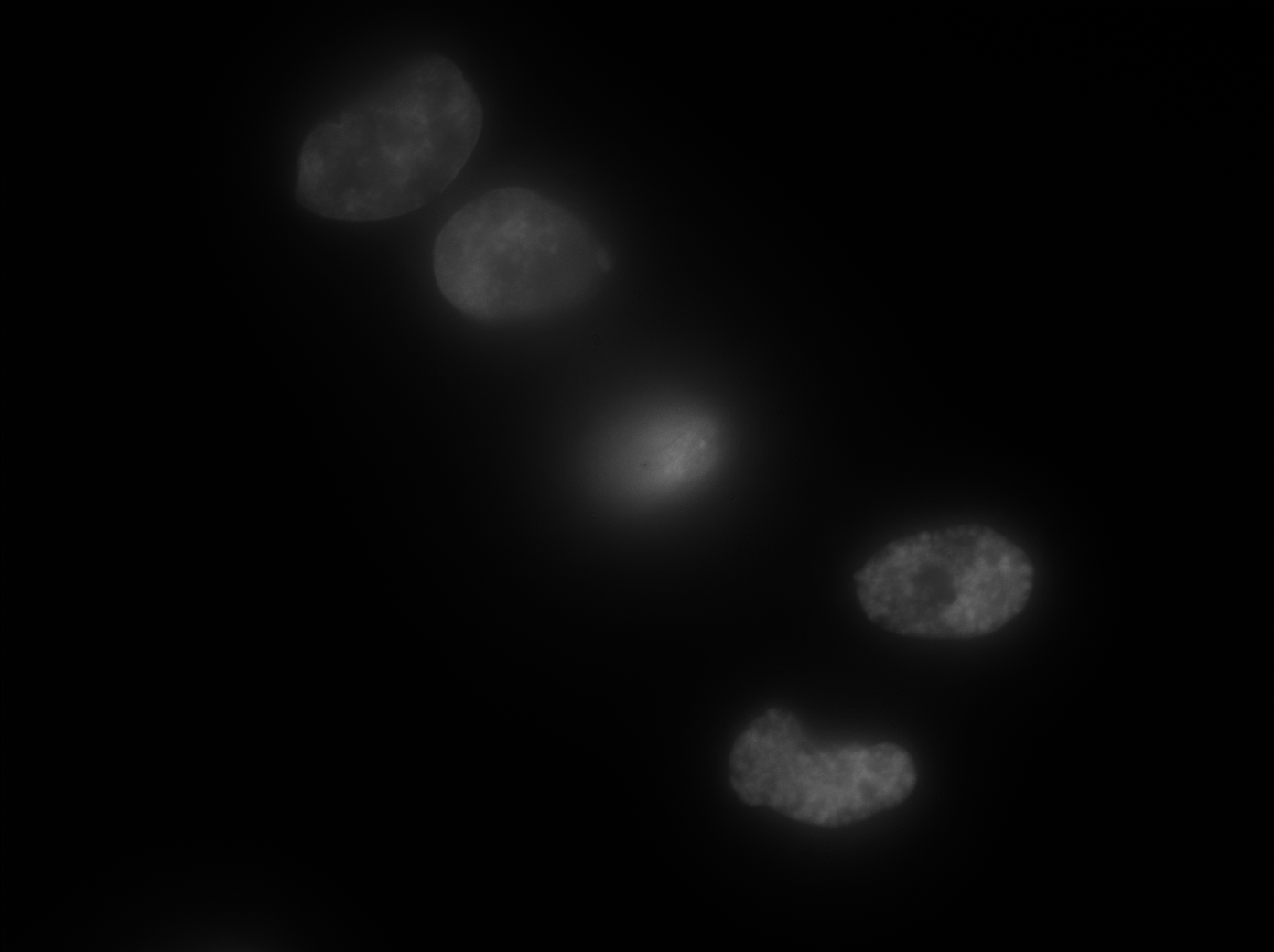

Supplement: Supplementary file 20 — Source data Fig. 6 part 1 [file 44319_2026_742_MOESM20_ESM.zip › Figure 6 Part 1/Fig 6abcd Cas9 TPGS1-KO acetylated tubulin atubulin/Cas9 R3 9-13-24 LT25.Project Maximum Z_XY1726767357_Z0_T0_C0.tif]

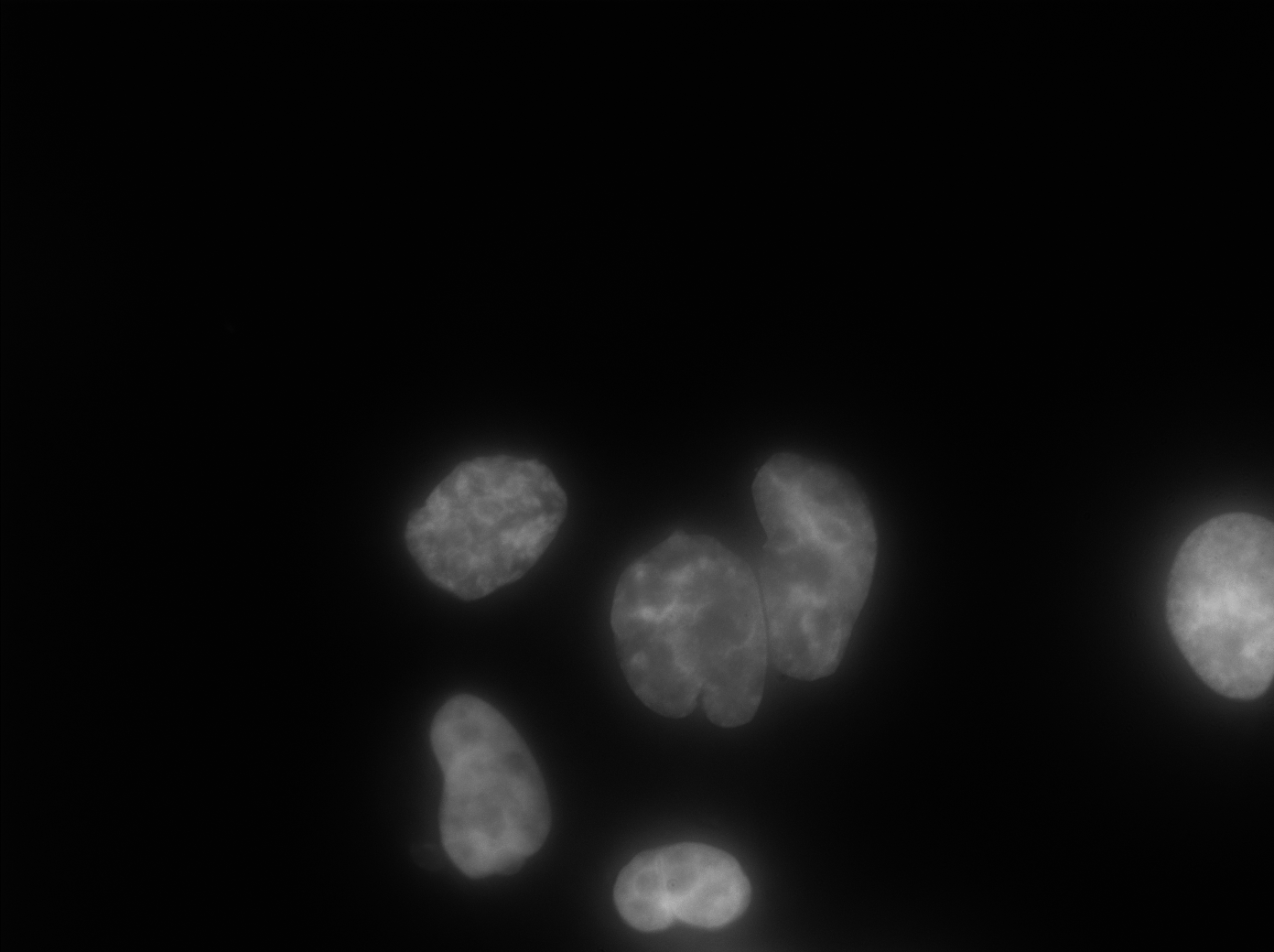

Supplement: Supplementary file 20 — Source data Fig. 6 part 1 [file 44319_2026_742_MOESM20_ESM.zip › Figure 6 Part 1/Fig 6abcd Cas9 TPGS1-KO acetylated tubulin atubulin/Cas9 R2 9-11-24 PA18.Project Maximum Z_XY1726180048_Z0_T0_C0.tif]

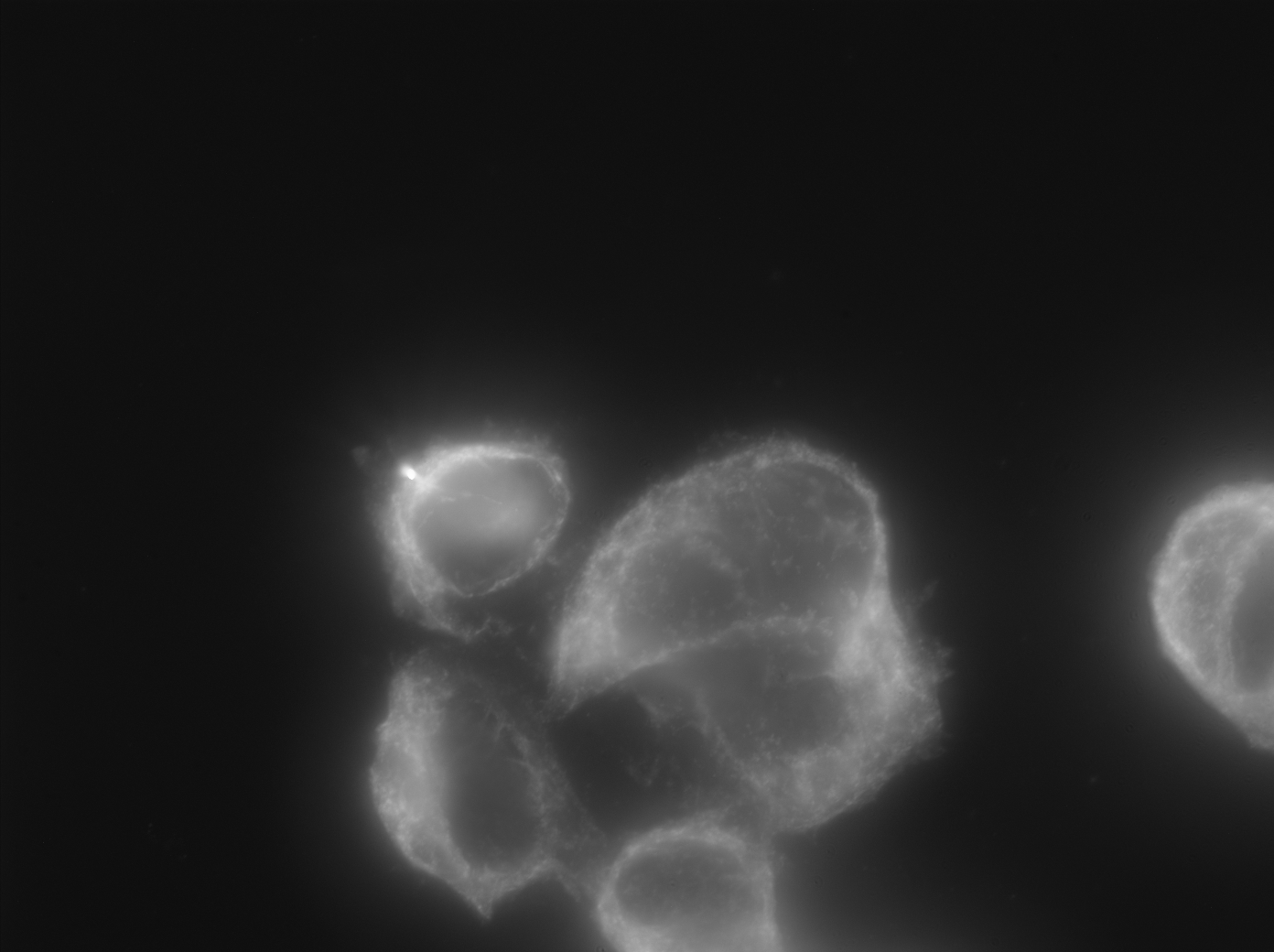

Supplement: Supplementary file 20 — Source data Fig. 6 part 1 [file 44319_2026_742_MOESM20_ESM.zip › Figure 6 Part 1/Fig 6abcd Cas9 TPGS1-KO acetylated tubulin atubulin/Cas9 R2 9-11-24 PA18.Project Maximum Z_XY1726180048_Z0_T0_C1.tif]

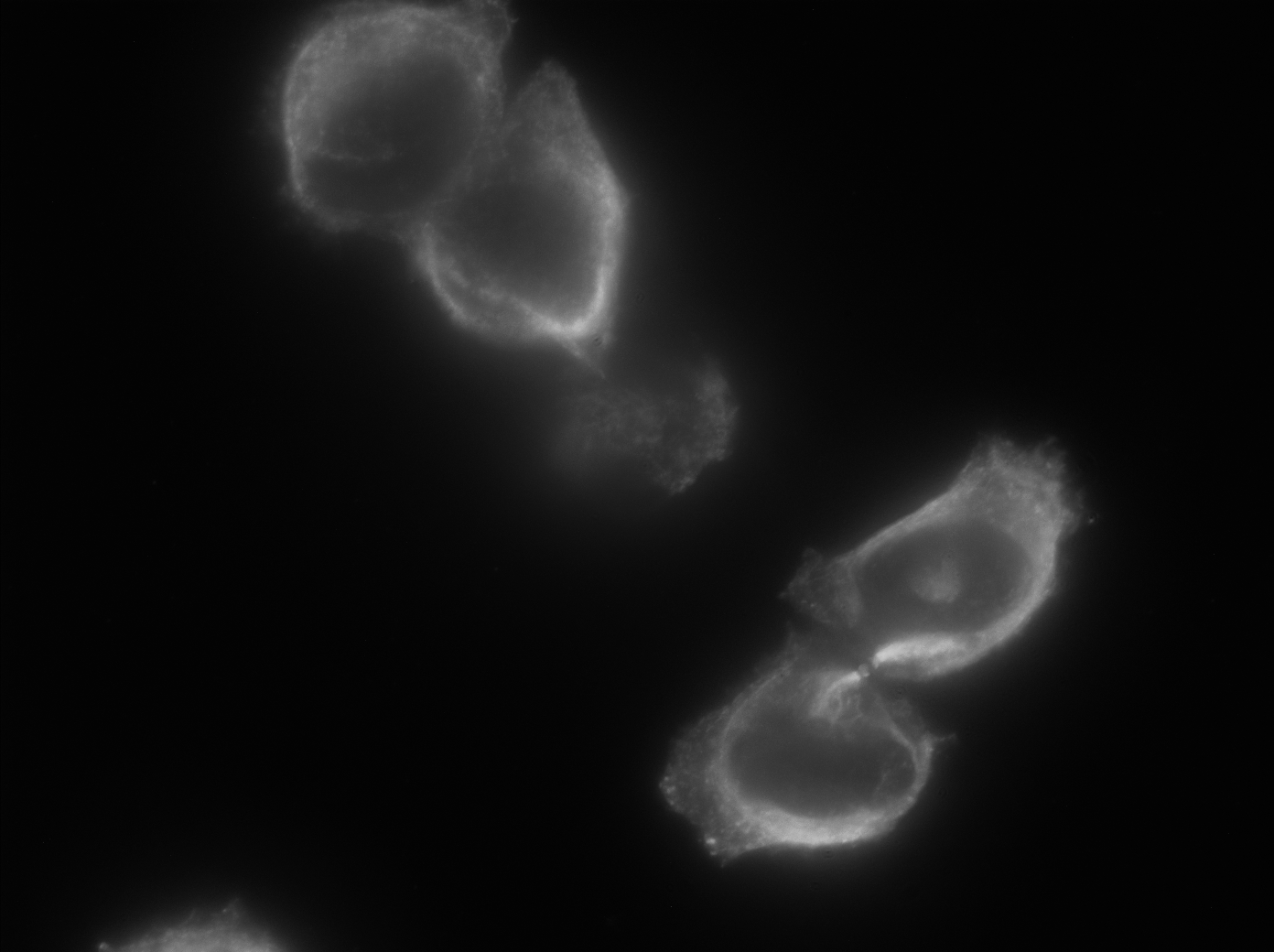

Supplement: Supplementary file 20 — Source data Fig. 6 part 1 [file 44319_2026_742_MOESM20_ESM.zip › Figure 6 Part 1/Fig 6abcd Cas9 TPGS1-KO acetylated tubulin atubulin/Cas9 R3 9-13-24 LT25.Project Maximum Z_XY1726767357_Z0_T0_C1.tif]

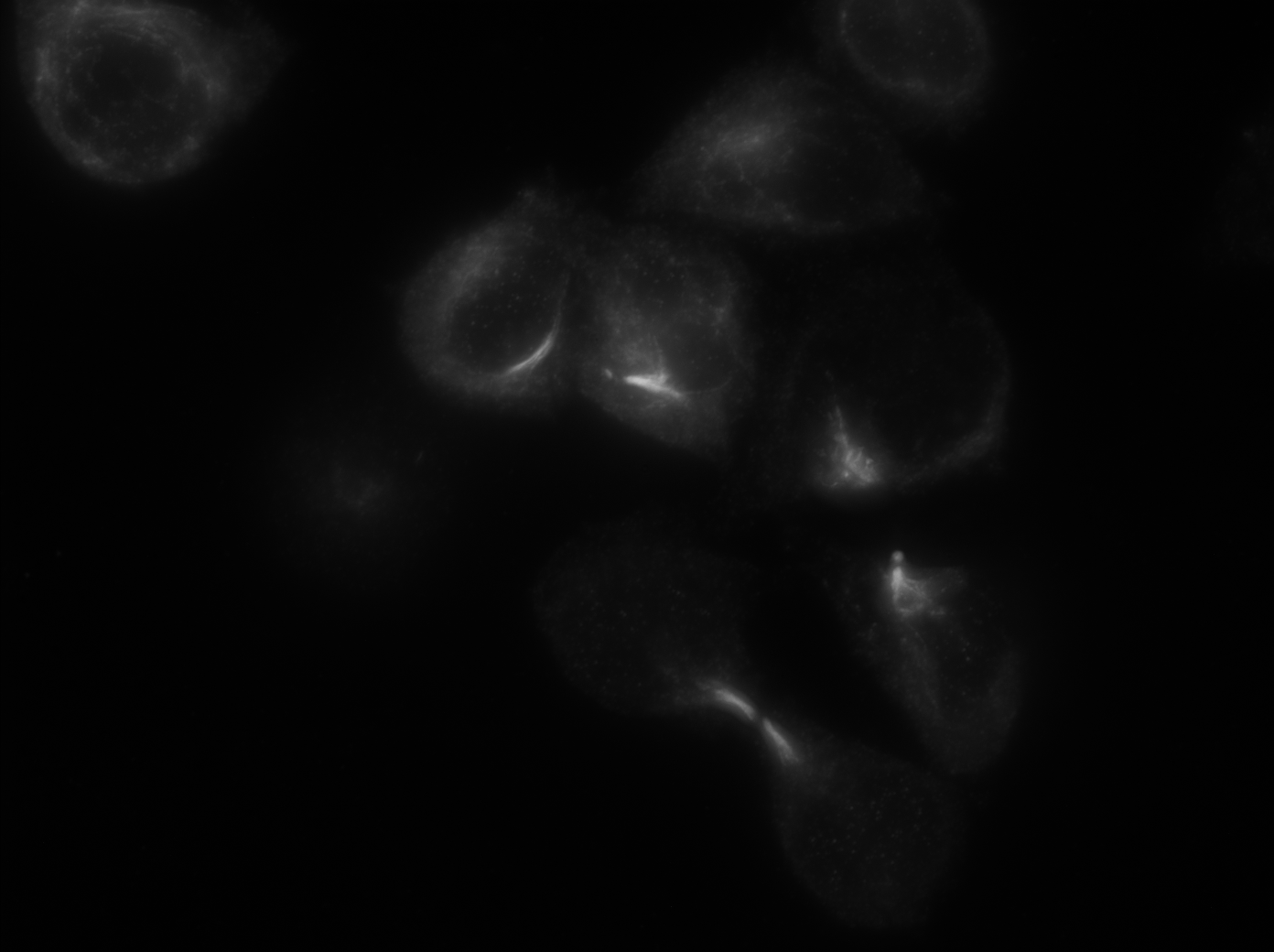

Supplement: Supplementary file 20 — Source data Fig. 6 part 1 [file 44319_2026_742_MOESM20_ESM.zip › Figure 6 Part 1/Fig 6abcd Cas9 TPGS1-KO acetylated tubulin atubulin/Cas9 R3 9-13-24 LT27 PA2PA3.Project Maximum Z_XY1726767575_Z0_T0_C2.tif]

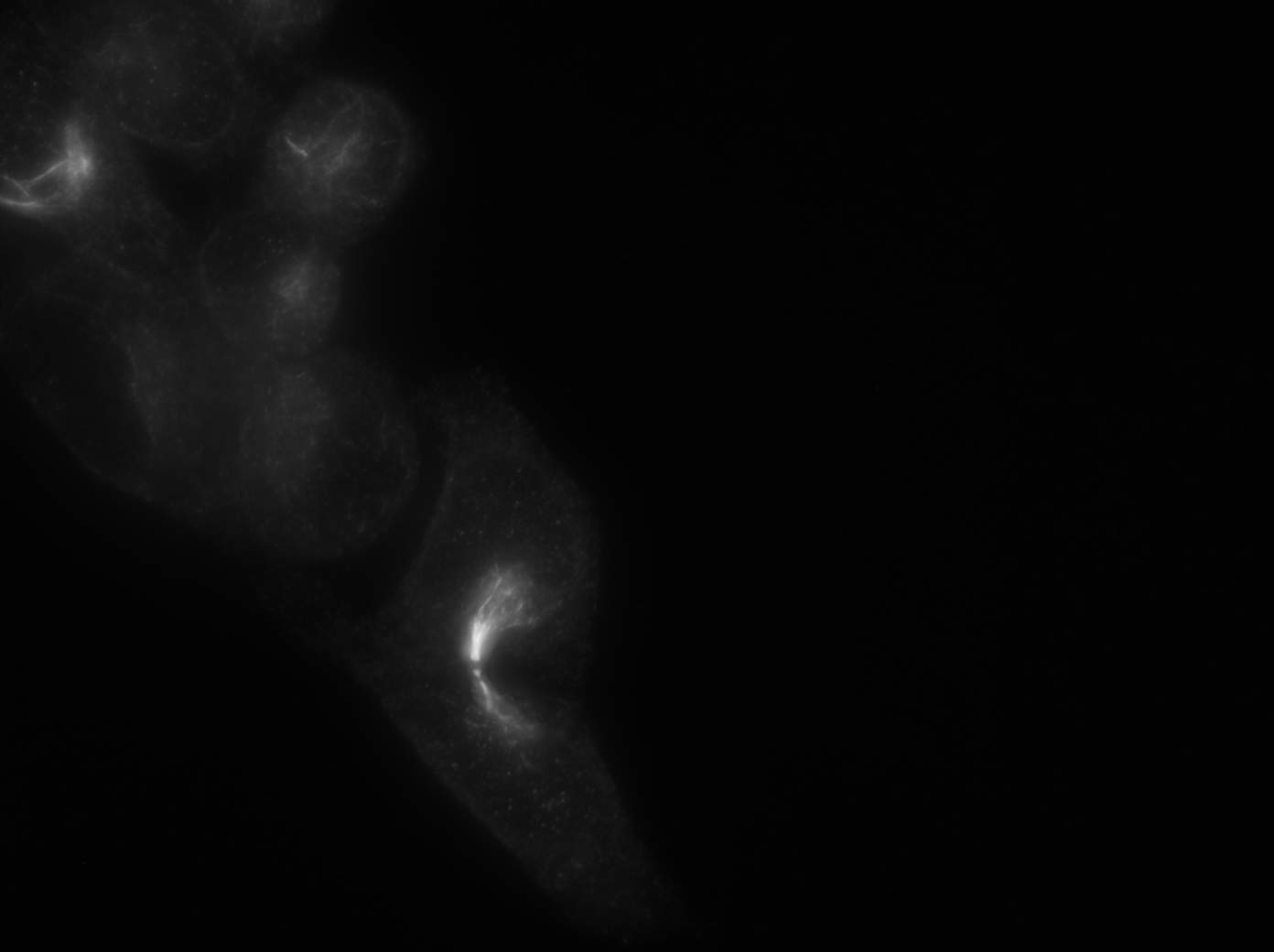

Supplement: Supplementary file 20 — Source data Fig. 6 part 1 [file 44319_2026_742_MOESM20_ESM.zip › Figure 6 Part 1/Fig 6abcd Cas9 TPGS1-KO acetylated tubulin atubulin/Cas9 R3 9-13-24 LT28.Project Maximum Z_XY1726767691_Z0_T0_C2.tif]

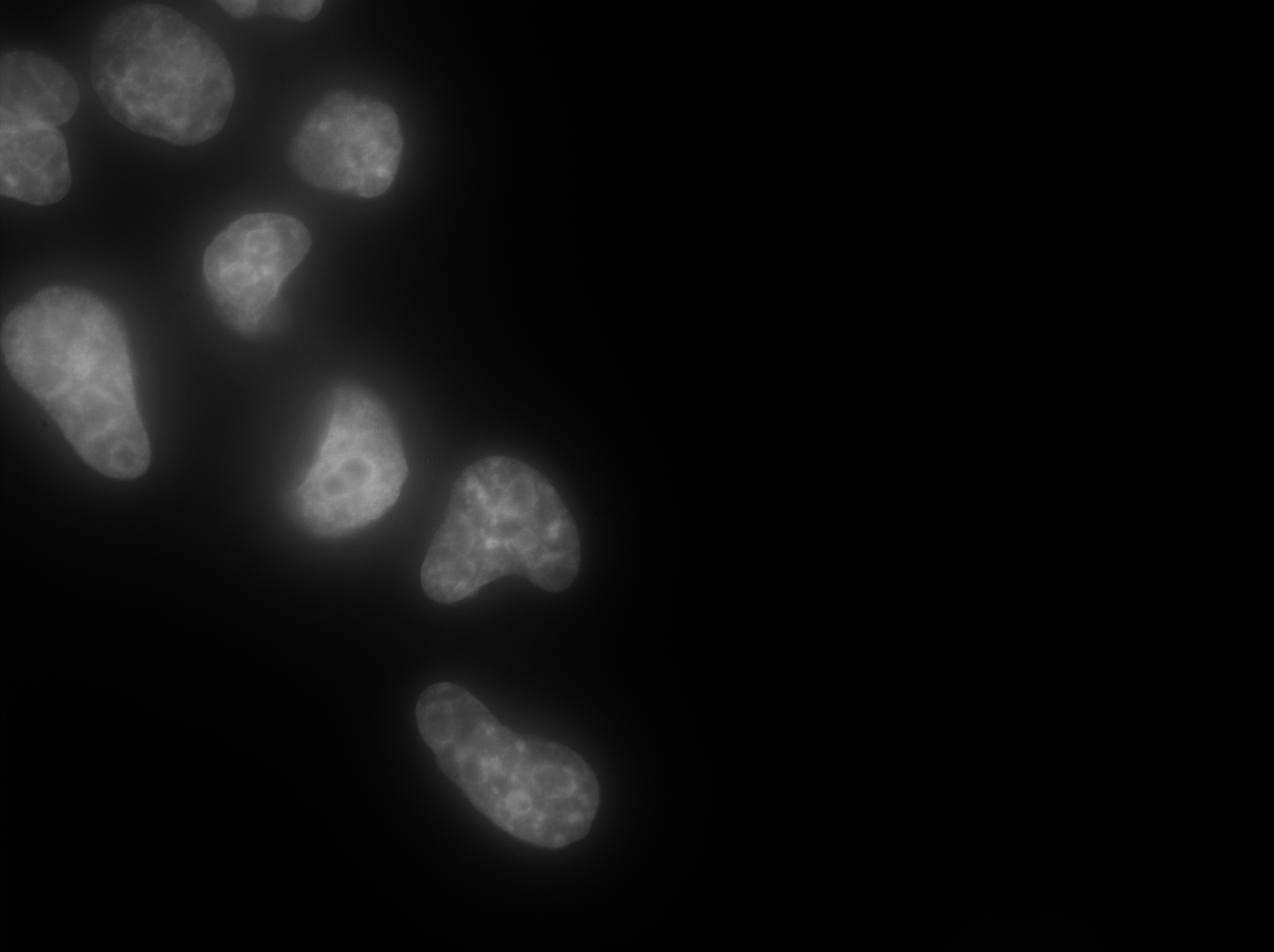

Supplement: Supplementary file 20 — Source data Fig. 6 part 1 [file 44319_2026_742_MOESM20_ESM.zip › Figure 6 Part 1/Fig 6abcd Cas9 TPGS1-KO acetylated tubulin atubulin/Cas9 R3 9-13-24 LT28.Project Maximum Z_XY1726767691_Z0_T0_C0.tif]

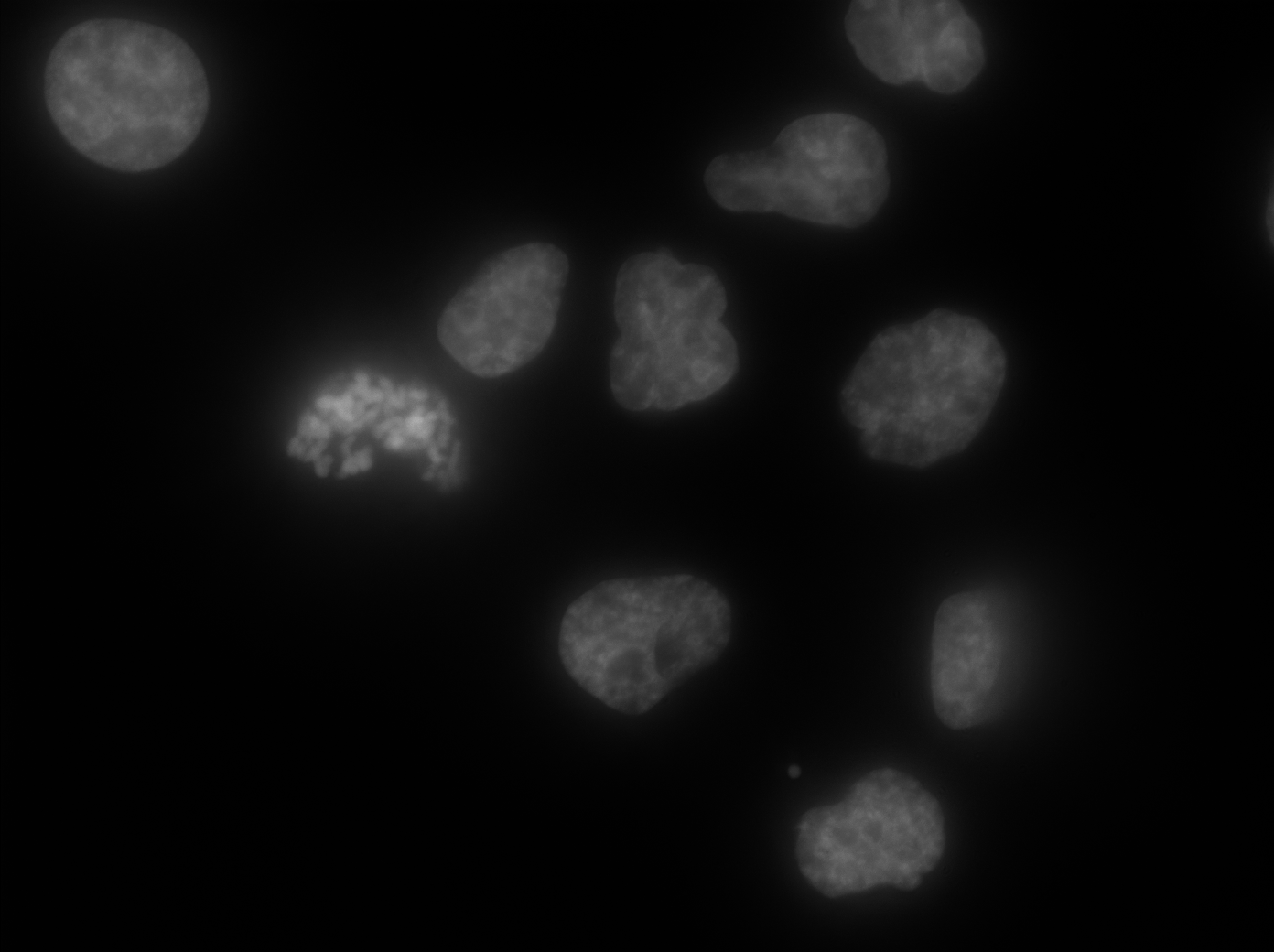

Supplement: Supplementary file 20 — Source data Fig. 6 part 1 [file 44319_2026_742_MOESM20_ESM.zip › Figure 6 Part 1/Fig 6abcd Cas9 TPGS1-KO acetylated tubulin atubulin/Cas9 R3 9-13-24 LT27 PA2PA3.Project Maximum Z_XY1726767575_Z0_T0_C0.tif]

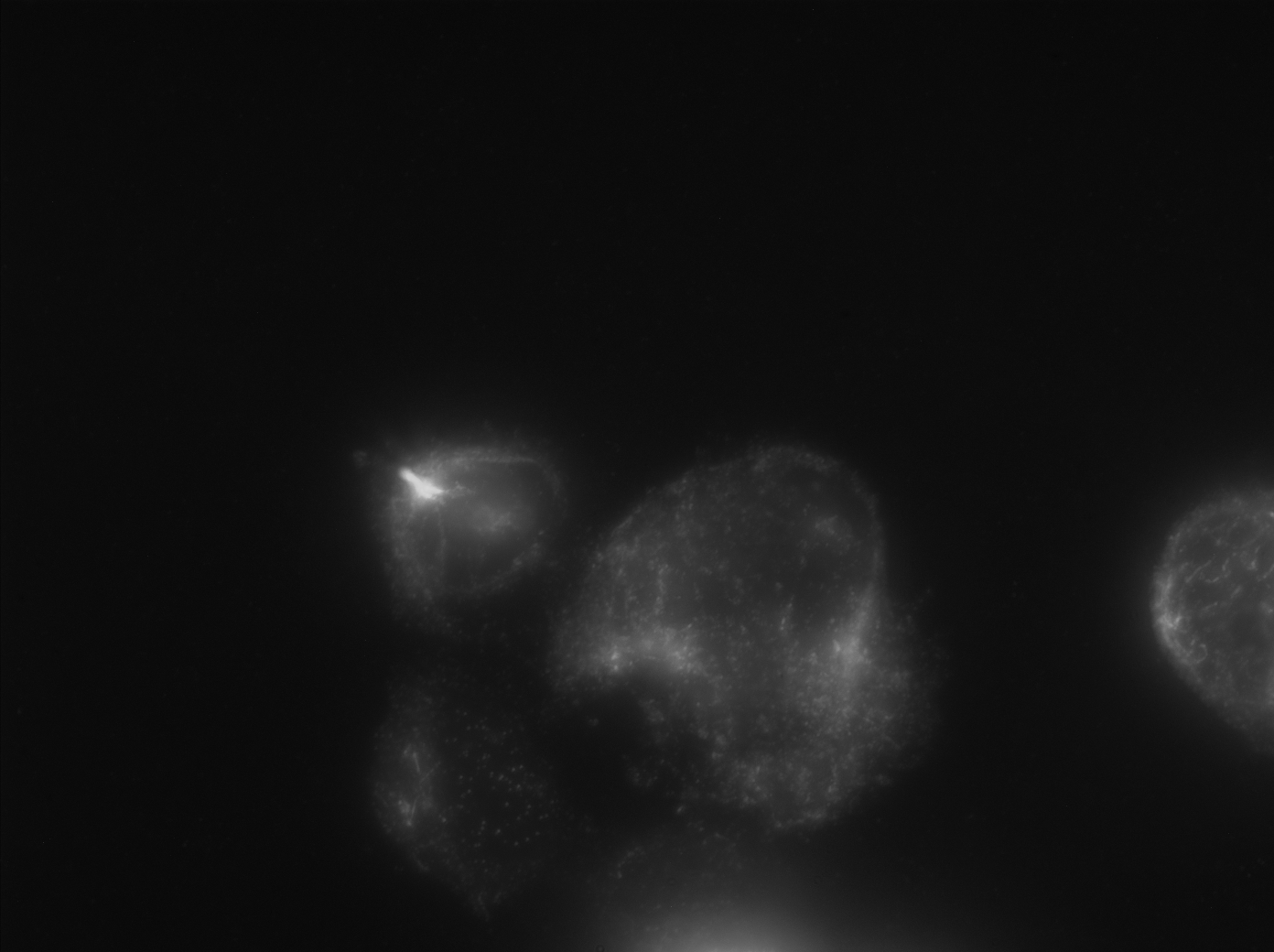

Supplement: Supplementary file 20 — Source data Fig. 6 part 1 [file 44319_2026_742_MOESM20_ESM.zip › Figure 6 Part 1/Fig 6abcd Cas9 TPGS1-KO acetylated tubulin atubulin/Cas9 R2 9-11-24 PA18.Project Maximum Z_XY1726180048_Z0_T0_C2.tif]

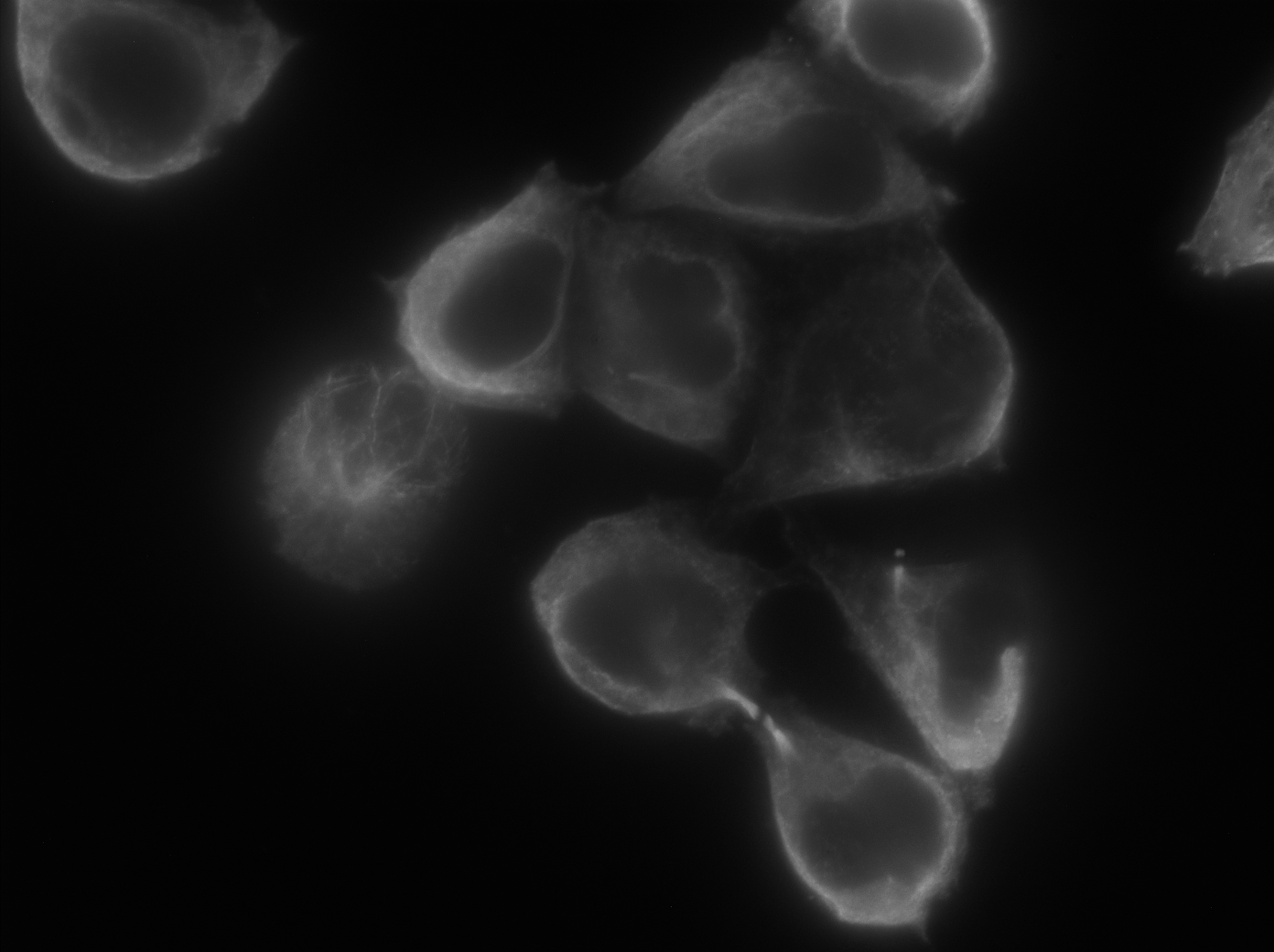

Supplement: Supplementary file 20 — Source data Fig. 6 part 1 [file 44319_2026_742_MOESM20_ESM.zip › Figure 6 Part 1/Fig 6abcd Cas9 TPGS1-KO acetylated tubulin atubulin/Cas9 R3 9-13-24 LT27 PA2PA3.Project Maximum Z_XY1726767575_Z0_T0_C1.tif]

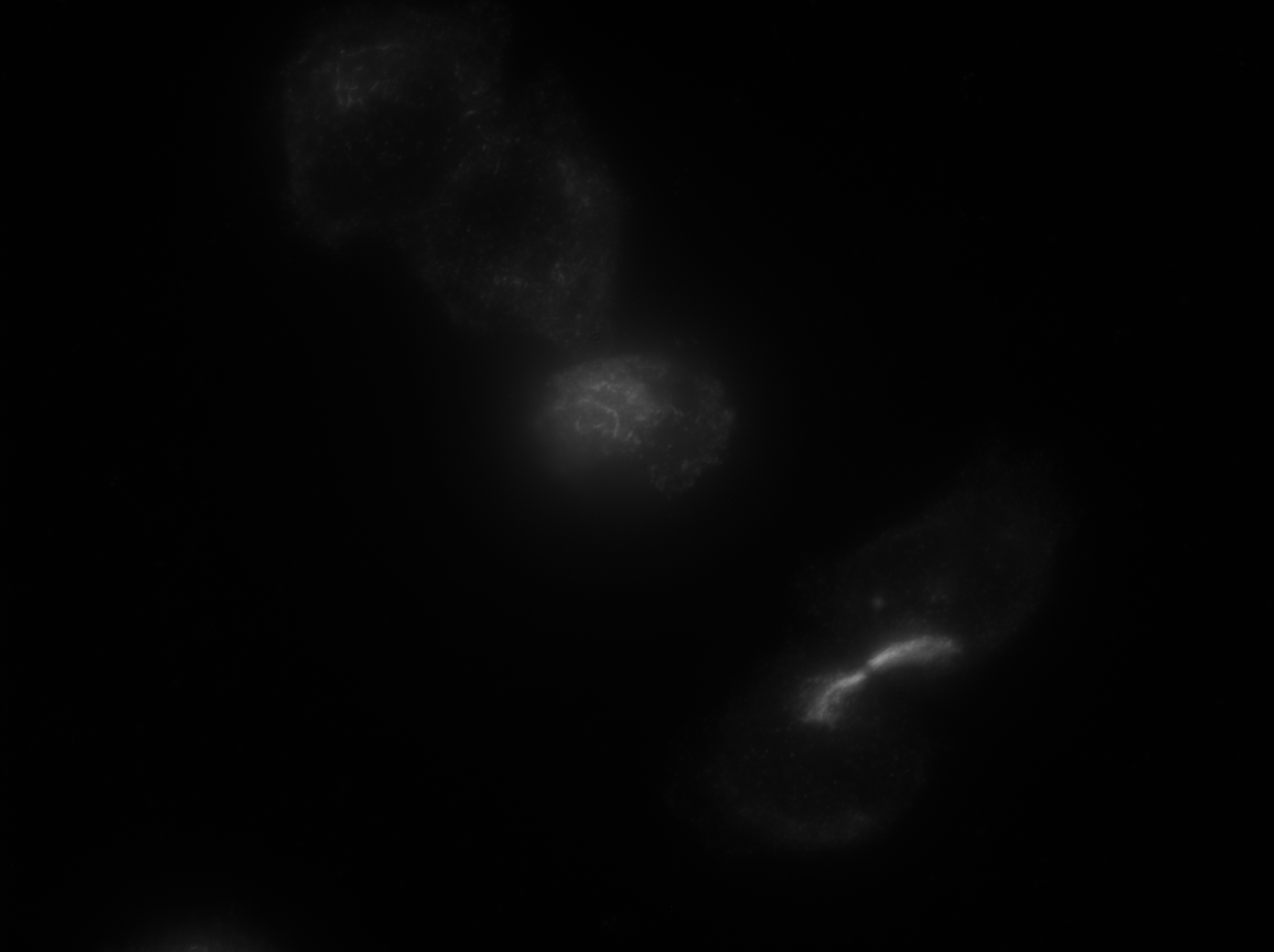

Supplement: Supplementary file 20 — Source data Fig. 6 part 1 [file 44319_2026_742_MOESM20_ESM.zip › Figure 6 Part 1/Fig 6abcd Cas9 TPGS1-KO acetylated tubulin atubulin/Cas9 R3 9-13-24 LT25.Project Maximum Z_XY1726767357_Z0_T0_C2.tif]

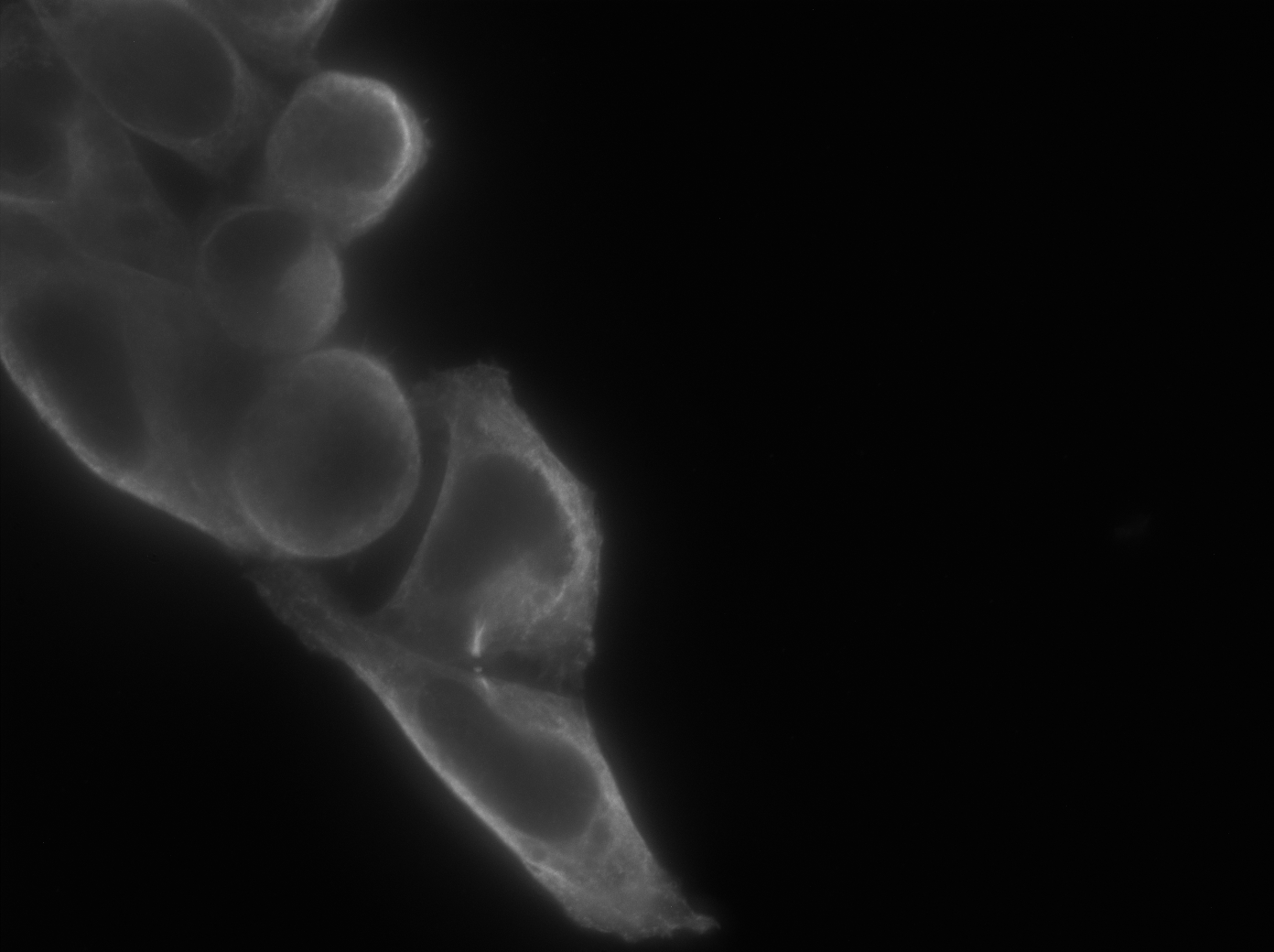

Supplement: Supplementary file 20 — Source data Fig. 6 part 1 [file 44319_2026_742_MOESM20_ESM.zip › Figure 6 Part 1/Fig 6abcd Cas9 TPGS1-KO acetylated tubulin atubulin/Cas9 R3 9-13-24 LT28.Project Maximum Z_XY1726767691_Z0_T0_C1.tif]

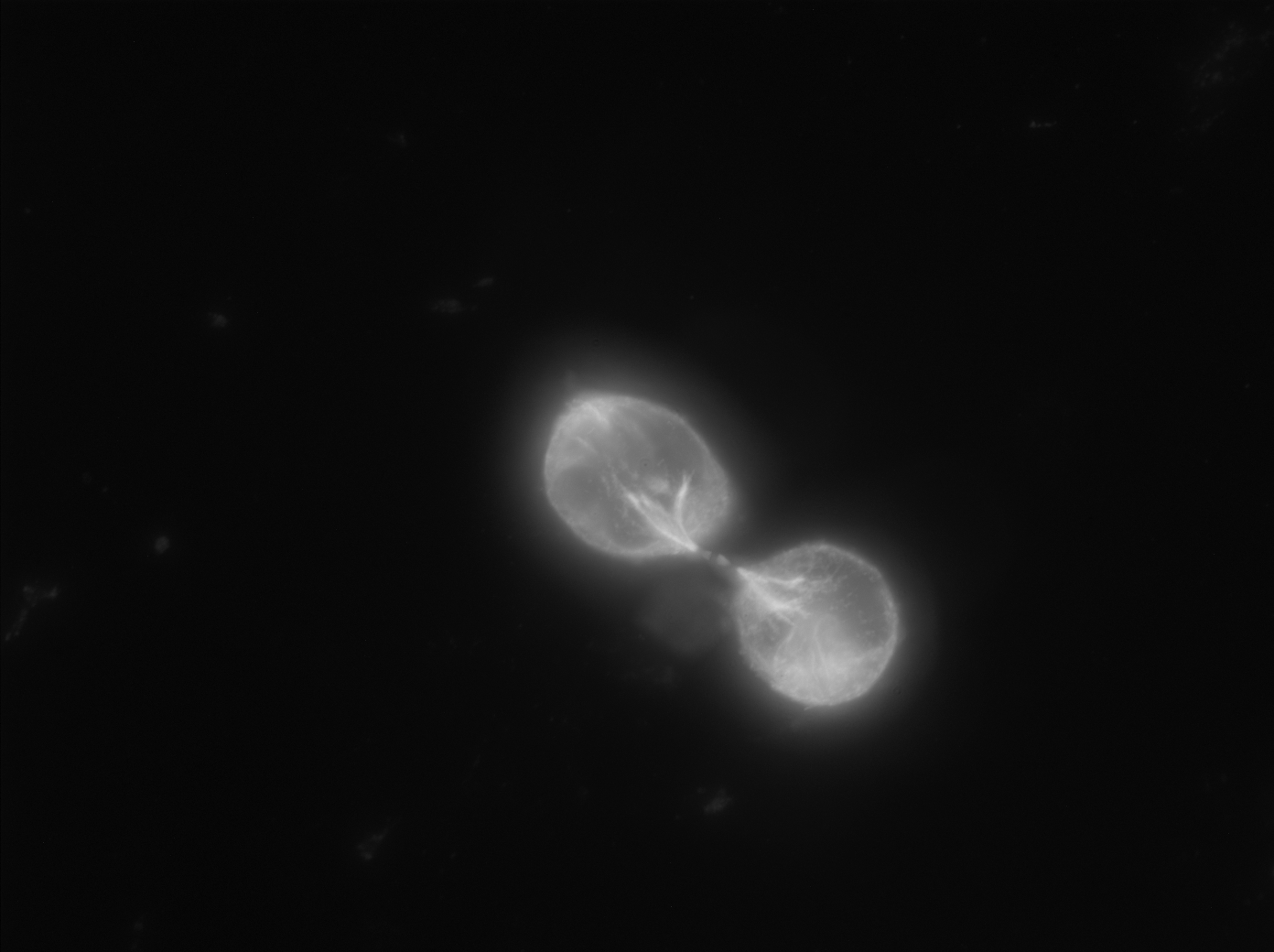

Supplement: Supplementary file 20 — Source data Fig. 6 part 1 [file 44319_2026_742_MOESM20_ESM.zip › Figure 6 Part 1/Fig 6abcd Cas9 TPGS1-KO acetylated tubulin atubulin/Cas9 R2 9-11-24 LT4.Project Maximum Z_XY1726172892_Z0_T0_C1.tif]

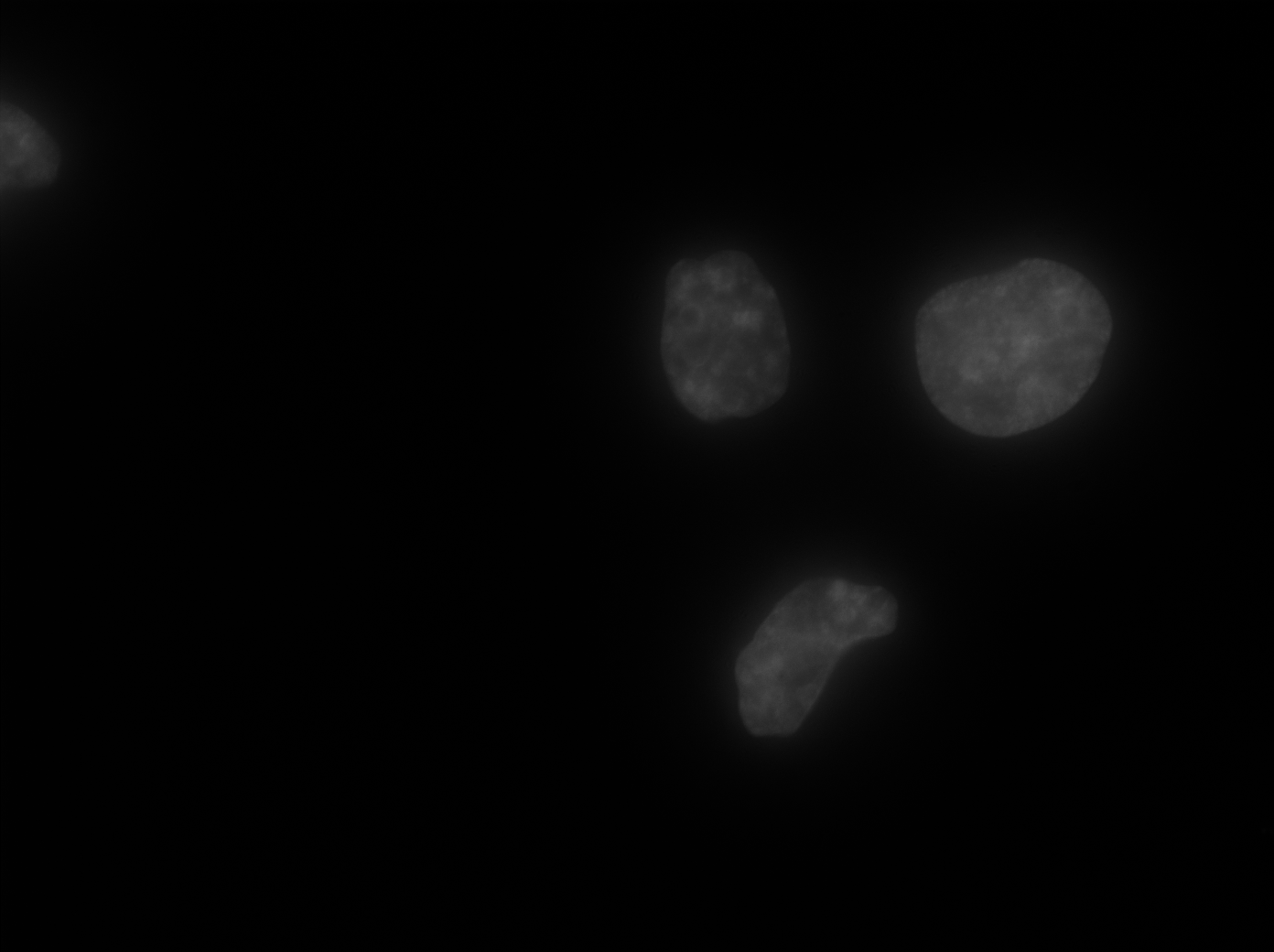

Supplement: Supplementary file 20 — Source data Fig. 6 part 1 [file 44319_2026_742_MOESM20_ESM.zip › Figure 6 Part 1/Fig 6abcd Cas9 TPGS1-KO acetylated tubulin atubulin/Cas9 R3 9-13-24 LT23.Project Maximum Z_XY1726767151_Z0_T0_C0.tif]

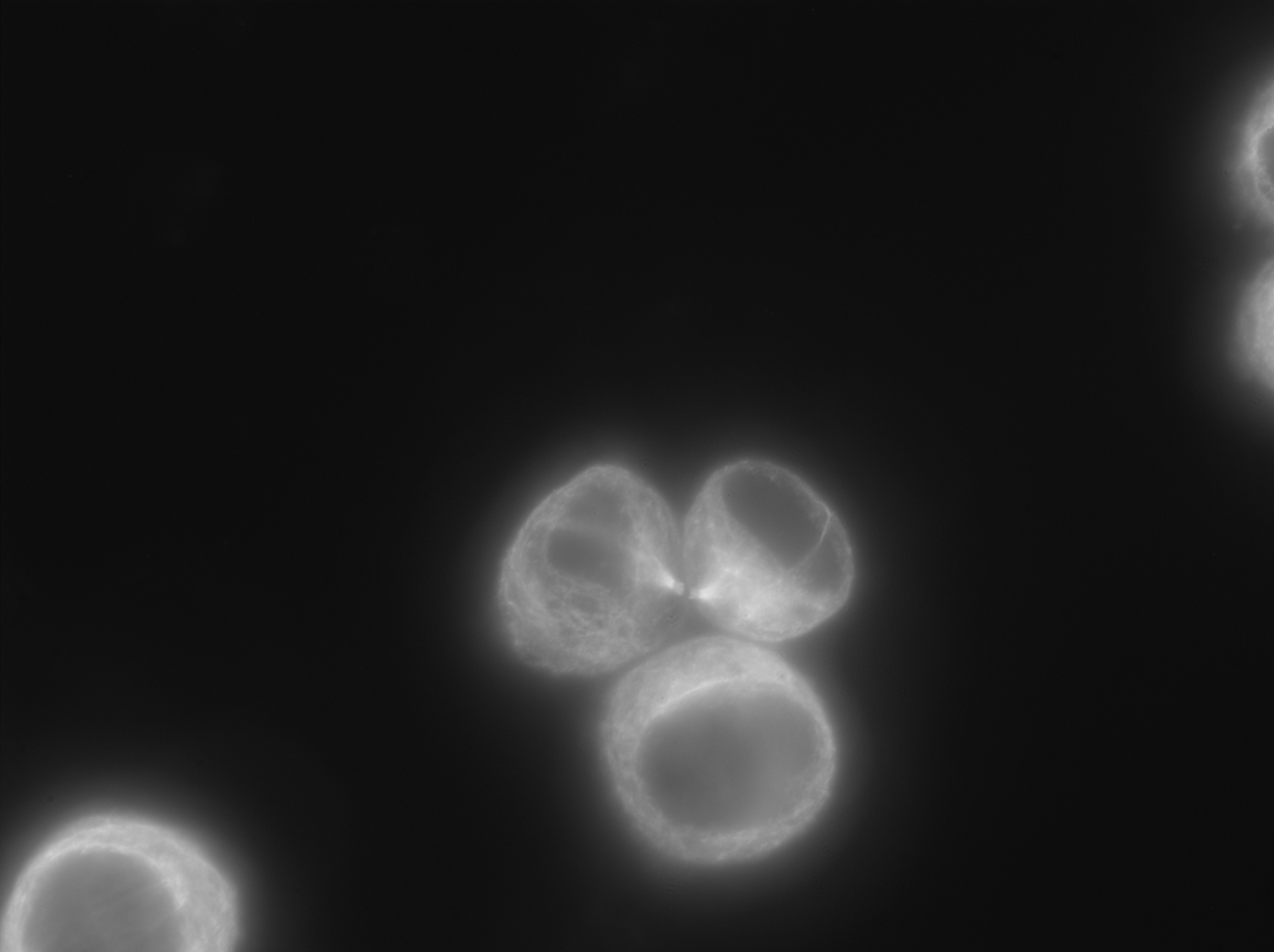

Supplement: Supplementary file 20 — Source data Fig. 6 part 1 [file 44319_2026_742_MOESM20_ESM.zip › Figure 6 Part 1/Fig 6abcd Cas9 TPGS1-KO acetylated tubulin atubulin/Cas9 R2 9-11-24 LT16.Project Maximum Z_XY1726178049_Z0_T0_C1.tif]

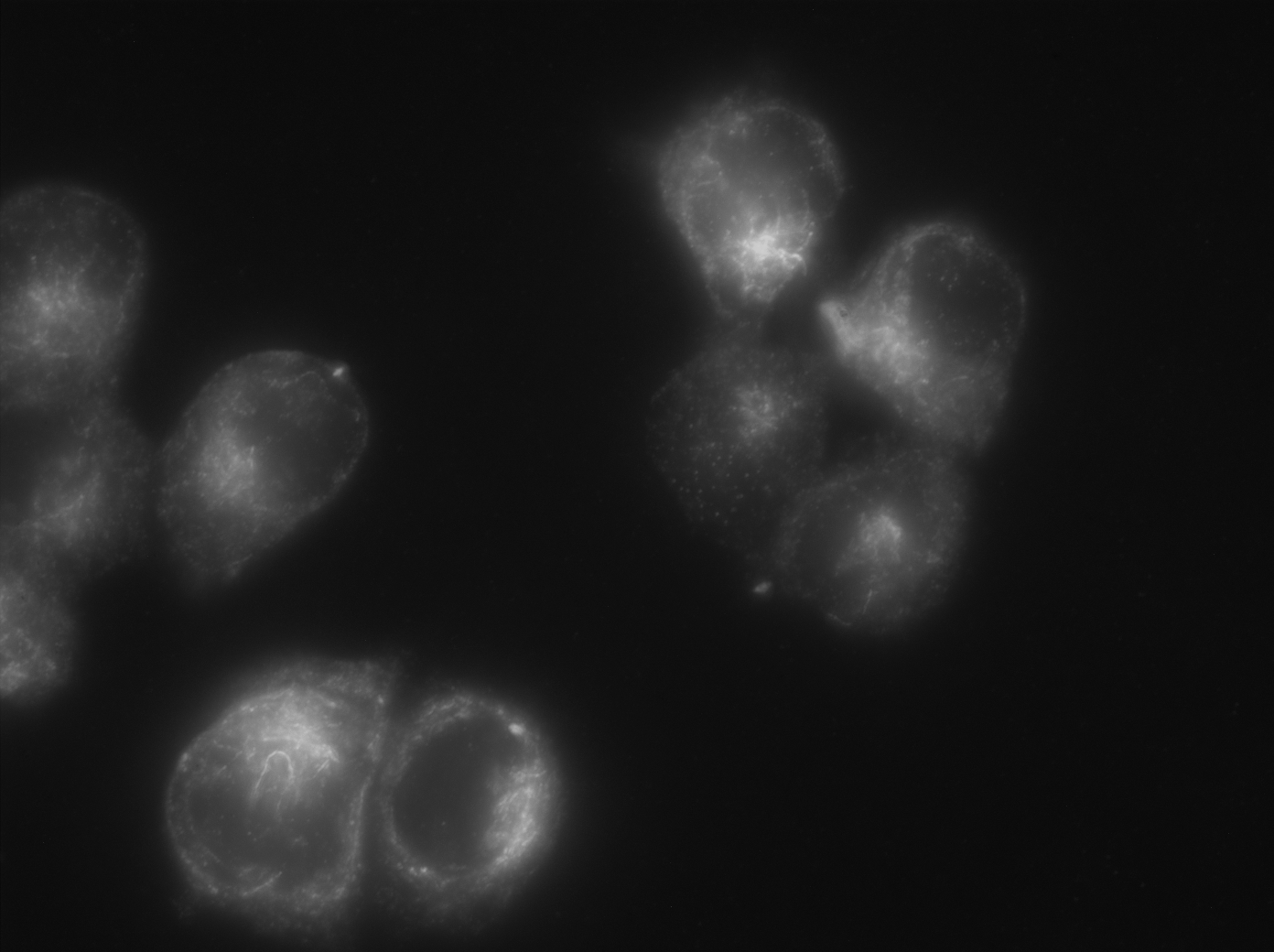

Supplement: Supplementary file 20 — Source data Fig. 6 part 1 [file 44319_2026_742_MOESM20_ESM.zip › Figure 6 Part 1/Fig 6abcd Cas9 TPGS1-KO acetylated tubulin atubulin/Cas9 R2 9-11-24 PA13PA14.Project Maximum Z_XY1726179490_Z0_T0_C2.tif]

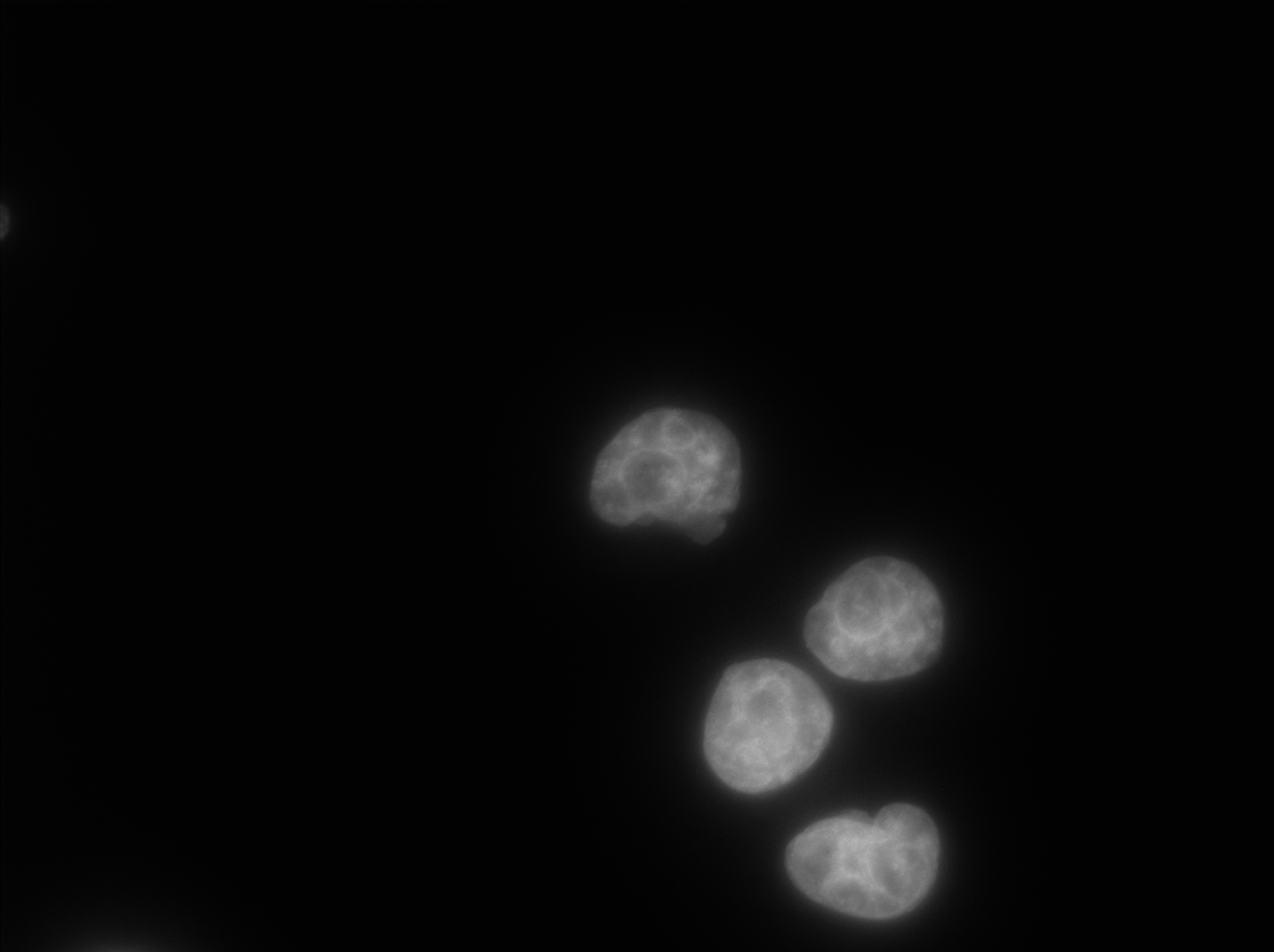

Supplement: Supplementary file 20 — Source data Fig. 6 part 1 [file 44319_2026_742_MOESM20_ESM.zip › Figure 6 Part 1/Fig 6abcd Cas9 TPGS1-KO acetylated tubulin atubulin/Cas9 R2 9-11-24 PA27.Project Maximum Z_XY1726181564_Z0_T0_C0.tif]

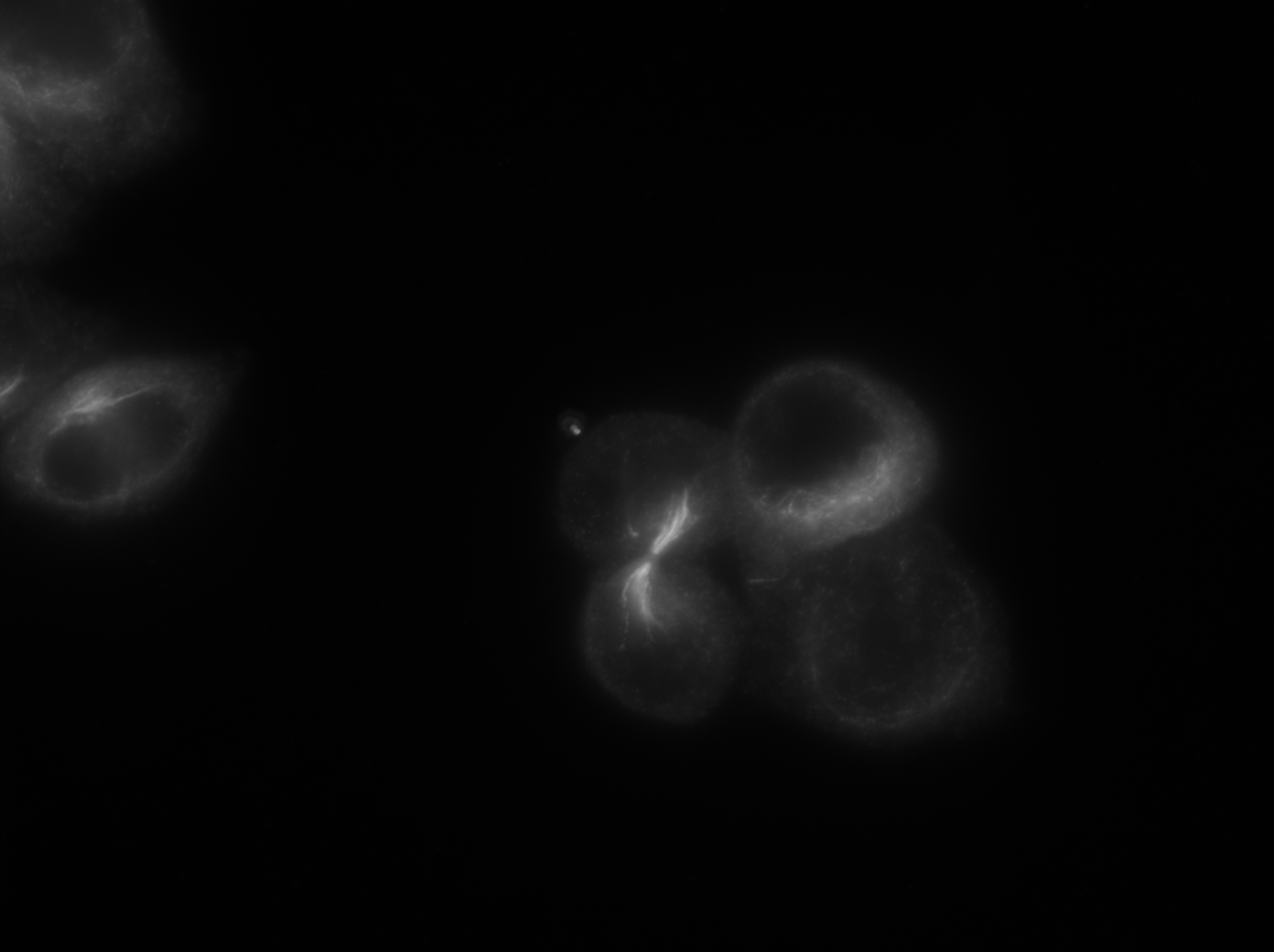

Supplement: Supplementary file 20 — Source data Fig. 6 part 1 [file 44319_2026_742_MOESM20_ESM.zip › Figure 6 Part 1/Fig 6abcd Cas9 TPGS1-KO acetylated tubulin atubulin/Cas9 R3 9-13-24 LT21.Project Maximum Z_XY1726766984_Z0_T0_C2.tif]

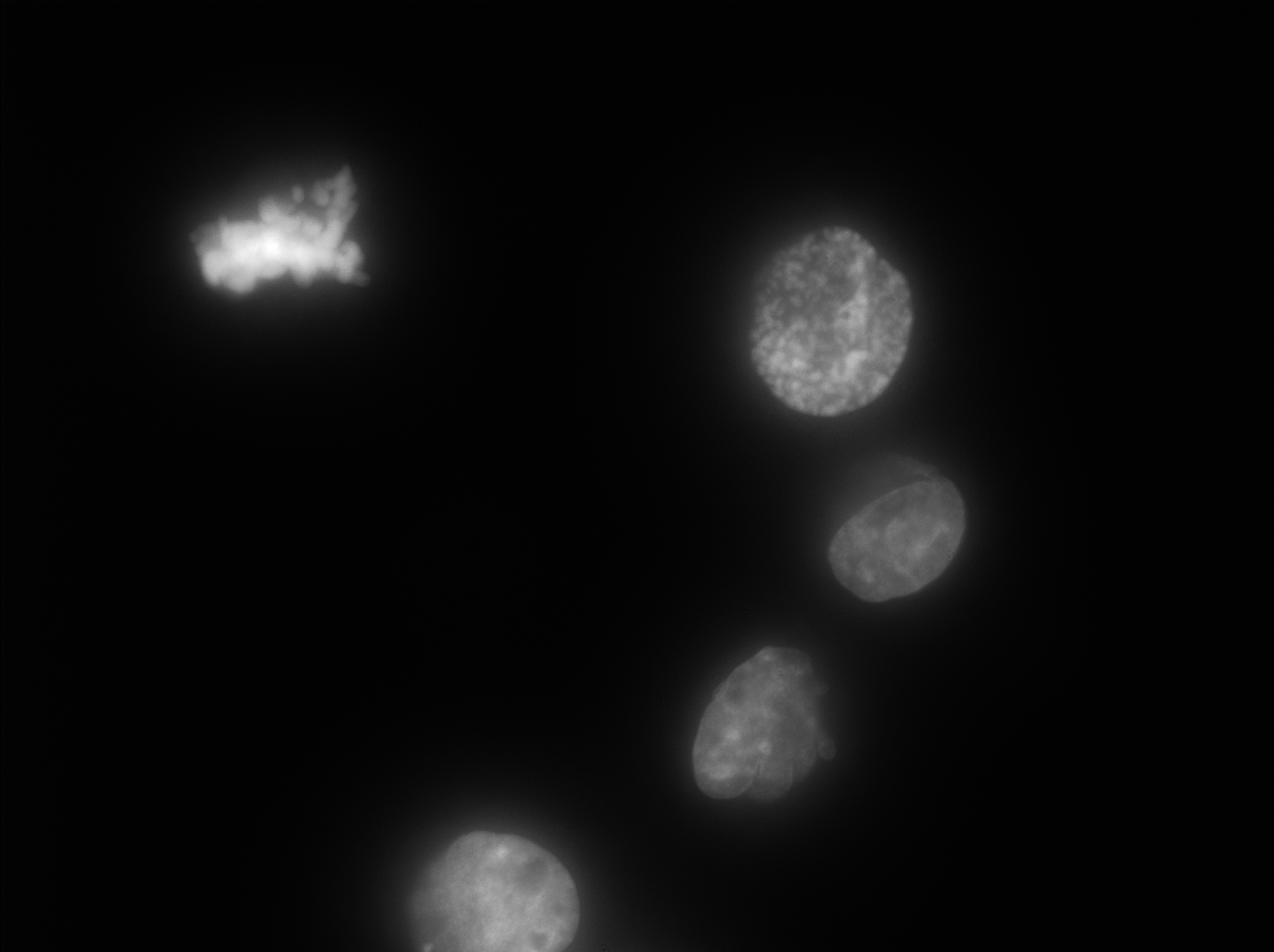

Supplement: Supplementary file 20 — Source data Fig. 6 part 1 [file 44319_2026_742_MOESM20_ESM.zip › Figure 6 Part 1/Fig 6abcd Cas9 TPGS1-KO acetylated tubulin atubulin/Cas9 R2 9-11-24 LT29.Project Maximum Z_XY1726181454_Z0_T0_C0.tif]

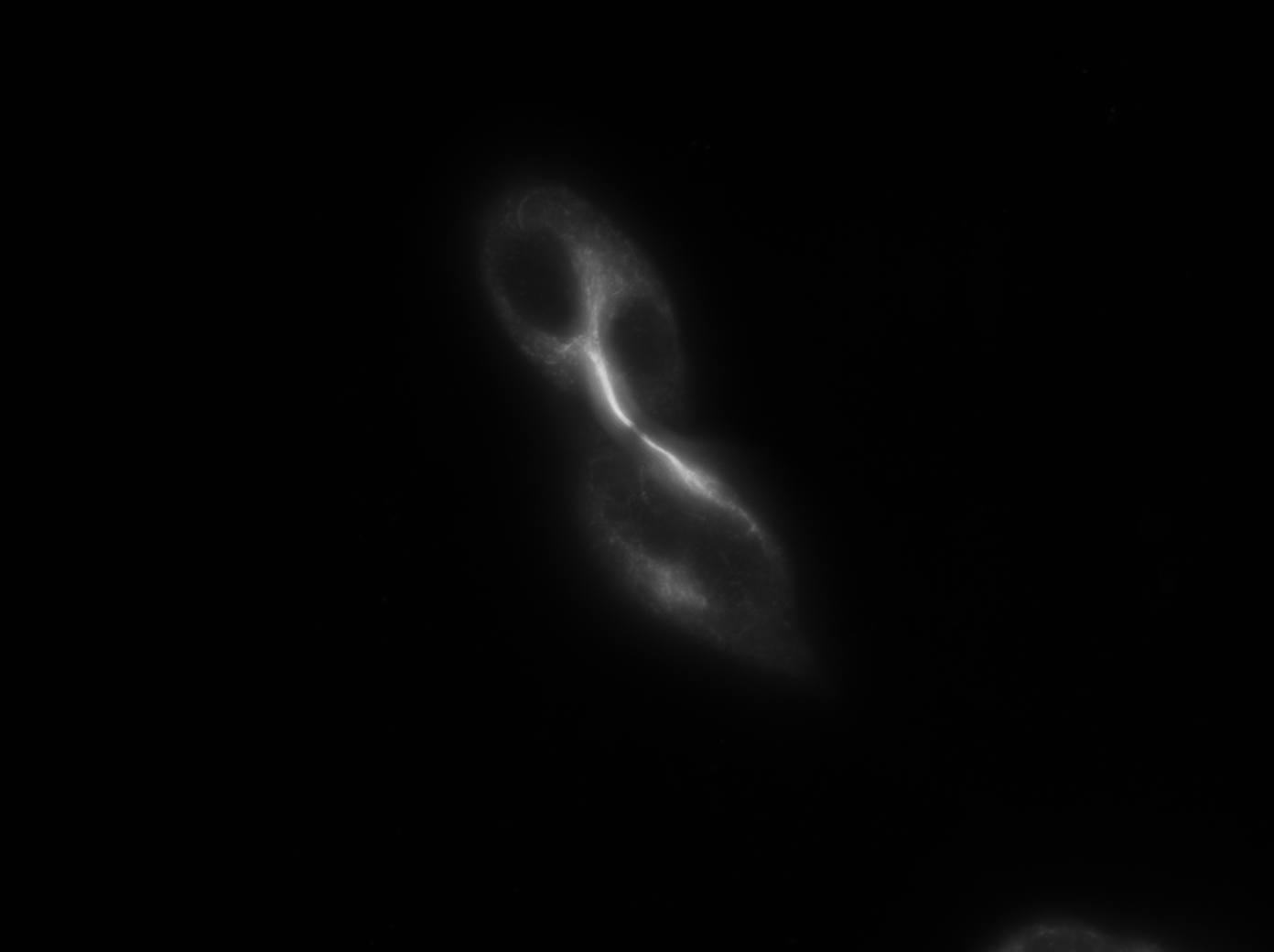

Supplement: Supplementary file 20 — Source data Fig. 6 part 1 [file 44319_2026_742_MOESM20_ESM.zip › Figure 6 Part 1/Fig 6abcd Cas9 TPGS1-KO acetylated tubulin atubulin/Cas9 R3 9-13-24 LT15.Project Maximum Z_XY1726766365_Z0_T0_C2.tif]

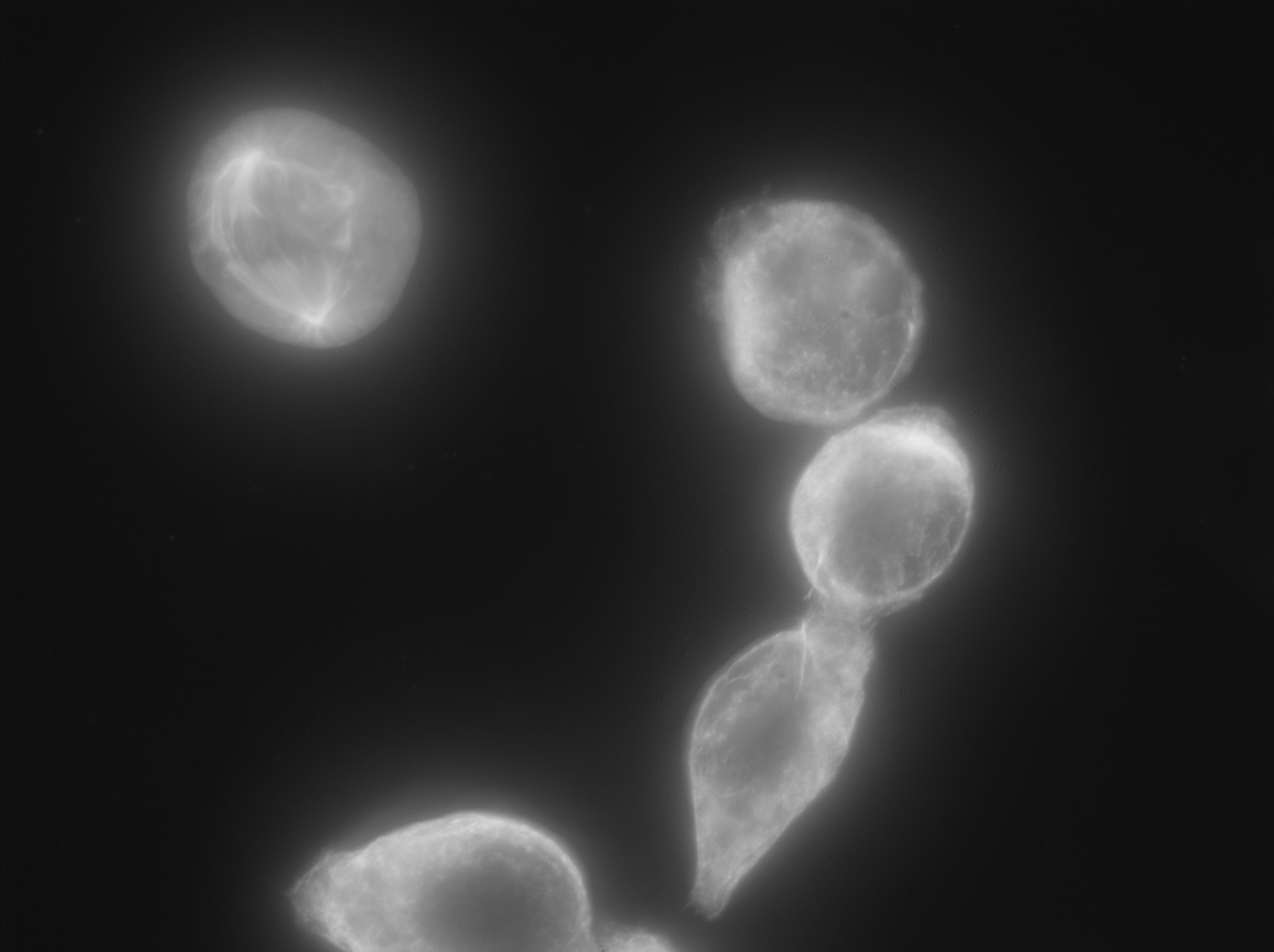

Supplement: Supplementary file 20 — Source data Fig. 6 part 1 [file 44319_2026_742_MOESM20_ESM.zip › Figure 6 Part 1/Fig 6abcd Cas9 TPGS1-KO acetylated tubulin atubulin/Cas9 R2 9-11-24 LT29.Project Maximum Z_XY1726181454_Z0_T0_C1.tif]

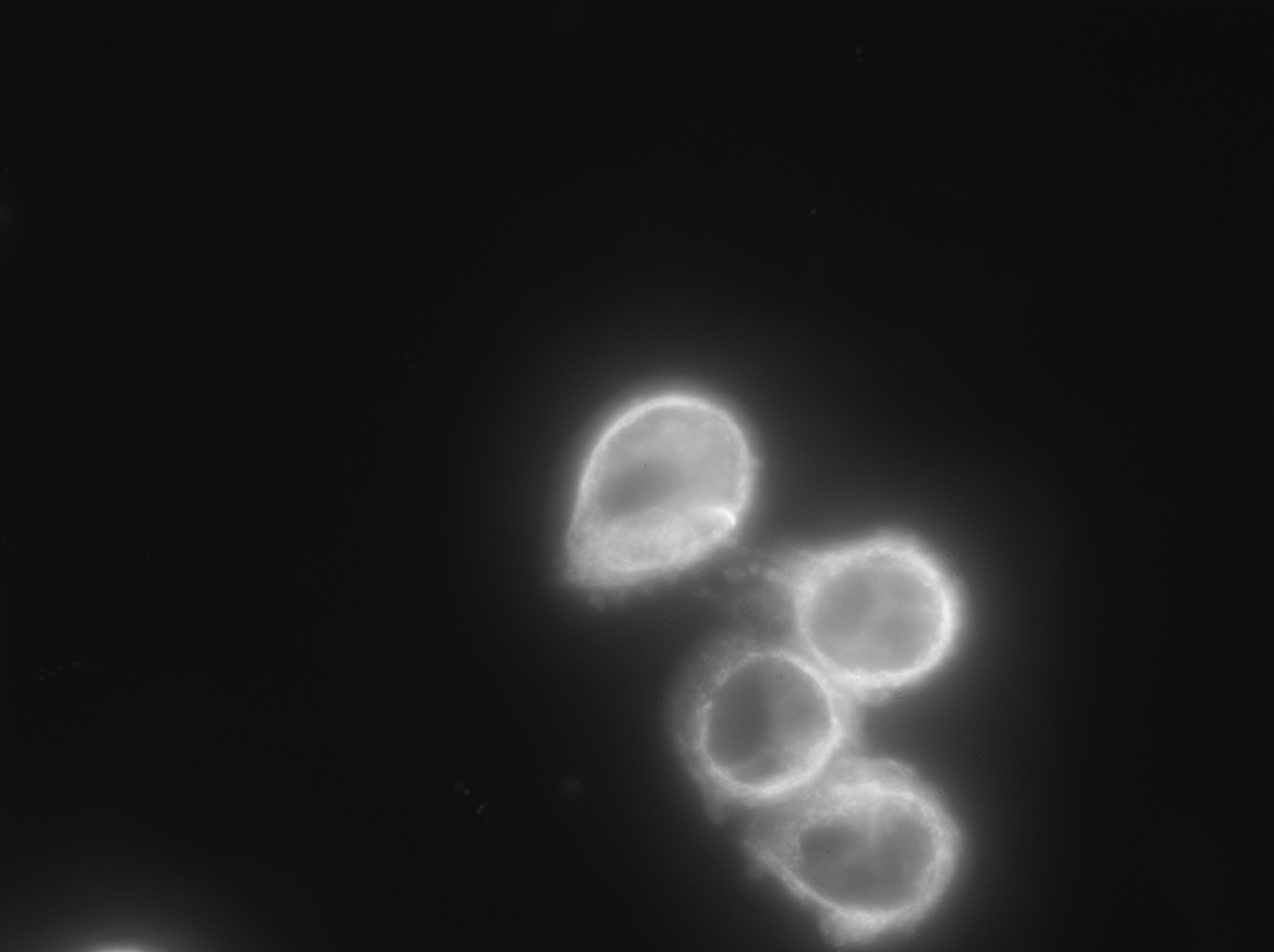

Supplement: Supplementary file 20 — Source data Fig. 6 part 1 [file 44319_2026_742_MOESM20_ESM.zip › Figure 6 Part 1/Fig 6abcd Cas9 TPGS1-KO acetylated tubulin atubulin/Cas9 R2 9-11-24 PA27.Project Maximum Z_XY1726181564_Z0_T0_C1.tif]

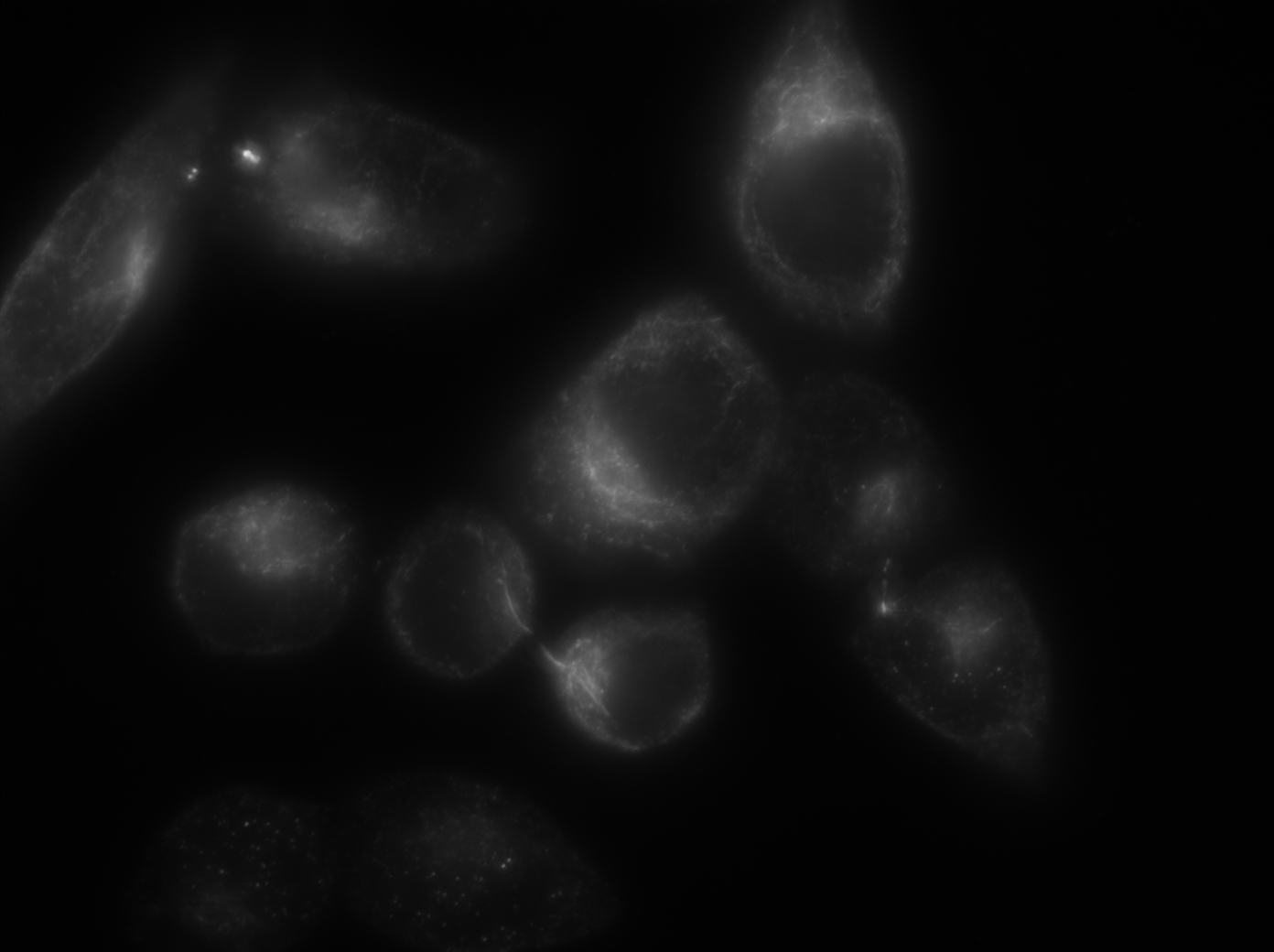

Supplement: Supplementary file 20 — Source data Fig. 6 part 1 [file 44319_2026_742_MOESM20_ESM.zip › Figure 6 Part 1/Fig 6abcd Cas9 TPGS1-KO acetylated tubulin atubulin/Cas9 R3 9-13-24 LT14.Project Maximum Z_XY1726766209_Z0_T0_C2.tif]

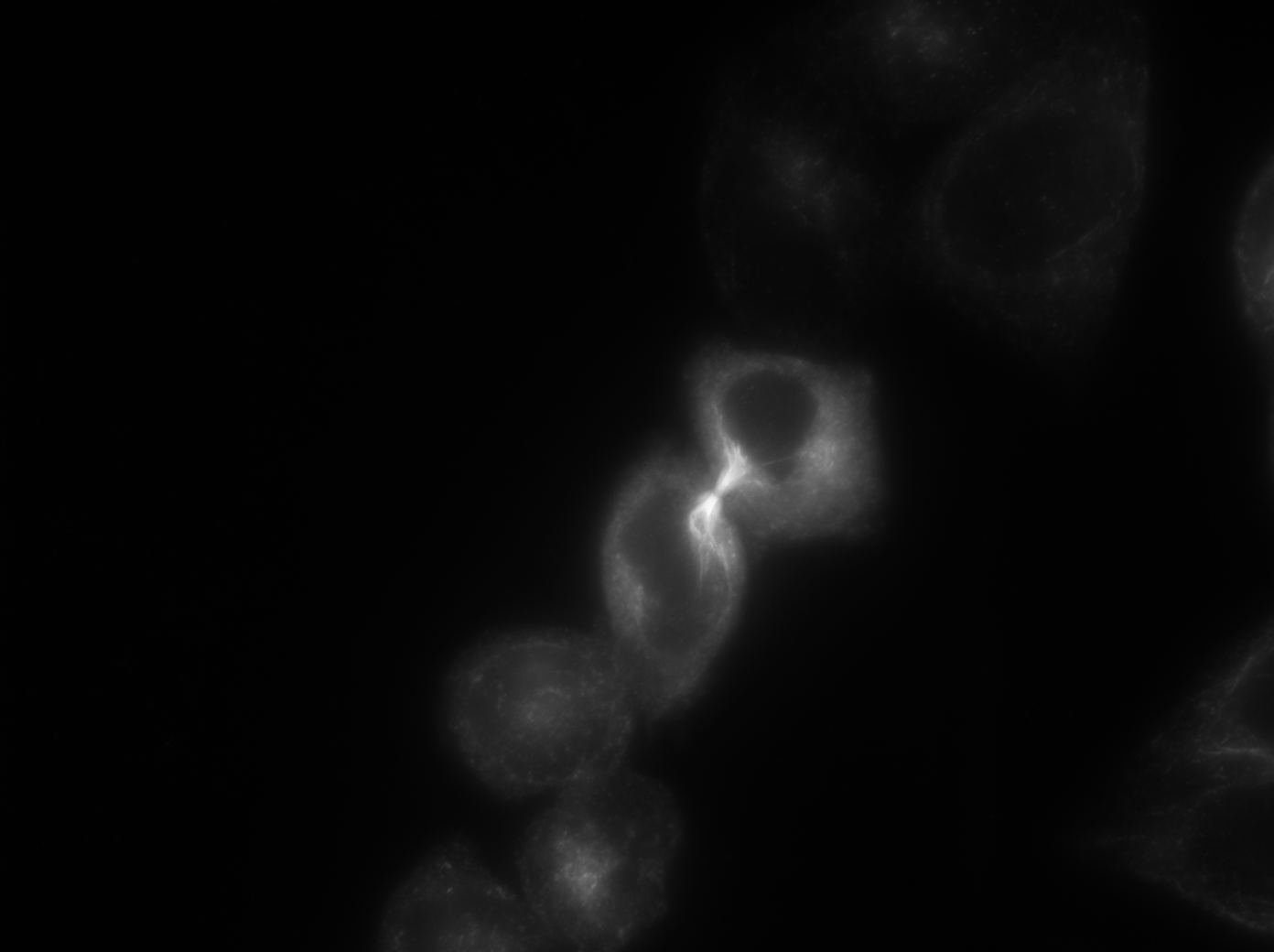

Supplement: Supplementary file 20 — Source data Fig. 6 part 1 [file 44319_2026_742_MOESM20_ESM.zip › Figure 6 Part 1/Fig 6abcd Cas9 TPGS1-KO acetylated tubulin atubulin/Cas9 R3 9-13-24 LT19.Project Maximum Z_XY1726766746_Z0_T0_C2.tif]

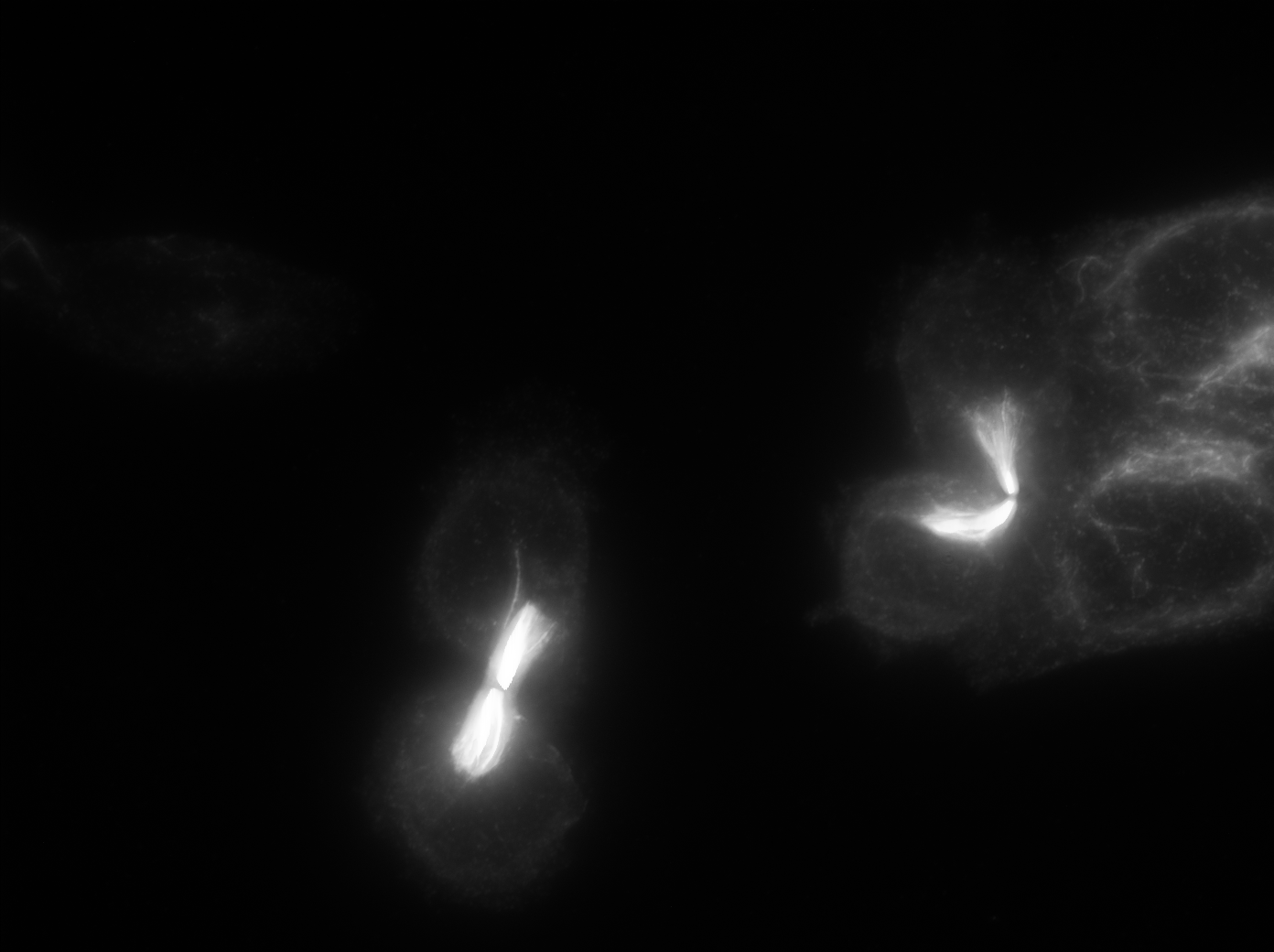

Supplement: Supplementary file 20 — Source data Fig. 6 part 1 [file 44319_2026_742_MOESM20_ESM.zip › Figure 6 Part 1/Fig 6abcd Cas9 TPGS1-KO acetylated tubulin atubulin/Cas9 R3 9-13-24 LT2LT3.Project Maximum Z_XY1726765382_Z0_T0_C2.tif]

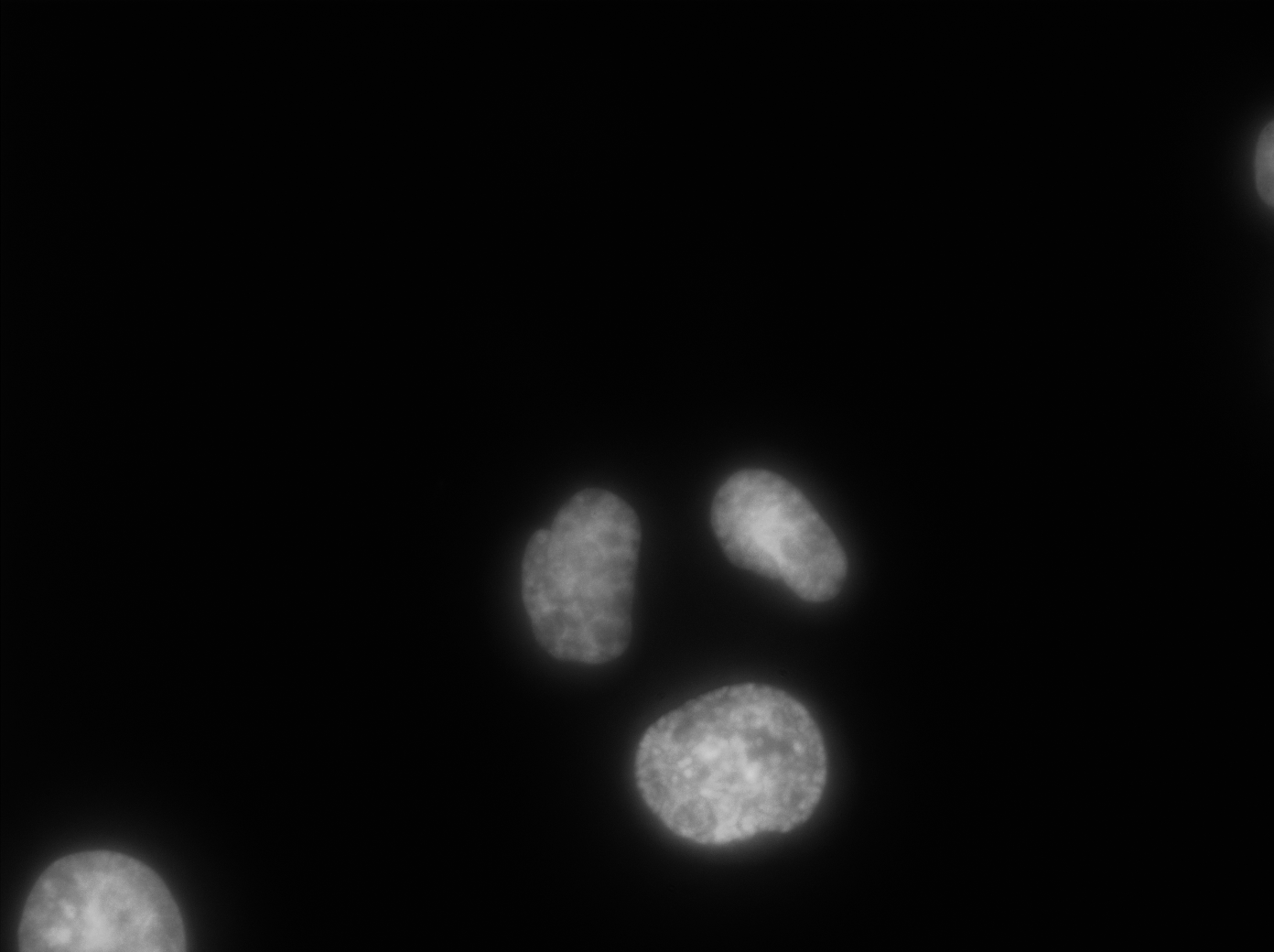

Supplement: Supplementary file 20 — Source data Fig. 6 part 1 [file 44319_2026_742_MOESM20_ESM.zip › Figure 6 Part 1/Fig 6abcd Cas9 TPGS1-KO acetylated tubulin atubulin/Cas9 R2 9-11-24 LT16.Project Maximum Z_XY1726178049_Z0_T0_C0.tif]

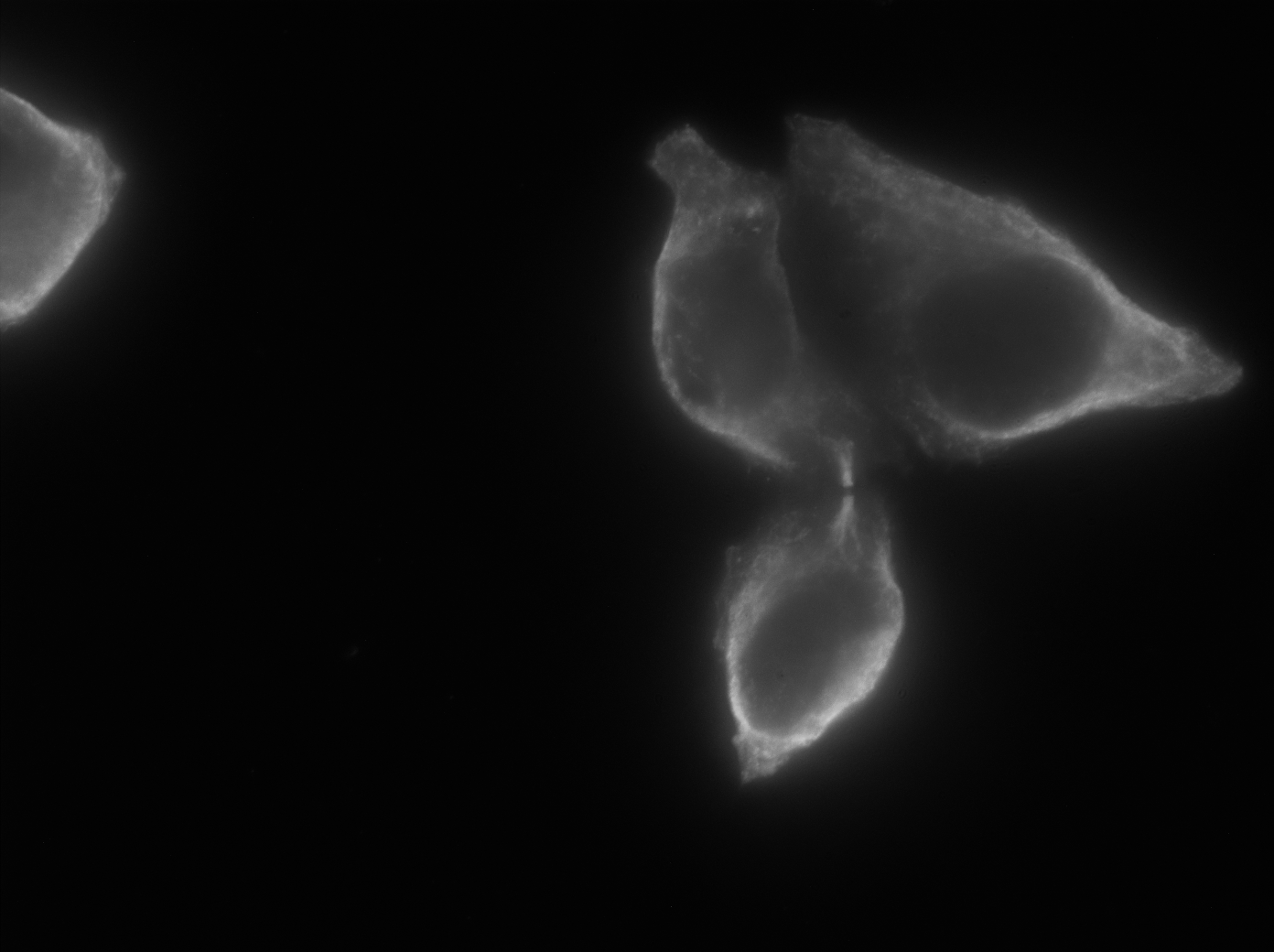

Supplement: Supplementary file 20 — Source data Fig. 6 part 1 [file 44319_2026_742_MOESM20_ESM.zip › Figure 6 Part 1/Fig 6abcd Cas9 TPGS1-KO acetylated tubulin atubulin/Cas9 R3 9-13-24 LT23.Project Maximum Z_XY1726767151_Z0_T0_C1.tif]

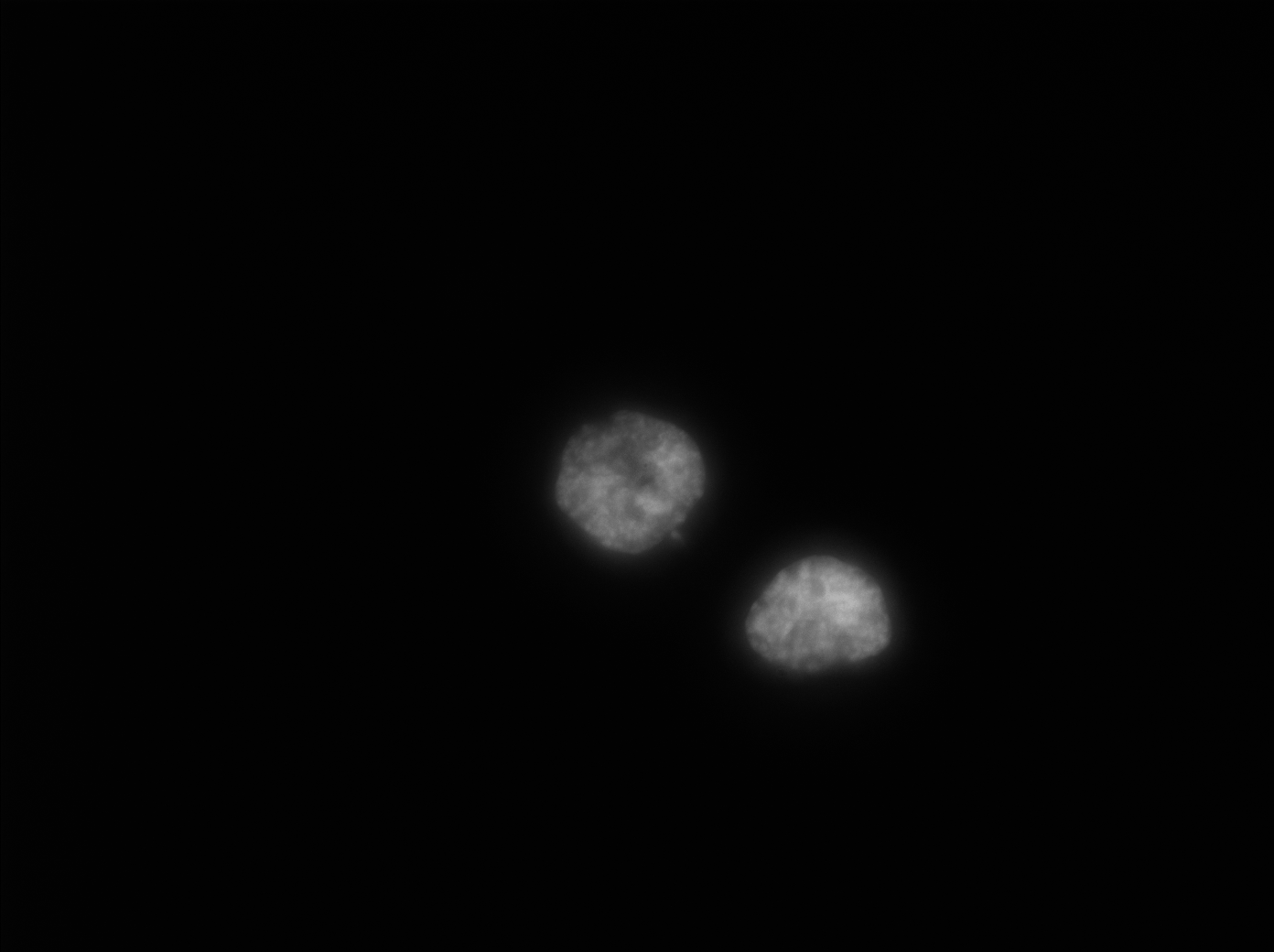

Supplement: Supplementary file 20 — Source data Fig. 6 part 1 [file 44319_2026_742_MOESM20_ESM.zip › Figure 6 Part 1/Fig 6abcd Cas9 TPGS1-KO acetylated tubulin atubulin/Cas9 R2 9-11-24 LT4.Project Maximum Z_XY1726172892_Z0_T0_C0.tif]

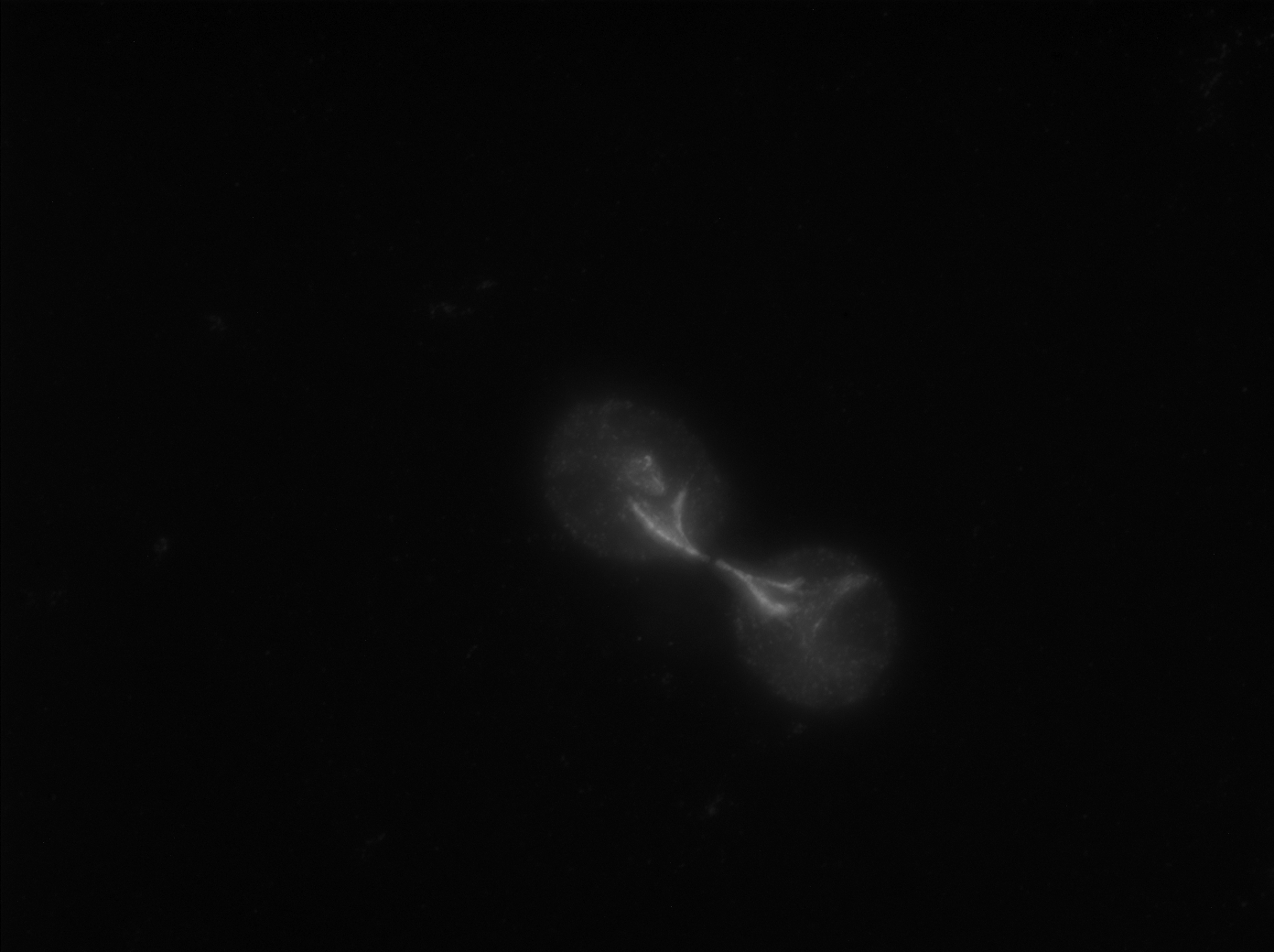

Supplement: Supplementary file 20 — Source data Fig. 6 part 1 [file 44319_2026_742_MOESM20_ESM.zip › Figure 6 Part 1/Fig 6abcd Cas9 TPGS1-KO acetylated tubulin atubulin/Cas9 R2 9-11-24 LT4.Project Maximum Z_XY1726172892_Z0_T0_C2.tif]

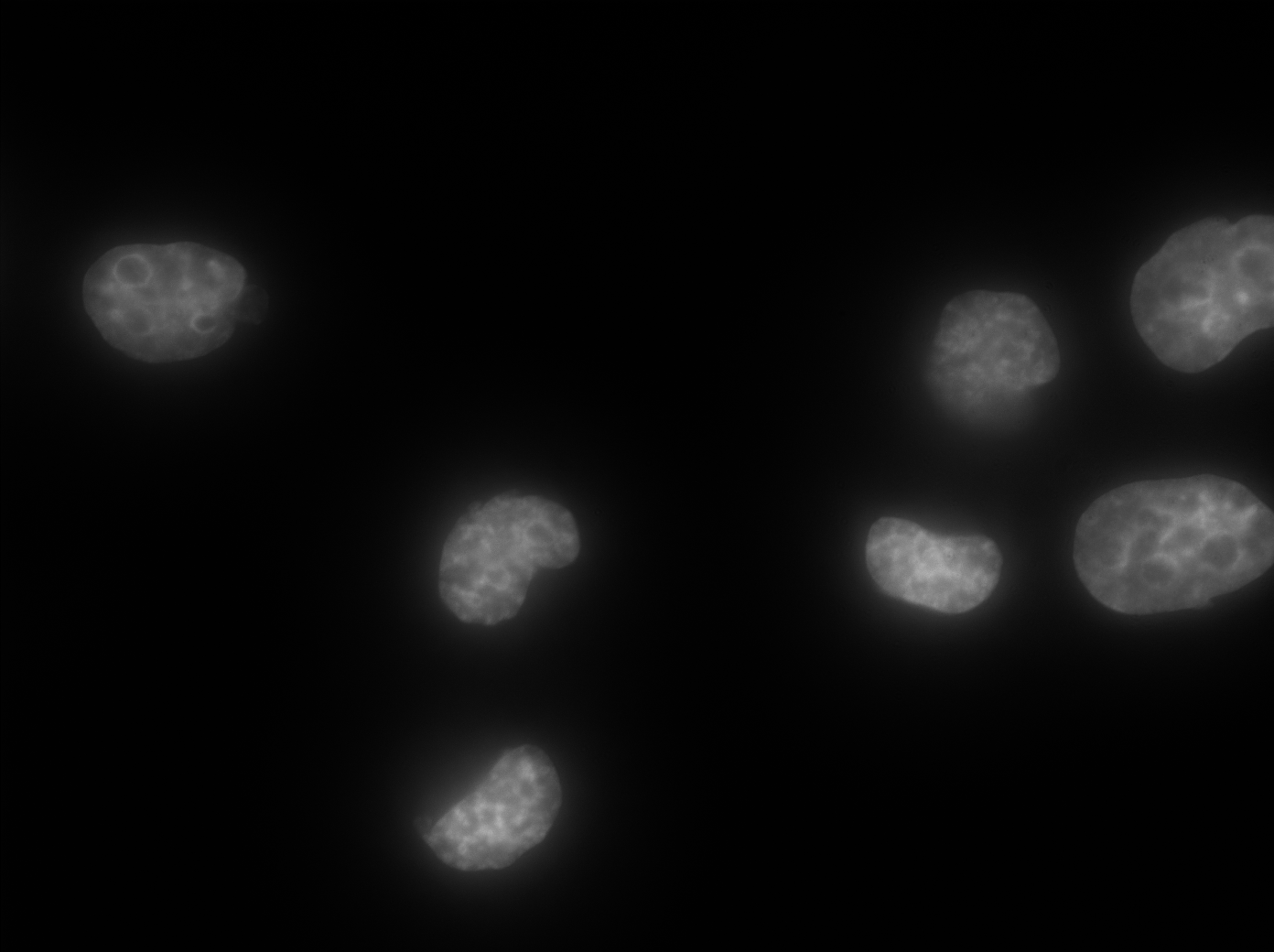

Supplement: Supplementary file 20 — Source data Fig. 6 part 1 [file 44319_2026_742_MOESM20_ESM.zip › Figure 6 Part 1/Fig 6abcd Cas9 TPGS1-KO acetylated tubulin atubulin/Cas9 R3 9-13-24 LT2LT3.Project Maximum Z_XY1726765382_Z0_T0_C0.tif]

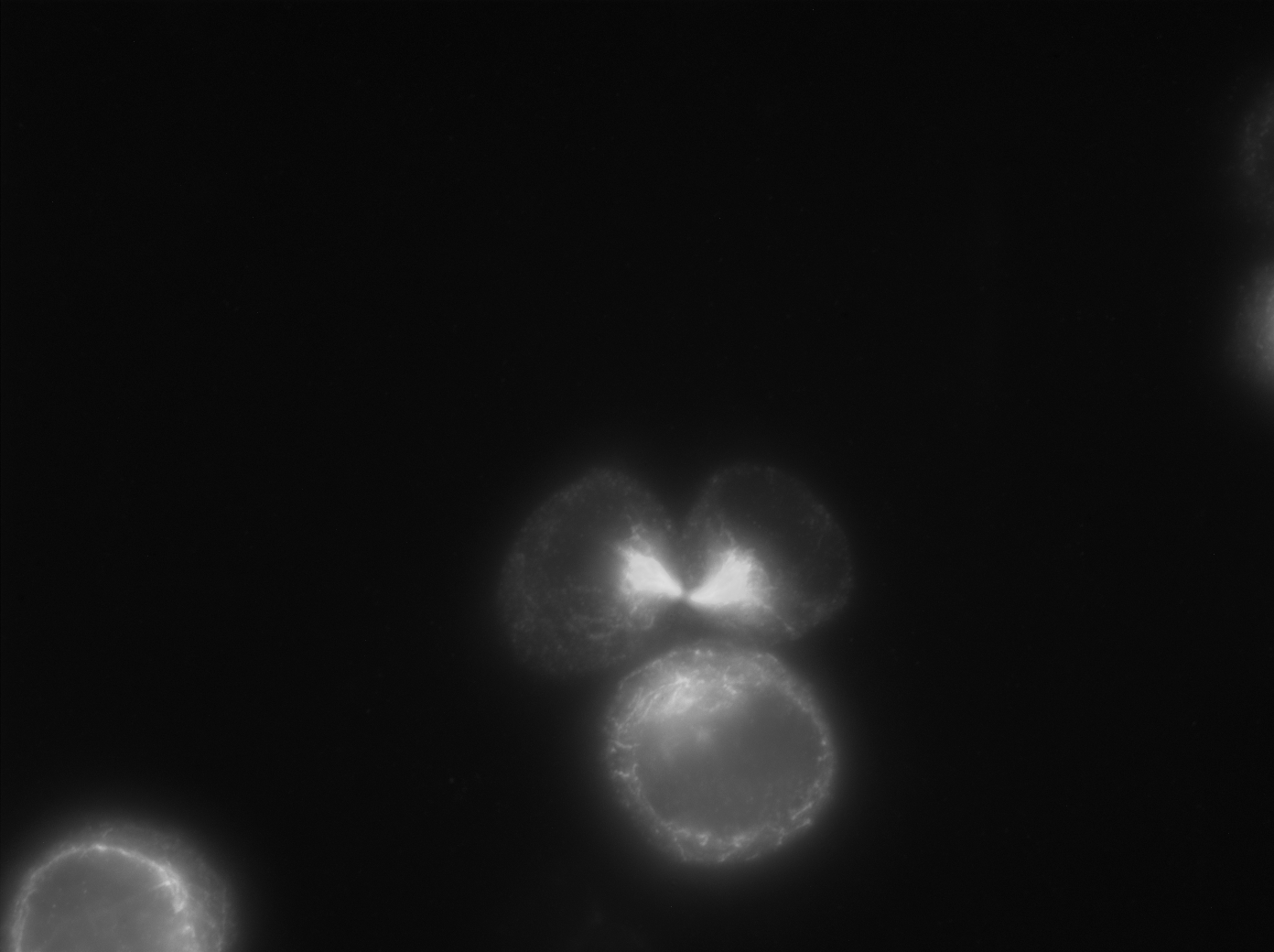

Supplement: Supplementary file 20 — Source data Fig. 6 part 1 [file 44319_2026_742_MOESM20_ESM.zip › Figure 6 Part 1/Fig 6abcd Cas9 TPGS1-KO acetylated tubulin atubulin/Cas9 R2 9-11-24 LT16.Project Maximum Z_XY1726178049_Z0_T0_C2.tif]

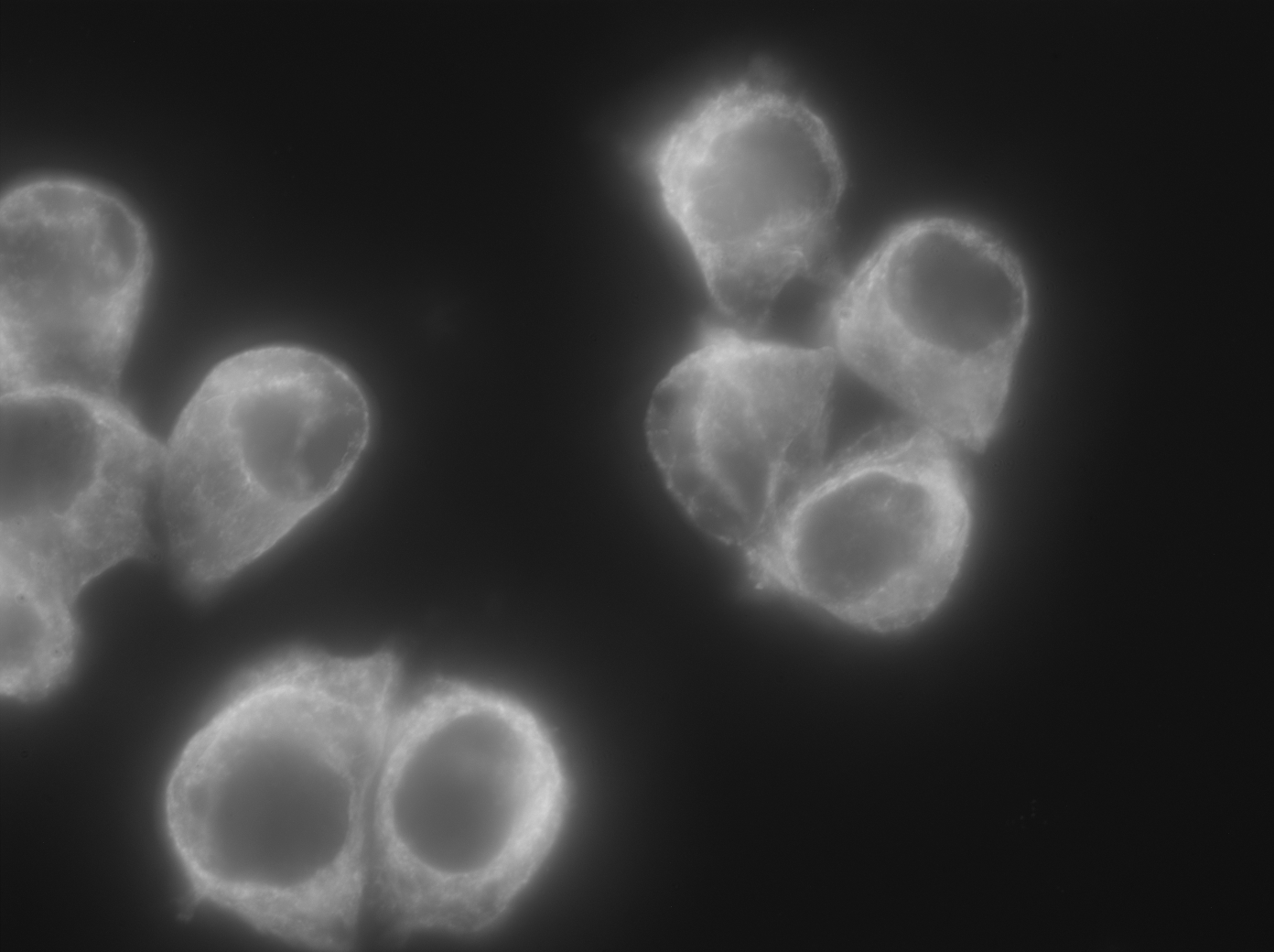

Supplement: Supplementary file 20 — Source data Fig. 6 part 1 [file 44319_2026_742_MOESM20_ESM.zip › Figure 6 Part 1/Fig 6abcd Cas9 TPGS1-KO acetylated tubulin atubulin/Cas9 R2 9-11-24 PA13PA14.Project Maximum Z_XY1726179490_Z0_T0_C1.tif]

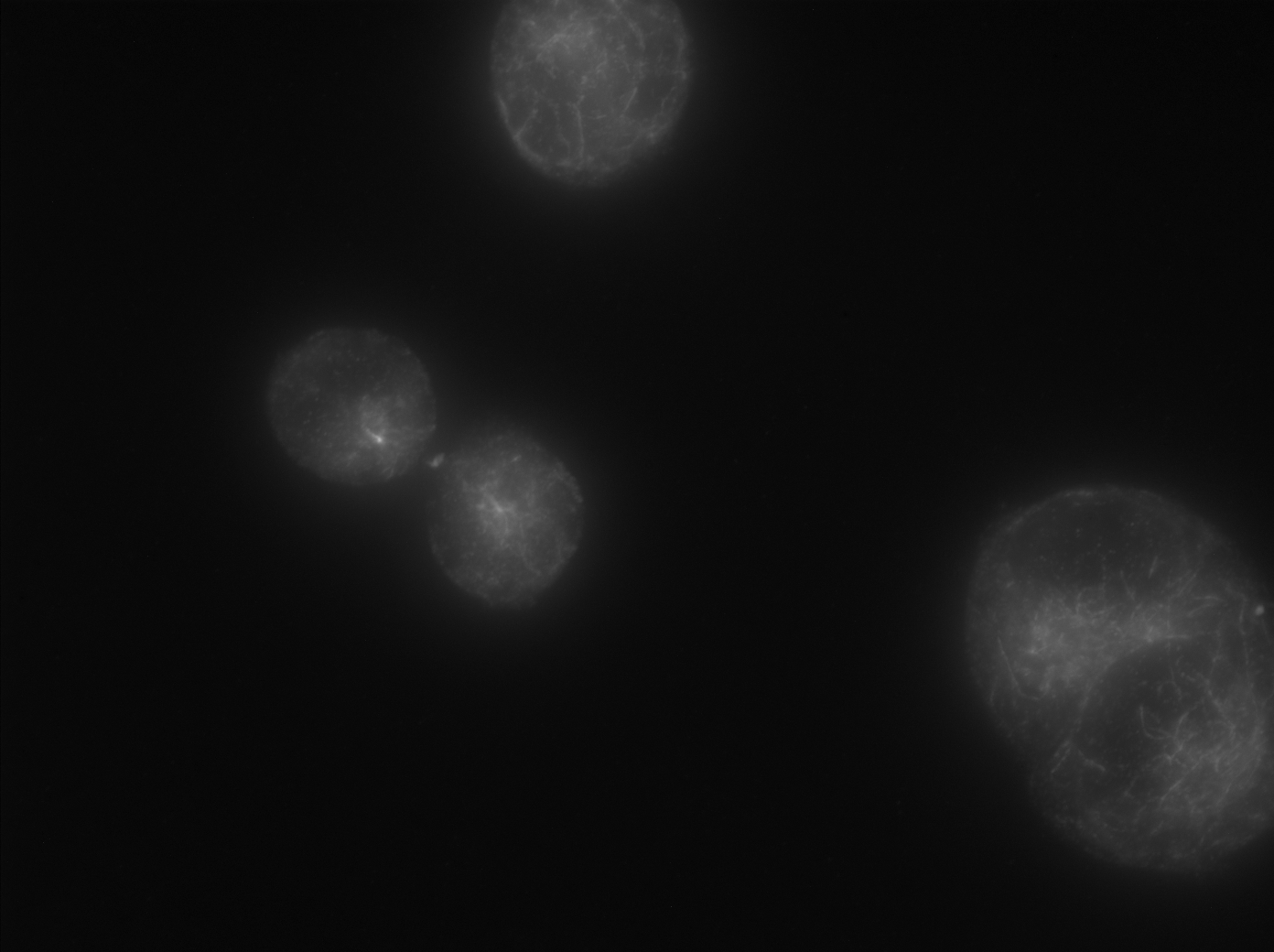

Supplement: Supplementary file 21 — Source data Fig. 6 part 2 [file 44319_2026_742_MOESM21_ESM.zip › Figure 6 Part 2/Fig 6abcd Cas9 TPGS1-KO acetylated tubulin atubulin part 2/TPGS1-KO R2 9-11-24 PA4.Project Maximum Z_XY1726260057_Z0_T0_C2.tif]

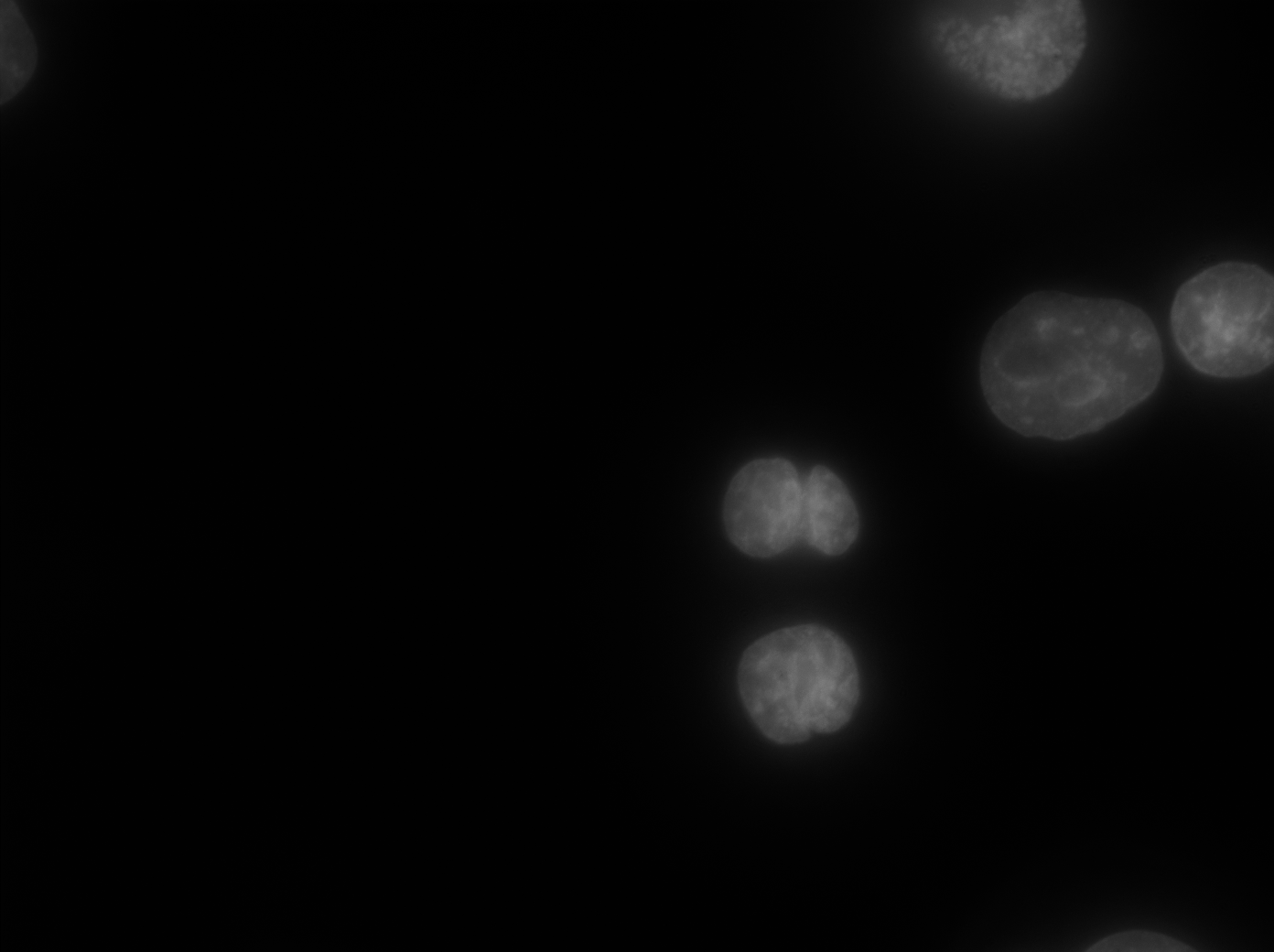

Supplement: Supplementary file 21 — Source data Fig. 6 part 2 [file 44319_2026_742_MOESM21_ESM.zip › Figure 6 Part 2/Fig 6abcd Cas9 TPGS1-KO acetylated tubulin atubulin part 2/TPGS1-KO R3 9-13-24 LT2.Project Maximum Z_XY1726760085_Z0_T0_C0.tif]

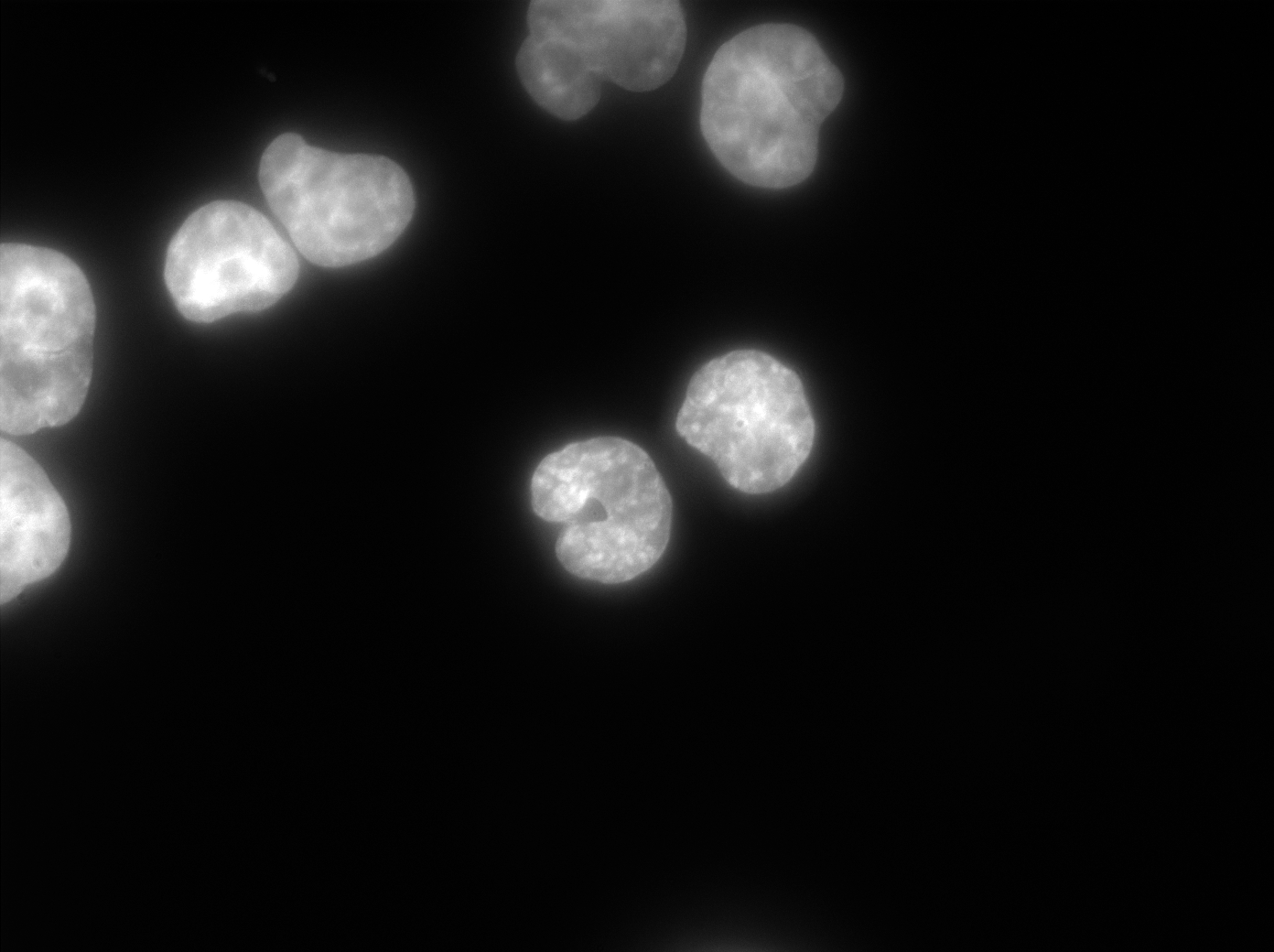

Supplement: Supplementary file 21 — Source data Fig. 6 part 2 [file 44319_2026_742_MOESM21_ESM.zip › Figure 6 Part 2/Fig 6abcd Cas9 TPGS1-KO acetylated tubulin atubulin part 2/TPGS1-KO R2 9-11-24 LT23.Project Maximum Z_XY1726268081_Z0_T0_C0.tif]

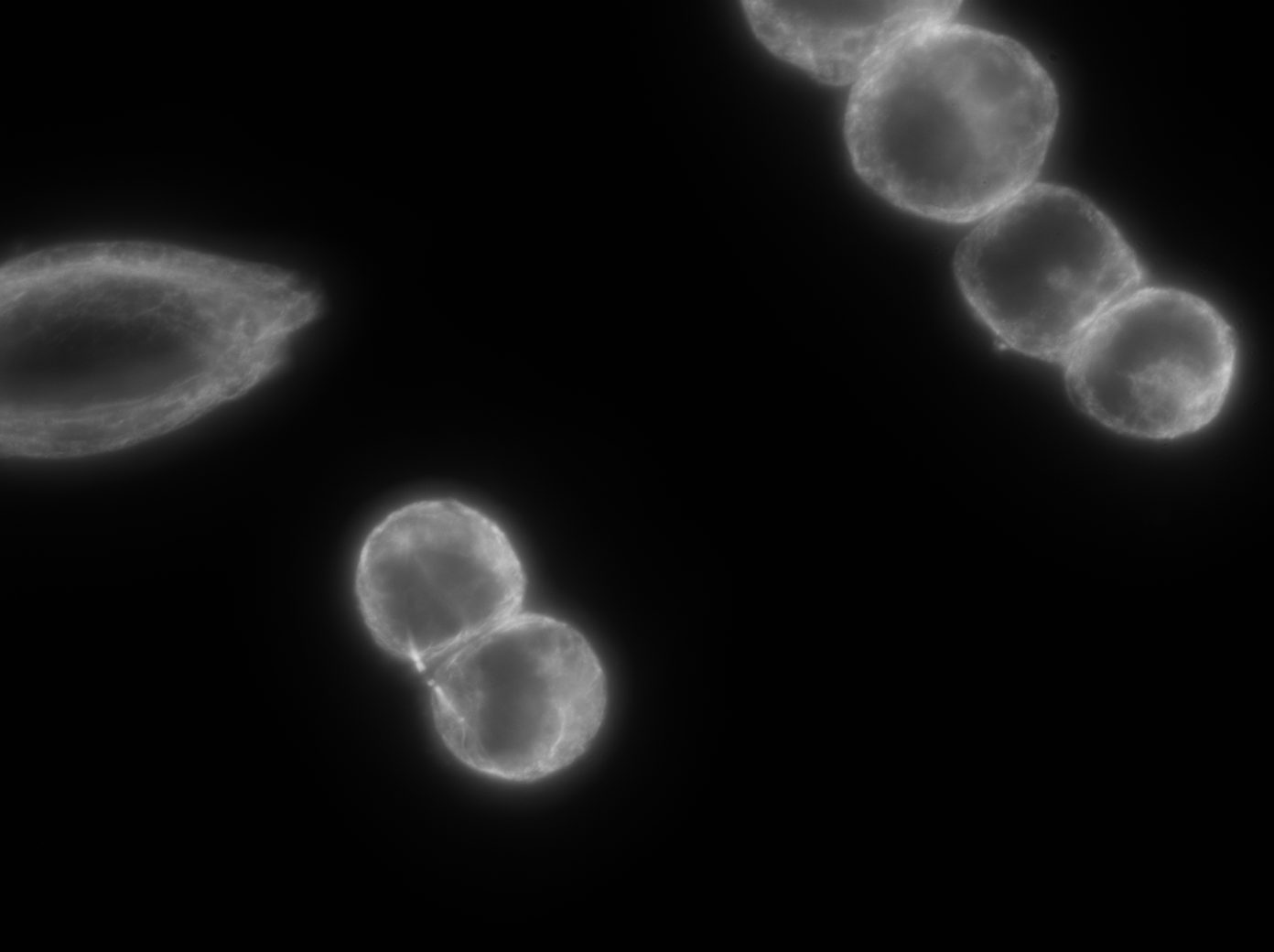

Supplement: Supplementary file 21 — Source data Fig. 6 part 2 [file 44319_2026_742_MOESM21_ESM.zip › Figure 6 Part 2/Fig 6abcd Cas9 TPGS1-KO acetylated tubulin atubulin part 2/TPGS1-KO R2 9-11-24 LT10 PA8.Project Maximum Z_XY1726261995_Z0_T0_C1.tif]

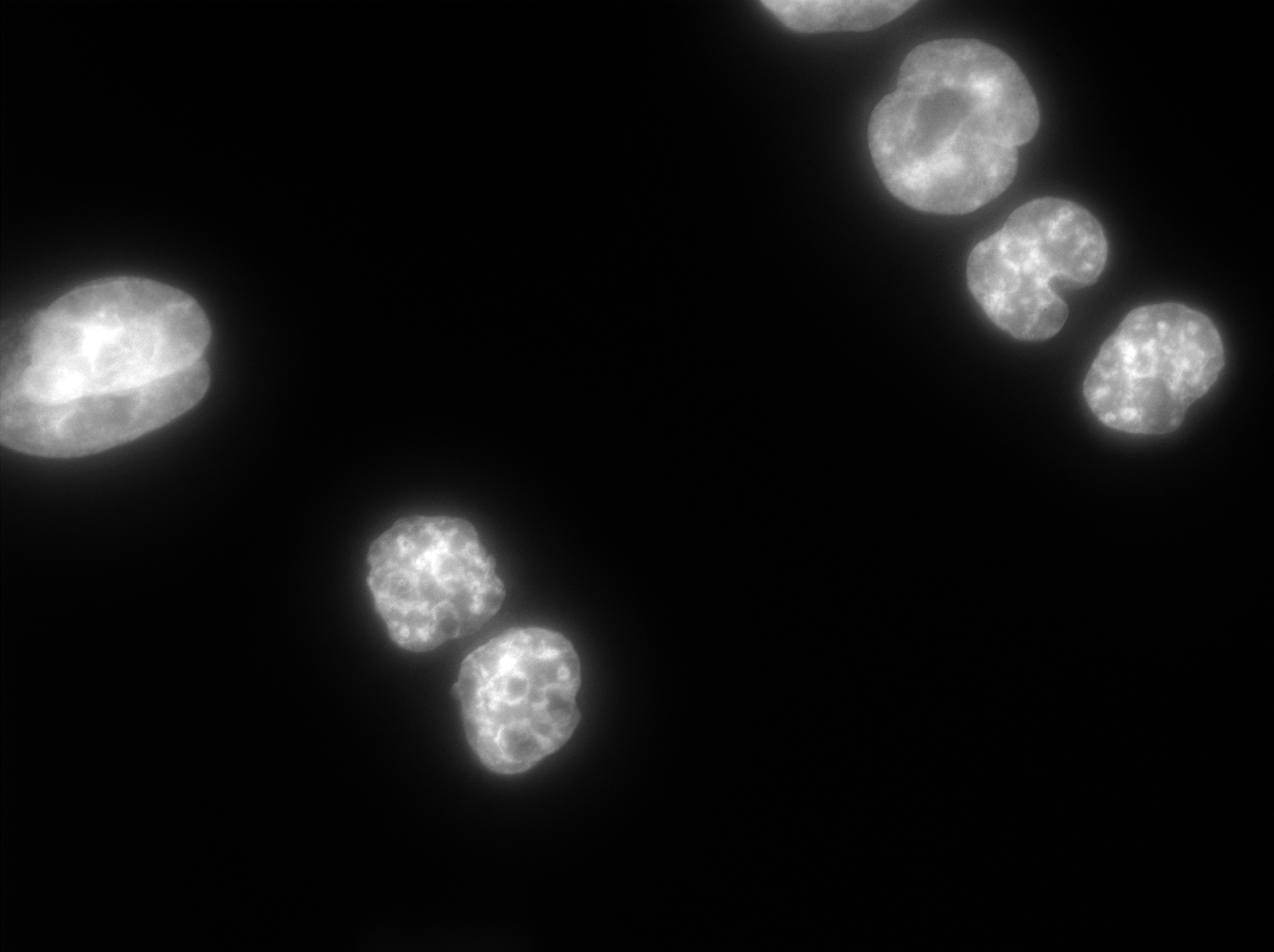

Supplement: Supplementary file 21 — Source data Fig. 6 part 2 [file 44319_2026_742_MOESM21_ESM.zip › Figure 6 Part 2/Fig 6abcd Cas9 TPGS1-KO acetylated tubulin atubulin part 2/TPGS1-KO R2 9-11-24 LT10 PA8.Project Maximum Z_XY1726261995_Z0_T0_C0.tif]

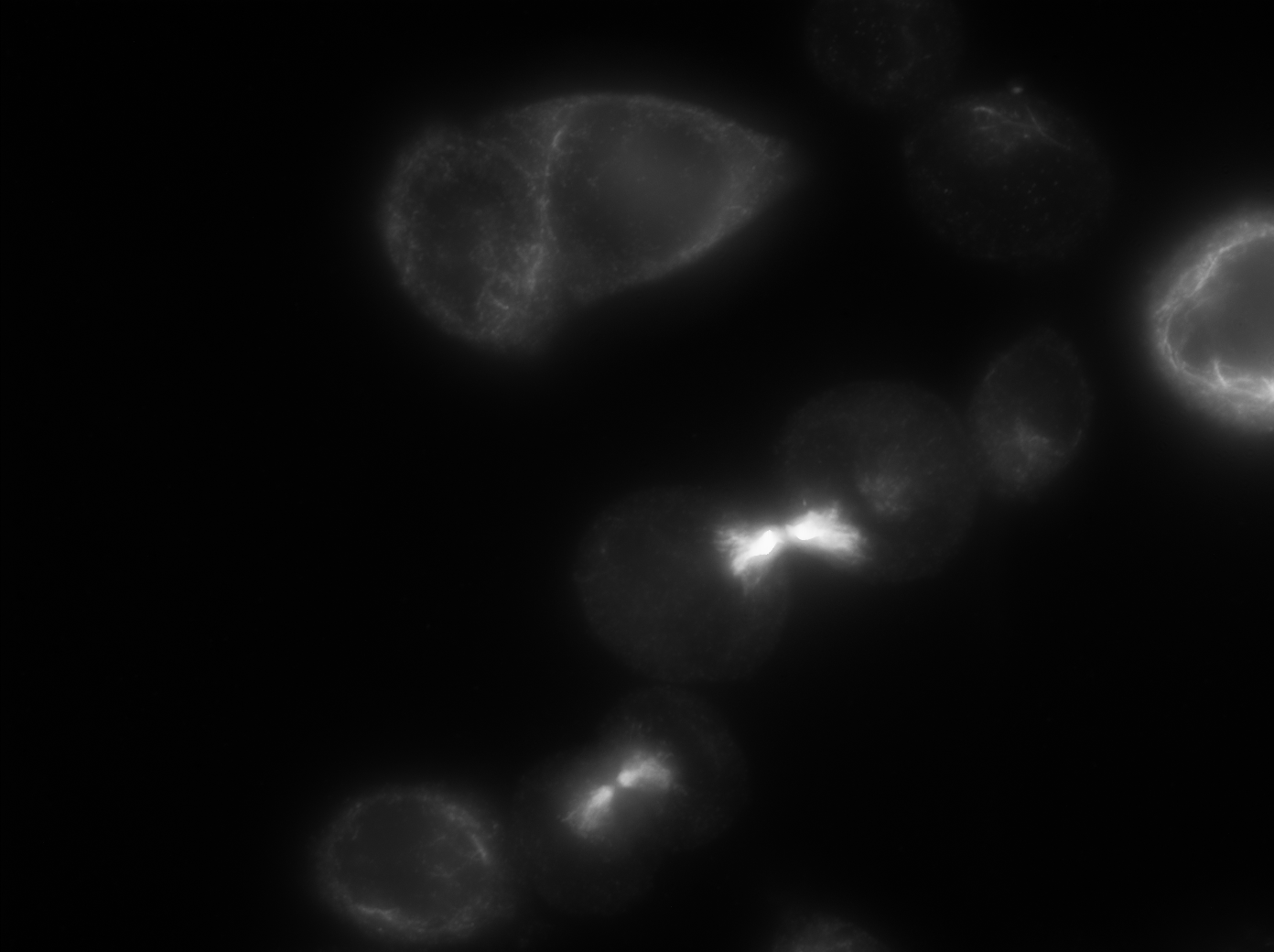

Supplement: Supplementary file 21 — Source data Fig. 6 part 2 [file 44319_2026_742_MOESM21_ESM.zip › Figure 6 Part 2/Fig 6abcd Cas9 TPGS1-KO acetylated tubulin atubulin part 2/TPGS1-KO R3 9-13-24 LT10.Project Maximum Z_XY1726760993_Z0_T0_C2.tif]

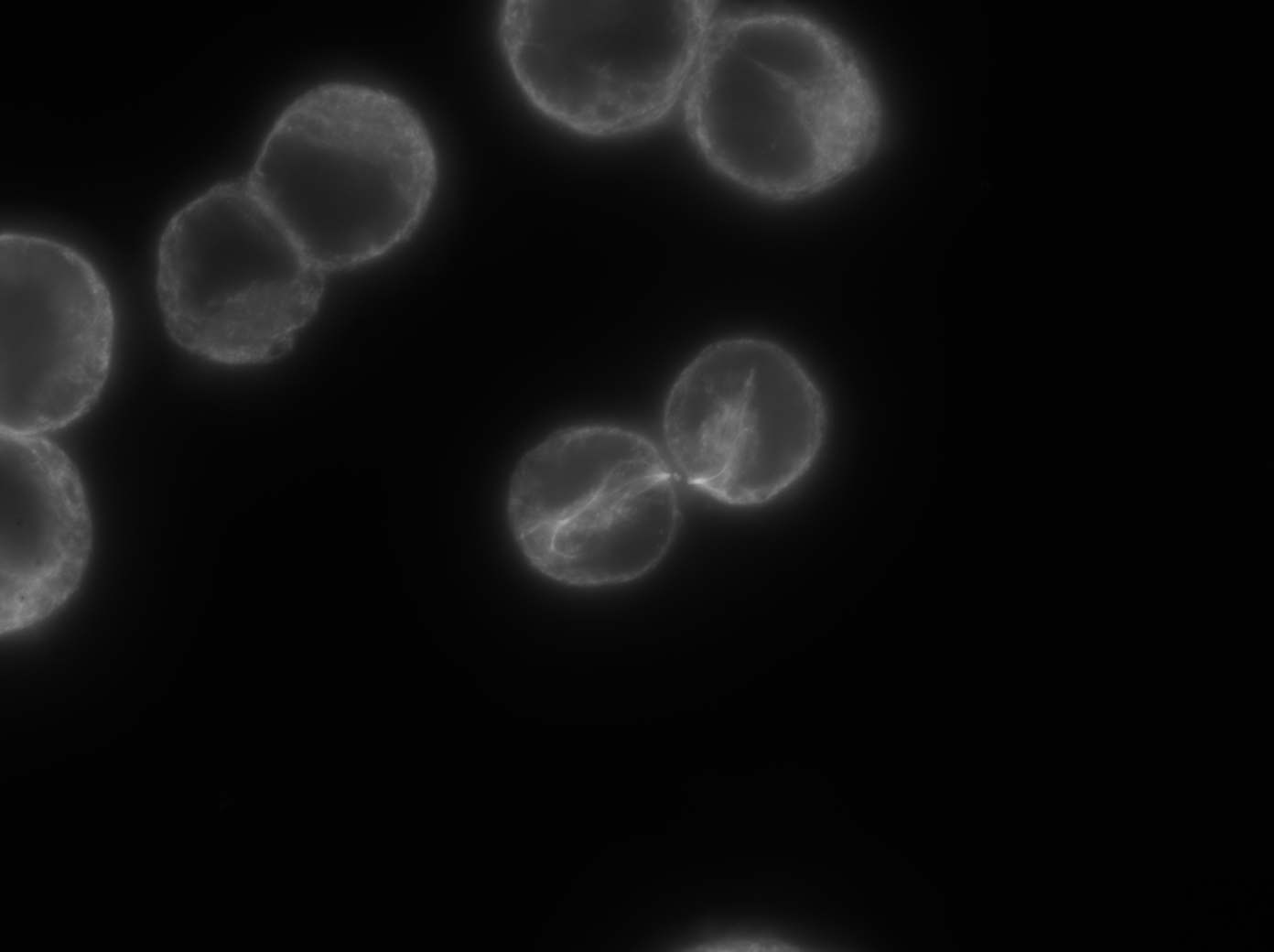

Supplement: Supplementary file 21 — Source data Fig. 6 part 2 [file 44319_2026_742_MOESM21_ESM.zip › Figure 6 Part 2/Fig 6abcd Cas9 TPGS1-KO acetylated tubulin atubulin part 2/TPGS1-KO R2 9-11-24 LT23.Project Maximum Z_XY1726268081_Z0_T0_C1.tif]

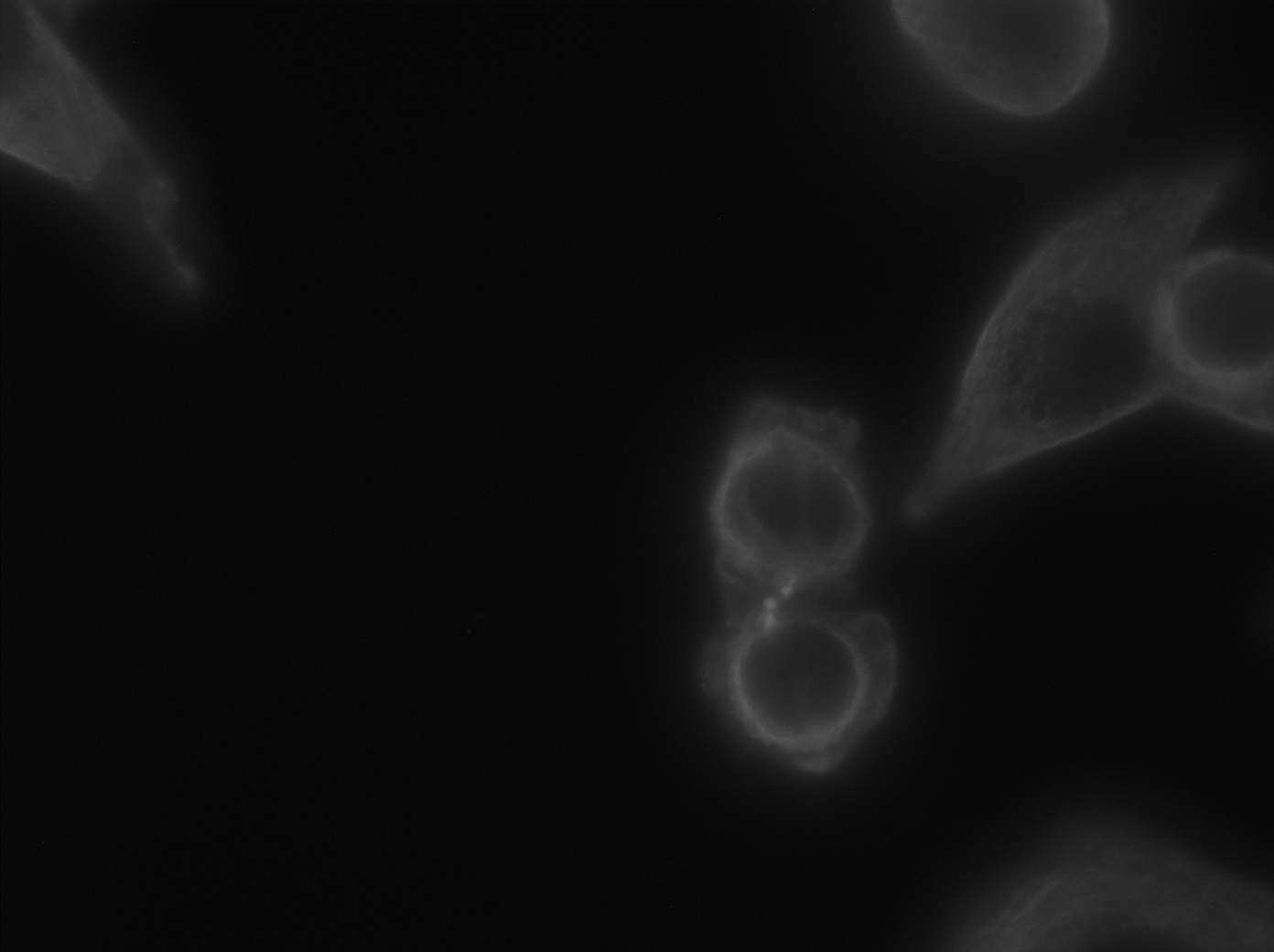

Supplement: Supplementary file 21 — Source data Fig. 6 part 2 [file 44319_2026_742_MOESM21_ESM.zip › Figure 6 Part 2/Fig 6abcd Cas9 TPGS1-KO acetylated tubulin atubulin part 2/TPGS1-KO R3 9-13-24 LT2.Project Maximum Z_XY1726760085_Z0_T0_C1.tif]

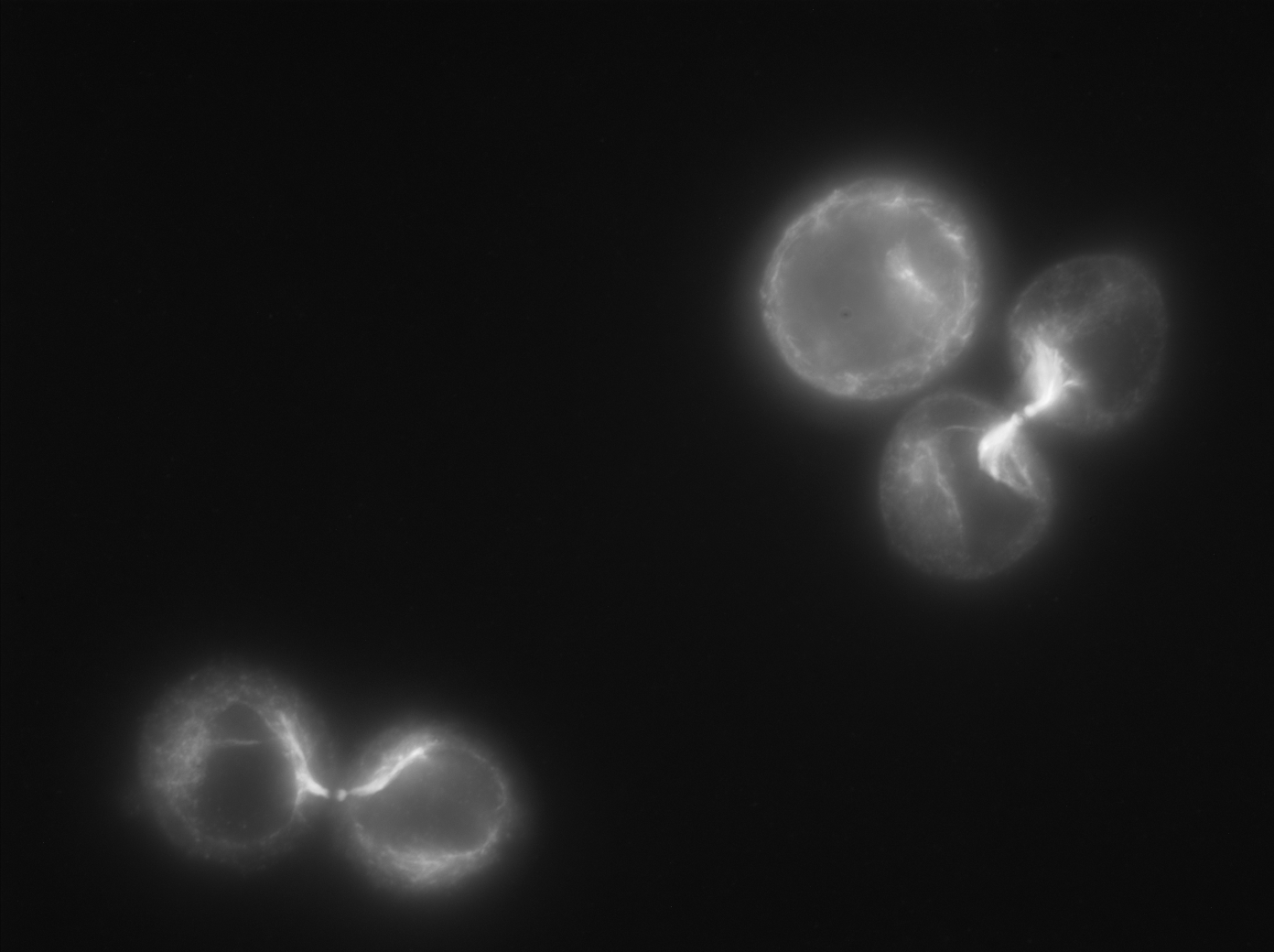

Supplement: Supplementary file 21 — Source data Fig. 6 part 2 [file 44319_2026_742_MOESM21_ESM.zip › Figure 6 Part 2/Fig 6abcd Cas9 TPGS1-KO acetylated tubulin atubulin part 2/TPGS1-KO R2 9-11-24 LT17LT18.Project Maximum Z_XY1726266302_Z0_T0_C2.tif]

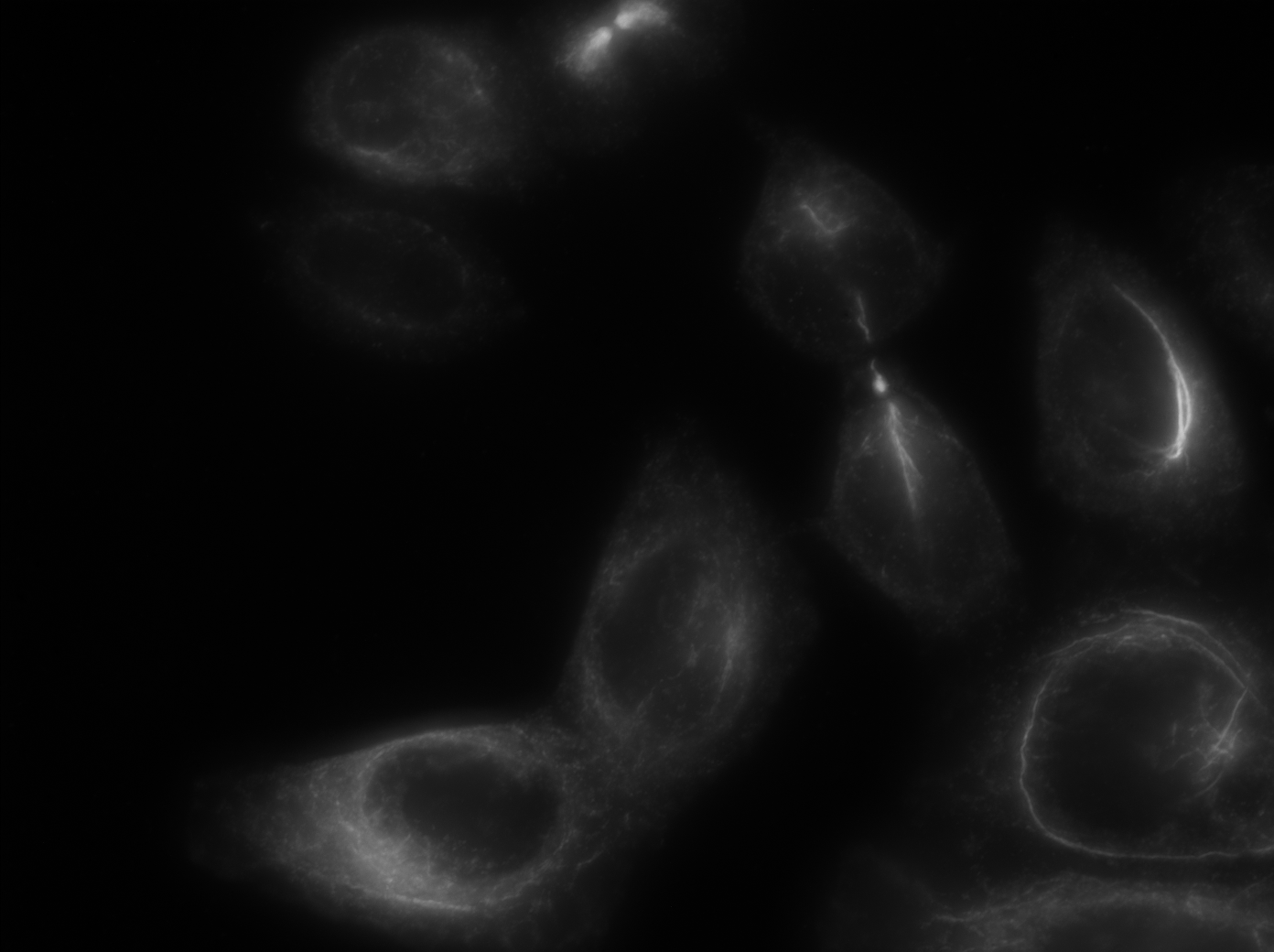

Supplement: Supplementary file 21 — Source data Fig. 6 part 2 [file 44319_2026_742_MOESM21_ESM.zip › Figure 6 Part 2/Fig 6abcd Cas9 TPGS1-KO acetylated tubulin atubulin part 2/TPGS1-KO R3 9-13-24 LT11.Project Maximum Z_XY1726761069_Z0_T0_C2.tif]

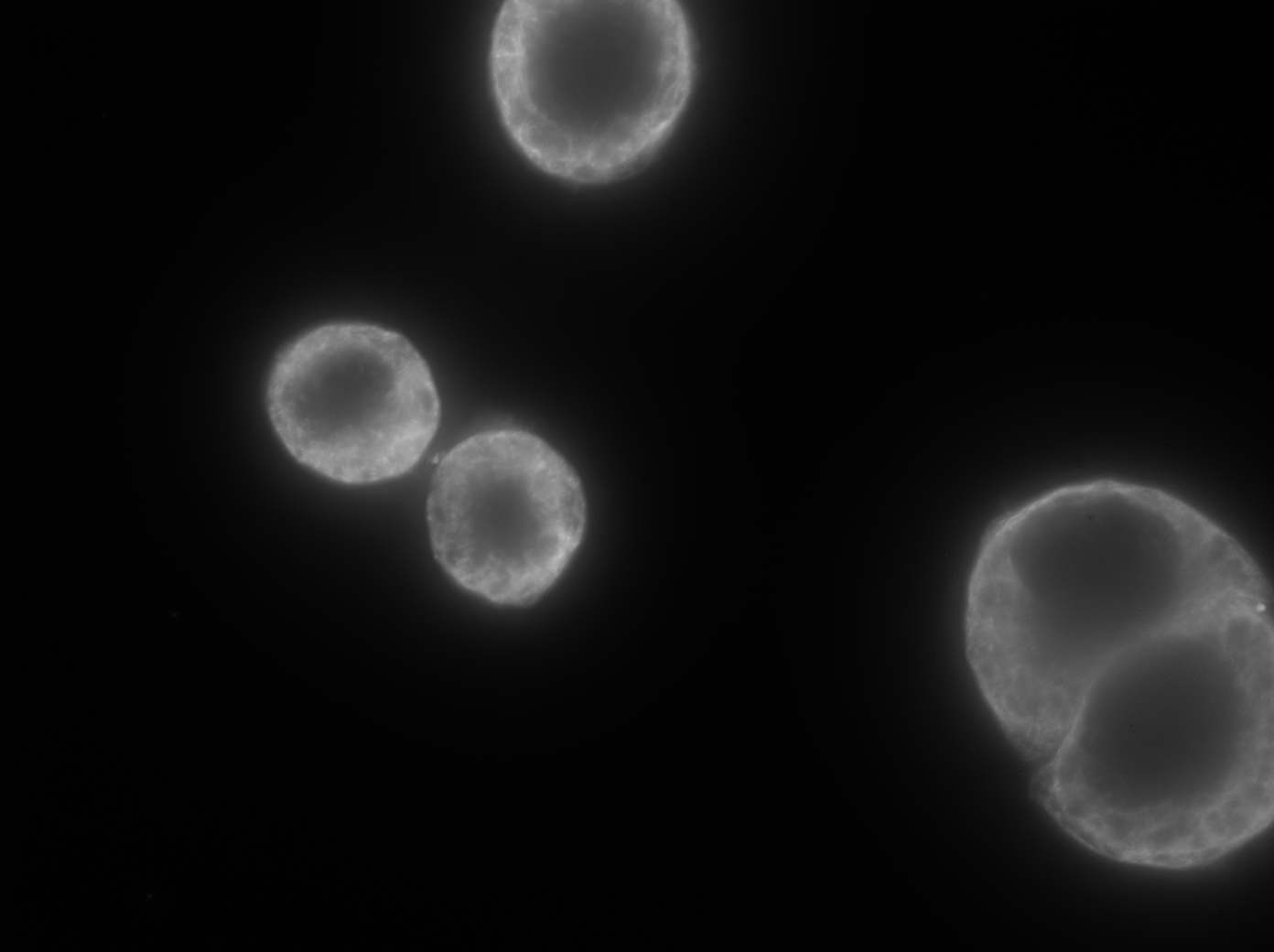

Supplement: Supplementary file 21 — Source data Fig. 6 part 2 [file 44319_2026_742_MOESM21_ESM.zip › Figure 6 Part 2/Fig 6abcd Cas9 TPGS1-KO acetylated tubulin atubulin part 2/TPGS1-KO R2 9-11-24 PA4.Project Maximum Z_XY1726260057_Z0_T0_C1.tif]

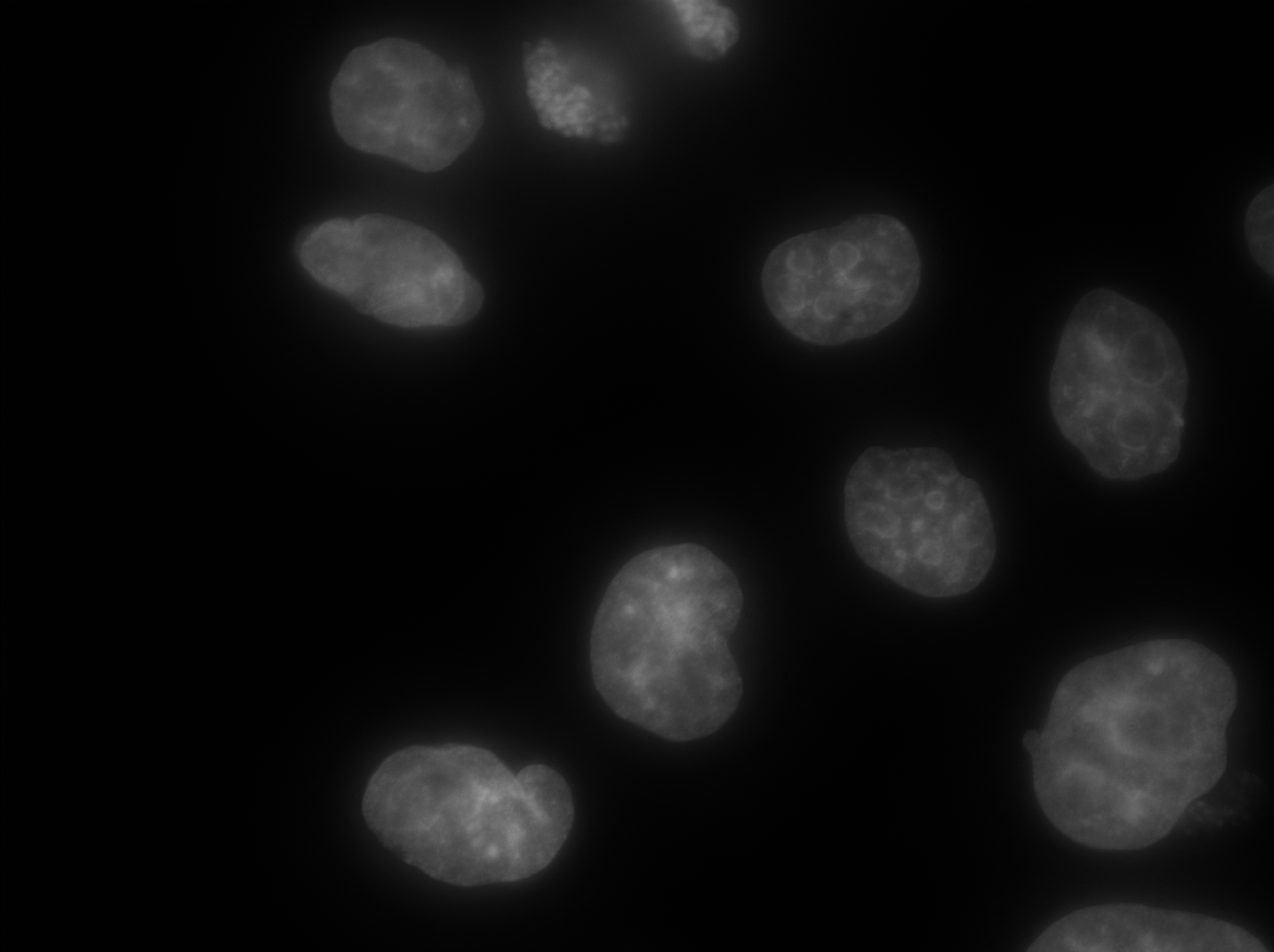

Supplement: Supplementary file 21 — Source data Fig. 6 part 2 [file 44319_2026_742_MOESM21_ESM.zip › Figure 6 Part 2/Fig 6abcd Cas9 TPGS1-KO acetylated tubulin atubulin part 2/TPGS1-KO R3 9-13-24 LT11.Project Maximum Z_XY1726761069_Z0_T0_C0.tif]

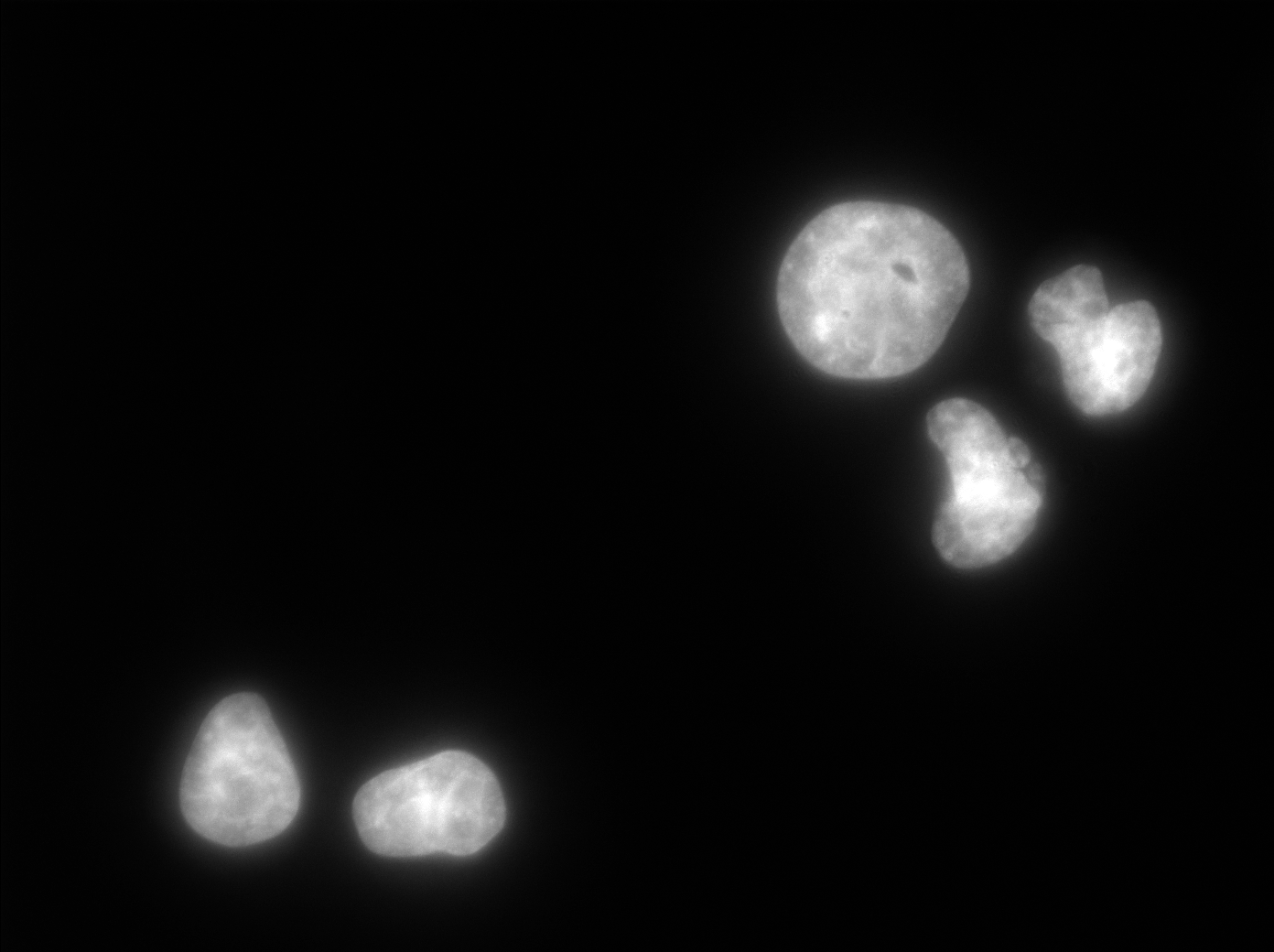

Supplement: Supplementary file 21 — Source data Fig. 6 part 2 [file 44319_2026_742_MOESM21_ESM.zip › Figure 6 Part 2/Fig 6abcd Cas9 TPGS1-KO acetylated tubulin atubulin part 2/TPGS1-KO R2 9-11-24 LT17LT18.Project Maximum Z_XY1726266302_Z0_T0_C0.tif]

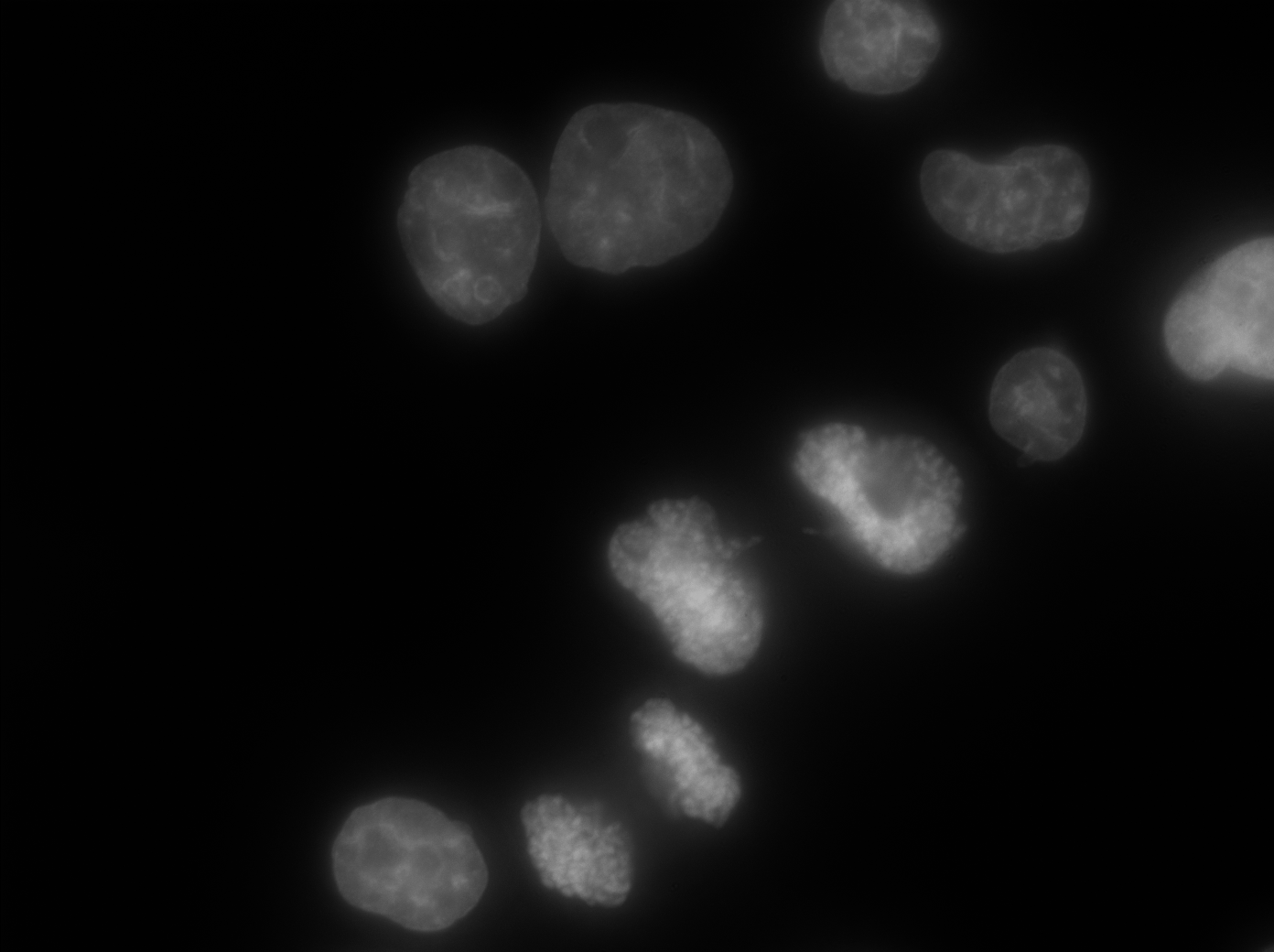

Supplement: Supplementary file 21 — Source data Fig. 6 part 2 [file 44319_2026_742_MOESM21_ESM.zip › Figure 6 Part 2/Fig 6abcd Cas9 TPGS1-KO acetylated tubulin atubulin part 2/TPGS1-KO R3 9-13-24 LT10.Project Maximum Z_XY1726760993_Z0_T0_C0.tif]
