# Supplementary figures and images for: TPGS1 regulates central spindle microtubule glutamylation and remodeling during telophase and abscission (part 22 of 36)
Source: EMBO Rep. 2026 Mar 23;27(8):1944–63. doi: 10.1038/s44319-026-00742-3 (PMC13121839; doi:10.1038/s44319-026-00742-3)

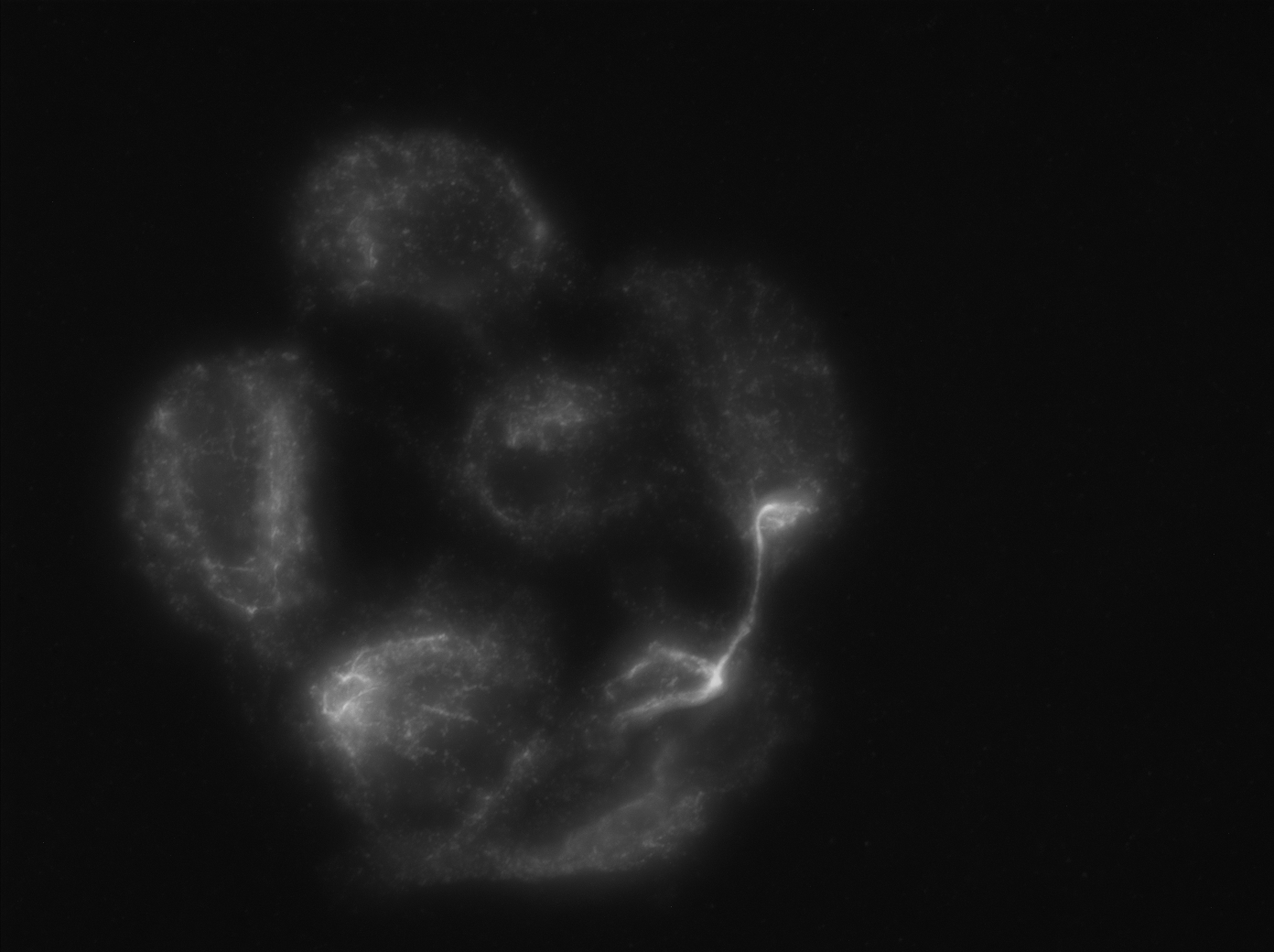

Supplement: Supplementary file 20 — Source data Fig. 6 part 1 [file 44319_2026_742_MOESM20_ESM.zip › Figure 6 Part 1/Fig 6abcd Cas9 TPGS1-KO acetylated tubulin atubulin/Cas9 R2 9-11-24 LT27.Project Maximum Z_XY1726181153_Z0_T0_C2.tif]

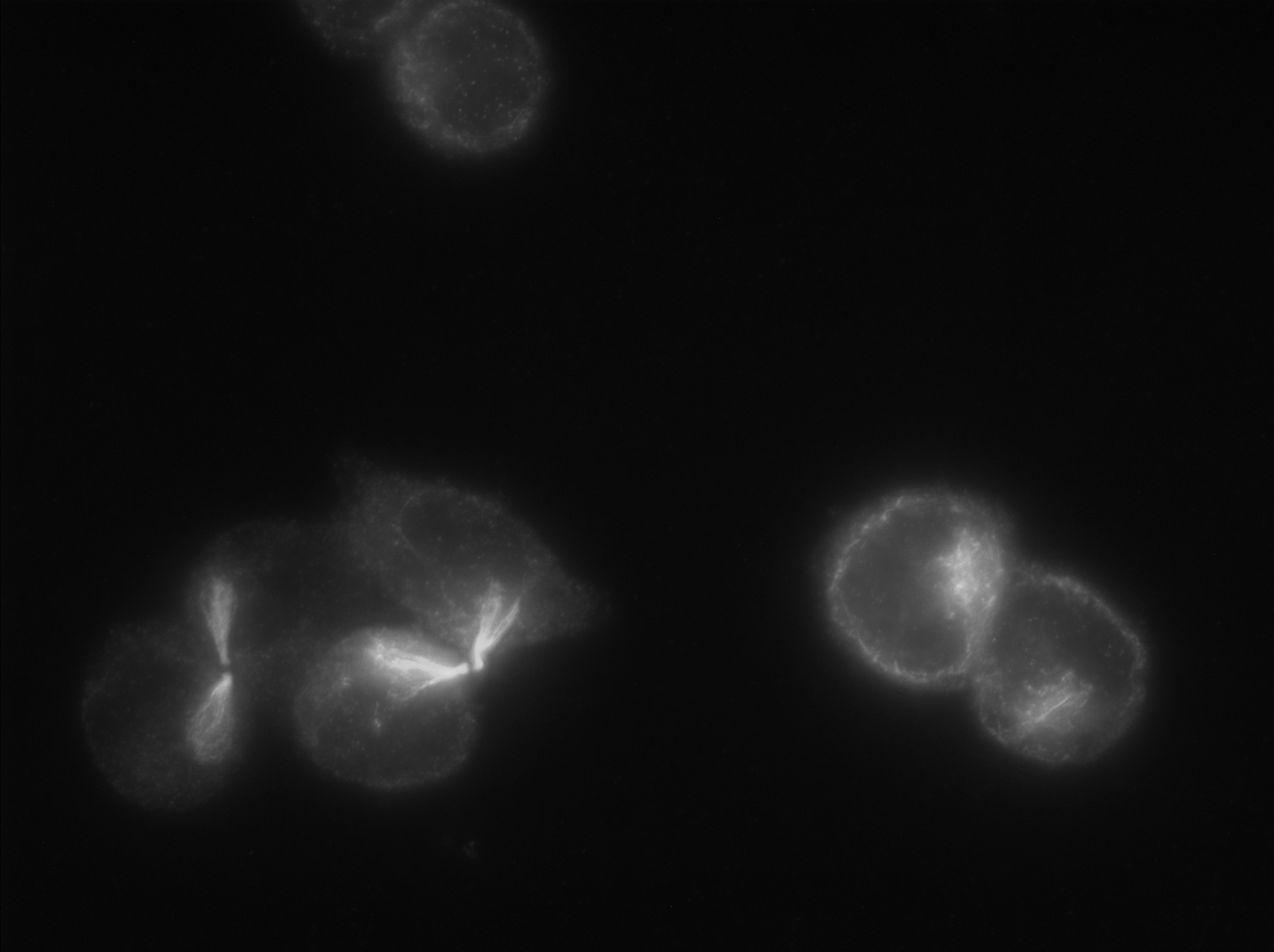

Supplement: Supplementary file 20 — Source data Fig. 6 part 1 [file 44319_2026_742_MOESM20_ESM.zip › Figure 6 Part 1/Fig 6abcd Cas9 TPGS1-KO acetylated tubulin atubulin/Cas9 R2 9-11-24 LT13LT14.Project Maximum Z_XY1726174301_Z0_T0_C2.tif]

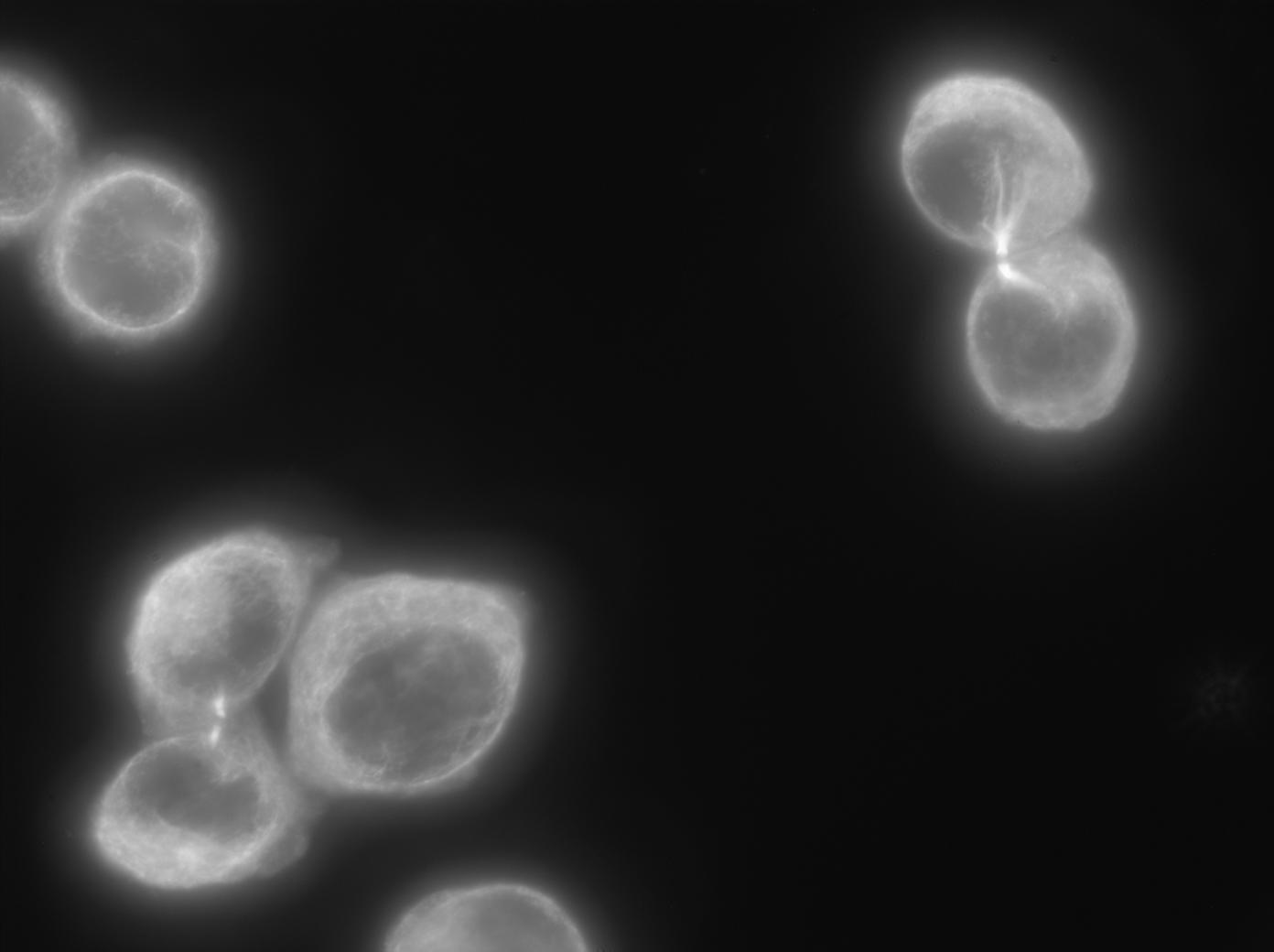

Supplement: Supplementary file 20 — Source data Fig. 6 part 1 [file 44319_2026_742_MOESM20_ESM.zip › Figure 6 Part 1/Fig 6abcd Cas9 TPGS1-KO acetylated tubulin atubulin/Cas9 R2 9-11-24 LT7LT8.Project Maximum Z_XY1726173377_Z0_T0_C1.tif]

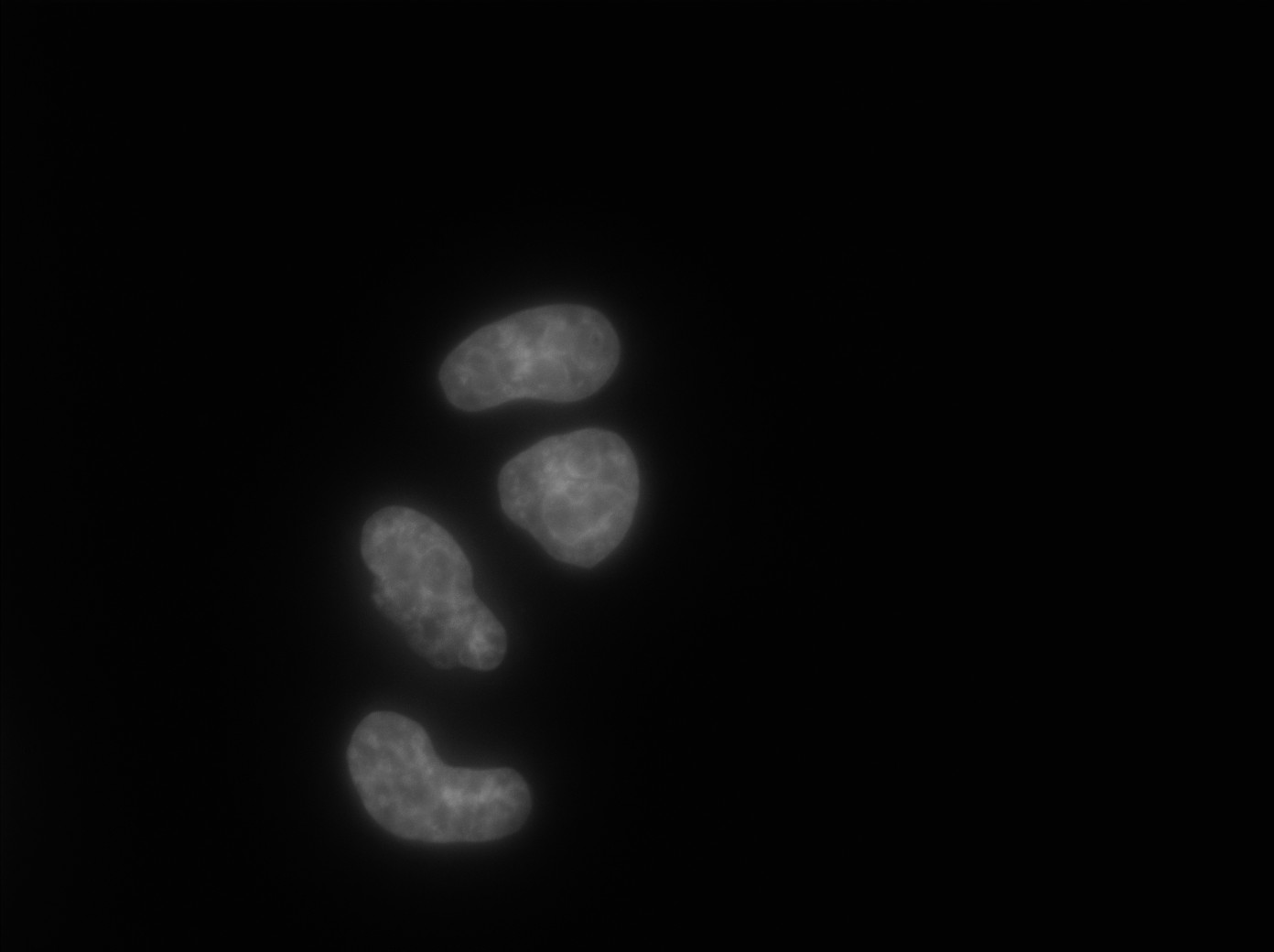

Supplement: Supplementary file 20 — Source data Fig. 6 part 1 [file 44319_2026_742_MOESM20_ESM.zip › Figure 6 Part 1/Fig 6abcd Cas9 TPGS1-KO acetylated tubulin atubulin/Cas9 R2 9-11-24 LT19 PA9.Project Maximum Z_XY1726178474_Z0_T0_C0.tif]

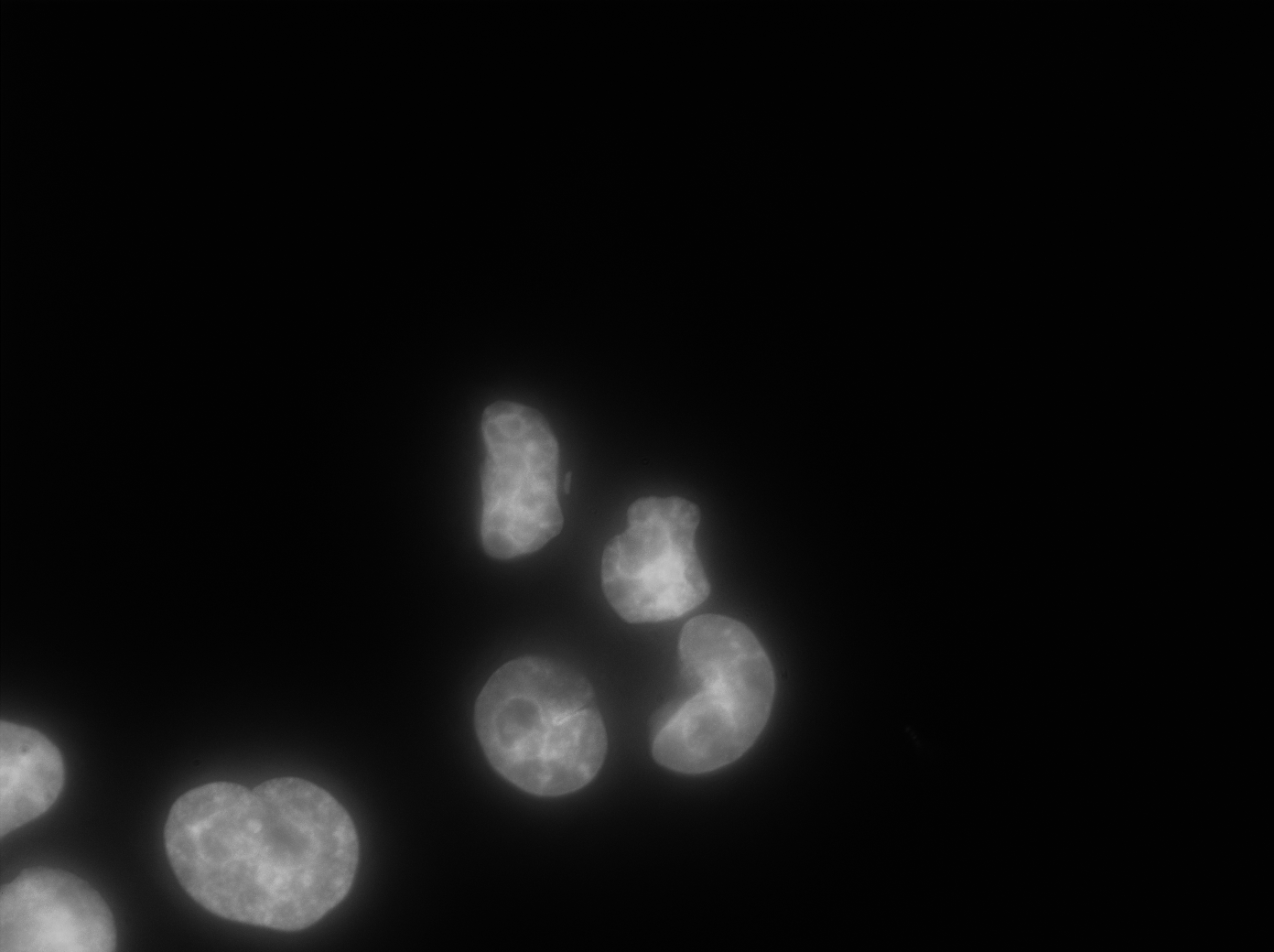

Supplement: Supplementary file 20 — Source data Fig. 6 part 1 [file 44319_2026_742_MOESM20_ESM.zip › Figure 6 Part 1/Fig 6abcd Cas9 TPGS1-KO acetylated tubulin atubulin/Cas9 R2 9-11-24 PA29.Project Maximum Z_XY1726181930_Z0_T0_C0.tif]

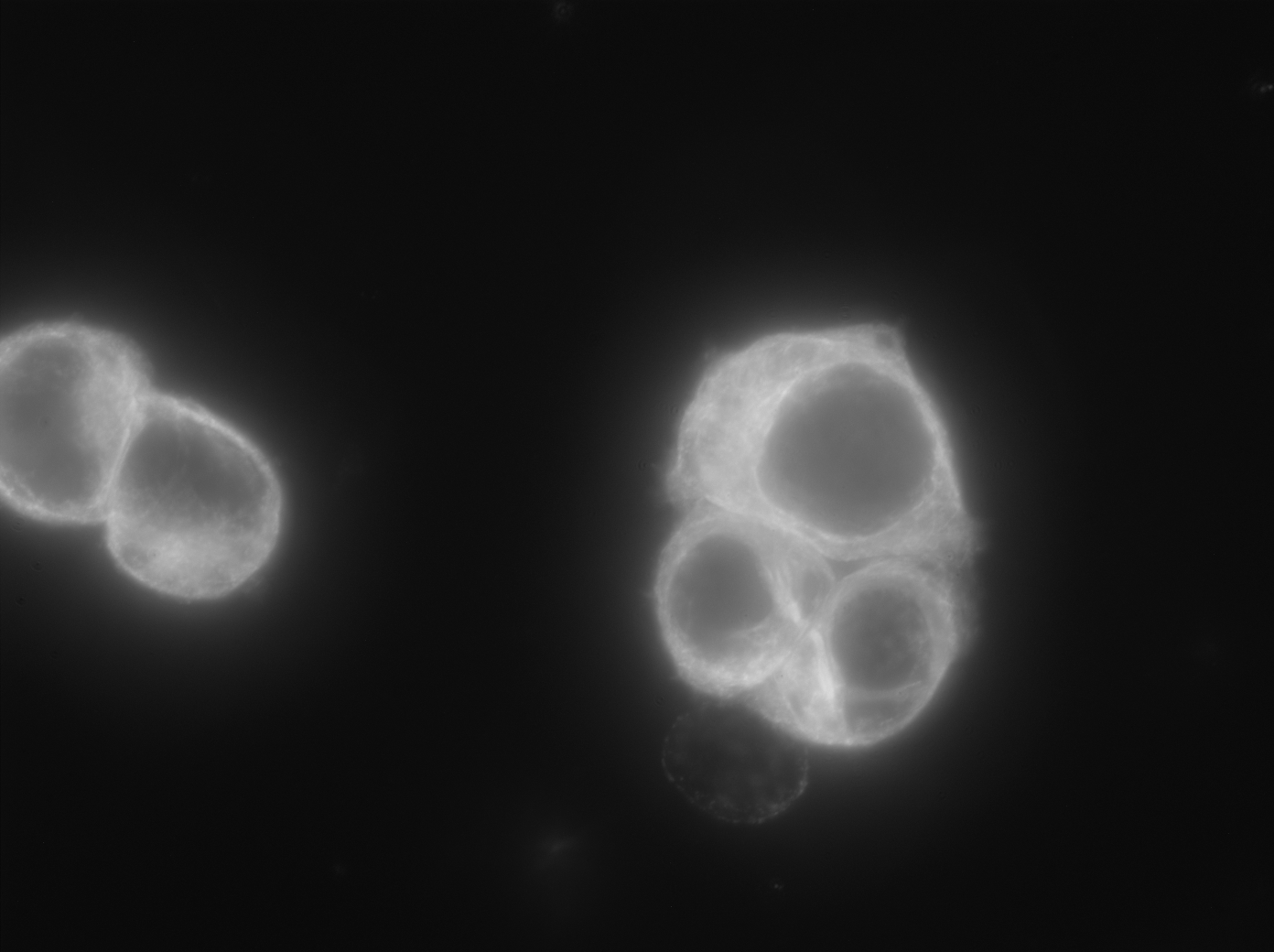

Supplement: Supplementary file 20 — Source data Fig. 6 part 1 [file 44319_2026_742_MOESM20_ESM.zip › Figure 6 Part 1/Fig 6abcd Cas9 TPGS1-KO acetylated tubulin atubulin/Cas9 R2 9-11-24 LT15.Project Maximum Z_XY1726174550_Z0_T0_C1.tif]

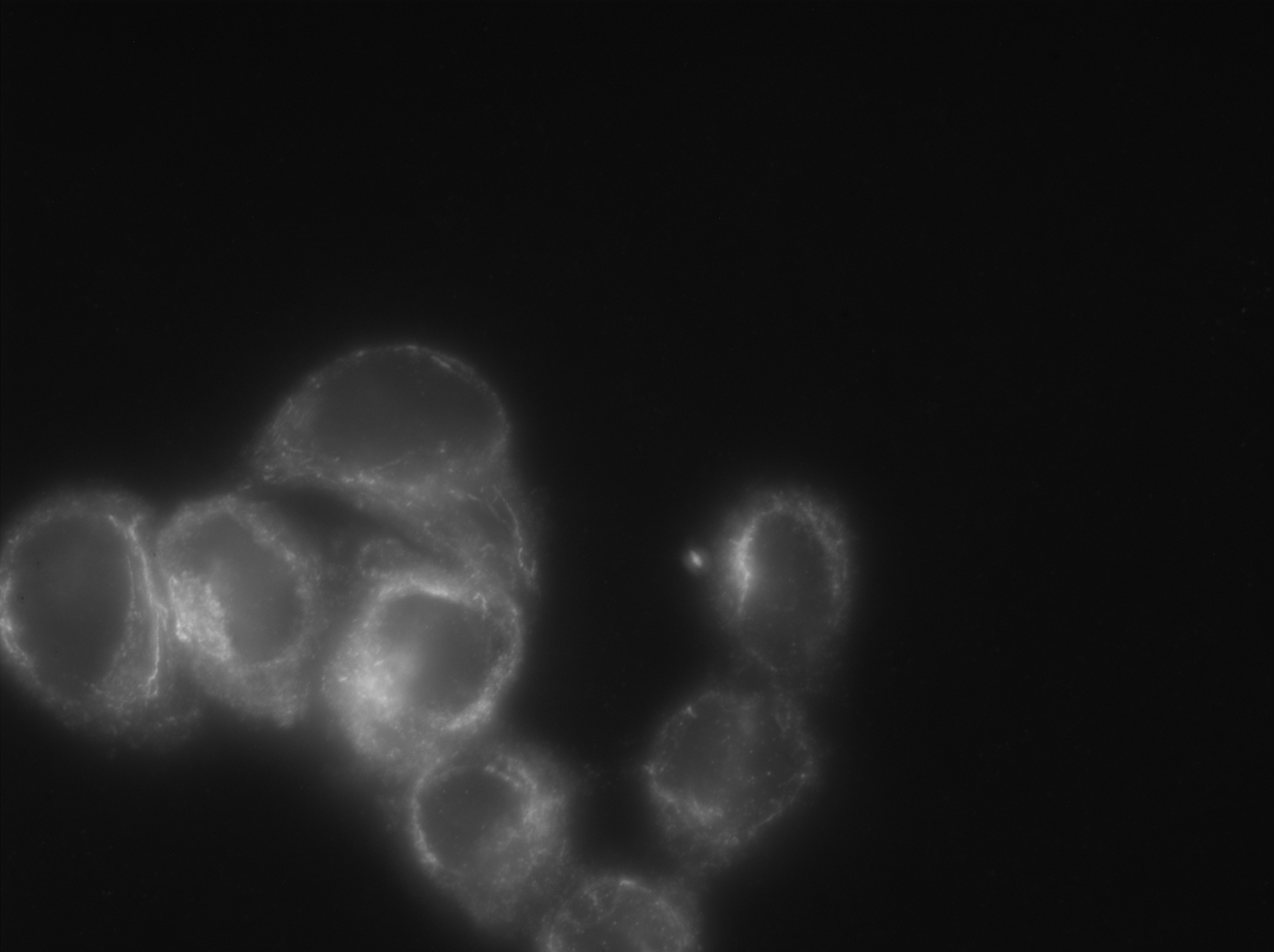

Supplement: Supplementary file 20 — Source data Fig. 6 part 1 [file 44319_2026_742_MOESM20_ESM.zip › Figure 6 Part 1/Fig 6abcd Cas9 TPGS1-KO acetylated tubulin atubulin/Cas9 R2 9-11-24 PA17.Project Maximum Z_XY1726179977_Z0_T0_C2.tif]

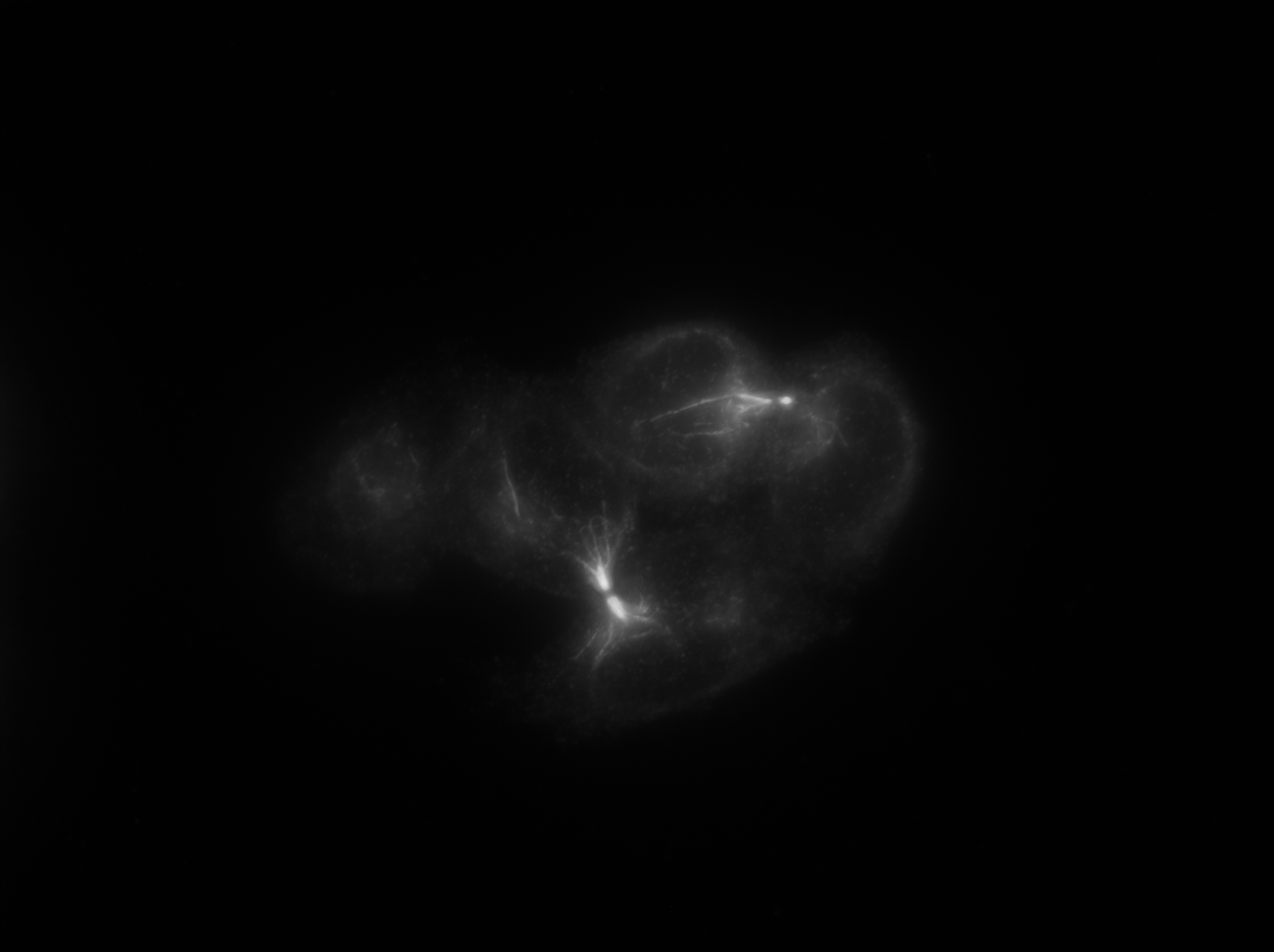

Supplement: Supplementary file 20 — Source data Fig. 6 part 1 [file 44319_2026_742_MOESM20_ESM.zip › Figure 6 Part 1/Fig 6abcd Cas9 TPGS1-KO acetylated tubulin atubulin/Cas9 R3 9-13-24 LT16LT17.Project Maximum Z_XY1726766464_Z0_T0_C2.tif]

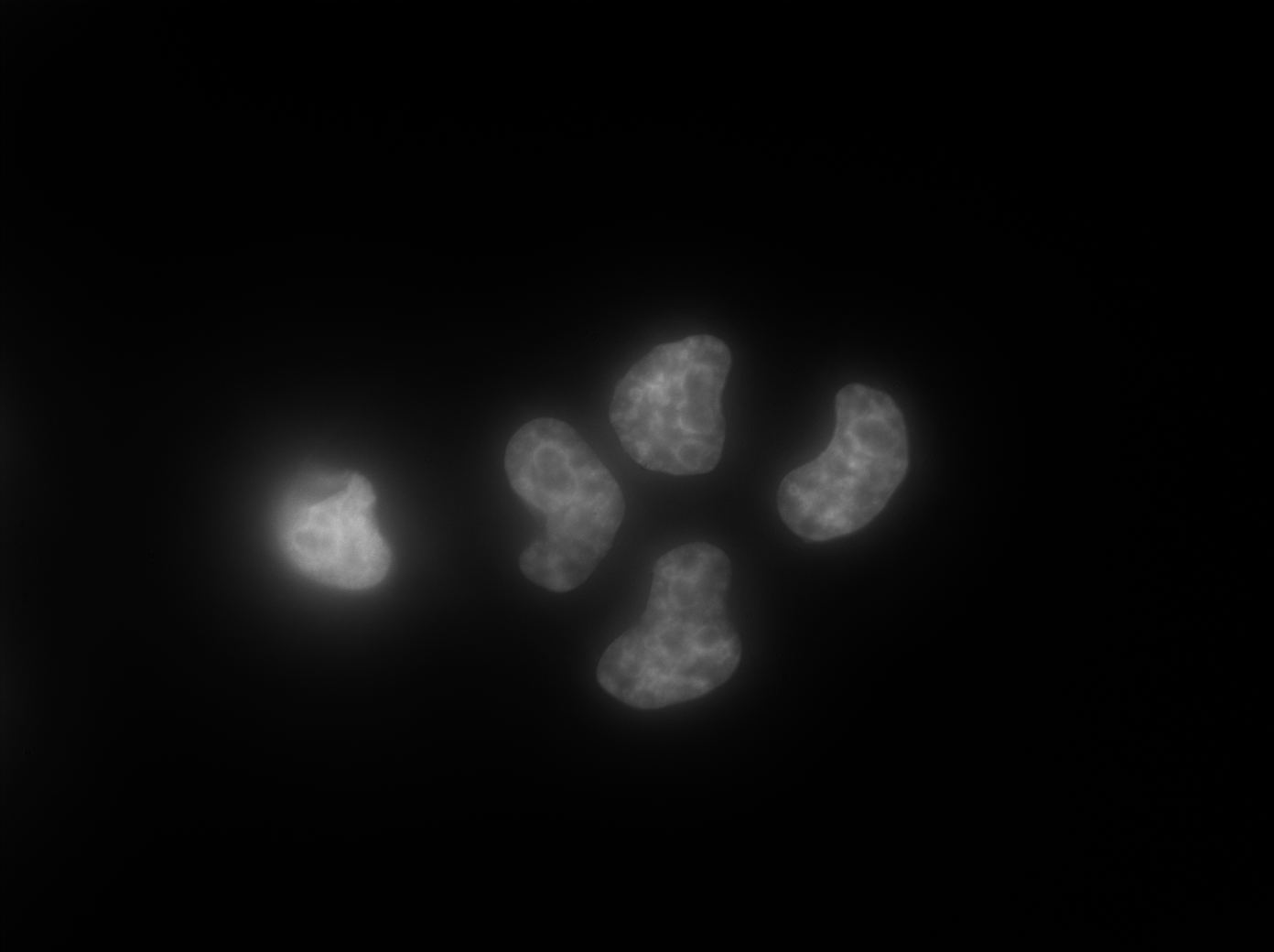

Supplement: Supplementary file 20 — Source data Fig. 6 part 1 [file 44319_2026_742_MOESM20_ESM.zip › Figure 6 Part 1/Fig 6abcd Cas9 TPGS1-KO acetylated tubulin atubulin/Cas9 R3 9-13-24 LT16LT17.Project Maximum Z_XY1726766464_Z0_T0_C0.tif]

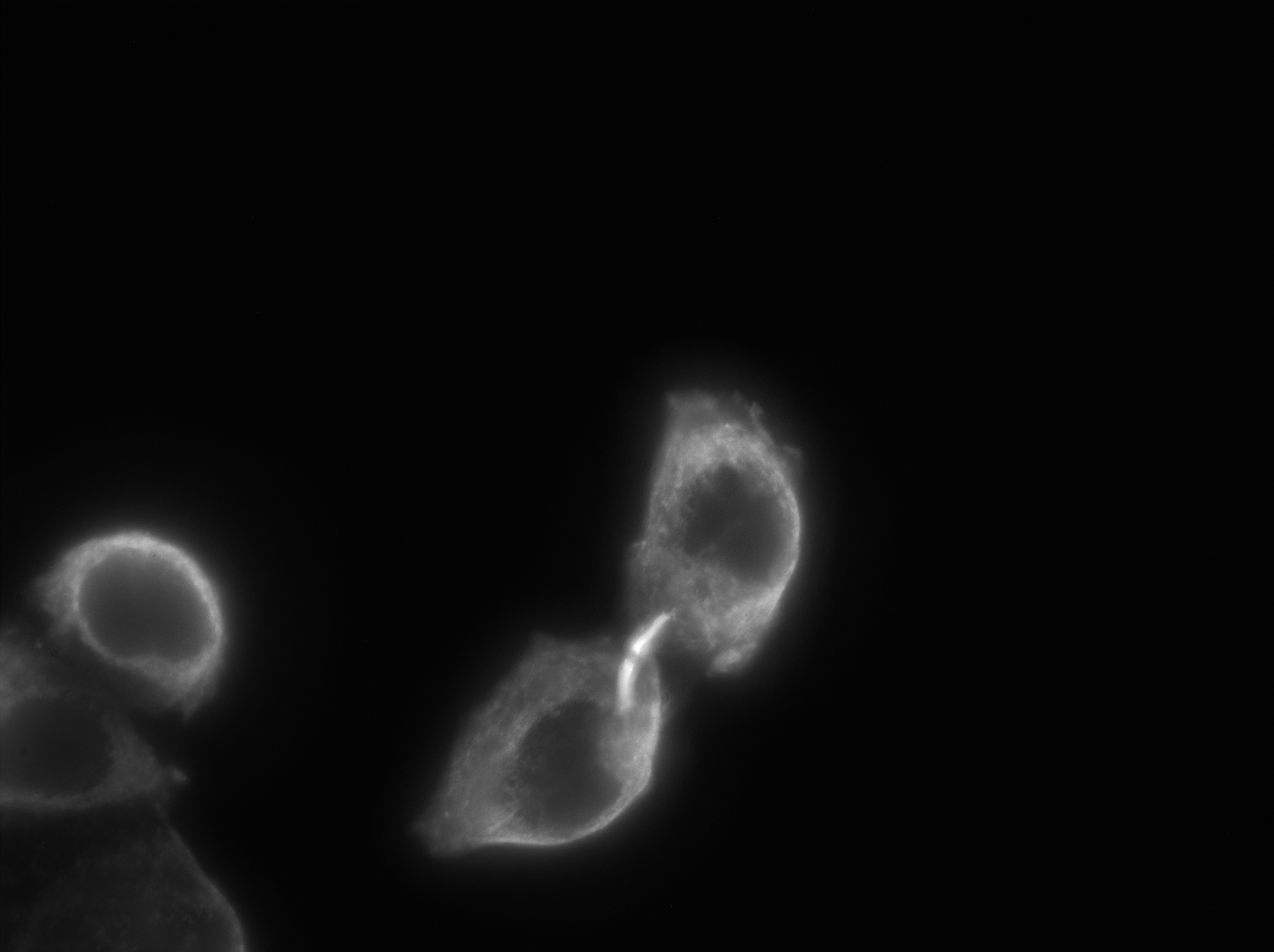

Supplement: Supplementary file 20 — Source data Fig. 6 part 1 [file 44319_2026_742_MOESM20_ESM.zip › Figure 6 Part 1/Fig 6abcd Cas9 TPGS1-KO acetylated tubulin atubulin/Cas9 R3 9-13-24 LT26.Project Maximum Z_XY1726767497_Z0_T0_C1.tif]

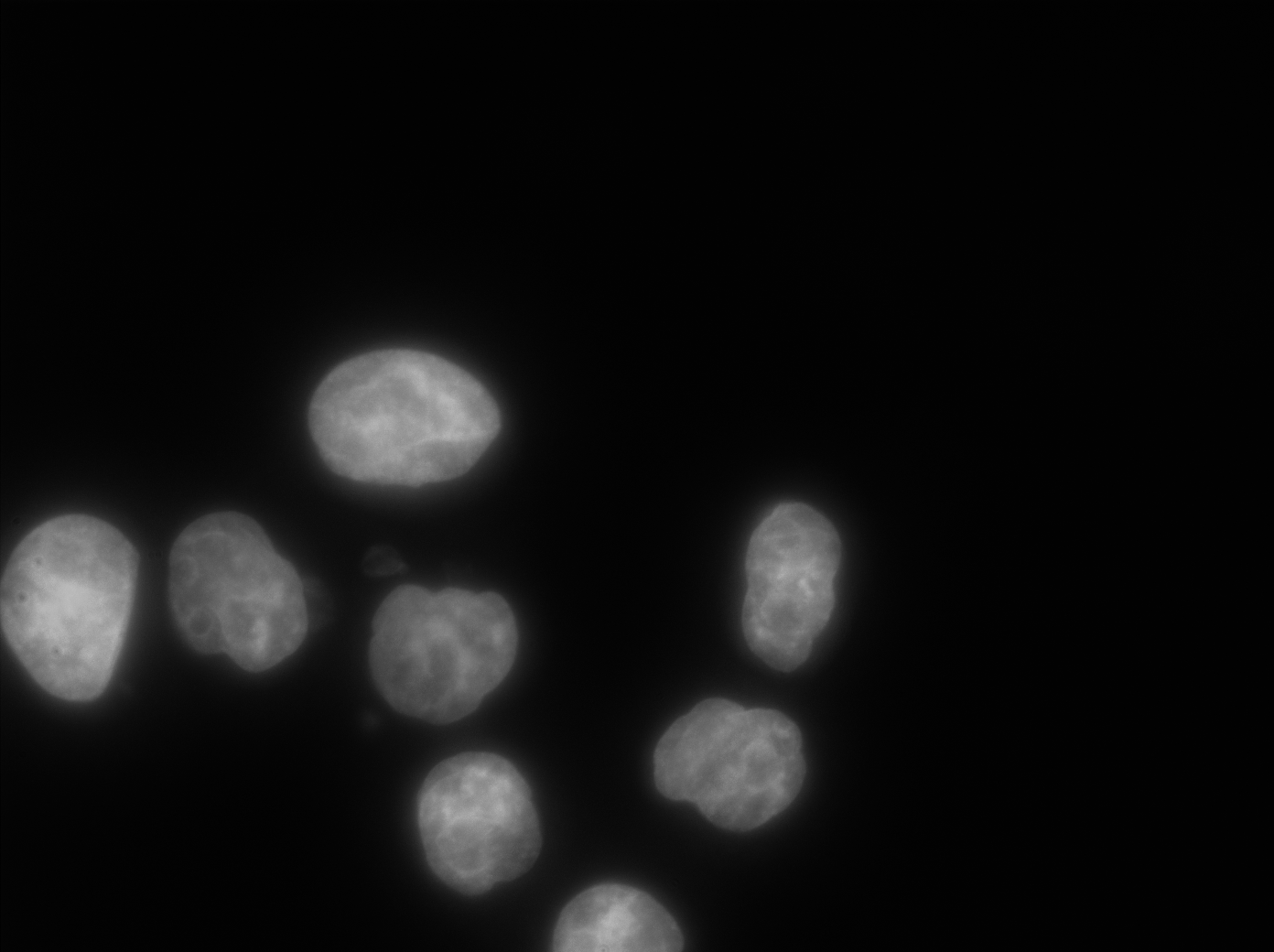

Supplement: Supplementary file 20 — Source data Fig. 6 part 1 [file 44319_2026_742_MOESM20_ESM.zip › Figure 6 Part 1/Fig 6abcd Cas9 TPGS1-KO acetylated tubulin atubulin/Cas9 R2 9-11-24 PA17.Project Maximum Z_XY1726179977_Z0_T0_C0.tif]

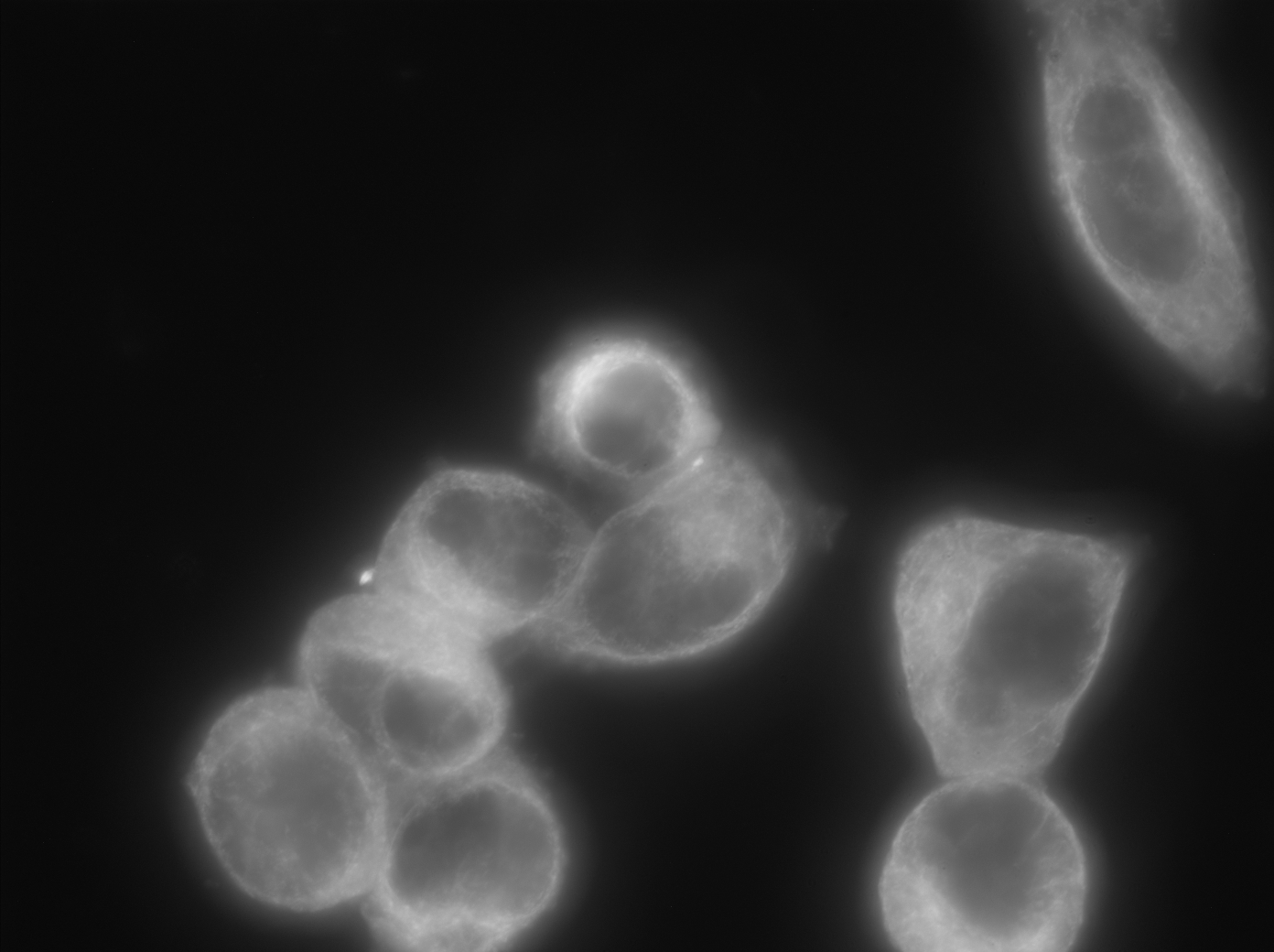

Supplement: Supplementary file 20 — Source data Fig. 6 part 1 [file 44319_2026_742_MOESM20_ESM.zip › Figure 6 Part 1/Fig 6abcd Cas9 TPGS1-KO acetylated tubulin atubulin/Cas9 R2 9-11-24 PA6PA7.Project Maximum Z_XY1726173793_Z0_T0_C1.tif]

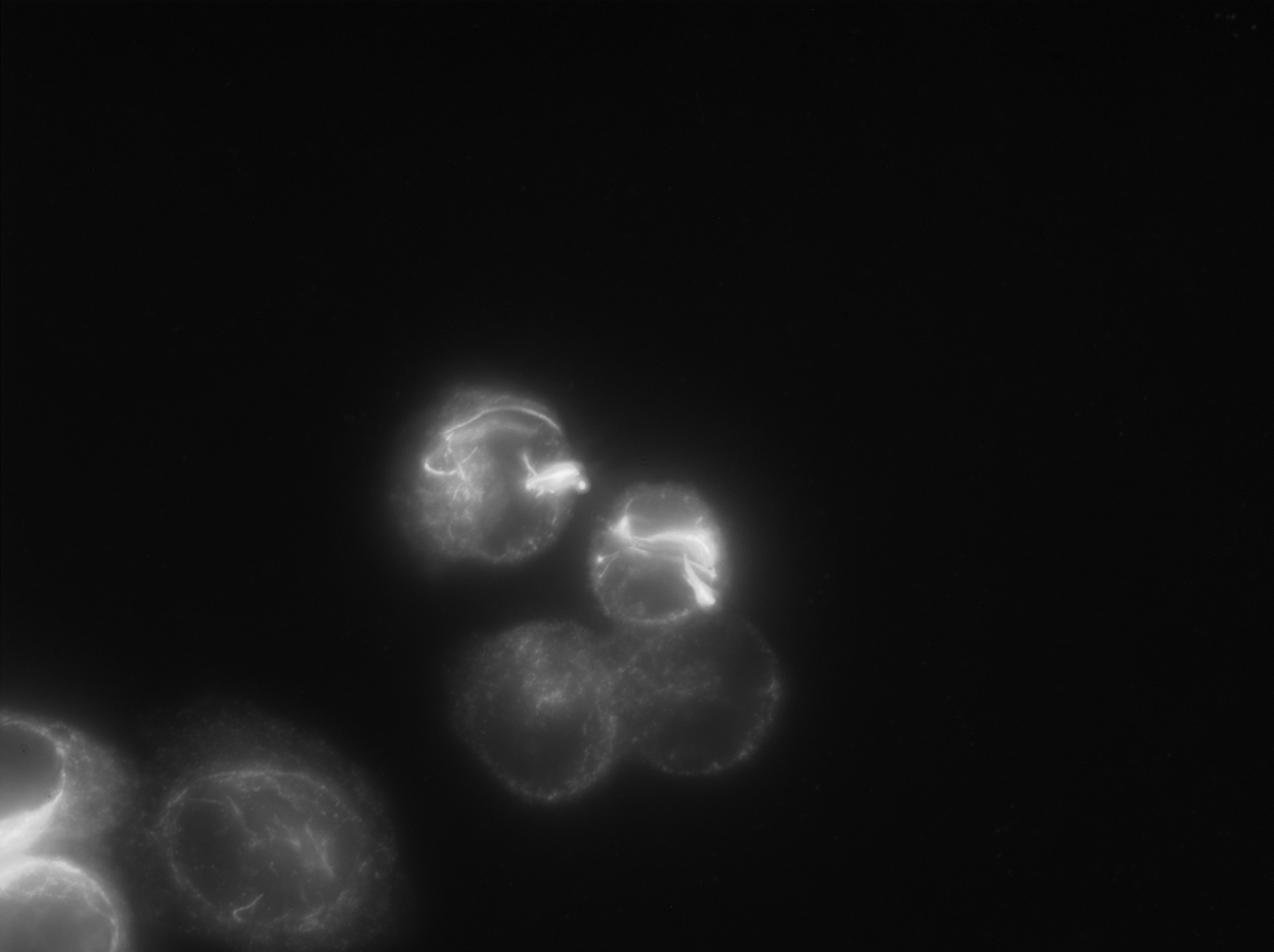

Supplement: Supplementary file 20 — Source data Fig. 6 part 1 [file 44319_2026_742_MOESM20_ESM.zip › Figure 6 Part 1/Fig 6abcd Cas9 TPGS1-KO acetylated tubulin atubulin/Cas9 R2 9-11-24 PA29.Project Maximum Z_XY1726181930_Z0_T0_C2.tif]

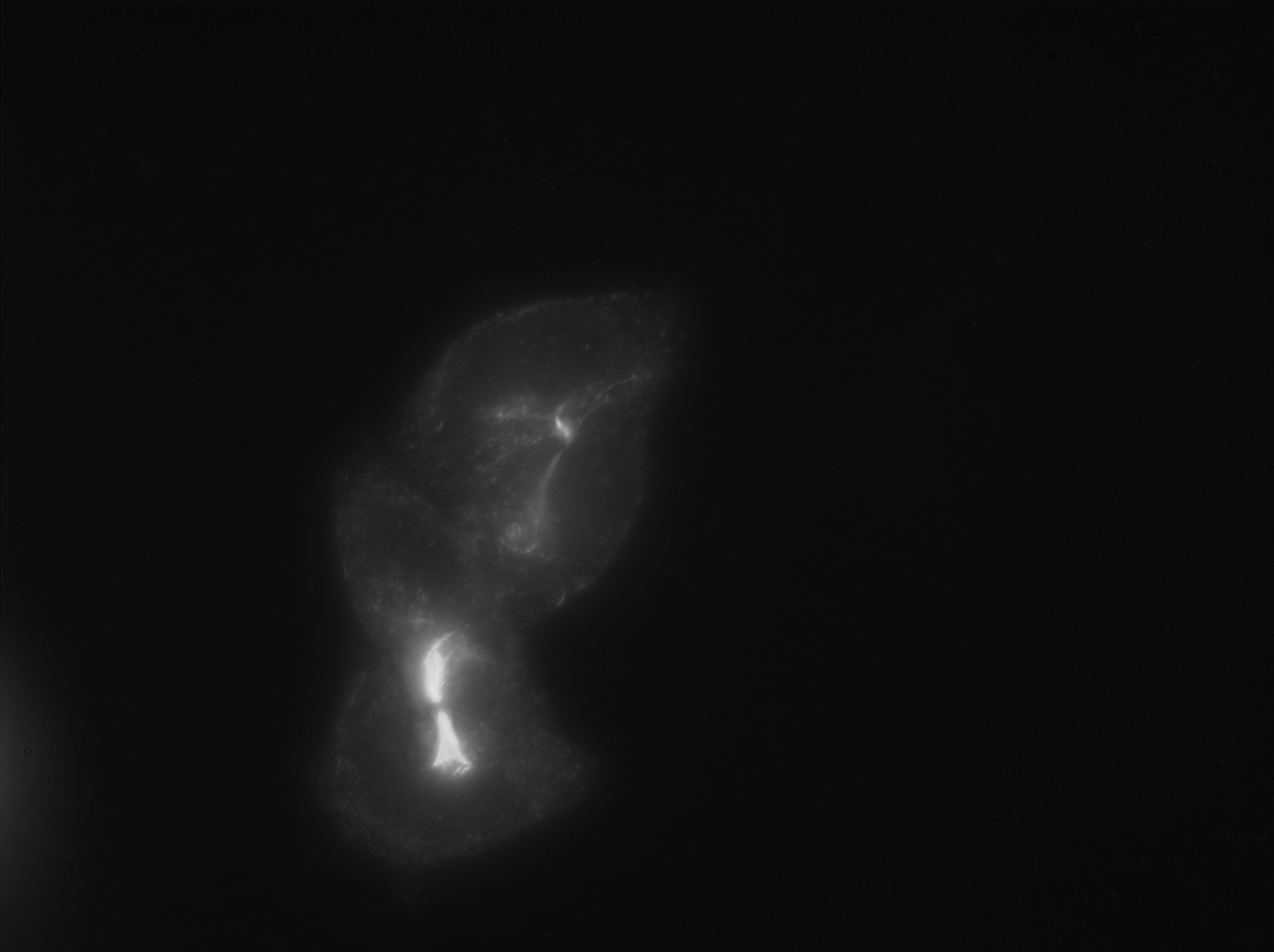

Supplement: Supplementary file 20 — Source data Fig. 6 part 1 [file 44319_2026_742_MOESM20_ESM.zip › Figure 6 Part 1/Fig 6abcd Cas9 TPGS1-KO acetylated tubulin atubulin/Cas9 R2 9-11-24 LT19 PA9.Project Maximum Z_XY1726178474_Z0_T0_C2.tif]

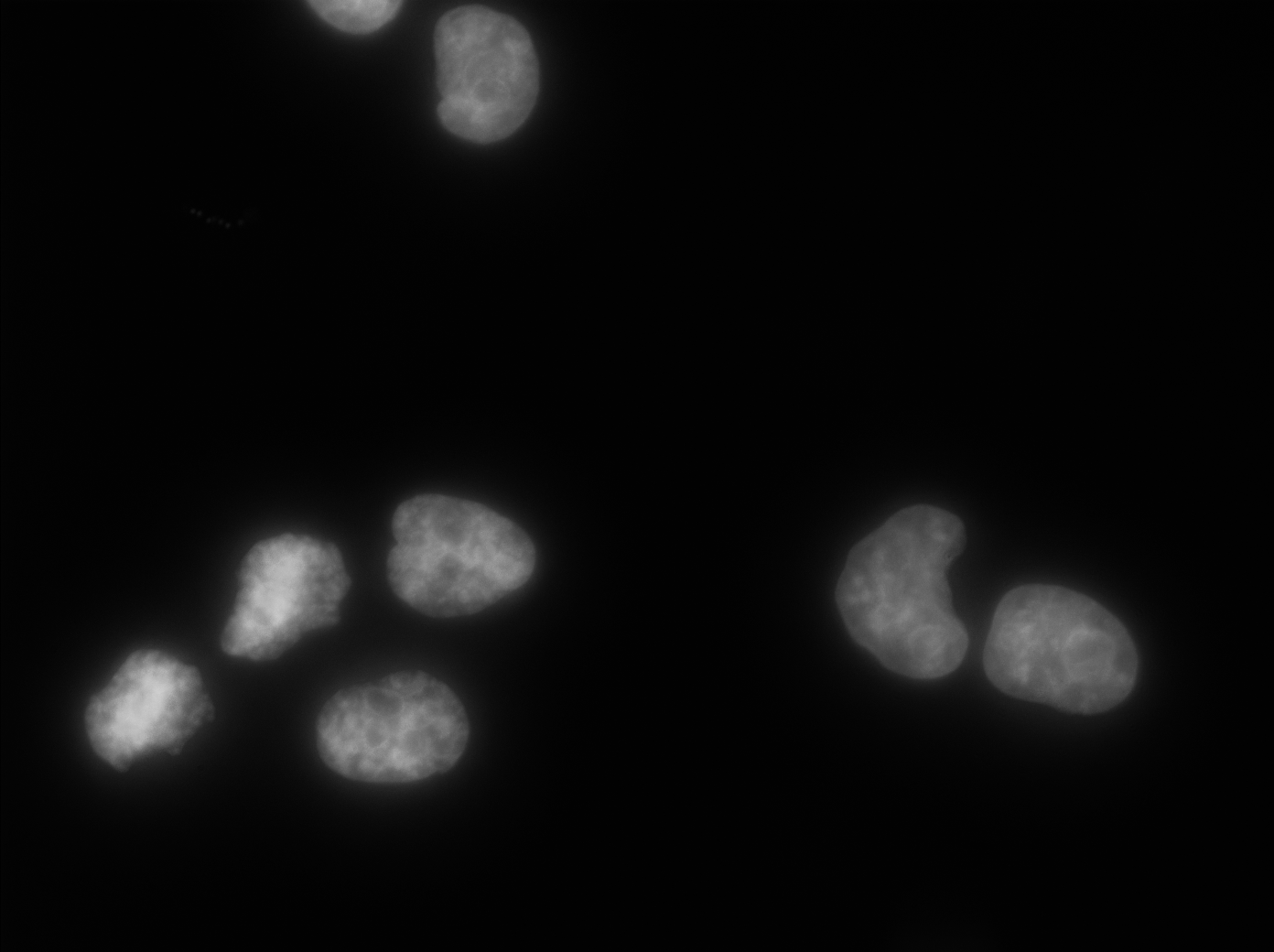

Supplement: Supplementary file 20 — Source data Fig. 6 part 1 [file 44319_2026_742_MOESM20_ESM.zip › Figure 6 Part 1/Fig 6abcd Cas9 TPGS1-KO acetylated tubulin atubulin/Cas9 R2 9-11-24 LT13LT14.Project Maximum Z_XY1726174301_Z0_T0_C0.tif]

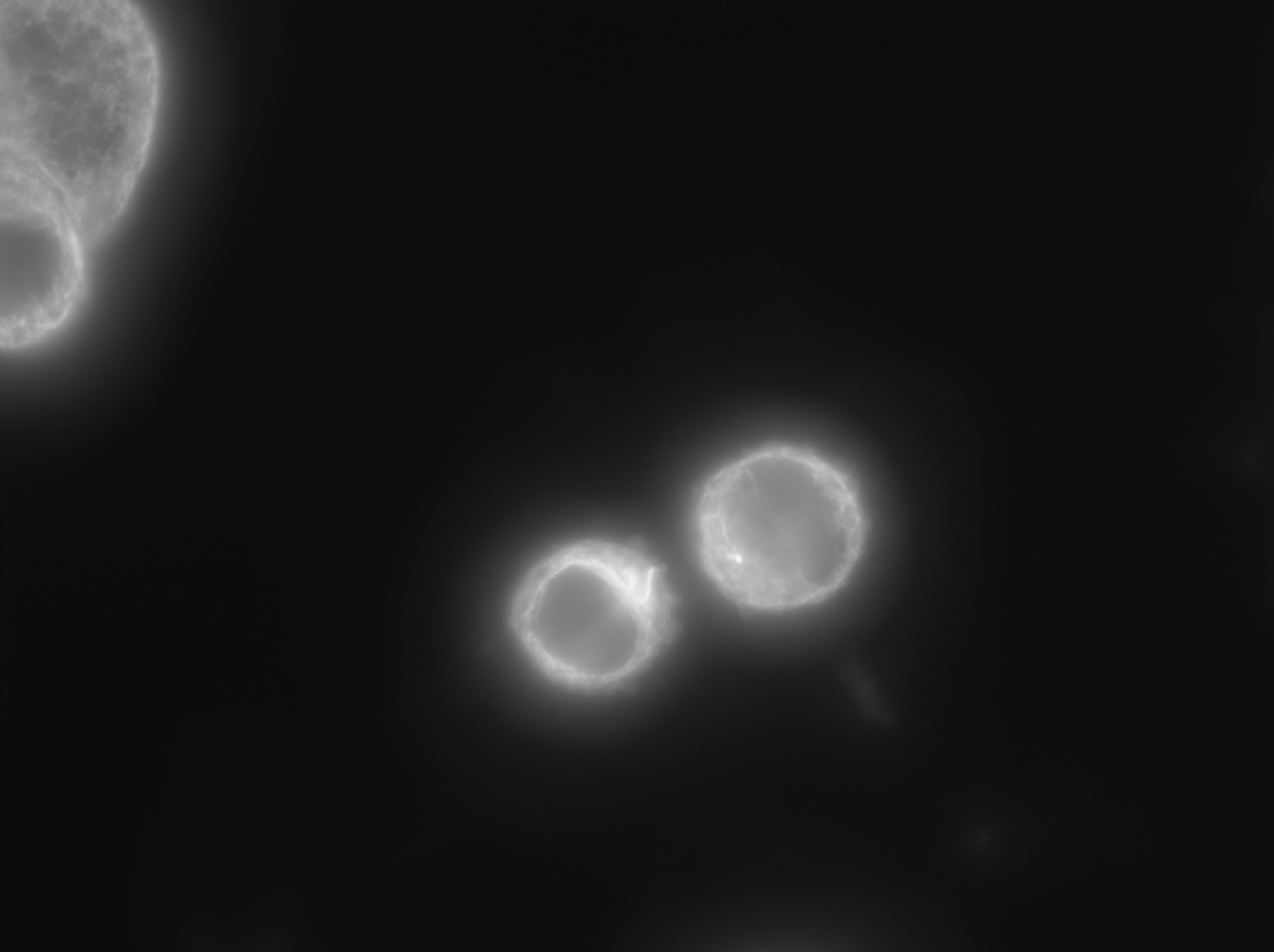

Supplement: Supplementary file 20 — Source data Fig. 6 part 1 [file 44319_2026_742_MOESM20_ESM.zip › Figure 6 Part 1/Fig 6abcd Cas9 TPGS1-KO acetylated tubulin atubulin/Cas9 R2 9-11-24 PA8.Project Maximum Z_XY1726178188_Z0_T0_C1.tif]

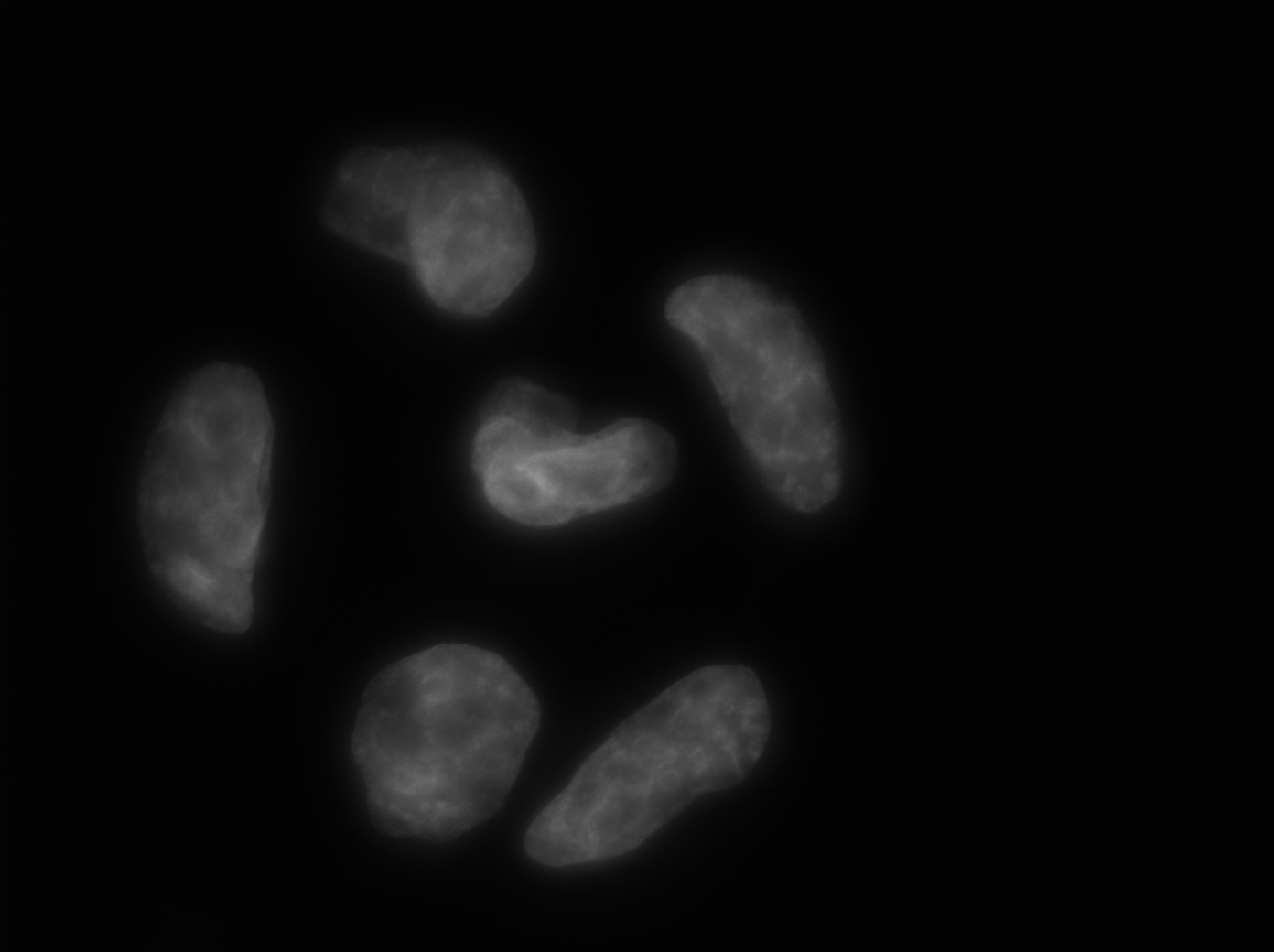

Supplement: Supplementary file 20 — Source data Fig. 6 part 1 [file 44319_2026_742_MOESM20_ESM.zip › Figure 6 Part 1/Fig 6abcd Cas9 TPGS1-KO acetylated tubulin atubulin/Cas9 R2 9-11-24 LT27.Project Maximum Z_XY1726181153_Z0_T0_C0.tif]

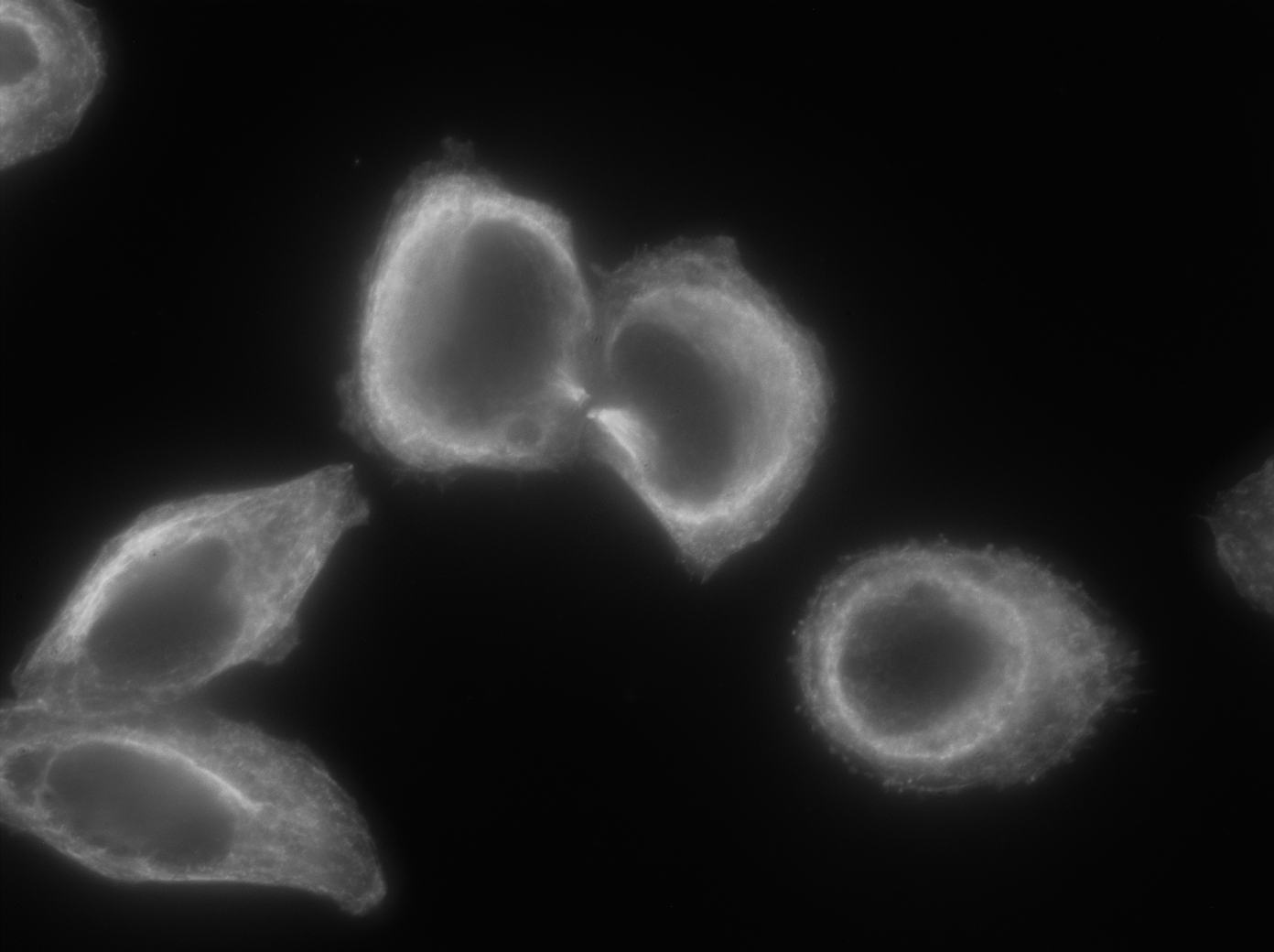

Supplement: Supplementary file 20 — Source data Fig. 6 part 1 [file 44319_2026_742_MOESM20_ESM.zip › Figure 6 Part 1/Fig 6abcd Cas9 TPGS1-KO acetylated tubulin atubulin/Cas9 R3 9-13-24 LT1.Project Maximum Z_XY1726765271_Z0_T0_C1.tif]

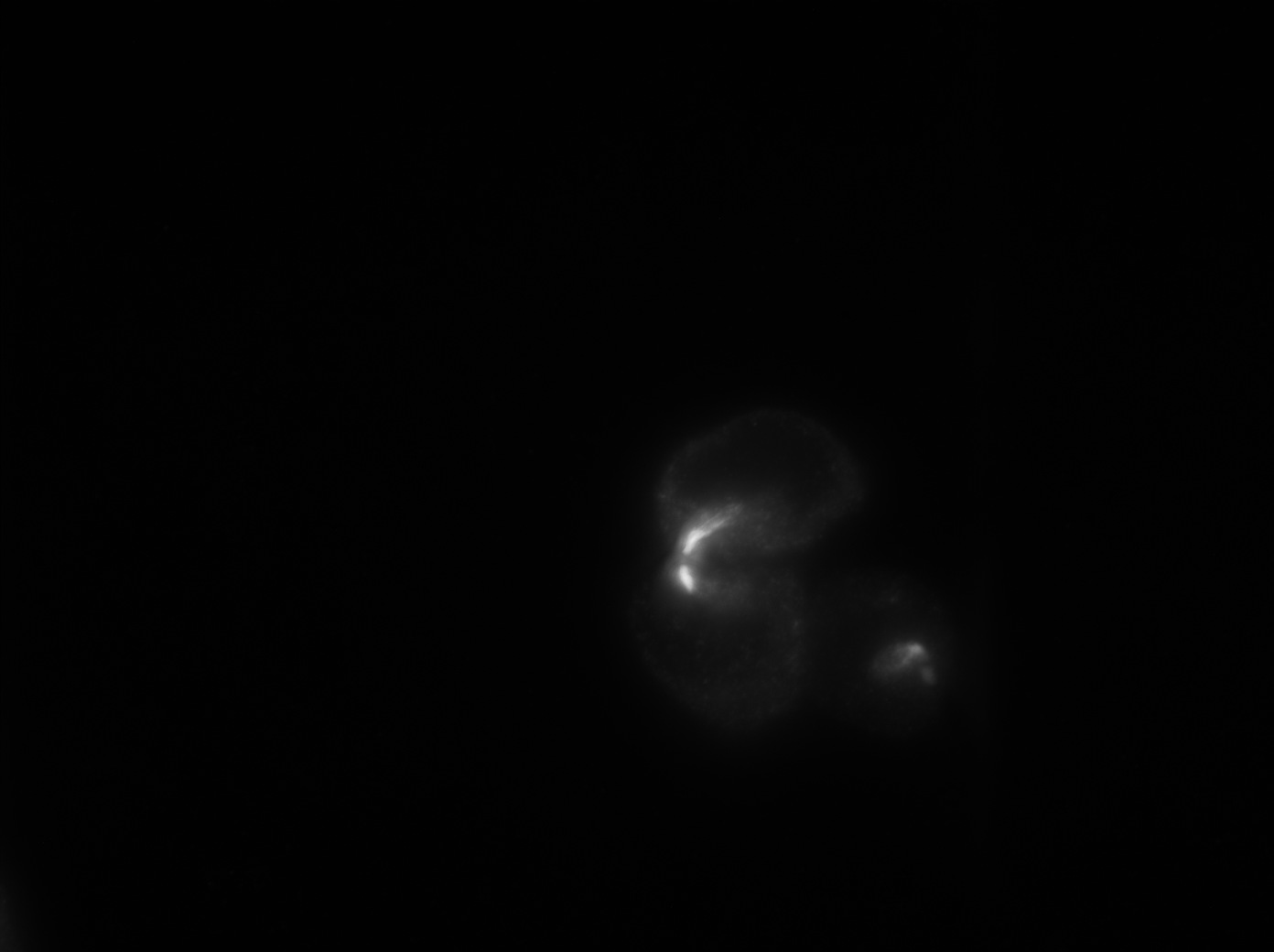

Supplement: Supplementary file 20 — Source data Fig. 6 part 1 [file 44319_2026_742_MOESM20_ESM.zip › Figure 6 Part 1/Fig 6abcd Cas9 TPGS1-KO acetylated tubulin atubulin/Cas9 R3 9-13-24 LT20.Project Maximum Z_XY1726766906_Z0_T0_C2.tif]

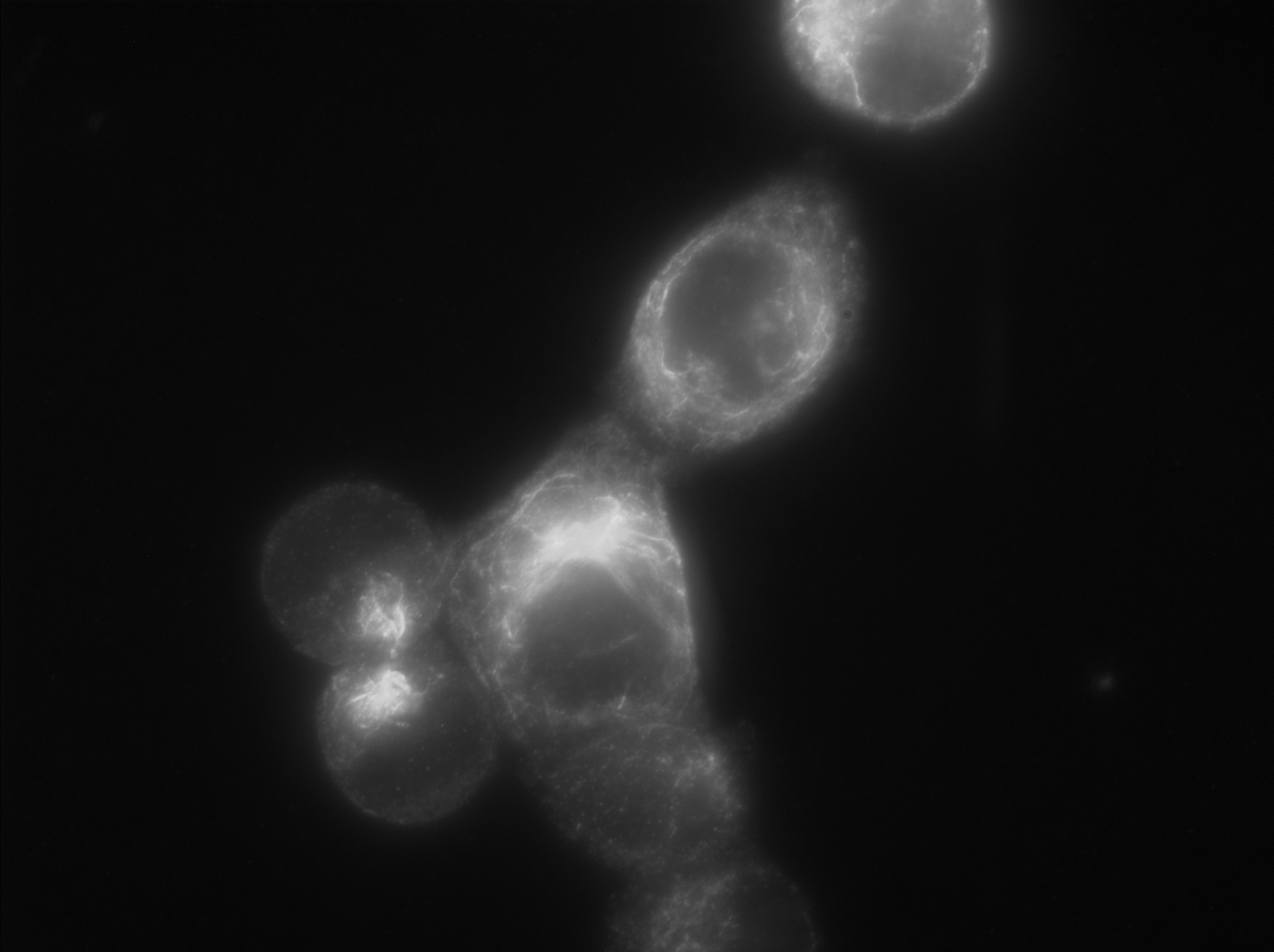

Supplement: Supplementary file 20 — Source data Fig. 6 part 1 [file 44319_2026_742_MOESM20_ESM.zip › Figure 6 Part 1/Fig 6abcd Cas9 TPGS1-KO acetylated tubulin atubulin/Cas9 R2 9-11-24 LT6.Project Maximum Z_XY1726173281_Z0_T0_C2.tif]

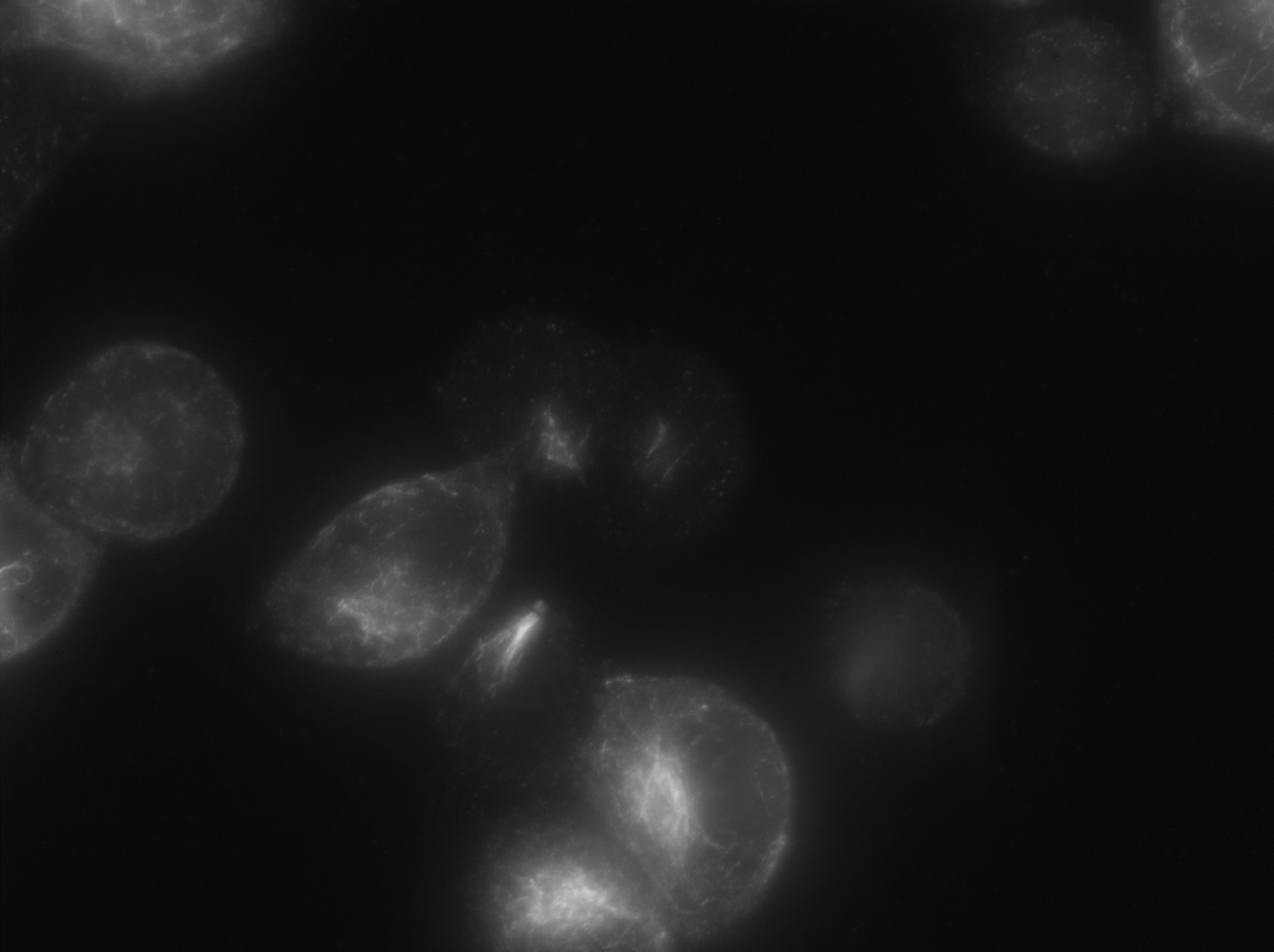

Supplement: Supplementary file 20 — Source data Fig. 6 part 1 [file 44319_2026_742_MOESM20_ESM.zip › Figure 6 Part 1/Fig 6abcd Cas9 TPGS1-KO acetylated tubulin atubulin/Cas9 R2 9-11-24 PA30.Project Maximum Z_XY1726182071_Z0_T0_C2.tif]

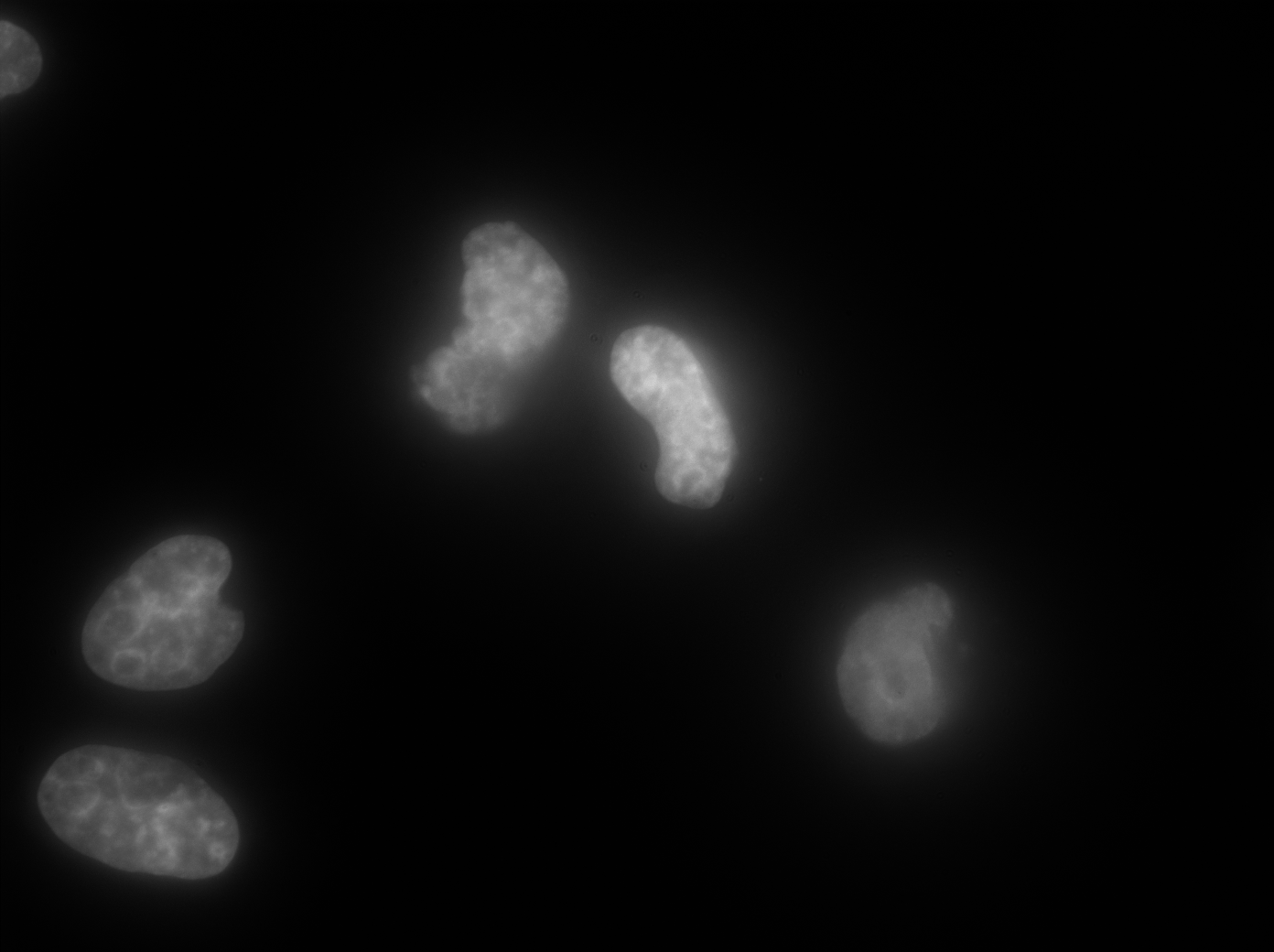

Supplement: Supplementary file 20 — Source data Fig. 6 part 1 [file 44319_2026_742_MOESM20_ESM.zip › Figure 6 Part 1/Fig 6abcd Cas9 TPGS1-KO acetylated tubulin atubulin/Cas9 R3 9-13-24 LT1.Project Maximum Z_XY1726765271_Z0_T0_C0.tif]

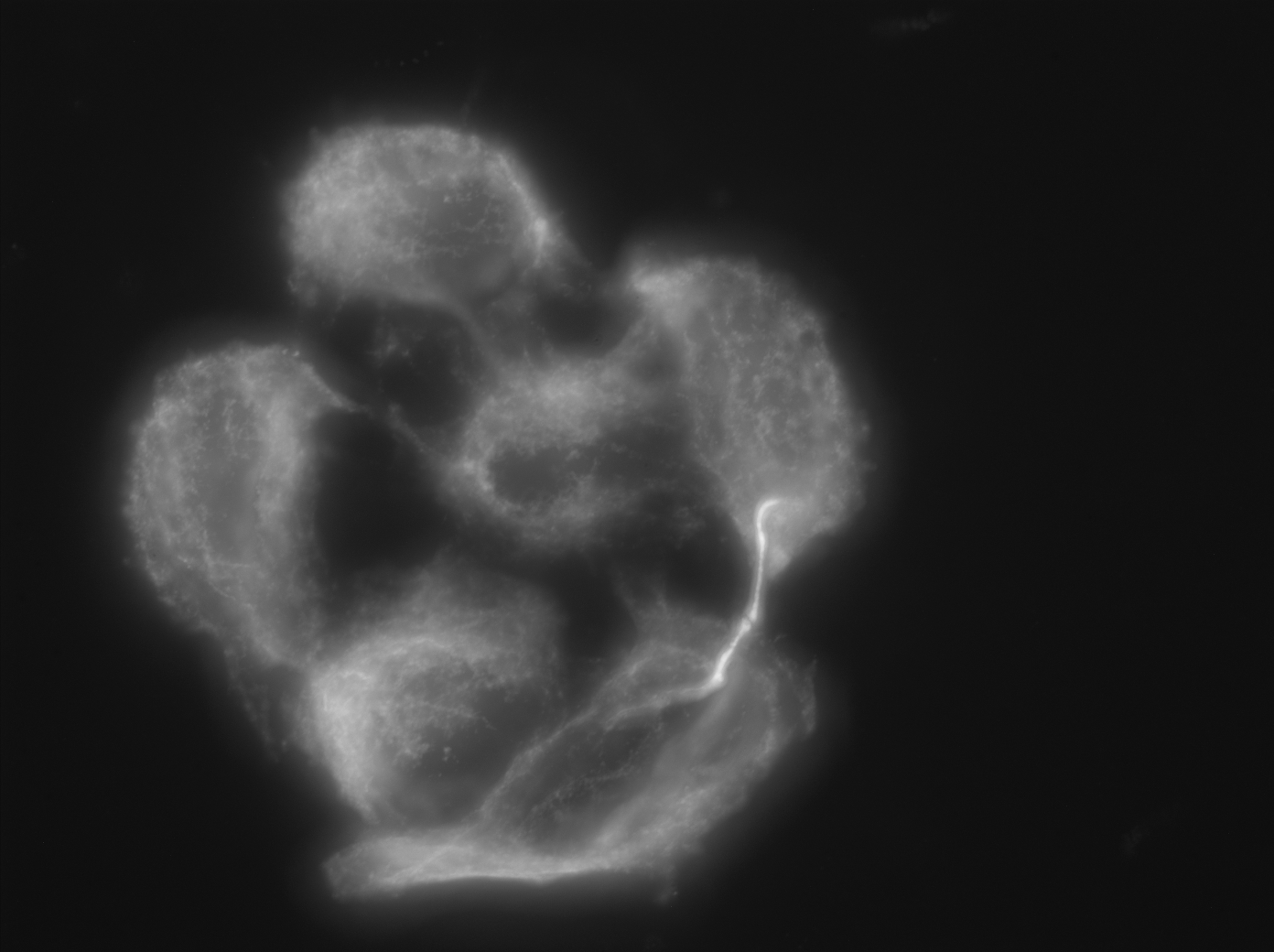

Supplement: Supplementary file 20 — Source data Fig. 6 part 1 [file 44319_2026_742_MOESM20_ESM.zip › Figure 6 Part 1/Fig 6abcd Cas9 TPGS1-KO acetylated tubulin atubulin/Cas9 R2 9-11-24 LT27.Project Maximum Z_XY1726181153_Z0_T0_C1.tif]

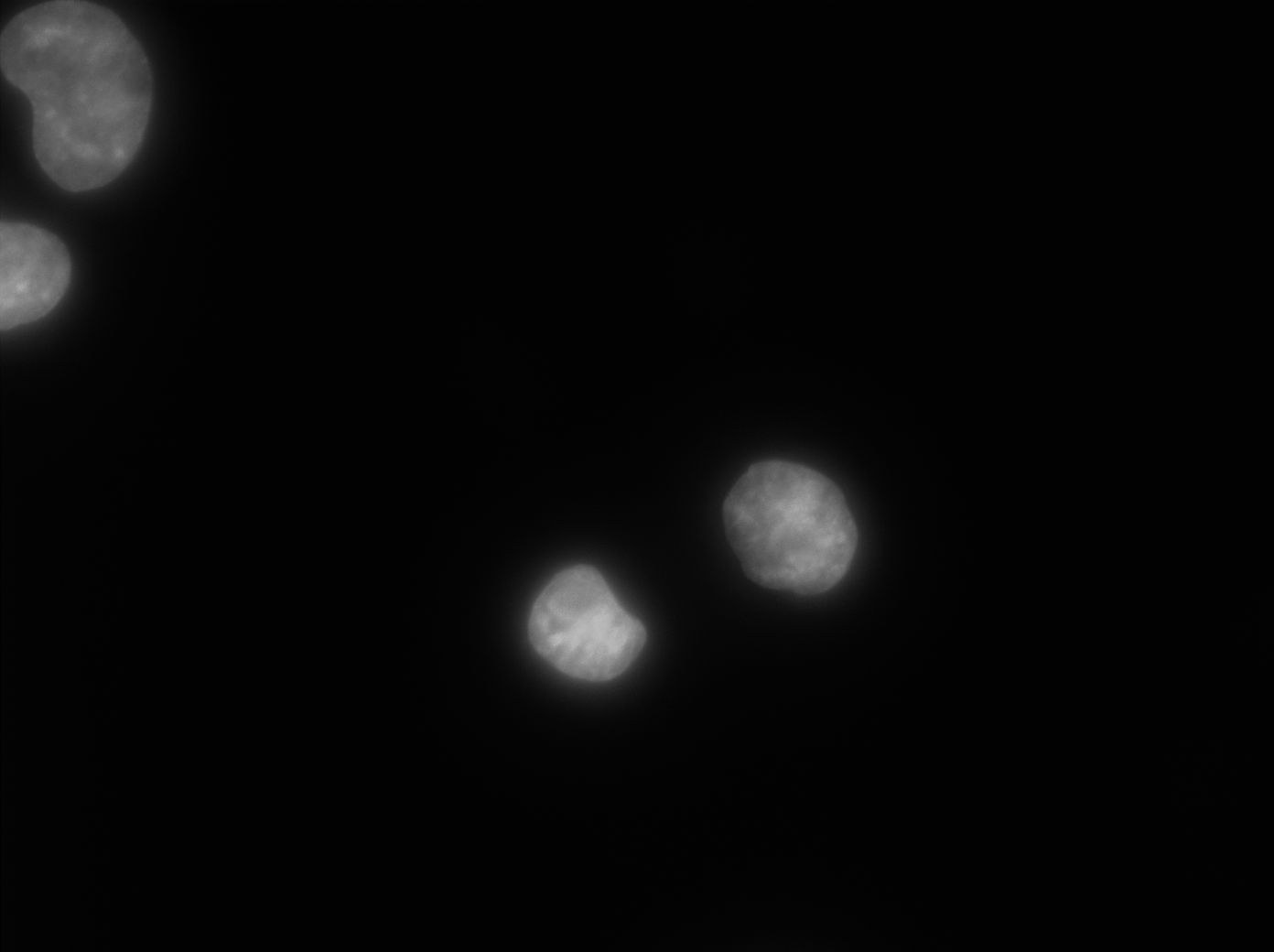

Supplement: Supplementary file 20 — Source data Fig. 6 part 1 [file 44319_2026_742_MOESM20_ESM.zip › Figure 6 Part 1/Fig 6abcd Cas9 TPGS1-KO acetylated tubulin atubulin/Cas9 R2 9-11-24 PA8.Project Maximum Z_XY1726178188_Z0_T0_C0.tif]

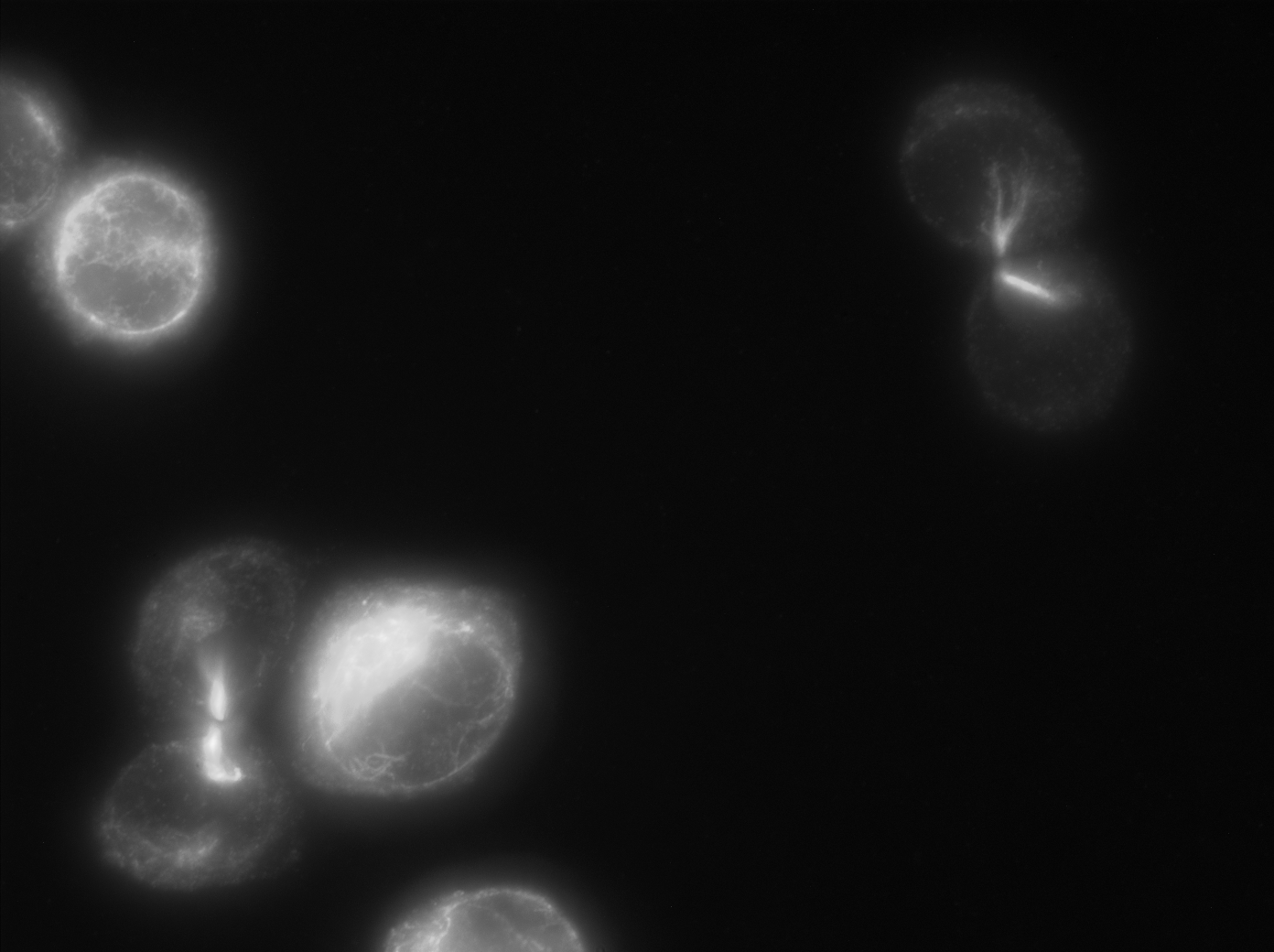

Supplement: Supplementary file 20 — Source data Fig. 6 part 1 [file 44319_2026_742_MOESM20_ESM.zip › Figure 6 Part 1/Fig 6abcd Cas9 TPGS1-KO acetylated tubulin atubulin/Cas9 R2 9-11-24 LT7LT8.Project Maximum Z_XY1726173377_Z0_T0_C2.tif]

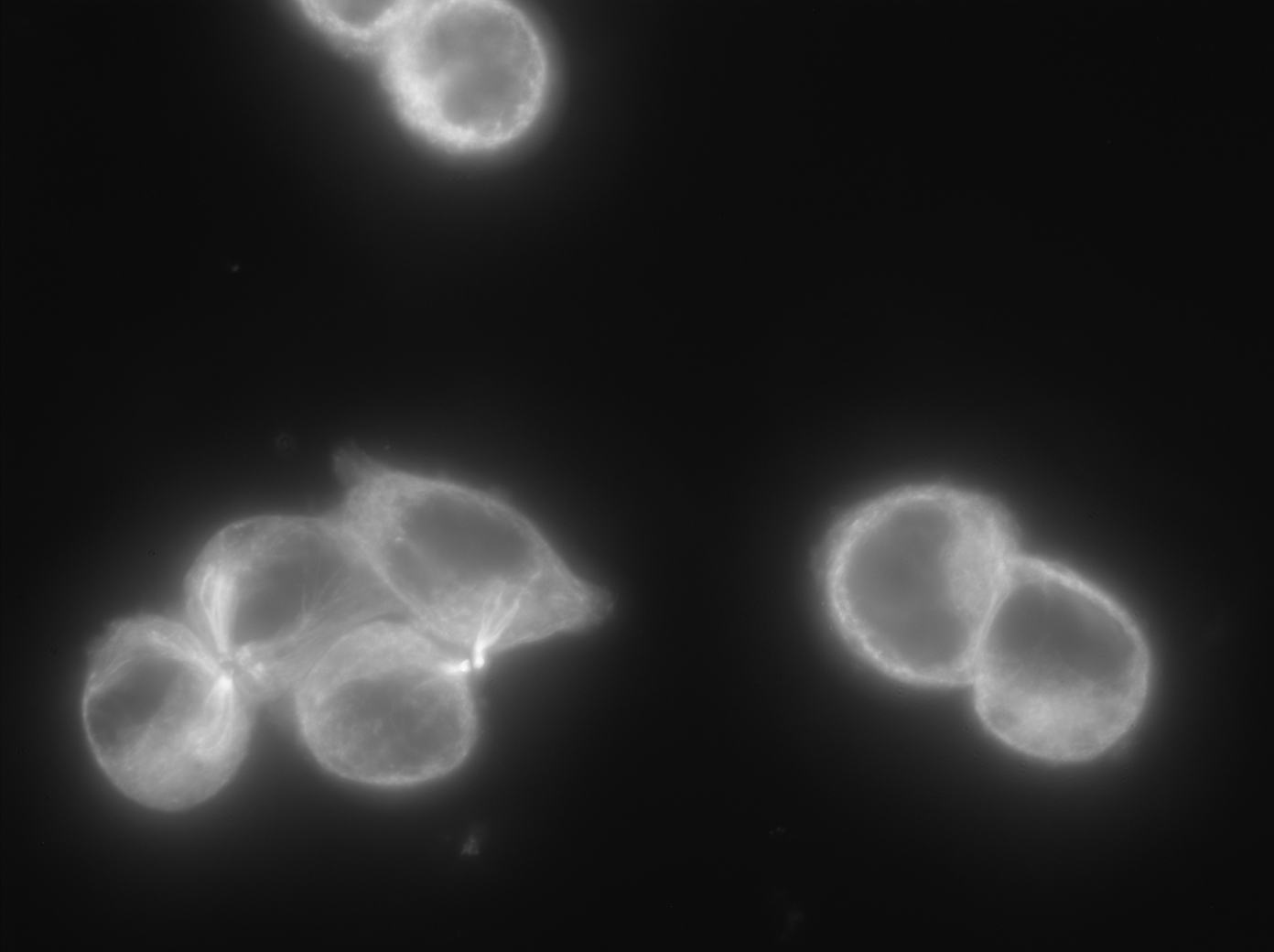

Supplement: Supplementary file 20 — Source data Fig. 6 part 1 [file 44319_2026_742_MOESM20_ESM.zip › Figure 6 Part 1/Fig 6abcd Cas9 TPGS1-KO acetylated tubulin atubulin/Cas9 R2 9-11-24 LT13LT14.Project Maximum Z_XY1726174301_Z0_T0_C1.tif]

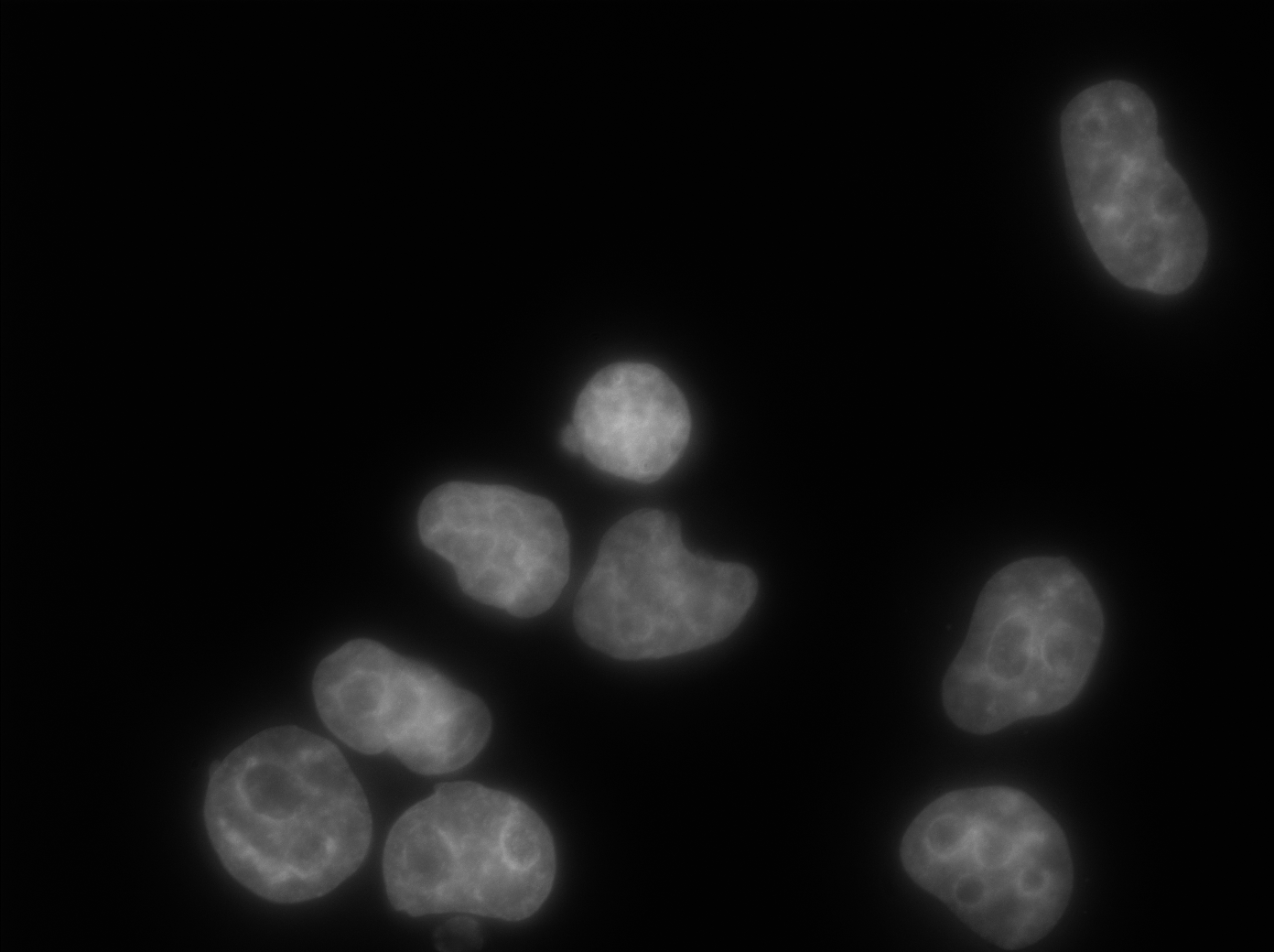

Supplement: Supplementary file 20 — Source data Fig. 6 part 1 [file 44319_2026_742_MOESM20_ESM.zip › Figure 6 Part 1/Fig 6abcd Cas9 TPGS1-KO acetylated tubulin atubulin/Cas9 R2 9-11-24 PA6PA7.Project Maximum Z_XY1726173793_Z0_T0_C0.tif]

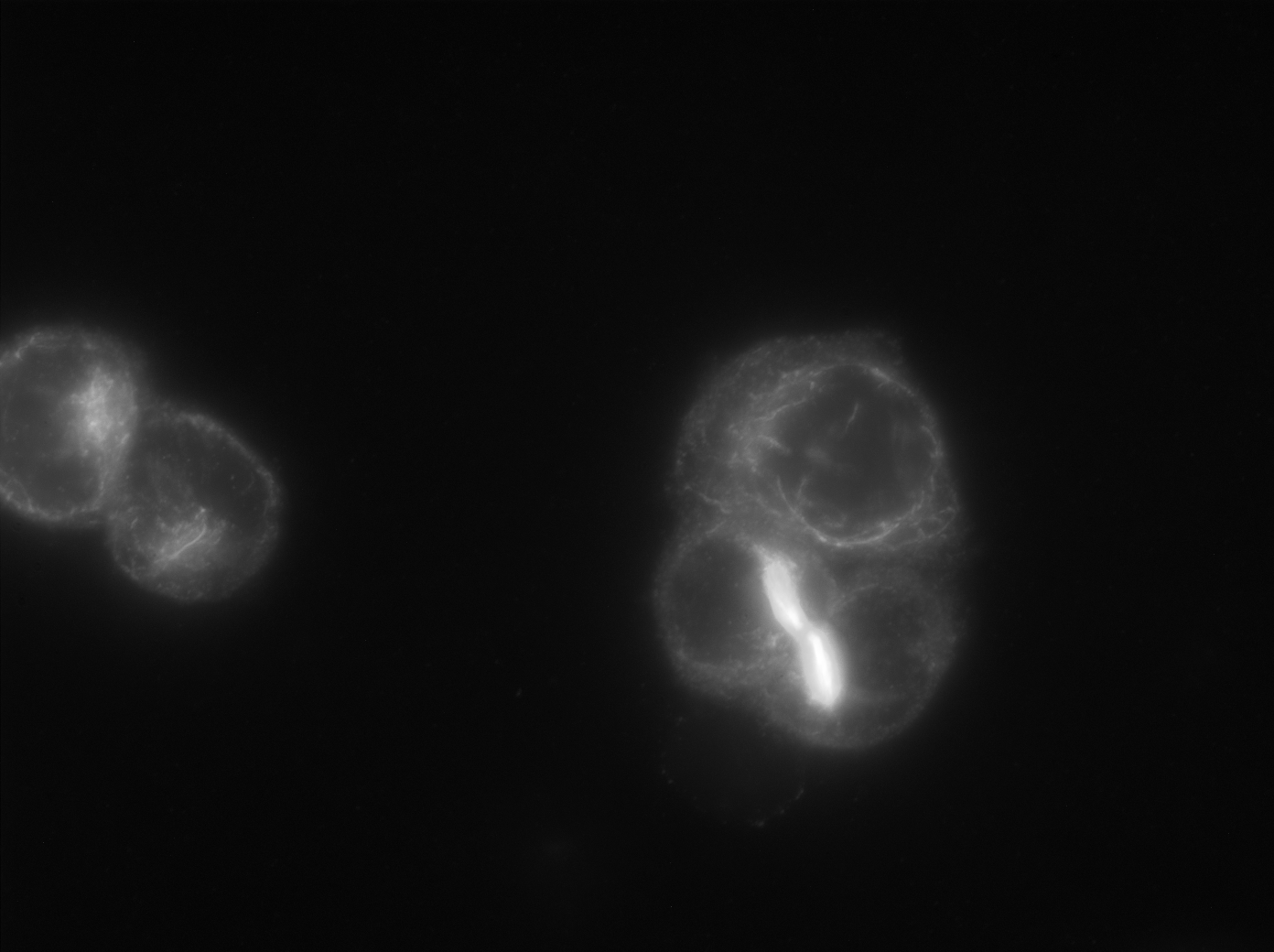

Supplement: Supplementary file 20 — Source data Fig. 6 part 1 [file 44319_2026_742_MOESM20_ESM.zip › Figure 6 Part 1/Fig 6abcd Cas9 TPGS1-KO acetylated tubulin atubulin/Cas9 R2 9-11-24 LT15.Project Maximum Z_XY1726174550_Z0_T0_C2.tif]

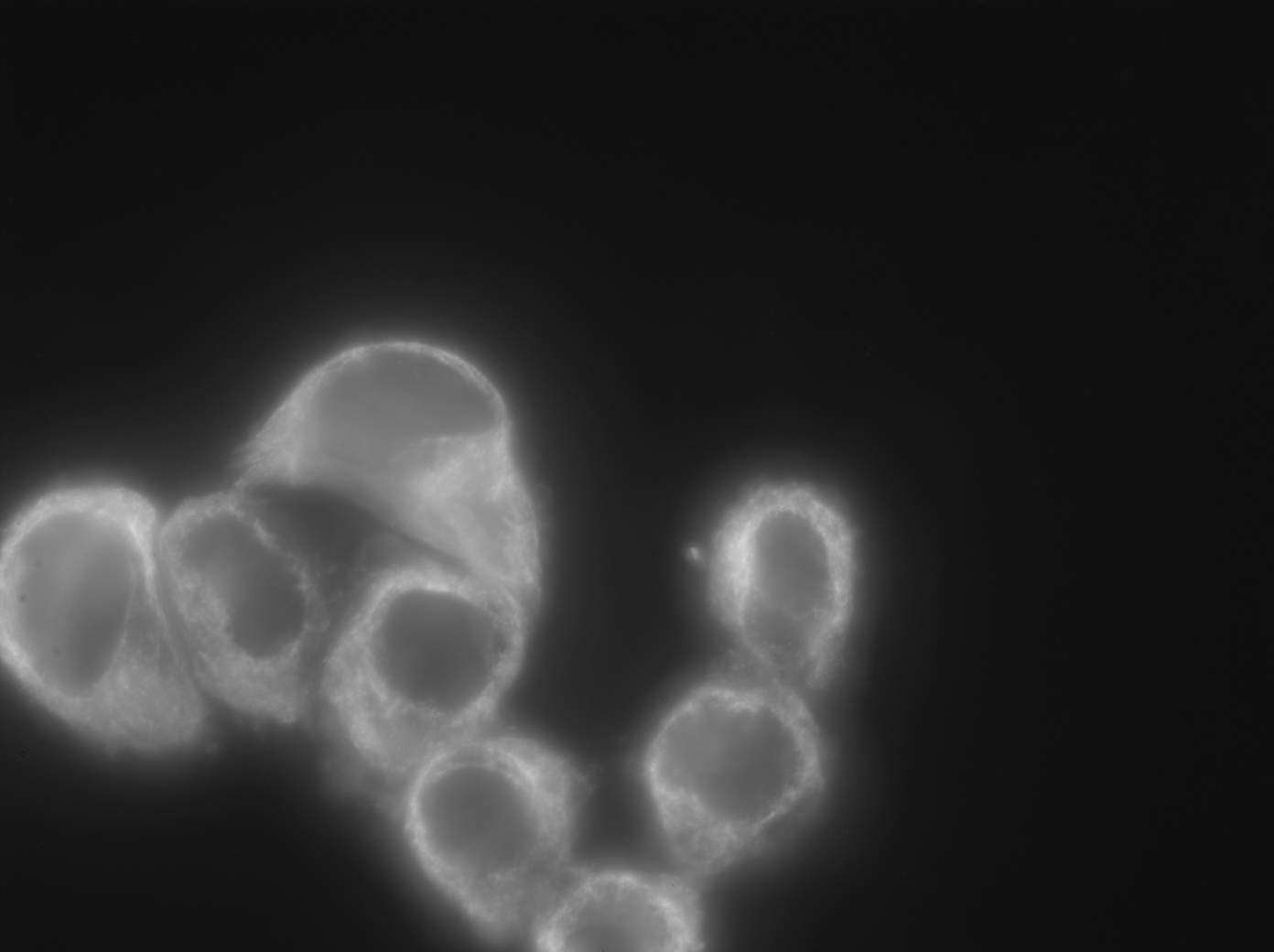

Supplement: Supplementary file 20 — Source data Fig. 6 part 1 [file 44319_2026_742_MOESM20_ESM.zip › Figure 6 Part 1/Fig 6abcd Cas9 TPGS1-KO acetylated tubulin atubulin/Cas9 R2 9-11-24 PA17.Project Maximum Z_XY1726179977_Z0_T0_C1.tif]

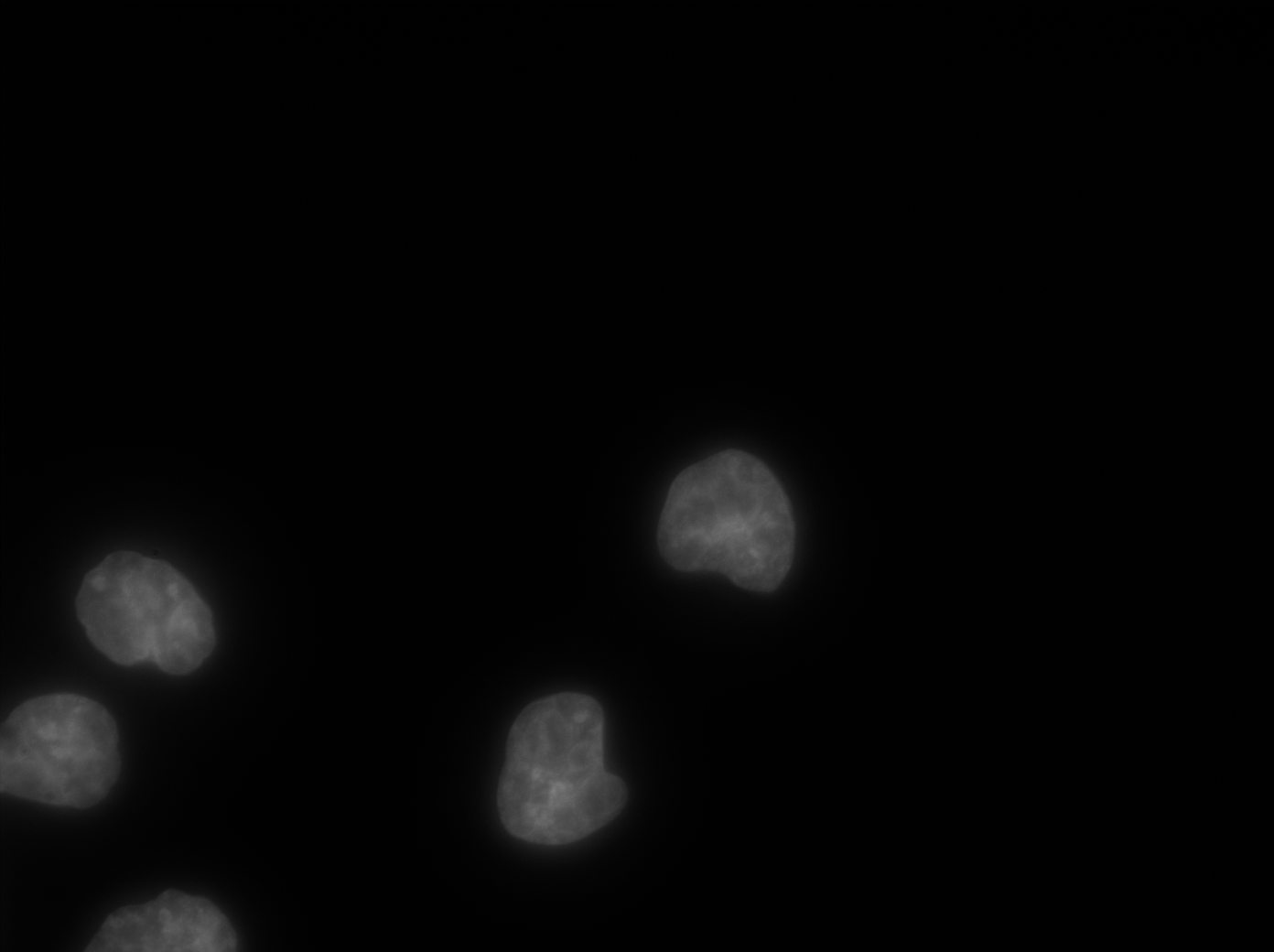

Supplement: Supplementary file 20 — Source data Fig. 6 part 1 [file 44319_2026_742_MOESM20_ESM.zip › Figure 6 Part 1/Fig 6abcd Cas9 TPGS1-KO acetylated tubulin atubulin/Cas9 R3 9-13-24 LT26.Project Maximum Z_XY1726767497_Z0_T0_C0.tif]

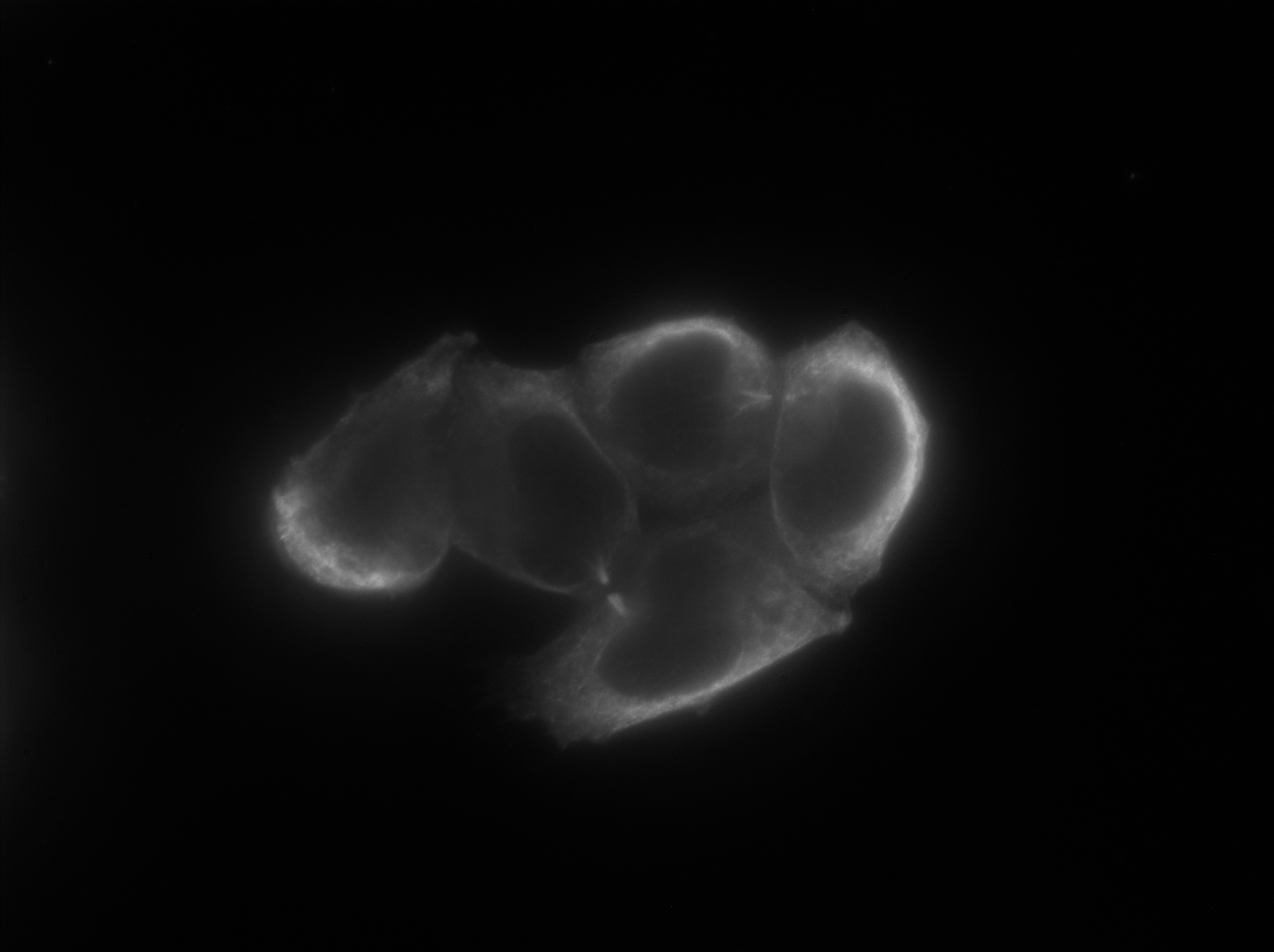

Supplement: Supplementary file 20 — Source data Fig. 6 part 1 [file 44319_2026_742_MOESM20_ESM.zip › Figure 6 Part 1/Fig 6abcd Cas9 TPGS1-KO acetylated tubulin atubulin/Cas9 R3 9-13-24 LT16LT17.Project Maximum Z_XY1726766464_Z0_T0_C1.tif]

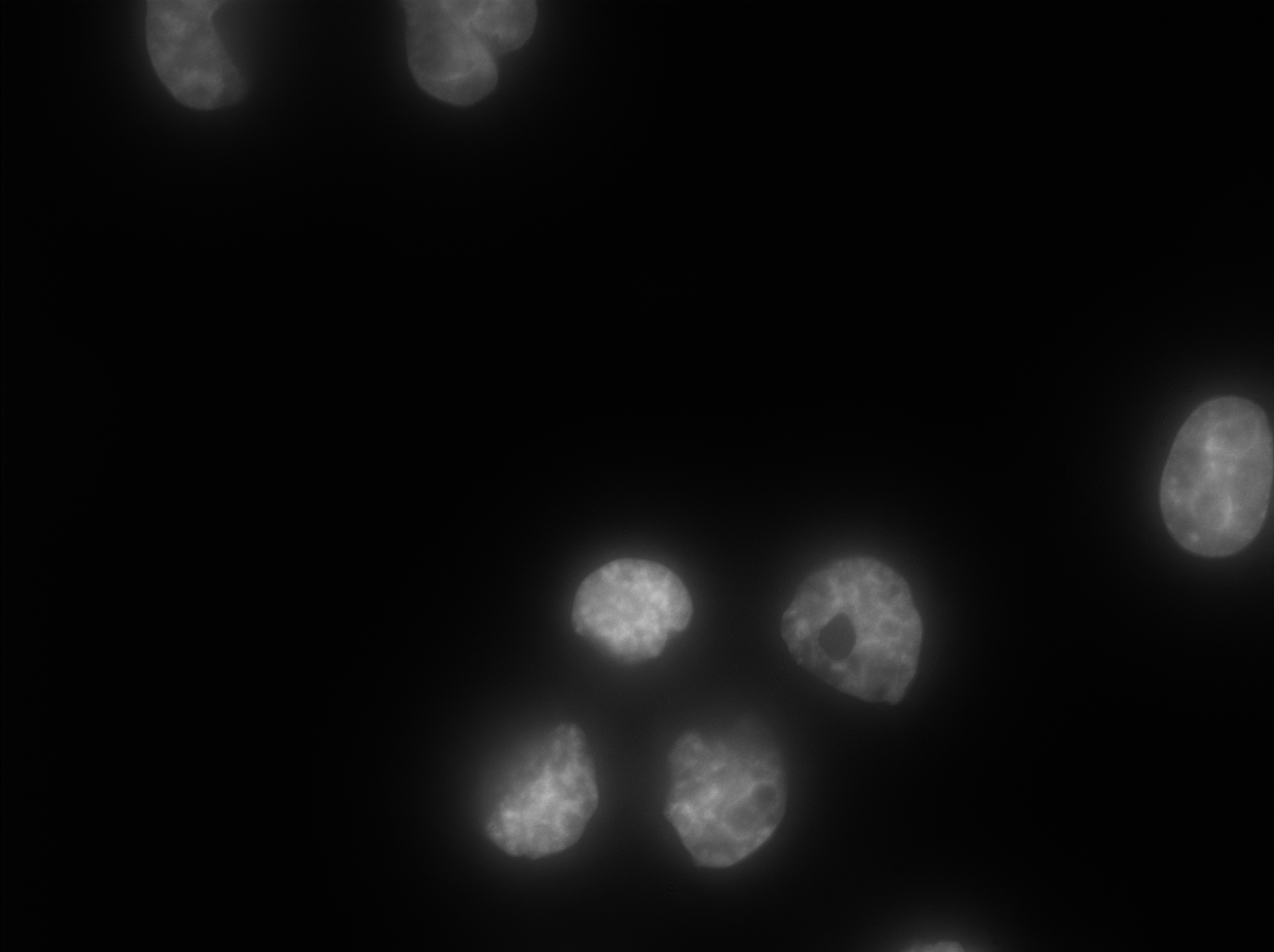

Supplement: Supplementary file 20 — Source data Fig. 6 part 1 [file 44319_2026_742_MOESM20_ESM.zip › Figure 6 Part 1/Fig 6abcd Cas9 TPGS1-KO acetylated tubulin atubulin/Cas9 R2 9-11-24 LT2.Project Maximum Z_XY1726172269_Z0_T0_C0.tif]

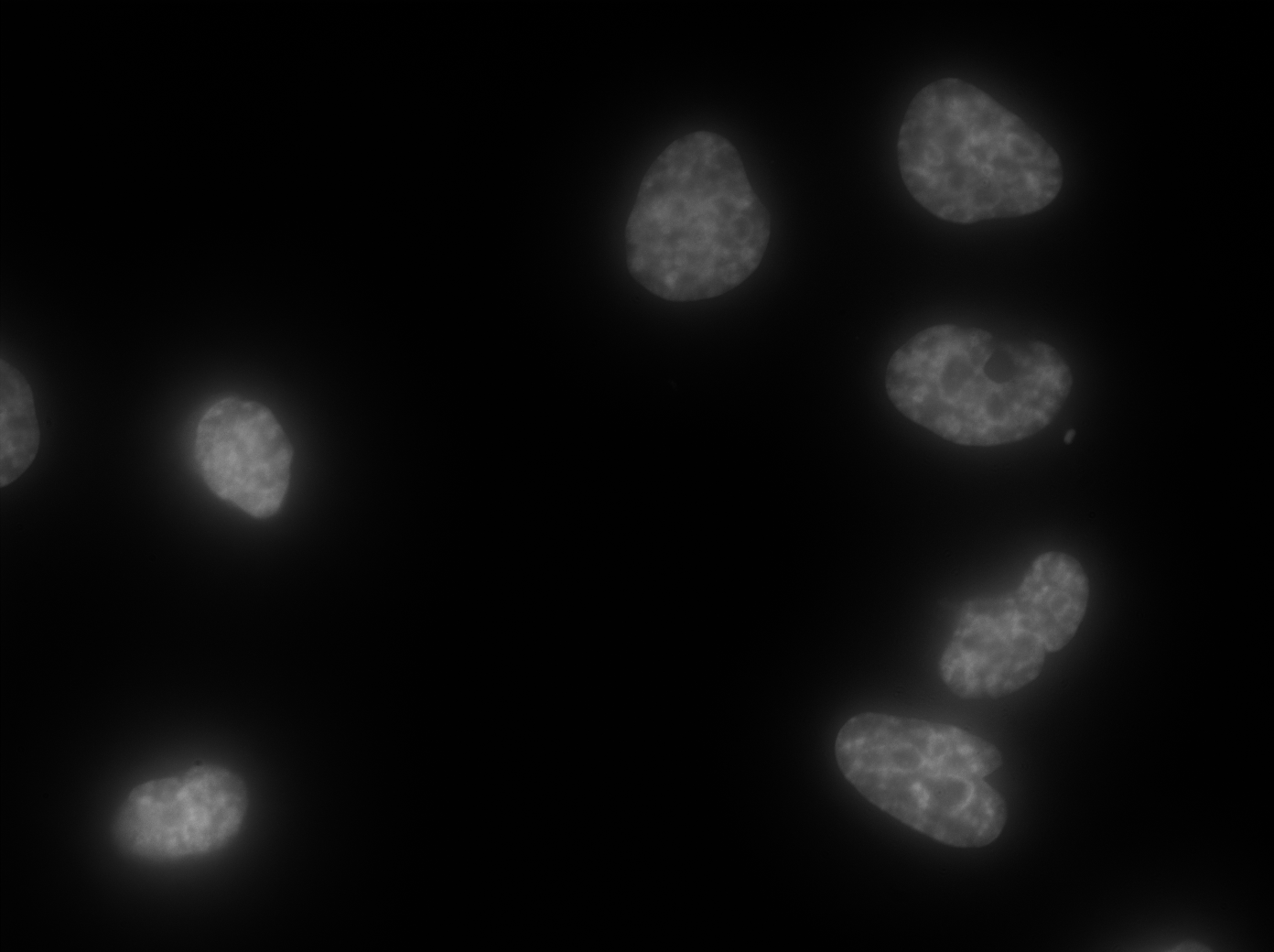

Supplement: Supplementary file 20 — Source data Fig. 6 part 1 [file 44319_2026_742_MOESM20_ESM.zip › Figure 6 Part 1/Fig 6abcd Cas9 TPGS1-KO acetylated tubulin atubulin/Cas9 R3 9-13-24 LT5LT6LT7.Project Maximum Z_XY1726765644_Z0_T0_C0.tif]

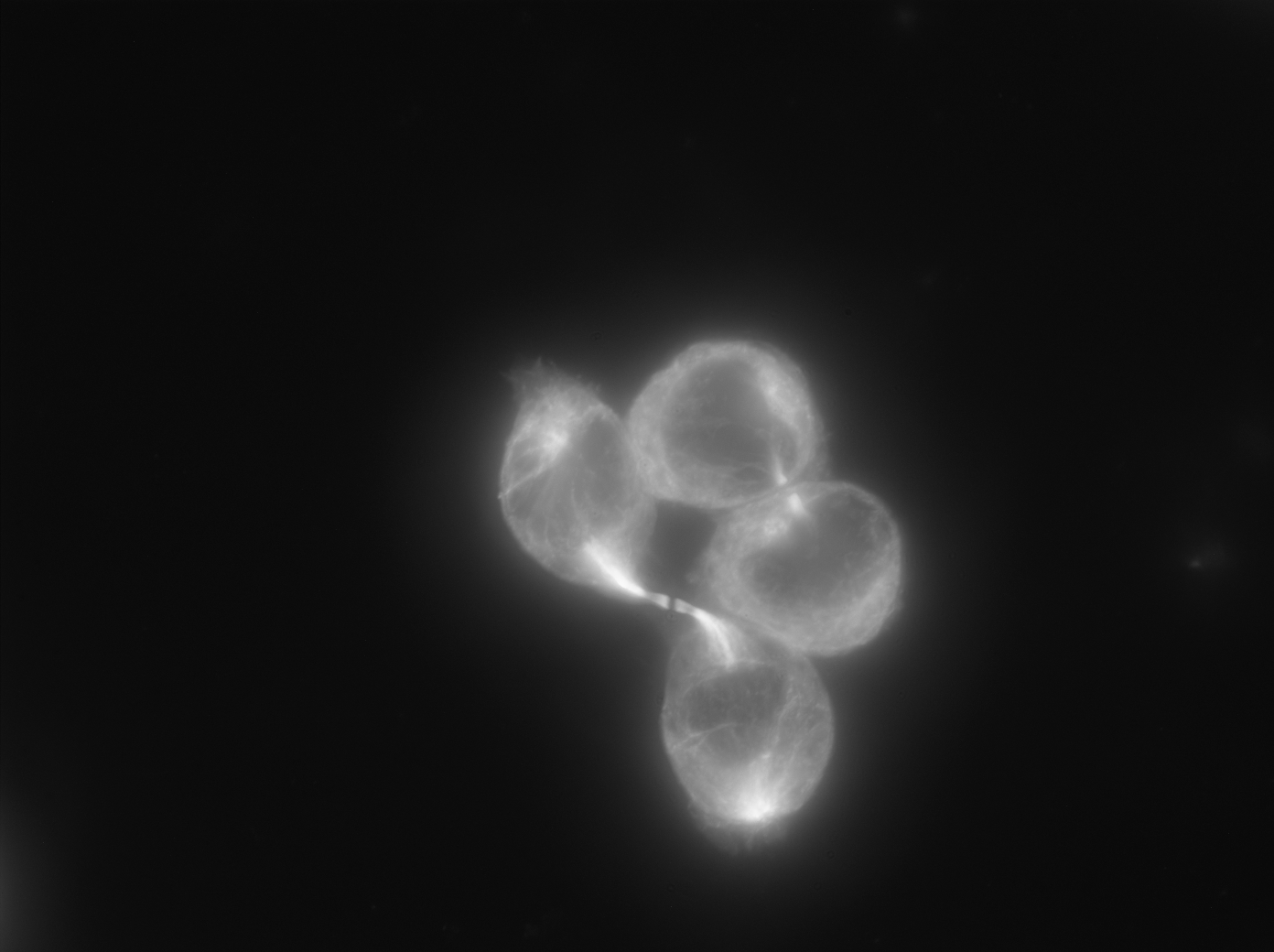

Supplement: Supplementary file 20 — Source data Fig. 6 part 1 [file 44319_2026_742_MOESM20_ESM.zip › Figure 6 Part 1/Fig 6abcd Cas9 TPGS1-KO acetylated tubulin atubulin/Cas9 R2 9-11-24 LT10LT11.Project Maximum Z_XY1726174037_Z0_T0_C1.tif]

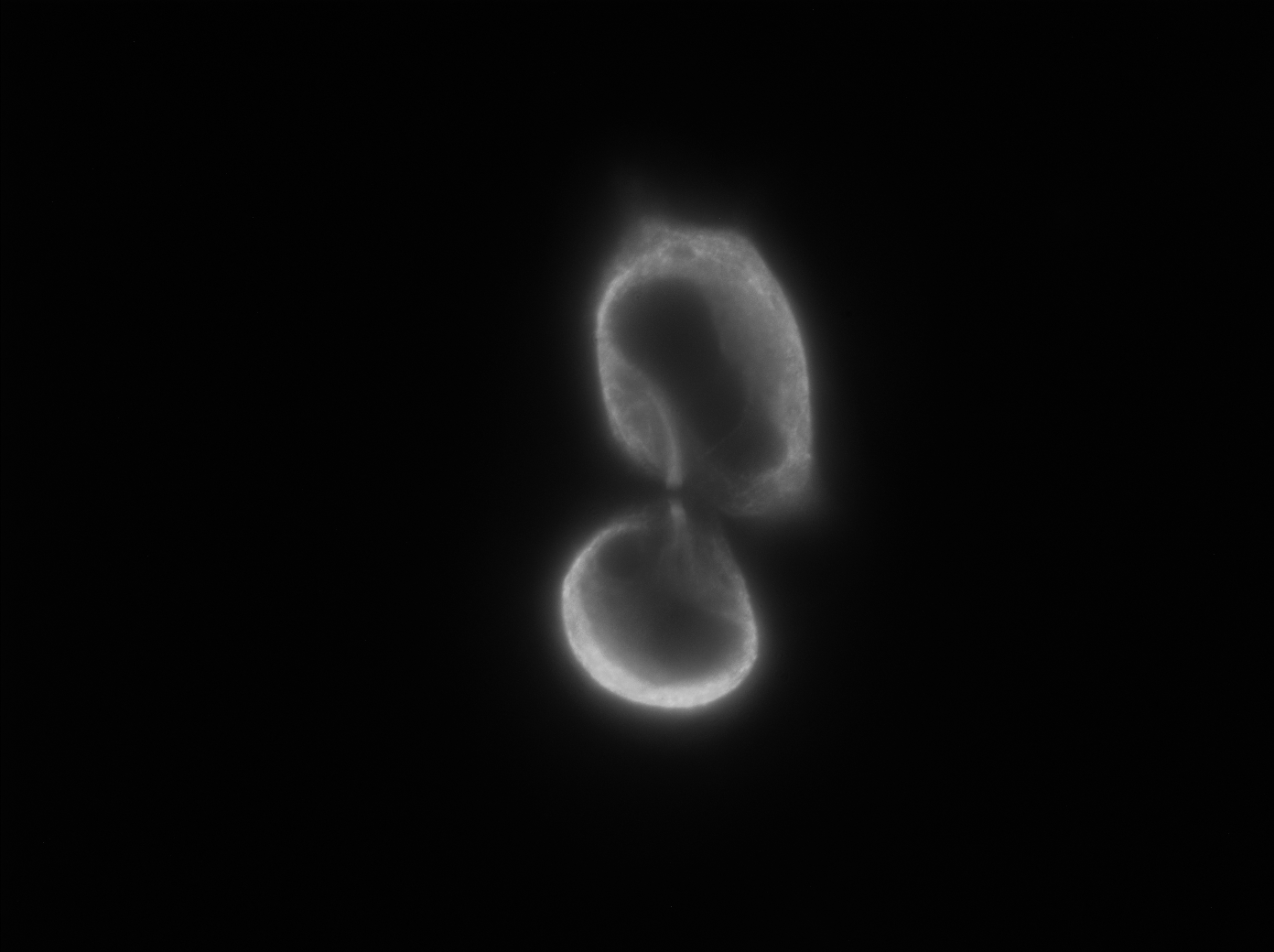

Supplement: Supplementary file 20 — Source data Fig. 6 part 1 [file 44319_2026_742_MOESM20_ESM.zip › Figure 6 Part 1/Fig 6abcd Cas9 TPGS1-KO acetylated tubulin atubulin/Cas9 R3 9-13-24 LT12.Project Maximum Z_XY1726765966_Z0_T0_C1.tif]

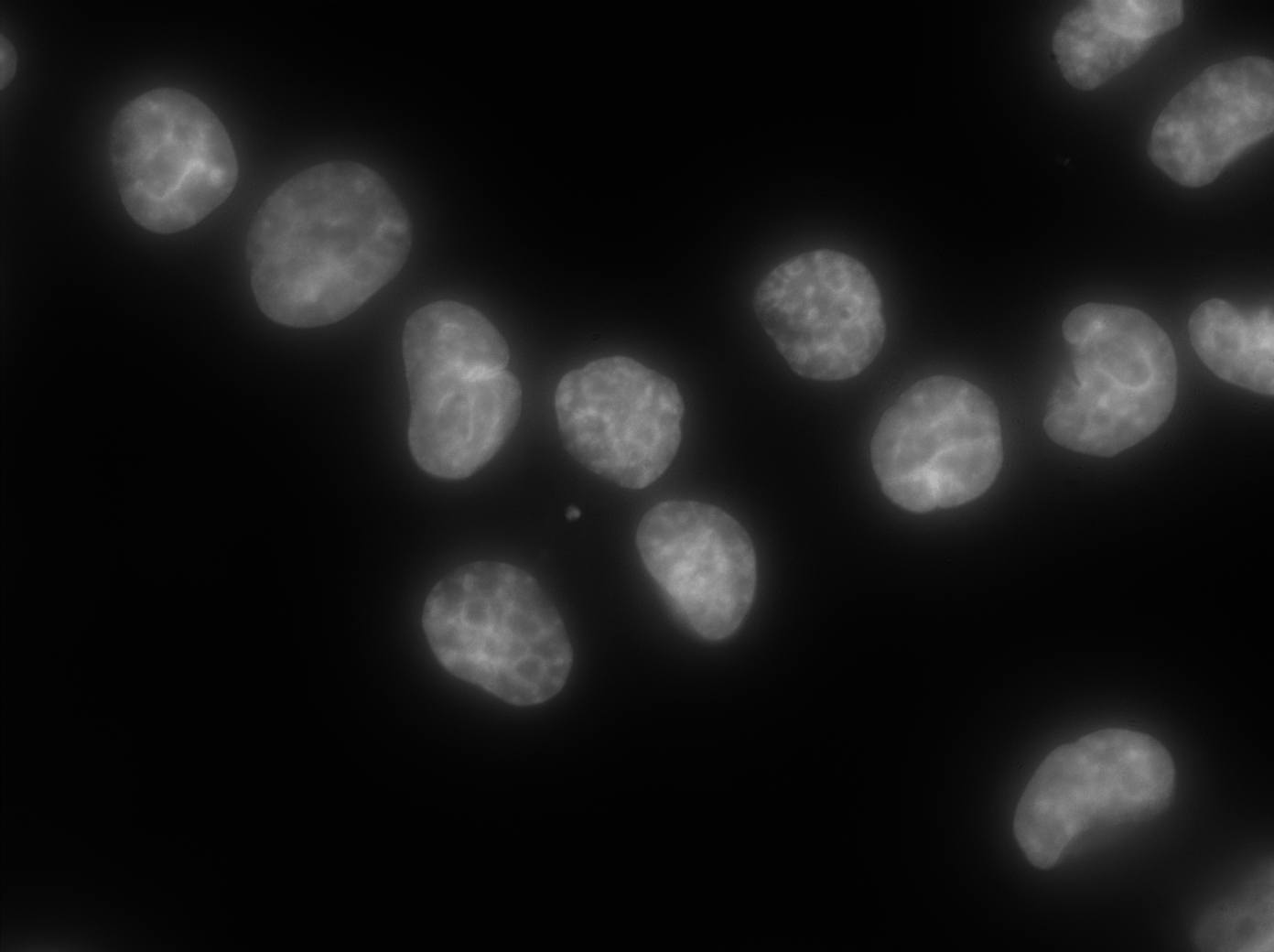

Supplement: Supplementary file 20 — Source data Fig. 6 part 1 [file 44319_2026_742_MOESM20_ESM.zip › Figure 6 Part 1/Fig 6abcd Cas9 TPGS1-KO acetylated tubulin atubulin/Cas9 R2 9-11-24 LT3.Project Maximum Z_XY1726172499_Z0_T0_C0.tif]

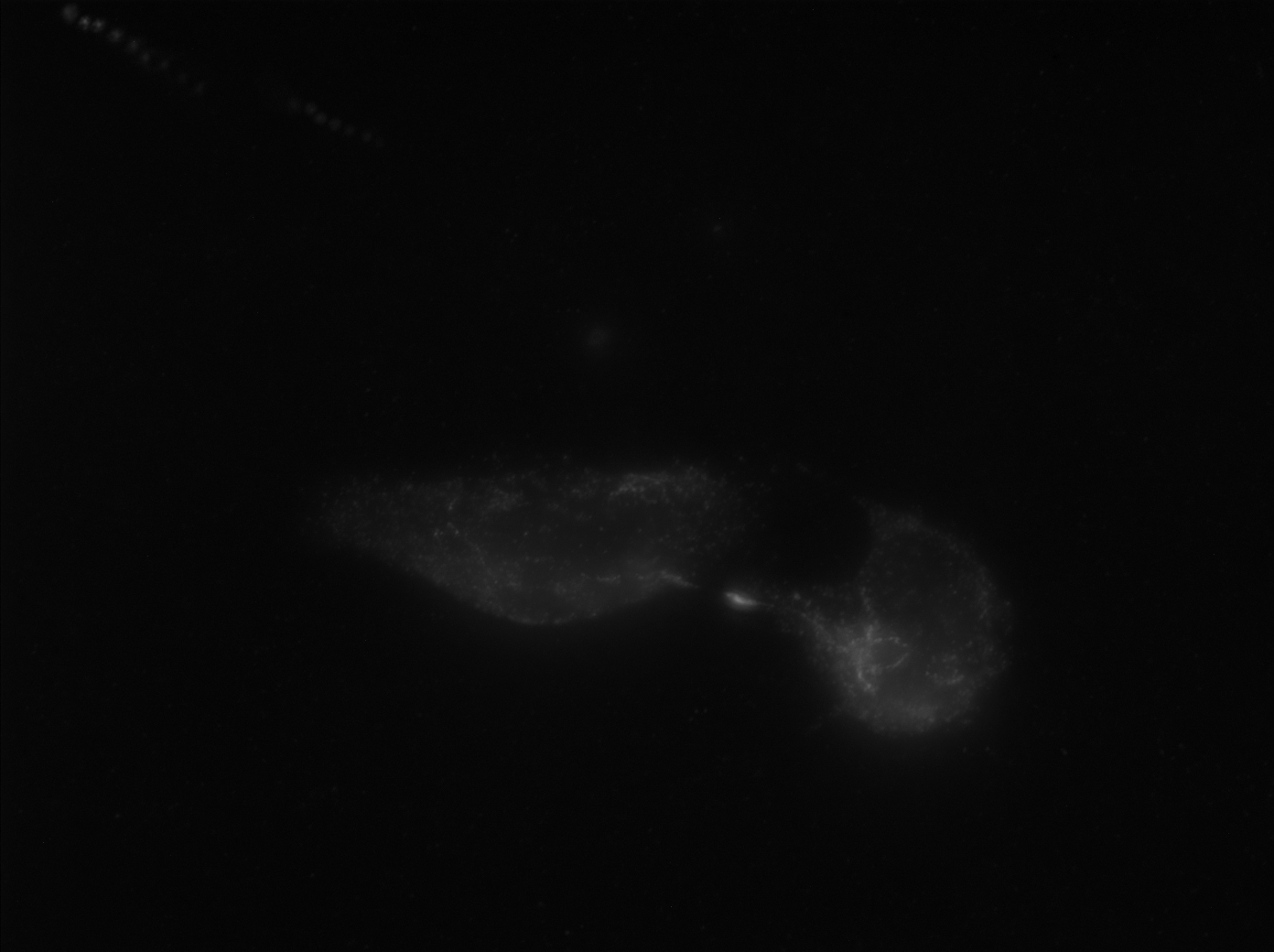

Supplement: Supplementary file 20 — Source data Fig. 6 part 1 [file 44319_2026_742_MOESM20_ESM.zip › Figure 6 Part 1/Fig 6abcd Cas9 TPGS1-KO acetylated tubulin atubulin/Cas9 R2 9-11-24 PA2.Project Maximum Z_XY1726172627_Z0_T0_C2.tif]

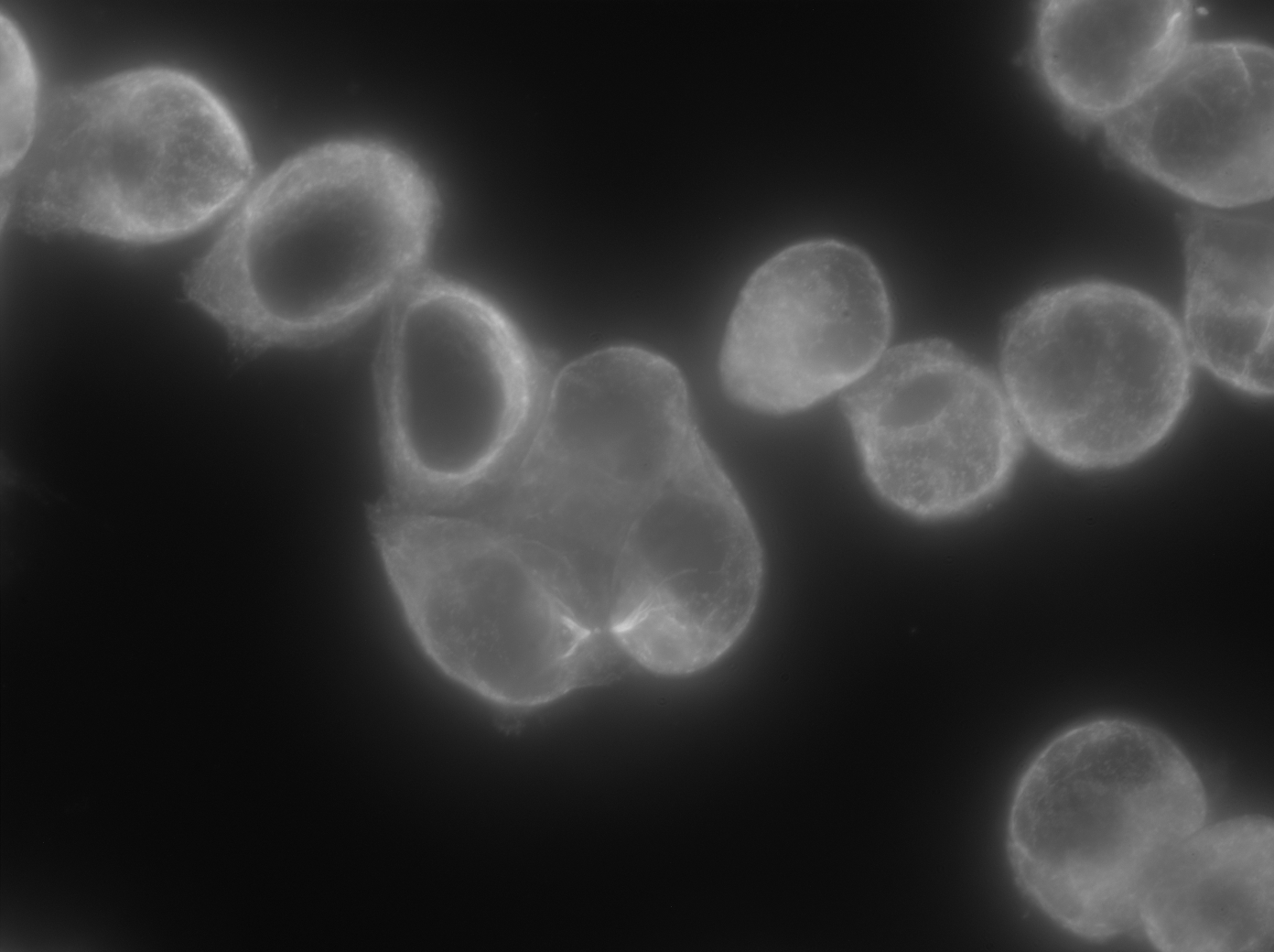

Supplement: Supplementary file 20 — Source data Fig. 6 part 1 [file 44319_2026_742_MOESM20_ESM.zip › Figure 6 Part 1/Fig 6abcd Cas9 TPGS1-KO acetylated tubulin atubulin/Cas9 R2 9-11-24 LT3.Project Maximum Z_XY1726172499_Z0_T0_C1.tif]

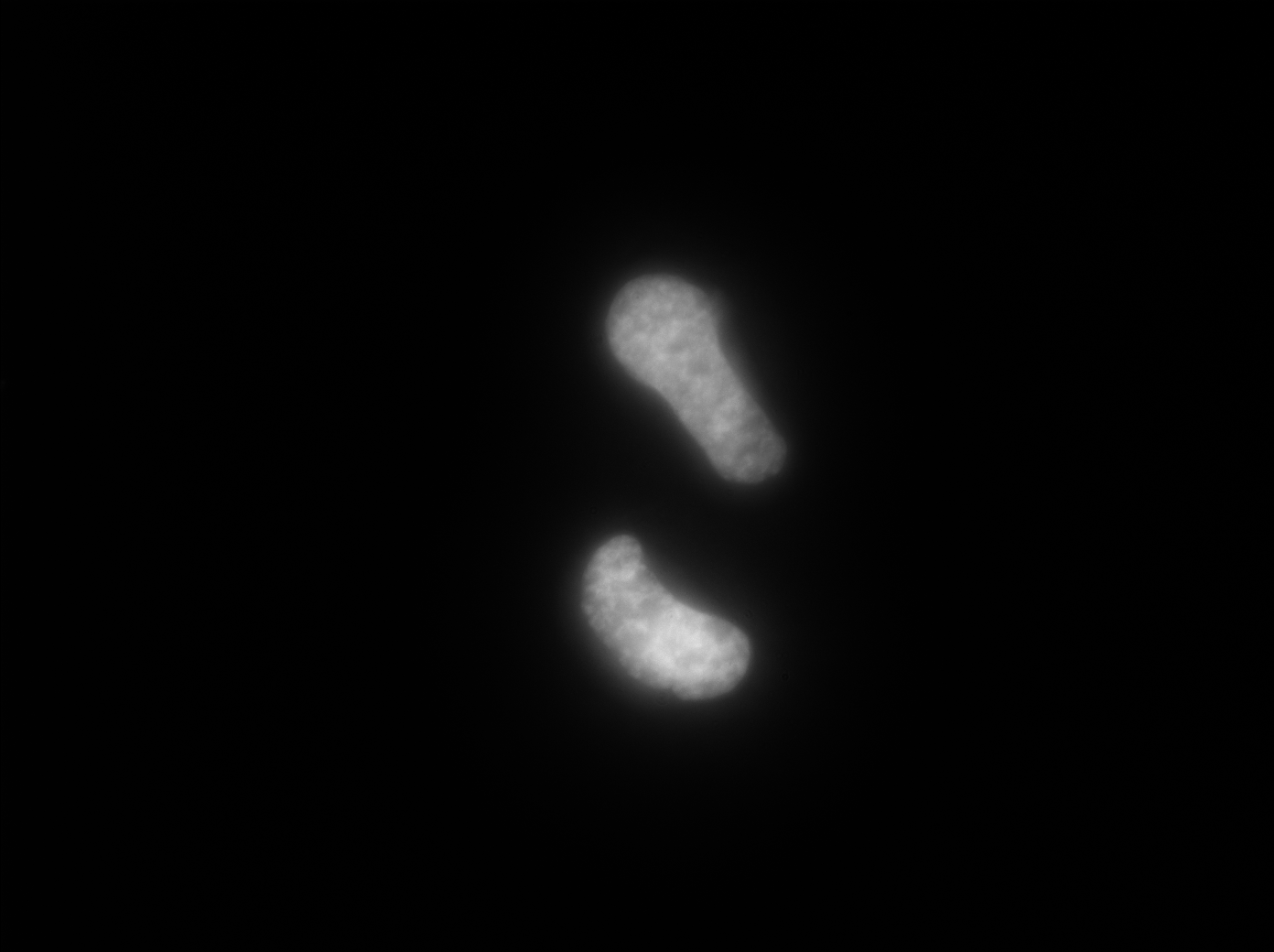

Supplement: Supplementary file 20 — Source data Fig. 6 part 1 [file 44319_2026_742_MOESM20_ESM.zip › Figure 6 Part 1/Fig 6abcd Cas9 TPGS1-KO acetylated tubulin atubulin/Cas9 R3 9-13-24 LT12.Project Maximum Z_XY1726765966_Z0_T0_C0.tif]

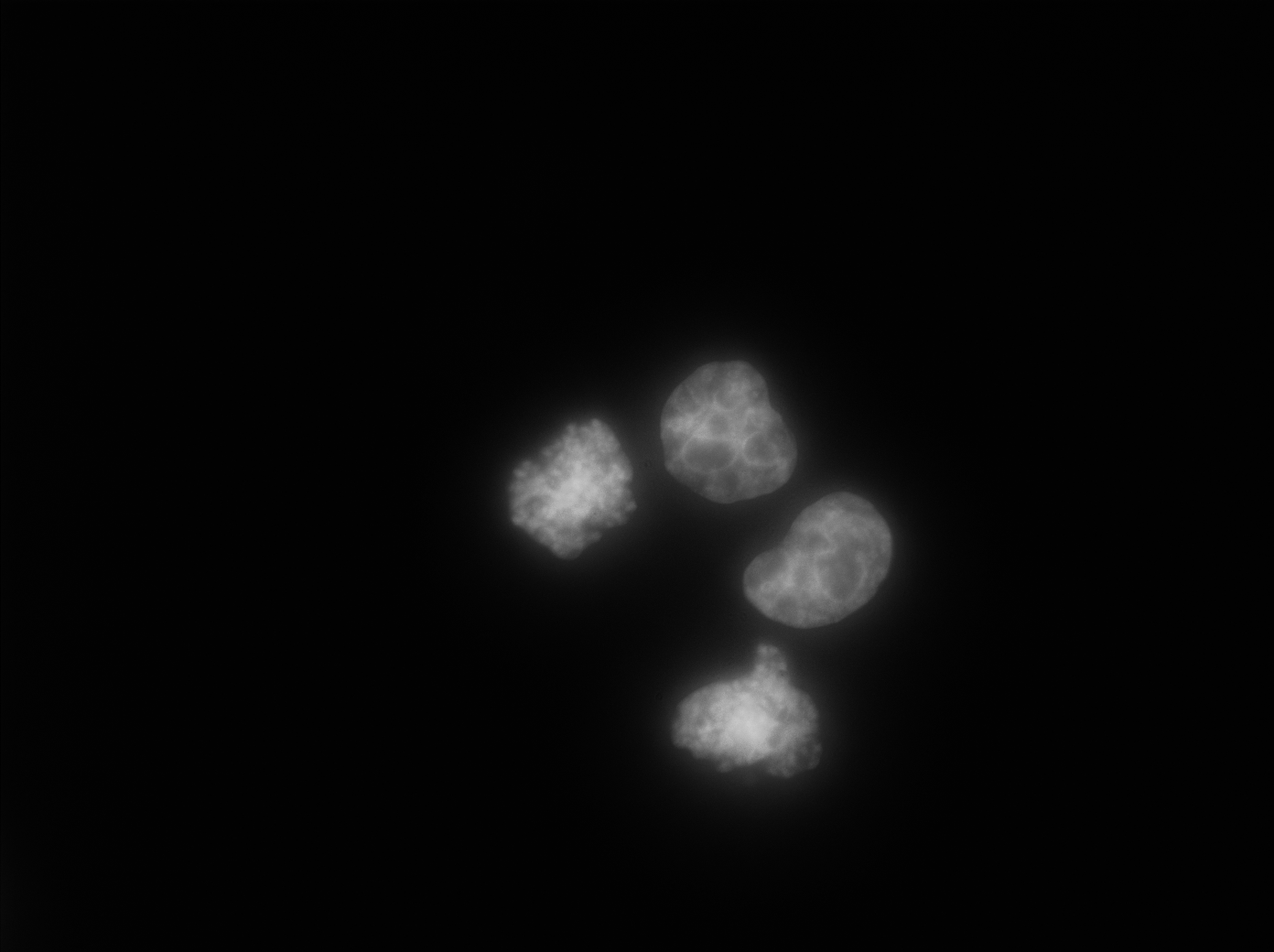

Supplement: Supplementary file 20 — Source data Fig. 6 part 1 [file 44319_2026_742_MOESM20_ESM.zip › Figure 6 Part 1/Fig 6abcd Cas9 TPGS1-KO acetylated tubulin atubulin/Cas9 R2 9-11-24 LT10LT11.Project Maximum Z_XY1726174037_Z0_T0_C0.tif]

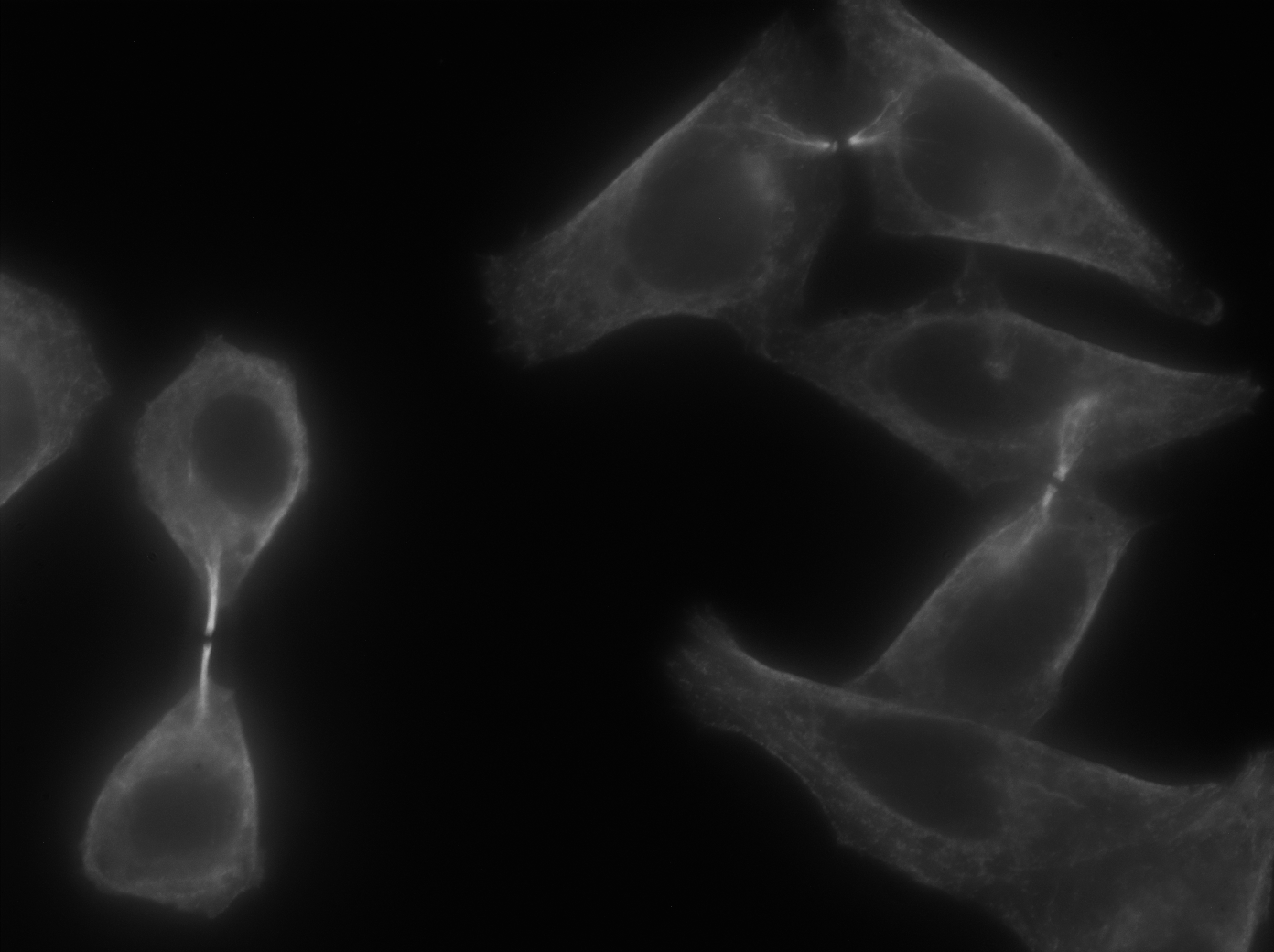

Supplement: Supplementary file 20 — Source data Fig. 6 part 1 [file 44319_2026_742_MOESM20_ESM.zip › Figure 6 Part 1/Fig 6abcd Cas9 TPGS1-KO acetylated tubulin atubulin/Cas9 R3 9-13-24 LT5LT6LT7.Project Maximum Z_XY1726765644_Z0_T0_C1.tif]

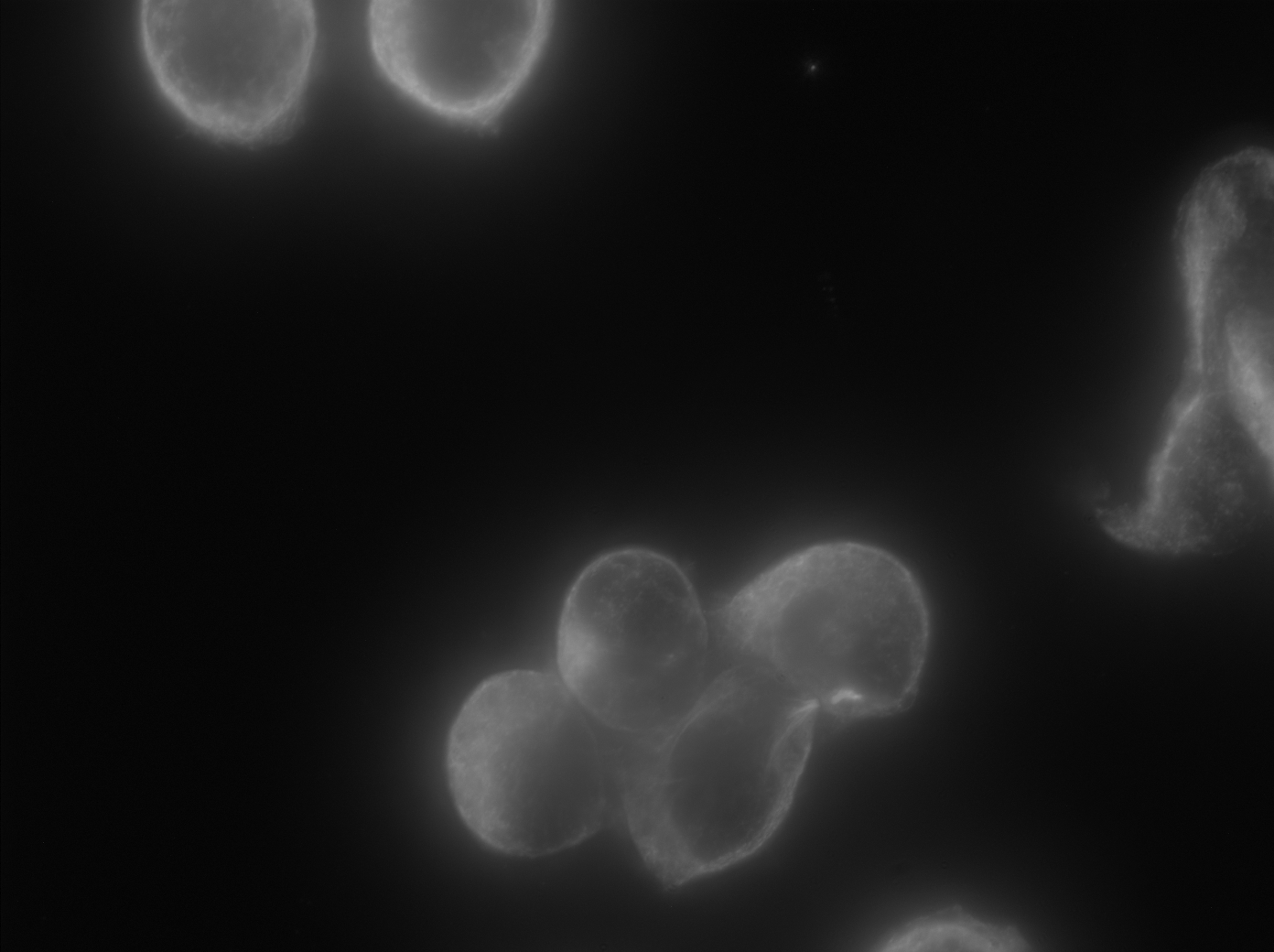

Supplement: Supplementary file 20 — Source data Fig. 6 part 1 [file 44319_2026_742_MOESM20_ESM.zip › Figure 6 Part 1/Fig 6abcd Cas9 TPGS1-KO acetylated tubulin atubulin/Cas9 R2 9-11-24 LT2.Project Maximum Z_XY1726172269_Z0_T0_C1.tif]

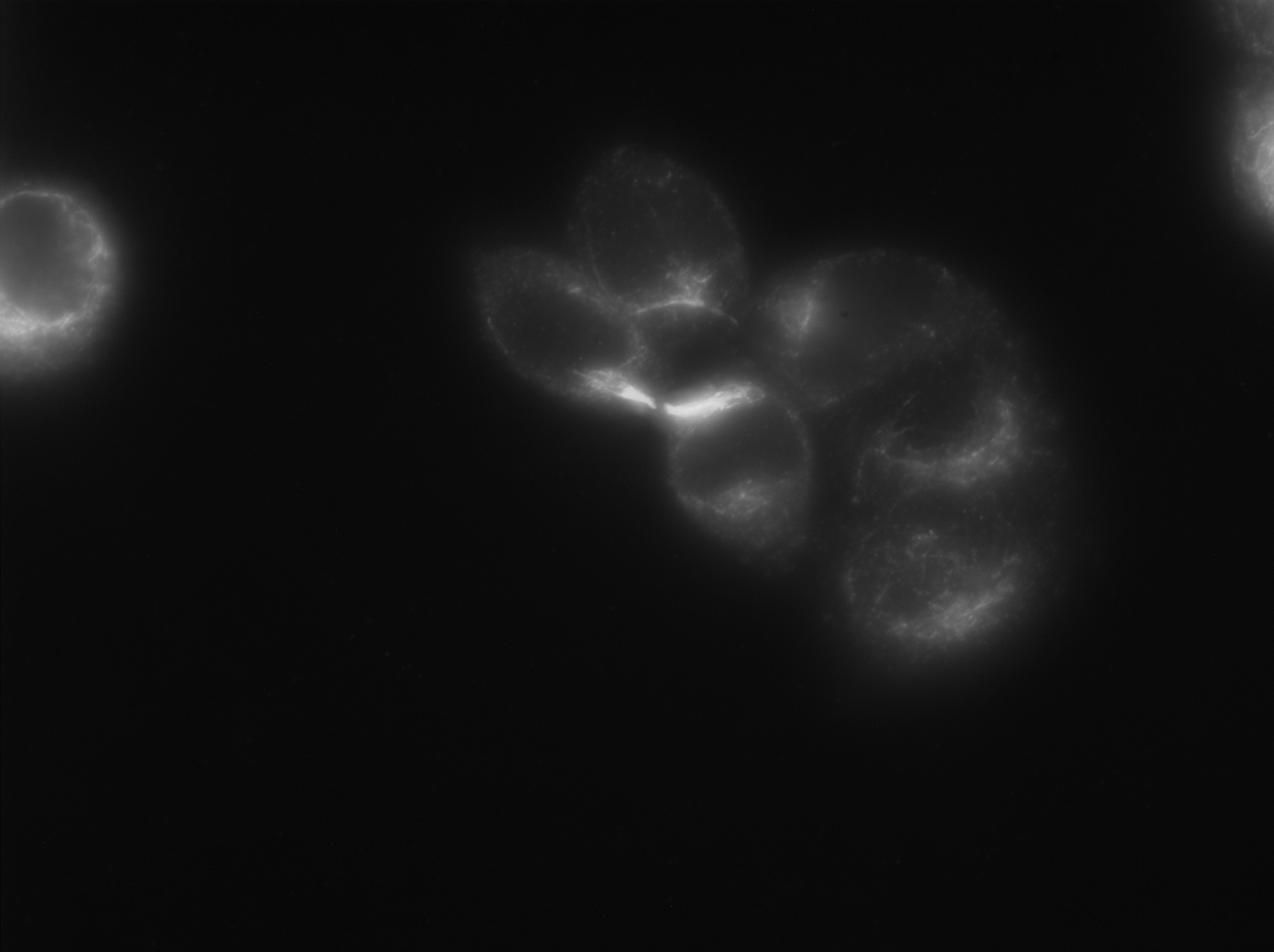

Supplement: Supplementary file 20 — Source data Fig. 6 part 1 [file 44319_2026_742_MOESM20_ESM.zip › Figure 6 Part 1/Fig 6abcd Cas9 TPGS1-KO acetylated tubulin atubulin/Cas9 R2 9-11-24 LT21 PA10.Project Maximum Z_XY1726178978_Z0_T0_C2.tif]

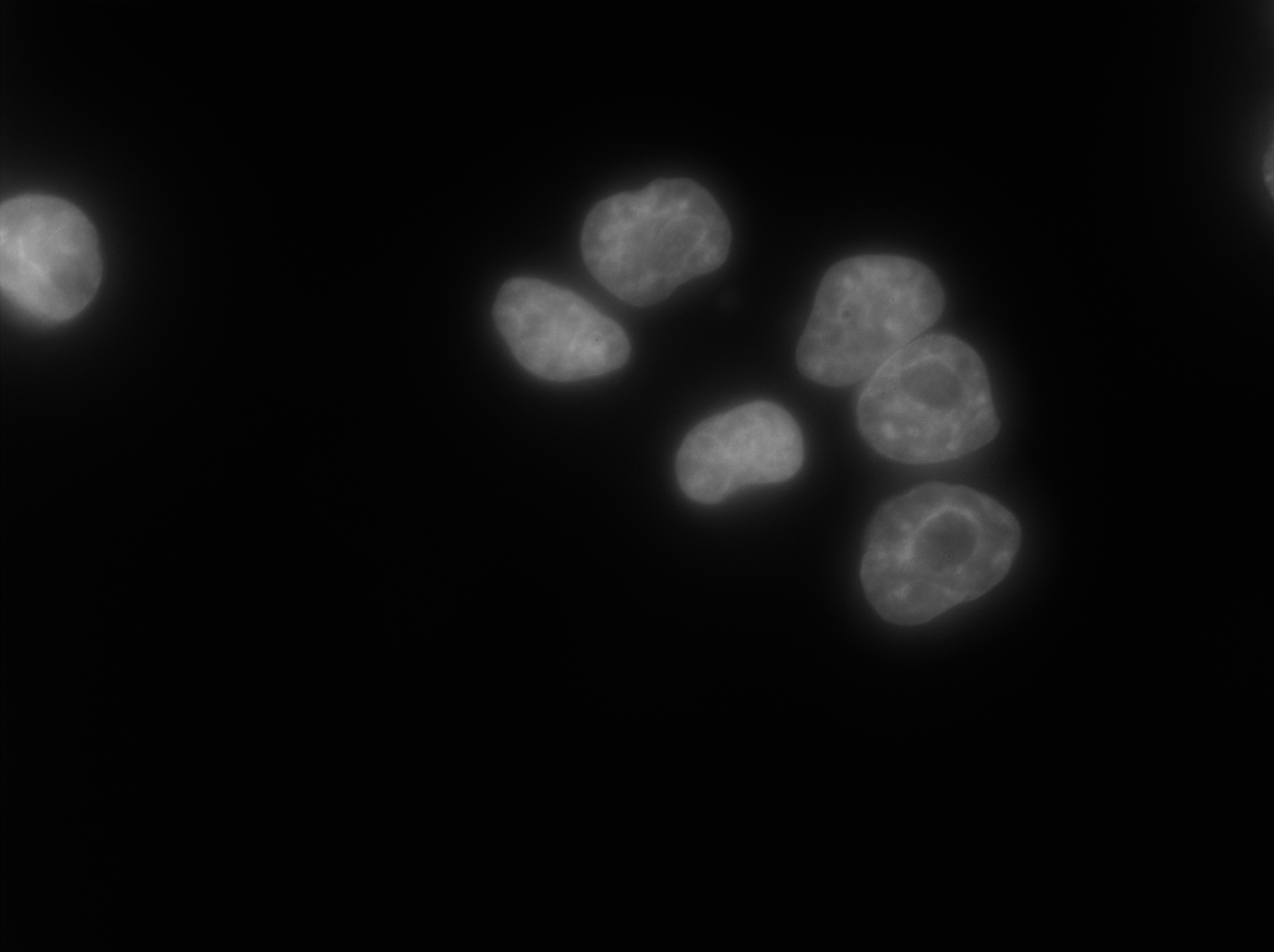

Supplement: Supplementary file 20 — Source data Fig. 6 part 1 [file 44319_2026_742_MOESM20_ESM.zip › Figure 6 Part 1/Fig 6abcd Cas9 TPGS1-KO acetylated tubulin atubulin/Cas9 R2 9-11-24 LT21 PA10.Project Maximum Z_XY1726178978_Z0_T0_C0.tif]

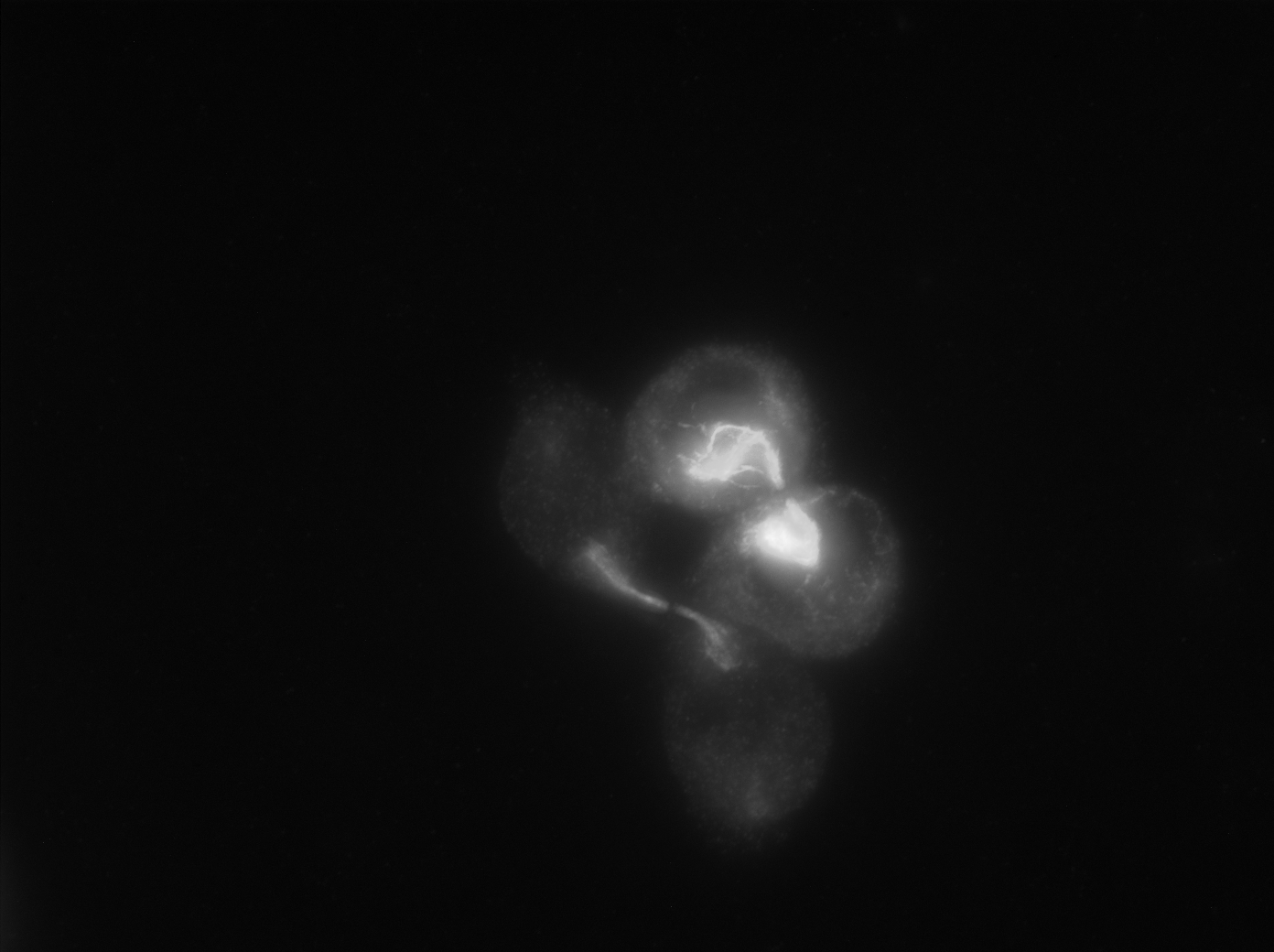

Supplement: Supplementary file 20 — Source data Fig. 6 part 1 [file 44319_2026_742_MOESM20_ESM.zip › Figure 6 Part 1/Fig 6abcd Cas9 TPGS1-KO acetylated tubulin atubulin/Cas9 R2 9-11-24 LT10LT11.Project Maximum Z_XY1726174037_Z0_T0_C2.tif]

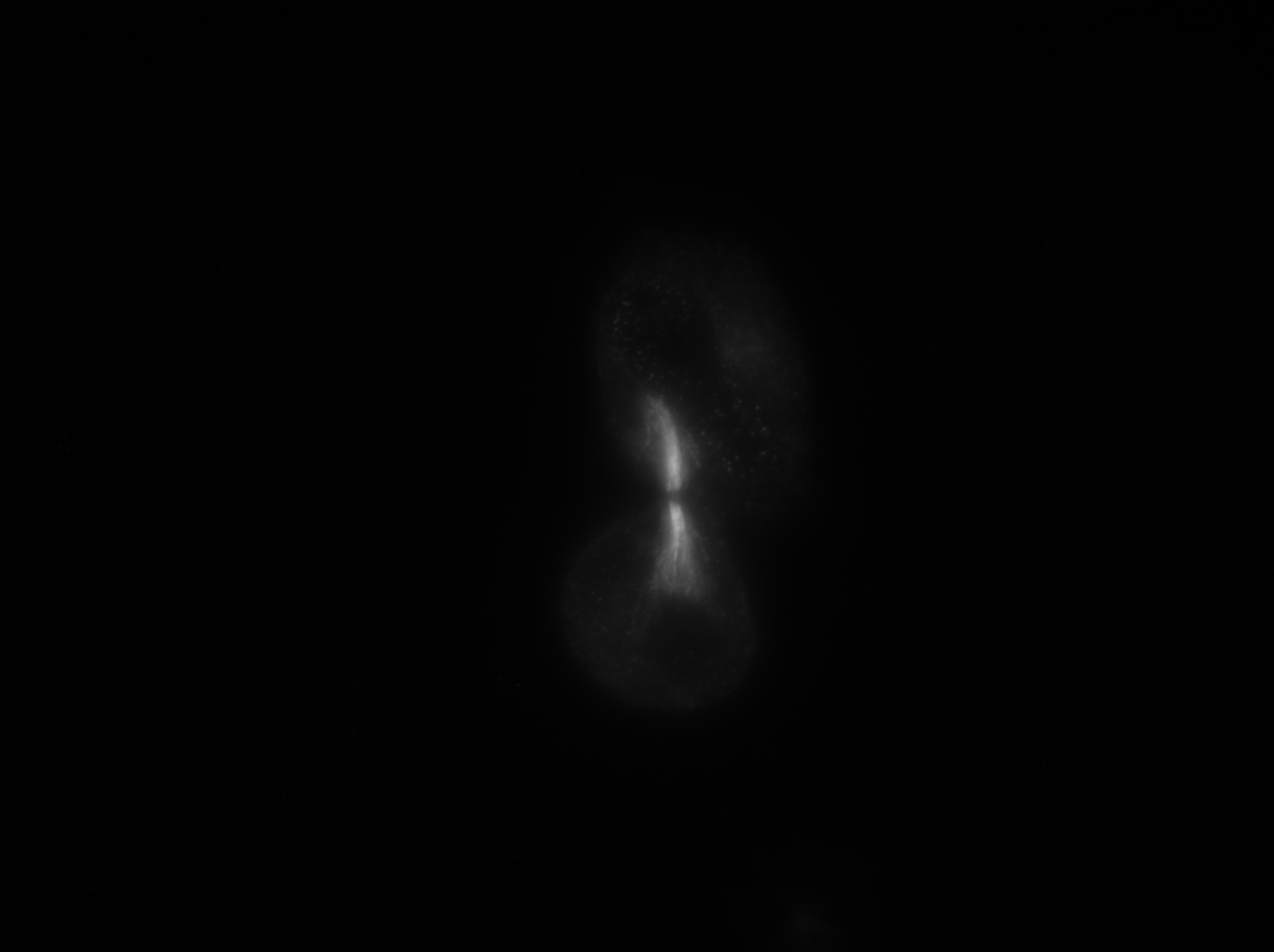

Supplement: Supplementary file 20 — Source data Fig. 6 part 1 [file 44319_2026_742_MOESM20_ESM.zip › Figure 6 Part 1/Fig 6abcd Cas9 TPGS1-KO acetylated tubulin atubulin/Cas9 R3 9-13-24 LT12.Project Maximum Z_XY1726765966_Z0_T0_C2.tif]

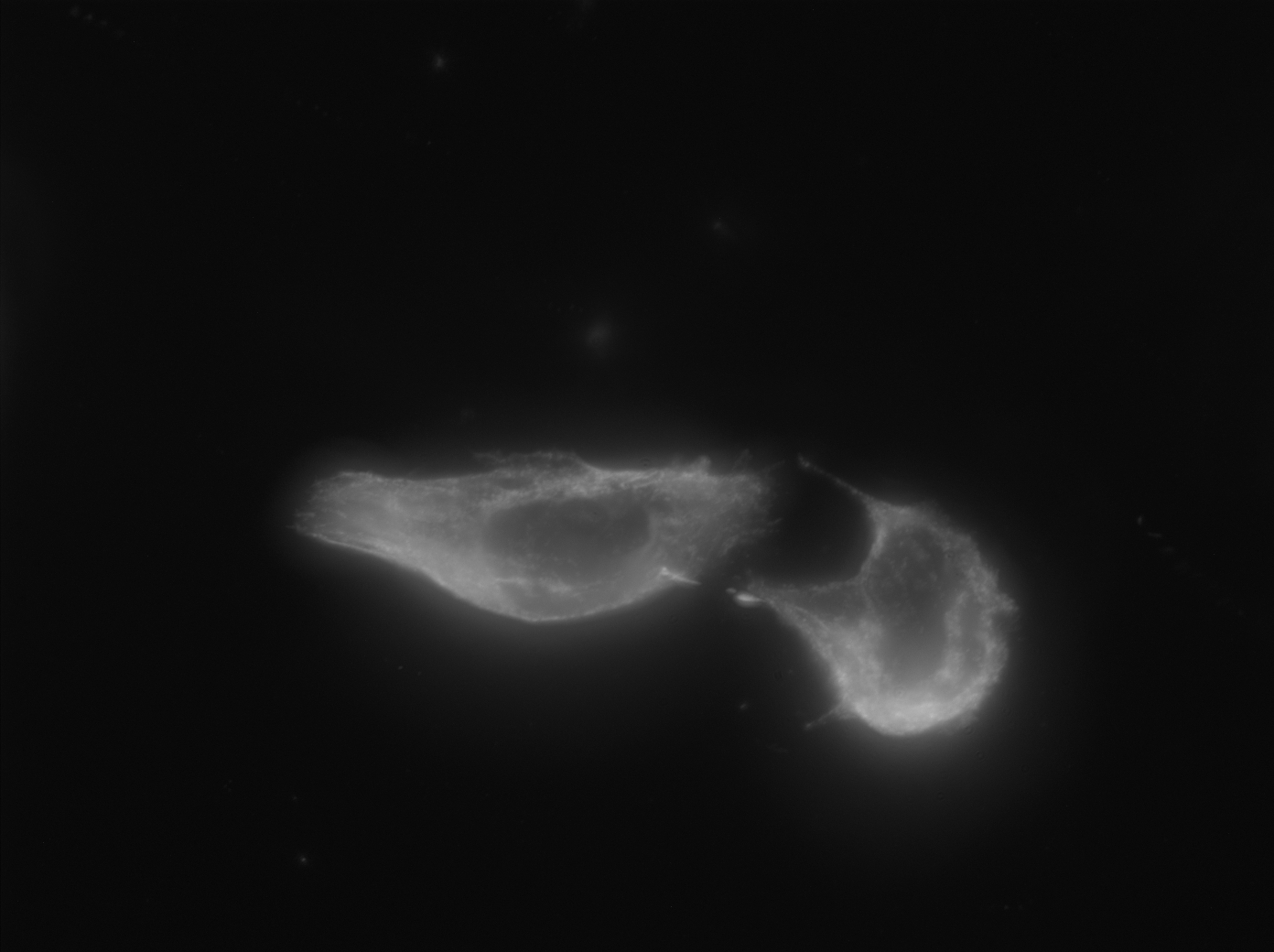

Supplement: Supplementary file 20 — Source data Fig. 6 part 1 [file 44319_2026_742_MOESM20_ESM.zip › Figure 6 Part 1/Fig 6abcd Cas9 TPGS1-KO acetylated tubulin atubulin/Cas9 R2 9-11-24 PA2.Project Maximum Z_XY1726172627_Z0_T0_C1.tif]

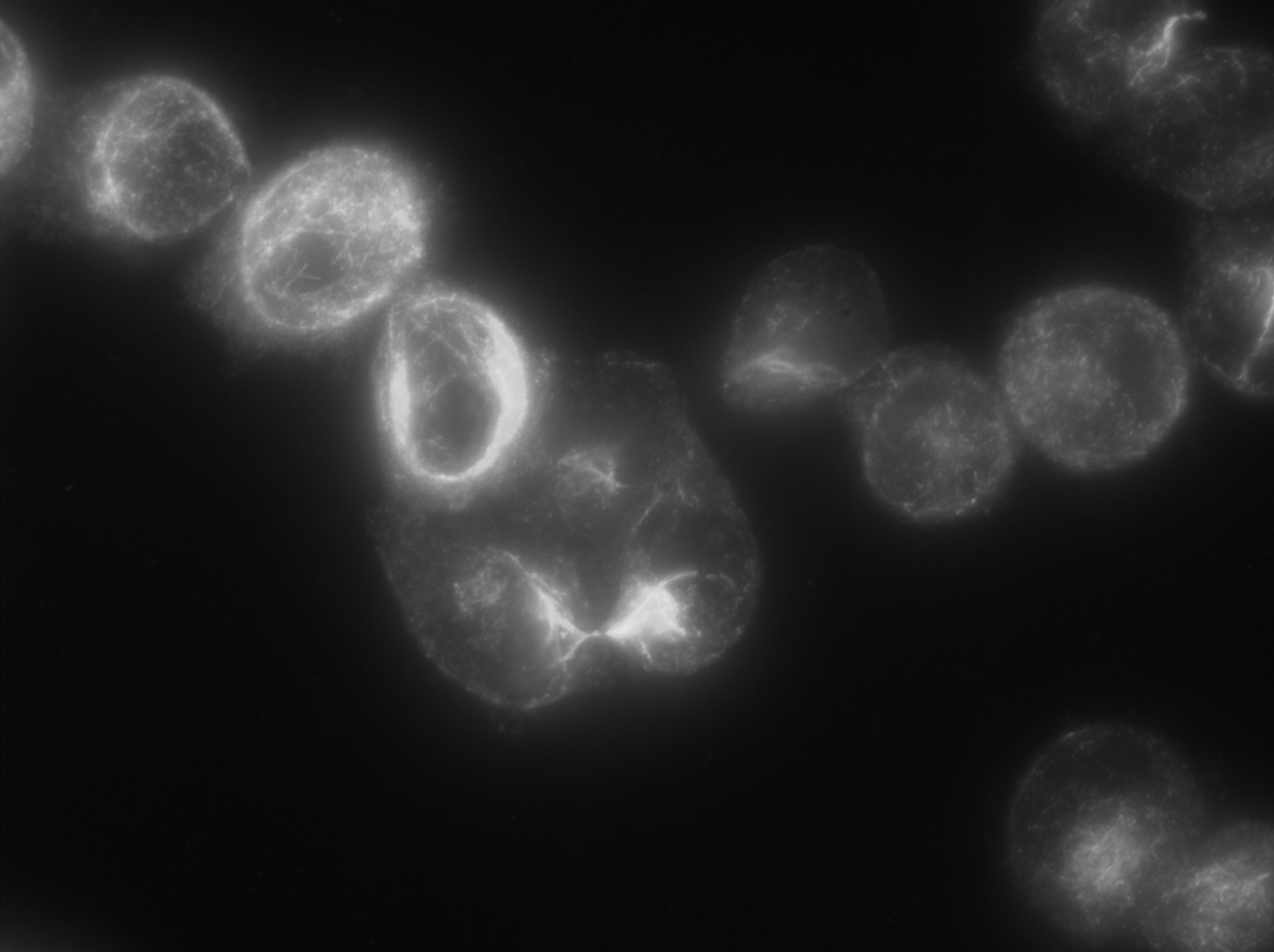

Supplement: Supplementary file 20 — Source data Fig. 6 part 1 [file 44319_2026_742_MOESM20_ESM.zip › Figure 6 Part 1/Fig 6abcd Cas9 TPGS1-KO acetylated tubulin atubulin/Cas9 R2 9-11-24 LT3.Project Maximum Z_XY1726172499_Z0_T0_C2.tif]

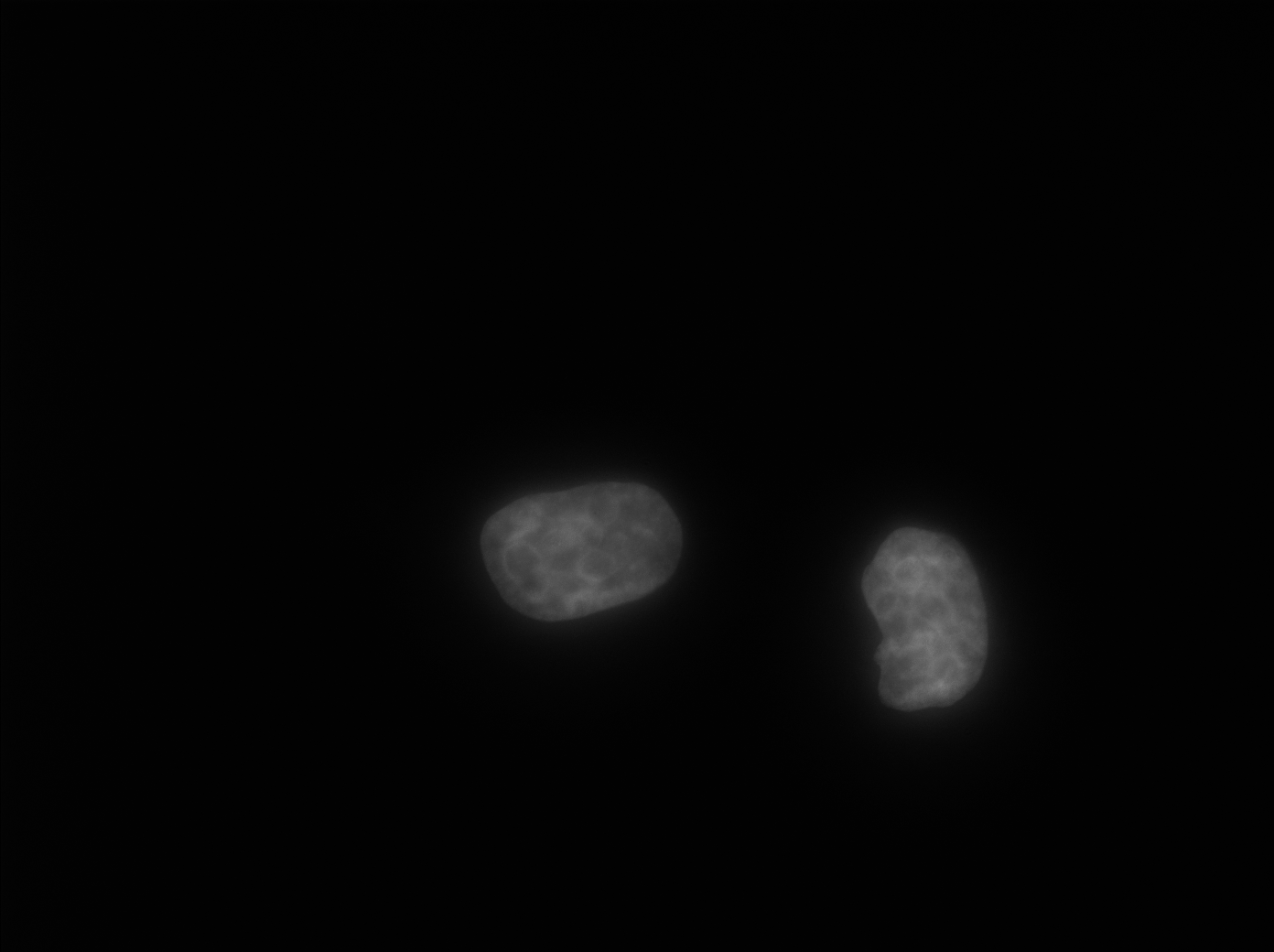

Supplement: Supplementary file 20 — Source data Fig. 6 part 1 [file 44319_2026_742_MOESM20_ESM.zip › Figure 6 Part 1/Fig 6abcd Cas9 TPGS1-KO acetylated tubulin atubulin/Cas9 R2 9-11-24 PA2.Project Maximum Z_XY1726172627_Z0_T0_C0.tif]

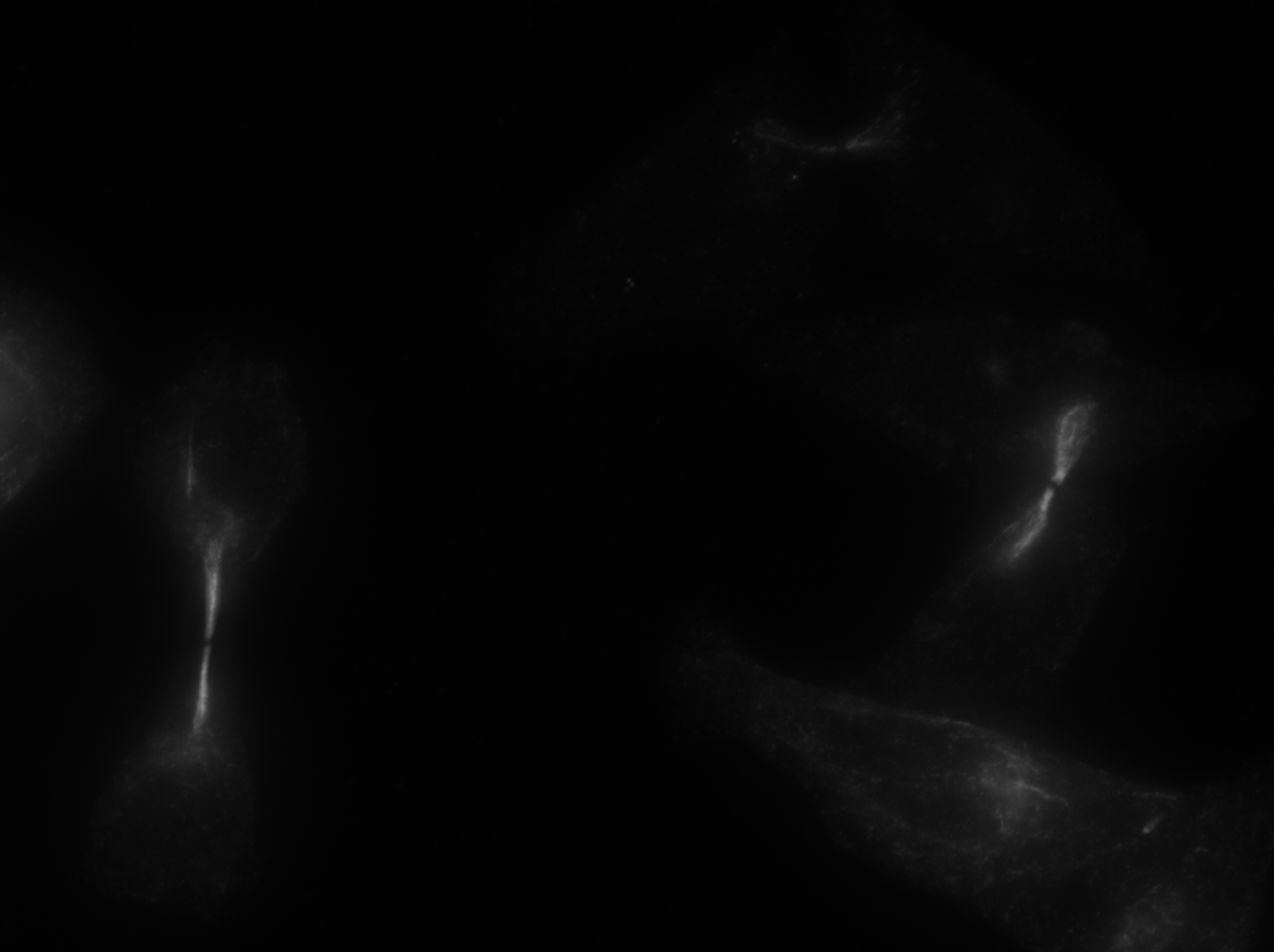

Supplement: Supplementary file 20 — Source data Fig. 6 part 1 [file 44319_2026_742_MOESM20_ESM.zip › Figure 6 Part 1/Fig 6abcd Cas9 TPGS1-KO acetylated tubulin atubulin/Cas9 R3 9-13-24 LT5LT6LT7.Project Maximum Z_XY1726765644_Z0_T0_C2.tif]

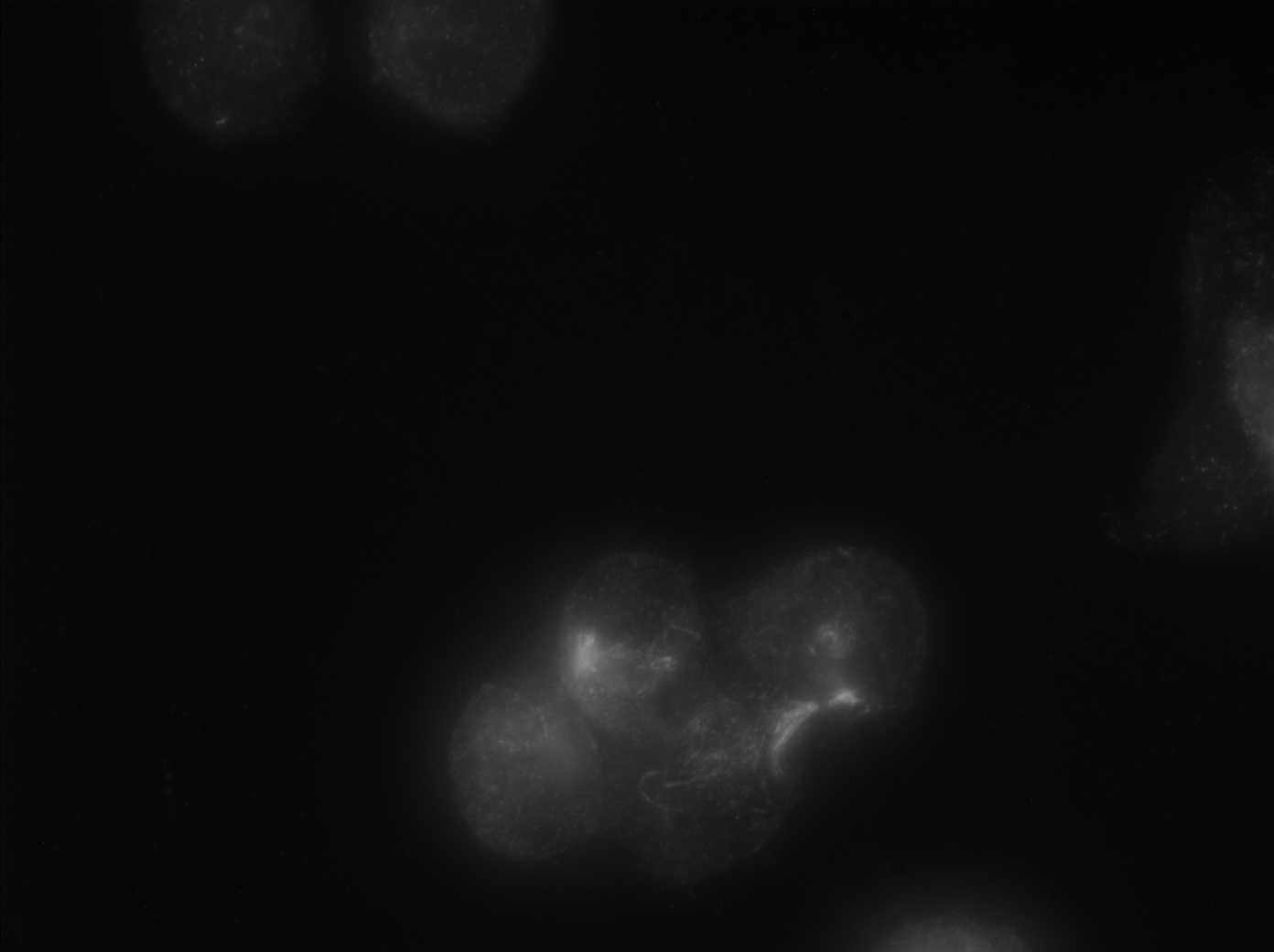

Supplement: Supplementary file 20 — Source data Fig. 6 part 1 [file 44319_2026_742_MOESM20_ESM.zip › Figure 6 Part 1/Fig 6abcd Cas9 TPGS1-KO acetylated tubulin atubulin/Cas9 R2 9-11-24 LT2.Project Maximum Z_XY1726172269_Z0_T0_C2.tif]

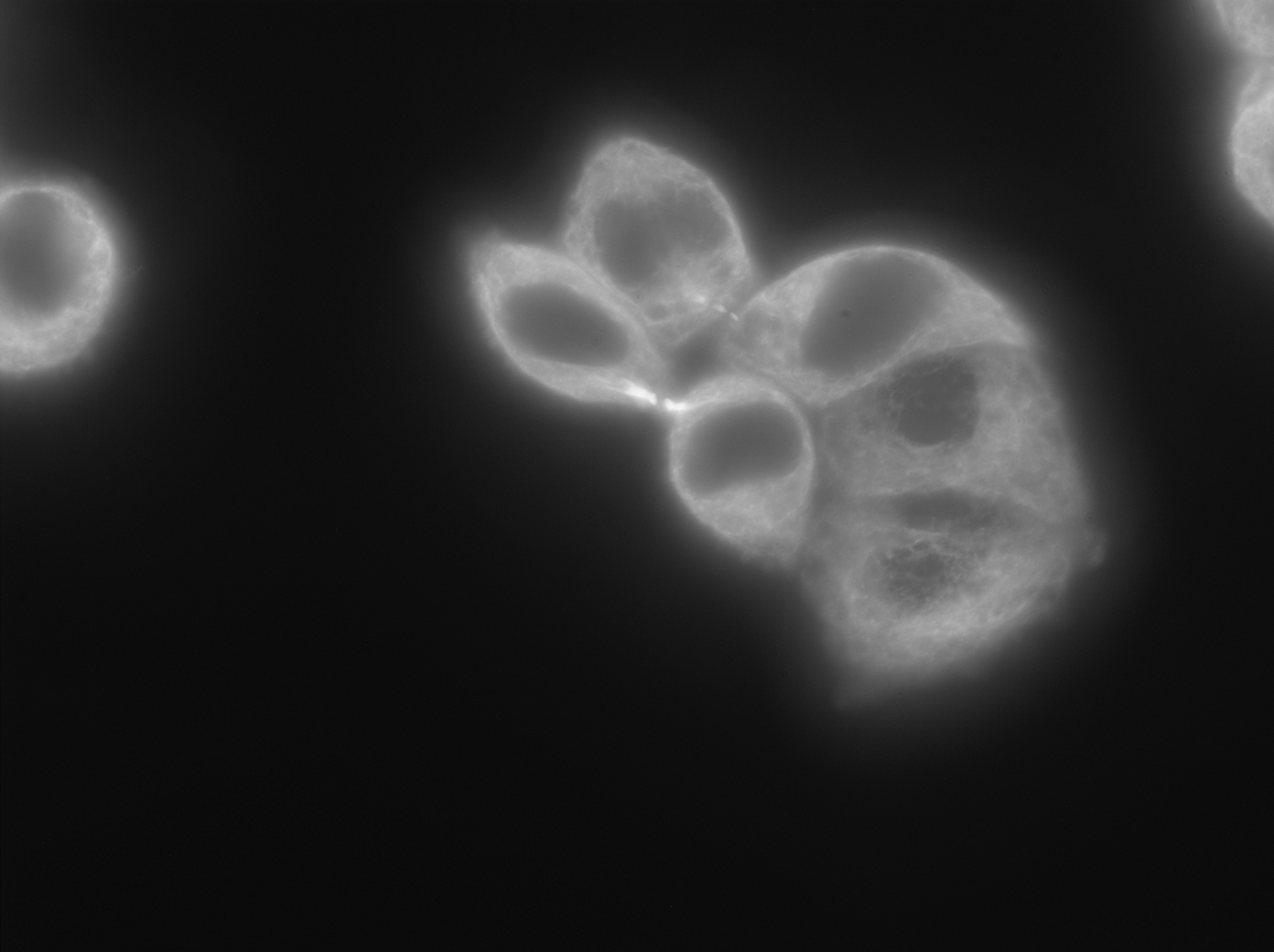

Supplement: Supplementary file 20 — Source data Fig. 6 part 1 [file 44319_2026_742_MOESM20_ESM.zip › Figure 6 Part 1/Fig 6abcd Cas9 TPGS1-KO acetylated tubulin atubulin/Cas9 R2 9-11-24 LT21 PA10.Project Maximum Z_XY1726178978_Z0_T0_C1.tif]

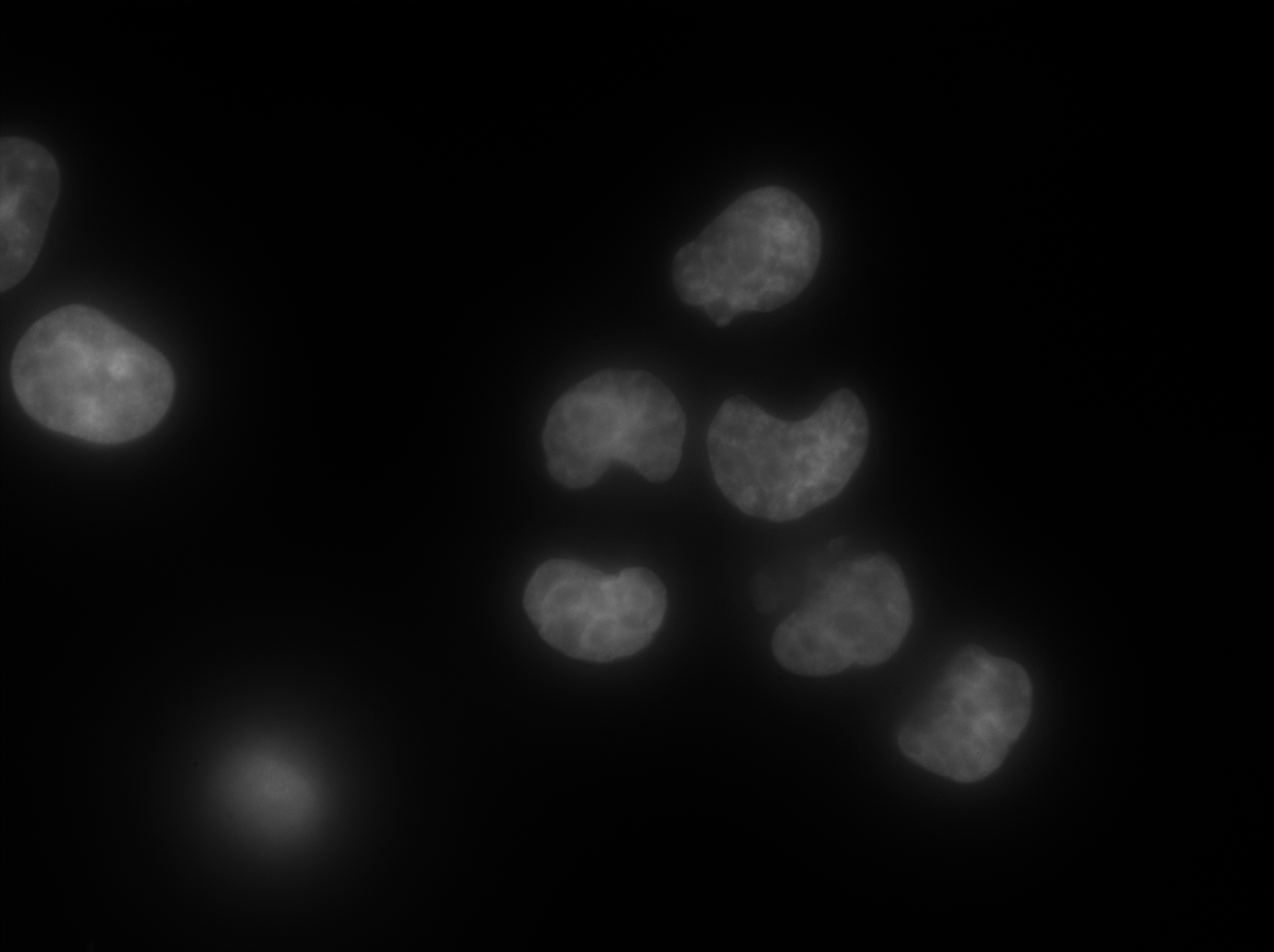

Supplement: Supplementary file 20 — Source data Fig. 6 part 1 [file 44319_2026_742_MOESM20_ESM.zip › Figure 6 Part 1/Fig 6abcd Cas9 TPGS1-KO acetylated tubulin atubulin/Cas9 R3 9-13-24 LT22.Project Maximum Z_XY1726767057_Z0_T0_C0.tif]

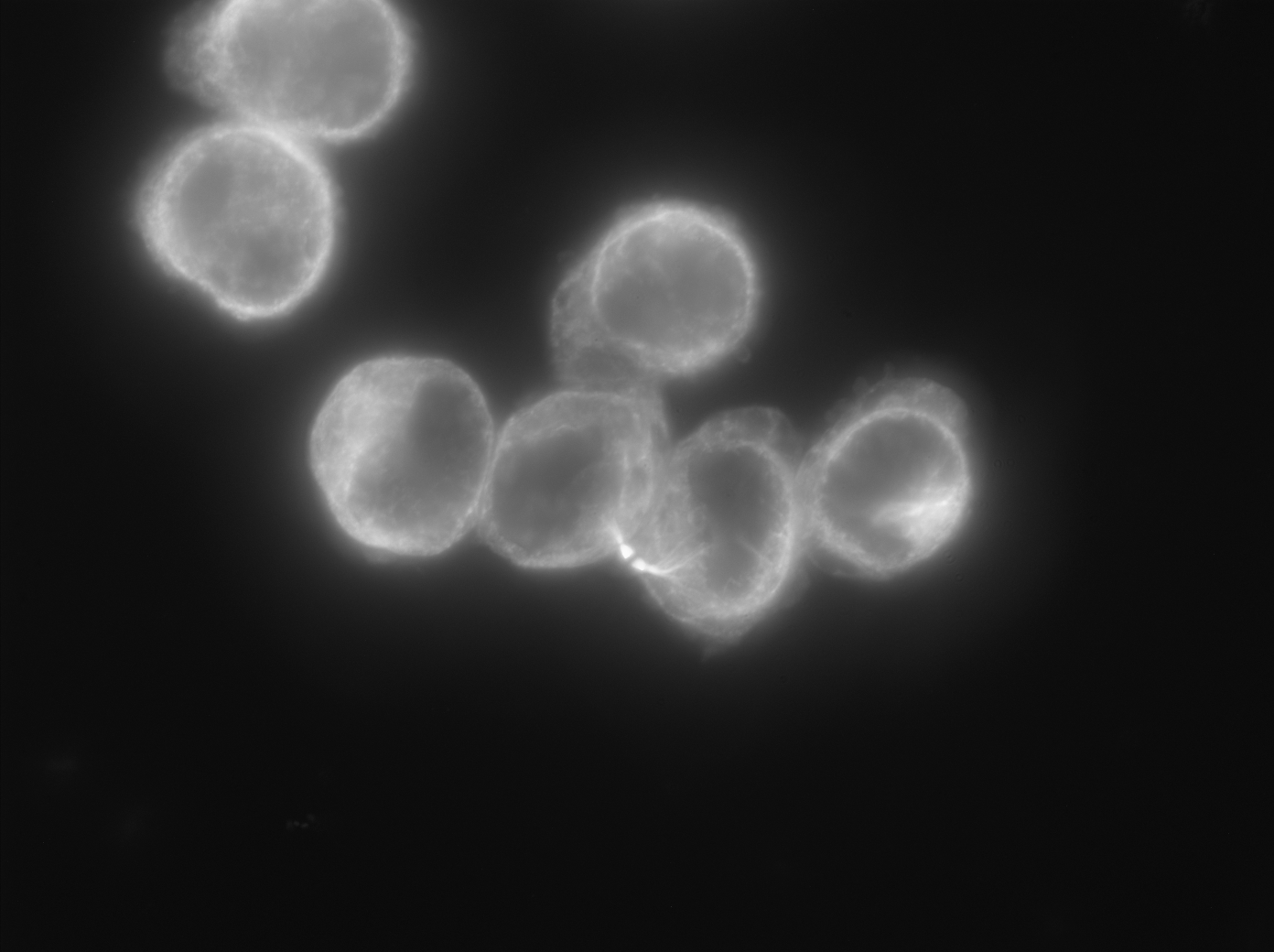

Supplement: Supplementary file 20 — Source data Fig. 6 part 1 [file 44319_2026_742_MOESM20_ESM.zip › Figure 6 Part 1/Fig 6abcd Cas9 TPGS1-KO acetylated tubulin atubulin/Cas9 R2 9-11-24 LT12.Project Maximum Z_XY1726174218_Z0_T0_C1.tif]

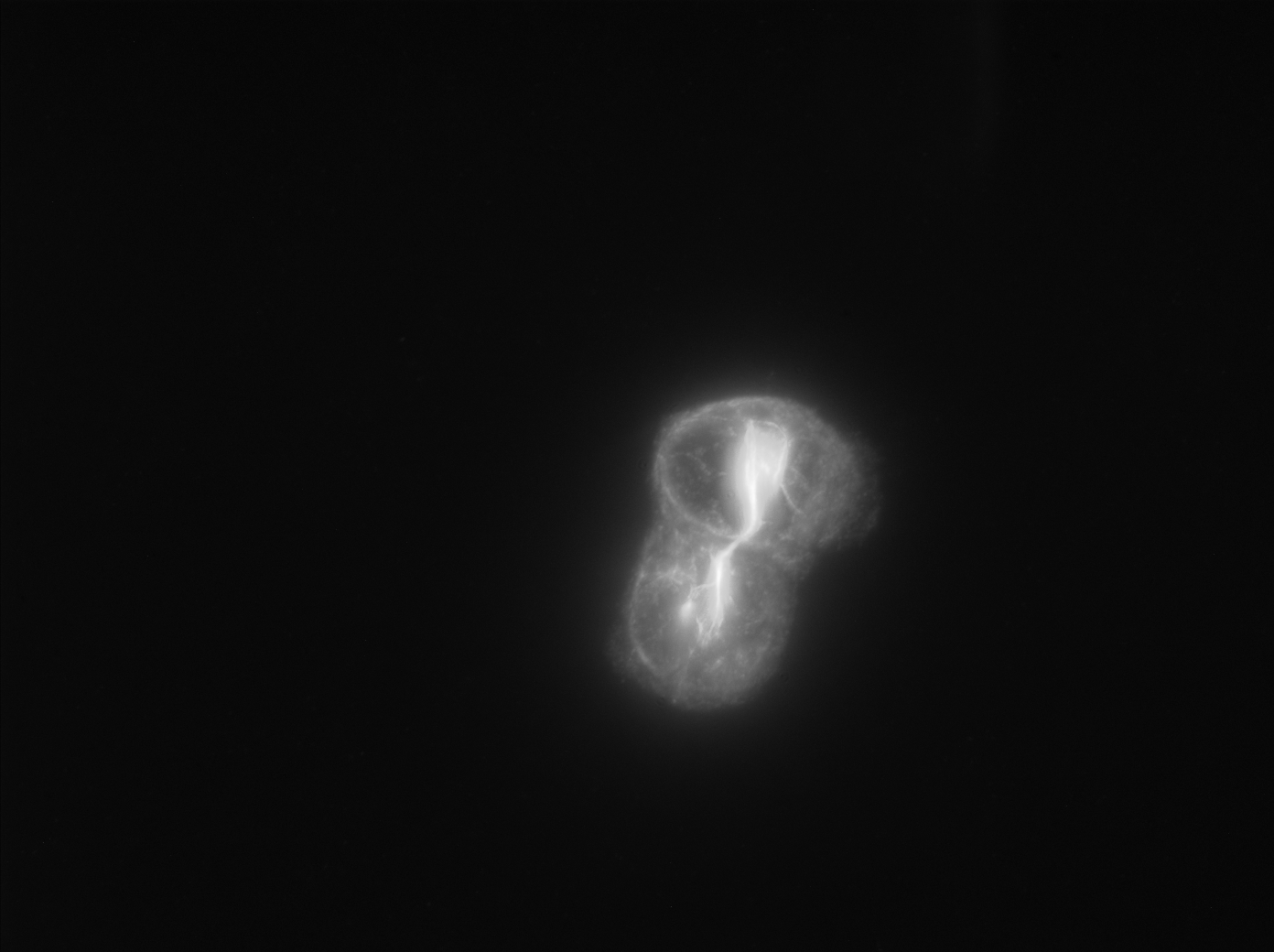

Supplement: Supplementary file 20 — Source data Fig. 6 part 1 [file 44319_2026_742_MOESM20_ESM.zip › Figure 6 Part 1/Fig 6abcd Cas9 TPGS1-KO acetylated tubulin atubulin/Cas9 R2 9-11-24 LT18.Project Maximum Z_XY1726178367_Z0_T0_C2.tif]

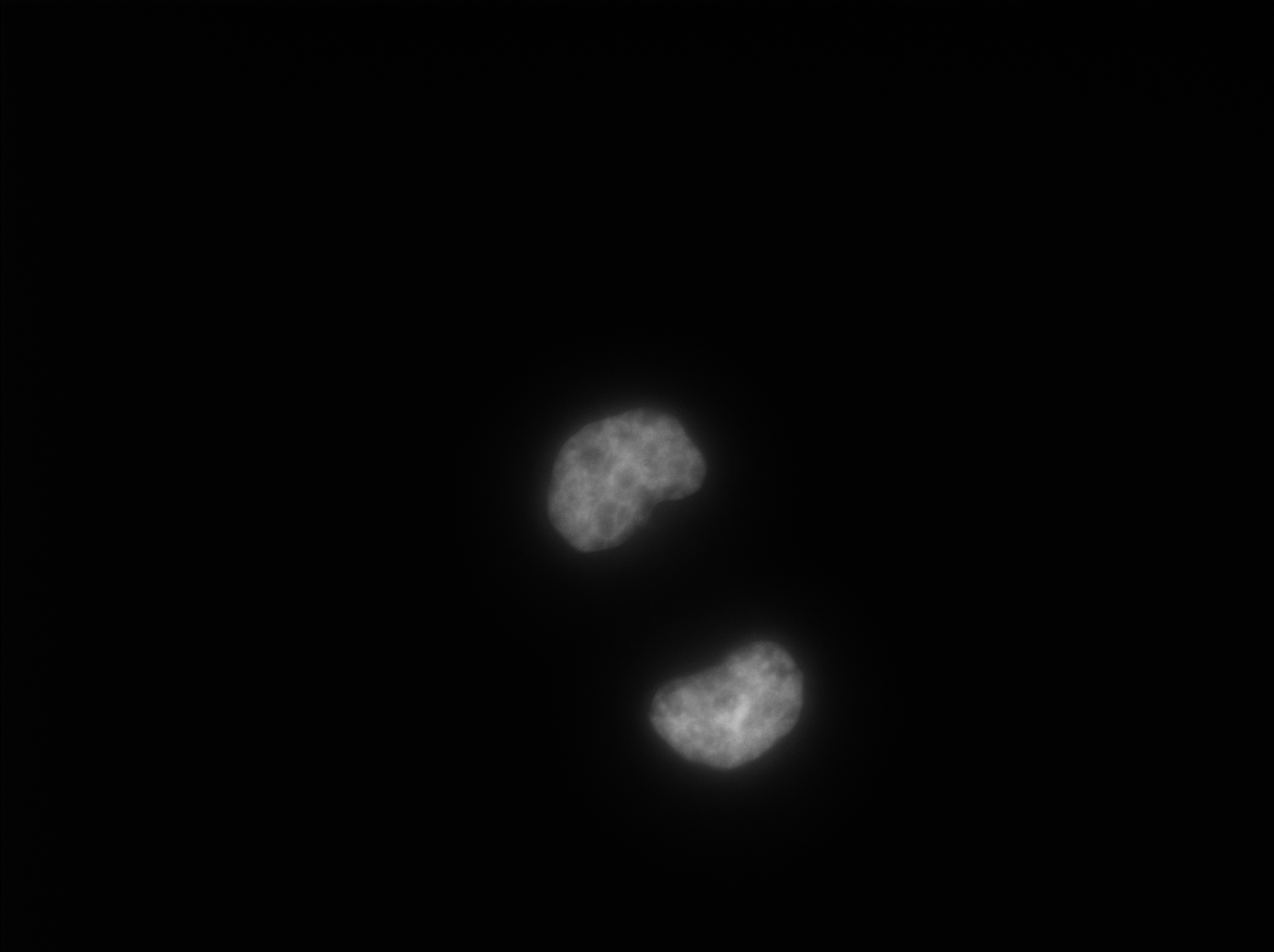

Supplement: Supplementary file 20 — Source data Fig. 6 part 1 [file 44319_2026_742_MOESM20_ESM.zip › Figure 6 Part 1/Fig 6abcd Cas9 TPGS1-KO acetylated tubulin atubulin/Cas9 R2 9-11-24 LT5.Project Maximum Z_XY1726173075_Z0_T0_C0.tif]

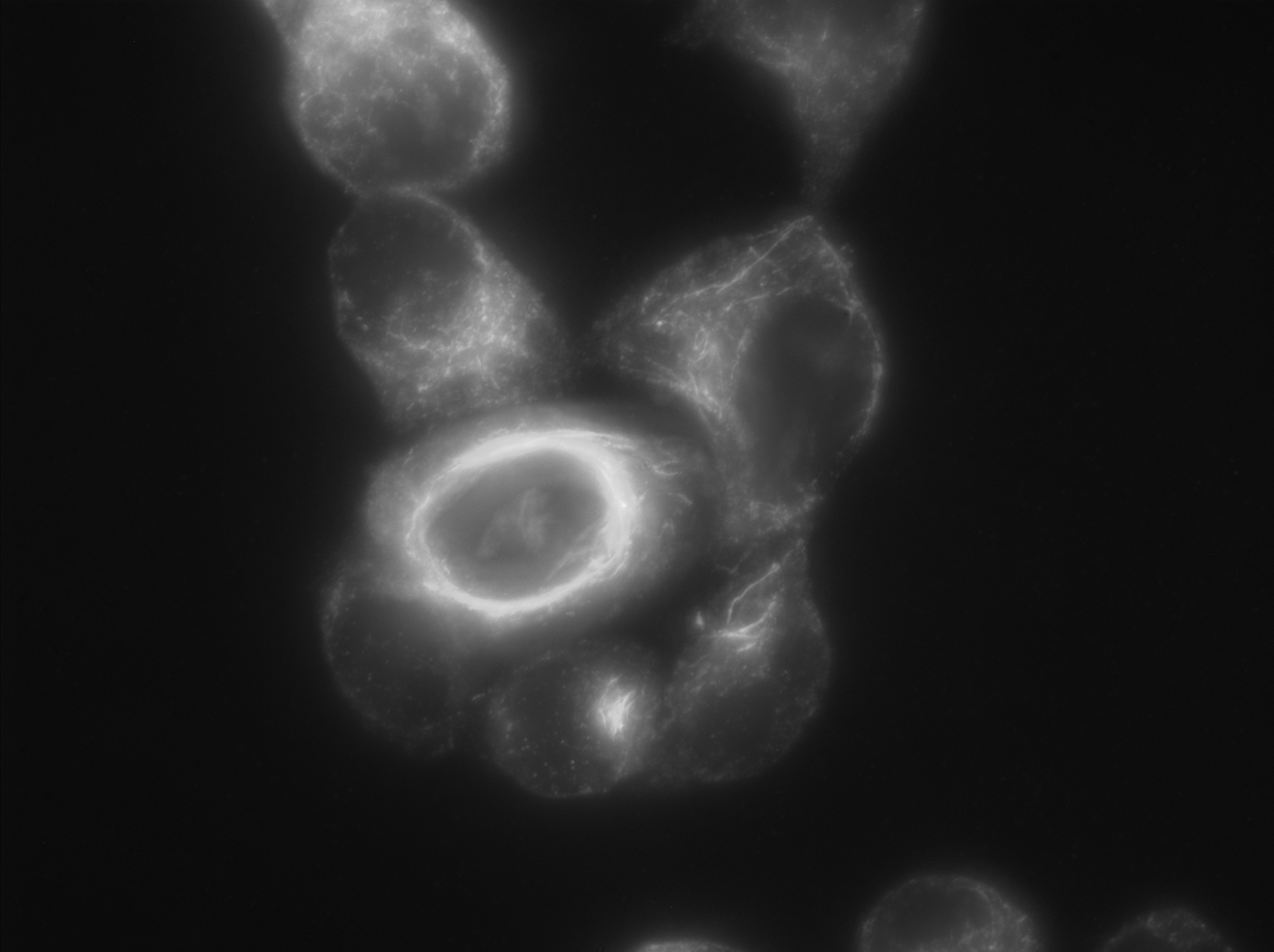

Supplement: Supplementary file 20 — Source data Fig. 6 part 1 [file 44319_2026_742_MOESM20_ESM.zip › Figure 6 Part 1/Fig 6abcd Cas9 TPGS1-KO acetylated tubulin atubulin/Cas9 R2 9-11-24 PA20.Project Maximum Z_XY1726180346_Z0_T0_C2.tif]

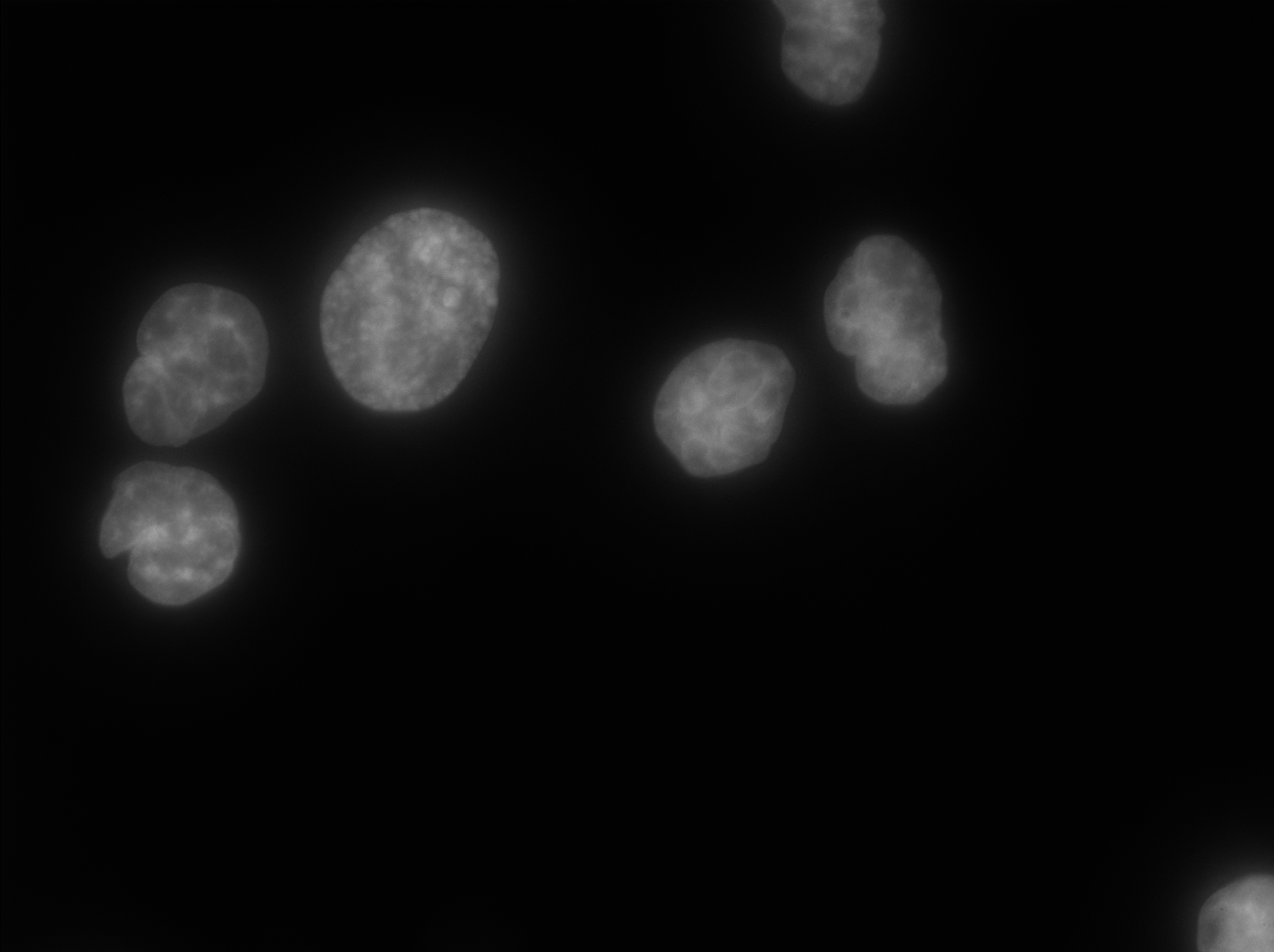

Supplement: Supplementary file 20 — Source data Fig. 6 part 1 [file 44319_2026_742_MOESM20_ESM.zip › Figure 6 Part 1/Fig 6abcd Cas9 TPGS1-KO acetylated tubulin atubulin/Cas9 R2 9-11-24 LT28.Project Maximum Z_XY1726181354_Z0_T0_C0.tif]

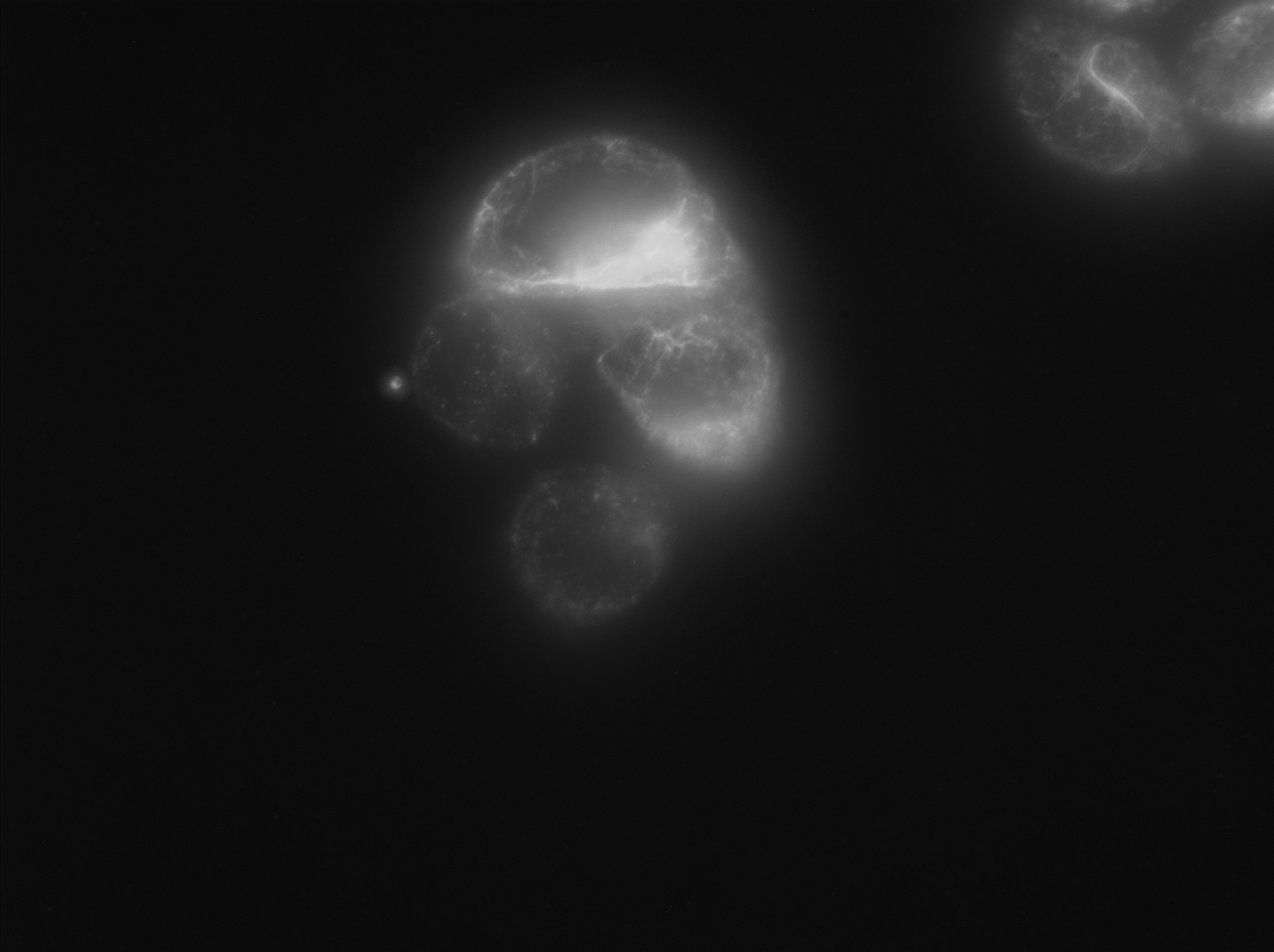

Supplement: Supplementary file 20 — Source data Fig. 6 part 1 [file 44319_2026_742_MOESM20_ESM.zip › Figure 6 Part 1/Fig 6abcd Cas9 TPGS1-KO acetylated tubulin atubulin/Cas9 R2 9-11-24 PA12.Project Maximum Z_XY1726179241_Z0_T0_C2.tif]

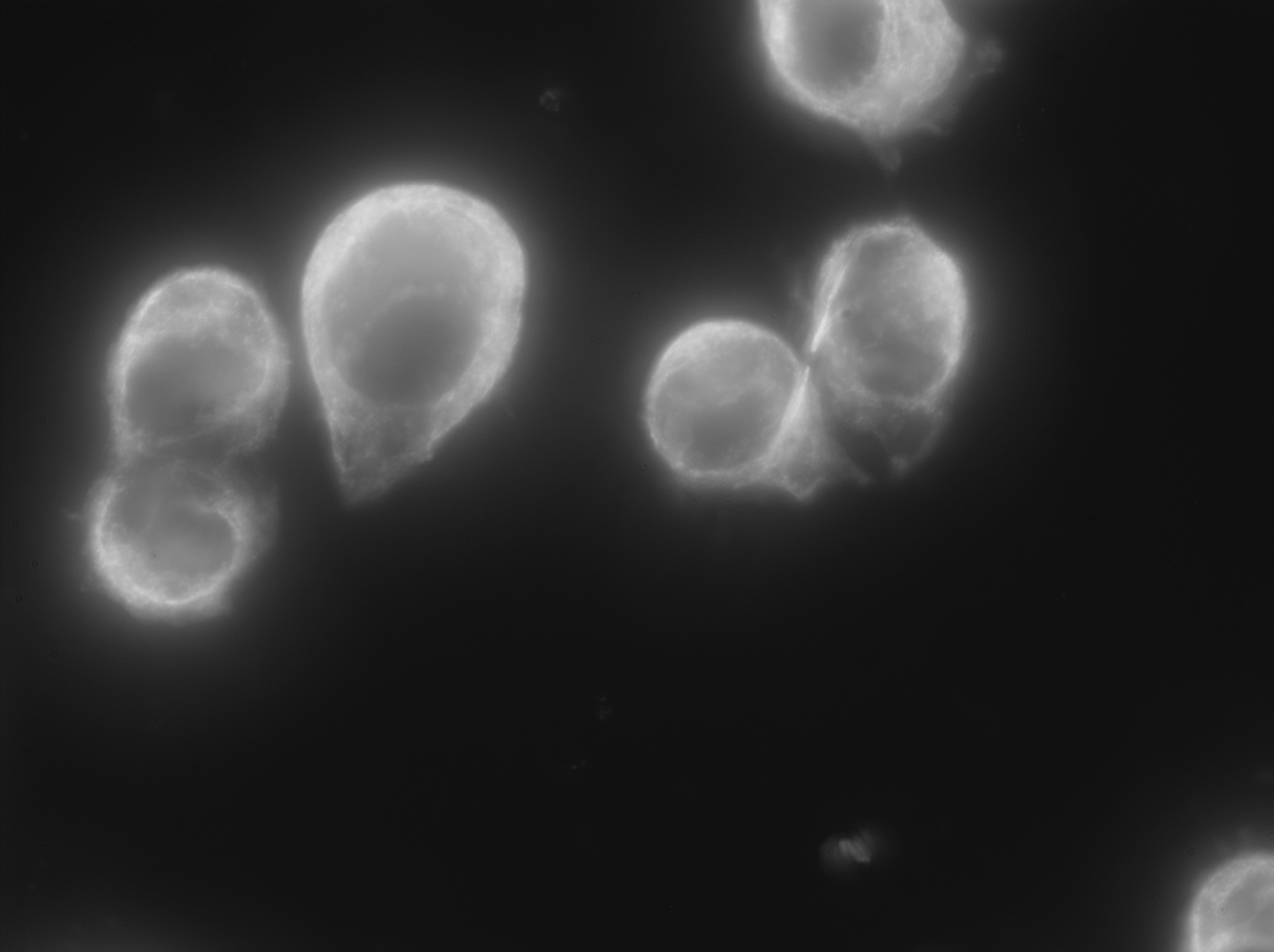

Supplement: Supplementary file 20 — Source data Fig. 6 part 1 [file 44319_2026_742_MOESM20_ESM.zip › Figure 6 Part 1/Fig 6abcd Cas9 TPGS1-KO acetylated tubulin atubulin/Cas9 R2 9-11-24 LT28.Project Maximum Z_XY1726181354_Z0_T0_C1.tif]

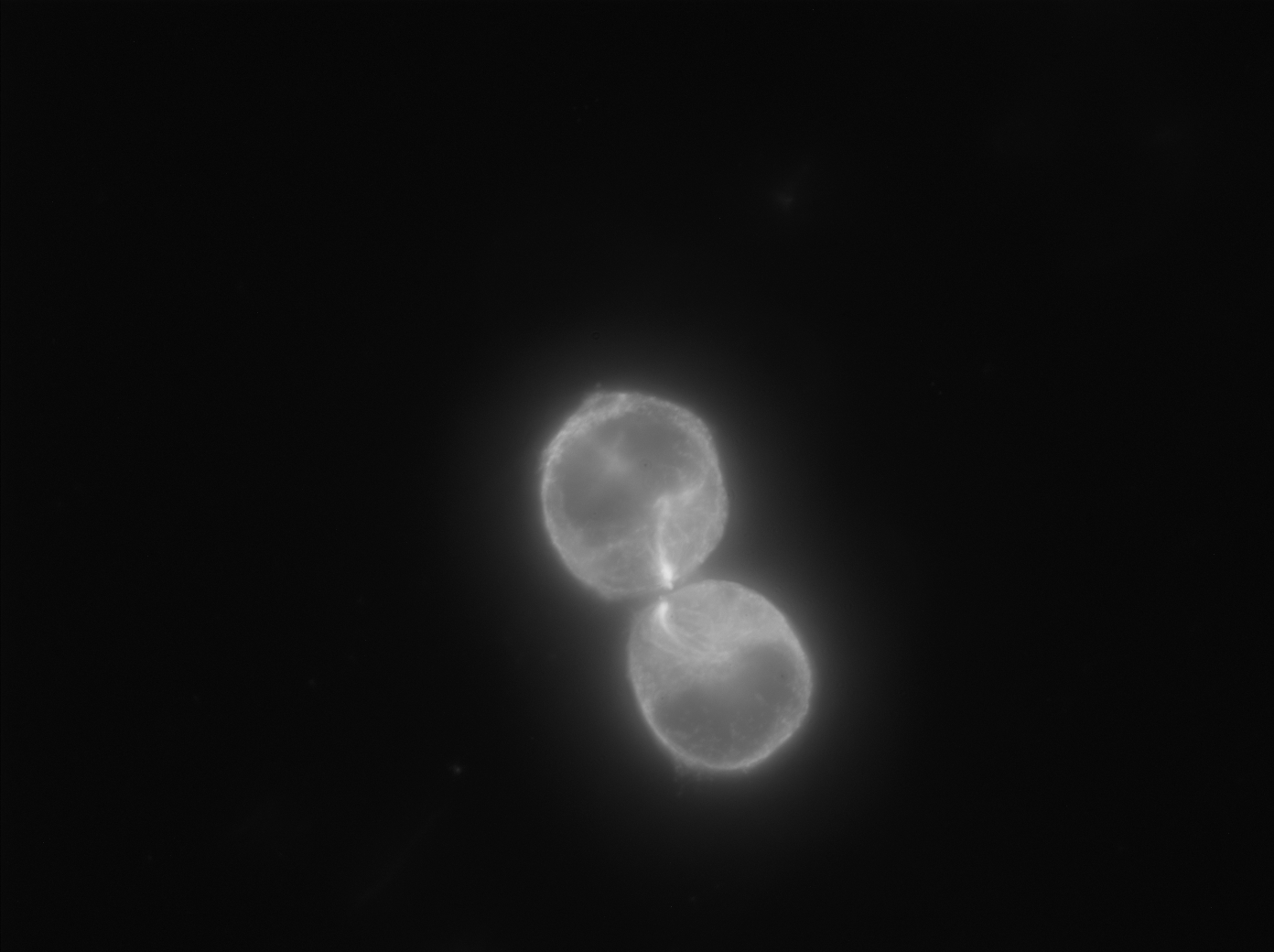

Supplement: Supplementary file 20 — Source data Fig. 6 part 1 [file 44319_2026_742_MOESM20_ESM.zip › Figure 6 Part 1/Fig 6abcd Cas9 TPGS1-KO acetylated tubulin atubulin/Cas9 R2 9-11-24 LT5.Project Maximum Z_XY1726173075_Z0_T0_C1.tif]

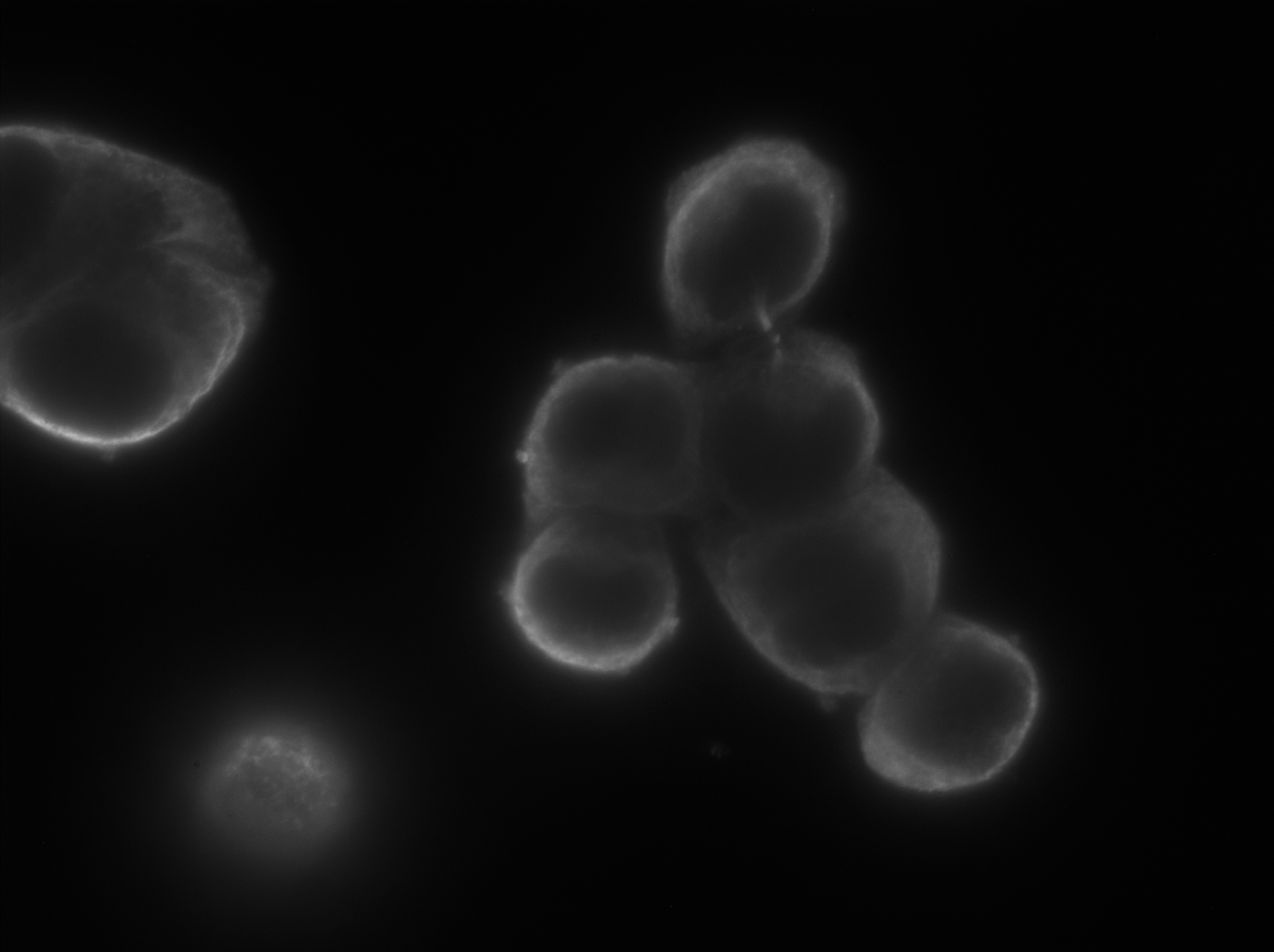

Supplement: Supplementary file 20 — Source data Fig. 6 part 1 [file 44319_2026_742_MOESM20_ESM.zip › Figure 6 Part 1/Fig 6abcd Cas9 TPGS1-KO acetylated tubulin atubulin/Cas9 R3 9-13-24 LT22.Project Maximum Z_XY1726767057_Z0_T0_C1.tif]

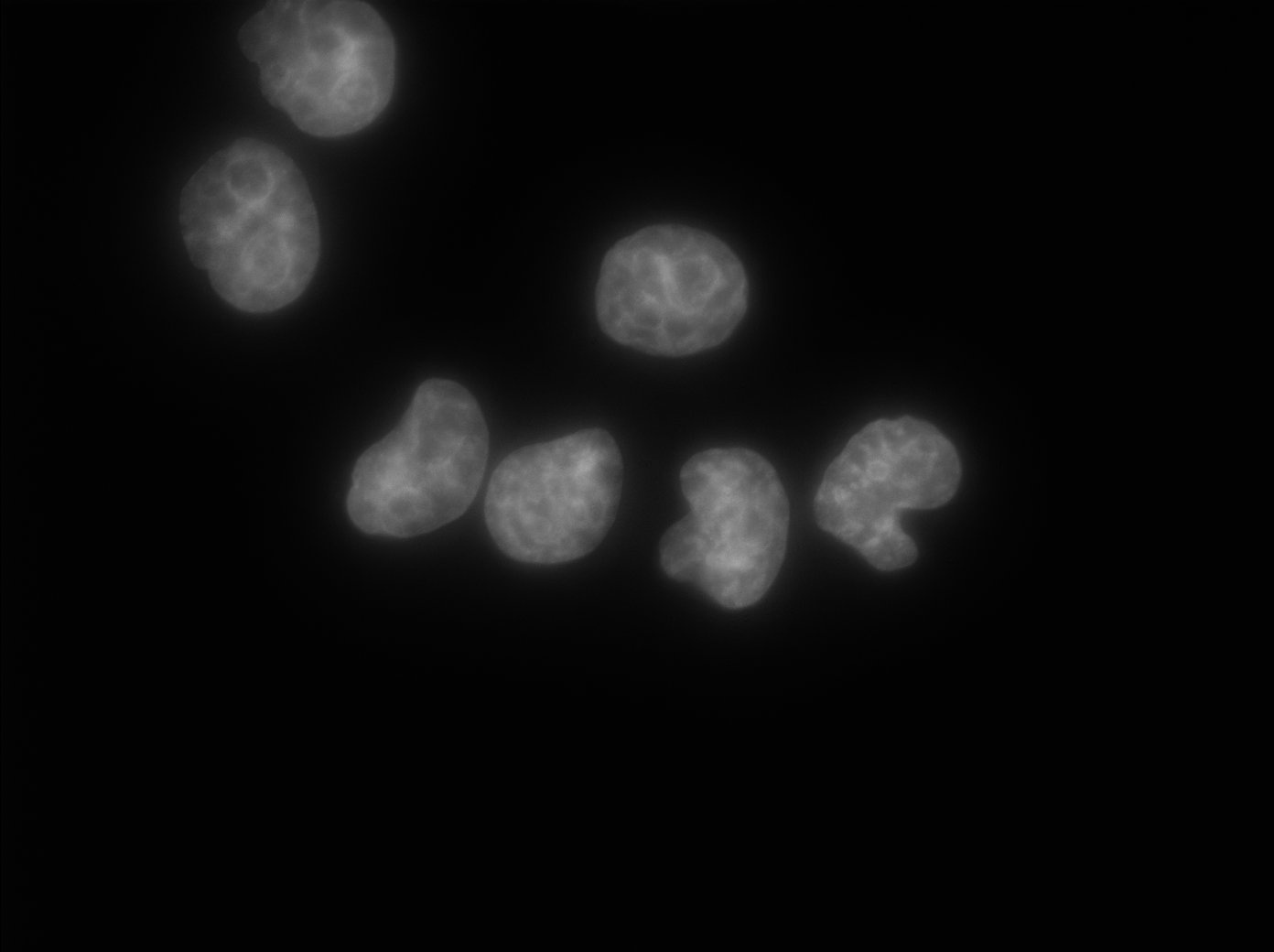

Supplement: Supplementary file 20 — Source data Fig. 6 part 1 [file 44319_2026_742_MOESM20_ESM.zip › Figure 6 Part 1/Fig 6abcd Cas9 TPGS1-KO acetylated tubulin atubulin/Cas9 R2 9-11-24 LT12.Project Maximum Z_XY1726174218_Z0_T0_C0.tif]

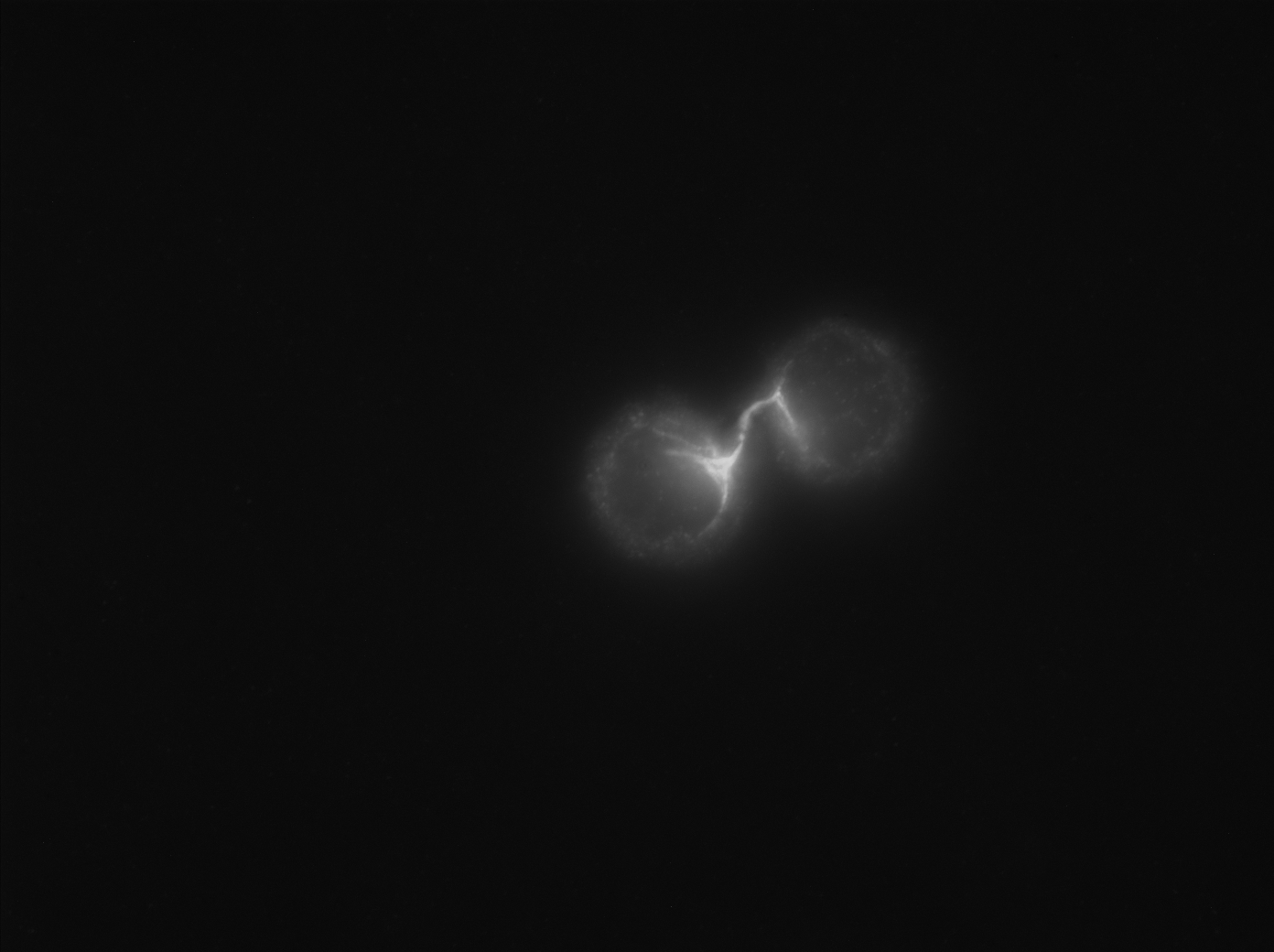

Supplement: Supplementary file 20 — Source data Fig. 6 part 1 [file 44319_2026_742_MOESM20_ESM.zip › Figure 6 Part 1/Fig 6abcd Cas9 TPGS1-KO acetylated tubulin atubulin/Cas9 R2 9-11-24 LT22.Project Maximum Z_XY1726179312_Z0_T0_C2.tif]

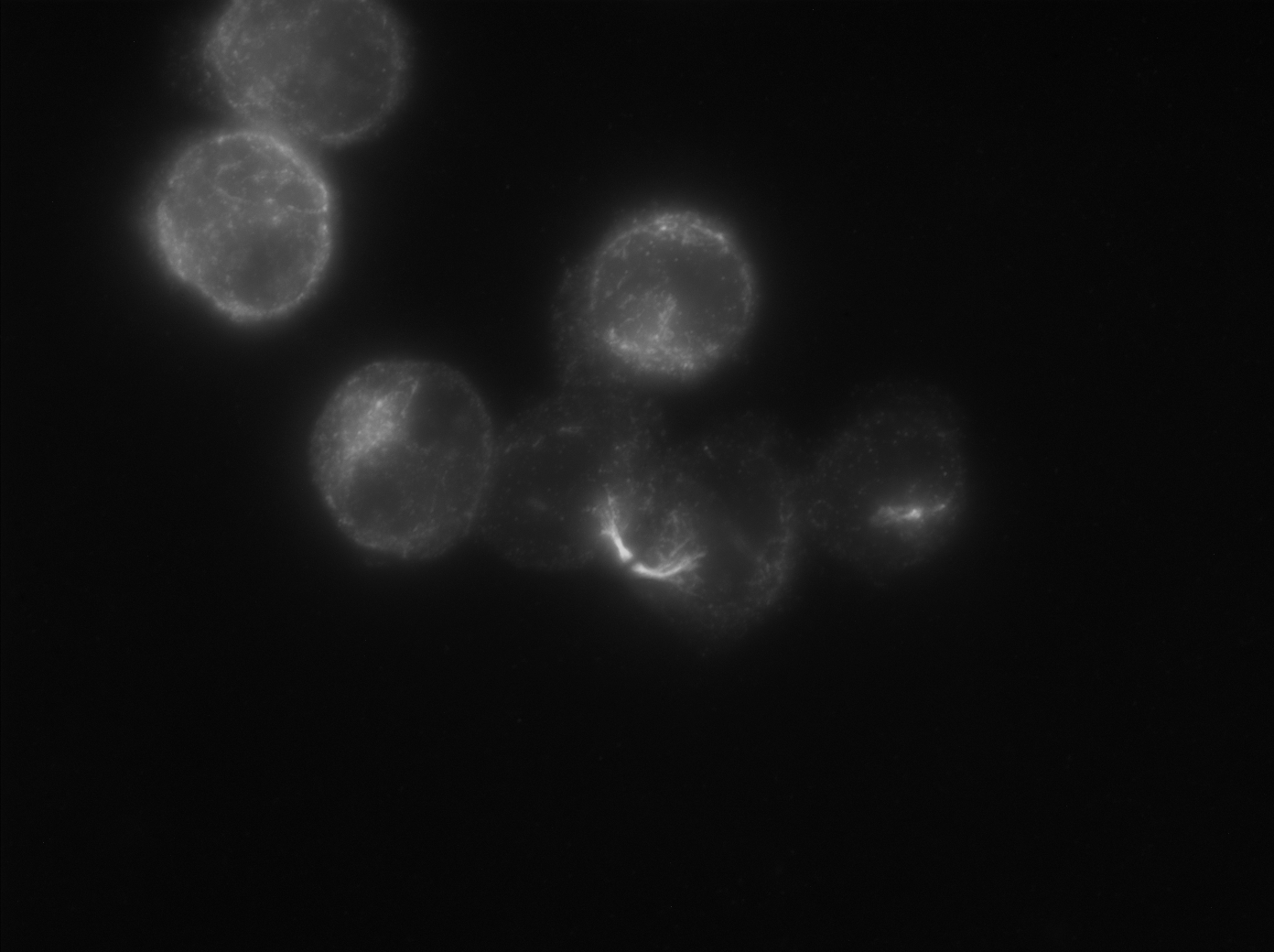

Supplement: Supplementary file 20 — Source data Fig. 6 part 1 [file 44319_2026_742_MOESM20_ESM.zip › Figure 6 Part 1/Fig 6abcd Cas9 TPGS1-KO acetylated tubulin atubulin/Cas9 R2 9-11-24 LT12.Project Maximum Z_XY1726174218_Z0_T0_C2.tif]

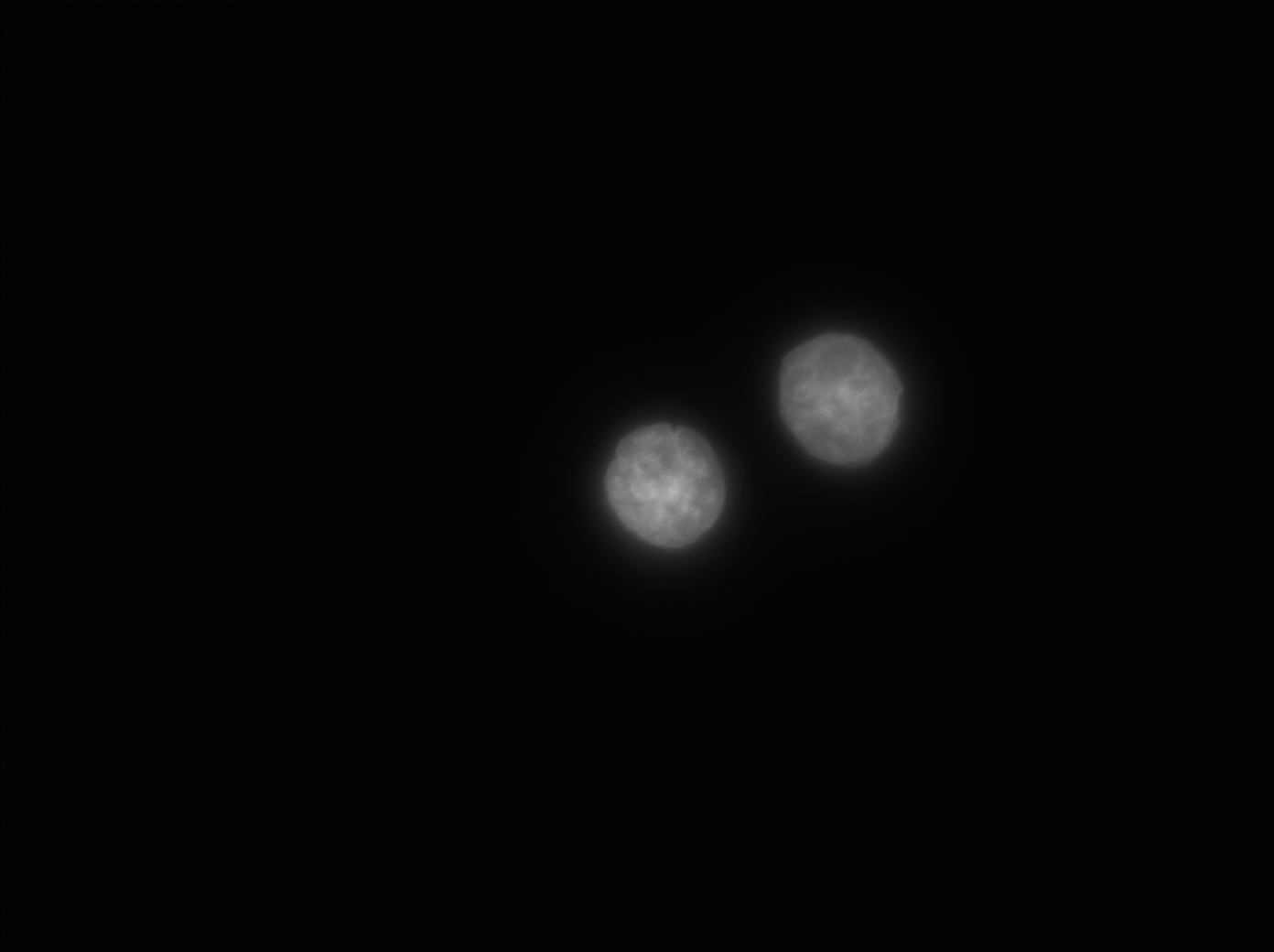

Supplement: Supplementary file 20 — Source data Fig. 6 part 1 [file 44319_2026_742_MOESM20_ESM.zip › Figure 6 Part 1/Fig 6abcd Cas9 TPGS1-KO acetylated tubulin atubulin/Cas9 R2 9-11-24 LT22.Project Maximum Z_XY1726179312_Z0_T0_C0.tif]

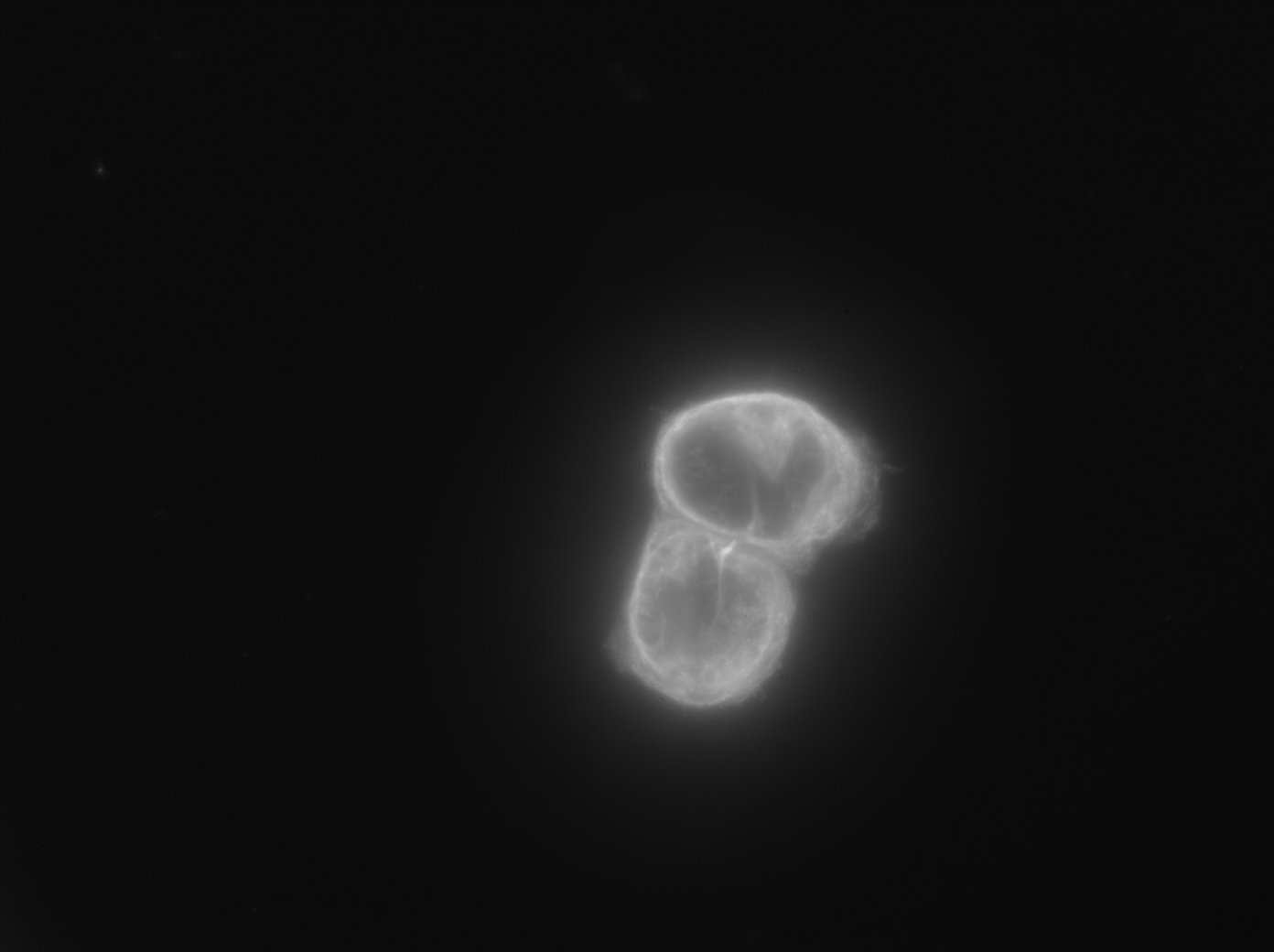

Supplement: Supplementary file 20 — Source data Fig. 6 part 1 [file 44319_2026_742_MOESM20_ESM.zip › Figure 6 Part 1/Fig 6abcd Cas9 TPGS1-KO acetylated tubulin atubulin/Cas9 R2 9-11-24 LT18.Project Maximum Z_XY1726178367_Z0_T0_C1.tif]

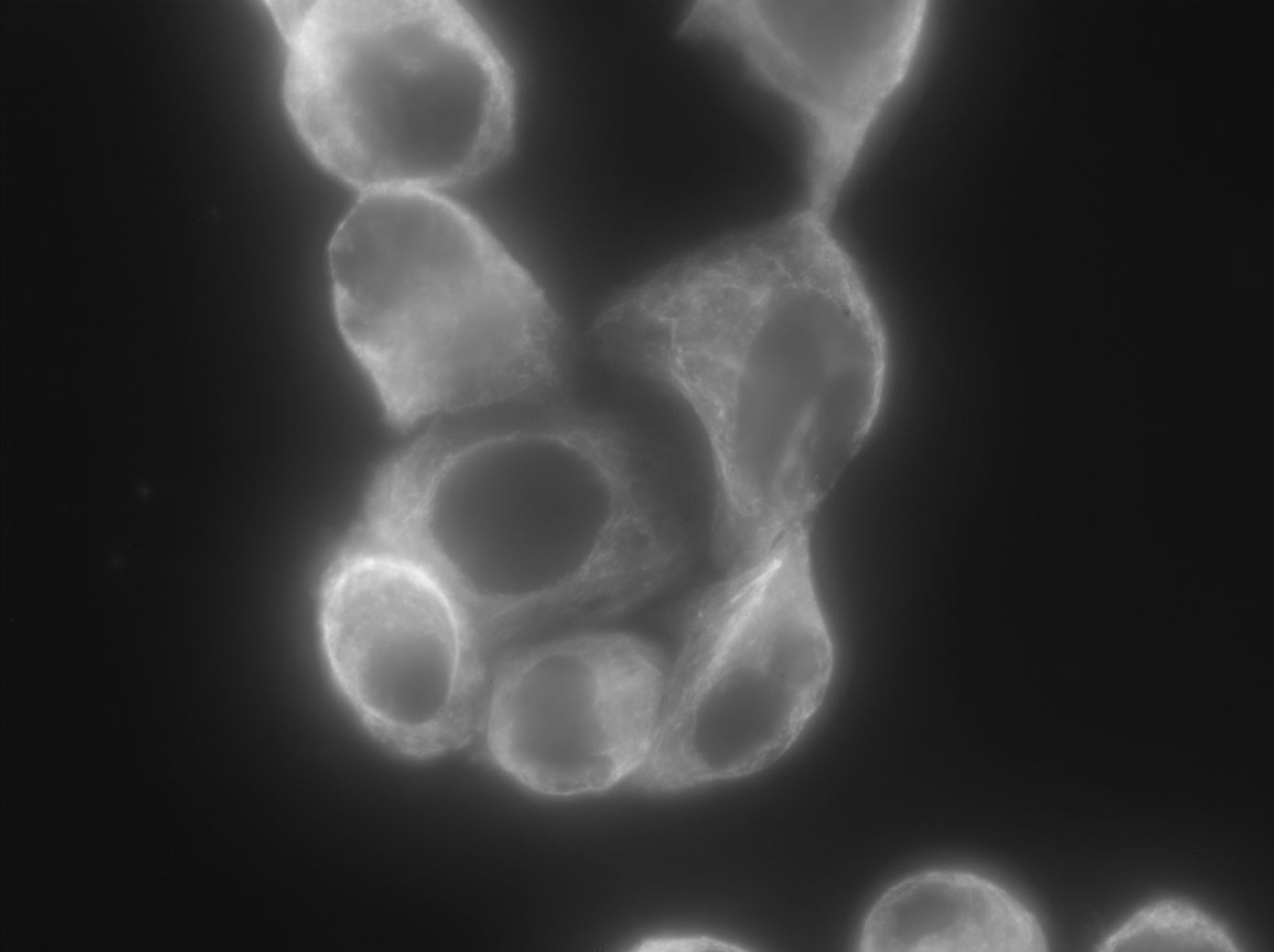

Supplement: Supplementary file 20 — Source data Fig. 6 part 1 [file 44319_2026_742_MOESM20_ESM.zip › Figure 6 Part 1/Fig 6abcd Cas9 TPGS1-KO acetylated tubulin atubulin/Cas9 R2 9-11-24 PA20.Project Maximum Z_XY1726180346_Z0_T0_C1.tif]

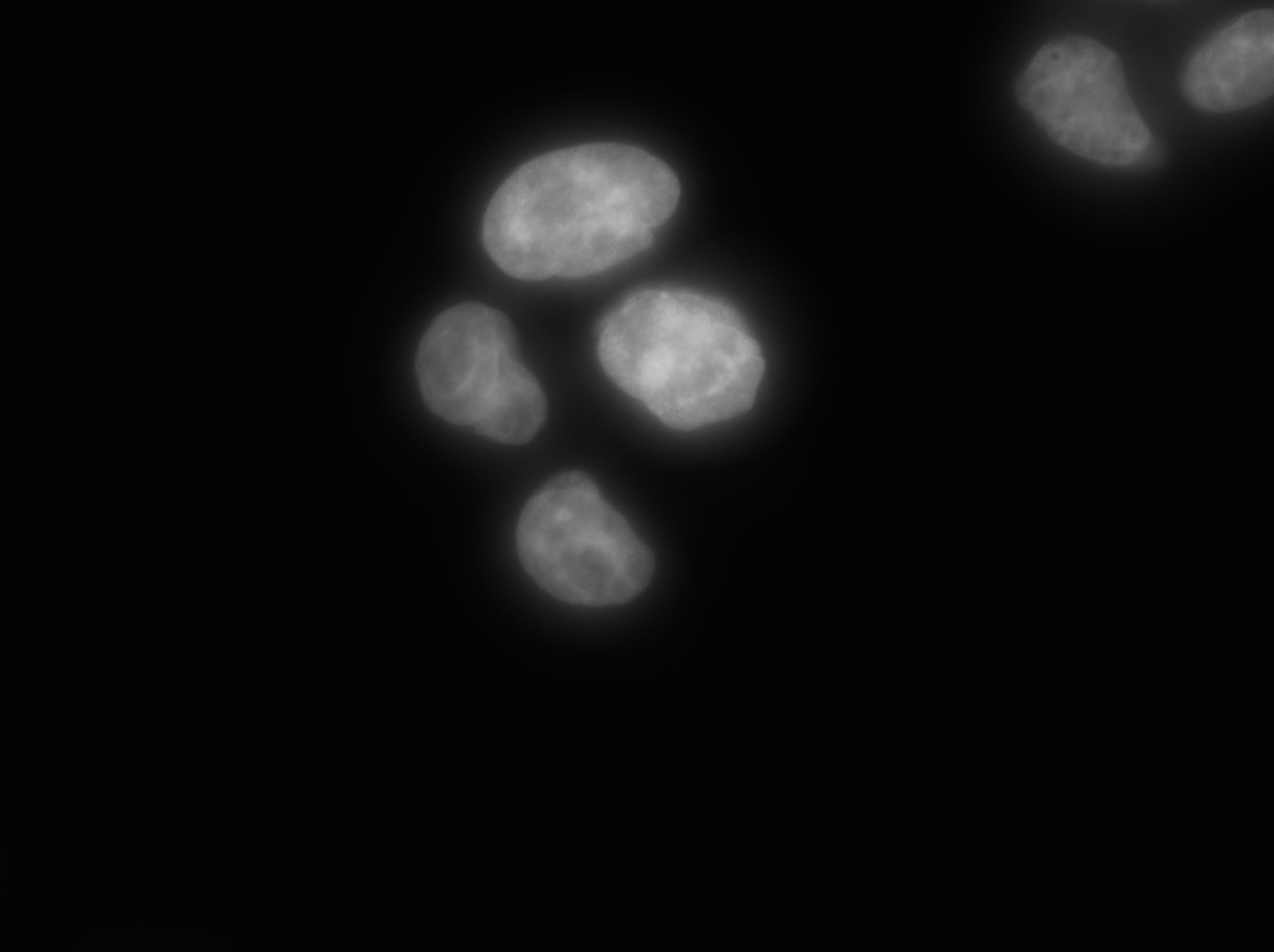

Supplement: Supplementary file 20 — Source data Fig. 6 part 1 [file 44319_2026_742_MOESM20_ESM.zip › Figure 6 Part 1/Fig 6abcd Cas9 TPGS1-KO acetylated tubulin atubulin/Cas9 R2 9-11-24 PA12.Project Maximum Z_XY1726179241_Z0_T0_C0.tif]

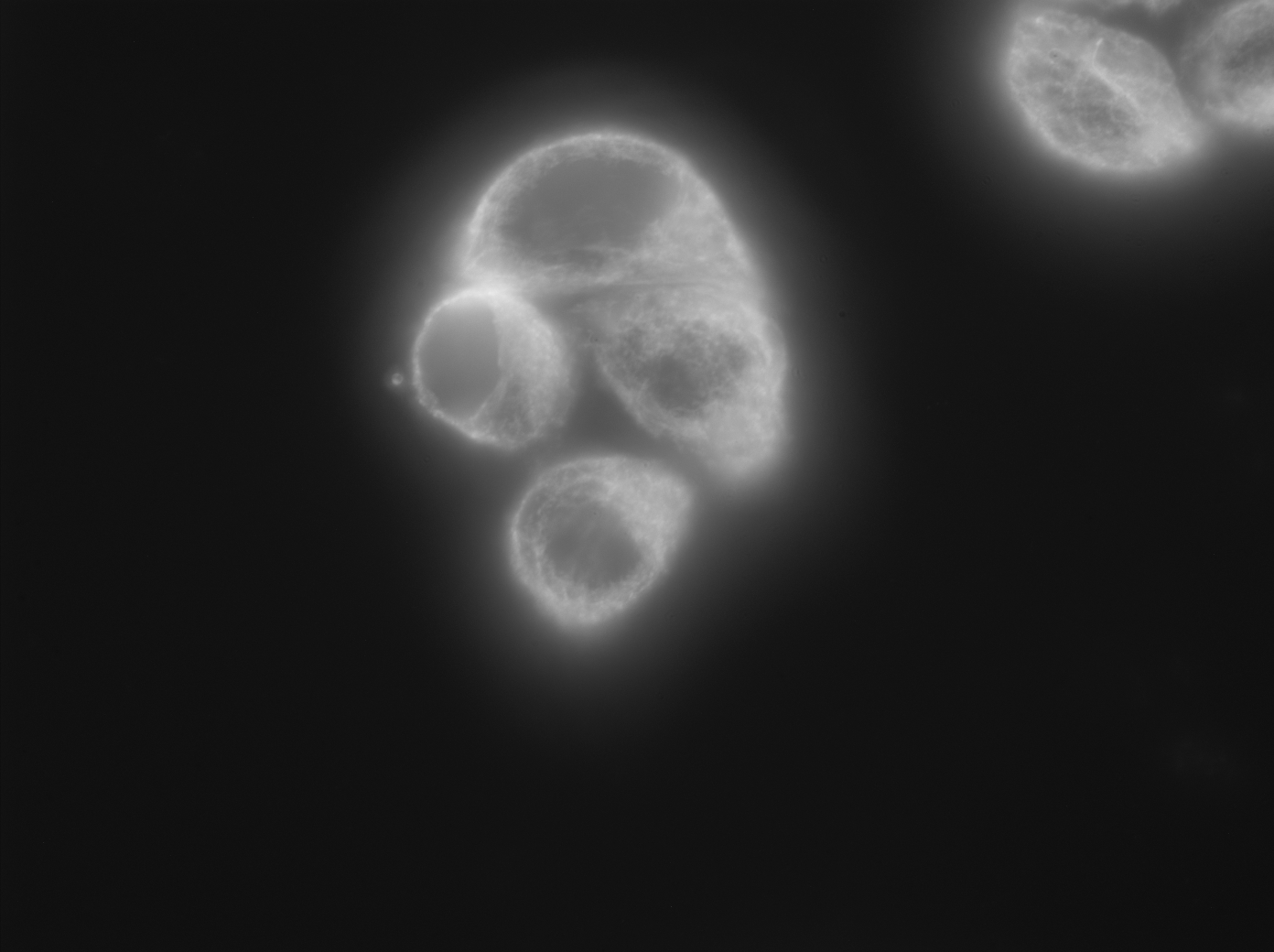

Supplement: Supplementary file 20 — Source data Fig. 6 part 1 [file 44319_2026_742_MOESM20_ESM.zip › Figure 6 Part 1/Fig 6abcd Cas9 TPGS1-KO acetylated tubulin atubulin/Cas9 R2 9-11-24 PA12.Project Maximum Z_XY1726179241_Z0_T0_C1.tif]

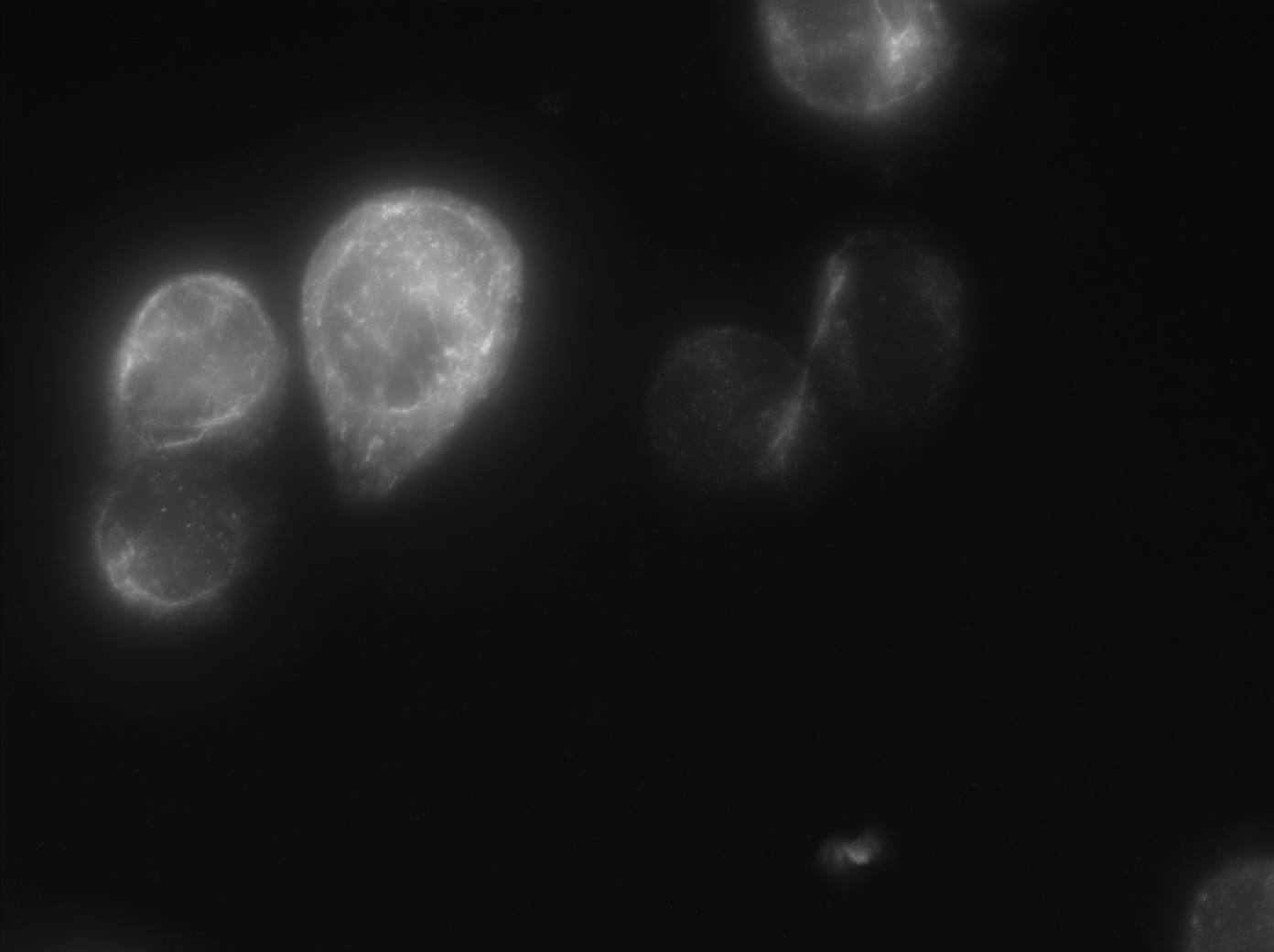

Supplement: Supplementary file 20 — Source data Fig. 6 part 1 [file 44319_2026_742_MOESM20_ESM.zip › Figure 6 Part 1/Fig 6abcd Cas9 TPGS1-KO acetylated tubulin atubulin/Cas9 R2 9-11-24 LT28.Project Maximum Z_XY1726181354_Z0_T0_C2.tif]

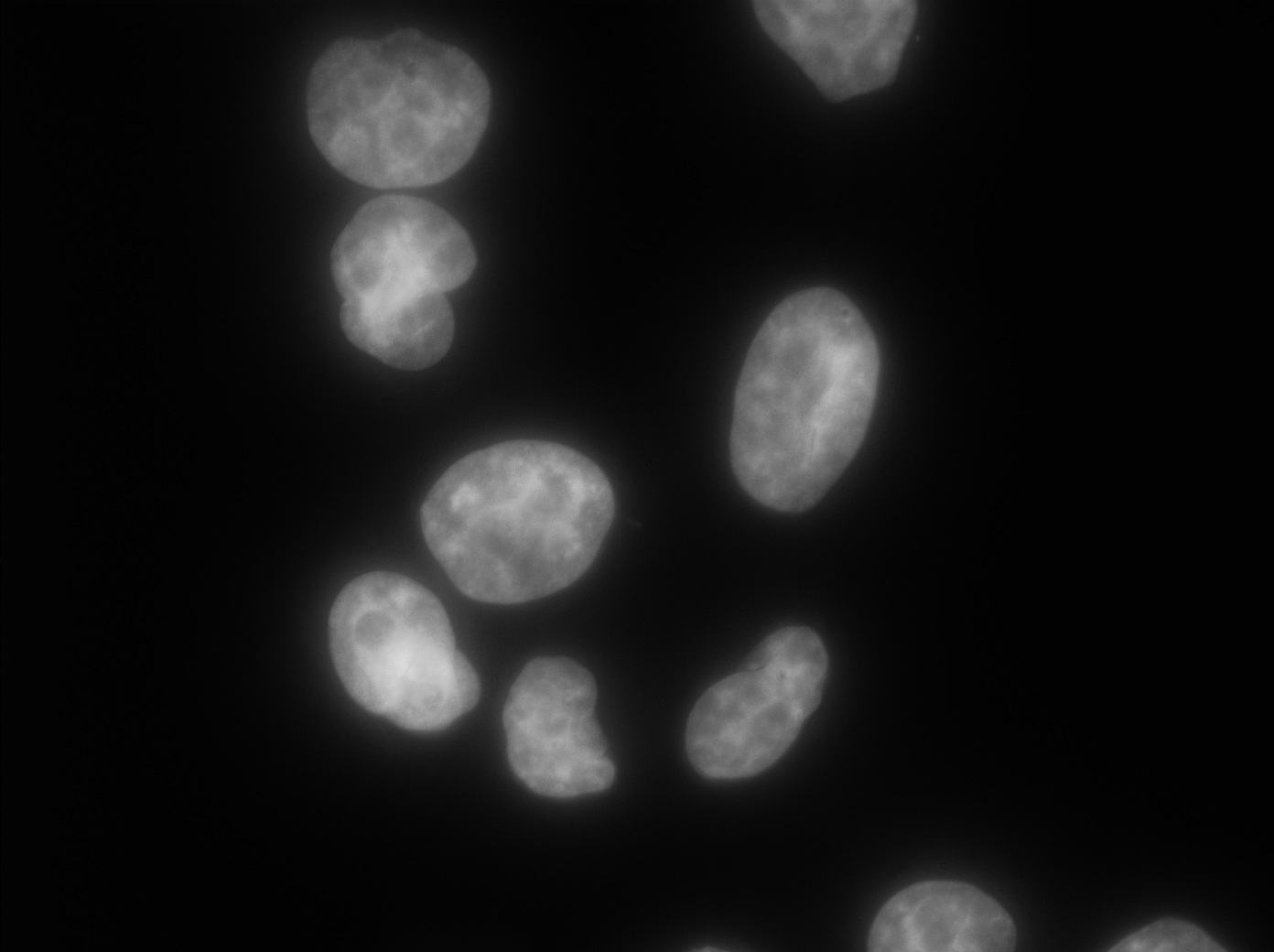

Supplement: Supplementary file 20 — Source data Fig. 6 part 1 [file 44319_2026_742_MOESM20_ESM.zip › Figure 6 Part 1/Fig 6abcd Cas9 TPGS1-KO acetylated tubulin atubulin/Cas9 R2 9-11-24 PA20.Project Maximum Z_XY1726180346_Z0_T0_C0.tif]

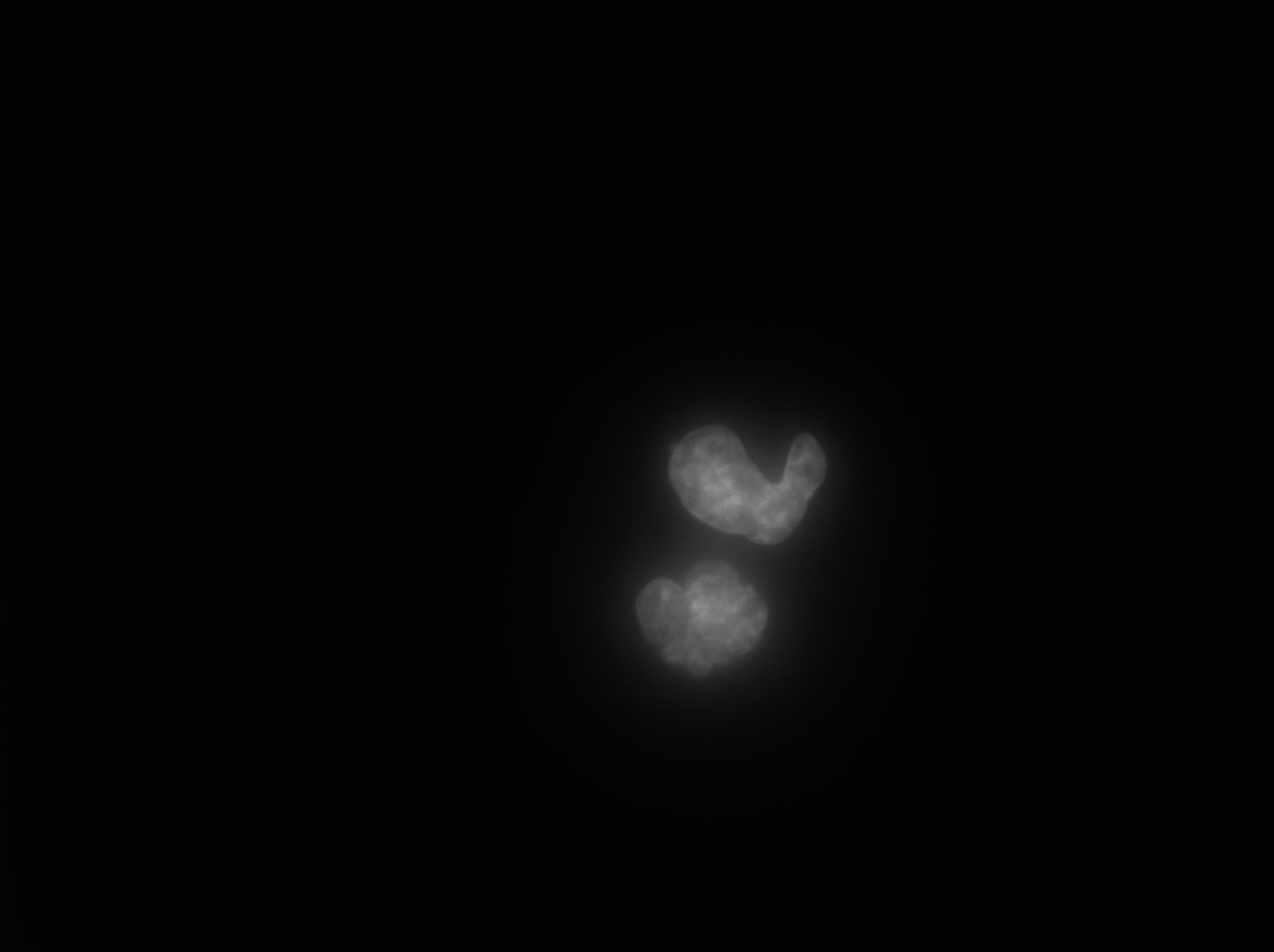

Supplement: Supplementary file 20 — Source data Fig. 6 part 1 [file 44319_2026_742_MOESM20_ESM.zip › Figure 6 Part 1/Fig 6abcd Cas9 TPGS1-KO acetylated tubulin atubulin/Cas9 R2 9-11-24 LT18.Project Maximum Z_XY1726178367_Z0_T0_C0.tif]

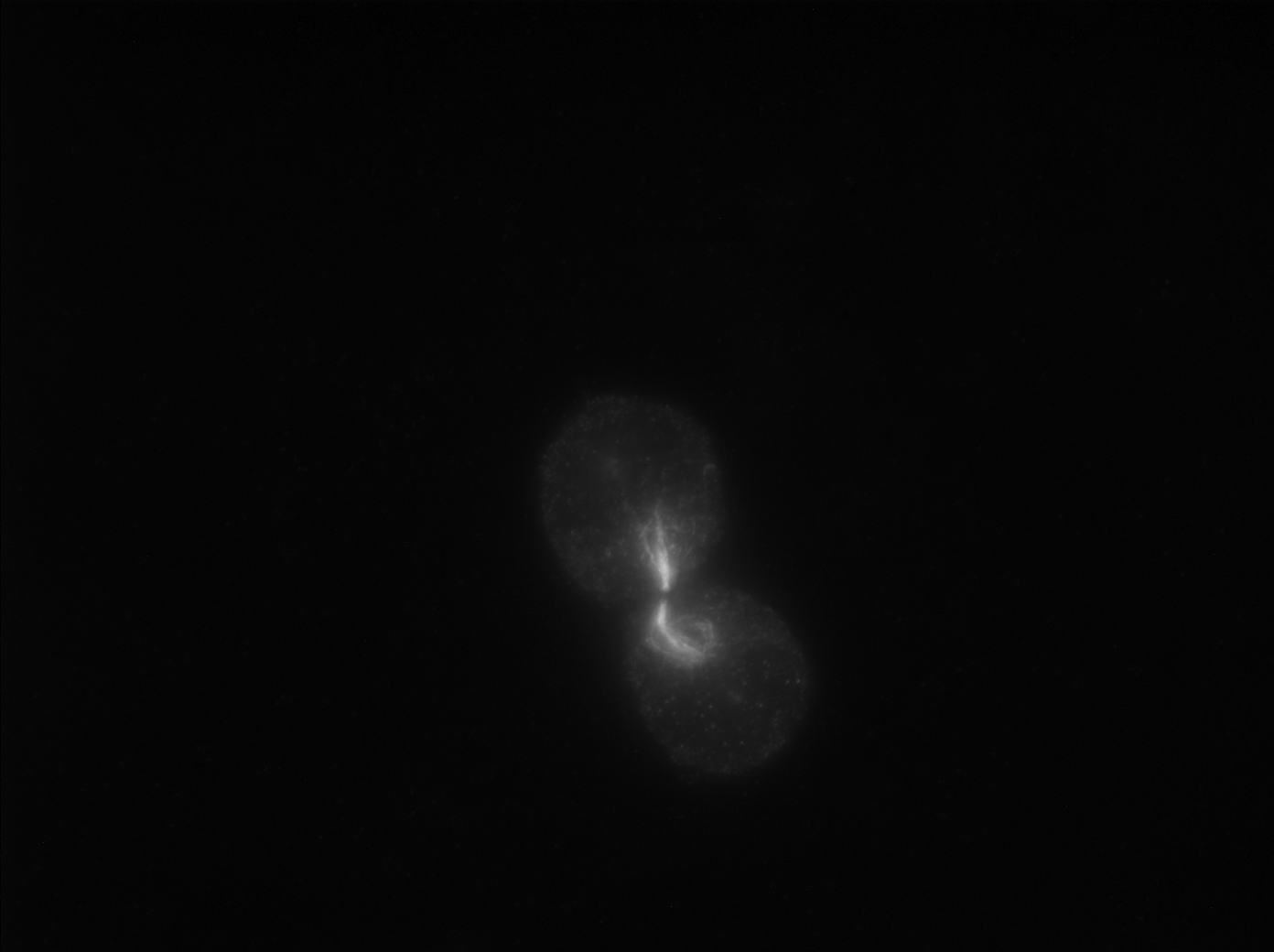

Supplement: Supplementary file 20 — Source data Fig. 6 part 1 [file 44319_2026_742_MOESM20_ESM.zip › Figure 6 Part 1/Fig 6abcd Cas9 TPGS1-KO acetylated tubulin atubulin/Cas9 R2 9-11-24 LT5.Project Maximum Z_XY1726173075_Z0_T0_C2.tif]

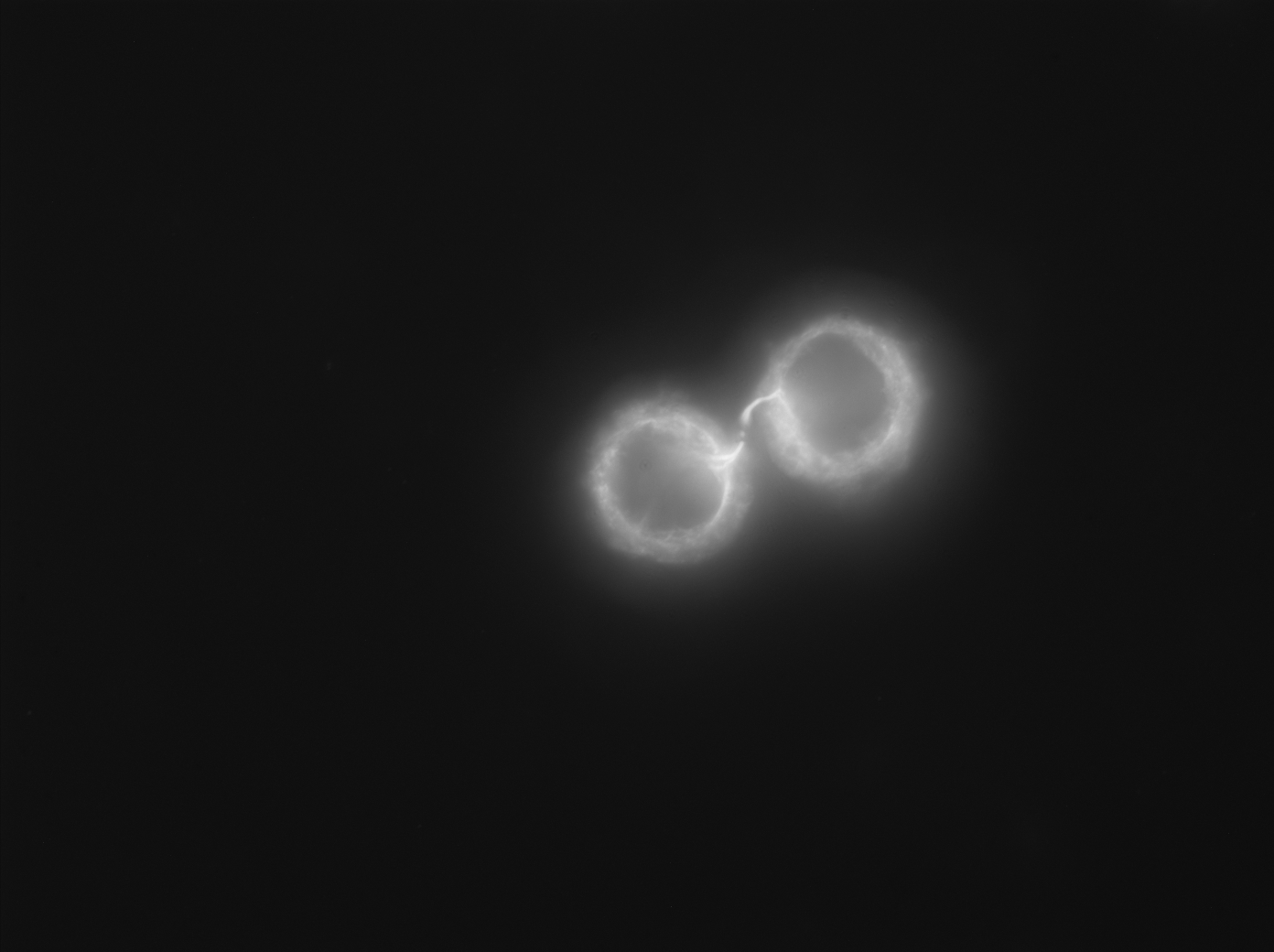

Supplement: Supplementary file 20 — Source data Fig. 6 part 1 [file 44319_2026_742_MOESM20_ESM.zip › Figure 6 Part 1/Fig 6abcd Cas9 TPGS1-KO acetylated tubulin atubulin/Cas9 R2 9-11-24 LT22.Project Maximum Z_XY1726179312_Z0_T0_C1.tif]

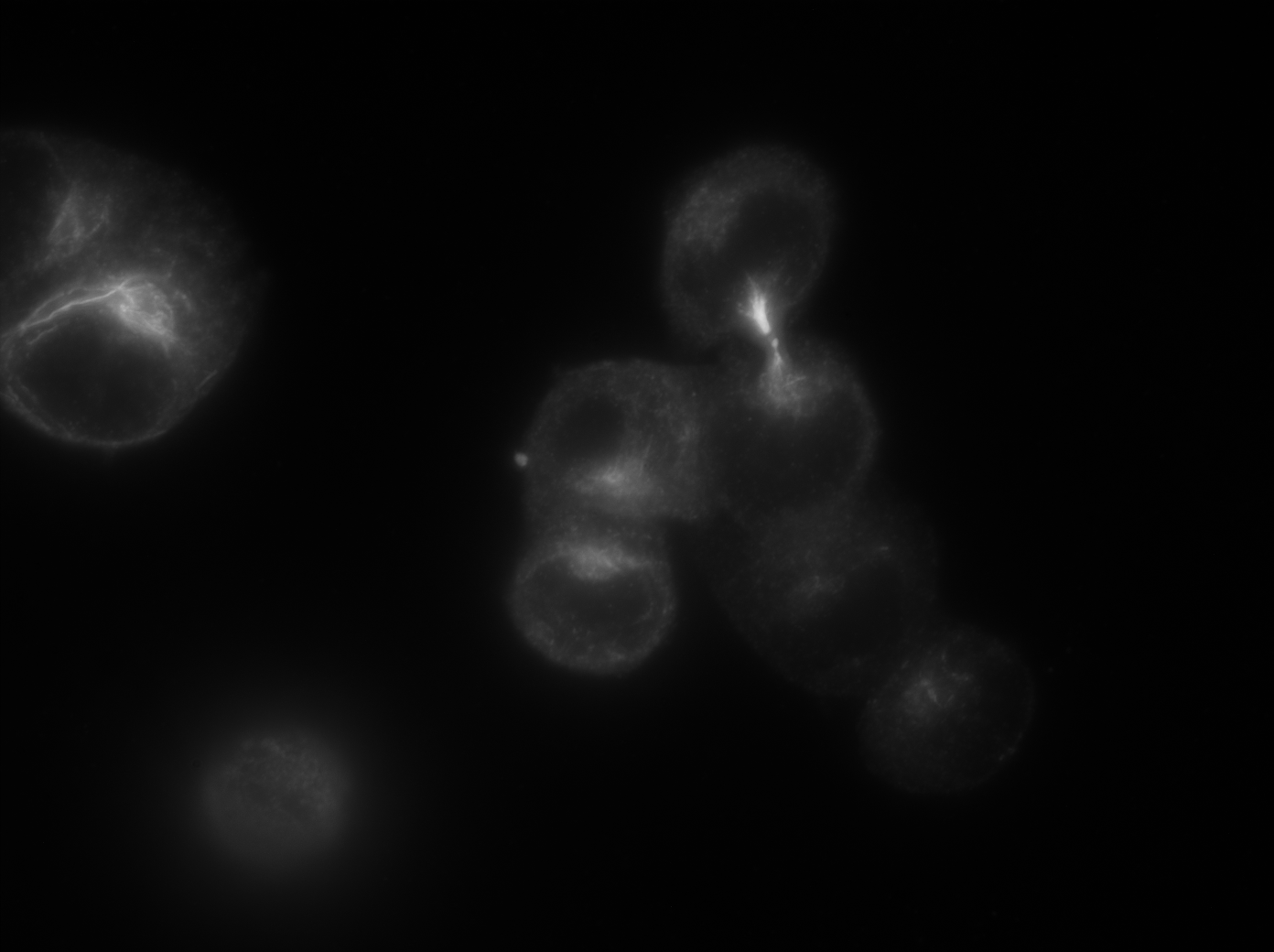

Supplement: Supplementary file 20 — Source data Fig. 6 part 1 [file 44319_2026_742_MOESM20_ESM.zip › Figure 6 Part 1/Fig 6abcd Cas9 TPGS1-KO acetylated tubulin atubulin/Cas9 R3 9-13-24 LT22.Project Maximum Z_XY1726767057_Z0_T0_C2.tif]

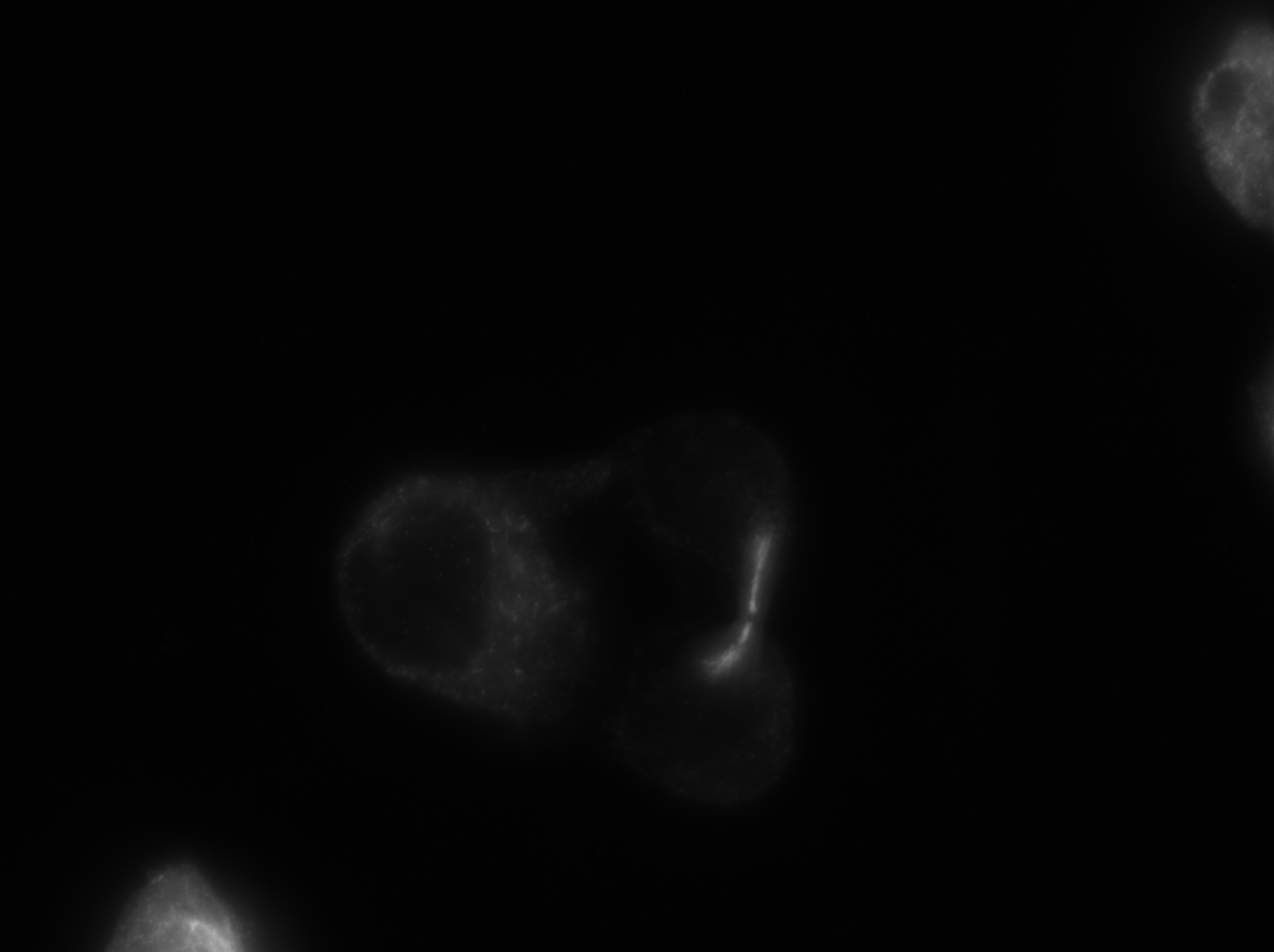

Supplement: Supplementary file 20 — Source data Fig. 6 part 1 [file 44319_2026_742_MOESM20_ESM.zip › Figure 6 Part 1/Fig 6abcd Cas9 TPGS1-KO acetylated tubulin atubulin/Cas9 R3 9-13-24 LT24.Project Maximum Z_XY1726767260_Z0_T0_C2.tif]

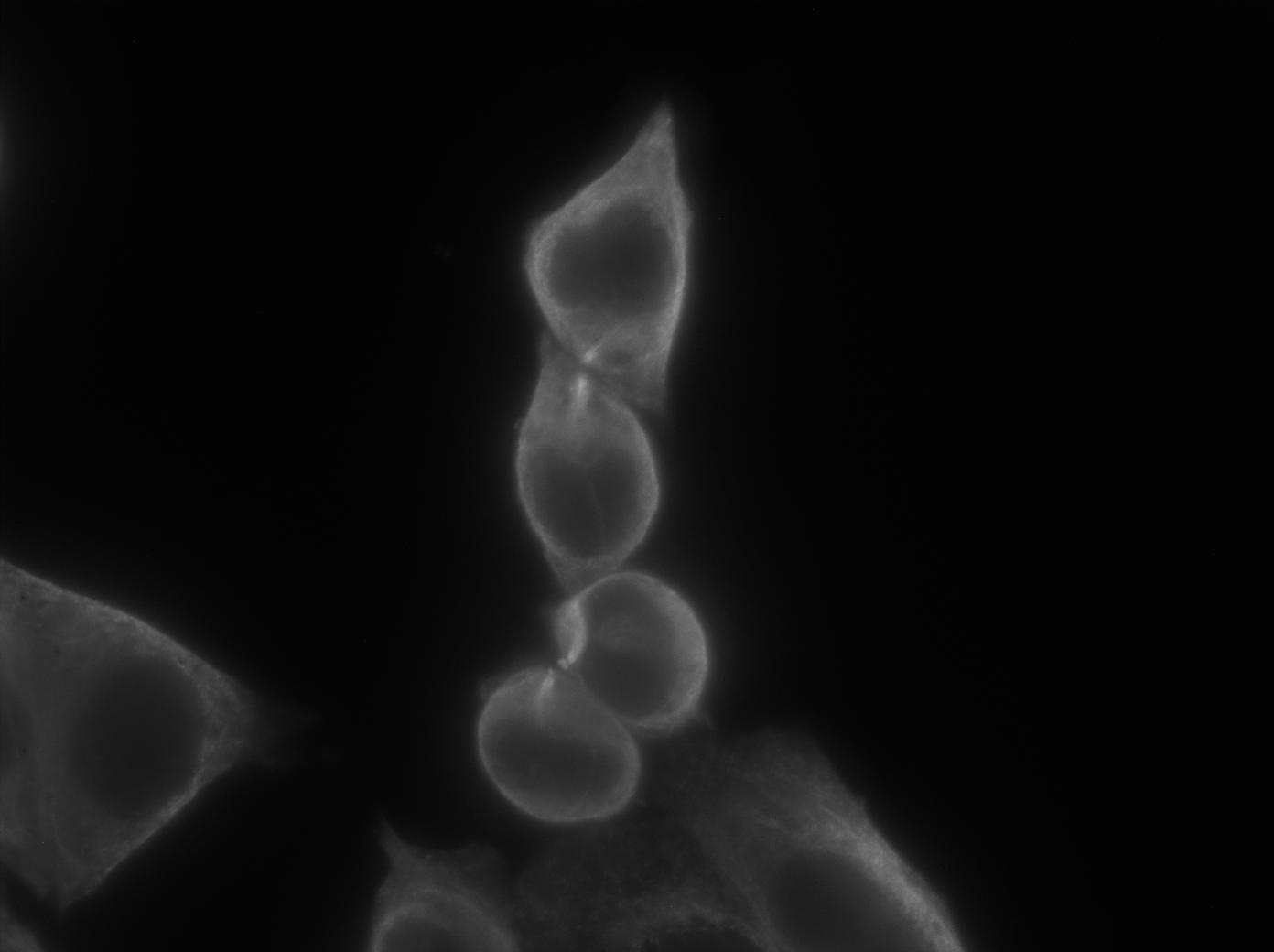

Supplement: Supplementary file 20 — Source data Fig. 6 part 1 [file 44319_2026_742_MOESM20_ESM.zip › Figure 6 Part 1/Fig 6abcd Cas9 TPGS1-KO acetylated tubulin atubulin/Cas9 R3 9-13-24 LT29LT30.Project Maximum Z_XY1726767787_Z0_T0_C1.tif]

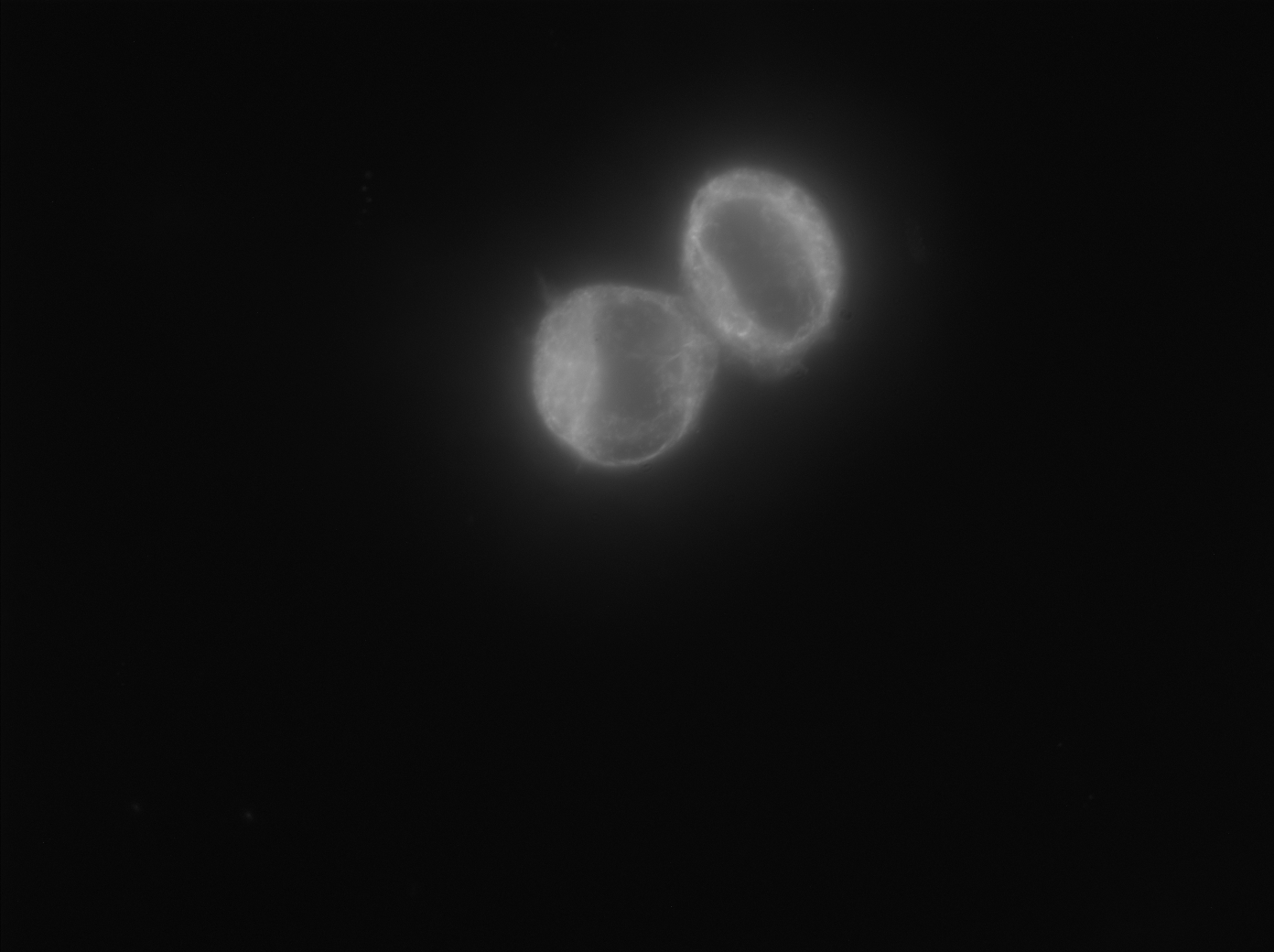

Supplement: Supplementary file 20 — Source data Fig. 6 part 1 [file 44319_2026_742_MOESM20_ESM.zip › Figure 6 Part 1/Fig 6abcd Cas9 TPGS1-KO acetylated tubulin atubulin/Cas9 R2 9-11-24 PA1.Project Maximum Z_XY1726172379_Z0_T0_C1.tif]

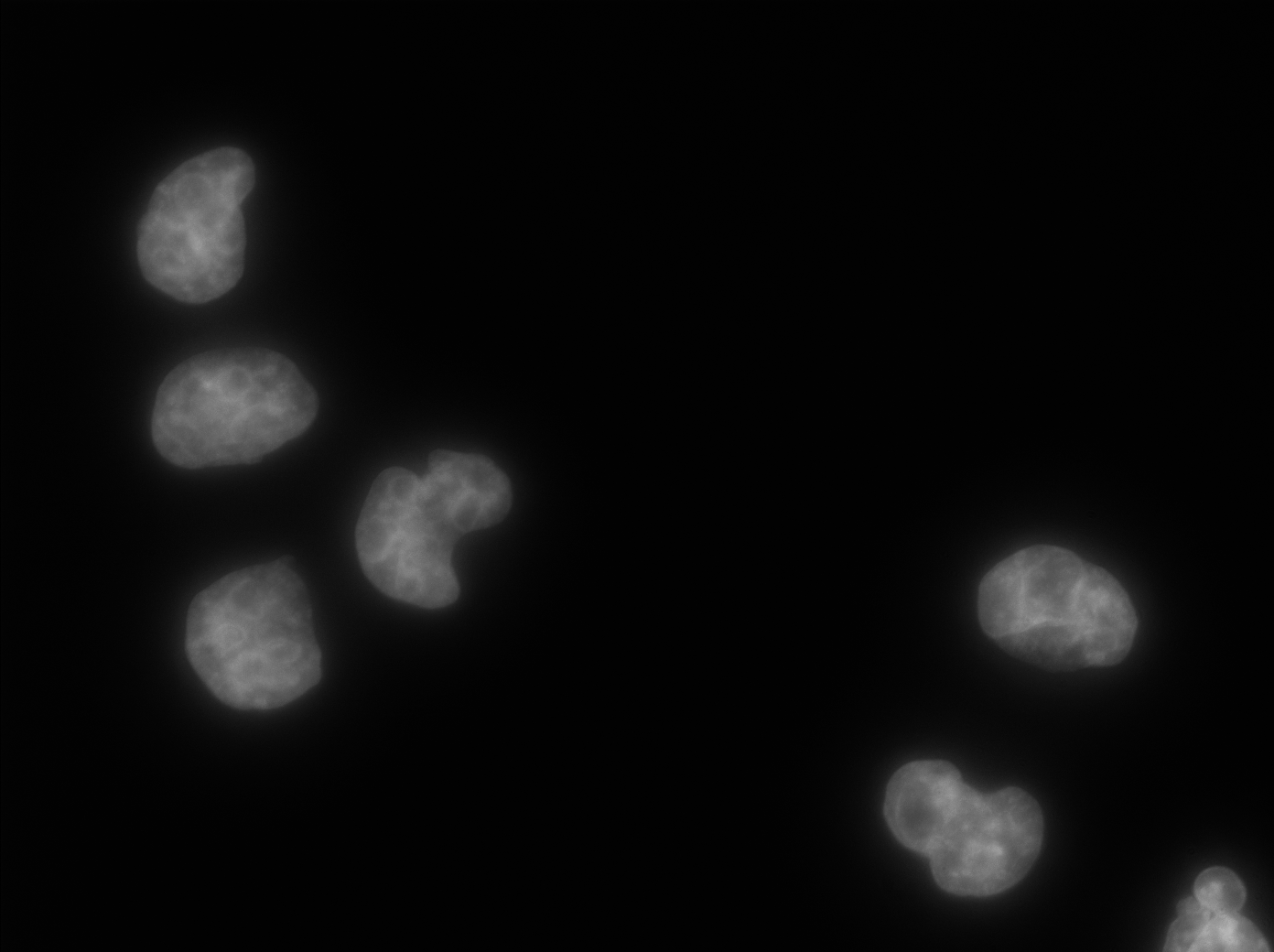

Supplement: Supplementary file 20 — Source data Fig. 6 part 1 [file 44319_2026_742_MOESM20_ESM.zip › Figure 6 Part 1/Fig 6abcd Cas9 TPGS1-KO acetylated tubulin atubulin/Cas9 R2 9-11-24 PA3PA4.Project Maximum Z_XY1726172775_Z0_T0_C0.tif]

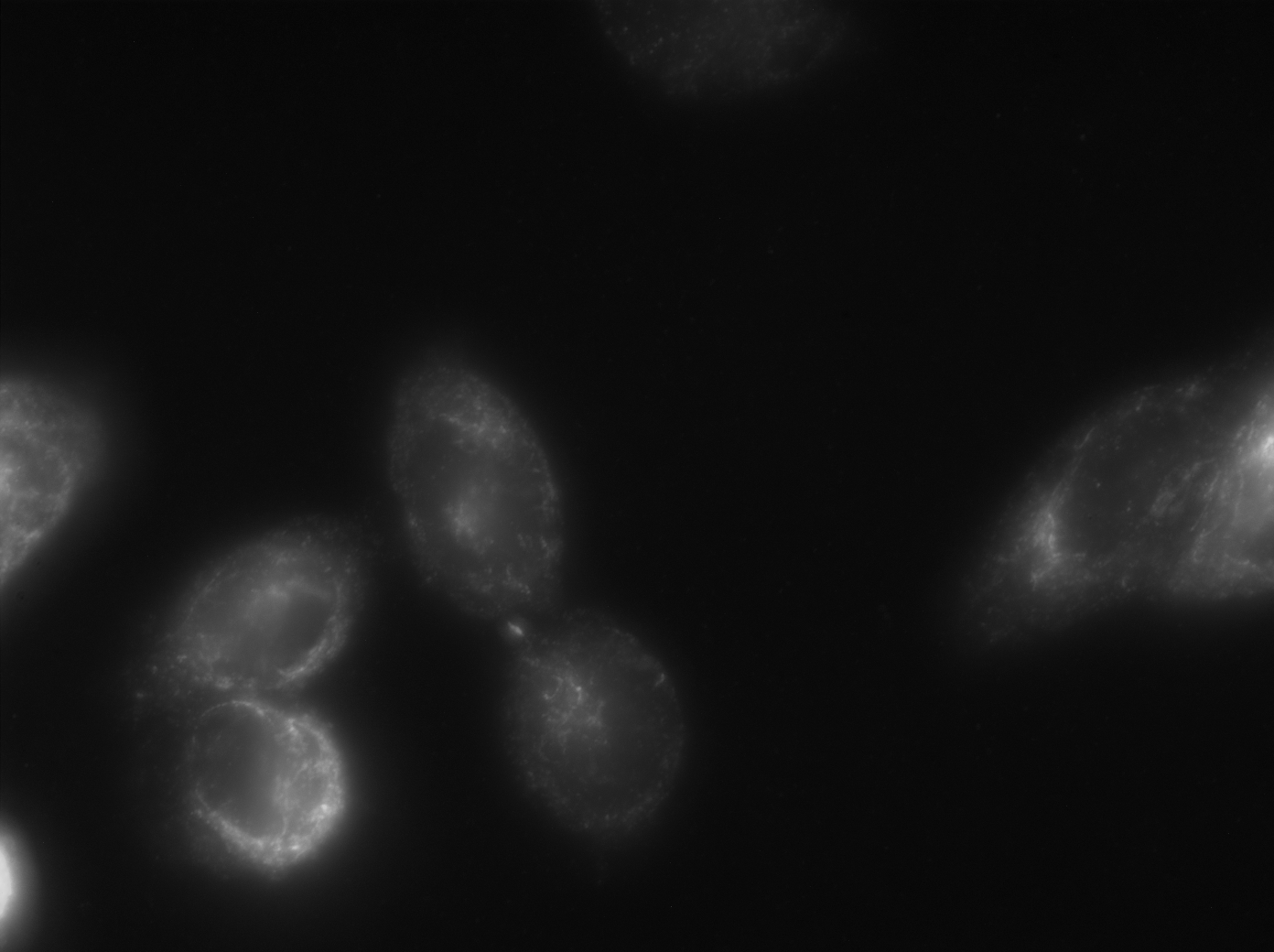

Supplement: Supplementary file 20 — Source data Fig. 6 part 1 [file 44319_2026_742_MOESM20_ESM.zip › Figure 6 Part 1/Fig 6abcd Cas9 TPGS1-KO acetylated tubulin atubulin/Cas9 R2 9-11-24 PA28.Project Maximum Z_XY1726181804_Z0_T0_C2.tif]

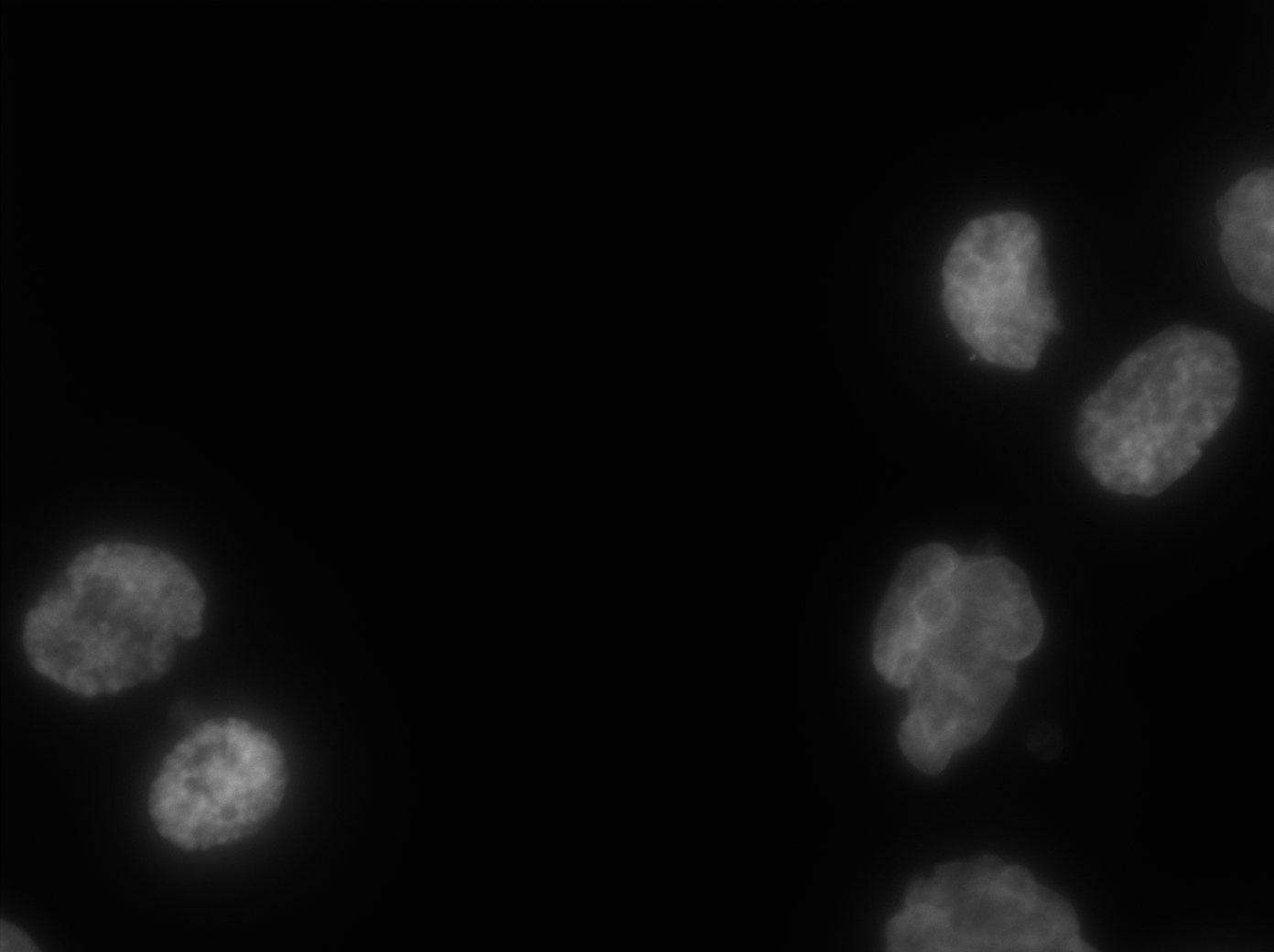

Supplement: Supplementary file 20 — Source data Fig. 6 part 1 [file 44319_2026_742_MOESM20_ESM.zip › Figure 6 Part 1/Fig 6abcd Cas9 TPGS1-KO acetylated tubulin atubulin/Cas9 R2 9-11-24 LT25 PA22PA23.Project Maximum Z_XY1726180658_Z0_T0_C0.tif]

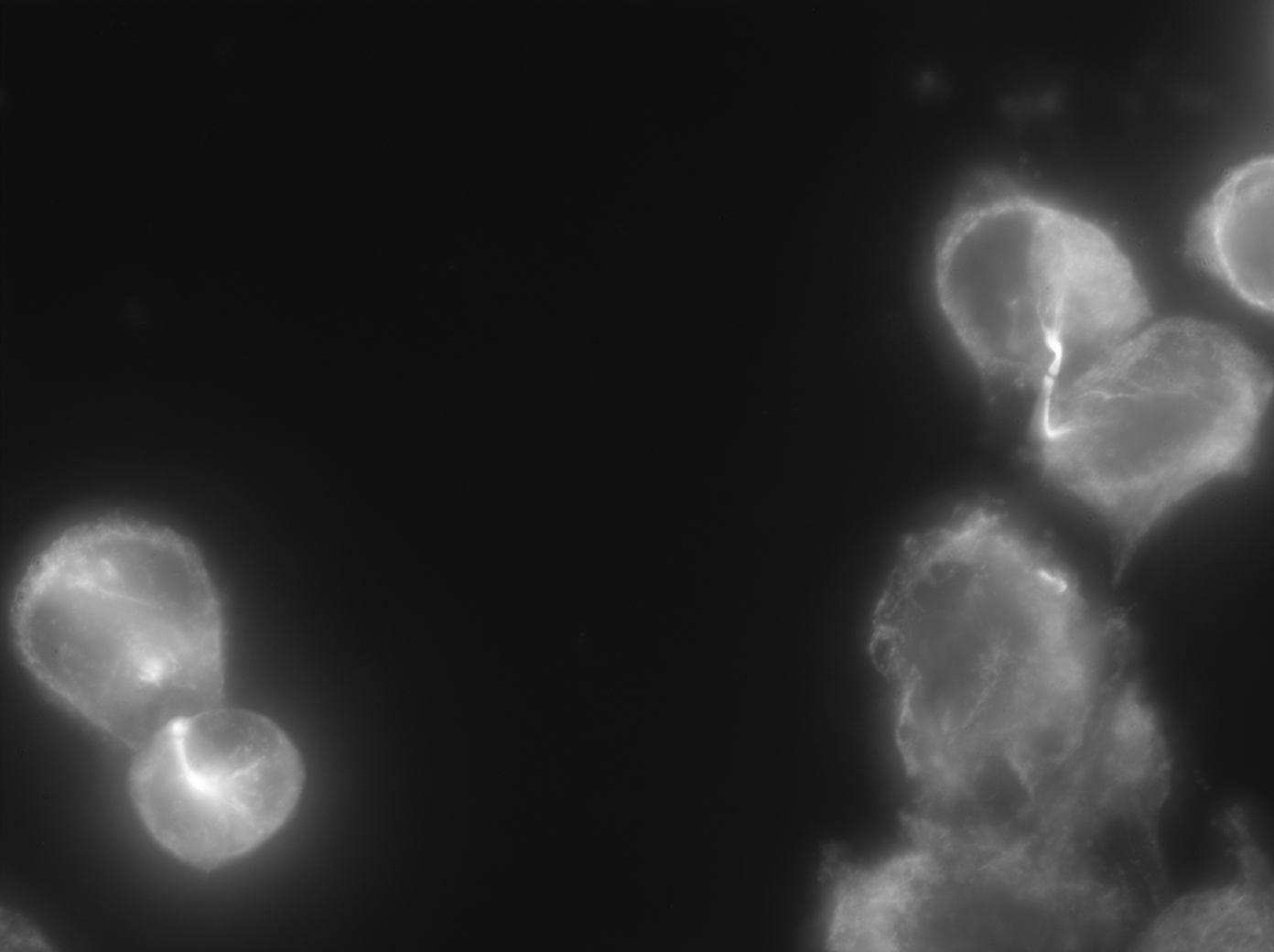

Supplement: Supplementary file 20 — Source data Fig. 6 part 1 [file 44319_2026_742_MOESM20_ESM.zip › Figure 6 Part 1/Fig 6abcd Cas9 TPGS1-KO acetylated tubulin atubulin/Cas9 R2 9-11-24 LT25 PA22PA23.Project Maximum Z_XY1726180658_Z0_T0_C1.tif]

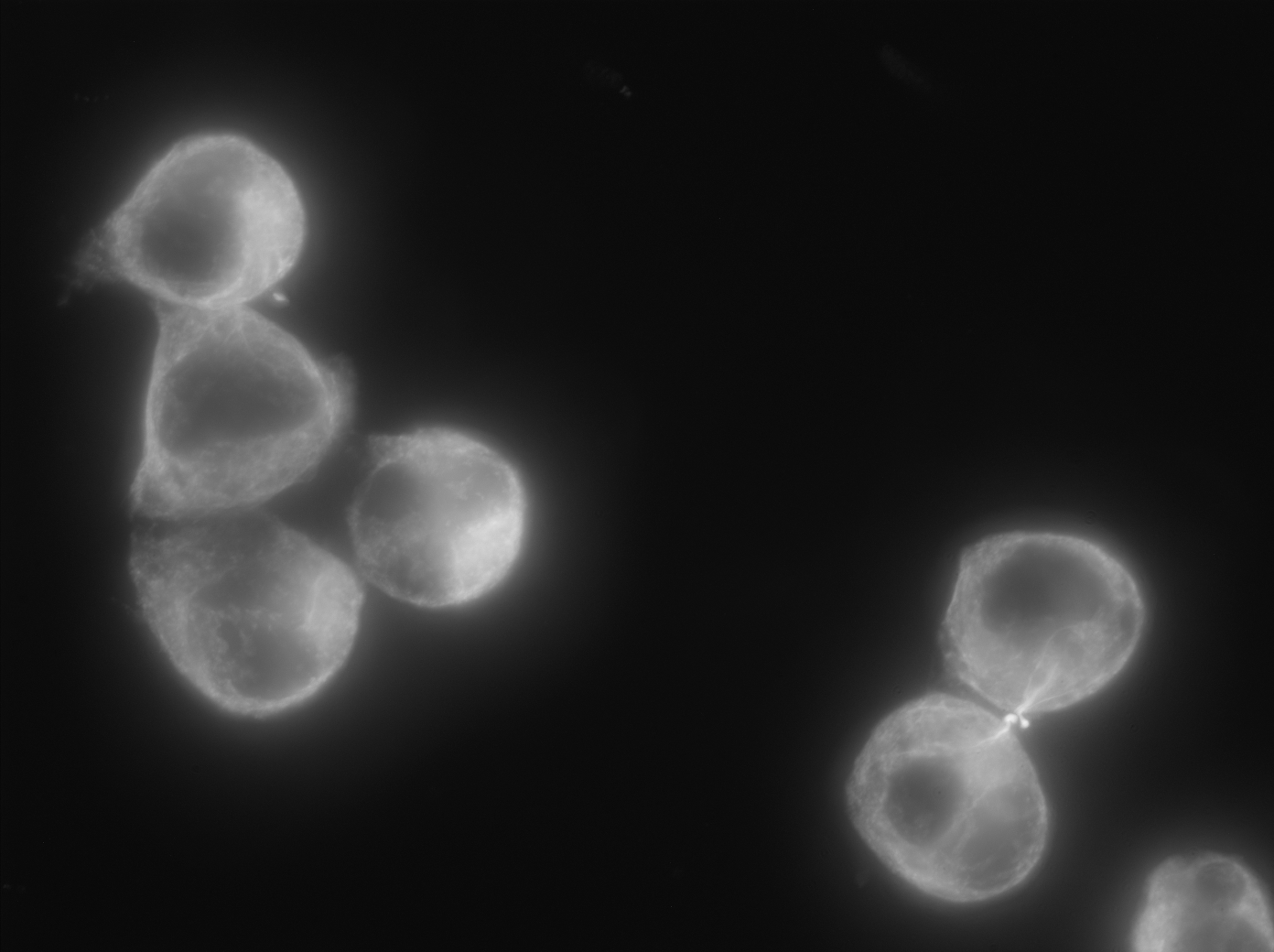

Supplement: Supplementary file 20 — Source data Fig. 6 part 1 [file 44319_2026_742_MOESM20_ESM.zip › Figure 6 Part 1/Fig 6abcd Cas9 TPGS1-KO acetylated tubulin atubulin/Cas9 R2 9-11-24 PA3PA4.Project Maximum Z_XY1726172775_Z0_T0_C1.tif]

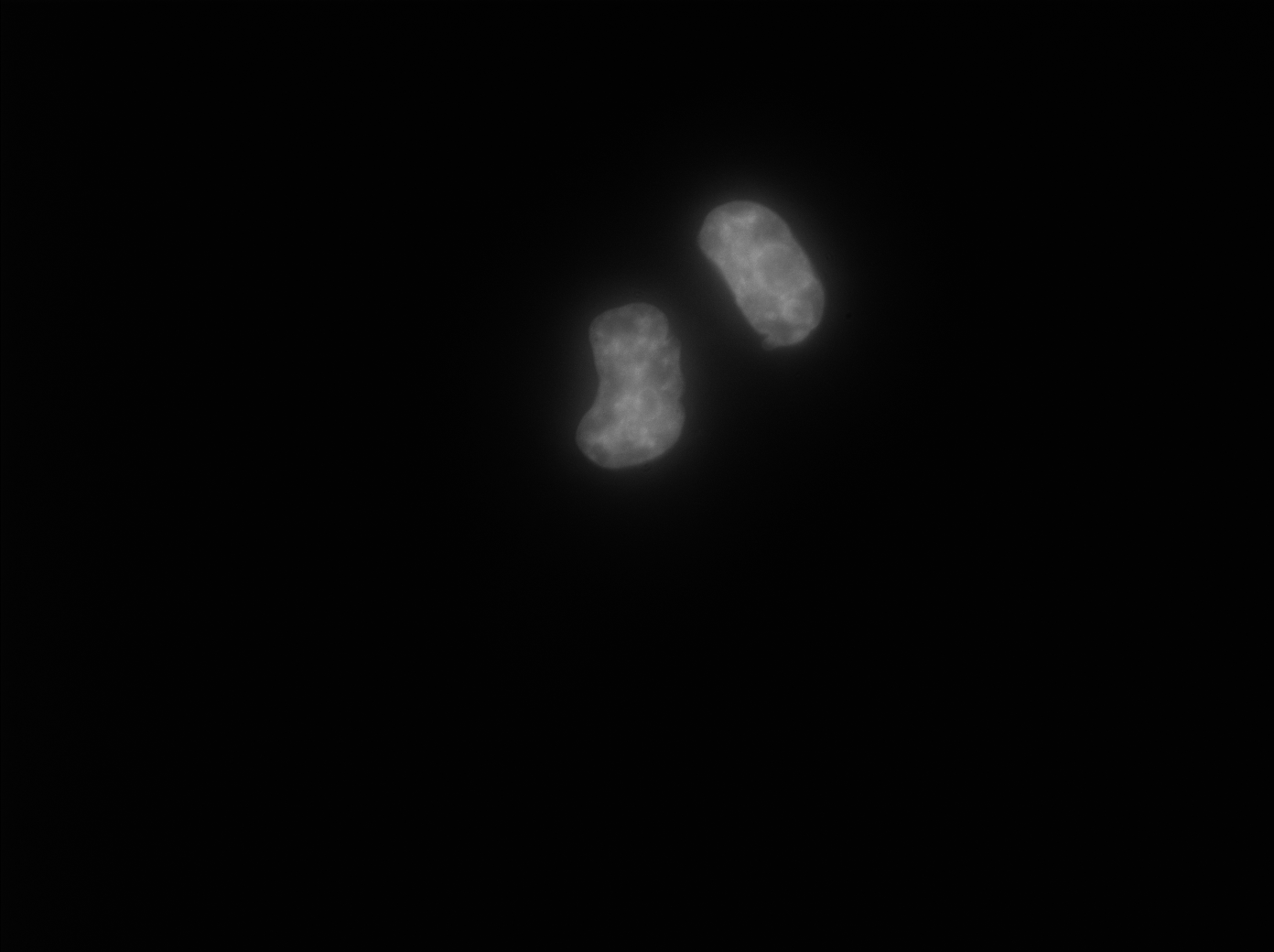

Supplement: Supplementary file 20 — Source data Fig. 6 part 1 [file 44319_2026_742_MOESM20_ESM.zip › Figure 6 Part 1/Fig 6abcd Cas9 TPGS1-KO acetylated tubulin atubulin/Cas9 R2 9-11-24 PA1.Project Maximum Z_XY1726172379_Z0_T0_C0.tif]

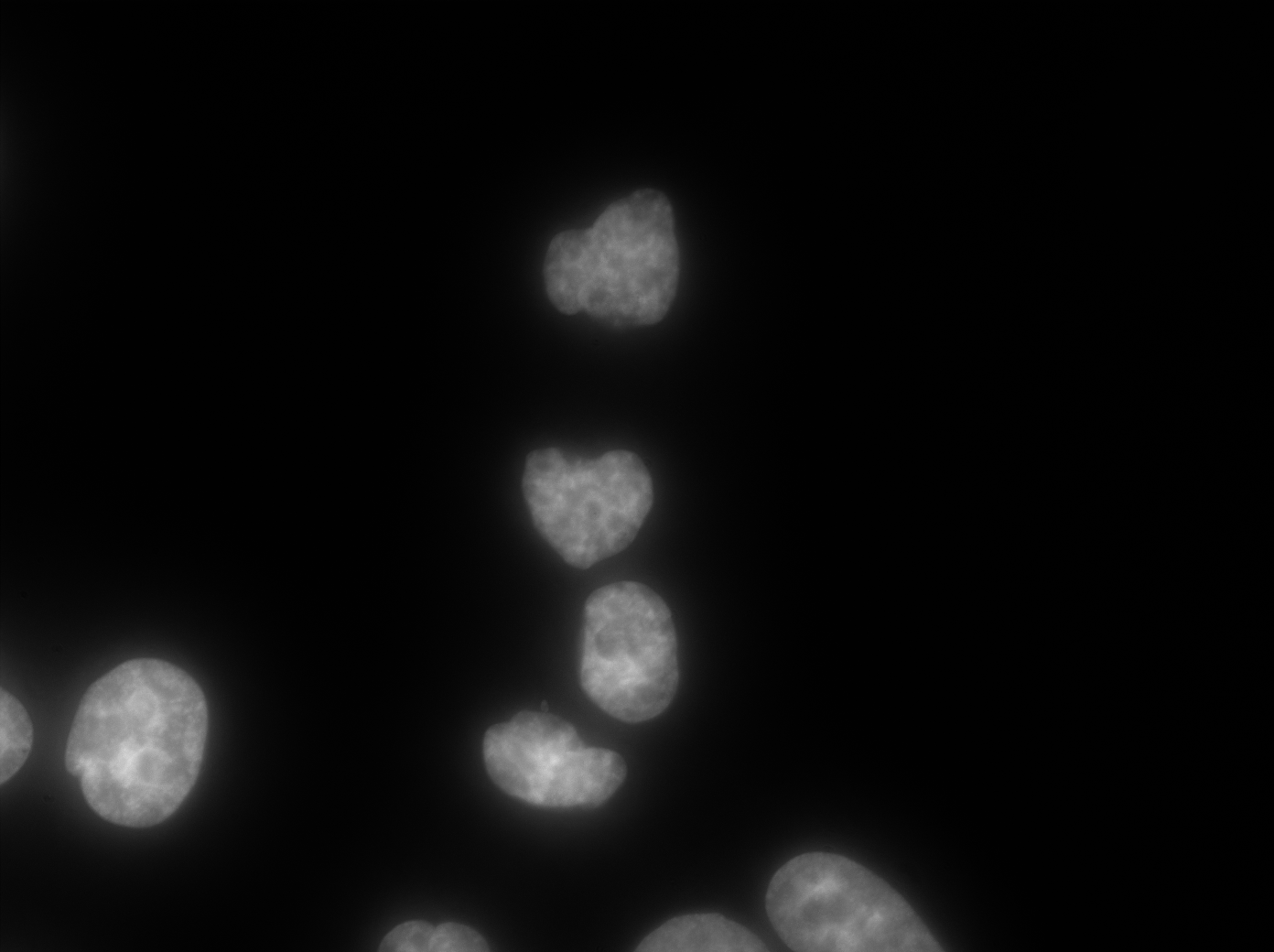

Supplement: Supplementary file 20 — Source data Fig. 6 part 1 [file 44319_2026_742_MOESM20_ESM.zip › Figure 6 Part 1/Fig 6abcd Cas9 TPGS1-KO acetylated tubulin atubulin/Cas9 R3 9-13-24 LT29LT30.Project Maximum Z_XY1726767787_Z0_T0_C0.tif]

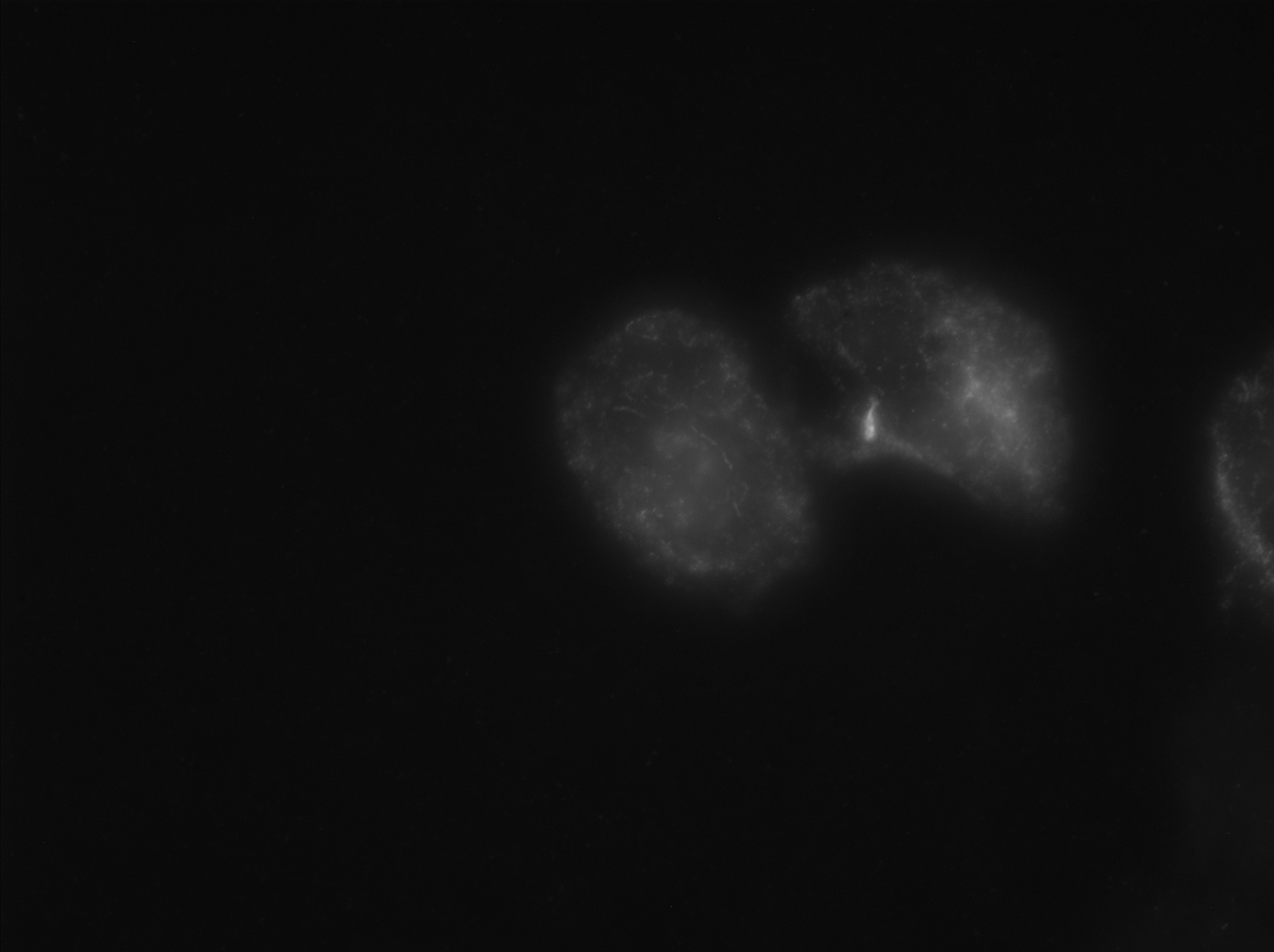

Supplement: Supplementary file 20 — Source data Fig. 6 part 1 [file 44319_2026_742_MOESM20_ESM.zip › Figure 6 Part 1/Fig 6abcd Cas9 TPGS1-KO acetylated tubulin atubulin/Cas9 R2 9-11-24 PA24.Project Maximum Z_XY1726180766_Z0_T0_C2.tif]

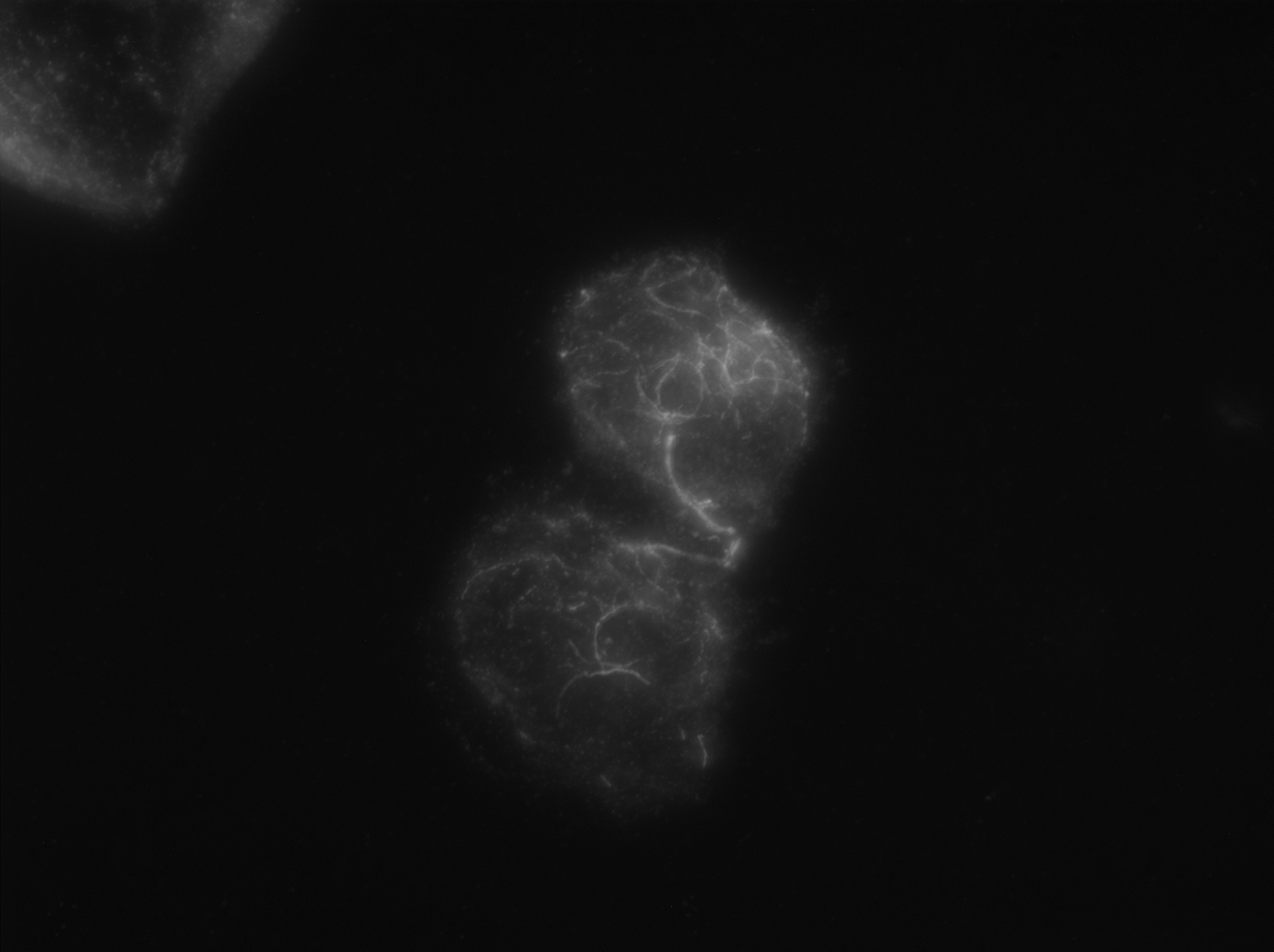

Supplement: Supplementary file 20 — Source data Fig. 6 part 1 [file 44319_2026_742_MOESM20_ESM.zip › Figure 6 Part 1/Fig 6abcd Cas9 TPGS1-KO acetylated tubulin atubulin/Cas9 R2 9-11-24 LT25.Project Maximum Z_XY1726180921_Z0_T0_C2.tif]

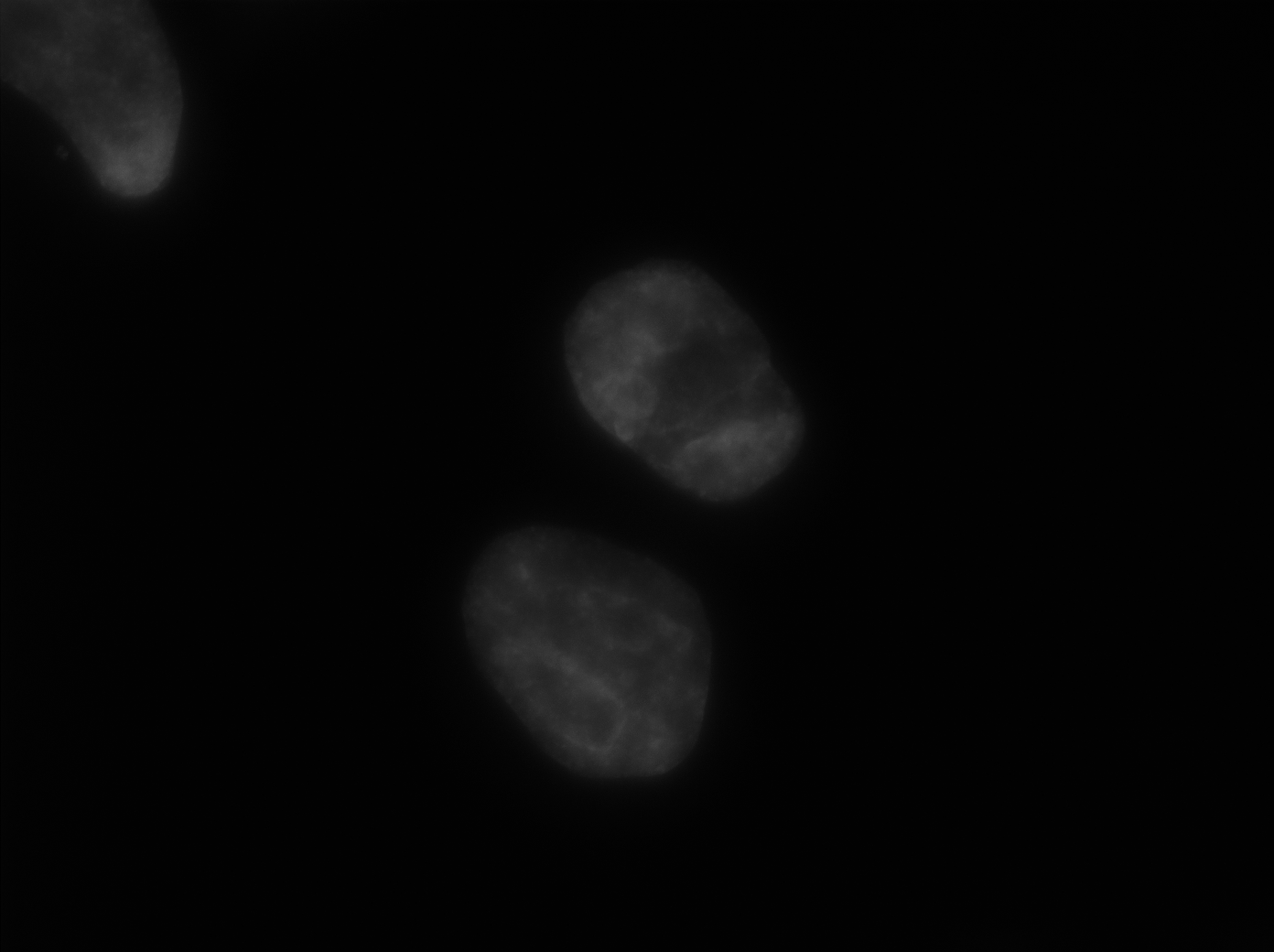

Supplement: Supplementary file 20 — Source data Fig. 6 part 1 [file 44319_2026_742_MOESM20_ESM.zip › Figure 6 Part 1/Fig 6abcd Cas9 TPGS1-KO acetylated tubulin atubulin/Cas9 R2 9-11-24 LT25.Project Maximum Z_XY1726180921_Z0_T0_C0.tif]

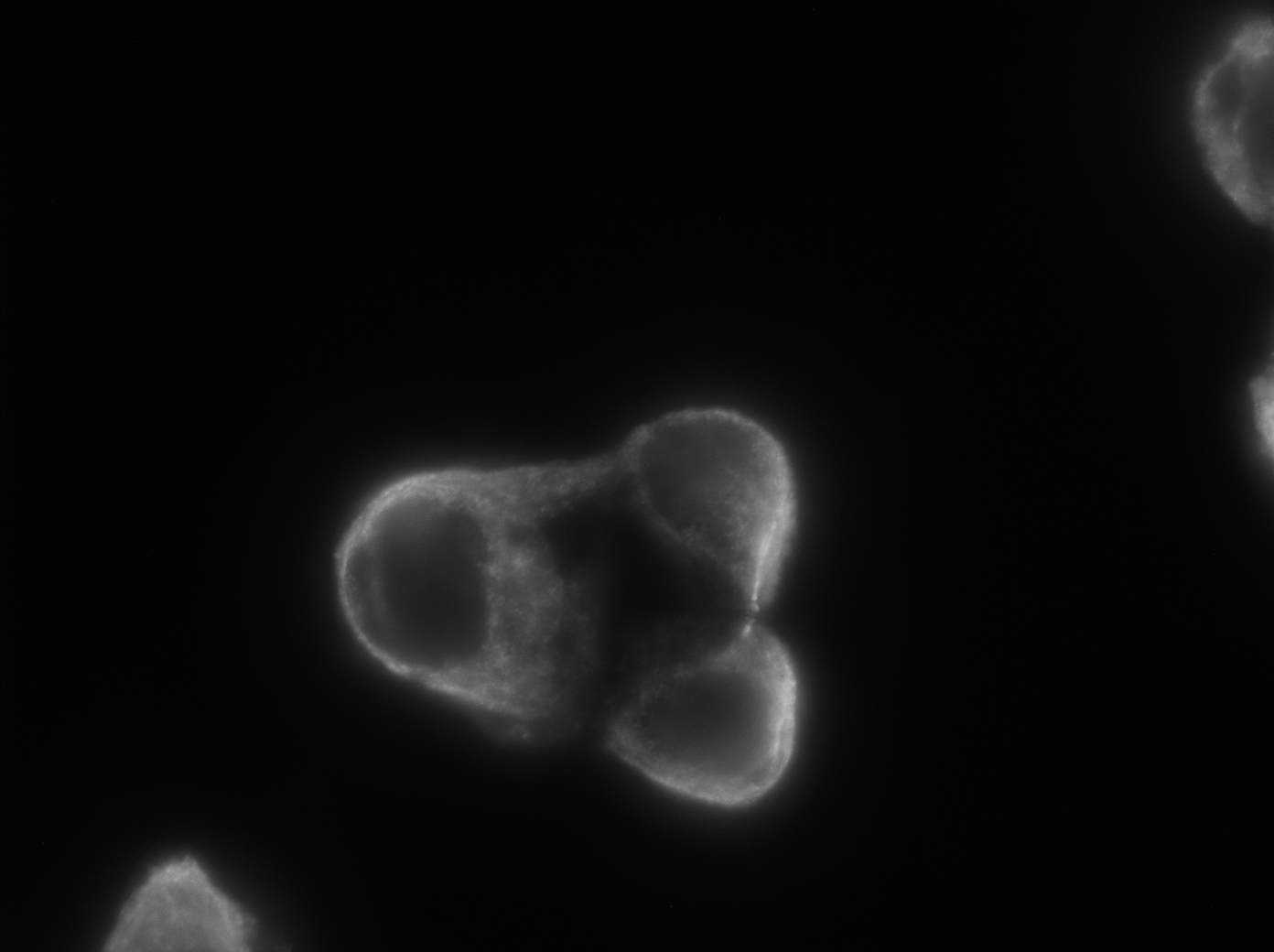

Supplement: Supplementary file 20 — Source data Fig. 6 part 1 [file 44319_2026_742_MOESM20_ESM.zip › Figure 6 Part 1/Fig 6abcd Cas9 TPGS1-KO acetylated tubulin atubulin/Cas9 R3 9-13-24 LT24.Project Maximum Z_XY1726767260_Z0_T0_C1.tif]

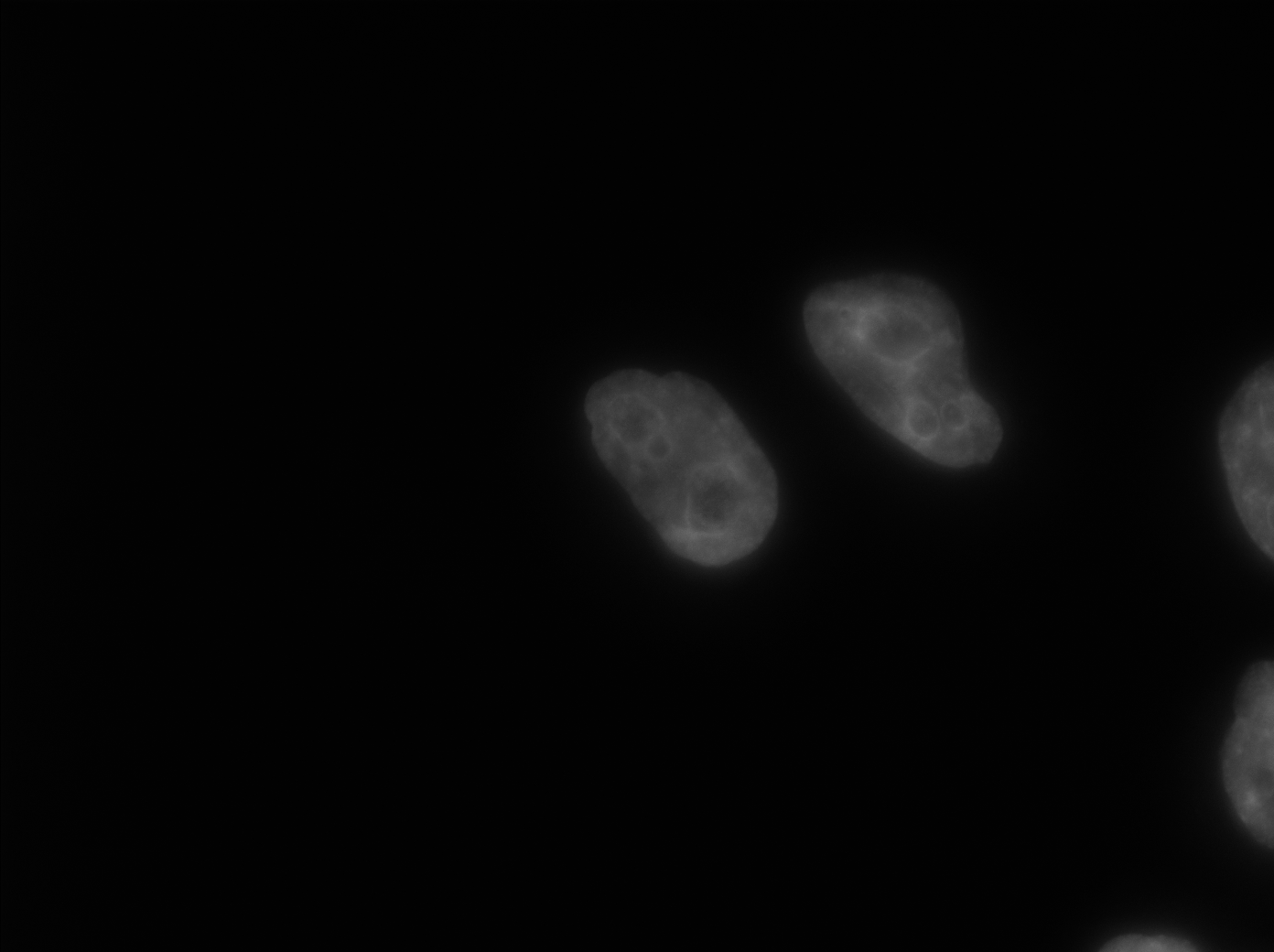

Supplement: Supplementary file 20 — Source data Fig. 6 part 1 [file 44319_2026_742_MOESM20_ESM.zip › Figure 6 Part 1/Fig 6abcd Cas9 TPGS1-KO acetylated tubulin atubulin/Cas9 R2 9-11-24 PA24.Project Maximum Z_XY1726180766_Z0_T0_C0.tif]

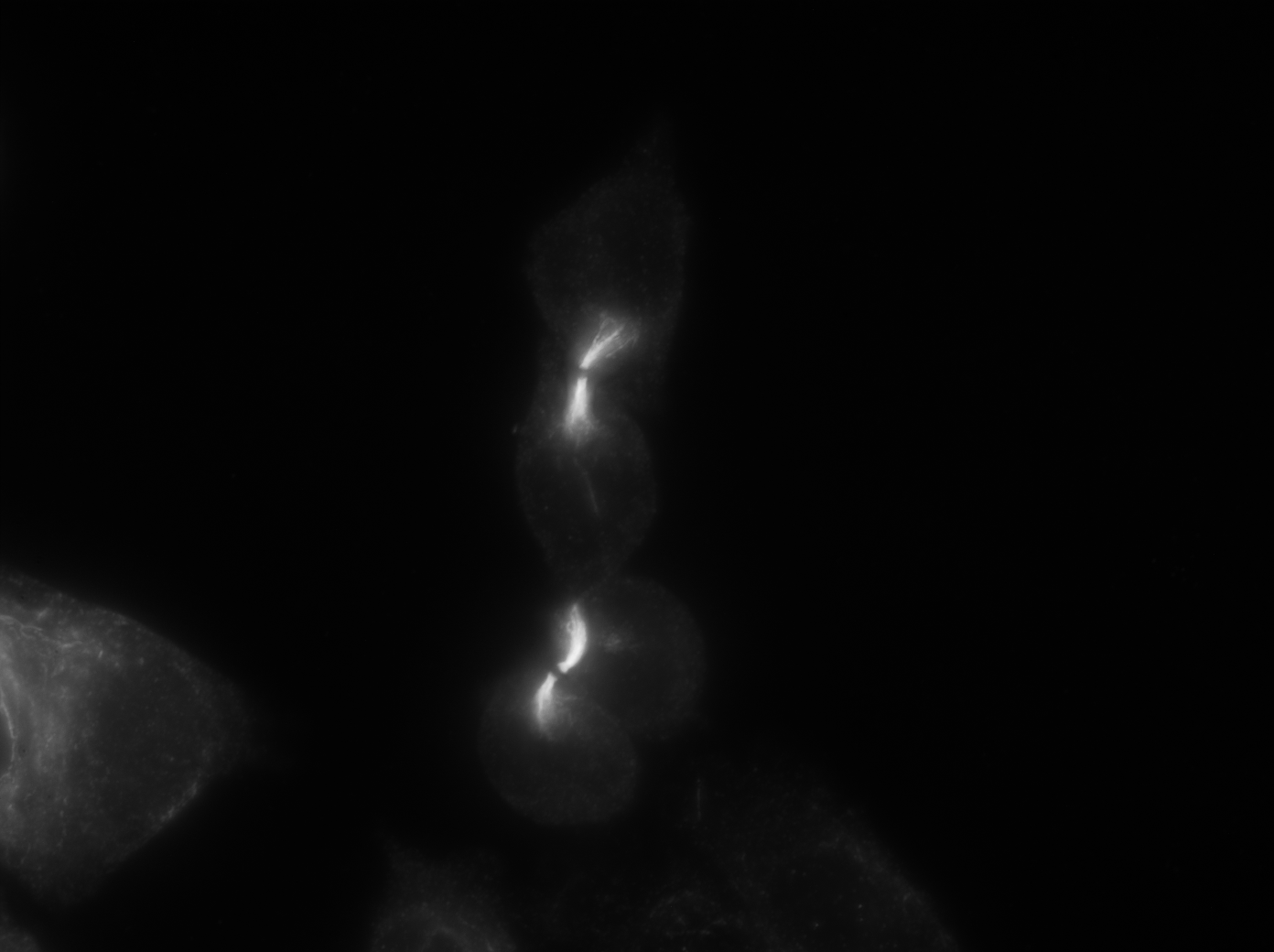

Supplement: Supplementary file 20 — Source data Fig. 6 part 1 [file 44319_2026_742_MOESM20_ESM.zip › Figure 6 Part 1/Fig 6abcd Cas9 TPGS1-KO acetylated tubulin atubulin/Cas9 R3 9-13-24 LT29LT30.Project Maximum Z_XY1726767787_Z0_T0_C2.tif]

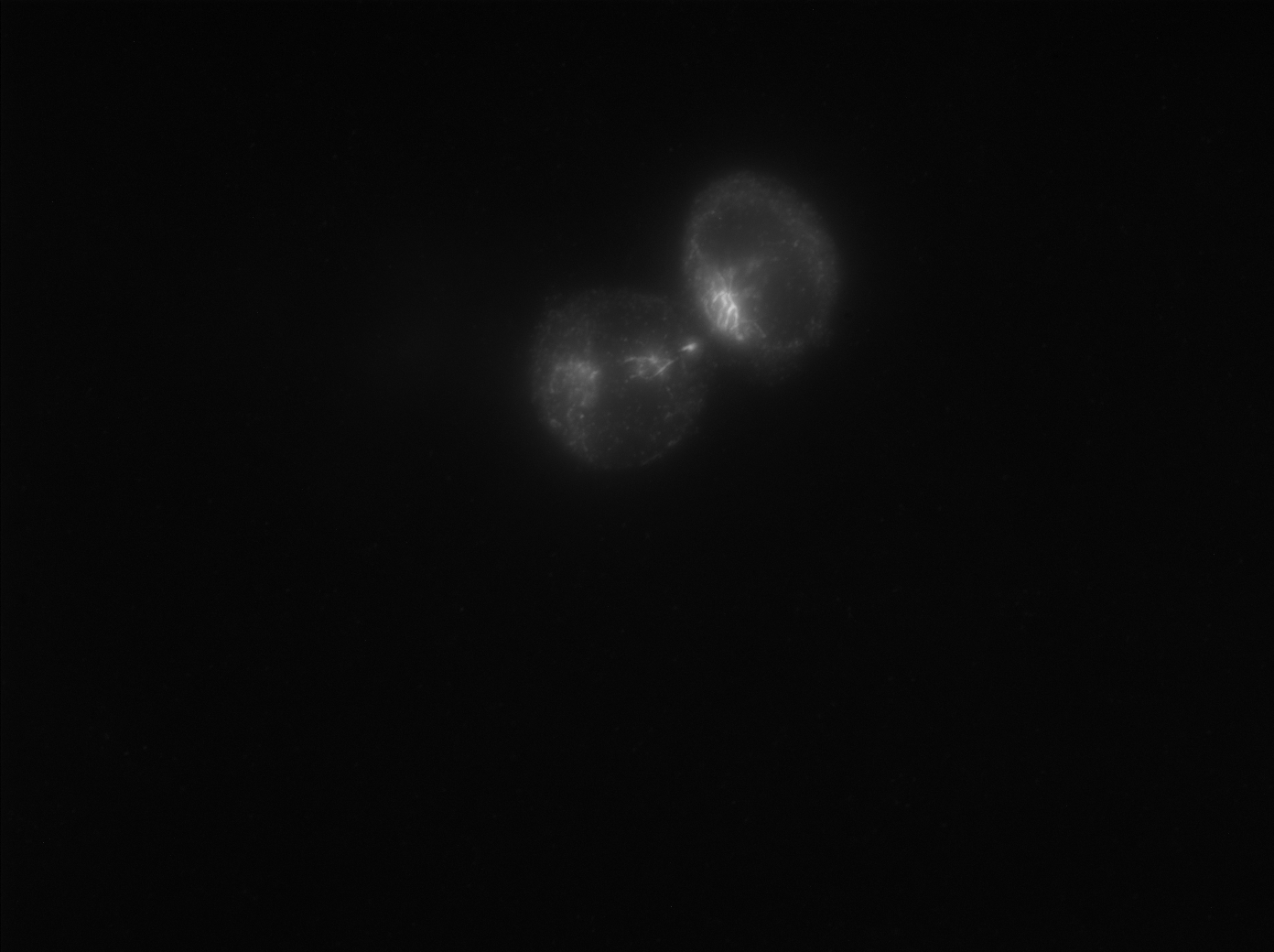

Supplement: Supplementary file 20 — Source data Fig. 6 part 1 [file 44319_2026_742_MOESM20_ESM.zip › Figure 6 Part 1/Fig 6abcd Cas9 TPGS1-KO acetylated tubulin atubulin/Cas9 R2 9-11-24 PA1.Project Maximum Z_XY1726172379_Z0_T0_C2.tif]

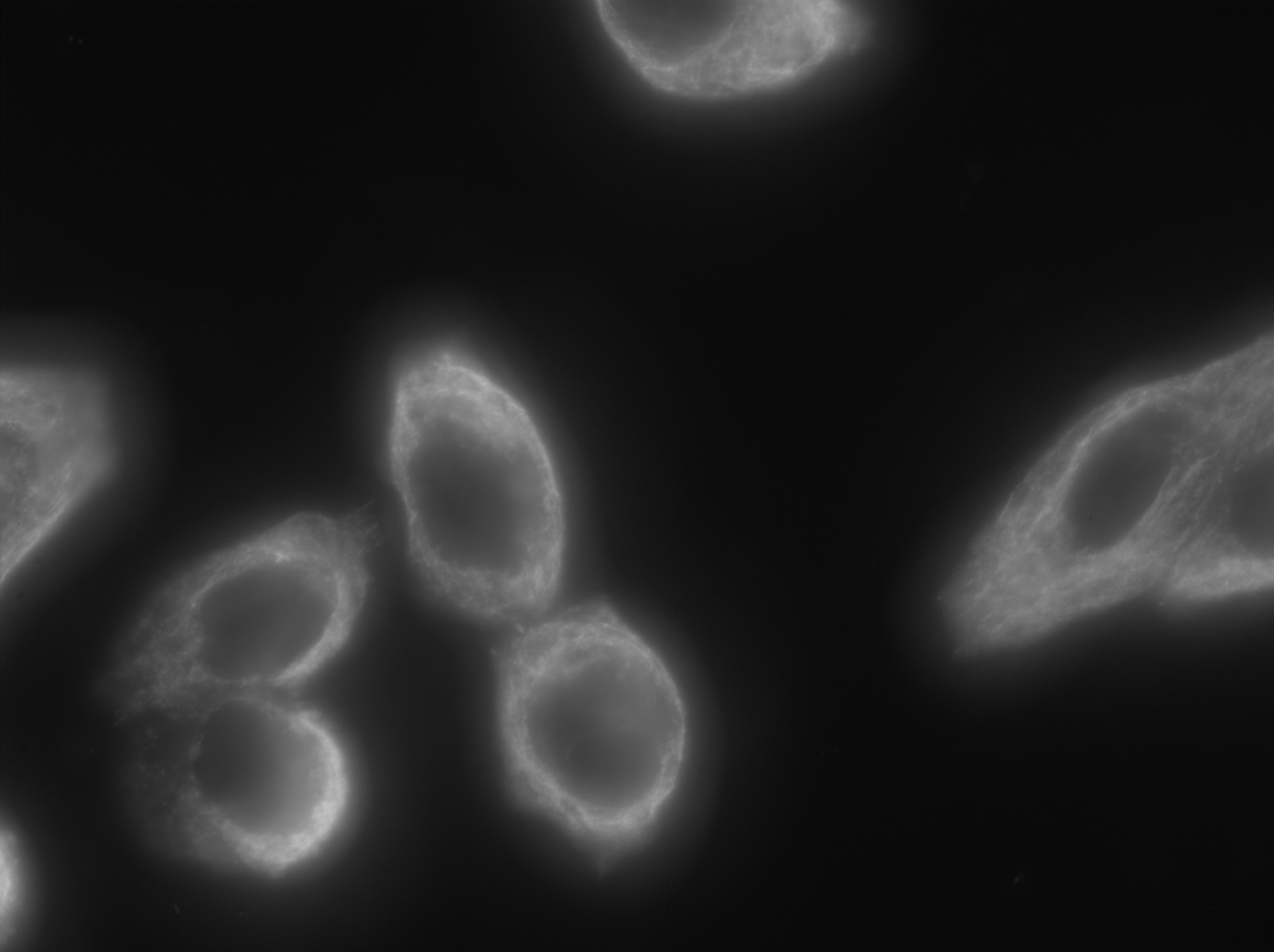

Supplement: Supplementary file 20 — Source data Fig. 6 part 1 [file 44319_2026_742_MOESM20_ESM.zip › Figure 6 Part 1/Fig 6abcd Cas9 TPGS1-KO acetylated tubulin atubulin/Cas9 R2 9-11-24 PA28.Project Maximum Z_XY1726181804_Z0_T0_C1.tif]

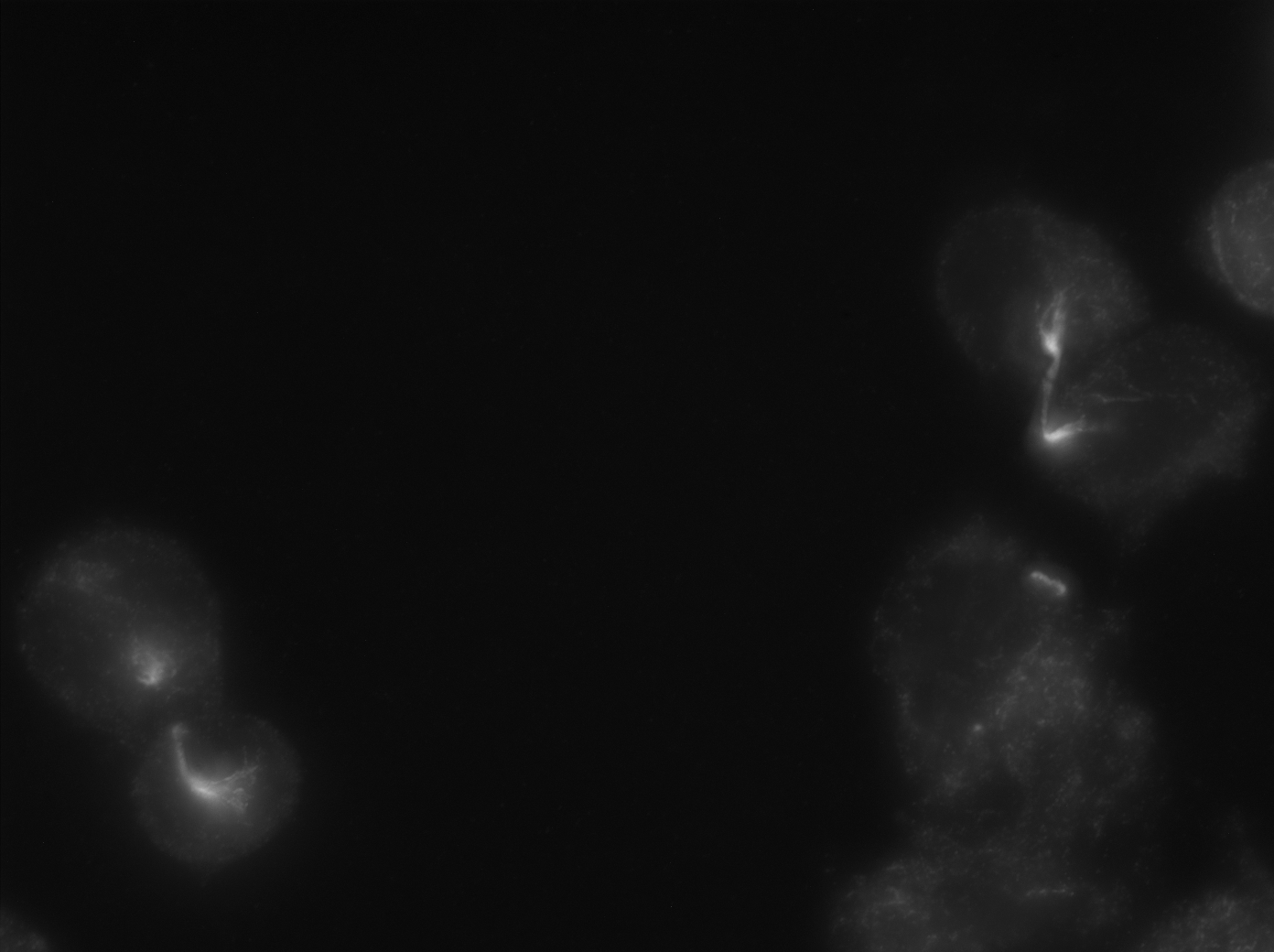

Supplement: Supplementary file 20 — Source data Fig. 6 part 1 [file 44319_2026_742_MOESM20_ESM.zip › Figure 6 Part 1/Fig 6abcd Cas9 TPGS1-KO acetylated tubulin atubulin/Cas9 R2 9-11-24 LT25 PA22PA23.Project Maximum Z_XY1726180658_Z0_T0_C2.tif]

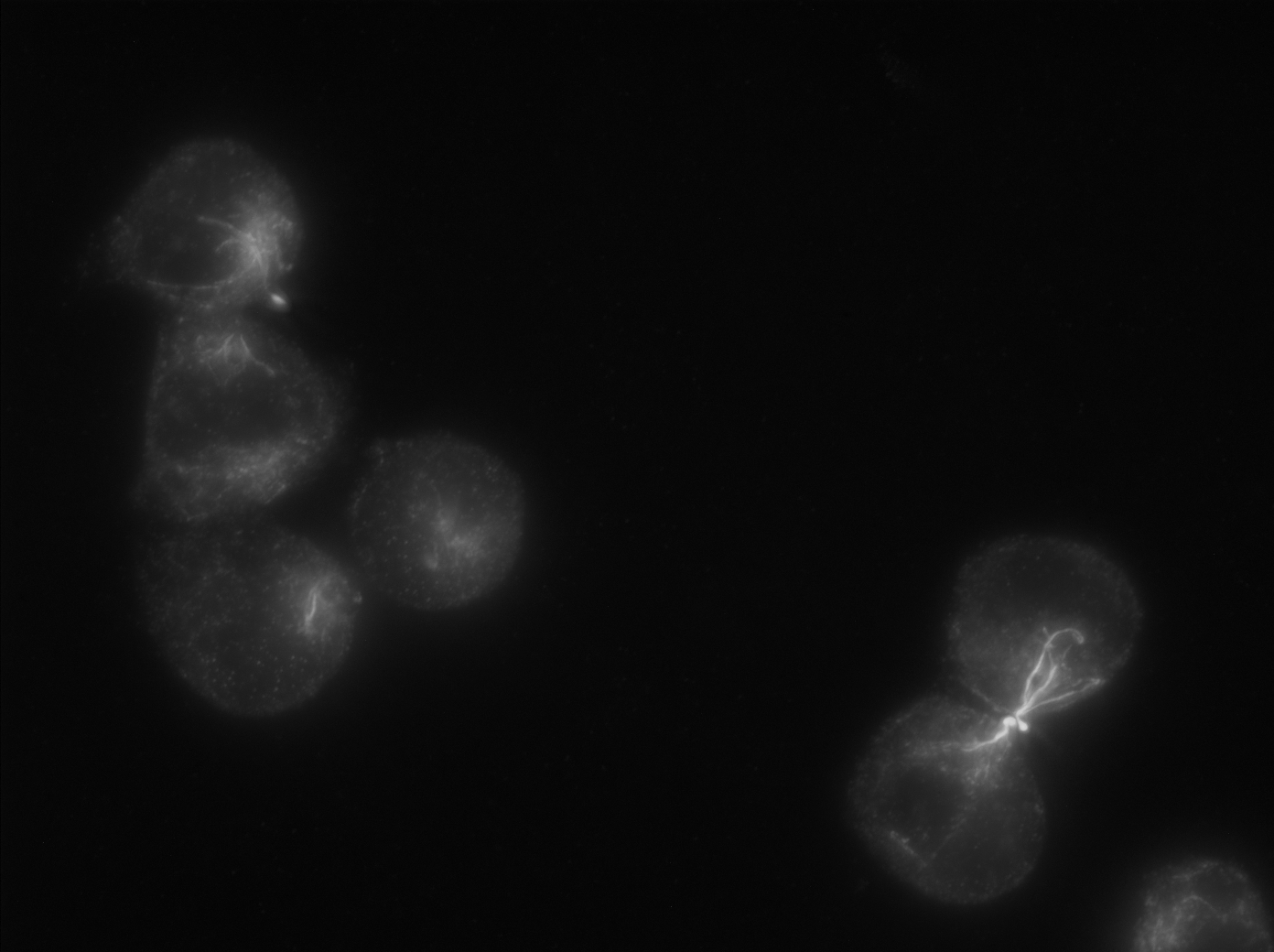

Supplement: Supplementary file 20 — Source data Fig. 6 part 1 [file 44319_2026_742_MOESM20_ESM.zip › Figure 6 Part 1/Fig 6abcd Cas9 TPGS1-KO acetylated tubulin atubulin/Cas9 R2 9-11-24 PA3PA4.Project Maximum Z_XY1726172775_Z0_T0_C2.tif]

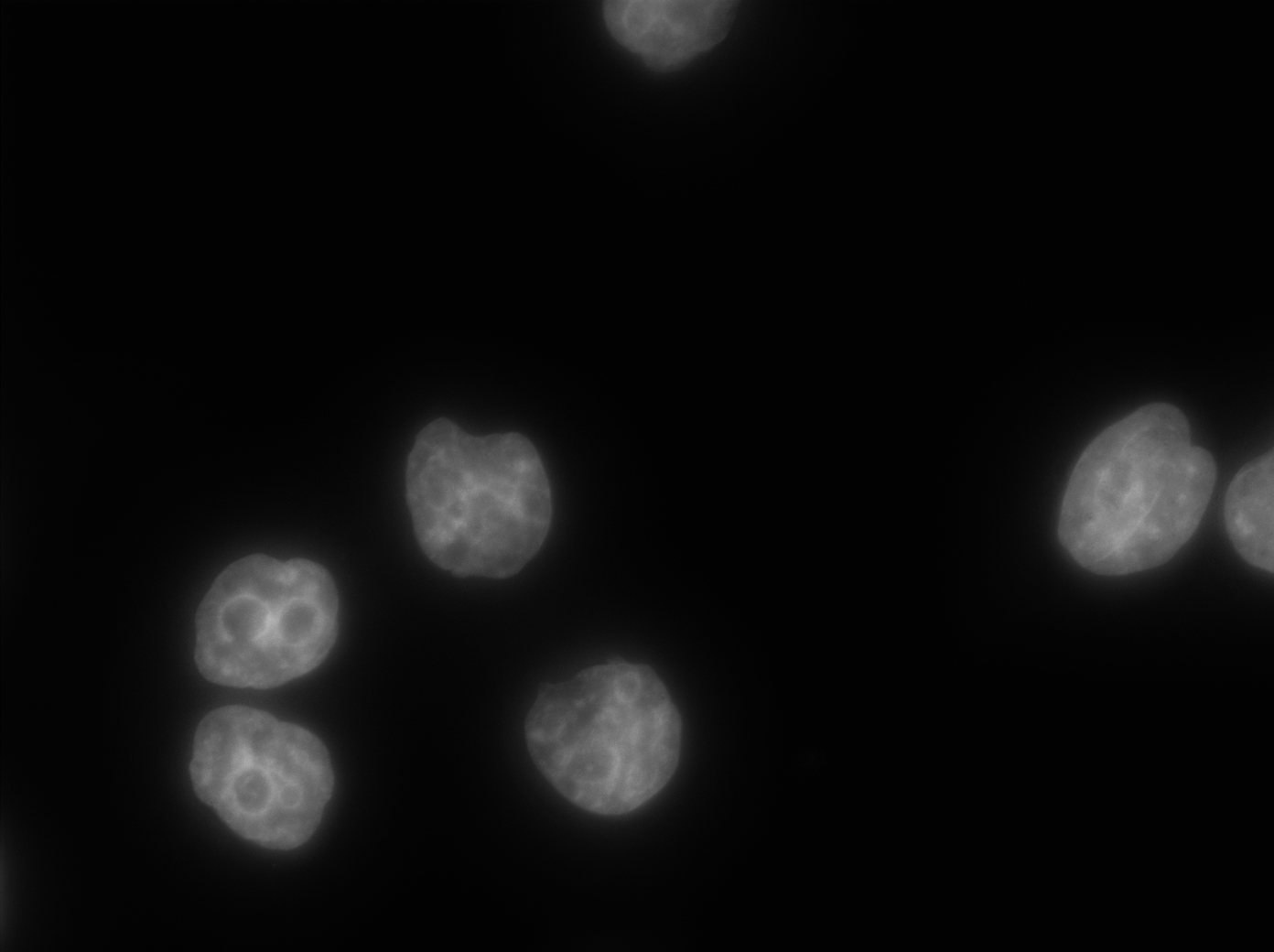

Supplement: Supplementary file 20 — Source data Fig. 6 part 1 [file 44319_2026_742_MOESM20_ESM.zip › Figure 6 Part 1/Fig 6abcd Cas9 TPGS1-KO acetylated tubulin atubulin/Cas9 R2 9-11-24 PA28.Project Maximum Z_XY1726181804_Z0_T0_C0.tif]

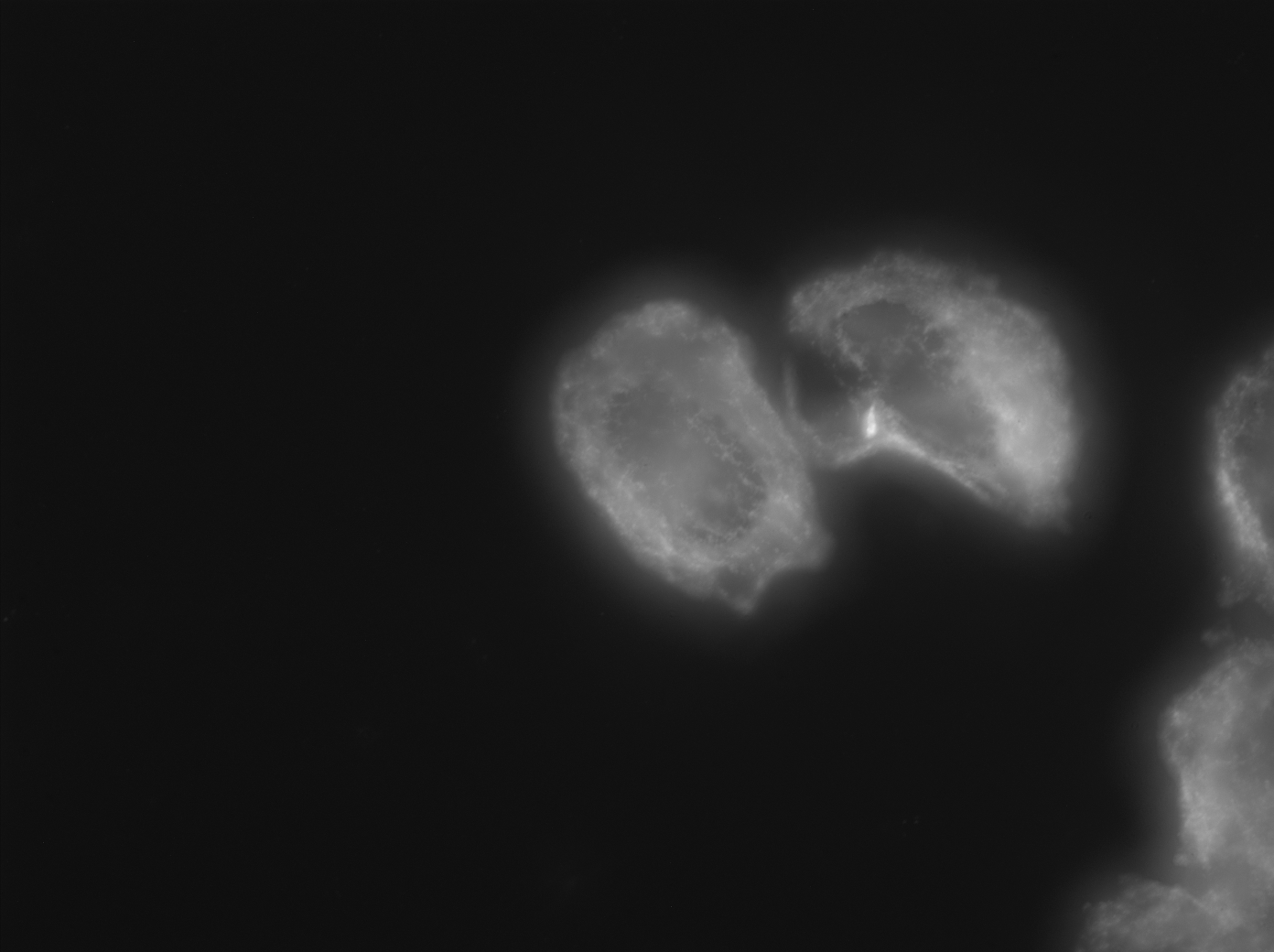

Supplement: Supplementary file 20 — Source data Fig. 6 part 1 [file 44319_2026_742_MOESM20_ESM.zip › Figure 6 Part 1/Fig 6abcd Cas9 TPGS1-KO acetylated tubulin atubulin/Cas9 R2 9-11-24 PA24.Project Maximum Z_XY1726180766_Z0_T0_C1.tif]

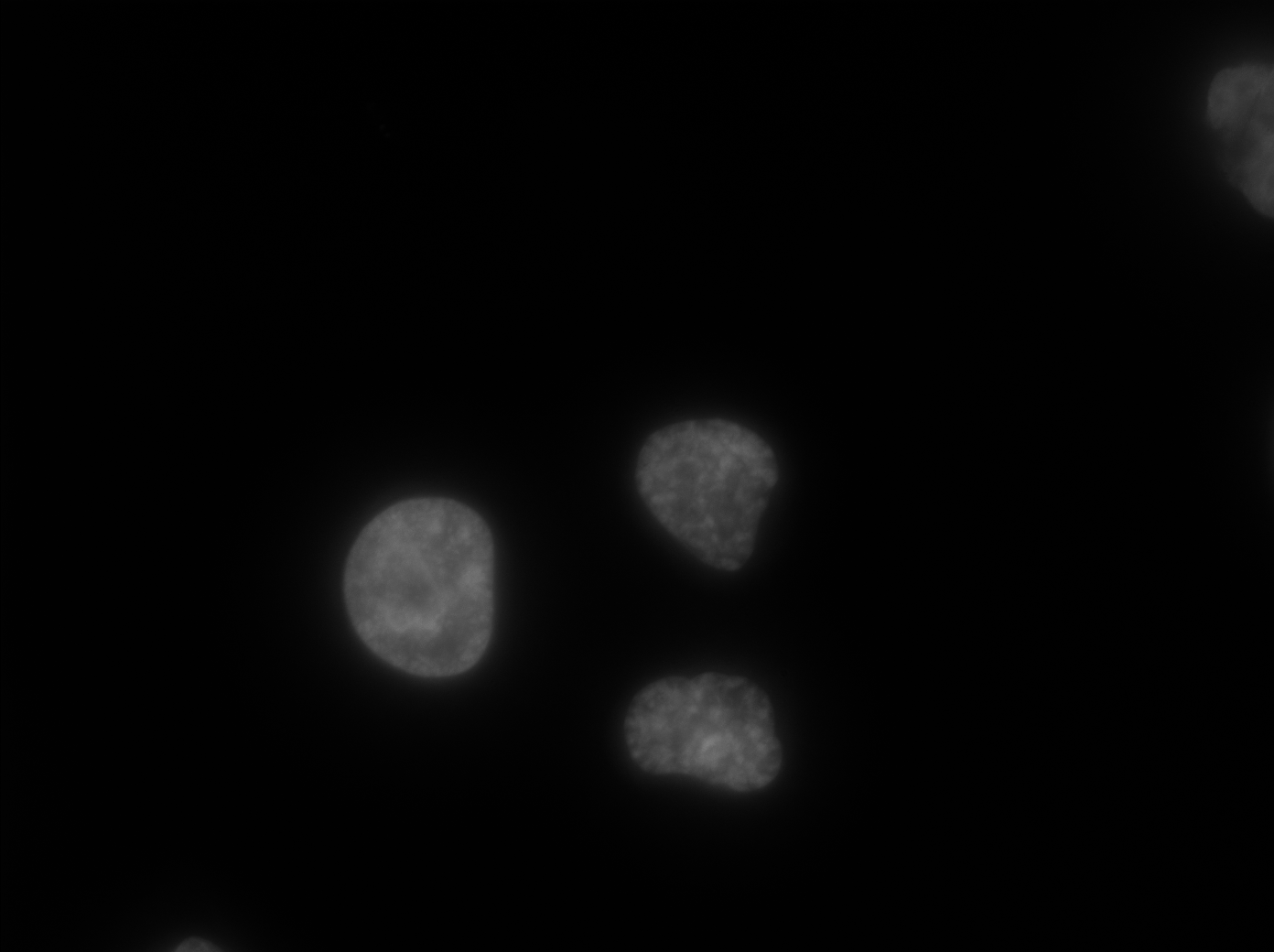

Supplement: Supplementary file 20 — Source data Fig. 6 part 1 [file 44319_2026_742_MOESM20_ESM.zip › Figure 6 Part 1/Fig 6abcd Cas9 TPGS1-KO acetylated tubulin atubulin/Cas9 R3 9-13-24 LT24.Project Maximum Z_XY1726767260_Z0_T0_C0.tif]

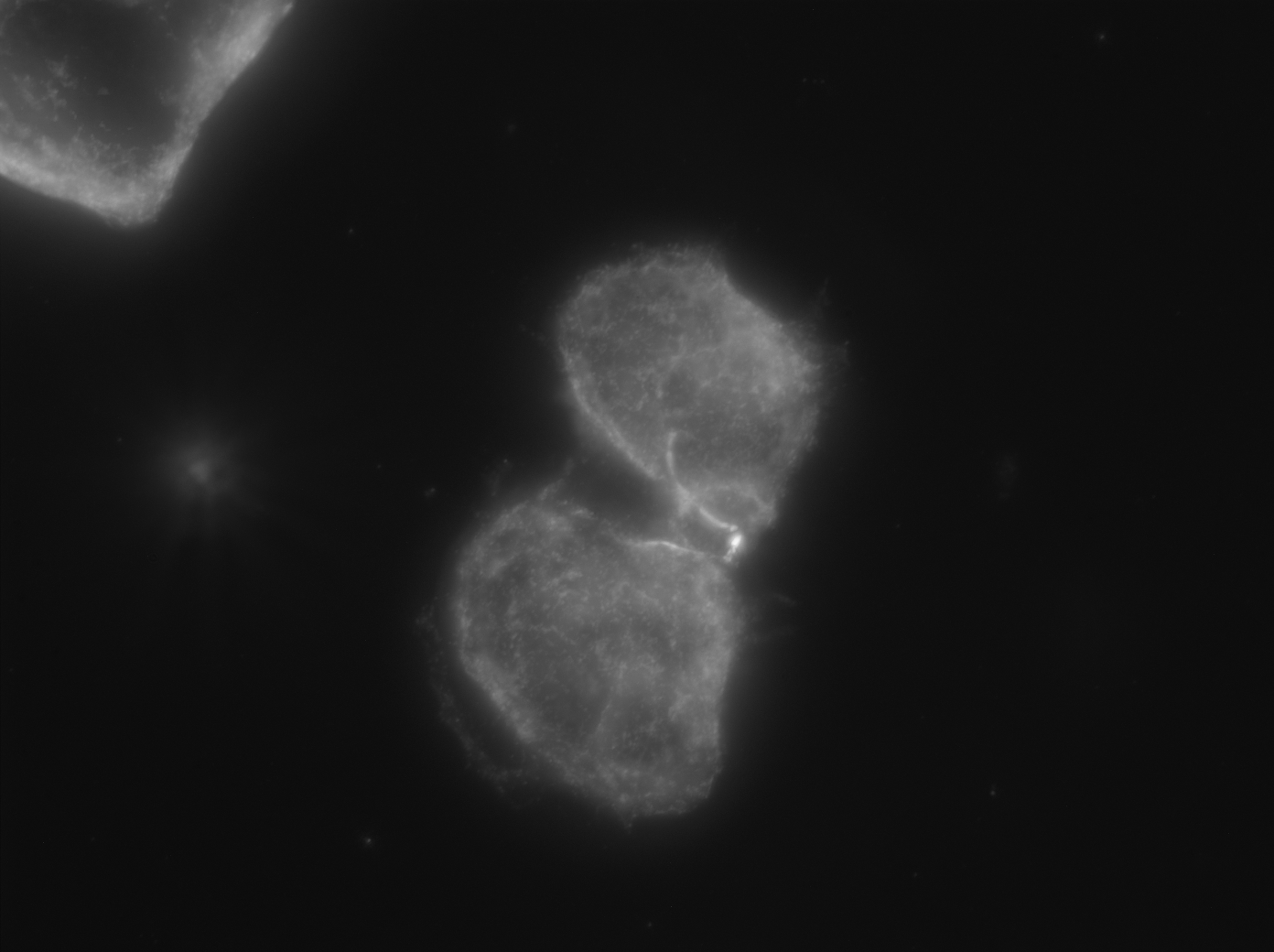

Supplement: Supplementary file 20 — Source data Fig. 6 part 1 [file 44319_2026_742_MOESM20_ESM.zip › Figure 6 Part 1/Fig 6abcd Cas9 TPGS1-KO acetylated tubulin atubulin/Cas9 R2 9-11-24 LT25.Project Maximum Z_XY1726180921_Z0_T0_C1.tif]
