# Supplementary figures and images for: TPGS1 regulates central spindle microtubule glutamylation and remodeling during telophase and abscission (part 25 of 36)
Source: EMBO Rep. 2026 Mar 23;27(8):1944–63. doi: 10.1038/s44319-026-00742-3 (PMC13121839; doi:10.1038/s44319-026-00742-3)

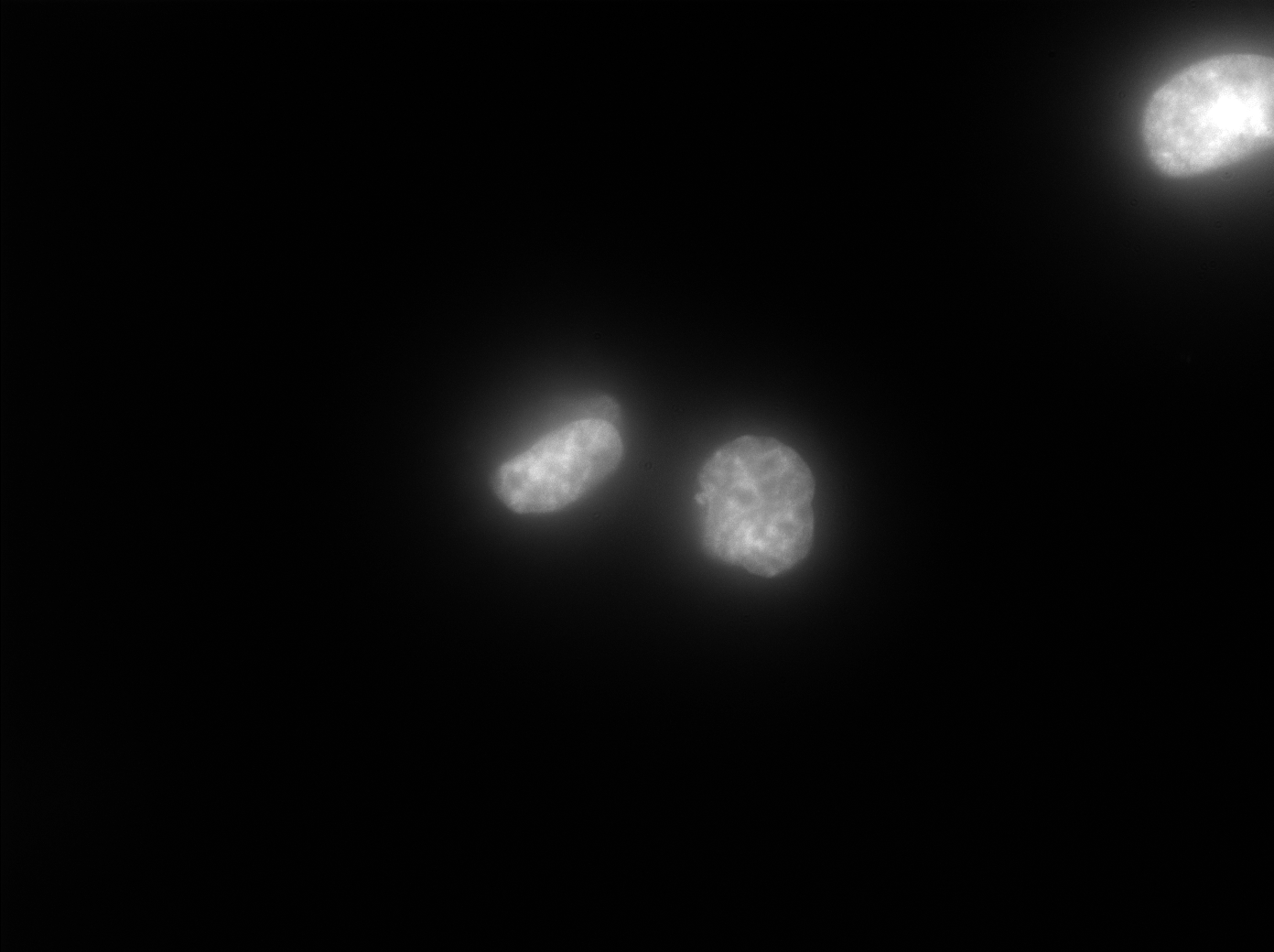

Supplement: Supplementary file 21 — Source data Fig. 6 part 2 [file 44319_2026_742_MOESM21_ESM.zip › Figure 6 Part 2/Fig 6abcd Cas9 TPGS1-KO acetylated tubulin atubulin part 2/TPGS1-KO R2 9-11-24 LT30.Project Maximum Z_XY1726270338_Z0_T0_C0.tif]

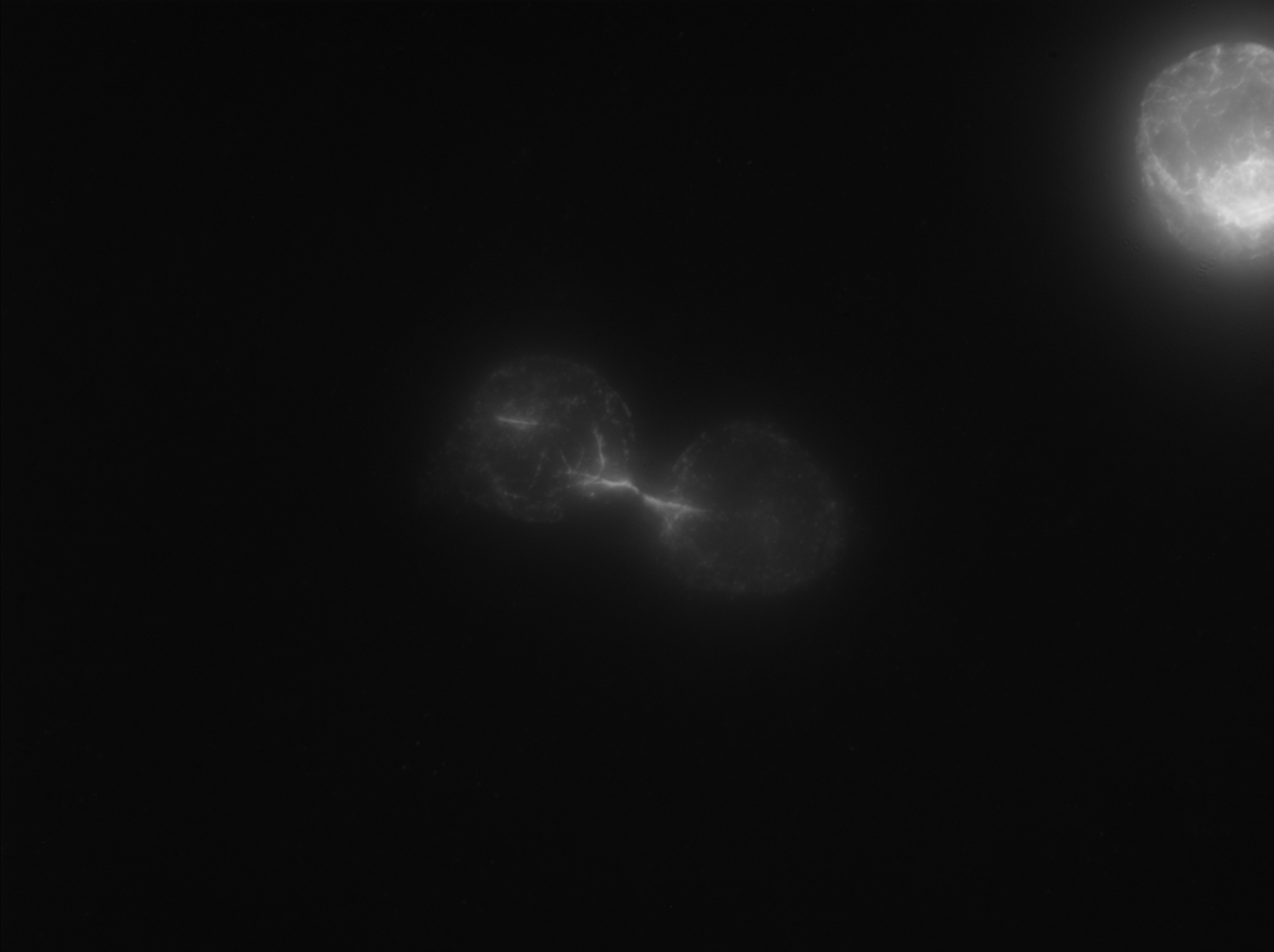

Supplement: Supplementary file 21 — Source data Fig. 6 part 2 [file 44319_2026_742_MOESM21_ESM.zip › Figure 6 Part 2/Fig 6abcd Cas9 TPGS1-KO acetylated tubulin atubulin part 2/TPGS1-KO R2 9-11-24 LT30.Project Maximum Z_XY1726270338_Z0_T0_C2.tif]

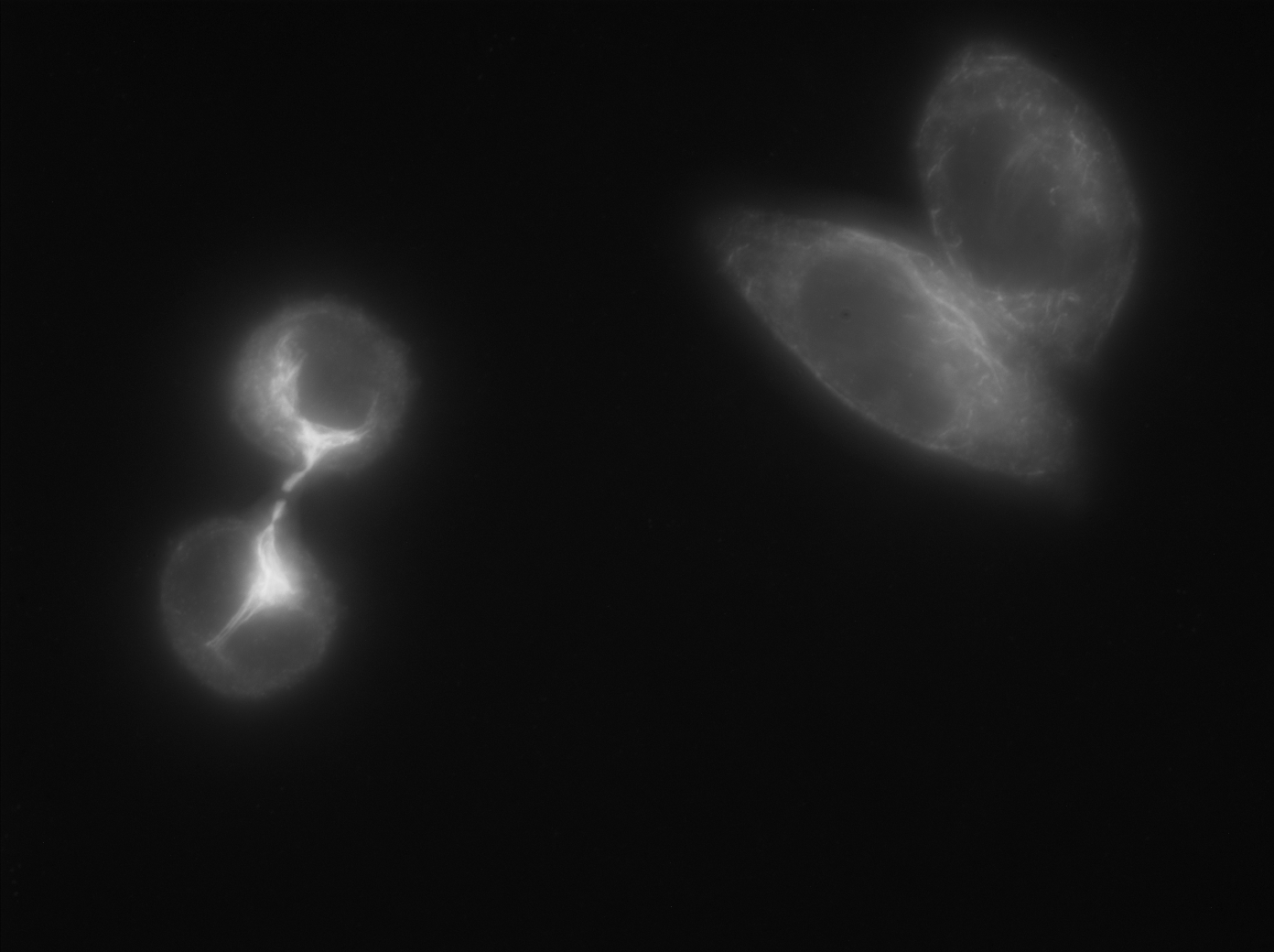

Supplement: Supplementary file 21 — Source data Fig. 6 part 2 [file 44319_2026_742_MOESM21_ESM.zip › Figure 6 Part 2/Fig 6abcd Cas9 TPGS1-KO acetylated tubulin atubulin part 2/TPGS1-KO R2 9-11-24 LT7.Project Maximum Z_XY1726261460_Z0_T0_C2.tif]

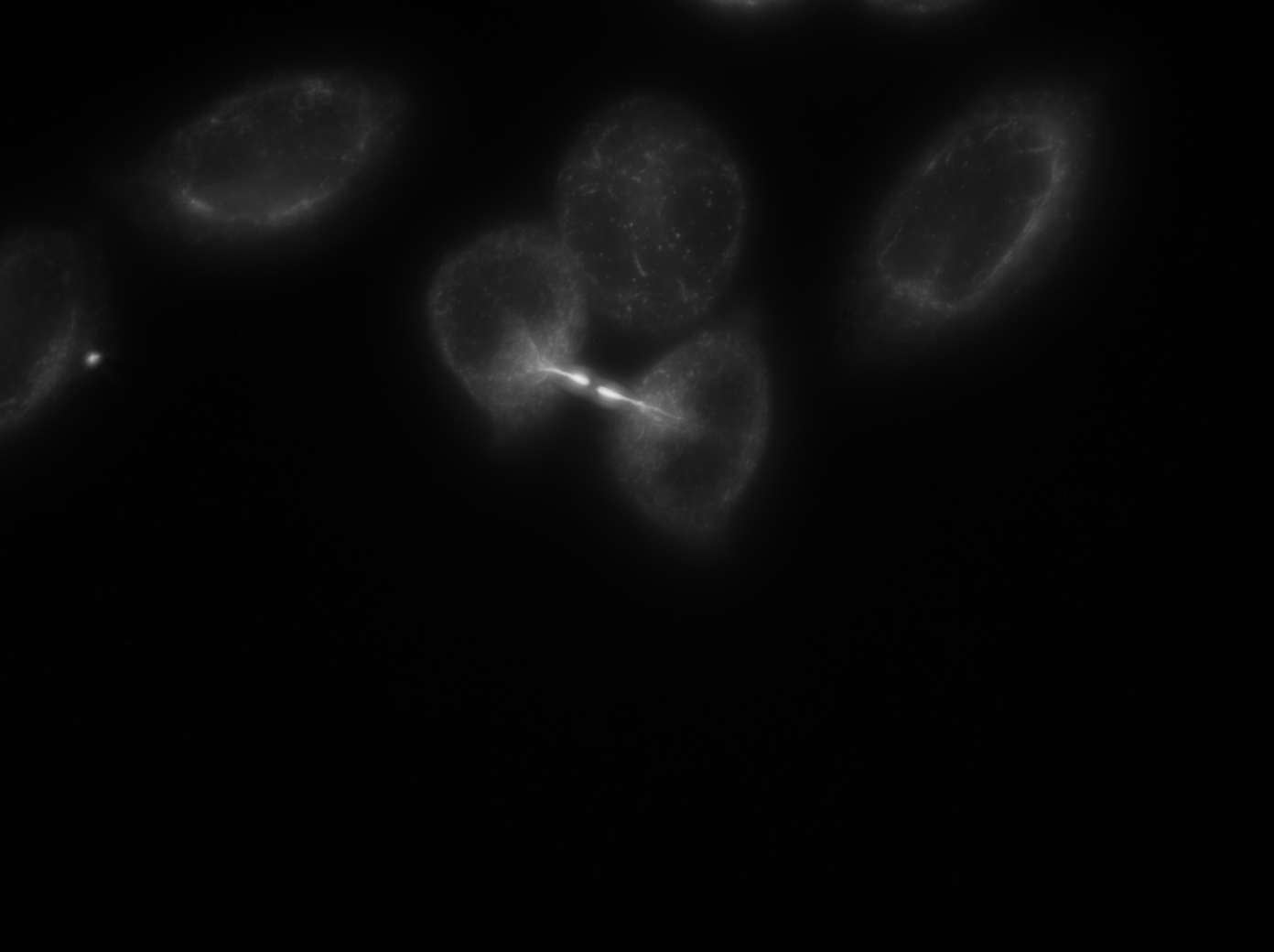

Supplement: Supplementary file 21 — Source data Fig. 6 part 2 [file 44319_2026_742_MOESM21_ESM.zip › Figure 6 Part 2/Fig 6abcd Cas9 TPGS1-KO acetylated tubulin atubulin part 2/TPGS1-KO R3 9-13-24 LT16.Project Maximum Z_XY1726763904_Z0_T0_C2.tif]

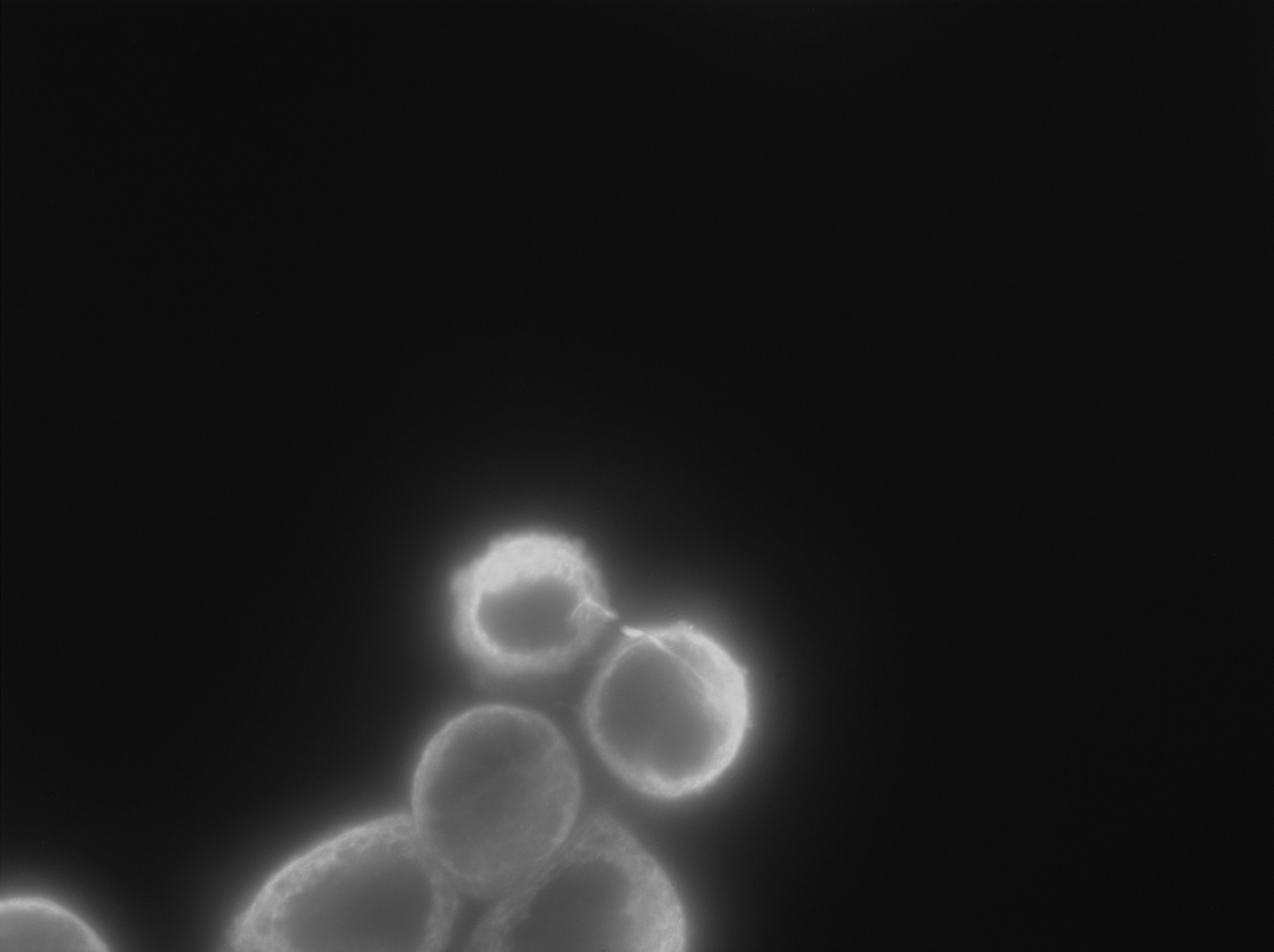

Supplement: Supplementary file 21 — Source data Fig. 6 part 2 [file 44319_2026_742_MOESM21_ESM.zip › Figure 6 Part 2/Fig 6abcd Cas9 TPGS1-KO acetylated tubulin atubulin part 2/TPGS1-KO R3 9-13-24 LT19.Project Maximum Z_XY1726764103_Z0_T0_C1.tif]

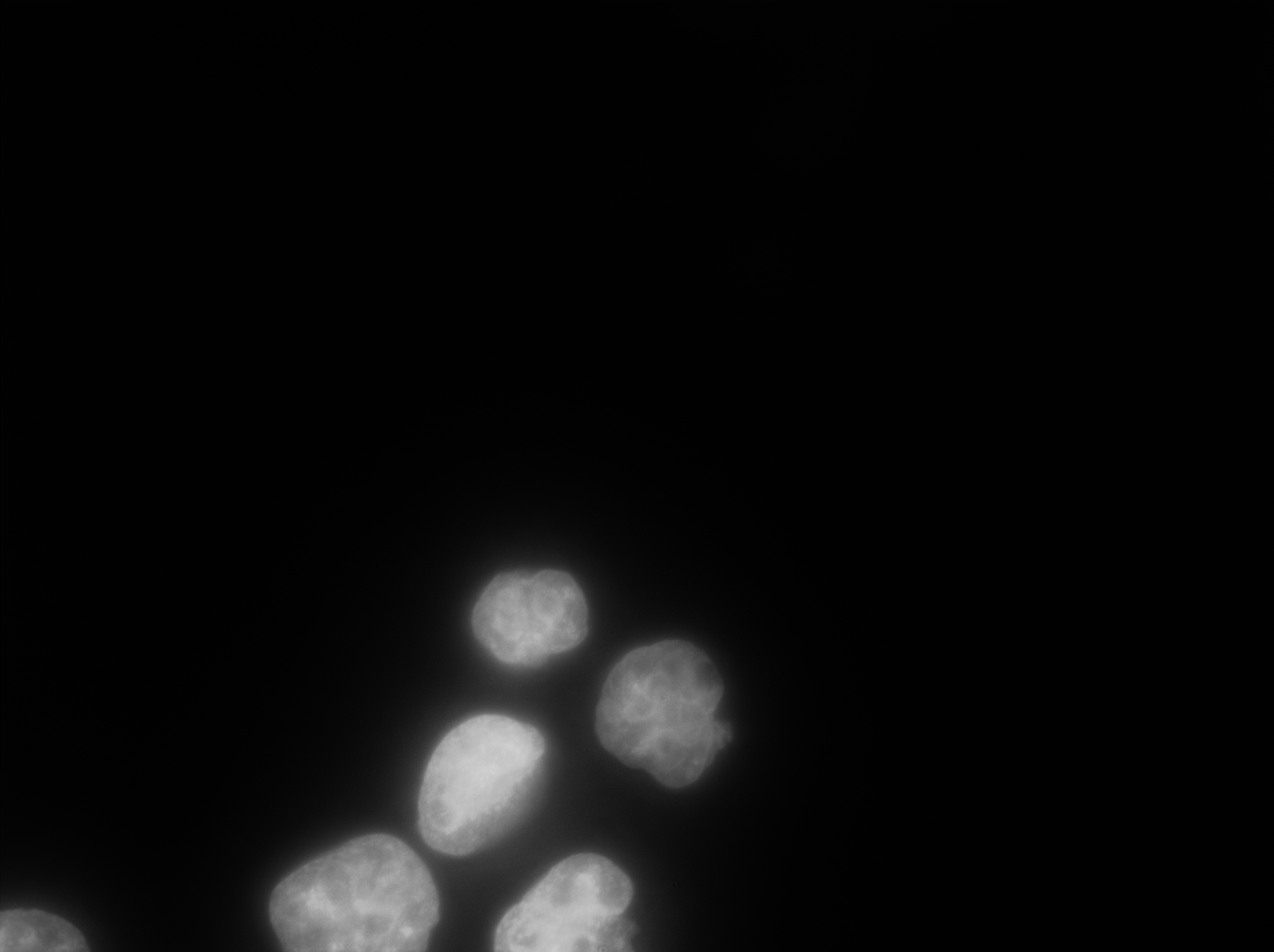

Supplement: Supplementary file 21 — Source data Fig. 6 part 2 [file 44319_2026_742_MOESM21_ESM.zip › Figure 6 Part 2/Fig 6abcd Cas9 TPGS1-KO acetylated tubulin atubulin part 2/TPGS1-KO R3 9-13-24 LT19.Project Maximum Z_XY1726764103_Z0_T0_C0.tif]

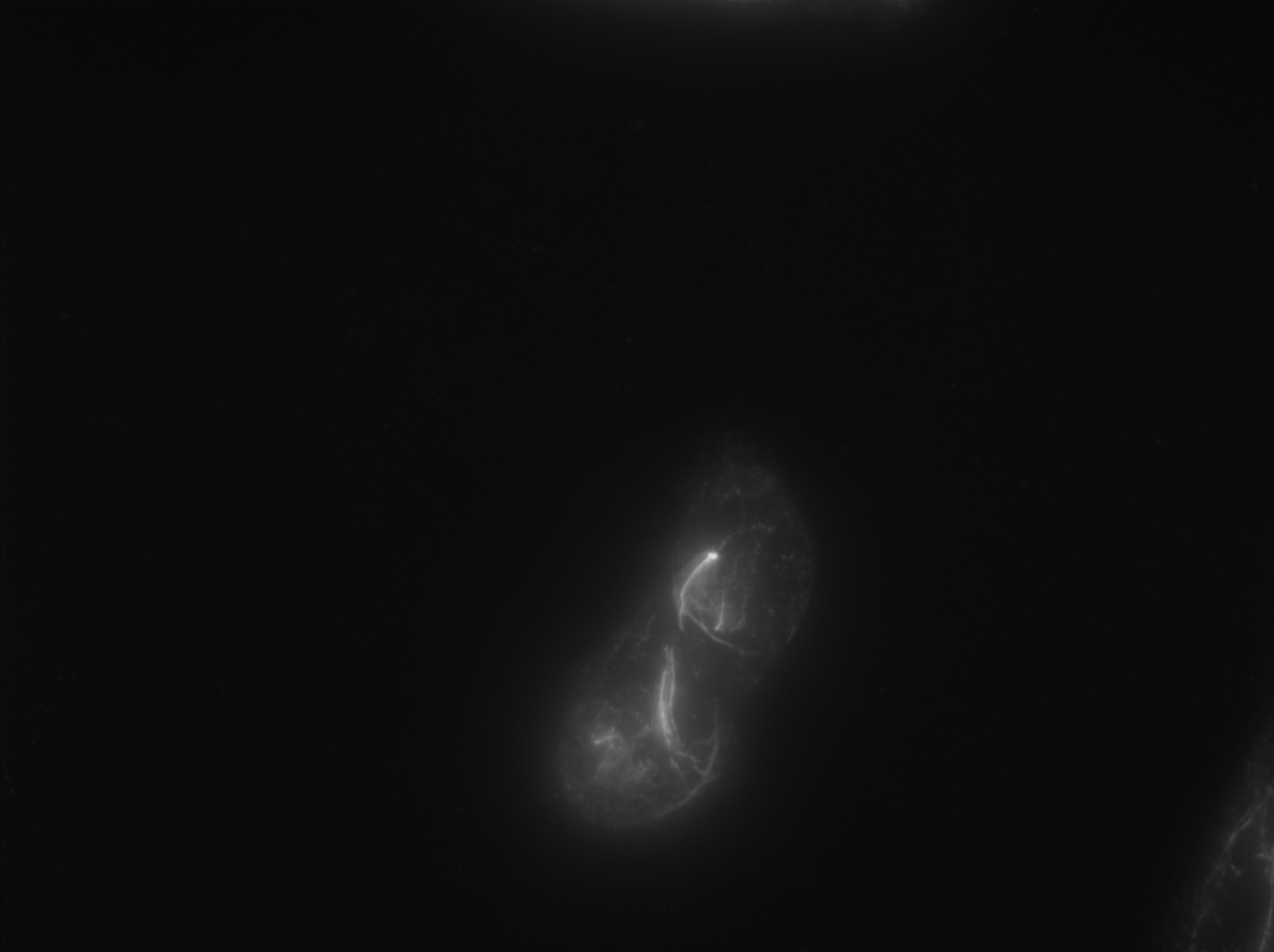

Supplement: Supplementary file 21 — Source data Fig. 6 part 2 [file 44319_2026_742_MOESM21_ESM.zip › Figure 6 Part 2/Fig 6abcd Cas9 TPGS1-KO acetylated tubulin atubulin part 2/TPGS1-KO R2 9-11-24 PA18.Project Maximum Z_XY1726268793_Z0_T0_C2.tif]

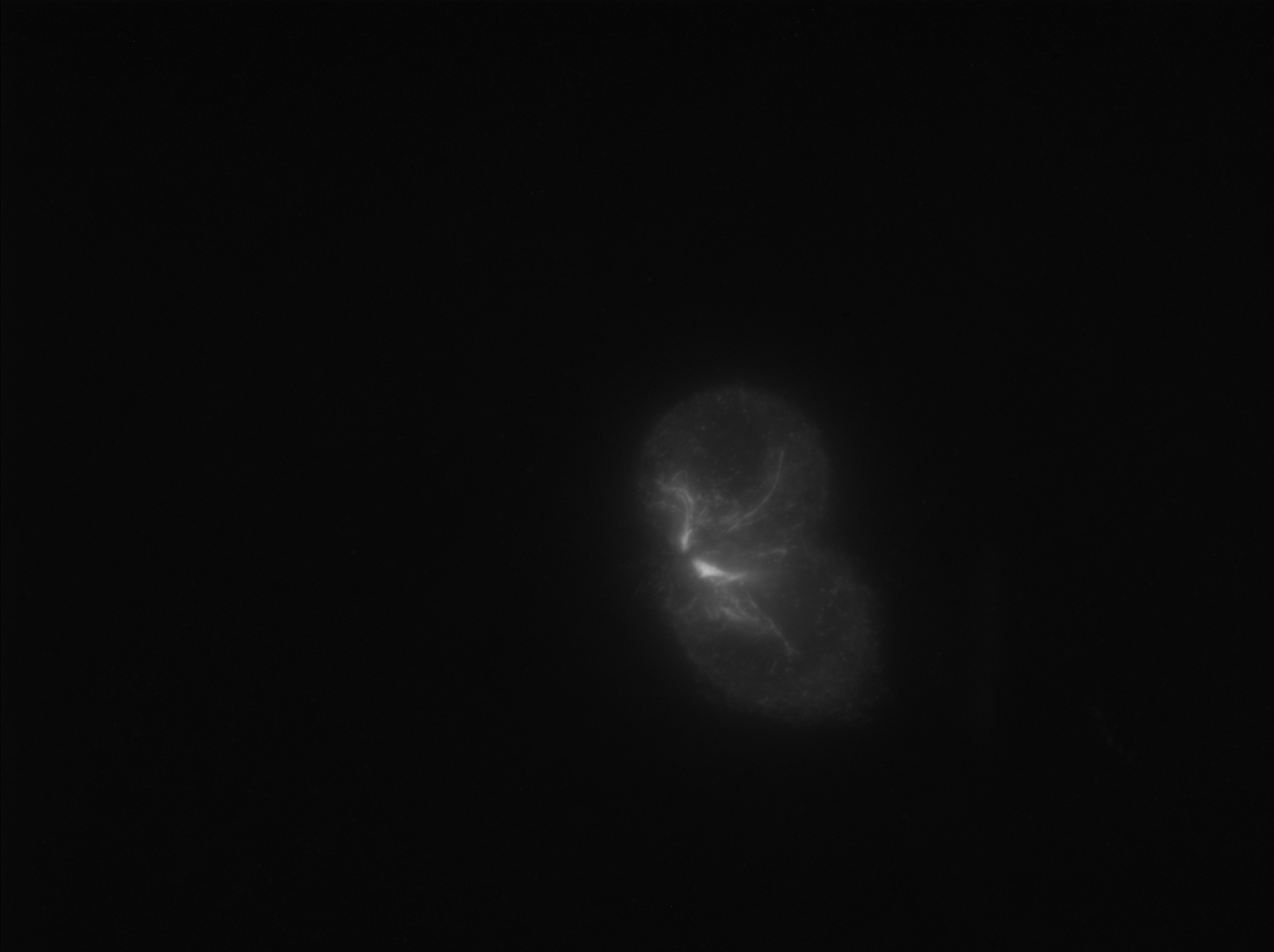

Supplement: Supplementary file 21 — Source data Fig. 6 part 2 [file 44319_2026_742_MOESM21_ESM.zip › Figure 6 Part 2/Fig 6abcd Cas9 TPGS1-KO acetylated tubulin atubulin part 2/TPGS1-KO R2 9-11-24 LT2.Project Maximum Z_XY1726259084_Z0_T0_C2.tif]

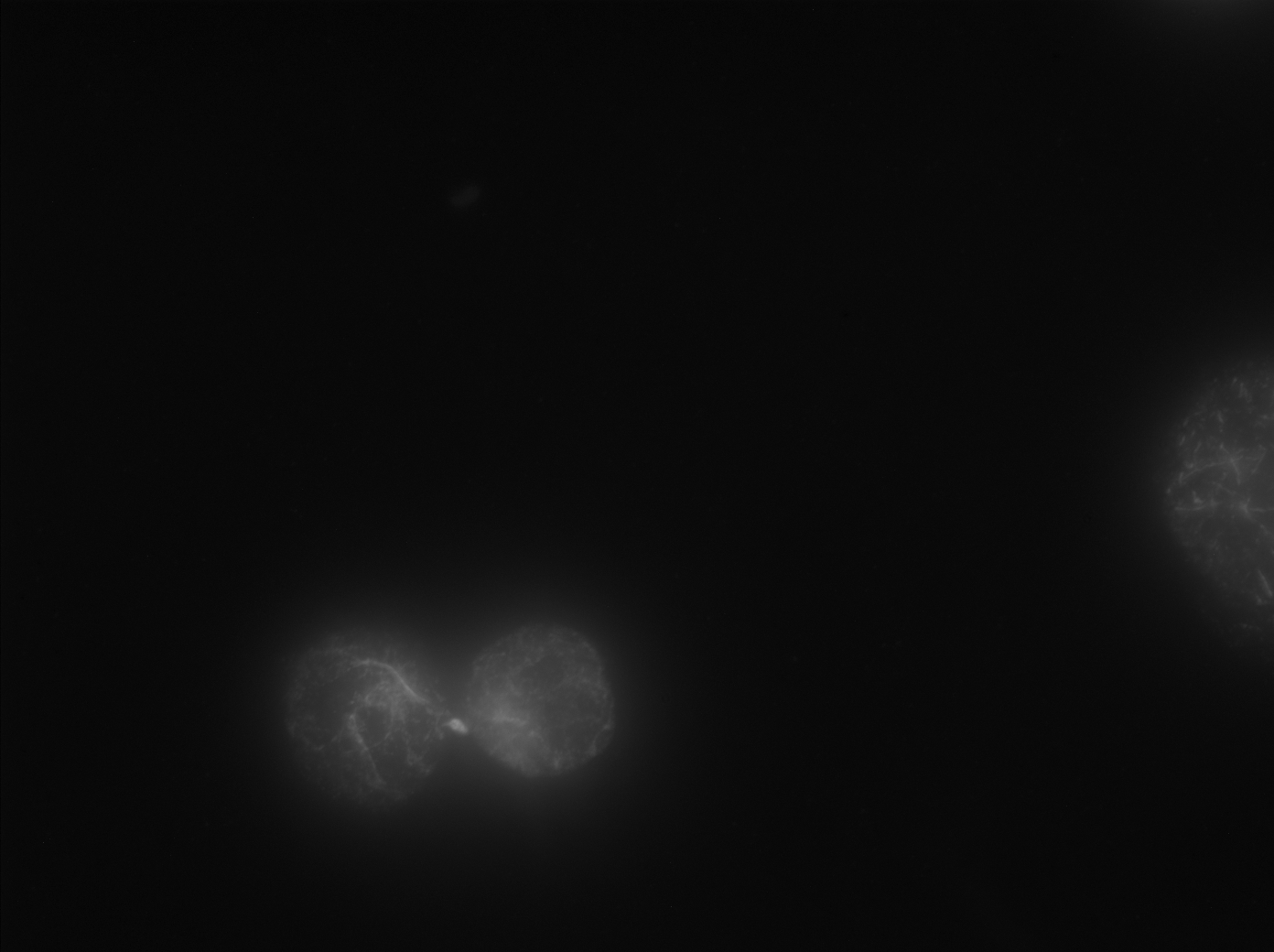

Supplement: Supplementary file 21 — Source data Fig. 6 part 2 [file 44319_2026_742_MOESM21_ESM.zip › Figure 6 Part 2/Fig 6abcd Cas9 TPGS1-KO acetylated tubulin atubulin part 2/TPGS1-KO R2 9-11-24 PA6.Project Maximum Z_XY1726261339_Z0_T0_C2.tif]

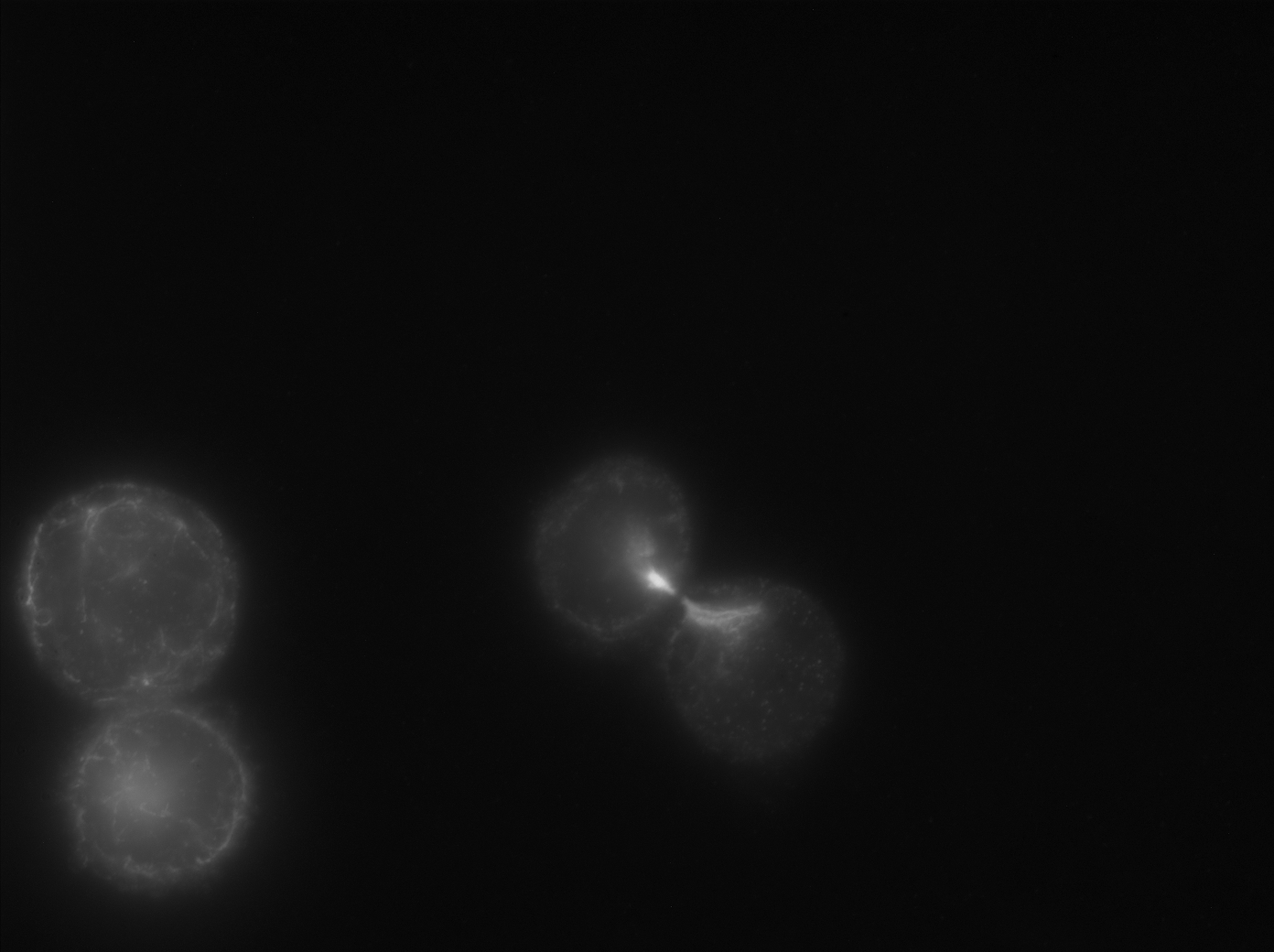

Supplement: Supplementary file 21 — Source data Fig. 6 part 2 [file 44319_2026_742_MOESM21_ESM.zip › Figure 6 Part 2/Fig 6abcd Cas9 TPGS1-KO acetylated tubulin atubulin part 2/TPGS1-KO R2 9-11-24 LT3.Project Maximum Z_XY1726259338_Z0_T0_C2.tif]

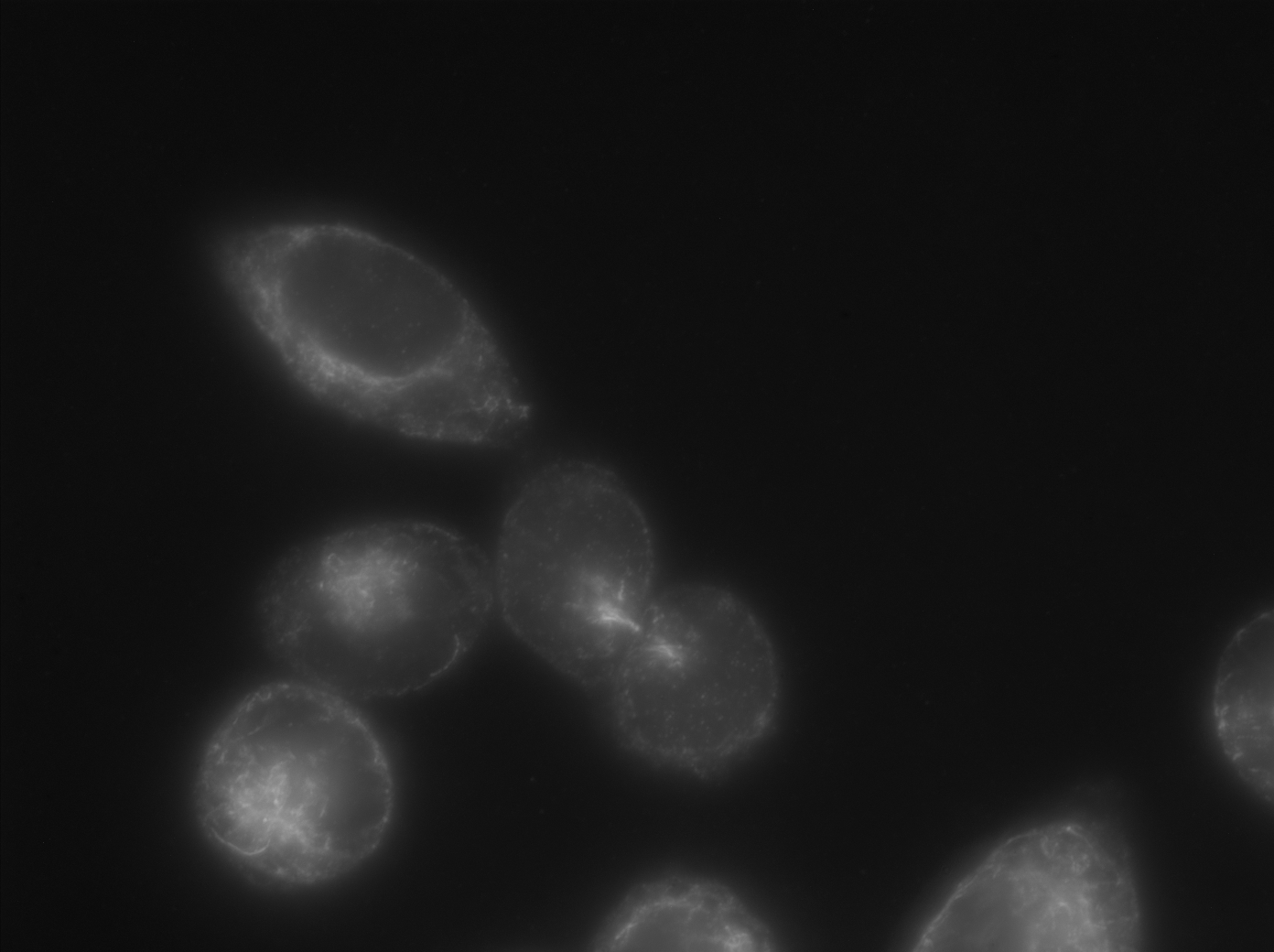

Supplement: Supplementary file 21 — Source data Fig. 6 part 2 [file 44319_2026_742_MOESM21_ESM.zip › Figure 6 Part 2/Fig 6abcd Cas9 TPGS1-KO acetylated tubulin atubulin part 2/TPGS1-KO R2 9-11-24 PA12.Project Maximum Z_XY1726265237_Z0_T0_C2.tif]

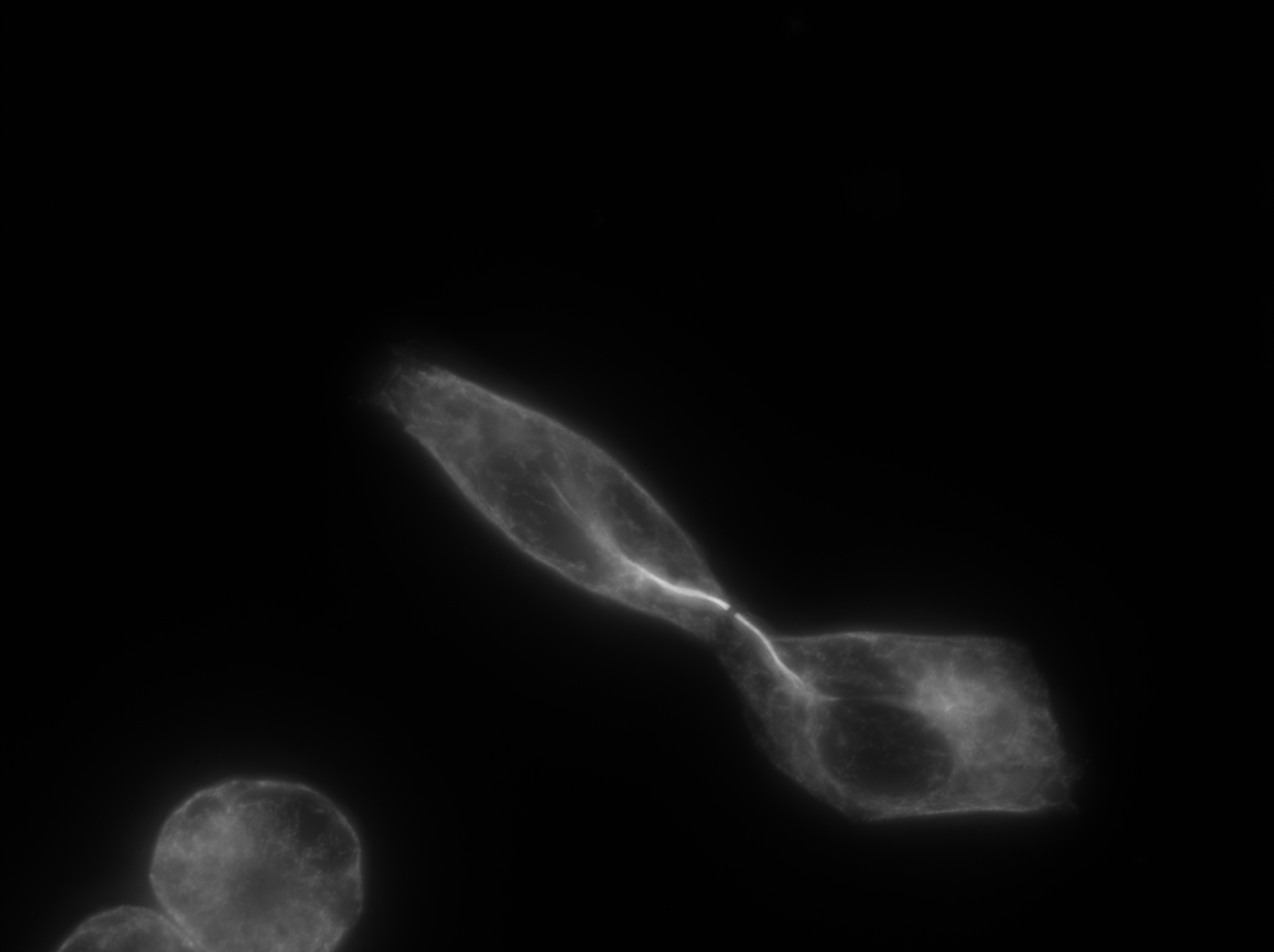

Supplement: Supplementary file 21 — Source data Fig. 6 part 2 [file 44319_2026_742_MOESM21_ESM.zip › Figure 6 Part 2/Fig 6abcd Cas9 TPGS1-KO acetylated tubulin atubulin part 2/TPGS1-KO R2 9-11-24 LT29.Project Maximum Z_XY1726269729_Z0_T0_C1.tif]

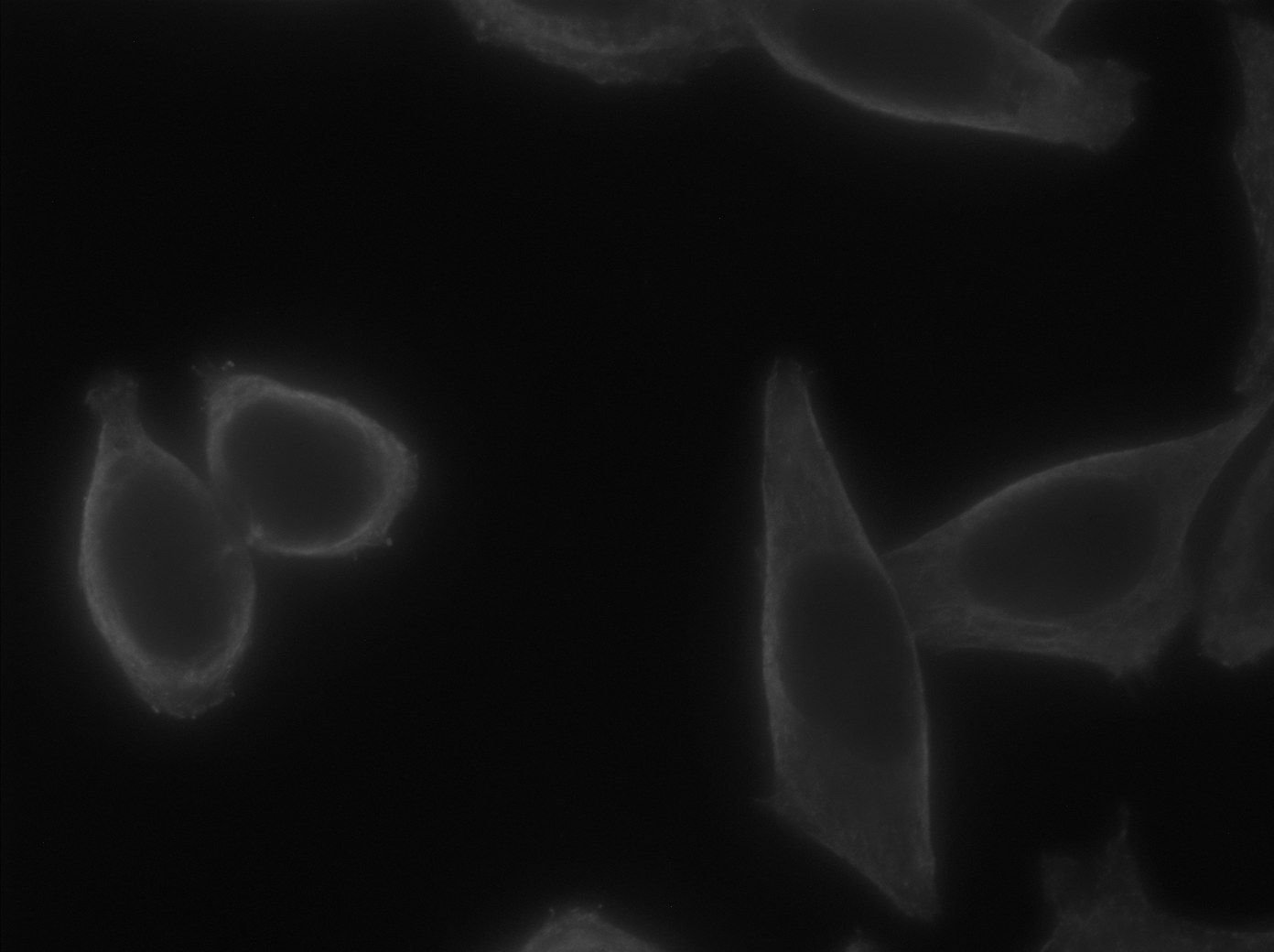

Supplement: Supplementary file 21 — Source data Fig. 6 part 2 [file 44319_2026_742_MOESM21_ESM.zip › Figure 6 Part 2/Fig 6abcd Cas9 TPGS1-KO acetylated tubulin atubulin part 2/TPGS1-KO R3 9-13-24 LT8 PA3.Project Maximum Z_XY1726760773_Z0_T0_C1.tif]

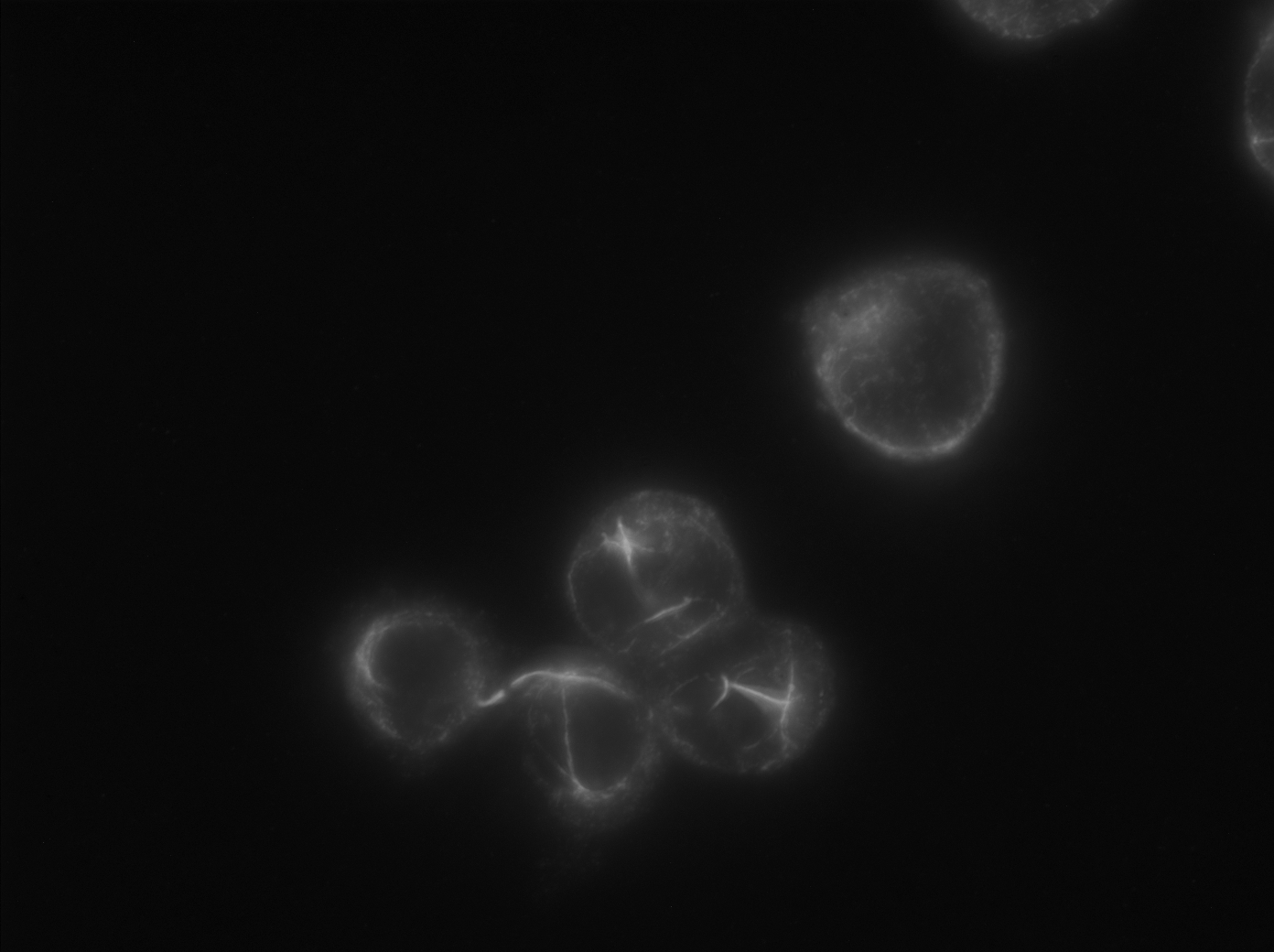

Supplement: Supplementary file 21 — Source data Fig. 6 part 2 [file 44319_2026_742_MOESM21_ESM.zip › Figure 6 Part 2/Fig 6abcd Cas9 TPGS1-KO acetylated tubulin atubulin part 2/TPGS1-KO R2 9-11-24 LT25.Project Maximum Z_XY1726268436_Z0_T0_C2.tif]

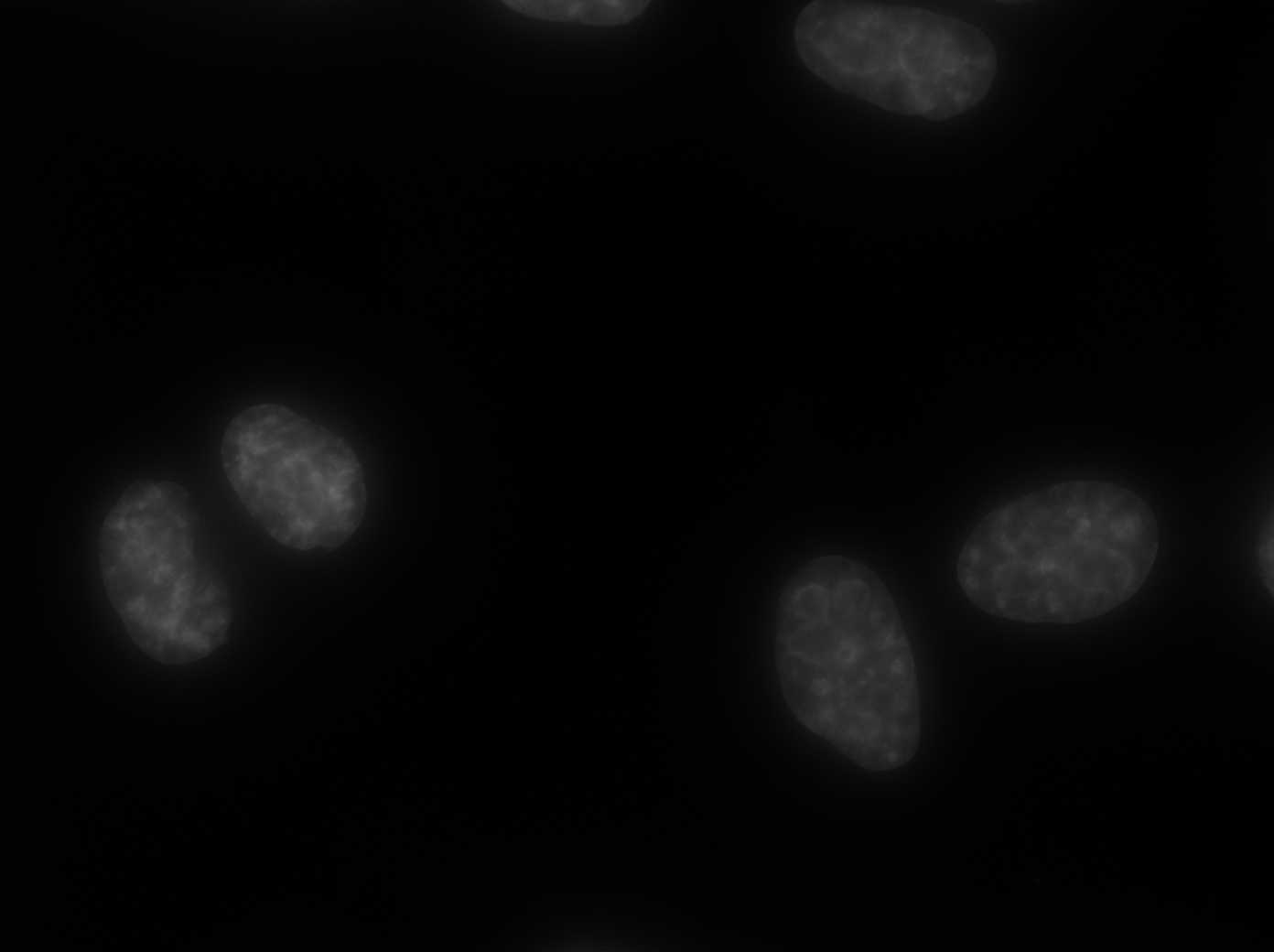

Supplement: Supplementary file 21 — Source data Fig. 6 part 2 [file 44319_2026_742_MOESM21_ESM.zip › Figure 6 Part 2/Fig 6abcd Cas9 TPGS1-KO acetylated tubulin atubulin part 2/TPGS1-KO R3 9-13-24 LT8 PA3.Project Maximum Z_XY1726760773_Z0_T0_C0.tif]

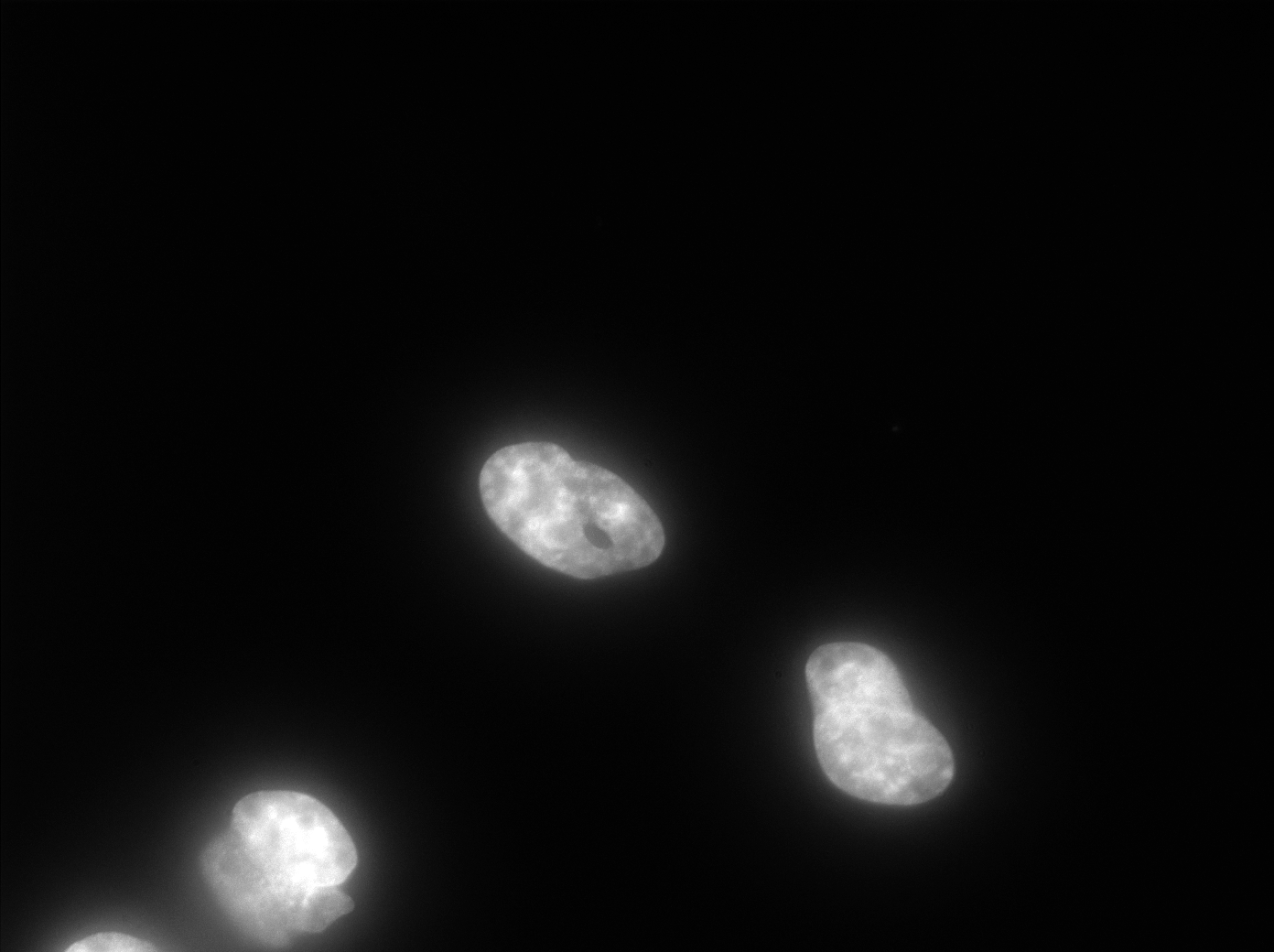

Supplement: Supplementary file 21 — Source data Fig. 6 part 2 [file 44319_2026_742_MOESM21_ESM.zip › Figure 6 Part 2/Fig 6abcd Cas9 TPGS1-KO acetylated tubulin atubulin part 2/TPGS1-KO R2 9-11-24 LT29.Project Maximum Z_XY1726269729_Z0_T0_C0.tif]

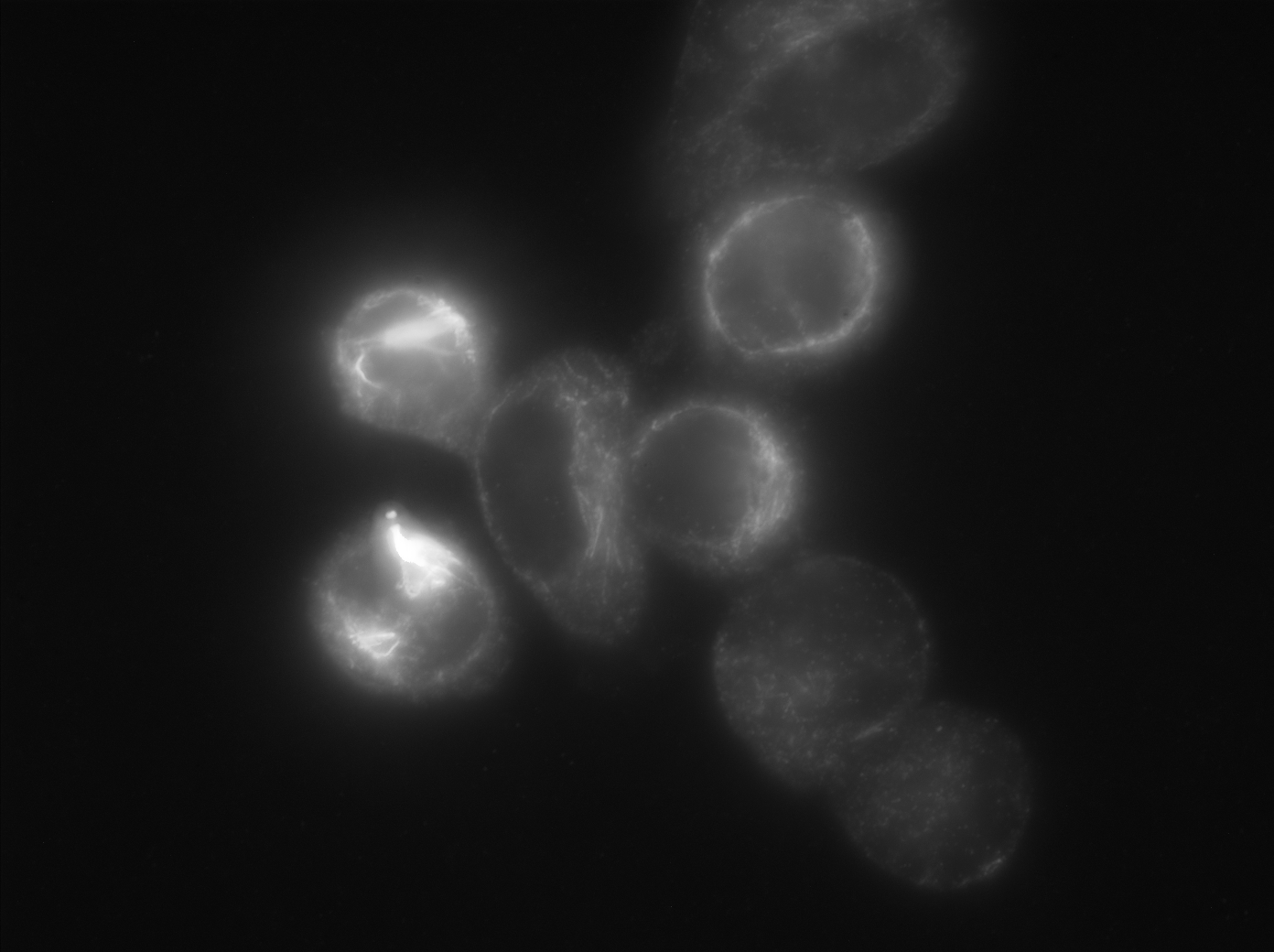

Supplement: Supplementary file 21 — Source data Fig. 6 part 2 [file 44319_2026_742_MOESM21_ESM.zip › Figure 6 Part 2/Fig 6abcd Cas9 TPGS1-KO acetylated tubulin atubulin part 2/TPGS1-KO R2 9-11-24 PA19.Project Maximum Z_XY1726269018_Z0_T0_C2.tif]

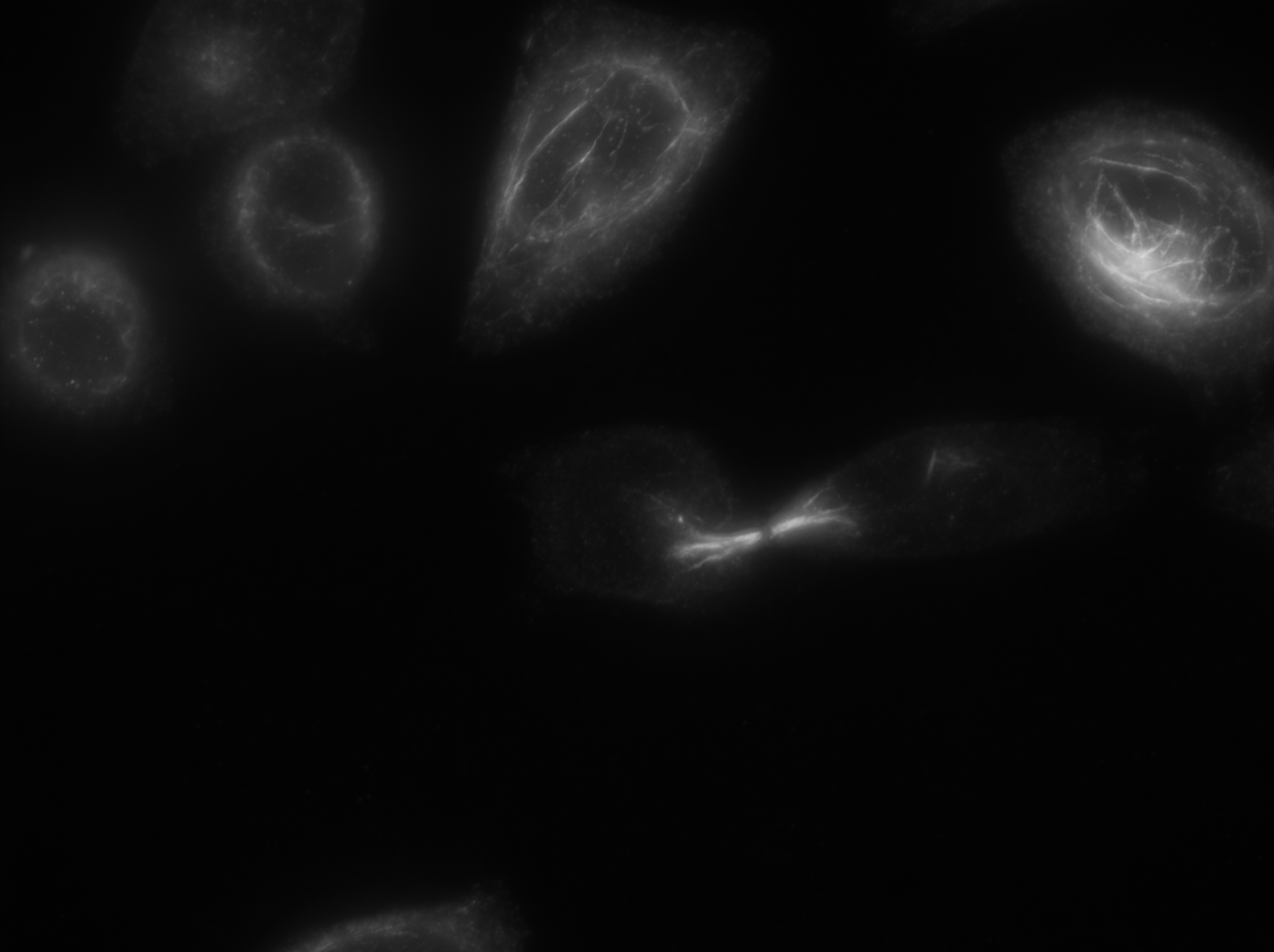

Supplement: Supplementary file 21 — Source data Fig. 6 part 2 [file 44319_2026_742_MOESM21_ESM.zip › Figure 6 Part 2/Fig 6abcd Cas9 TPGS1-KO acetylated tubulin atubulin part 2/TPGS1-KO R3 9-13-24 LT27.Project Maximum Z_XY1726764803_Z0_T0_C2.tif]

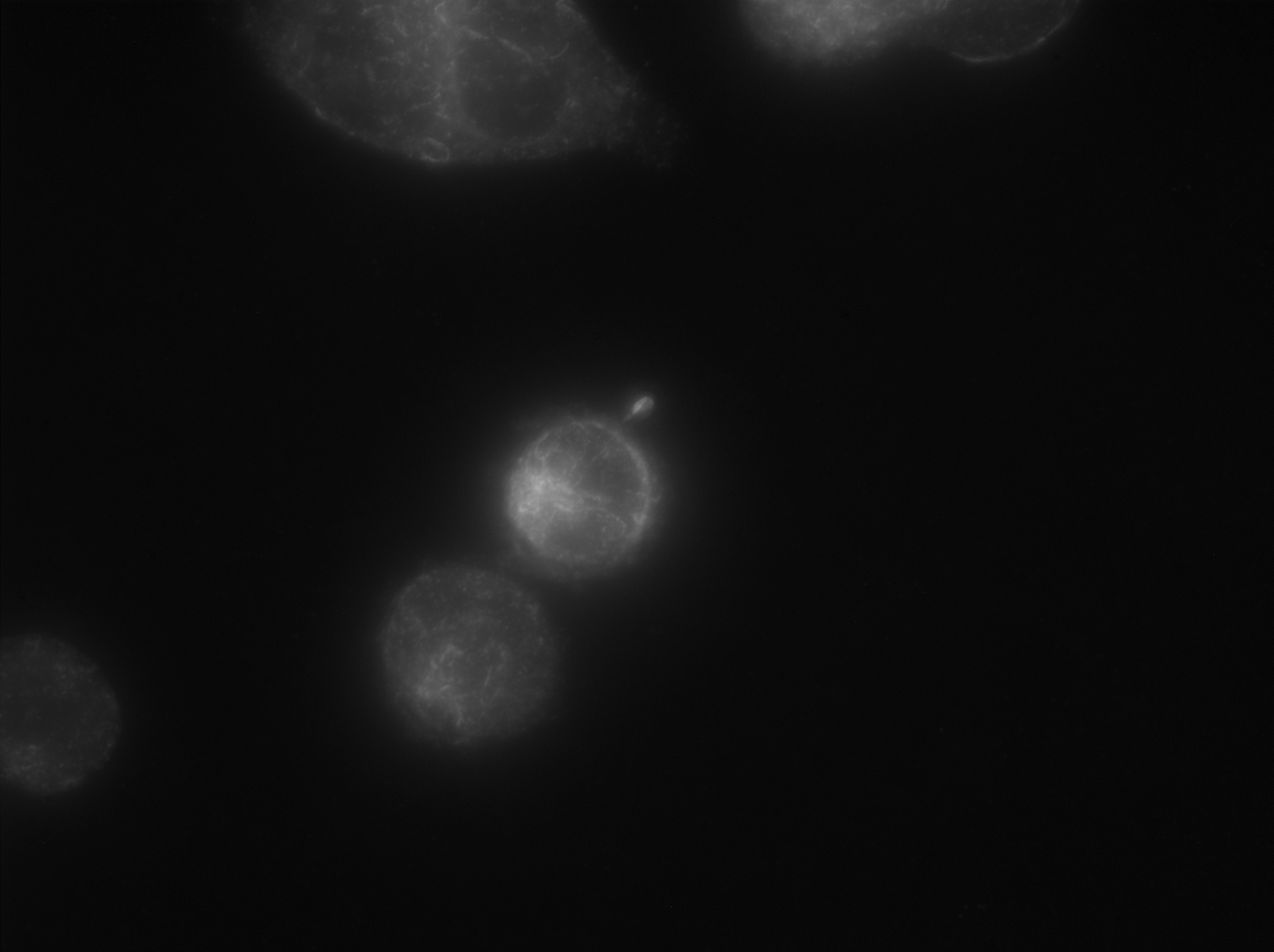

Supplement: Supplementary file 21 — Source data Fig. 6 part 2 [file 44319_2026_742_MOESM21_ESM.zip › Figure 6 Part 2/Fig 6abcd Cas9 TPGS1-KO acetylated tubulin atubulin part 2/TPGS1-KO R2 9-11-24 PA2.Project Maximum Z_XY1726259232_Z0_T0_C2.tif]

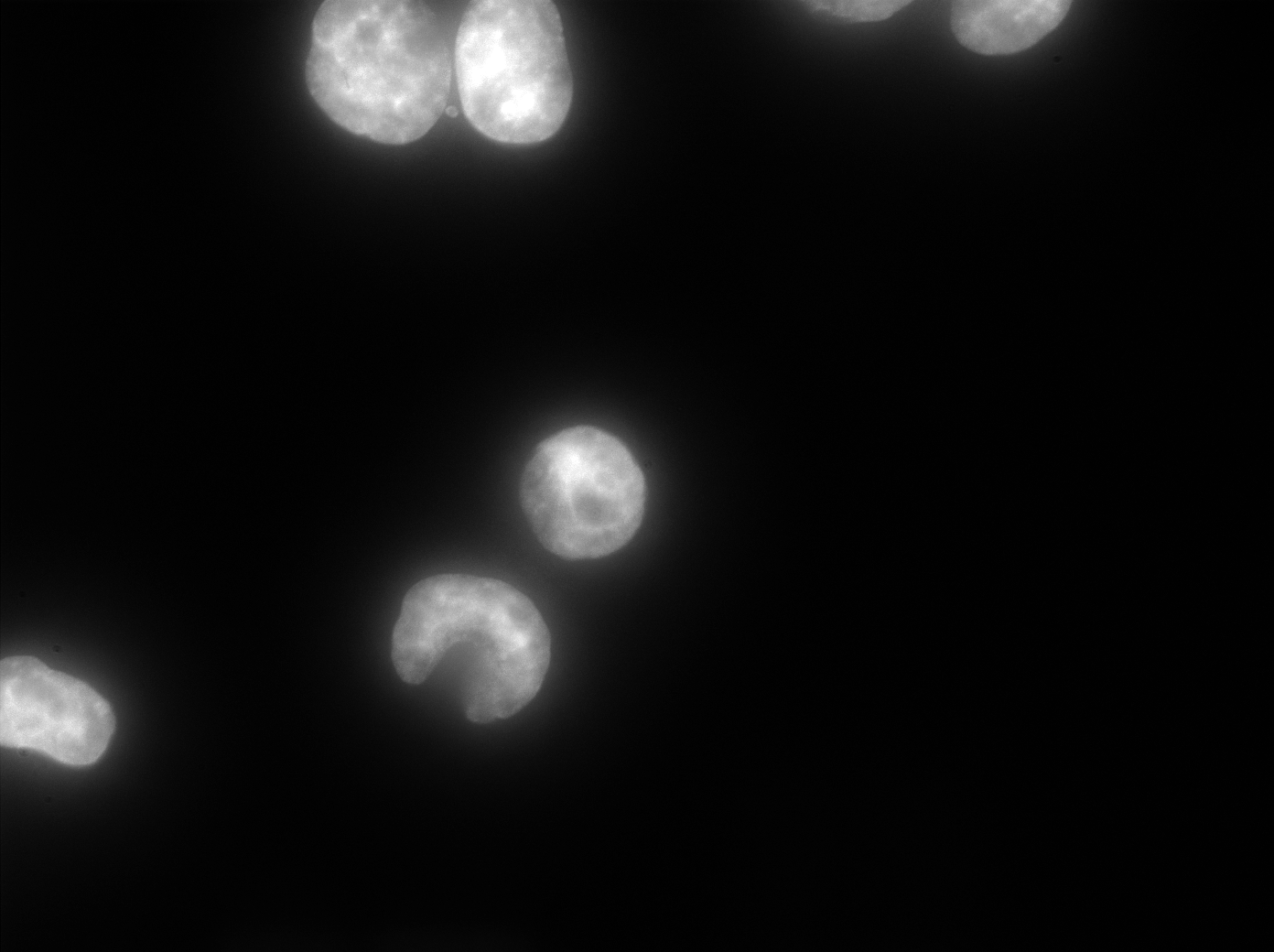

Supplement: Supplementary file 21 — Source data Fig. 6 part 2 [file 44319_2026_742_MOESM21_ESM.zip › Figure 6 Part 2/Fig 6abcd Cas9 TPGS1-KO acetylated tubulin atubulin part 2/TPGS1-KO R2 9-11-24 PA2.Project Maximum Z_XY1726259232_Z0_T0_C0.tif]

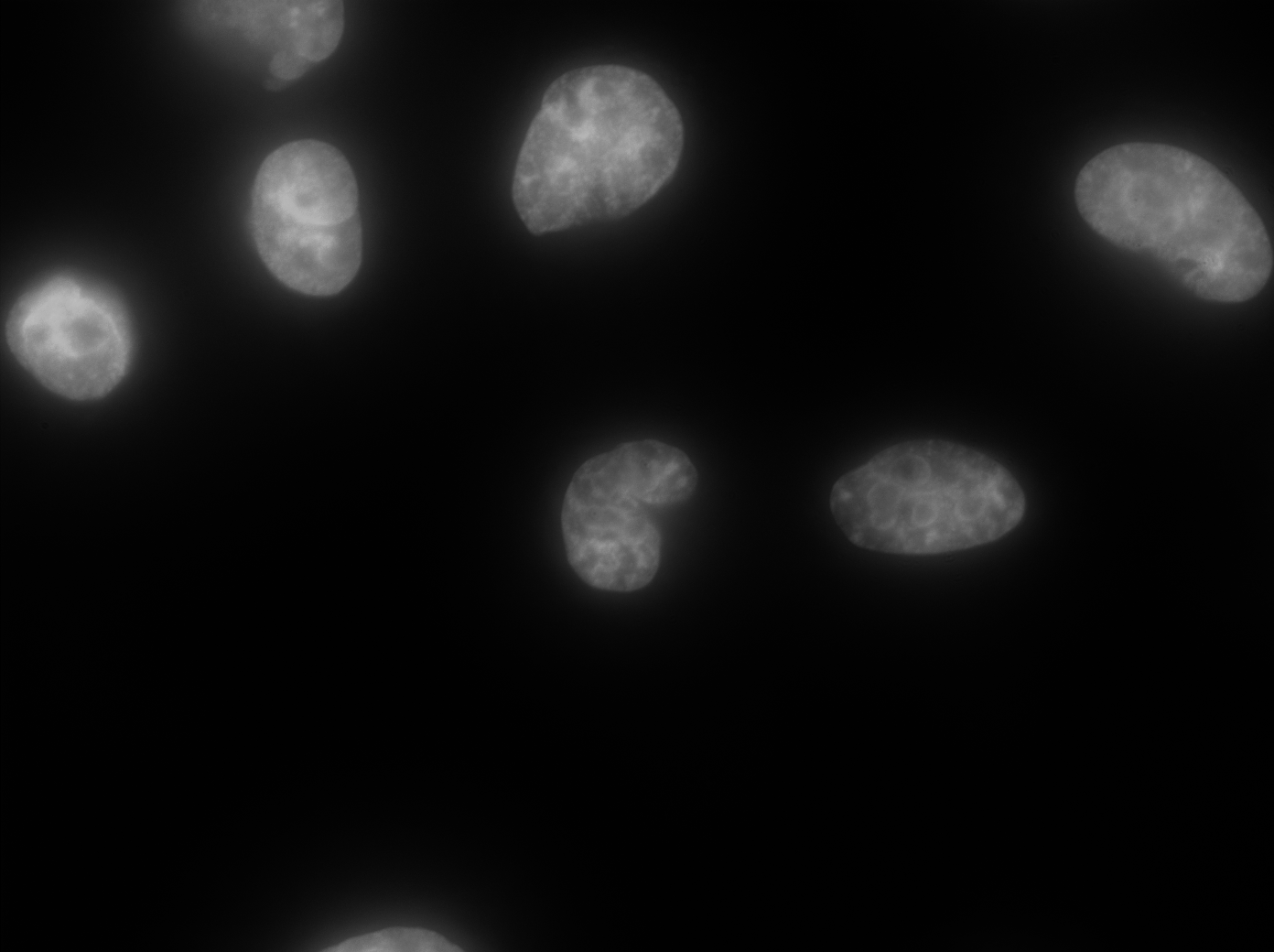

Supplement: Supplementary file 21 — Source data Fig. 6 part 2 [file 44319_2026_742_MOESM21_ESM.zip › Figure 6 Part 2/Fig 6abcd Cas9 TPGS1-KO acetylated tubulin atubulin part 2/TPGS1-KO R3 9-13-24 LT27.Project Maximum Z_XY1726764803_Z0_T0_C0.tif]

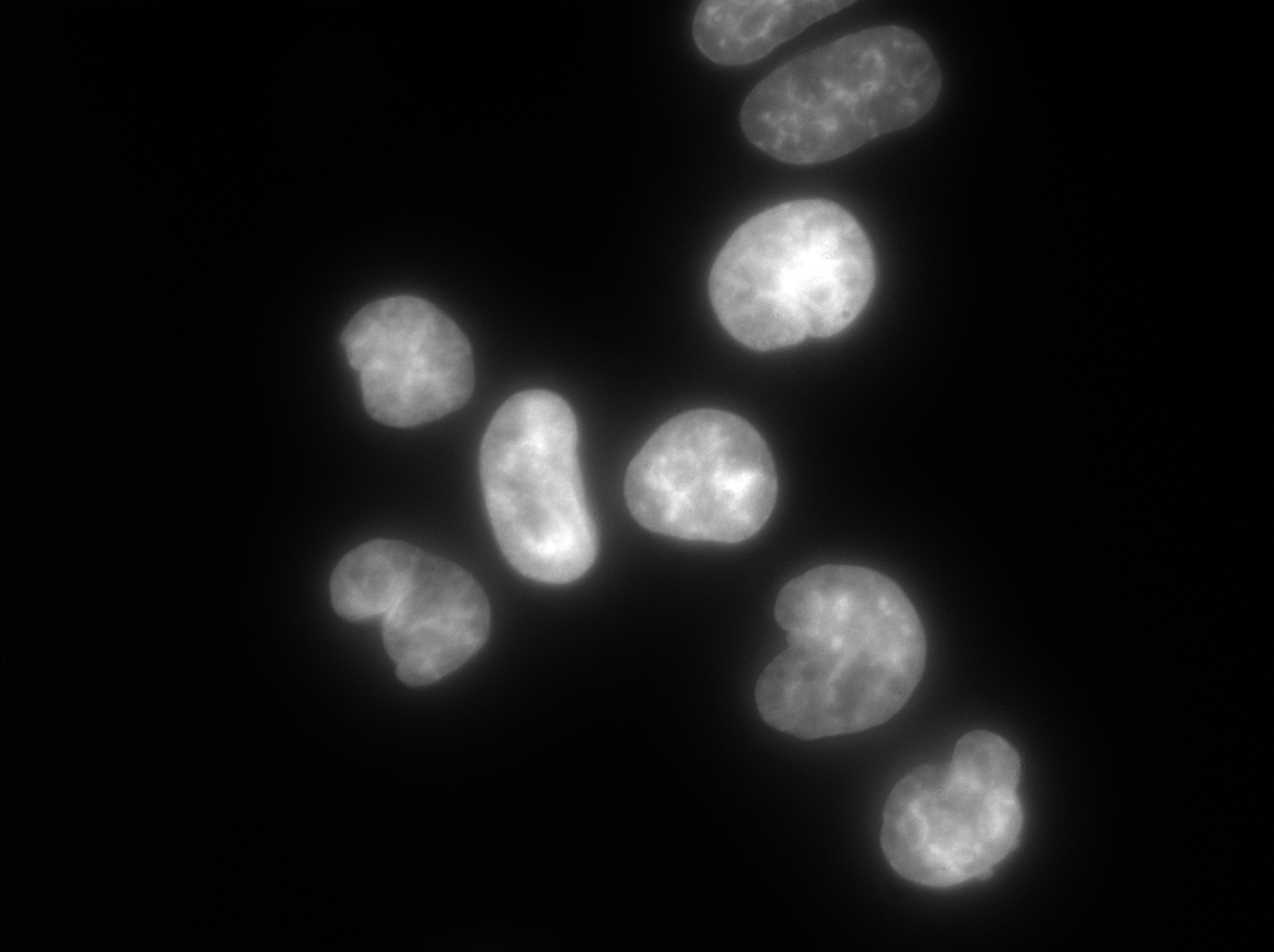

Supplement: Supplementary file 21 — Source data Fig. 6 part 2 [file 44319_2026_742_MOESM21_ESM.zip › Figure 6 Part 2/Fig 6abcd Cas9 TPGS1-KO acetylated tubulin atubulin part 2/TPGS1-KO R2 9-11-24 PA19.Project Maximum Z_XY1726269018_Z0_T0_C0.tif]

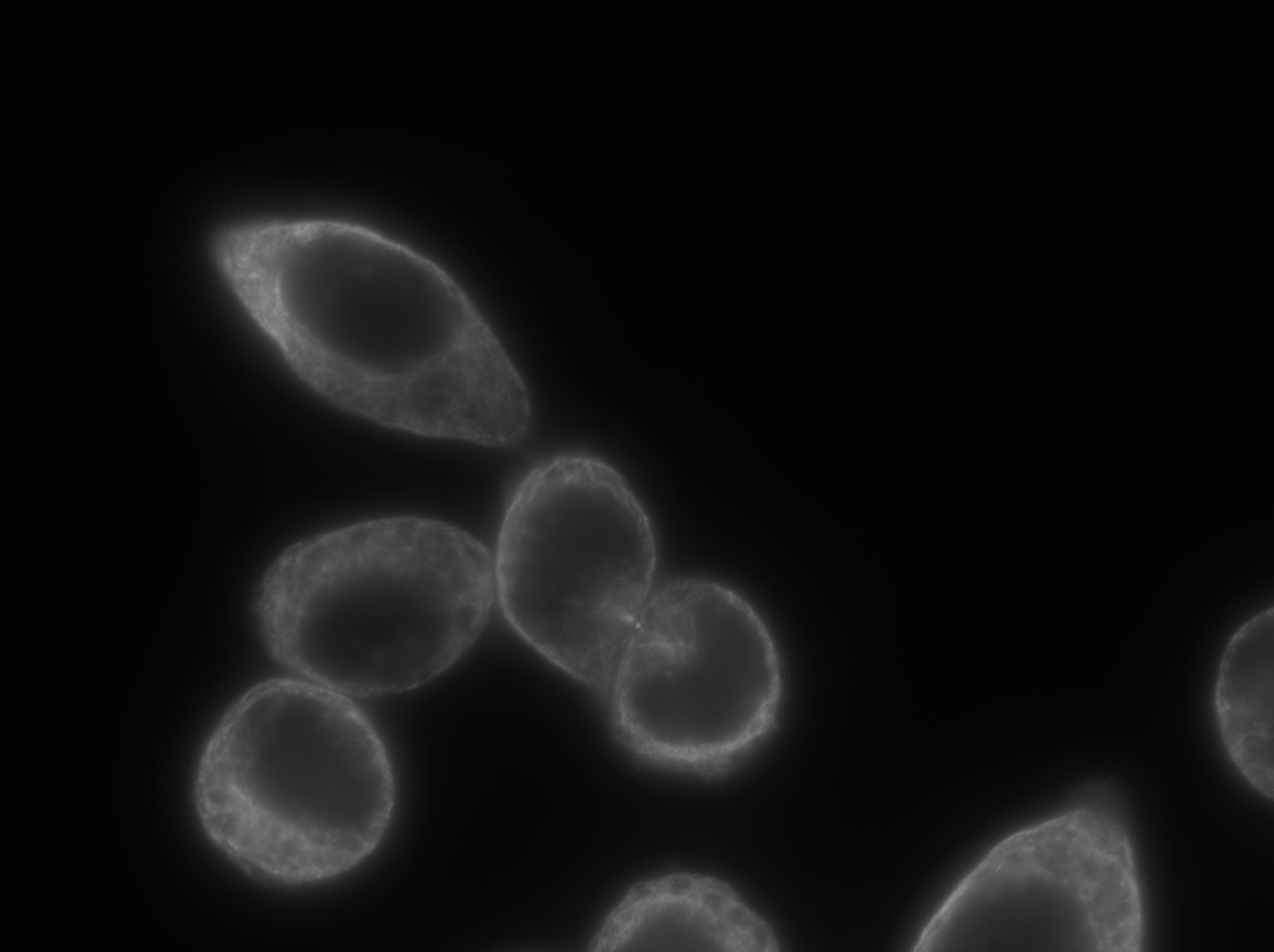

Supplement: Supplementary file 21 — Source data Fig. 6 part 2 [file 44319_2026_742_MOESM21_ESM.zip › Figure 6 Part 2/Fig 6abcd Cas9 TPGS1-KO acetylated tubulin atubulin part 2/TPGS1-KO R2 9-11-24 PA12.Project Maximum Z_XY1726265237_Z0_T0_C1.tif]

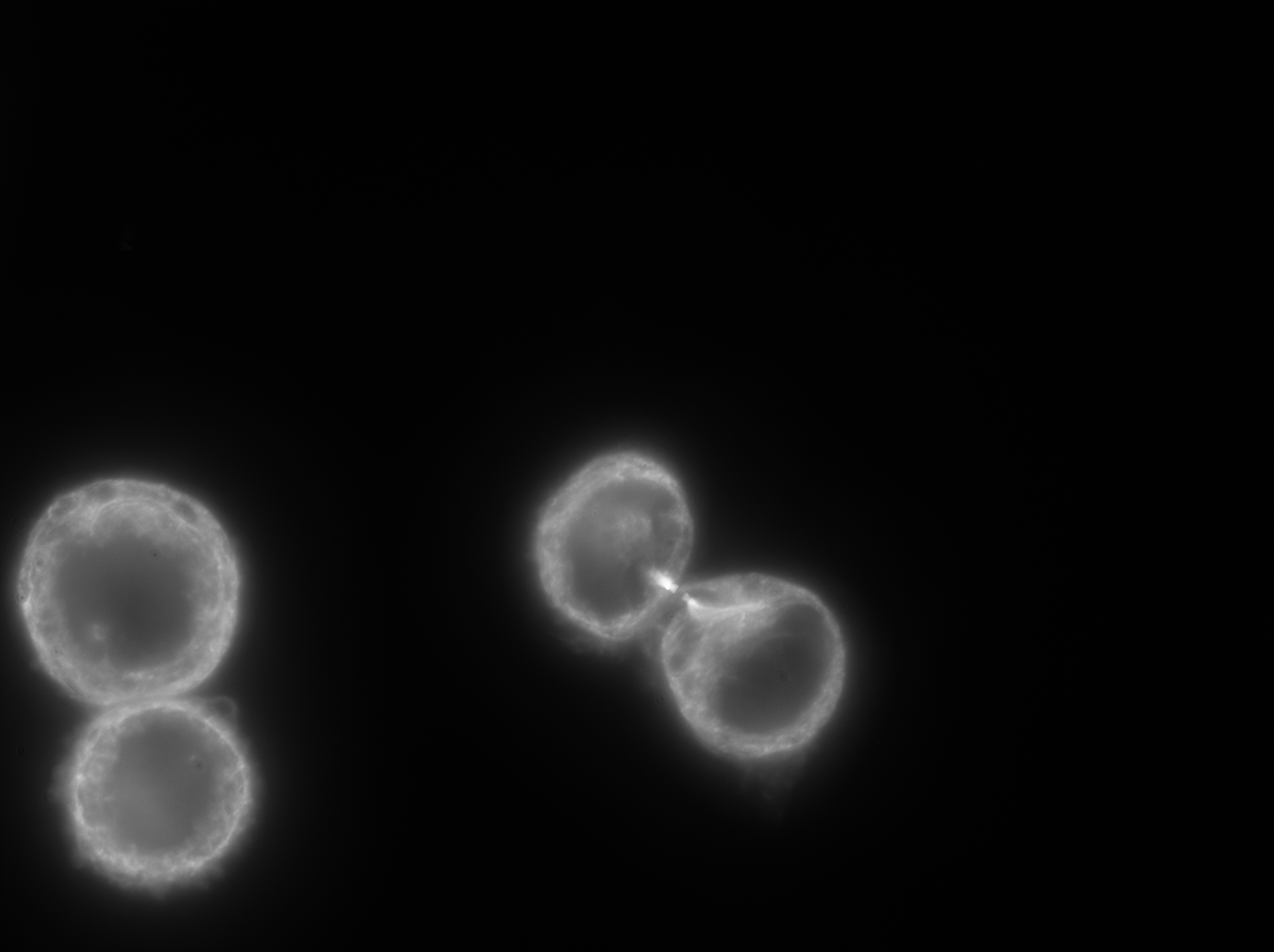

Supplement: Supplementary file 21 — Source data Fig. 6 part 2 [file 44319_2026_742_MOESM21_ESM.zip › Figure 6 Part 2/Fig 6abcd Cas9 TPGS1-KO acetylated tubulin atubulin part 2/TPGS1-KO R2 9-11-24 LT3.Project Maximum Z_XY1726259338_Z0_T0_C1.tif]

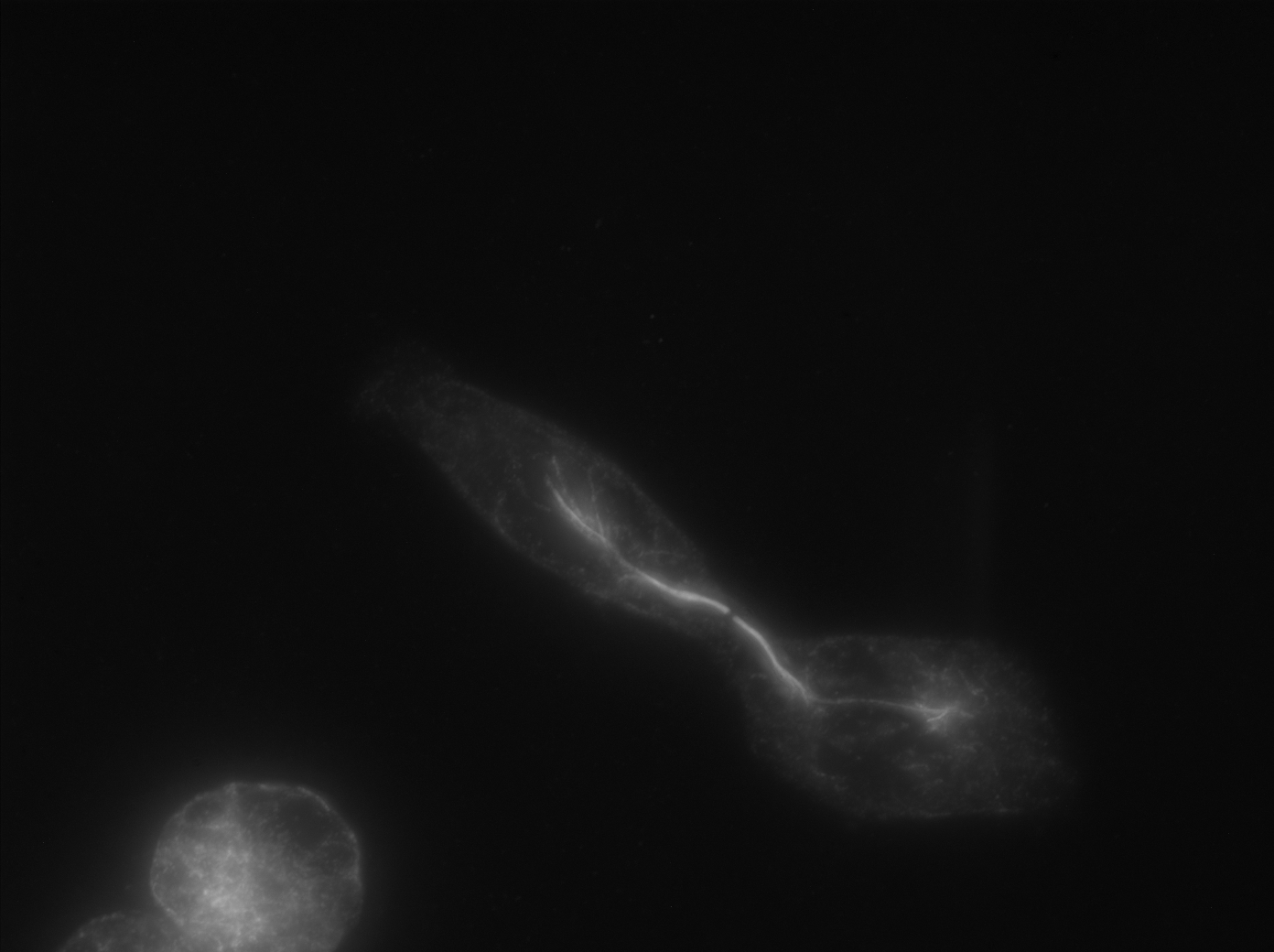

Supplement: Supplementary file 21 — Source data Fig. 6 part 2 [file 44319_2026_742_MOESM21_ESM.zip › Figure 6 Part 2/Fig 6abcd Cas9 TPGS1-KO acetylated tubulin atubulin part 2/TPGS1-KO R2 9-11-24 LT29.Project Maximum Z_XY1726269729_Z0_T0_C2.tif]

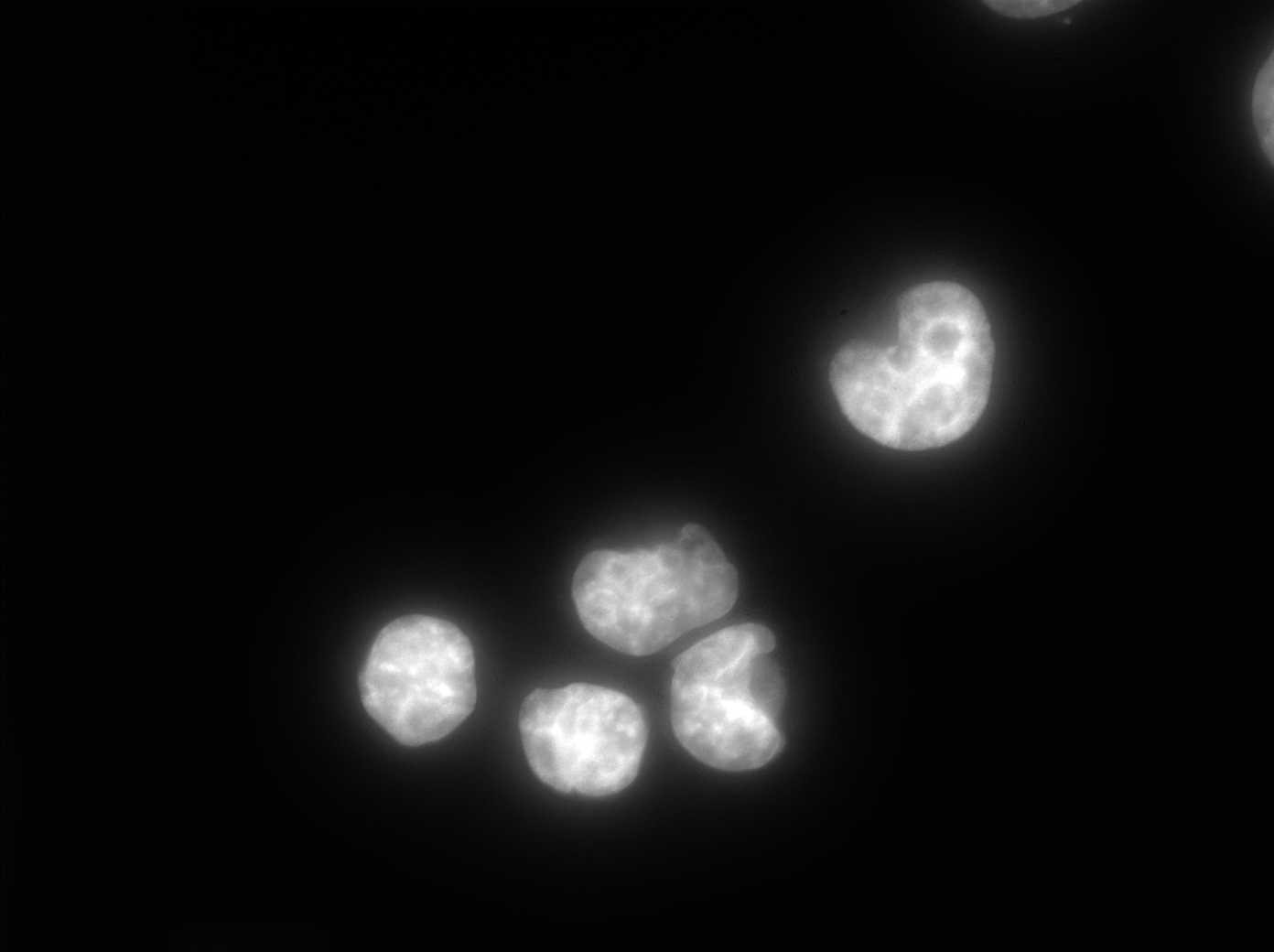

Supplement: Supplementary file 21 — Source data Fig. 6 part 2 [file 44319_2026_742_MOESM21_ESM.zip › Figure 6 Part 2/Fig 6abcd Cas9 TPGS1-KO acetylated tubulin atubulin part 2/TPGS1-KO R2 9-11-24 LT25.Project Maximum Z_XY1726268436_Z0_T0_C0.tif]

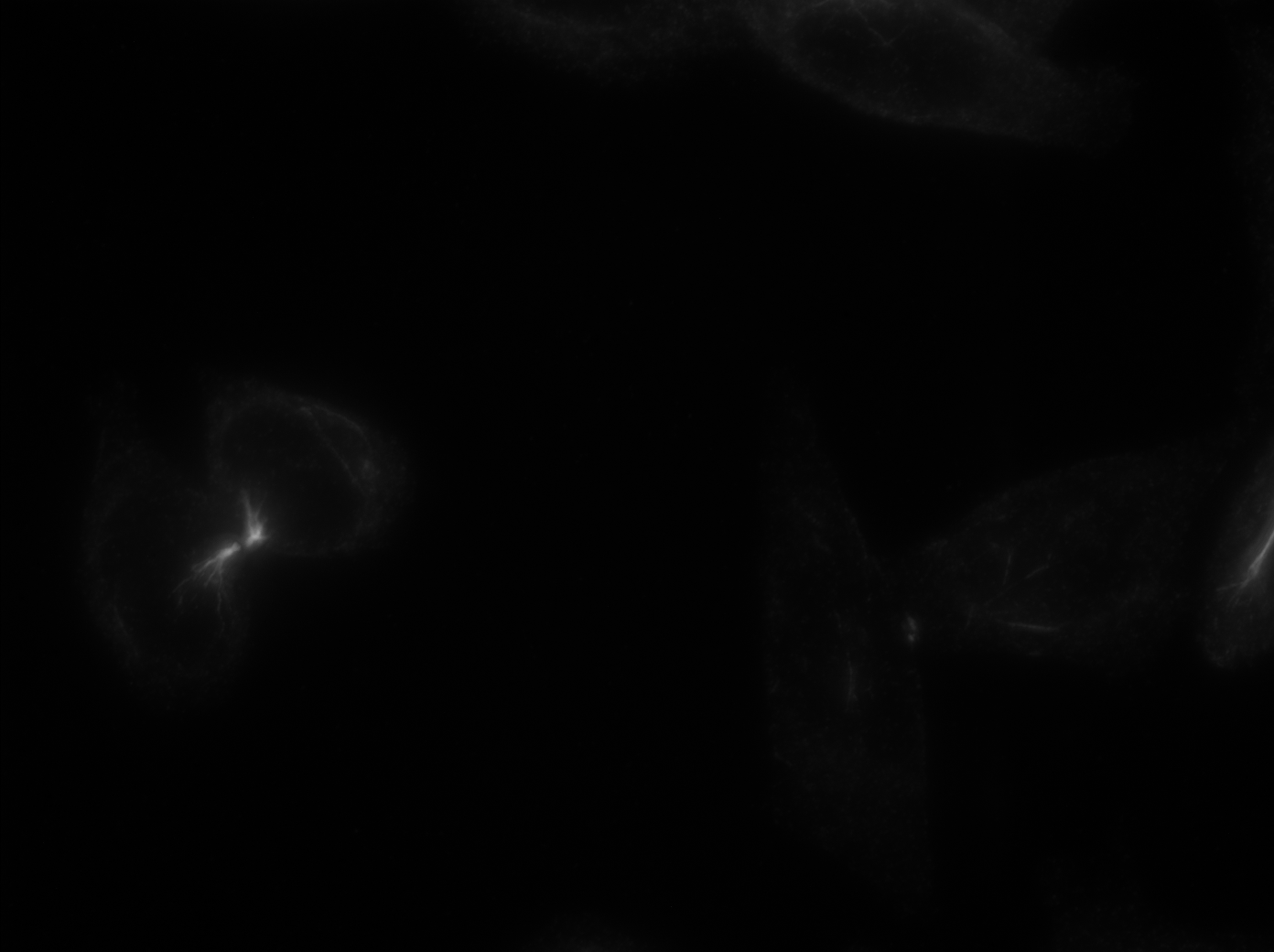

Supplement: Supplementary file 21 — Source data Fig. 6 part 2 [file 44319_2026_742_MOESM21_ESM.zip › Figure 6 Part 2/Fig 6abcd Cas9 TPGS1-KO acetylated tubulin atubulin part 2/TPGS1-KO R3 9-13-24 LT8 PA3.Project Maximum Z_XY1726760773_Z0_T0_C2.tif]

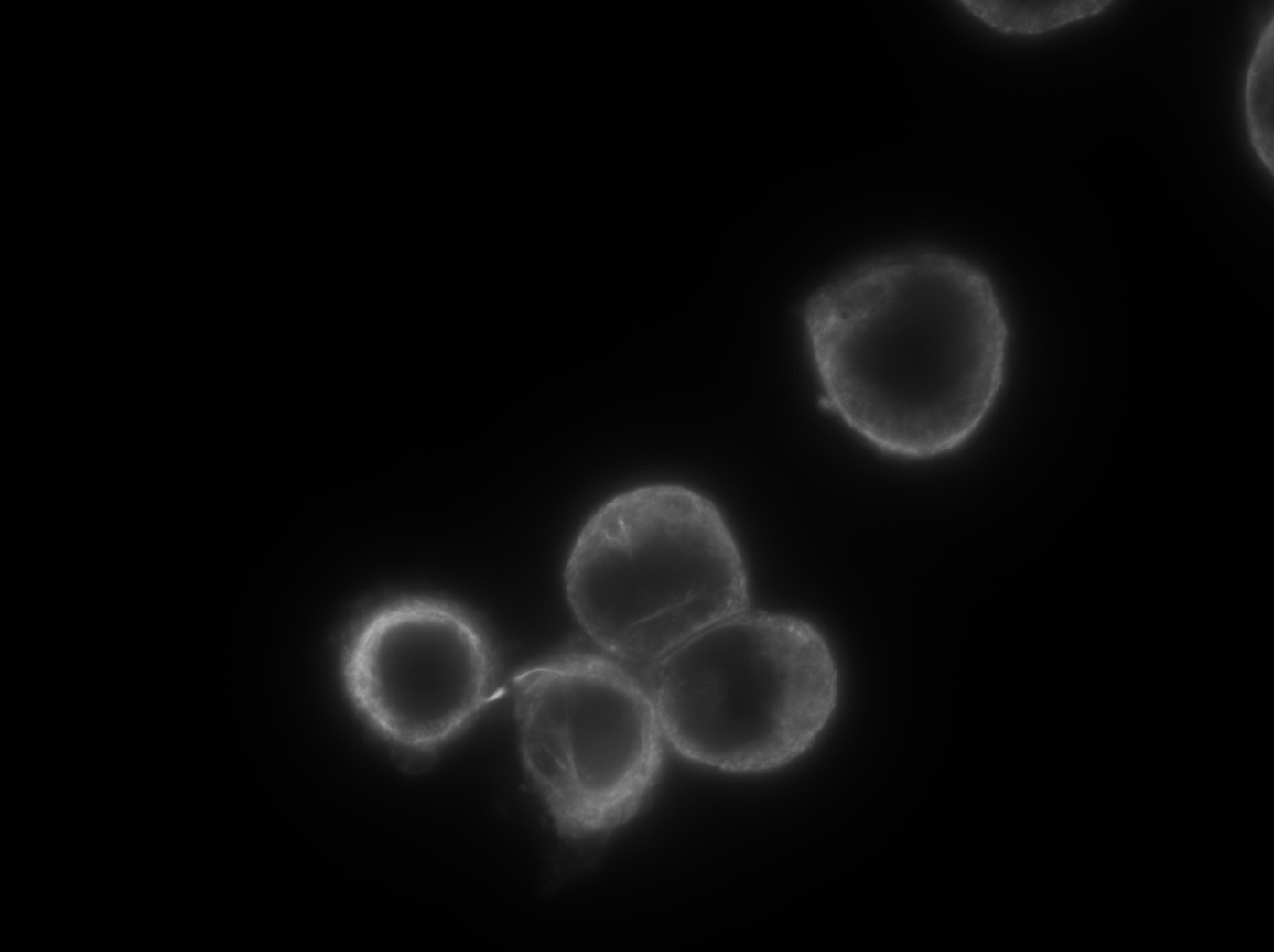

Supplement: Supplementary file 21 — Source data Fig. 6 part 2 [file 44319_2026_742_MOESM21_ESM.zip › Figure 6 Part 2/Fig 6abcd Cas9 TPGS1-KO acetylated tubulin atubulin part 2/TPGS1-KO R2 9-11-24 LT25.Project Maximum Z_XY1726268436_Z0_T0_C1.tif]

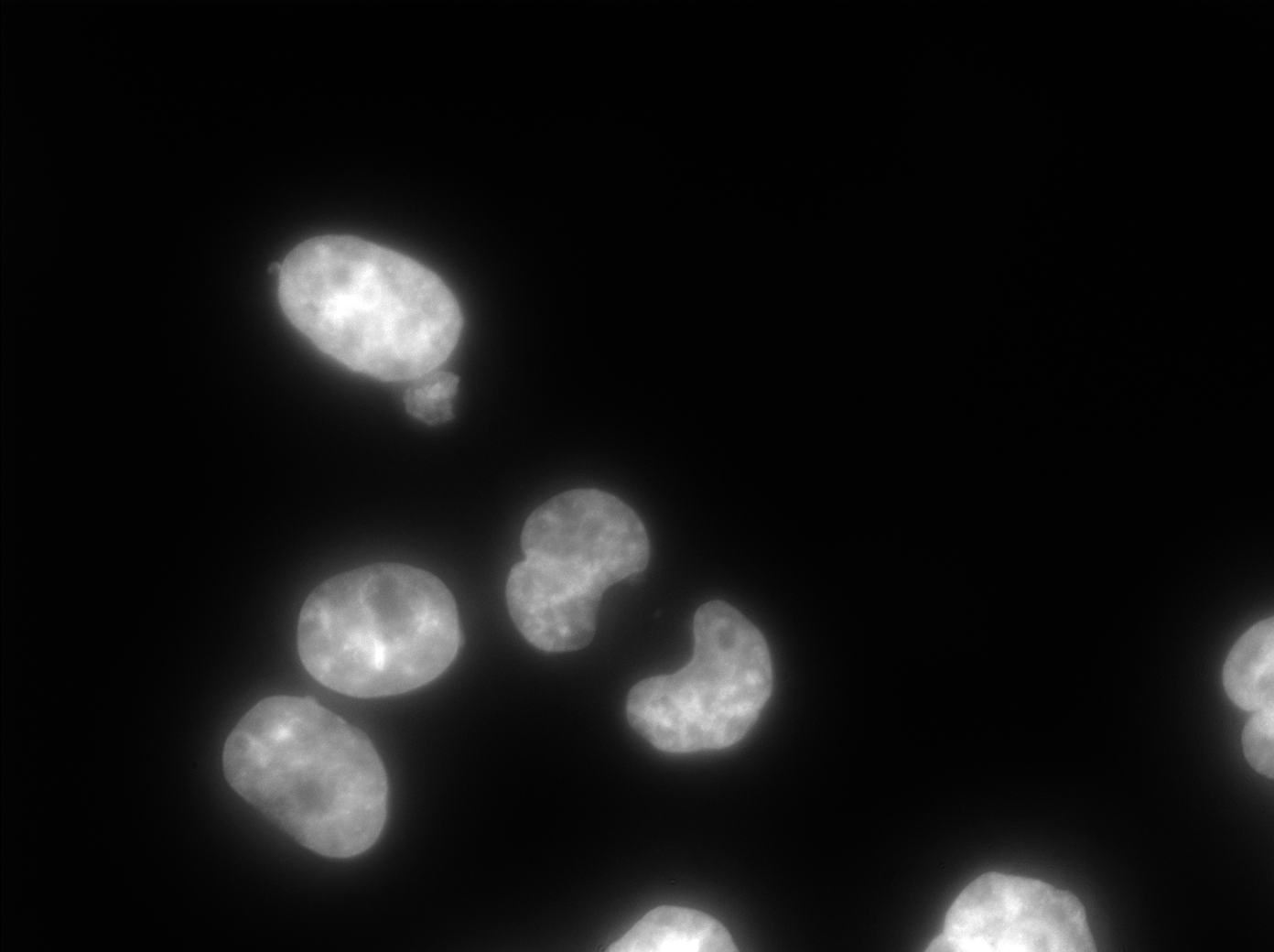

Supplement: Supplementary file 21 — Source data Fig. 6 part 2 [file 44319_2026_742_MOESM21_ESM.zip › Figure 6 Part 2/Fig 6abcd Cas9 TPGS1-KO acetylated tubulin atubulin part 2/TPGS1-KO R2 9-11-24 PA12.Project Maximum Z_XY1726265237_Z0_T0_C0.tif]

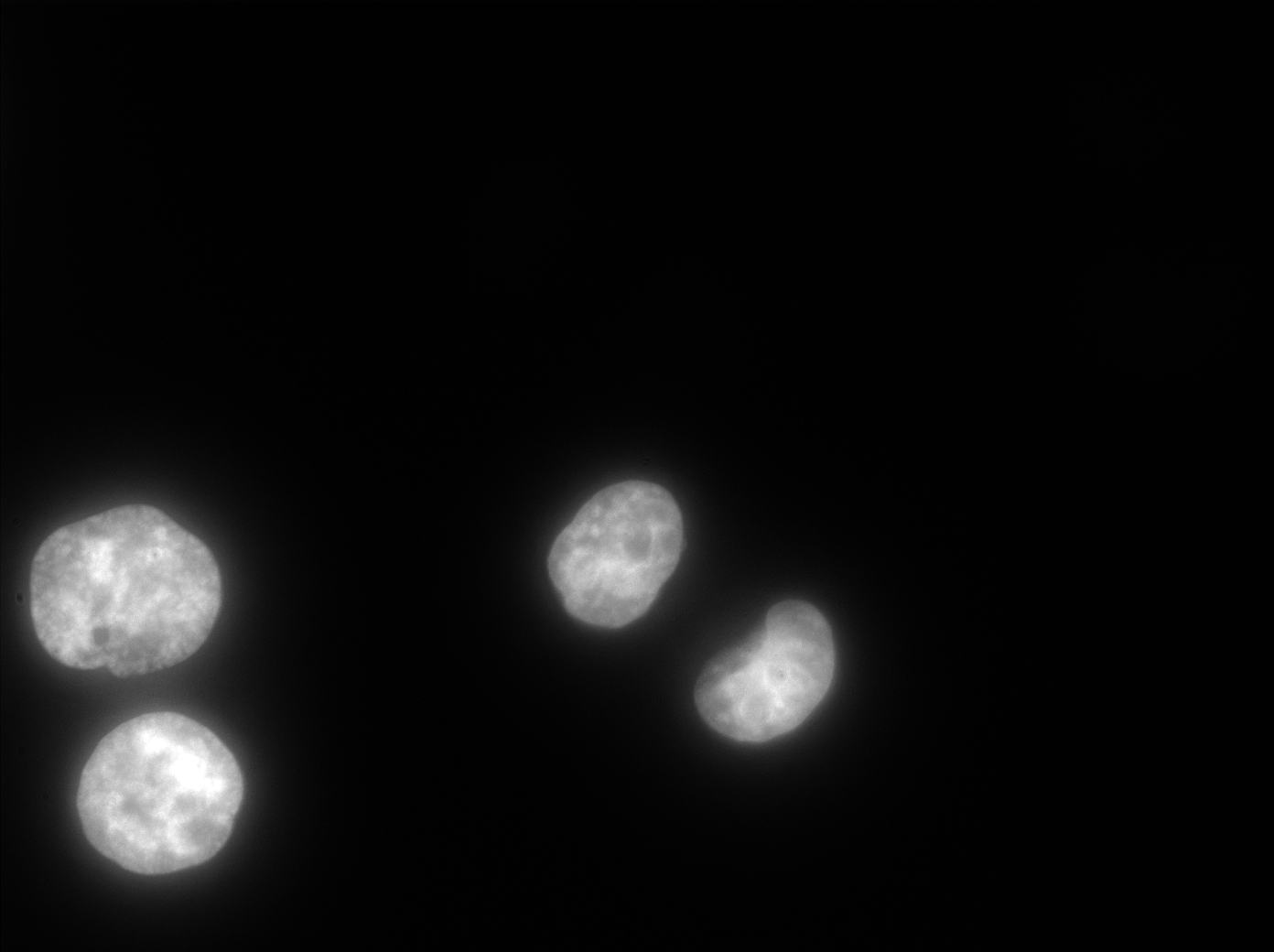

Supplement: Supplementary file 21 — Source data Fig. 6 part 2 [file 44319_2026_742_MOESM21_ESM.zip › Figure 6 Part 2/Fig 6abcd Cas9 TPGS1-KO acetylated tubulin atubulin part 2/TPGS1-KO R2 9-11-24 LT3.Project Maximum Z_XY1726259338_Z0_T0_C0.tif]

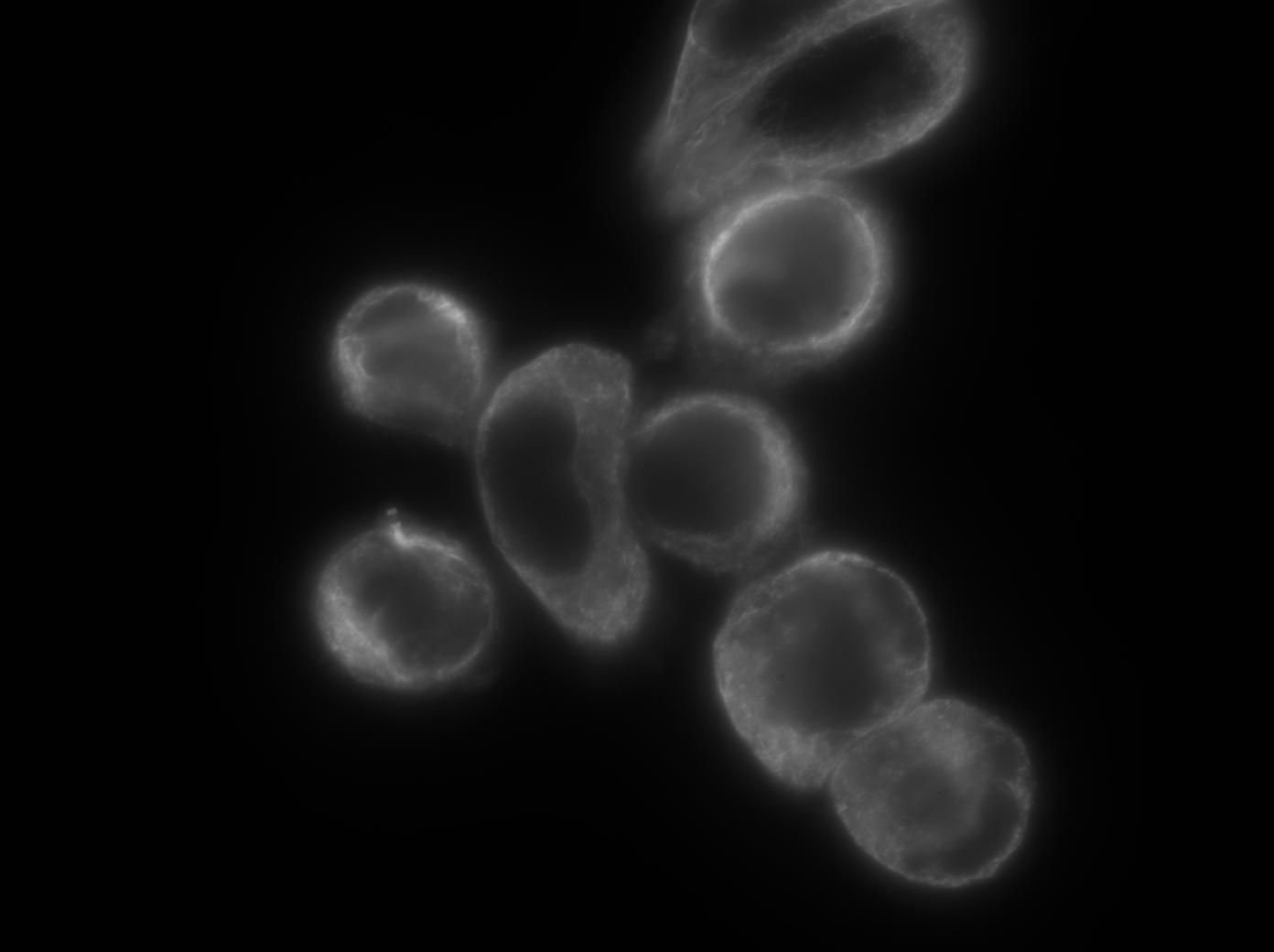

Supplement: Supplementary file 21 — Source data Fig. 6 part 2 [file 44319_2026_742_MOESM21_ESM.zip › Figure 6 Part 2/Fig 6abcd Cas9 TPGS1-KO acetylated tubulin atubulin part 2/TPGS1-KO R2 9-11-24 PA19.Project Maximum Z_XY1726269018_Z0_T0_C1.tif]

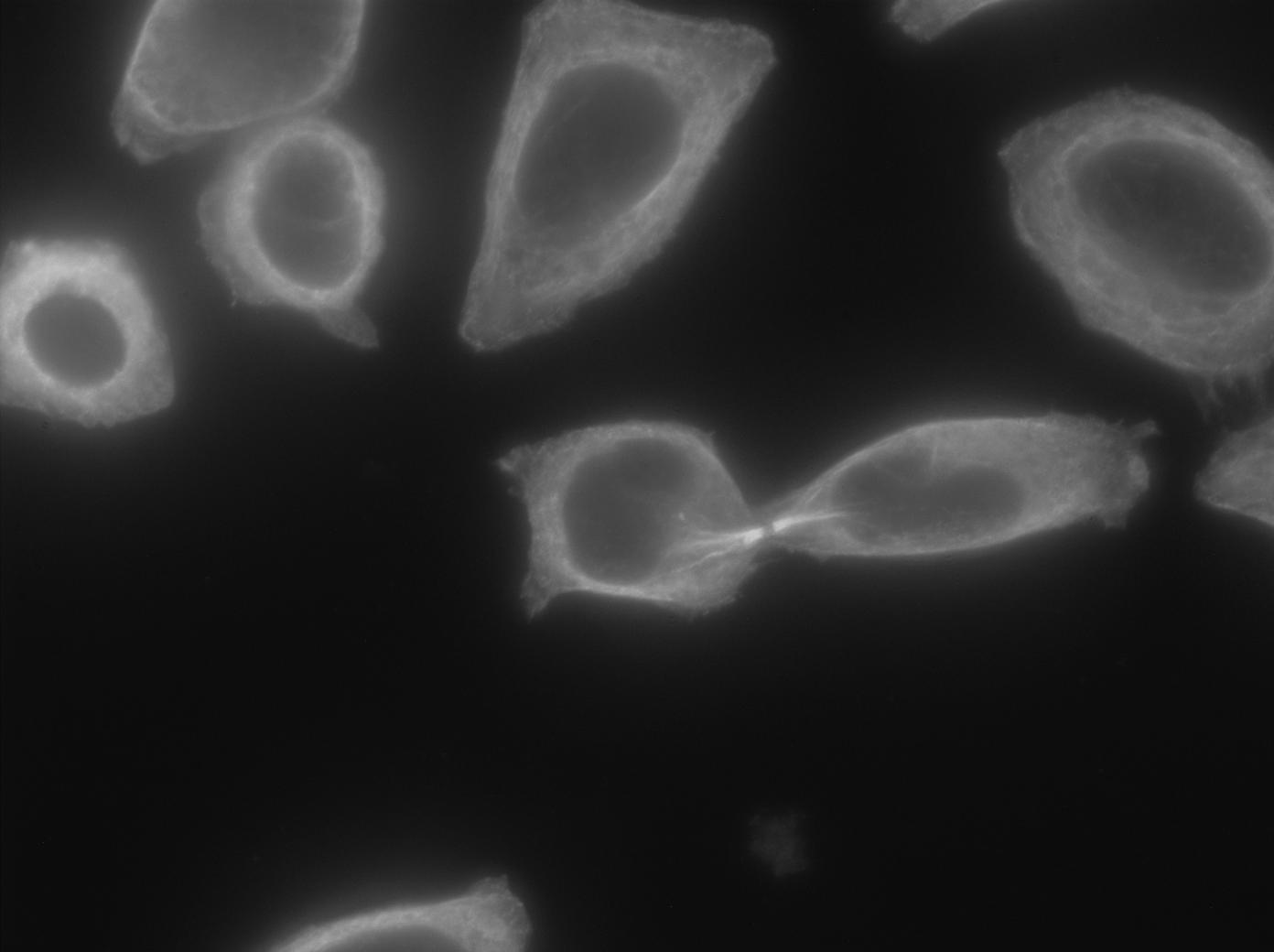

Supplement: Supplementary file 21 — Source data Fig. 6 part 2 [file 44319_2026_742_MOESM21_ESM.zip › Figure 6 Part 2/Fig 6abcd Cas9 TPGS1-KO acetylated tubulin atubulin part 2/TPGS1-KO R3 9-13-24 LT27.Project Maximum Z_XY1726764803_Z0_T0_C1.tif]

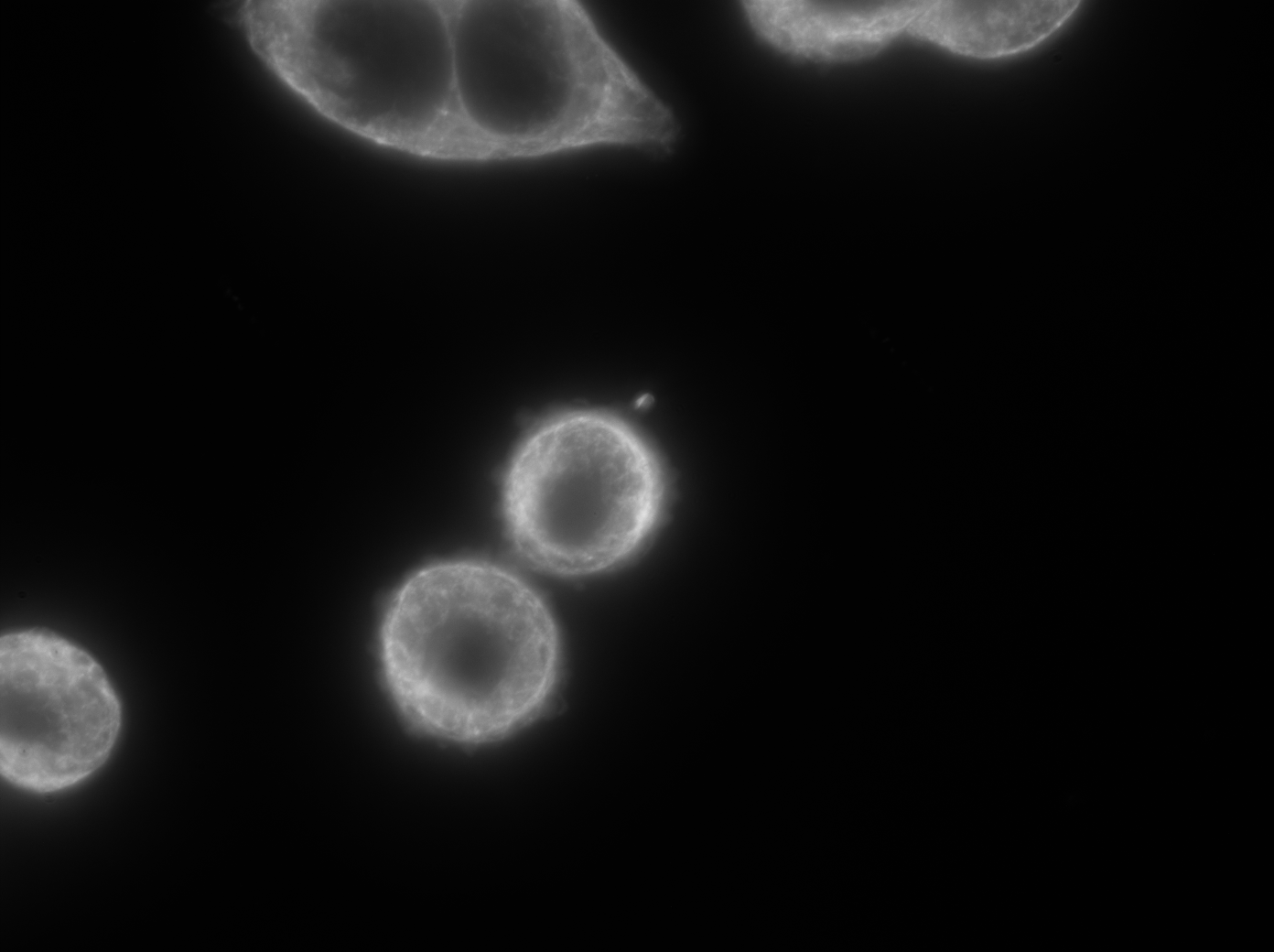

Supplement: Supplementary file 21 — Source data Fig. 6 part 2 [file 44319_2026_742_MOESM21_ESM.zip › Figure 6 Part 2/Fig 6abcd Cas9 TPGS1-KO acetylated tubulin atubulin part 2/TPGS1-KO R2 9-11-24 PA2.Project Maximum Z_XY1726259232_Z0_T0_C1.tif]

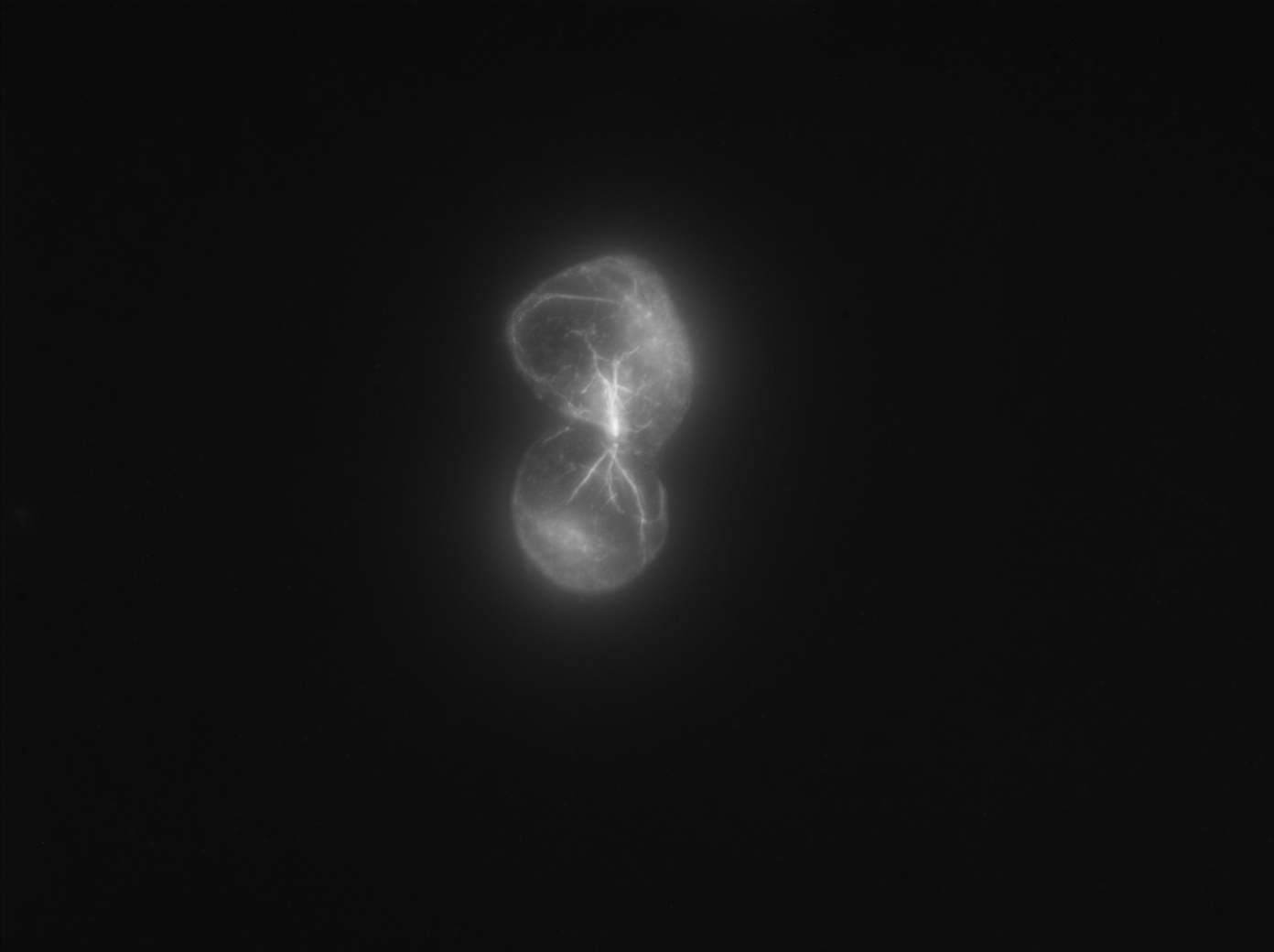

Supplement: Supplementary file 21 — Source data Fig. 6 part 2 [file 44319_2026_742_MOESM21_ESM.zip › Figure 6 Part 2/Fig 6abcd Cas9 TPGS1-KO acetylated tubulin atubulin part 2/TPGS1-KO R2 9-11-24 LT19.Project Maximum Z_XY1726266398_Z0_T0_C2.tif]

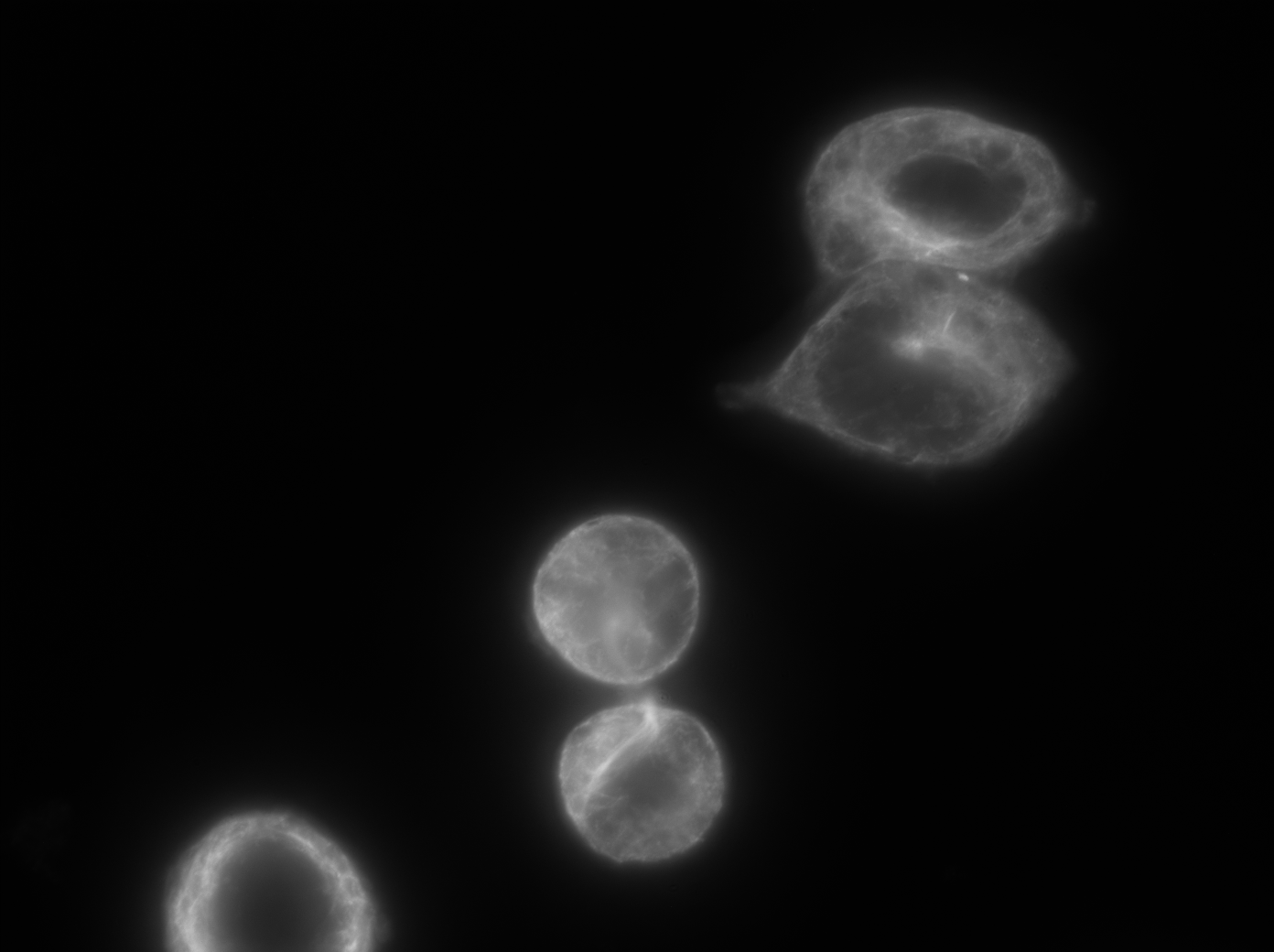

Supplement: Supplementary file 21 — Source data Fig. 6 part 2 [file 44319_2026_742_MOESM21_ESM.zip › Figure 6 Part 2/Fig 6abcd Cas9 TPGS1-KO acetylated tubulin atubulin part 2/TPGS1-KO R2 9-11-24 LT6 PA5.Project Maximum Z_XY1726261251_Z0_T0_C1.tif]

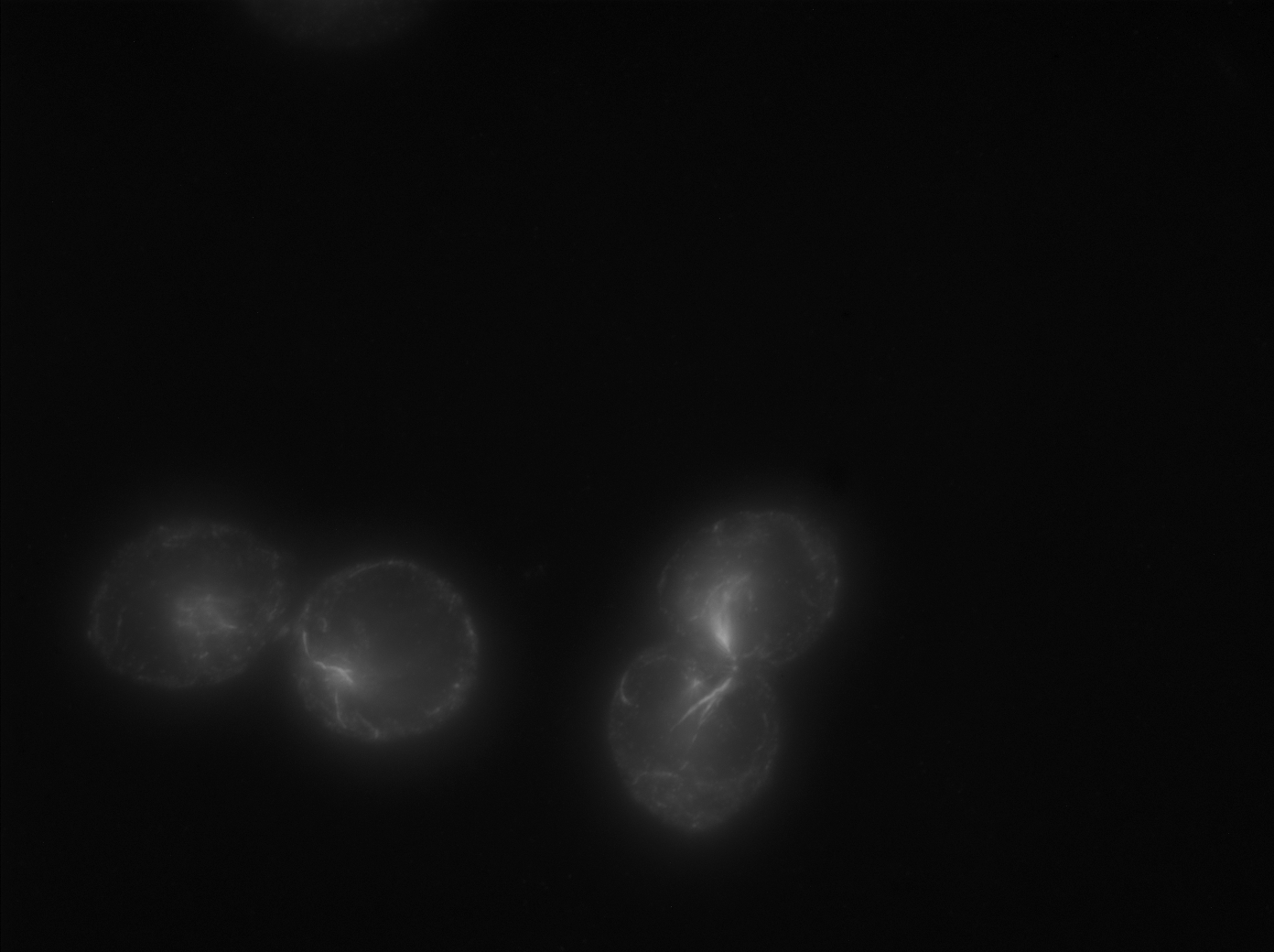

Supplement: Supplementary file 21 — Source data Fig. 6 part 2 [file 44319_2026_742_MOESM21_ESM.zip › Figure 6 Part 2/Fig 6abcd Cas9 TPGS1-KO acetylated tubulin atubulin part 2/TPGS1-KO R2 9-11-24 LT4.Project Maximum Z_XY1726259553_Z0_T0_C2.tif]

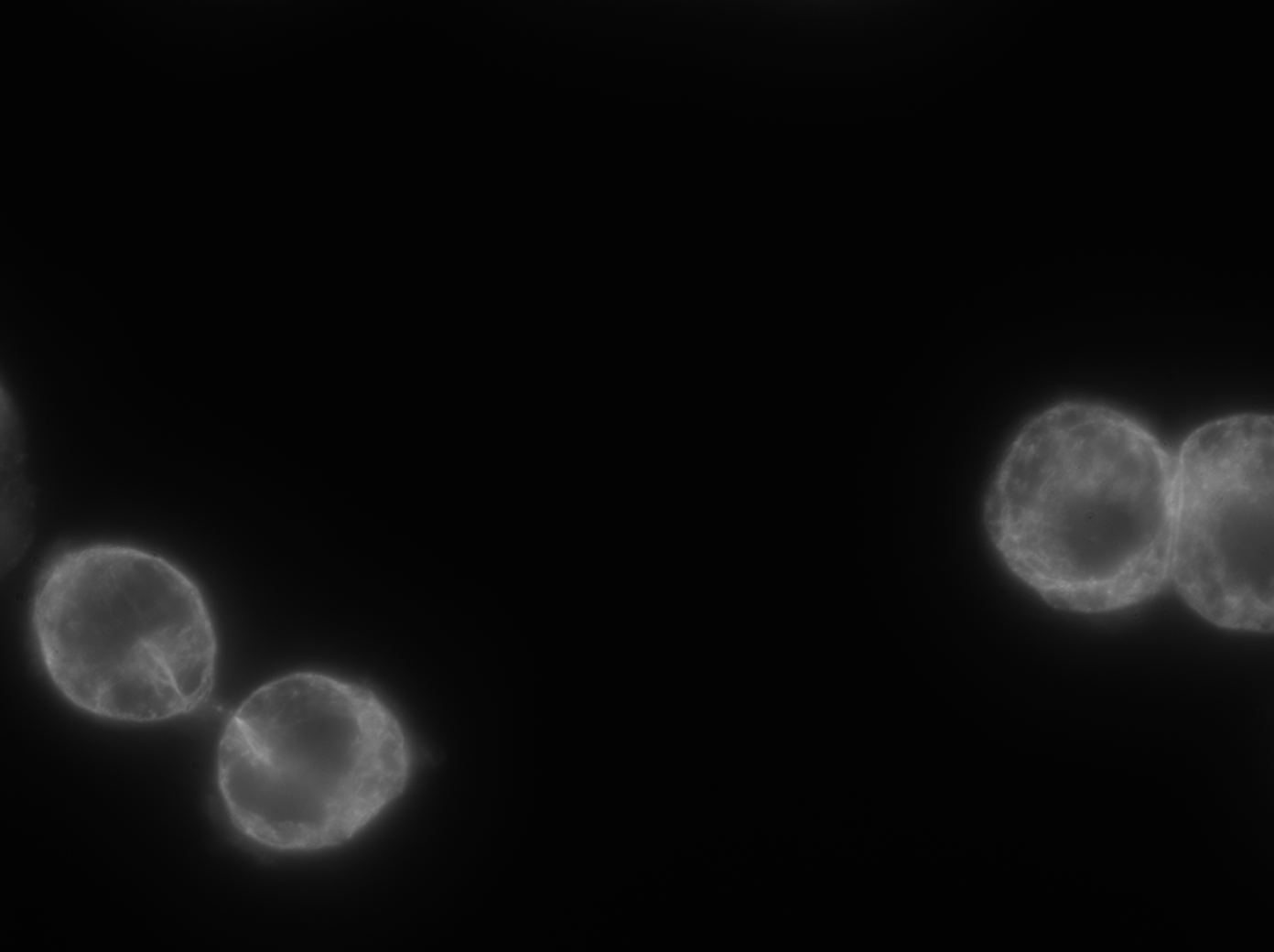

Supplement: Supplementary file 21 — Source data Fig. 6 part 2 [file 44319_2026_742_MOESM21_ESM.zip › Figure 6 Part 2/Fig 6abcd Cas9 TPGS1-KO acetylated tubulin atubulin part 2/TPGS1-KO R2 9-11-24 PA13.Project Maximum Z_XY1726265351_Z0_T0_C1.tif]

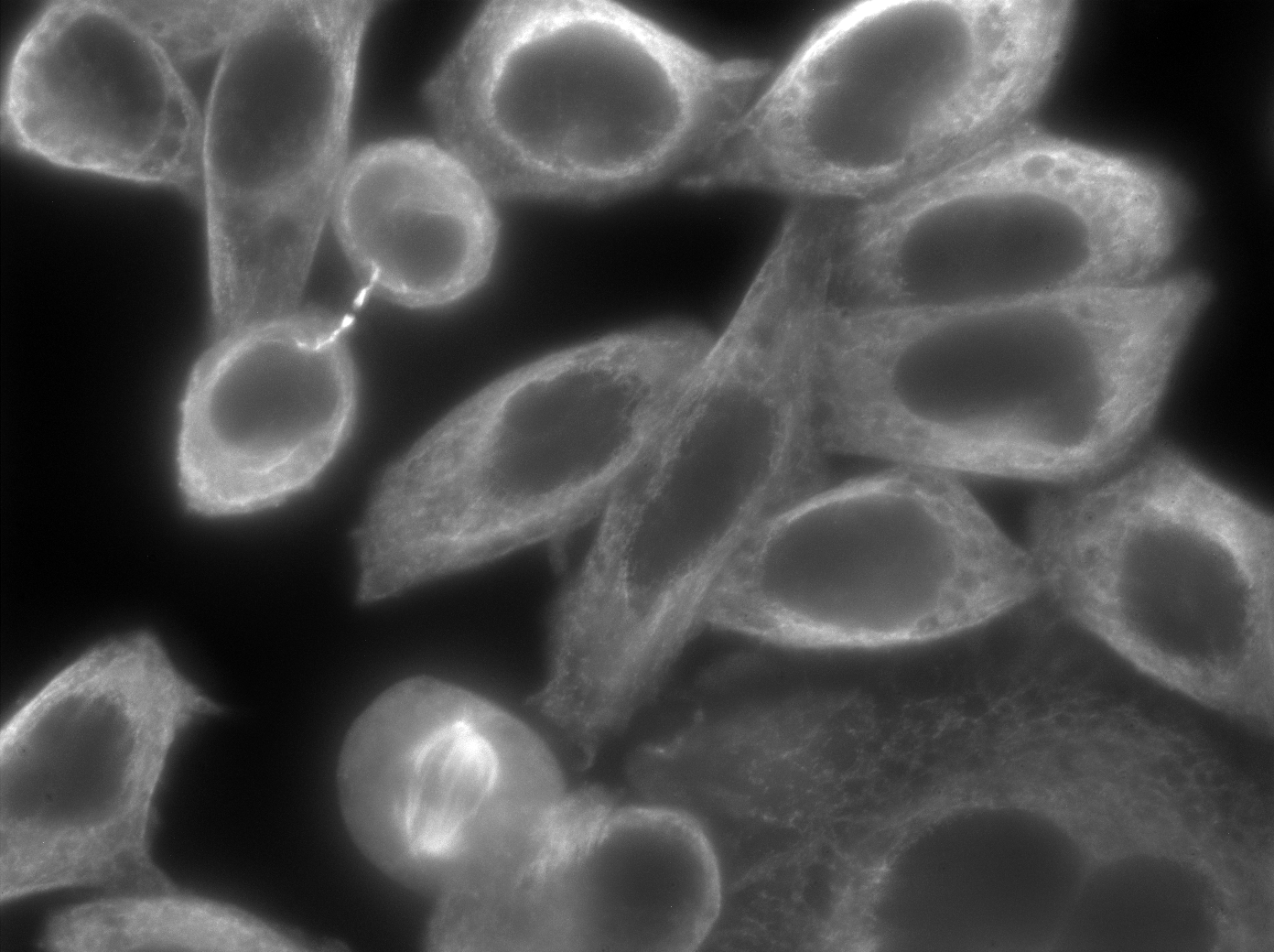

Supplement: Supplementary file 21 — Source data Fig. 6 part 2 [file 44319_2026_742_MOESM21_ESM.zip › Figure 6 Part 2/Fig 6abcd Cas9 TPGS1-KO acetylated tubulin atubulin part 2/TPGS1-KO R3 9-13-24 LT30.Project Maximum Z_XY1726765116_Z0_T0_C1.tif]

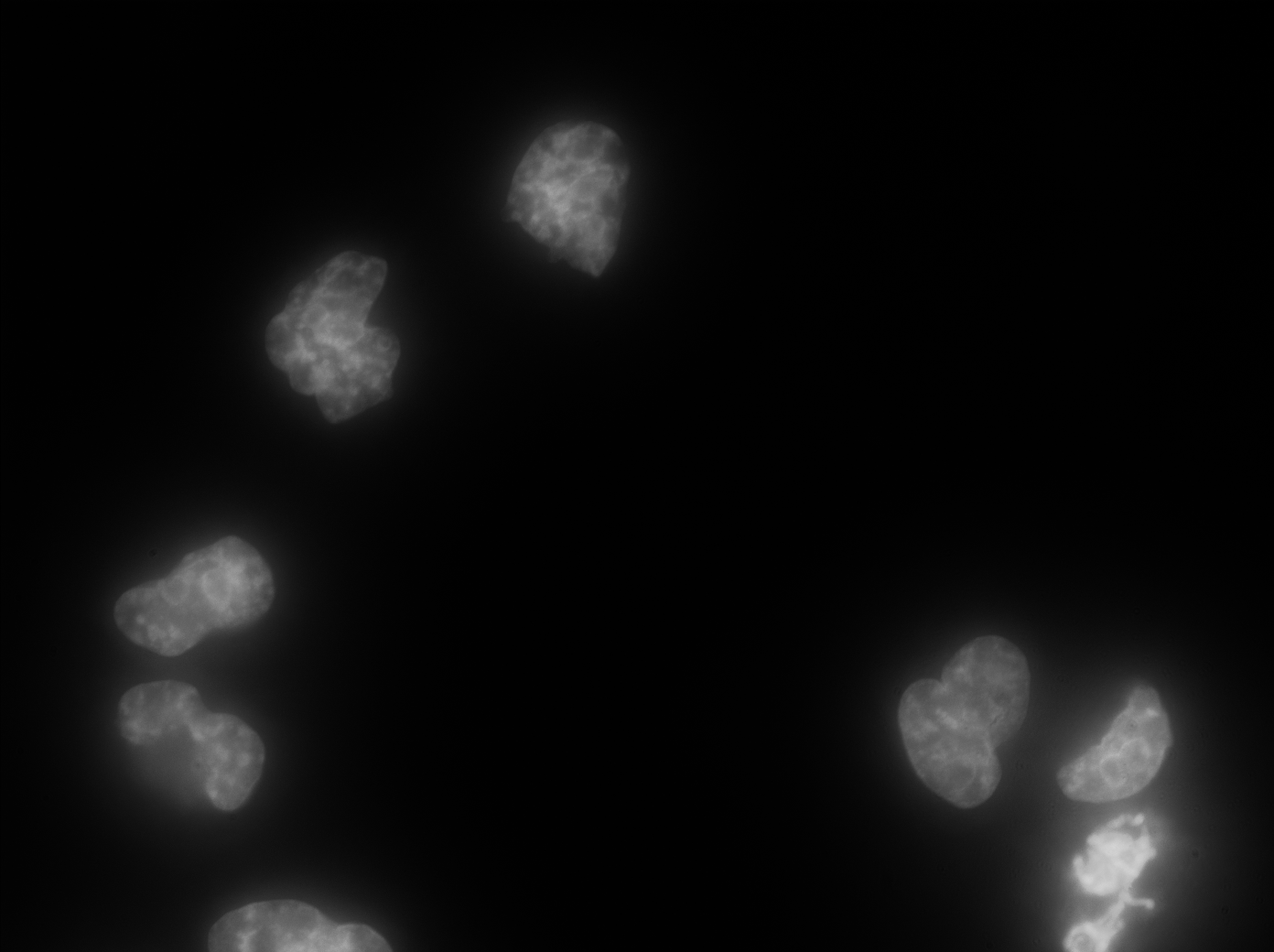

Supplement: Supplementary file 21 — Source data Fig. 6 part 2 [file 44319_2026_742_MOESM21_ESM.zip › Figure 6 Part 2/Fig 6abcd Cas9 TPGS1-KO acetylated tubulin atubulin part 2/TPGS1-KO R3 9-13-24 LT24LT25LT26.Project Maximum Z_XY1726764690_Z0_T0_C0.tif]

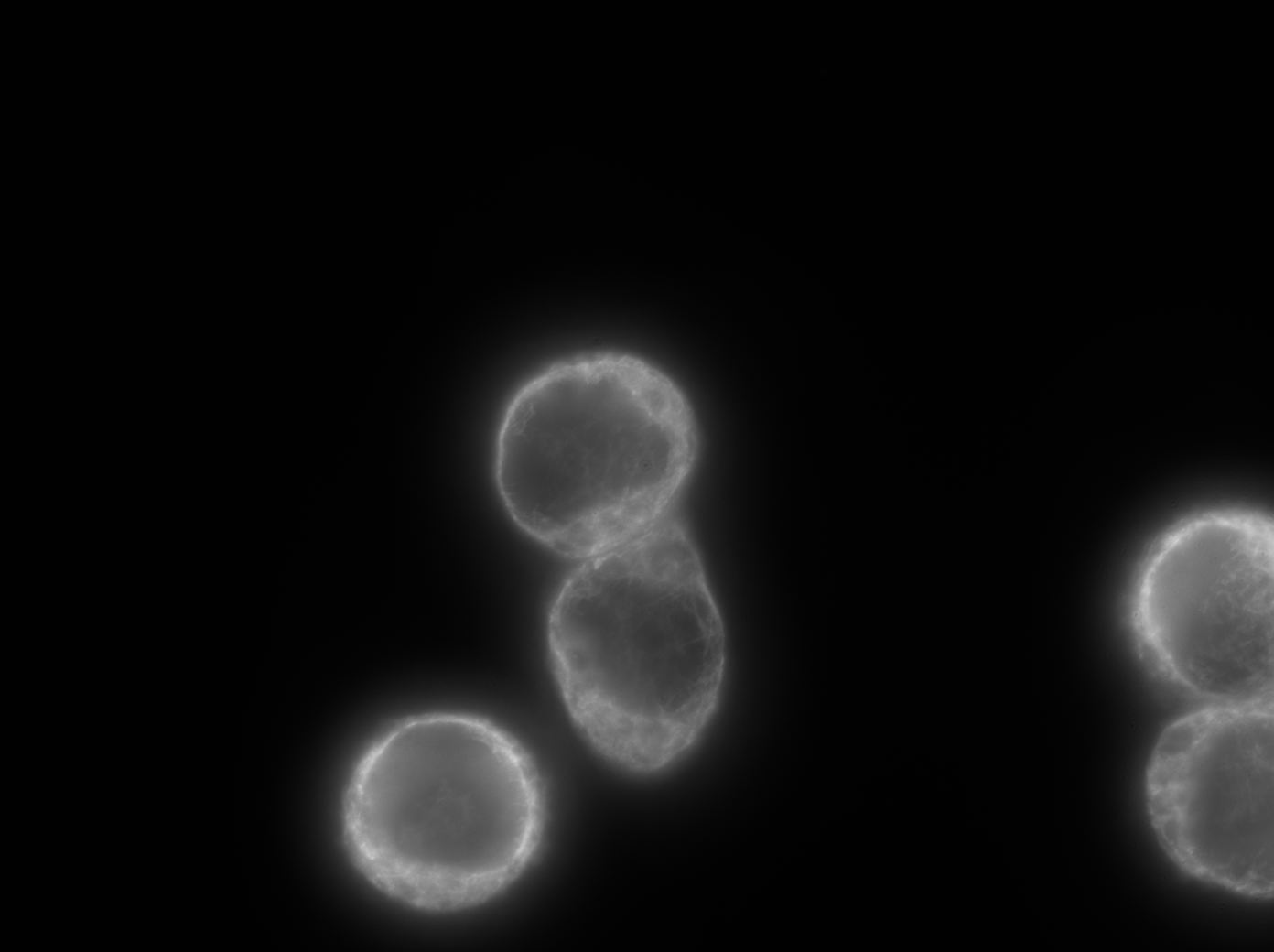

Supplement: Supplementary file 21 — Source data Fig. 6 part 2 [file 44319_2026_742_MOESM21_ESM.zip › Figure 6 Part 2/Fig 6abcd Cas9 TPGS1-KO acetylated tubulin atubulin part 2/TPGS1-KO R2 9-11-24 PA7.Project Maximum Z_XY1726261664_Z0_T0_C1.tif]

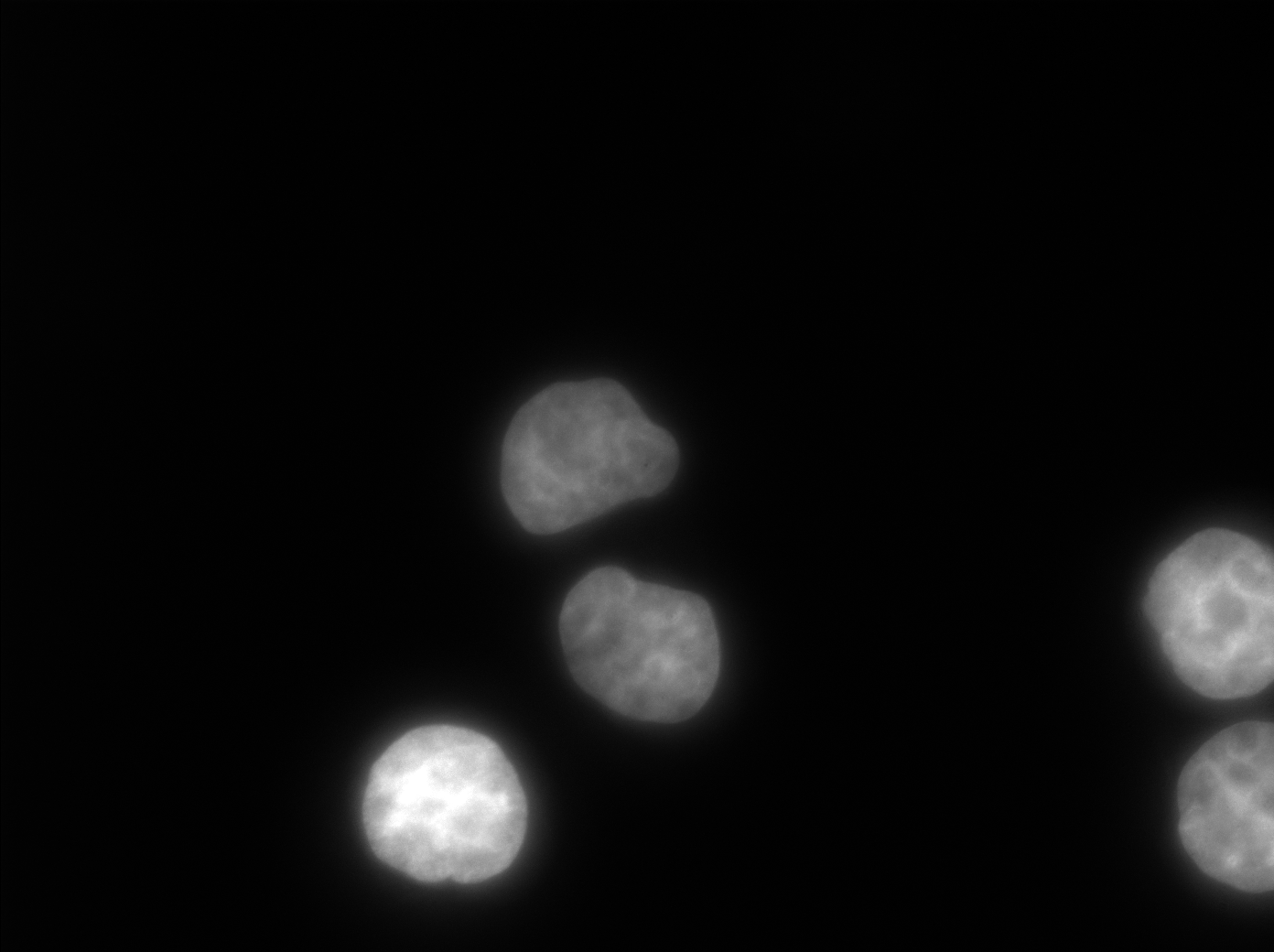

Supplement: Supplementary file 21 — Source data Fig. 6 part 2 [file 44319_2026_742_MOESM21_ESM.zip › Figure 6 Part 2/Fig 6abcd Cas9 TPGS1-KO acetylated tubulin atubulin part 2/TPGS1-KO R2 9-11-24 PA7.Project Maximum Z_XY1726261664_Z0_T0_C0.tif]

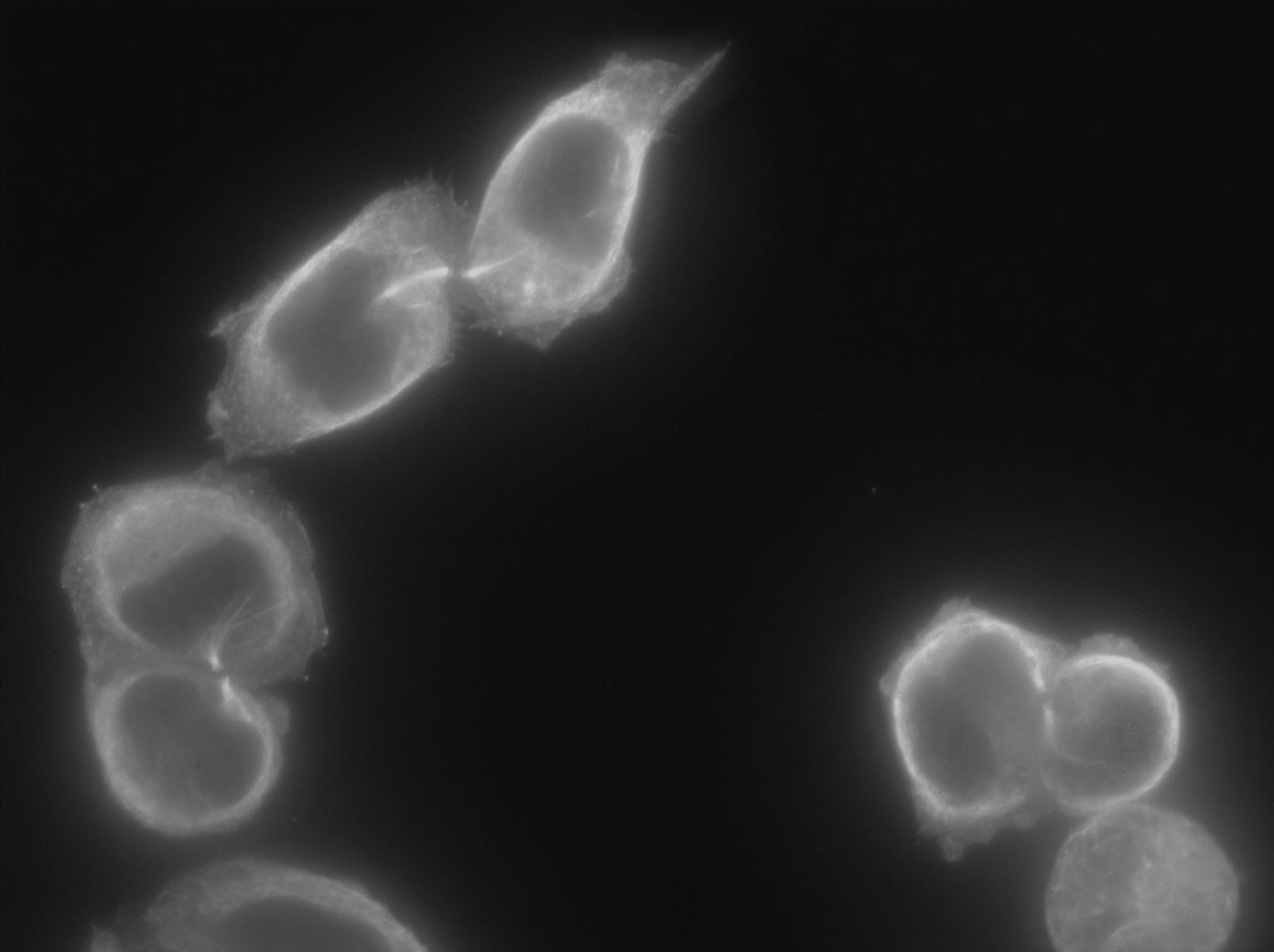

Supplement: Supplementary file 21 — Source data Fig. 6 part 2 [file 44319_2026_742_MOESM21_ESM.zip › Figure 6 Part 2/Fig 6abcd Cas9 TPGS1-KO acetylated tubulin atubulin part 2/TPGS1-KO R3 9-13-24 LT24LT25LT26.Project Maximum Z_XY1726764690_Z0_T0_C1.tif]

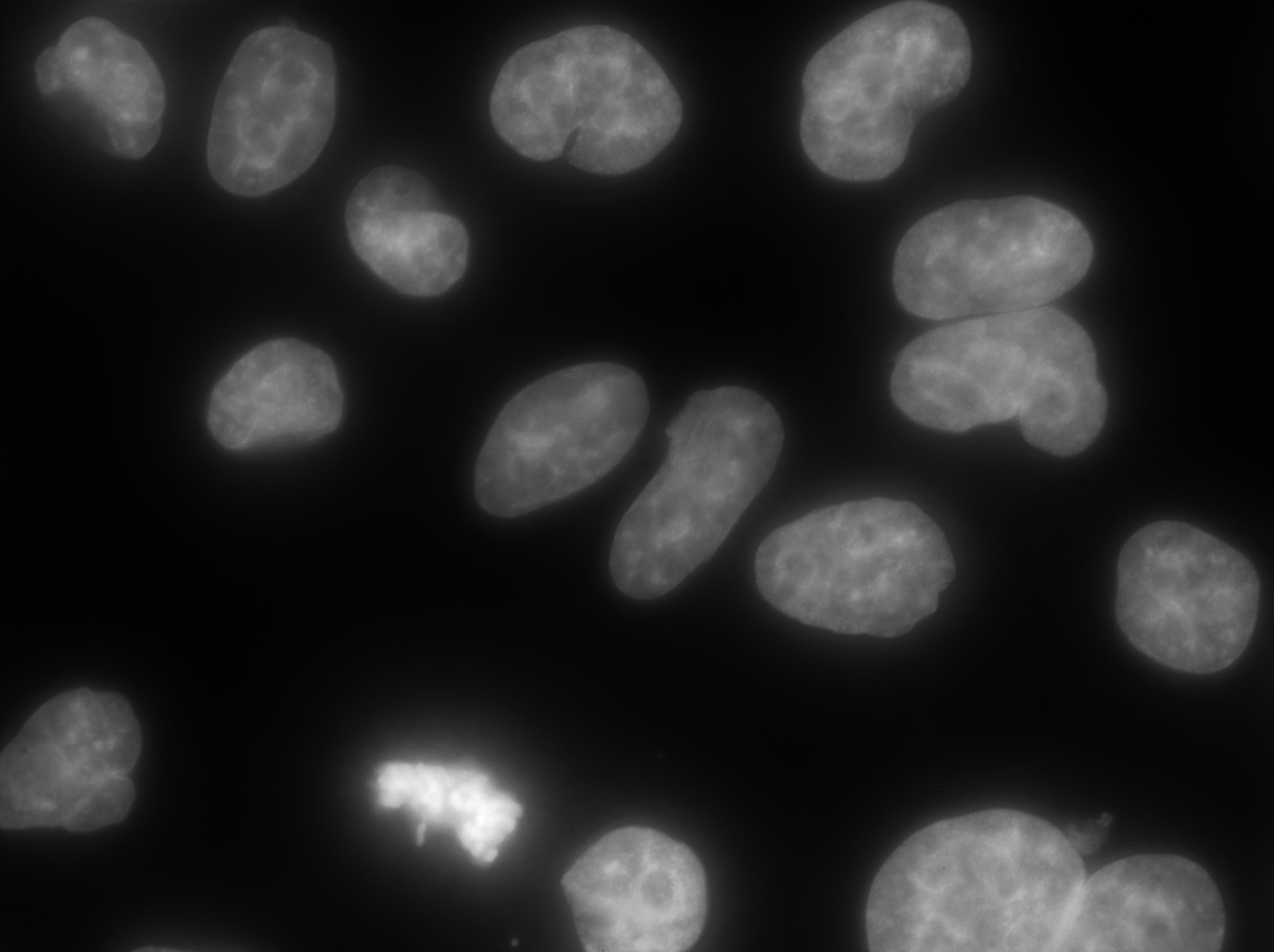

Supplement: Supplementary file 21 — Source data Fig. 6 part 2 [file 44319_2026_742_MOESM21_ESM.zip › Figure 6 Part 2/Fig 6abcd Cas9 TPGS1-KO acetylated tubulin atubulin part 2/TPGS1-KO R3 9-13-24 LT30.Project Maximum Z_XY1726765116_Z0_T0_C0.tif]

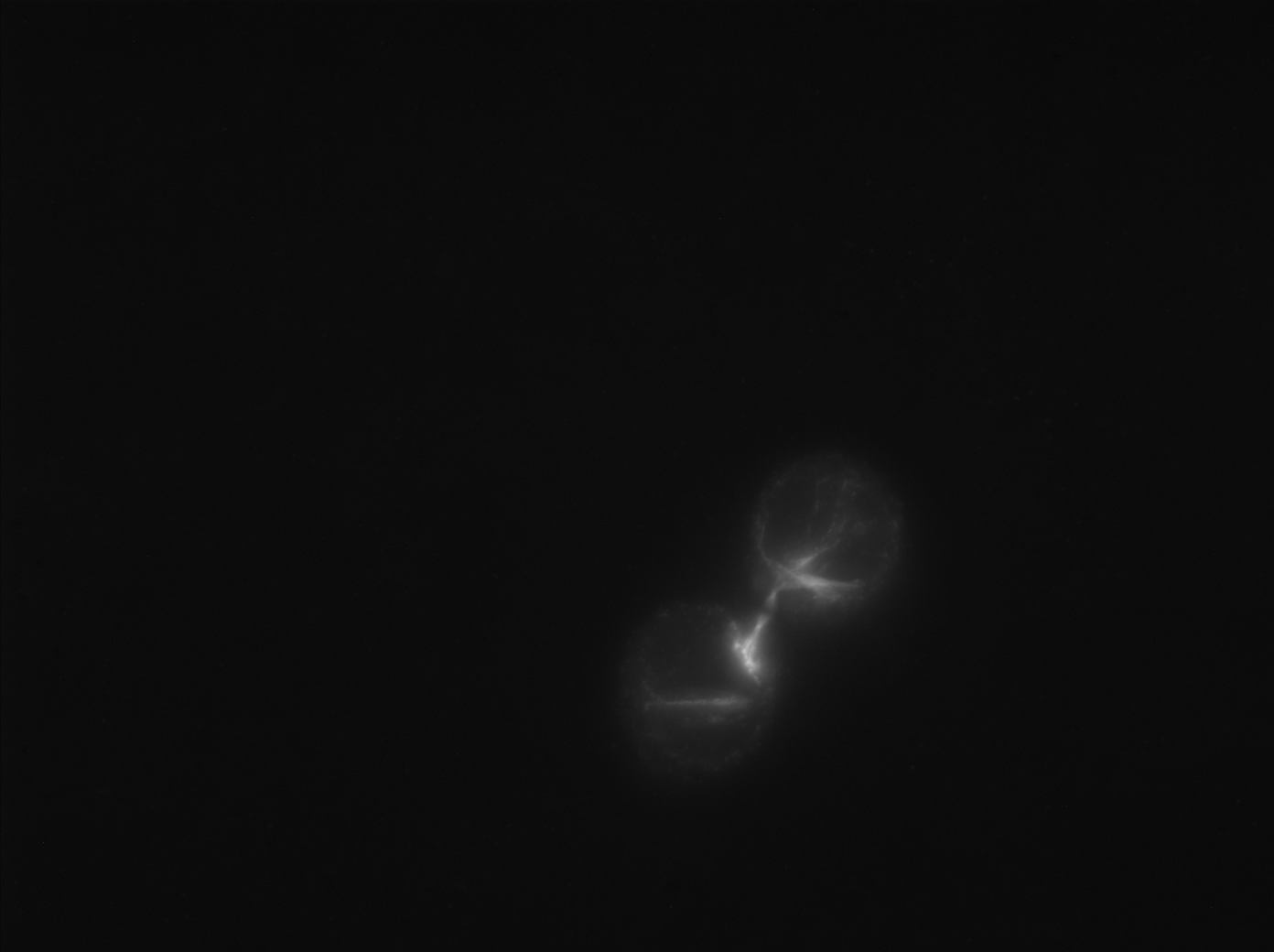

Supplement: Supplementary file 21 — Source data Fig. 6 part 2 [file 44319_2026_742_MOESM21_ESM.zip › Figure 6 Part 2/Fig 6abcd Cas9 TPGS1-KO acetylated tubulin atubulin part 2/TPGS1-KO R2 9-11-24 LT24.Project Maximum Z_XY1726268192_Z0_T0_C2.tif]

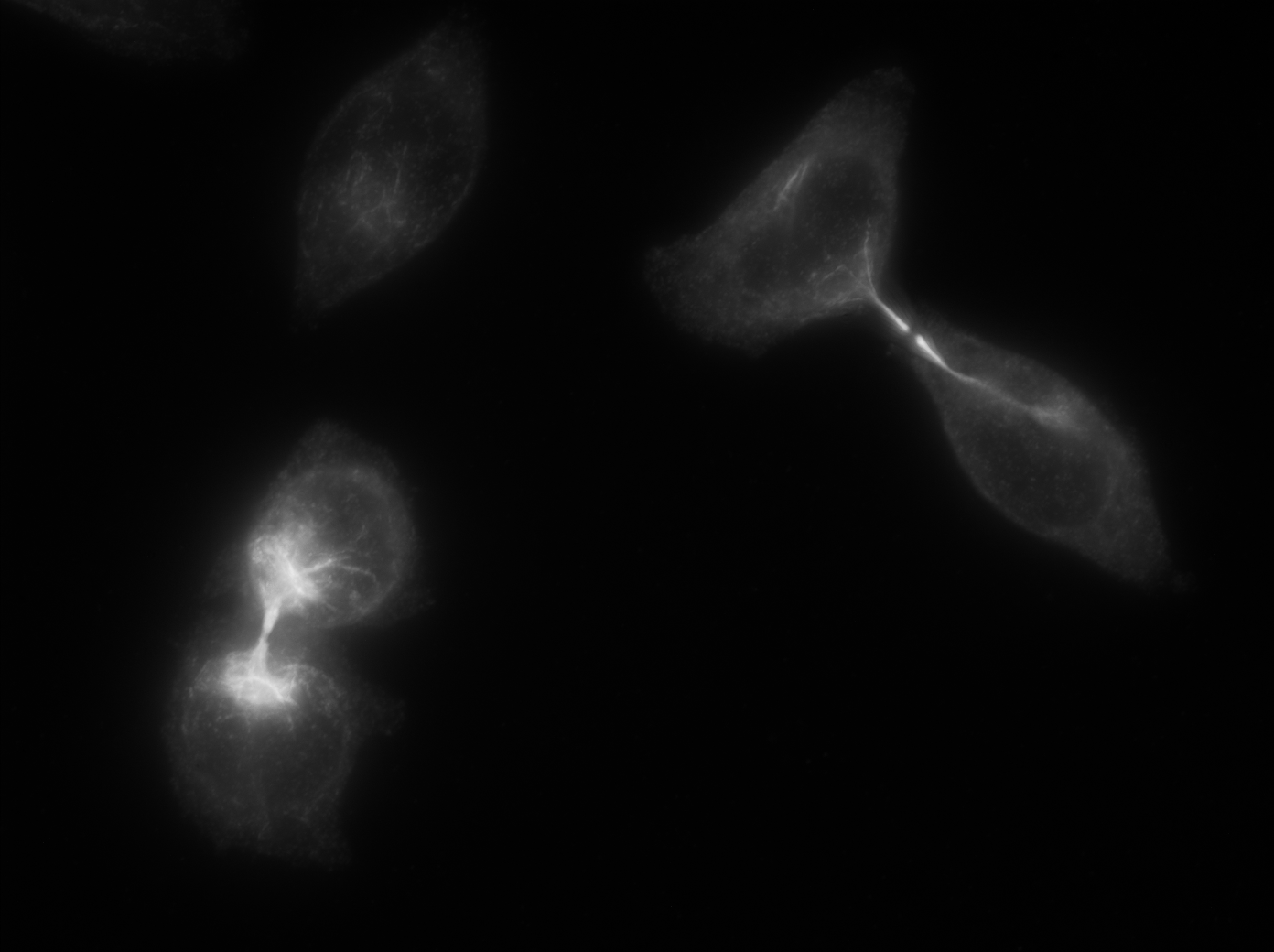

Supplement: Supplementary file 21 — Source data Fig. 6 part 2 [file 44319_2026_742_MOESM21_ESM.zip › Figure 6 Part 2/Fig 6abcd Cas9 TPGS1-KO acetylated tubulin atubulin part 2/TPGS1-KO R3 9-13-24 LT12LT13.Project Maximum Z_XY1726761185_Z0_T0_C2.tif]

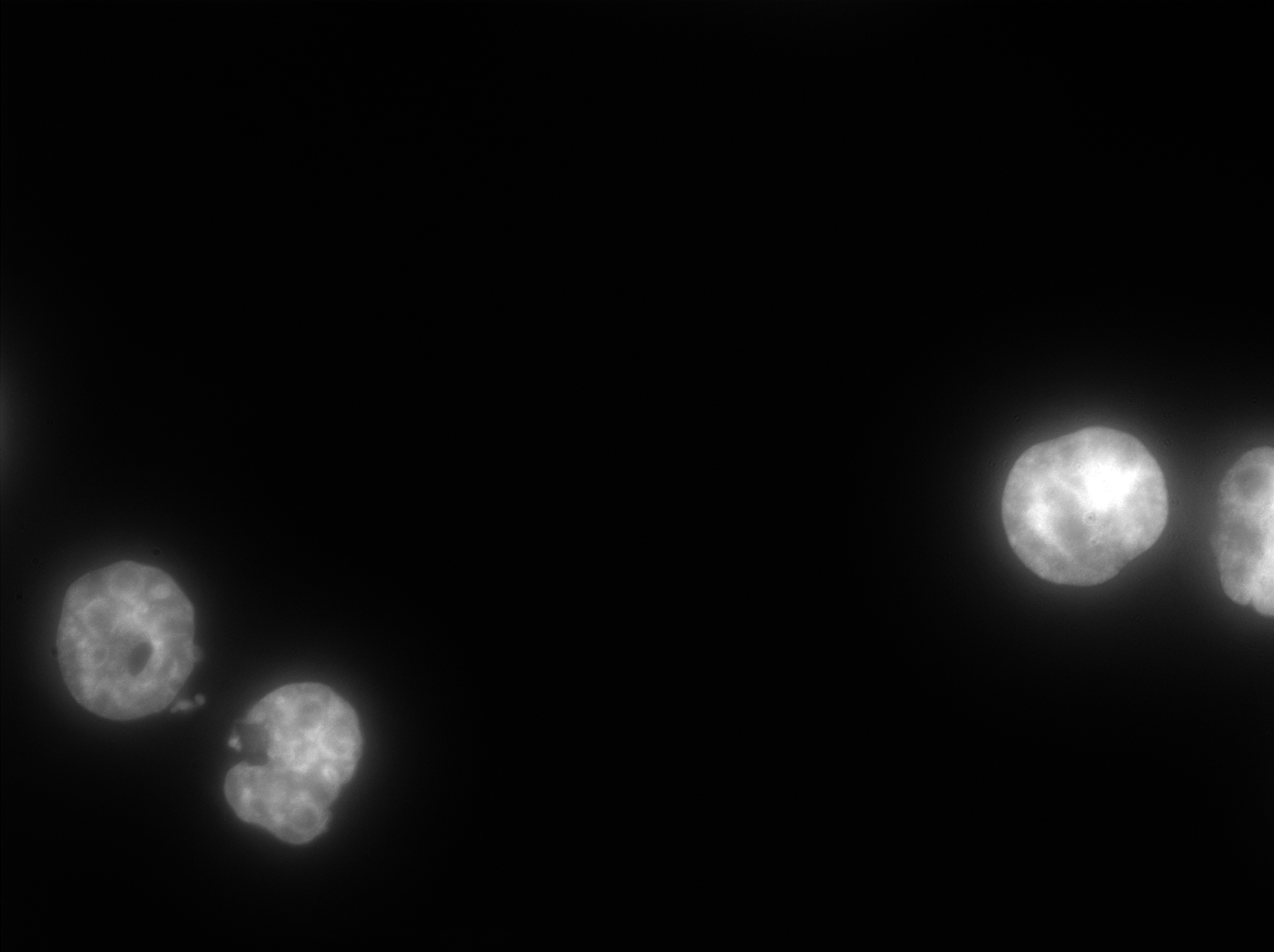

Supplement: Supplementary file 21 — Source data Fig. 6 part 2 [file 44319_2026_742_MOESM21_ESM.zip › Figure 6 Part 2/Fig 6abcd Cas9 TPGS1-KO acetylated tubulin atubulin part 2/TPGS1-KO R2 9-11-24 PA13.Project Maximum Z_XY1726265351_Z0_T0_C0.tif]

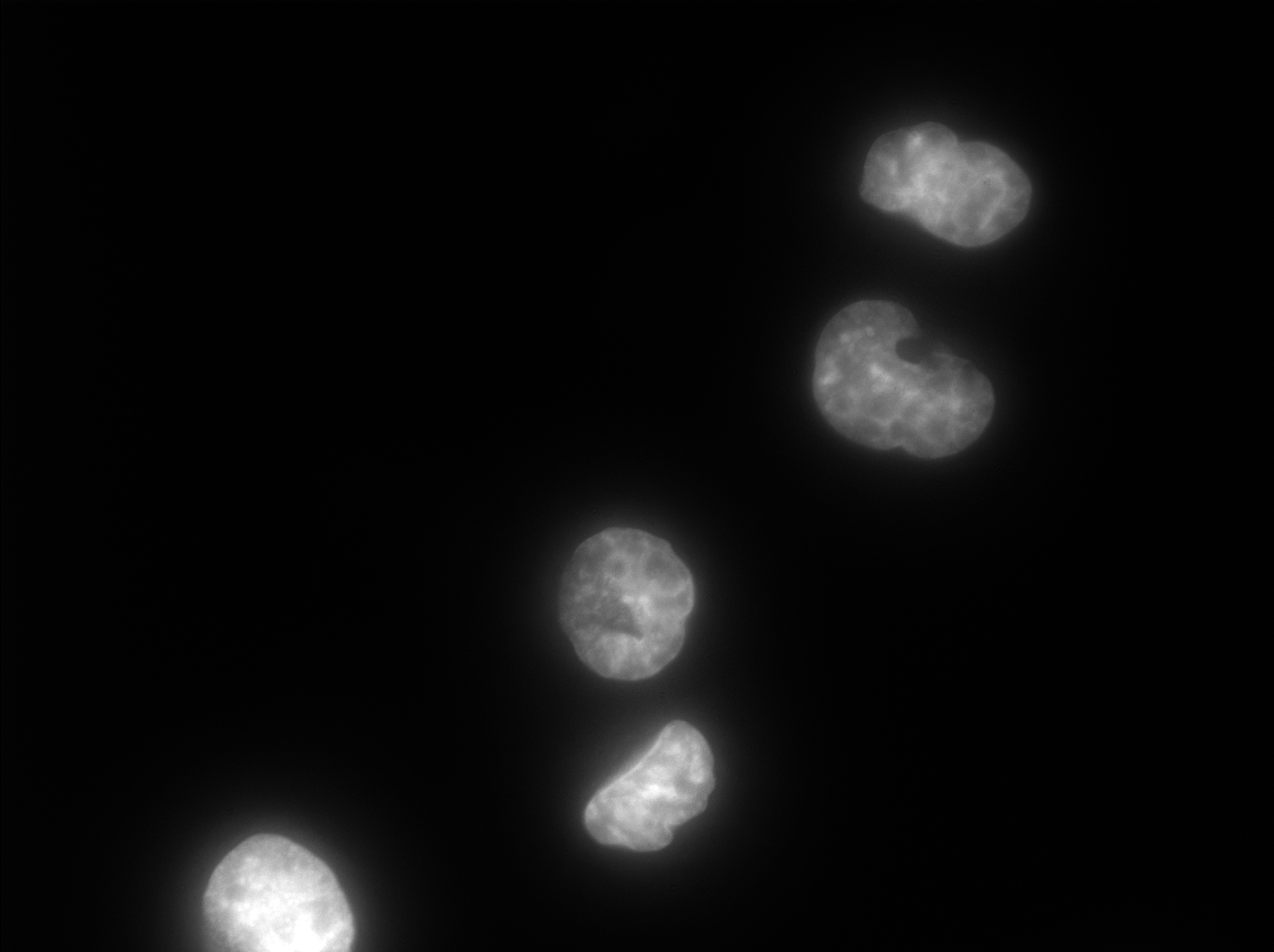

Supplement: Supplementary file 21 — Source data Fig. 6 part 2 [file 44319_2026_742_MOESM21_ESM.zip › Figure 6 Part 2/Fig 6abcd Cas9 TPGS1-KO acetylated tubulin atubulin part 2/TPGS1-KO R2 9-11-24 LT6 PA5.Project Maximum Z_XY1726261251_Z0_T0_C0.tif]

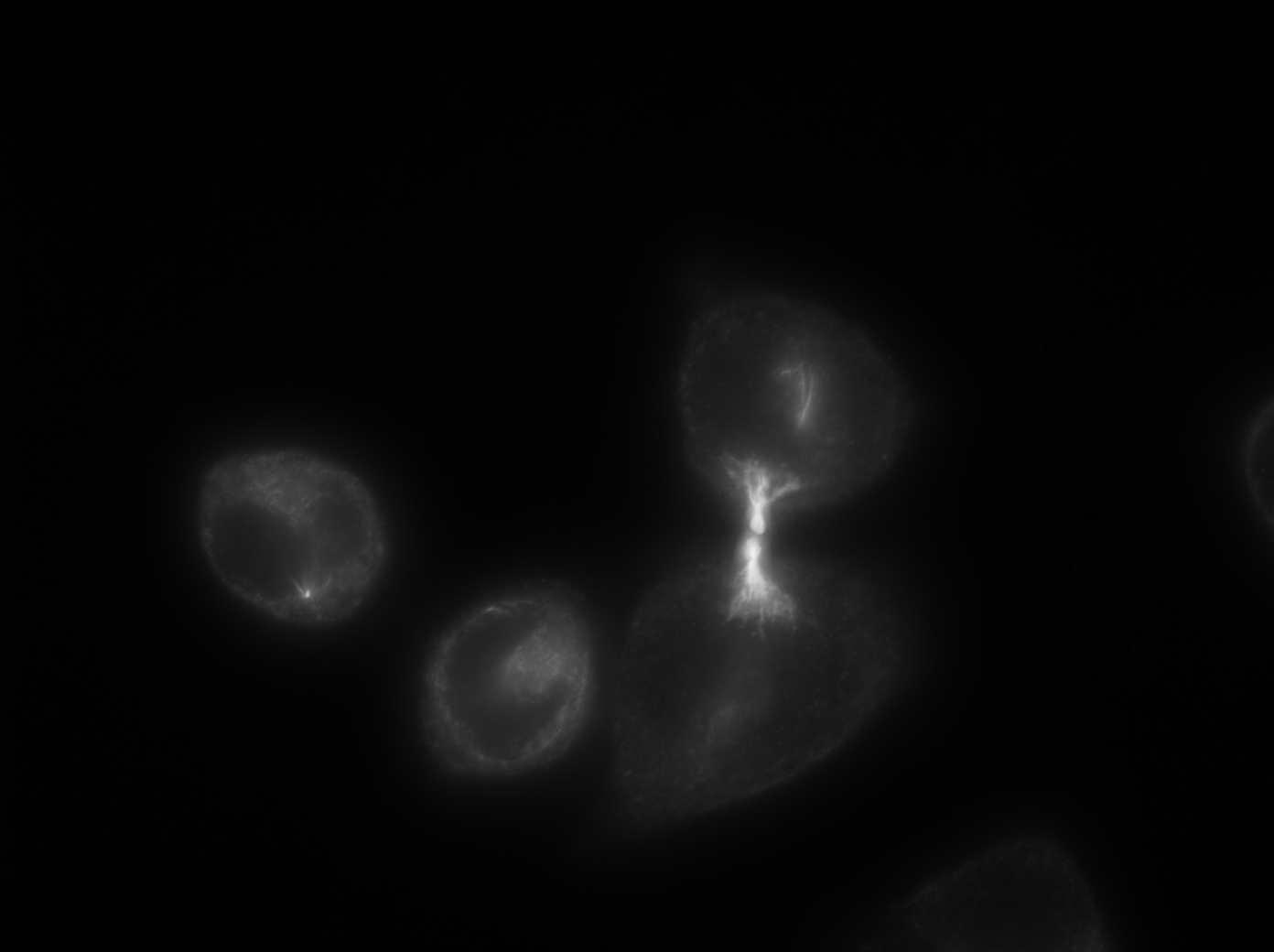

Supplement: Supplementary file 21 — Source data Fig. 6 part 2 [file 44319_2026_742_MOESM21_ESM.zip › Figure 6 Part 2/Fig 6abcd Cas9 TPGS1-KO acetylated tubulin atubulin part 2/TPGS1-KO R3 9-13-24 LT3.Project Maximum Z_XY1726760259_Z0_T0_C2.tif]

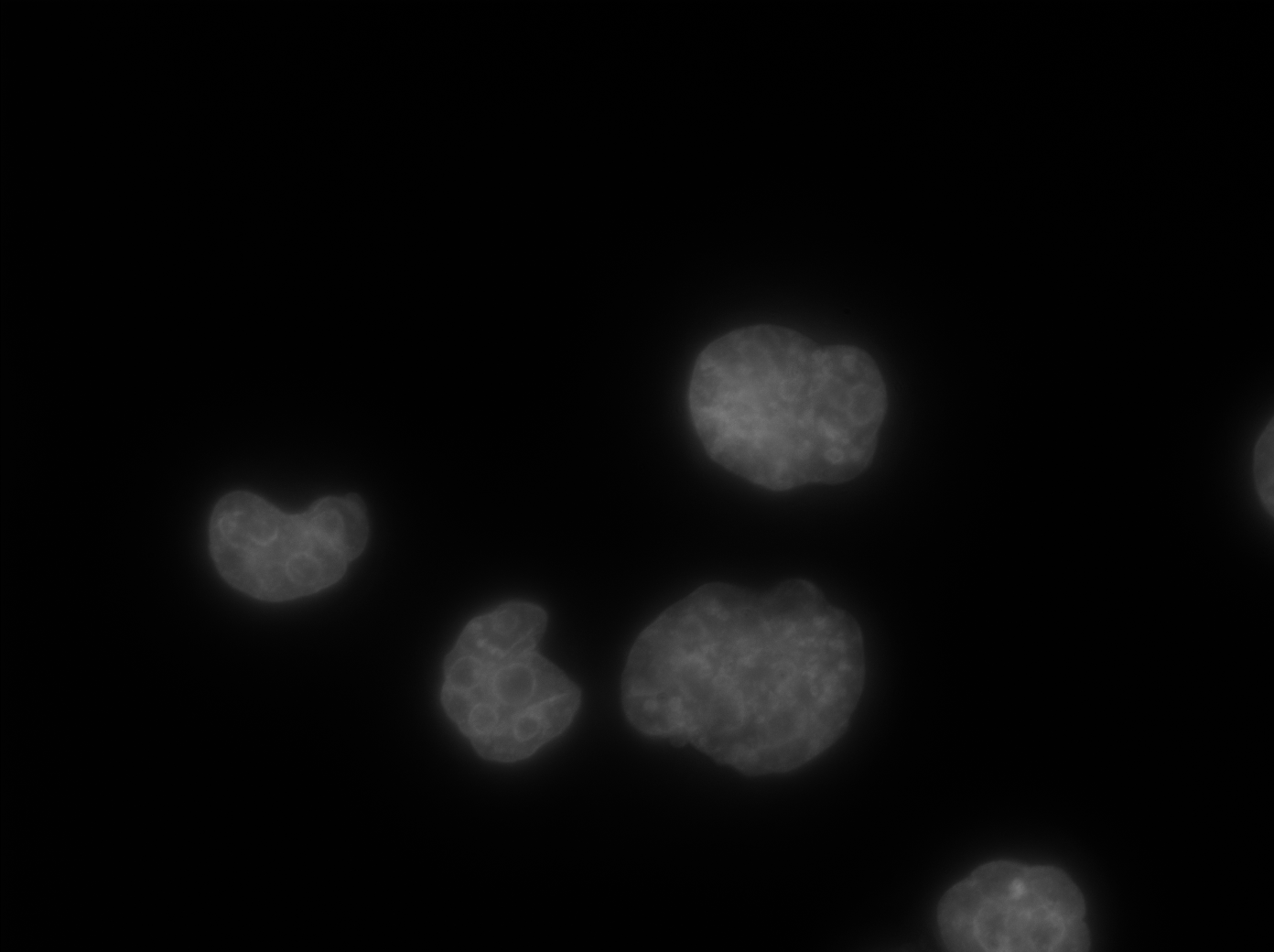

Supplement: Supplementary file 21 — Source data Fig. 6 part 2 [file 44319_2026_742_MOESM21_ESM.zip › Figure 6 Part 2/Fig 6abcd Cas9 TPGS1-KO acetylated tubulin atubulin part 2/TPGS1-KO R3 9-13-24 LT3.Project Maximum Z_XY1726760259_Z0_T0_C0.tif]

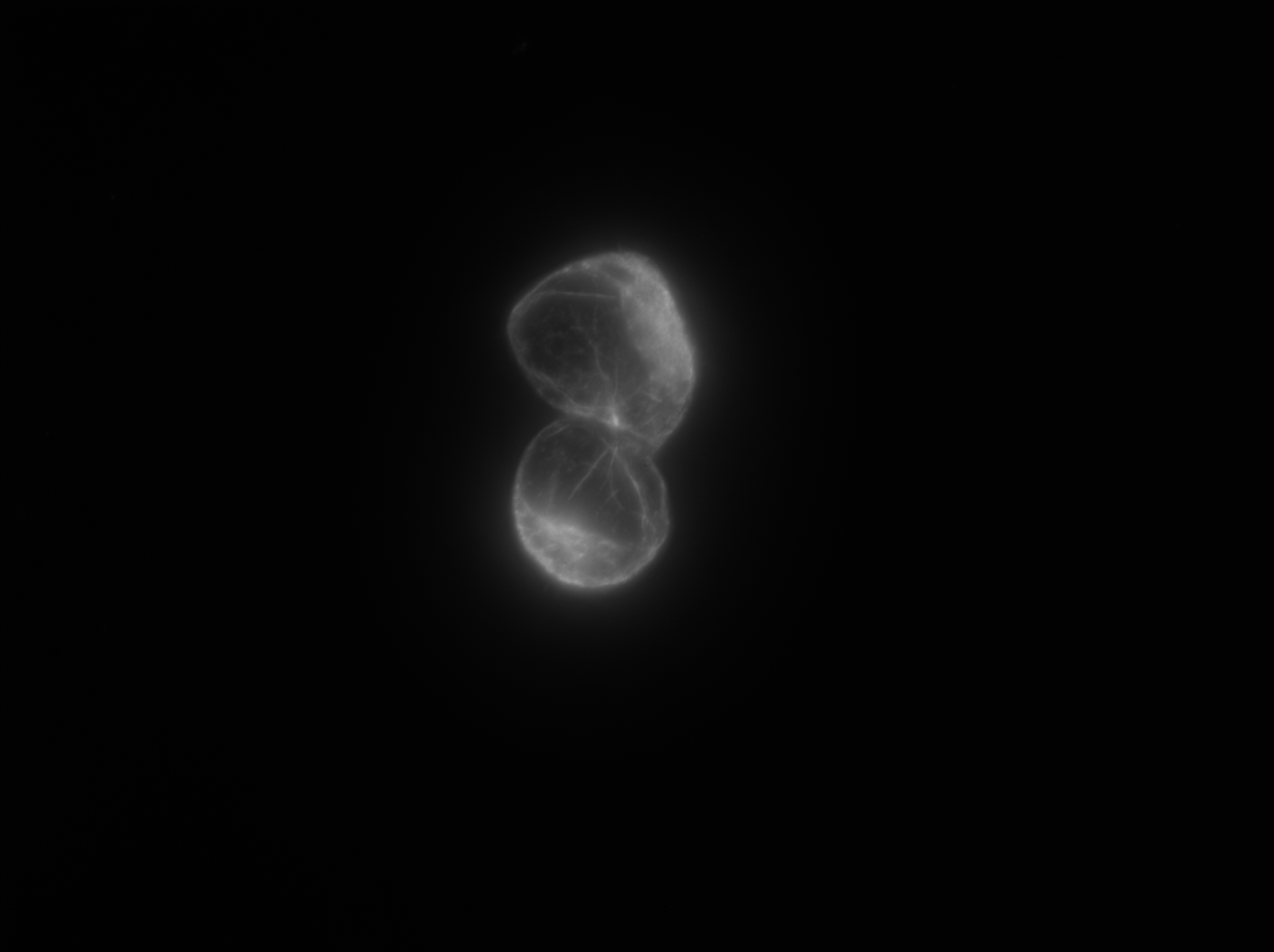

Supplement: Supplementary file 21 — Source data Fig. 6 part 2 [file 44319_2026_742_MOESM21_ESM.zip › Figure 6 Part 2/Fig 6abcd Cas9 TPGS1-KO acetylated tubulin atubulin part 2/TPGS1-KO R2 9-11-24 LT19.Project Maximum Z_XY1726266398_Z0_T0_C1.tif]

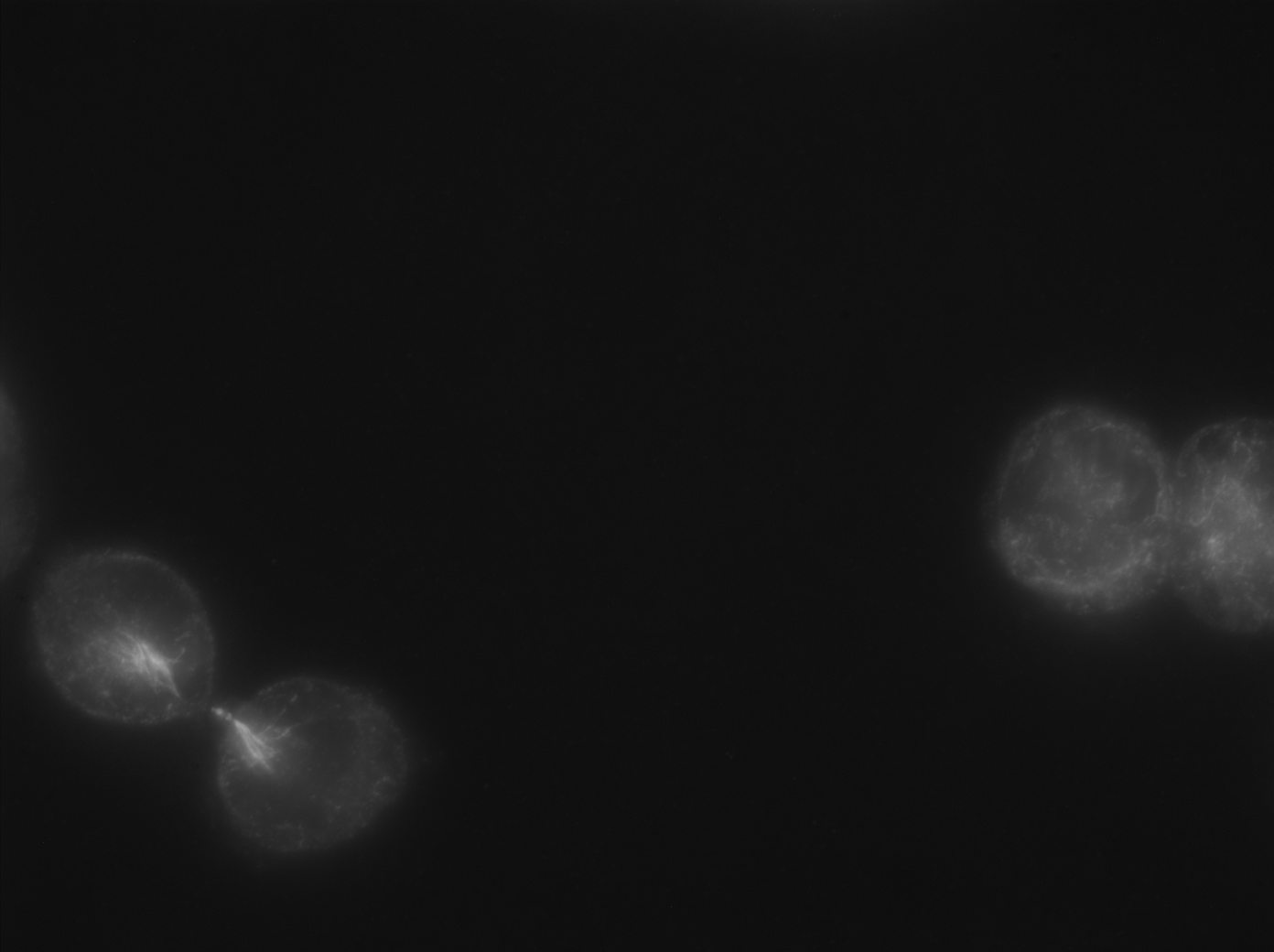

Supplement: Supplementary file 21 — Source data Fig. 6 part 2 [file 44319_2026_742_MOESM21_ESM.zip › Figure 6 Part 2/Fig 6abcd Cas9 TPGS1-KO acetylated tubulin atubulin part 2/TPGS1-KO R2 9-11-24 PA13.Project Maximum Z_XY1726265351_Z0_T0_C2.tif]

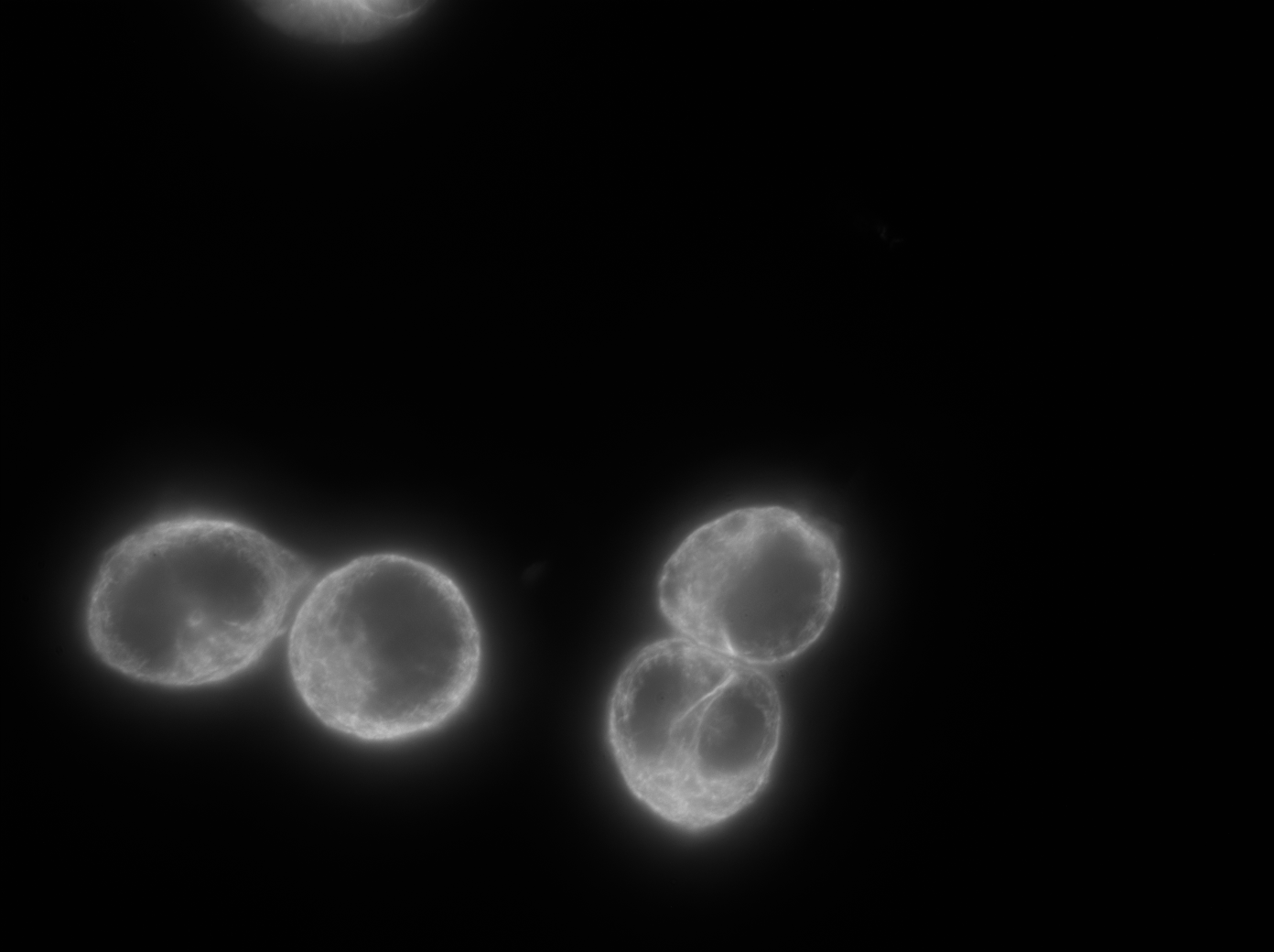

Supplement: Supplementary file 21 — Source data Fig. 6 part 2 [file 44319_2026_742_MOESM21_ESM.zip › Figure 6 Part 2/Fig 6abcd Cas9 TPGS1-KO acetylated tubulin atubulin part 2/TPGS1-KO R2 9-11-24 LT4.Project Maximum Z_XY1726259553_Z0_T0_C1.tif]

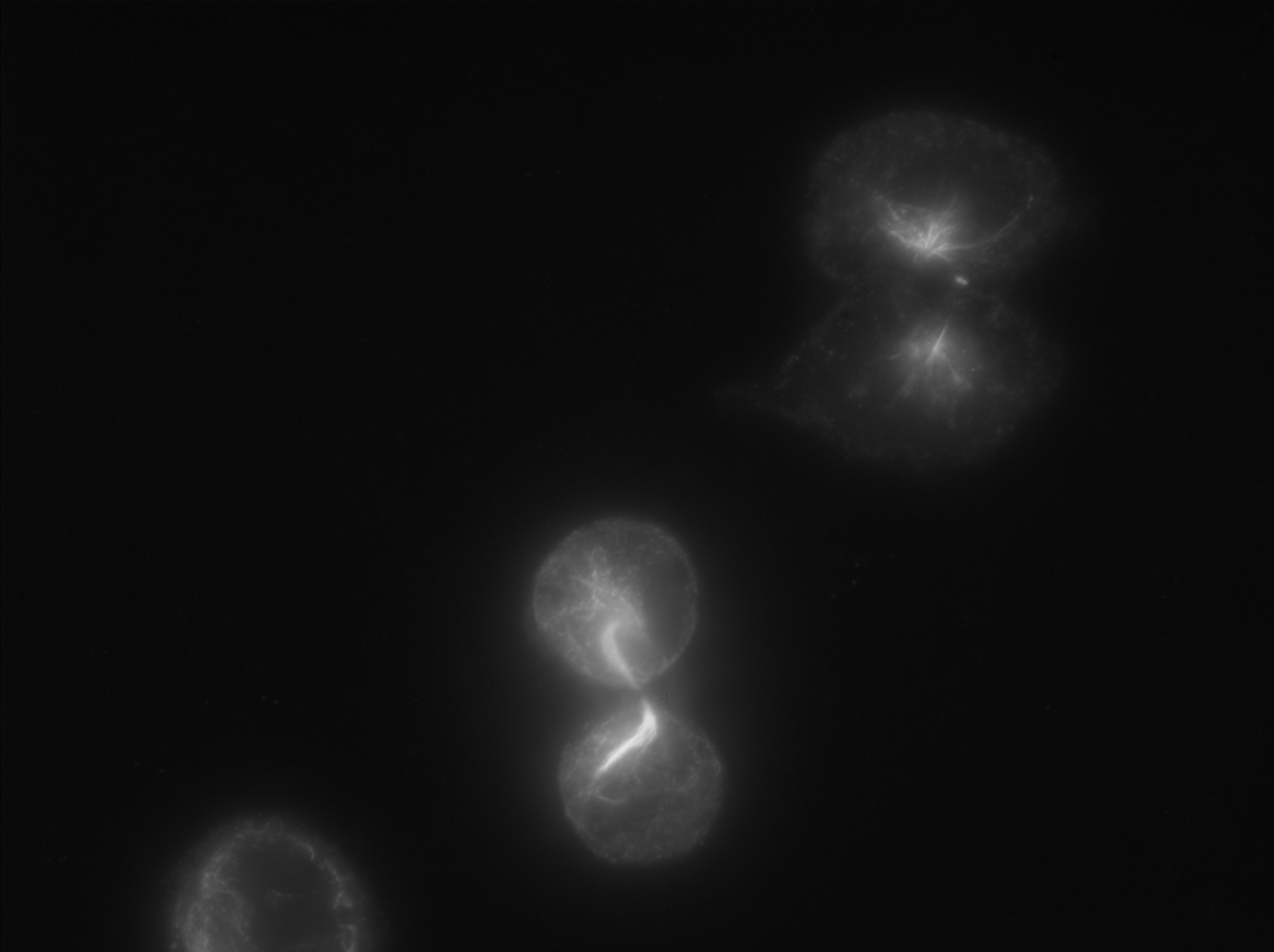

Supplement: Supplementary file 21 — Source data Fig. 6 part 2 [file 44319_2026_742_MOESM21_ESM.zip › Figure 6 Part 2/Fig 6abcd Cas9 TPGS1-KO acetylated tubulin atubulin part 2/TPGS1-KO R2 9-11-24 LT6 PA5.Project Maximum Z_XY1726261251_Z0_T0_C2.tif]

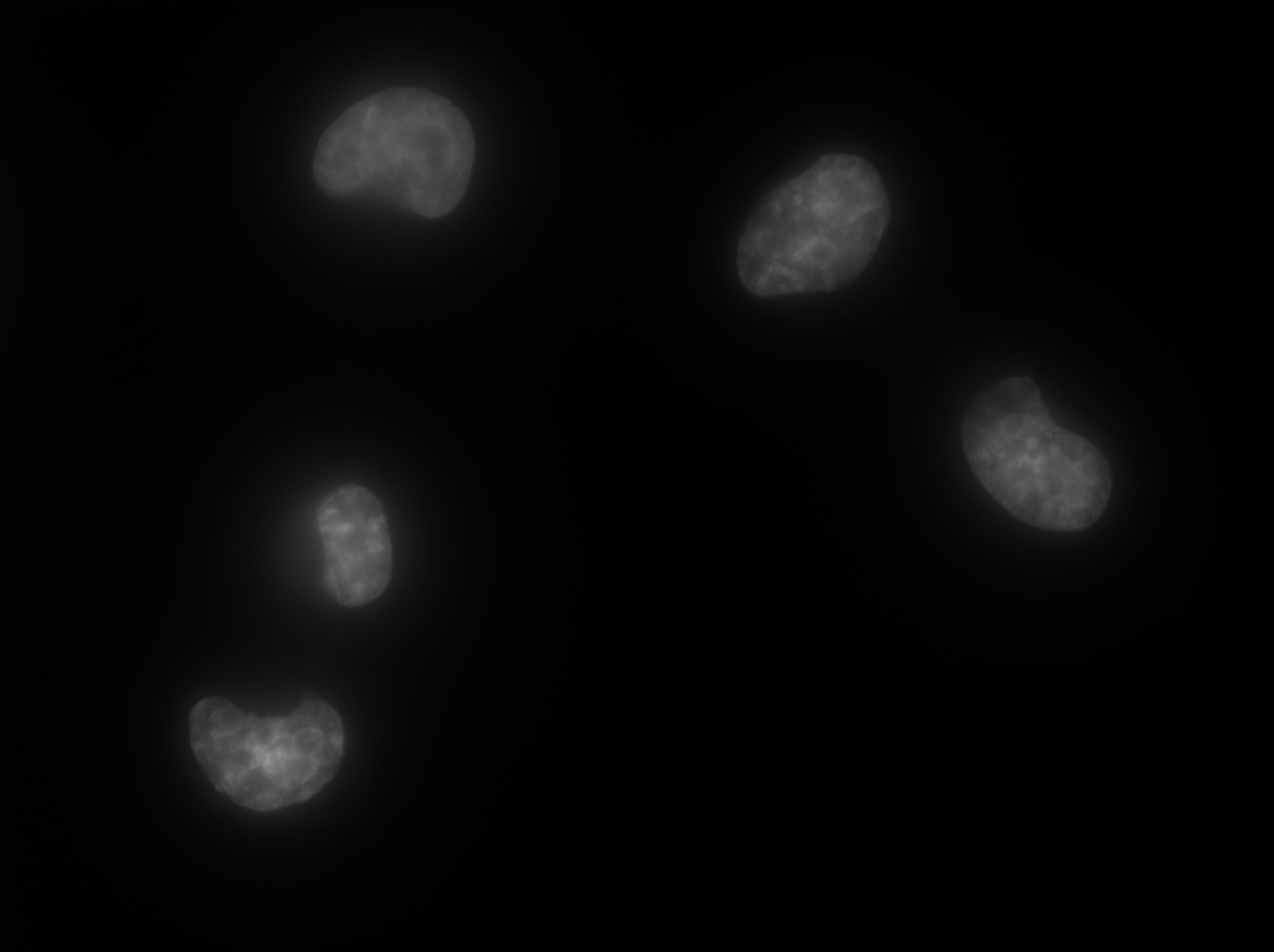

Supplement: Supplementary file 21 — Source data Fig. 6 part 2 [file 44319_2026_742_MOESM21_ESM.zip › Figure 6 Part 2/Fig 6abcd Cas9 TPGS1-KO acetylated tubulin atubulin part 2/TPGS1-KO R3 9-13-24 LT12LT13.Project Maximum Z_XY1726761185_Z0_T0_C0.tif]

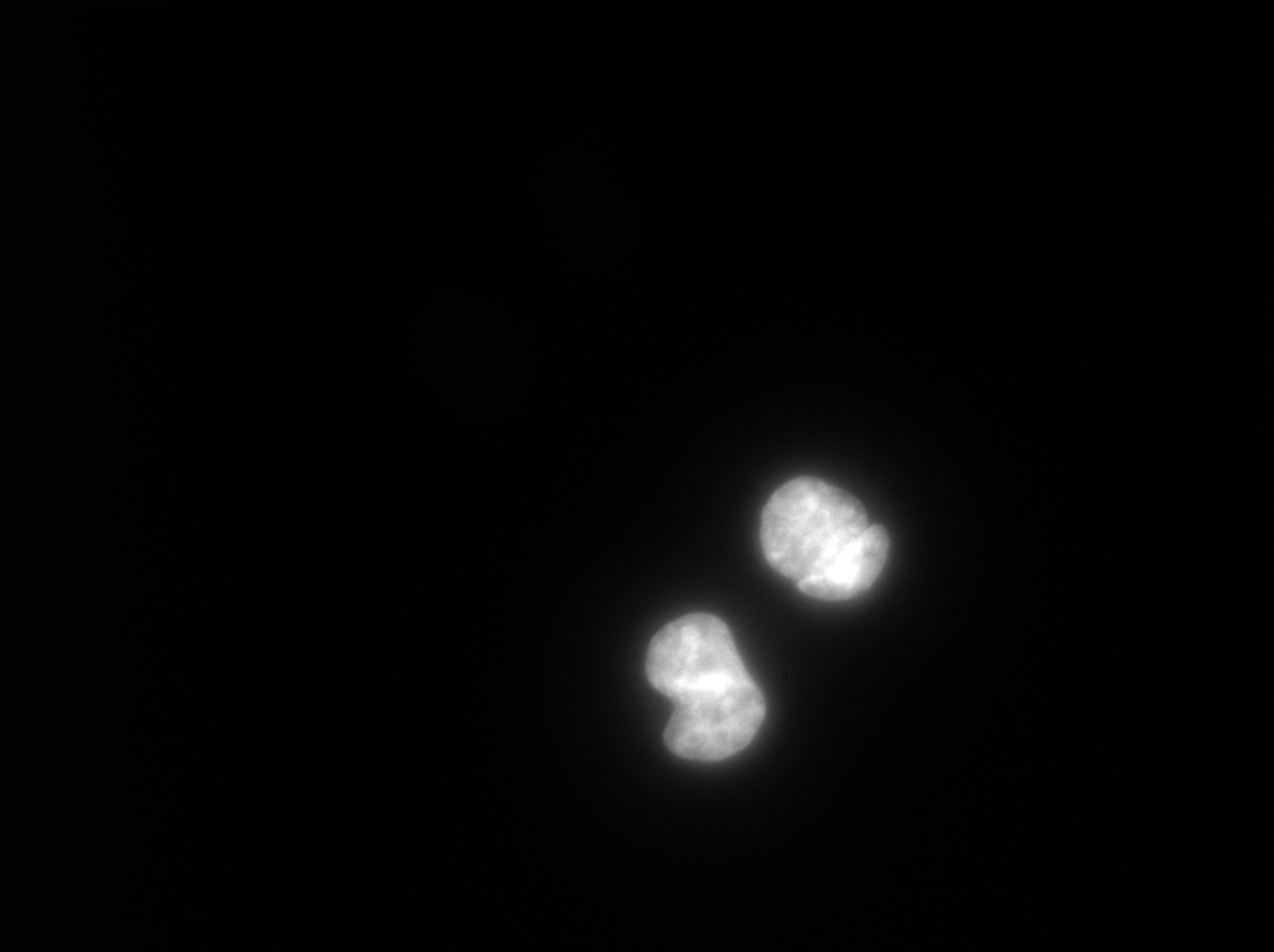

Supplement: Supplementary file 21 — Source data Fig. 6 part 2 [file 44319_2026_742_MOESM21_ESM.zip › Figure 6 Part 2/Fig 6abcd Cas9 TPGS1-KO acetylated tubulin atubulin part 2/TPGS1-KO R2 9-11-24 LT24.Project Maximum Z_XY1726268192_Z0_T0_C0.tif]

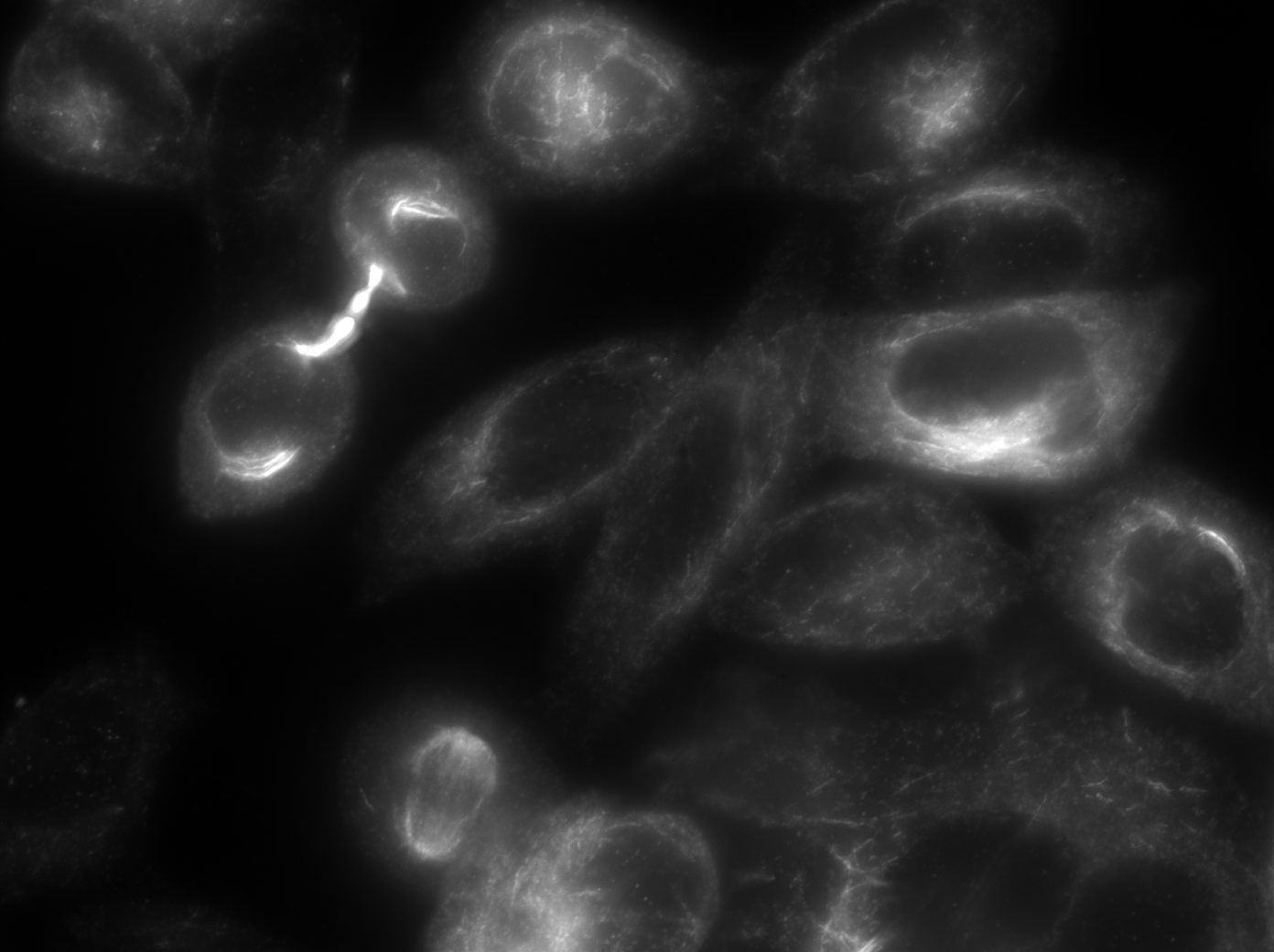

Supplement: Supplementary file 21 — Source data Fig. 6 part 2 [file 44319_2026_742_MOESM21_ESM.zip › Figure 6 Part 2/Fig 6abcd Cas9 TPGS1-KO acetylated tubulin atubulin part 2/TPGS1-KO R3 9-13-24 LT30.Project Maximum Z_XY1726765116_Z0_T0_C2.tif]

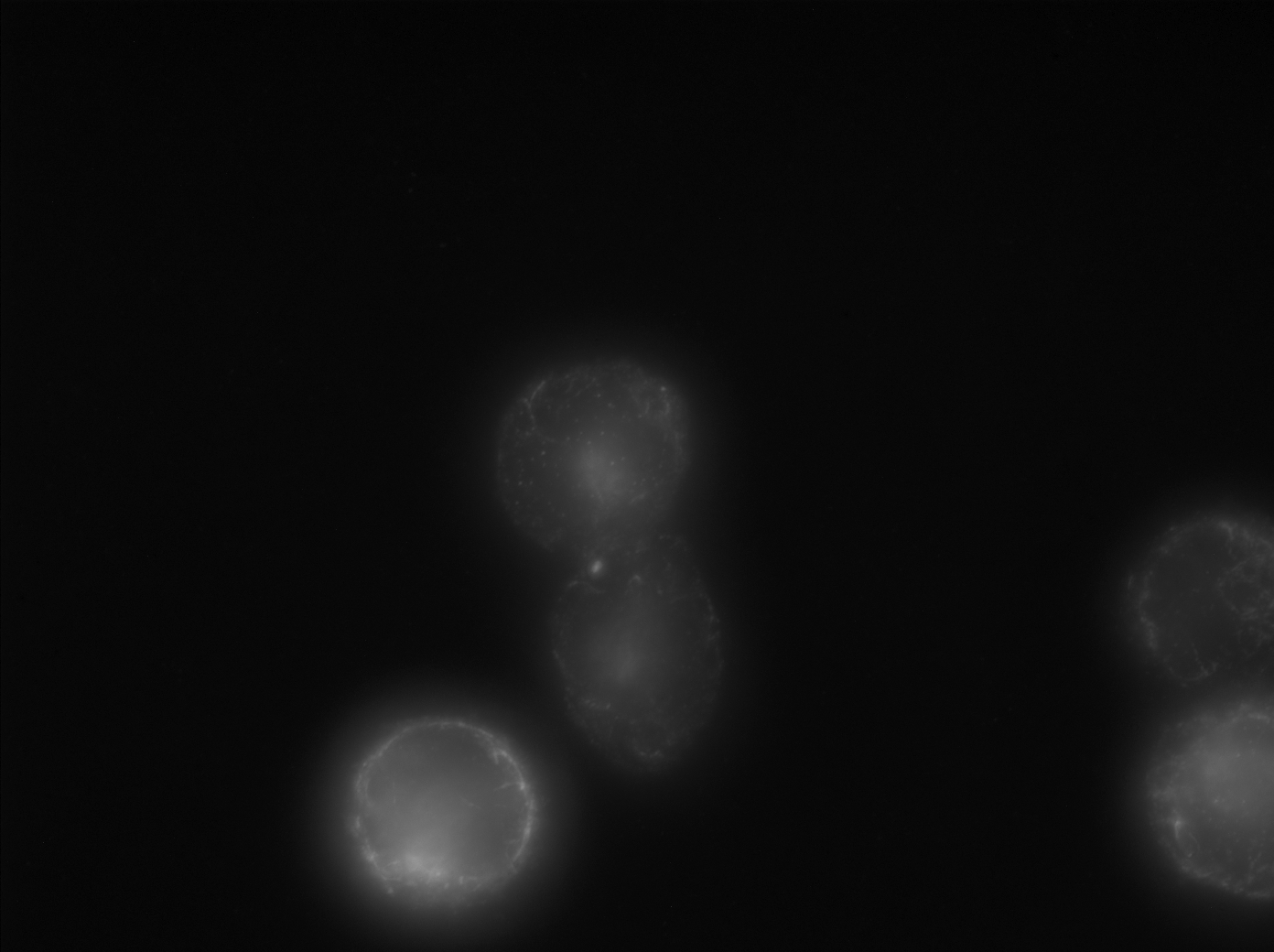

Supplement: Supplementary file 21 — Source data Fig. 6 part 2 [file 44319_2026_742_MOESM21_ESM.zip › Figure 6 Part 2/Fig 6abcd Cas9 TPGS1-KO acetylated tubulin atubulin part 2/TPGS1-KO R2 9-11-24 PA7.Project Maximum Z_XY1726261664_Z0_T0_C2.tif]

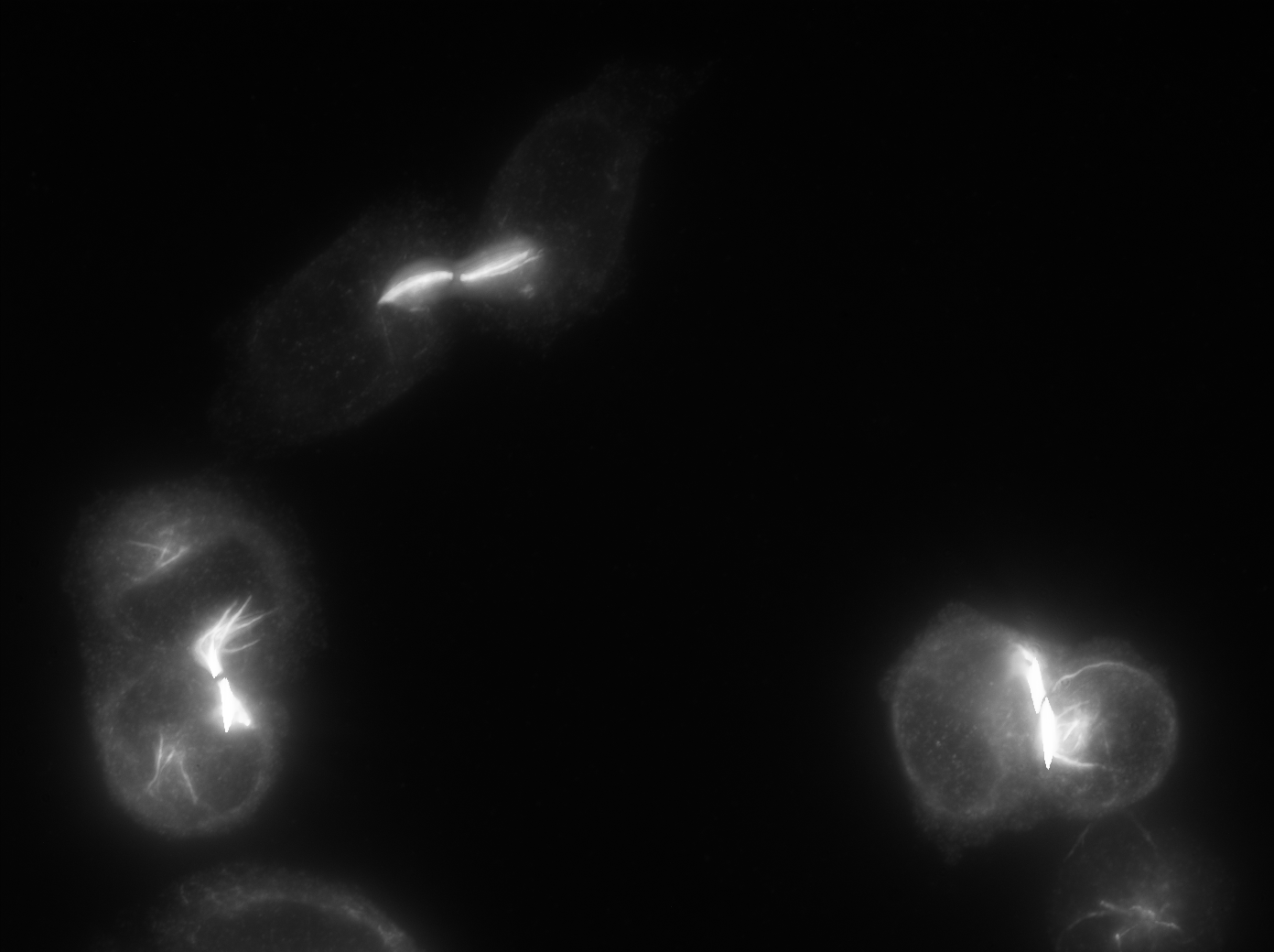

Supplement: Supplementary file 21 — Source data Fig. 6 part 2 [file 44319_2026_742_MOESM21_ESM.zip › Figure 6 Part 2/Fig 6abcd Cas9 TPGS1-KO acetylated tubulin atubulin part 2/TPGS1-KO R3 9-13-24 LT24LT25LT26.Project Maximum Z_XY1726764690_Z0_T0_C2.tif]

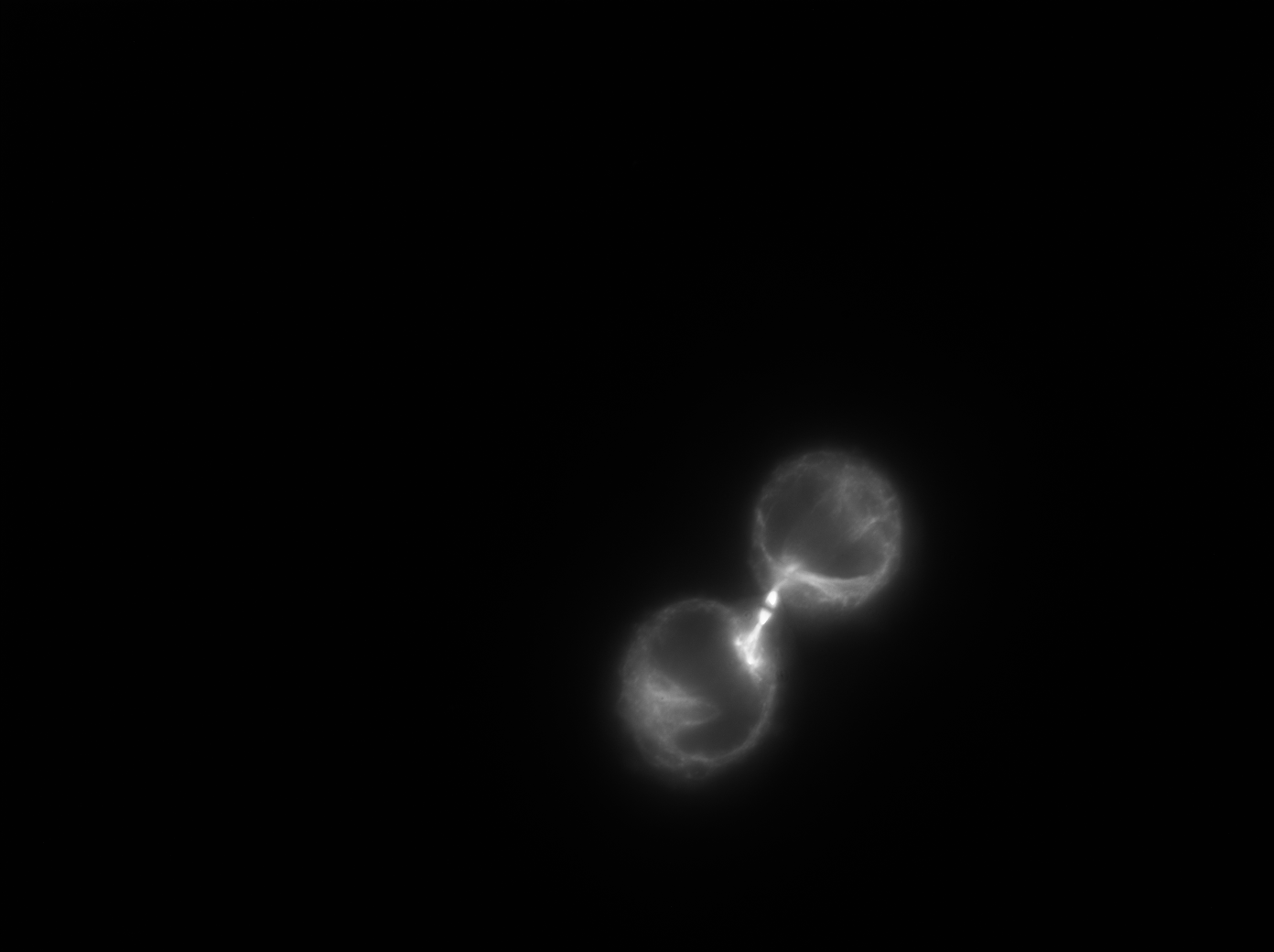

Supplement: Supplementary file 21 — Source data Fig. 6 part 2 [file 44319_2026_742_MOESM21_ESM.zip › Figure 6 Part 2/Fig 6abcd Cas9 TPGS1-KO acetylated tubulin atubulin part 2/TPGS1-KO R2 9-11-24 LT24.Project Maximum Z_XY1726268192_Z0_T0_C1.tif]

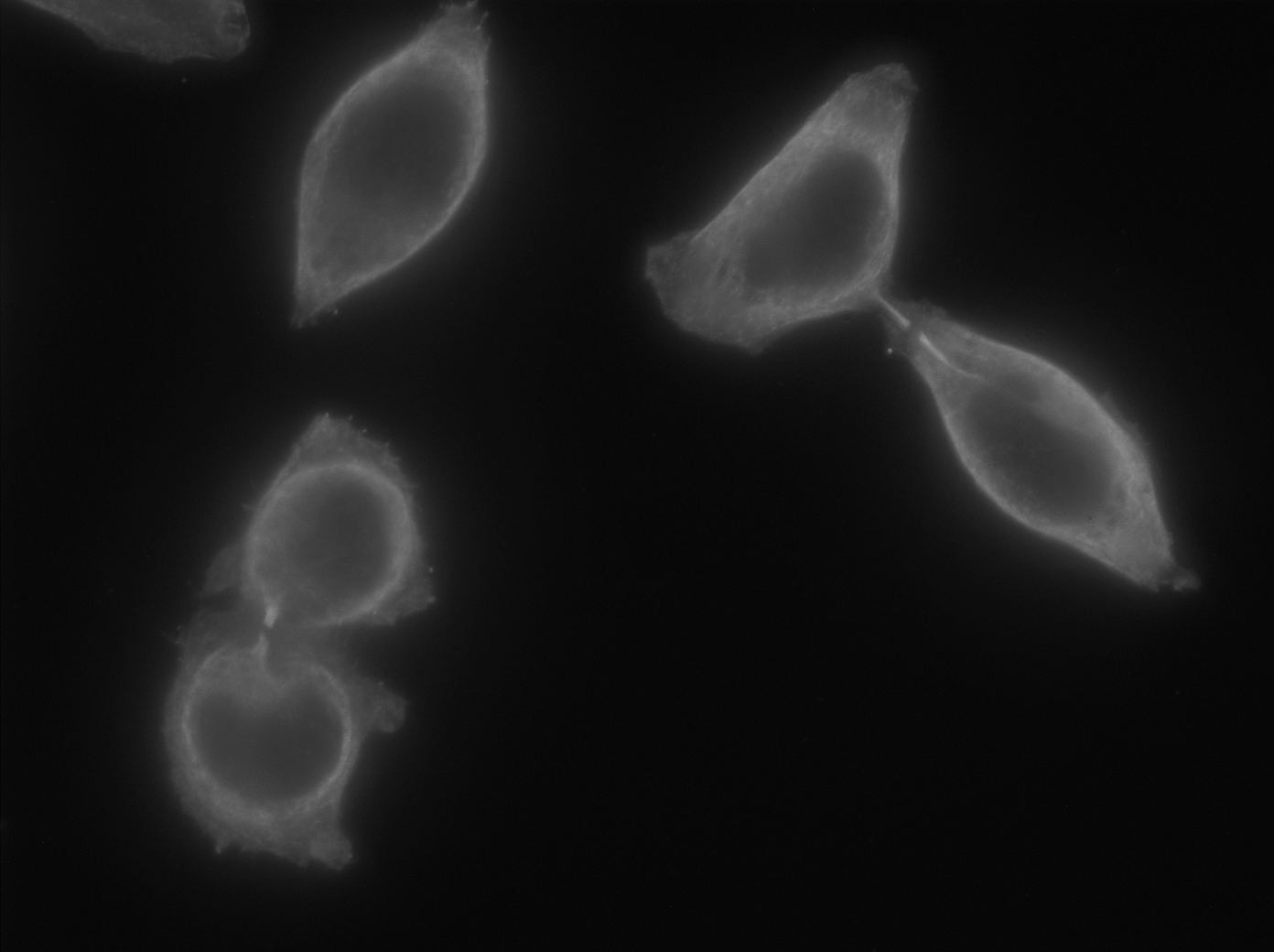

Supplement: Supplementary file 21 — Source data Fig. 6 part 2 [file 44319_2026_742_MOESM21_ESM.zip › Figure 6 Part 2/Fig 6abcd Cas9 TPGS1-KO acetylated tubulin atubulin part 2/TPGS1-KO R3 9-13-24 LT12LT13.Project Maximum Z_XY1726761185_Z0_T0_C1.tif]

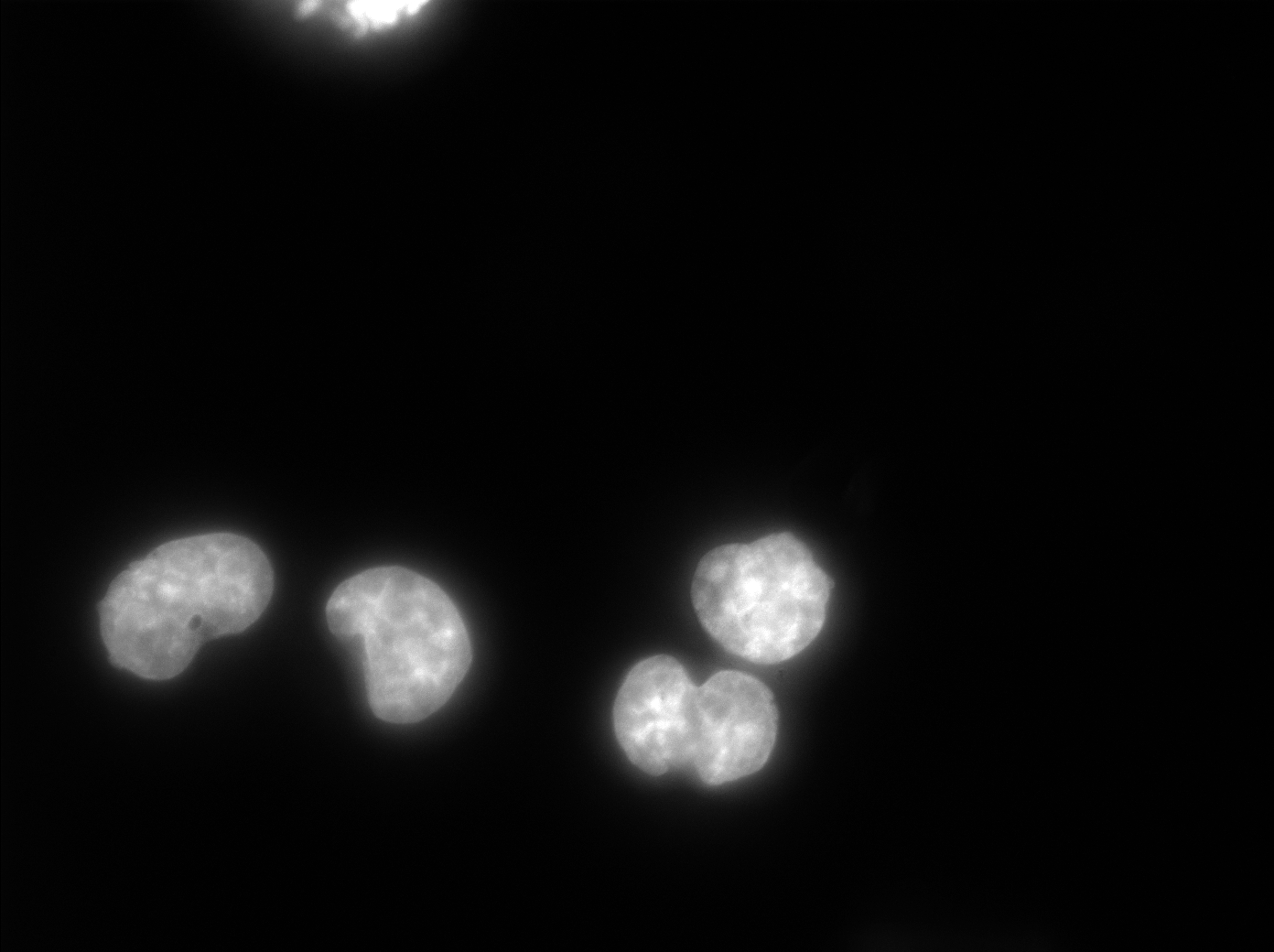

Supplement: Supplementary file 21 — Source data Fig. 6 part 2 [file 44319_2026_742_MOESM21_ESM.zip › Figure 6 Part 2/Fig 6abcd Cas9 TPGS1-KO acetylated tubulin atubulin part 2/TPGS1-KO R2 9-11-24 LT4.Project Maximum Z_XY1726259553_Z0_T0_C0.tif]

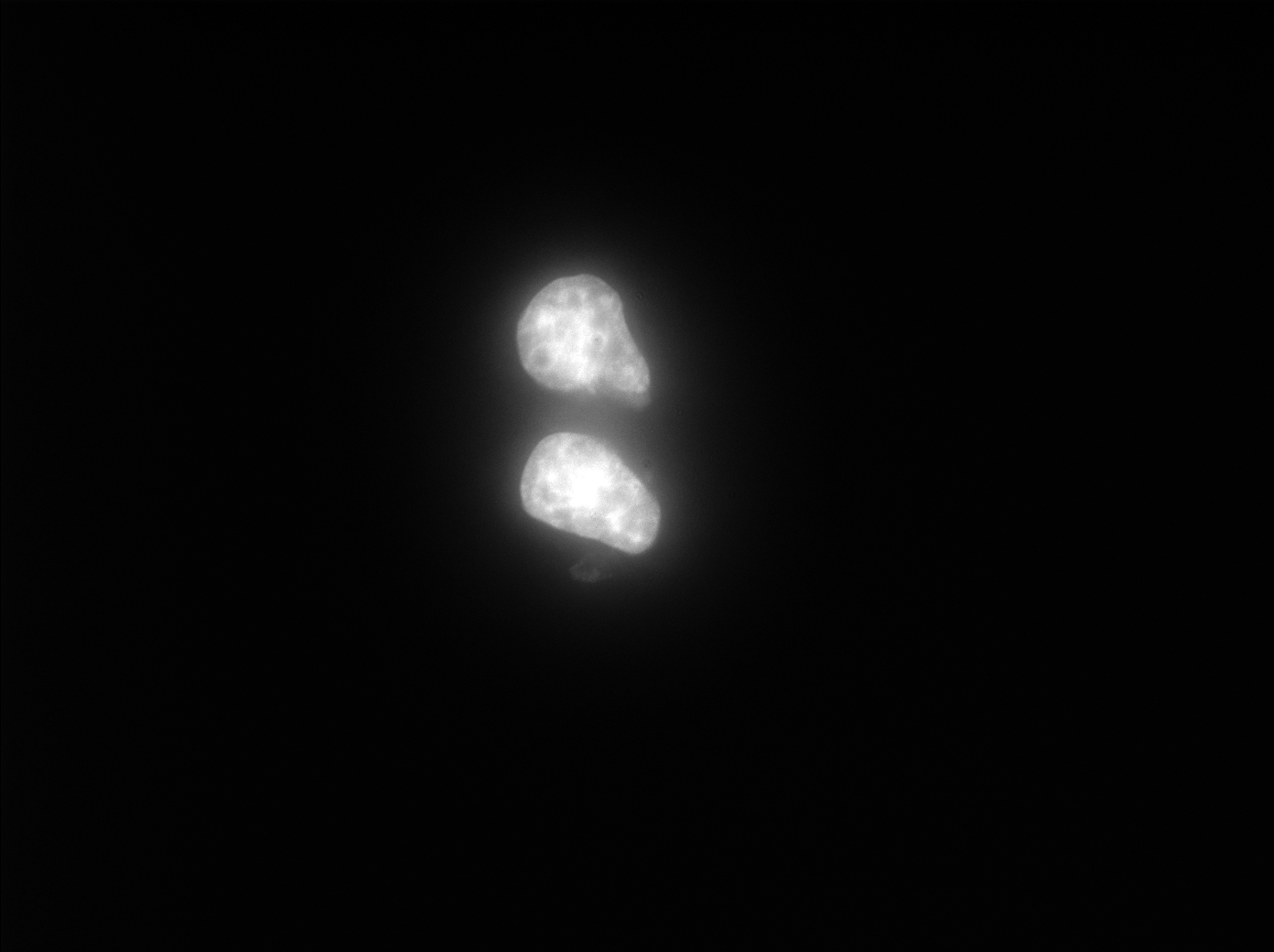

Supplement: Supplementary file 21 — Source data Fig. 6 part 2 [file 44319_2026_742_MOESM21_ESM.zip › Figure 6 Part 2/Fig 6abcd Cas9 TPGS1-KO acetylated tubulin atubulin part 2/TPGS1-KO R2 9-11-24 LT19.Project Maximum Z_XY1726266398_Z0_T0_C0.tif]

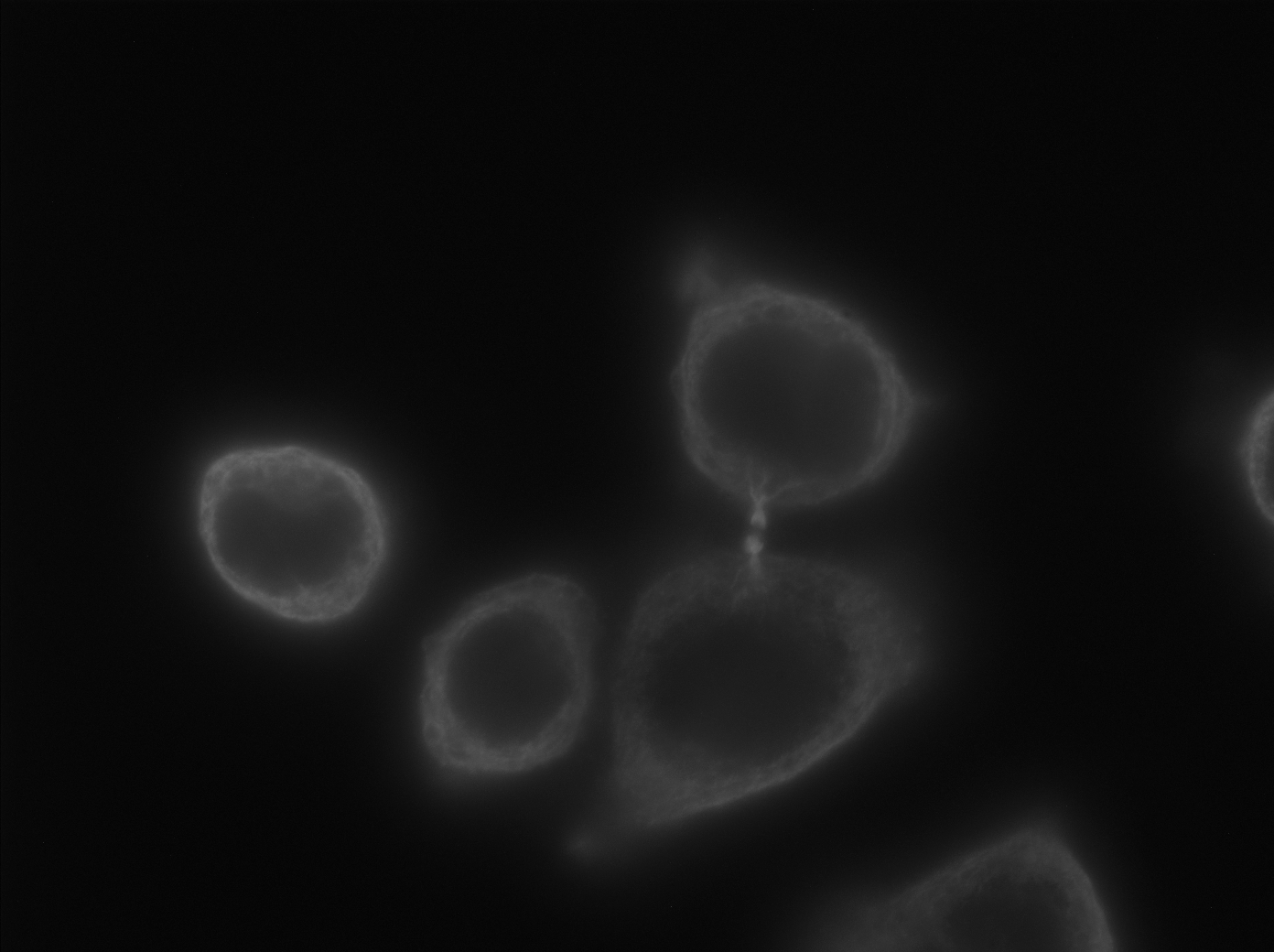

Supplement: Supplementary file 21 — Source data Fig. 6 part 2 [file 44319_2026_742_MOESM21_ESM.zip › Figure 6 Part 2/Fig 6abcd Cas9 TPGS1-KO acetylated tubulin atubulin part 2/TPGS1-KO R3 9-13-24 LT3.Project Maximum Z_XY1726760259_Z0_T0_C1.tif]

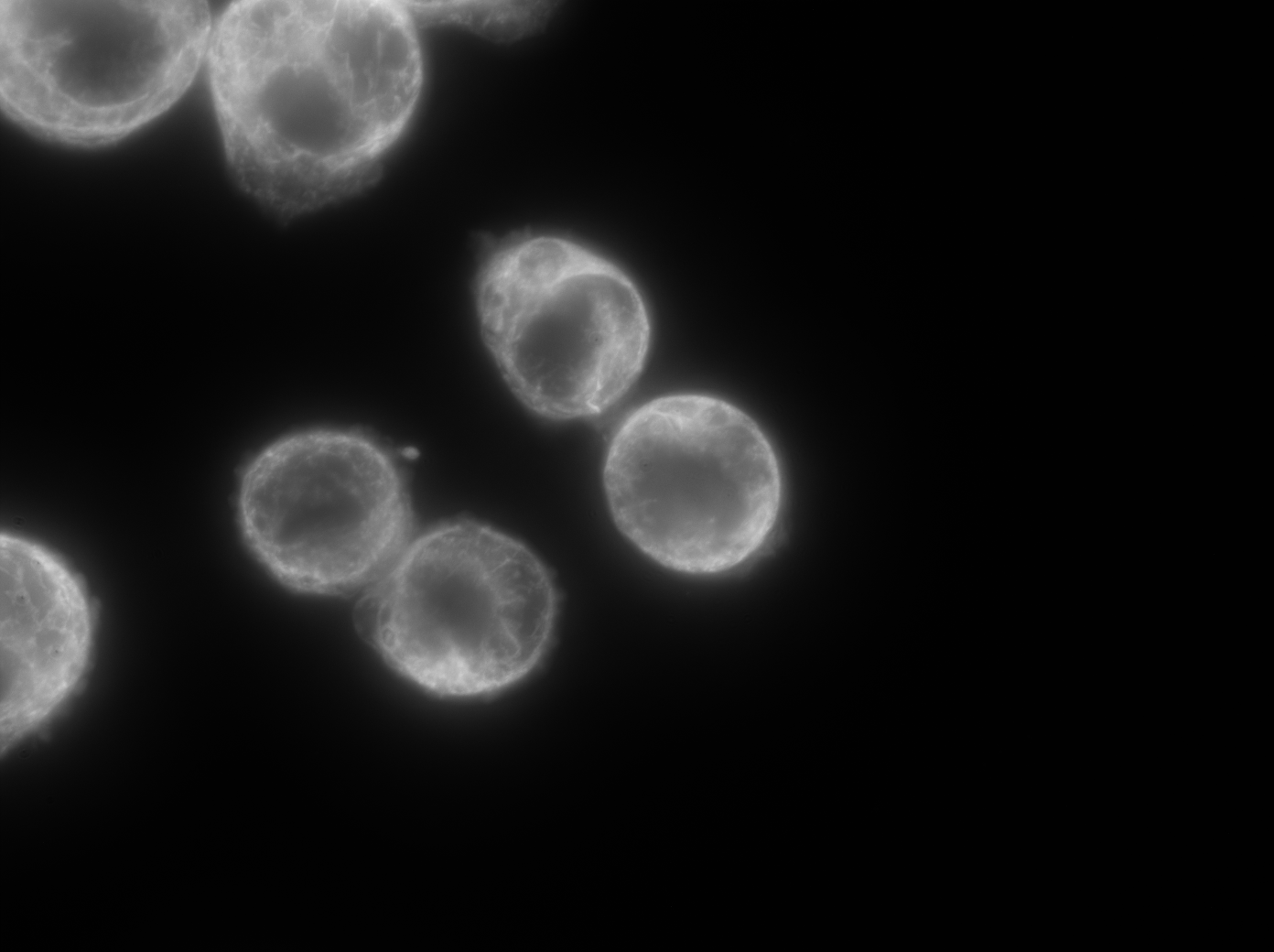

Supplement: Supplementary file 21 — Source data Fig. 6 part 2 [file 44319_2026_742_MOESM21_ESM.zip › Figure 6 Part 2/Fig 6abcd Cas9 TPGS1-KO acetylated tubulin atubulin part 2/TPGS1-KO R2 9-11-24 PA1.Project Maximum Z_XY1726258742_Z0_T0_C1.tif]

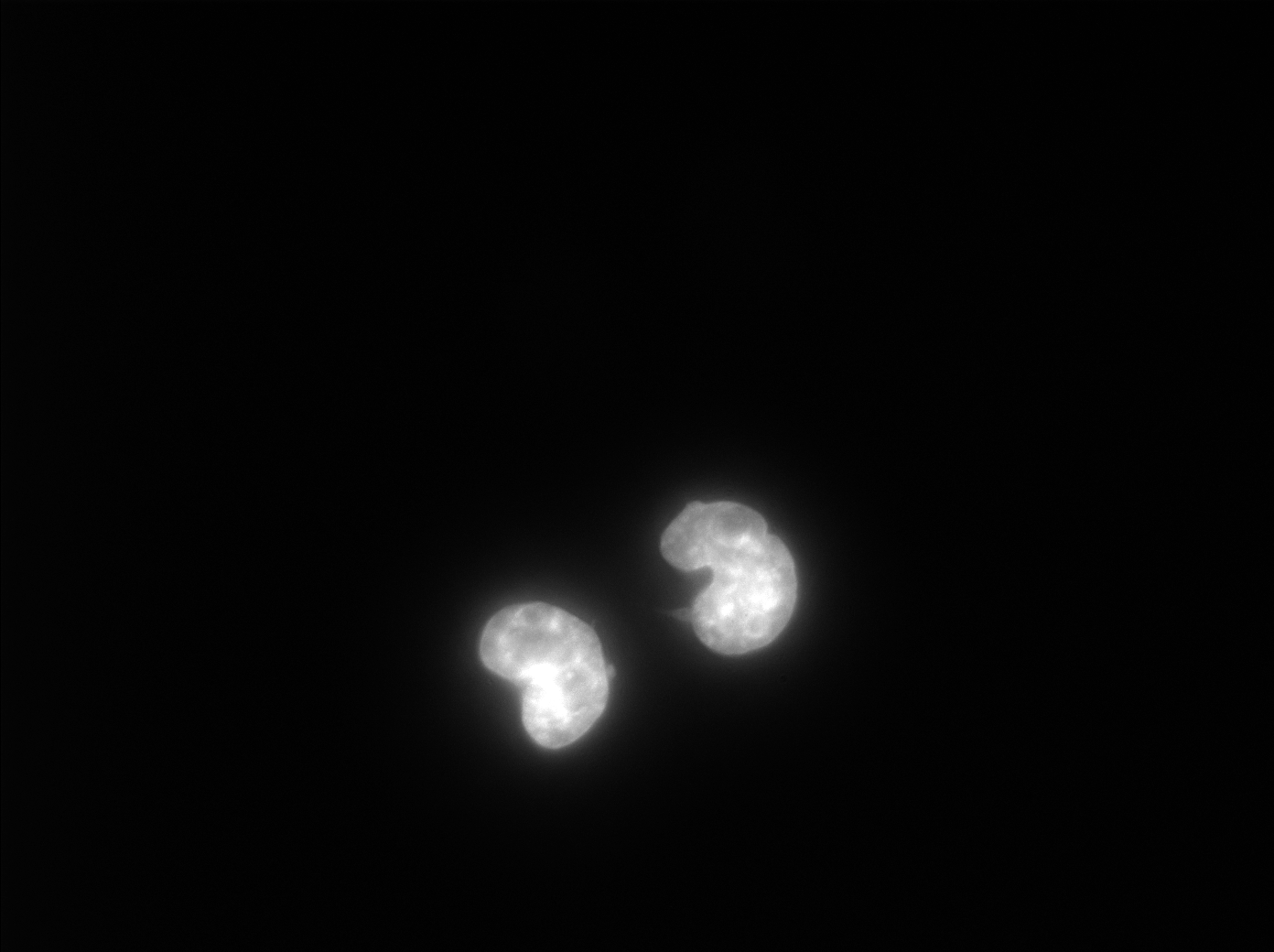

Supplement: Supplementary file 21 — Source data Fig. 6 part 2 [file 44319_2026_742_MOESM21_ESM.zip › Figure 6 Part 2/Fig 6abcd Cas9 TPGS1-KO acetylated tubulin atubulin part 2/TPGS1-KO R2 9-11-24 LT1.Project Maximum Z_XY1726258922_Z0_T0_C0.tif]

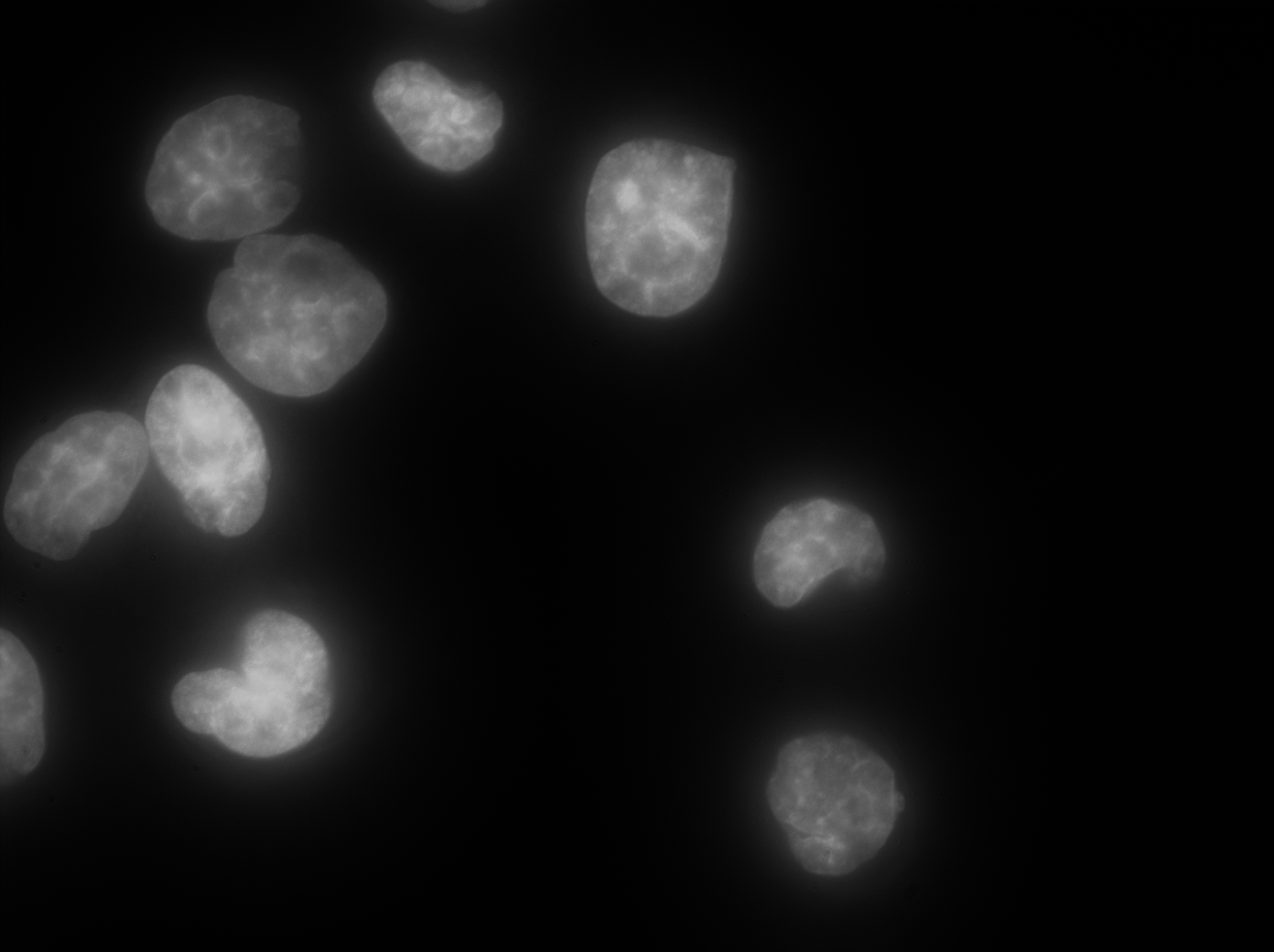

Supplement: Supplementary file 21 — Source data Fig. 6 part 2 [file 44319_2026_742_MOESM21_ESM.zip › Figure 6 Part 2/Fig 6abcd Cas9 TPGS1-KO acetylated tubulin atubulin part 2/TPGS1-KO R3 9-13-24 LT22.Project Maximum Z_XY1726764435_Z0_T0_C0.tif]

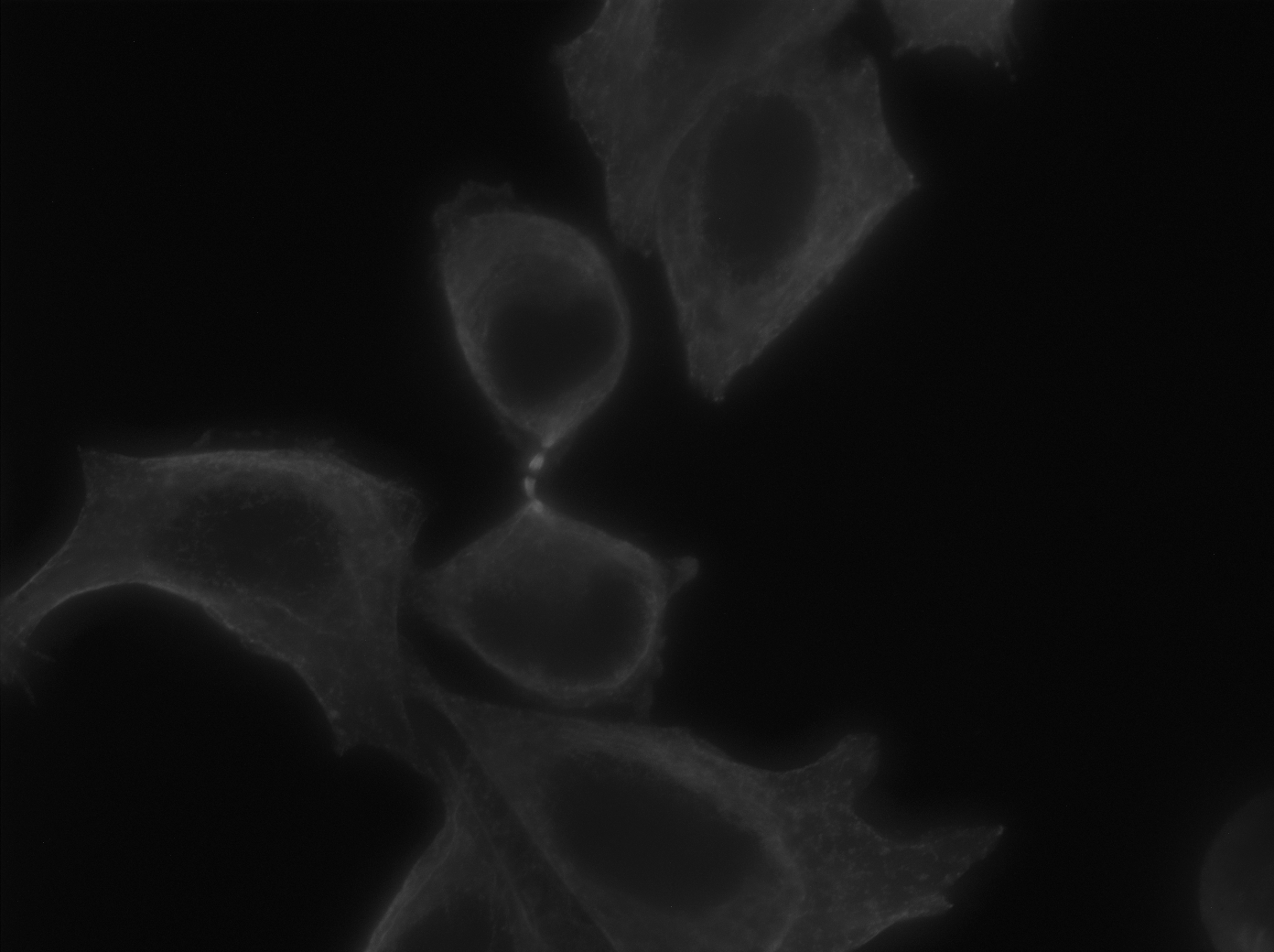

Supplement: Supplementary file 21 — Source data Fig. 6 part 2 [file 44319_2026_742_MOESM21_ESM.zip › Figure 6 Part 2/Fig 6abcd Cas9 TPGS1-KO acetylated tubulin atubulin part 2/TPGS1-KO R3 9-13-24 LT5.Project Maximum Z_XY1726760507_Z0_T0_C1.tif]

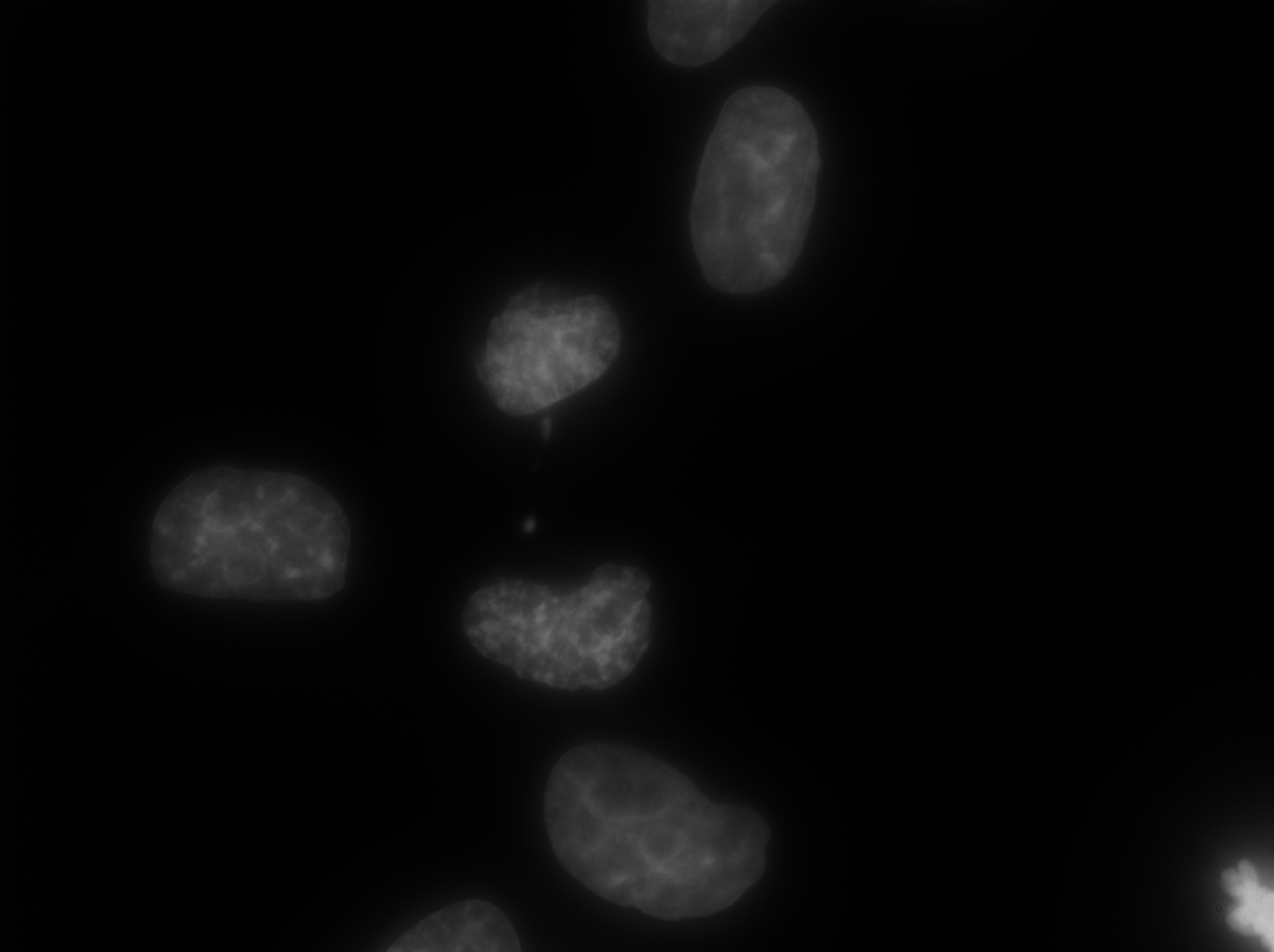

Supplement: Supplementary file 21 — Source data Fig. 6 part 2 [file 44319_2026_742_MOESM21_ESM.zip › Figure 6 Part 2/Fig 6abcd Cas9 TPGS1-KO acetylated tubulin atubulin part 2/TPGS1-KO R3 9-13-24 LT5.Project Maximum Z_XY1726760507_Z0_T0_C0.tif]

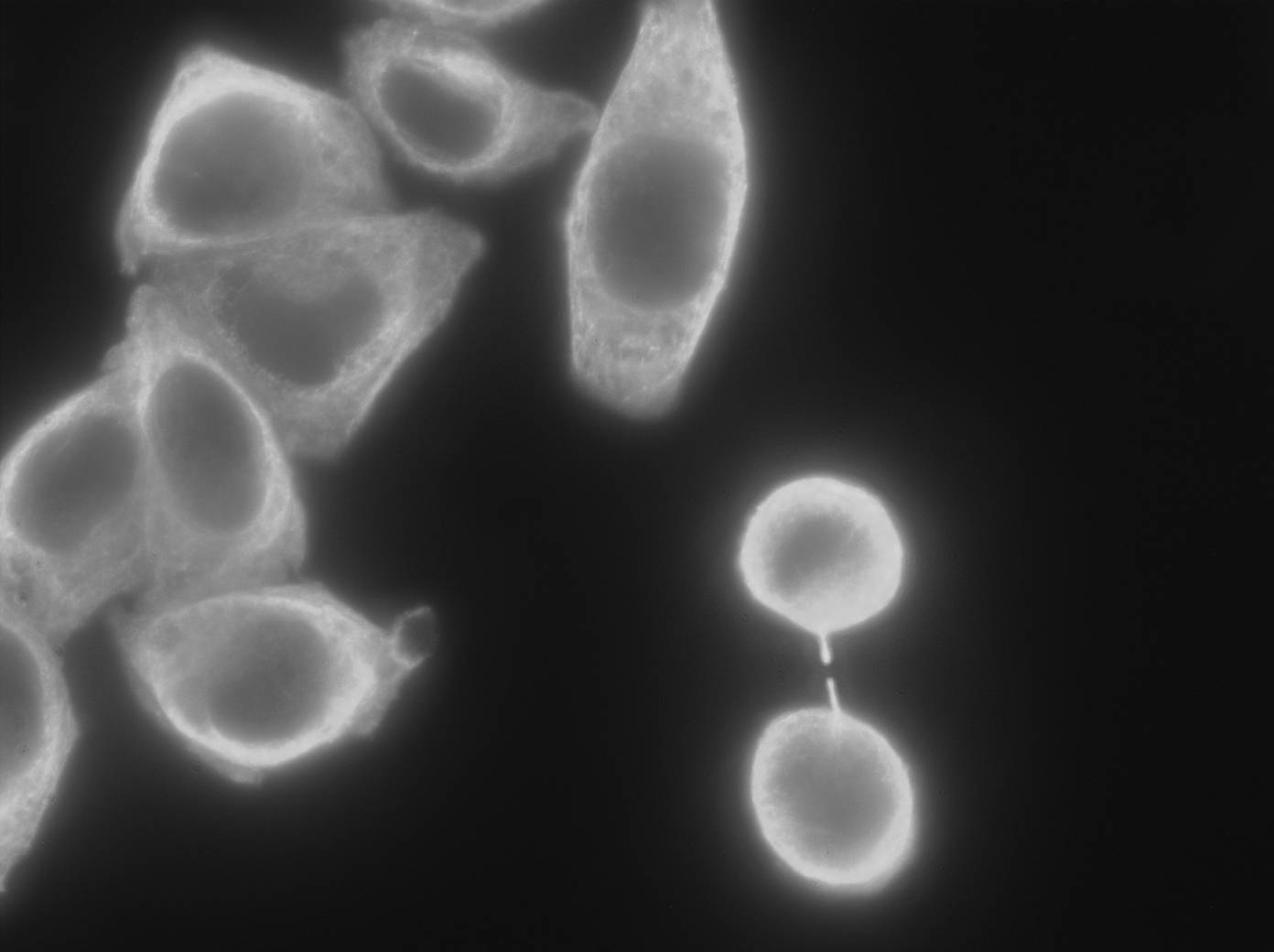

Supplement: Supplementary file 21 — Source data Fig. 6 part 2 [file 44319_2026_742_MOESM21_ESM.zip › Figure 6 Part 2/Fig 6abcd Cas9 TPGS1-KO acetylated tubulin atubulin part 2/TPGS1-KO R3 9-13-24 LT22.Project Maximum Z_XY1726764435_Z0_T0_C1.tif]

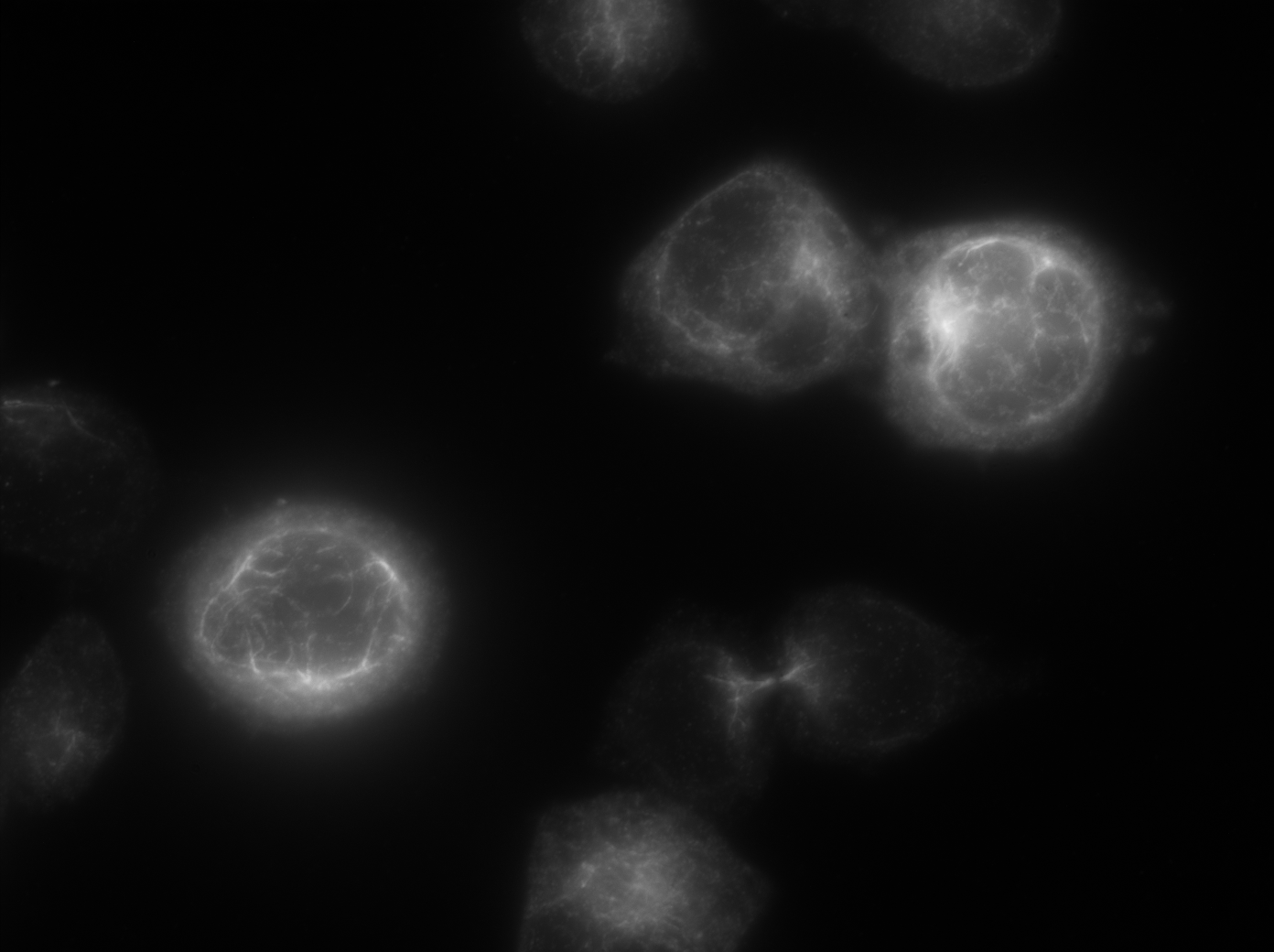

Supplement: Supplementary file 21 — Source data Fig. 6 part 2 [file 44319_2026_742_MOESM21_ESM.zip › Figure 6 Part 2/Fig 6abcd Cas9 TPGS1-KO acetylated tubulin atubulin part 2/TPGS1-KO R3 9-13-24 LT9.Project Maximum Z_XY1726760912_Z0_T0_C2.tif]

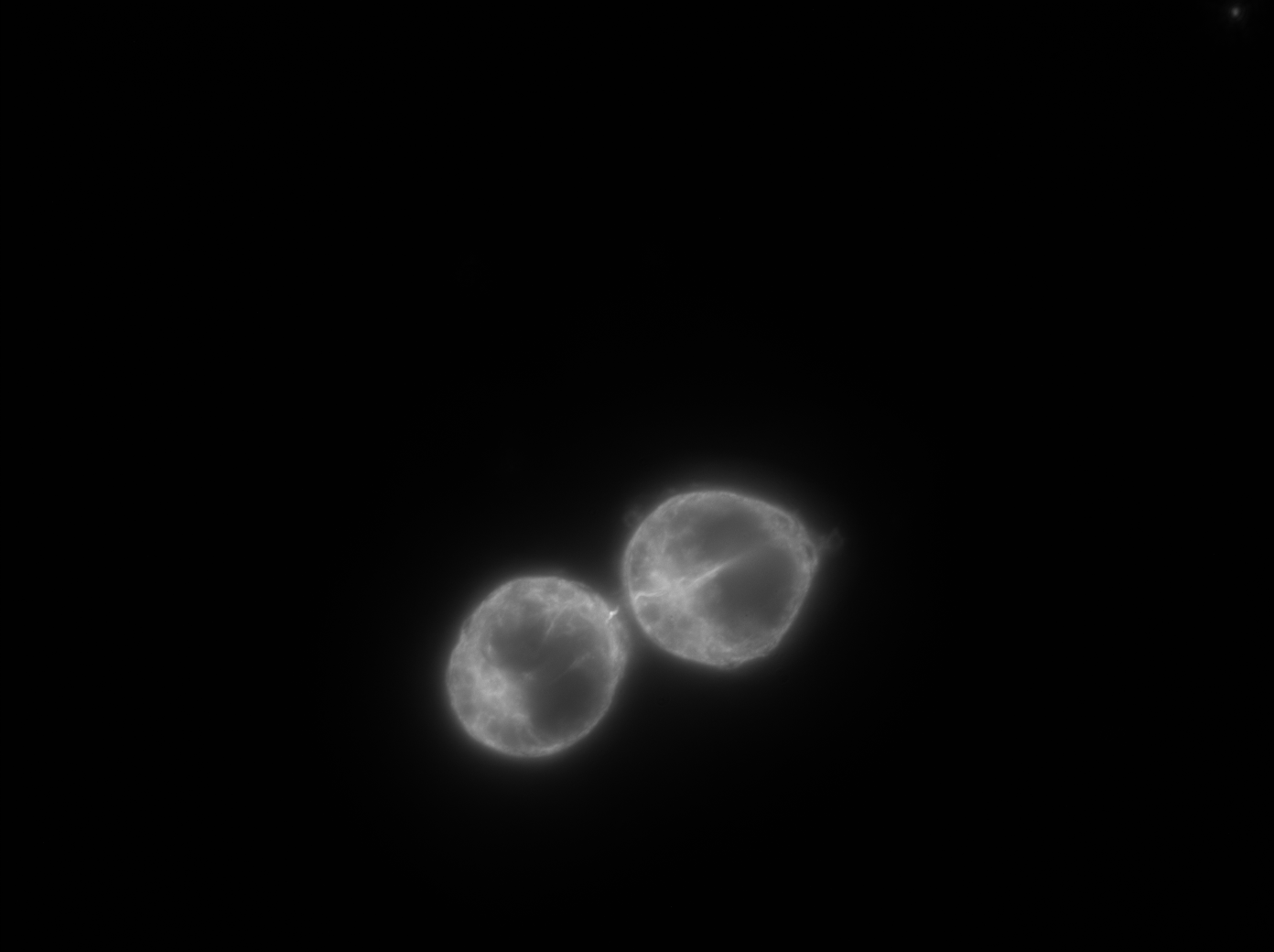

Supplement: Supplementary file 21 — Source data Fig. 6 part 2 [file 44319_2026_742_MOESM21_ESM.zip › Figure 6 Part 2/Fig 6abcd Cas9 TPGS1-KO acetylated tubulin atubulin part 2/TPGS1-KO R2 9-11-24 LT1.Project Maximum Z_XY1726258922_Z0_T0_C1.tif]

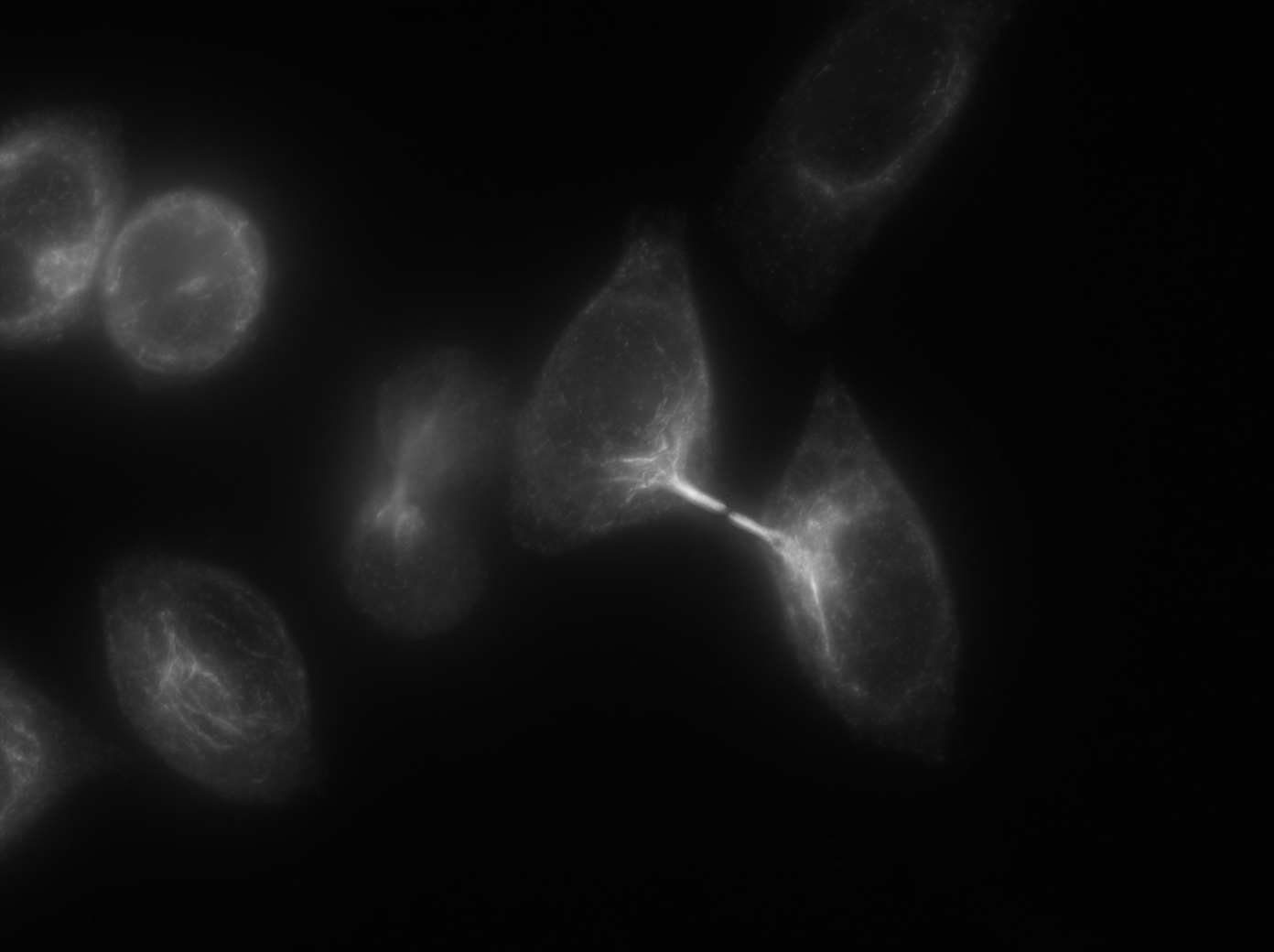

Supplement: Supplementary file 21 — Source data Fig. 6 part 2 [file 44319_2026_742_MOESM21_ESM.zip › Figure 6 Part 2/Fig 6abcd Cas9 TPGS1-KO acetylated tubulin atubulin part 2/TPGS1-KO R3 9-13-24 LT28.Project Maximum Z_XY1726764895_Z0_T0_C2.tif]

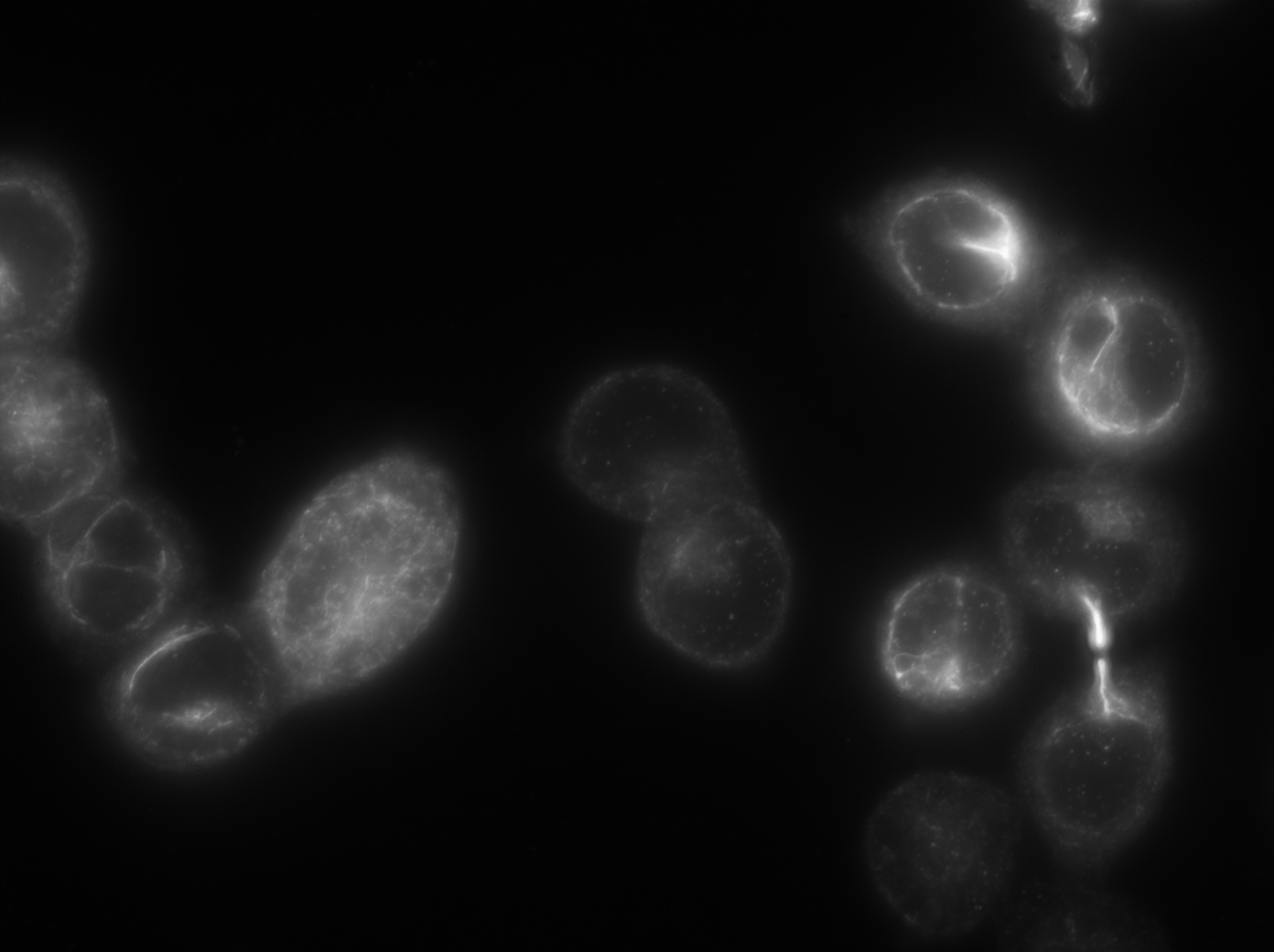

Supplement: Supplementary file 21 — Source data Fig. 6 part 2 [file 44319_2026_742_MOESM21_ESM.zip › Figure 6 Part 2/Fig 6abcd Cas9 TPGS1-KO acetylated tubulin atubulin part 2/TPGS1-KO R3 9-13-24 LT21.Project Maximum Z_XY1726764309_Z0_T0_C2.tif]

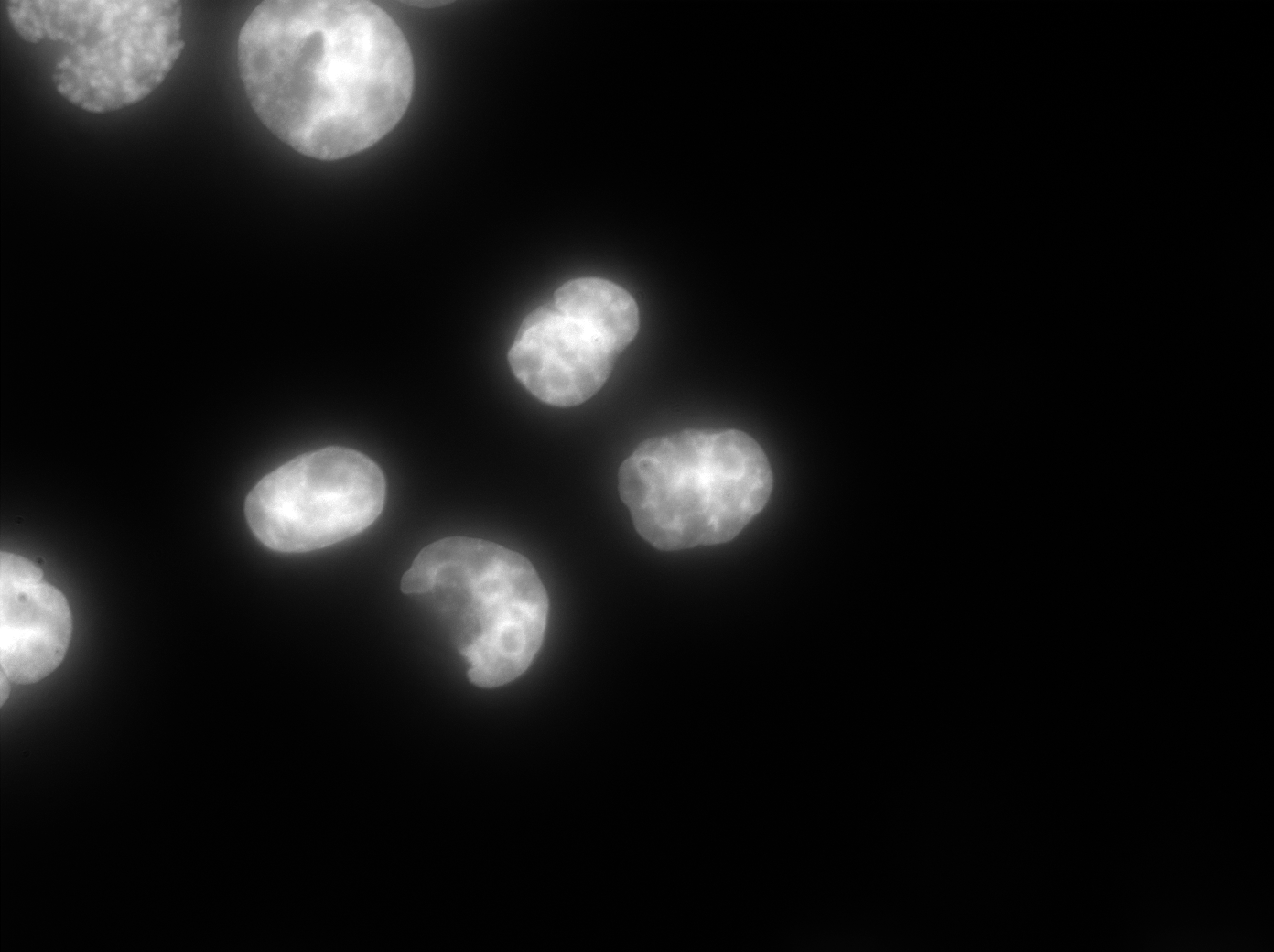

Supplement: Supplementary file 21 — Source data Fig. 6 part 2 [file 44319_2026_742_MOESM21_ESM.zip › Figure 6 Part 2/Fig 6abcd Cas9 TPGS1-KO acetylated tubulin atubulin part 2/TPGS1-KO R2 9-11-24 PA1.Project Maximum Z_XY1726258742_Z0_T0_C0.tif]

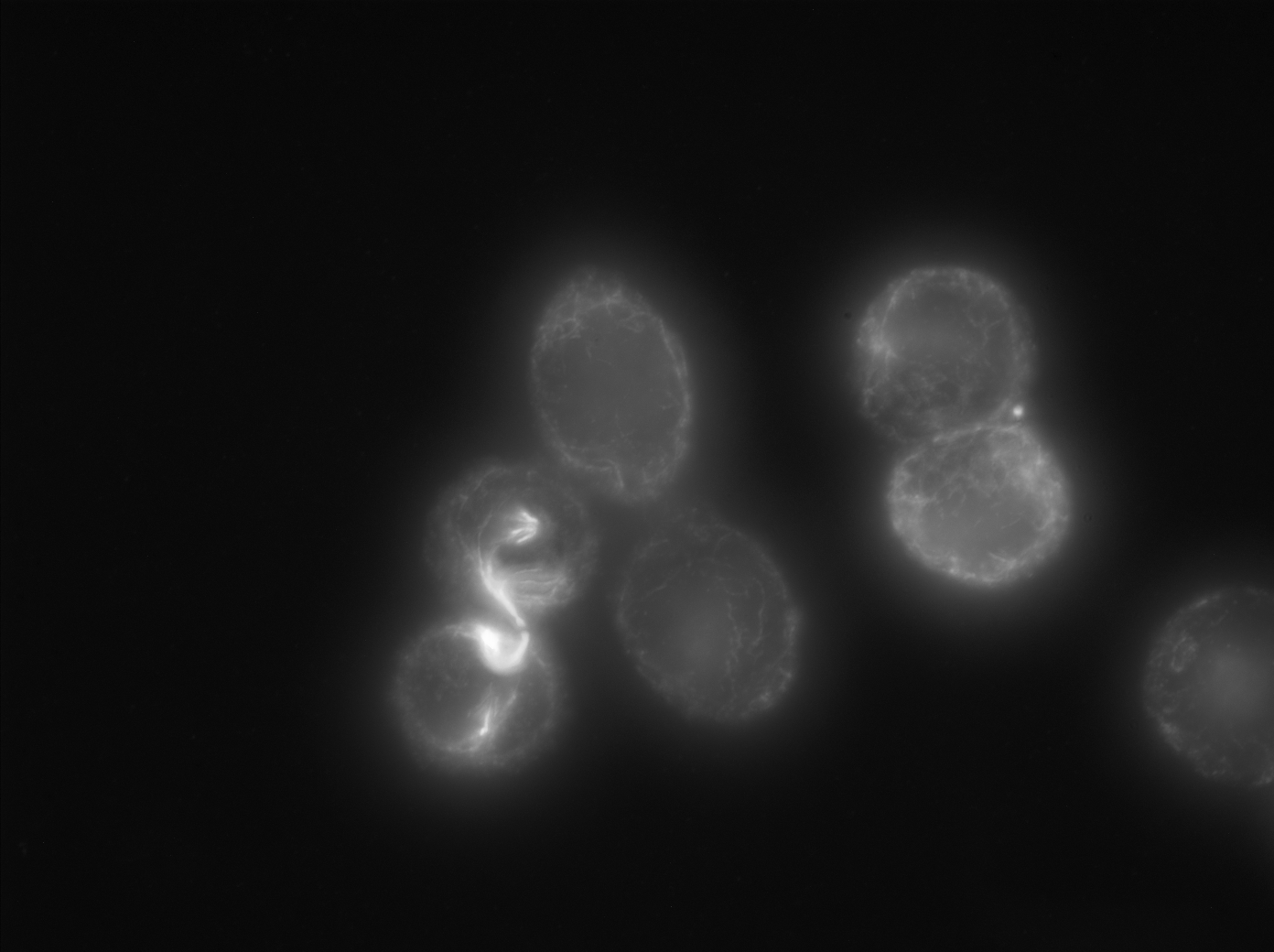

Supplement: Supplementary file 21 — Source data Fig. 6 part 2 [file 44319_2026_742_MOESM21_ESM.zip › Figure 6 Part 2/Fig 6abcd Cas9 TPGS1-KO acetylated tubulin atubulin part 2/TPGS1-KO R2 9-11-24 LT13 PA11.Project Maximum Z_XY1726262832_Z0_T0_C2.tif]

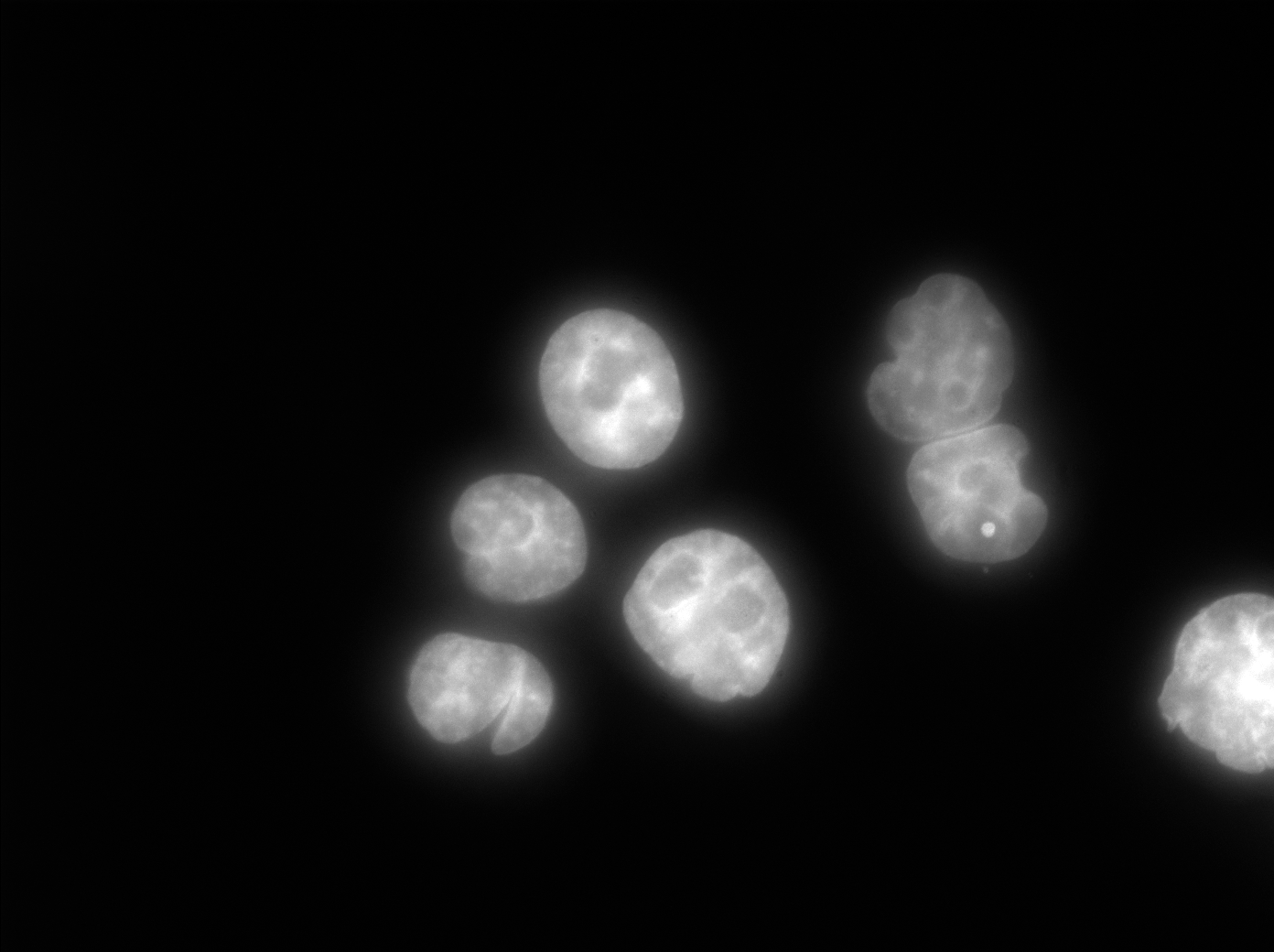

Supplement: Supplementary file 21 — Source data Fig. 6 part 2 [file 44319_2026_742_MOESM21_ESM.zip › Figure 6 Part 2/Fig 6abcd Cas9 TPGS1-KO acetylated tubulin atubulin part 2/TPGS1-KO R2 9-11-24 LT13 PA11.Project Maximum Z_XY1726262832_Z0_T0_C0.tif]

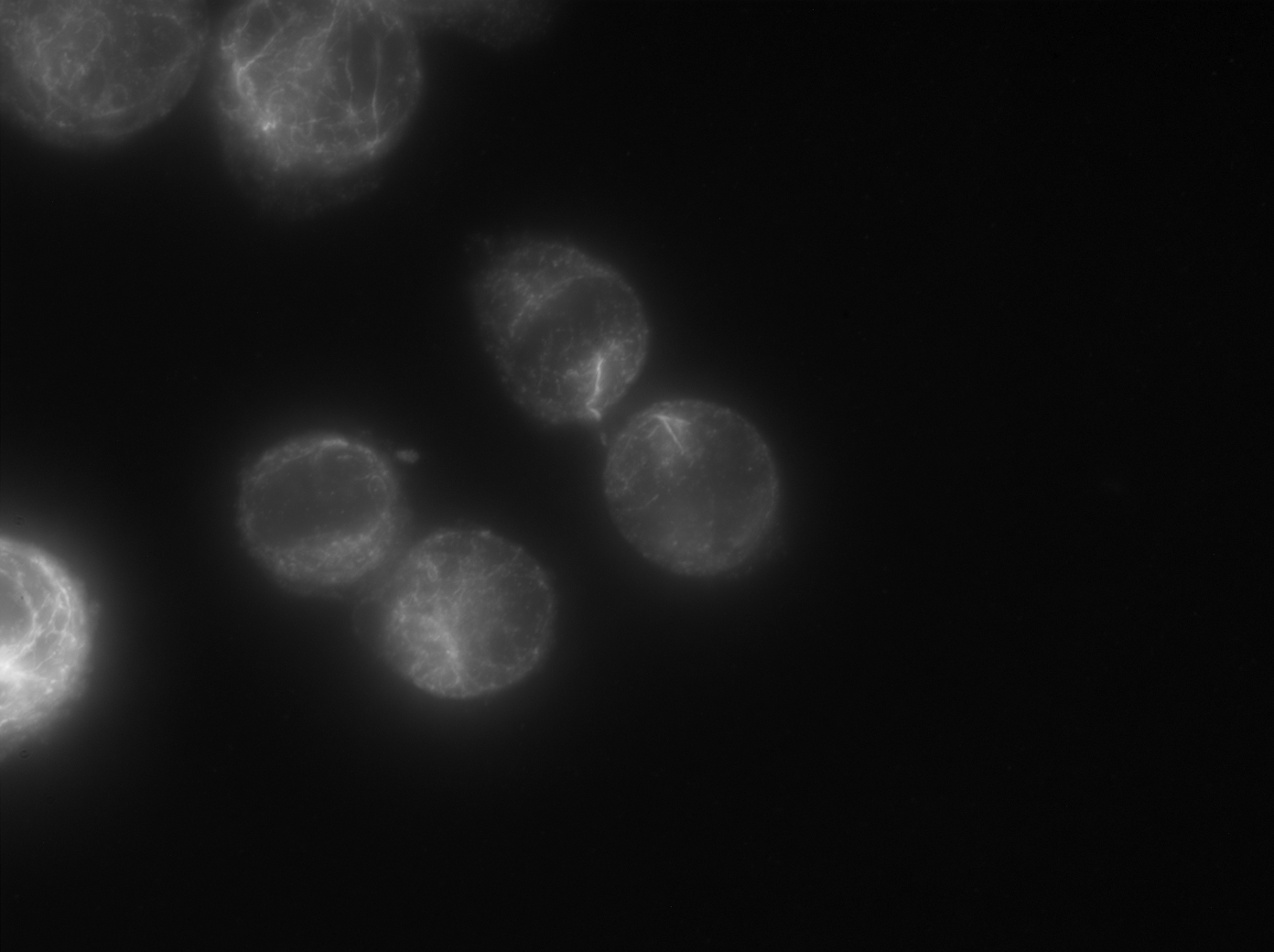

Supplement: Supplementary file 21 — Source data Fig. 6 part 2 [file 44319_2026_742_MOESM21_ESM.zip › Figure 6 Part 2/Fig 6abcd Cas9 TPGS1-KO acetylated tubulin atubulin part 2/TPGS1-KO R2 9-11-24 PA1.Project Maximum Z_XY1726258742_Z0_T0_C2.tif]

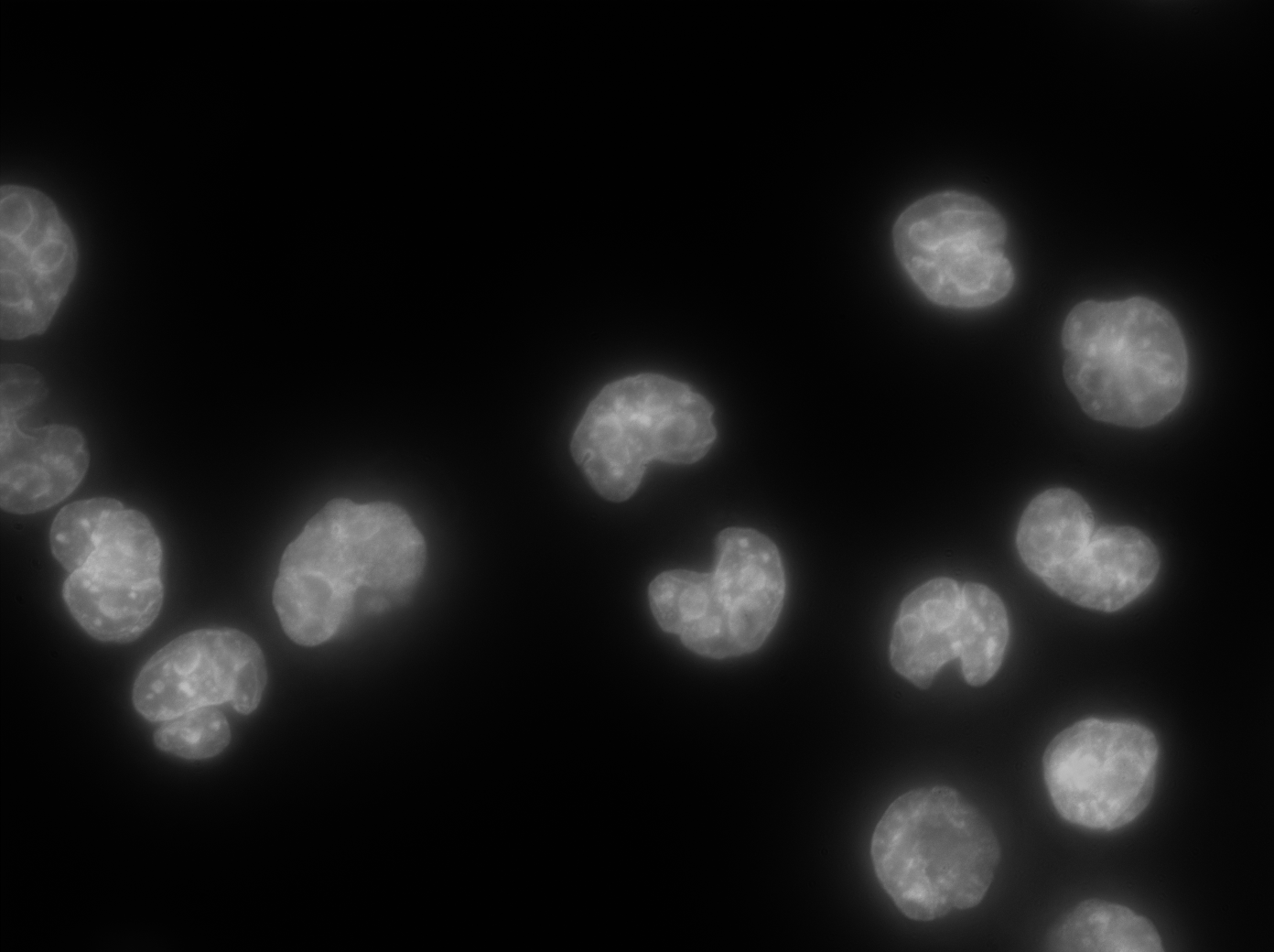

Supplement: Supplementary file 21 — Source data Fig. 6 part 2 [file 44319_2026_742_MOESM21_ESM.zip › Figure 6 Part 2/Fig 6abcd Cas9 TPGS1-KO acetylated tubulin atubulin part 2/TPGS1-KO R3 9-13-24 LT21.Project Maximum Z_XY1726764309_Z0_T0_C0.tif]

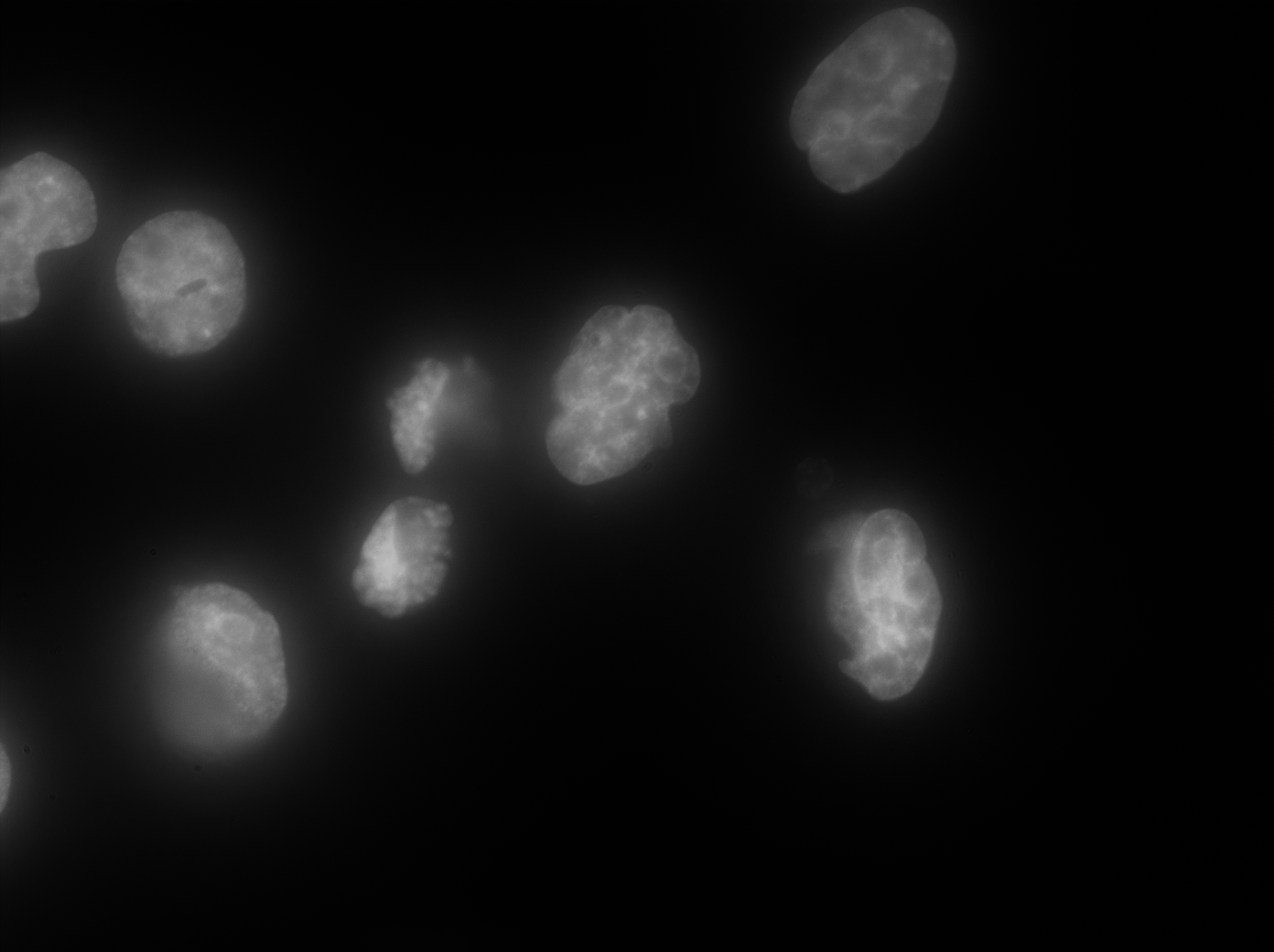

Supplement: Supplementary file 21 — Source data Fig. 6 part 2 [file 44319_2026_742_MOESM21_ESM.zip › Figure 6 Part 2/Fig 6abcd Cas9 TPGS1-KO acetylated tubulin atubulin part 2/TPGS1-KO R3 9-13-24 LT28.Project Maximum Z_XY1726764895_Z0_T0_C0.tif]

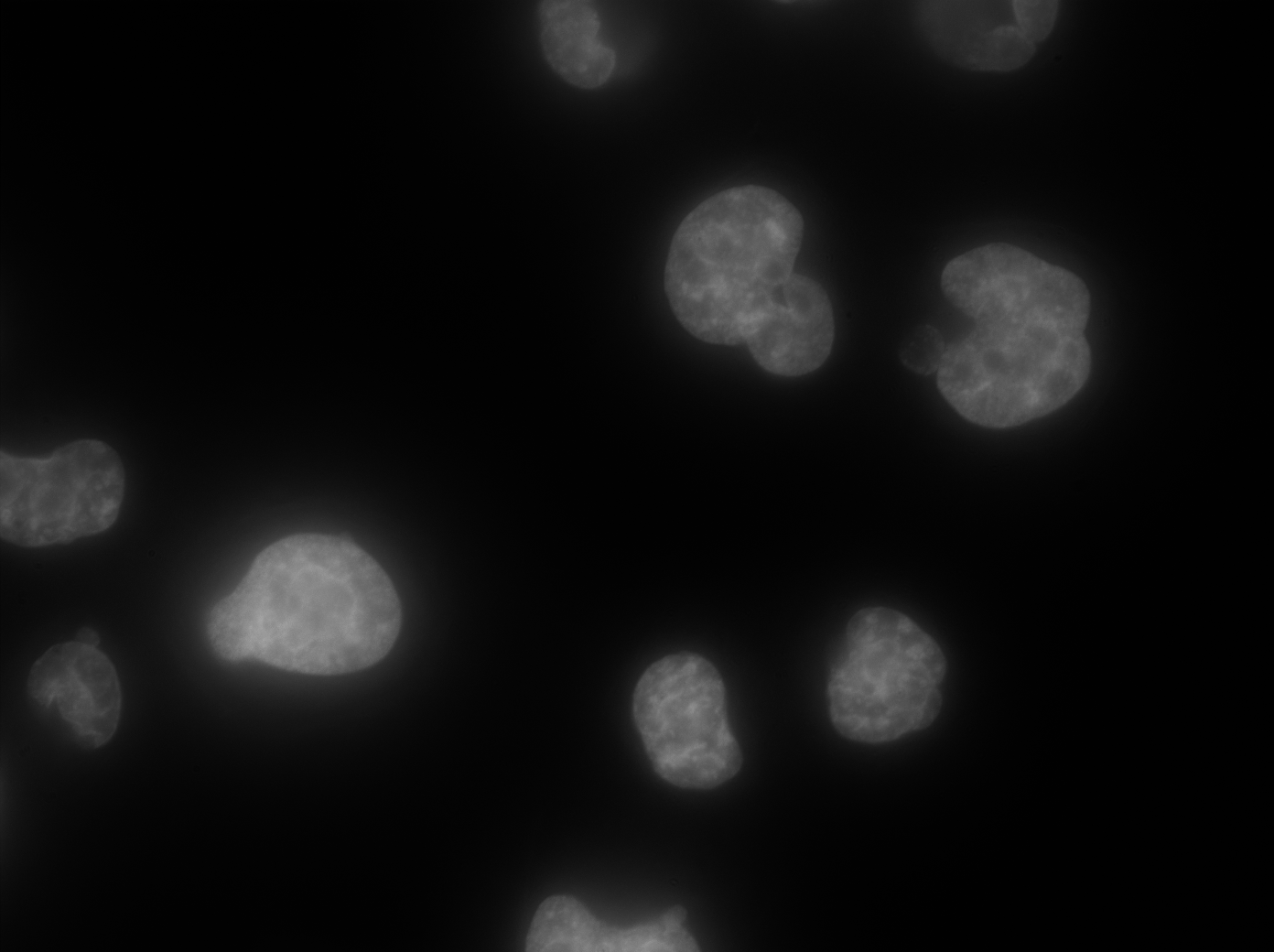

Supplement: Supplementary file 21 — Source data Fig. 6 part 2 [file 44319_2026_742_MOESM21_ESM.zip › Figure 6 Part 2/Fig 6abcd Cas9 TPGS1-KO acetylated tubulin atubulin part 2/TPGS1-KO R3 9-13-24 LT9.Project Maximum Z_XY1726760912_Z0_T0_C0.tif]

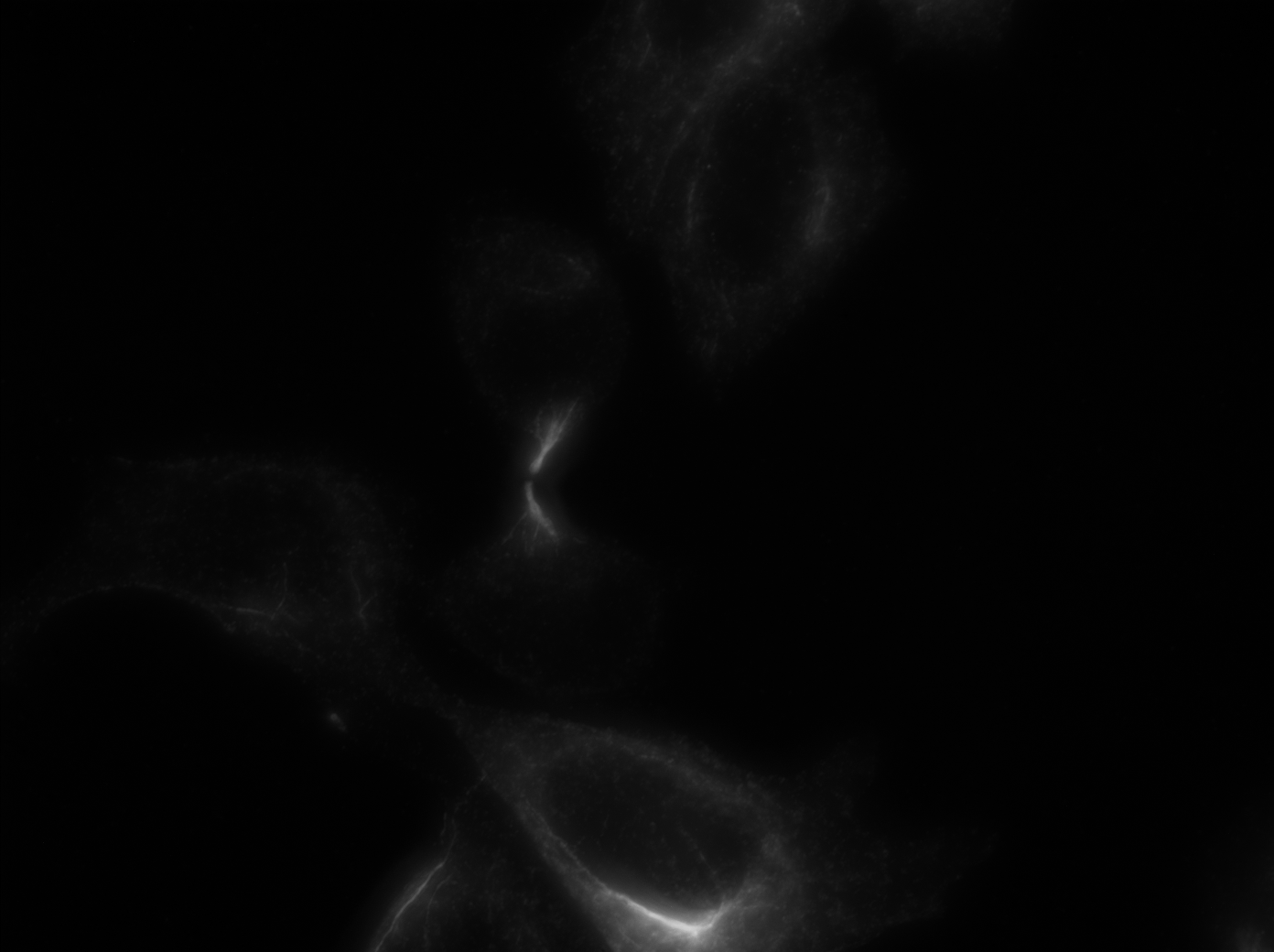

Supplement: Supplementary file 21 — Source data Fig. 6 part 2 [file 44319_2026_742_MOESM21_ESM.zip › Figure 6 Part 2/Fig 6abcd Cas9 TPGS1-KO acetylated tubulin atubulin part 2/TPGS1-KO R3 9-13-24 LT5.Project Maximum Z_XY1726760507_Z0_T0_C2.tif]

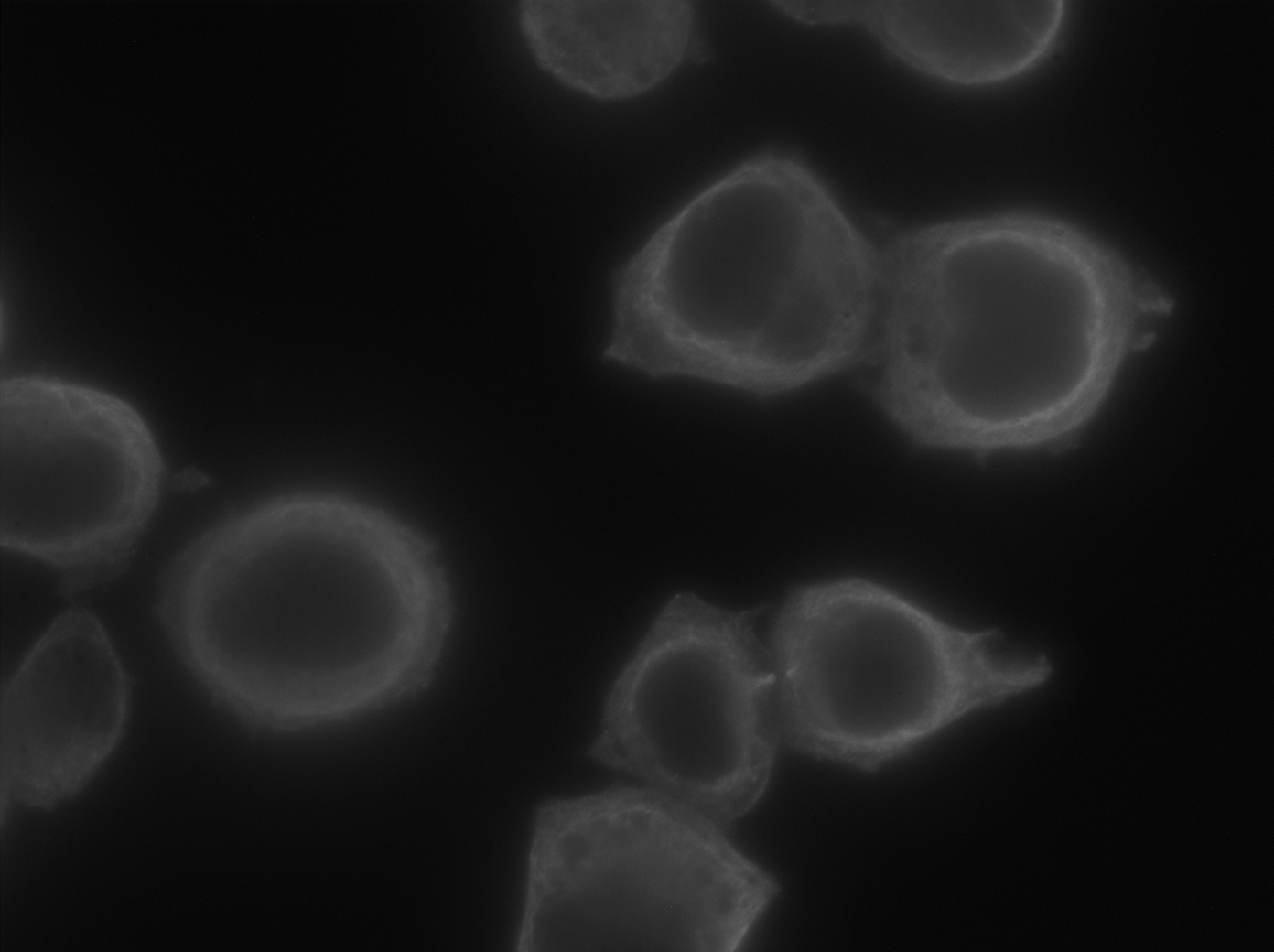

Supplement: Supplementary file 21 — Source data Fig. 6 part 2 [file 44319_2026_742_MOESM21_ESM.zip › Figure 6 Part 2/Fig 6abcd Cas9 TPGS1-KO acetylated tubulin atubulin part 2/TPGS1-KO R3 9-13-24 LT9.Project Maximum Z_XY1726760912_Z0_T0_C1.tif]

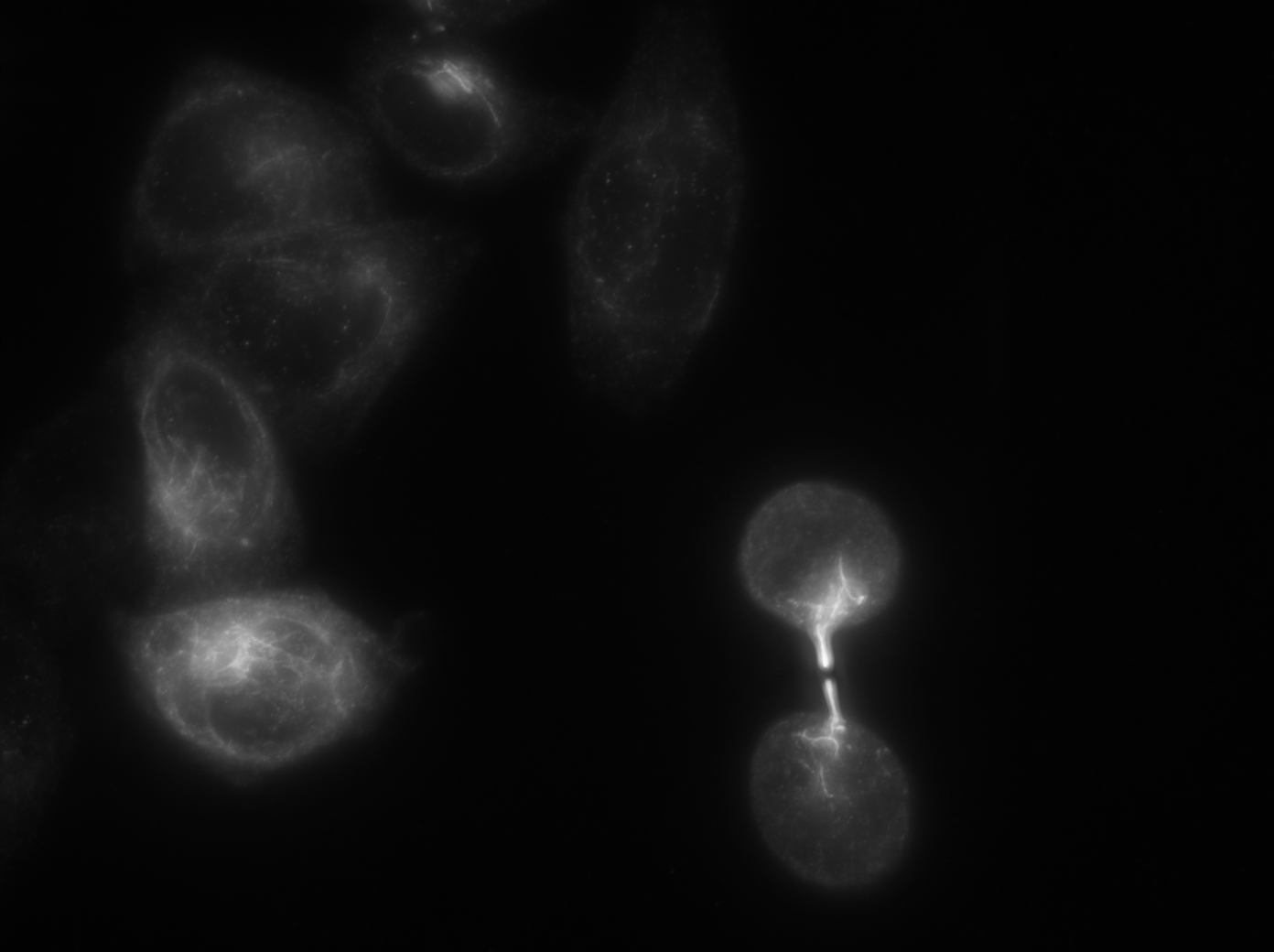

Supplement: Supplementary file 21 — Source data Fig. 6 part 2 [file 44319_2026_742_MOESM21_ESM.zip › Figure 6 Part 2/Fig 6abcd Cas9 TPGS1-KO acetylated tubulin atubulin part 2/TPGS1-KO R3 9-13-24 LT22.Project Maximum Z_XY1726764435_Z0_T0_C2.tif]

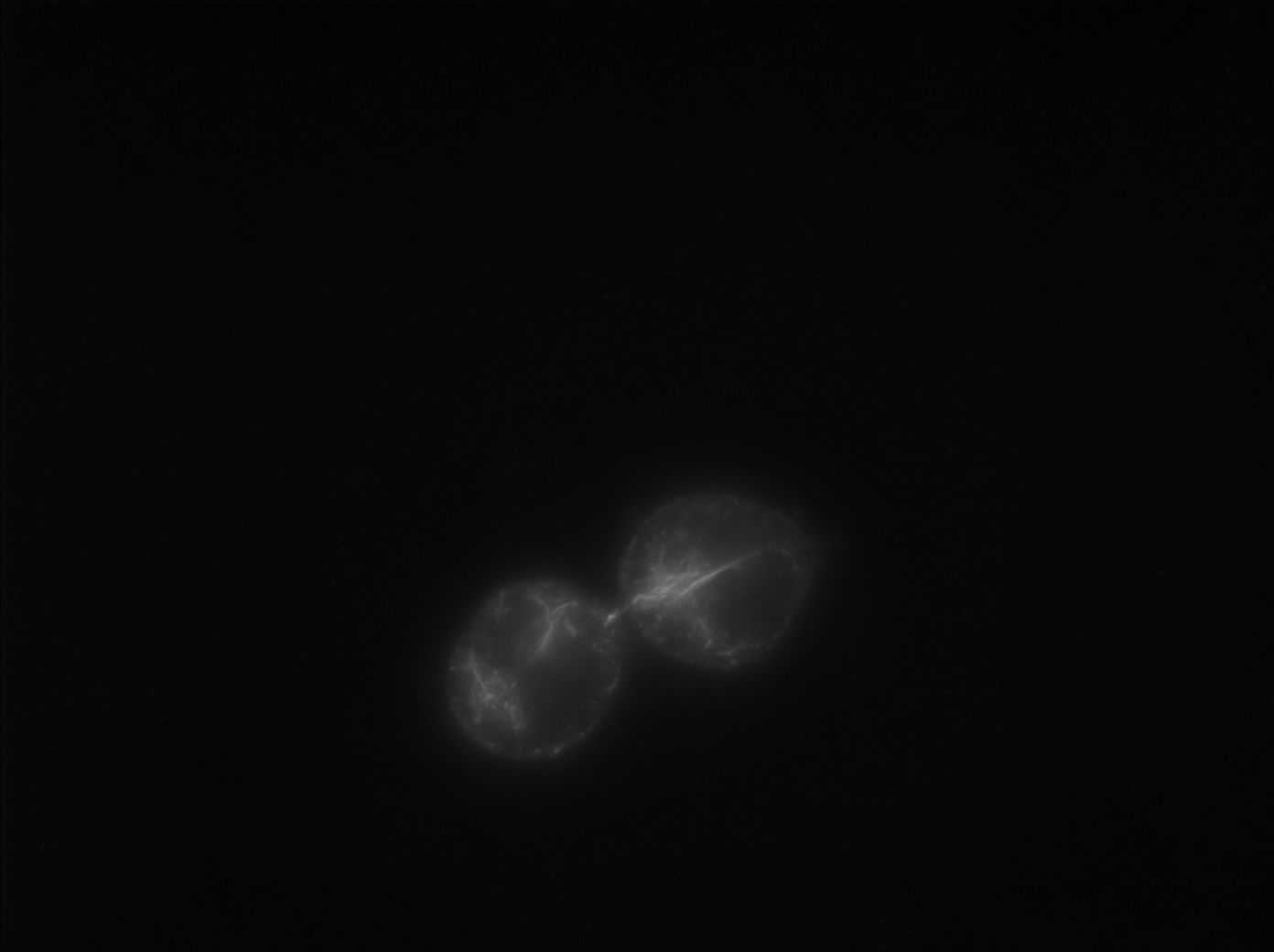

Supplement: Supplementary file 21 — Source data Fig. 6 part 2 [file 44319_2026_742_MOESM21_ESM.zip › Figure 6 Part 2/Fig 6abcd Cas9 TPGS1-KO acetylated tubulin atubulin part 2/TPGS1-KO R2 9-11-24 LT1.Project Maximum Z_XY1726258922_Z0_T0_C2.tif]

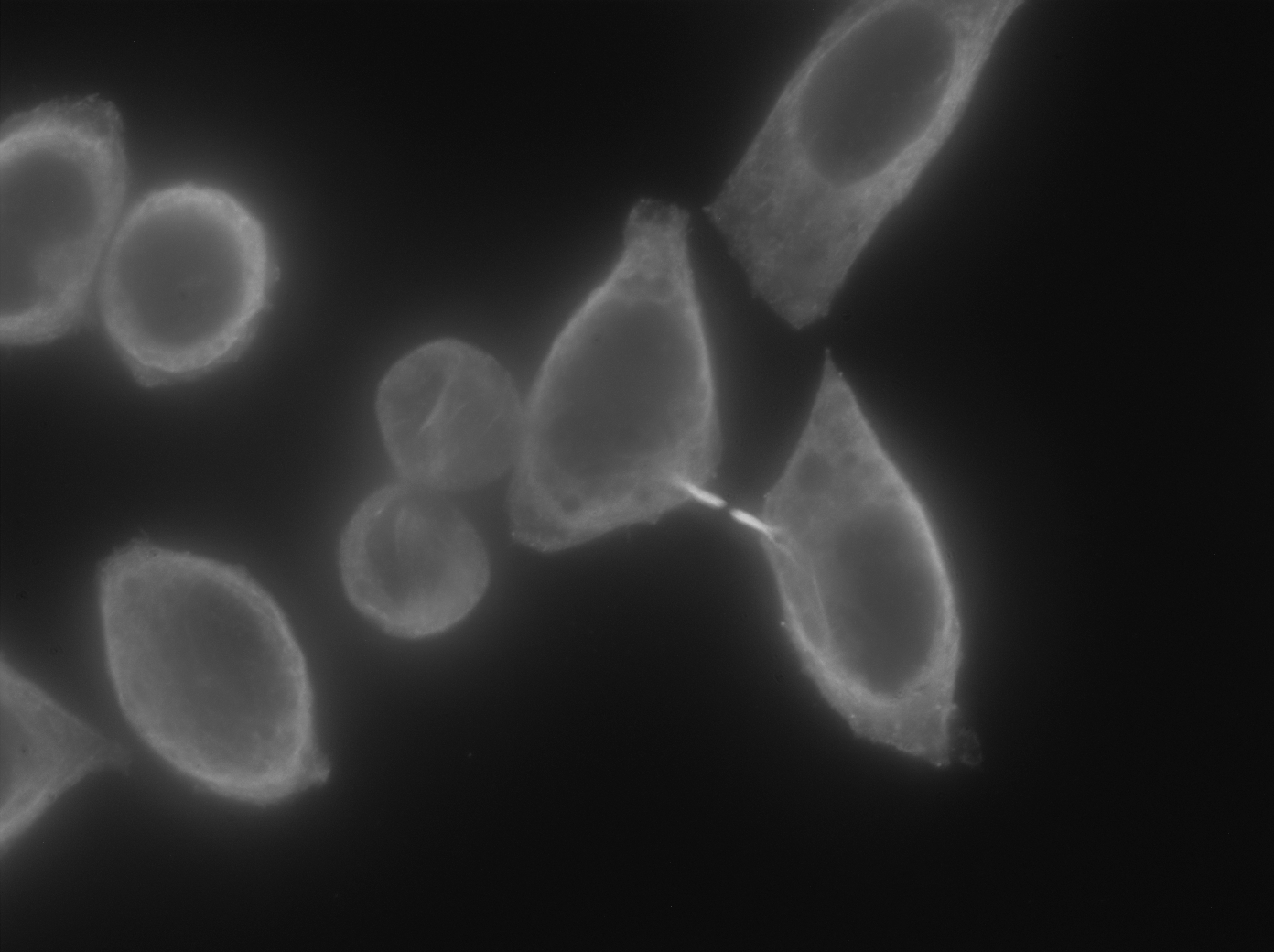

Supplement: Supplementary file 21 — Source data Fig. 6 part 2 [file 44319_2026_742_MOESM21_ESM.zip › Figure 6 Part 2/Fig 6abcd Cas9 TPGS1-KO acetylated tubulin atubulin part 2/TPGS1-KO R3 9-13-24 LT28.Project Maximum Z_XY1726764895_Z0_T0_C1.tif]

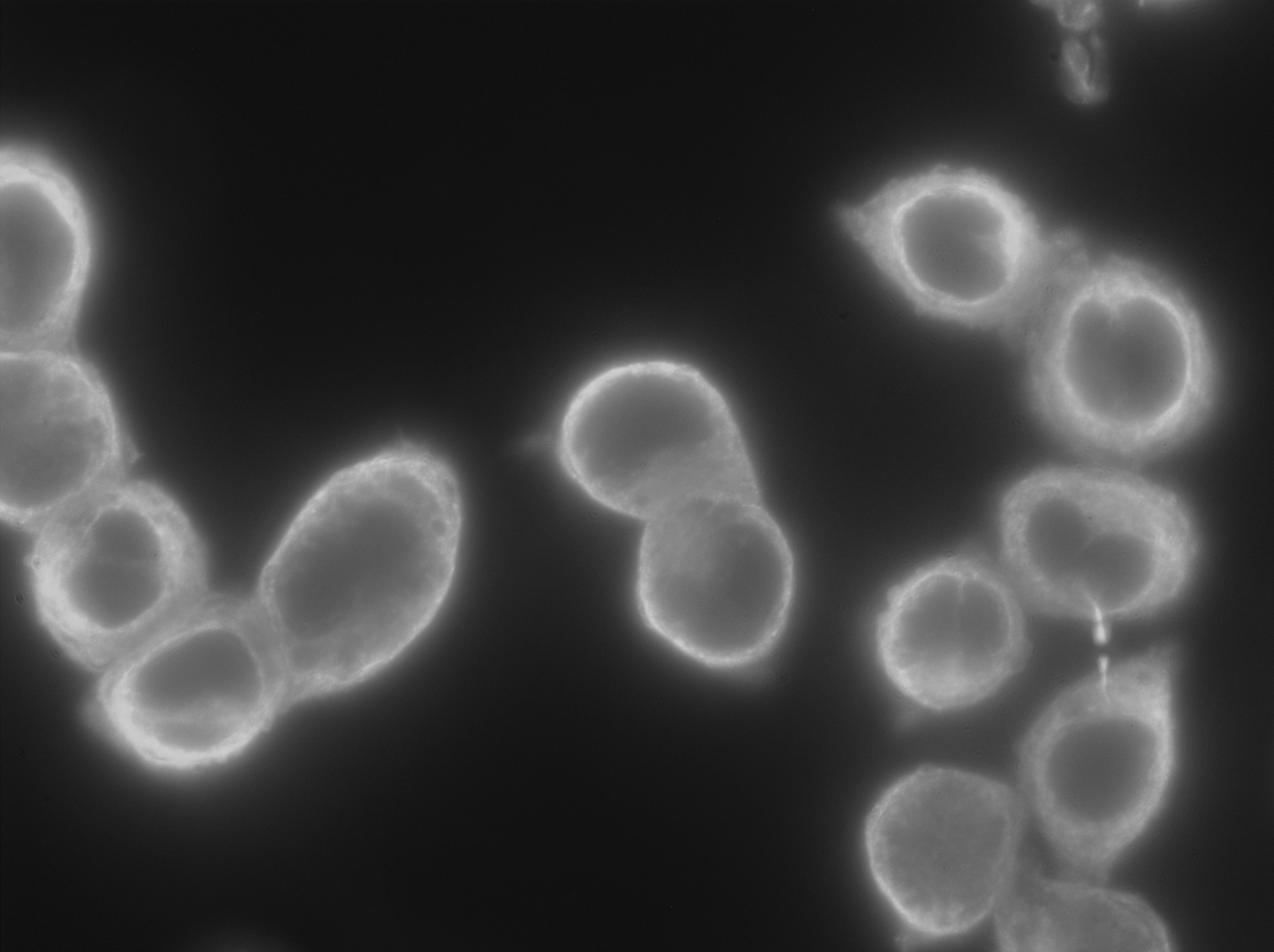

Supplement: Supplementary file 21 — Source data Fig. 6 part 2 [file 44319_2026_742_MOESM21_ESM.zip › Figure 6 Part 2/Fig 6abcd Cas9 TPGS1-KO acetylated tubulin atubulin part 2/TPGS1-KO R3 9-13-24 LT21.Project Maximum Z_XY1726764309_Z0_T0_C1.tif]

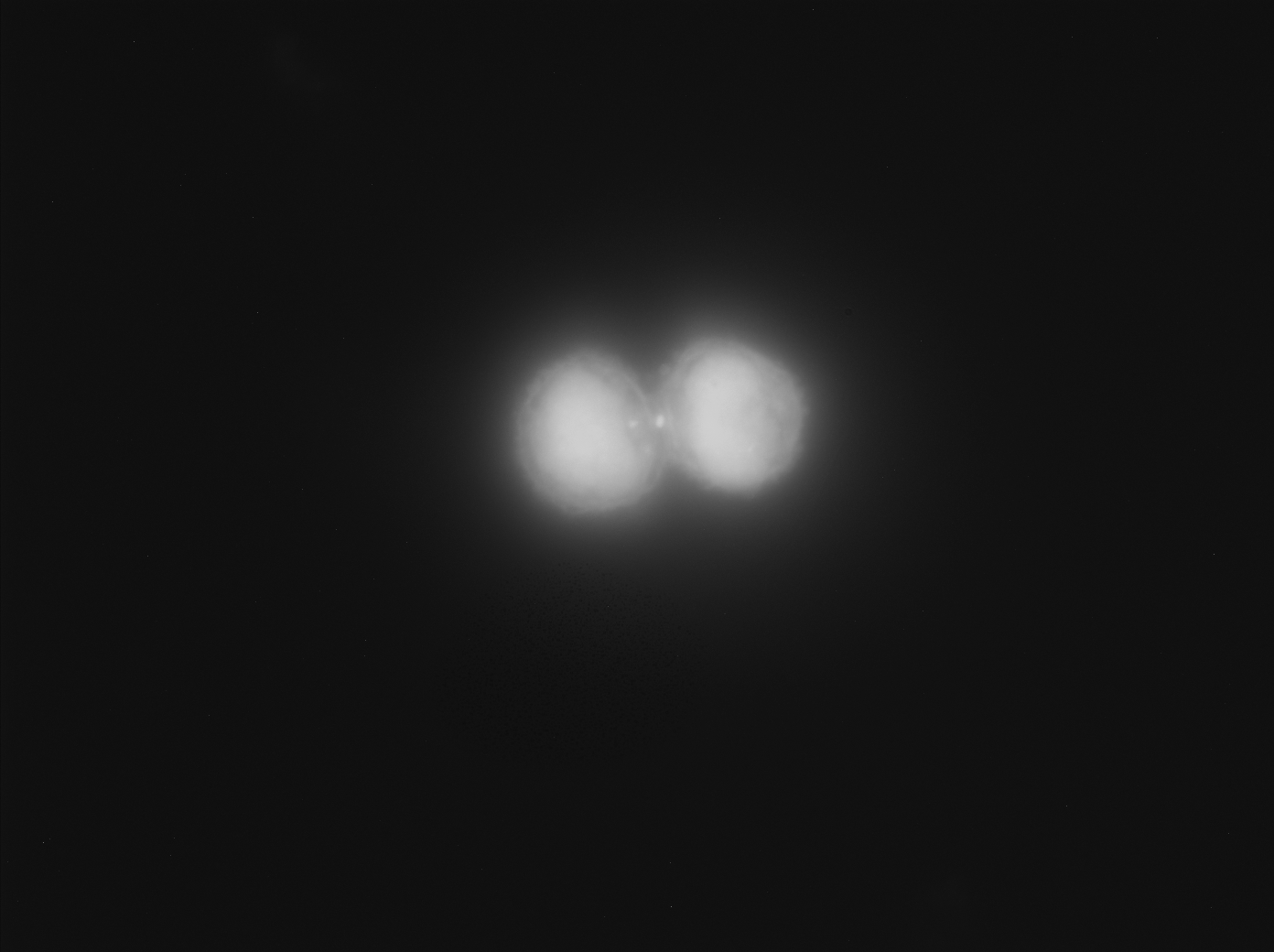

Supplement: Supplementary file 22 — Source data Fig. 6 part 3 [file 44319_2026_742_MOESM22_ESM.zip › Figure 6 Part 3/Fig 6efg TPGS1-KO TPGS1 rescue experiments/R1/TPGS1-KO EYFP only actub R1 7-31-25 ET4.Project Maximum Z_XY1754335973_Z0_T0_C1.tif]

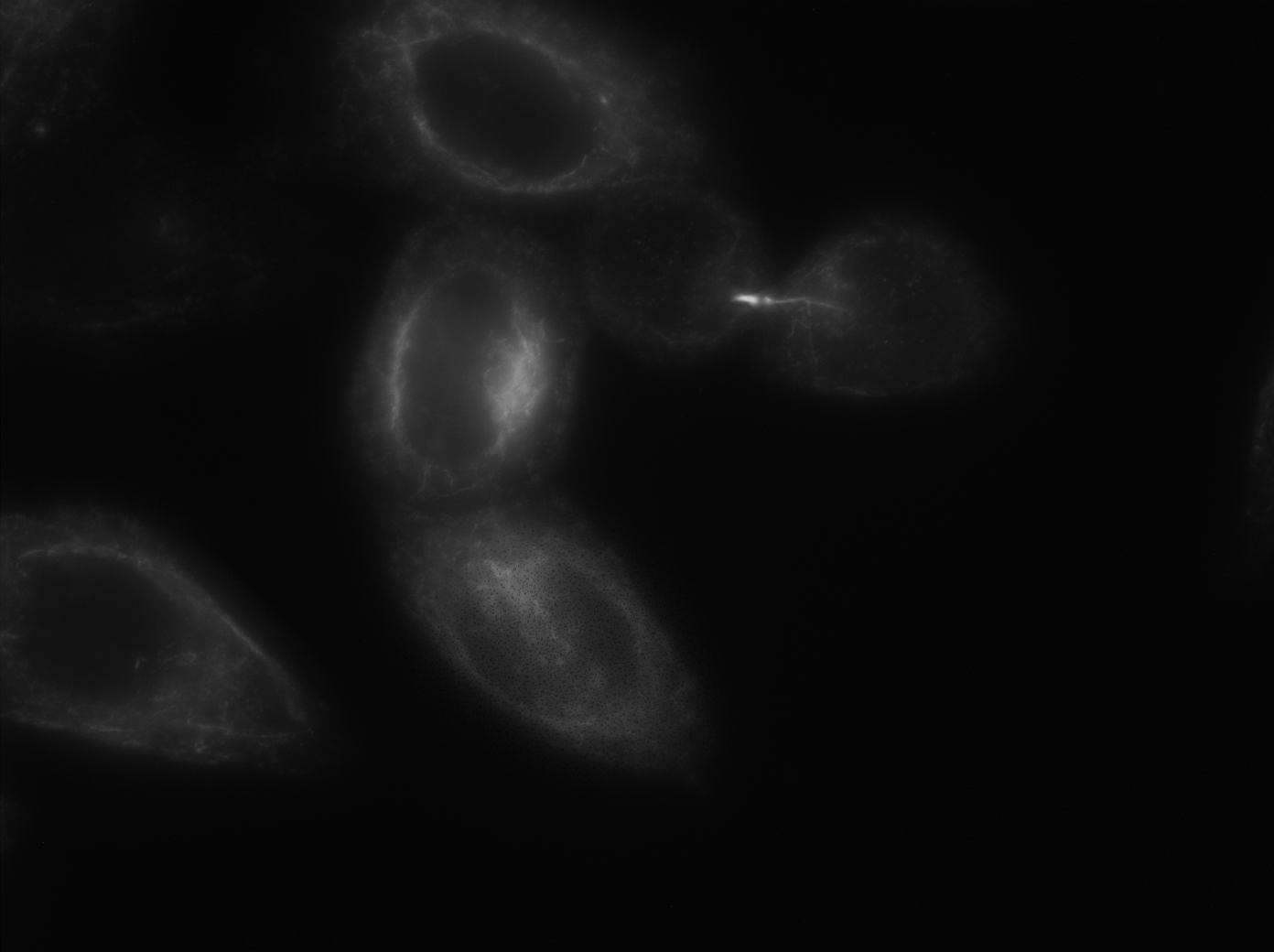

Supplement: Supplementary file 22 — Source data Fig. 6 part 3 [file 44319_2026_742_MOESM22_ESM.zip › Figure 6 Part 3/Fig 6efg TPGS1-KO TPGS1 rescue experiments/R1/TPGS1-KO TPGS1-3UTR-EYFP actub R1 7-31-25 ET1.Project Maximum Z_XY1753981618_Z0_T0_C2.tif]

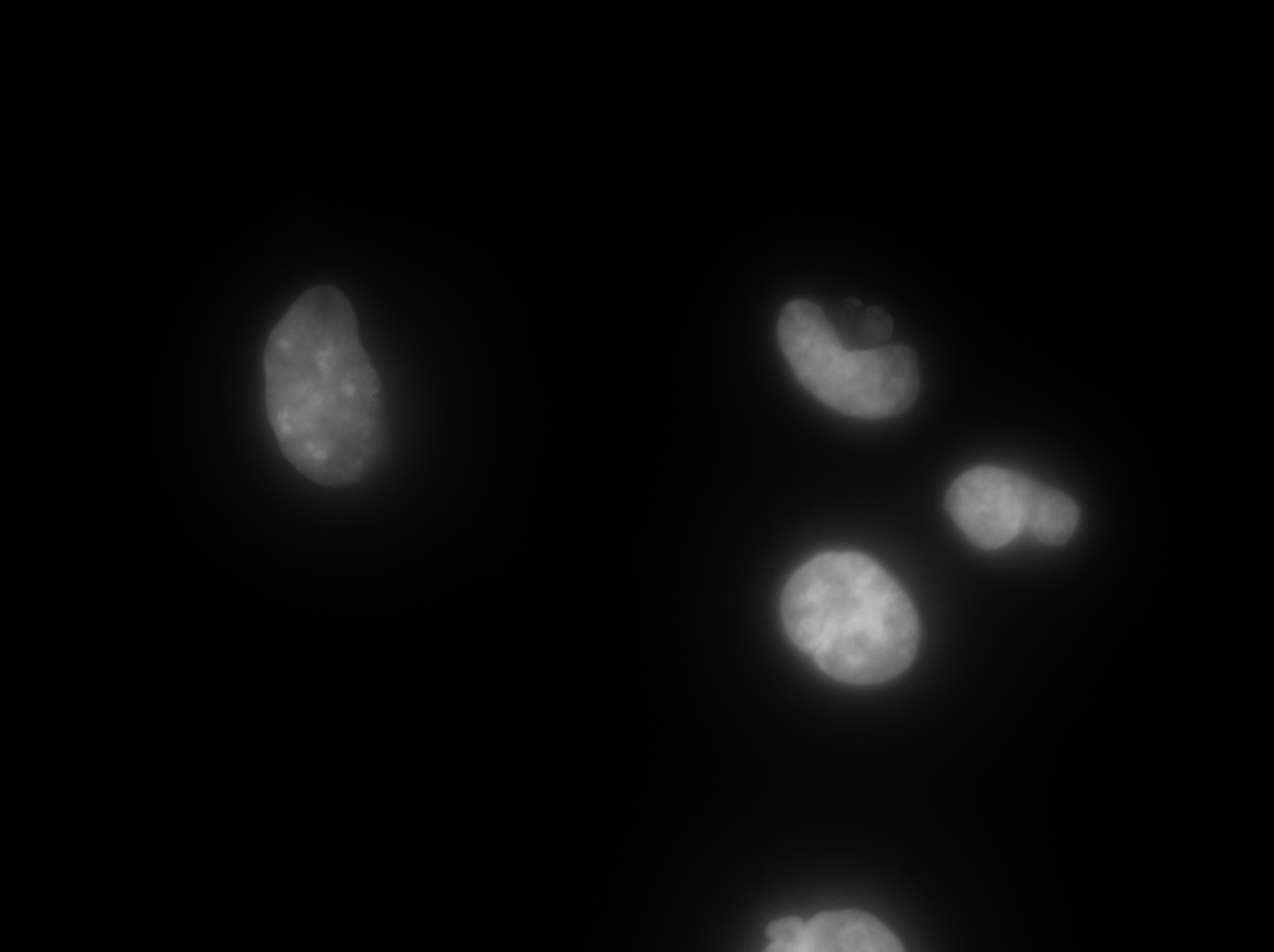

Supplement: Supplementary file 22 — Source data Fig. 6 part 3 [file 44319_2026_742_MOESM22_ESM.zip › Figure 6 Part 3/Fig 6efg TPGS1-KO TPGS1 rescue experiments/R1/TPGS1-KO EYFP only actub R1 7-31-25 LT4.Project Maximum Z_XY1754336072_Z0_T0_C0.tif]

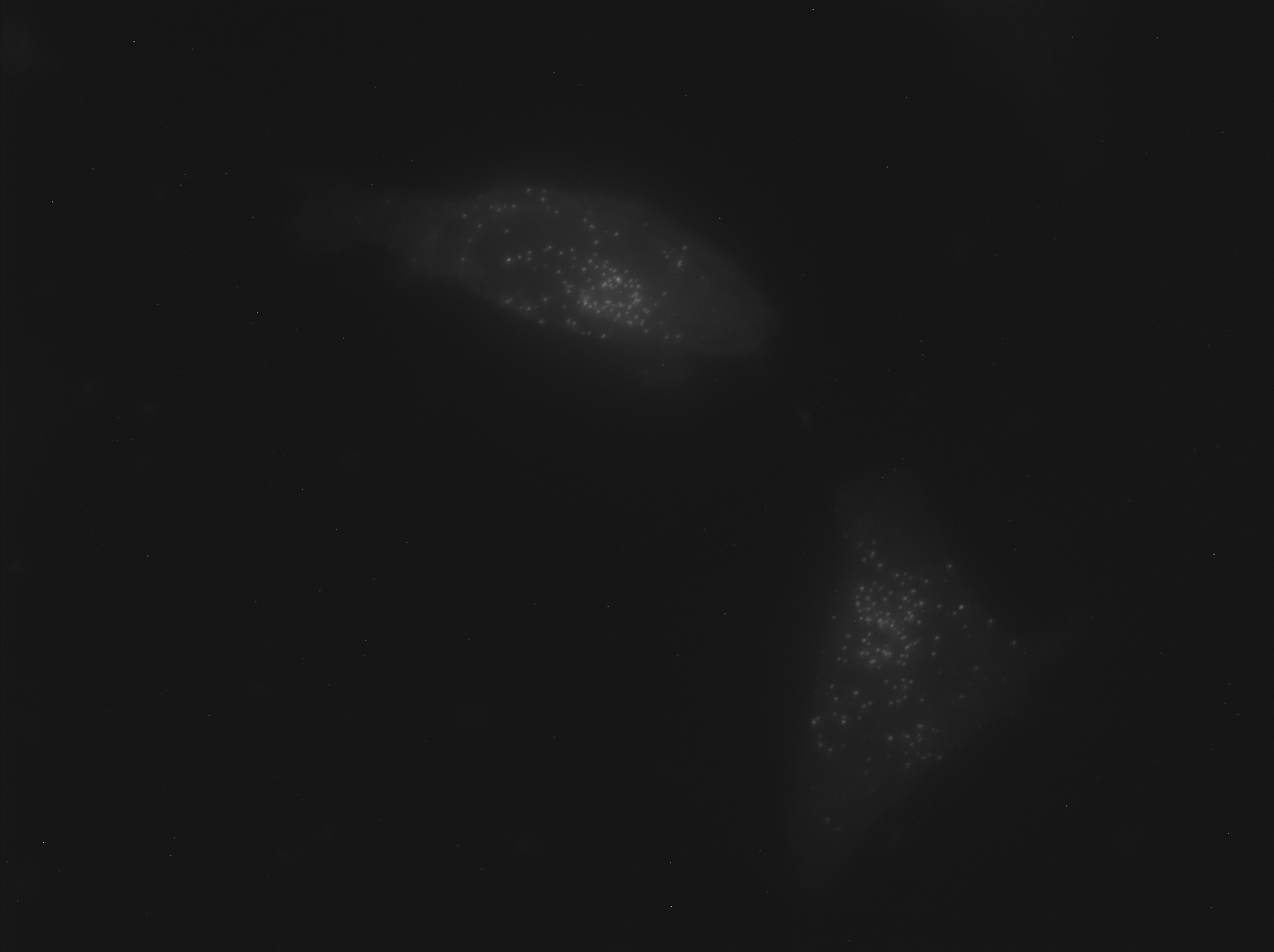

Supplement: Supplementary file 22 — Source data Fig. 6 part 3 [file 44319_2026_742_MOESM22_ESM.zip › Figure 6 Part 3/Fig 6efg TPGS1-KO TPGS1 rescue experiments/R1/TPGS1-KO TPGS1-3UTR-EYFP actub R1 7-31-25 LT6.Project Maximum Z_XY1753988729_Z0_T0_C1.tif]

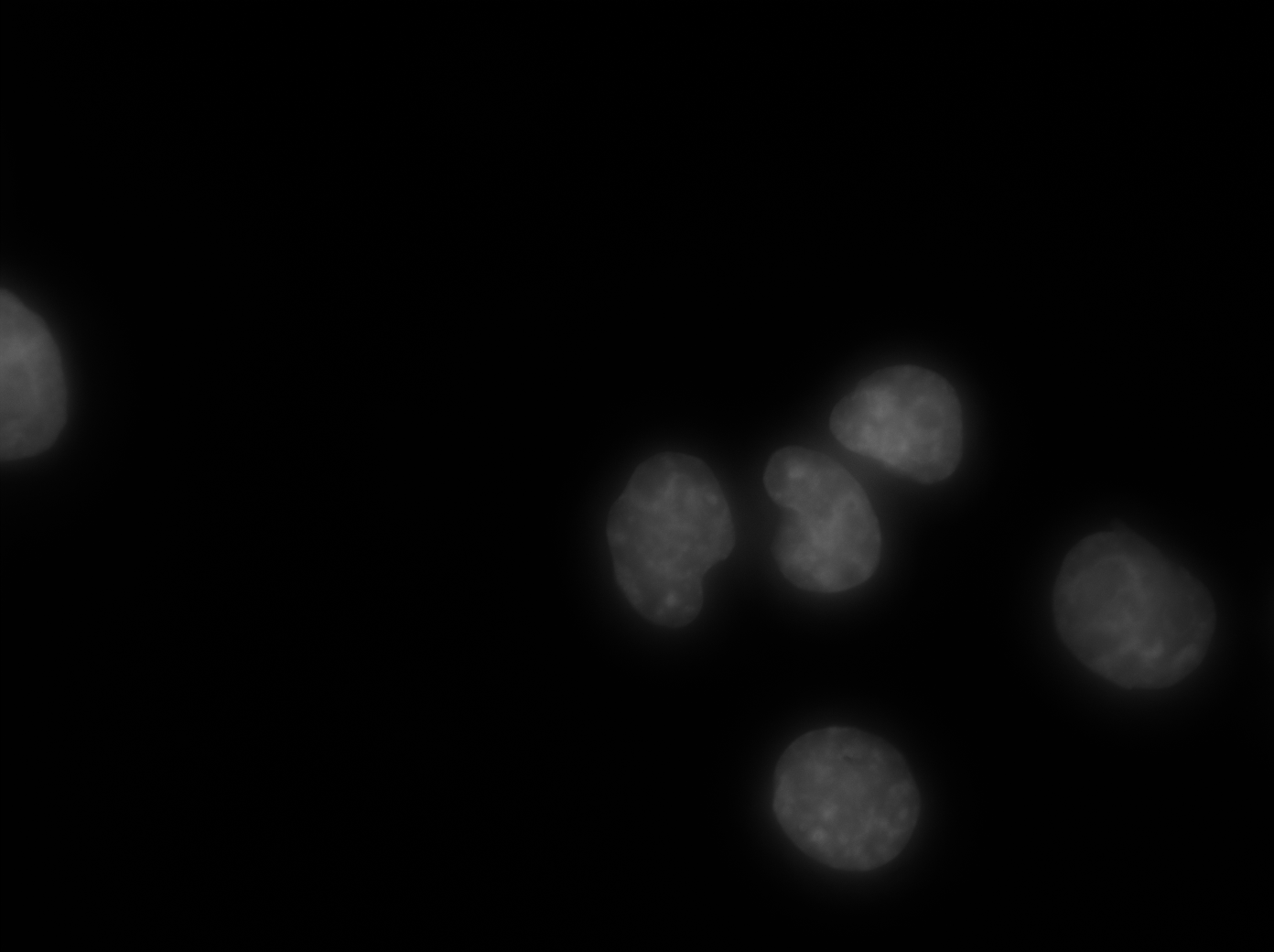

Supplement: Supplementary file 22 — Source data Fig. 6 part 3 [file 44319_2026_742_MOESM22_ESM.zip › Figure 6 Part 3/Fig 6efg TPGS1-KO TPGS1 rescue experiments/R1/TPGS1-KO TPGS1-3UTR-EYFP actub R1 7-31-25 LT10.Project Maximum Z_XY1753992791_Z0_T0_C0.tif]

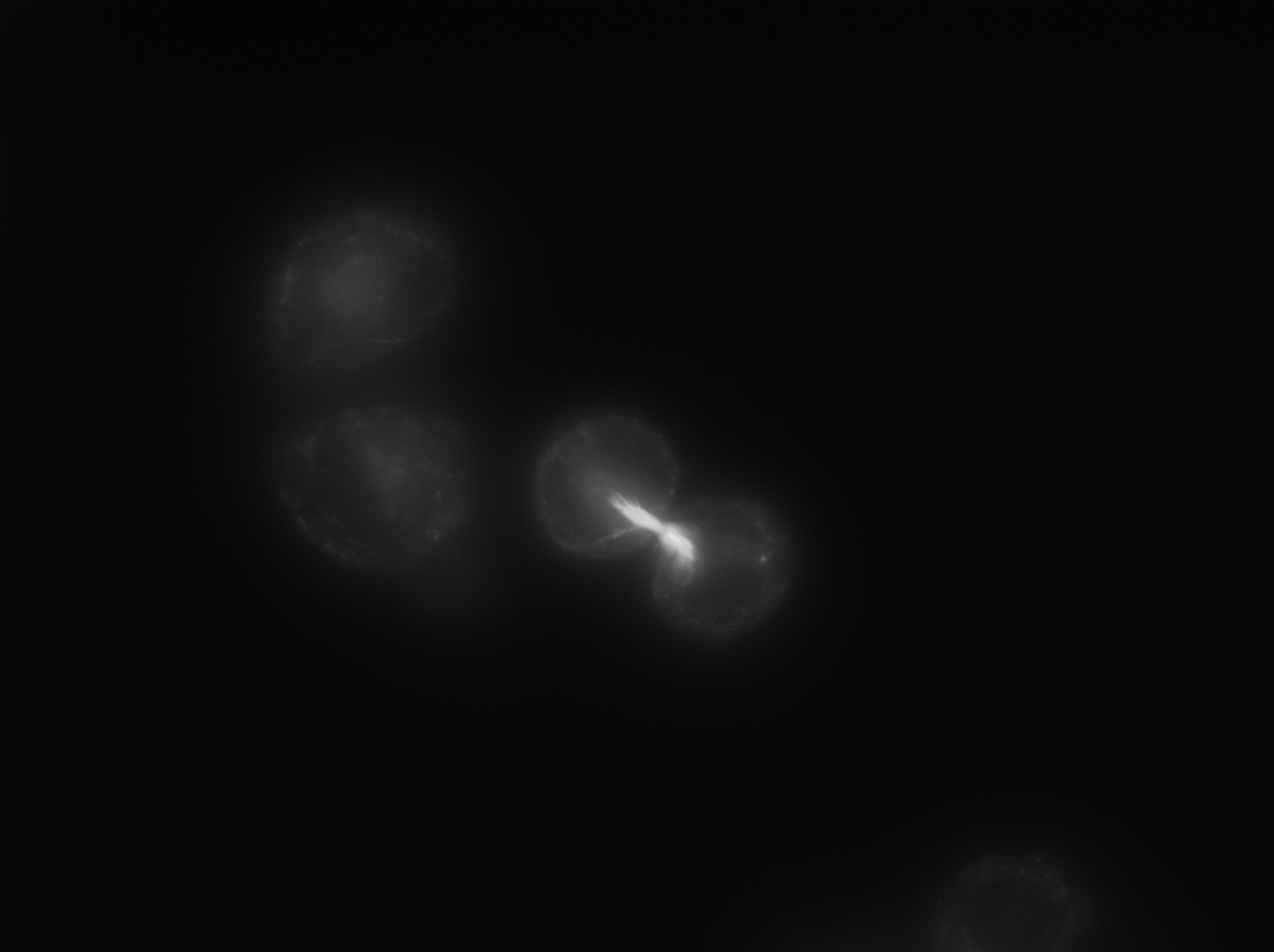

Supplement: Supplementary file 22 — Source data Fig. 6 part 3 [file 44319_2026_742_MOESM22_ESM.zip › Figure 6 Part 3/Fig 6efg TPGS1-KO TPGS1 rescue experiments/R1/TPGS1-KO EYFP only actub R1 7-31-25 ET8.Project Maximum Z_XY1754339329_Z0_T0_C2.tif]

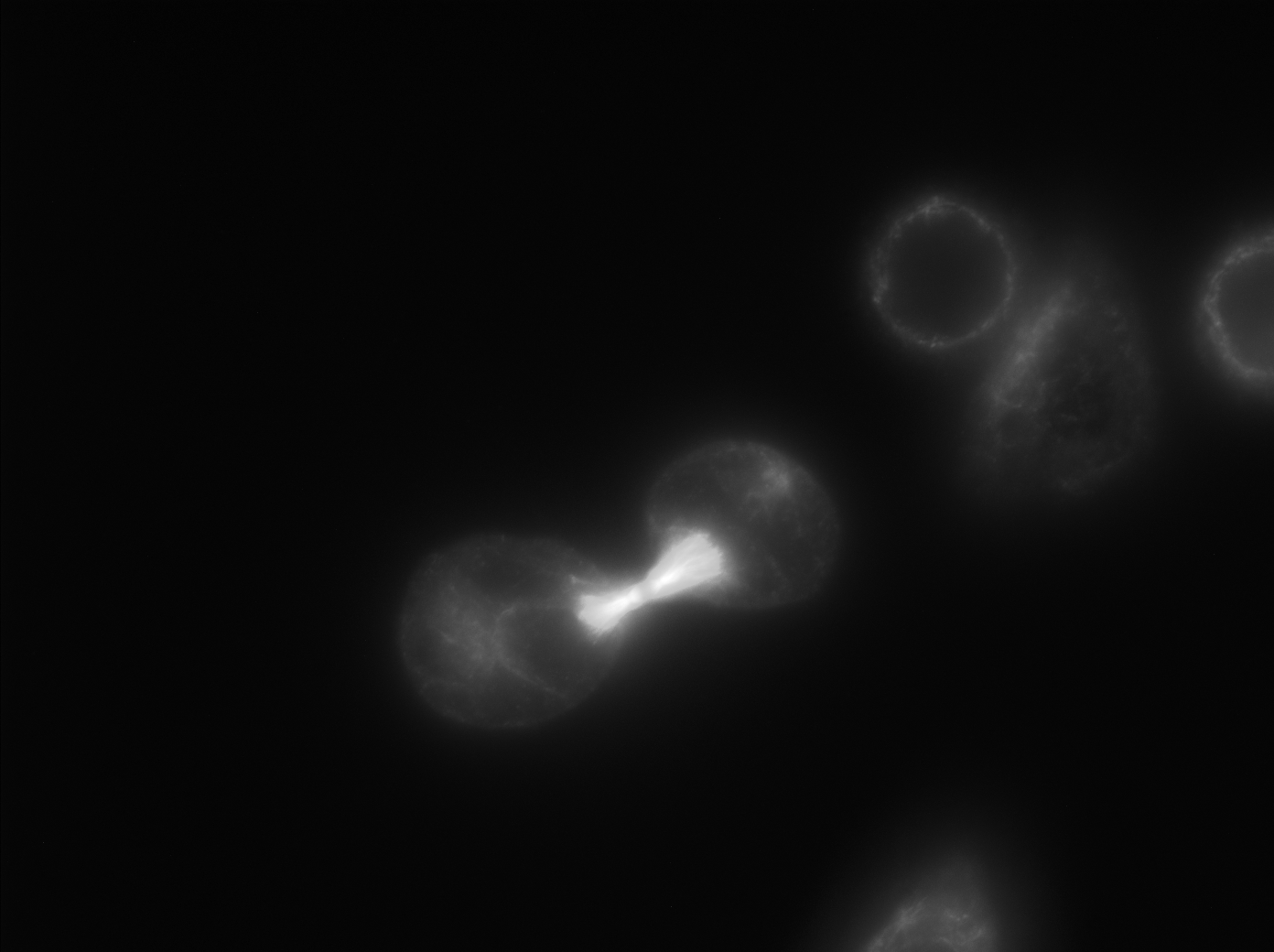

Supplement: Supplementary file 22 — Source data Fig. 6 part 3 [file 44319_2026_742_MOESM22_ESM.zip › Figure 6 Part 3/Fig 6efg TPGS1-KO TPGS1 rescue experiments/R1/TPGS1-KO EYFP only actub R1 7-31-25 ET9.Project Maximum Z_XY1754339444_Z0_T0_C2.tif]

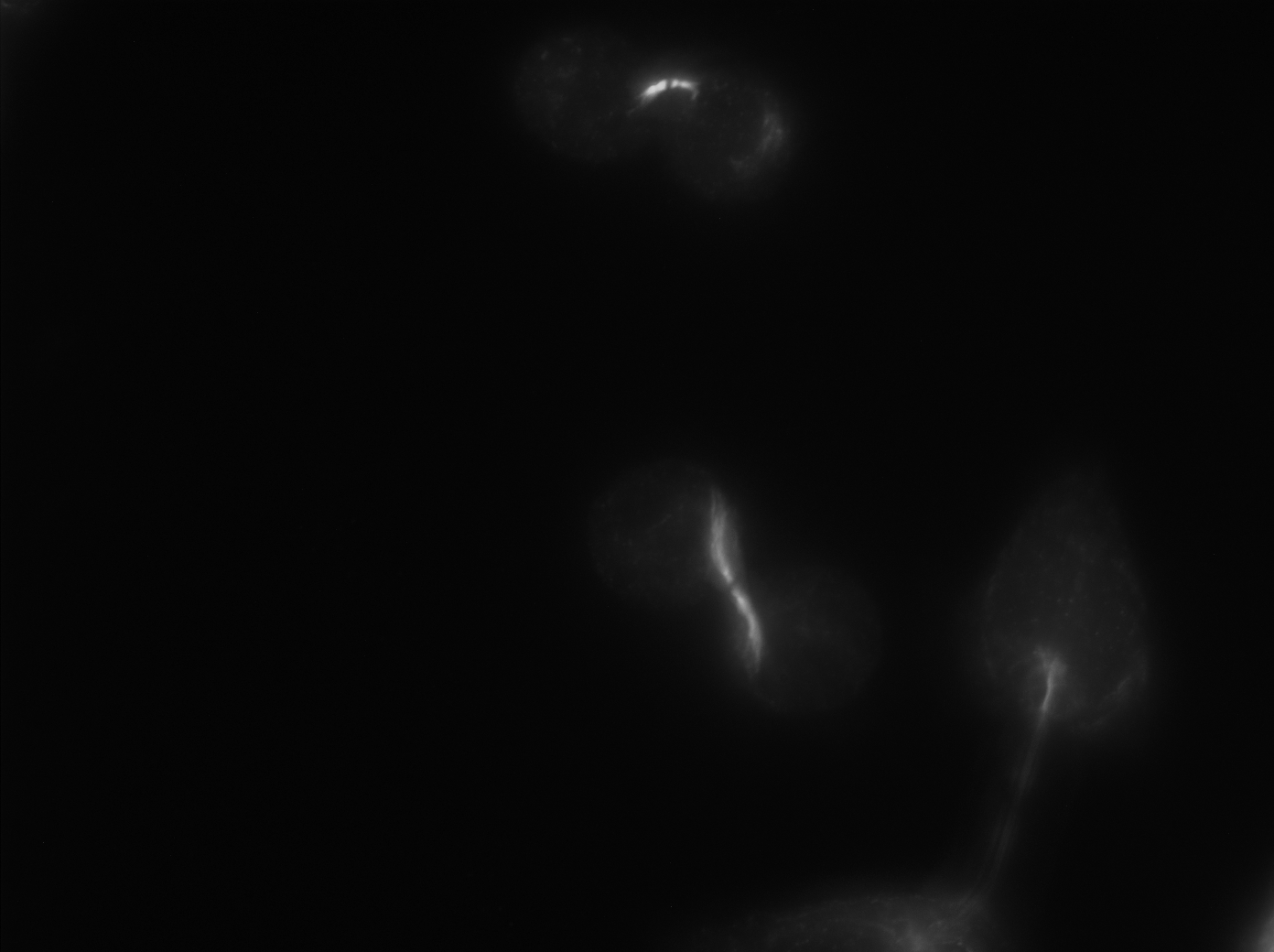

Supplement: Supplementary file 22 — Source data Fig. 6 part 3 [file 44319_2026_742_MOESM22_ESM.zip › Figure 6 Part 3/Fig 6efg TPGS1-KO TPGS1 rescue experiments/R1/TPGS1-KO TPGS1-3UTR-EYFP actub R1 7-31-25 ET9.Project Maximum Z_XY1753992196_Z0_T0_C2.tif]

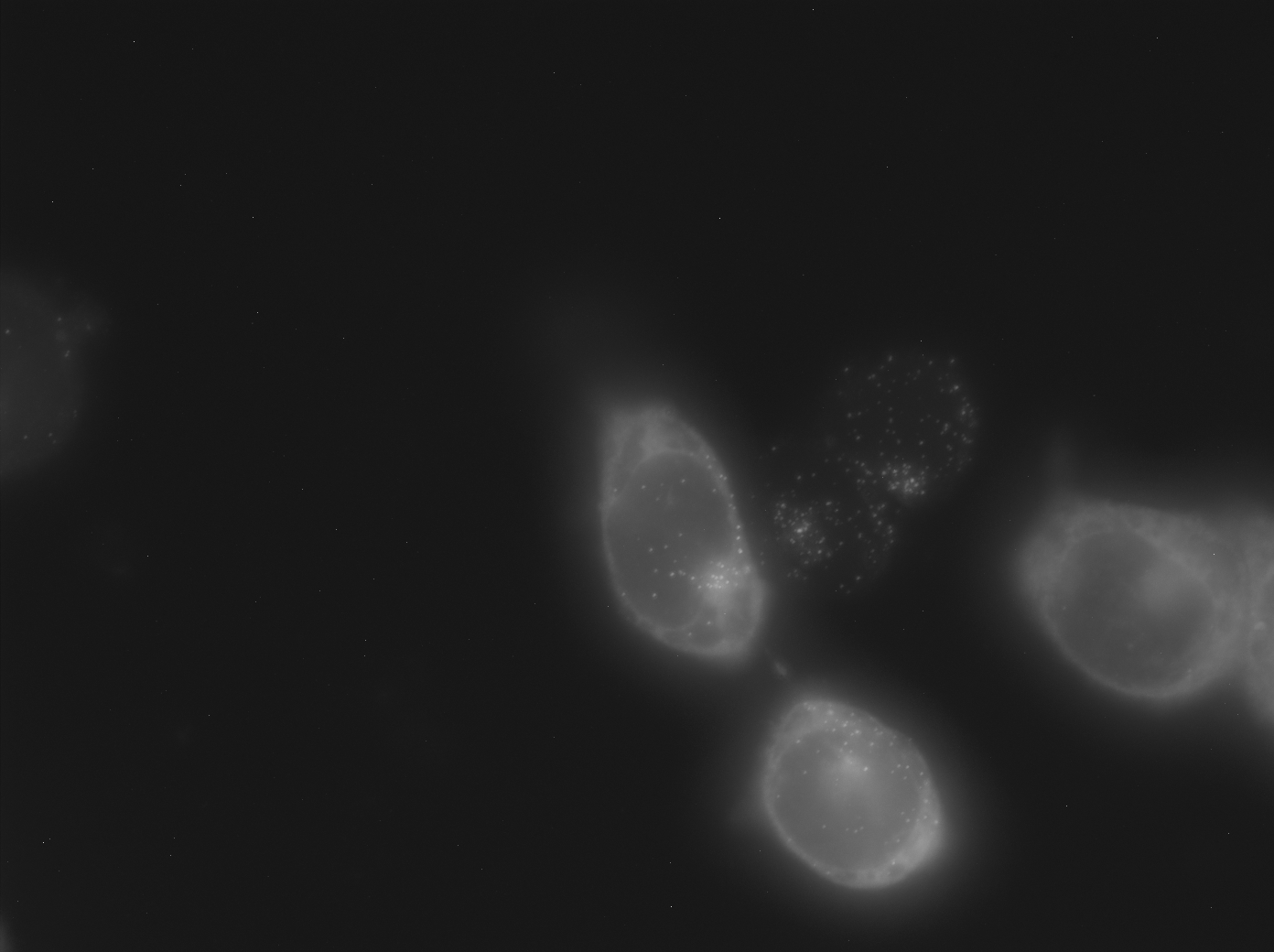

Supplement: Supplementary file 22 — Source data Fig. 6 part 3 [file 44319_2026_742_MOESM22_ESM.zip › Figure 6 Part 3/Fig 6efg TPGS1-KO TPGS1 rescue experiments/R1/TPGS1-KO TPGS1-3UTR-EYFP actub R1 7-31-25 LT10.Project Maximum Z_XY1753992791_Z0_T0_C1.tif]

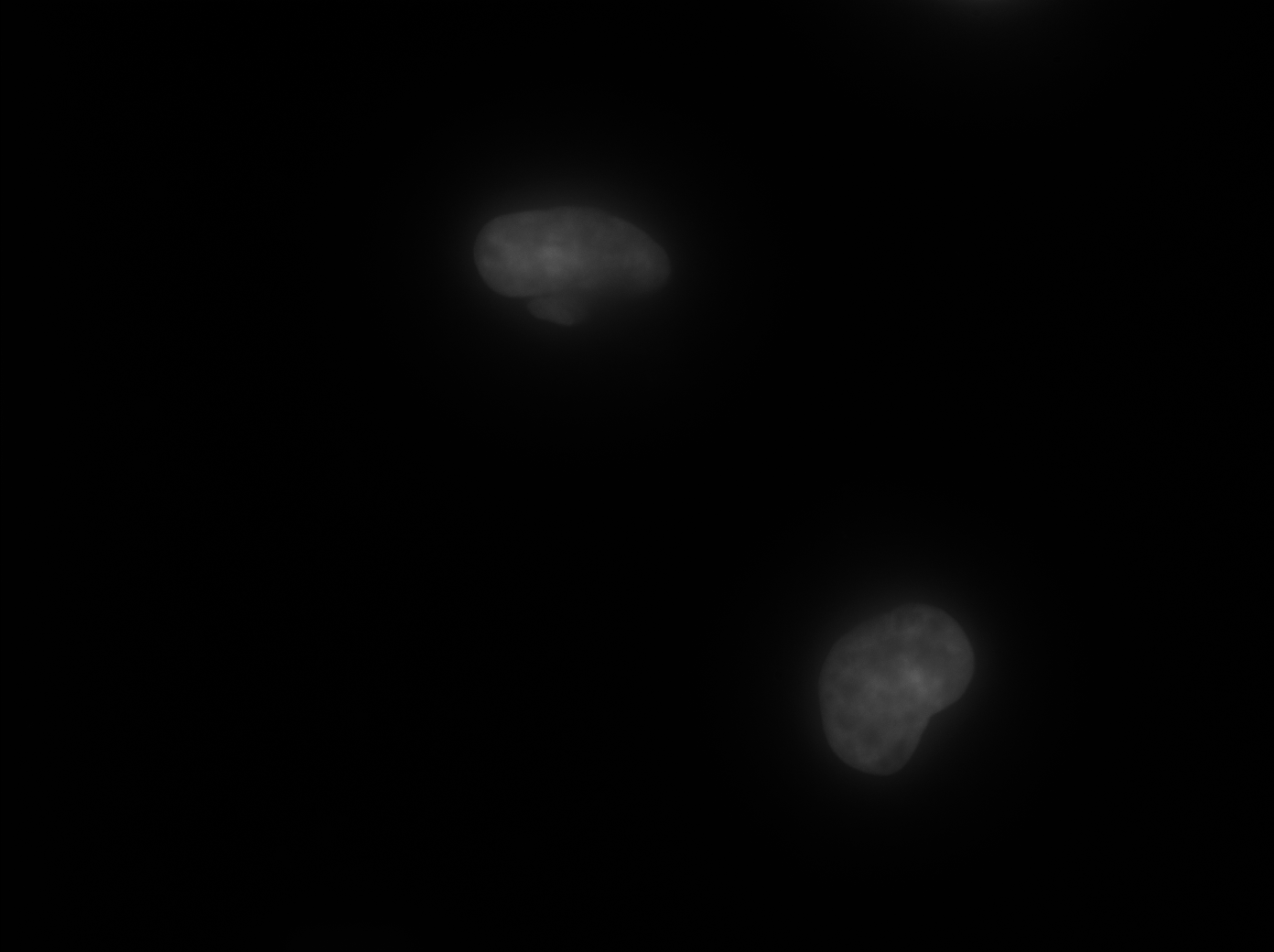

Supplement: Supplementary file 22 — Source data Fig. 6 part 3 [file 44319_2026_742_MOESM22_ESM.zip › Figure 6 Part 3/Fig 6efg TPGS1-KO TPGS1 rescue experiments/R1/TPGS1-KO TPGS1-3UTR-EYFP actub R1 7-31-25 LT6.Project Maximum Z_XY1753988729_Z0_T0_C0.tif]

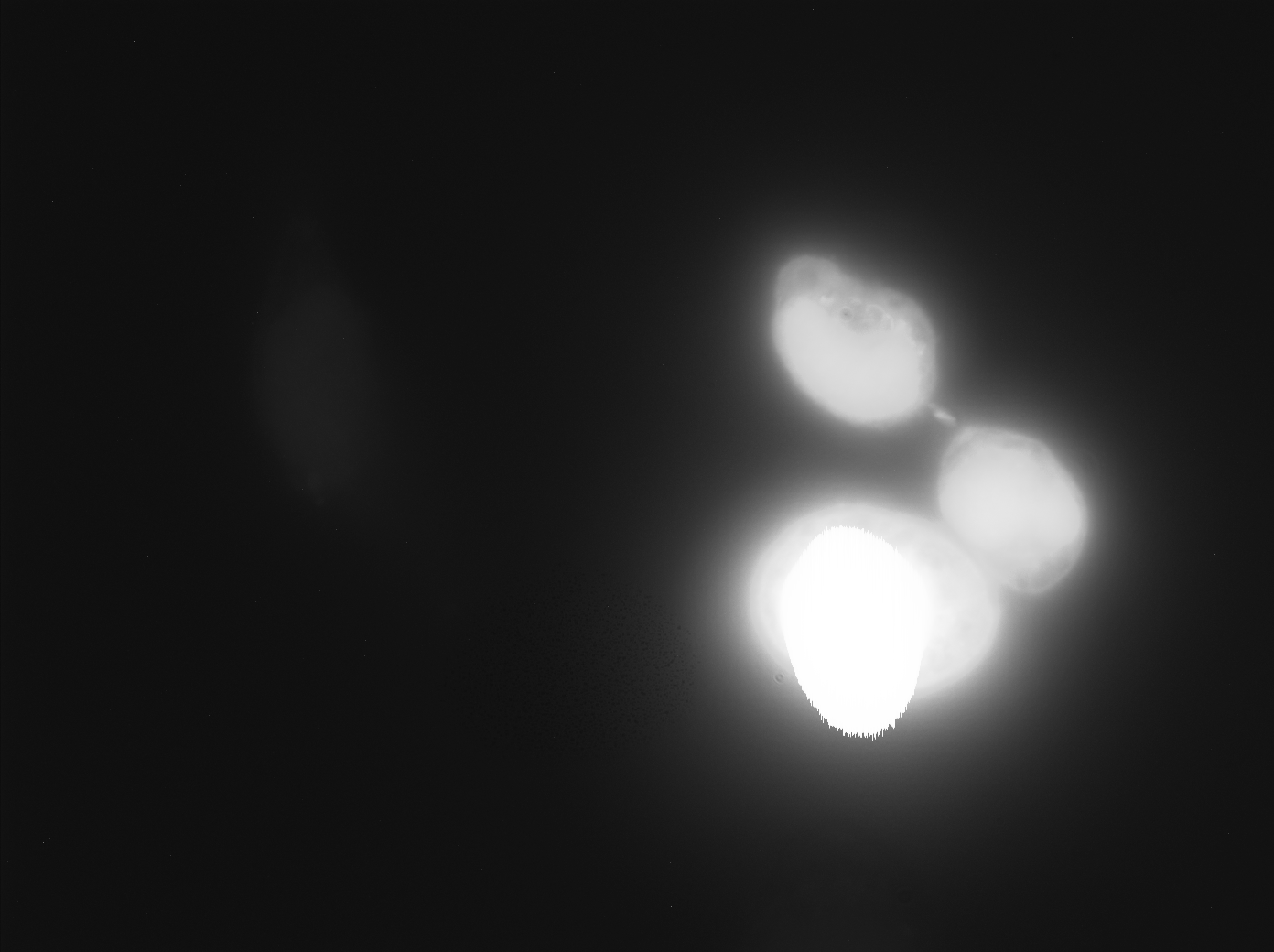

Supplement: Supplementary file 22 — Source data Fig. 6 part 3 [file 44319_2026_742_MOESM22_ESM.zip › Figure 6 Part 3/Fig 6efg TPGS1-KO TPGS1 rescue experiments/R1/TPGS1-KO EYFP only actub R1 7-31-25 LT4.Project Maximum Z_XY1754336072_Z0_T0_C1.tif]

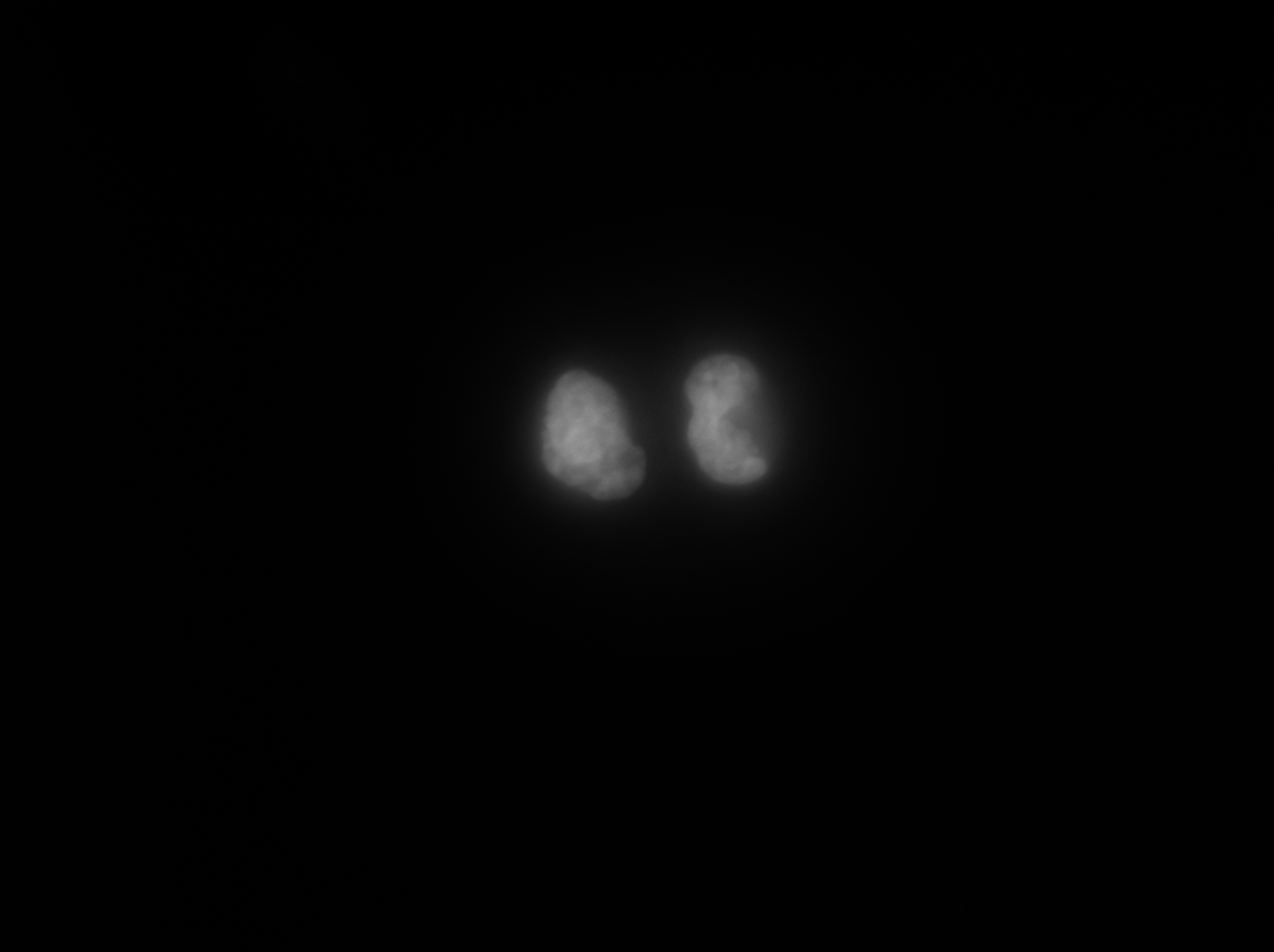

Supplement: Supplementary file 22 — Source data Fig. 6 part 3 [file 44319_2026_742_MOESM22_ESM.zip › Figure 6 Part 3/Fig 6efg TPGS1-KO TPGS1 rescue experiments/R1/TPGS1-KO EYFP only actub R1 7-31-25 ET4.Project Maximum Z_XY1754335973_Z0_T0_C0.tif]

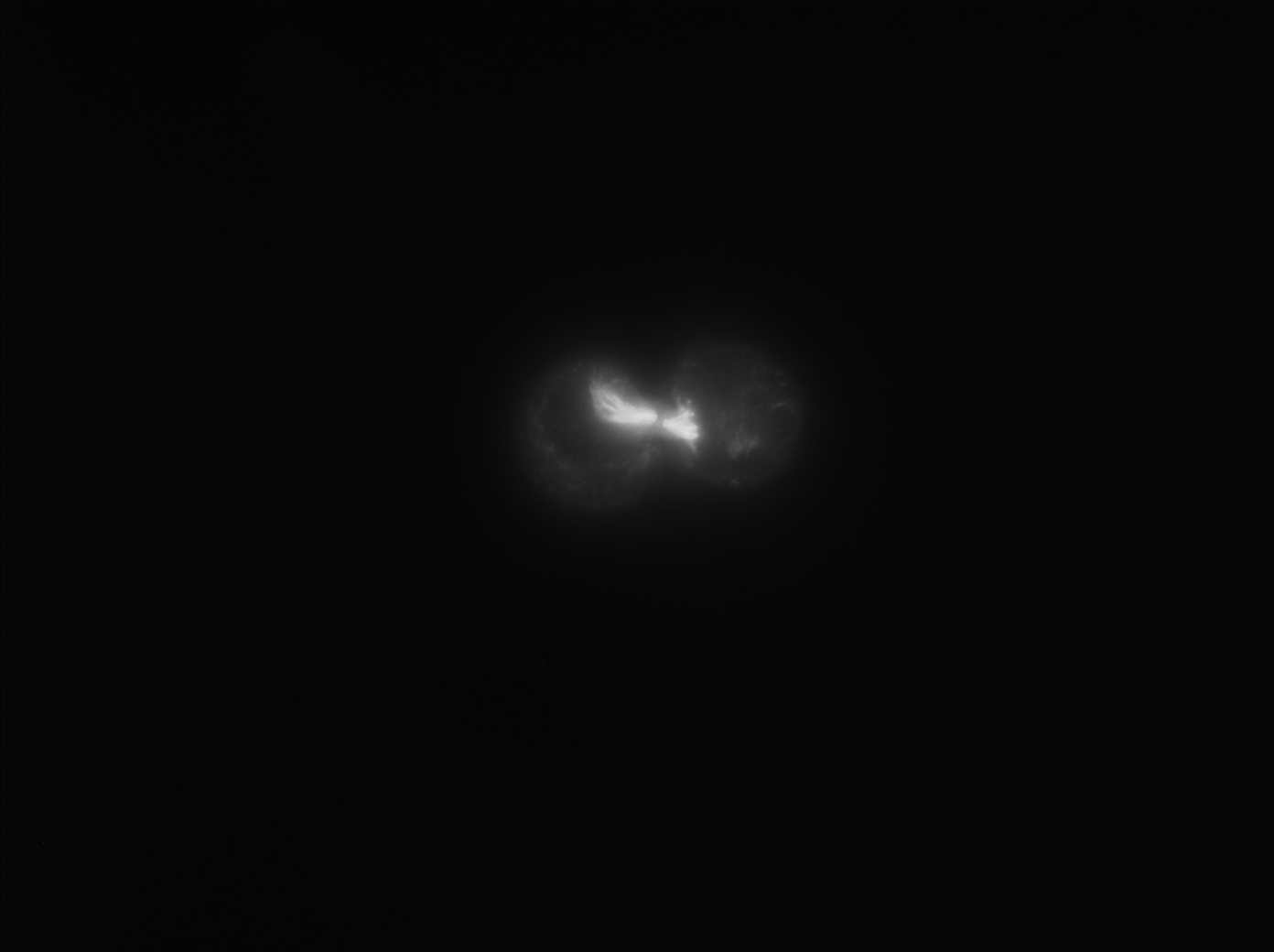

Supplement: Supplementary file 22 — Source data Fig. 6 part 3 [file 44319_2026_742_MOESM22_ESM.zip › Figure 6 Part 3/Fig 6efg TPGS1-KO TPGS1 rescue experiments/R1/TPGS1-KO EYFP only actub R1 7-31-25 ET4.Project Maximum Z_XY1754335973_Z0_T0_C2.tif]

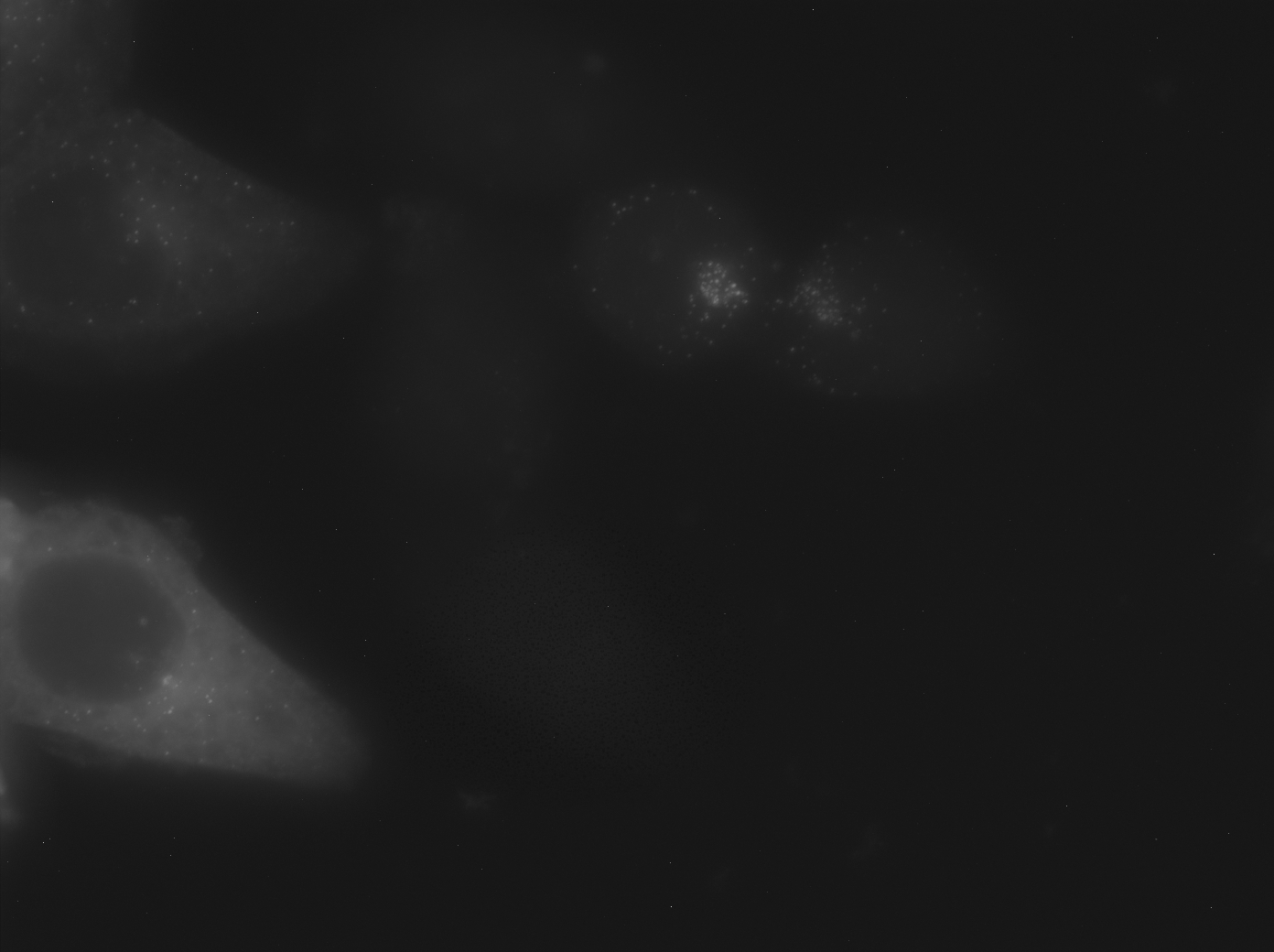

Supplement: Supplementary file 22 — Source data Fig. 6 part 3 [file 44319_2026_742_MOESM22_ESM.zip › Figure 6 Part 3/Fig 6efg TPGS1-KO TPGS1 rescue experiments/R1/TPGS1-KO TPGS1-3UTR-EYFP actub R1 7-31-25 ET1.Project Maximum Z_XY1753981618_Z0_T0_C1.tif]
