# Supplementary figures and images for: TPGS1 regulates central spindle microtubule glutamylation and remodeling during telophase and abscission (part 24 of 36)
Source: EMBO Rep. 2026 Mar 23;27(8):1944–63. doi: 10.1038/s44319-026-00742-3 (PMC13121839; doi:10.1038/s44319-026-00742-3)

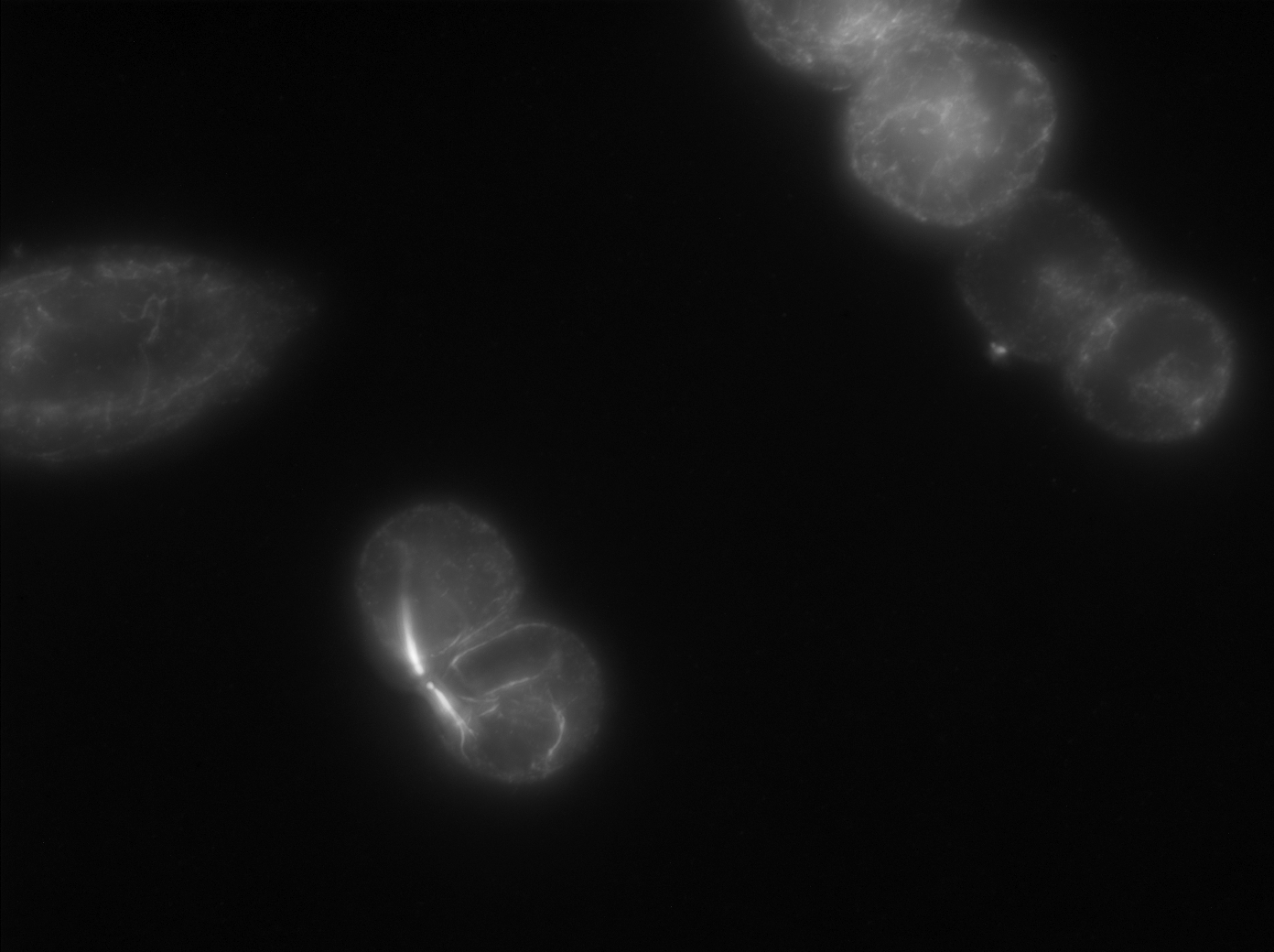

Supplement: Supplementary file 21 — Source data Fig. 6 part 2 [file 44319_2026_742_MOESM21_ESM.zip › Figure 6 Part 2/Fig 6abcd Cas9 TPGS1-KO acetylated tubulin atubulin part 2/TPGS1-KO R2 9-11-24 LT10 PA8.Project Maximum Z_XY1726261995_Z0_T0_C2.tif]

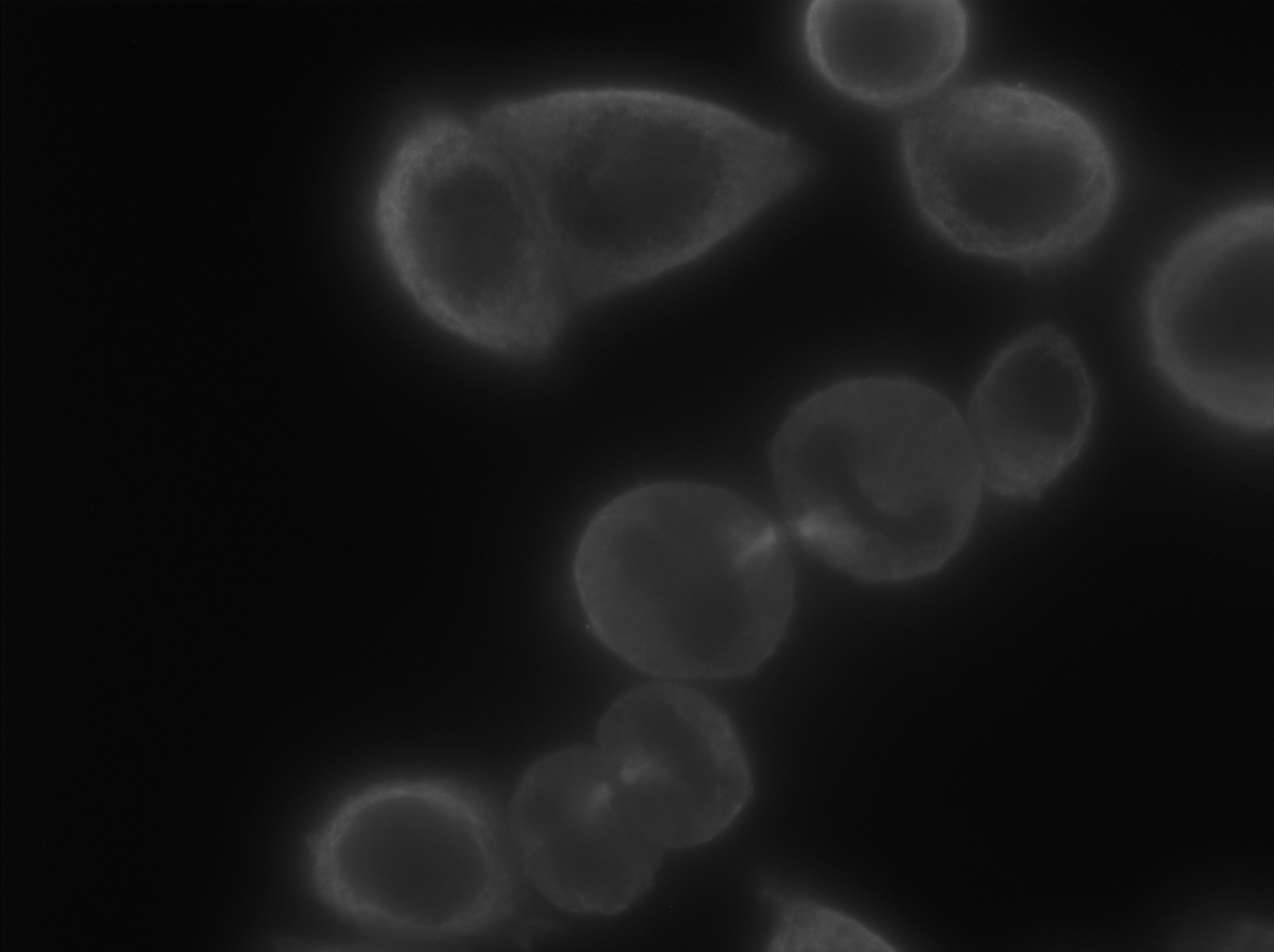

Supplement: Supplementary file 21 — Source data Fig. 6 part 2 [file 44319_2026_742_MOESM21_ESM.zip › Figure 6 Part 2/Fig 6abcd Cas9 TPGS1-KO acetylated tubulin atubulin part 2/TPGS1-KO R3 9-13-24 LT10.Project Maximum Z_XY1726760993_Z0_T0_C1.tif]

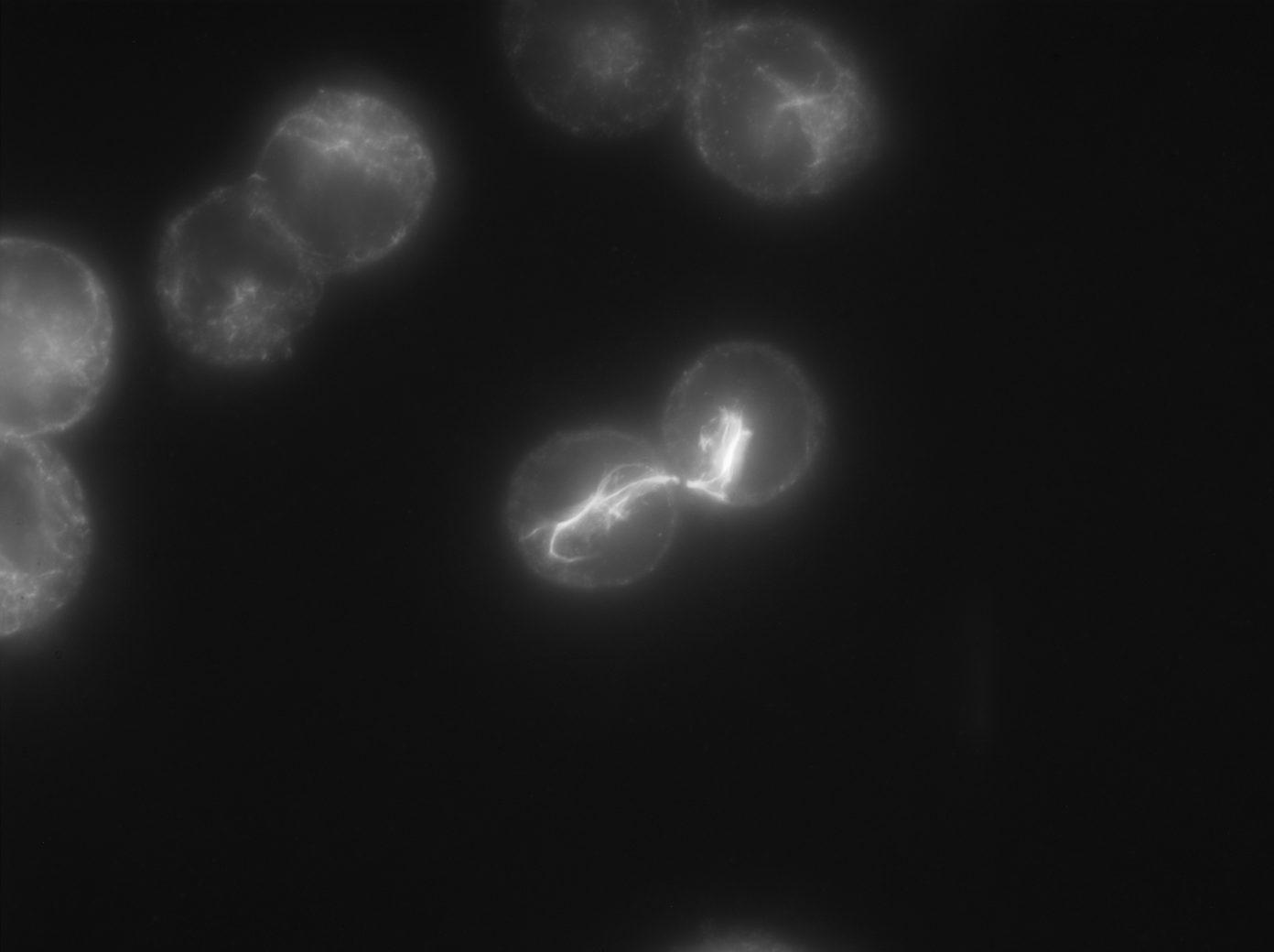

Supplement: Supplementary file 21 — Source data Fig. 6 part 2 [file 44319_2026_742_MOESM21_ESM.zip › Figure 6 Part 2/Fig 6abcd Cas9 TPGS1-KO acetylated tubulin atubulin part 2/TPGS1-KO R2 9-11-24 LT23.Project Maximum Z_XY1726268081_Z0_T0_C2.tif]

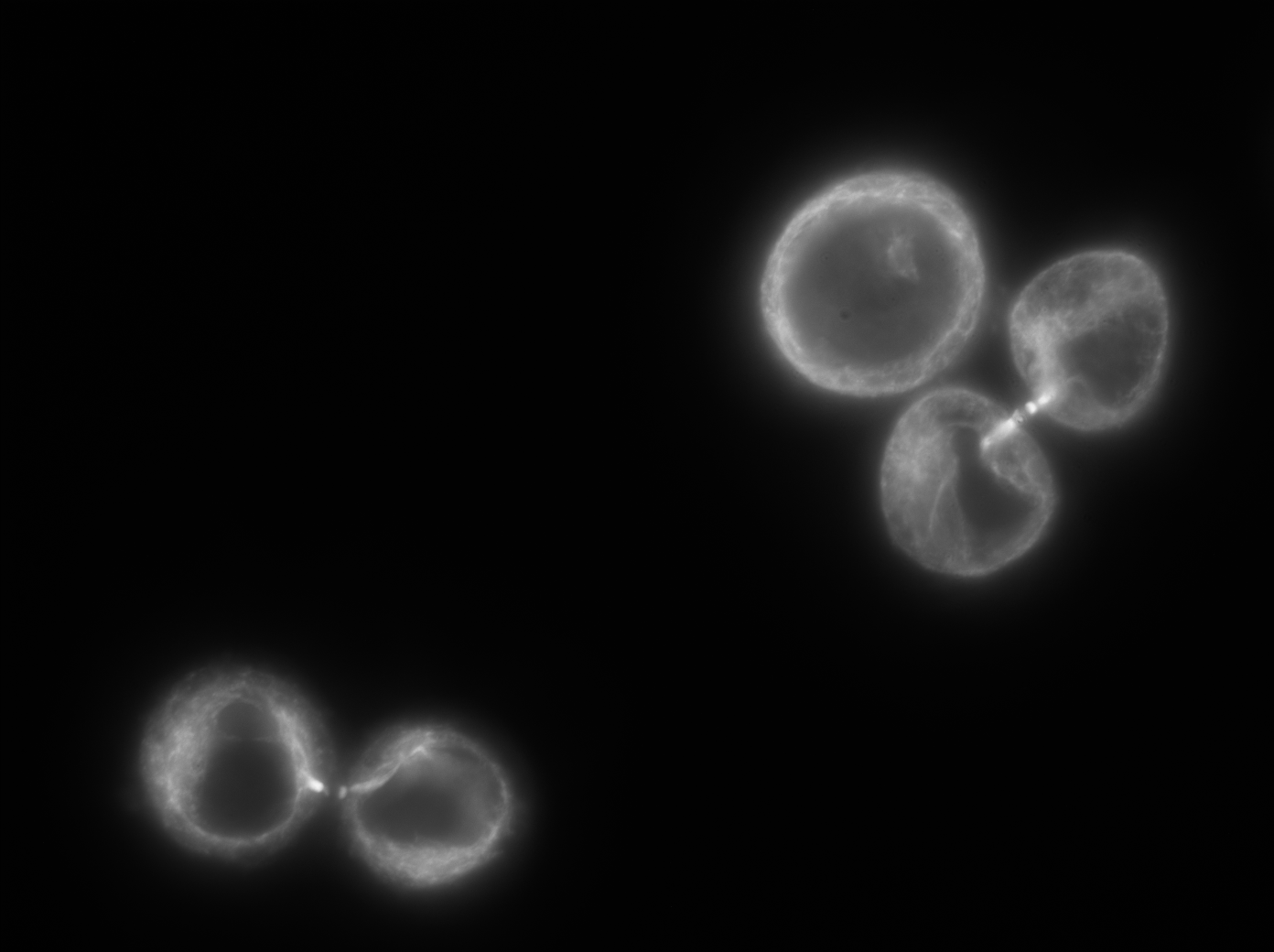

Supplement: Supplementary file 21 — Source data Fig. 6 part 2 [file 44319_2026_742_MOESM21_ESM.zip › Figure 6 Part 2/Fig 6abcd Cas9 TPGS1-KO acetylated tubulin atubulin part 2/TPGS1-KO R2 9-11-24 LT17LT18.Project Maximum Z_XY1726266302_Z0_T0_C1.tif]

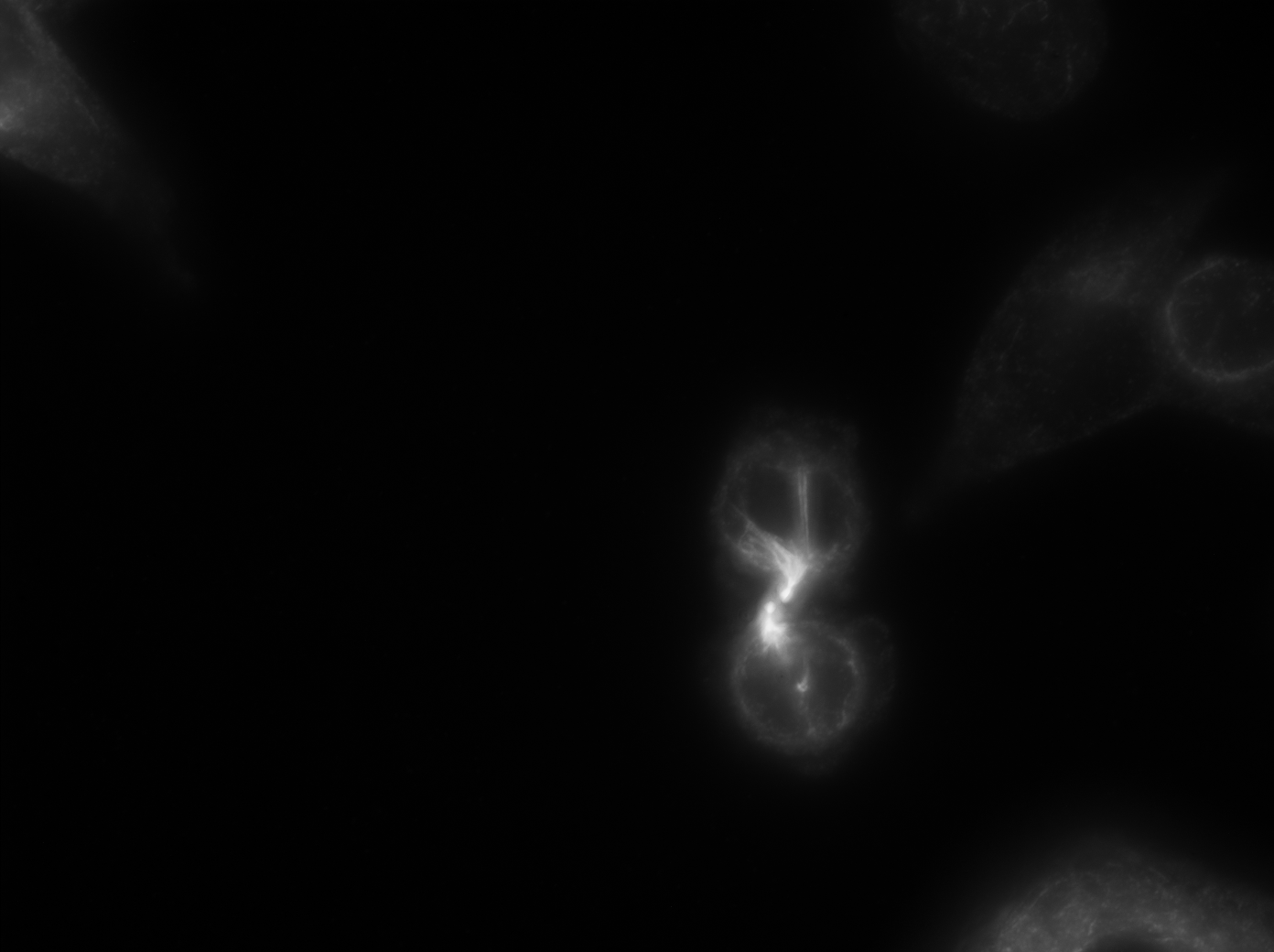

Supplement: Supplementary file 21 — Source data Fig. 6 part 2 [file 44319_2026_742_MOESM21_ESM.zip › Figure 6 Part 2/Fig 6abcd Cas9 TPGS1-KO acetylated tubulin atubulin part 2/TPGS1-KO R3 9-13-24 LT2.Project Maximum Z_XY1726760085_Z0_T0_C2.tif]

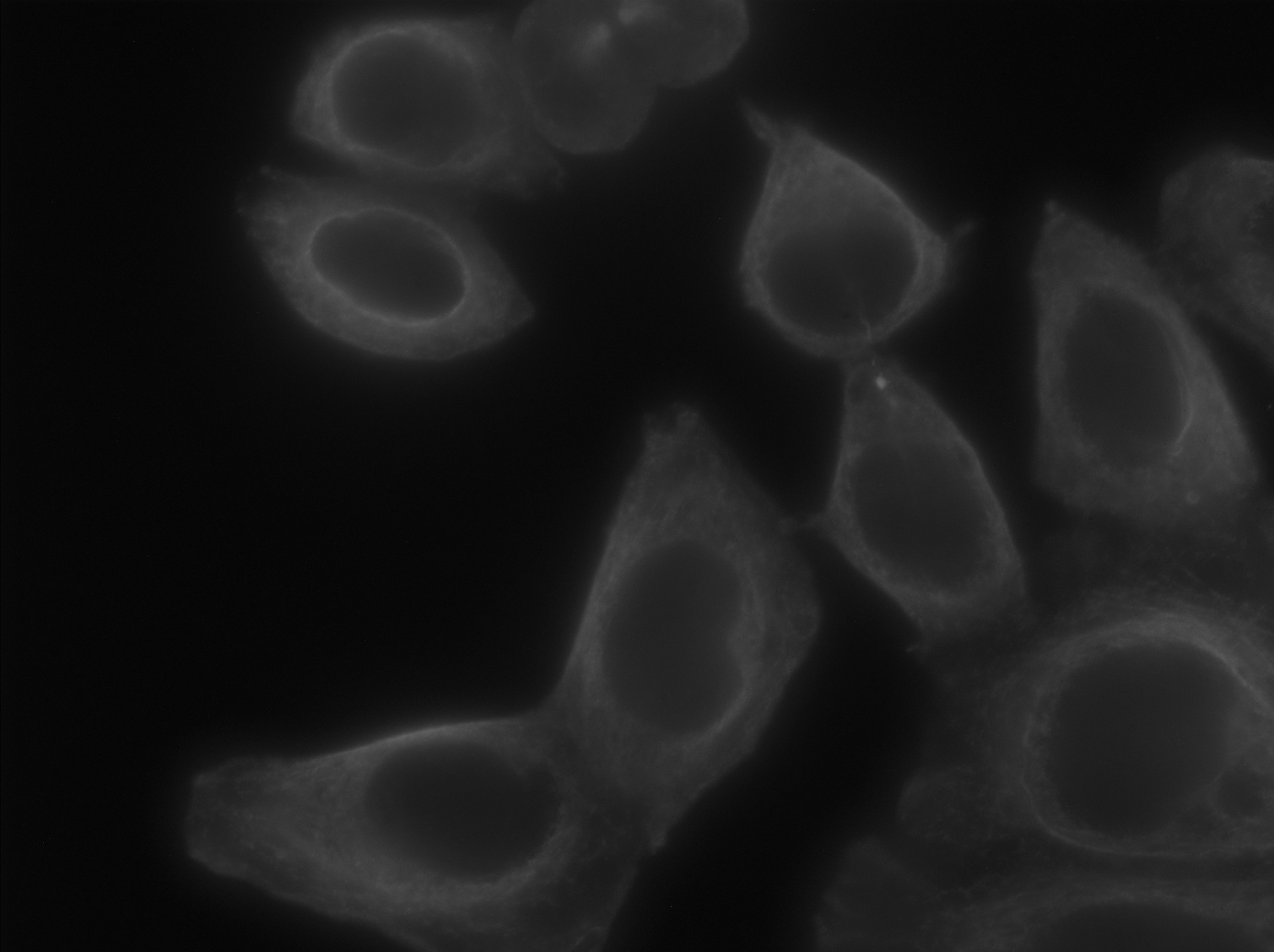

Supplement: Supplementary file 21 — Source data Fig. 6 part 2 [file 44319_2026_742_MOESM21_ESM.zip › Figure 6 Part 2/Fig 6abcd Cas9 TPGS1-KO acetylated tubulin atubulin part 2/TPGS1-KO R3 9-13-24 LT11.Project Maximum Z_XY1726761069_Z0_T0_C1.tif]

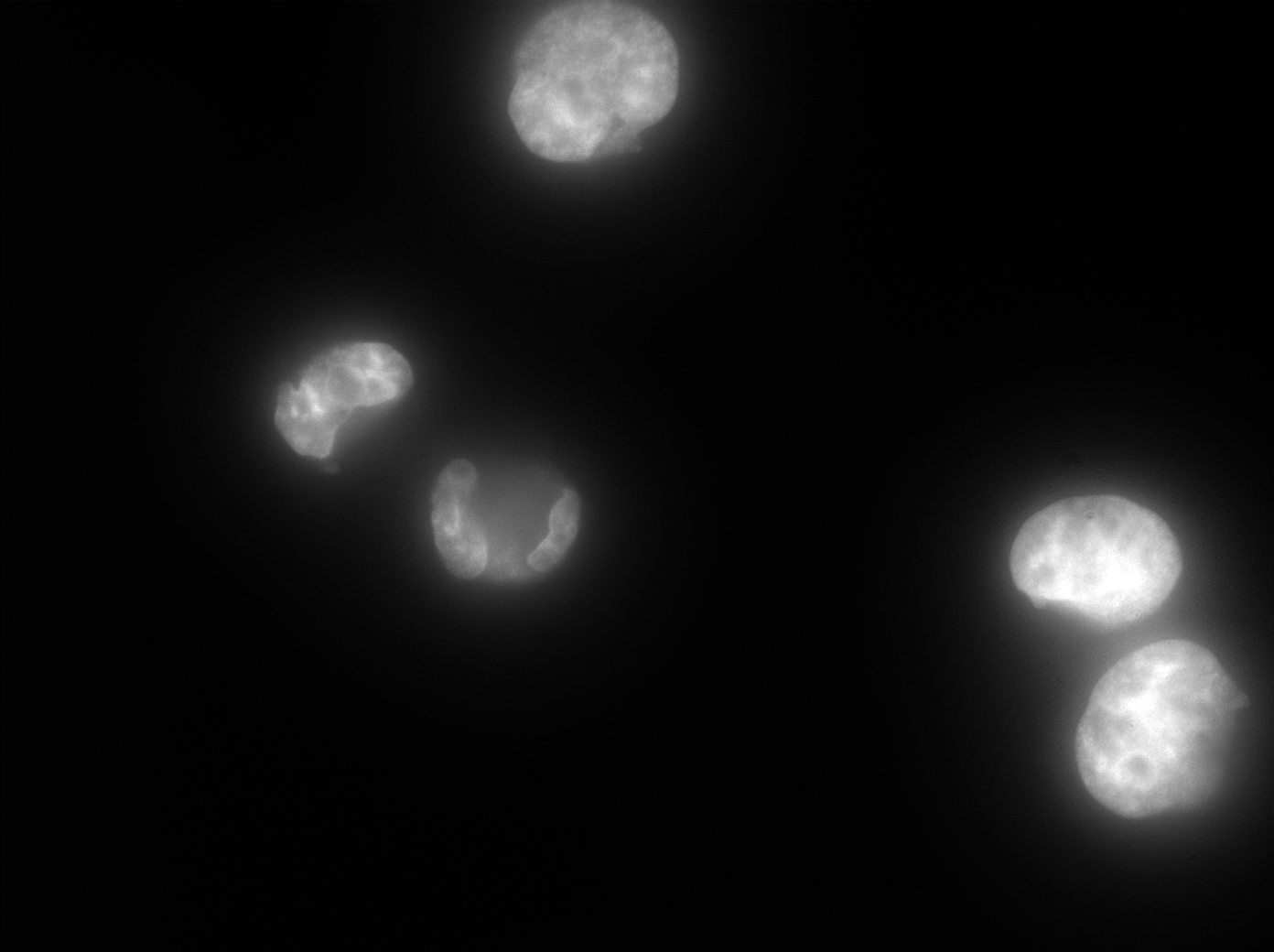

Supplement: Supplementary file 21 — Source data Fig. 6 part 2 [file 44319_2026_742_MOESM21_ESM.zip › Figure 6 Part 2/Fig 6abcd Cas9 TPGS1-KO acetylated tubulin atubulin part 2/TPGS1-KO R2 9-11-24 PA4.Project Maximum Z_XY1726260057_Z0_T0_C0.tif]

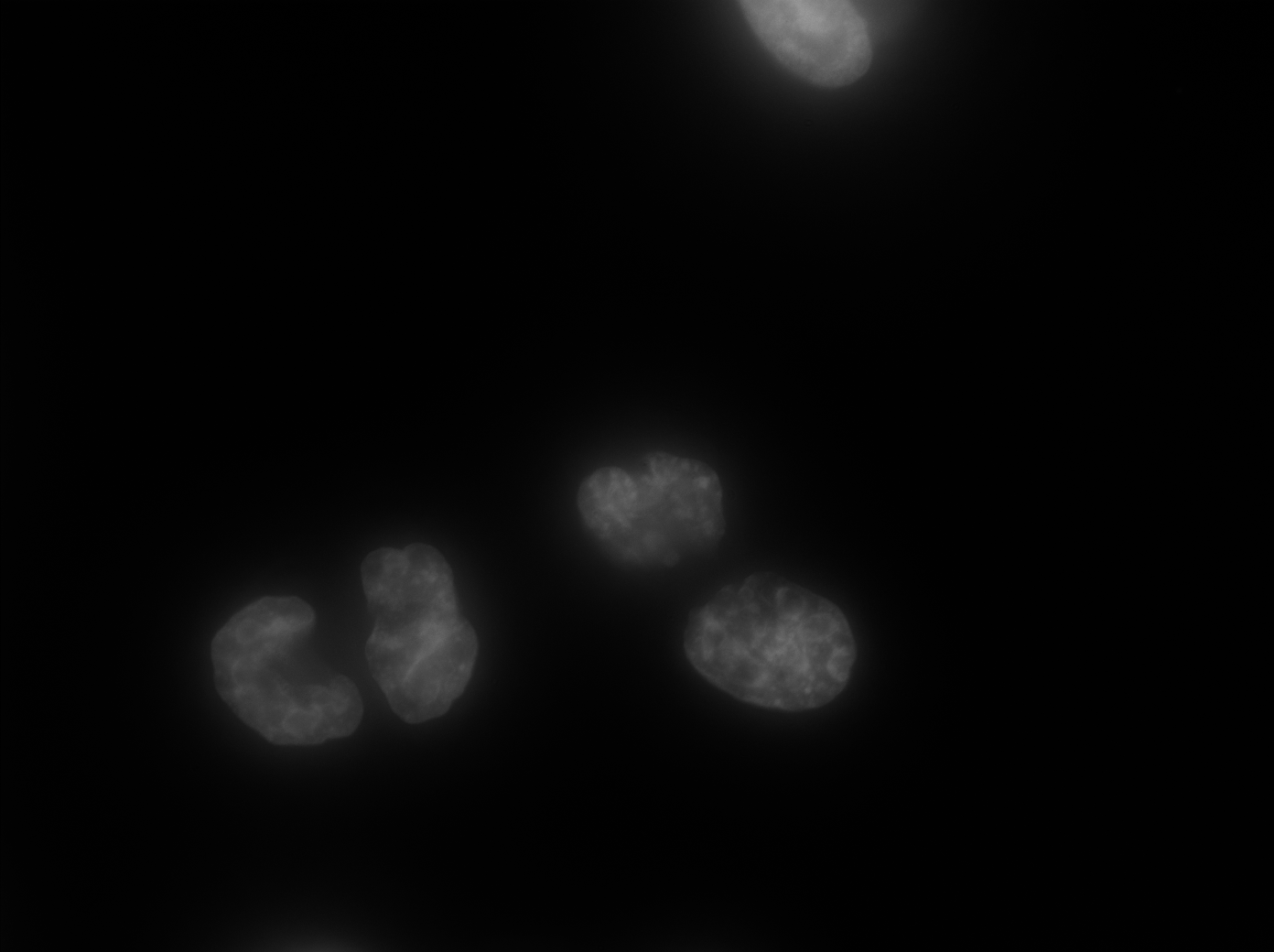

Supplement: Supplementary file 21 — Source data Fig. 6 part 2 [file 44319_2026_742_MOESM21_ESM.zip › Figure 6 Part 2/Fig 6abcd Cas9 TPGS1-KO acetylated tubulin atubulin part 2/TPGS1-KO R3 9-13-24 LT1.Project Maximum Z_XY1726759987_Z0_T0_C0.tif]

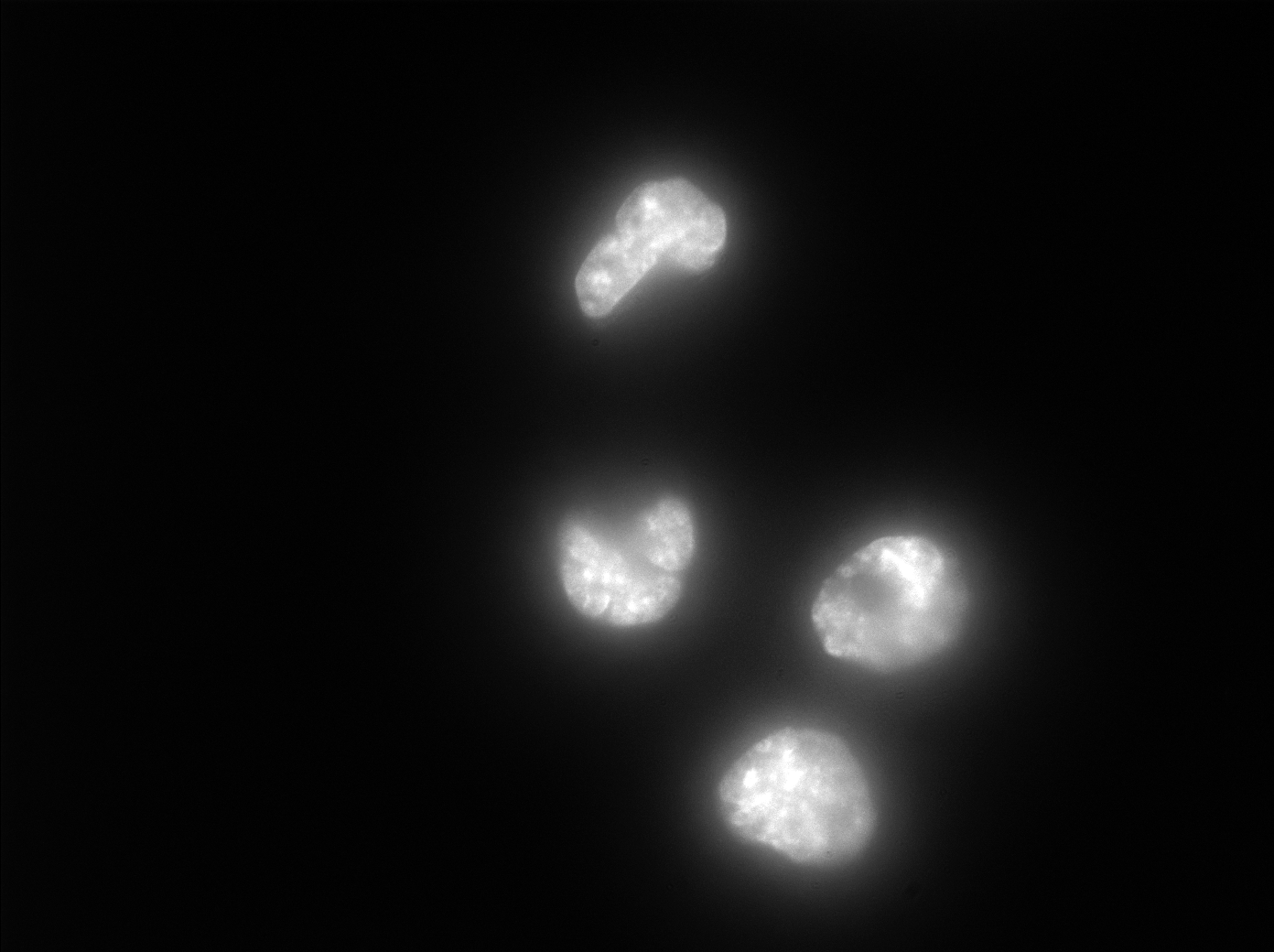

Supplement: Supplementary file 21 — Source data Fig. 6 part 2 [file 44319_2026_742_MOESM21_ESM.zip › Figure 6 Part 2/Fig 6abcd Cas9 TPGS1-KO acetylated tubulin atubulin part 2/TPGS1-KO R2 9-11-24 LT27 PA20.Project Maximum Z_XY1726269402_Z0_T0_C0.tif]

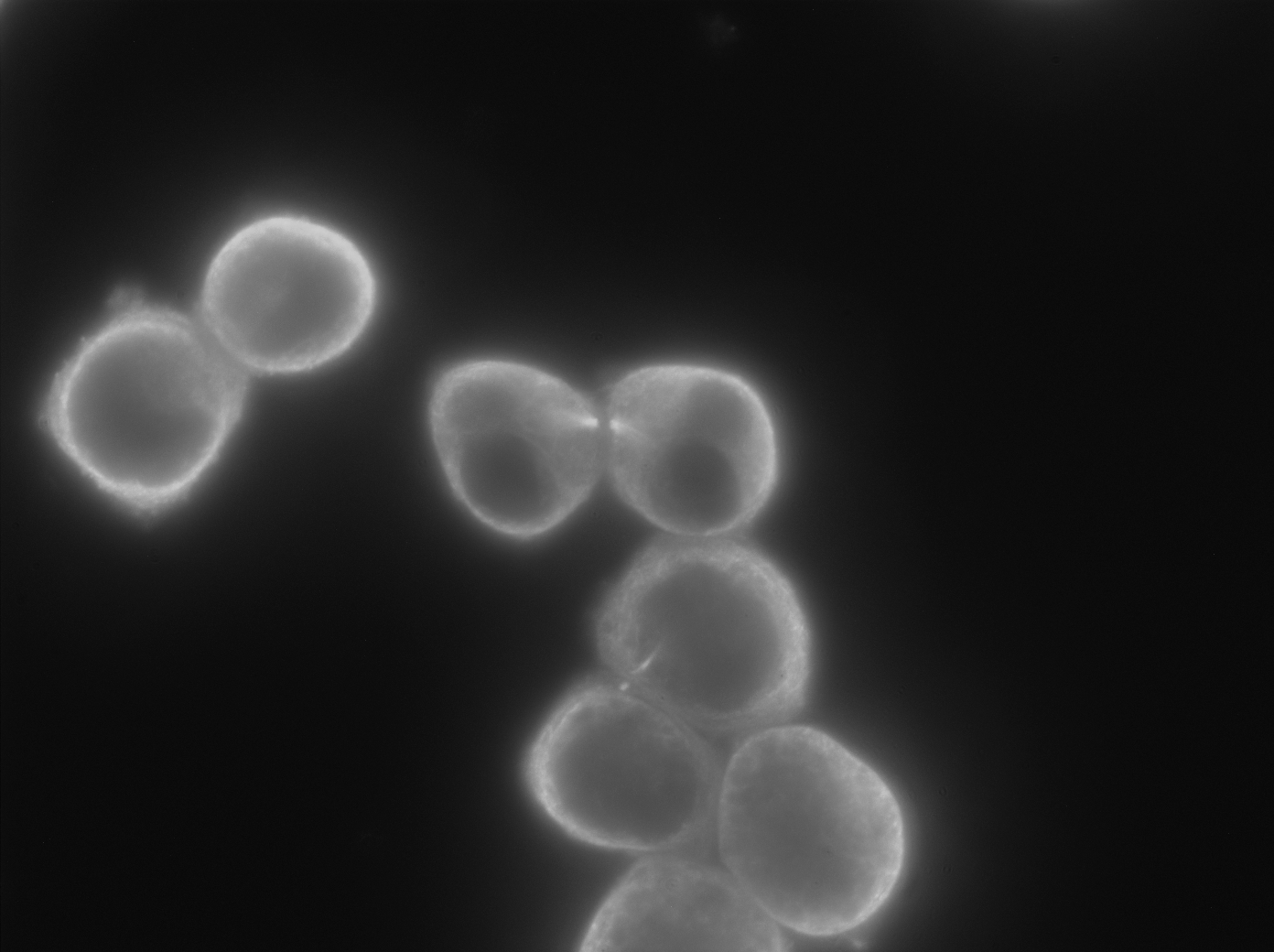

Supplement: Supplementary file 21 — Source data Fig. 6 part 2 [file 44319_2026_742_MOESM21_ESM.zip › Figure 6 Part 2/Fig 6abcd Cas9 TPGS1-KO acetylated tubulin atubulin part 2/TPGS1-KO R3 9-13-24 LT17LT18.Project Maximum Z_XY1726763998_Z0_T0_C1.tif]

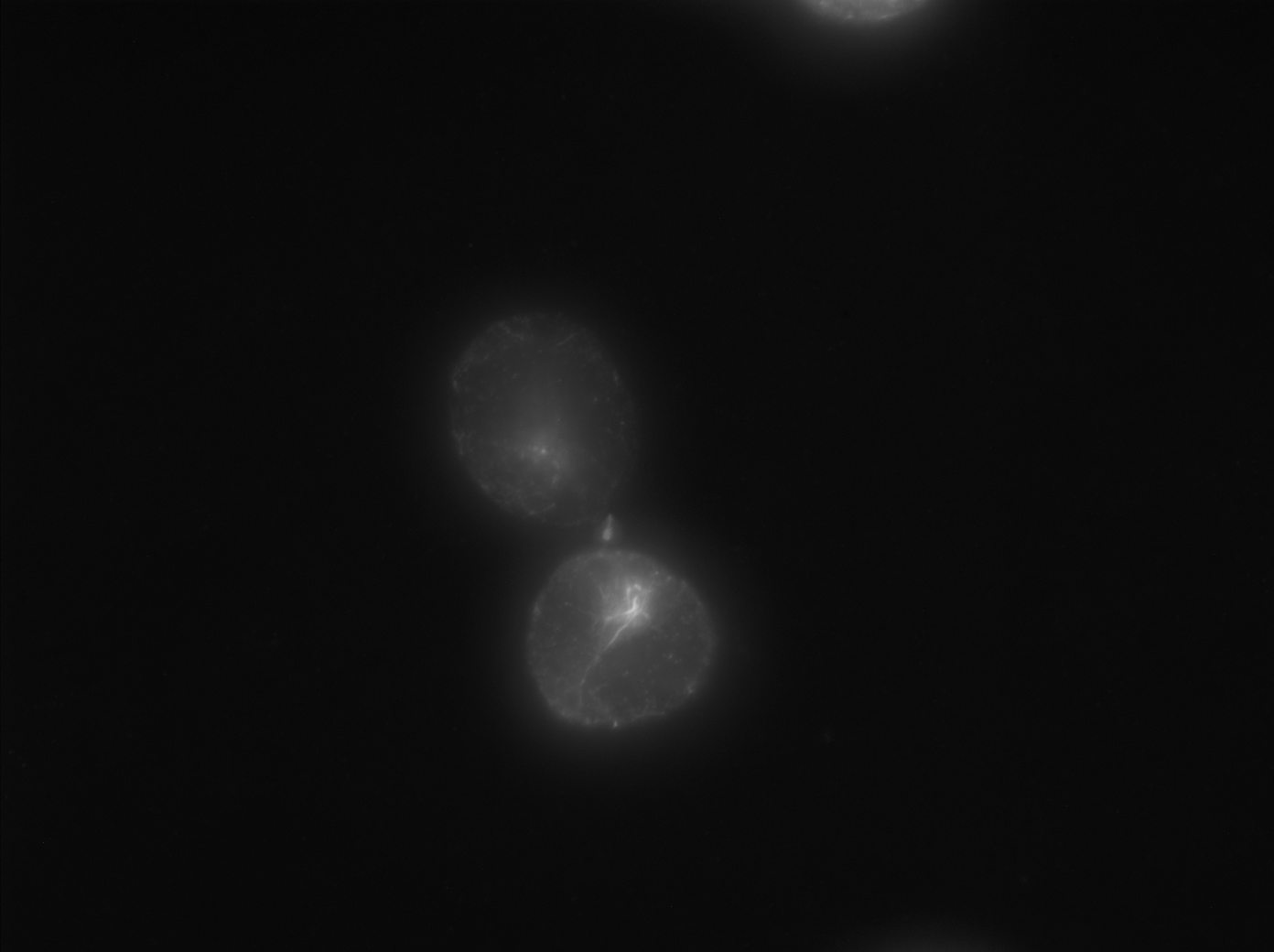

Supplement: Supplementary file 21 — Source data Fig. 6 part 2 [file 44319_2026_742_MOESM21_ESM.zip › Figure 6 Part 2/Fig 6abcd Cas9 TPGS1-KO acetylated tubulin atubulin part 2/TPGS1-KO R2 9-11-24 PA10.Project Maximum Z_XY1726262600_Z0_T0_C2.tif]

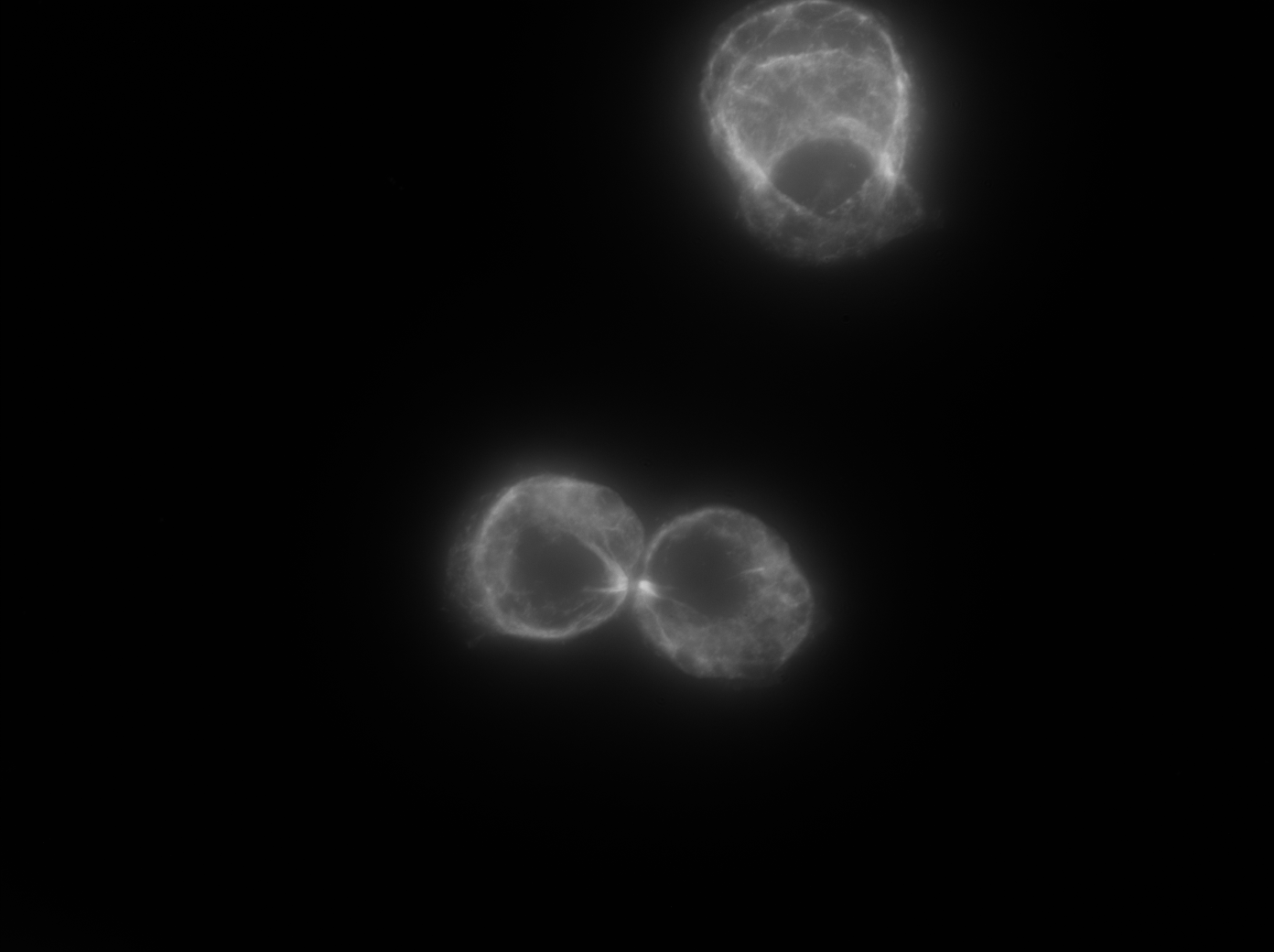

Supplement: Supplementary file 21 — Source data Fig. 6 part 2 [file 44319_2026_742_MOESM21_ESM.zip › Figure 6 Part 2/Fig 6abcd Cas9 TPGS1-KO acetylated tubulin atubulin part 2/TPGS1-KO R2 9-11-24 LT28.Project Maximum Z_XY1726269506_Z0_T0_C1.tif]

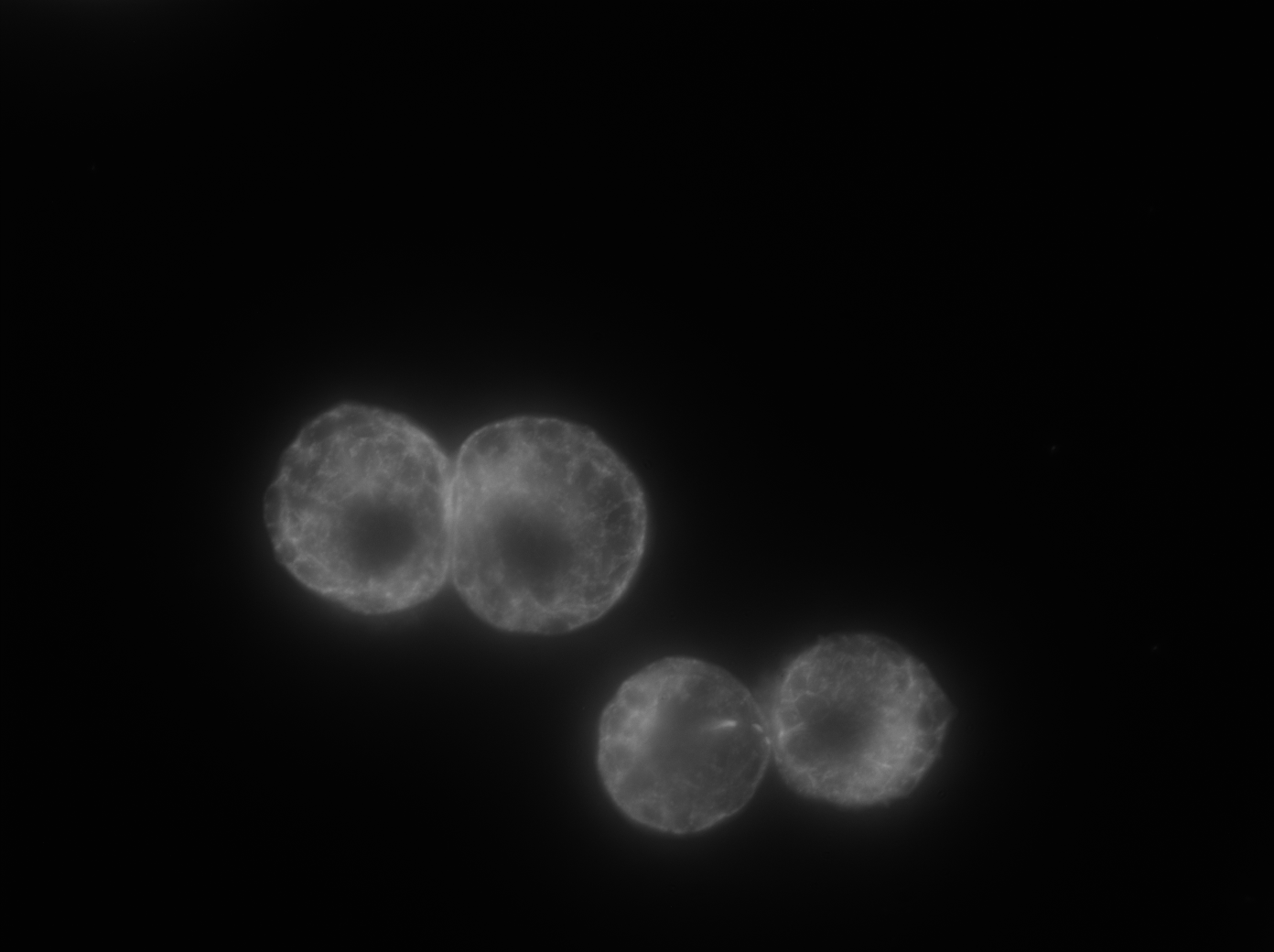

Supplement: Supplementary file 21 — Source data Fig. 6 part 2 [file 44319_2026_742_MOESM21_ESM.zip › Figure 6 Part 2/Fig 6abcd Cas9 TPGS1-KO acetylated tubulin atubulin part 2/TPGS1-KO R2 9-11-24 PA14.Project Maximum Z_XY1726265426_Z0_T0_C1.tif]

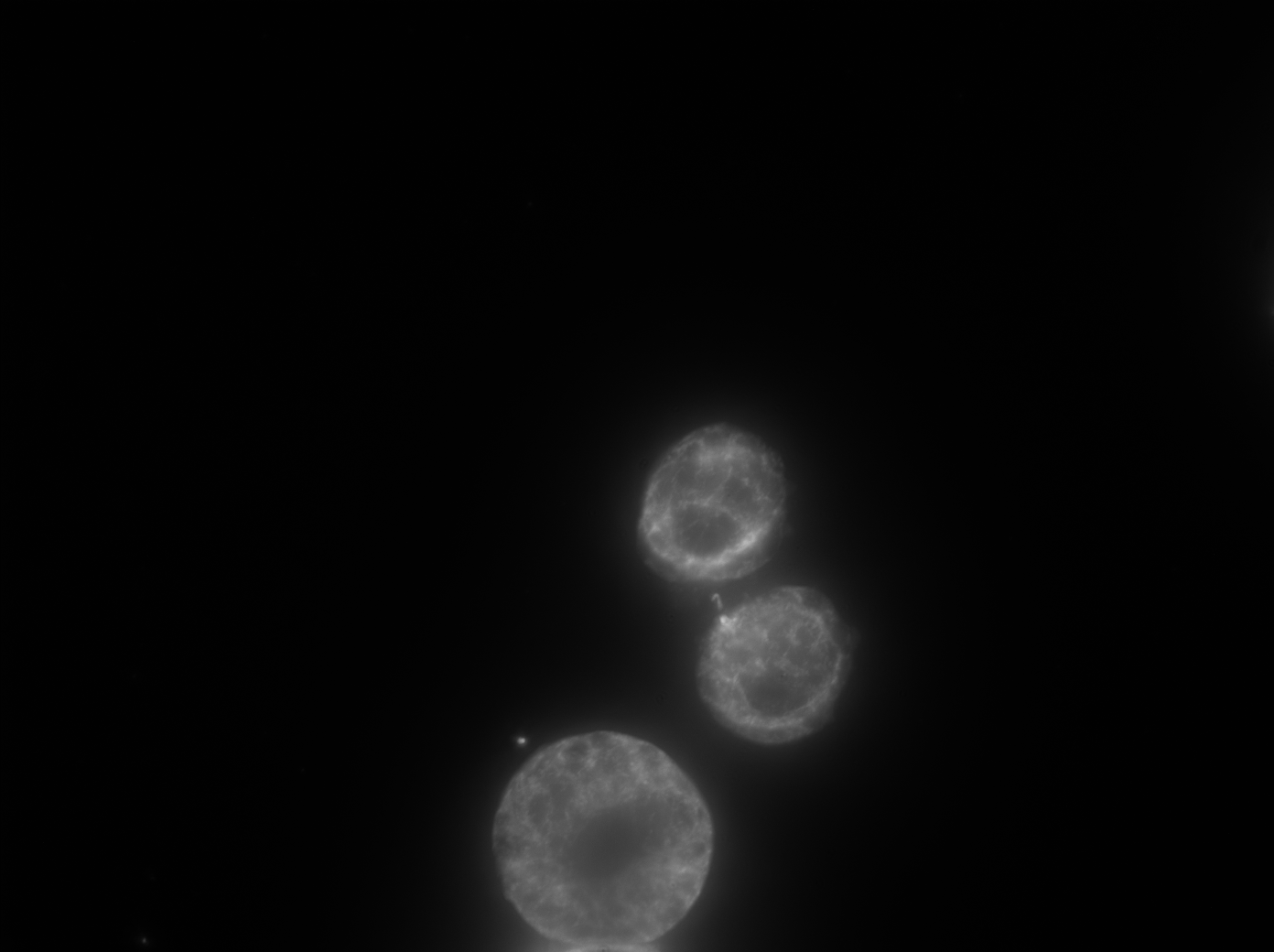

Supplement: Supplementary file 21 — Source data Fig. 6 part 2 [file 44319_2026_742_MOESM21_ESM.zip › Figure 6 Part 2/Fig 6abcd Cas9 TPGS1-KO acetylated tubulin atubulin part 2/TPGS1-KO R2 9-11-24 LT5.Project Maximum Z_XY1726259988_Z0_T0_C1.tif]

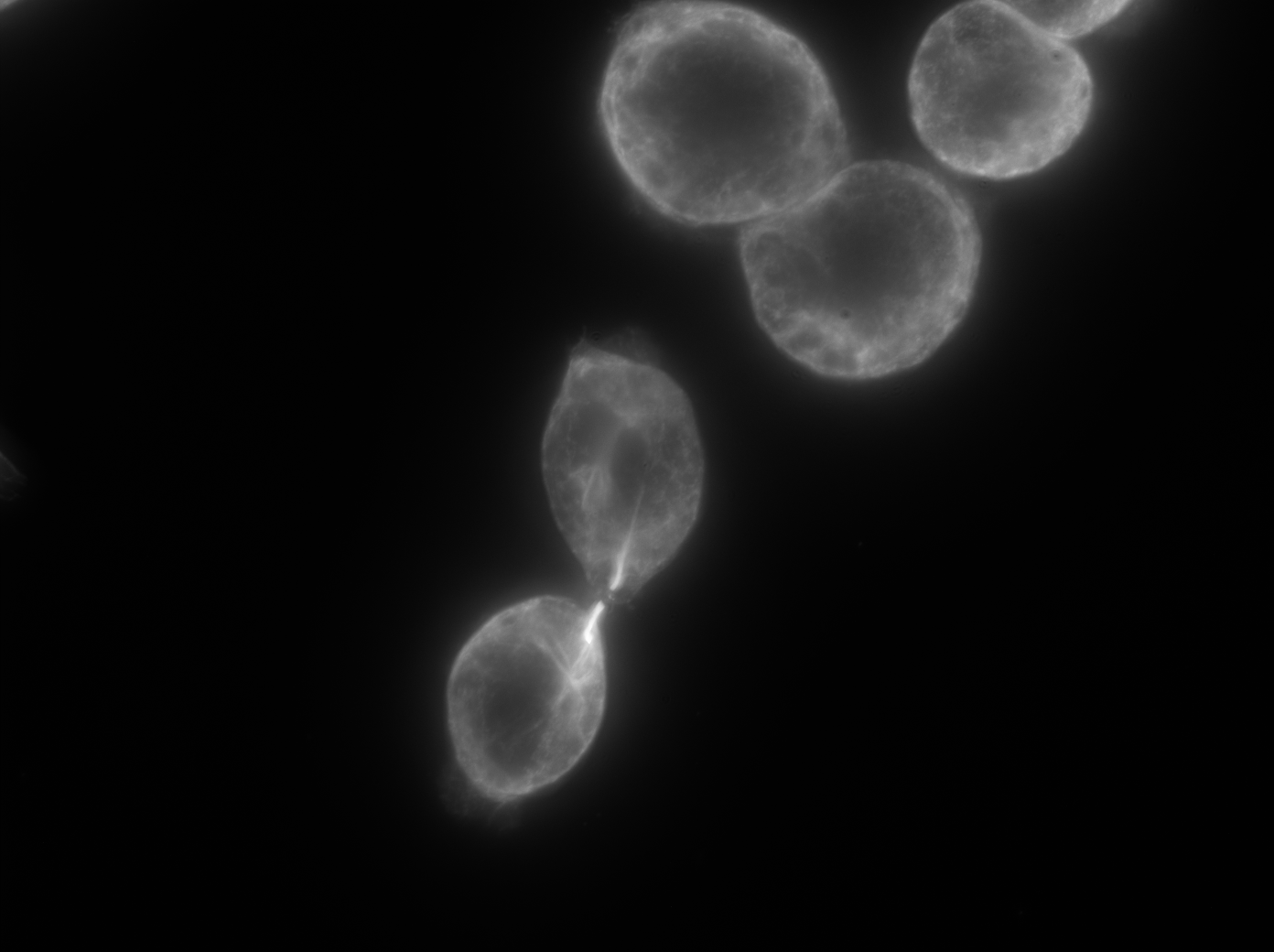

Supplement: Supplementary file 21 — Source data Fig. 6 part 2 [file 44319_2026_742_MOESM21_ESM.zip › Figure 6 Part 2/Fig 6abcd Cas9 TPGS1-KO acetylated tubulin atubulin part 2/TPGS1-KO R2 9-11-24 LT12.Project Maximum Z_XY1726262255_Z0_T0_C1.tif]

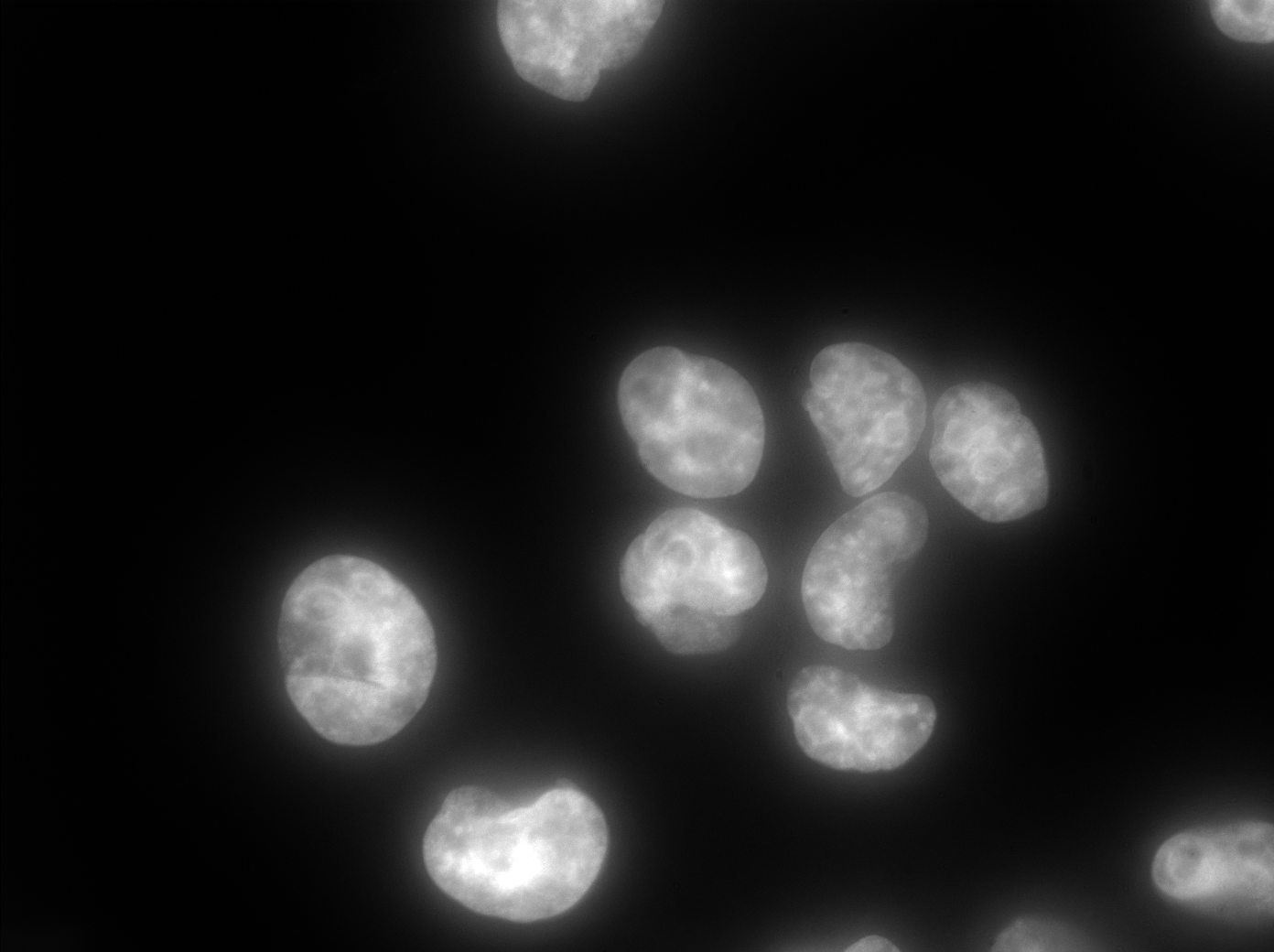

Supplement: Supplementary file 21 — Source data Fig. 6 part 2 [file 44319_2026_742_MOESM21_ESM.zip › Figure 6 Part 2/Fig 6abcd Cas9 TPGS1-KO acetylated tubulin atubulin part 2/TPGS1-KO R2 9-11-24 LT16 PA15.Project Maximum Z_XY1726265624_Z0_T0_C0.tif]

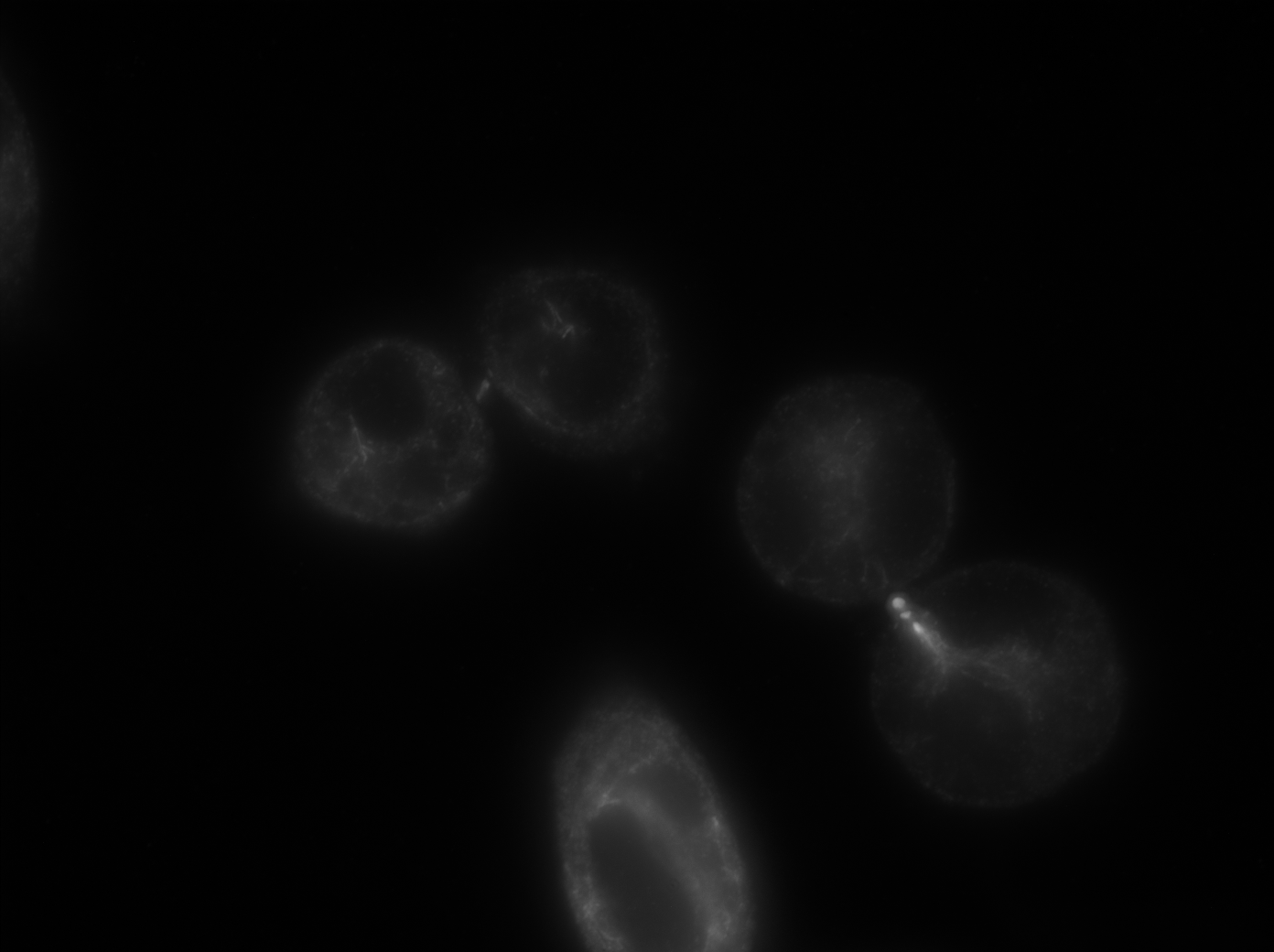

Supplement: Supplementary file 21 — Source data Fig. 6 part 2 [file 44319_2026_742_MOESM21_ESM.zip › Figure 6 Part 2/Fig 6abcd Cas9 TPGS1-KO acetylated tubulin atubulin part 2/TPGS1-KO R3 9-13-24 PA1PA2.Project Maximum Z_XY1726760183_Z0_T0_C2.tif]

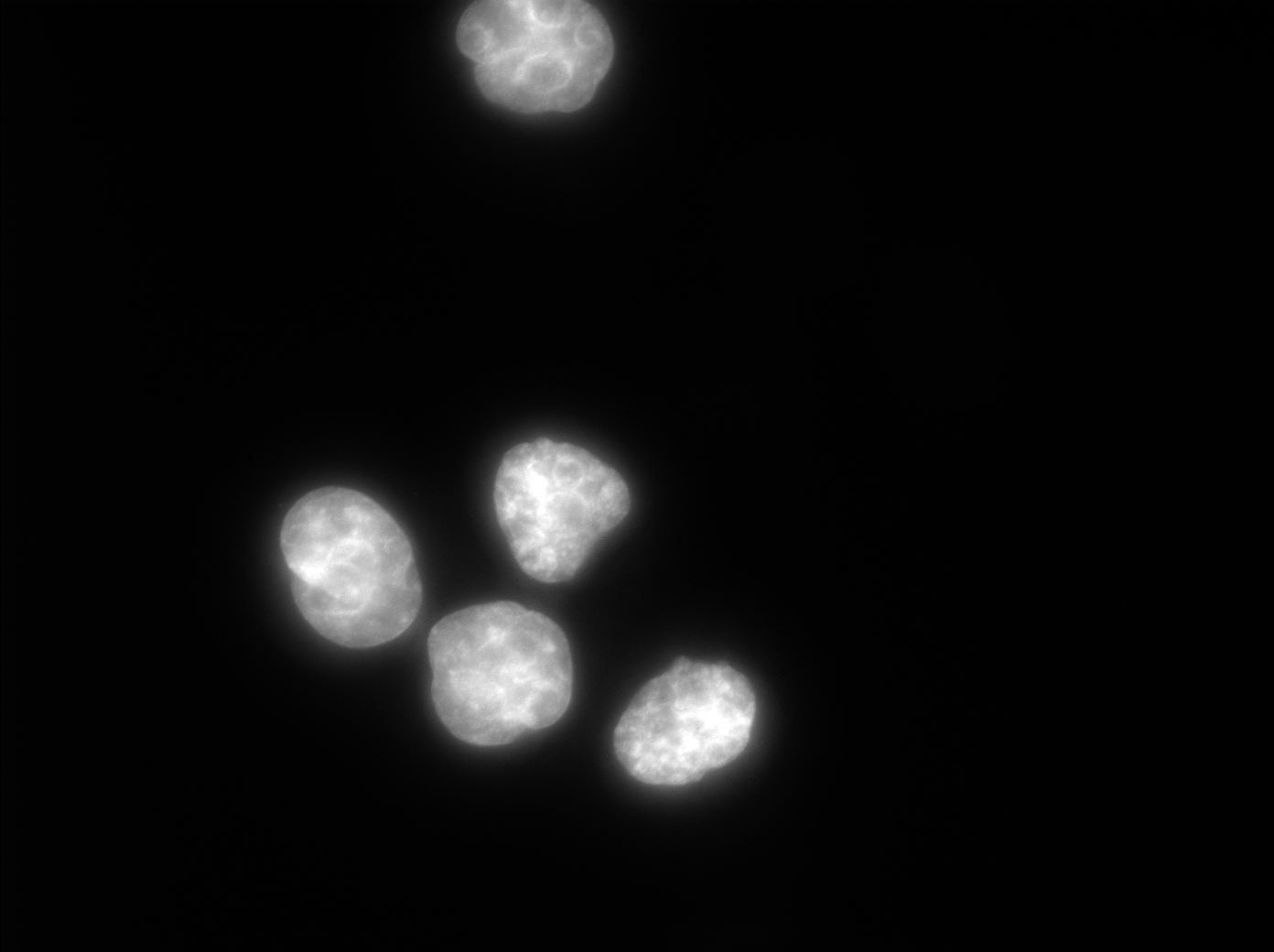

Supplement: Supplementary file 21 — Source data Fig. 6 part 2 [file 44319_2026_742_MOESM21_ESM.zip › Figure 6 Part 2/Fig 6abcd Cas9 TPGS1-KO acetylated tubulin atubulin part 2/TPGS1-KO R2 9-11-24 LT11.Project Maximum Z_XY1726262113_Z0_T0_C0.tif]

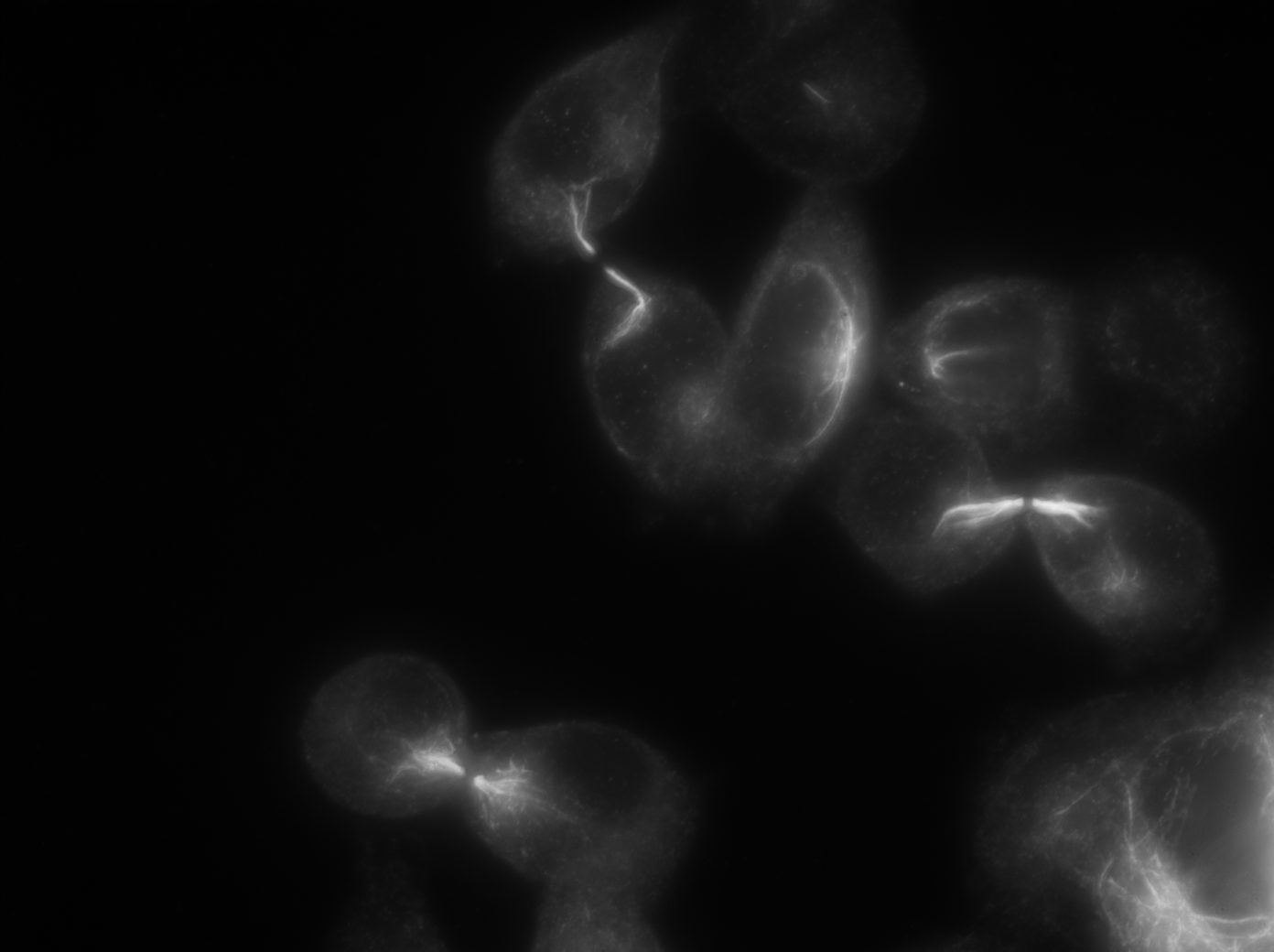

Supplement: Supplementary file 21 — Source data Fig. 6 part 2 [file 44319_2026_742_MOESM21_ESM.zip › Figure 6 Part 2/Fig 6abcd Cas9 TPGS1-KO acetylated tubulin atubulin part 2/TPGS1-KO R3 9-13-24 LT13LT14LT15.Project Maximum Z_XY1726763813_Z0_T0_C2.tif]

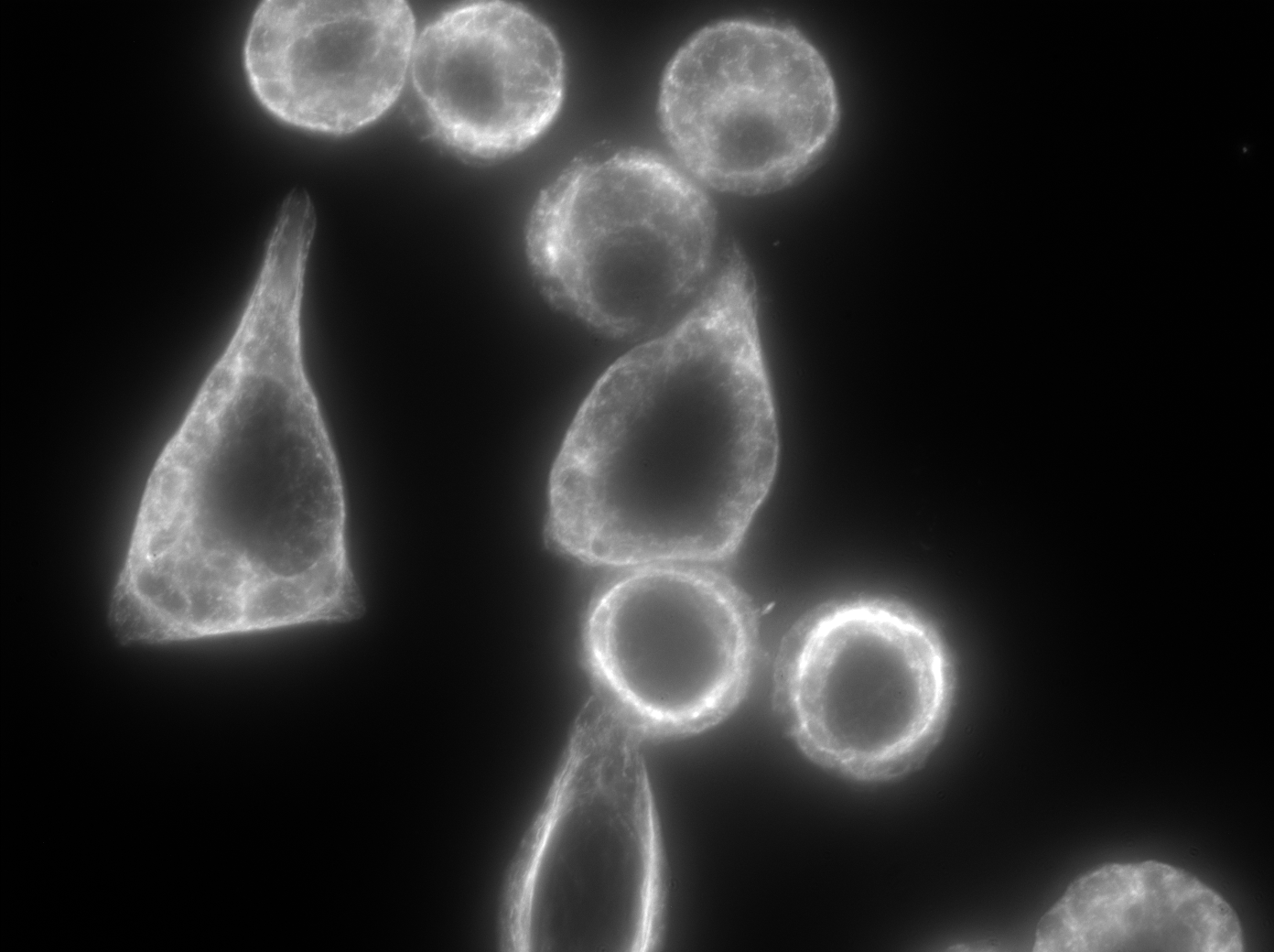

Supplement: Supplementary file 21 — Source data Fig. 6 part 2 [file 44319_2026_742_MOESM21_ESM.zip › Figure 6 Part 2/Fig 6abcd Cas9 TPGS1-KO acetylated tubulin atubulin part 2/TPGS1-KO R2 9-11-24 PA16.Project Maximum Z_XY1726266634_Z0_T0_C1.tif]

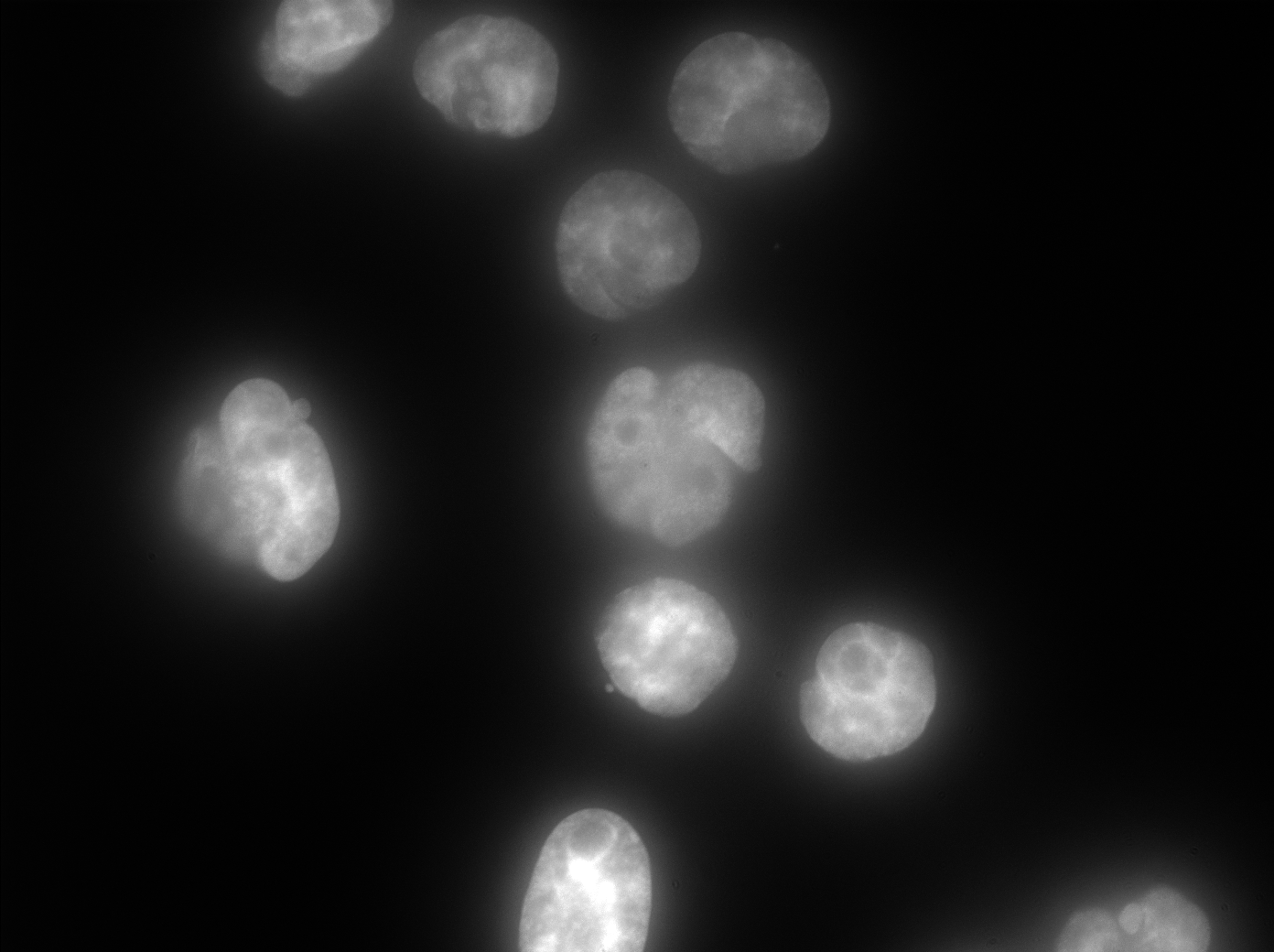

Supplement: Supplementary file 21 — Source data Fig. 6 part 2 [file 44319_2026_742_MOESM21_ESM.zip › Figure 6 Part 2/Fig 6abcd Cas9 TPGS1-KO acetylated tubulin atubulin part 2/TPGS1-KO R2 9-11-24 PA16.Project Maximum Z_XY1726266634_Z0_T0_C0.tif]

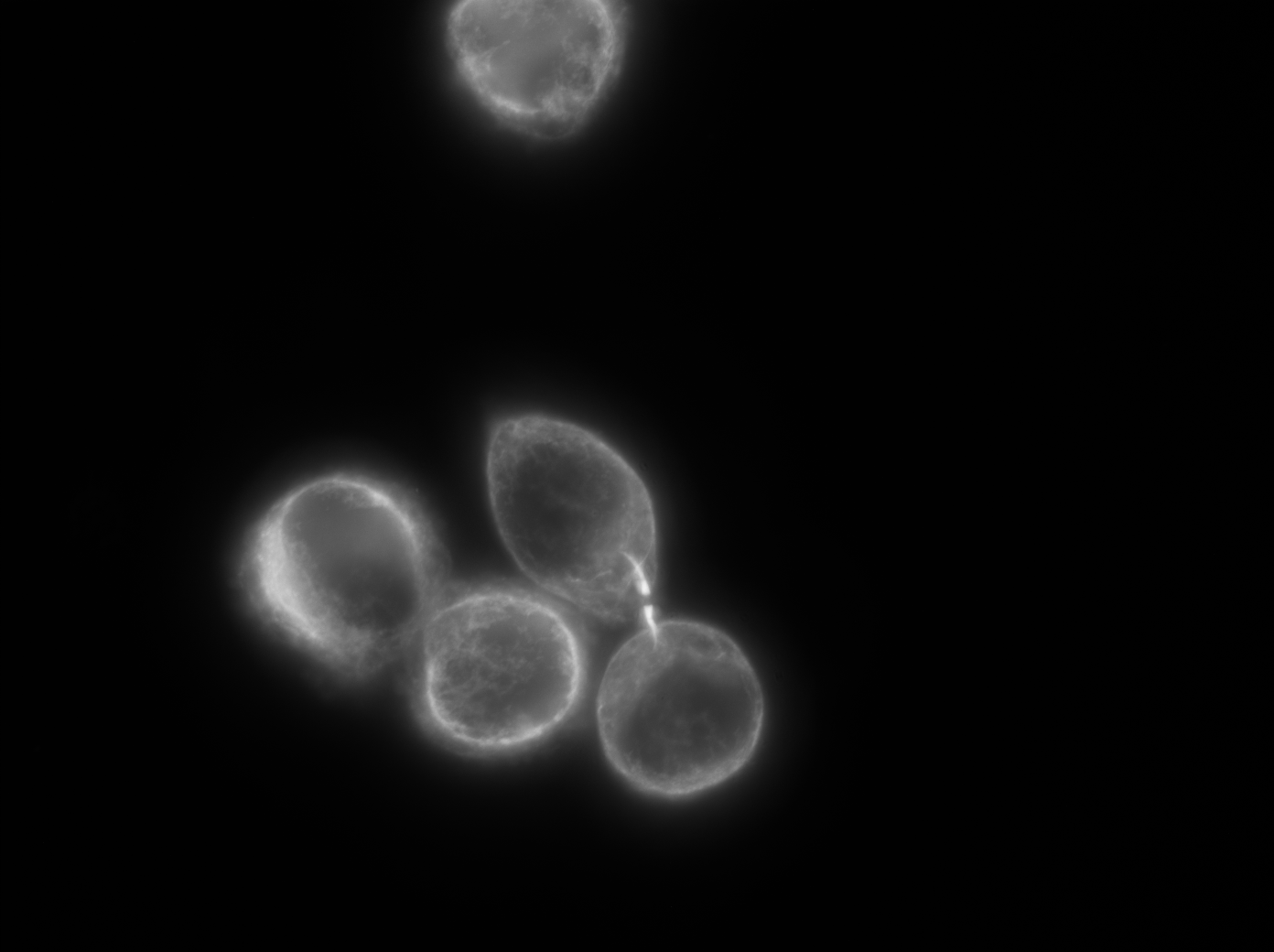

Supplement: Supplementary file 21 — Source data Fig. 6 part 2 [file 44319_2026_742_MOESM21_ESM.zip › Figure 6 Part 2/Fig 6abcd Cas9 TPGS1-KO acetylated tubulin atubulin part 2/TPGS1-KO R2 9-11-24 LT11.Project Maximum Z_XY1726262113_Z0_T0_C1.tif]

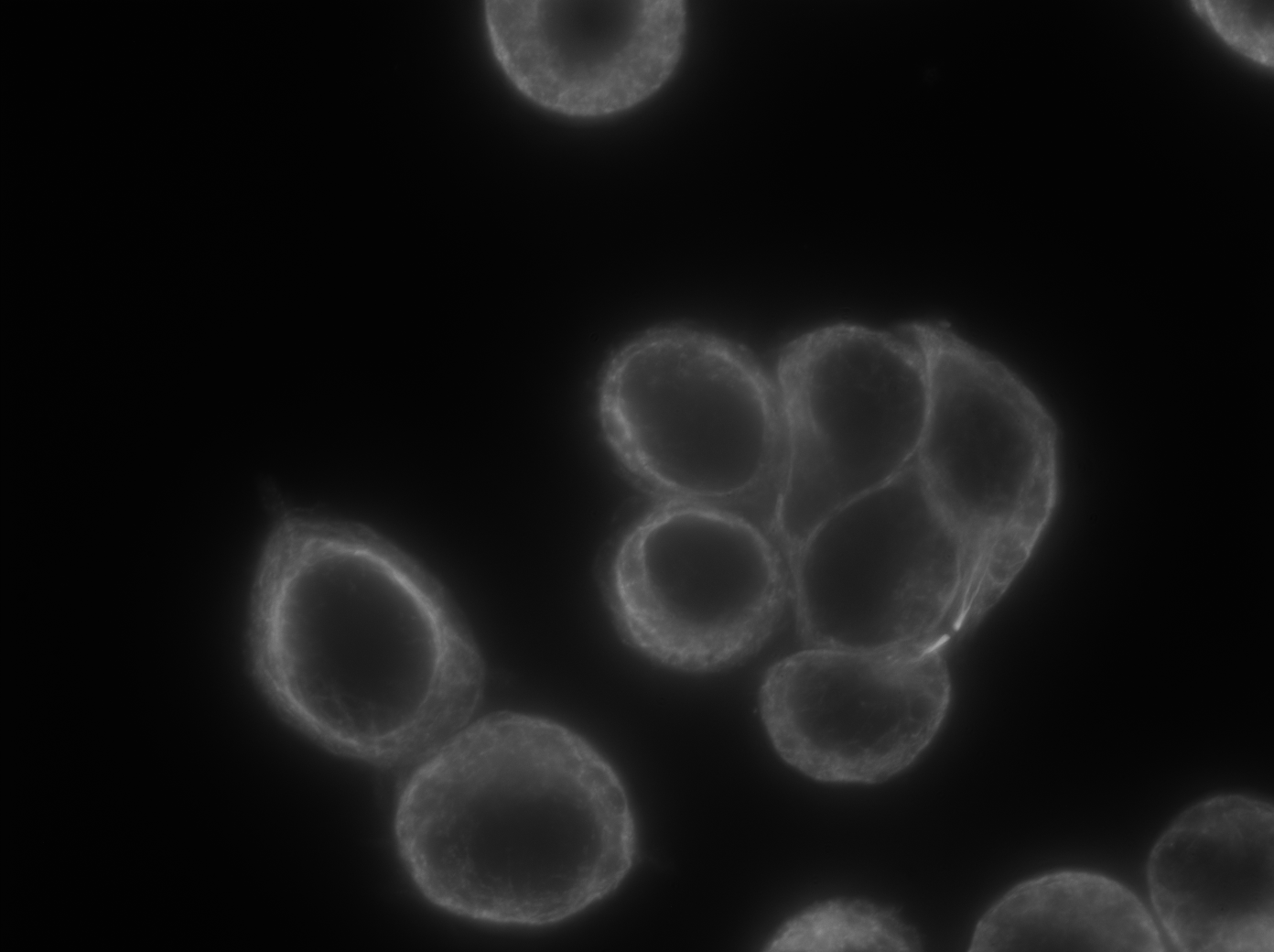

Supplement: Supplementary file 21 — Source data Fig. 6 part 2 [file 44319_2026_742_MOESM21_ESM.zip › Figure 6 Part 2/Fig 6abcd Cas9 TPGS1-KO acetylated tubulin atubulin part 2/TPGS1-KO R2 9-11-24 LT16 PA15.Project Maximum Z_XY1726265624_Z0_T0_C1.tif]

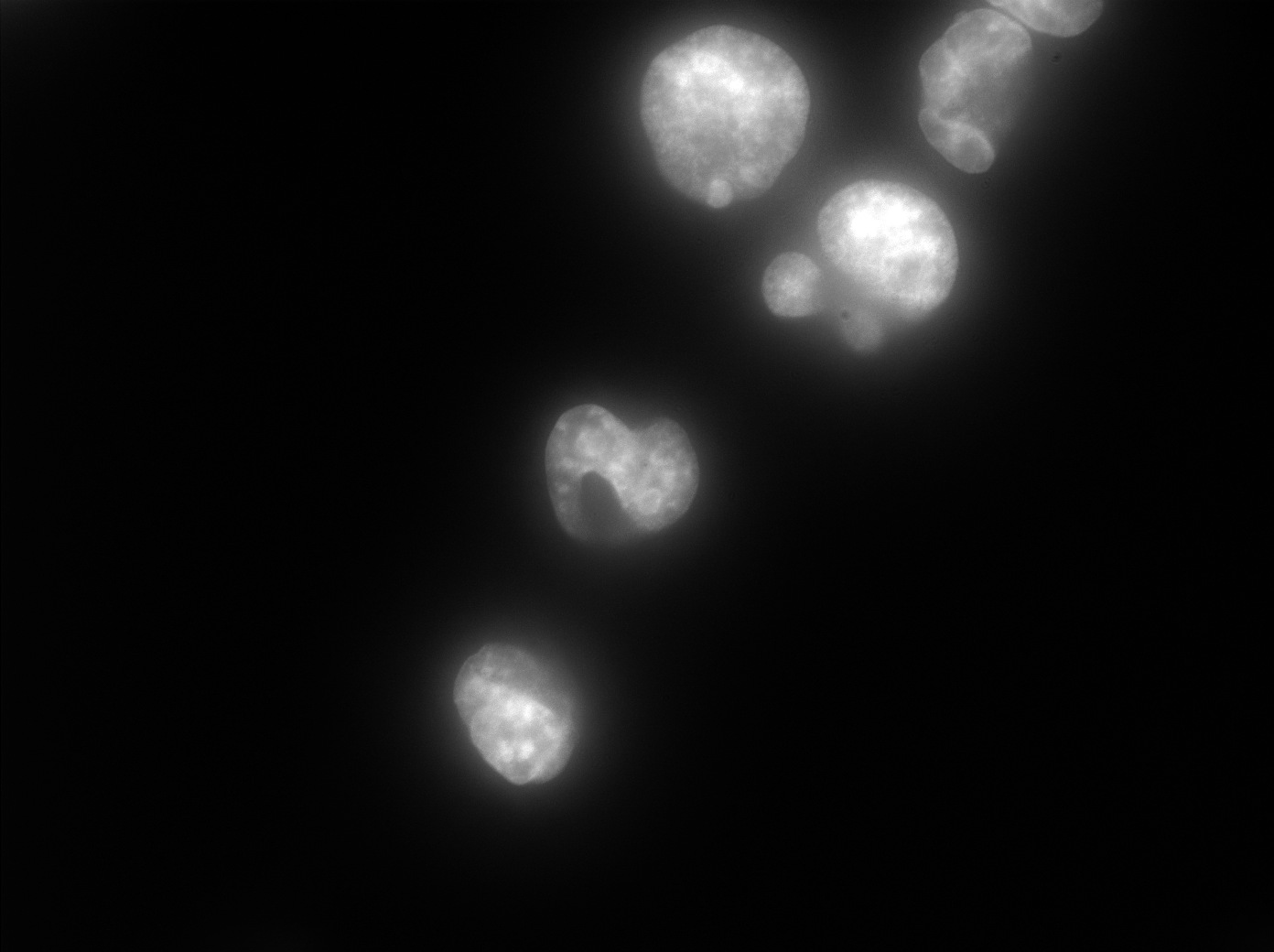

Supplement: Supplementary file 21 — Source data Fig. 6 part 2 [file 44319_2026_742_MOESM21_ESM.zip › Figure 6 Part 2/Fig 6abcd Cas9 TPGS1-KO acetylated tubulin atubulin part 2/TPGS1-KO R2 9-11-24 LT12.Project Maximum Z_XY1726262255_Z0_T0_C0.tif]

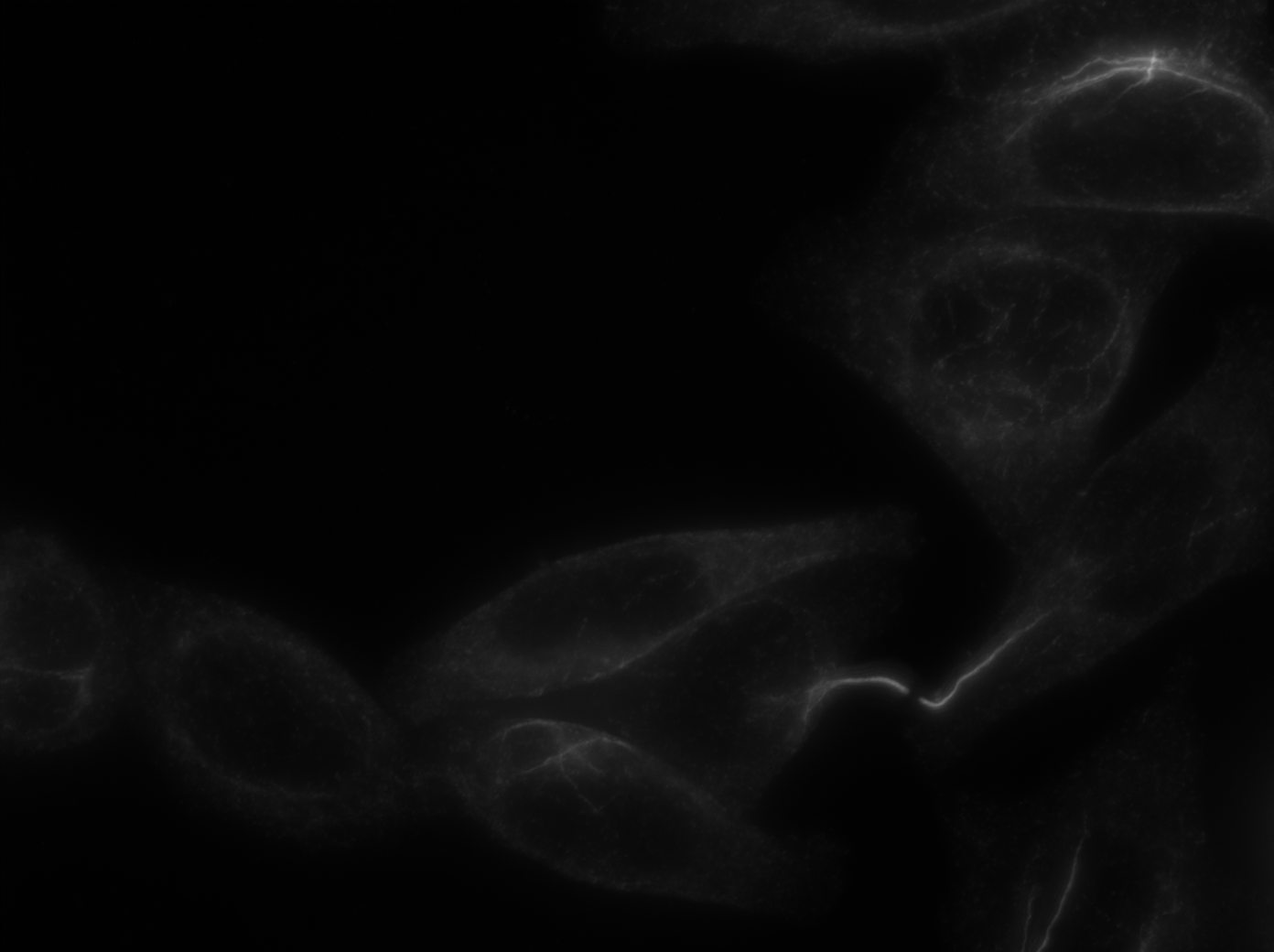

Supplement: Supplementary file 21 — Source data Fig. 6 part 2 [file 44319_2026_742_MOESM21_ESM.zip › Figure 6 Part 2/Fig 6abcd Cas9 TPGS1-KO acetylated tubulin atubulin part 2/TPGS1-KO R3 9-13-24 LT7.Project Maximum Z_XY1726760685_Z0_T0_C2.tif]

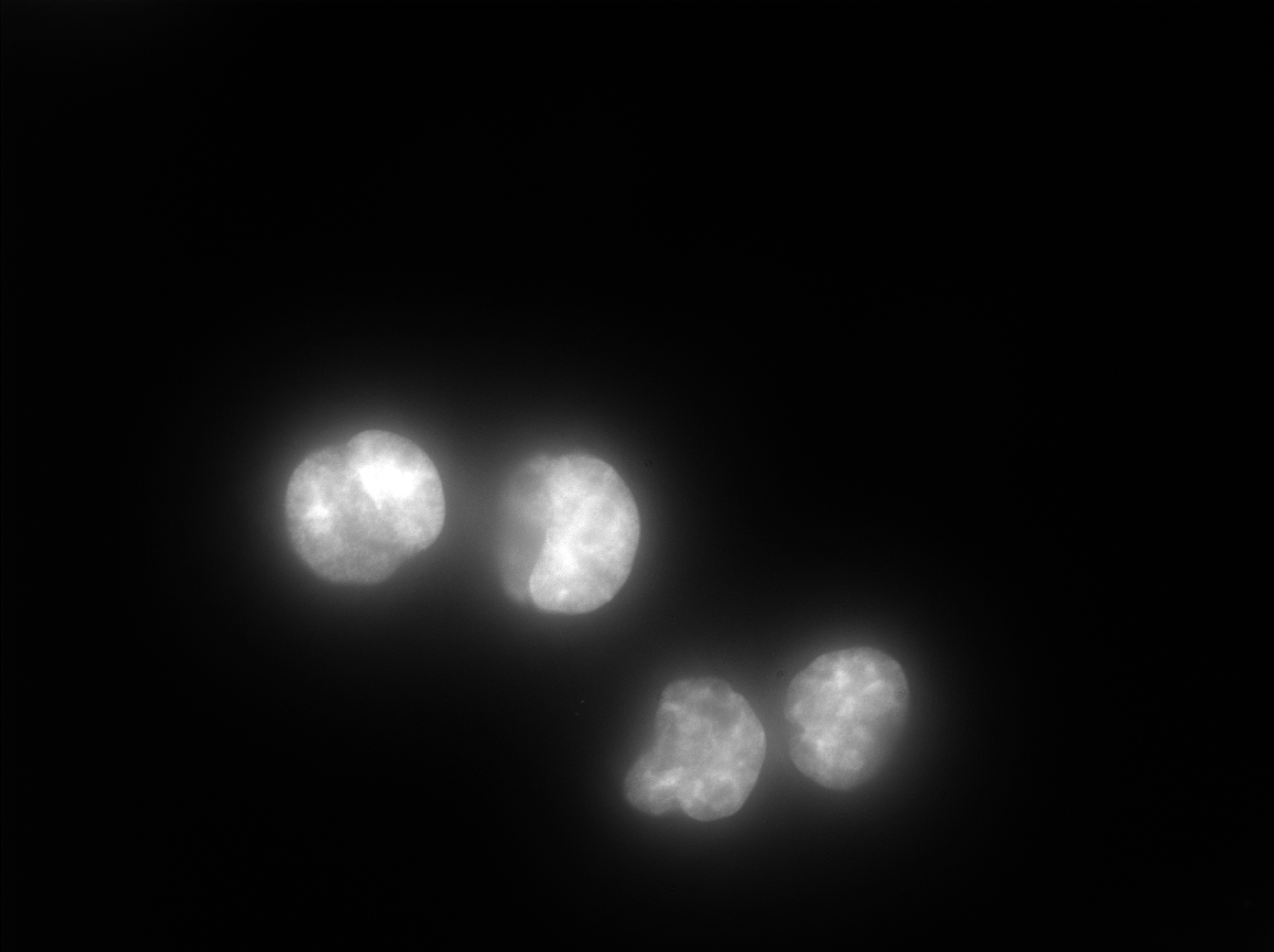

Supplement: Supplementary file 21 — Source data Fig. 6 part 2 [file 44319_2026_742_MOESM21_ESM.zip › Figure 6 Part 2/Fig 6abcd Cas9 TPGS1-KO acetylated tubulin atubulin part 2/TPGS1-KO R2 9-11-24 PA14.Project Maximum Z_XY1726265426_Z0_T0_C0.tif]

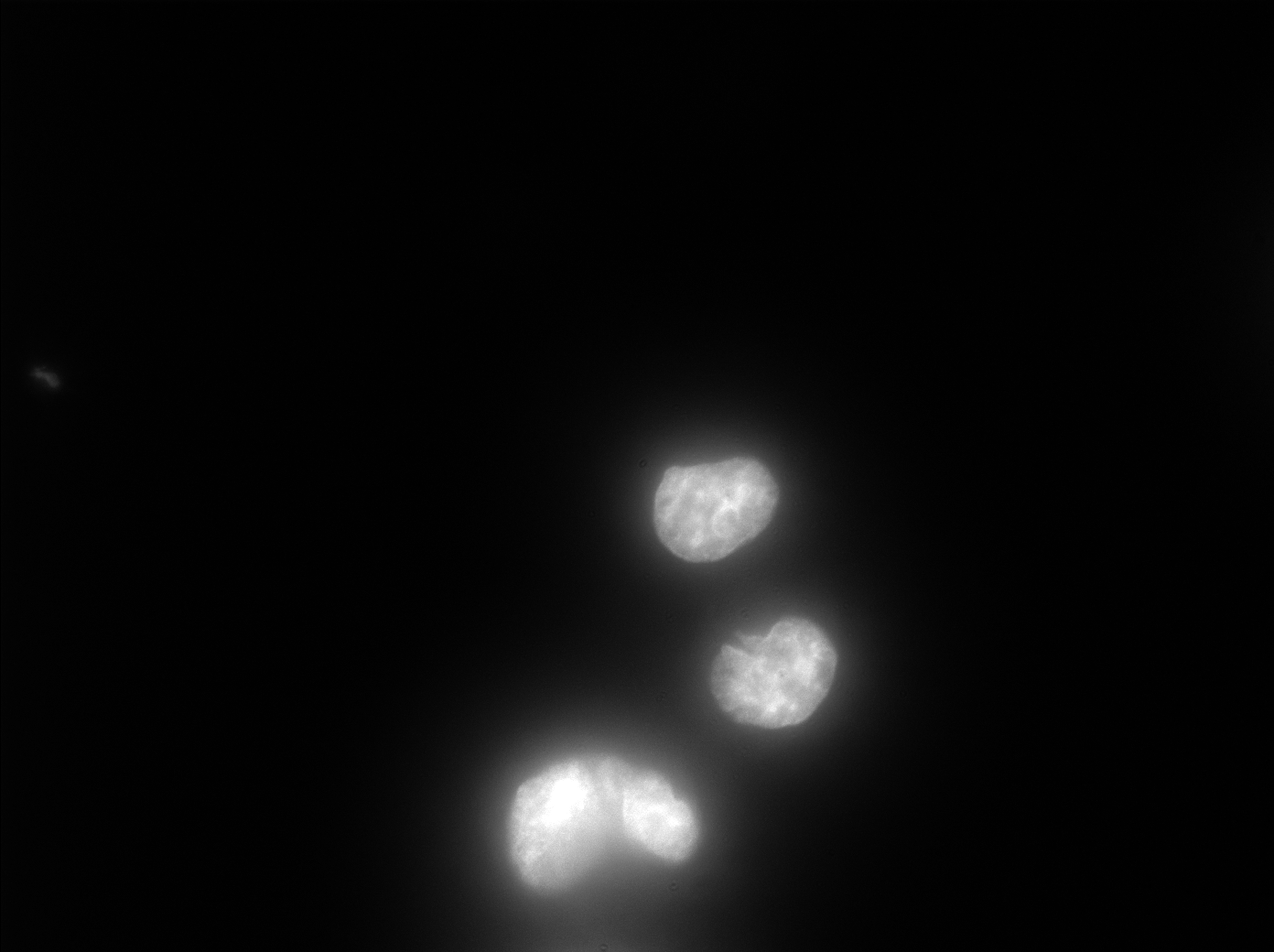

Supplement: Supplementary file 21 — Source data Fig. 6 part 2 [file 44319_2026_742_MOESM21_ESM.zip › Figure 6 Part 2/Fig 6abcd Cas9 TPGS1-KO acetylated tubulin atubulin part 2/TPGS1-KO R2 9-11-24 LT5.Project Maximum Z_XY1726259988_Z0_T0_C0.tif]

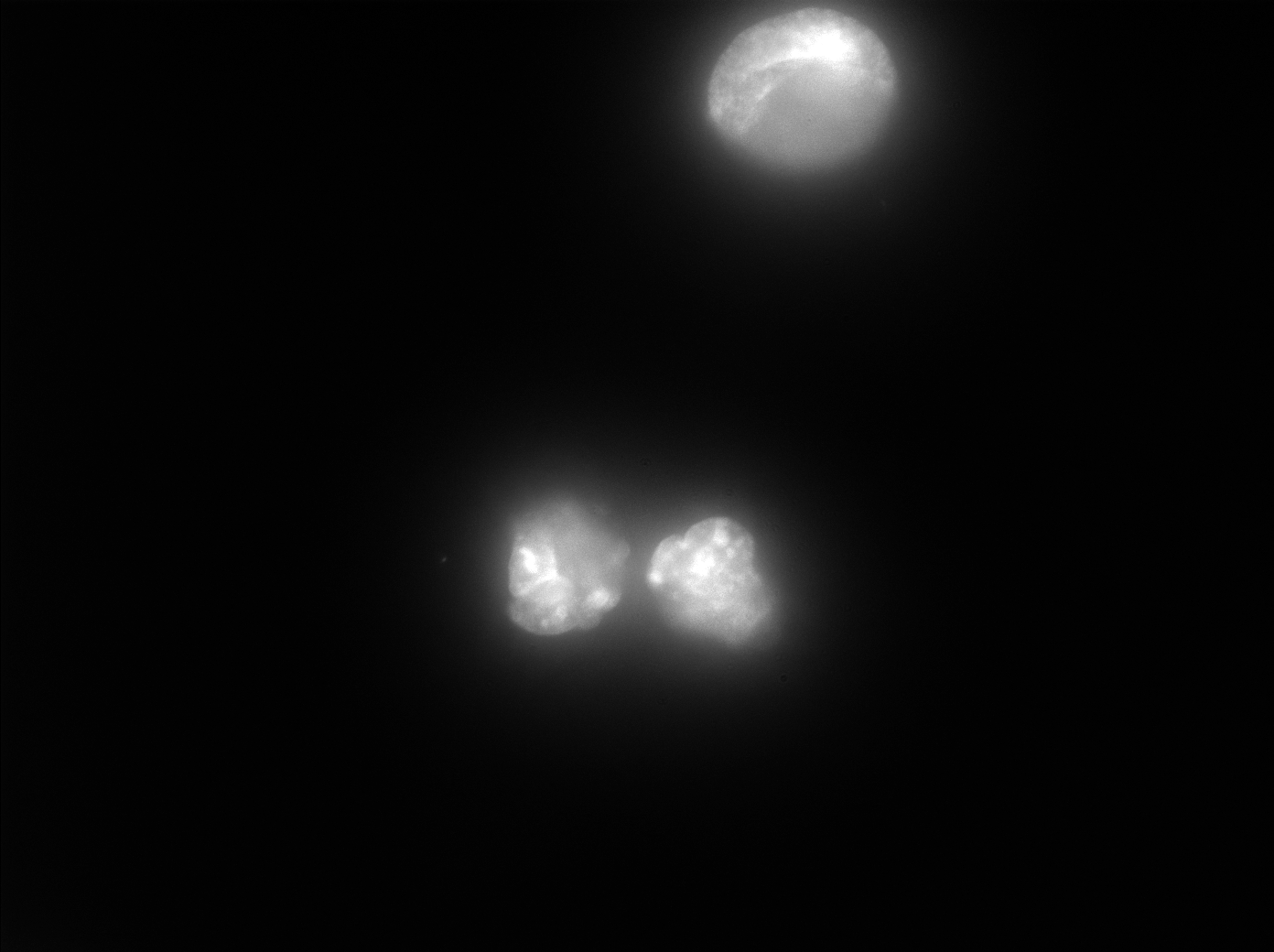

Supplement: Supplementary file 21 — Source data Fig. 6 part 2 [file 44319_2026_742_MOESM21_ESM.zip › Figure 6 Part 2/Fig 6abcd Cas9 TPGS1-KO acetylated tubulin atubulin part 2/TPGS1-KO R2 9-11-24 LT28.Project Maximum Z_XY1726269506_Z0_T0_C0.tif]

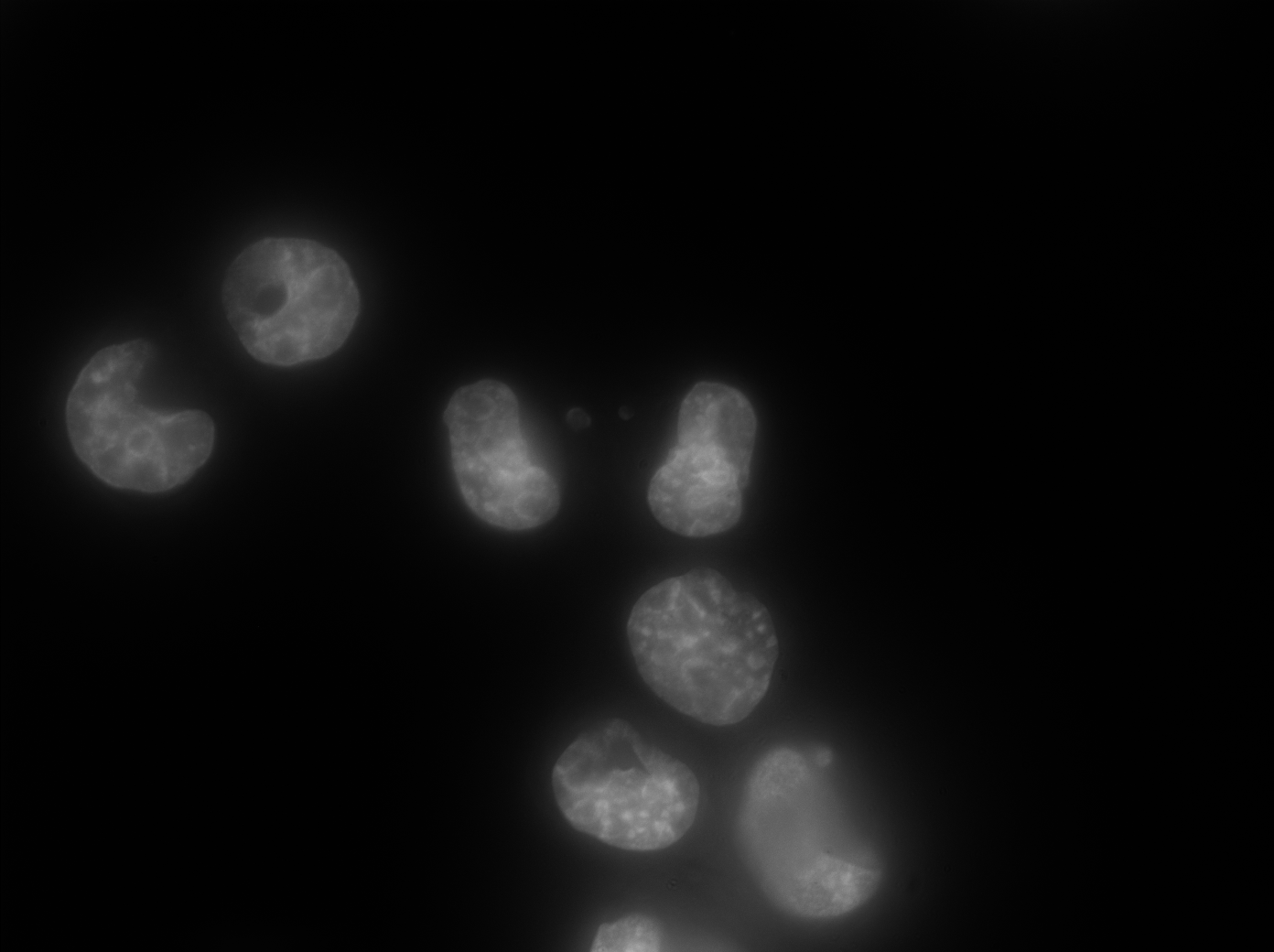

Supplement: Supplementary file 21 — Source data Fig. 6 part 2 [file 44319_2026_742_MOESM21_ESM.zip › Figure 6 Part 2/Fig 6abcd Cas9 TPGS1-KO acetylated tubulin atubulin part 2/TPGS1-KO R3 9-13-24 LT17LT18.Project Maximum Z_XY1726763998_Z0_T0_C0.tif]

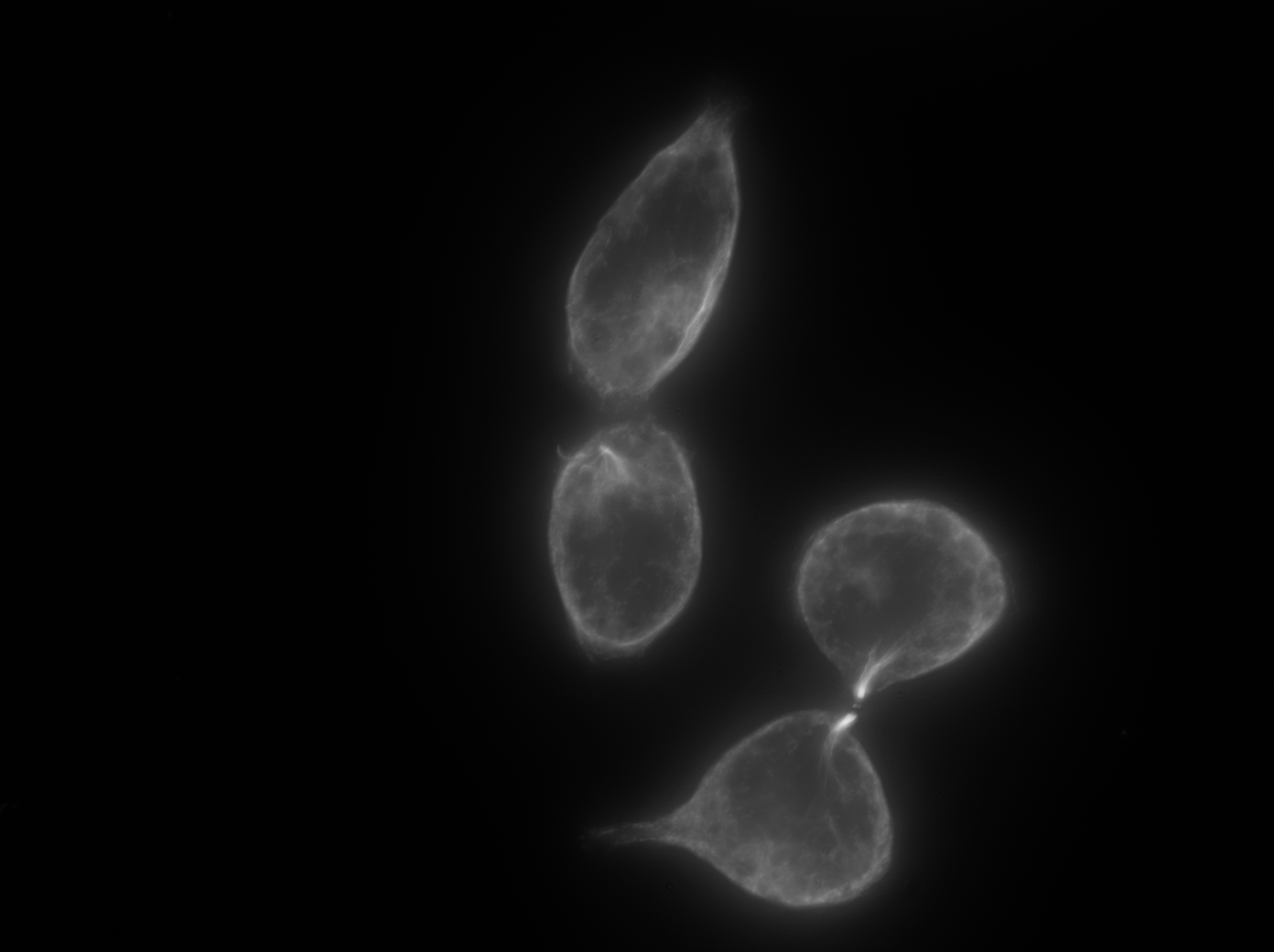

Supplement: Supplementary file 21 — Source data Fig. 6 part 2 [file 44319_2026_742_MOESM21_ESM.zip › Figure 6 Part 2/Fig 6abcd Cas9 TPGS1-KO acetylated tubulin atubulin part 2/TPGS1-KO R2 9-11-24 LT27 PA20.Project Maximum Z_XY1726269402_Z0_T0_C1.tif]

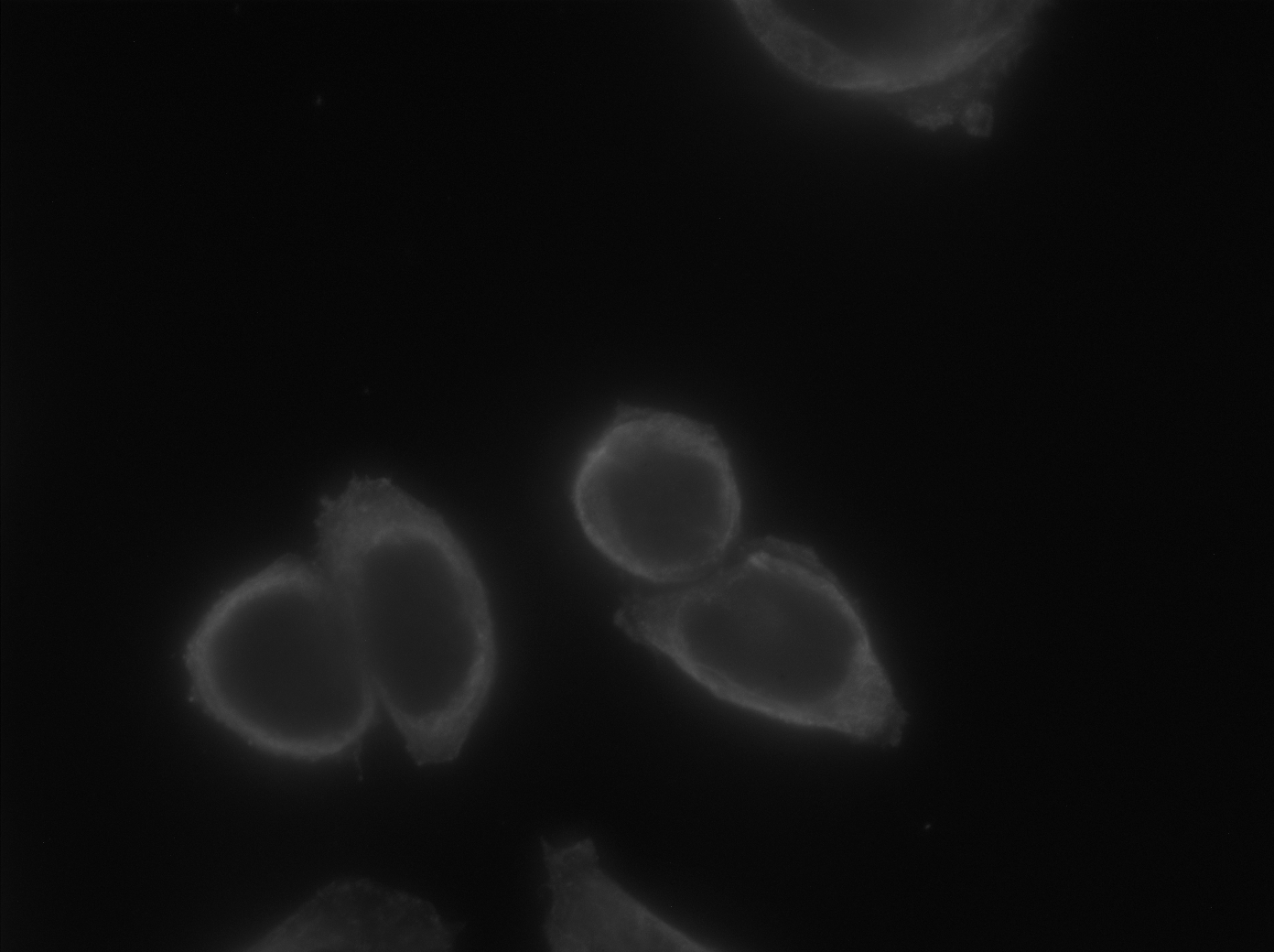

Supplement: Supplementary file 21 — Source data Fig. 6 part 2 [file 44319_2026_742_MOESM21_ESM.zip › Figure 6 Part 2/Fig 6abcd Cas9 TPGS1-KO acetylated tubulin atubulin part 2/TPGS1-KO R3 9-13-24 LT1.Project Maximum Z_XY1726759987_Z0_T0_C1.tif]

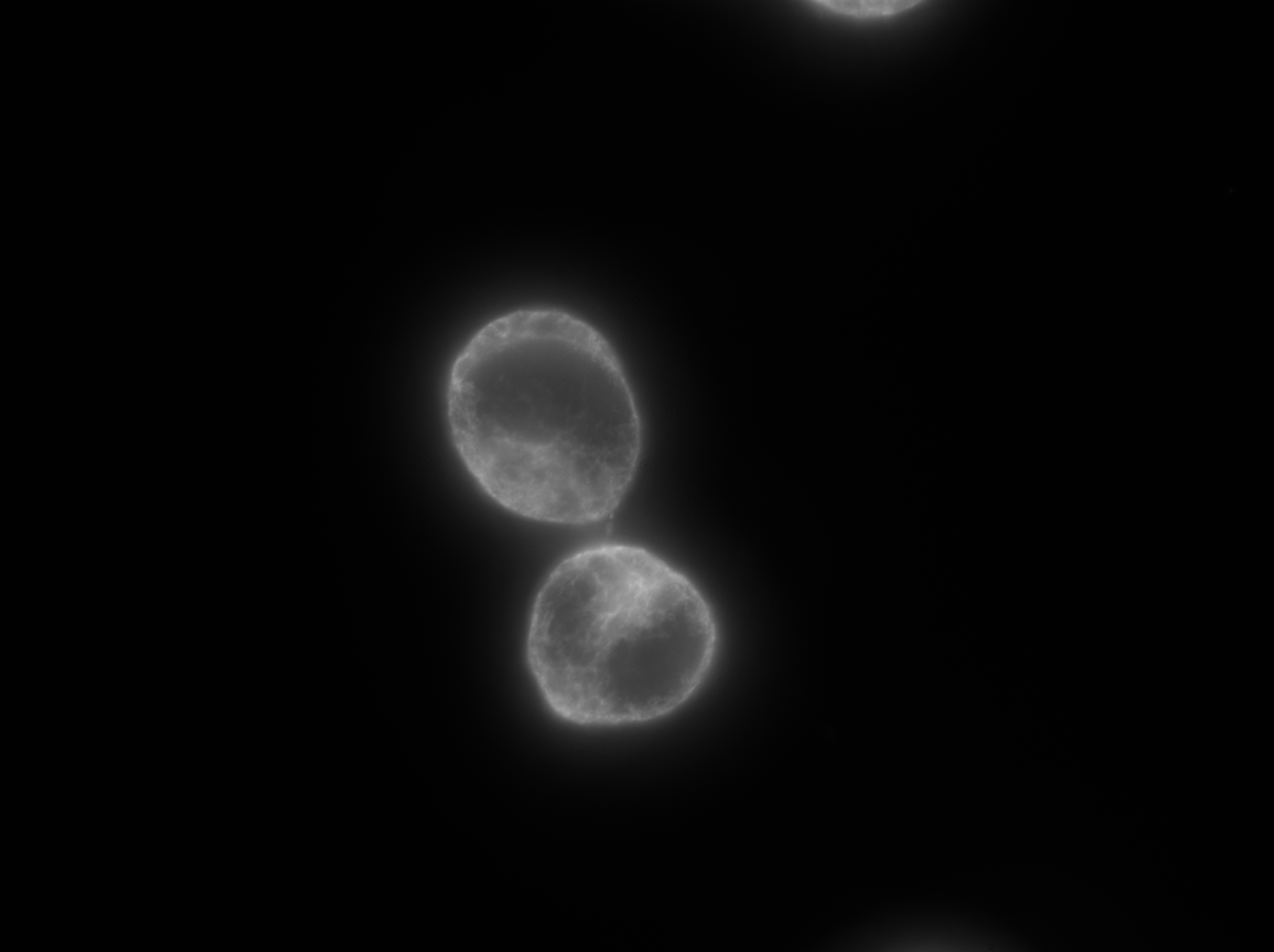

Supplement: Supplementary file 21 — Source data Fig. 6 part 2 [file 44319_2026_742_MOESM21_ESM.zip › Figure 6 Part 2/Fig 6abcd Cas9 TPGS1-KO acetylated tubulin atubulin part 2/TPGS1-KO R2 9-11-24 PA10.Project Maximum Z_XY1726262600_Z0_T0_C1.tif]

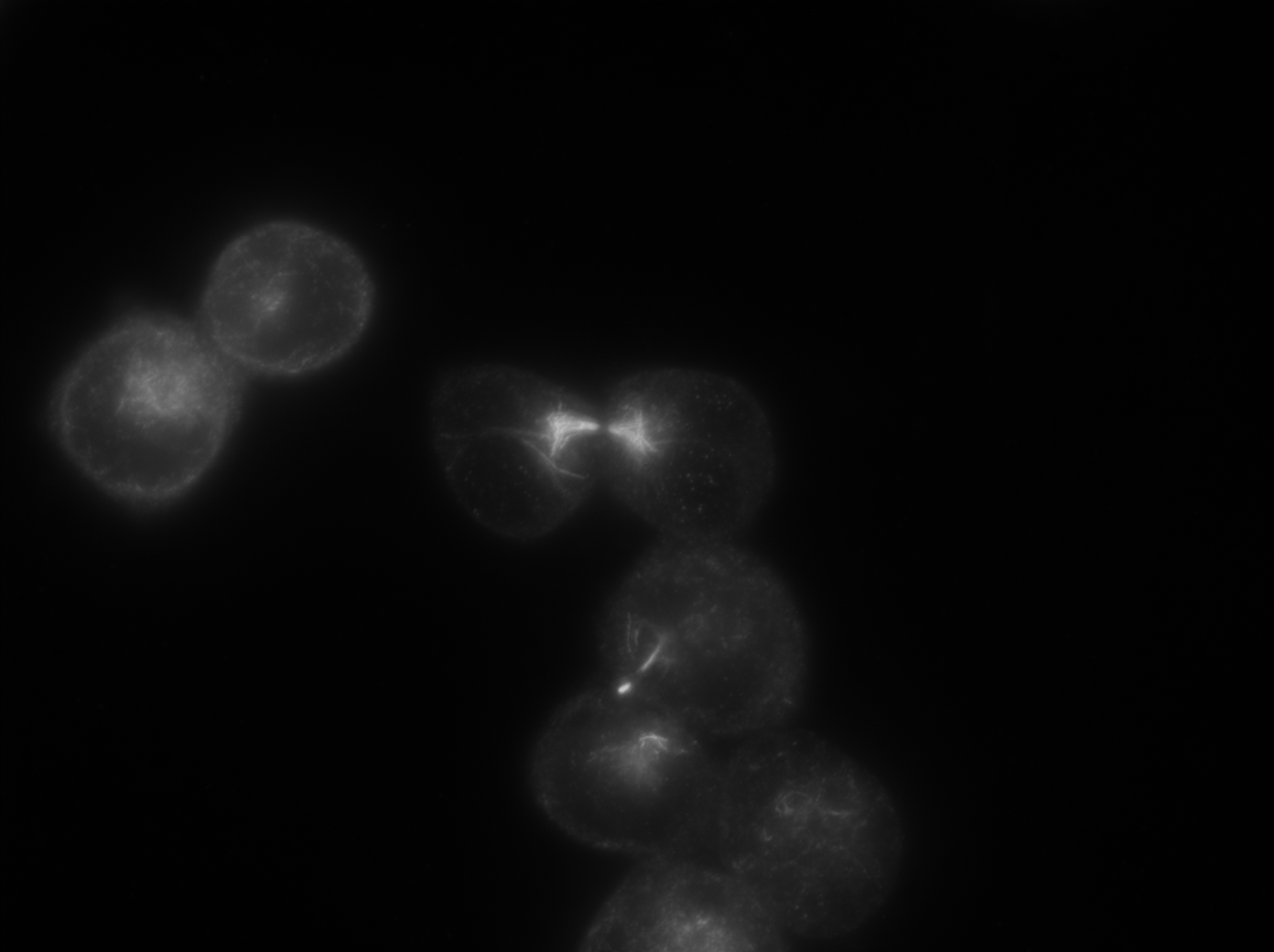

Supplement: Supplementary file 21 — Source data Fig. 6 part 2 [file 44319_2026_742_MOESM21_ESM.zip › Figure 6 Part 2/Fig 6abcd Cas9 TPGS1-KO acetylated tubulin atubulin part 2/TPGS1-KO R3 9-13-24 LT17LT18.Project Maximum Z_XY1726763998_Z0_T0_C2.tif]

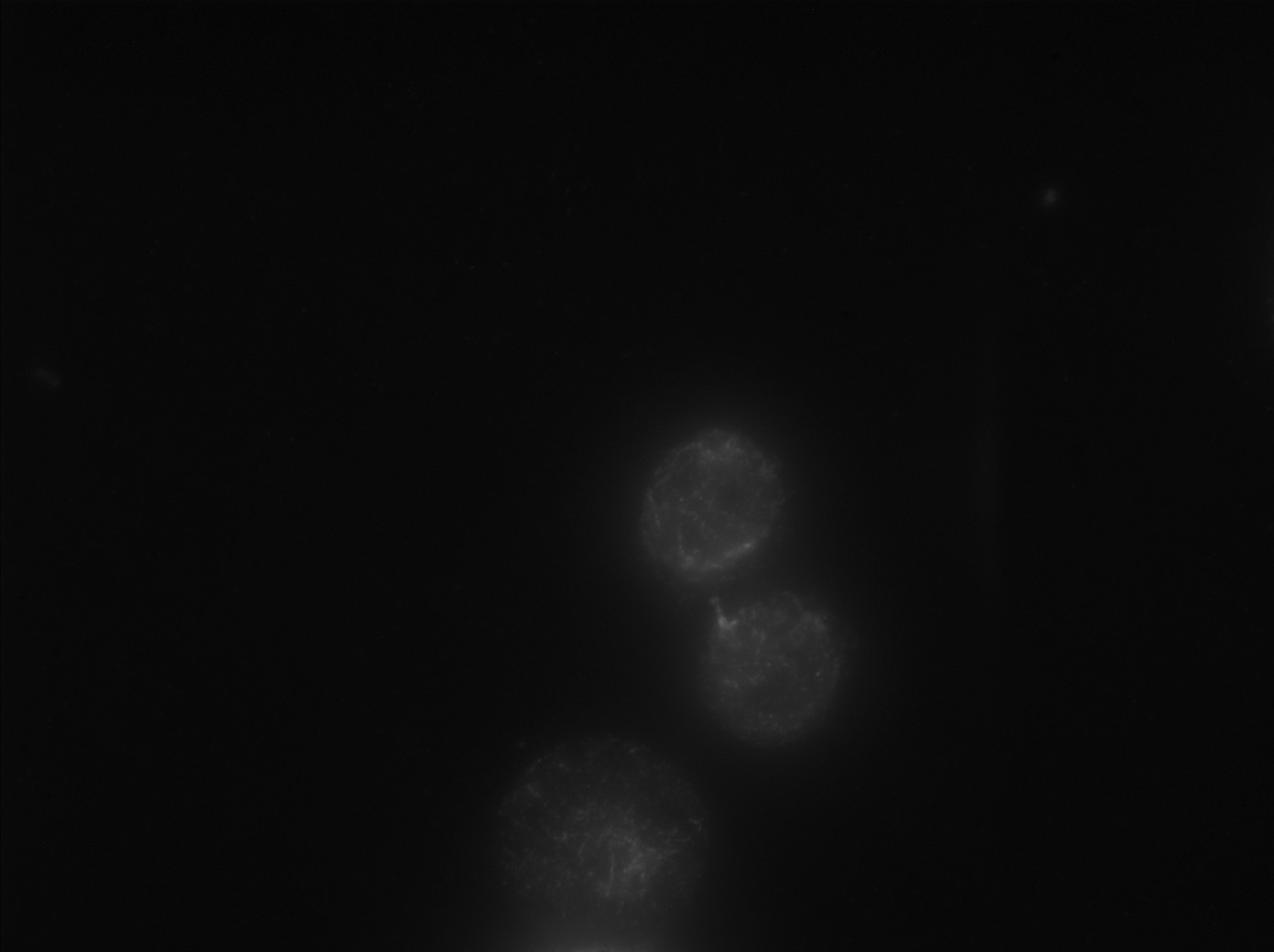

Supplement: Supplementary file 21 — Source data Fig. 6 part 2 [file 44319_2026_742_MOESM21_ESM.zip › Figure 6 Part 2/Fig 6abcd Cas9 TPGS1-KO acetylated tubulin atubulin part 2/TPGS1-KO R2 9-11-24 LT5.Project Maximum Z_XY1726259988_Z0_T0_C2.tif]

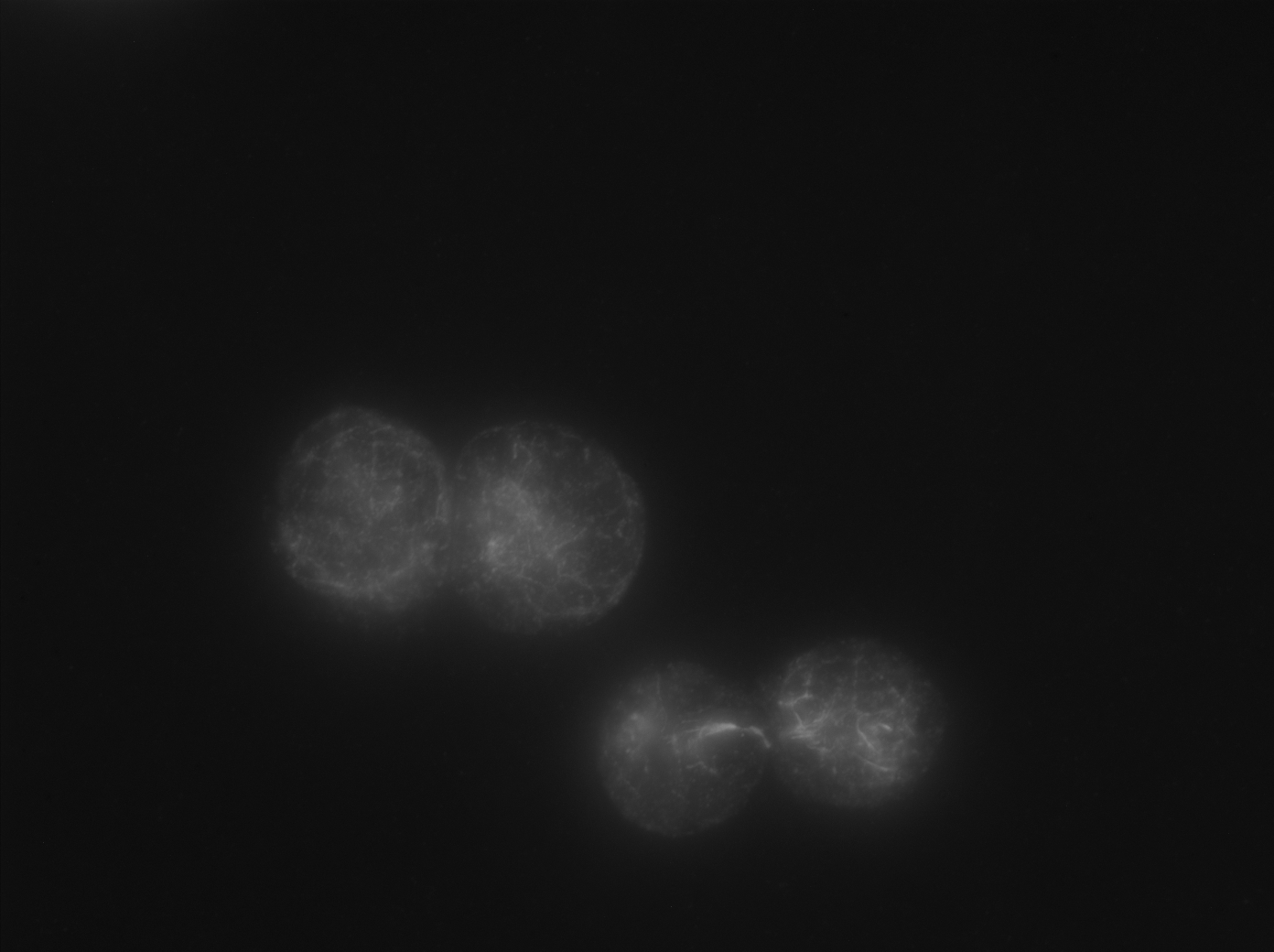

Supplement: Supplementary file 21 — Source data Fig. 6 part 2 [file 44319_2026_742_MOESM21_ESM.zip › Figure 6 Part 2/Fig 6abcd Cas9 TPGS1-KO acetylated tubulin atubulin part 2/TPGS1-KO R2 9-11-24 PA14.Project Maximum Z_XY1726265426_Z0_T0_C2.tif]

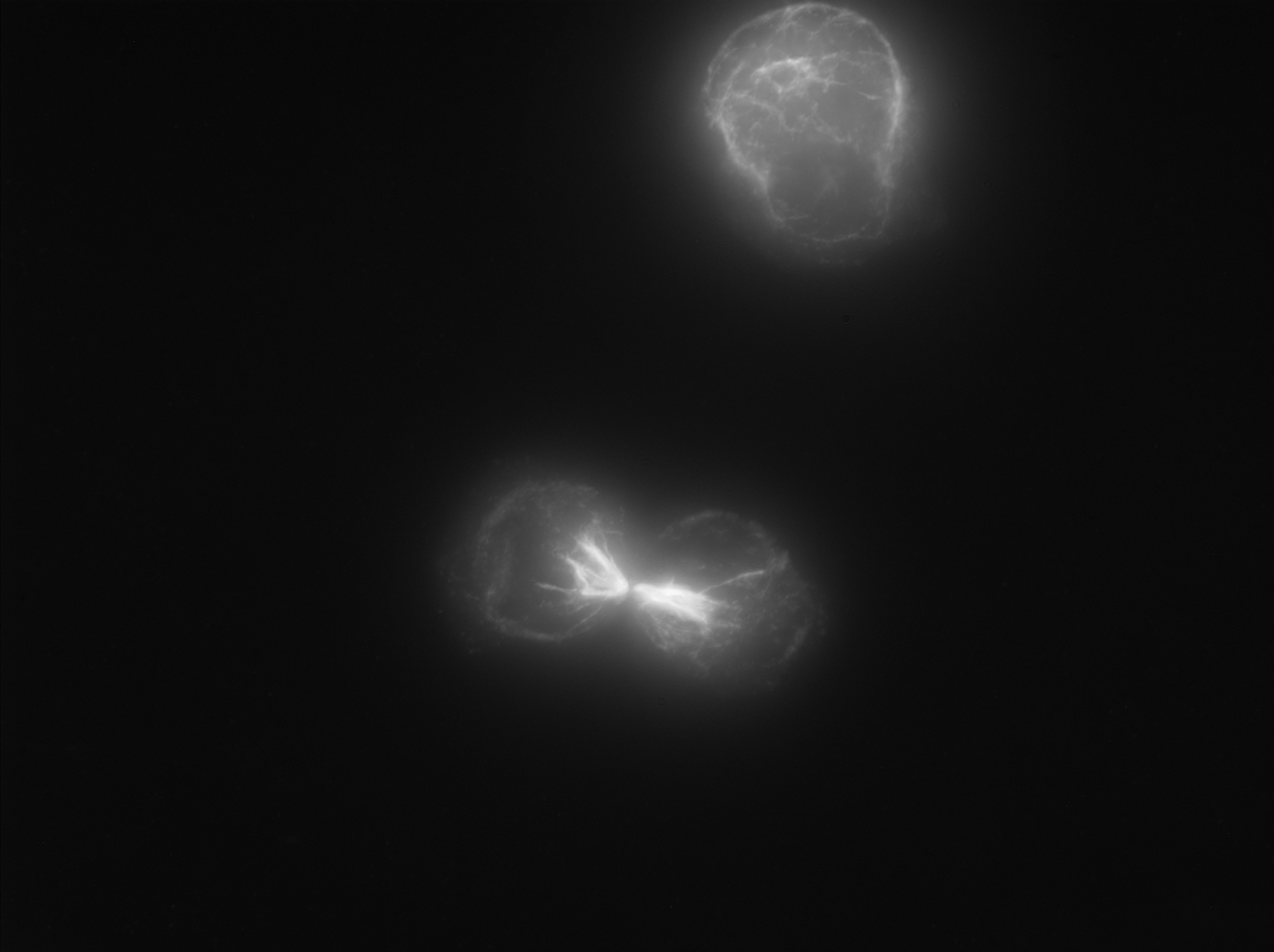

Supplement: Supplementary file 21 — Source data Fig. 6 part 2 [file 44319_2026_742_MOESM21_ESM.zip › Figure 6 Part 2/Fig 6abcd Cas9 TPGS1-KO acetylated tubulin atubulin part 2/TPGS1-KO R2 9-11-24 LT28.Project Maximum Z_XY1726269506_Z0_T0_C2.tif]

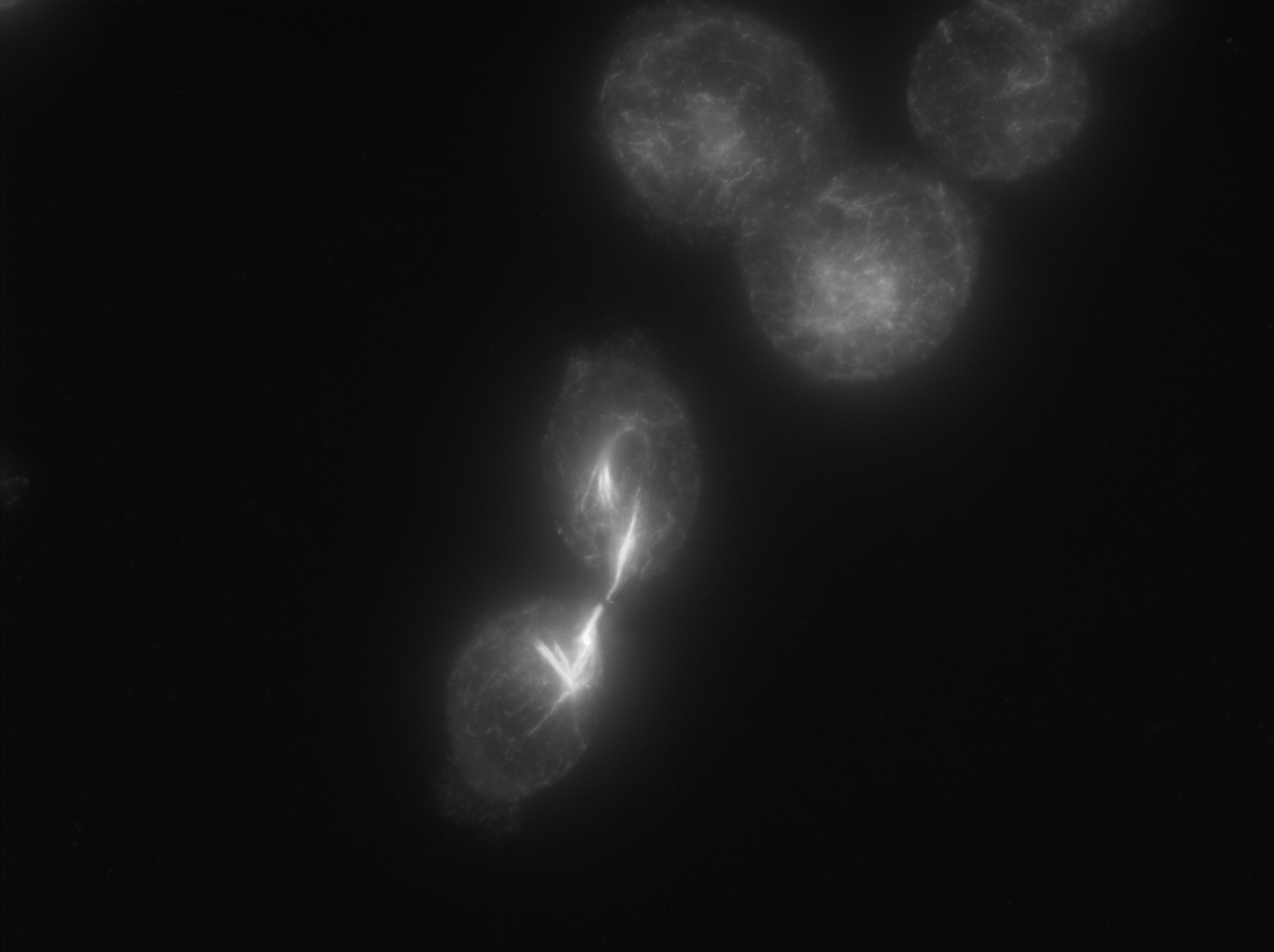

Supplement: Supplementary file 21 — Source data Fig. 6 part 2 [file 44319_2026_742_MOESM21_ESM.zip › Figure 6 Part 2/Fig 6abcd Cas9 TPGS1-KO acetylated tubulin atubulin part 2/TPGS1-KO R2 9-11-24 LT12.Project Maximum Z_XY1726262255_Z0_T0_C2.tif]

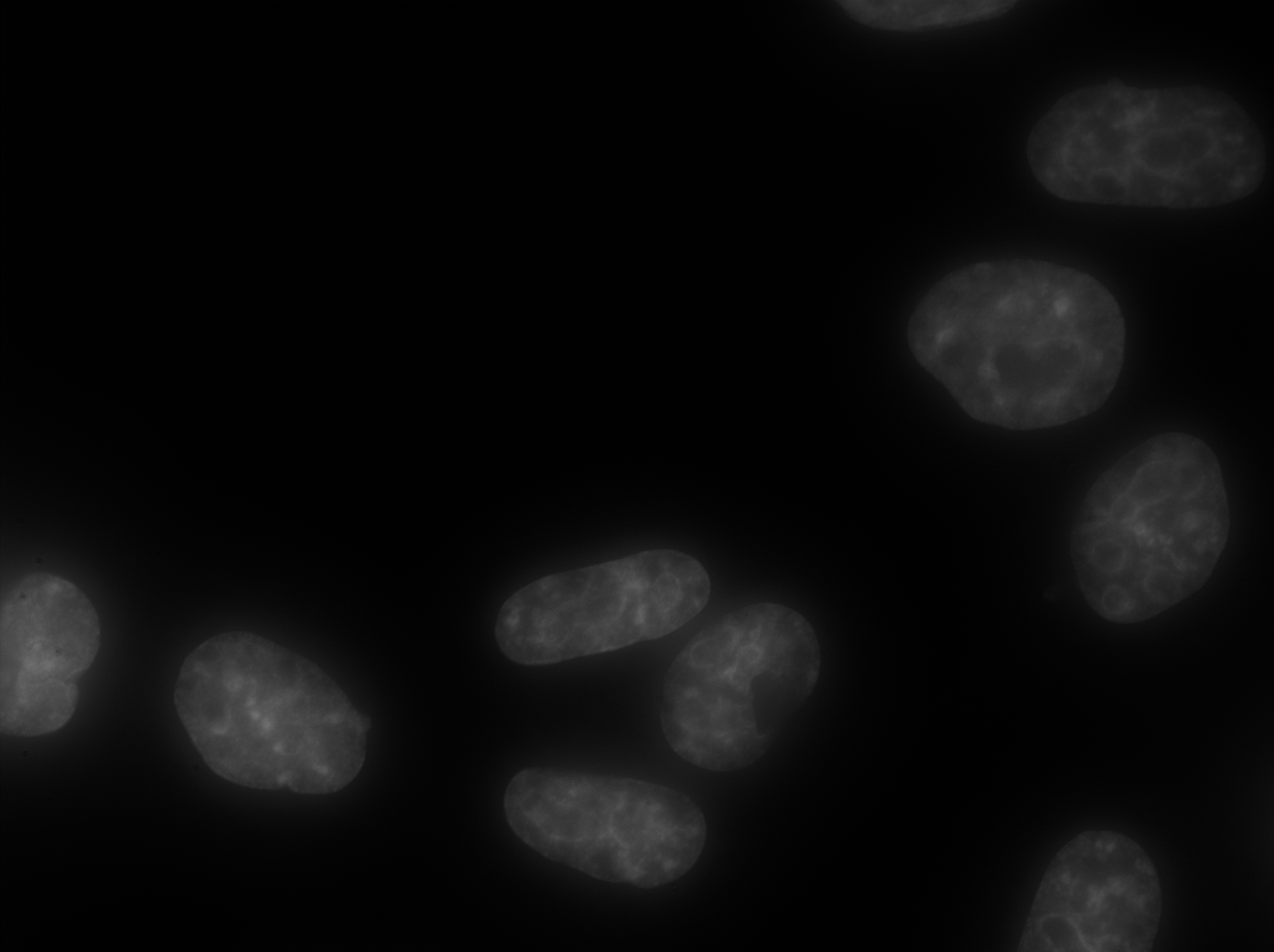

Supplement: Supplementary file 21 — Source data Fig. 6 part 2 [file 44319_2026_742_MOESM21_ESM.zip › Figure 6 Part 2/Fig 6abcd Cas9 TPGS1-KO acetylated tubulin atubulin part 2/TPGS1-KO R3 9-13-24 LT7.Project Maximum Z_XY1726760685_Z0_T0_C0.tif]

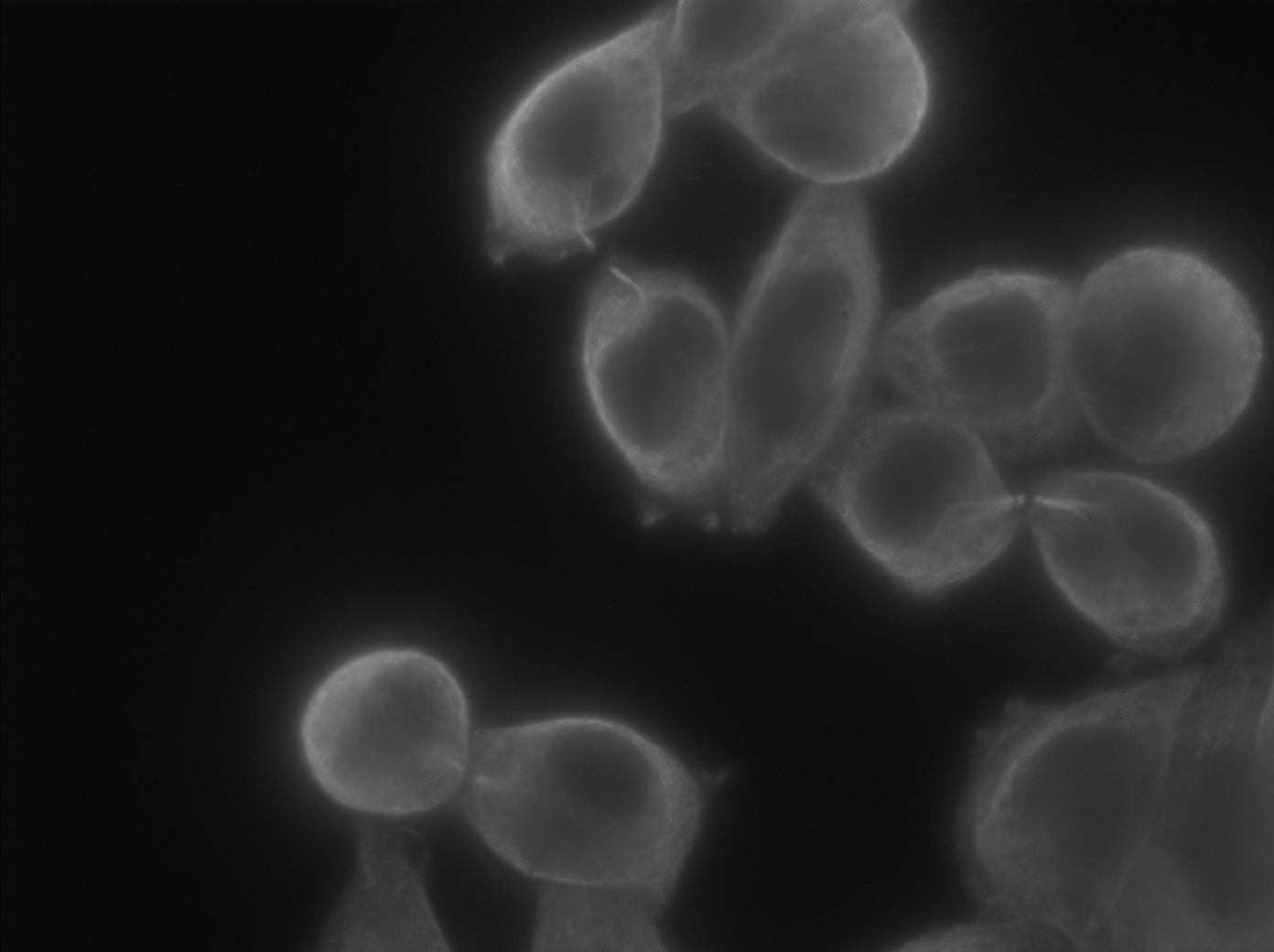

Supplement: Supplementary file 21 — Source data Fig. 6 part 2 [file 44319_2026_742_MOESM21_ESM.zip › Figure 6 Part 2/Fig 6abcd Cas9 TPGS1-KO acetylated tubulin atubulin part 2/TPGS1-KO R3 9-13-24 LT13LT14LT15.Project Maximum Z_XY1726763813_Z0_T0_C1.tif]

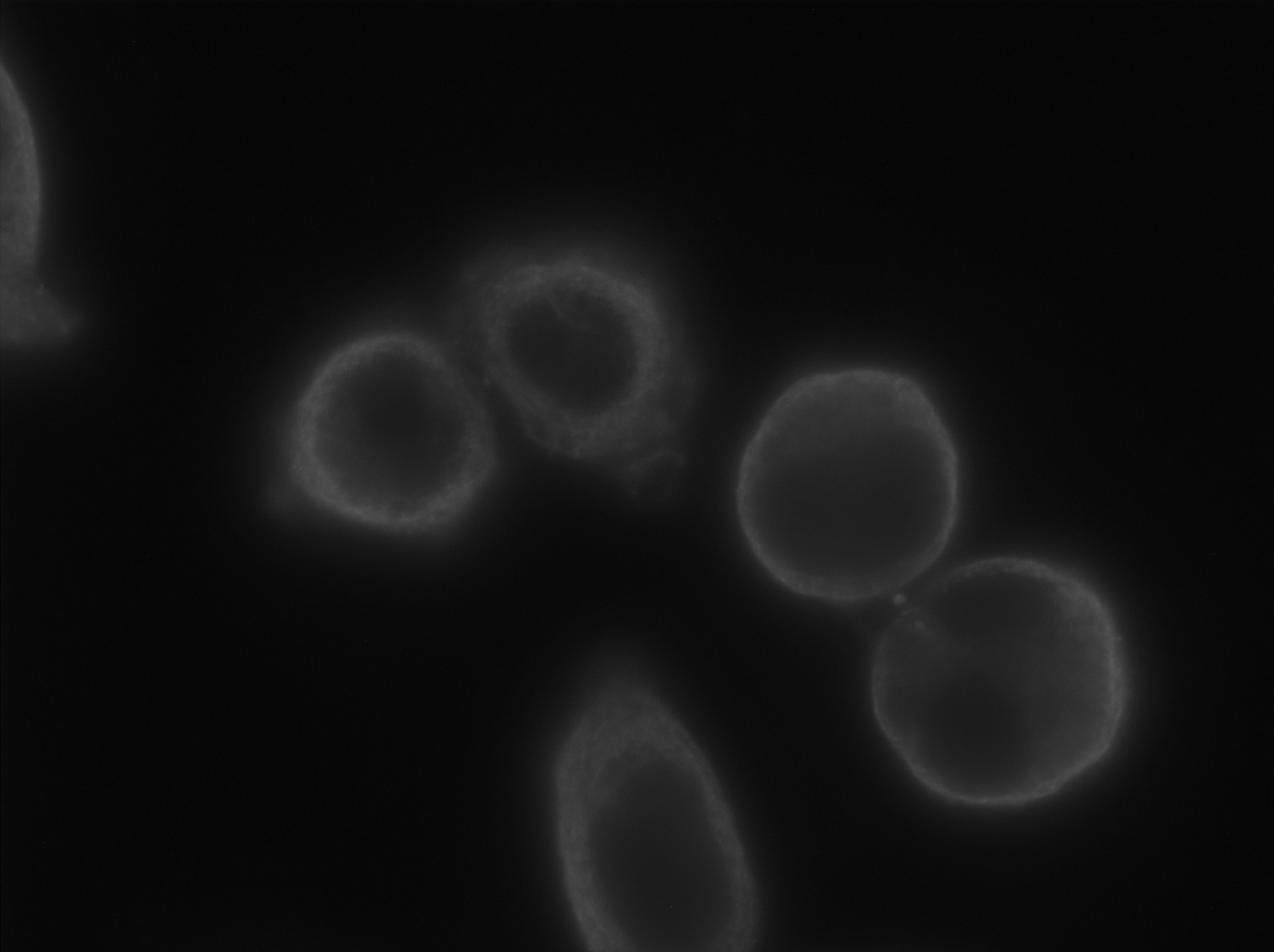

Supplement: Supplementary file 21 — Source data Fig. 6 part 2 [file 44319_2026_742_MOESM21_ESM.zip › Figure 6 Part 2/Fig 6abcd Cas9 TPGS1-KO acetylated tubulin atubulin part 2/TPGS1-KO R3 9-13-24 PA1PA2.Project Maximum Z_XY1726760183_Z0_T0_C1.tif]

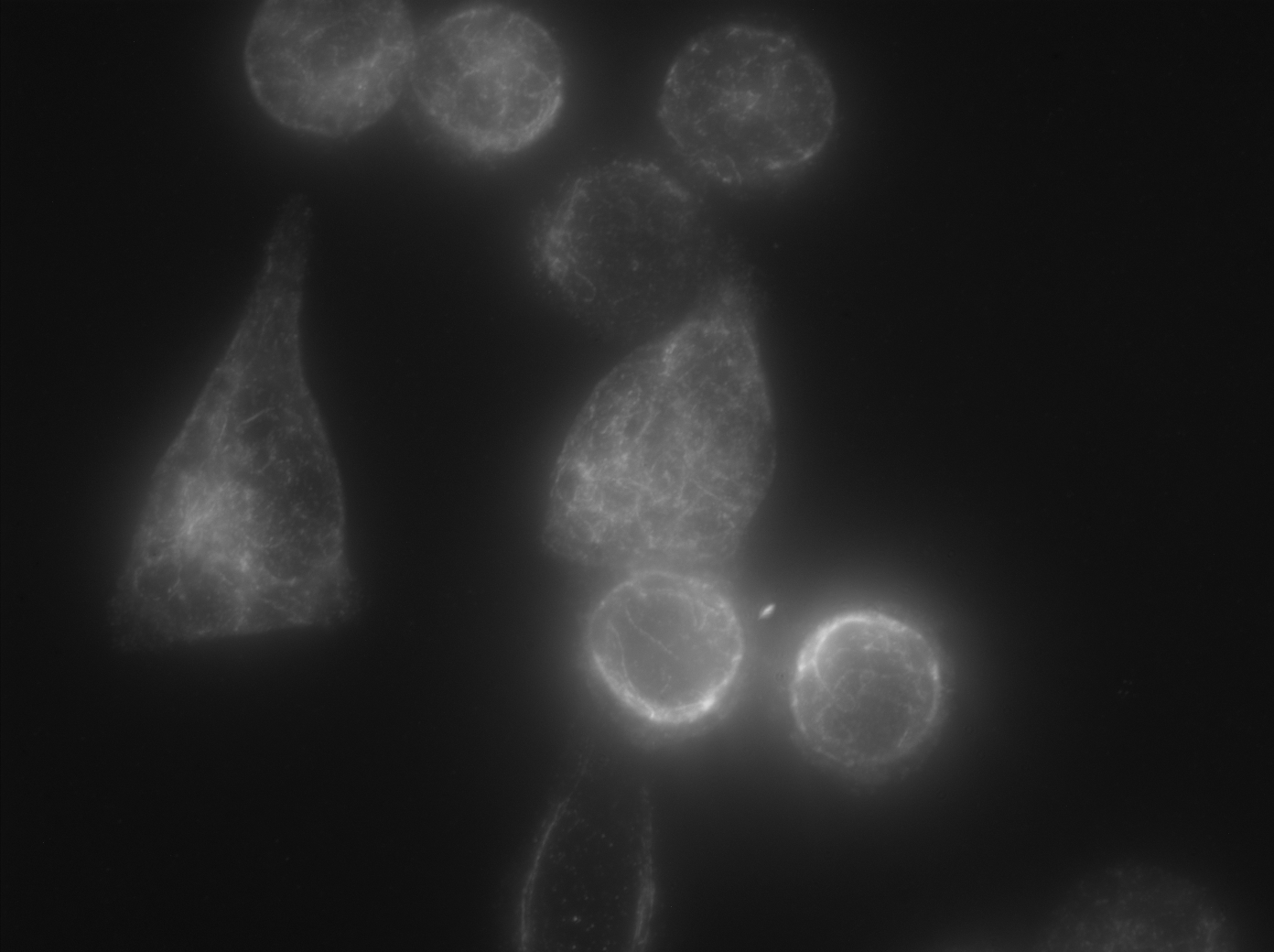

Supplement: Supplementary file 21 — Source data Fig. 6 part 2 [file 44319_2026_742_MOESM21_ESM.zip › Figure 6 Part 2/Fig 6abcd Cas9 TPGS1-KO acetylated tubulin atubulin part 2/TPGS1-KO R2 9-11-24 PA16.Project Maximum Z_XY1726266634_Z0_T0_C2.tif]

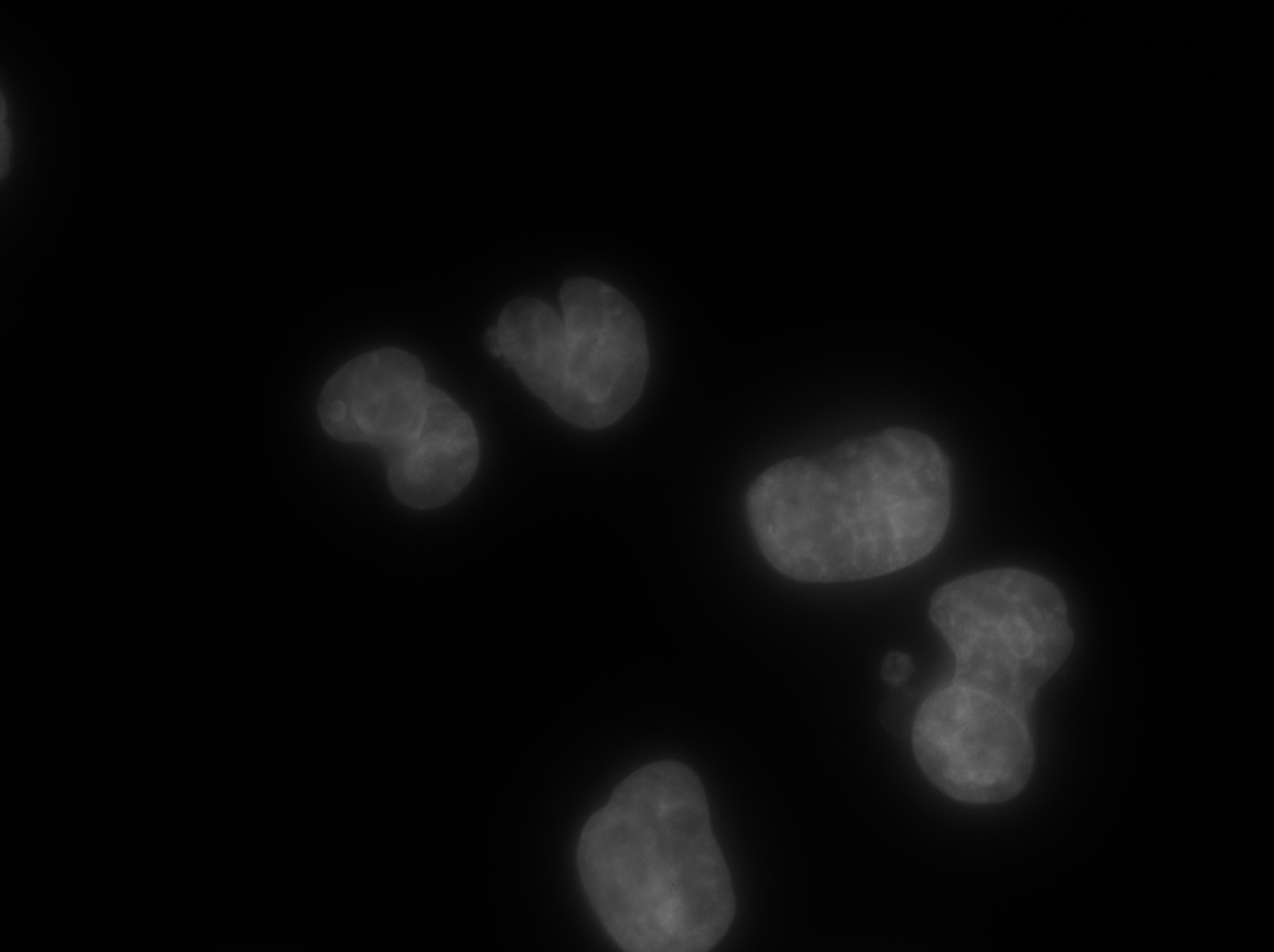

Supplement: Supplementary file 21 — Source data Fig. 6 part 2 [file 44319_2026_742_MOESM21_ESM.zip › Figure 6 Part 2/Fig 6abcd Cas9 TPGS1-KO acetylated tubulin atubulin part 2/TPGS1-KO R3 9-13-24 PA1PA2.Project Maximum Z_XY1726760183_Z0_T0_C0.tif]

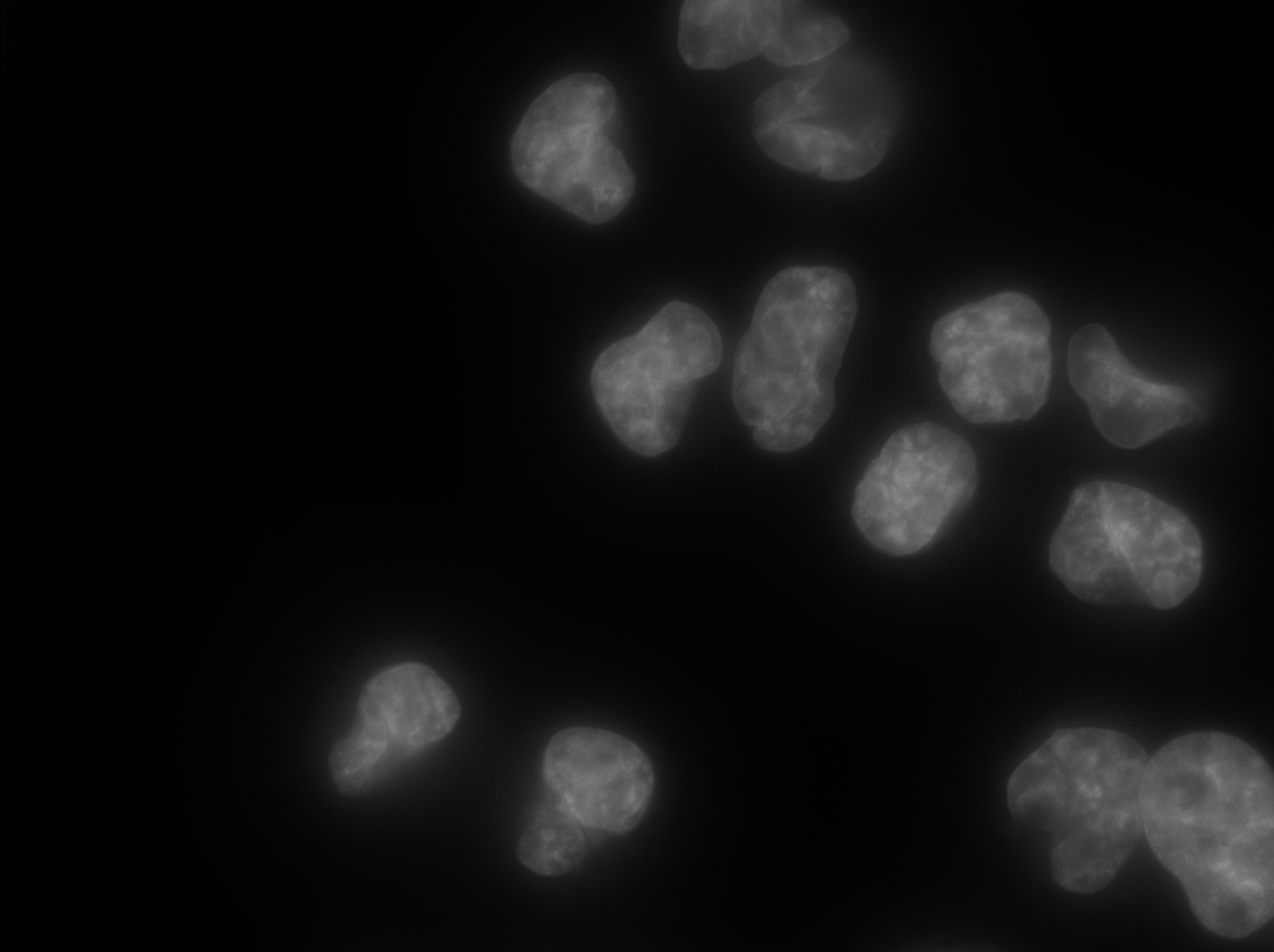

Supplement: Supplementary file 21 — Source data Fig. 6 part 2 [file 44319_2026_742_MOESM21_ESM.zip › Figure 6 Part 2/Fig 6abcd Cas9 TPGS1-KO acetylated tubulin atubulin part 2/TPGS1-KO R3 9-13-24 LT13LT14LT15.Project Maximum Z_XY1726763813_Z0_T0_C0.tif]

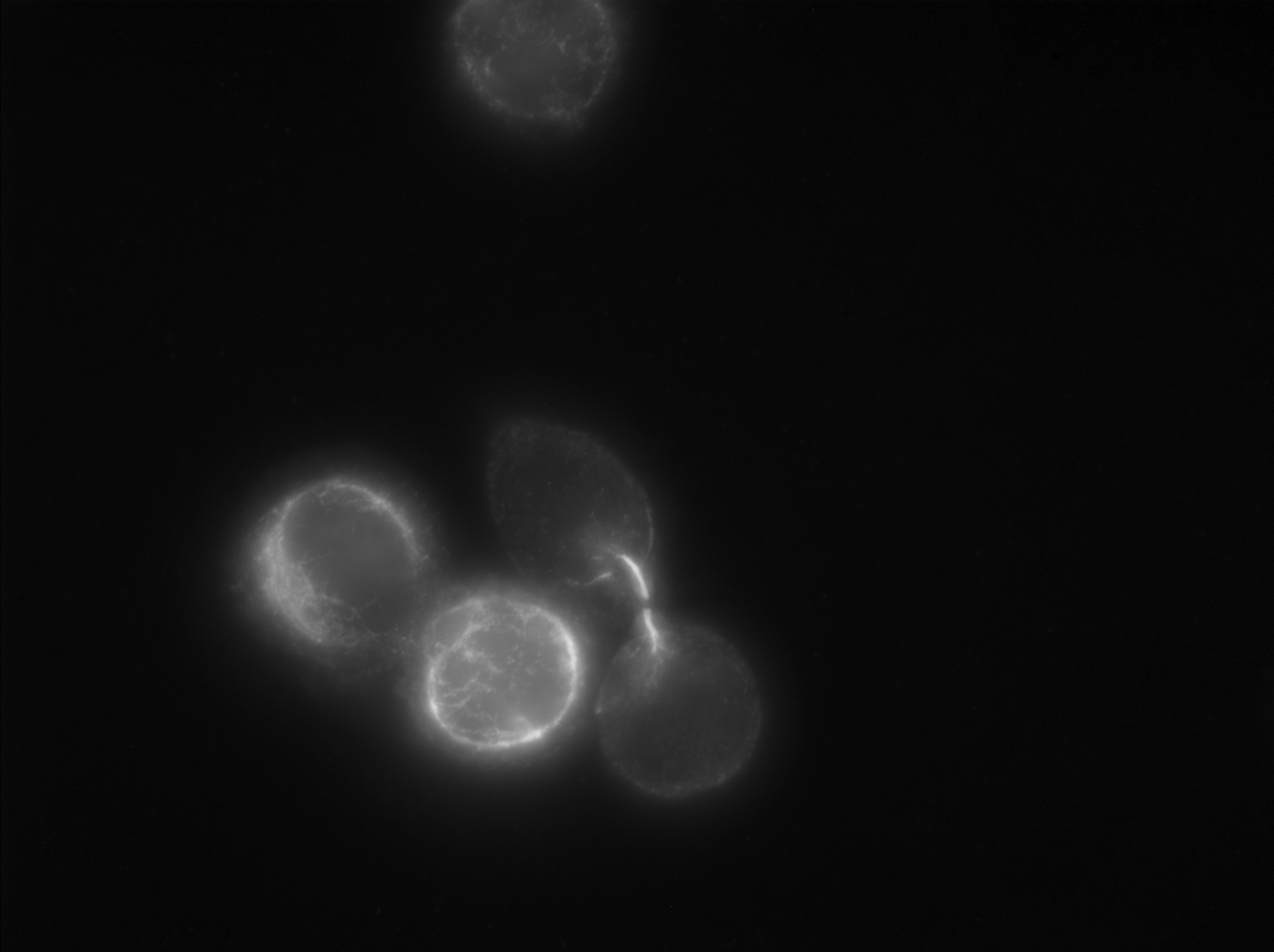

Supplement: Supplementary file 21 — Source data Fig. 6 part 2 [file 44319_2026_742_MOESM21_ESM.zip › Figure 6 Part 2/Fig 6abcd Cas9 TPGS1-KO acetylated tubulin atubulin part 2/TPGS1-KO R2 9-11-24 LT11.Project Maximum Z_XY1726262113_Z0_T0_C2.tif]

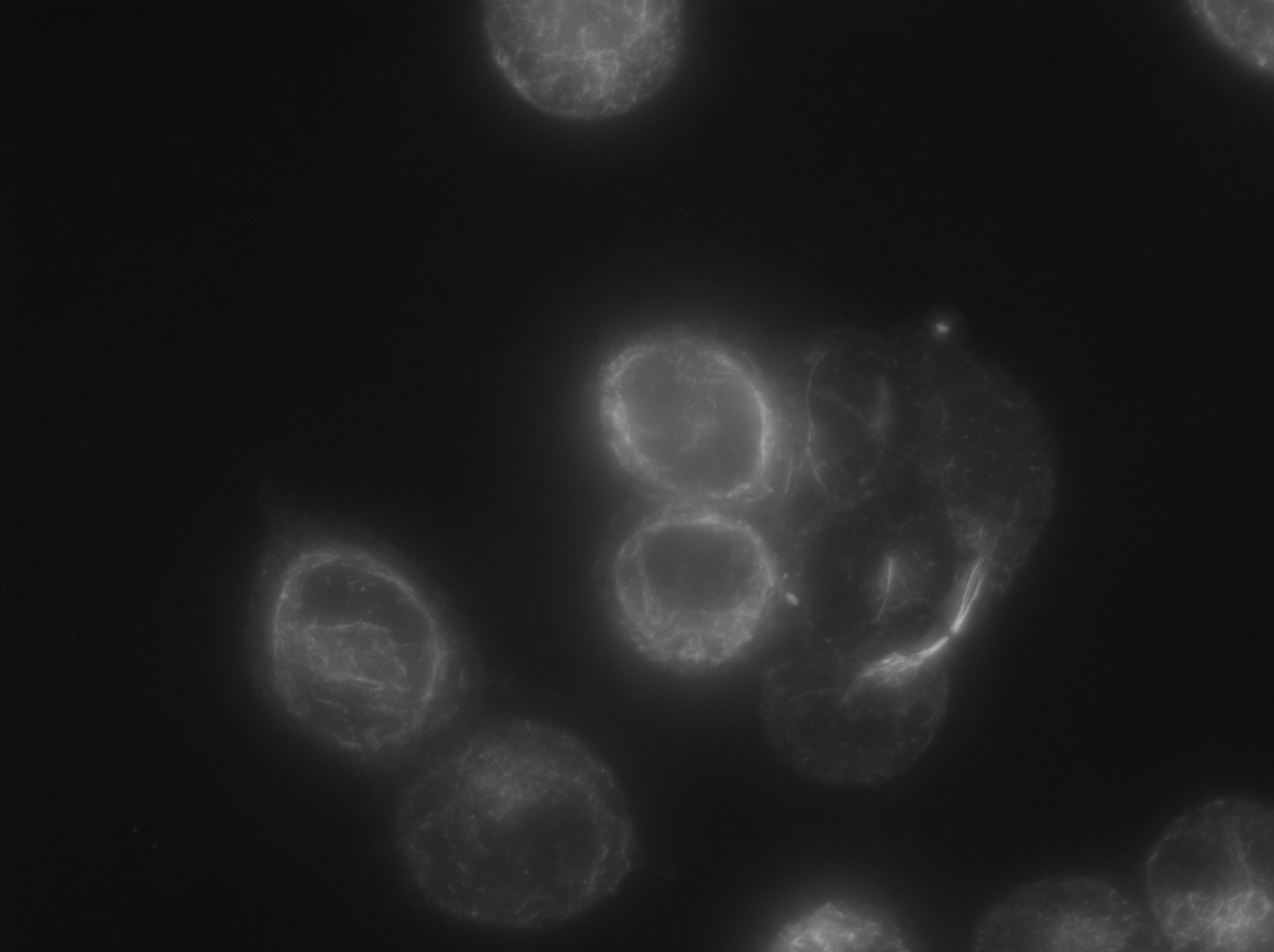

Supplement: Supplementary file 21 — Source data Fig. 6 part 2 [file 44319_2026_742_MOESM21_ESM.zip › Figure 6 Part 2/Fig 6abcd Cas9 TPGS1-KO acetylated tubulin atubulin part 2/TPGS1-KO R2 9-11-24 LT16 PA15.Project Maximum Z_XY1726265624_Z0_T0_C2.tif]

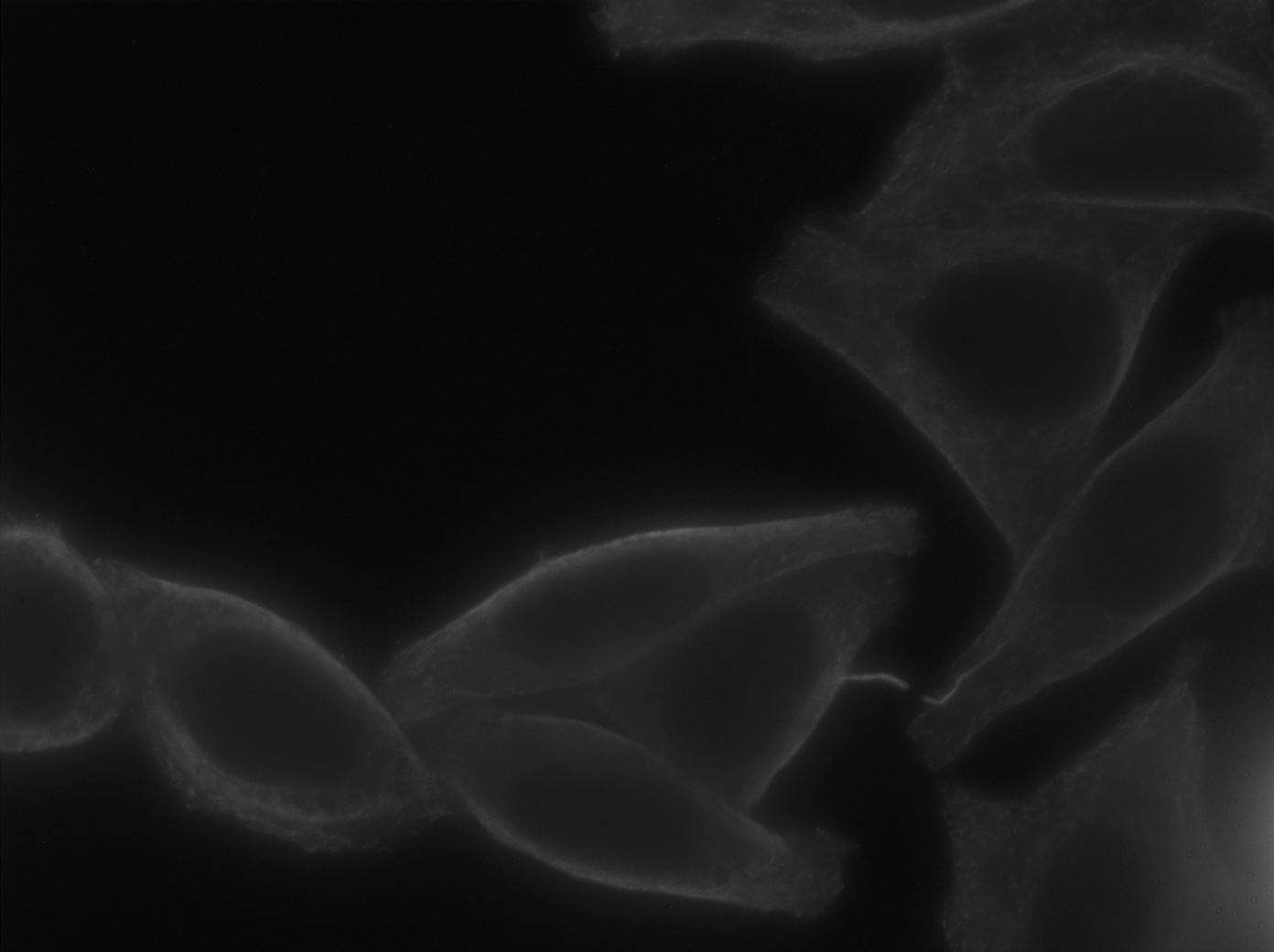

Supplement: Supplementary file 21 — Source data Fig. 6 part 2 [file 44319_2026_742_MOESM21_ESM.zip › Figure 6 Part 2/Fig 6abcd Cas9 TPGS1-KO acetylated tubulin atubulin part 2/TPGS1-KO R3 9-13-24 LT7.Project Maximum Z_XY1726760685_Z0_T0_C1.tif]

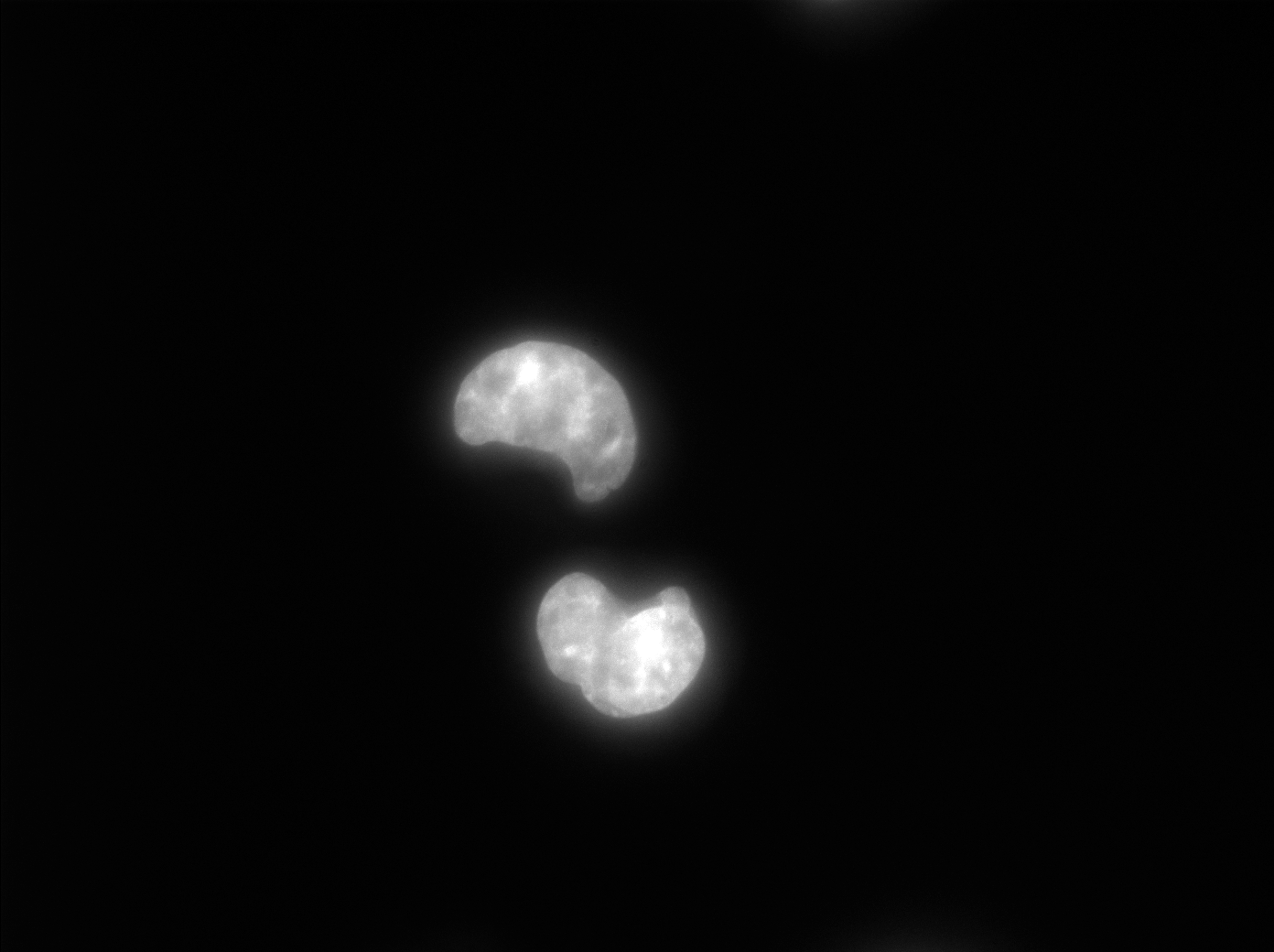

Supplement: Supplementary file 21 — Source data Fig. 6 part 2 [file 44319_2026_742_MOESM21_ESM.zip › Figure 6 Part 2/Fig 6abcd Cas9 TPGS1-KO acetylated tubulin atubulin part 2/TPGS1-KO R2 9-11-24 PA10.Project Maximum Z_XY1726262600_Z0_T0_C0.tif]

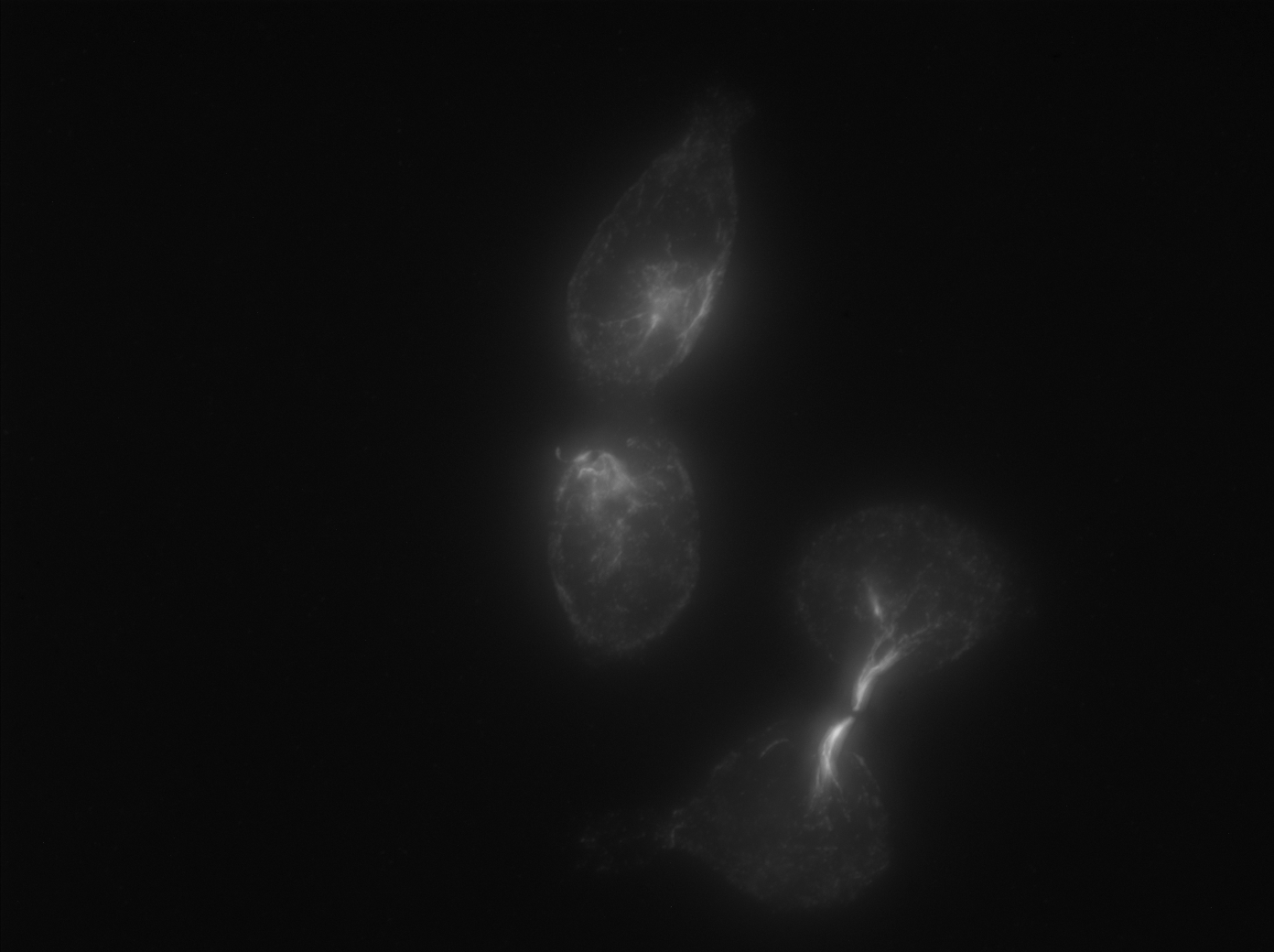

Supplement: Supplementary file 21 — Source data Fig. 6 part 2 [file 44319_2026_742_MOESM21_ESM.zip › Figure 6 Part 2/Fig 6abcd Cas9 TPGS1-KO acetylated tubulin atubulin part 2/TPGS1-KO R2 9-11-24 LT27 PA20.Project Maximum Z_XY1726269402_Z0_T0_C2.tif]

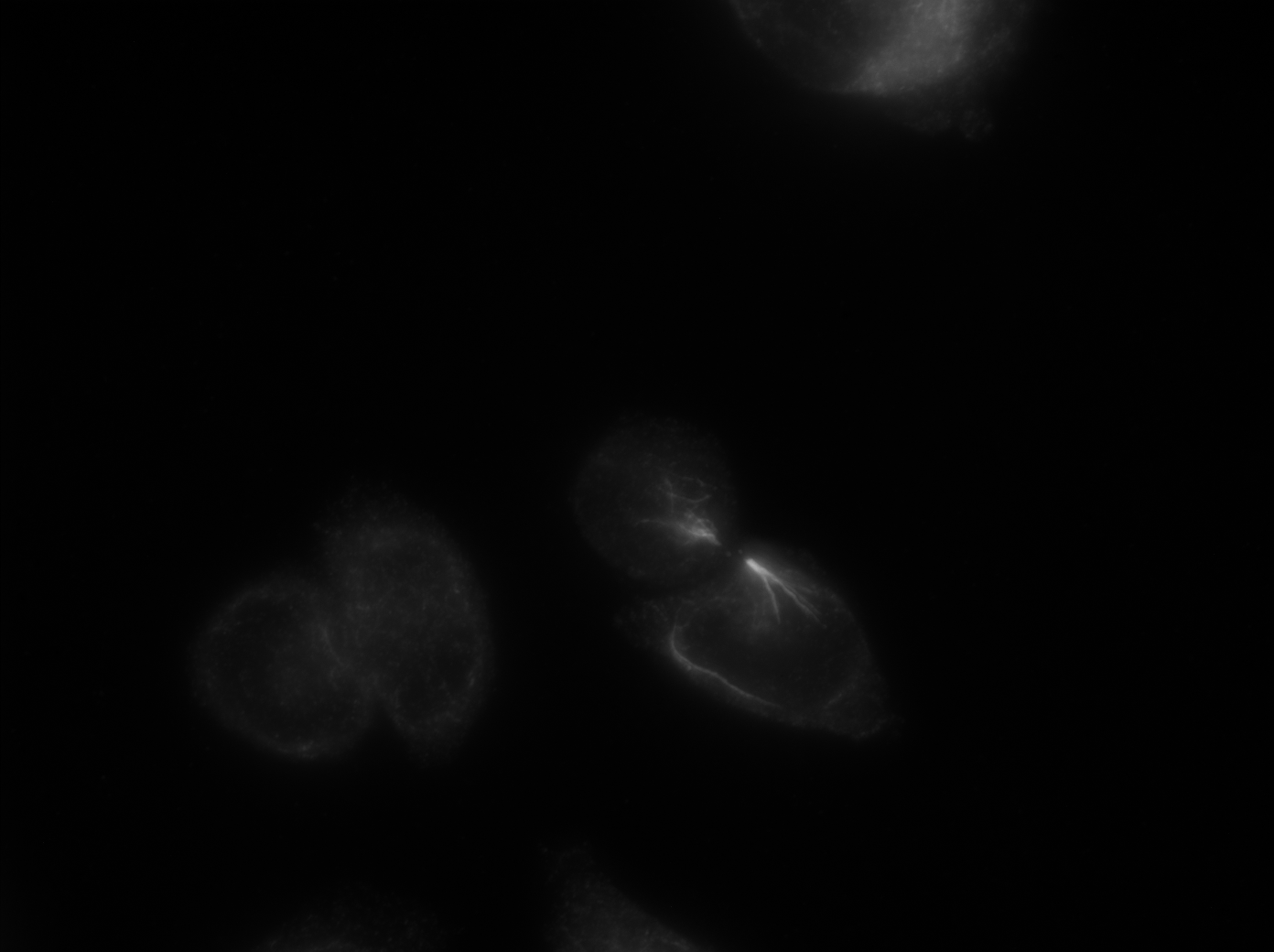

Supplement: Supplementary file 21 — Source data Fig. 6 part 2 [file 44319_2026_742_MOESM21_ESM.zip › Figure 6 Part 2/Fig 6abcd Cas9 TPGS1-KO acetylated tubulin atubulin part 2/TPGS1-KO R3 9-13-24 LT1.Project Maximum Z_XY1726759987_Z0_T0_C2.tif]

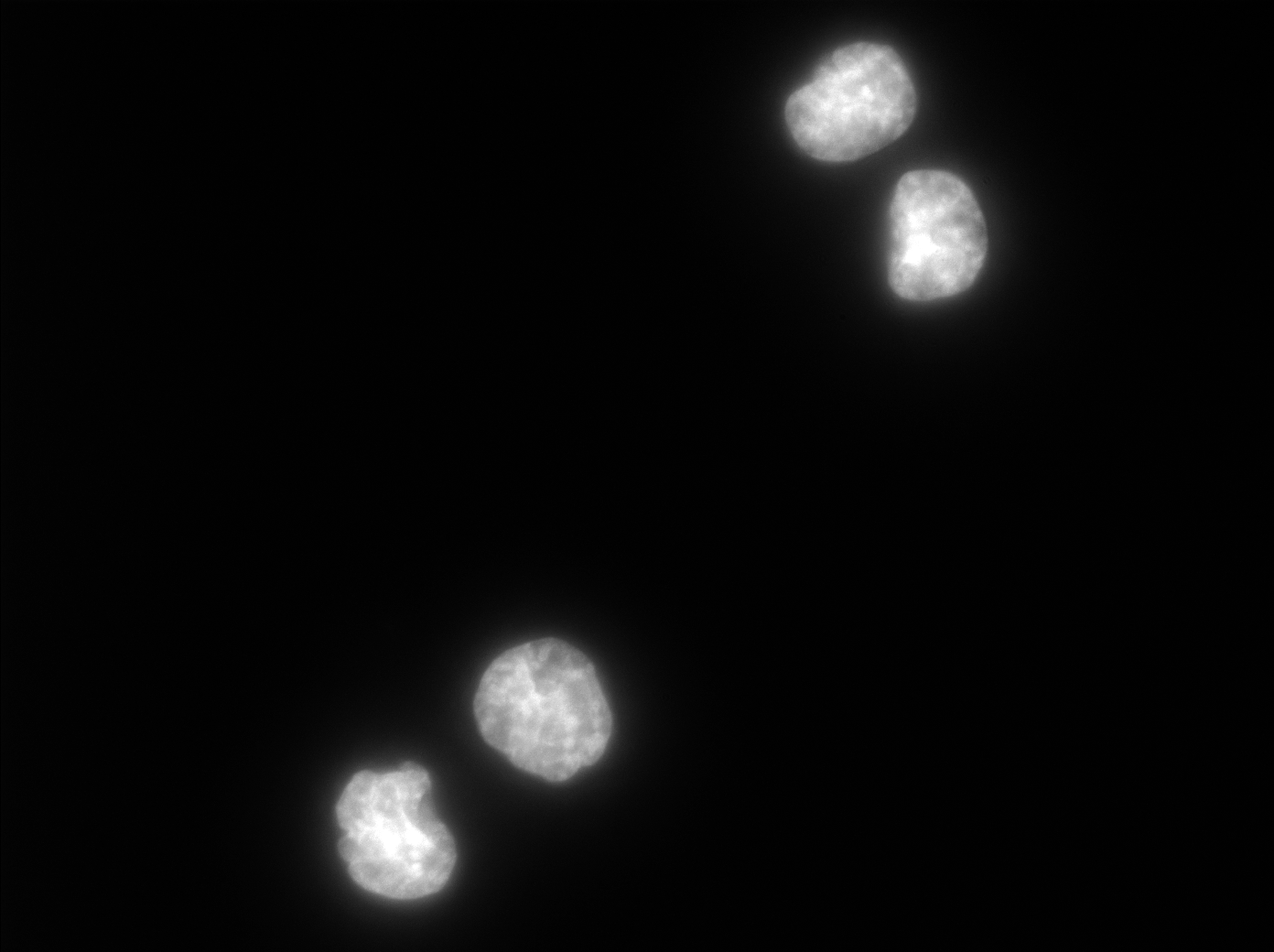

Supplement: Supplementary file 21 — Source data Fig. 6 part 2 [file 44319_2026_742_MOESM21_ESM.zip › Figure 6 Part 2/Fig 6abcd Cas9 TPGS1-KO acetylated tubulin atubulin part 2/TPGS1-KO R2 9-11-24 LT14LT15.Project Maximum Z_XY1726265117_Z0_T0_C0.tif]

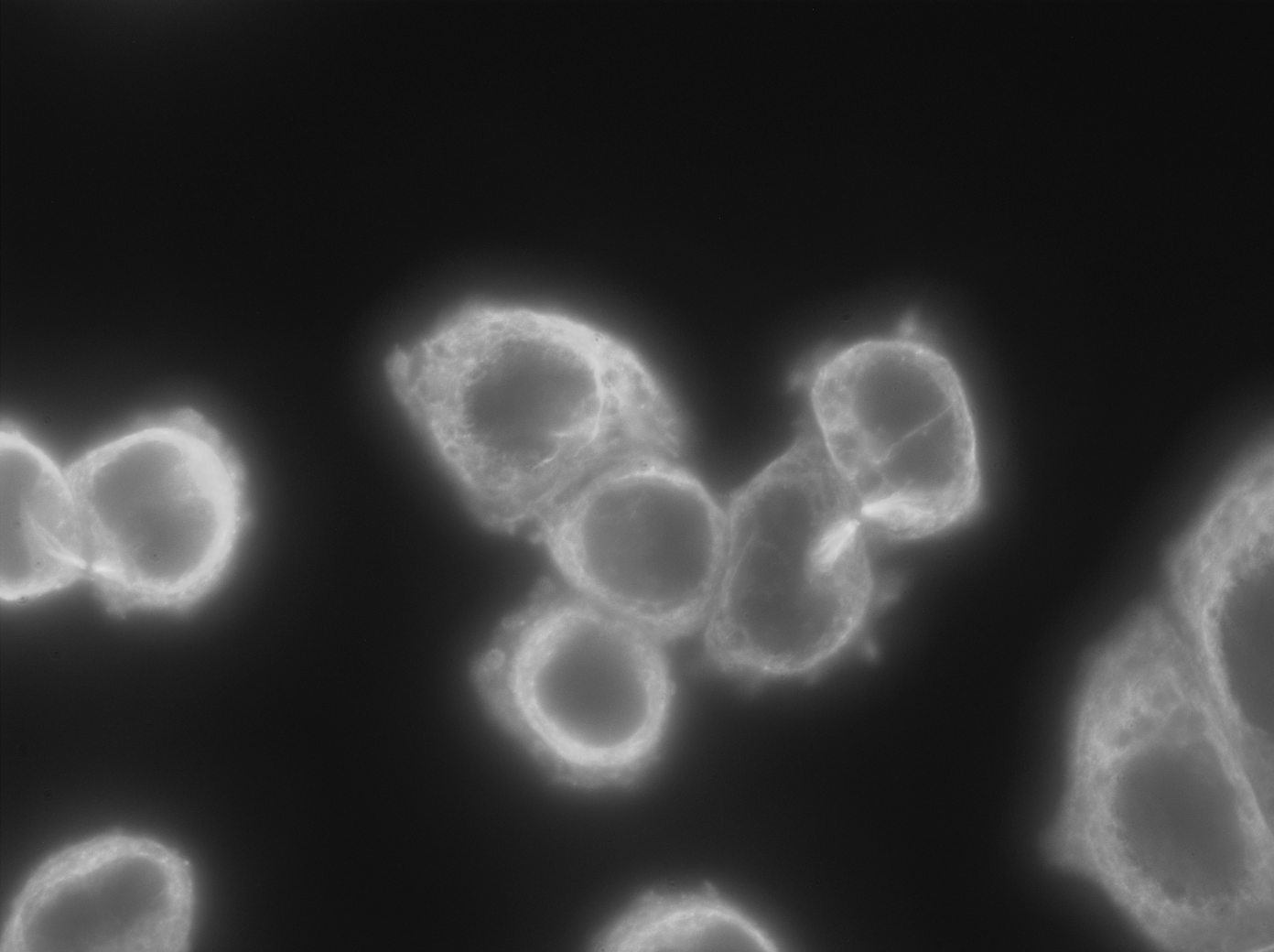

Supplement: Supplementary file 21 — Source data Fig. 6 part 2 [file 44319_2026_742_MOESM21_ESM.zip › Figure 6 Part 2/Fig 6abcd Cas9 TPGS1-KO acetylated tubulin atubulin part 2/TPGS1-KO R3 9-13-24 LT20 PA4.Project Maximum Z_XY1726764207_Z0_T0_C1.tif]

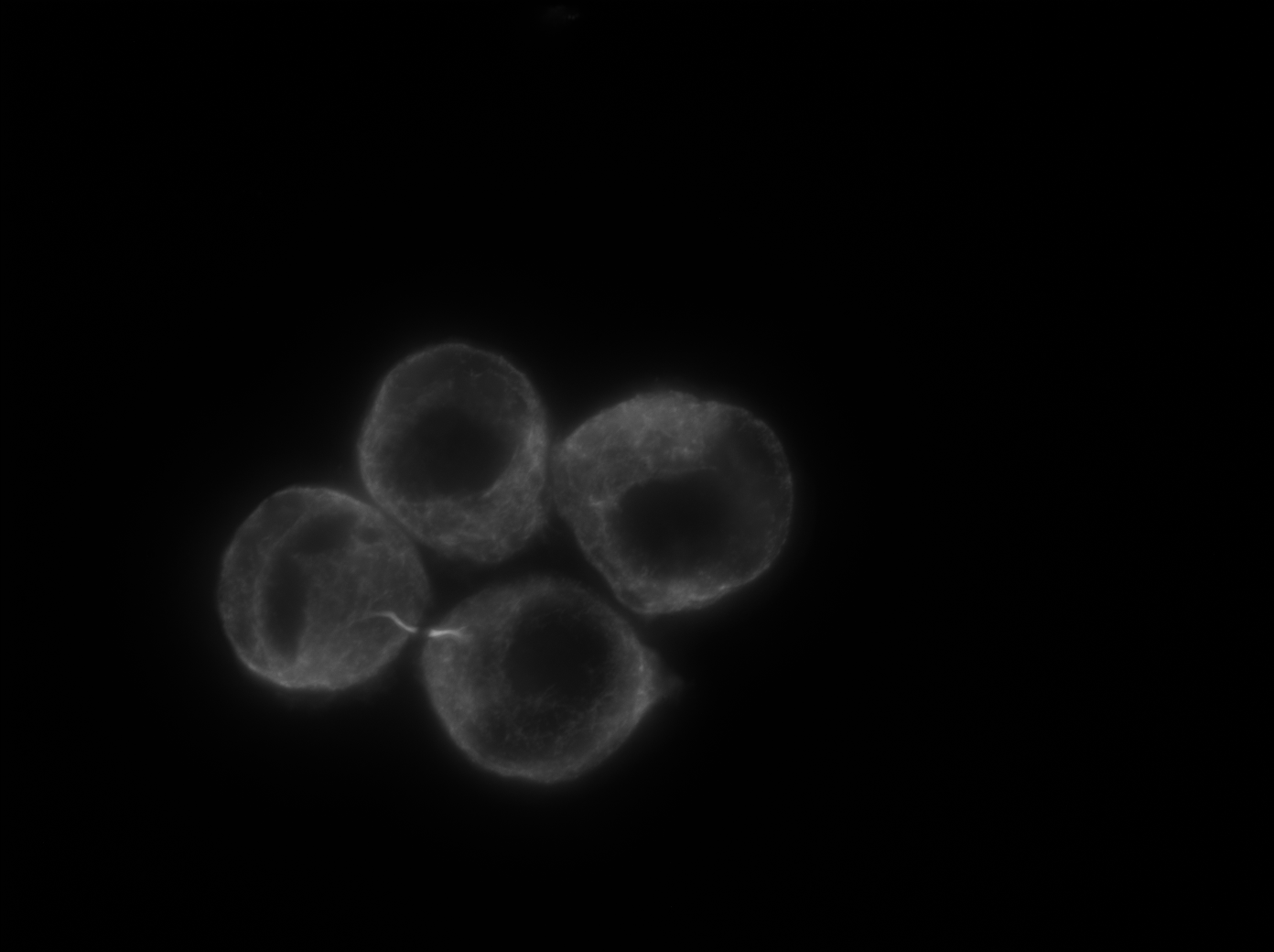

Supplement: Supplementary file 21 — Source data Fig. 6 part 2 [file 44319_2026_742_MOESM21_ESM.zip › Figure 6 Part 2/Fig 6abcd Cas9 TPGS1-KO acetylated tubulin atubulin part 2/TPGS1-KO R2 9-11-24 LT26 PA17.Project Maximum Z_XY1726268563_Z0_T0_C1.tif]

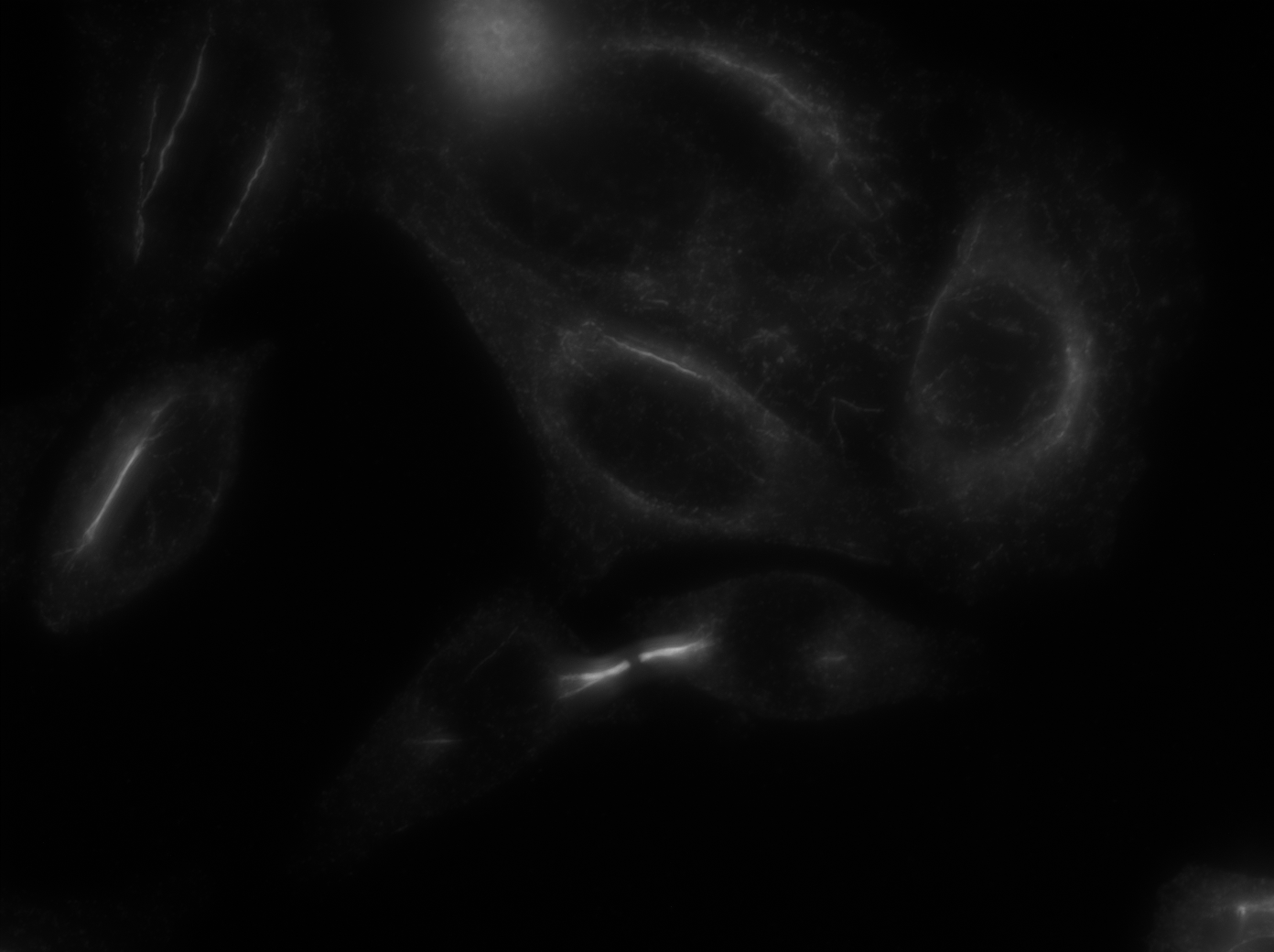

Supplement: Supplementary file 21 — Source data Fig. 6 part 2 [file 44319_2026_742_MOESM21_ESM.zip › Figure 6 Part 2/Fig 6abcd Cas9 TPGS1-KO acetylated tubulin atubulin part 2/TPGS1-KO R3 9-13-24 LT6.Project Maximum Z_XY1726760598_Z0_T0_C2.tif]

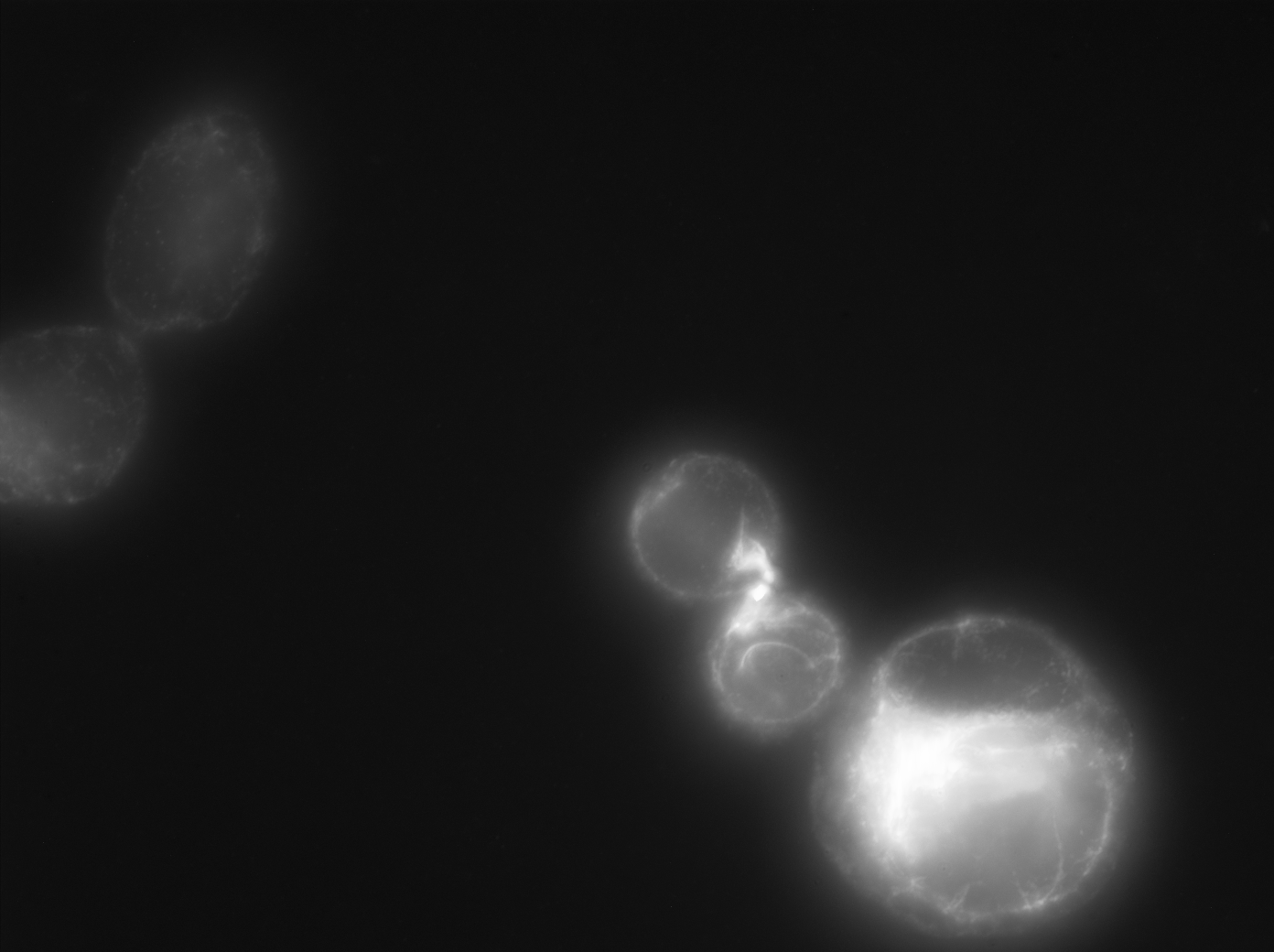

Supplement: Supplementary file 21 — Source data Fig. 6 part 2 [file 44319_2026_742_MOESM21_ESM.zip › Figure 6 Part 2/Fig 6abcd Cas9 TPGS1-KO acetylated tubulin atubulin part 2/TPGS1-KO R2 9-11-24 LT21.Project Maximum Z_XY1726267755_Z0_T0_C2.tif]

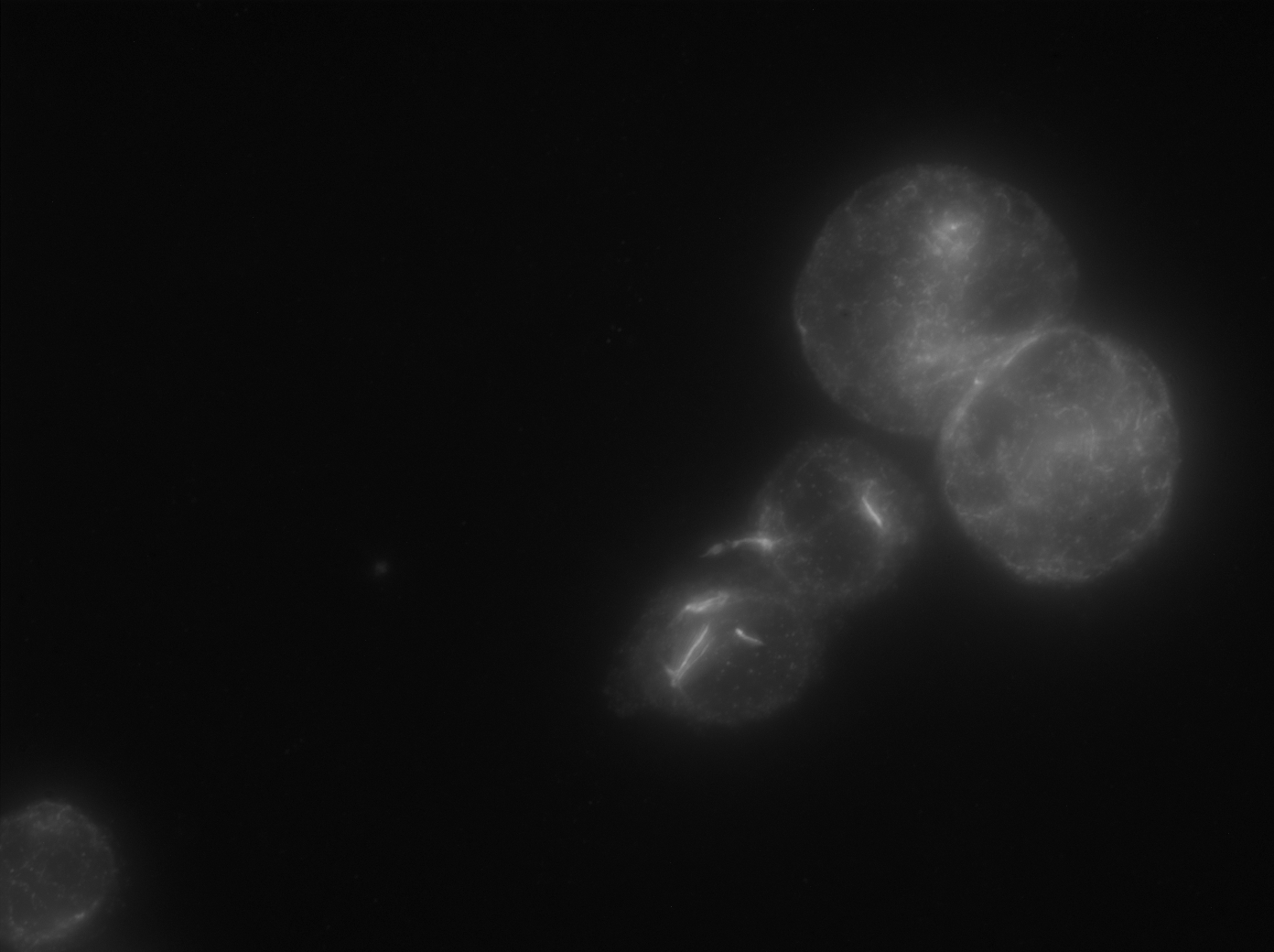

Supplement: Supplementary file 21 — Source data Fig. 6 part 2 [file 44319_2026_742_MOESM21_ESM.zip › Figure 6 Part 2/Fig 6abcd Cas9 TPGS1-KO acetylated tubulin atubulin part 2/TPGS1-KO R2 9-11-24 PA3.Project Maximum Z_XY1726259750_Z0_T0_C2.tif]

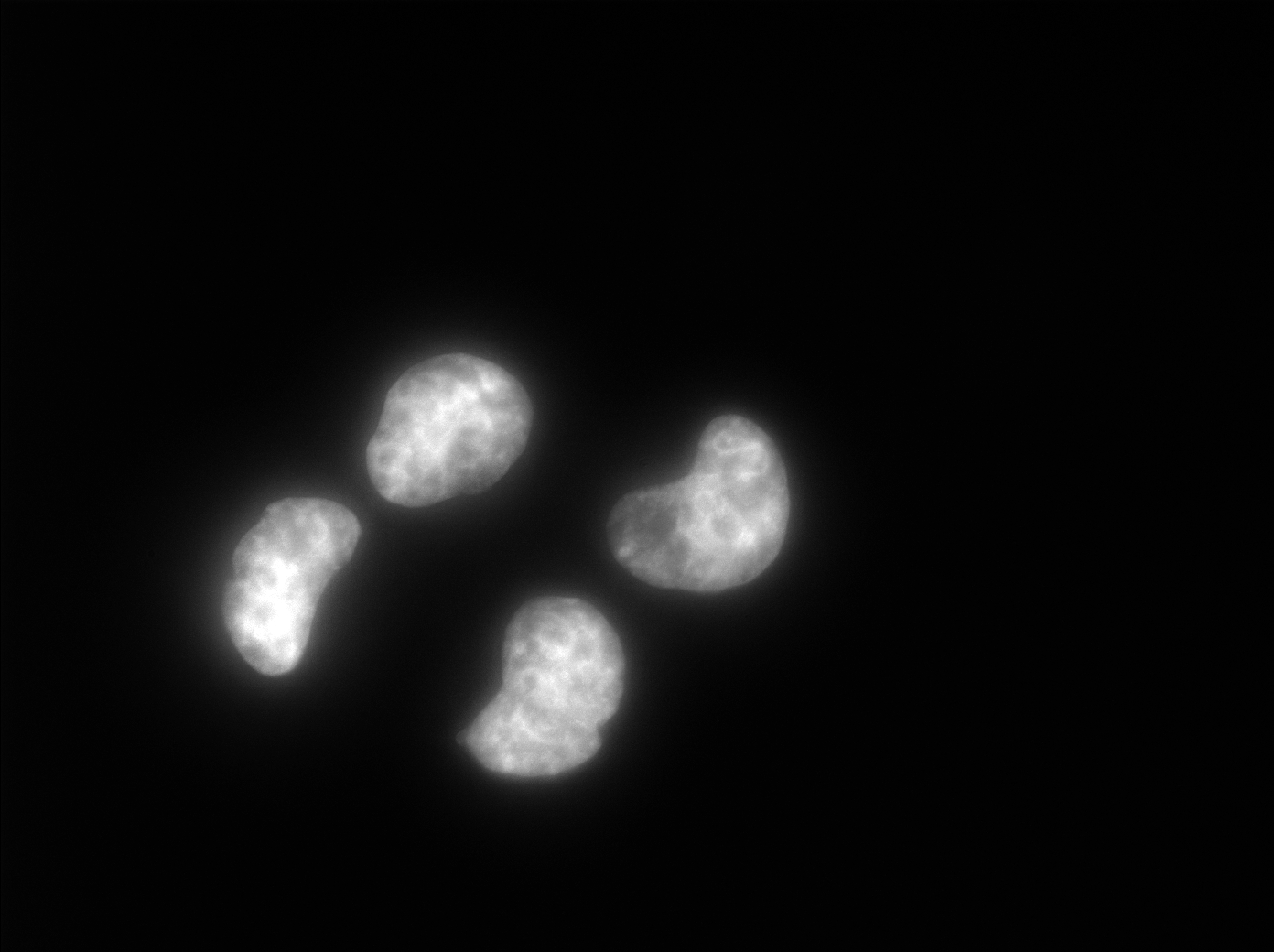

Supplement: Supplementary file 21 — Source data Fig. 6 part 2 [file 44319_2026_742_MOESM21_ESM.zip › Figure 6 Part 2/Fig 6abcd Cas9 TPGS1-KO acetylated tubulin atubulin part 2/TPGS1-KO R2 9-11-24 LT26 PA17.Project Maximum Z_XY1726268563_Z0_T0_C0.tif]

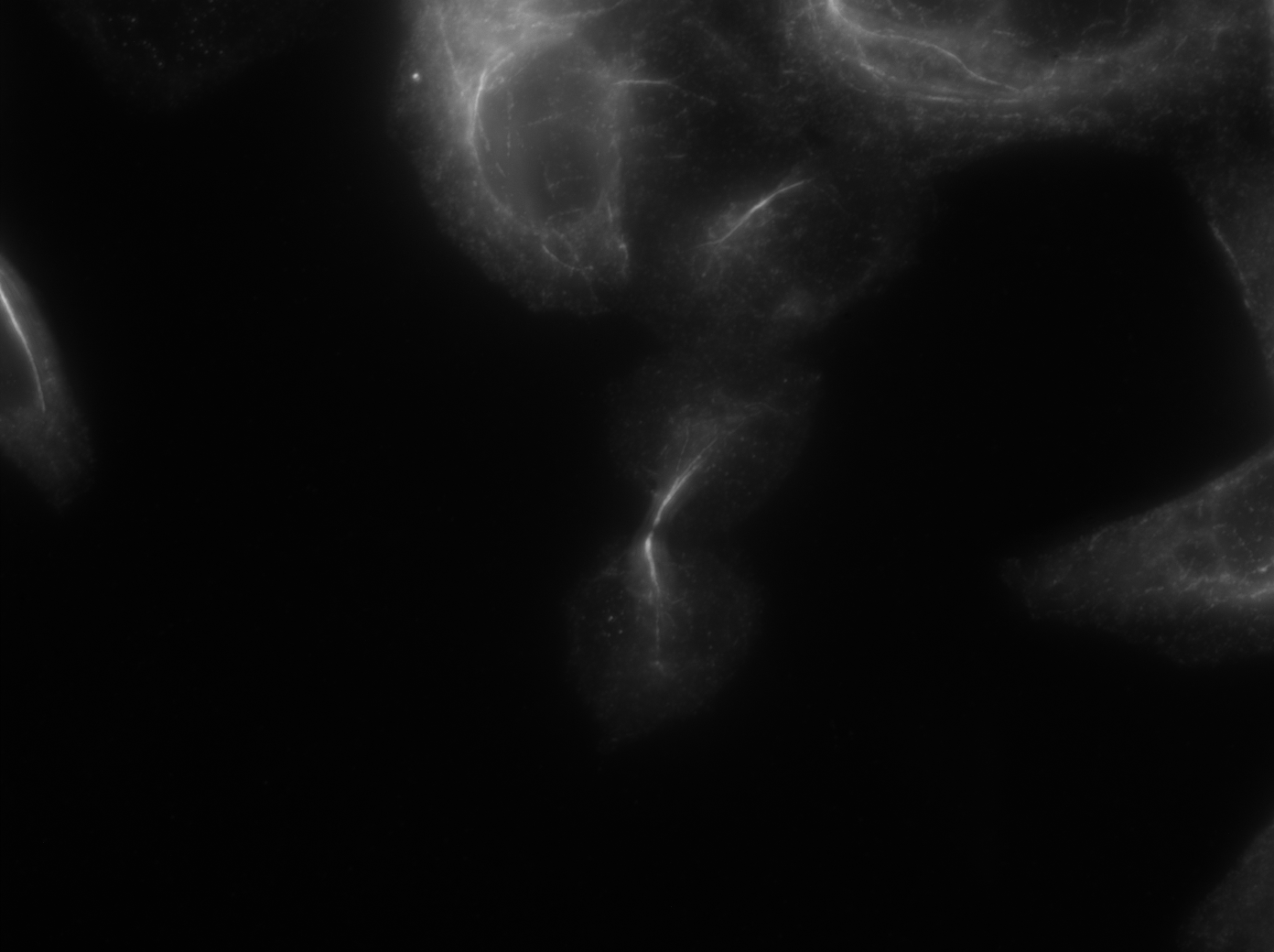

Supplement: Supplementary file 21 — Source data Fig. 6 part 2 [file 44319_2026_742_MOESM21_ESM.zip › Figure 6 Part 2/Fig 6abcd Cas9 TPGS1-KO acetylated tubulin atubulin part 2/TPGS1-KO R3 9-13-24 LT29.Project Maximum Z_XY1726764993_Z0_T0_C2.tif]

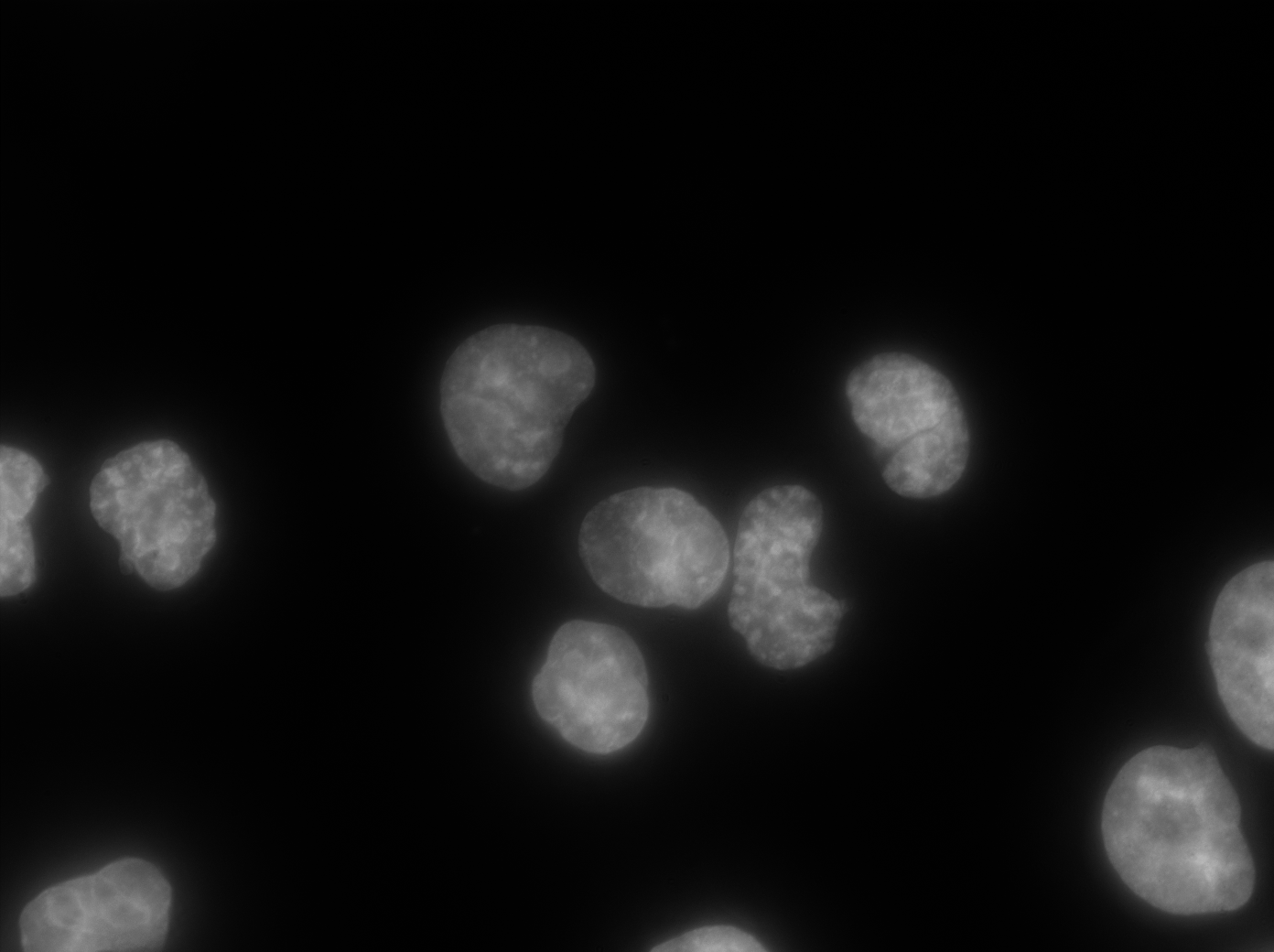

Supplement: Supplementary file 21 — Source data Fig. 6 part 2 [file 44319_2026_742_MOESM21_ESM.zip › Figure 6 Part 2/Fig 6abcd Cas9 TPGS1-KO acetylated tubulin atubulin part 2/TPGS1-KO R3 9-13-24 LT20 PA4.Project Maximum Z_XY1726764207_Z0_T0_C0.tif]

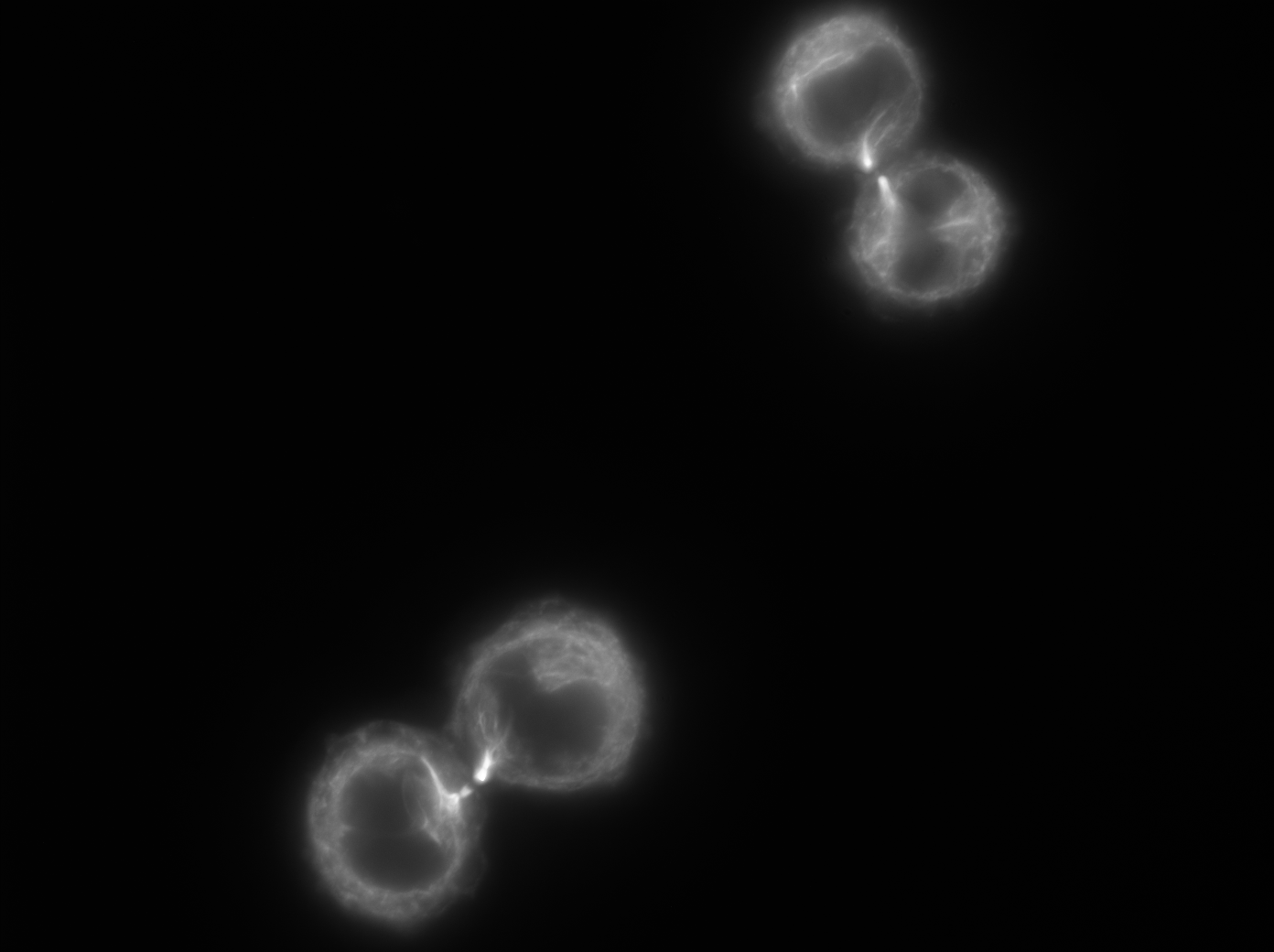

Supplement: Supplementary file 21 — Source data Fig. 6 part 2 [file 44319_2026_742_MOESM21_ESM.zip › Figure 6 Part 2/Fig 6abcd Cas9 TPGS1-KO acetylated tubulin atubulin part 2/TPGS1-KO R2 9-11-24 LT14LT15.Project Maximum Z_XY1726265117_Z0_T0_C1.tif]

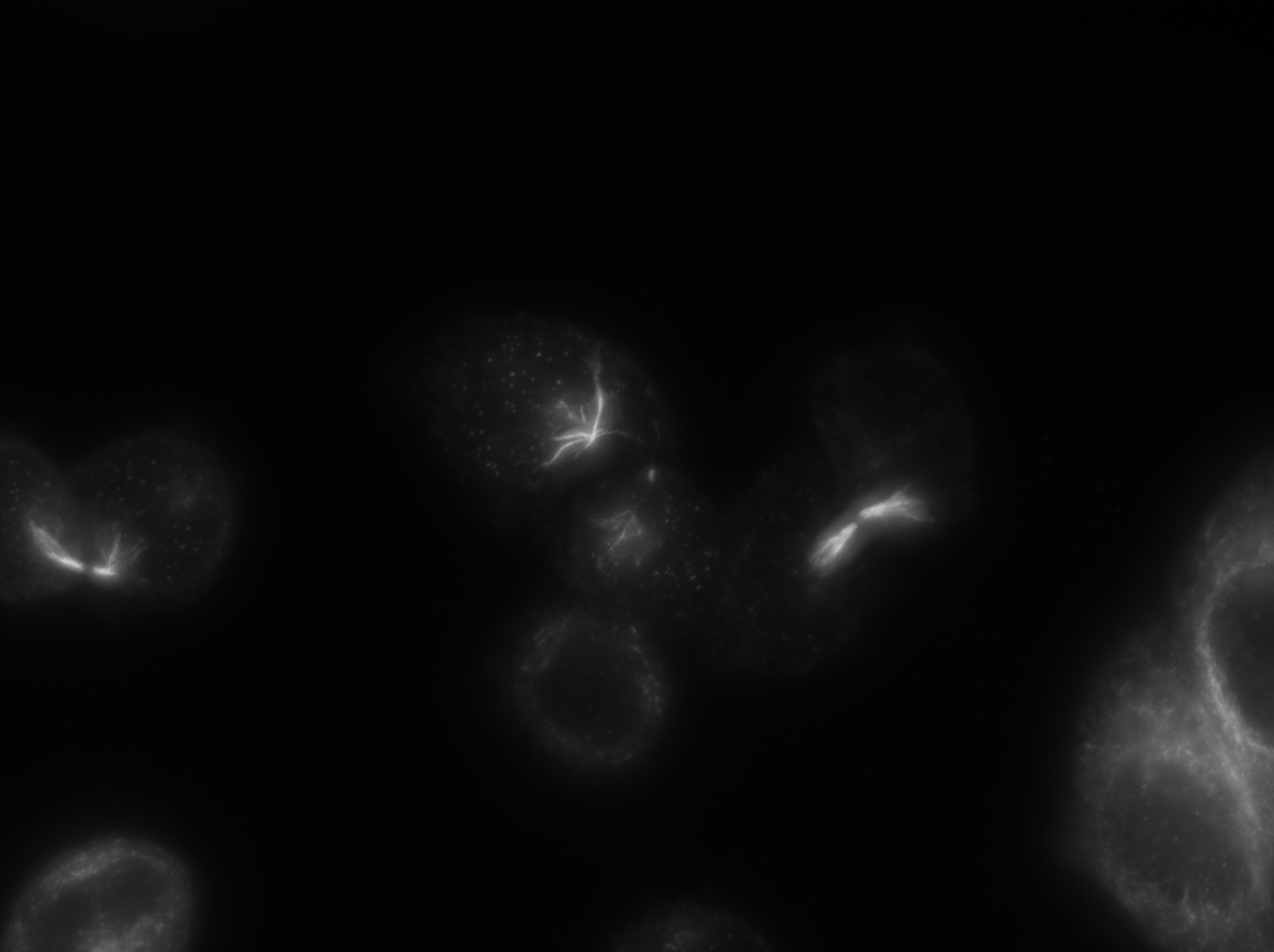

Supplement: Supplementary file 21 — Source data Fig. 6 part 2 [file 44319_2026_742_MOESM21_ESM.zip › Figure 6 Part 2/Fig 6abcd Cas9 TPGS1-KO acetylated tubulin atubulin part 2/TPGS1-KO R3 9-13-24 LT20 PA4.Project Maximum Z_XY1726764207_Z0_T0_C2.tif]

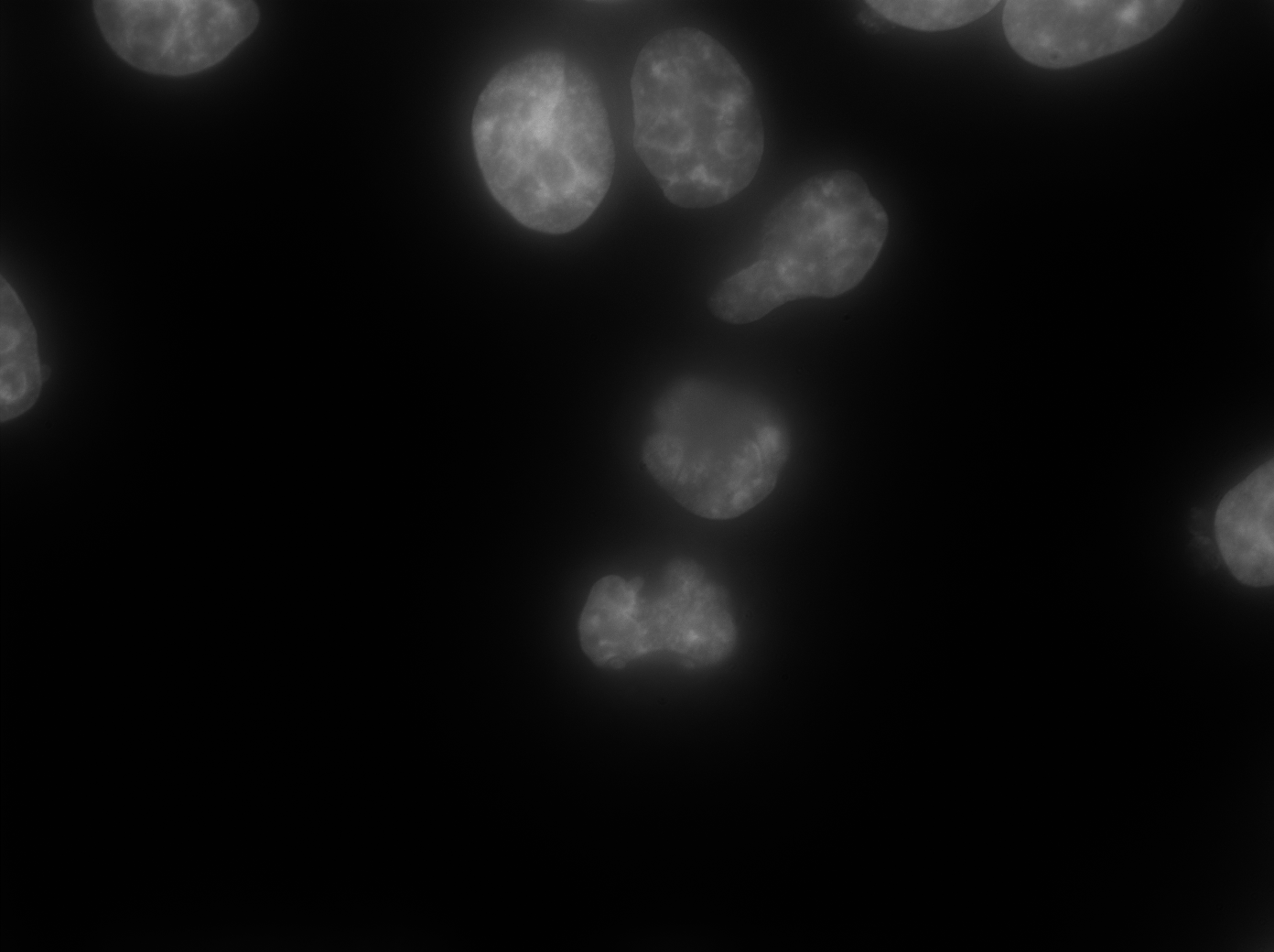

Supplement: Supplementary file 21 — Source data Fig. 6 part 2 [file 44319_2026_742_MOESM21_ESM.zip › Figure 6 Part 2/Fig 6abcd Cas9 TPGS1-KO acetylated tubulin atubulin part 2/TPGS1-KO R3 9-13-24 LT29.Project Maximum Z_XY1726764993_Z0_T0_C0.tif]

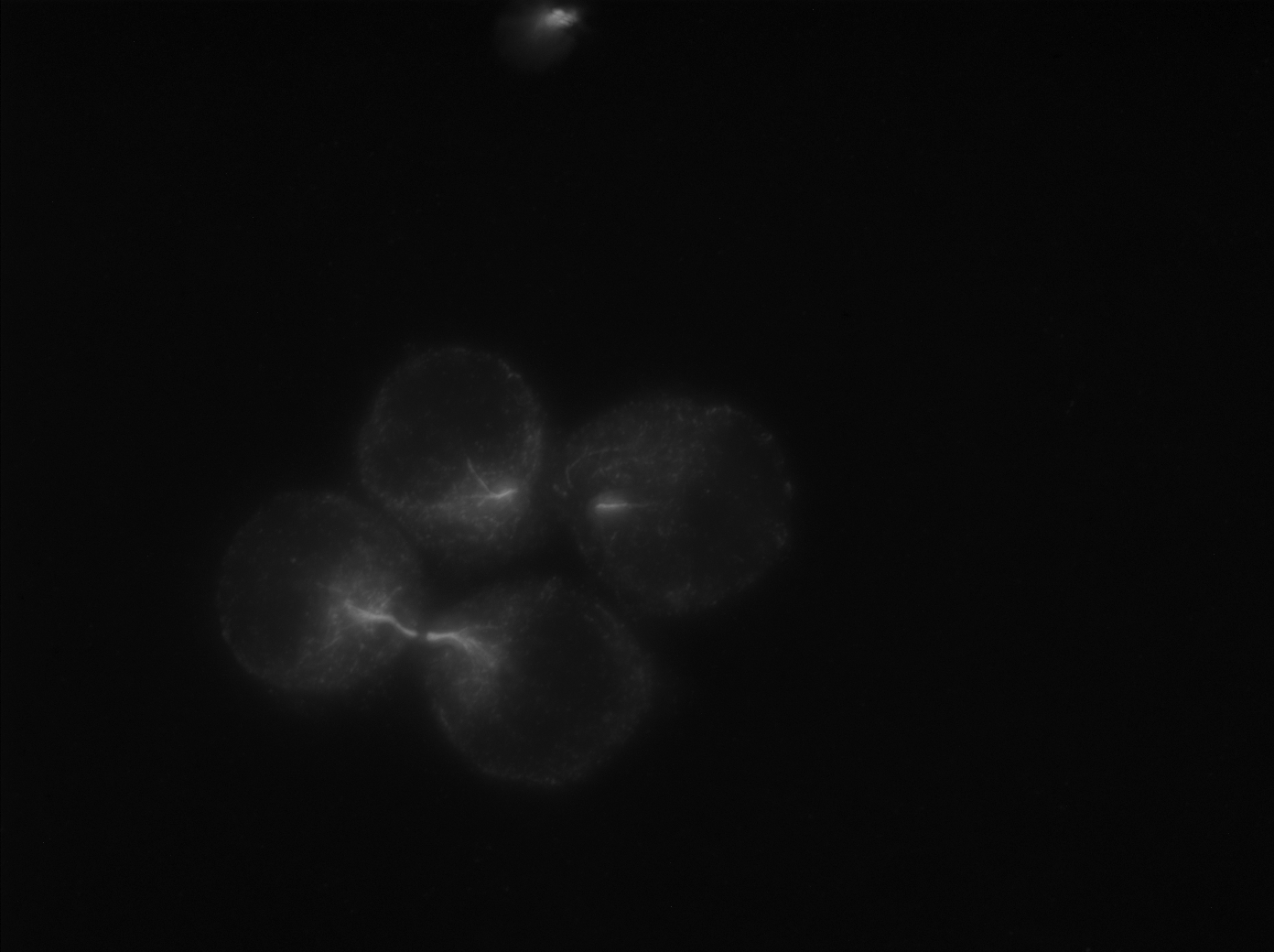

Supplement: Supplementary file 21 — Source data Fig. 6 part 2 [file 44319_2026_742_MOESM21_ESM.zip › Figure 6 Part 2/Fig 6abcd Cas9 TPGS1-KO acetylated tubulin atubulin part 2/TPGS1-KO R2 9-11-24 LT26 PA17.Project Maximum Z_XY1726268563_Z0_T0_C2.tif]

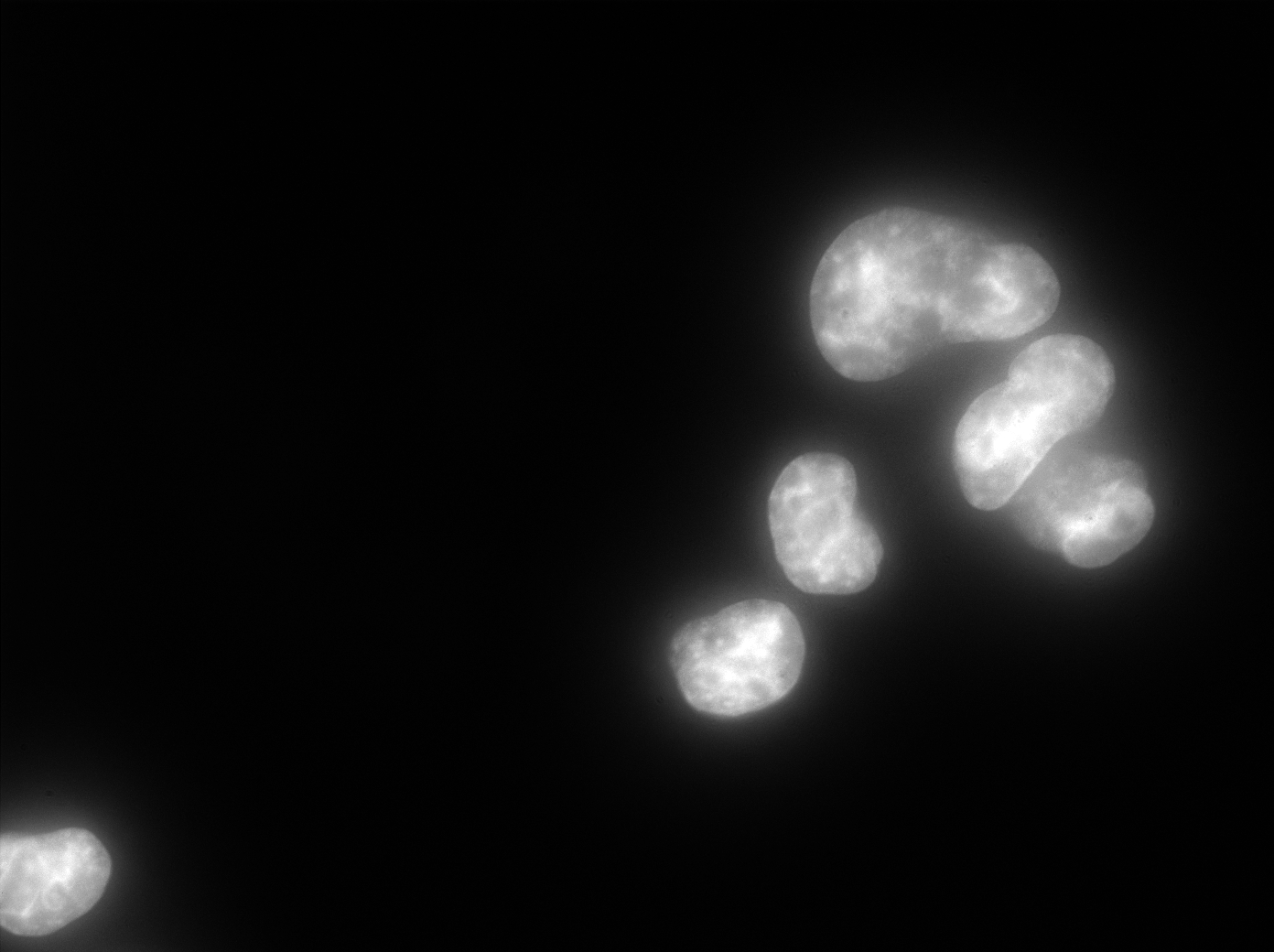

Supplement: Supplementary file 21 — Source data Fig. 6 part 2 [file 44319_2026_742_MOESM21_ESM.zip › Figure 6 Part 2/Fig 6abcd Cas9 TPGS1-KO acetylated tubulin atubulin part 2/TPGS1-KO R2 9-11-24 PA3.Project Maximum Z_XY1726259750_Z0_T0_C0.tif]

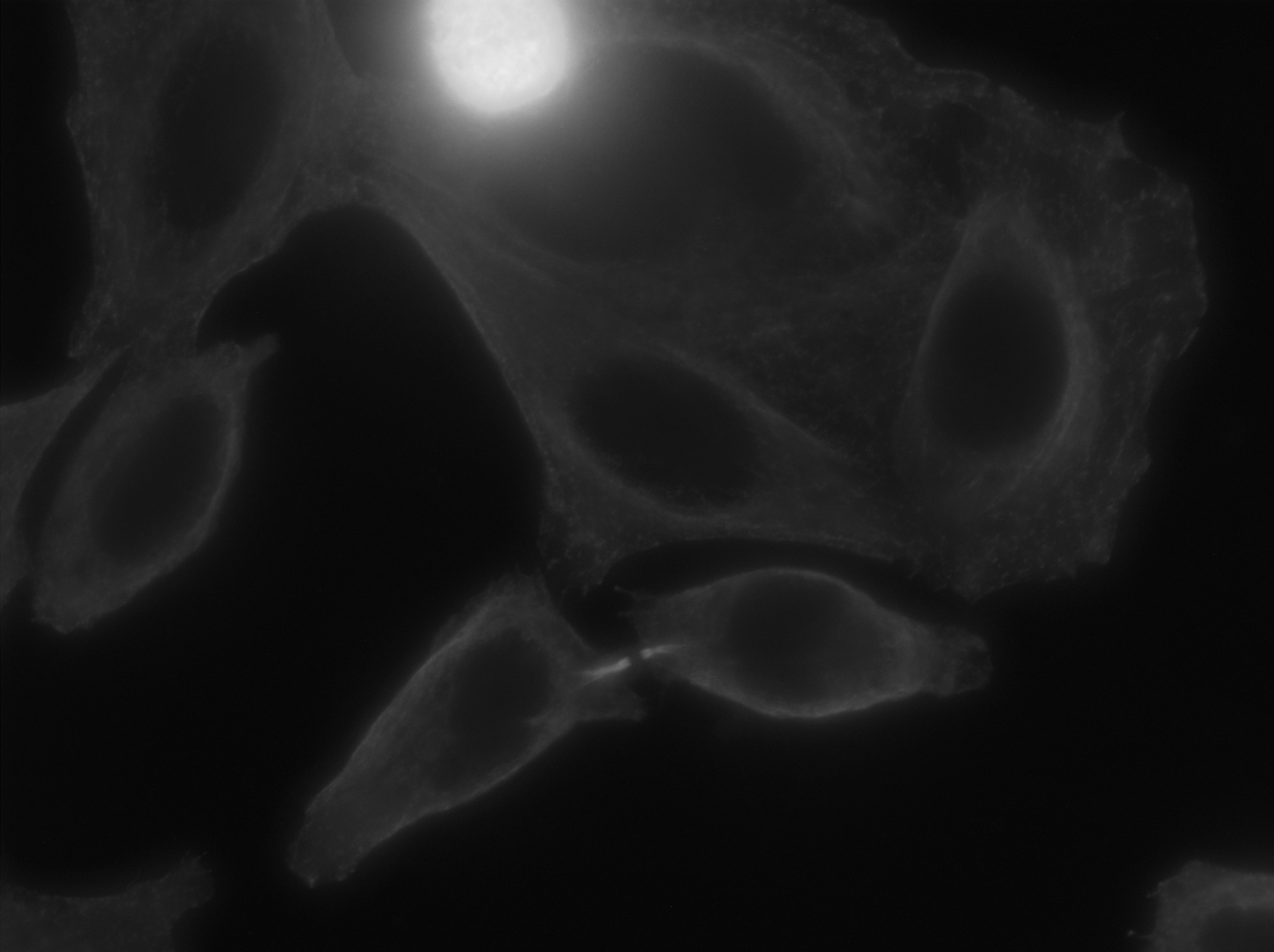

Supplement: Supplementary file 21 — Source data Fig. 6 part 2 [file 44319_2026_742_MOESM21_ESM.zip › Figure 6 Part 2/Fig 6abcd Cas9 TPGS1-KO acetylated tubulin atubulin part 2/TPGS1-KO R3 9-13-24 LT6.Project Maximum Z_XY1726760598_Z0_T0_C1.tif]

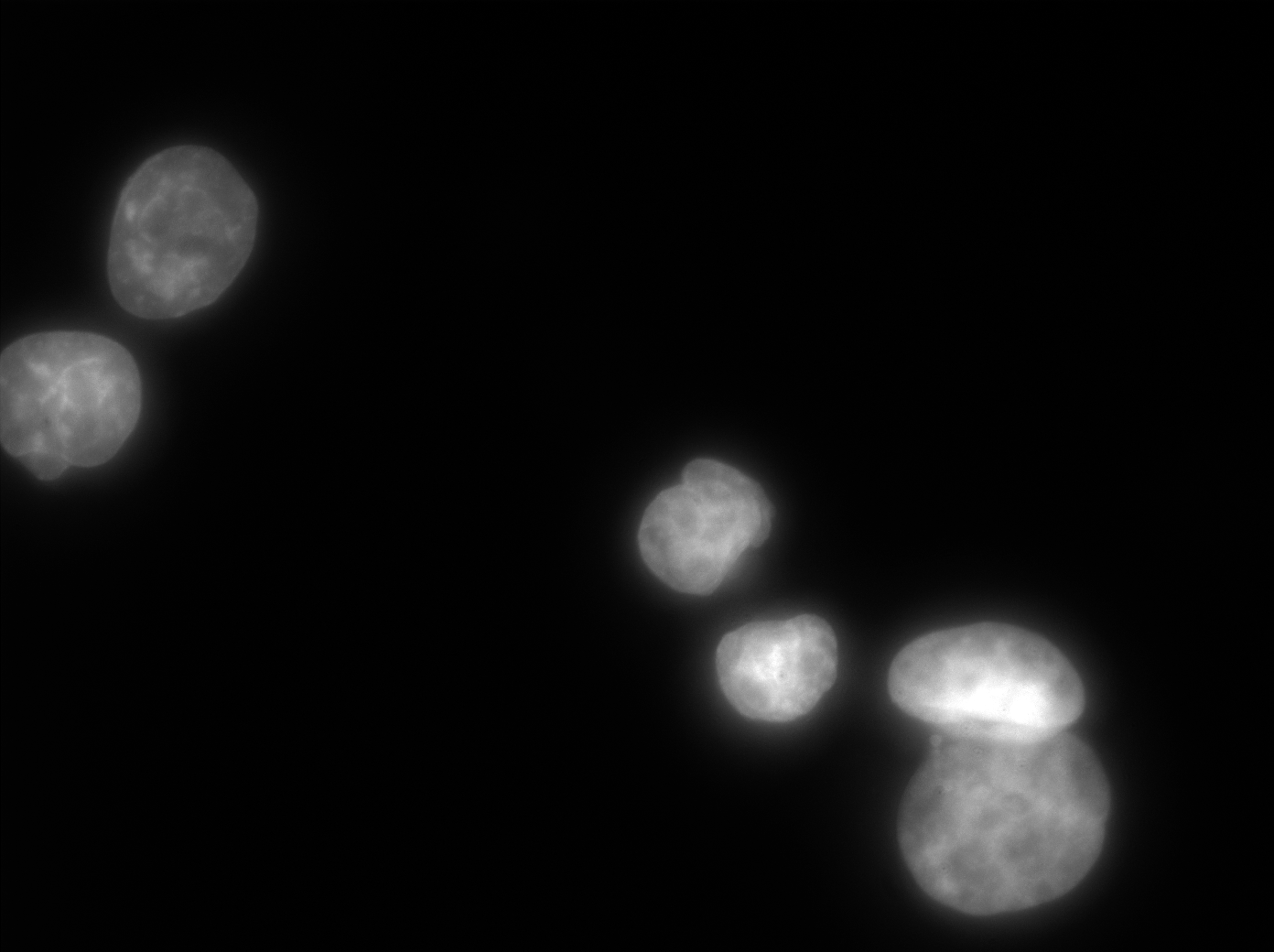

Supplement: Supplementary file 21 — Source data Fig. 6 part 2 [file 44319_2026_742_MOESM21_ESM.zip › Figure 6 Part 2/Fig 6abcd Cas9 TPGS1-KO acetylated tubulin atubulin part 2/TPGS1-KO R2 9-11-24 LT21.Project Maximum Z_XY1726267755_Z0_T0_C0.tif]

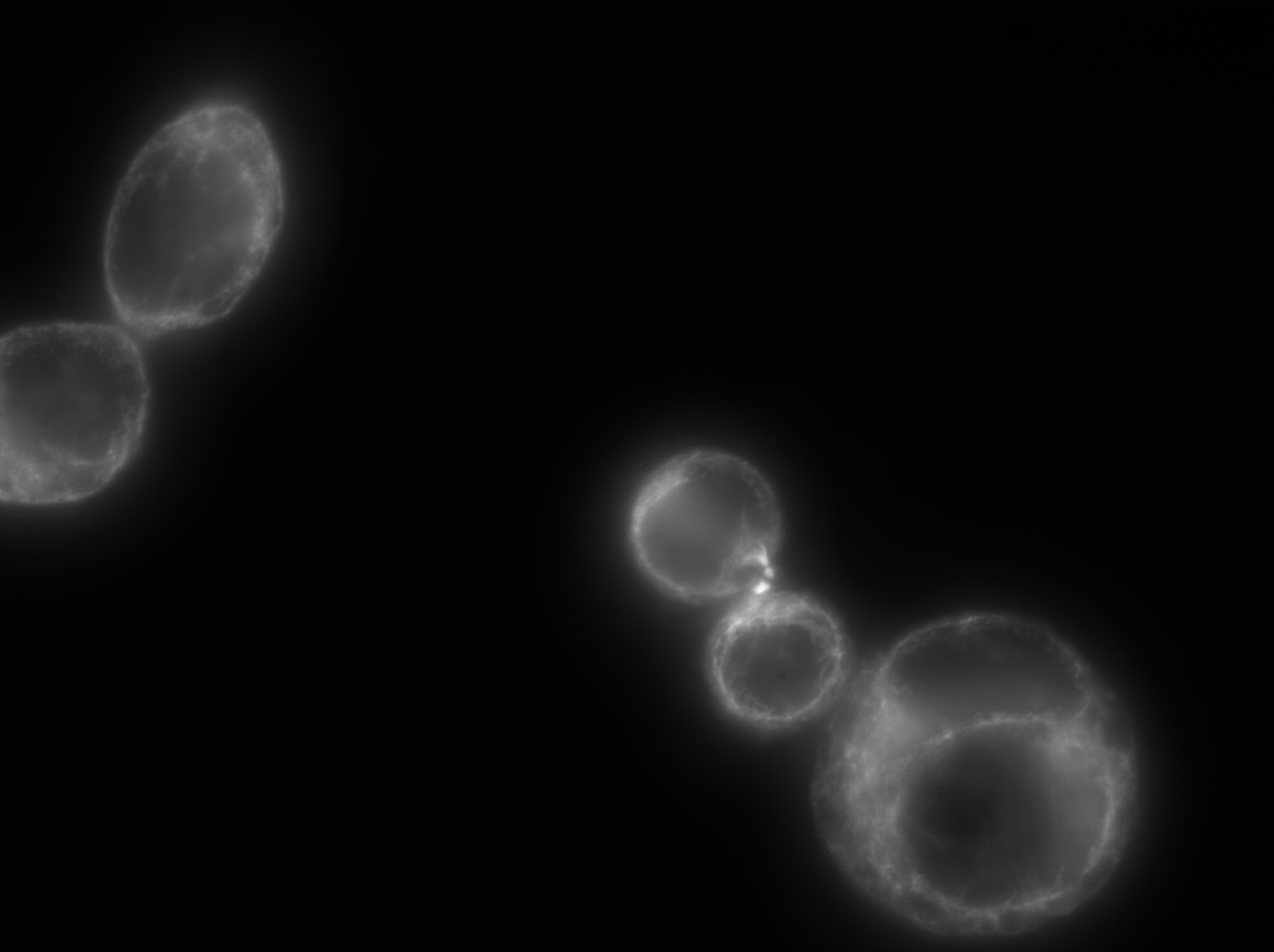

Supplement: Supplementary file 21 — Source data Fig. 6 part 2 [file 44319_2026_742_MOESM21_ESM.zip › Figure 6 Part 2/Fig 6abcd Cas9 TPGS1-KO acetylated tubulin atubulin part 2/TPGS1-KO R2 9-11-24 LT21.Project Maximum Z_XY1726267755_Z0_T0_C1.tif]

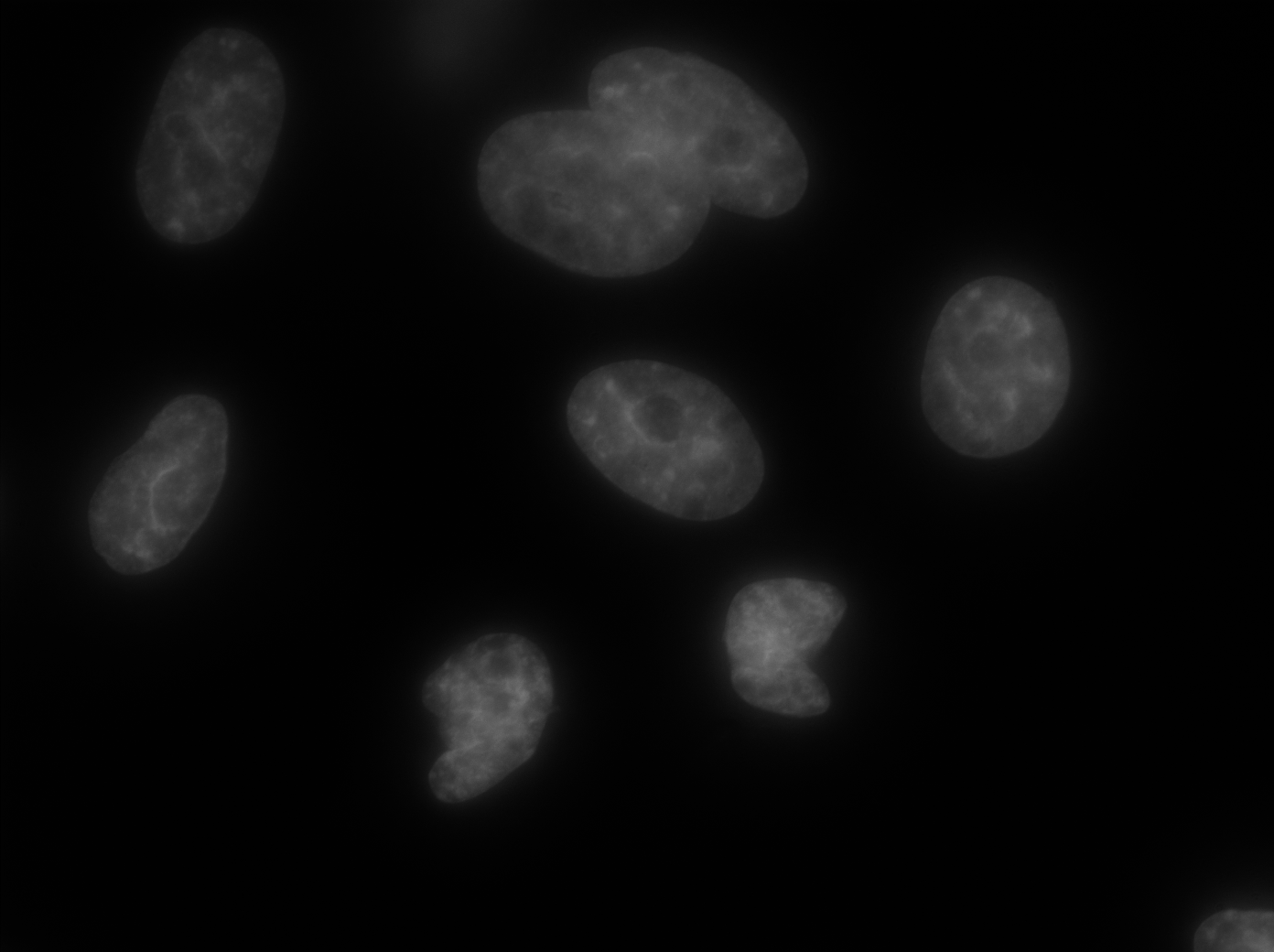

Supplement: Supplementary file 21 — Source data Fig. 6 part 2 [file 44319_2026_742_MOESM21_ESM.zip › Figure 6 Part 2/Fig 6abcd Cas9 TPGS1-KO acetylated tubulin atubulin part 2/TPGS1-KO R3 9-13-24 LT6.Project Maximum Z_XY1726760598_Z0_T0_C0.tif]

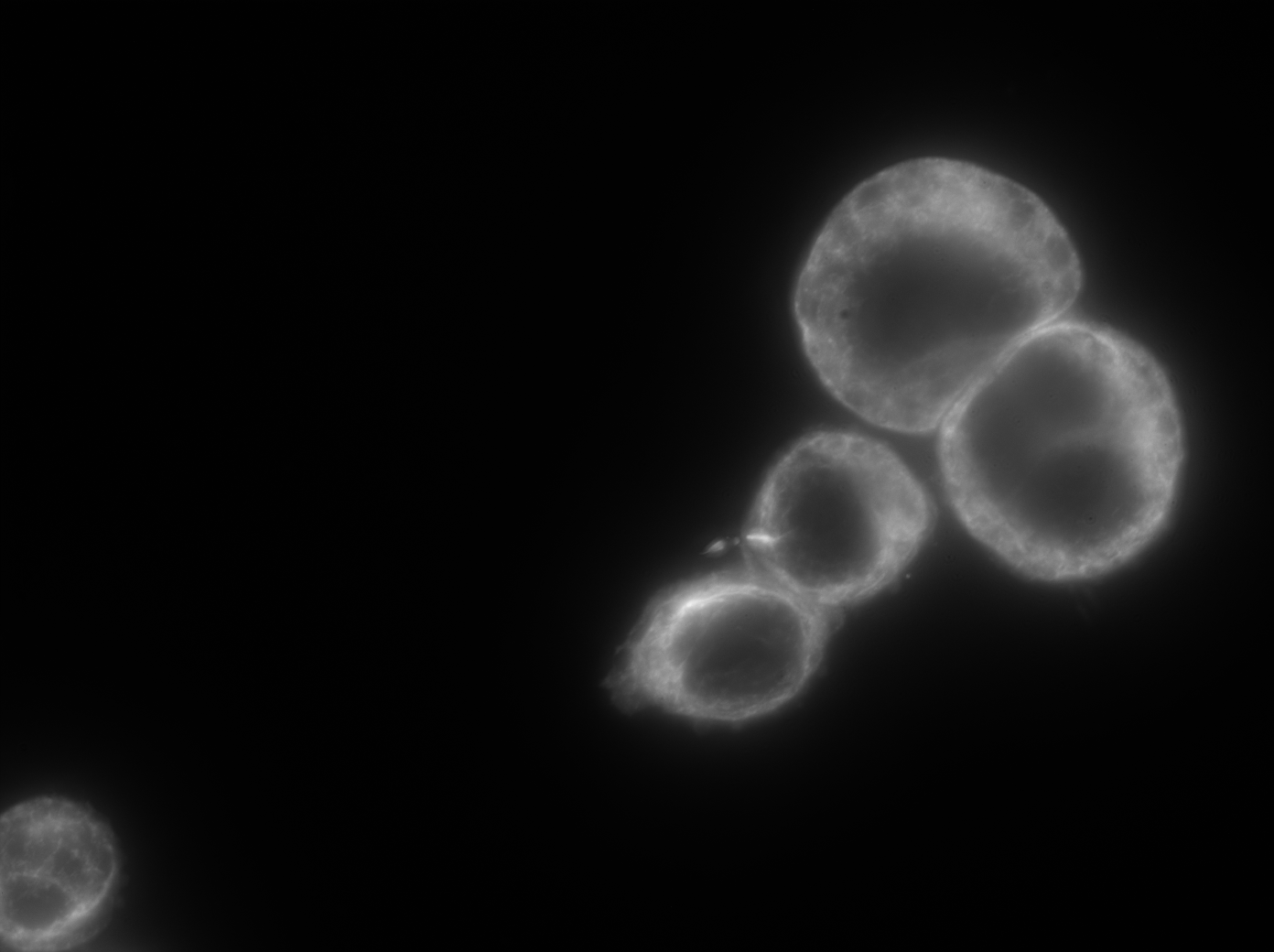

Supplement: Supplementary file 21 — Source data Fig. 6 part 2 [file 44319_2026_742_MOESM21_ESM.zip › Figure 6 Part 2/Fig 6abcd Cas9 TPGS1-KO acetylated tubulin atubulin part 2/TPGS1-KO R2 9-11-24 PA3.Project Maximum Z_XY1726259750_Z0_T0_C1.tif]

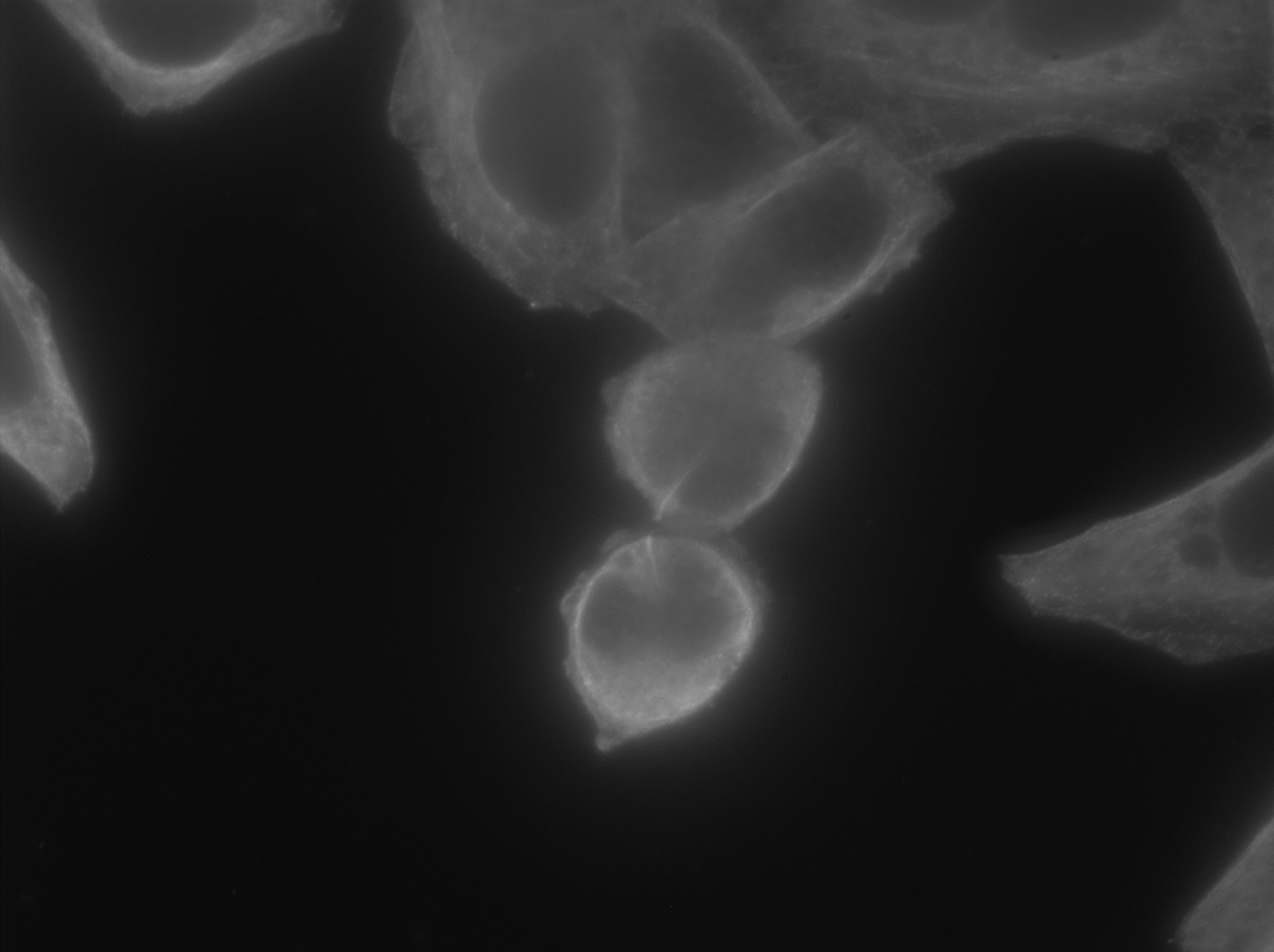

Supplement: Supplementary file 21 — Source data Fig. 6 part 2 [file 44319_2026_742_MOESM21_ESM.zip › Figure 6 Part 2/Fig 6abcd Cas9 TPGS1-KO acetylated tubulin atubulin part 2/TPGS1-KO R3 9-13-24 LT29.Project Maximum Z_XY1726764993_Z0_T0_C1.tif]

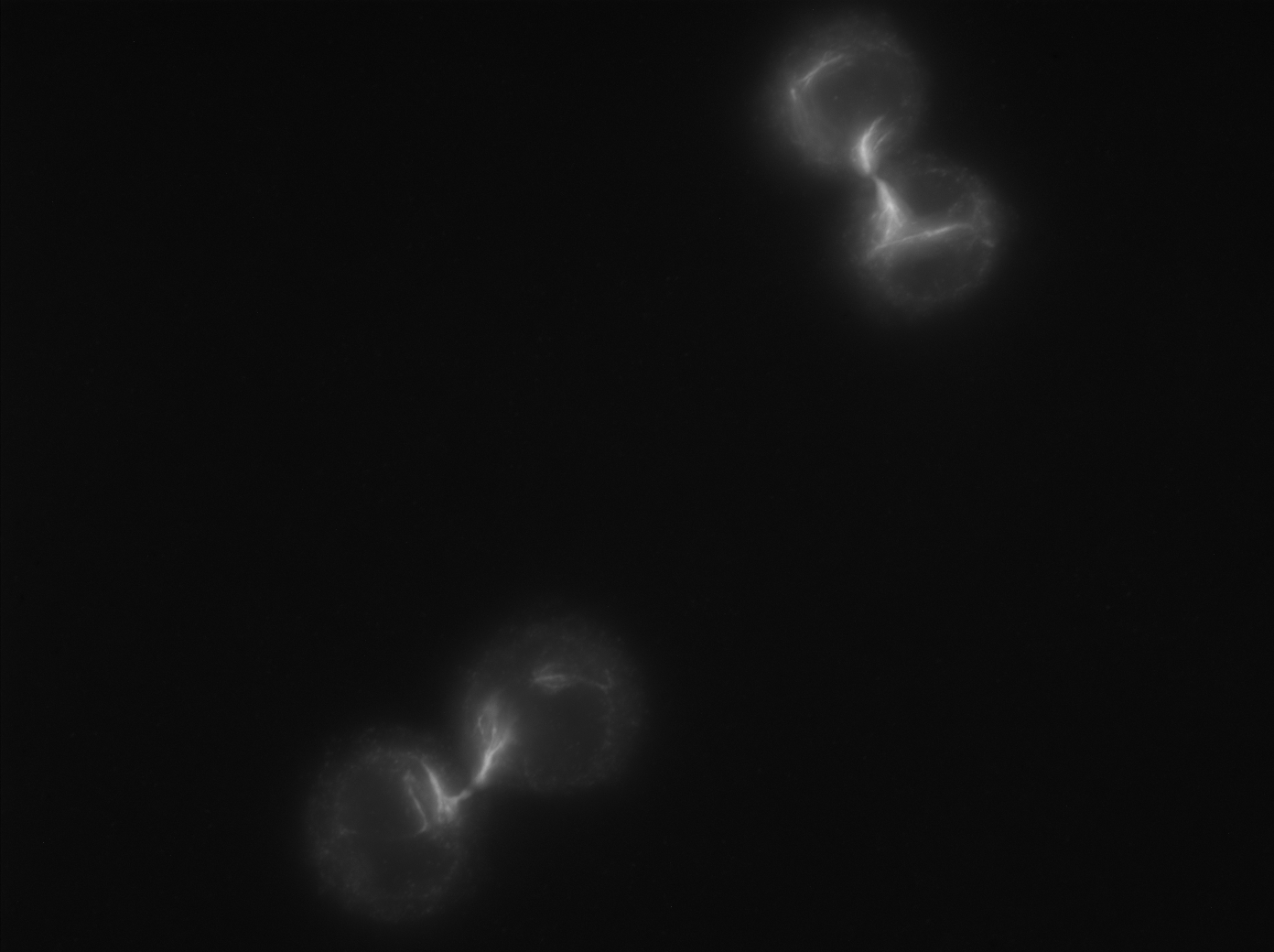

Supplement: Supplementary file 21 — Source data Fig. 6 part 2 [file 44319_2026_742_MOESM21_ESM.zip › Figure 6 Part 2/Fig 6abcd Cas9 TPGS1-KO acetylated tubulin atubulin part 2/TPGS1-KO R2 9-11-24 LT14LT15.Project Maximum Z_XY1726265117_Z0_T0_C2.tif]

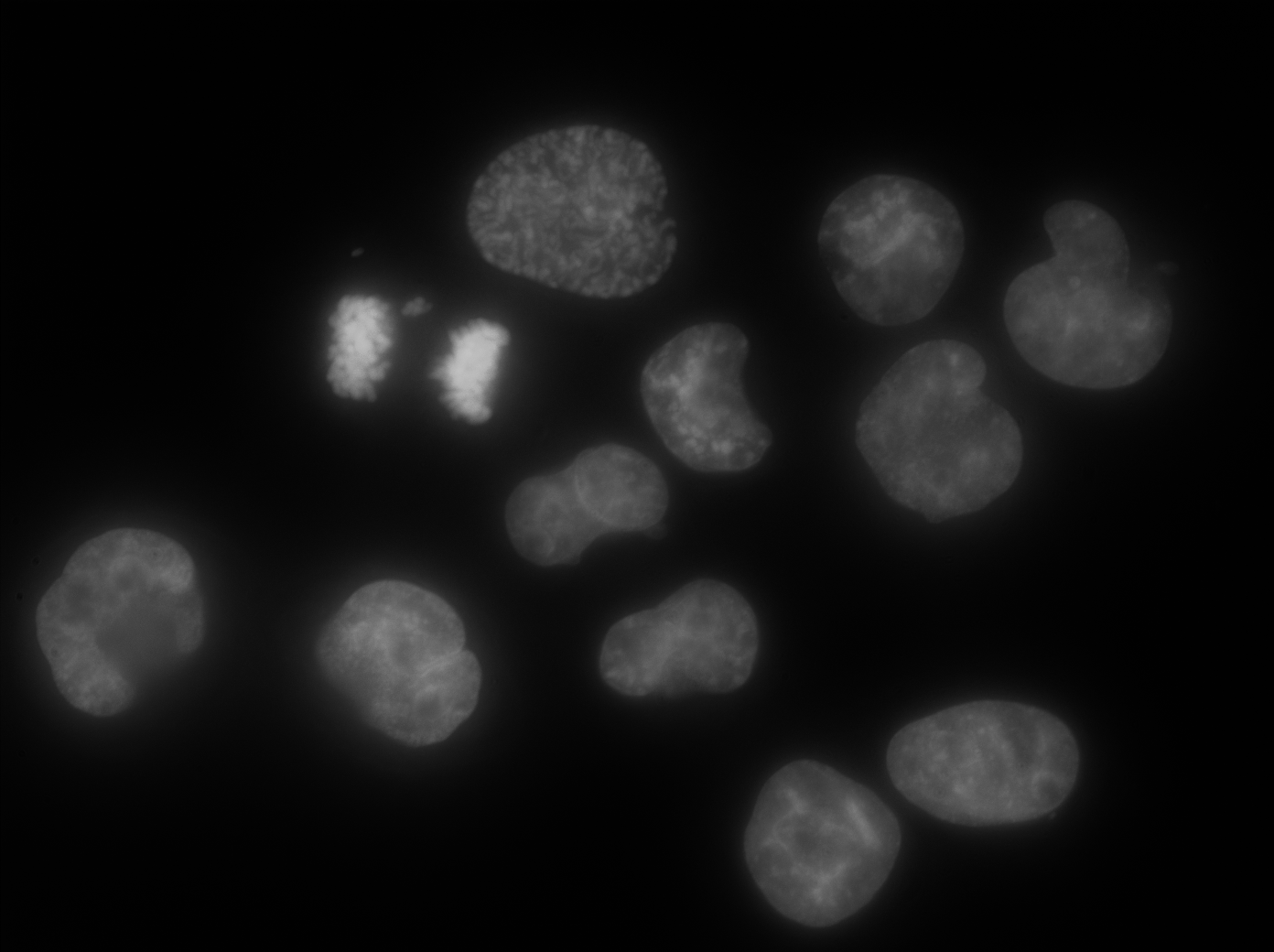

Supplement: Supplementary file 21 — Source data Fig. 6 part 2 [file 44319_2026_742_MOESM21_ESM.zip › Figure 6 Part 2/Fig 6abcd Cas9 TPGS1-KO acetylated tubulin atubulin part 2/TPGS1-KO R3 9-13-24 LT4.Project Maximum Z_XY1726760376_Z0_T0_C0.tif]

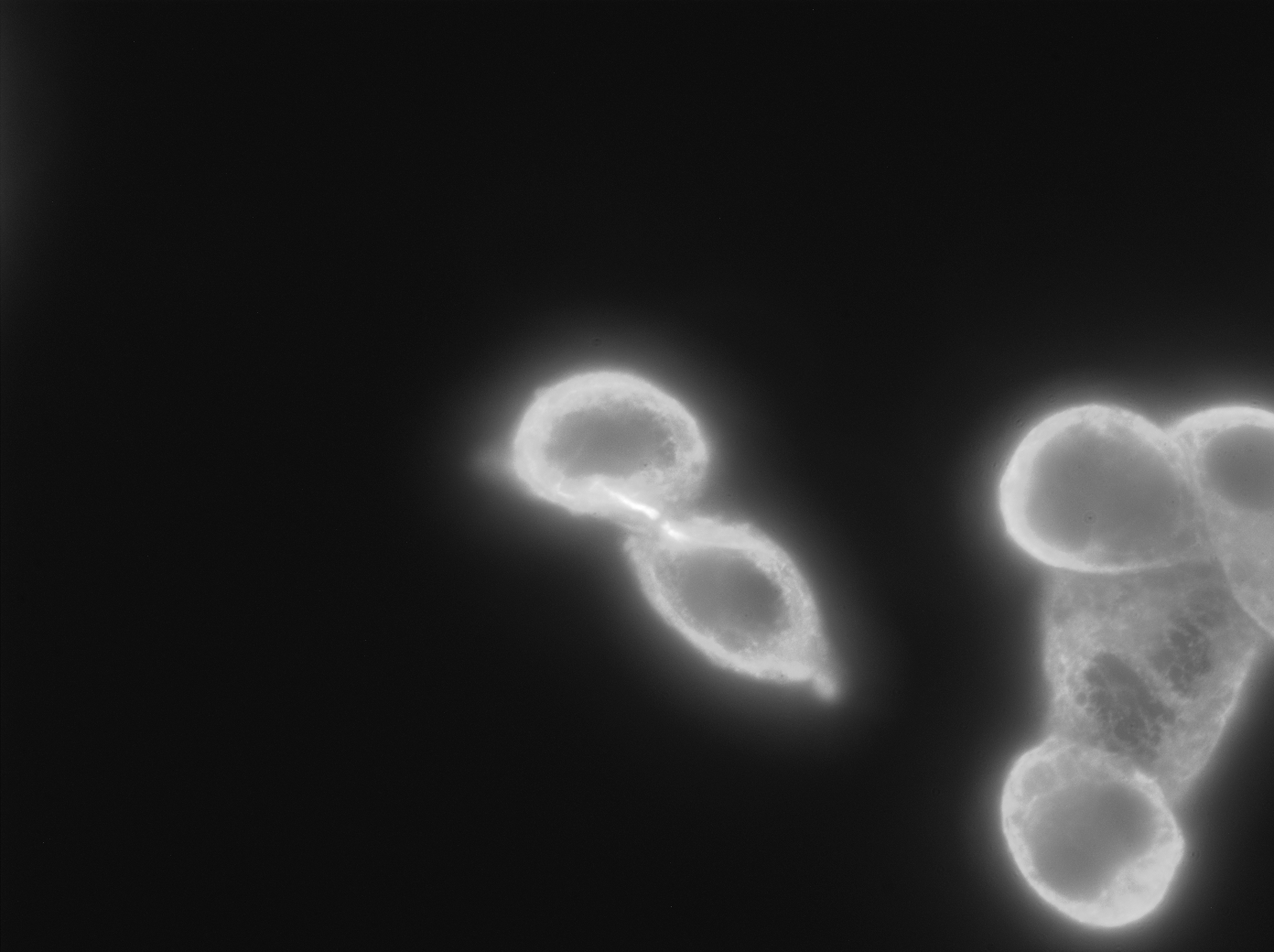

Supplement: Supplementary file 21 — Source data Fig. 6 part 2 [file 44319_2026_742_MOESM21_ESM.zip › Figure 6 Part 2/Fig 6abcd Cas9 TPGS1-KO acetylated tubulin atubulin part 2/TPGS1-KO R3 9-13-24 LT23.Project Maximum Z_XY1726764560_Z0_T0_C1.tif]

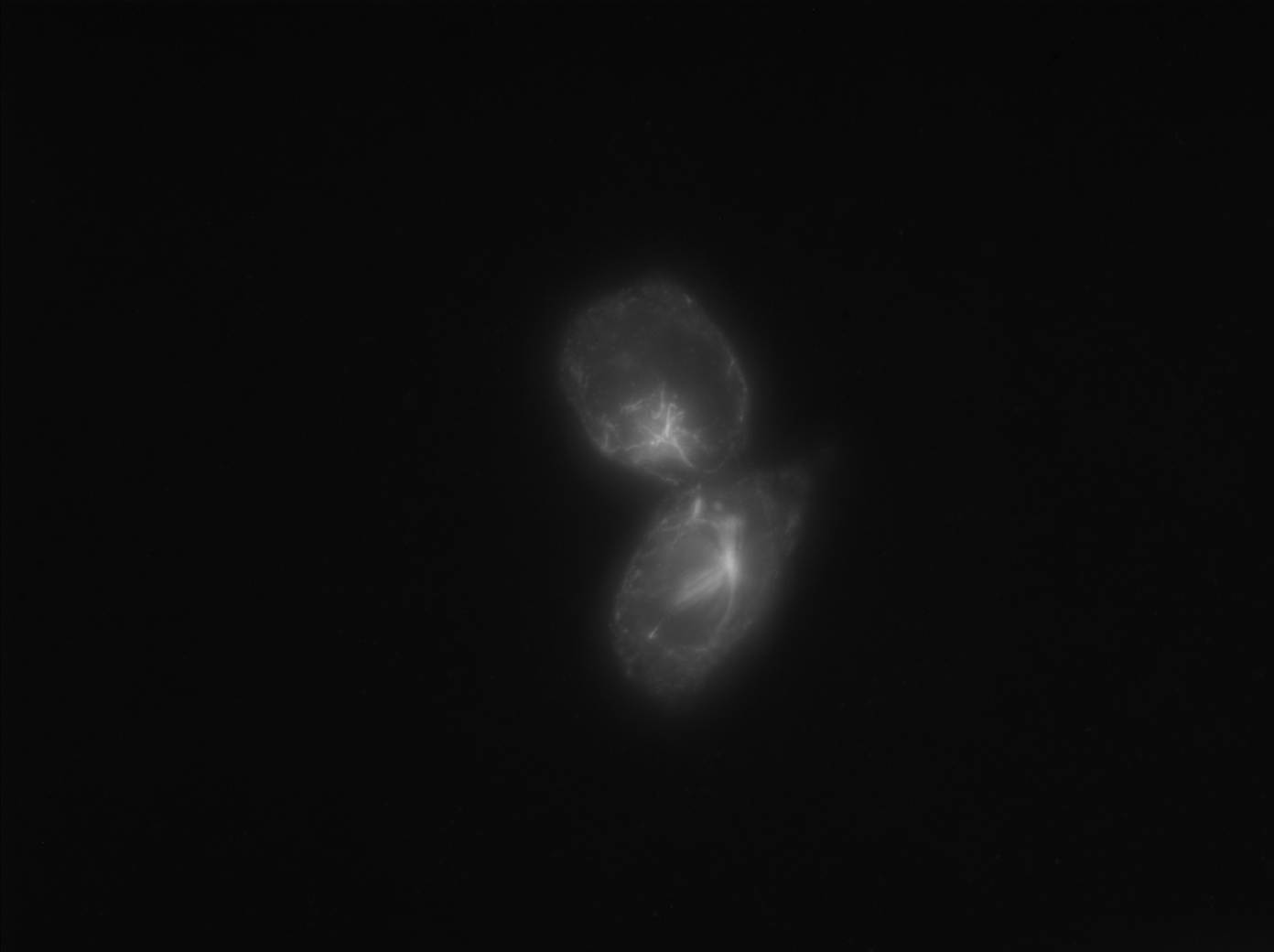

Supplement: Supplementary file 21 — Source data Fig. 6 part 2 [file 44319_2026_742_MOESM21_ESM.zip › Figure 6 Part 2/Fig 6abcd Cas9 TPGS1-KO acetylated tubulin atubulin part 2/TPGS1-KO R2 9-11-24 PA9.Project Maximum Z_XY1726262450_Z0_T0_C2.tif]

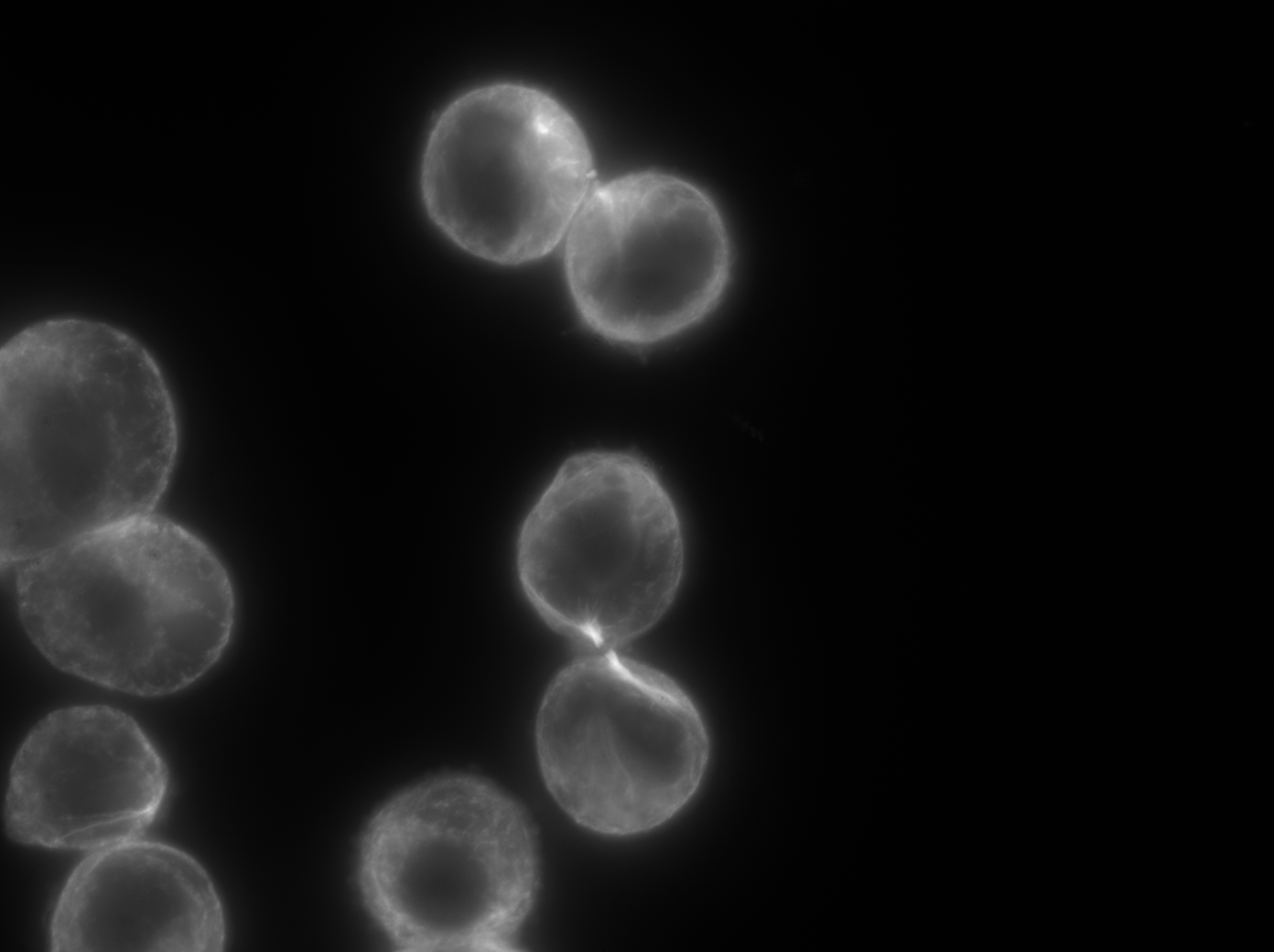

Supplement: Supplementary file 21 — Source data Fig. 6 part 2 [file 44319_2026_742_MOESM21_ESM.zip › Figure 6 Part 2/Fig 6abcd Cas9 TPGS1-KO acetylated tubulin atubulin part 2/TPGS1-KO R2 9-11-24 LT8LT9.Project Maximum Z_XY1726261890_Z0_T0_C1.tif]

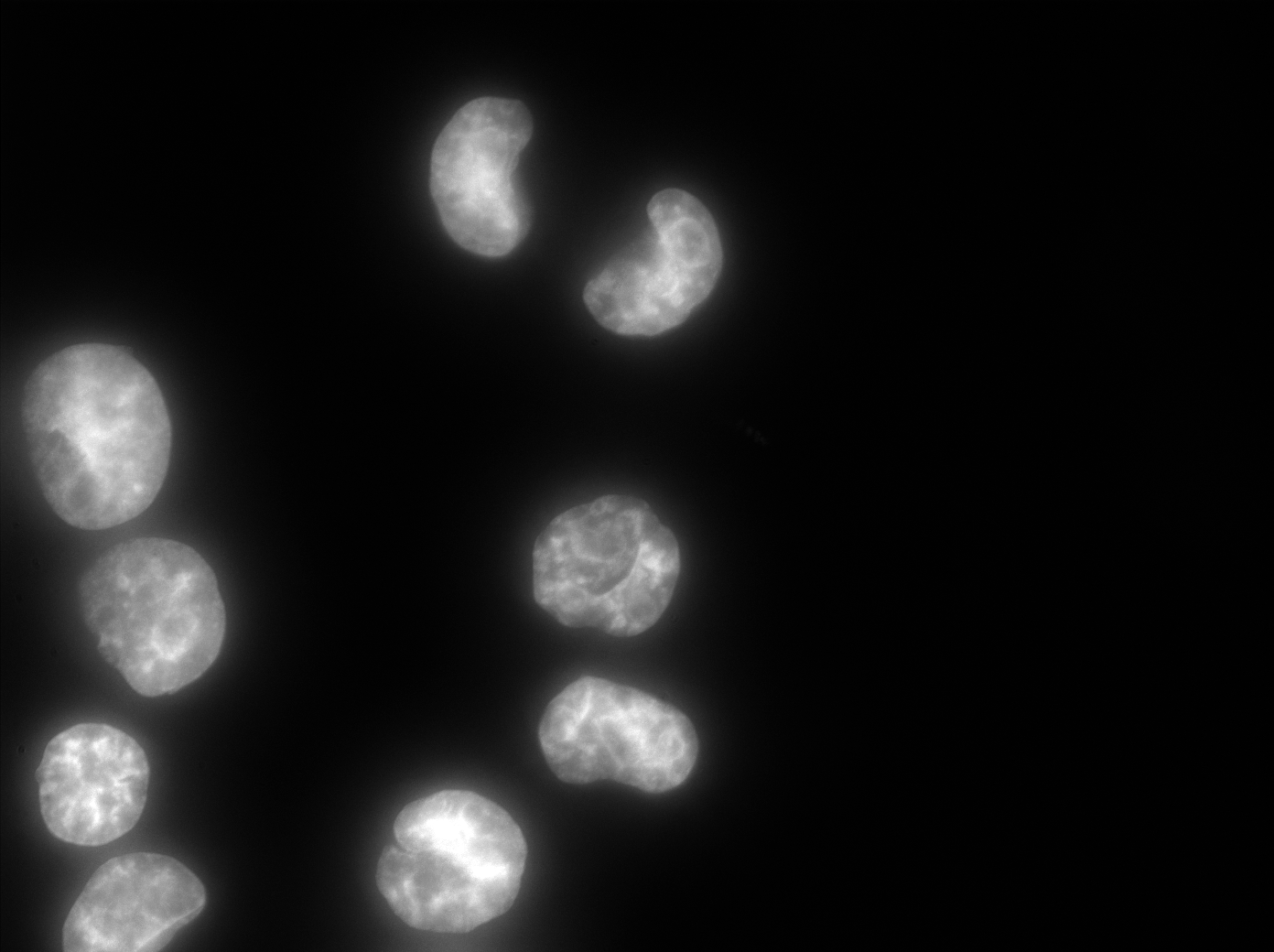

Supplement: Supplementary file 21 — Source data Fig. 6 part 2 [file 44319_2026_742_MOESM21_ESM.zip › Figure 6 Part 2/Fig 6abcd Cas9 TPGS1-KO acetylated tubulin atubulin part 2/TPGS1-KO R2 9-11-24 LT8LT9.Project Maximum Z_XY1726261890_Z0_T0_C0.tif]

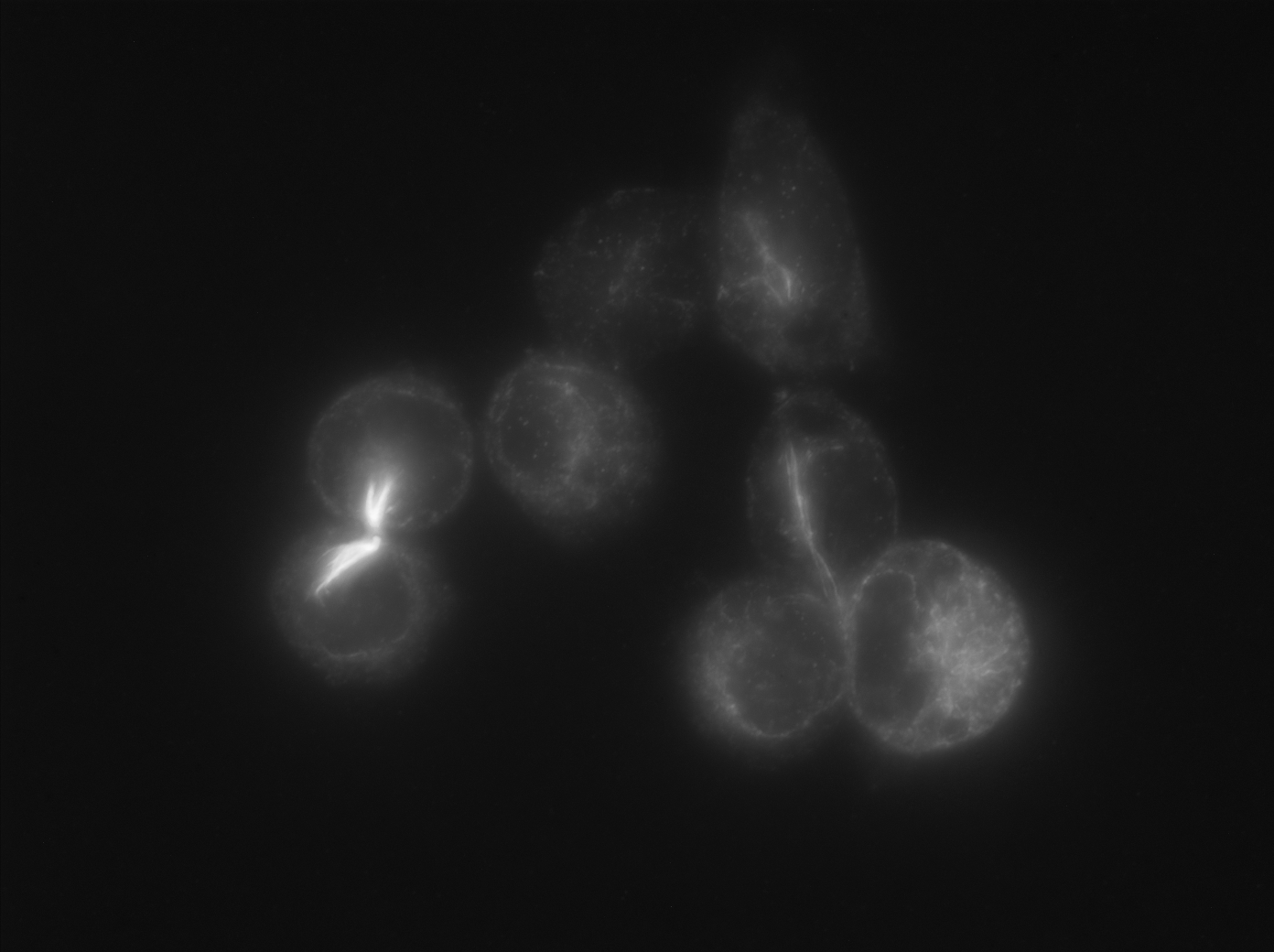

Supplement: Supplementary file 21 — Source data Fig. 6 part 2 [file 44319_2026_742_MOESM21_ESM.zip › Figure 6 Part 2/Fig 6abcd Cas9 TPGS1-KO acetylated tubulin atubulin part 2/TPGS1-KO R2 9-11-24 LT22.Project Maximum Z_XY1726267969_Z0_T0_C2.tif]

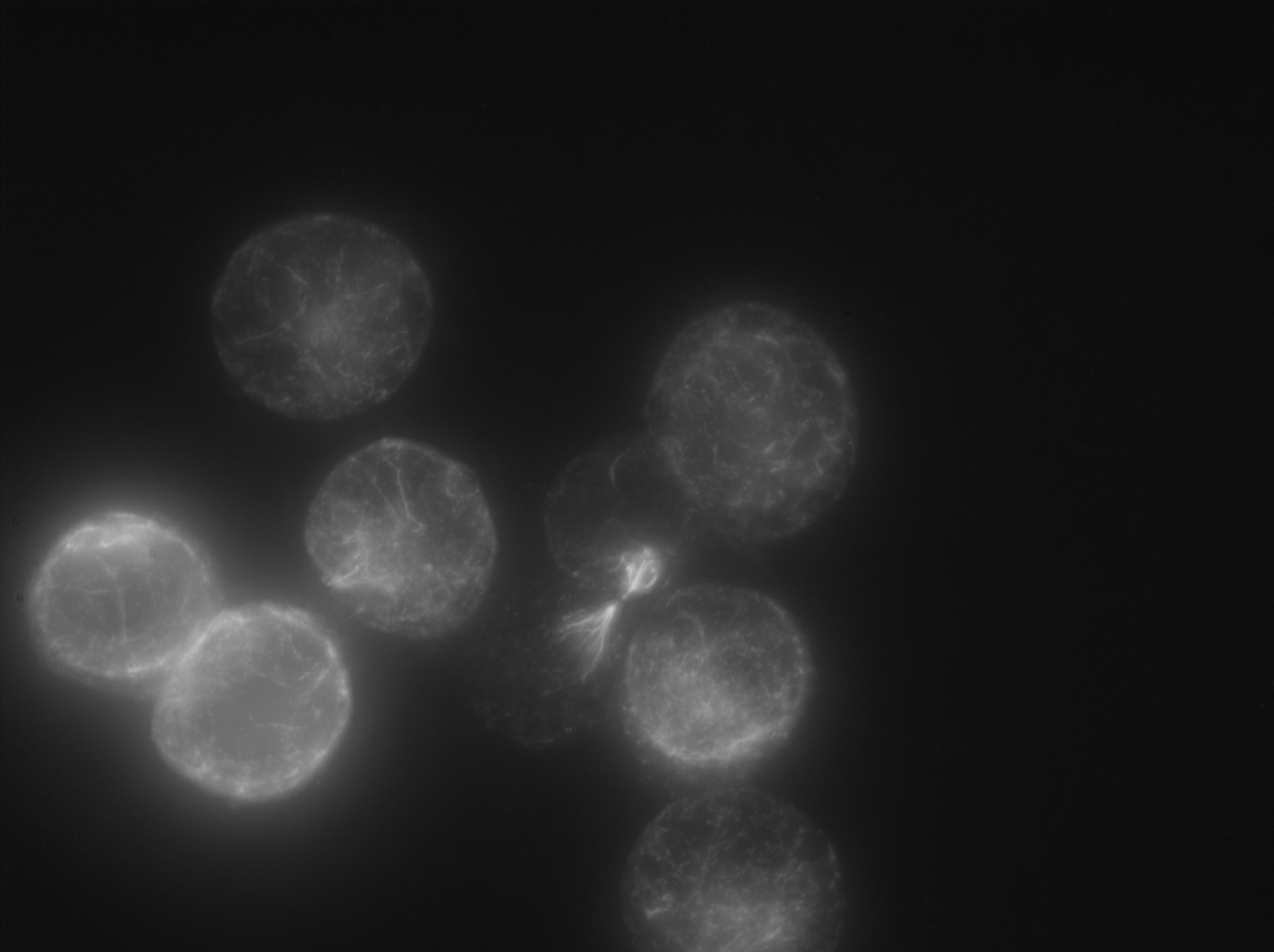

Supplement: Supplementary file 21 — Source data Fig. 6 part 2 [file 44319_2026_742_MOESM21_ESM.zip › Figure 6 Part 2/Fig 6abcd Cas9 TPGS1-KO acetylated tubulin atubulin part 2/TPGS1-KO R2 9-11-24 LT20.Project Maximum Z_XY1726266998_Z0_T0_C2.tif]

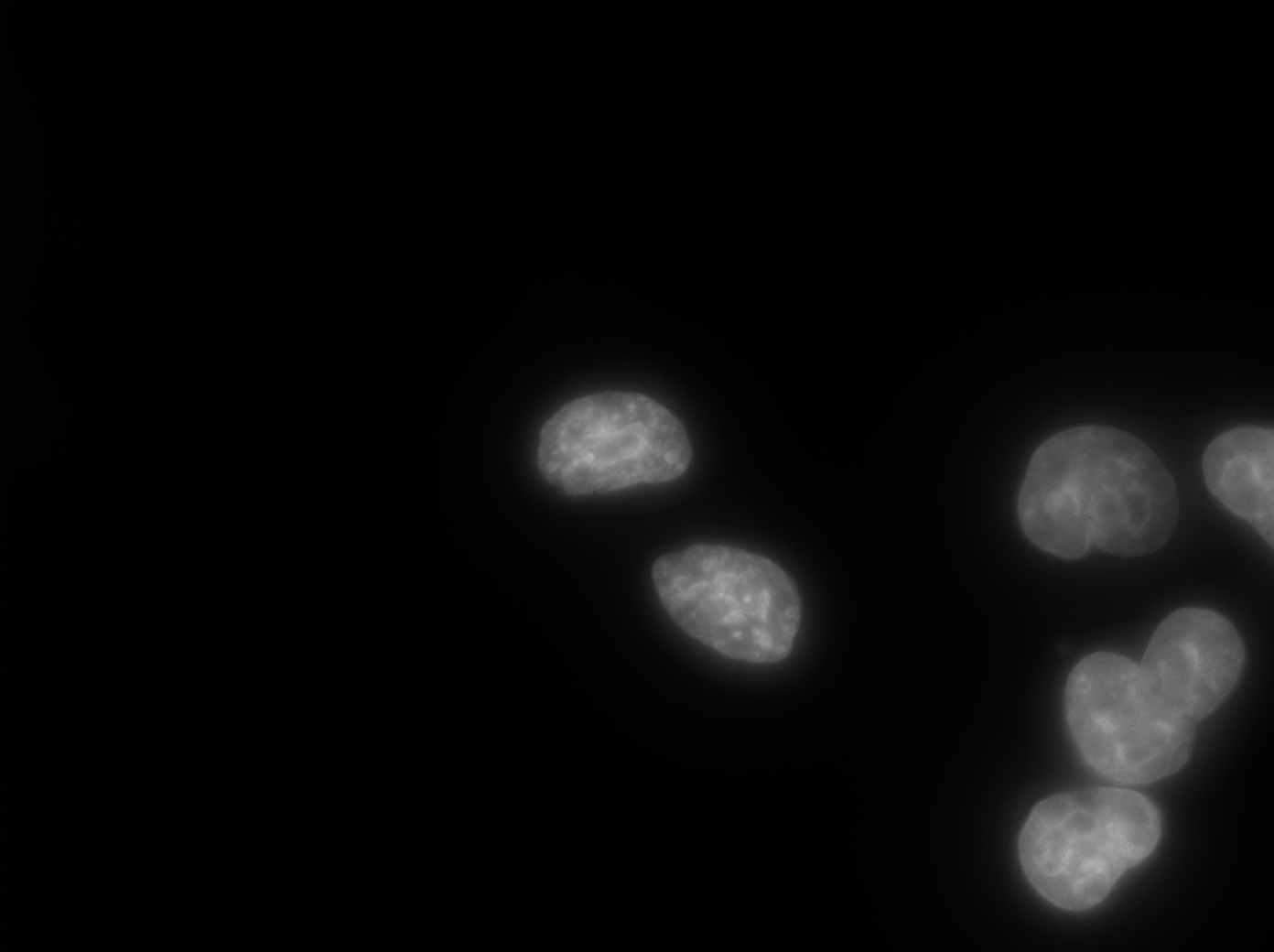

Supplement: Supplementary file 21 — Source data Fig. 6 part 2 [file 44319_2026_742_MOESM21_ESM.zip › Figure 6 Part 2/Fig 6abcd Cas9 TPGS1-KO acetylated tubulin atubulin part 2/TPGS1-KO R3 9-13-24 LT23.Project Maximum Z_XY1726764560_Z0_T0_C0.tif]

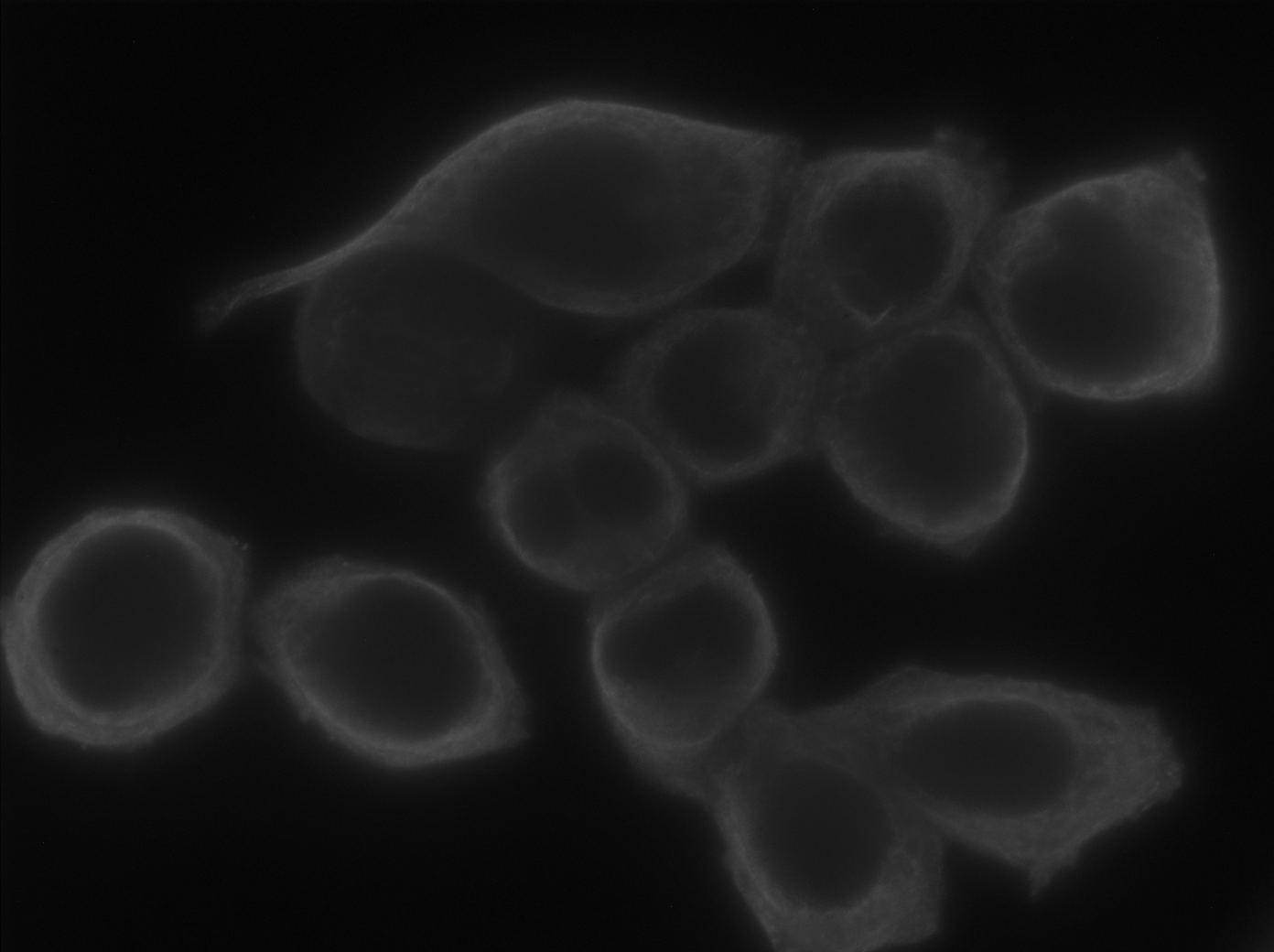

Supplement: Supplementary file 21 — Source data Fig. 6 part 2 [file 44319_2026_742_MOESM21_ESM.zip › Figure 6 Part 2/Fig 6abcd Cas9 TPGS1-KO acetylated tubulin atubulin part 2/TPGS1-KO R3 9-13-24 LT4.Project Maximum Z_XY1726760376_Z0_T0_C1.tif]

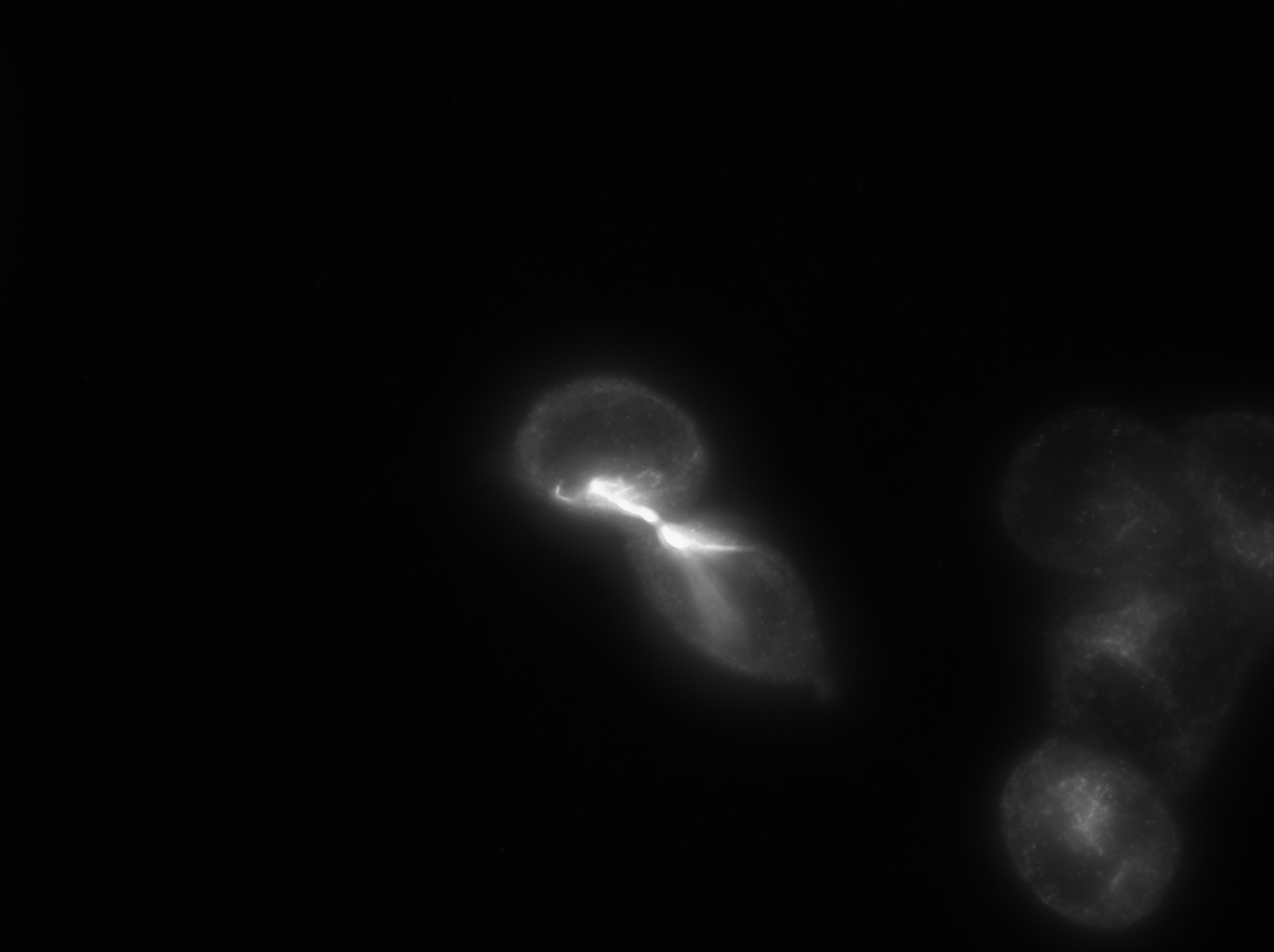

Supplement: Supplementary file 21 — Source data Fig. 6 part 2 [file 44319_2026_742_MOESM21_ESM.zip › Figure 6 Part 2/Fig 6abcd Cas9 TPGS1-KO acetylated tubulin atubulin part 2/TPGS1-KO R3 9-13-24 LT23.Project Maximum Z_XY1726764560_Z0_T0_C2.tif]

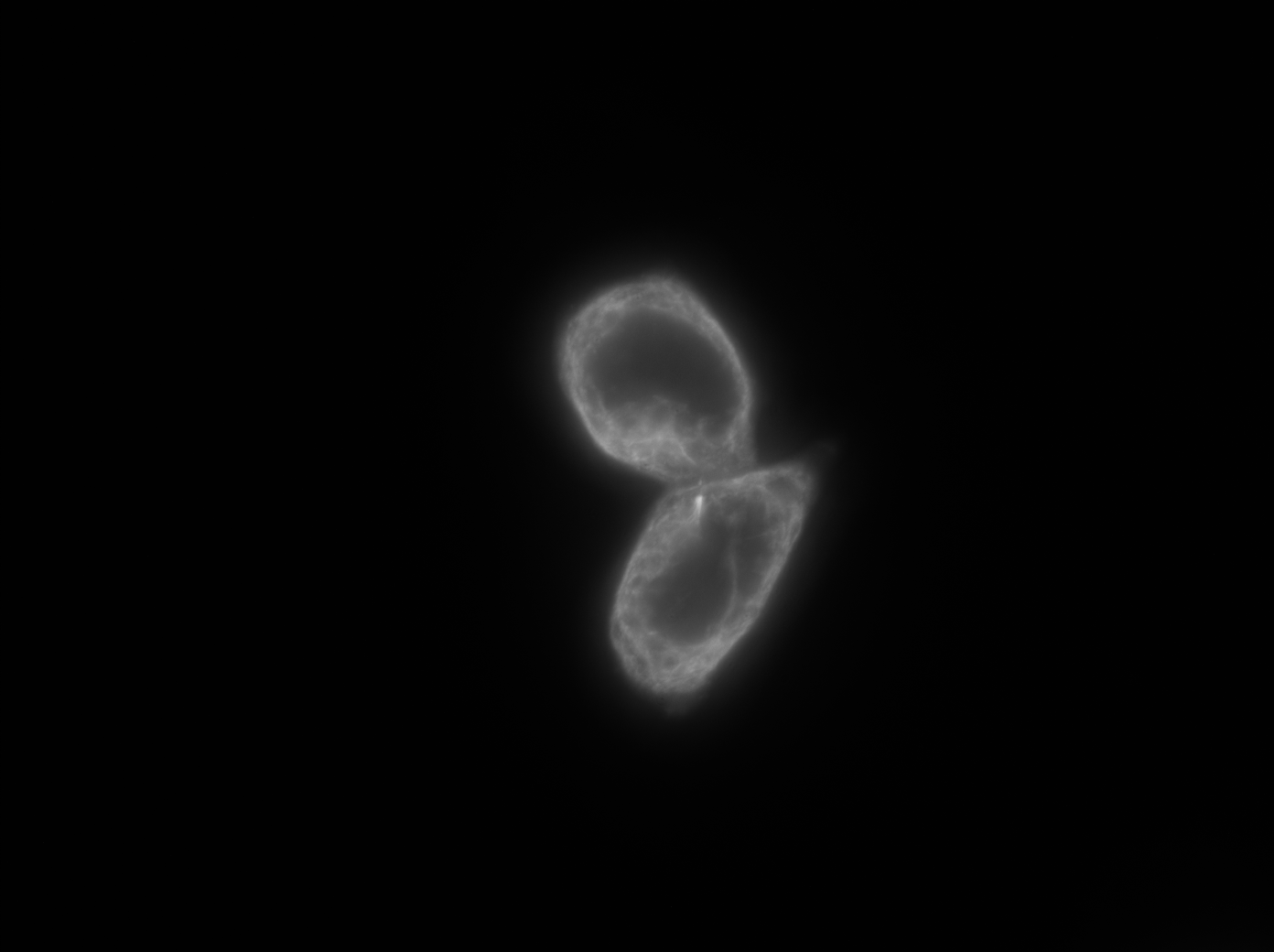

Supplement: Supplementary file 21 — Source data Fig. 6 part 2 [file 44319_2026_742_MOESM21_ESM.zip › Figure 6 Part 2/Fig 6abcd Cas9 TPGS1-KO acetylated tubulin atubulin part 2/TPGS1-KO R2 9-11-24 PA9.Project Maximum Z_XY1726262450_Z0_T0_C1.tif]

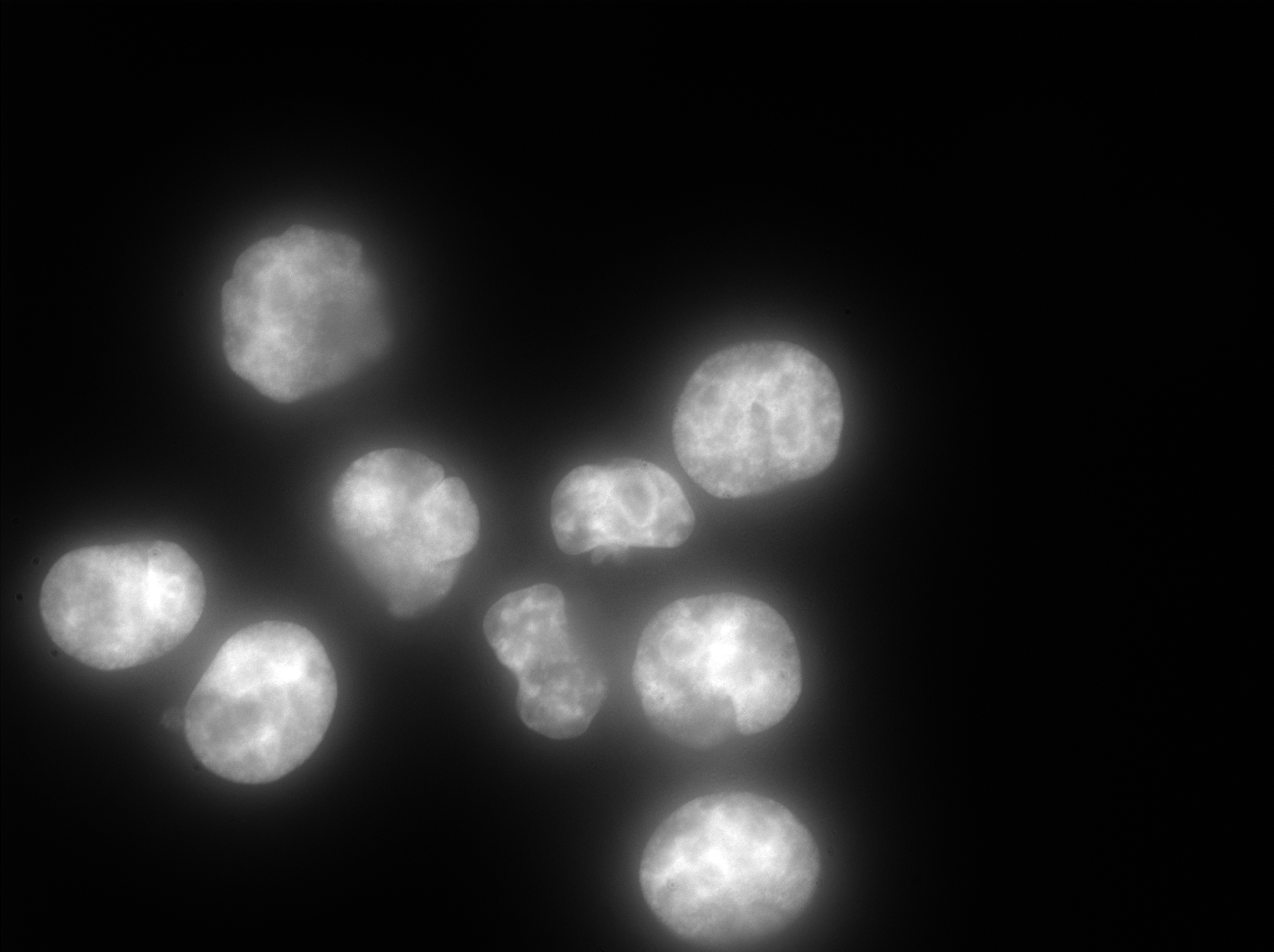

Supplement: Supplementary file 21 — Source data Fig. 6 part 2 [file 44319_2026_742_MOESM21_ESM.zip › Figure 6 Part 2/Fig 6abcd Cas9 TPGS1-KO acetylated tubulin atubulin part 2/TPGS1-KO R2 9-11-24 LT20.Project Maximum Z_XY1726266998_Z0_T0_C0.tif]

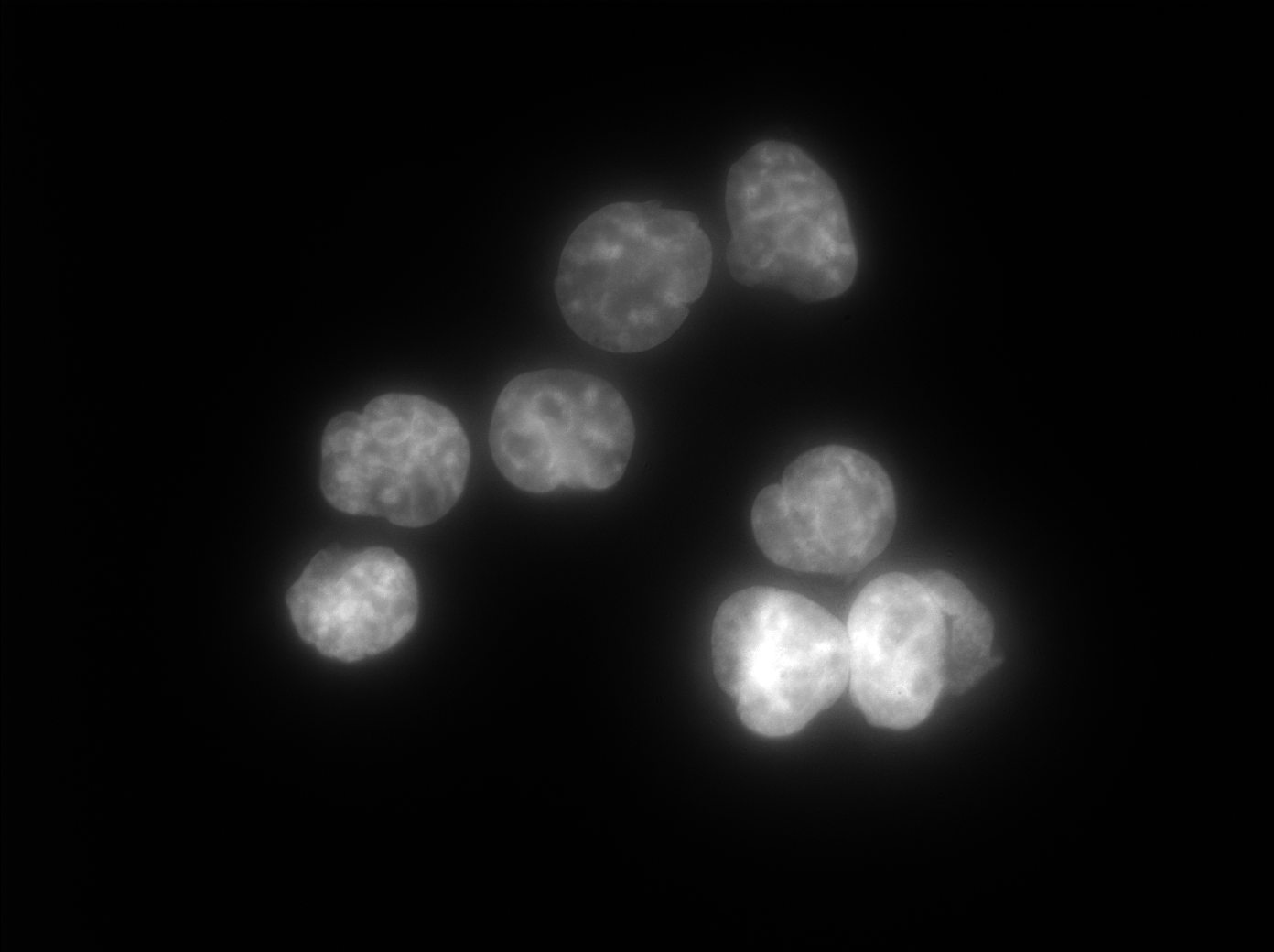

Supplement: Supplementary file 21 — Source data Fig. 6 part 2 [file 44319_2026_742_MOESM21_ESM.zip › Figure 6 Part 2/Fig 6abcd Cas9 TPGS1-KO acetylated tubulin atubulin part 2/TPGS1-KO R2 9-11-24 LT22.Project Maximum Z_XY1726267969_Z0_T0_C0.tif]

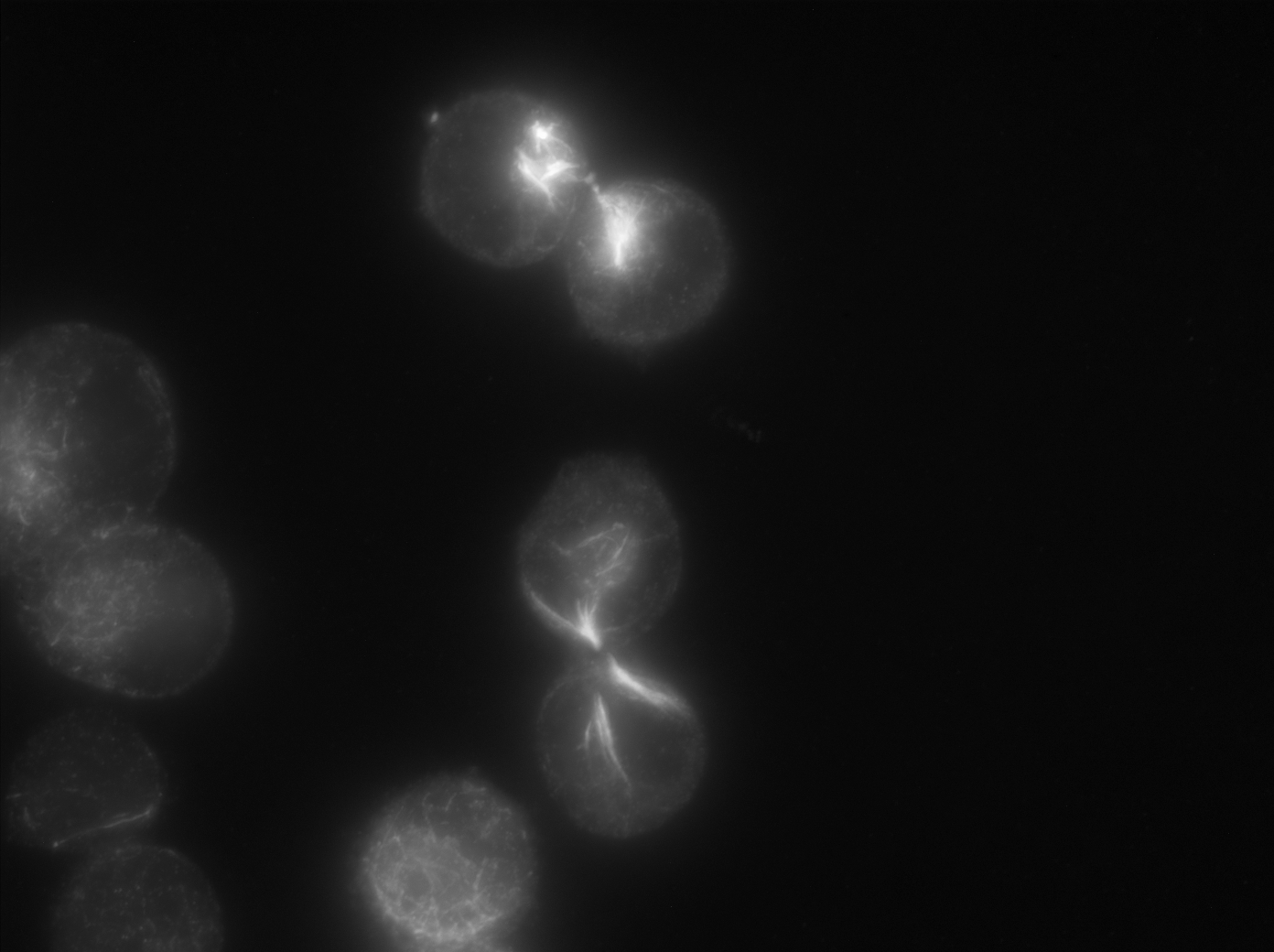

Supplement: Supplementary file 21 — Source data Fig. 6 part 2 [file 44319_2026_742_MOESM21_ESM.zip › Figure 6 Part 2/Fig 6abcd Cas9 TPGS1-KO acetylated tubulin atubulin part 2/TPGS1-KO R2 9-11-24 LT8LT9.Project Maximum Z_XY1726261890_Z0_T0_C2.tif]

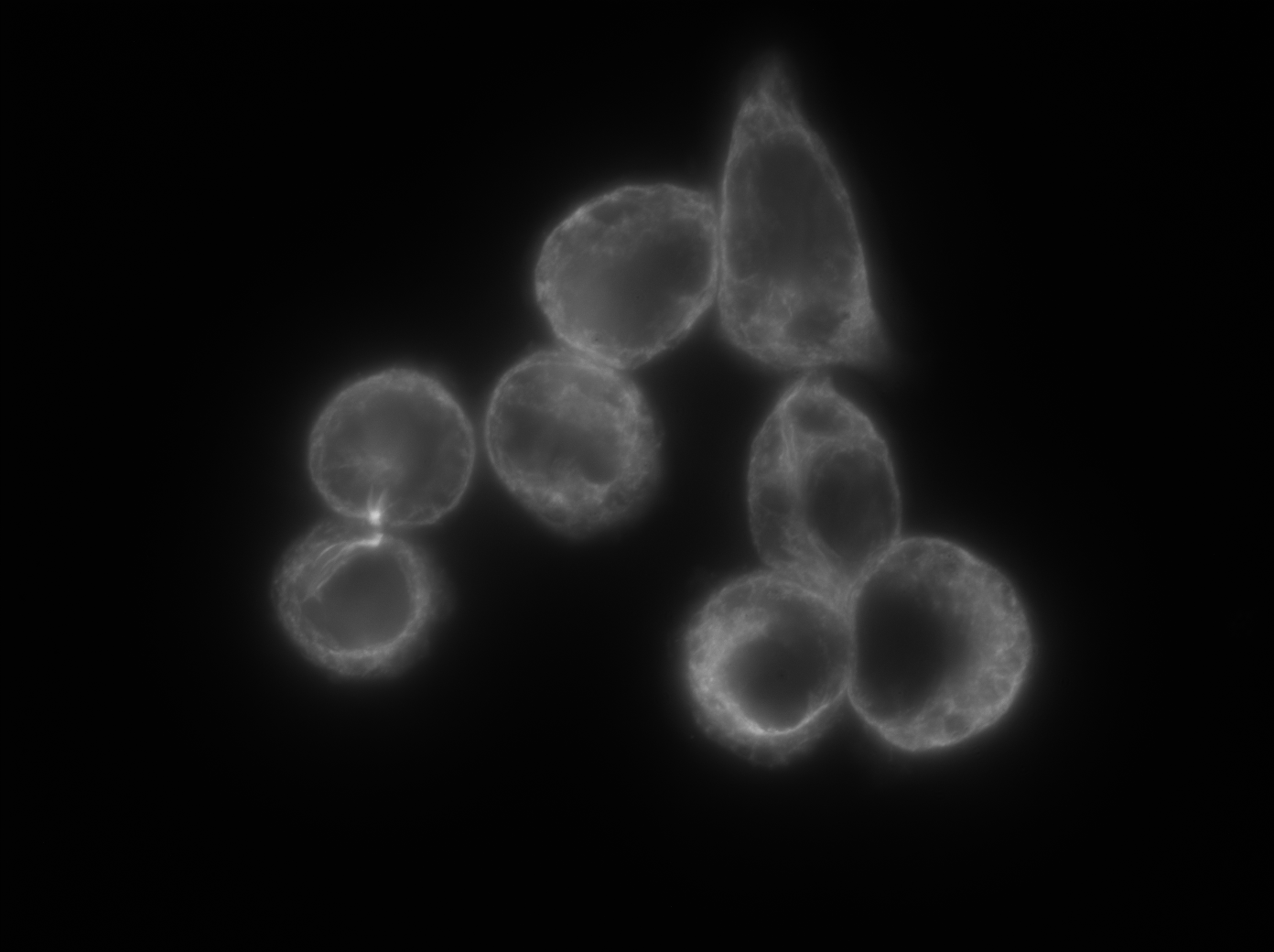

Supplement: Supplementary file 21 — Source data Fig. 6 part 2 [file 44319_2026_742_MOESM21_ESM.zip › Figure 6 Part 2/Fig 6abcd Cas9 TPGS1-KO acetylated tubulin atubulin part 2/TPGS1-KO R2 9-11-24 LT22.Project Maximum Z_XY1726267969_Z0_T0_C1.tif]

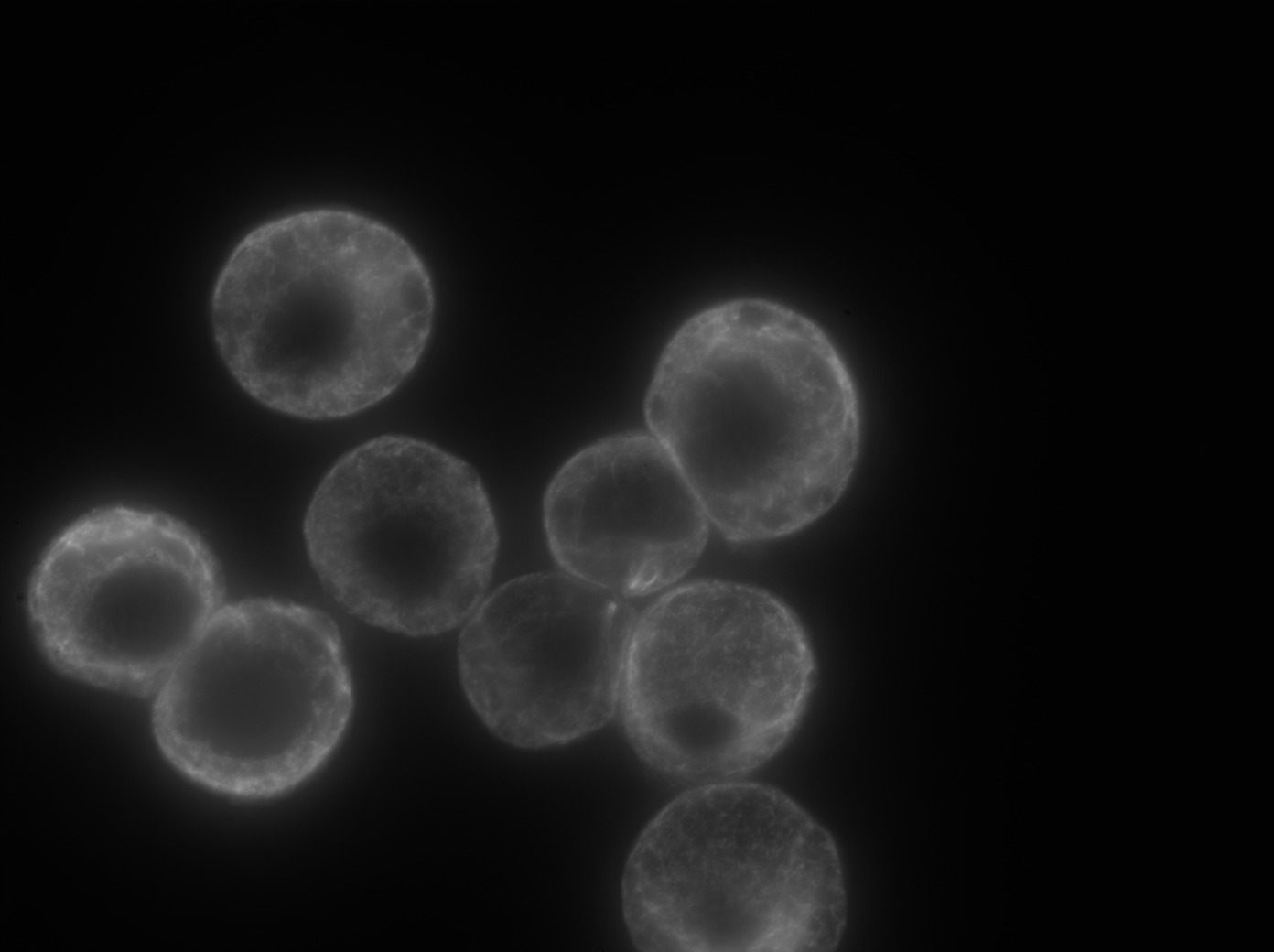

Supplement: Supplementary file 21 — Source data Fig. 6 part 2 [file 44319_2026_742_MOESM21_ESM.zip › Figure 6 Part 2/Fig 6abcd Cas9 TPGS1-KO acetylated tubulin atubulin part 2/TPGS1-KO R2 9-11-24 LT20.Project Maximum Z_XY1726266998_Z0_T0_C1.tif]

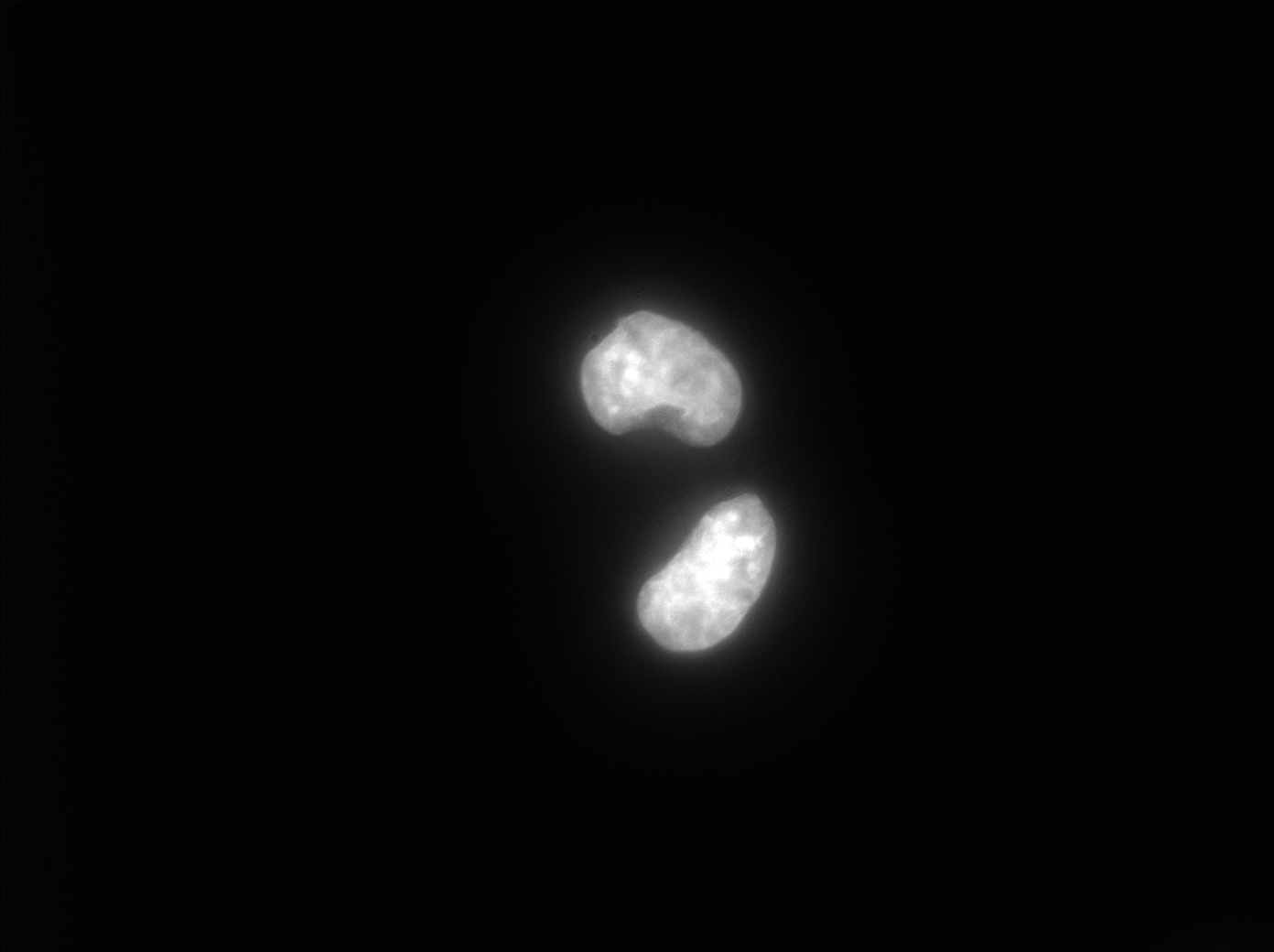

Supplement: Supplementary file 21 — Source data Fig. 6 part 2 [file 44319_2026_742_MOESM21_ESM.zip › Figure 6 Part 2/Fig 6abcd Cas9 TPGS1-KO acetylated tubulin atubulin part 2/TPGS1-KO R2 9-11-24 PA9.Project Maximum Z_XY1726262450_Z0_T0_C0.tif]

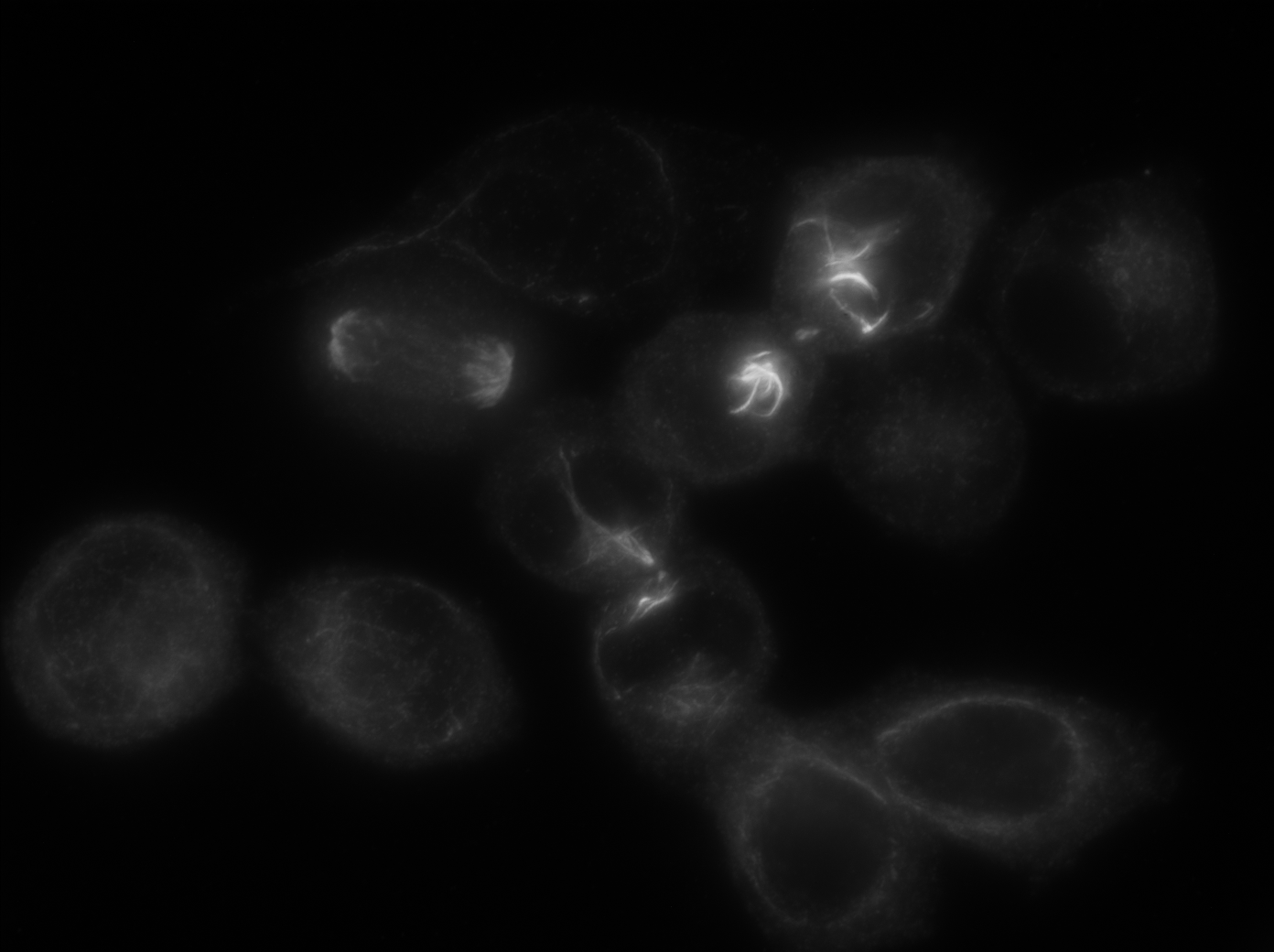

Supplement: Supplementary file 21 — Source data Fig. 6 part 2 [file 44319_2026_742_MOESM21_ESM.zip › Figure 6 Part 2/Fig 6abcd Cas9 TPGS1-KO acetylated tubulin atubulin part 2/TPGS1-KO R3 9-13-24 LT4.Project Maximum Z_XY1726760376_Z0_T0_C2.tif]

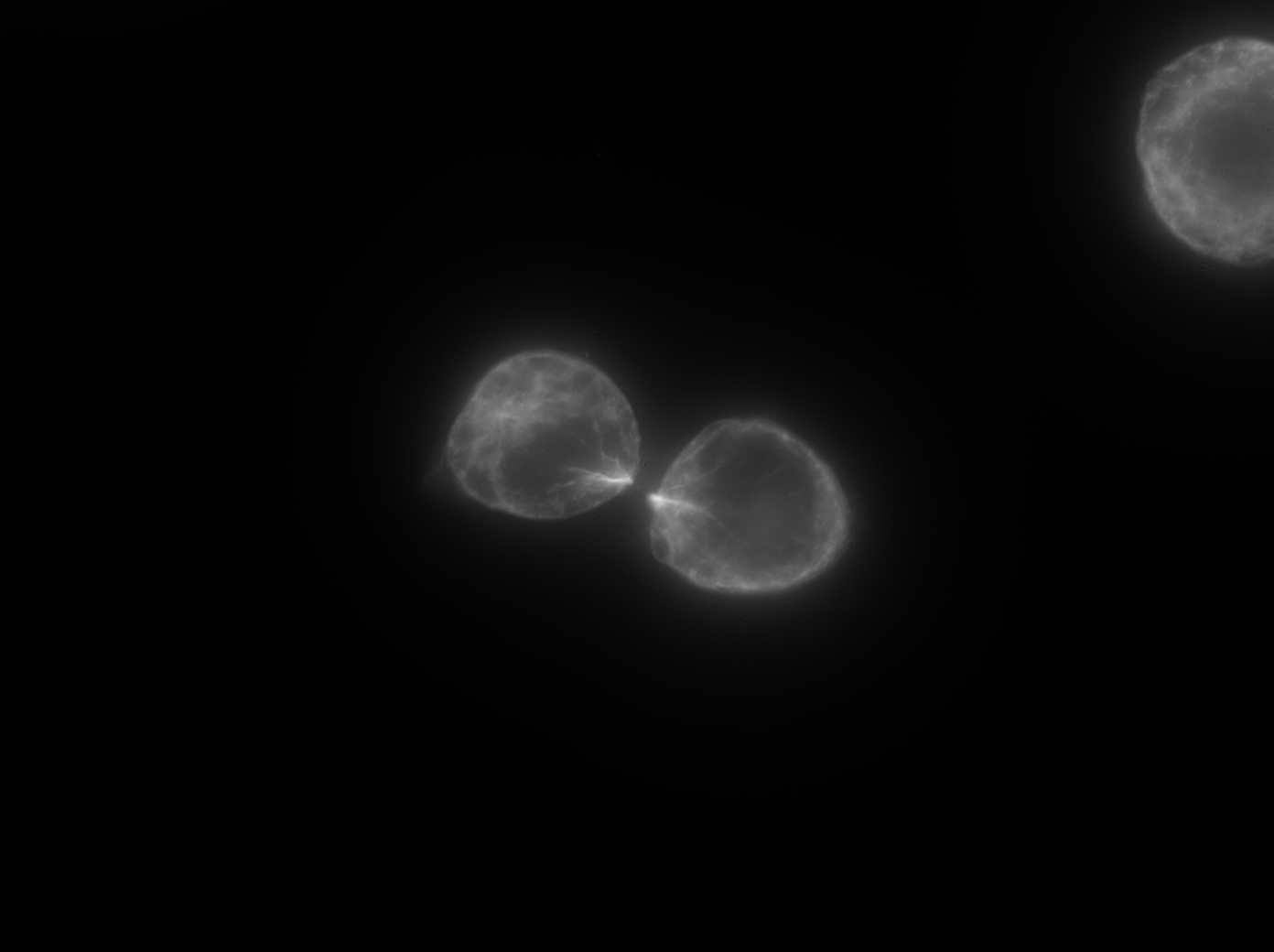

Supplement: Supplementary file 21 — Source data Fig. 6 part 2 [file 44319_2026_742_MOESM21_ESM.zip › Figure 6 Part 2/Fig 6abcd Cas9 TPGS1-KO acetylated tubulin atubulin part 2/TPGS1-KO R2 9-11-24 LT30.Project Maximum Z_XY1726270338_Z0_T0_C1.tif]

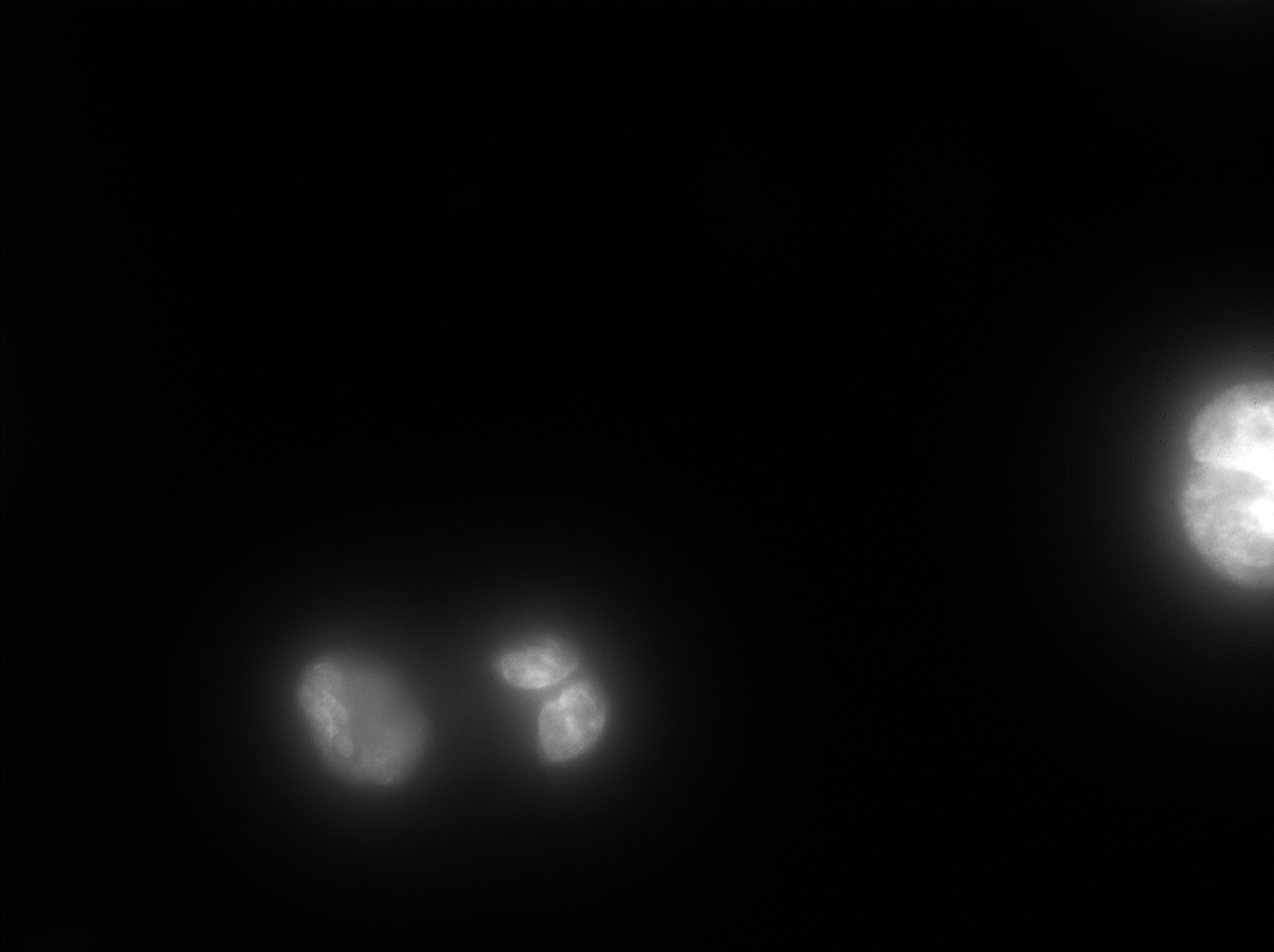

Supplement: Supplementary file 21 — Source data Fig. 6 part 2 [file 44319_2026_742_MOESM21_ESM.zip › Figure 6 Part 2/Fig 6abcd Cas9 TPGS1-KO acetylated tubulin atubulin part 2/TPGS1-KO R2 9-11-24 PA6.Project Maximum Z_XY1726261339_Z0_T0_C0.tif]

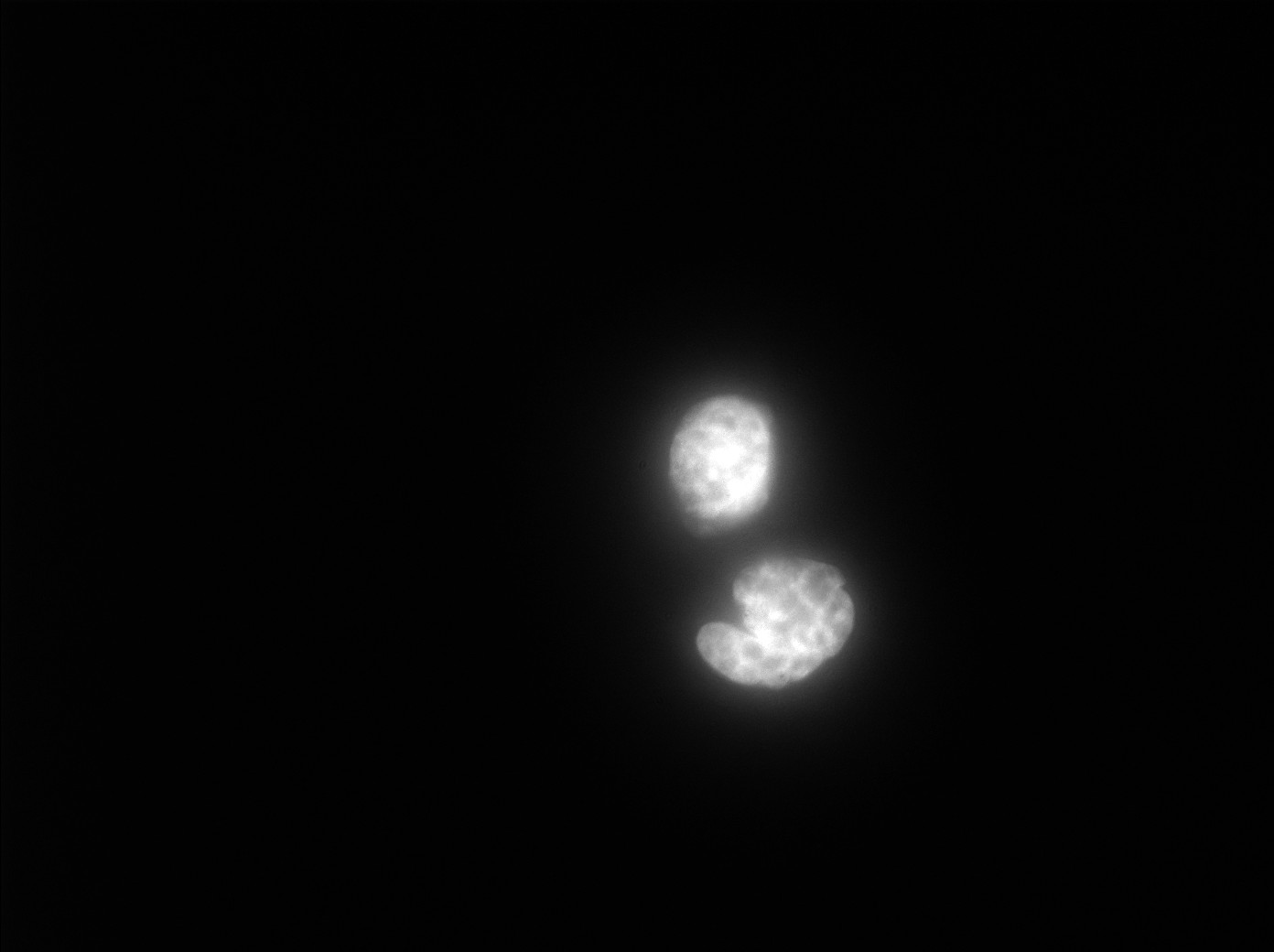

Supplement: Supplementary file 21 — Source data Fig. 6 part 2 [file 44319_2026_742_MOESM21_ESM.zip › Figure 6 Part 2/Fig 6abcd Cas9 TPGS1-KO acetylated tubulin atubulin part 2/TPGS1-KO R2 9-11-24 LT2.Project Maximum Z_XY1726259084_Z0_T0_C0.tif]

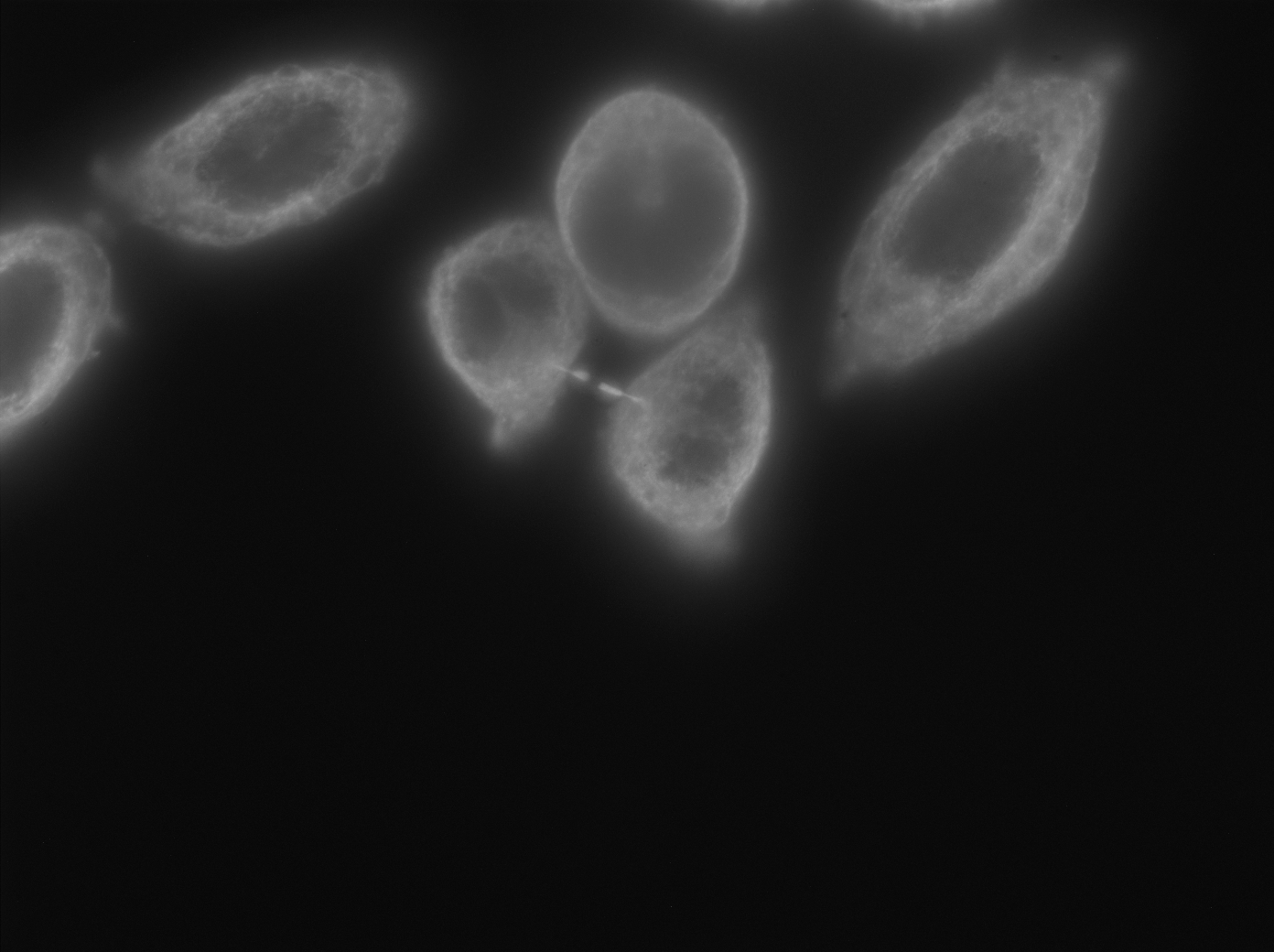

Supplement: Supplementary file 21 — Source data Fig. 6 part 2 [file 44319_2026_742_MOESM21_ESM.zip › Figure 6 Part 2/Fig 6abcd Cas9 TPGS1-KO acetylated tubulin atubulin part 2/TPGS1-KO R3 9-13-24 LT16.Project Maximum Z_XY1726763904_Z0_T0_C1.tif]

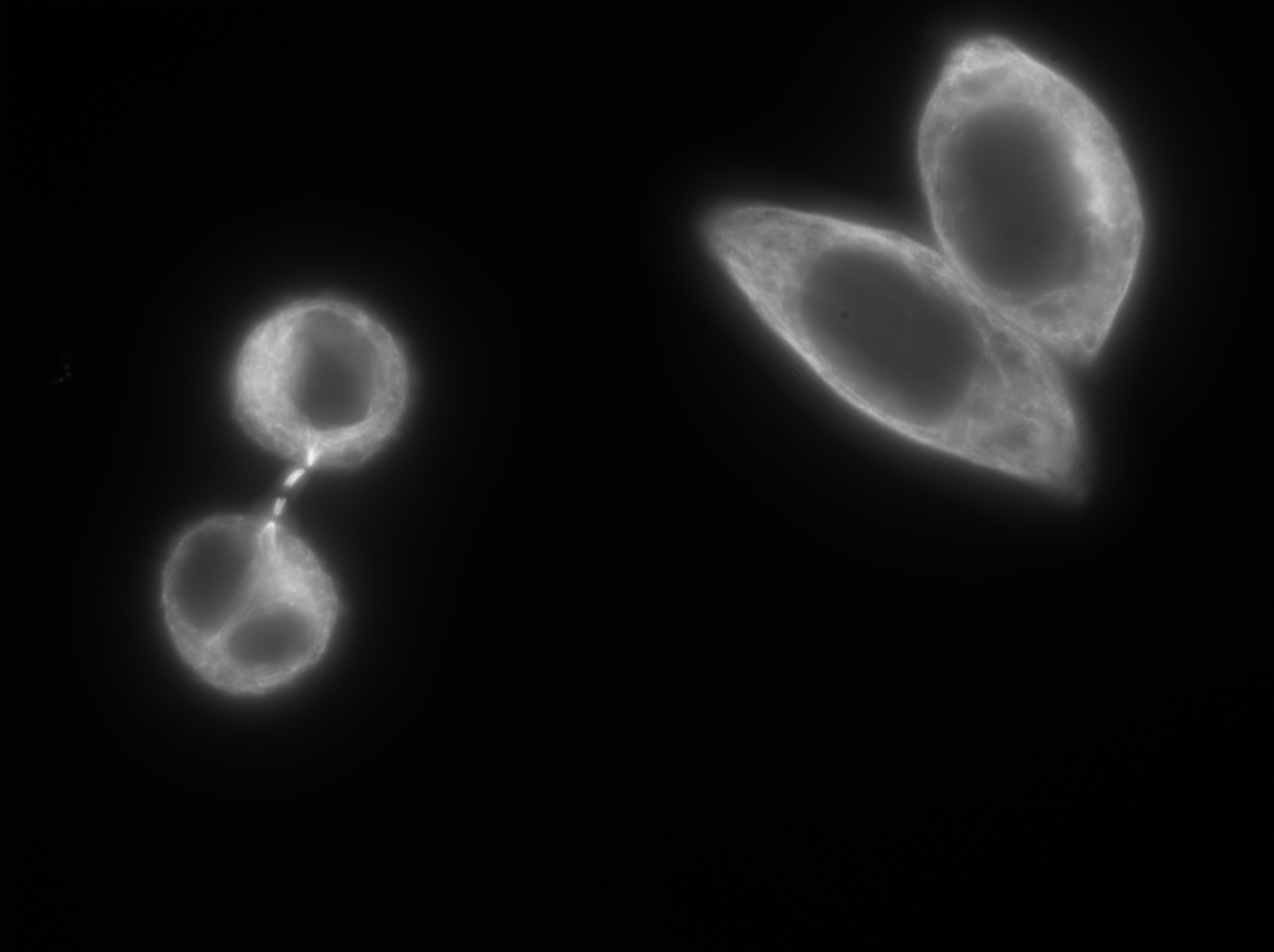

Supplement: Supplementary file 21 — Source data Fig. 6 part 2 [file 44319_2026_742_MOESM21_ESM.zip › Figure 6 Part 2/Fig 6abcd Cas9 TPGS1-KO acetylated tubulin atubulin part 2/TPGS1-KO R2 9-11-24 LT7.Project Maximum Z_XY1726261460_Z0_T0_C1.tif]

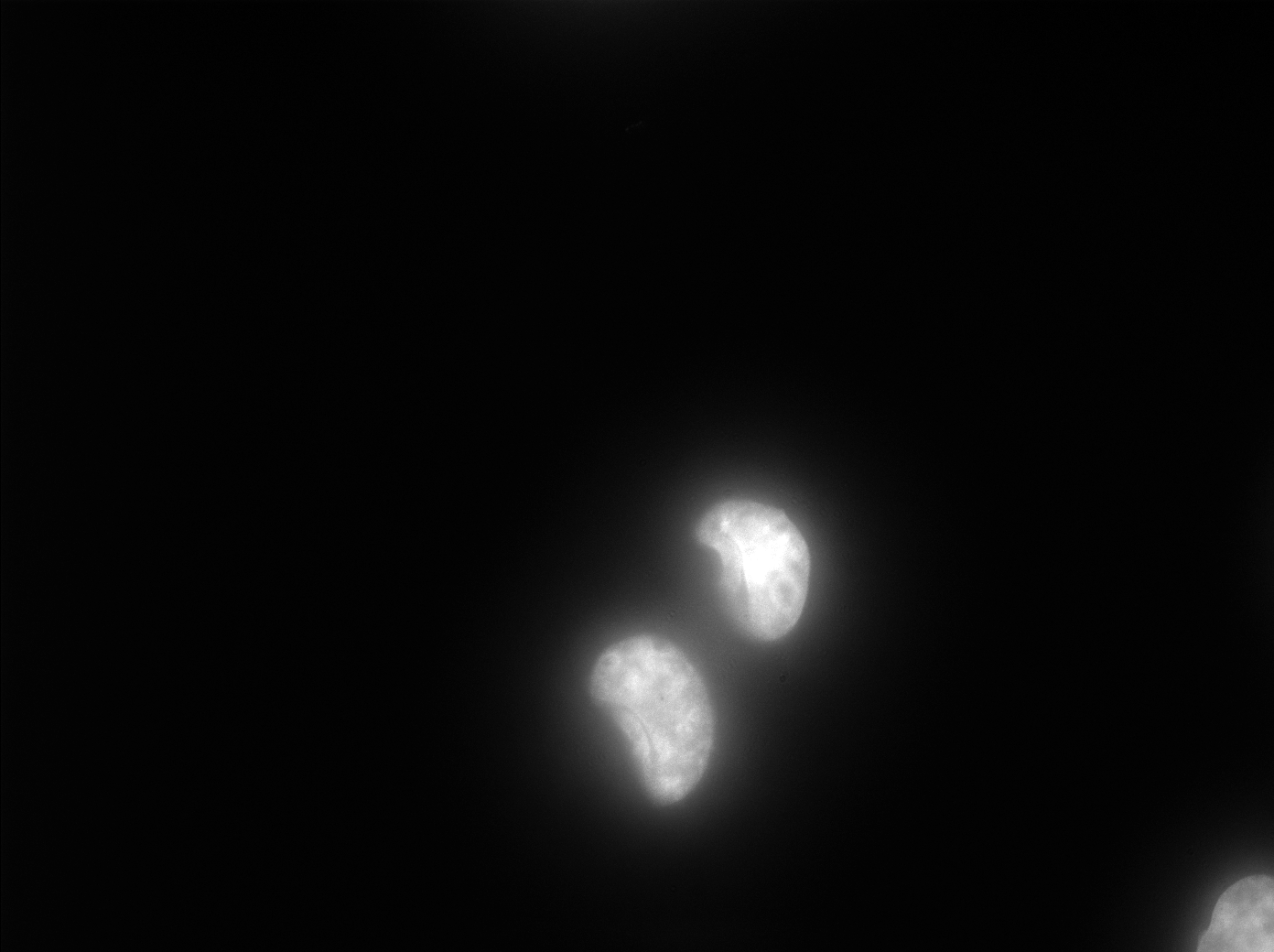

Supplement: Supplementary file 21 — Source data Fig. 6 part 2 [file 44319_2026_742_MOESM21_ESM.zip › Figure 6 Part 2/Fig 6abcd Cas9 TPGS1-KO acetylated tubulin atubulin part 2/TPGS1-KO R2 9-11-24 PA18.Project Maximum Z_XY1726268793_Z0_T0_C0.tif]

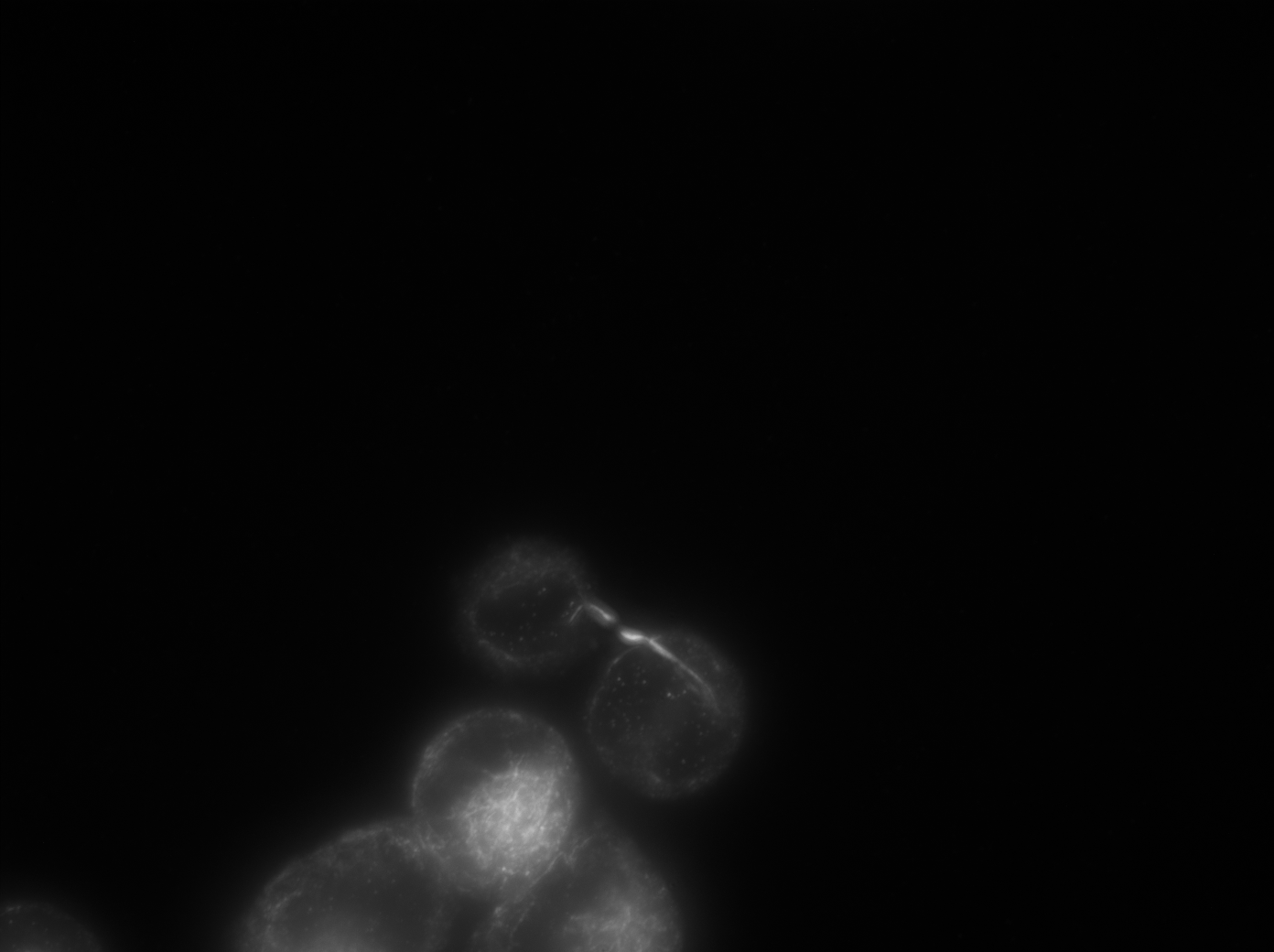

Supplement: Supplementary file 21 — Source data Fig. 6 part 2 [file 44319_2026_742_MOESM21_ESM.zip › Figure 6 Part 2/Fig 6abcd Cas9 TPGS1-KO acetylated tubulin atubulin part 2/TPGS1-KO R3 9-13-24 LT19.Project Maximum Z_XY1726764103_Z0_T0_C2.tif]

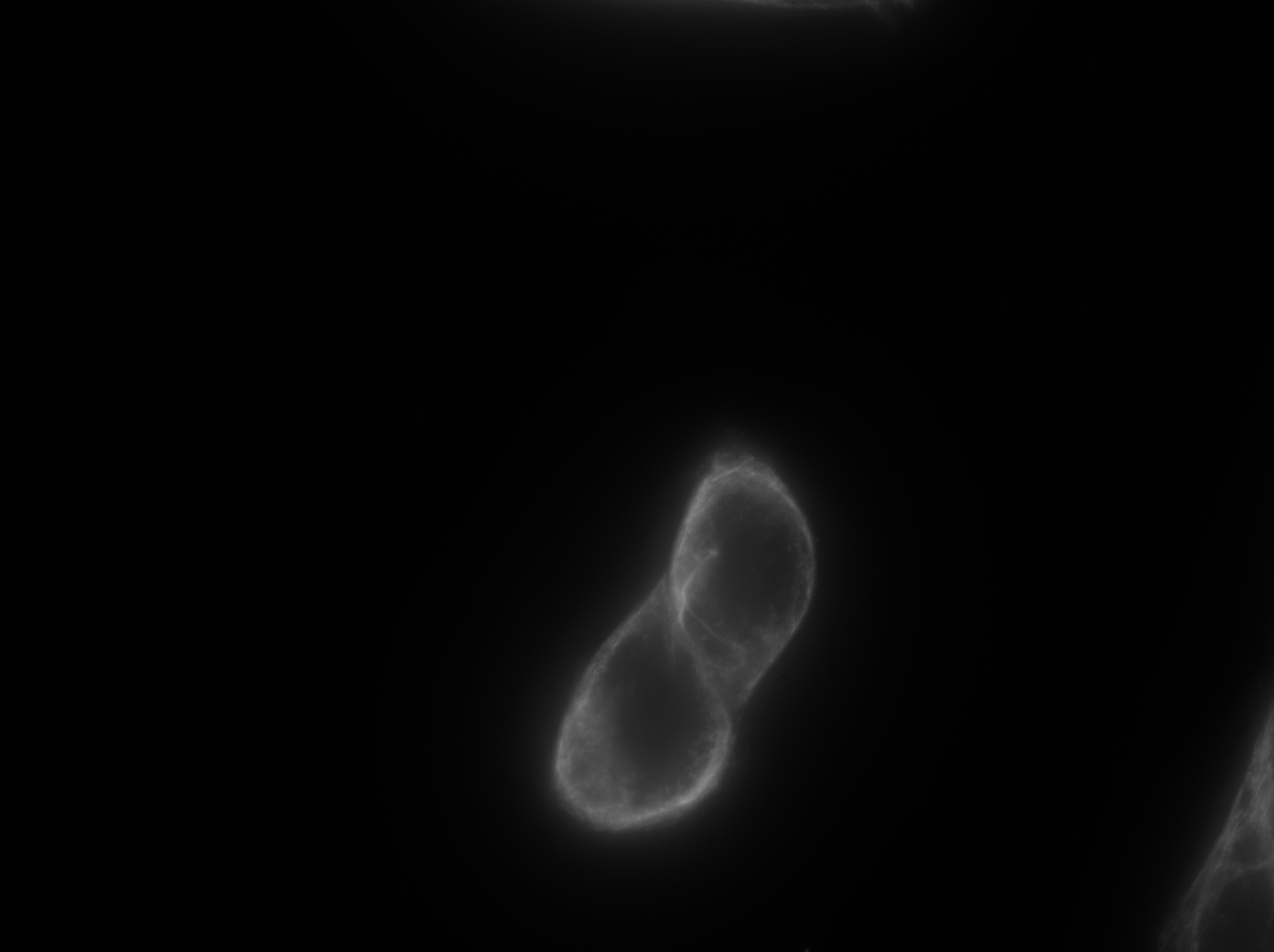

Supplement: Supplementary file 21 — Source data Fig. 6 part 2 [file 44319_2026_742_MOESM21_ESM.zip › Figure 6 Part 2/Fig 6abcd Cas9 TPGS1-KO acetylated tubulin atubulin part 2/TPGS1-KO R2 9-11-24 PA18.Project Maximum Z_XY1726268793_Z0_T0_C1.tif]

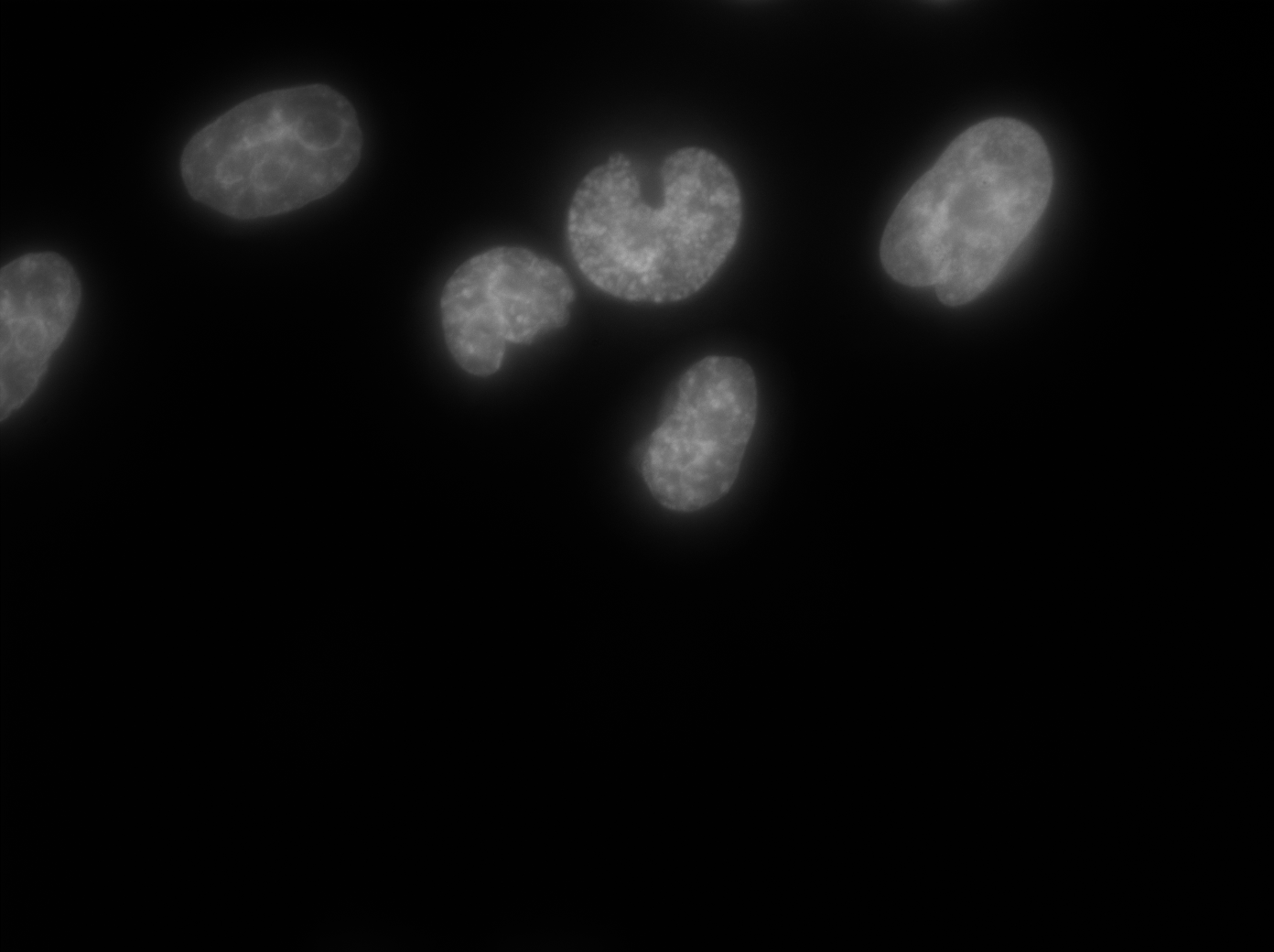

Supplement: Supplementary file 21 — Source data Fig. 6 part 2 [file 44319_2026_742_MOESM21_ESM.zip › Figure 6 Part 2/Fig 6abcd Cas9 TPGS1-KO acetylated tubulin atubulin part 2/TPGS1-KO R3 9-13-24 LT16.Project Maximum Z_XY1726763904_Z0_T0_C0.tif]

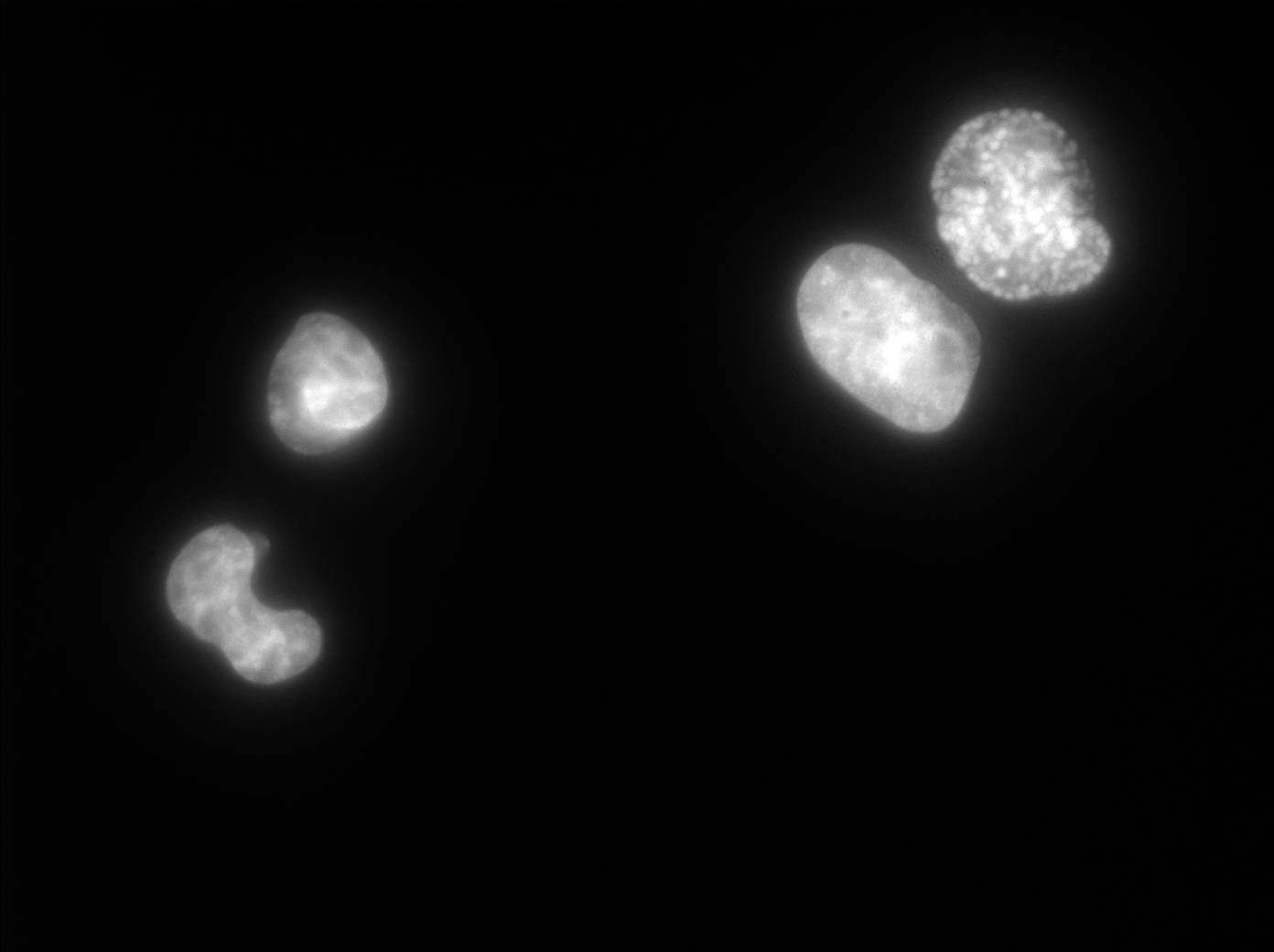

Supplement: Supplementary file 21 — Source data Fig. 6 part 2 [file 44319_2026_742_MOESM21_ESM.zip › Figure 6 Part 2/Fig 6abcd Cas9 TPGS1-KO acetylated tubulin atubulin part 2/TPGS1-KO R2 9-11-24 LT7.Project Maximum Z_XY1726261460_Z0_T0_C0.tif]

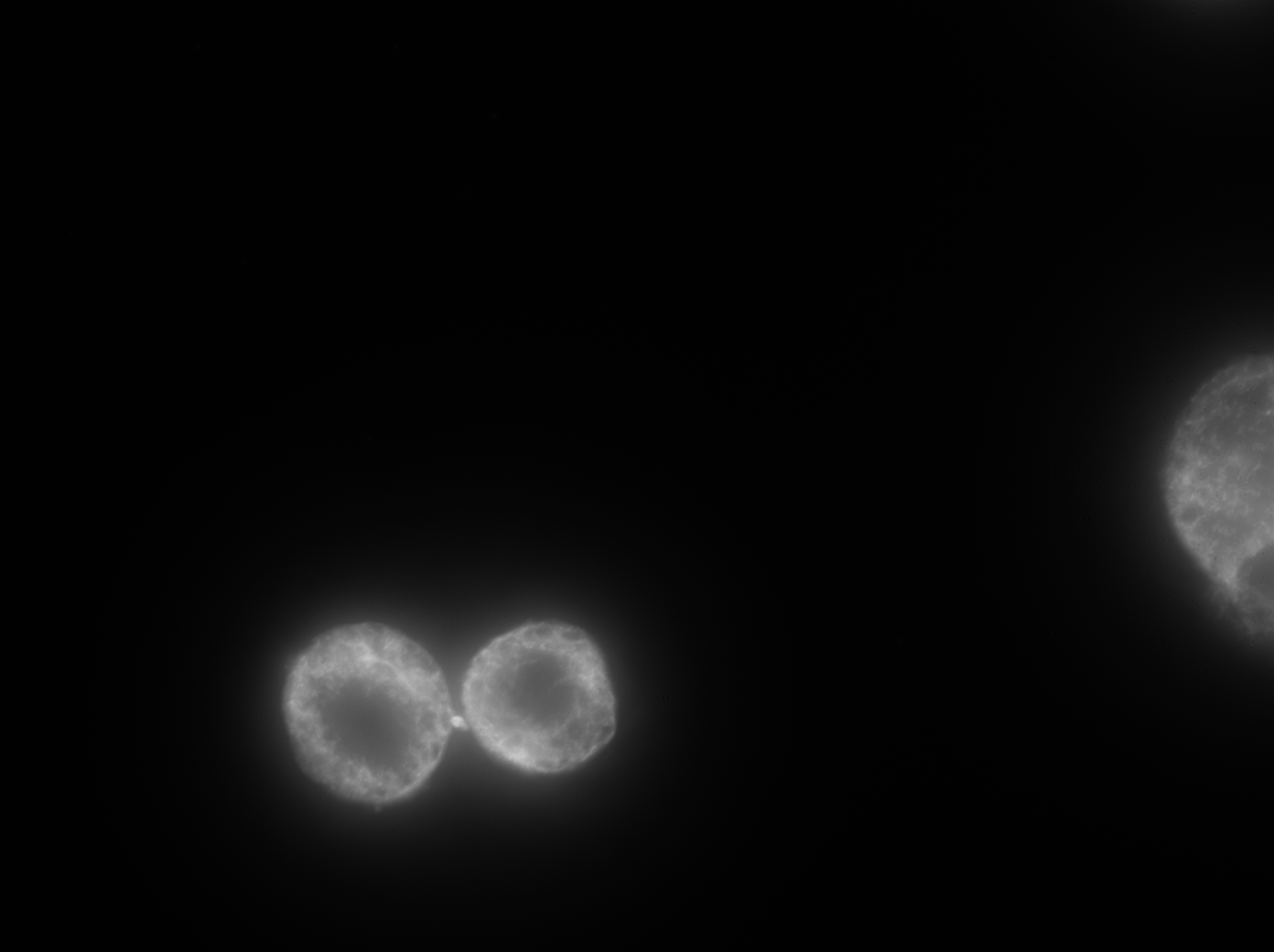

Supplement: Supplementary file 21 — Source data Fig. 6 part 2 [file 44319_2026_742_MOESM21_ESM.zip › Figure 6 Part 2/Fig 6abcd Cas9 TPGS1-KO acetylated tubulin atubulin part 2/TPGS1-KO R2 9-11-24 PA6.Project Maximum Z_XY1726261339_Z0_T0_C1.tif]

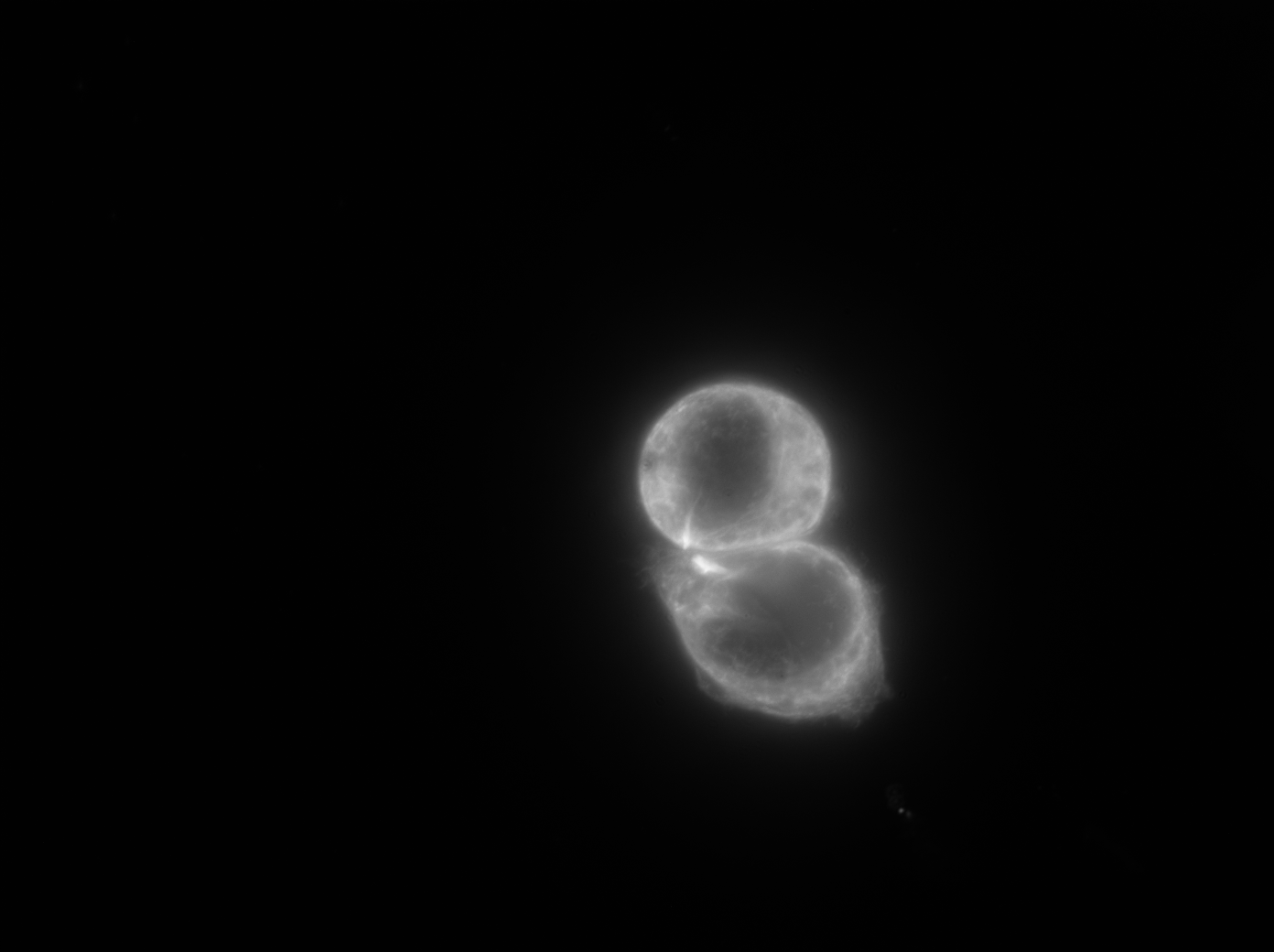

Supplement: Supplementary file 21 — Source data Fig. 6 part 2 [file 44319_2026_742_MOESM21_ESM.zip › Figure 6 Part 2/Fig 6abcd Cas9 TPGS1-KO acetylated tubulin atubulin part 2/TPGS1-KO R2 9-11-24 LT2.Project Maximum Z_XY1726259084_Z0_T0_C1.tif]
