# Supplementary figures and images for: TPGS1 regulates central spindle microtubule glutamylation and remodeling during telophase and abscission (part 27 of 36)
Source: EMBO Rep. 2026 Mar 23;27(8):1944–63. doi: 10.1038/s44319-026-00742-3 (PMC13121839; doi:10.1038/s44319-026-00742-3)

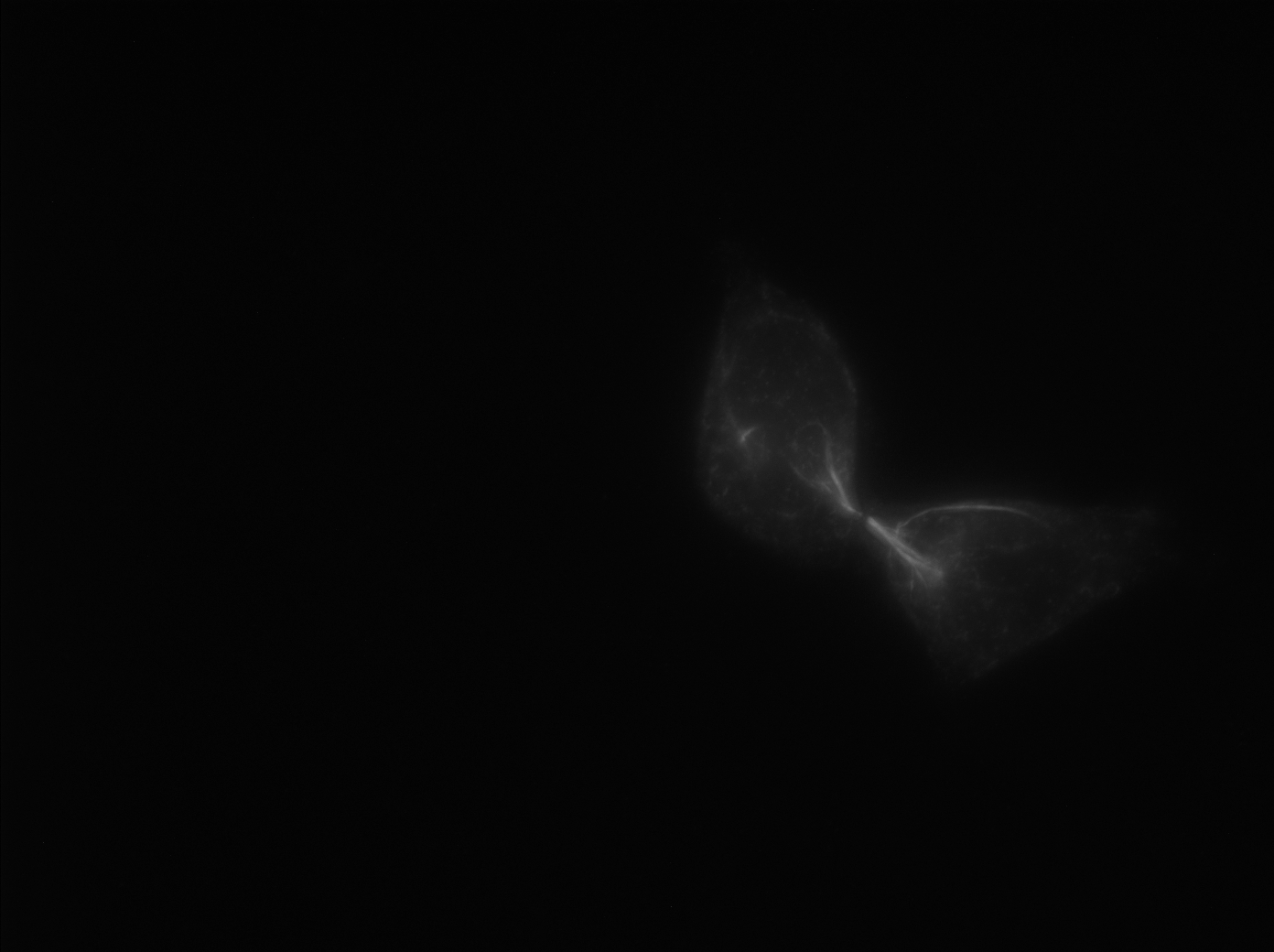

Supplement: Supplementary file 22 — Source data Fig. 6 part 3 [file 44319_2026_742_MOESM22_ESM.zip › Figure 6 Part 3/Fig 6efg TPGS1-KO TPGS1 rescue experiments/R1/TPGS1-KO TPGS1-3UTR-EYFP actub R1 7-31-25 LT8.Project Maximum Z_XY1753991165_Z0_T0_C2.tif]

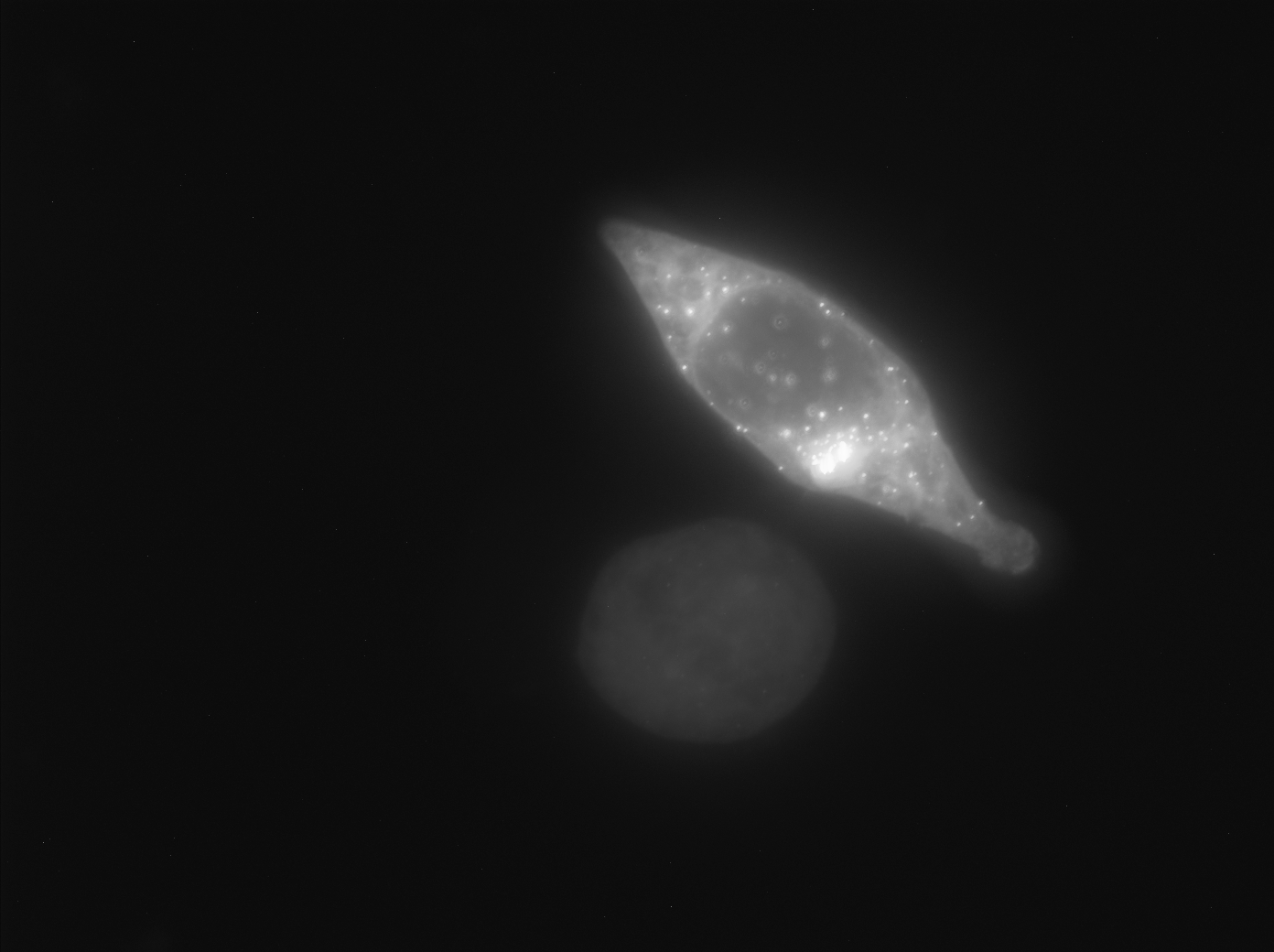

Supplement: Supplementary file 22 — Source data Fig. 6 part 3 [file 44319_2026_742_MOESM22_ESM.zip › Figure 6 Part 3/Fig 6efg TPGS1-KO TPGS1 rescue experiments/R1/TPGS1-KO TPGS1-3UTR-EYFP actub R1 7-31-25 M1 I1.Project Maximum Z_XY1753992586_Z0_T0_C1.tif]

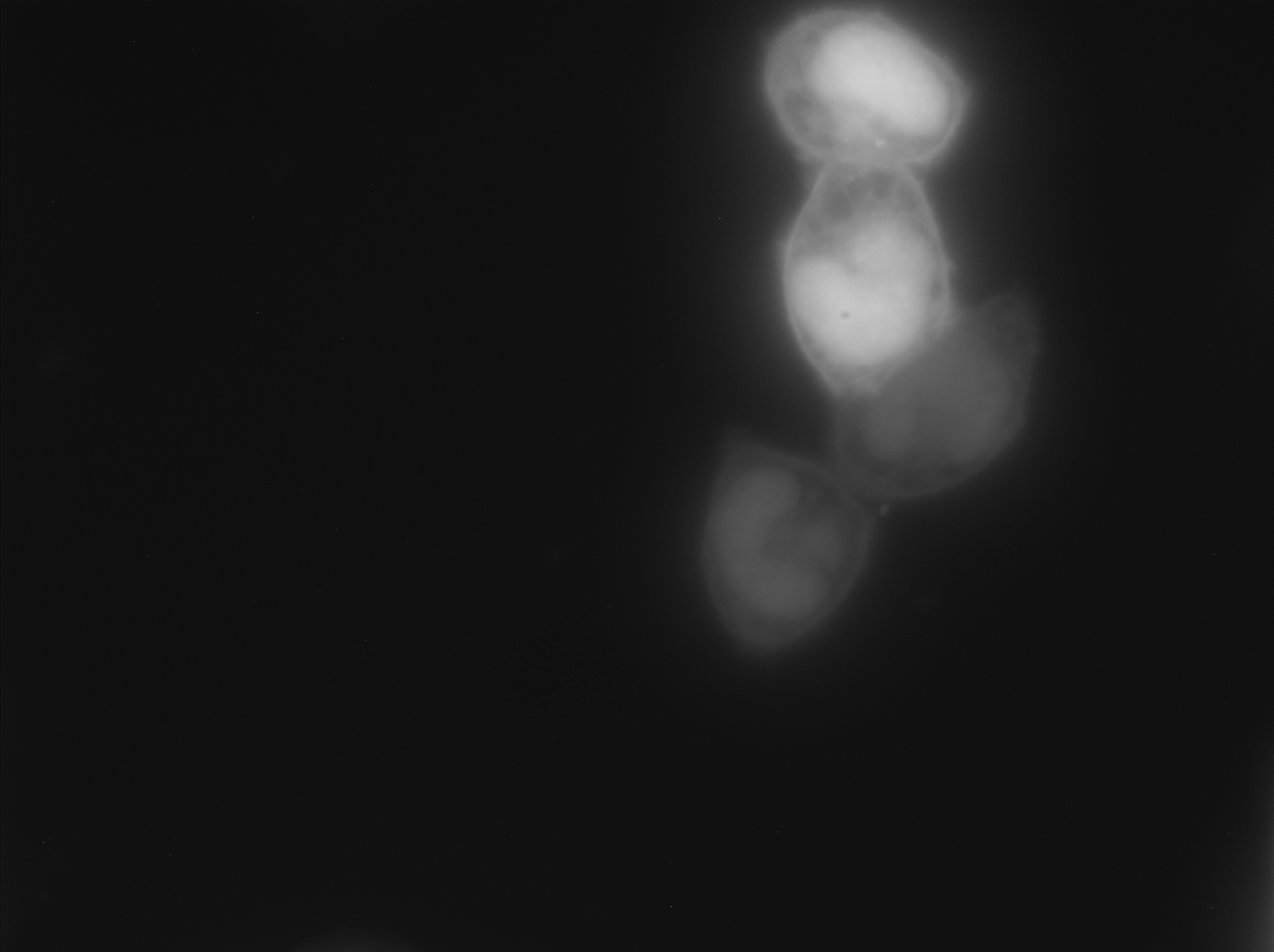

Supplement: Supplementary file 22 — Source data Fig. 6 part 3 [file 44319_2026_742_MOESM22_ESM.zip › Figure 6 Part 3/Fig 6efg TPGS1-KO TPGS1 rescue experiments/R1/TPGS1-KO EYFP only actub R1 7-31-25 LT7.Project Maximum Z_XY1754336696_Z0_T0_C1.tif]

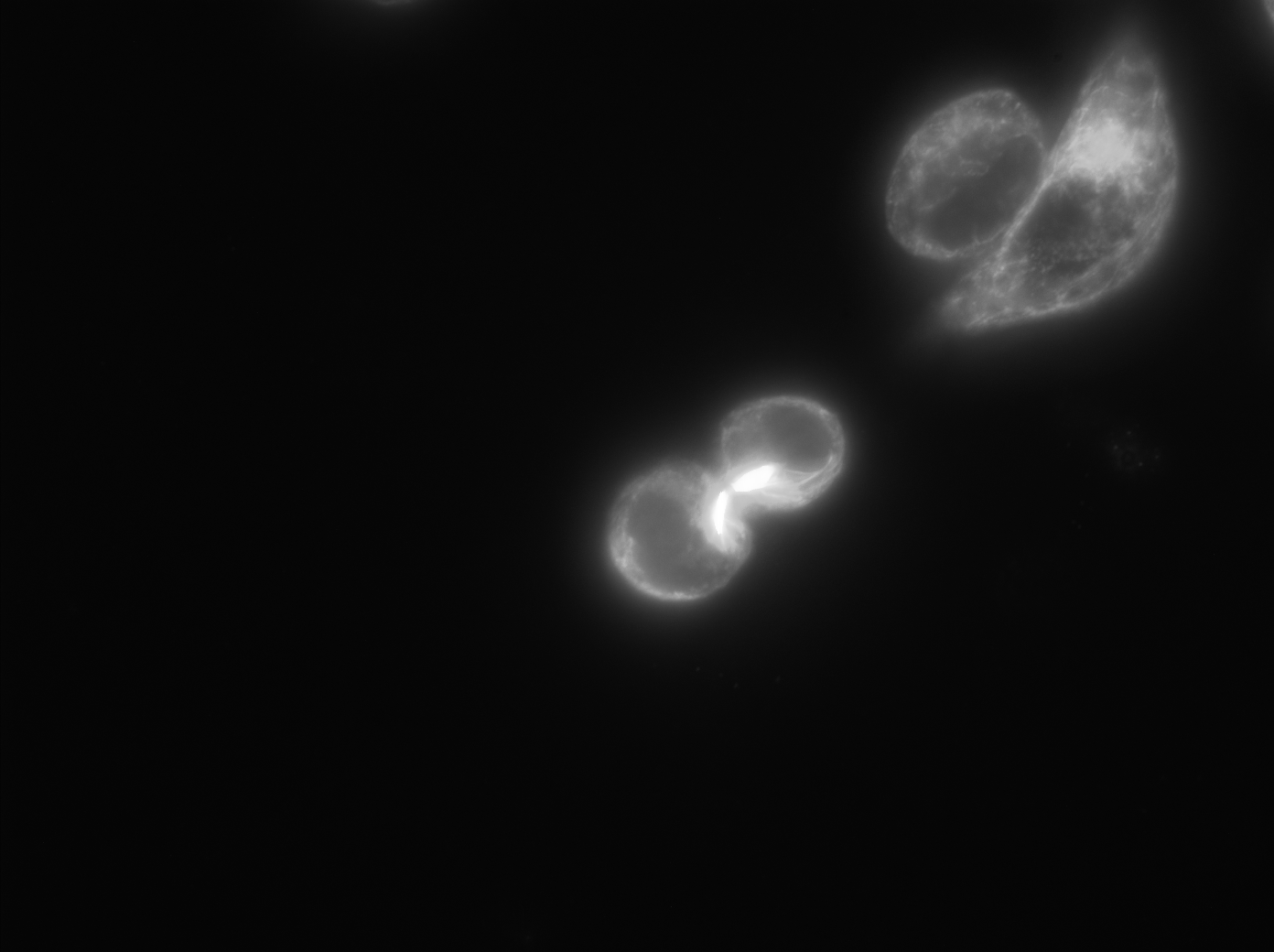

Supplement: Supplementary file 23 — Source data Fig. 6 part 4 [file 44319_2026_742_MOESM23_ESM.zip › Figure 6 Part 4/Fig 6efg TPGS1-KO TPGS1 rescue experiments part 2/R2R3/TPGS1-KO EYFP-only actub 7-31-25 R3 ET9.Project Maximum Z_XY1756495193_Z0_T0_C2.tif]

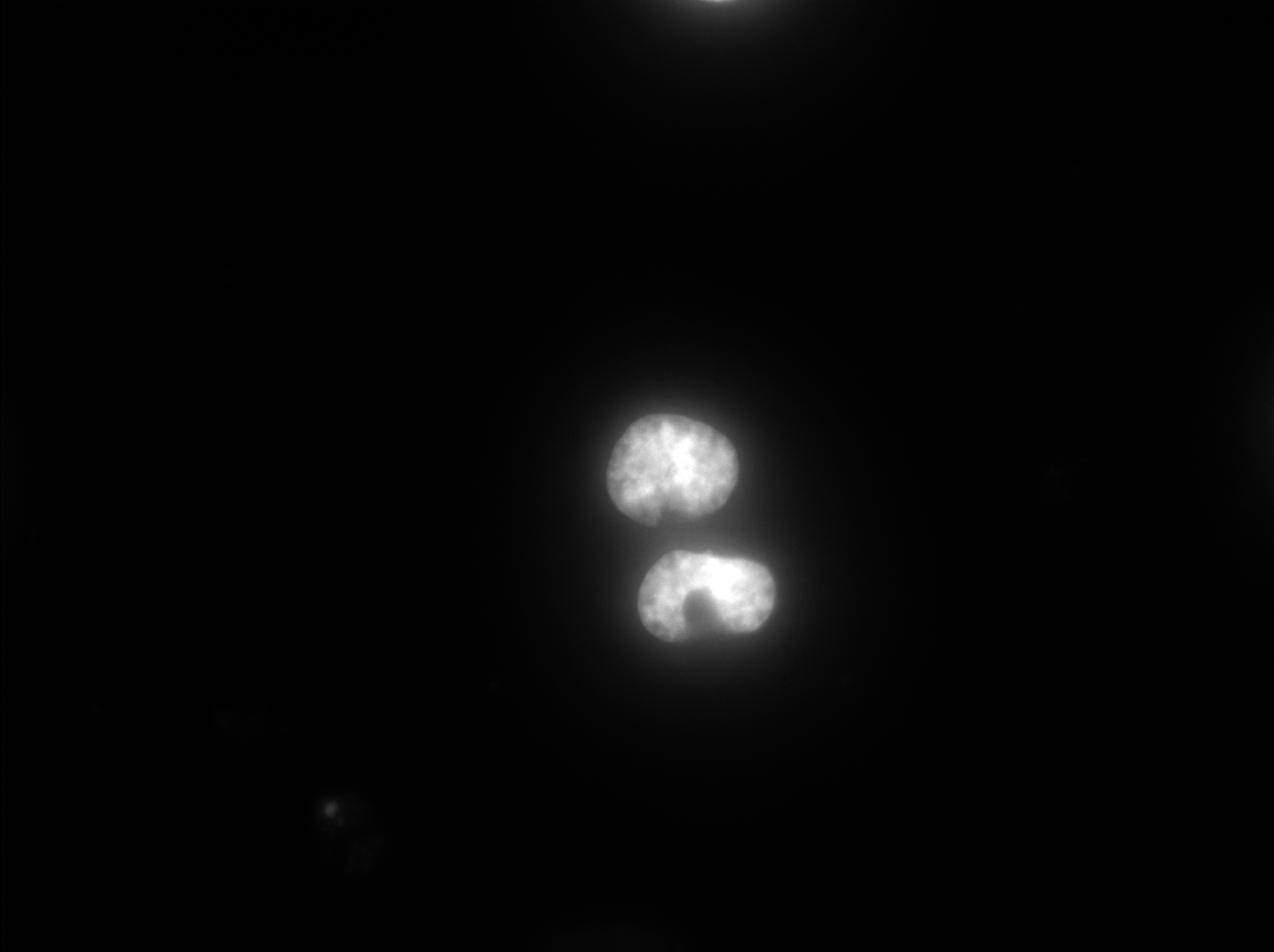

Supplement: Supplementary file 23 — Source data Fig. 6 part 4 [file 44319_2026_742_MOESM23_ESM.zip › Figure 6 Part 4/Fig 6efg TPGS1-KO TPGS1 rescue experiments part 2/R2R3/TPGS1-KO EYFP-only actub 7-31-25 R3 ET6.Project Maximum Z_XY1756494172_Z0_T0_C0.tif]

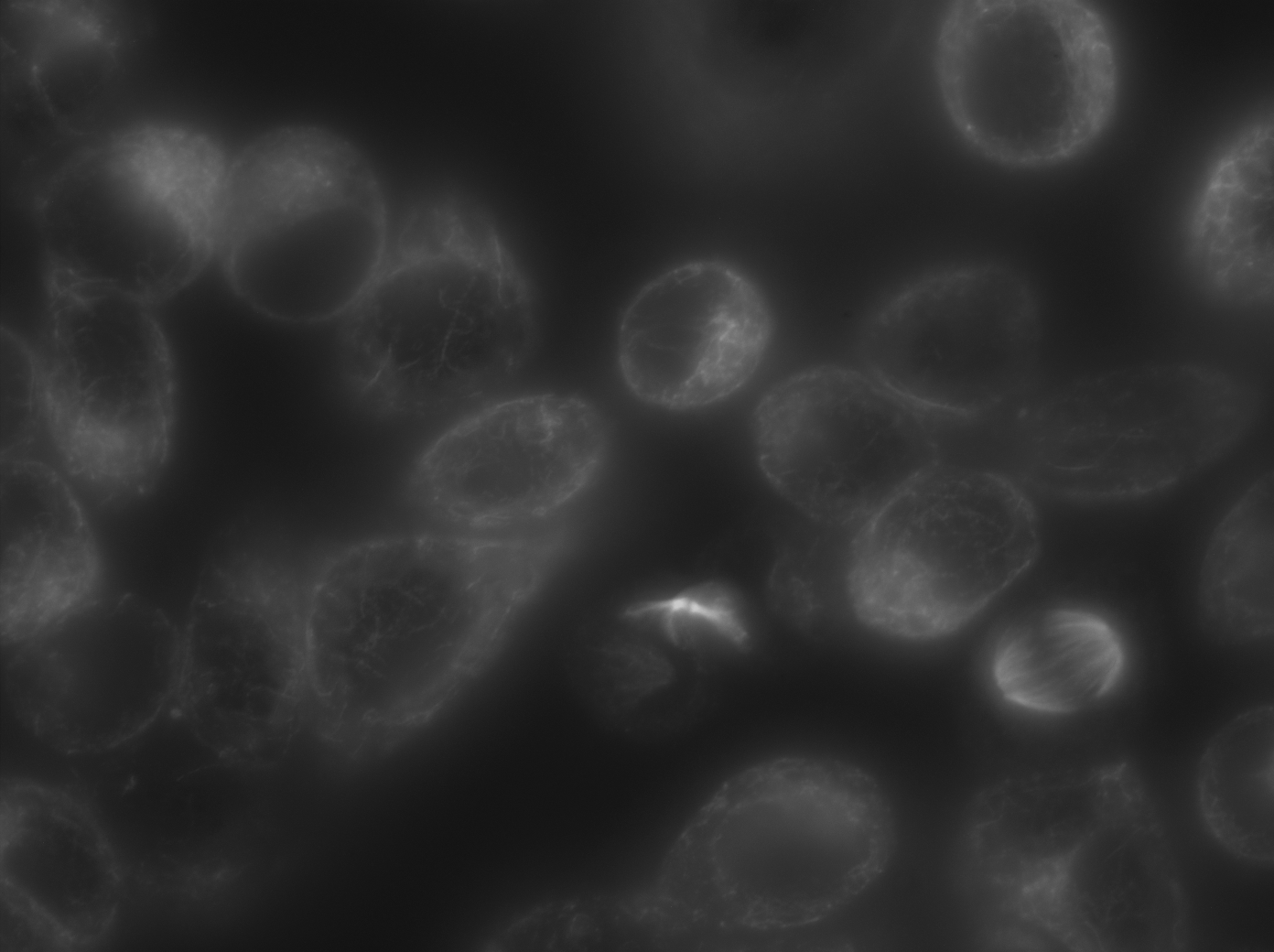

Supplement: Supplementary file 23 — Source data Fig. 6 part 4 [file 44319_2026_742_MOESM23_ESM.zip › Figure 6 Part 4/Fig 6efg TPGS1-KO TPGS1 rescue experiments part 2/R2R3/TPGS1-KO TPGS1-EYFP-3'UTR actub 7-31-25 R2 ET5.Project Maximum Z_XY1756410606_Z0_T0_C2.tif]

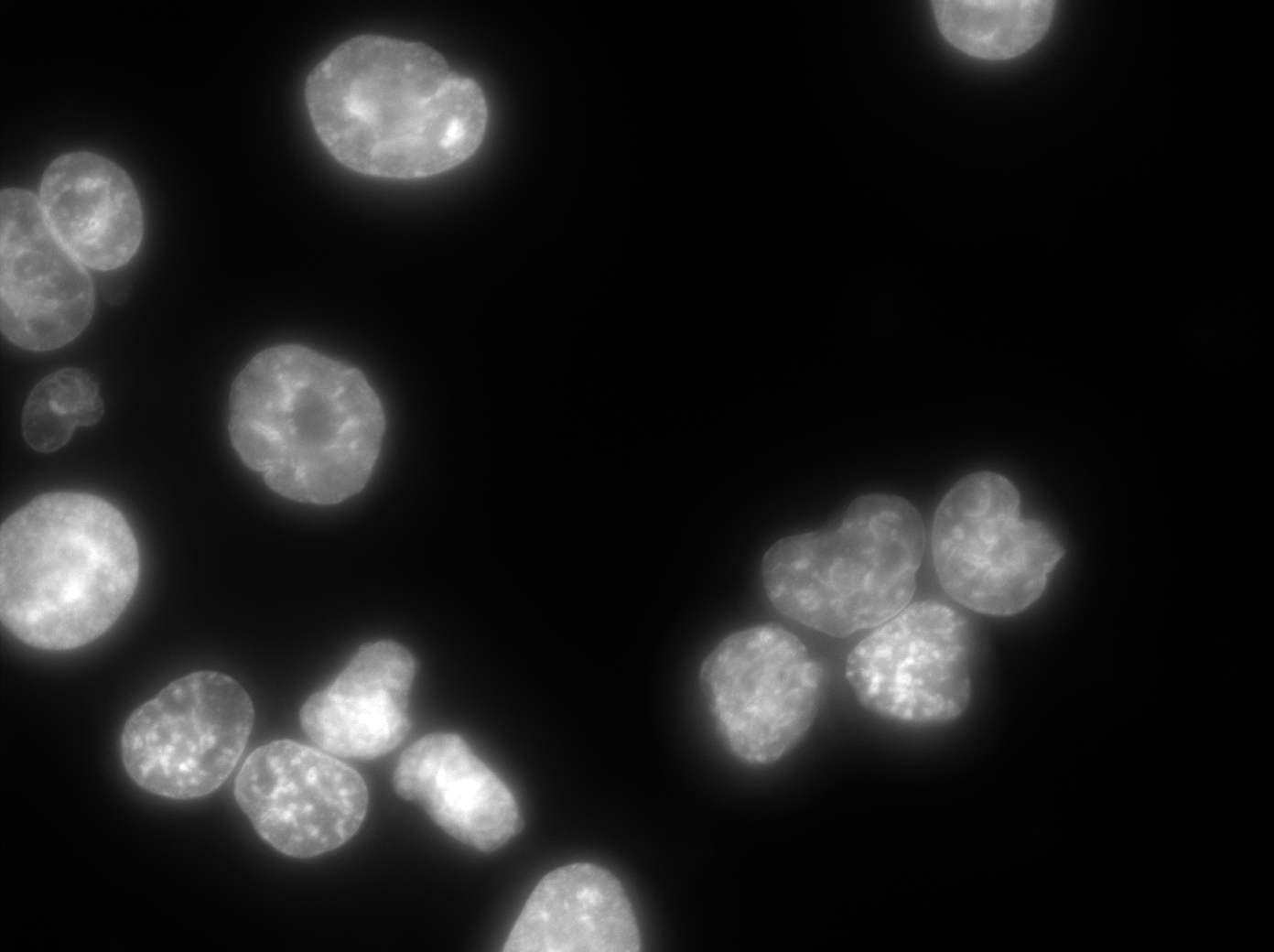

Supplement: Supplementary file 23 — Source data Fig. 6 part 4 [file 44319_2026_742_MOESM23_ESM.zip › Figure 6 Part 4/Fig 6efg TPGS1-KO TPGS1 rescue experiments part 2/R2R3/TPGS1-KO EYFP-only actub 7-31-25 R3 LT8.Project Maximum Z_XY1756496000_Z0_T0_C0.tif]

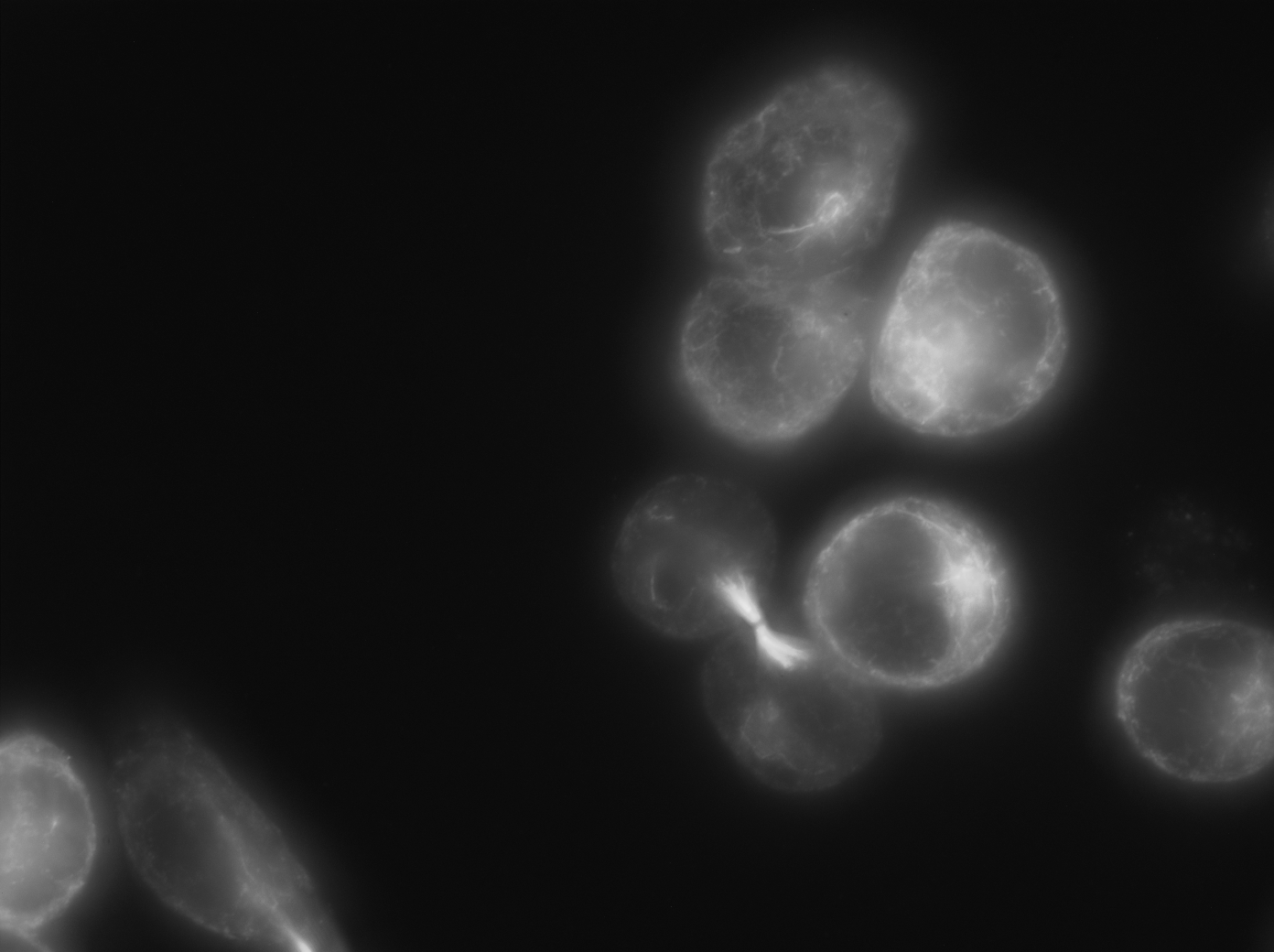

Supplement: Supplementary file 23 — Source data Fig. 6 part 4 [file 44319_2026_742_MOESM23_ESM.zip › Figure 6 Part 4/Fig 6efg TPGS1-KO TPGS1 rescue experiments part 2/R2R3/TPGS1-KO EYFP-only actub 7-31-25 R2 ET6.Project Maximum Z_XY1756415325_Z0_T0_C2.tif]

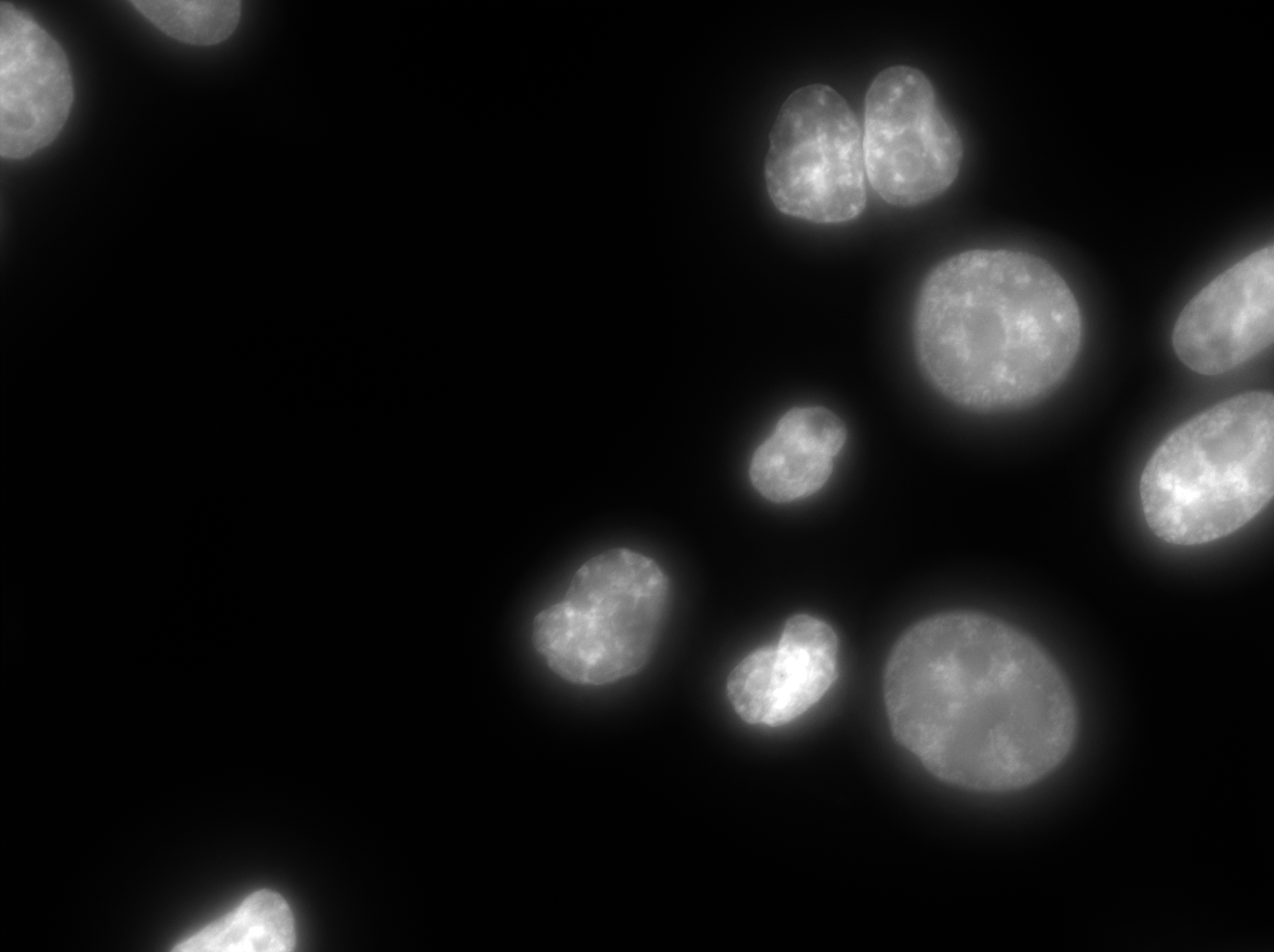

Supplement: Supplementary file 23 — Source data Fig. 6 part 4 [file 44319_2026_742_MOESM23_ESM.zip › Figure 6 Part 4/Fig 6efg TPGS1-KO TPGS1 rescue experiments part 2/R2R3/TPGS1-KO EYFP-only actub 7-31-25 R3 LT9.Project Maximum Z_XY1756496535_Z0_T0_C0.tif]

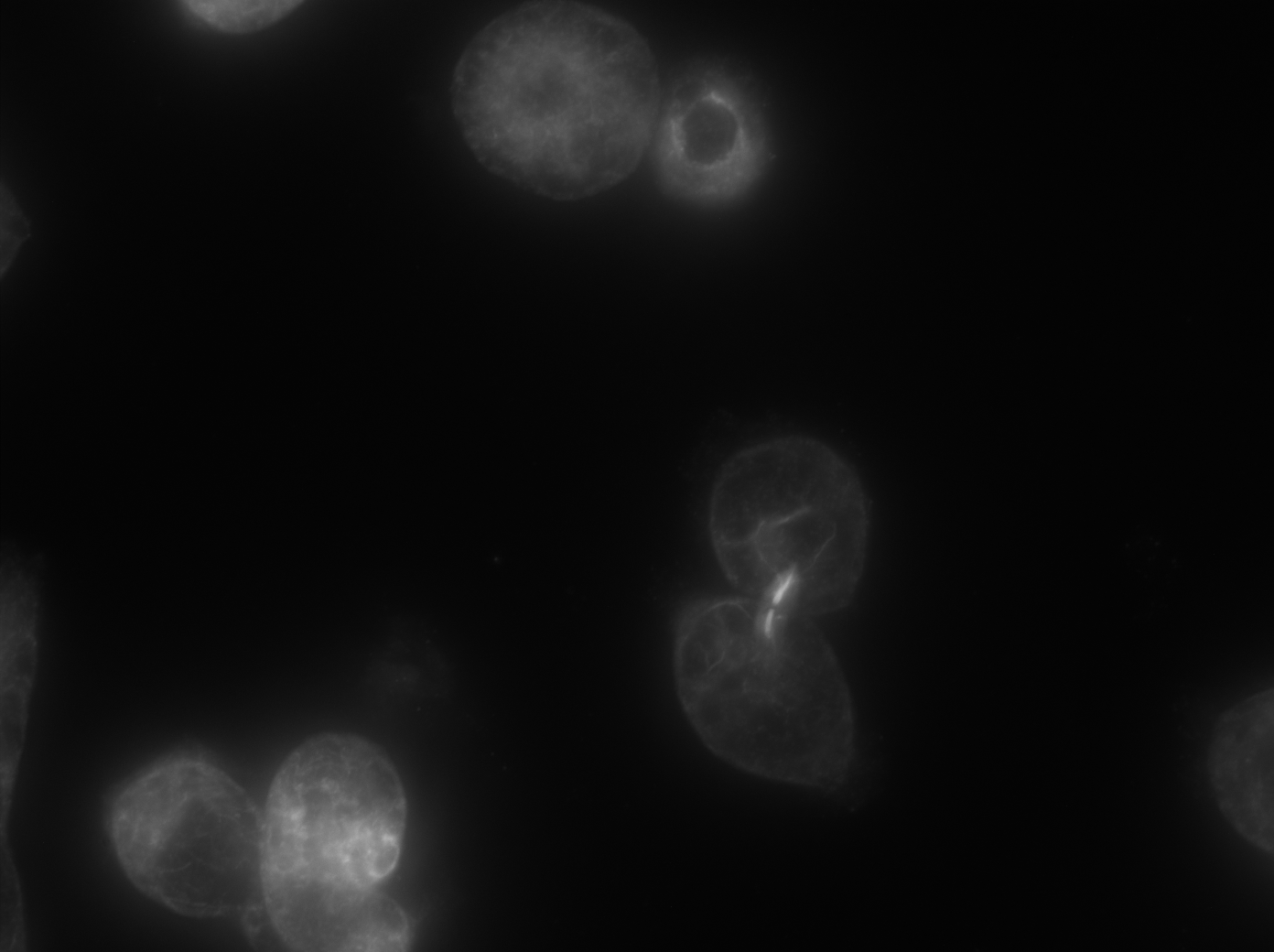

Supplement: Supplementary file 23 — Source data Fig. 6 part 4 [file 44319_2026_742_MOESM23_ESM.zip › Figure 6 Part 4/Fig 6efg TPGS1-KO TPGS1 rescue experiments part 2/R2R3/TPGS1-KO EYFP-only actub 7-31-25 R3 LT6.Project Maximum Z_XY1756493784_Z0_T0_C2.tif]

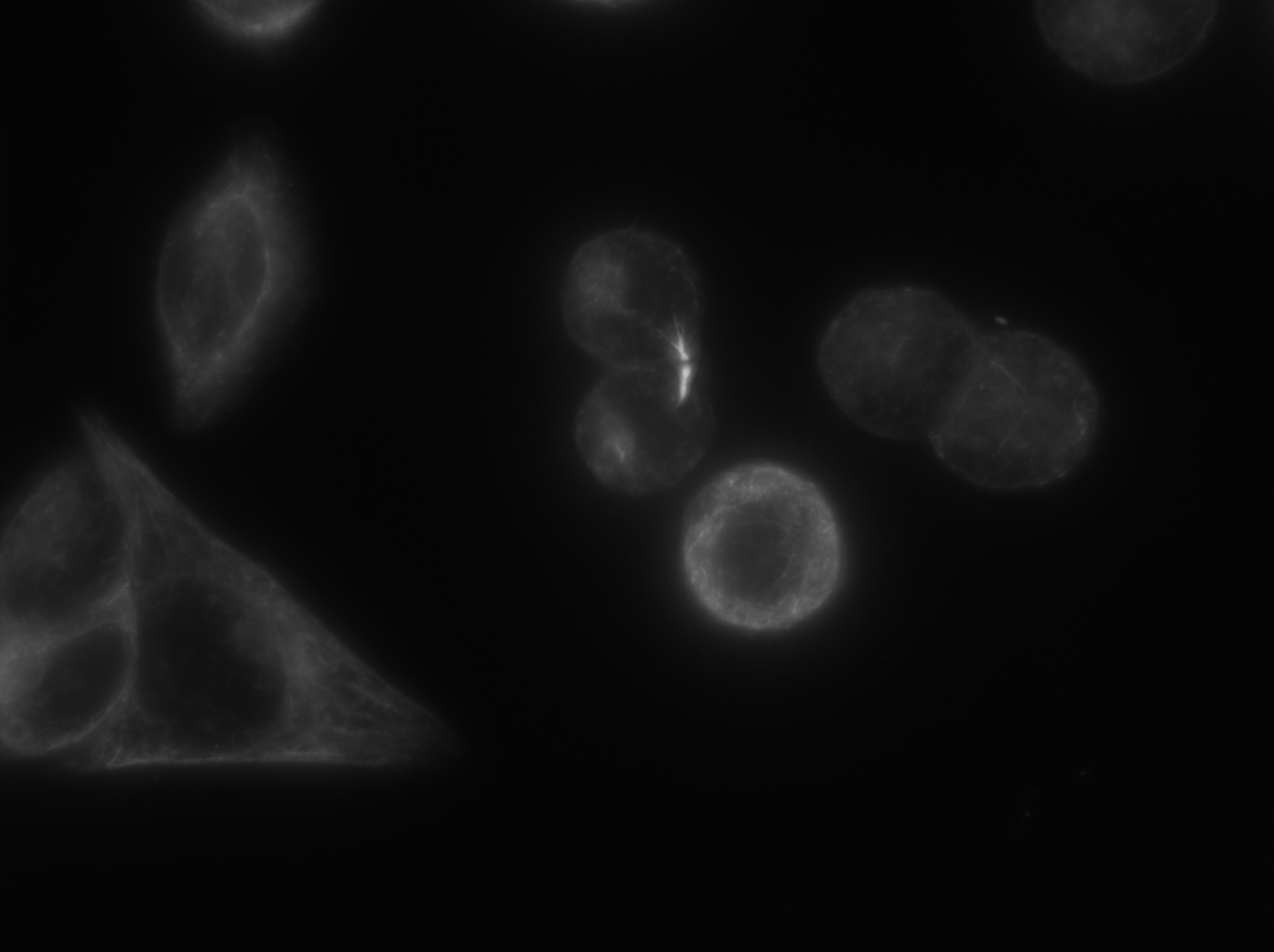

Supplement: Supplementary file 23 — Source data Fig. 6 part 4 [file 44319_2026_742_MOESM23_ESM.zip › Figure 6 Part 4/Fig 6efg TPGS1-KO TPGS1 rescue experiments part 2/R2R3/TPGS1-KO EYFP-only actub 7-31-25 R3 ET10.Project Maximum Z_XY1756495480_Z0_T0_C2.tif]

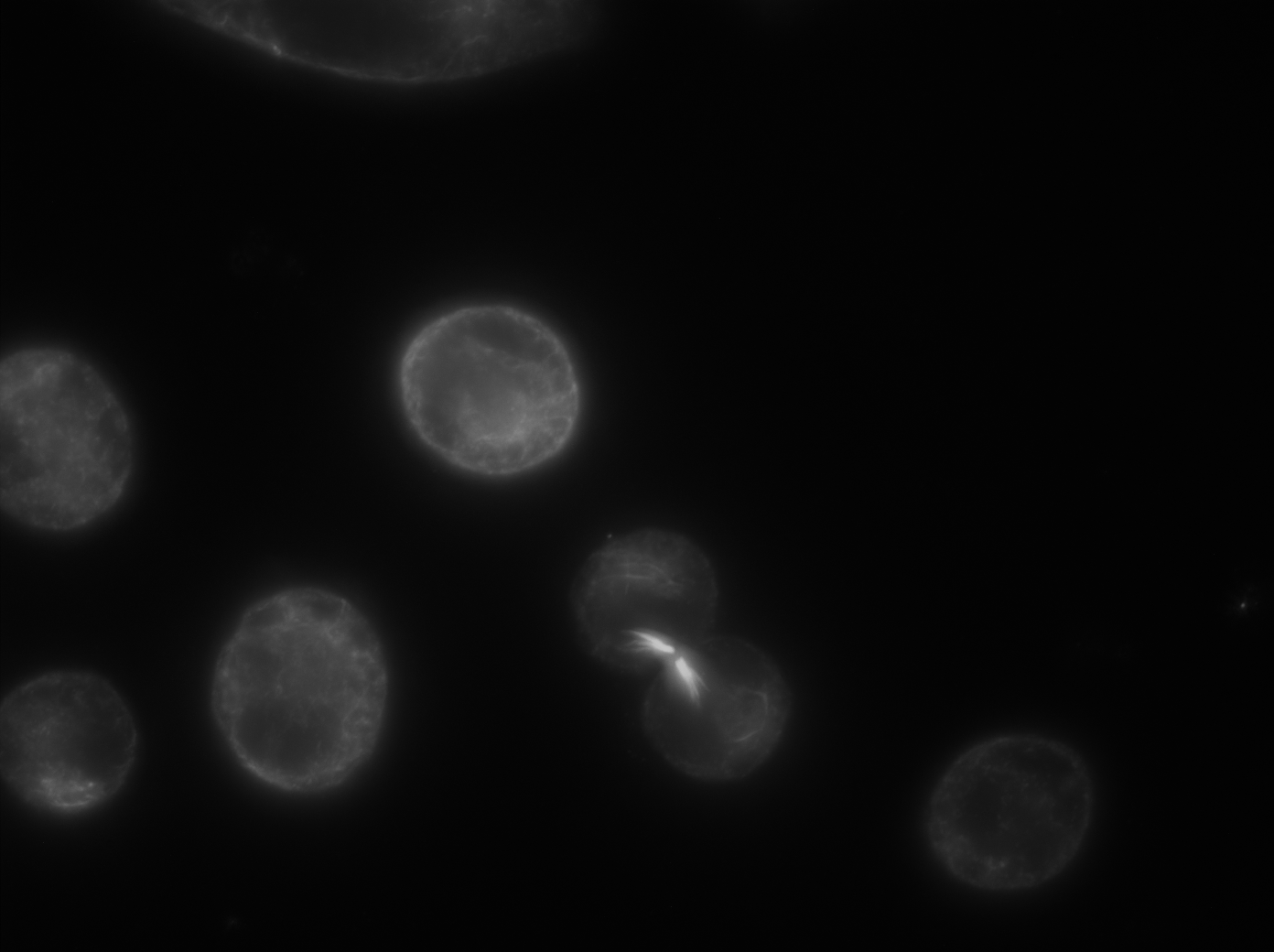

Supplement: Supplementary file 23 — Source data Fig. 6 part 4 [file 44319_2026_742_MOESM23_ESM.zip › Figure 6 Part 4/Fig 6efg TPGS1-KO TPGS1 rescue experiments part 2/R2R3/TPGS1-KO EYFP-only actub 7-31-25 R3 ET8.Project Maximum Z_XY1756494896_Z0_T0_C2.tif]

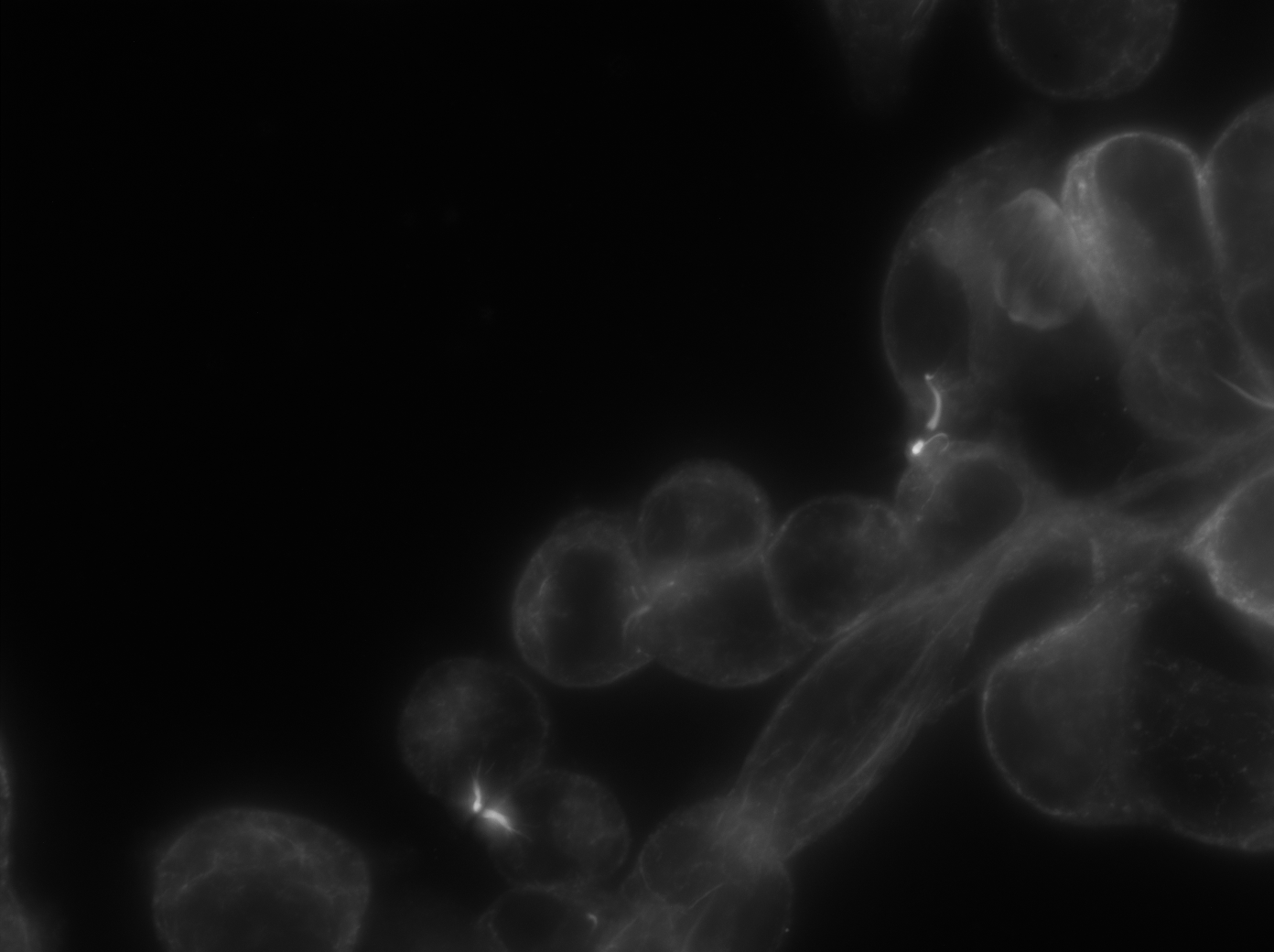

Supplement: Supplementary file 23 — Source data Fig. 6 part 4 [file 44319_2026_742_MOESM23_ESM.zip › Figure 6 Part 4/Fig 6efg TPGS1-KO TPGS1 rescue experiments part 2/R2R3/TPGS1-KO TPGS1-EYFP-3'UTR actub 7-31-25 R3 LT3 ET4.Project Maximum Z_XY1756500418_Z0_T0_C2.tif]

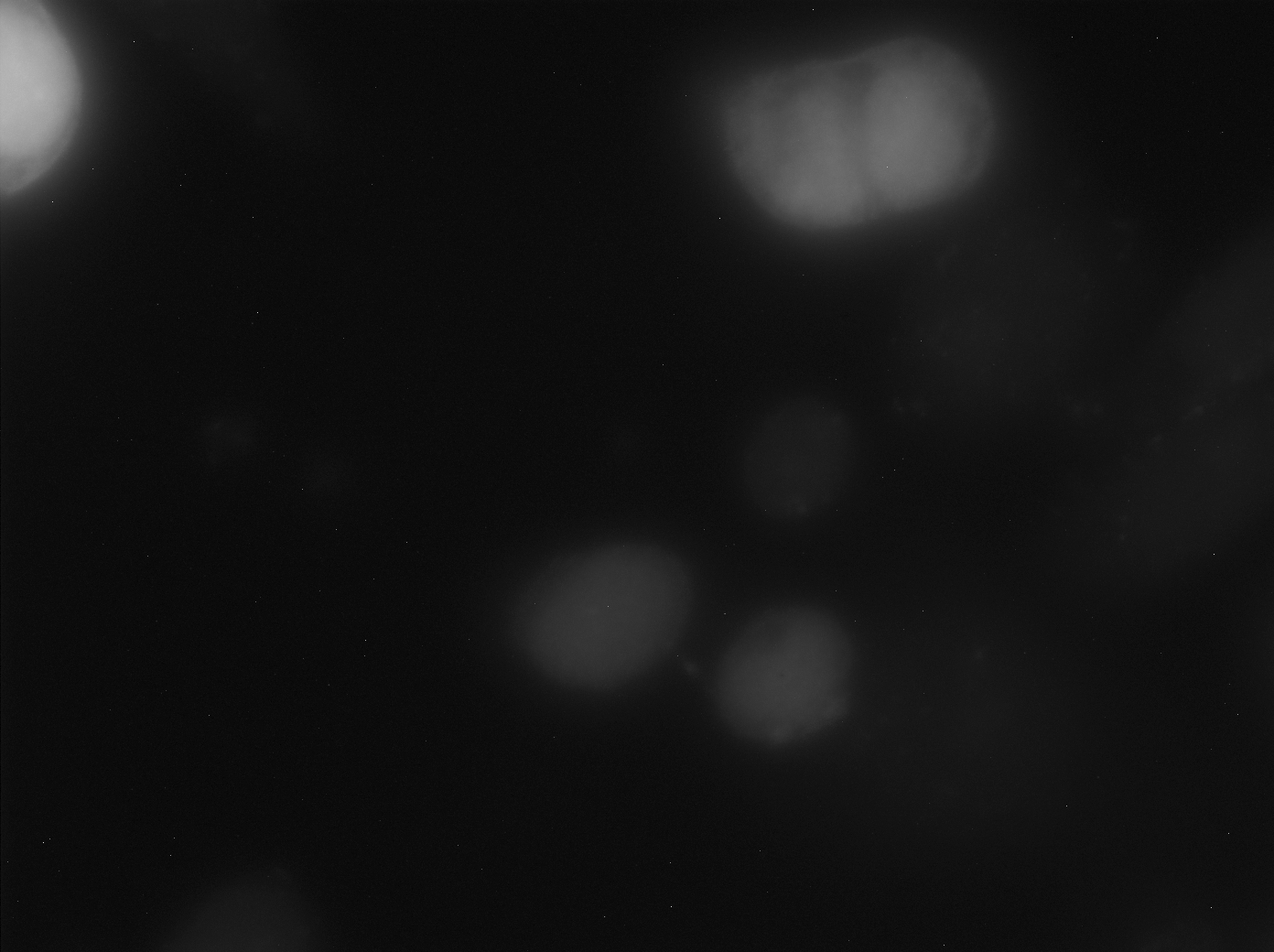

Supplement: Supplementary file 23 — Source data Fig. 6 part 4 [file 44319_2026_742_MOESM23_ESM.zip › Figure 6 Part 4/Fig 6efg TPGS1-KO TPGS1 rescue experiments part 2/R2R3/TPGS1-KO EYFP-only actub 7-31-25 R3 LT9.Project Maximum Z_XY1756496535_Z0_T0_C1.tif]

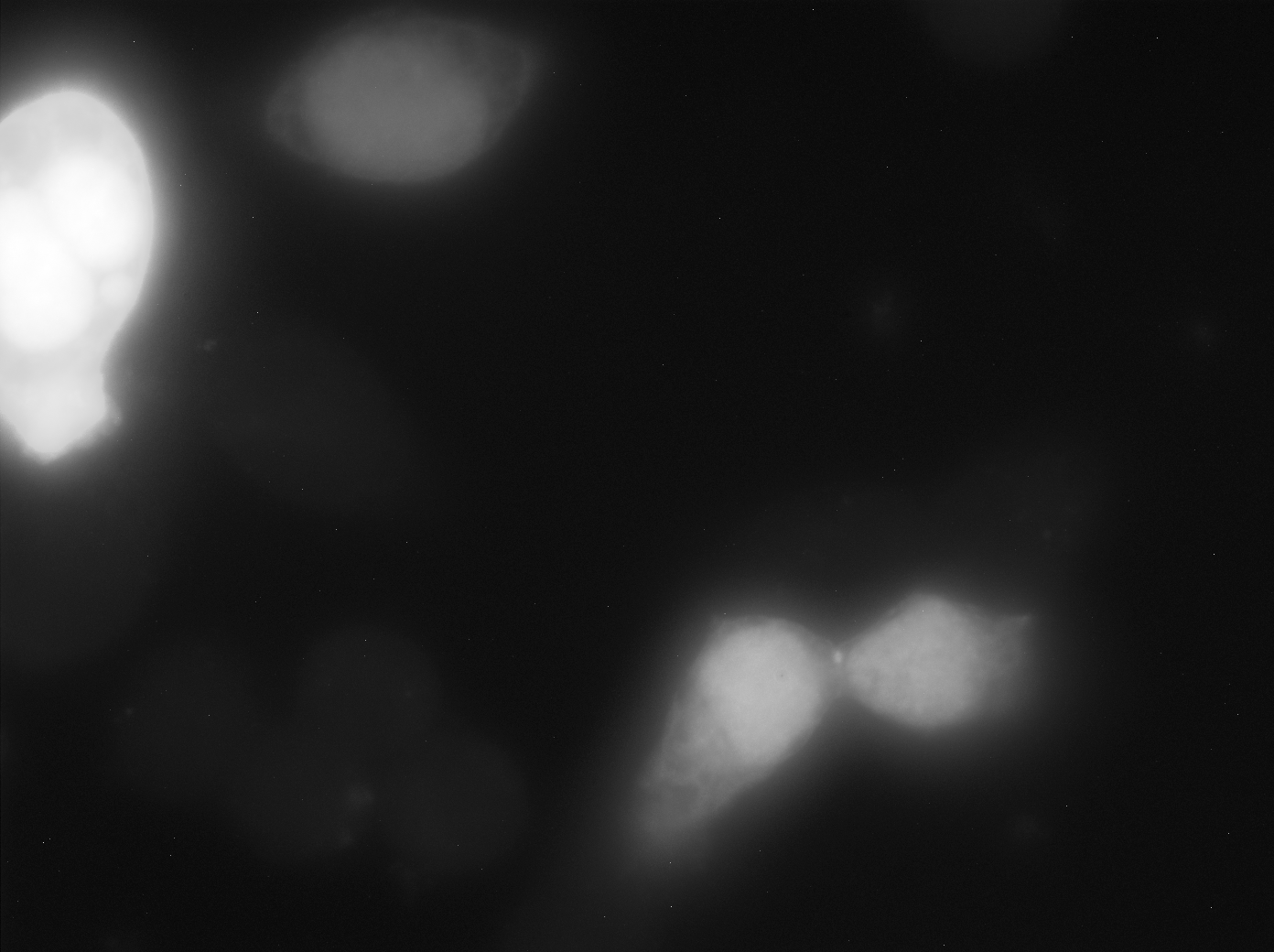

Supplement: Supplementary file 23 — Source data Fig. 6 part 4 [file 44319_2026_742_MOESM23_ESM.zip › Figure 6 Part 4/Fig 6efg TPGS1-KO TPGS1 rescue experiments part 2/R2R3/TPGS1-KO EYFP-only actub 7-31-25 R3 LT8.Project Maximum Z_XY1756496000_Z0_T0_C1.tif]

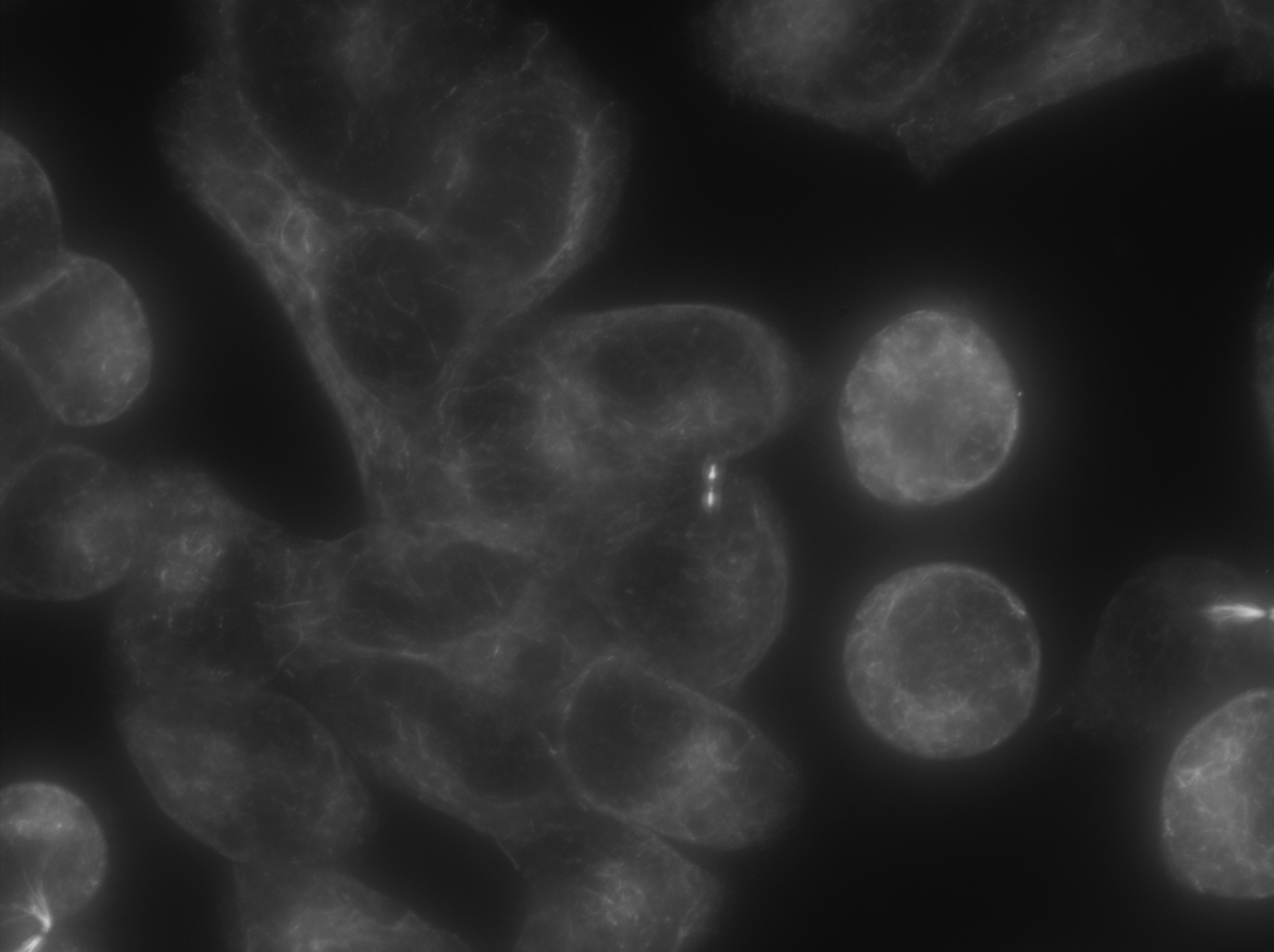

Supplement: Supplementary file 23 — Source data Fig. 6 part 4 [file 44319_2026_742_MOESM23_ESM.zip › Figure 6 Part 4/Fig 6efg TPGS1-KO TPGS1 rescue experiments part 2/R2R3/TPGS1-KO TPGS1-EYFP-3'UTR actub 7-31-25 R2 LT2.Project Maximum Z_XY1756407015_Z0_T0_C2.tif]

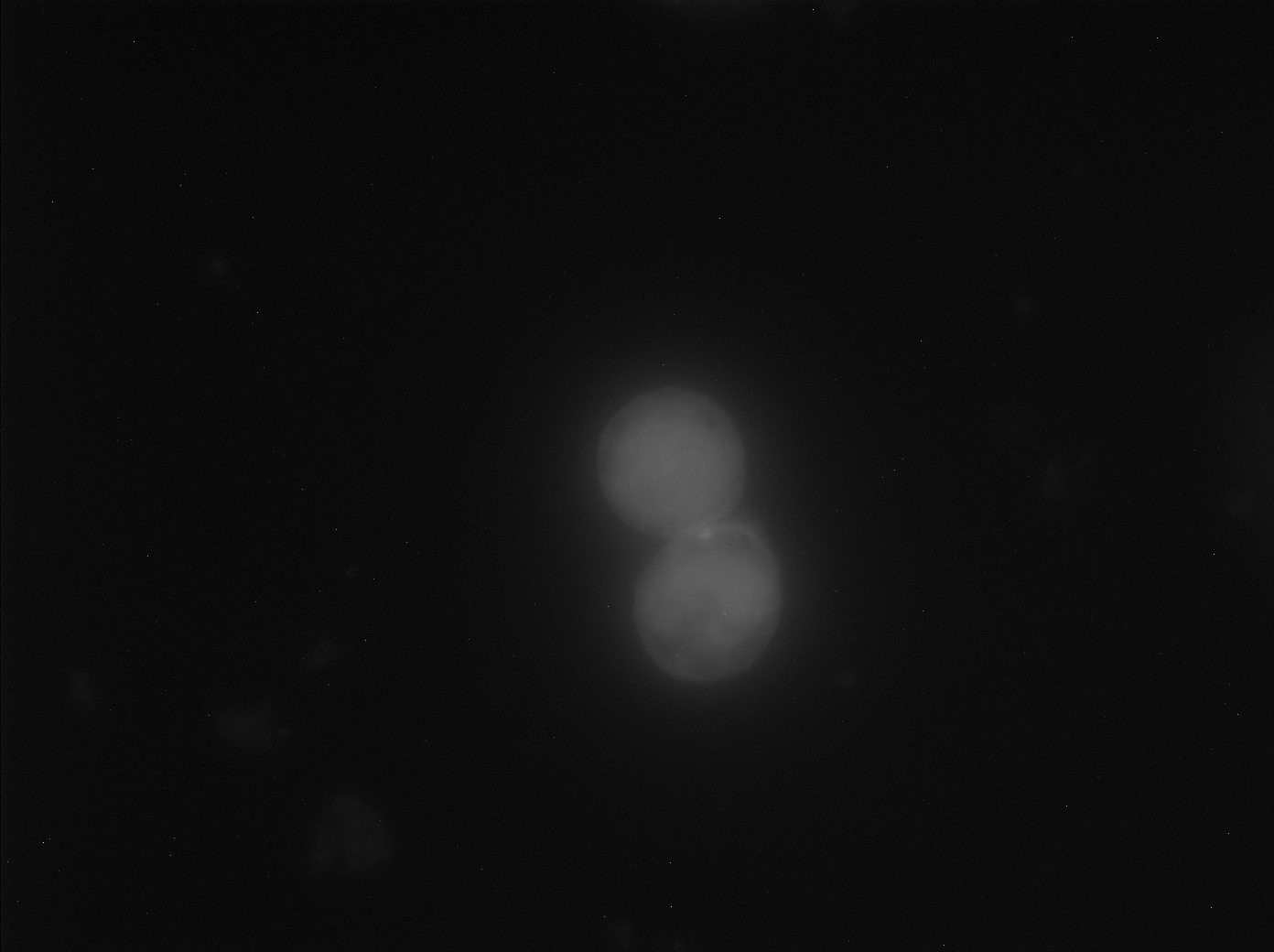

Supplement: Supplementary file 23 — Source data Fig. 6 part 4 [file 44319_2026_742_MOESM23_ESM.zip › Figure 6 Part 4/Fig 6efg TPGS1-KO TPGS1 rescue experiments part 2/R2R3/TPGS1-KO EYFP-only actub 7-31-25 R3 ET6.Project Maximum Z_XY1756494172_Z0_T0_C1.tif]

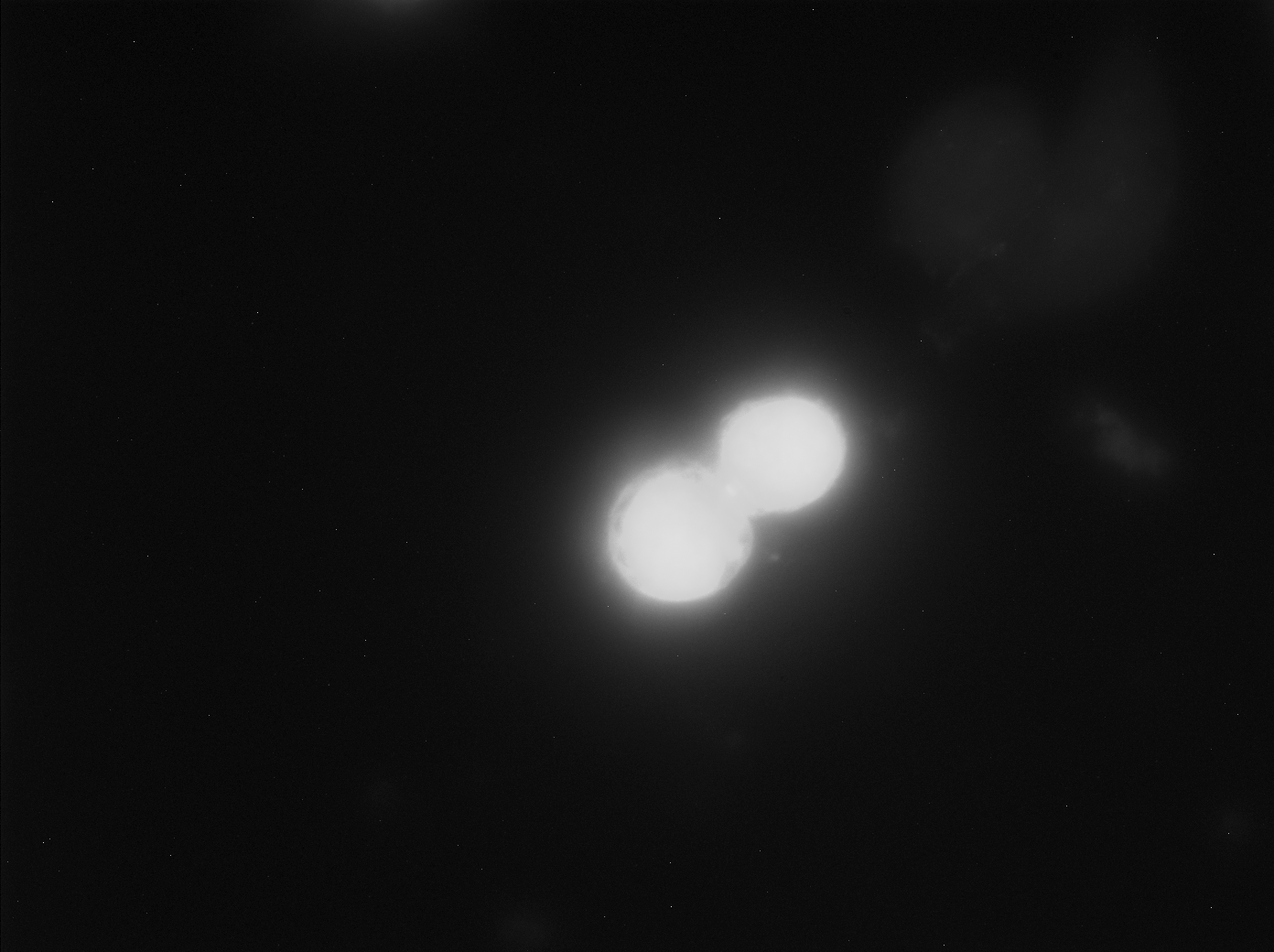

Supplement: Supplementary file 23 — Source data Fig. 6 part 4 [file 44319_2026_742_MOESM23_ESM.zip › Figure 6 Part 4/Fig 6efg TPGS1-KO TPGS1 rescue experiments part 2/R2R3/TPGS1-KO EYFP-only actub 7-31-25 R3 ET9.Project Maximum Z_XY1756495193_Z0_T0_C1.tif]

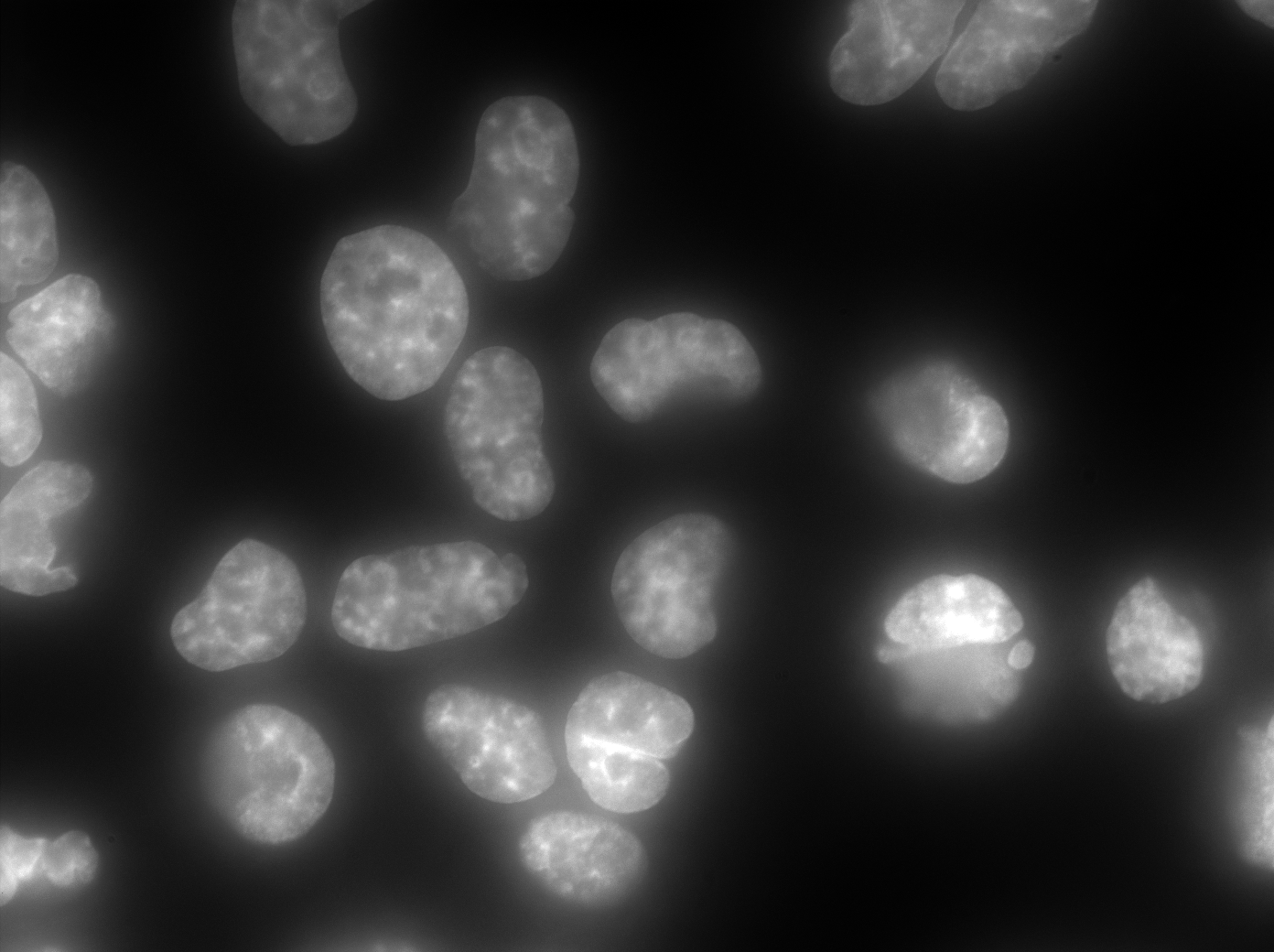

Supplement: Supplementary file 23 — Source data Fig. 6 part 4 [file 44319_2026_742_MOESM23_ESM.zip › Figure 6 Part 4/Fig 6efg TPGS1-KO TPGS1 rescue experiments part 2/R2R3/TPGS1-KO TPGS1-EYFP-3'UTR actub 7-31-25 R2 LT2.Project Maximum Z_XY1756407015_Z0_T0_C0.tif]

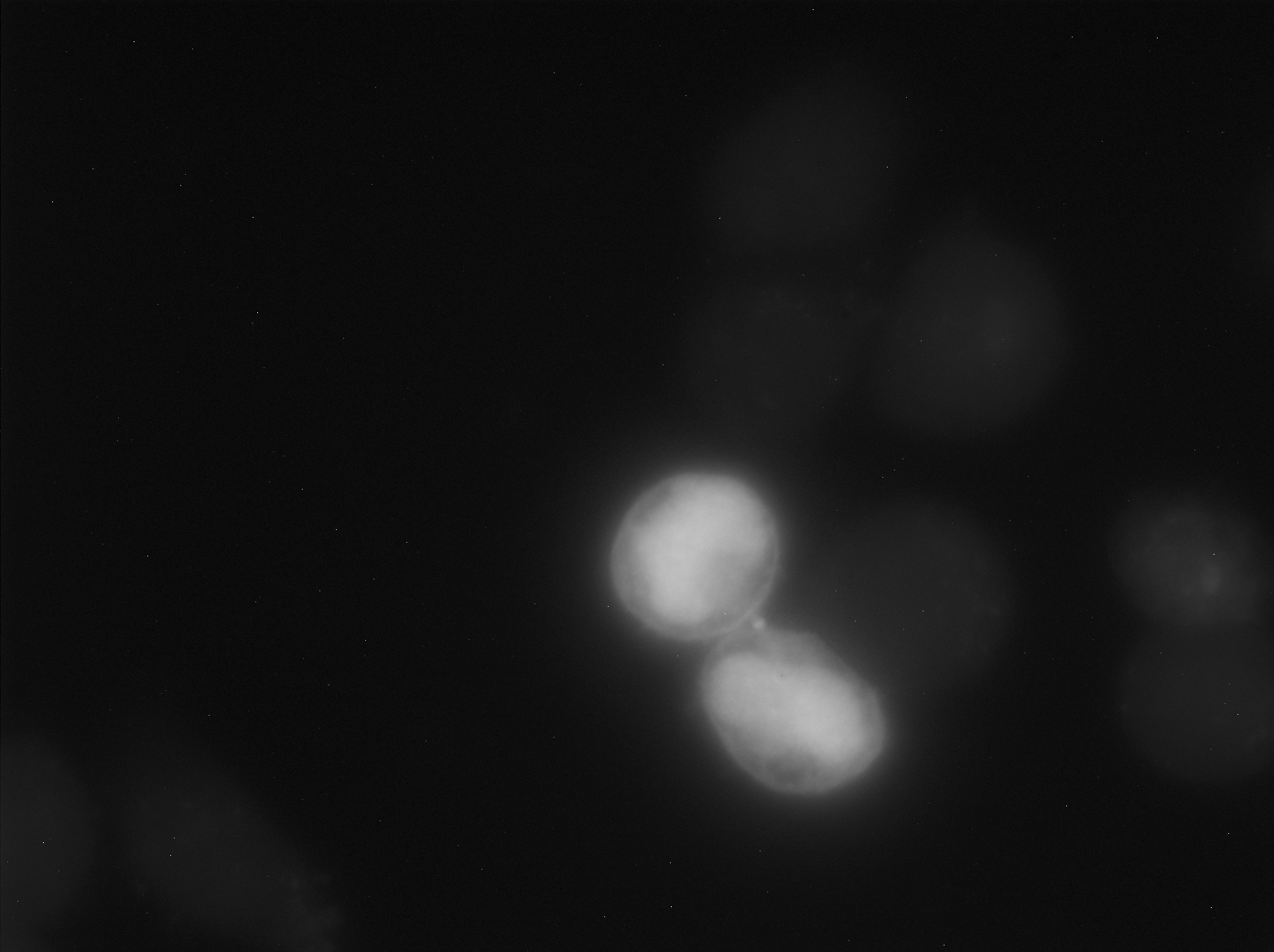

Supplement: Supplementary file 23 — Source data Fig. 6 part 4 [file 44319_2026_742_MOESM23_ESM.zip › Figure 6 Part 4/Fig 6efg TPGS1-KO TPGS1 rescue experiments part 2/R2R3/TPGS1-KO EYFP-only actub 7-31-25 R2 ET6.Project Maximum Z_XY1756415325_Z0_T0_C1.tif]

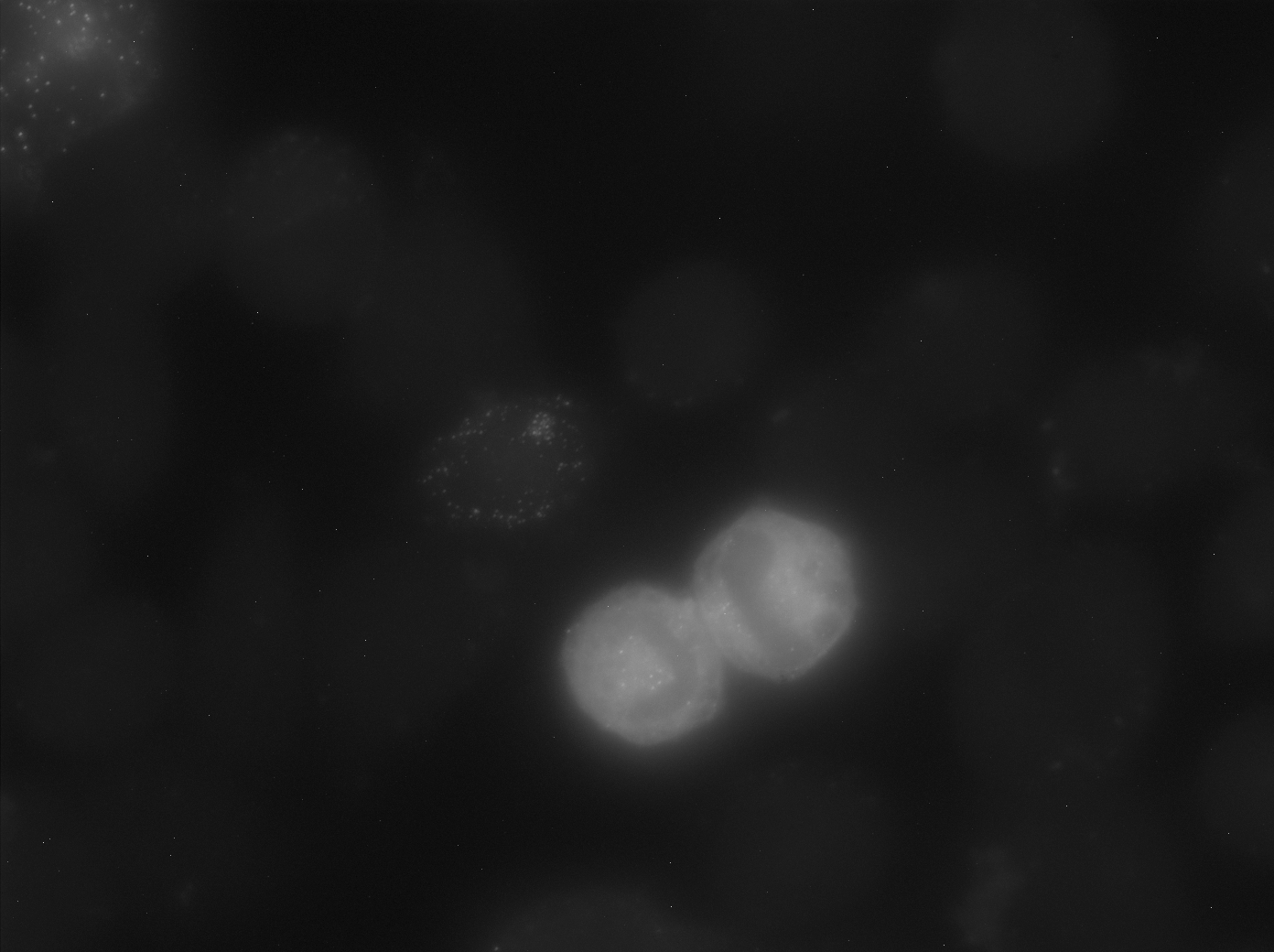

Supplement: Supplementary file 23 — Source data Fig. 6 part 4 [file 44319_2026_742_MOESM23_ESM.zip › Figure 6 Part 4/Fig 6efg TPGS1-KO TPGS1 rescue experiments part 2/R2R3/TPGS1-KO TPGS1-EYFP-3'UTR actub 7-31-25 R2 ET5.Project Maximum Z_XY1756410606_Z0_T0_C1.tif]

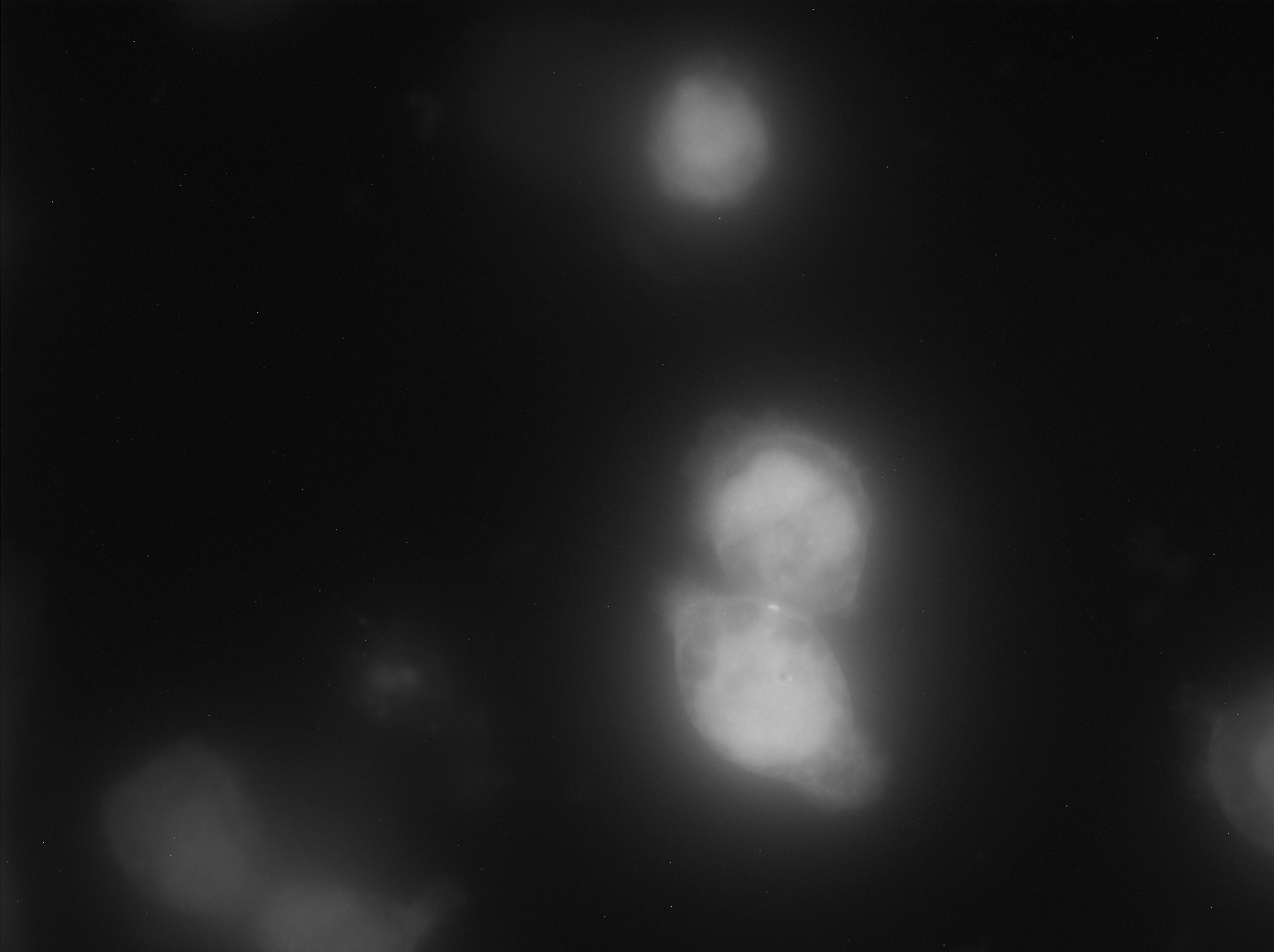

Supplement: Supplementary file 23 — Source data Fig. 6 part 4 [file 44319_2026_742_MOESM23_ESM.zip › Figure 6 Part 4/Fig 6efg TPGS1-KO TPGS1 rescue experiments part 2/R2R3/TPGS1-KO EYFP-only actub 7-31-25 R3 LT6.Project Maximum Z_XY1756493784_Z0_T0_C1.tif]

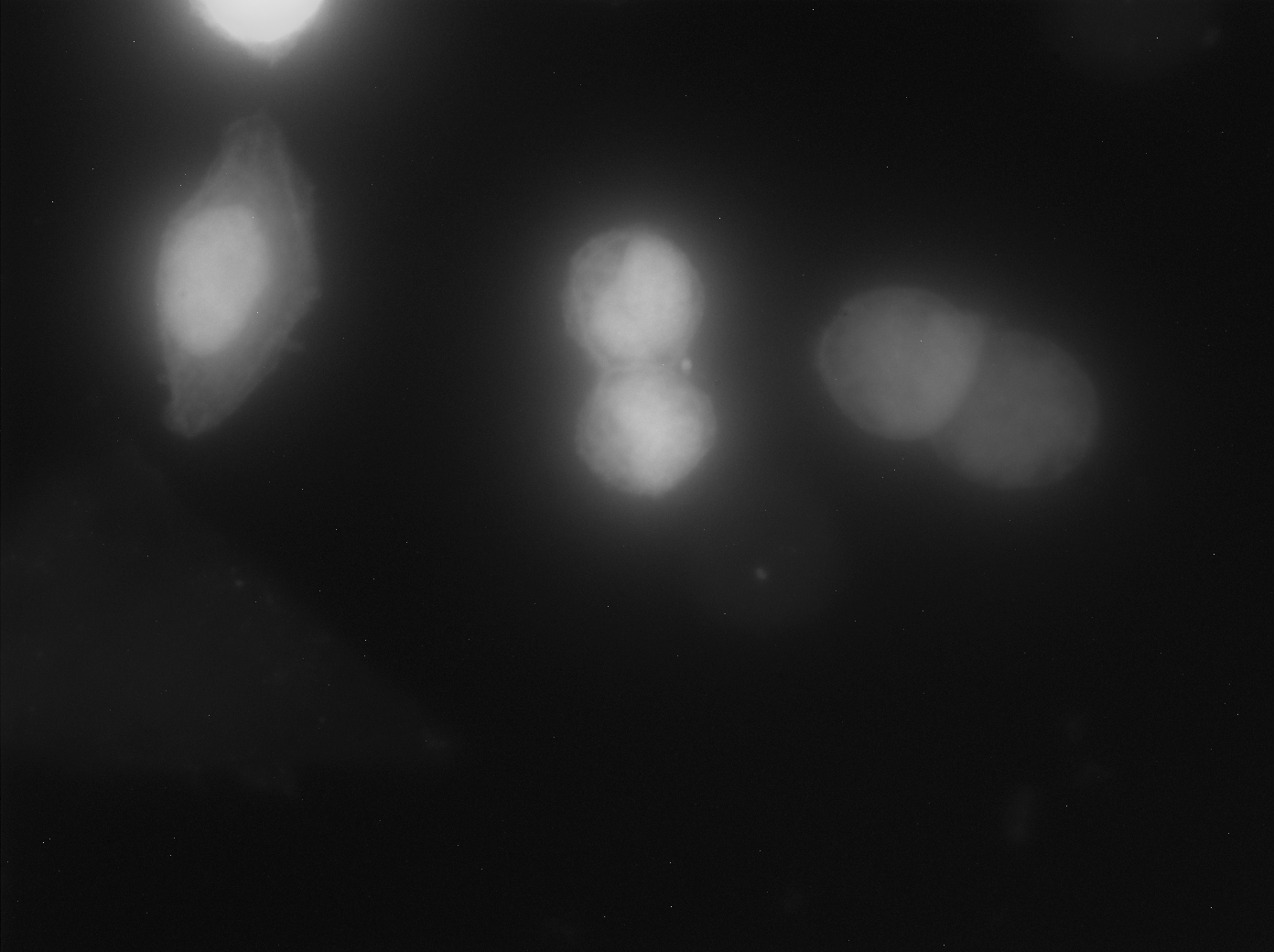

Supplement: Supplementary file 23 — Source data Fig. 6 part 4 [file 44319_2026_742_MOESM23_ESM.zip › Figure 6 Part 4/Fig 6efg TPGS1-KO TPGS1 rescue experiments part 2/R2R3/TPGS1-KO EYFP-only actub 7-31-25 R3 ET10.Project Maximum Z_XY1756495480_Z0_T0_C1.tif]

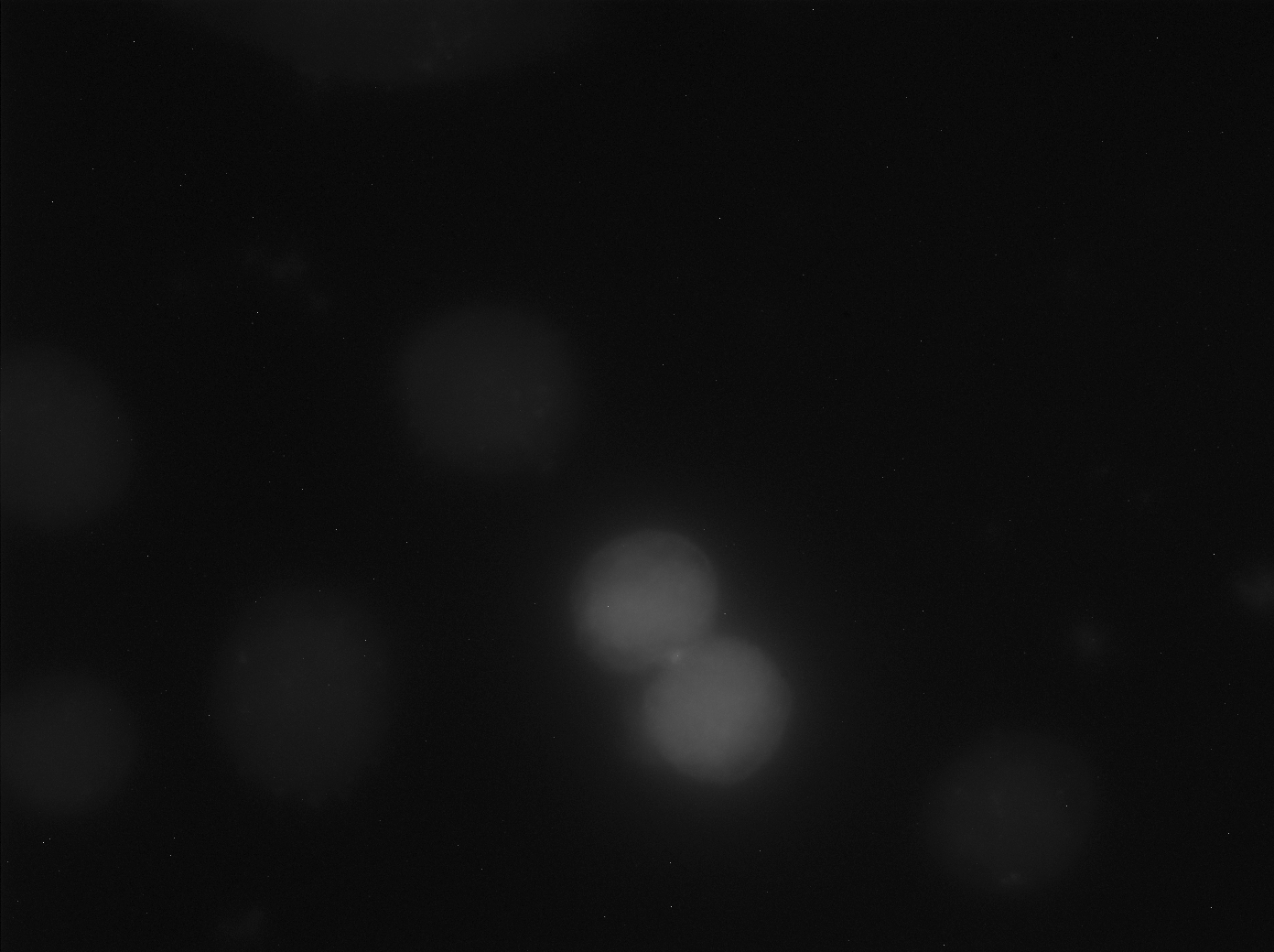

Supplement: Supplementary file 23 — Source data Fig. 6 part 4 [file 44319_2026_742_MOESM23_ESM.zip › Figure 6 Part 4/Fig 6efg TPGS1-KO TPGS1 rescue experiments part 2/R2R3/TPGS1-KO EYFP-only actub 7-31-25 R3 ET8.Project Maximum Z_XY1756494896_Z0_T0_C1.tif]

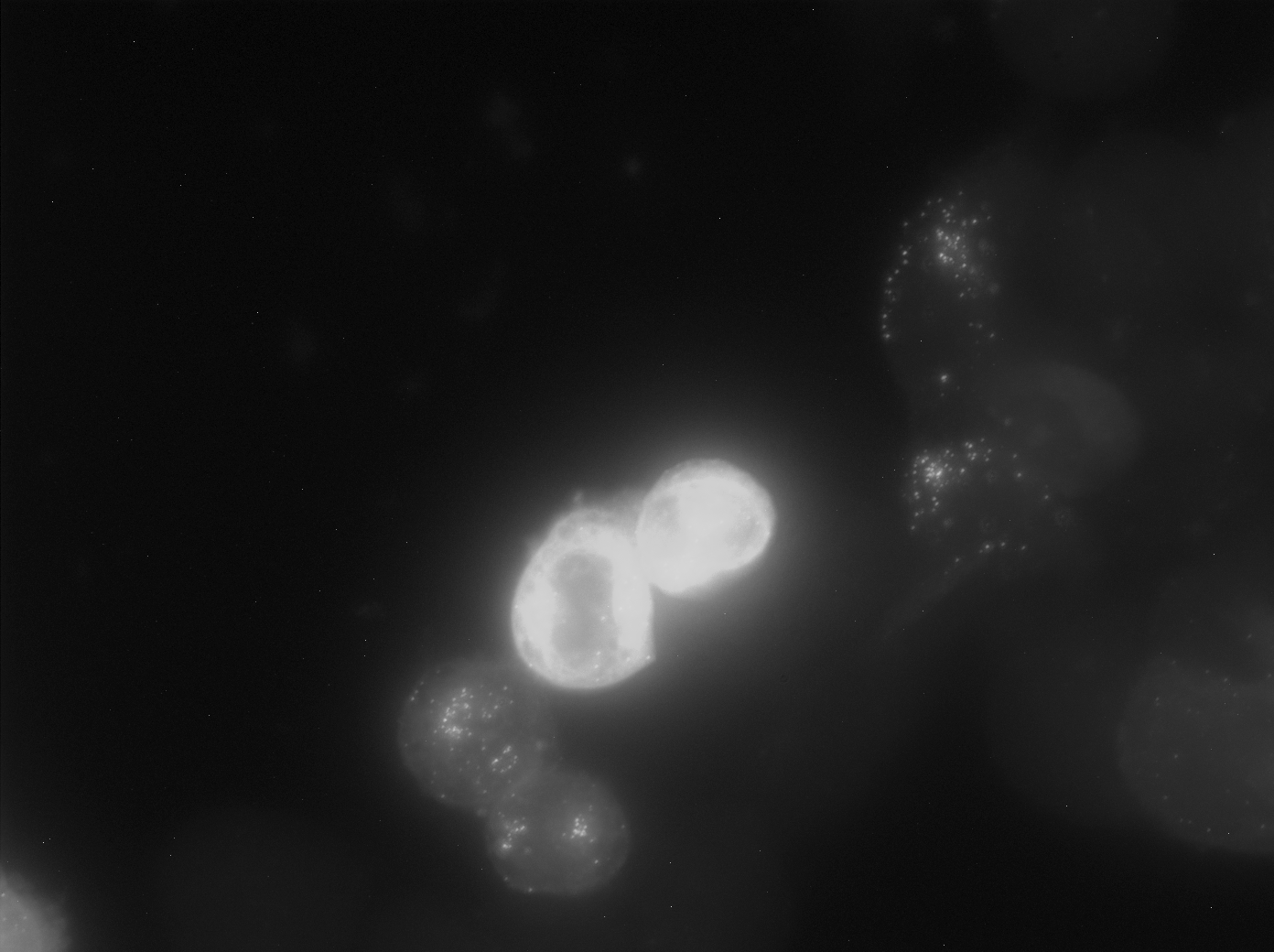

Supplement: Supplementary file 23 — Source data Fig. 6 part 4 [file 44319_2026_742_MOESM23_ESM.zip › Figure 6 Part 4/Fig 6efg TPGS1-KO TPGS1 rescue experiments part 2/R2R3/TPGS1-KO TPGS1-EYFP-3'UTR actub 7-31-25 R3 LT3 ET4.Project Maximum Z_XY1756500418_Z0_T0_C1.tif]

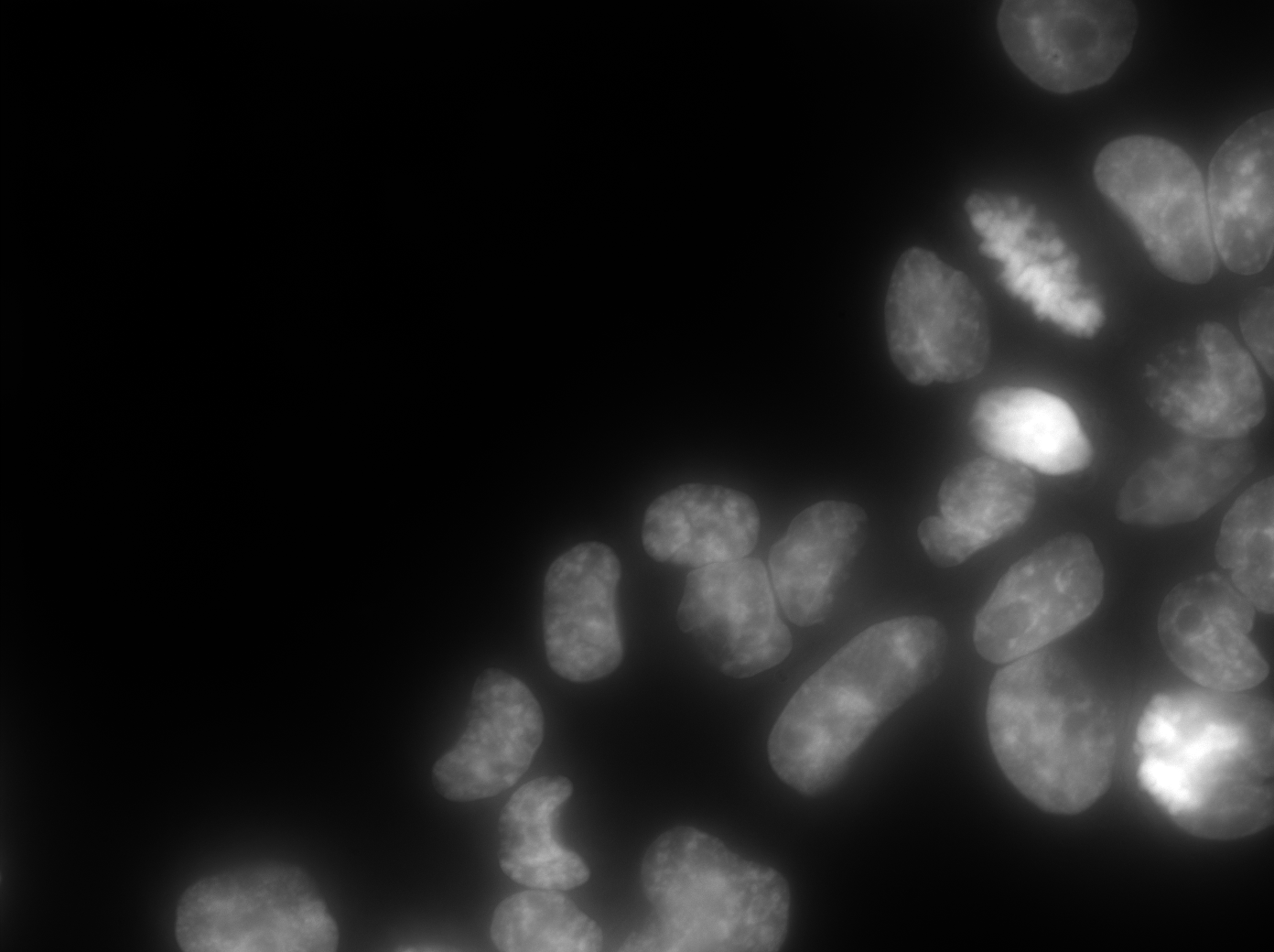

Supplement: Supplementary file 23 — Source data Fig. 6 part 4 [file 44319_2026_742_MOESM23_ESM.zip › Figure 6 Part 4/Fig 6efg TPGS1-KO TPGS1 rescue experiments part 2/R2R3/TPGS1-KO TPGS1-EYFP-3'UTR actub 7-31-25 R3 LT3 ET4.Project Maximum Z_XY1756500418_Z0_T0_C0.tif]

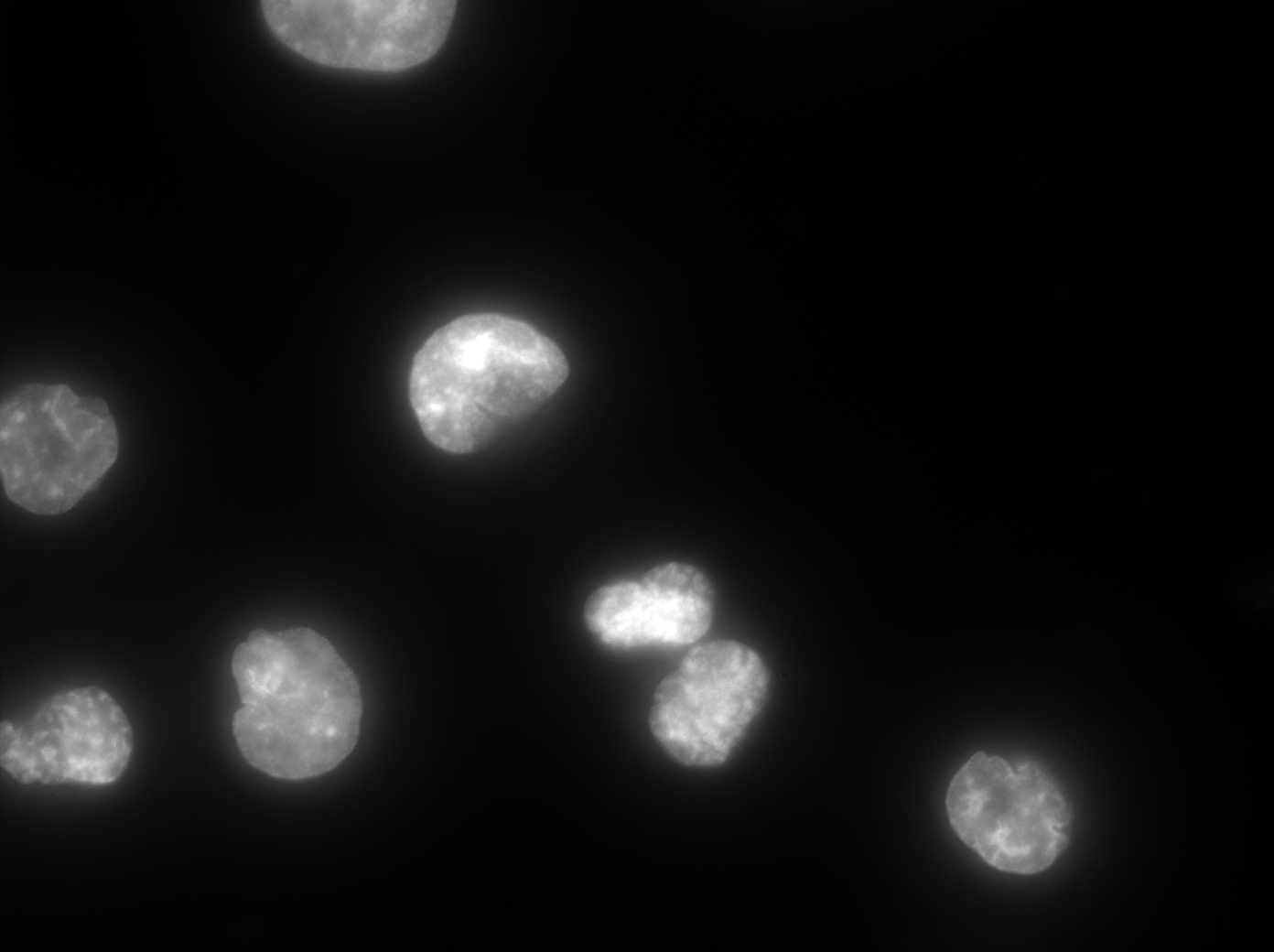

Supplement: Supplementary file 23 — Source data Fig. 6 part 4 [file 44319_2026_742_MOESM23_ESM.zip › Figure 6 Part 4/Fig 6efg TPGS1-KO TPGS1 rescue experiments part 2/R2R3/TPGS1-KO EYFP-only actub 7-31-25 R3 ET8.Project Maximum Z_XY1756494896_Z0_T0_C0.tif]

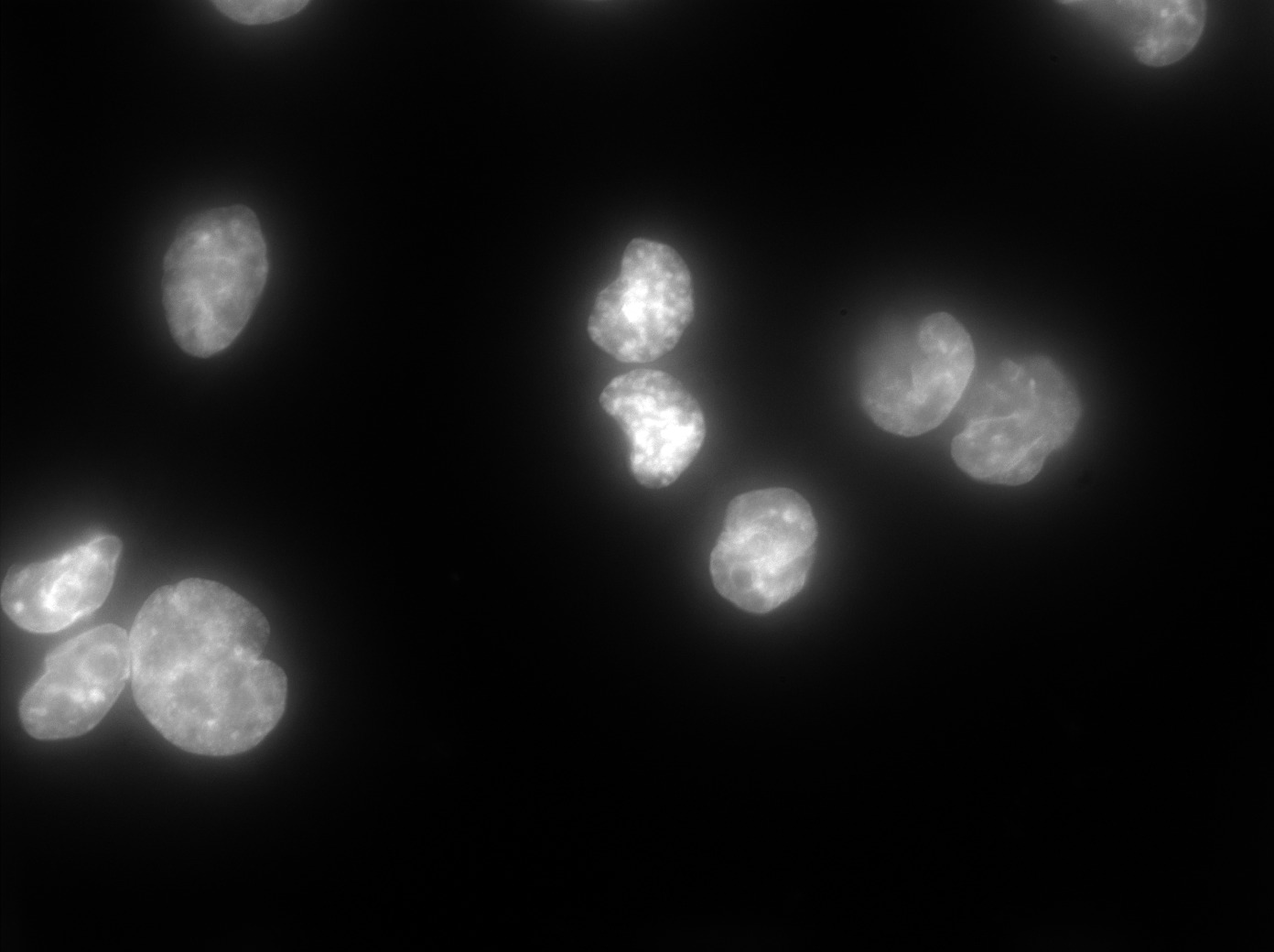

Supplement: Supplementary file 23 — Source data Fig. 6 part 4 [file 44319_2026_742_MOESM23_ESM.zip › Figure 6 Part 4/Fig 6efg TPGS1-KO TPGS1 rescue experiments part 2/R2R3/TPGS1-KO EYFP-only actub 7-31-25 R3 ET10.Project Maximum Z_XY1756495480_Z0_T0_C0.tif]

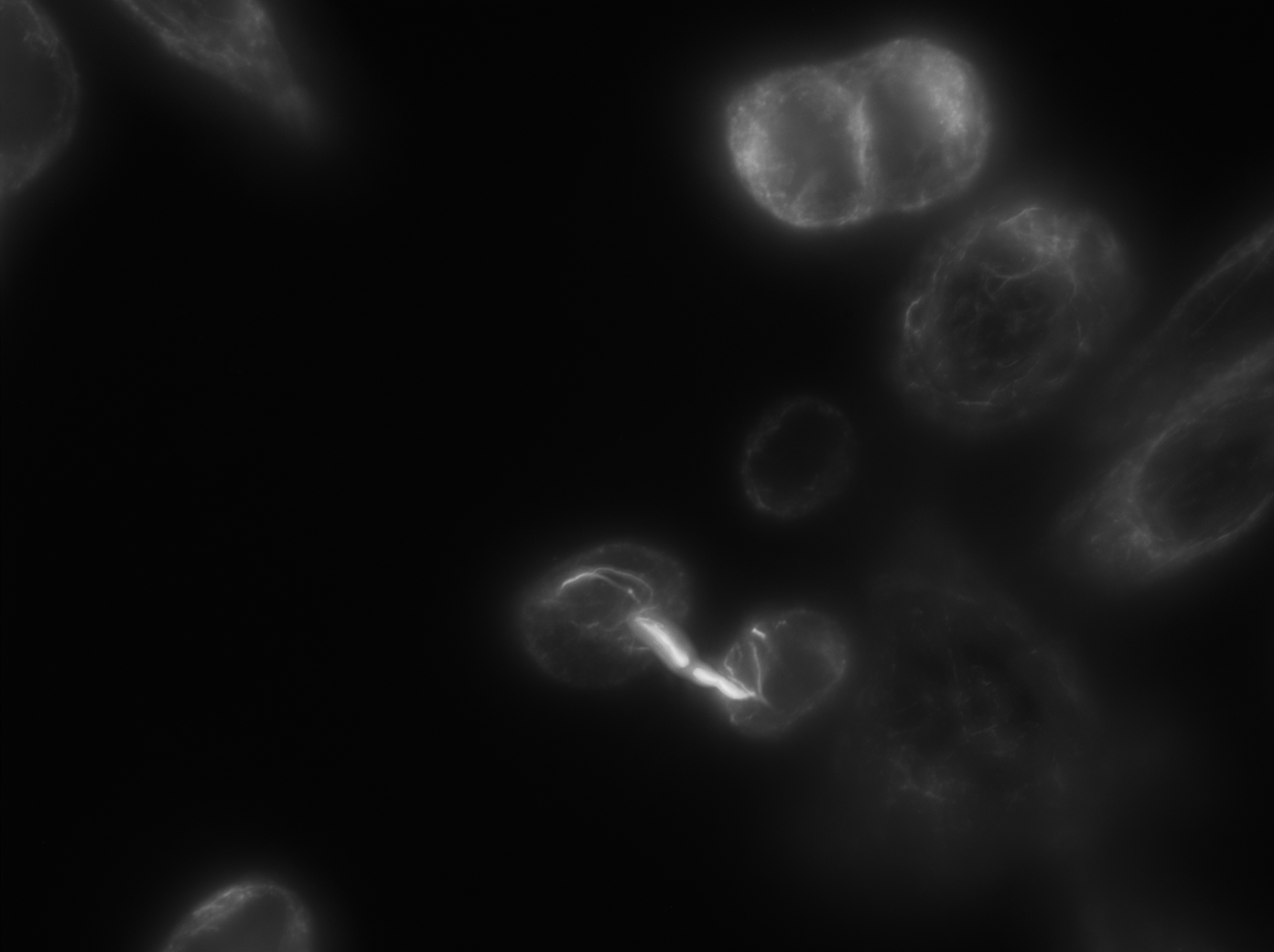

Supplement: Supplementary file 23 — Source data Fig. 6 part 4 [file 44319_2026_742_MOESM23_ESM.zip › Figure 6 Part 4/Fig 6efg TPGS1-KO TPGS1 rescue experiments part 2/R2R3/TPGS1-KO EYFP-only actub 7-31-25 R3 LT9.Project Maximum Z_XY1756496535_Z0_T0_C2.tif]

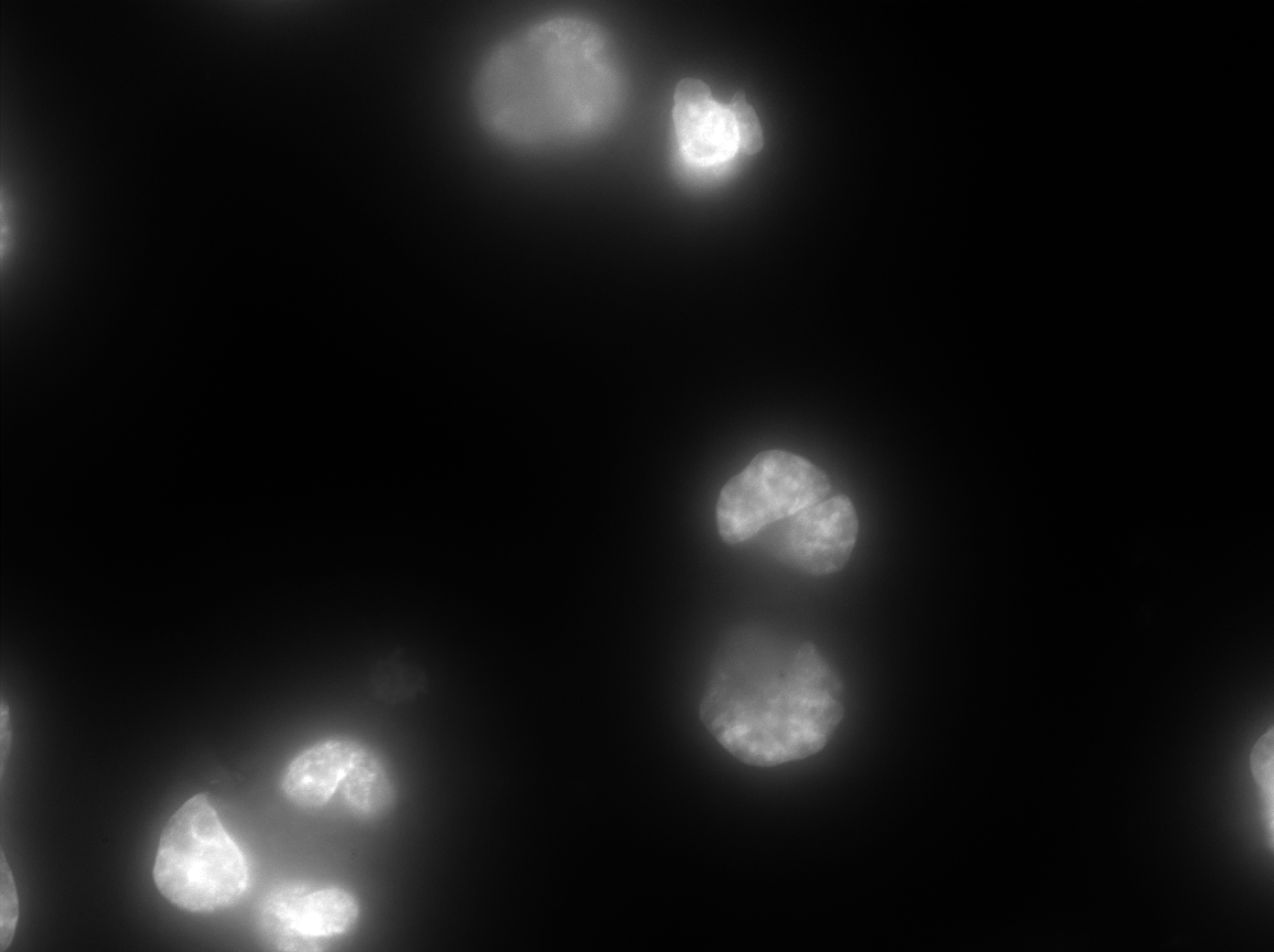

Supplement: Supplementary file 23 — Source data Fig. 6 part 4 [file 44319_2026_742_MOESM23_ESM.zip › Figure 6 Part 4/Fig 6efg TPGS1-KO TPGS1 rescue experiments part 2/R2R3/TPGS1-KO EYFP-only actub 7-31-25 R3 LT6.Project Maximum Z_XY1756493784_Z0_T0_C0.tif]

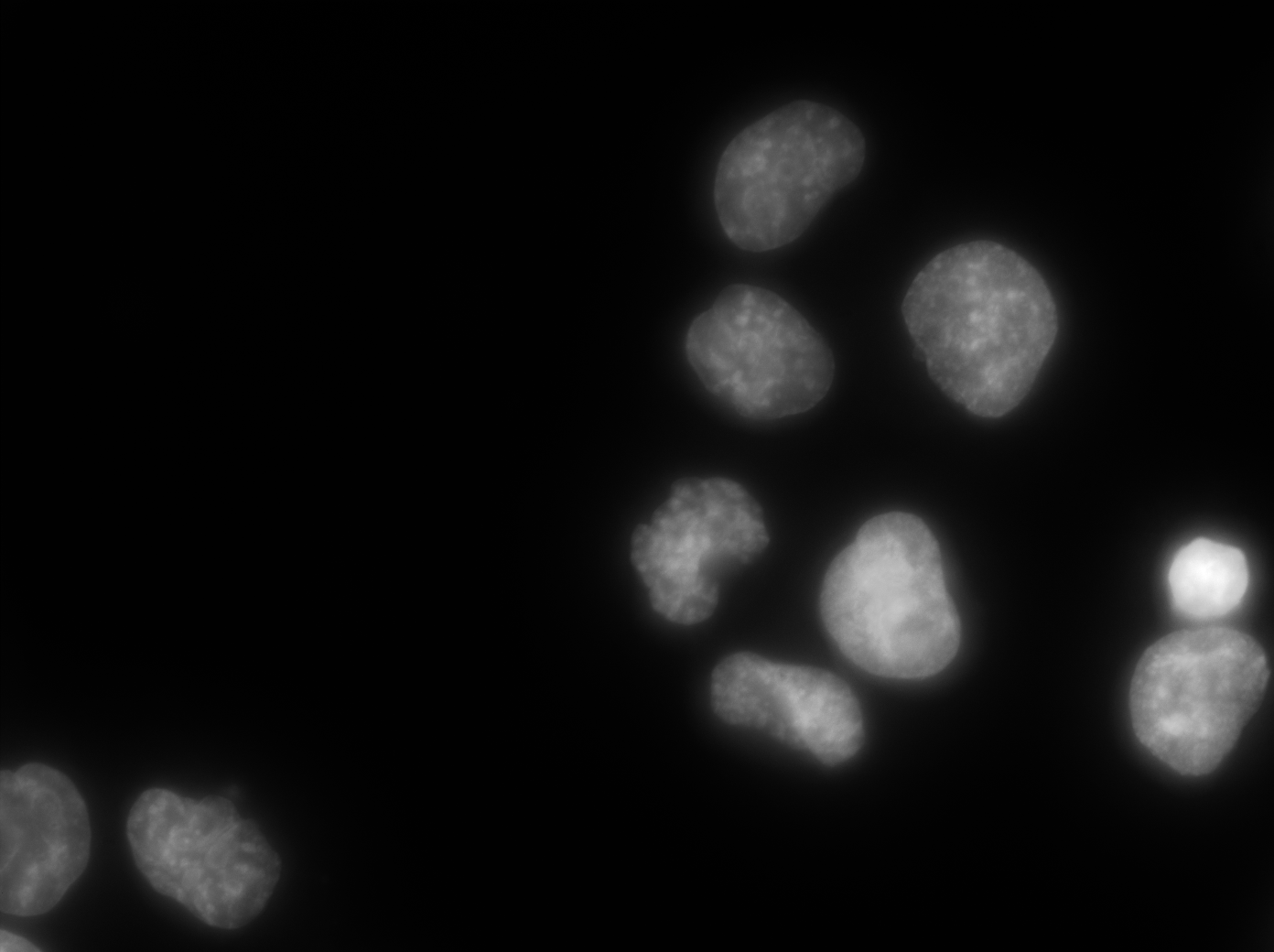

Supplement: Supplementary file 23 — Source data Fig. 6 part 4 [file 44319_2026_742_MOESM23_ESM.zip › Figure 6 Part 4/Fig 6efg TPGS1-KO TPGS1 rescue experiments part 2/R2R3/TPGS1-KO EYFP-only actub 7-31-25 R2 ET6.Project Maximum Z_XY1756415325_Z0_T0_C0.tif]

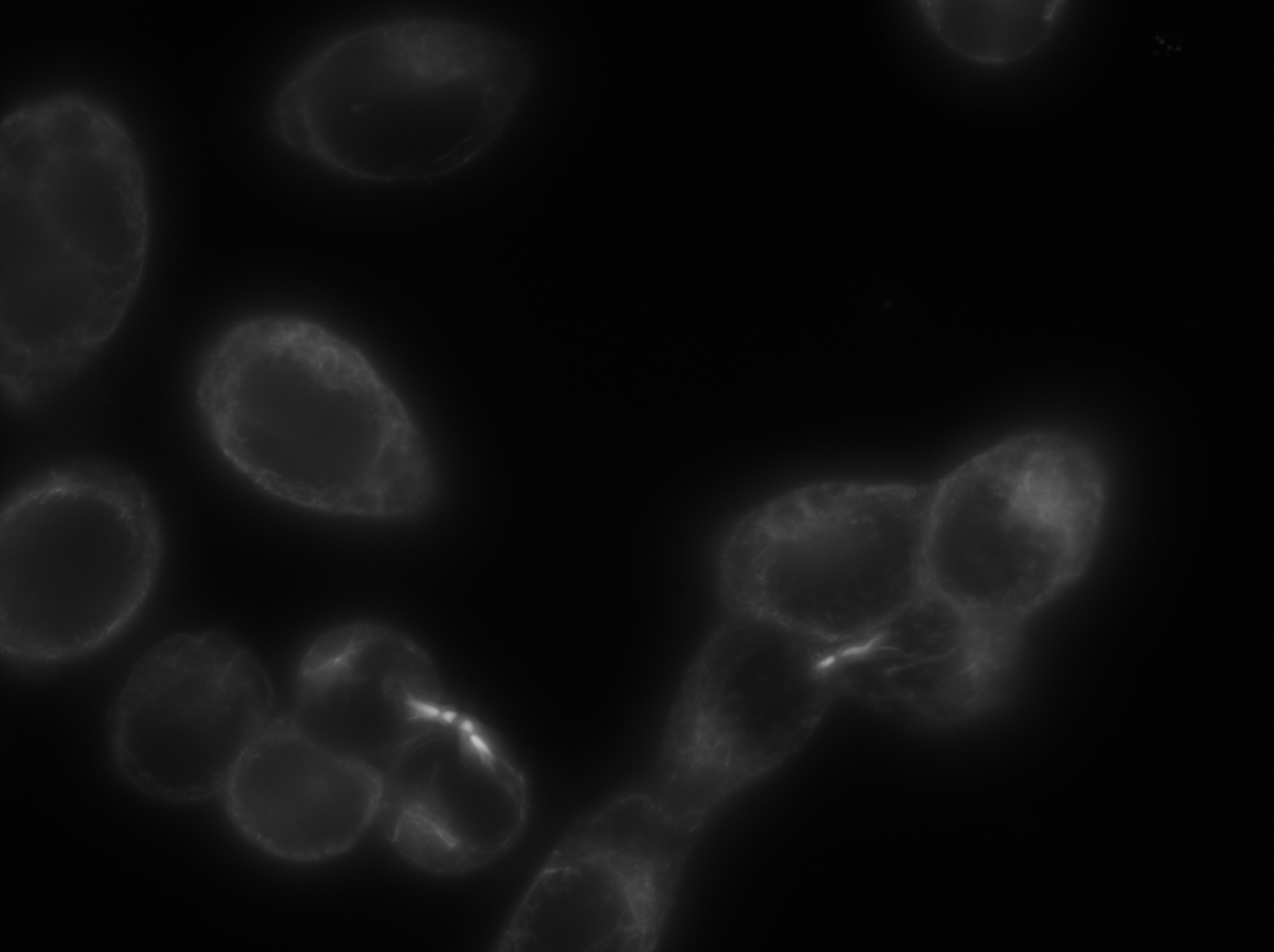

Supplement: Supplementary file 23 — Source data Fig. 6 part 4 [file 44319_2026_742_MOESM23_ESM.zip › Figure 6 Part 4/Fig 6efg TPGS1-KO TPGS1 rescue experiments part 2/R2R3/TPGS1-KO EYFP-only actub 7-31-25 R3 LT8.Project Maximum Z_XY1756496000_Z0_T0_C2.tif]

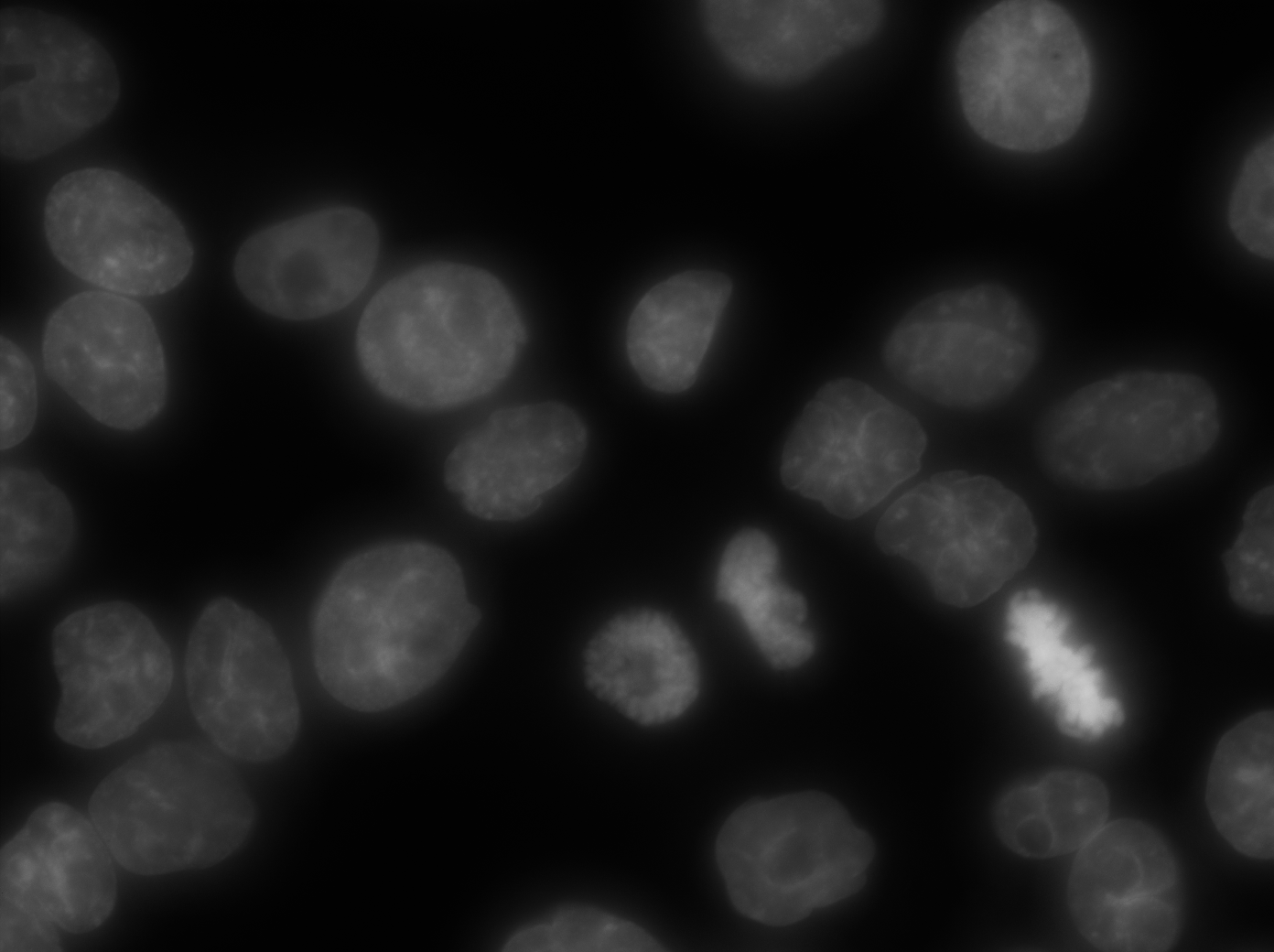

Supplement: Supplementary file 23 — Source data Fig. 6 part 4 [file 44319_2026_742_MOESM23_ESM.zip › Figure 6 Part 4/Fig 6efg TPGS1-KO TPGS1 rescue experiments part 2/R2R3/TPGS1-KO TPGS1-EYFP-3'UTR actub 7-31-25 R2 ET5.Project Maximum Z_XY1756410606_Z0_T0_C0.tif]

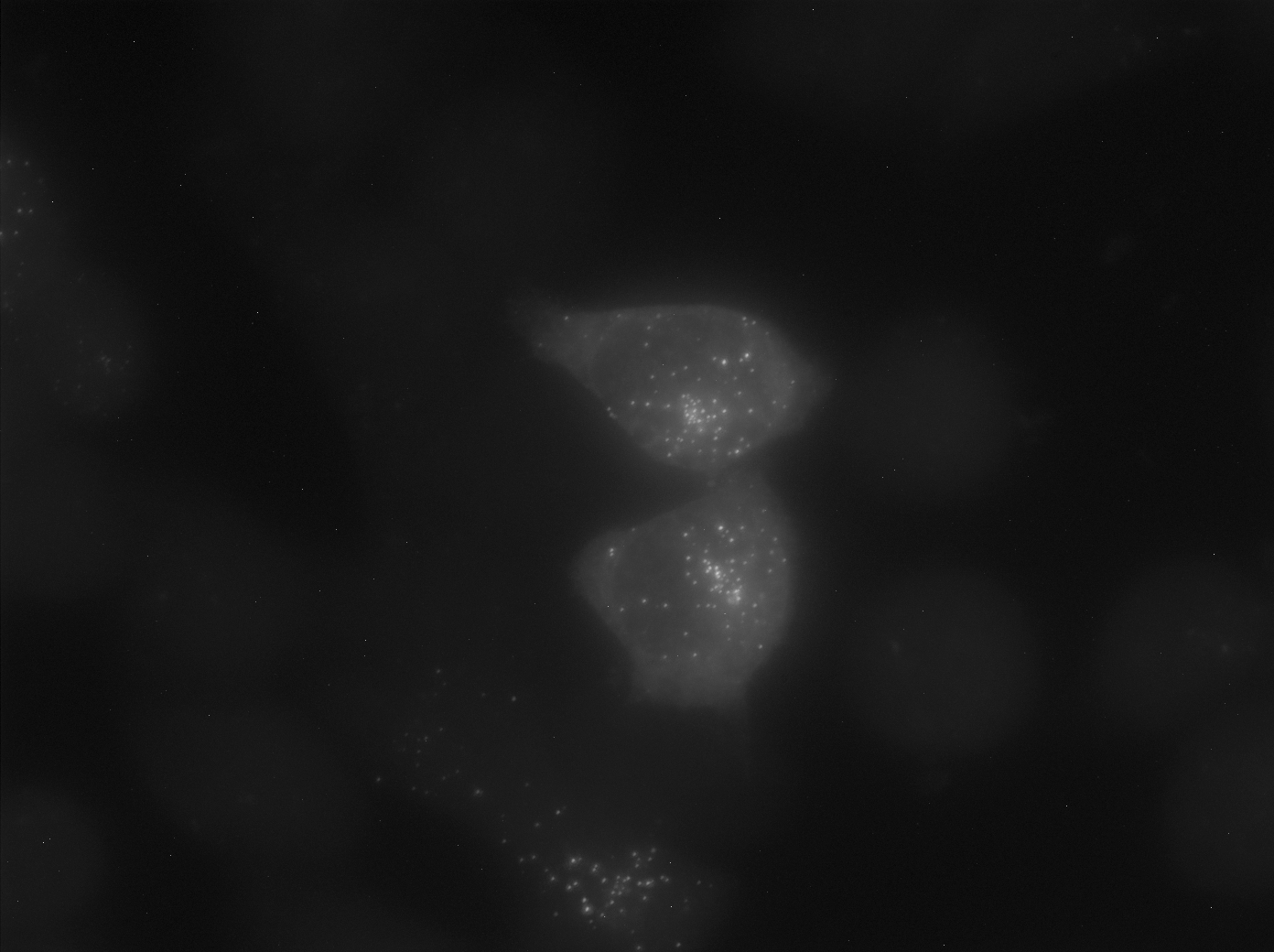

Supplement: Supplementary file 23 — Source data Fig. 6 part 4 [file 44319_2026_742_MOESM23_ESM.zip › Figure 6 Part 4/Fig 6efg TPGS1-KO TPGS1 rescue experiments part 2/R2R3/TPGS1-KO TPGS1-EYFP-3'UTR actub 7-31-25 R2 LT2.Project Maximum Z_XY1756407015_Z0_T0_C1.tif]

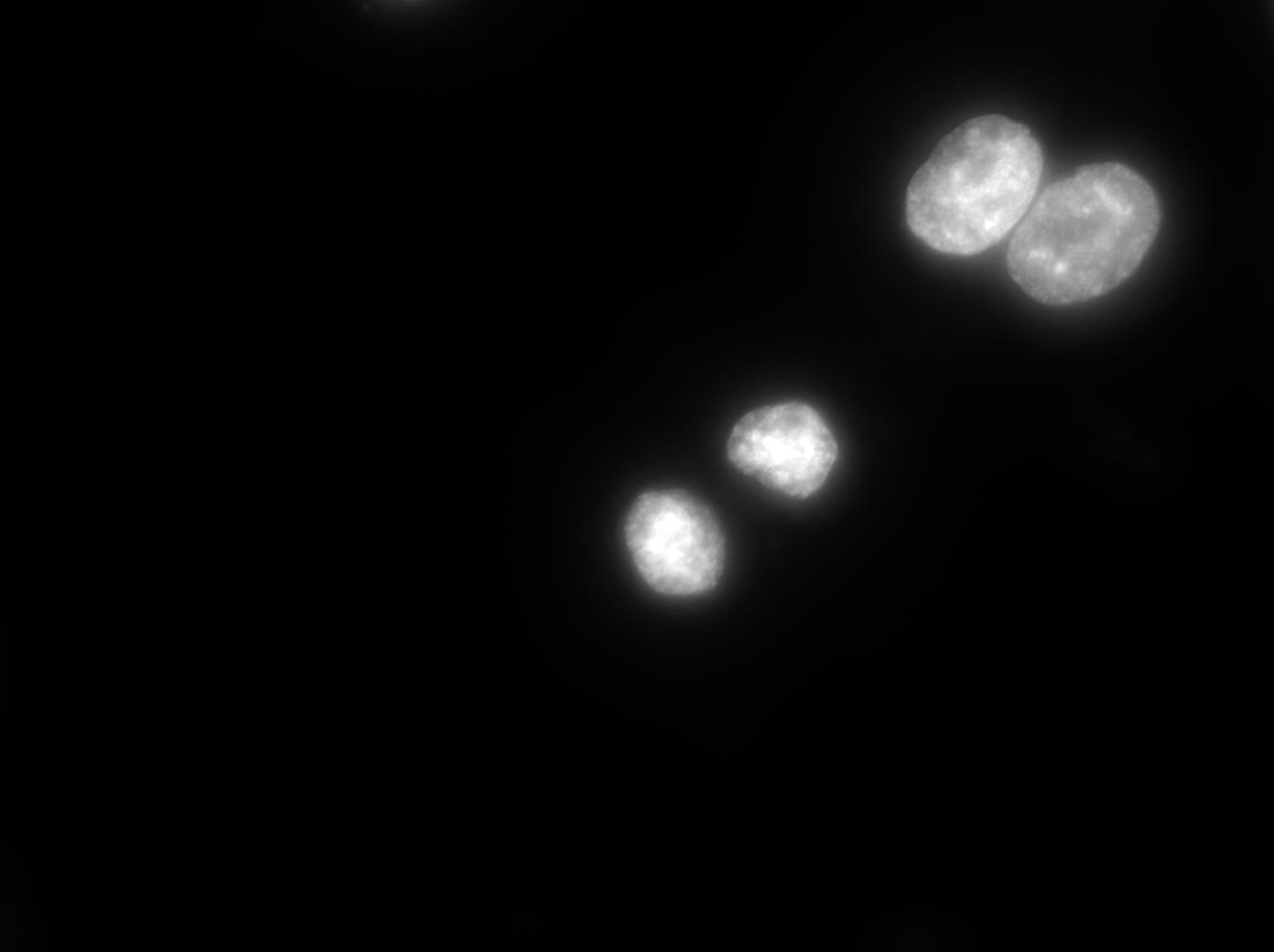

Supplement: Supplementary file 23 — Source data Fig. 6 part 4 [file 44319_2026_742_MOESM23_ESM.zip › Figure 6 Part 4/Fig 6efg TPGS1-KO TPGS1 rescue experiments part 2/R2R3/TPGS1-KO EYFP-only actub 7-31-25 R3 ET9.Project Maximum Z_XY1756495193_Z0_T0_C0.tif]

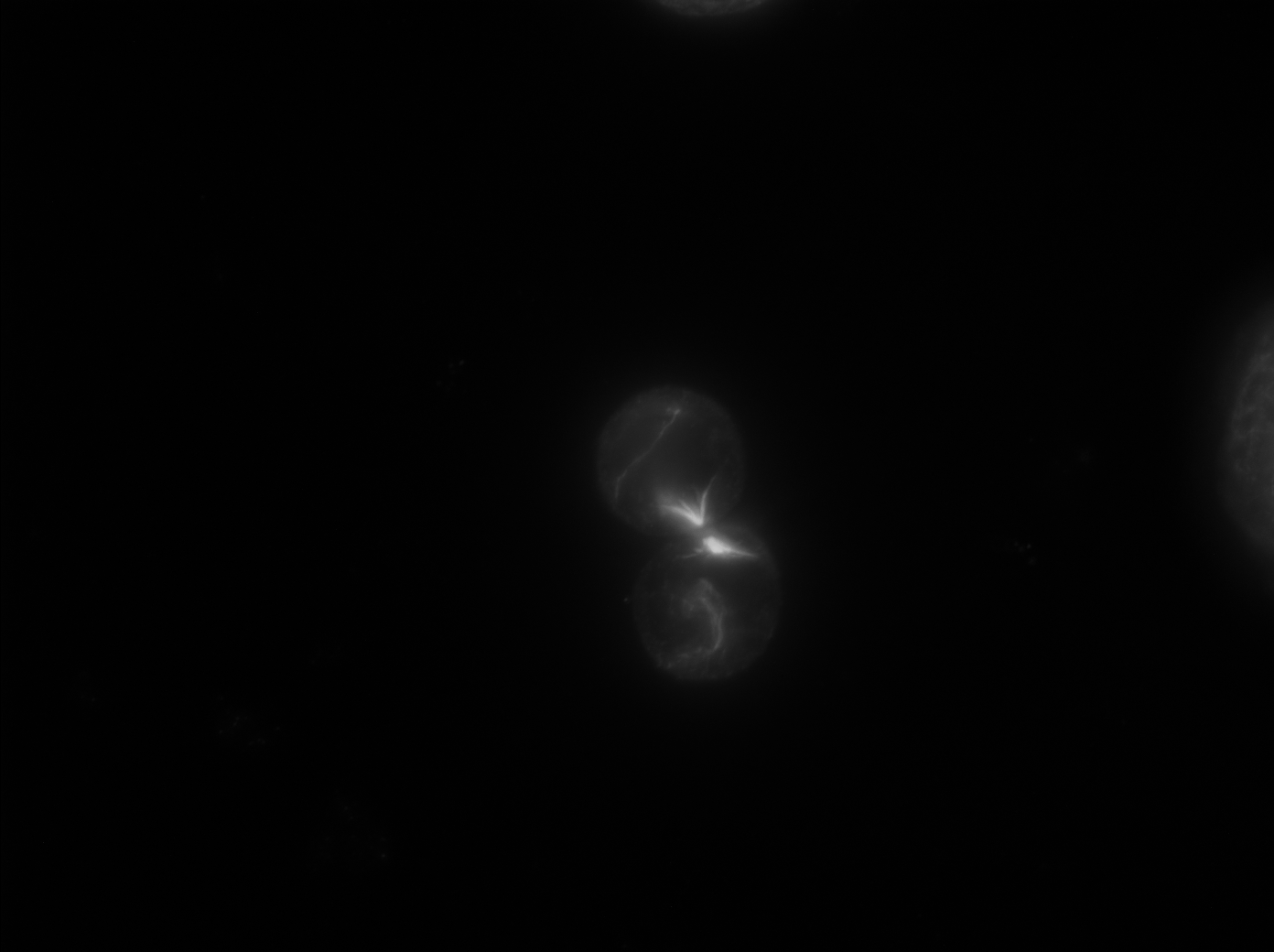

Supplement: Supplementary file 23 — Source data Fig. 6 part 4 [file 44319_2026_742_MOESM23_ESM.zip › Figure 6 Part 4/Fig 6efg TPGS1-KO TPGS1 rescue experiments part 2/R2R3/TPGS1-KO EYFP-only actub 7-31-25 R3 ET6.Project Maximum Z_XY1756494172_Z0_T0_C2.tif]

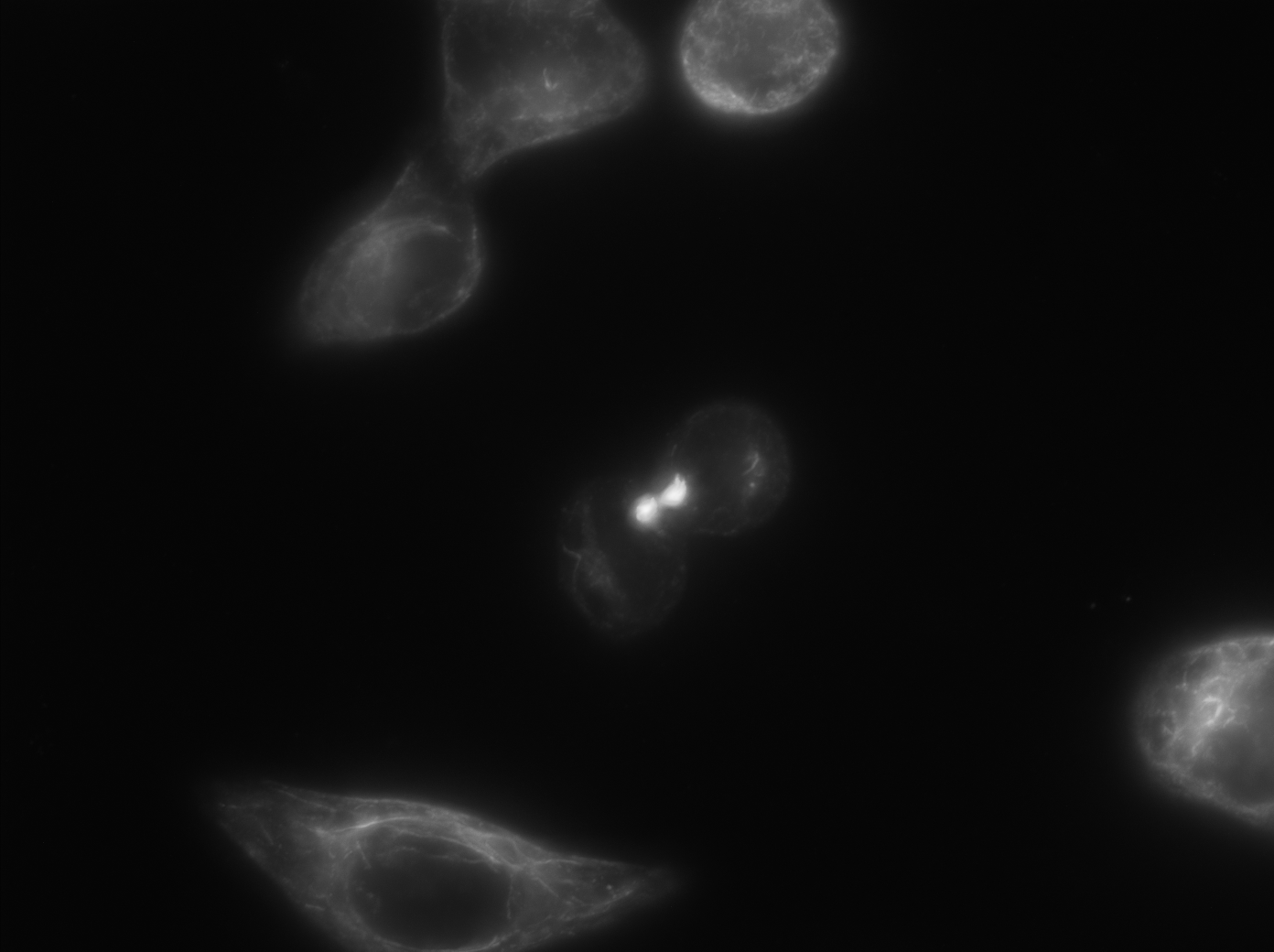

Supplement: Supplementary file 23 — Source data Fig. 6 part 4 [file 44319_2026_742_MOESM23_ESM.zip › Figure 6 Part 4/Fig 6efg TPGS1-KO TPGS1 rescue experiments part 2/R2R3/TPGS1-KO EYFP-only actub 7-31-25 R3 ET1.Project Maximum Z_XY1756491656_Z0_T0_C2.tif]

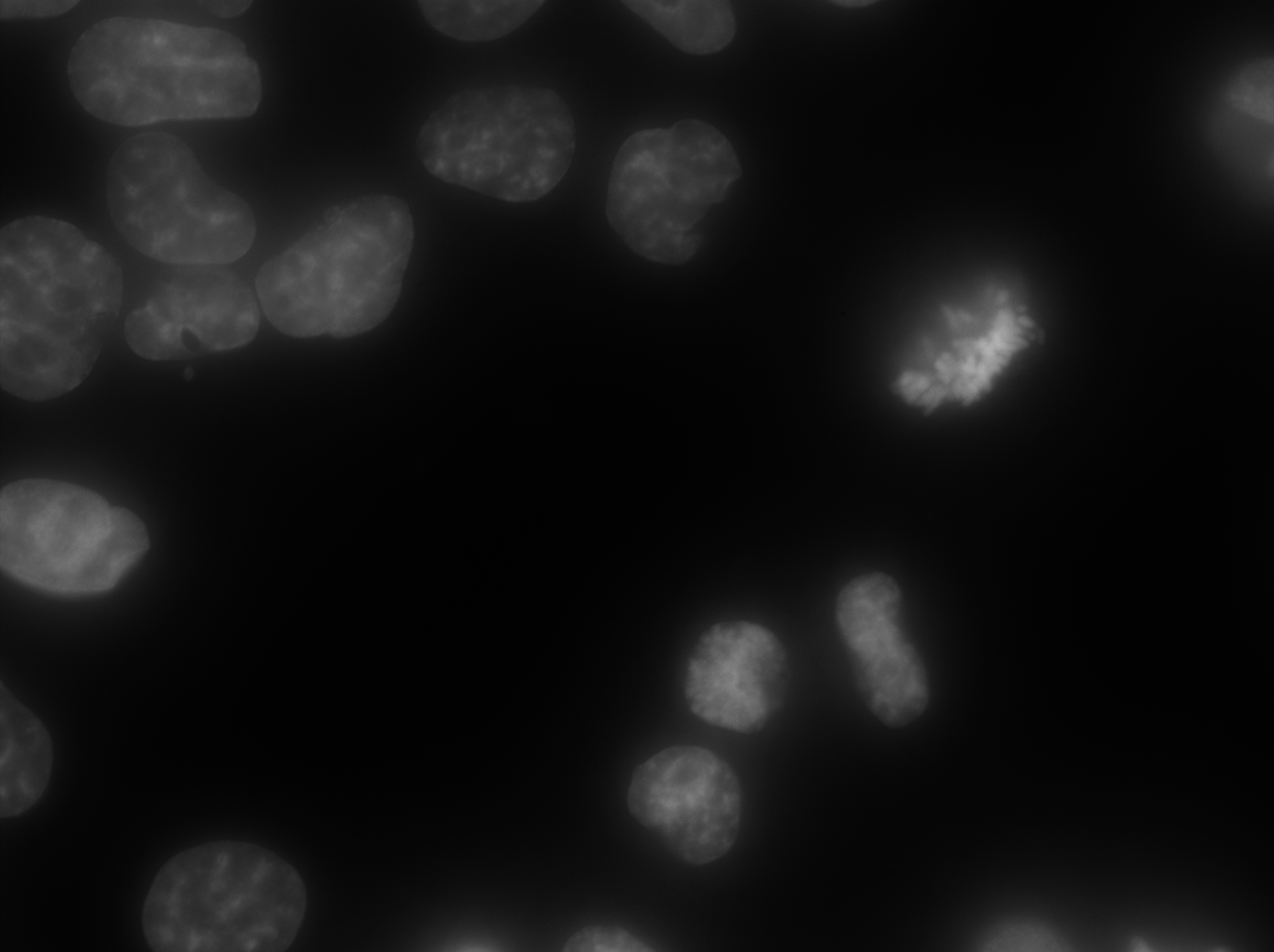

Supplement: Supplementary file 23 — Source data Fig. 6 part 4 [file 44319_2026_742_MOESM23_ESM.zip › Figure 6 Part 4/Fig 6efg TPGS1-KO TPGS1 rescue experiments part 2/R2R3/TPGS1-KO TPGS1-EYFP-3'UTR actub 7-31-25 R2 ET3.Project Maximum Z_XY1756408369_Z0_T0_C0.tif]

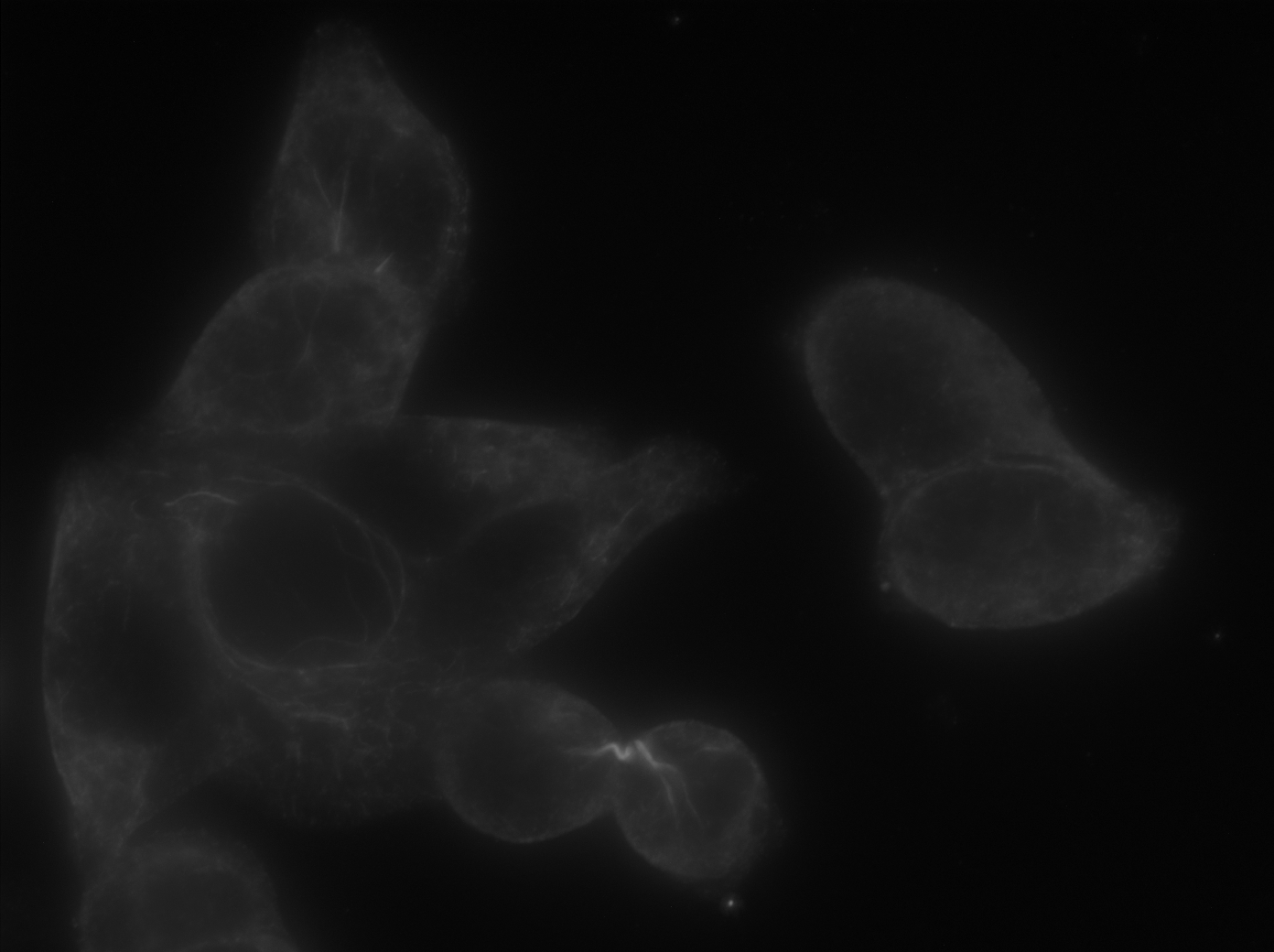

Supplement: Supplementary file 23 — Source data Fig. 6 part 4 [file 44319_2026_742_MOESM23_ESM.zip › Figure 6 Part 4/Fig 6efg TPGS1-KO TPGS1 rescue experiments part 2/R2R3/TPGS1-KO EYFP-only actub 7-31-25 R2 LT8.Project Maximum Z_XY1756416035_Z0_T0_C2.tif]

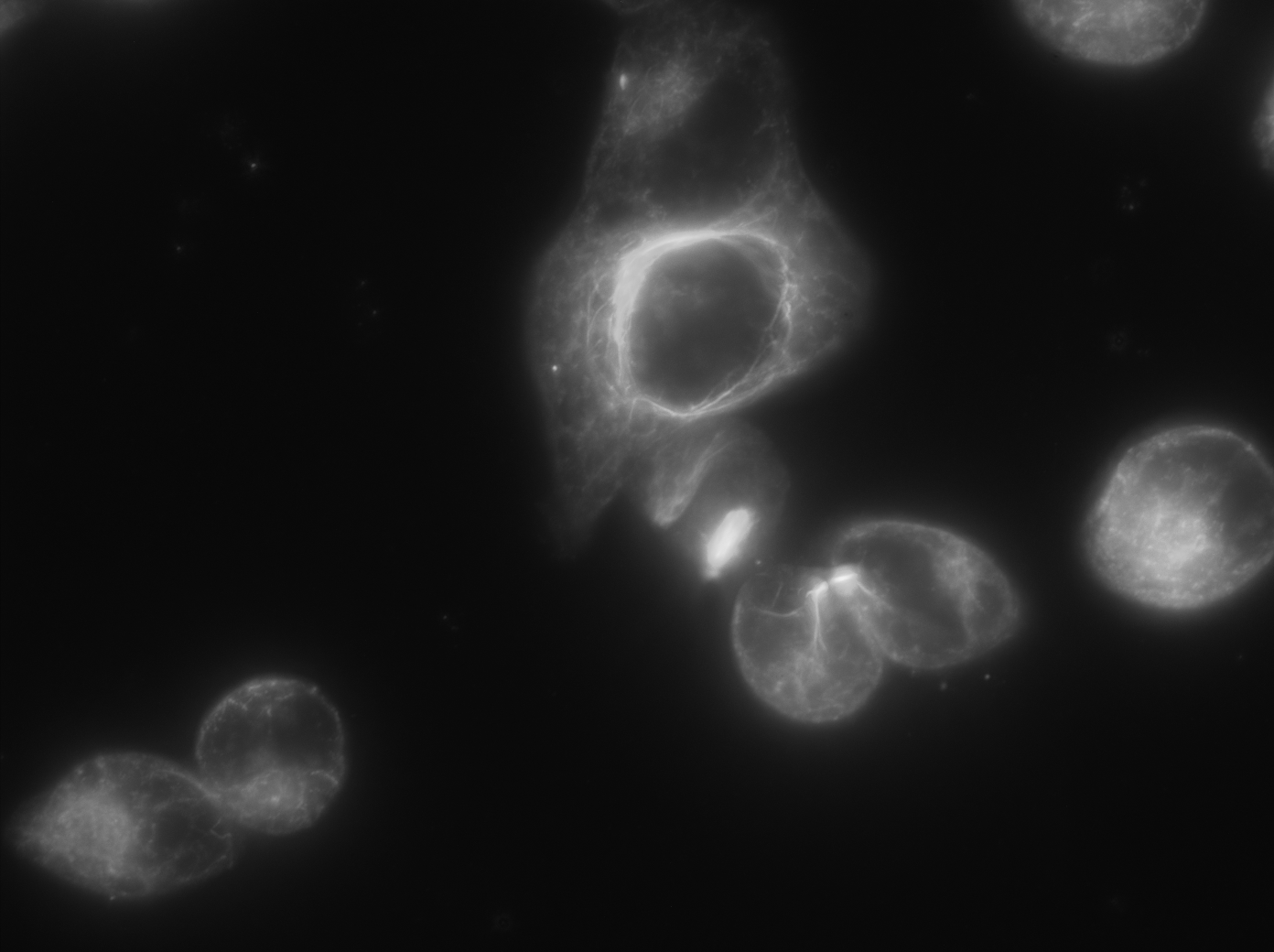

Supplement: Supplementary file 23 — Source data Fig. 6 part 4 [file 44319_2026_742_MOESM23_ESM.zip › Figure 6 Part 4/Fig 6efg TPGS1-KO TPGS1 rescue experiments part 2/R2R3/TPGS1-KO TPGS1-EYFP-3'UTR actub 7-31-25 R3 LT4.Project Maximum Z_XY1756500919_Z0_T0_C2.tif]

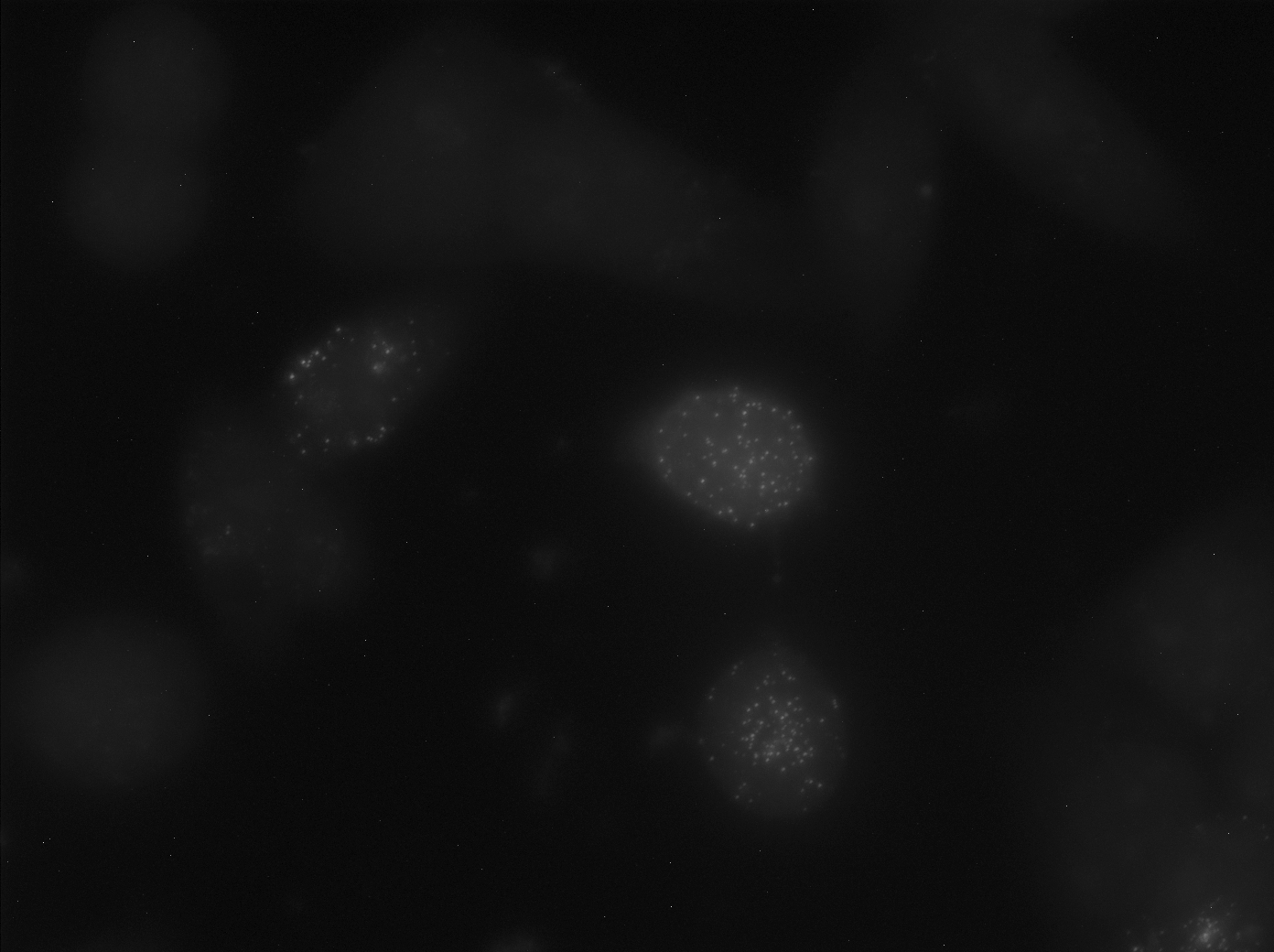

Supplement: Supplementary file 23 — Source data Fig. 6 part 4 [file 44319_2026_742_MOESM23_ESM.zip › Figure 6 Part 4/Fig 6efg TPGS1-KO TPGS1 rescue experiments part 2/R2R3/TPGS1-KO TPGS1-EYFP-3'UTR actub 7-31-25 R3 LT9.Project Maximum Z_XY1756502312_Z0_T0_C1.tif]

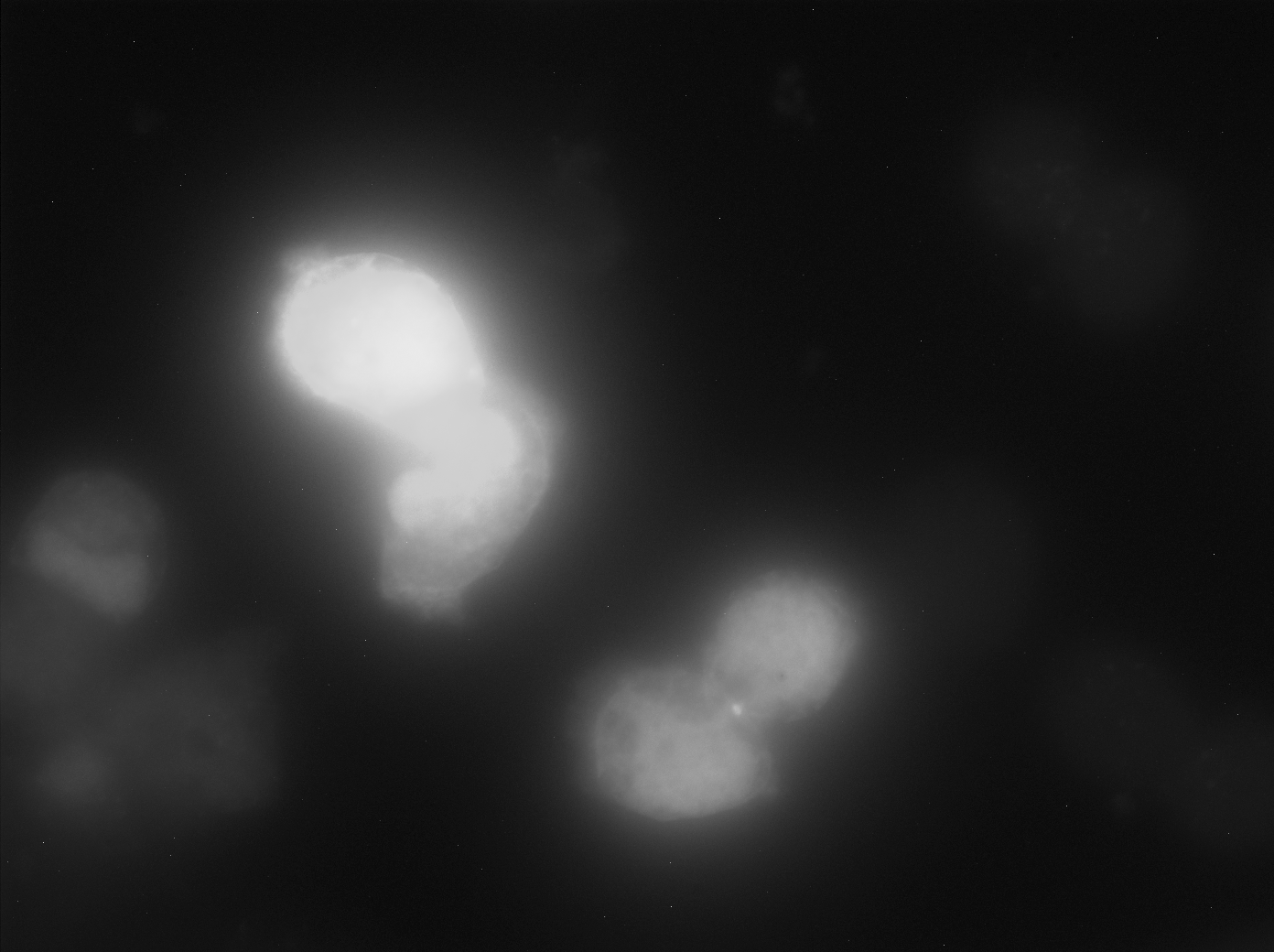

Supplement: Supplementary file 23 — Source data Fig. 6 part 4 [file 44319_2026_742_MOESM23_ESM.zip › Figure 6 Part 4/Fig 6efg TPGS1-KO TPGS1 rescue experiments part 2/R2R3/TPGS1-KO EYFP-only actub 7-31-25 R3 LT3.Project Maximum Z_XY1756492484_Z0_T0_C1.tif]

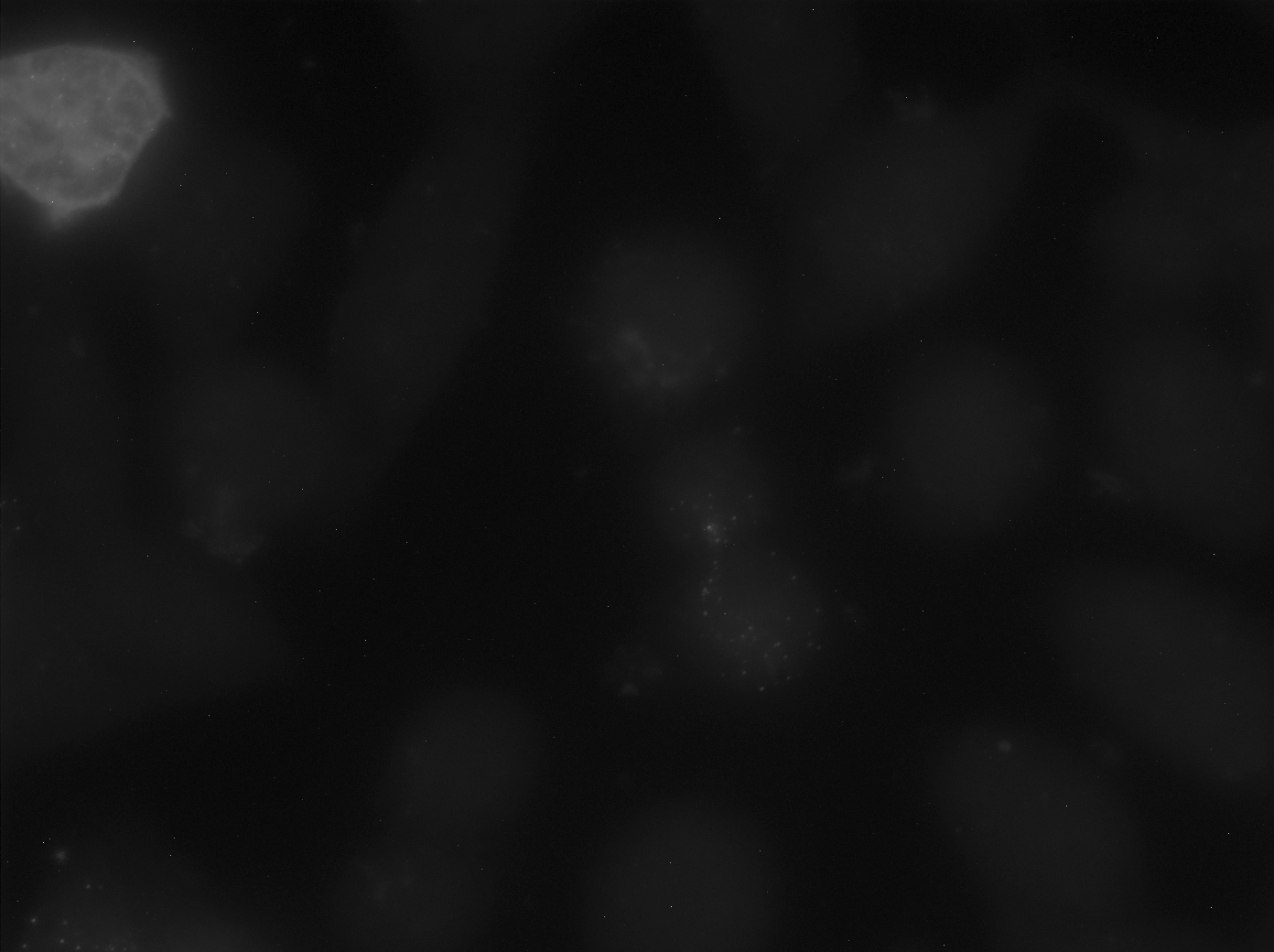

Supplement: Supplementary file 23 — Source data Fig. 6 part 4 [file 44319_2026_742_MOESM23_ESM.zip › Figure 6 Part 4/Fig 6efg TPGS1-KO TPGS1 rescue experiments part 2/R2R3/TPGS1-KO TPGS1-EYFP-3'UTR actub 7-31-25 R3 ET6.Project Maximum Z_XY1756501524_Z0_T0_C1.tif]

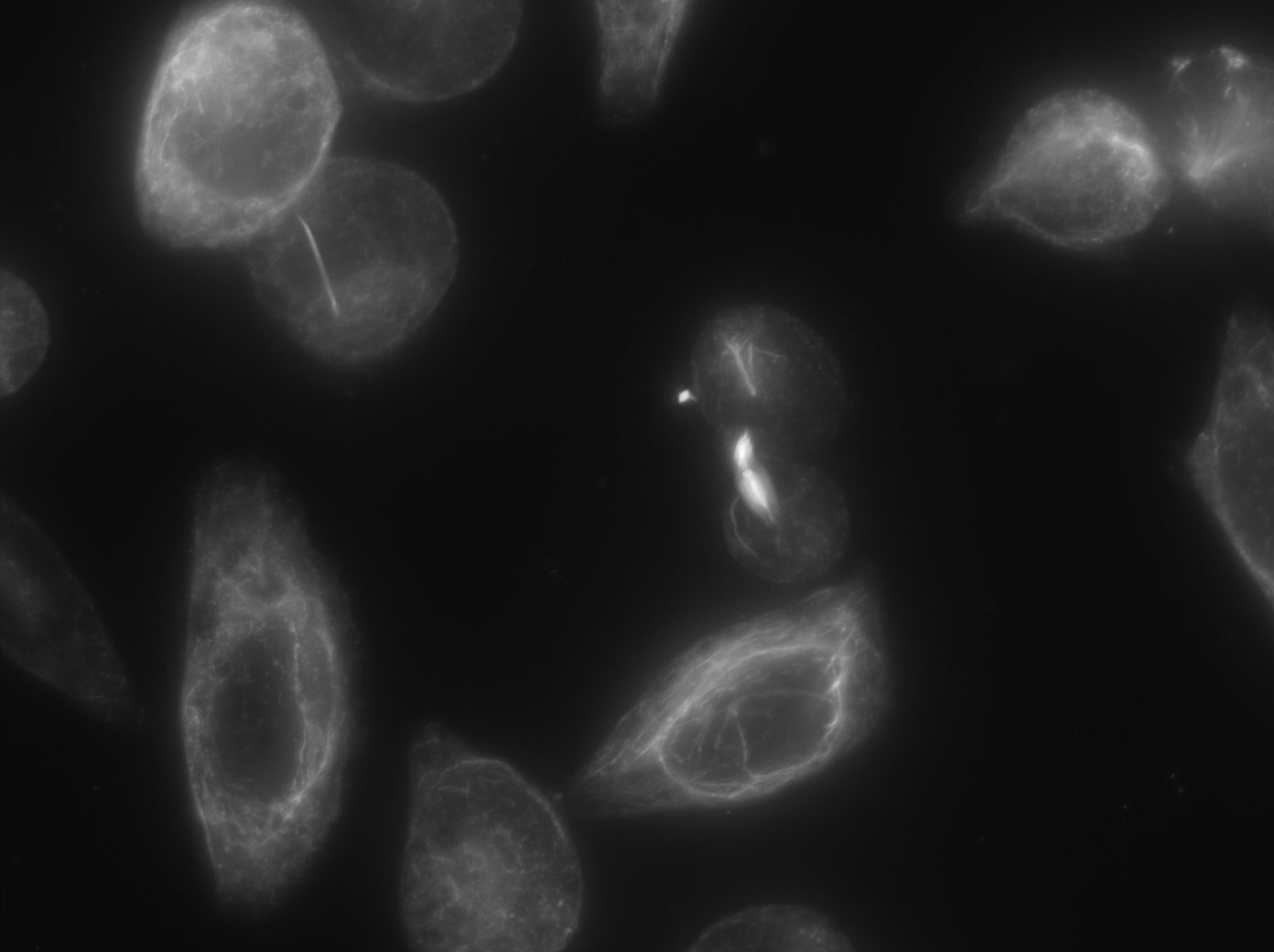

Supplement: Supplementary file 23 — Source data Fig. 6 part 4 [file 44319_2026_742_MOESM23_ESM.zip › Figure 6 Part 4/Fig 6efg TPGS1-KO TPGS1 rescue experiments part 2/R2R3/TPGS1-KO TPGS1-EYFP-3'UTR actub 7-31-25 R3 ET7.Project Maximum Z_XY1756503229_Z0_T0_C2.tif]

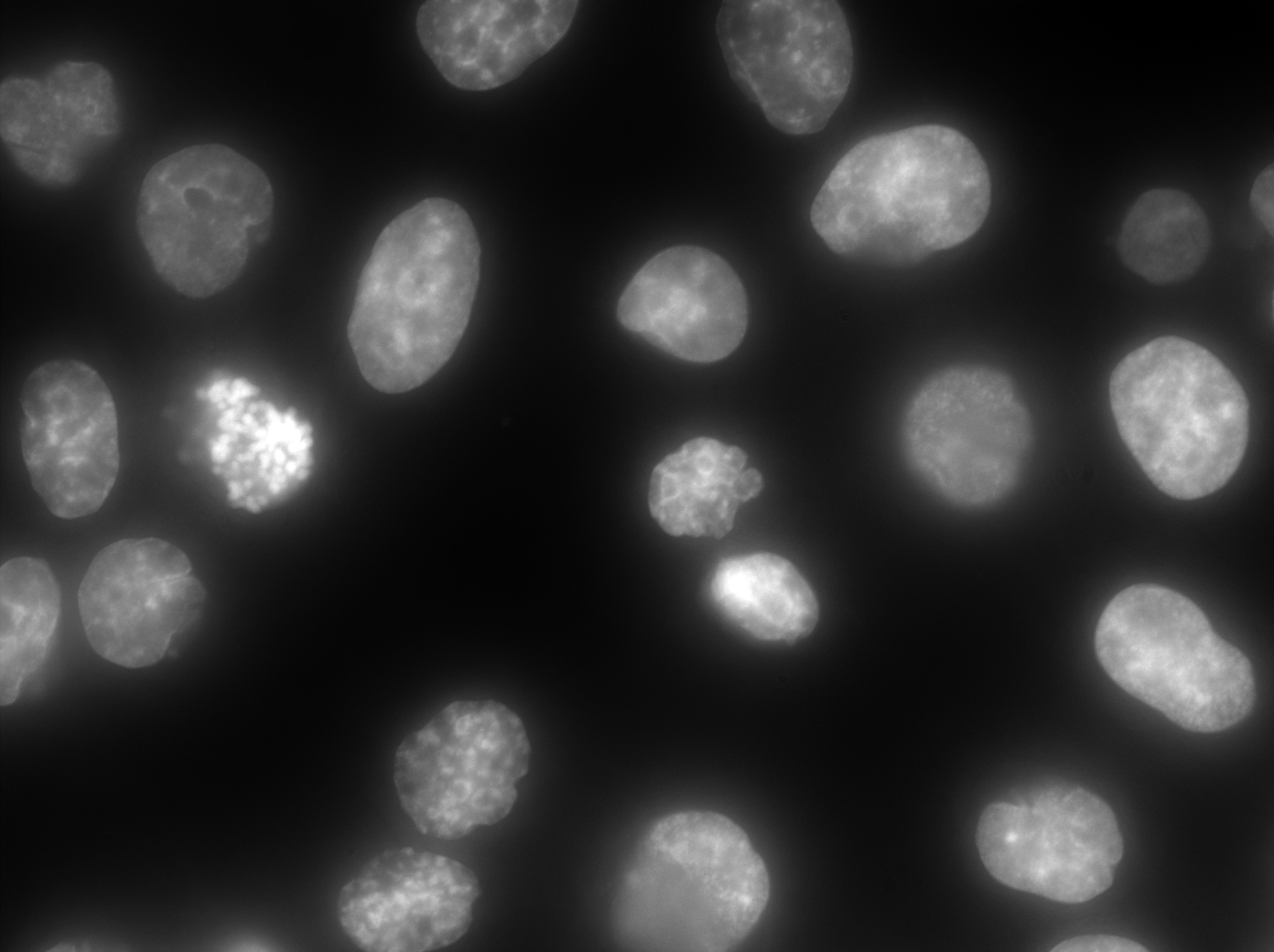

Supplement: Supplementary file 23 — Source data Fig. 6 part 4 [file 44319_2026_742_MOESM23_ESM.zip › Figure 6 Part 4/Fig 6efg TPGS1-KO TPGS1 rescue experiments part 2/R2R3/TPGS1-KO TPGS1-EYFP-3'UTR actub 7-31-25 R3 ET6.Project Maximum Z_XY1756501524_Z0_T0_C0.tif]

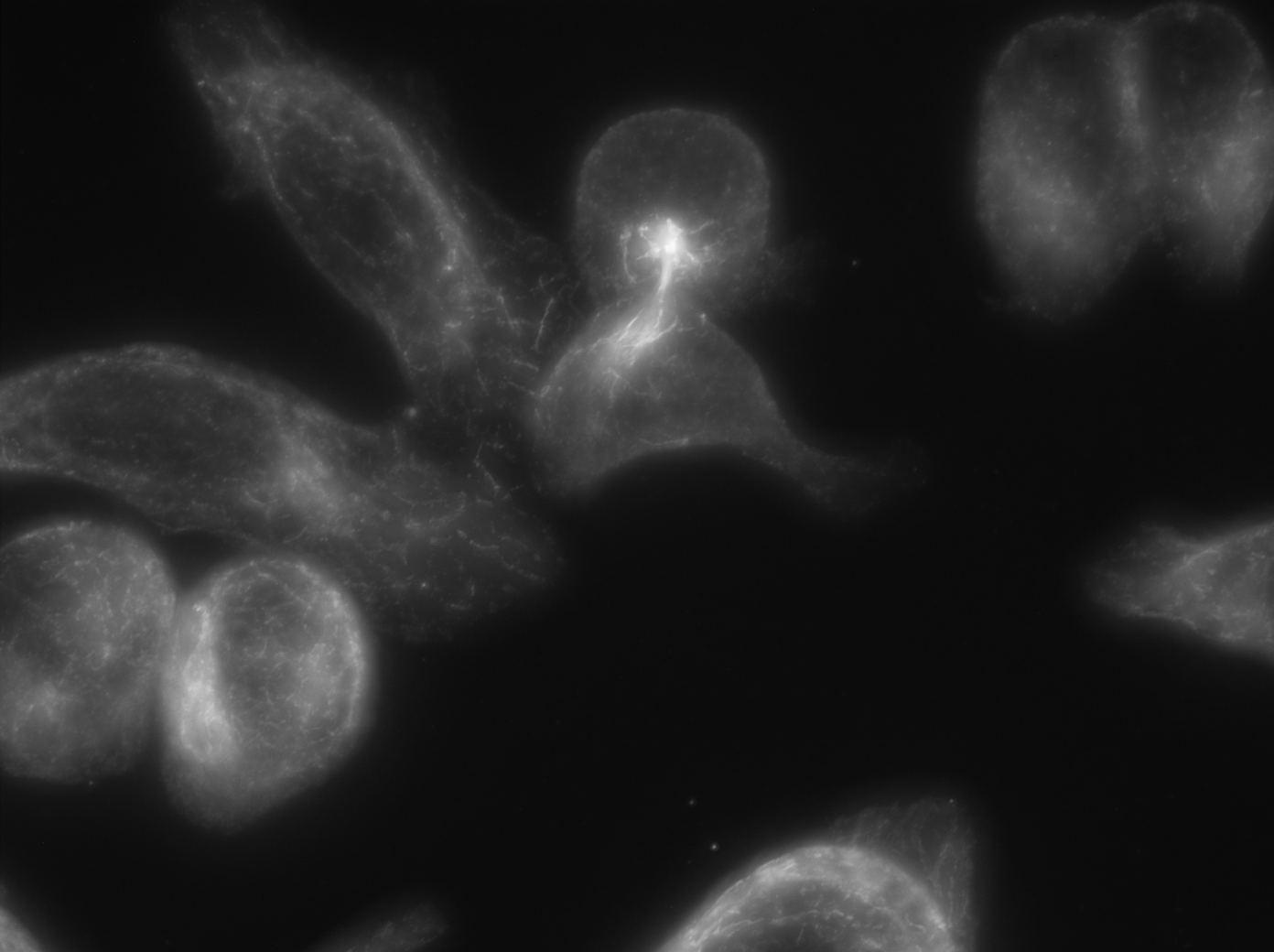

Supplement: Supplementary file 23 — Source data Fig. 6 part 4 [file 44319_2026_742_MOESM23_ESM.zip › Figure 6 Part 4/Fig 6efg TPGS1-KO TPGS1 rescue experiments part 2/R2R3/TPGS1-KO EYFP-only actub 7-31-25 R2 LT2.Project Maximum Z_XY1756413914_Z0_T0_C2.tif]

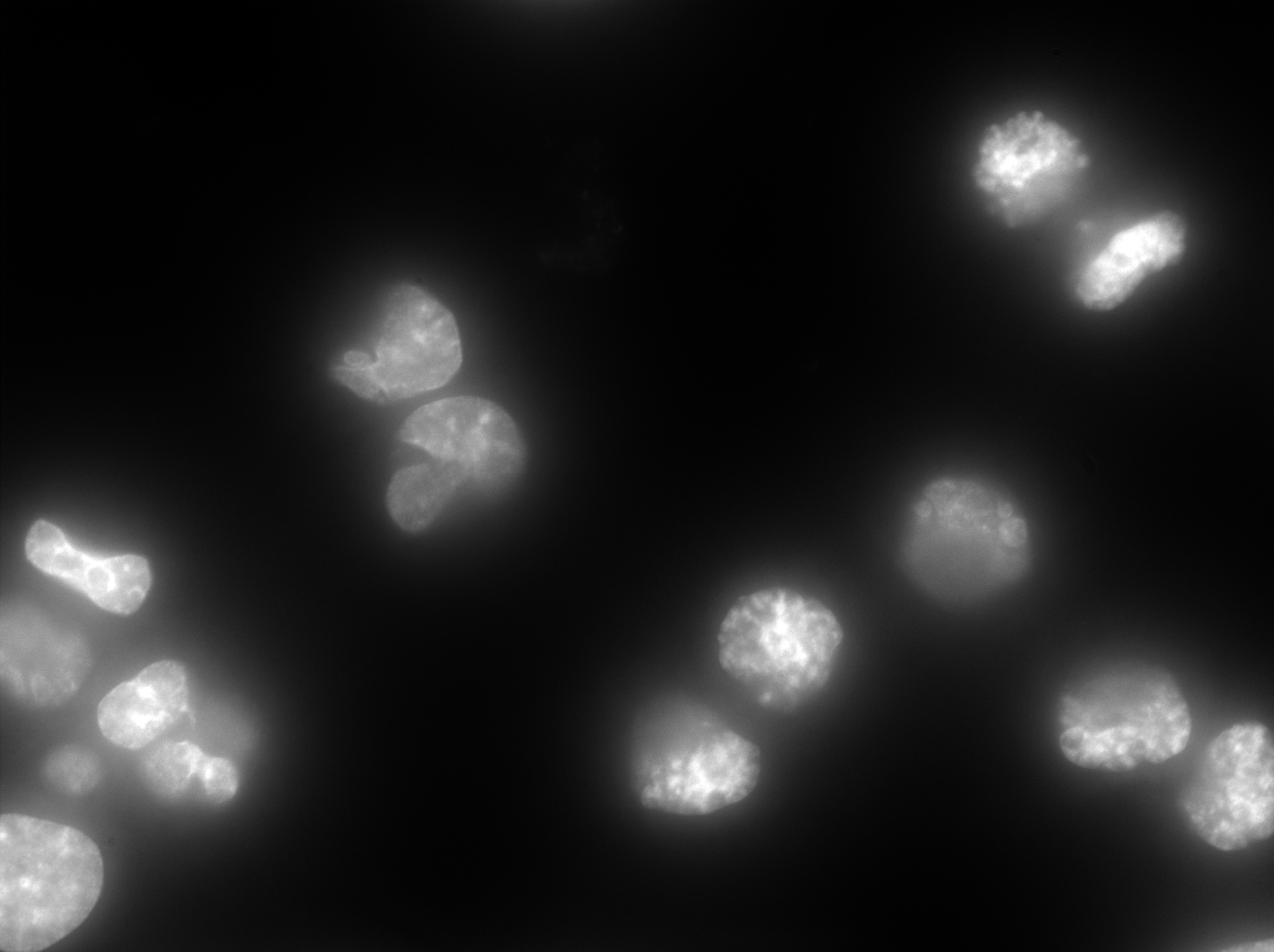

Supplement: Supplementary file 23 — Source data Fig. 6 part 4 [file 44319_2026_742_MOESM23_ESM.zip › Figure 6 Part 4/Fig 6efg TPGS1-KO TPGS1 rescue experiments part 2/R2R3/TPGS1-KO EYFP-only actub 7-31-25 R3 LT3.Project Maximum Z_XY1756492484_Z0_T0_C0.tif]

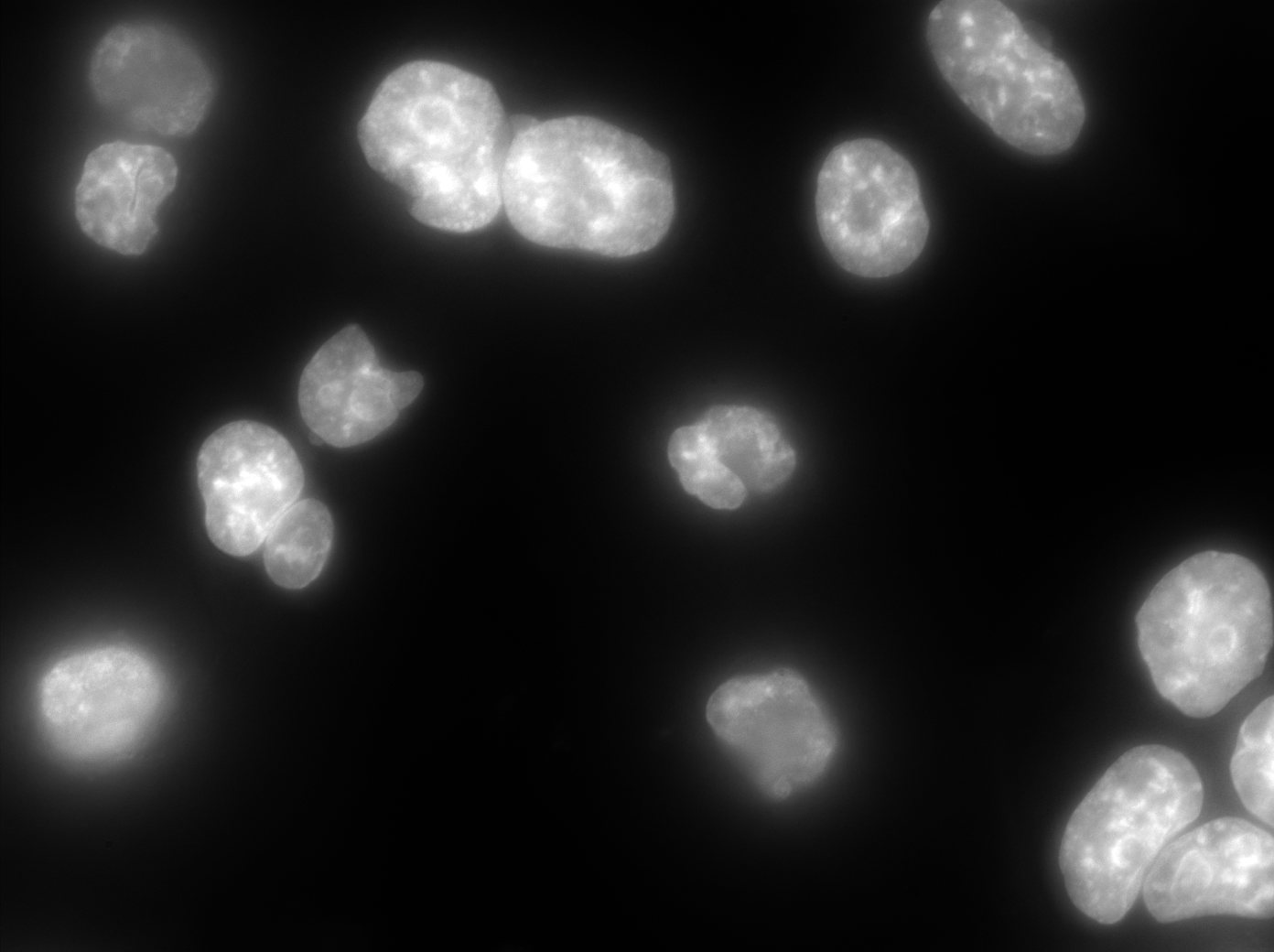

Supplement: Supplementary file 23 — Source data Fig. 6 part 4 [file 44319_2026_742_MOESM23_ESM.zip › Figure 6 Part 4/Fig 6efg TPGS1-KO TPGS1 rescue experiments part 2/R2R3/TPGS1-KO TPGS1-EYFP-3'UTR actub 7-31-25 R3 LT9.Project Maximum Z_XY1756502312_Z0_T0_C0.tif]

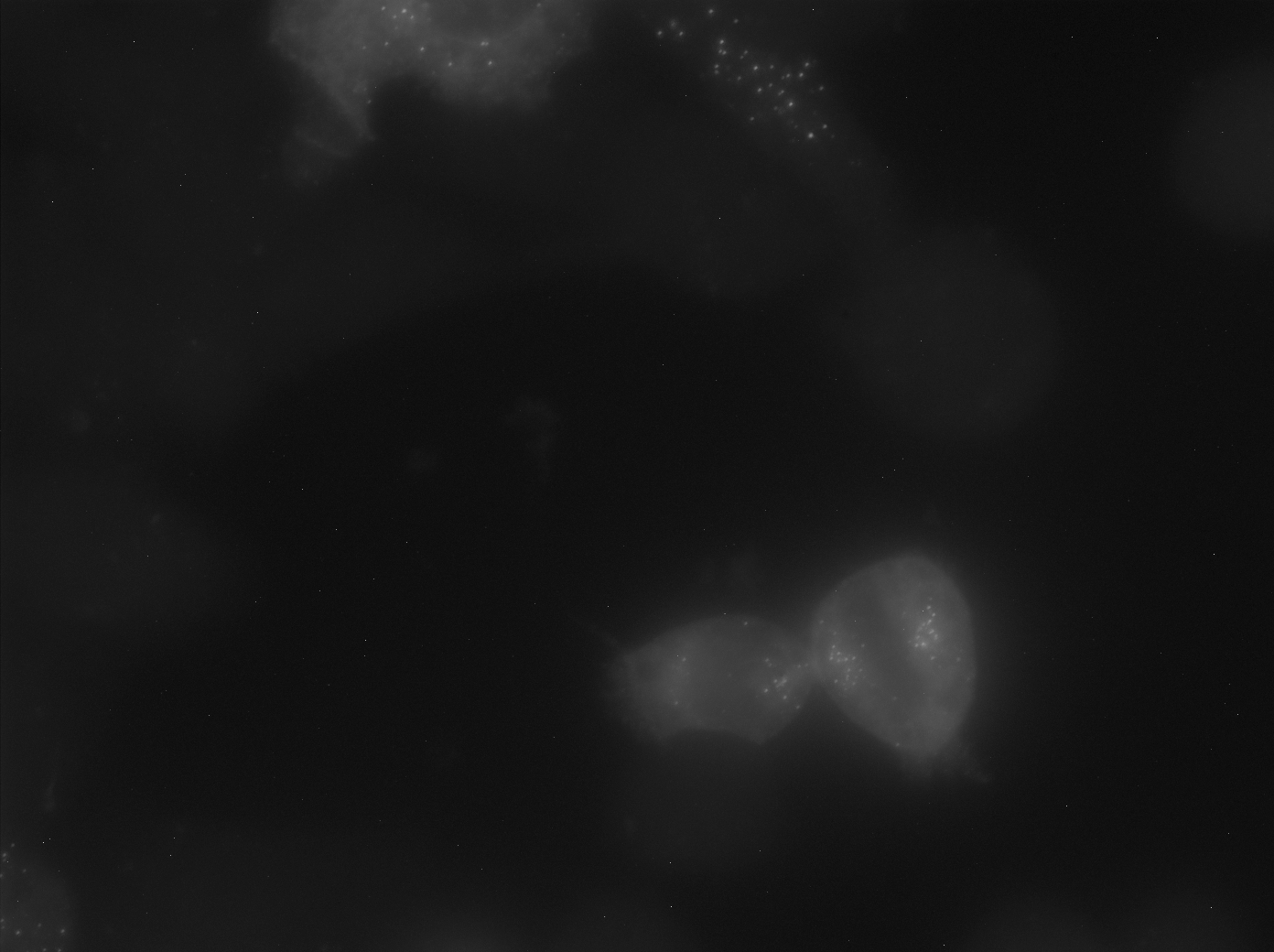

Supplement: Supplementary file 23 — Source data Fig. 6 part 4 [file 44319_2026_742_MOESM23_ESM.zip › Figure 6 Part 4/Fig 6efg TPGS1-KO TPGS1 rescue experiments part 2/R2R3/TPGS1-KO TPGS1-EYFP-3'UTR actub 7-31-25 R2 ET3.Project Maximum Z_XY1756408369_Z0_T0_C1.tif]

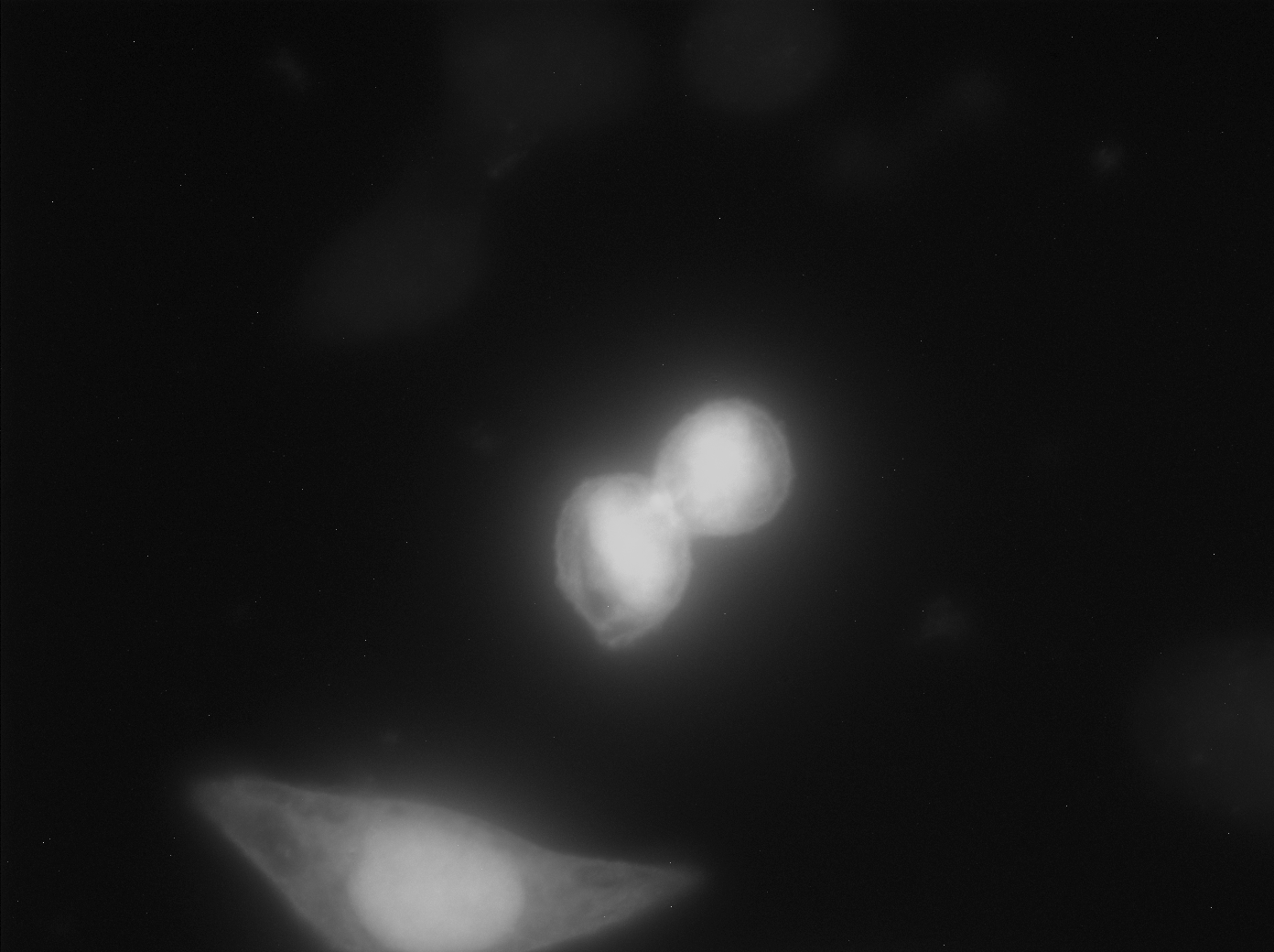

Supplement: Supplementary file 23 — Source data Fig. 6 part 4 [file 44319_2026_742_MOESM23_ESM.zip › Figure 6 Part 4/Fig 6efg TPGS1-KO TPGS1 rescue experiments part 2/R2R3/TPGS1-KO EYFP-only actub 7-31-25 R3 ET1.Project Maximum Z_XY1756491656_Z0_T0_C1.tif]

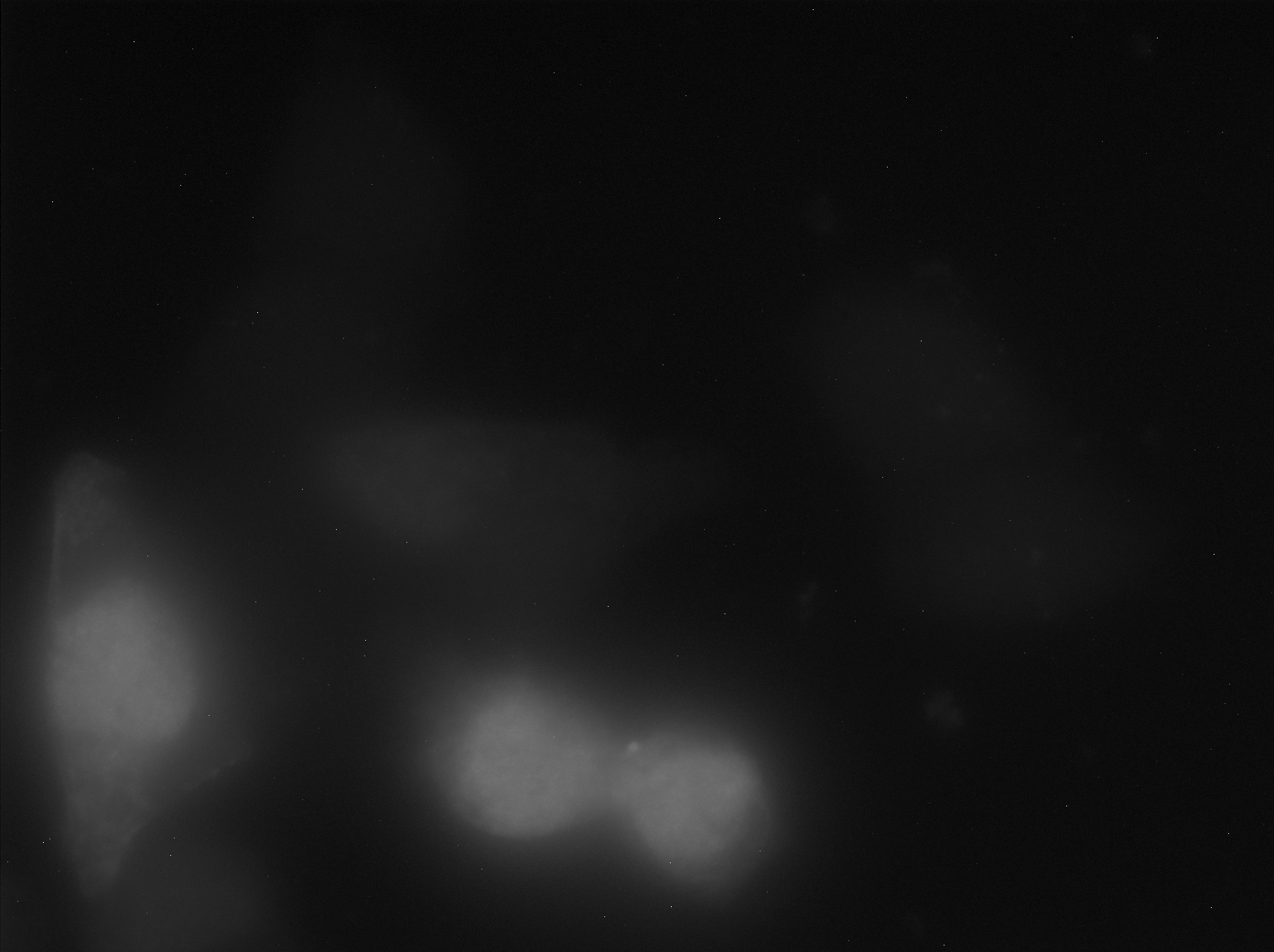

Supplement: Supplementary file 23 — Source data Fig. 6 part 4 [file 44319_2026_742_MOESM23_ESM.zip › Figure 6 Part 4/Fig 6efg TPGS1-KO TPGS1 rescue experiments part 2/R2R3/TPGS1-KO EYFP-only actub 7-31-25 R2 LT8.Project Maximum Z_XY1756416035_Z0_T0_C1.tif]

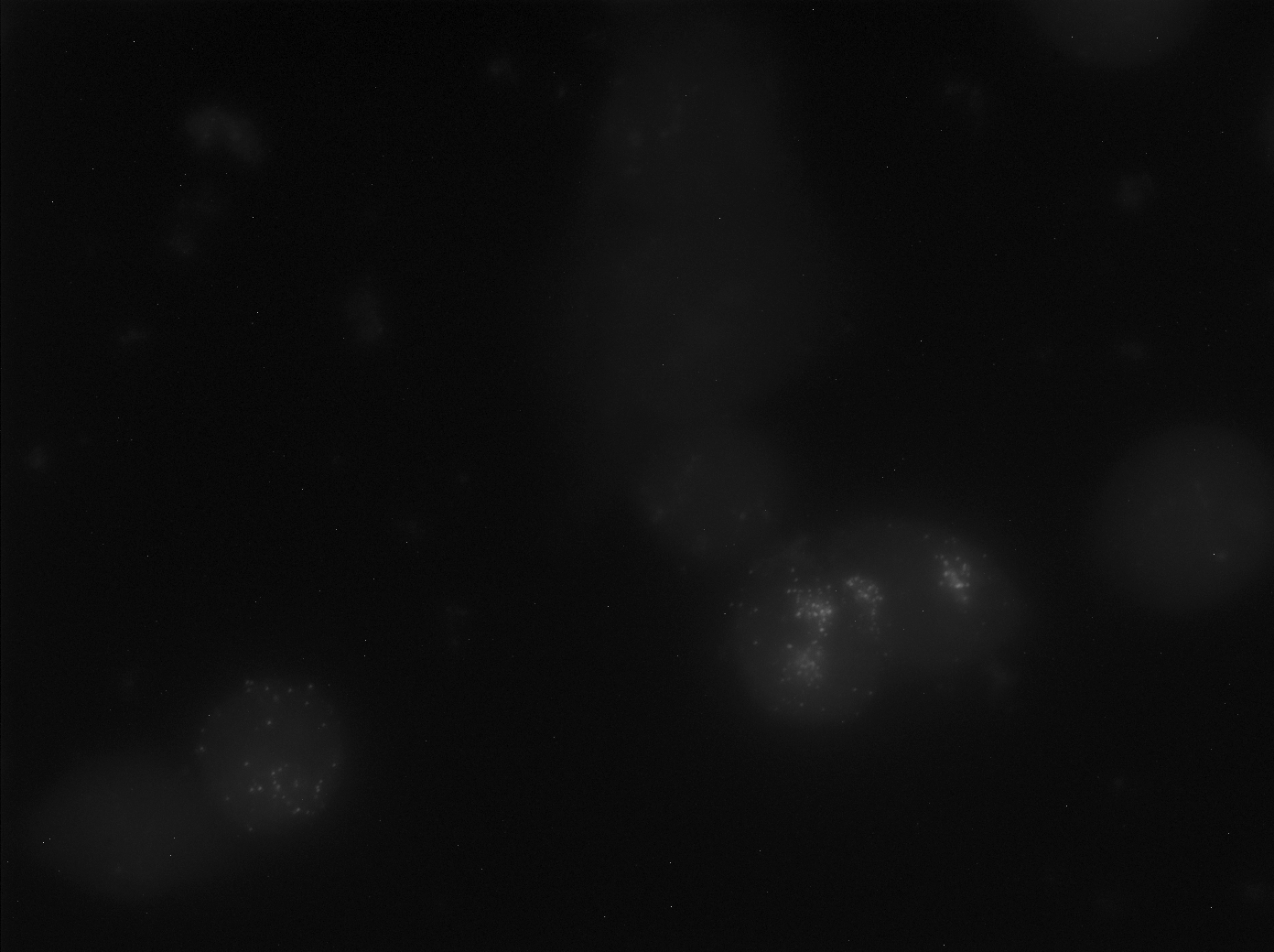

Supplement: Supplementary file 23 — Source data Fig. 6 part 4 [file 44319_2026_742_MOESM23_ESM.zip › Figure 6 Part 4/Fig 6efg TPGS1-KO TPGS1 rescue experiments part 2/R2R3/TPGS1-KO TPGS1-EYFP-3'UTR actub 7-31-25 R3 LT4.Project Maximum Z_XY1756500919_Z0_T0_C1.tif]

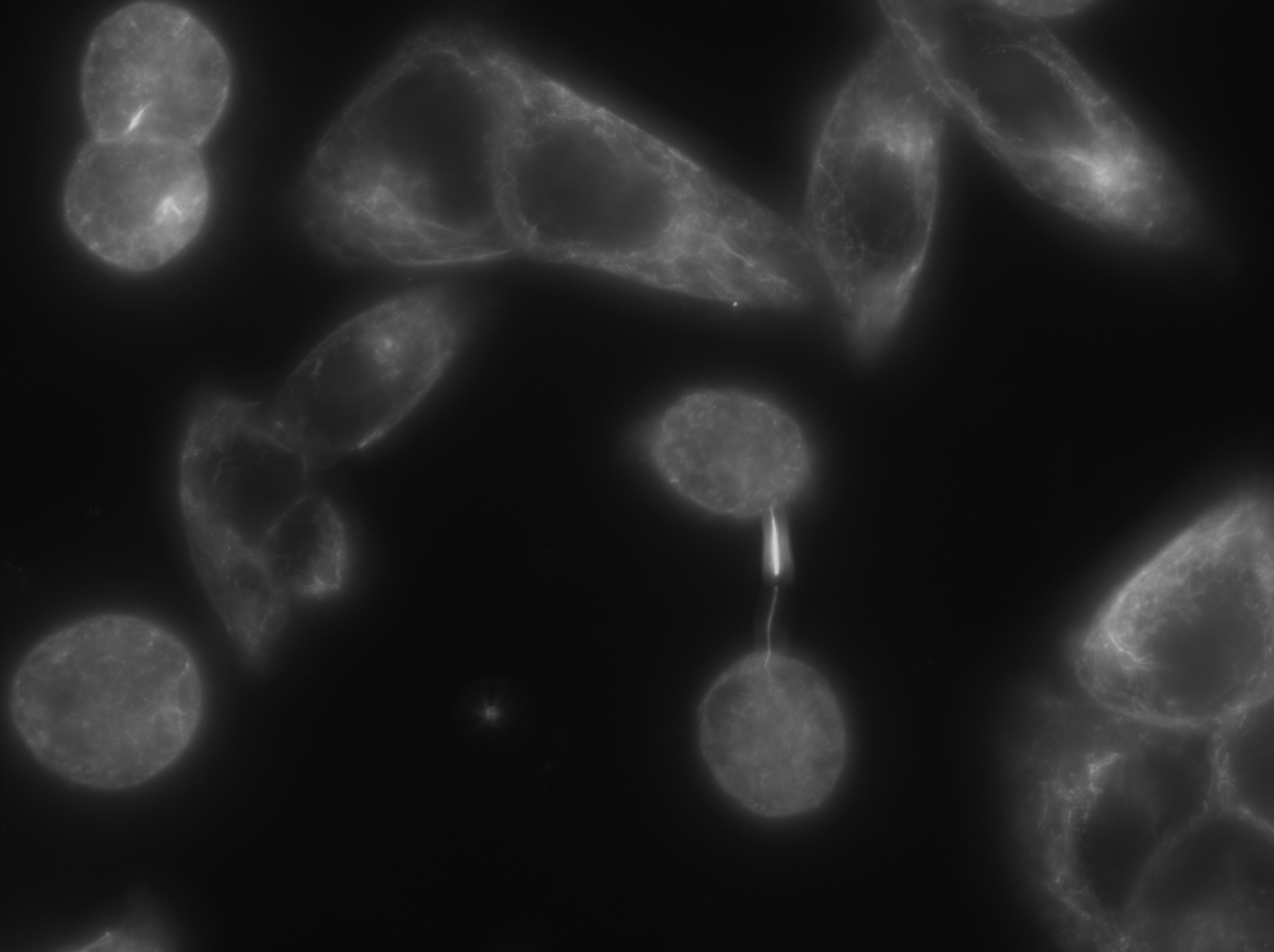

Supplement: Supplementary file 23 — Source data Fig. 6 part 4 [file 44319_2026_742_MOESM23_ESM.zip › Figure 6 Part 4/Fig 6efg TPGS1-KO TPGS1 rescue experiments part 2/R2R3/TPGS1-KO TPGS1-EYFP-3'UTR actub 7-31-25 R3 LT9.Project Maximum Z_XY1756502312_Z0_T0_C2.tif]

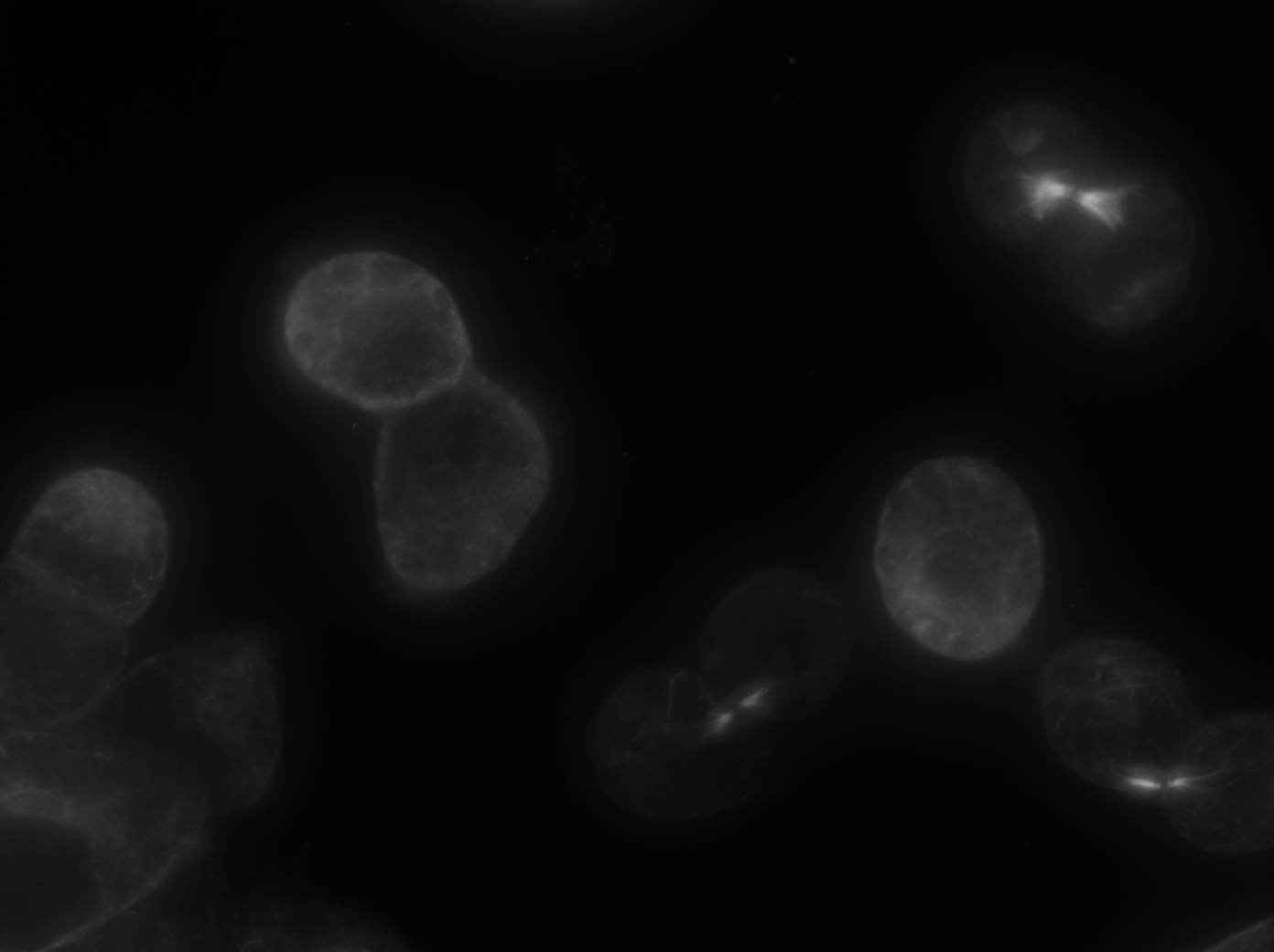

Supplement: Supplementary file 23 — Source data Fig. 6 part 4 [file 44319_2026_742_MOESM23_ESM.zip › Figure 6 Part 4/Fig 6efg TPGS1-KO TPGS1 rescue experiments part 2/R2R3/TPGS1-KO EYFP-only actub 7-31-25 R3 LT3.Project Maximum Z_XY1756492484_Z0_T0_C2.tif]

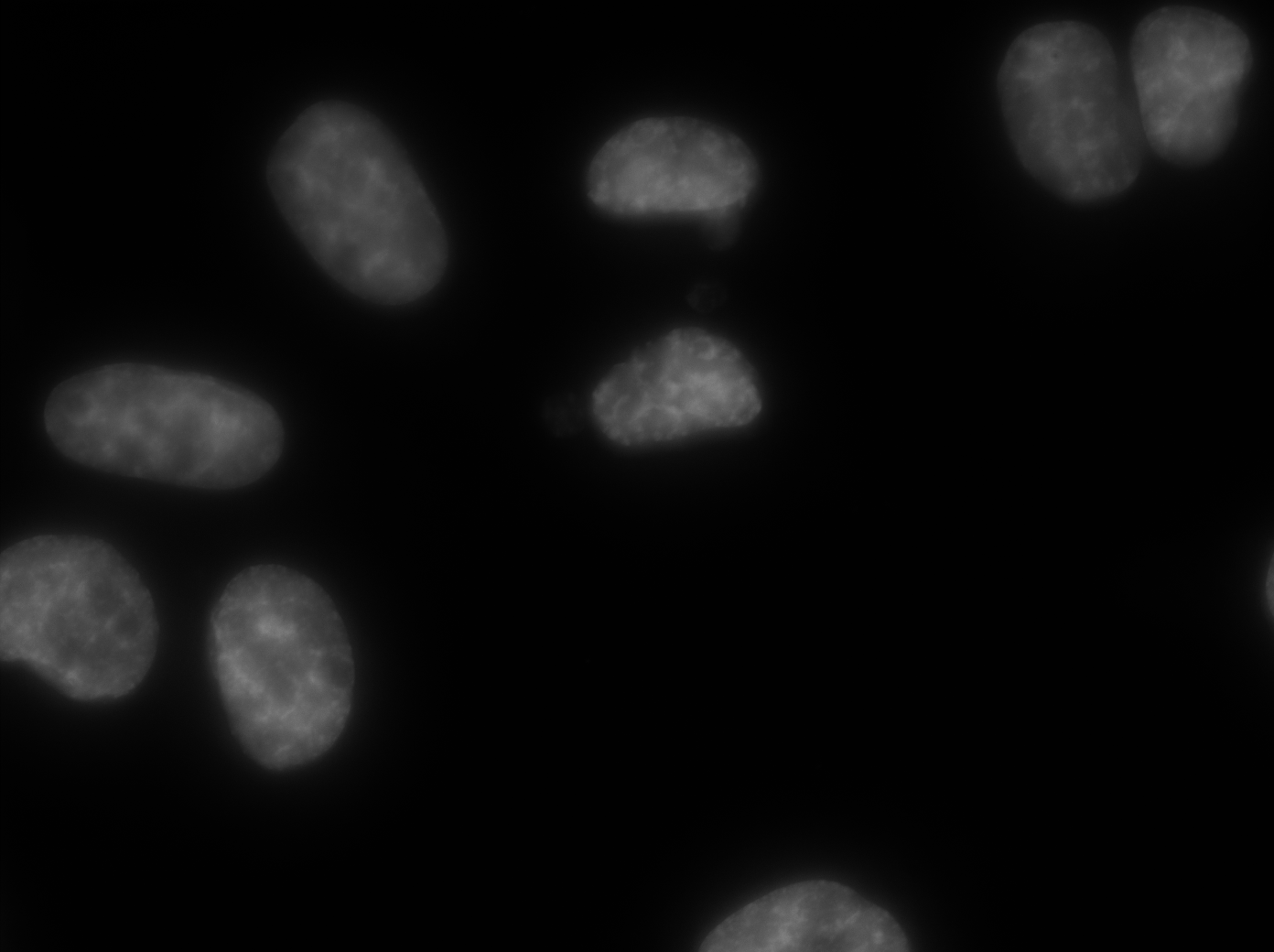

Supplement: Supplementary file 23 — Source data Fig. 6 part 4 [file 44319_2026_742_MOESM23_ESM.zip › Figure 6 Part 4/Fig 6efg TPGS1-KO TPGS1 rescue experiments part 2/R2R3/TPGS1-KO EYFP-only actub 7-31-25 R2 LT2.Project Maximum Z_XY1756413914_Z0_T0_C0.tif]

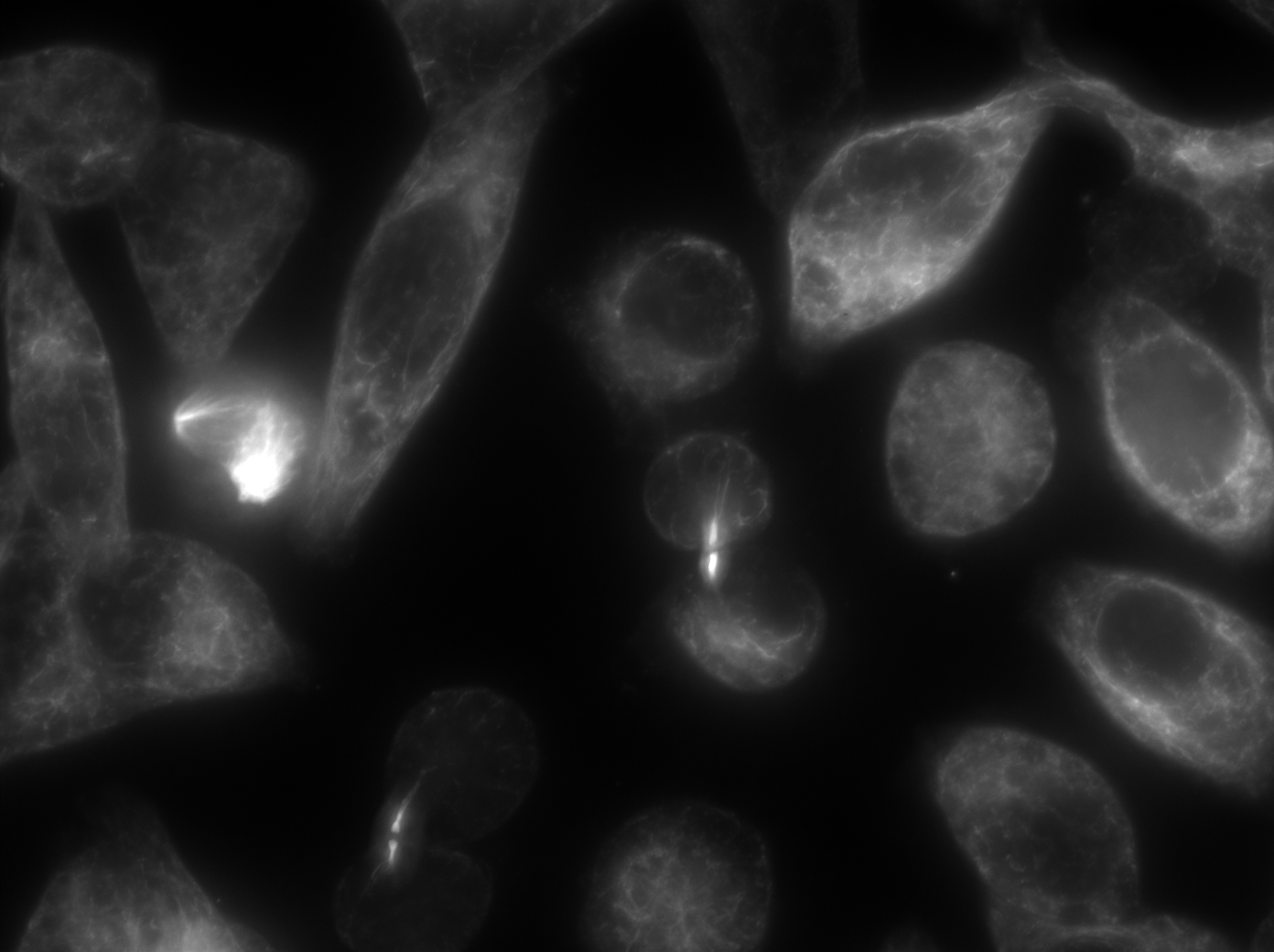

Supplement: Supplementary file 23 — Source data Fig. 6 part 4 [file 44319_2026_742_MOESM23_ESM.zip › Figure 6 Part 4/Fig 6efg TPGS1-KO TPGS1 rescue experiments part 2/R2R3/TPGS1-KO TPGS1-EYFP-3'UTR actub 7-31-25 R3 ET6.Project Maximum Z_XY1756501524_Z0_T0_C2.tif]

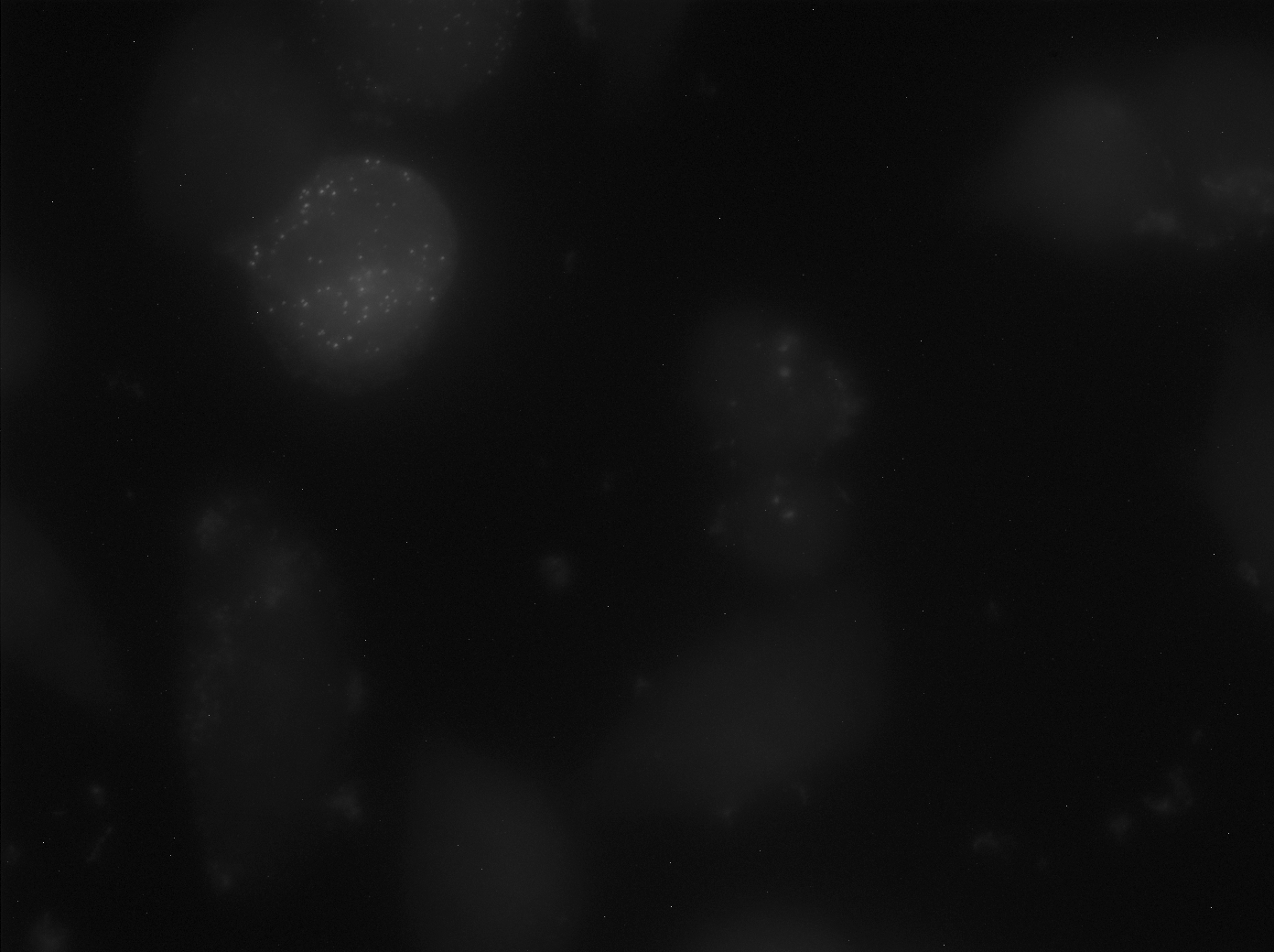

Supplement: Supplementary file 23 — Source data Fig. 6 part 4 [file 44319_2026_742_MOESM23_ESM.zip › Figure 6 Part 4/Fig 6efg TPGS1-KO TPGS1 rescue experiments part 2/R2R3/TPGS1-KO TPGS1-EYFP-3'UTR actub 7-31-25 R3 ET7.Project Maximum Z_XY1756503229_Z0_T0_C1.tif]

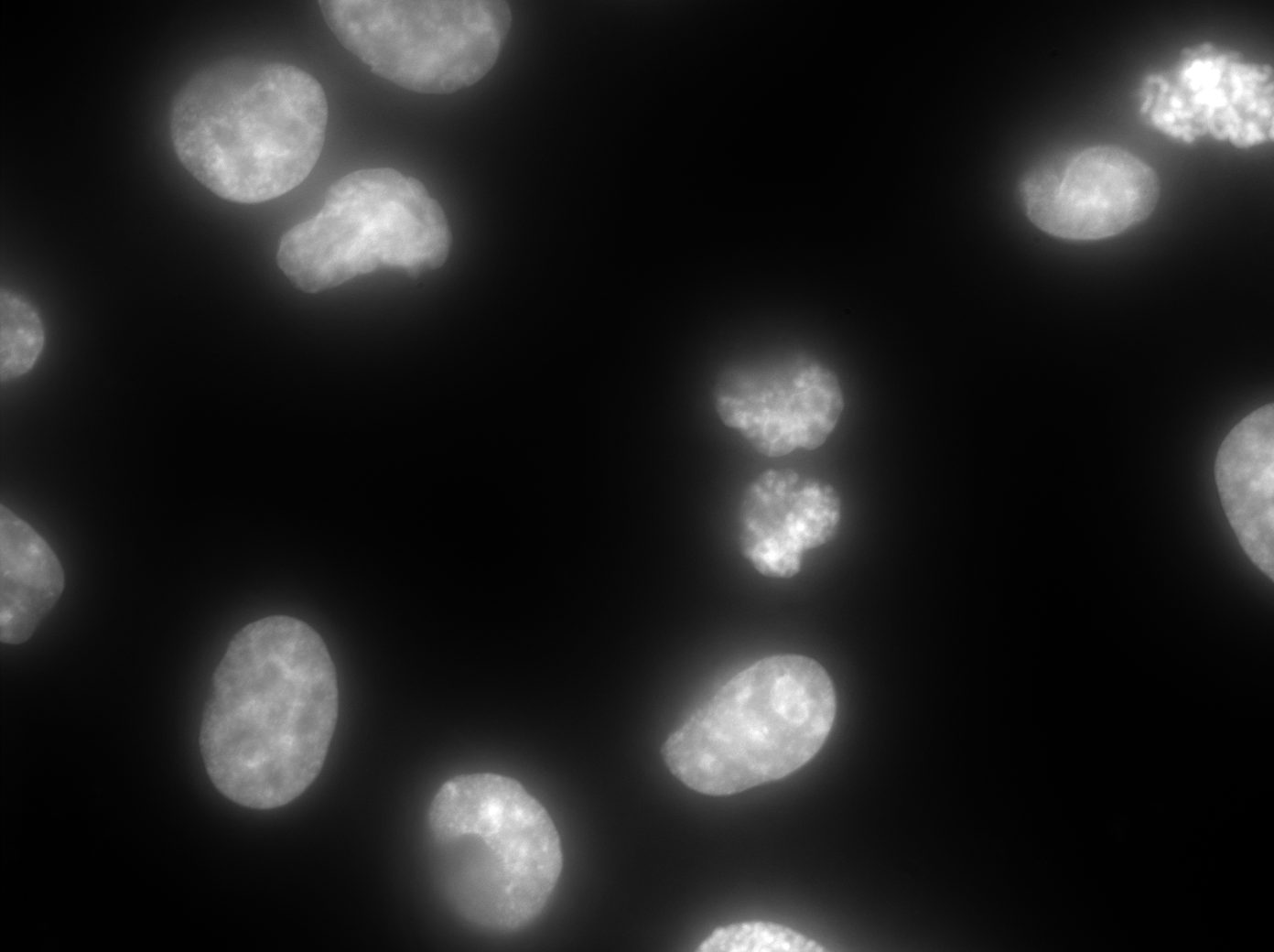

Supplement: Supplementary file 23 — Source data Fig. 6 part 4 [file 44319_2026_742_MOESM23_ESM.zip › Figure 6 Part 4/Fig 6efg TPGS1-KO TPGS1 rescue experiments part 2/R2R3/TPGS1-KO TPGS1-EYFP-3'UTR actub 7-31-25 R3 ET7.Project Maximum Z_XY1756503229_Z0_T0_C0.tif]

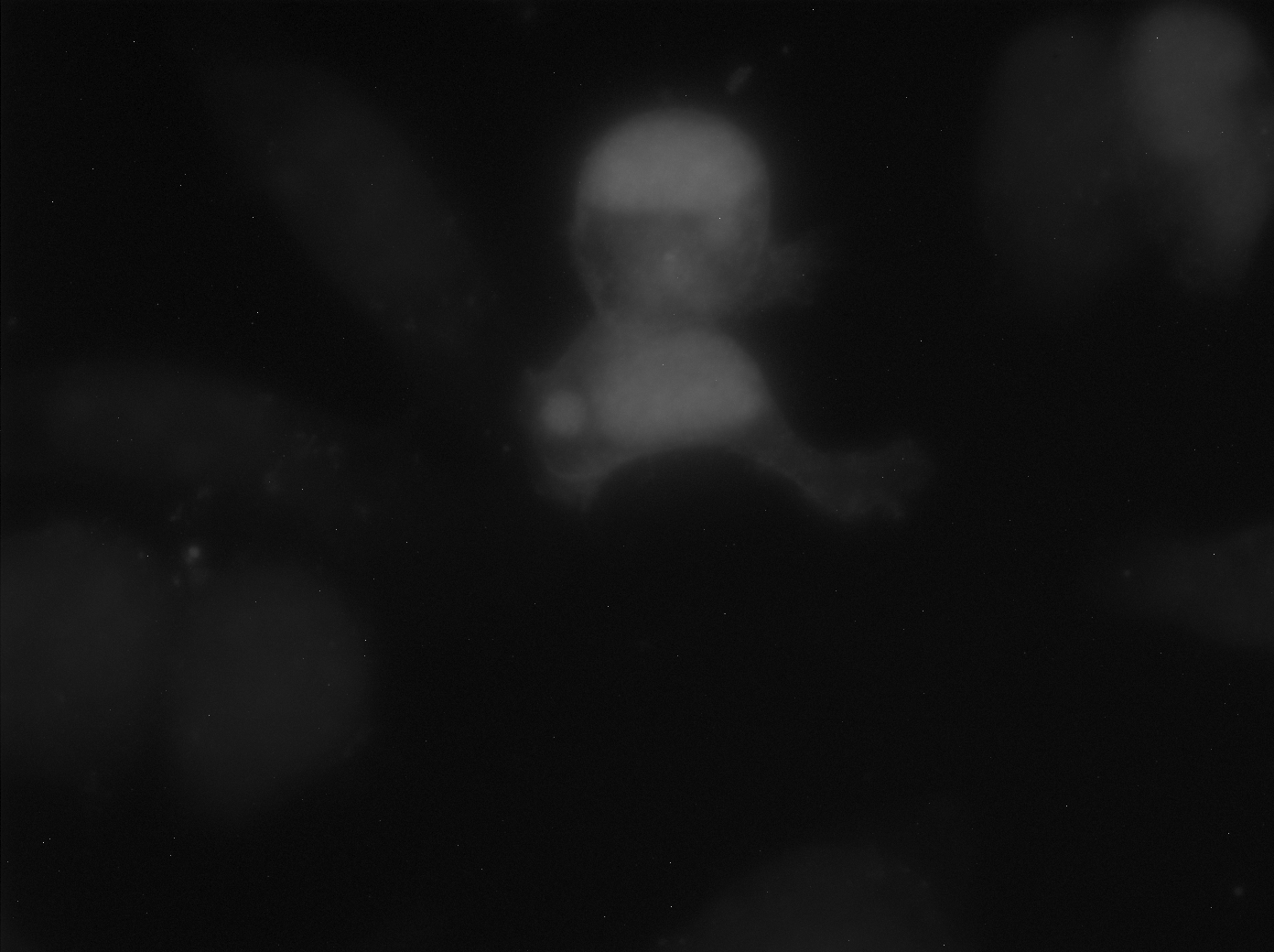

Supplement: Supplementary file 23 — Source data Fig. 6 part 4 [file 44319_2026_742_MOESM23_ESM.zip › Figure 6 Part 4/Fig 6efg TPGS1-KO TPGS1 rescue experiments part 2/R2R3/TPGS1-KO EYFP-only actub 7-31-25 R2 LT2.Project Maximum Z_XY1756413914_Z0_T0_C1.tif]

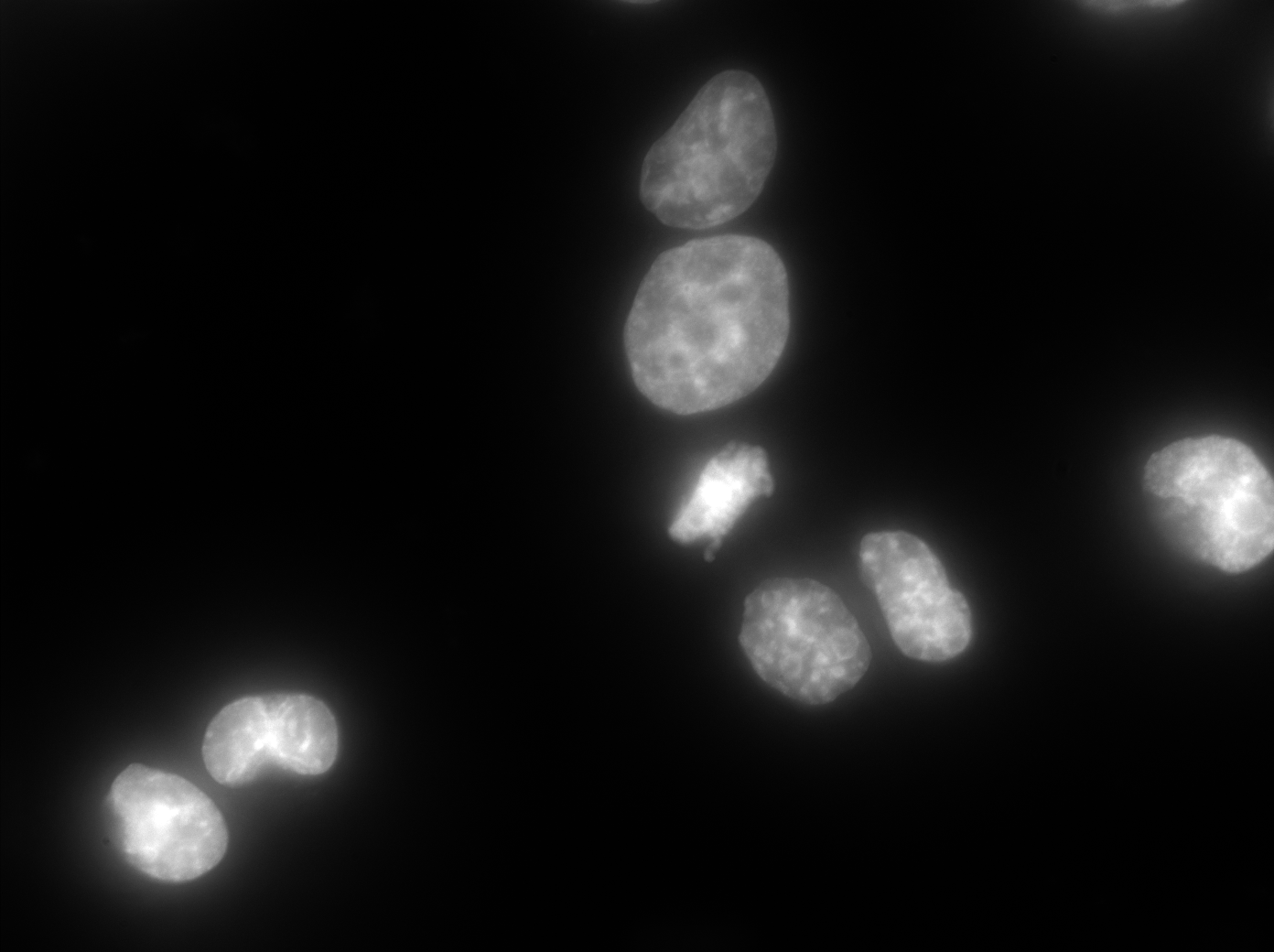

Supplement: Supplementary file 23 — Source data Fig. 6 part 4 [file 44319_2026_742_MOESM23_ESM.zip › Figure 6 Part 4/Fig 6efg TPGS1-KO TPGS1 rescue experiments part 2/R2R3/TPGS1-KO TPGS1-EYFP-3'UTR actub 7-31-25 R3 LT4.Project Maximum Z_XY1756500919_Z0_T0_C0.tif]

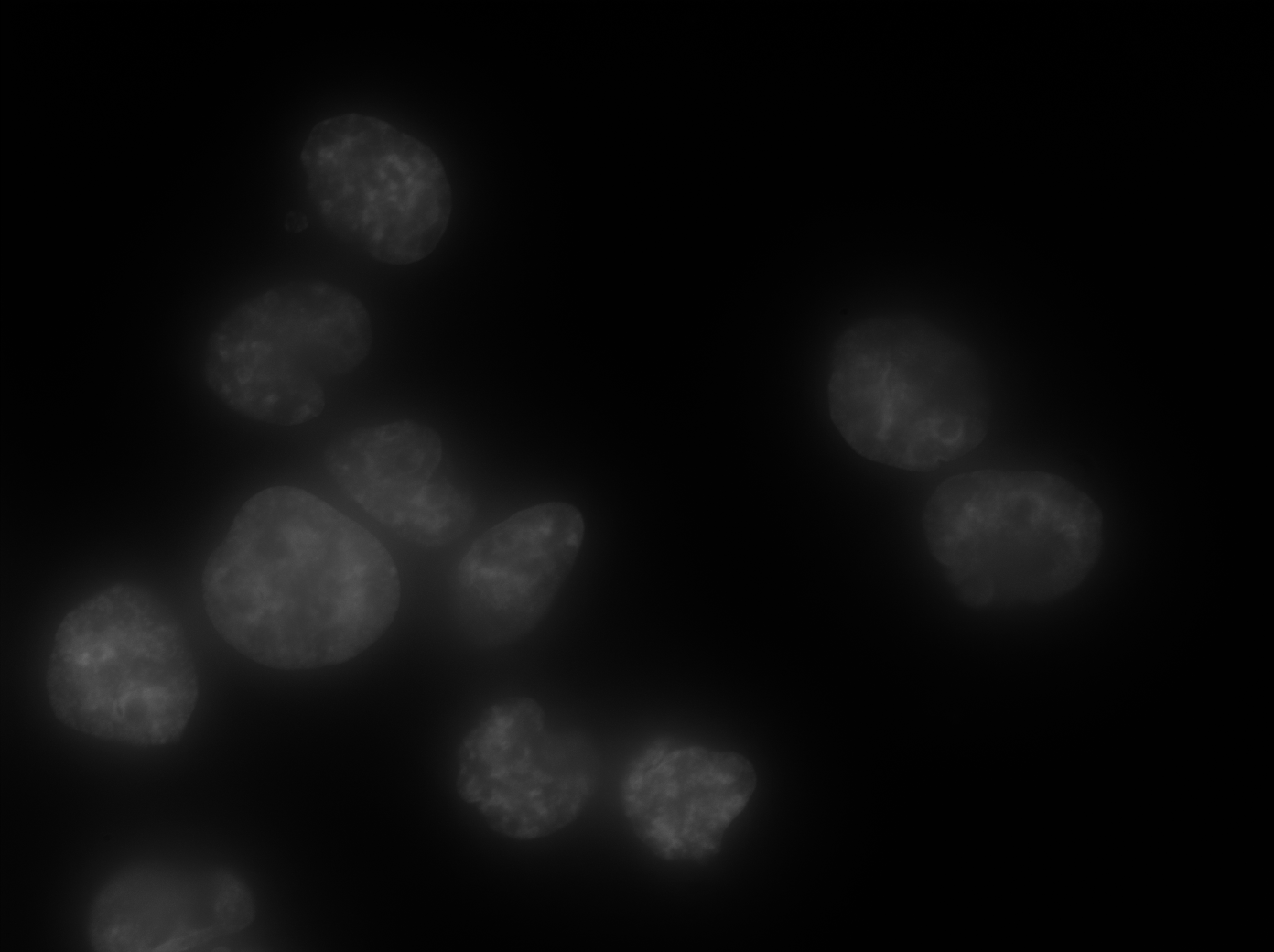

Supplement: Supplementary file 23 — Source data Fig. 6 part 4 [file 44319_2026_742_MOESM23_ESM.zip › Figure 6 Part 4/Fig 6efg TPGS1-KO TPGS1 rescue experiments part 2/R2R3/TPGS1-KO EYFP-only actub 7-31-25 R2 LT8.Project Maximum Z_XY1756416035_Z0_T0_C0.tif]

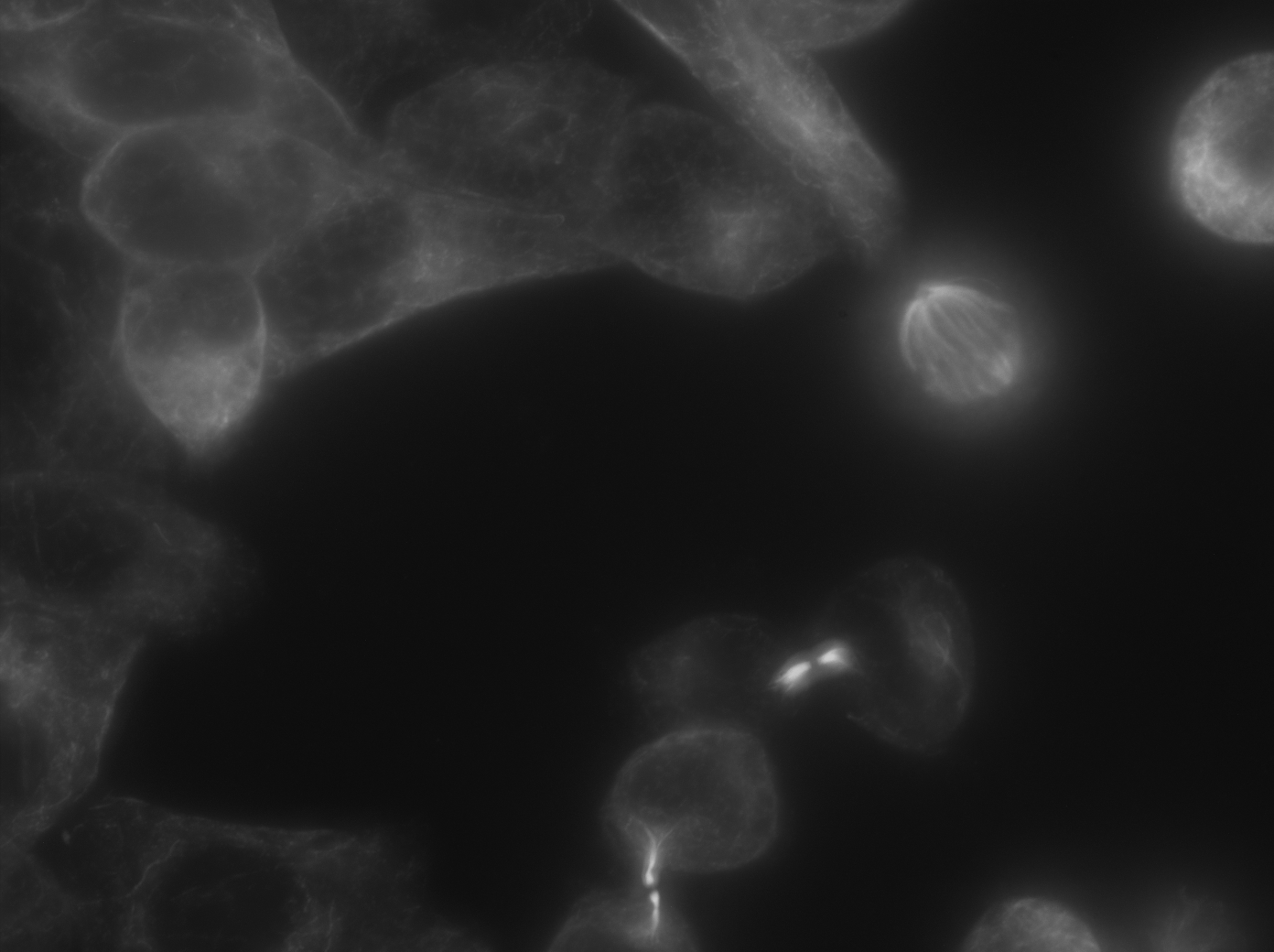

Supplement: Supplementary file 23 — Source data Fig. 6 part 4 [file 44319_2026_742_MOESM23_ESM.zip › Figure 6 Part 4/Fig 6efg TPGS1-KO TPGS1 rescue experiments part 2/R2R3/TPGS1-KO TPGS1-EYFP-3'UTR actub 7-31-25 R2 ET3.Project Maximum Z_XY1756408369_Z0_T0_C2.tif]

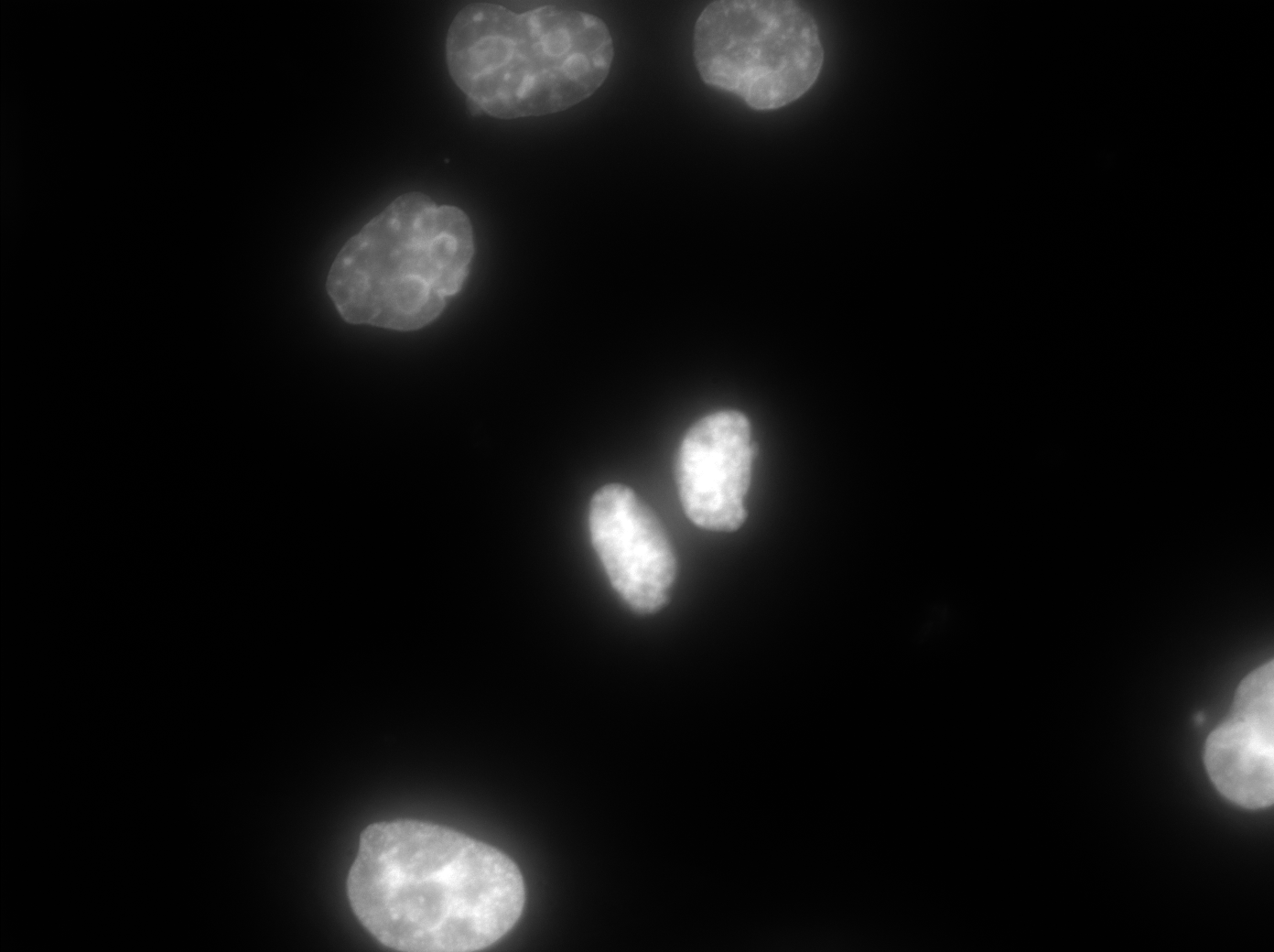

Supplement: Supplementary file 23 — Source data Fig. 6 part 4 [file 44319_2026_742_MOESM23_ESM.zip › Figure 6 Part 4/Fig 6efg TPGS1-KO TPGS1 rescue experiments part 2/R2R3/TPGS1-KO EYFP-only actub 7-31-25 R3 ET1.Project Maximum Z_XY1756491656_Z0_T0_C0.tif]

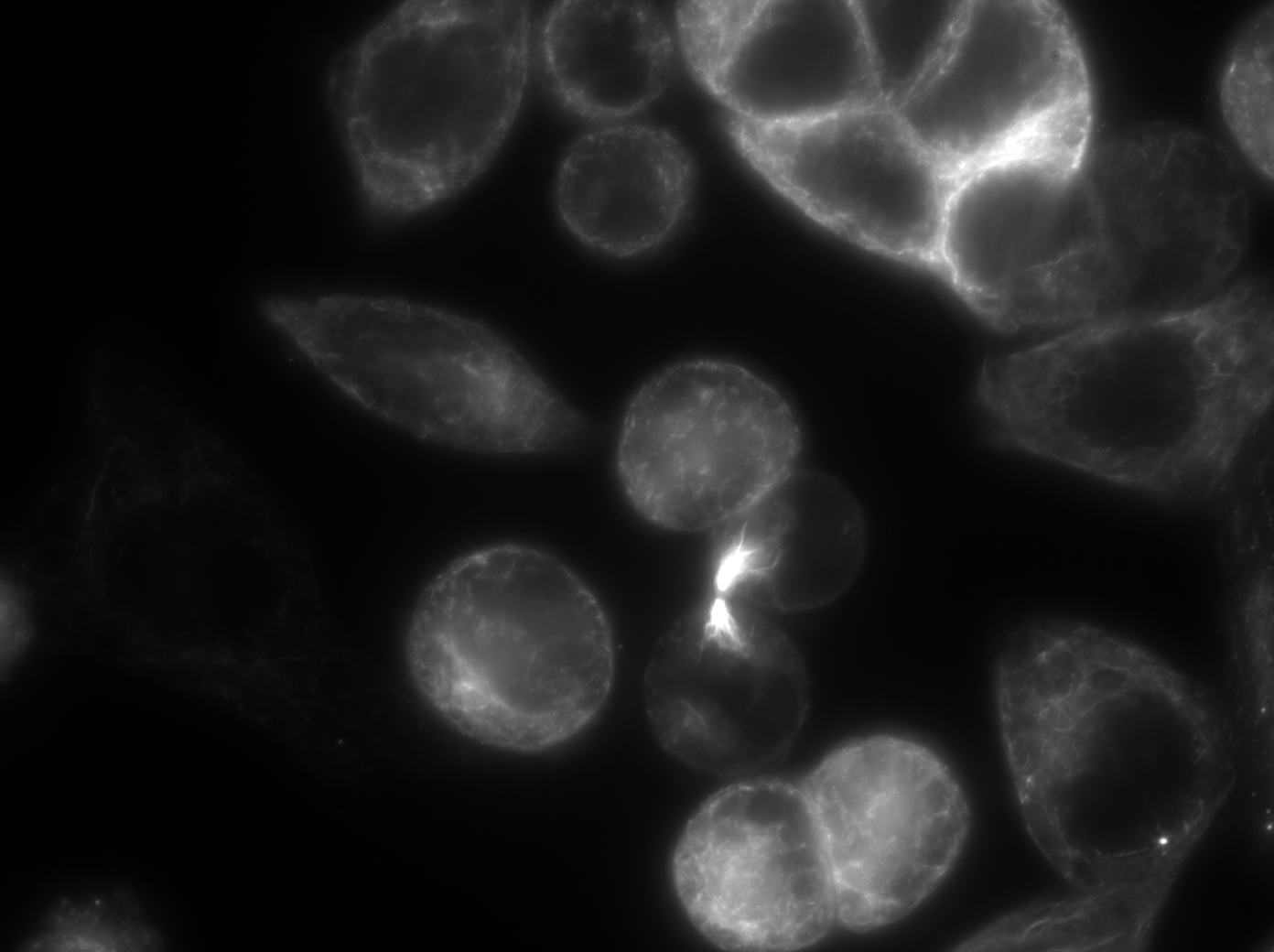

Supplement: Supplementary file 23 — Source data Fig. 6 part 4 [file 44319_2026_742_MOESM23_ESM.zip › Figure 6 Part 4/Fig 6efg TPGS1-KO TPGS1 rescue experiments part 2/R2R3/TPGS1-KO TPGS1-EYFP-3'UTR actub 7-31-25 R3 ET2.Project Maximum Z_XY1756499014_Z0_T0_C2.tif]

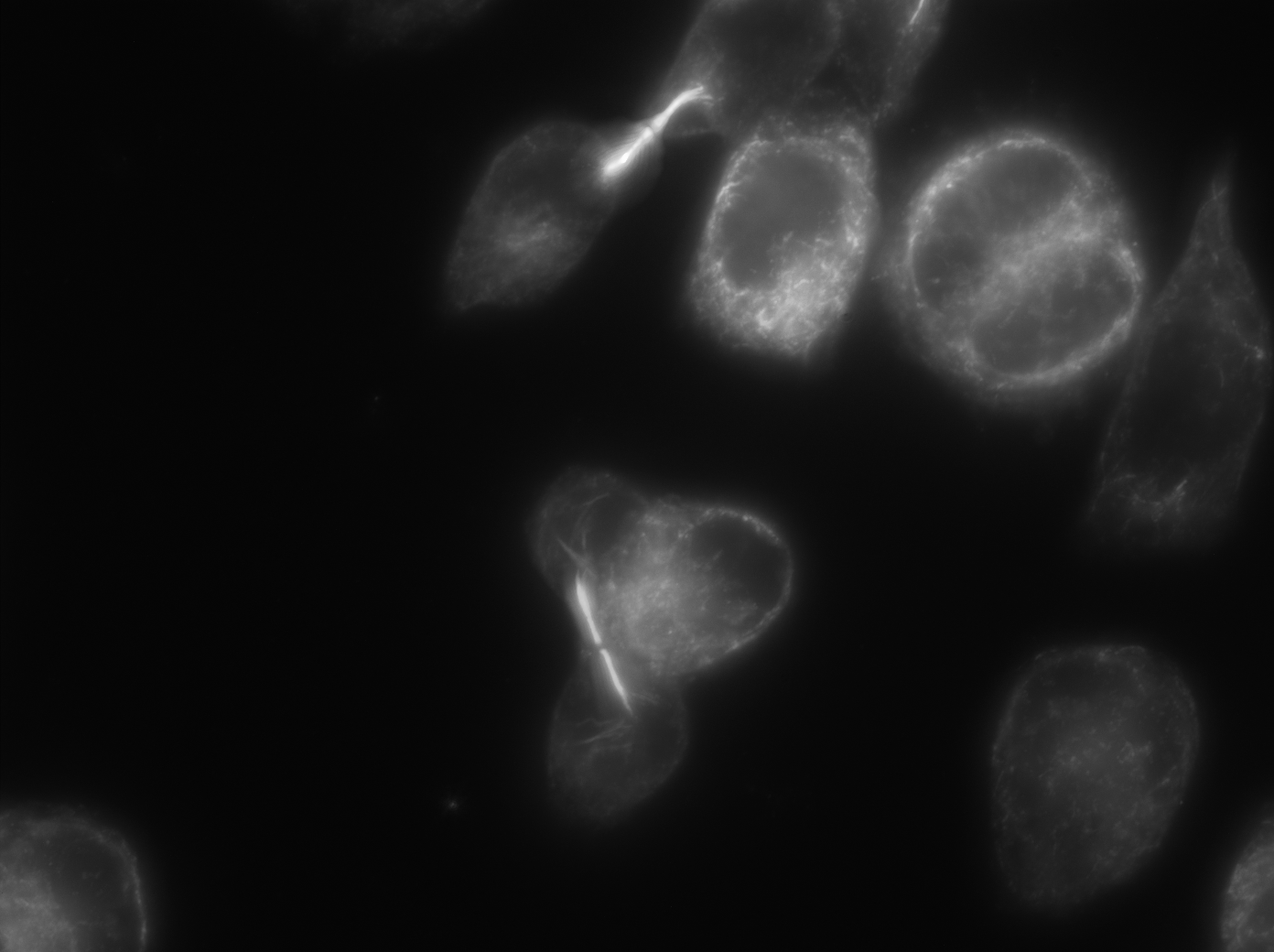

Supplement: Supplementary file 23 — Source data Fig. 6 part 4 [file 44319_2026_742_MOESM23_ESM.zip › Figure 6 Part 4/Fig 6efg TPGS1-KO TPGS1 rescue experiments part 2/R2R3/TPGS1-KO EYFP-only actub 7-31-25 R3 LT2.Project Maximum Z_XY1756491400_Z0_T0_C2.tif]

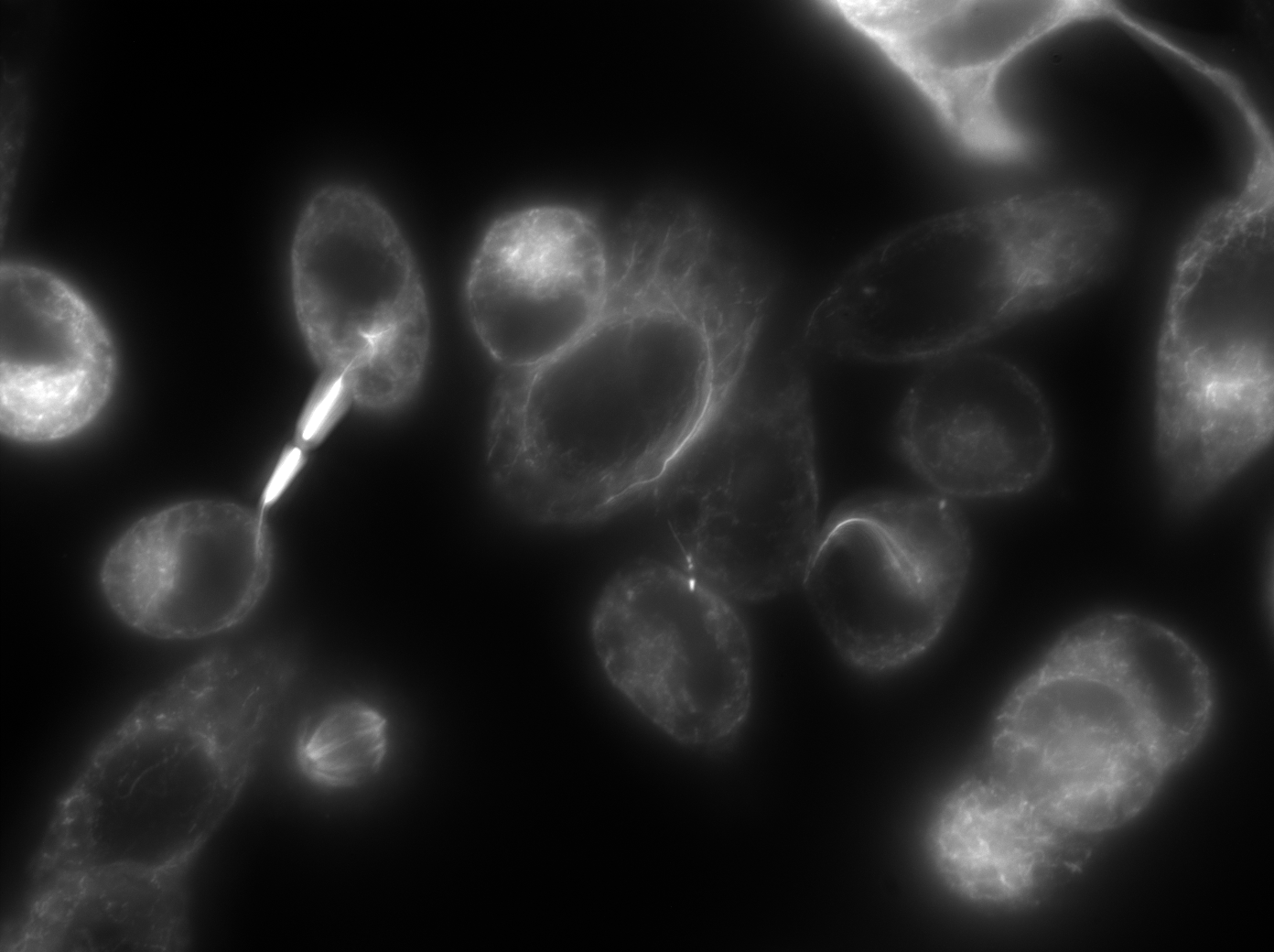

Supplement: Supplementary file 23 — Source data Fig. 6 part 4 [file 44319_2026_742_MOESM23_ESM.zip › Figure 6 Part 4/Fig 6efg TPGS1-KO TPGS1 rescue experiments part 2/R2R3/TPGS1-KO TPGS1-EYFP-3'UTR actub 7-31-25 R2 LT9.Project Maximum Z_XY1756411917_Z0_T0_C2.tif]

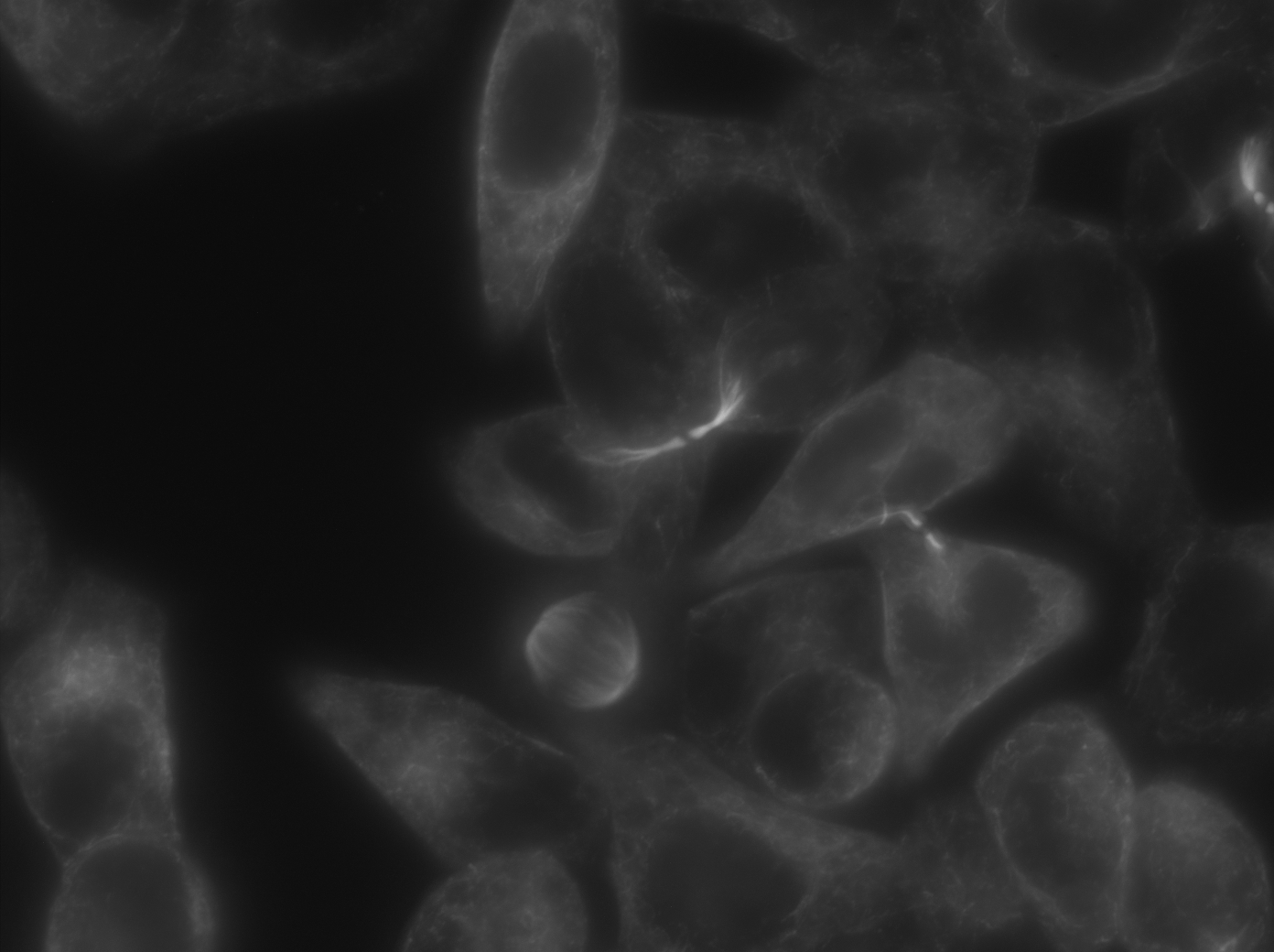

Supplement: Supplementary file 23 — Source data Fig. 6 part 4 [file 44319_2026_742_MOESM23_ESM.zip › Figure 6 Part 4/Fig 6efg TPGS1-KO TPGS1 rescue experiments part 2/R2R3/TPGS1-KO TPGS1-EYFP-3'UTR actub 7-31-25 R2 LT4.Project Maximum Z_XY1756407822_Z0_T0_C2.tif]

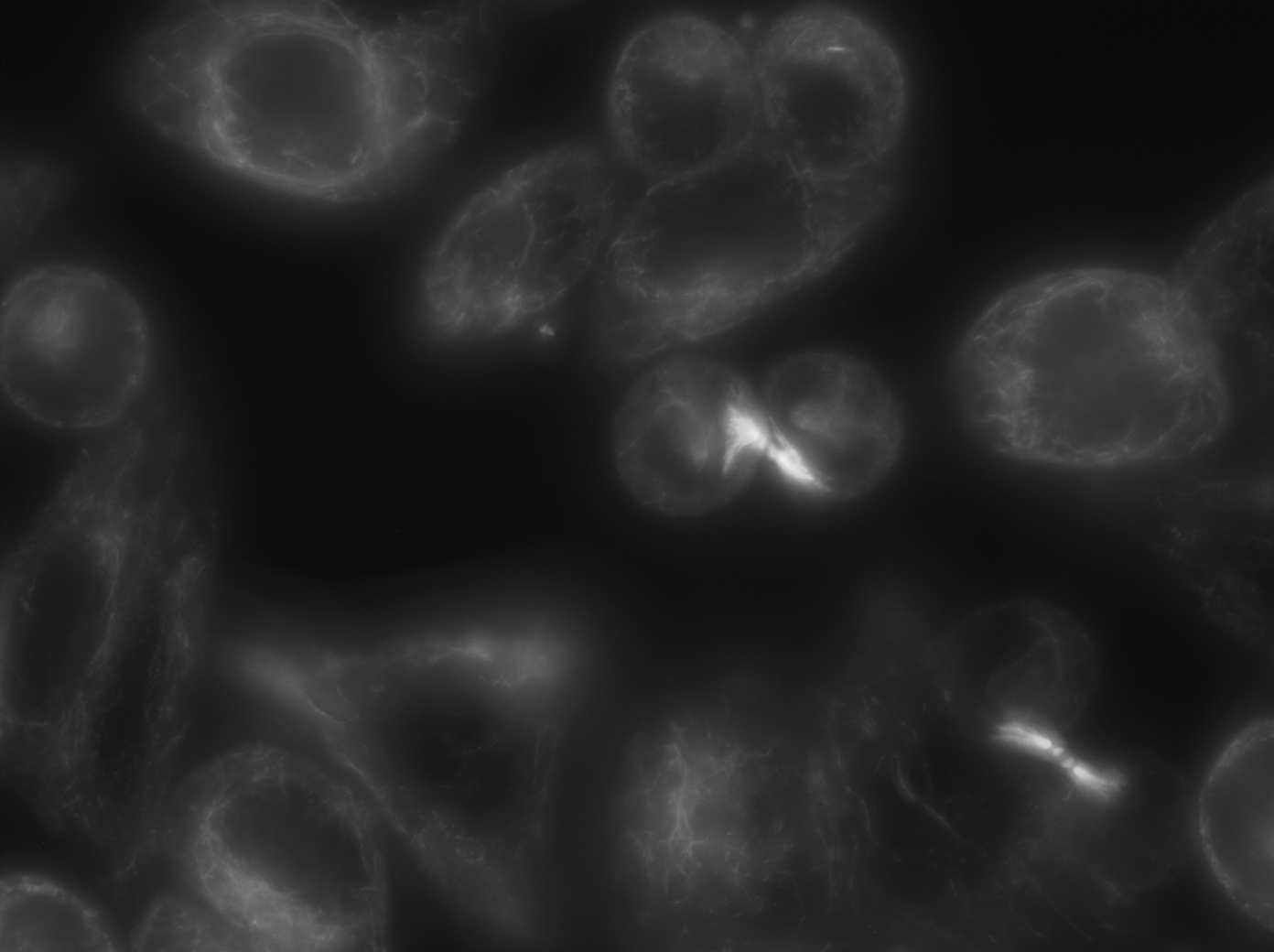

Supplement: Supplementary file 23 — Source data Fig. 6 part 4 [file 44319_2026_742_MOESM23_ESM.zip › Figure 6 Part 4/Fig 6efg TPGS1-KO TPGS1 rescue experiments part 2/R2R3/TPGS1-KO TPGS1-EYFP-3'UTR actub 7-31-25 R2 ET4.Project Maximum Z_XY1756408521_Z0_T0_C2.tif]

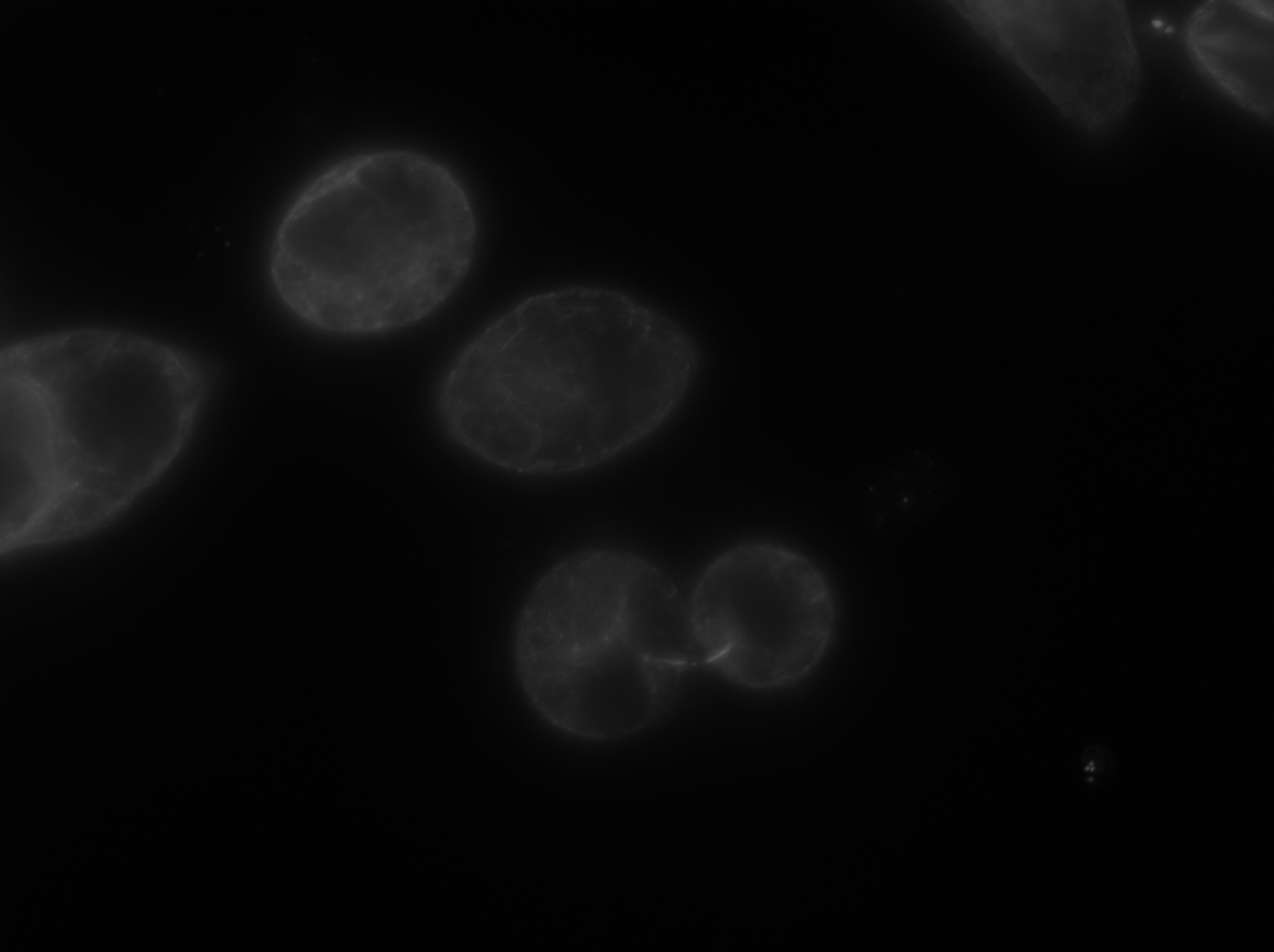

Supplement: Supplementary file 23 — Source data Fig. 6 part 4 [file 44319_2026_742_MOESM23_ESM.zip › Figure 6 Part 4/Fig 6efg TPGS1-KO TPGS1 rescue experiments part 2/R2R3/TPGS1-KO EYFP-only actub 7-31-25 R3 ET3.Project Maximum Z_XY1756492702_Z0_T0_C2.tif]

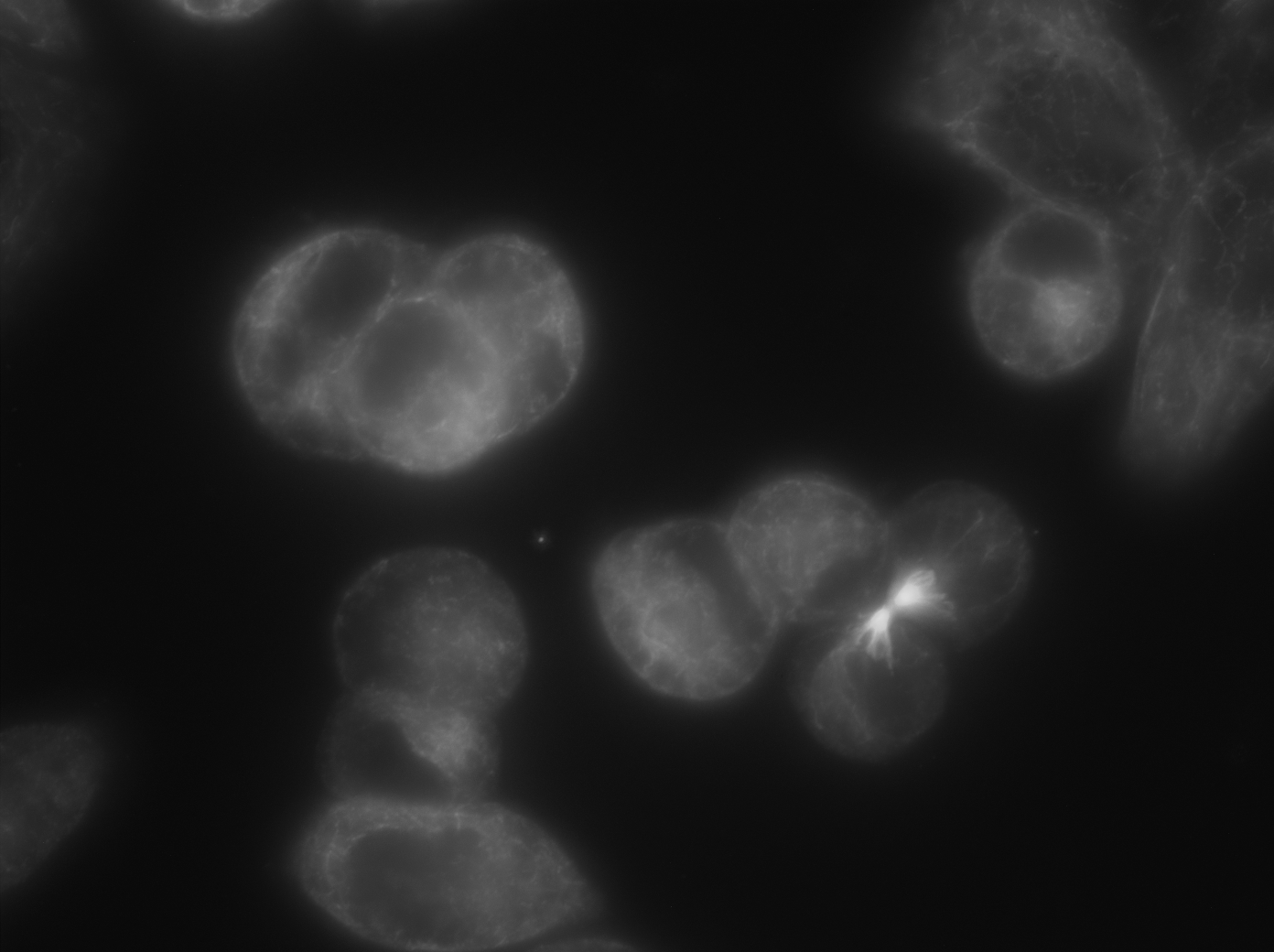

Supplement: Supplementary file 23 — Source data Fig. 6 part 4 [file 44319_2026_742_MOESM23_ESM.zip › Figure 6 Part 4/Fig 6efg TPGS1-KO TPGS1 rescue experiments part 2/R2R3/TPGS1-KO TPGS1-EYFP-3'UTR actub 7-31-25 R2 ET1.Project Maximum Z_XY1756407202_Z0_T0_C2.tif]

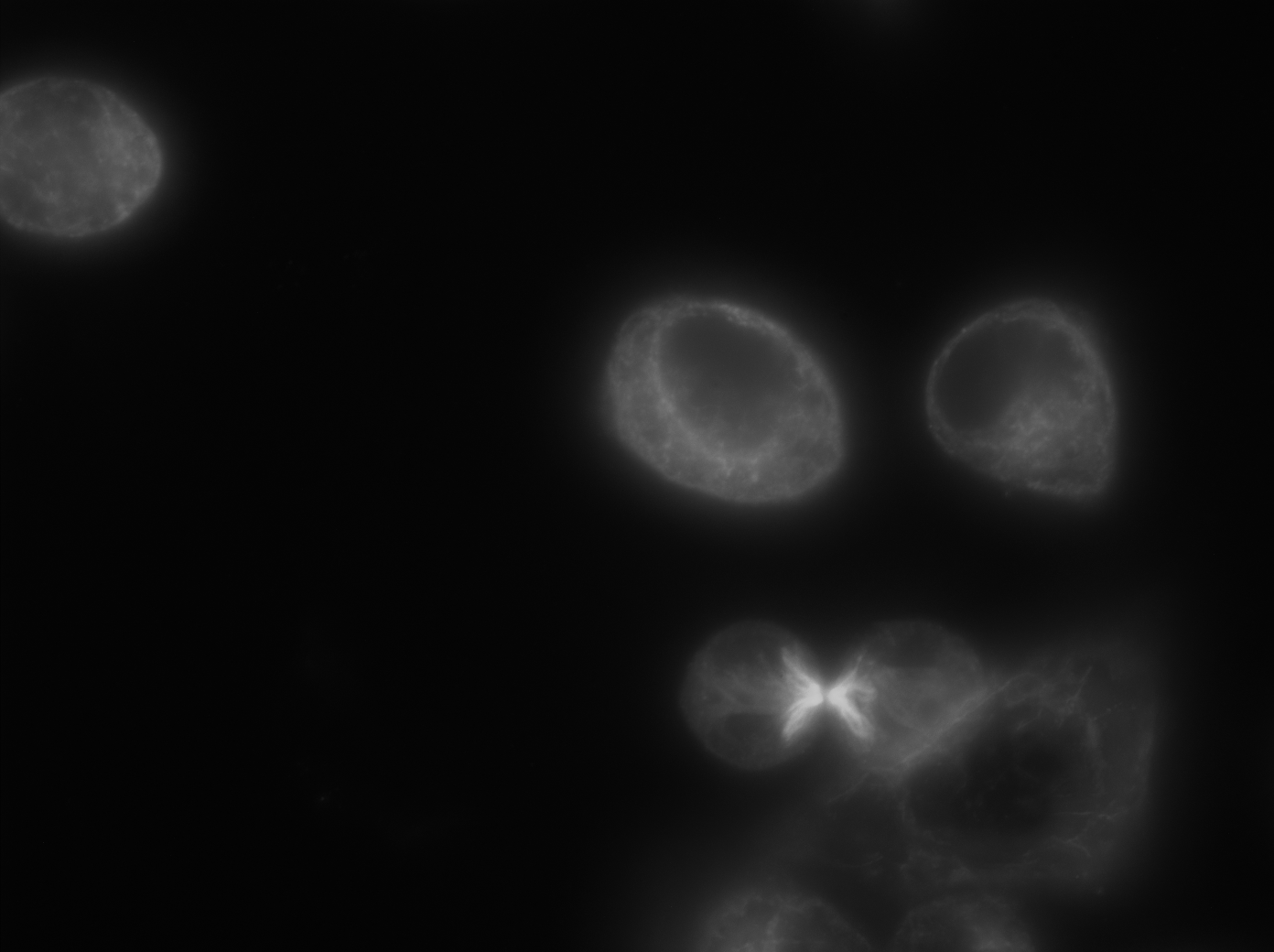

Supplement: Supplementary file 23 — Source data Fig. 6 part 4 [file 44319_2026_742_MOESM23_ESM.zip › Figure 6 Part 4/Fig 6efg TPGS1-KO TPGS1 rescue experiments part 2/R2R3/TPGS1-KO EYFP-only actub 7-31-25 R3 ET5.Project Maximum Z_XY1756493643_Z0_T0_C2.tif]

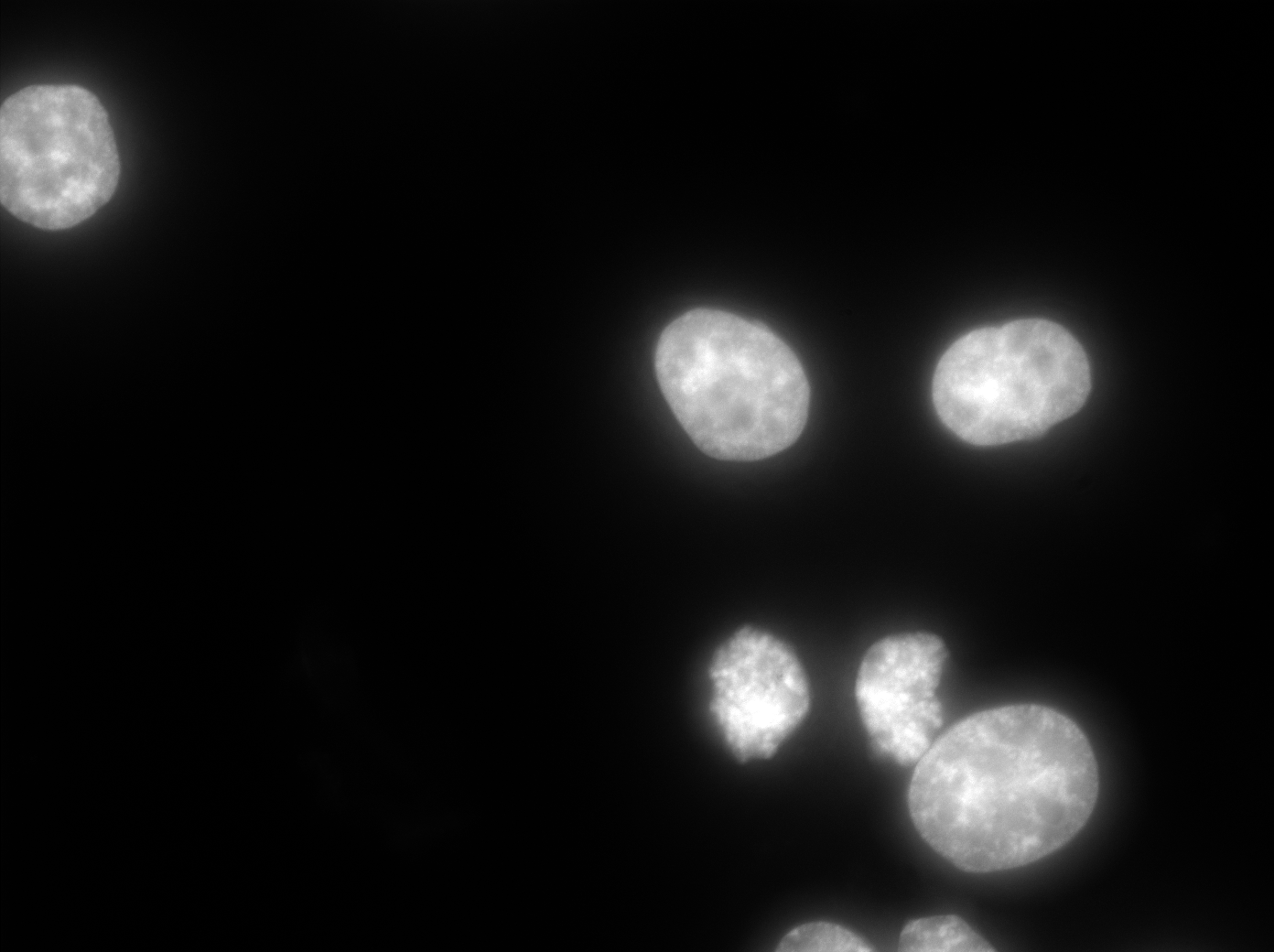

Supplement: Supplementary file 23 — Source data Fig. 6 part 4 [file 44319_2026_742_MOESM23_ESM.zip › Figure 6 Part 4/Fig 6efg TPGS1-KO TPGS1 rescue experiments part 2/R2R3/TPGS1-KO EYFP-only actub 7-31-25 R3 ET5.Project Maximum Z_XY1756493643_Z0_T0_C0.tif]

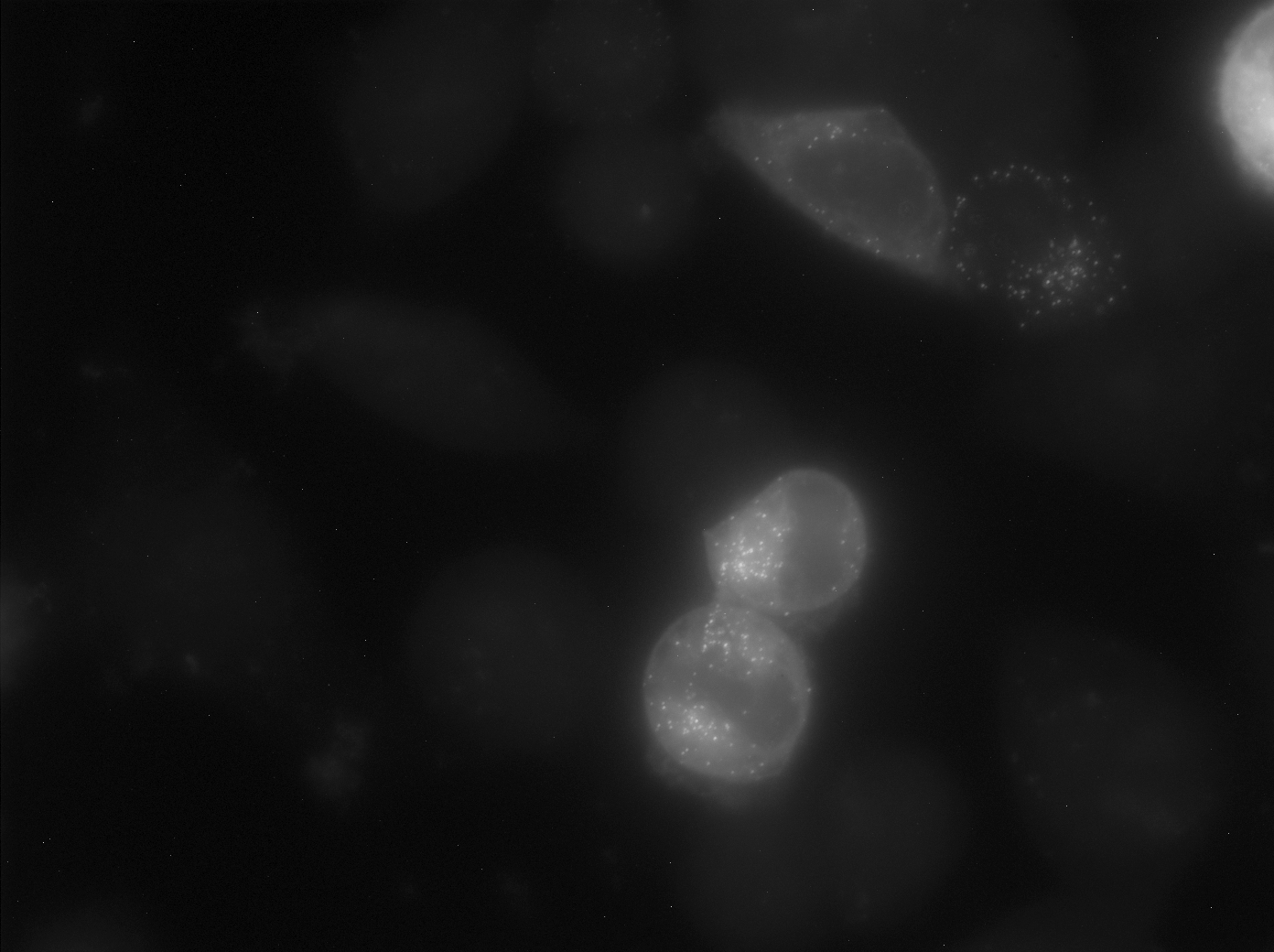

Supplement: Supplementary file 23 — Source data Fig. 6 part 4 [file 44319_2026_742_MOESM23_ESM.zip › Figure 6 Part 4/Fig 6efg TPGS1-KO TPGS1 rescue experiments part 2/R2R3/TPGS1-KO TPGS1-EYFP-3'UTR actub 7-31-25 R3 ET2.Project Maximum Z_XY1756499014_Z0_T0_C1.tif]

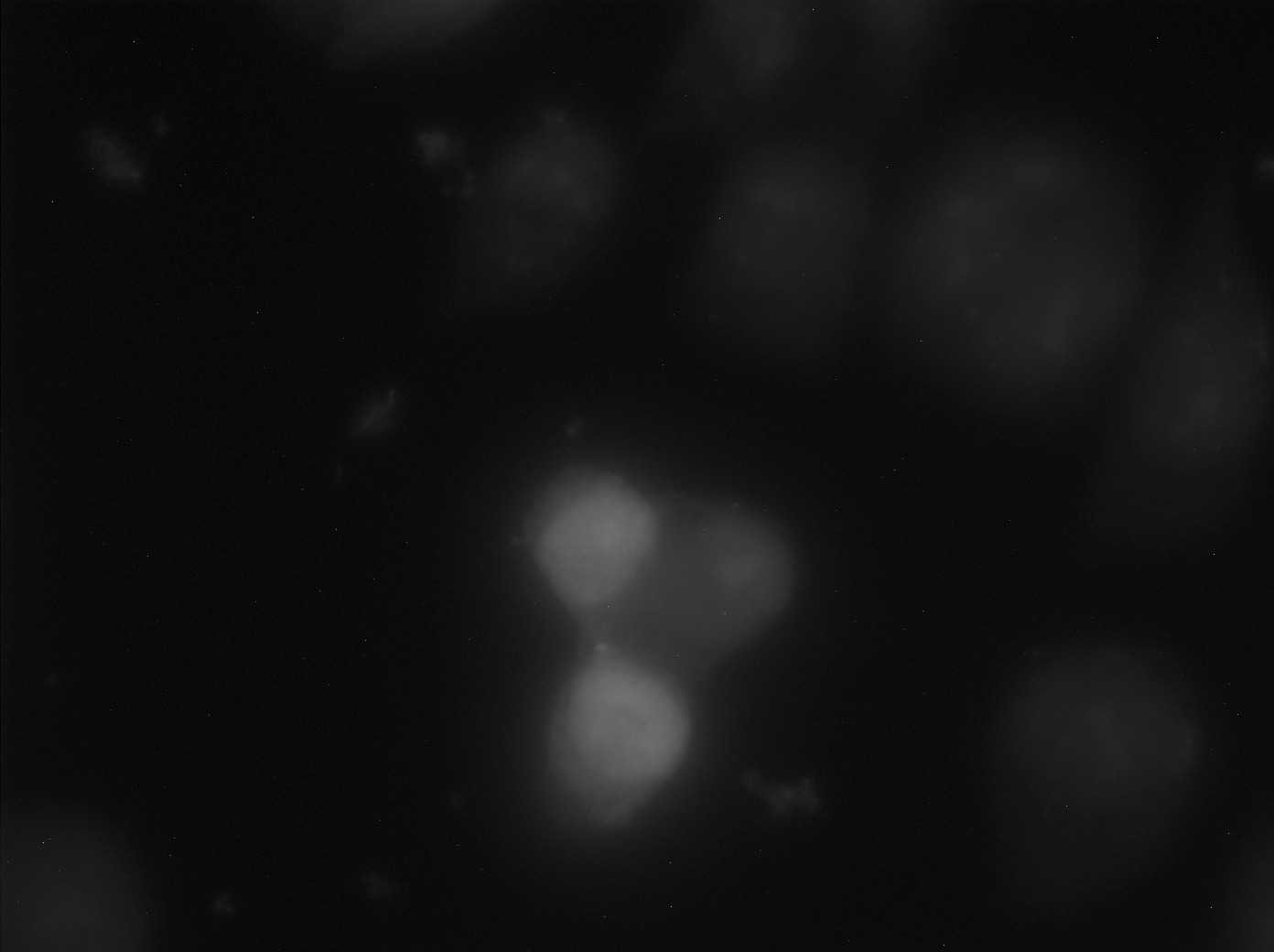

Supplement: Supplementary file 23 — Source data Fig. 6 part 4 [file 44319_2026_742_MOESM23_ESM.zip › Figure 6 Part 4/Fig 6efg TPGS1-KO TPGS1 rescue experiments part 2/R2R3/TPGS1-KO EYFP-only actub 7-31-25 R3 LT2.Project Maximum Z_XY1756491400_Z0_T0_C1.tif]

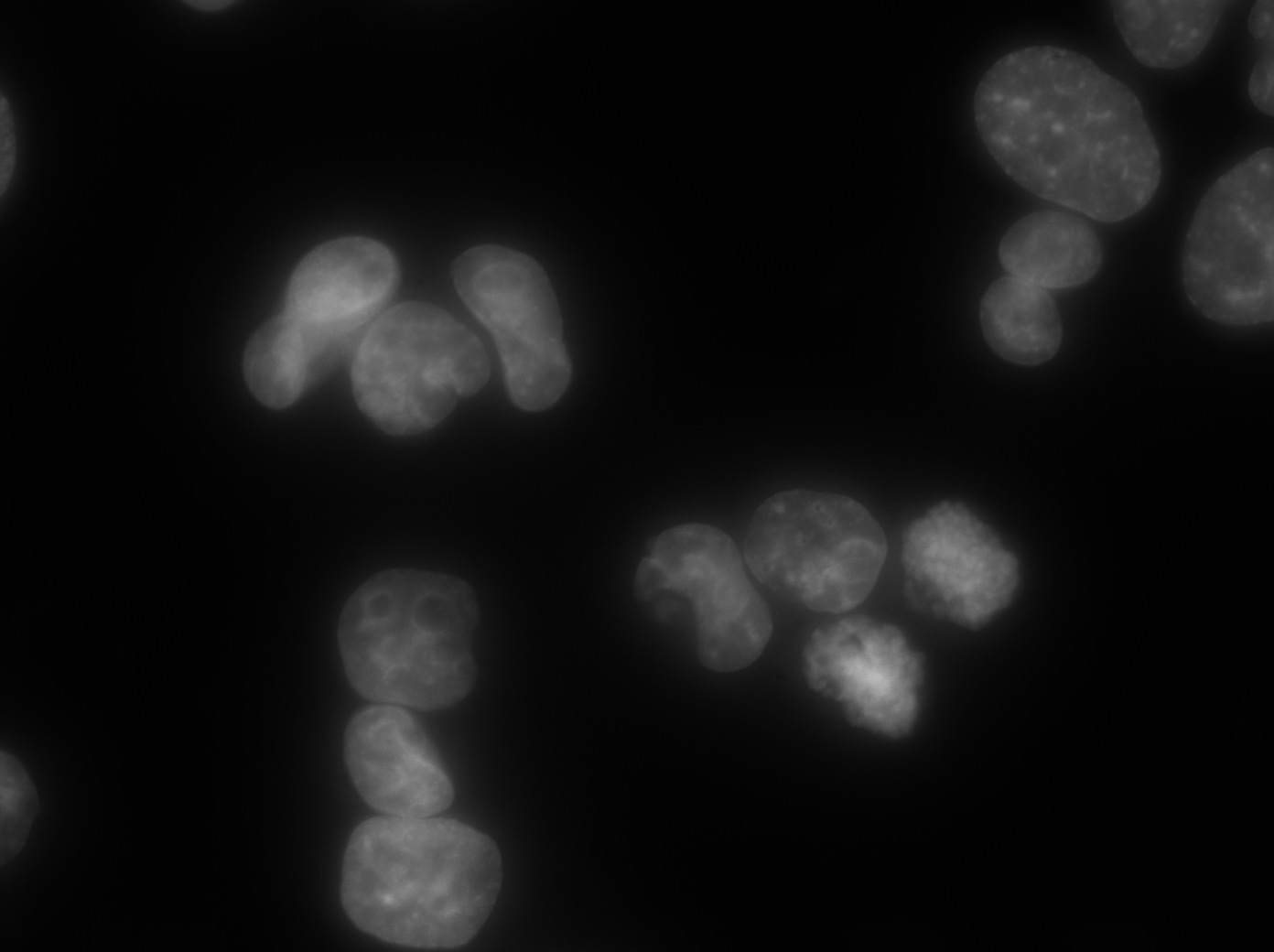

Supplement: Supplementary file 23 — Source data Fig. 6 part 4 [file 44319_2026_742_MOESM23_ESM.zip › Figure 6 Part 4/Fig 6efg TPGS1-KO TPGS1 rescue experiments part 2/R2R3/TPGS1-KO TPGS1-EYFP-3'UTR actub 7-31-25 R2 ET1.Project Maximum Z_XY1756407202_Z0_T0_C0.tif]

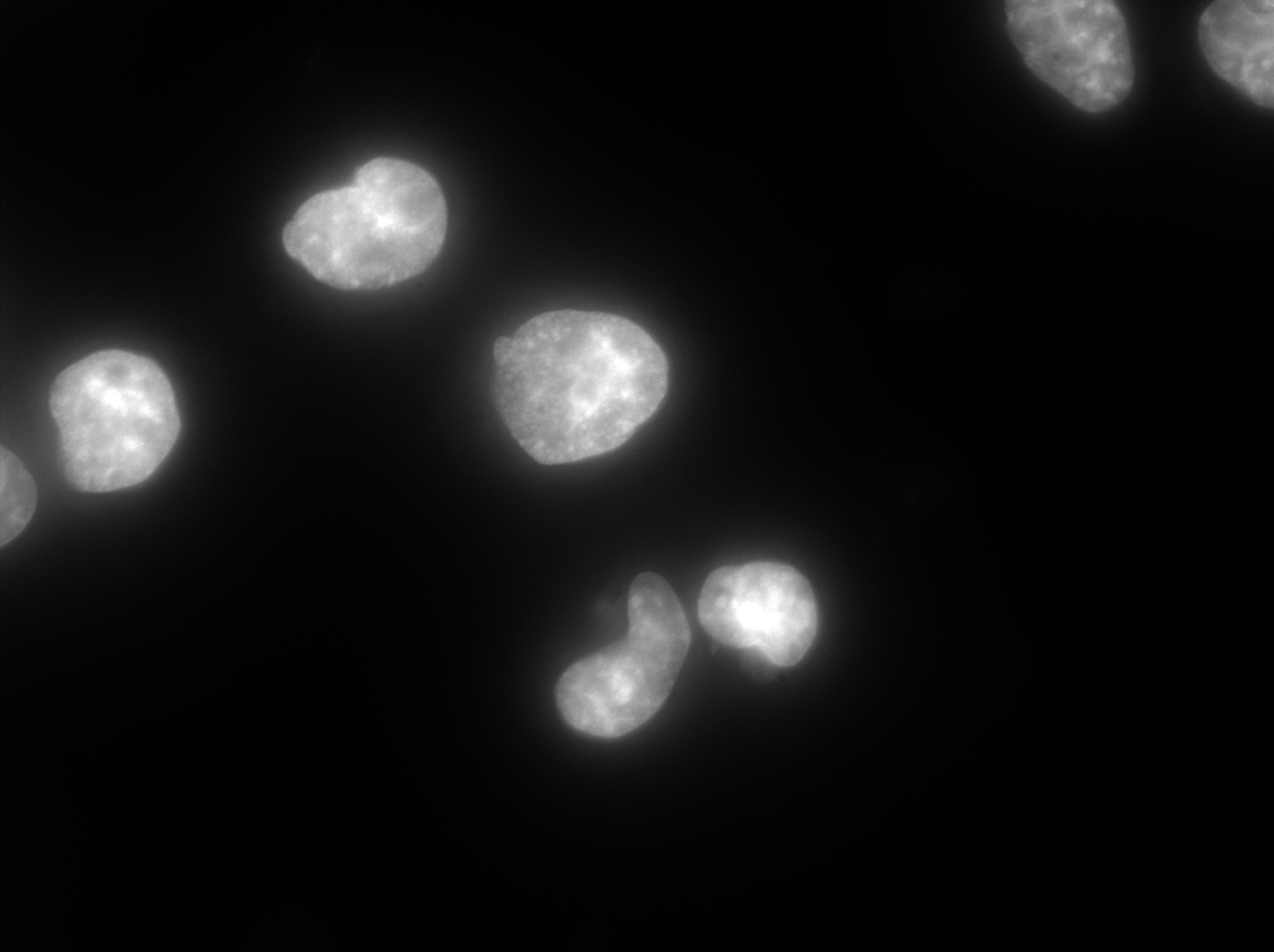

Supplement: Supplementary file 23 — Source data Fig. 6 part 4 [file 44319_2026_742_MOESM23_ESM.zip › Figure 6 Part 4/Fig 6efg TPGS1-KO TPGS1 rescue experiments part 2/R2R3/TPGS1-KO EYFP-only actub 7-31-25 R3 ET3.Project Maximum Z_XY1756492702_Z0_T0_C0.tif]

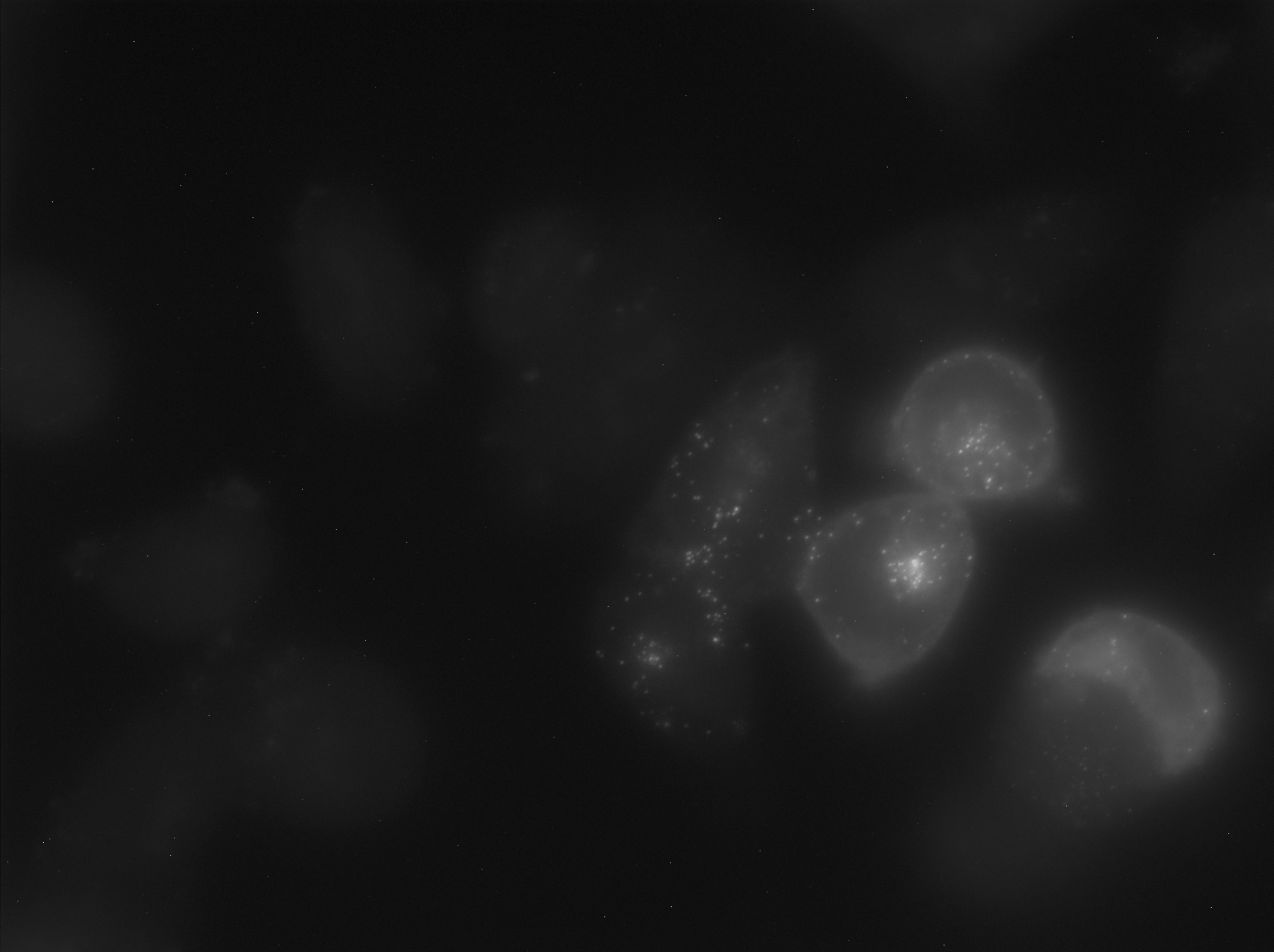

Supplement: Supplementary file 23 — Source data Fig. 6 part 4 [file 44319_2026_742_MOESM23_ESM.zip › Figure 6 Part 4/Fig 6efg TPGS1-KO TPGS1 rescue experiments part 2/R2R3/TPGS1-KO TPGS1-EYFP-3'UTR actub 7-31-25 R2 LT9.Project Maximum Z_XY1756411917_Z0_T0_C1.tif]

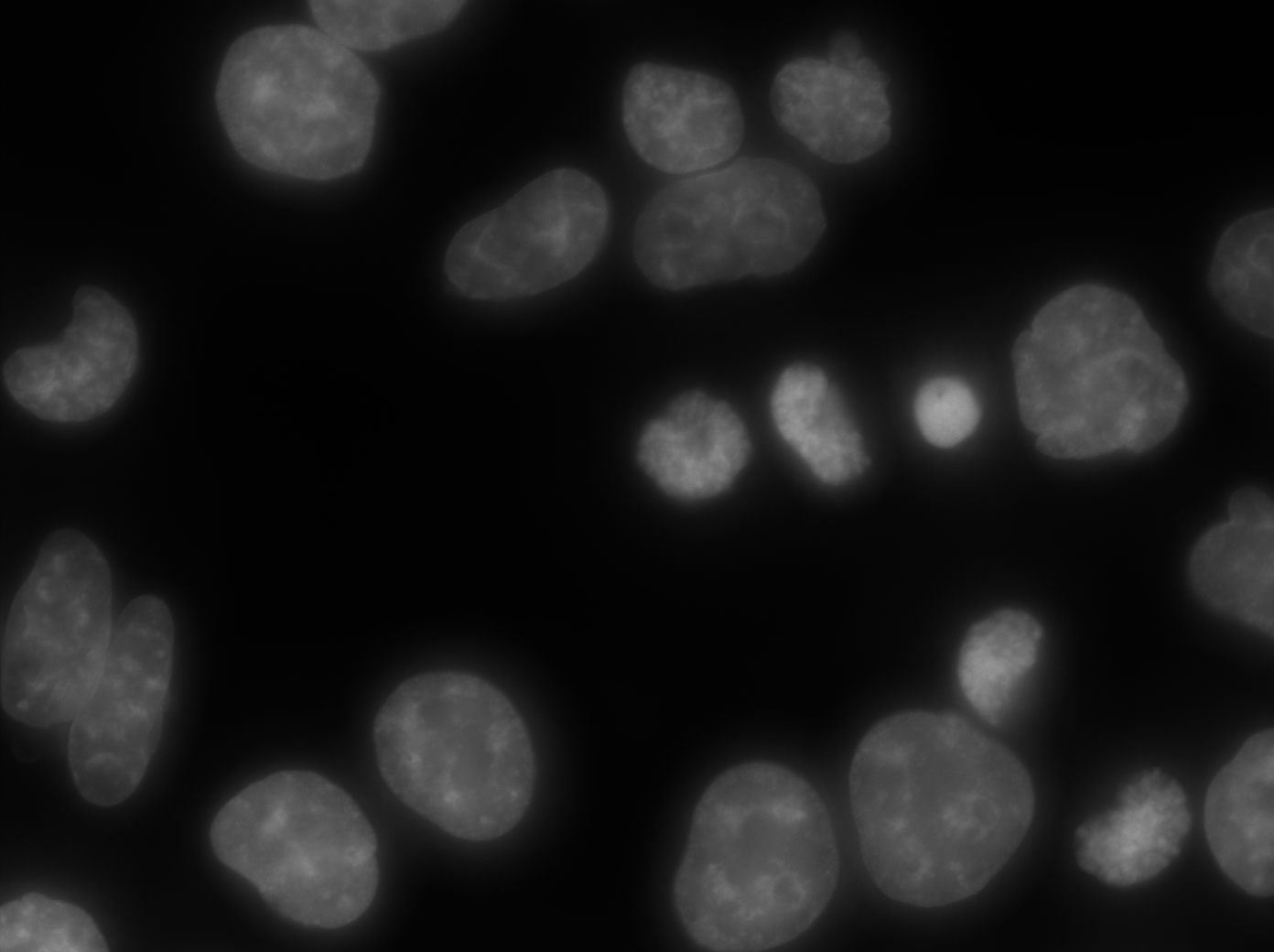

Supplement: Supplementary file 23 — Source data Fig. 6 part 4 [file 44319_2026_742_MOESM23_ESM.zip › Figure 6 Part 4/Fig 6efg TPGS1-KO TPGS1 rescue experiments part 2/R2R3/TPGS1-KO TPGS1-EYFP-3'UTR actub 7-31-25 R2 ET4.Project Maximum Z_XY1756408521_Z0_T0_C0.tif]

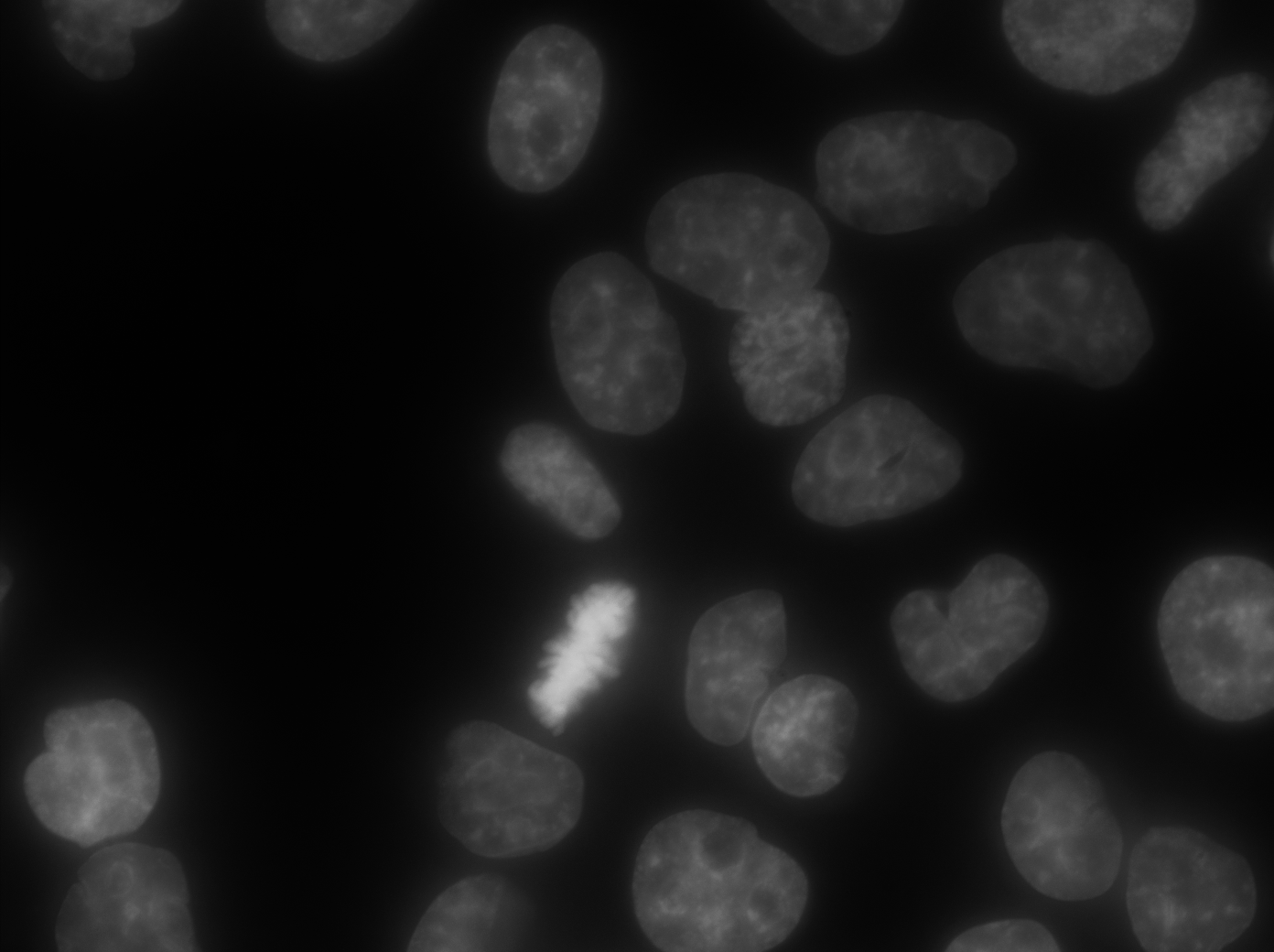

Supplement: Supplementary file 23 — Source data Fig. 6 part 4 [file 44319_2026_742_MOESM23_ESM.zip › Figure 6 Part 4/Fig 6efg TPGS1-KO TPGS1 rescue experiments part 2/R2R3/TPGS1-KO TPGS1-EYFP-3'UTR actub 7-31-25 R2 LT4.Project Maximum Z_XY1756407822_Z0_T0_C0.tif]

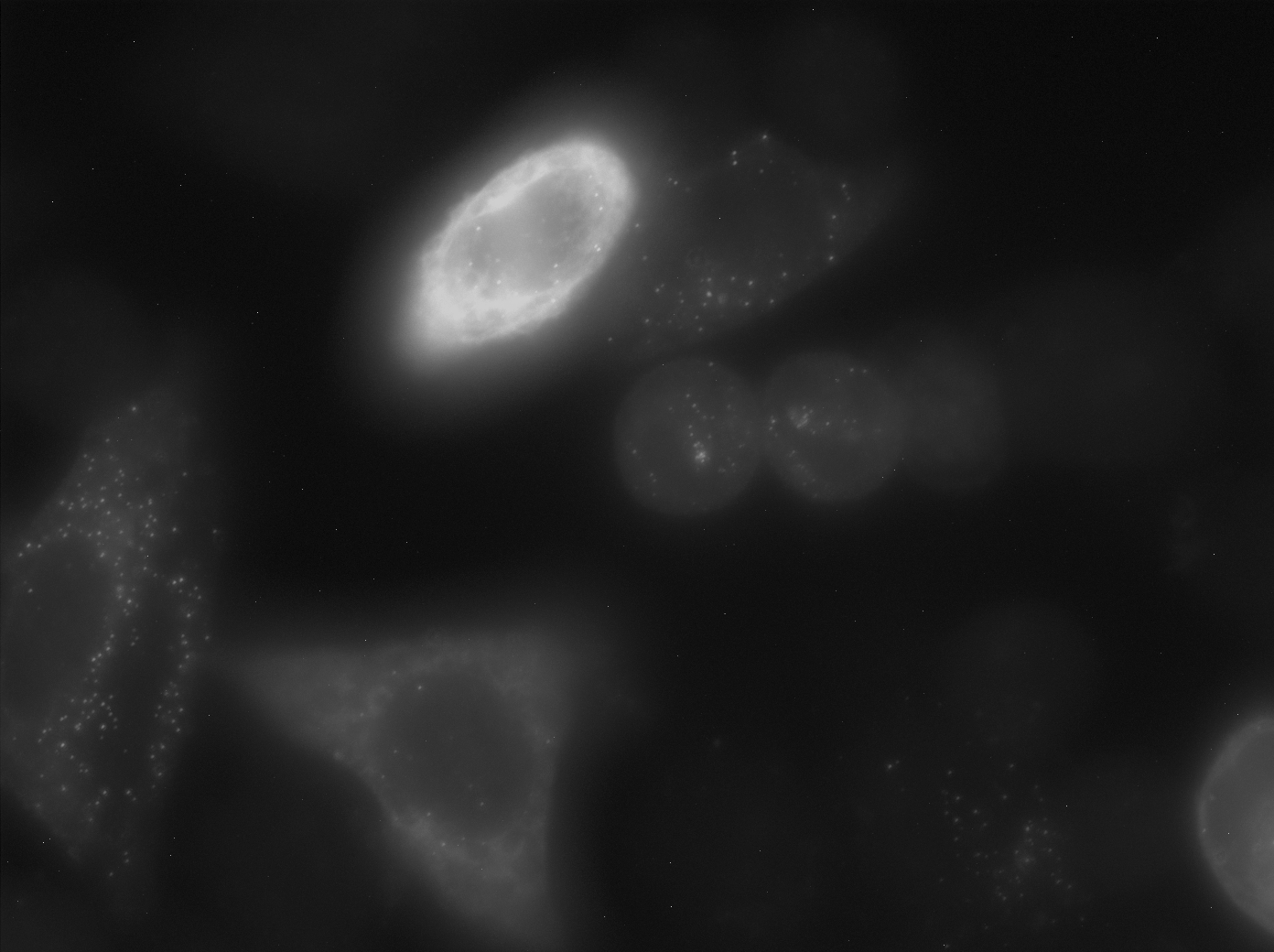

Supplement: Supplementary file 23 — Source data Fig. 6 part 4 [file 44319_2026_742_MOESM23_ESM.zip › Figure 6 Part 4/Fig 6efg TPGS1-KO TPGS1 rescue experiments part 2/R2R3/TPGS1-KO TPGS1-EYFP-3'UTR actub 7-31-25 R2 ET4.Project Maximum Z_XY1756408521_Z0_T0_C1.tif]

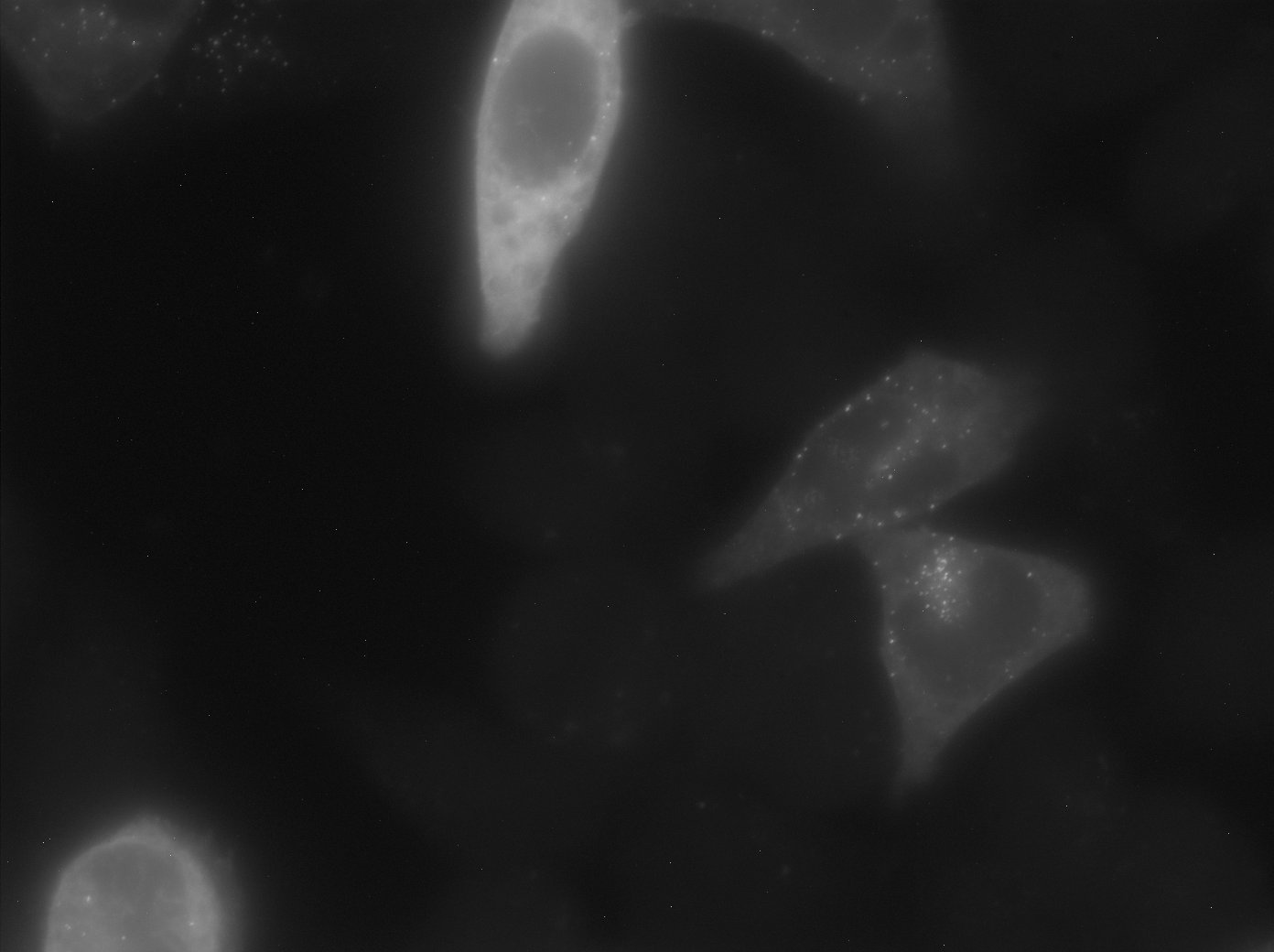

Supplement: Supplementary file 23 — Source data Fig. 6 part 4 [file 44319_2026_742_MOESM23_ESM.zip › Figure 6 Part 4/Fig 6efg TPGS1-KO TPGS1 rescue experiments part 2/R2R3/TPGS1-KO TPGS1-EYFP-3'UTR actub 7-31-25 R2 LT4.Project Maximum Z_XY1756407822_Z0_T0_C1.tif]

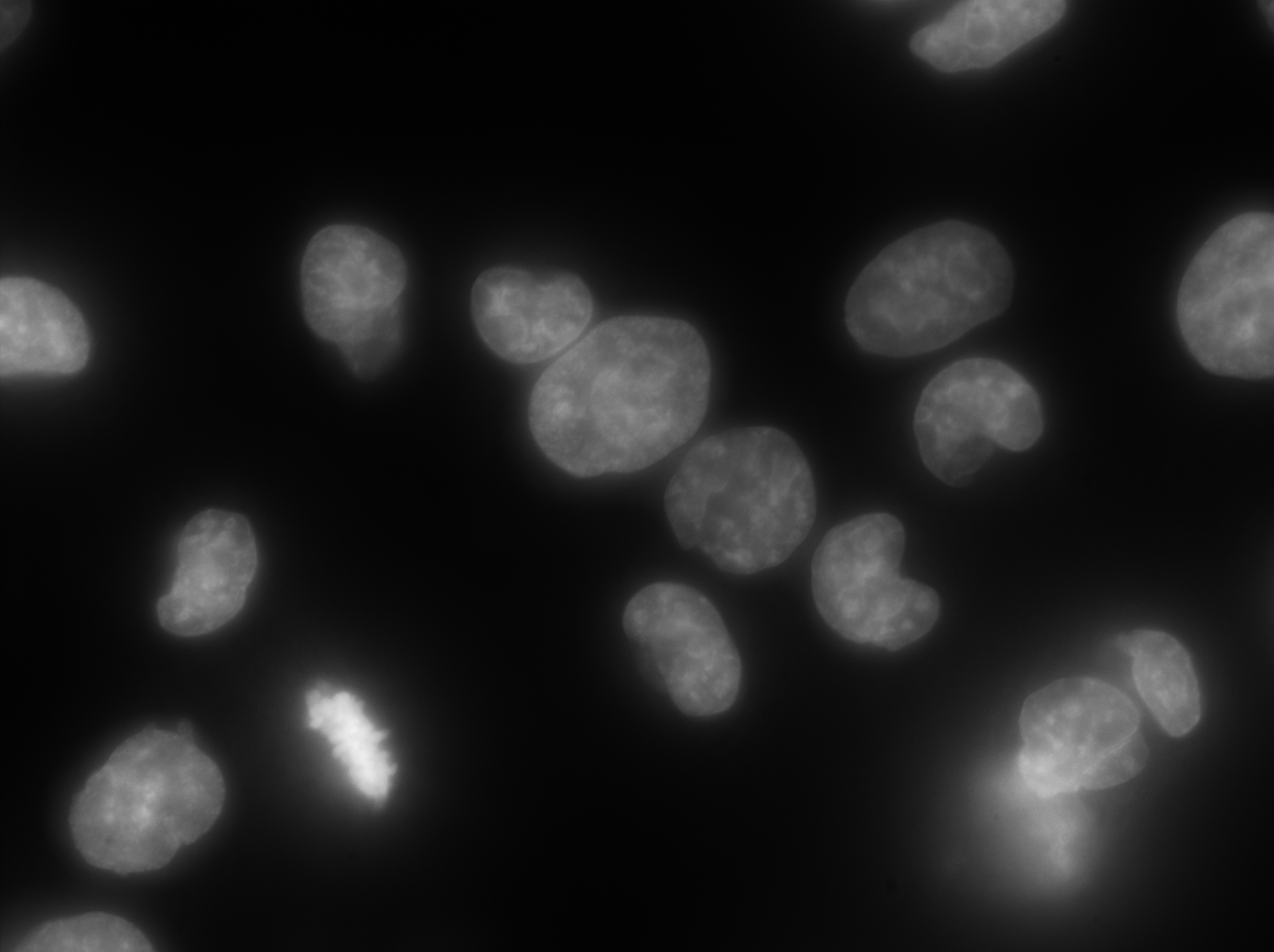

Supplement: Supplementary file 23 — Source data Fig. 6 part 4 [file 44319_2026_742_MOESM23_ESM.zip › Figure 6 Part 4/Fig 6efg TPGS1-KO TPGS1 rescue experiments part 2/R2R3/TPGS1-KO TPGS1-EYFP-3'UTR actub 7-31-25 R2 LT9.Project Maximum Z_XY1756411917_Z0_T0_C0.tif]

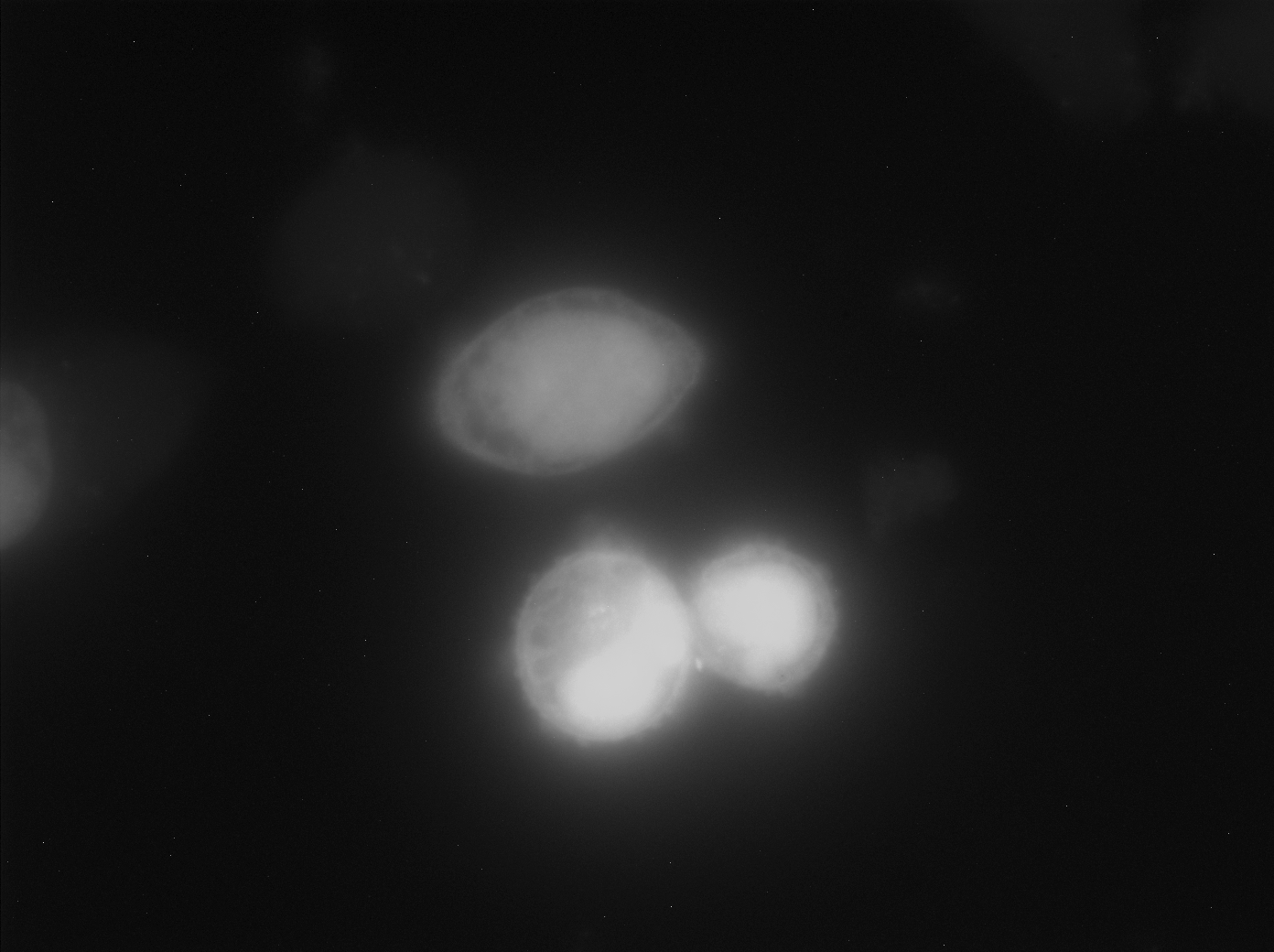

Supplement: Supplementary file 23 — Source data Fig. 6 part 4 [file 44319_2026_742_MOESM23_ESM.zip › Figure 6 Part 4/Fig 6efg TPGS1-KO TPGS1 rescue experiments part 2/R2R3/TPGS1-KO EYFP-only actub 7-31-25 R3 ET3.Project Maximum Z_XY1756492702_Z0_T0_C1.tif]

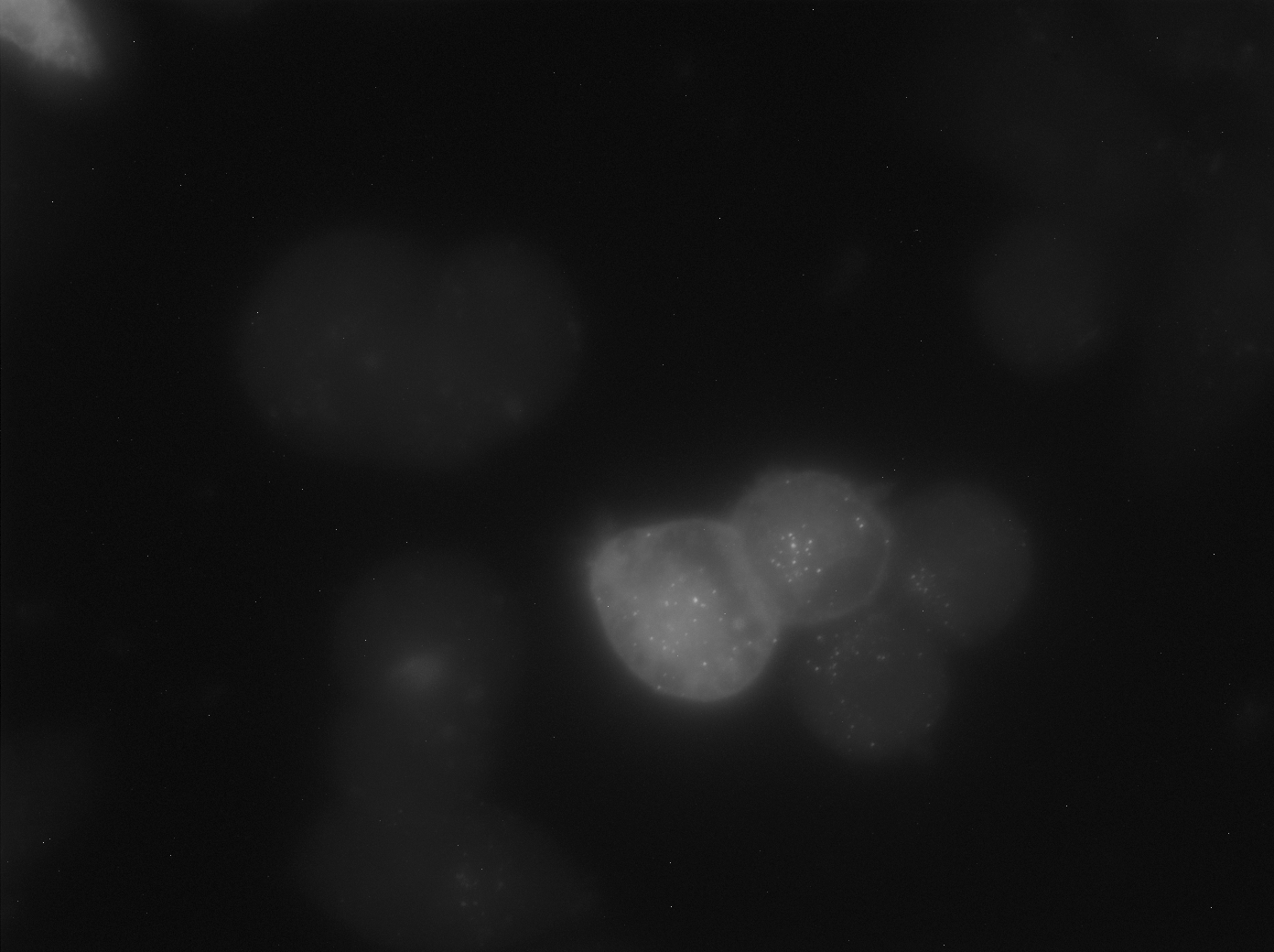

Supplement: Supplementary file 23 — Source data Fig. 6 part 4 [file 44319_2026_742_MOESM23_ESM.zip › Figure 6 Part 4/Fig 6efg TPGS1-KO TPGS1 rescue experiments part 2/R2R3/TPGS1-KO TPGS1-EYFP-3'UTR actub 7-31-25 R2 ET1.Project Maximum Z_XY1756407202_Z0_T0_C1.tif]

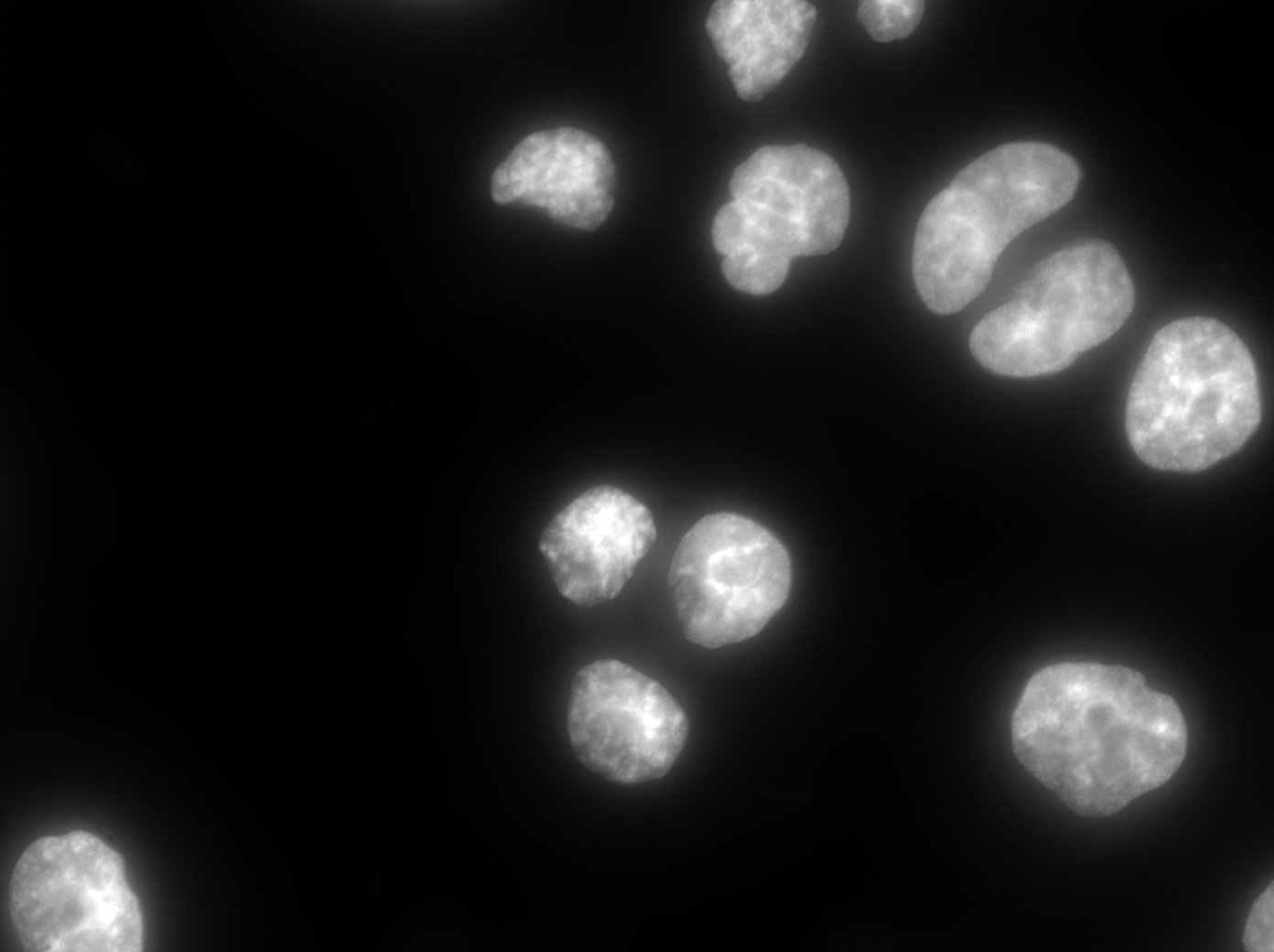

Supplement: Supplementary file 23 — Source data Fig. 6 part 4 [file 44319_2026_742_MOESM23_ESM.zip › Figure 6 Part 4/Fig 6efg TPGS1-KO TPGS1 rescue experiments part 2/R2R3/TPGS1-KO EYFP-only actub 7-31-25 R3 LT2.Project Maximum Z_XY1756491400_Z0_T0_C0.tif]

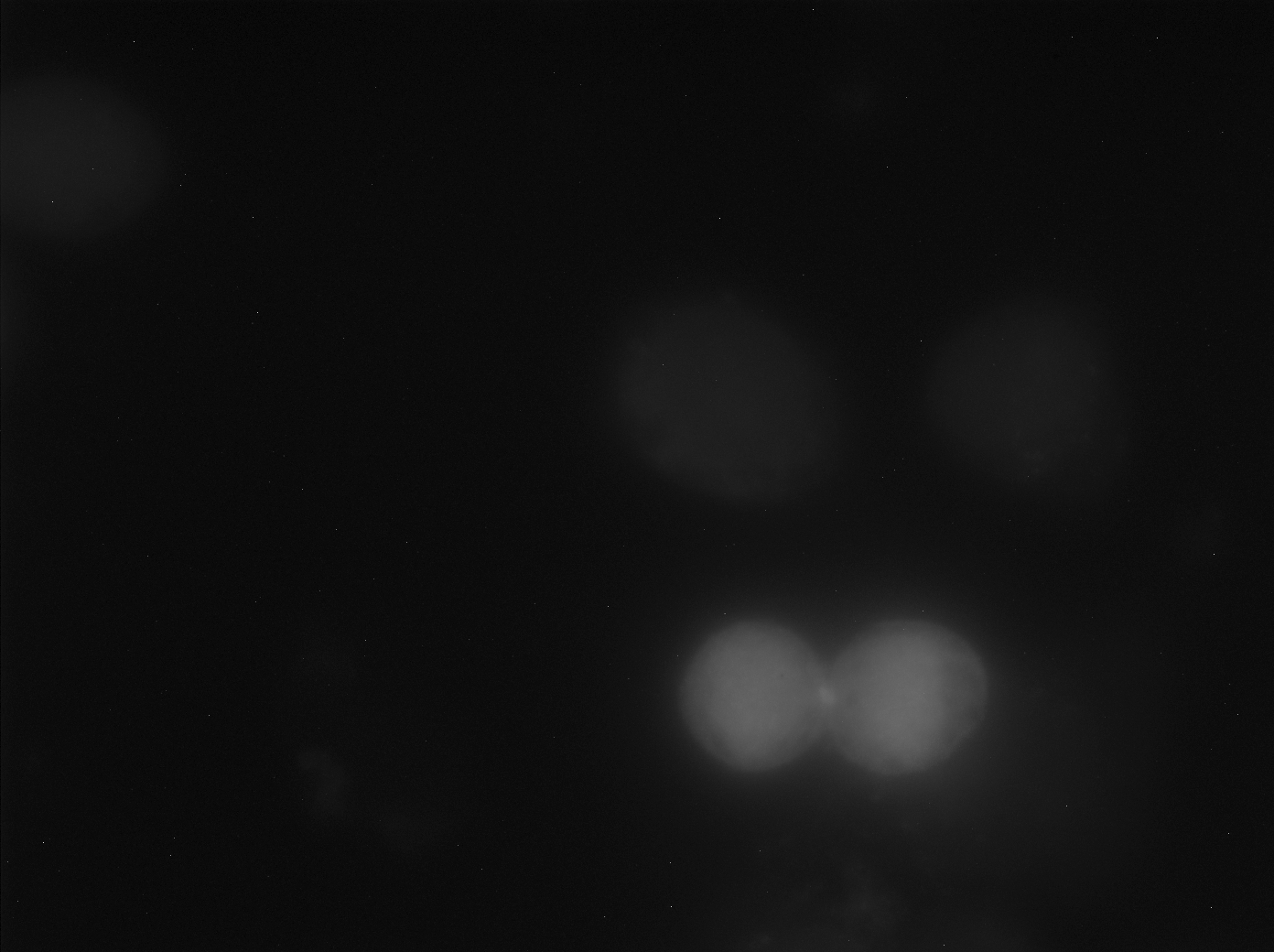

Supplement: Supplementary file 23 — Source data Fig. 6 part 4 [file 44319_2026_742_MOESM23_ESM.zip › Figure 6 Part 4/Fig 6efg TPGS1-KO TPGS1 rescue experiments part 2/R2R3/TPGS1-KO EYFP-only actub 7-31-25 R3 ET5.Project Maximum Z_XY1756493643_Z0_T0_C1.tif]

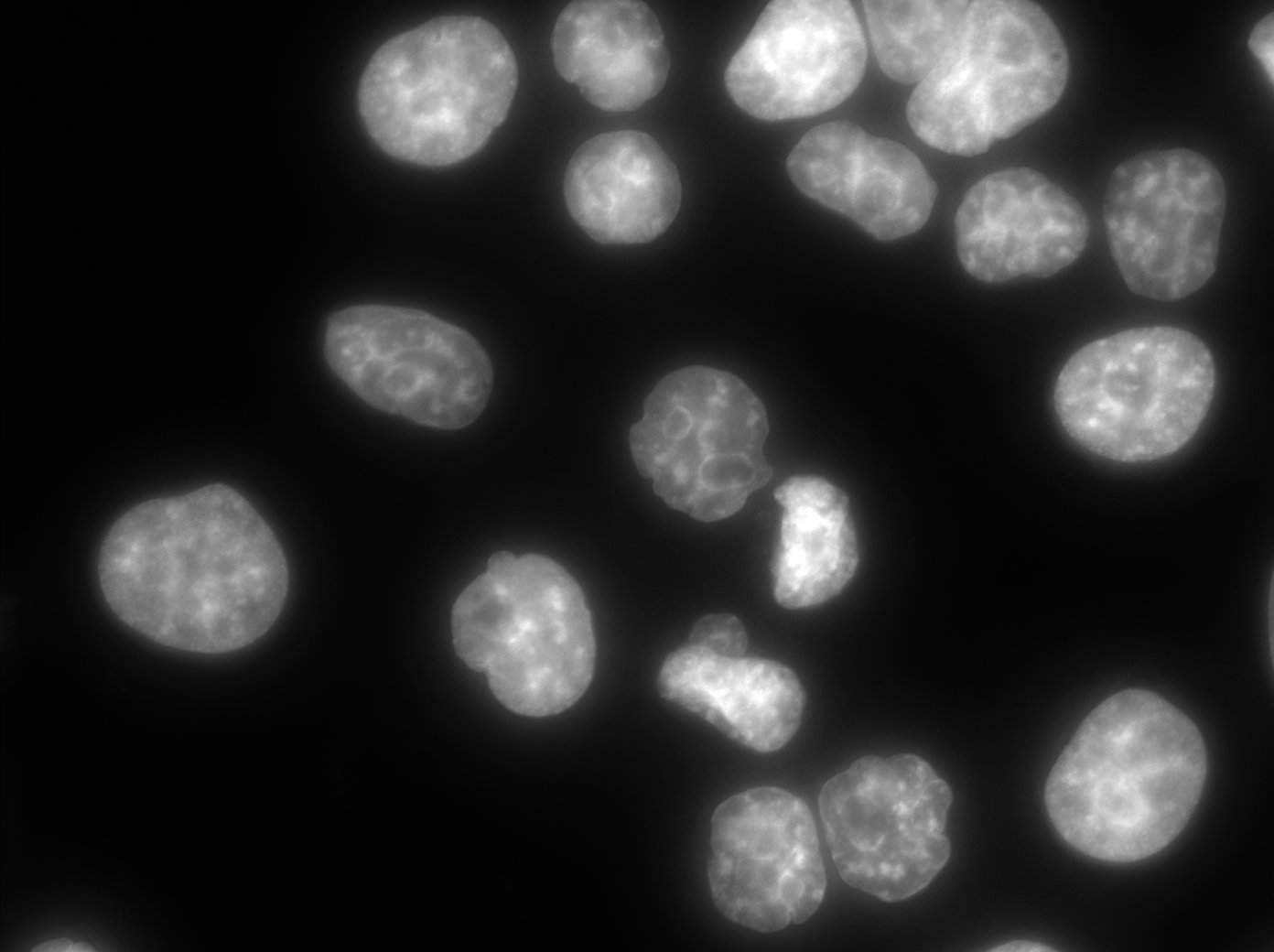

Supplement: Supplementary file 23 — Source data Fig. 6 part 4 [file 44319_2026_742_MOESM23_ESM.zip › Figure 6 Part 4/Fig 6efg TPGS1-KO TPGS1 rescue experiments part 2/R2R3/TPGS1-KO TPGS1-EYFP-3'UTR actub 7-31-25 R3 ET2.Project Maximum Z_XY1756499014_Z0_T0_C0.tif]

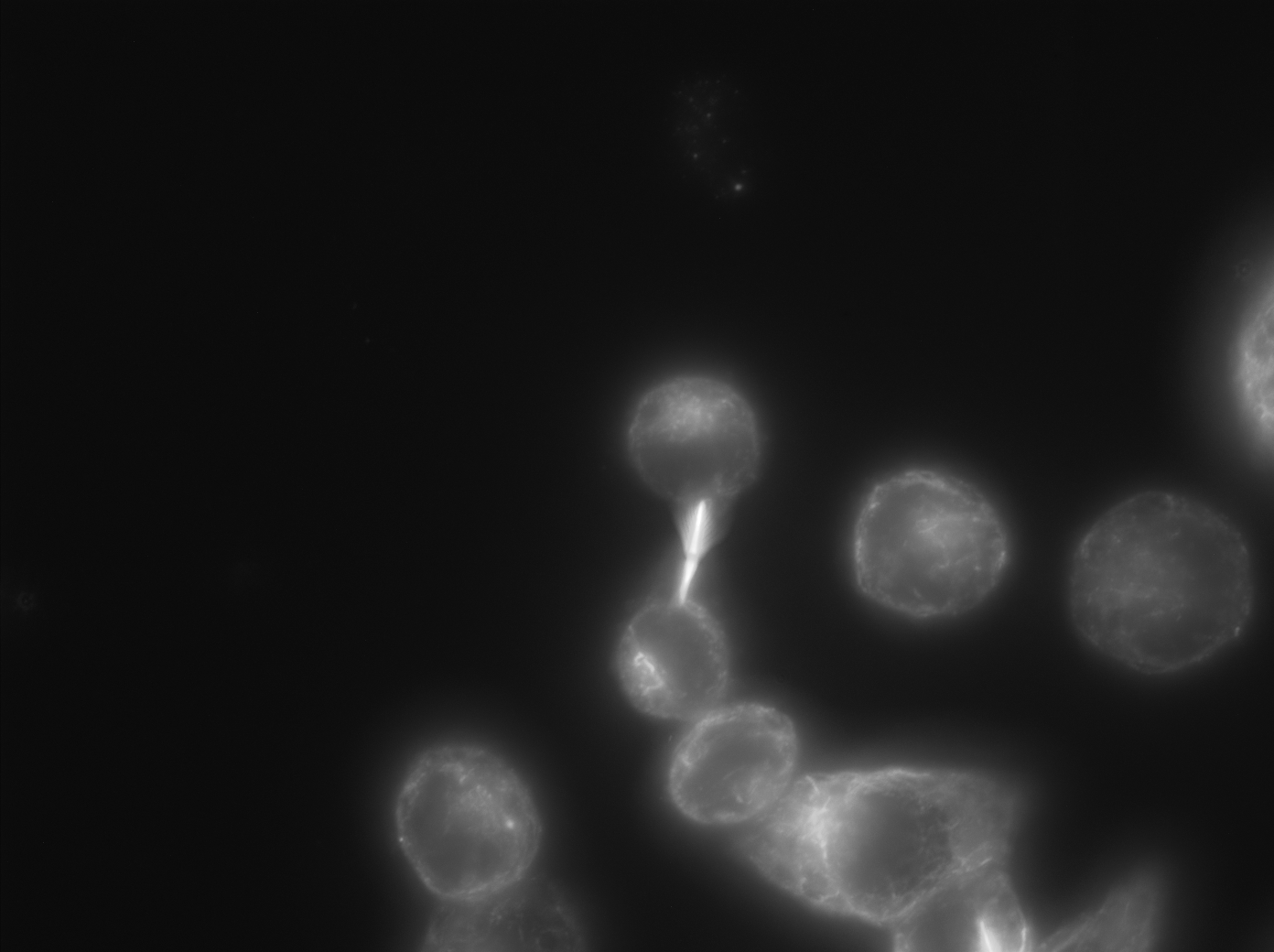

Supplement: Supplementary file 23 — Source data Fig. 6 part 4 [file 44319_2026_742_MOESM23_ESM.zip › Figure 6 Part 4/Fig 6efg TPGS1-KO TPGS1 rescue experiments part 2/R2R3/TPGS1-KO EYFP-only actub 7-31-25 R2 ET9.Project Maximum Z_XY1756416411_Z0_T0_C2.tif]

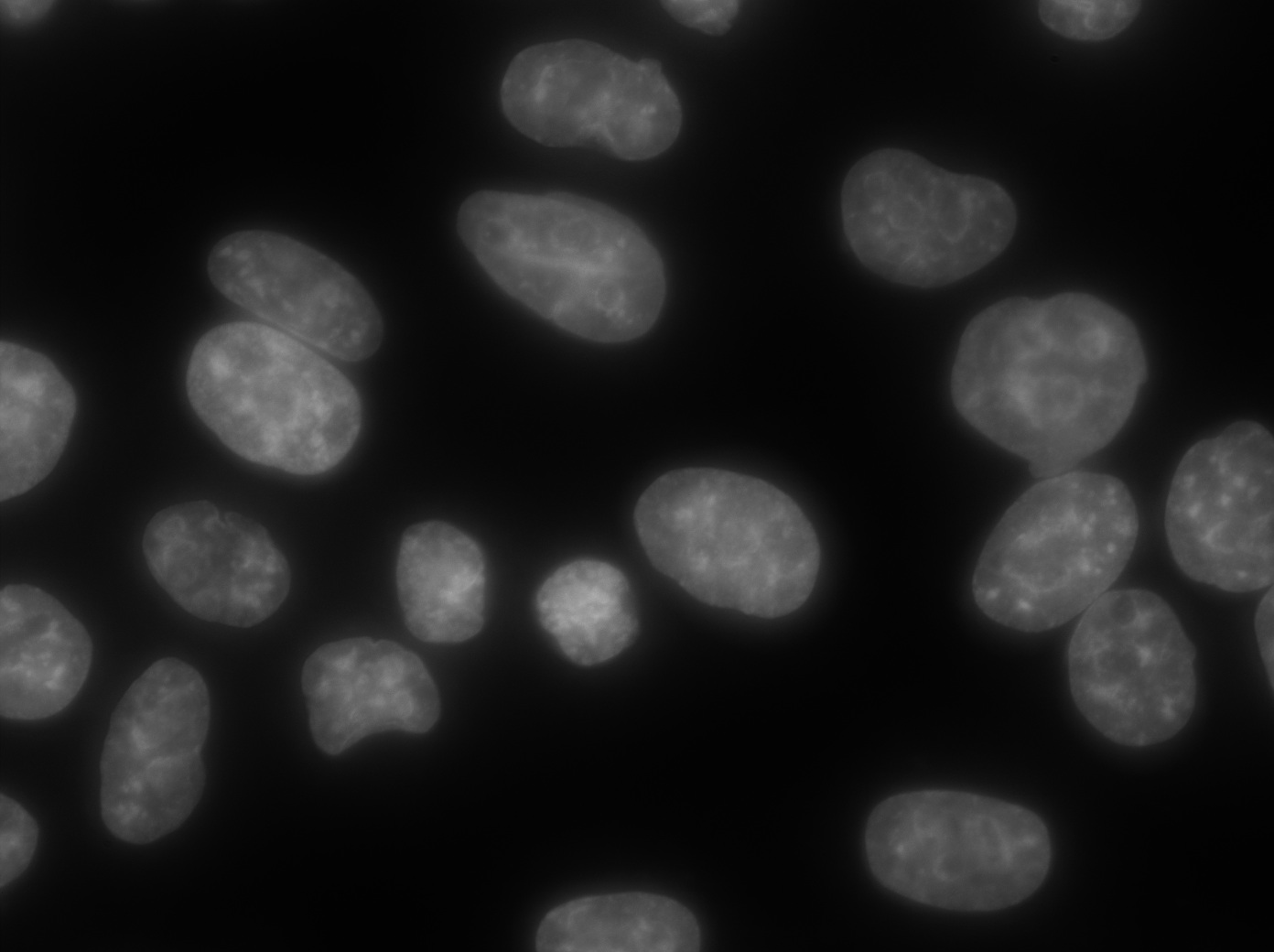

Supplement: Supplementary file 23 — Source data Fig. 6 part 4 [file 44319_2026_742_MOESM23_ESM.zip › Figure 6 Part 4/Fig 6efg TPGS1-KO TPGS1 rescue experiments part 2/R2R3/TPGS1-KO TPGS1-EYFP-3'UTR actub 7-31-25 R2 LT8.Project Maximum Z_XY1756411785_Z0_T0_C0.tif]

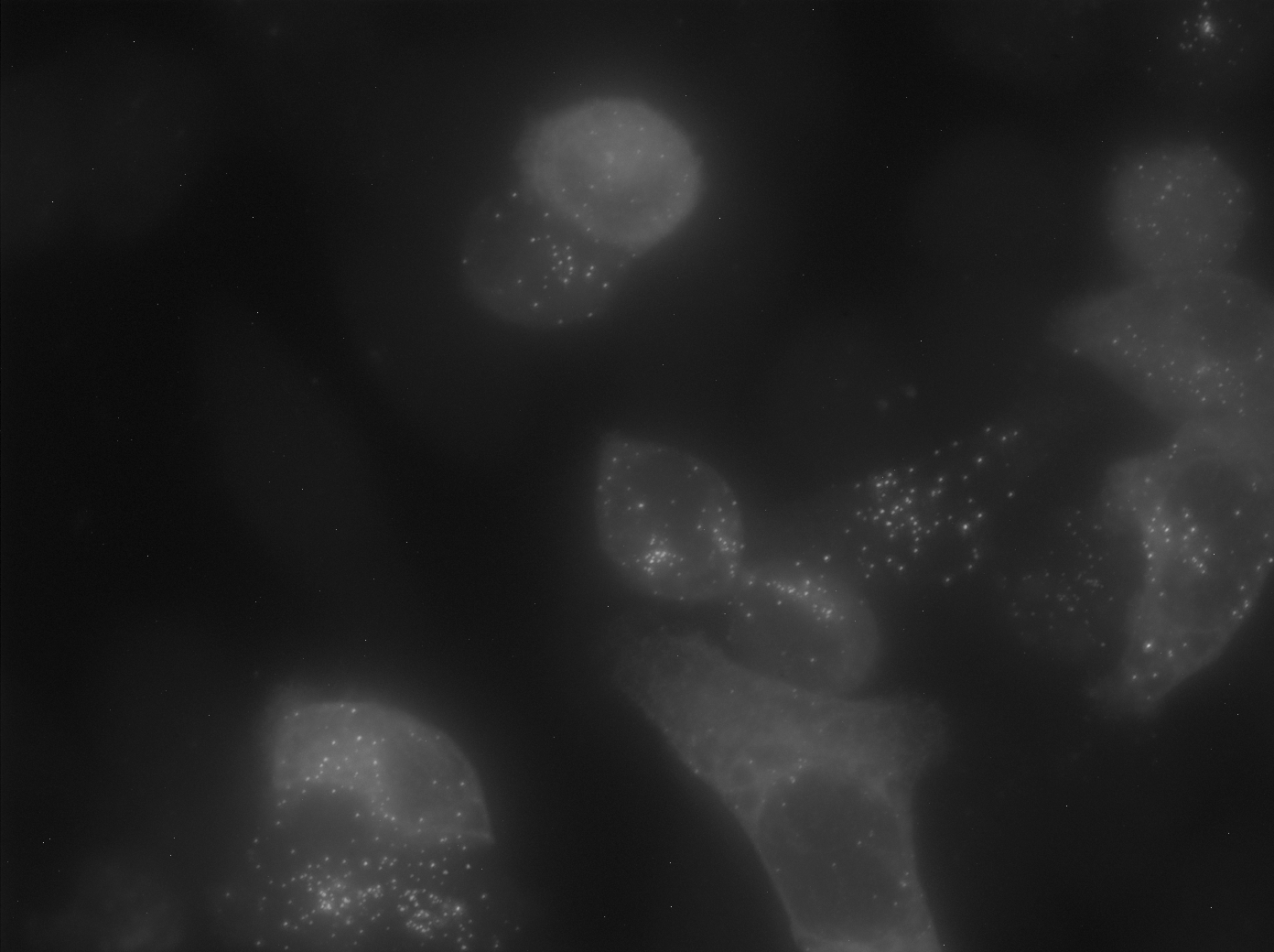

Supplement: Supplementary file 23 — Source data Fig. 6 part 4 [file 44319_2026_742_MOESM23_ESM.zip › Figure 6 Part 4/Fig 6efg TPGS1-KO TPGS1 rescue experiments part 2/R2R3/TPGS1-KO TPGS1-EYFP-3'UTR actub 7-31-25 R2 LT3.Project Maximum Z_XY1756407610_Z0_T0_C1.tif]

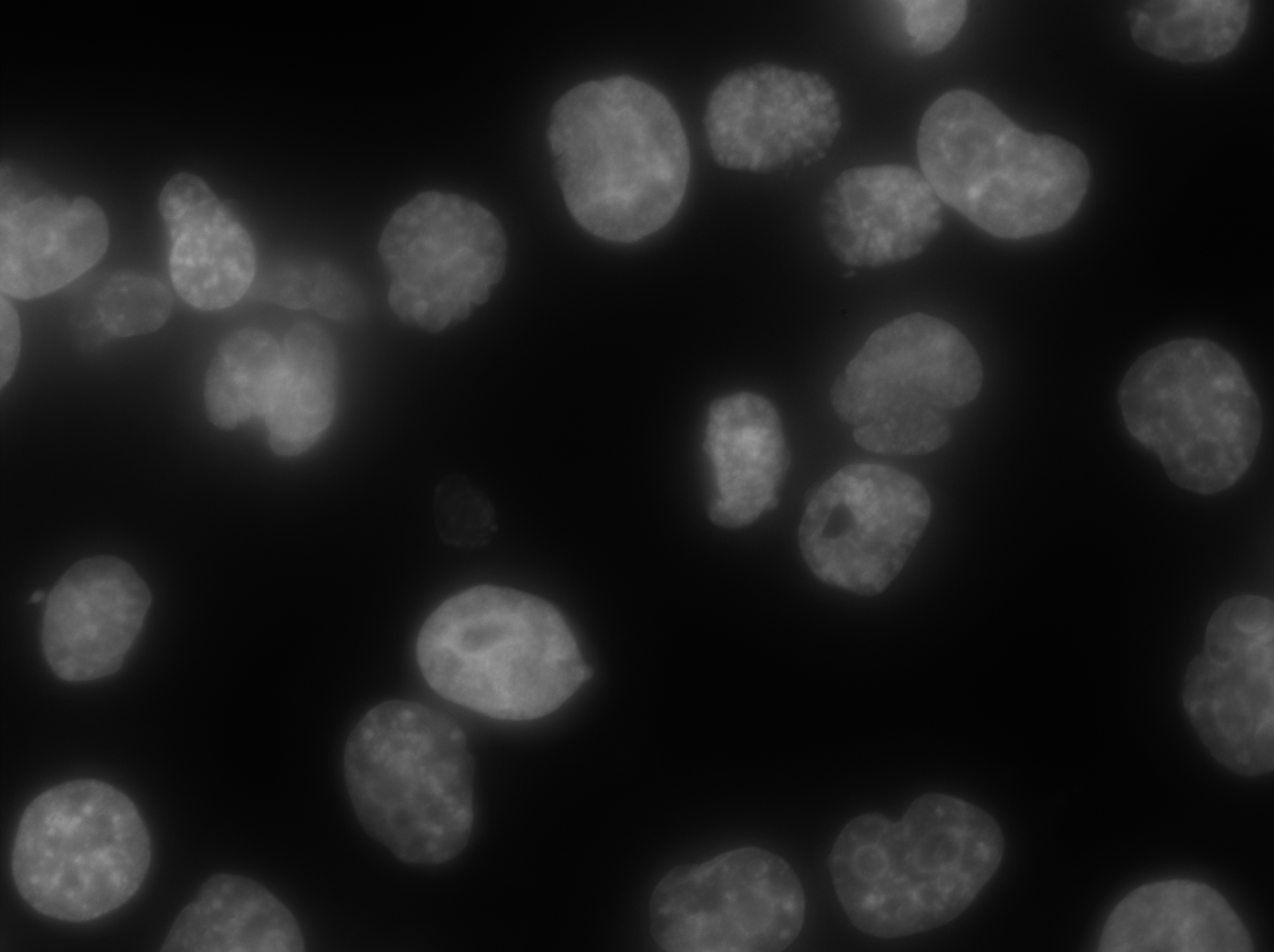

Supplement: Supplementary file 23 — Source data Fig. 6 part 4 [file 44319_2026_742_MOESM23_ESM.zip › Figure 6 Part 4/Fig 6efg TPGS1-KO TPGS1 rescue experiments part 2/R2R3/TPGS1-KO TPGS1-EYFP-3'UTR actub 7-31-25 R2 ET6.Project Maximum Z_XY1756411247_Z0_T0_C0.tif]

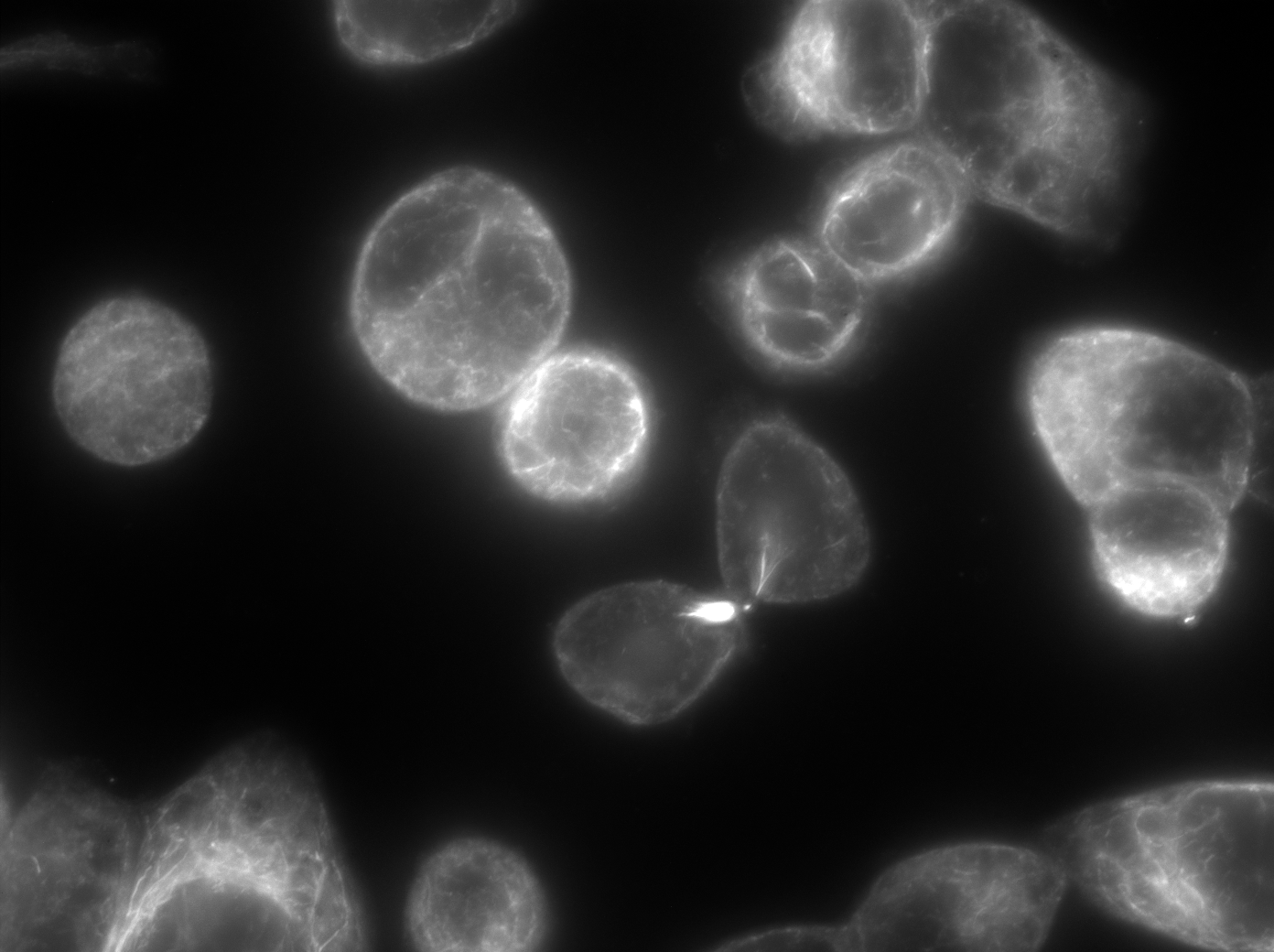

Supplement: Supplementary file 23 — Source data Fig. 6 part 4 [file 44319_2026_742_MOESM23_ESM.zip › Figure 6 Part 4/Fig 6efg TPGS1-KO TPGS1 rescue experiments part 2/R2R3/TPGS1-KO TPGS1-EYFP-3'UTR actub 7-31-25 R3 LT8.Project Maximum Z_XY1756501925_Z0_T0_C2.tif]

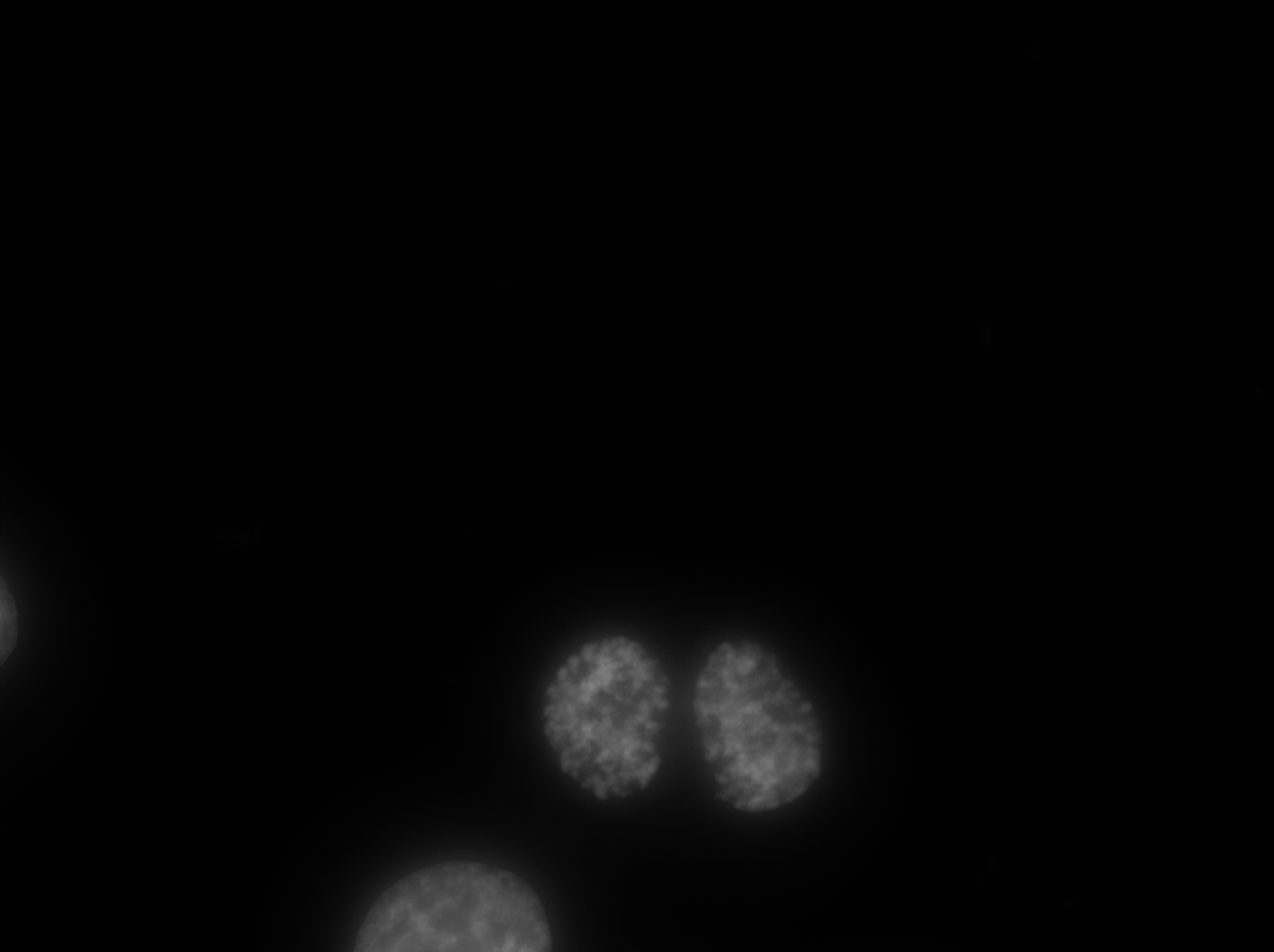

Supplement: Supplementary file 23 — Source data Fig. 6 part 4 [file 44319_2026_742_MOESM23_ESM.zip › Figure 6 Part 4/Fig 6efg TPGS1-KO TPGS1 rescue experiments part 2/R2R3/TPGS1-KO EYFP-only actub 7-31-25 R2 ET3.Project Maximum Z_XY1756414255_Z0_T0_C0.tif]

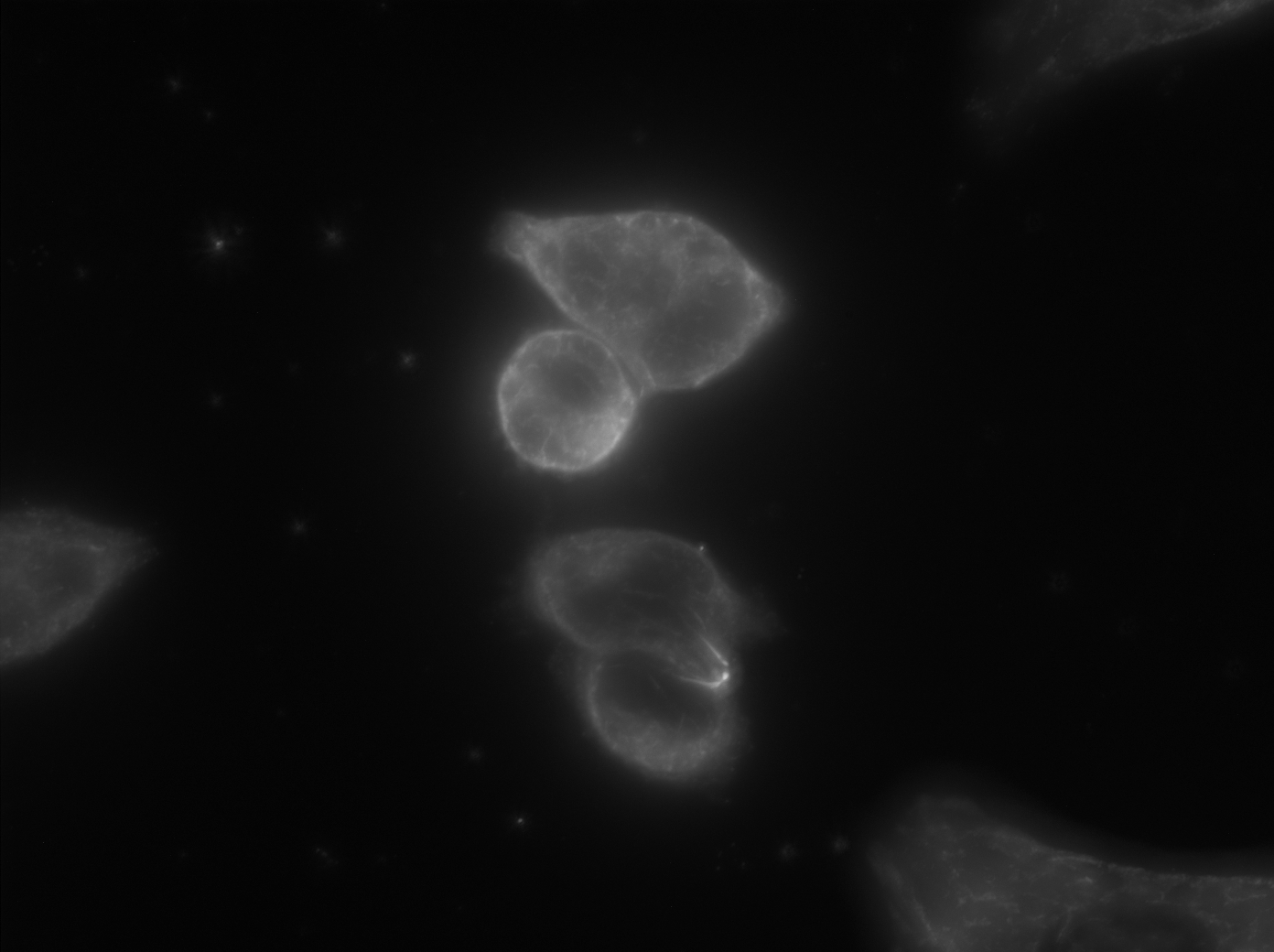

Supplement: Supplementary file 23 — Source data Fig. 6 part 4 [file 44319_2026_742_MOESM23_ESM.zip › Figure 6 Part 4/Fig 6efg TPGS1-KO TPGS1 rescue experiments part 2/R2R3/TPGS1-KO EYFP-only actub 7-31-25 R2 LT7.Project Maximum Z_XY1756415824_Z0_T0_C2.tif]

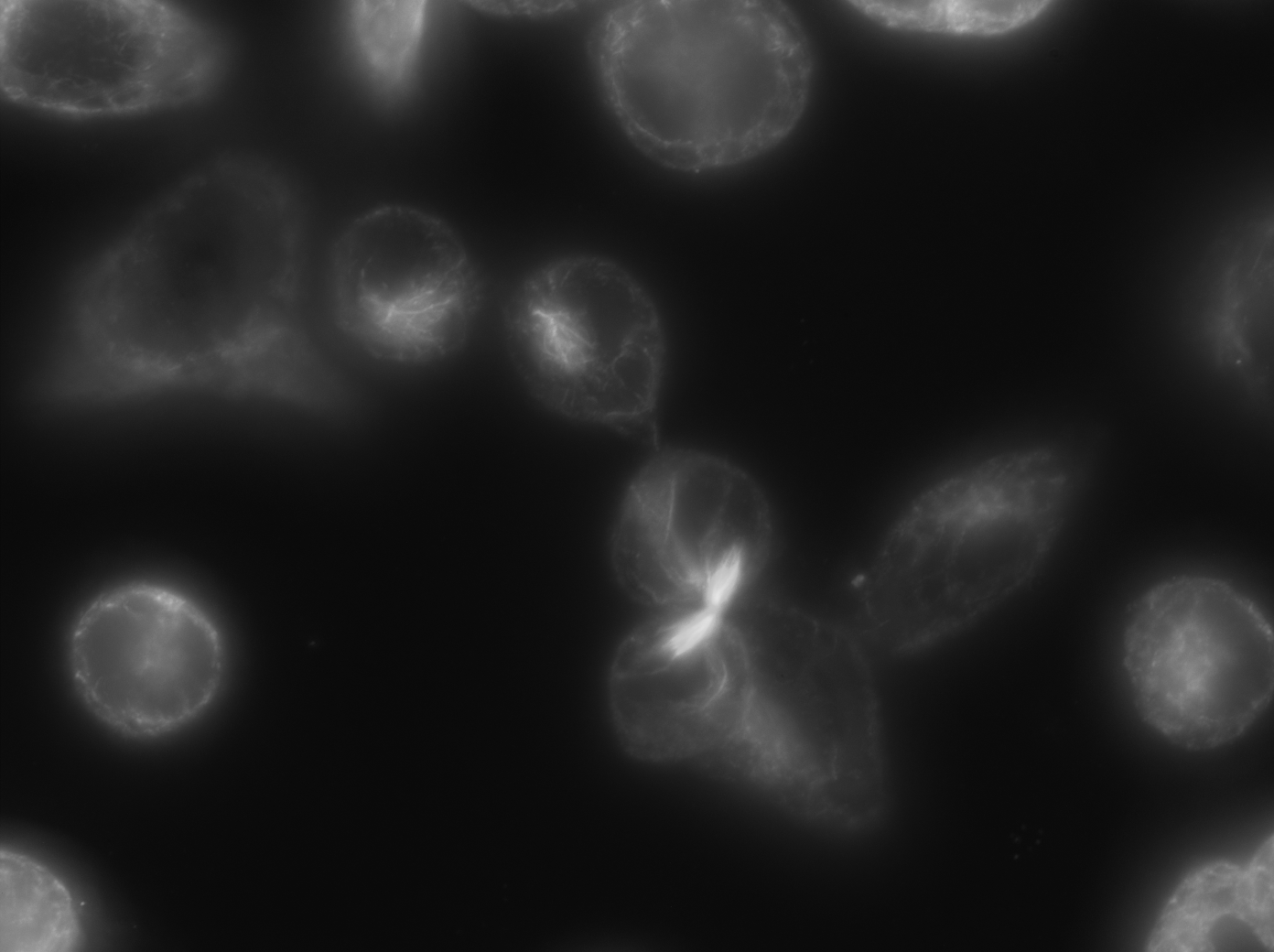

Supplement: Supplementary file 23 — Source data Fig. 6 part 4 [file 44319_2026_742_MOESM23_ESM.zip › Figure 6 Part 4/Fig 6efg TPGS1-KO TPGS1 rescue experiments part 2/R2R3/TPGS1-KO TPGS1-EYFP-3'UTR actub 7-31-25 R3 ET5.Project Maximum Z_XY1756501355_Z0_T0_C2.tif]

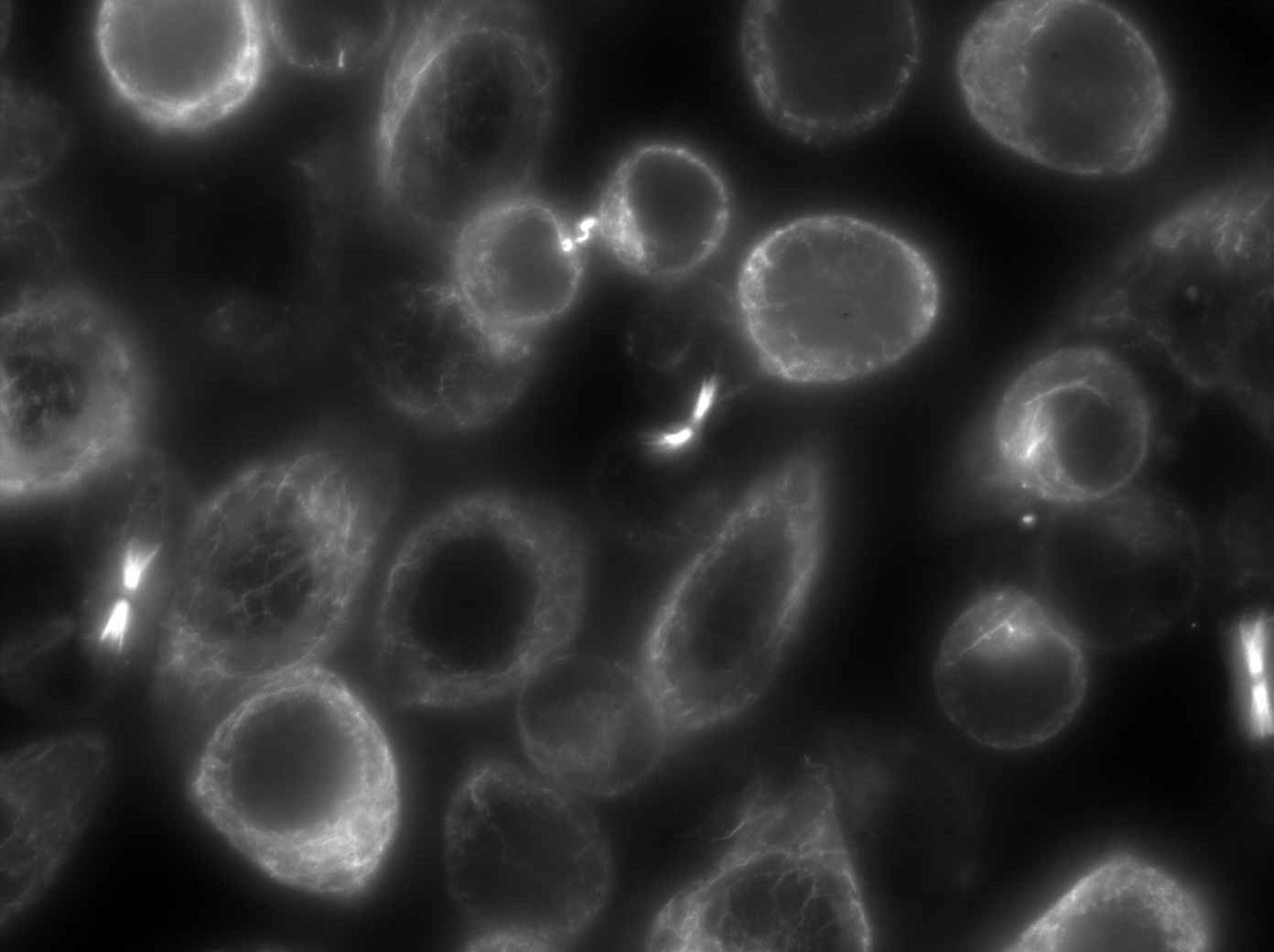

Supplement: Supplementary file 23 — Source data Fig. 6 part 4 [file 44319_2026_742_MOESM23_ESM.zip › Figure 6 Part 4/Fig 6efg TPGS1-KO TPGS1 rescue experiments part 2/R2R3/TPGS1-KO TPGS1-EYFP-3'UTR actub 7-31-25 R2 ET2.Project Maximum Z_XY1756407950_Z0_T0_C2.tif]

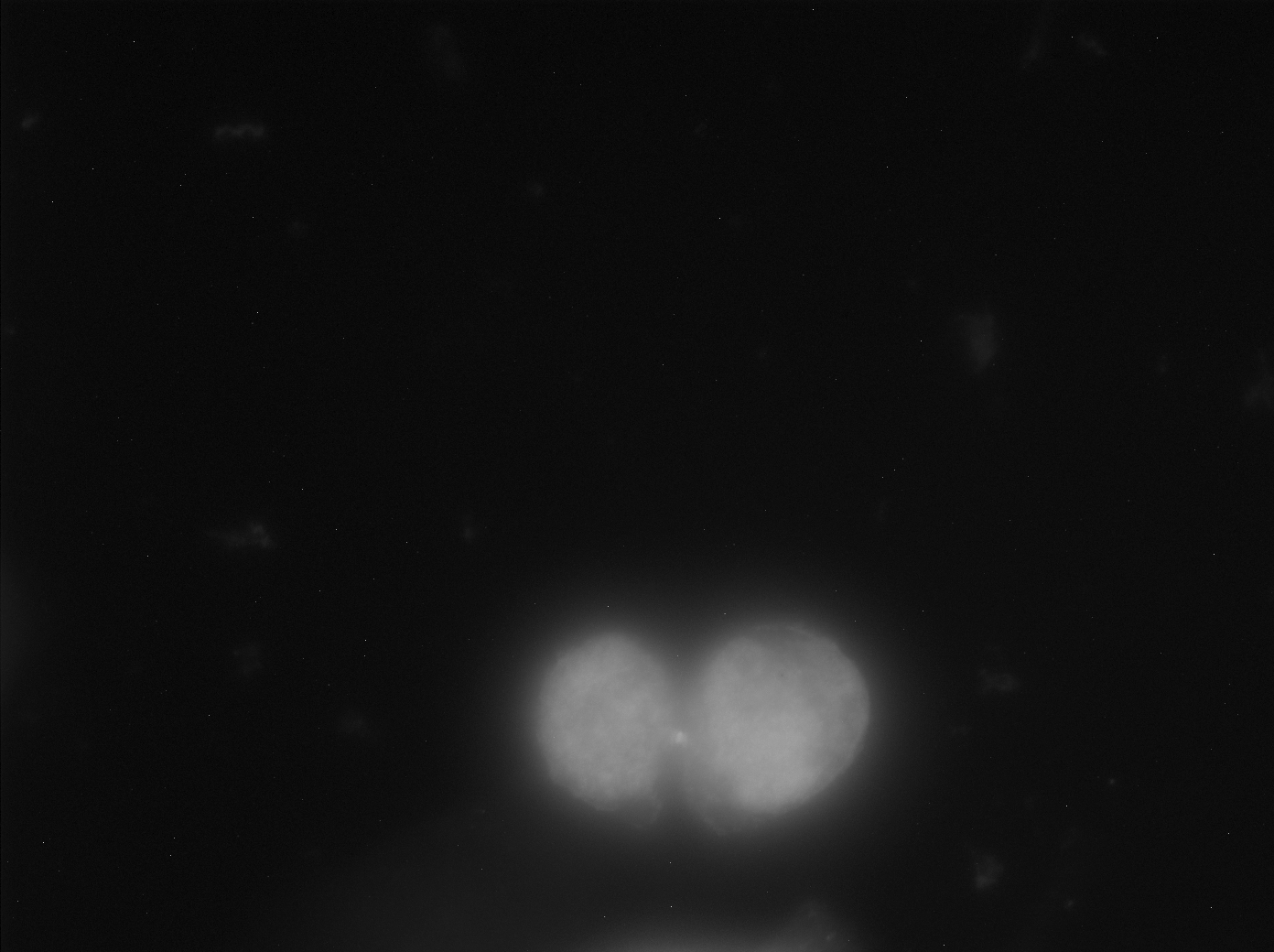

Supplement: Supplementary file 23 — Source data Fig. 6 part 4 [file 44319_2026_742_MOESM23_ESM.zip › Figure 6 Part 4/Fig 6efg TPGS1-KO TPGS1 rescue experiments part 2/R2R3/TPGS1-KO EYFP-only actub 7-31-25 R2 ET3.Project Maximum Z_XY1756414255_Z0_T0_C1.tif]

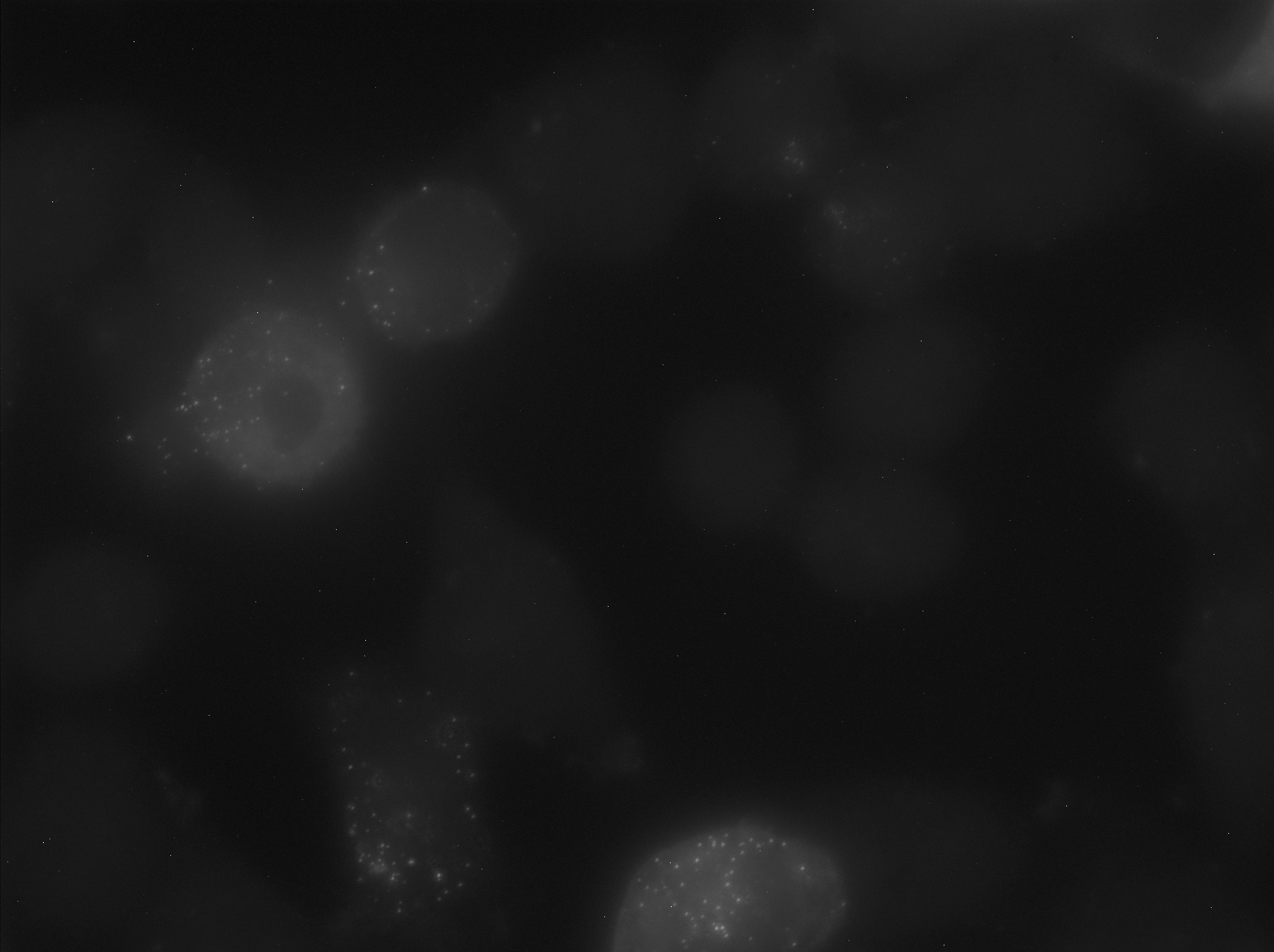

Supplement: Supplementary file 23 — Source data Fig. 6 part 4 [file 44319_2026_742_MOESM23_ESM.zip › Figure 6 Part 4/Fig 6efg TPGS1-KO TPGS1 rescue experiments part 2/R2R3/TPGS1-KO TPGS1-EYFP-3'UTR actub 7-31-25 R2 ET6.Project Maximum Z_XY1756411247_Z0_T0_C1.tif]

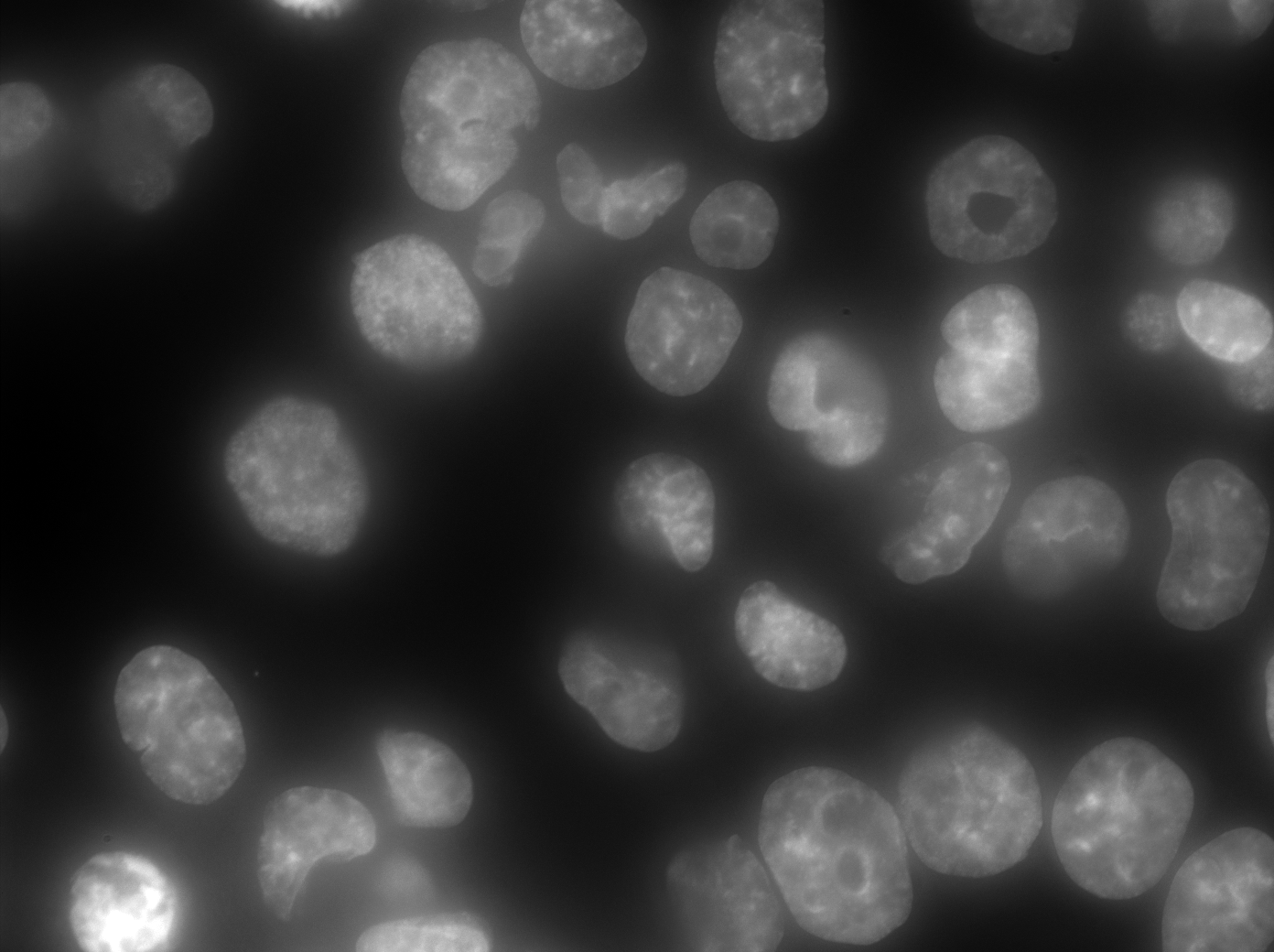

Supplement: Supplementary file 23 — Source data Fig. 6 part 4 [file 44319_2026_742_MOESM23_ESM.zip › Figure 6 Part 4/Fig 6efg TPGS1-KO TPGS1 rescue experiments part 2/R2R3/TPGS1-KO TPGS1-EYFP-3'UTR actub 7-31-25 R2 LT3.Project Maximum Z_XY1756407610_Z0_T0_C0.tif]

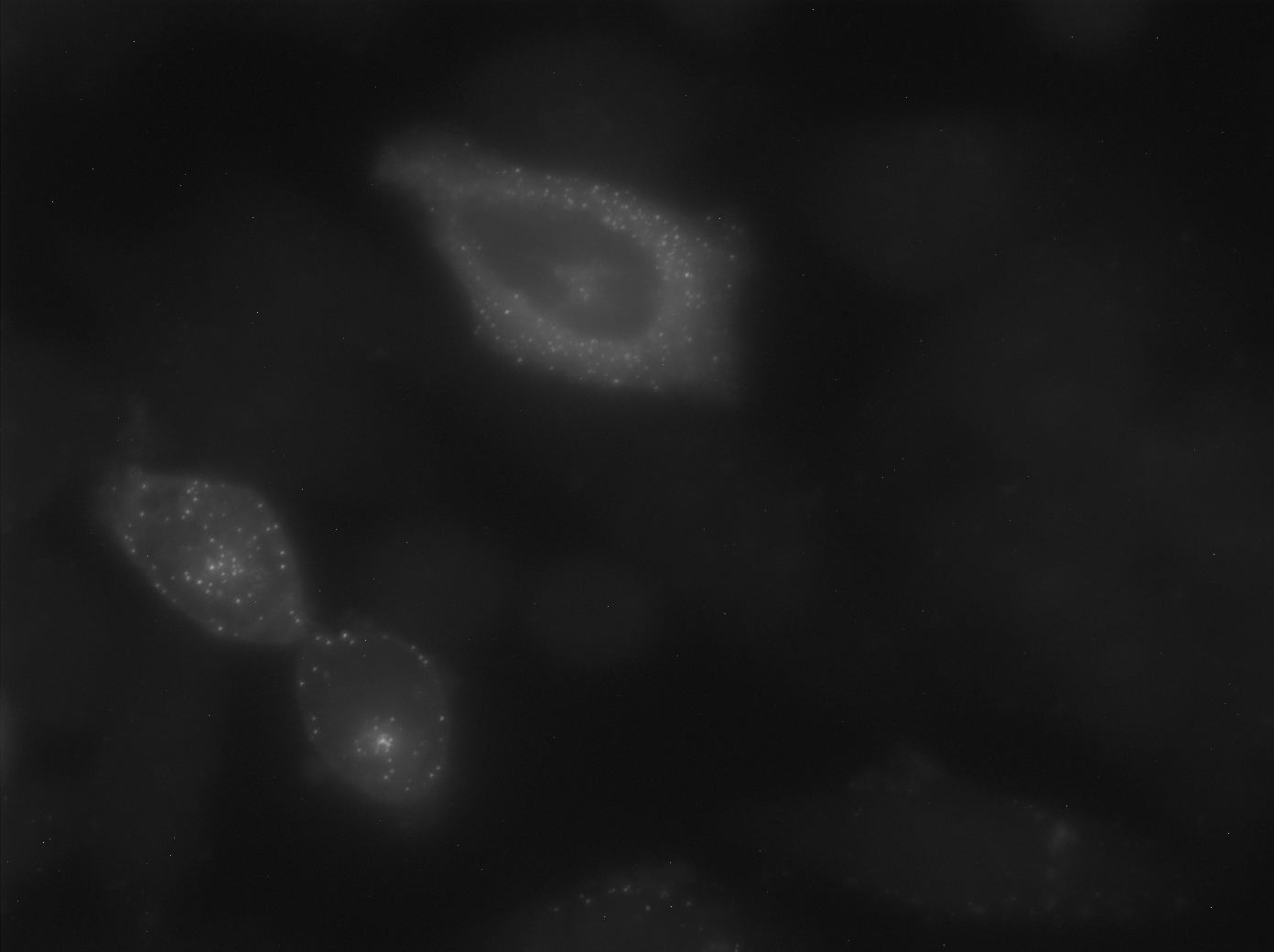

Supplement: Supplementary file 23 — Source data Fig. 6 part 4 [file 44319_2026_742_MOESM23_ESM.zip › Figure 6 Part 4/Fig 6efg TPGS1-KO TPGS1 rescue experiments part 2/R2R3/TPGS1-KO TPGS1-EYFP-3'UTR actub 7-31-25 R2 LT8.Project Maximum Z_XY1756411785_Z0_T0_C1.tif]
