# Supplementary figures and images for: TPGS1 regulates central spindle microtubule glutamylation and remodeling during telophase and abscission (part 26 of 36)
Source: EMBO Rep. 2026 Mar 23;27(8):1944–63. doi: 10.1038/s44319-026-00742-3 (PMC13121839; doi:10.1038/s44319-026-00742-3)

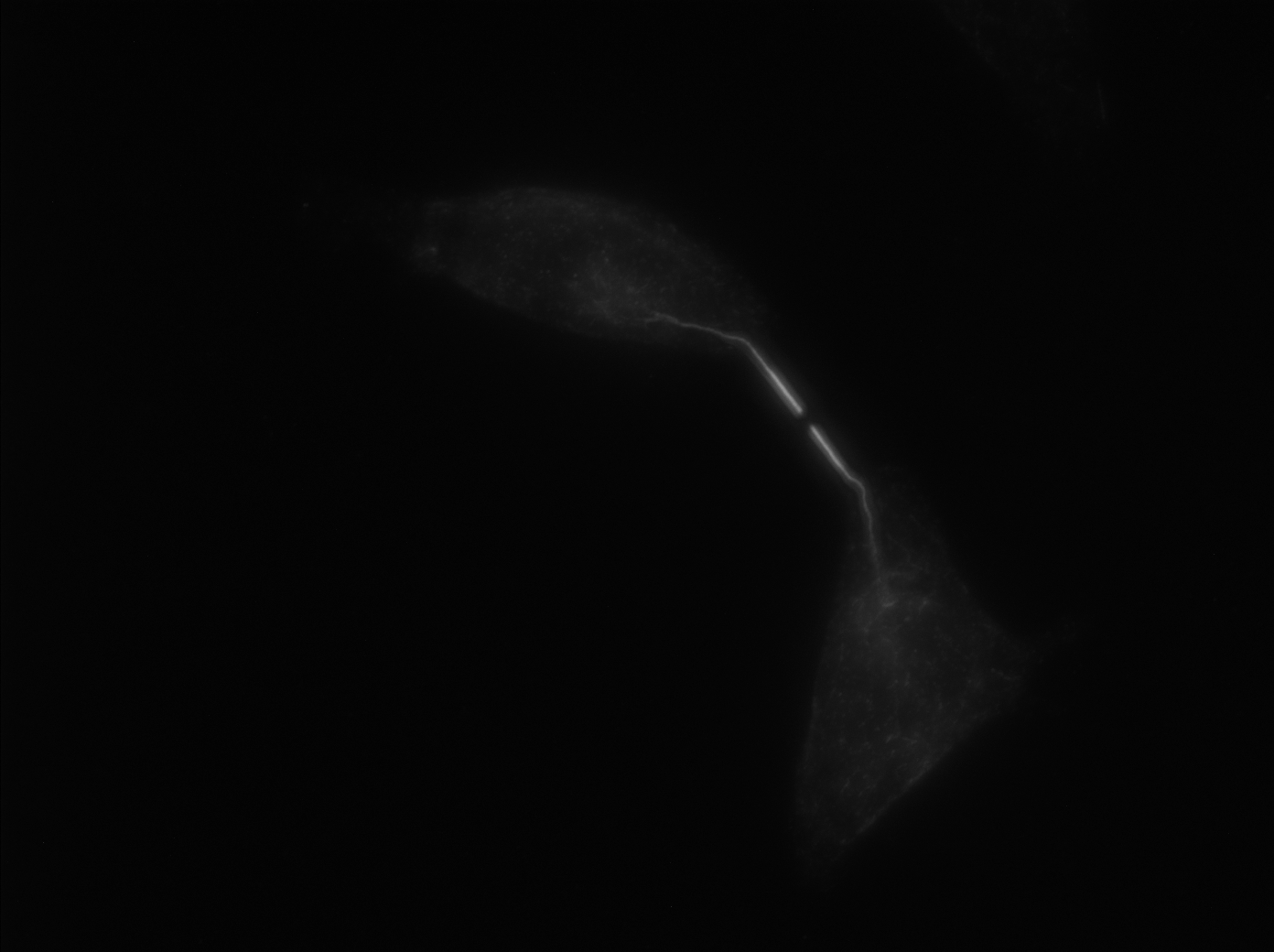

Supplement: Supplementary file 22 — Source data Fig. 6 part 3 [file 44319_2026_742_MOESM22_ESM.zip › Figure 6 Part 3/Fig 6efg TPGS1-KO TPGS1 rescue experiments/R1/TPGS1-KO TPGS1-3UTR-EYFP actub R1 7-31-25 LT6.Project Maximum Z_XY1753988729_Z0_T0_C2.tif]

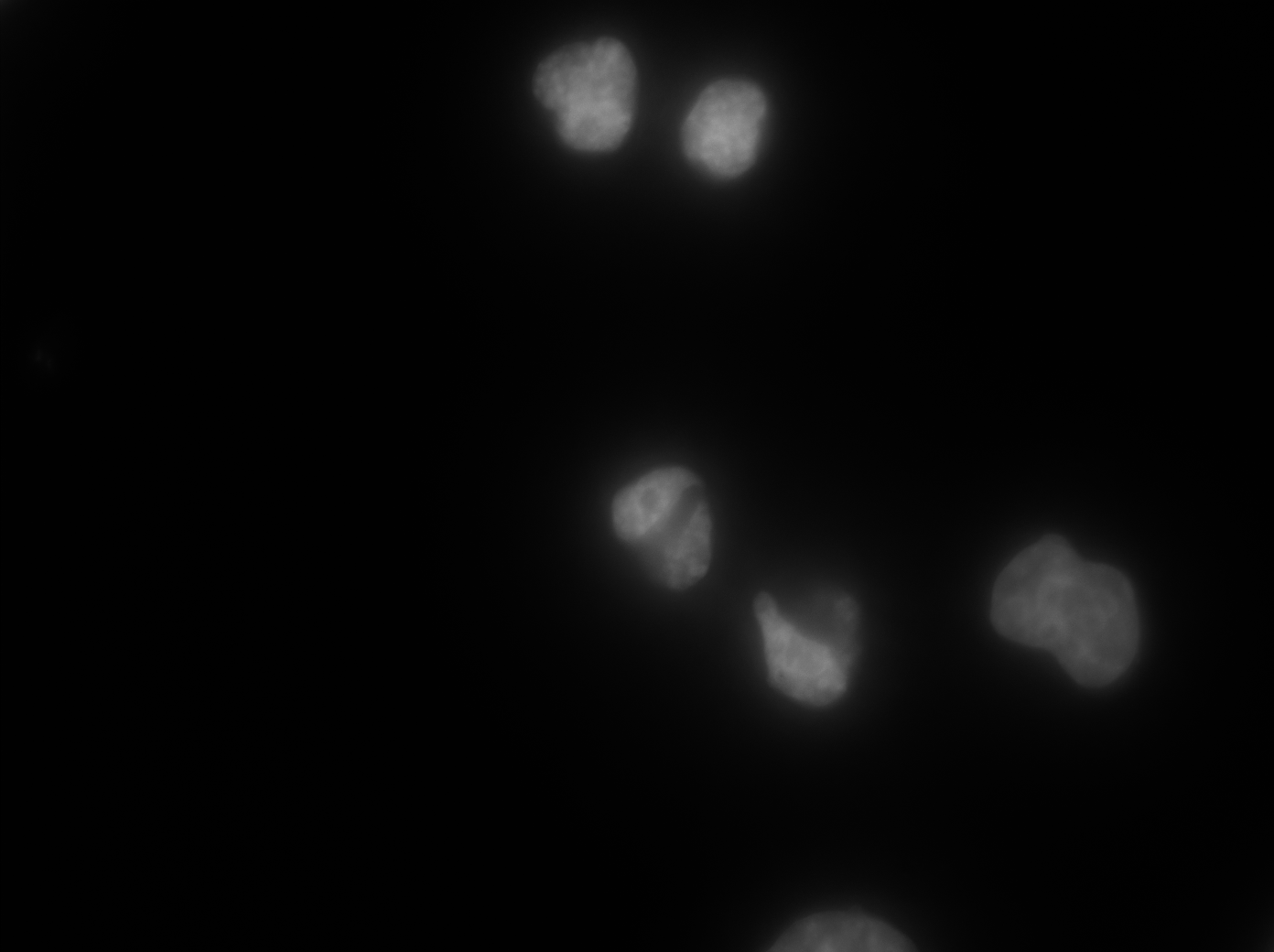

Supplement: Supplementary file 22 — Source data Fig. 6 part 3 [file 44319_2026_742_MOESM22_ESM.zip › Figure 6 Part 3/Fig 6efg TPGS1-KO TPGS1 rescue experiments/R1/TPGS1-KO TPGS1-3UTR-EYFP actub R1 7-31-25 ET9.Project Maximum Z_XY1753992196_Z0_T0_C0.tif]

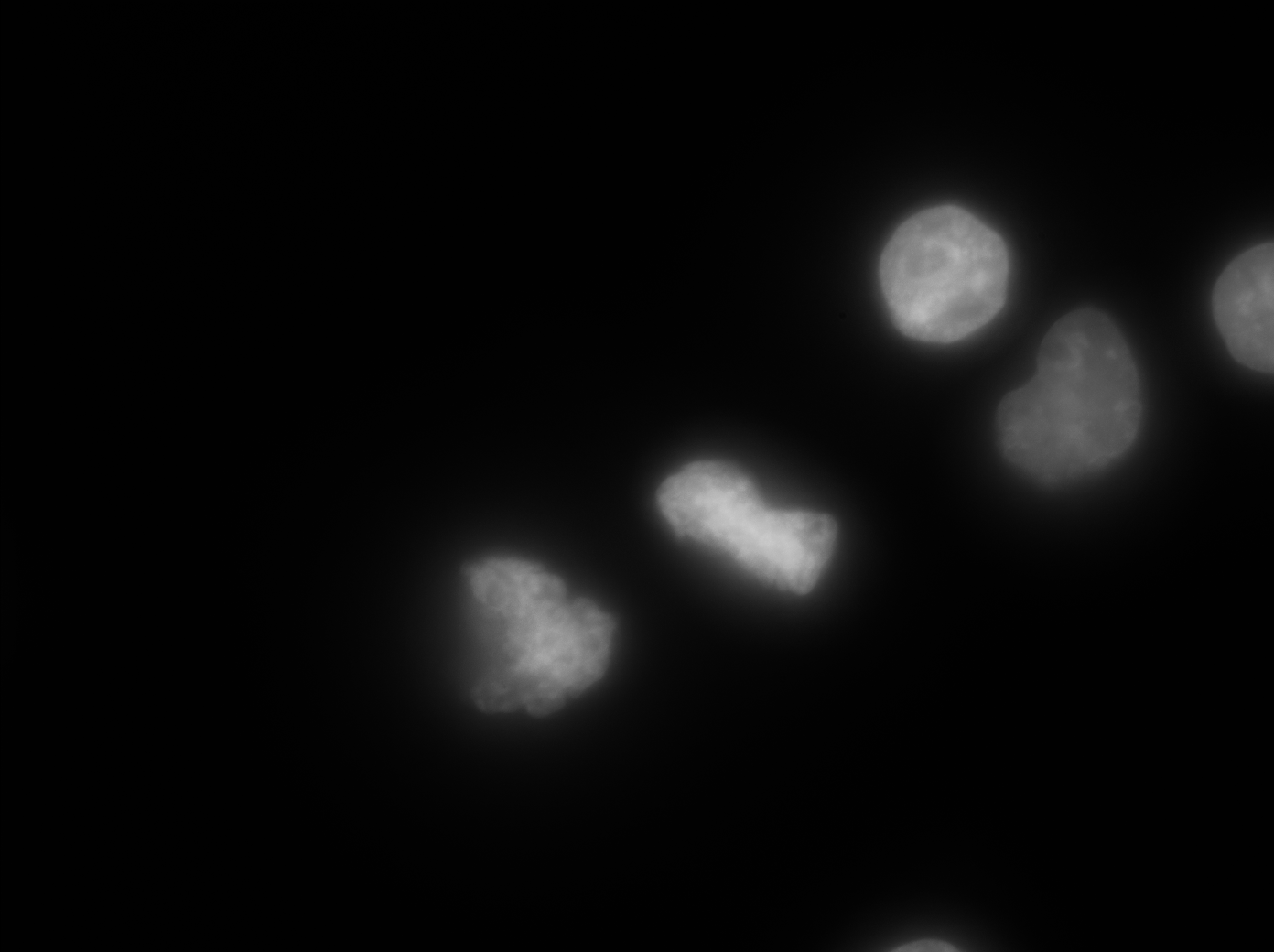

Supplement: Supplementary file 22 — Source data Fig. 6 part 3 [file 44319_2026_742_MOESM22_ESM.zip › Figure 6 Part 3/Fig 6efg TPGS1-KO TPGS1 rescue experiments/R1/TPGS1-KO EYFP only actub R1 7-31-25 ET9.Project Maximum Z_XY1754339444_Z0_T0_C0.tif]

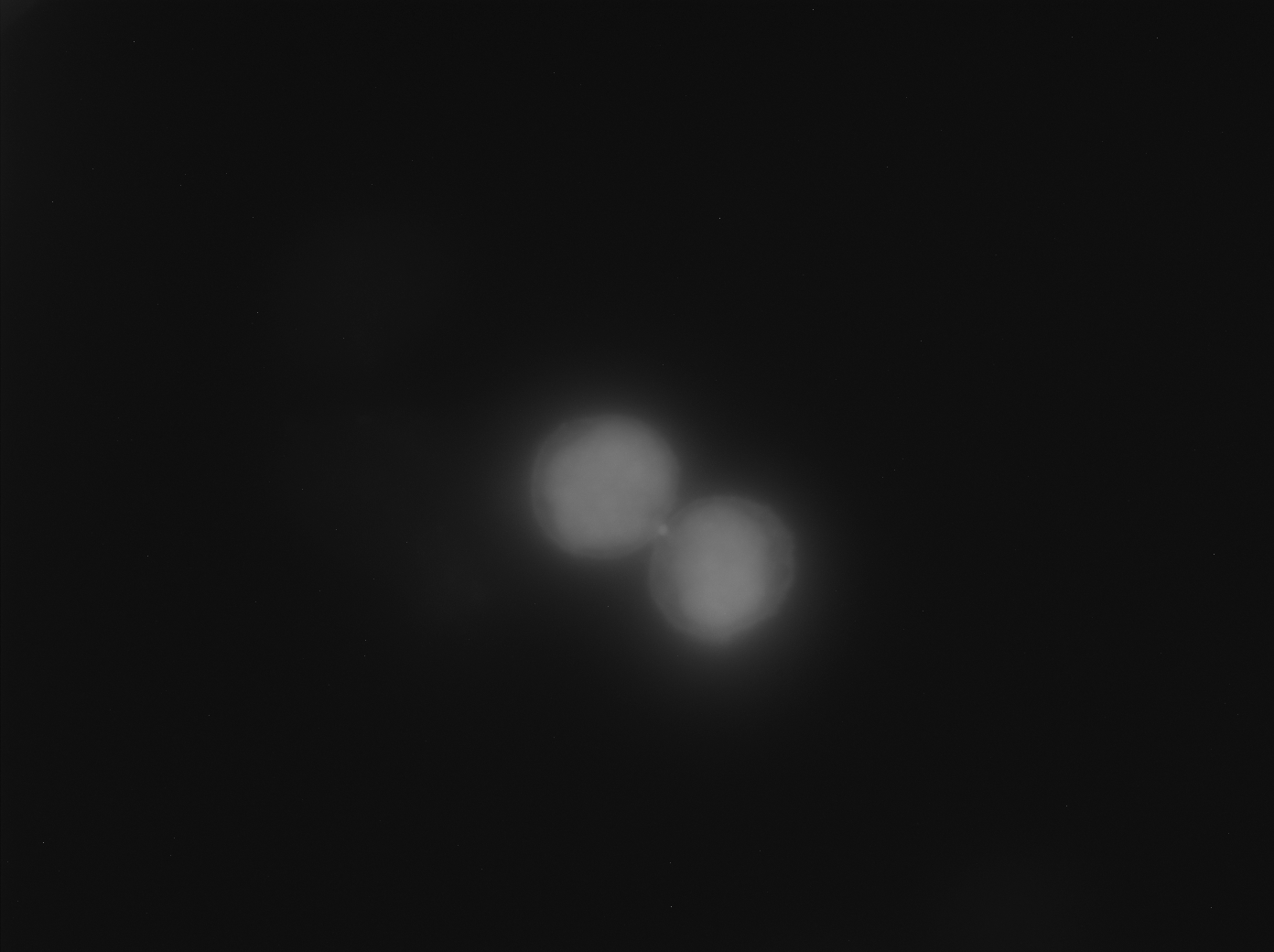

Supplement: Supplementary file 22 — Source data Fig. 6 part 3 [file 44319_2026_742_MOESM22_ESM.zip › Figure 6 Part 3/Fig 6efg TPGS1-KO TPGS1 rescue experiments/R1/TPGS1-KO EYFP only actub R1 7-31-25 ET8.Project Maximum Z_XY1754339329_Z0_T0_C1.tif]

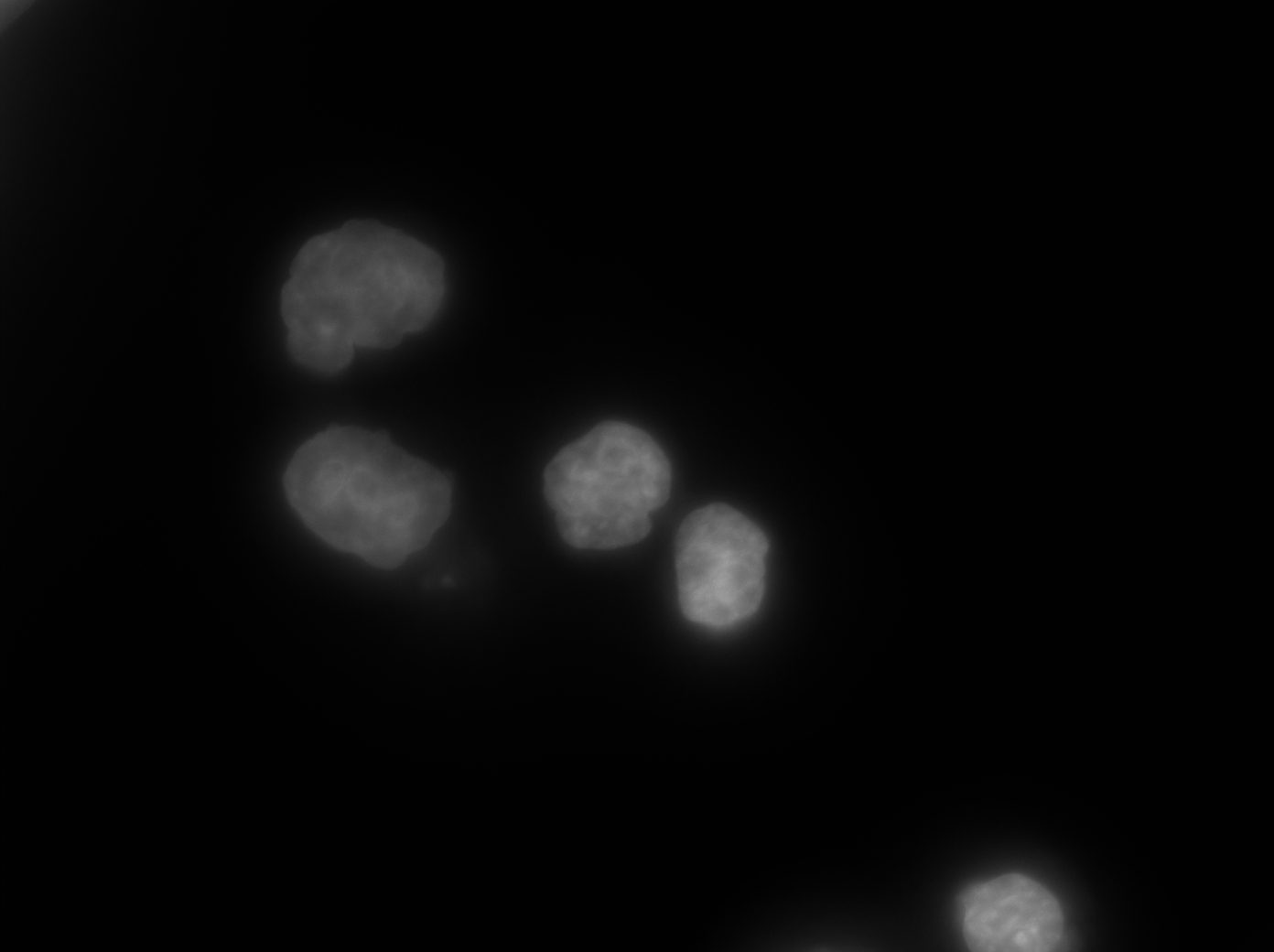

Supplement: Supplementary file 22 — Source data Fig. 6 part 3 [file 44319_2026_742_MOESM22_ESM.zip › Figure 6 Part 3/Fig 6efg TPGS1-KO TPGS1 rescue experiments/R1/TPGS1-KO EYFP only actub R1 7-31-25 ET8.Project Maximum Z_XY1754339329_Z0_T0_C0.tif]

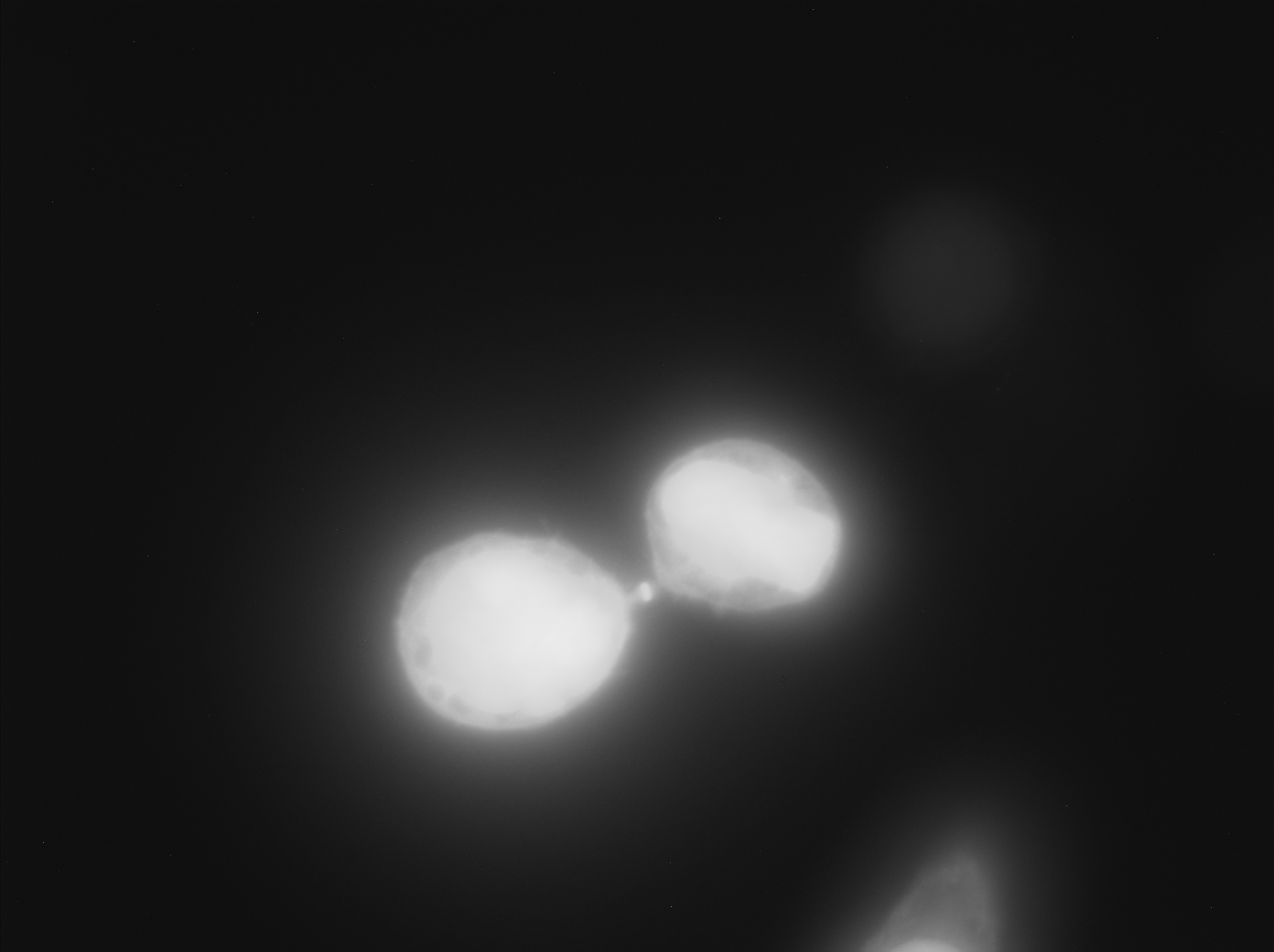

Supplement: Supplementary file 22 — Source data Fig. 6 part 3 [file 44319_2026_742_MOESM22_ESM.zip › Figure 6 Part 3/Fig 6efg TPGS1-KO TPGS1 rescue experiments/R1/TPGS1-KO EYFP only actub R1 7-31-25 ET9.Project Maximum Z_XY1754339444_Z0_T0_C1.tif]

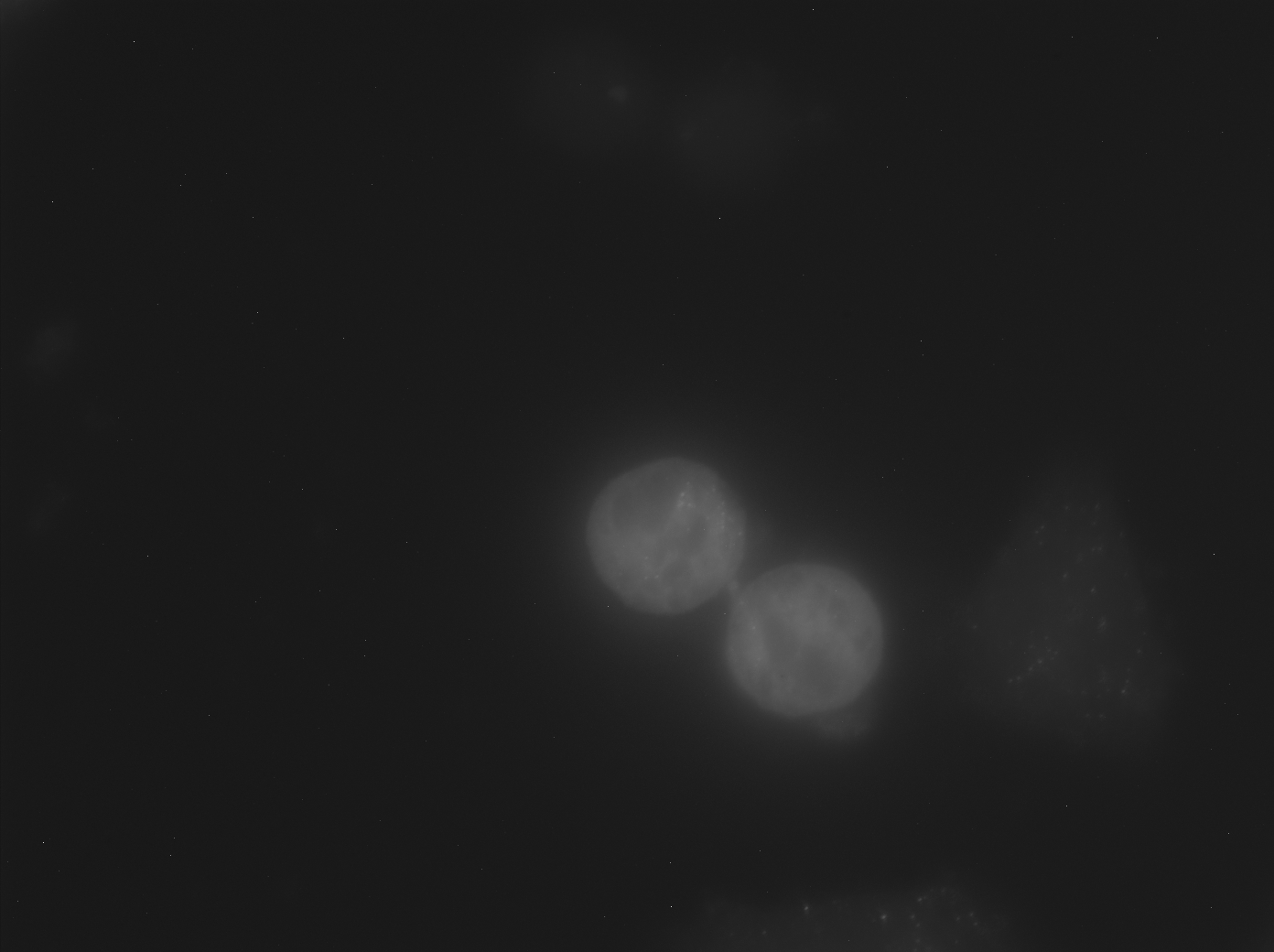

Supplement: Supplementary file 22 — Source data Fig. 6 part 3 [file 44319_2026_742_MOESM22_ESM.zip › Figure 6 Part 3/Fig 6efg TPGS1-KO TPGS1 rescue experiments/R1/TPGS1-KO TPGS1-3UTR-EYFP actub R1 7-31-25 ET9.Project Maximum Z_XY1753992196_Z0_T0_C1.tif]

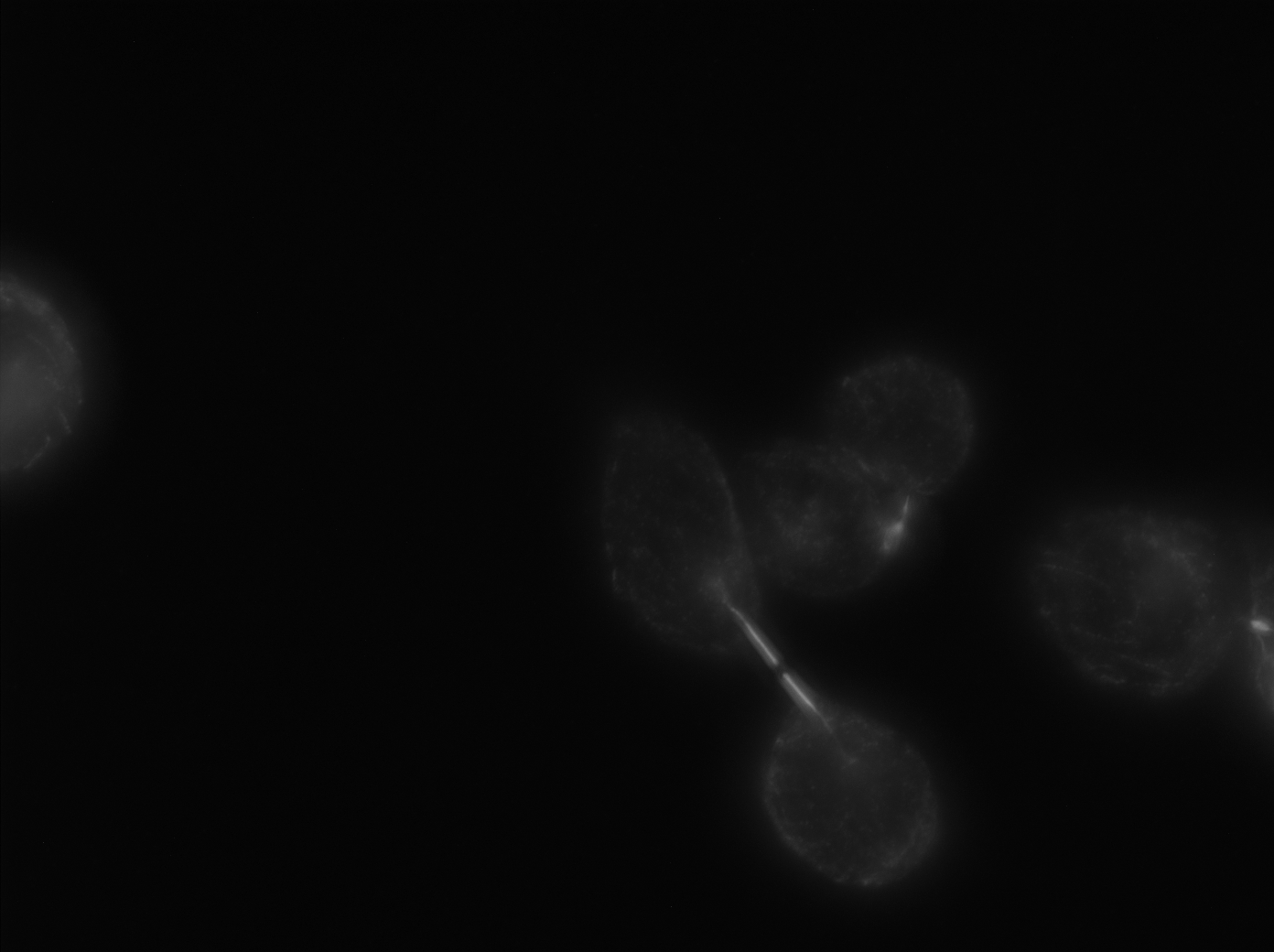

Supplement: Supplementary file 22 — Source data Fig. 6 part 3 [file 44319_2026_742_MOESM22_ESM.zip › Figure 6 Part 3/Fig 6efg TPGS1-KO TPGS1 rescue experiments/R1/TPGS1-KO TPGS1-3UTR-EYFP actub R1 7-31-25 LT10.Project Maximum Z_XY1753992791_Z0_T0_C2.tif]

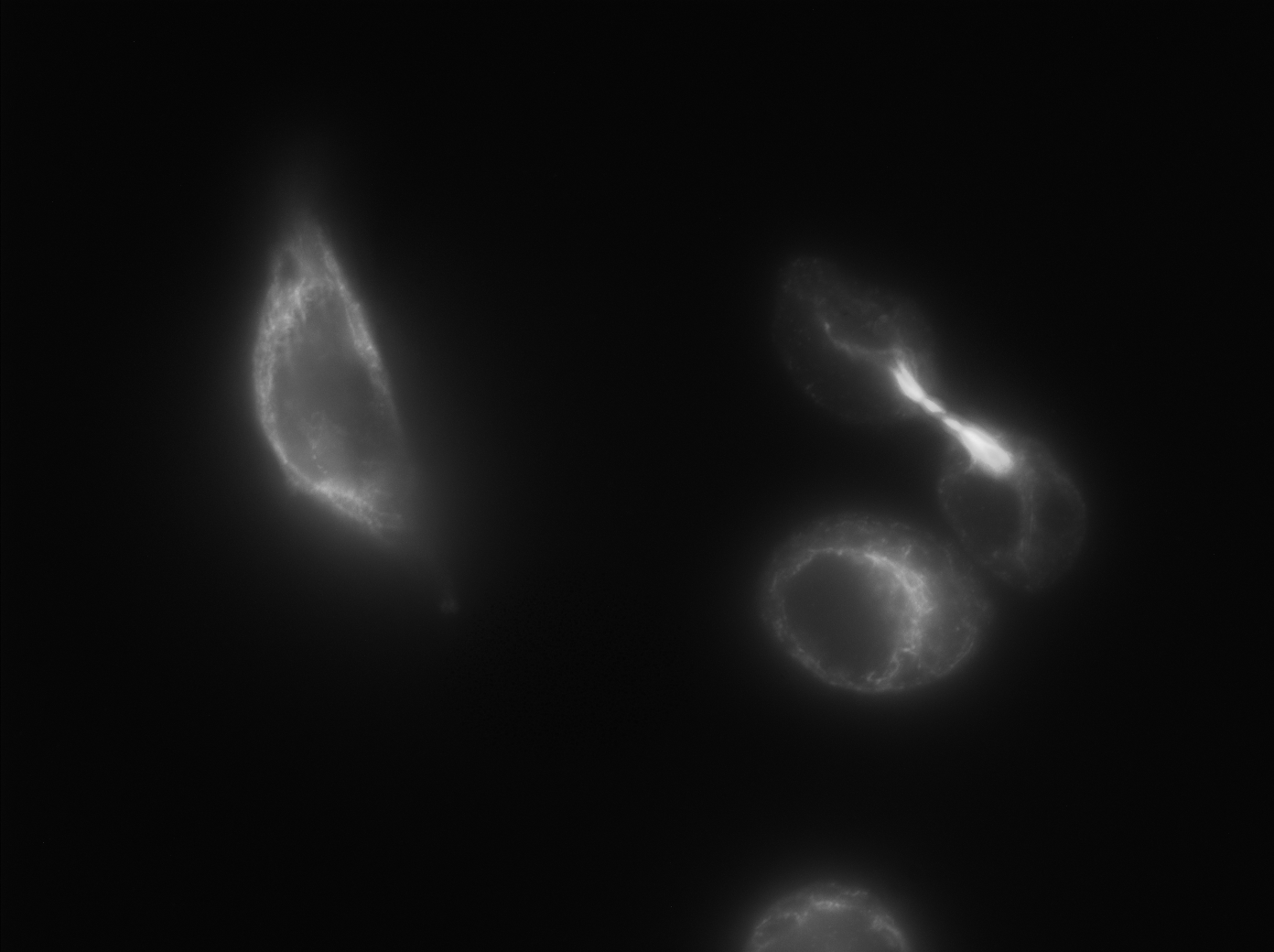

Supplement: Supplementary file 22 — Source data Fig. 6 part 3 [file 44319_2026_742_MOESM22_ESM.zip › Figure 6 Part 3/Fig 6efg TPGS1-KO TPGS1 rescue experiments/R1/TPGS1-KO EYFP only actub R1 7-31-25 LT4.Project Maximum Z_XY1754336072_Z0_T0_C2.tif]

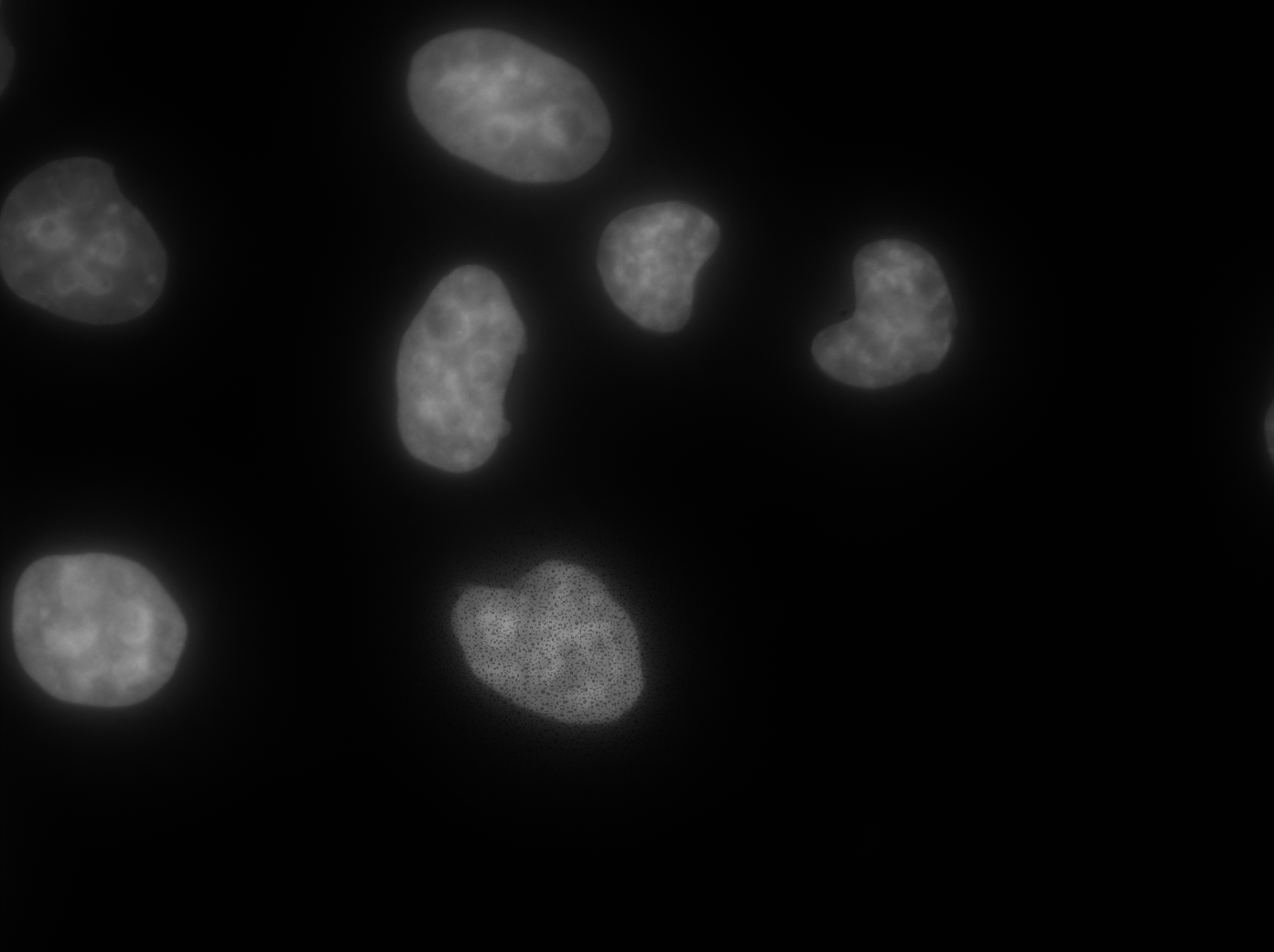

Supplement: Supplementary file 22 — Source data Fig. 6 part 3 [file 44319_2026_742_MOESM22_ESM.zip › Figure 6 Part 3/Fig 6efg TPGS1-KO TPGS1 rescue experiments/R1/TPGS1-KO TPGS1-3UTR-EYFP actub R1 7-31-25 ET1.Project Maximum Z_XY1753981618_Z0_T0_C0.tif]

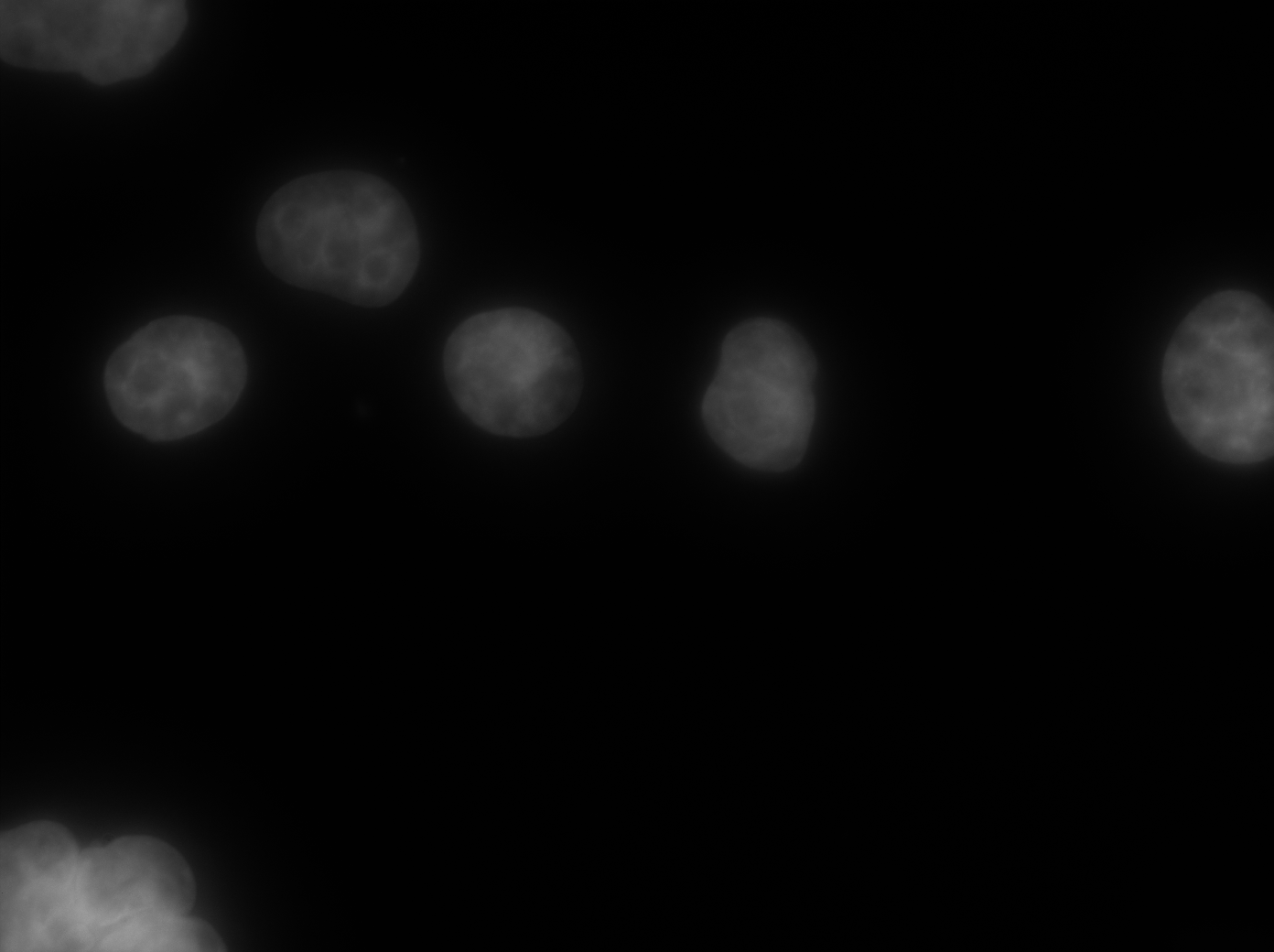

Supplement: Supplementary file 22 — Source data Fig. 6 part 3 [file 44319_2026_742_MOESM22_ESM.zip › Figure 6 Part 3/Fig 6efg TPGS1-KO TPGS1 rescue experiments/R1/TPGS1-KO TPGS1-3UTR-EYFP actub R1 7-31-25 LT7.Project Maximum Z_XY1753989093_Z0_T0_C0.tif]

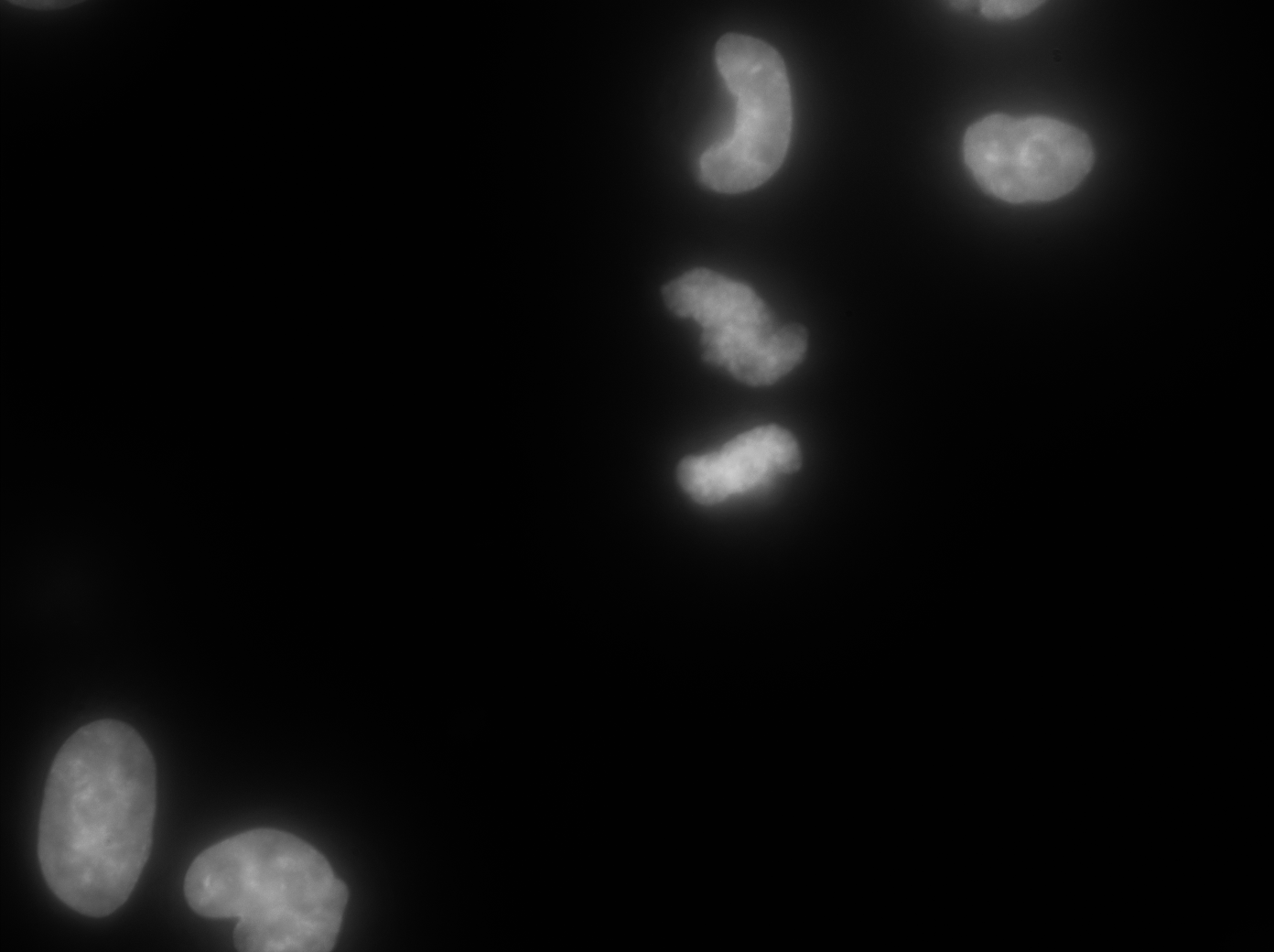

Supplement: Supplementary file 22 — Source data Fig. 6 part 3 [file 44319_2026_742_MOESM22_ESM.zip › Figure 6 Part 3/Fig 6efg TPGS1-KO TPGS1 rescue experiments/R1/TPGS1-KO EYFP only actub R1 7-31-25 ET6.Project Maximum Z_XY1754336574_Z0_T0_C0.tif]

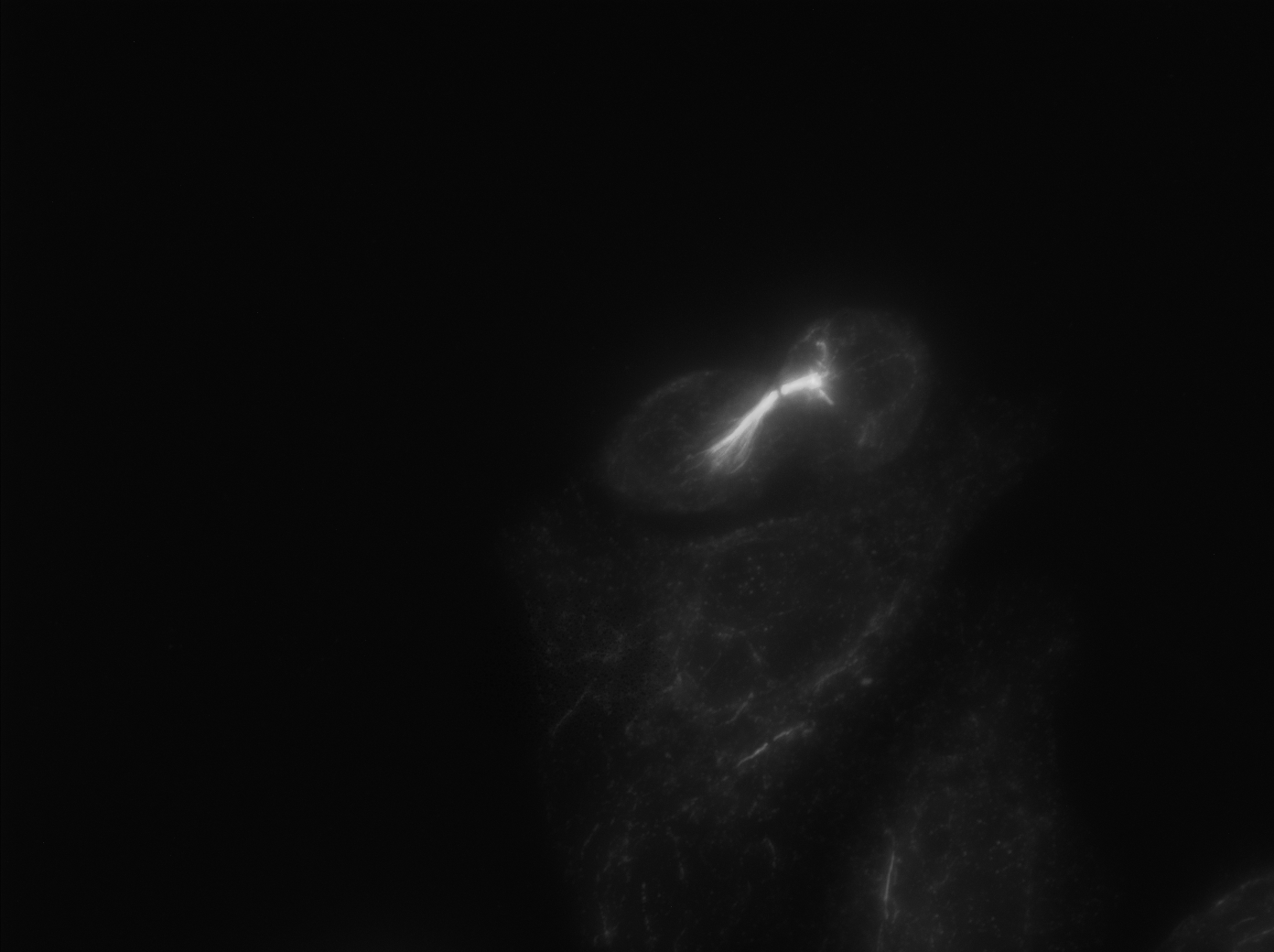

Supplement: Supplementary file 22 — Source data Fig. 6 part 3 [file 44319_2026_742_MOESM22_ESM.zip › Figure 6 Part 3/Fig 6efg TPGS1-KO TPGS1 rescue experiments/R1/TPGS1-KO EYFP only actub R1 7-31-25 LT5.Project Maximum Z_XY1754336364_Z0_T0_C2.tif]

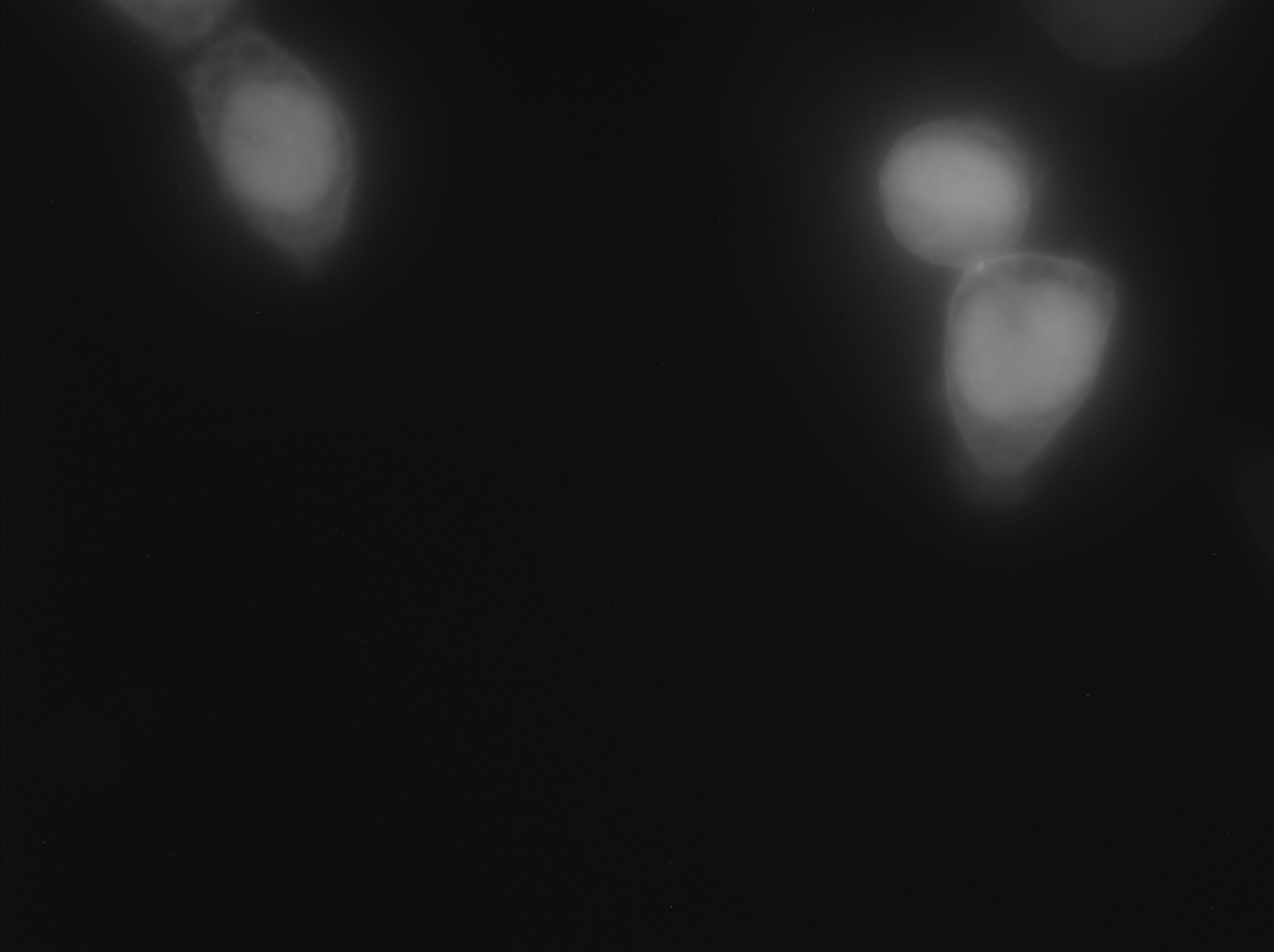

Supplement: Supplementary file 22 — Source data Fig. 6 part 3 [file 44319_2026_742_MOESM22_ESM.zip › Figure 6 Part 3/Fig 6efg TPGS1-KO TPGS1 rescue experiments/R1/TPGS1-KO EYFP only actub R1 7-31-25 LT9.Project Maximum Z_XY1754338968_Z0_T0_C1.tif]

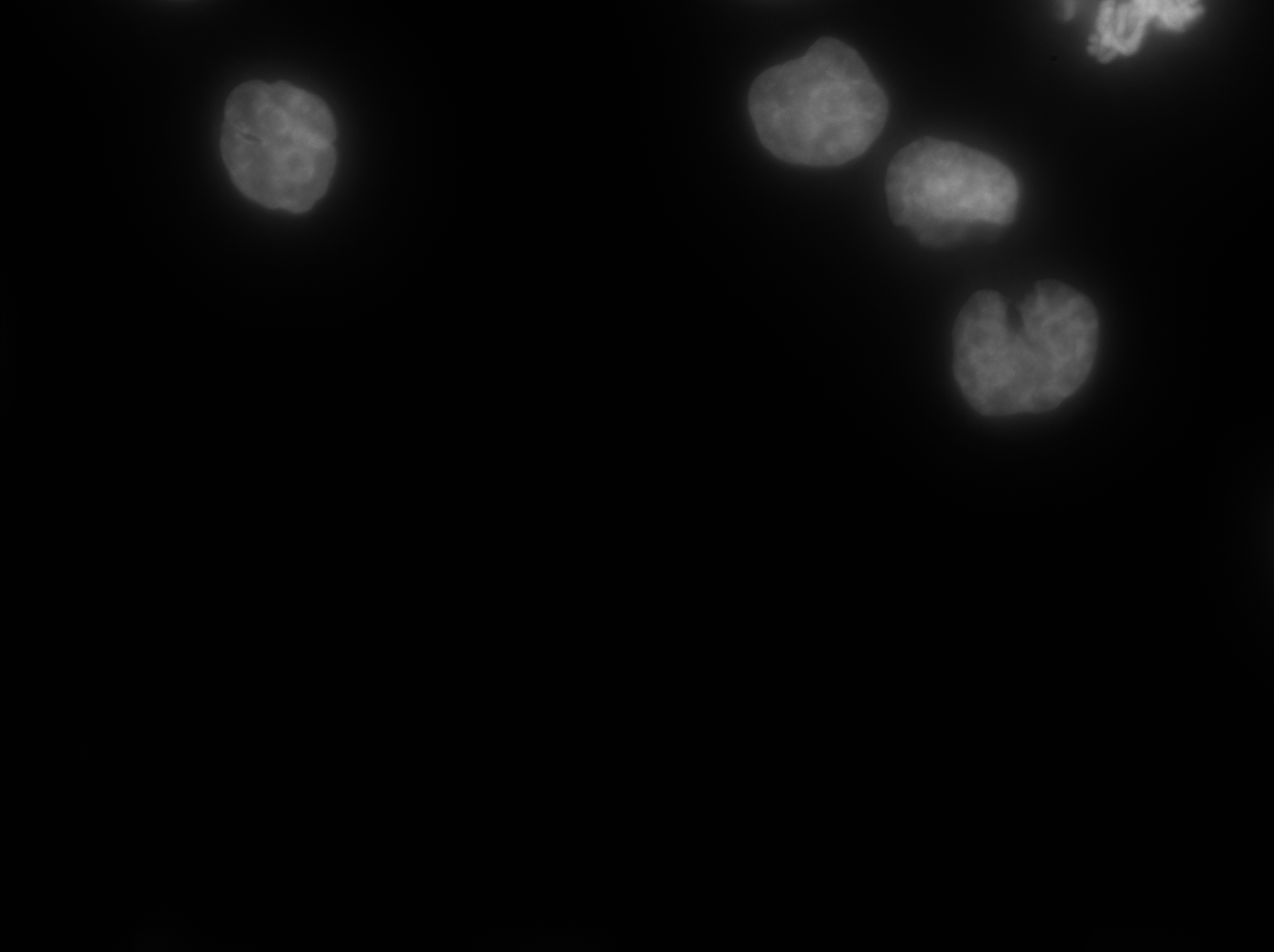

Supplement: Supplementary file 22 — Source data Fig. 6 part 3 [file 44319_2026_742_MOESM22_ESM.zip › Figure 6 Part 3/Fig 6efg TPGS1-KO TPGS1 rescue experiments/R1/TPGS1-KO EYFP only actub R1 7-31-25 LT9.Project Maximum Z_XY1754338968_Z0_T0_C0.tif]

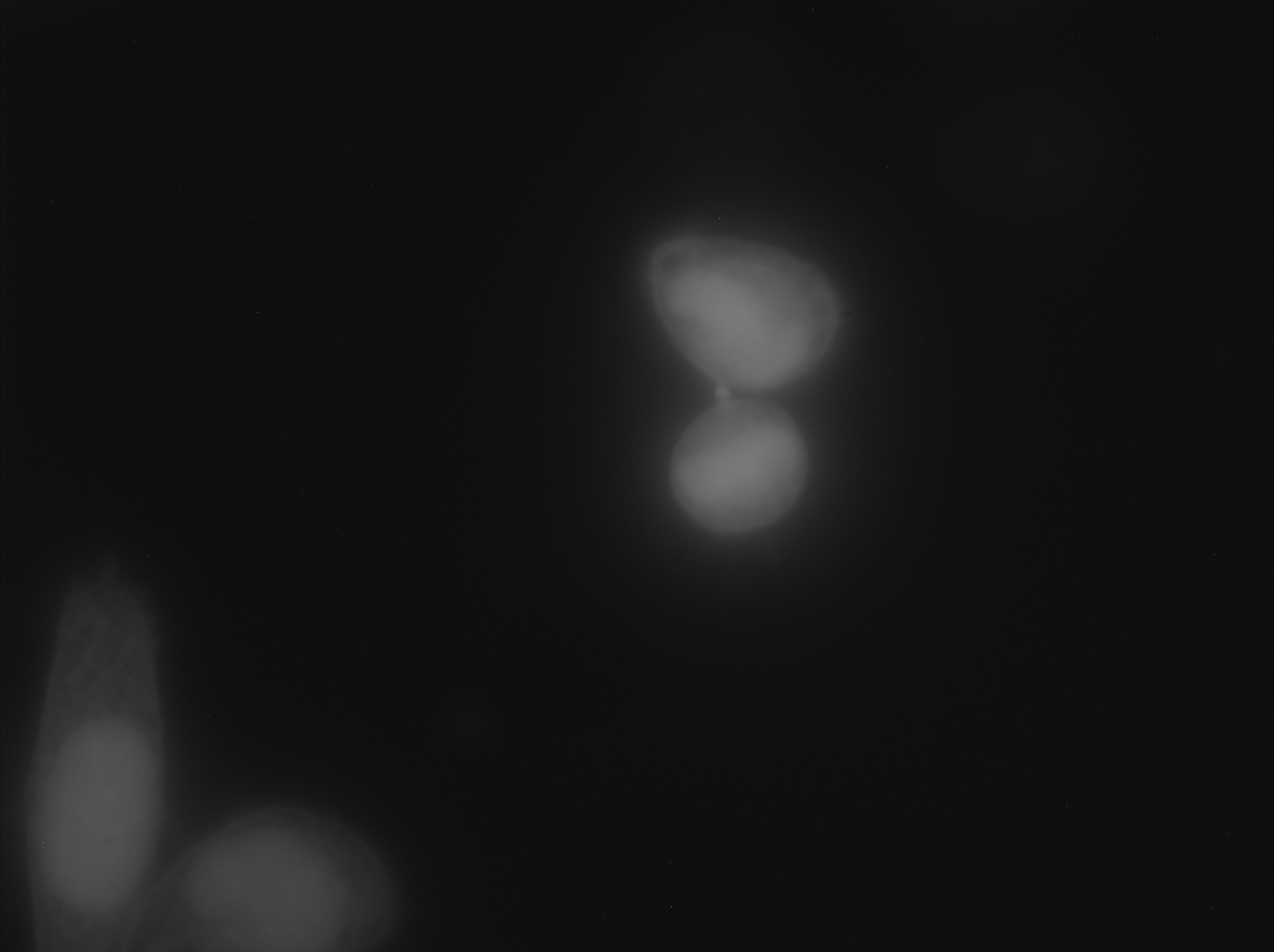

Supplement: Supplementary file 22 — Source data Fig. 6 part 3 [file 44319_2026_742_MOESM22_ESM.zip › Figure 6 Part 3/Fig 6efg TPGS1-KO TPGS1 rescue experiments/R1/TPGS1-KO EYFP only actub R1 7-31-25 ET6.Project Maximum Z_XY1754336574_Z0_T0_C1.tif]

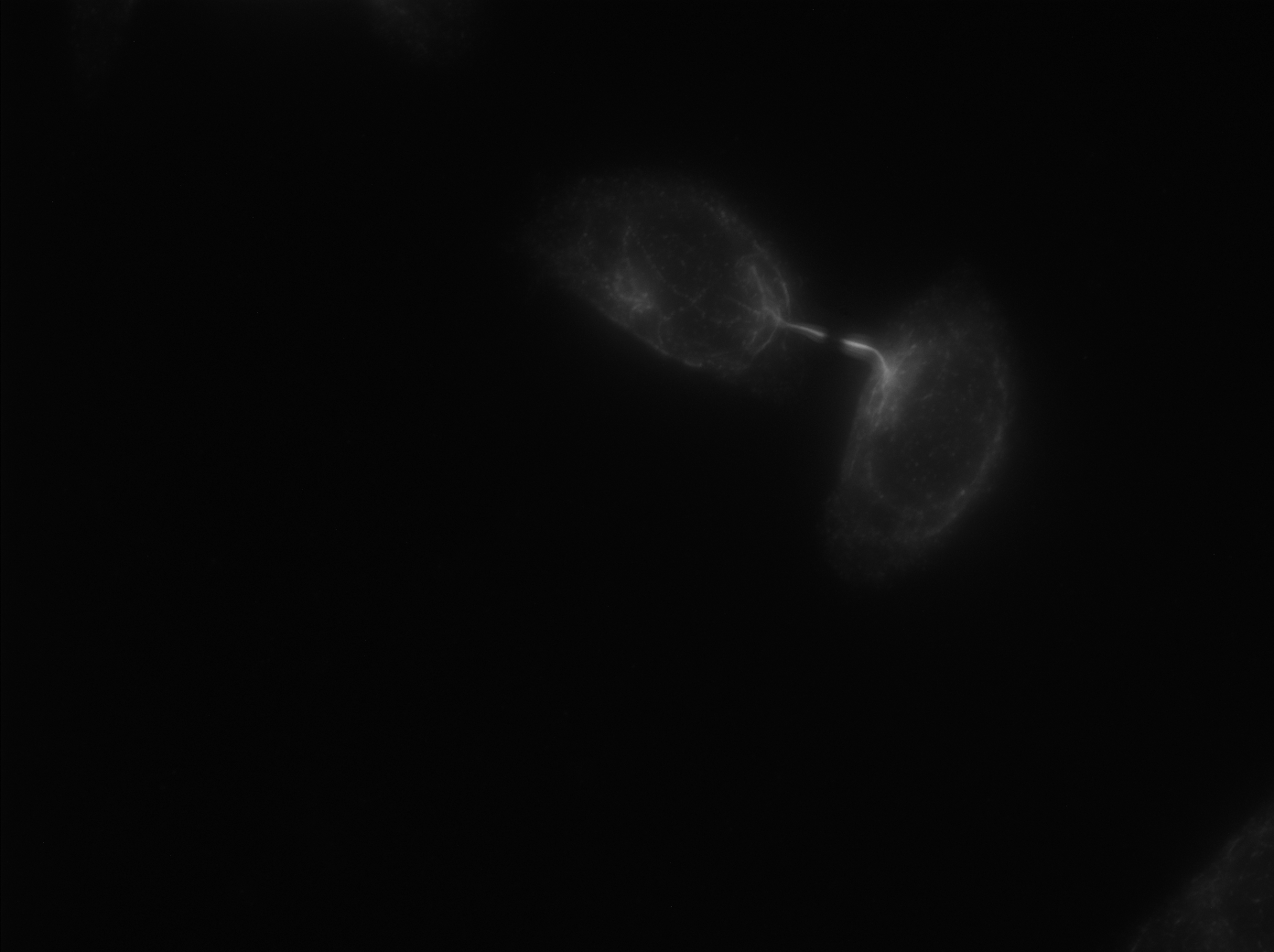

Supplement: Supplementary file 22 — Source data Fig. 6 part 3 [file 44319_2026_742_MOESM22_ESM.zip › Figure 6 Part 3/Fig 6efg TPGS1-KO TPGS1 rescue experiments/R1/TPGS1-KO TPGS1-3UTR-EYFP actub R1 7-31-25 LT2.Project Maximum Z_XY1753987675_Z0_T0_C2.tif]

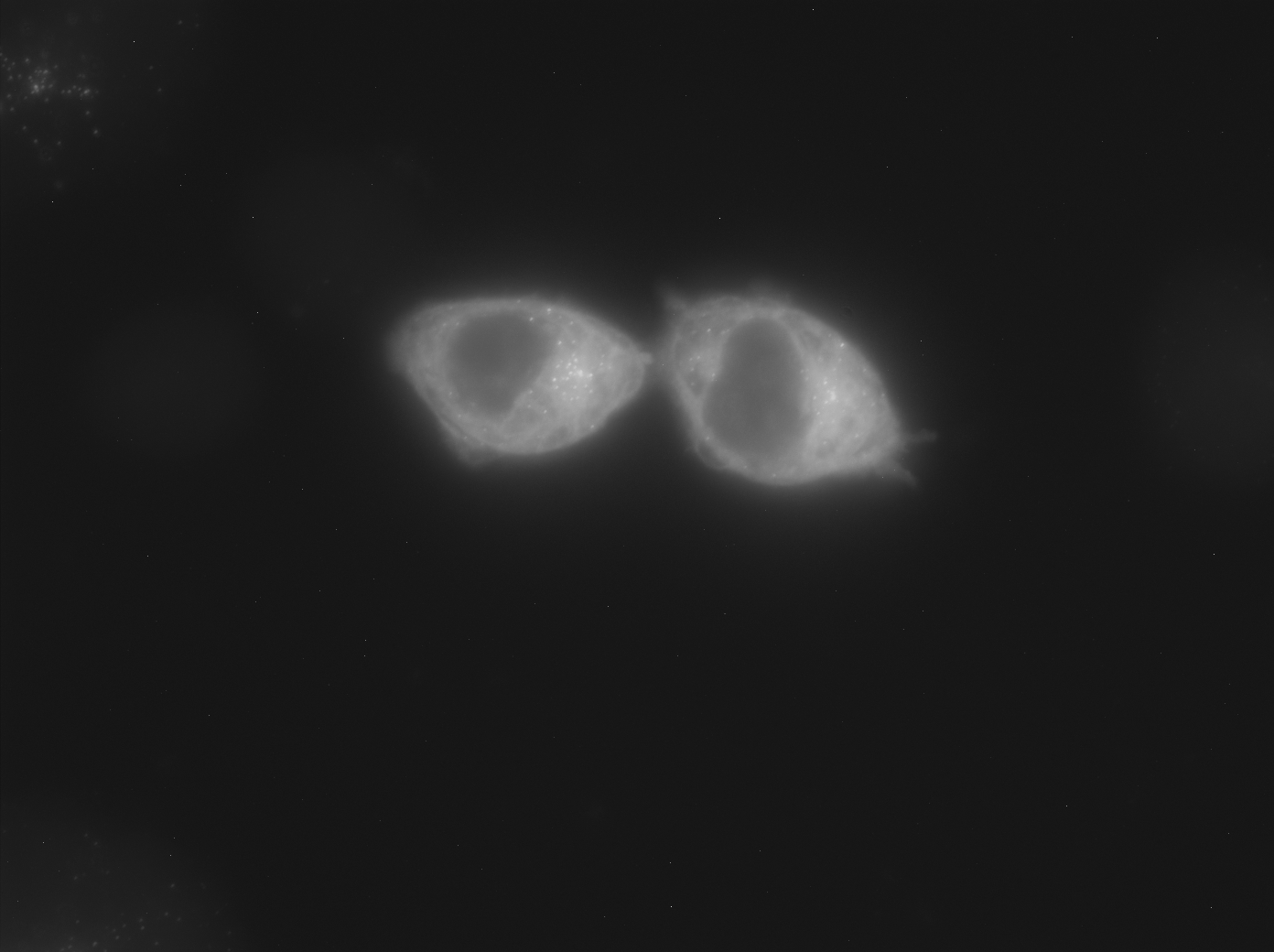

Supplement: Supplementary file 22 — Source data Fig. 6 part 3 [file 44319_2026_742_MOESM22_ESM.zip › Figure 6 Part 3/Fig 6efg TPGS1-KO TPGS1 rescue experiments/R1/TPGS1-KO TPGS1-3UTR-EYFP actub R1 7-31-25 LT7.Project Maximum Z_XY1753989093_Z0_T0_C1.tif]

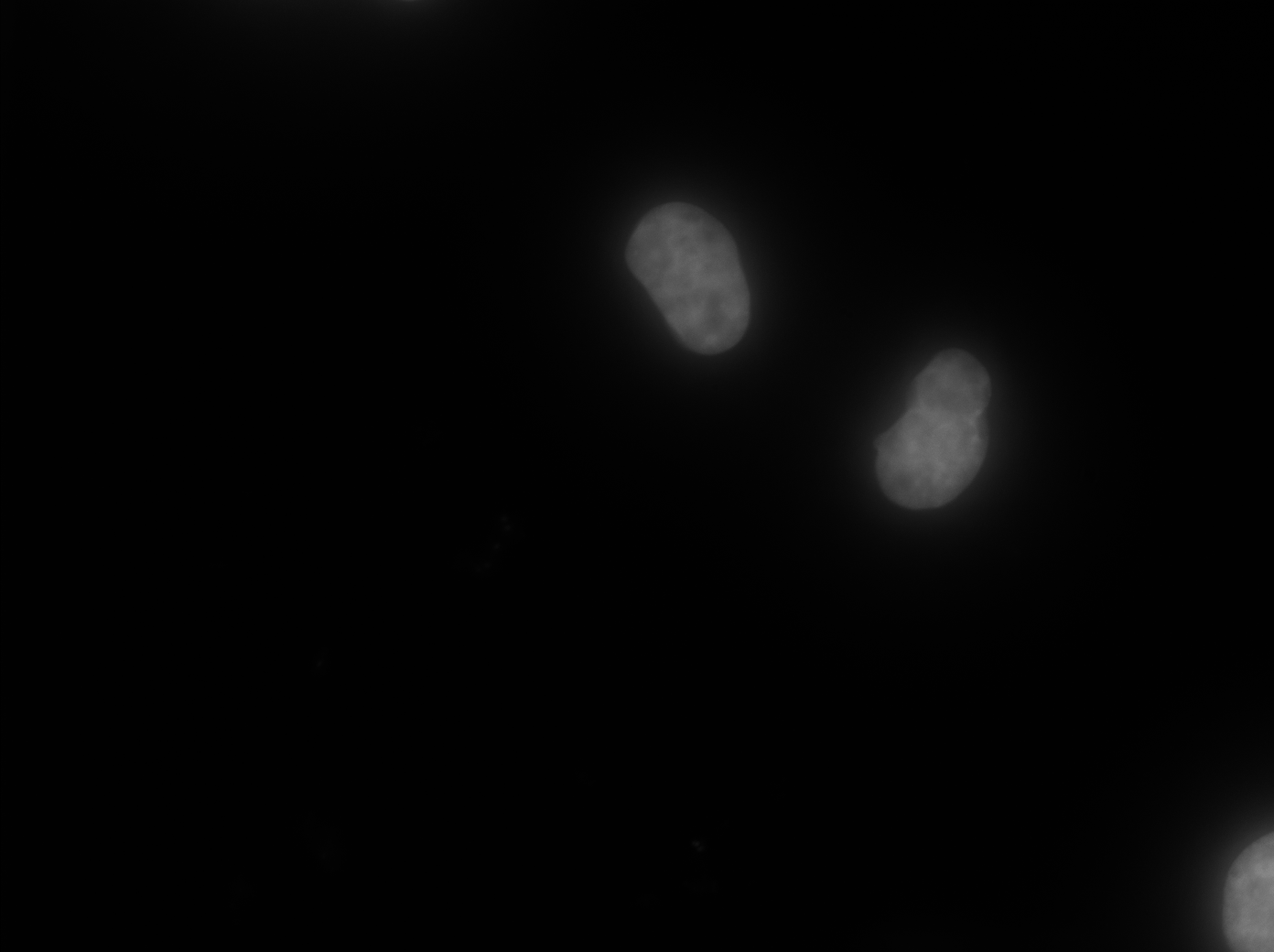

Supplement: Supplementary file 22 — Source data Fig. 6 part 3 [file 44319_2026_742_MOESM22_ESM.zip › Figure 6 Part 3/Fig 6efg TPGS1-KO TPGS1 rescue experiments/R1/TPGS1-KO TPGS1-3UTR-EYFP actub R1 7-31-25 LT2.Project Maximum Z_XY1753987675_Z0_T0_C0.tif]

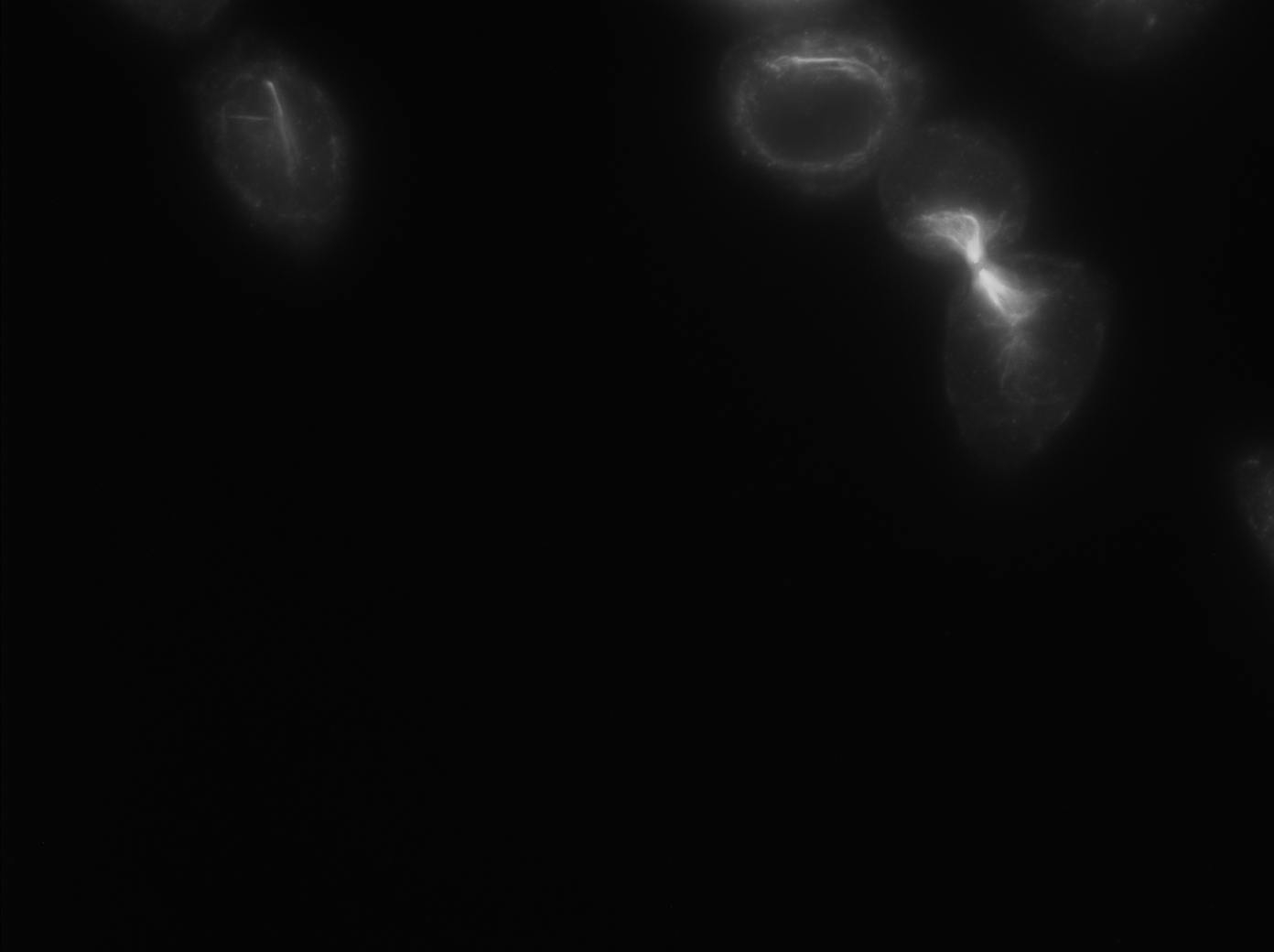

Supplement: Supplementary file 22 — Source data Fig. 6 part 3 [file 44319_2026_742_MOESM22_ESM.zip › Figure 6 Part 3/Fig 6efg TPGS1-KO TPGS1 rescue experiments/R1/TPGS1-KO EYFP only actub R1 7-31-25 LT9.Project Maximum Z_XY1754338968_Z0_T0_C2.tif]

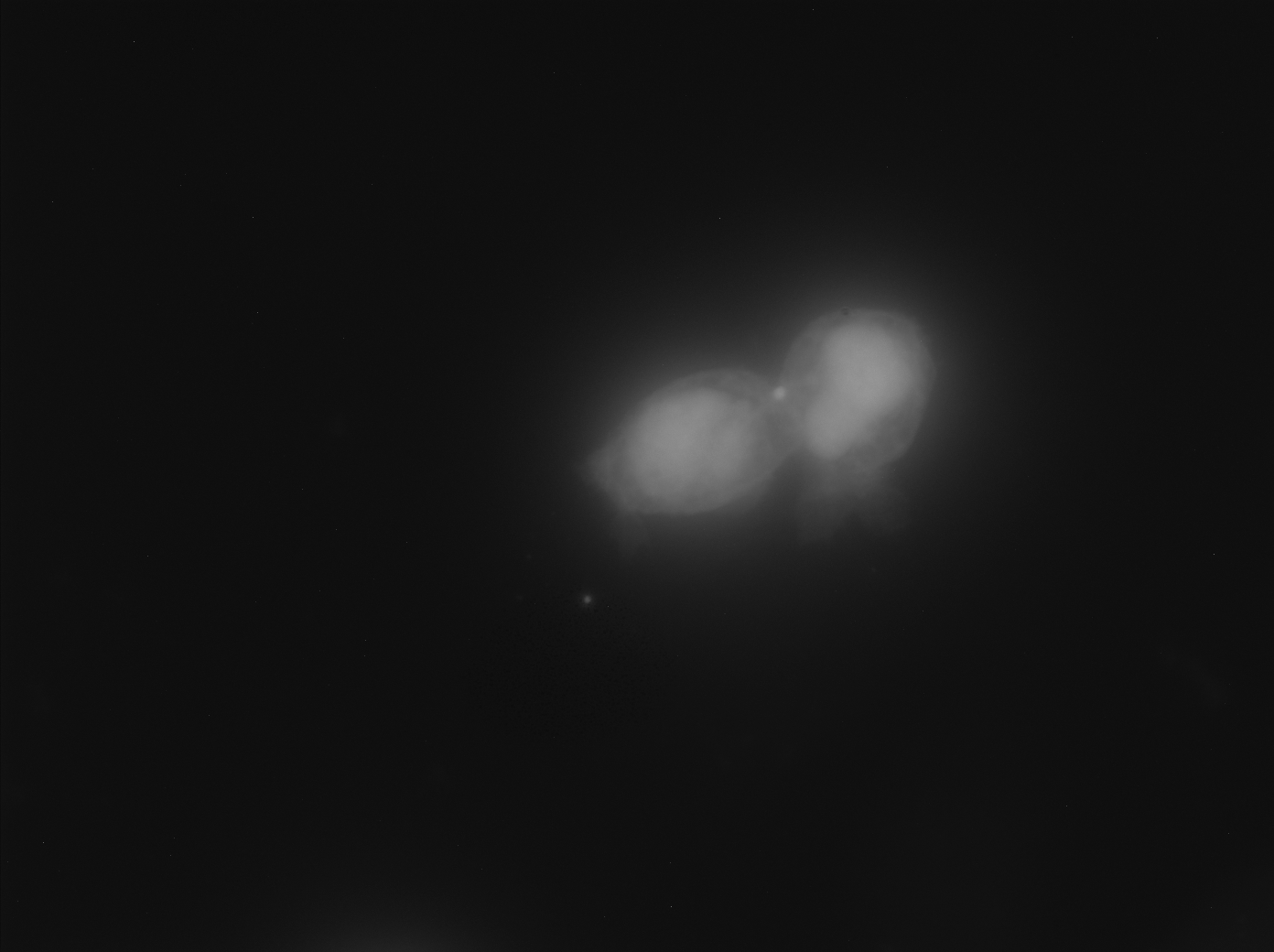

Supplement: Supplementary file 22 — Source data Fig. 6 part 3 [file 44319_2026_742_MOESM22_ESM.zip › Figure 6 Part 3/Fig 6efg TPGS1-KO TPGS1 rescue experiments/R1/TPGS1-KO EYFP only actub R1 7-31-25 LT5.Project Maximum Z_XY1754336364_Z0_T0_C1.tif]

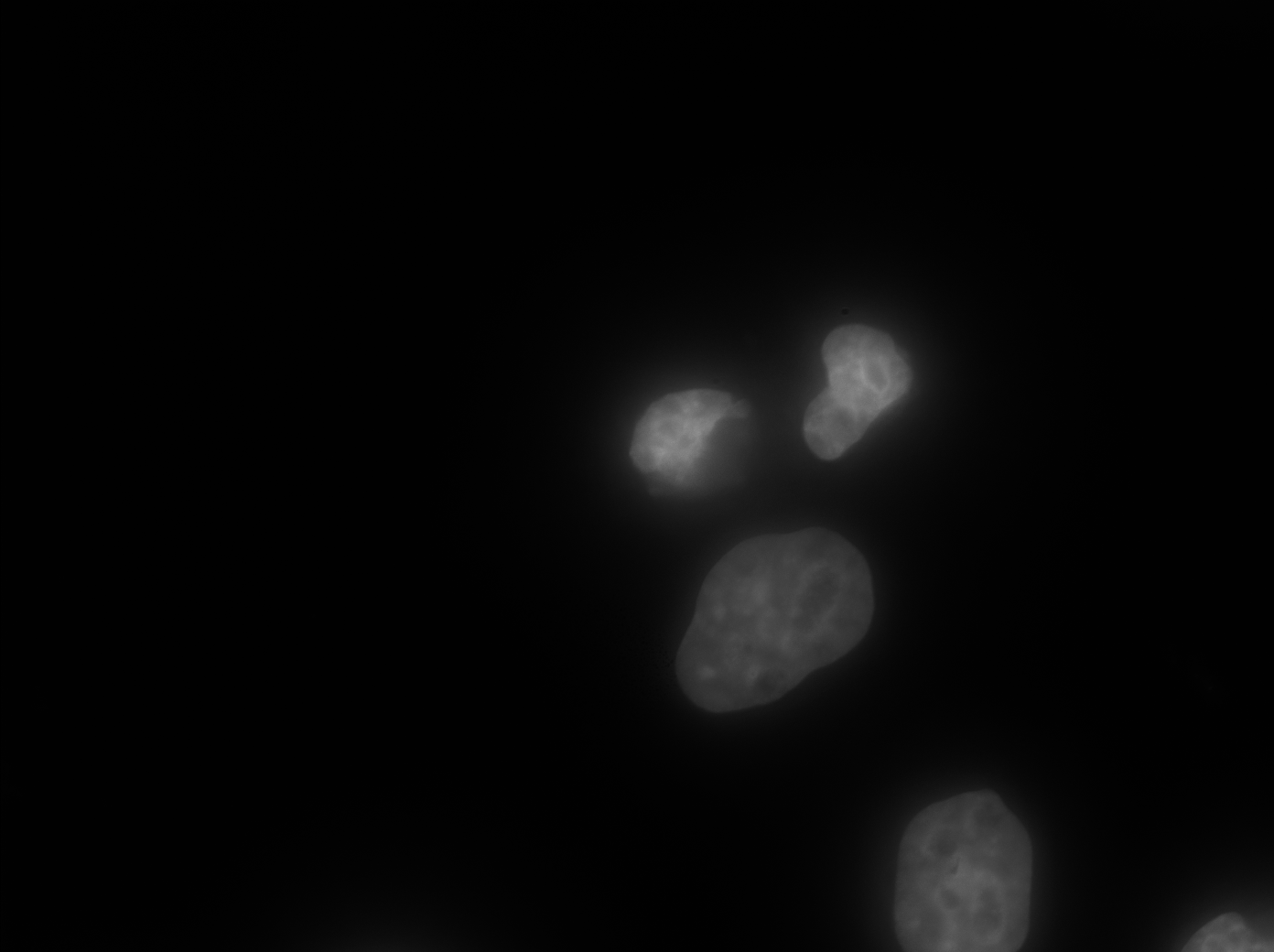

Supplement: Supplementary file 22 — Source data Fig. 6 part 3 [file 44319_2026_742_MOESM22_ESM.zip › Figure 6 Part 3/Fig 6efg TPGS1-KO TPGS1 rescue experiments/R1/TPGS1-KO EYFP only actub R1 7-31-25 LT5.Project Maximum Z_XY1754336364_Z0_T0_C0.tif]

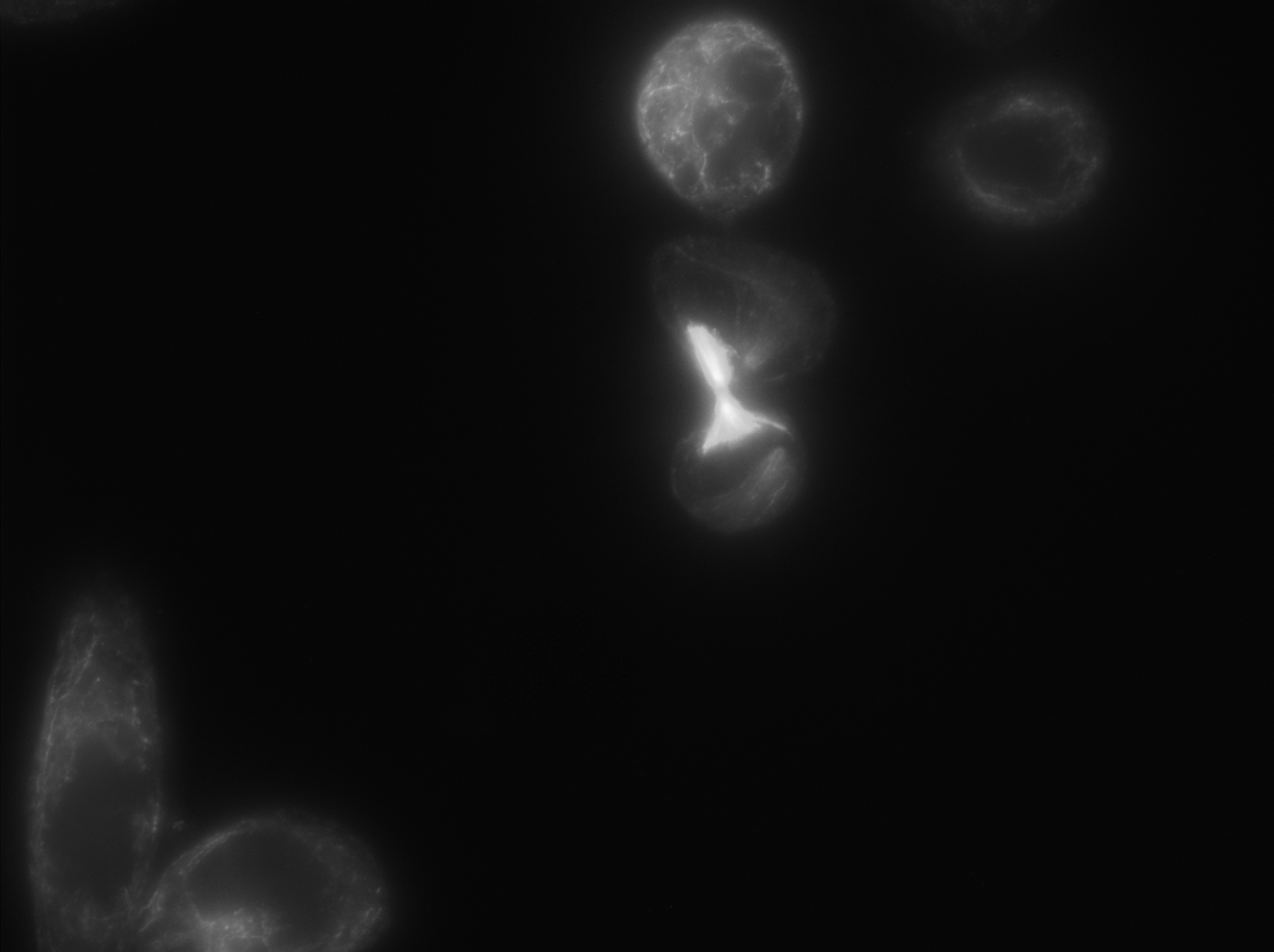

Supplement: Supplementary file 22 — Source data Fig. 6 part 3 [file 44319_2026_742_MOESM22_ESM.zip › Figure 6 Part 3/Fig 6efg TPGS1-KO TPGS1 rescue experiments/R1/TPGS1-KO EYFP only actub R1 7-31-25 ET6.Project Maximum Z_XY1754336574_Z0_T0_C2.tif]

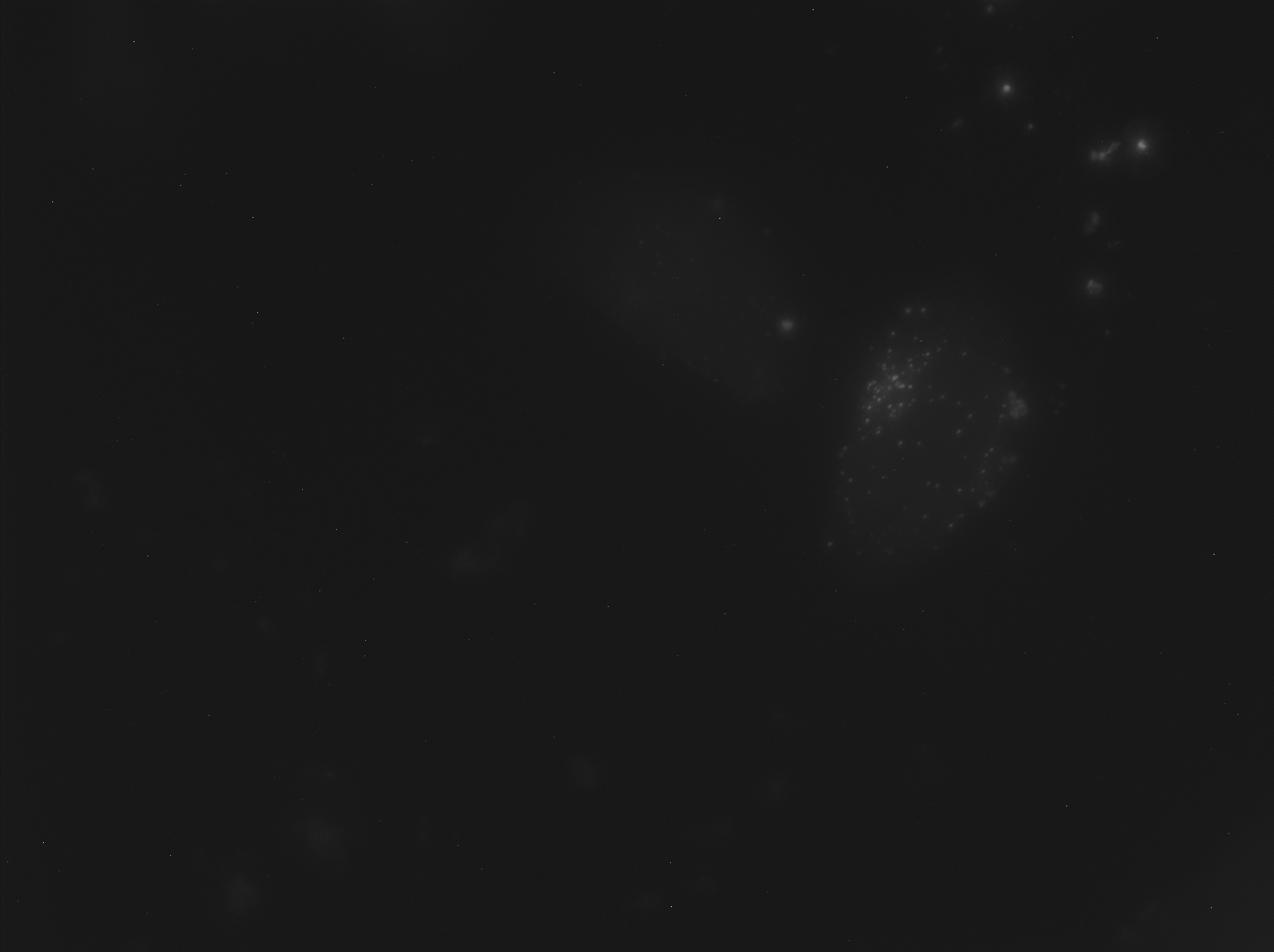

Supplement: Supplementary file 22 — Source data Fig. 6 part 3 [file 44319_2026_742_MOESM22_ESM.zip › Figure 6 Part 3/Fig 6efg TPGS1-KO TPGS1 rescue experiments/R1/TPGS1-KO TPGS1-3UTR-EYFP actub R1 7-31-25 LT2.Project Maximum Z_XY1753987675_Z0_T0_C1.tif]

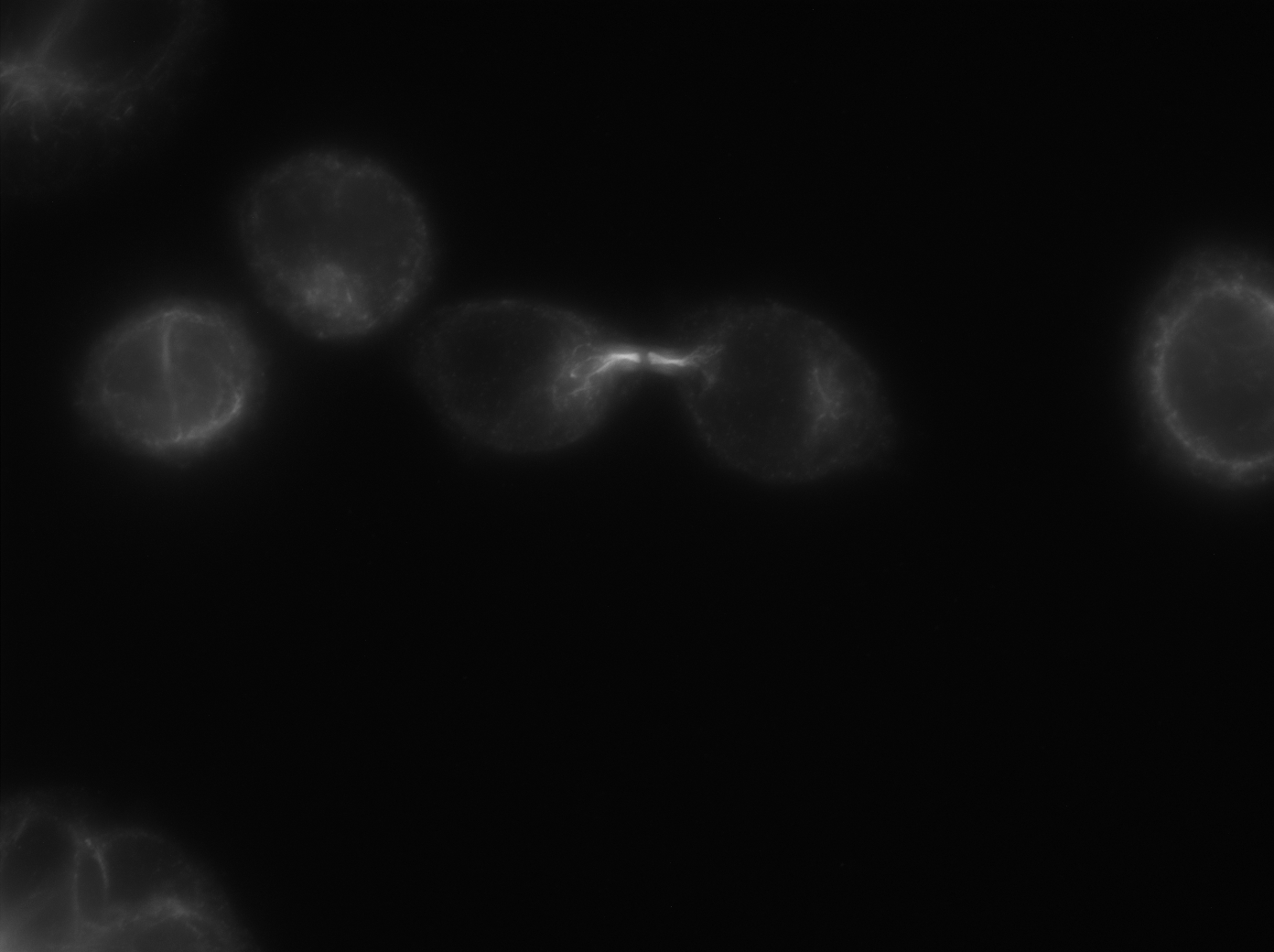

Supplement: Supplementary file 22 — Source data Fig. 6 part 3 [file 44319_2026_742_MOESM22_ESM.zip › Figure 6 Part 3/Fig 6efg TPGS1-KO TPGS1 rescue experiments/R1/TPGS1-KO TPGS1-3UTR-EYFP actub R1 7-31-25 LT7.Project Maximum Z_XY1753989093_Z0_T0_C2.tif]

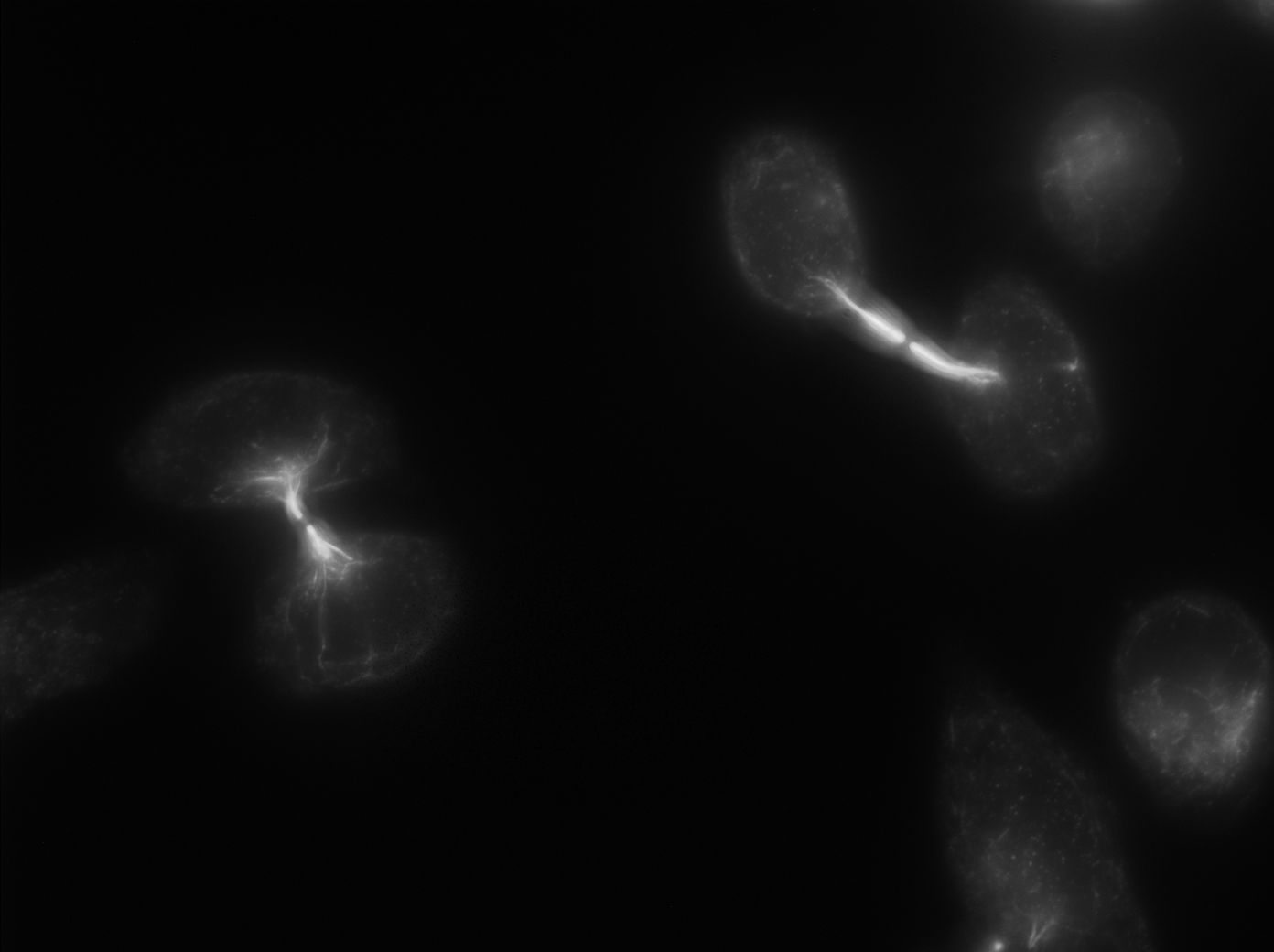

Supplement: Supplementary file 22 — Source data Fig. 6 part 3 [file 44319_2026_742_MOESM22_ESM.zip › Figure 6 Part 3/Fig 6efg TPGS1-KO TPGS1 rescue experiments/R1/TPGS1-KO EYFP only actub R1 7-31-25 LT1.Project Maximum Z_XY1754334969_Z0_T0_C2.tif]

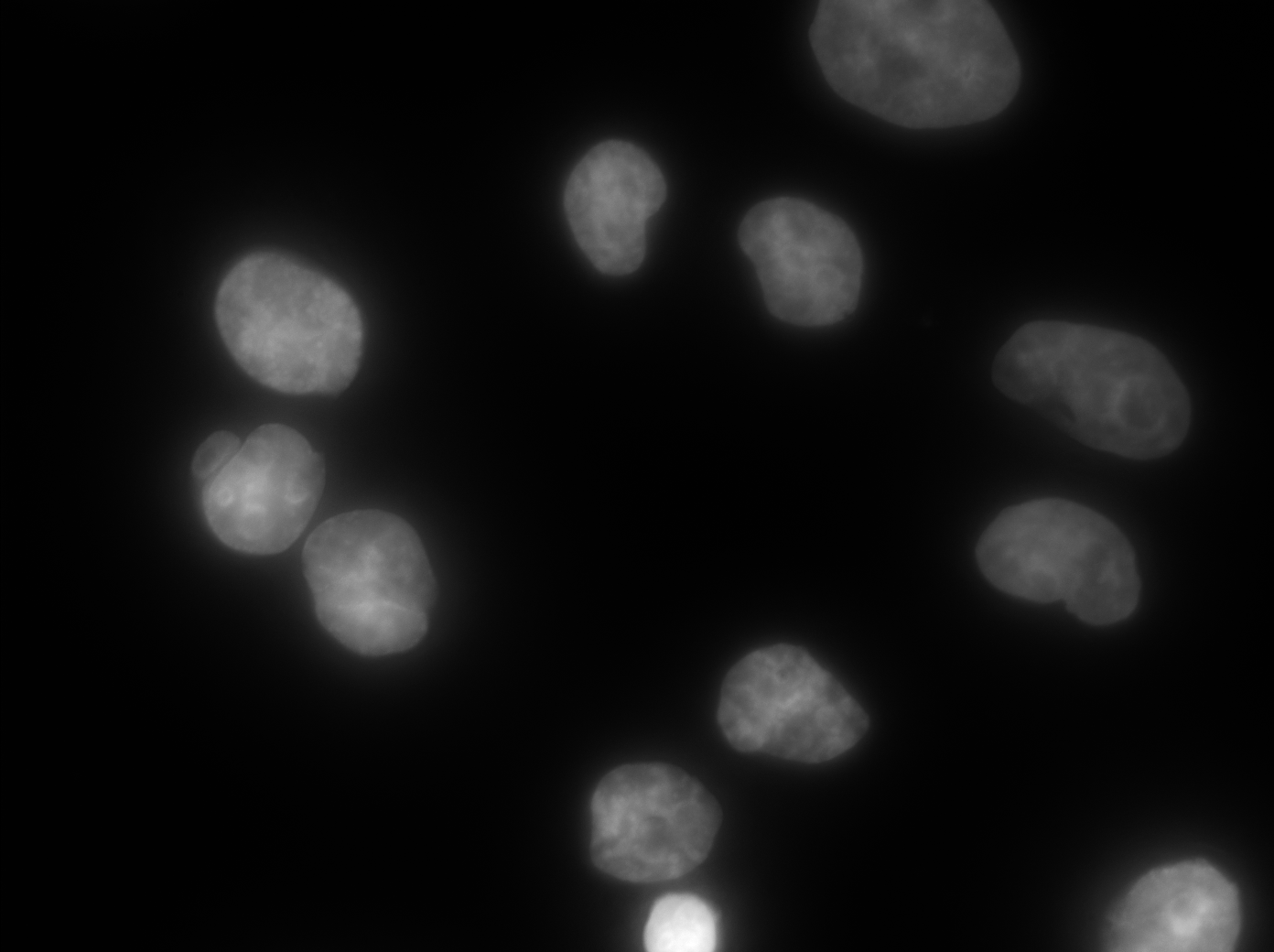

Supplement: Supplementary file 22 — Source data Fig. 6 part 3 [file 44319_2026_742_MOESM22_ESM.zip › Figure 6 Part 3/Fig 6efg TPGS1-KO TPGS1 rescue experiments/R1/TPGS1-KO TPGS1-3UTR-EYFP actub R1 7-31-25 LT4LT5.Project Maximum Z_XY1753988414_Z0_T0_C0.tif]

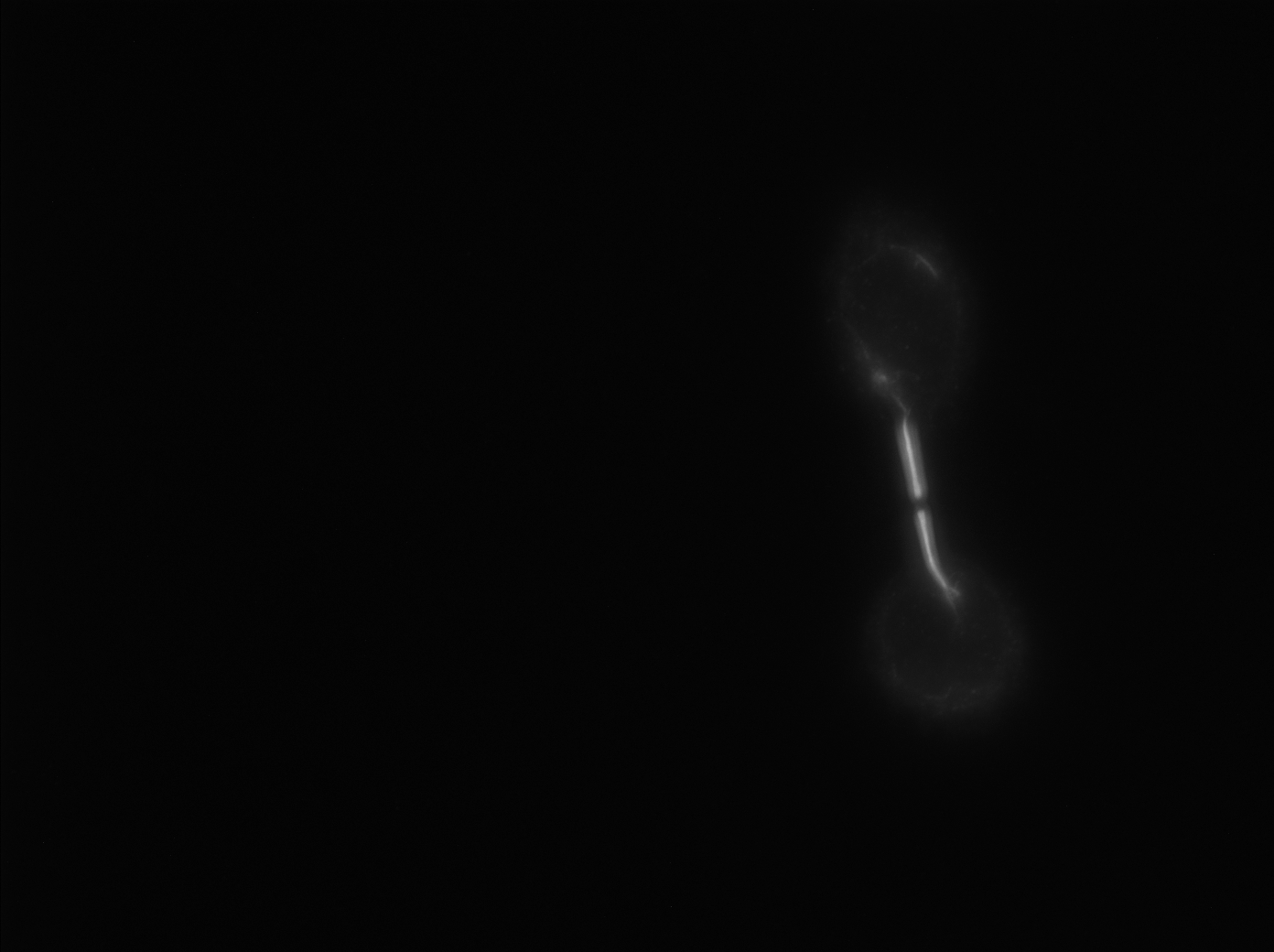

Supplement: Supplementary file 22 — Source data Fig. 6 part 3 [file 44319_2026_742_MOESM22_ESM.zip › Figure 6 Part 3/Fig 6efg TPGS1-KO TPGS1 rescue experiments/R1/TPGS1-KO TPGS1-3UTR-EYFP actub R1 7-31-25 LT3.Project Maximum Z_XY1753988228_Z0_T0_C2.tif]

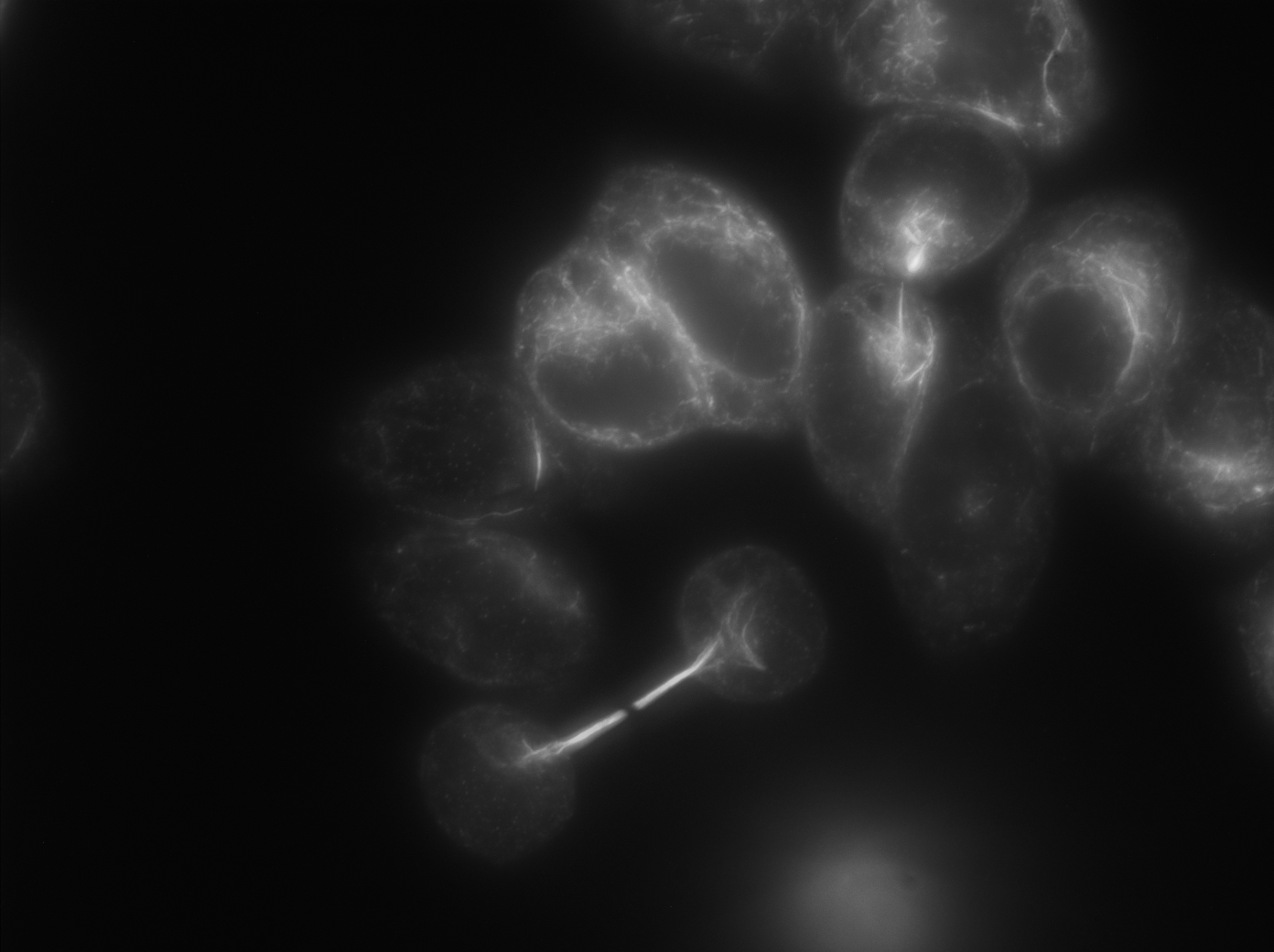

Supplement: Supplementary file 22 — Source data Fig. 6 part 3 [file 44319_2026_742_MOESM22_ESM.zip › Figure 6 Part 3/Fig 6efg TPGS1-KO TPGS1 rescue experiments/R1/TPGS1-KO EYFP only actub R1 7-31-25 LT10 ET7.Project Maximum Z_XY1754339080_Z0_T0_C2.tif]

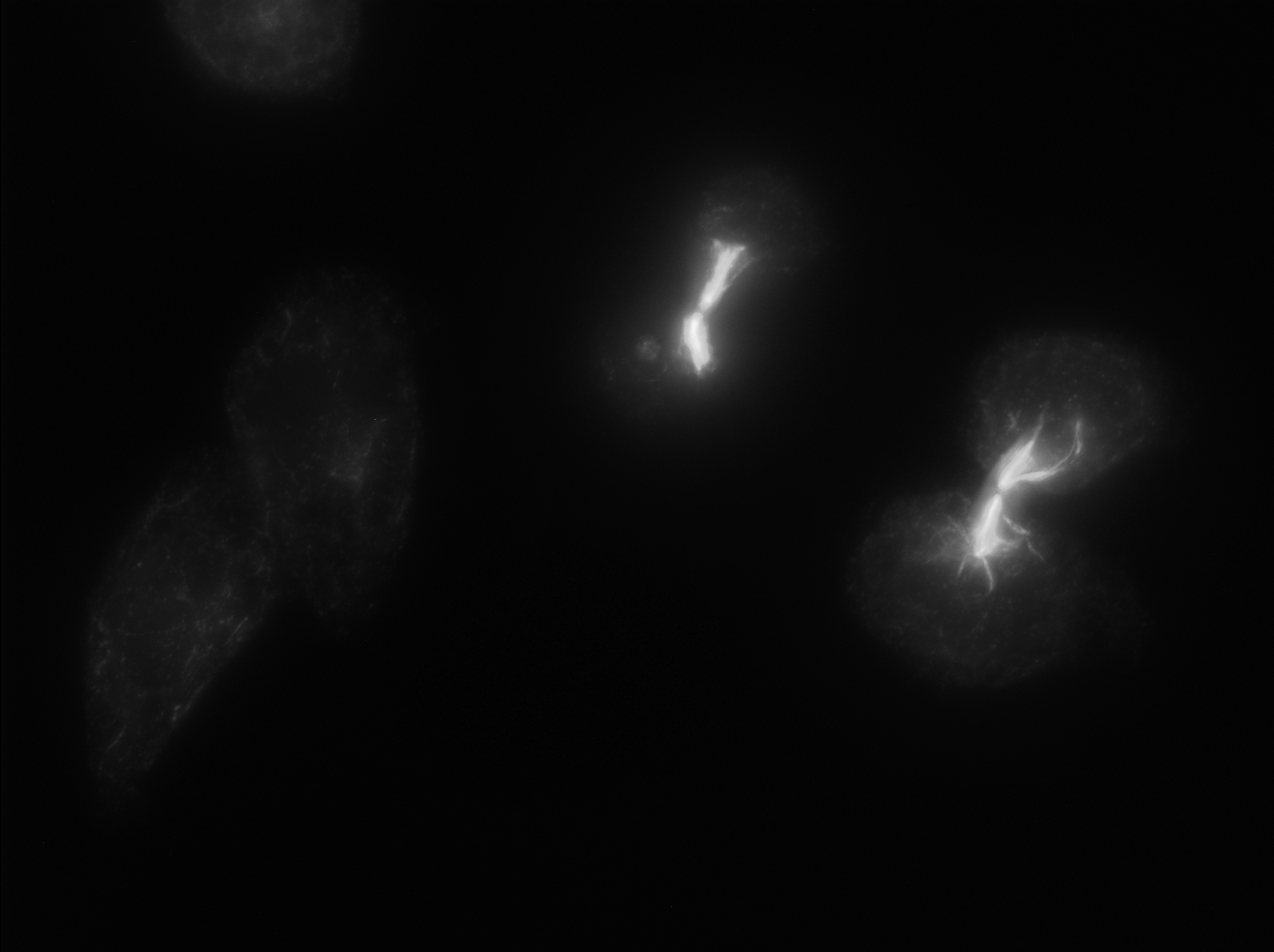

Supplement: Supplementary file 22 — Source data Fig. 6 part 3 [file 44319_2026_742_MOESM22_ESM.zip › Figure 6 Part 3/Fig 6efg TPGS1-KO TPGS1 rescue experiments/R1/TPGS1-KO EYFP only actub R1 7-31-25 LT3 ET1.Project Maximum Z_XY1754335501_Z0_T0_C2.tif]

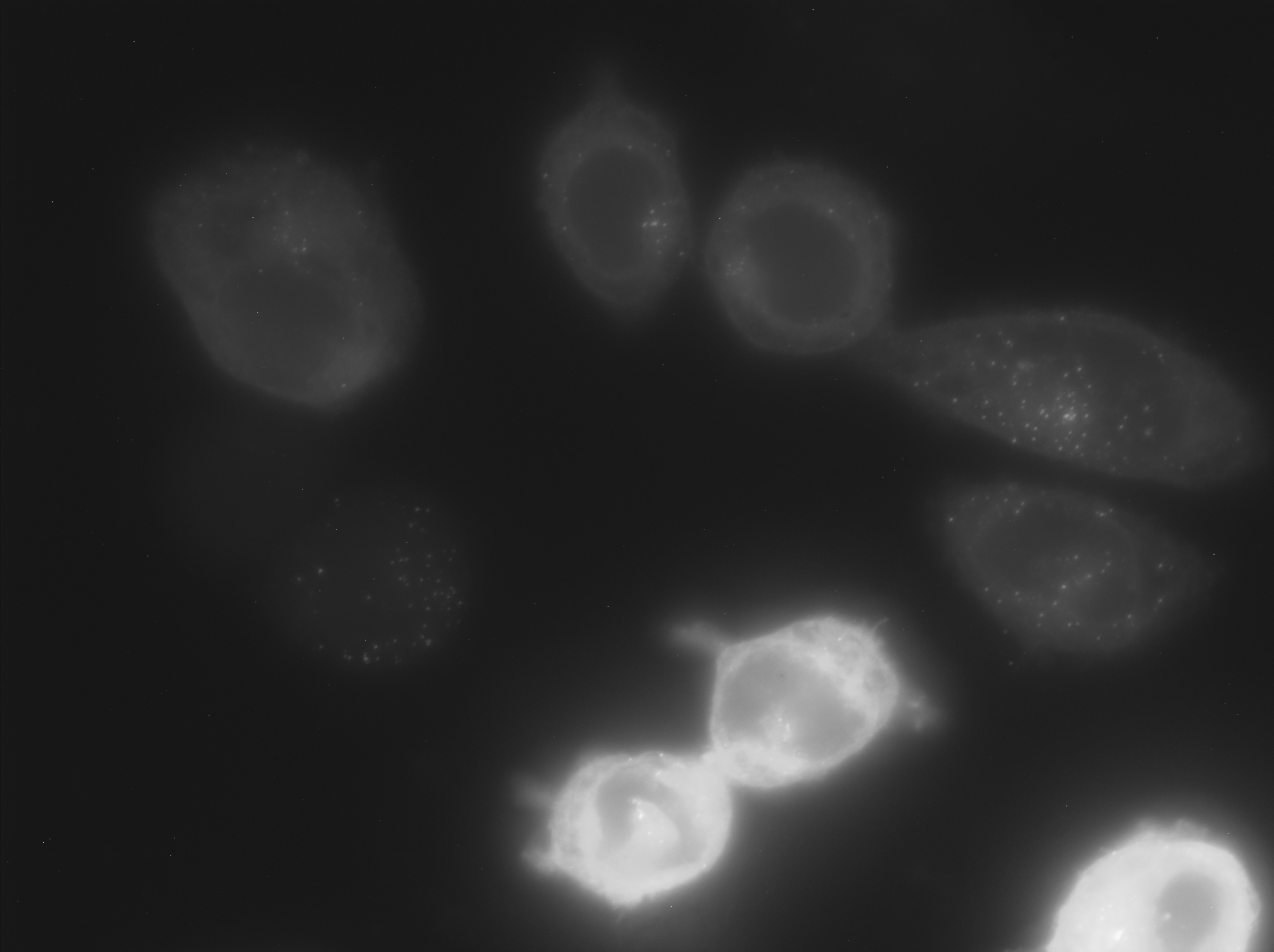

Supplement: Supplementary file 22 — Source data Fig. 6 part 3 [file 44319_2026_742_MOESM22_ESM.zip › Figure 6 Part 3/Fig 6efg TPGS1-KO TPGS1 rescue experiments/R1/TPGS1-KO TPGS1-3UTR-EYFP actub R1 7-31-25 LT4LT5.Project Maximum Z_XY1753988414_Z0_T0_C1.tif]

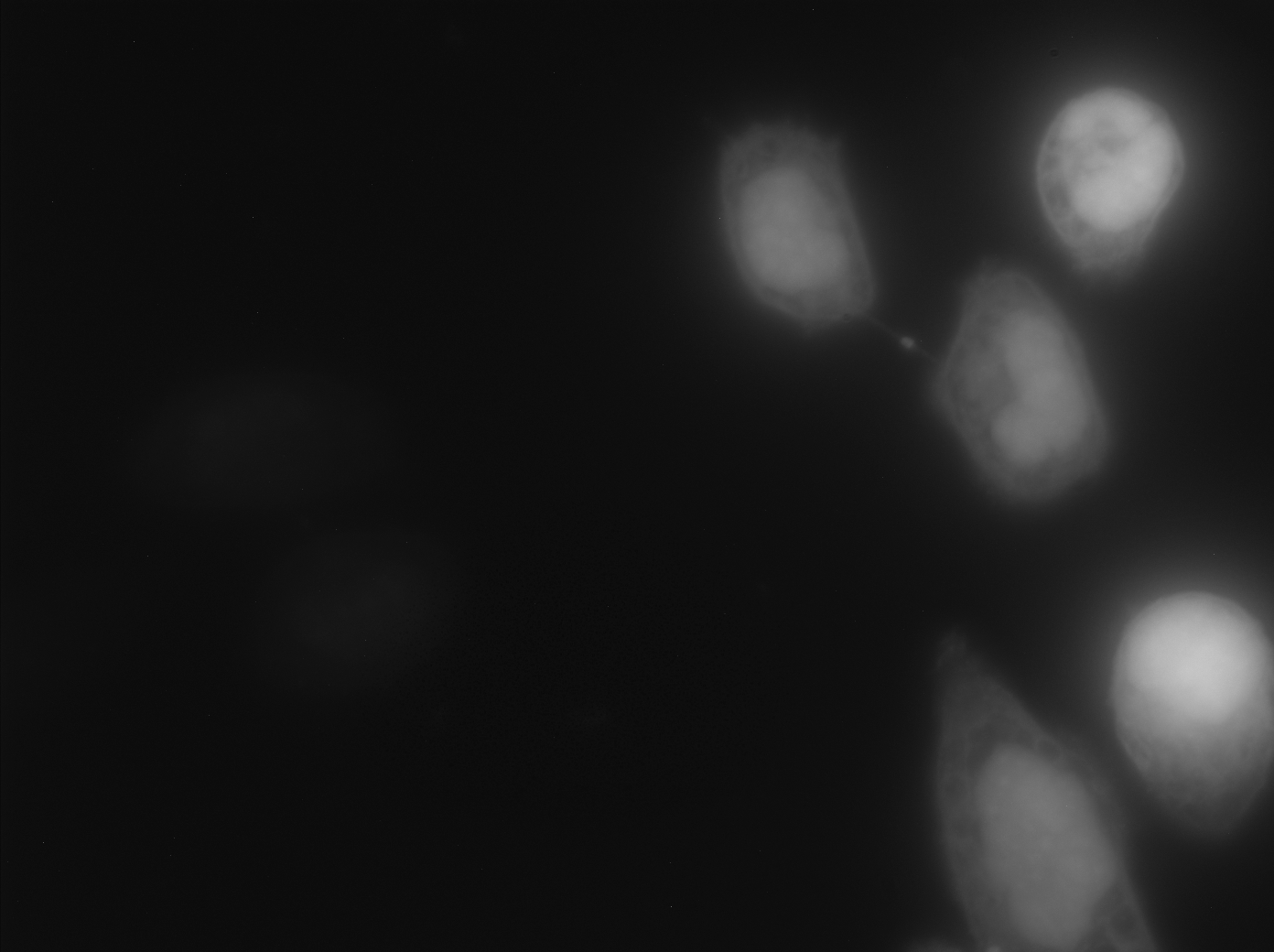

Supplement: Supplementary file 22 — Source data Fig. 6 part 3 [file 44319_2026_742_MOESM22_ESM.zip › Figure 6 Part 3/Fig 6efg TPGS1-KO TPGS1 rescue experiments/R1/TPGS1-KO EYFP only actub R1 7-31-25 LT1.Project Maximum Z_XY1754334969_Z0_T0_C1.tif]

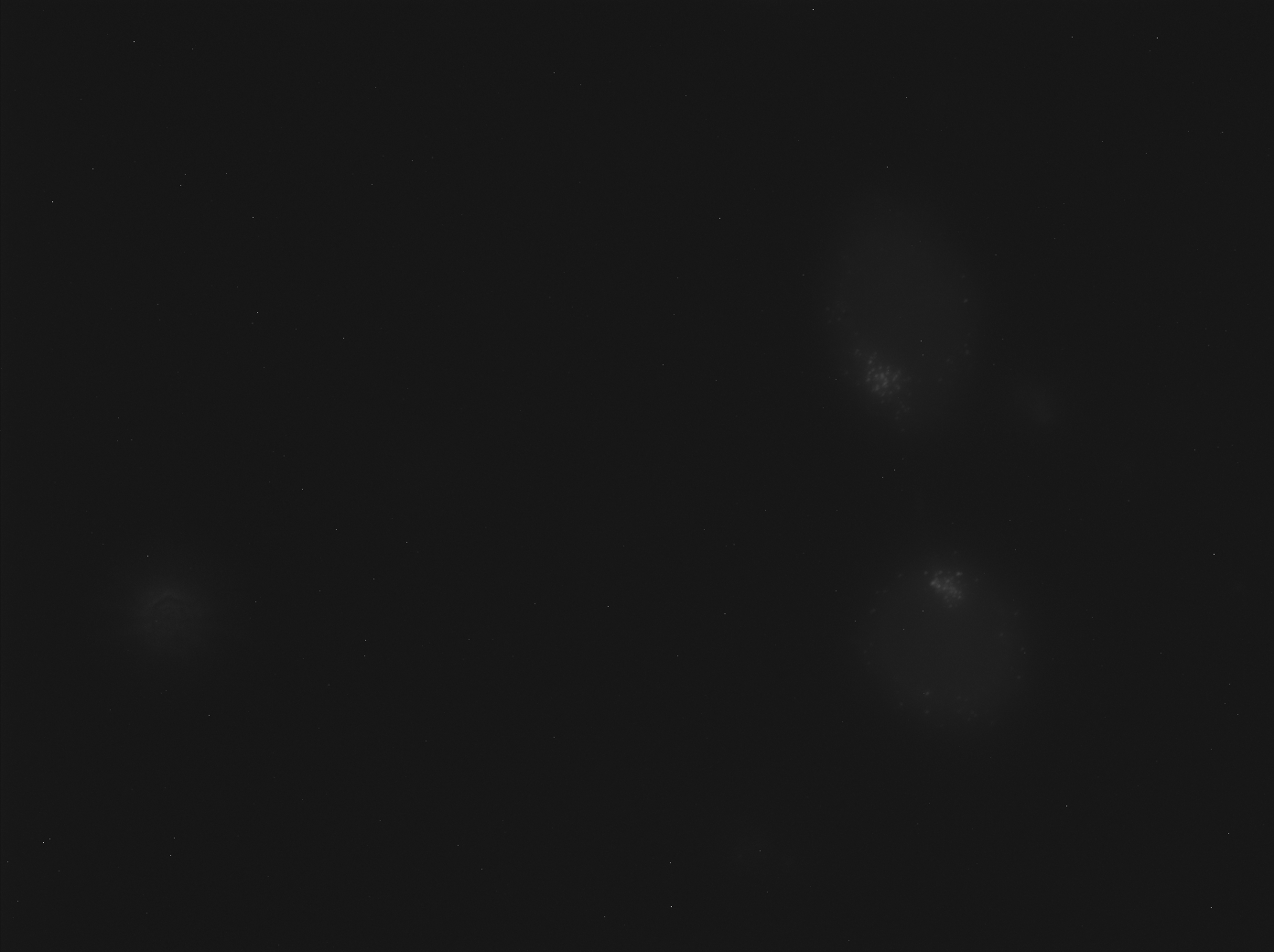

Supplement: Supplementary file 22 — Source data Fig. 6 part 3 [file 44319_2026_742_MOESM22_ESM.zip › Figure 6 Part 3/Fig 6efg TPGS1-KO TPGS1 rescue experiments/R1/TPGS1-KO TPGS1-3UTR-EYFP actub R1 7-31-25 LT3.Project Maximum Z_XY1753988228_Z0_T0_C1.tif]

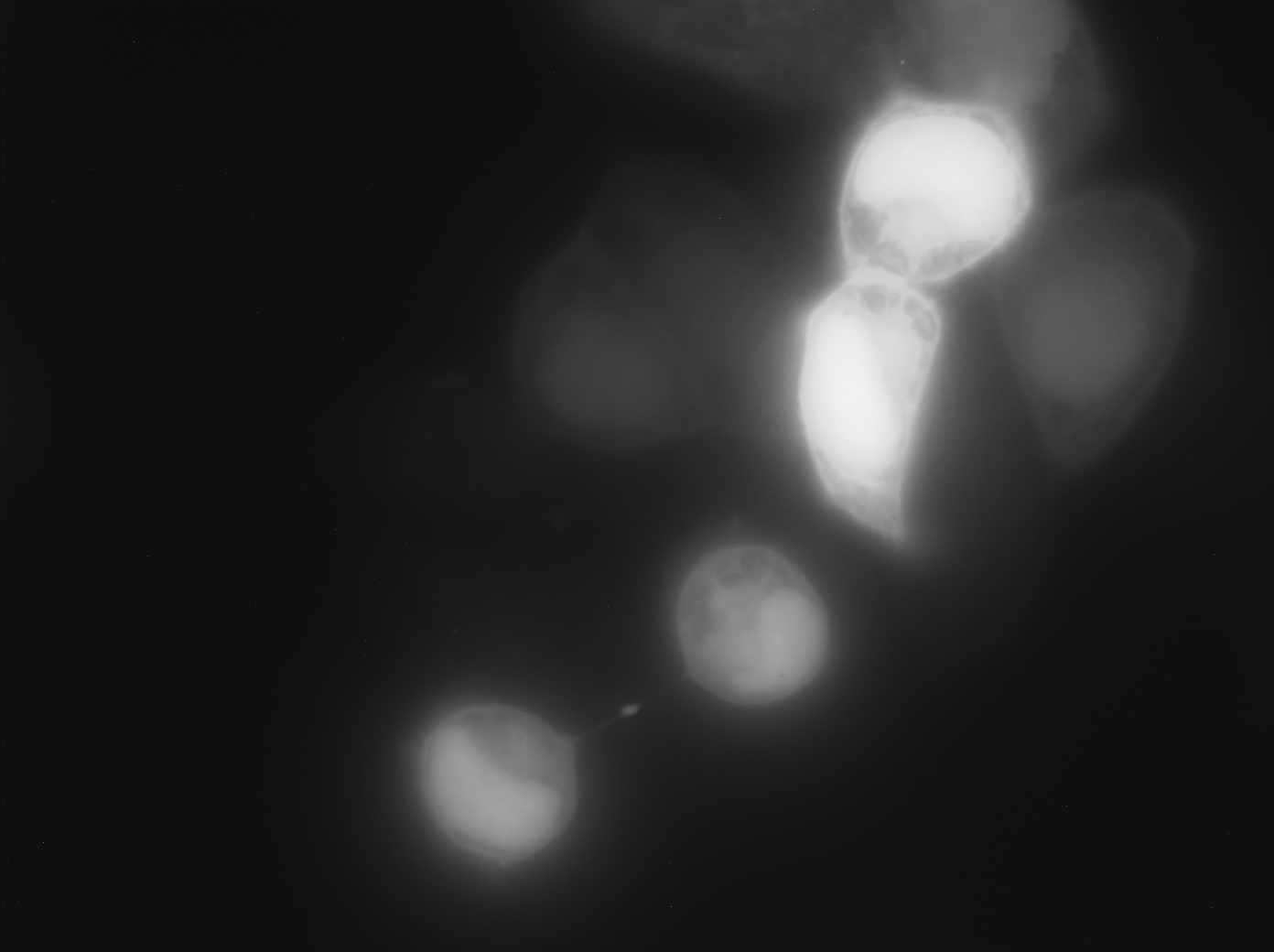

Supplement: Supplementary file 22 — Source data Fig. 6 part 3 [file 44319_2026_742_MOESM22_ESM.zip › Figure 6 Part 3/Fig 6efg TPGS1-KO TPGS1 rescue experiments/R1/TPGS1-KO EYFP only actub R1 7-31-25 LT10 ET7.Project Maximum Z_XY1754339080_Z0_T0_C1.tif]

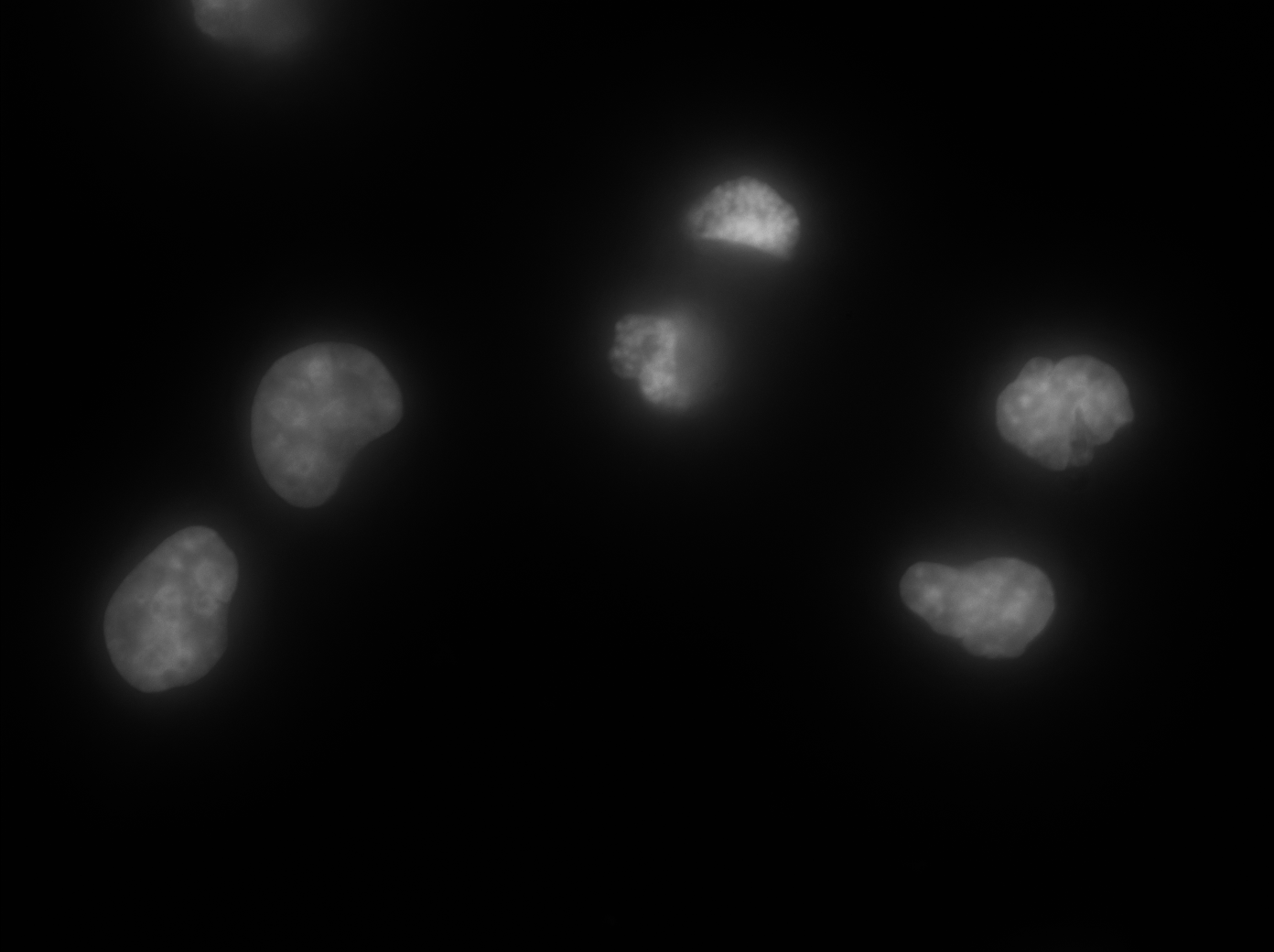

Supplement: Supplementary file 22 — Source data Fig. 6 part 3 [file 44319_2026_742_MOESM22_ESM.zip › Figure 6 Part 3/Fig 6efg TPGS1-KO TPGS1 rescue experiments/R1/TPGS1-KO EYFP only actub R1 7-31-25 LT3 ET1.Project Maximum Z_XY1754335501_Z0_T0_C0.tif]

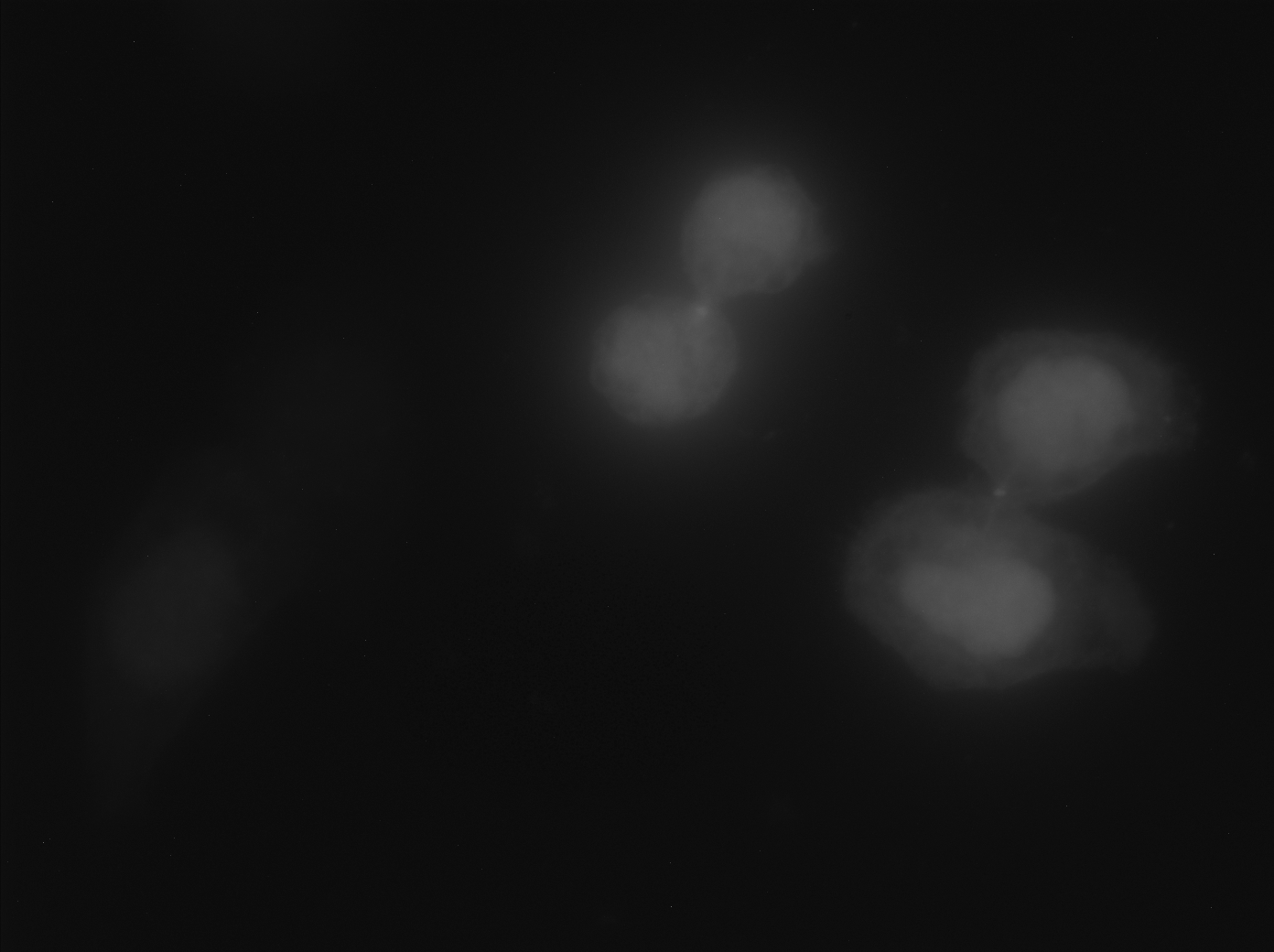

Supplement: Supplementary file 22 — Source data Fig. 6 part 3 [file 44319_2026_742_MOESM22_ESM.zip › Figure 6 Part 3/Fig 6efg TPGS1-KO TPGS1 rescue experiments/R1/TPGS1-KO EYFP only actub R1 7-31-25 LT3 ET1.Project Maximum Z_XY1754335501_Z0_T0_C1.tif]

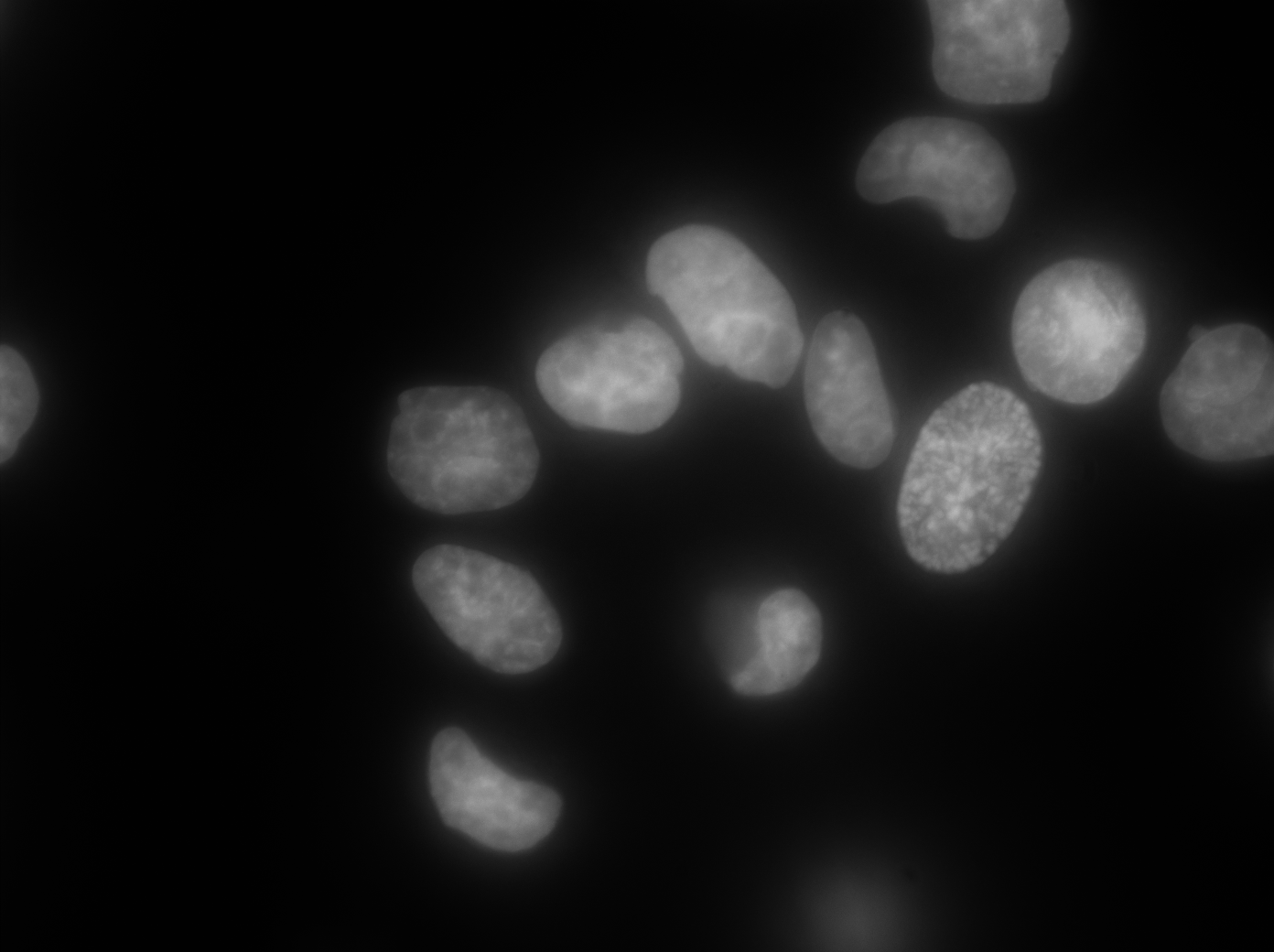

Supplement: Supplementary file 22 — Source data Fig. 6 part 3 [file 44319_2026_742_MOESM22_ESM.zip › Figure 6 Part 3/Fig 6efg TPGS1-KO TPGS1 rescue experiments/R1/TPGS1-KO EYFP only actub R1 7-31-25 LT10 ET7.Project Maximum Z_XY1754339080_Z0_T0_C0.tif]

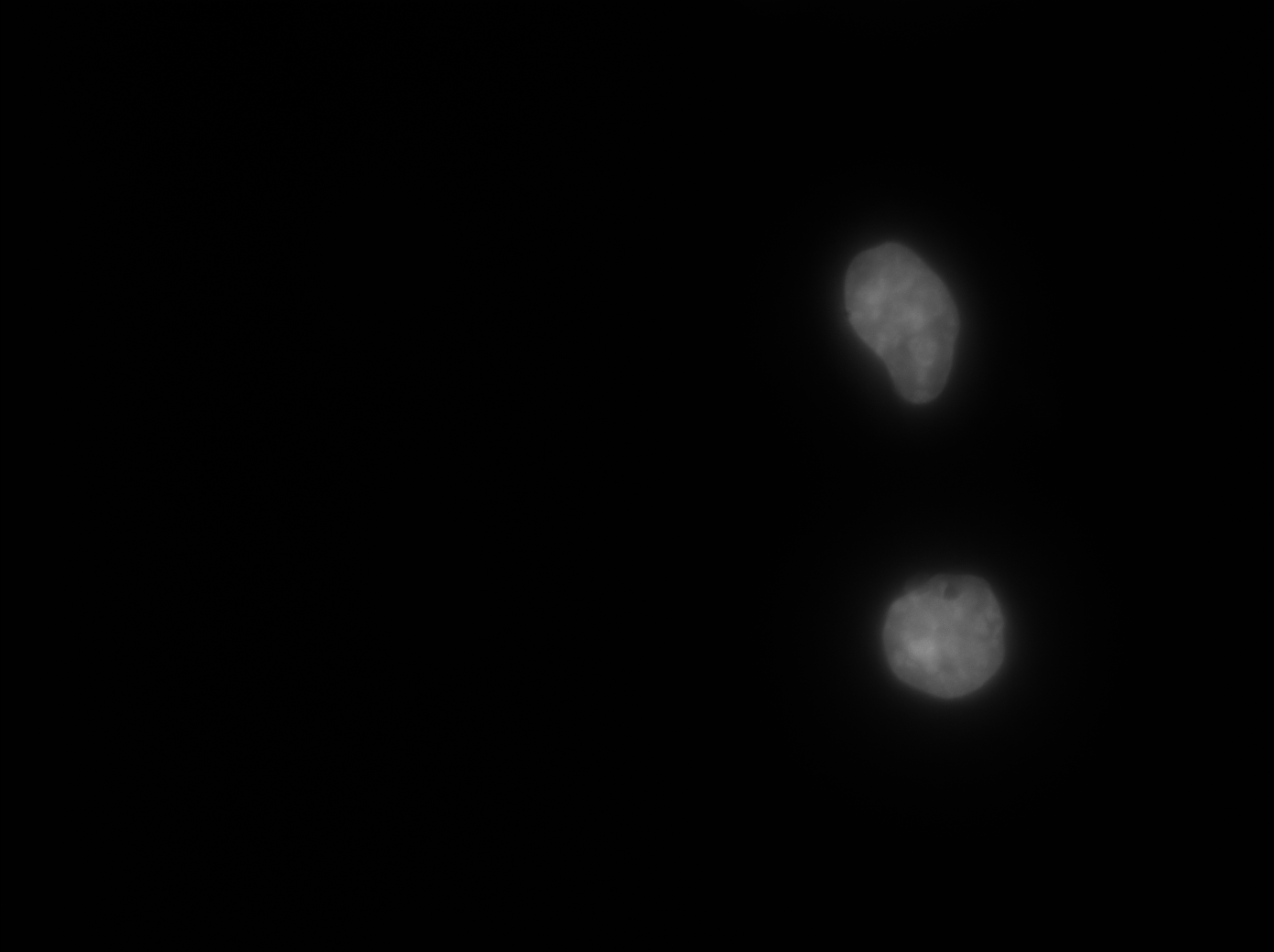

Supplement: Supplementary file 22 — Source data Fig. 6 part 3 [file 44319_2026_742_MOESM22_ESM.zip › Figure 6 Part 3/Fig 6efg TPGS1-KO TPGS1 rescue experiments/R1/TPGS1-KO TPGS1-3UTR-EYFP actub R1 7-31-25 LT3.Project Maximum Z_XY1753988228_Z0_T0_C0.tif]

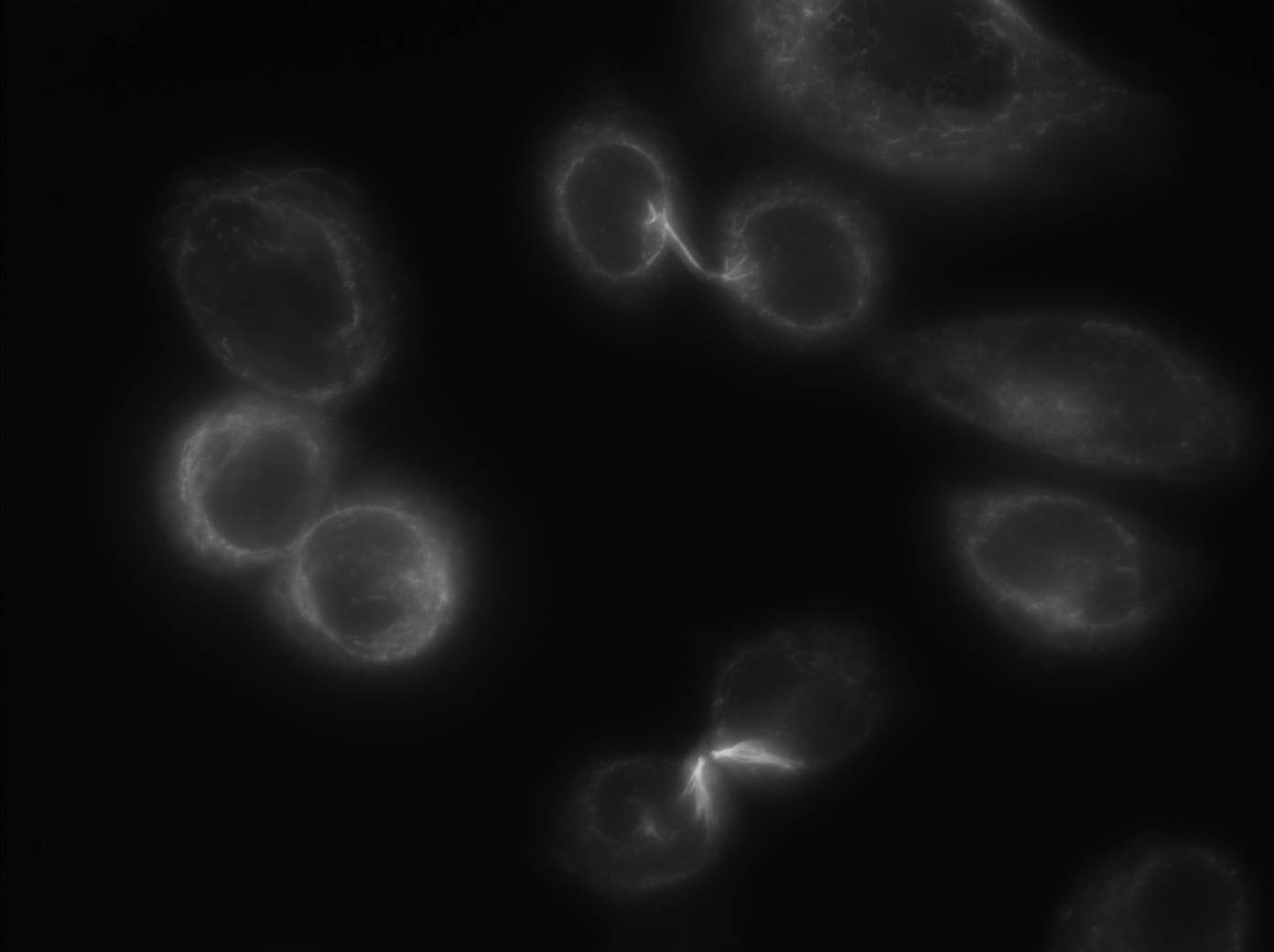

Supplement: Supplementary file 22 — Source data Fig. 6 part 3 [file 44319_2026_742_MOESM22_ESM.zip › Figure 6 Part 3/Fig 6efg TPGS1-KO TPGS1 rescue experiments/R1/TPGS1-KO TPGS1-3UTR-EYFP actub R1 7-31-25 LT4LT5.Project Maximum Z_XY1753988414_Z0_T0_C2.tif]

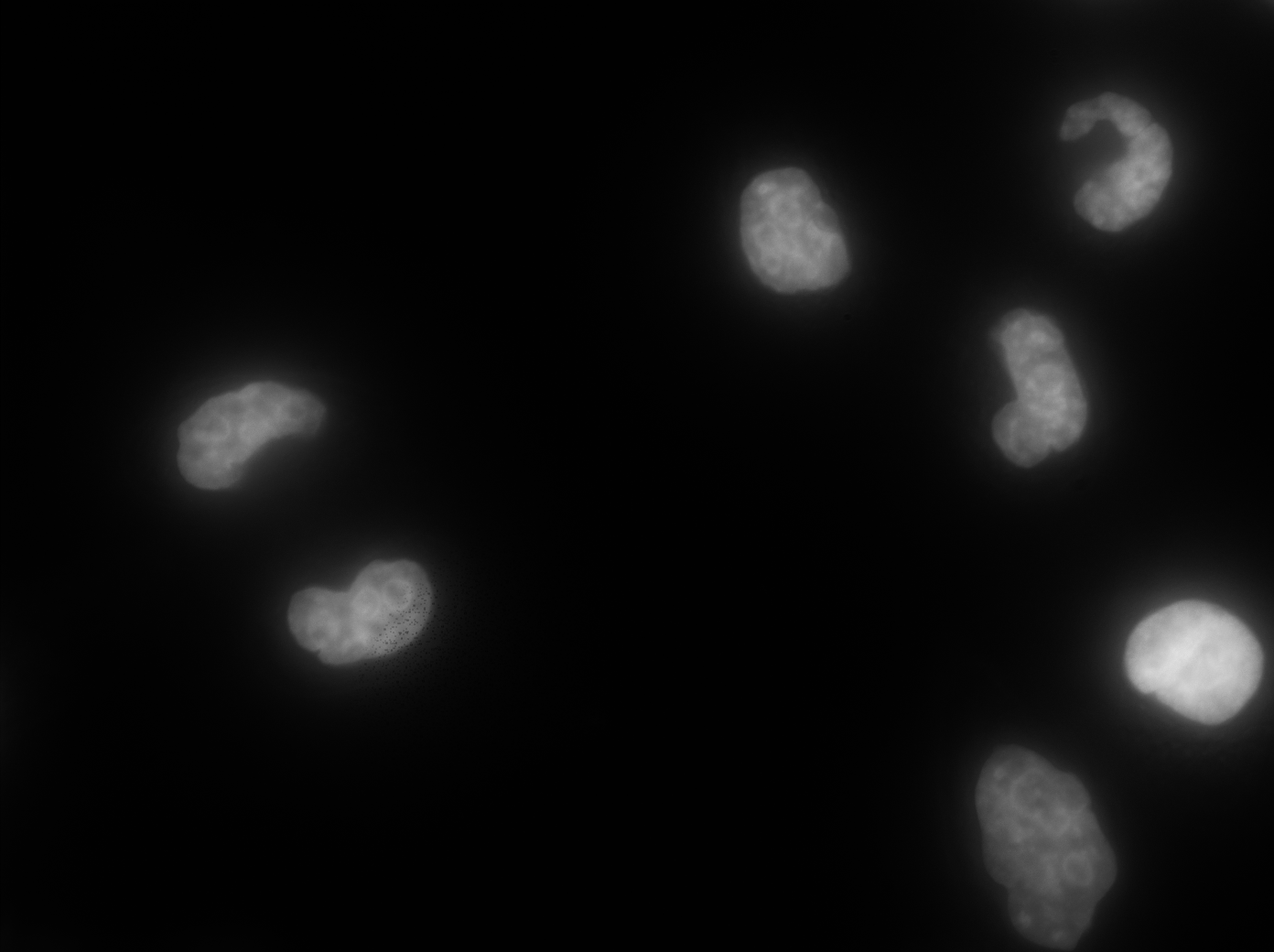

Supplement: Supplementary file 22 — Source data Fig. 6 part 3 [file 44319_2026_742_MOESM22_ESM.zip › Figure 6 Part 3/Fig 6efg TPGS1-KO TPGS1 rescue experiments/R1/TPGS1-KO EYFP only actub R1 7-31-25 LT1.Project Maximum Z_XY1754334969_Z0_T0_C0.tif]

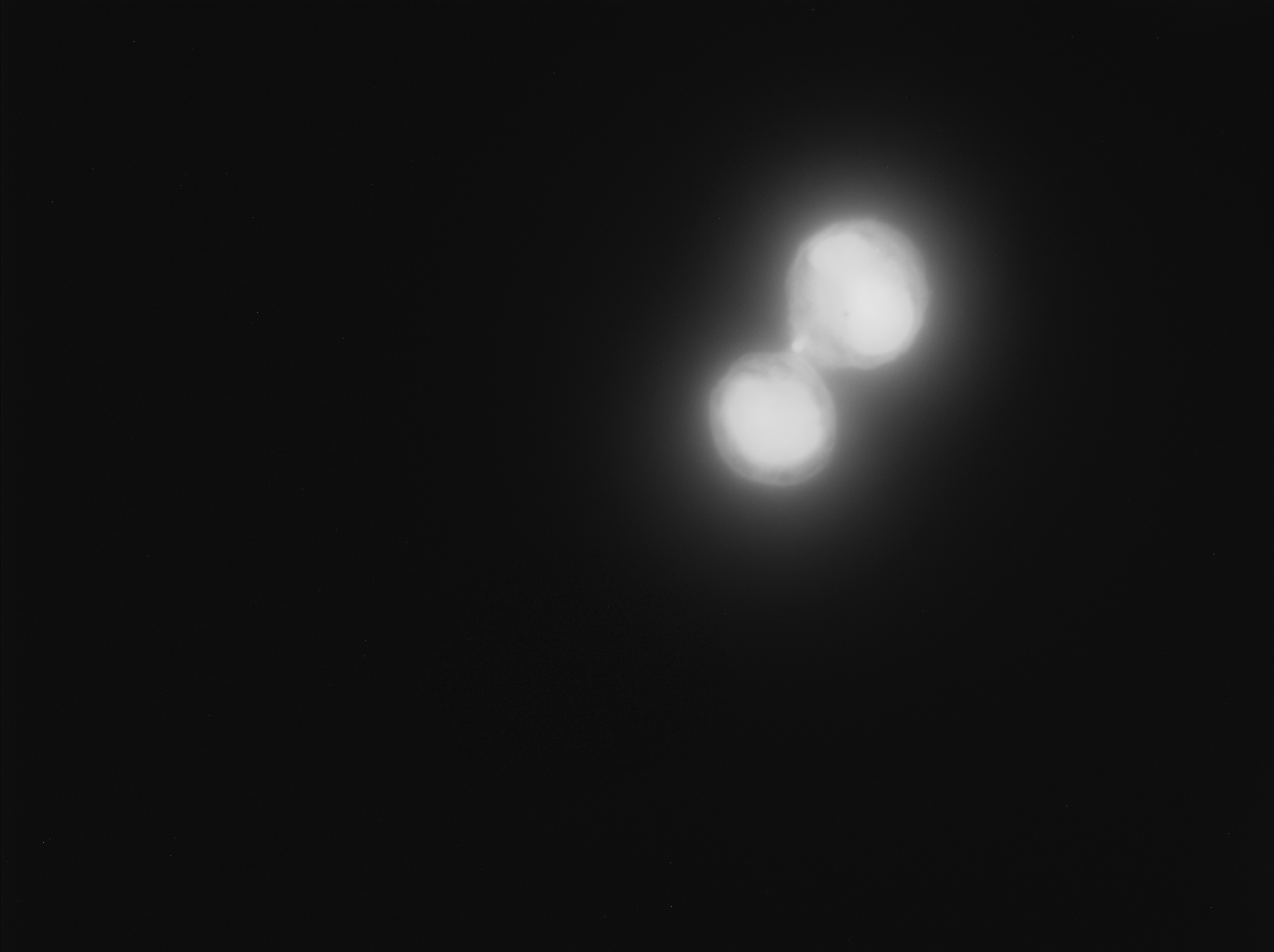

Supplement: Supplementary file 22 — Source data Fig. 6 part 3 [file 44319_2026_742_MOESM22_ESM.zip › Figure 6 Part 3/Fig 6efg TPGS1-KO TPGS1 rescue experiments/R1/TPGS1-KO EYFP only actub R1 7-31-25 ET2.Project Maximum Z_XY1754335621_Z0_T0_C1.tif]

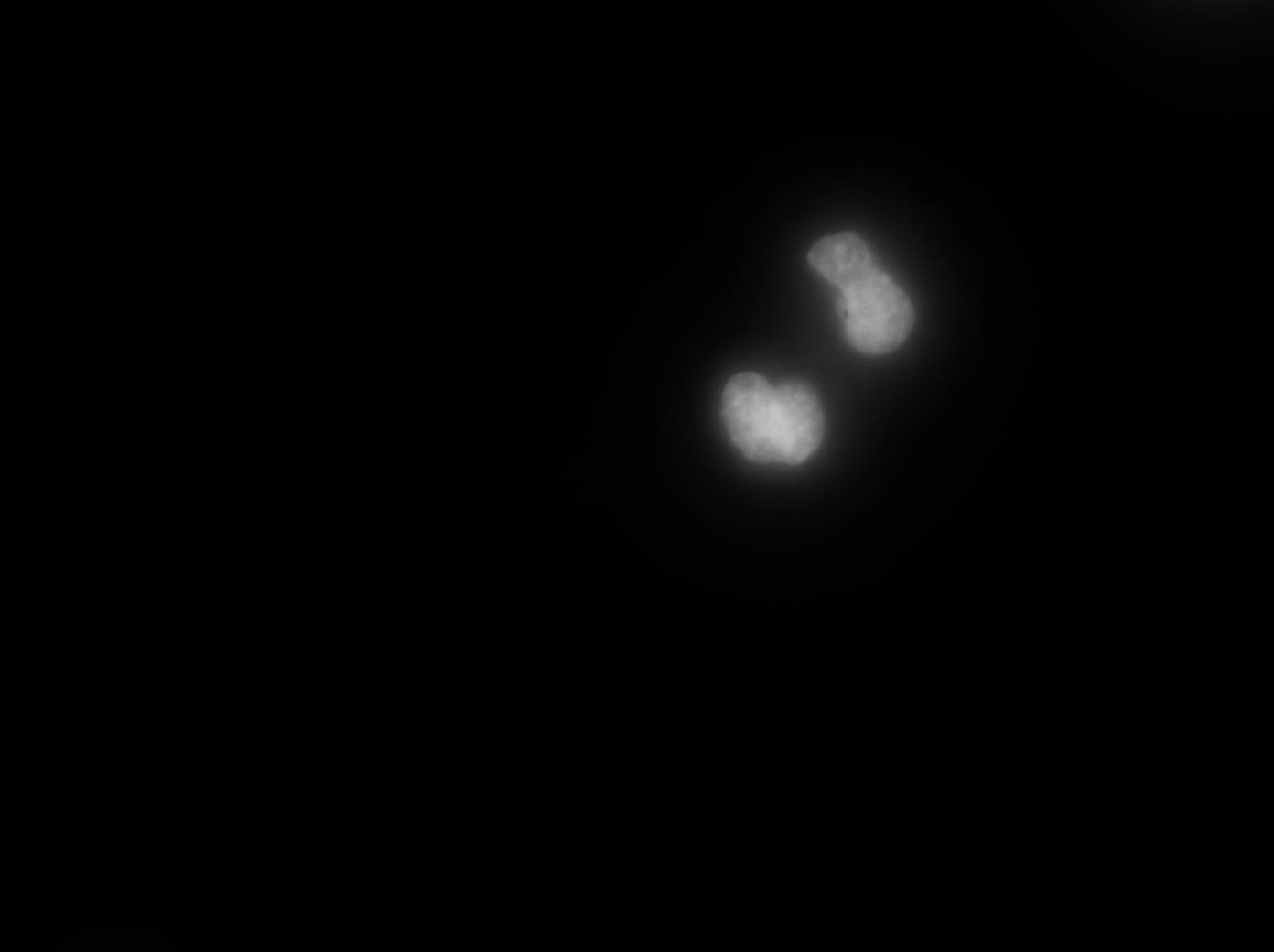

Supplement: Supplementary file 22 — Source data Fig. 6 part 3 [file 44319_2026_742_MOESM22_ESM.zip › Figure 6 Part 3/Fig 6efg TPGS1-KO TPGS1 rescue experiments/R1/TPGS1-KO EYFP only actub R1 7-31-25 ET2.Project Maximum Z_XY1754335621_Z0_T0_C0.tif]

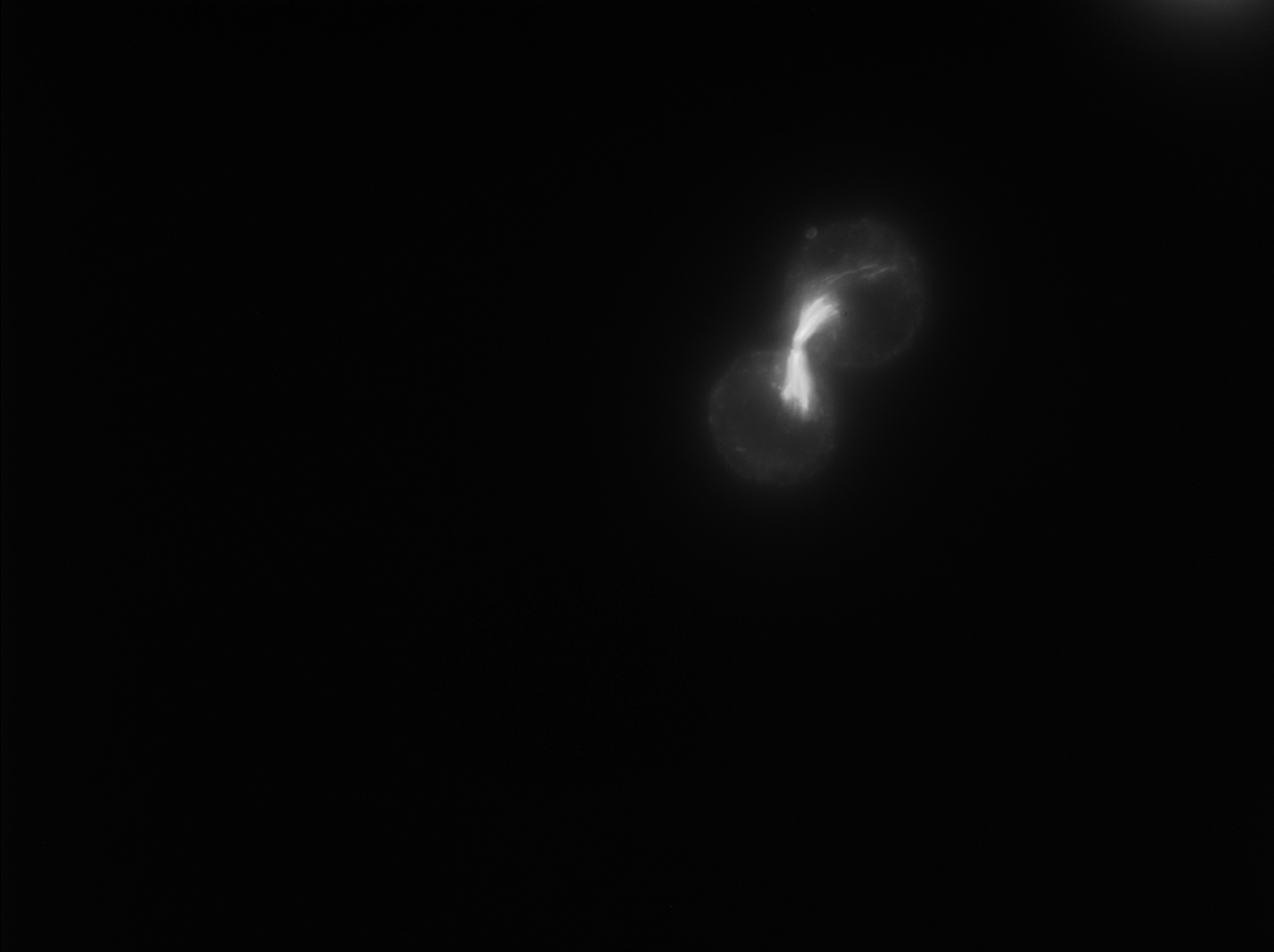

Supplement: Supplementary file 22 — Source data Fig. 6 part 3 [file 44319_2026_742_MOESM22_ESM.zip › Figure 6 Part 3/Fig 6efg TPGS1-KO TPGS1 rescue experiments/R1/TPGS1-KO EYFP only actub R1 7-31-25 ET2.Project Maximum Z_XY1754335621_Z0_T0_C2.tif]

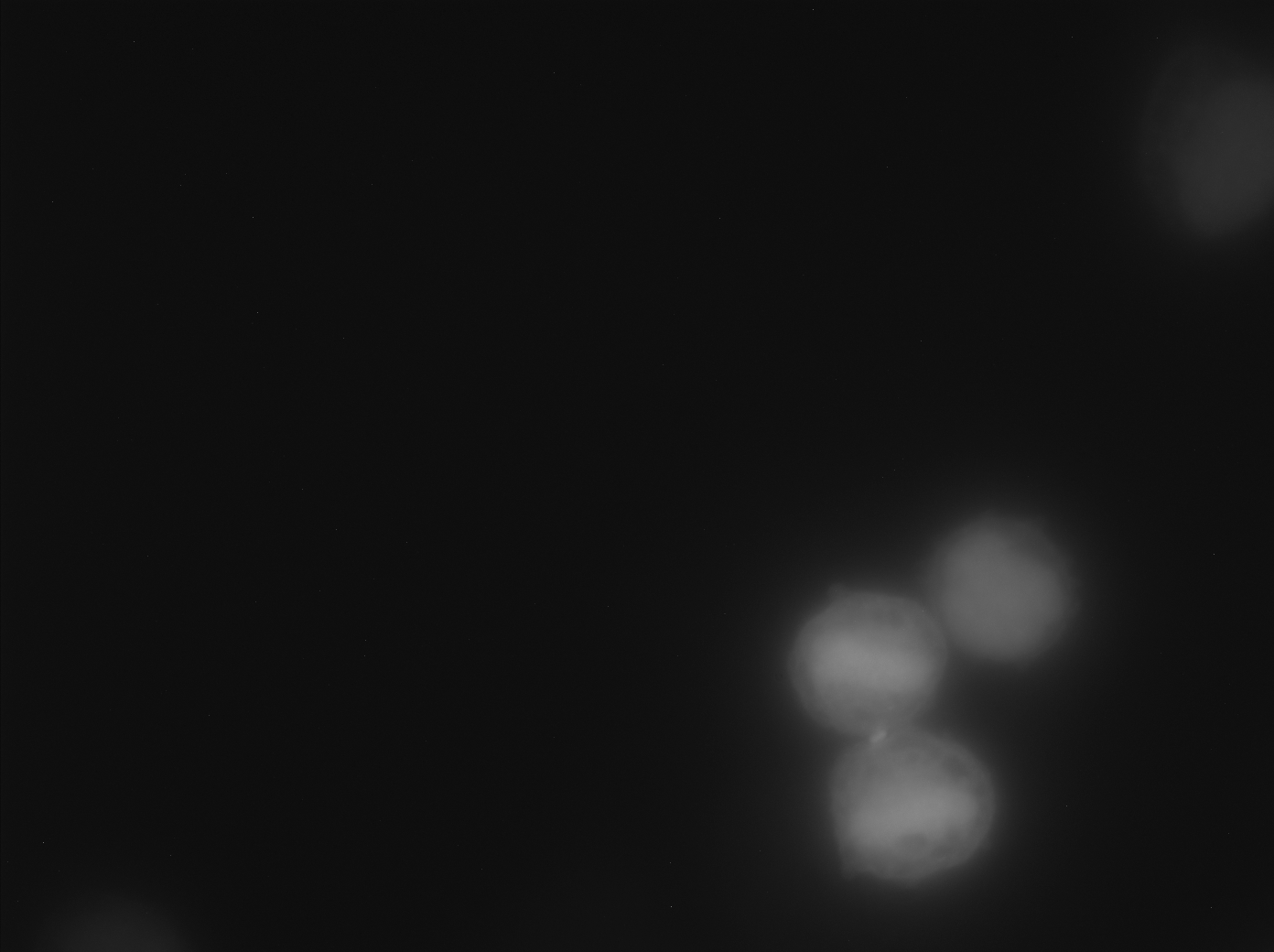

Supplement: Supplementary file 22 — Source data Fig. 6 part 3 [file 44319_2026_742_MOESM22_ESM.zip › Figure 6 Part 3/Fig 6efg TPGS1-KO TPGS1 rescue experiments/R1/TPGS1-KO EYFP only actub R1 7-31-25 ET10.Project Maximum Z_XY1754339543_Z0_T0_C1.tif]

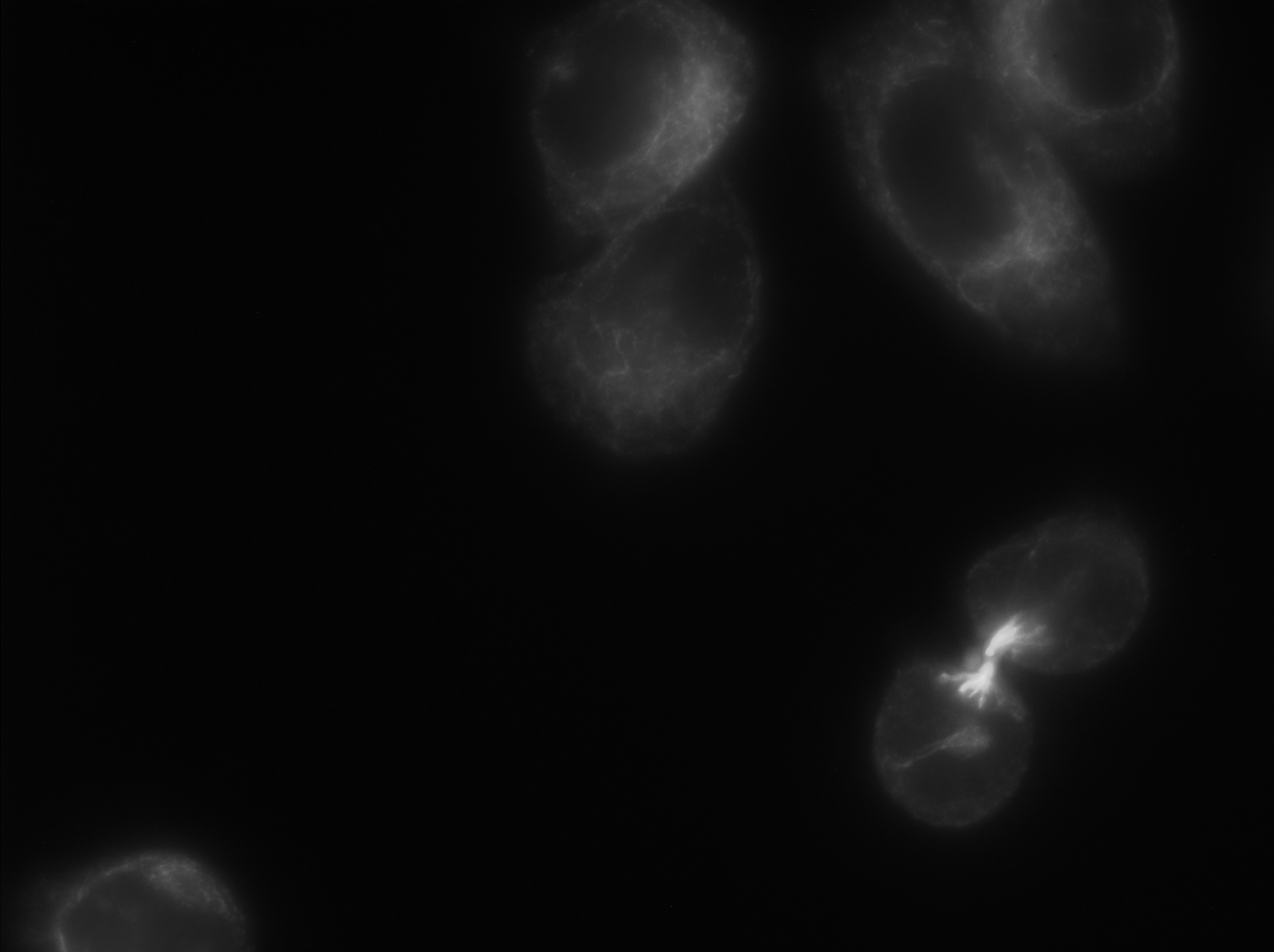

Supplement: Supplementary file 22 — Source data Fig. 6 part 3 [file 44319_2026_742_MOESM22_ESM.zip › Figure 6 Part 3/Fig 6efg TPGS1-KO TPGS1 rescue experiments/R1/TPGS1-KO TPGS1-3UTR-EYFP actub R1 7-31-25 ET5.Project Maximum Z_XY1753991318_Z0_T0_C2.tif]

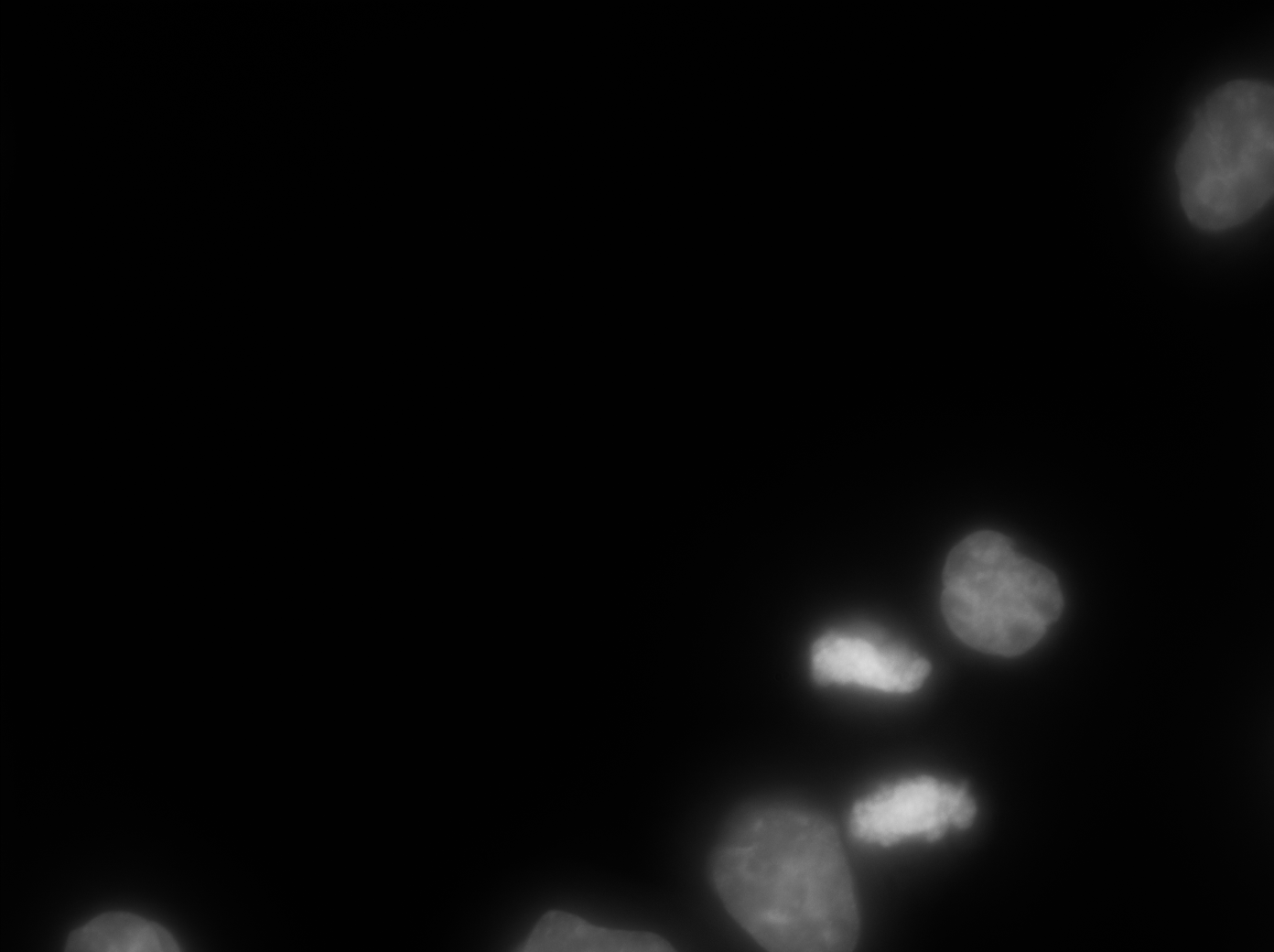

Supplement: Supplementary file 22 — Source data Fig. 6 part 3 [file 44319_2026_742_MOESM22_ESM.zip › Figure 6 Part 3/Fig 6efg TPGS1-KO TPGS1 rescue experiments/R1/TPGS1-KO EYFP only actub R1 7-31-25 ET10.Project Maximum Z_XY1754339543_Z0_T0_C0.tif]

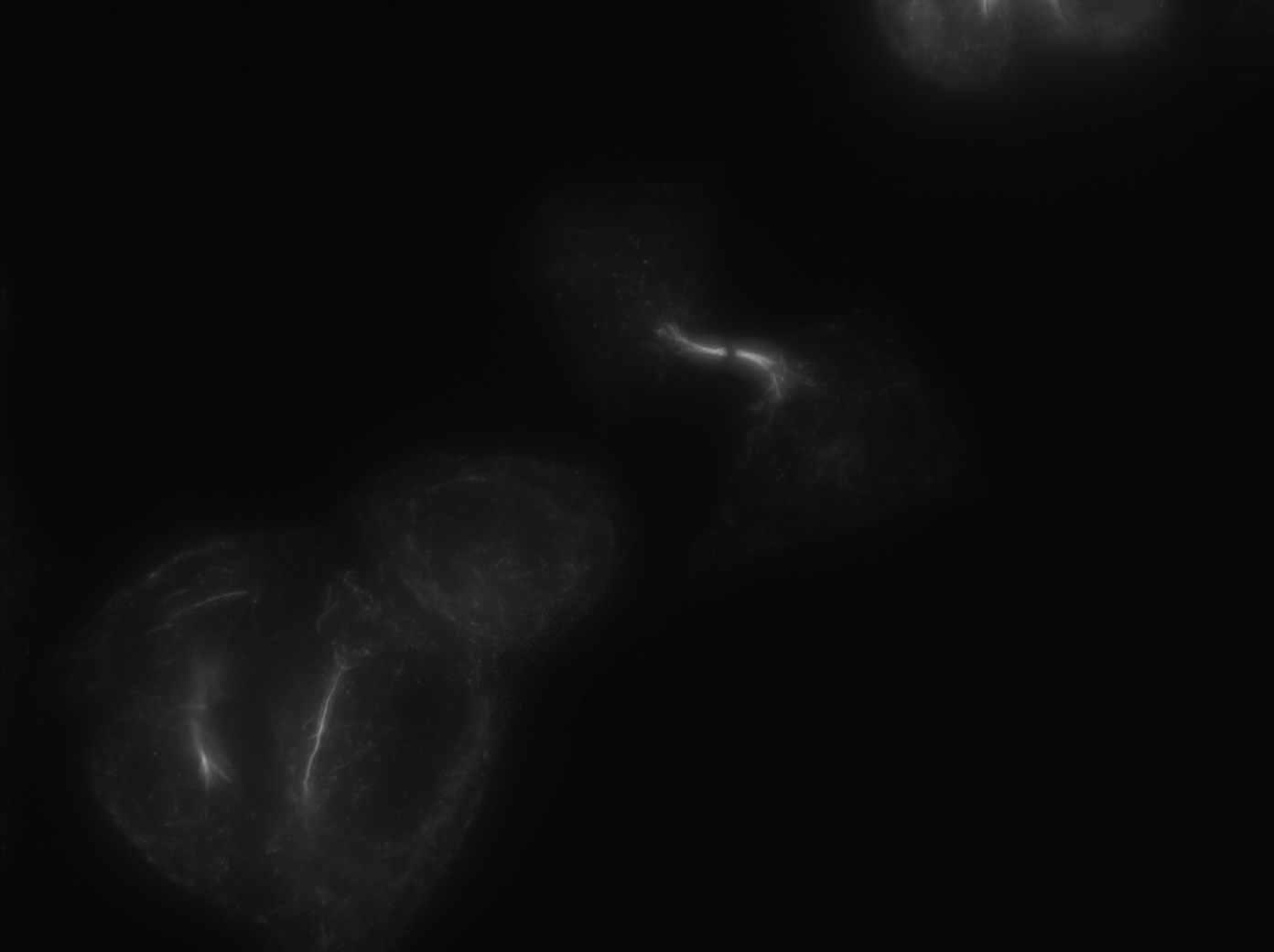

Supplement: Supplementary file 22 — Source data Fig. 6 part 3 [file 44319_2026_742_MOESM22_ESM.zip › Figure 6 Part 3/Fig 6efg TPGS1-KO TPGS1 rescue experiments/R1/TPGS1-KO EYFP only actub R1 7-31-25 LT6.Project Maximum Z_XY1754336444_Z0_T0_C2.tif]

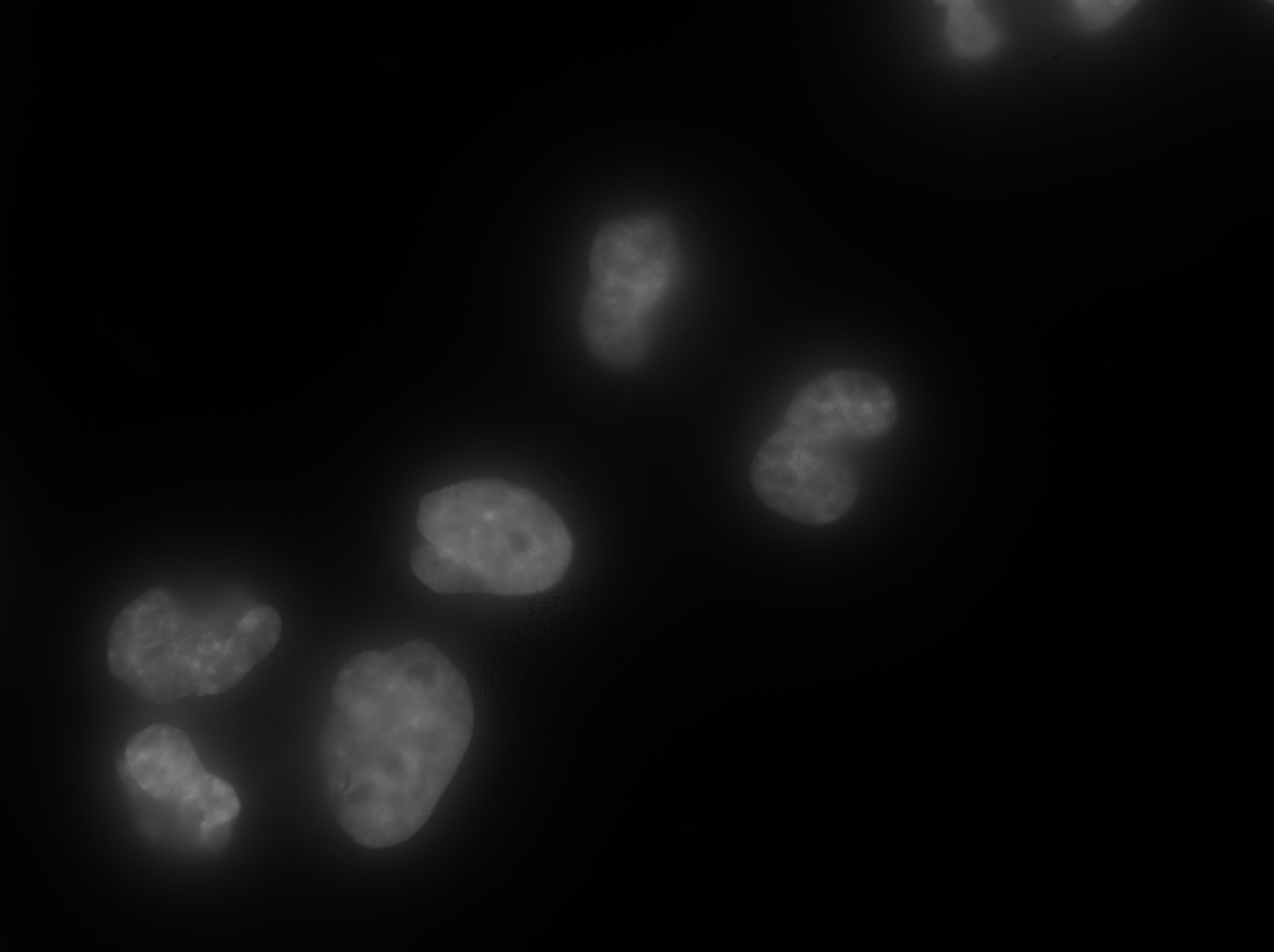

Supplement: Supplementary file 22 — Source data Fig. 6 part 3 [file 44319_2026_742_MOESM22_ESM.zip › Figure 6 Part 3/Fig 6efg TPGS1-KO TPGS1 rescue experiments/R1/TPGS1-KO EYFP only actub R1 7-31-25 LT6.Project Maximum Z_XY1754336444_Z0_T0_C0.tif]

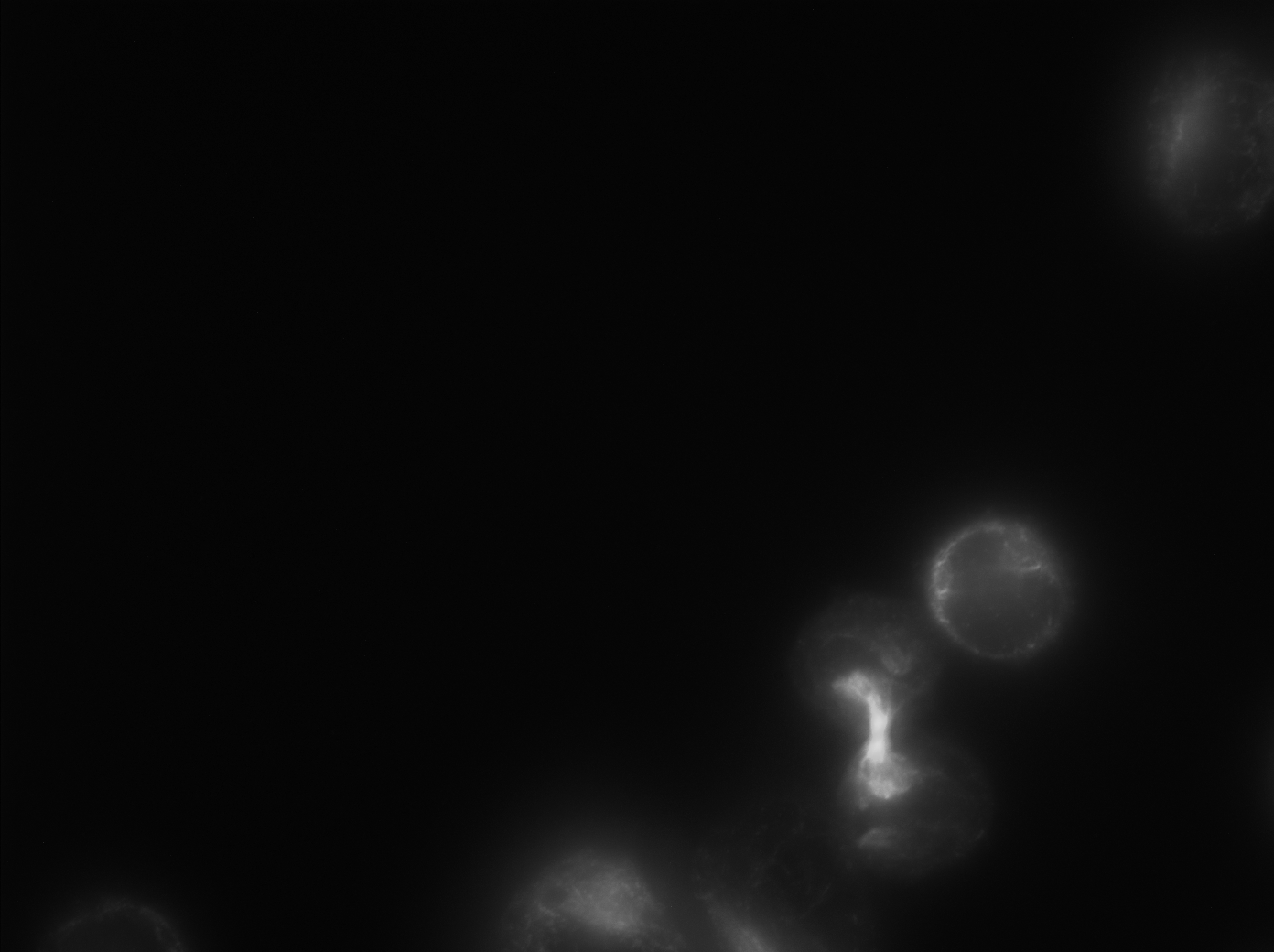

Supplement: Supplementary file 22 — Source data Fig. 6 part 3 [file 44319_2026_742_MOESM22_ESM.zip › Figure 6 Part 3/Fig 6efg TPGS1-KO TPGS1 rescue experiments/R1/TPGS1-KO EYFP only actub R1 7-31-25 ET10.Project Maximum Z_XY1754339543_Z0_T0_C2.tif]

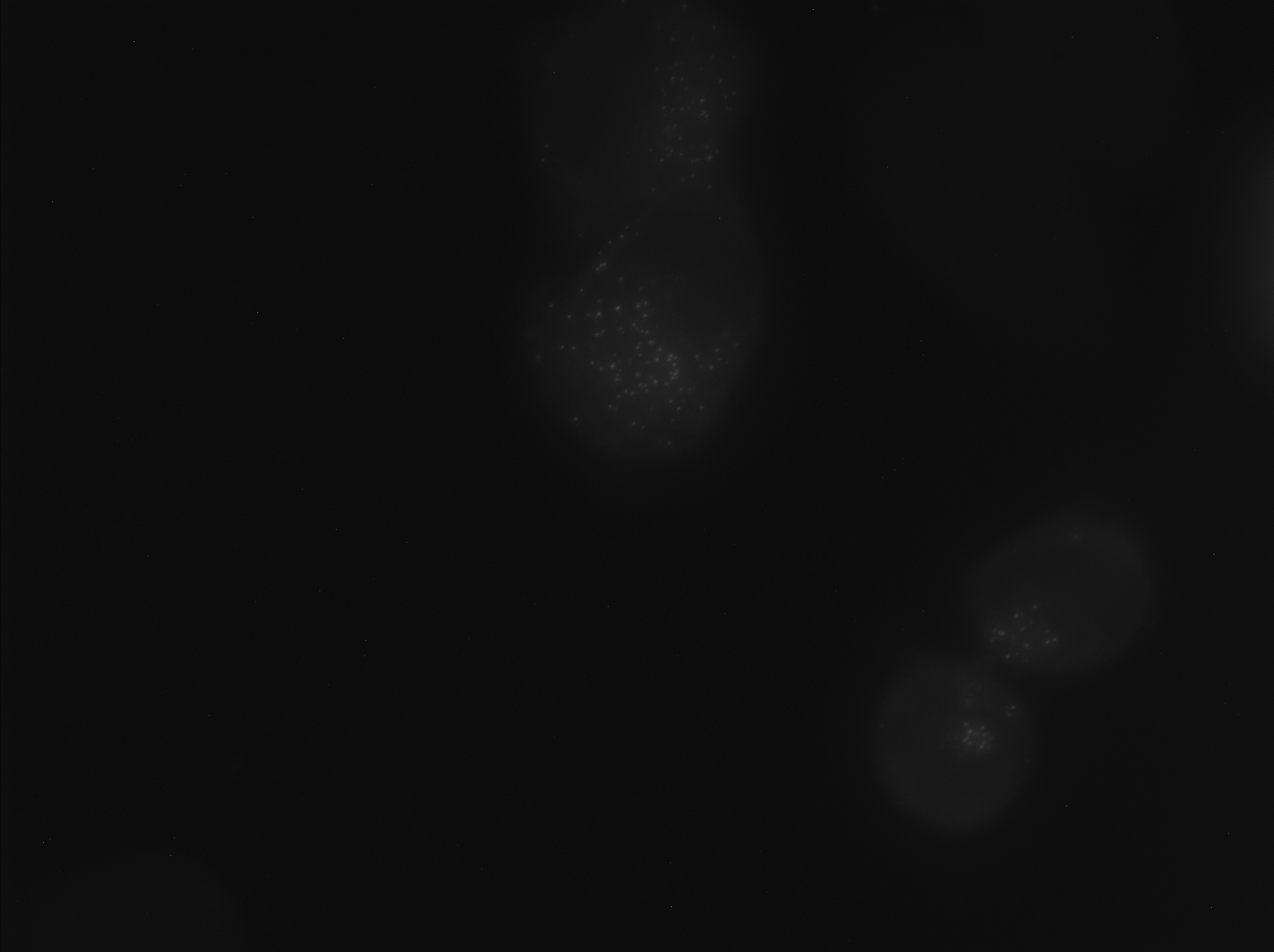

Supplement: Supplementary file 22 — Source data Fig. 6 part 3 [file 44319_2026_742_MOESM22_ESM.zip › Figure 6 Part 3/Fig 6efg TPGS1-KO TPGS1 rescue experiments/R1/TPGS1-KO TPGS1-3UTR-EYFP actub R1 7-31-25 ET5.Project Maximum Z_XY1753991318_Z0_T0_C1.tif]

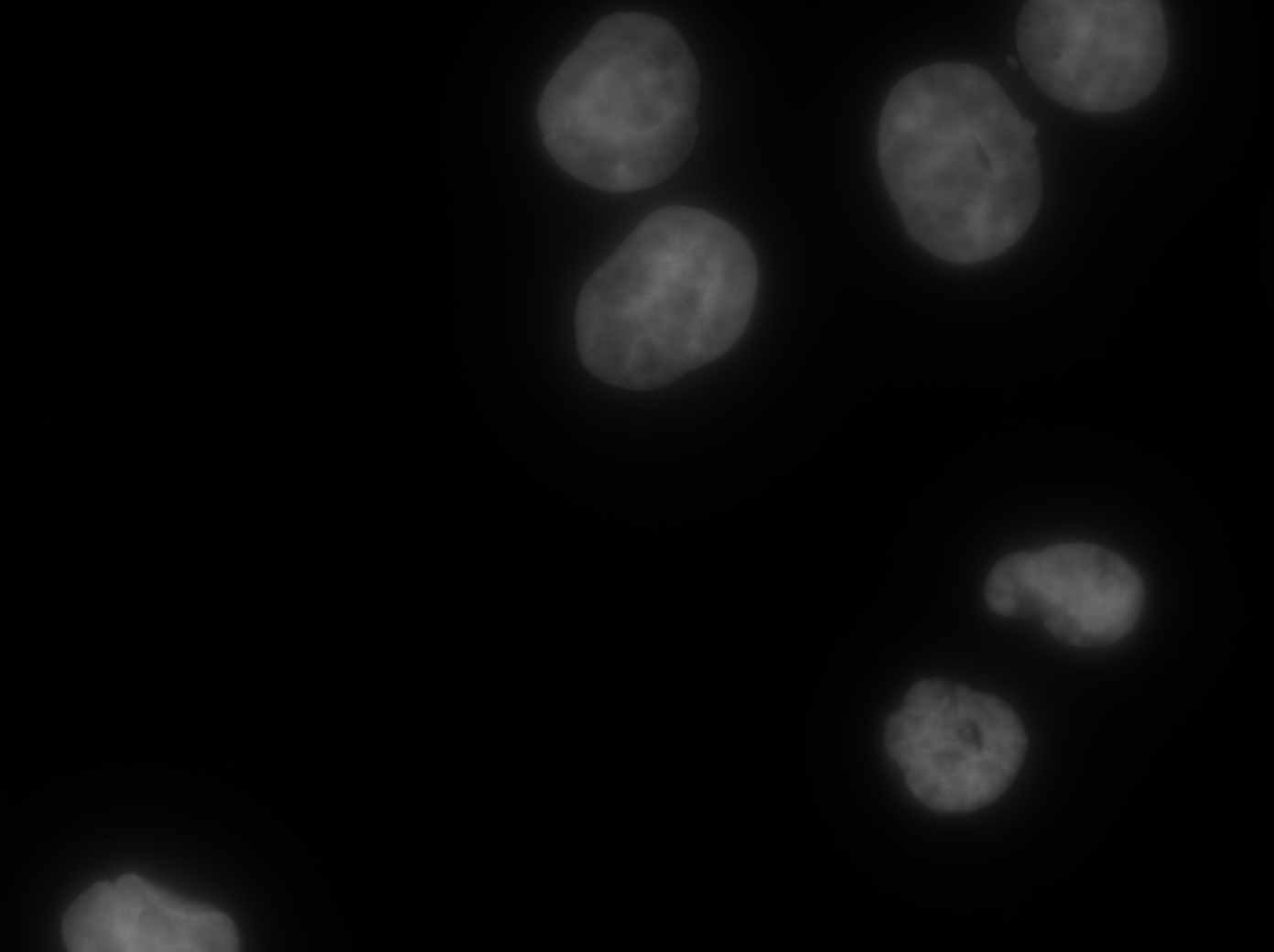

Supplement: Supplementary file 22 — Source data Fig. 6 part 3 [file 44319_2026_742_MOESM22_ESM.zip › Figure 6 Part 3/Fig 6efg TPGS1-KO TPGS1 rescue experiments/R1/TPGS1-KO TPGS1-3UTR-EYFP actub R1 7-31-25 ET5.Project Maximum Z_XY1753991318_Z0_T0_C0.tif]

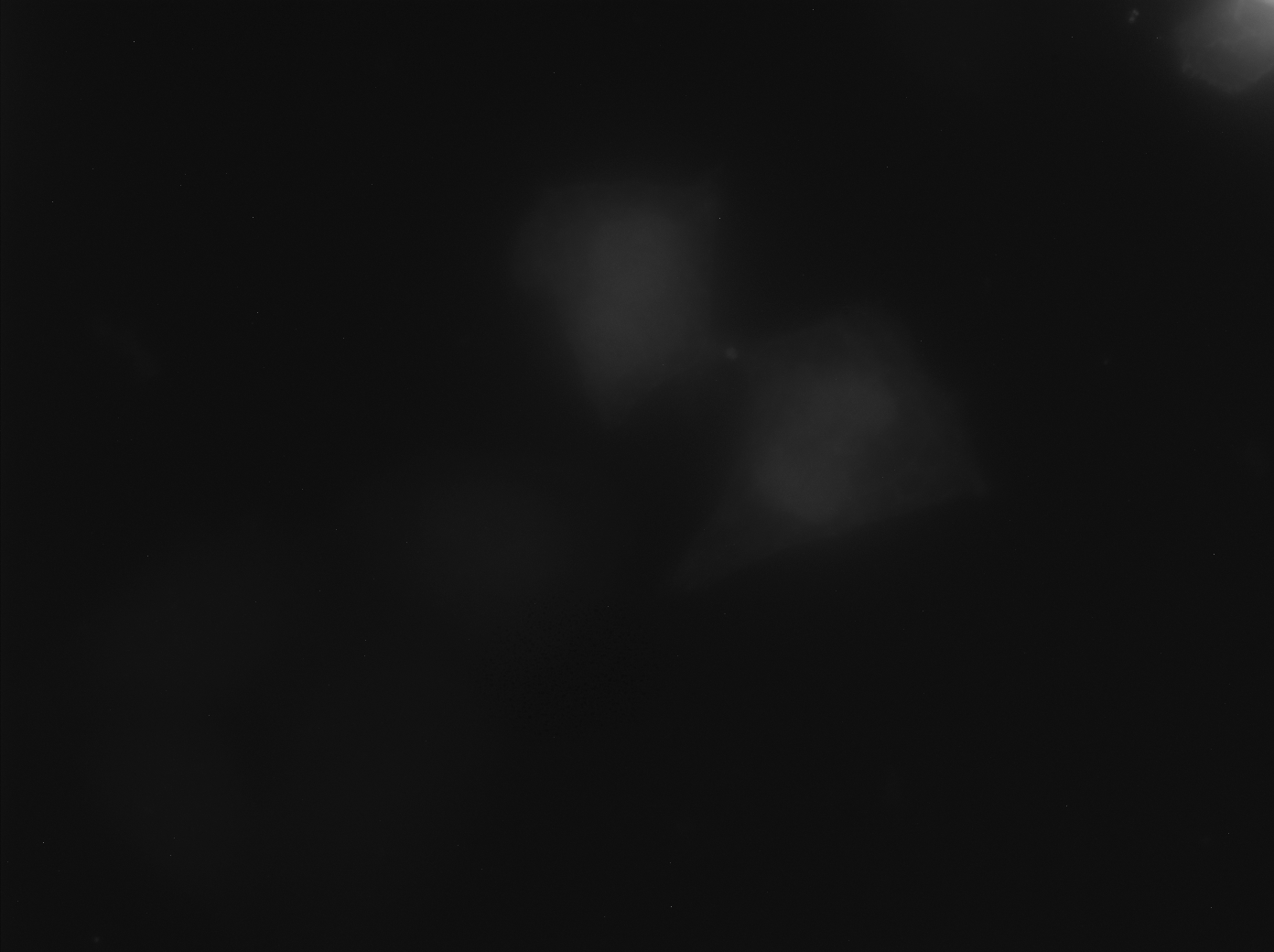

Supplement: Supplementary file 22 — Source data Fig. 6 part 3 [file 44319_2026_742_MOESM22_ESM.zip › Figure 6 Part 3/Fig 6efg TPGS1-KO TPGS1 rescue experiments/R1/TPGS1-KO EYFP only actub R1 7-31-25 LT6.Project Maximum Z_XY1754336444_Z0_T0_C1.tif]

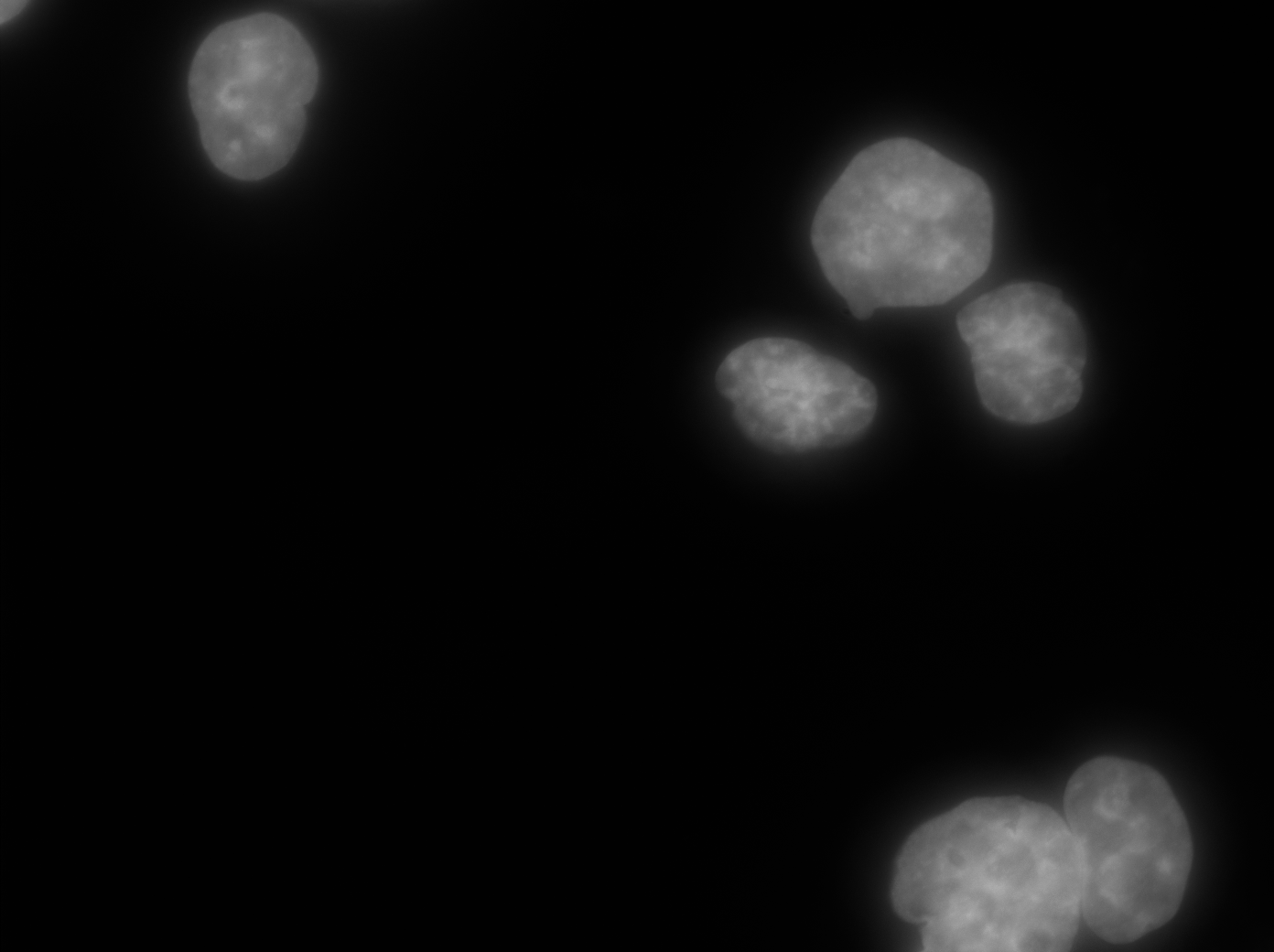

Supplement: Supplementary file 22 — Source data Fig. 6 part 3 [file 44319_2026_742_MOESM22_ESM.zip › Figure 6 Part 3/Fig 6efg TPGS1-KO TPGS1 rescue experiments/R1/TPGS1-KO TPGS1-3UTR-EYFP actub R1 7-31-25 LT1.Project Maximum Z_XY1753981191_Z0_T0_C0.tif]

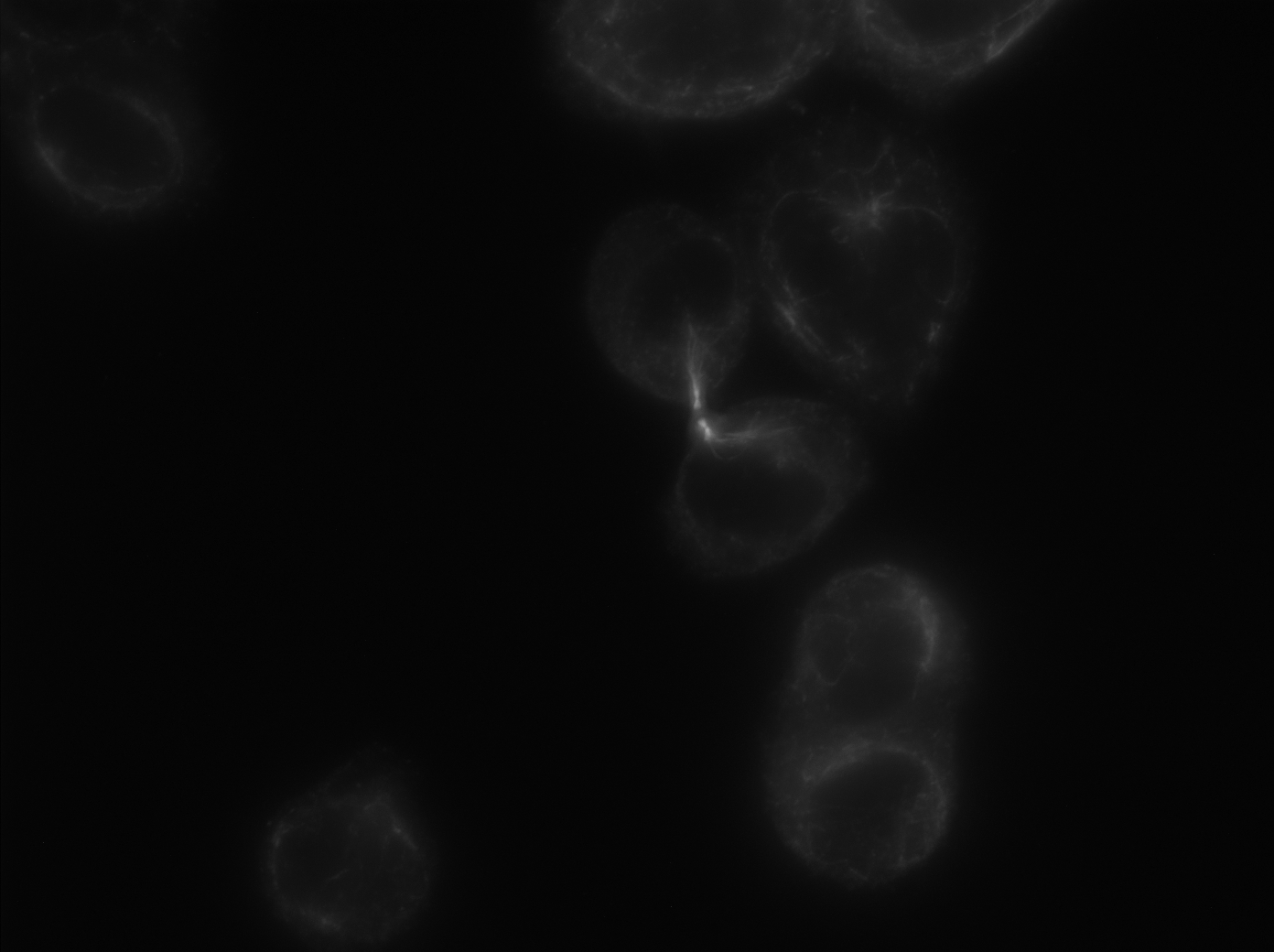

Supplement: Supplementary file 22 — Source data Fig. 6 part 3 [file 44319_2026_742_MOESM22_ESM.zip › Figure 6 Part 3/Fig 6efg TPGS1-KO TPGS1 rescue experiments/R1/TPGS1-KO EYFP only actub R1 7-31-25 LT8.Project Maximum Z_XY1754338859_Z0_T0_C2.tif]

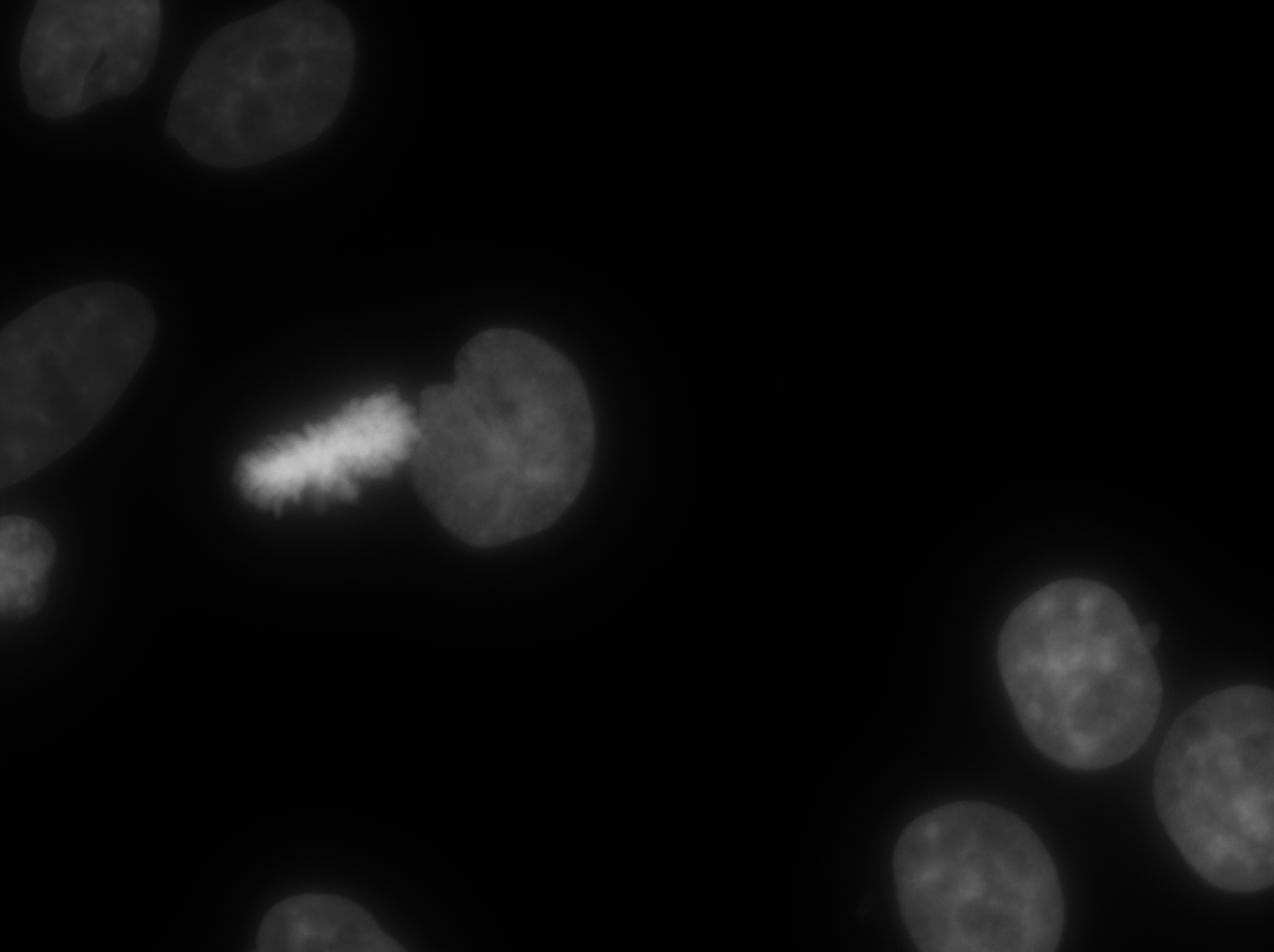

Supplement: Supplementary file 22 — Source data Fig. 6 part 3 [file 44319_2026_742_MOESM22_ESM.zip › Figure 6 Part 3/Fig 6efg TPGS1-KO TPGS1 rescue experiments/R1/TPGS1-KO TPGS1-3UTR-EYFP actub R1 7-31-25 M2.Project Maximum Z_XY1753993001_Z0_T0_C0.tif]

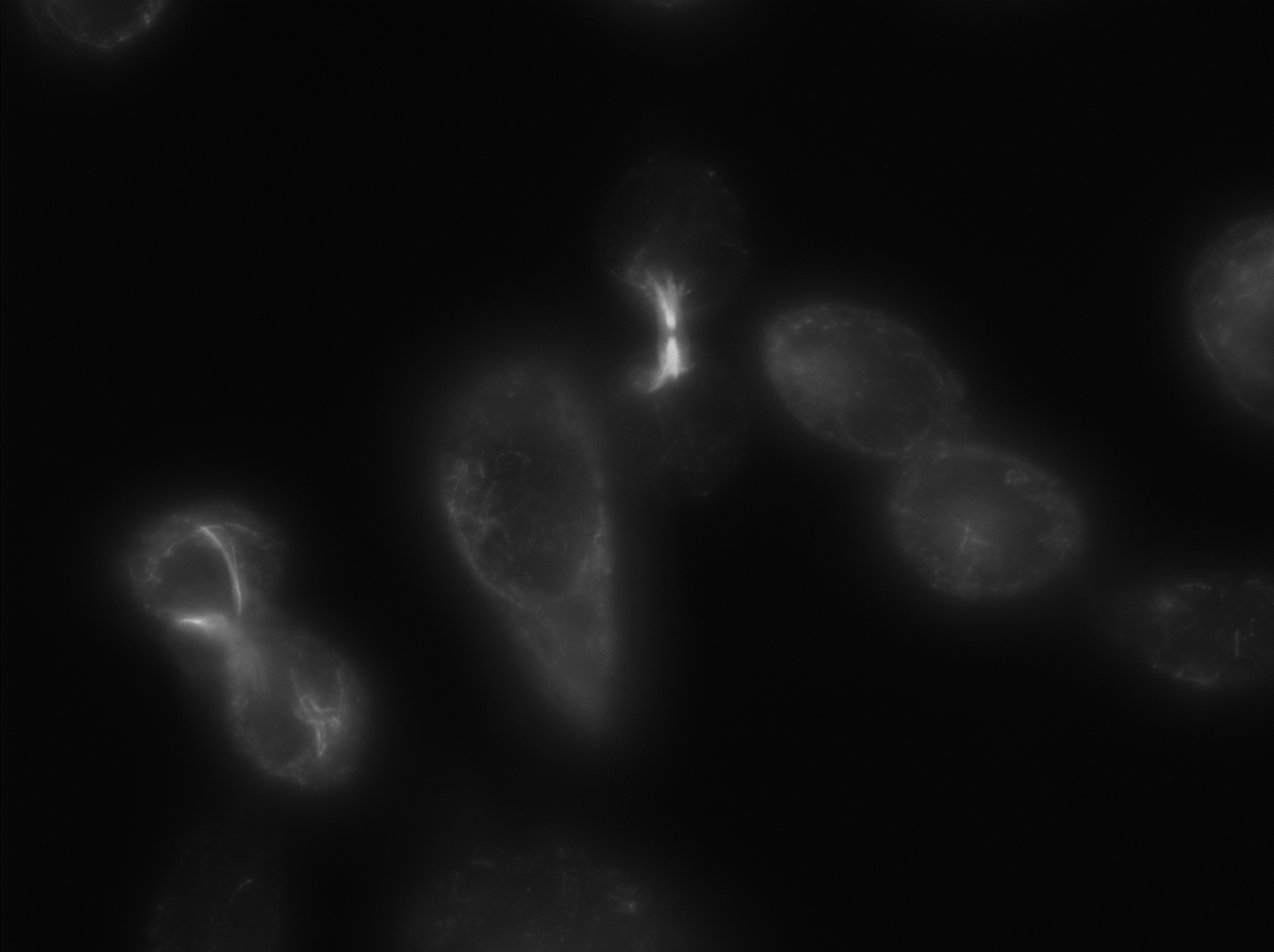

Supplement: Supplementary file 22 — Source data Fig. 6 part 3 [file 44319_2026_742_MOESM22_ESM.zip › Figure 6 Part 3/Fig 6efg TPGS1-KO TPGS1 rescue experiments/R1/TPGS1-KO TPGS1-3UTR-EYFP actub R1 7-31-25 ET7.Project Maximum Z_XY1753991843_Z0_T0_C2.tif]

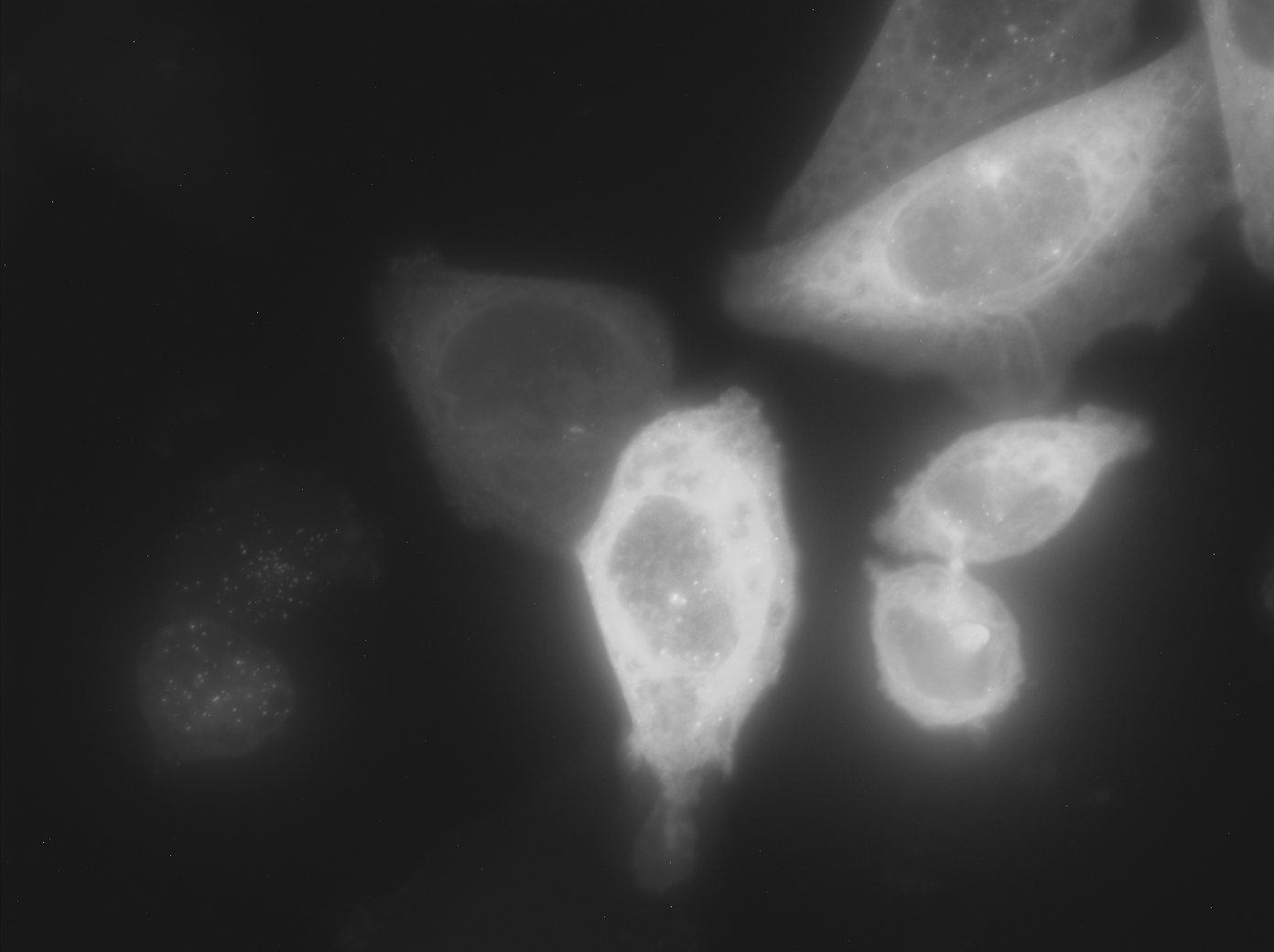

Supplement: Supplementary file 22 — Source data Fig. 6 part 3 [file 44319_2026_742_MOESM22_ESM.zip › Figure 6 Part 3/Fig 6efg TPGS1-KO TPGS1 rescue experiments/R1/TPGS1-KO TPGS1-3UTR-EYFP actub R1 7-31-25 ET2.Project Maximum Z_XY1753987827_Z0_T0_C1.tif]

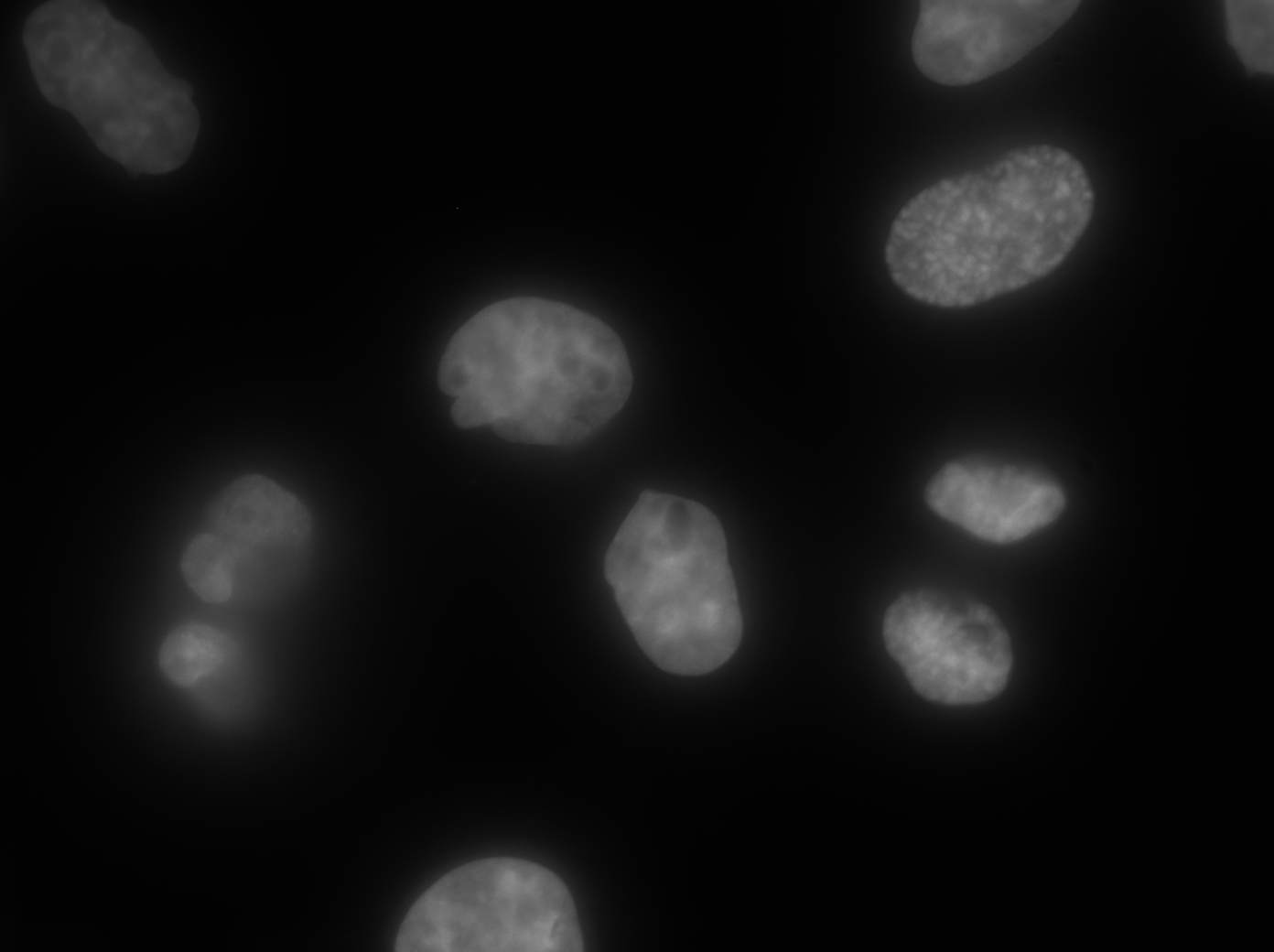

Supplement: Supplementary file 22 — Source data Fig. 6 part 3 [file 44319_2026_742_MOESM22_ESM.zip › Figure 6 Part 3/Fig 6efg TPGS1-KO TPGS1 rescue experiments/R1/TPGS1-KO TPGS1-3UTR-EYFP actub R1 7-31-25 ET2.Project Maximum Z_XY1753987827_Z0_T0_C0.tif]

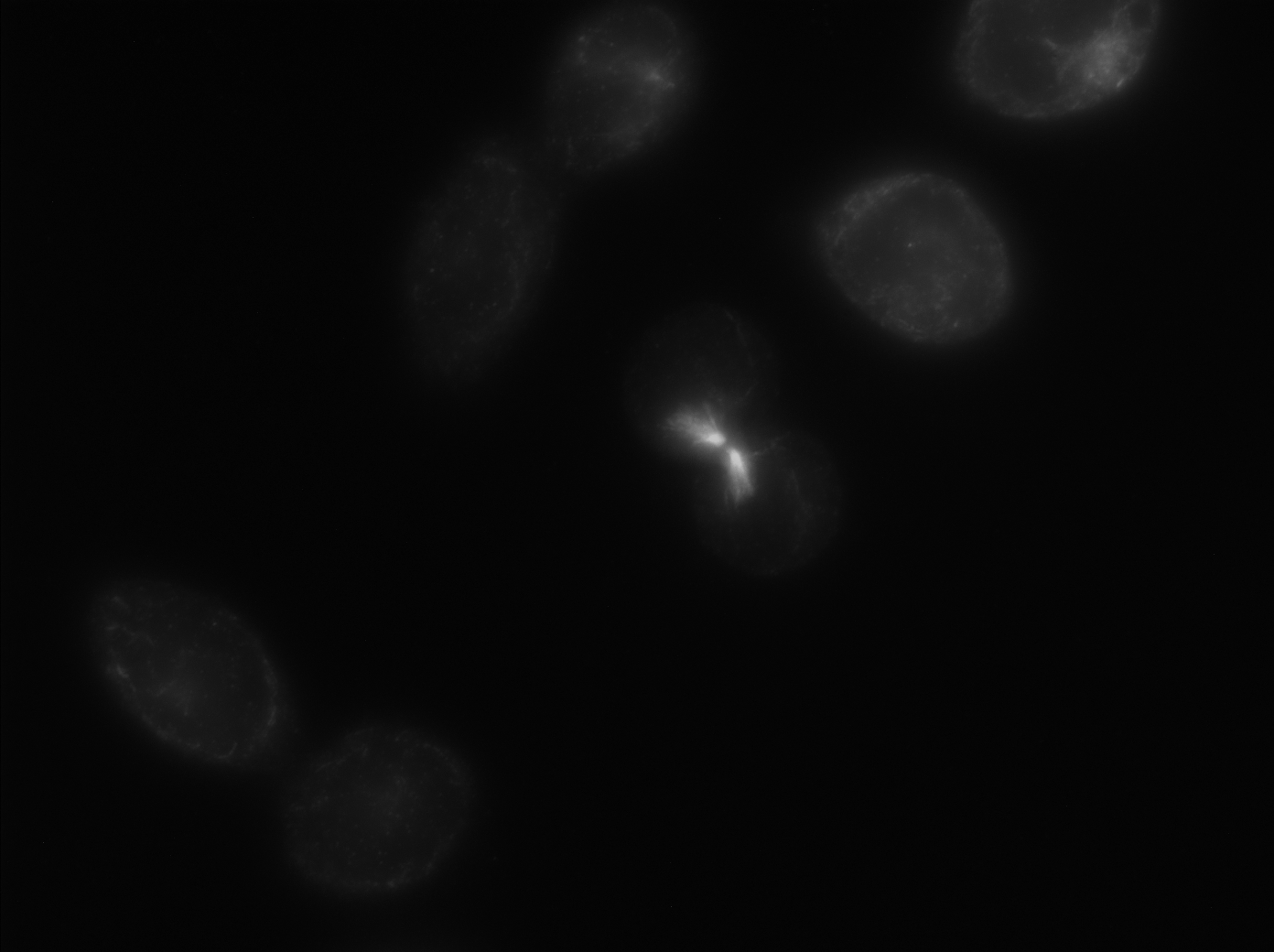

Supplement: Supplementary file 22 — Source data Fig. 6 part 3 [file 44319_2026_742_MOESM22_ESM.zip › Figure 6 Part 3/Fig 6efg TPGS1-KO TPGS1 rescue experiments/R1/TPGS1-KO TPGS1-3UTR-EYFP actub R1 7-31-25 ET3.Project Maximum Z_XY1753988552_Z0_T0_C2.tif]

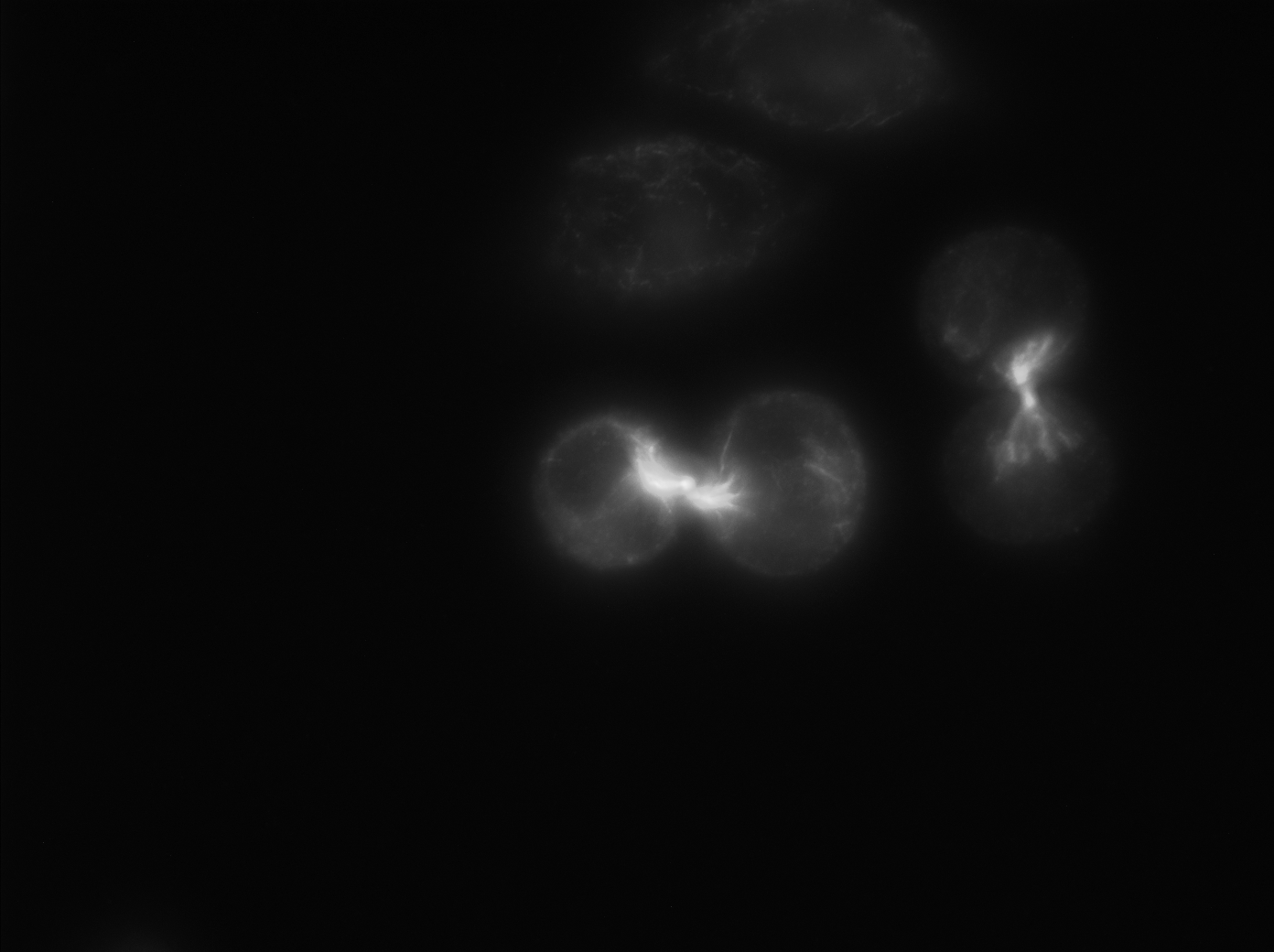

Supplement: Supplementary file 22 — Source data Fig. 6 part 3 [file 44319_2026_742_MOESM22_ESM.zip › Figure 6 Part 3/Fig 6efg TPGS1-KO TPGS1 rescue experiments/R1/TPGS1-KO TPGS1-3UTR-EYFP actub R1 7-31-25 ET10.Project Maximum Z_XY1753994658_Z0_T0_C2.tif]

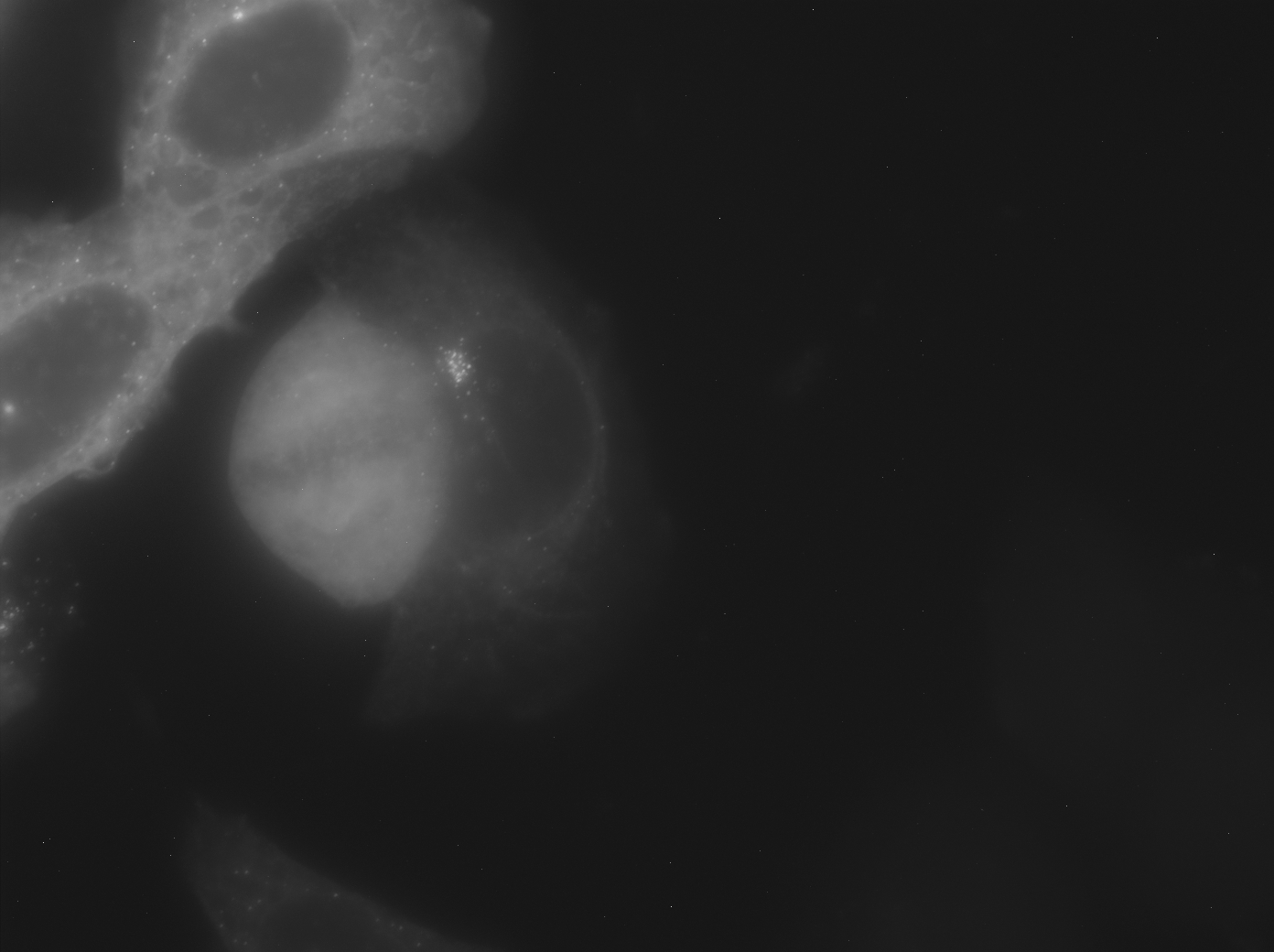

Supplement: Supplementary file 22 — Source data Fig. 6 part 3 [file 44319_2026_742_MOESM22_ESM.zip › Figure 6 Part 3/Fig 6efg TPGS1-KO TPGS1 rescue experiments/R1/TPGS1-KO TPGS1-3UTR-EYFP actub R1 7-31-25 M2.Project Maximum Z_XY1753993001_Z0_T0_C1.tif]

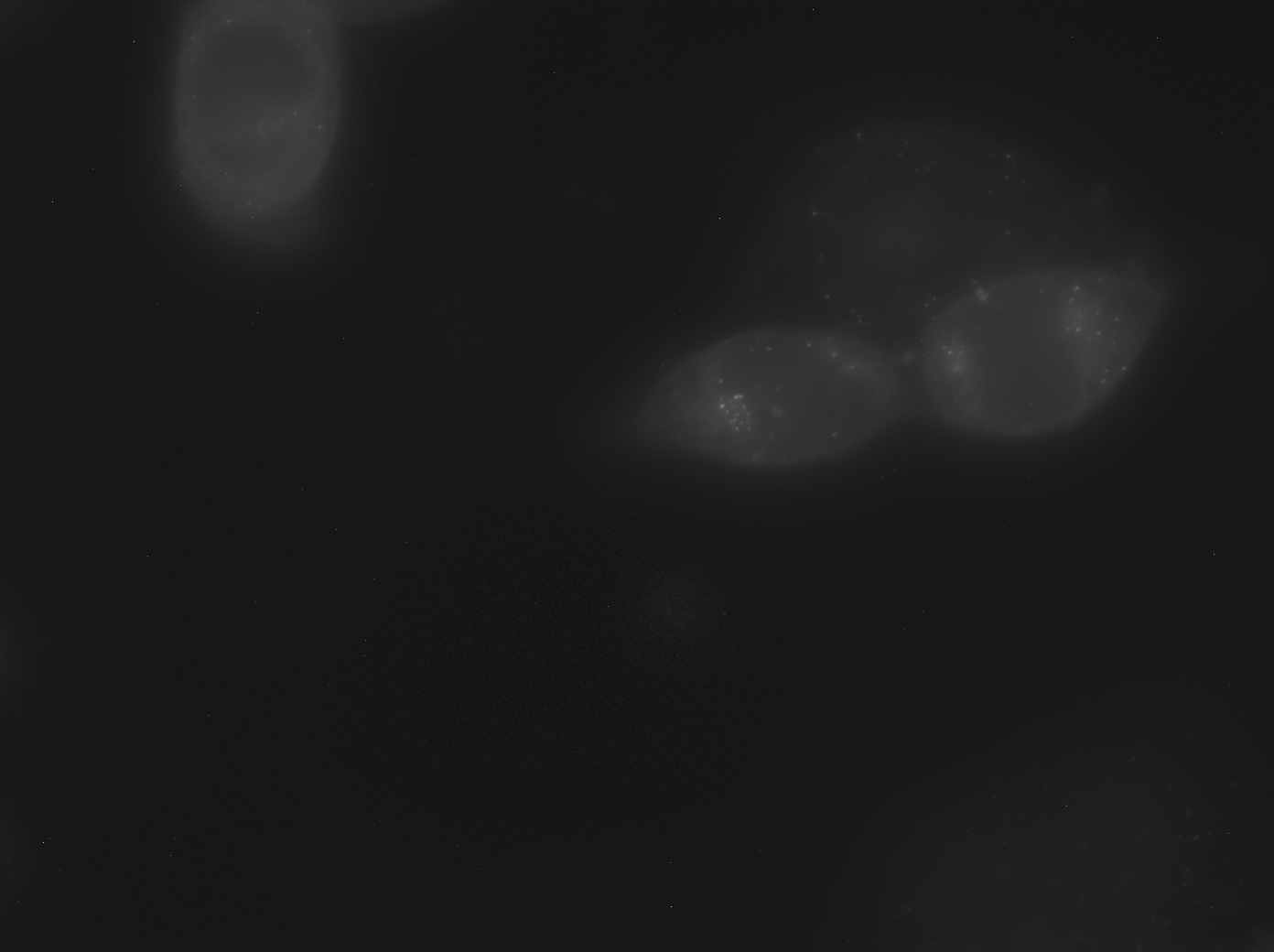

Supplement: Supplementary file 22 — Source data Fig. 6 part 3 [file 44319_2026_742_MOESM22_ESM.zip › Figure 6 Part 3/Fig 6efg TPGS1-KO TPGS1 rescue experiments/R1/TPGS1-KO TPGS1-3UTR-EYFP actub R1 7-31-25 LT1.Project Maximum Z_XY1753981191_Z0_T0_C1.tif]

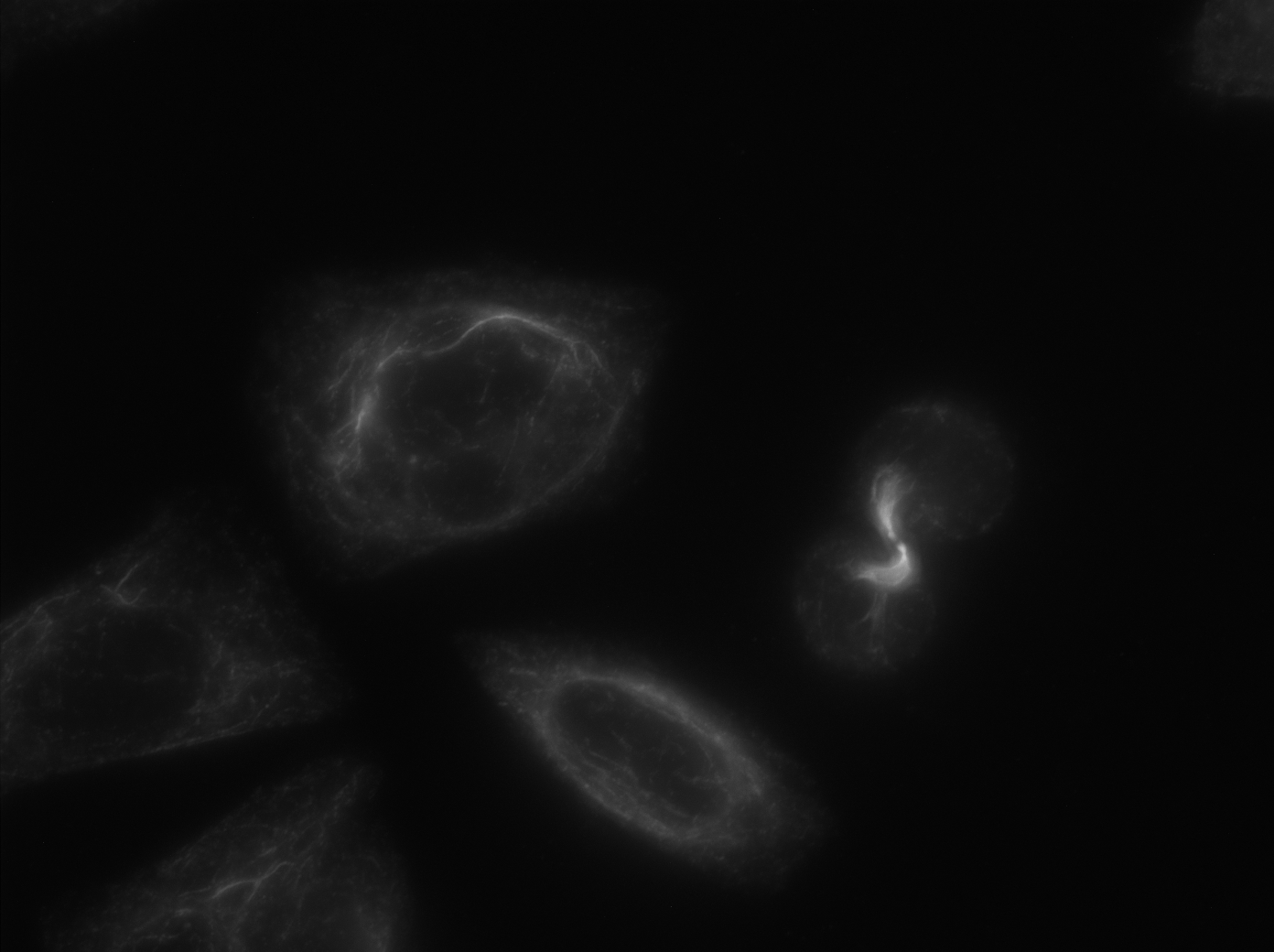

Supplement: Supplementary file 22 — Source data Fig. 6 part 3 [file 44319_2026_742_MOESM22_ESM.zip › Figure 6 Part 3/Fig 6efg TPGS1-KO TPGS1 rescue experiments/R1/TPGS1-KO TPGS1-3UTR-EYFP actub R1 7-31-25 ET4.Project Maximum Z_XY1753988880_Z0_T0_C2.tif]

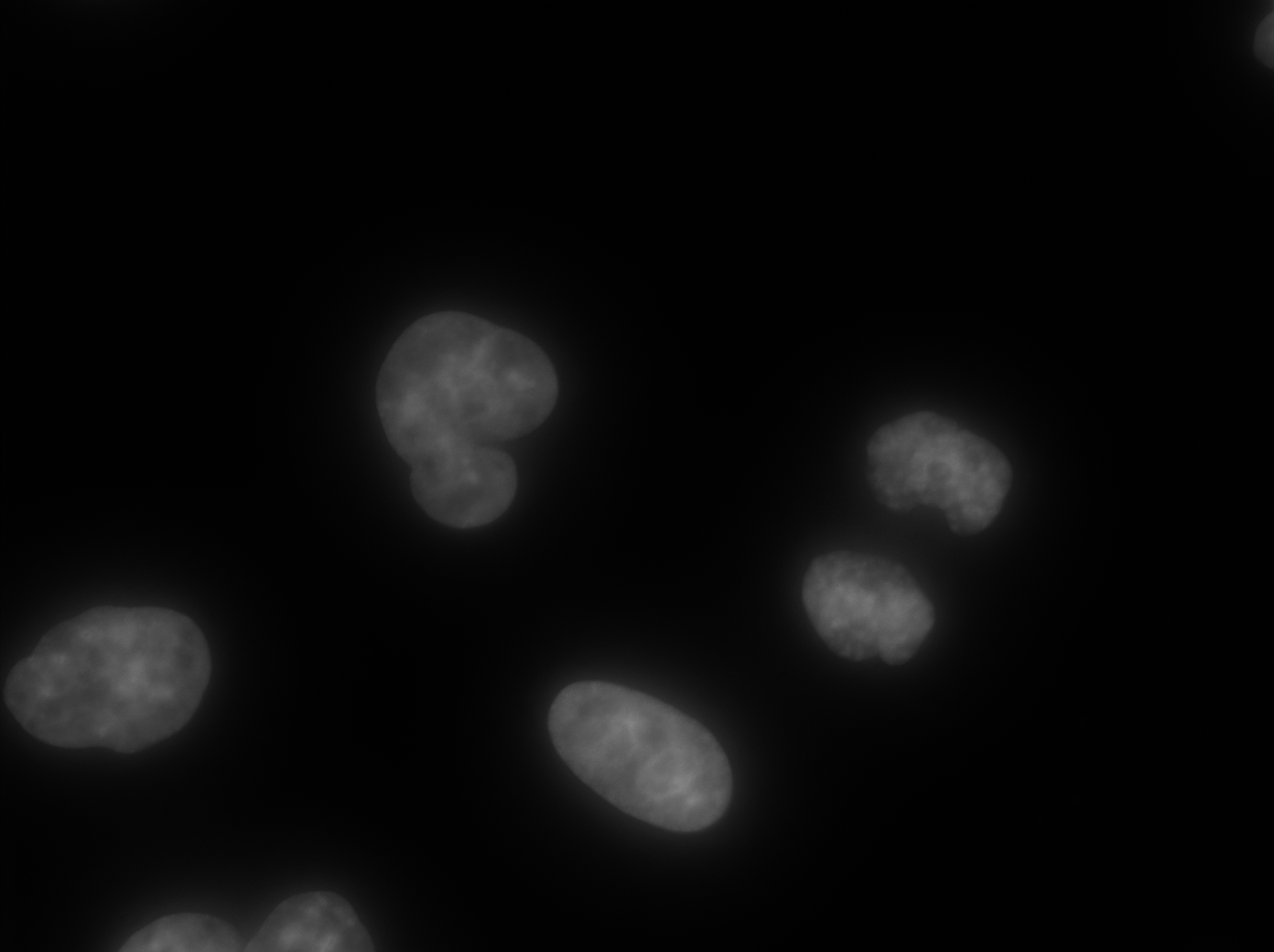

Supplement: Supplementary file 22 — Source data Fig. 6 part 3 [file 44319_2026_742_MOESM22_ESM.zip › Figure 6 Part 3/Fig 6efg TPGS1-KO TPGS1 rescue experiments/R1/TPGS1-KO TPGS1-3UTR-EYFP actub R1 7-31-25 ET4.Project Maximum Z_XY1753988880_Z0_T0_C0.tif]

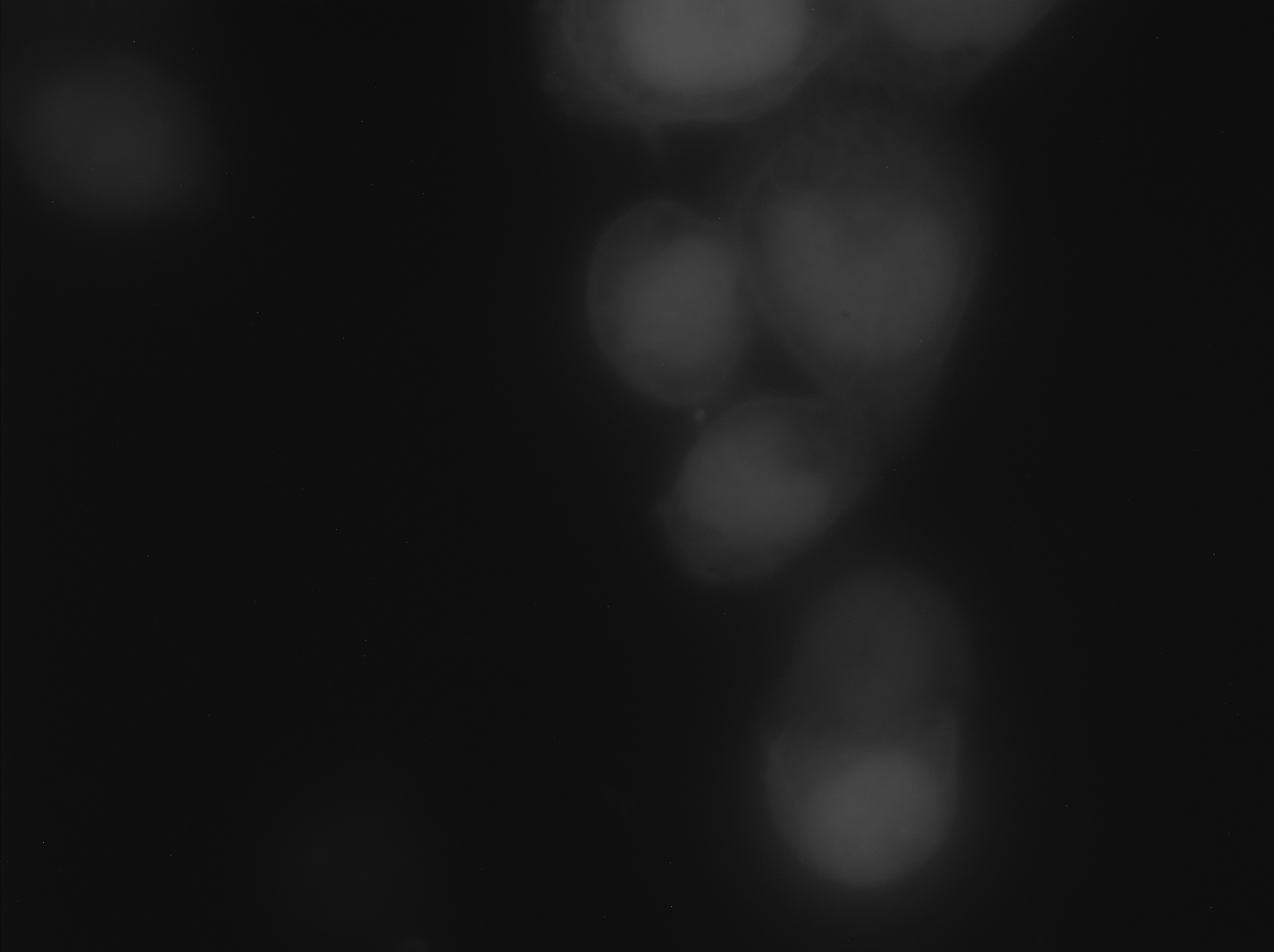

Supplement: Supplementary file 22 — Source data Fig. 6 part 3 [file 44319_2026_742_MOESM22_ESM.zip › Figure 6 Part 3/Fig 6efg TPGS1-KO TPGS1 rescue experiments/R1/TPGS1-KO EYFP only actub R1 7-31-25 LT8.Project Maximum Z_XY1754338859_Z0_T0_C1.tif]

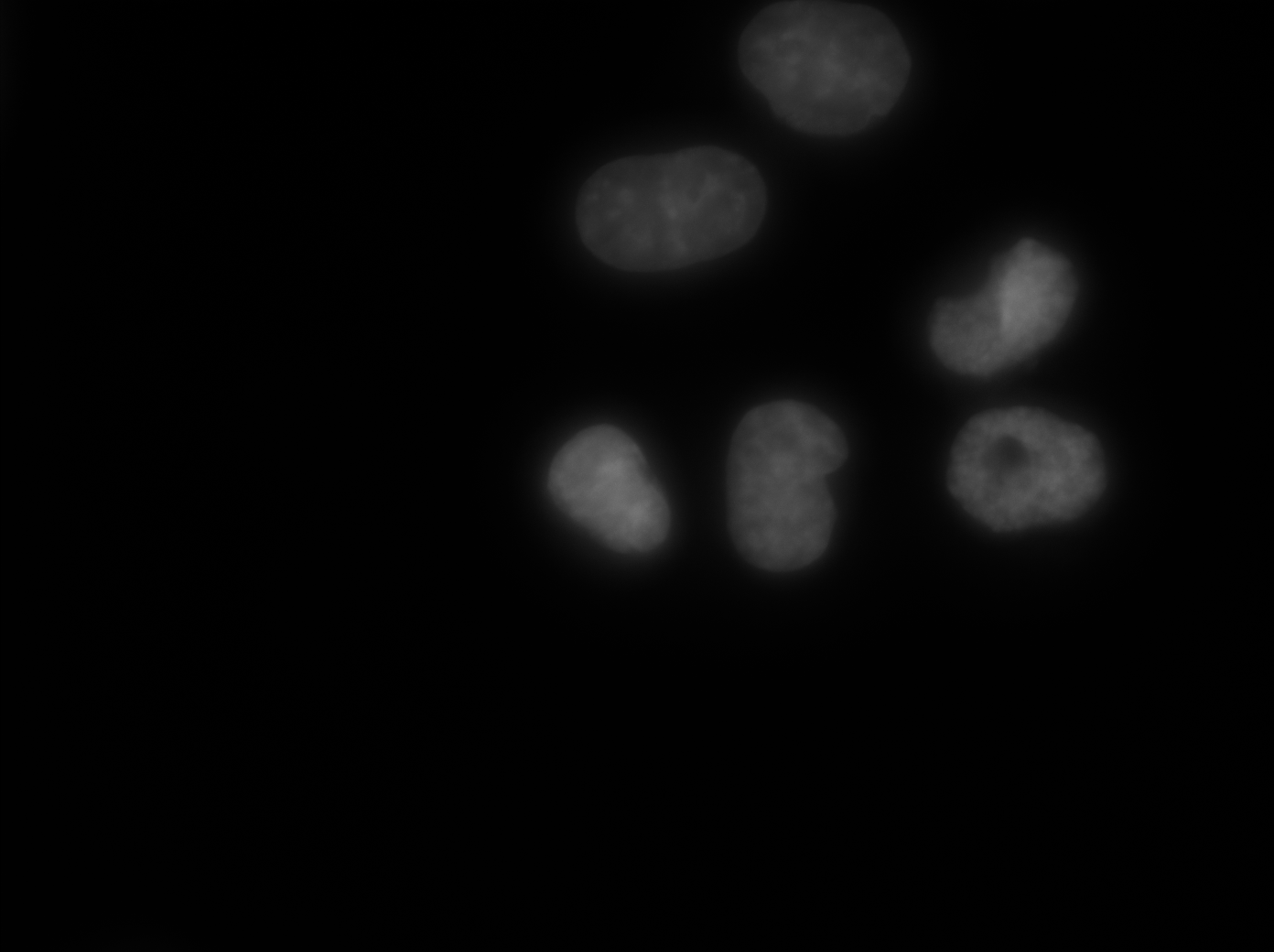

Supplement: Supplementary file 22 — Source data Fig. 6 part 3 [file 44319_2026_742_MOESM22_ESM.zip › Figure 6 Part 3/Fig 6efg TPGS1-KO TPGS1 rescue experiments/R1/TPGS1-KO TPGS1-3UTR-EYFP actub R1 7-31-25 ET10.Project Maximum Z_XY1753994658_Z0_T0_C0.tif]

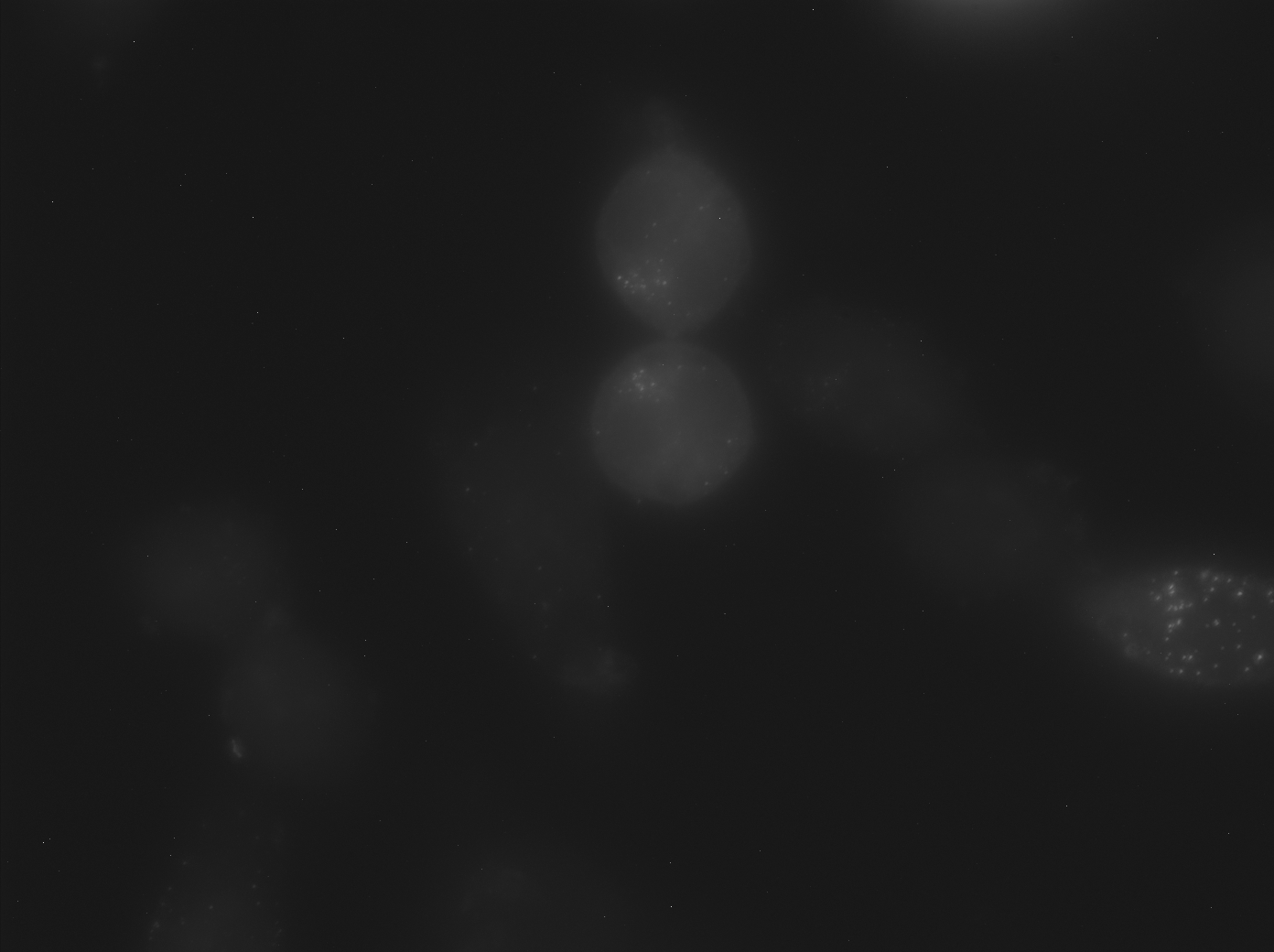

Supplement: Supplementary file 22 — Source data Fig. 6 part 3 [file 44319_2026_742_MOESM22_ESM.zip › Figure 6 Part 3/Fig 6efg TPGS1-KO TPGS1 rescue experiments/R1/TPGS1-KO TPGS1-3UTR-EYFP actub R1 7-31-25 ET7.Project Maximum Z_XY1753991843_Z0_T0_C1.tif]

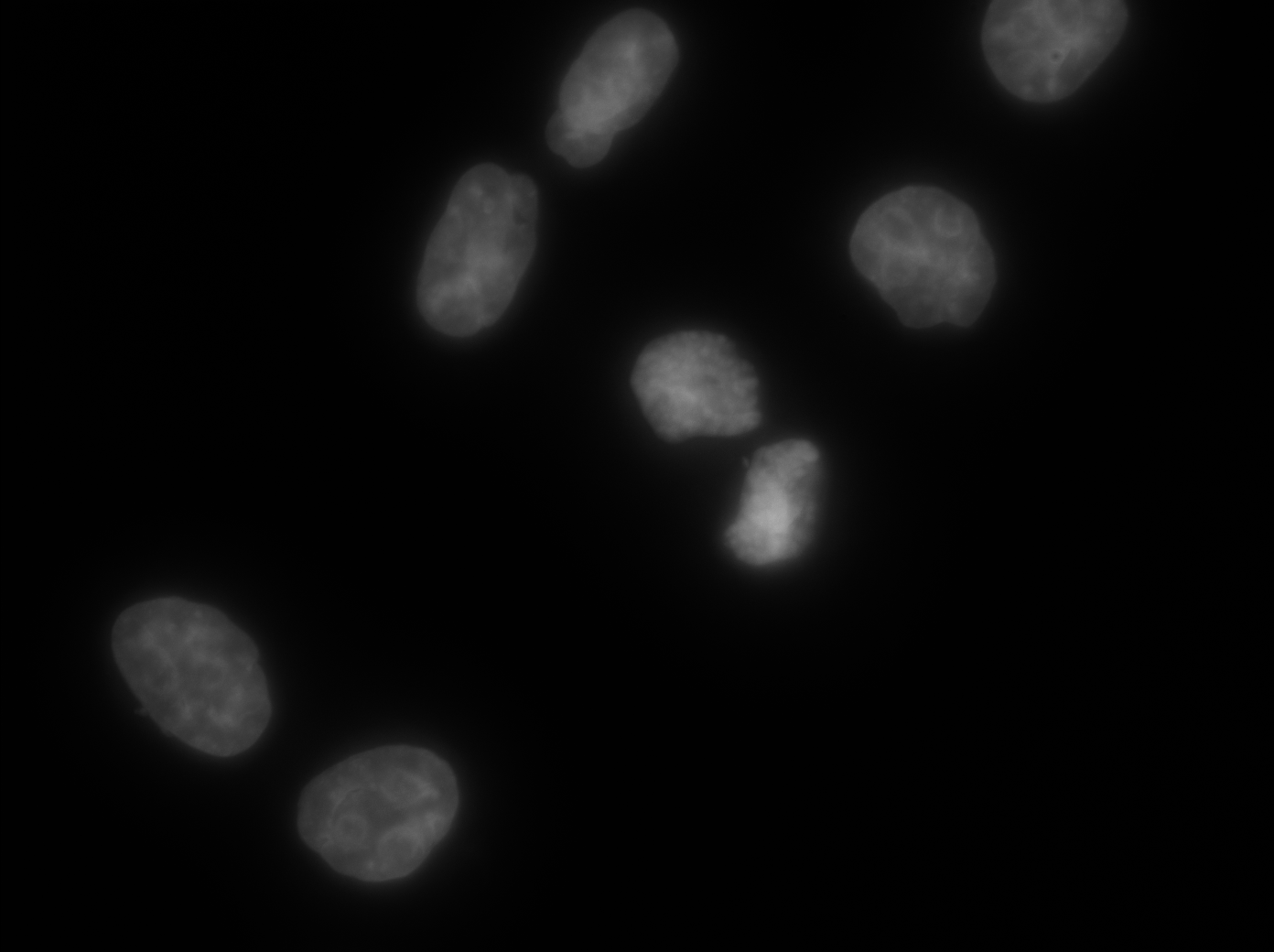

Supplement: Supplementary file 22 — Source data Fig. 6 part 3 [file 44319_2026_742_MOESM22_ESM.zip › Figure 6 Part 3/Fig 6efg TPGS1-KO TPGS1 rescue experiments/R1/TPGS1-KO TPGS1-3UTR-EYFP actub R1 7-31-25 ET3.Project Maximum Z_XY1753988552_Z0_T0_C0.tif]

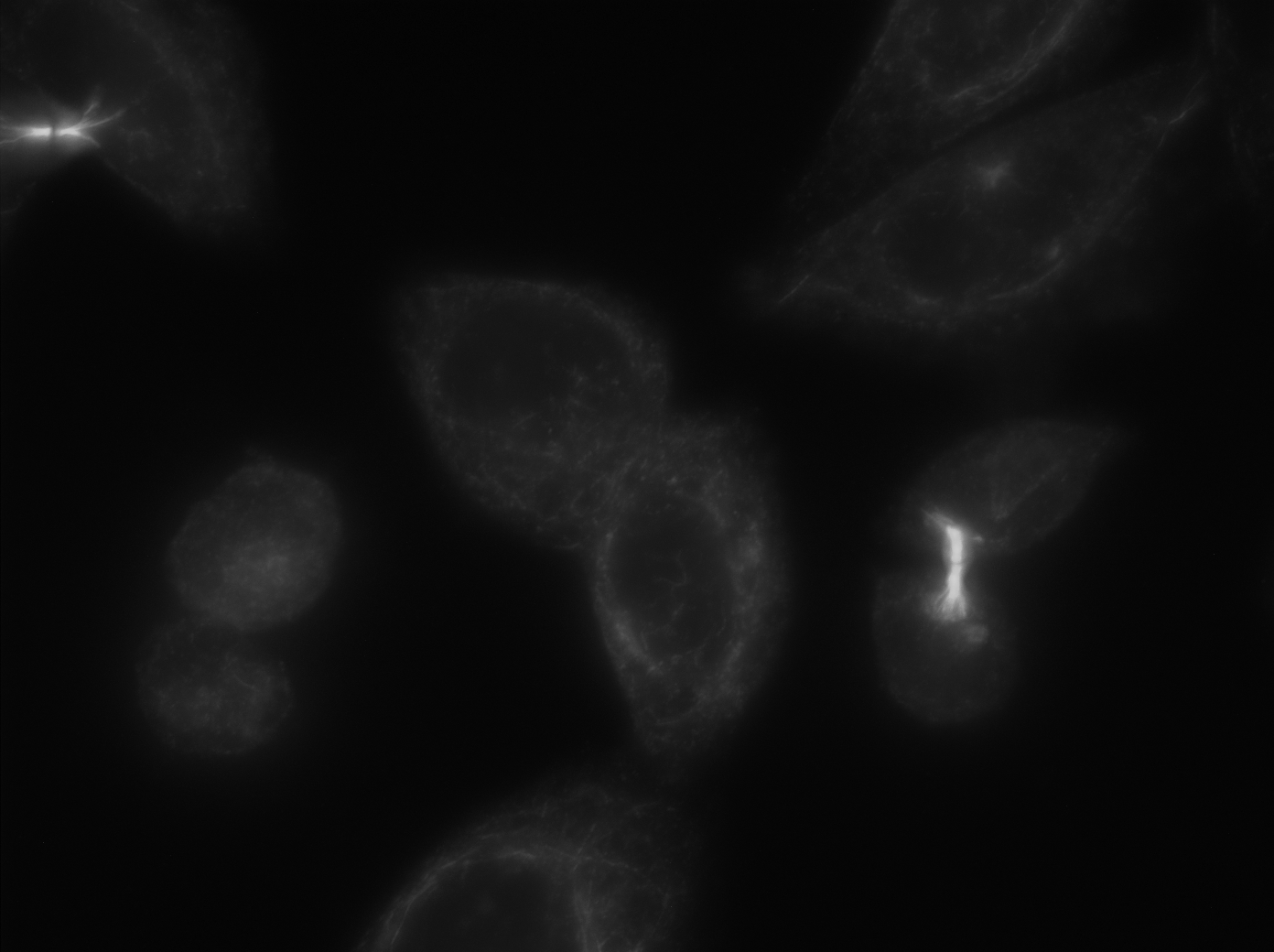

Supplement: Supplementary file 22 — Source data Fig. 6 part 3 [file 44319_2026_742_MOESM22_ESM.zip › Figure 6 Part 3/Fig 6efg TPGS1-KO TPGS1 rescue experiments/R1/TPGS1-KO TPGS1-3UTR-EYFP actub R1 7-31-25 ET2.Project Maximum Z_XY1753987827_Z0_T0_C2.tif]

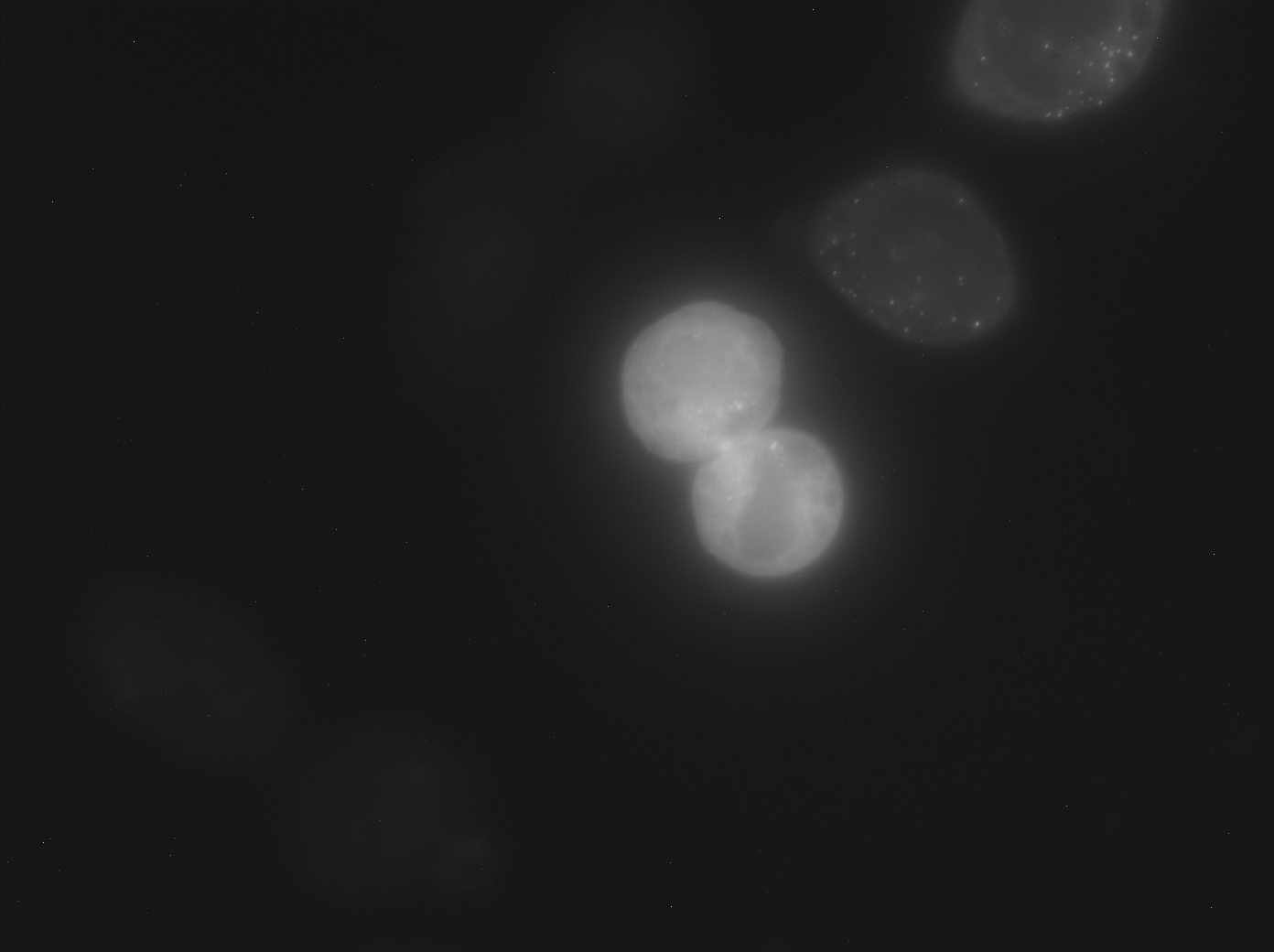

Supplement: Supplementary file 22 — Source data Fig. 6 part 3 [file 44319_2026_742_MOESM22_ESM.zip › Figure 6 Part 3/Fig 6efg TPGS1-KO TPGS1 rescue experiments/R1/TPGS1-KO TPGS1-3UTR-EYFP actub R1 7-31-25 ET3.Project Maximum Z_XY1753988552_Z0_T0_C1.tif]

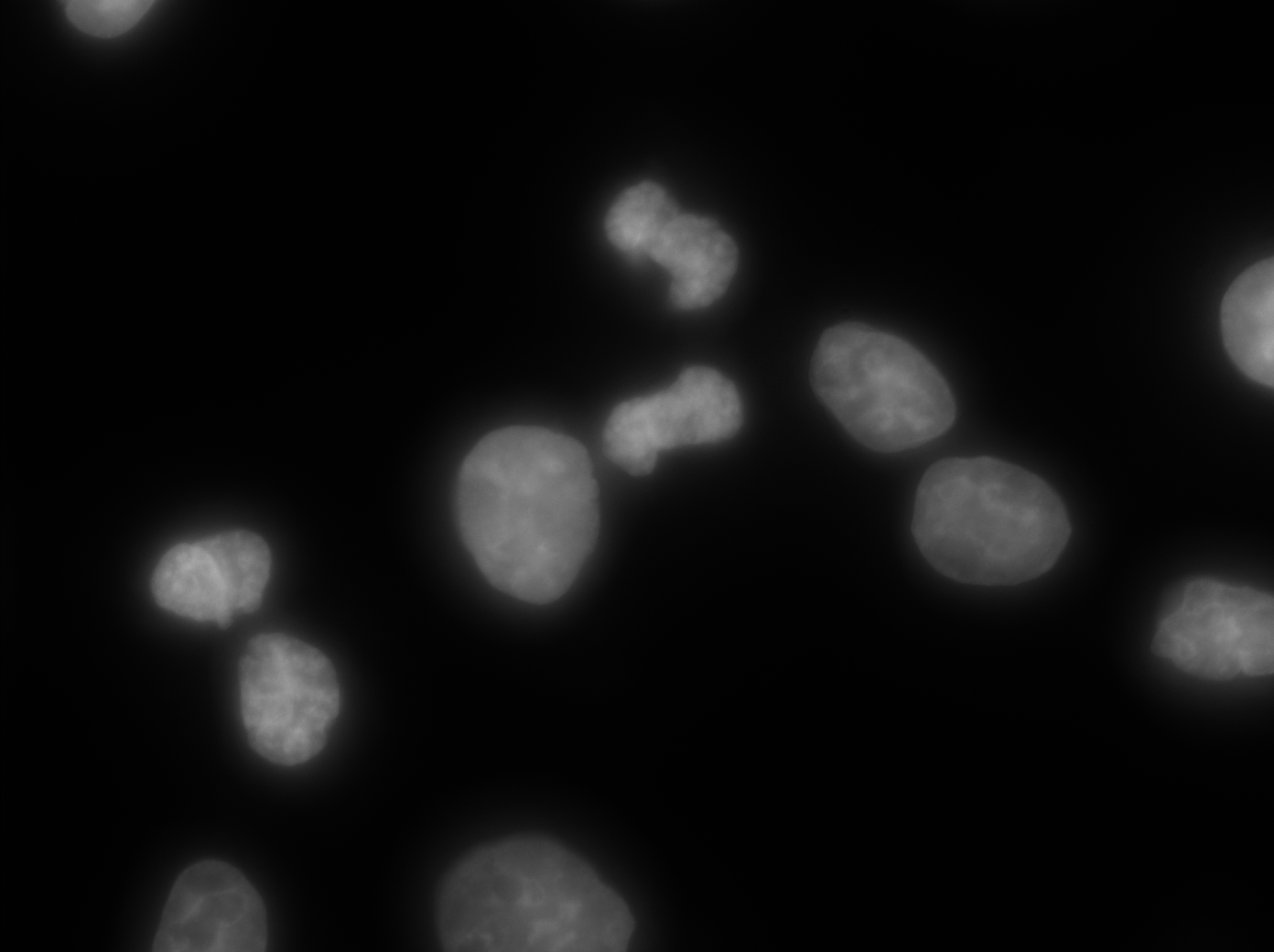

Supplement: Supplementary file 22 — Source data Fig. 6 part 3 [file 44319_2026_742_MOESM22_ESM.zip › Figure 6 Part 3/Fig 6efg TPGS1-KO TPGS1 rescue experiments/R1/TPGS1-KO TPGS1-3UTR-EYFP actub R1 7-31-25 ET7.Project Maximum Z_XY1753991843_Z0_T0_C0.tif]

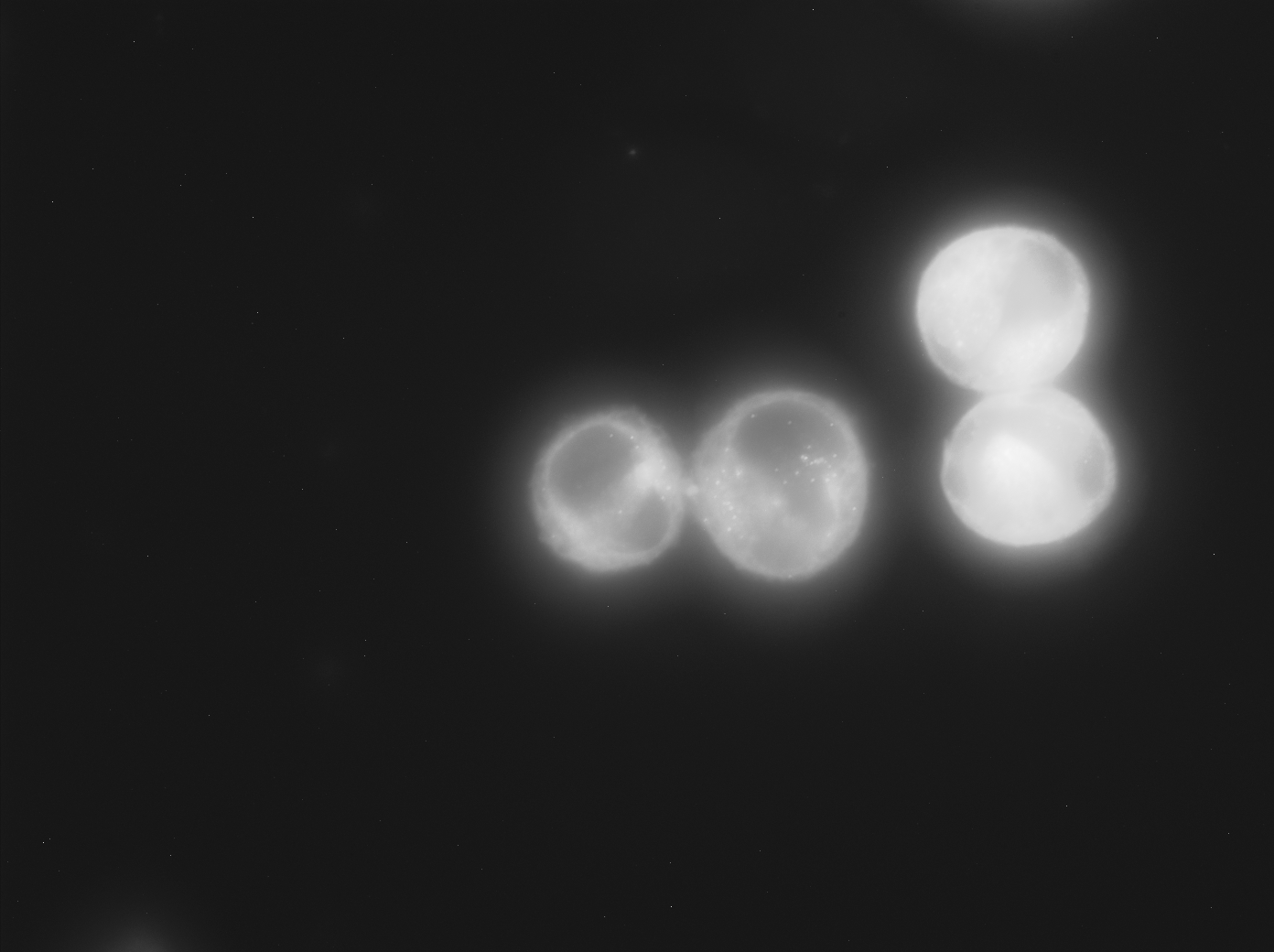

Supplement: Supplementary file 22 — Source data Fig. 6 part 3 [file 44319_2026_742_MOESM22_ESM.zip › Figure 6 Part 3/Fig 6efg TPGS1-KO TPGS1 rescue experiments/R1/TPGS1-KO TPGS1-3UTR-EYFP actub R1 7-31-25 ET10.Project Maximum Z_XY1753994658_Z0_T0_C1.tif]

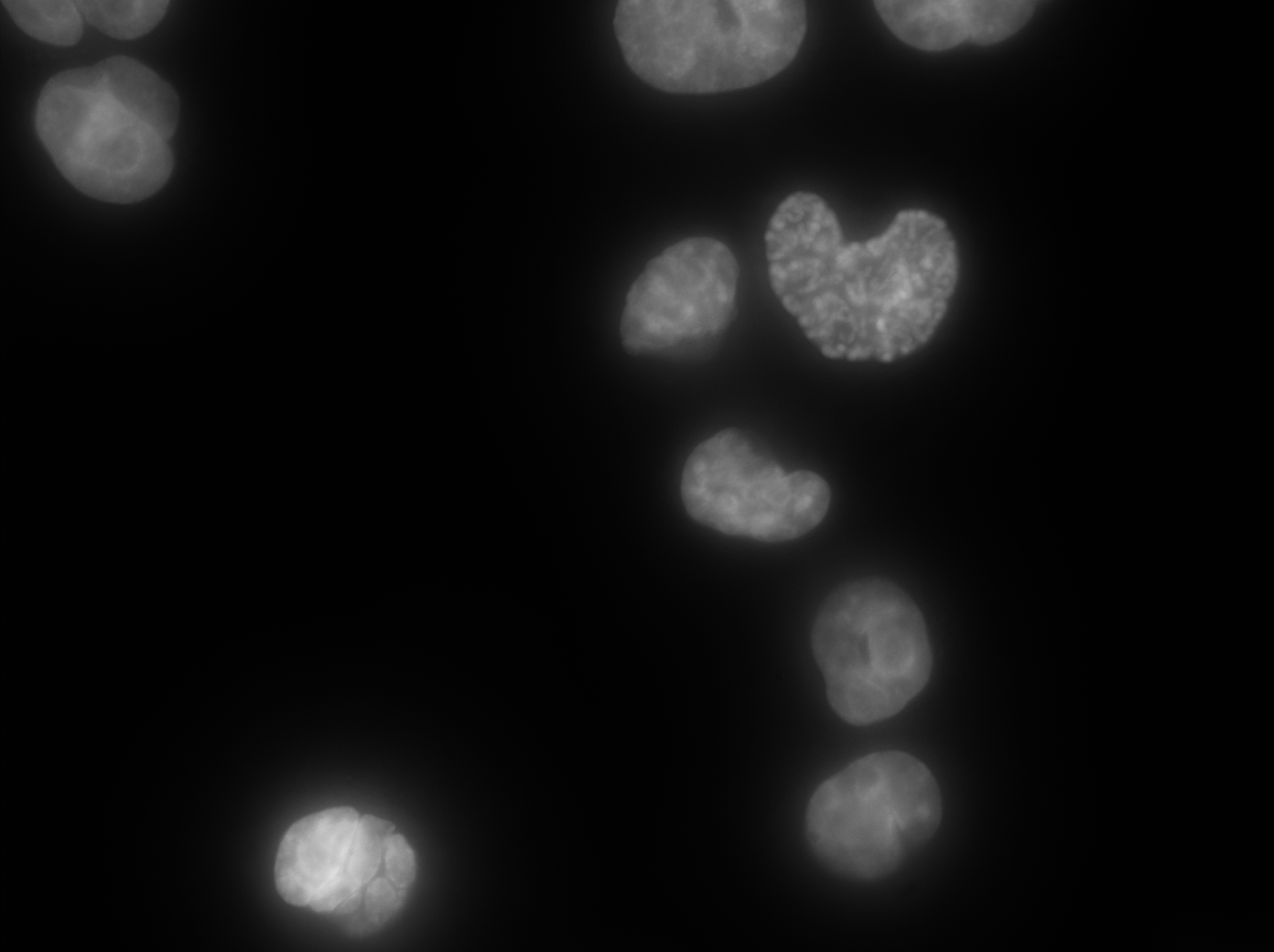

Supplement: Supplementary file 22 — Source data Fig. 6 part 3 [file 44319_2026_742_MOESM22_ESM.zip › Figure 6 Part 3/Fig 6efg TPGS1-KO TPGS1 rescue experiments/R1/TPGS1-KO EYFP only actub R1 7-31-25 LT8.Project Maximum Z_XY1754338859_Z0_T0_C0.tif]

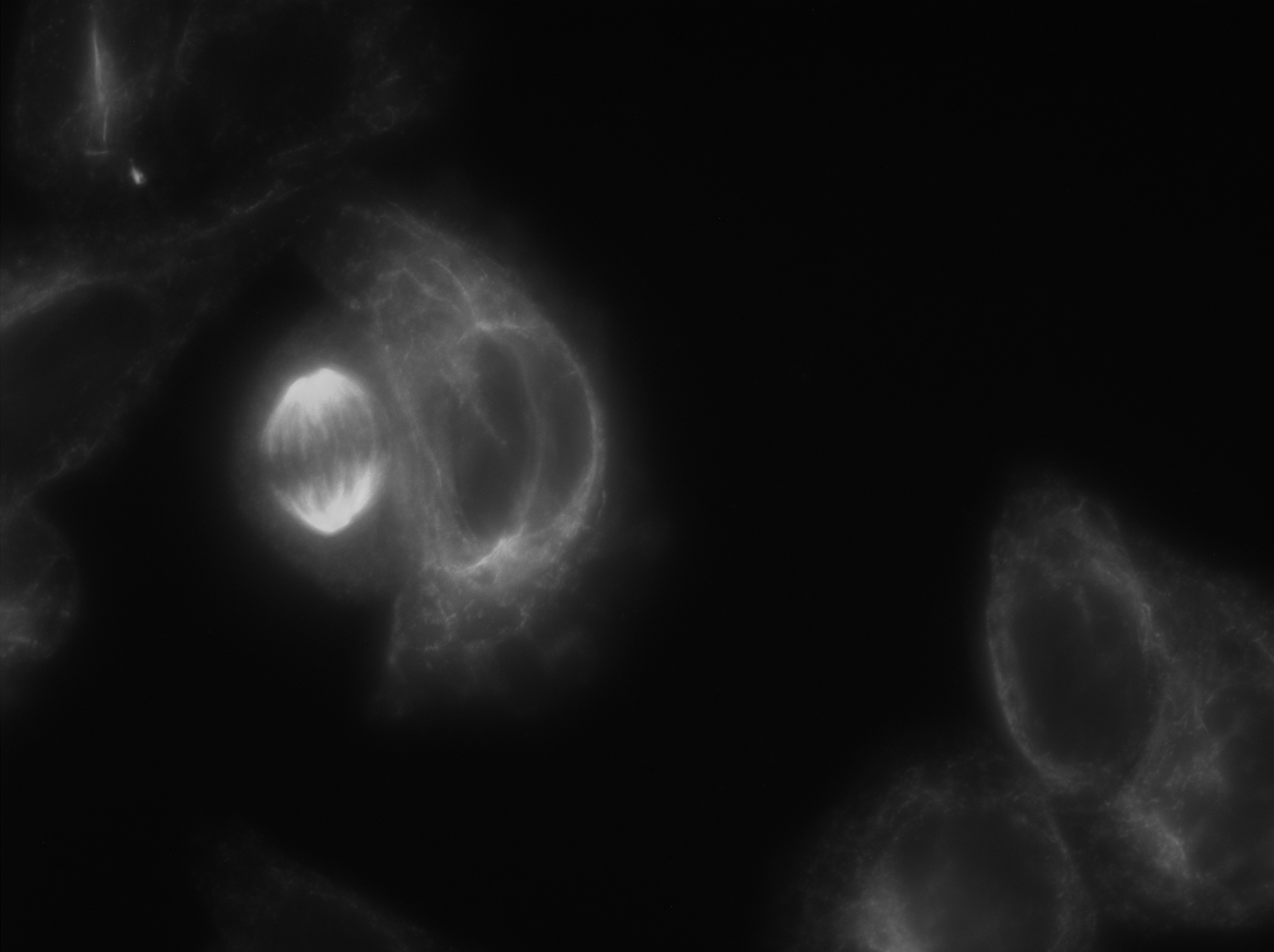

Supplement: Supplementary file 22 — Source data Fig. 6 part 3 [file 44319_2026_742_MOESM22_ESM.zip › Figure 6 Part 3/Fig 6efg TPGS1-KO TPGS1 rescue experiments/R1/TPGS1-KO TPGS1-3UTR-EYFP actub R1 7-31-25 M2.Project Maximum Z_XY1753993001_Z0_T0_C2.tif]

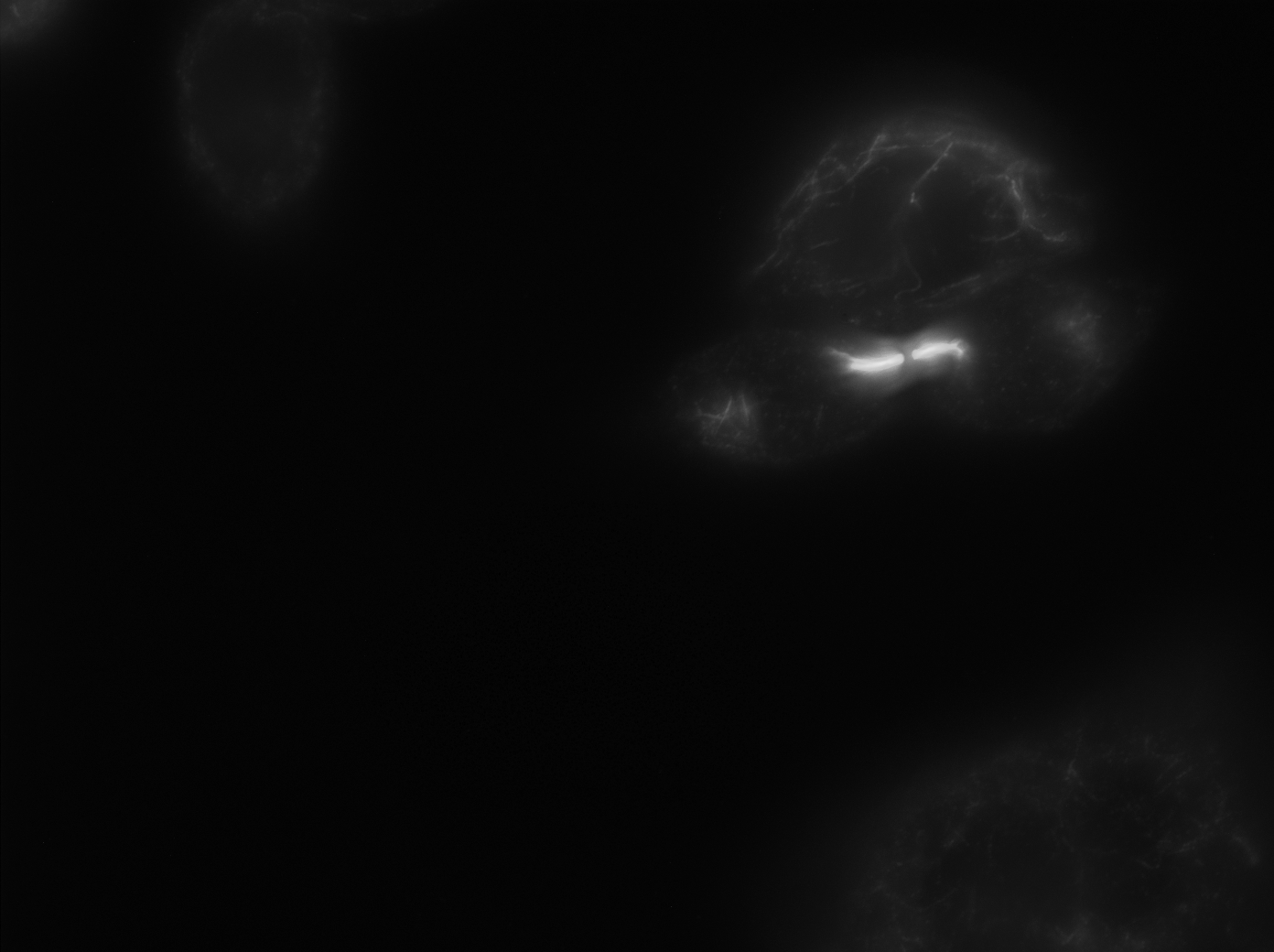

Supplement: Supplementary file 22 — Source data Fig. 6 part 3 [file 44319_2026_742_MOESM22_ESM.zip › Figure 6 Part 3/Fig 6efg TPGS1-KO TPGS1 rescue experiments/R1/TPGS1-KO TPGS1-3UTR-EYFP actub R1 7-31-25 LT1.Project Maximum Z_XY1753981191_Z0_T0_C2.tif]

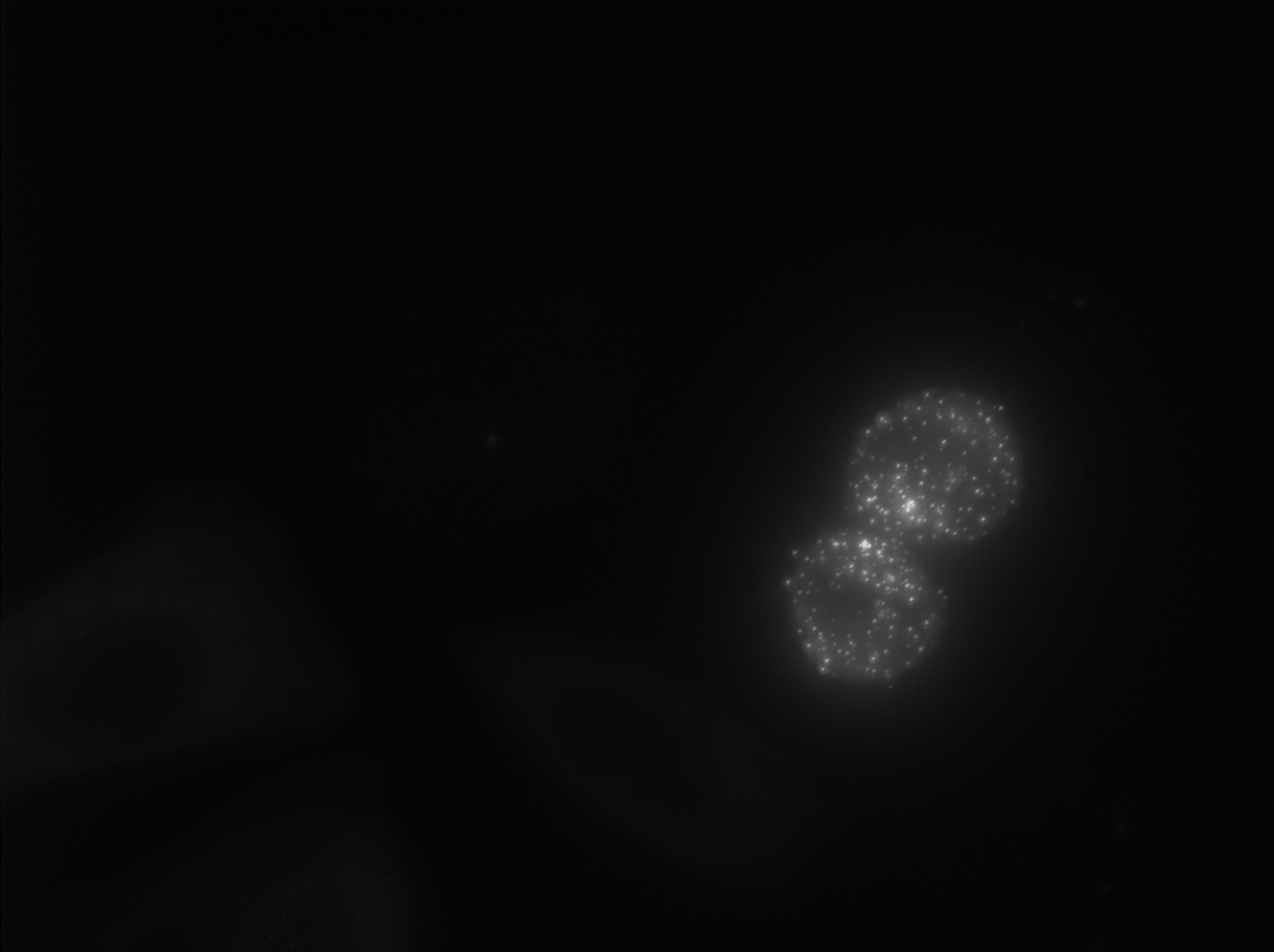

Supplement: Supplementary file 22 — Source data Fig. 6 part 3 [file 44319_2026_742_MOESM22_ESM.zip › Figure 6 Part 3/Fig 6efg TPGS1-KO TPGS1 rescue experiments/R1/TPGS1-KO TPGS1-3UTR-EYFP actub R1 7-31-25 ET4.Project Maximum Z_XY1753988880_Z0_T0_C1.tif]

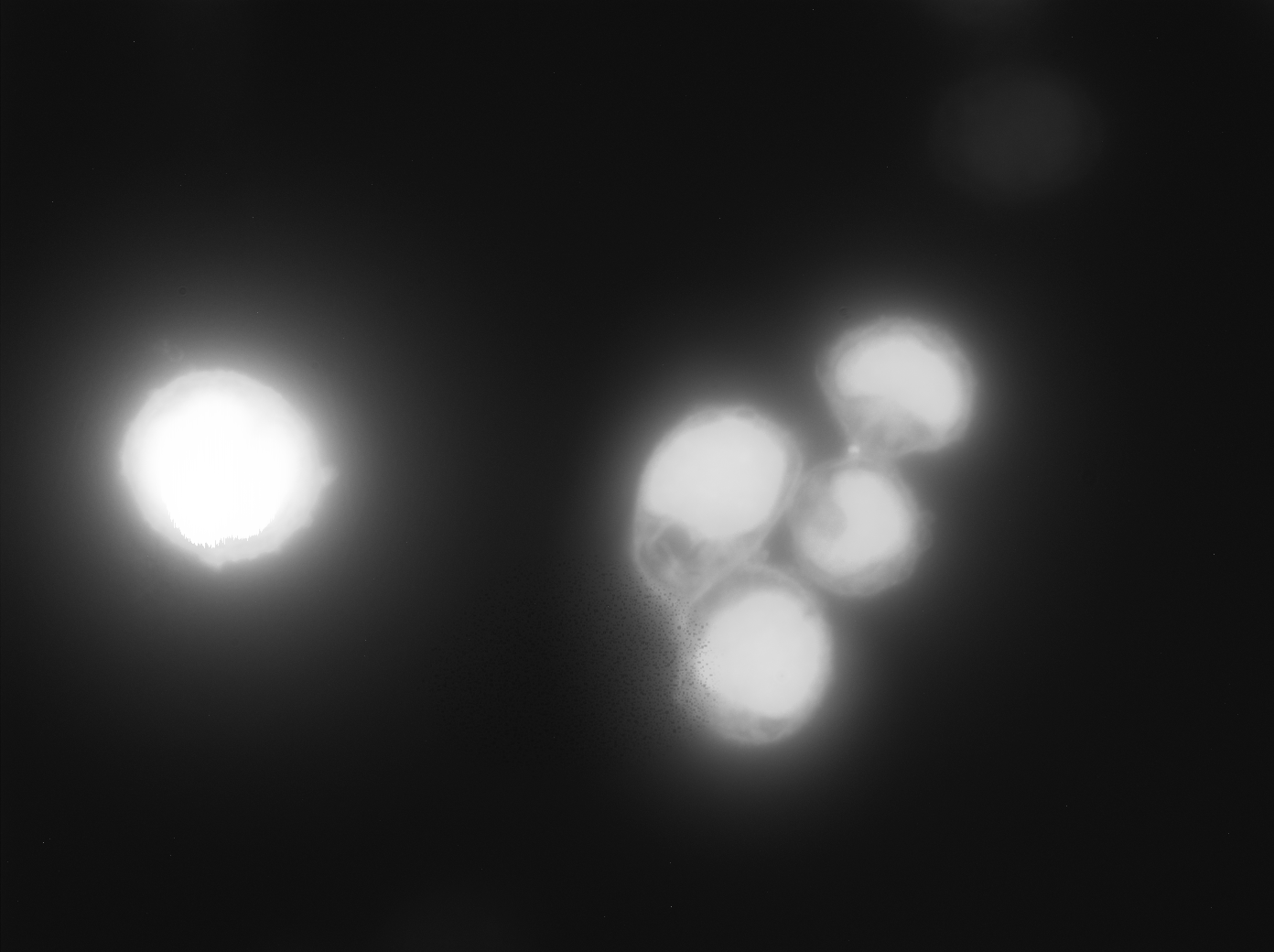

Supplement: Supplementary file 22 — Source data Fig. 6 part 3 [file 44319_2026_742_MOESM22_ESM.zip › Figure 6 Part 3/Fig 6efg TPGS1-KO TPGS1 rescue experiments/R1/TPGS1-KO EYFP only actub R1 7-31-25 ET3.Project Maximum Z_XY1754335811_Z0_T0_C1.tif]

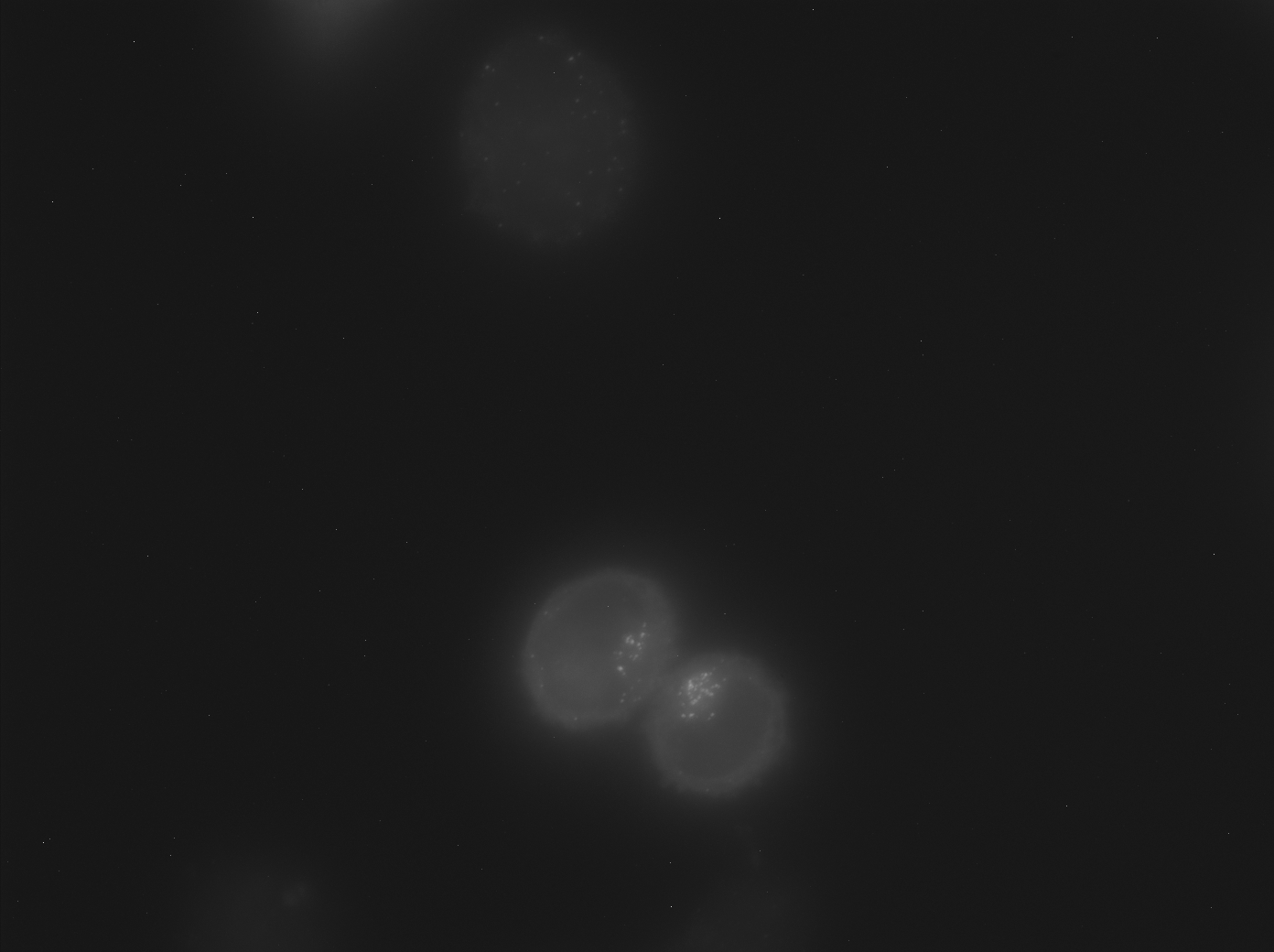

Supplement: Supplementary file 22 — Source data Fig. 6 part 3 [file 44319_2026_742_MOESM22_ESM.zip › Figure 6 Part 3/Fig 6efg TPGS1-KO TPGS1 rescue experiments/R1/TPGS1-KO TPGS1-3UTR-EYFP actub R1 7-31-25 ET8.Project Maximum Z_XY1753992062_Z0_T0_C1.tif]

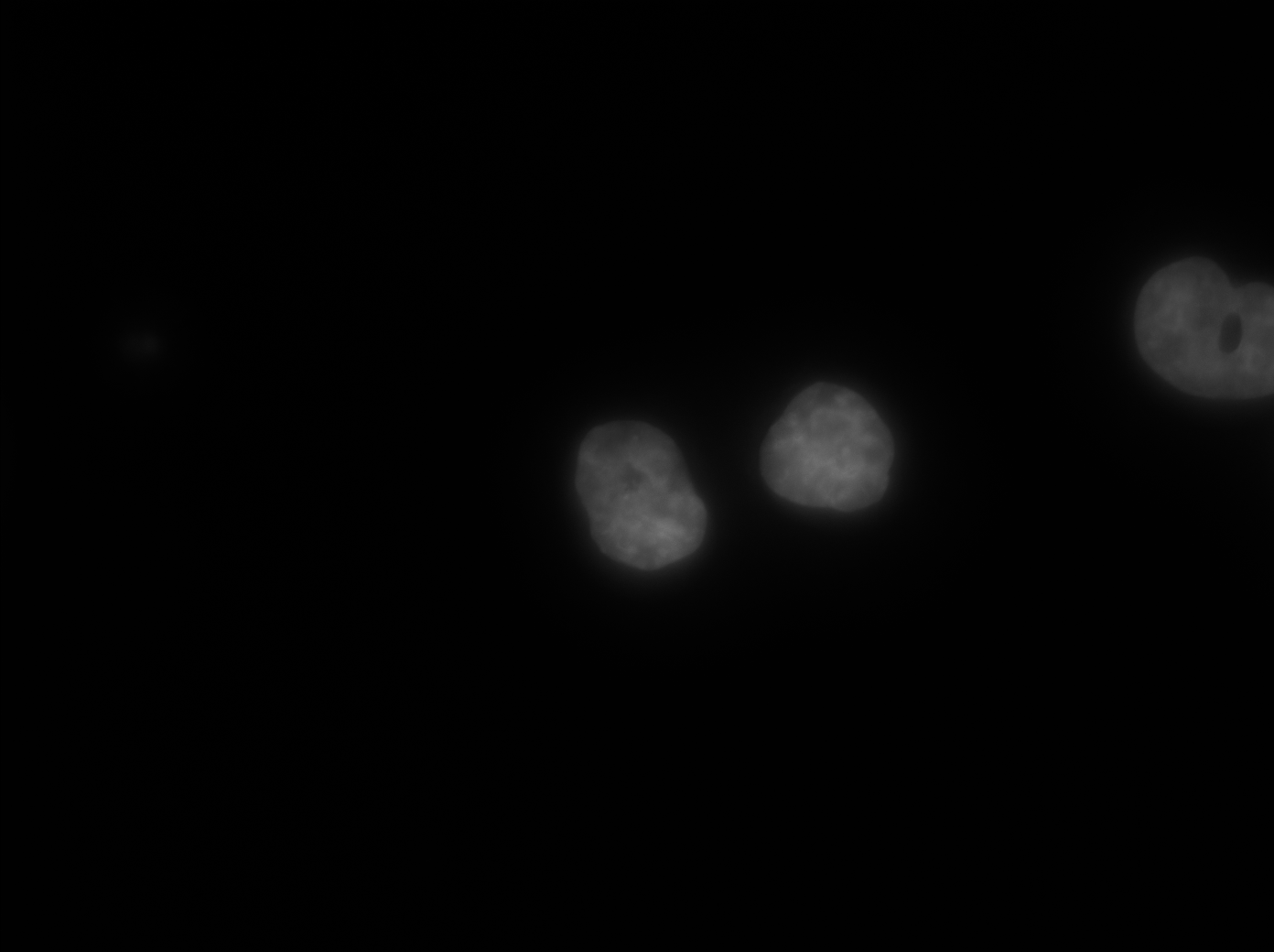

Supplement: Supplementary file 22 — Source data Fig. 6 part 3 [file 44319_2026_742_MOESM22_ESM.zip › Figure 6 Part 3/Fig 6efg TPGS1-KO TPGS1 rescue experiments/R1/TPGS1-KO TPGS1-3UTR-EYFP actub R1 7-31-25 LT9.Project Maximum Z_XY1753992358_Z0_T0_C0.tif]

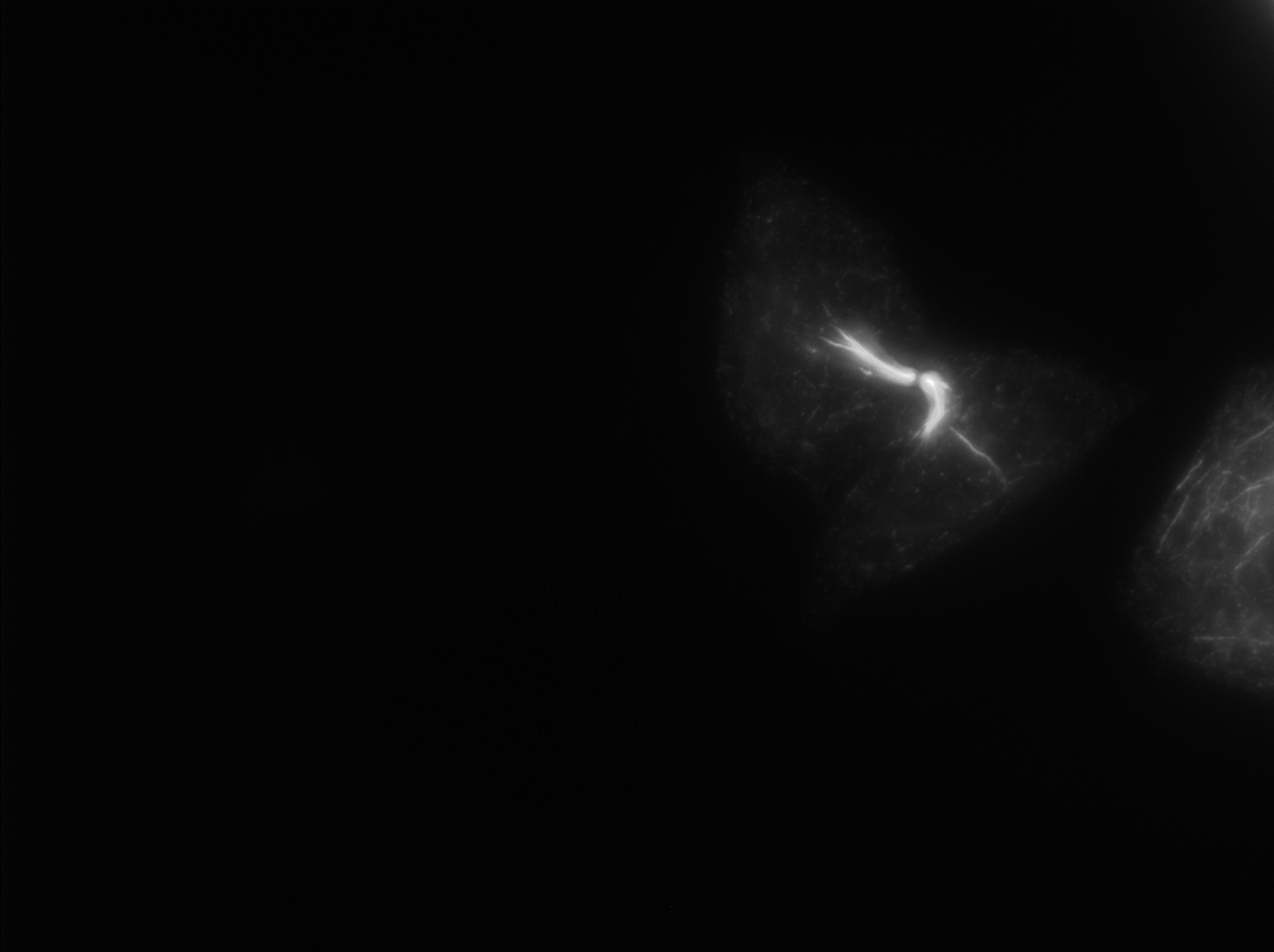

Supplement: Supplementary file 22 — Source data Fig. 6 part 3 [file 44319_2026_742_MOESM22_ESM.zip › Figure 6 Part 3/Fig 6efg TPGS1-KO TPGS1 rescue experiments/R1/TPGS1-KO EYFP only actub R1 7-31-25 LT2.Project Maximum Z_XY1754335102_Z0_T0_C2.tif]

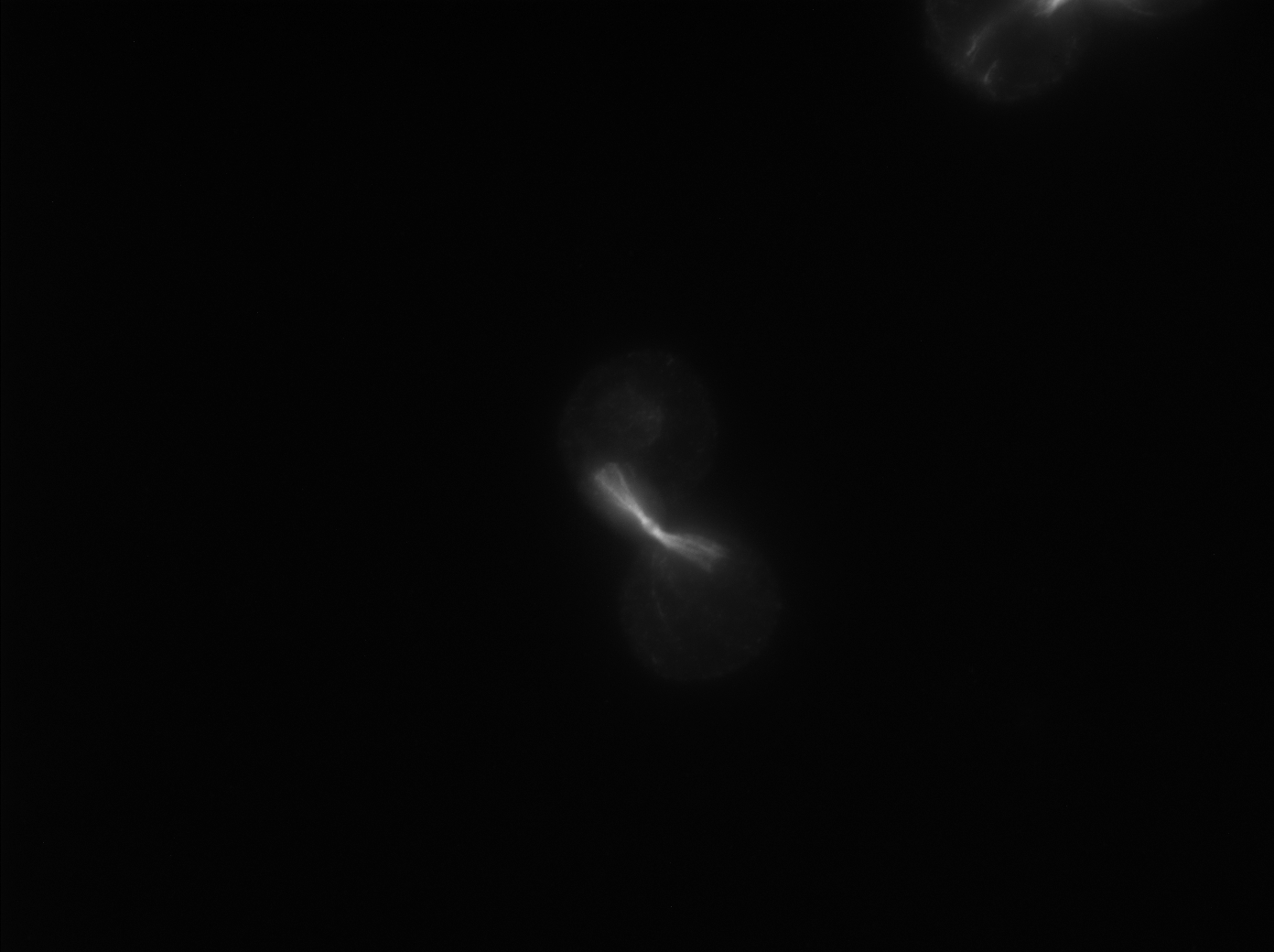

Supplement: Supplementary file 22 — Source data Fig. 6 part 3 [file 44319_2026_742_MOESM22_ESM.zip › Figure 6 Part 3/Fig 6efg TPGS1-KO TPGS1 rescue experiments/R1/TPGS1-KO TPGS1-3UTR-EYFP actub R1 7-31-25 ET6.Project Maximum Z_XY1753991490_Z0_T0_C2.tif]

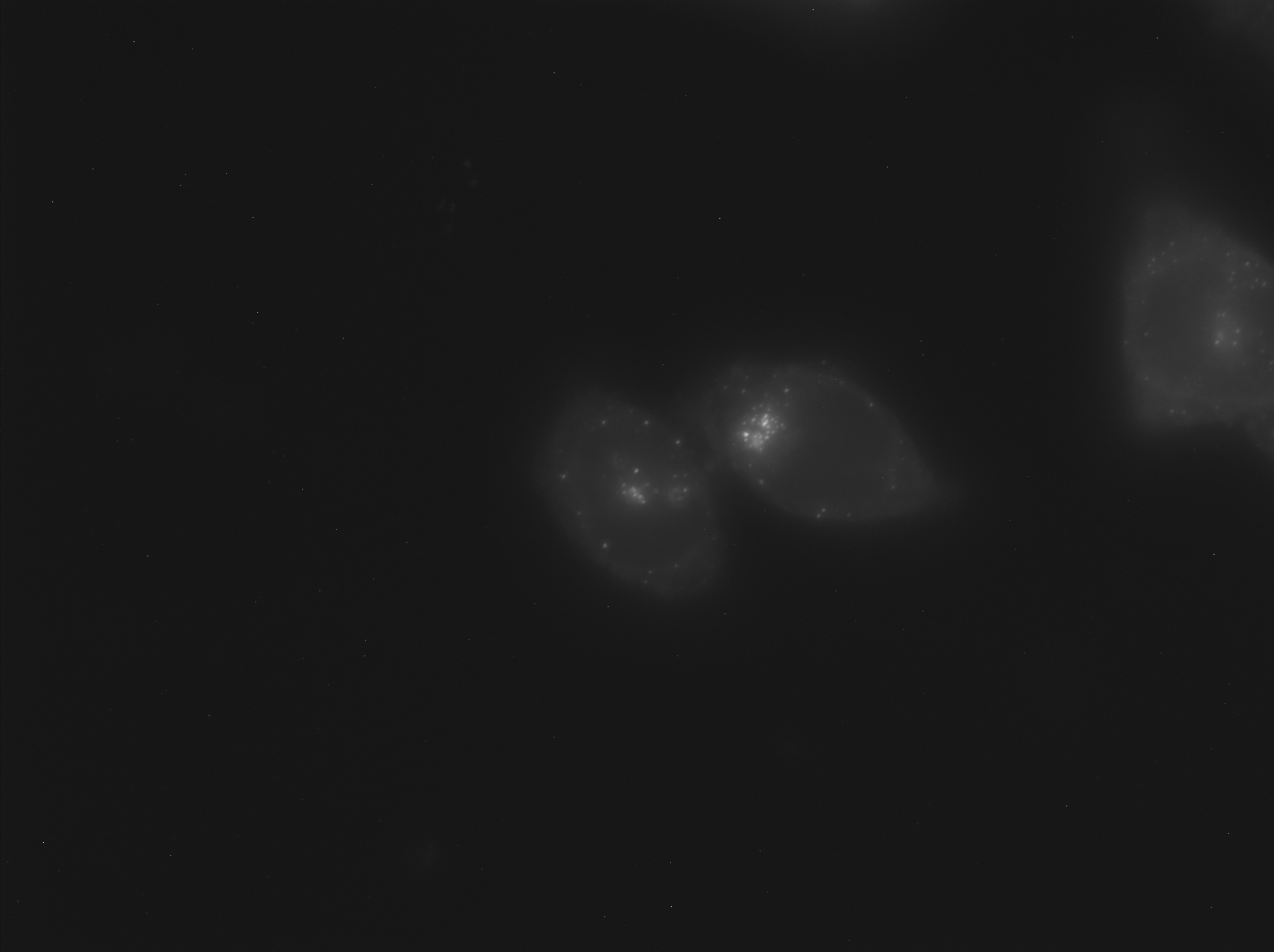

Supplement: Supplementary file 22 — Source data Fig. 6 part 3 [file 44319_2026_742_MOESM22_ESM.zip › Figure 6 Part 3/Fig 6efg TPGS1-KO TPGS1 rescue experiments/R1/TPGS1-KO TPGS1-3UTR-EYFP actub R1 7-31-25 LT9.Project Maximum Z_XY1753992358_Z0_T0_C1.tif]

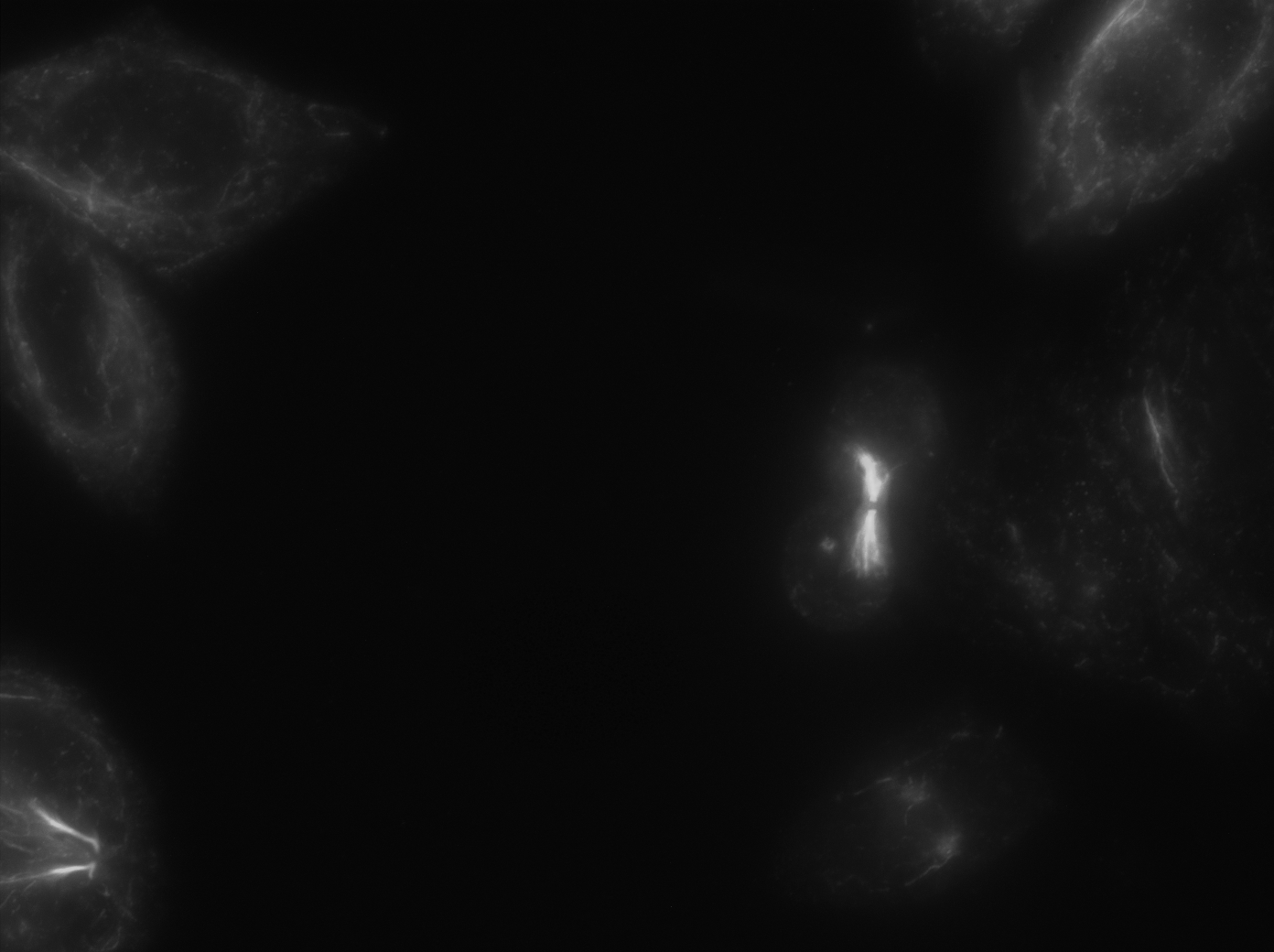

Supplement: Supplementary file 22 — Source data Fig. 6 part 3 [file 44319_2026_742_MOESM22_ESM.zip › Figure 6 Part 3/Fig 6efg TPGS1-KO TPGS1 rescue experiments/R1/TPGS1-KO EYFP only actub R1 7-31-25 ET5.Project Maximum Z_XY1754336188_Z0_T0_C2.tif]

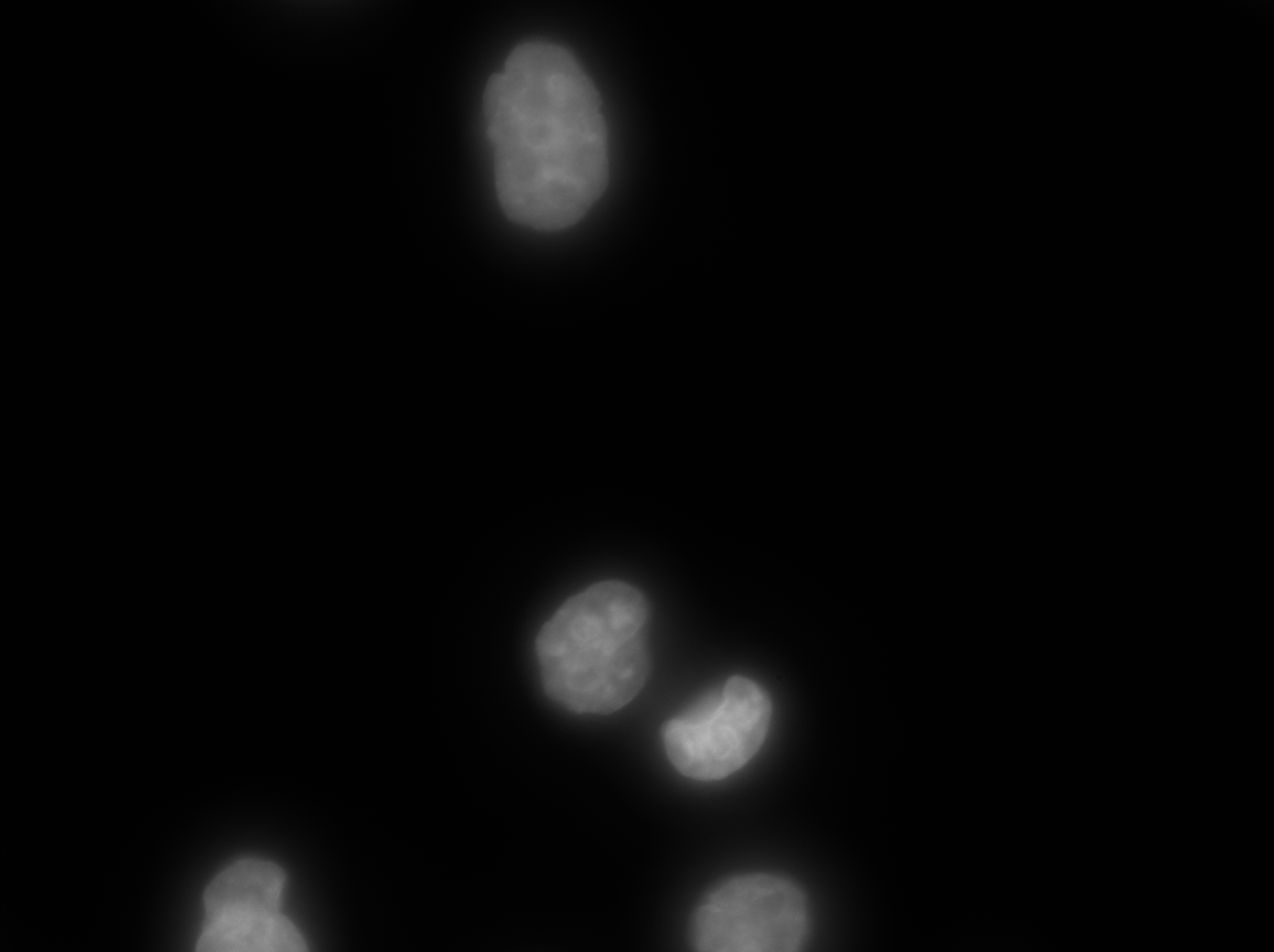

Supplement: Supplementary file 22 — Source data Fig. 6 part 3 [file 44319_2026_742_MOESM22_ESM.zip › Figure 6 Part 3/Fig 6efg TPGS1-KO TPGS1 rescue experiments/R1/TPGS1-KO TPGS1-3UTR-EYFP actub R1 7-31-25 ET8.Project Maximum Z_XY1753992062_Z0_T0_C0.tif]

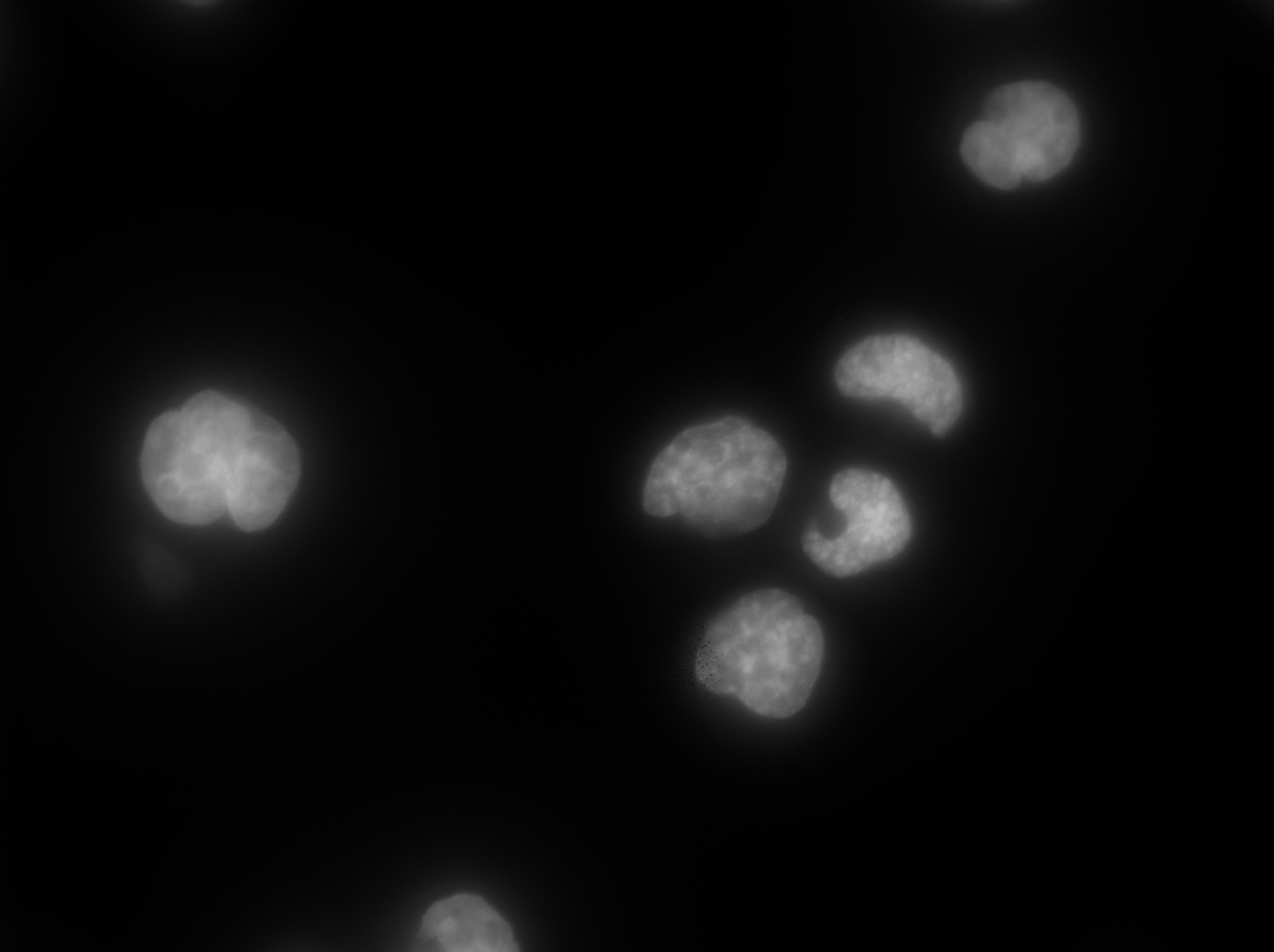

Supplement: Supplementary file 22 — Source data Fig. 6 part 3 [file 44319_2026_742_MOESM22_ESM.zip › Figure 6 Part 3/Fig 6efg TPGS1-KO TPGS1 rescue experiments/R1/TPGS1-KO EYFP only actub R1 7-31-25 ET3.Project Maximum Z_XY1754335811_Z0_T0_C0.tif]

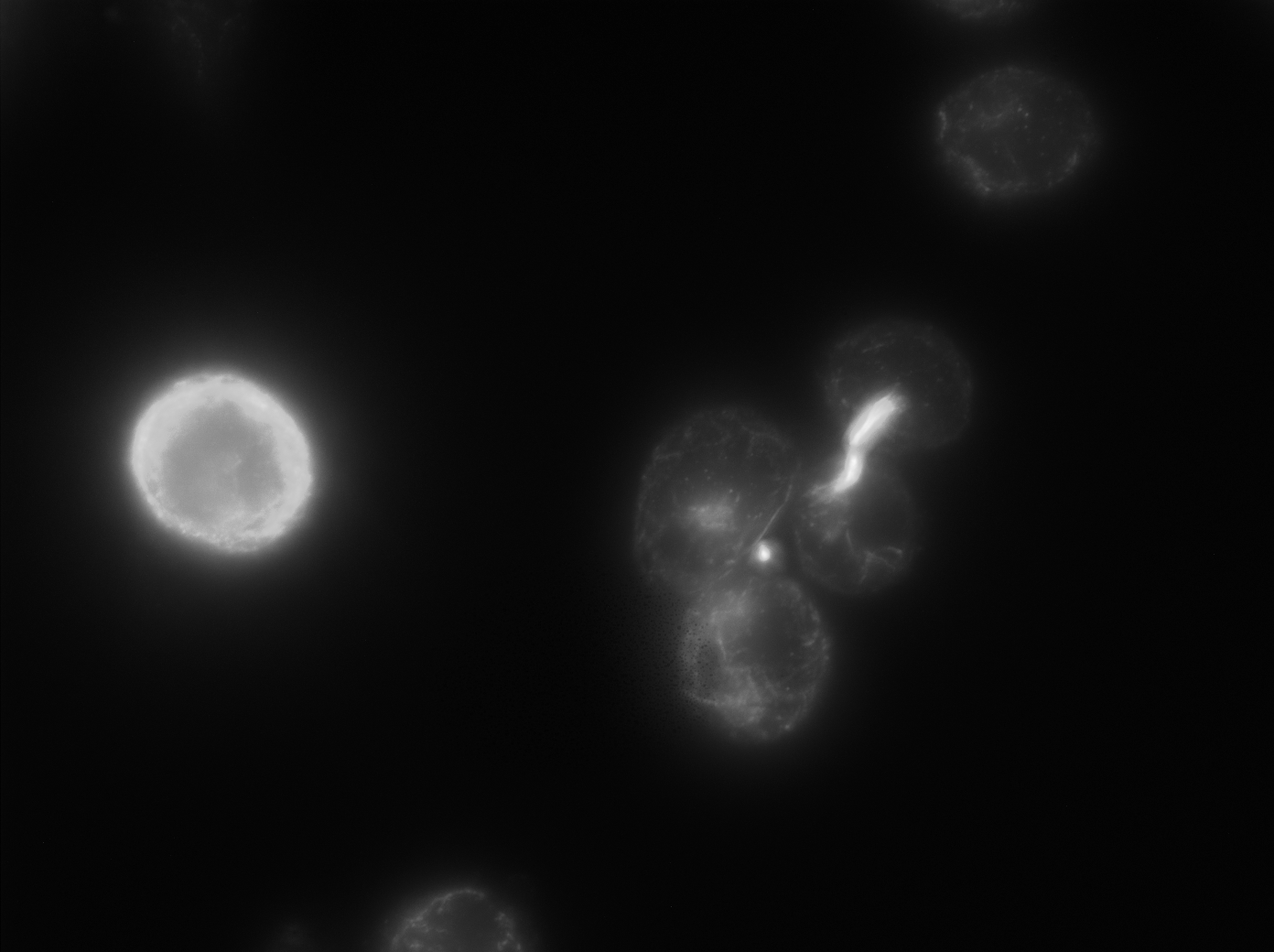

Supplement: Supplementary file 22 — Source data Fig. 6 part 3 [file 44319_2026_742_MOESM22_ESM.zip › Figure 6 Part 3/Fig 6efg TPGS1-KO TPGS1 rescue experiments/R1/TPGS1-KO EYFP only actub R1 7-31-25 ET3.Project Maximum Z_XY1754335811_Z0_T0_C2.tif]

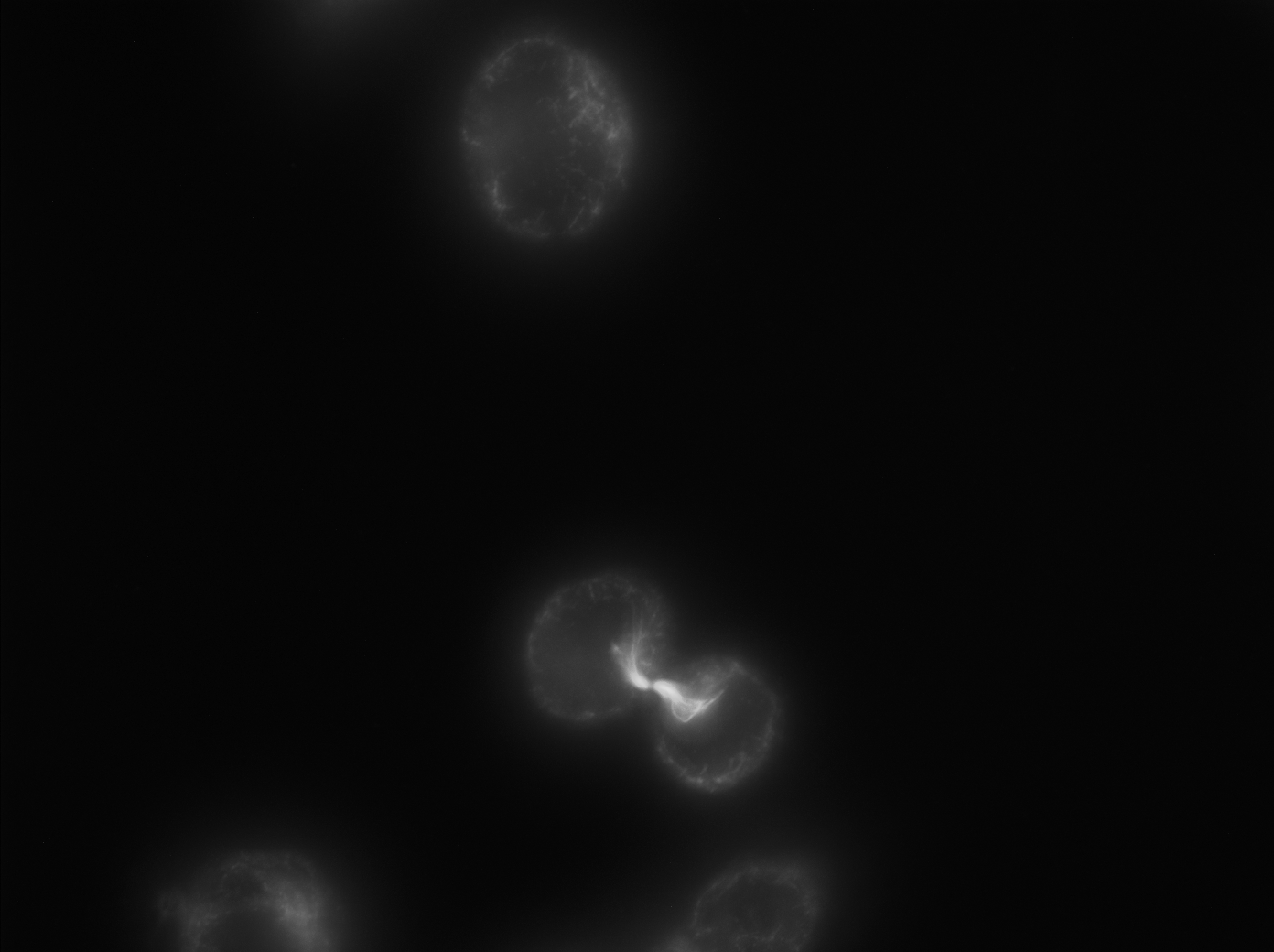

Supplement: Supplementary file 22 — Source data Fig. 6 part 3 [file 44319_2026_742_MOESM22_ESM.zip › Figure 6 Part 3/Fig 6efg TPGS1-KO TPGS1 rescue experiments/R1/TPGS1-KO TPGS1-3UTR-EYFP actub R1 7-31-25 ET8.Project Maximum Z_XY1753992062_Z0_T0_C2.tif]

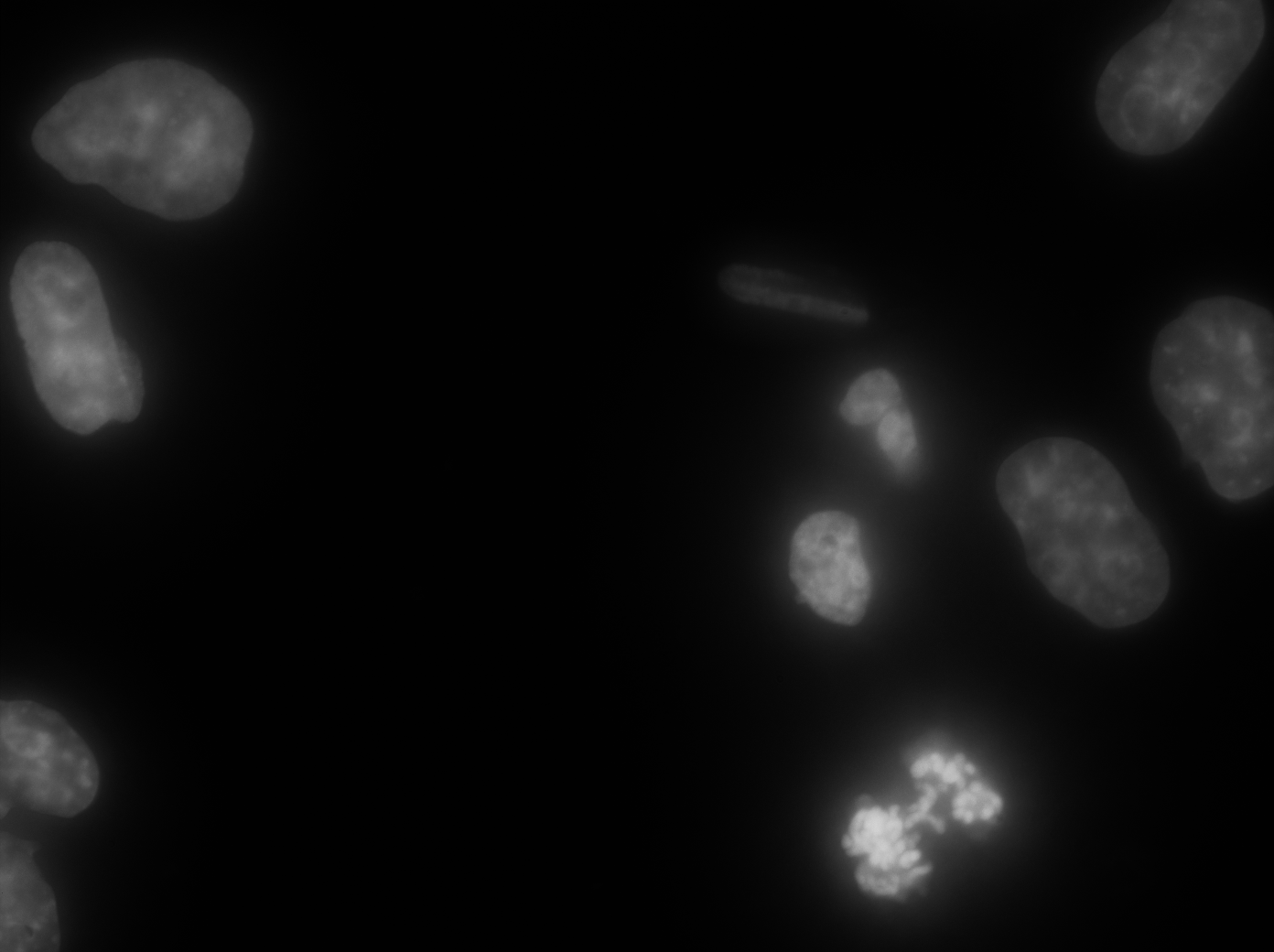

Supplement: Supplementary file 22 — Source data Fig. 6 part 3 [file 44319_2026_742_MOESM22_ESM.zip › Figure 6 Part 3/Fig 6efg TPGS1-KO TPGS1 rescue experiments/R1/TPGS1-KO EYFP only actub R1 7-31-25 ET5.Project Maximum Z_XY1754336188_Z0_T0_C0.tif]

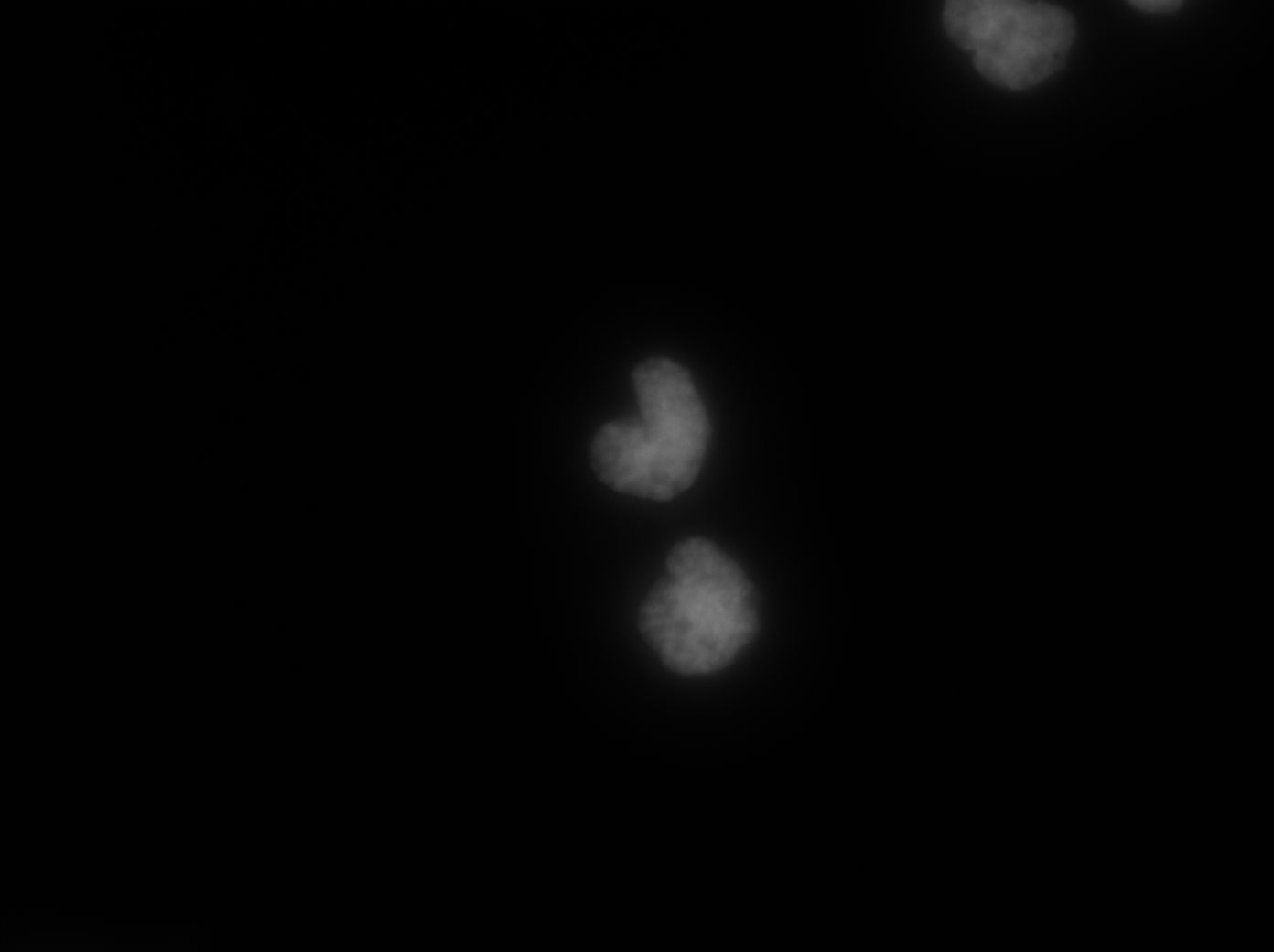

Supplement: Supplementary file 22 — Source data Fig. 6 part 3 [file 44319_2026_742_MOESM22_ESM.zip › Figure 6 Part 3/Fig 6efg TPGS1-KO TPGS1 rescue experiments/R1/TPGS1-KO TPGS1-3UTR-EYFP actub R1 7-31-25 ET6.Project Maximum Z_XY1753991490_Z0_T0_C0.tif]

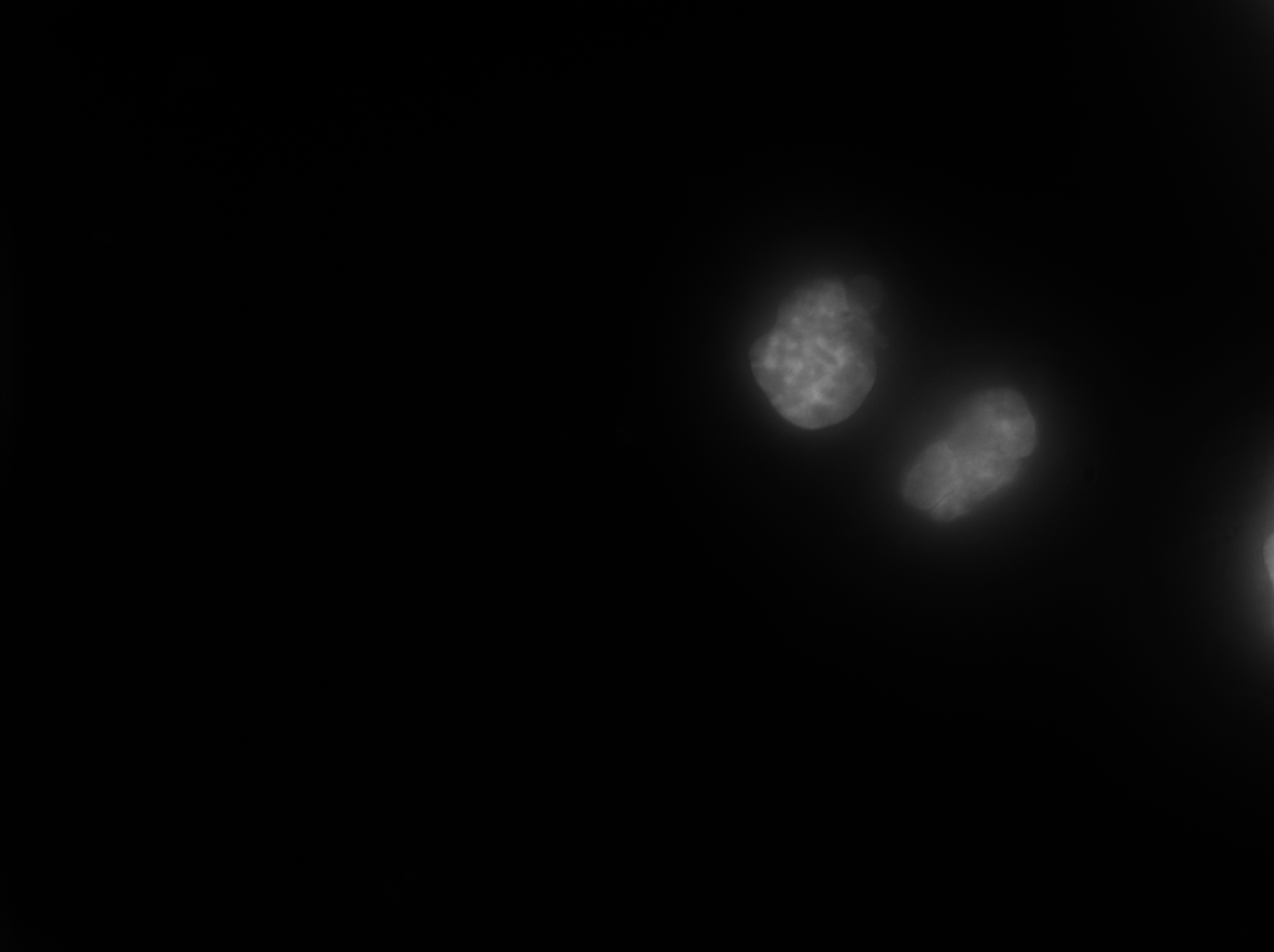

Supplement: Supplementary file 22 — Source data Fig. 6 part 3 [file 44319_2026_742_MOESM22_ESM.zip › Figure 6 Part 3/Fig 6efg TPGS1-KO TPGS1 rescue experiments/R1/TPGS1-KO EYFP only actub R1 7-31-25 LT2.Project Maximum Z_XY1754335102_Z0_T0_C0.tif]

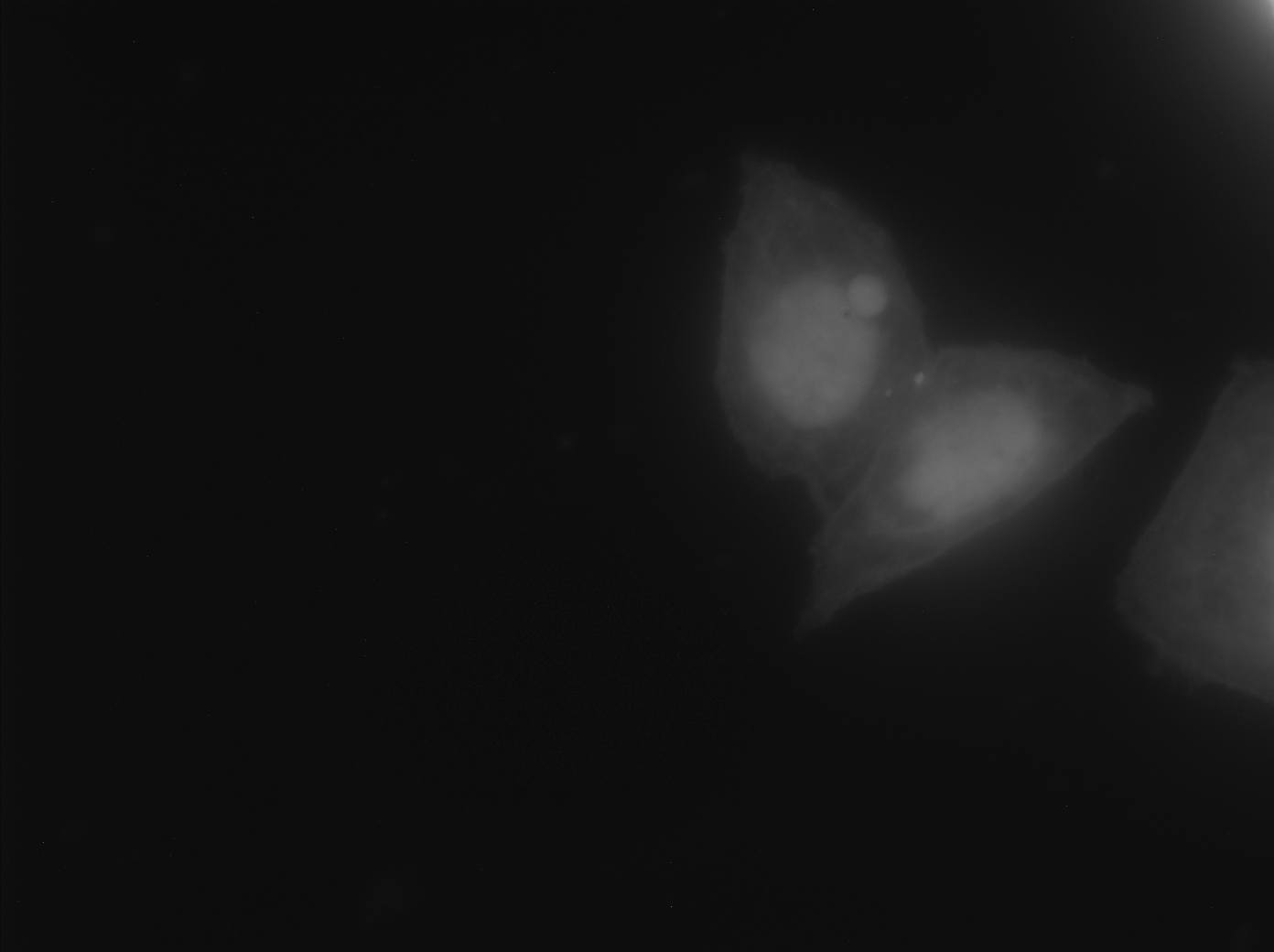

Supplement: Supplementary file 22 — Source data Fig. 6 part 3 [file 44319_2026_742_MOESM22_ESM.zip › Figure 6 Part 3/Fig 6efg TPGS1-KO TPGS1 rescue experiments/R1/TPGS1-KO EYFP only actub R1 7-31-25 LT2.Project Maximum Z_XY1754335102_Z0_T0_C1.tif]

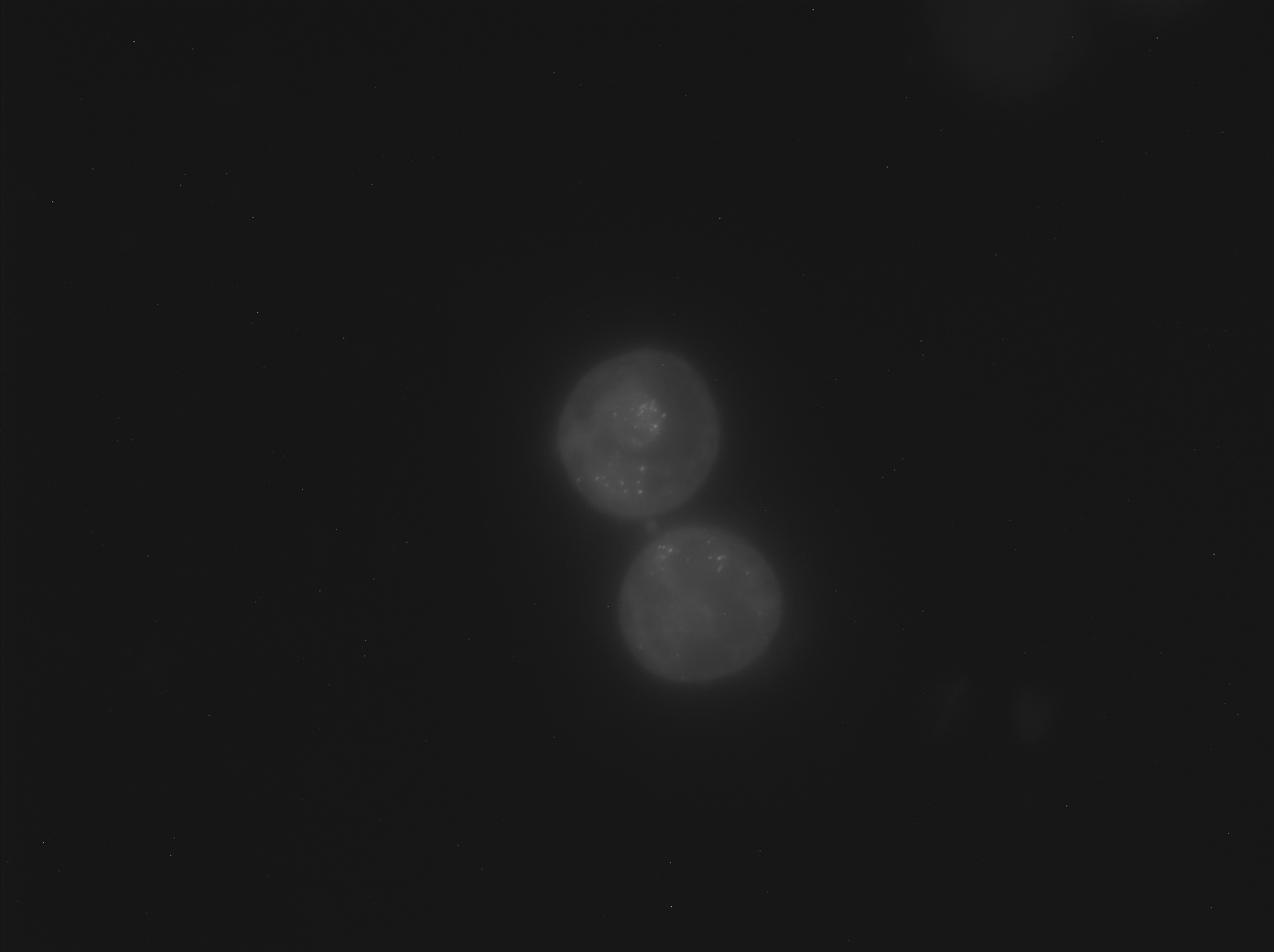

Supplement: Supplementary file 22 — Source data Fig. 6 part 3 [file 44319_2026_742_MOESM22_ESM.zip › Figure 6 Part 3/Fig 6efg TPGS1-KO TPGS1 rescue experiments/R1/TPGS1-KO TPGS1-3UTR-EYFP actub R1 7-31-25 ET6.Project Maximum Z_XY1753991490_Z0_T0_C1.tif]

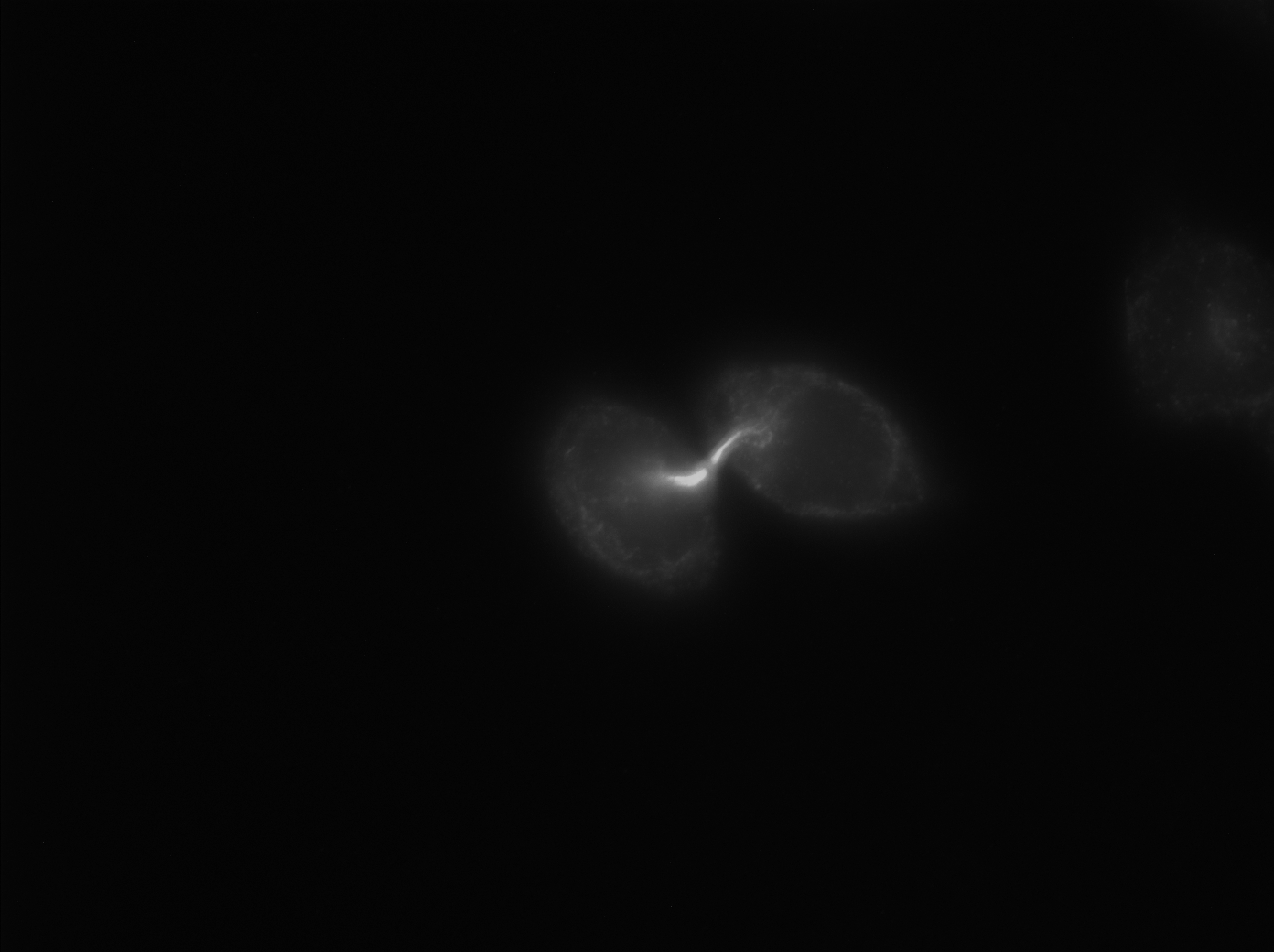

Supplement: Supplementary file 22 — Source data Fig. 6 part 3 [file 44319_2026_742_MOESM22_ESM.zip › Figure 6 Part 3/Fig 6efg TPGS1-KO TPGS1 rescue experiments/R1/TPGS1-KO TPGS1-3UTR-EYFP actub R1 7-31-25 LT9.Project Maximum Z_XY1753992358_Z0_T0_C2.tif]

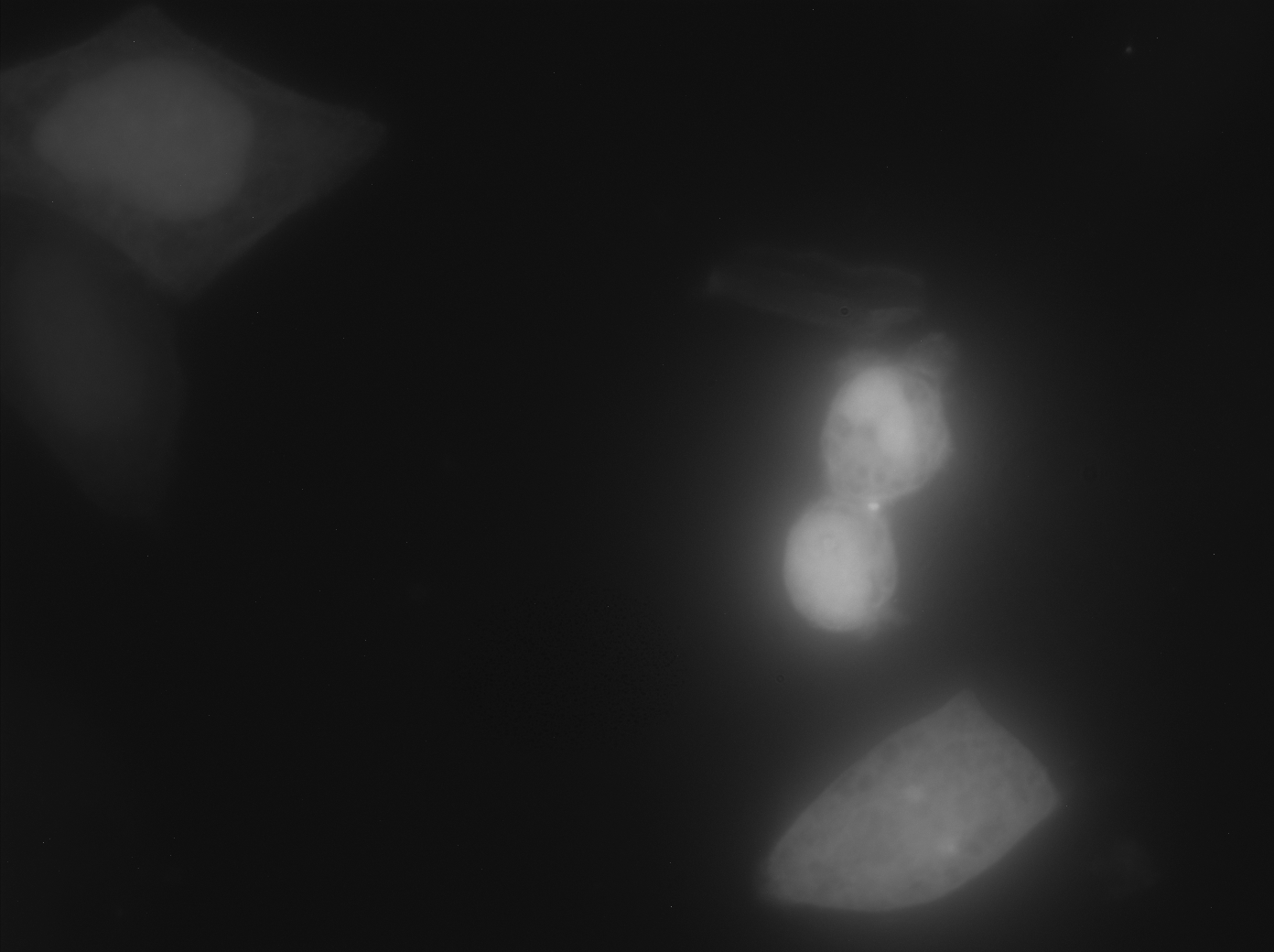

Supplement: Supplementary file 22 — Source data Fig. 6 part 3 [file 44319_2026_742_MOESM22_ESM.zip › Figure 6 Part 3/Fig 6efg TPGS1-KO TPGS1 rescue experiments/R1/TPGS1-KO EYFP only actub R1 7-31-25 ET5.Project Maximum Z_XY1754336188_Z0_T0_C1.tif]

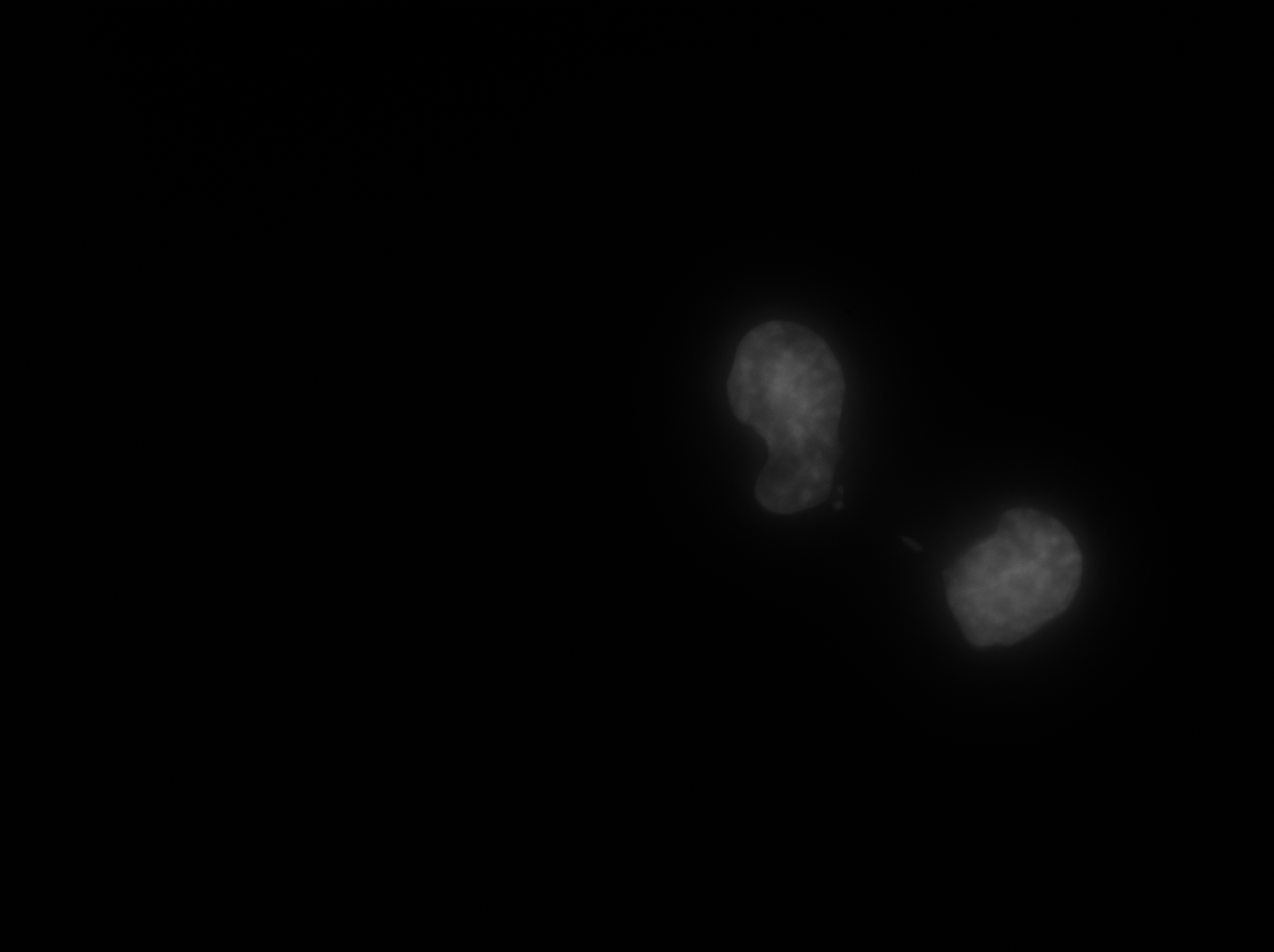

Supplement: Supplementary file 22 — Source data Fig. 6 part 3 [file 44319_2026_742_MOESM22_ESM.zip › Figure 6 Part 3/Fig 6efg TPGS1-KO TPGS1 rescue experiments/R1/TPGS1-KO TPGS1-3UTR-EYFP actub R1 7-31-25 LT8.Project Maximum Z_XY1753991165_Z0_T0_C0.tif]

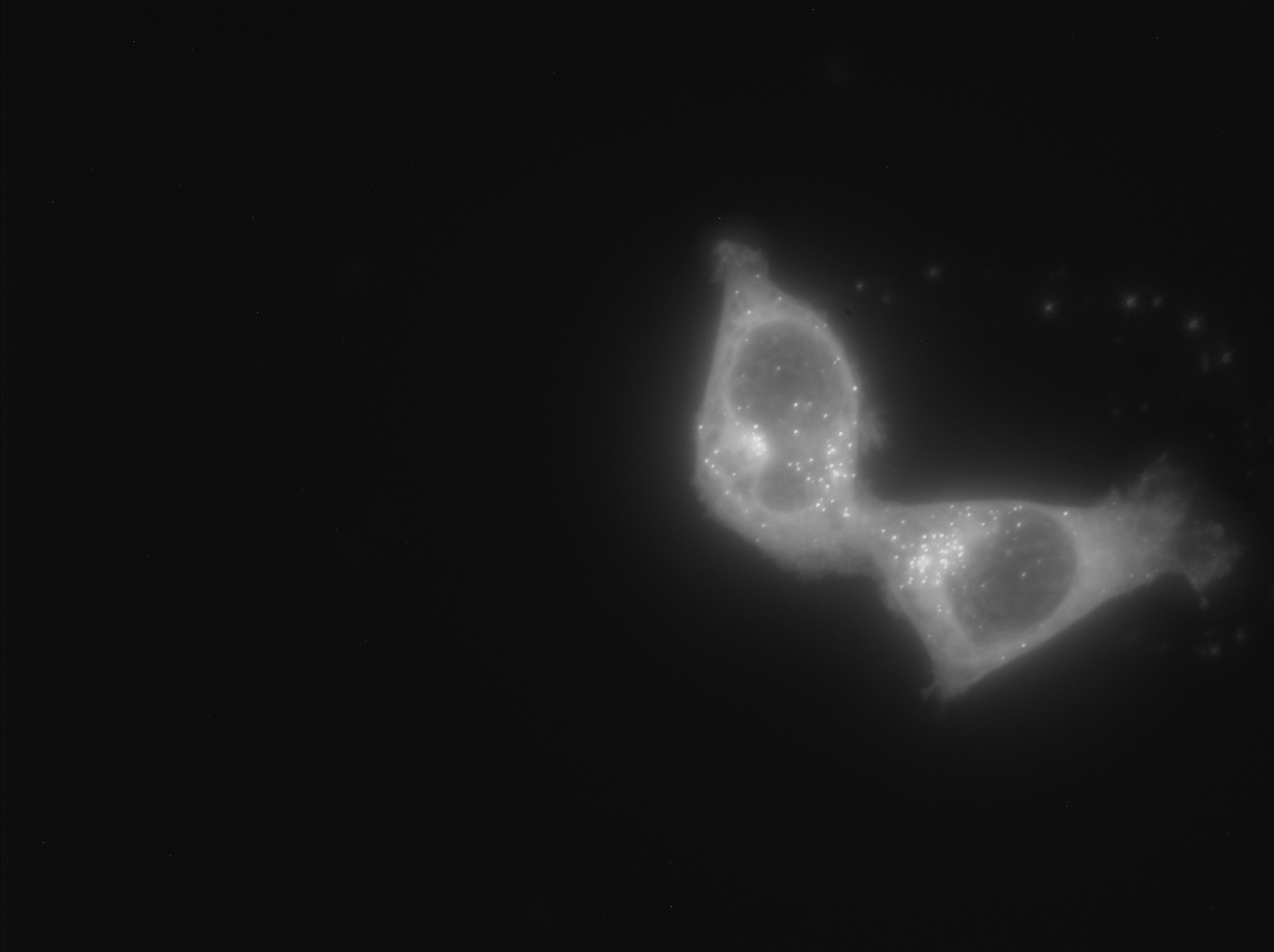

Supplement: Supplementary file 22 — Source data Fig. 6 part 3 [file 44319_2026_742_MOESM22_ESM.zip › Figure 6 Part 3/Fig 6efg TPGS1-KO TPGS1 rescue experiments/R1/TPGS1-KO TPGS1-3UTR-EYFP actub R1 7-31-25 LT8.Project Maximum Z_XY1753991165_Z0_T0_C1.tif]

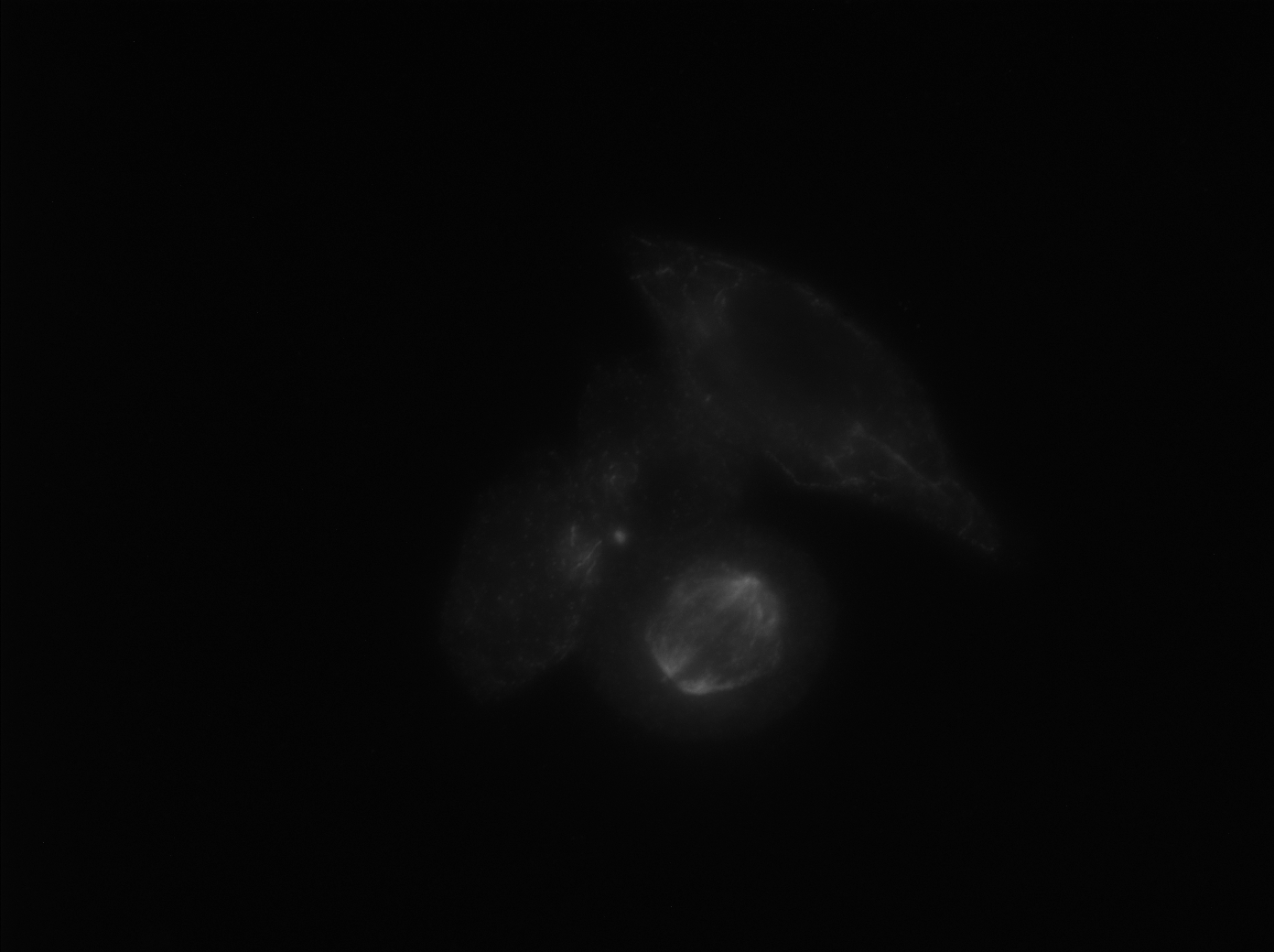

Supplement: Supplementary file 22 — Source data Fig. 6 part 3 [file 44319_2026_742_MOESM22_ESM.zip › Figure 6 Part 3/Fig 6efg TPGS1-KO TPGS1 rescue experiments/R1/TPGS1-KO TPGS1-3UTR-EYFP actub R1 7-31-25 M1 I1.Project Maximum Z_XY1753992586_Z0_T0_C2.tif]

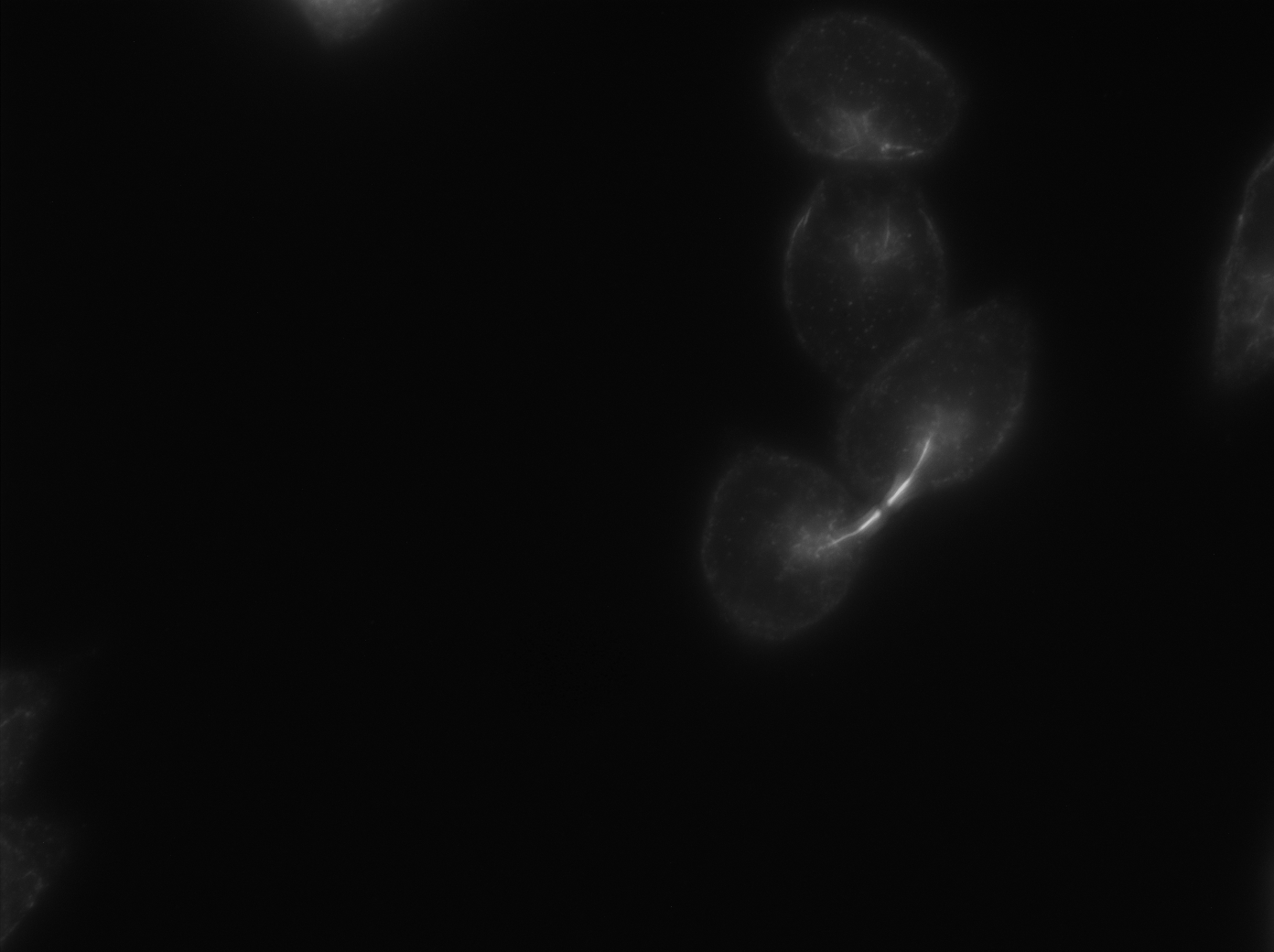

Supplement: Supplementary file 22 — Source data Fig. 6 part 3 [file 44319_2026_742_MOESM22_ESM.zip › Figure 6 Part 3/Fig 6efg TPGS1-KO TPGS1 rescue experiments/R1/TPGS1-KO EYFP only actub R1 7-31-25 LT7.Project Maximum Z_XY1754336696_Z0_T0_C2.tif]

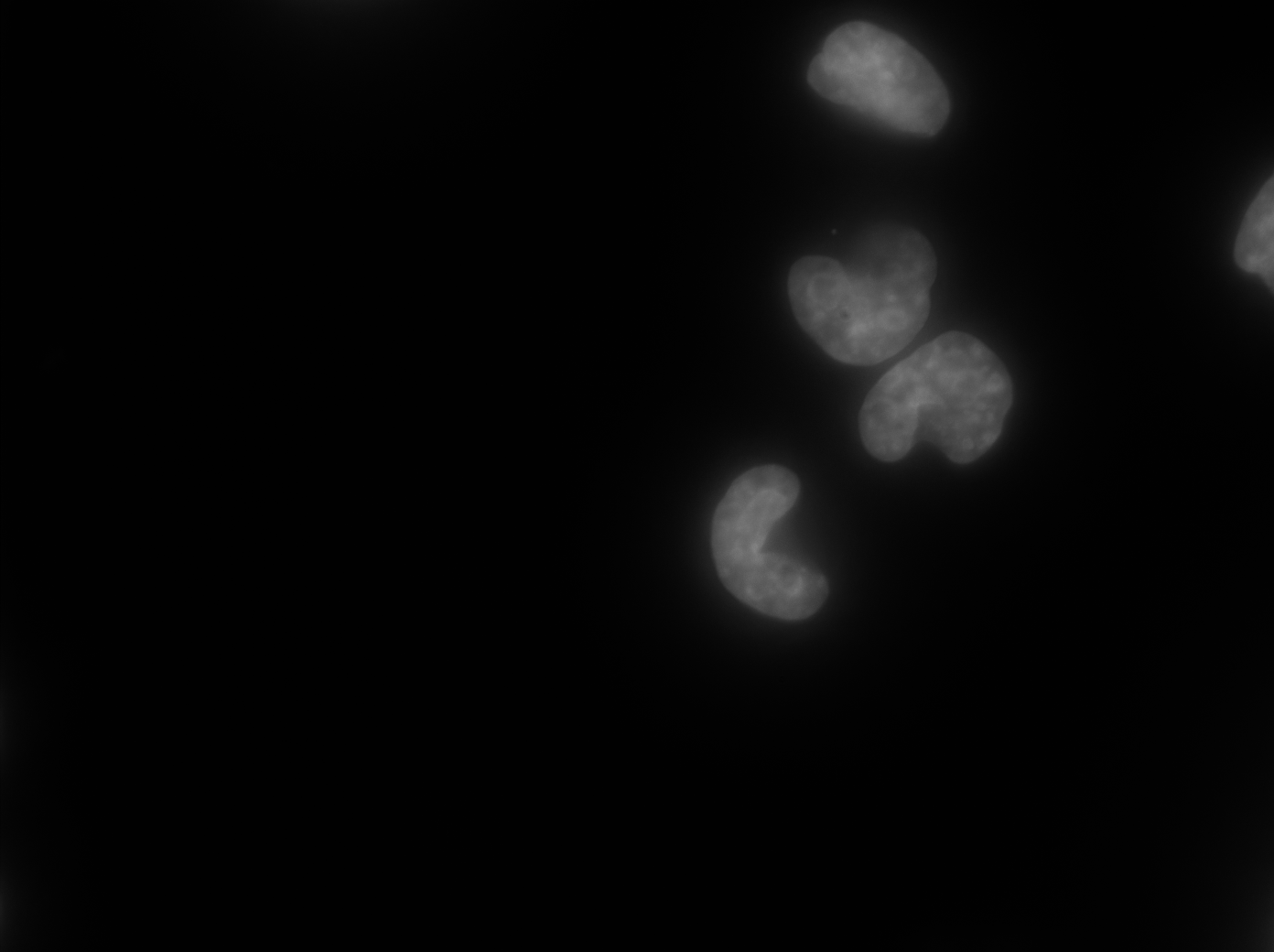

Supplement: Supplementary file 22 — Source data Fig. 6 part 3 [file 44319_2026_742_MOESM22_ESM.zip › Figure 6 Part 3/Fig 6efg TPGS1-KO TPGS1 rescue experiments/R1/TPGS1-KO EYFP only actub R1 7-31-25 LT7.Project Maximum Z_XY1754336696_Z0_T0_C0.tif]

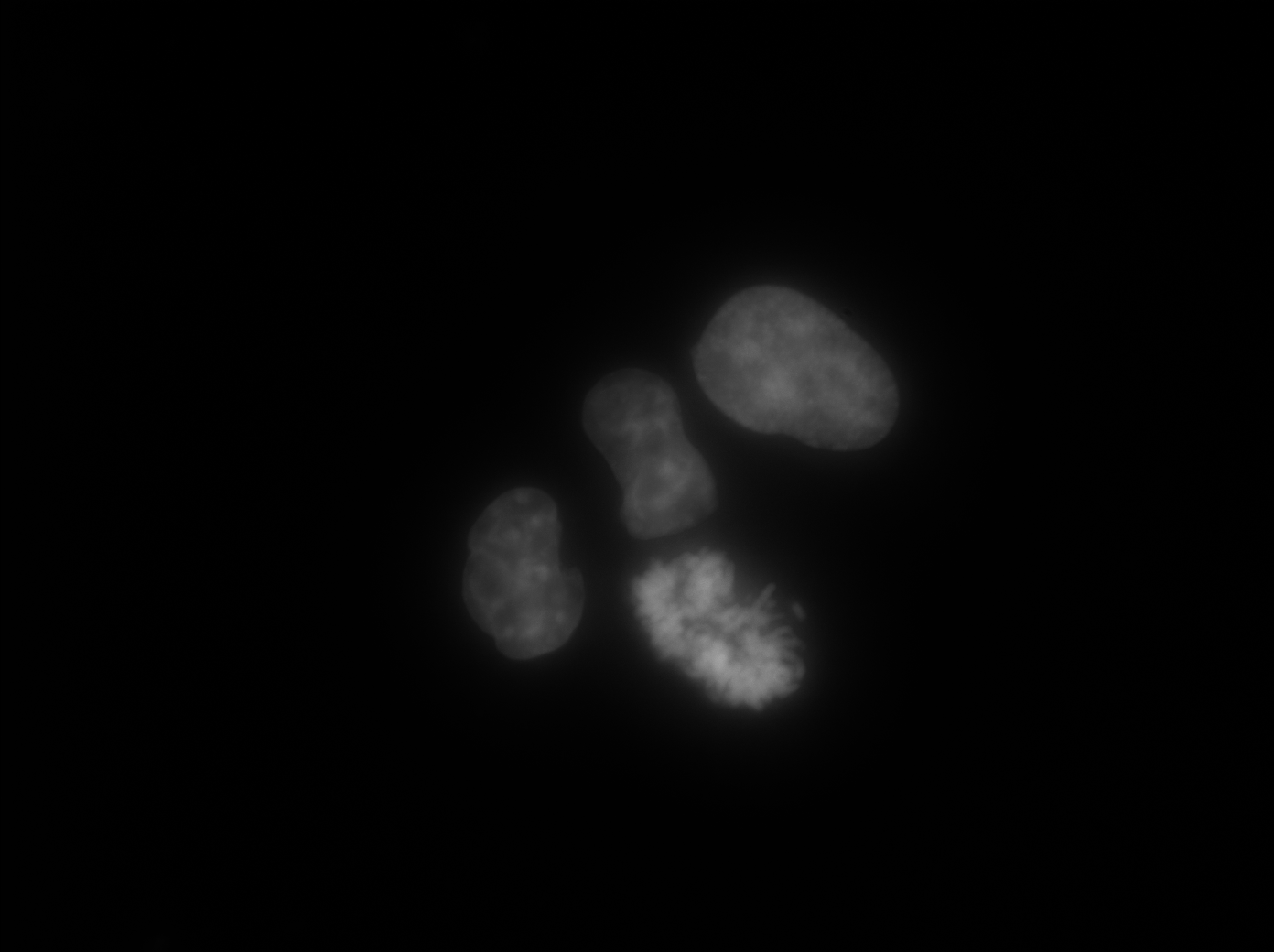

Supplement: Supplementary file 22 — Source data Fig. 6 part 3 [file 44319_2026_742_MOESM22_ESM.zip › Figure 6 Part 3/Fig 6efg TPGS1-KO TPGS1 rescue experiments/R1/TPGS1-KO TPGS1-3UTR-EYFP actub R1 7-31-25 M1 I1.Project Maximum Z_XY1753992586_Z0_T0_C0.tif]
