# Supplementary figures and images for: TPGS1 regulates central spindle microtubule glutamylation and remodeling during telophase and abscission (part 29 of 36)
Source: EMBO Rep. 2026 Mar 23;27(8):1944–63. doi: 10.1038/s44319-026-00742-3 (PMC13121839; doi:10.1038/s44319-026-00742-3)

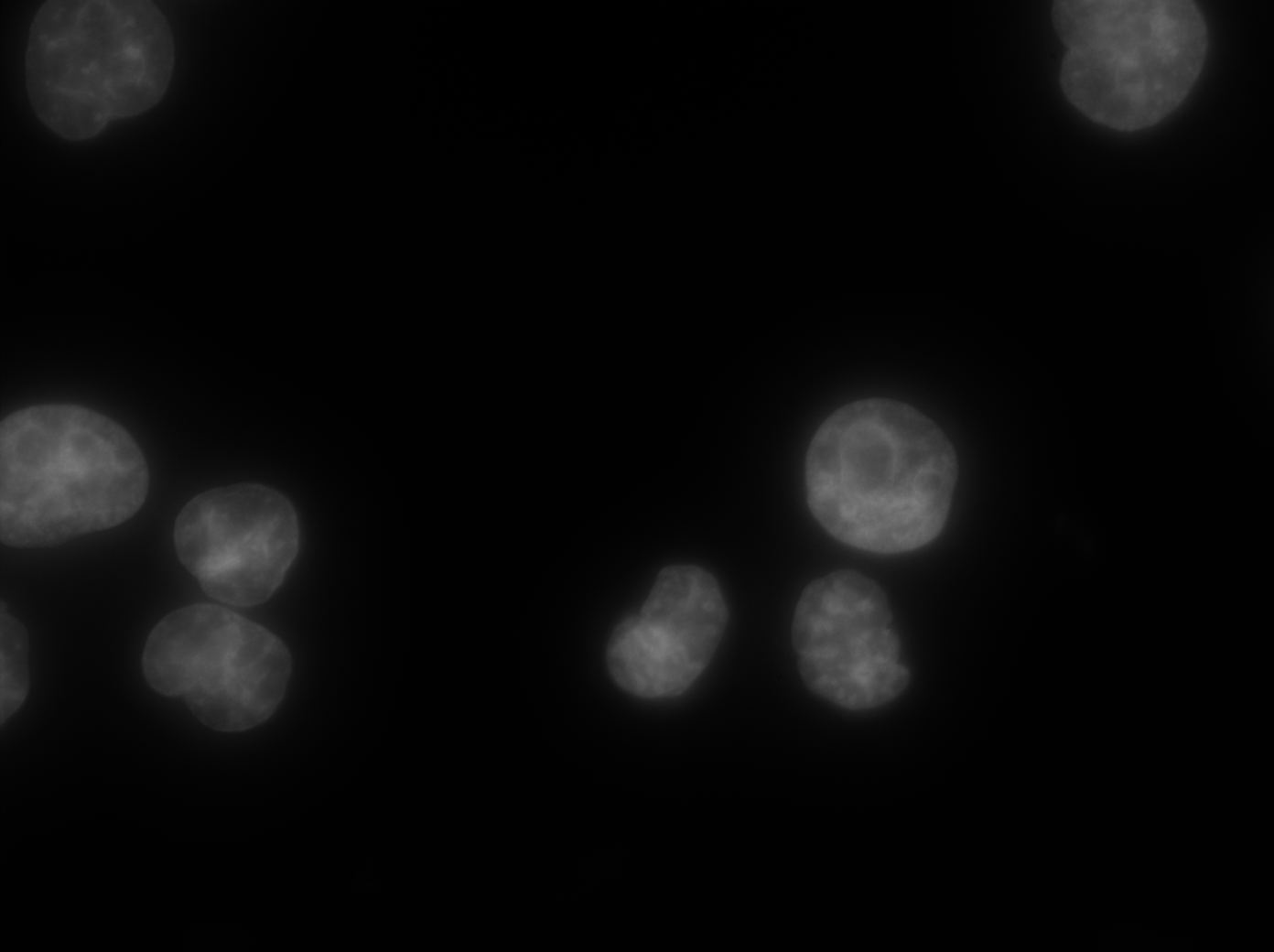

Supplement: Supplementary file 23 — Source data Fig. 6 part 4 [file 44319_2026_742_MOESM23_ESM.zip › Figure 6 Part 4/Fig 6efg TPGS1-KO TPGS1 rescue experiments part 2/R2R3/TPGS1-KO EYFP-only actub 7-31-25 R2 ET10.Project Maximum Z_XY1756416721_Z0_T0_C0.tif]

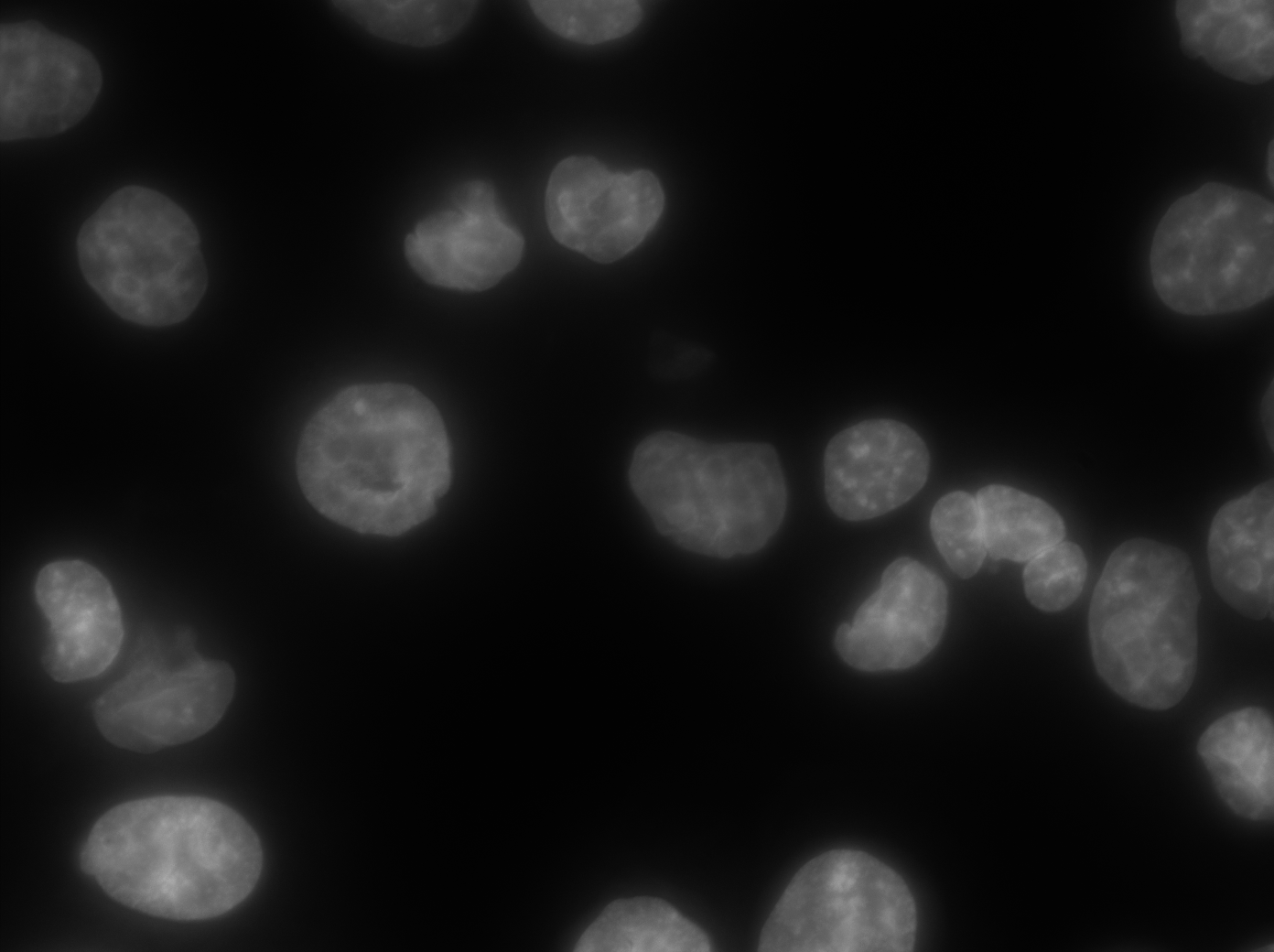

Supplement: Supplementary file 23 — Source data Fig. 6 part 4 [file 44319_2026_742_MOESM23_ESM.zip › Figure 6 Part 4/Fig 6efg TPGS1-KO TPGS1 rescue experiments part 2/R2R3/TPGS1-KO TPGS1-EYFP-3'UTR actub 7-31-25 R2 LT1.Project Maximum Z_XY1756406667_Z0_T0_C0.tif]

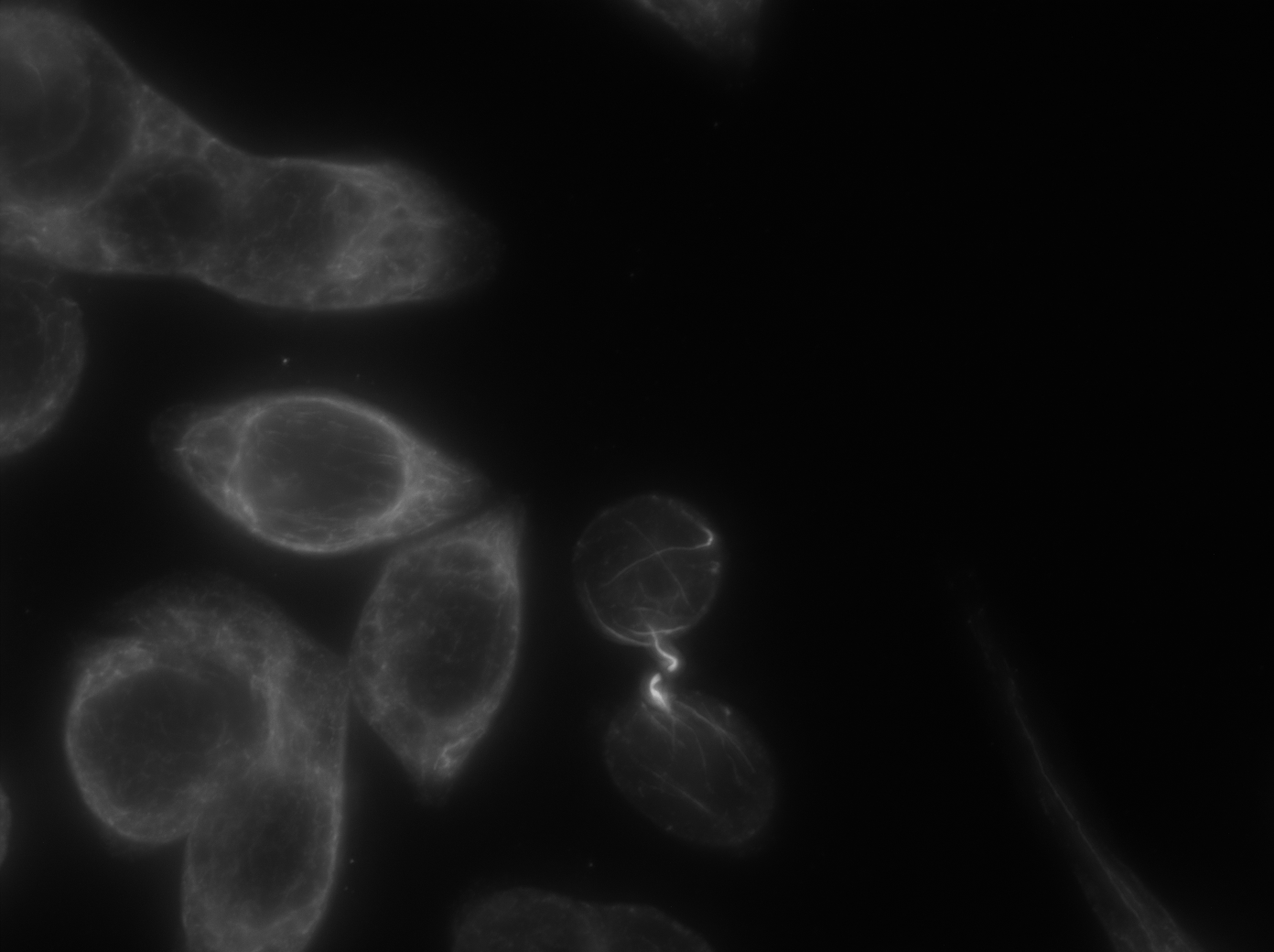

Supplement: Supplementary file 23 — Source data Fig. 6 part 4 [file 44319_2026_742_MOESM23_ESM.zip › Figure 6 Part 4/Fig 6efg TPGS1-KO TPGS1 rescue experiments part 2/R2R3/TPGS1-KO TPGS1-EYFP-3'UTR actub 7-31-25 R3 LT2.Project Maximum Z_XY1756500278_Z0_T0_C2.tif]

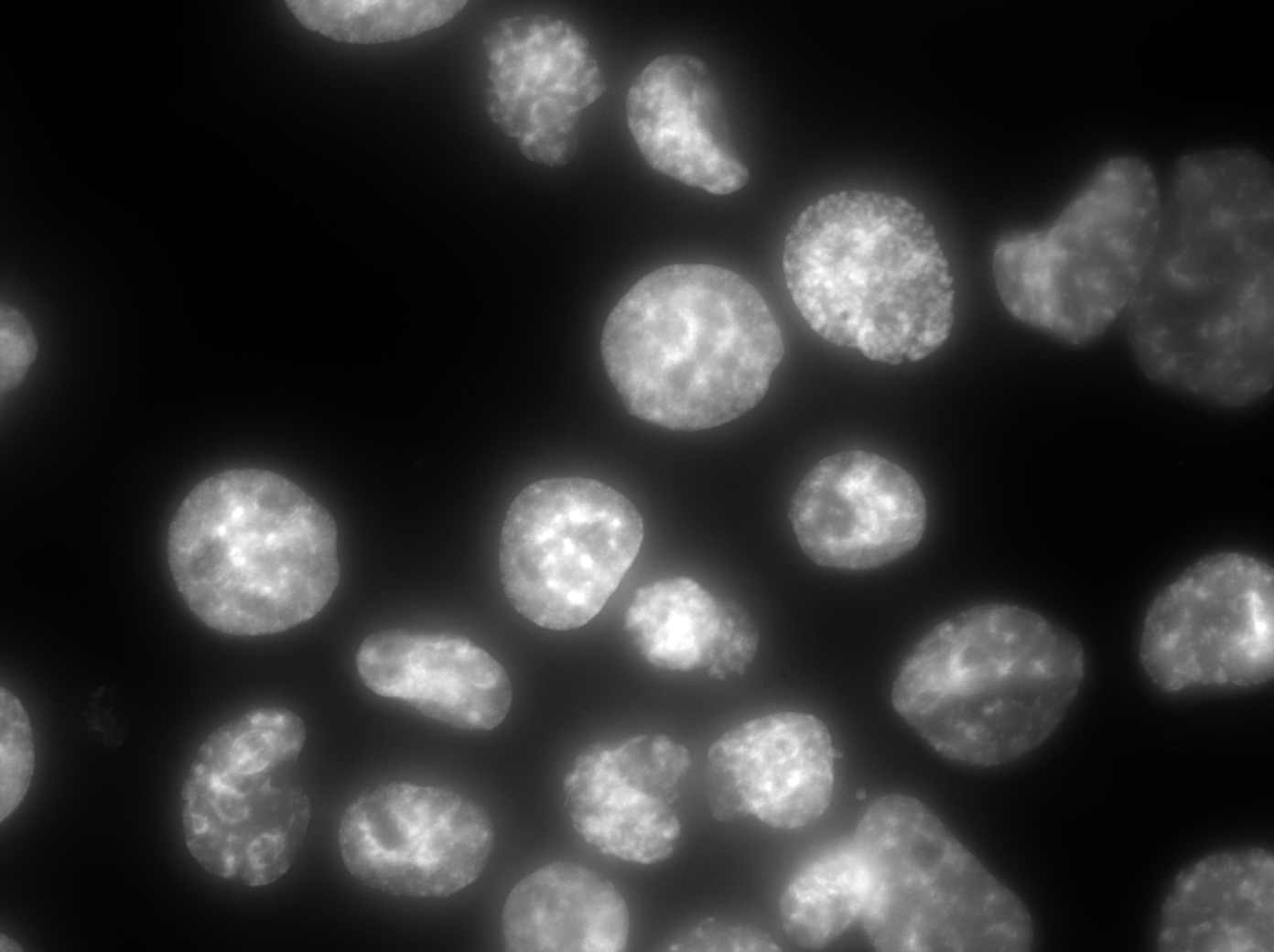

Supplement: Supplementary file 24 — Source data Fig. 6 part 5 [file 44319_2026_742_MOESM24_ESM.zip › Figure 6 Part 5/Fig 6efg TPGS1-KO TPGS1 rescue experiments part 3/R2R3/TPGS1-KO untransfected actub 7-31-25 R3 LT8LT9.Project Maximum Z_XY1756505786_Z0_T0_C0.tif]

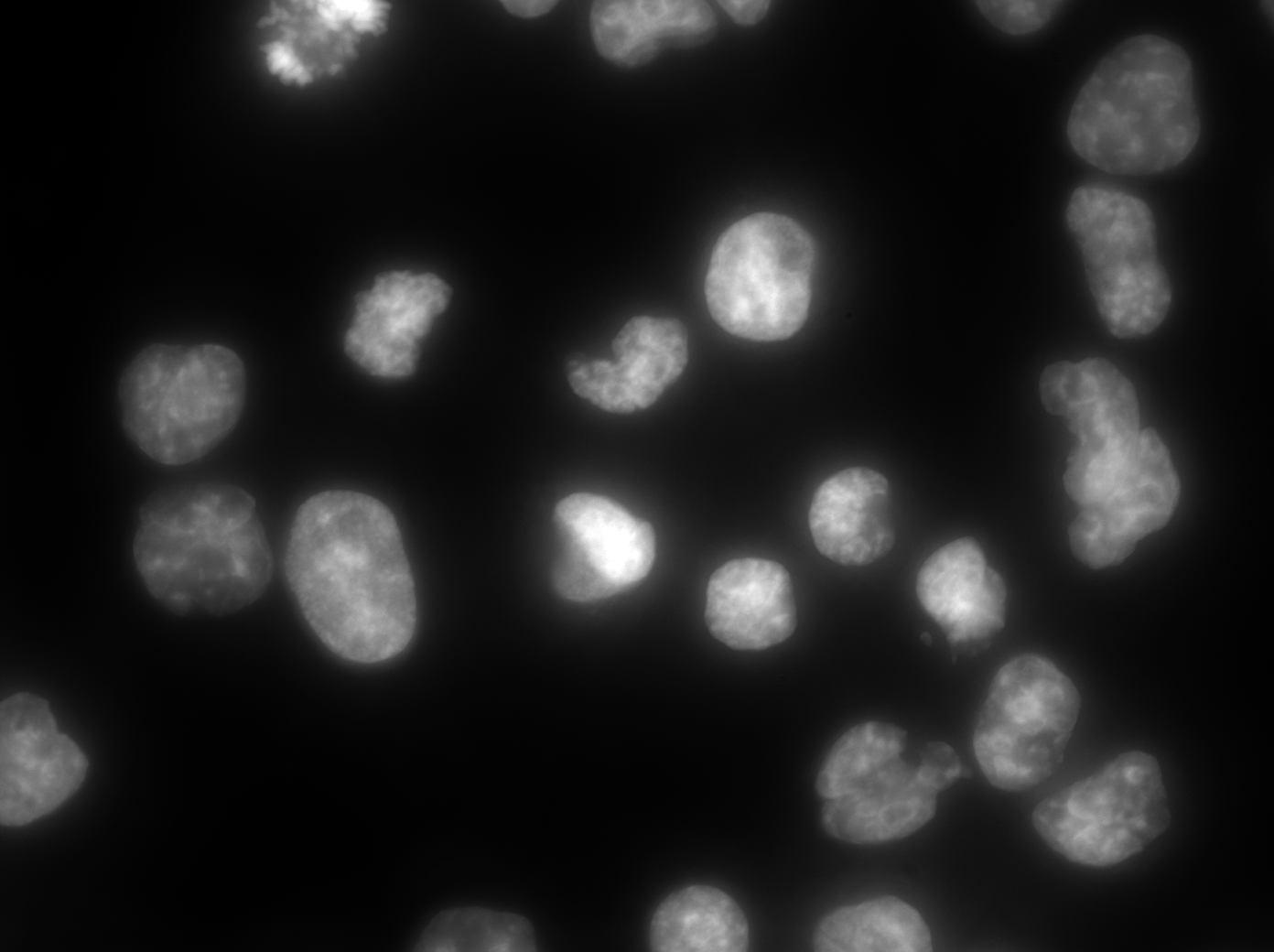

Supplement: Supplementary file 24 — Source data Fig. 6 part 5 [file 44319_2026_742_MOESM24_ESM.zip › Figure 6 Part 5/Fig 6efg TPGS1-KO TPGS1 rescue experiments part 3/R2R3/TPGS1-KO Untransfected actub 7-31-25 R2 LT7LT8.Project Maximum Z_XY1756412808_Z0_T0_C0.tif]

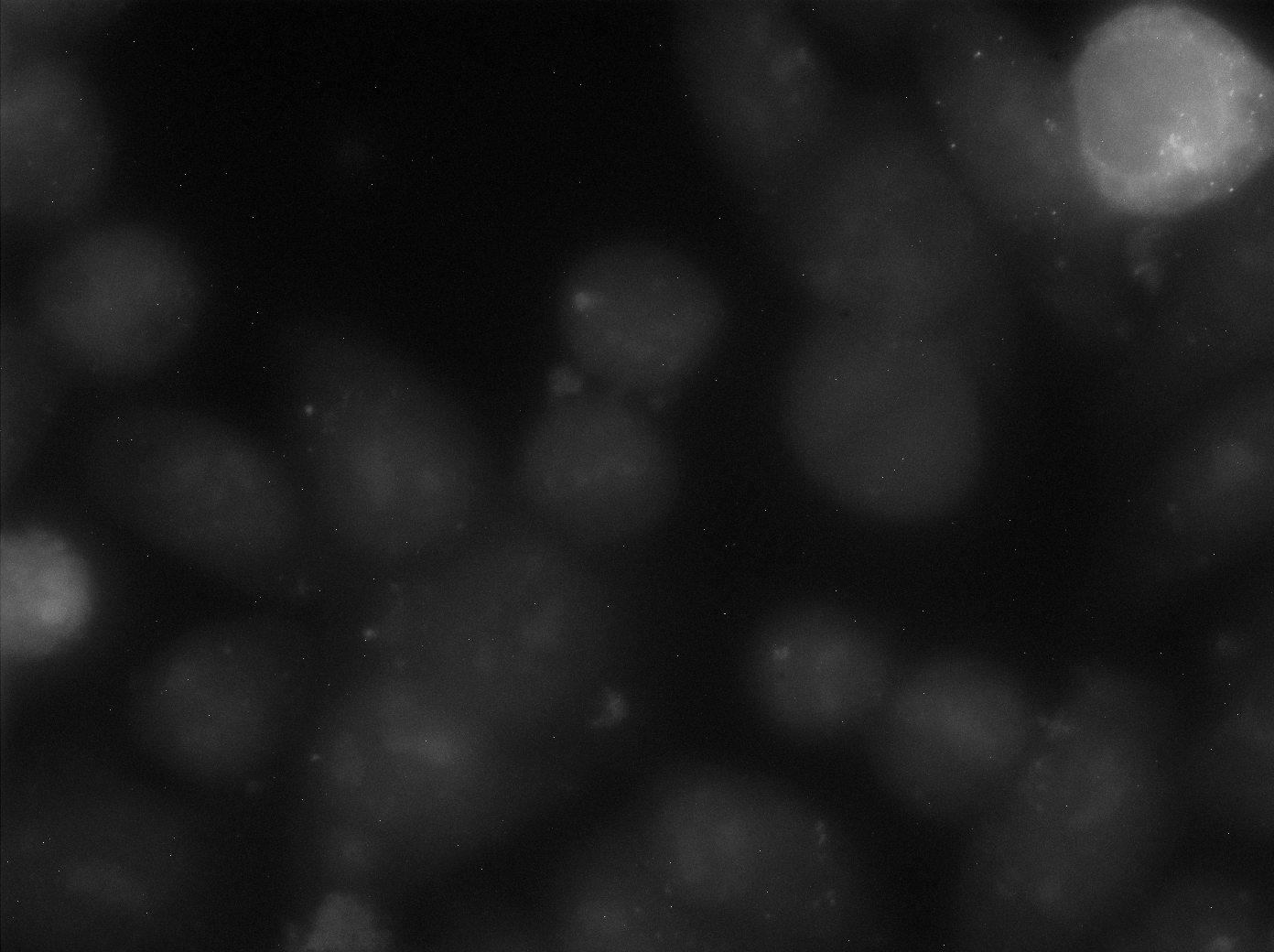

Supplement: Supplementary file 24 — Source data Fig. 6 part 5 [file 44319_2026_742_MOESM24_ESM.zip › Figure 6 Part 5/Fig 6efg TPGS1-KO TPGS1 rescue experiments part 3/R2R3/TPGS1-KO Untransfected actub 7-31-25 R2 ET2 LT2.Project Maximum Z_XY1756406133_Z0_T0_C1.tif]

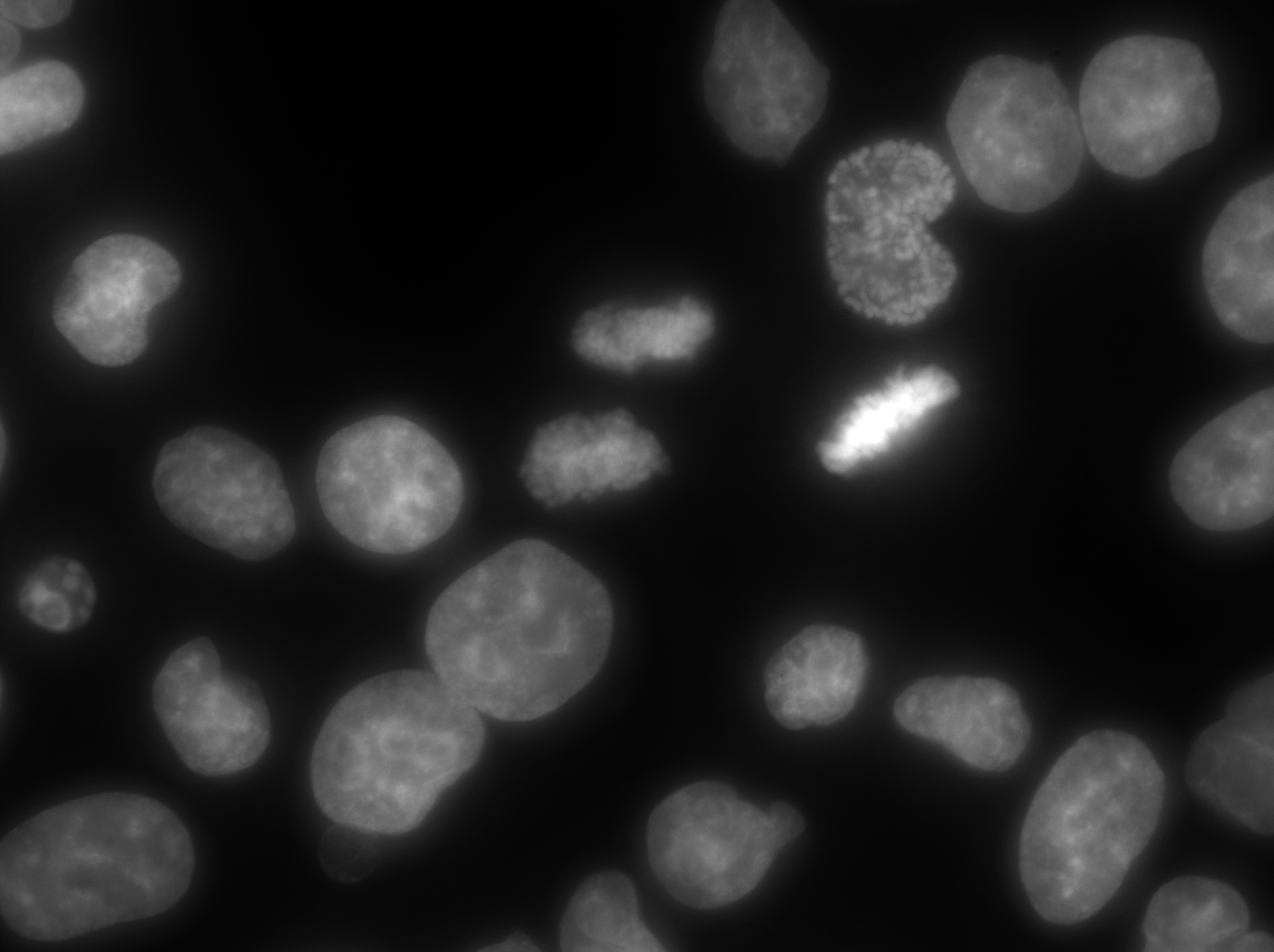

Supplement: Supplementary file 24 — Source data Fig. 6 part 5 [file 44319_2026_742_MOESM24_ESM.zip › Figure 6 Part 5/Fig 6efg TPGS1-KO TPGS1 rescue experiments part 3/R2R3/TPGS1-KO Untransfected actub 7-31-25 R2 ET2 LT2.Project Maximum Z_XY1756406133_Z0_T0_C0.tif]

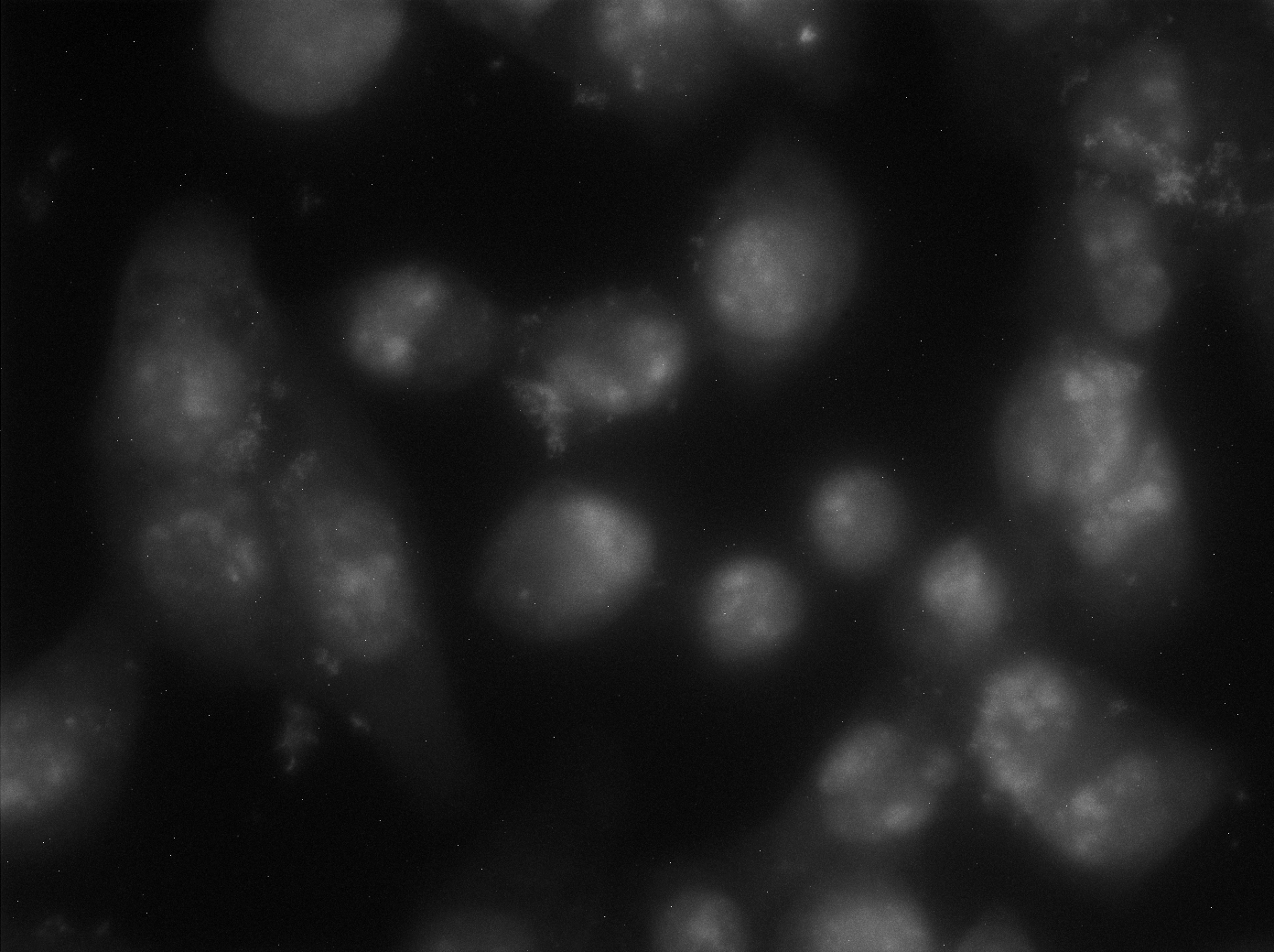

Supplement: Supplementary file 24 — Source data Fig. 6 part 5 [file 44319_2026_742_MOESM24_ESM.zip › Figure 6 Part 5/Fig 6efg TPGS1-KO TPGS1 rescue experiments part 3/R2R3/TPGS1-KO Untransfected actub 7-31-25 R2 LT7LT8.Project Maximum Z_XY1756412808_Z0_T0_C1.tif]

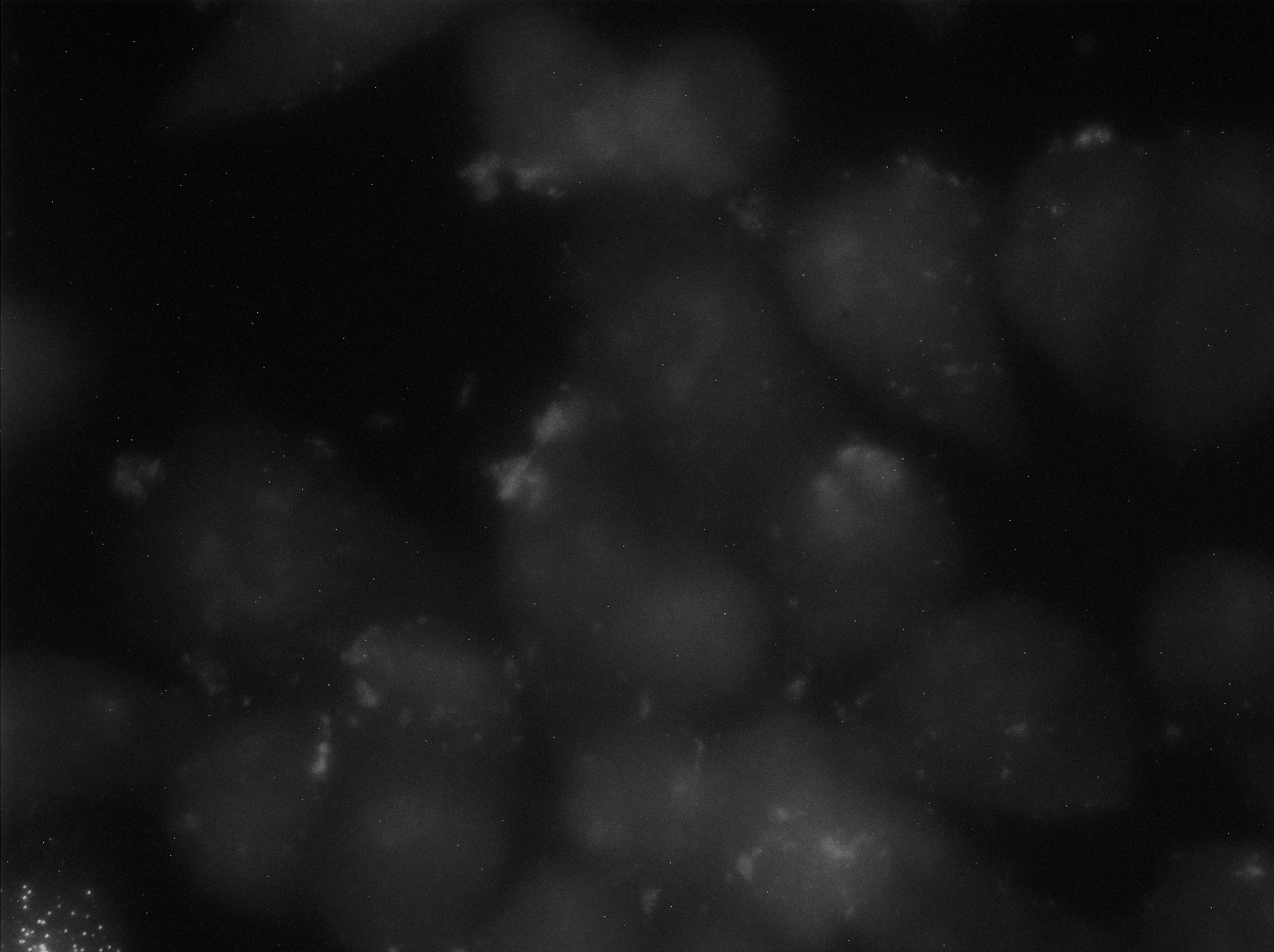

Supplement: Supplementary file 24 — Source data Fig. 6 part 5 [file 44319_2026_742_MOESM24_ESM.zip › Figure 6 Part 5/Fig 6efg TPGS1-KO TPGS1 rescue experiments part 3/R2R3/TPGS1-KO untransfected actub 7-31-25 R3 LT8LT9.Project Maximum Z_XY1756505786_Z0_T0_C1.tif]

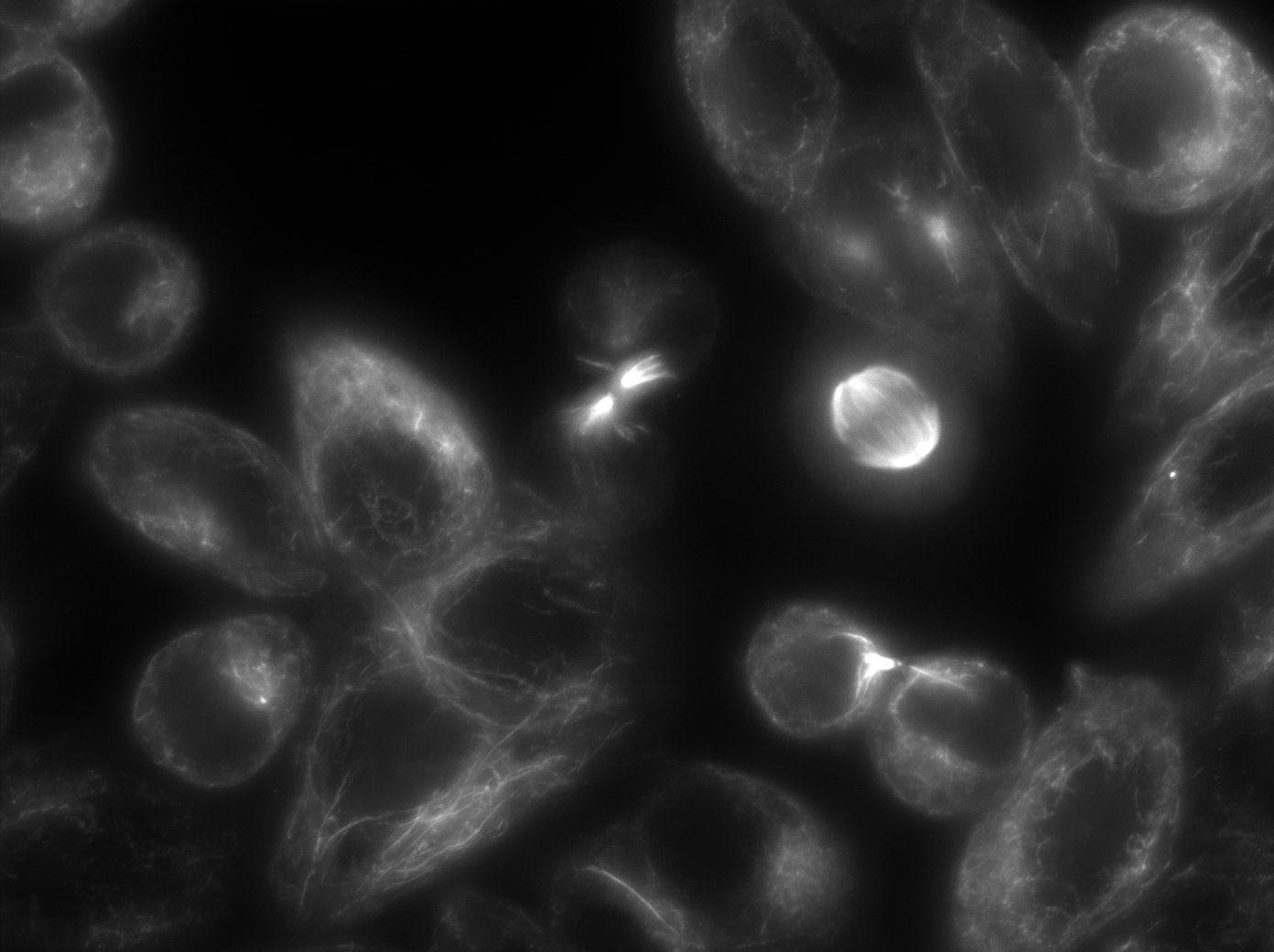

Supplement: Supplementary file 24 — Source data Fig. 6 part 5 [file 44319_2026_742_MOESM24_ESM.zip › Figure 6 Part 5/Fig 6efg TPGS1-KO TPGS1 rescue experiments part 3/R2R3/TPGS1-KO Untransfected actub 7-31-25 R2 ET2 LT2.Project Maximum Z_XY1756406133_Z0_T0_C2.tif]

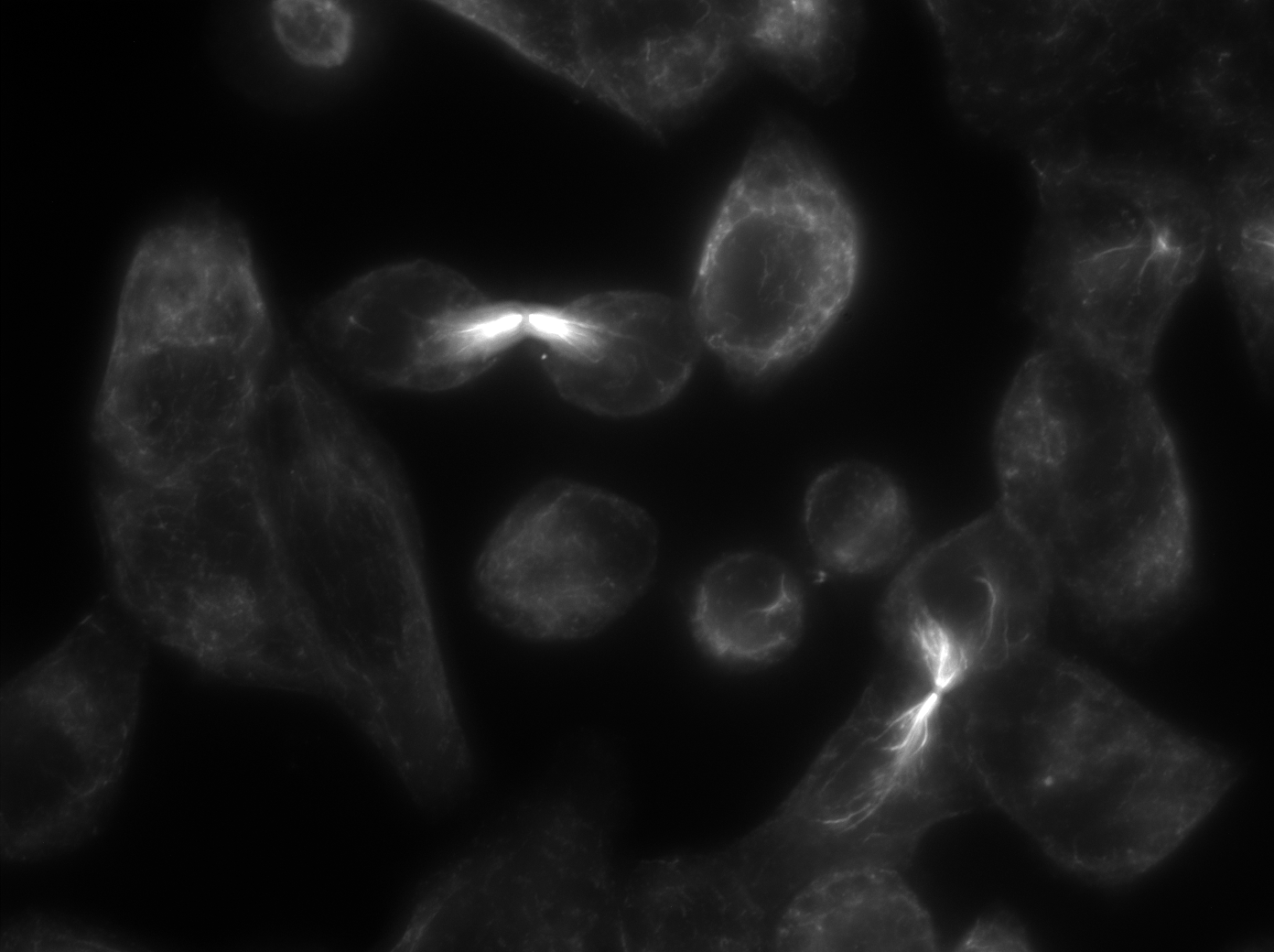

Supplement: Supplementary file 24 — Source data Fig. 6 part 5 [file 44319_2026_742_MOESM24_ESM.zip › Figure 6 Part 5/Fig 6efg TPGS1-KO TPGS1 rescue experiments part 3/R2R3/TPGS1-KO Untransfected actub 7-31-25 R2 LT7LT8.Project Maximum Z_XY1756412808_Z0_T0_C2.tif]

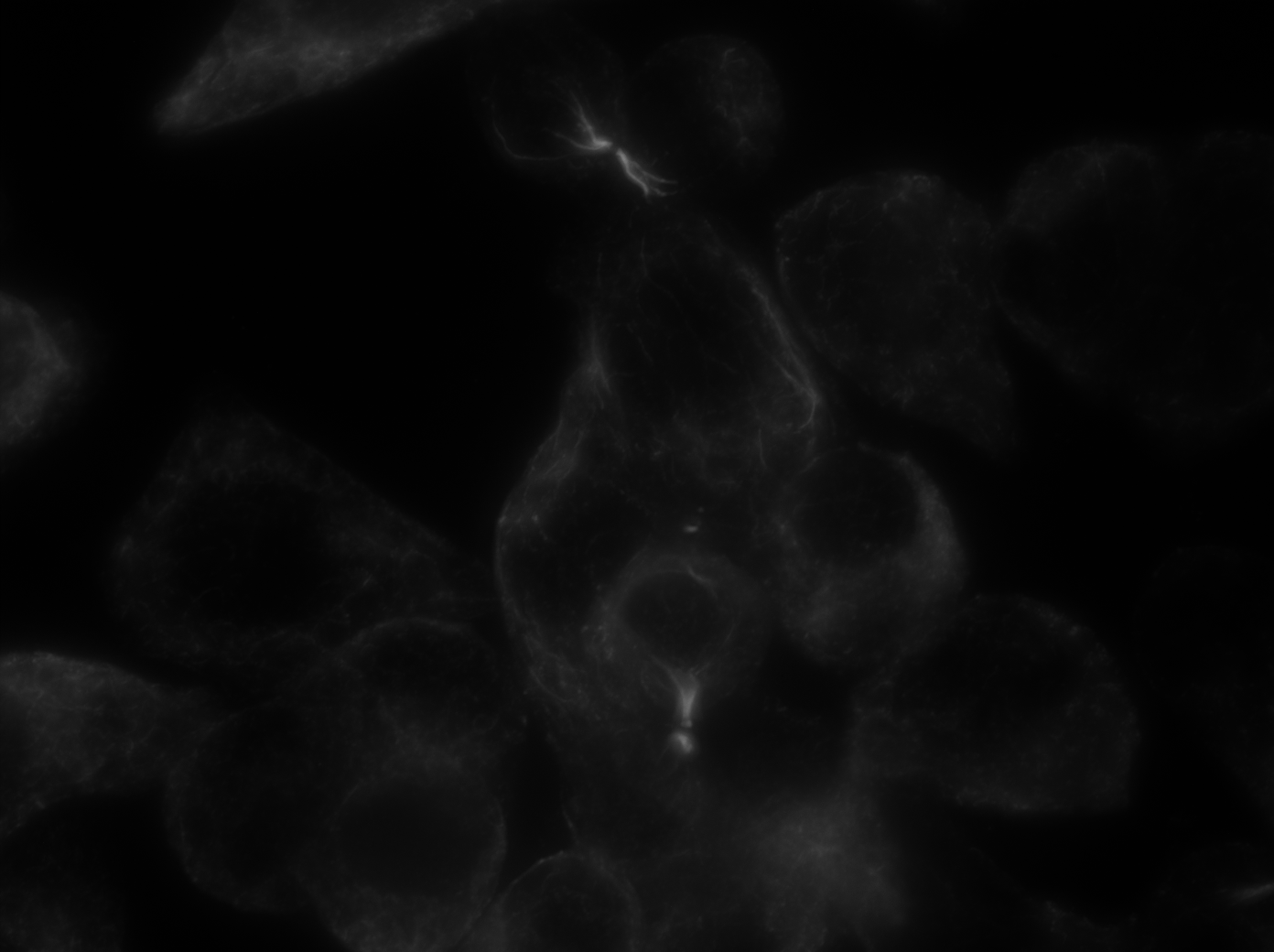

Supplement: Supplementary file 24 — Source data Fig. 6 part 5 [file 44319_2026_742_MOESM24_ESM.zip › Figure 6 Part 5/Fig 6efg TPGS1-KO TPGS1 rescue experiments part 3/R2R3/TPGS1-KO untransfected actub 7-31-25 R3 LT8LT9.Project Maximum Z_XY1756505786_Z0_T0_C2.tif]

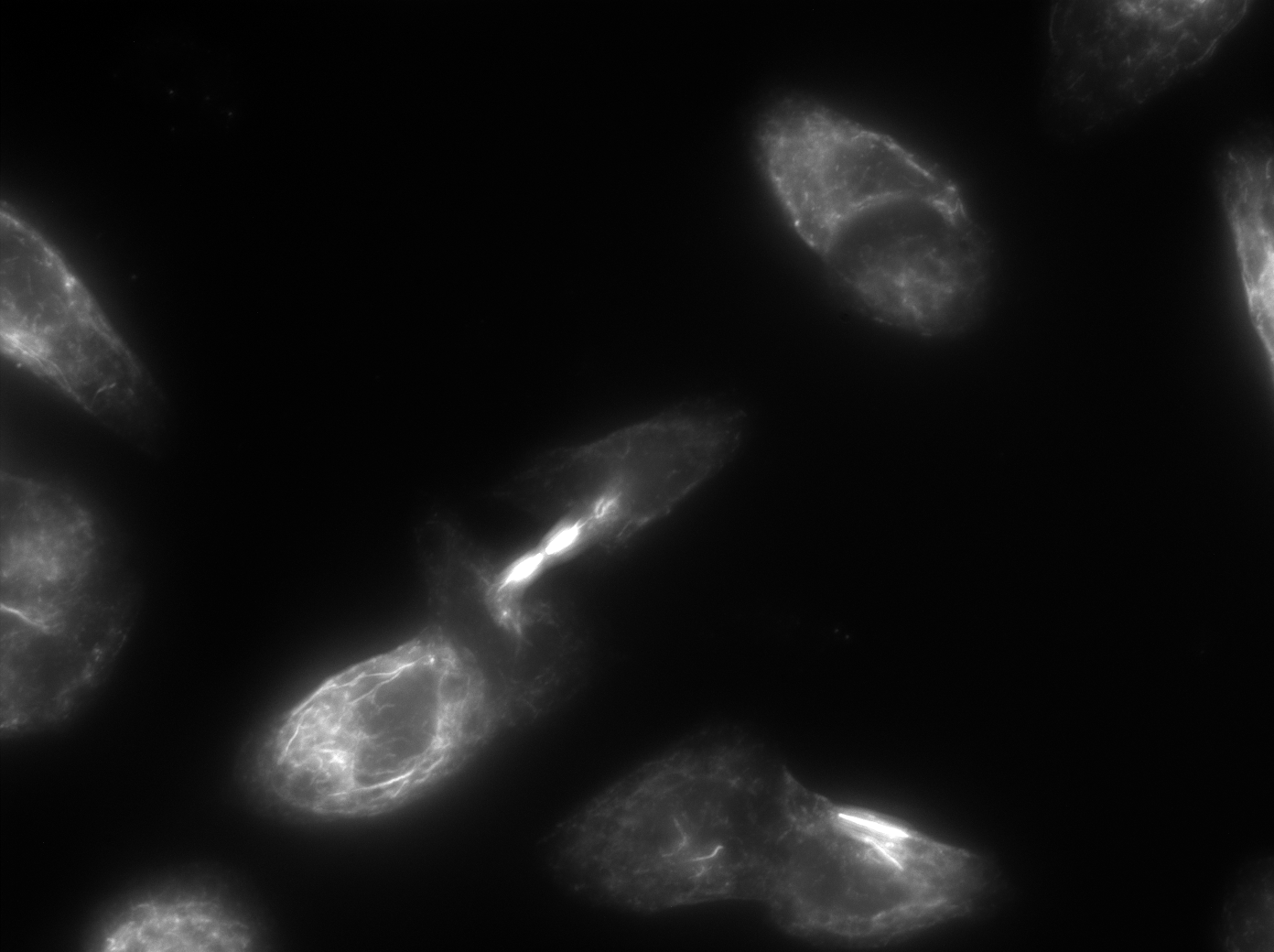

Supplement: Supplementary file 24 — Source data Fig. 6 part 5 [file 44319_2026_742_MOESM24_ESM.zip › Figure 6 Part 5/Fig 6efg TPGS1-KO TPGS1 rescue experiments part 3/R2R3/TPGS1-KO Untransfected actub 7-31-25 R2 LT6.Project Maximum Z_XY1756412699_Z0_T0_C2.tif]

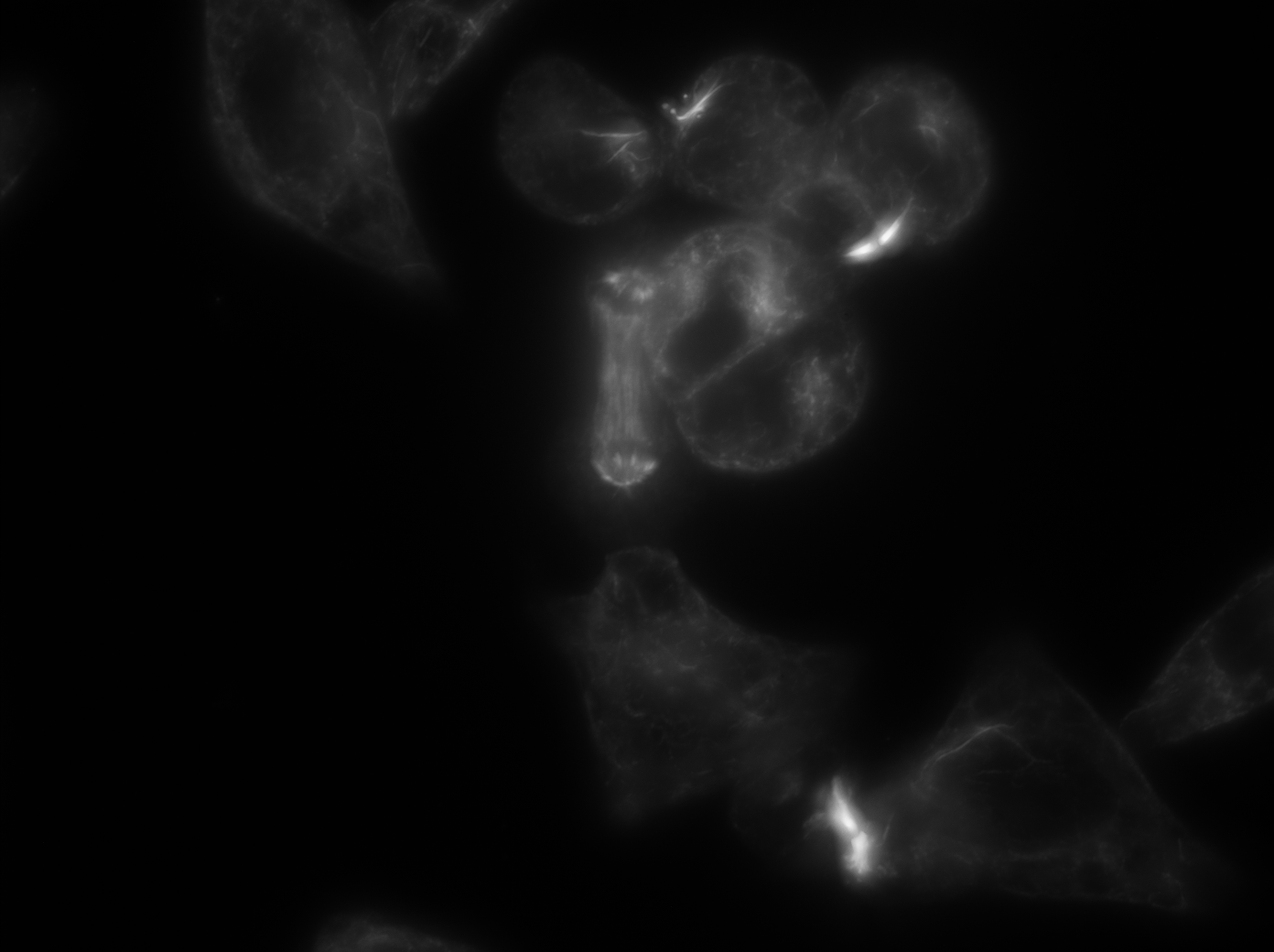

Supplement: Supplementary file 24 — Source data Fig. 6 part 5 [file 44319_2026_742_MOESM24_ESM.zip › Figure 6 Part 5/Fig 6efg TPGS1-KO TPGS1 rescue experiments part 3/R2R3/TPGS1-KO untransfected actub 7-31-25 R3 LT10 ET8.Project Maximum Z_XY1756506015_Z0_T0_C2.tif]

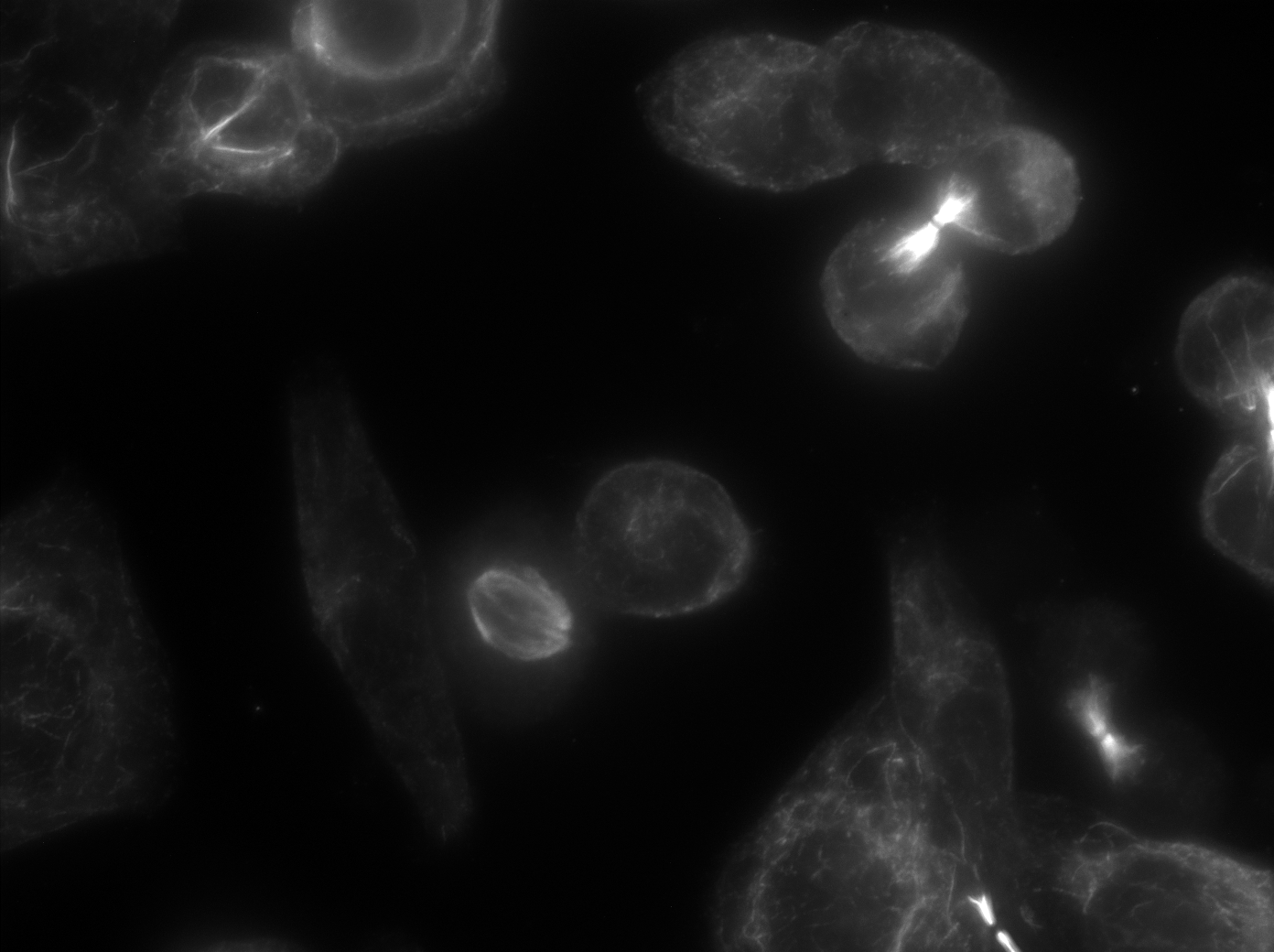

Supplement: Supplementary file 24 — Source data Fig. 6 part 5 [file 44319_2026_742_MOESM24_ESM.zip › Figure 6 Part 5/Fig 6efg TPGS1-KO TPGS1 rescue experiments part 3/R2R3/TPGS1-KO Untransfected actub 7-31-25 R2 ET6ET7.Project Maximum Z_XY1756413060_Z0_T0_C2.tif]

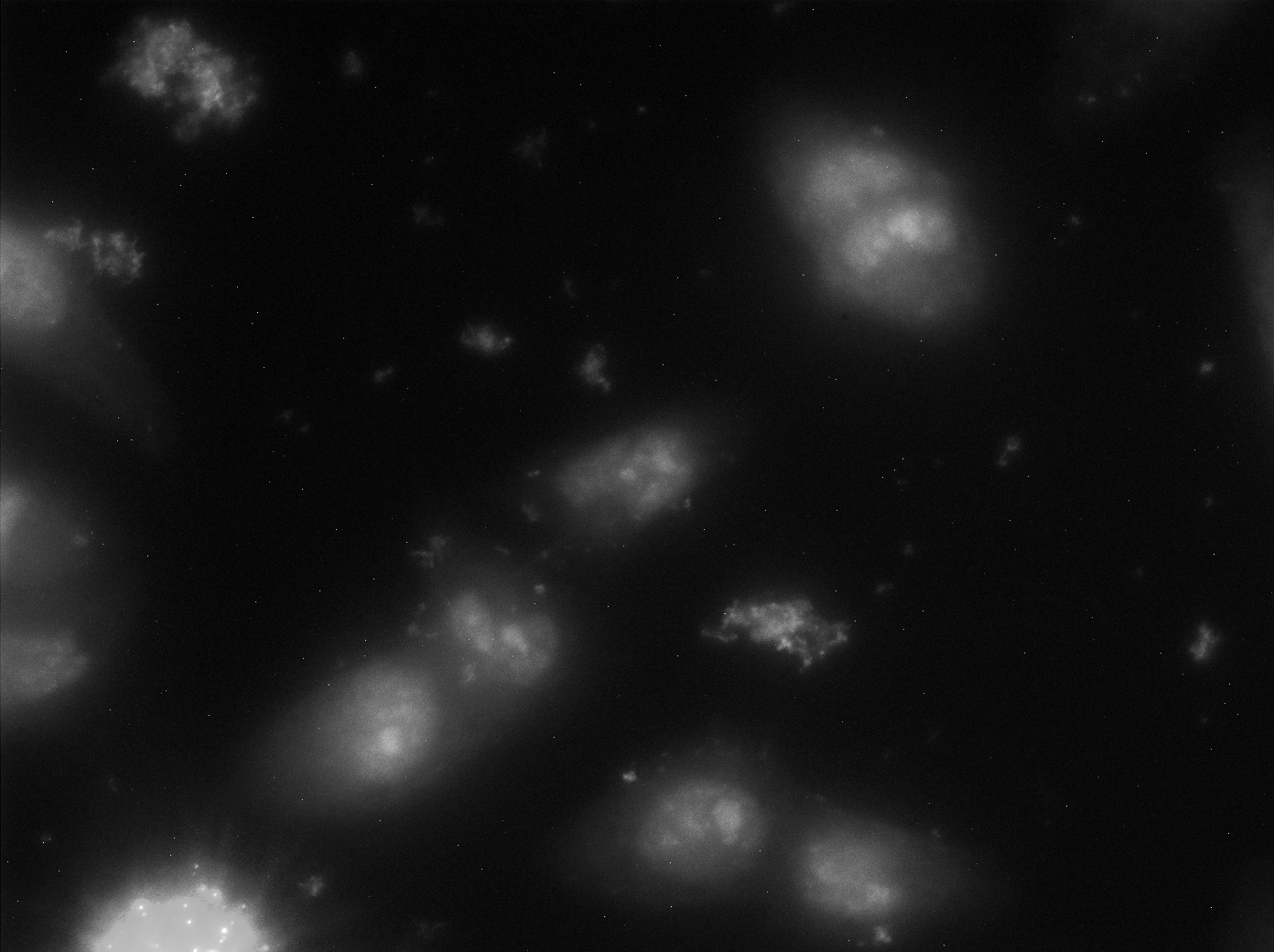

Supplement: Supplementary file 24 — Source data Fig. 6 part 5 [file 44319_2026_742_MOESM24_ESM.zip › Figure 6 Part 5/Fig 6efg TPGS1-KO TPGS1 rescue experiments part 3/R2R3/TPGS1-KO Untransfected actub 7-31-25 R2 LT6.Project Maximum Z_XY1756412699_Z0_T0_C1.tif]

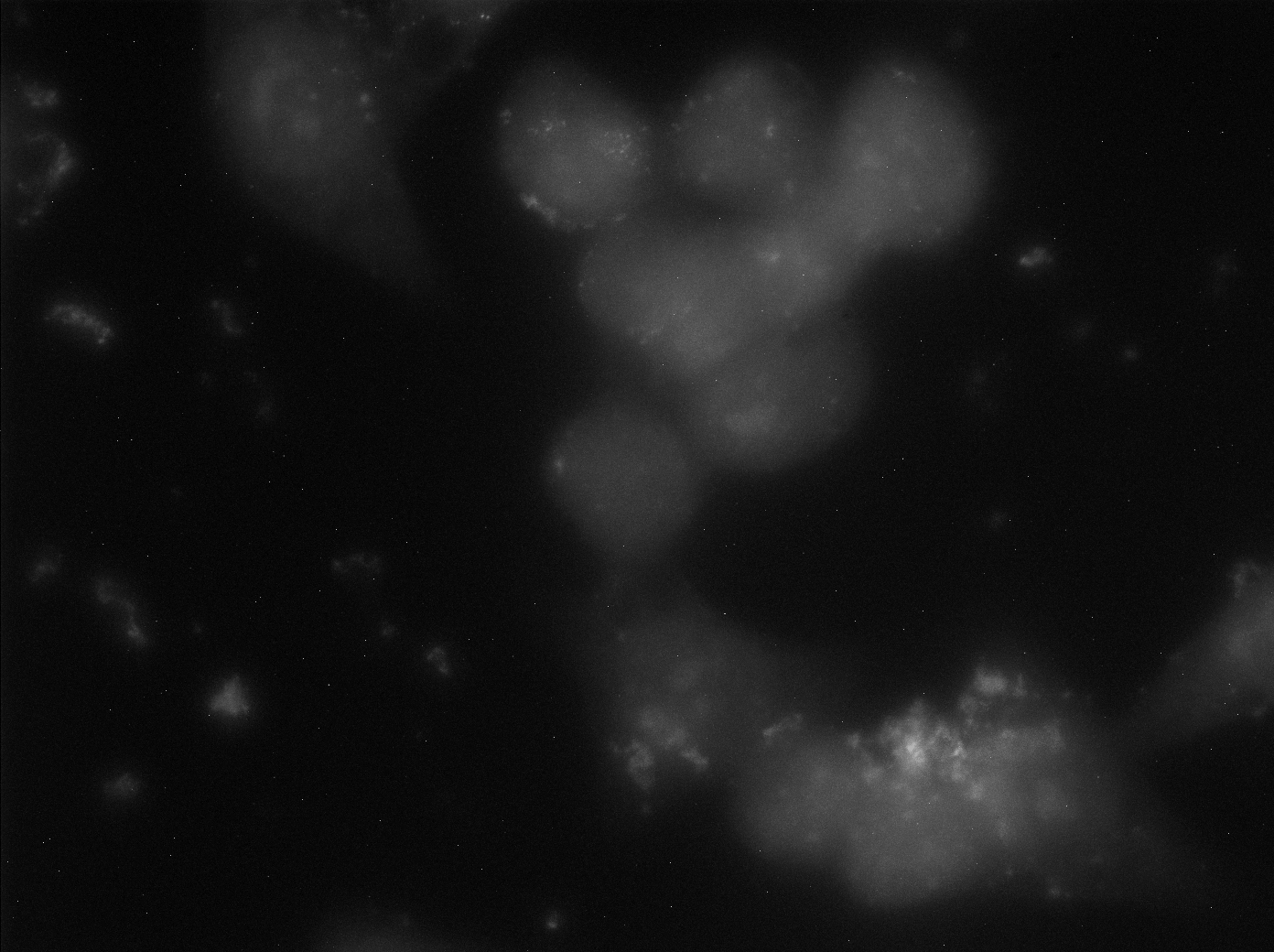

Supplement: Supplementary file 24 — Source data Fig. 6 part 5 [file 44319_2026_742_MOESM24_ESM.zip › Figure 6 Part 5/Fig 6efg TPGS1-KO TPGS1 rescue experiments part 3/R2R3/TPGS1-KO untransfected actub 7-31-25 R3 LT10 ET8.Project Maximum Z_XY1756506015_Z0_T0_C1.tif]

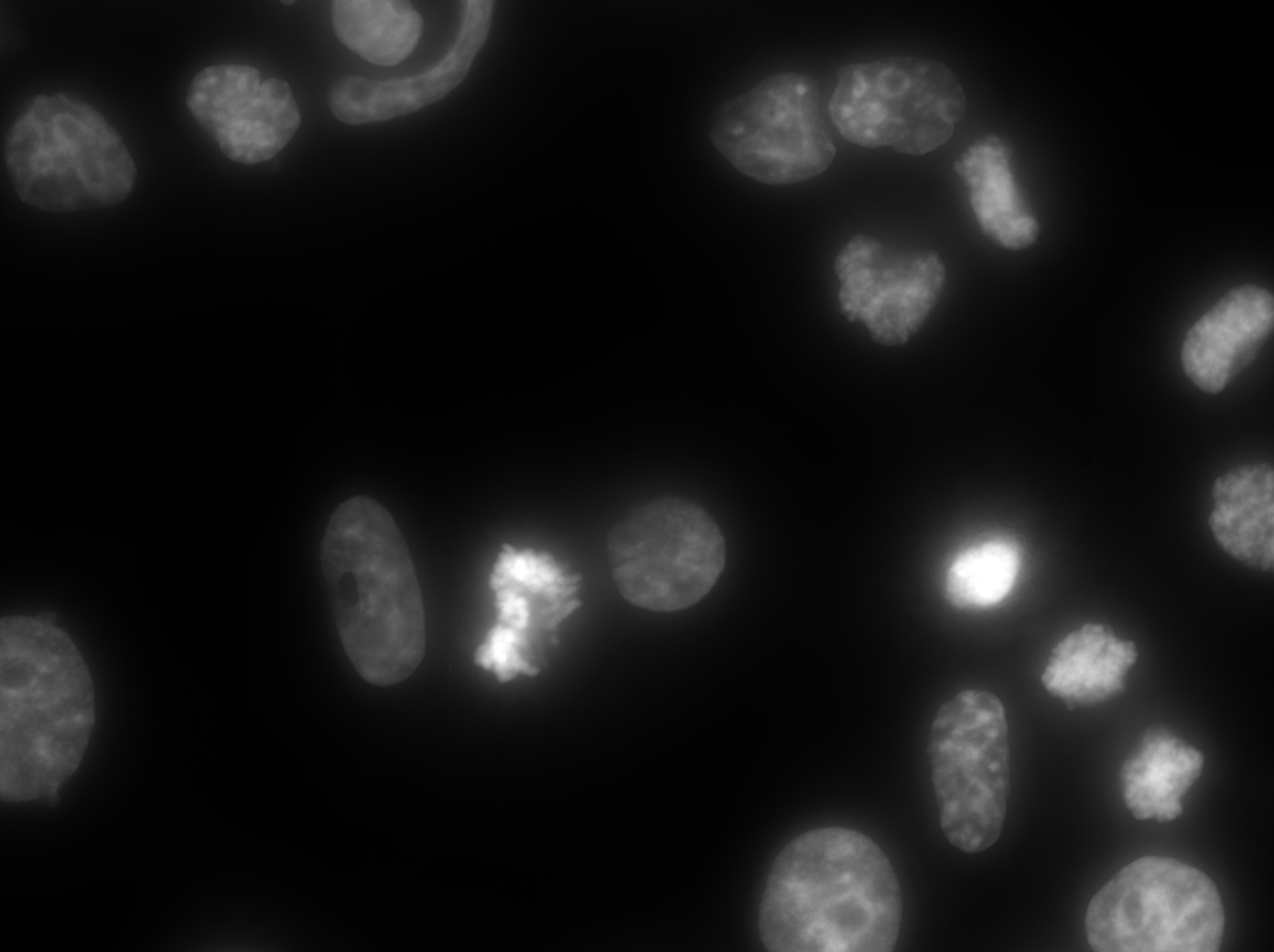

Supplement: Supplementary file 24 — Source data Fig. 6 part 5 [file 44319_2026_742_MOESM24_ESM.zip › Figure 6 Part 5/Fig 6efg TPGS1-KO TPGS1 rescue experiments part 3/R2R3/TPGS1-KO Untransfected actub 7-31-25 R2 ET6ET7.Project Maximum Z_XY1756413060_Z0_T0_C0.tif]

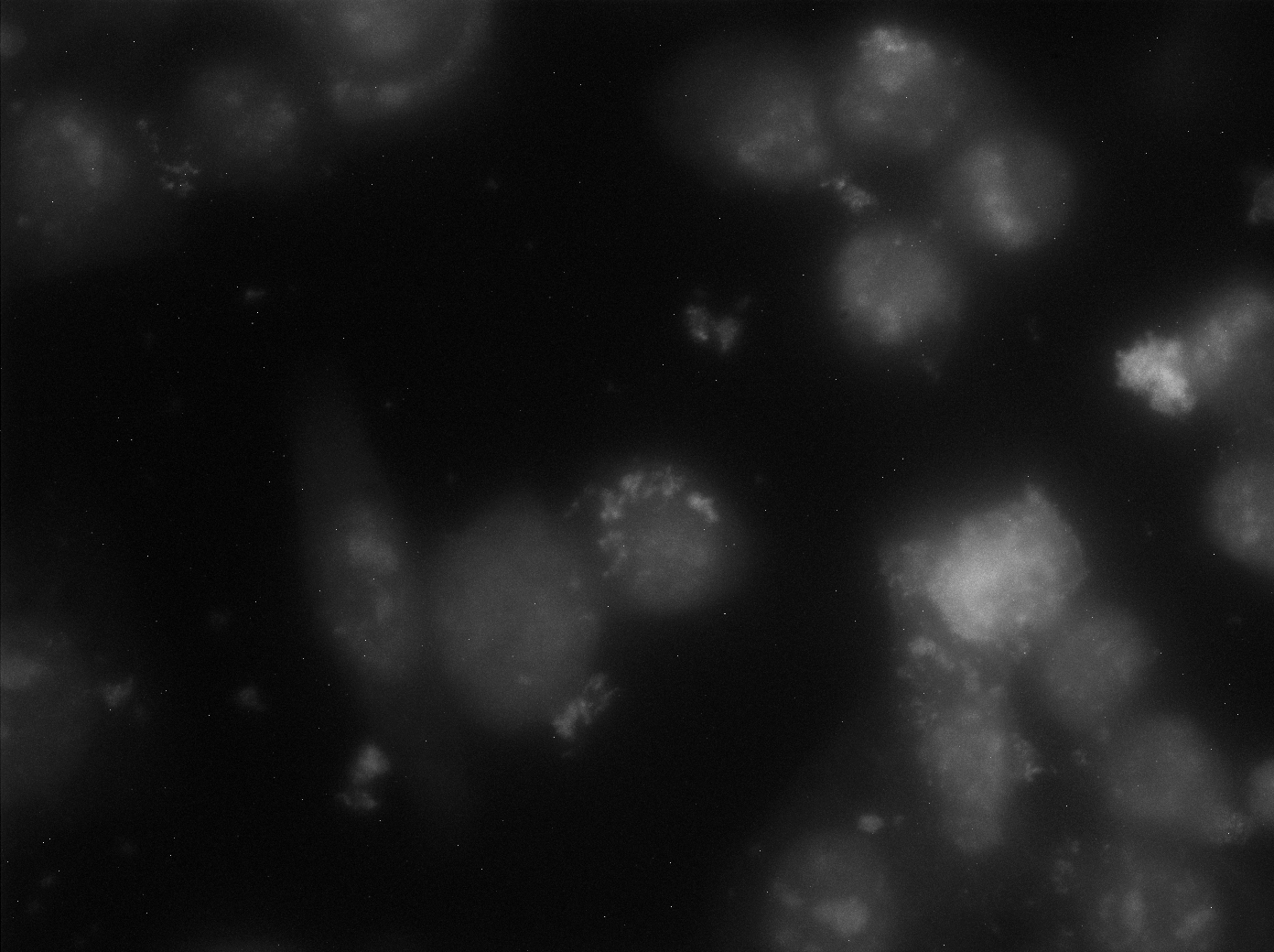

Supplement: Supplementary file 24 — Source data Fig. 6 part 5 [file 44319_2026_742_MOESM24_ESM.zip › Figure 6 Part 5/Fig 6efg TPGS1-KO TPGS1 rescue experiments part 3/R2R3/TPGS1-KO Untransfected actub 7-31-25 R2 ET6ET7.Project Maximum Z_XY1756413060_Z0_T0_C1.tif]

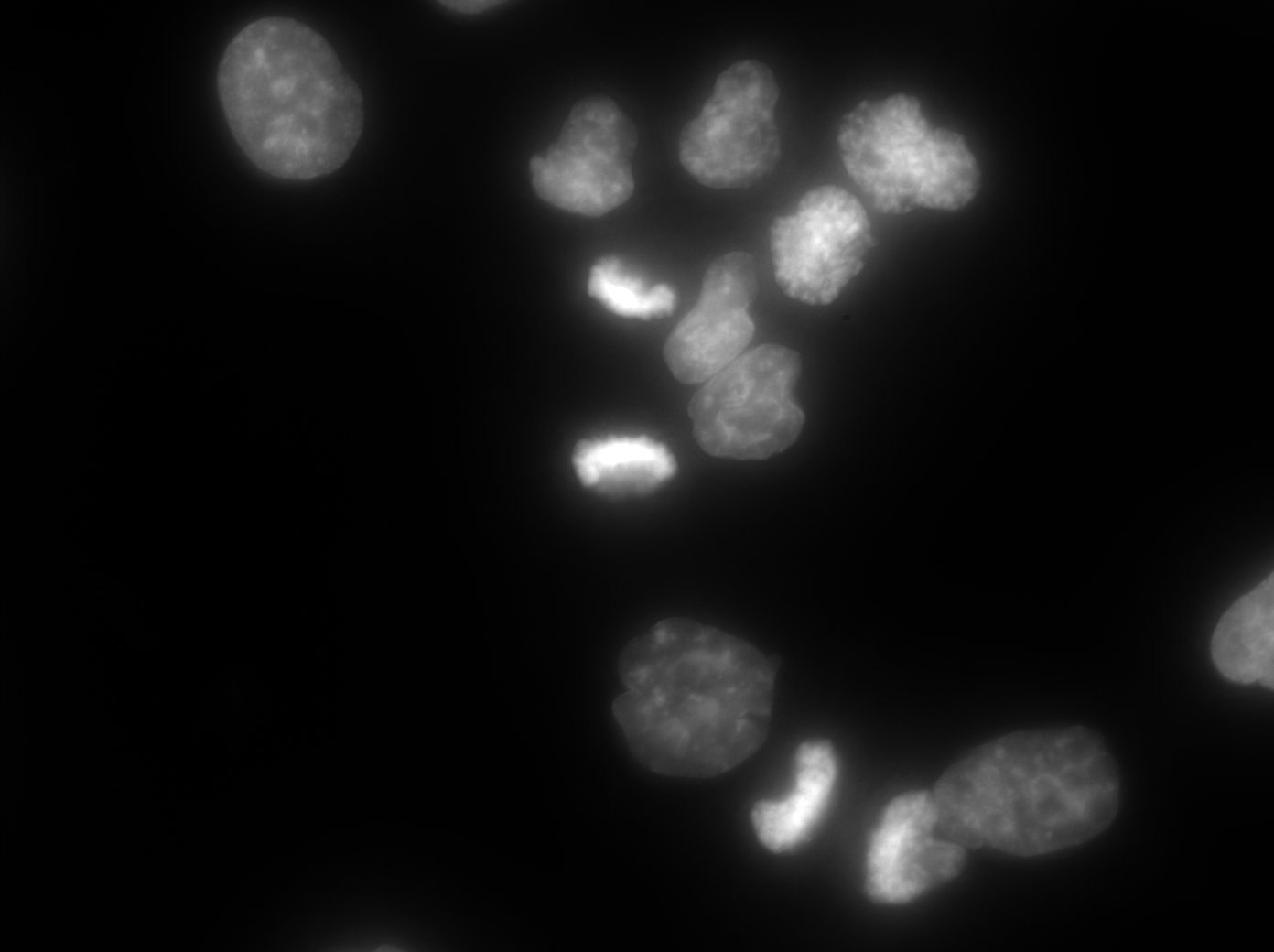

Supplement: Supplementary file 24 — Source data Fig. 6 part 5 [file 44319_2026_742_MOESM24_ESM.zip › Figure 6 Part 5/Fig 6efg TPGS1-KO TPGS1 rescue experiments part 3/R2R3/TPGS1-KO untransfected actub 7-31-25 R3 LT10 ET8.Project Maximum Z_XY1756506015_Z0_T0_C0.tif]

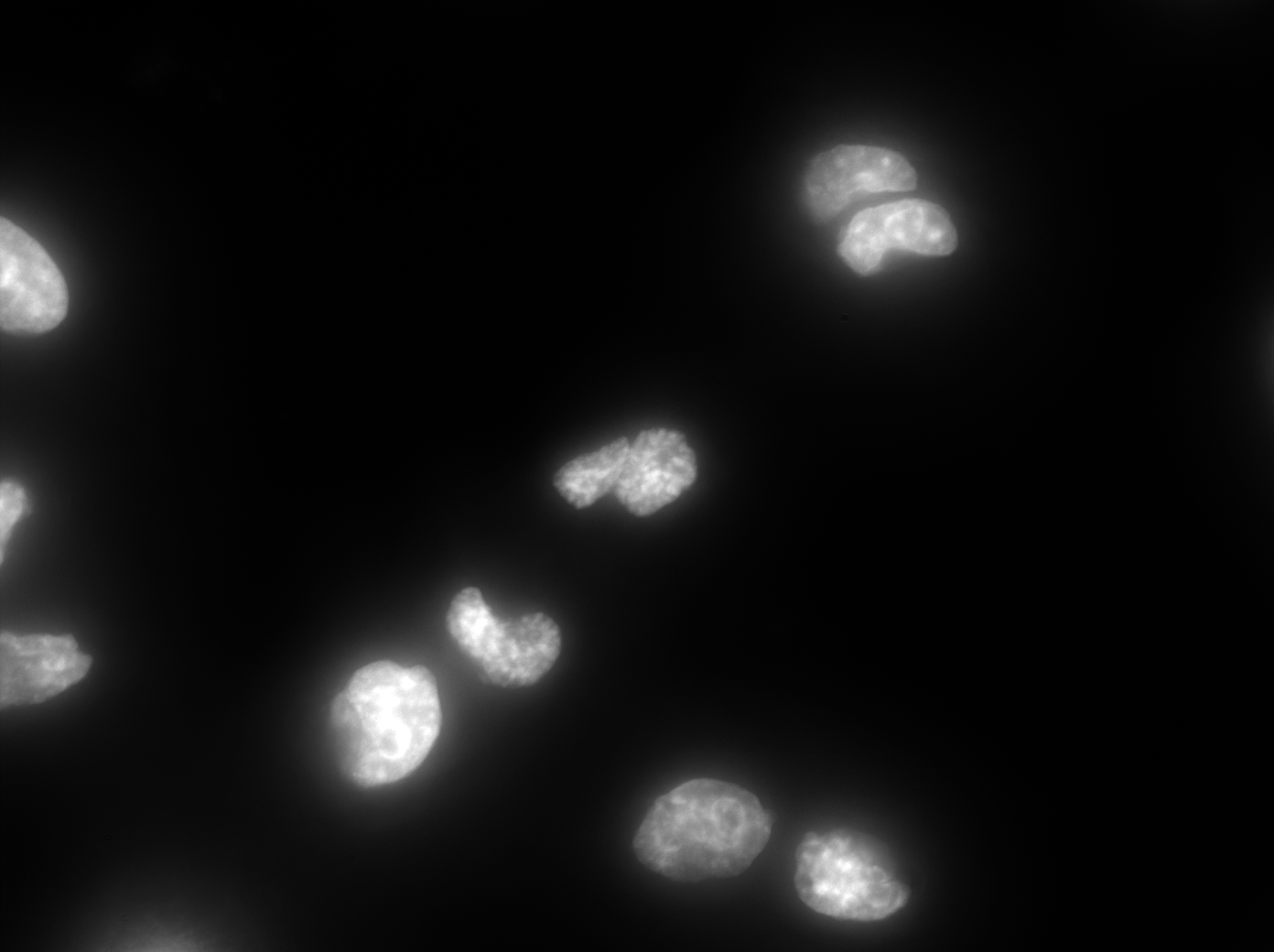

Supplement: Supplementary file 24 — Source data Fig. 6 part 5 [file 44319_2026_742_MOESM24_ESM.zip › Figure 6 Part 5/Fig 6efg TPGS1-KO TPGS1 rescue experiments part 3/R2R3/TPGS1-KO Untransfected actub 7-31-25 R2 LT6.Project Maximum Z_XY1756412699_Z0_T0_C0.tif]

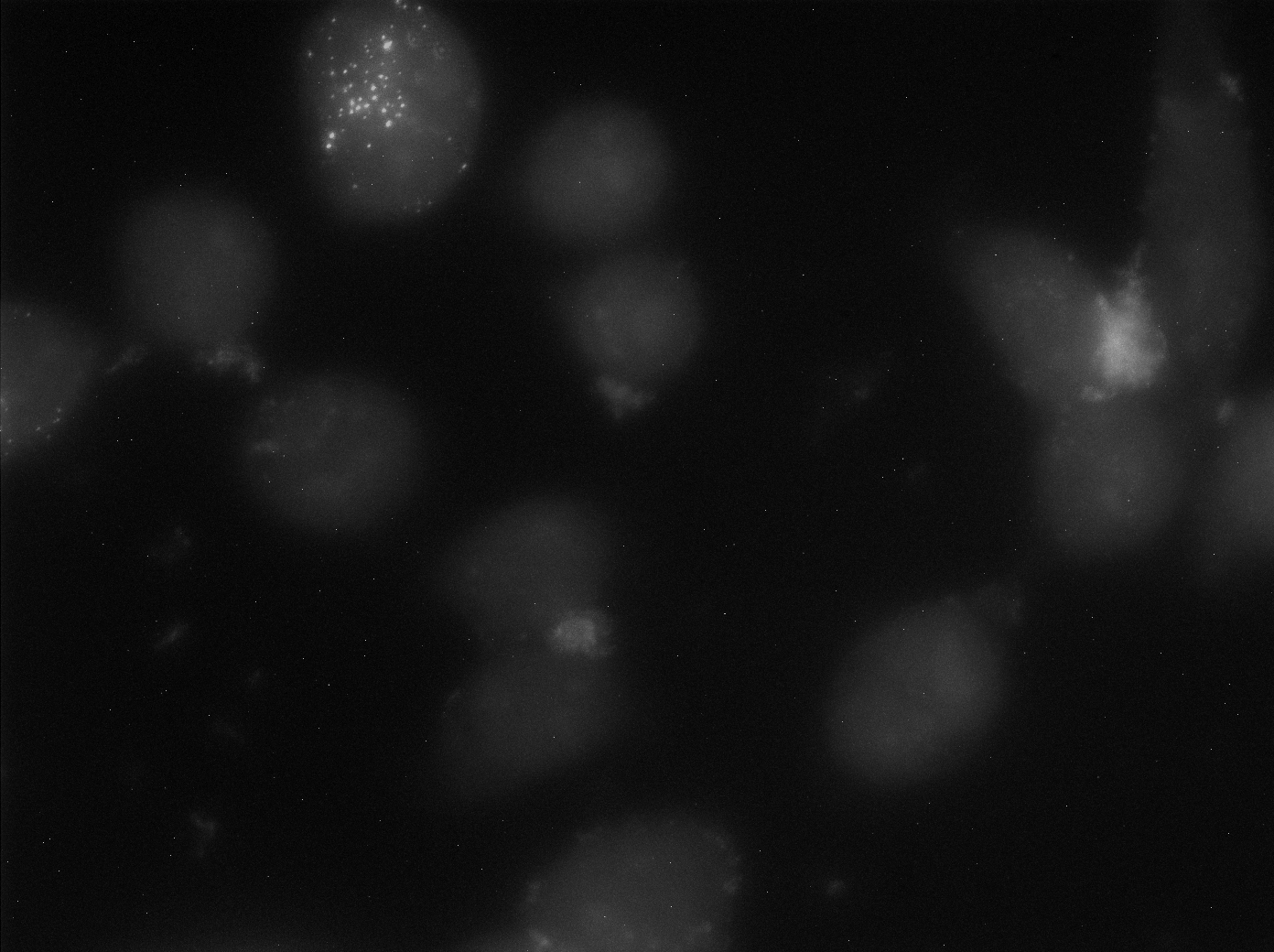

Supplement: Supplementary file 24 — Source data Fig. 6 part 5 [file 44319_2026_742_MOESM24_ESM.zip › Figure 6 Part 5/Fig 6efg TPGS1-KO TPGS1 rescue experiments part 3/R2R3/TPGS1-KO untransfected actub 7-31-25 R3 LT4LT5.Project Maximum Z_XY1756503383_Z0_T0_C1.tif]

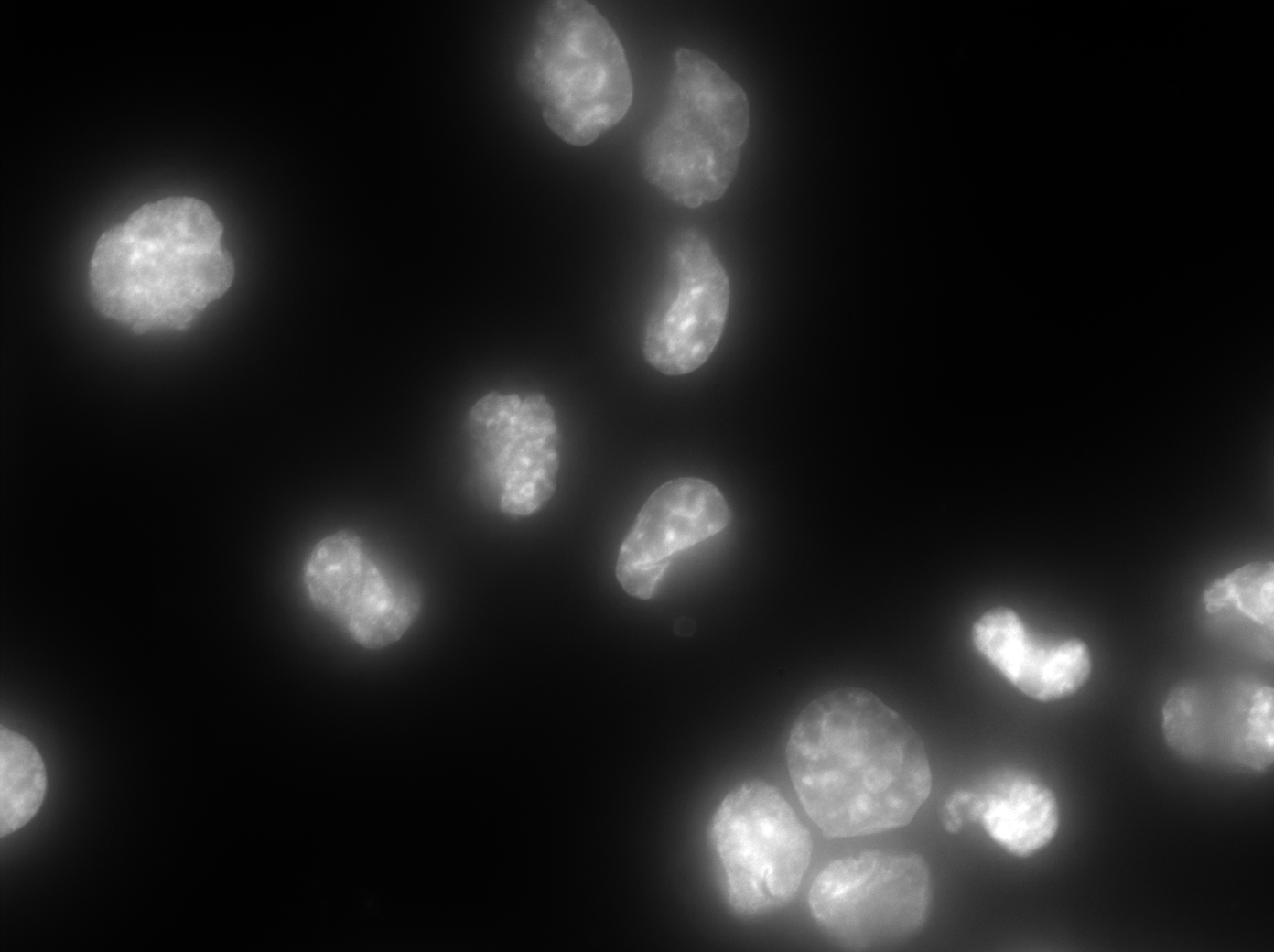

Supplement: Supplementary file 24 — Source data Fig. 6 part 5 [file 44319_2026_742_MOESM24_ESM.zip › Figure 6 Part 5/Fig 6efg TPGS1-KO TPGS1 rescue experiments part 3/R2R3/TPGS1-KO untransfected actub 7-31-25 R3 ET2 LT3.Project Maximum Z_XY1756502995_Z0_T0_C0.tif]

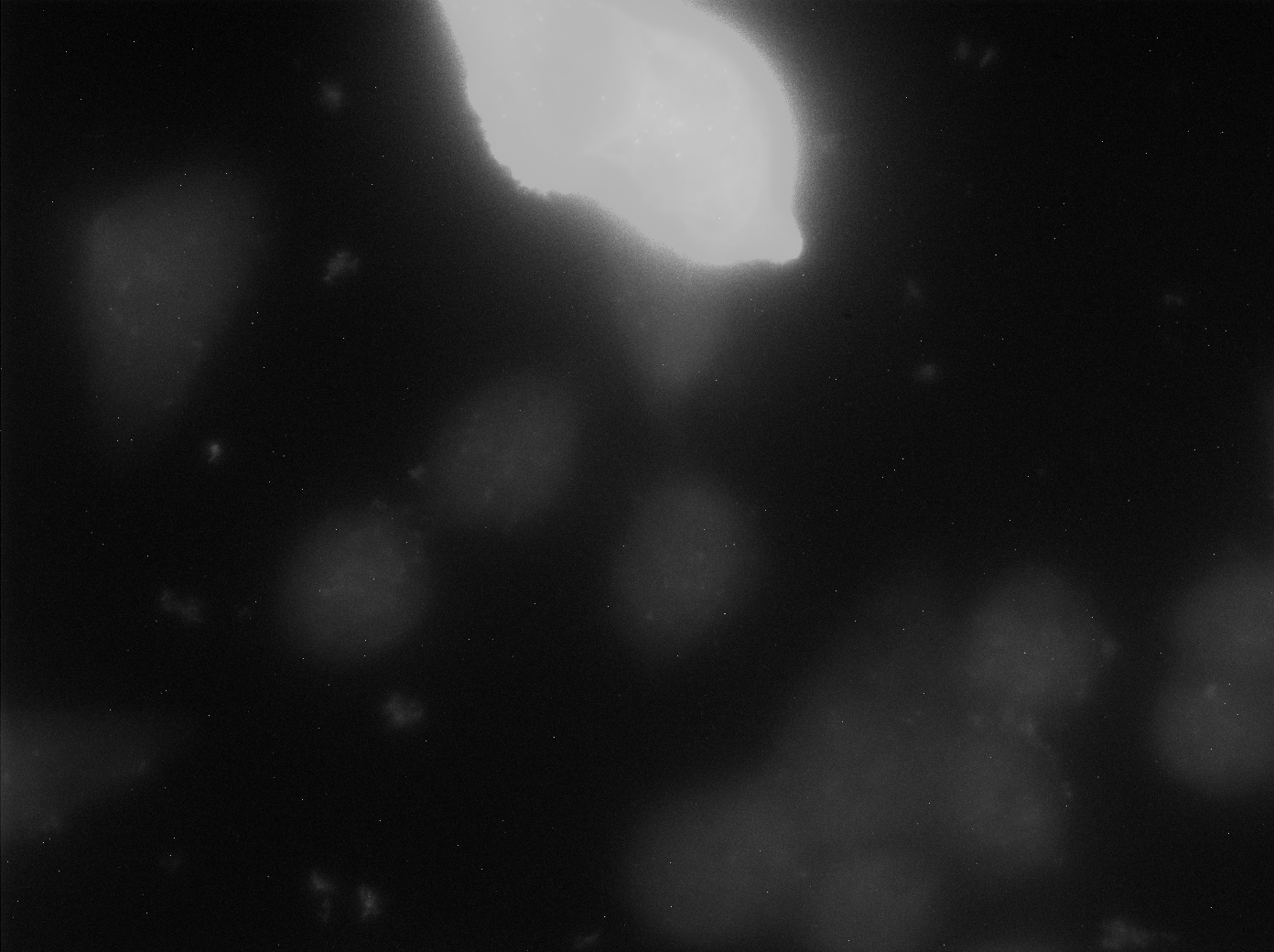

Supplement: Supplementary file 24 — Source data Fig. 6 part 5 [file 44319_2026_742_MOESM24_ESM.zip › Figure 6 Part 5/Fig 6efg TPGS1-KO TPGS1 rescue experiments part 3/R2R3/TPGS1-KO untransfected actub 7-31-25 R3 ET2 LT3.Project Maximum Z_XY1756502995_Z0_T0_C1.tif]

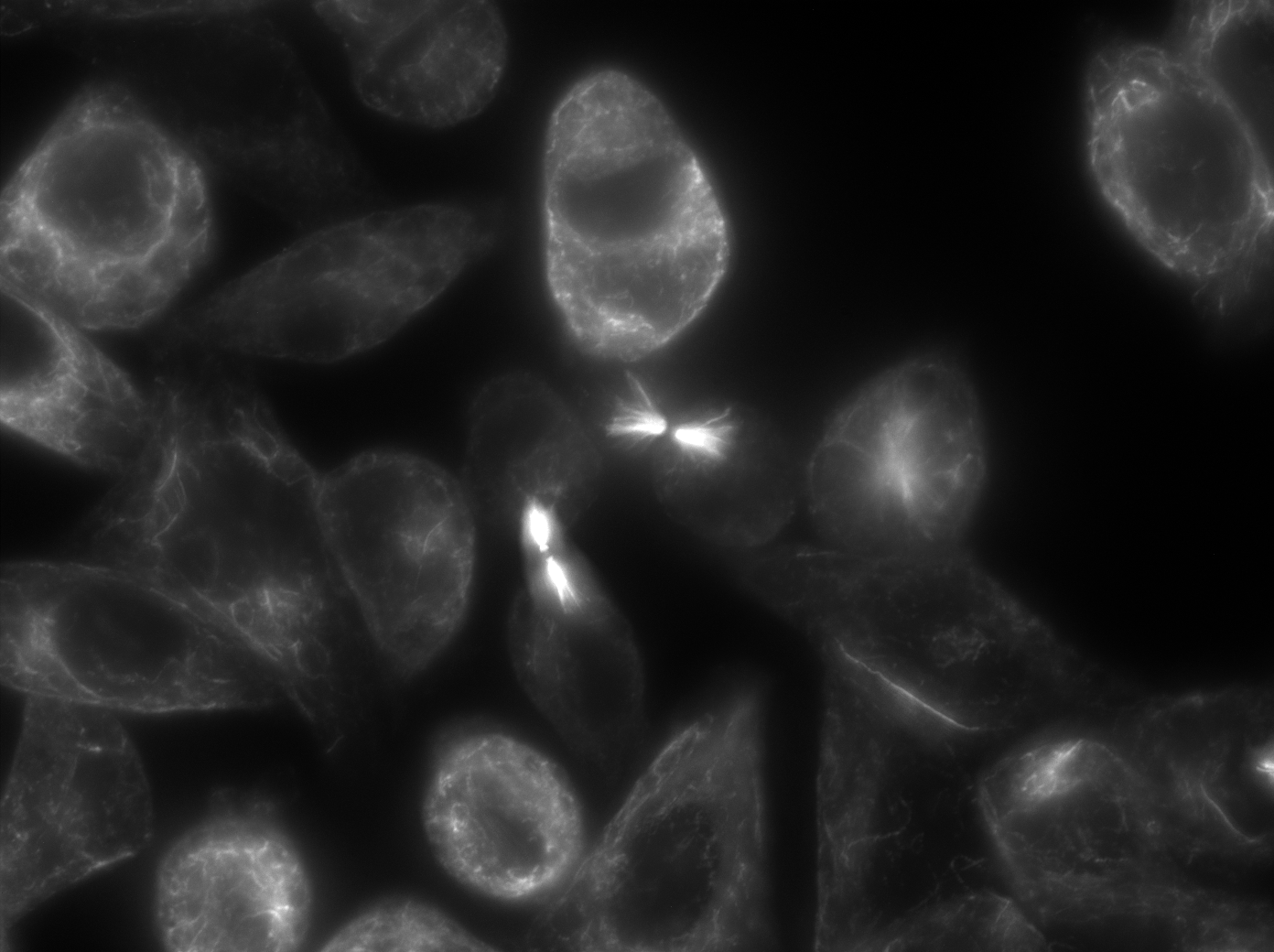

Supplement: Supplementary file 24 — Source data Fig. 6 part 5 [file 44319_2026_742_MOESM24_ESM.zip › Figure 6 Part 5/Fig 6efg TPGS1-KO TPGS1 rescue experiments part 3/R2R3/TPGS1-KO Untransfected actub 7-31-25 R2 ET4 LT4.Project Maximum Z_XY1756406399_Z0_T0_C2.tif]

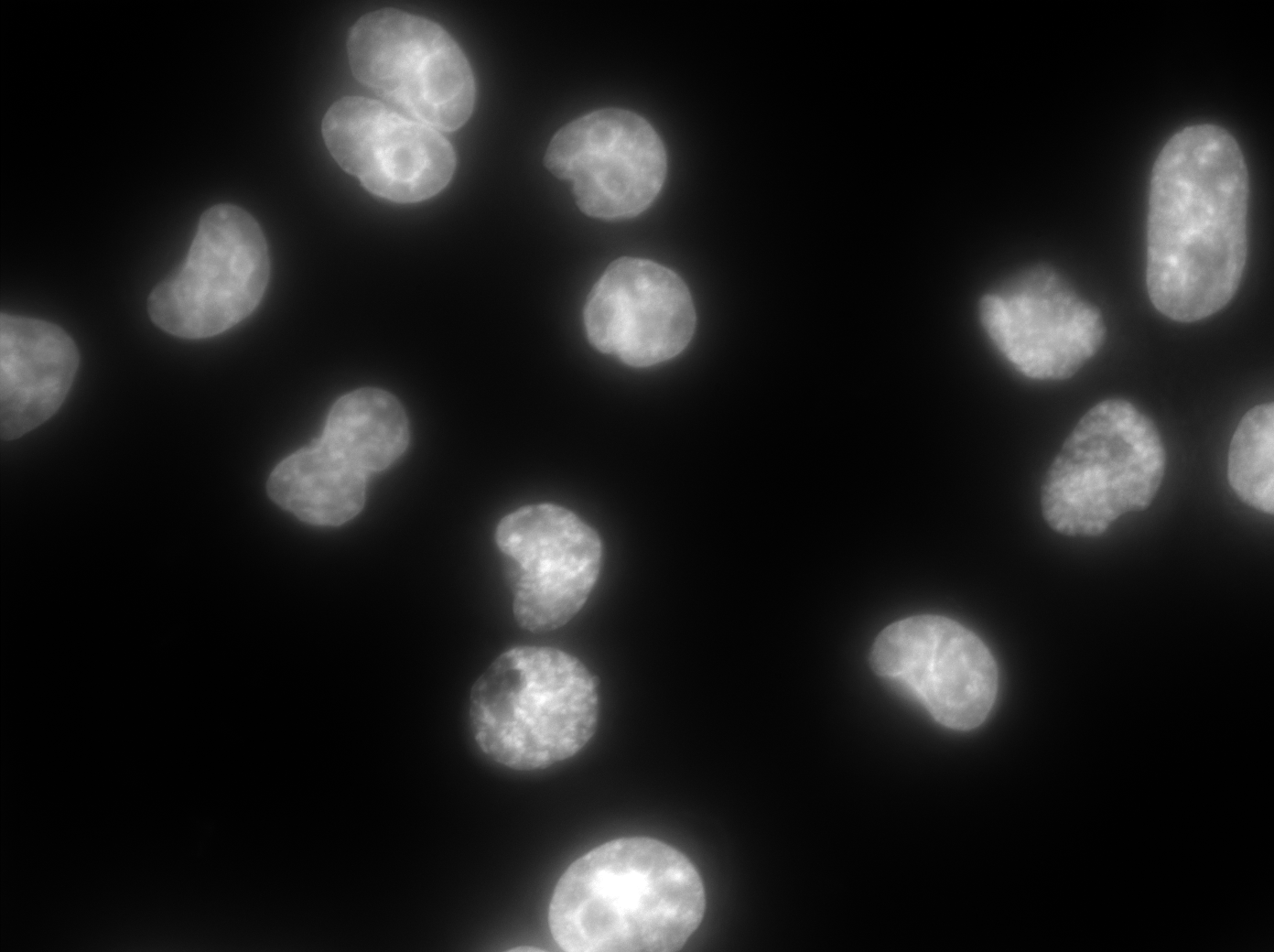

Supplement: Supplementary file 24 — Source data Fig. 6 part 5 [file 44319_2026_742_MOESM24_ESM.zip › Figure 6 Part 5/Fig 6efg TPGS1-KO TPGS1 rescue experiments part 3/R2R3/TPGS1-KO untransfected actub 7-31-25 R3 LT4LT5.Project Maximum Z_XY1756503383_Z0_T0_C0.tif]

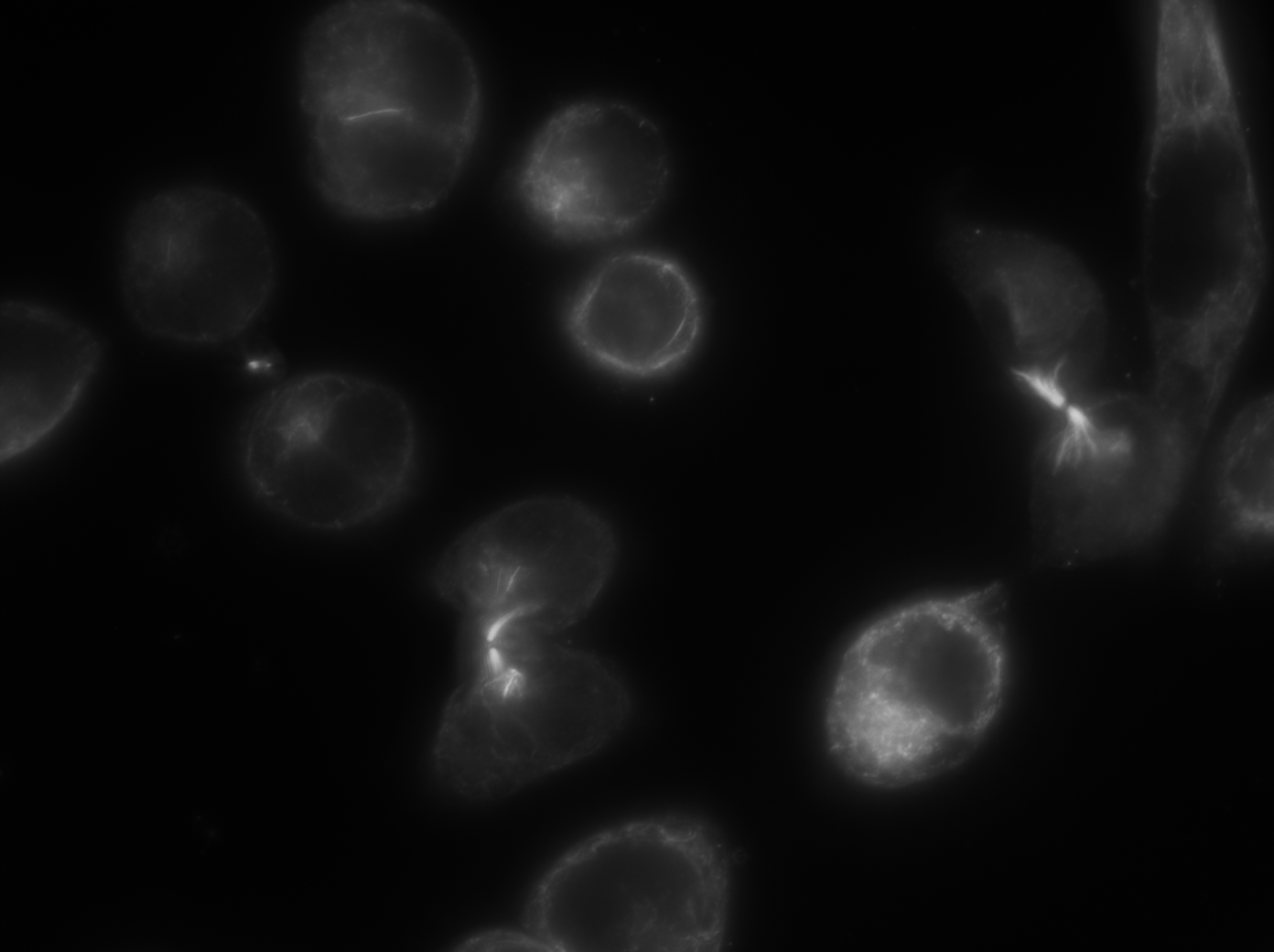

Supplement: Supplementary file 24 — Source data Fig. 6 part 5 [file 44319_2026_742_MOESM24_ESM.zip › Figure 6 Part 5/Fig 6efg TPGS1-KO TPGS1 rescue experiments part 3/R2R3/TPGS1-KO untransfected actub 7-31-25 R3 LT4LT5.Project Maximum Z_XY1756503383_Z0_T0_C2.tif]

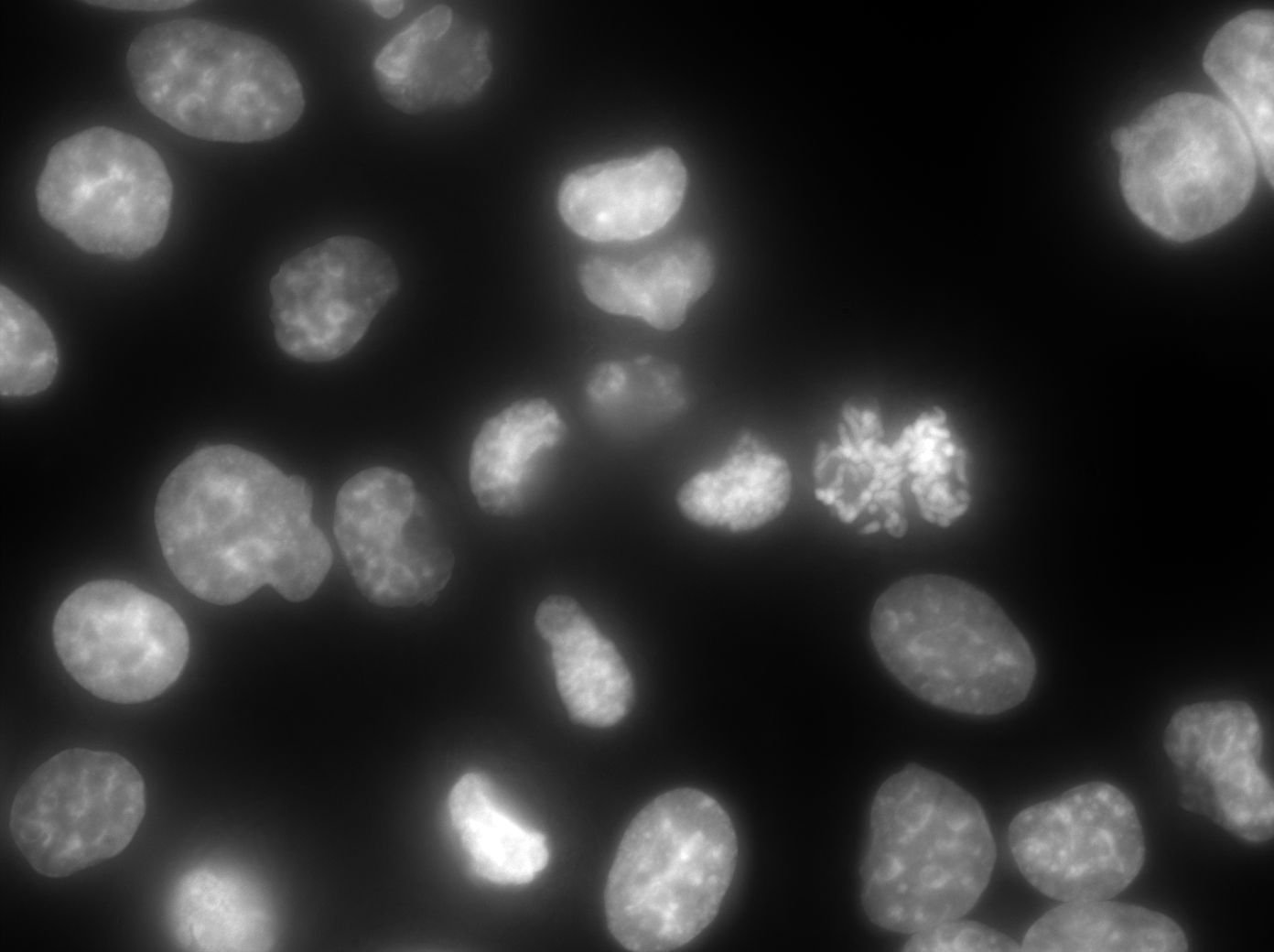

Supplement: Supplementary file 24 — Source data Fig. 6 part 5 [file 44319_2026_742_MOESM24_ESM.zip › Figure 6 Part 5/Fig 6efg TPGS1-KO TPGS1 rescue experiments part 3/R2R3/TPGS1-KO Untransfected actub 7-31-25 R2 ET4 LT4.Project Maximum Z_XY1756406399_Z0_T0_C0.tif]

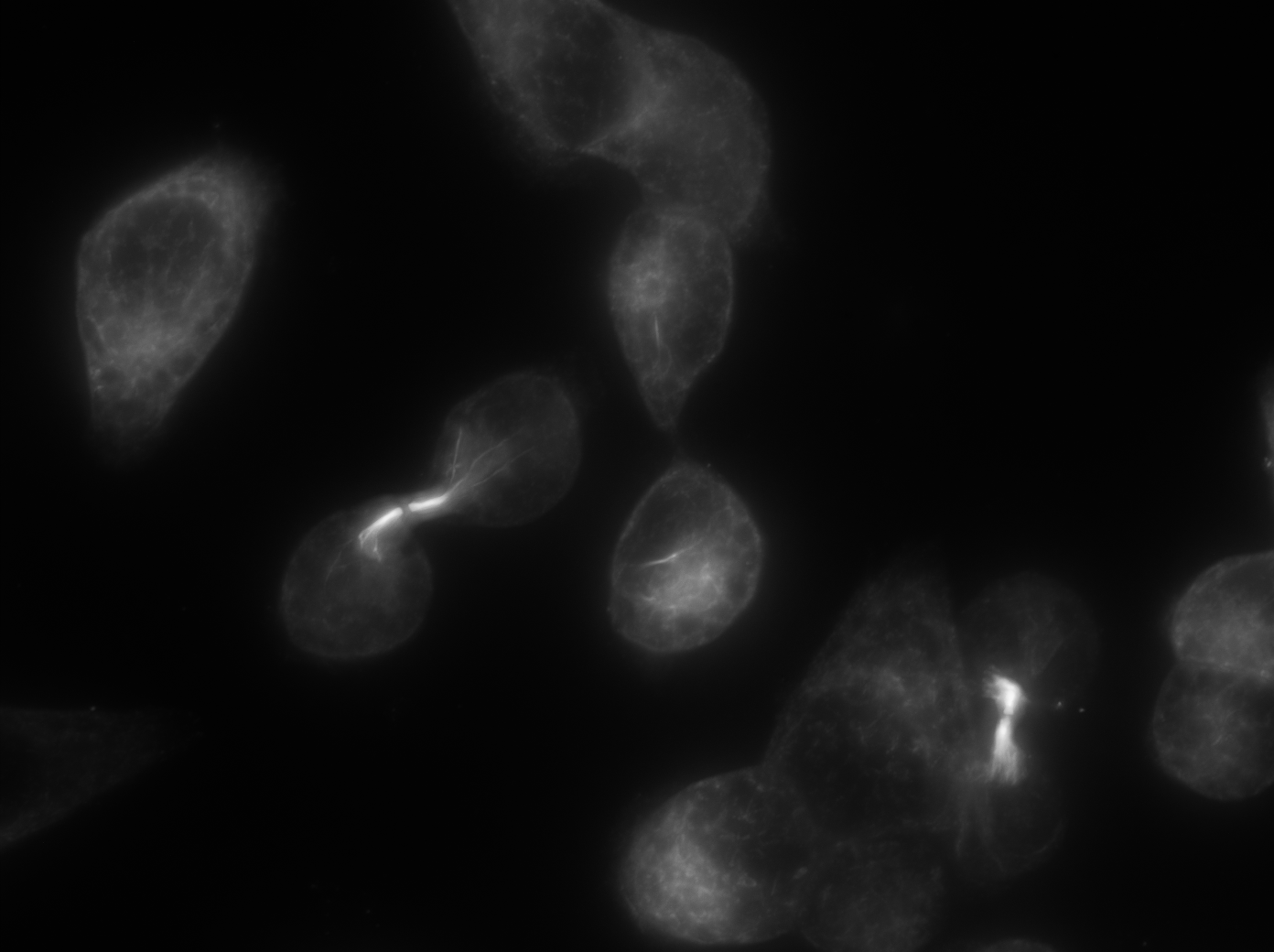

Supplement: Supplementary file 24 — Source data Fig. 6 part 5 [file 44319_2026_742_MOESM24_ESM.zip › Figure 6 Part 5/Fig 6efg TPGS1-KO TPGS1 rescue experiments part 3/R2R3/TPGS1-KO untransfected actub 7-31-25 R3 ET2 LT3.Project Maximum Z_XY1756502995_Z0_T0_C2.tif]

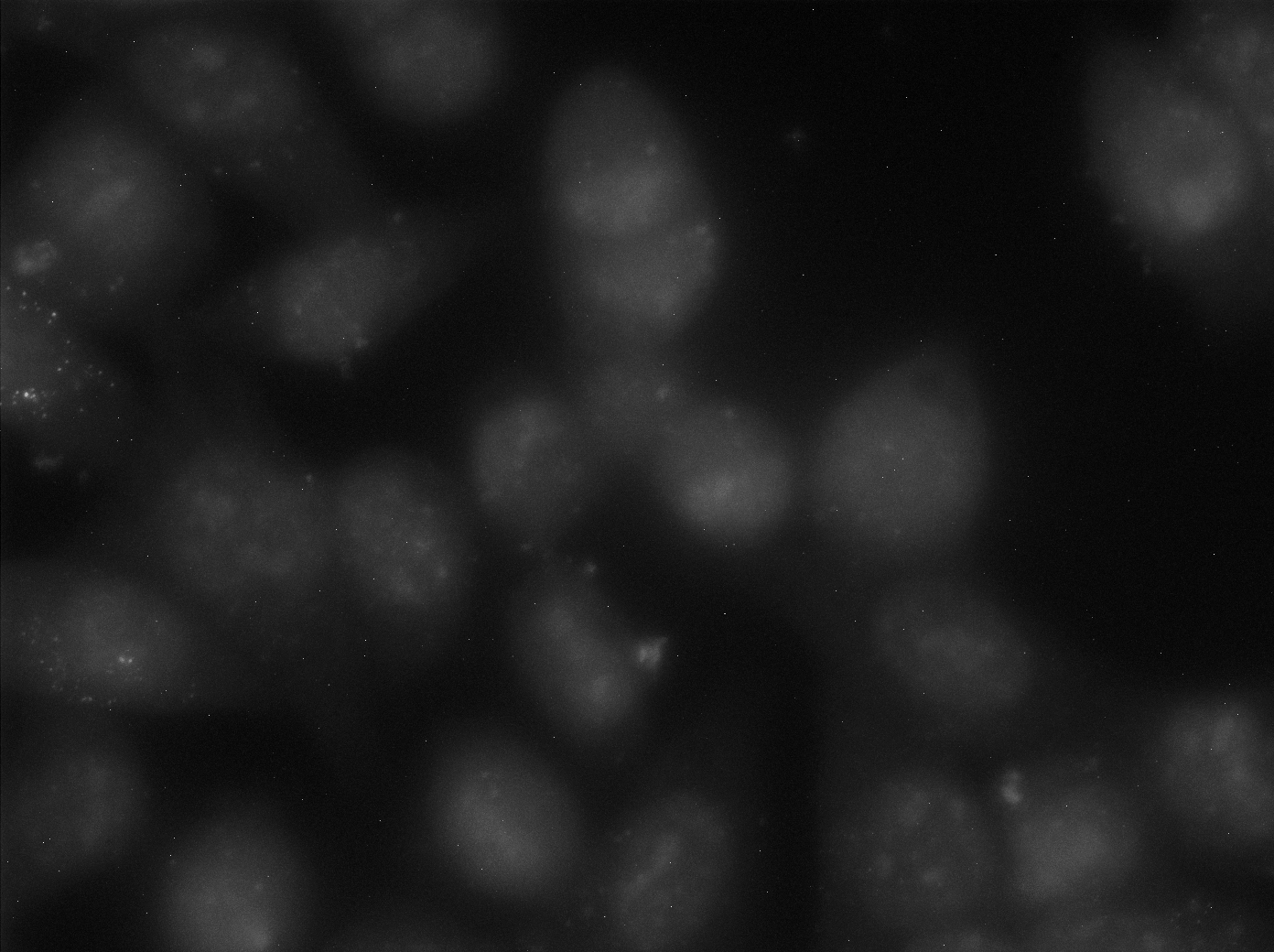

Supplement: Supplementary file 24 — Source data Fig. 6 part 5 [file 44319_2026_742_MOESM24_ESM.zip › Figure 6 Part 5/Fig 6efg TPGS1-KO TPGS1 rescue experiments part 3/R2R3/TPGS1-KO Untransfected actub 7-31-25 R2 ET4 LT4.Project Maximum Z_XY1756406399_Z0_T0_C1.tif]

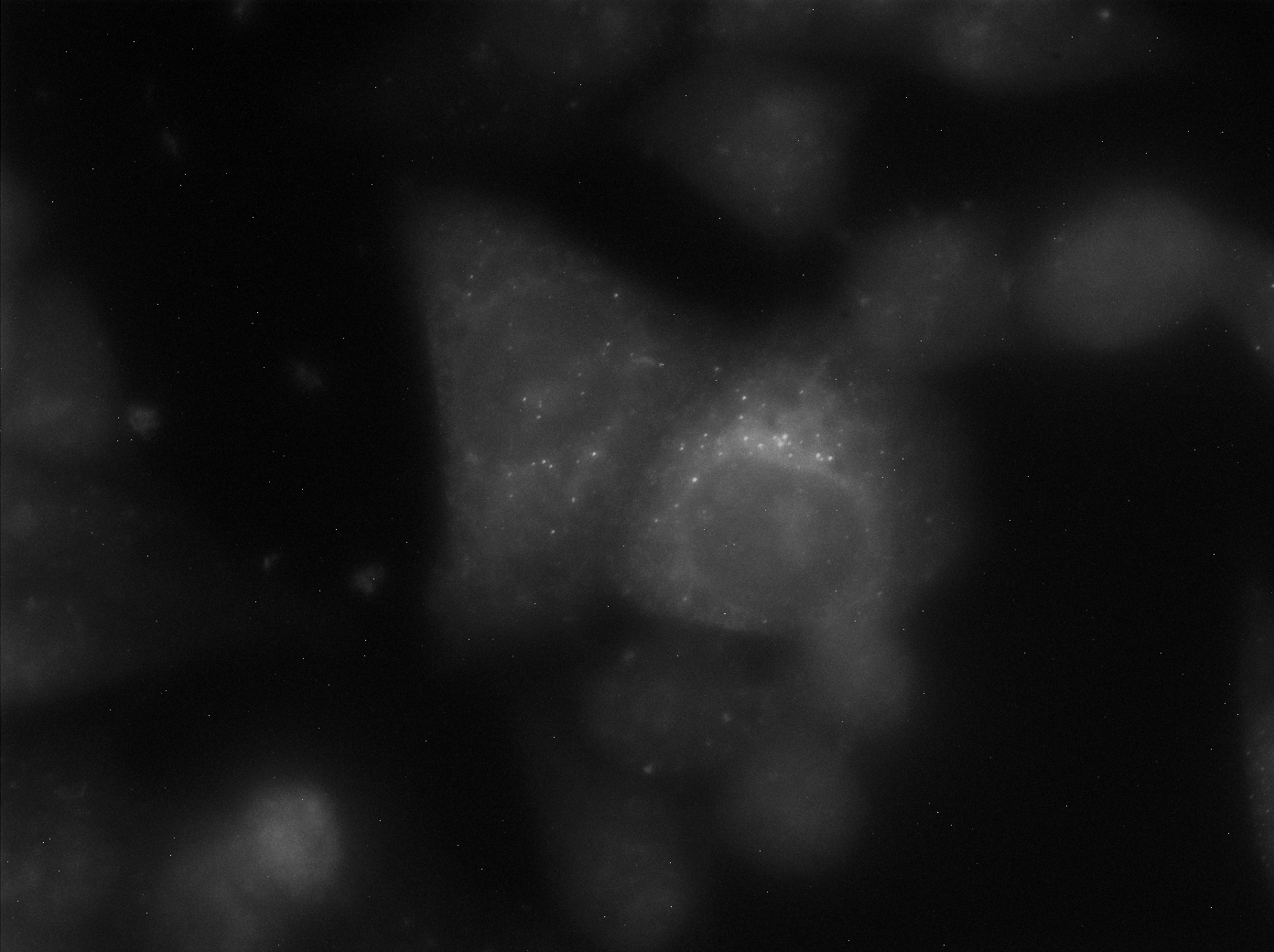

Supplement: Supplementary file 24 — Source data Fig. 6 part 5 [file 44319_2026_742_MOESM24_ESM.zip › Figure 6 Part 5/Fig 6efg TPGS1-KO TPGS1 rescue experiments part 3/R2R3/TPGS1-KO Untransfected actub 7-31-25 R2 ET3 LT3.Project Maximum Z_XY1756406318_Z0_T0_C1.tif]

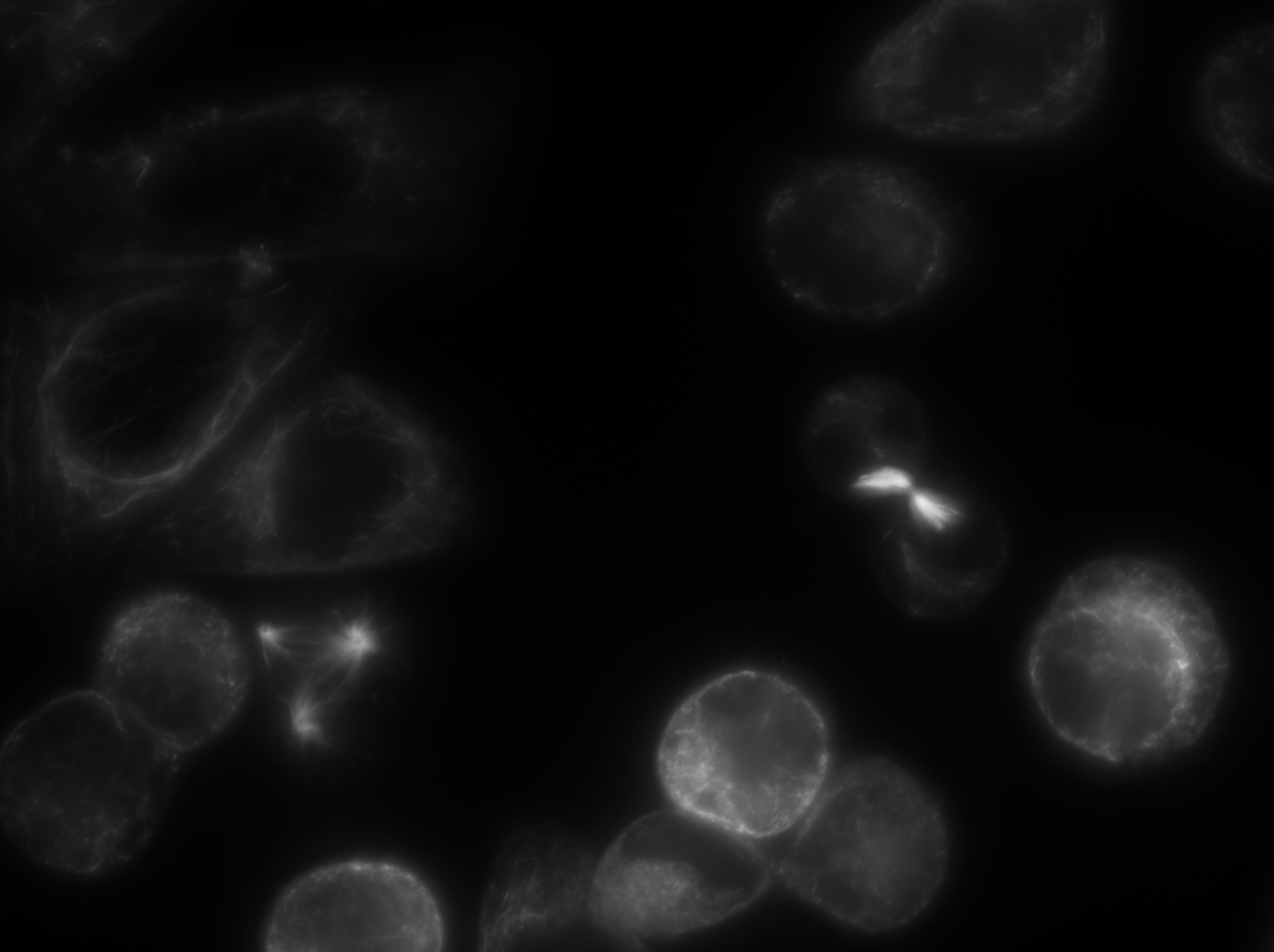

Supplement: Supplementary file 24 — Source data Fig. 6 part 5 [file 44319_2026_742_MOESM24_ESM.zip › Figure 6 Part 5/Fig 6efg TPGS1-KO TPGS1 rescue experiments part 3/R2R3/TPGS1-KO untransfected actub 7-31-25 R3 ET7.Project Maximum Z_XY1756505656_Z0_T0_C2.tif]

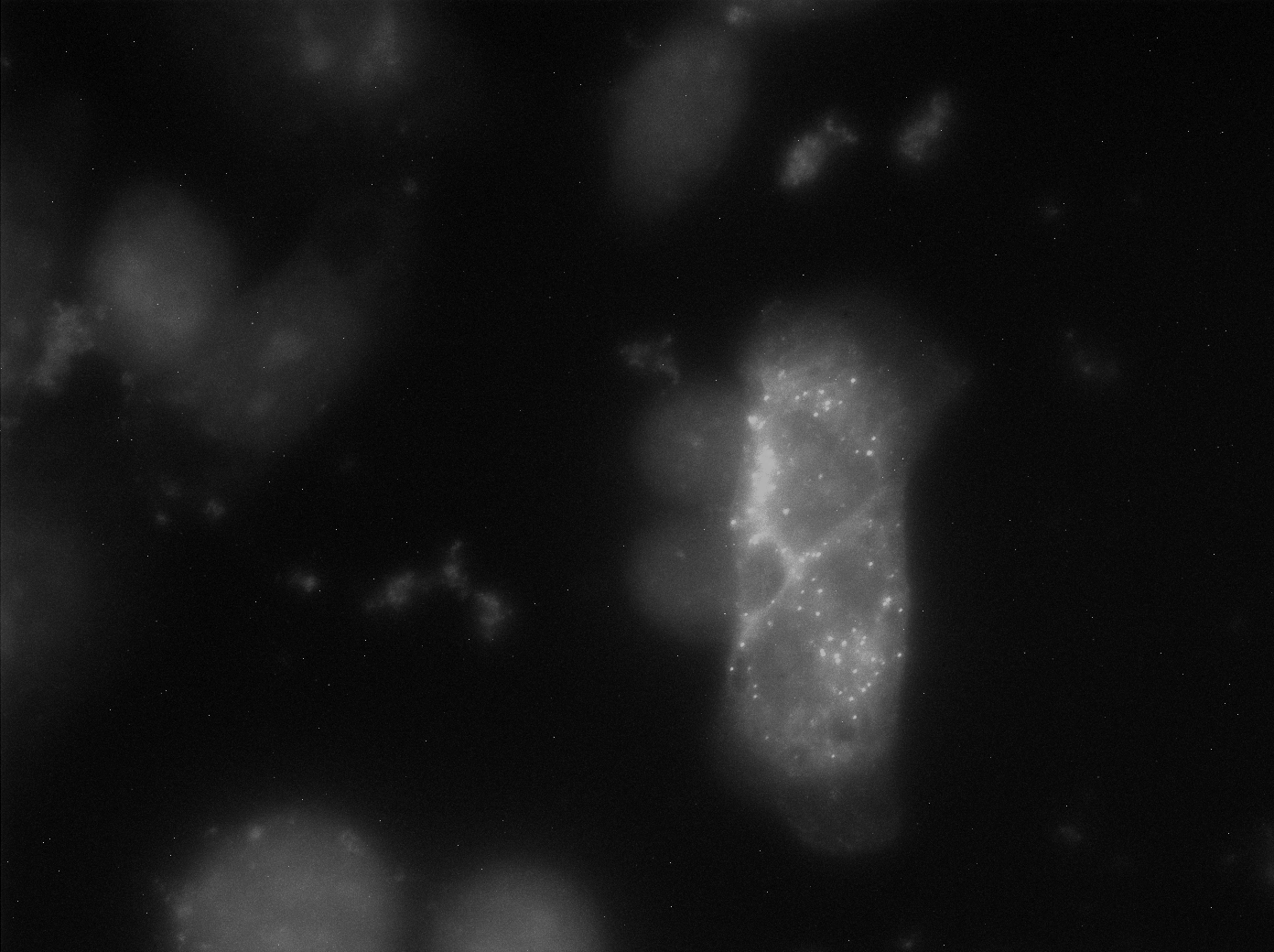

Supplement: Supplementary file 24 — Source data Fig. 6 part 5 [file 44319_2026_742_MOESM24_ESM.zip › Figure 6 Part 5/Fig 6efg TPGS1-KO TPGS1 rescue experiments part 3/R2R3/TPGS1-KO untransfected actub 7-31-25 R3 ET9.Project Maximum Z_XY1756506174_Z0_T0_C1.tif]

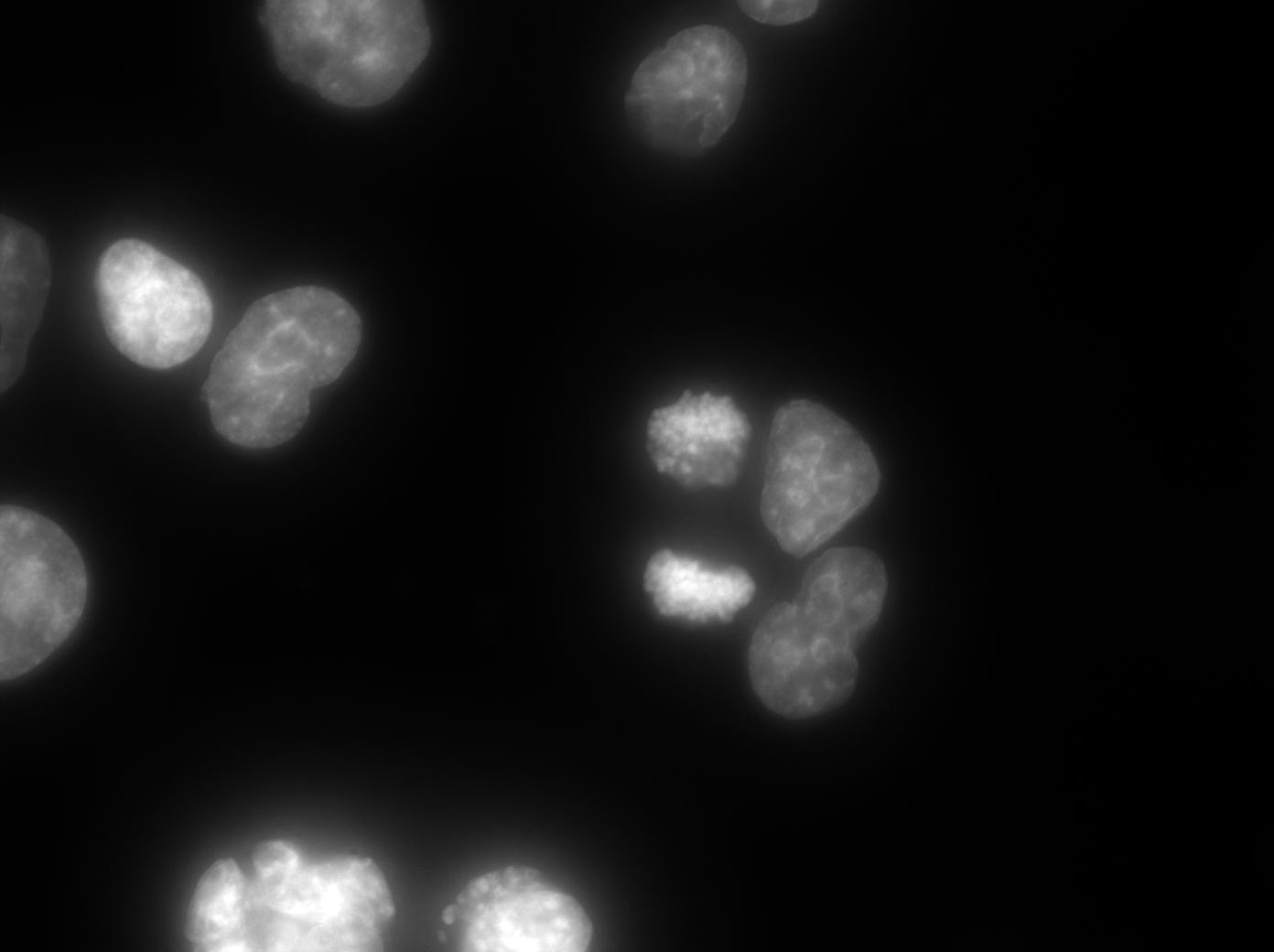

Supplement: Supplementary file 24 — Source data Fig. 6 part 5 [file 44319_2026_742_MOESM24_ESM.zip › Figure 6 Part 5/Fig 6efg TPGS1-KO TPGS1 rescue experiments part 3/R2R3/TPGS1-KO untransfected actub 7-31-25 R3 ET9.Project Maximum Z_XY1756506174_Z0_T0_C0.tif]

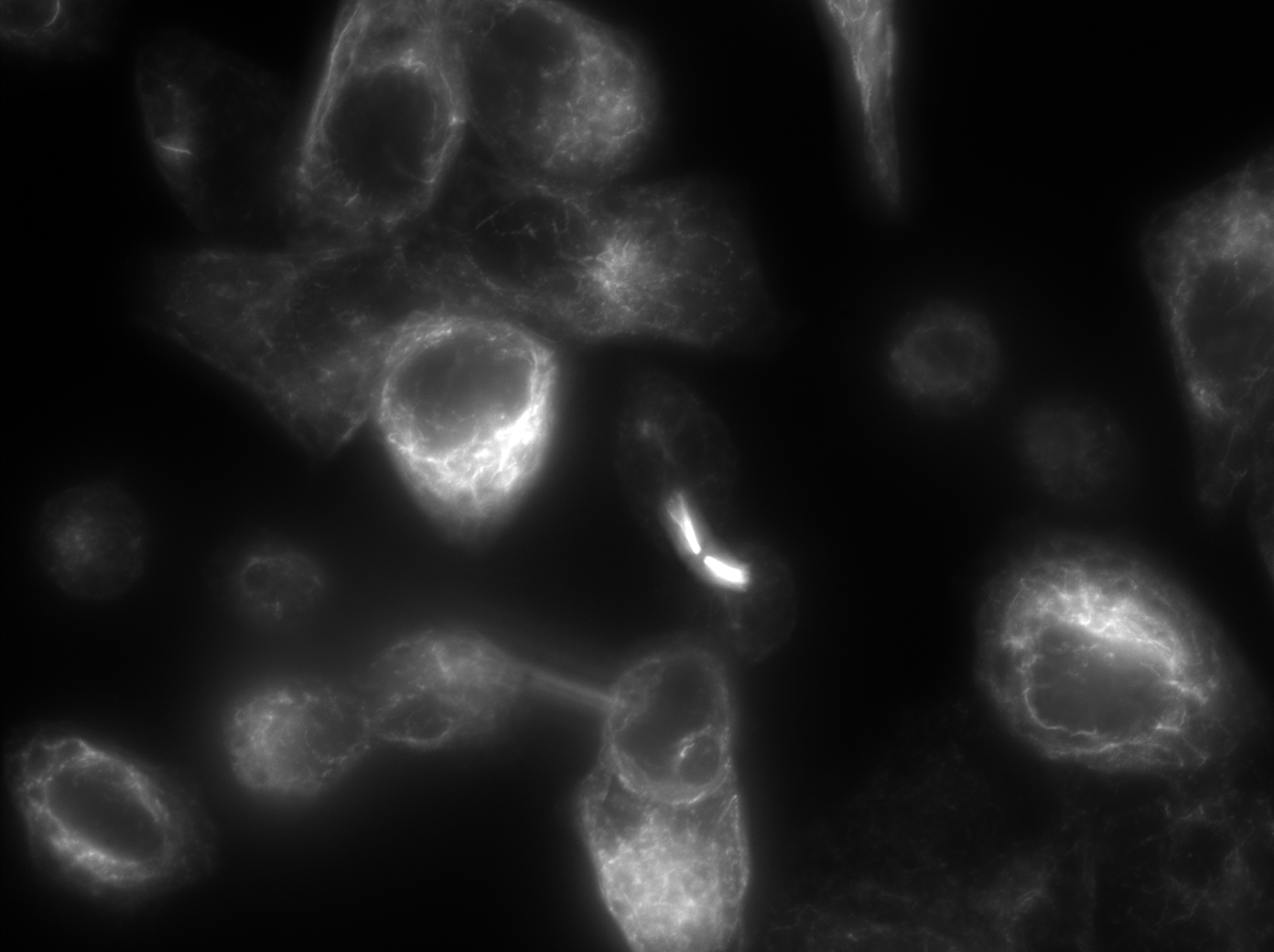

Supplement: Supplementary file 24 — Source data Fig. 6 part 5 [file 44319_2026_742_MOESM24_ESM.zip › Figure 6 Part 5/Fig 6efg TPGS1-KO TPGS1 rescue experiments part 3/R2R3/TPGS1-KO Untransfected actub 7-31-25 R2 ET10.Project Maximum Z_XY1756413287_Z0_T0_C2.tif]

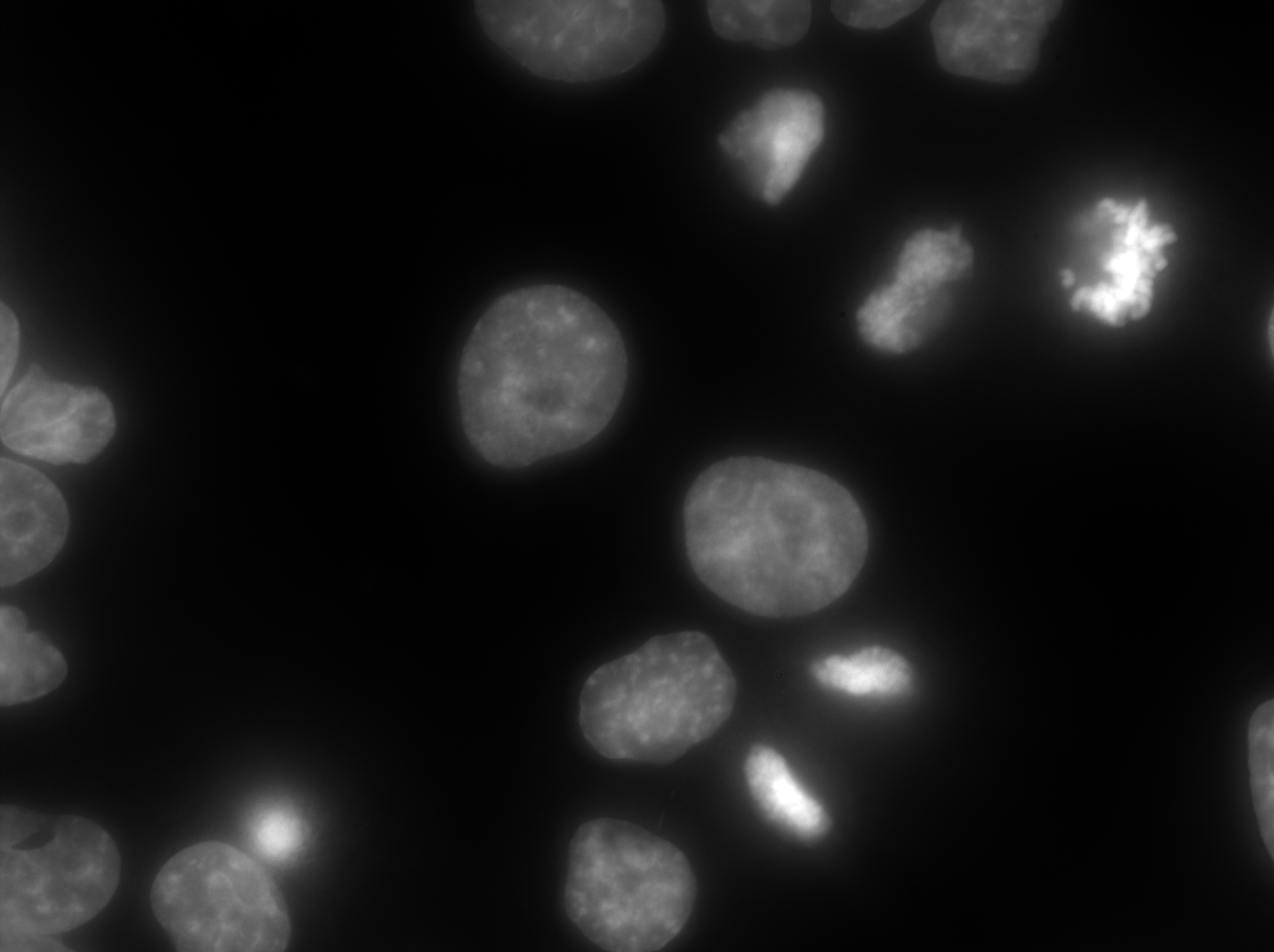

Supplement: Supplementary file 24 — Source data Fig. 6 part 5 [file 44319_2026_742_MOESM24_ESM.zip › Figure 6 Part 5/Fig 6efg TPGS1-KO TPGS1 rescue experiments part 3/R2R3/TPGS1-KO Untransfected actub 7-31-25 R2 ET3 LT3.Project Maximum Z_XY1756406318_Z0_T0_C0.tif]

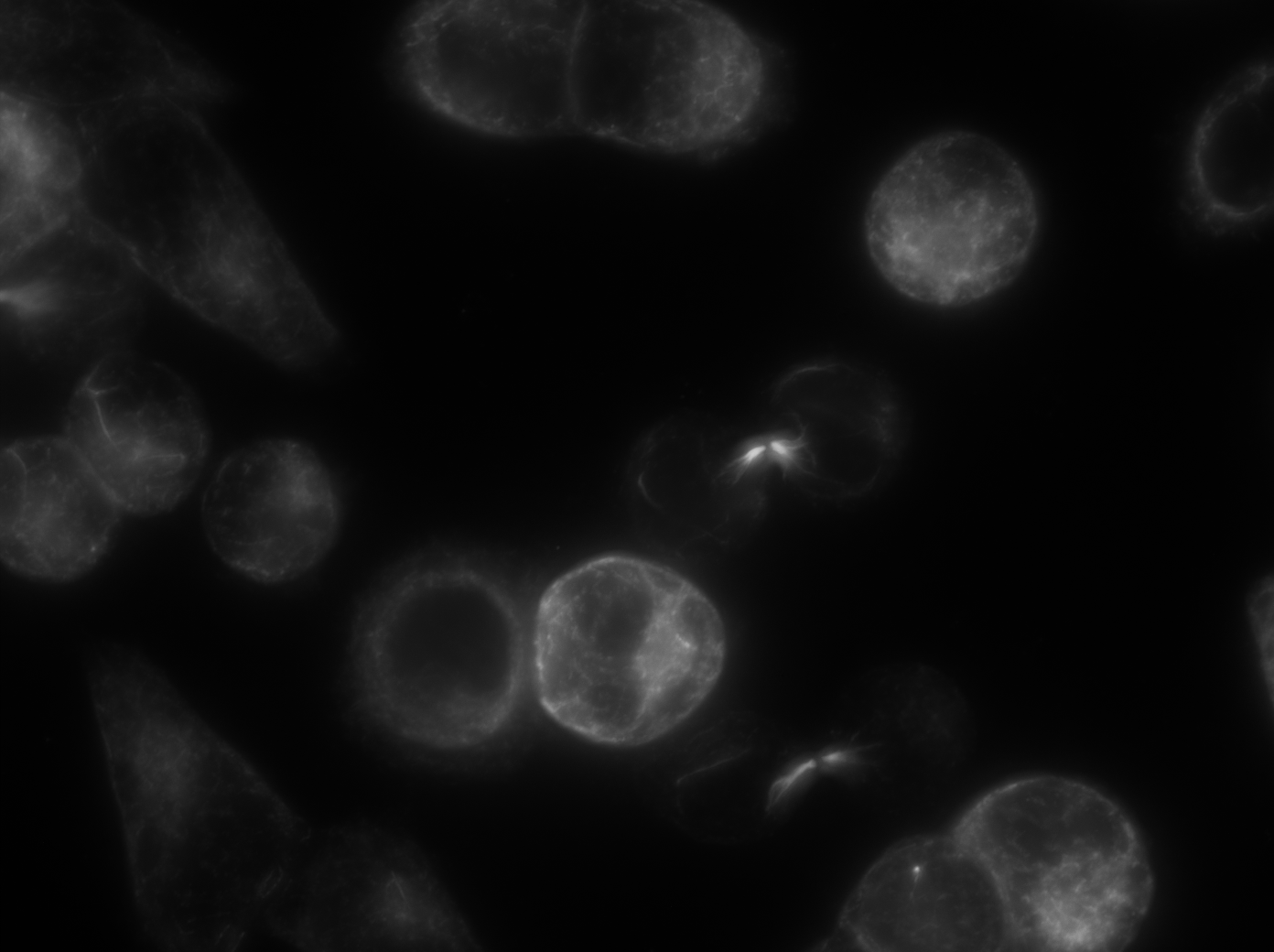

Supplement: Supplementary file 24 — Source data Fig. 6 part 5 [file 44319_2026_742_MOESM24_ESM.zip › Figure 6 Part 5/Fig 6efg TPGS1-KO TPGS1 rescue experiments part 3/R2R3/TPGS1-KO untransfected actub 7-31-25 R3 ET4ET5.Project Maximum Z_XY1756503767_Z0_T0_C2.tif]

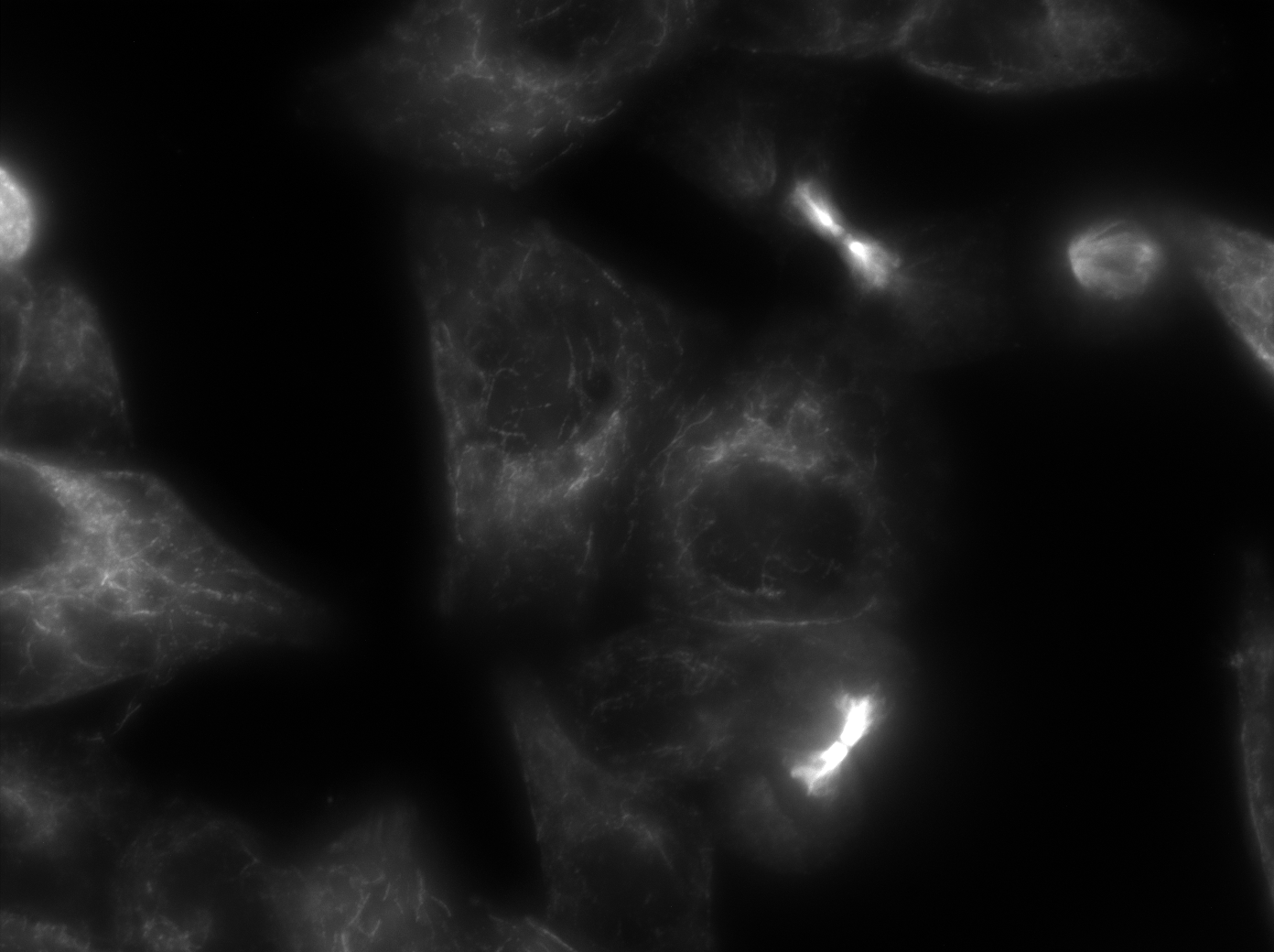

Supplement: Supplementary file 24 — Source data Fig. 6 part 5 [file 44319_2026_742_MOESM24_ESM.zip › Figure 6 Part 5/Fig 6efg TPGS1-KO TPGS1 rescue experiments part 3/R2R3/TPGS1-KO Untransfected actub 7-31-25 R2 ET3 LT3.Project Maximum Z_XY1756406318_Z0_T0_C2.tif]

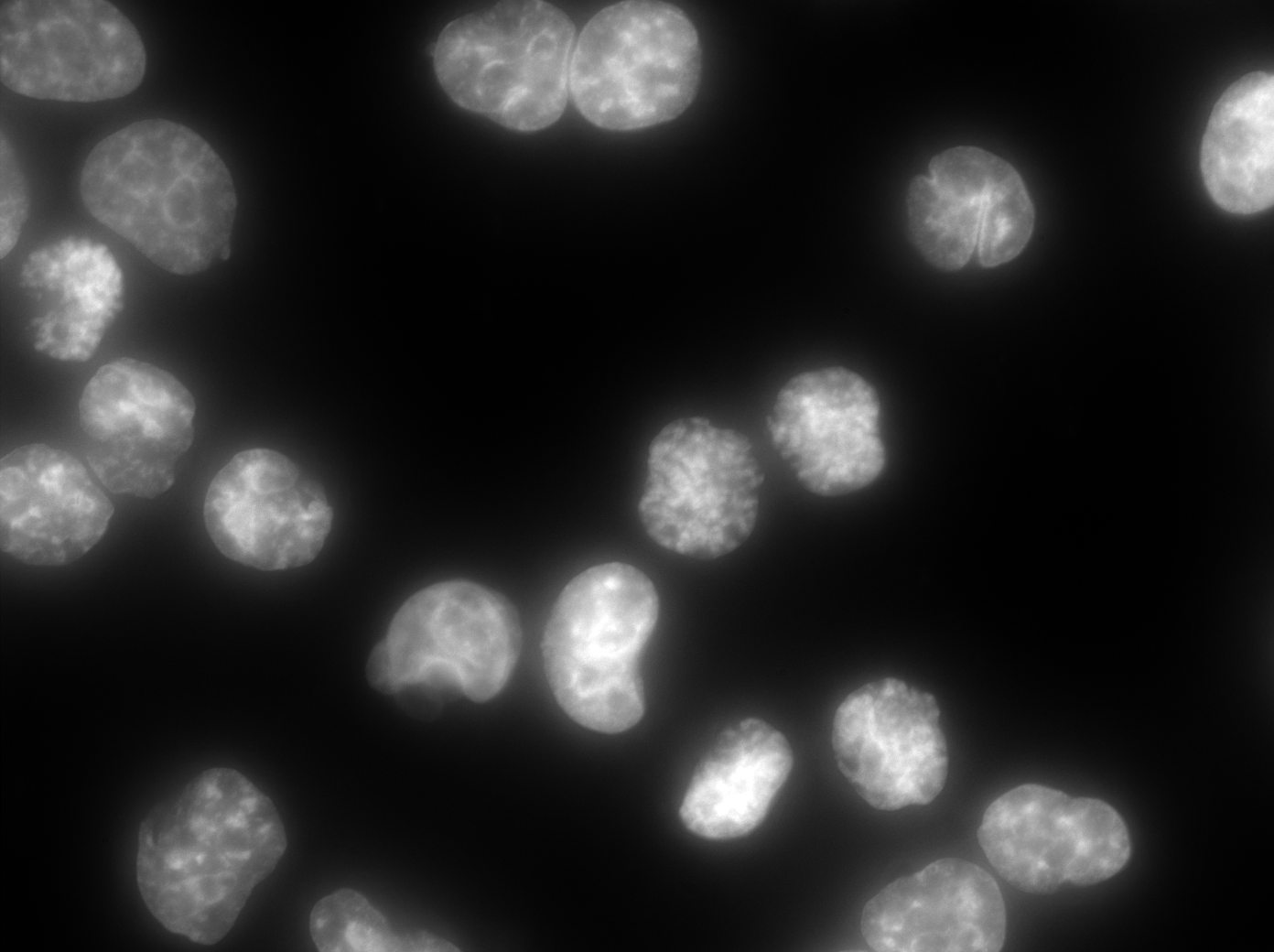

Supplement: Supplementary file 24 — Source data Fig. 6 part 5 [file 44319_2026_742_MOESM24_ESM.zip › Figure 6 Part 5/Fig 6efg TPGS1-KO TPGS1 rescue experiments part 3/R2R3/TPGS1-KO untransfected actub 7-31-25 R3 ET4ET5.Project Maximum Z_XY1756503767_Z0_T0_C0.tif]

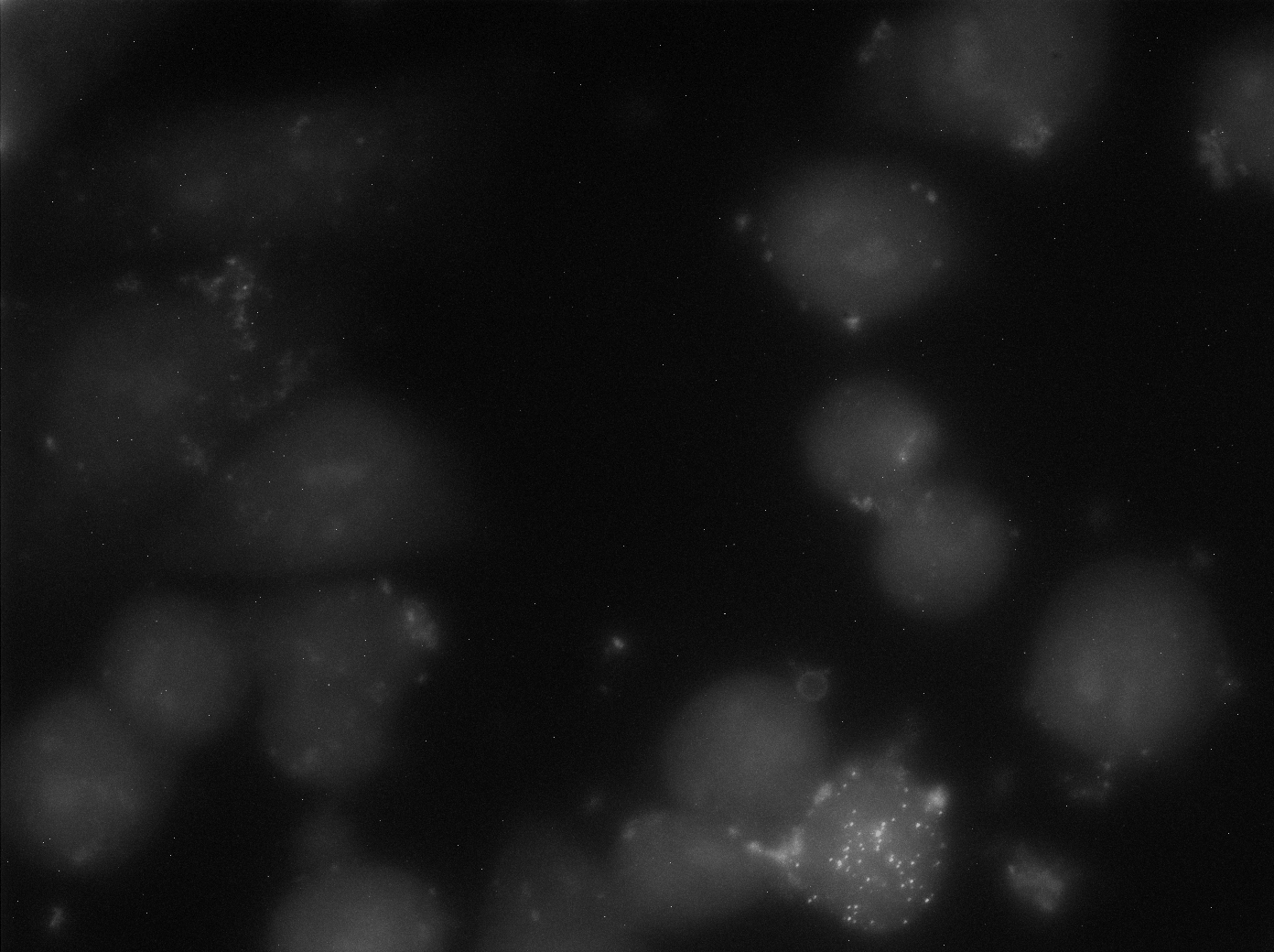

Supplement: Supplementary file 24 — Source data Fig. 6 part 5 [file 44319_2026_742_MOESM24_ESM.zip › Figure 6 Part 5/Fig 6efg TPGS1-KO TPGS1 rescue experiments part 3/R2R3/TPGS1-KO untransfected actub 7-31-25 R3 ET7.Project Maximum Z_XY1756505656_Z0_T0_C1.tif]

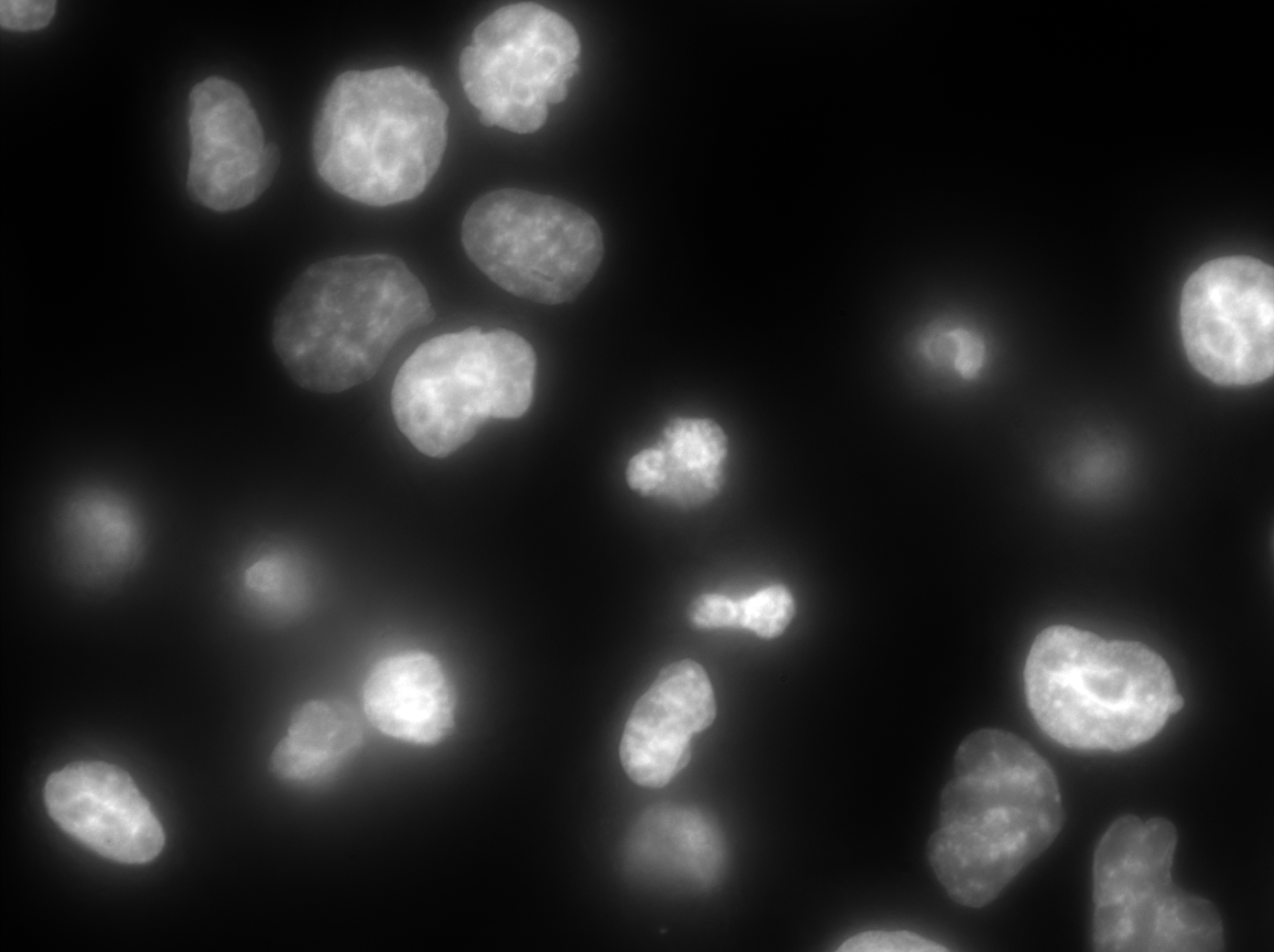

Supplement: Supplementary file 24 — Source data Fig. 6 part 5 [file 44319_2026_742_MOESM24_ESM.zip › Figure 6 Part 5/Fig 6efg TPGS1-KO TPGS1 rescue experiments part 3/R2R3/TPGS1-KO Untransfected actub 7-31-25 R2 ET10.Project Maximum Z_XY1756413287_Z0_T0_C0.tif]

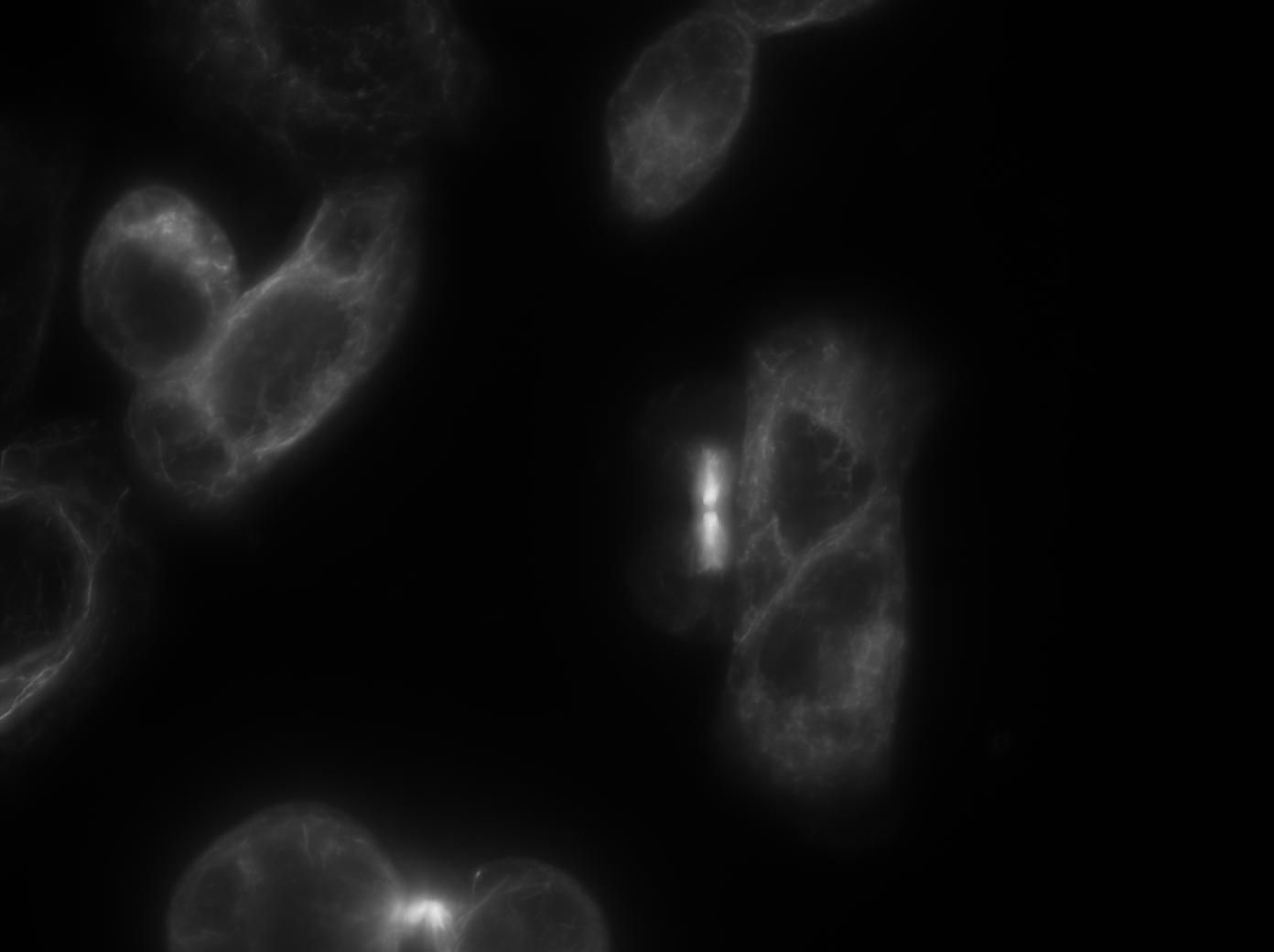

Supplement: Supplementary file 24 — Source data Fig. 6 part 5 [file 44319_2026_742_MOESM24_ESM.zip › Figure 6 Part 5/Fig 6efg TPGS1-KO TPGS1 rescue experiments part 3/R2R3/TPGS1-KO untransfected actub 7-31-25 R3 ET9.Project Maximum Z_XY1756506174_Z0_T0_C2.tif]

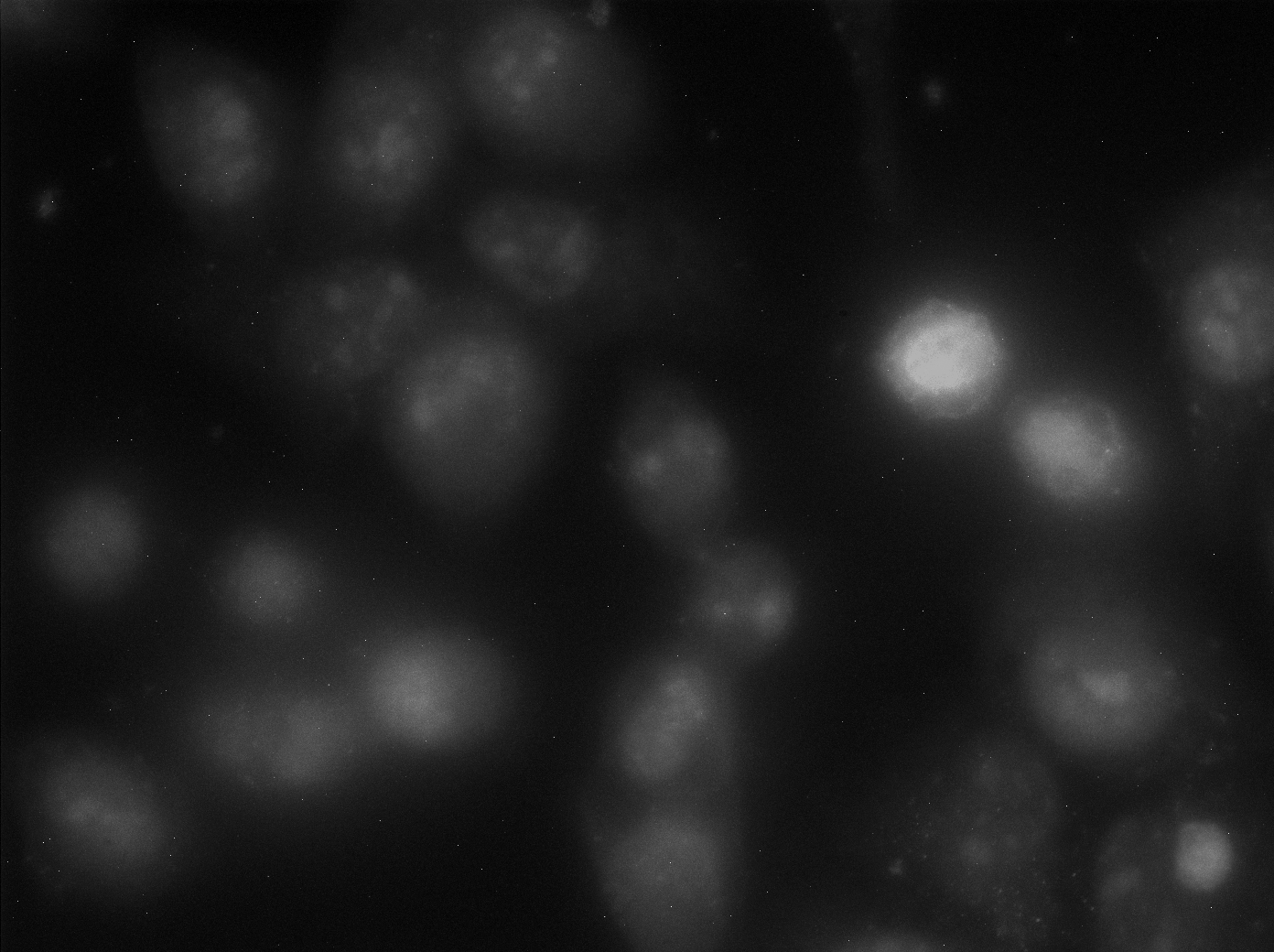

Supplement: Supplementary file 24 — Source data Fig. 6 part 5 [file 44319_2026_742_MOESM24_ESM.zip › Figure 6 Part 5/Fig 6efg TPGS1-KO TPGS1 rescue experiments part 3/R2R3/TPGS1-KO Untransfected actub 7-31-25 R2 ET10.Project Maximum Z_XY1756413287_Z0_T0_C1.tif]

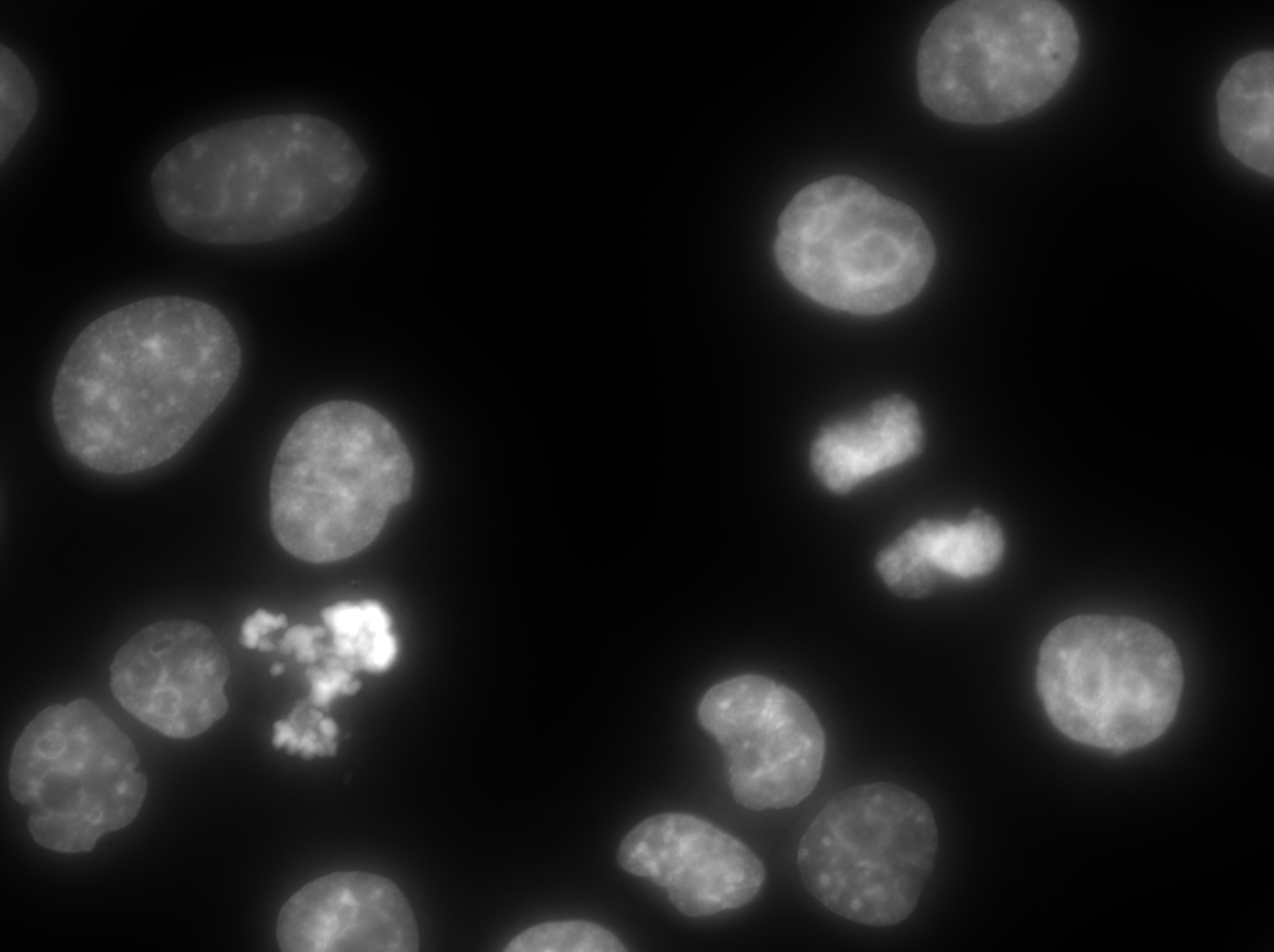

Supplement: Supplementary file 24 — Source data Fig. 6 part 5 [file 44319_2026_742_MOESM24_ESM.zip › Figure 6 Part 5/Fig 6efg TPGS1-KO TPGS1 rescue experiments part 3/R2R3/TPGS1-KO untransfected actub 7-31-25 R3 ET7.Project Maximum Z_XY1756505656_Z0_T0_C0.tif]

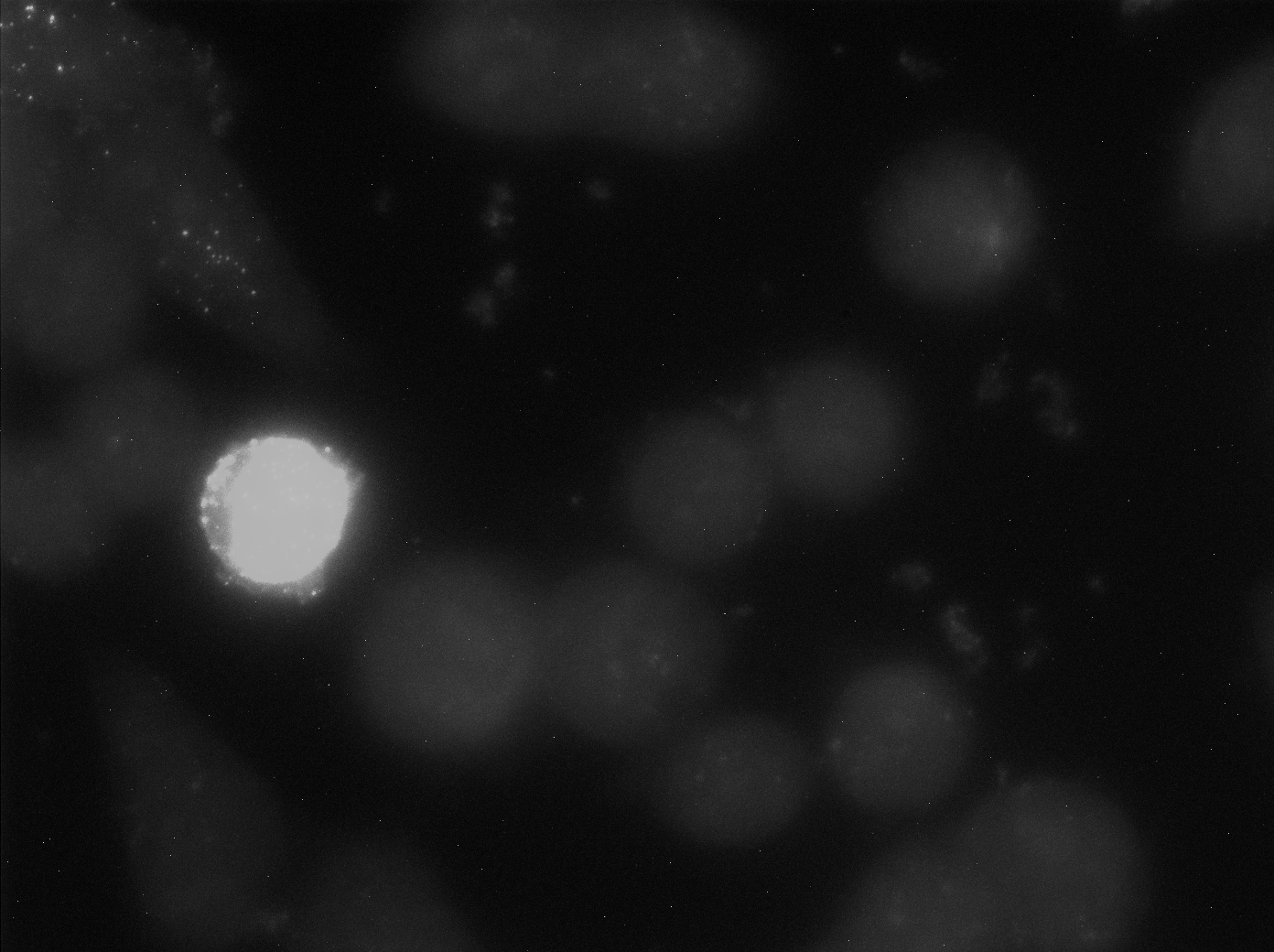

Supplement: Supplementary file 24 — Source data Fig. 6 part 5 [file 44319_2026_742_MOESM24_ESM.zip › Figure 6 Part 5/Fig 6efg TPGS1-KO TPGS1 rescue experiments part 3/R2R3/TPGS1-KO untransfected actub 7-31-25 R3 ET4ET5.Project Maximum Z_XY1756503767_Z0_T0_C1.tif]

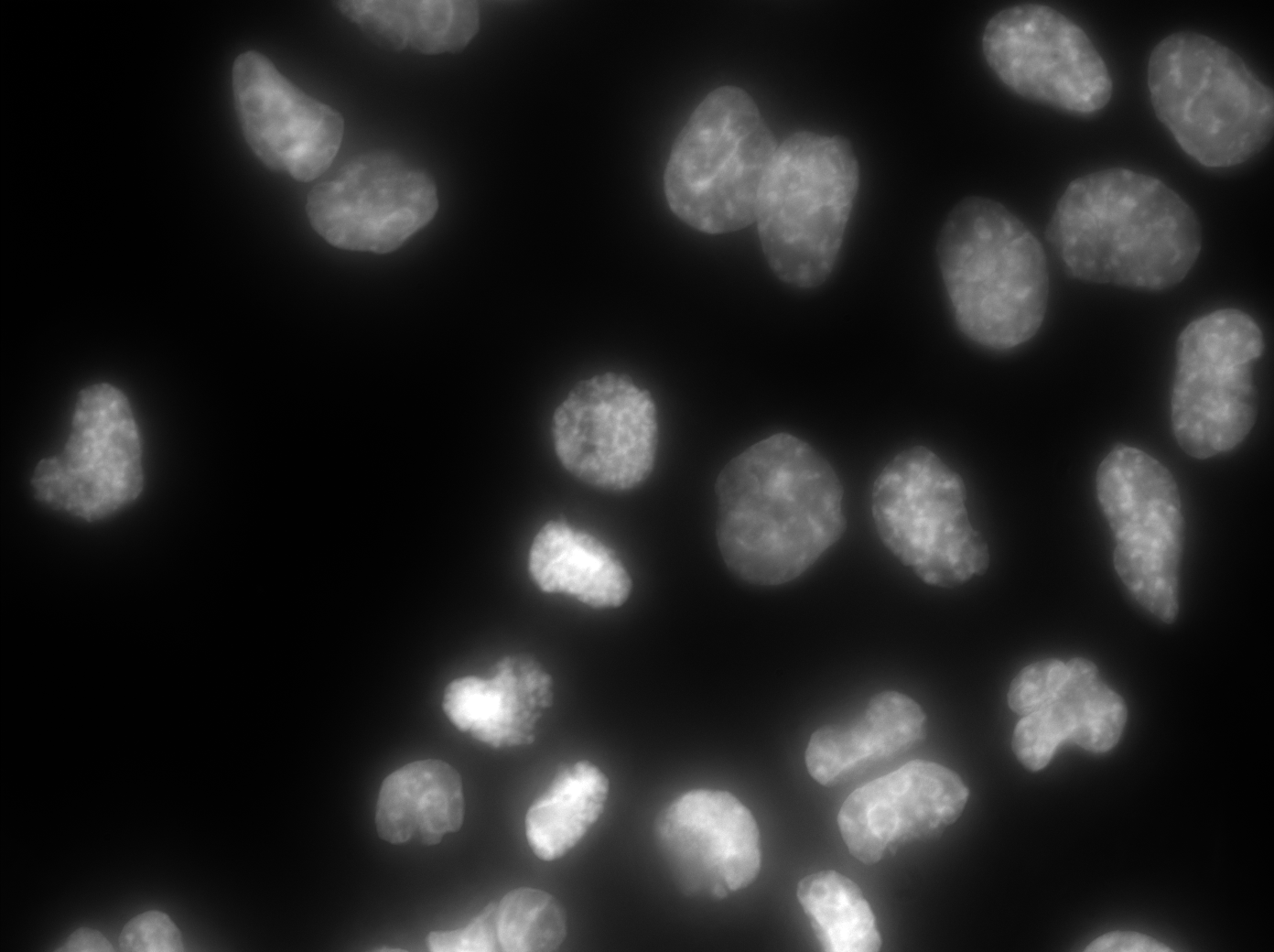

Supplement: Supplementary file 24 — Source data Fig. 6 part 5 [file 44319_2026_742_MOESM24_ESM.zip › Figure 6 Part 5/Fig 6efg TPGS1-KO TPGS1 rescue experiments part 3/R2R3/TPGS1-KO Untransfected actub 7-31-25 R2 LT9LT10 ET5.Project Maximum Z_XY1756412925_Z0_T0_C0.tif]

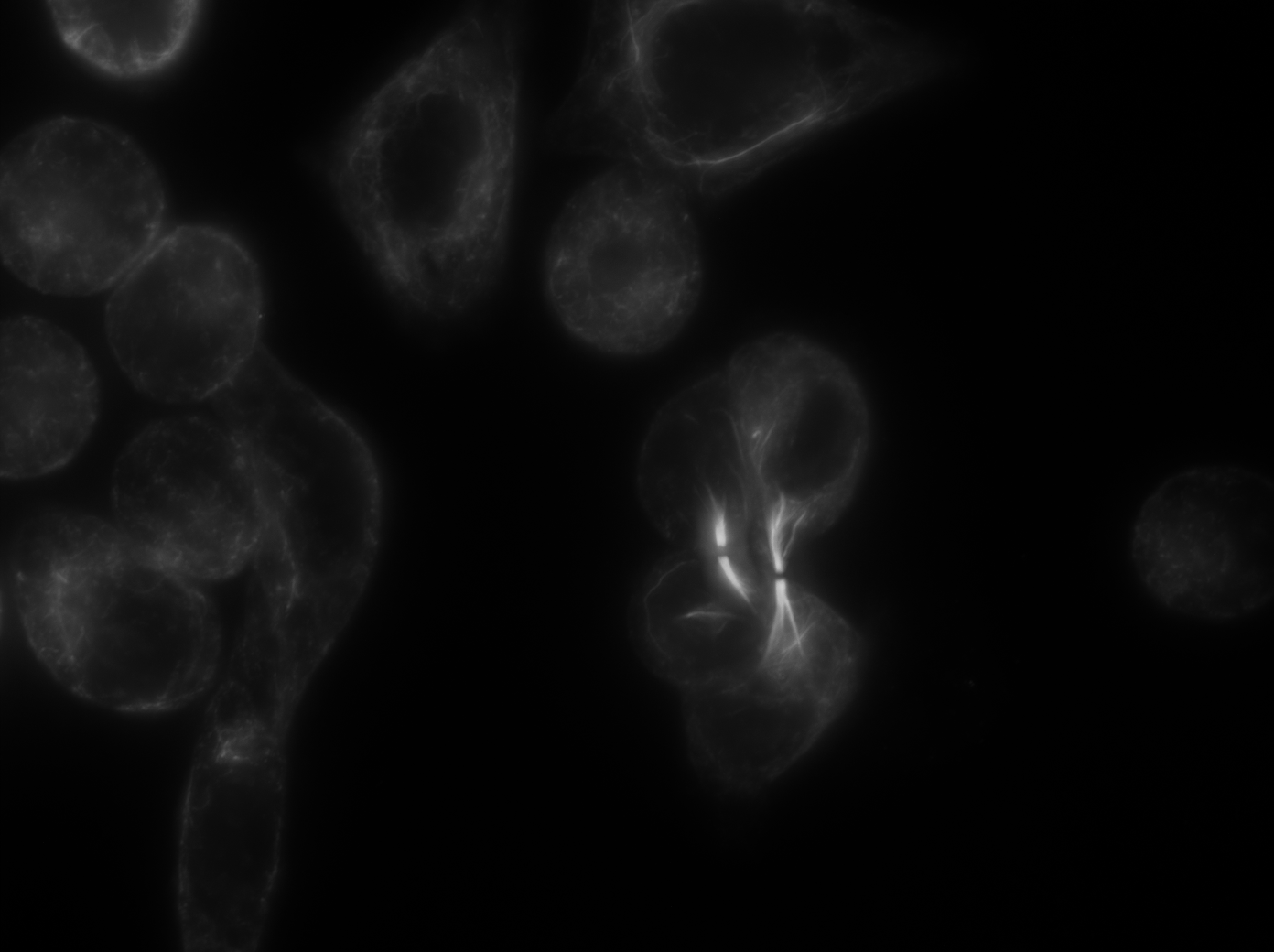

Supplement: Supplementary file 24 — Source data Fig. 6 part 5 [file 44319_2026_742_MOESM24_ESM.zip › Figure 6 Part 5/Fig 6efg TPGS1-KO TPGS1 rescue experiments part 3/R2R3/TPGS1-KO untransfected actub 7-31-25 R3 LT6LT7.Project Maximum Z_XY1756505497_Z0_T0_C2.tif]

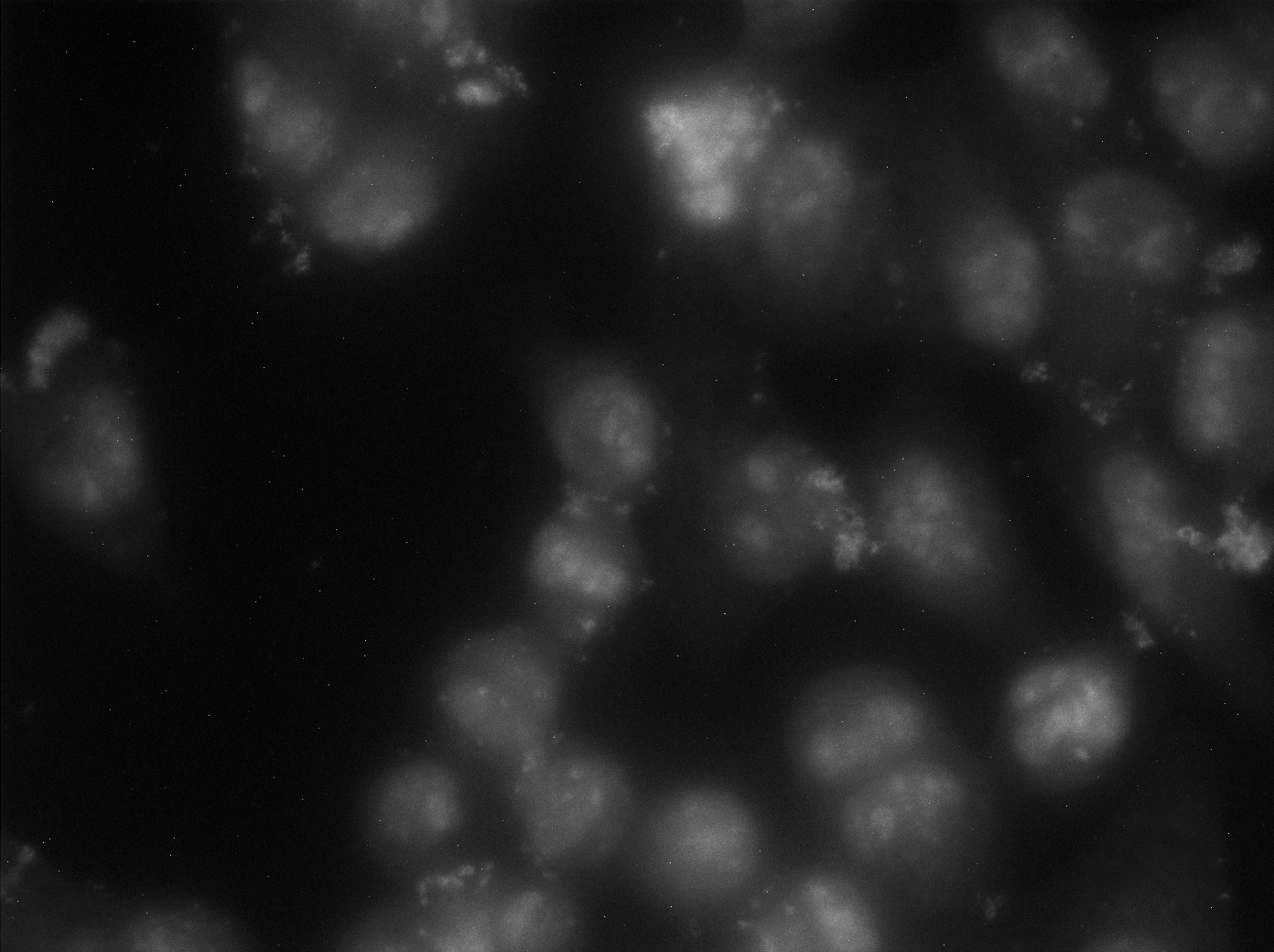

Supplement: Supplementary file 24 — Source data Fig. 6 part 5 [file 44319_2026_742_MOESM24_ESM.zip › Figure 6 Part 5/Fig 6efg TPGS1-KO TPGS1 rescue experiments part 3/R2R3/TPGS1-KO Untransfected actub 7-31-25 R2 LT9LT10 ET5.Project Maximum Z_XY1756412925_Z0_T0_C1.tif]

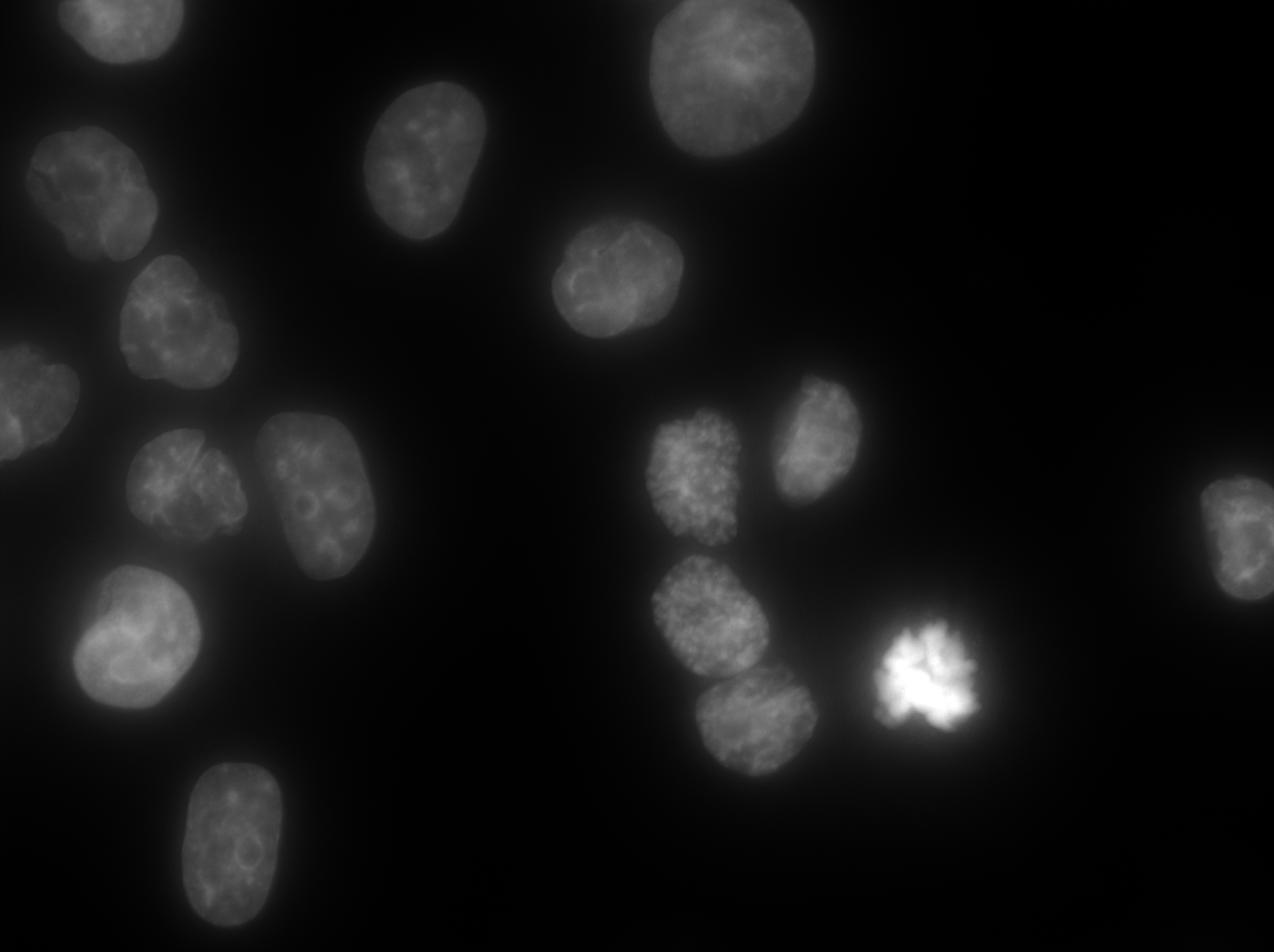

Supplement: Supplementary file 24 — Source data Fig. 6 part 5 [file 44319_2026_742_MOESM24_ESM.zip › Figure 6 Part 5/Fig 6efg TPGS1-KO TPGS1 rescue experiments part 3/R2R3/TPGS1-KO untransfected actub 7-31-25 R3 LT6LT7.Project Maximum Z_XY1756505497_Z0_T0_C0.tif]

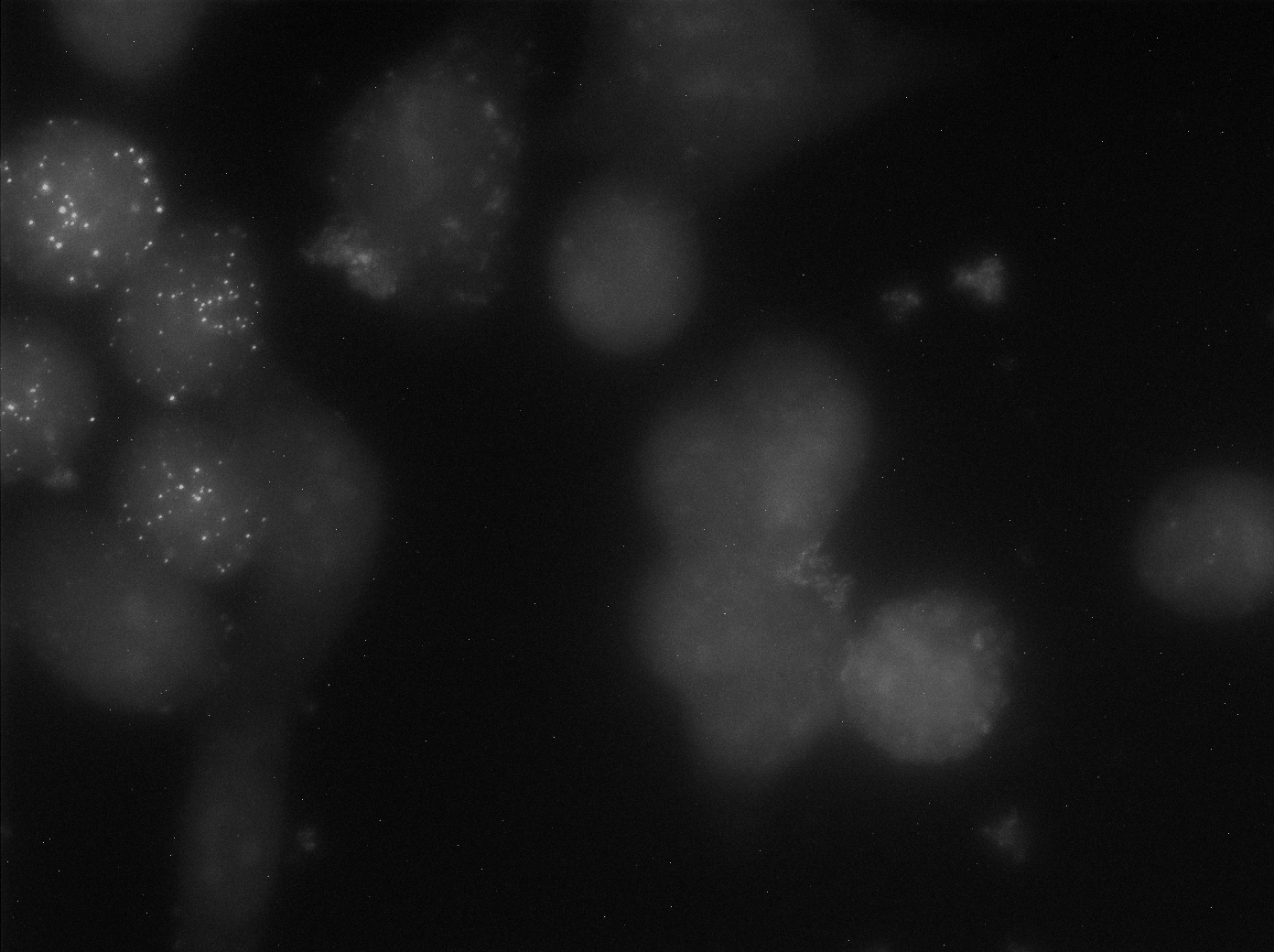

Supplement: Supplementary file 24 — Source data Fig. 6 part 5 [file 44319_2026_742_MOESM24_ESM.zip › Figure 6 Part 5/Fig 6efg TPGS1-KO TPGS1 rescue experiments part 3/R2R3/TPGS1-KO untransfected actub 7-31-25 R3 LT6LT7.Project Maximum Z_XY1756505497_Z0_T0_C1.tif]

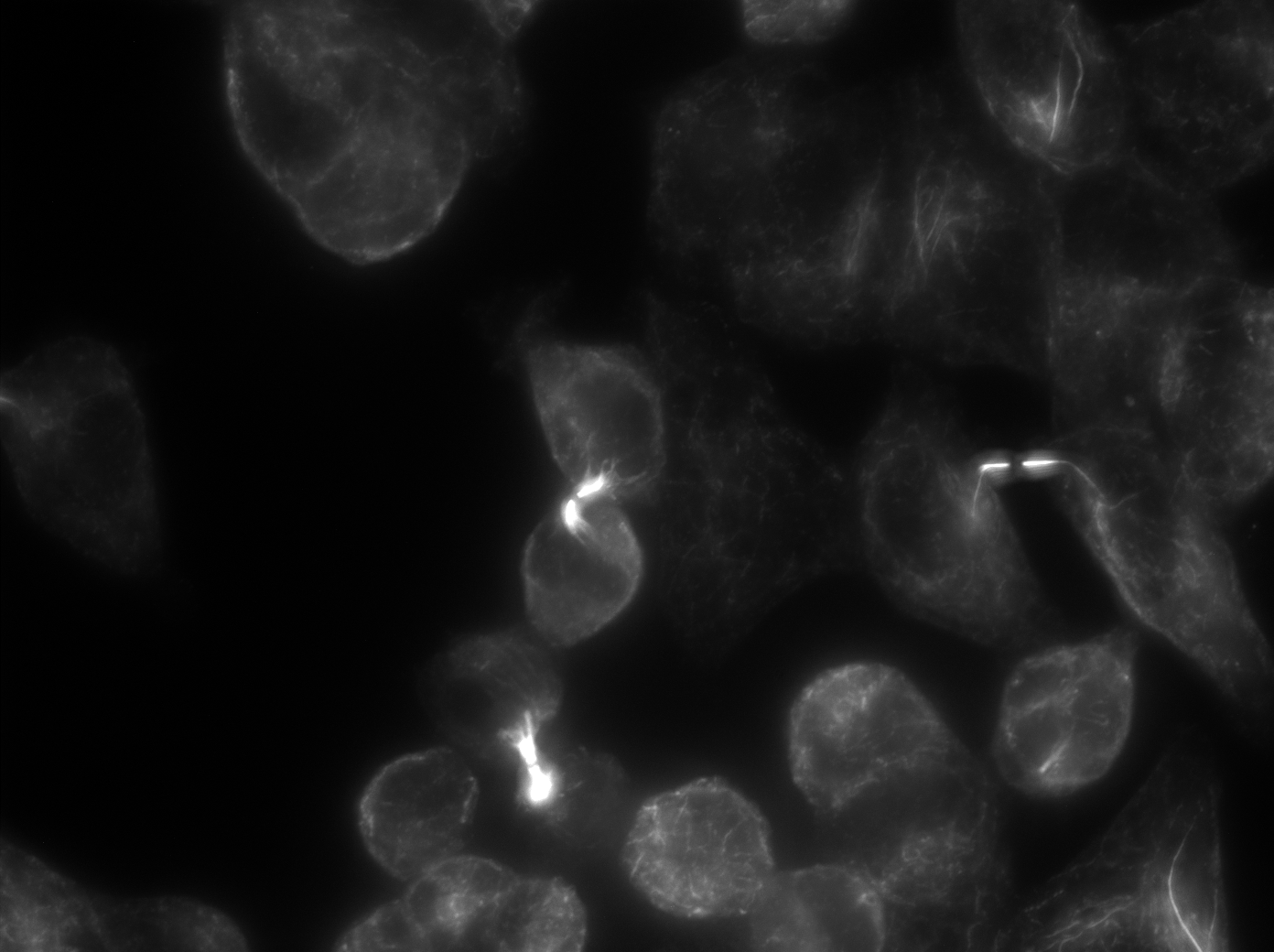

Supplement: Supplementary file 24 — Source data Fig. 6 part 5 [file 44319_2026_742_MOESM24_ESM.zip › Figure 6 Part 5/Fig 6efg TPGS1-KO TPGS1 rescue experiments part 3/R2R3/TPGS1-KO Untransfected actub 7-31-25 R2 LT9LT10 ET5.Project Maximum Z_XY1756412925_Z0_T0_C2.tif]

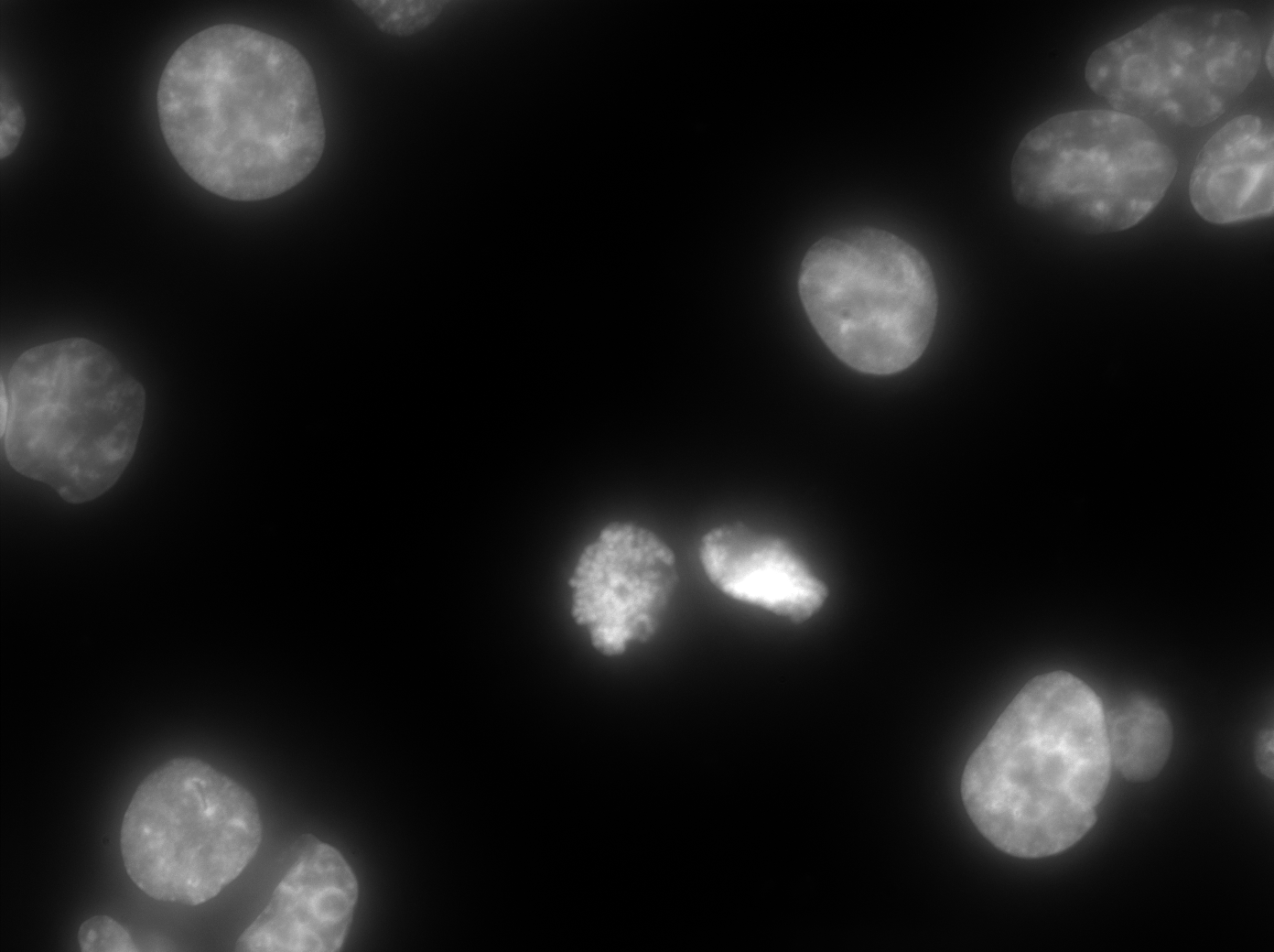

Supplement: Supplementary file 24 — Source data Fig. 6 part 5 [file 44319_2026_742_MOESM24_ESM.zip › Figure 6 Part 5/Fig 6efg TPGS1-KO TPGS1 rescue experiments part 3/R2R3/TPGS1-KO untransfected actub 7-31-25 R3 ET6.Project Maximum Z_XY1756505234_Z0_T0_C0.tif]

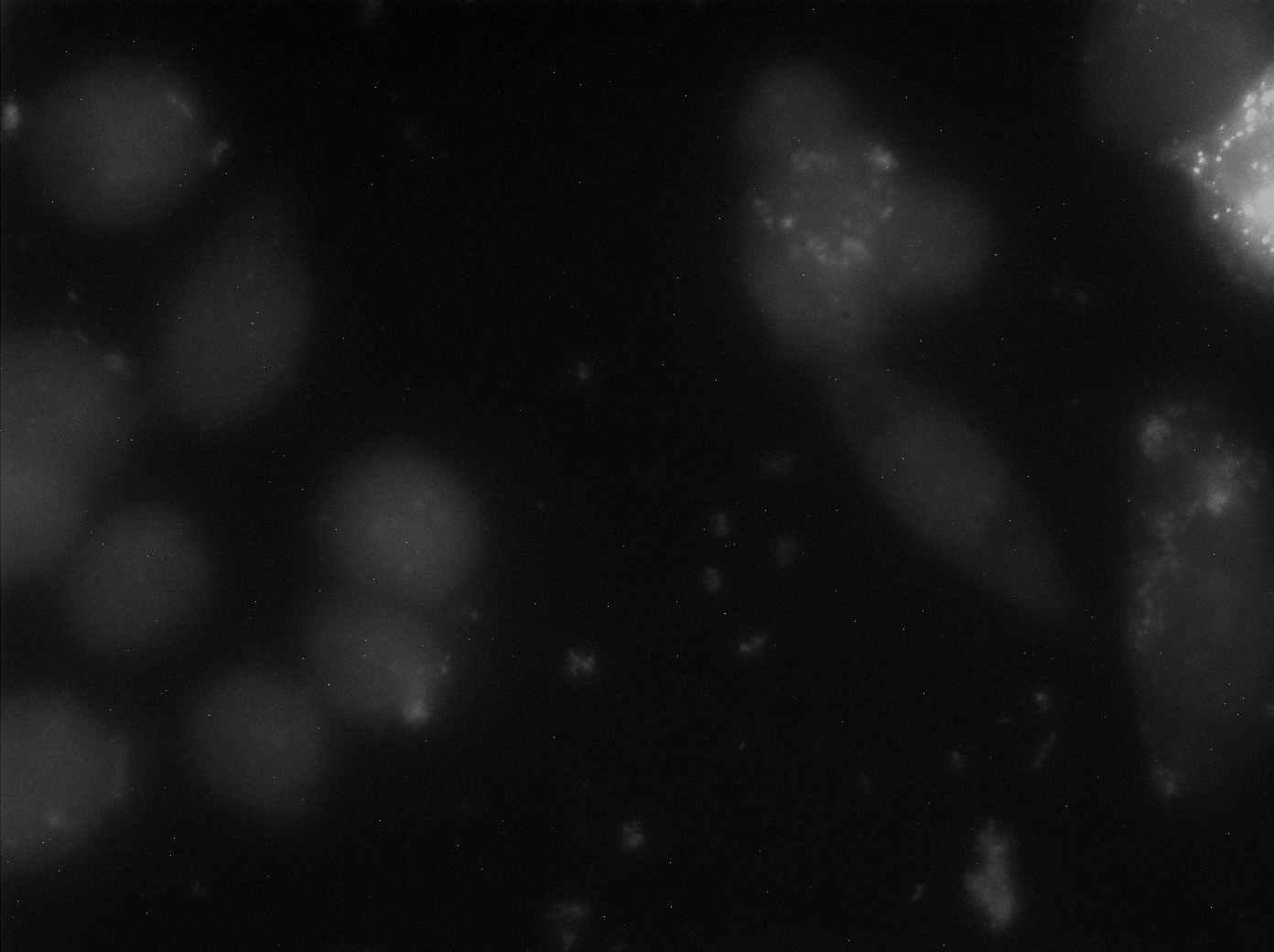

Supplement: Supplementary file 24 — Source data Fig. 6 part 5 [file 44319_2026_742_MOESM24_ESM.zip › Figure 6 Part 5/Fig 6efg TPGS1-KO TPGS1 rescue experiments part 3/R2R3/TPGS1-KO untransfected actub 7-31-25 R3 ET3.Project Maximum Z_XY1756503133_Z0_T0_C1.tif]

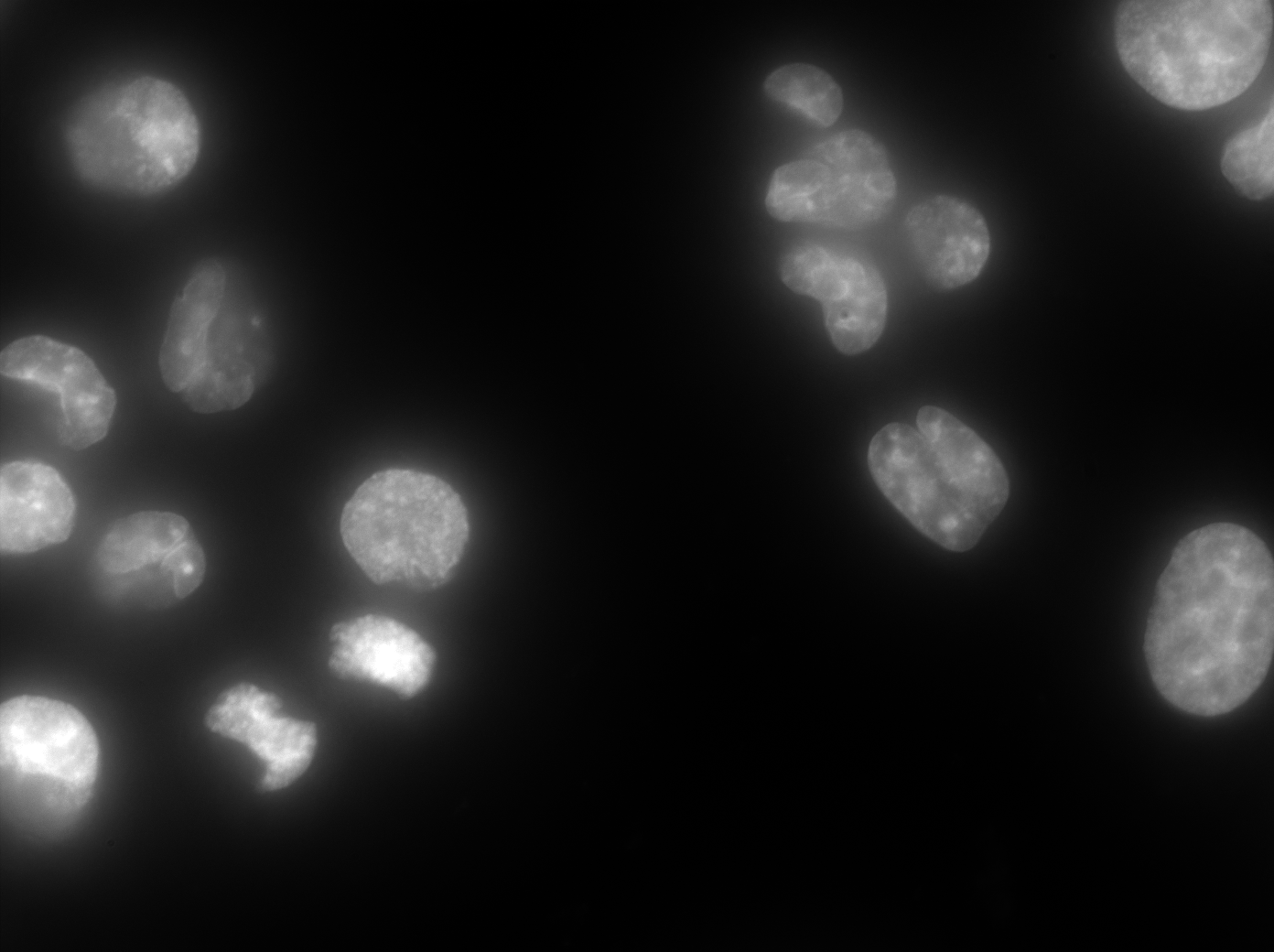

Supplement: Supplementary file 24 — Source data Fig. 6 part 5 [file 44319_2026_742_MOESM24_ESM.zip › Figure 6 Part 5/Fig 6efg TPGS1-KO TPGS1 rescue experiments part 3/R2R3/TPGS1-KO untransfected actub 7-31-25 R3 ET3.Project Maximum Z_XY1756503133_Z0_T0_C0.tif]

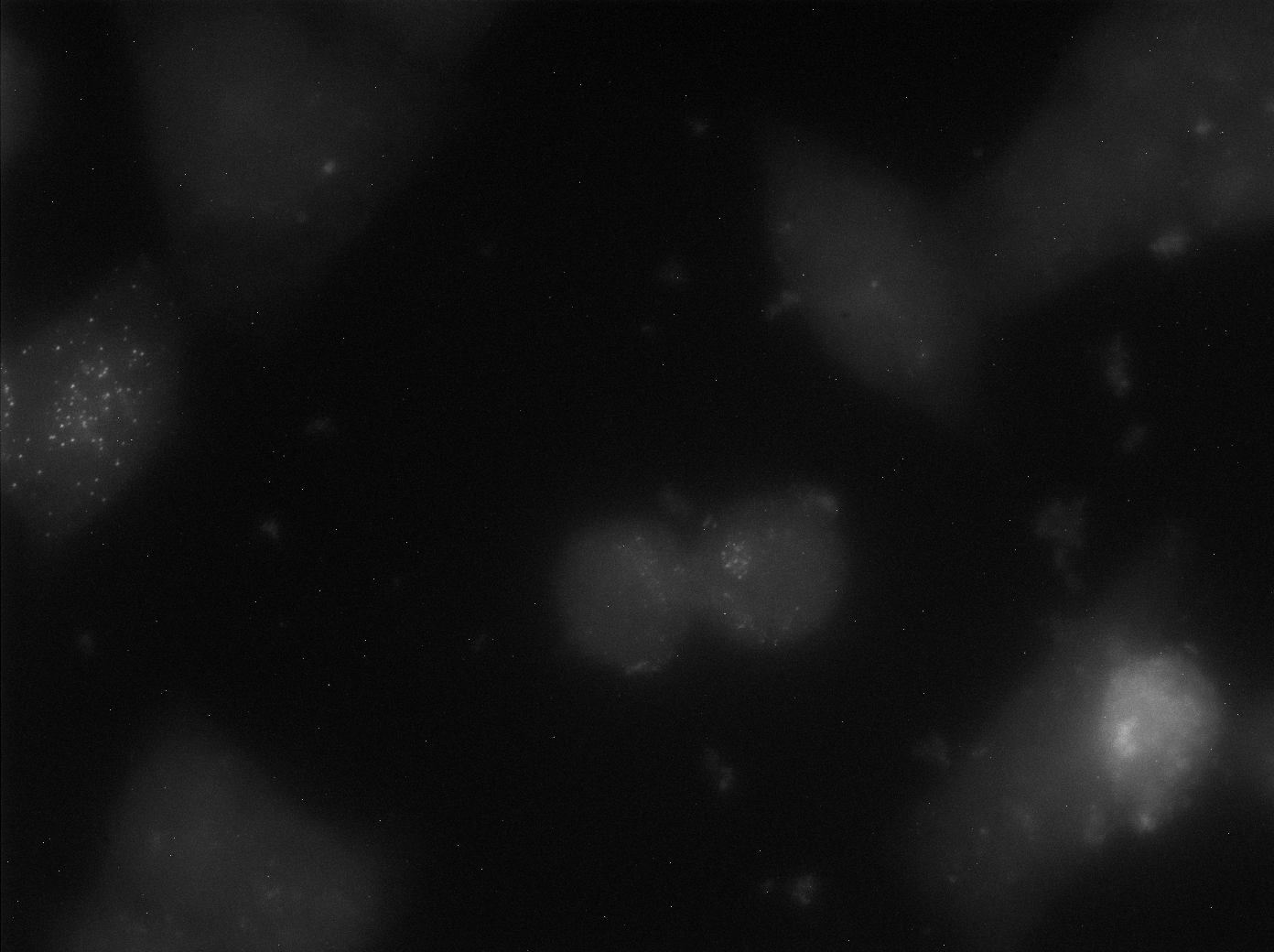

Supplement: Supplementary file 24 — Source data Fig. 6 part 5 [file 44319_2026_742_MOESM24_ESM.zip › Figure 6 Part 5/Fig 6efg TPGS1-KO TPGS1 rescue experiments part 3/R2R3/TPGS1-KO untransfected actub 7-31-25 R3 ET6.Project Maximum Z_XY1756505234_Z0_T0_C1.tif]

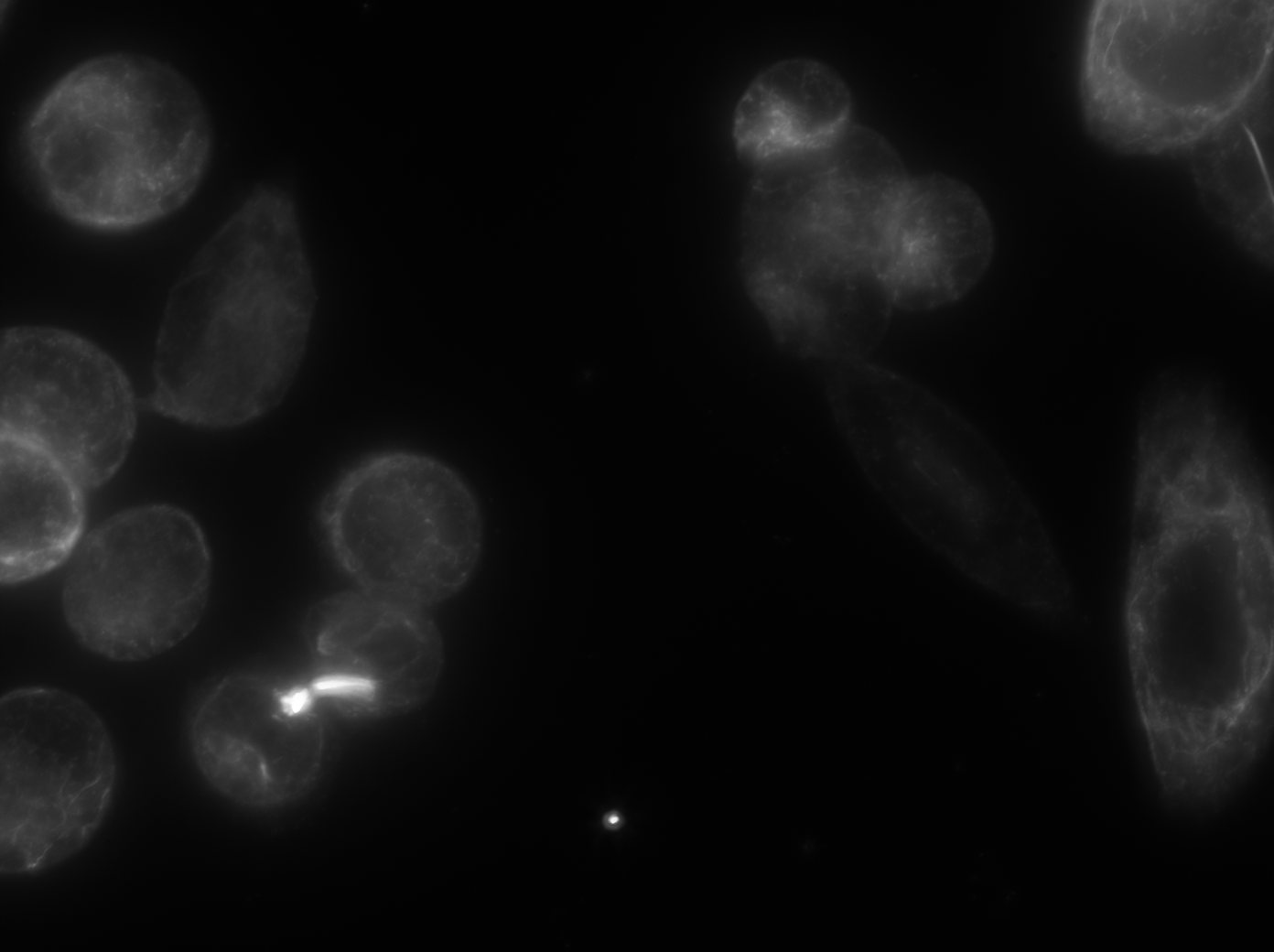

Supplement: Supplementary file 24 — Source data Fig. 6 part 5 [file 44319_2026_742_MOESM24_ESM.zip › Figure 6 Part 5/Fig 6efg TPGS1-KO TPGS1 rescue experiments part 3/R2R3/TPGS1-KO untransfected actub 7-31-25 R3 ET3.Project Maximum Z_XY1756503133_Z0_T0_C2.tif]

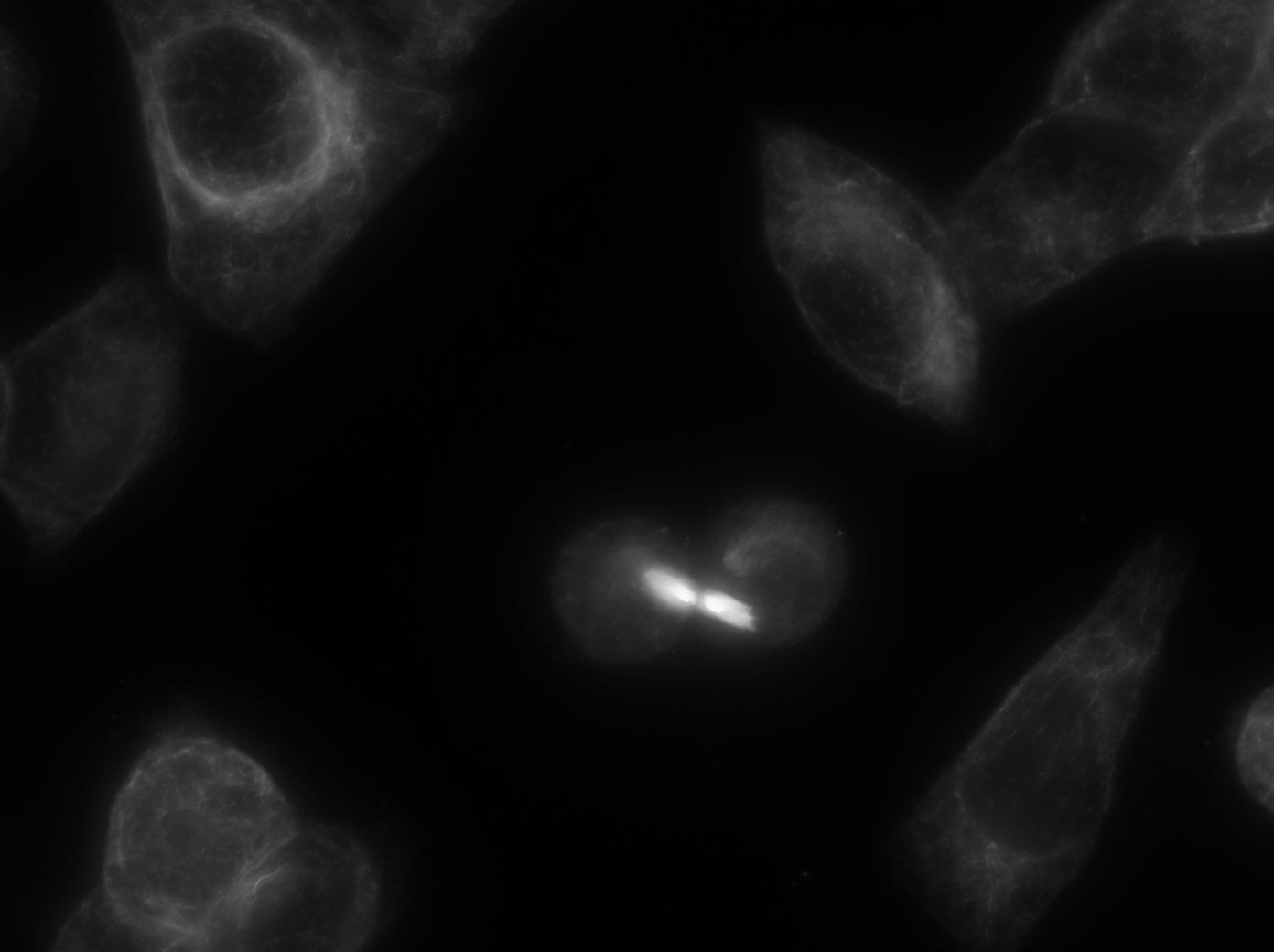

Supplement: Supplementary file 24 — Source data Fig. 6 part 5 [file 44319_2026_742_MOESM24_ESM.zip › Figure 6 Part 5/Fig 6efg TPGS1-KO TPGS1 rescue experiments part 3/R2R3/TPGS1-KO untransfected actub 7-31-25 R3 ET6.Project Maximum Z_XY1756505234_Z0_T0_C2.tif]

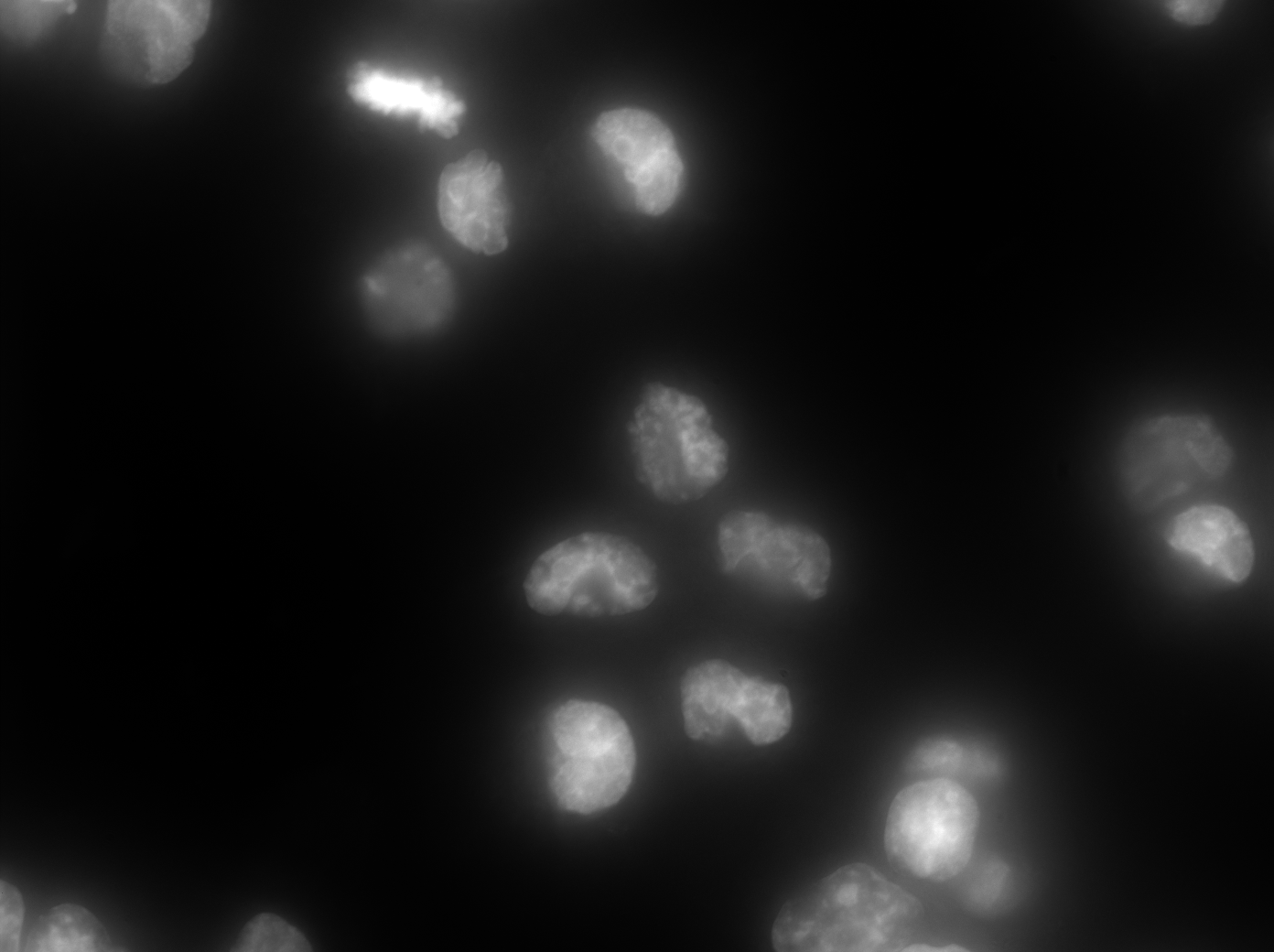

Supplement: Supplementary file 24 — Source data Fig. 6 part 5 [file 44319_2026_742_MOESM24_ESM.zip › Figure 6 Part 5/Fig 6efg TPGS1-KO TPGS1 rescue experiments part 3/R2R3/TPGS1-KO untransfected actub 7-31-25 R3 LT1LT2.Project Maximum Z_XY1756502737_Z0_T0_C0.tif]

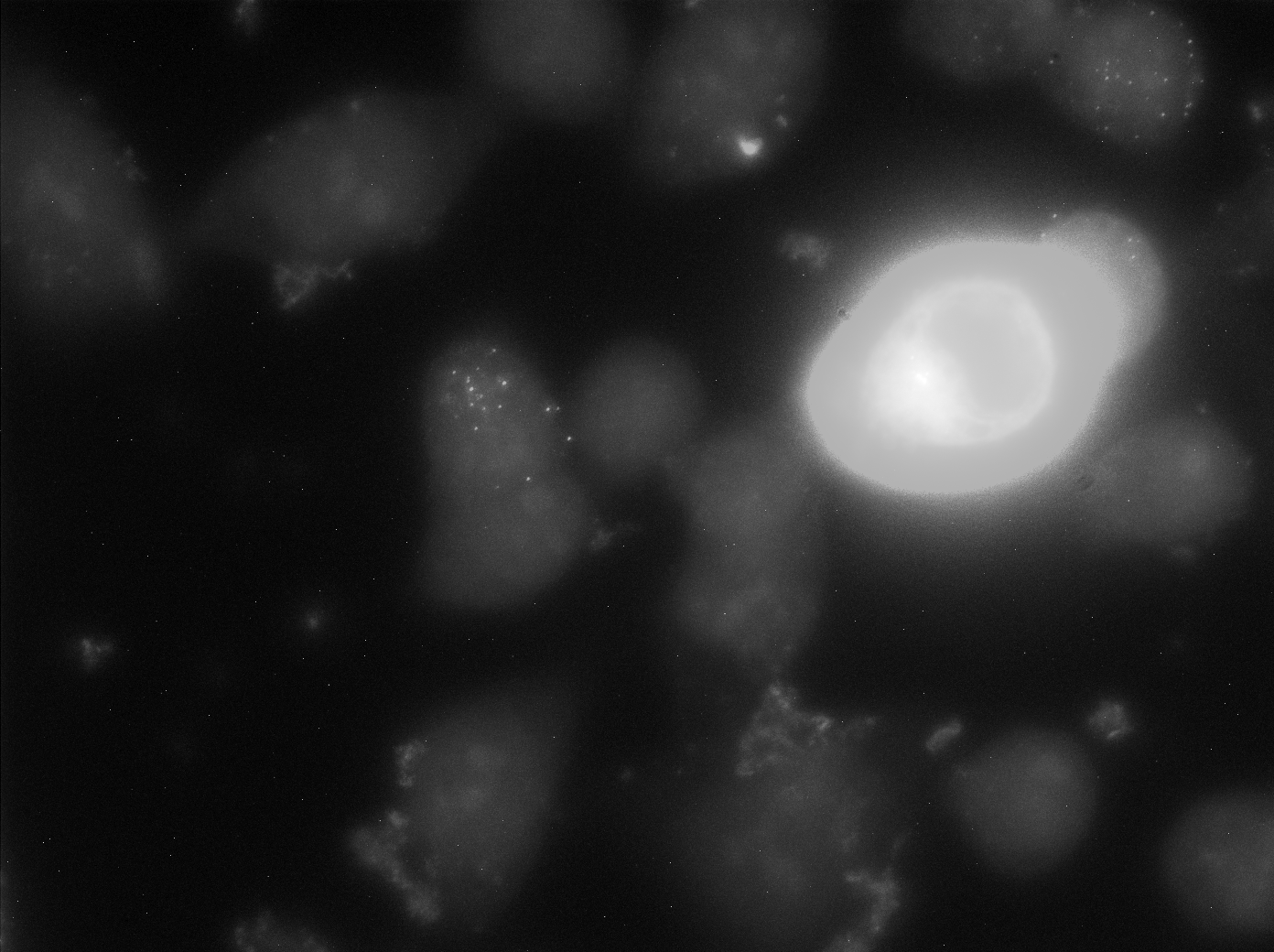

Supplement: Supplementary file 24 — Source data Fig. 6 part 5 [file 44319_2026_742_MOESM24_ESM.zip › Figure 6 Part 5/Fig 6efg TPGS1-KO TPGS1 rescue experiments part 3/R2R3/TPGS1-KO untransfected actub 7-31-25 R3 ET1.Project Maximum Z_XY1756502864_Z0_T0_C1.tif]

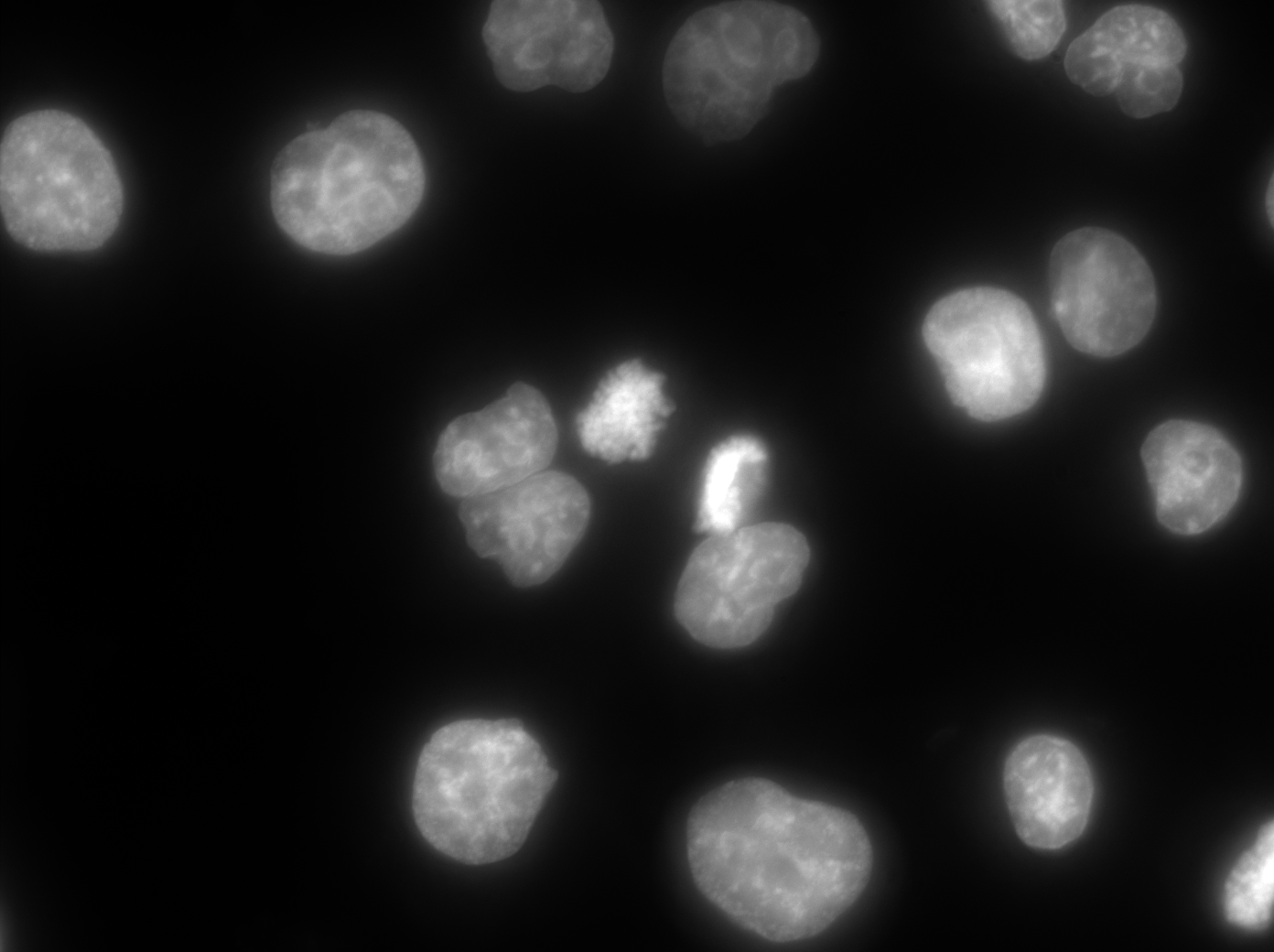

Supplement: Supplementary file 24 — Source data Fig. 6 part 5 [file 44319_2026_742_MOESM24_ESM.zip › Figure 6 Part 5/Fig 6efg TPGS1-KO TPGS1 rescue experiments part 3/R2R3/TPGS1-KO untransfected actub 7-31-25 R3 ET1.Project Maximum Z_XY1756502864_Z0_T0_C0.tif]

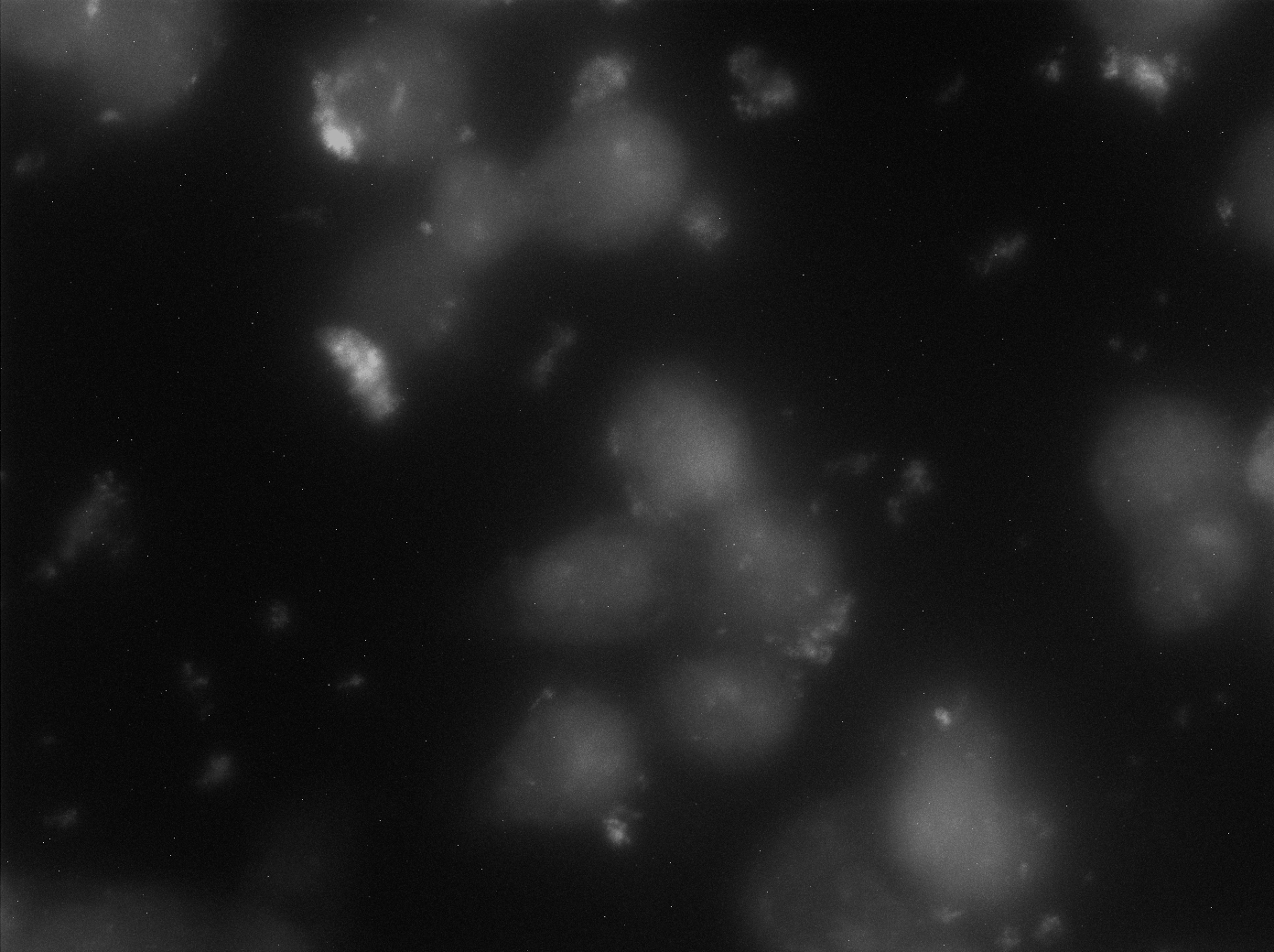

Supplement: Supplementary file 24 — Source data Fig. 6 part 5 [file 44319_2026_742_MOESM24_ESM.zip › Figure 6 Part 5/Fig 6efg TPGS1-KO TPGS1 rescue experiments part 3/R2R3/TPGS1-KO untransfected actub 7-31-25 R3 LT1LT2.Project Maximum Z_XY1756502737_Z0_T0_C1.tif]

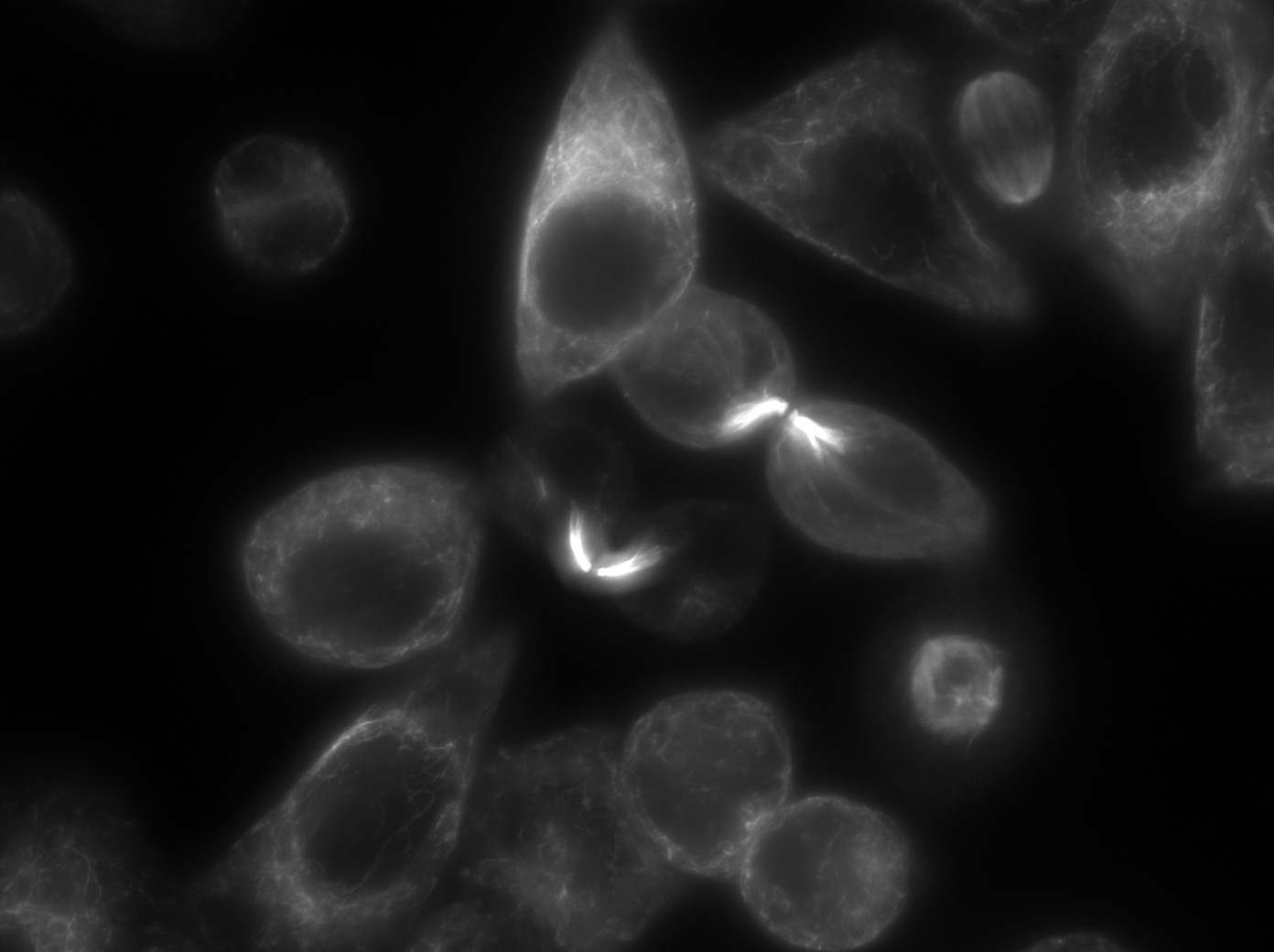

Supplement: Supplementary file 24 — Source data Fig. 6 part 5 [file 44319_2026_742_MOESM24_ESM.zip › Figure 6 Part 5/Fig 6efg TPGS1-KO TPGS1 rescue experiments part 3/R2R3/TPGS1-KO Untransfected actub 7-31-25 R2 ET1 LT1.Project Maximum Z_XY1756406015_Z0_T0_C2.tif]

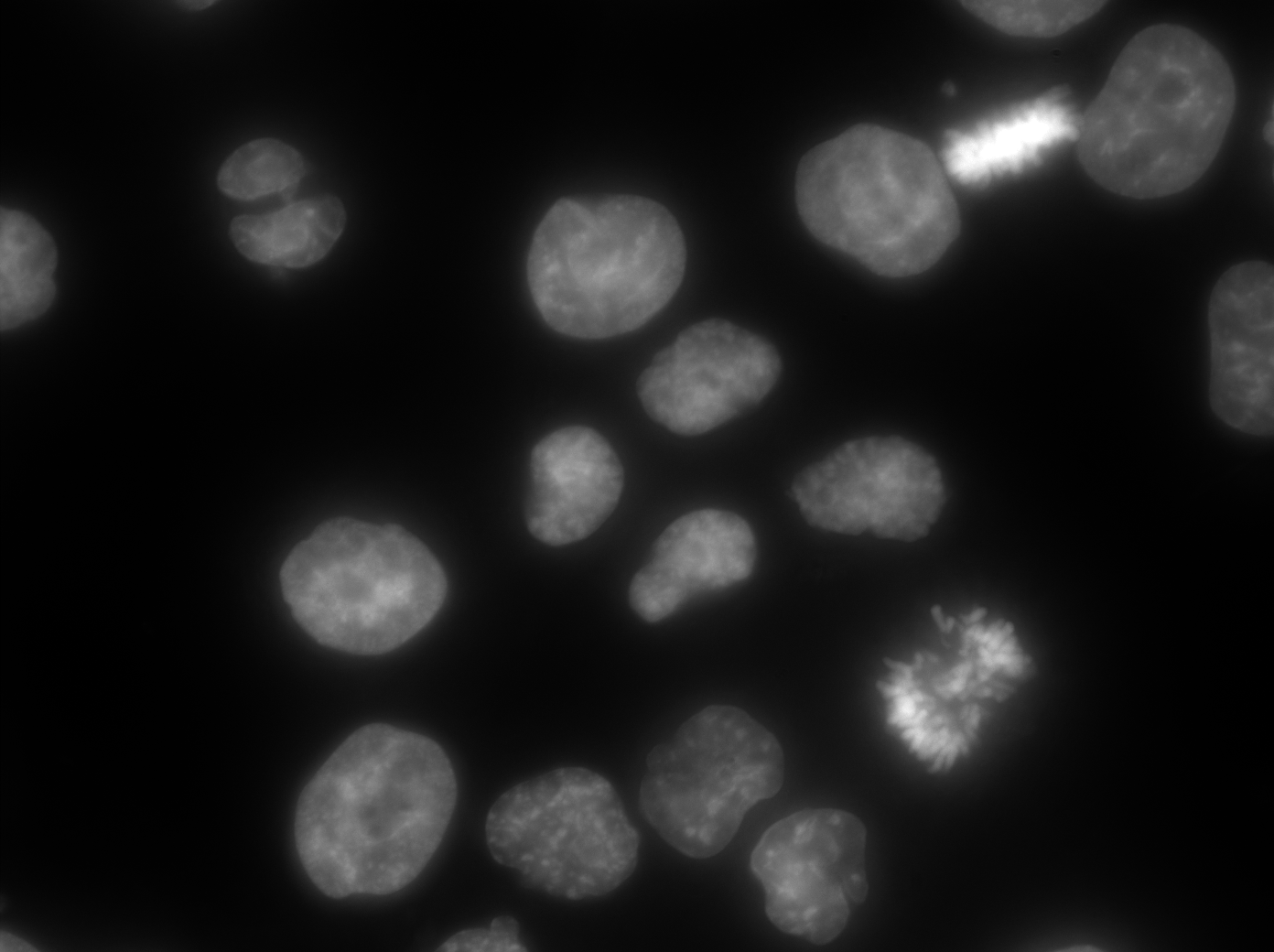

Supplement: Supplementary file 24 — Source data Fig. 6 part 5 [file 44319_2026_742_MOESM24_ESM.zip › Figure 6 Part 5/Fig 6efg TPGS1-KO TPGS1 rescue experiments part 3/R2R3/TPGS1-KO Untransfected actub 7-31-25 R2 ET1 LT1.Project Maximum Z_XY1756406015_Z0_T0_C0.tif]

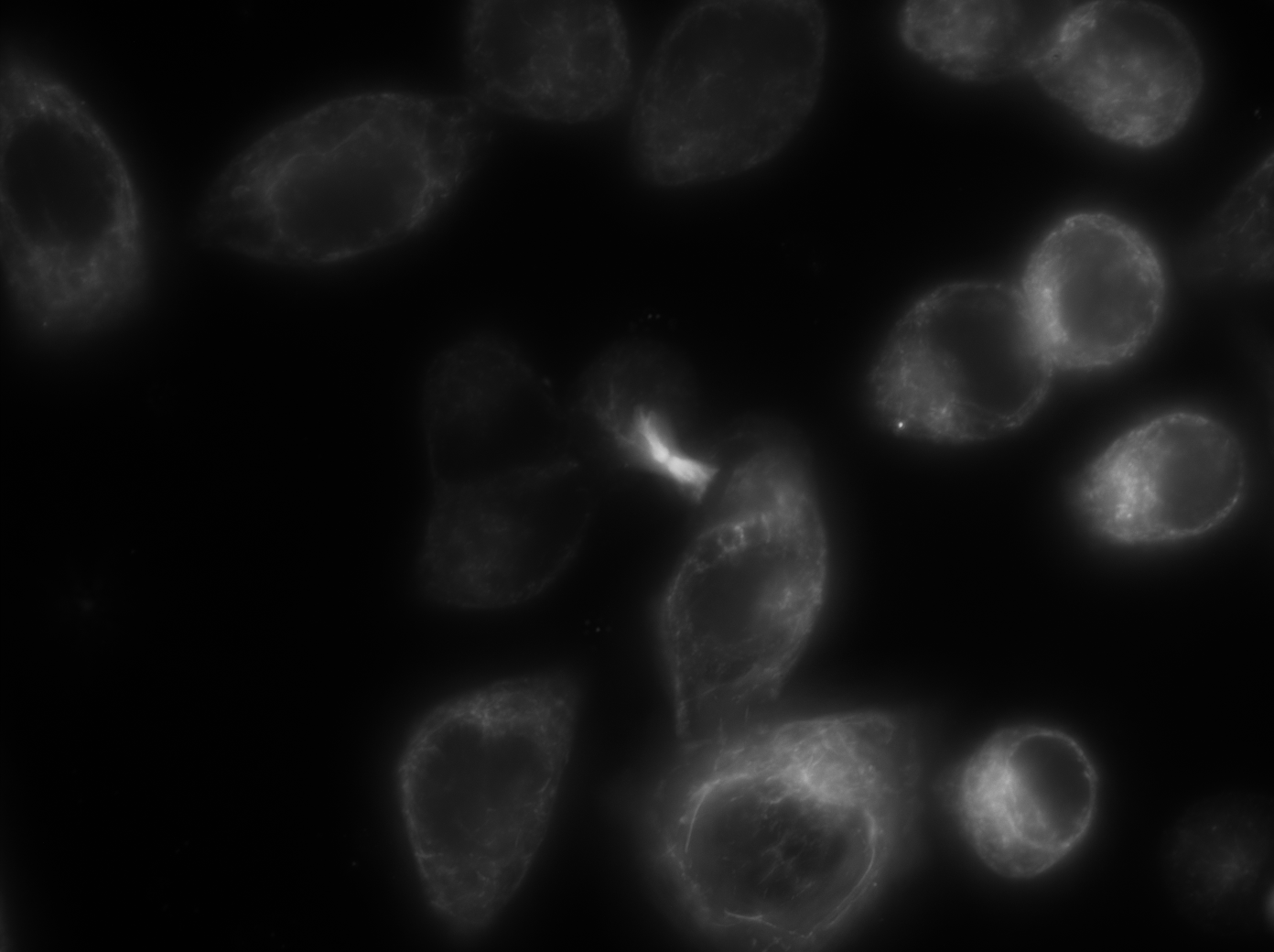

Supplement: Supplementary file 24 — Source data Fig. 6 part 5 [file 44319_2026_742_MOESM24_ESM.zip › Figure 6 Part 5/Fig 6efg TPGS1-KO TPGS1 rescue experiments part 3/R2R3/TPGS1-KO untransfected actub 7-31-25 R3 ET1.Project Maximum Z_XY1756502864_Z0_T0_C2.tif]

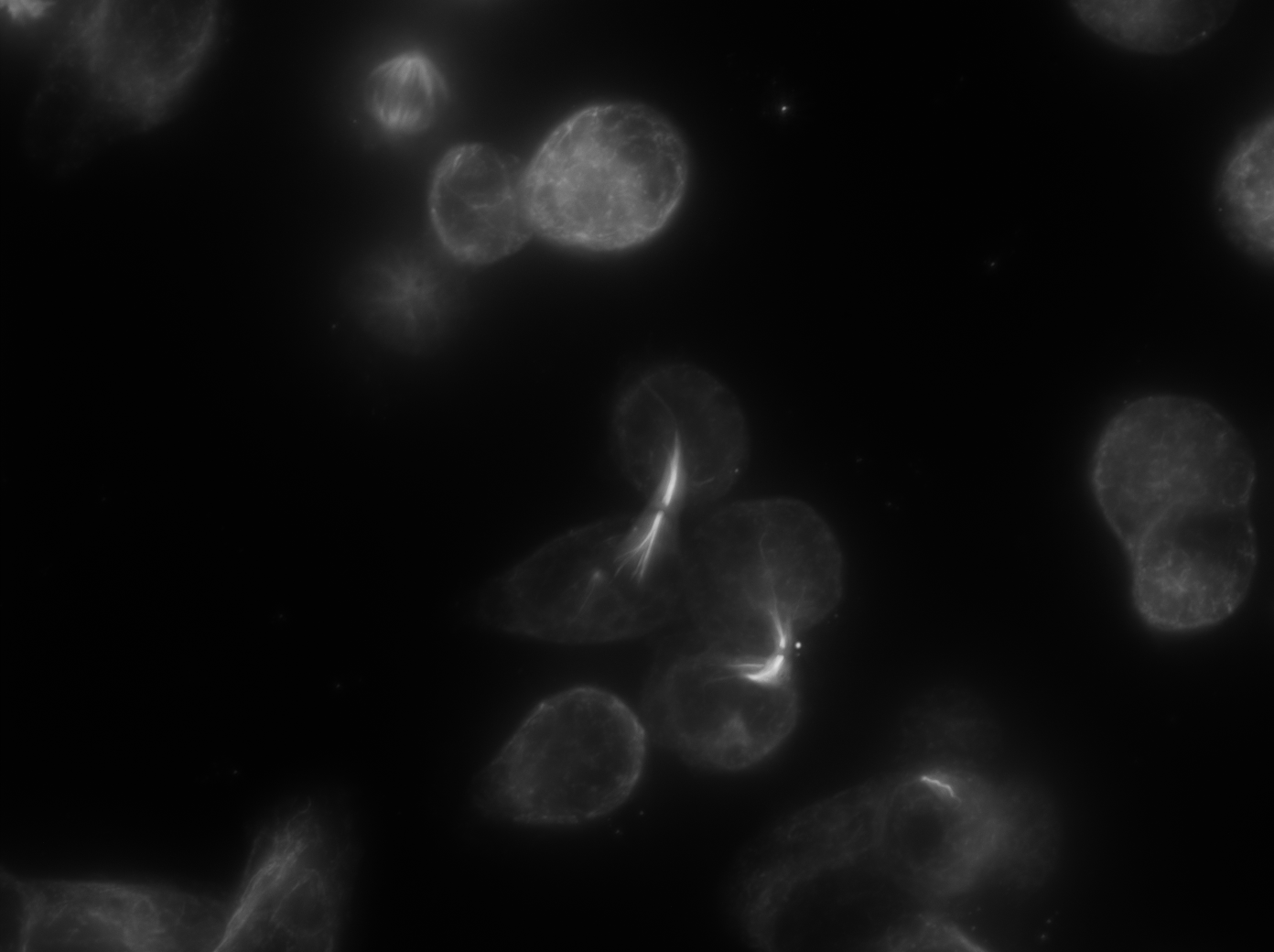

Supplement: Supplementary file 24 — Source data Fig. 6 part 5 [file 44319_2026_742_MOESM24_ESM.zip › Figure 6 Part 5/Fig 6efg TPGS1-KO TPGS1 rescue experiments part 3/R2R3/TPGS1-KO untransfected actub 7-31-25 R3 LT1LT2.Project Maximum Z_XY1756502737_Z0_T0_C2.tif]

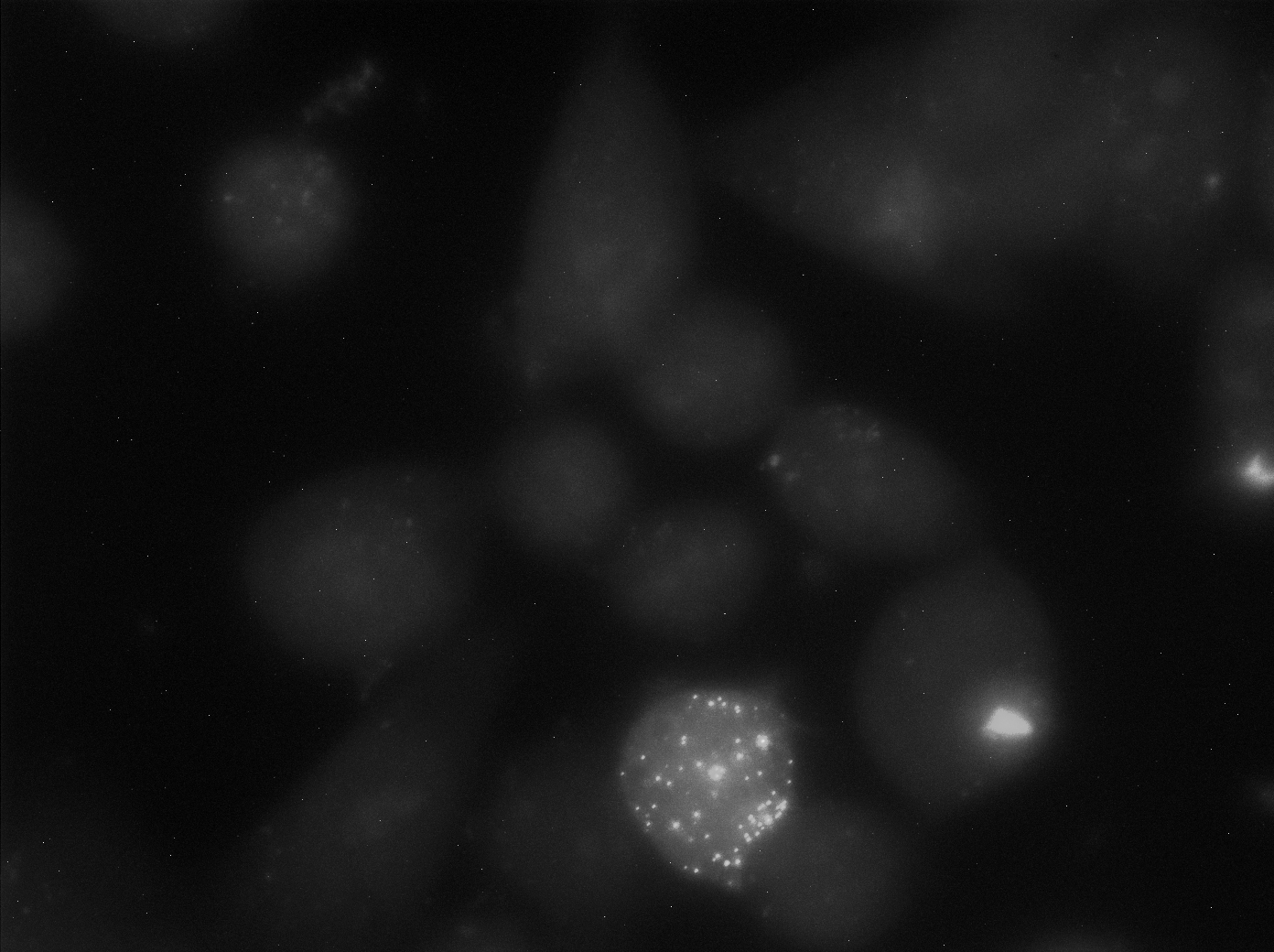

Supplement: Supplementary file 24 — Source data Fig. 6 part 5 [file 44319_2026_742_MOESM24_ESM.zip › Figure 6 Part 5/Fig 6efg TPGS1-KO TPGS1 rescue experiments part 3/R2R3/TPGS1-KO Untransfected actub 7-31-25 R2 ET1 LT1.Project Maximum Z_XY1756406015_Z0_T0_C1.tif]

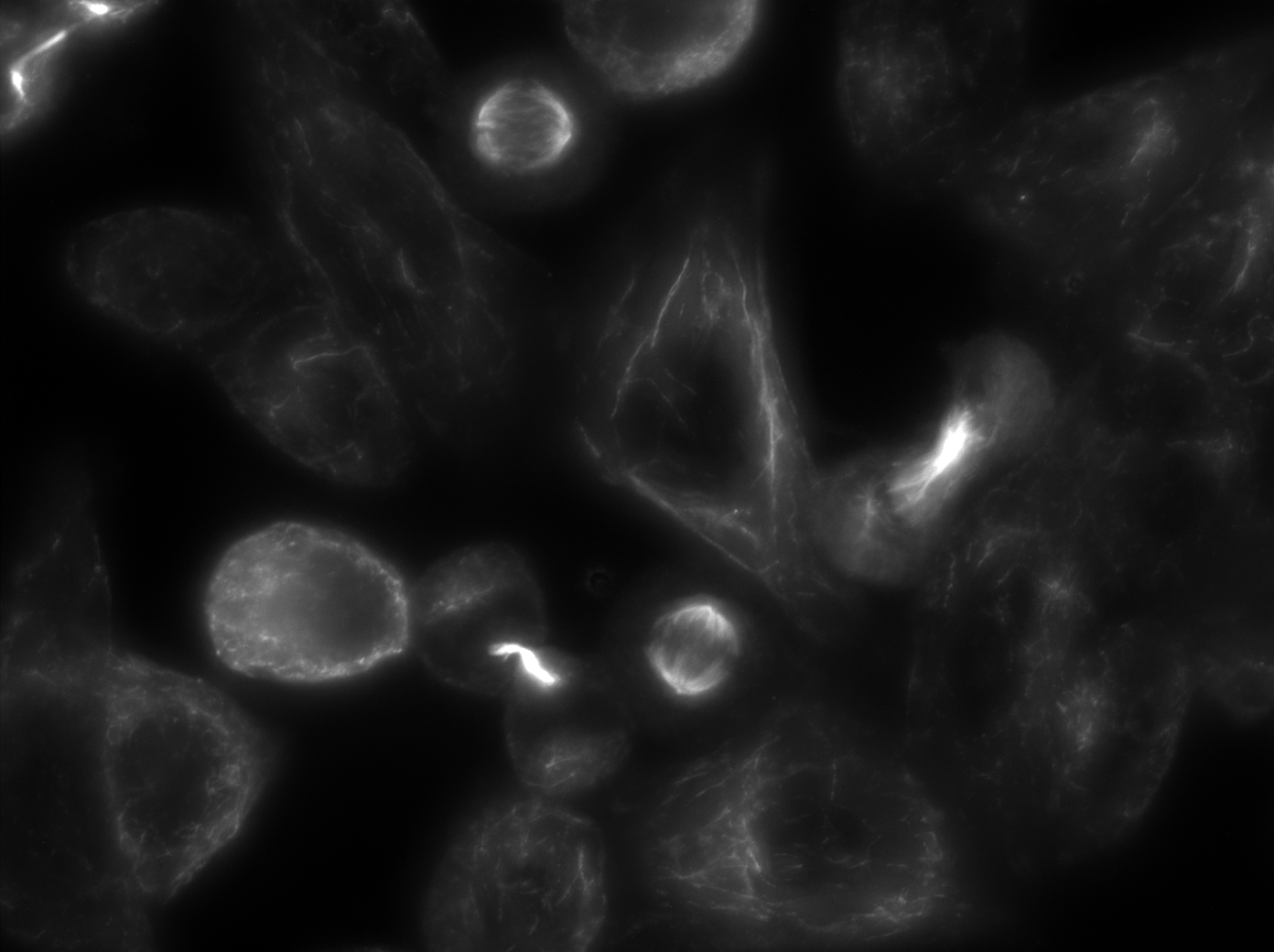

Supplement: Supplementary file 24 — Source data Fig. 6 part 5 [file 44319_2026_742_MOESM24_ESM.zip › Figure 6 Part 5/Fig 6efg TPGS1-KO TPGS1 rescue experiments part 3/R2R3/TPGS1-KO Untransfected actub 7-31-25 R2 ET8ET9.Project Maximum Z_XY1756413188_Z0_T0_C2.tif]

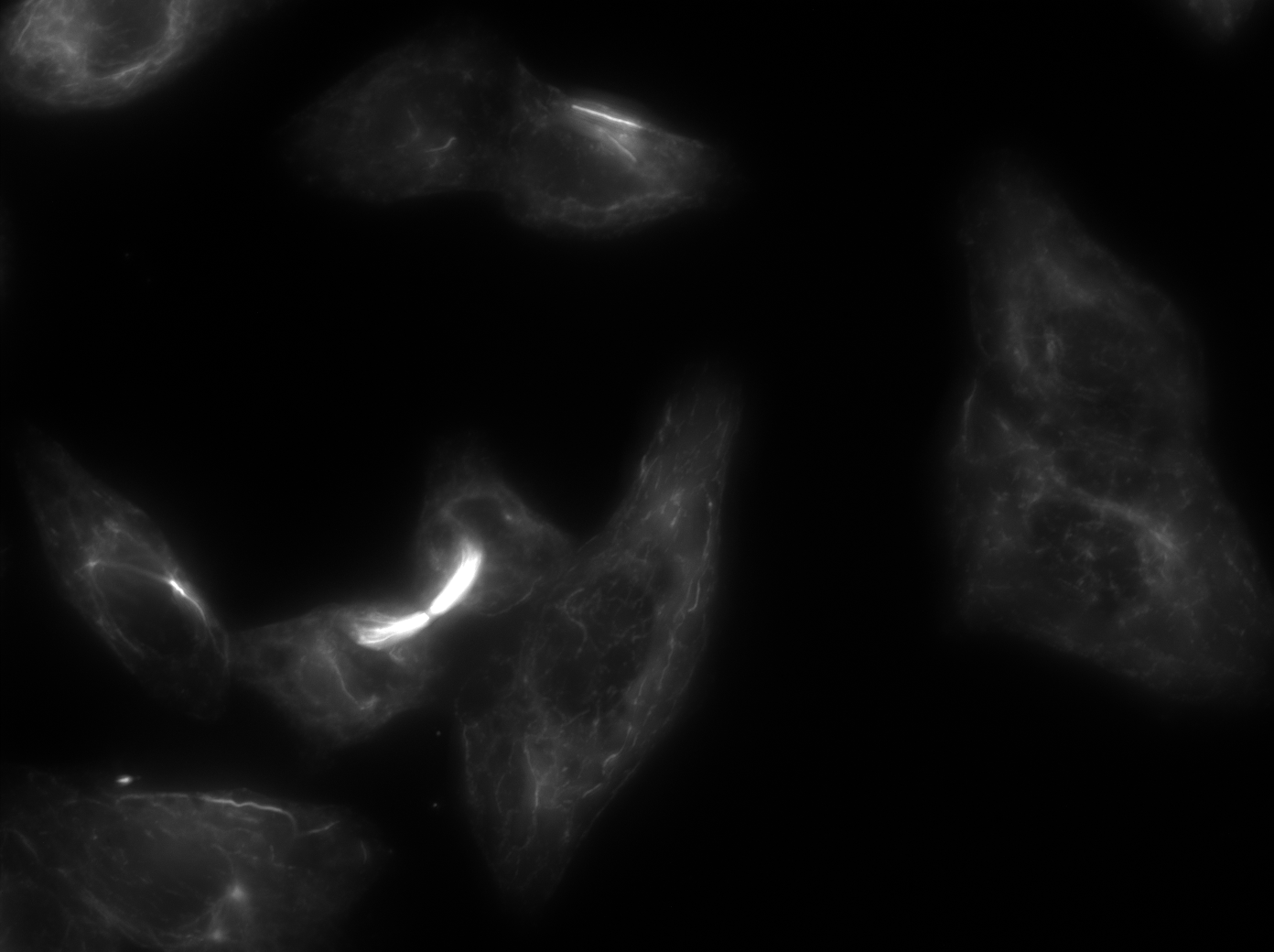

Supplement: Supplementary file 24 — Source data Fig. 6 part 5 [file 44319_2026_742_MOESM24_ESM.zip › Figure 6 Part 5/Fig 6efg TPGS1-KO TPGS1 rescue experiments part 3/R2R3/TPGS1-KO Untransfected actub 7-31-25 R2 LT5.Project Maximum Z_XY1756412619_Z0_T0_C2.tif]

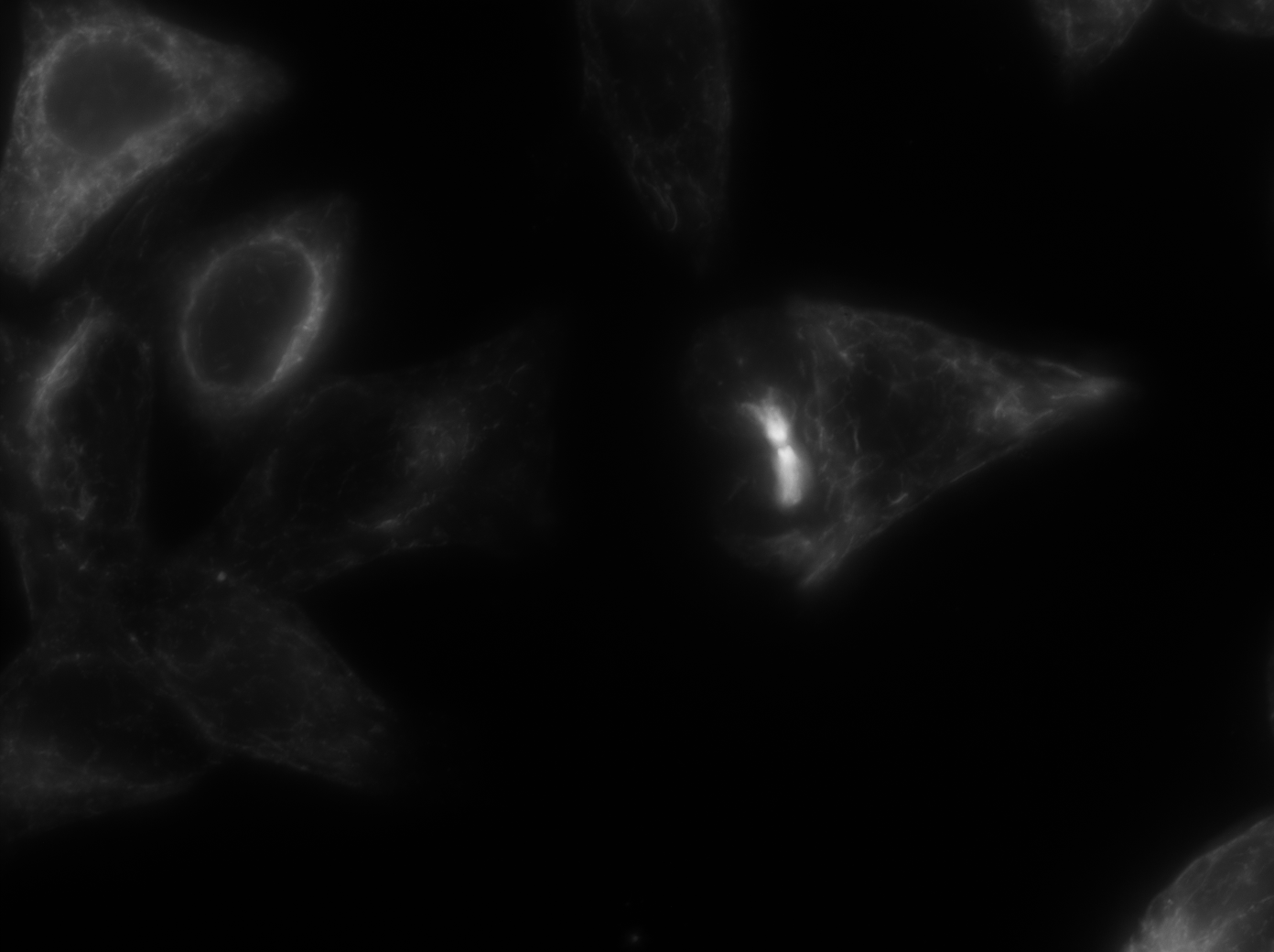

Supplement: Supplementary file 24 — Source data Fig. 6 part 5 [file 44319_2026_742_MOESM24_ESM.zip › Figure 6 Part 5/Fig 6efg TPGS1-KO TPGS1 rescue experiments part 3/R2R3/TPGS1-KO untransfected actub 7-31-25 R3 ET10.Project Maximum Z_XY1756506270_Z0_T0_C2.tif]

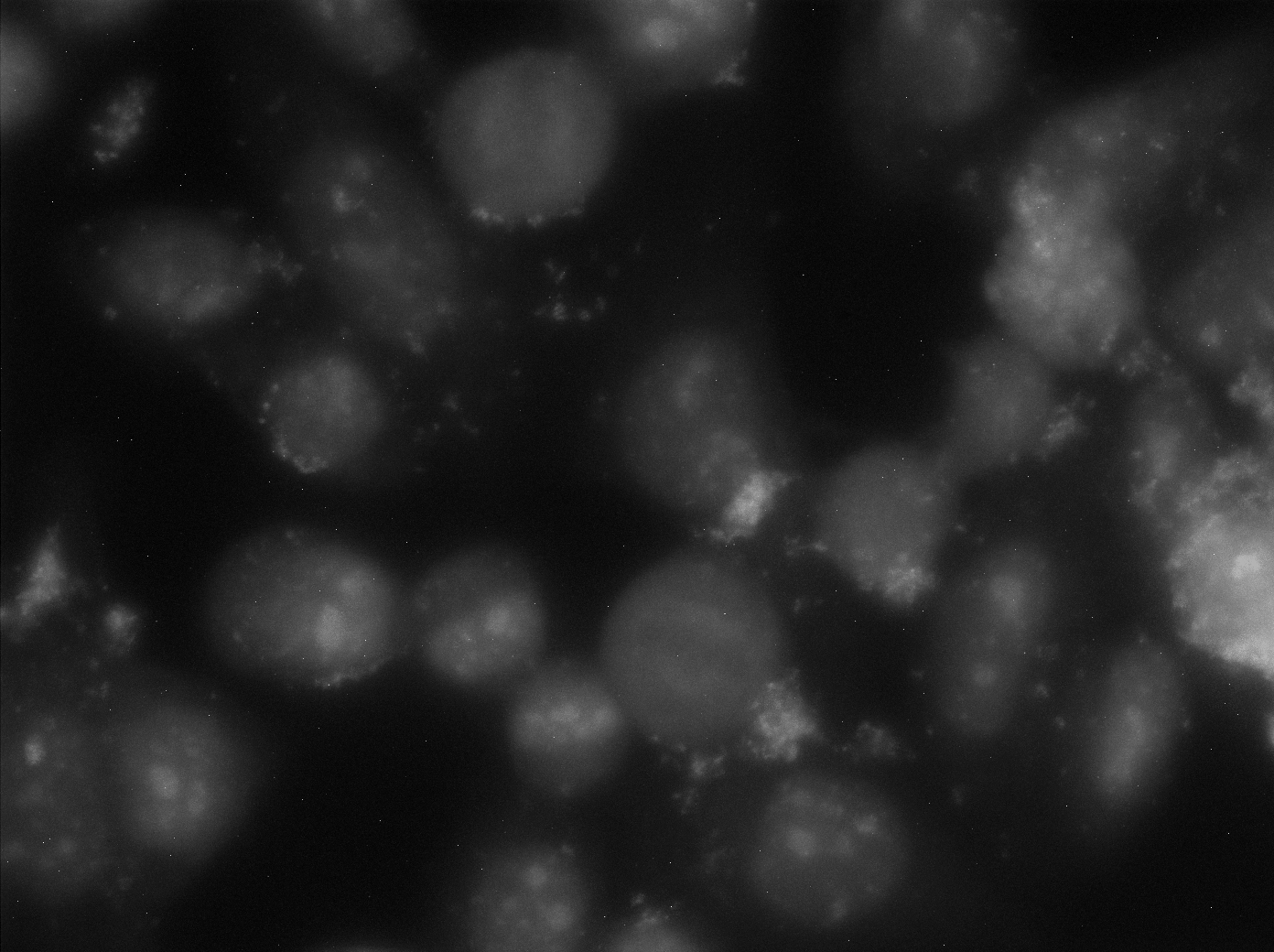

Supplement: Supplementary file 24 — Source data Fig. 6 part 5 [file 44319_2026_742_MOESM24_ESM.zip › Figure 6 Part 5/Fig 6efg TPGS1-KO TPGS1 rescue experiments part 3/R2R3/TPGS1-KO Untransfected actub 7-31-25 R2 ET8ET9.Project Maximum Z_XY1756413188_Z0_T0_C1.tif]

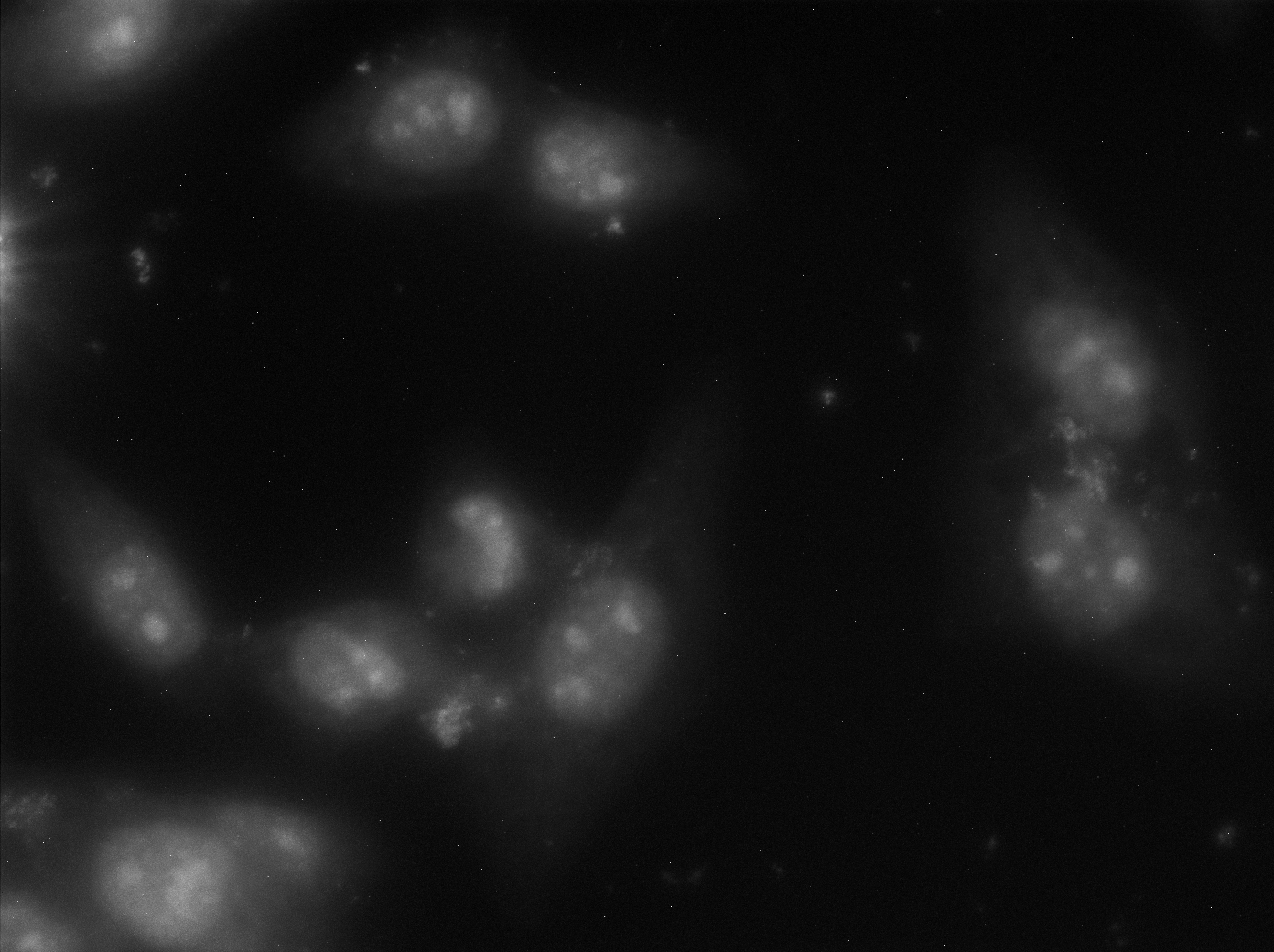

Supplement: Supplementary file 24 — Source data Fig. 6 part 5 [file 44319_2026_742_MOESM24_ESM.zip › Figure 6 Part 5/Fig 6efg TPGS1-KO TPGS1 rescue experiments part 3/R2R3/TPGS1-KO Untransfected actub 7-31-25 R2 LT5.Project Maximum Z_XY1756412619_Z0_T0_C1.tif]

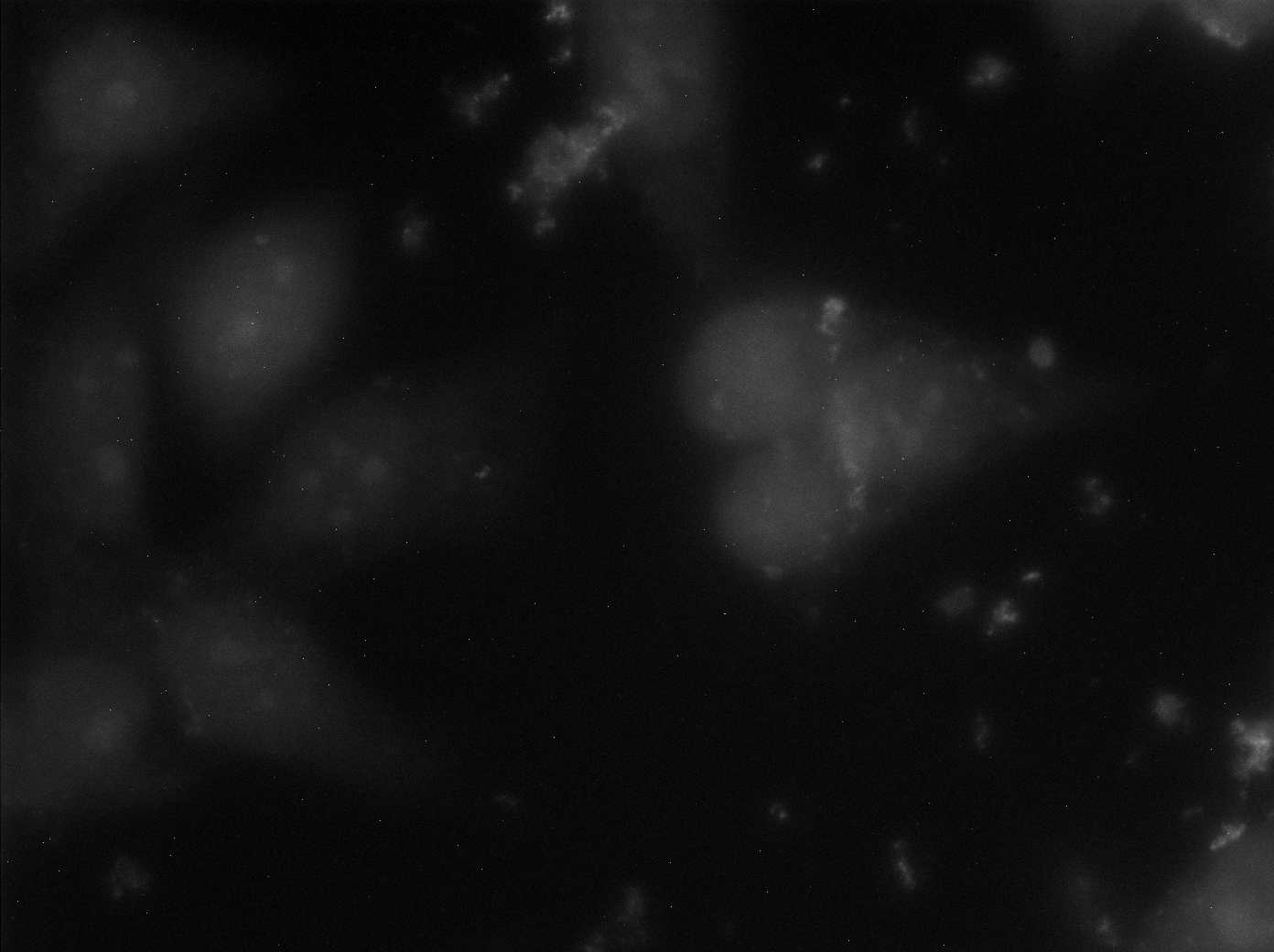

Supplement: Supplementary file 24 — Source data Fig. 6 part 5 [file 44319_2026_742_MOESM24_ESM.zip › Figure 6 Part 5/Fig 6efg TPGS1-KO TPGS1 rescue experiments part 3/R2R3/TPGS1-KO untransfected actub 7-31-25 R3 ET10.Project Maximum Z_XY1756506270_Z0_T0_C1.tif]

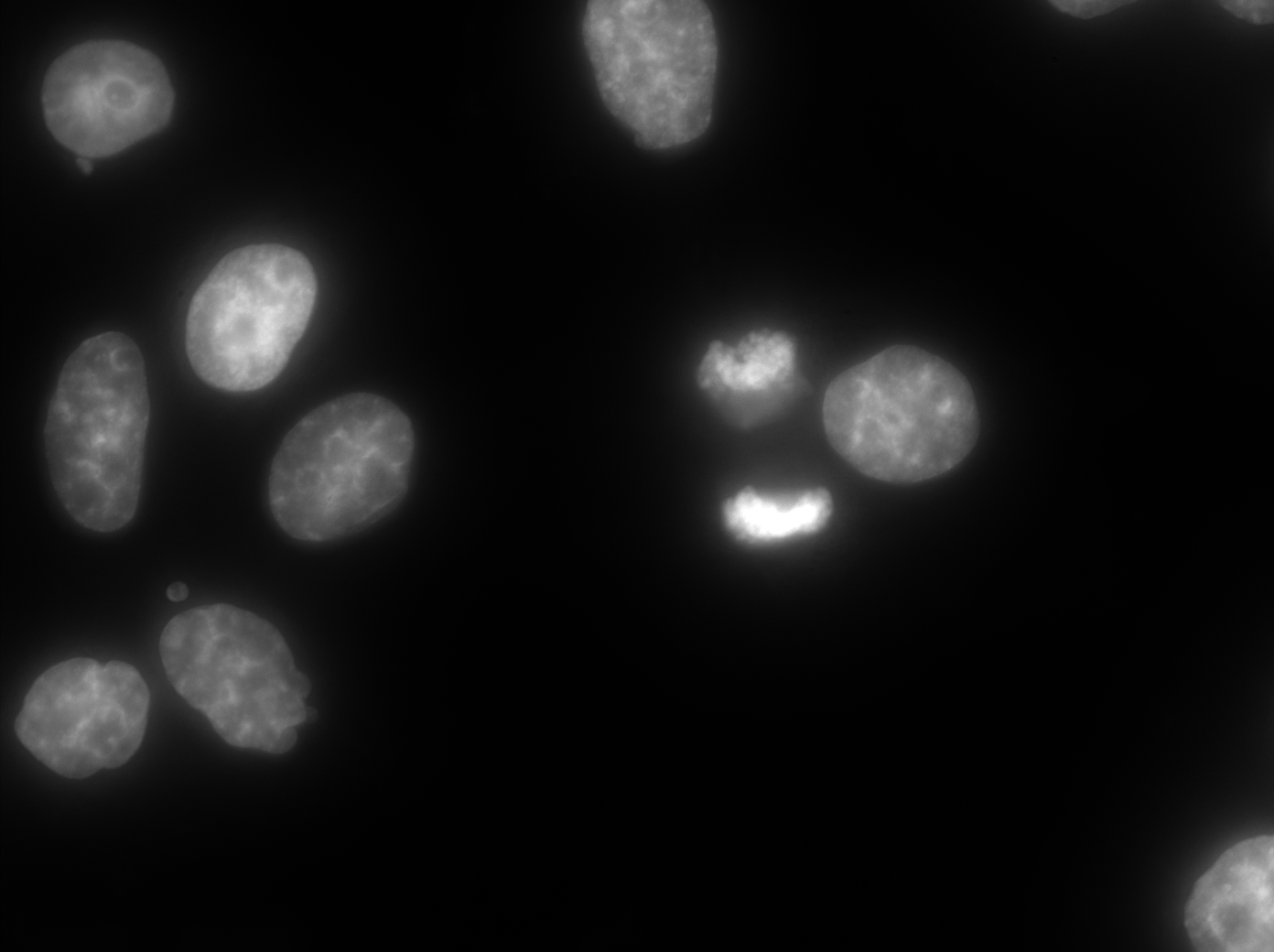

Supplement: Supplementary file 24 — Source data Fig. 6 part 5 [file 44319_2026_742_MOESM24_ESM.zip › Figure 6 Part 5/Fig 6efg TPGS1-KO TPGS1 rescue experiments part 3/R2R3/TPGS1-KO untransfected actub 7-31-25 R3 ET10.Project Maximum Z_XY1756506270_Z0_T0_C0.tif]

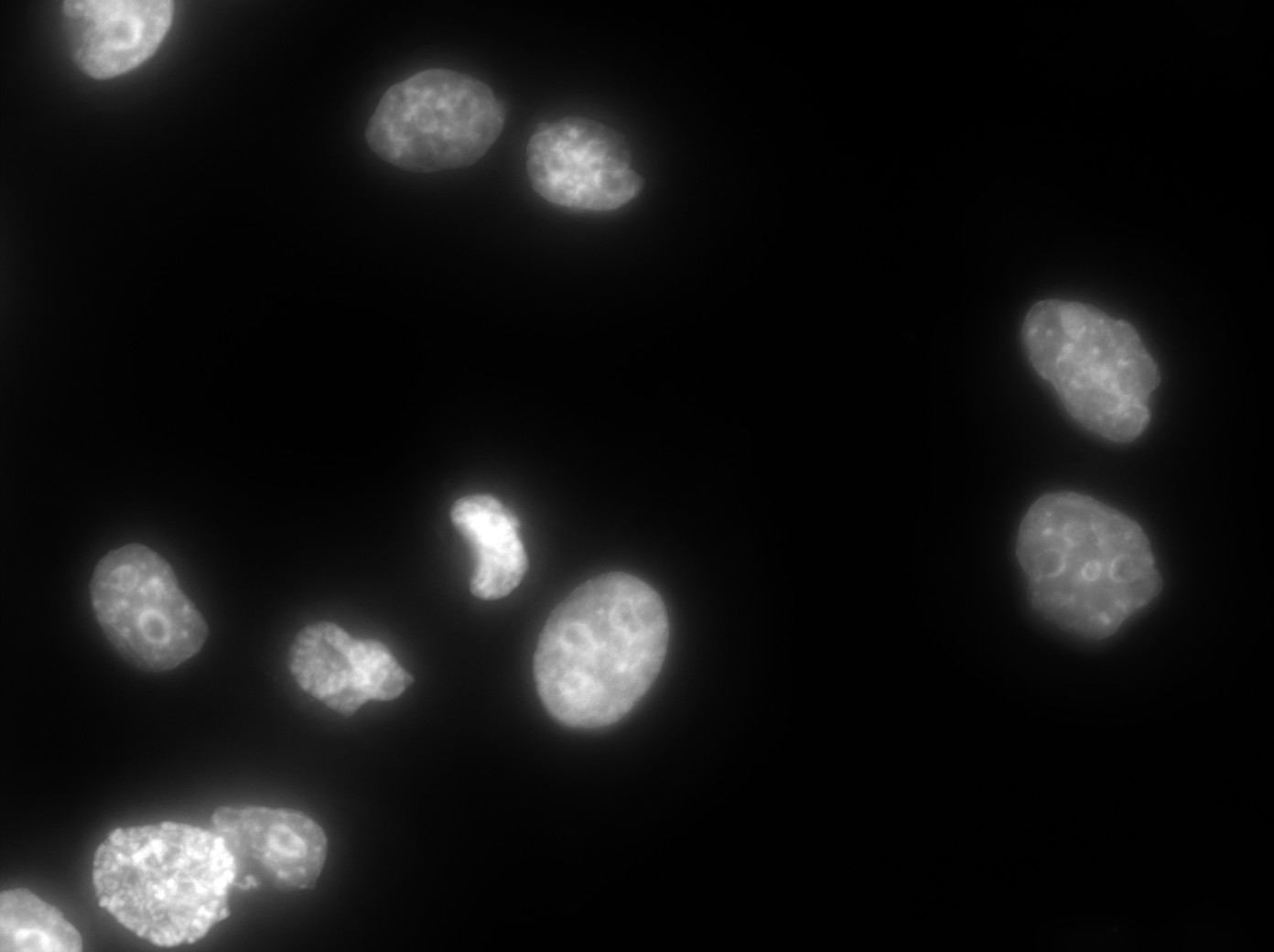

Supplement: Supplementary file 24 — Source data Fig. 6 part 5 [file 44319_2026_742_MOESM24_ESM.zip › Figure 6 Part 5/Fig 6efg TPGS1-KO TPGS1 rescue experiments part 3/R2R3/TPGS1-KO Untransfected actub 7-31-25 R2 LT5.Project Maximum Z_XY1756412619_Z0_T0_C0.tif]

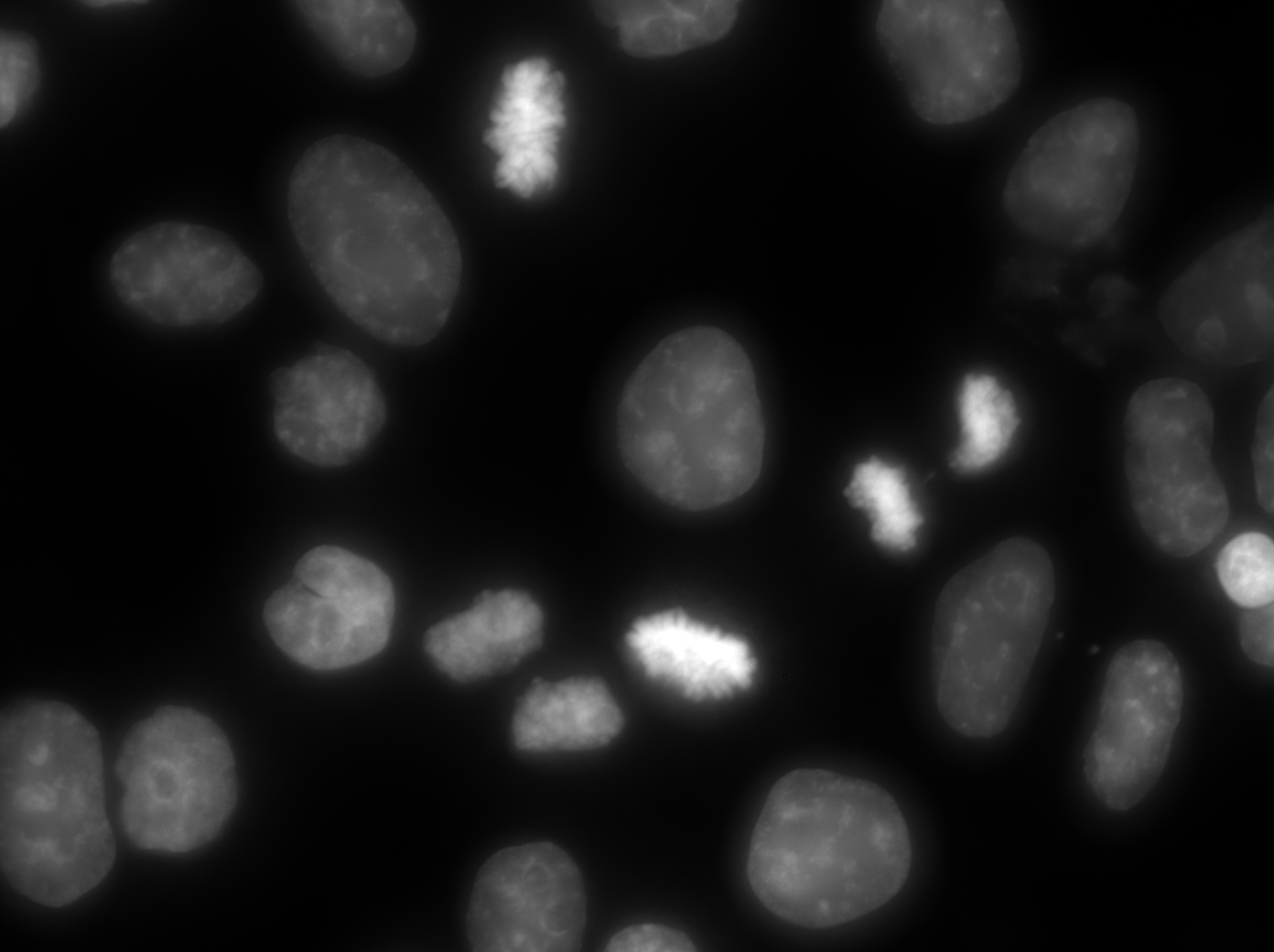

Supplement: Supplementary file 24 — Source data Fig. 6 part 5 [file 44319_2026_742_MOESM24_ESM.zip › Figure 6 Part 5/Fig 6efg TPGS1-KO TPGS1 rescue experiments part 3/R2R3/TPGS1-KO Untransfected actub 7-31-25 R2 ET8ET9.Project Maximum Z_XY1756413188_Z0_T0_C0.tif]

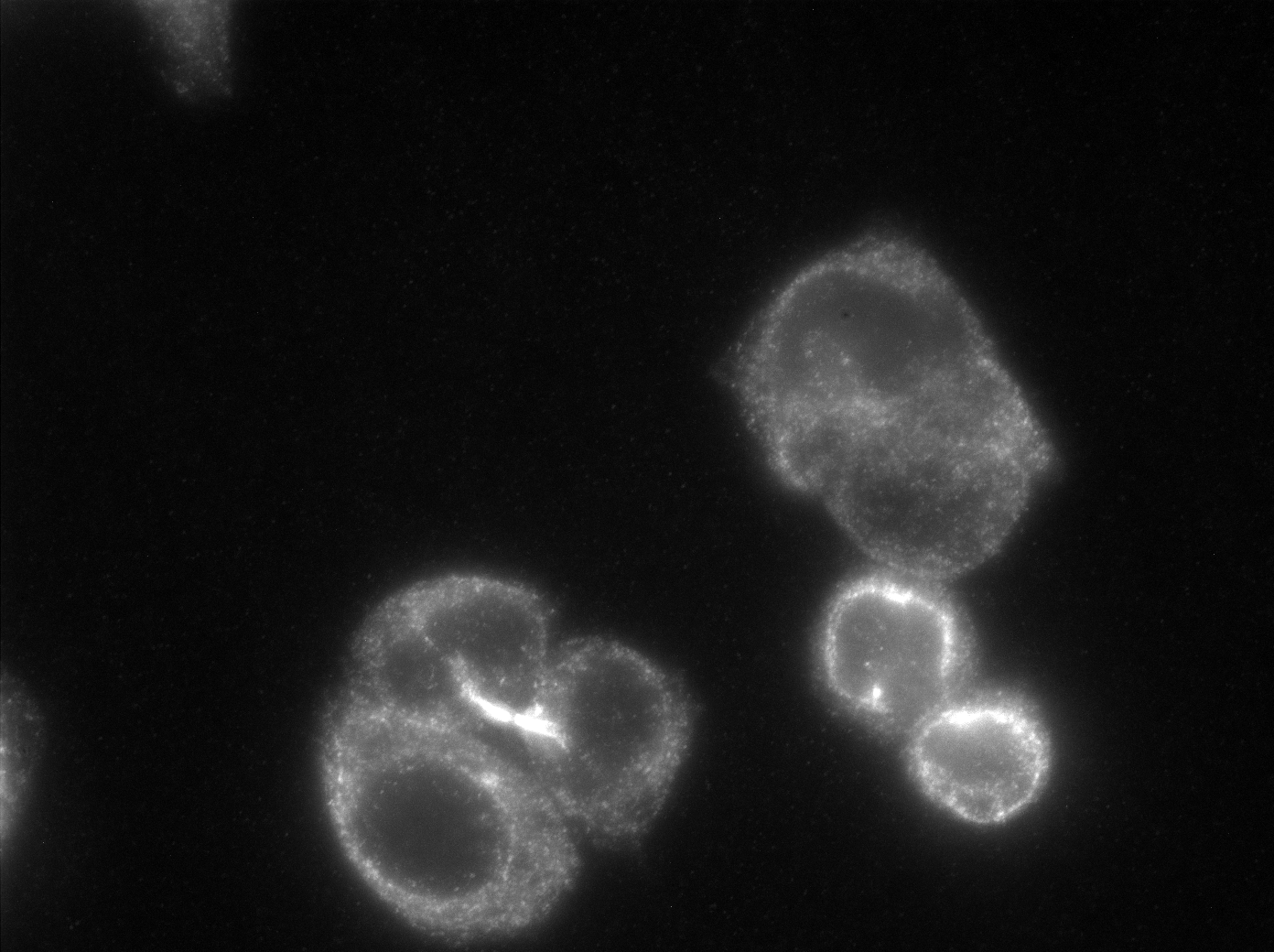

Supplement: Supplementary file 25 — Source data Fig. 7 part 1 [file 44319_2026_742_MOESM25_ESM.zip › Figure 7 Part 1/Fig 7acd Cas9 and TPGS1-ko rGT335 atubulin/Cas9 GT335recomb atub 3-24-25 R3 LT2.Project Maximum Z_XY1742848702_Z0_T0_C2.tif]

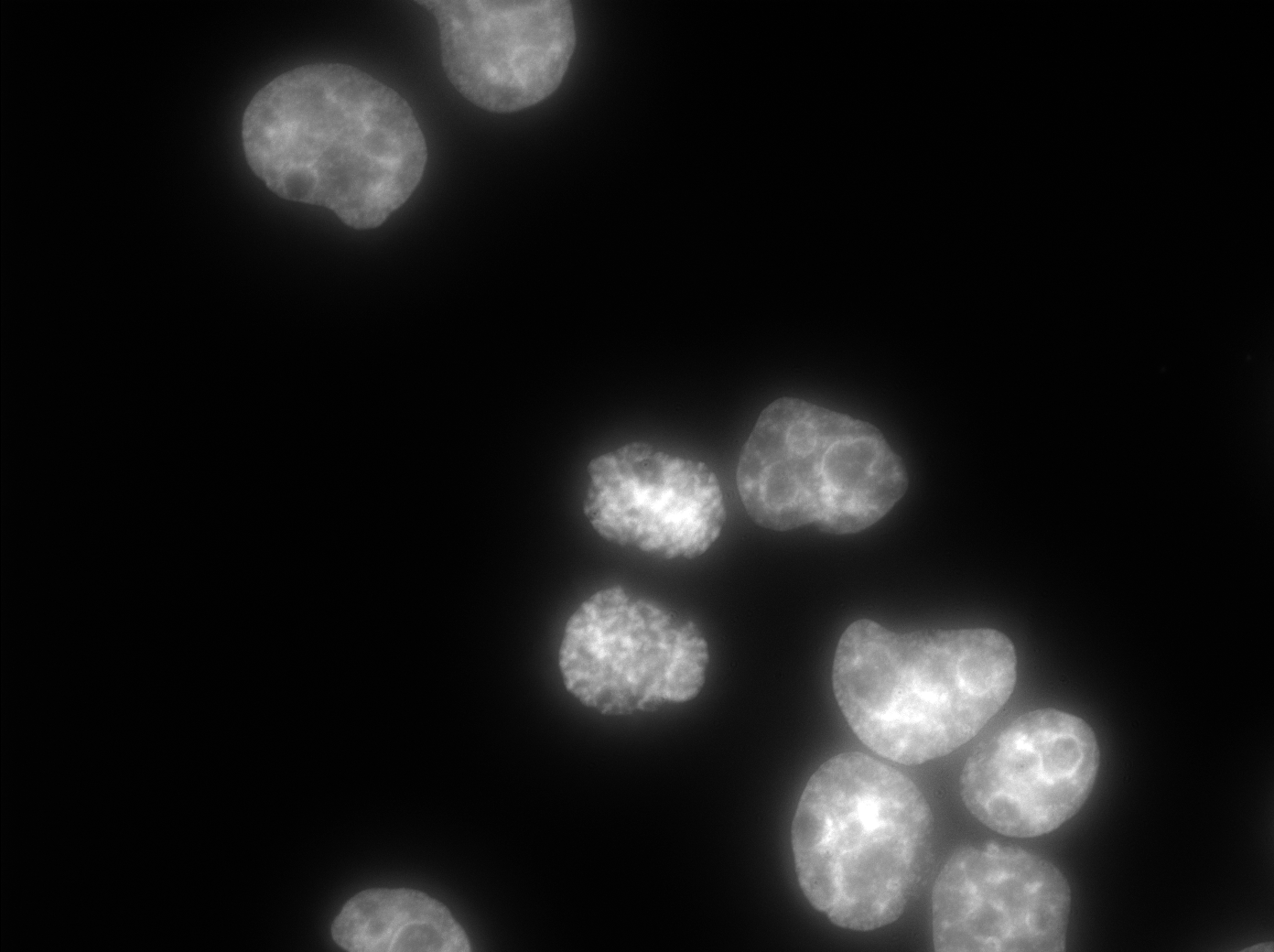

Supplement: Supplementary file 25 — Source data Fig. 7 part 1 [file 44319_2026_742_MOESM25_ESM.zip › Figure 7 Part 1/Fig 7acd Cas9 and TPGS1-ko rGT335 atubulin/Cas9 GT335recomb atub 3-24-25 R2 ET6.Project Maximum Z_XY1742846357_Z0_T0_C0.tif]

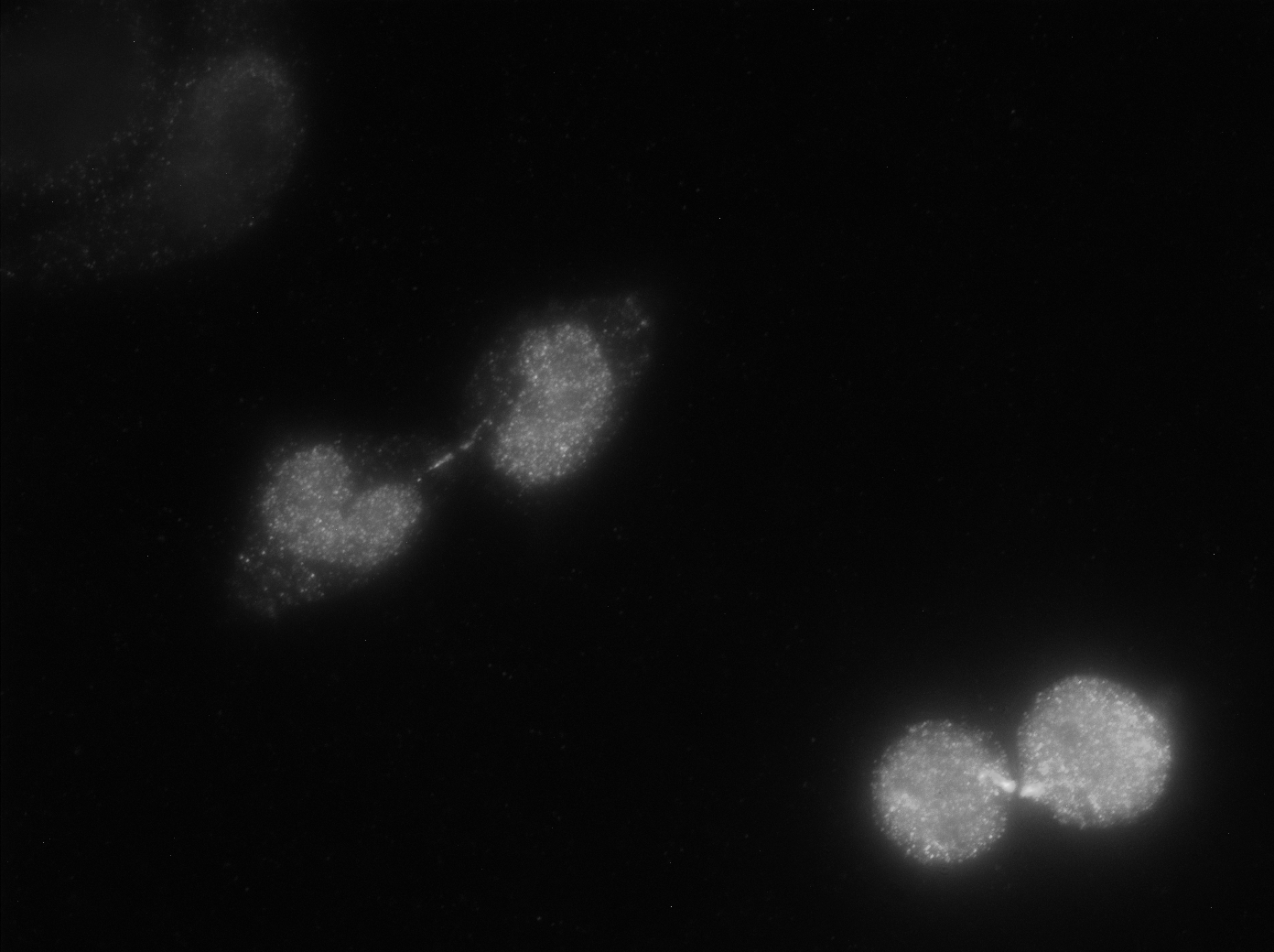

Supplement: Supplementary file 25 — Source data Fig. 7 part 1 [file 44319_2026_742_MOESM25_ESM.zip › Figure 7 Part 1/Fig 7acd Cas9 and TPGS1-ko rGT335 atubulin/Cas9 GT335recomb atub 3-24-25 R1 ET10 LT9.Project Maximum Z_XY1742836532_Z0_T0_C1.tif]

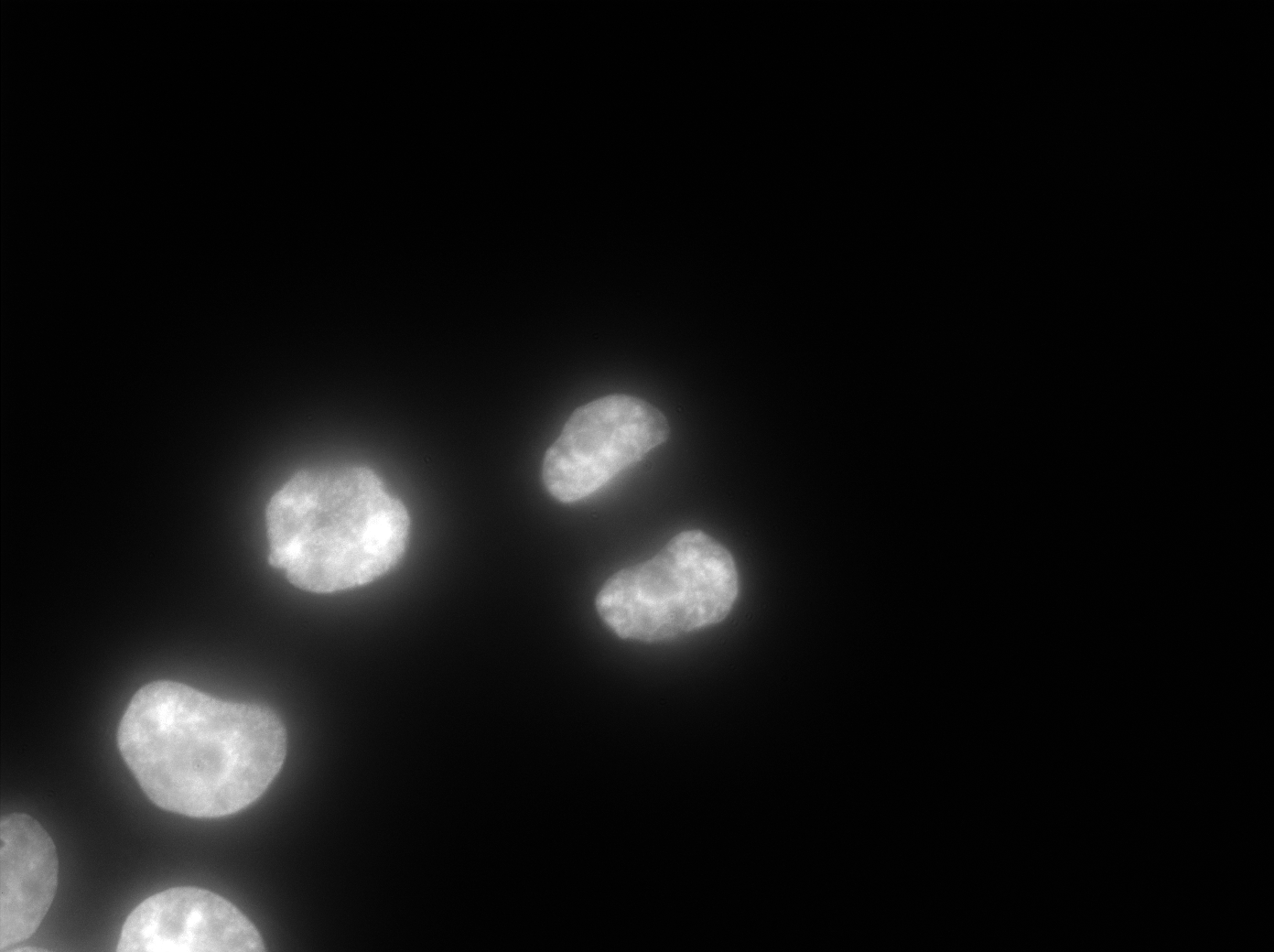

Supplement: Supplementary file 25 — Source data Fig. 7 part 1 [file 44319_2026_742_MOESM25_ESM.zip › Figure 7 Part 1/Fig 7acd Cas9 and TPGS1-ko rGT335 atubulin/Cas9 GT335recomb atub 3-24-25 R3 ET9.Project Maximum Z_XY1742850787_Z0_T0_C0.tif]

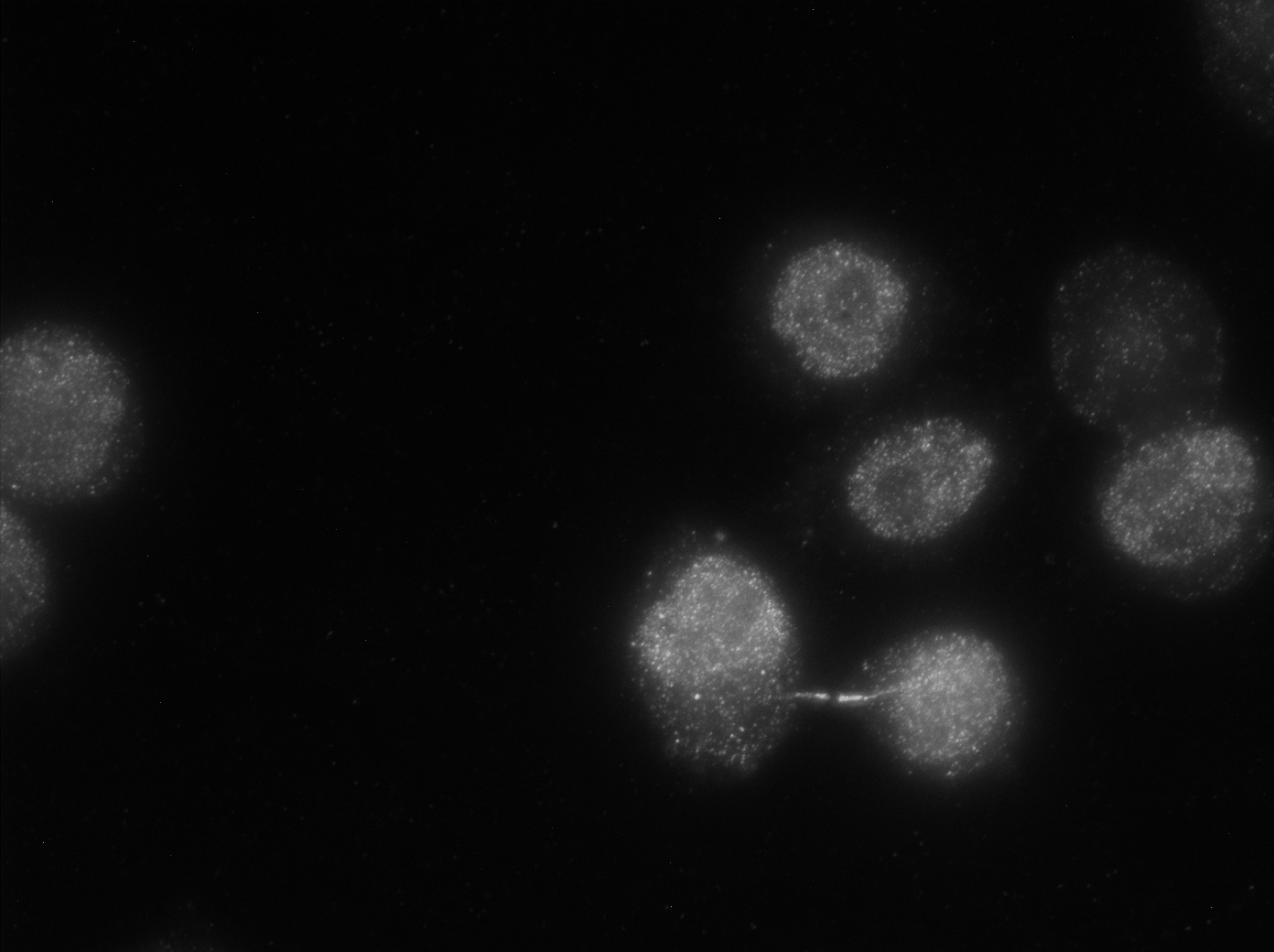

Supplement: Supplementary file 25 — Source data Fig. 7 part 1 [file 44319_2026_742_MOESM25_ESM.zip › Figure 7 Part 1/Fig 7acd Cas9 and TPGS1-ko rGT335 atubulin/Cas9 GT335recomb atub 3-24-25 R1 LT2.Project Maximum Z_XY1742834932_Z0_T0_C1.tif]

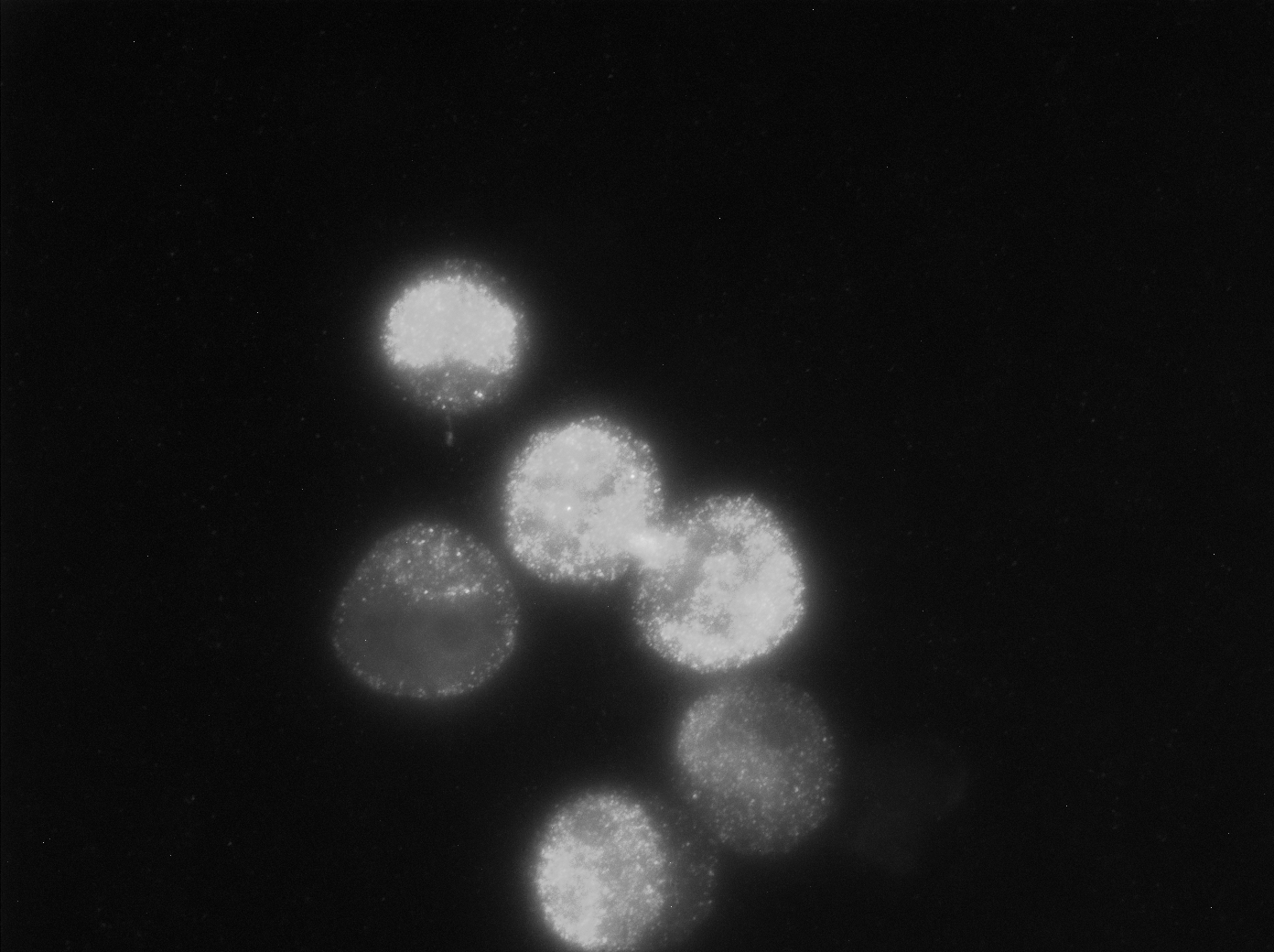

Supplement: Supplementary file 25 — Source data Fig. 7 part 1 [file 44319_2026_742_MOESM25_ESM.zip › Figure 7 Part 1/Fig 7acd Cas9 and TPGS1-ko rGT335 atubulin/Cas9 GT335recomb atub 3-24-25 R2 ET2.Project Maximum Z_XY1742845267_Z0_T0_C1.tif]

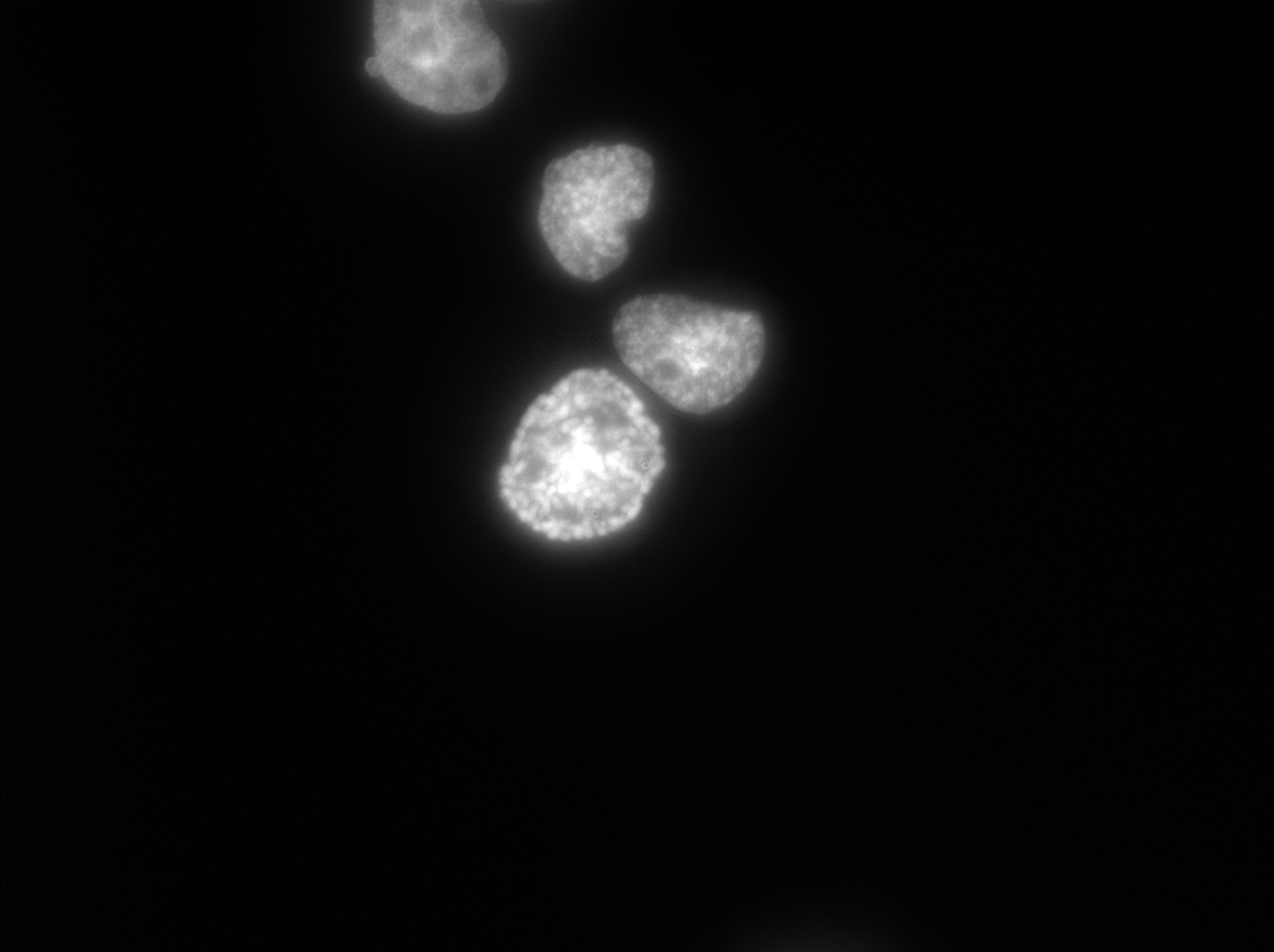

Supplement: Supplementary file 25 — Source data Fig. 7 part 1 [file 44319_2026_742_MOESM25_ESM.zip › Figure 7 Part 1/Fig 7acd Cas9 and TPGS1-ko rGT335 atubulin/Cas9 GT335recomb atub 3-24-25 R2 LT3 P1.Project Maximum Z_XY1742846092_Z0_T0_C0.tif]

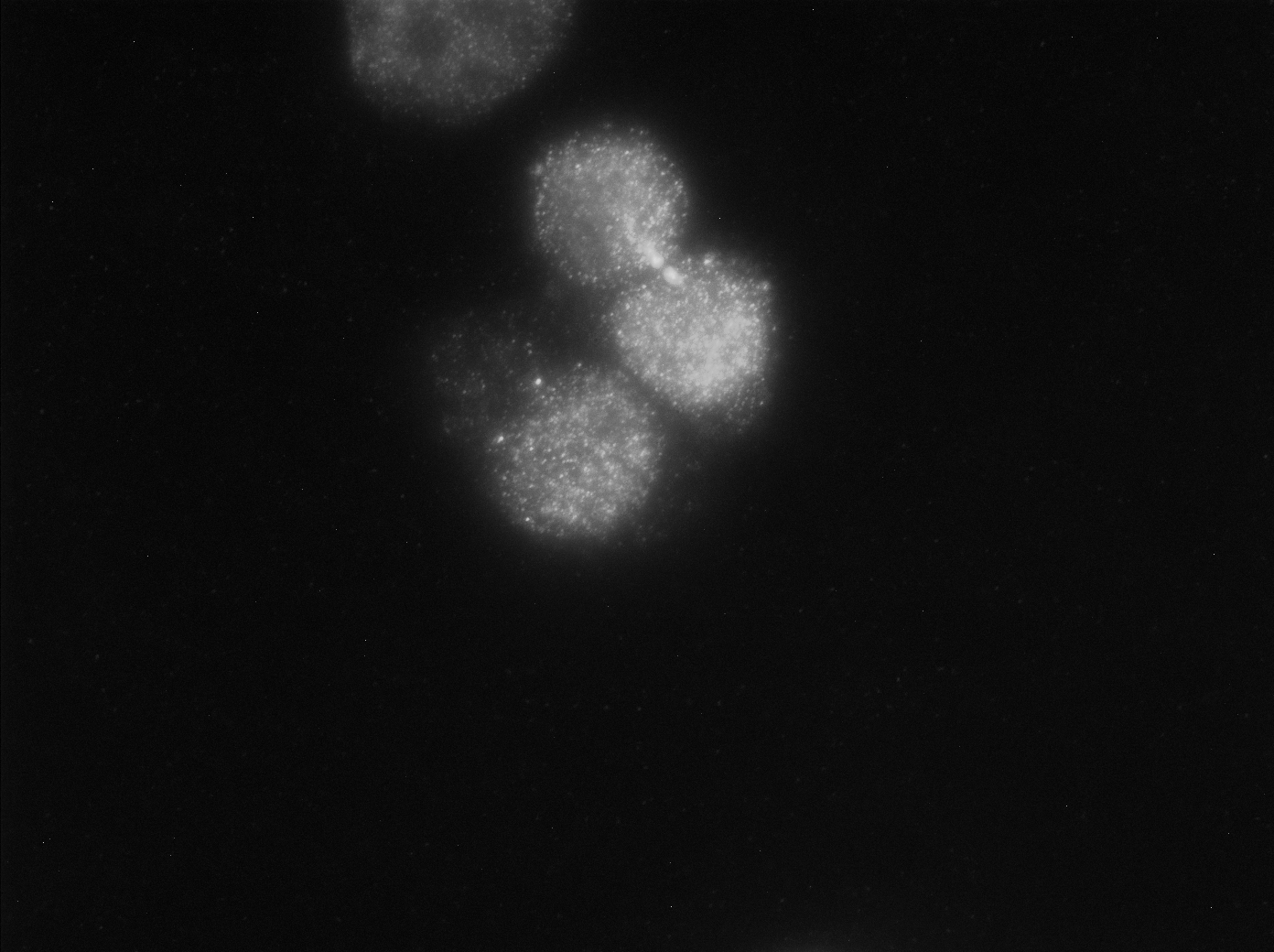

Supplement: Supplementary file 25 — Source data Fig. 7 part 1 [file 44319_2026_742_MOESM25_ESM.zip › Figure 7 Part 1/Fig 7acd Cas9 and TPGS1-ko rGT335 atubulin/Cas9 GT335recomb atub 3-24-25 R2 LT3 P1.Project Maximum Z_XY1742846092_Z0_T0_C1.tif]

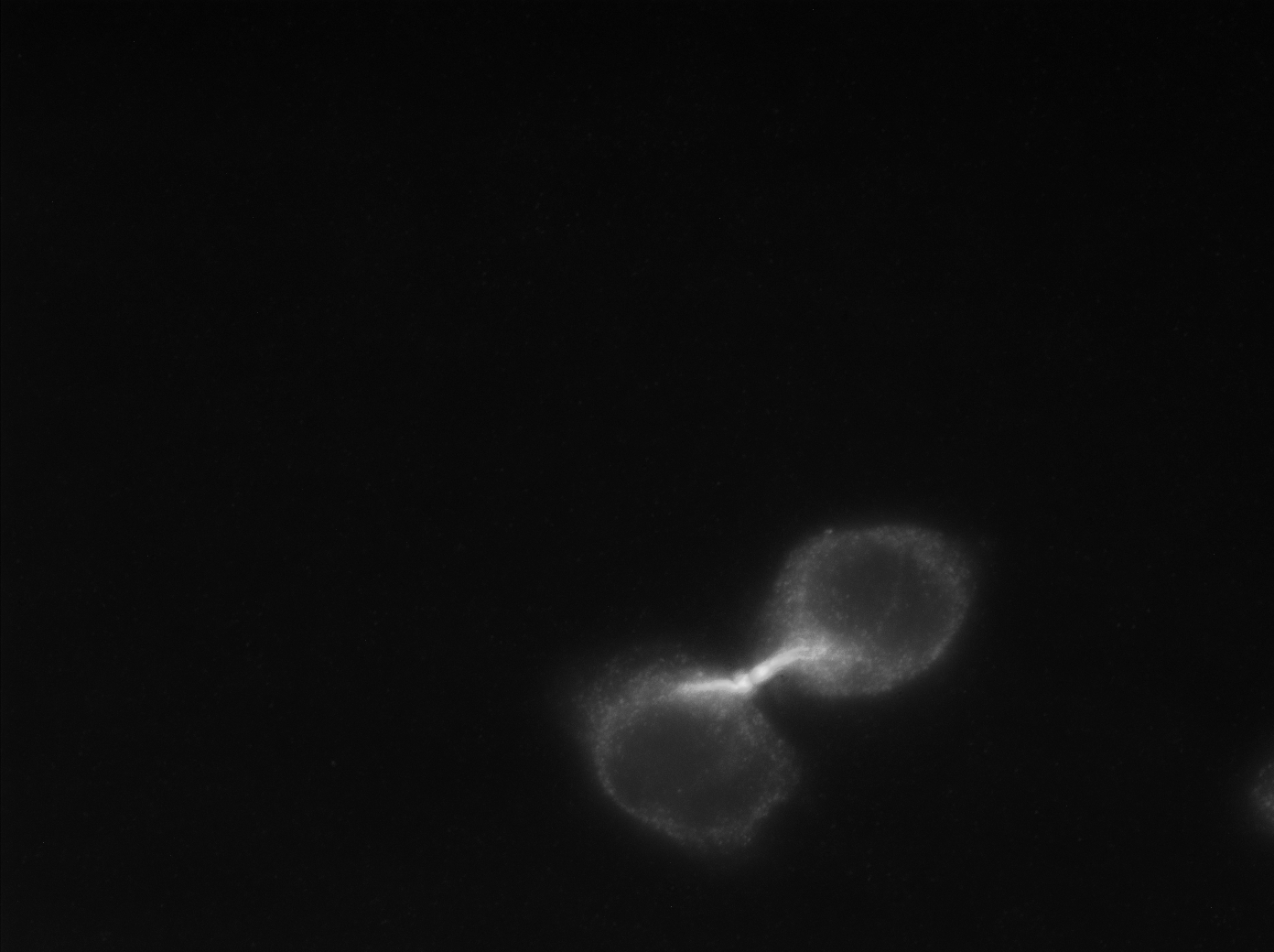

Supplement: Supplementary file 25 — Source data Fig. 7 part 1 [file 44319_2026_742_MOESM25_ESM.zip › Figure 7 Part 1/Fig 7acd Cas9 and TPGS1-ko rGT335 atubulin/Cas9 GT335recomb atub 3-24-25 R2 LT9.Project Maximum Z_XY1742847464_Z0_T0_C2.tif]

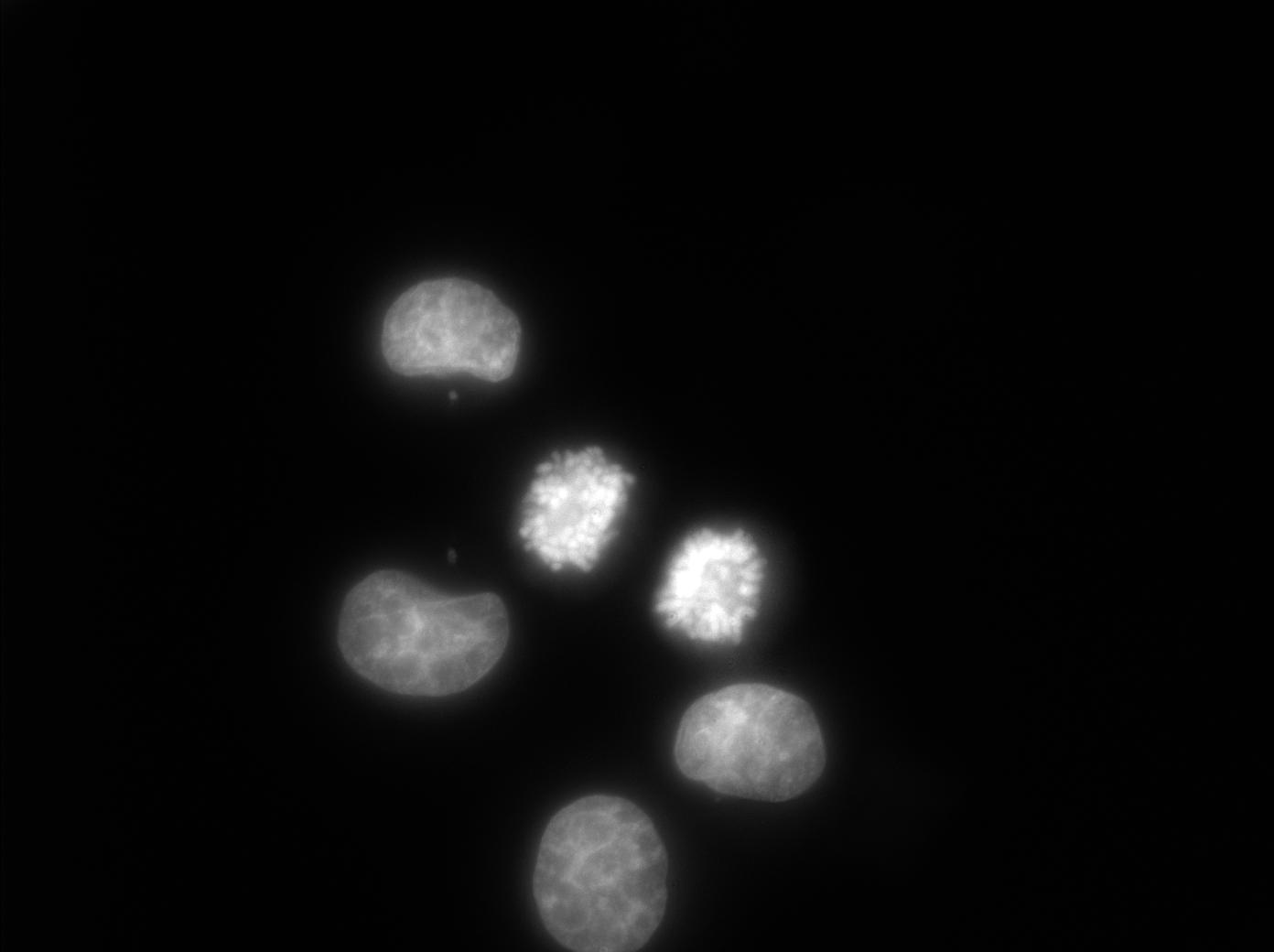

Supplement: Supplementary file 25 — Source data Fig. 7 part 1 [file 44319_2026_742_MOESM25_ESM.zip › Figure 7 Part 1/Fig 7acd Cas9 and TPGS1-ko rGT335 atubulin/Cas9 GT335recomb atub 3-24-25 R2 ET2.Project Maximum Z_XY1742845267_Z0_T0_C0.tif]

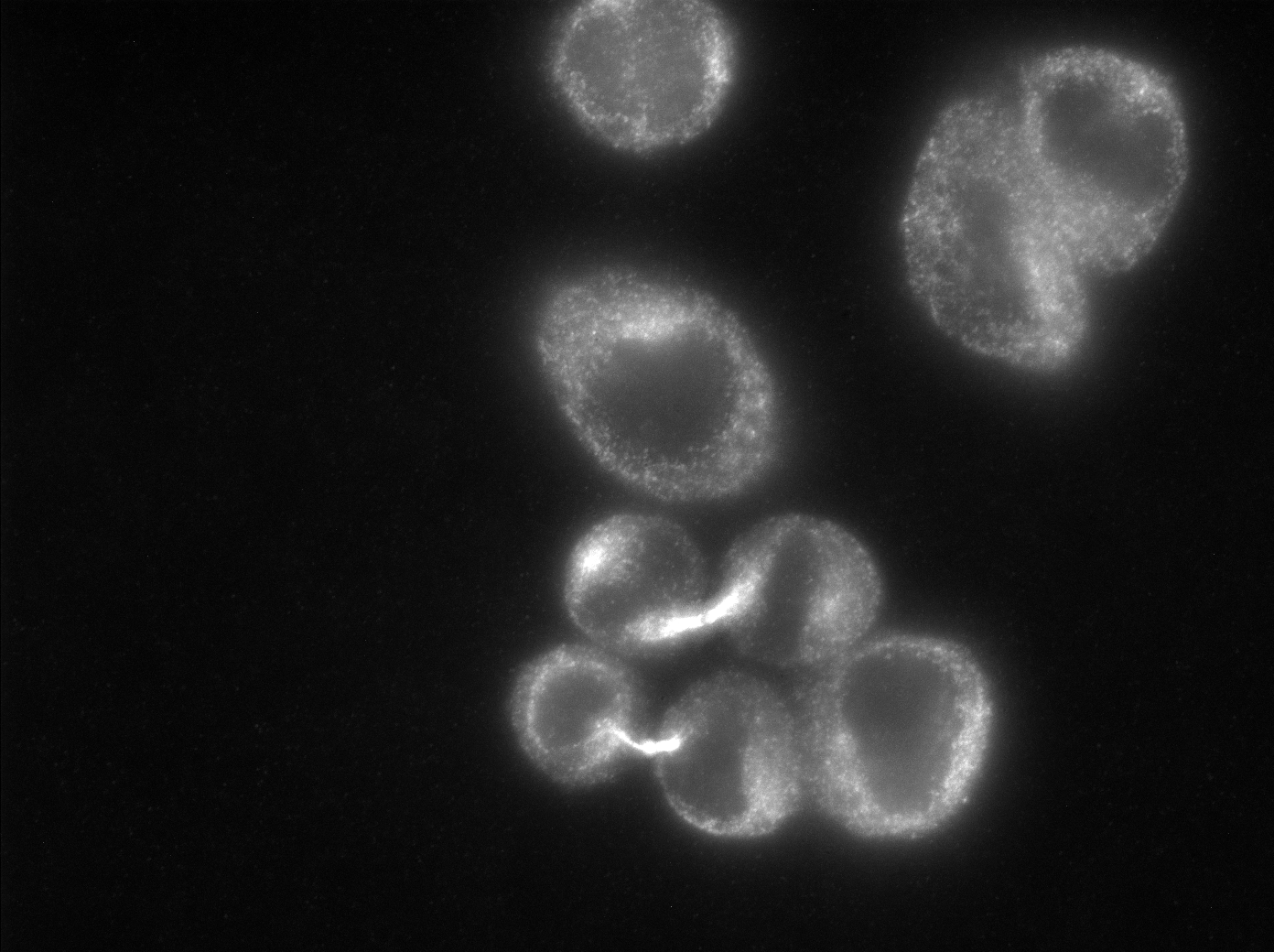

Supplement: Supplementary file 25 — Source data Fig. 7 part 1 [file 44319_2026_742_MOESM25_ESM.zip › Figure 7 Part 1/Fig 7acd Cas9 and TPGS1-ko rGT335 atubulin/Cas9 GT335recomb atub 3-24-25 R1 ET8 PA3.Project Maximum Z_XY1742836284_Z0_T0_C2.tif]

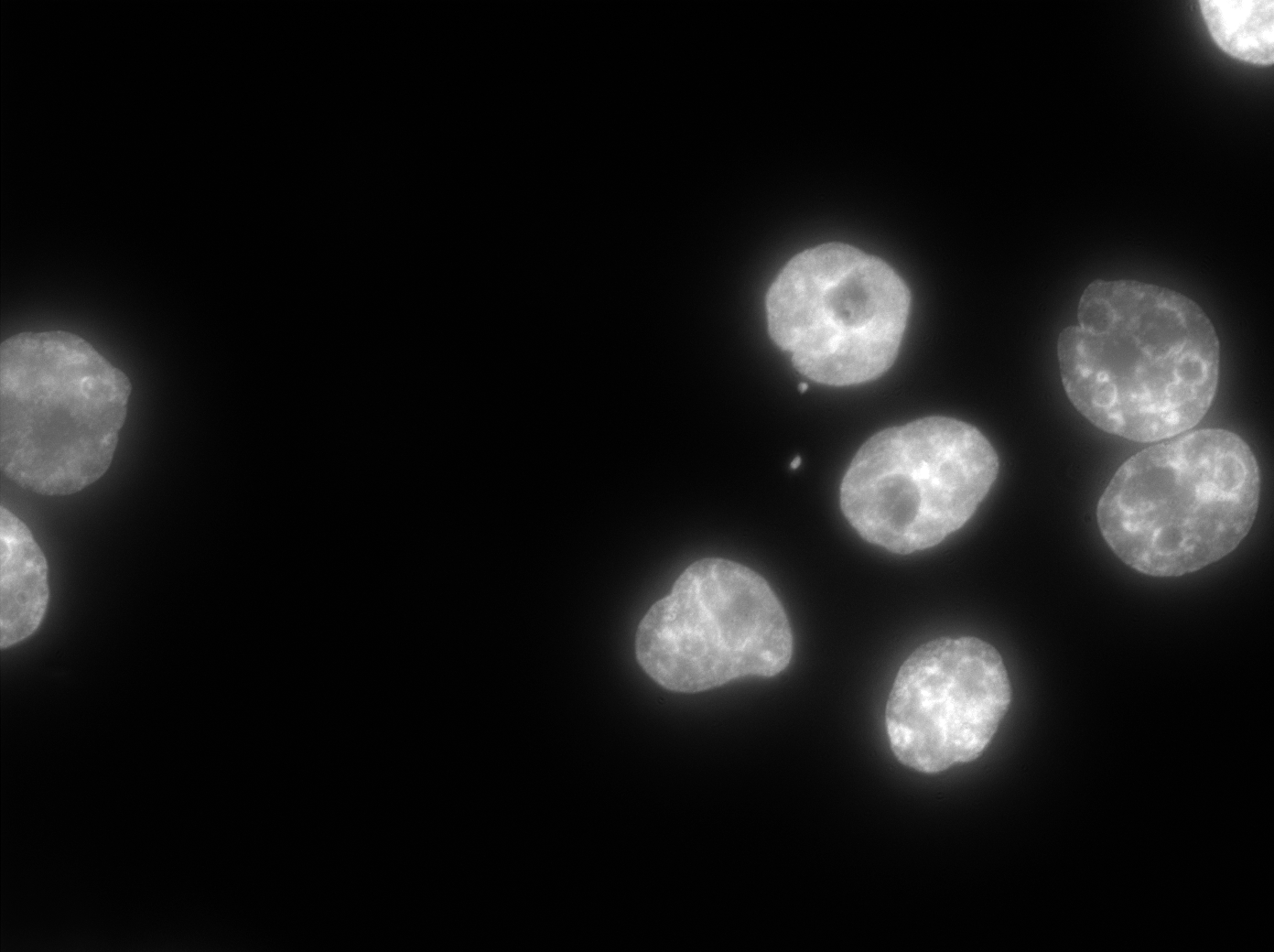

Supplement: Supplementary file 25 — Source data Fig. 7 part 1 [file 44319_2026_742_MOESM25_ESM.zip › Figure 7 Part 1/Fig 7acd Cas9 and TPGS1-ko rGT335 atubulin/Cas9 GT335recomb atub 3-24-25 R1 LT2.Project Maximum Z_XY1742834932_Z0_T0_C0.tif]

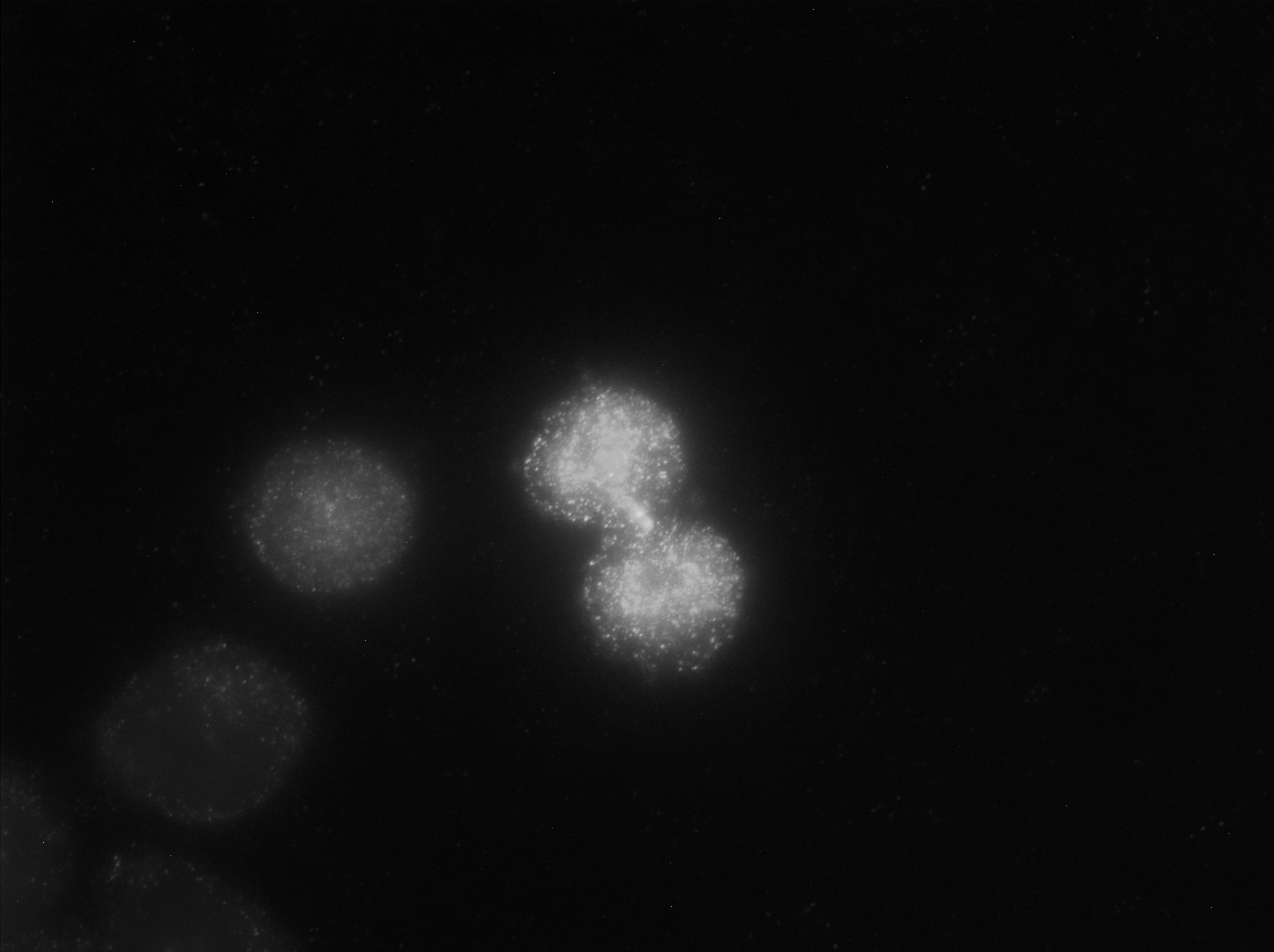

Supplement: Supplementary file 25 — Source data Fig. 7 part 1 [file 44319_2026_742_MOESM25_ESM.zip › Figure 7 Part 1/Fig 7acd Cas9 and TPGS1-ko rGT335 atubulin/Cas9 GT335recomb atub 3-24-25 R3 ET9.Project Maximum Z_XY1742850787_Z0_T0_C1.tif]

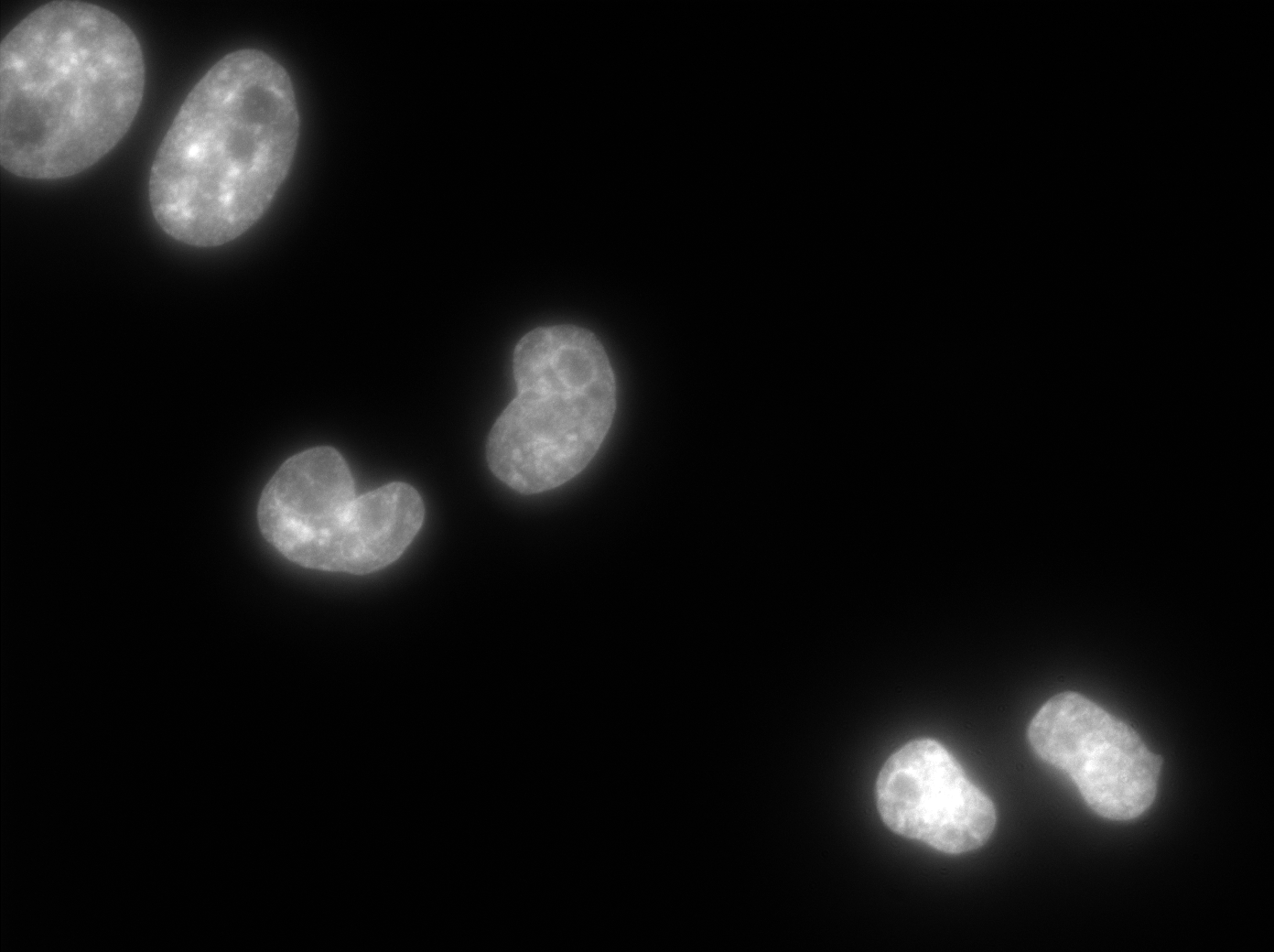

Supplement: Supplementary file 25 — Source data Fig. 7 part 1 [file 44319_2026_742_MOESM25_ESM.zip › Figure 7 Part 1/Fig 7acd Cas9 and TPGS1-ko rGT335 atubulin/Cas9 GT335recomb atub 3-24-25 R1 ET10 LT9.Project Maximum Z_XY1742836532_Z0_T0_C0.tif]

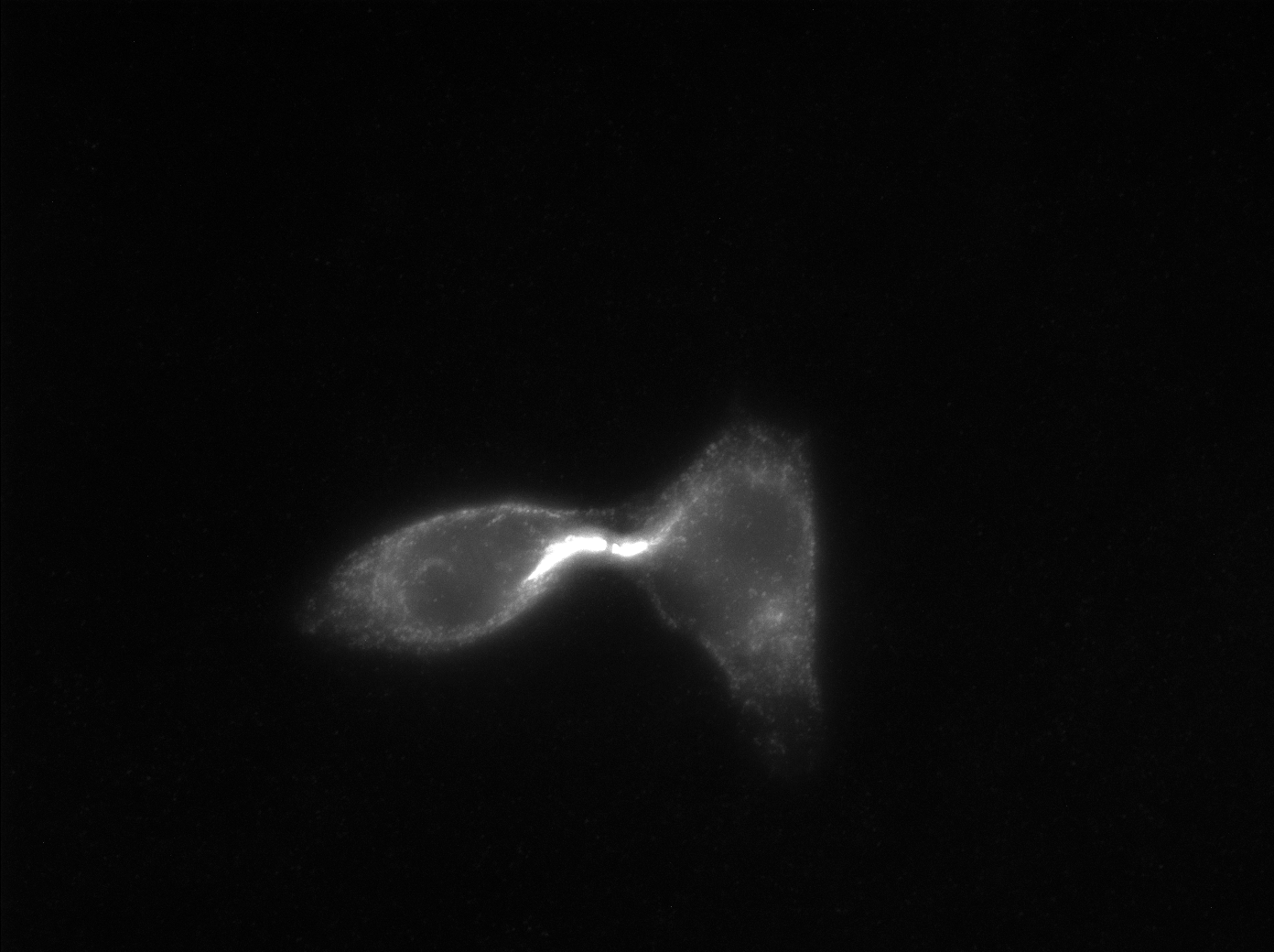

Supplement: Supplementary file 25 — Source data Fig. 7 part 1 [file 44319_2026_742_MOESM25_ESM.zip › Figure 7 Part 1/Fig 7acd Cas9 and TPGS1-ko rGT335 atubulin/Cas9 GT335recomb atub 3-24-25 R1 LT10.Project Maximum Z_XY1742836712_Z0_T0_C2.tif]

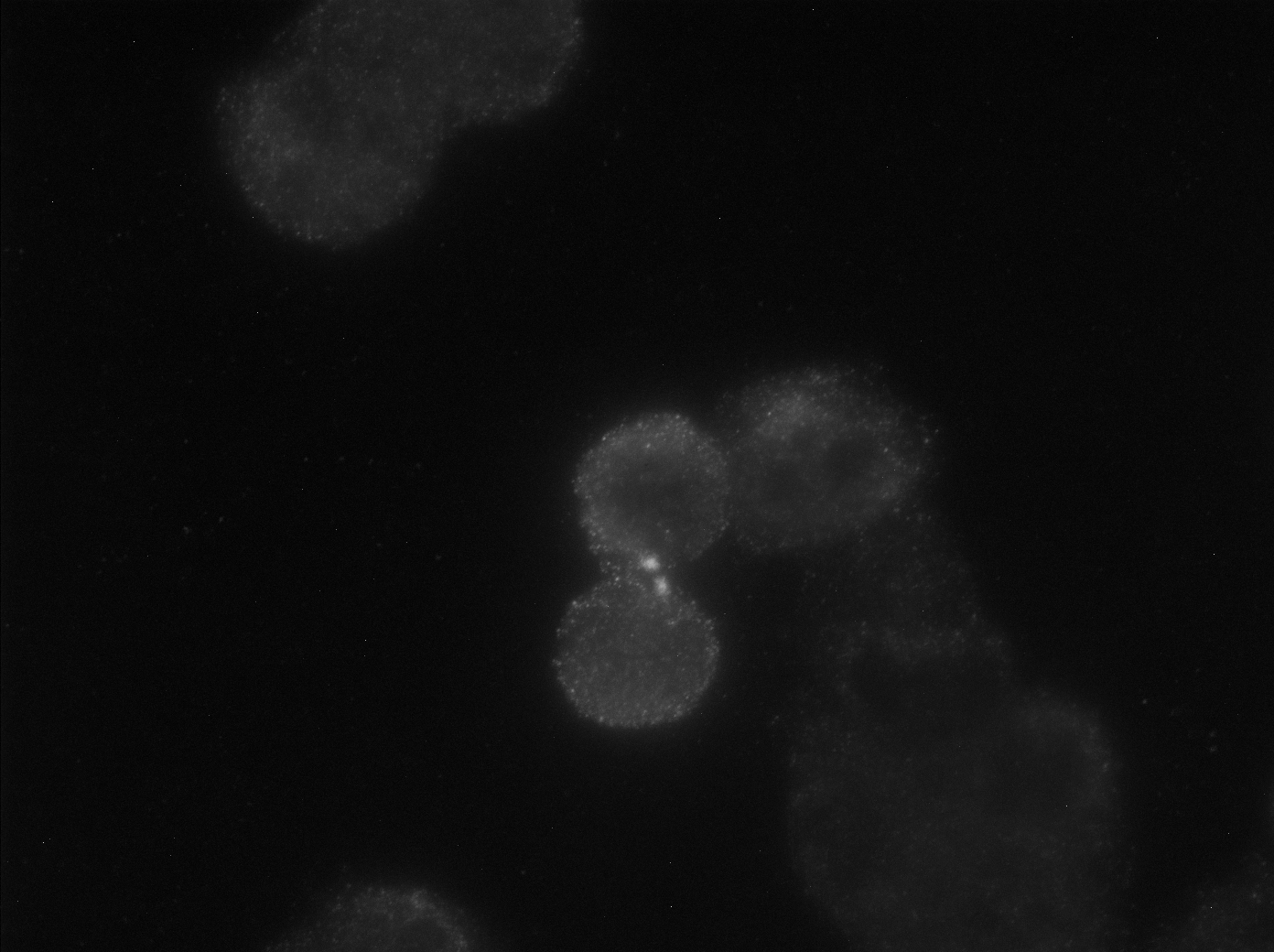

Supplement: Supplementary file 25 — Source data Fig. 7 part 1 [file 44319_2026_742_MOESM25_ESM.zip › Figure 7 Part 1/Fig 7acd Cas9 and TPGS1-ko rGT335 atubulin/Cas9 GT335recomb atub 3-24-25 R2 ET6.Project Maximum Z_XY1742846357_Z0_T0_C1.tif]

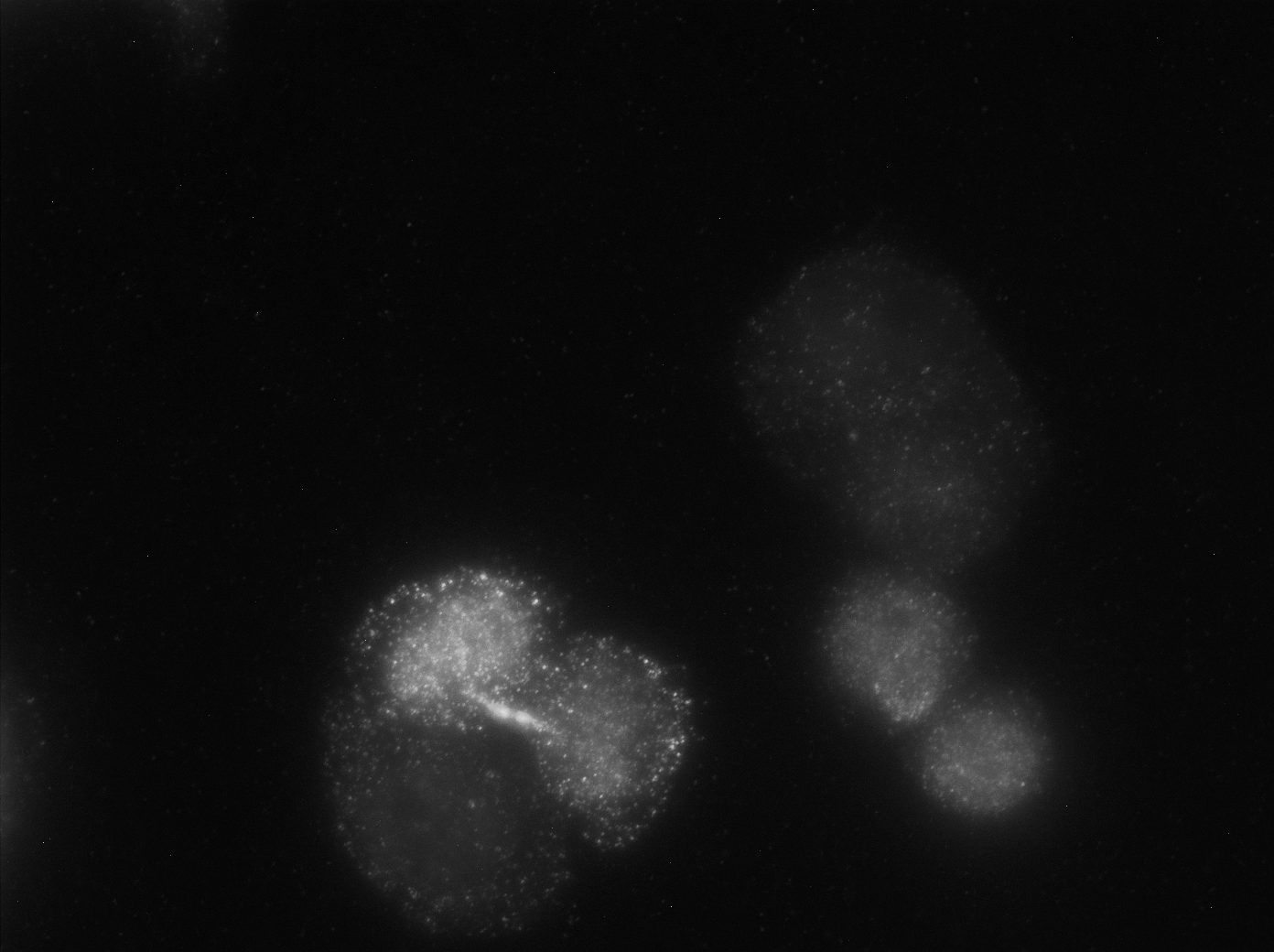

Supplement: Supplementary file 25 — Source data Fig. 7 part 1 [file 44319_2026_742_MOESM25_ESM.zip › Figure 7 Part 1/Fig 7acd Cas9 and TPGS1-ko rGT335 atubulin/Cas9 GT335recomb atub 3-24-25 R3 LT2.Project Maximum Z_XY1742848702_Z0_T0_C1.tif]

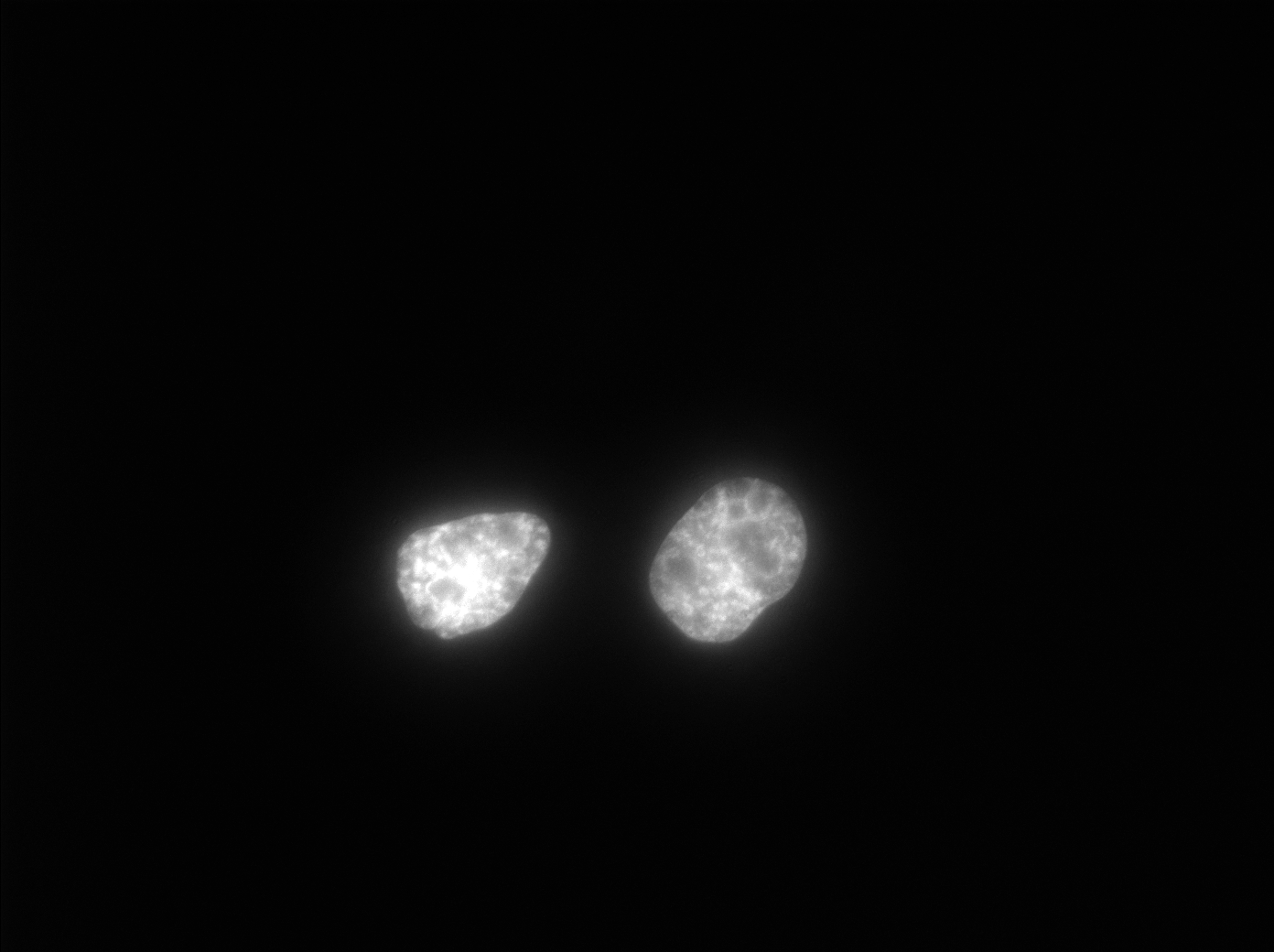

Supplement: Supplementary file 25 — Source data Fig. 7 part 1 [file 44319_2026_742_MOESM25_ESM.zip › Figure 7 Part 1/Fig 7acd Cas9 and TPGS1-ko rGT335 atubulin/Cas9 GT335recomb atub 3-24-25 R1 LT10.Project Maximum Z_XY1742836712_Z0_T0_C0.tif]

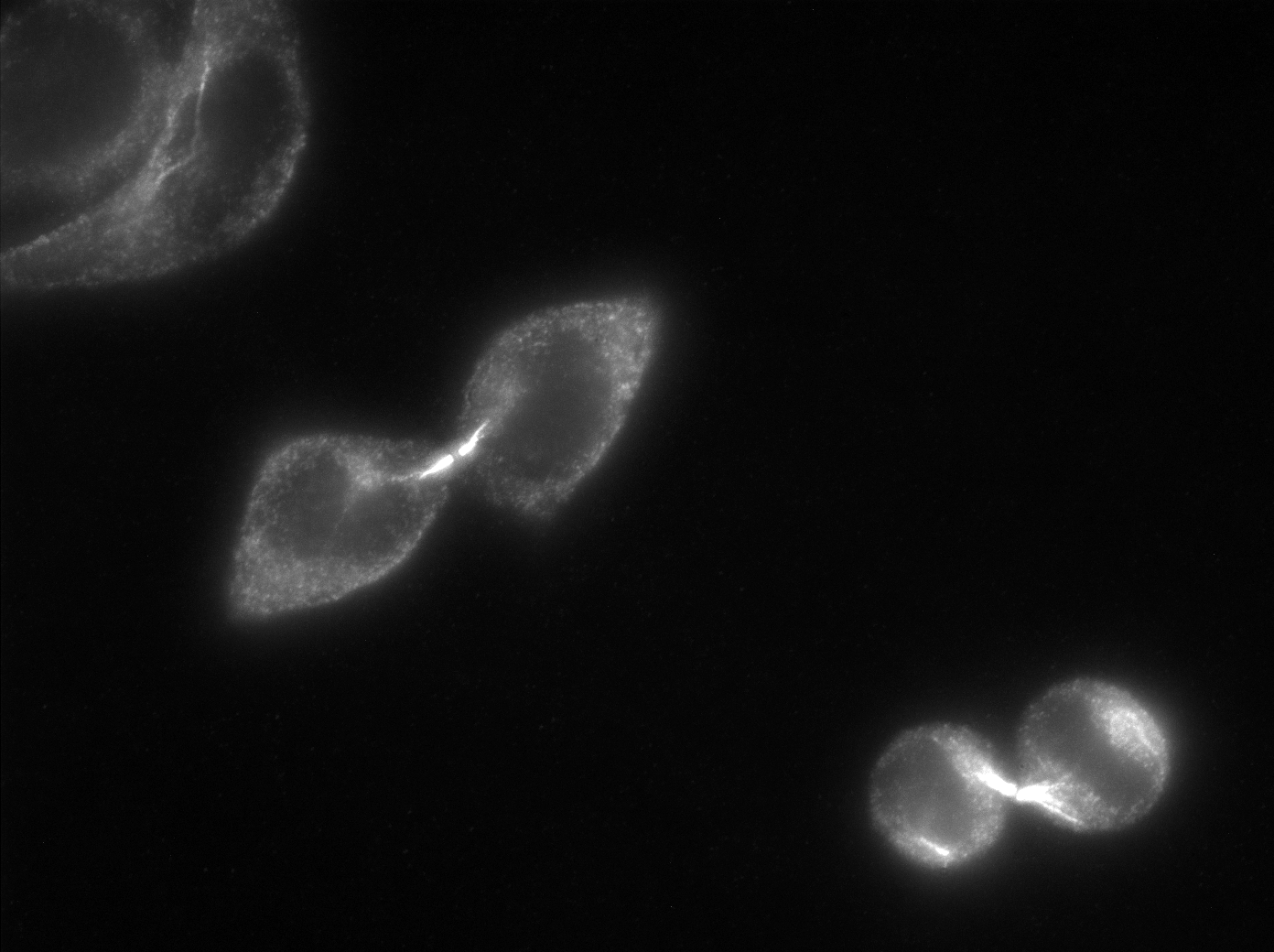

Supplement: Supplementary file 25 — Source data Fig. 7 part 1 [file 44319_2026_742_MOESM25_ESM.zip › Figure 7 Part 1/Fig 7acd Cas9 and TPGS1-ko rGT335 atubulin/Cas9 GT335recomb atub 3-24-25 R1 ET10 LT9.Project Maximum Z_XY1742836532_Z0_T0_C2.tif]

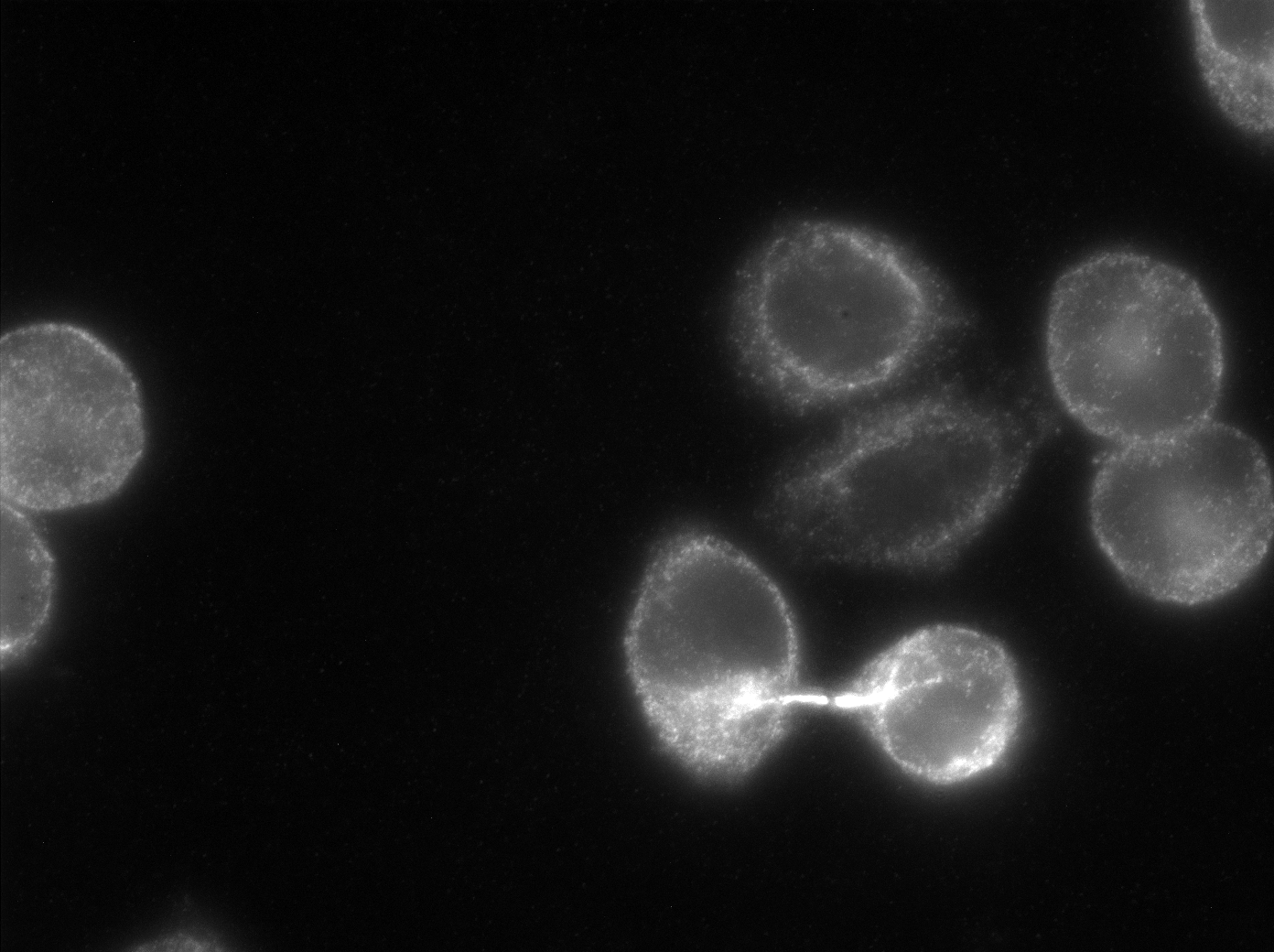

Supplement: Supplementary file 25 — Source data Fig. 7 part 1 [file 44319_2026_742_MOESM25_ESM.zip › Figure 7 Part 1/Fig 7acd Cas9 and TPGS1-ko rGT335 atubulin/Cas9 GT335recomb atub 3-24-25 R1 LT2.Project Maximum Z_XY1742834932_Z0_T0_C2.tif]

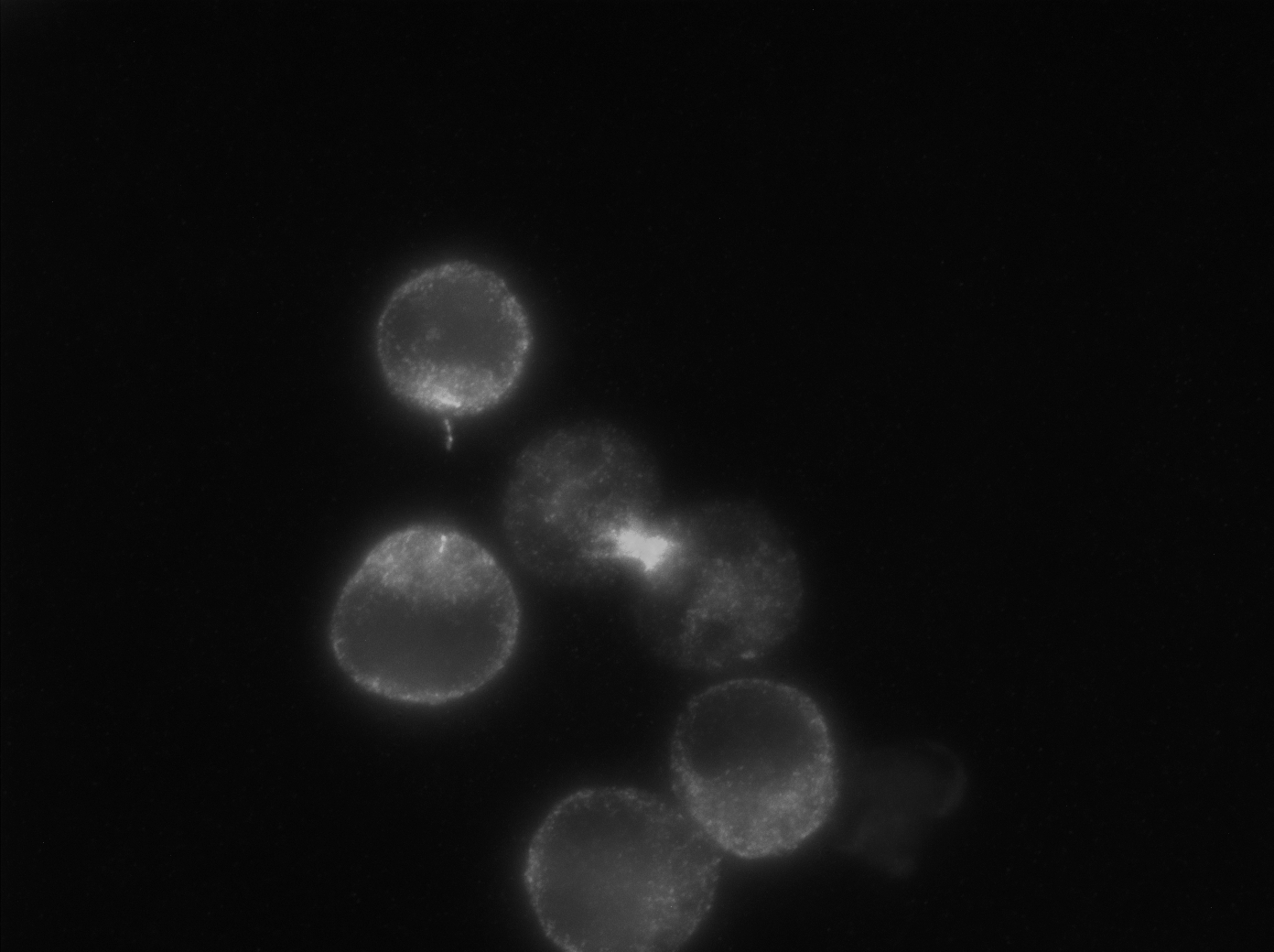

Supplement: Supplementary file 25 — Source data Fig. 7 part 1 [file 44319_2026_742_MOESM25_ESM.zip › Figure 7 Part 1/Fig 7acd Cas9 and TPGS1-ko rGT335 atubulin/Cas9 GT335recomb atub 3-24-25 R2 ET2.Project Maximum Z_XY1742845267_Z0_T0_C2.tif]

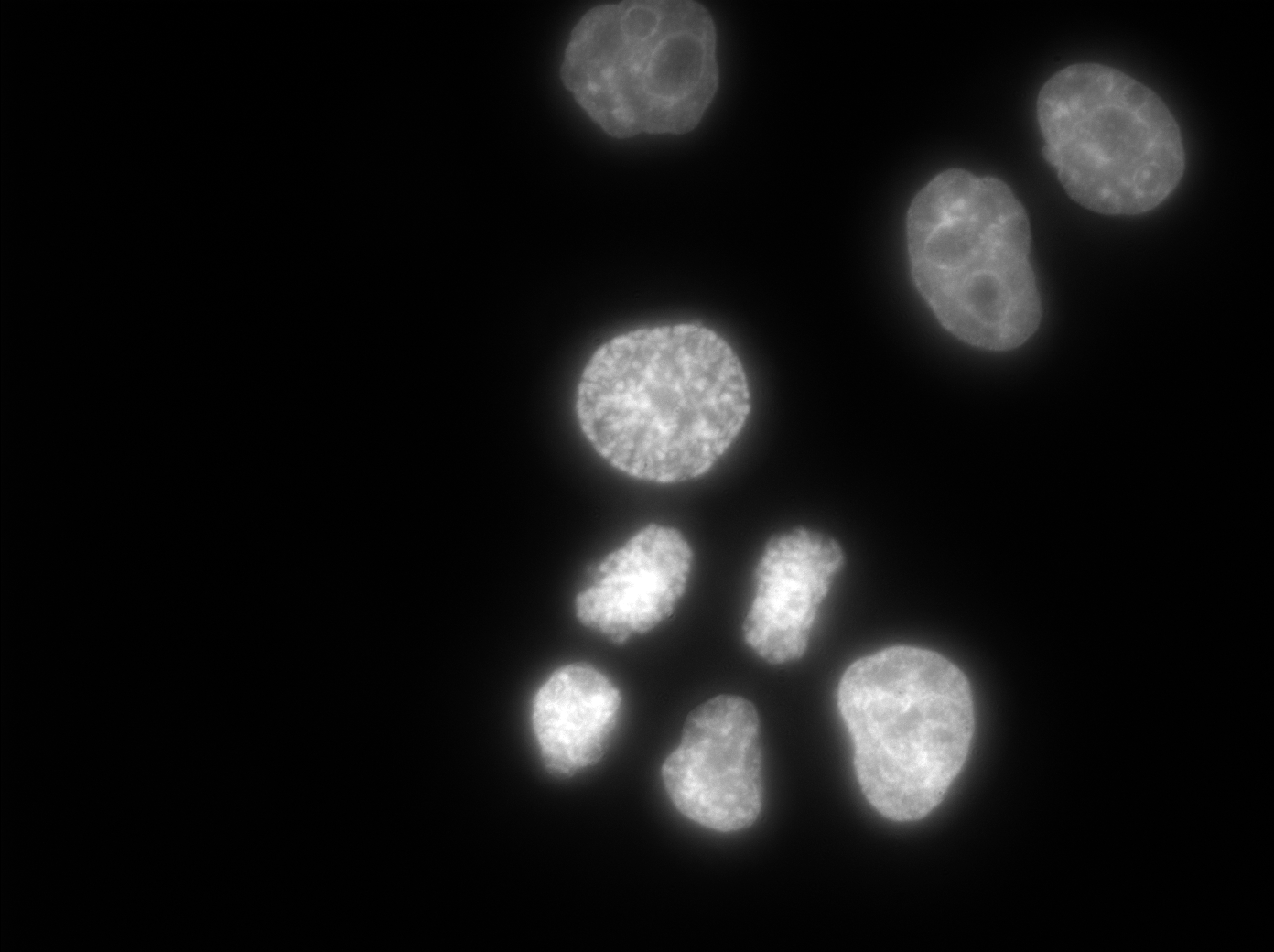

Supplement: Supplementary file 25 — Source data Fig. 7 part 1 [file 44319_2026_742_MOESM25_ESM.zip › Figure 7 Part 1/Fig 7acd Cas9 and TPGS1-ko rGT335 atubulin/Cas9 GT335recomb atub 3-24-25 R1 ET8 PA3.Project Maximum Z_XY1742836284_Z0_T0_C0.tif]

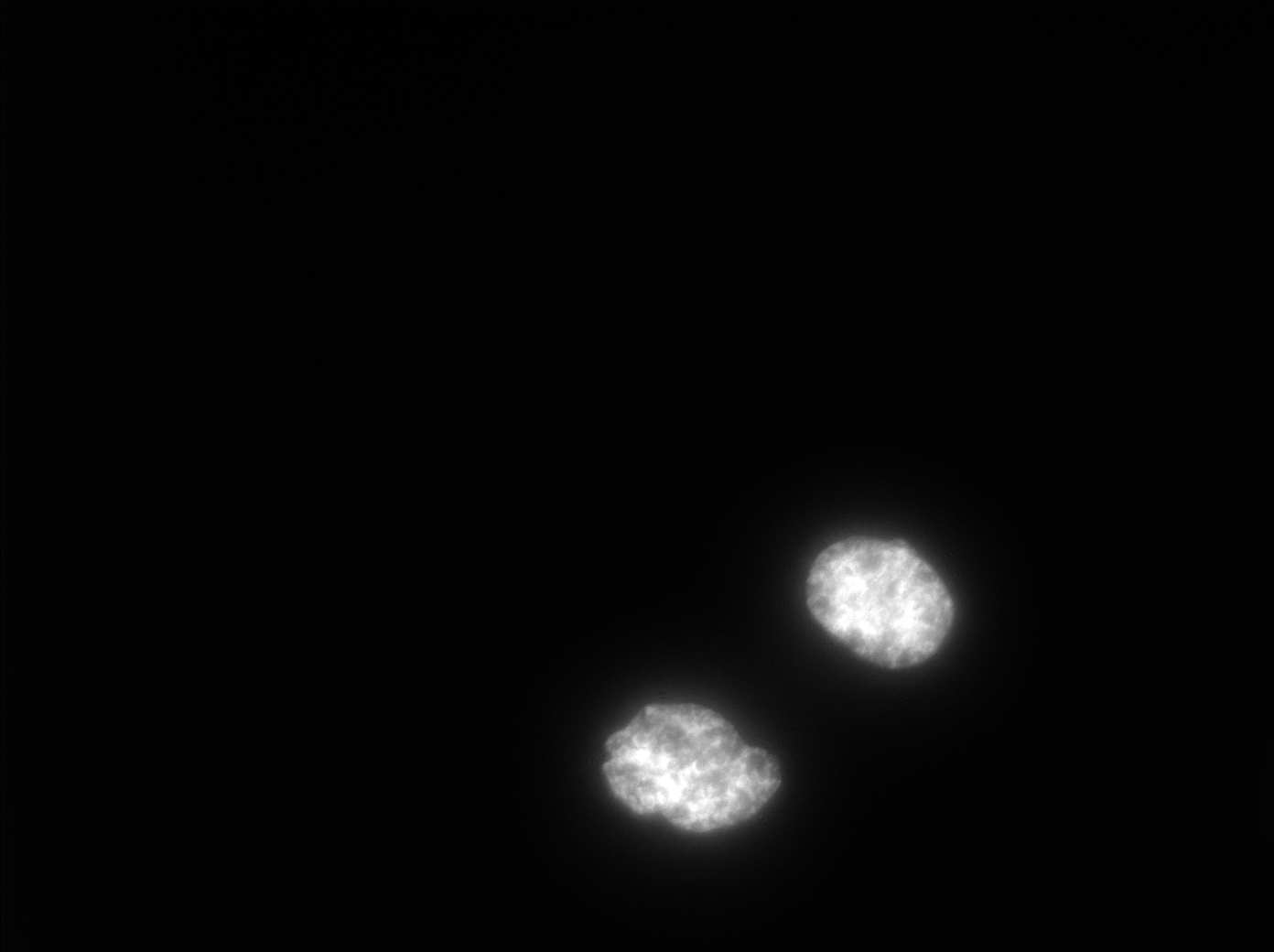

Supplement: Supplementary file 25 — Source data Fig. 7 part 1 [file 44319_2026_742_MOESM25_ESM.zip › Figure 7 Part 1/Fig 7acd Cas9 and TPGS1-ko rGT335 atubulin/Cas9 GT335recomb atub 3-24-25 R2 LT9.Project Maximum Z_XY1742847464_Z0_T0_C0.tif]

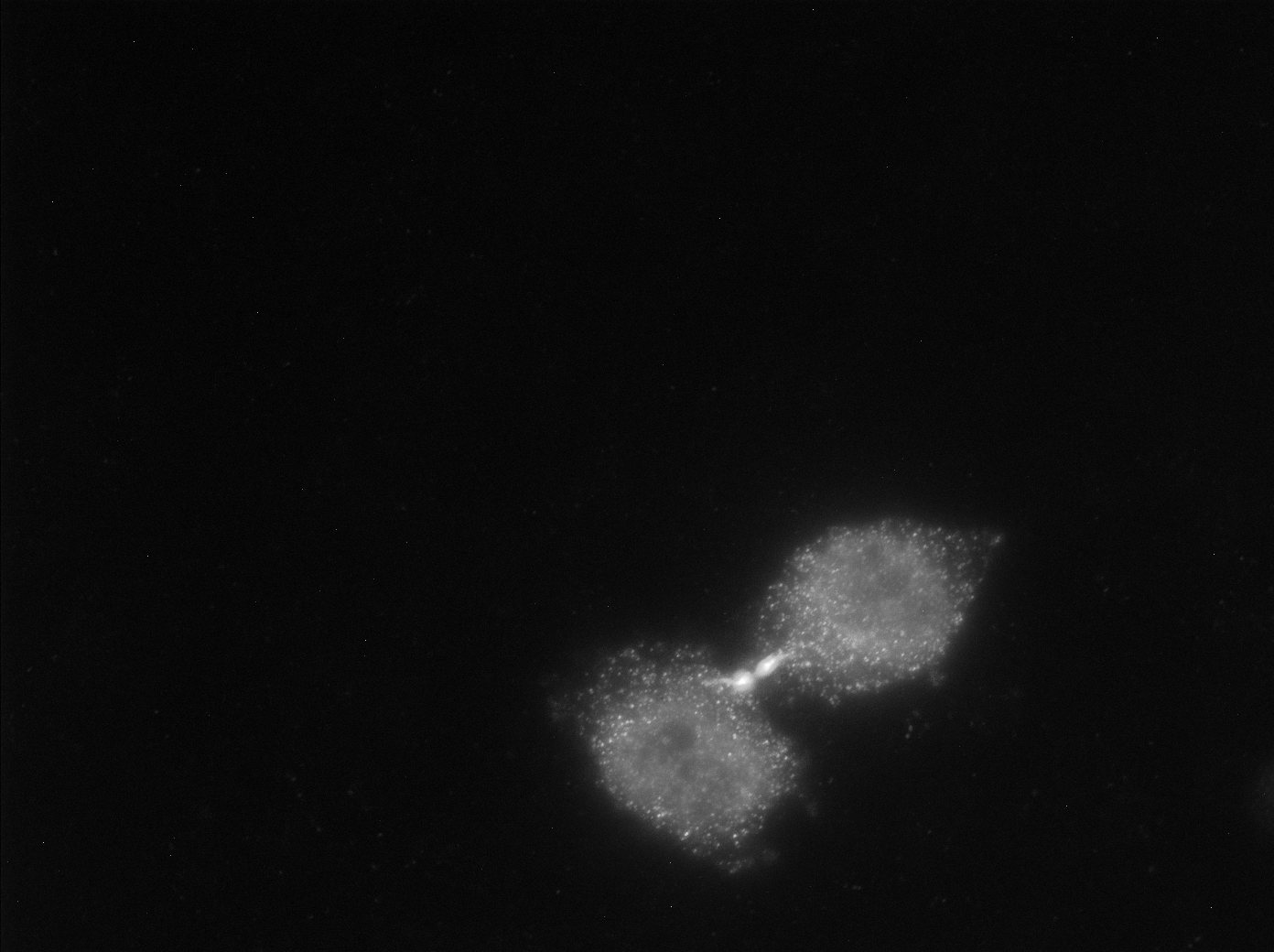

Supplement: Supplementary file 25 — Source data Fig. 7 part 1 [file 44319_2026_742_MOESM25_ESM.zip › Figure 7 Part 1/Fig 7acd Cas9 and TPGS1-ko rGT335 atubulin/Cas9 GT335recomb atub 3-24-25 R2 LT9.Project Maximum Z_XY1742847464_Z0_T0_C1.tif]

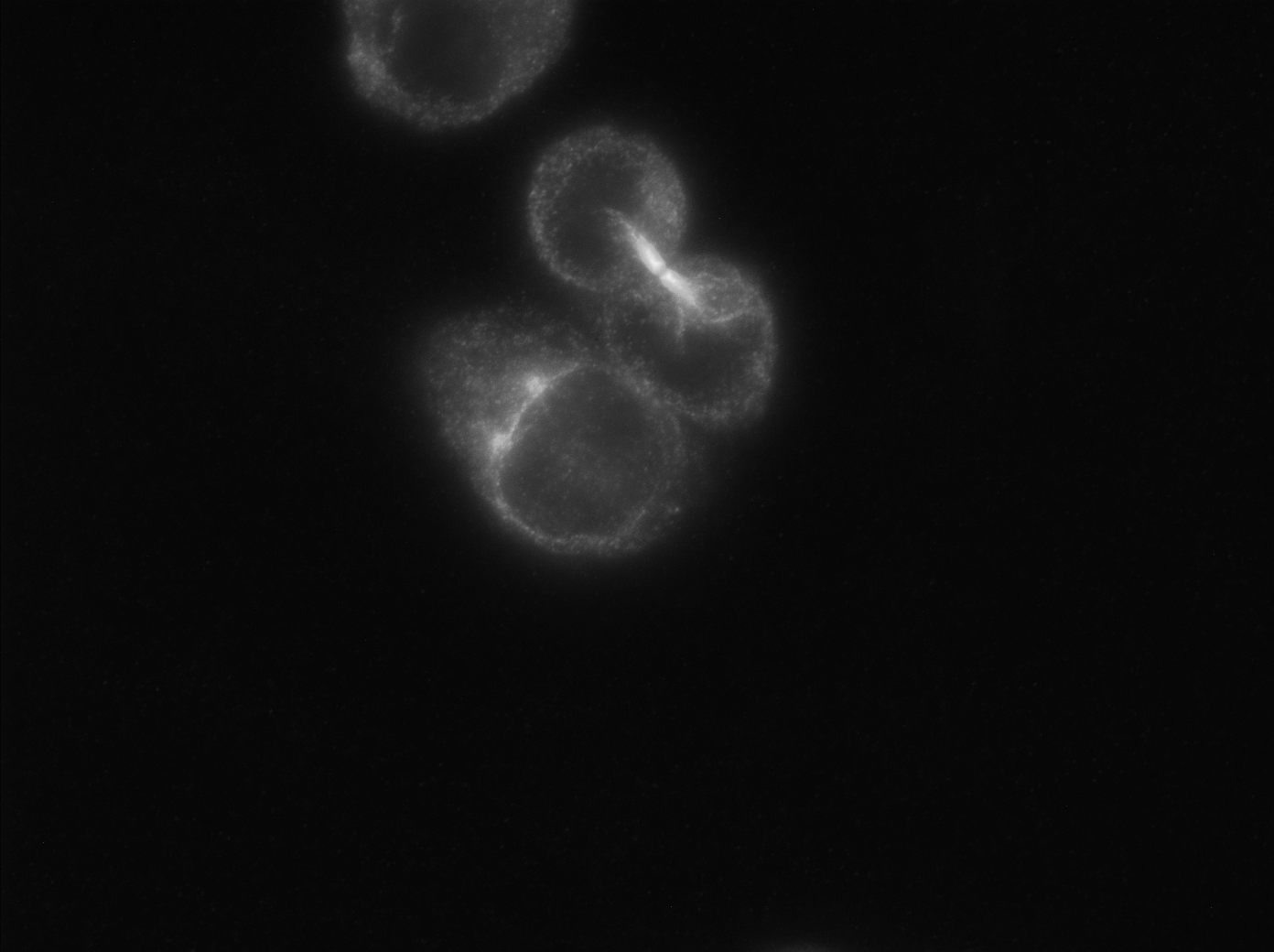

Supplement: Supplementary file 25 — Source data Fig. 7 part 1 [file 44319_2026_742_MOESM25_ESM.zip › Figure 7 Part 1/Fig 7acd Cas9 and TPGS1-ko rGT335 atubulin/Cas9 GT335recomb atub 3-24-25 R2 LT3 P1.Project Maximum Z_XY1742846092_Z0_T0_C2.tif]
