# Supplementary figures and images for: TPGS1 regulates central spindle microtubule glutamylation and remodeling during telophase and abscission (part 28 of 36)
Source: EMBO Rep. 2026 Mar 23;27(8):1944–63. doi: 10.1038/s44319-026-00742-3 (PMC13121839; doi:10.1038/s44319-026-00742-3)

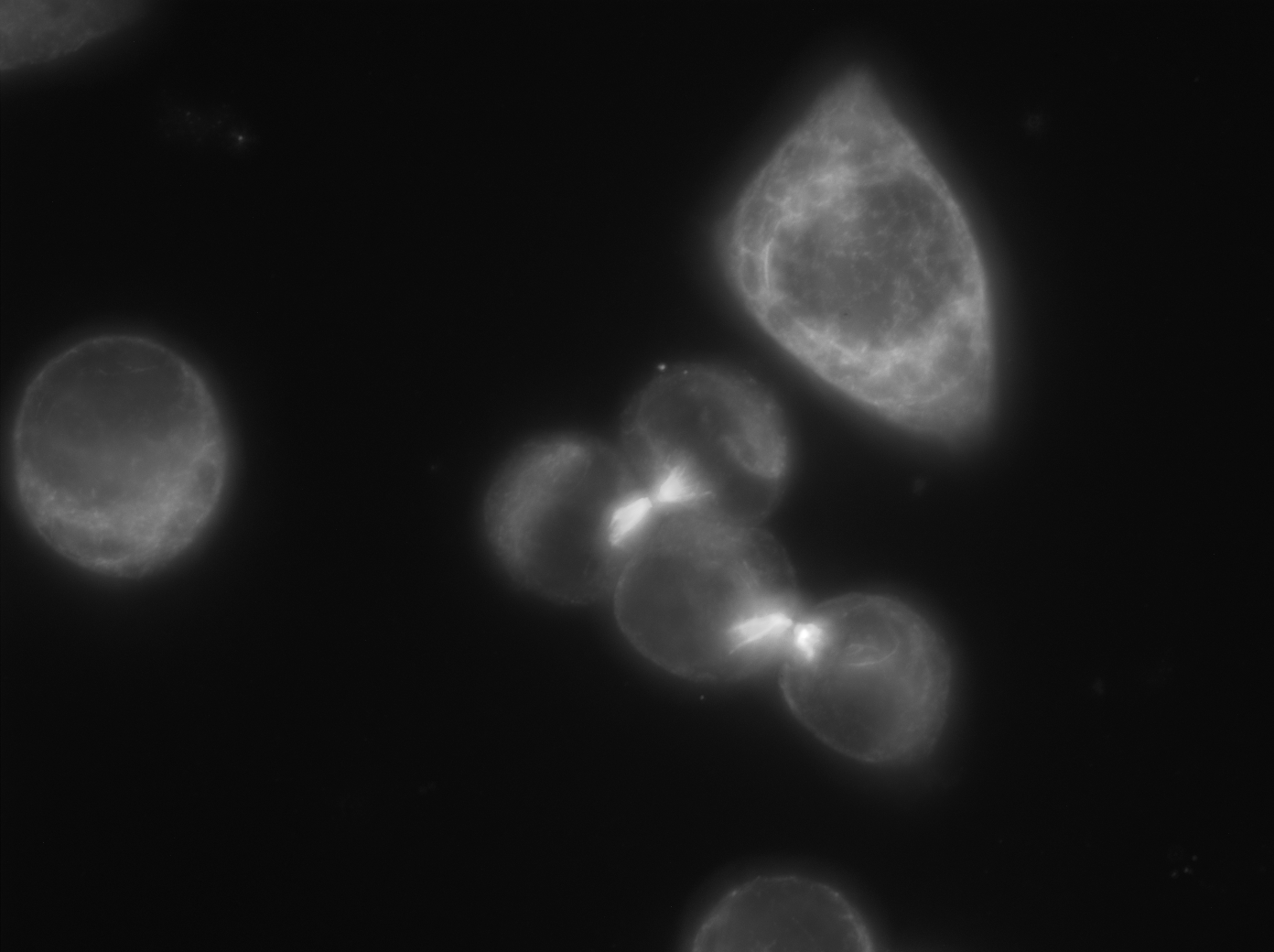

Supplement: Supplementary file 23 — Source data Fig. 6 part 4 [file 44319_2026_742_MOESM23_ESM.zip › Figure 6 Part 4/Fig 6efg TPGS1-KO TPGS1 rescue experiments part 2/R2R3/TPGS1-KO EYFP-only actub 7-31-25 R2 ET4.Project Maximum Z_XY1756414878_Z0_T0_C2.tif]

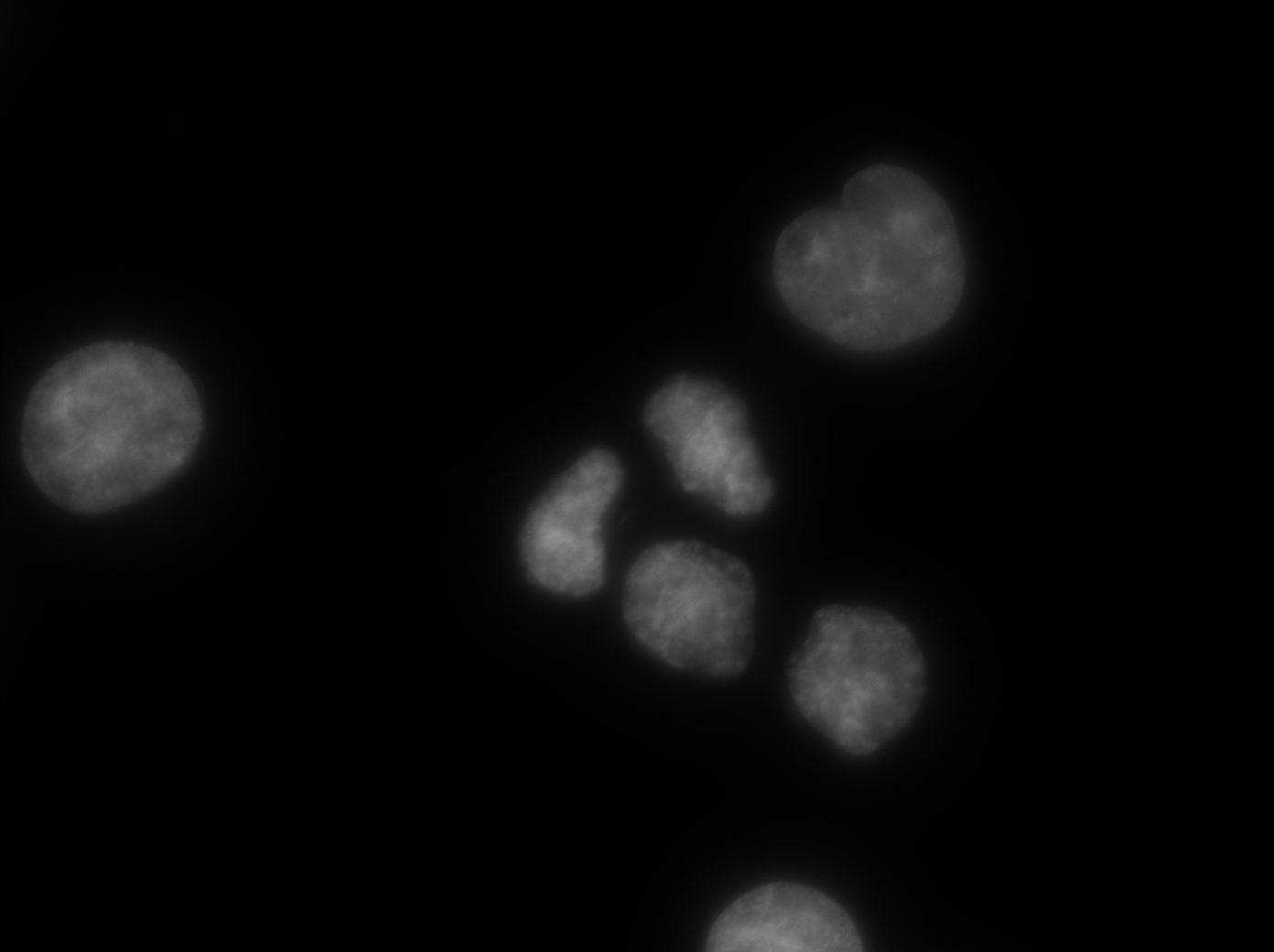

Supplement: Supplementary file 23 — Source data Fig. 6 part 4 [file 44319_2026_742_MOESM23_ESM.zip › Figure 6 Part 4/Fig 6efg TPGS1-KO TPGS1 rescue experiments part 2/R2R3/TPGS1-KO EYFP-only actub 7-31-25 R2 ET4.Project Maximum Z_XY1756414878_Z0_T0_C0.tif]

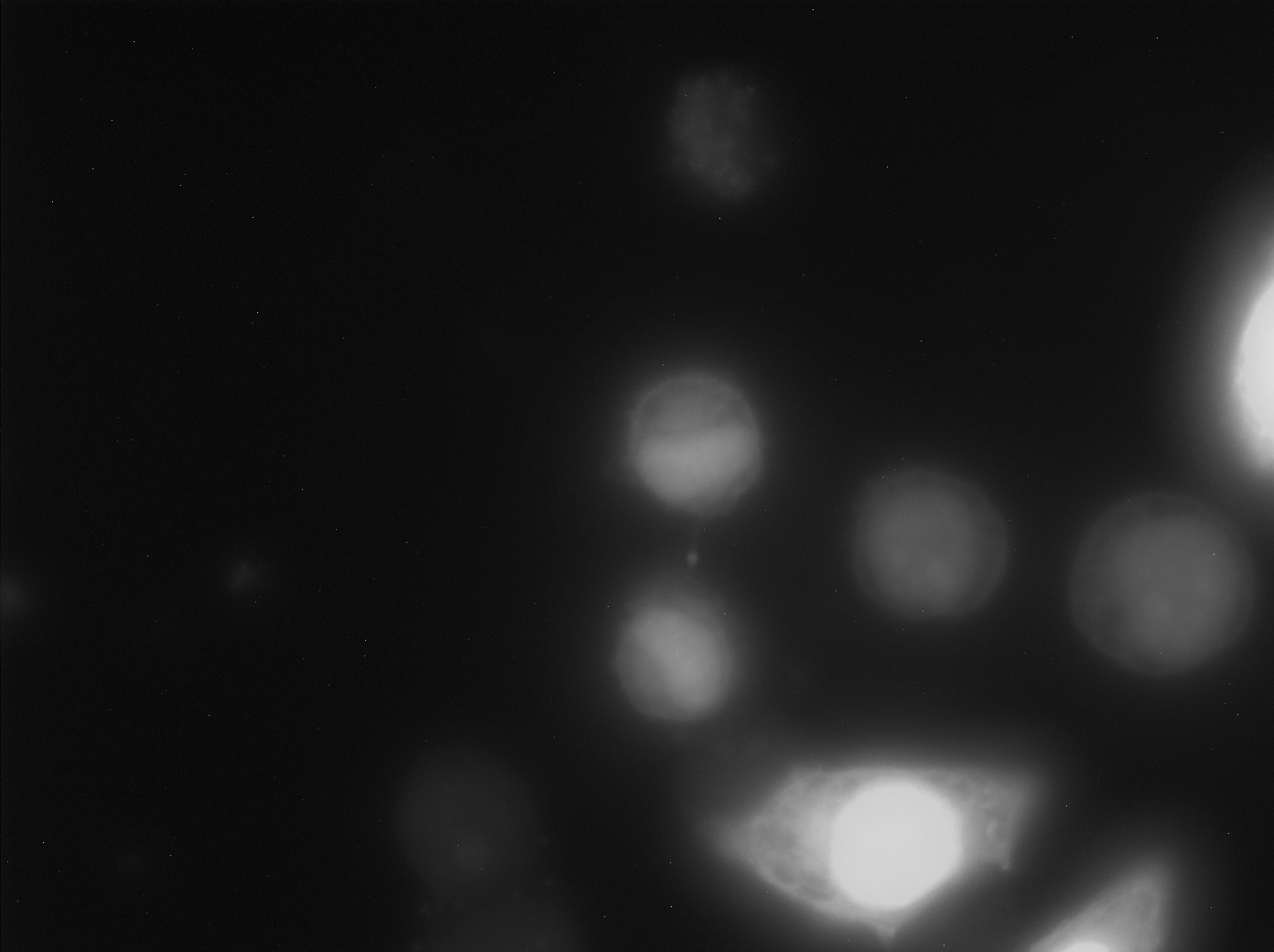

Supplement: Supplementary file 23 — Source data Fig. 6 part 4 [file 44319_2026_742_MOESM23_ESM.zip › Figure 6 Part 4/Fig 6efg TPGS1-KO TPGS1 rescue experiments part 2/R2R3/TPGS1-KO EYFP-only actub 7-31-25 R2 ET9.Project Maximum Z_XY1756416411_Z0_T0_C1.tif]

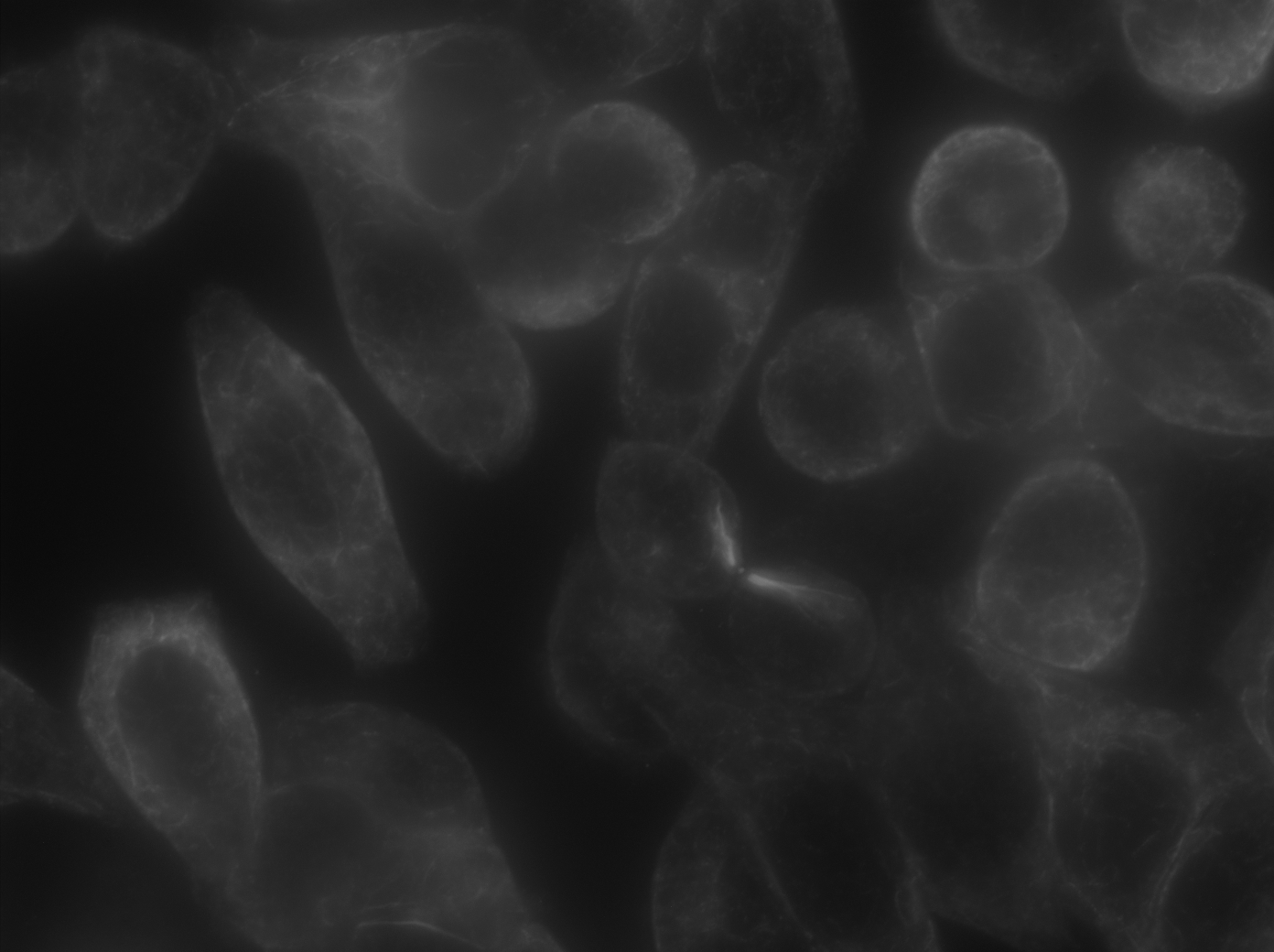

Supplement: Supplementary file 23 — Source data Fig. 6 part 4 [file 44319_2026_742_MOESM23_ESM.zip › Figure 6 Part 4/Fig 6efg TPGS1-KO TPGS1 rescue experiments part 2/R2R3/TPGS1-KO TPGS1-EYFP-3'UTR actub 7-31-25 R2 LT3.Project Maximum Z_XY1756407610_Z0_T0_C2.tif]

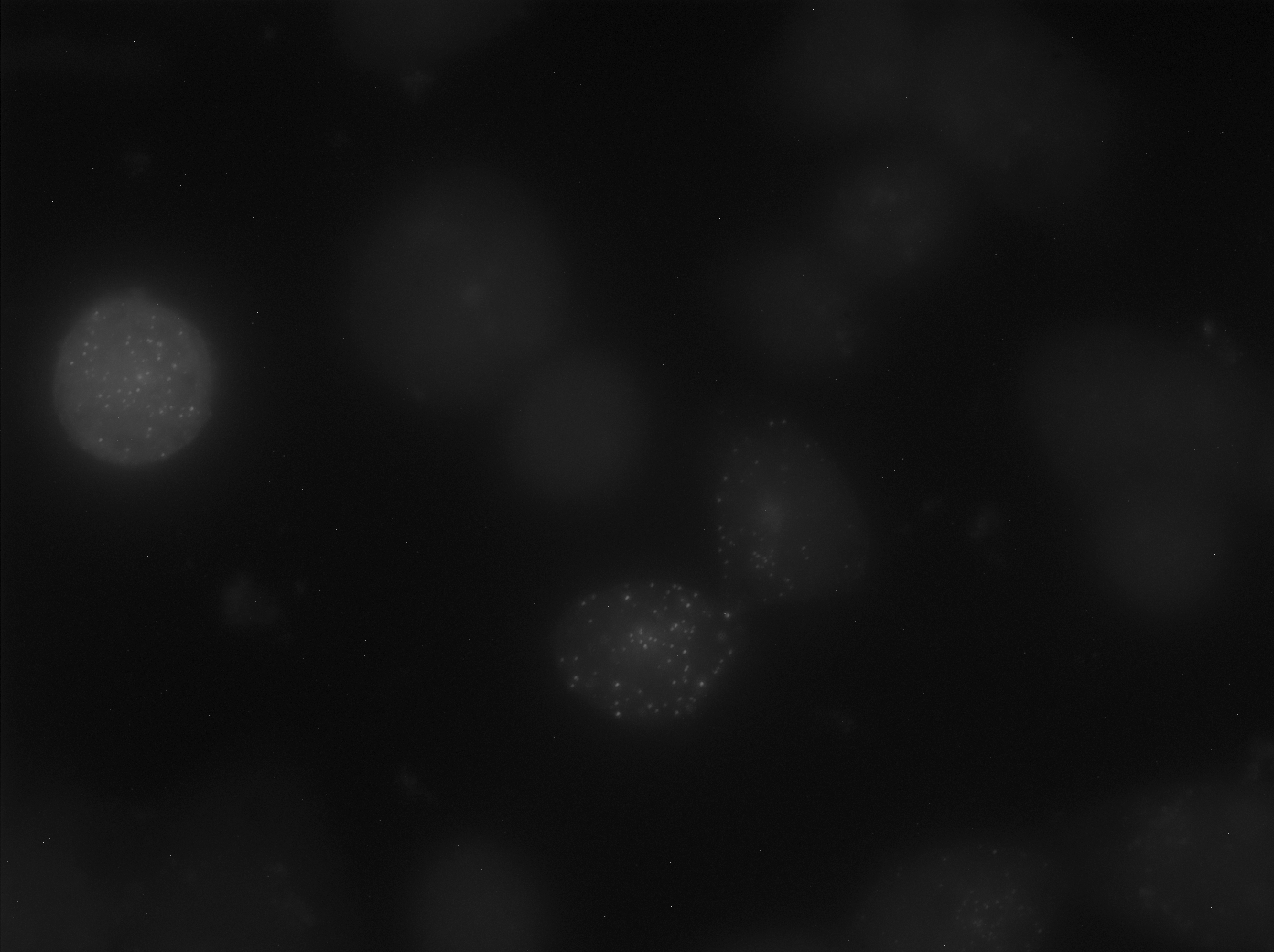

Supplement: Supplementary file 23 — Source data Fig. 6 part 4 [file 44319_2026_742_MOESM23_ESM.zip › Figure 6 Part 4/Fig 6efg TPGS1-KO TPGS1 rescue experiments part 2/R2R3/TPGS1-KO TPGS1-EYFP-3'UTR actub 7-31-25 R3 LT8.Project Maximum Z_XY1756501925_Z0_T0_C1.tif]

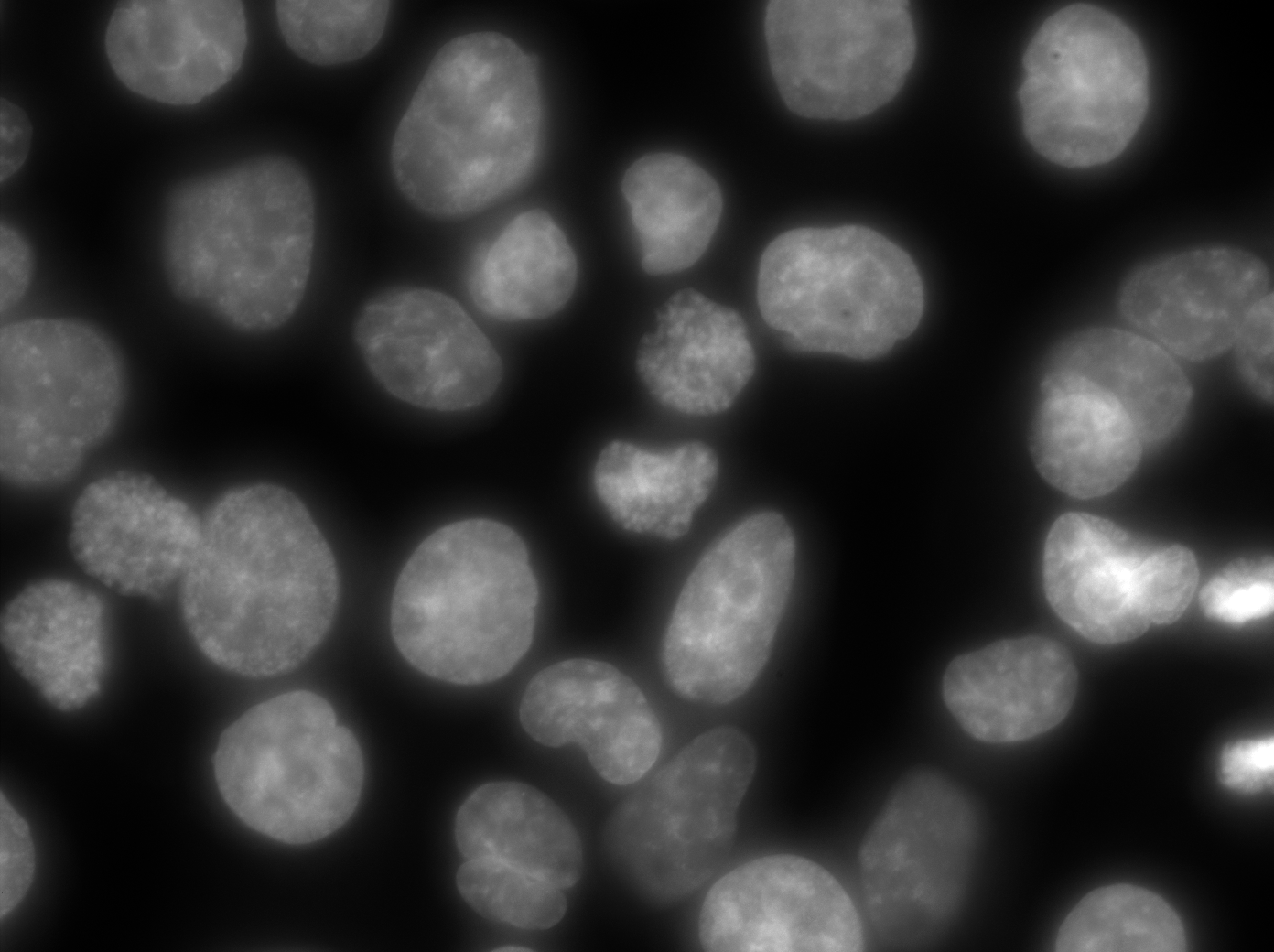

Supplement: Supplementary file 23 — Source data Fig. 6 part 4 [file 44319_2026_742_MOESM23_ESM.zip › Figure 6 Part 4/Fig 6efg TPGS1-KO TPGS1 rescue experiments part 2/R2R3/TPGS1-KO TPGS1-EYFP-3'UTR actub 7-31-25 R2 ET2.Project Maximum Z_XY1756407950_Z0_T0_C0.tif]

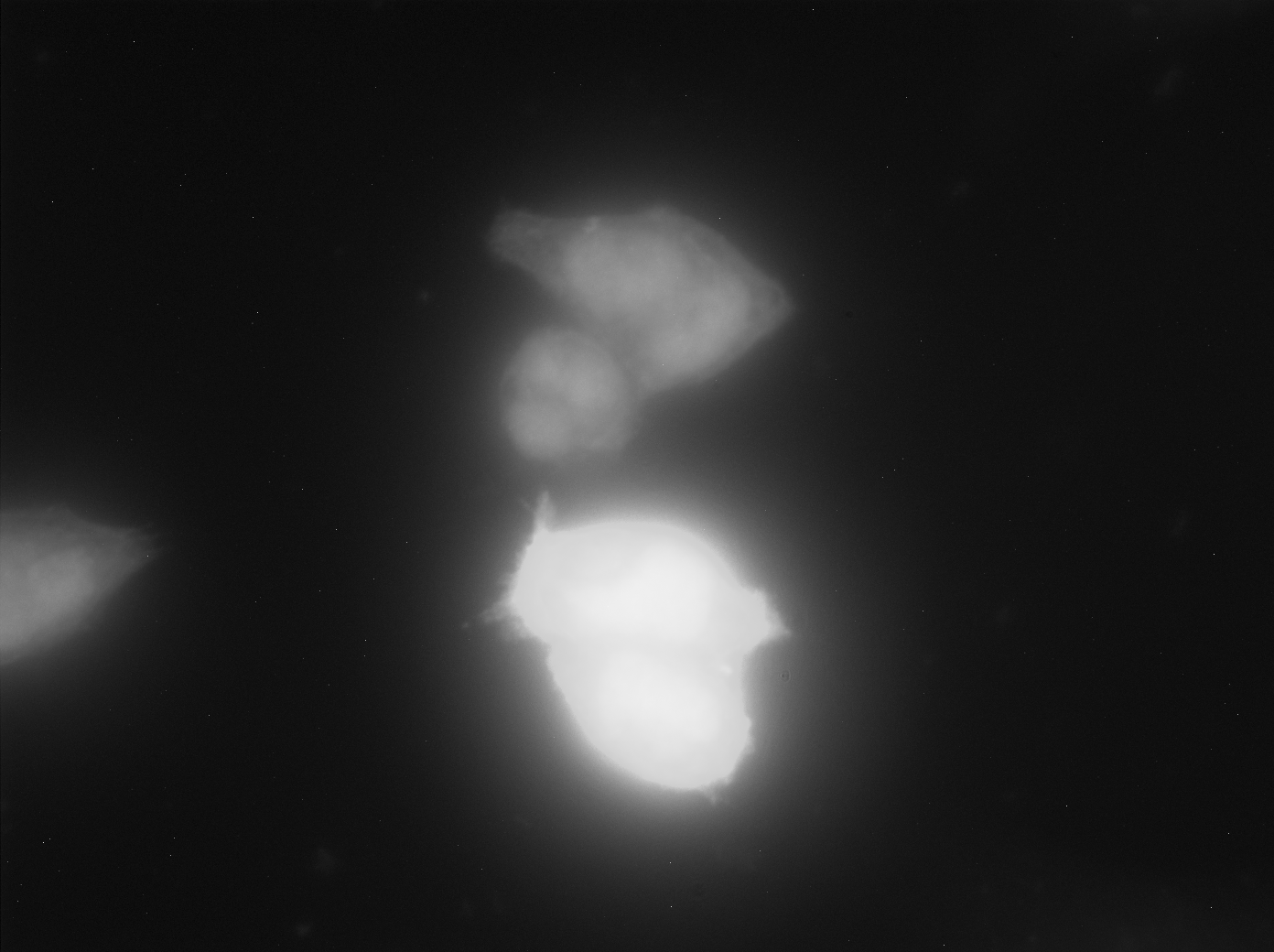

Supplement: Supplementary file 23 — Source data Fig. 6 part 4 [file 44319_2026_742_MOESM23_ESM.zip › Figure 6 Part 4/Fig 6efg TPGS1-KO TPGS1 rescue experiments part 2/R2R3/TPGS1-KO EYFP-only actub 7-31-25 R2 LT7.Project Maximum Z_XY1756415824_Z0_T0_C1.tif]

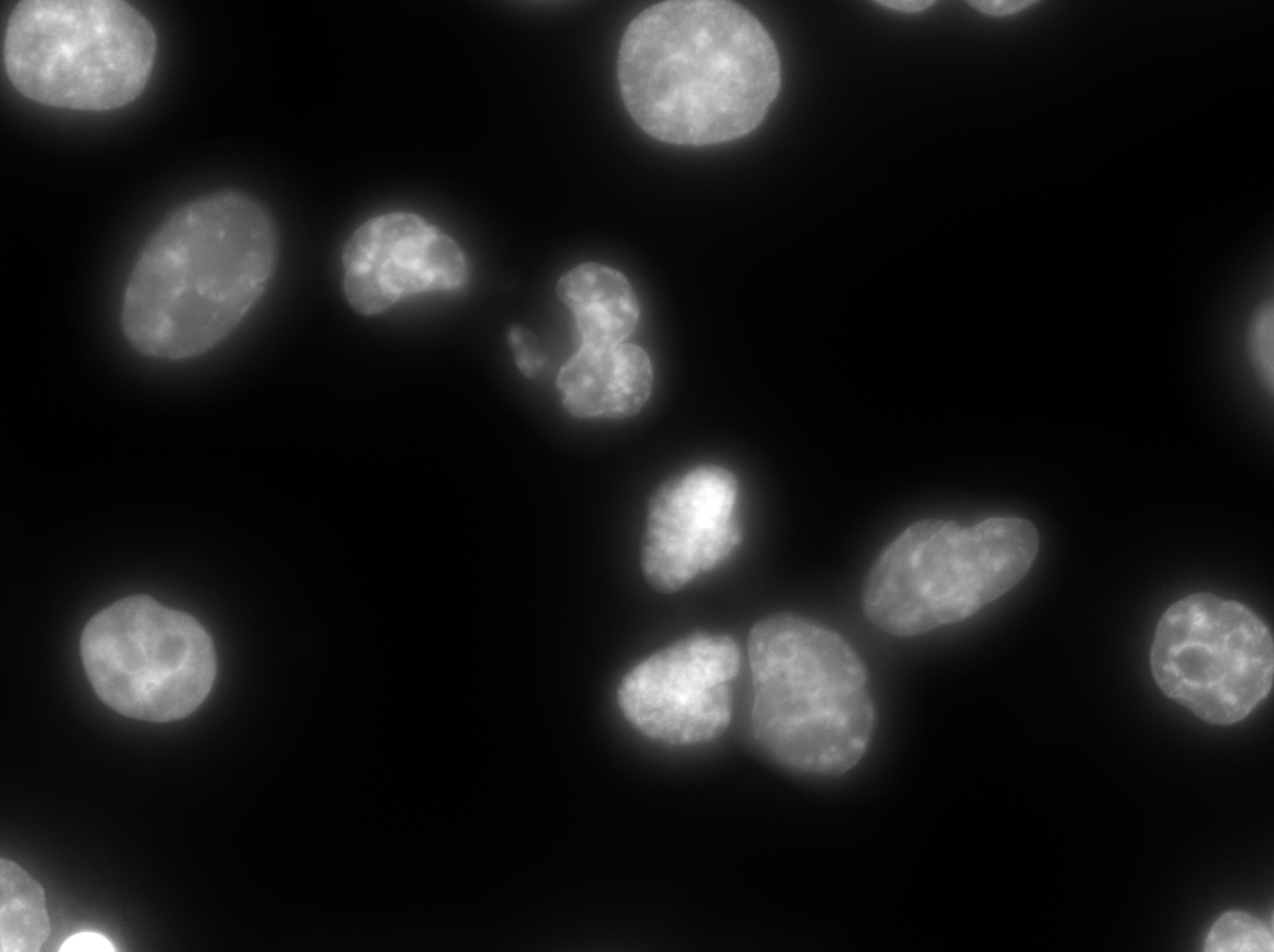

Supplement: Supplementary file 23 — Source data Fig. 6 part 4 [file 44319_2026_742_MOESM23_ESM.zip › Figure 6 Part 4/Fig 6efg TPGS1-KO TPGS1 rescue experiments part 2/R2R3/TPGS1-KO TPGS1-EYFP-3'UTR actub 7-31-25 R3 ET5.Project Maximum Z_XY1756501355_Z0_T0_C0.tif]

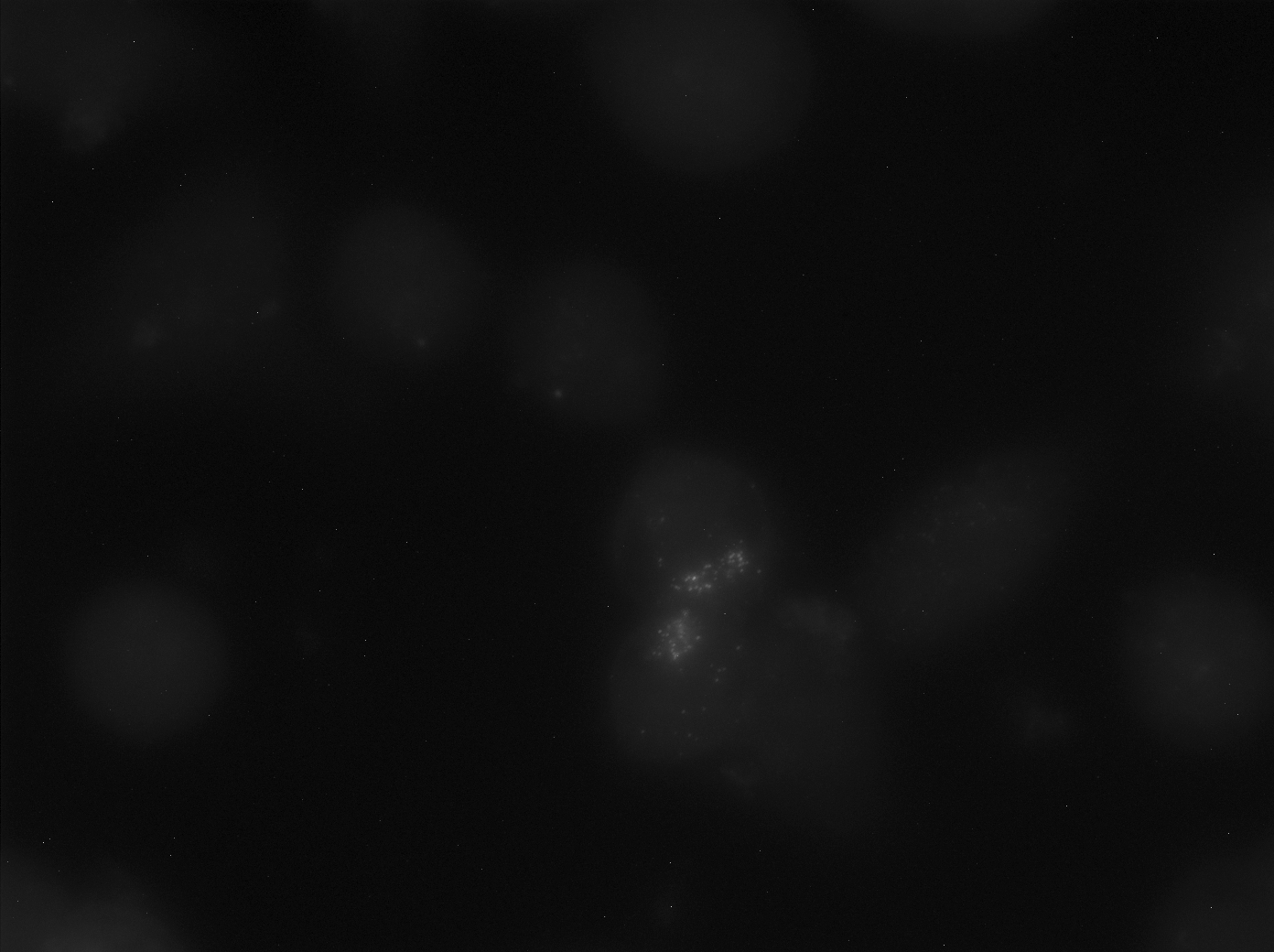

Supplement: Supplementary file 23 — Source data Fig. 6 part 4 [file 44319_2026_742_MOESM23_ESM.zip › Figure 6 Part 4/Fig 6efg TPGS1-KO TPGS1 rescue experiments part 2/R2R3/TPGS1-KO TPGS1-EYFP-3'UTR actub 7-31-25 R3 ET5.Project Maximum Z_XY1756501355_Z0_T0_C1.tif]

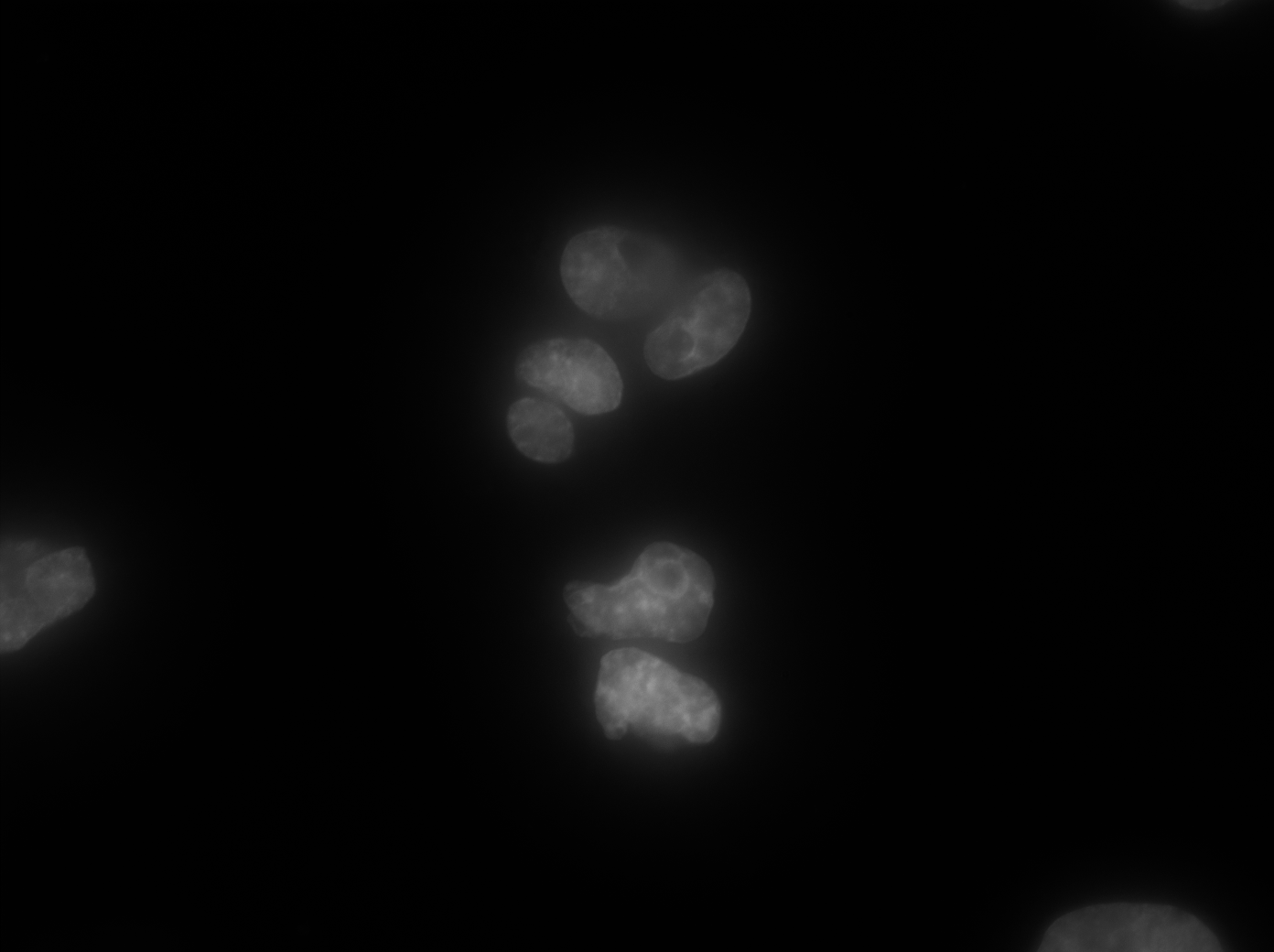

Supplement: Supplementary file 23 — Source data Fig. 6 part 4 [file 44319_2026_742_MOESM23_ESM.zip › Figure 6 Part 4/Fig 6efg TPGS1-KO TPGS1 rescue experiments part 2/R2R3/TPGS1-KO EYFP-only actub 7-31-25 R2 LT7.Project Maximum Z_XY1756415824_Z0_T0_C0.tif]

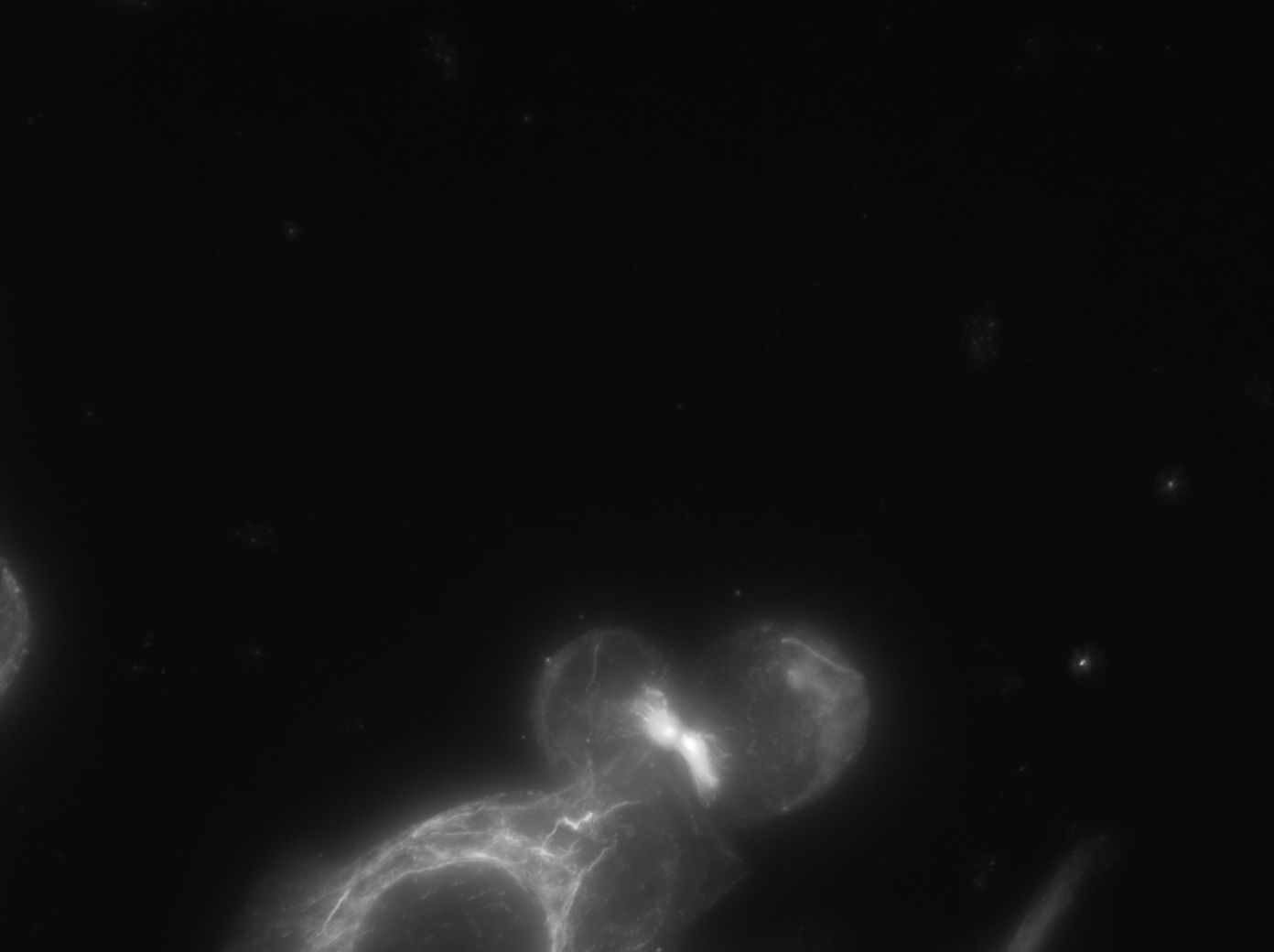

Supplement: Supplementary file 23 — Source data Fig. 6 part 4 [file 44319_2026_742_MOESM23_ESM.zip › Figure 6 Part 4/Fig 6efg TPGS1-KO TPGS1 rescue experiments part 2/R2R3/TPGS1-KO EYFP-only actub 7-31-25 R2 ET3.Project Maximum Z_XY1756414255_Z0_T0_C2.tif]

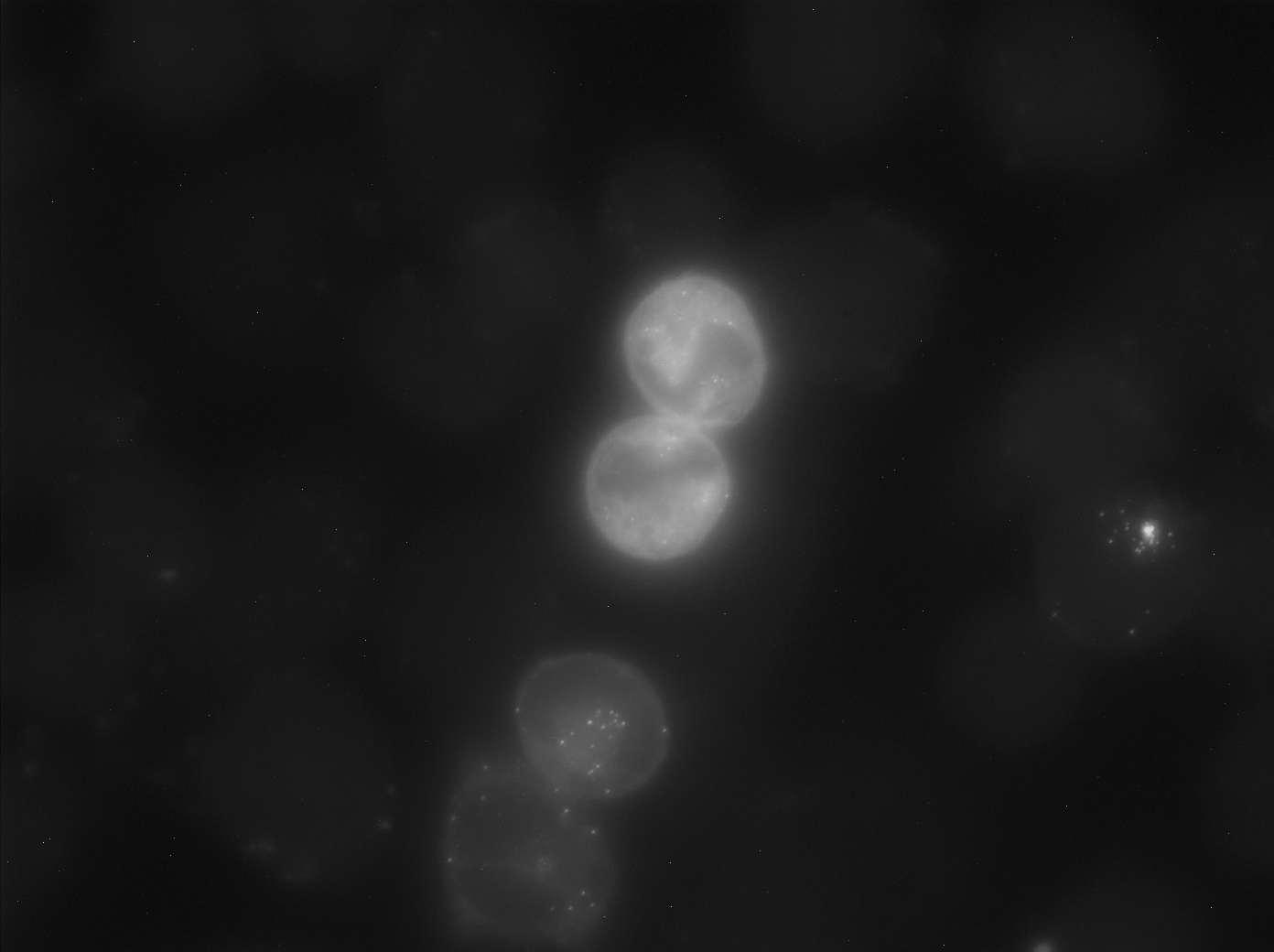

Supplement: Supplementary file 23 — Source data Fig. 6 part 4 [file 44319_2026_742_MOESM23_ESM.zip › Figure 6 Part 4/Fig 6efg TPGS1-KO TPGS1 rescue experiments part 2/R2R3/TPGS1-KO TPGS1-EYFP-3'UTR actub 7-31-25 R2 ET2.Project Maximum Z_XY1756407950_Z0_T0_C1.tif]

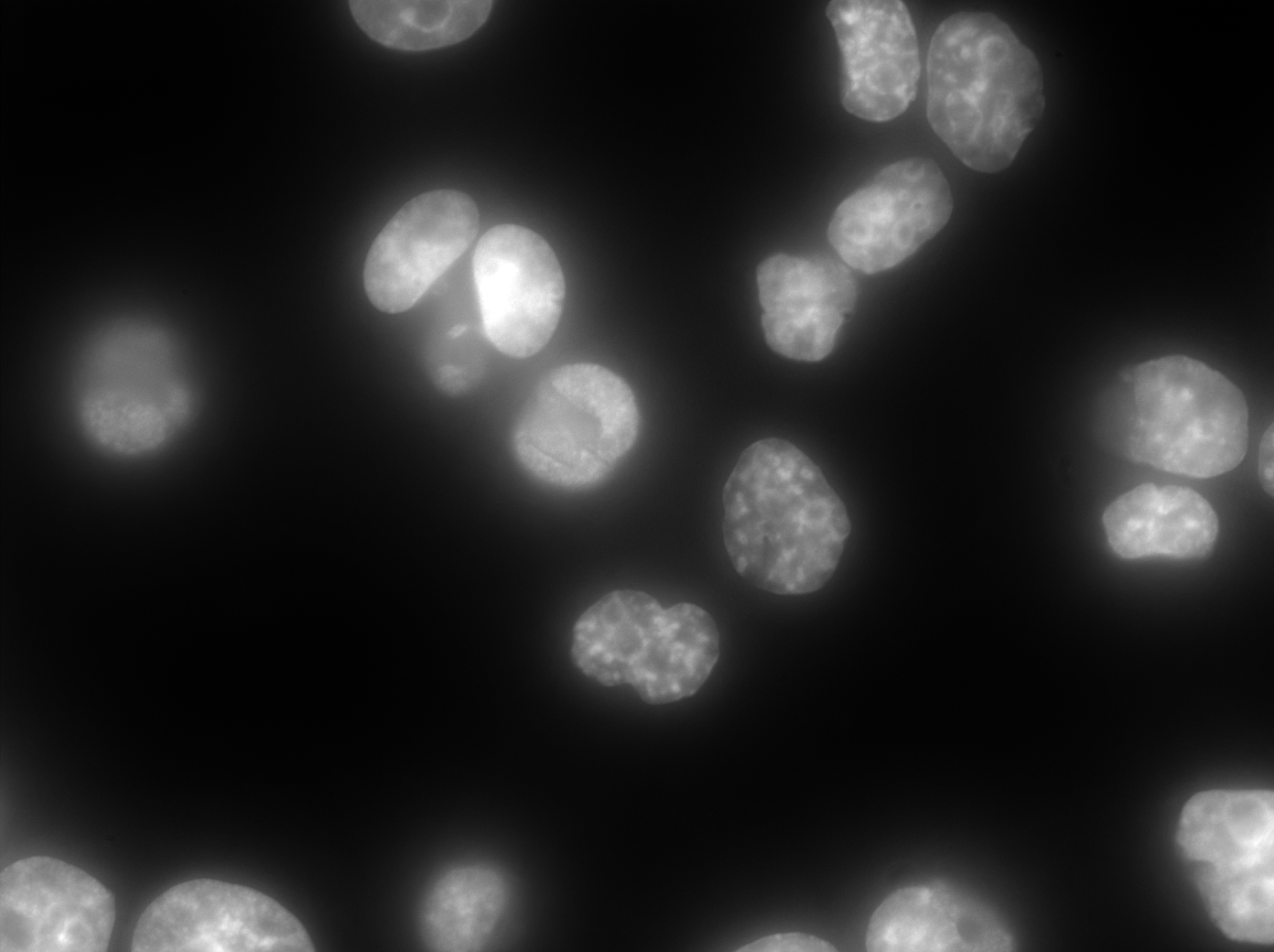

Supplement: Supplementary file 23 — Source data Fig. 6 part 4 [file 44319_2026_742_MOESM23_ESM.zip › Figure 6 Part 4/Fig 6efg TPGS1-KO TPGS1 rescue experiments part 2/R2R3/TPGS1-KO TPGS1-EYFP-3'UTR actub 7-31-25 R3 LT8.Project Maximum Z_XY1756501925_Z0_T0_C0.tif]

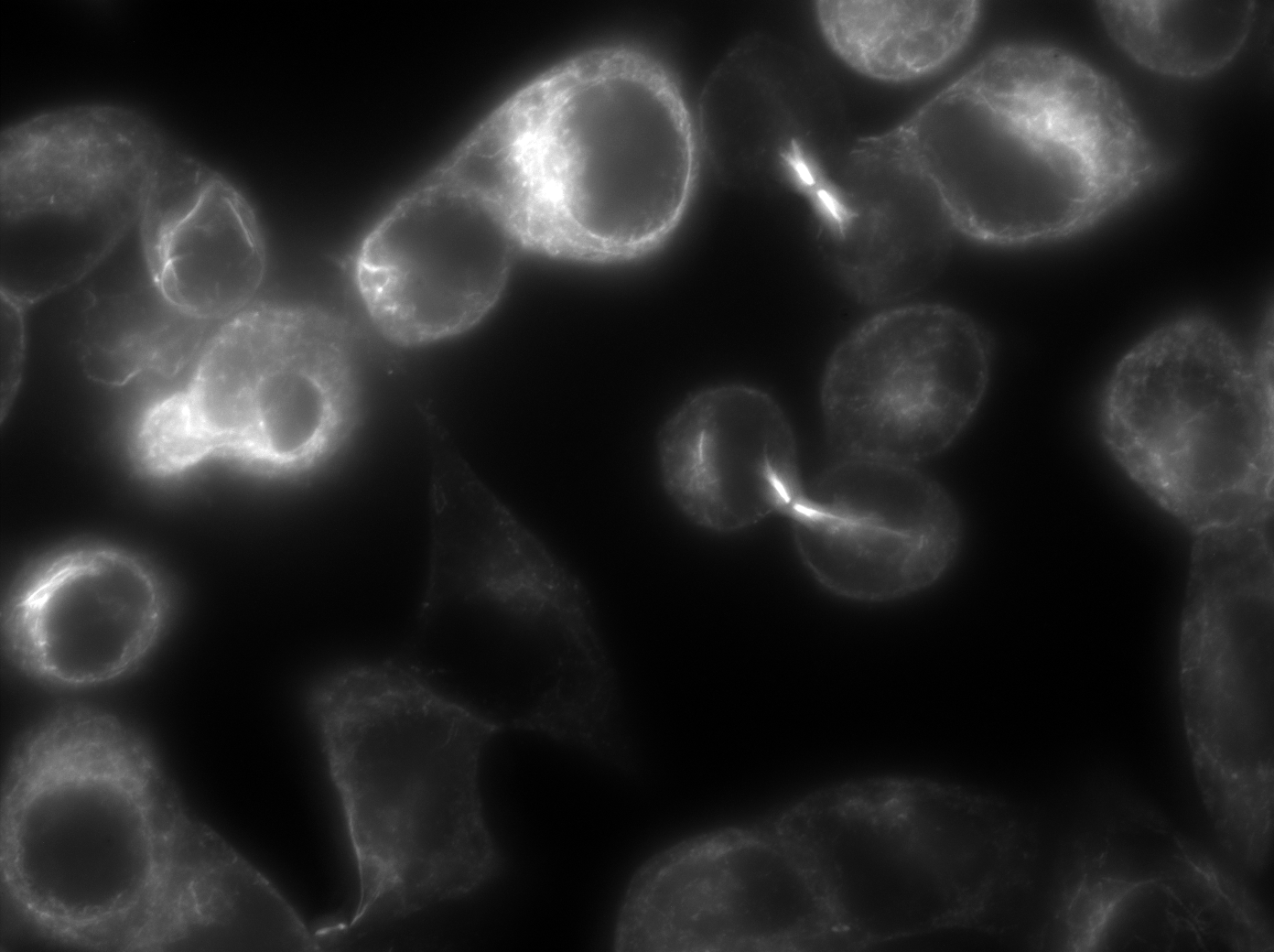

Supplement: Supplementary file 23 — Source data Fig. 6 part 4 [file 44319_2026_742_MOESM23_ESM.zip › Figure 6 Part 4/Fig 6efg TPGS1-KO TPGS1 rescue experiments part 2/R2R3/TPGS1-KO TPGS1-EYFP-3'UTR actub 7-31-25 R2 ET6.Project Maximum Z_XY1756411247_Z0_T0_C2.tif]

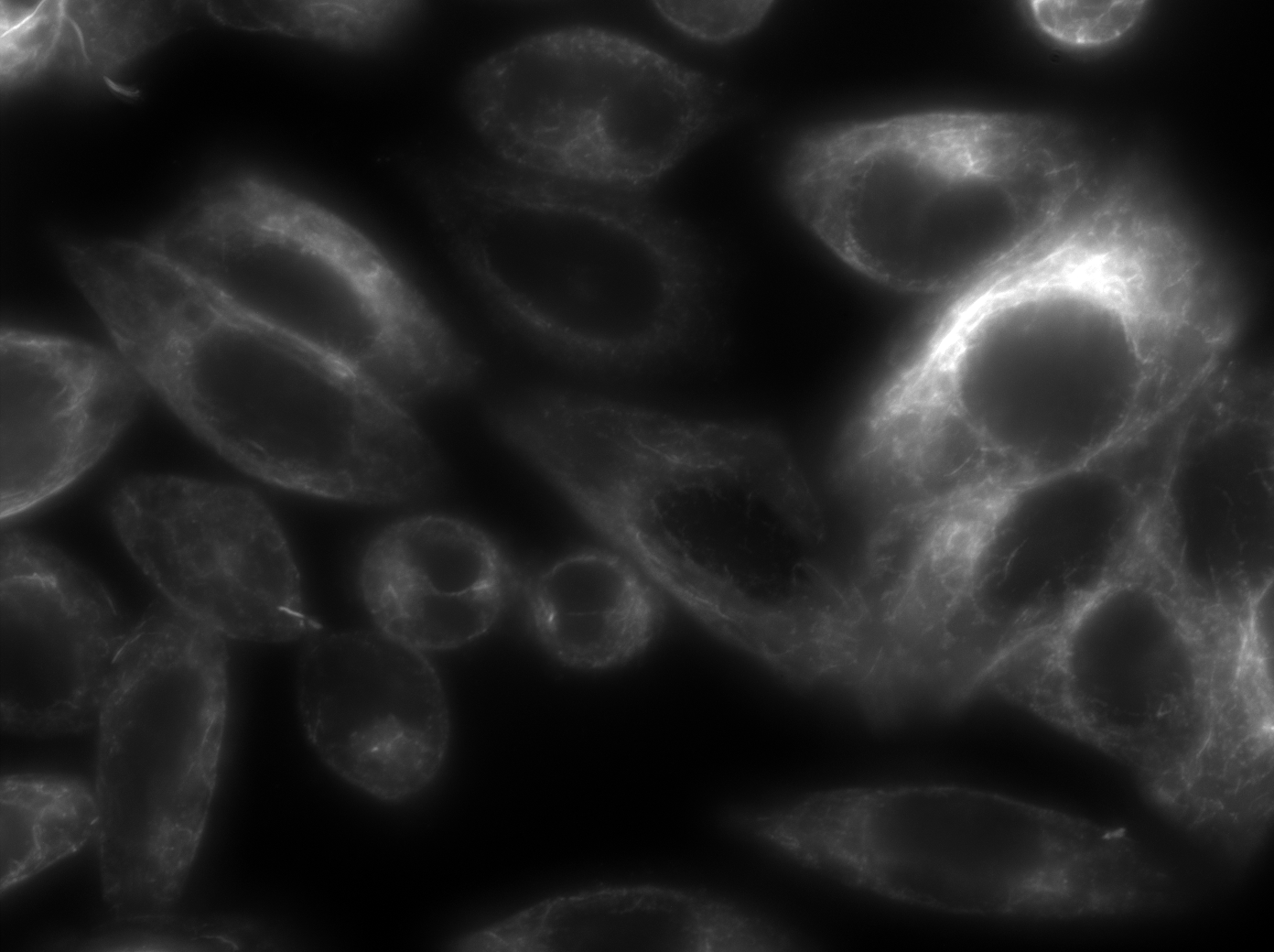

Supplement: Supplementary file 23 — Source data Fig. 6 part 4 [file 44319_2026_742_MOESM23_ESM.zip › Figure 6 Part 4/Fig 6efg TPGS1-KO TPGS1 rescue experiments part 2/R2R3/TPGS1-KO TPGS1-EYFP-3'UTR actub 7-31-25 R2 LT8.Project Maximum Z_XY1756411785_Z0_T0_C2.tif]

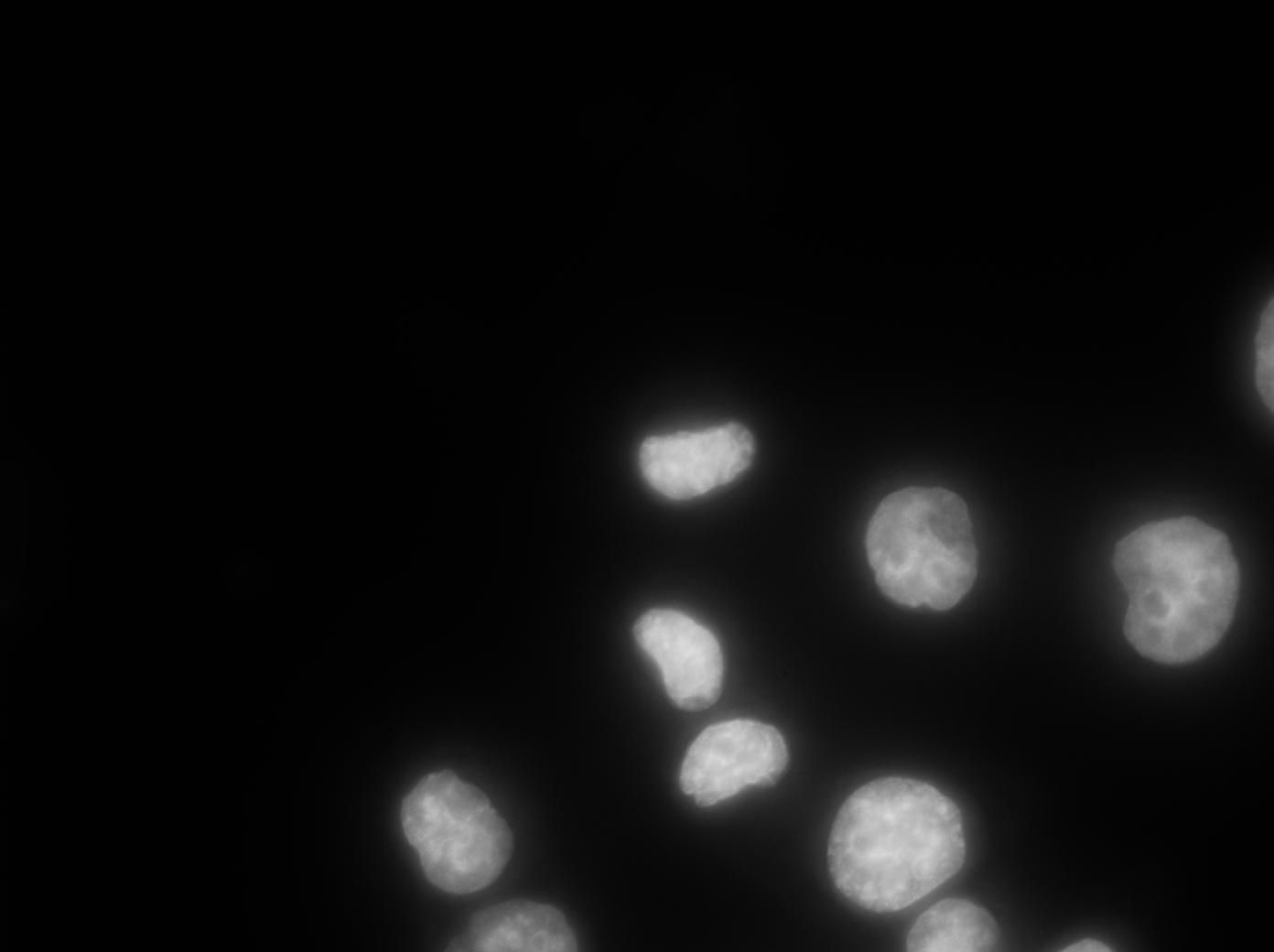

Supplement: Supplementary file 23 — Source data Fig. 6 part 4 [file 44319_2026_742_MOESM23_ESM.zip › Figure 6 Part 4/Fig 6efg TPGS1-KO TPGS1 rescue experiments part 2/R2R3/TPGS1-KO EYFP-only actub 7-31-25 R2 ET9.Project Maximum Z_XY1756416411_Z0_T0_C0.tif]

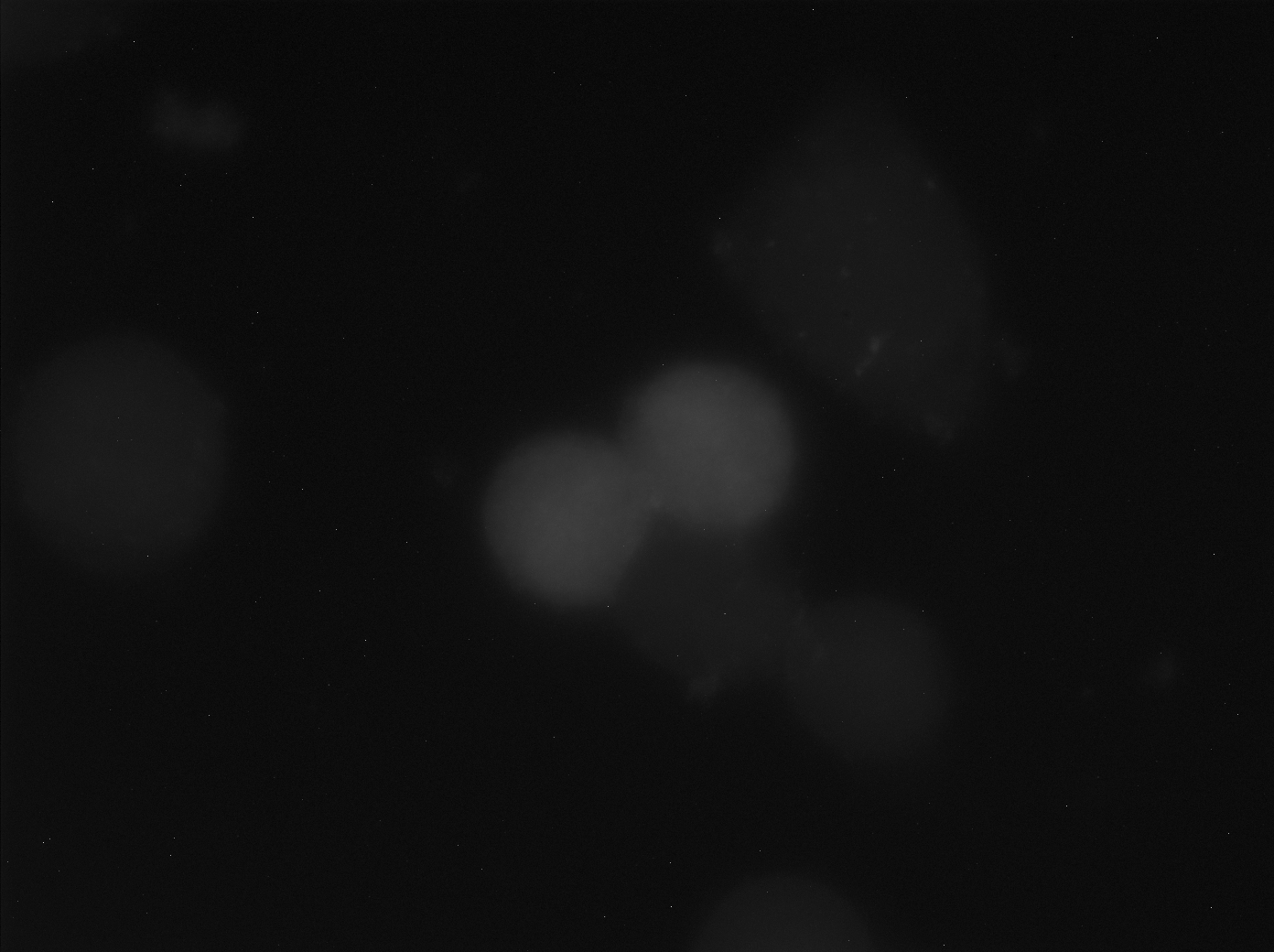

Supplement: Supplementary file 23 — Source data Fig. 6 part 4 [file 44319_2026_742_MOESM23_ESM.zip › Figure 6 Part 4/Fig 6efg TPGS1-KO TPGS1 rescue experiments part 2/R2R3/TPGS1-KO EYFP-only actub 7-31-25 R2 ET4.Project Maximum Z_XY1756414878_Z0_T0_C1.tif]

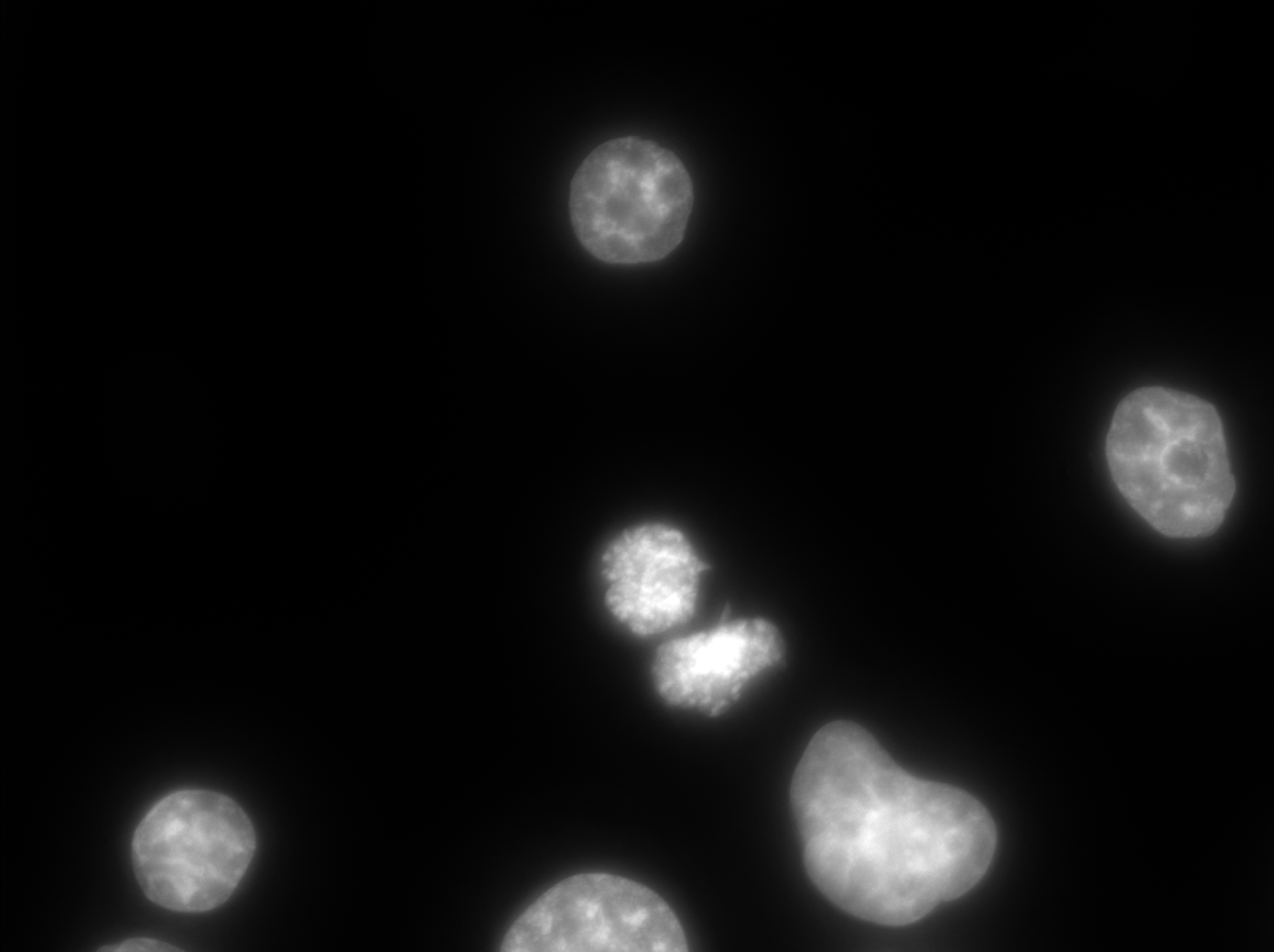

Supplement: Supplementary file 23 — Source data Fig. 6 part 4 [file 44319_2026_742_MOESM23_ESM.zip › Figure 6 Part 4/Fig 6efg TPGS1-KO TPGS1 rescue experiments part 2/R2R3/TPGS1-KO EYFP-only actub 7-31-25 R3 ET2.Project Maximum Z_XY1756492327_Z0_T0_C0.tif]

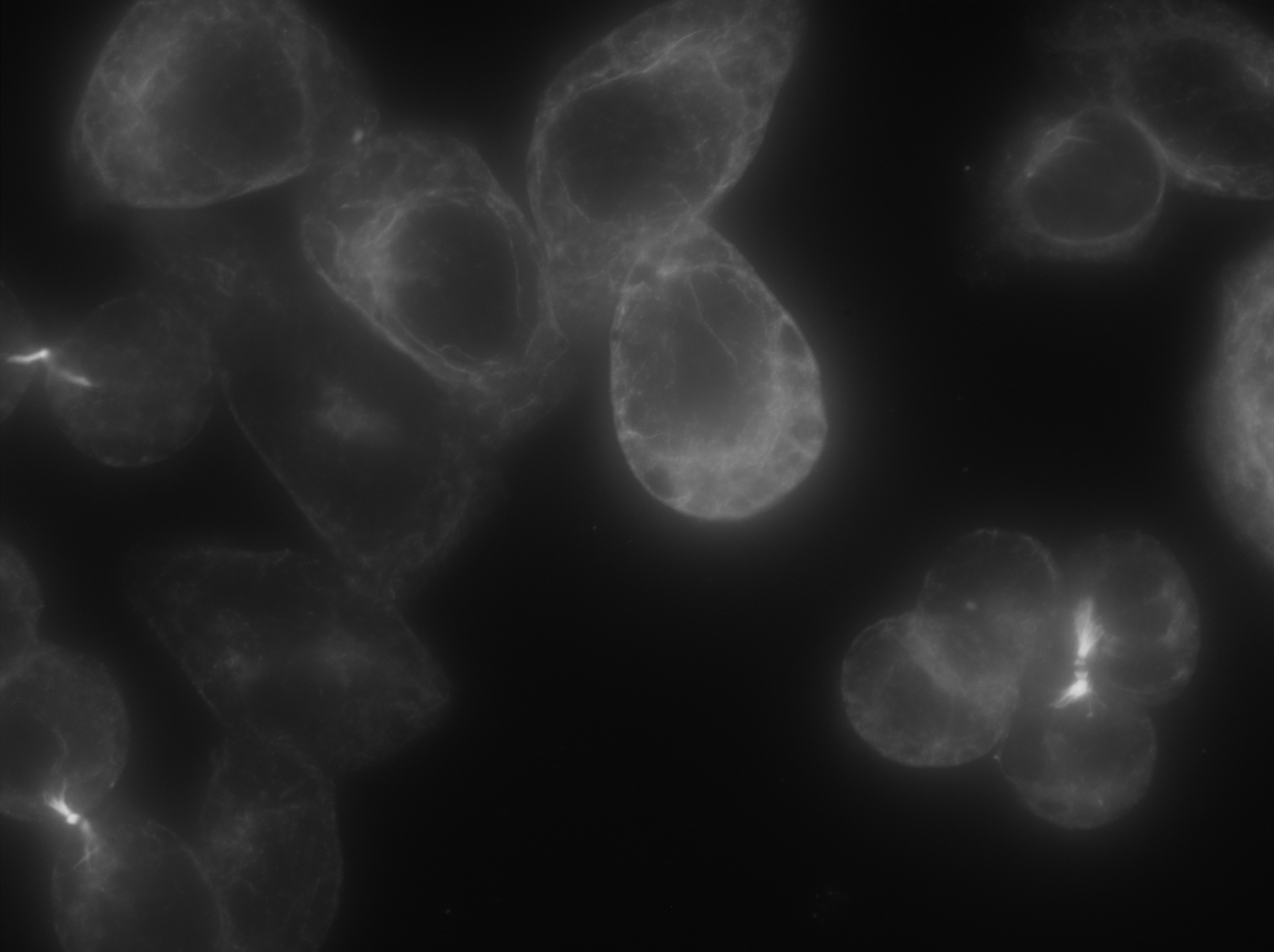

Supplement: Supplementary file 23 — Source data Fig. 6 part 4 [file 44319_2026_742_MOESM23_ESM.zip › Figure 6 Part 4/Fig 6efg TPGS1-KO TPGS1 rescue experiments part 2/R2R3/TPGS1-KO EYFP-only actub 7-31-25 R2 ET7.Project Maximum Z_XY1756415514_Z0_T0_C2.tif]

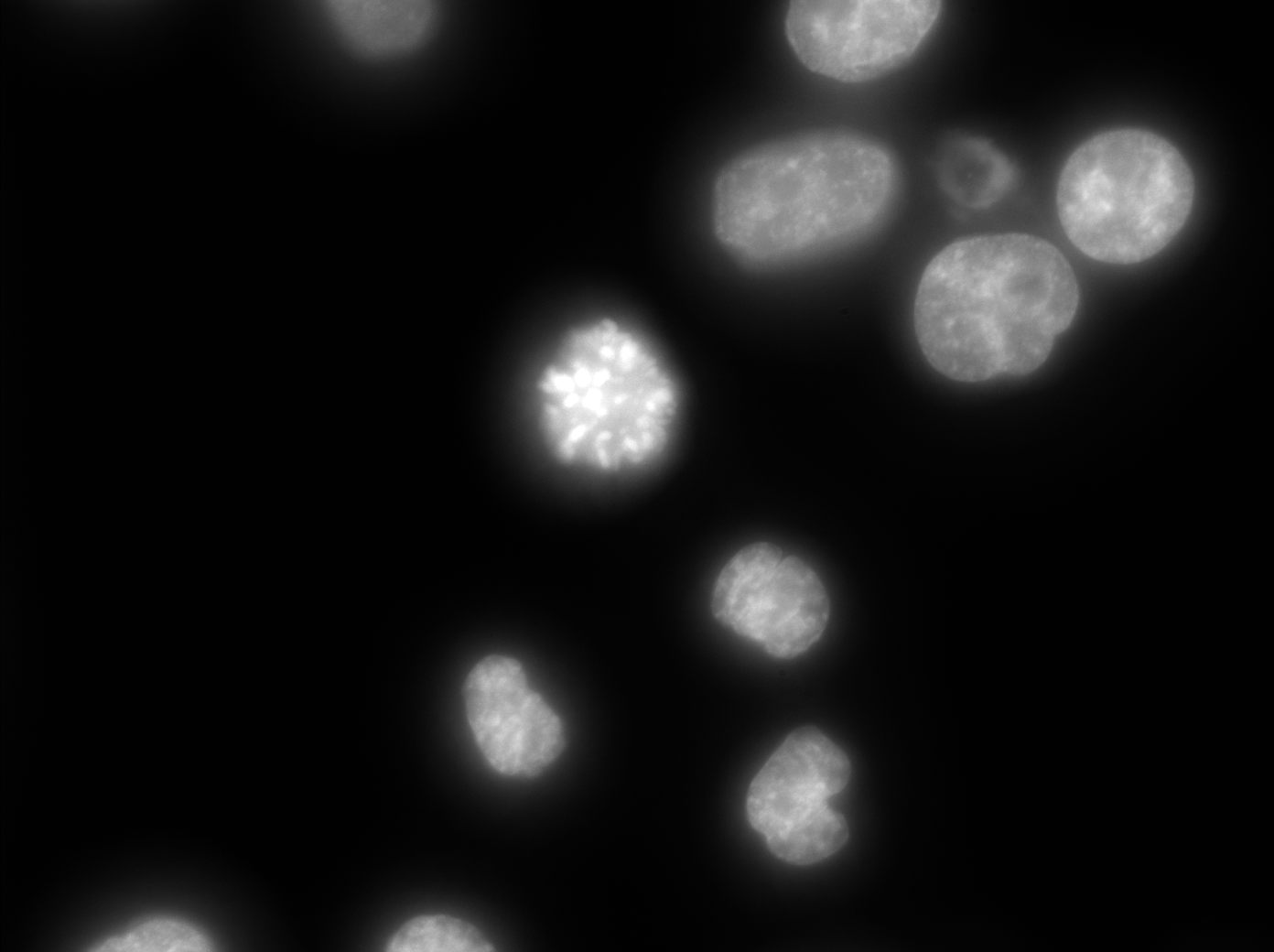

Supplement: Supplementary file 23 — Source data Fig. 6 part 4 [file 44319_2026_742_MOESM23_ESM.zip › Figure 6 Part 4/Fig 6efg TPGS1-KO TPGS1 rescue experiments part 2/R2R3/TPGS1-KO EYFP-only actub 7-31-25 R3 LT5.Project Maximum Z_XY1756493505_Z0_T0_C0.tif]

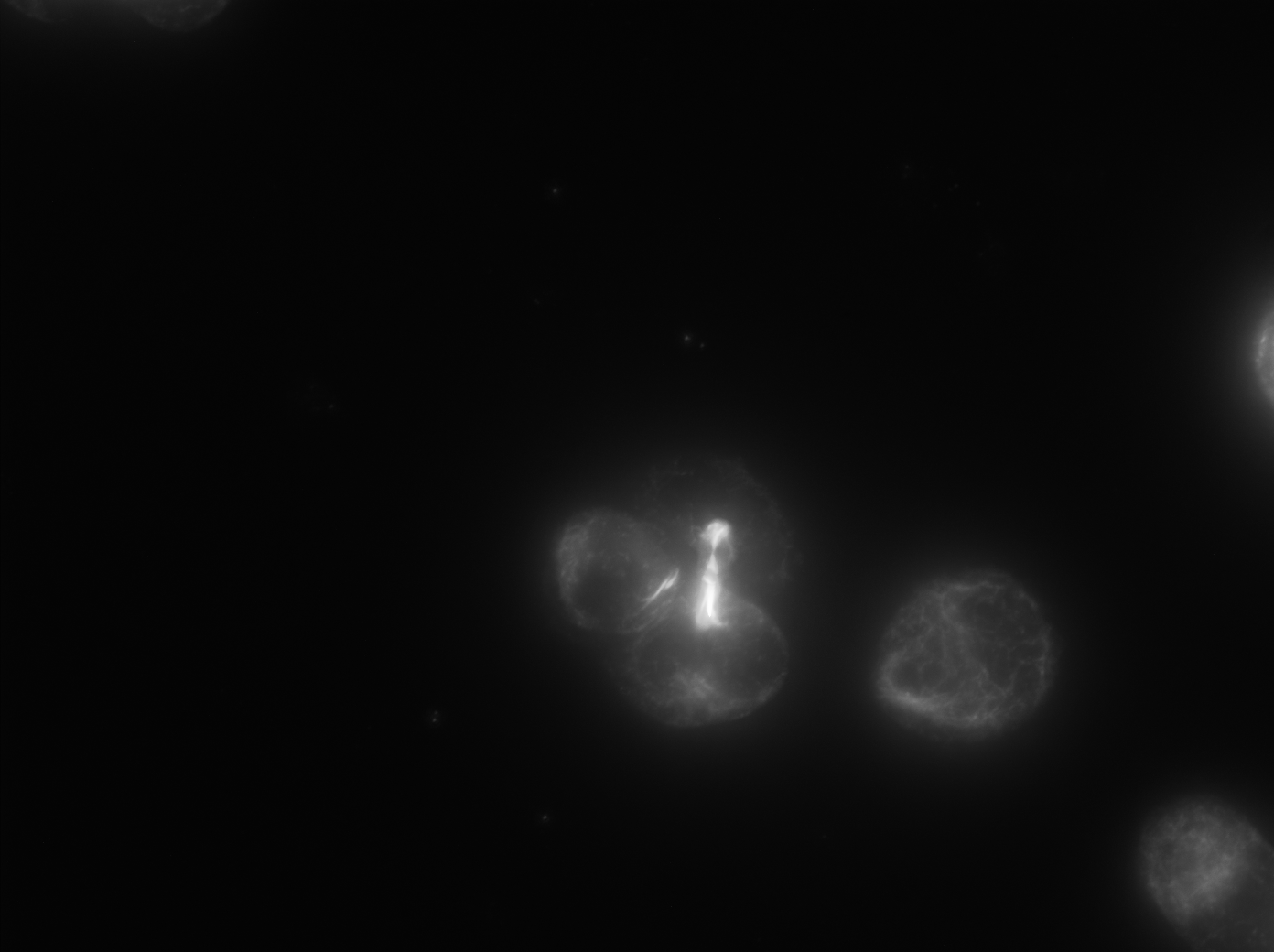

Supplement: Supplementary file 23 — Source data Fig. 6 part 4 [file 44319_2026_742_MOESM23_ESM.zip › Figure 6 Part 4/Fig 6efg TPGS1-KO TPGS1 rescue experiments part 2/R2R3/TPGS1-KO EYFP-only actub 7-31-25 R3 LT10.Project Maximum Z_XY1756496715_Z0_T0_C2.tif]

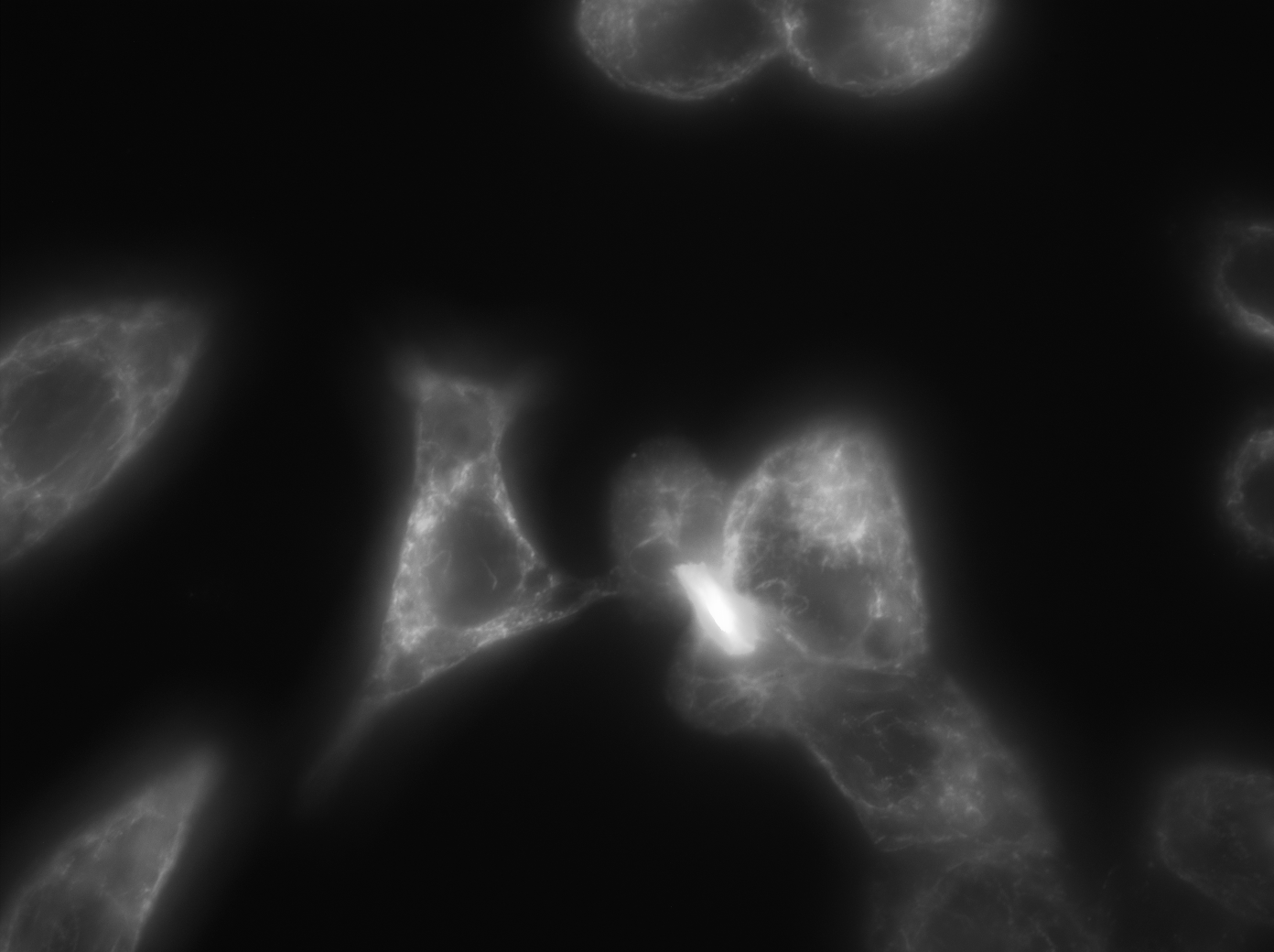

Supplement: Supplementary file 23 — Source data Fig. 6 part 4 [file 44319_2026_742_MOESM23_ESM.zip › Figure 6 Part 4/Fig 6efg TPGS1-KO TPGS1 rescue experiments part 2/R2R3/TPGS1-KO TPGS1-EYFP-3'UTR actub 7-31-25 R3 ET1.Project Maximum Z_XY1756498603_Z0_T0_C2.tif]

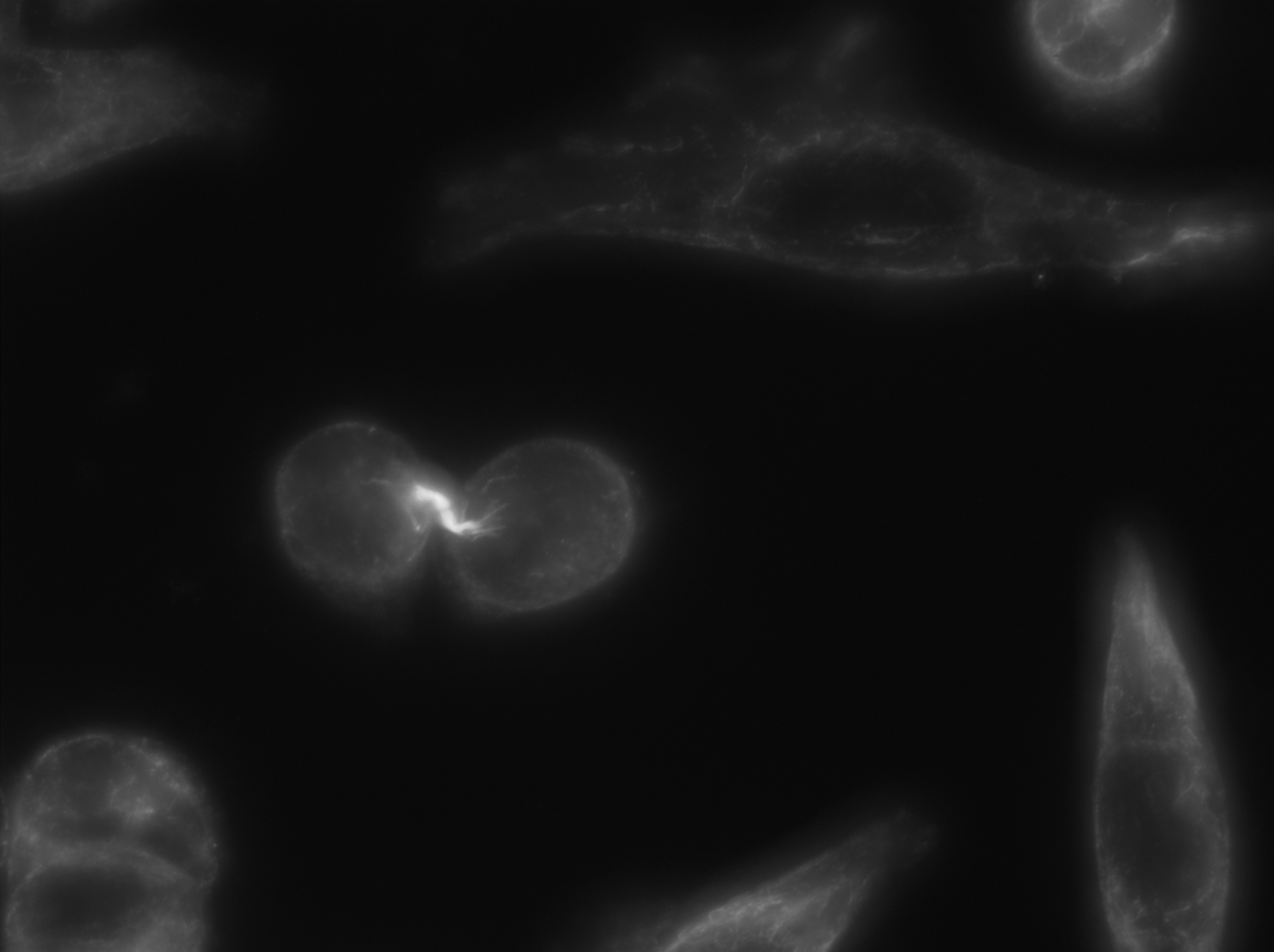

Supplement: Supplementary file 23 — Source data Fig. 6 part 4 [file 44319_2026_742_MOESM23_ESM.zip › Figure 6 Part 4/Fig 6efg TPGS1-KO TPGS1 rescue experiments part 2/R2R3/TPGS1-KO EYFP-only actub 7-31-25 R2 LT6.Project Maximum Z_XY1756415028_Z0_T0_C2.tif]

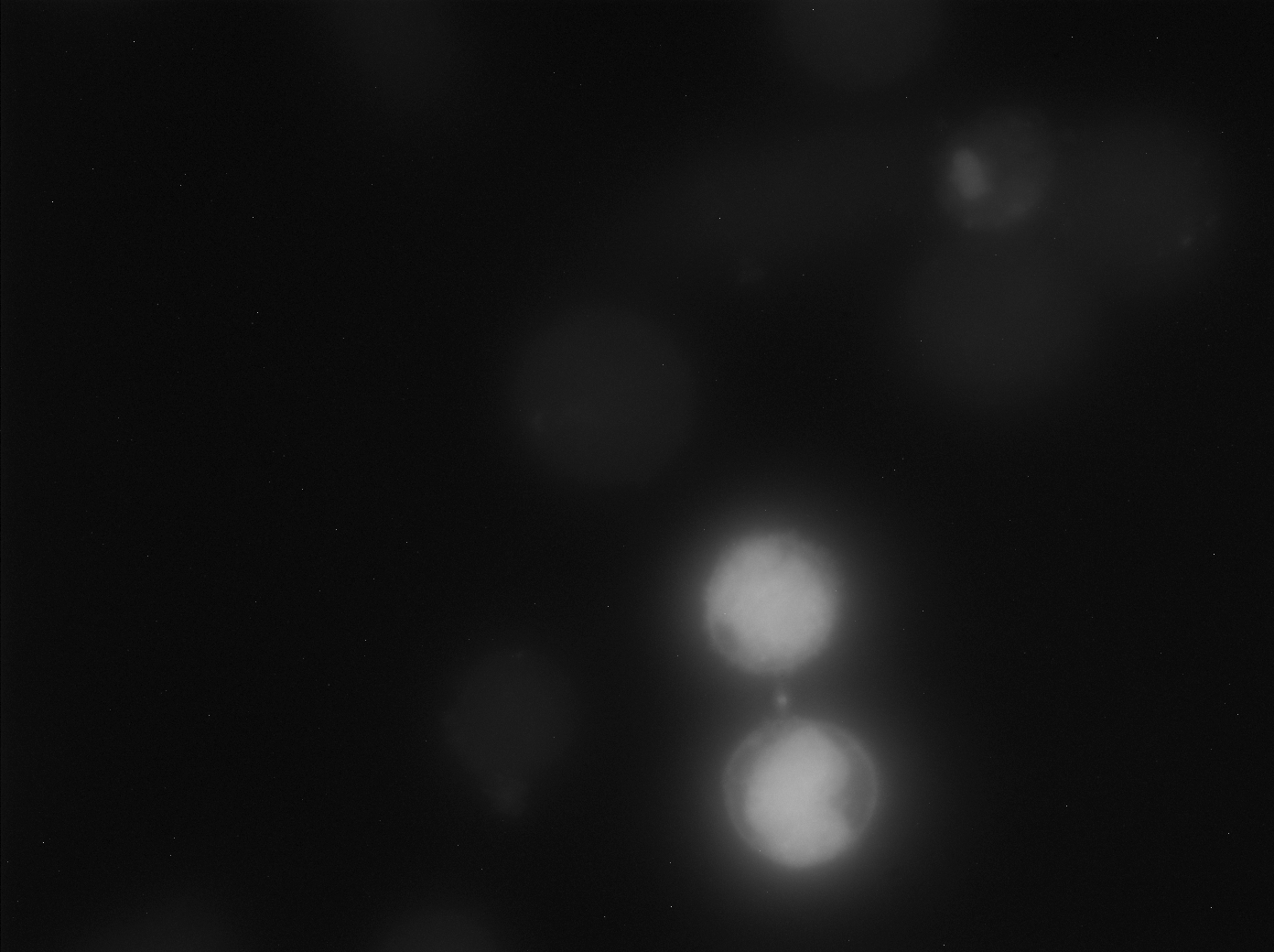

Supplement: Supplementary file 23 — Source data Fig. 6 part 4 [file 44319_2026_742_MOESM23_ESM.zip › Figure 6 Part 4/Fig 6efg TPGS1-KO TPGS1 rescue experiments part 2/R2R3/TPGS1-KO EYFP-only actub 7-31-25 R3 LT5.Project Maximum Z_XY1756493505_Z0_T0_C1.tif]

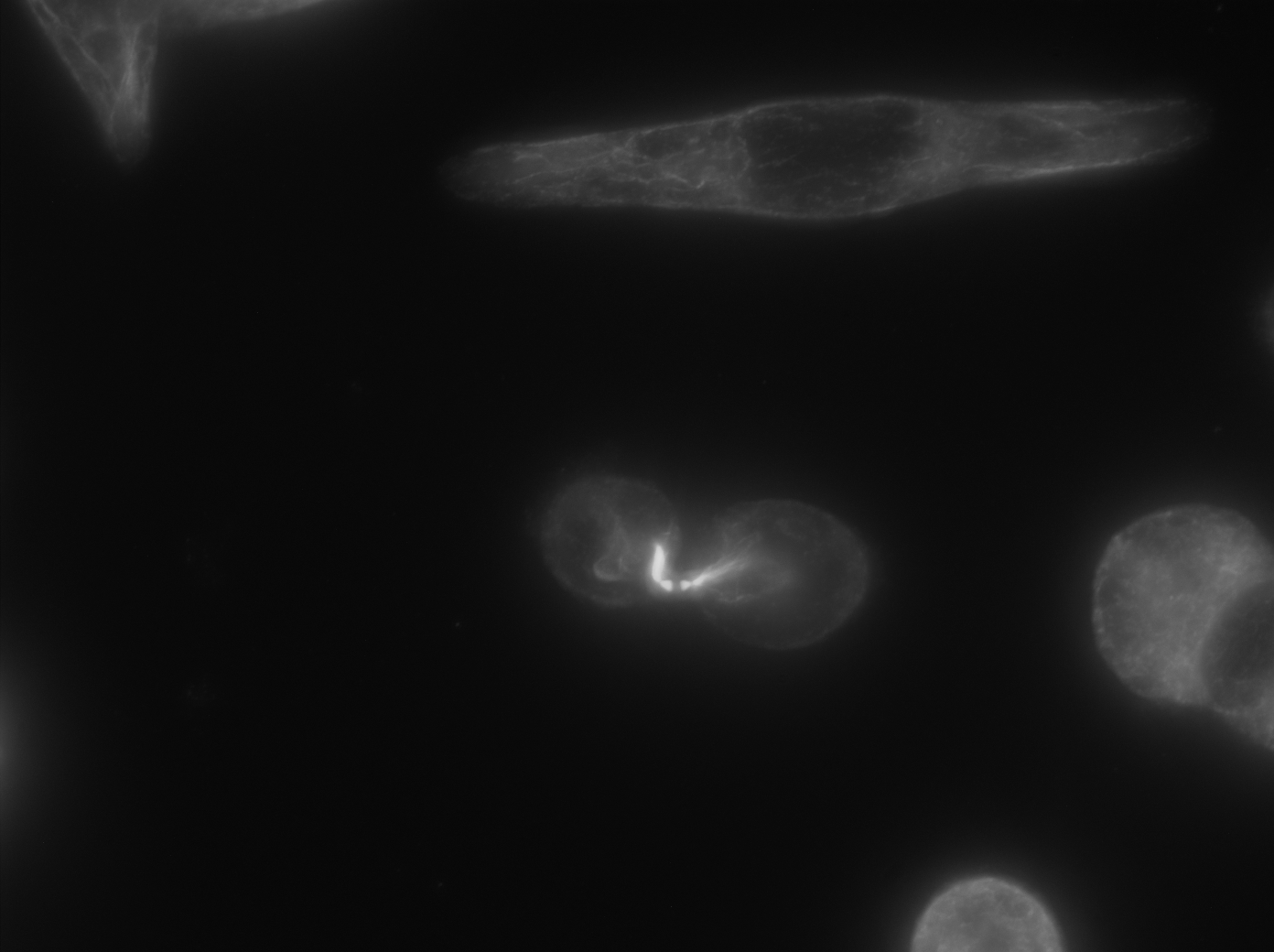

Supplement: Supplementary file 23 — Source data Fig. 6 part 4 [file 44319_2026_742_MOESM23_ESM.zip › Figure 6 Part 4/Fig 6efg TPGS1-KO TPGS1 rescue experiments part 2/R2R3/TPGS1-KO EYFP-only actub 7-31-25 R2 ET8.Project Maximum Z_XY1756416166_Z0_T0_C2.tif]

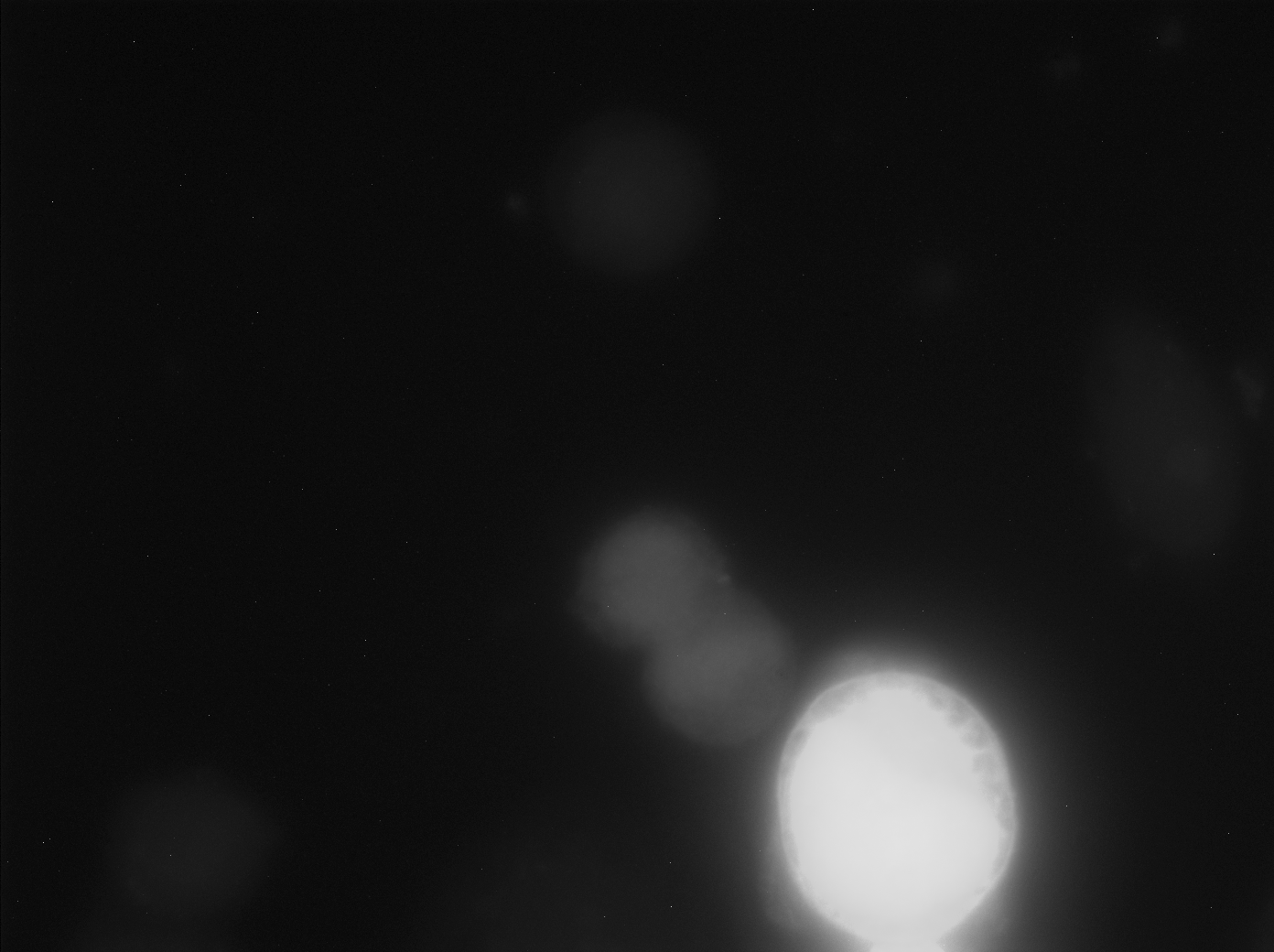

Supplement: Supplementary file 23 — Source data Fig. 6 part 4 [file 44319_2026_742_MOESM23_ESM.zip › Figure 6 Part 4/Fig 6efg TPGS1-KO TPGS1 rescue experiments part 2/R2R3/TPGS1-KO EYFP-only actub 7-31-25 R3 ET2.Project Maximum Z_XY1756492327_Z0_T0_C1.tif]

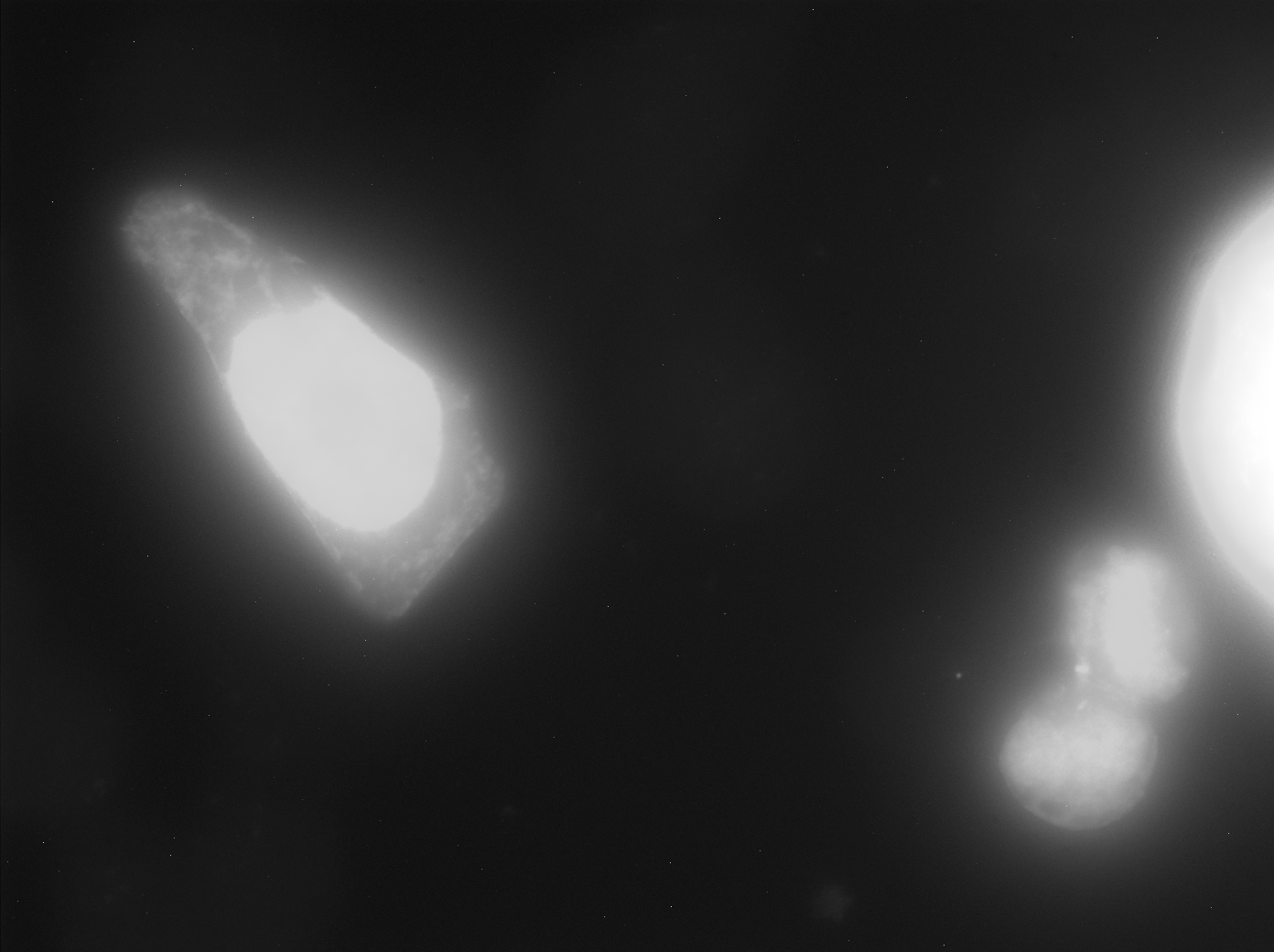

Supplement: Supplementary file 23 — Source data Fig. 6 part 4 [file 44319_2026_742_MOESM23_ESM.zip › Figure 6 Part 4/Fig 6efg TPGS1-KO TPGS1 rescue experiments part 2/R2R3/TPGS1-KO EYFP-only actub 7-31-25 R2 ET7.Project Maximum Z_XY1756415514_Z0_T0_C1.tif]

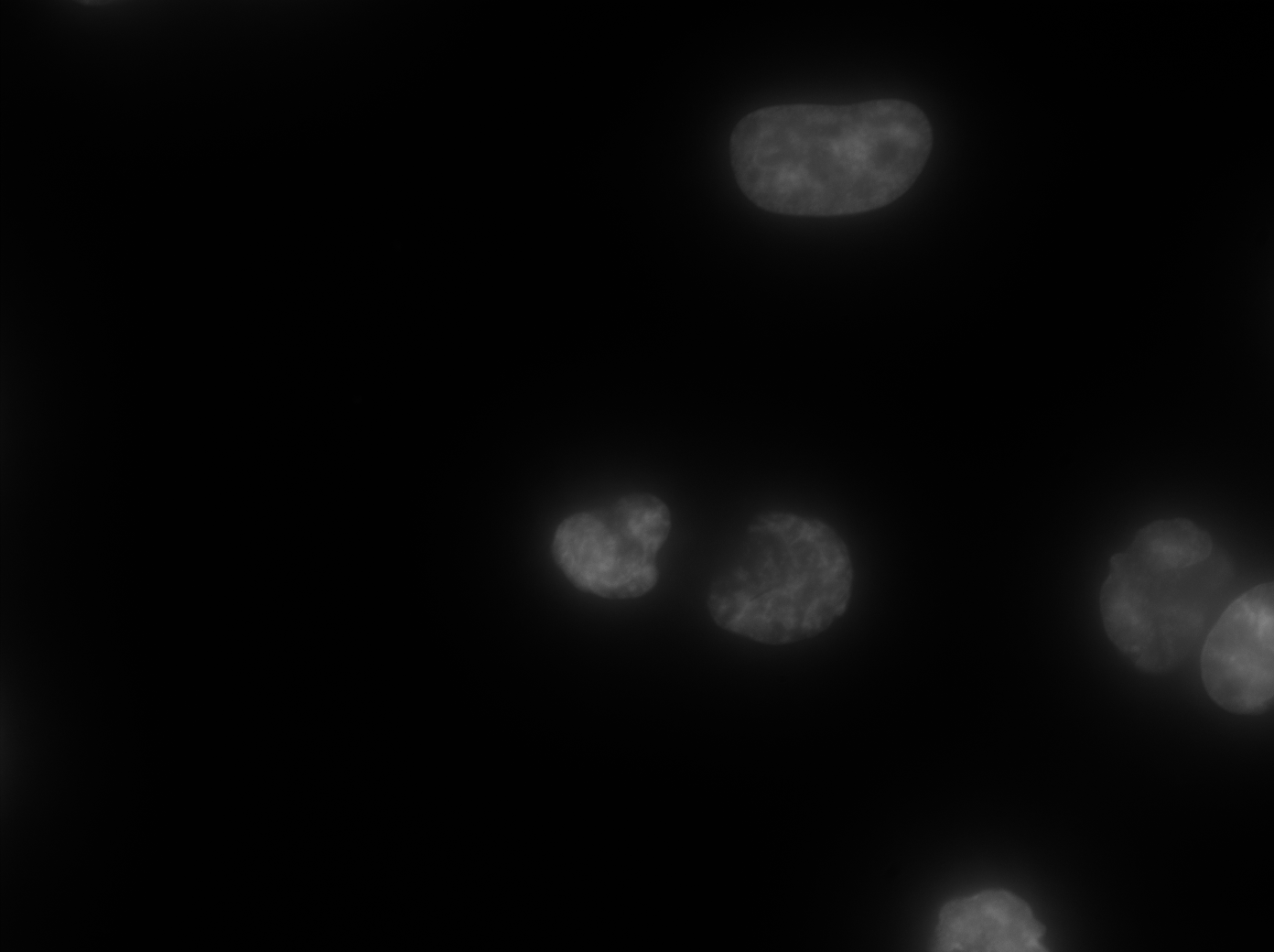

Supplement: Supplementary file 23 — Source data Fig. 6 part 4 [file 44319_2026_742_MOESM23_ESM.zip › Figure 6 Part 4/Fig 6efg TPGS1-KO TPGS1 rescue experiments part 2/R2R3/TPGS1-KO EYFP-only actub 7-31-25 R2 ET8.Project Maximum Z_XY1756416166_Z0_T0_C0.tif]

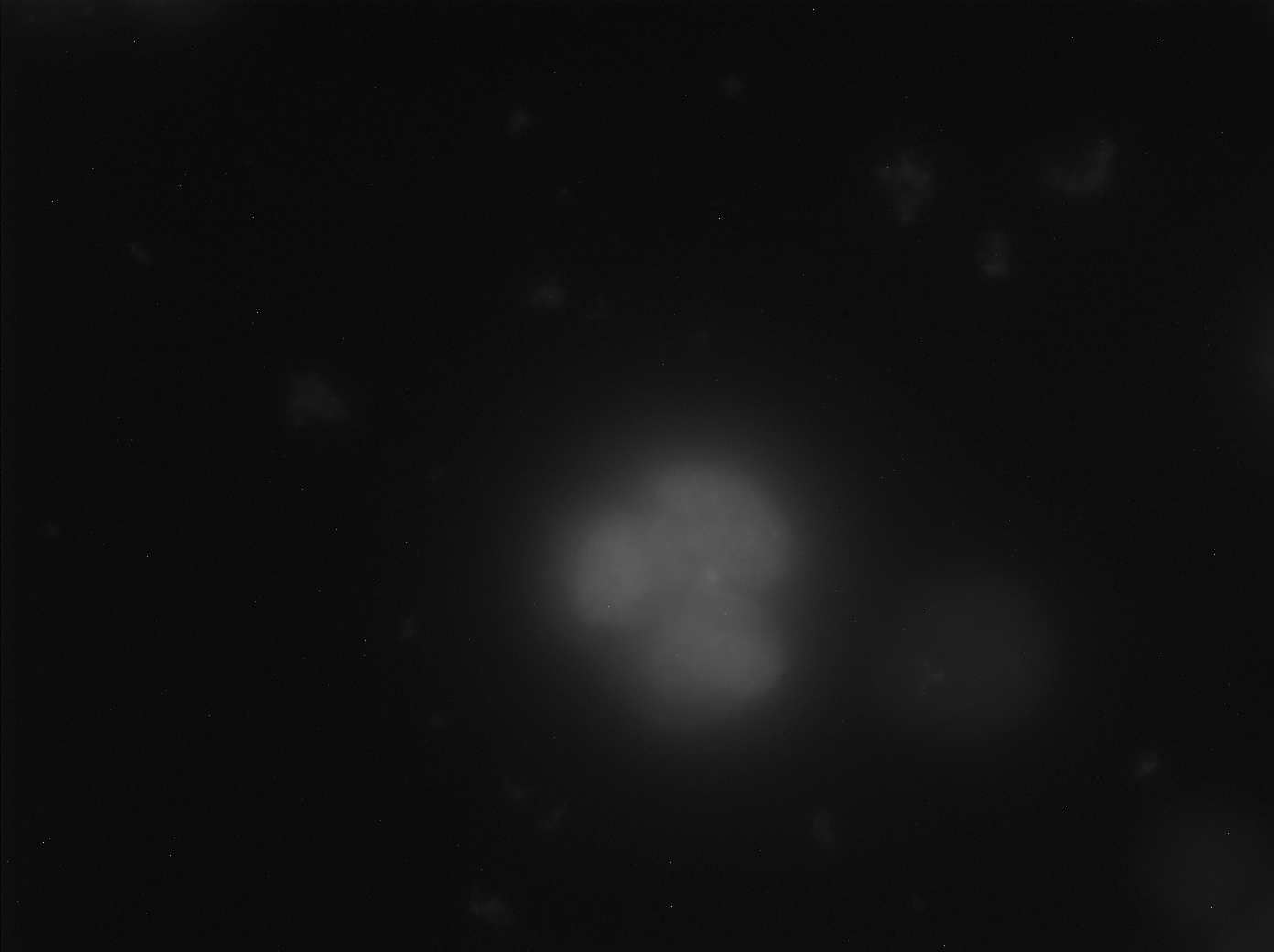

Supplement: Supplementary file 23 — Source data Fig. 6 part 4 [file 44319_2026_742_MOESM23_ESM.zip › Figure 6 Part 4/Fig 6efg TPGS1-KO TPGS1 rescue experiments part 2/R2R3/TPGS1-KO EYFP-only actub 7-31-25 R3 LT10.Project Maximum Z_XY1756496715_Z0_T0_C1.tif]

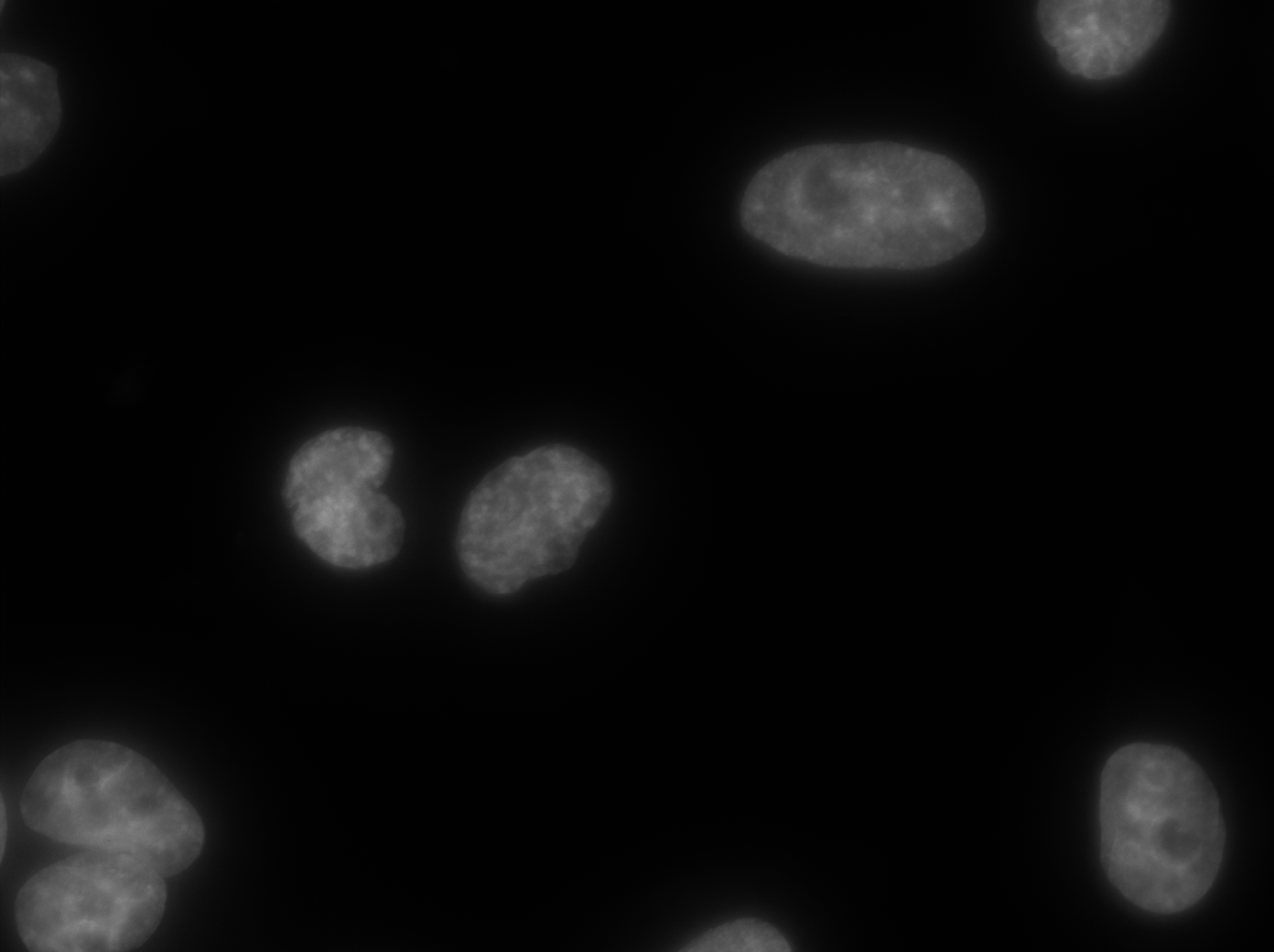

Supplement: Supplementary file 23 — Source data Fig. 6 part 4 [file 44319_2026_742_MOESM23_ESM.zip › Figure 6 Part 4/Fig 6efg TPGS1-KO TPGS1 rescue experiments part 2/R2R3/TPGS1-KO EYFP-only actub 7-31-25 R2 LT6.Project Maximum Z_XY1756415028_Z0_T0_C0.tif]

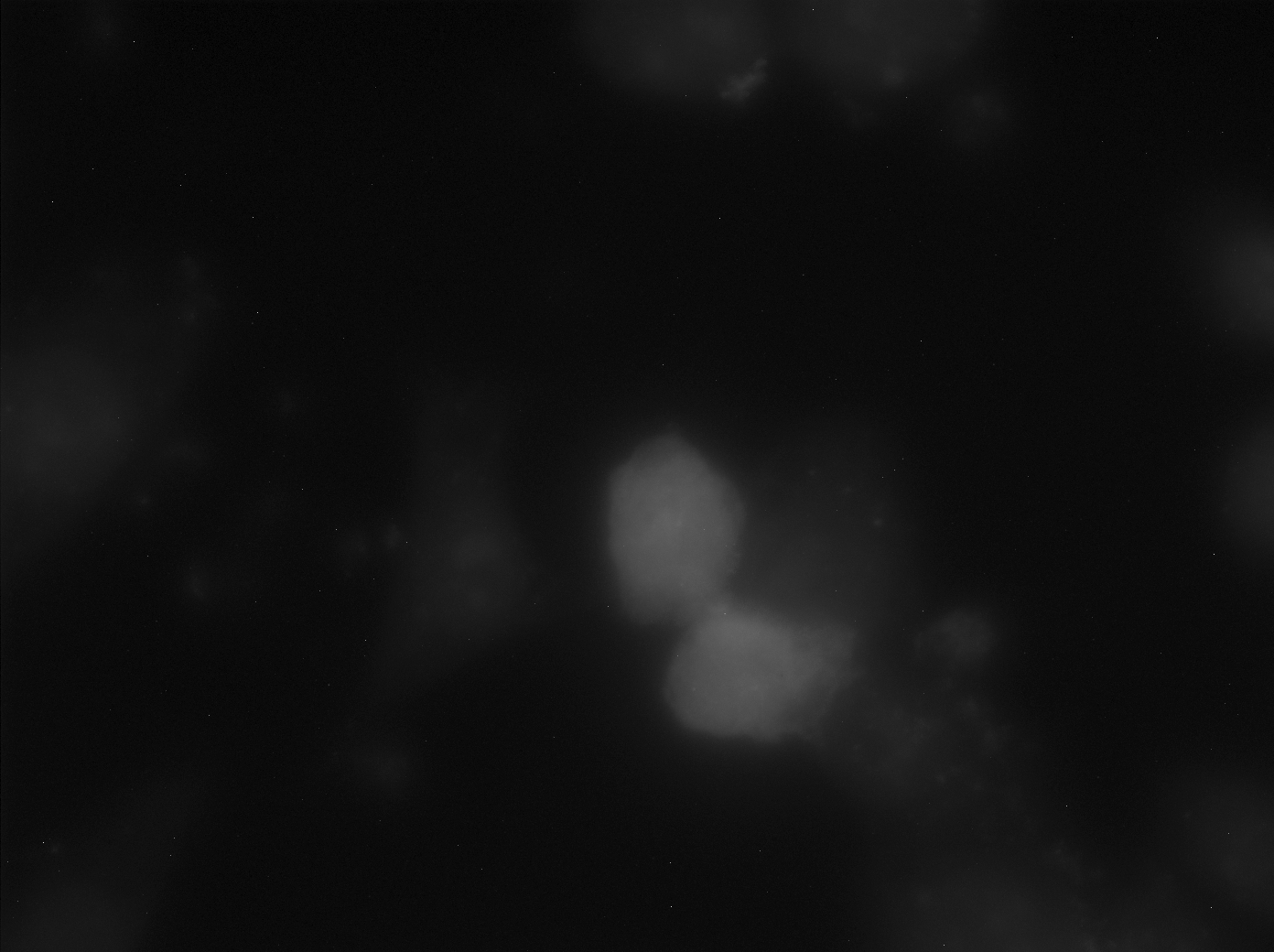

Supplement: Supplementary file 23 — Source data Fig. 6 part 4 [file 44319_2026_742_MOESM23_ESM.zip › Figure 6 Part 4/Fig 6efg TPGS1-KO TPGS1 rescue experiments part 2/R2R3/TPGS1-KO TPGS1-EYFP-3'UTR actub 7-31-25 R3 ET1.Project Maximum Z_XY1756498603_Z0_T0_C1.tif]

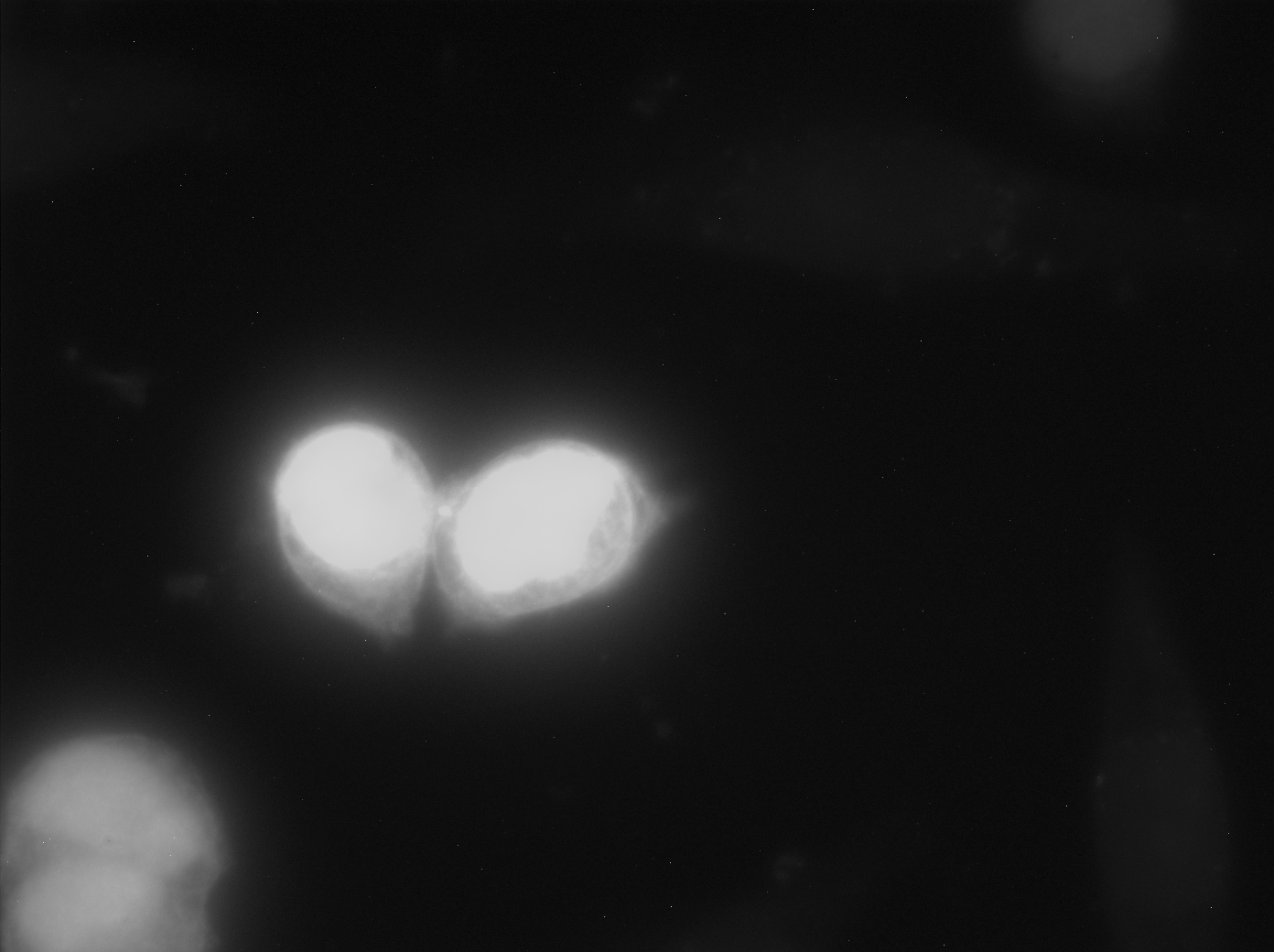

Supplement: Supplementary file 23 — Source data Fig. 6 part 4 [file 44319_2026_742_MOESM23_ESM.zip › Figure 6 Part 4/Fig 6efg TPGS1-KO TPGS1 rescue experiments part 2/R2R3/TPGS1-KO EYFP-only actub 7-31-25 R2 LT6.Project Maximum Z_XY1756415028_Z0_T0_C1.tif]

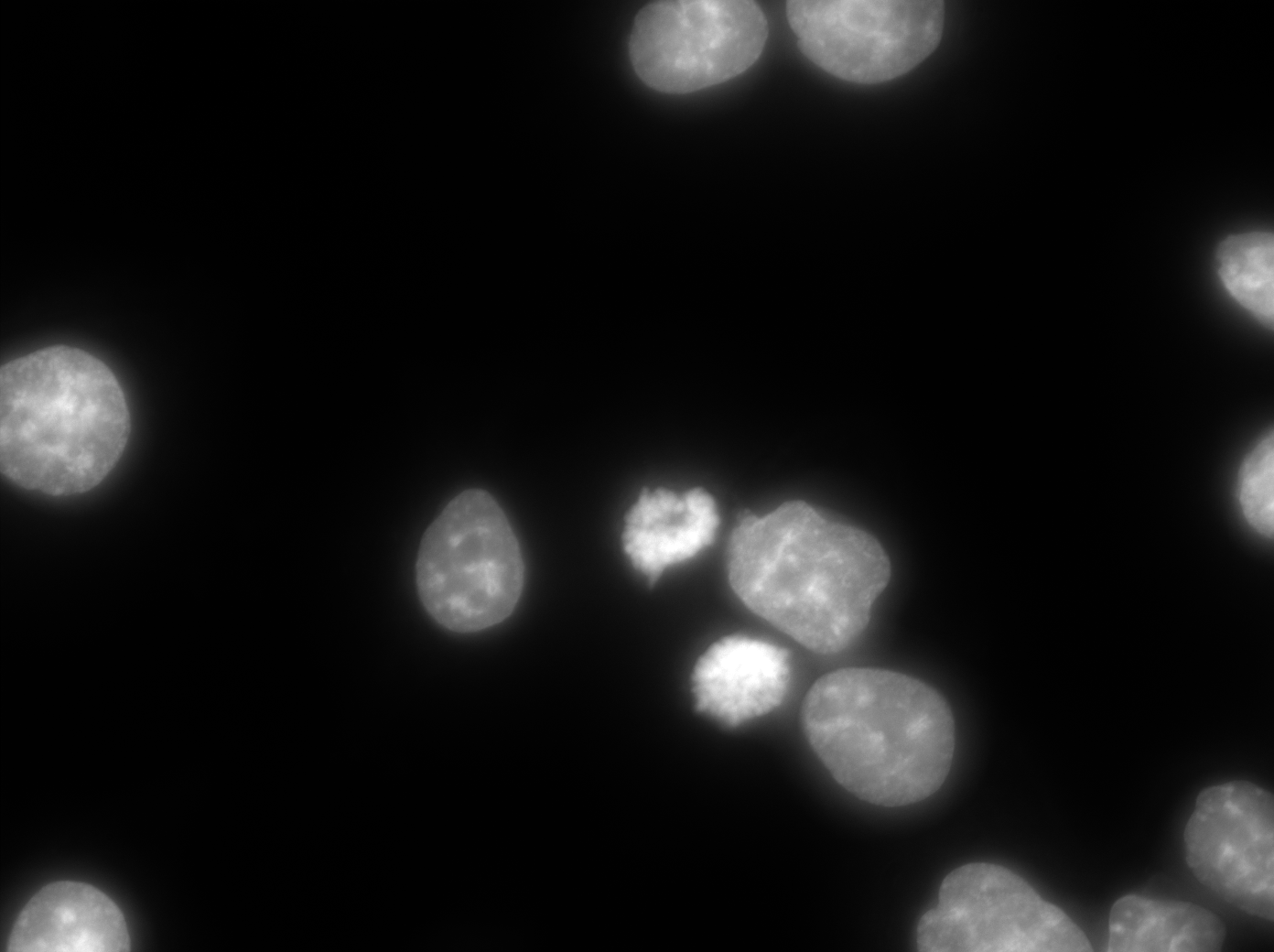

Supplement: Supplementary file 23 — Source data Fig. 6 part 4 [file 44319_2026_742_MOESM23_ESM.zip › Figure 6 Part 4/Fig 6efg TPGS1-KO TPGS1 rescue experiments part 2/R2R3/TPGS1-KO TPGS1-EYFP-3'UTR actub 7-31-25 R3 ET1.Project Maximum Z_XY1756498603_Z0_T0_C0.tif]

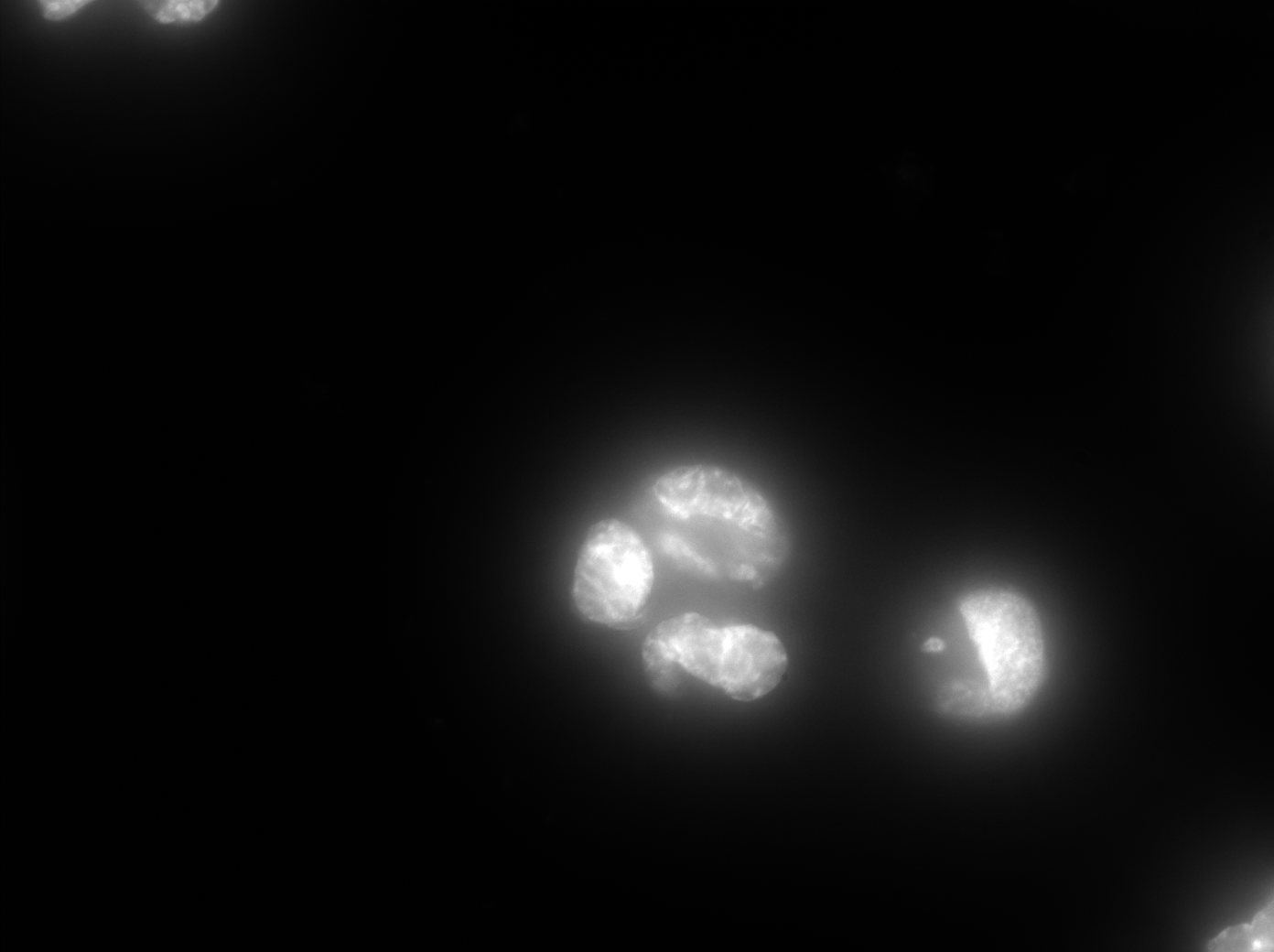

Supplement: Supplementary file 23 — Source data Fig. 6 part 4 [file 44319_2026_742_MOESM23_ESM.zip › Figure 6 Part 4/Fig 6efg TPGS1-KO TPGS1 rescue experiments part 2/R2R3/TPGS1-KO EYFP-only actub 7-31-25 R3 LT10.Project Maximum Z_XY1756496715_Z0_T0_C0.tif]

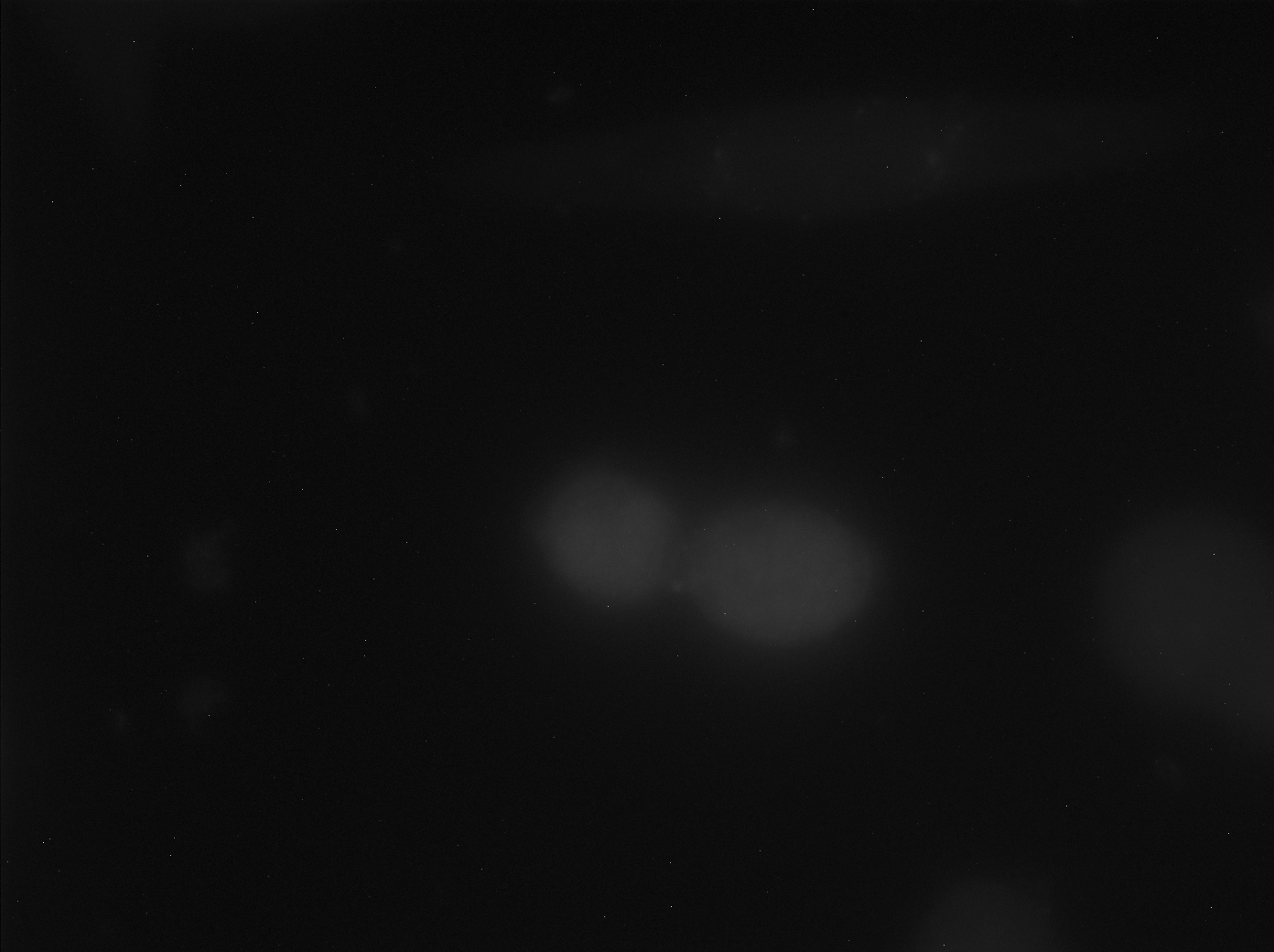

Supplement: Supplementary file 23 — Source data Fig. 6 part 4 [file 44319_2026_742_MOESM23_ESM.zip › Figure 6 Part 4/Fig 6efg TPGS1-KO TPGS1 rescue experiments part 2/R2R3/TPGS1-KO EYFP-only actub 7-31-25 R2 ET8.Project Maximum Z_XY1756416166_Z0_T0_C1.tif]

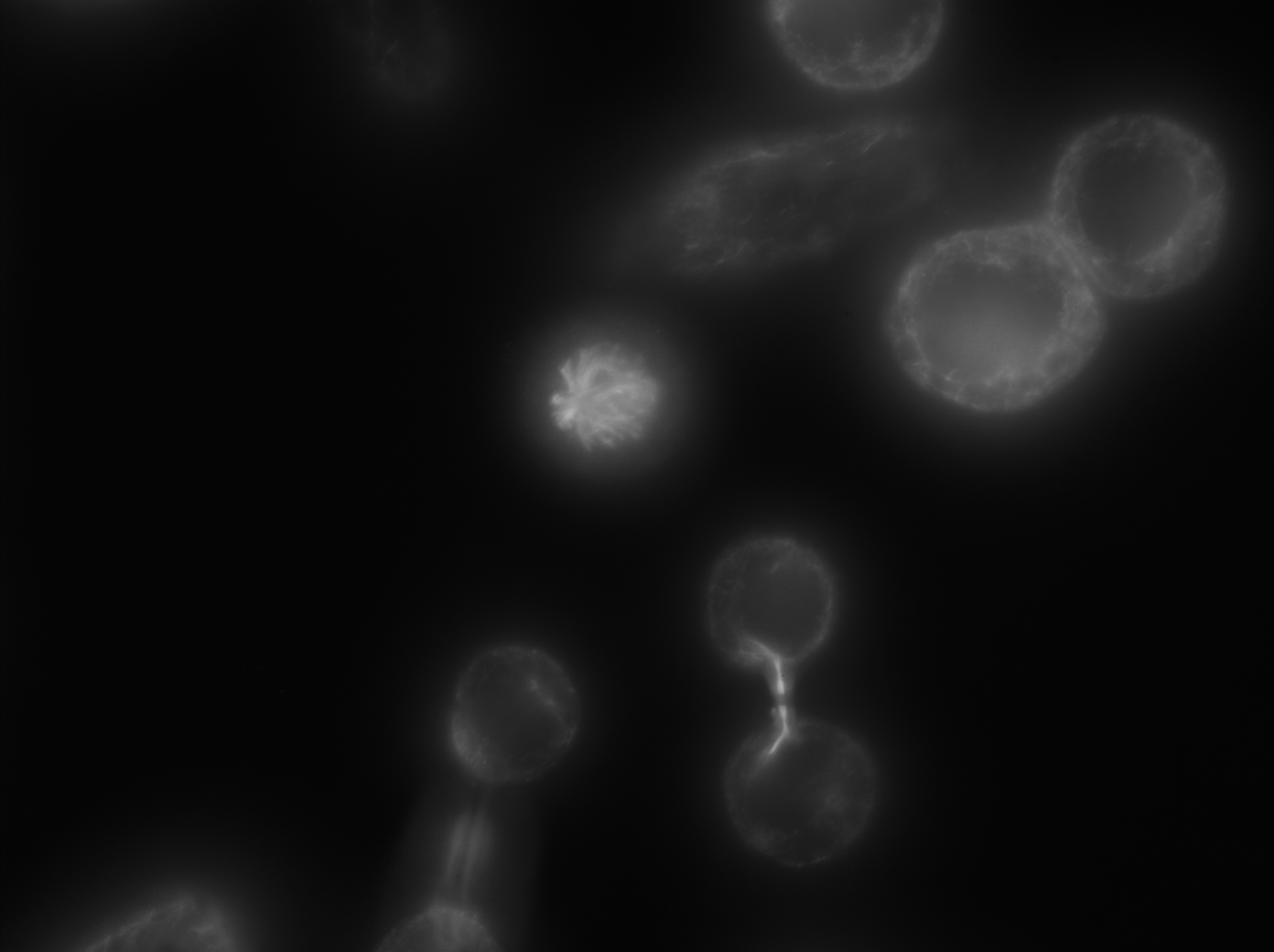

Supplement: Supplementary file 23 — Source data Fig. 6 part 4 [file 44319_2026_742_MOESM23_ESM.zip › Figure 6 Part 4/Fig 6efg TPGS1-KO TPGS1 rescue experiments part 2/R2R3/TPGS1-KO EYFP-only actub 7-31-25 R3 LT5.Project Maximum Z_XY1756493505_Z0_T0_C2.tif]

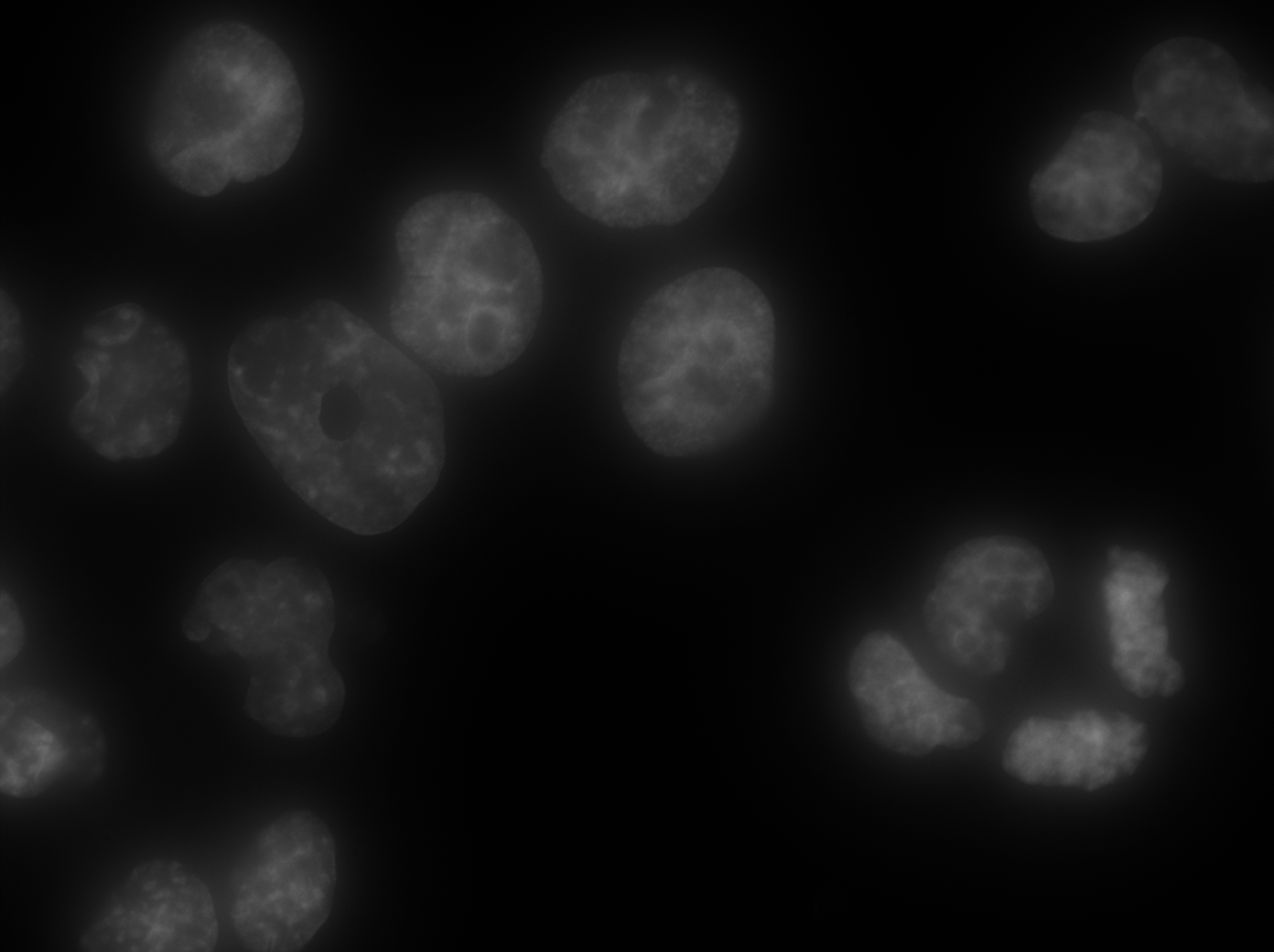

Supplement: Supplementary file 23 — Source data Fig. 6 part 4 [file 44319_2026_742_MOESM23_ESM.zip › Figure 6 Part 4/Fig 6efg TPGS1-KO TPGS1 rescue experiments part 2/R2R3/TPGS1-KO EYFP-only actub 7-31-25 R2 ET7.Project Maximum Z_XY1756415514_Z0_T0_C0.tif]

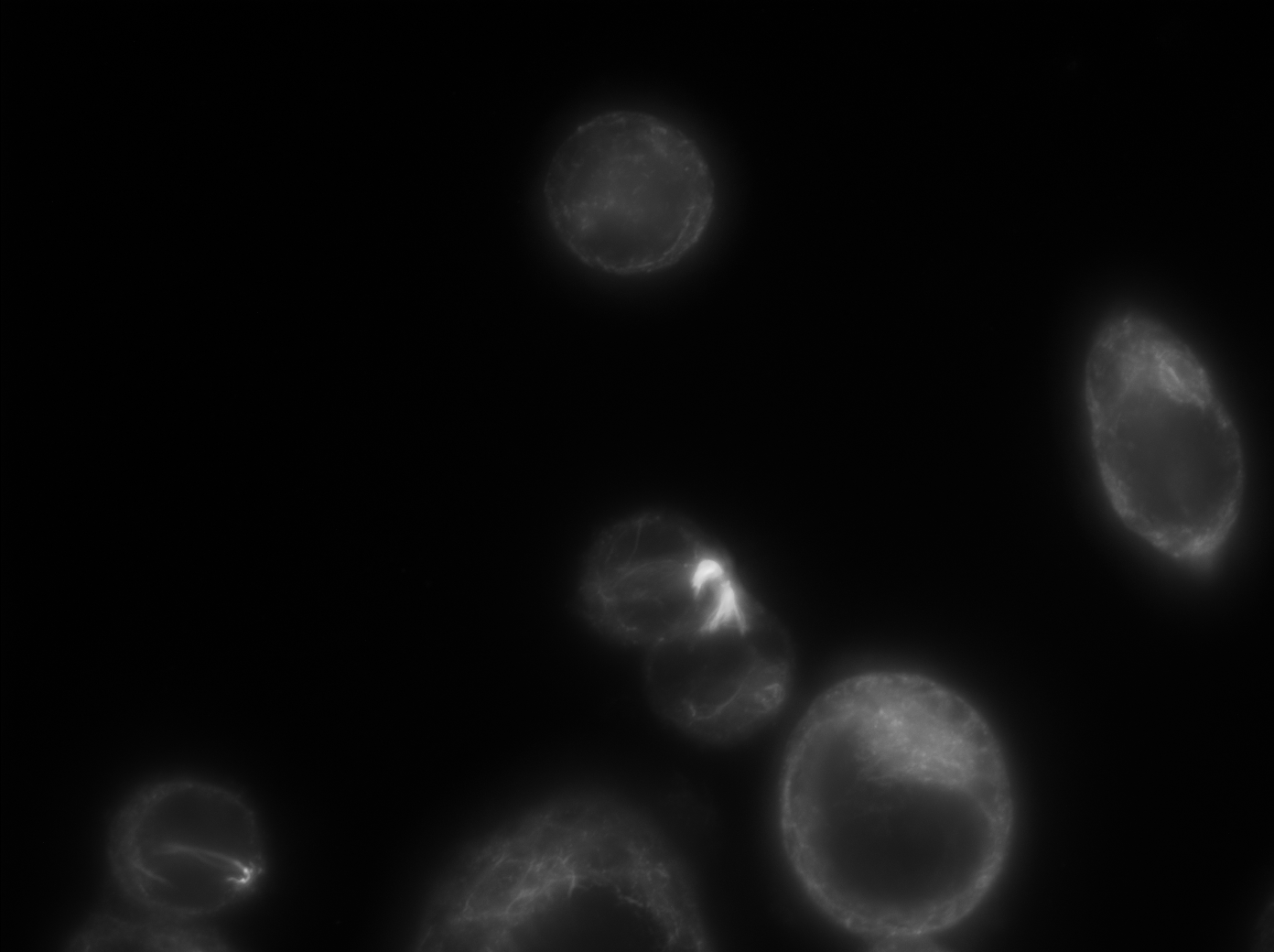

Supplement: Supplementary file 23 — Source data Fig. 6 part 4 [file 44319_2026_742_MOESM23_ESM.zip › Figure 6 Part 4/Fig 6efg TPGS1-KO TPGS1 rescue experiments part 2/R2R3/TPGS1-KO EYFP-only actub 7-31-25 R3 ET2.Project Maximum Z_XY1756492327_Z0_T0_C2.tif]

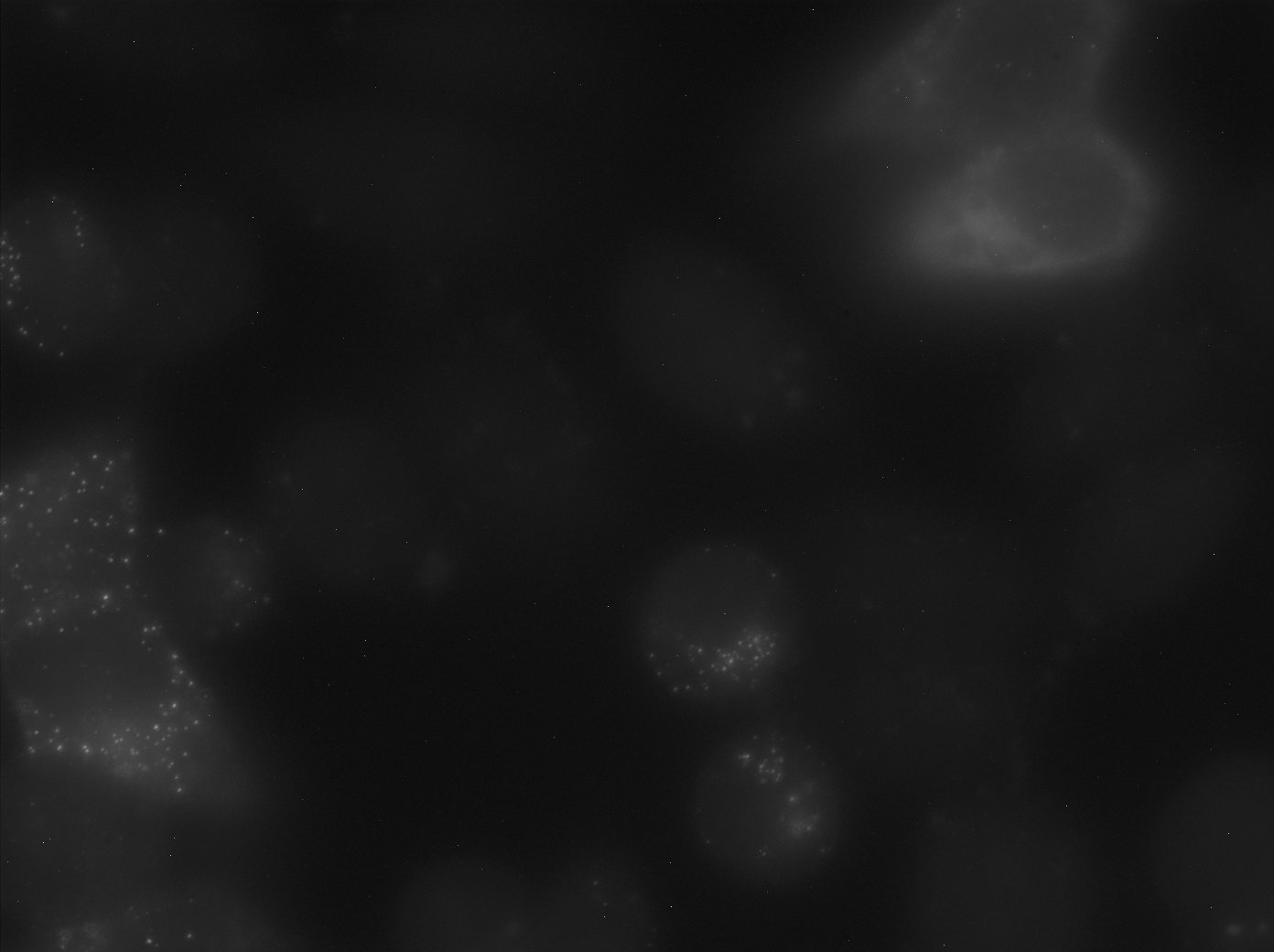

Supplement: Supplementary file 23 — Source data Fig. 6 part 4 [file 44319_2026_742_MOESM23_ESM.zip › Figure 6 Part 4/Fig 6efg TPGS1-KO TPGS1 rescue experiments part 2/R2R3/TPGS1-KO TPGS1-EYFP-3'UTR actub 7-31-25 R2 ET7.Project Maximum Z_XY1756411606_Z0_T0_C1.tif]

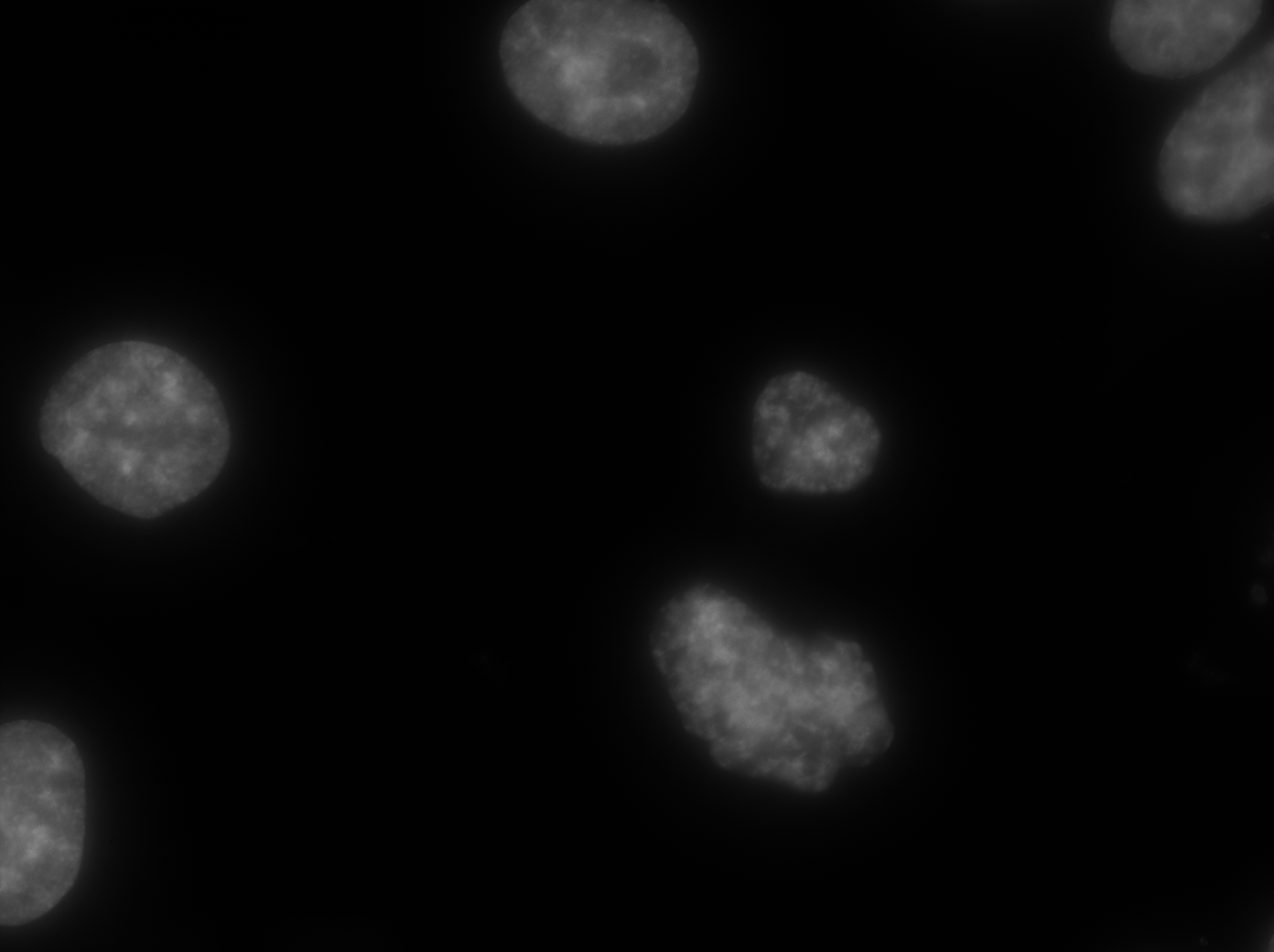

Supplement: Supplementary file 23 — Source data Fig. 6 part 4 [file 44319_2026_742_MOESM23_ESM.zip › Figure 6 Part 4/Fig 6efg TPGS1-KO TPGS1 rescue experiments part 2/R2R3/TPGS1-KO EYFP-only actub 7-31-25 R2 UNKOWN.Project Maximum Z_XY1756414147_Z0_T0_C0.tif]

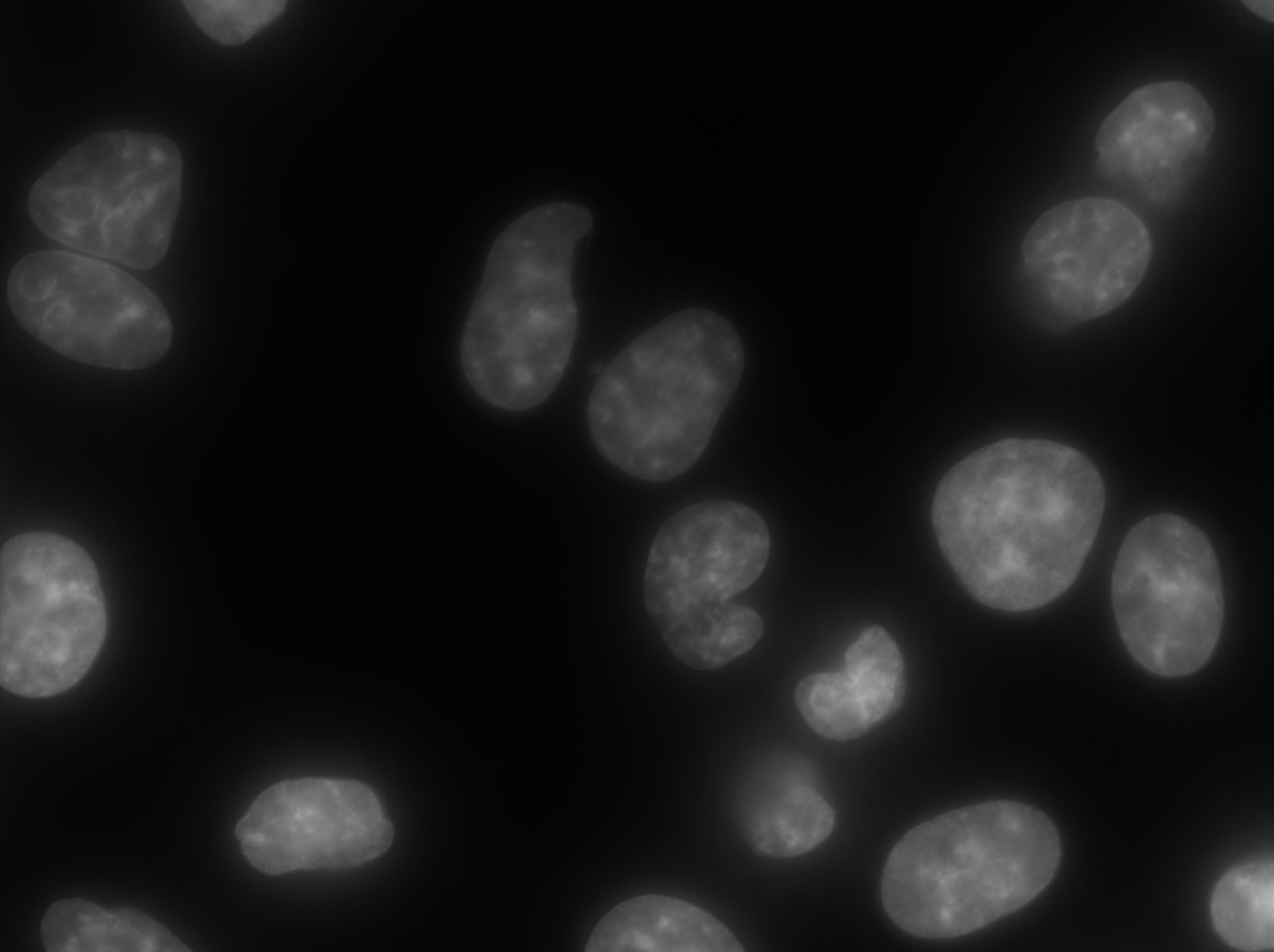

Supplement: Supplementary file 23 — Source data Fig. 6 part 4 [file 44319_2026_742_MOESM23_ESM.zip › Figure 6 Part 4/Fig 6efg TPGS1-KO TPGS1 rescue experiments part 2/R2R3/TPGS1-KO TPGS1-EYFP-3'UTR actub 7-31-25 R2 LT10.Project Maximum Z_XY1756412394_Z0_T0_C0.tif]

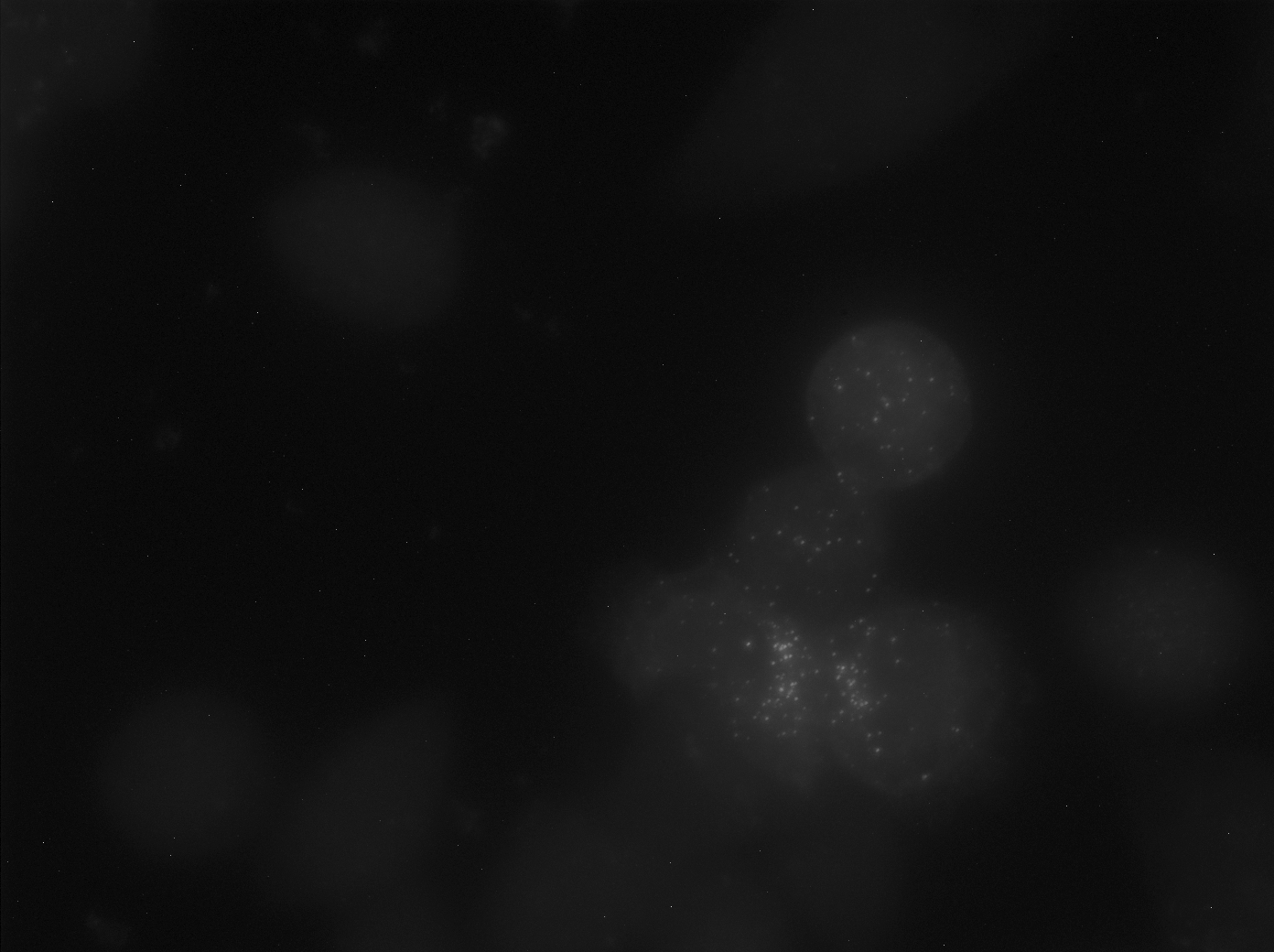

Supplement: Supplementary file 23 — Source data Fig. 6 part 4 [file 44319_2026_742_MOESM23_ESM.zip › Figure 6 Part 4/Fig 6efg TPGS1-KO TPGS1 rescue experiments part 2/R2R3/TPGS1-KO TPGS1-EYFP-3'UTR actub 7-31-25 R3 LT6.Project Maximum Z_XY1756501222_Z0_T0_C1.tif]

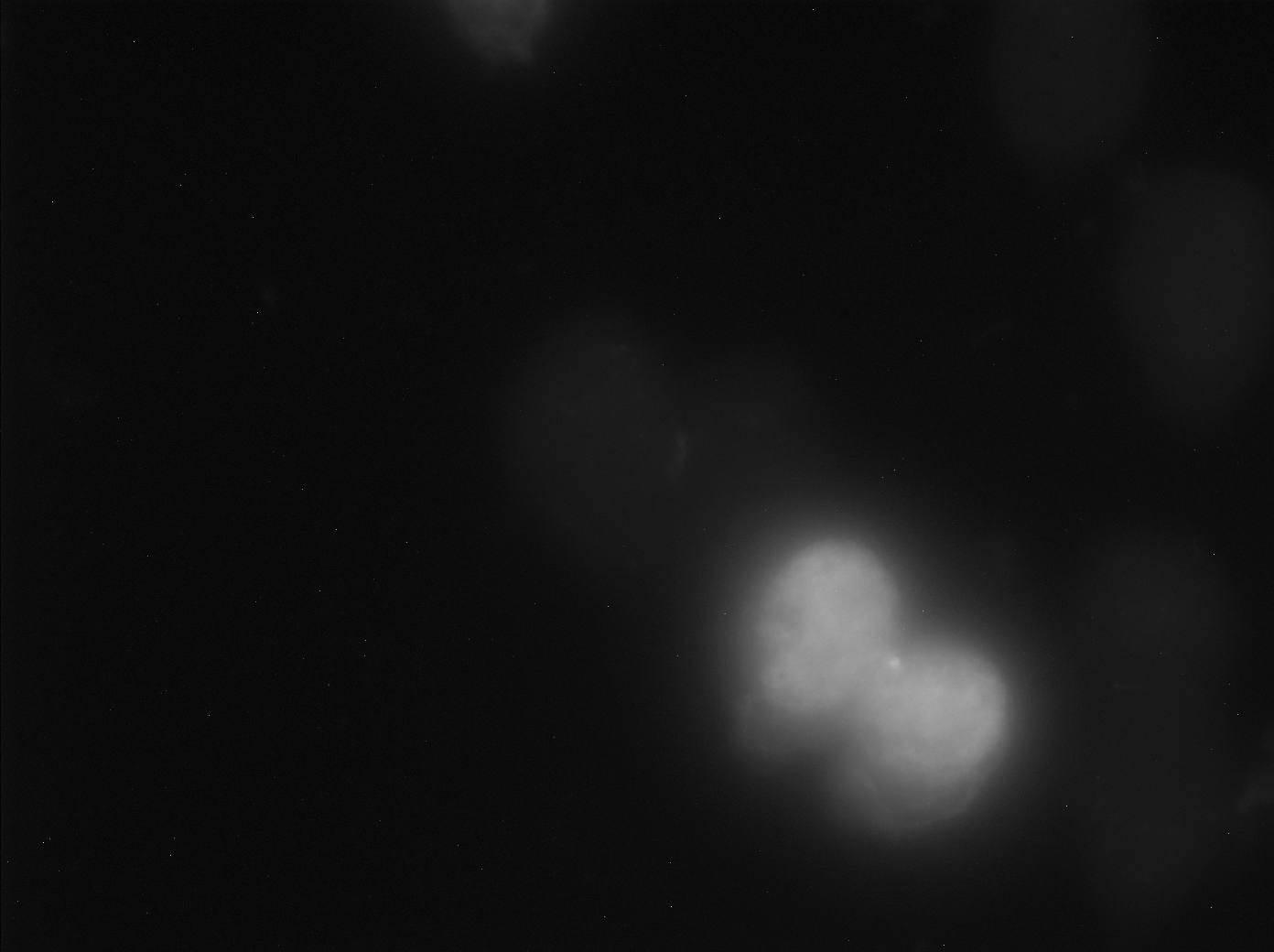

Supplement: Supplementary file 23 — Source data Fig. 6 part 4 [file 44319_2026_742_MOESM23_ESM.zip › Figure 6 Part 4/Fig 6efg TPGS1-KO TPGS1 rescue experiments part 2/R2R3/TPGS1-KO EYFP-only actub 7-31-25 R2 LT5.Project Maximum Z_XY1756414729_Z0_T0_C1.tif]

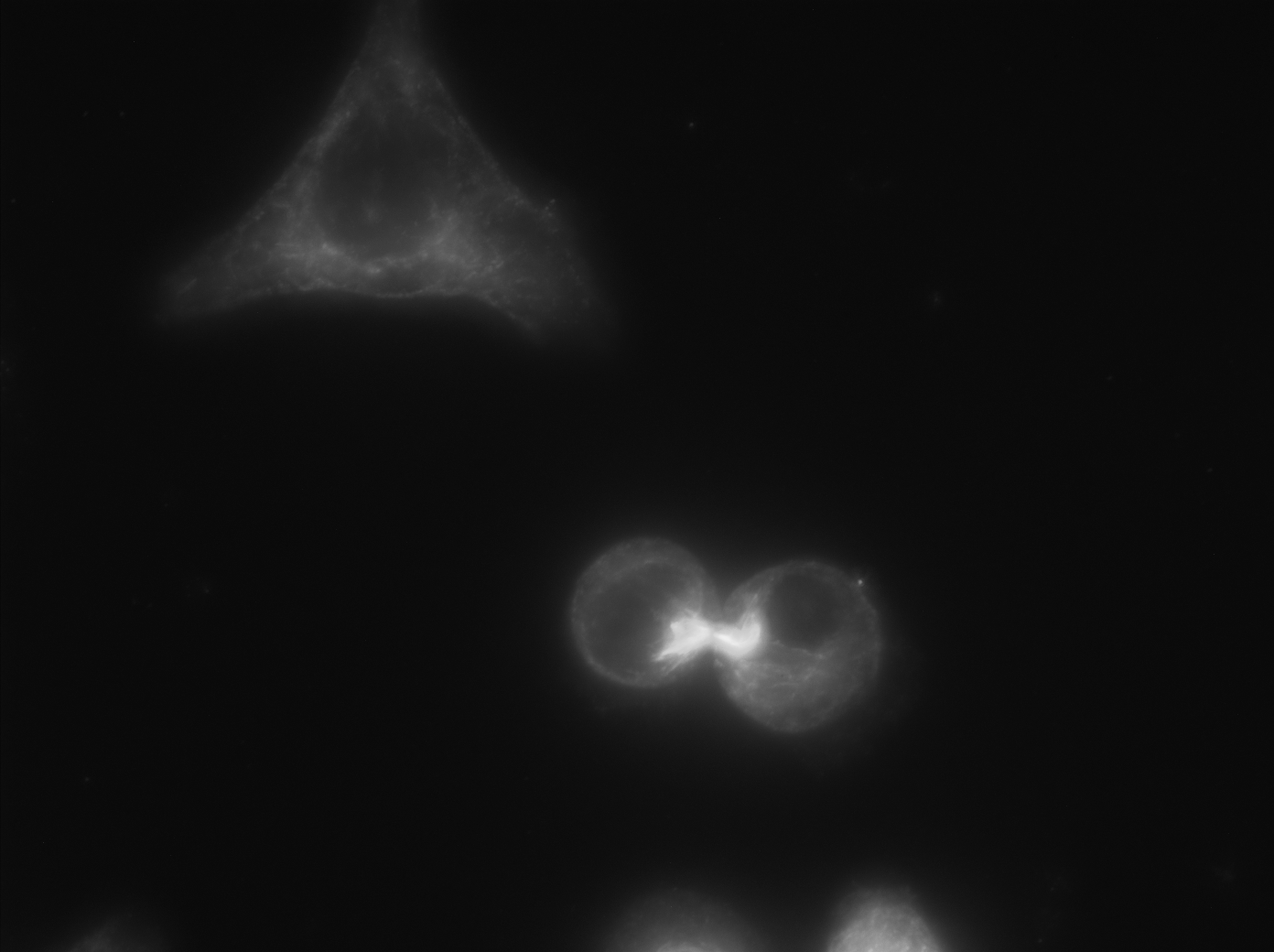

Supplement: Supplementary file 23 — Source data Fig. 6 part 4 [file 44319_2026_742_MOESM23_ESM.zip › Figure 6 Part 4/Fig 6efg TPGS1-KO TPGS1 rescue experiments part 2/R2R3/TPGS1-KO EYFP-only actub 7-31-25 R2 ET5.Project Maximum Z_XY1756415216_Z0_T0_C2.tif]

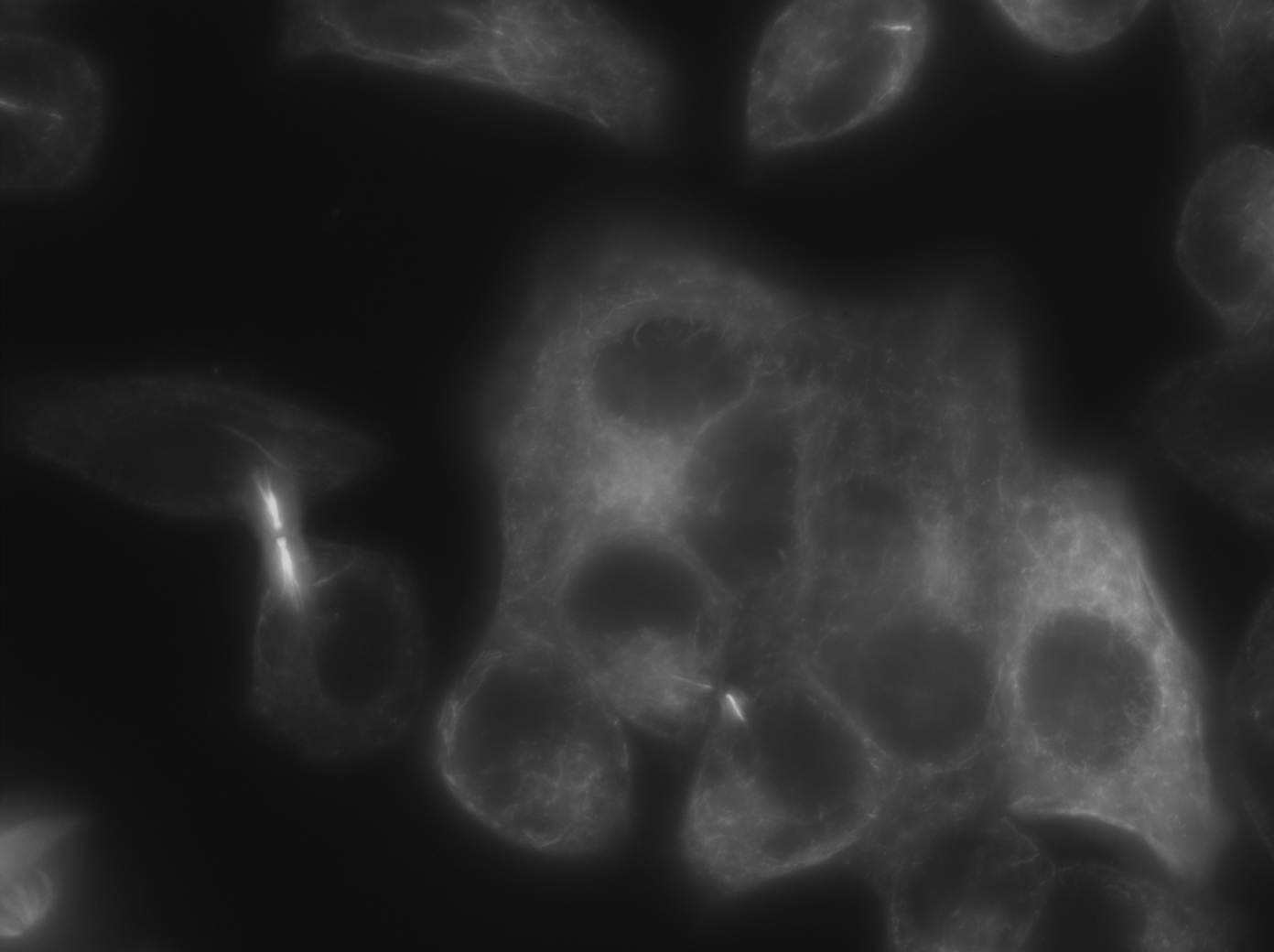

Supplement: Supplementary file 23 — Source data Fig. 6 part 4 [file 44319_2026_742_MOESM23_ESM.zip › Figure 6 Part 4/Fig 6efg TPGS1-KO TPGS1 rescue experiments part 2/R2R3/TPGS1-KO TPGS1-EYFP-3'UTR actub 7-31-25 R2 LT5.Project Maximum Z_XY1756408791_Z0_T0_C2.tif]

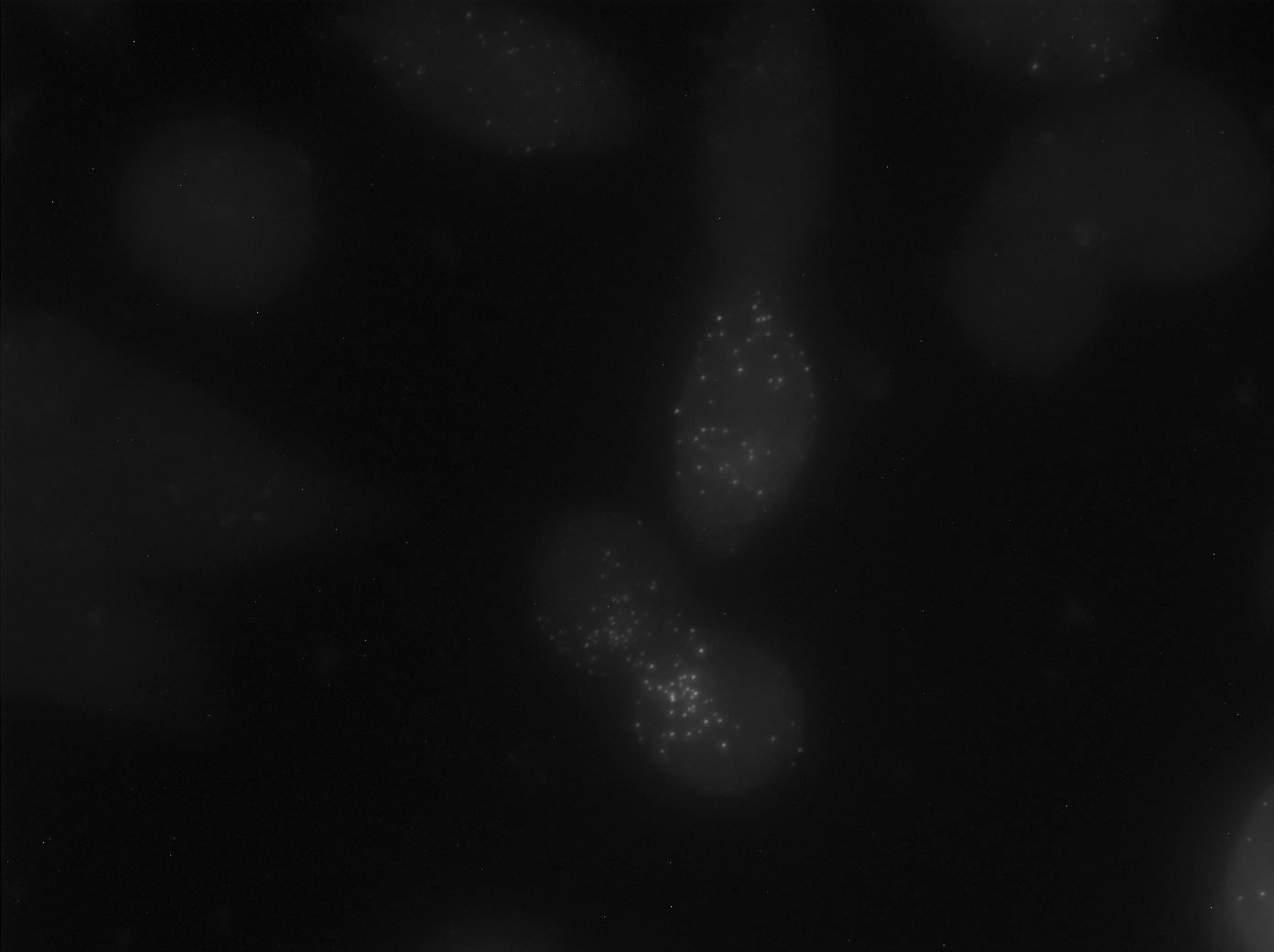

Supplement: Supplementary file 23 — Source data Fig. 6 part 4 [file 44319_2026_742_MOESM23_ESM.zip › Figure 6 Part 4/Fig 6efg TPGS1-KO TPGS1 rescue experiments part 2/R2R3/TPGS1-KO TPGS1-EYFP-3'UTR actub 7-31-25 R3 LT1.Project Maximum Z_XY1756500057_Z0_T0_C1.tif]

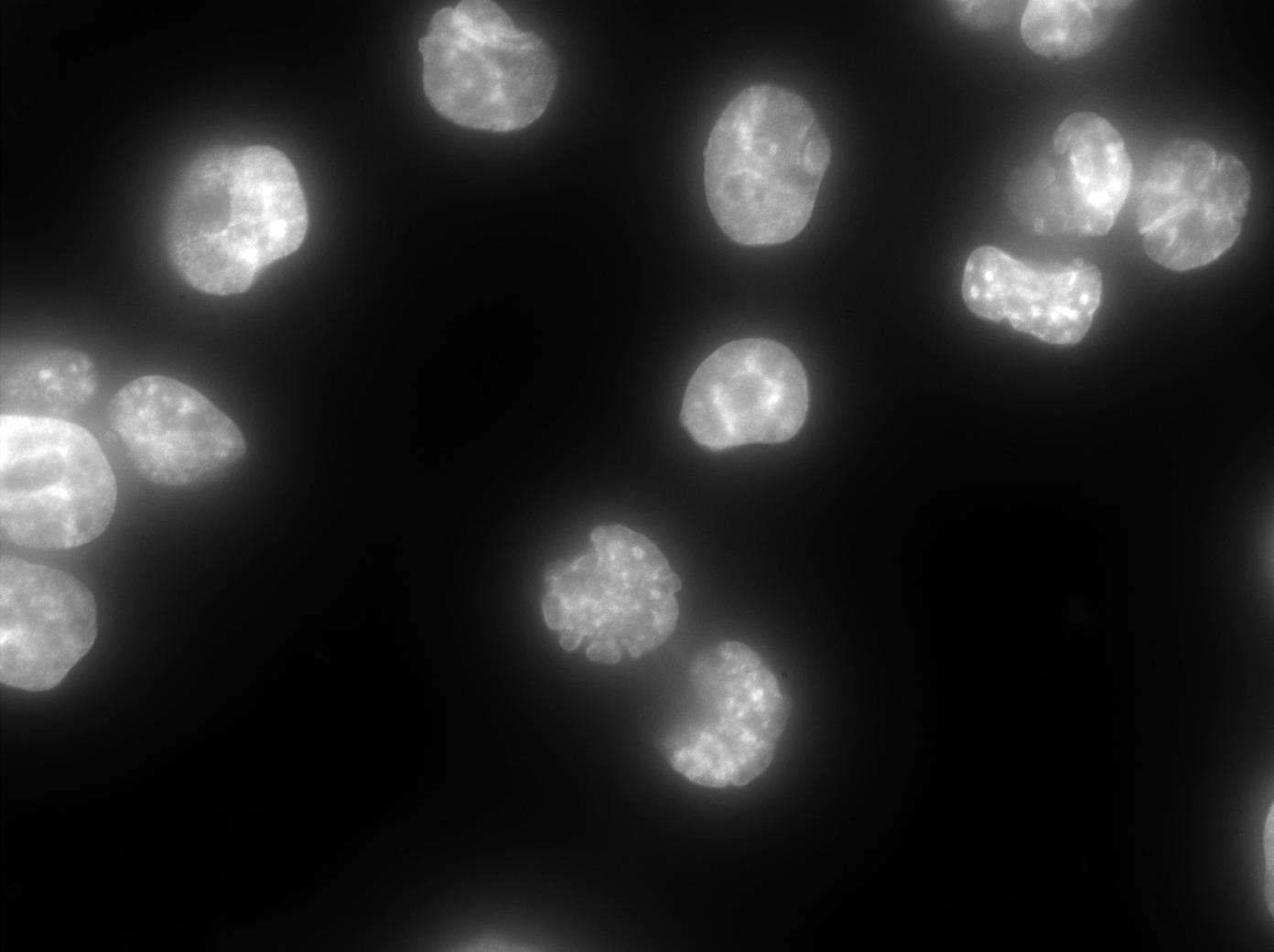

Supplement: Supplementary file 23 — Source data Fig. 6 part 4 [file 44319_2026_742_MOESM23_ESM.zip › Figure 6 Part 4/Fig 6efg TPGS1-KO TPGS1 rescue experiments part 2/R2R3/TPGS1-KO TPGS1-EYFP-3'UTR actub 7-31-25 R3 LT1.Project Maximum Z_XY1756500057_Z0_T0_C0.tif]

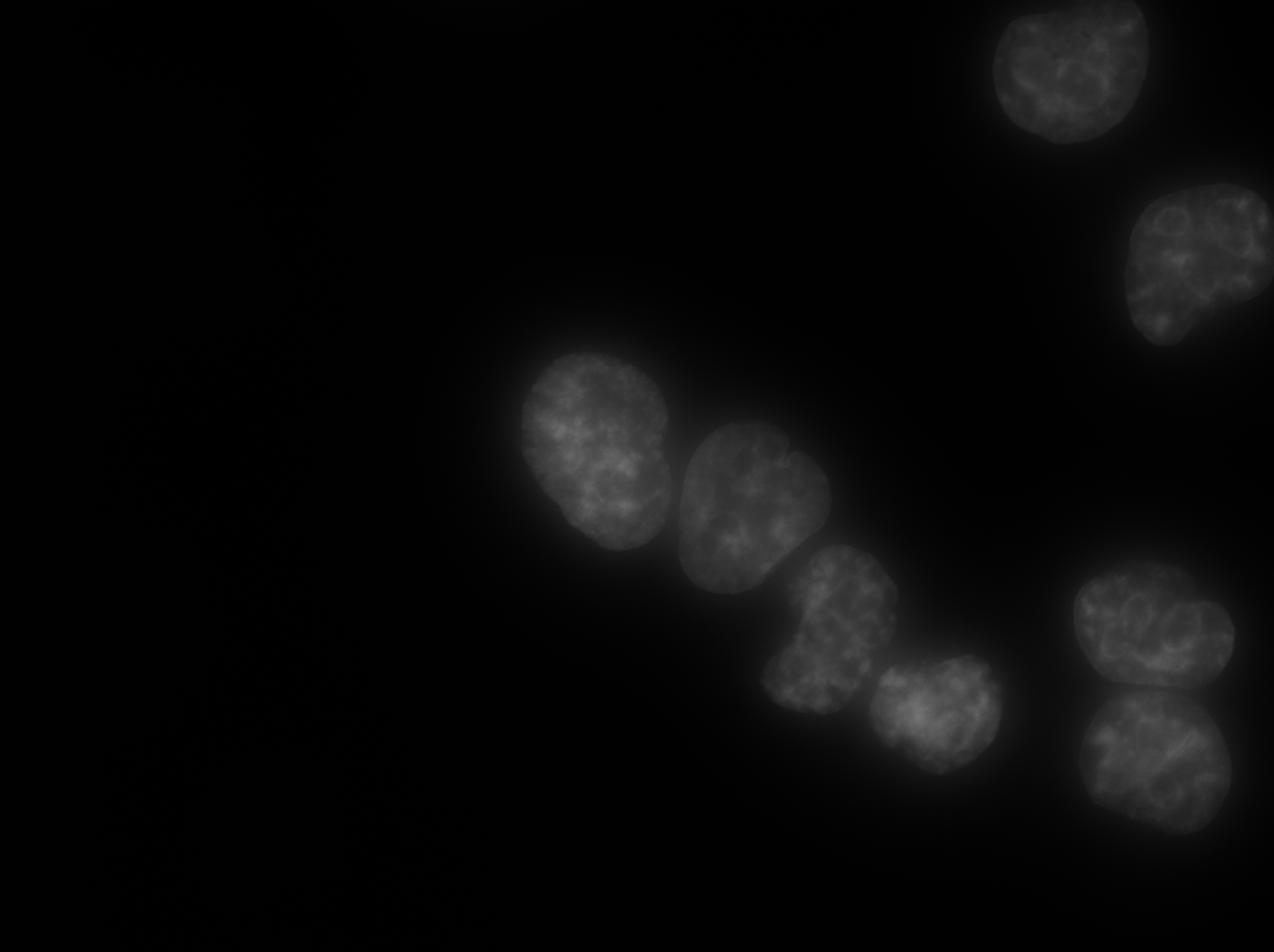

Supplement: Supplementary file 23 — Source data Fig. 6 part 4 [file 44319_2026_742_MOESM23_ESM.zip › Figure 6 Part 4/Fig 6efg TPGS1-KO TPGS1 rescue experiments part 2/R2R3/TPGS1-KO EYFP-only actub 7-31-25 R2 LT5.Project Maximum Z_XY1756414729_Z0_T0_C0.tif]

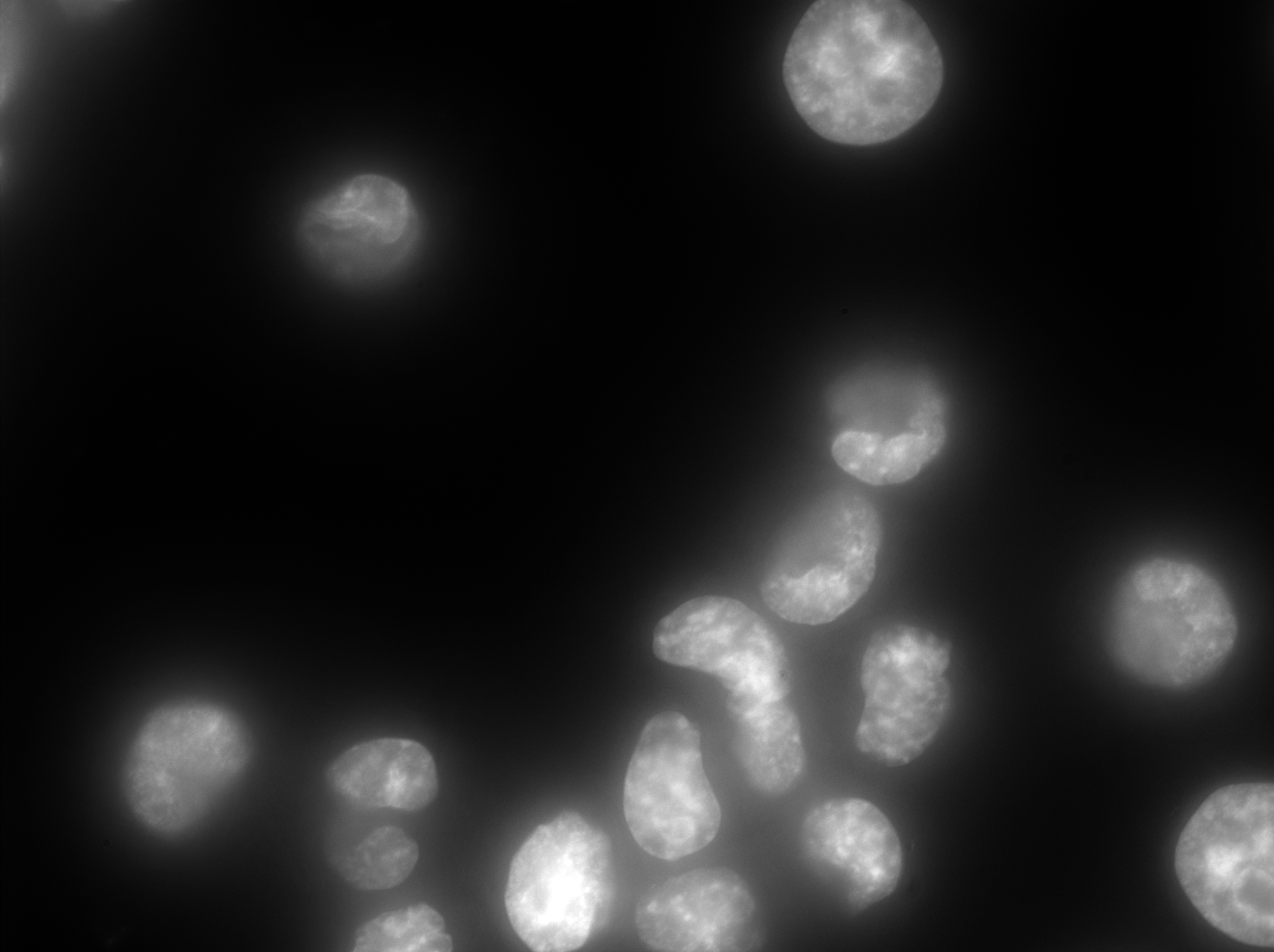

Supplement: Supplementary file 23 — Source data Fig. 6 part 4 [file 44319_2026_742_MOESM23_ESM.zip › Figure 6 Part 4/Fig 6efg TPGS1-KO TPGS1 rescue experiments part 2/R2R3/TPGS1-KO TPGS1-EYFP-3'UTR actub 7-31-25 R3 LT6.Project Maximum Z_XY1756501222_Z0_T0_C0.tif]

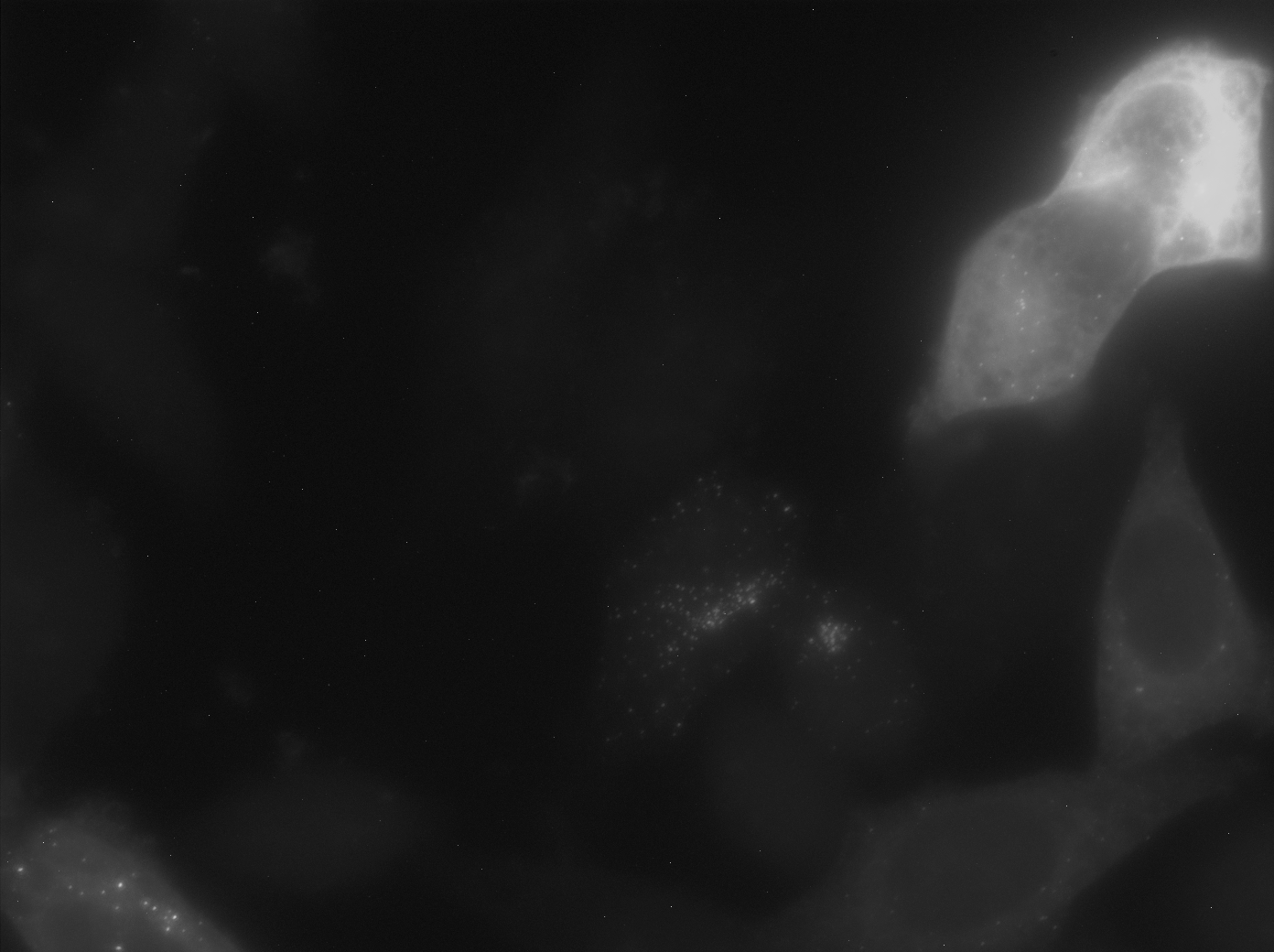

Supplement: Supplementary file 23 — Source data Fig. 6 part 4 [file 44319_2026_742_MOESM23_ESM.zip › Figure 6 Part 4/Fig 6efg TPGS1-KO TPGS1 rescue experiments part 2/R2R3/TPGS1-KO TPGS1-EYFP-3'UTR actub 7-31-25 R2 LT10.Project Maximum Z_XY1756412394_Z0_T0_C1.tif]

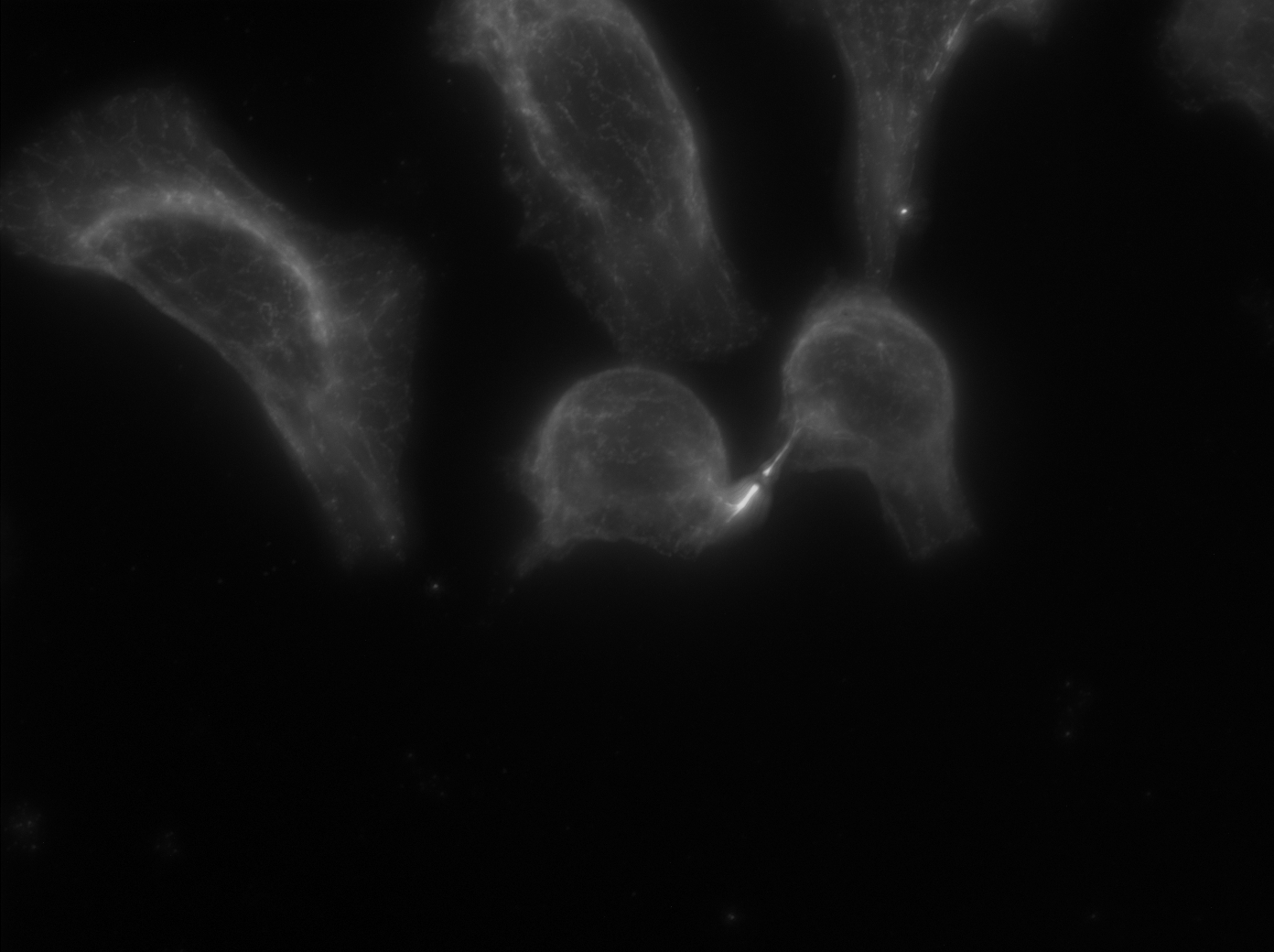

Supplement: Supplementary file 23 — Source data Fig. 6 part 4 [file 44319_2026_742_MOESM23_ESM.zip › Figure 6 Part 4/Fig 6efg TPGS1-KO TPGS1 rescue experiments part 2/R2R3/TPGS1-KO EYFP-only actub 7-31-25 R2 LT3.Project Maximum Z_XY1756414428_Z0_T0_C2.tif]

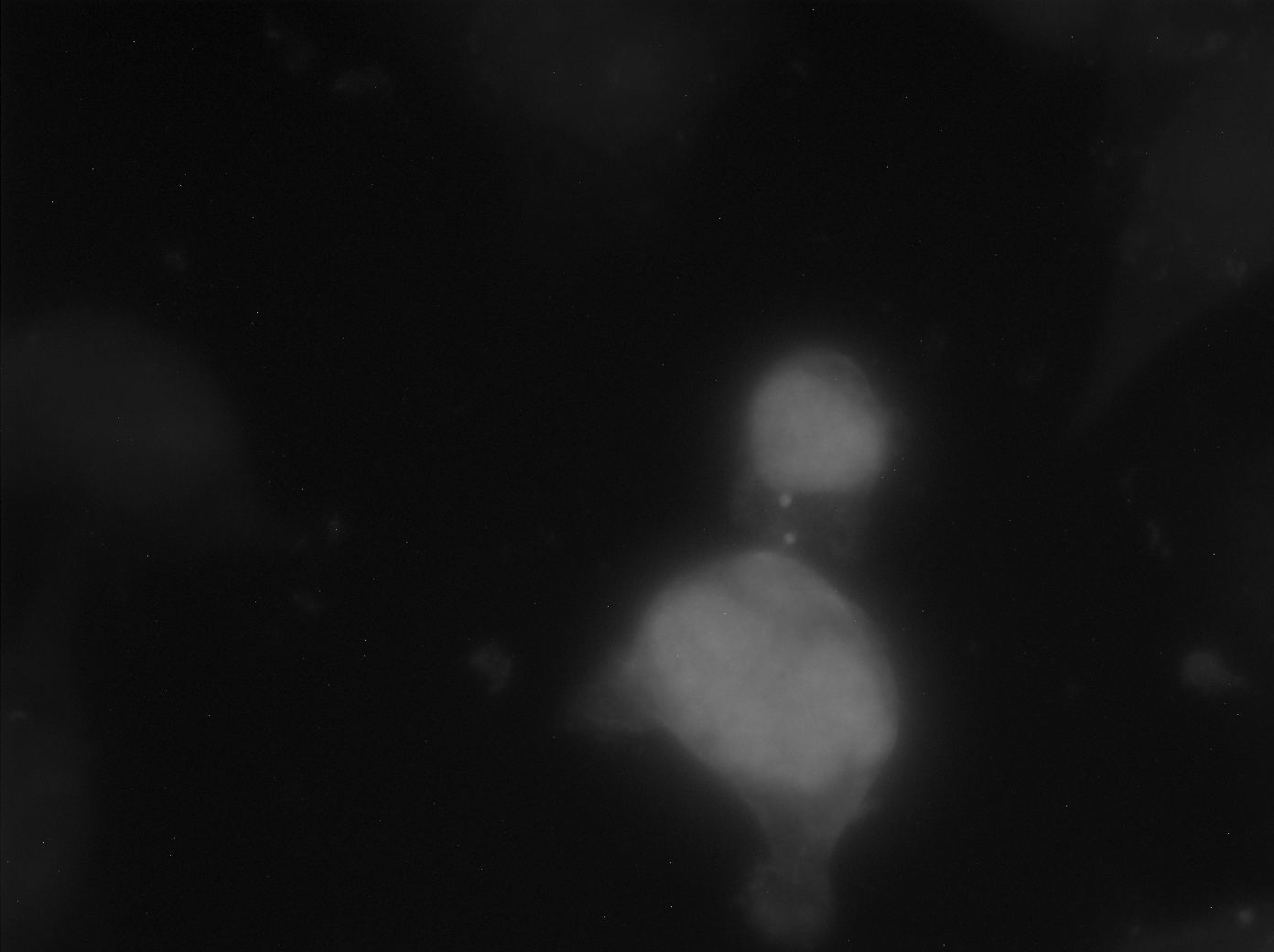

Supplement: Supplementary file 23 — Source data Fig. 6 part 4 [file 44319_2026_742_MOESM23_ESM.zip › Figure 6 Part 4/Fig 6efg TPGS1-KO TPGS1 rescue experiments part 2/R2R3/TPGS1-KO EYFP-only actub 7-31-25 R2 UNKOWN.Project Maximum Z_XY1756414147_Z0_T0_C1.tif]

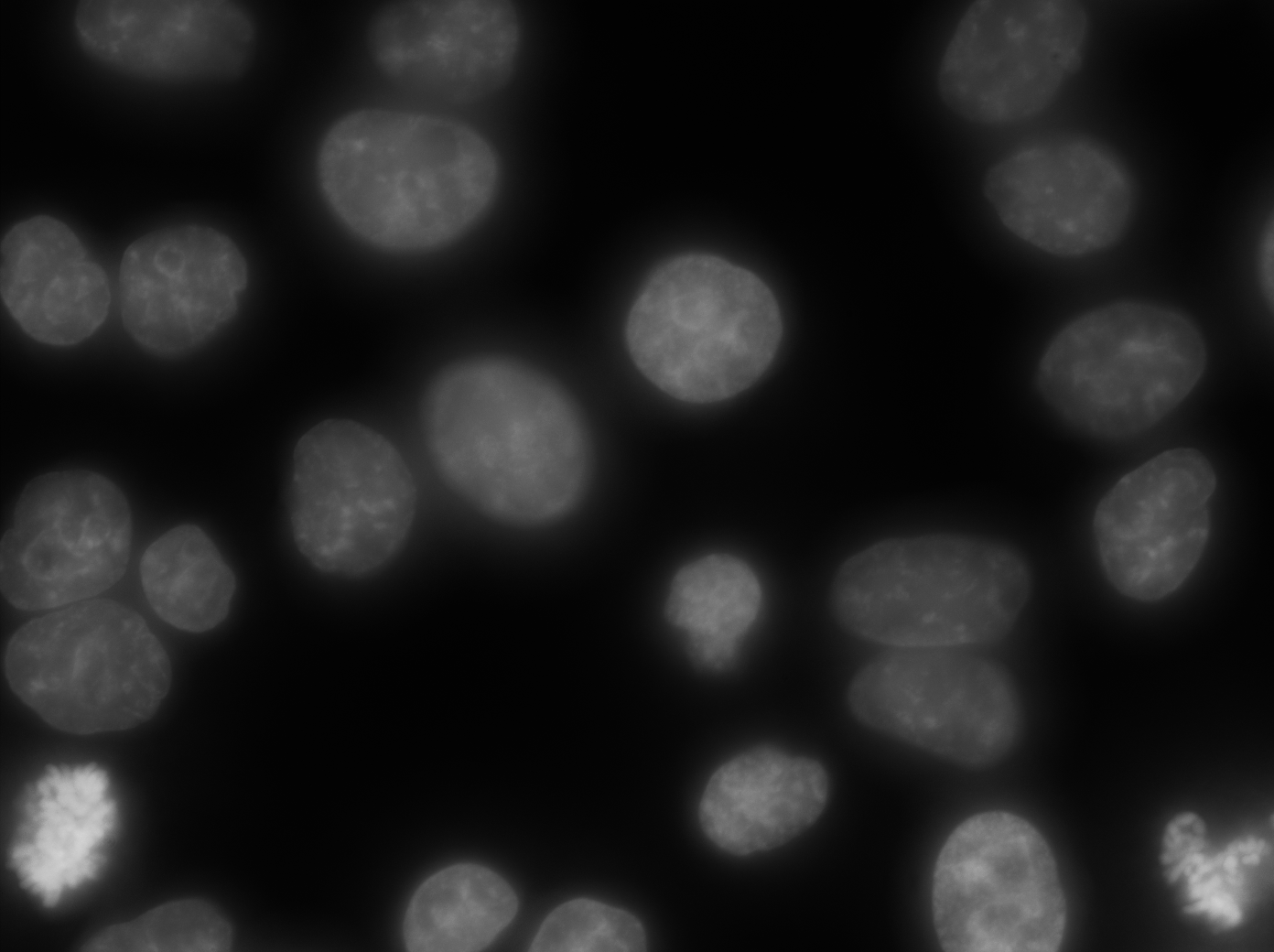

Supplement: Supplementary file 23 — Source data Fig. 6 part 4 [file 44319_2026_742_MOESM23_ESM.zip › Figure 6 Part 4/Fig 6efg TPGS1-KO TPGS1 rescue experiments part 2/R2R3/TPGS1-KO TPGS1-EYFP-3'UTR actub 7-31-25 R2 ET7.Project Maximum Z_XY1756411606_Z0_T0_C0.tif]

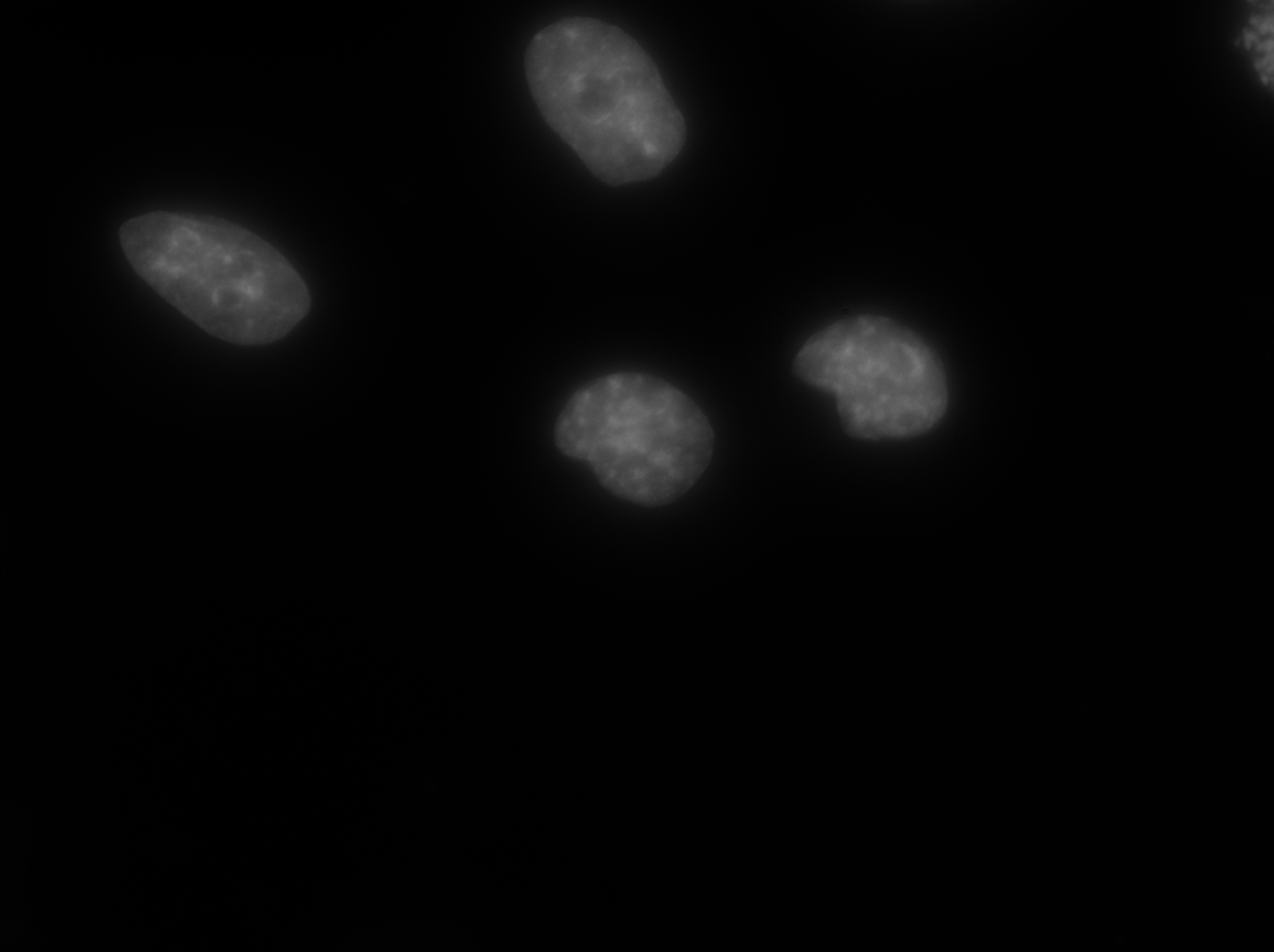

Supplement: Supplementary file 23 — Source data Fig. 6 part 4 [file 44319_2026_742_MOESM23_ESM.zip › Figure 6 Part 4/Fig 6efg TPGS1-KO TPGS1 rescue experiments part 2/R2R3/TPGS1-KO EYFP-only actub 7-31-25 R2 LT3.Project Maximum Z_XY1756414428_Z0_T0_C0.tif]

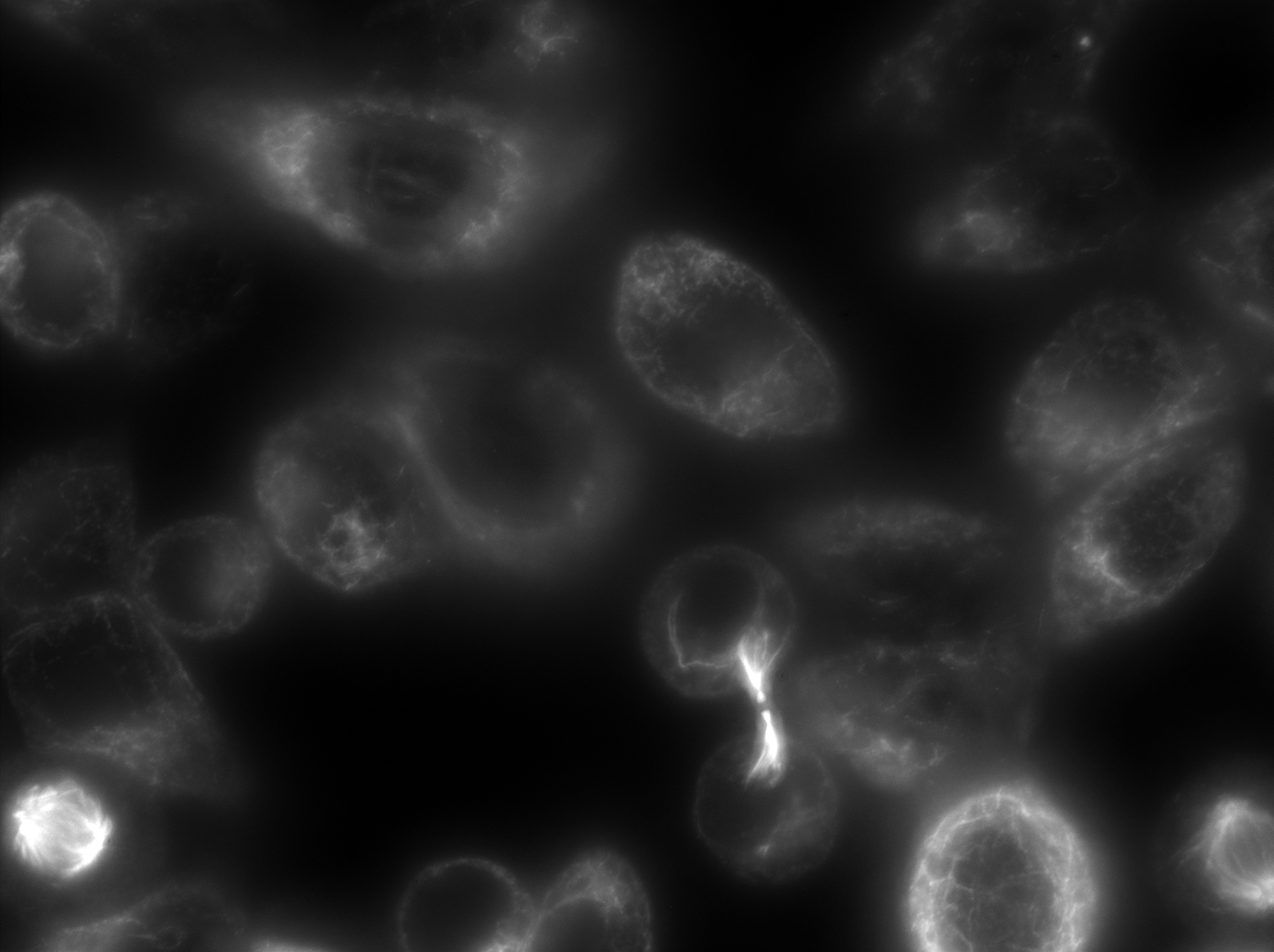

Supplement: Supplementary file 23 — Source data Fig. 6 part 4 [file 44319_2026_742_MOESM23_ESM.zip › Figure 6 Part 4/Fig 6efg TPGS1-KO TPGS1 rescue experiments part 2/R2R3/TPGS1-KO TPGS1-EYFP-3'UTR actub 7-31-25 R2 ET7.Project Maximum Z_XY1756411606_Z0_T0_C2.tif]

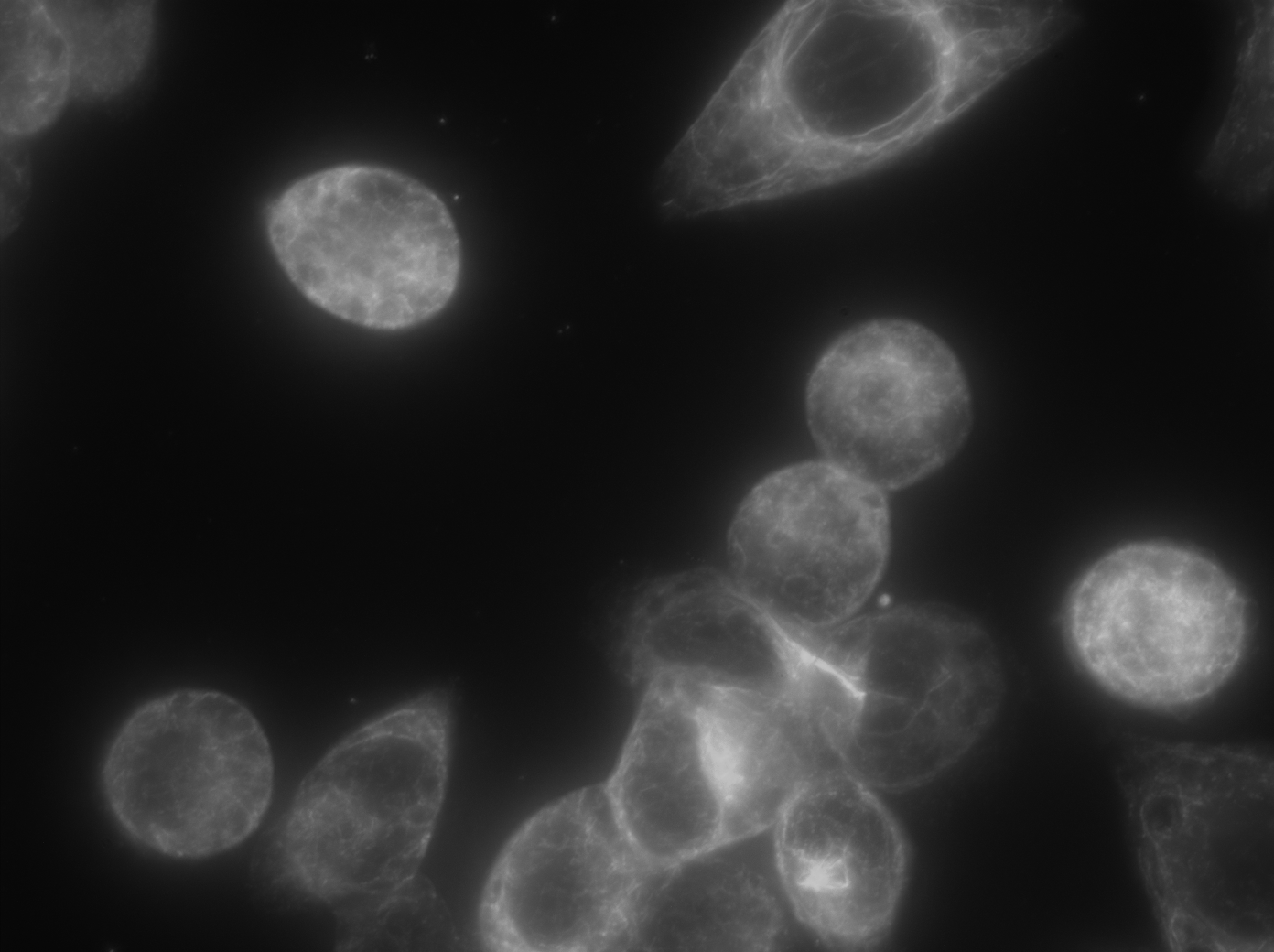

Supplement: Supplementary file 23 — Source data Fig. 6 part 4 [file 44319_2026_742_MOESM23_ESM.zip › Figure 6 Part 4/Fig 6efg TPGS1-KO TPGS1 rescue experiments part 2/R2R3/TPGS1-KO TPGS1-EYFP-3'UTR actub 7-31-25 R3 LT6.Project Maximum Z_XY1756501222_Z0_T0_C2.tif]

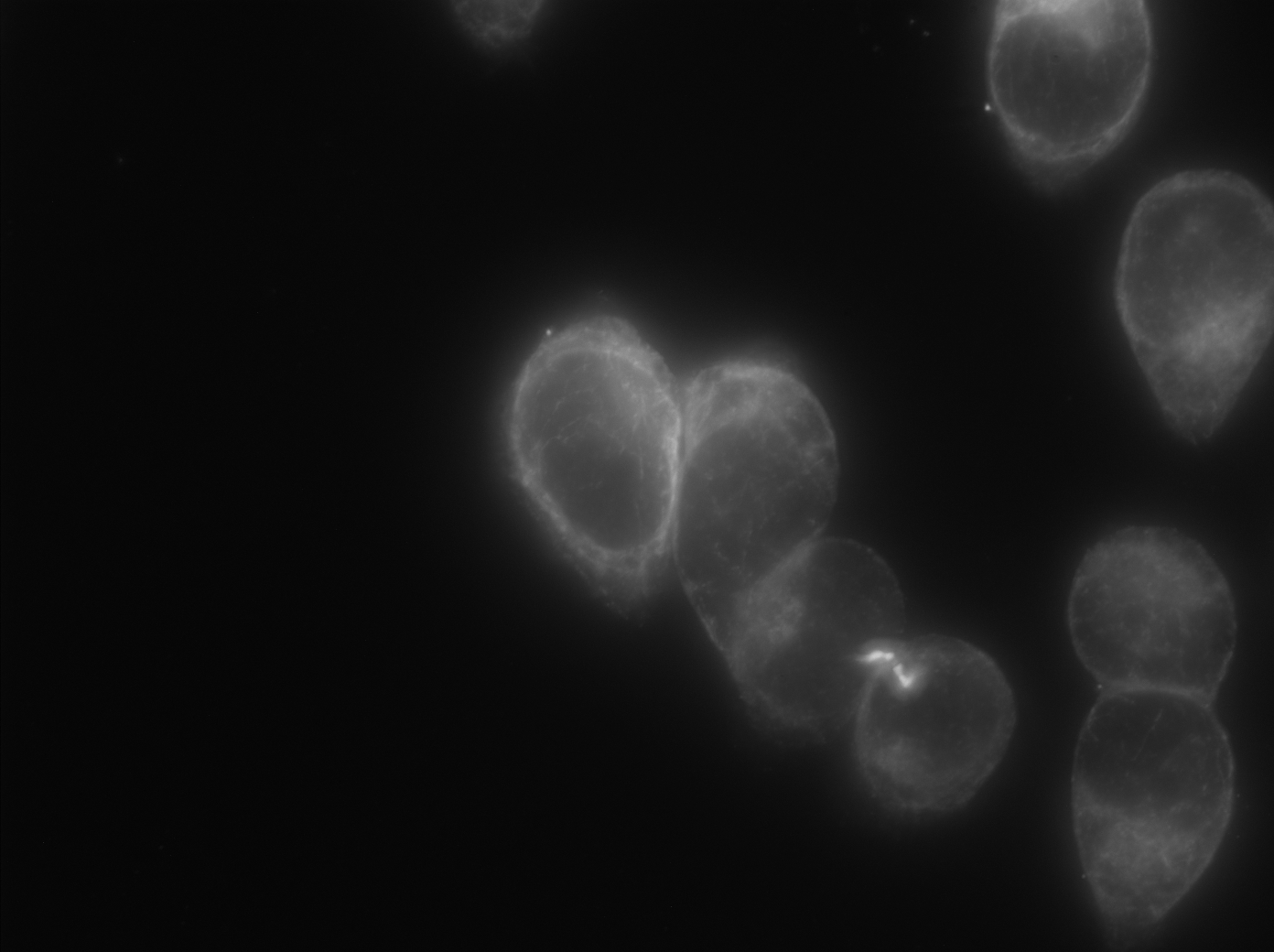

Supplement: Supplementary file 23 — Source data Fig. 6 part 4 [file 44319_2026_742_MOESM23_ESM.zip › Figure 6 Part 4/Fig 6efg TPGS1-KO TPGS1 rescue experiments part 2/R2R3/TPGS1-KO EYFP-only actub 7-31-25 R2 LT5.Project Maximum Z_XY1756414729_Z0_T0_C2.tif]

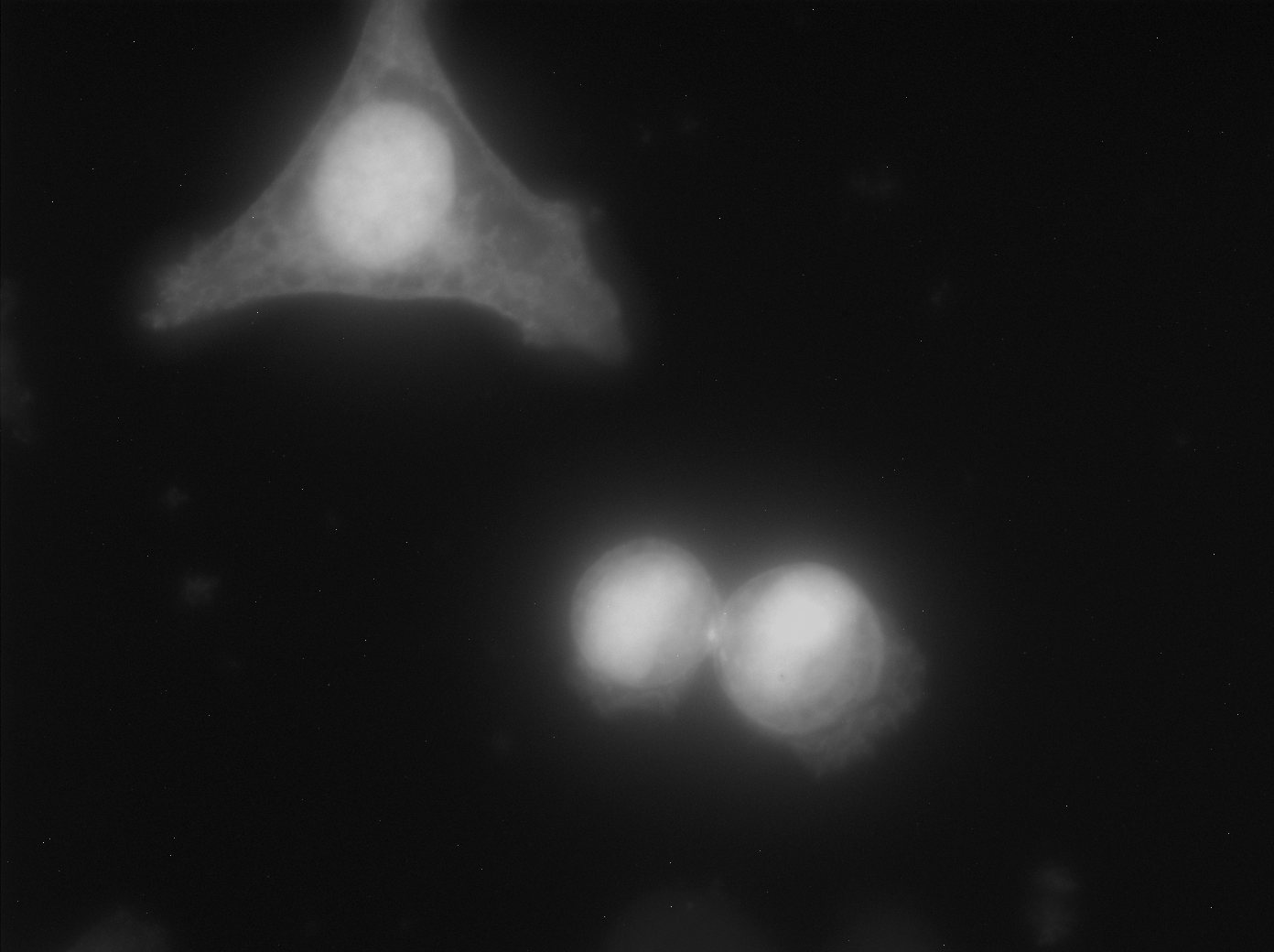

Supplement: Supplementary file 23 — Source data Fig. 6 part 4 [file 44319_2026_742_MOESM23_ESM.zip › Figure 6 Part 4/Fig 6efg TPGS1-KO TPGS1 rescue experiments part 2/R2R3/TPGS1-KO EYFP-only actub 7-31-25 R2 ET5.Project Maximum Z_XY1756415216_Z0_T0_C1.tif]

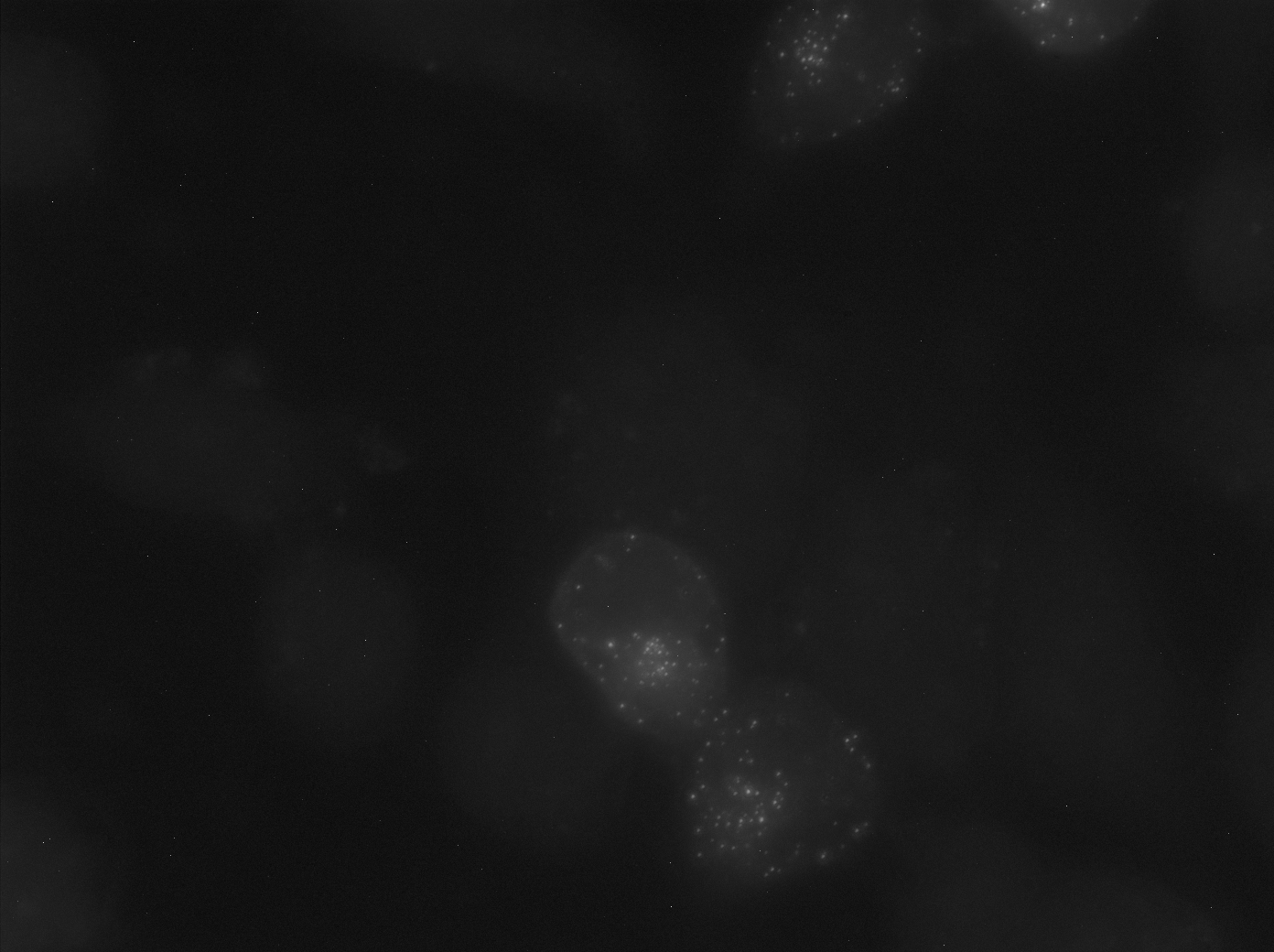

Supplement: Supplementary file 23 — Source data Fig. 6 part 4 [file 44319_2026_742_MOESM23_ESM.zip › Figure 6 Part 4/Fig 6efg TPGS1-KO TPGS1 rescue experiments part 2/R2R3/TPGS1-KO TPGS1-EYFP-3'UTR actub 7-31-25 R2 LT5.Project Maximum Z_XY1756408791_Z0_T0_C1.tif]

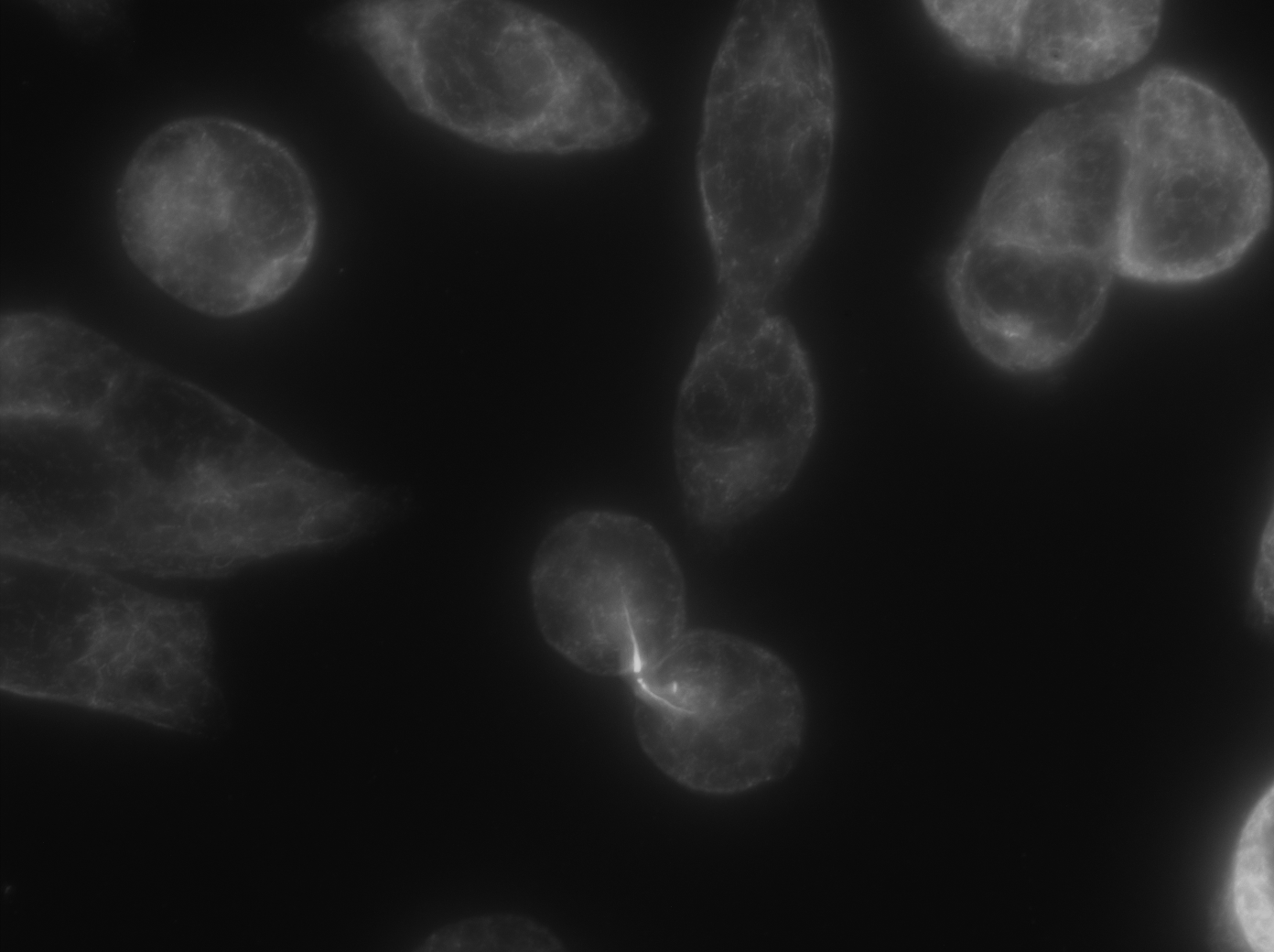

Supplement: Supplementary file 23 — Source data Fig. 6 part 4 [file 44319_2026_742_MOESM23_ESM.zip › Figure 6 Part 4/Fig 6efg TPGS1-KO TPGS1 rescue experiments part 2/R2R3/TPGS1-KO TPGS1-EYFP-3'UTR actub 7-31-25 R3 LT1.Project Maximum Z_XY1756500057_Z0_T0_C2.tif]

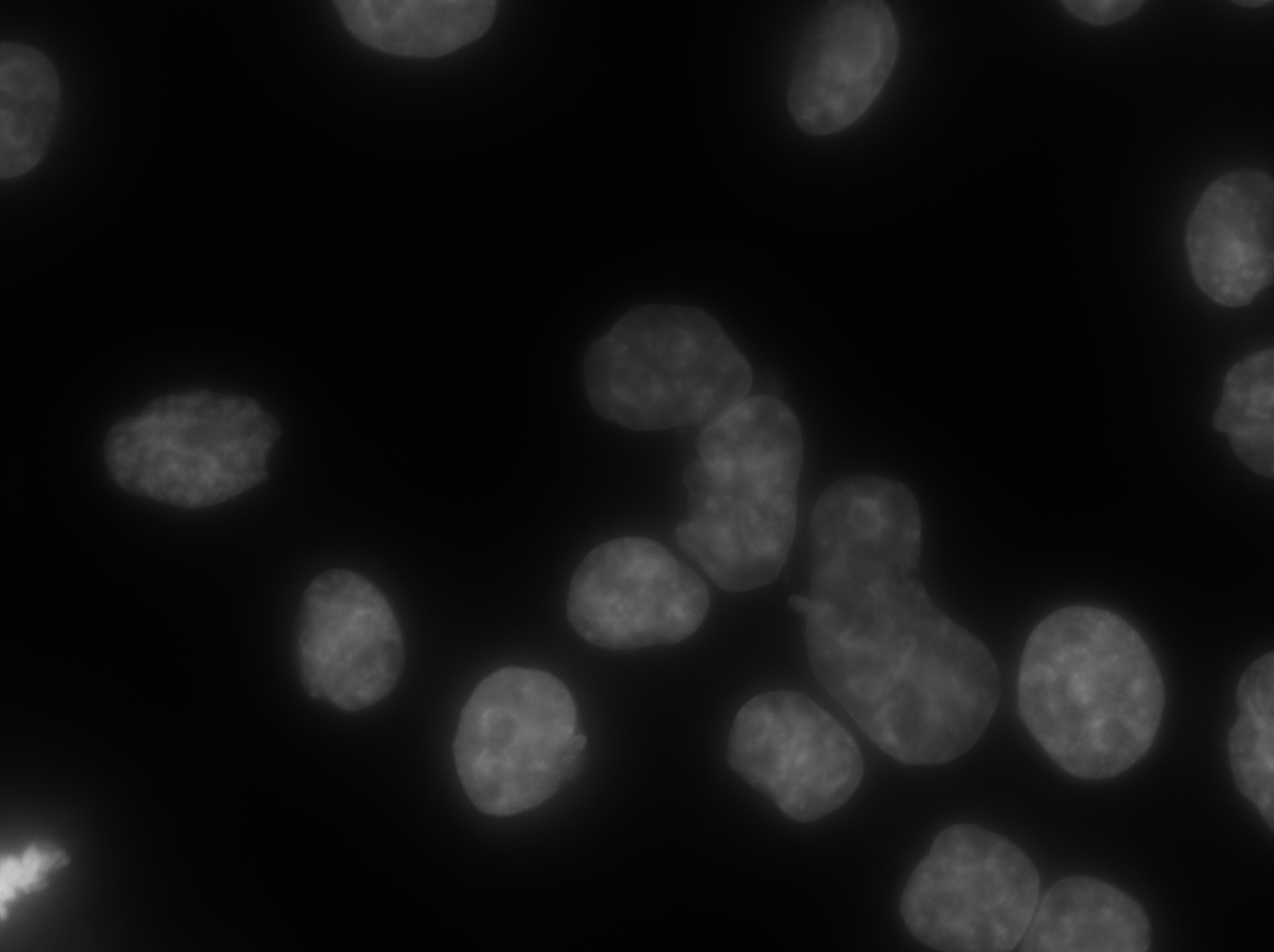

Supplement: Supplementary file 23 — Source data Fig. 6 part 4 [file 44319_2026_742_MOESM23_ESM.zip › Figure 6 Part 4/Fig 6efg TPGS1-KO TPGS1 rescue experiments part 2/R2R3/TPGS1-KO TPGS1-EYFP-3'UTR actub 7-31-25 R2 LT5.Project Maximum Z_XY1756408791_Z0_T0_C0.tif]

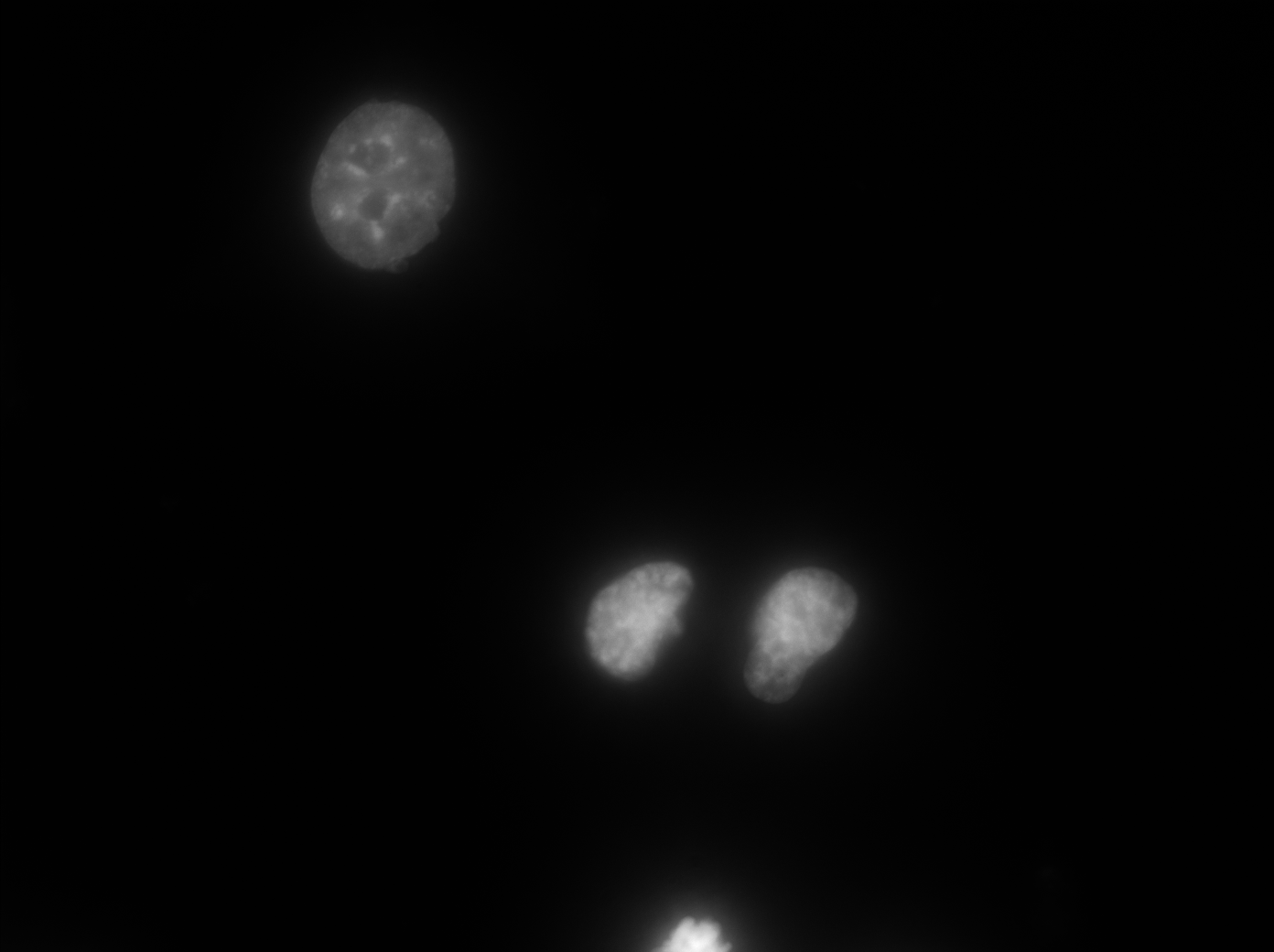

Supplement: Supplementary file 23 — Source data Fig. 6 part 4 [file 44319_2026_742_MOESM23_ESM.zip › Figure 6 Part 4/Fig 6efg TPGS1-KO TPGS1 rescue experiments part 2/R2R3/TPGS1-KO EYFP-only actub 7-31-25 R2 ET5.Project Maximum Z_XY1756415216_Z0_T0_C0.tif]

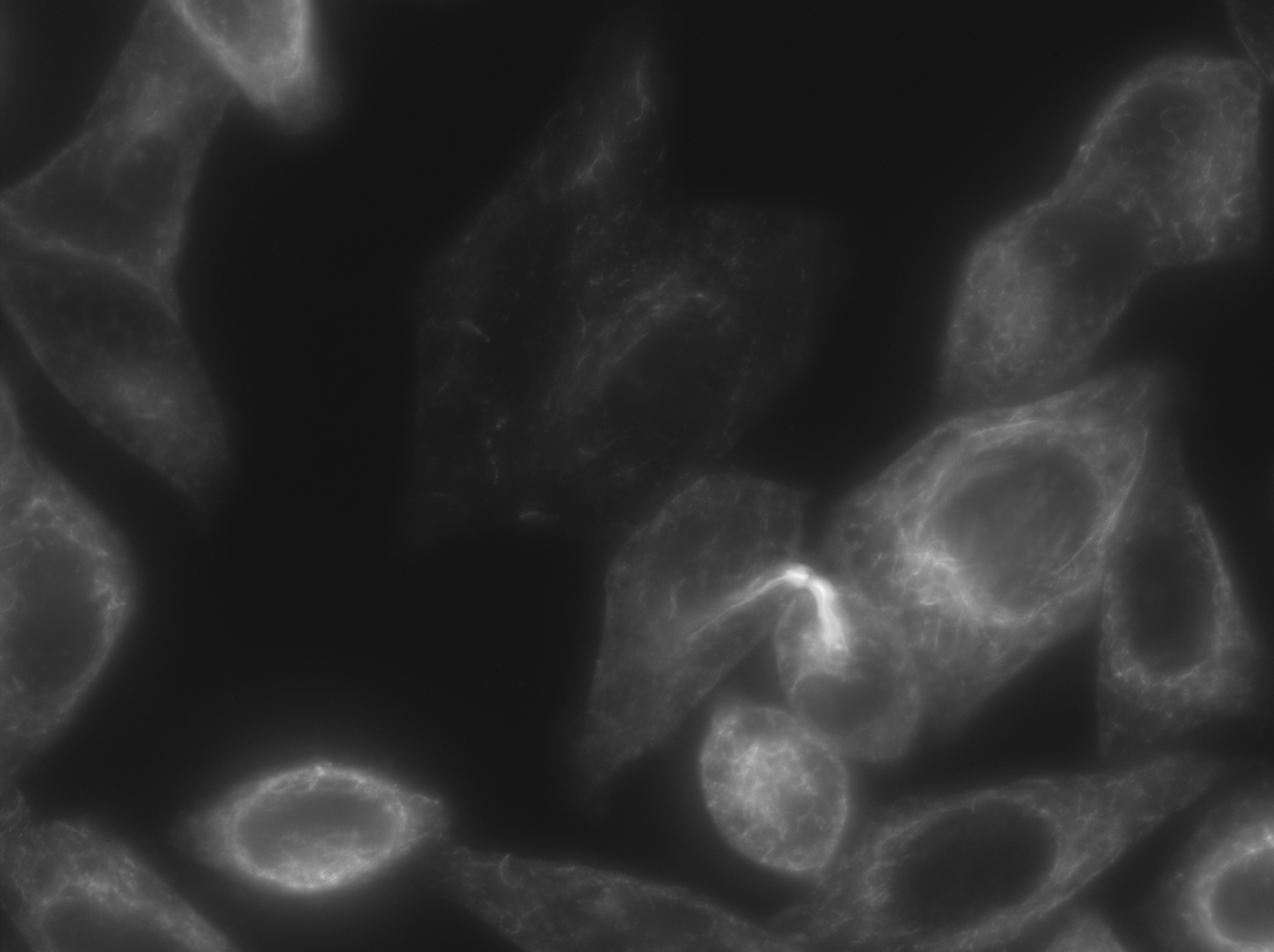

Supplement: Supplementary file 23 — Source data Fig. 6 part 4 [file 44319_2026_742_MOESM23_ESM.zip › Figure 6 Part 4/Fig 6efg TPGS1-KO TPGS1 rescue experiments part 2/R2R3/TPGS1-KO TPGS1-EYFP-3'UTR actub 7-31-25 R2 LT10.Project Maximum Z_XY1756412394_Z0_T0_C2.tif]

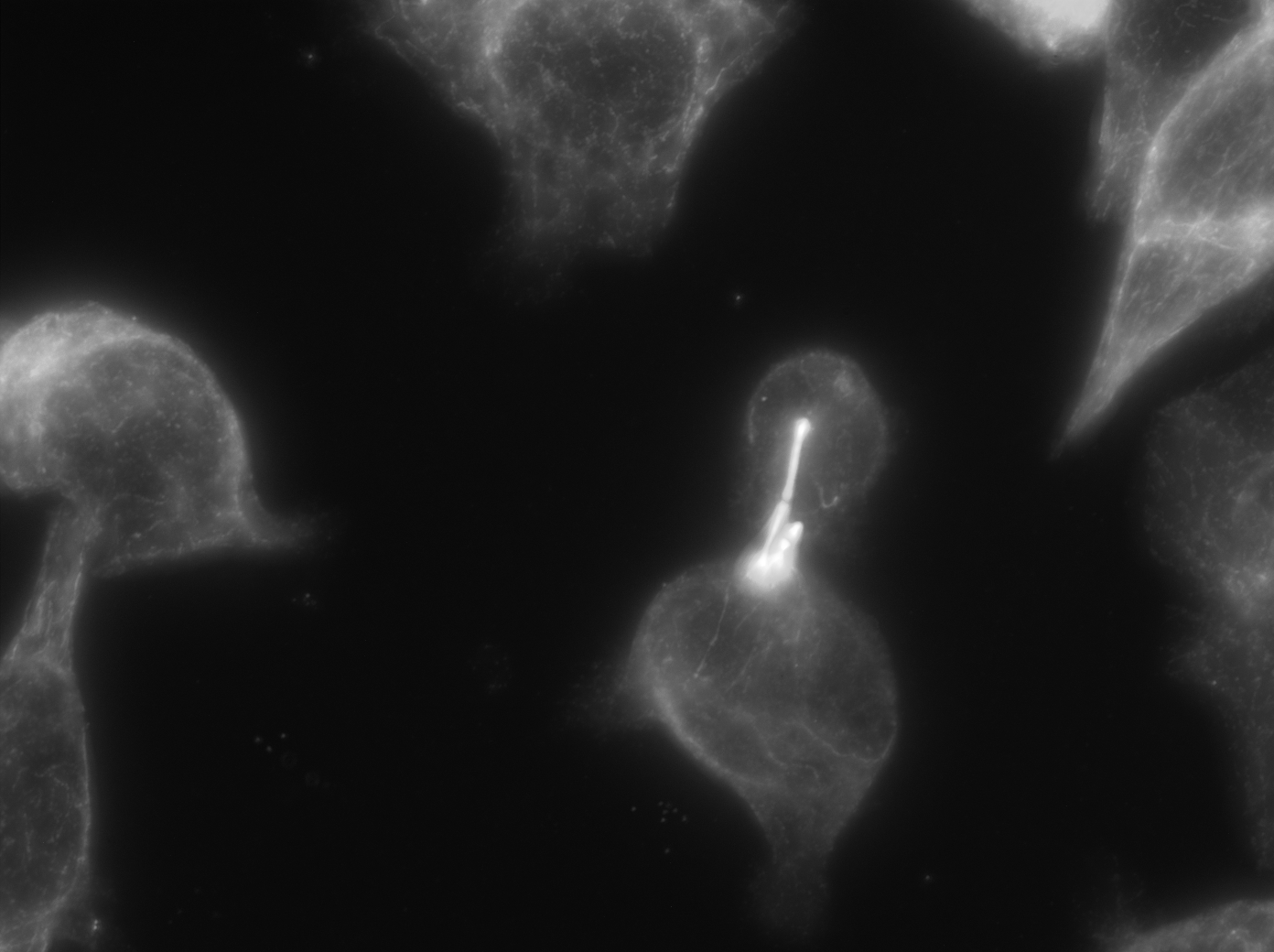

Supplement: Supplementary file 23 — Source data Fig. 6 part 4 [file 44319_2026_742_MOESM23_ESM.zip › Figure 6 Part 4/Fig 6efg TPGS1-KO TPGS1 rescue experiments part 2/R2R3/TPGS1-KO EYFP-only actub 7-31-25 R2 UNKOWN.Project Maximum Z_XY1756414147_Z0_T0_C2.tif]

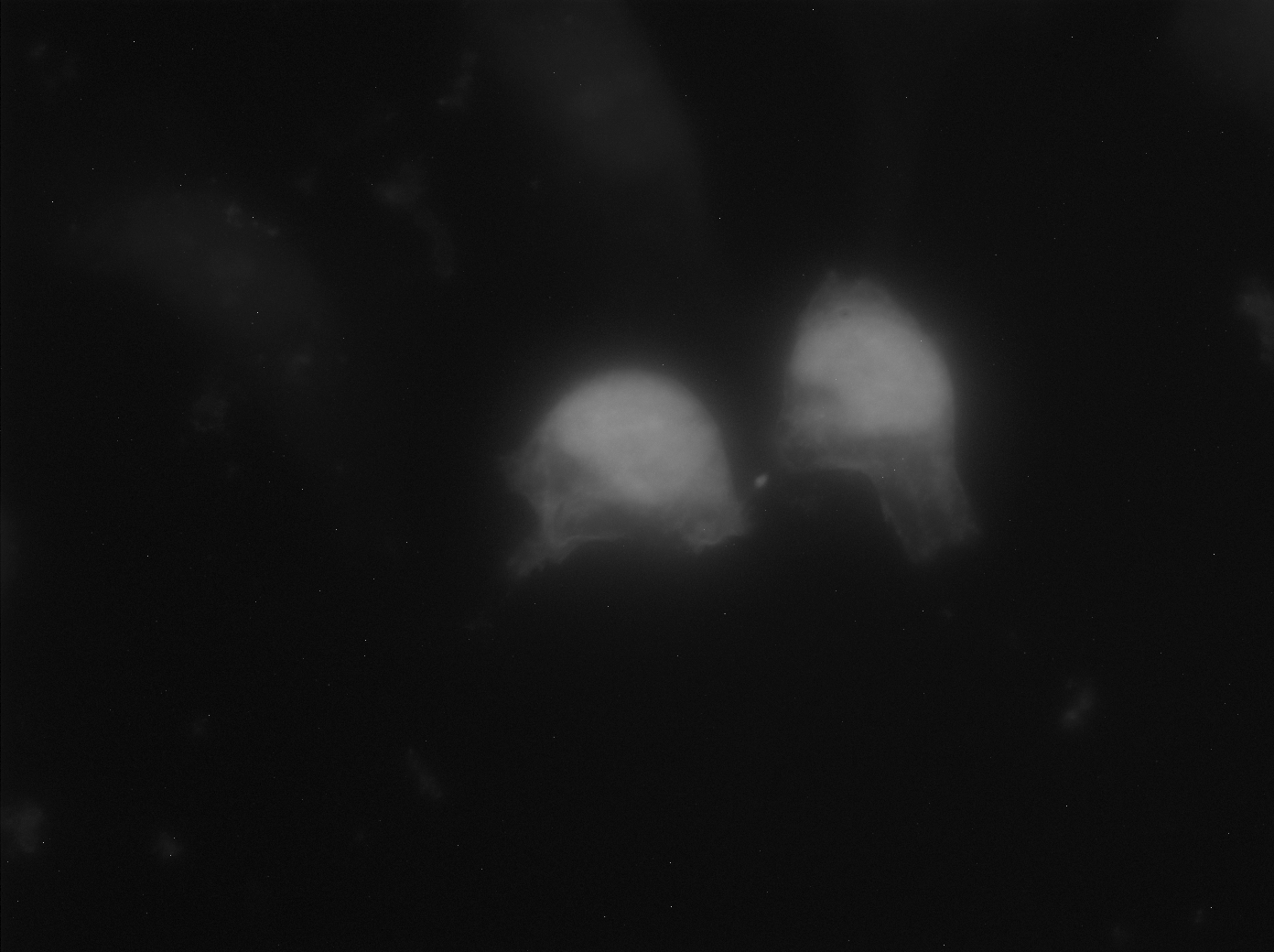

Supplement: Supplementary file 23 — Source data Fig. 6 part 4 [file 44319_2026_742_MOESM23_ESM.zip › Figure 6 Part 4/Fig 6efg TPGS1-KO TPGS1 rescue experiments part 2/R2R3/TPGS1-KO EYFP-only actub 7-31-25 R2 LT3.Project Maximum Z_XY1756414428_Z0_T0_C1.tif]

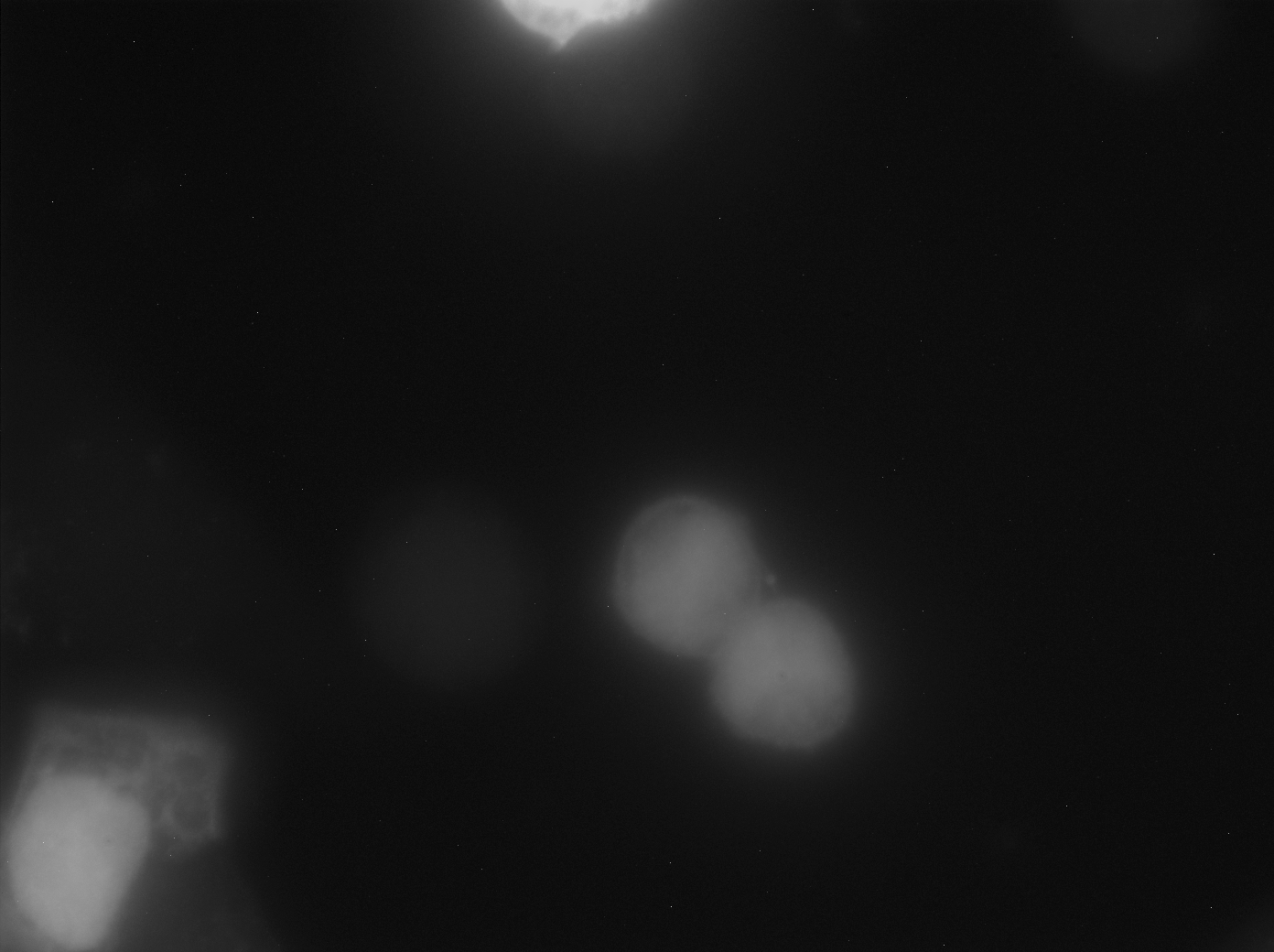

Supplement: Supplementary file 23 — Source data Fig. 6 part 4 [file 44319_2026_742_MOESM23_ESM.zip › Figure 6 Part 4/Fig 6efg TPGS1-KO TPGS1 rescue experiments part 2/R2R3/TPGS1-KO EYFP-only actub 7-31-25 R3 ET4.Project Maximum Z_XY1756493379_Z0_T0_C1.tif]

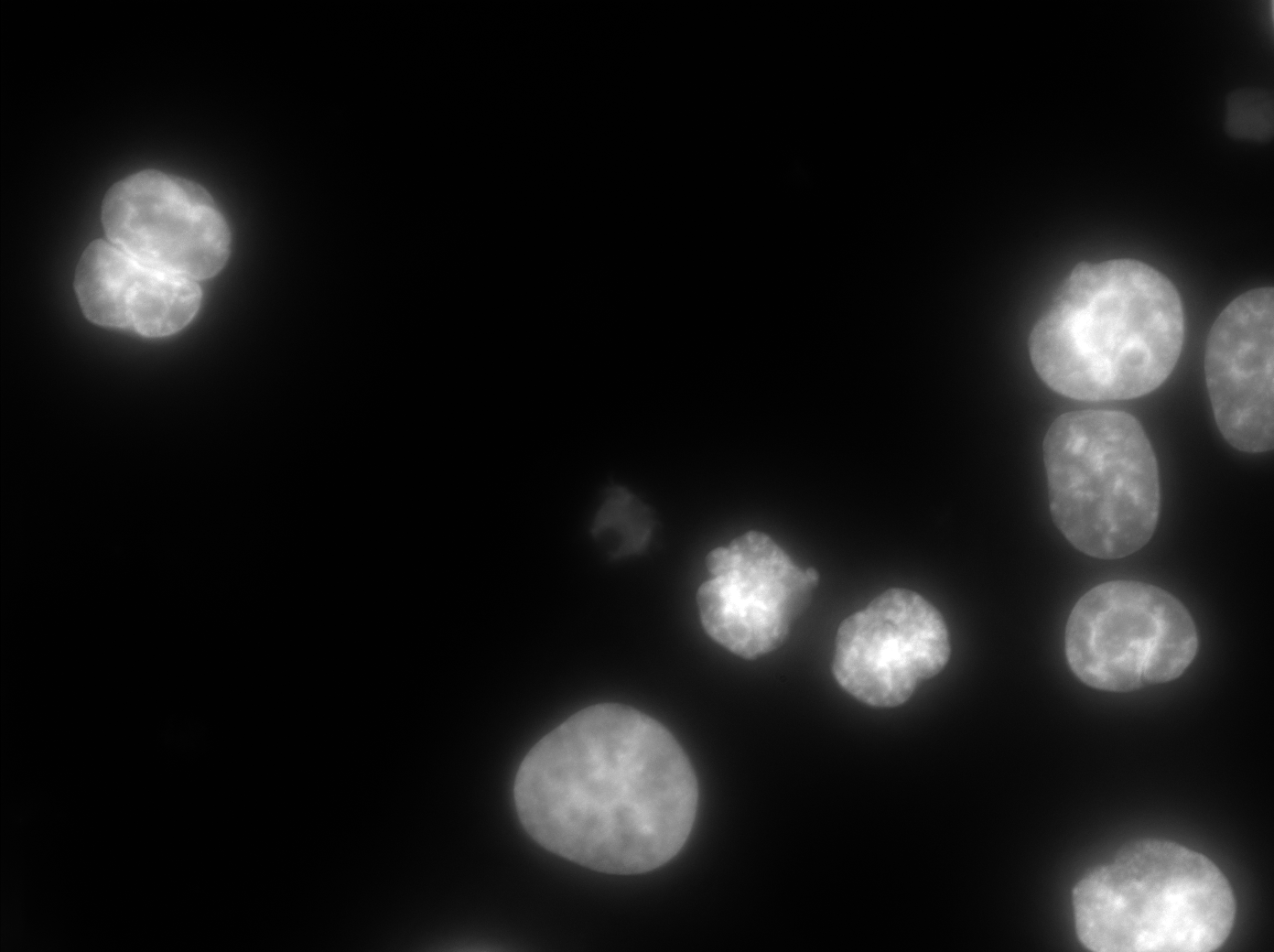

Supplement: Supplementary file 23 — Source data Fig. 6 part 4 [file 44319_2026_742_MOESM23_ESM.zip › Figure 6 Part 4/Fig 6efg TPGS1-KO TPGS1 rescue experiments part 2/R2R3/TPGS1-KO TPGS1-EYFP-3'UTR actub 7-31-25 R3 ET3.Project Maximum Z_XY1756499529_Z0_T0_C0.tif]

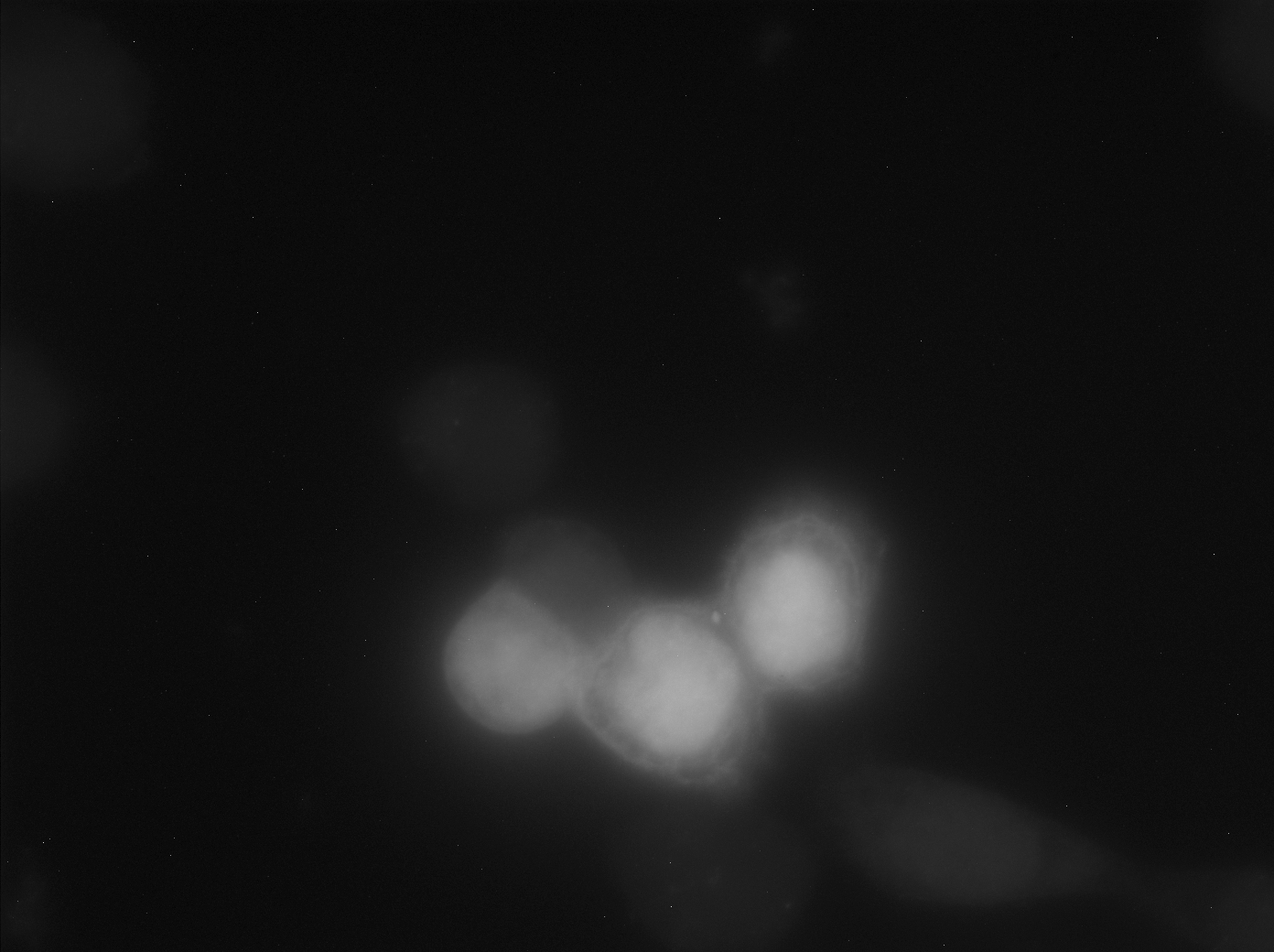

Supplement: Supplementary file 23 — Source data Fig. 6 part 4 [file 44319_2026_742_MOESM23_ESM.zip › Figure 6 Part 4/Fig 6efg TPGS1-KO TPGS1 rescue experiments part 2/R2R3/TPGS1-KO EYFP-only actub 7-31-25 R3 LT4.Project Maximum Z_XY1756492876_Z0_T0_C1.tif]

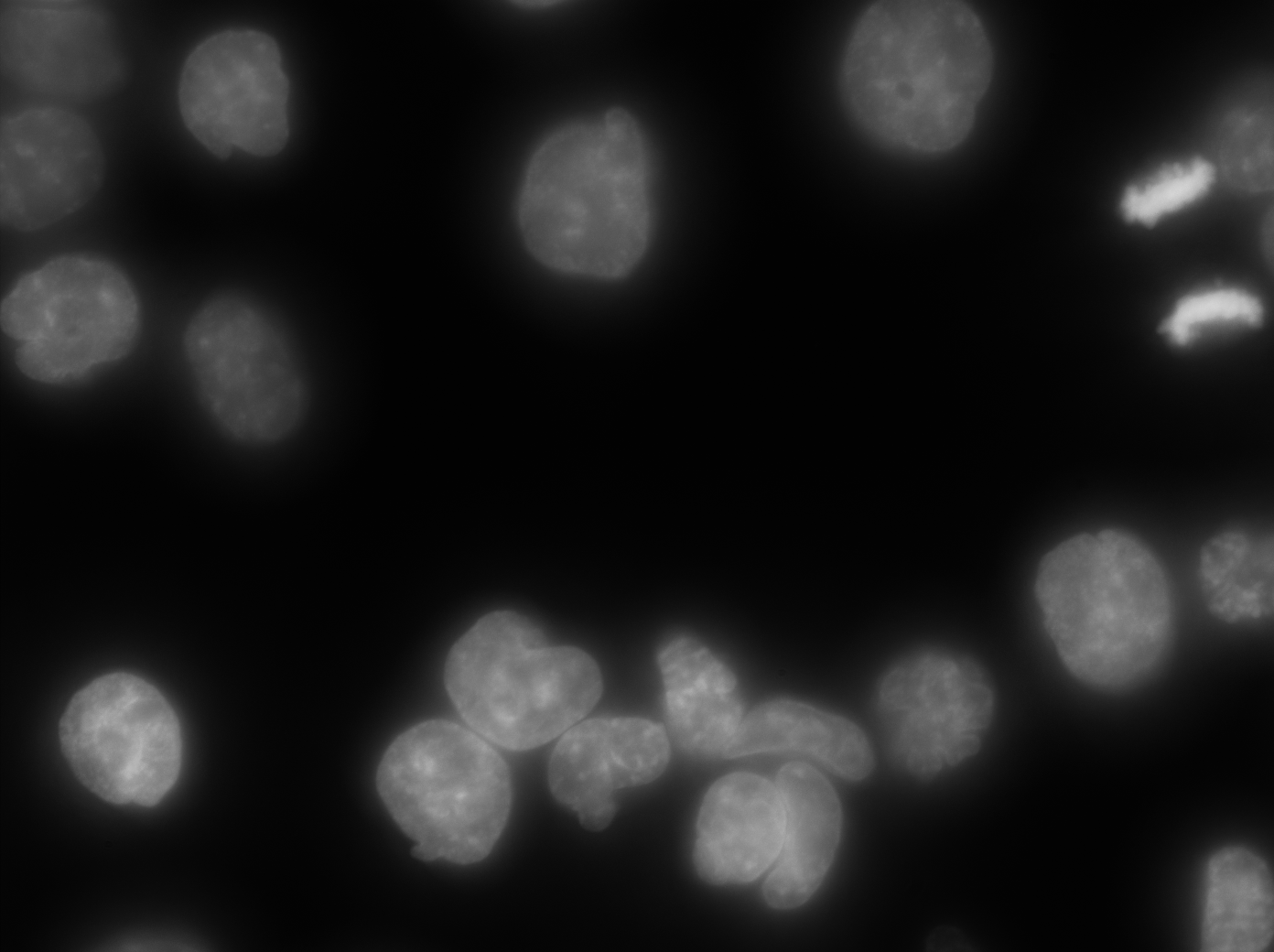

Supplement: Supplementary file 23 — Source data Fig. 6 part 4 [file 44319_2026_742_MOESM23_ESM.zip › Figure 6 Part 4/Fig 6efg TPGS1-KO TPGS1 rescue experiments part 2/R2R3/TPGS1-KO TPGS1-EYFP-3'UTR actub 7-31-25 R2 LT7.Project Maximum Z_XY1756411060_Z0_T0_C0.tif]

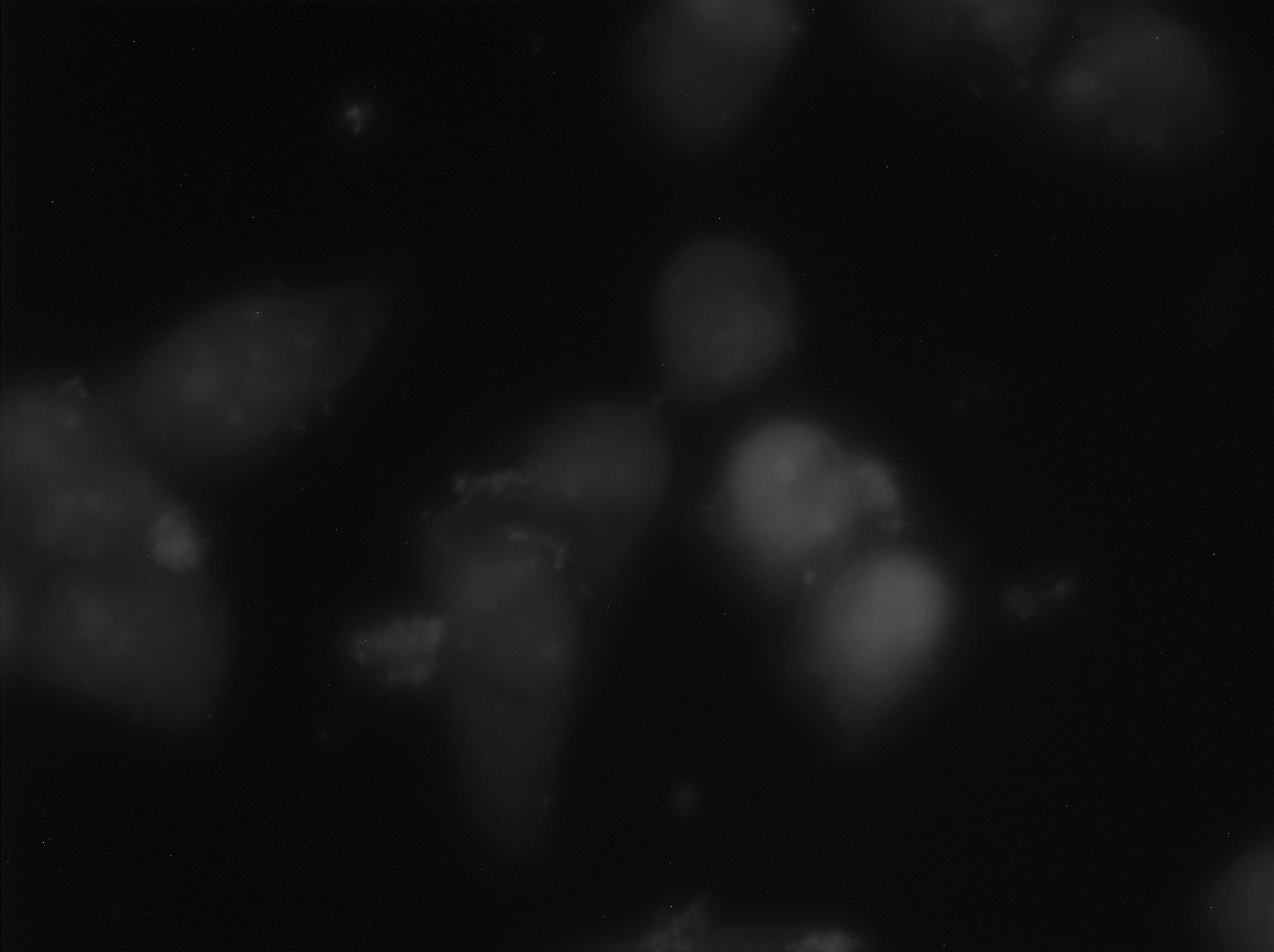

Supplement: Supplementary file 23 — Source data Fig. 6 part 4 [file 44319_2026_742_MOESM23_ESM.zip › Figure 6 Part 4/Fig 6efg TPGS1-KO TPGS1 rescue experiments part 2/R2R3/TPGS1-KO EYFP-only actub 7-31-25 R3 LT1.Project Maximum Z_XY1756490934_Z0_T0_C1.tif]

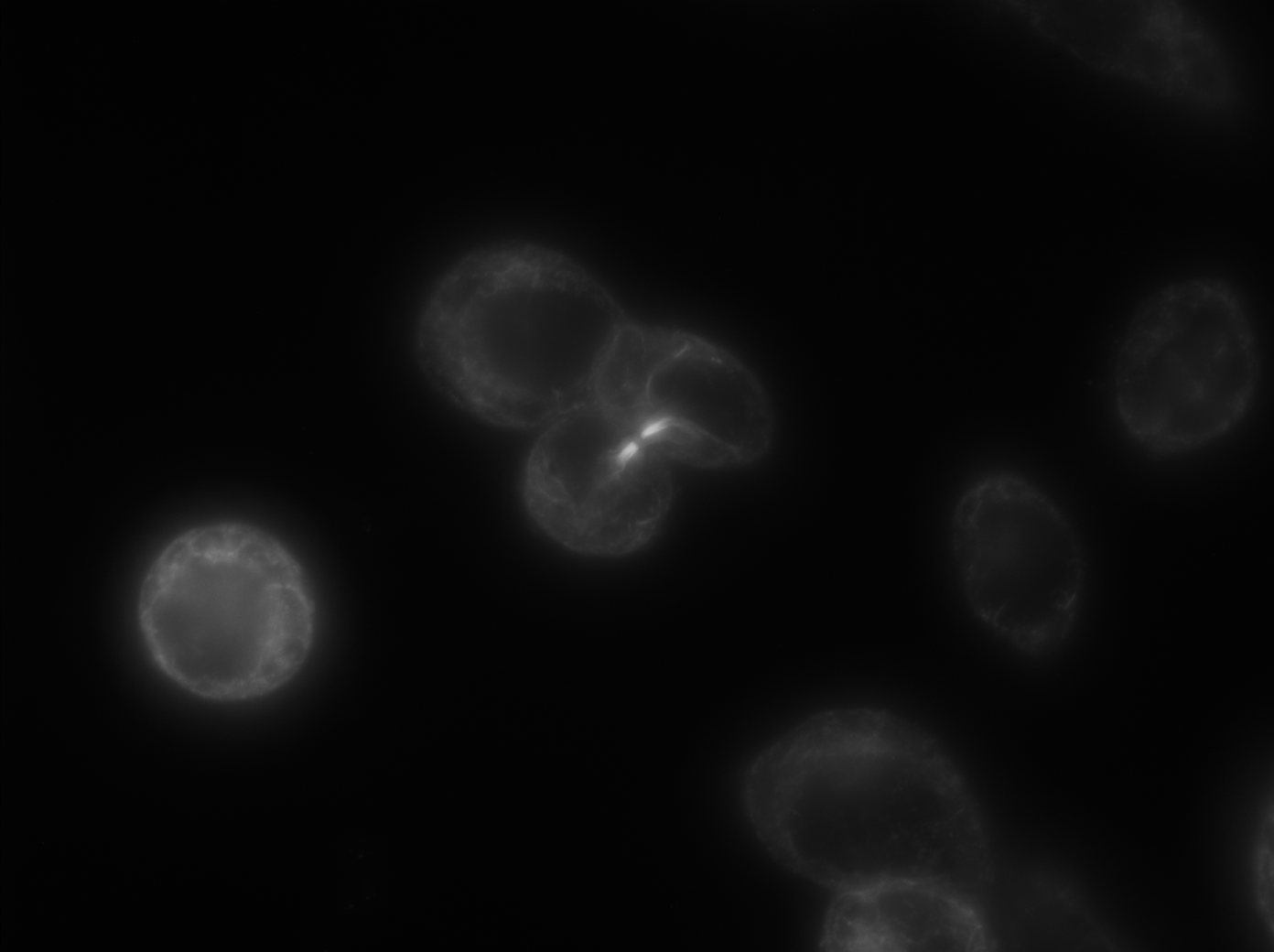

Supplement: Supplementary file 23 — Source data Fig. 6 part 4 [file 44319_2026_742_MOESM23_ESM.zip › Figure 6 Part 4/Fig 6efg TPGS1-KO TPGS1 rescue experiments part 2/R2R3/TPGS1-KO EYFP-only actub 7-31-25 R3 LT7.Project Maximum Z_XY1756494741_Z0_T0_C2.tif]

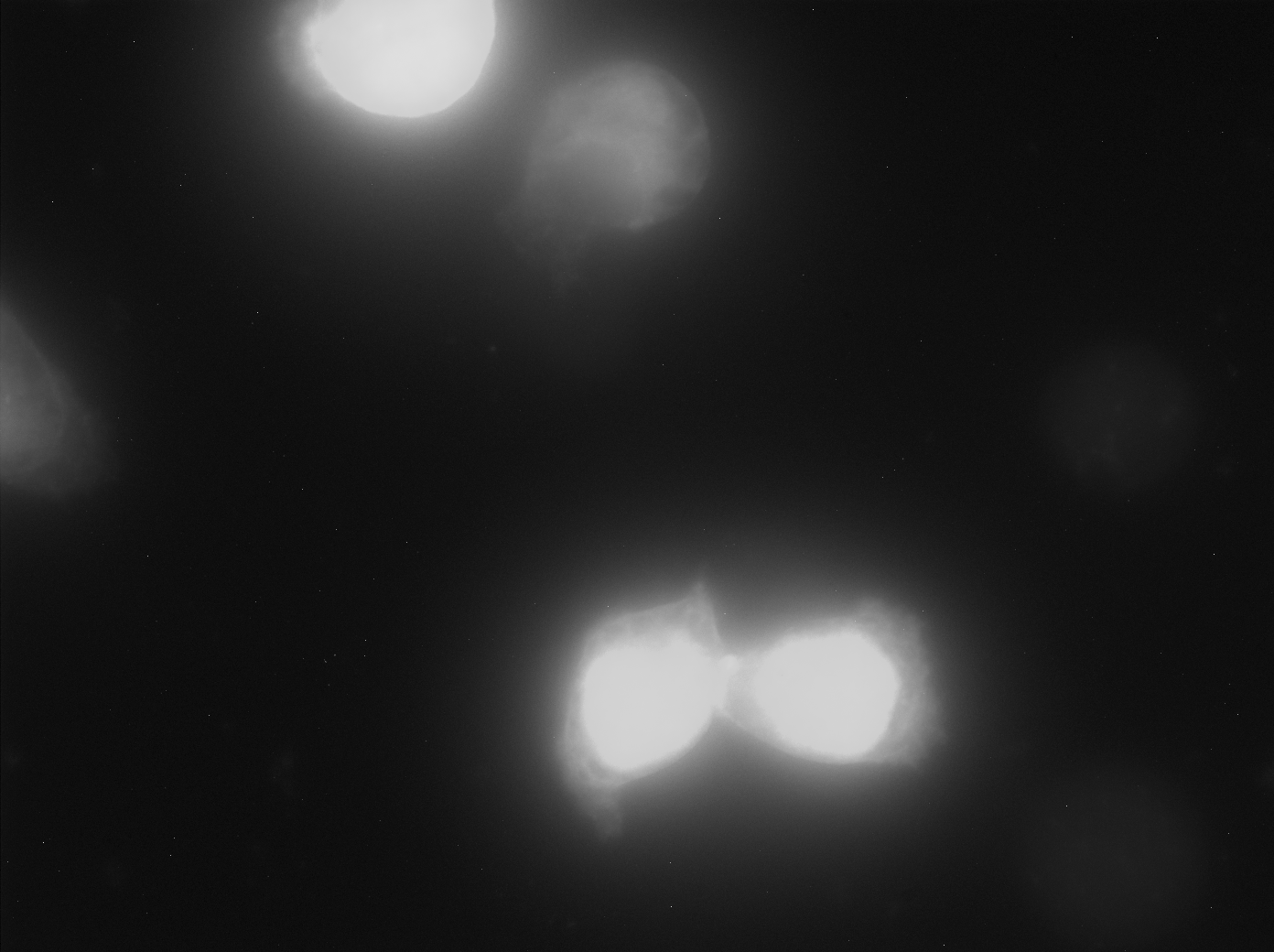

Supplement: Supplementary file 23 — Source data Fig. 6 part 4 [file 44319_2026_742_MOESM23_ESM.zip › Figure 6 Part 4/Fig 6efg TPGS1-KO TPGS1 rescue experiments part 2/R2R3/TPGS1-KO EYFP-only actub 7-31-25 R2 LT9.Project Maximum Z_XY1756416573_Z0_T0_C1.tif]

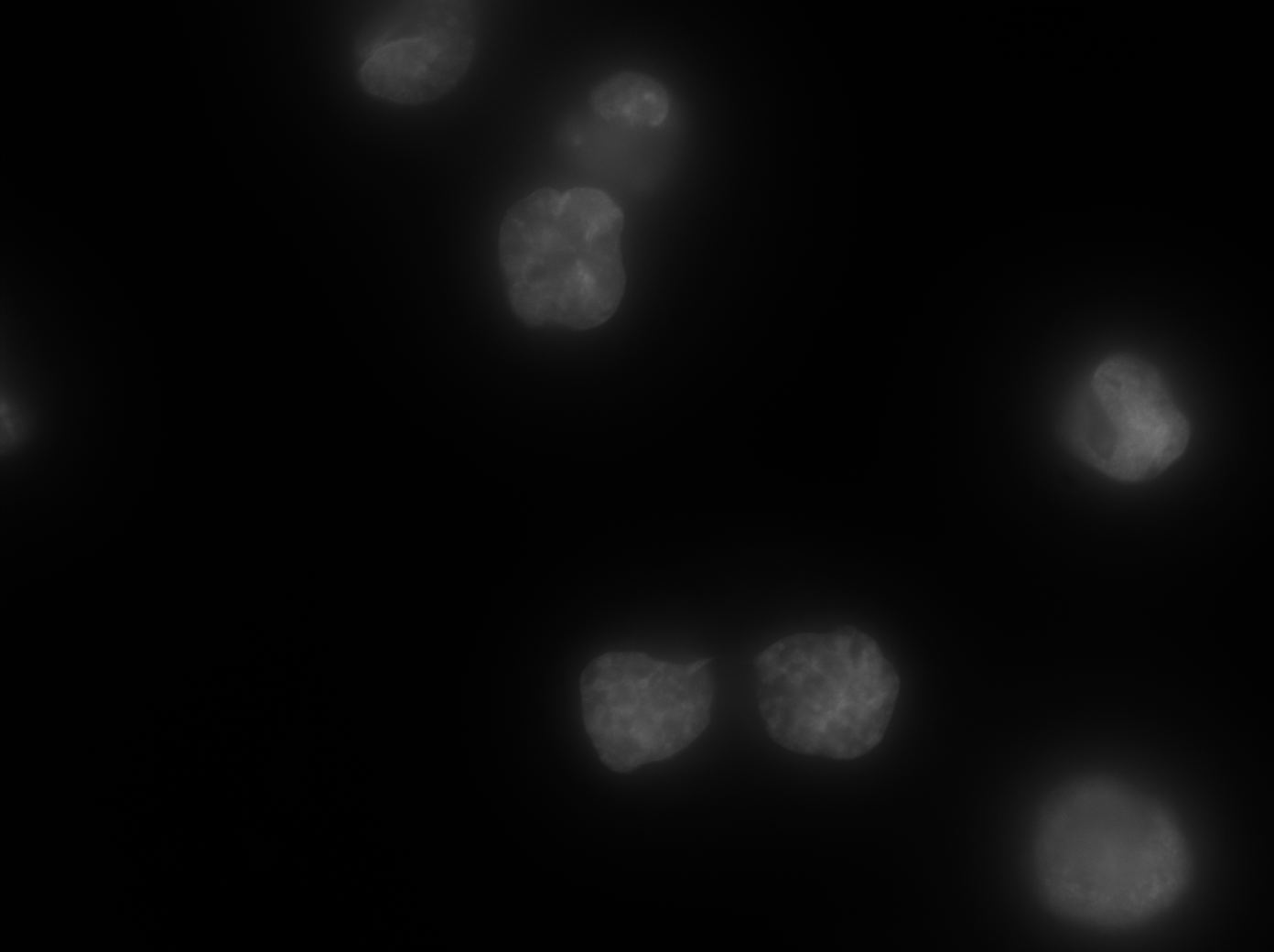

Supplement: Supplementary file 23 — Source data Fig. 6 part 4 [file 44319_2026_742_MOESM23_ESM.zip › Figure 6 Part 4/Fig 6efg TPGS1-KO TPGS1 rescue experiments part 2/R2R3/TPGS1-KO EYFP-only actub 7-31-25 R2 LT9.Project Maximum Z_XY1756416573_Z0_T0_C0.tif]

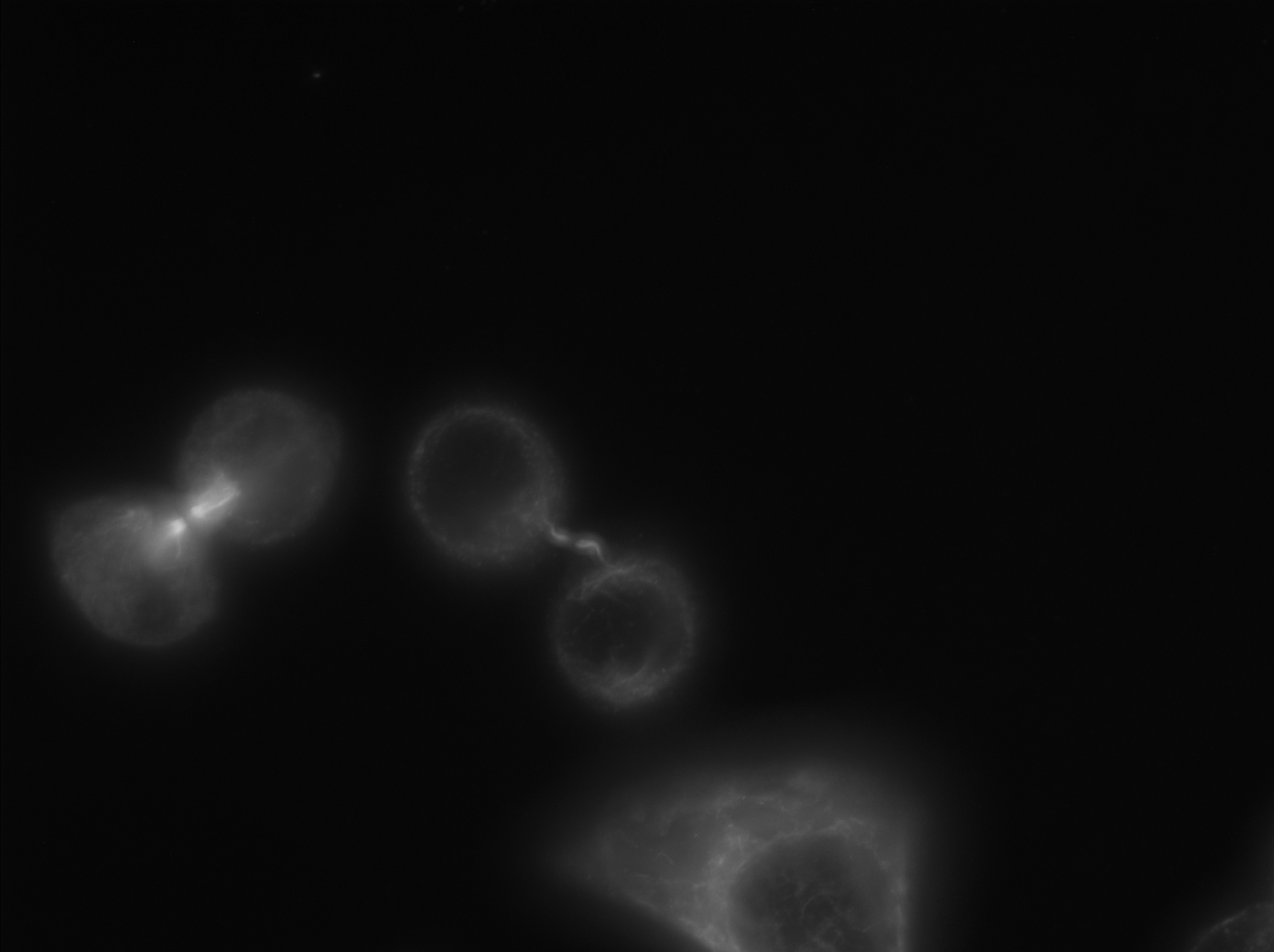

Supplement: Supplementary file 23 — Source data Fig. 6 part 4 [file 44319_2026_742_MOESM23_ESM.zip › Figure 6 Part 4/Fig 6efg TPGS1-KO TPGS1 rescue experiments part 2/R2R3/TPGS1-KO EYFP-only actub 7-31-25 R2 LT10.Project Maximum Z_XY1756416876_Z0_T0_C2.tif]

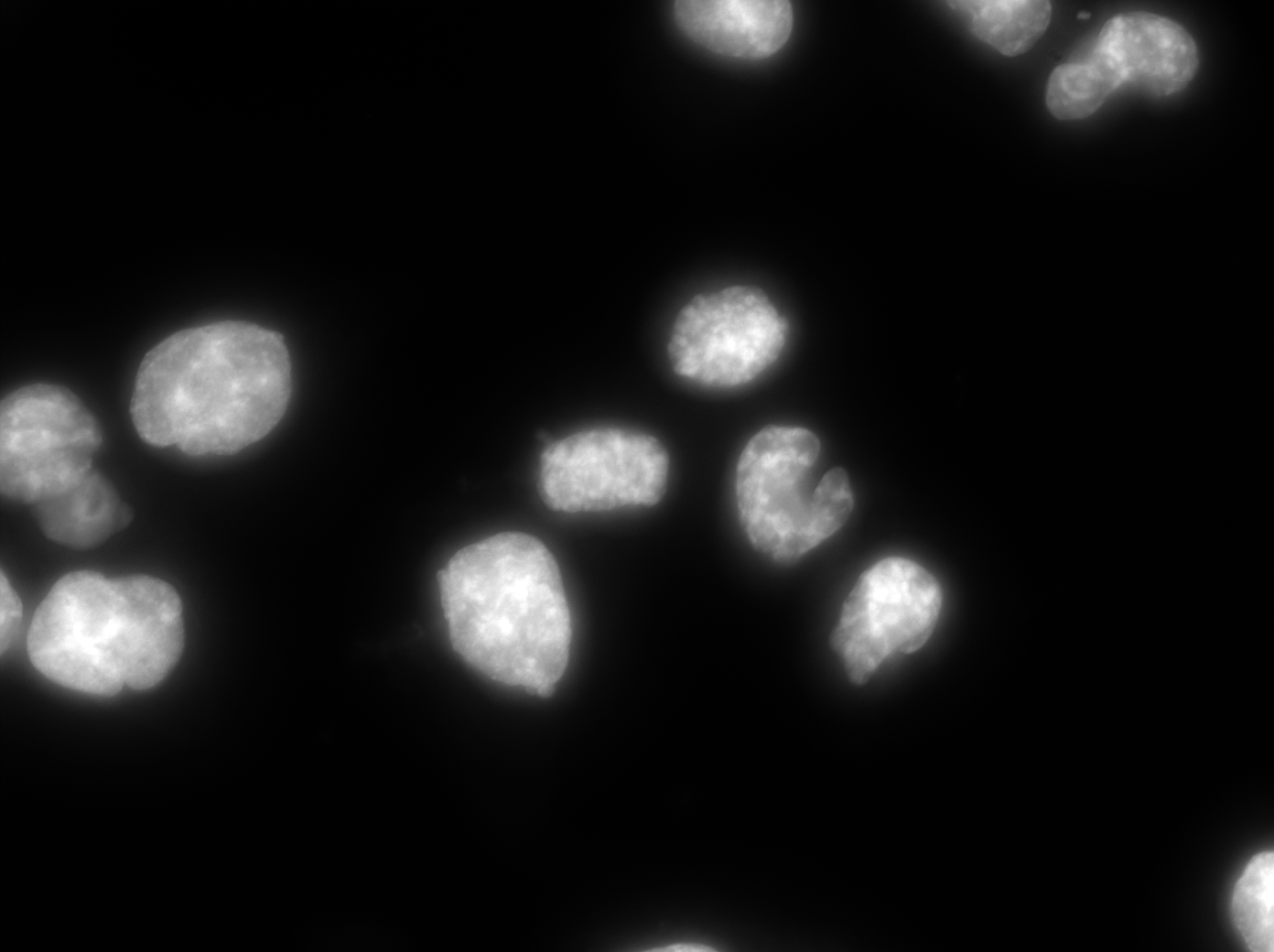

Supplement: Supplementary file 23 — Source data Fig. 6 part 4 [file 44319_2026_742_MOESM23_ESM.zip › Figure 6 Part 4/Fig 6efg TPGS1-KO TPGS1 rescue experiments part 2/R2R3/TPGS1-KO EYFP-only actub 7-31-25 R3 LT1.Project Maximum Z_XY1756490934_Z0_T0_C0.tif]

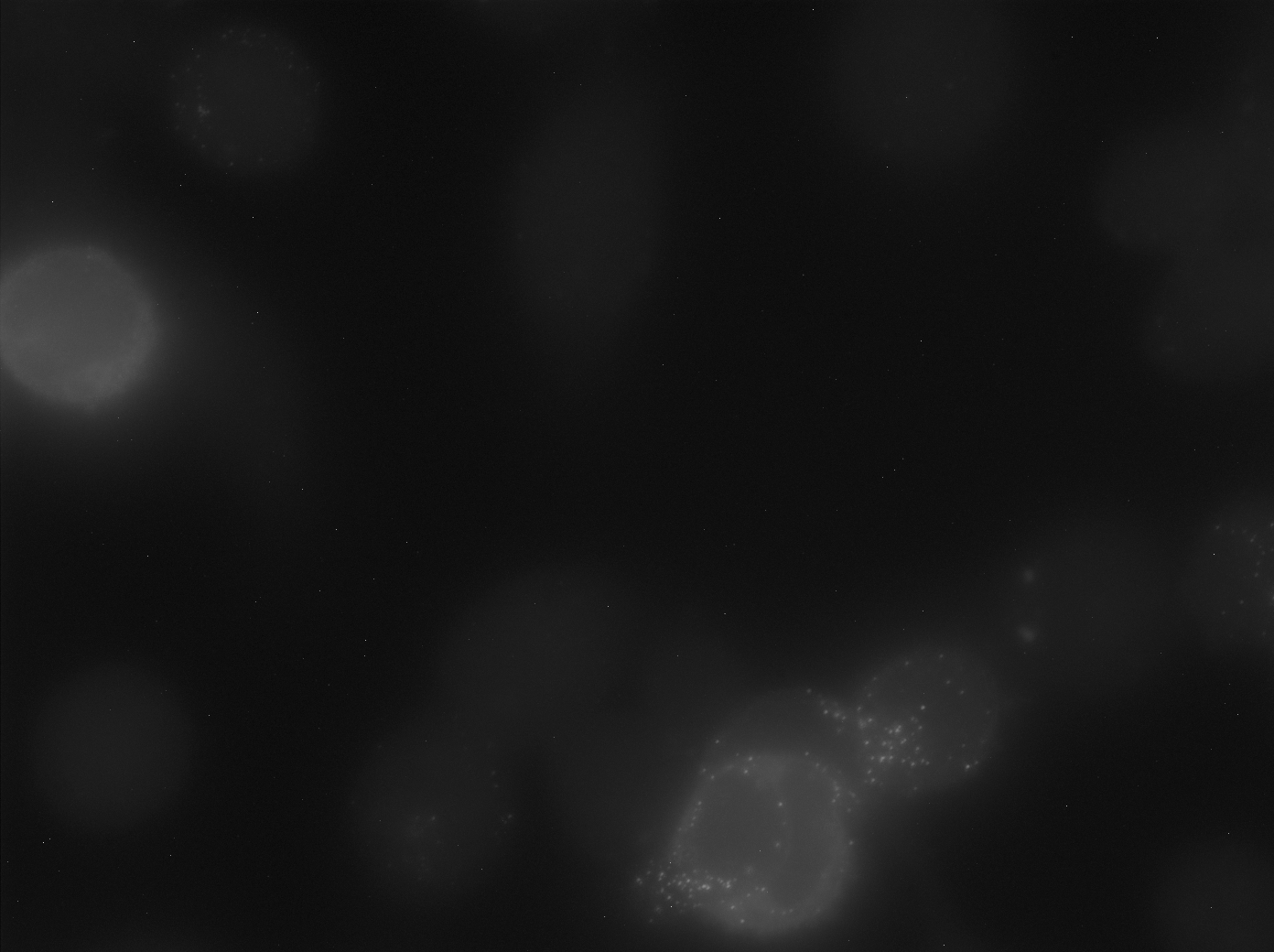

Supplement: Supplementary file 23 — Source data Fig. 6 part 4 [file 44319_2026_742_MOESM23_ESM.zip › Figure 6 Part 4/Fig 6efg TPGS1-KO TPGS1 rescue experiments part 2/R2R3/TPGS1-KO TPGS1-EYFP-3'UTR actub 7-31-25 R2 LT7.Project Maximum Z_XY1756411060_Z0_T0_C1.tif]

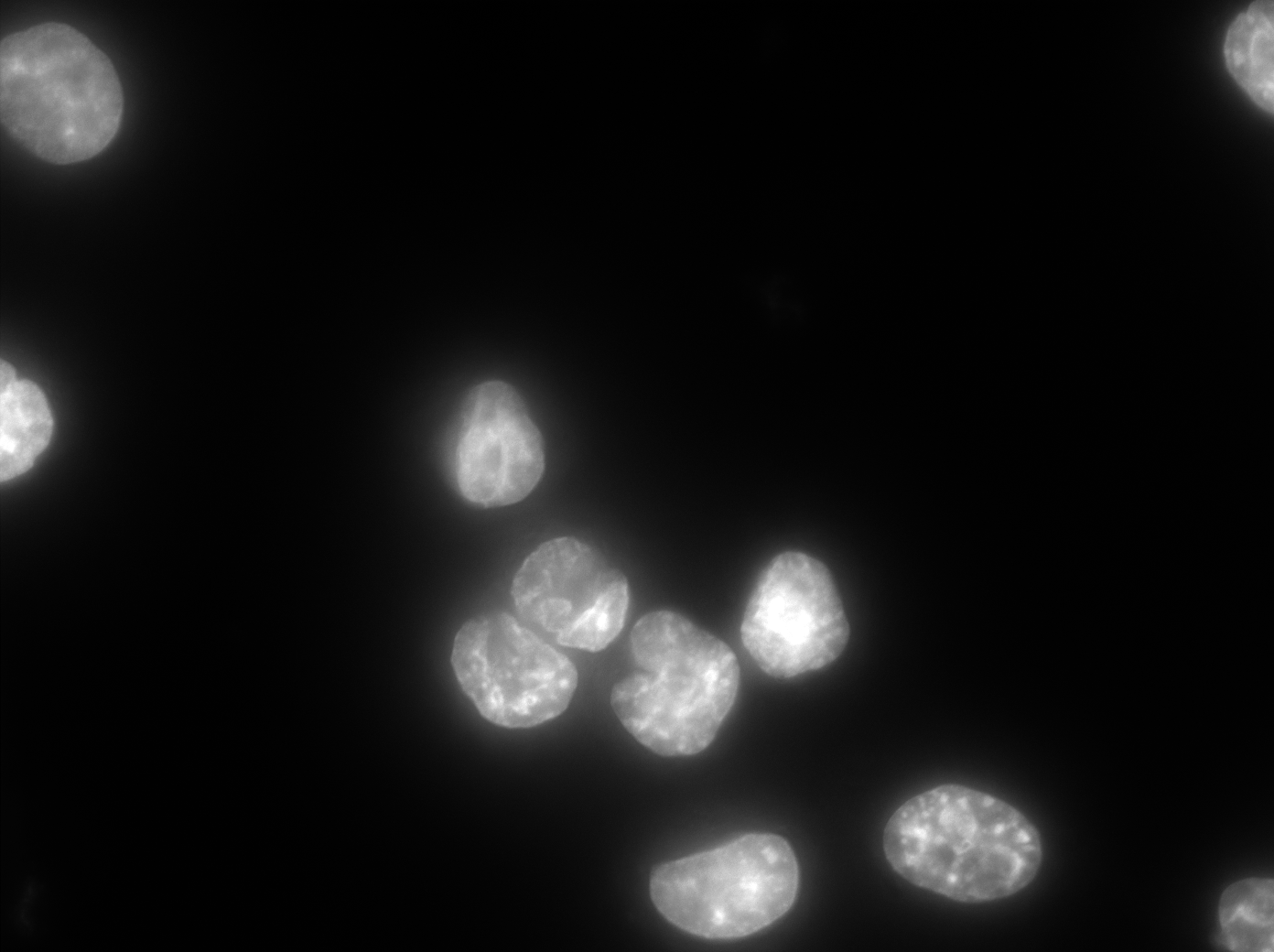

Supplement: Supplementary file 23 — Source data Fig. 6 part 4 [file 44319_2026_742_MOESM23_ESM.zip › Figure 6 Part 4/Fig 6efg TPGS1-KO TPGS1 rescue experiments part 2/R2R3/TPGS1-KO EYFP-only actub 7-31-25 R3 LT4.Project Maximum Z_XY1756492876_Z0_T0_C0.tif]

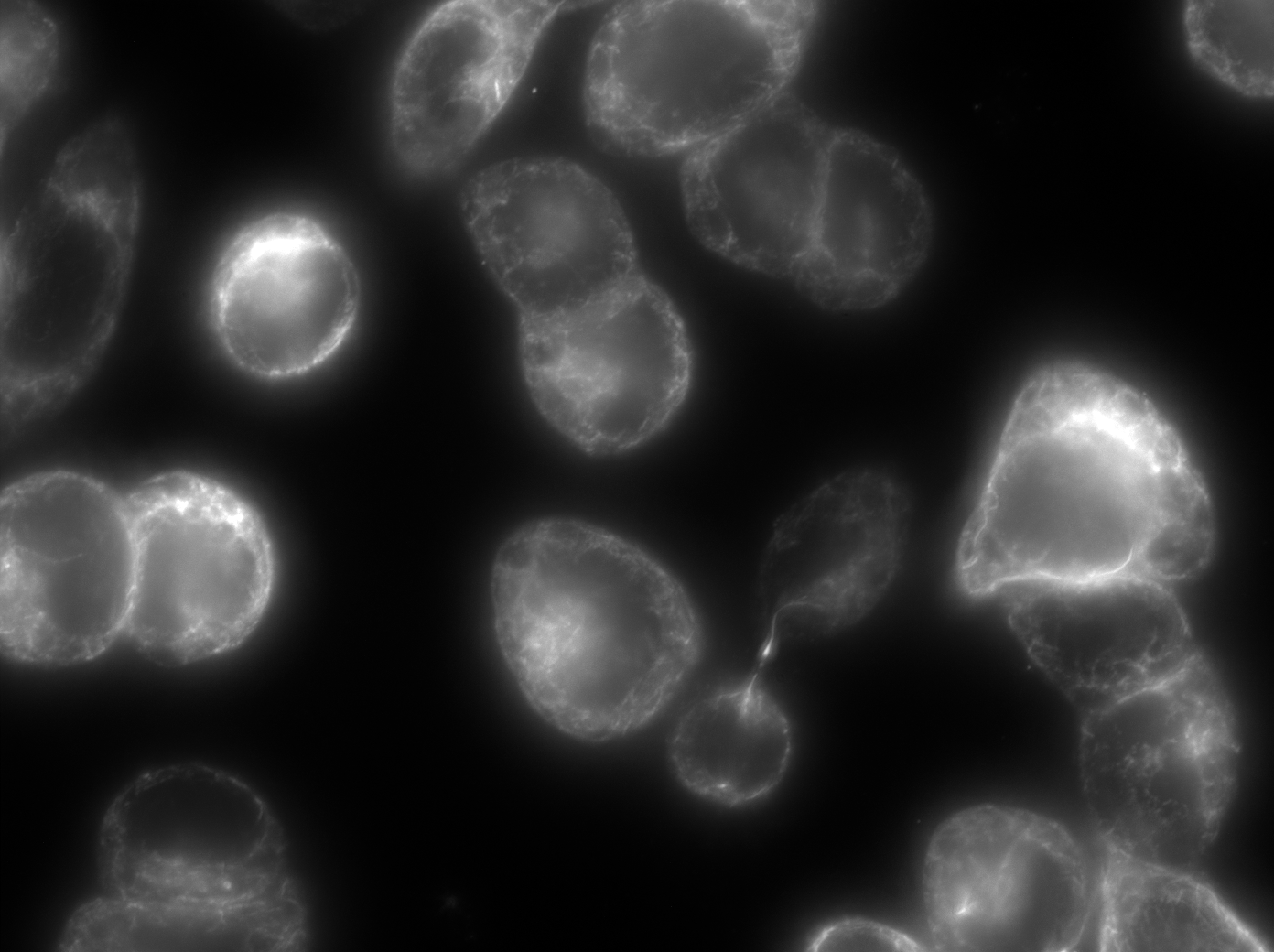

Supplement: Supplementary file 23 — Source data Fig. 6 part 4 [file 44319_2026_742_MOESM23_ESM.zip › Figure 6 Part 4/Fig 6efg TPGS1-KO TPGS1 rescue experiments part 2/R2R3/TPGS1-KO TPGS1-EYFP-3'UTR actub 7-31-25 R3 LT5.Project Maximum Z_XY1756501059_Z0_T0_C2.tif]

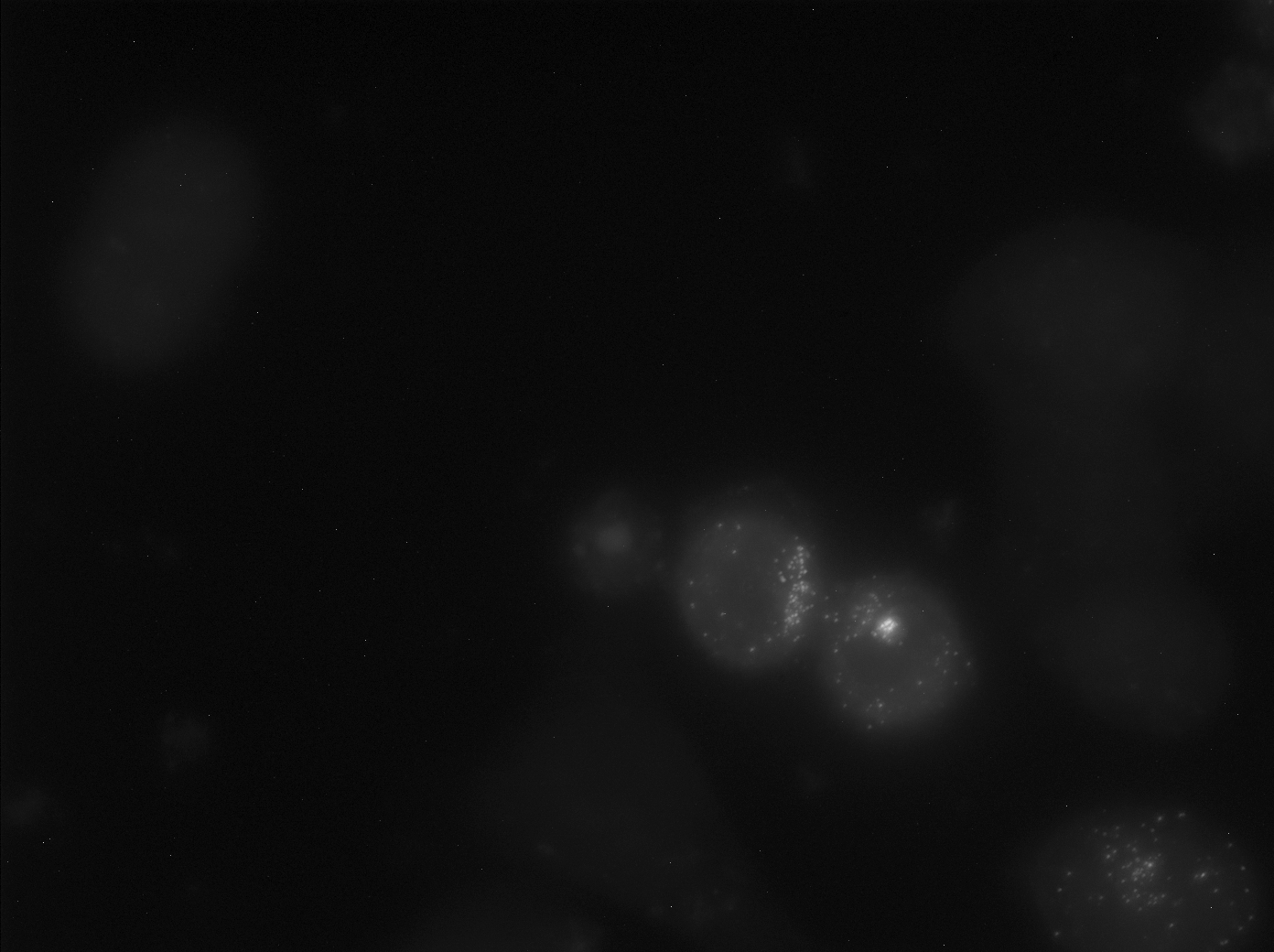

Supplement: Supplementary file 23 — Source data Fig. 6 part 4 [file 44319_2026_742_MOESM23_ESM.zip › Figure 6 Part 4/Fig 6efg TPGS1-KO TPGS1 rescue experiments part 2/R2R3/TPGS1-KO TPGS1-EYFP-3'UTR actub 7-31-25 R3 ET3.Project Maximum Z_XY1756499529_Z0_T0_C1.tif]

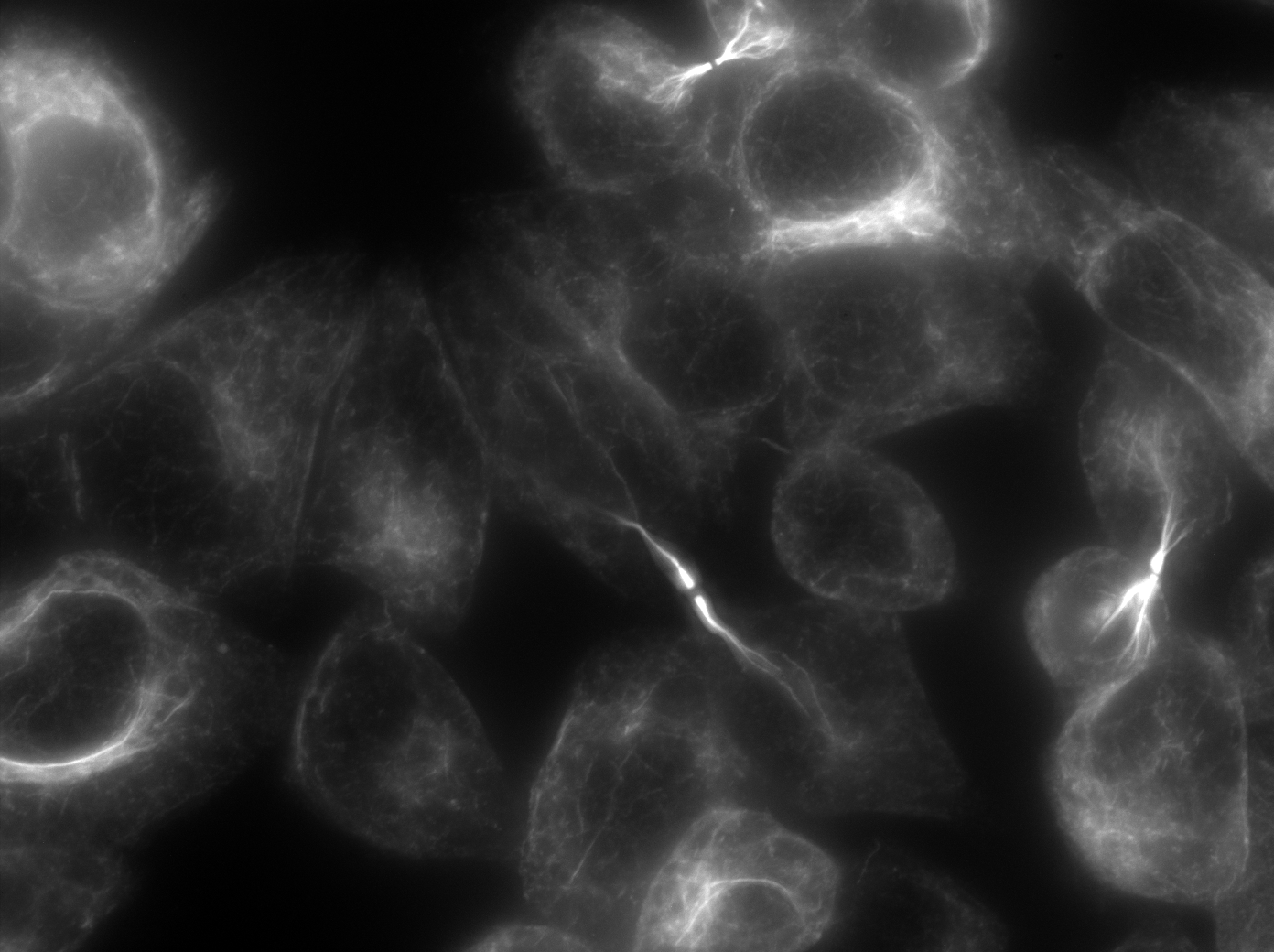

Supplement: Supplementary file 23 — Source data Fig. 6 part 4 [file 44319_2026_742_MOESM23_ESM.zip › Figure 6 Part 4/Fig 6efg TPGS1-KO TPGS1 rescue experiments part 2/R2R3/TPGS1-KO TPGS1-EYFP-3'UTR actub 7-31-25 R2 LT6.Project Maximum Z_XY1756410864_Z0_T0_C2.tif]

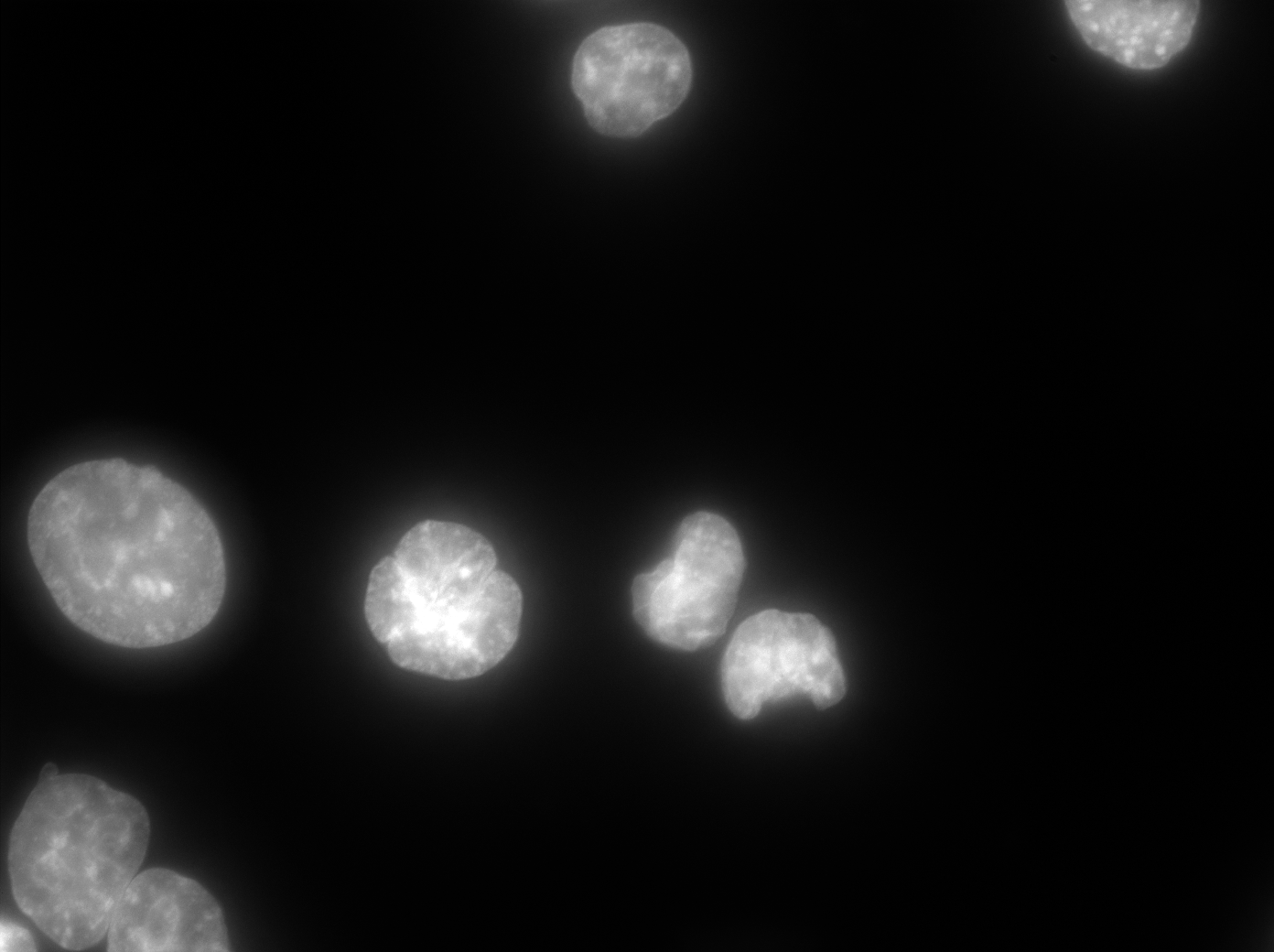

Supplement: Supplementary file 23 — Source data Fig. 6 part 4 [file 44319_2026_742_MOESM23_ESM.zip › Figure 6 Part 4/Fig 6efg TPGS1-KO TPGS1 rescue experiments part 2/R2R3/TPGS1-KO EYFP-only actub 7-31-25 R3 ET4.Project Maximum Z_XY1756493379_Z0_T0_C0.tif]

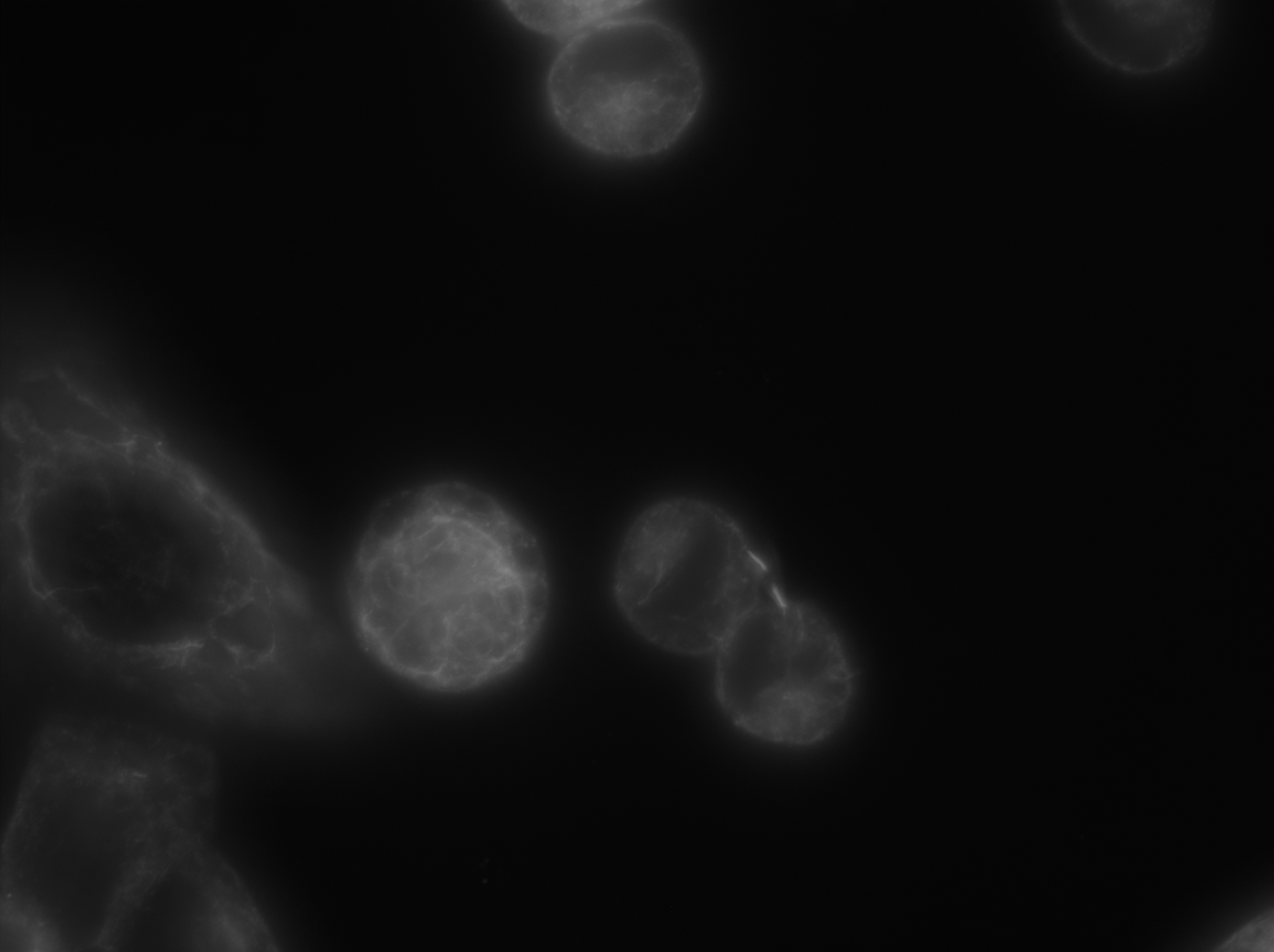

Supplement: Supplementary file 23 — Source data Fig. 6 part 4 [file 44319_2026_742_MOESM23_ESM.zip › Figure 6 Part 4/Fig 6efg TPGS1-KO TPGS1 rescue experiments part 2/R2R3/TPGS1-KO EYFP-only actub 7-31-25 R3 ET4.Project Maximum Z_XY1756493379_Z0_T0_C2.tif]

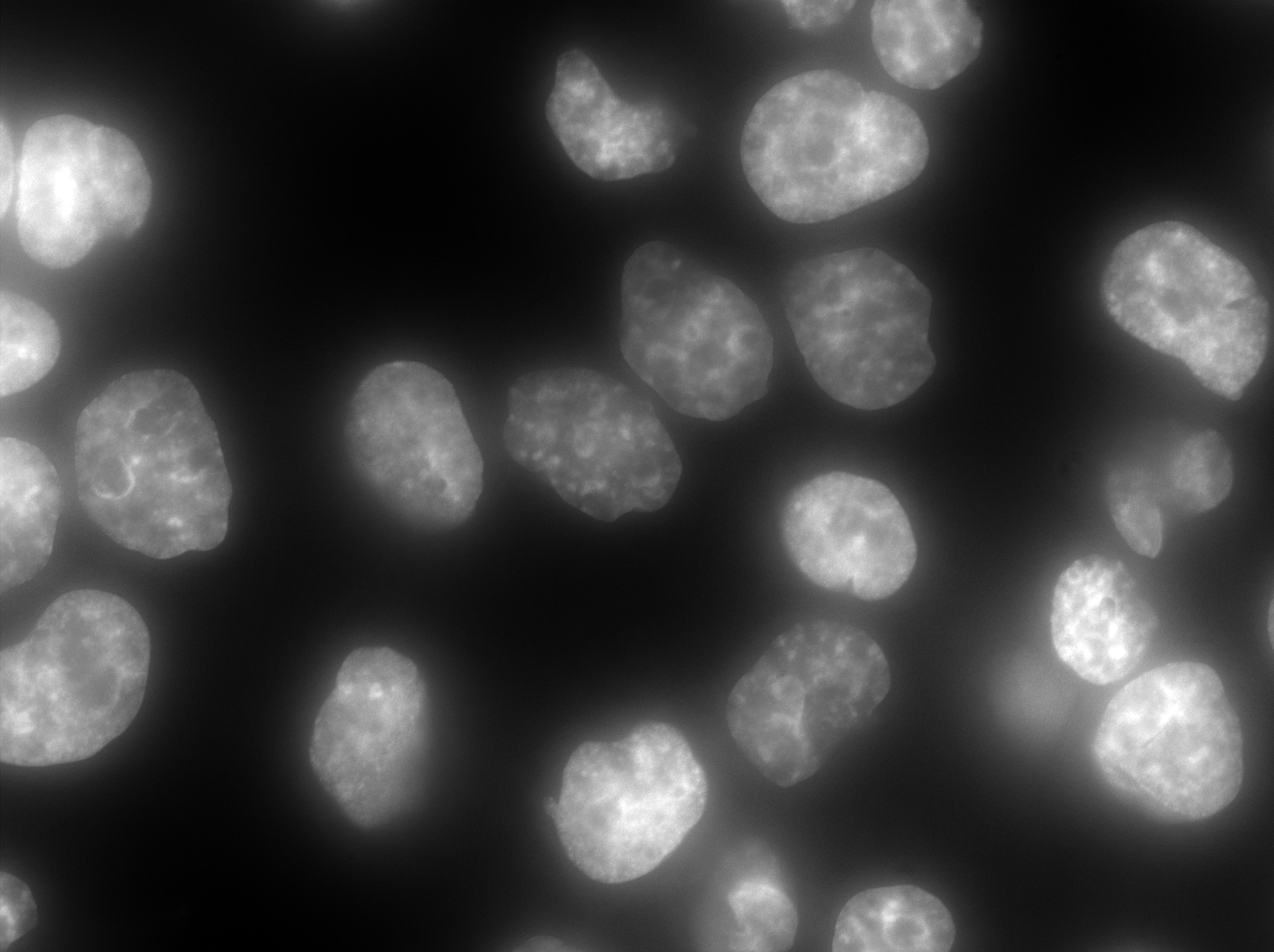

Supplement: Supplementary file 23 — Source data Fig. 6 part 4 [file 44319_2026_742_MOESM23_ESM.zip › Figure 6 Part 4/Fig 6efg TPGS1-KO TPGS1 rescue experiments part 2/R2R3/TPGS1-KO TPGS1-EYFP-3'UTR actub 7-31-25 R2 LT6.Project Maximum Z_XY1756410864_Z0_T0_C0.tif]

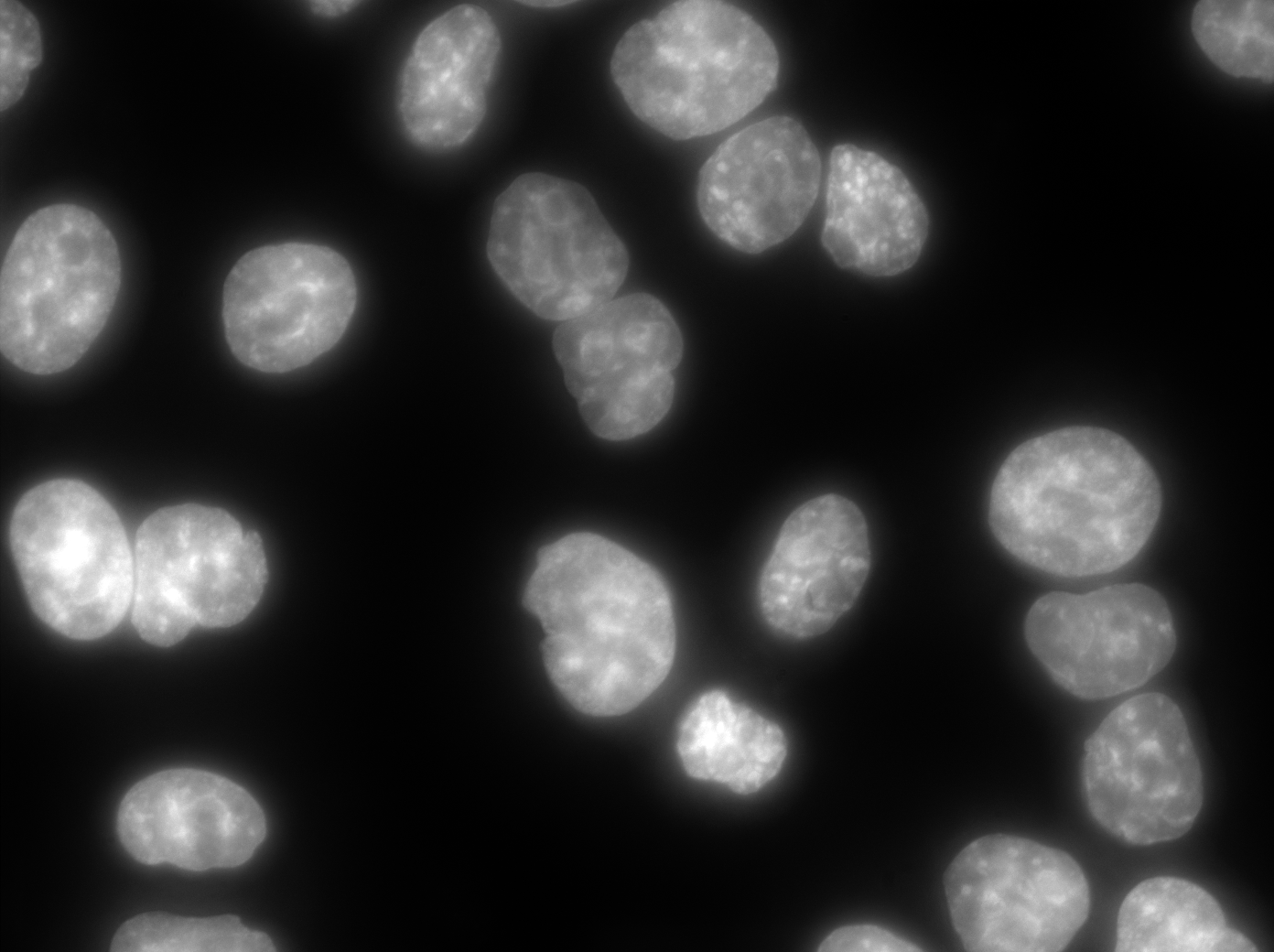

Supplement: Supplementary file 23 — Source data Fig. 6 part 4 [file 44319_2026_742_MOESM23_ESM.zip › Figure 6 Part 4/Fig 6efg TPGS1-KO TPGS1 rescue experiments part 2/R2R3/TPGS1-KO TPGS1-EYFP-3'UTR actub 7-31-25 R3 LT5.Project Maximum Z_XY1756501059_Z0_T0_C0.tif]

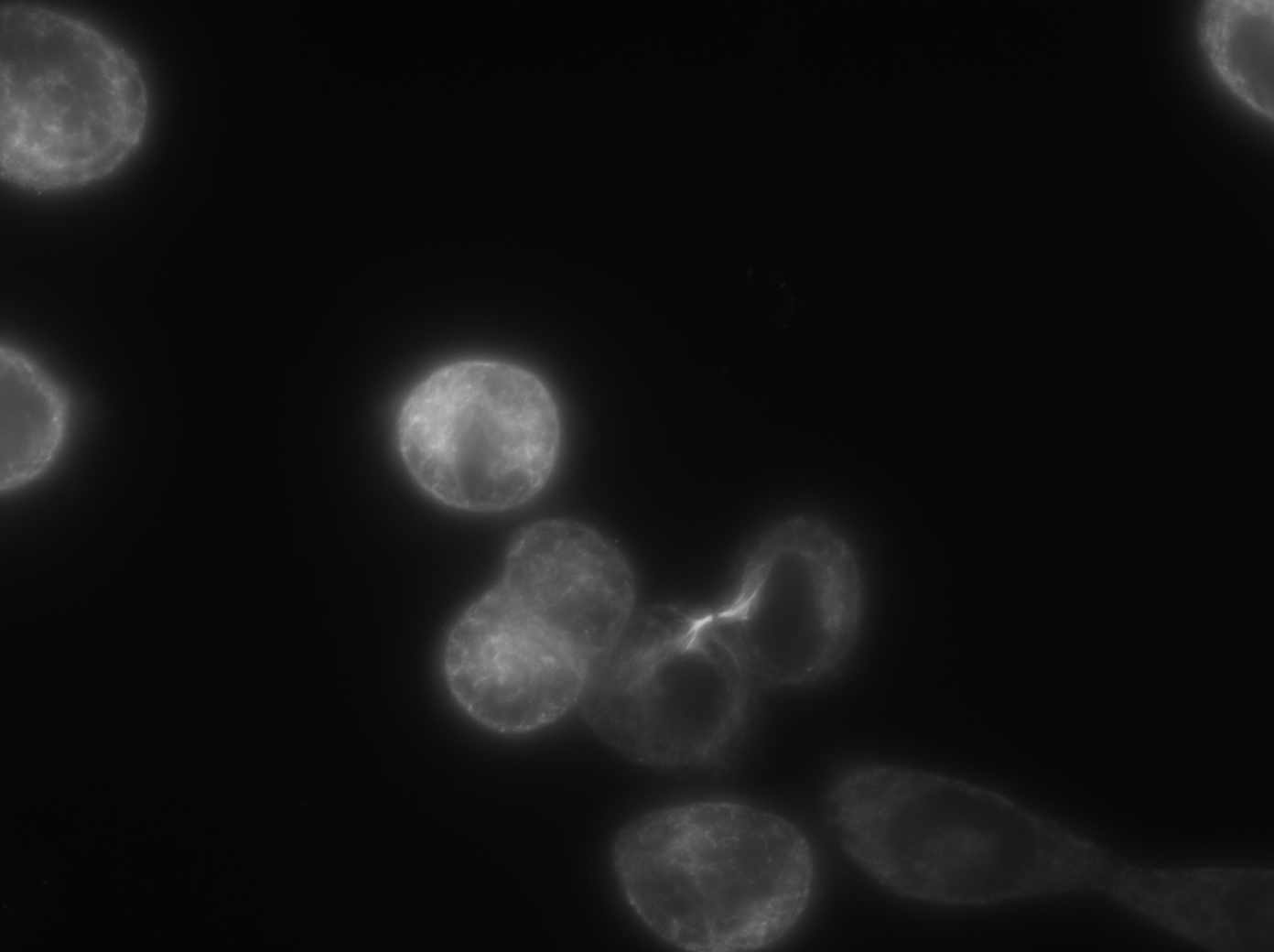

Supplement: Supplementary file 23 — Source data Fig. 6 part 4 [file 44319_2026_742_MOESM23_ESM.zip › Figure 6 Part 4/Fig 6efg TPGS1-KO TPGS1 rescue experiments part 2/R2R3/TPGS1-KO EYFP-only actub 7-31-25 R3 LT4.Project Maximum Z_XY1756492876_Z0_T0_C2.tif]

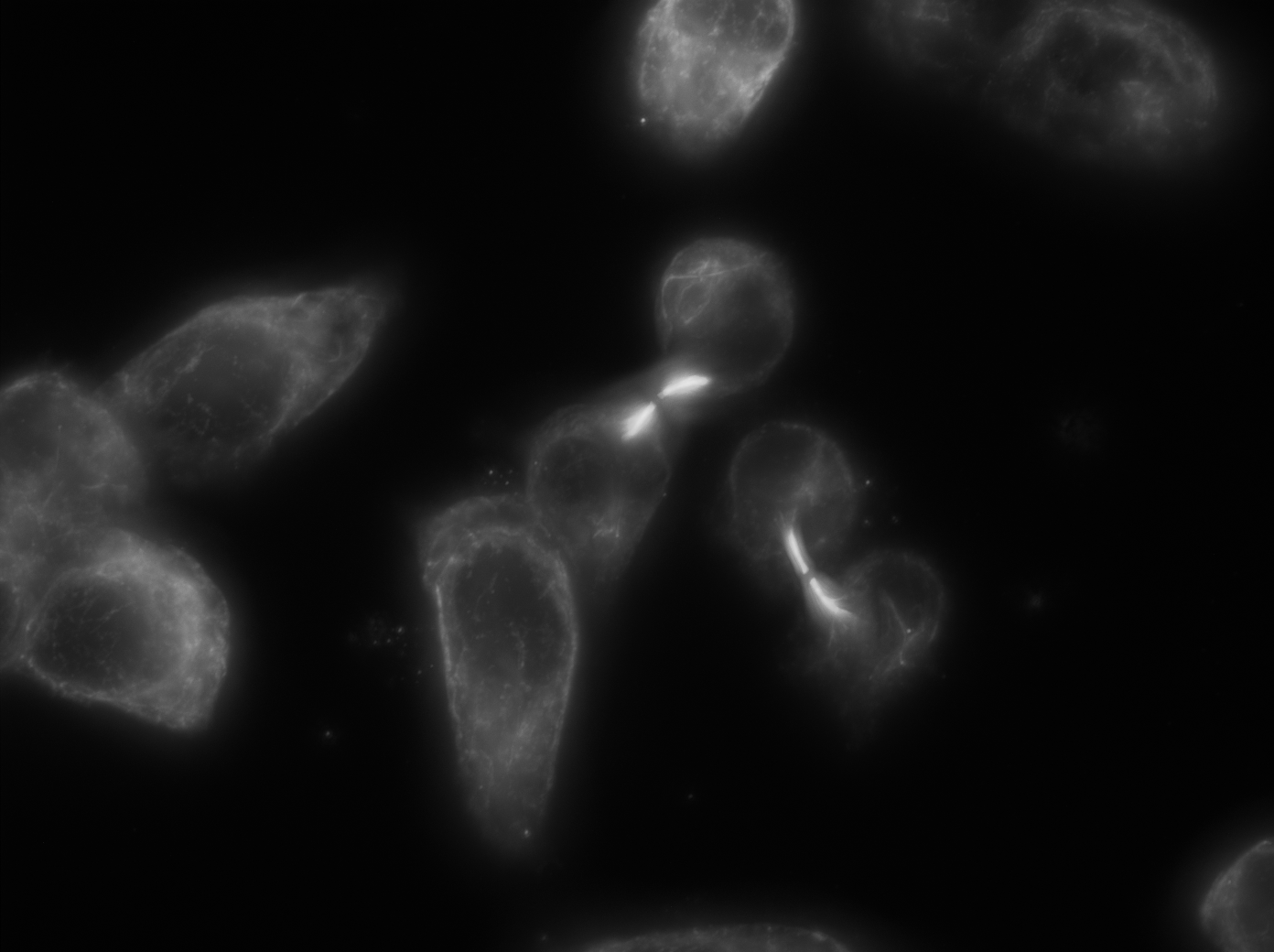

Supplement: Supplementary file 23 — Source data Fig. 6 part 4 [file 44319_2026_742_MOESM23_ESM.zip › Figure 6 Part 4/Fig 6efg TPGS1-KO TPGS1 rescue experiments part 2/R2R3/TPGS1-KO EYFP-only actub 7-31-25 R3 LT1.Project Maximum Z_XY1756490934_Z0_T0_C2.tif]

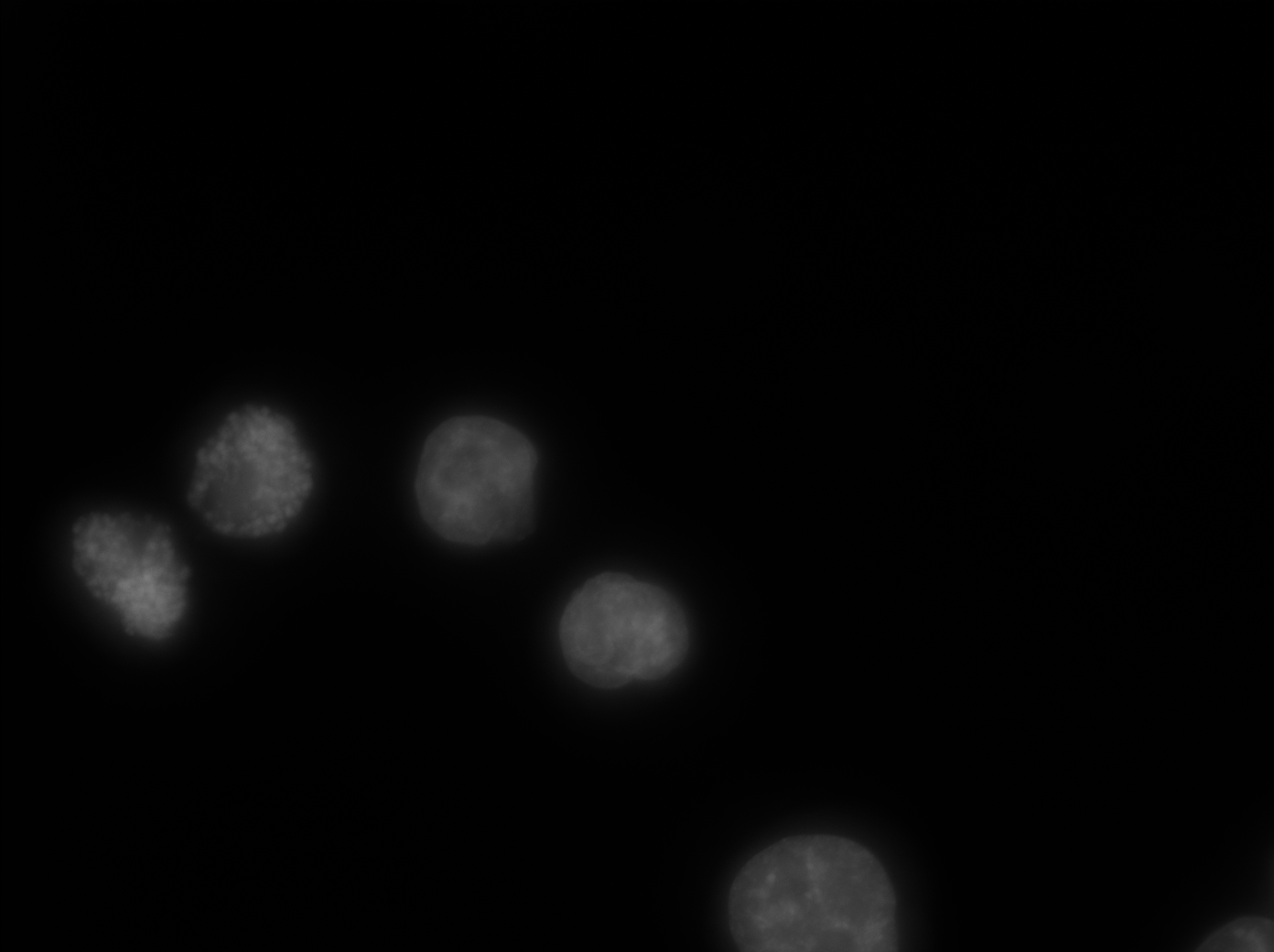

Supplement: Supplementary file 23 — Source data Fig. 6 part 4 [file 44319_2026_742_MOESM23_ESM.zip › Figure 6 Part 4/Fig 6efg TPGS1-KO TPGS1 rescue experiments part 2/R2R3/TPGS1-KO EYFP-only actub 7-31-25 R2 LT10.Project Maximum Z_XY1756416876_Z0_T0_C0.tif]

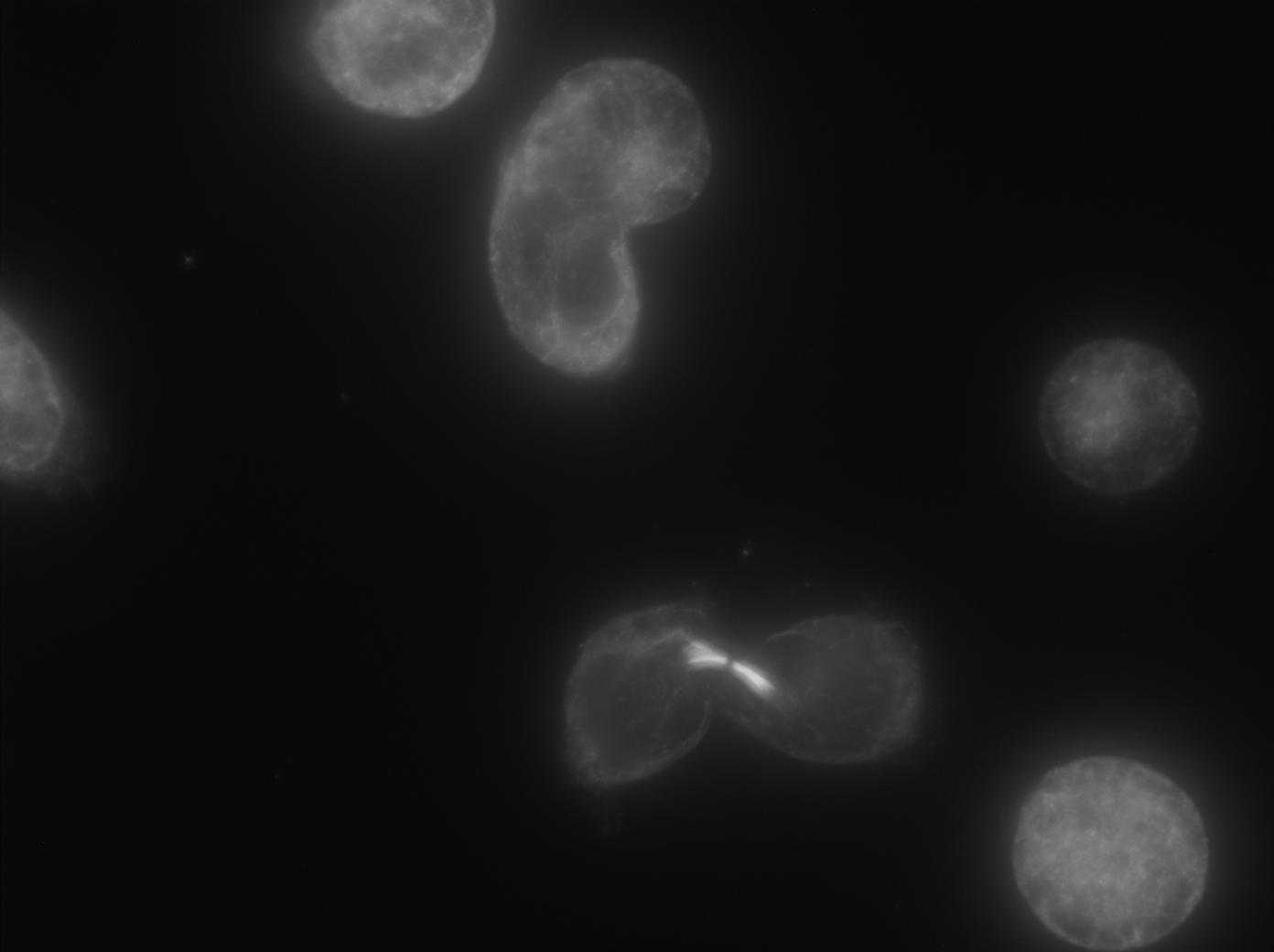

Supplement: Supplementary file 23 — Source data Fig. 6 part 4 [file 44319_2026_742_MOESM23_ESM.zip › Figure 6 Part 4/Fig 6efg TPGS1-KO TPGS1 rescue experiments part 2/R2R3/TPGS1-KO EYFP-only actub 7-31-25 R2 LT9.Project Maximum Z_XY1756416573_Z0_T0_C2.tif]

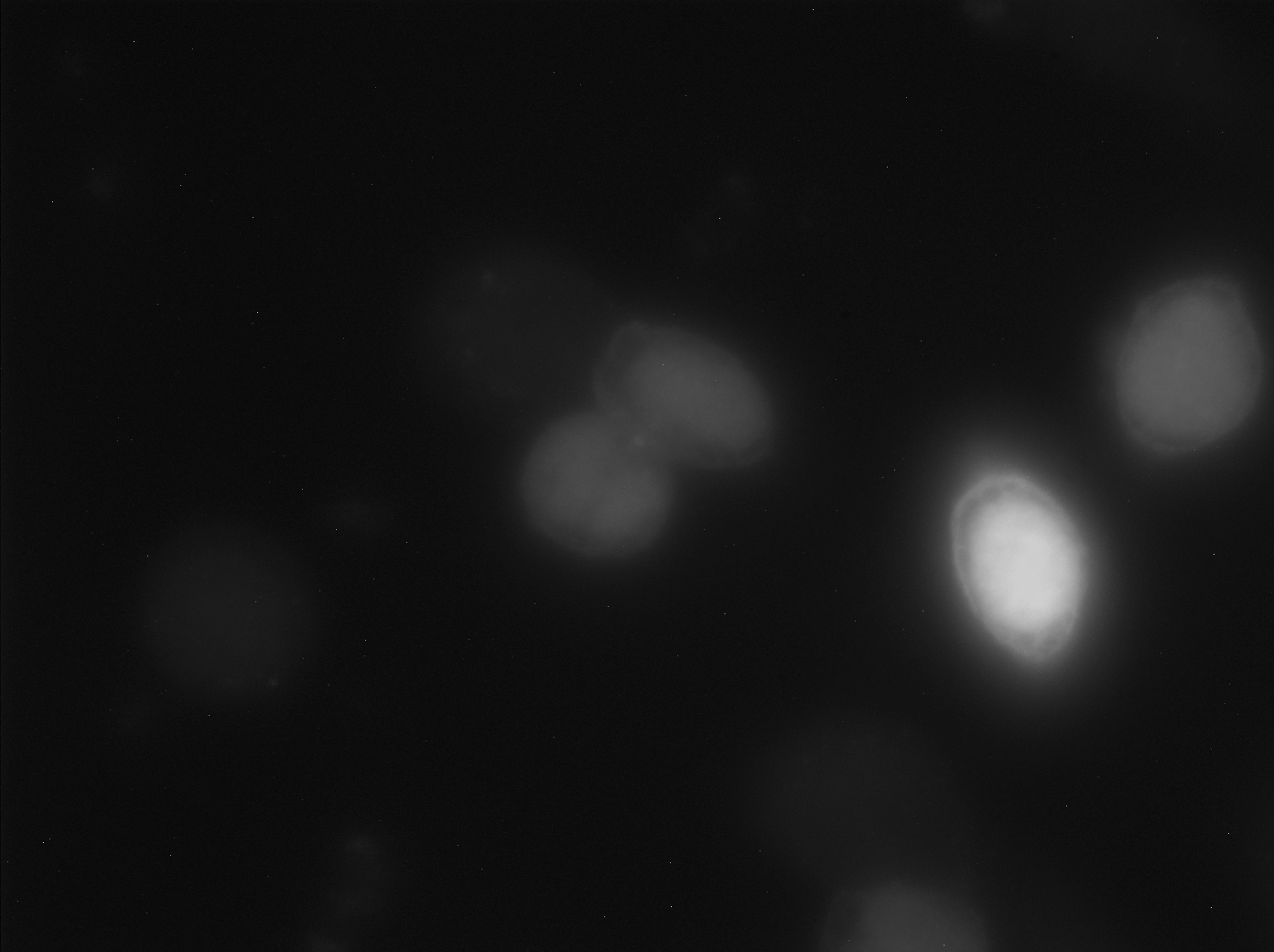

Supplement: Supplementary file 23 — Source data Fig. 6 part 4 [file 44319_2026_742_MOESM23_ESM.zip › Figure 6 Part 4/Fig 6efg TPGS1-KO TPGS1 rescue experiments part 2/R2R3/TPGS1-KO EYFP-only actub 7-31-25 R3 LT7.Project Maximum Z_XY1756494741_Z0_T0_C1.tif]

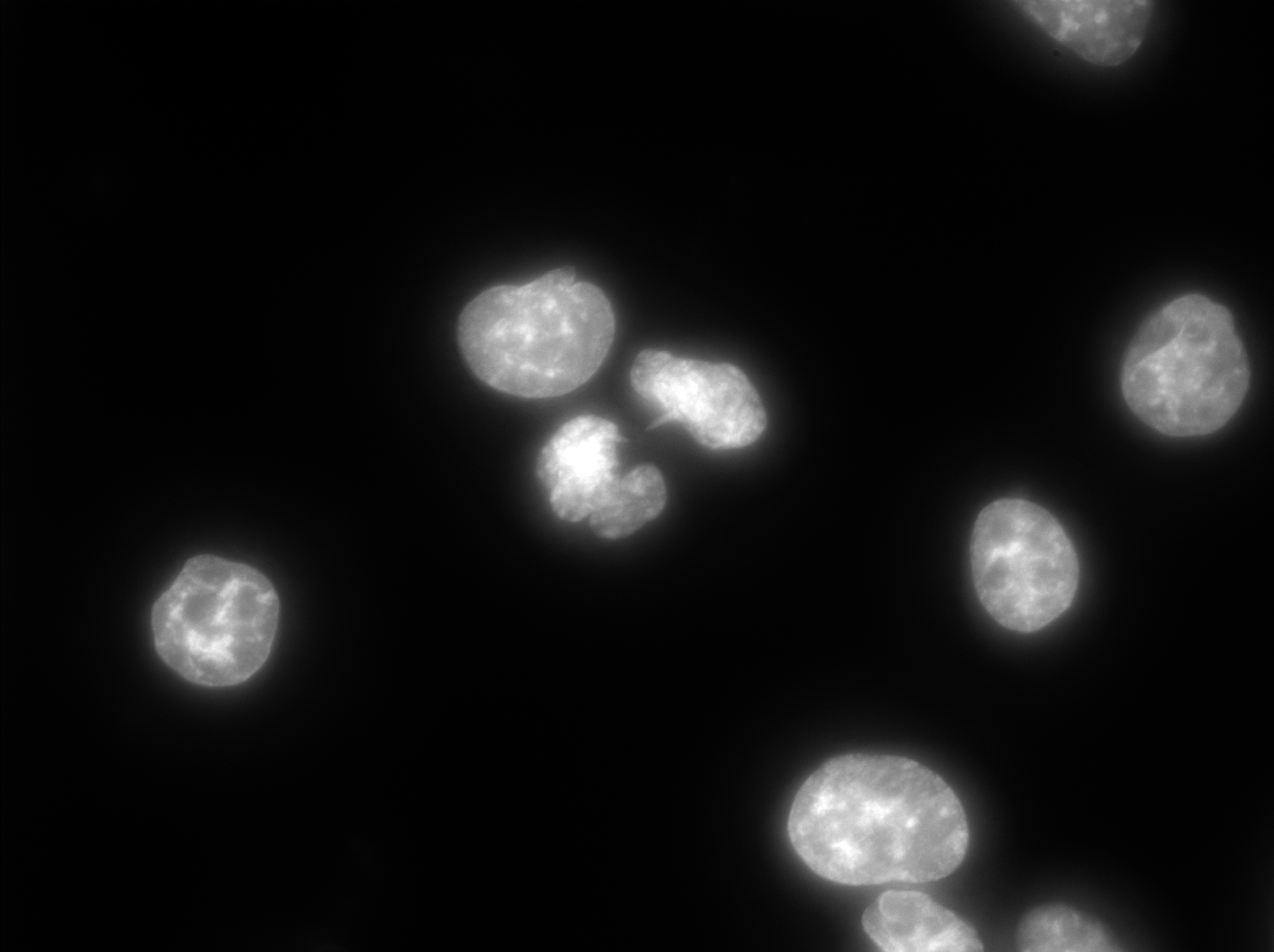

Supplement: Supplementary file 23 — Source data Fig. 6 part 4 [file 44319_2026_742_MOESM23_ESM.zip › Figure 6 Part 4/Fig 6efg TPGS1-KO TPGS1 rescue experiments part 2/R2R3/TPGS1-KO EYFP-only actub 7-31-25 R3 LT7.Project Maximum Z_XY1756494741_Z0_T0_C0.tif]

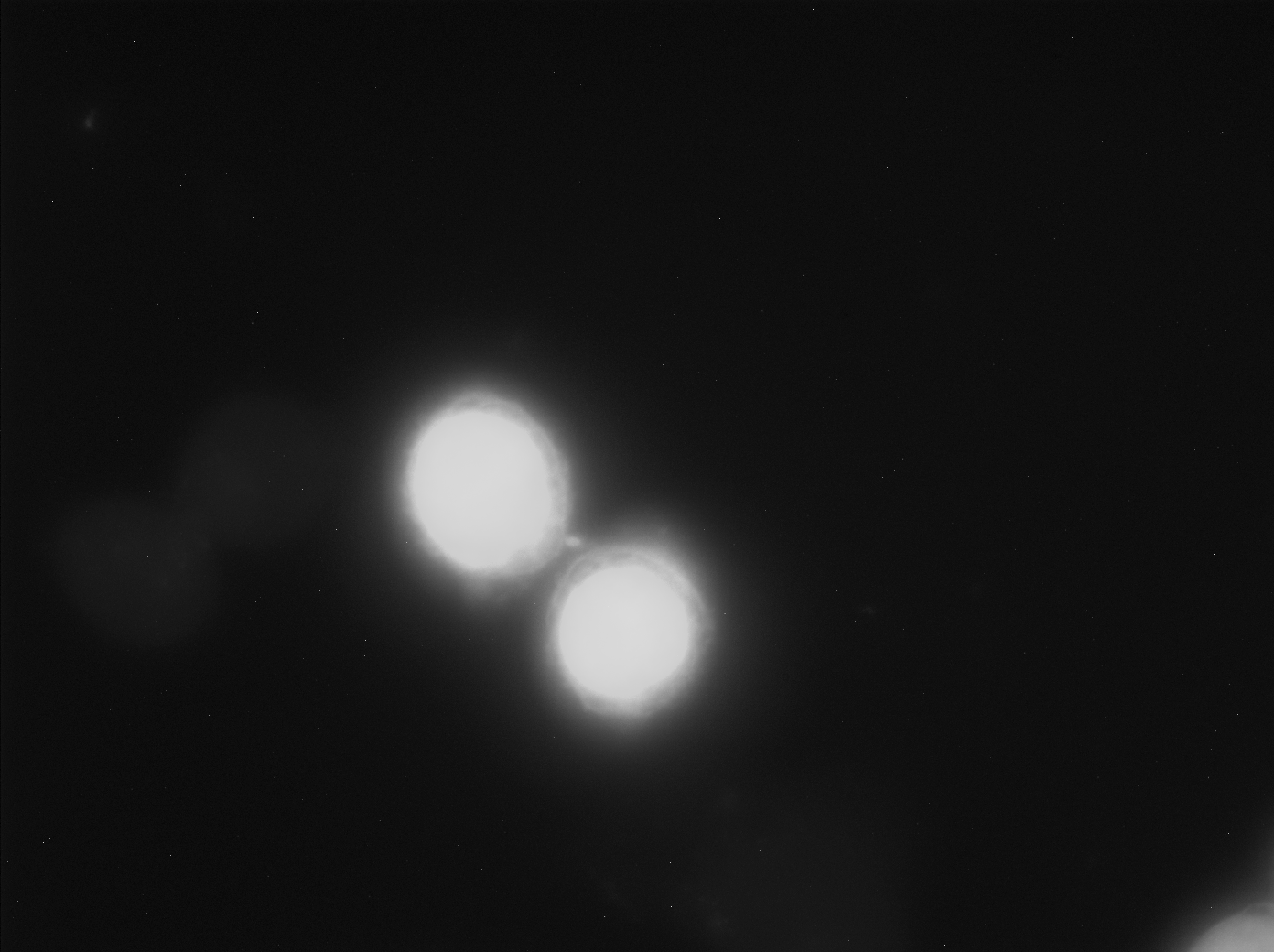

Supplement: Supplementary file 23 — Source data Fig. 6 part 4 [file 44319_2026_742_MOESM23_ESM.zip › Figure 6 Part 4/Fig 6efg TPGS1-KO TPGS1 rescue experiments part 2/R2R3/TPGS1-KO EYFP-only actub 7-31-25 R2 LT10.Project Maximum Z_XY1756416876_Z0_T0_C1.tif]

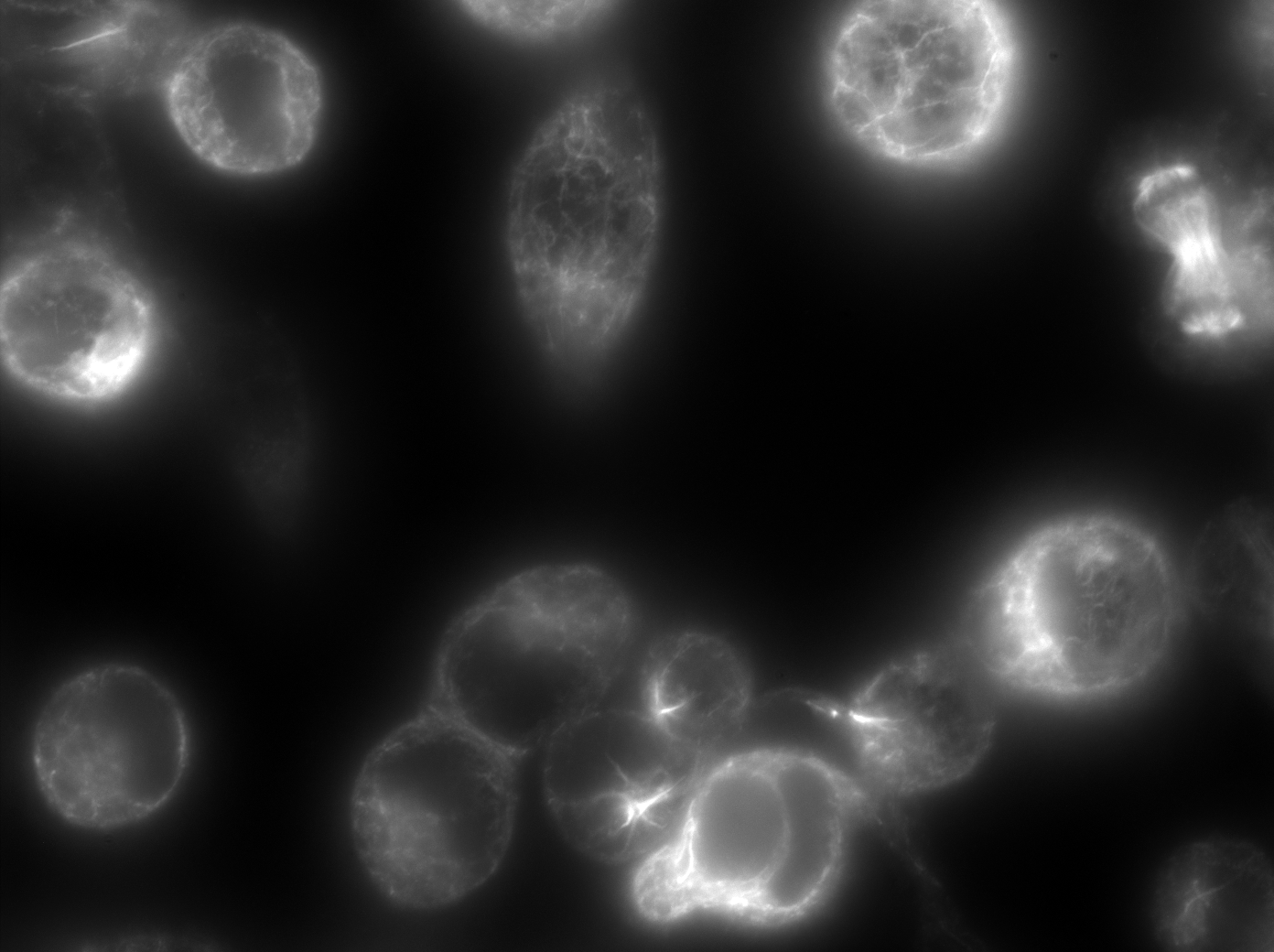

Supplement: Supplementary file 23 — Source data Fig. 6 part 4 [file 44319_2026_742_MOESM23_ESM.zip › Figure 6 Part 4/Fig 6efg TPGS1-KO TPGS1 rescue experiments part 2/R2R3/TPGS1-KO TPGS1-EYFP-3'UTR actub 7-31-25 R2 LT7.Project Maximum Z_XY1756411060_Z0_T0_C2.tif]

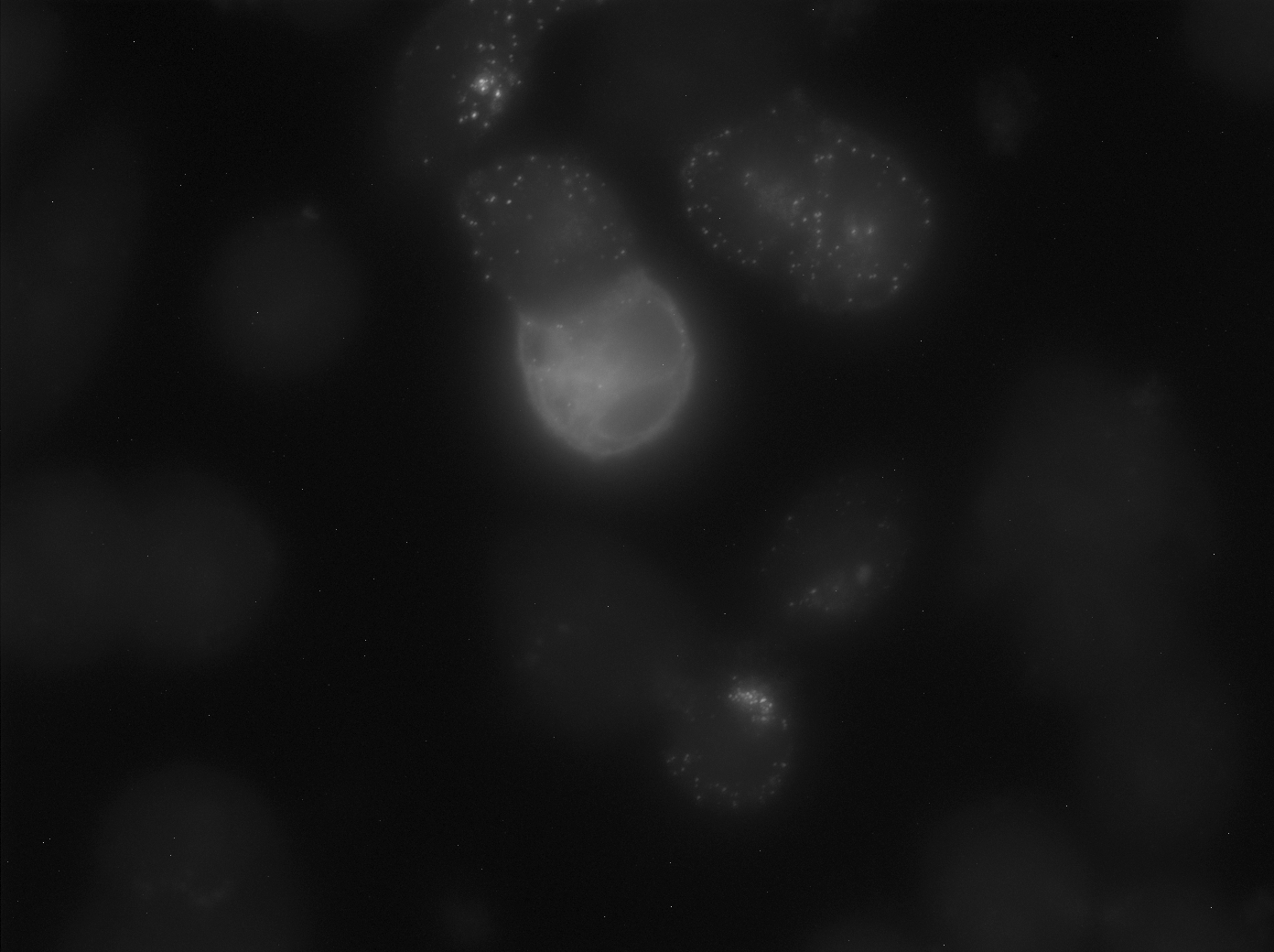

Supplement: Supplementary file 23 — Source data Fig. 6 part 4 [file 44319_2026_742_MOESM23_ESM.zip › Figure 6 Part 4/Fig 6efg TPGS1-KO TPGS1 rescue experiments part 2/R2R3/TPGS1-KO TPGS1-EYFP-3'UTR actub 7-31-25 R3 LT5.Project Maximum Z_XY1756501059_Z0_T0_C1.tif]

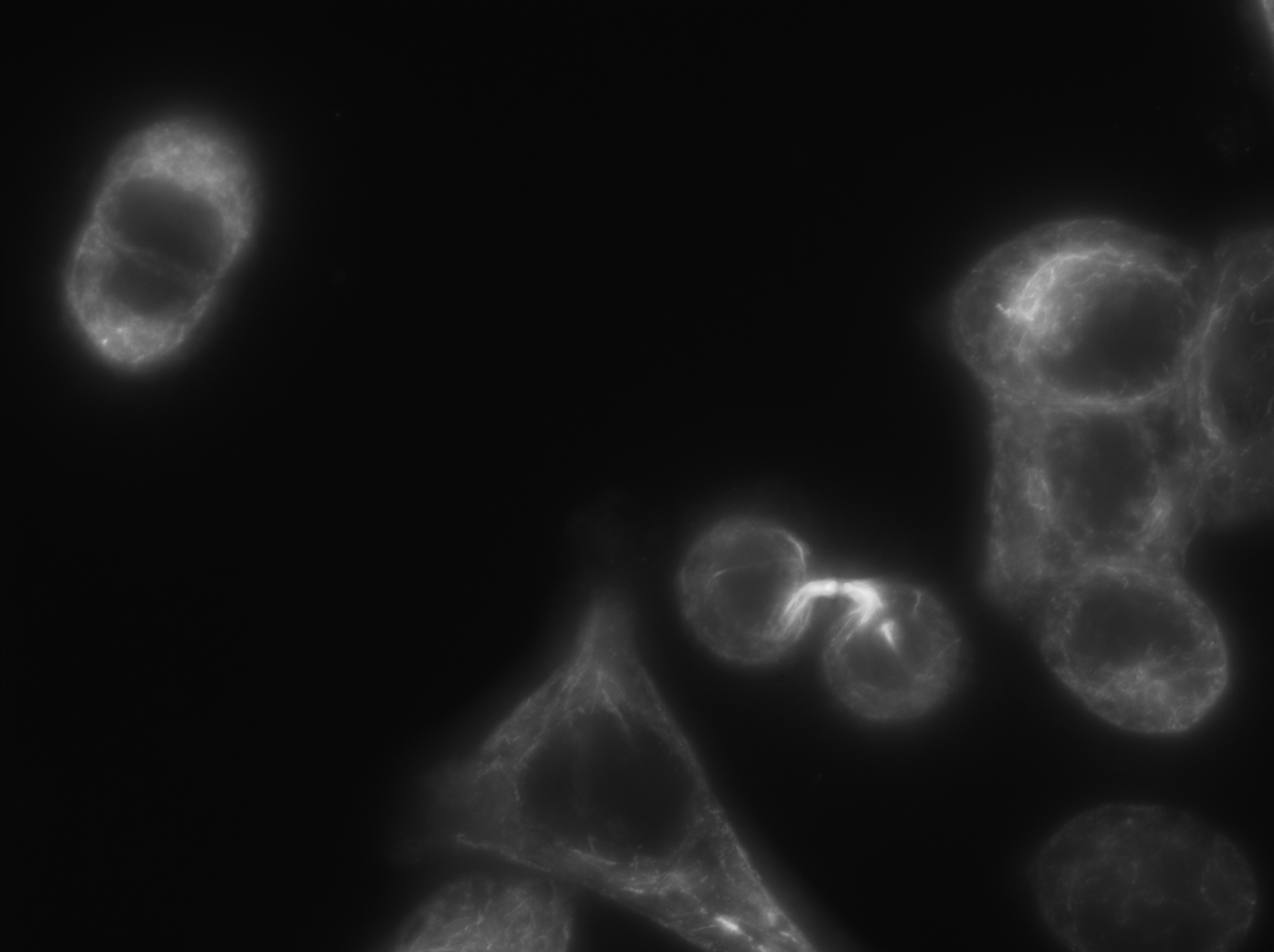

Supplement: Supplementary file 23 — Source data Fig. 6 part 4 [file 44319_2026_742_MOESM23_ESM.zip › Figure 6 Part 4/Fig 6efg TPGS1-KO TPGS1 rescue experiments part 2/R2R3/TPGS1-KO TPGS1-EYFP-3'UTR actub 7-31-25 R3 ET3.Project Maximum Z_XY1756499529_Z0_T0_C2.tif]

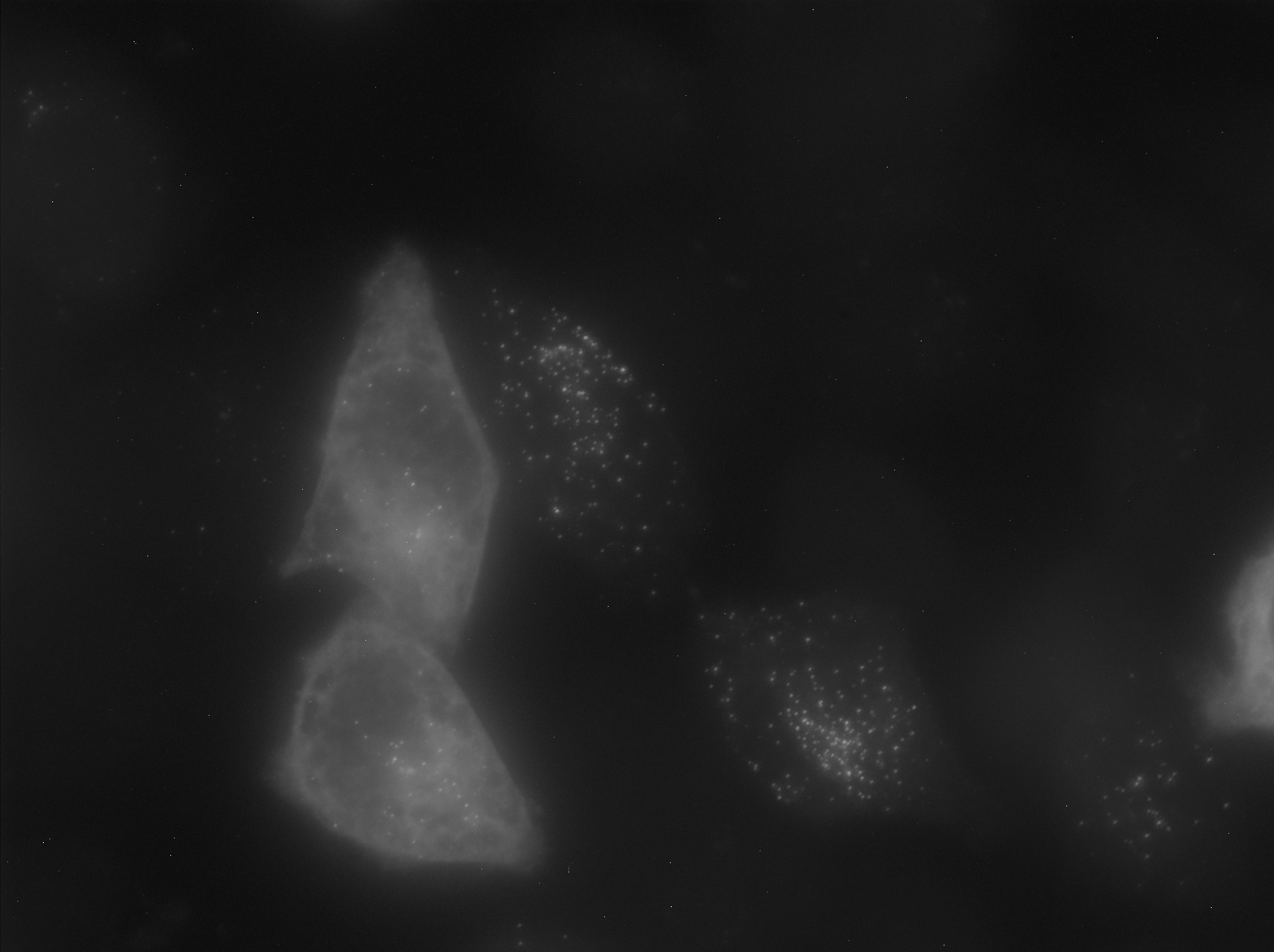

Supplement: Supplementary file 23 — Source data Fig. 6 part 4 [file 44319_2026_742_MOESM23_ESM.zip › Figure 6 Part 4/Fig 6efg TPGS1-KO TPGS1 rescue experiments part 2/R2R3/TPGS1-KO TPGS1-EYFP-3'UTR actub 7-31-25 R2 LT6.Project Maximum Z_XY1756410864_Z0_T0_C1.tif]

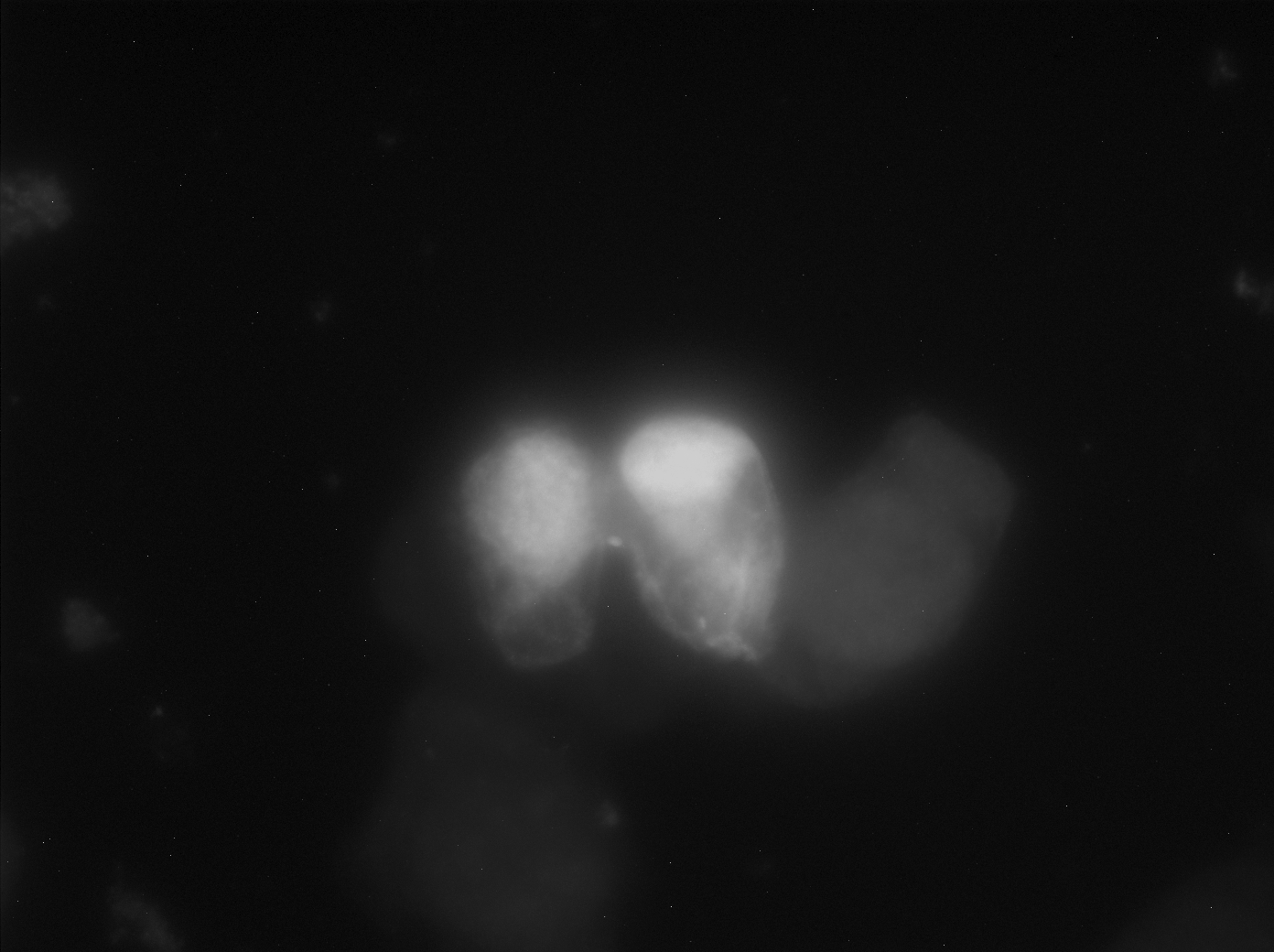

Supplement: Supplementary file 23 — Source data Fig. 6 part 4 [file 44319_2026_742_MOESM23_ESM.zip › Figure 6 Part 4/Fig 6efg TPGS1-KO TPGS1 rescue experiments part 2/R2R3/TPGS1-KO EYFP-only actub 7-31-25 R2 LT4.Project Maximum Z_XY1756414542_Z0_T0_C1.tif]

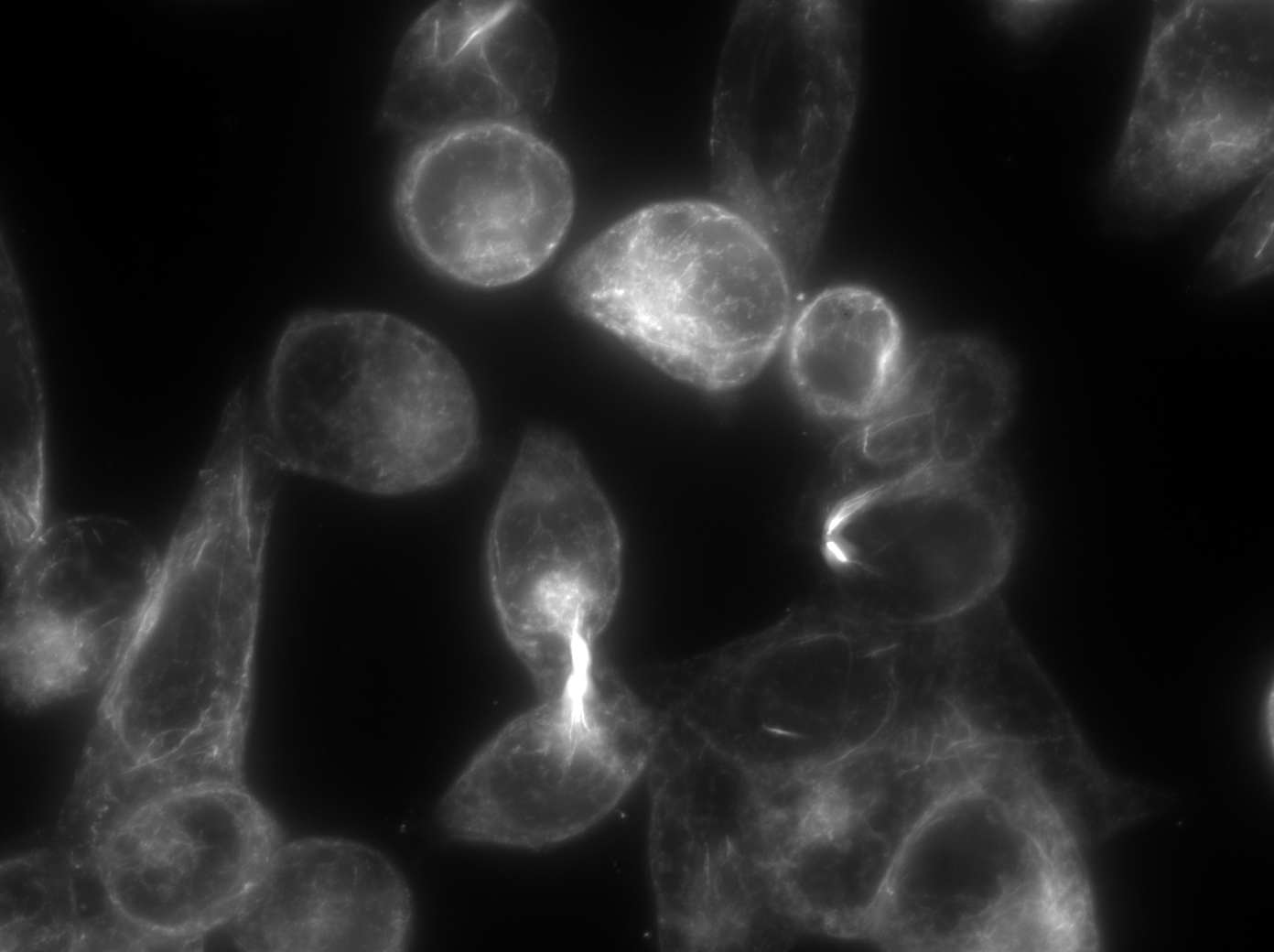

Supplement: Supplementary file 23 — Source data Fig. 6 part 4 [file 44319_2026_742_MOESM23_ESM.zip › Figure 6 Part 4/Fig 6efg TPGS1-KO TPGS1 rescue experiments part 2/R2R3/TPGS1-KO TPGS1-EYFP-3'UTR actub 7-31-25 R3 LT10.Project Maximum Z_XY1756503533_Z0_T0_C2.tif]

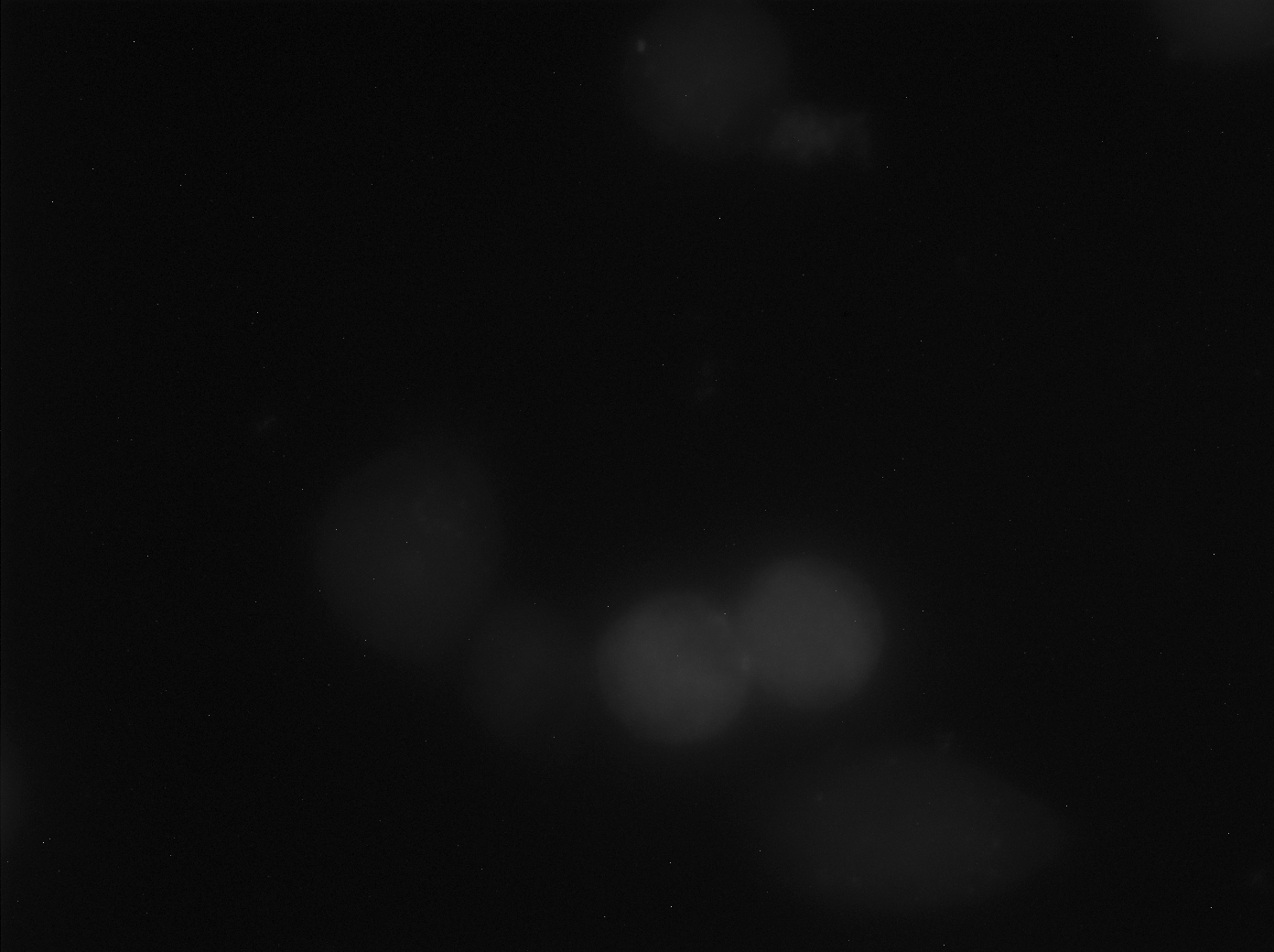

Supplement: Supplementary file 23 — Source data Fig. 6 part 4 [file 44319_2026_742_MOESM23_ESM.zip › Figure 6 Part 4/Fig 6efg TPGS1-KO TPGS1 rescue experiments part 2/R2R3/TPGS1-KO EYFP-only actub 7-31-25 R3 ET7.Project Maximum Z_XY1756494520_Z0_T0_C1.tif]

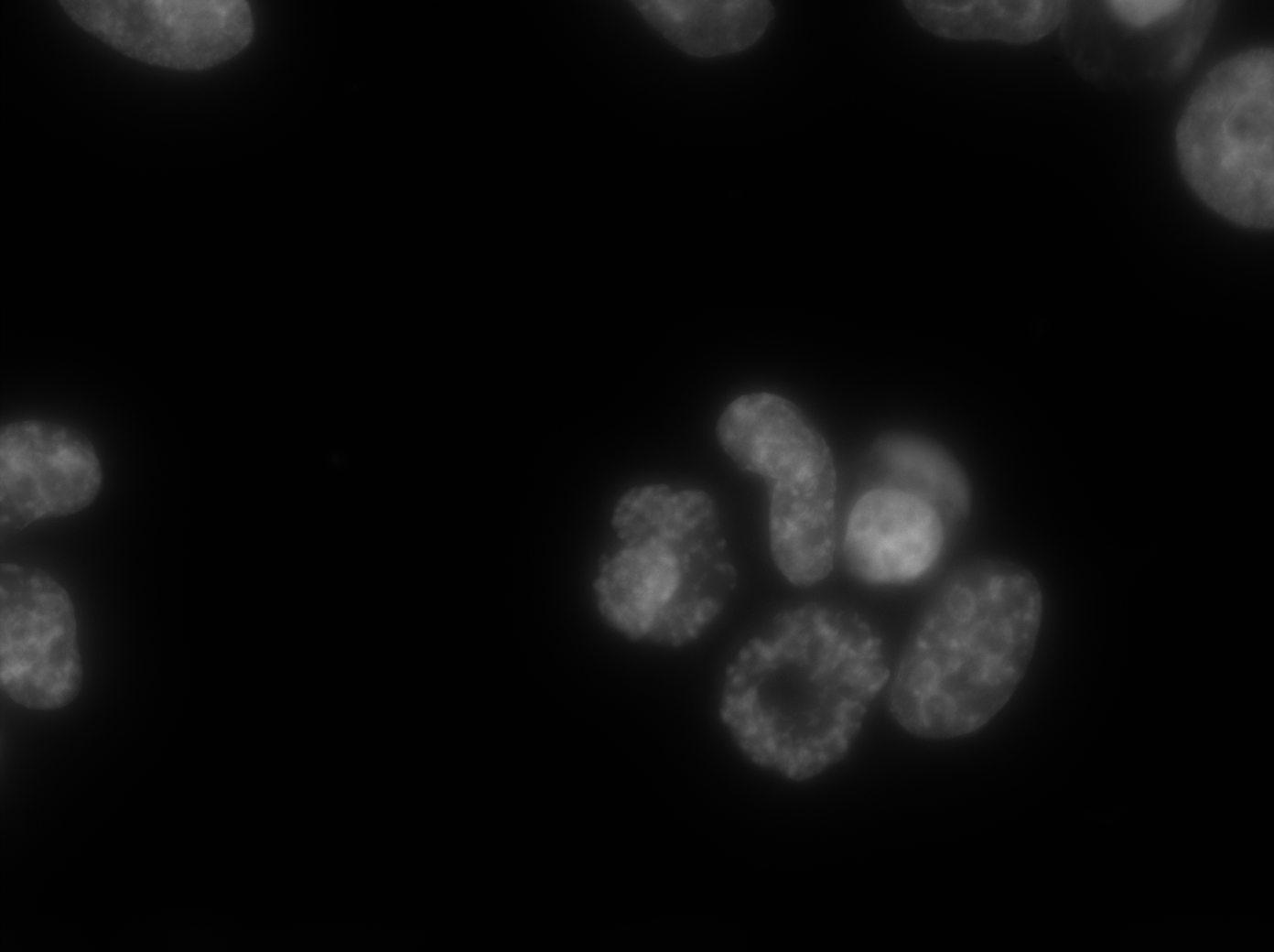

Supplement: Supplementary file 23 — Source data Fig. 6 part 4 [file 44319_2026_742_MOESM23_ESM.zip › Figure 6 Part 4/Fig 6efg TPGS1-KO TPGS1 rescue experiments part 2/R2R3/TPGS1-KO EYFP-only actub 7-31-25 R2 ET2.Project Maximum Z_XY1756413990_Z0_T0_C0.tif]

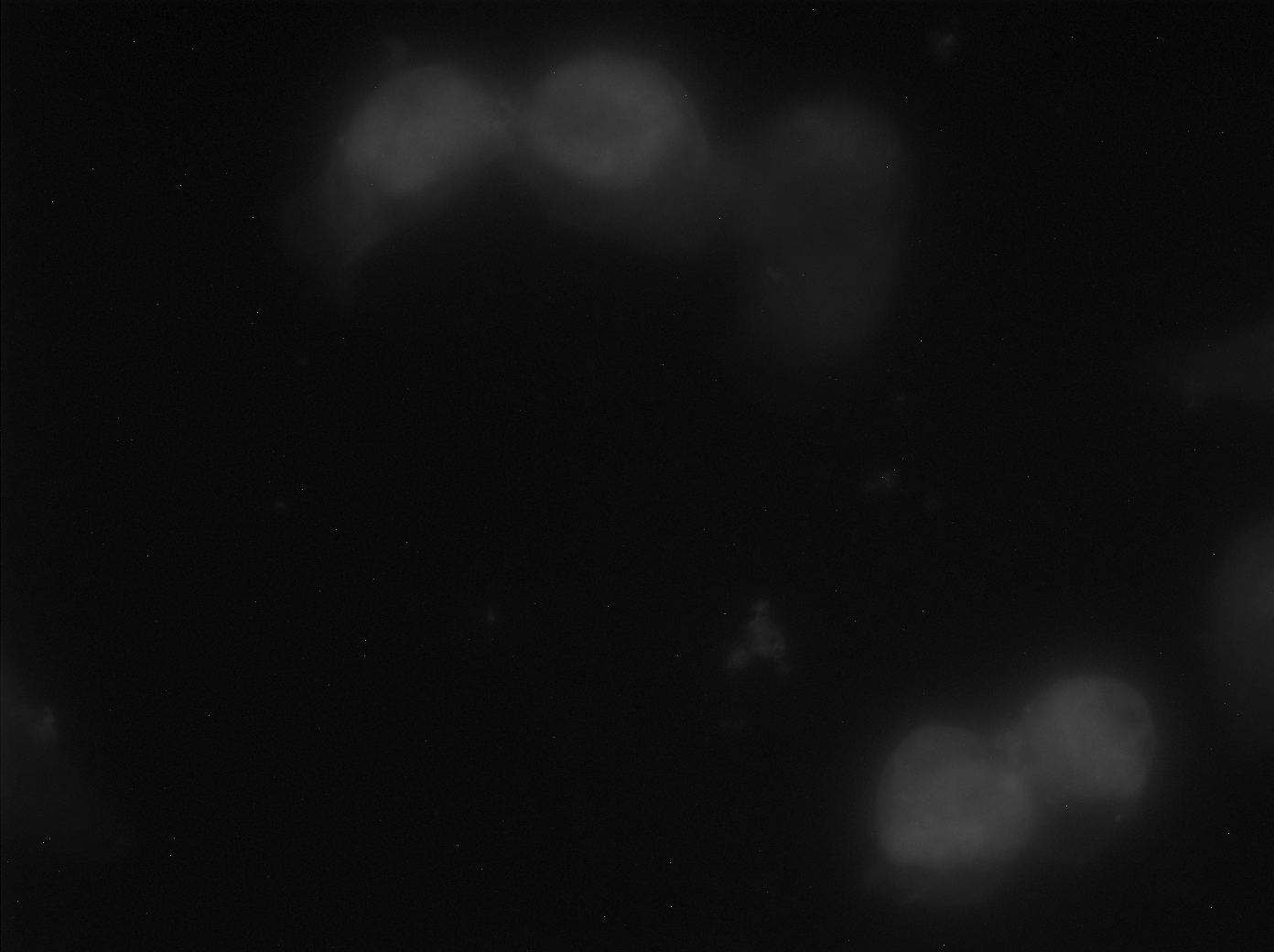

Supplement: Supplementary file 23 — Source data Fig. 6 part 4 [file 44319_2026_742_MOESM23_ESM.zip › Figure 6 Part 4/Fig 6efg TPGS1-KO TPGS1 rescue experiments part 2/R2R3/TPGS1-KO EYFP-only actub 7-31-25 R2 ET1 LT1.Project Maximum Z_XY1756413701_Z0_T0_C1.tif]
