# Supplementary figures and images for: TPGS1 regulates central spindle microtubule glutamylation and remodeling during telophase and abscission (part 31 of 36)
Source: EMBO Rep. 2026 Mar 23;27(8):1944–63. doi: 10.1038/s44319-026-00742-3 (PMC13121839; doi:10.1038/s44319-026-00742-3)

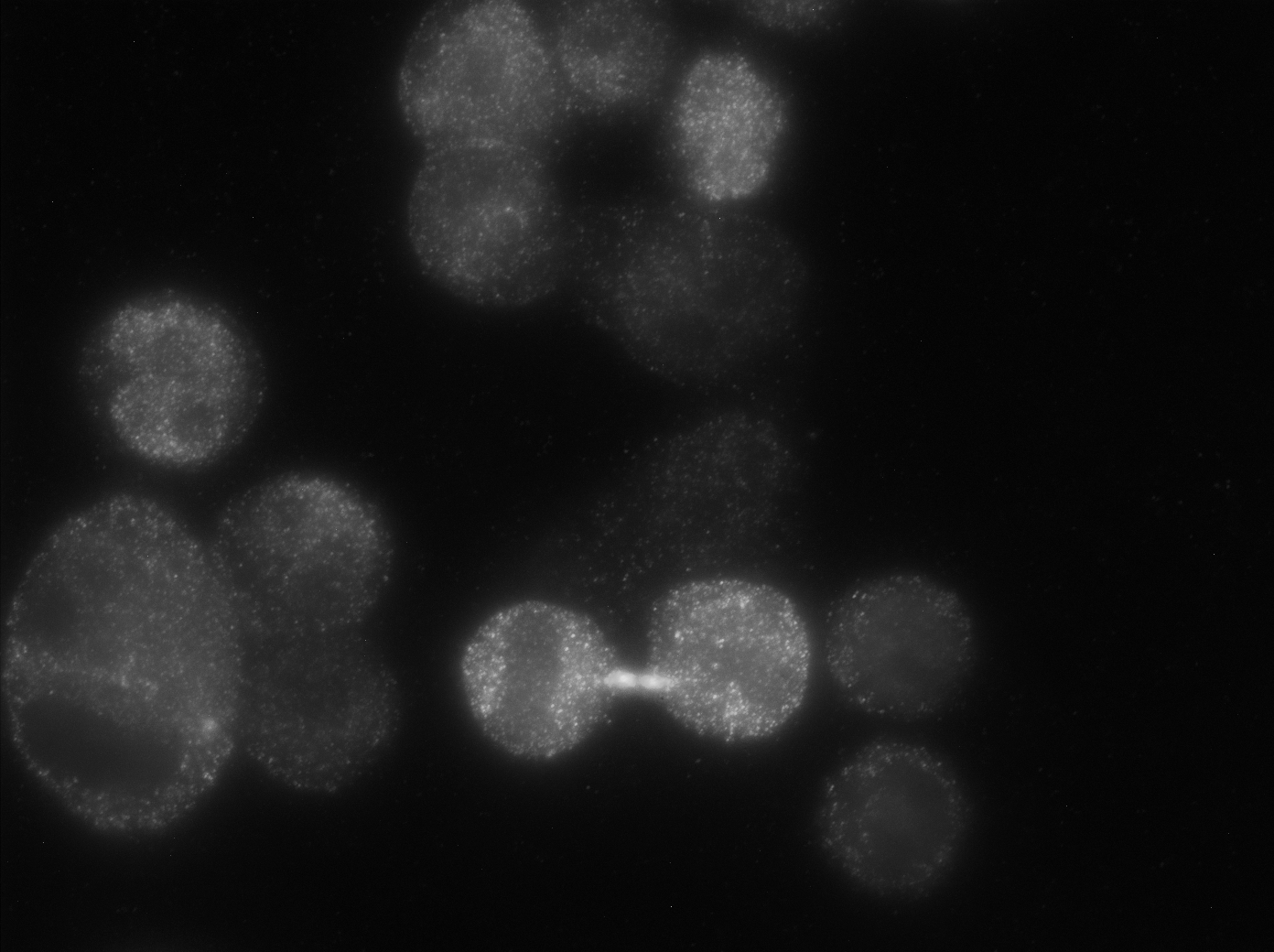

Supplement: Supplementary file 25 — Source data Fig. 7 part 1 [file 44319_2026_742_MOESM25_ESM.zip › Figure 7 Part 1/Fig 7acd Cas9 and TPGS1-ko rGT335 atubulin/Cas9 GT335recomb atub 3-24-25 R1 ET7.Project Maximum Z_XY1742836085_Z0_T0_C1.tif]

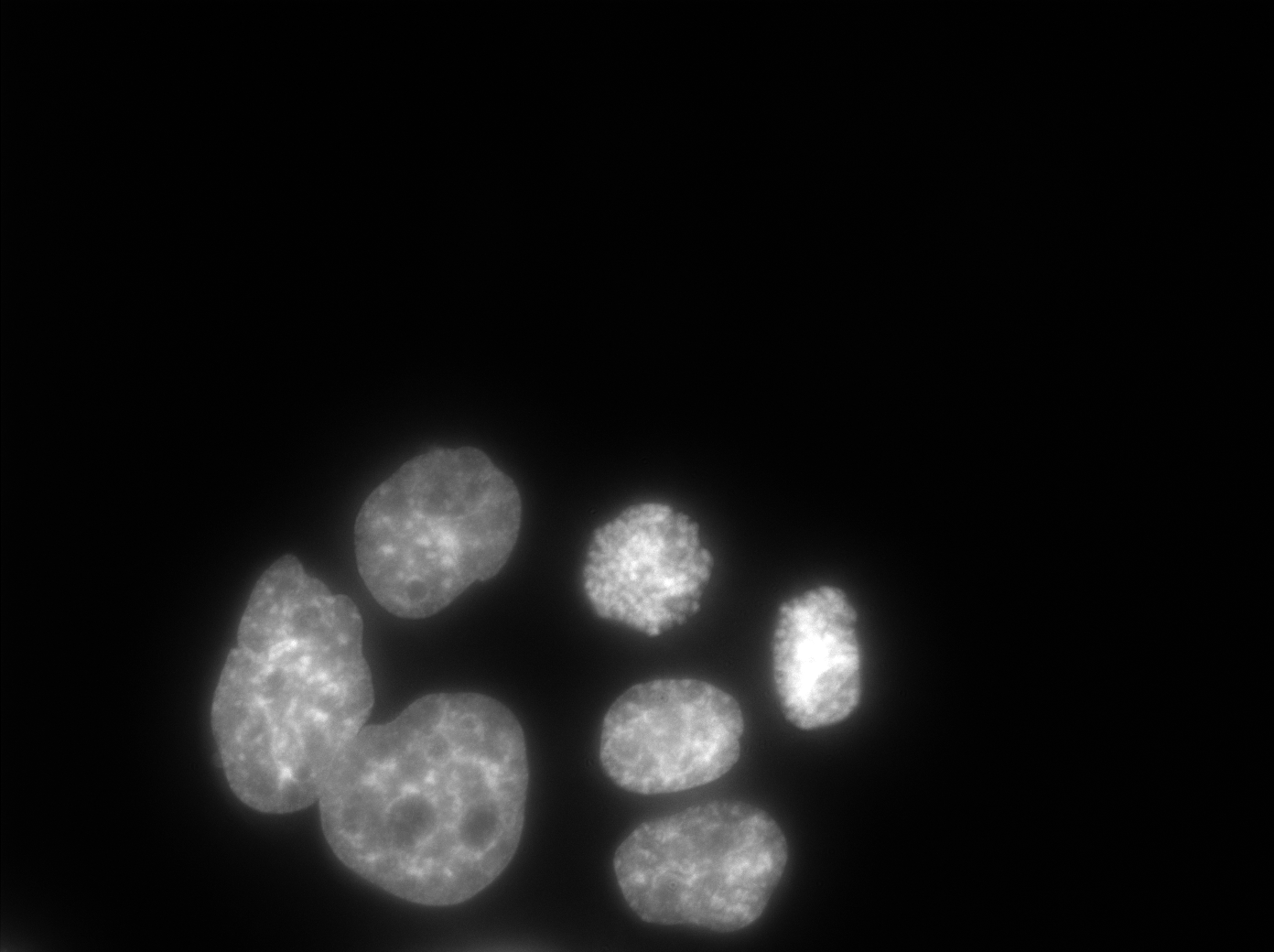

Supplement: Supplementary file 25 — Source data Fig. 7 part 1 [file 44319_2026_742_MOESM25_ESM.zip › Figure 7 Part 1/Fig 7acd Cas9 and TPGS1-ko rGT335 atubulin/Cas9 GT335recomb atub 3-24-25 R2 ET10.Project Maximum Z_XY1742847558_Z0_T0_C0.tif]

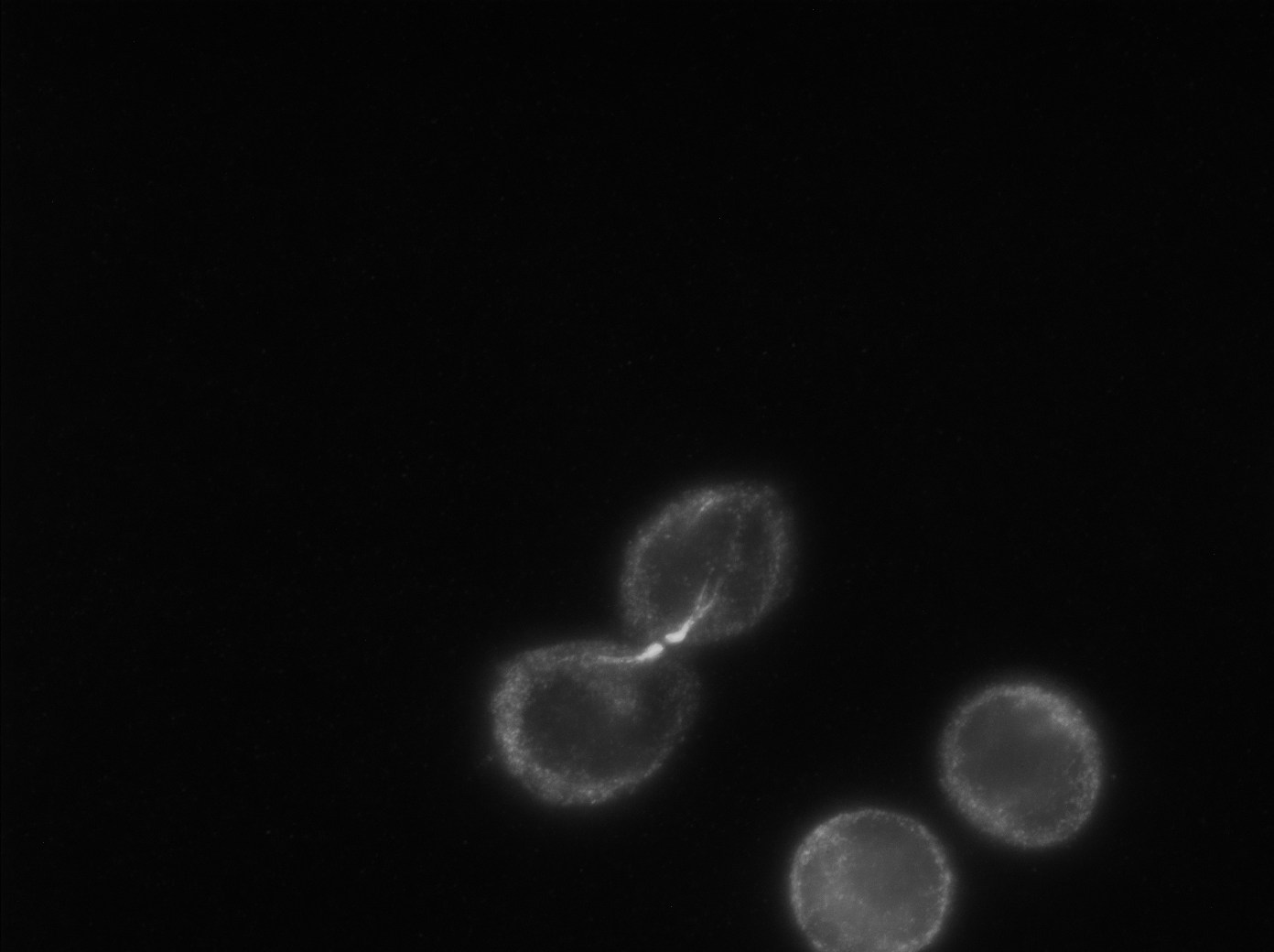

Supplement: Supplementary file 25 — Source data Fig. 7 part 1 [file 44319_2026_742_MOESM25_ESM.zip › Figure 7 Part 1/Fig 7acd Cas9 and TPGS1-ko rGT335 atubulin/Cas9 GT335recomb atub 3-24-25 R2 LT5.Project Maximum Z_XY1742846466_Z0_T0_C2.tif]

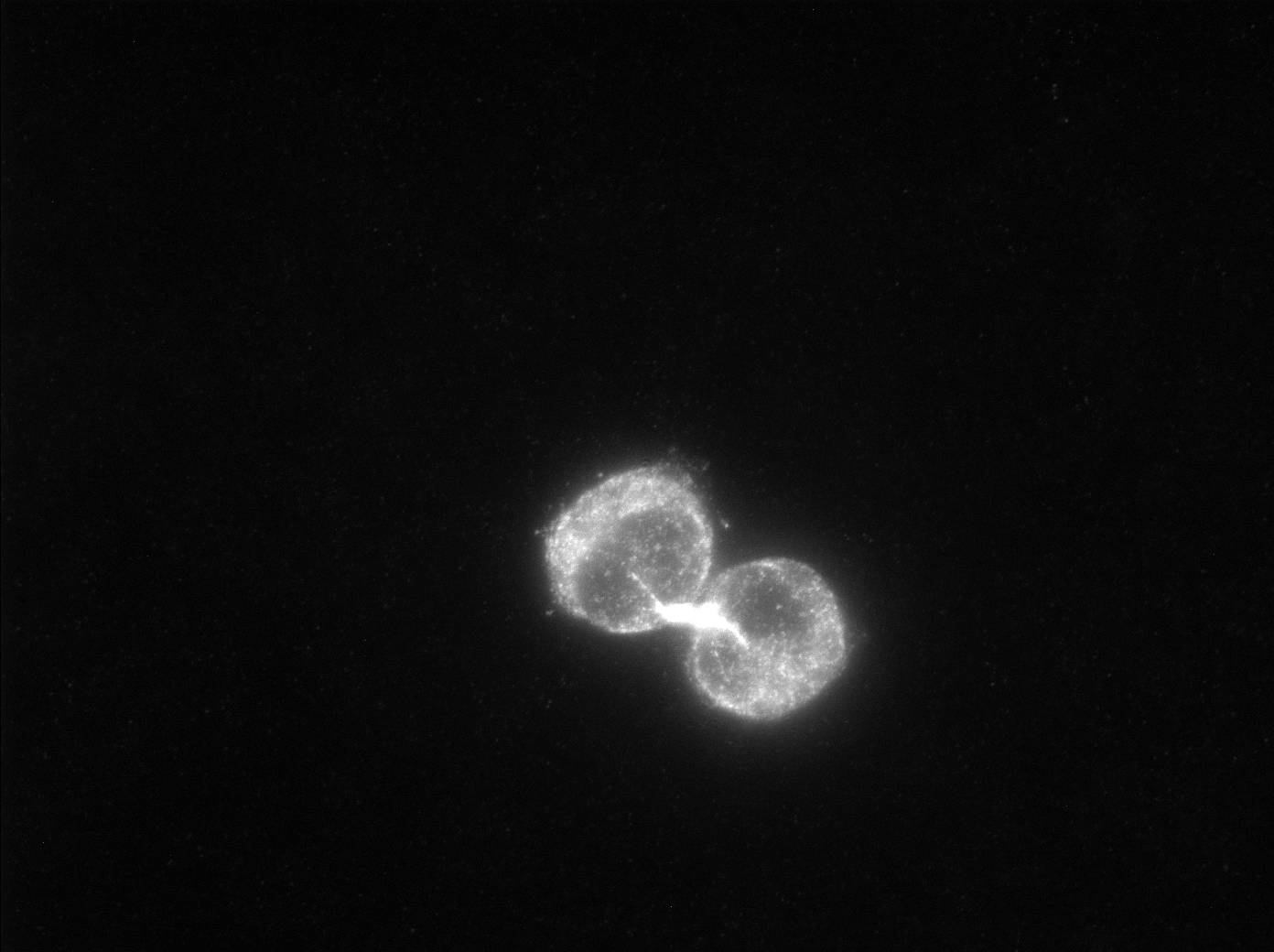

Supplement: Supplementary file 25 — Source data Fig. 7 part 1 [file 44319_2026_742_MOESM25_ESM.zip › Figure 7 Part 1/Fig 7acd Cas9 and TPGS1-ko rGT335 atubulin/Cas9 GT335recomb atub 3-24-25 R1 ET4.Project Maximum Z_XY1742835443_Z0_T0_C2.tif]

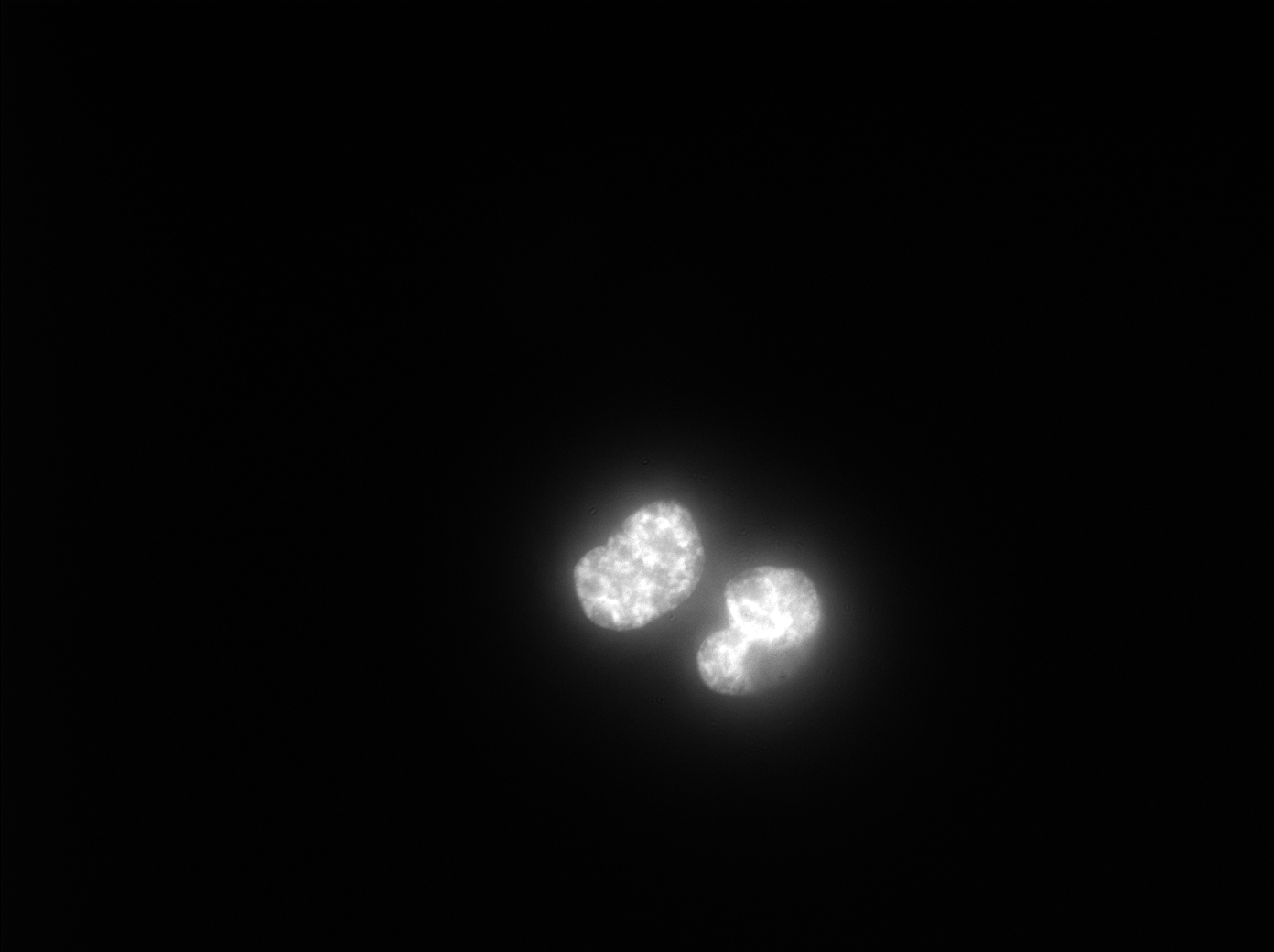

Supplement: Supplementary file 25 — Source data Fig. 7 part 1 [file 44319_2026_742_MOESM25_ESM.zip › Figure 7 Part 1/Fig 7acd Cas9 and TPGS1-ko rGT335 atubulin/Cas9 GT335recomb atub 3-24-25 R1 ET4.Project Maximum Z_XY1742835443_Z0_T0_C0.tif]

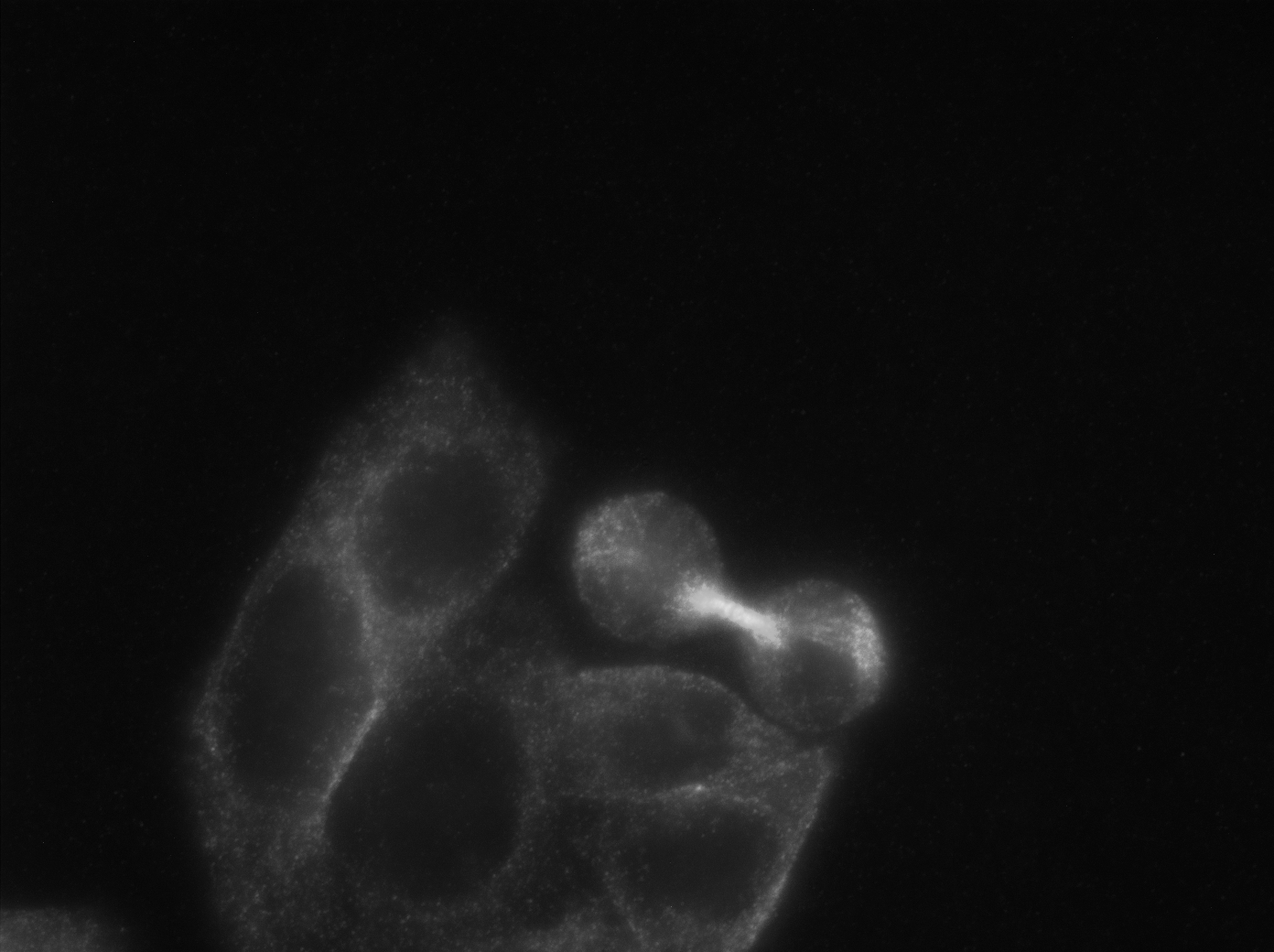

Supplement: Supplementary file 25 — Source data Fig. 7 part 1 [file 44319_2026_742_MOESM25_ESM.zip › Figure 7 Part 1/Fig 7acd Cas9 and TPGS1-ko rGT335 atubulin/Cas9 GT335recomb atub 3-24-25 R2 ET10.Project Maximum Z_XY1742847558_Z0_T0_C2.tif]

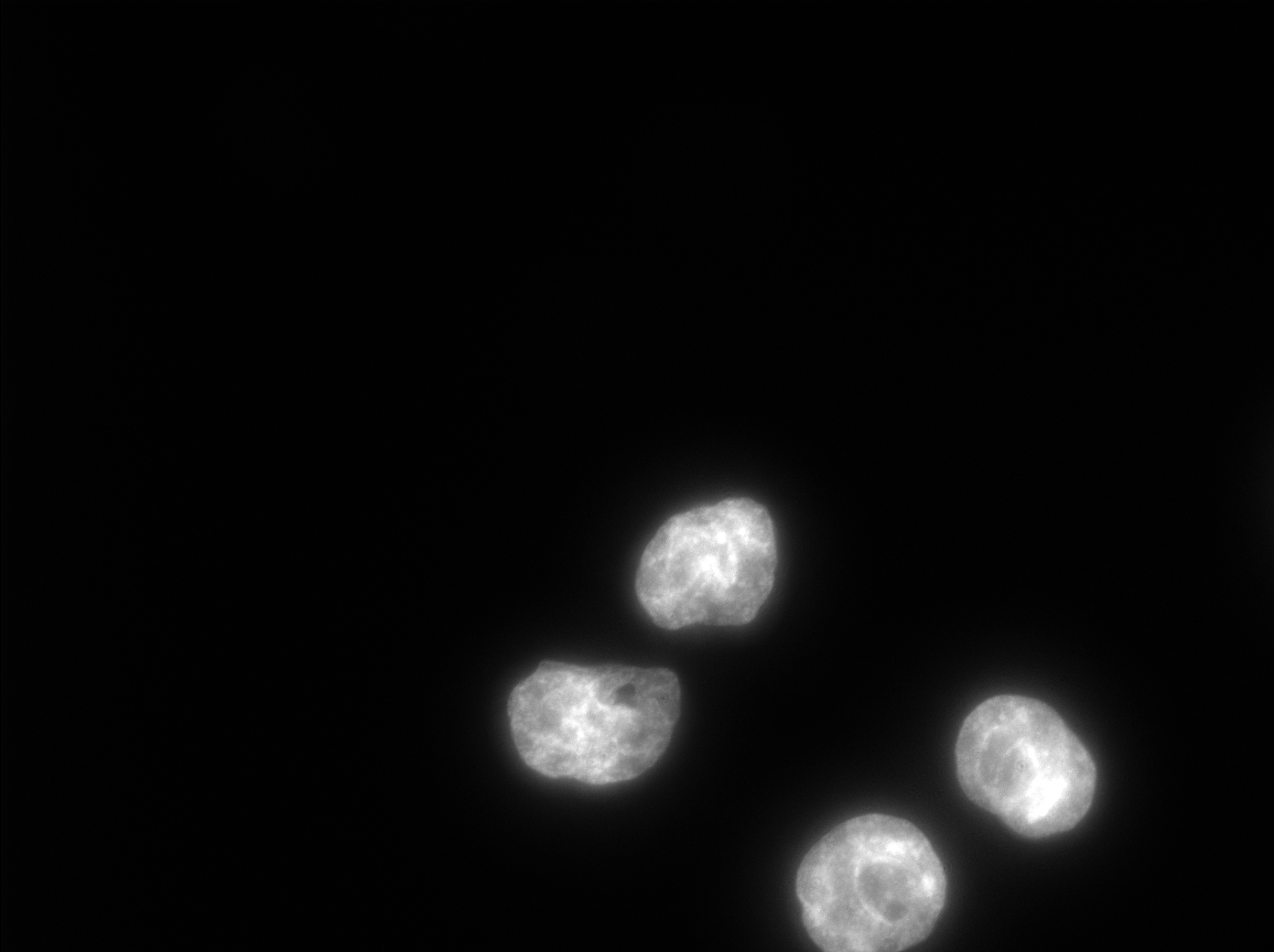

Supplement: Supplementary file 25 — Source data Fig. 7 part 1 [file 44319_2026_742_MOESM25_ESM.zip › Figure 7 Part 1/Fig 7acd Cas9 and TPGS1-ko rGT335 atubulin/Cas9 GT335recomb atub 3-24-25 R2 LT5.Project Maximum Z_XY1742846466_Z0_T0_C0.tif]

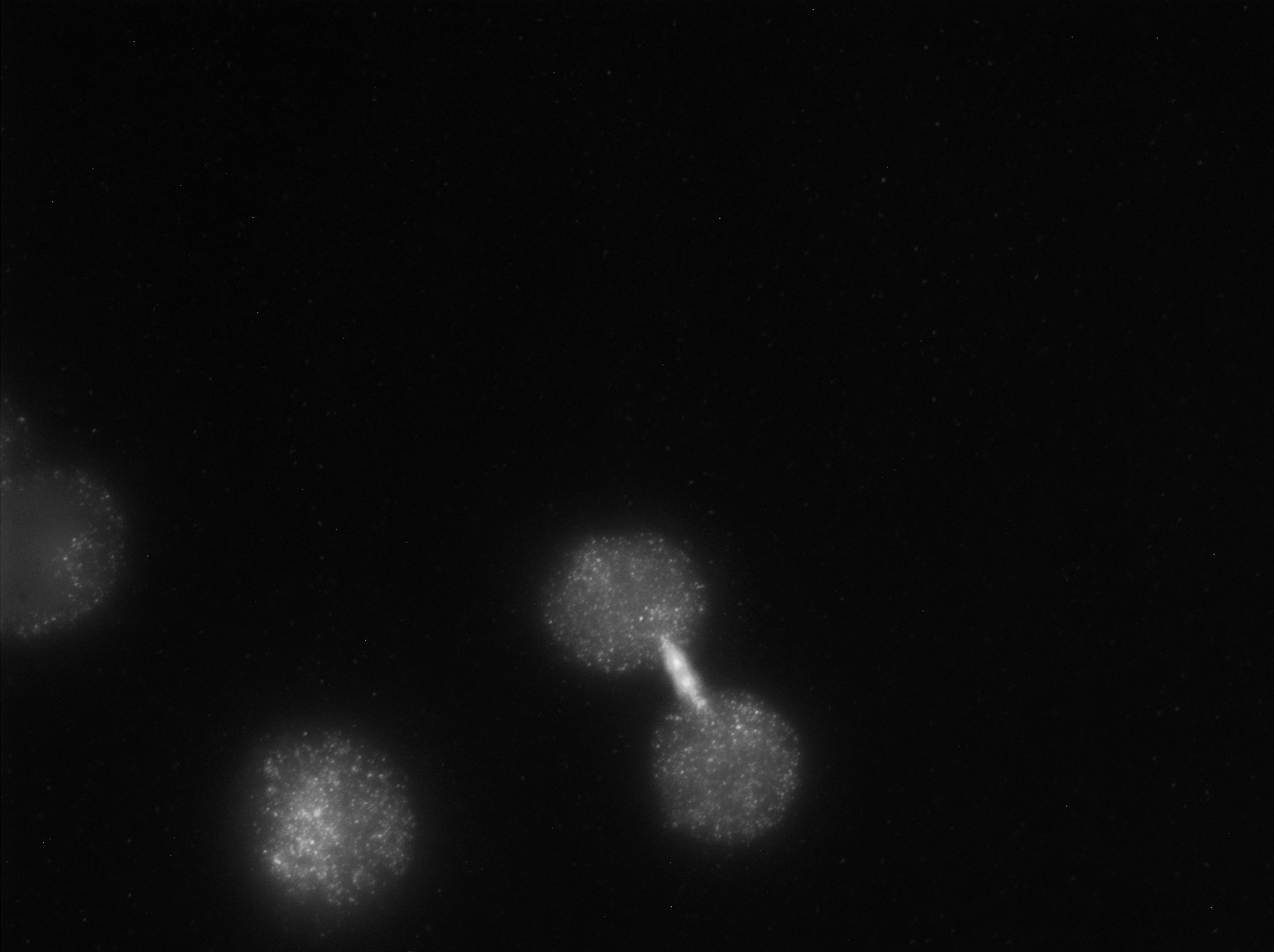

Supplement: Supplementary file 25 — Source data Fig. 7 part 1 [file 44319_2026_742_MOESM25_ESM.zip › Figure 7 Part 1/Fig 7acd Cas9 and TPGS1-ko rGT335 atubulin/Cas9 GT335recomb atub 3-24-25 R3 ET1.Project Maximum Z_XY1742848799_Z0_T0_C1.tif]

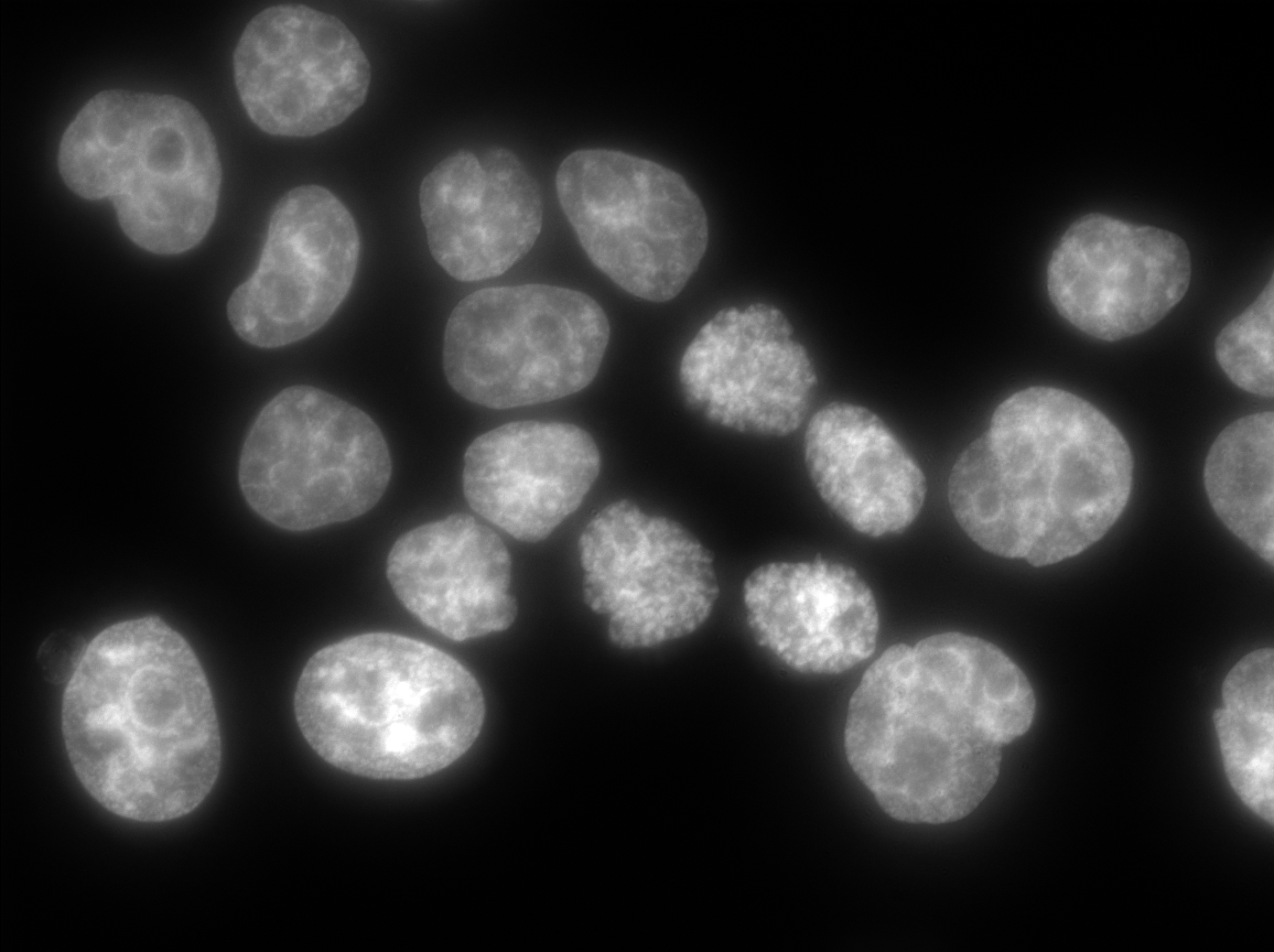

Supplement: Supplementary file 25 — Source data Fig. 7 part 1 [file 44319_2026_742_MOESM25_ESM.zip › Figure 7 Part 1/Fig 7acd Cas9 and TPGS1-ko rGT335 atubulin/Cas9 GT335recomb atub 3-24-25 R1 LT7LT8.Project Maximum Z_XY1742836175_Z0_T0_C0.tif]

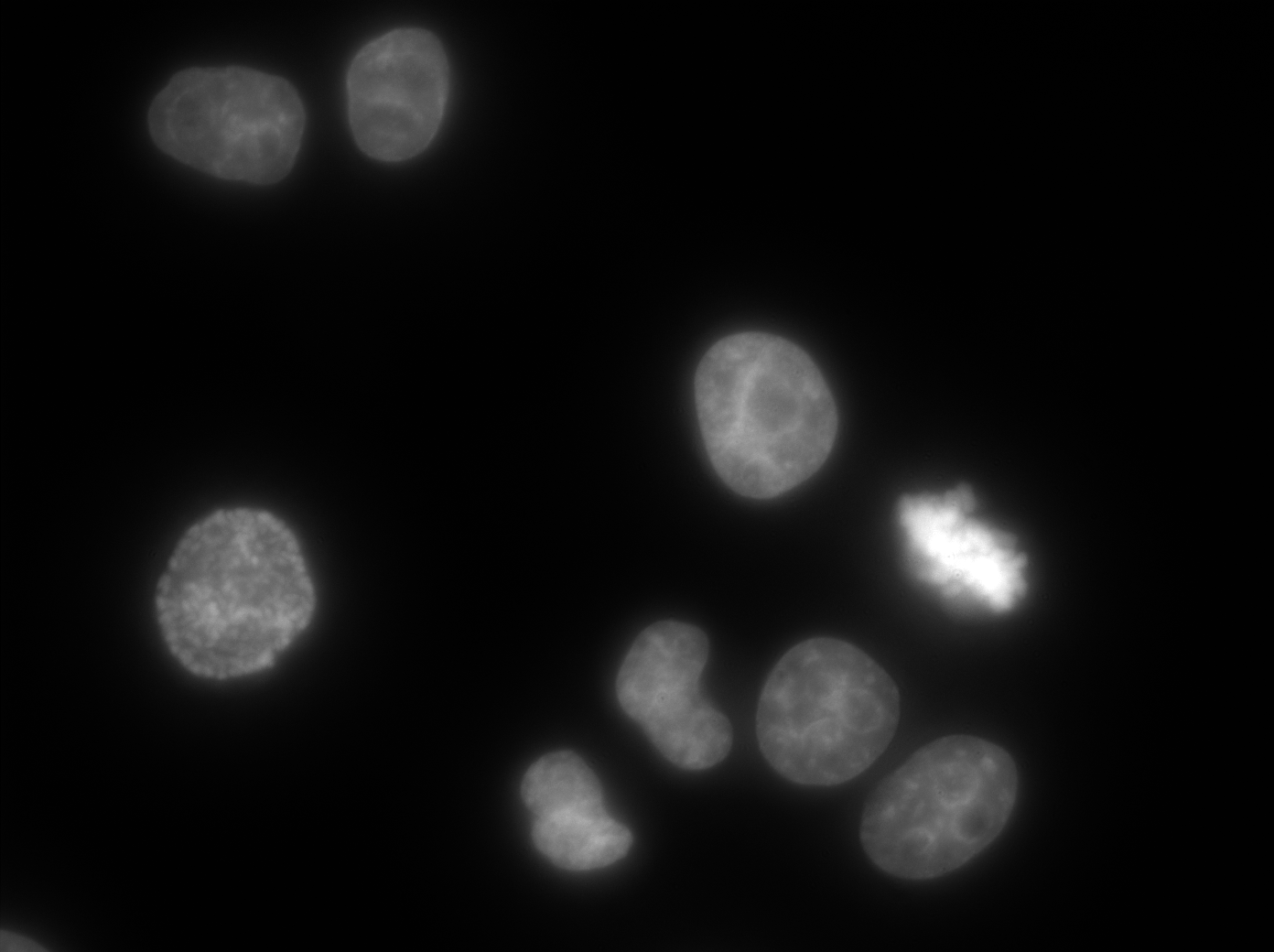

Supplement: Supplementary file 25 — Source data Fig. 7 part 1 [file 44319_2026_742_MOESM25_ESM.zip › Figure 7 Part 1/Fig 7acd Cas9 and TPGS1-ko rGT335 atubulin/Cas9 GT335recomb atub 3-24-25 R3 LT1 M1.Project Maximum Z_XY1742848535_Z0_T0_C0.tif]

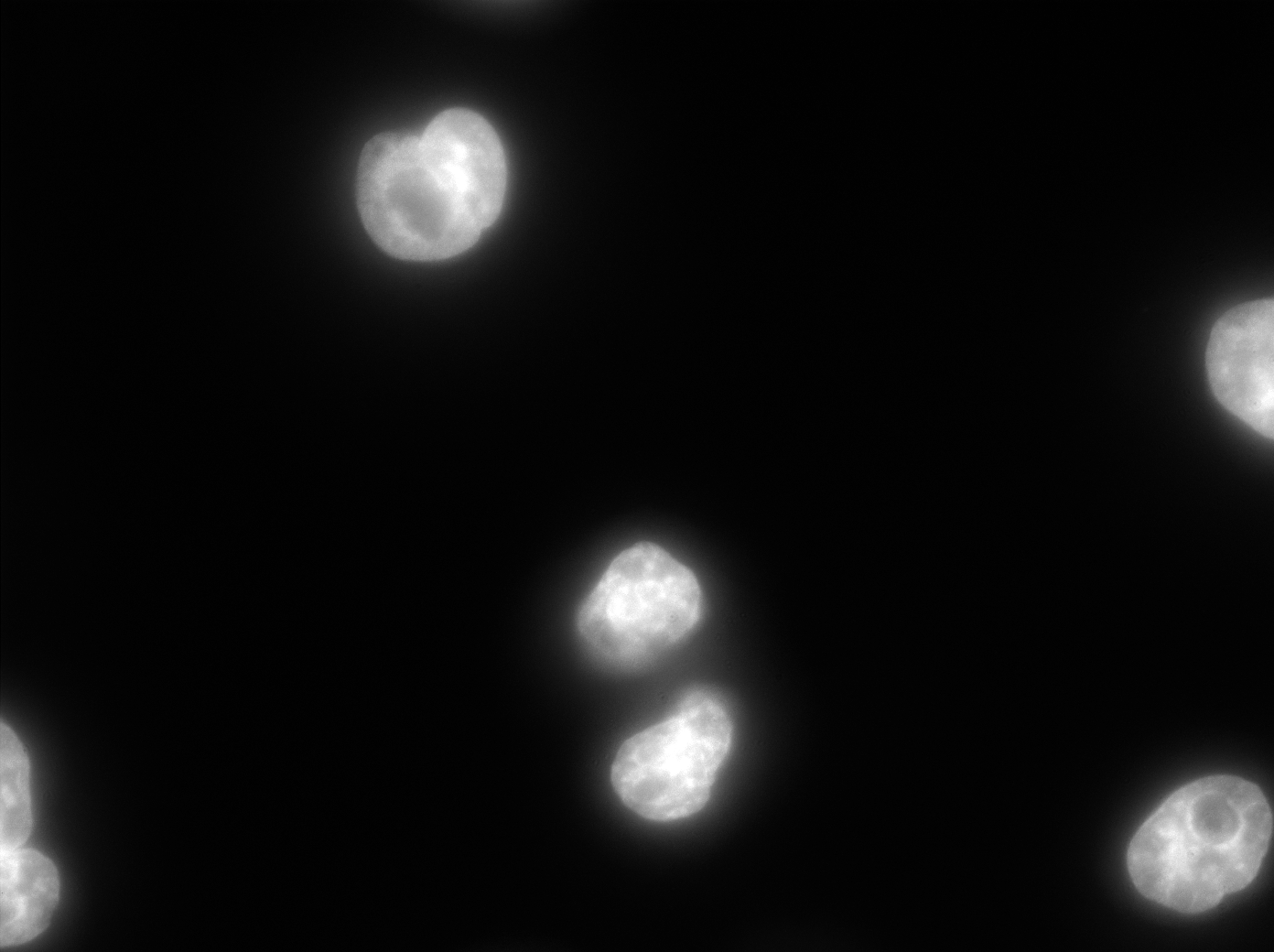

Supplement: Supplementary file 25 — Source data Fig. 7 part 1 [file 44319_2026_742_MOESM25_ESM.zip › Figure 7 Part 1/Fig 7acd Cas9 and TPGS1-ko rGT335 atubulin/Cas9 GT335recomb atub 3-24-25 R3 LT5.Project Maximum Z_XY1742849372_Z0_T0_C0.tif]

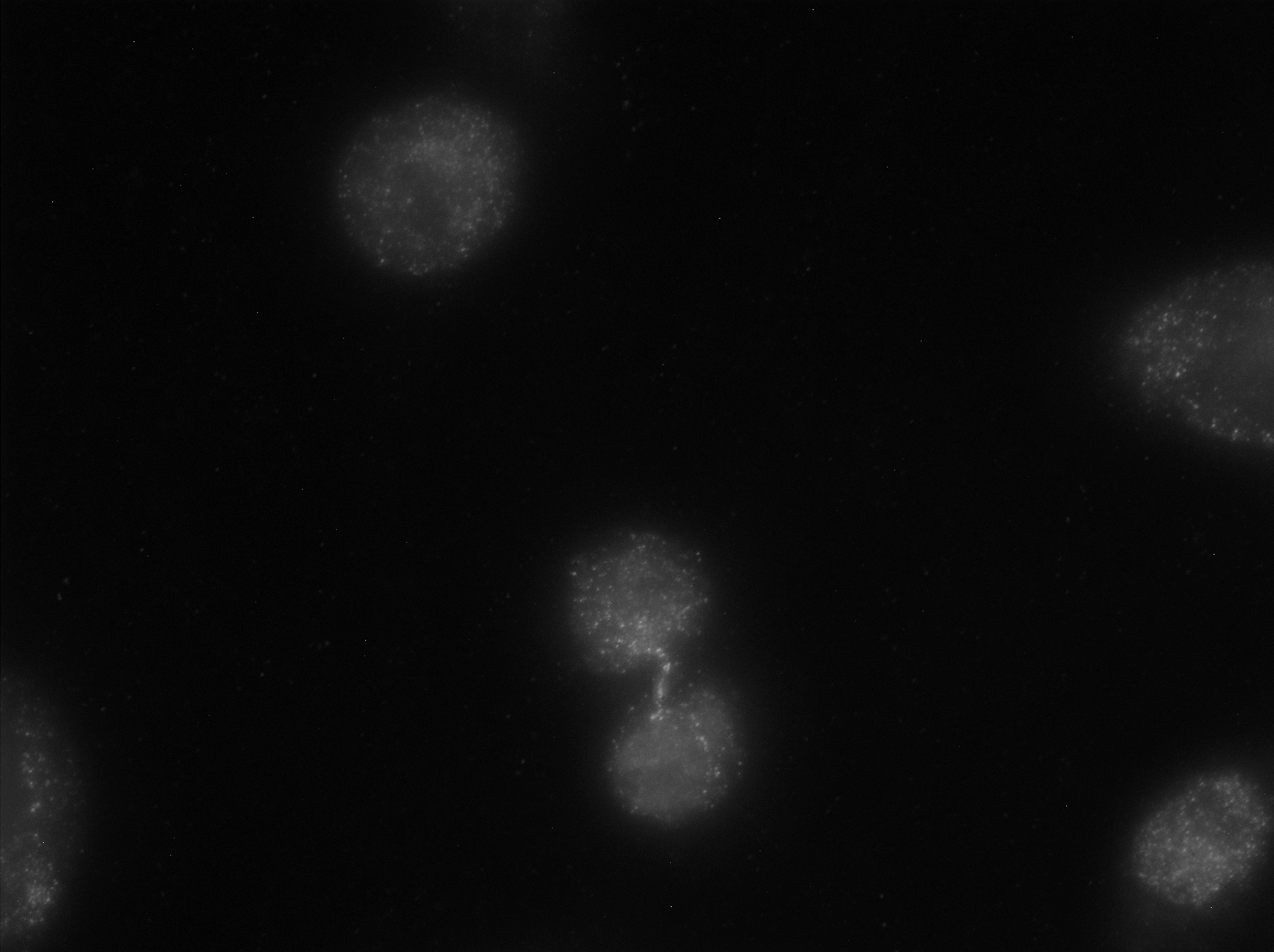

Supplement: Supplementary file 25 — Source data Fig. 7 part 1 [file 44319_2026_742_MOESM25_ESM.zip › Figure 7 Part 1/Fig 7acd Cas9 and TPGS1-ko rGT335 atubulin/Cas9 GT335recomb atub 3-24-25 R3 LT5.Project Maximum Z_XY1742849372_Z0_T0_C1.tif]

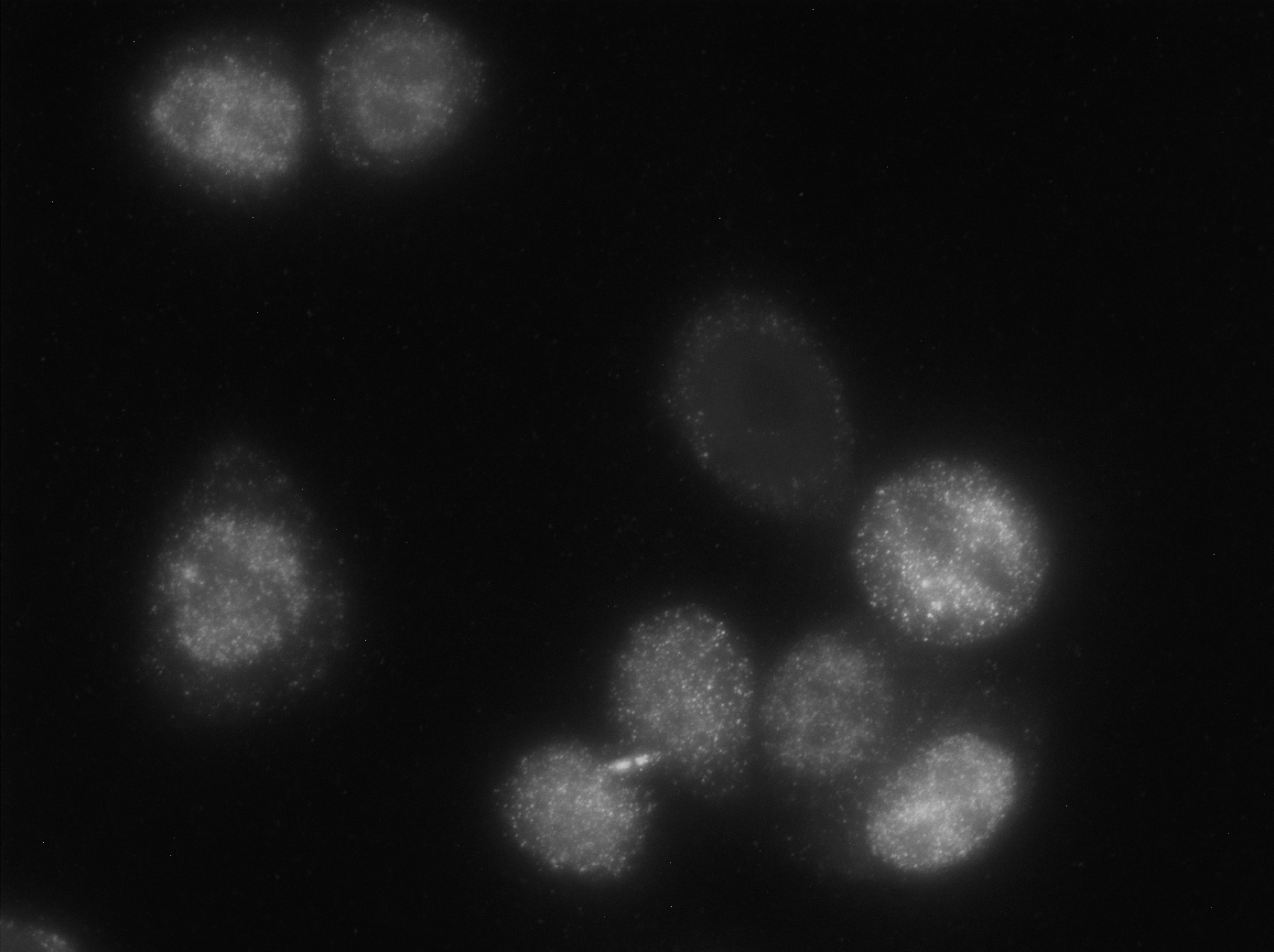

Supplement: Supplementary file 25 — Source data Fig. 7 part 1 [file 44319_2026_742_MOESM25_ESM.zip › Figure 7 Part 1/Fig 7acd Cas9 and TPGS1-ko rGT335 atubulin/Cas9 GT335recomb atub 3-24-25 R3 LT1 M1.Project Maximum Z_XY1742848535_Z0_T0_C1.tif]

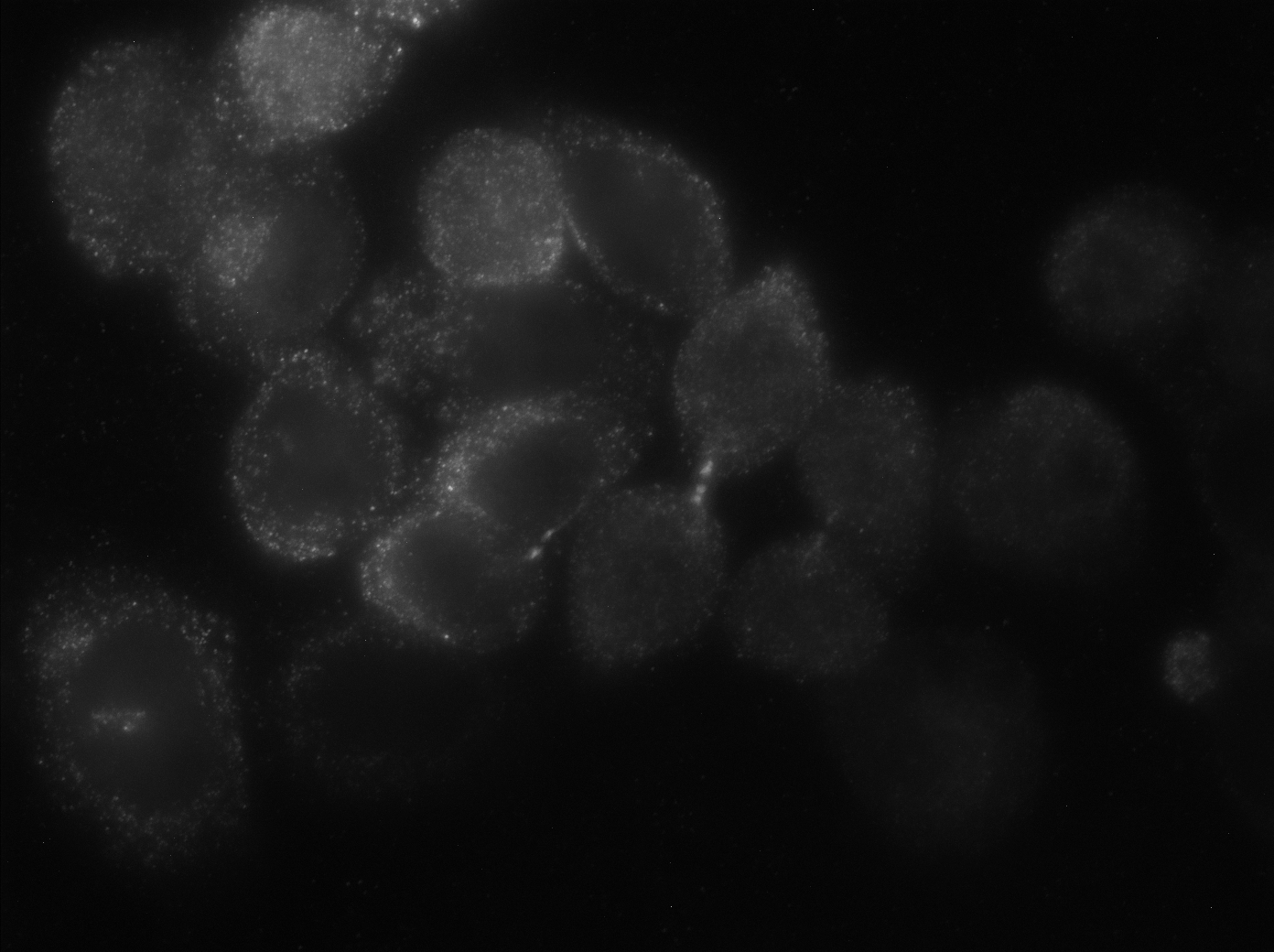

Supplement: Supplementary file 25 — Source data Fig. 7 part 1 [file 44319_2026_742_MOESM25_ESM.zip › Figure 7 Part 1/Fig 7acd Cas9 and TPGS1-ko rGT335 atubulin/Cas9 GT335recomb atub 3-24-25 R1 LT7LT8.Project Maximum Z_XY1742836175_Z0_T0_C1.tif]

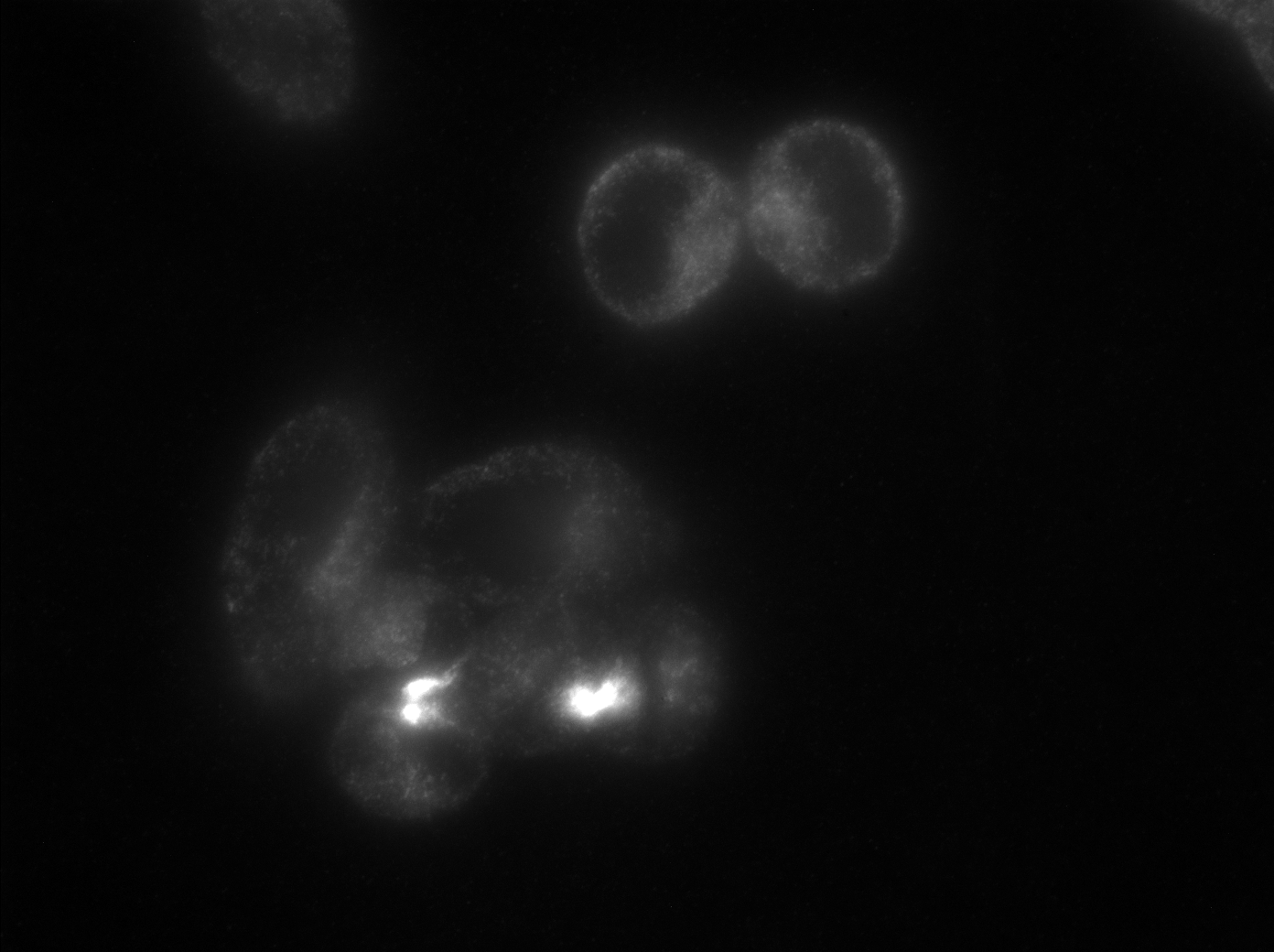

Supplement: Supplementary file 25 — Source data Fig. 7 part 1 [file 44319_2026_742_MOESM25_ESM.zip › Figure 7 Part 1/Fig 7acd Cas9 and TPGS1-ko rGT335 atubulin/Cas9 GT335recomb atub 3-24-25 R3 ET6ET7.Project Maximum Z_XY1742850373_Z0_T0_C2.tif]

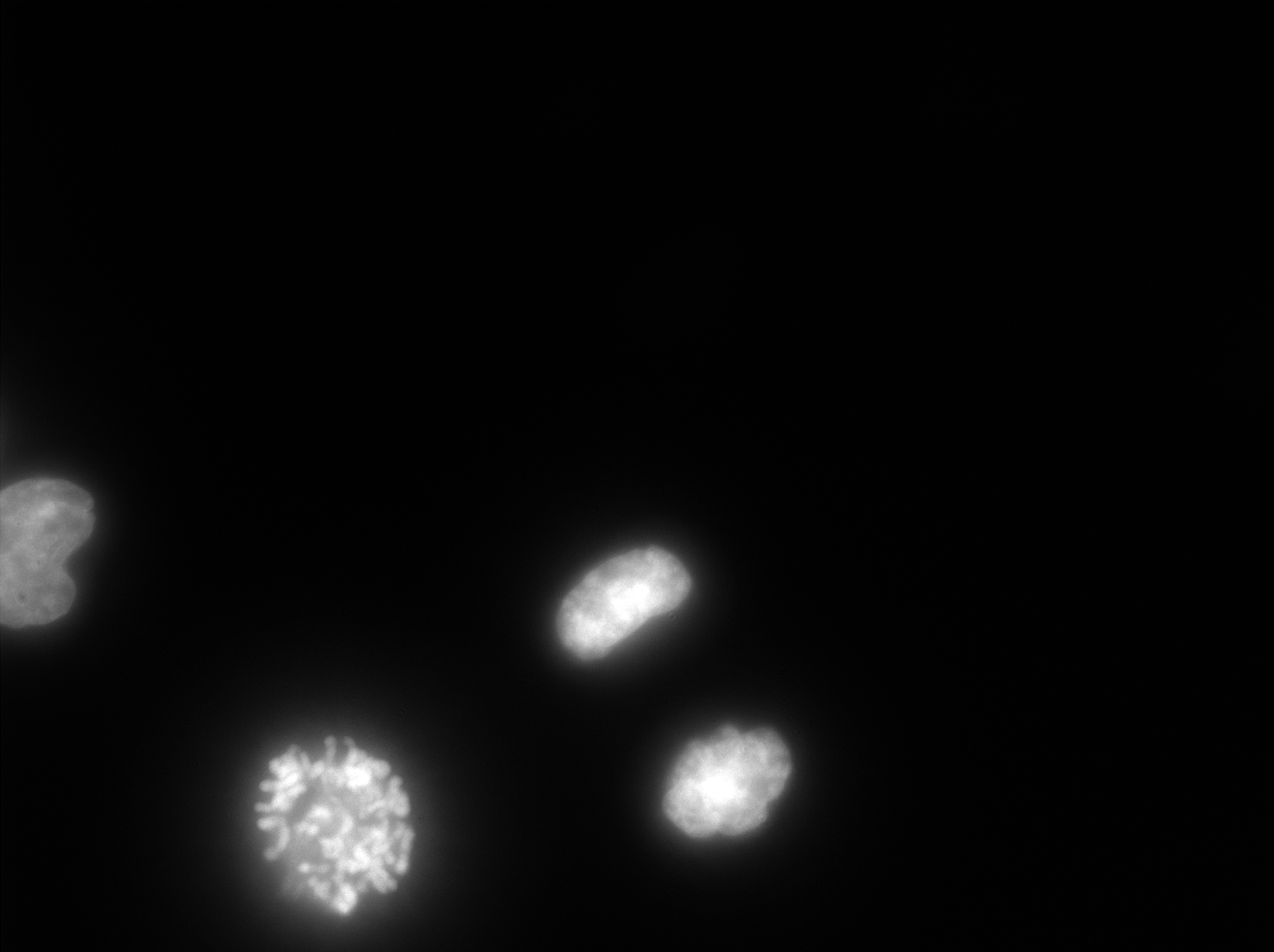

Supplement: Supplementary file 25 — Source data Fig. 7 part 1 [file 44319_2026_742_MOESM25_ESM.zip › Figure 7 Part 1/Fig 7acd Cas9 and TPGS1-ko rGT335 atubulin/Cas9 GT335recomb atub 3-24-25 R3 ET1.Project Maximum Z_XY1742848799_Z0_T0_C0.tif]

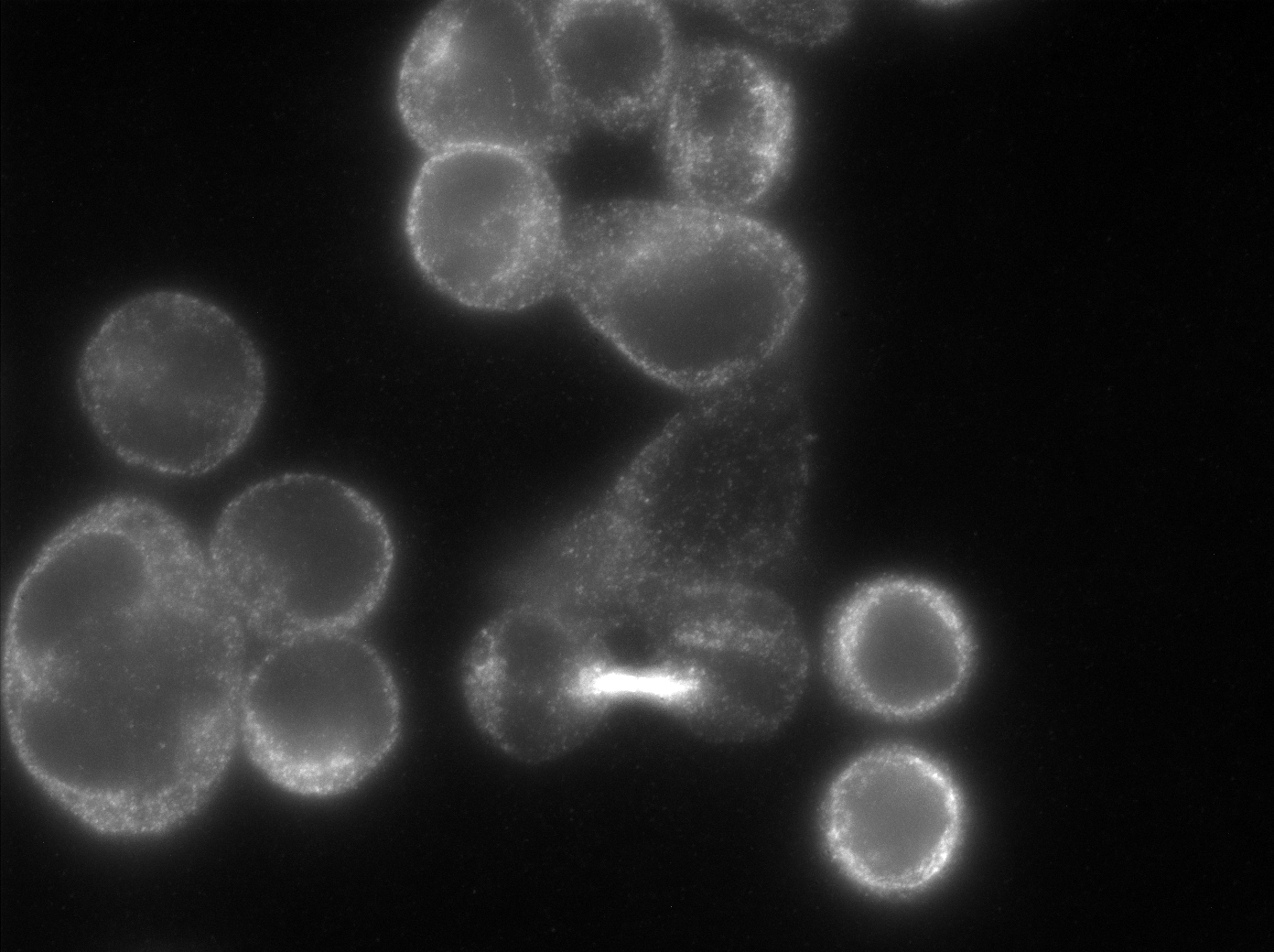

Supplement: Supplementary file 25 — Source data Fig. 7 part 1 [file 44319_2026_742_MOESM25_ESM.zip › Figure 7 Part 1/Fig 7acd Cas9 and TPGS1-ko rGT335 atubulin/Cas9 GT335recomb atub 3-24-25 R1 ET7.Project Maximum Z_XY1742836085_Z0_T0_C2.tif]

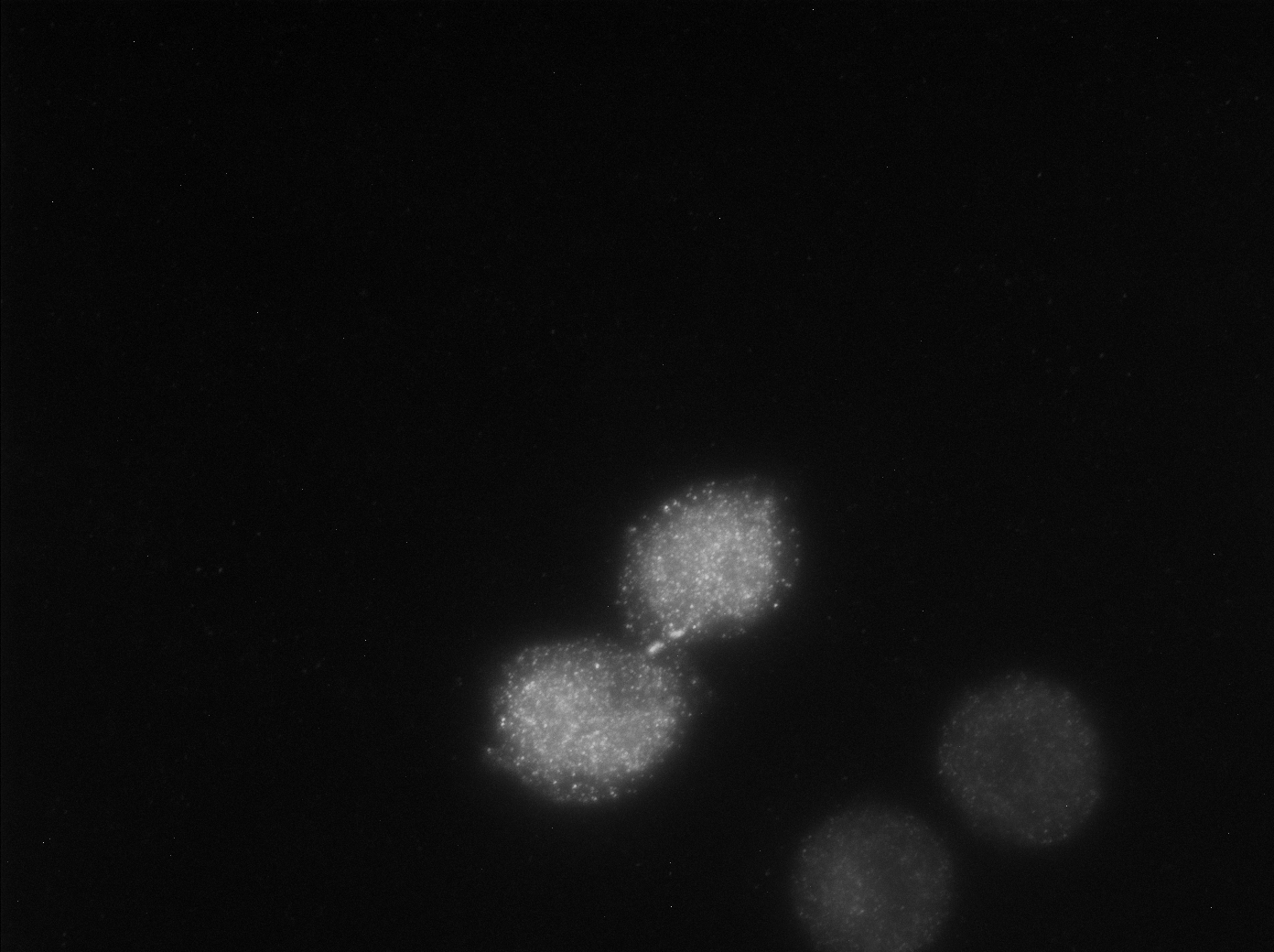

Supplement: Supplementary file 25 — Source data Fig. 7 part 1 [file 44319_2026_742_MOESM25_ESM.zip › Figure 7 Part 1/Fig 7acd Cas9 and TPGS1-ko rGT335 atubulin/Cas9 GT335recomb atub 3-24-25 R2 LT5.Project Maximum Z_XY1742846466_Z0_T0_C1.tif]

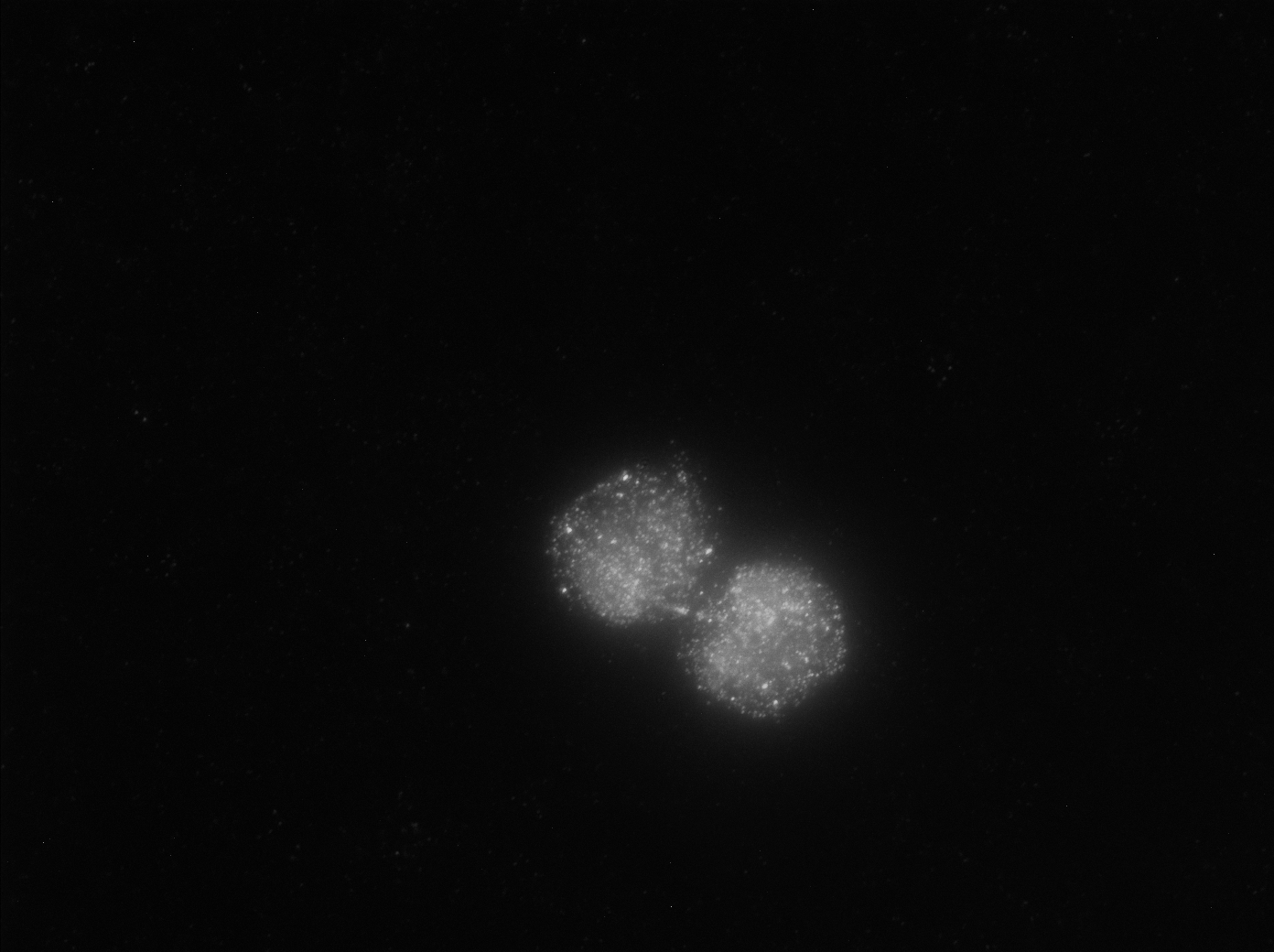

Supplement: Supplementary file 25 — Source data Fig. 7 part 1 [file 44319_2026_742_MOESM25_ESM.zip › Figure 7 Part 1/Fig 7acd Cas9 and TPGS1-ko rGT335 atubulin/Cas9 GT335recomb atub 3-24-25 R1 ET4.Project Maximum Z_XY1742835443_Z0_T0_C1.tif]

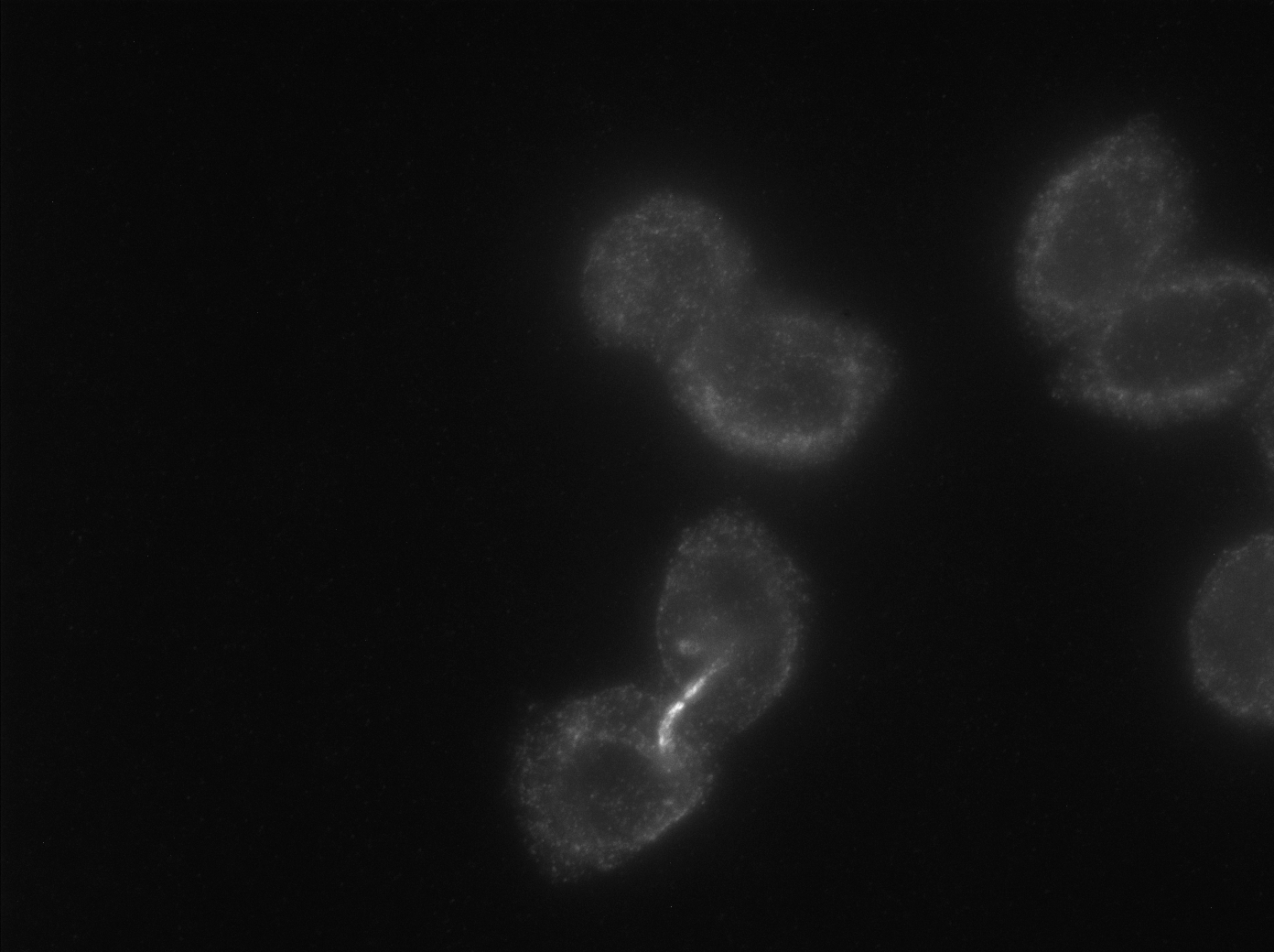

Supplement: Supplementary file 25 — Source data Fig. 7 part 1 [file 44319_2026_742_MOESM25_ESM.zip › Figure 7 Part 1/Fig 7acd Cas9 and TPGS1-ko rGT335 atubulin/Cas9 GT335recomb atub 3-24-25 R3 LT3.Project Maximum Z_XY1742849044_Z0_T0_C2.tif]

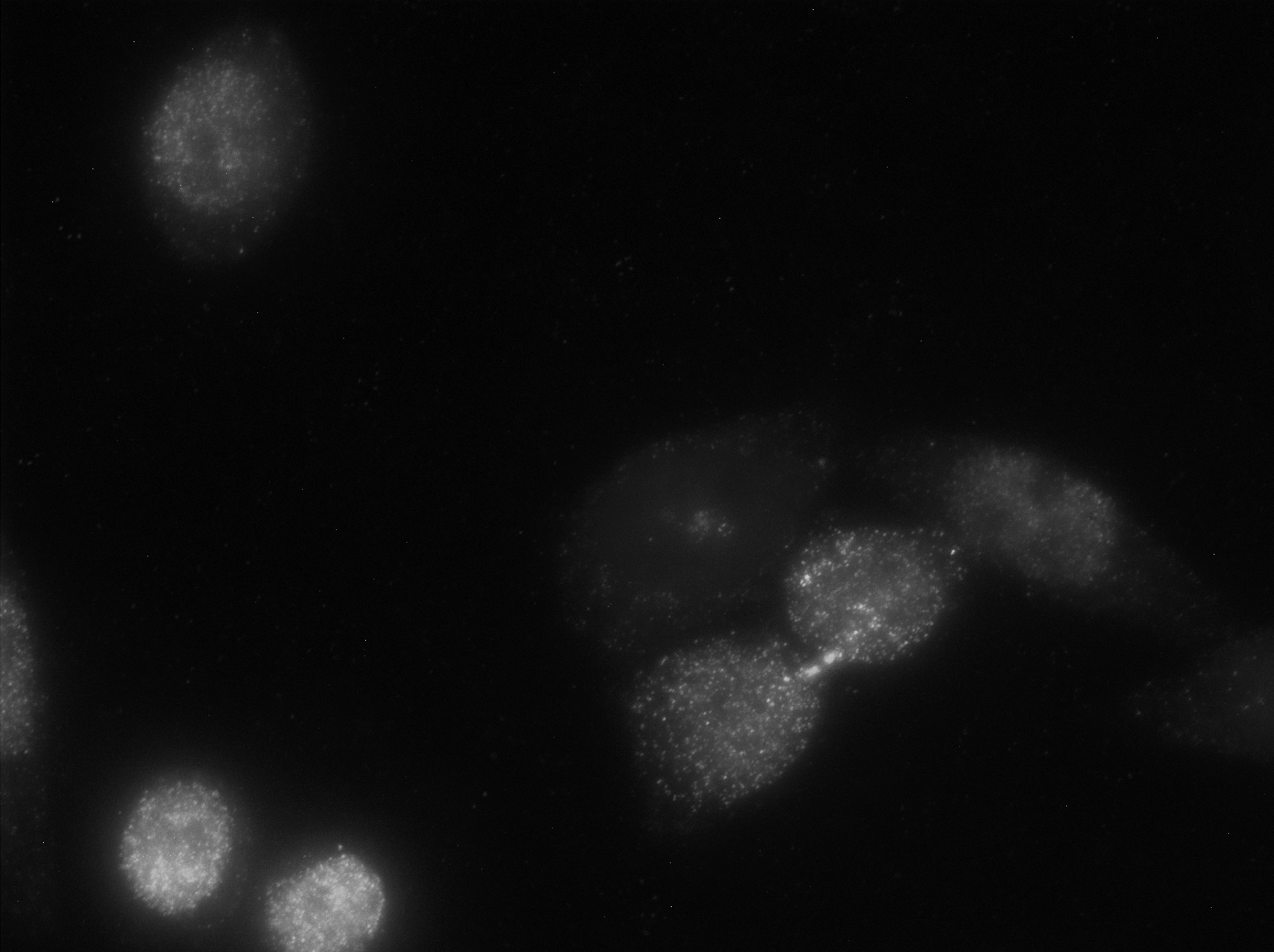

Supplement: Supplementary file 25 — Source data Fig. 7 part 1 [file 44319_2026_742_MOESM25_ESM.zip › Figure 7 Part 1/Fig 7acd Cas9 and TPGS1-ko rGT335 atubulin/Cas9 GT335recomb atub 3-24-25 R3 LT6.Project Maximum Z_XY1742849538_Z0_T0_C1.tif]

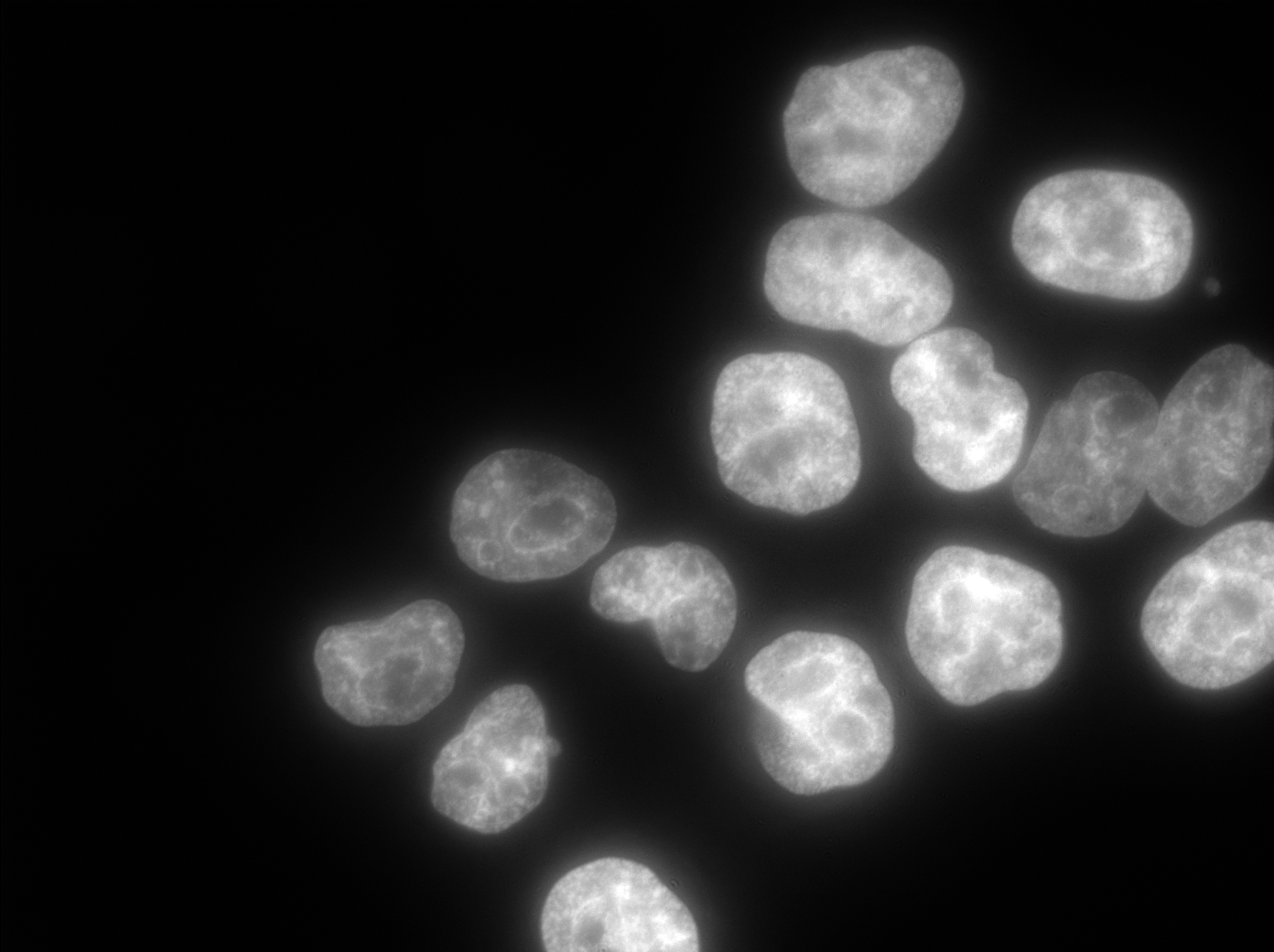

Supplement: Supplementary file 25 — Source data Fig. 7 part 1 [file 44319_2026_742_MOESM25_ESM.zip › Figure 7 Part 1/Fig 7acd Cas9 and TPGS1-ko rGT335 atubulin/Cas9 GT335recomb atub 3-24-25 R1 LT6.Project Maximum Z_XY1742835798_Z0_T0_C0.tif]

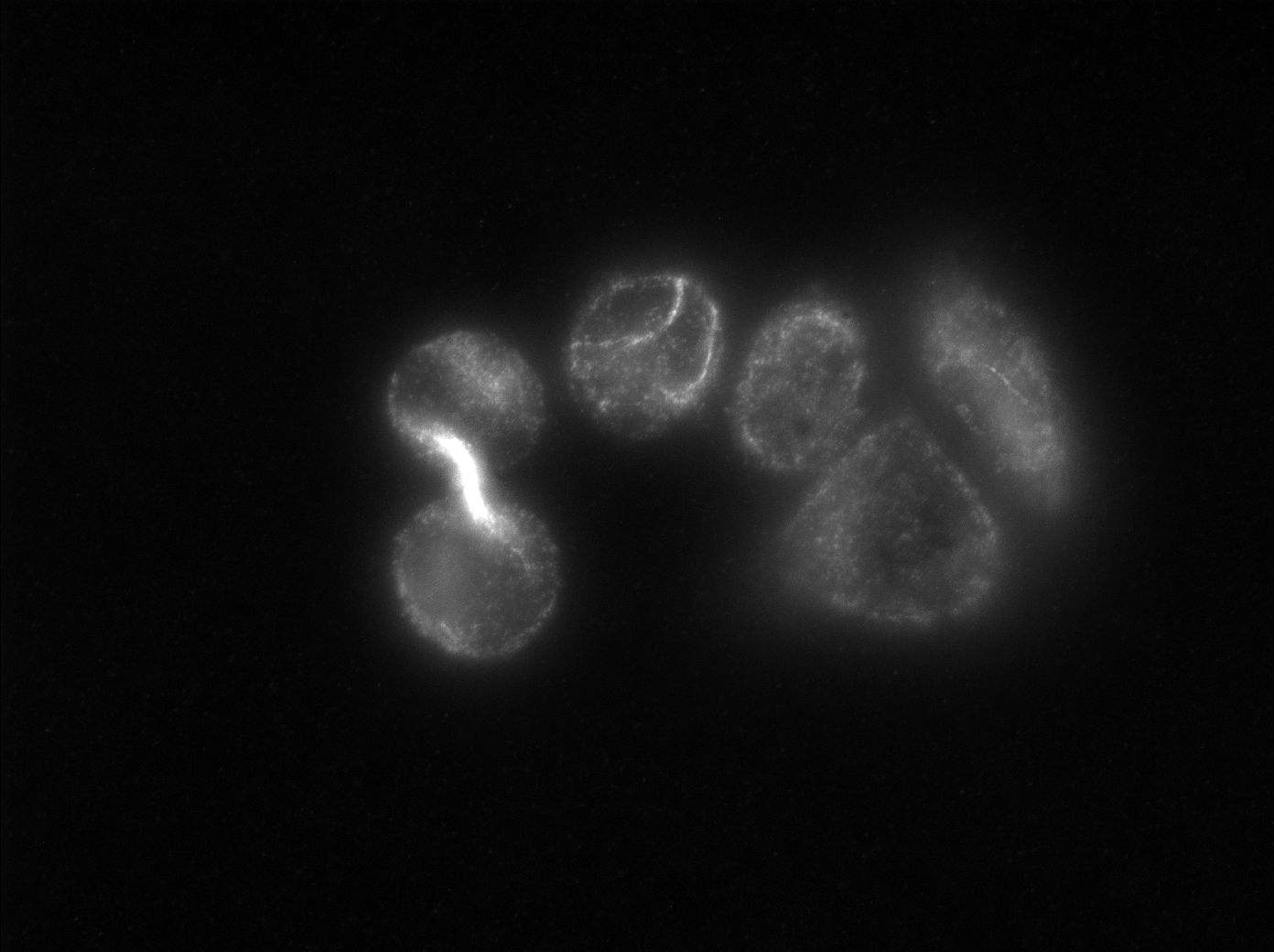

Supplement: Supplementary file 25 — Source data Fig. 7 part 1 [file 44319_2026_742_MOESM25_ESM.zip › Figure 7 Part 1/Fig 7acd Cas9 and TPGS1-ko rGT335 atubulin/Cas9 GT335recomb atub 3-24-25 R1 ET1.Project Maximum Z_XY1742834592_Z0_T0_C2.tif]

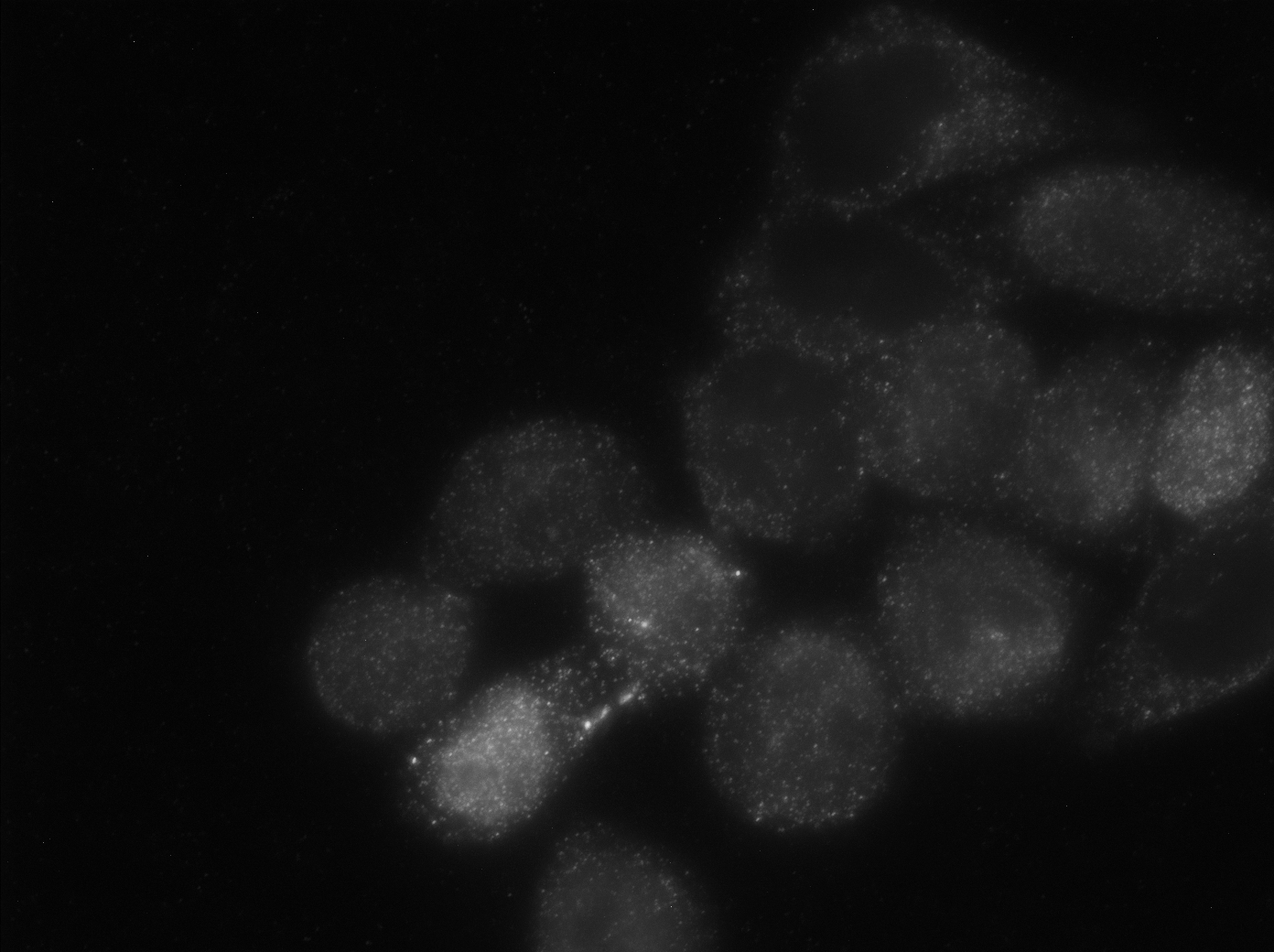

Supplement: Supplementary file 25 — Source data Fig. 7 part 1 [file 44319_2026_742_MOESM25_ESM.zip › Figure 7 Part 1/Fig 7acd Cas9 and TPGS1-ko rGT335 atubulin/Cas9 GT335recomb atub 3-24-25 R1 LT6.Project Maximum Z_XY1742835798_Z0_T0_C1.tif]

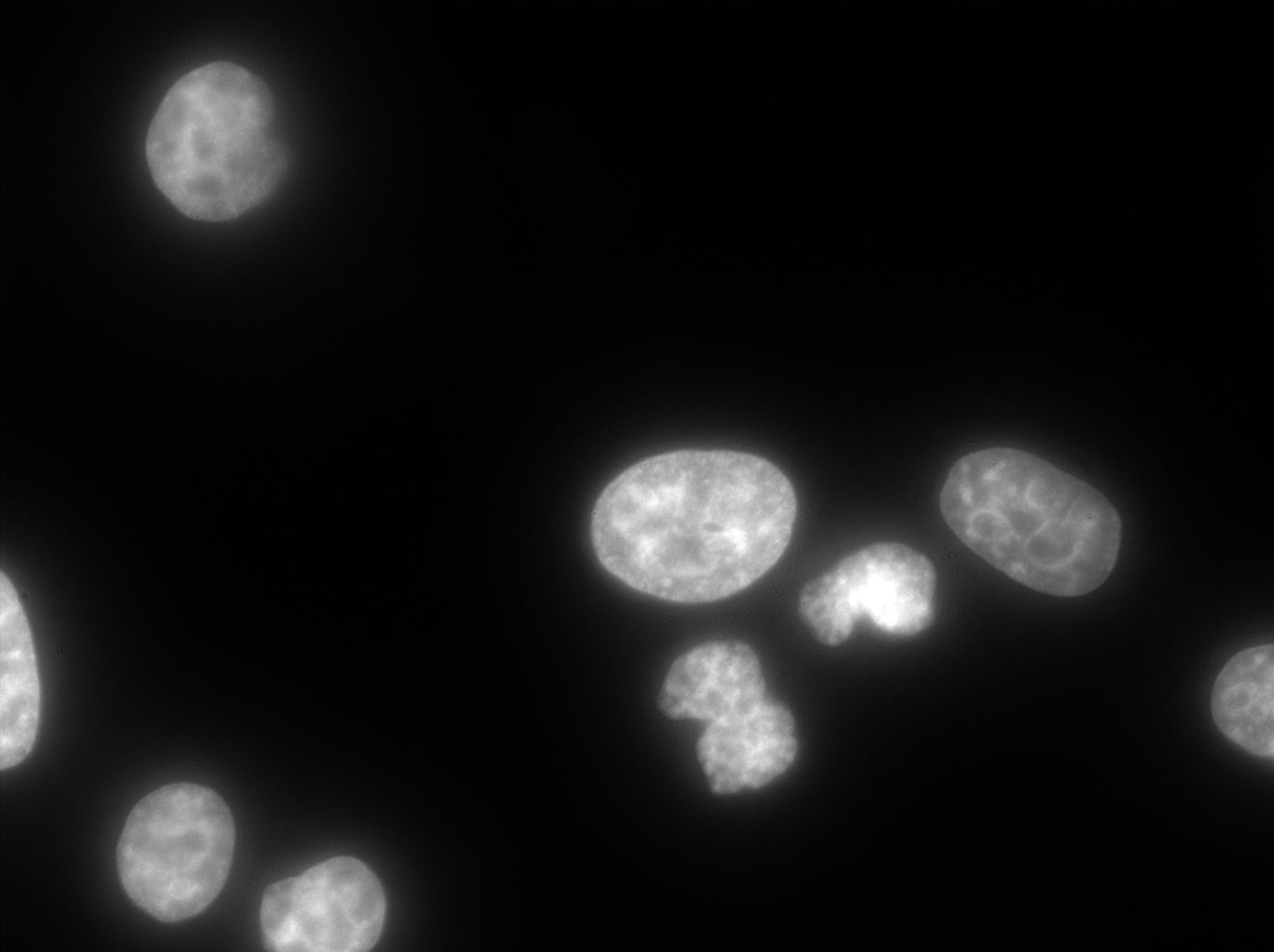

Supplement: Supplementary file 25 — Source data Fig. 7 part 1 [file 44319_2026_742_MOESM25_ESM.zip › Figure 7 Part 1/Fig 7acd Cas9 and TPGS1-ko rGT335 atubulin/Cas9 GT335recomb atub 3-24-25 R3 LT6.Project Maximum Z_XY1742849538_Z0_T0_C0.tif]

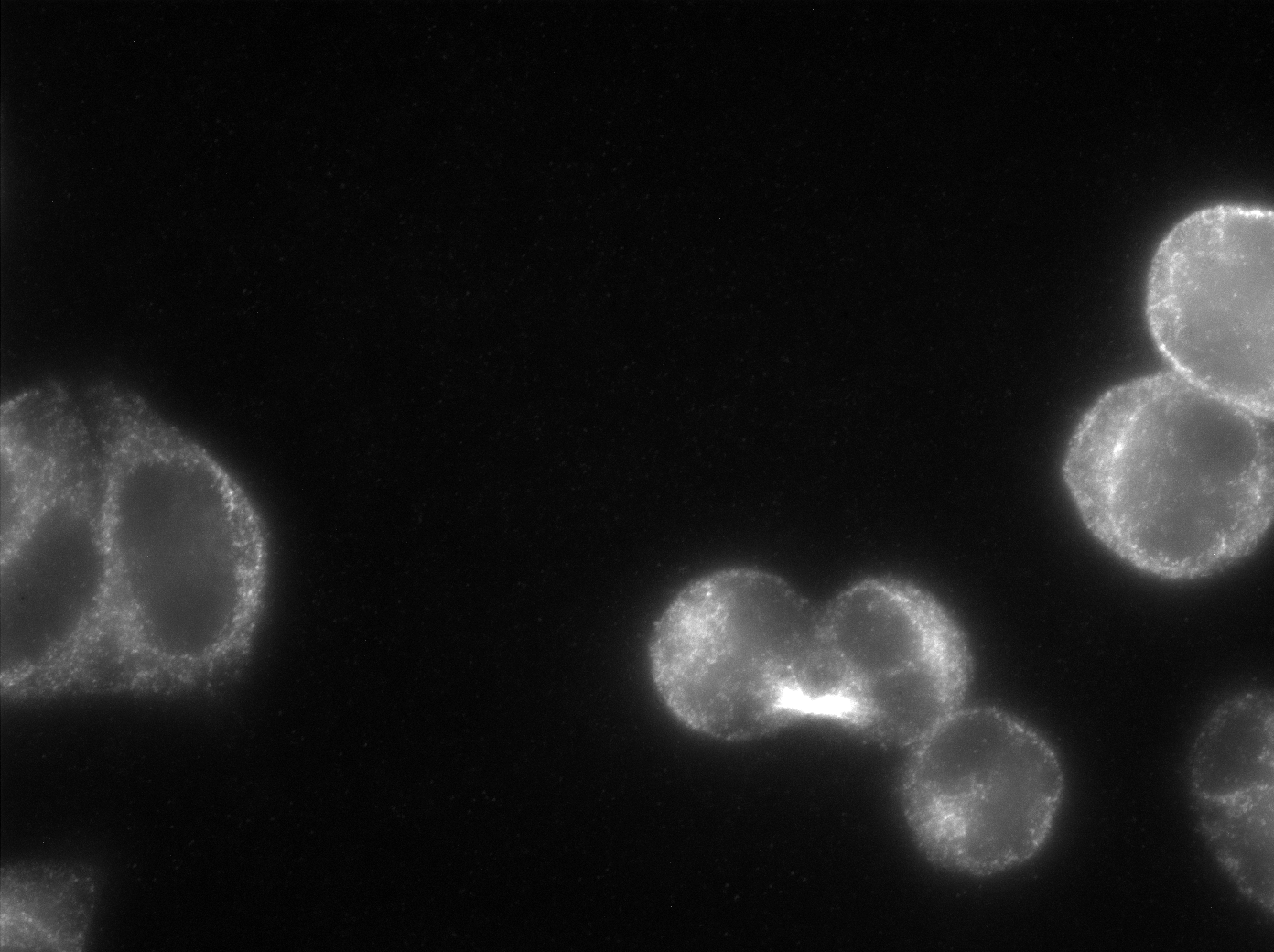

Supplement: Supplementary file 25 — Source data Fig. 7 part 1 [file 44319_2026_742_MOESM25_ESM.zip › Figure 7 Part 1/Fig 7acd Cas9 and TPGS1-ko rGT335 atubulin/Cas9 GT335recomb atub 3-24-25 R1 ET2.Project Maximum Z_XY1742835016_Z0_T0_C2.tif]

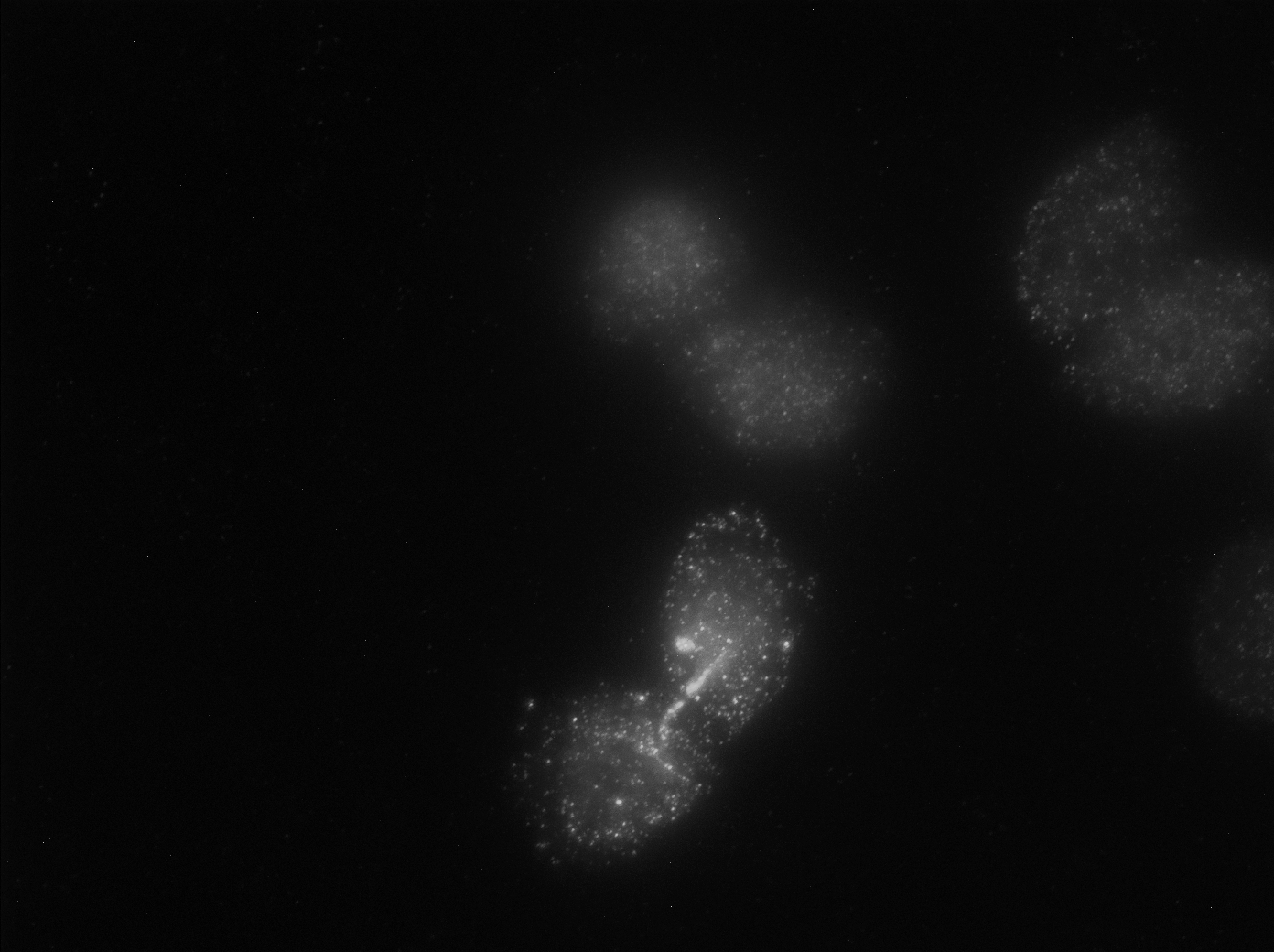

Supplement: Supplementary file 25 — Source data Fig. 7 part 1 [file 44319_2026_742_MOESM25_ESM.zip › Figure 7 Part 1/Fig 7acd Cas9 and TPGS1-ko rGT335 atubulin/Cas9 GT335recomb atub 3-24-25 R3 LT3.Project Maximum Z_XY1742849044_Z0_T0_C1.tif]

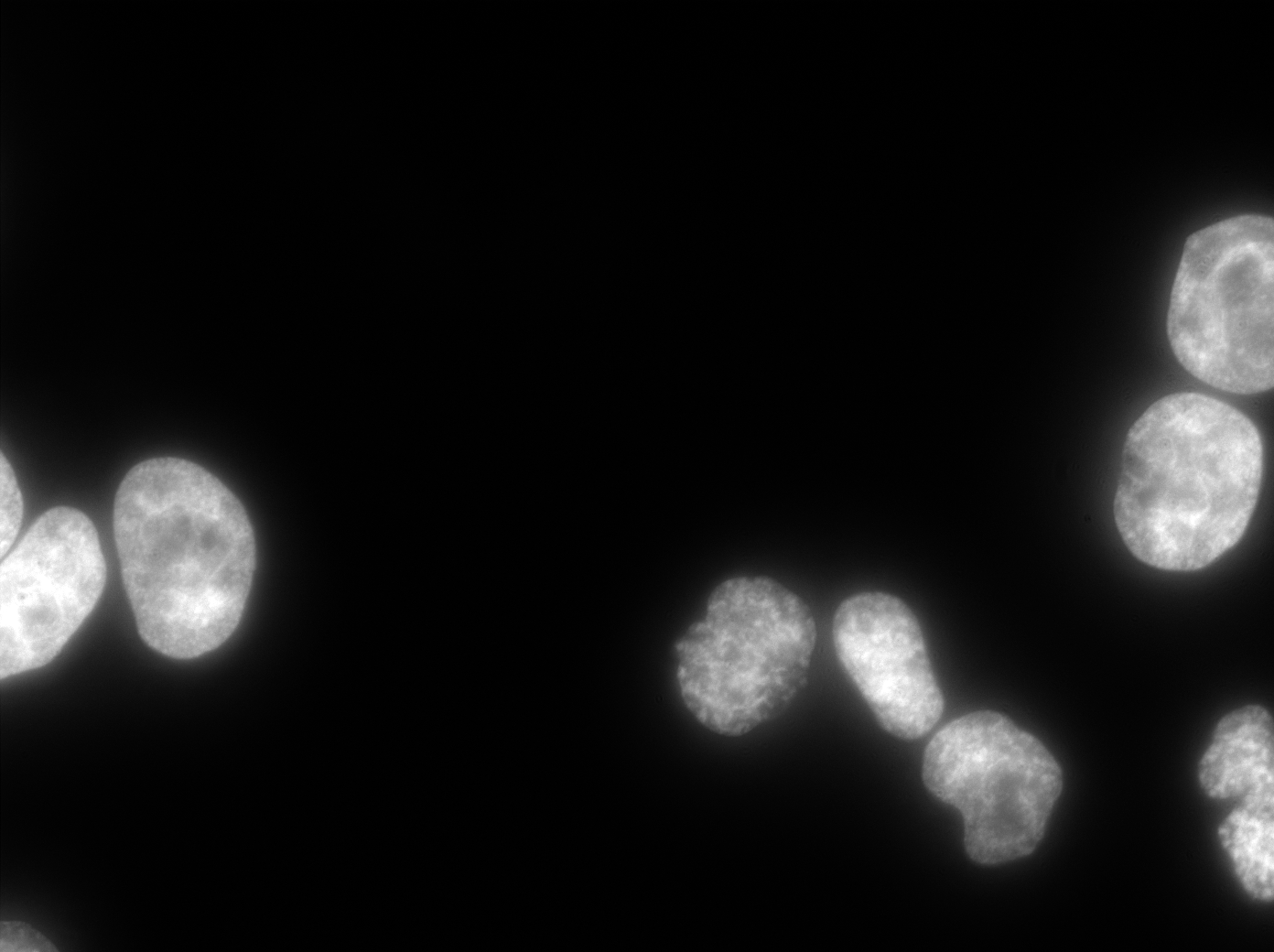

Supplement: Supplementary file 25 — Source data Fig. 7 part 1 [file 44319_2026_742_MOESM25_ESM.zip › Figure 7 Part 1/Fig 7acd Cas9 and TPGS1-ko rGT335 atubulin/Cas9 GT335recomb atub 3-24-25 R1 ET2.Project Maximum Z_XY1742835016_Z0_T0_C0.tif]

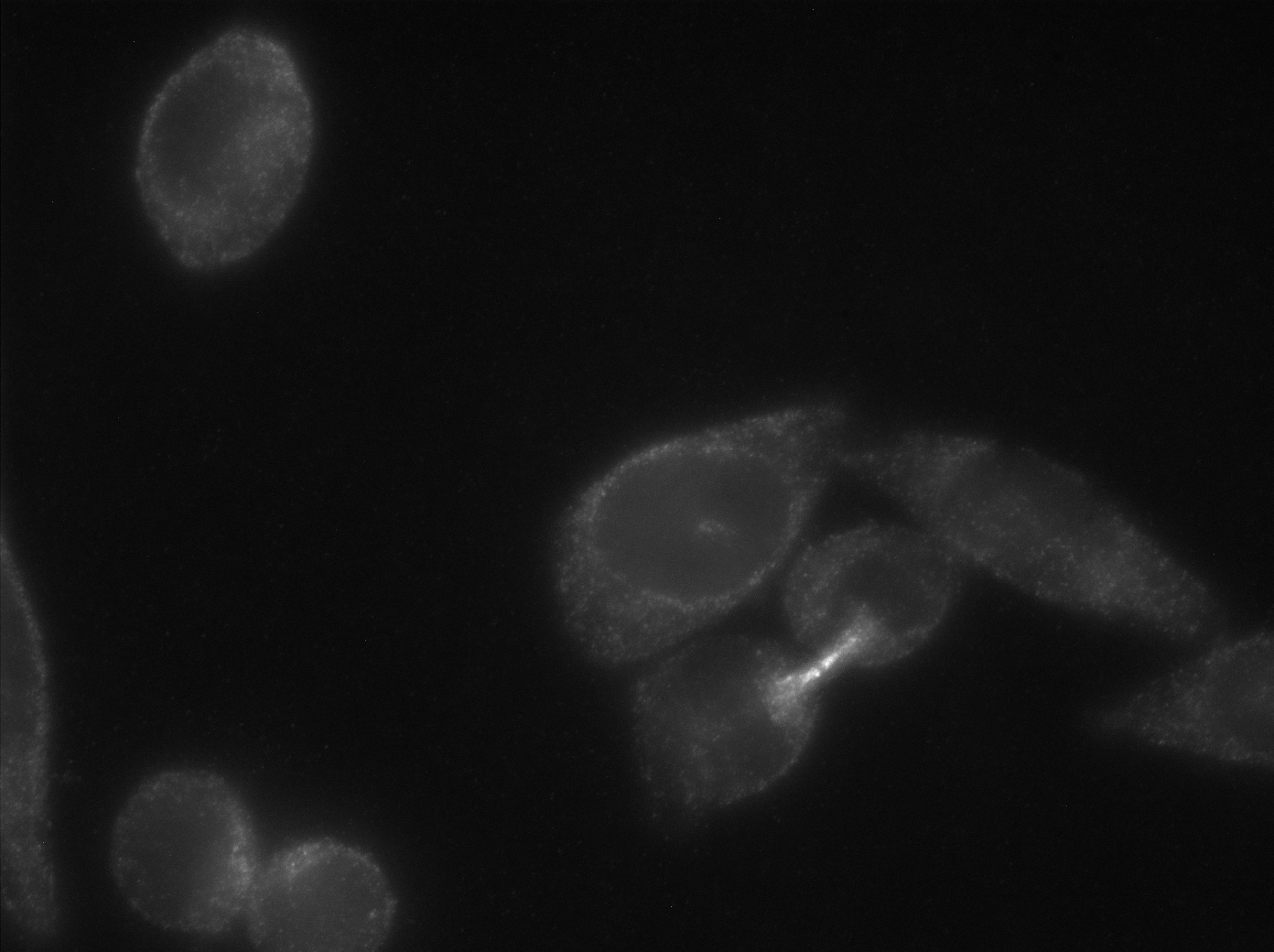

Supplement: Supplementary file 25 — Source data Fig. 7 part 1 [file 44319_2026_742_MOESM25_ESM.zip › Figure 7 Part 1/Fig 7acd Cas9 and TPGS1-ko rGT335 atubulin/Cas9 GT335recomb atub 3-24-25 R3 LT6.Project Maximum Z_XY1742849538_Z0_T0_C2.tif]

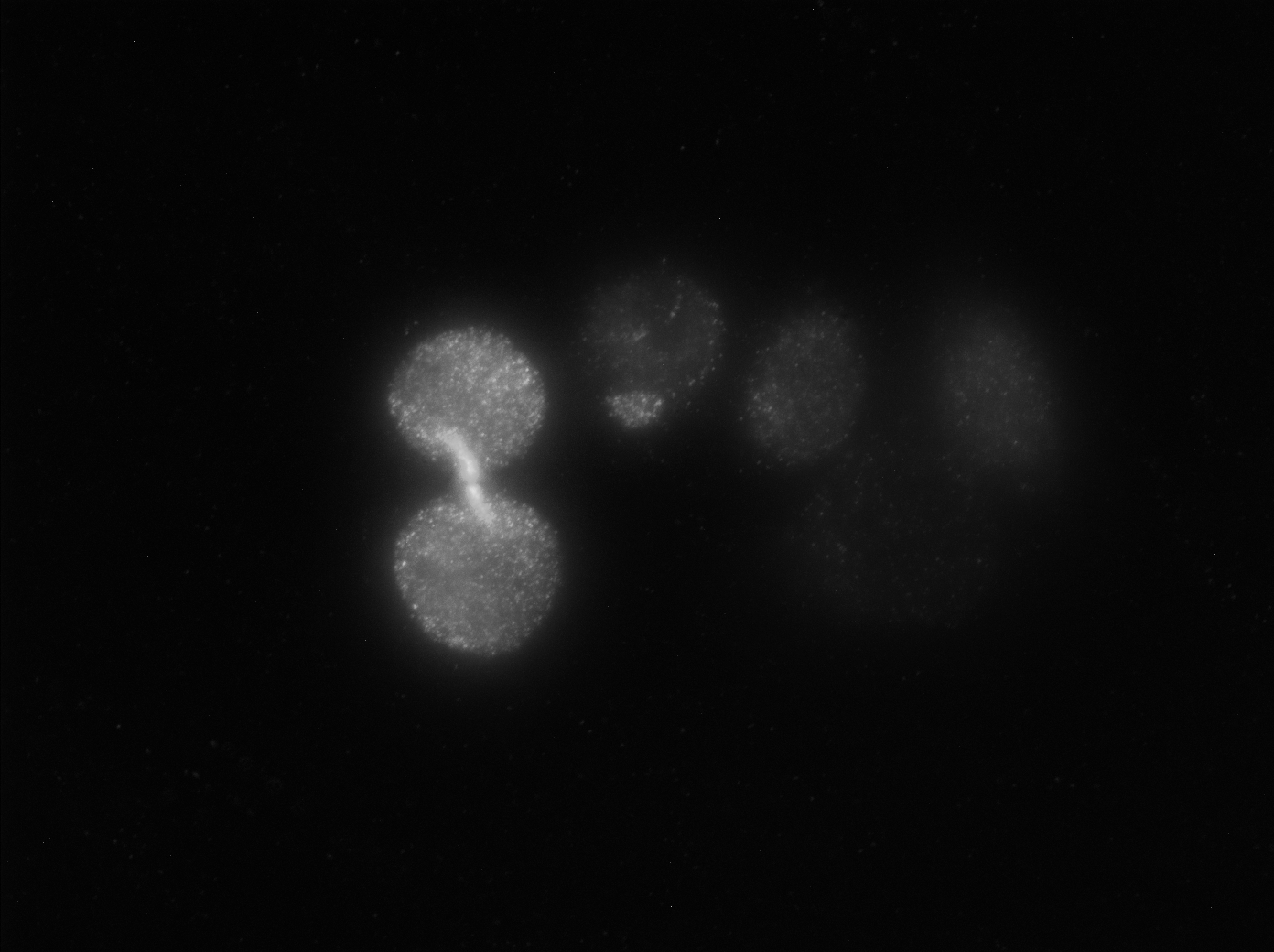

Supplement: Supplementary file 25 — Source data Fig. 7 part 1 [file 44319_2026_742_MOESM25_ESM.zip › Figure 7 Part 1/Fig 7acd Cas9 and TPGS1-ko rGT335 atubulin/Cas9 GT335recomb atub 3-24-25 R1 ET1.Project Maximum Z_XY1742834592_Z0_T0_C1.tif]

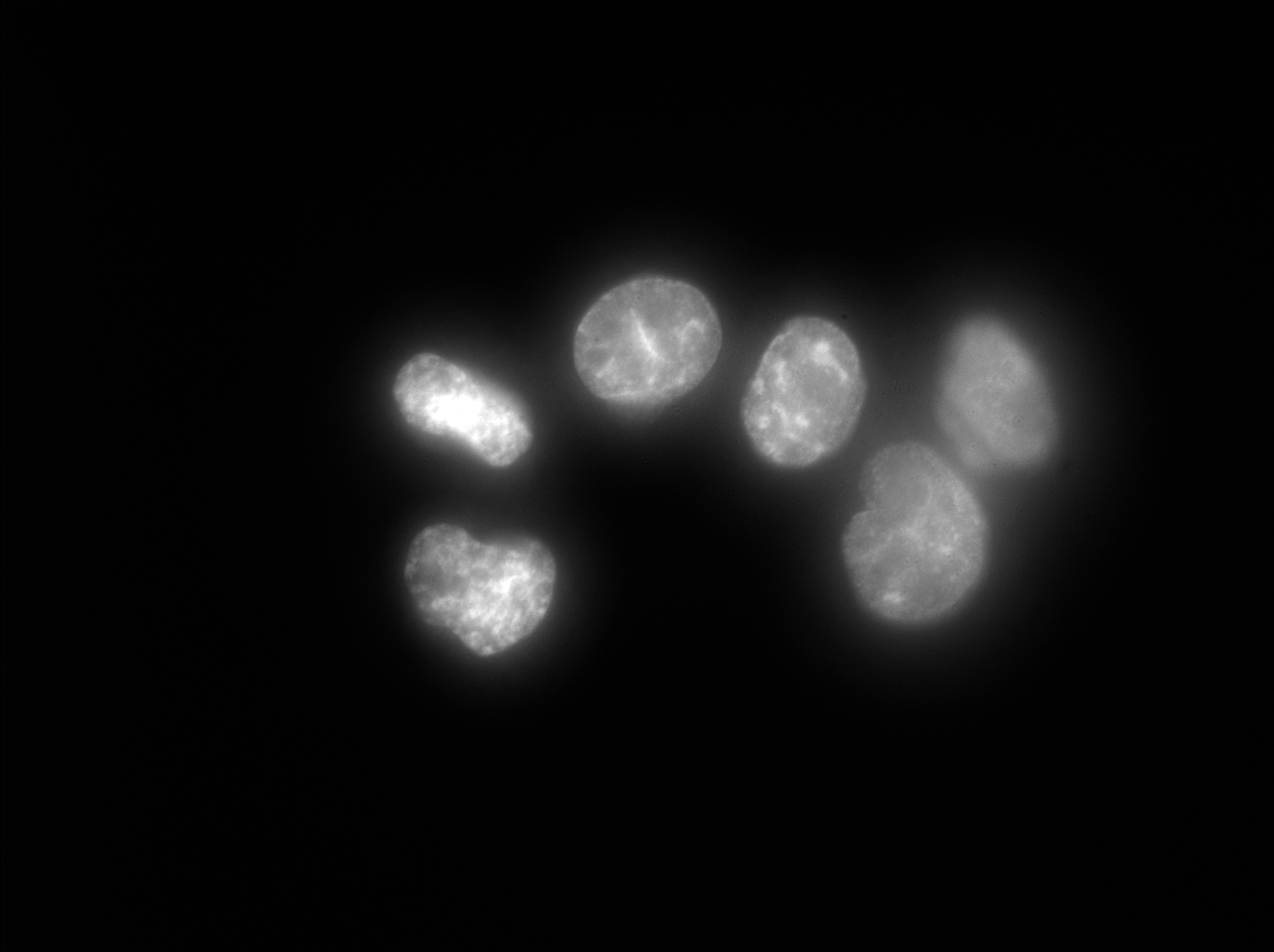

Supplement: Supplementary file 25 — Source data Fig. 7 part 1 [file 44319_2026_742_MOESM25_ESM.zip › Figure 7 Part 1/Fig 7acd Cas9 and TPGS1-ko rGT335 atubulin/Cas9 GT335recomb atub 3-24-25 R1 ET1.Project Maximum Z_XY1742834592_Z0_T0_C0.tif]

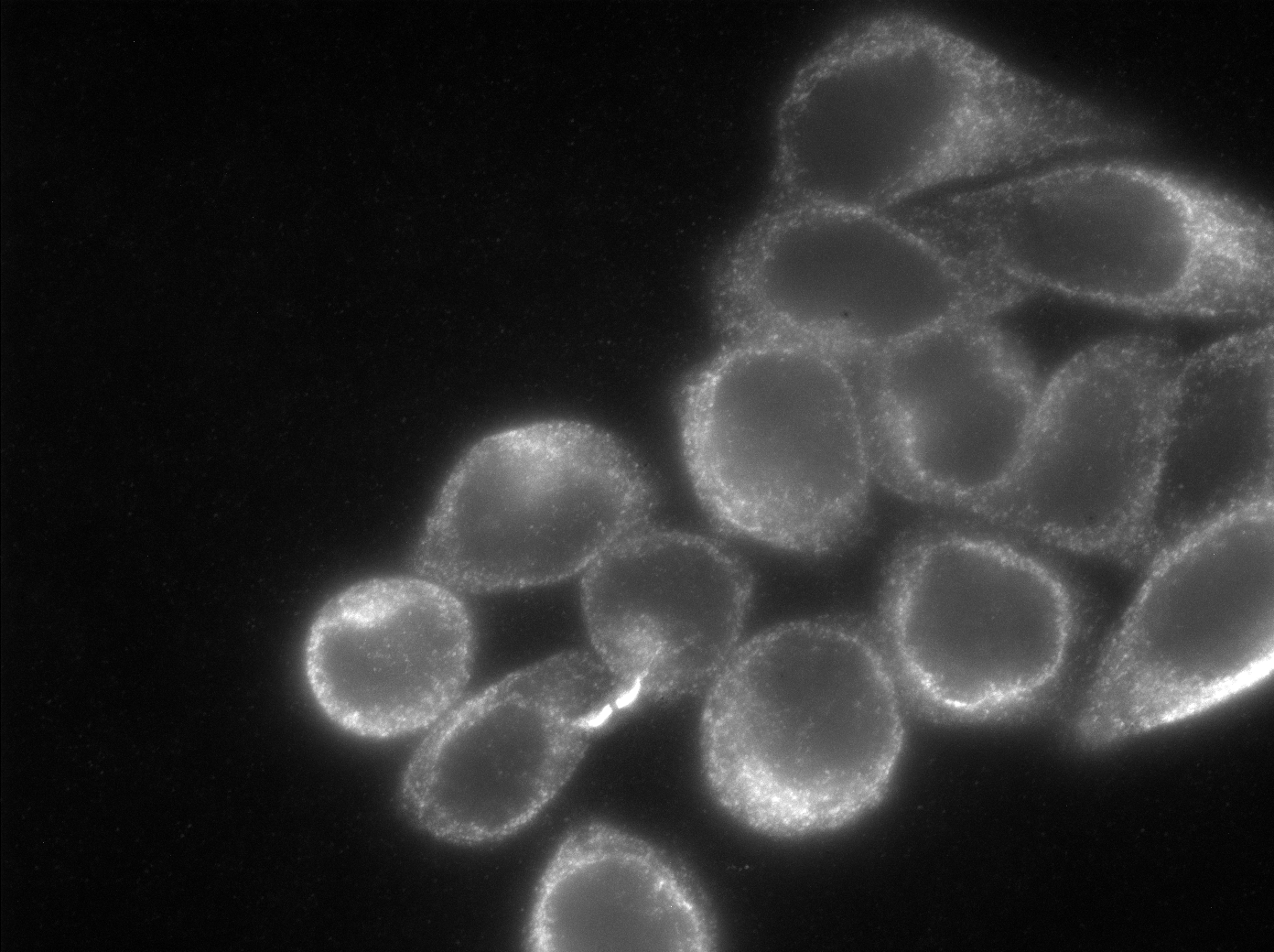

Supplement: Supplementary file 25 — Source data Fig. 7 part 1 [file 44319_2026_742_MOESM25_ESM.zip › Figure 7 Part 1/Fig 7acd Cas9 and TPGS1-ko rGT335 atubulin/Cas9 GT335recomb atub 3-24-25 R1 LT6.Project Maximum Z_XY1742835798_Z0_T0_C2.tif]

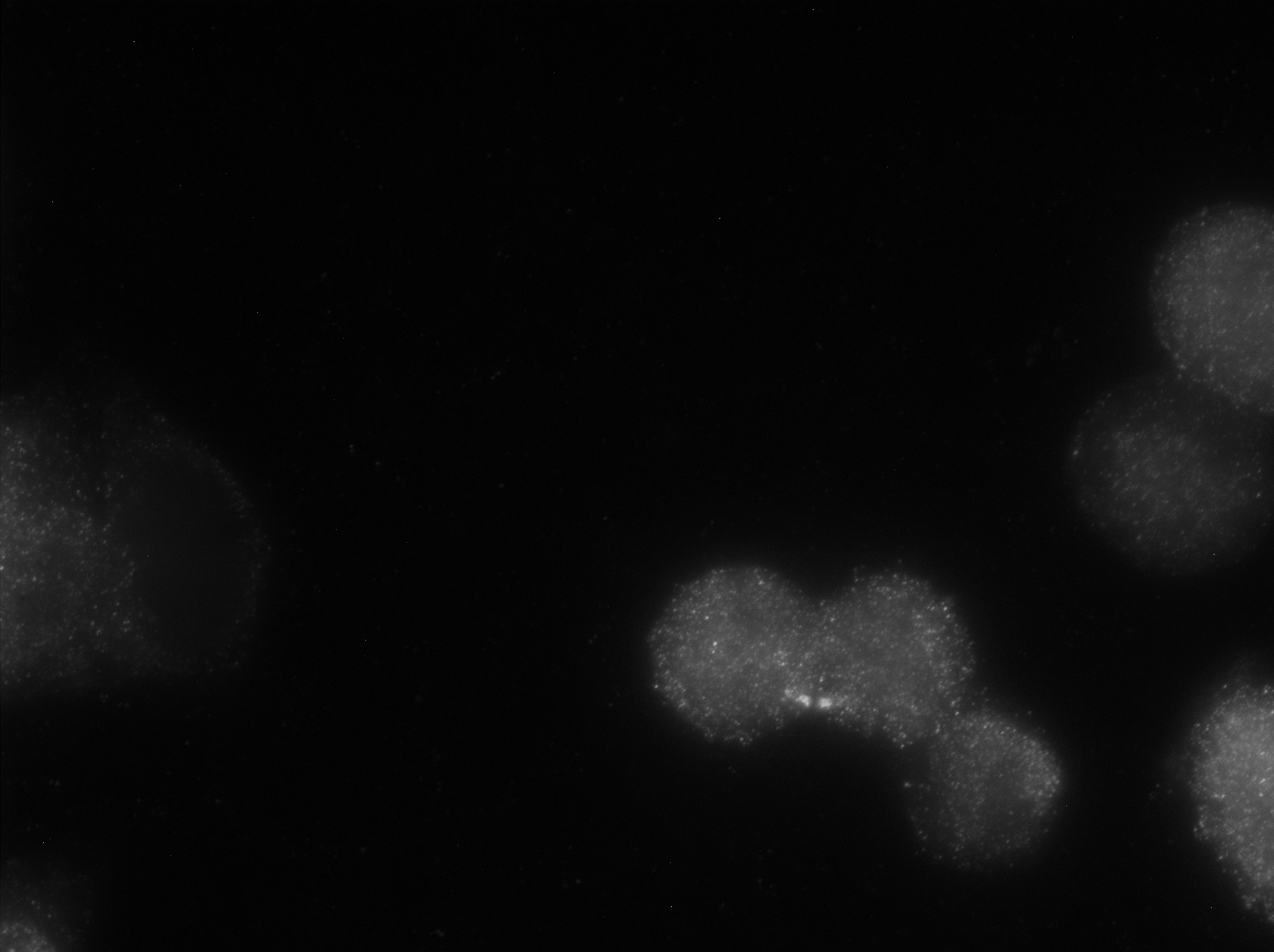

Supplement: Supplementary file 25 — Source data Fig. 7 part 1 [file 44319_2026_742_MOESM25_ESM.zip › Figure 7 Part 1/Fig 7acd Cas9 and TPGS1-ko rGT335 atubulin/Cas9 GT335recomb atub 3-24-25 R1 ET2.Project Maximum Z_XY1742835016_Z0_T0_C1.tif]

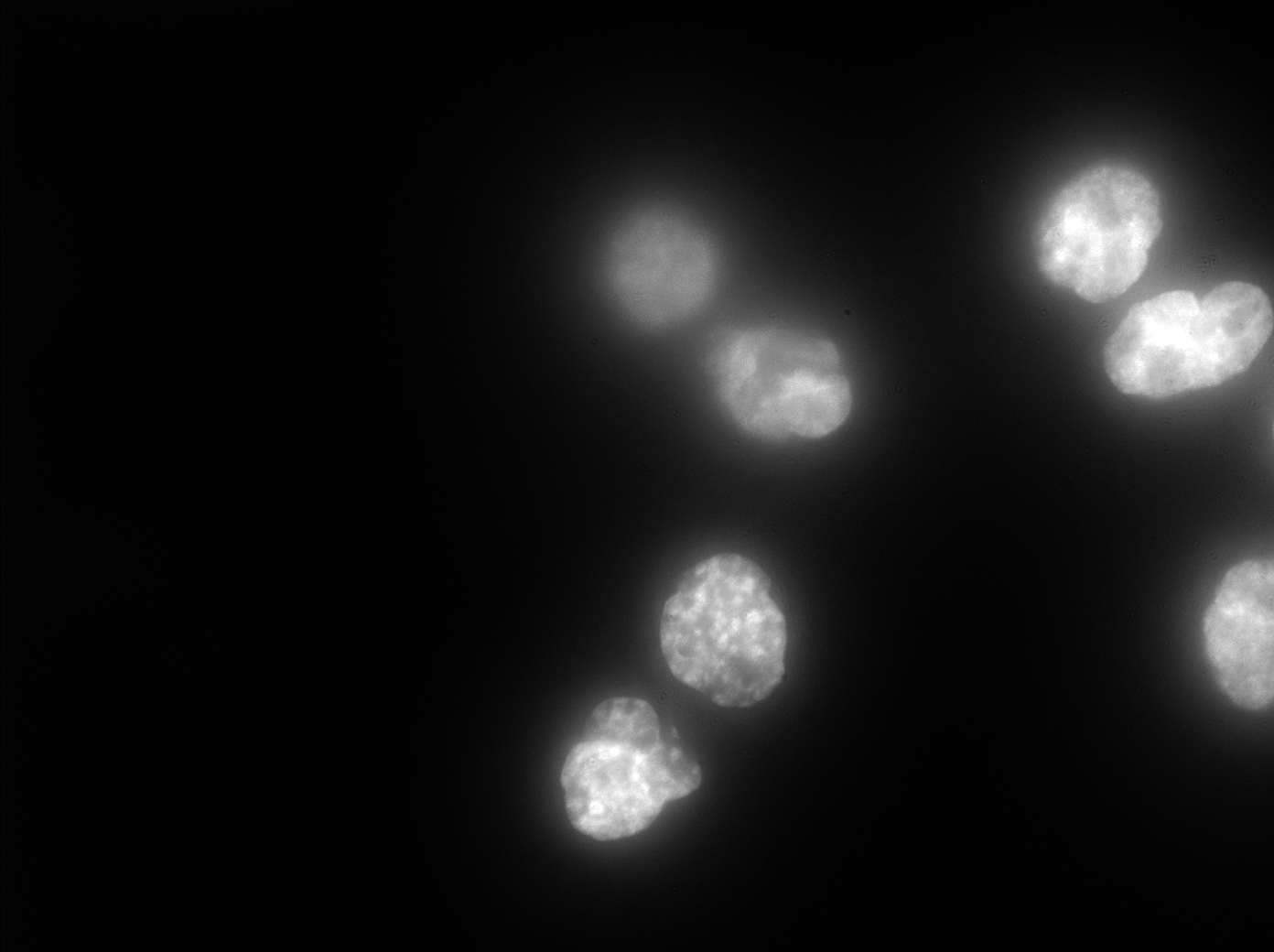

Supplement: Supplementary file 25 — Source data Fig. 7 part 1 [file 44319_2026_742_MOESM25_ESM.zip › Figure 7 Part 1/Fig 7acd Cas9 and TPGS1-ko rGT335 atubulin/Cas9 GT335recomb atub 3-24-25 R3 LT3.Project Maximum Z_XY1742849044_Z0_T0_C0.tif]

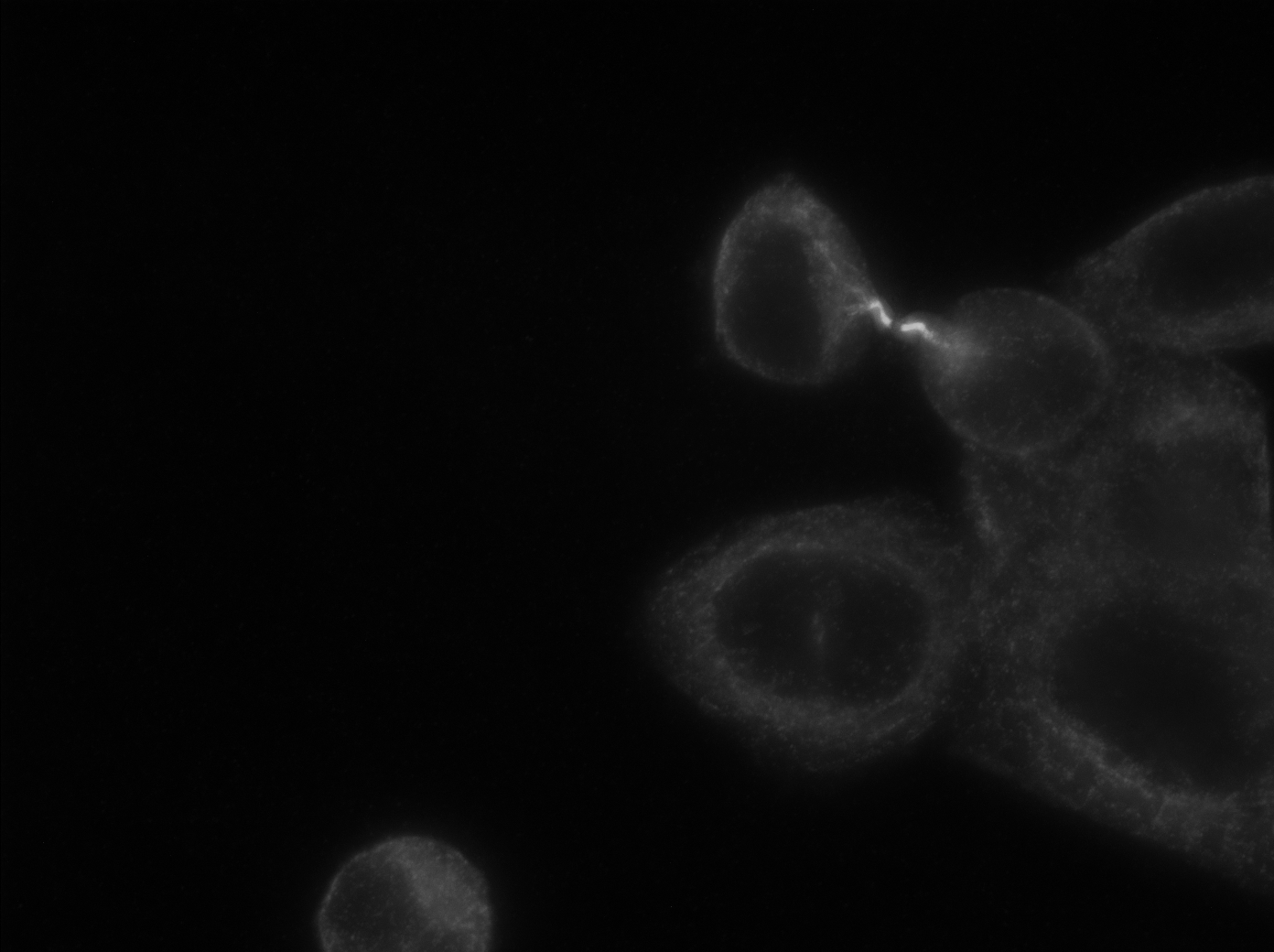

Supplement: Supplementary file 26 — Source data Fig. 7 part 2 [file 44319_2026_742_MOESM26_ESM.zip › Figure 7 Part 2/Fig 7acd Cas9 and TPGS1-ko rGT335 atubulin part 2/TPGS1-KO GT335recomb atub 3-24-25 R1 LT3.Project Maximum Z_XY1742839310_Z0_T0_C2.tif]

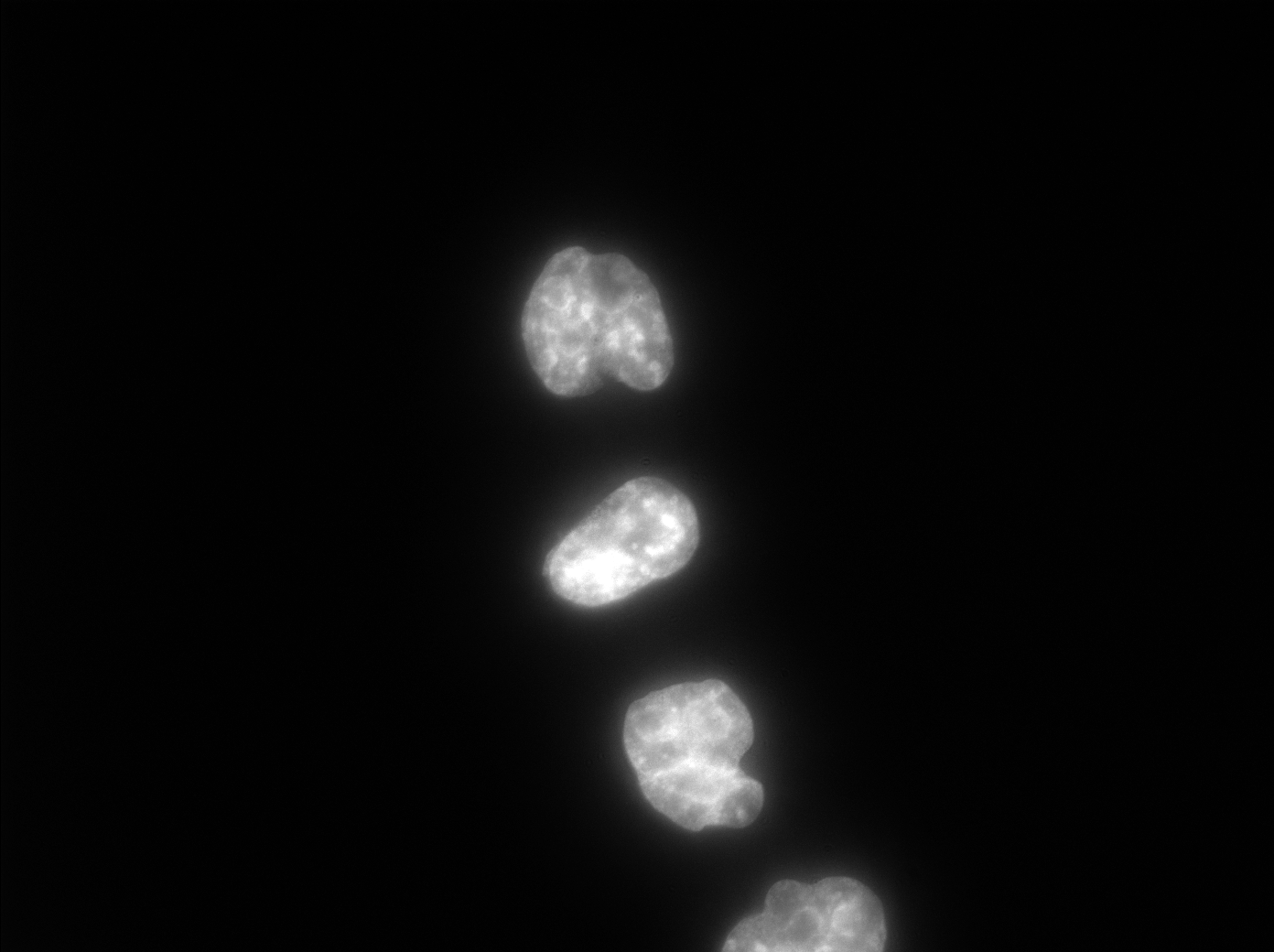

Supplement: Supplementary file 26 — Source data Fig. 7 part 2 [file 44319_2026_742_MOESM26_ESM.zip › Figure 7 Part 2/Fig 7acd Cas9 and TPGS1-ko rGT335 atubulin part 2/TPGS1-KO GT335recomb atub 3-24-25 R3 LT3.Project Maximum Z_XY1742852844_Z0_T0_C0.tif]

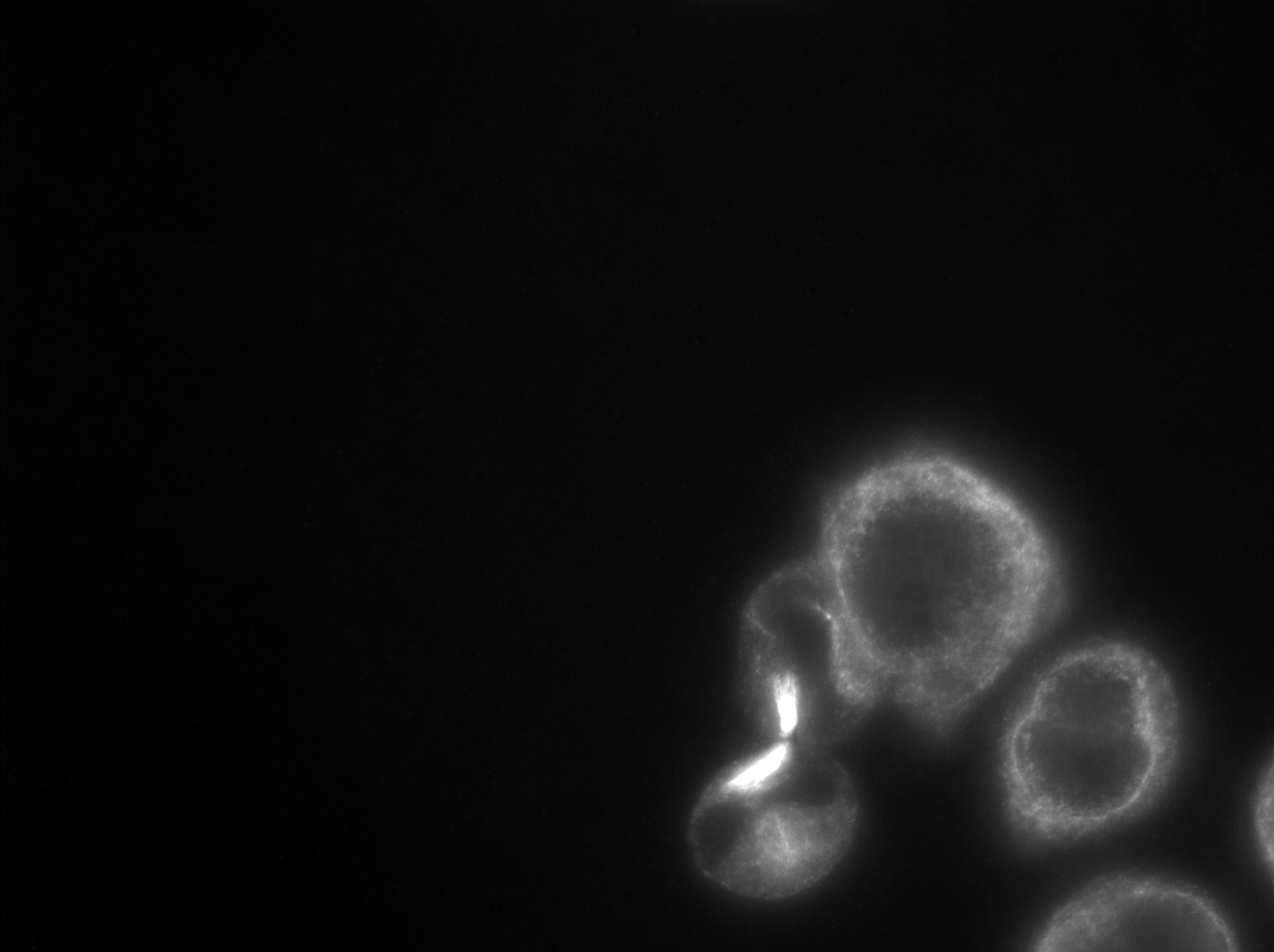

Supplement: Supplementary file 26 — Source data Fig. 7 part 2 [file 44319_2026_742_MOESM26_ESM.zip › Figure 7 Part 2/Fig 7acd Cas9 and TPGS1-ko rGT335 atubulin part 2/TPGS1-KO GT335recomb atub 3-24-25 R3 ET4.Project Maximum Z_XY1742852466_Z0_T0_C2.tif]

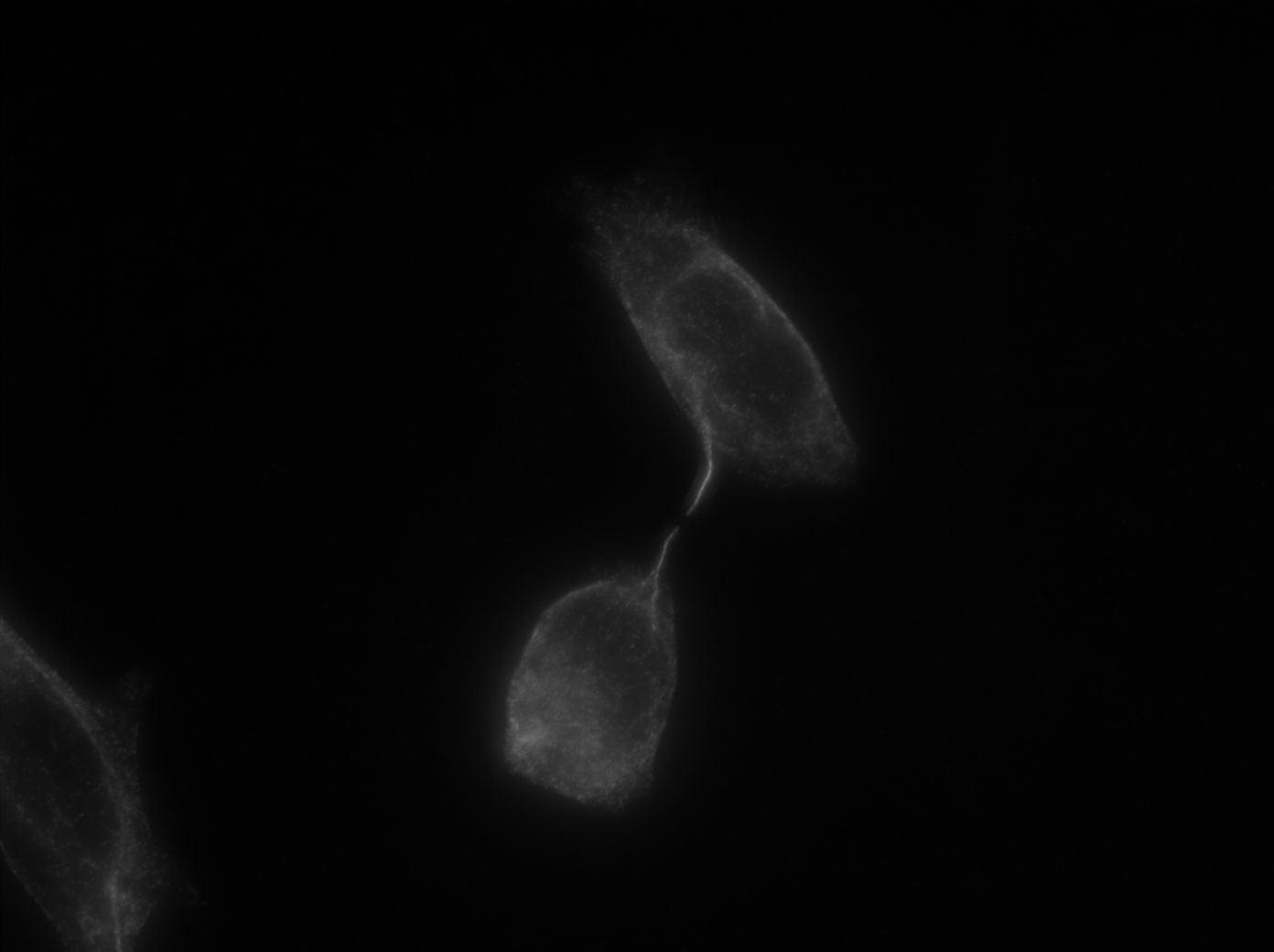

Supplement: Supplementary file 26 — Source data Fig. 7 part 2 [file 44319_2026_742_MOESM26_ESM.zip › Figure 7 Part 2/Fig 7acd Cas9 and TPGS1-ko rGT335 atubulin part 2/TPGS1-KO GT335recomb atub 3-24-25 R2 LT6.Project Maximum Z_XY1742842272_Z0_T0_C2.tif]

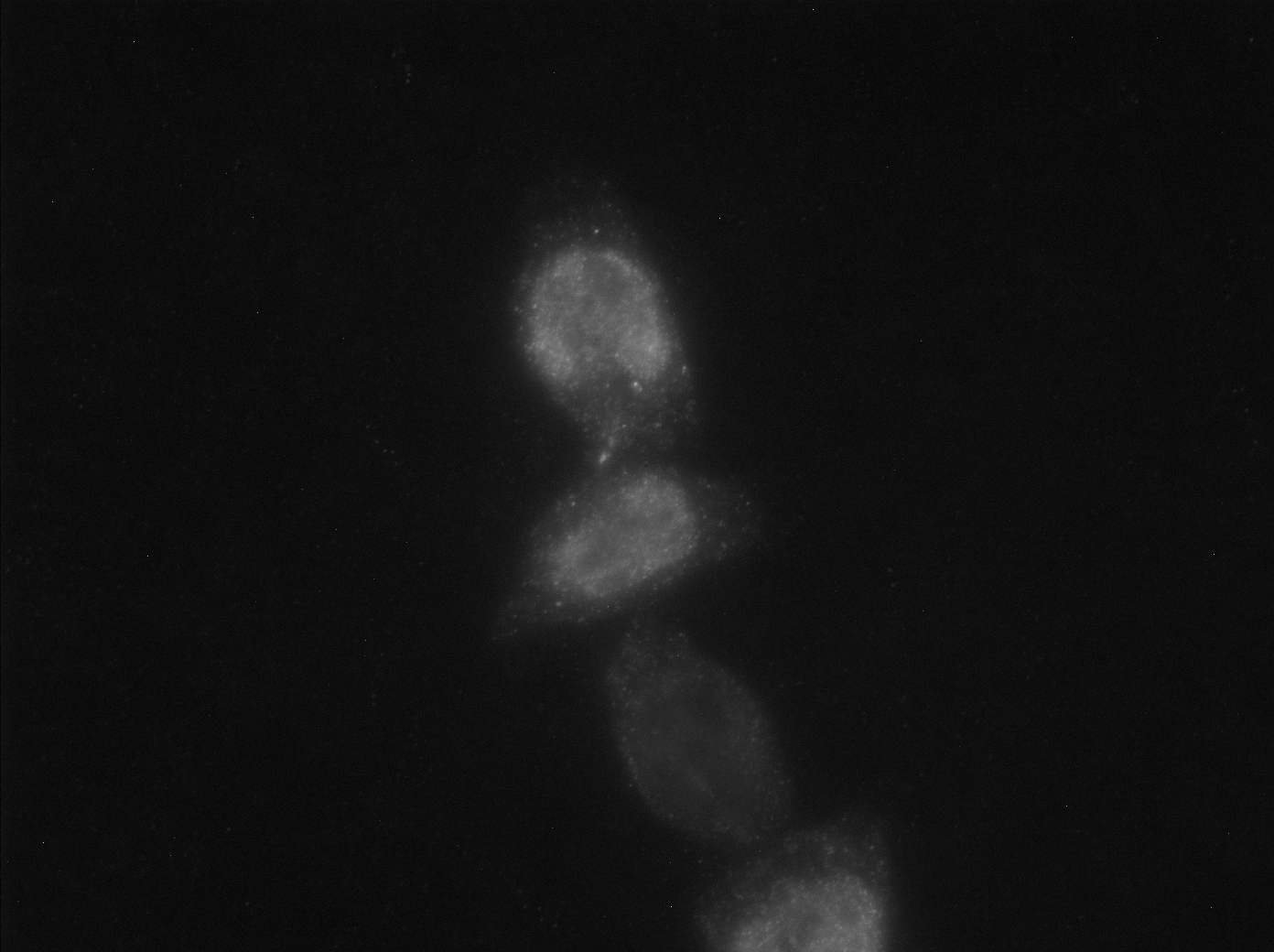

Supplement: Supplementary file 26 — Source data Fig. 7 part 2 [file 44319_2026_742_MOESM26_ESM.zip › Figure 7 Part 2/Fig 7acd Cas9 and TPGS1-ko rGT335 atubulin part 2/TPGS1-KO GT335recomb atub 3-24-25 R3 LT3.Project Maximum Z_XY1742852844_Z0_T0_C1.tif]

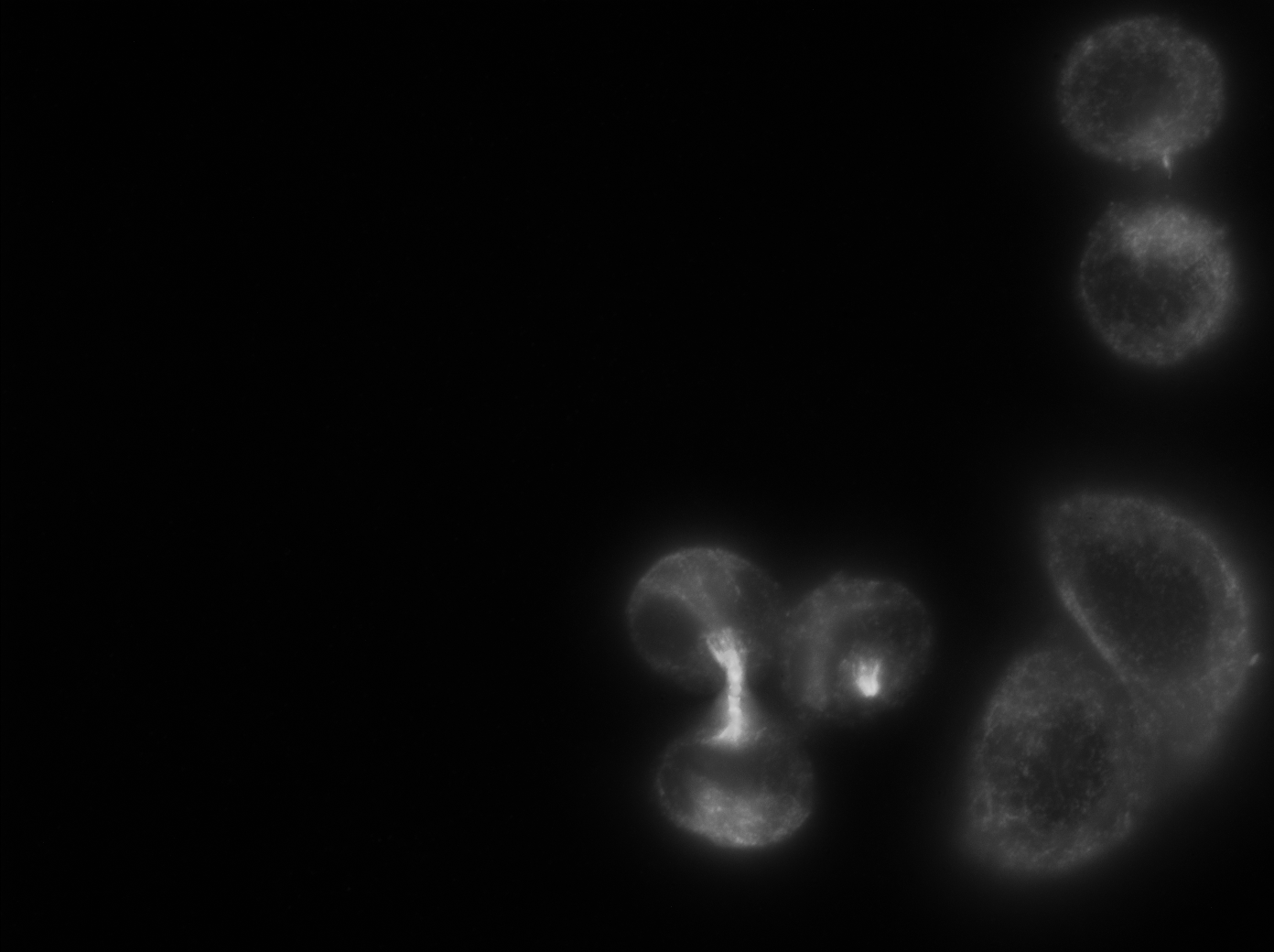

Supplement: Supplementary file 26 — Source data Fig. 7 part 2 [file 44319_2026_742_MOESM26_ESM.zip › Figure 7 Part 2/Fig 7acd Cas9 and TPGS1-ko rGT335 atubulin part 2/TPGS1-KO GT335recomb atub 3-24-25 R2 ET7.Project Maximum Z_XY1742841723_Z0_T0_C2.tif]

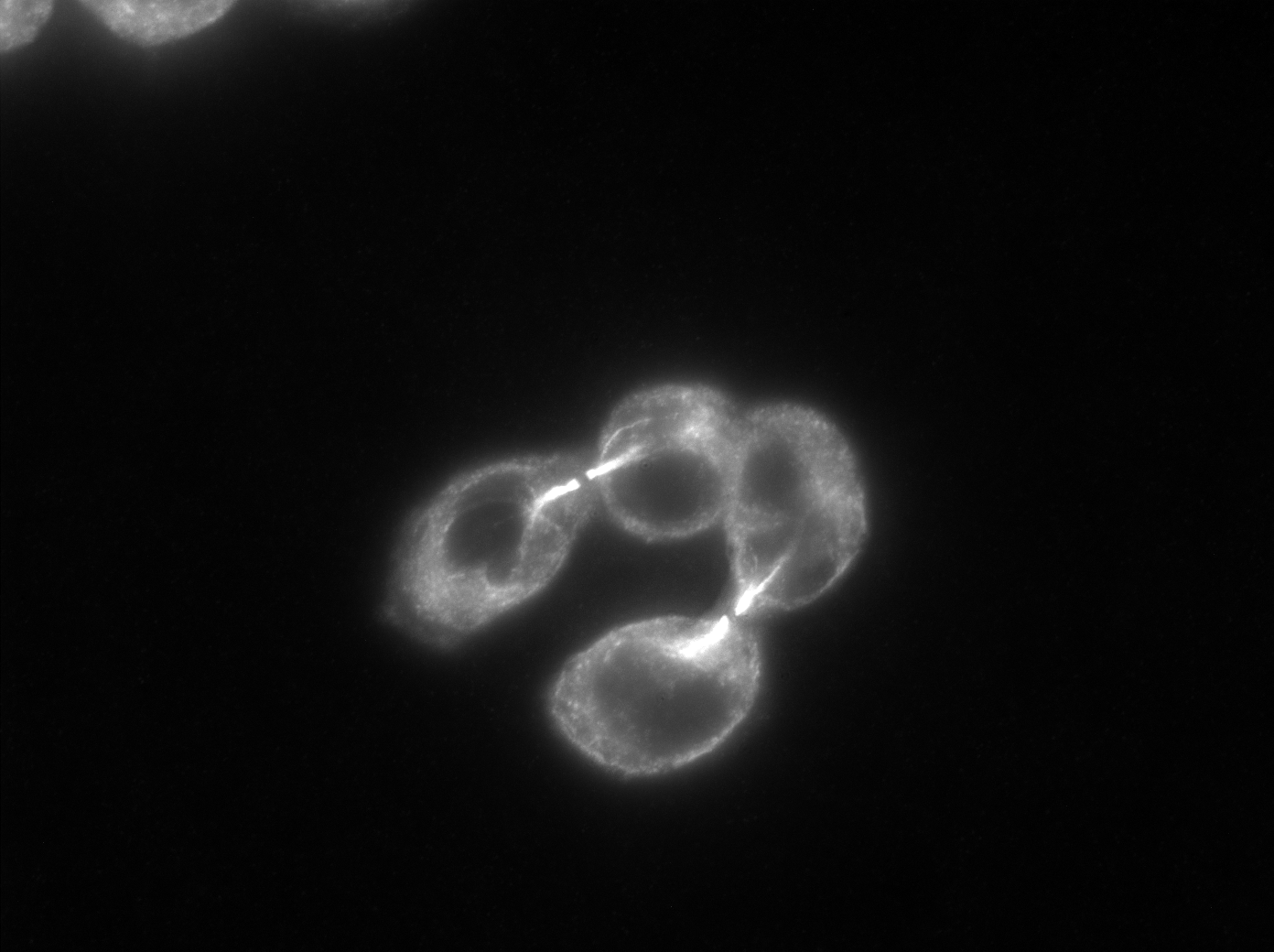

Supplement: Supplementary file 26 — Source data Fig. 7 part 2 [file 44319_2026_742_MOESM26_ESM.zip › Figure 7 Part 2/Fig 7acd Cas9 and TPGS1-ko rGT335 atubulin part 2/TPGS1-KO GT335recomb atub 3-24-25 R3 LT5LT6.Project Maximum Z_XY1742853153_Z0_T0_C2.tif]

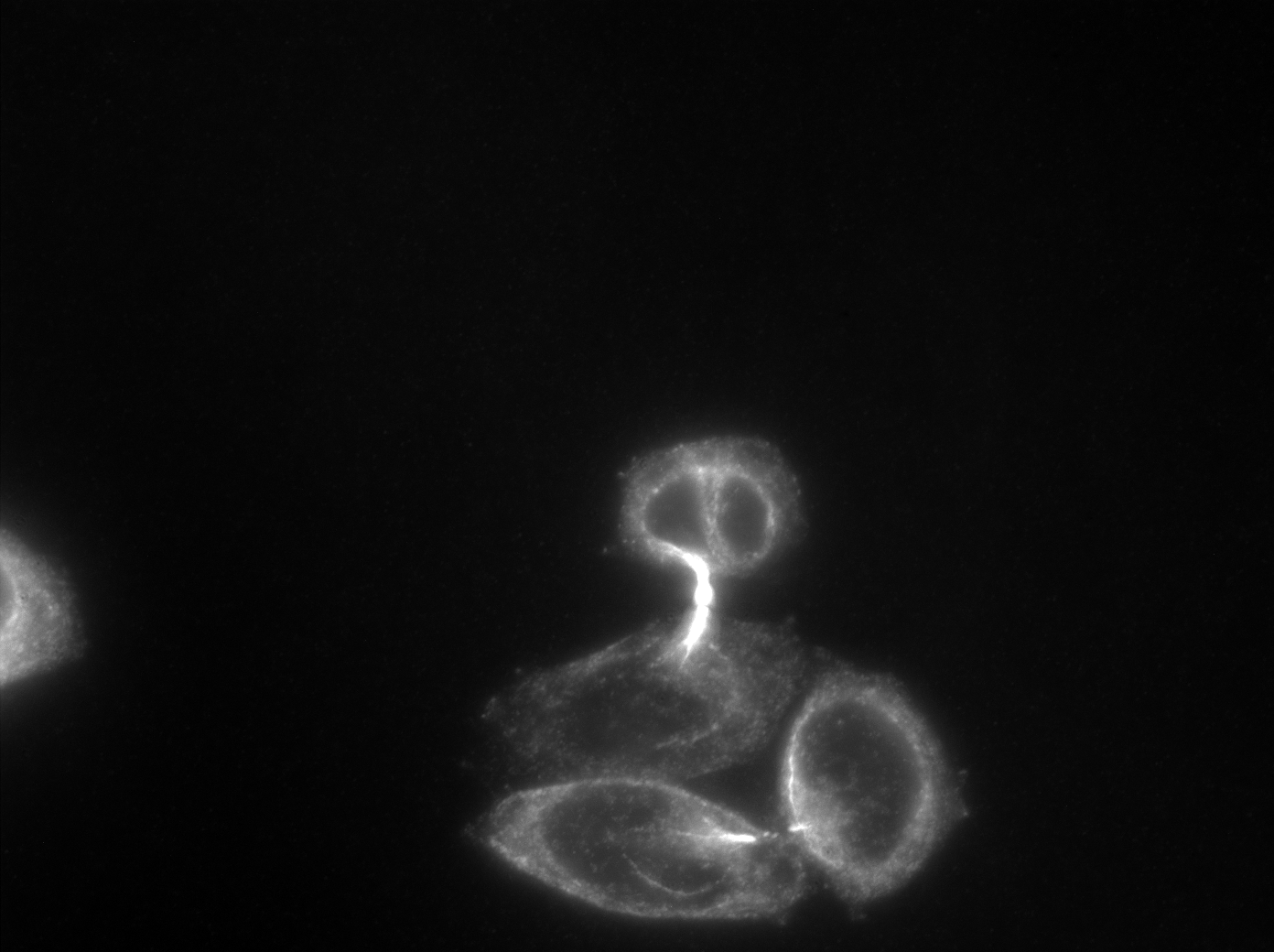

Supplement: Supplementary file 26 — Source data Fig. 7 part 2 [file 44319_2026_742_MOESM26_ESM.zip › Figure 7 Part 2/Fig 7acd Cas9 and TPGS1-ko rGT335 atubulin part 2/TPGS1-KO GT335recomb atub 3-24-25 R3 LT2.Project Maximum Z_XY1742851926_Z0_T0_C2.tif]

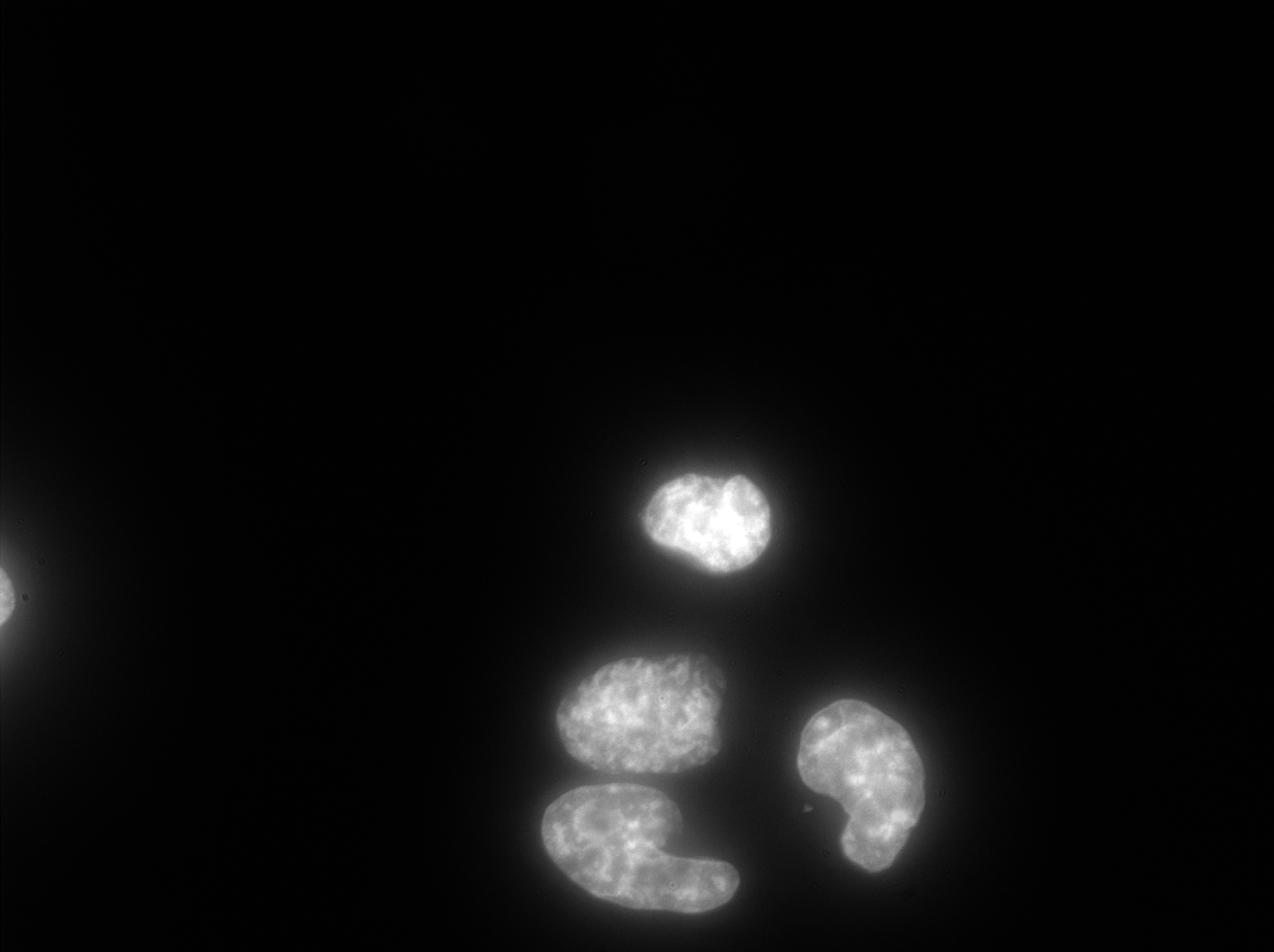

Supplement: Supplementary file 26 — Source data Fig. 7 part 2 [file 44319_2026_742_MOESM26_ESM.zip › Figure 7 Part 2/Fig 7acd Cas9 and TPGS1-ko rGT335 atubulin part 2/TPGS1-KO GT335recomb atub 3-24-25 R3 LT2.Project Maximum Z_XY1742851926_Z0_T0_C0.tif]

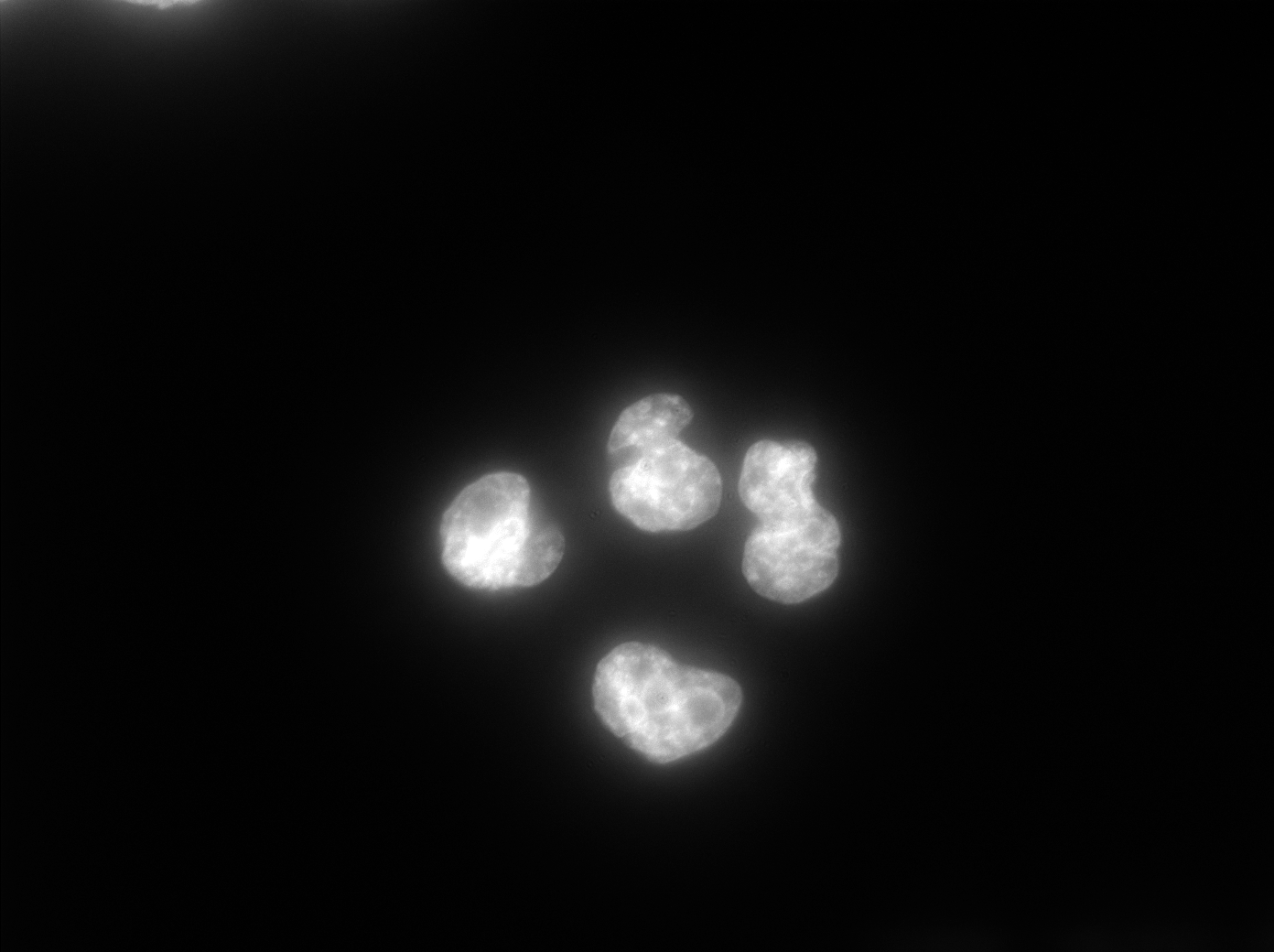

Supplement: Supplementary file 26 — Source data Fig. 7 part 2 [file 44319_2026_742_MOESM26_ESM.zip › Figure 7 Part 2/Fig 7acd Cas9 and TPGS1-ko rGT335 atubulin part 2/TPGS1-KO GT335recomb atub 3-24-25 R3 LT5LT6.Project Maximum Z_XY1742853153_Z0_T0_C0.tif]

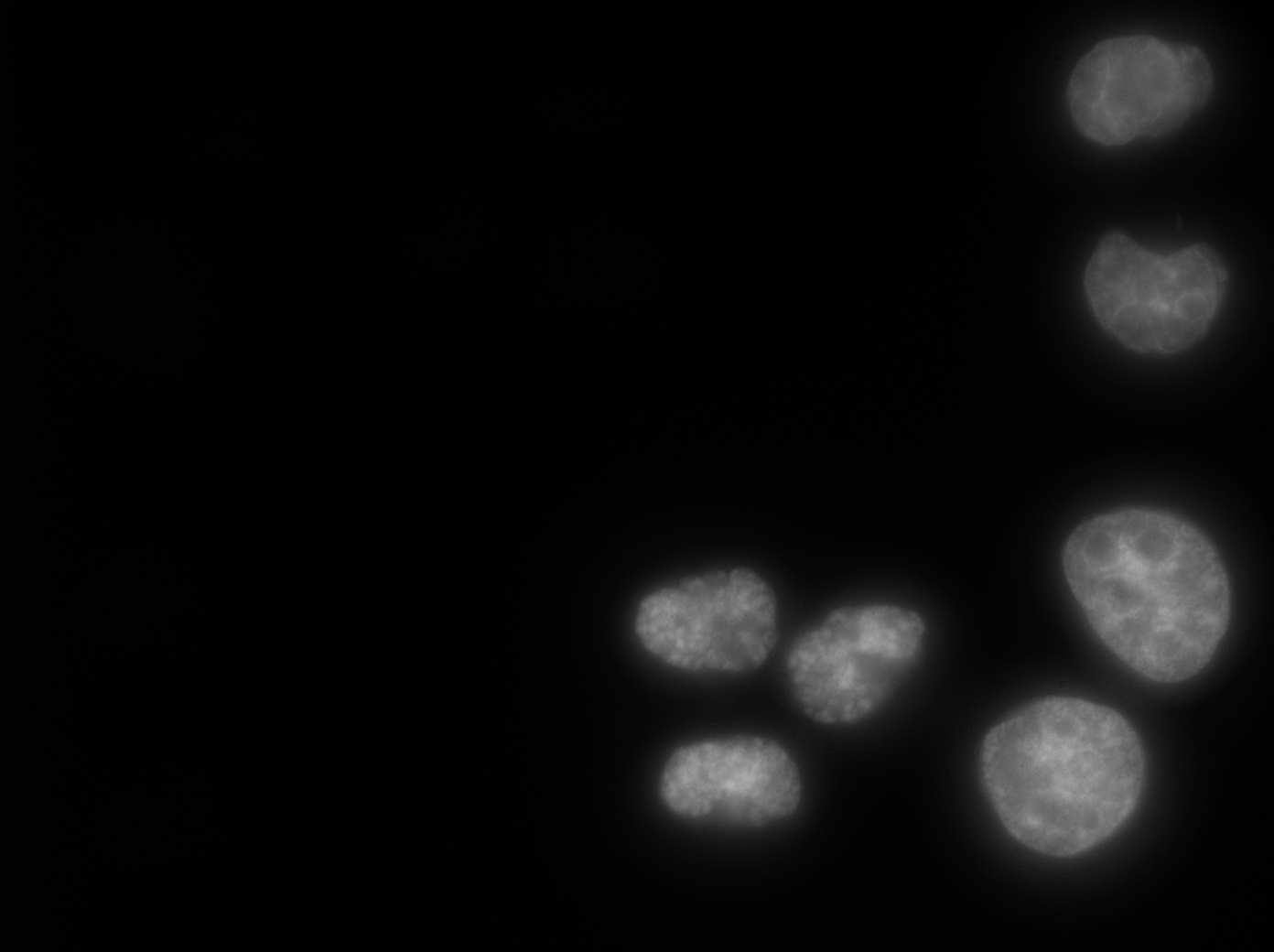

Supplement: Supplementary file 26 — Source data Fig. 7 part 2 [file 44319_2026_742_MOESM26_ESM.zip › Figure 7 Part 2/Fig 7acd Cas9 and TPGS1-ko rGT335 atubulin part 2/TPGS1-KO GT335recomb atub 3-24-25 R2 ET7.Project Maximum Z_XY1742841723_Z0_T0_C0.tif]

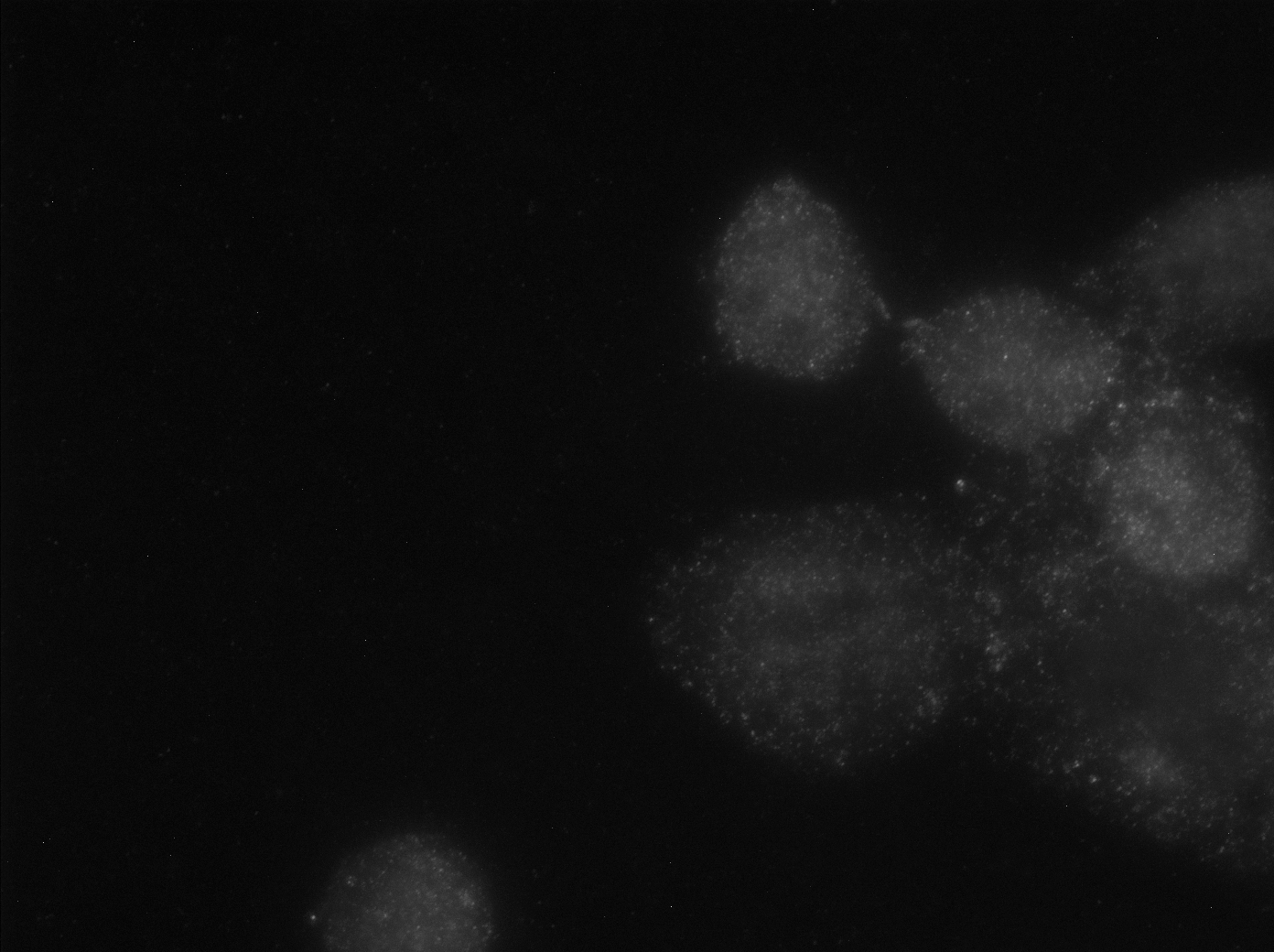

Supplement: Supplementary file 26 — Source data Fig. 7 part 2 [file 44319_2026_742_MOESM26_ESM.zip › Figure 7 Part 2/Fig 7acd Cas9 and TPGS1-ko rGT335 atubulin part 2/TPGS1-KO GT335recomb atub 3-24-25 R1 LT3.Project Maximum Z_XY1742839310_Z0_T0_C1.tif]

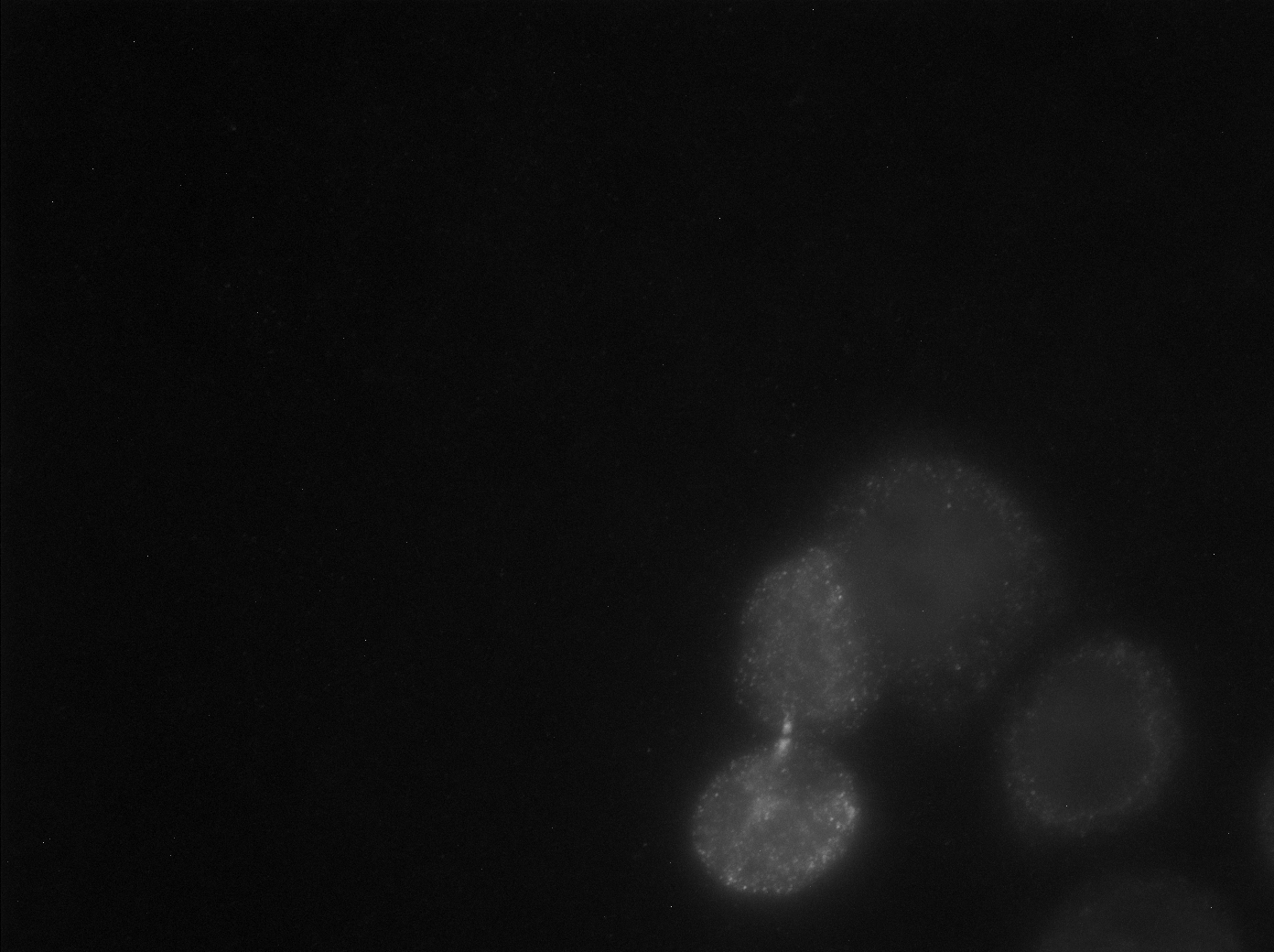

Supplement: Supplementary file 26 — Source data Fig. 7 part 2 [file 44319_2026_742_MOESM26_ESM.zip › Figure 7 Part 2/Fig 7acd Cas9 and TPGS1-ko rGT335 atubulin part 2/TPGS1-KO GT335recomb atub 3-24-25 R3 ET4.Project Maximum Z_XY1742852466_Z0_T0_C1.tif]

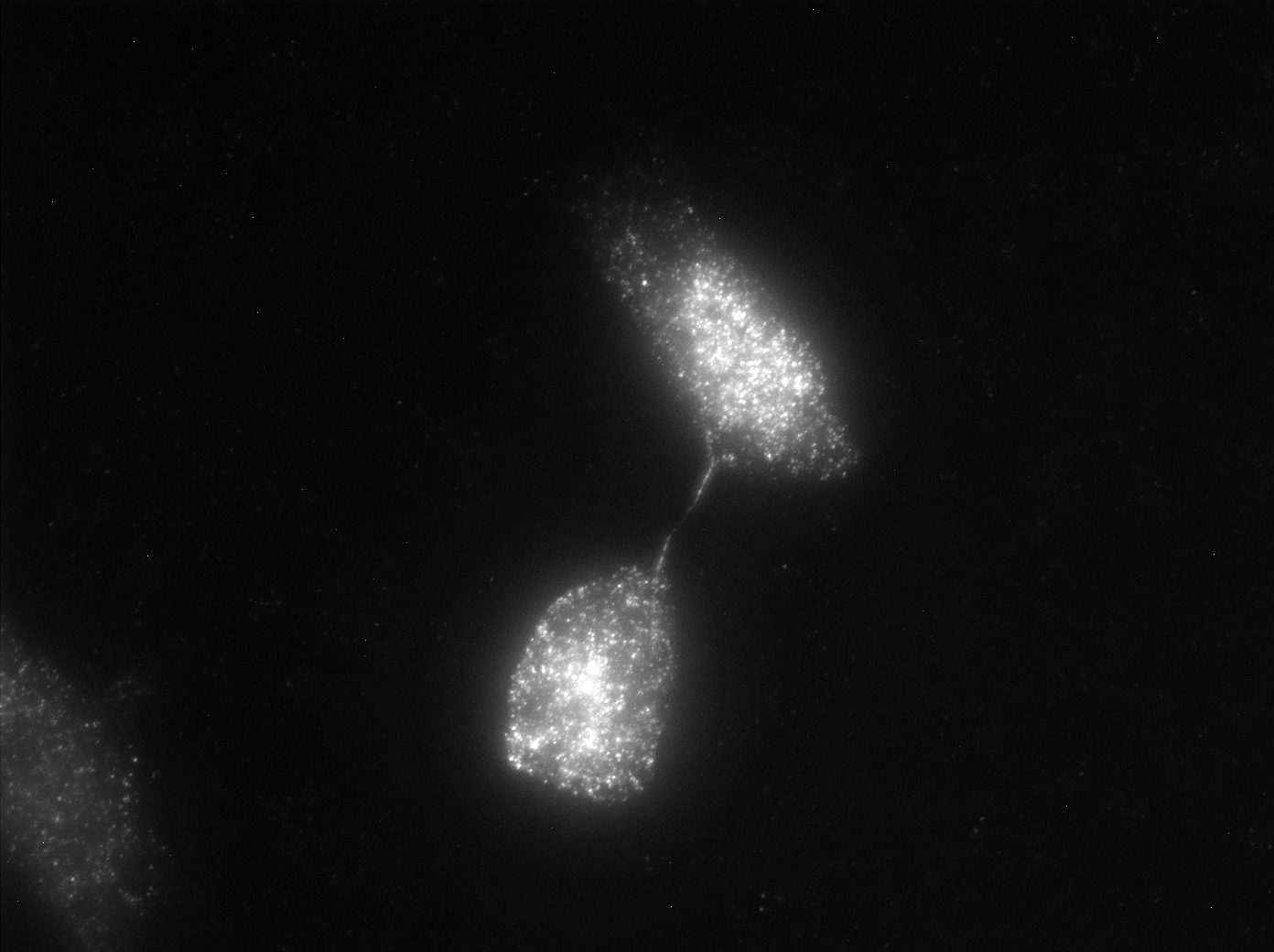

Supplement: Supplementary file 26 — Source data Fig. 7 part 2 [file 44319_2026_742_MOESM26_ESM.zip › Figure 7 Part 2/Fig 7acd Cas9 and TPGS1-ko rGT335 atubulin part 2/TPGS1-KO GT335recomb atub 3-24-25 R2 LT6.Project Maximum Z_XY1742842272_Z0_T0_C1.tif]

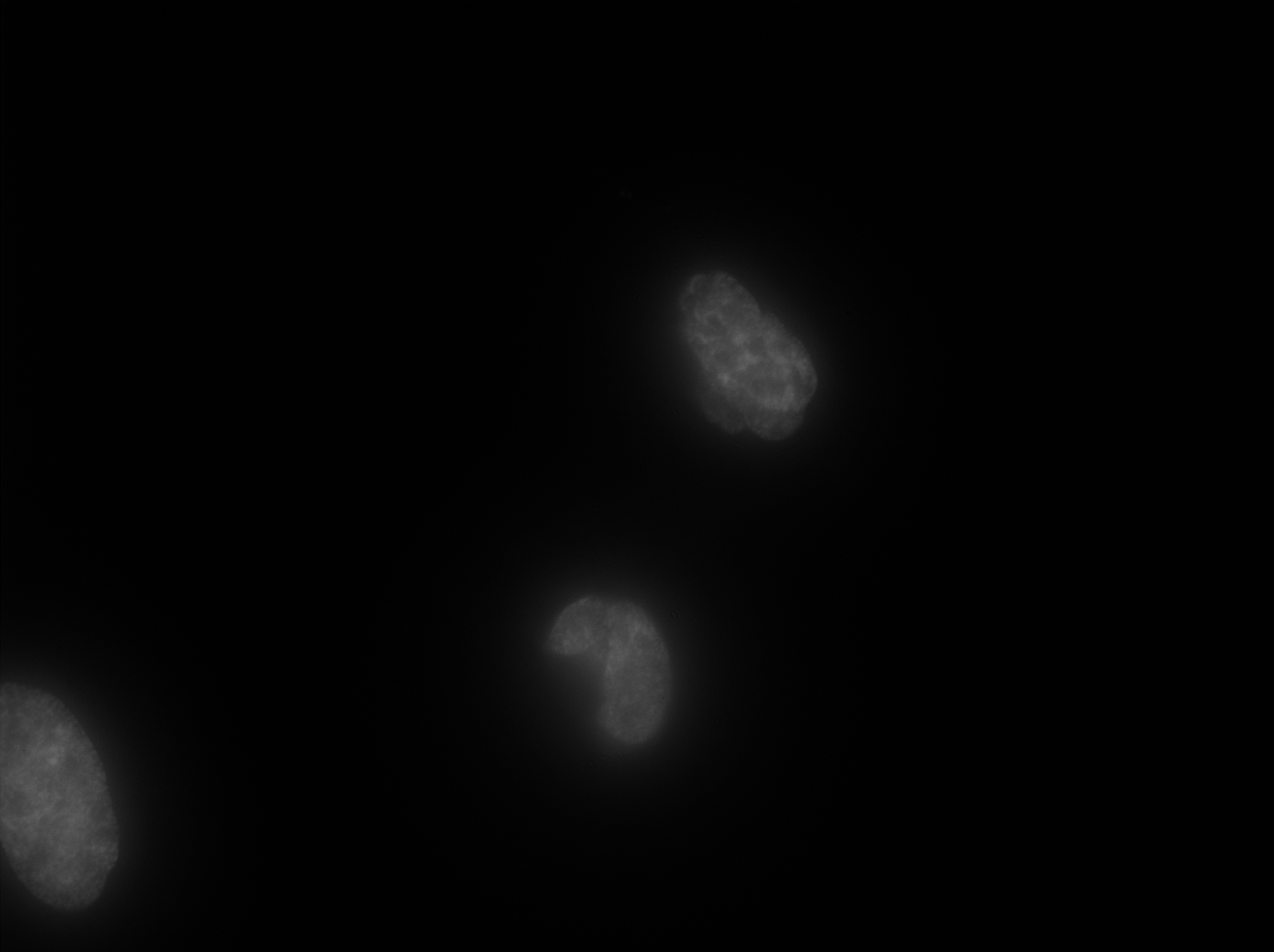

Supplement: Supplementary file 26 — Source data Fig. 7 part 2 [file 44319_2026_742_MOESM26_ESM.zip › Figure 7 Part 2/Fig 7acd Cas9 and TPGS1-ko rGT335 atubulin part 2/TPGS1-KO GT335recomb atub 3-24-25 R2 LT6.Project Maximum Z_XY1742842272_Z0_T0_C0.tif]

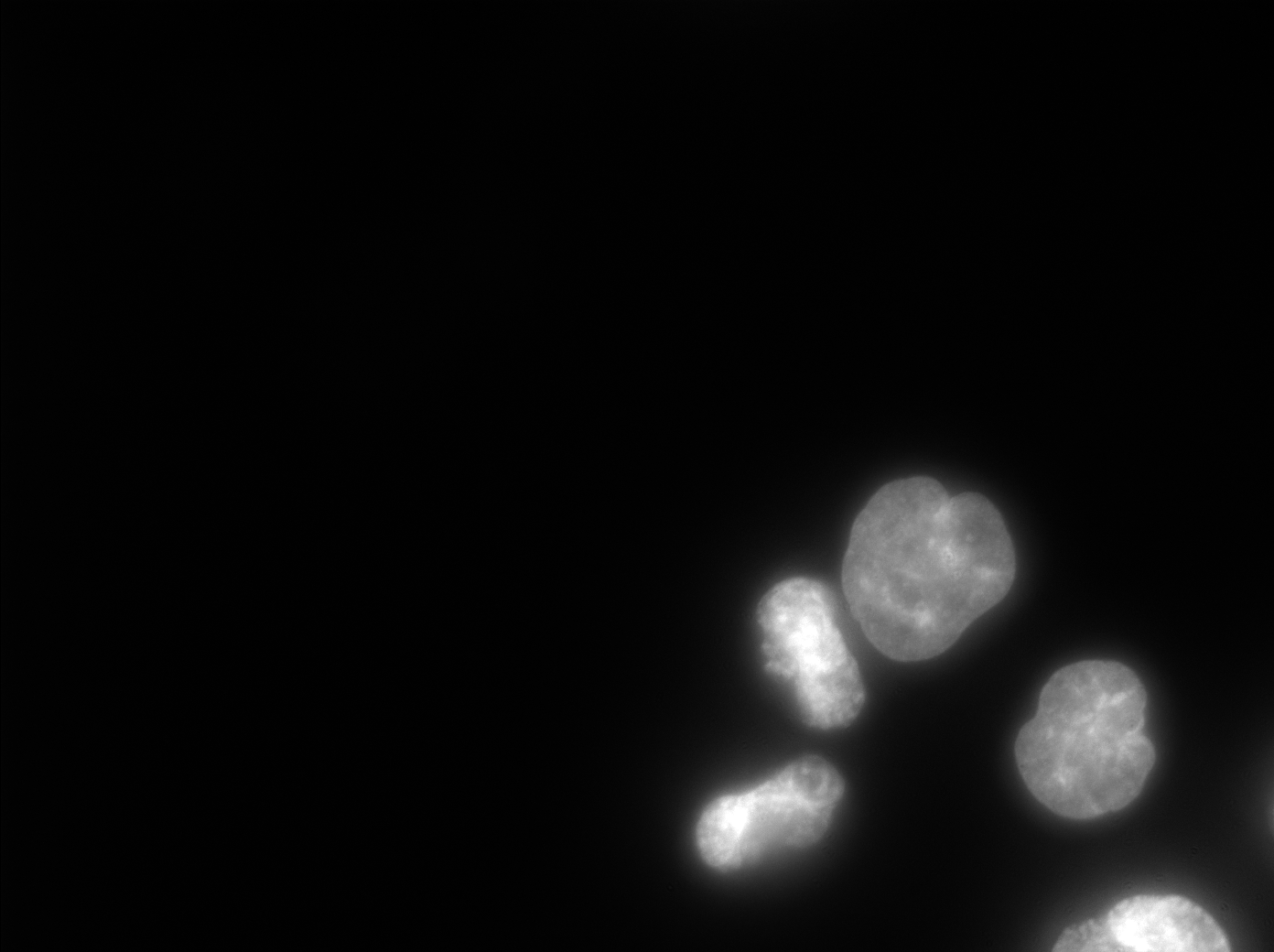

Supplement: Supplementary file 26 — Source data Fig. 7 part 2 [file 44319_2026_742_MOESM26_ESM.zip › Figure 7 Part 2/Fig 7acd Cas9 and TPGS1-ko rGT335 atubulin part 2/TPGS1-KO GT335recomb atub 3-24-25 R3 ET4.Project Maximum Z_XY1742852466_Z0_T0_C0.tif]

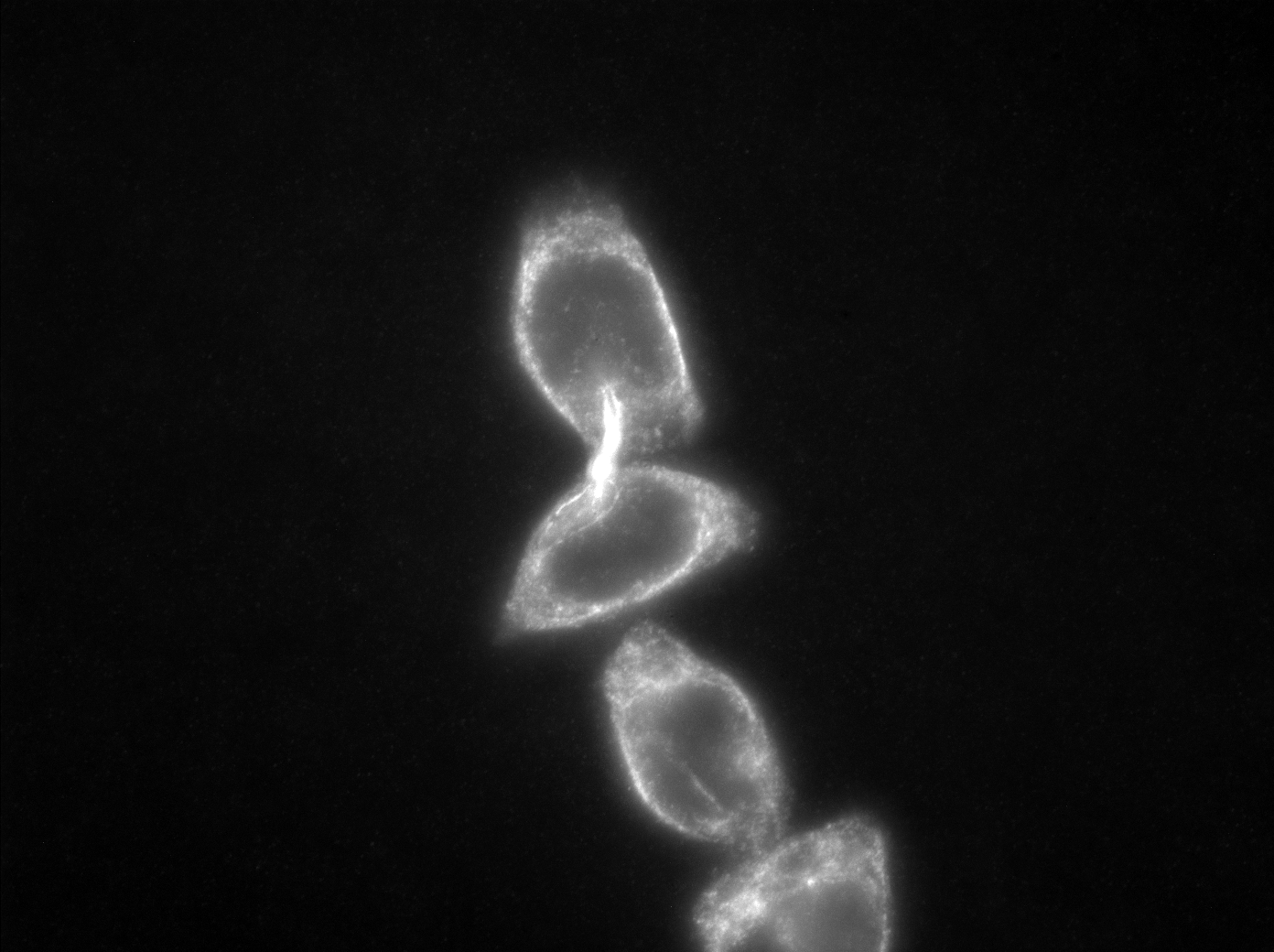

Supplement: Supplementary file 26 — Source data Fig. 7 part 2 [file 44319_2026_742_MOESM26_ESM.zip › Figure 7 Part 2/Fig 7acd Cas9 and TPGS1-ko rGT335 atubulin part 2/TPGS1-KO GT335recomb atub 3-24-25 R3 LT3.Project Maximum Z_XY1742852844_Z0_T0_C2.tif]

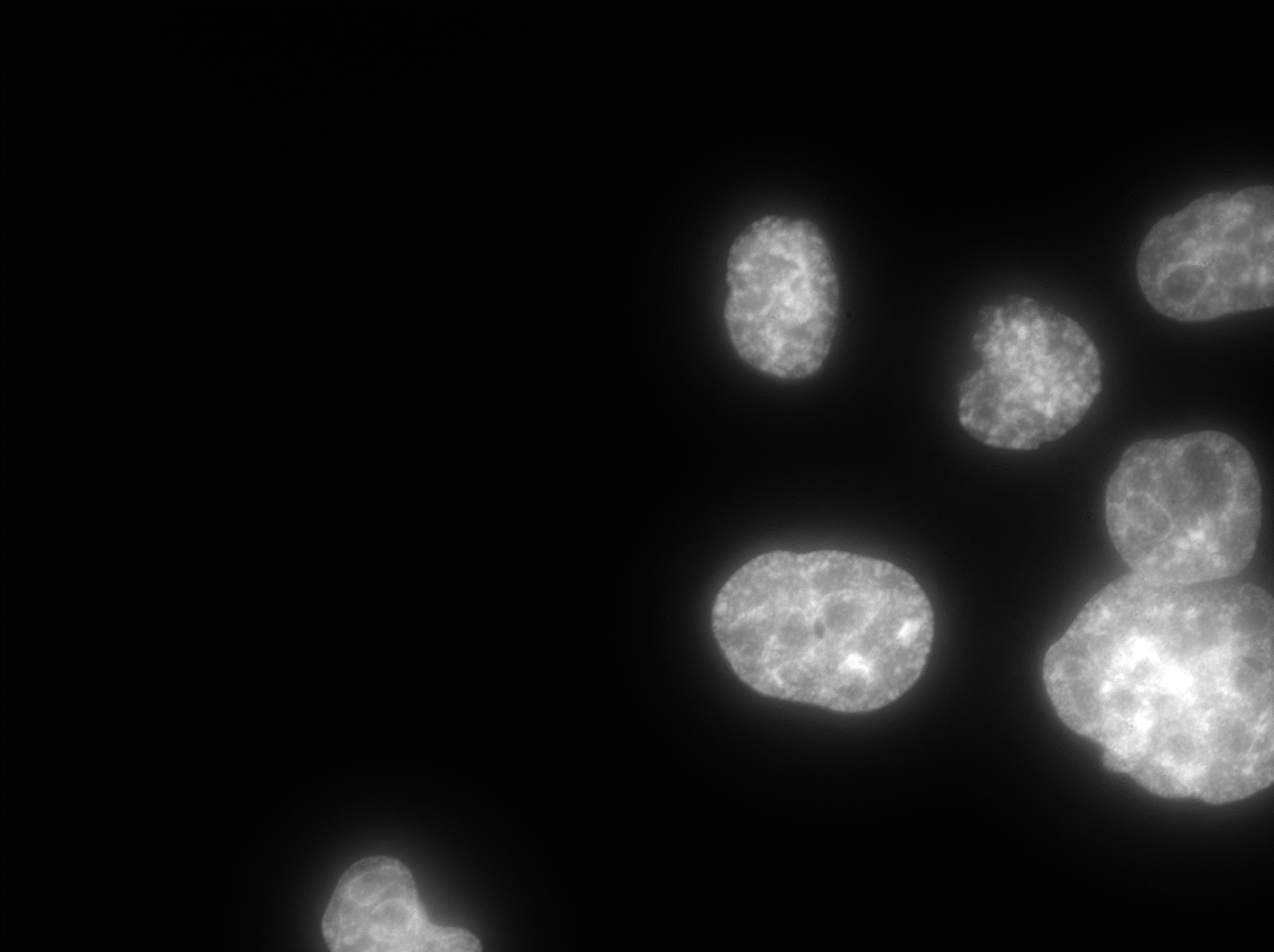

Supplement: Supplementary file 26 — Source data Fig. 7 part 2 [file 44319_2026_742_MOESM26_ESM.zip › Figure 7 Part 2/Fig 7acd Cas9 and TPGS1-ko rGT335 atubulin part 2/TPGS1-KO GT335recomb atub 3-24-25 R1 LT3.Project Maximum Z_XY1742839310_Z0_T0_C0.tif]

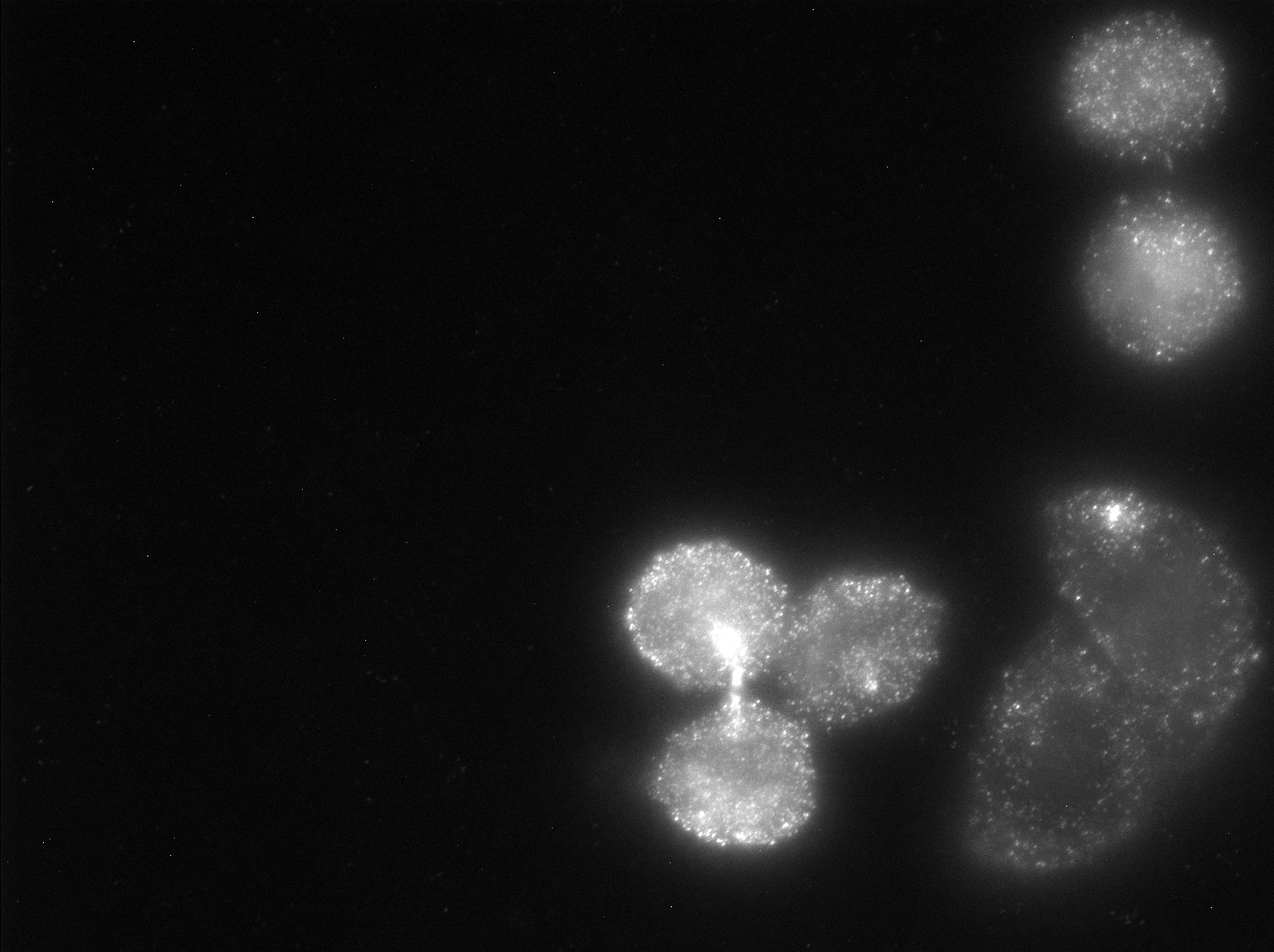

Supplement: Supplementary file 26 — Source data Fig. 7 part 2 [file 44319_2026_742_MOESM26_ESM.zip › Figure 7 Part 2/Fig 7acd Cas9 and TPGS1-ko rGT335 atubulin part 2/TPGS1-KO GT335recomb atub 3-24-25 R2 ET7.Project Maximum Z_XY1742841723_Z0_T0_C1.tif]

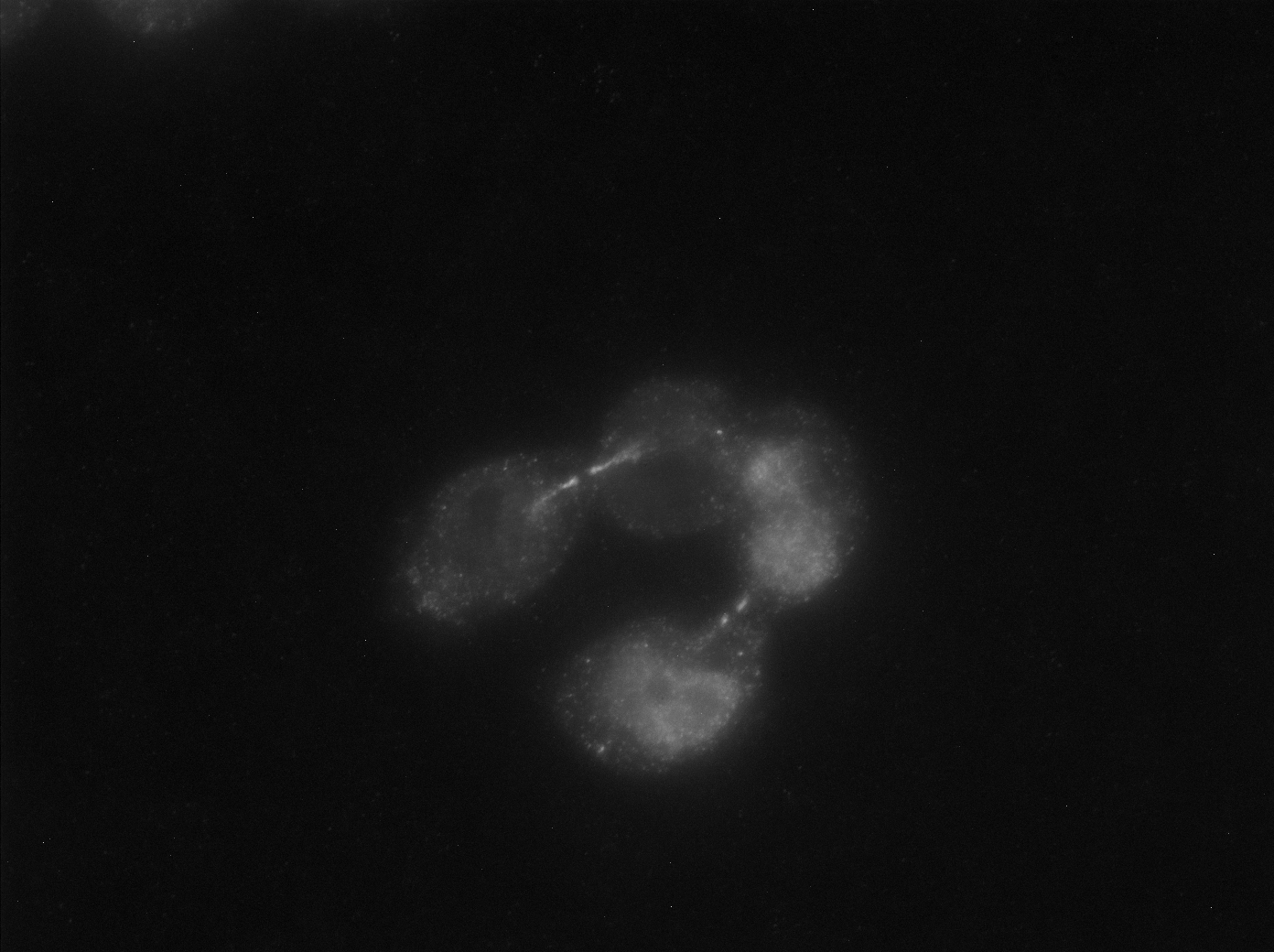

Supplement: Supplementary file 26 — Source data Fig. 7 part 2 [file 44319_2026_742_MOESM26_ESM.zip › Figure 7 Part 2/Fig 7acd Cas9 and TPGS1-ko rGT335 atubulin part 2/TPGS1-KO GT335recomb atub 3-24-25 R3 LT5LT6.Project Maximum Z_XY1742853153_Z0_T0_C1.tif]

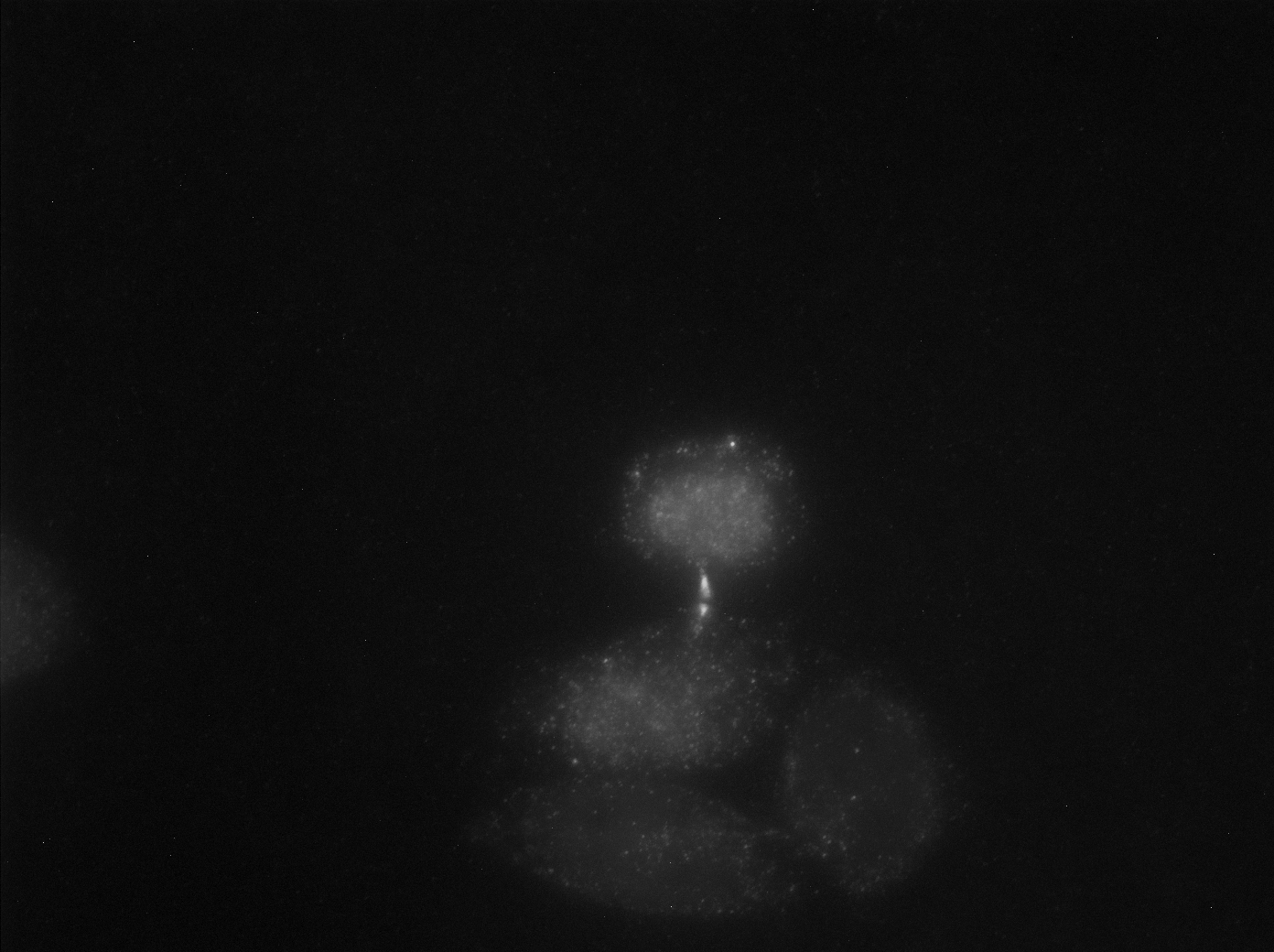

Supplement: Supplementary file 26 — Source data Fig. 7 part 2 [file 44319_2026_742_MOESM26_ESM.zip › Figure 7 Part 2/Fig 7acd Cas9 and TPGS1-ko rGT335 atubulin part 2/TPGS1-KO GT335recomb atub 3-24-25 R3 LT2.Project Maximum Z_XY1742851926_Z0_T0_C1.tif]

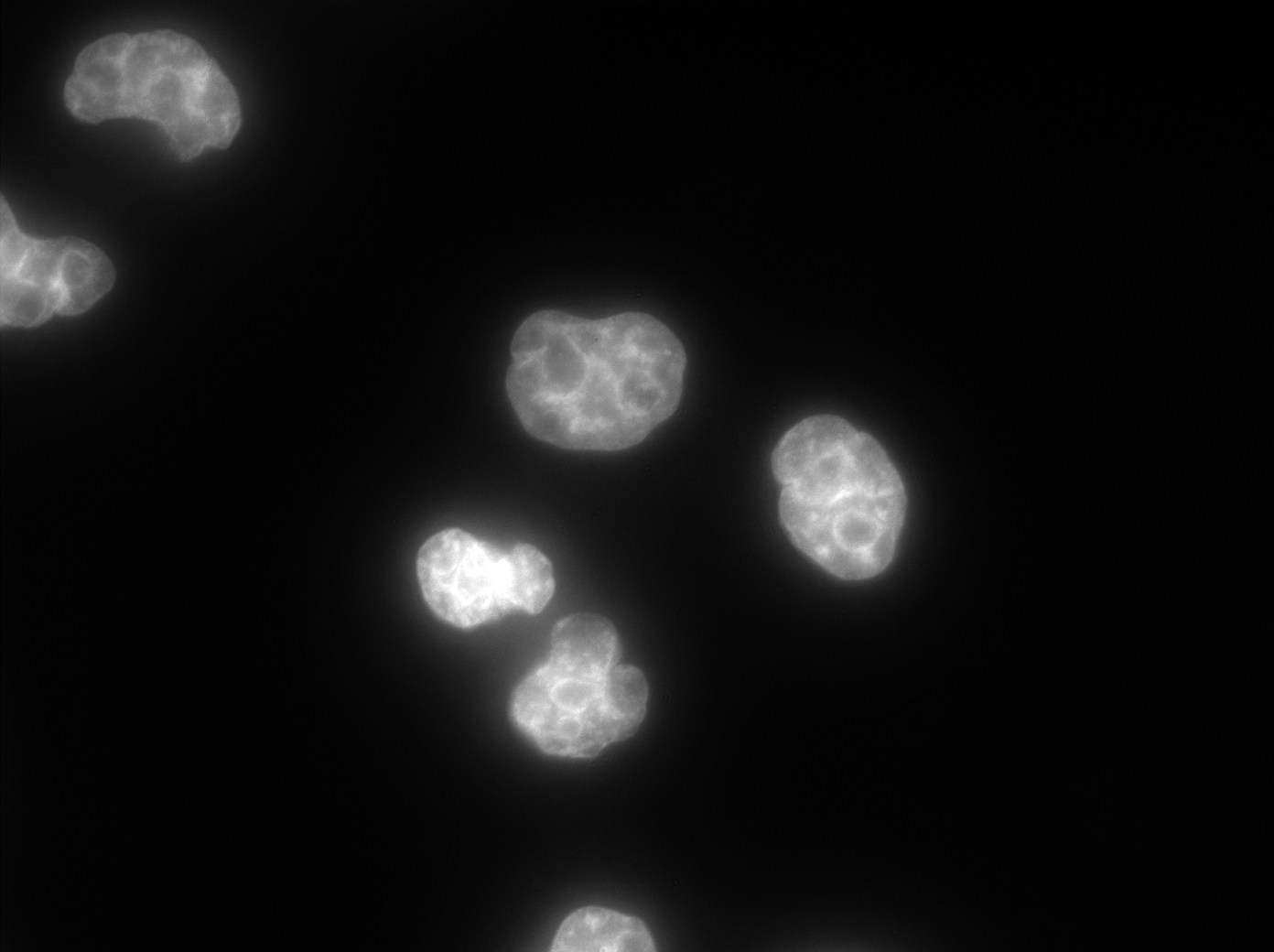

Supplement: Supplementary file 26 — Source data Fig. 7 part 2 [file 44319_2026_742_MOESM26_ESM.zip › Figure 7 Part 2/Fig 7acd Cas9 and TPGS1-ko rGT335 atubulin part 2/TPGS1-KO GT335recomb atub 3-24-25 R3 ET10.Project Maximum Z_XY1742853950_Z0_T0_C0.tif]

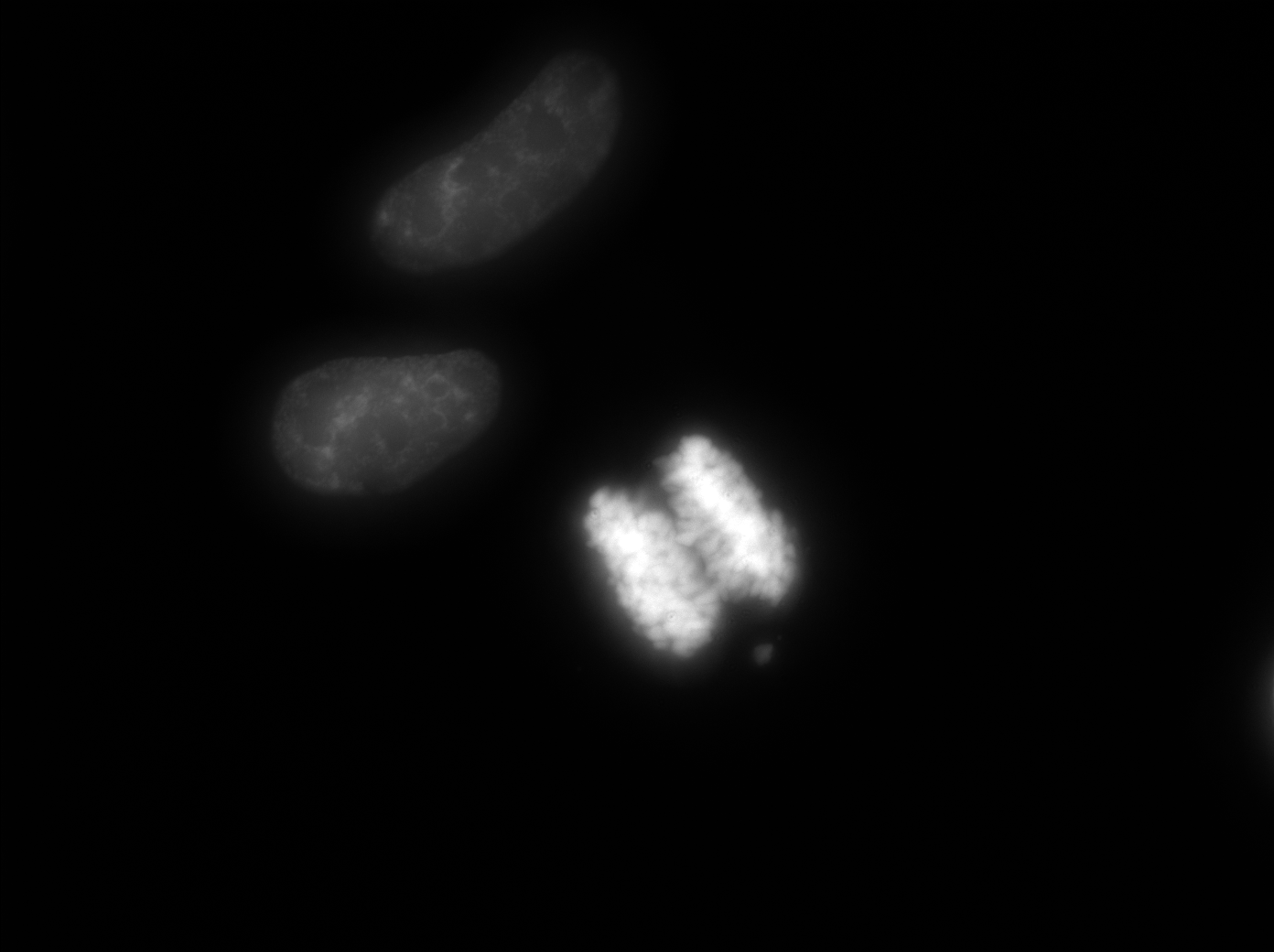

Supplement: Supplementary file 26 — Source data Fig. 7 part 2 [file 44319_2026_742_MOESM26_ESM.zip › Figure 7 Part 2/Fig 7acd Cas9 and TPGS1-ko rGT335 atubulin part 2/TPGS1-KO GT335recomb atub 3-24-25 R1 A1.Project Maximum Z_XY1742838798_Z0_T0_C0.tif]

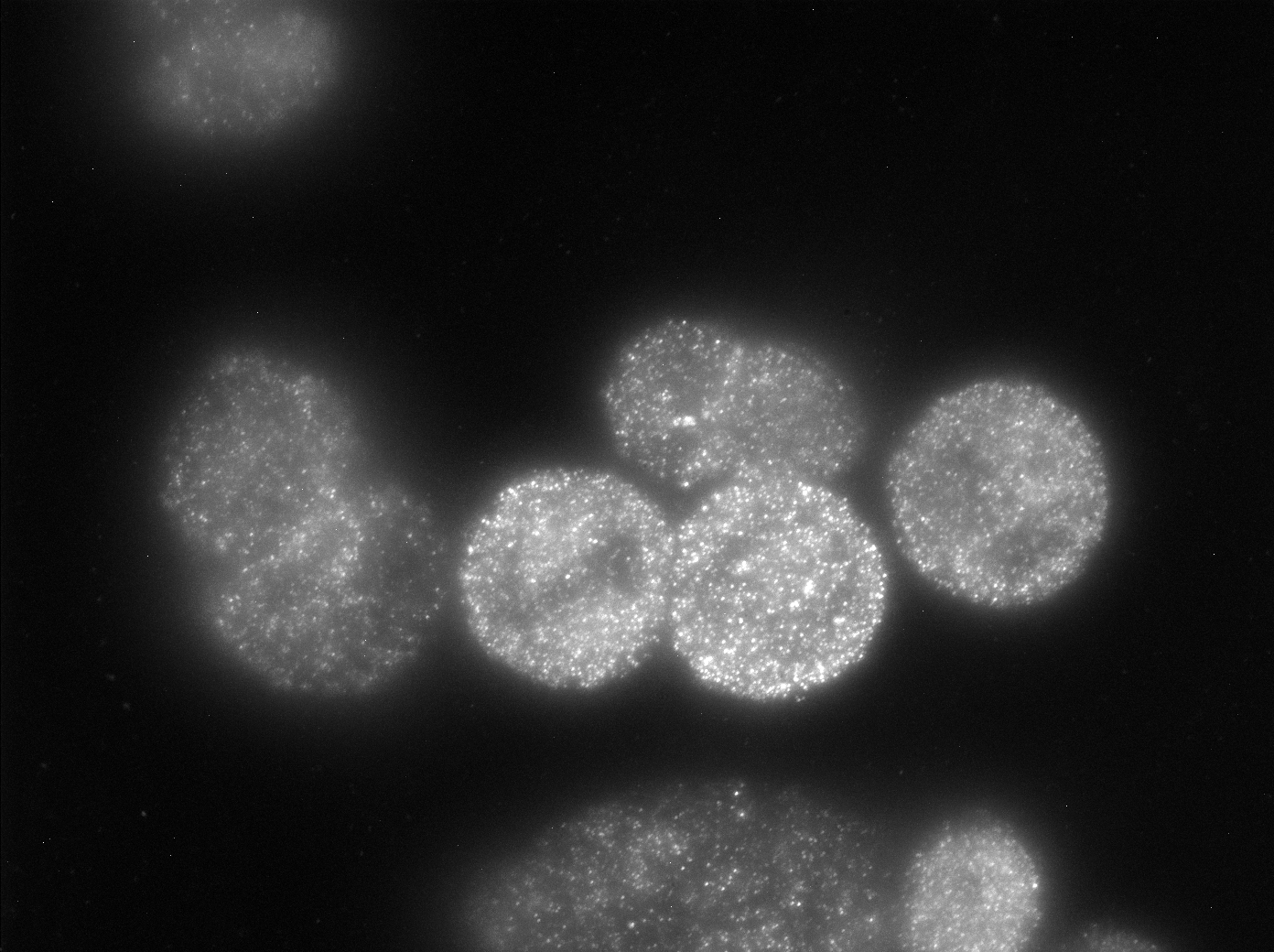

Supplement: Supplementary file 26 — Source data Fig. 7 part 2 [file 44319_2026_742_MOESM26_ESM.zip › Figure 7 Part 2/Fig 7acd Cas9 and TPGS1-ko rGT335 atubulin part 2/TPGS1-KO GT335recomb atub 3-24-25 R2 ET2 M1M2.Project Maximum Z_XY1742841221_Z0_T0_C1.tif]

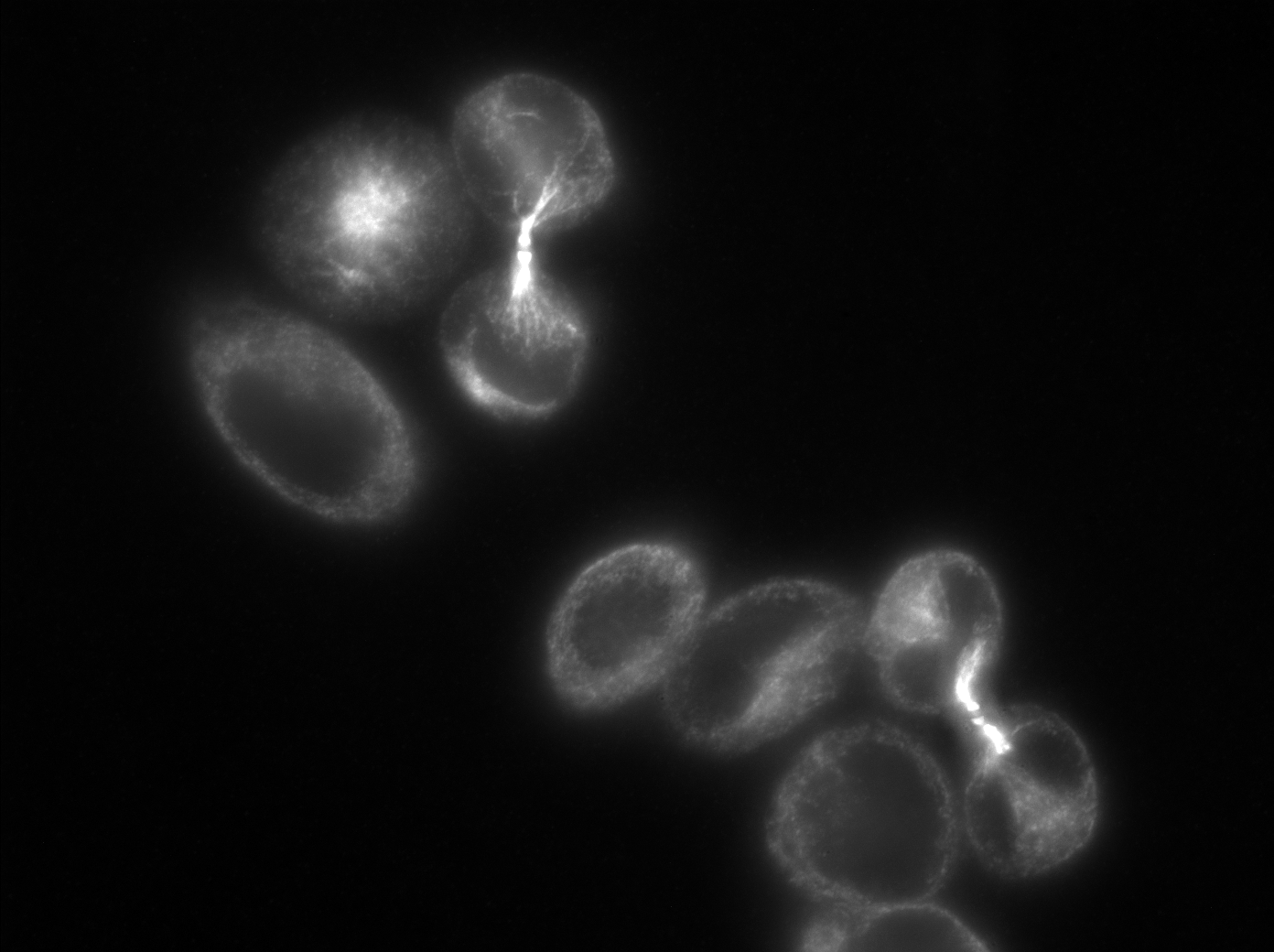

Supplement: Supplementary file 26 — Source data Fig. 7 part 2 [file 44319_2026_742_MOESM26_ESM.zip › Figure 7 Part 2/Fig 7acd Cas9 and TPGS1-ko rGT335 atubulin part 2/TPGS1-KO GT335recomb atub 3-24-25 R3 ET1ET2.Project Maximum Z_XY1742851506_Z0_T0_C2.tif]

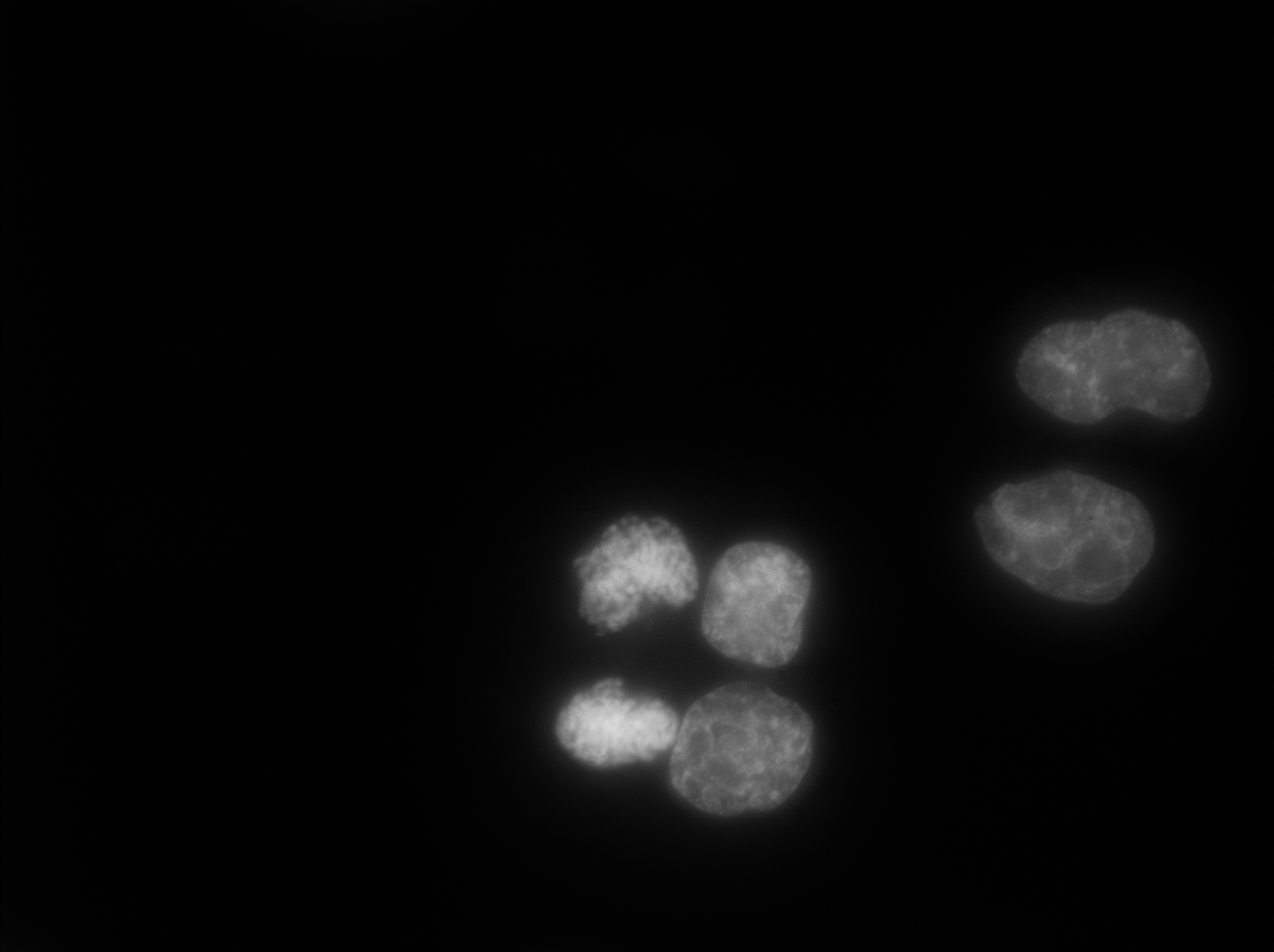

Supplement: Supplementary file 26 — Source data Fig. 7 part 2 [file 44319_2026_742_MOESM26_ESM.zip › Figure 7 Part 2/Fig 7acd Cas9 and TPGS1-ko rGT335 atubulin part 2/TPGS1-KO GT335recomb atub 3-24-25 R2 ET1 LT1.Project Maximum Z_XY1742841132_Z0_T0_C0.tif]

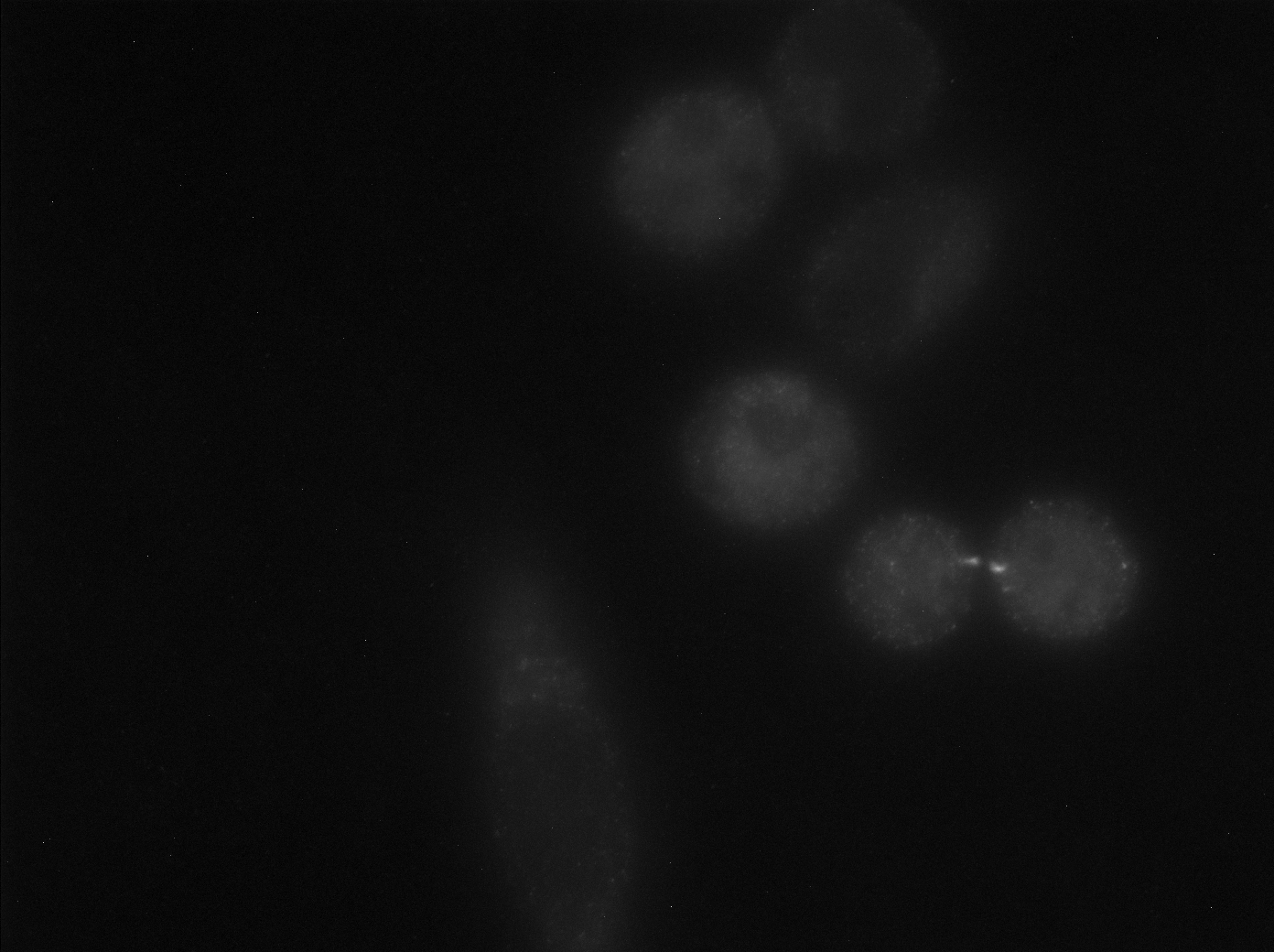

Supplement: Supplementary file 26 — Source data Fig. 7 part 2 [file 44319_2026_742_MOESM26_ESM.zip › Figure 7 Part 2/Fig 7acd Cas9 and TPGS1-ko rGT335 atubulin part 2/TPGS1-KO GT335recomb atub 3-24-25 R3 ET5.Project Maximum Z_XY1742852582_Z0_T0_C1.tif]

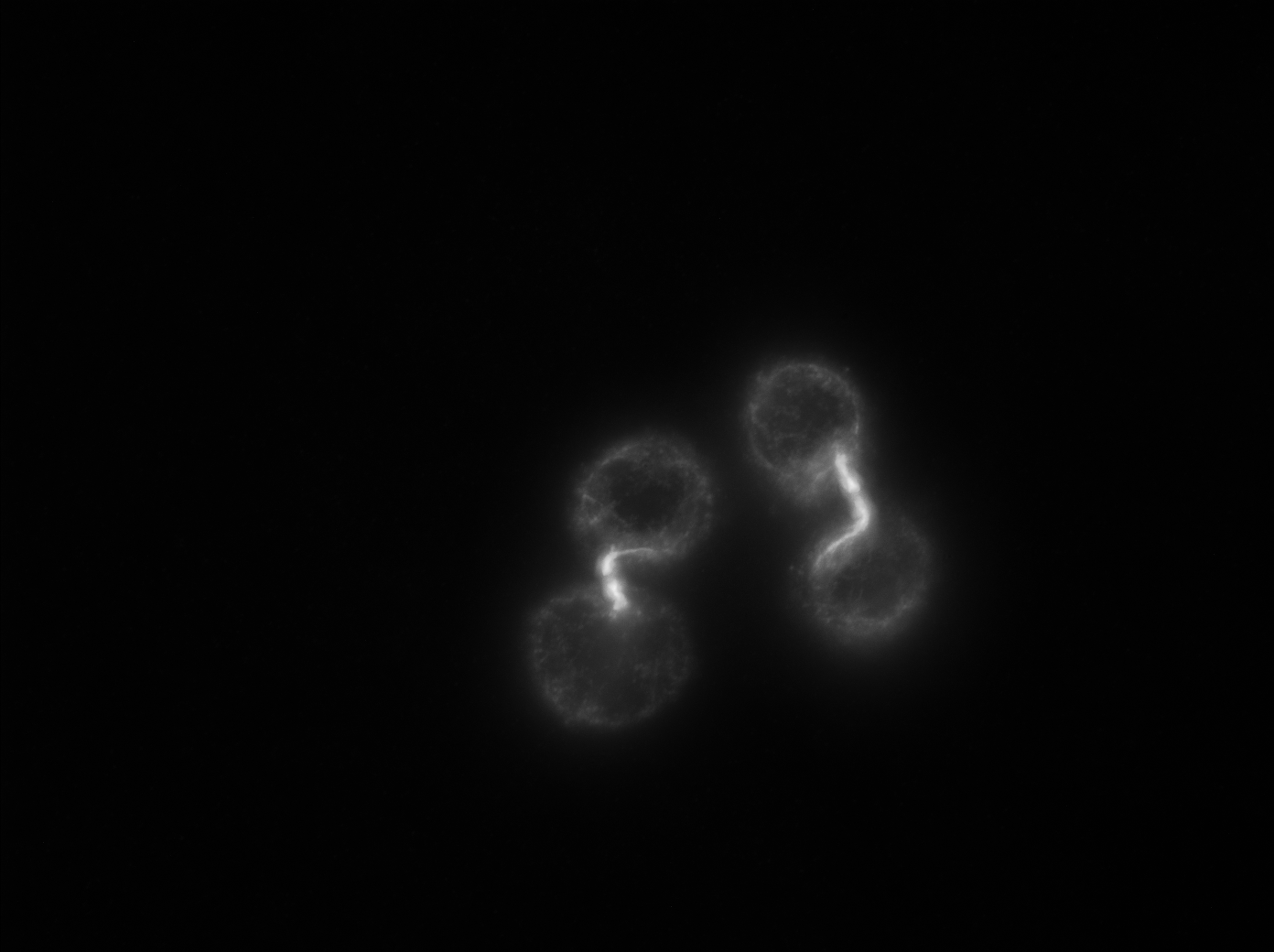

Supplement: Supplementary file 26 — Source data Fig. 7 part 2 [file 44319_2026_742_MOESM26_ESM.zip › Figure 7 Part 2/Fig 7acd Cas9 and TPGS1-ko rGT335 atubulin part 2/TPGS1-KO GT335recomb atub 3-24-25 R2 LT7LT8.Project Maximum Z_XY1742842515_Z0_T0_C2.tif]

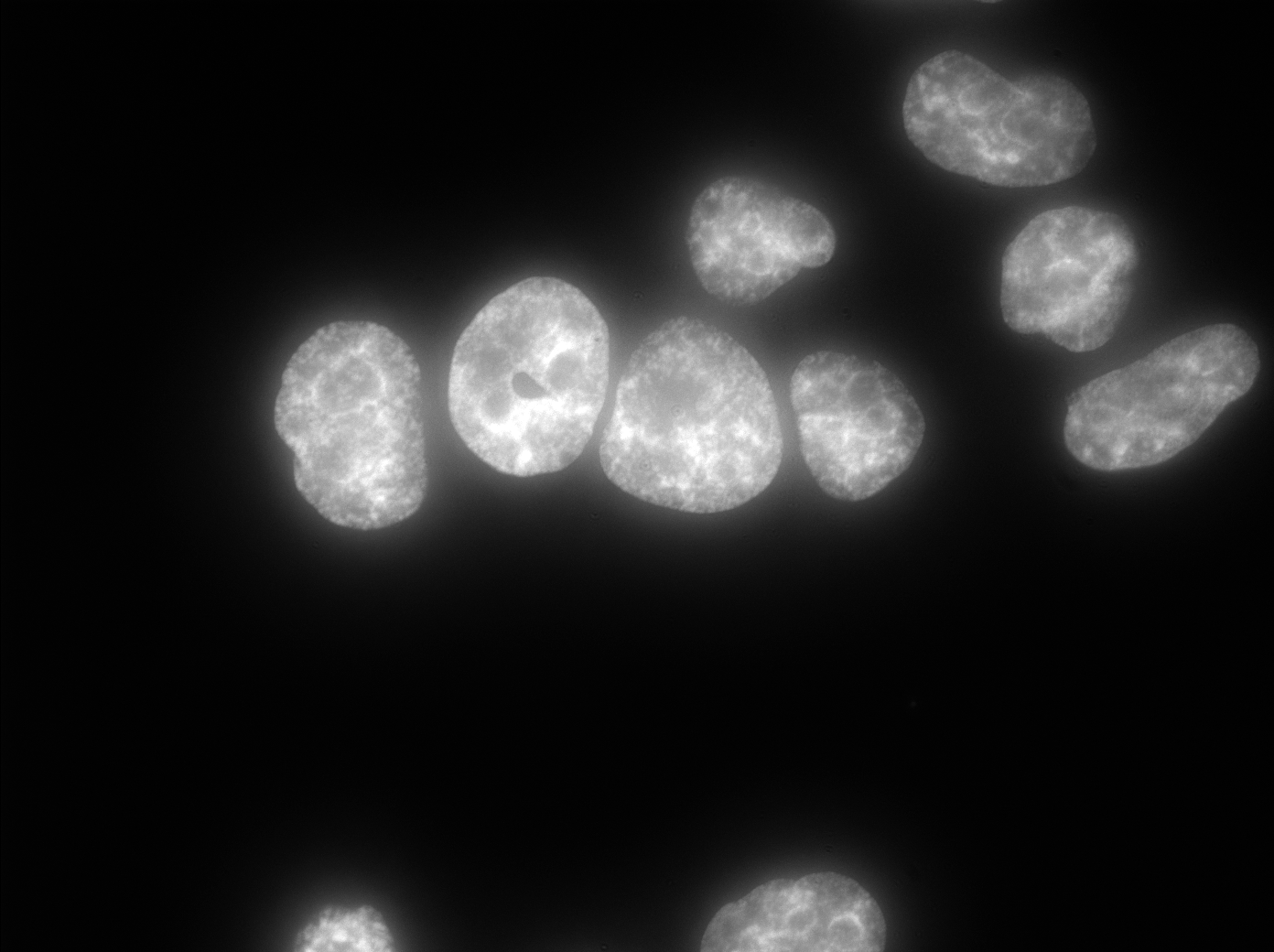

Supplement: Supplementary file 26 — Source data Fig. 7 part 2 [file 44319_2026_742_MOESM26_ESM.zip › Figure 7 Part 2/Fig 7acd Cas9 and TPGS1-ko rGT335 atubulin part 2/TPGS1-KO GT335recomb atub 3-24-25 R1 LT2.Project Maximum Z_XY1742839123_Z0_T0_C0.tif]

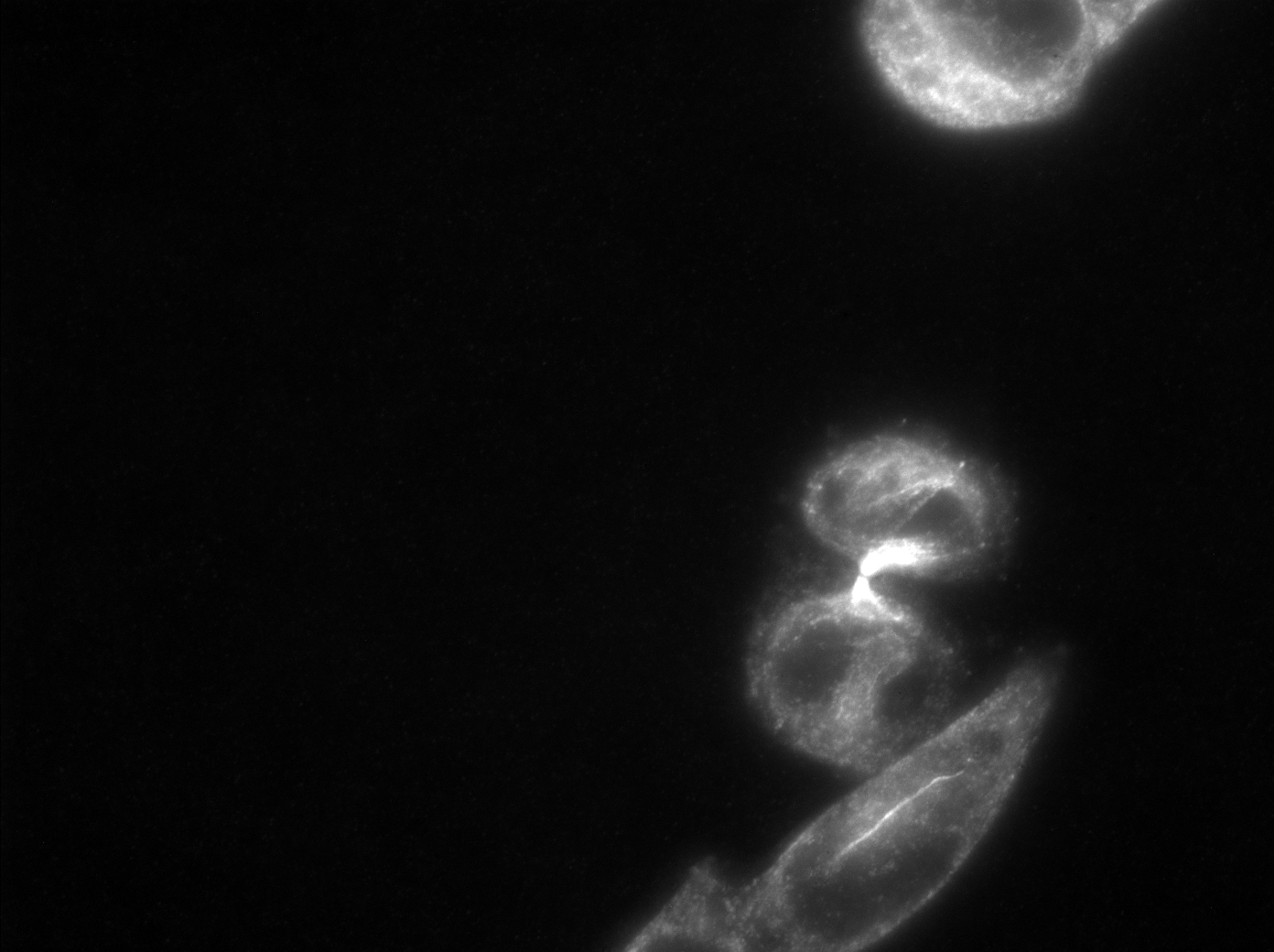

Supplement: Supplementary file 26 — Source data Fig. 7 part 2 [file 44319_2026_742_MOESM26_ESM.zip › Figure 7 Part 2/Fig 7acd Cas9 and TPGS1-ko rGT335 atubulin part 2/TPGS1-KO GT335recomb atub 3-24-25 R3 ET9.Project Maximum Z_XY1742853876_Z0_T0_C2.tif]

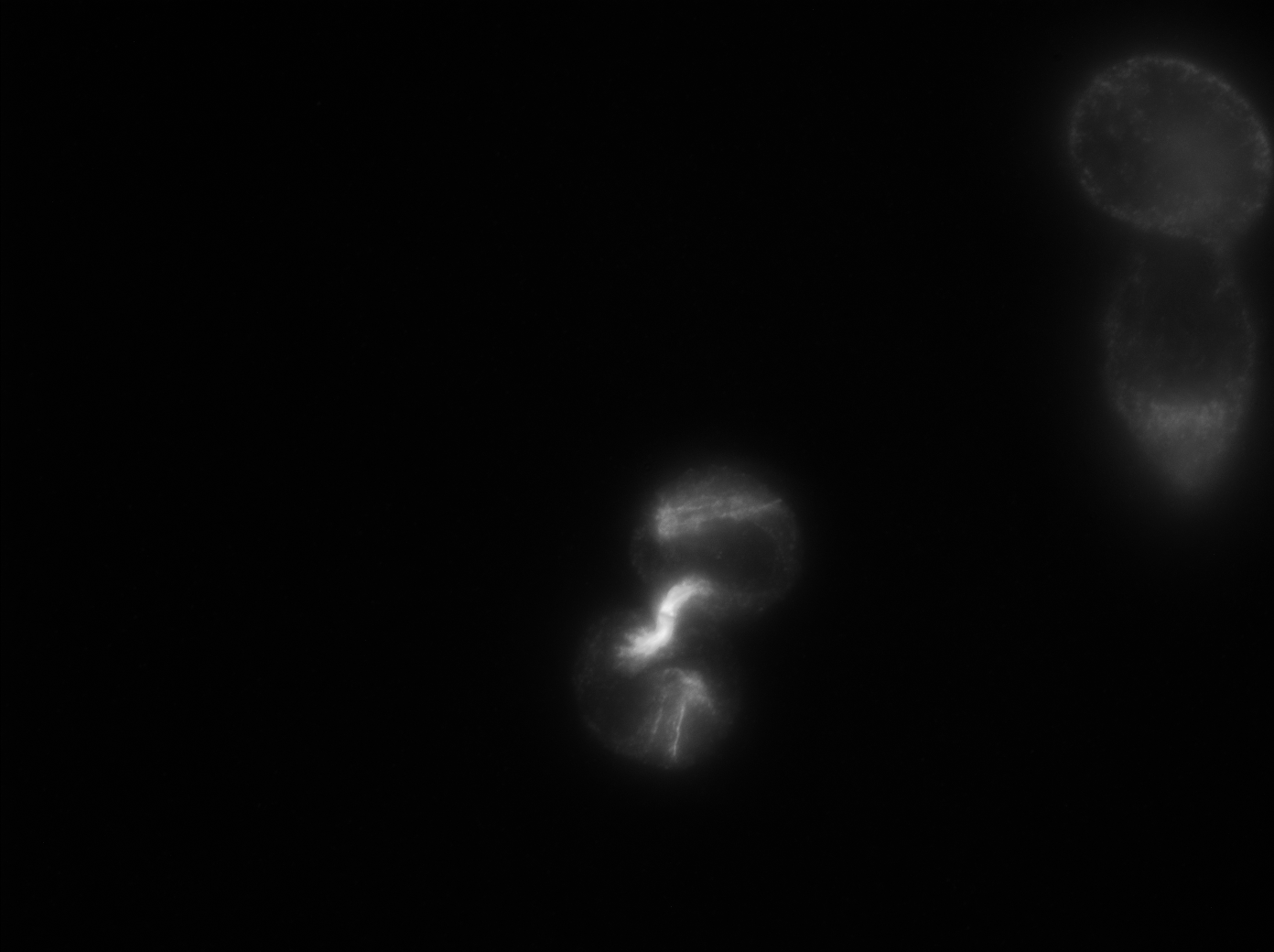

Supplement: Supplementary file 26 — Source data Fig. 7 part 2 [file 44319_2026_742_MOESM26_ESM.zip › Figure 7 Part 2/Fig 7acd Cas9 and TPGS1-ko rGT335 atubulin part 2/TPGS1-KO GT335recomb atub 3-24-25 R2 ET3.Project Maximum Z_XY1742841344_Z0_T0_C2.tif]

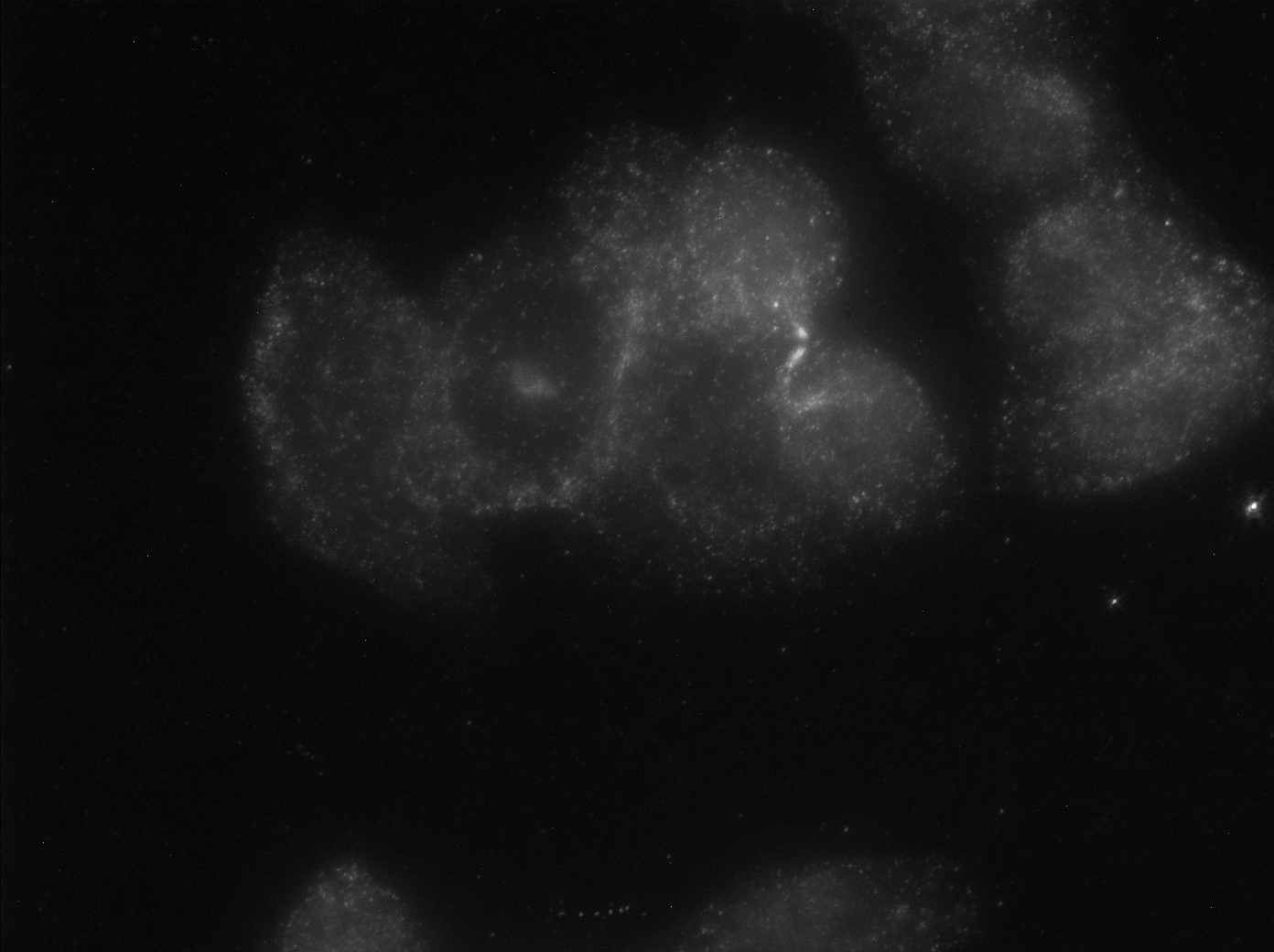

Supplement: Supplementary file 26 — Source data Fig. 7 part 2 [file 44319_2026_742_MOESM26_ESM.zip › Figure 7 Part 2/Fig 7acd Cas9 and TPGS1-ko rGT335 atubulin part 2/TPGS1-KO GT335recomb atub 3-24-25 R1 LT2.Project Maximum Z_XY1742839123_Z0_T0_C1.tif]

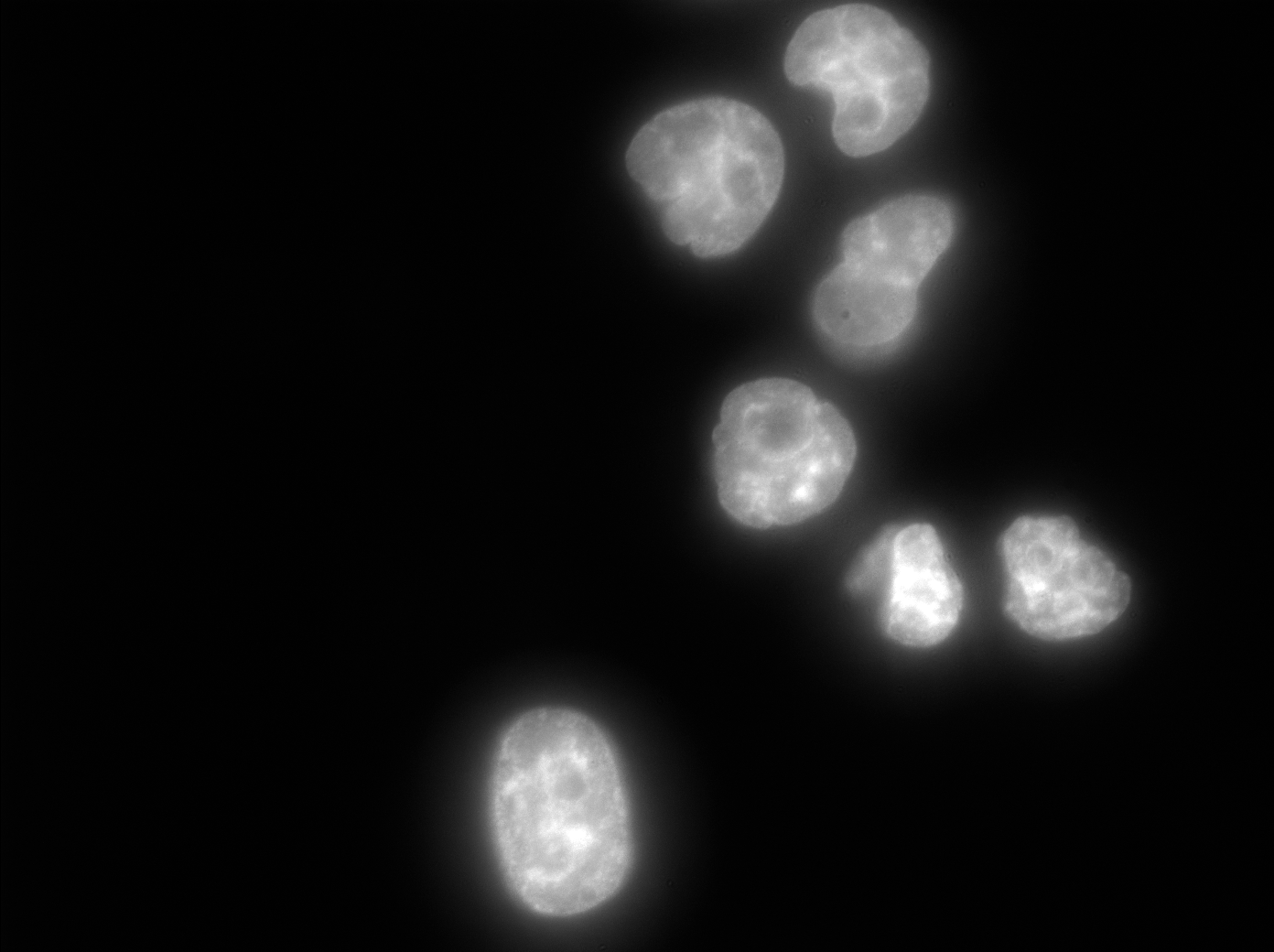

Supplement: Supplementary file 26 — Source data Fig. 7 part 2 [file 44319_2026_742_MOESM26_ESM.zip › Figure 7 Part 2/Fig 7acd Cas9 and TPGS1-ko rGT335 atubulin part 2/TPGS1-KO GT335recomb atub 3-24-25 R3 ET5.Project Maximum Z_XY1742852582_Z0_T0_C0.tif]

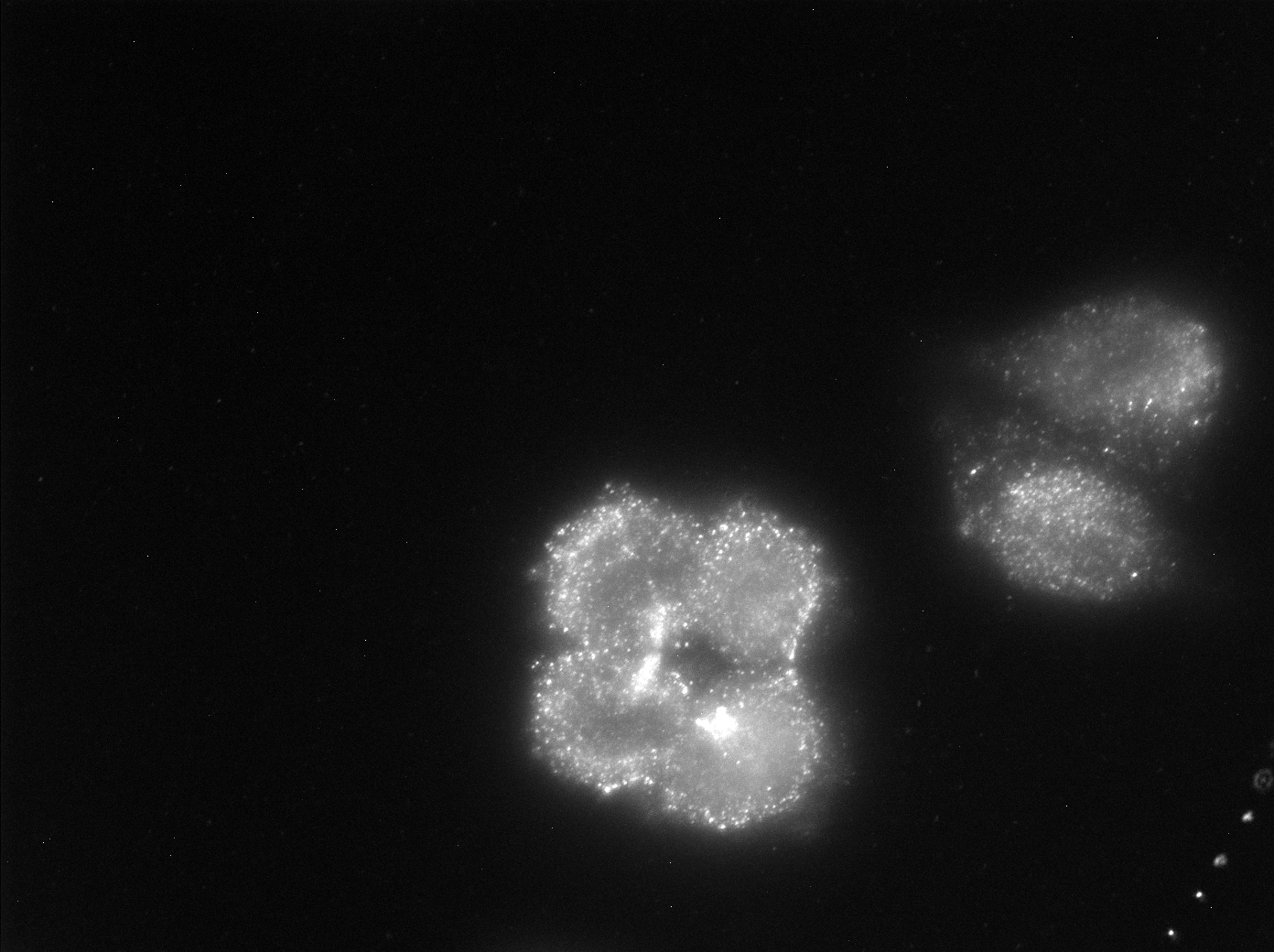

Supplement: Supplementary file 26 — Source data Fig. 7 part 2 [file 44319_2026_742_MOESM26_ESM.zip › Figure 7 Part 2/Fig 7acd Cas9 and TPGS1-ko rGT335 atubulin part 2/TPGS1-KO GT335recomb atub 3-24-25 R2 ET1 LT1.Project Maximum Z_XY1742841132_Z0_T0_C1.tif]

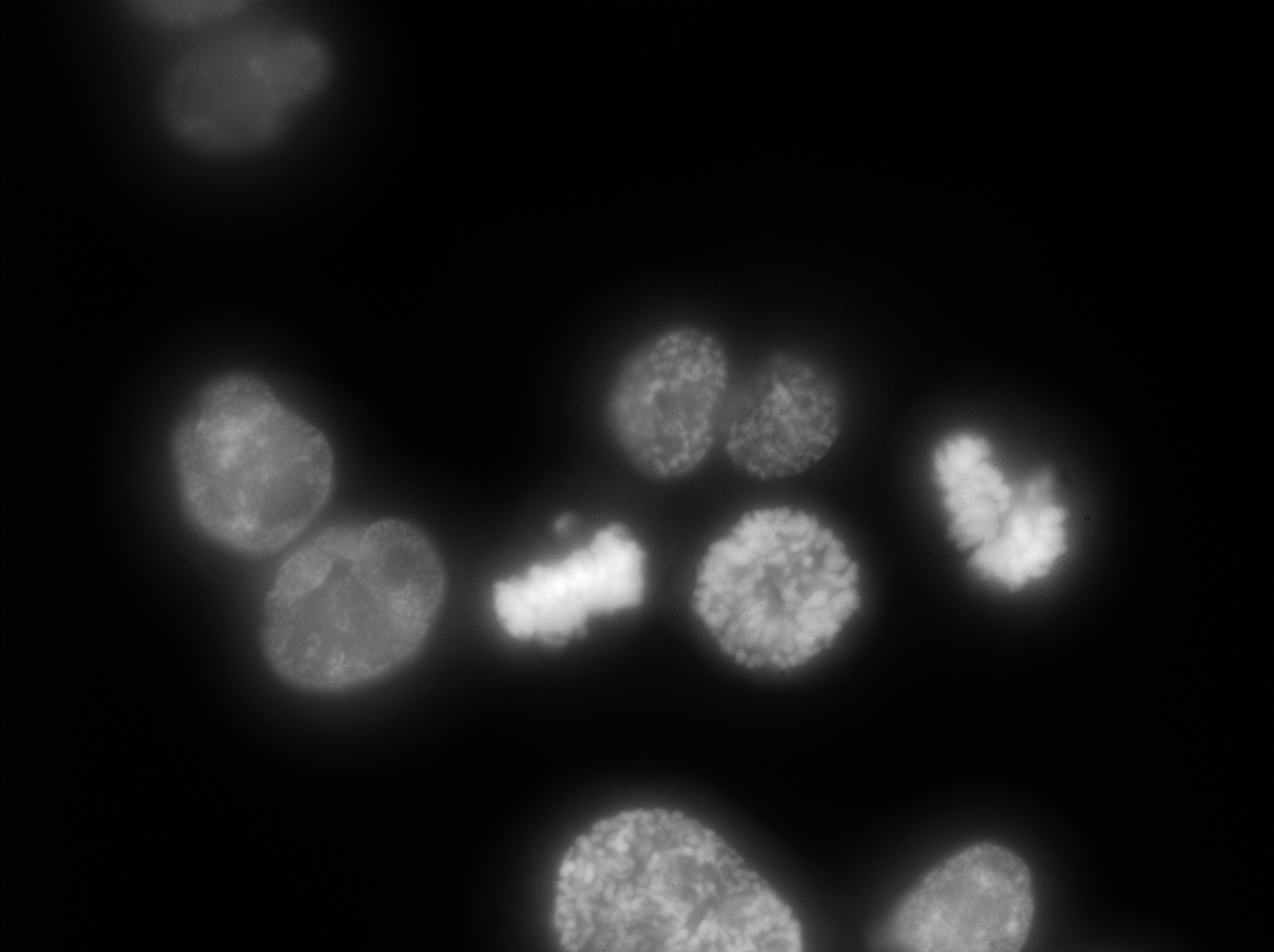

Supplement: Supplementary file 26 — Source data Fig. 7 part 2 [file 44319_2026_742_MOESM26_ESM.zip › Figure 7 Part 2/Fig 7acd Cas9 and TPGS1-ko rGT335 atubulin part 2/TPGS1-KO GT335recomb atub 3-24-25 R2 ET2 M1M2.Project Maximum Z_XY1742841221_Z0_T0_C0.tif]

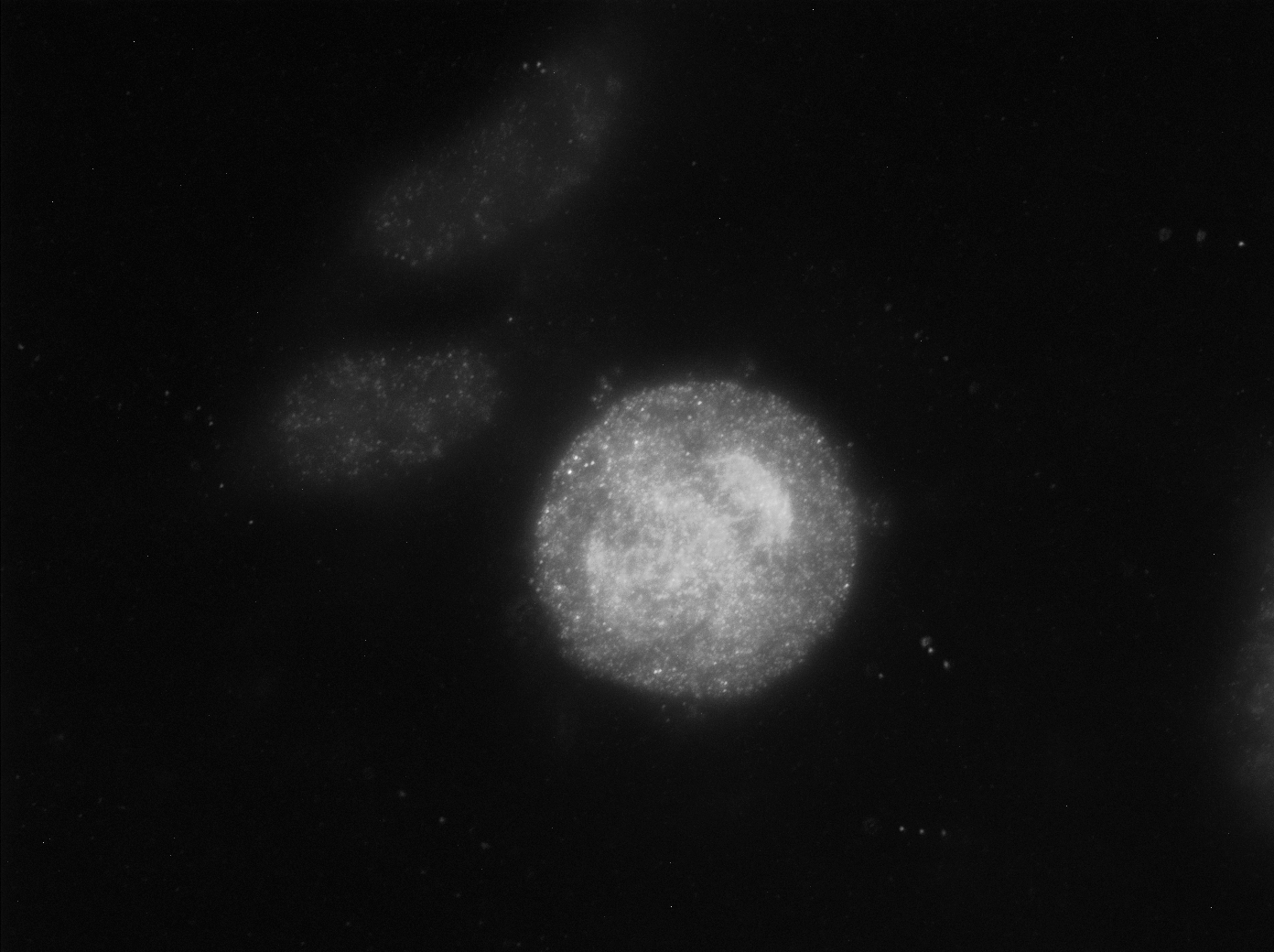

Supplement: Supplementary file 26 — Source data Fig. 7 part 2 [file 44319_2026_742_MOESM26_ESM.zip › Figure 7 Part 2/Fig 7acd Cas9 and TPGS1-ko rGT335 atubulin part 2/TPGS1-KO GT335recomb atub 3-24-25 R1 A1.Project Maximum Z_XY1742838798_Z0_T0_C1.tif]

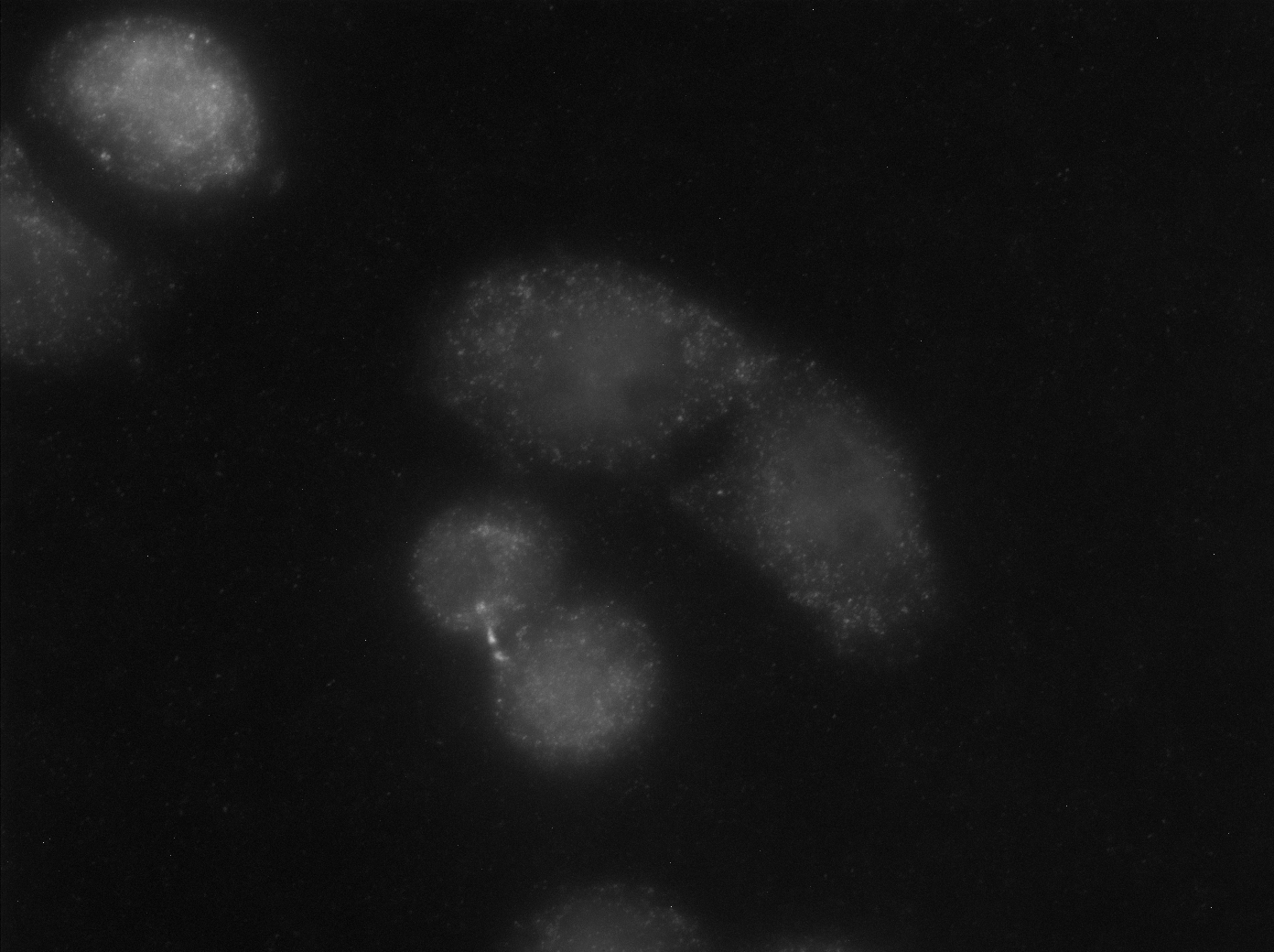

Supplement: Supplementary file 26 — Source data Fig. 7 part 2 [file 44319_2026_742_MOESM26_ESM.zip › Figure 7 Part 2/Fig 7acd Cas9 and TPGS1-ko rGT335 atubulin part 2/TPGS1-KO GT335recomb atub 3-24-25 R3 ET10.Project Maximum Z_XY1742853950_Z0_T0_C1.tif]

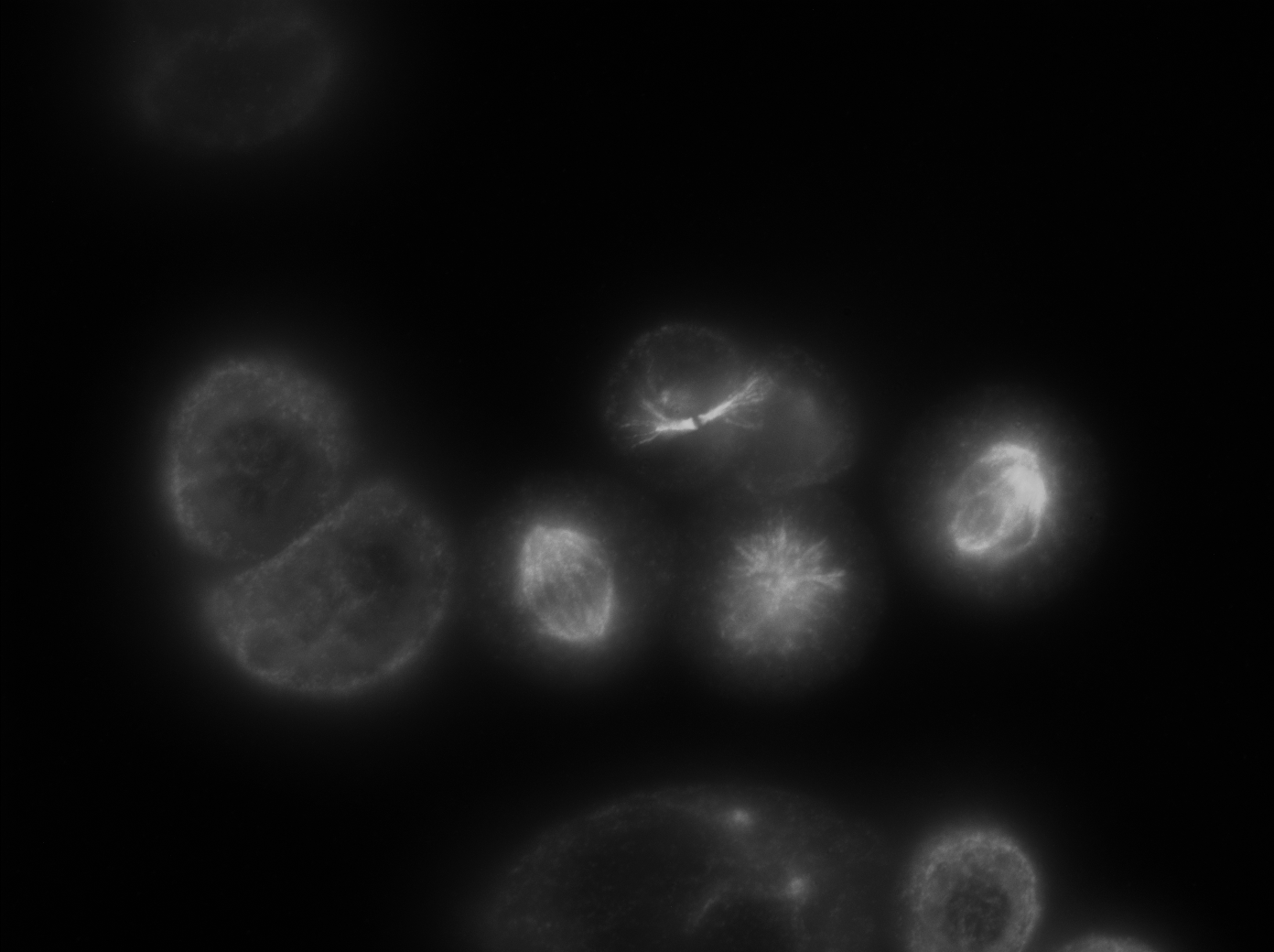

Supplement: Supplementary file 26 — Source data Fig. 7 part 2 [file 44319_2026_742_MOESM26_ESM.zip › Figure 7 Part 2/Fig 7acd Cas9 and TPGS1-ko rGT335 atubulin part 2/TPGS1-KO GT335recomb atub 3-24-25 R2 ET2 M1M2.Project Maximum Z_XY1742841221_Z0_T0_C2.tif]

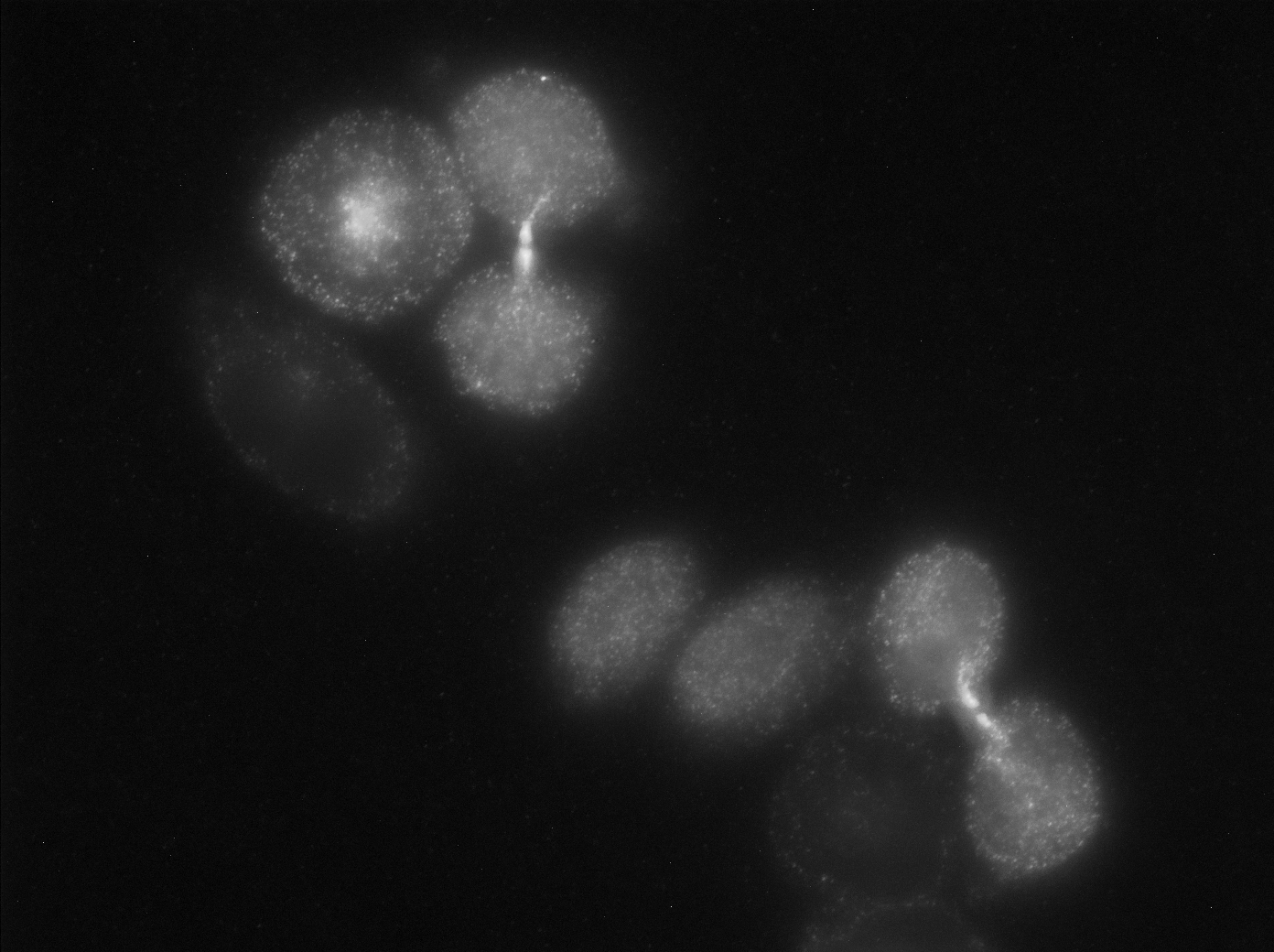

Supplement: Supplementary file 26 — Source data Fig. 7 part 2 [file 44319_2026_742_MOESM26_ESM.zip › Figure 7 Part 2/Fig 7acd Cas9 and TPGS1-ko rGT335 atubulin part 2/TPGS1-KO GT335recomb atub 3-24-25 R3 ET1ET2.Project Maximum Z_XY1742851506_Z0_T0_C1.tif]

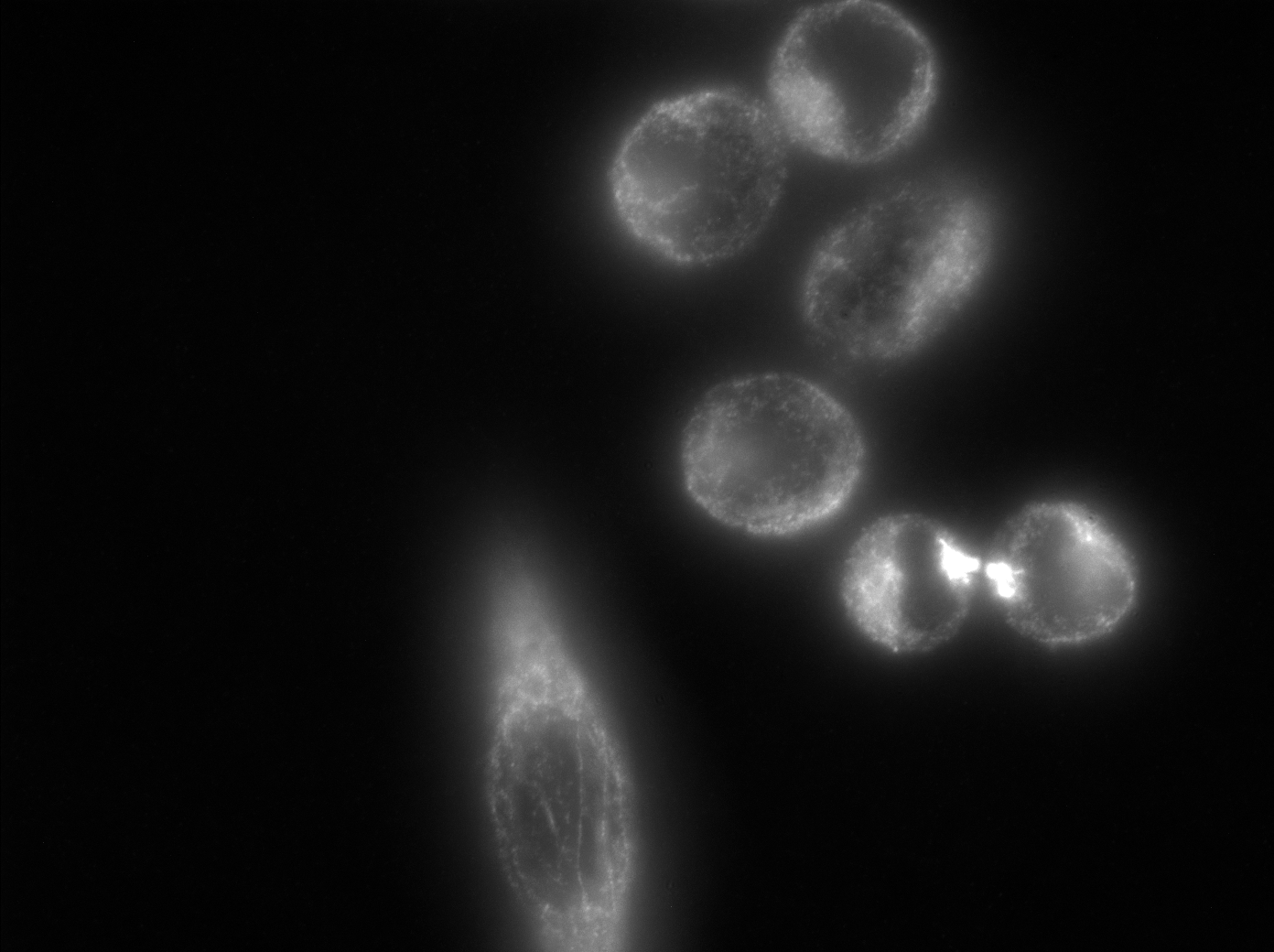

Supplement: Supplementary file 26 — Source data Fig. 7 part 2 [file 44319_2026_742_MOESM26_ESM.zip › Figure 7 Part 2/Fig 7acd Cas9 and TPGS1-ko rGT335 atubulin part 2/TPGS1-KO GT335recomb atub 3-24-25 R3 ET5.Project Maximum Z_XY1742852582_Z0_T0_C2.tif]

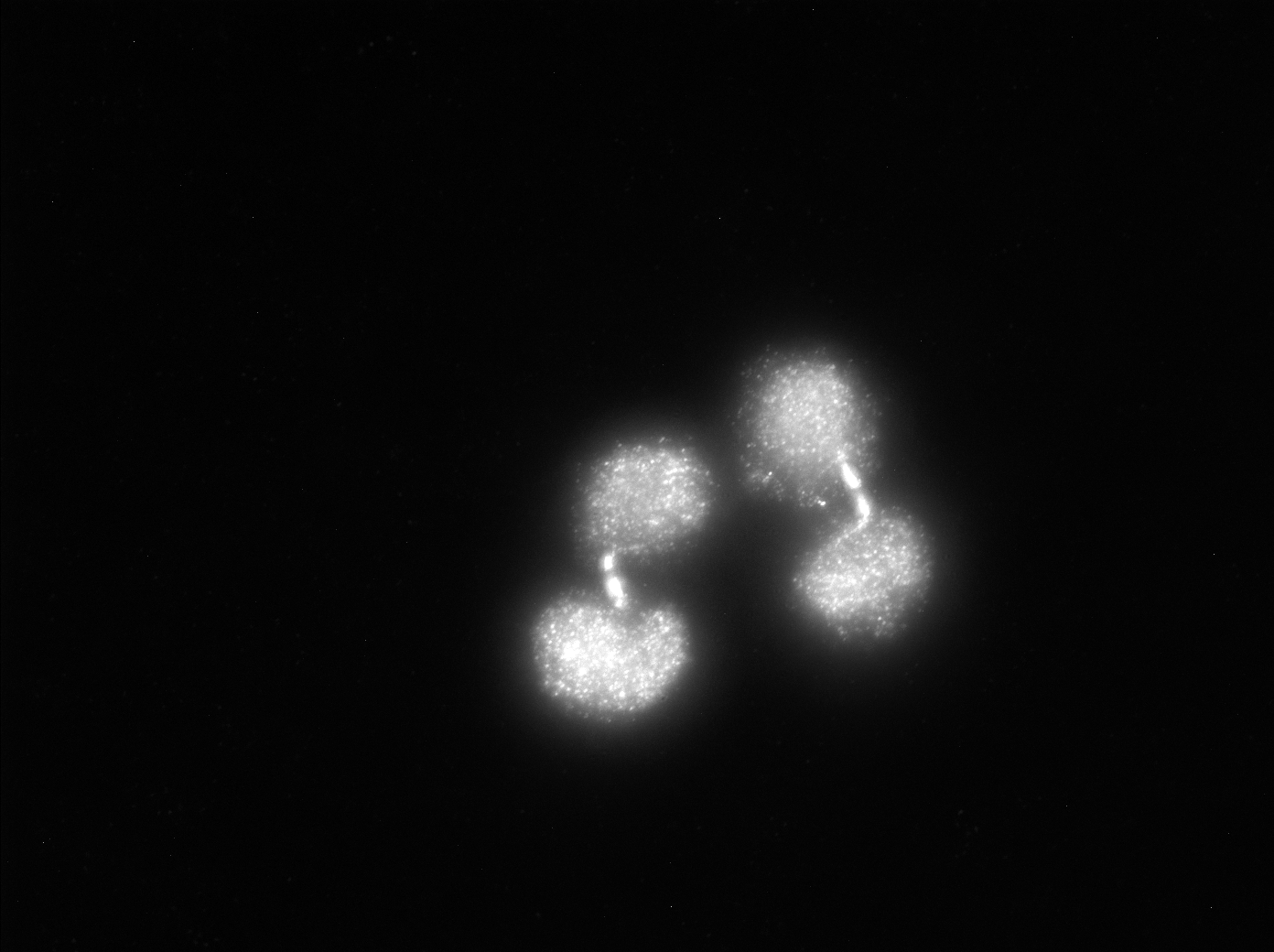

Supplement: Supplementary file 26 — Source data Fig. 7 part 2 [file 44319_2026_742_MOESM26_ESM.zip › Figure 7 Part 2/Fig 7acd Cas9 and TPGS1-ko rGT335 atubulin part 2/TPGS1-KO GT335recomb atub 3-24-25 R2 LT7LT8.Project Maximum Z_XY1742842515_Z0_T0_C1.tif]

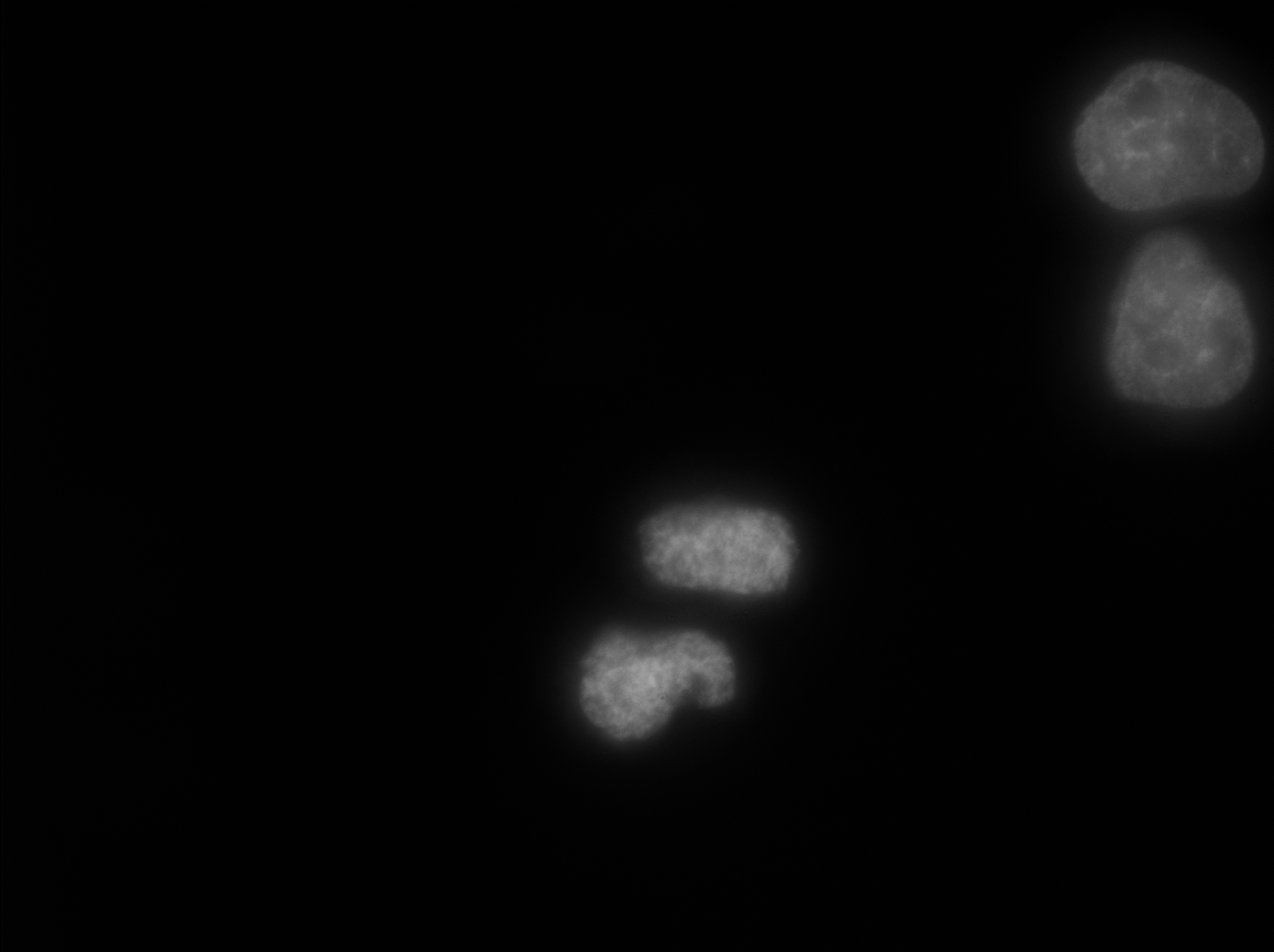

Supplement: Supplementary file 26 — Source data Fig. 7 part 2 [file 44319_2026_742_MOESM26_ESM.zip › Figure 7 Part 2/Fig 7acd Cas9 and TPGS1-ko rGT335 atubulin part 2/TPGS1-KO GT335recomb atub 3-24-25 R2 ET3.Project Maximum Z_XY1742841344_Z0_T0_C0.tif]

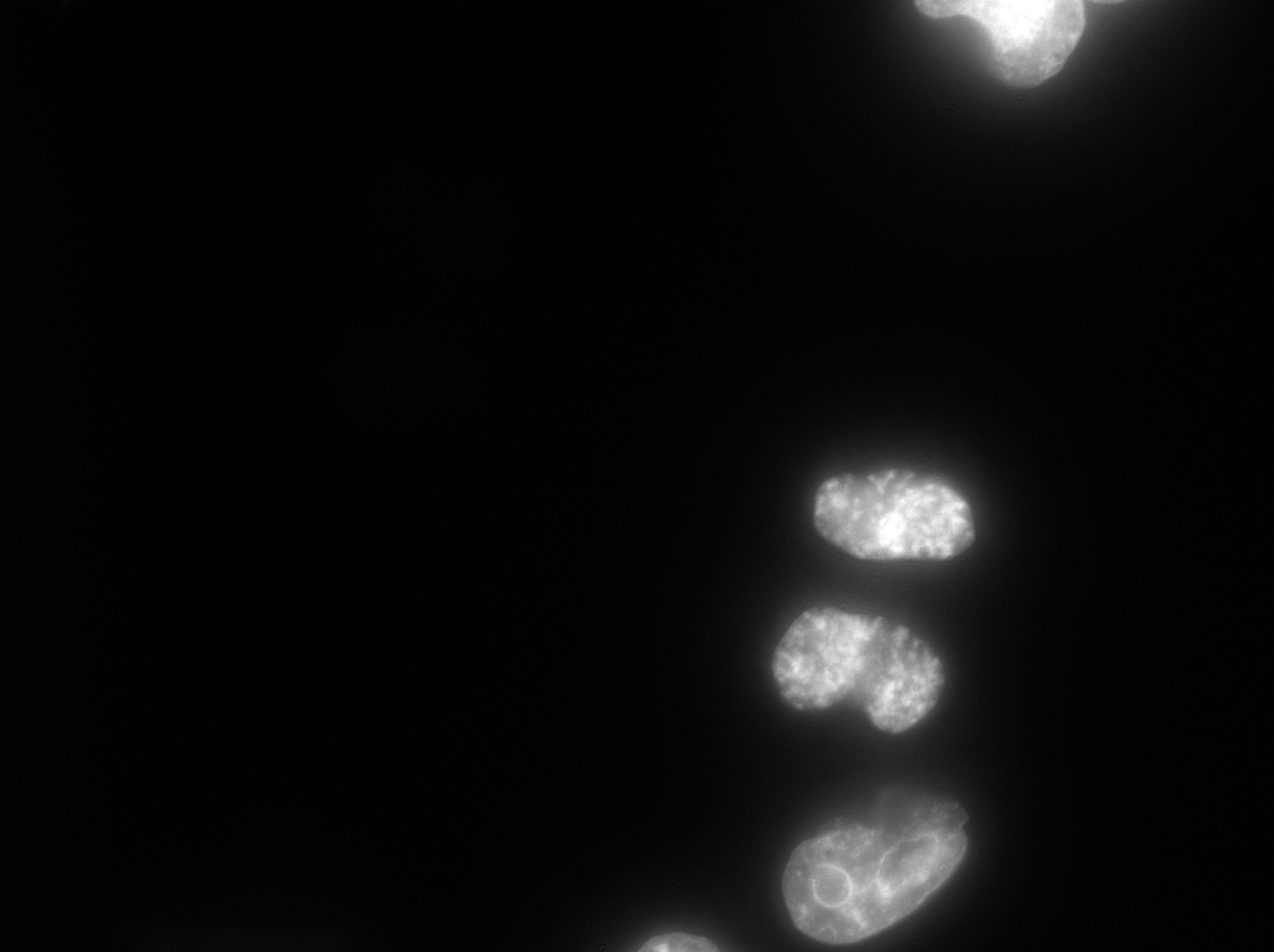

Supplement: Supplementary file 26 — Source data Fig. 7 part 2 [file 44319_2026_742_MOESM26_ESM.zip › Figure 7 Part 2/Fig 7acd Cas9 and TPGS1-ko rGT335 atubulin part 2/TPGS1-KO GT335recomb atub 3-24-25 R3 ET9.Project Maximum Z_XY1742853876_Z0_T0_C0.tif]

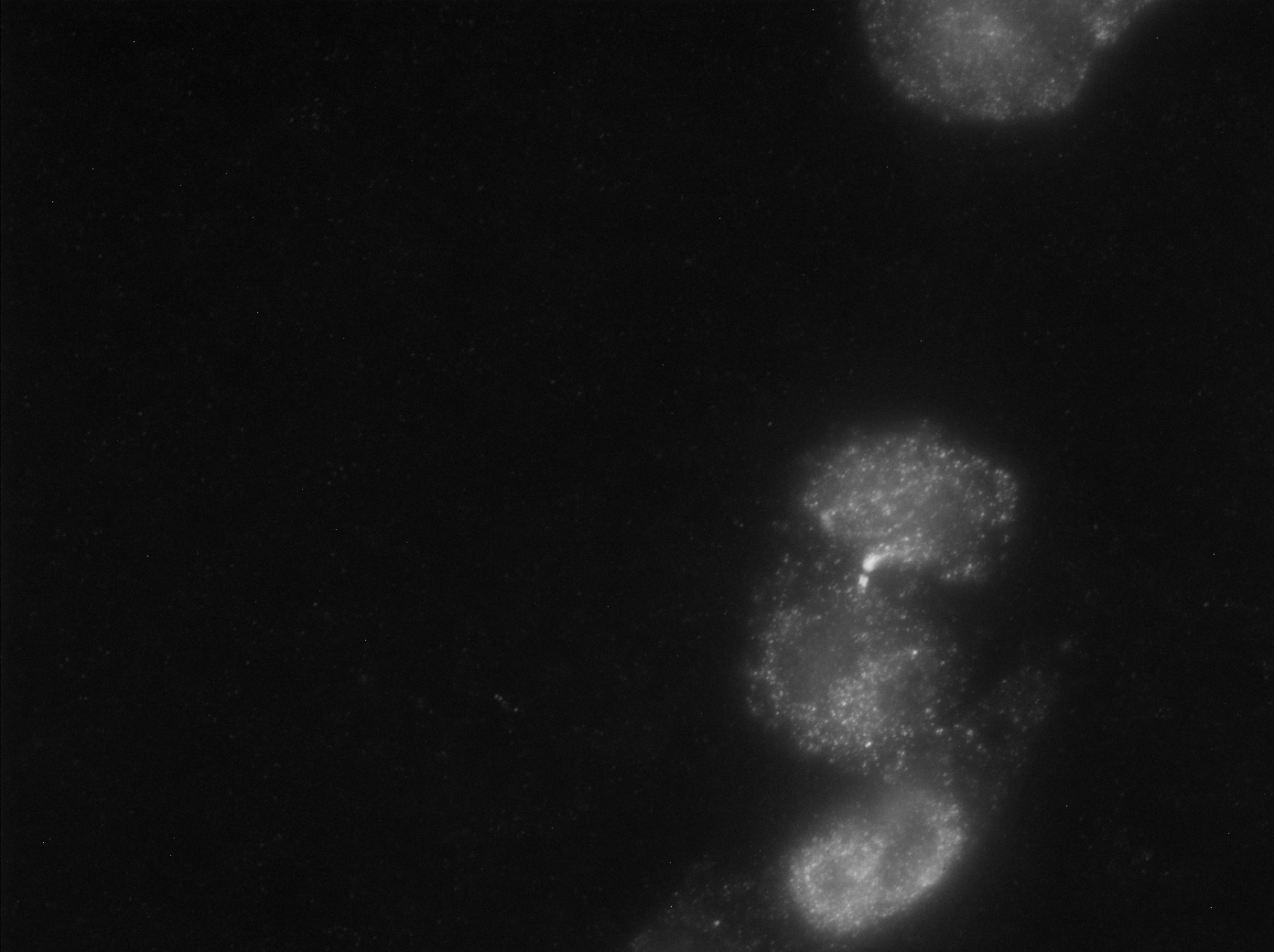

Supplement: Supplementary file 26 — Source data Fig. 7 part 2 [file 44319_2026_742_MOESM26_ESM.zip › Figure 7 Part 2/Fig 7acd Cas9 and TPGS1-ko rGT335 atubulin part 2/TPGS1-KO GT335recomb atub 3-24-25 R3 ET9.Project Maximum Z_XY1742853876_Z0_T0_C1.tif]

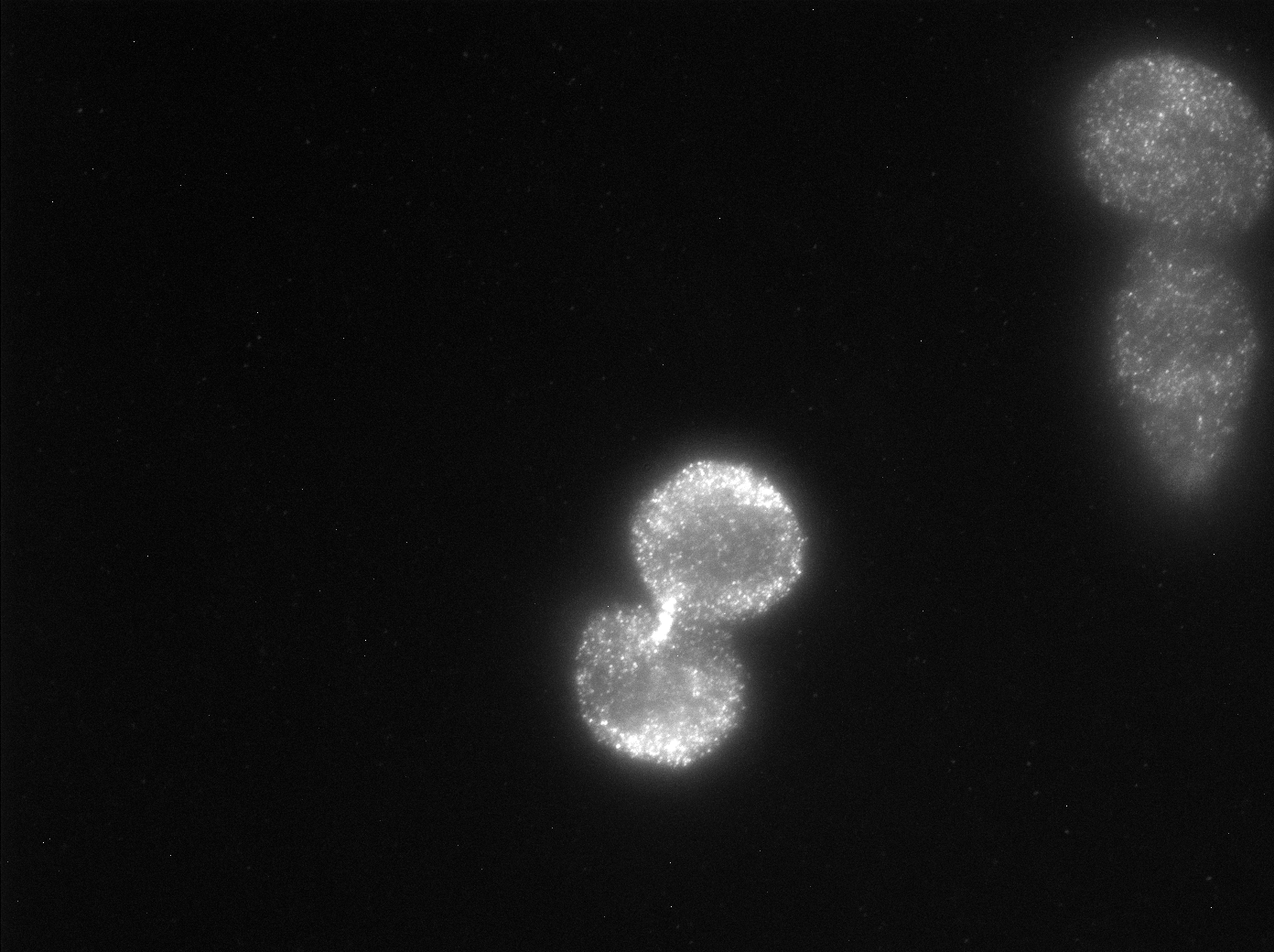

Supplement: Supplementary file 26 — Source data Fig. 7 part 2 [file 44319_2026_742_MOESM26_ESM.zip › Figure 7 Part 2/Fig 7acd Cas9 and TPGS1-ko rGT335 atubulin part 2/TPGS1-KO GT335recomb atub 3-24-25 R2 ET3.Project Maximum Z_XY1742841344_Z0_T0_C1.tif]

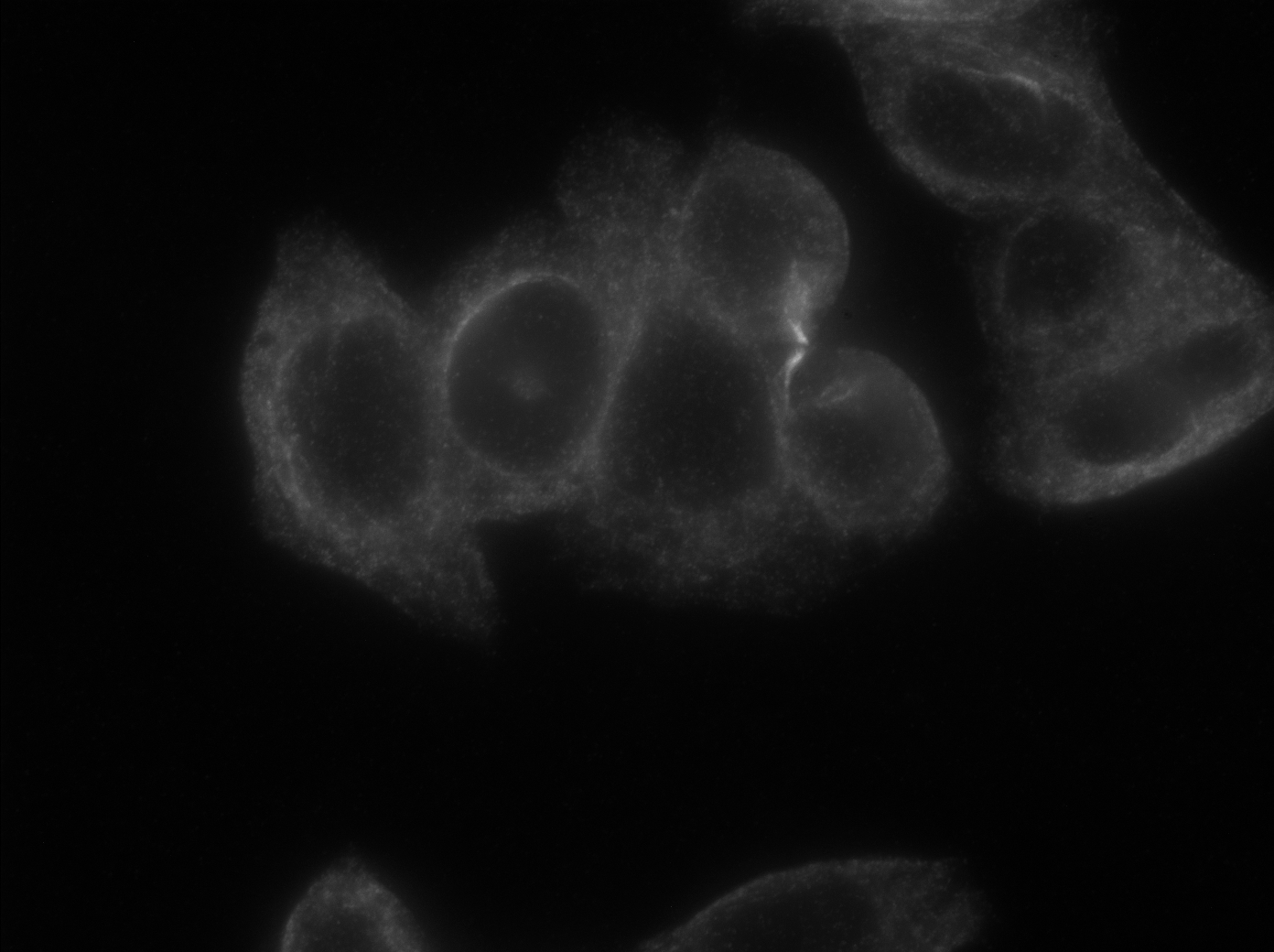

Supplement: Supplementary file 26 — Source data Fig. 7 part 2 [file 44319_2026_742_MOESM26_ESM.zip › Figure 7 Part 2/Fig 7acd Cas9 and TPGS1-ko rGT335 atubulin part 2/TPGS1-KO GT335recomb atub 3-24-25 R1 LT2.Project Maximum Z_XY1742839123_Z0_T0_C2.tif]

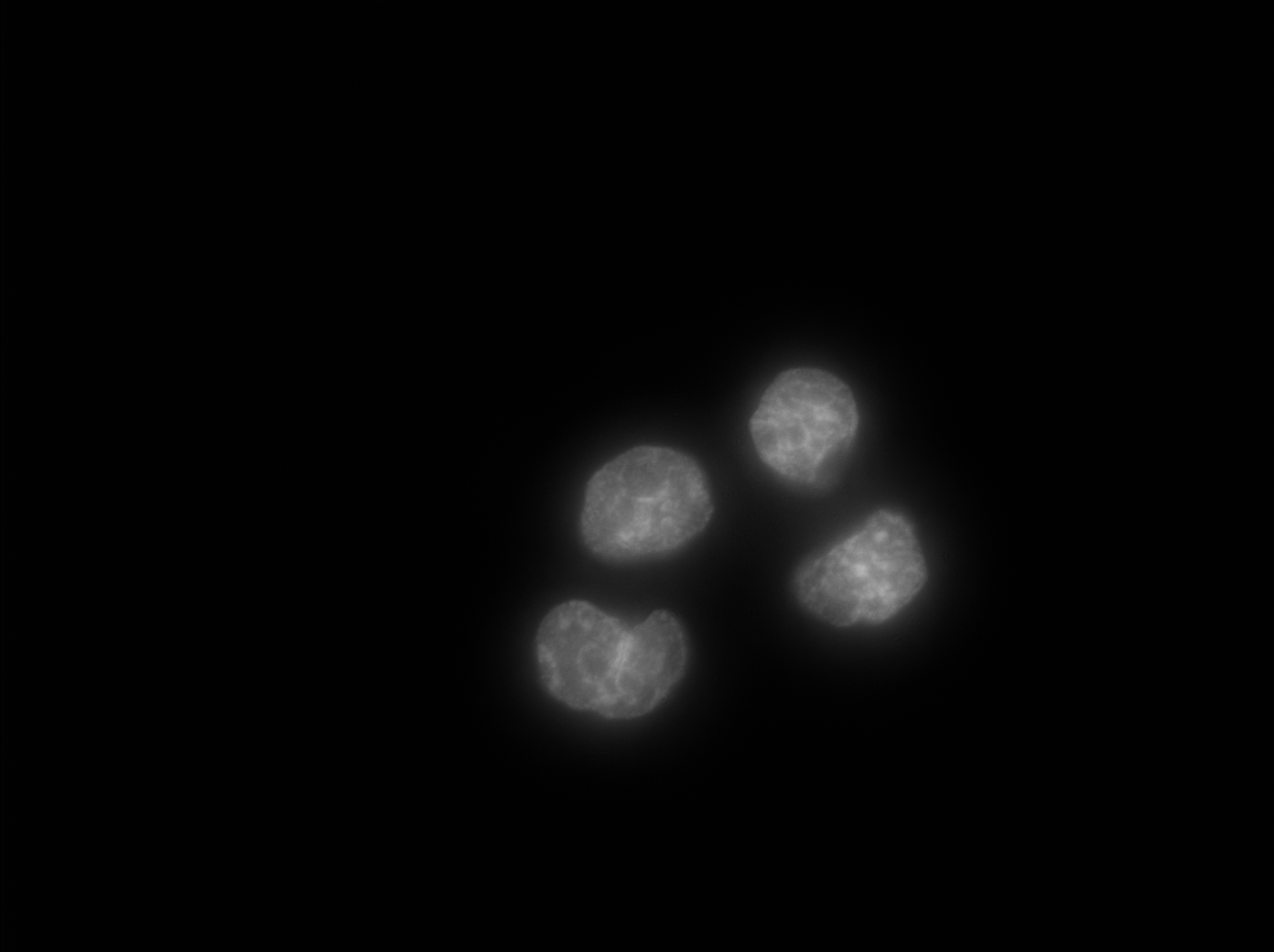

Supplement: Supplementary file 26 — Source data Fig. 7 part 2 [file 44319_2026_742_MOESM26_ESM.zip › Figure 7 Part 2/Fig 7acd Cas9 and TPGS1-ko rGT335 atubulin part 2/TPGS1-KO GT335recomb atub 3-24-25 R2 LT7LT8.Project Maximum Z_XY1742842515_Z0_T0_C0.tif]

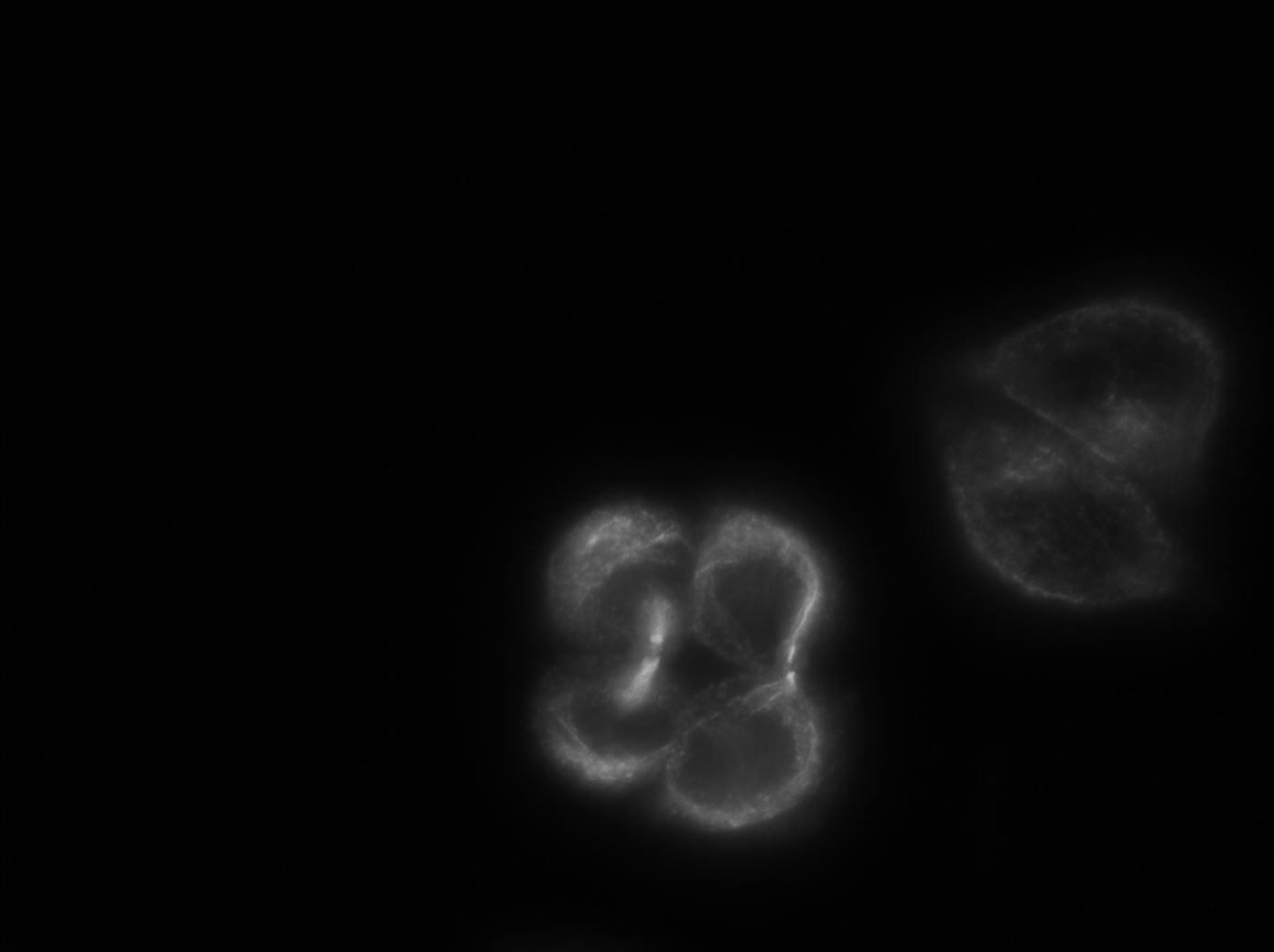

Supplement: Supplementary file 26 — Source data Fig. 7 part 2 [file 44319_2026_742_MOESM26_ESM.zip › Figure 7 Part 2/Fig 7acd Cas9 and TPGS1-ko rGT335 atubulin part 2/TPGS1-KO GT335recomb atub 3-24-25 R2 ET1 LT1.Project Maximum Z_XY1742841132_Z0_T0_C2.tif]

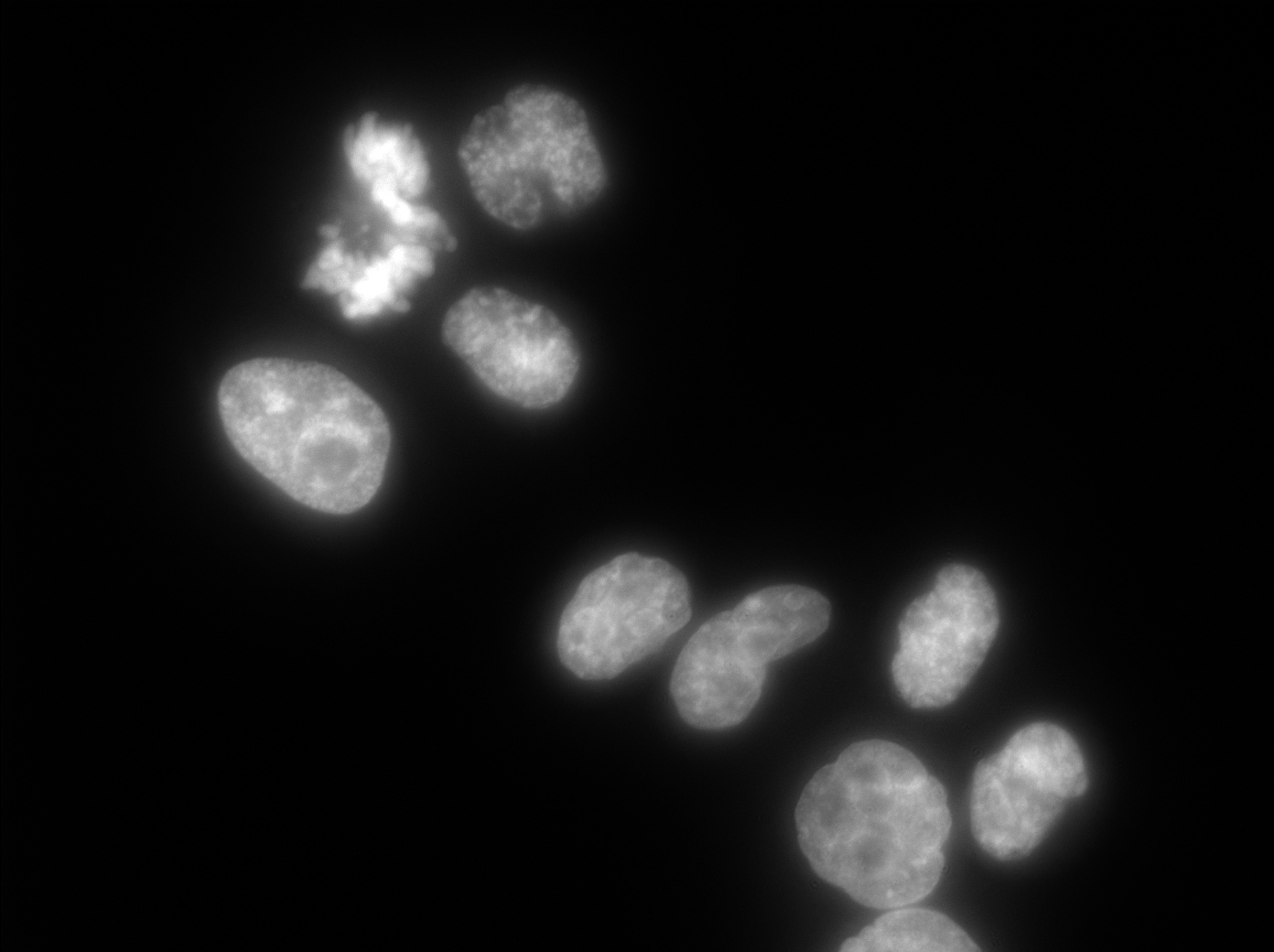

Supplement: Supplementary file 26 — Source data Fig. 7 part 2 [file 44319_2026_742_MOESM26_ESM.zip › Figure 7 Part 2/Fig 7acd Cas9 and TPGS1-ko rGT335 atubulin part 2/TPGS1-KO GT335recomb atub 3-24-25 R3 ET1ET2.Project Maximum Z_XY1742851506_Z0_T0_C0.tif]

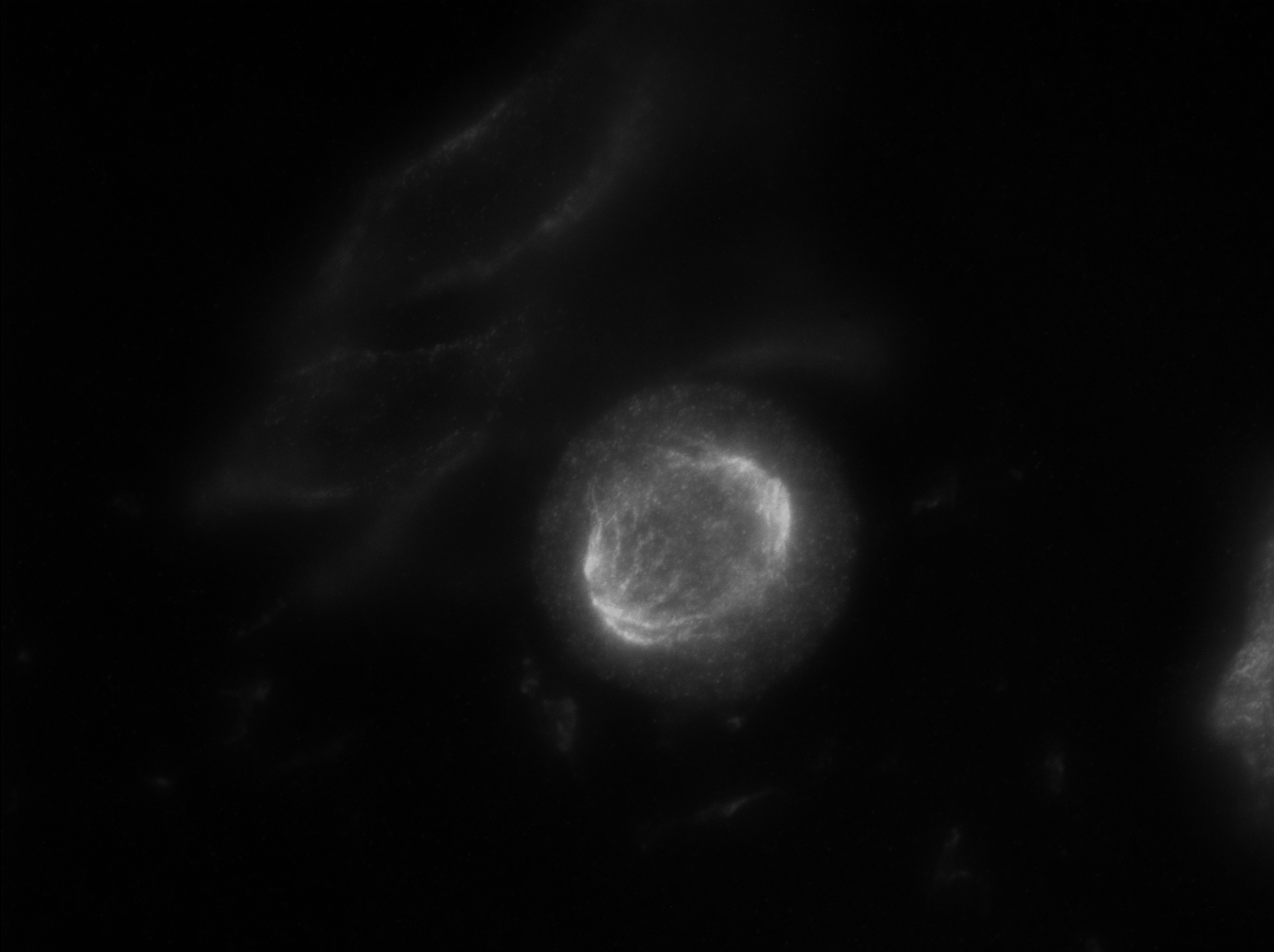

Supplement: Supplementary file 26 — Source data Fig. 7 part 2 [file 44319_2026_742_MOESM26_ESM.zip › Figure 7 Part 2/Fig 7acd Cas9 and TPGS1-ko rGT335 atubulin part 2/TPGS1-KO GT335recomb atub 3-24-25 R1 A1.Project Maximum Z_XY1742838798_Z0_T0_C2.tif]

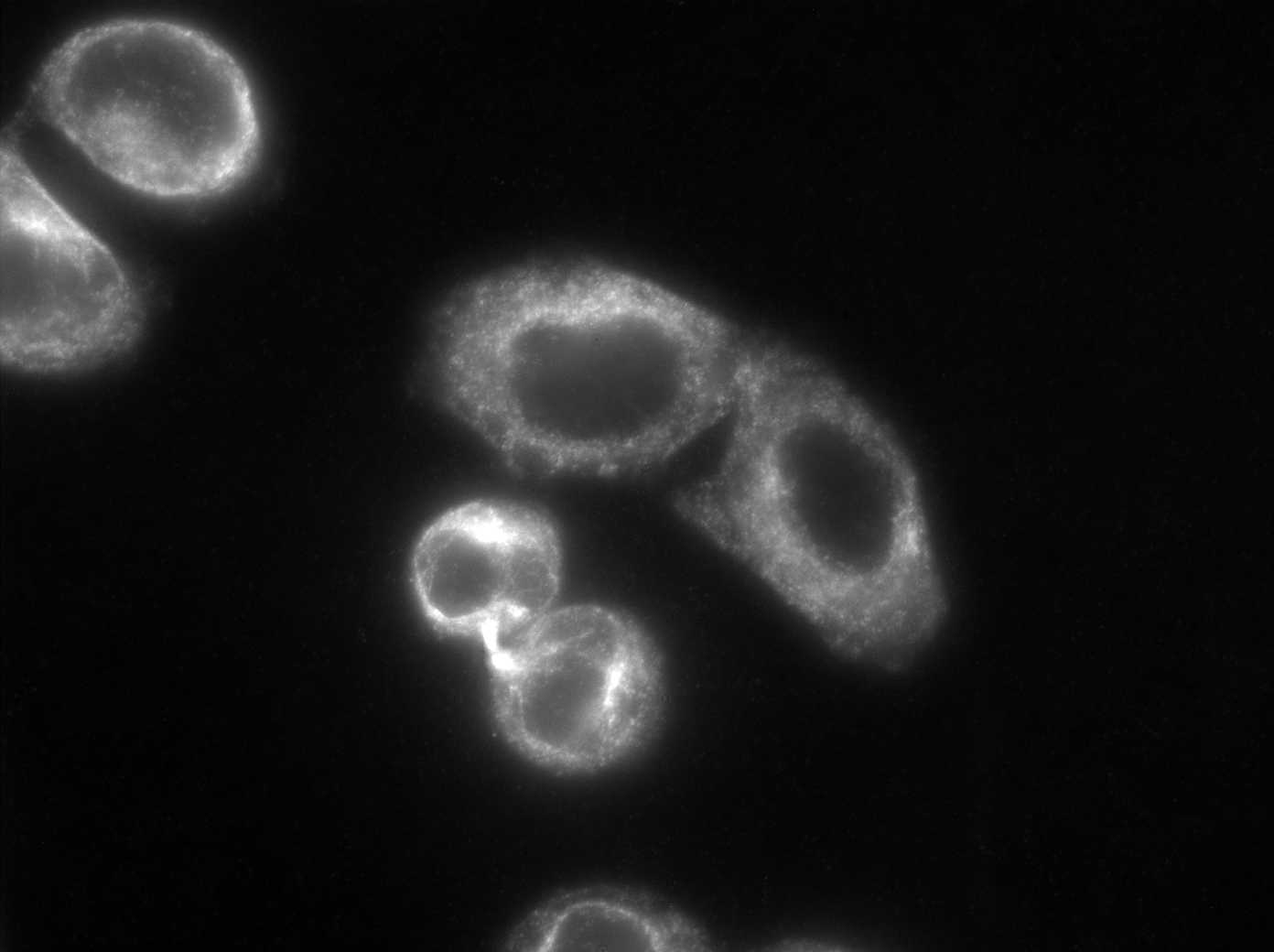

Supplement: Supplementary file 26 — Source data Fig. 7 part 2 [file 44319_2026_742_MOESM26_ESM.zip › Figure 7 Part 2/Fig 7acd Cas9 and TPGS1-ko rGT335 atubulin part 2/TPGS1-KO GT335recomb atub 3-24-25 R3 ET10.Project Maximum Z_XY1742853950_Z0_T0_C2.tif]

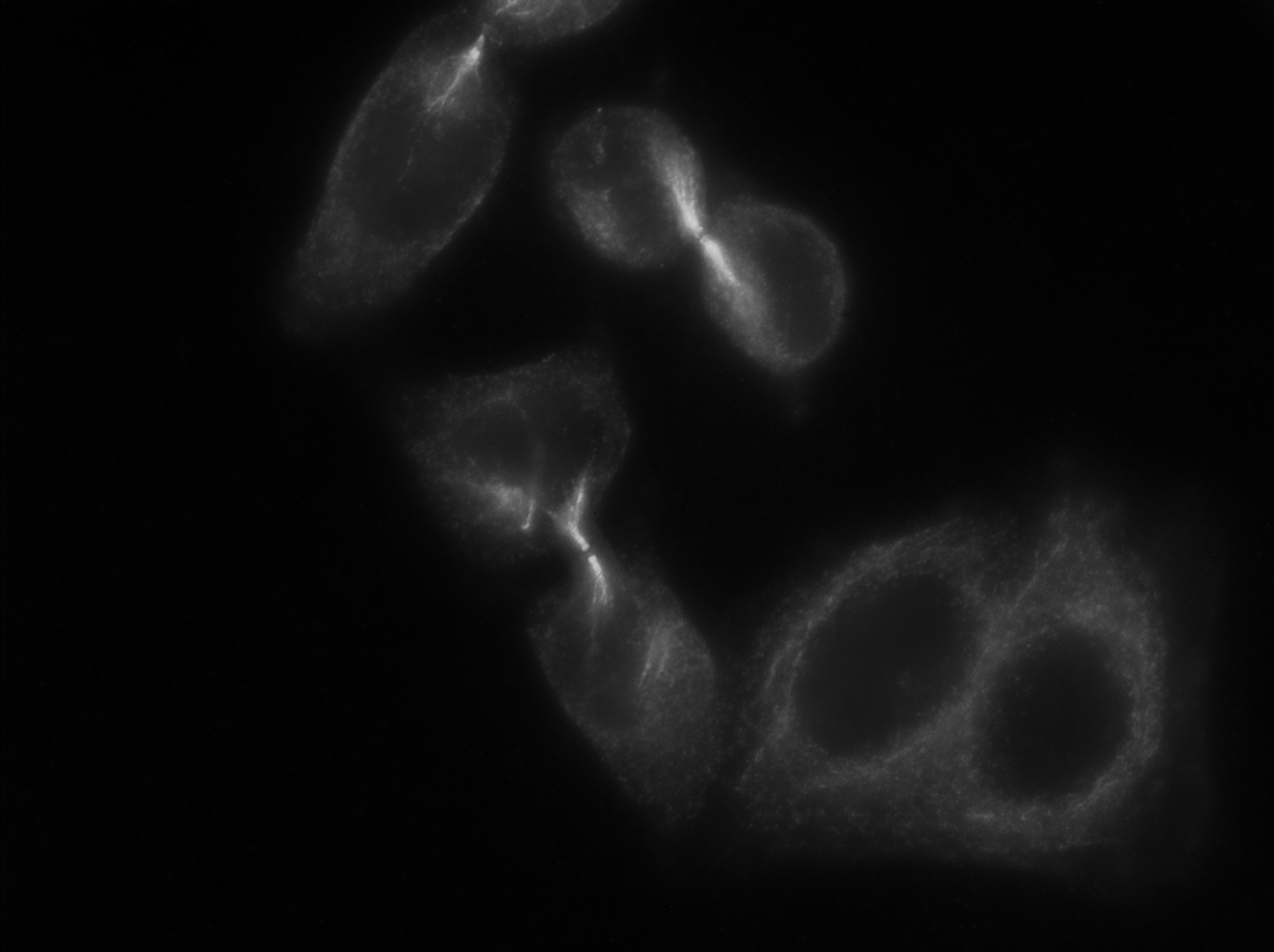

Supplement: Supplementary file 26 — Source data Fig. 7 part 2 [file 44319_2026_742_MOESM26_ESM.zip › Figure 7 Part 2/Fig 7acd Cas9 and TPGS1-ko rGT335 atubulin part 2/TPGS1-KO GT335recomb atub 3-24-25 R1 ET9 LT10.Project Maximum Z_XY1742840753_Z0_T0_C2.tif]

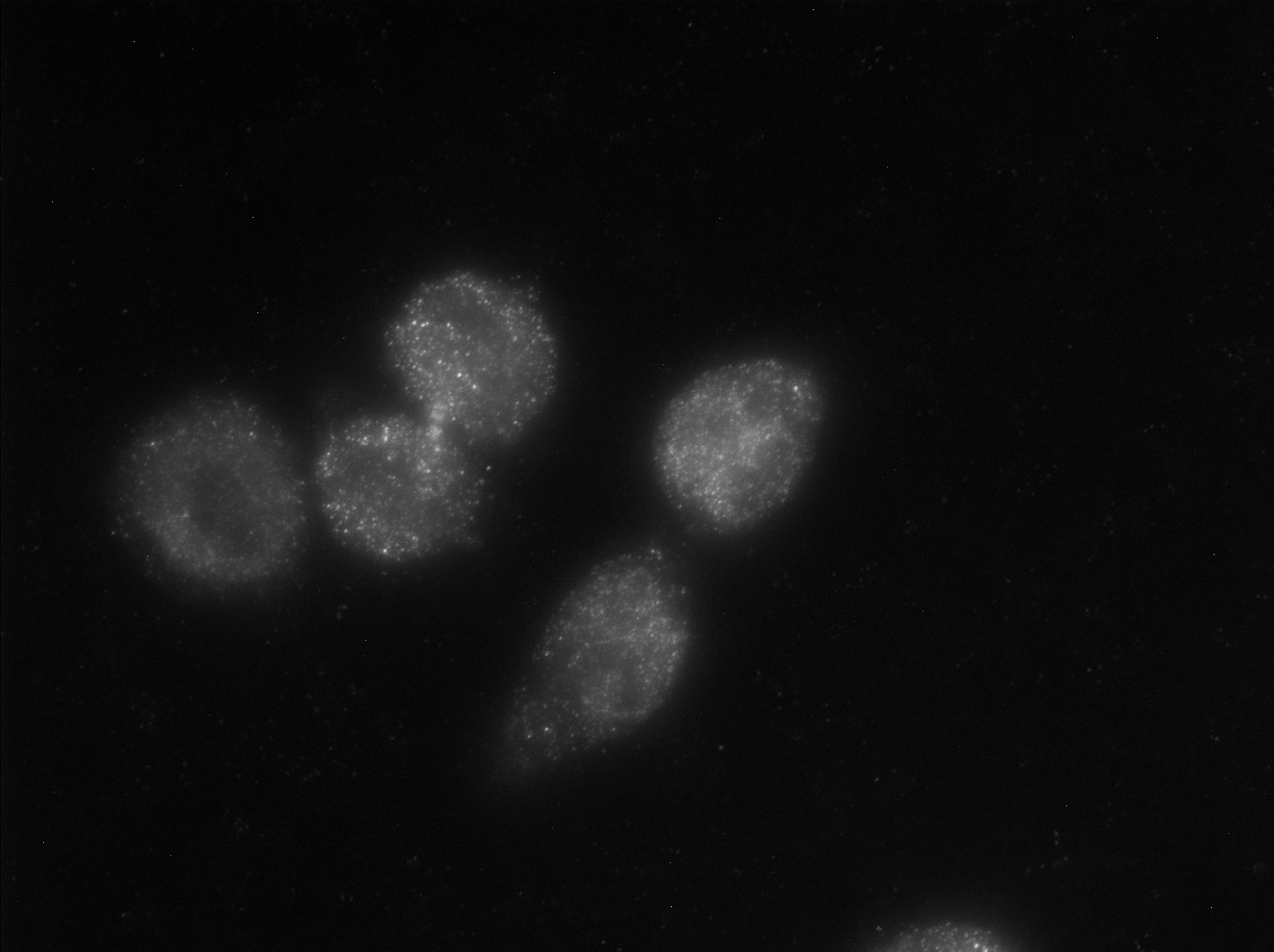

Supplement: Supplementary file 26 — Source data Fig. 7 part 2 [file 44319_2026_742_MOESM26_ESM.zip › Figure 7 Part 2/Fig 7acd Cas9 and TPGS1-ko rGT335 atubulin part 2/TPGS1-KO GT335recomb atub 3-24-25 R1 ET7.Project Maximum Z_XY1742840554_Z0_T0_C1.tif]

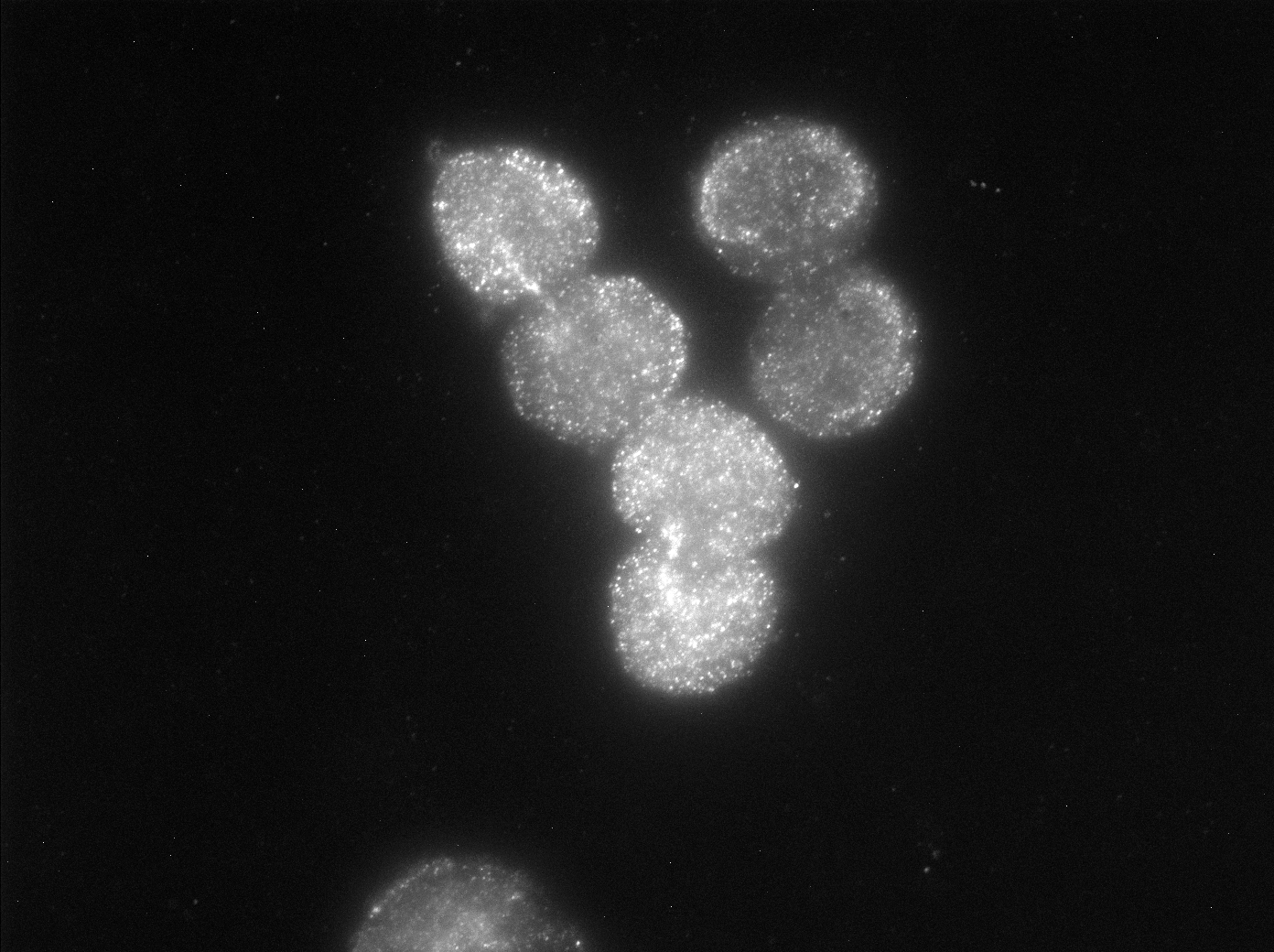

Supplement: Supplementary file 26 — Source data Fig. 7 part 2 [file 44319_2026_742_MOESM26_ESM.zip › Figure 7 Part 2/Fig 7acd Cas9 and TPGS1-ko rGT335 atubulin part 2/TPGS1-KO GT335recomb atub 3-24-25 R2 ET5ET6.Project Maximum Z_XY1742841534_Z0_T0_C1.tif]

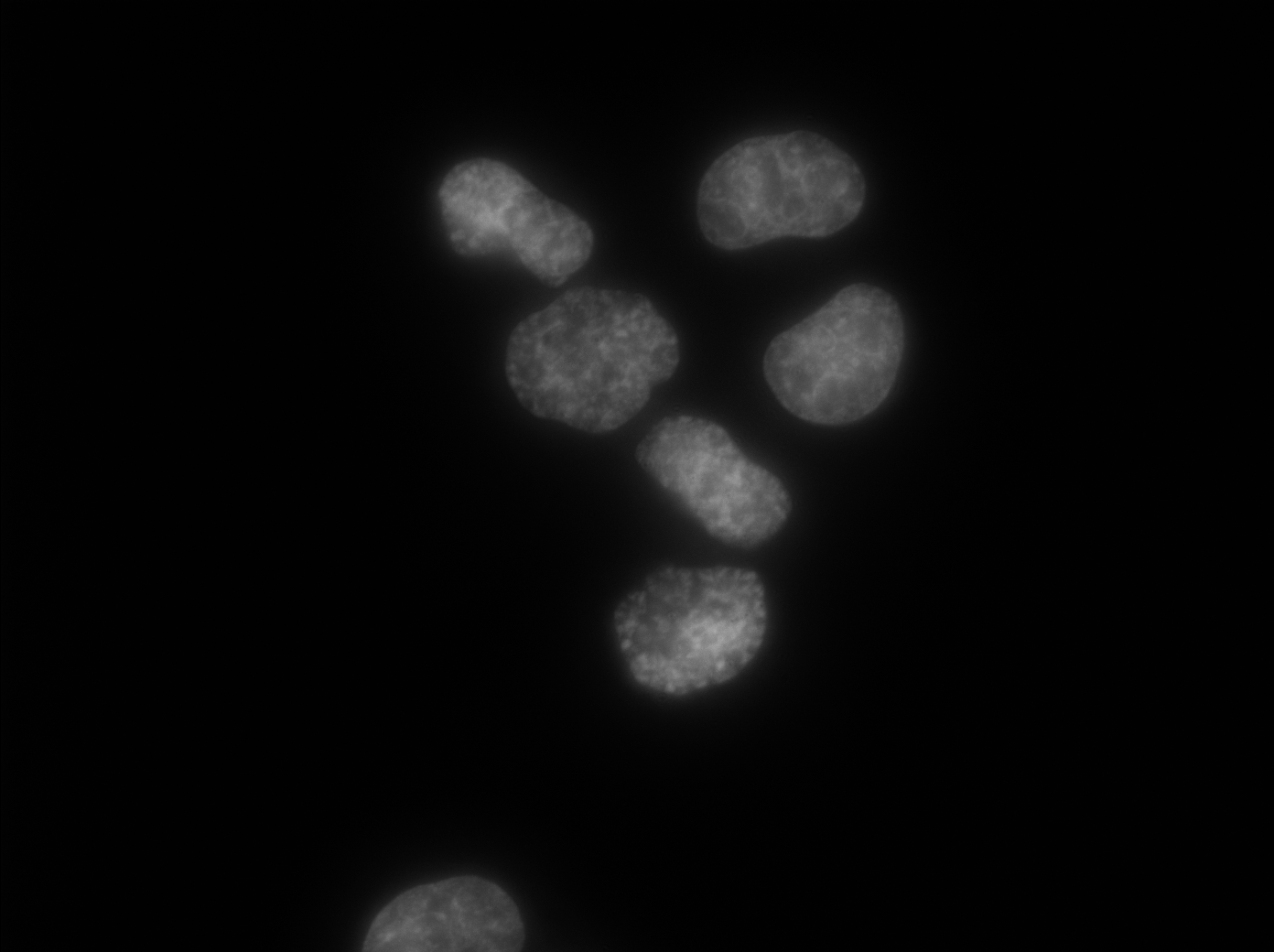

Supplement: Supplementary file 26 — Source data Fig. 7 part 2 [file 44319_2026_742_MOESM26_ESM.zip › Figure 7 Part 2/Fig 7acd Cas9 and TPGS1-ko rGT335 atubulin part 2/TPGS1-KO GT335recomb atub 3-24-25 R2 ET5ET6.Project Maximum Z_XY1742841534_Z0_T0_C0.tif]

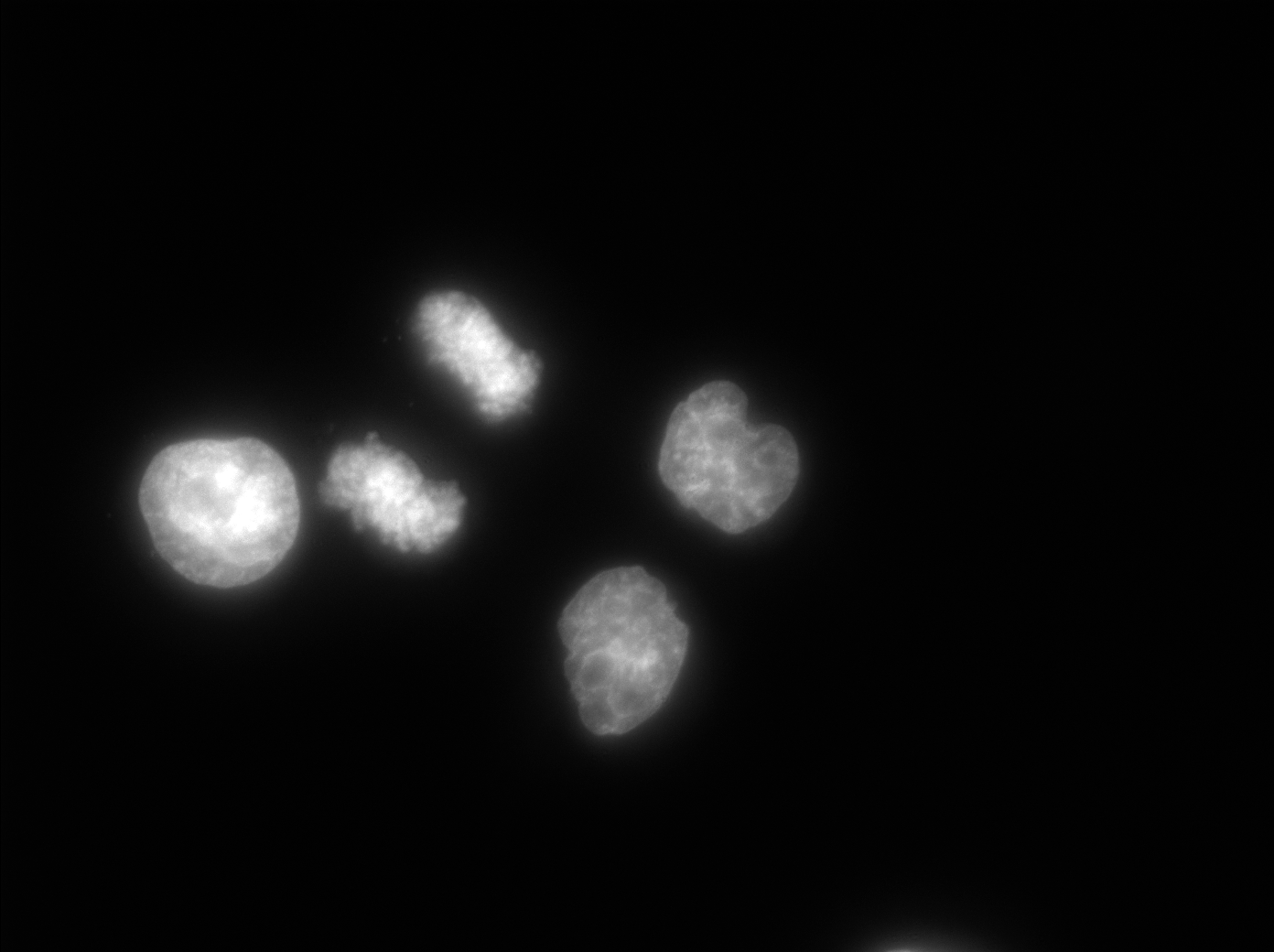

Supplement: Supplementary file 26 — Source data Fig. 7 part 2 [file 44319_2026_742_MOESM26_ESM.zip › Figure 7 Part 2/Fig 7acd Cas9 and TPGS1-ko rGT335 atubulin part 2/TPGS1-KO GT335recomb atub 3-24-25 R1 ET7.Project Maximum Z_XY1742840554_Z0_T0_C0.tif]

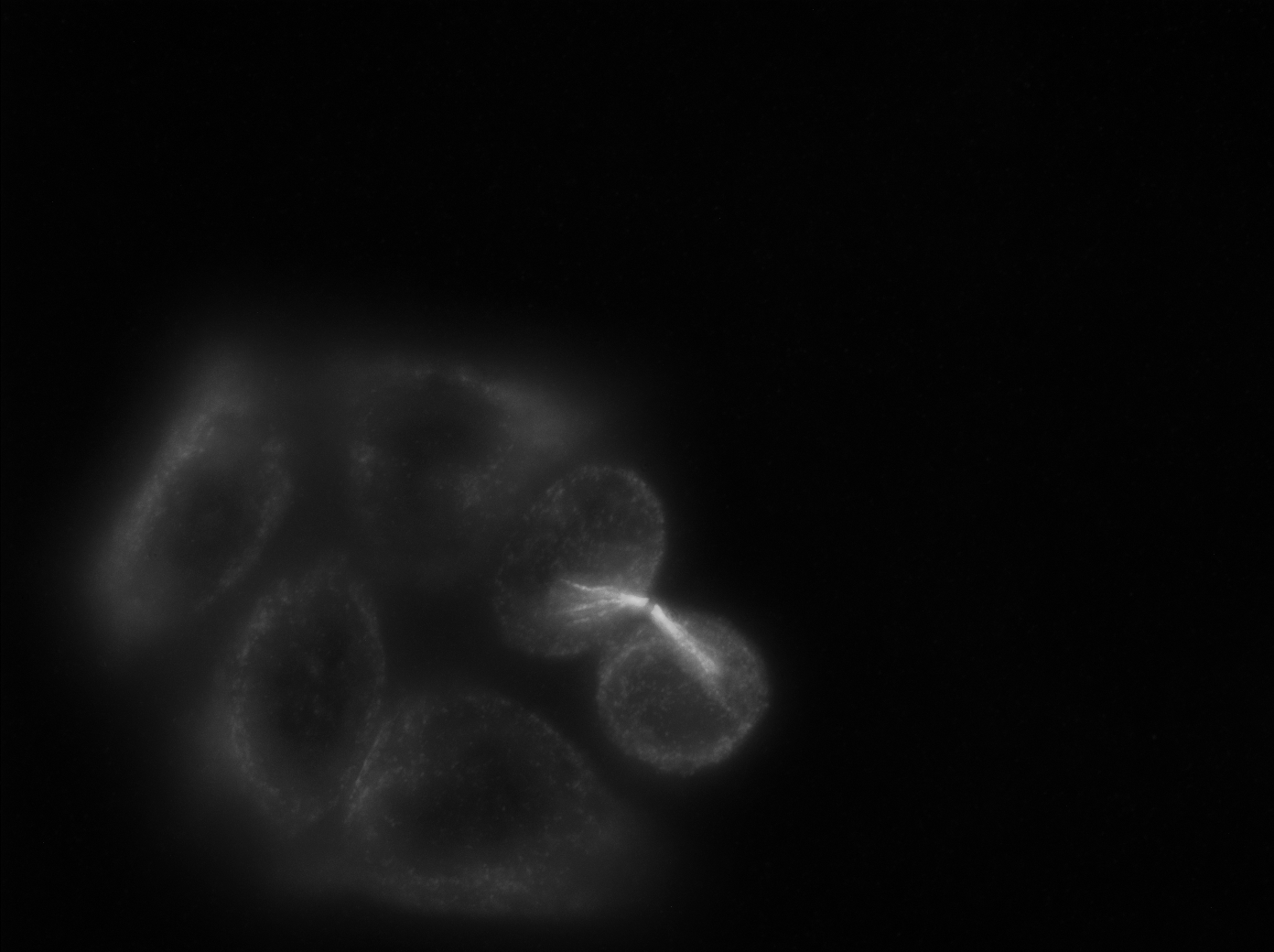

Supplement: Supplementary file 26 — Source data Fig. 7 part 2 [file 44319_2026_742_MOESM26_ESM.zip › Figure 7 Part 2/Fig 7acd Cas9 and TPGS1-ko rGT335 atubulin part 2/TPGS1-KO GT335recomb atub 3-24-25 R1 LT1.Project Maximum Z_XY1742838929_Z0_T0_C2.tif]

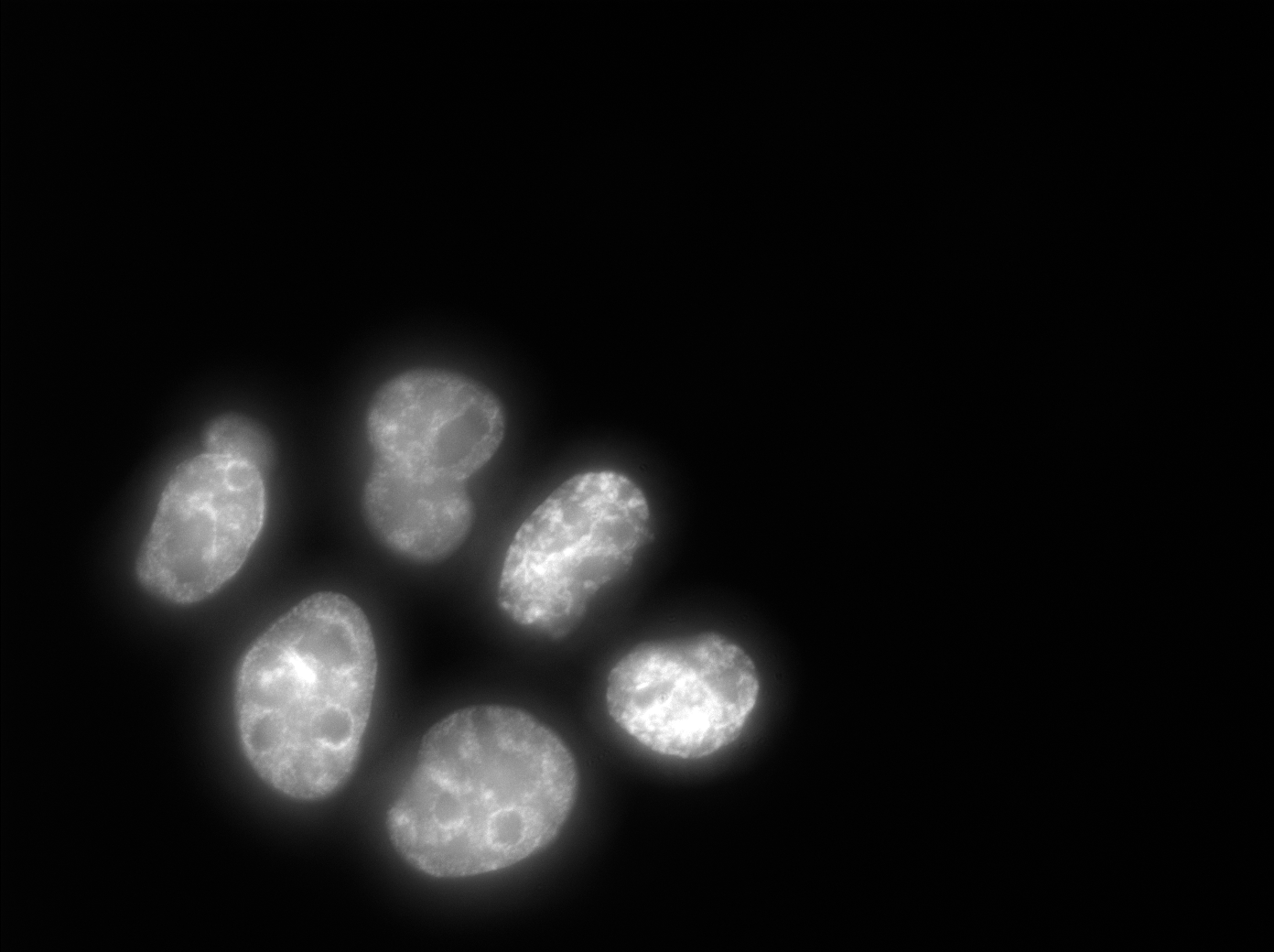

Supplement: Supplementary file 26 — Source data Fig. 7 part 2 [file 44319_2026_742_MOESM26_ESM.zip › Figure 7 Part 2/Fig 7acd Cas9 and TPGS1-ko rGT335 atubulin part 2/TPGS1-KO GT335recomb atub 3-24-25 R1 LT1.Project Maximum Z_XY1742838929_Z0_T0_C0.tif]

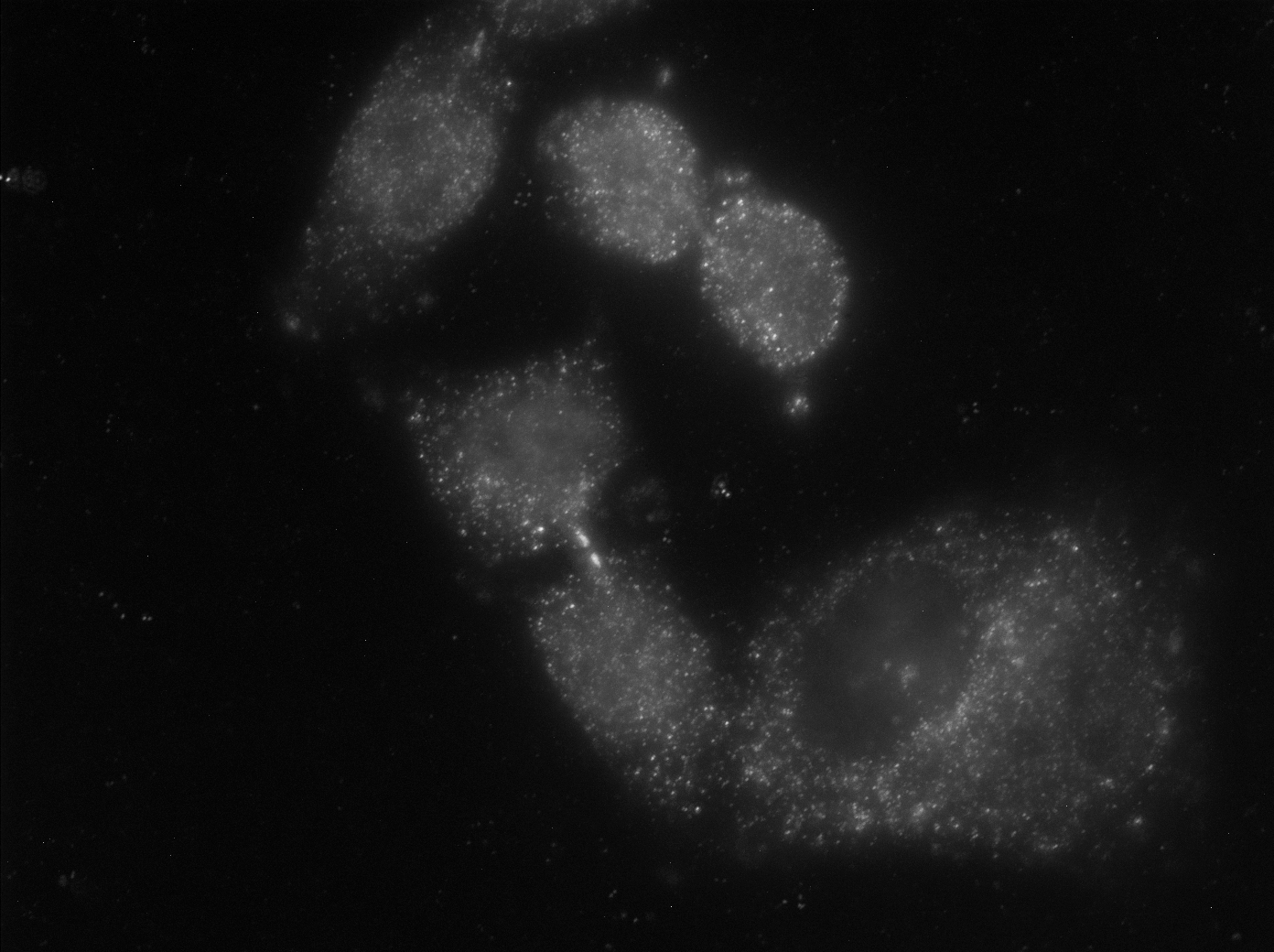

Supplement: Supplementary file 26 — Source data Fig. 7 part 2 [file 44319_2026_742_MOESM26_ESM.zip › Figure 7 Part 2/Fig 7acd Cas9 and TPGS1-ko rGT335 atubulin part 2/TPGS1-KO GT335recomb atub 3-24-25 R1 ET9 LT10.Project Maximum Z_XY1742840753_Z0_T0_C1.tif]

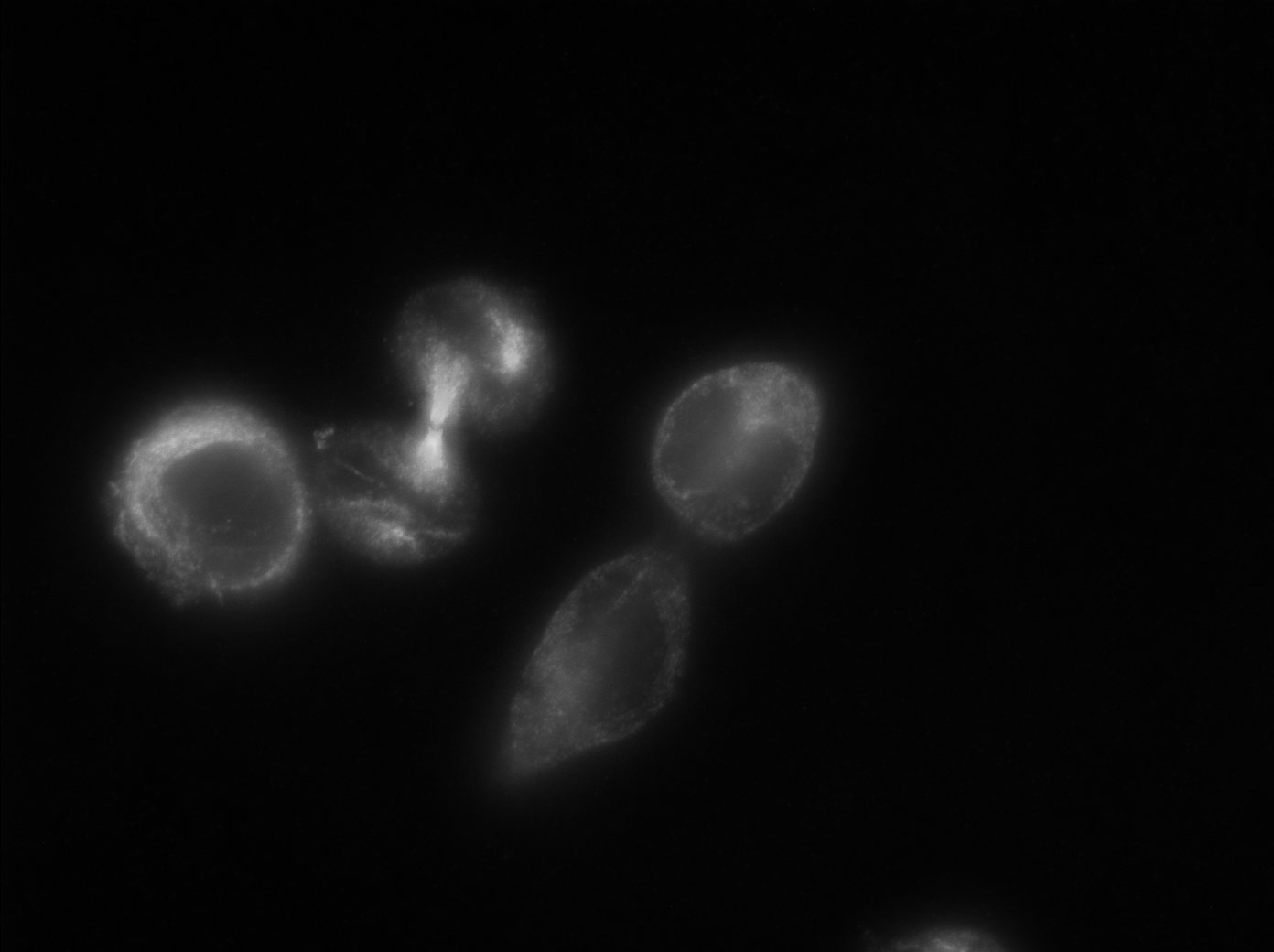

Supplement: Supplementary file 26 — Source data Fig. 7 part 2 [file 44319_2026_742_MOESM26_ESM.zip › Figure 7 Part 2/Fig 7acd Cas9 and TPGS1-ko rGT335 atubulin part 2/TPGS1-KO GT335recomb atub 3-24-25 R1 ET7.Project Maximum Z_XY1742840554_Z0_T0_C2.tif]

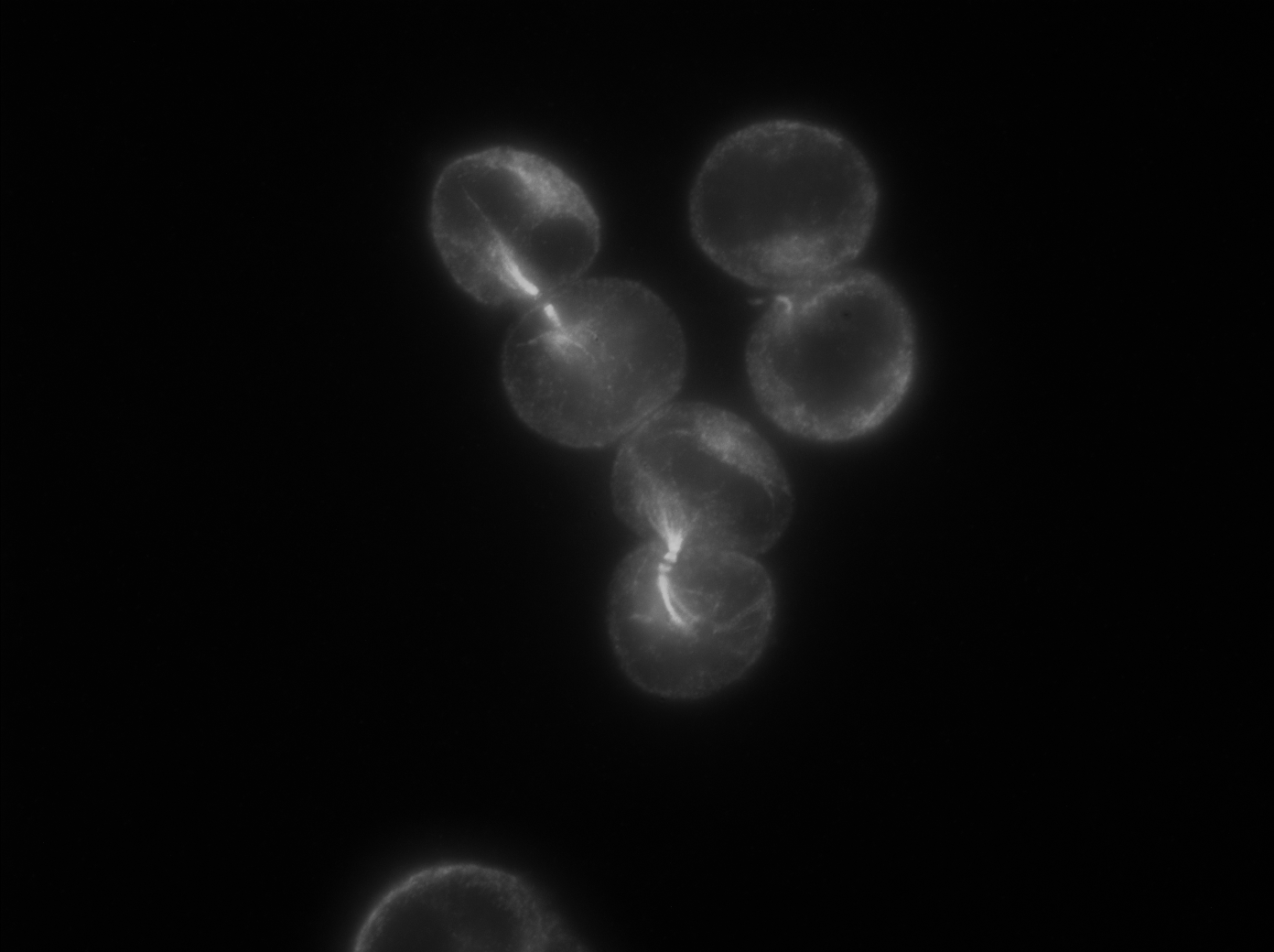

Supplement: Supplementary file 26 — Source data Fig. 7 part 2 [file 44319_2026_742_MOESM26_ESM.zip › Figure 7 Part 2/Fig 7acd Cas9 and TPGS1-ko rGT335 atubulin part 2/TPGS1-KO GT335recomb atub 3-24-25 R2 ET5ET6.Project Maximum Z_XY1742841534_Z0_T0_C2.tif]

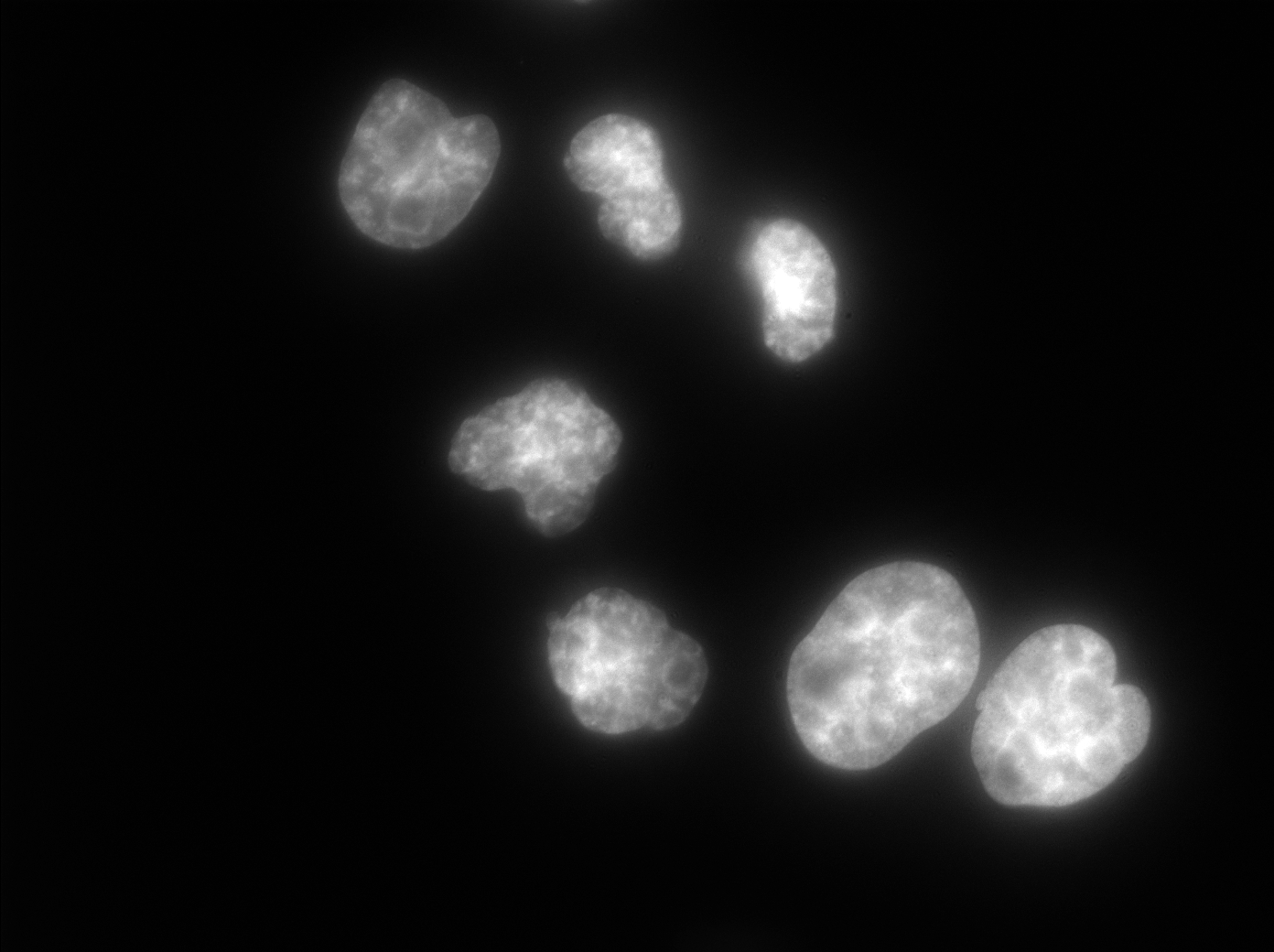

Supplement: Supplementary file 26 — Source data Fig. 7 part 2 [file 44319_2026_742_MOESM26_ESM.zip › Figure 7 Part 2/Fig 7acd Cas9 and TPGS1-ko rGT335 atubulin part 2/TPGS1-KO GT335recomb atub 3-24-25 R1 ET9 LT10.Project Maximum Z_XY1742840753_Z0_T0_C0.tif]

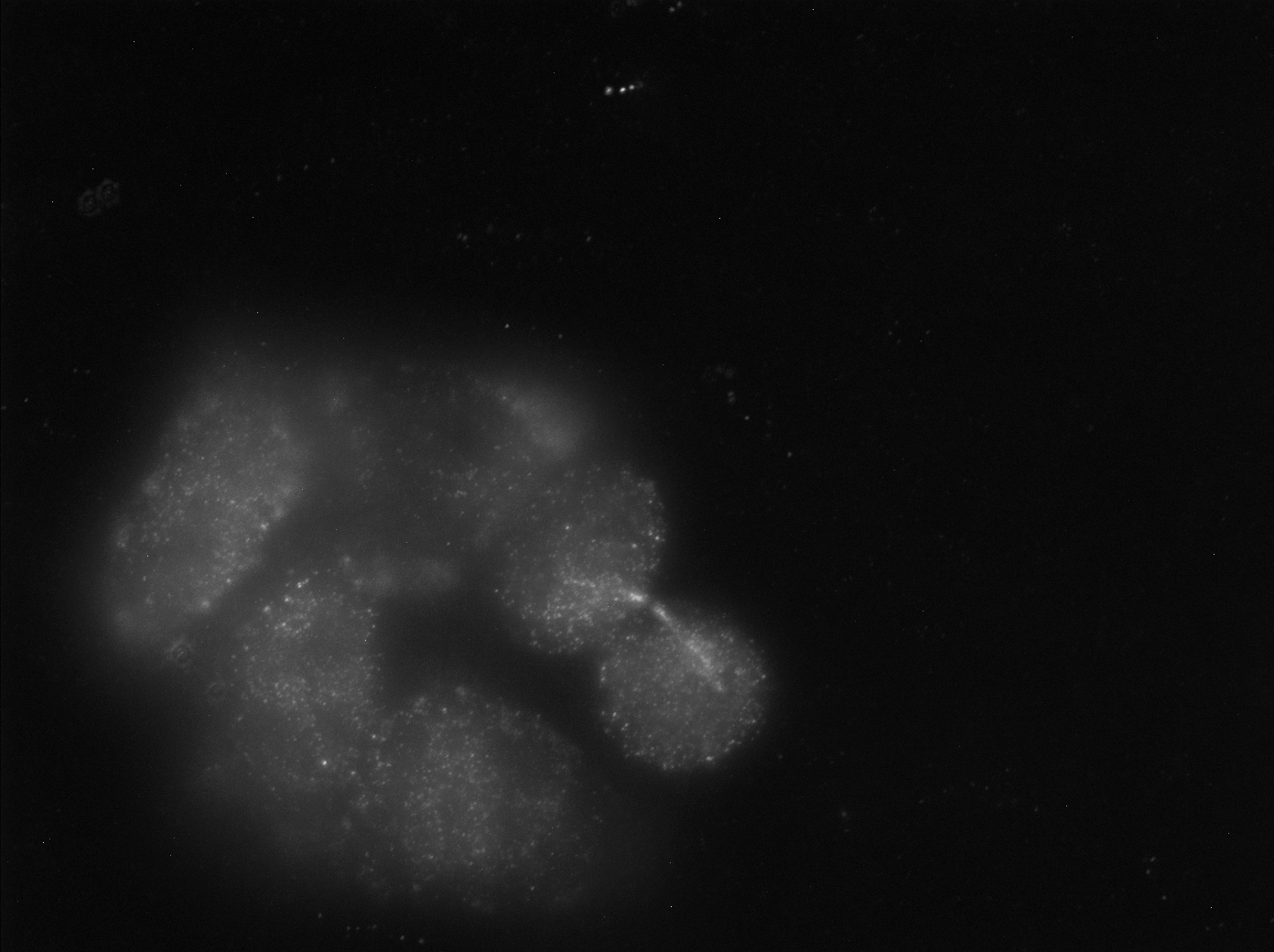

Supplement: Supplementary file 26 — Source data Fig. 7 part 2 [file 44319_2026_742_MOESM26_ESM.zip › Figure 7 Part 2/Fig 7acd Cas9 and TPGS1-ko rGT335 atubulin part 2/TPGS1-KO GT335recomb atub 3-24-25 R1 LT1.Project Maximum Z_XY1742838929_Z0_T0_C1.tif]

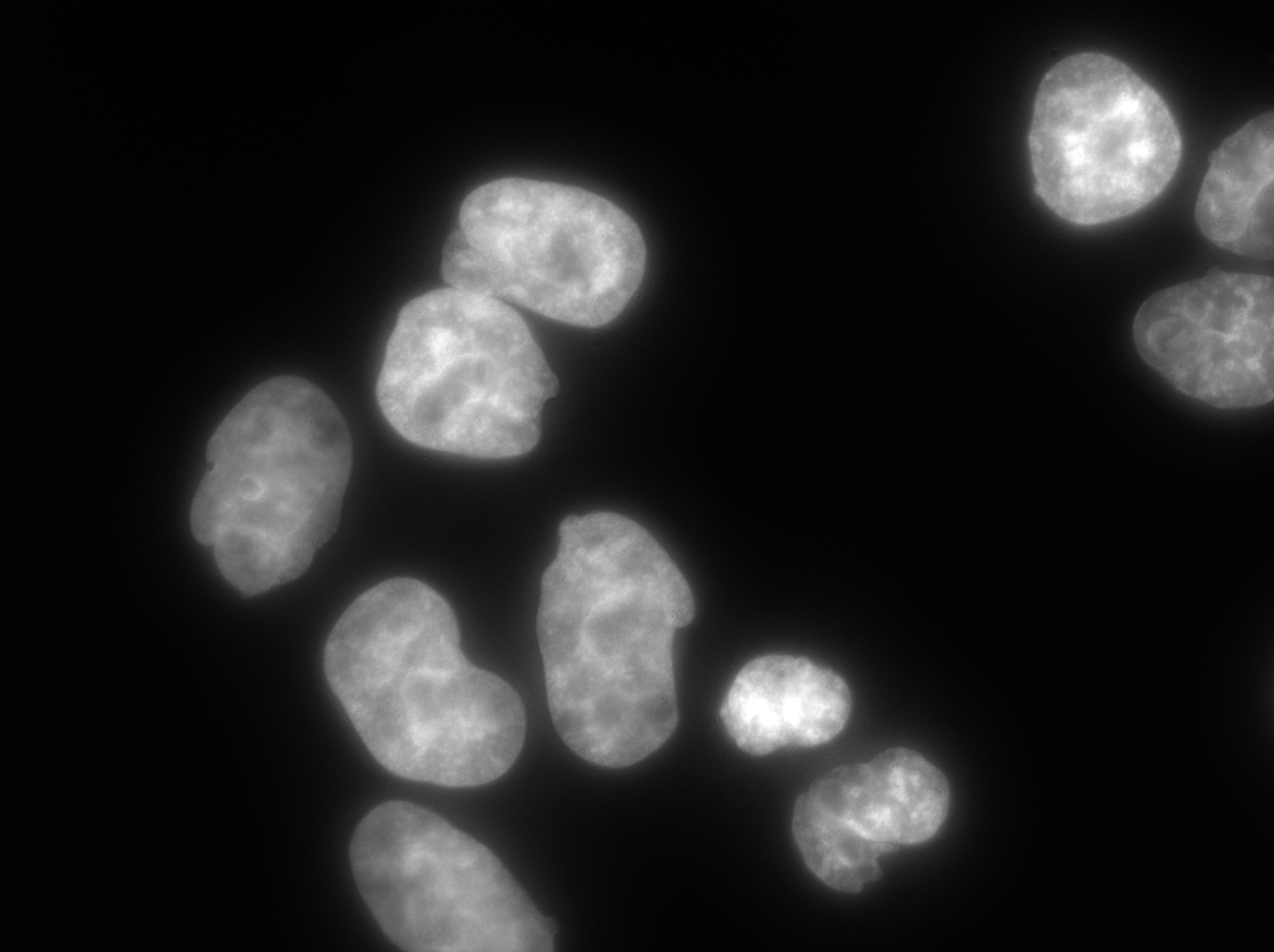

Supplement: Supplementary file 26 — Source data Fig. 7 part 2 [file 44319_2026_742_MOESM26_ESM.zip › Figure 7 Part 2/Fig 7acd Cas9 and TPGS1-ko rGT335 atubulin part 2/TPGS1-KO GT335recomb atub 3-24-25 R1 ET4.Project Maximum Z_XY1742840170_Z0_T0_C0.tif]

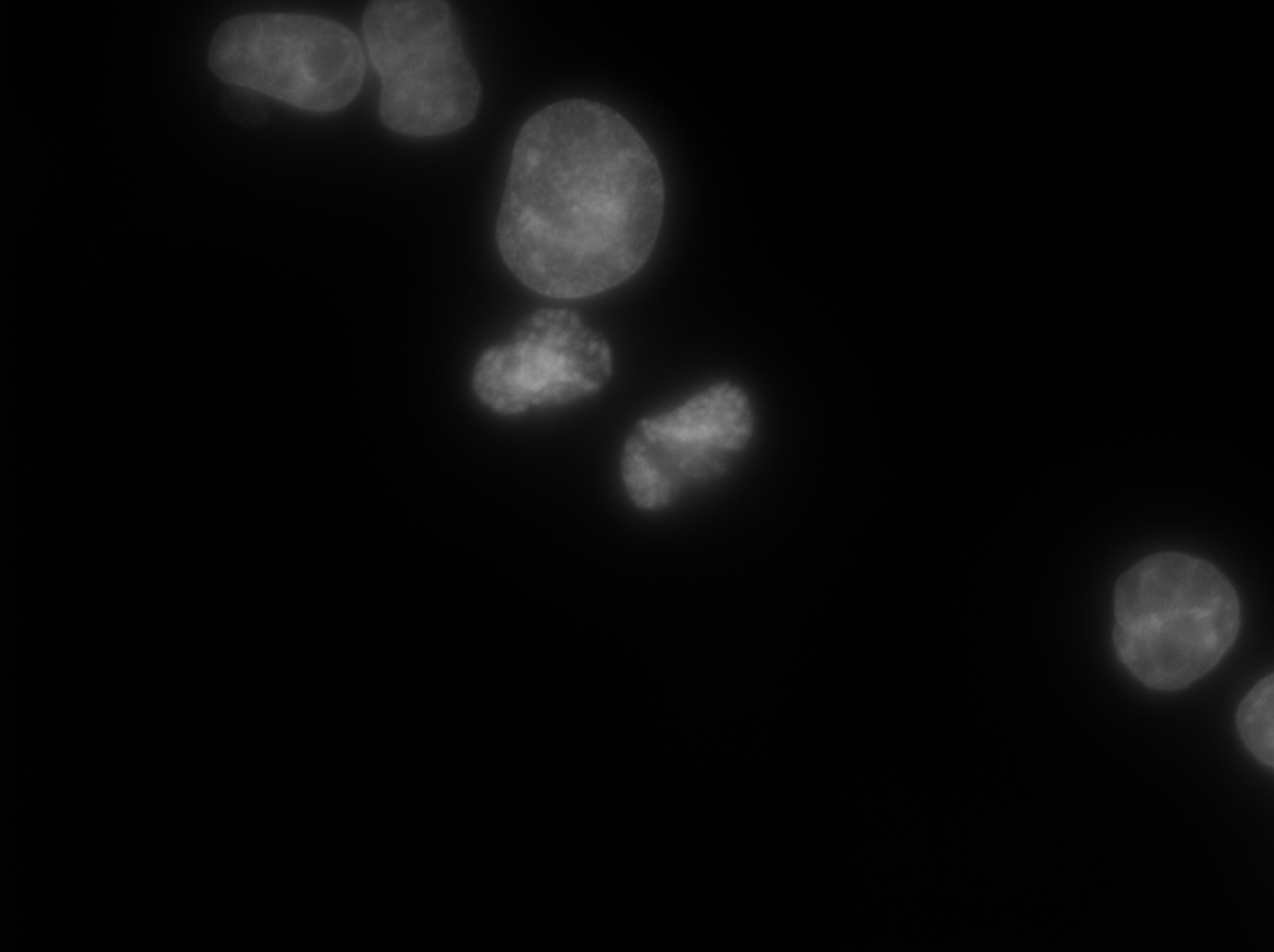

Supplement: Supplementary file 26 — Source data Fig. 7 part 2 [file 44319_2026_742_MOESM26_ESM.zip › Figure 7 Part 2/Fig 7acd Cas9 and TPGS1-ko rGT335 atubulin part 2/TPGS1-KO GT335recomb atub 3-24-25 R2 ET8.Project Maximum Z_XY1742842061_Z0_T0_C0.tif]

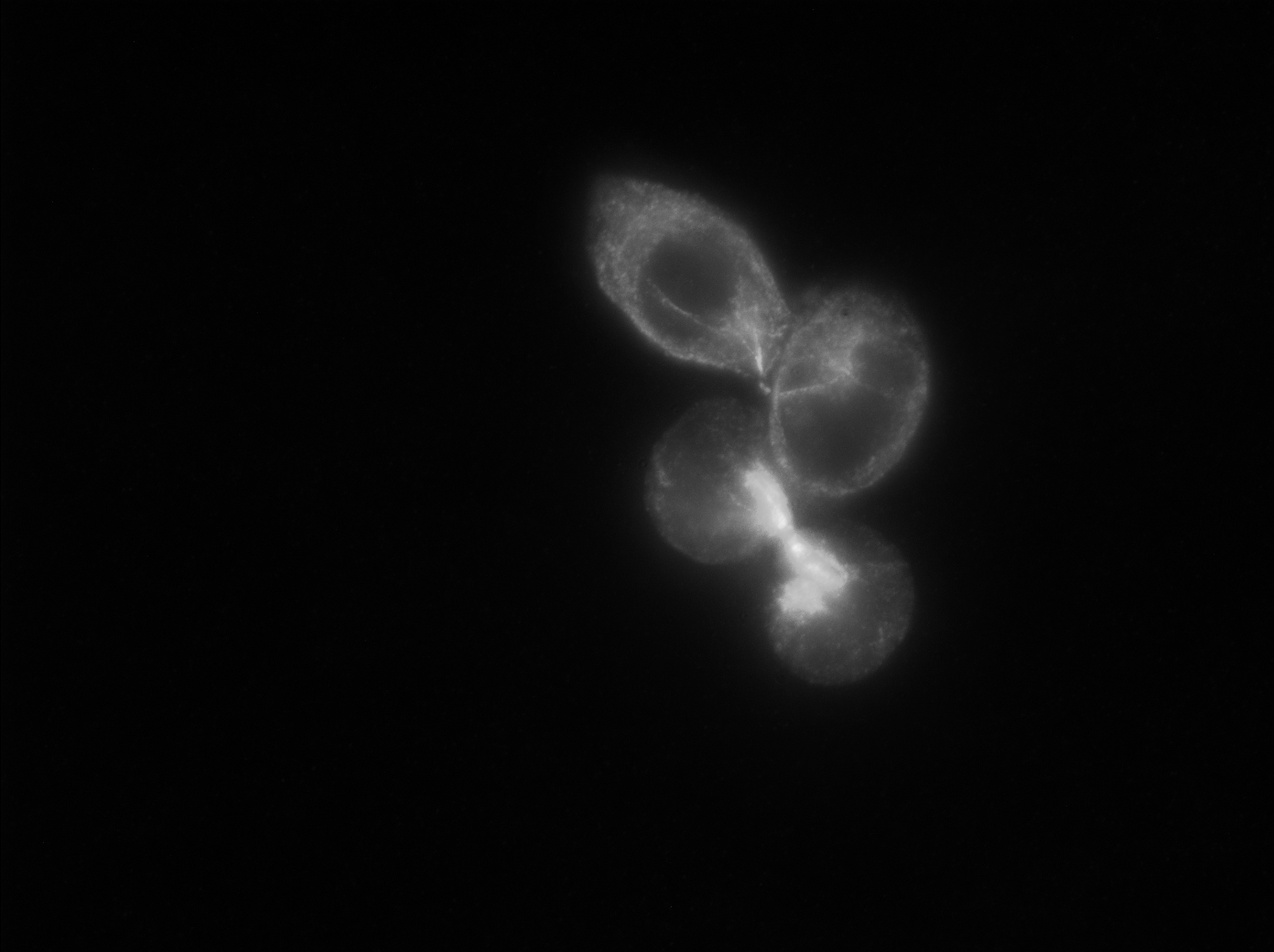

Supplement: Supplementary file 26 — Source data Fig. 7 part 2 [file 44319_2026_742_MOESM26_ESM.zip › Figure 7 Part 2/Fig 7acd Cas9 and TPGS1-ko rGT335 atubulin part 2/TPGS1-KO GT335recomb atub 3-24-25 R1 ET3 LT9.Project Maximum Z_XY1742840057_Z0_T0_C2.tif]
