# Supplementary figures and images for: TPGS1 regulates central spindle microtubule glutamylation and remodeling during telophase and abscission (part 30 of 36)
Source: EMBO Rep. 2026 Mar 23;27(8):1944–63. doi: 10.1038/s44319-026-00742-3 (PMC13121839; doi:10.1038/s44319-026-00742-3)

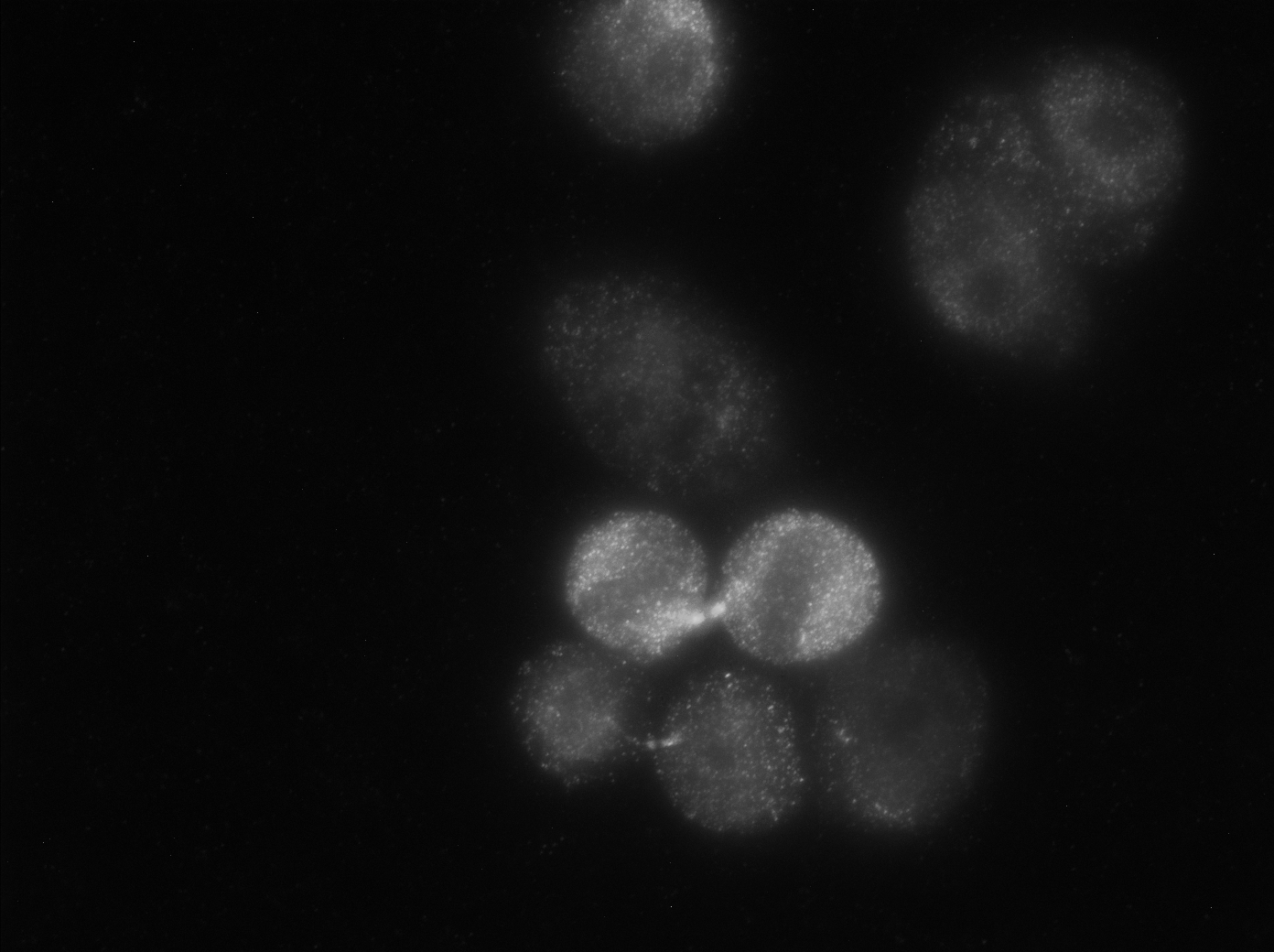

Supplement: Supplementary file 25 — Source data Fig. 7 part 1 [file 44319_2026_742_MOESM25_ESM.zip › Figure 7 Part 1/Fig 7acd Cas9 and TPGS1-ko rGT335 atubulin/Cas9 GT335recomb atub 3-24-25 R1 ET8 PA3.Project Maximum Z_XY1742836284_Z0_T0_C1.tif]

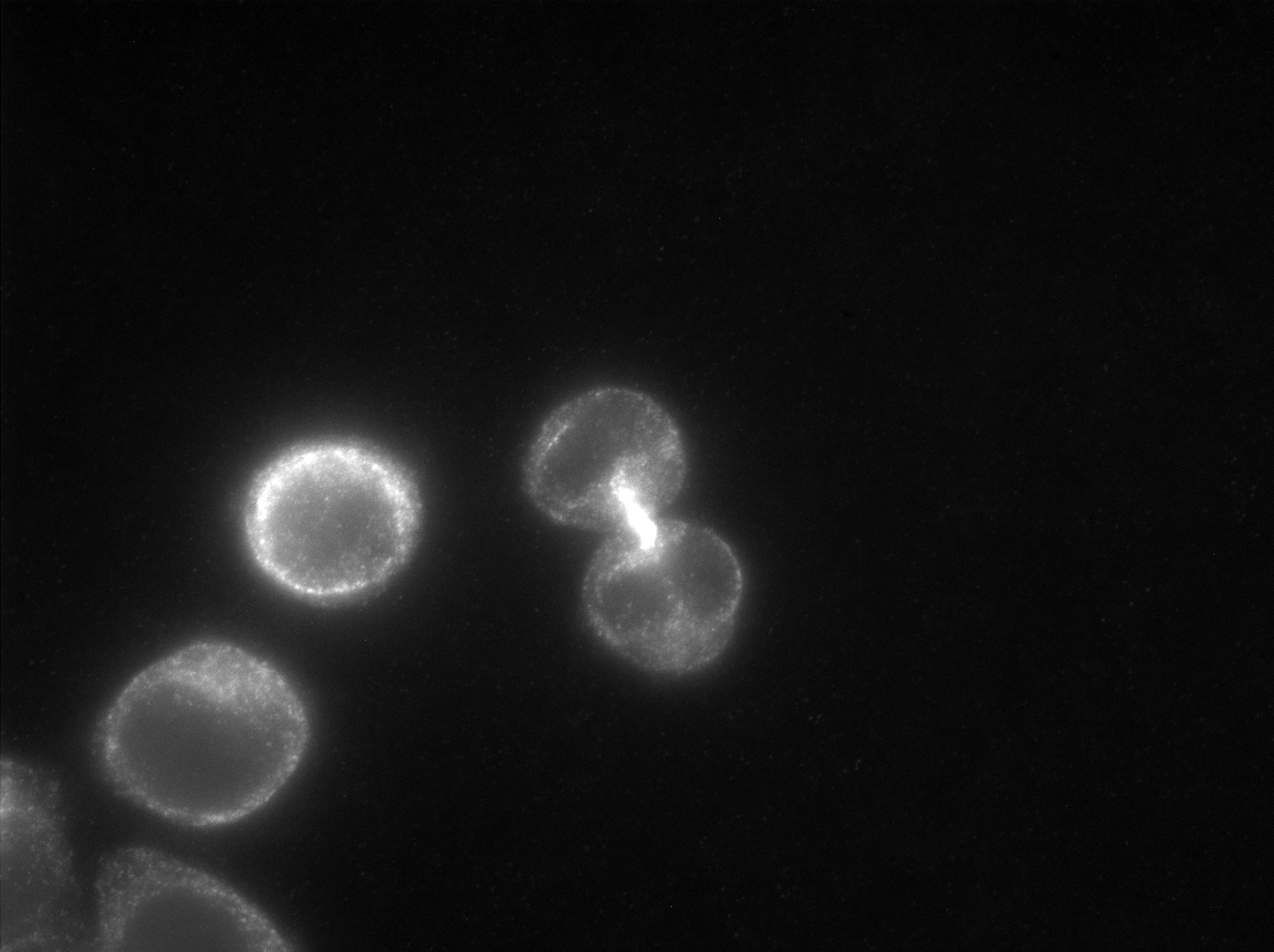

Supplement: Supplementary file 25 — Source data Fig. 7 part 1 [file 44319_2026_742_MOESM25_ESM.zip › Figure 7 Part 1/Fig 7acd Cas9 and TPGS1-ko rGT335 atubulin/Cas9 GT335recomb atub 3-24-25 R3 ET9.Project Maximum Z_XY1742850787_Z0_T0_C2.tif]

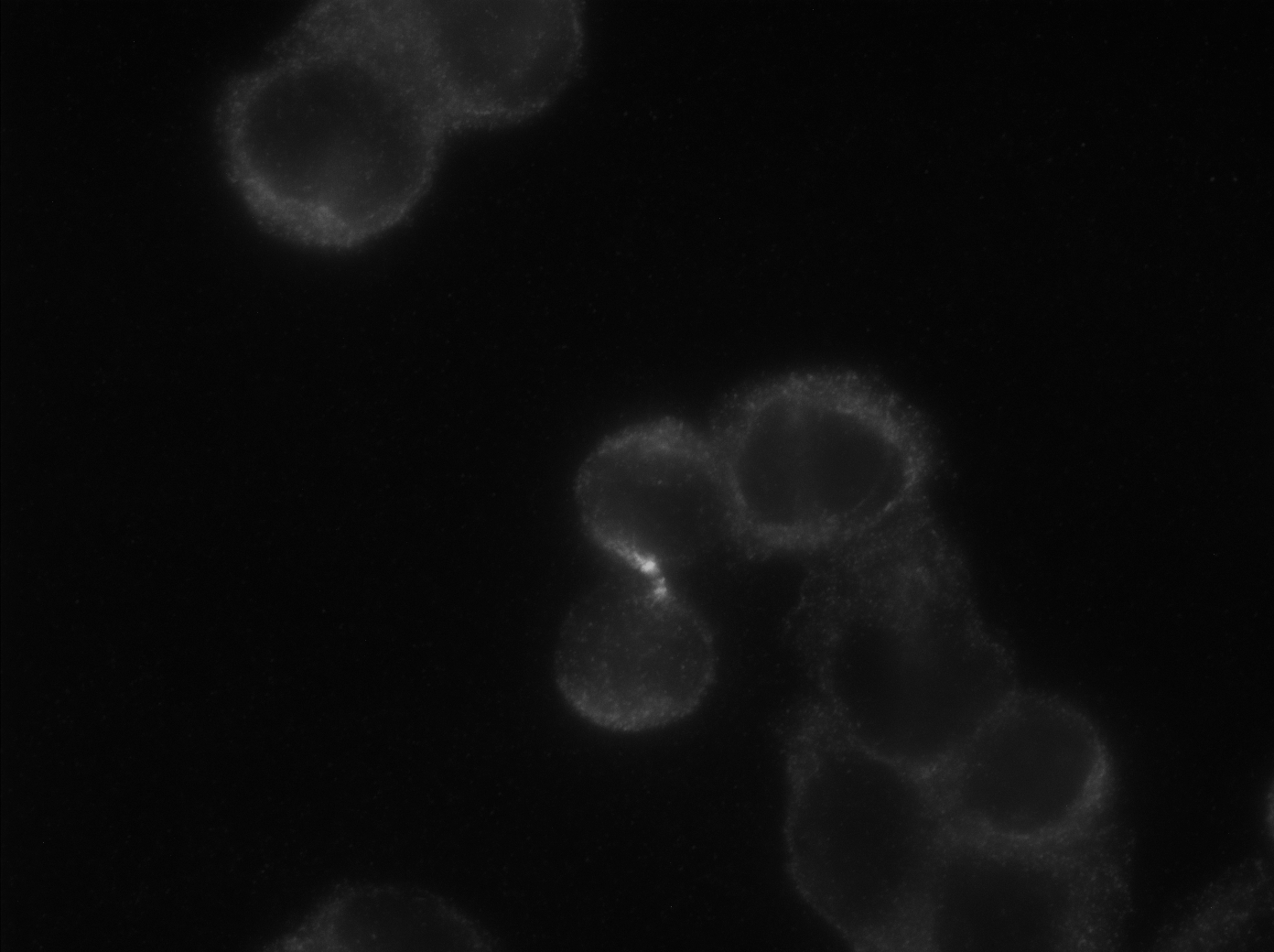

Supplement: Supplementary file 25 — Source data Fig. 7 part 1 [file 44319_2026_742_MOESM25_ESM.zip › Figure 7 Part 1/Fig 7acd Cas9 and TPGS1-ko rGT335 atubulin/Cas9 GT335recomb atub 3-24-25 R2 ET6.Project Maximum Z_XY1742846357_Z0_T0_C2.tif]

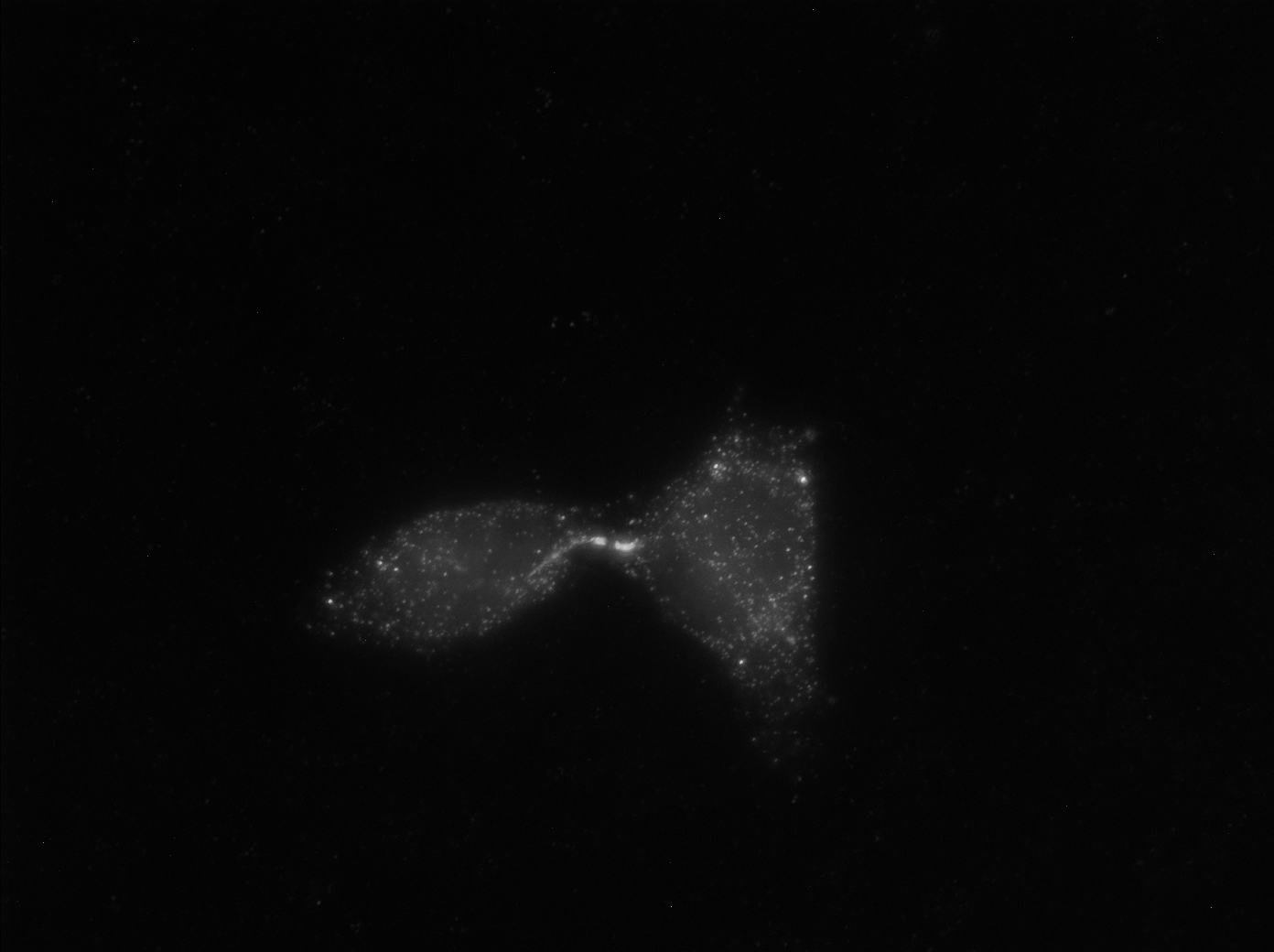

Supplement: Supplementary file 25 — Source data Fig. 7 part 1 [file 44319_2026_742_MOESM25_ESM.zip › Figure 7 Part 1/Fig 7acd Cas9 and TPGS1-ko rGT335 atubulin/Cas9 GT335recomb atub 3-24-25 R1 LT10.Project Maximum Z_XY1742836712_Z0_T0_C1.tif]

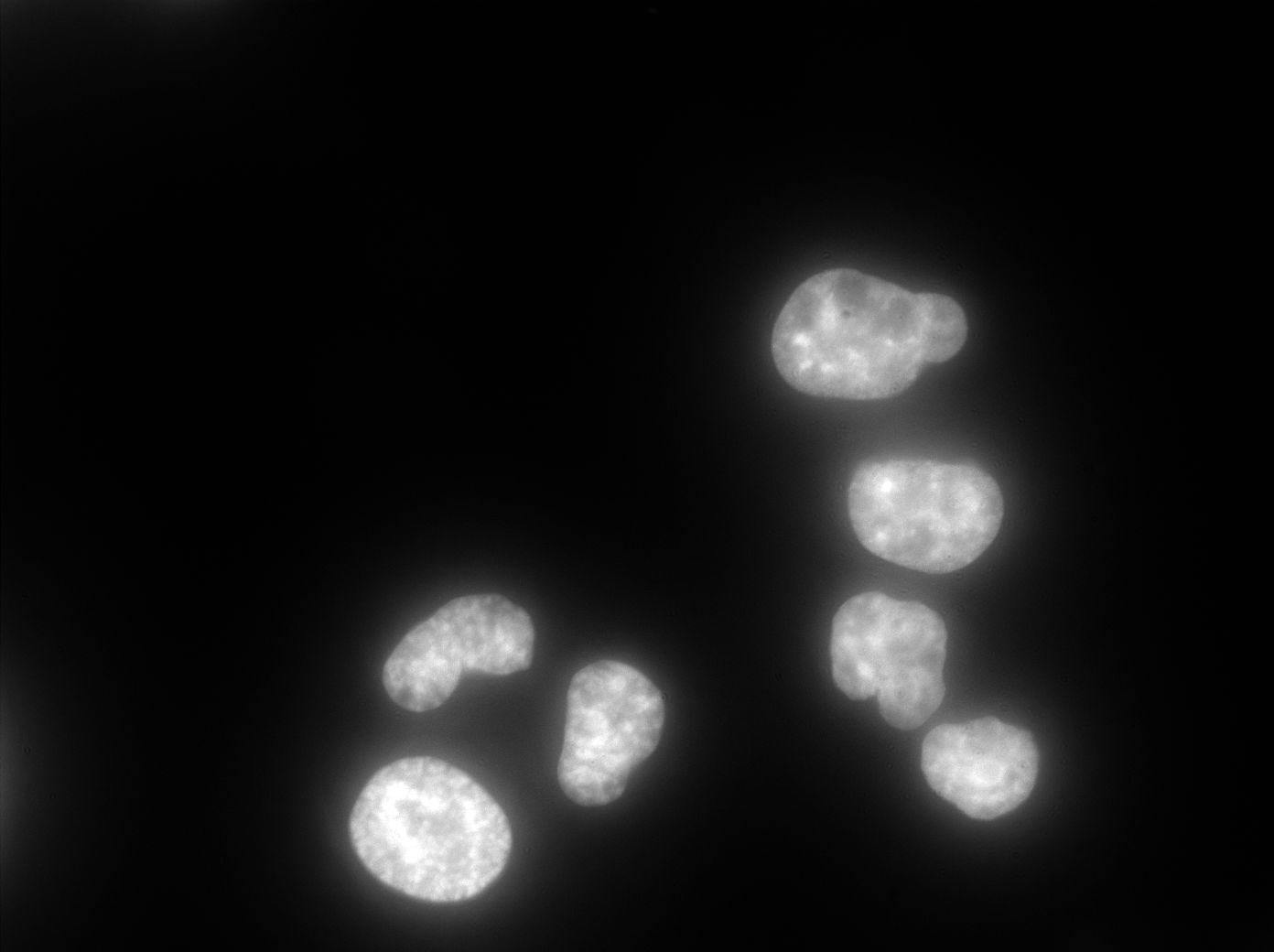

Supplement: Supplementary file 25 — Source data Fig. 7 part 1 [file 44319_2026_742_MOESM25_ESM.zip › Figure 7 Part 1/Fig 7acd Cas9 and TPGS1-ko rGT335 atubulin/Cas9 GT335recomb atub 3-24-25 R3 LT2.Project Maximum Z_XY1742848702_Z0_T0_C0.tif]

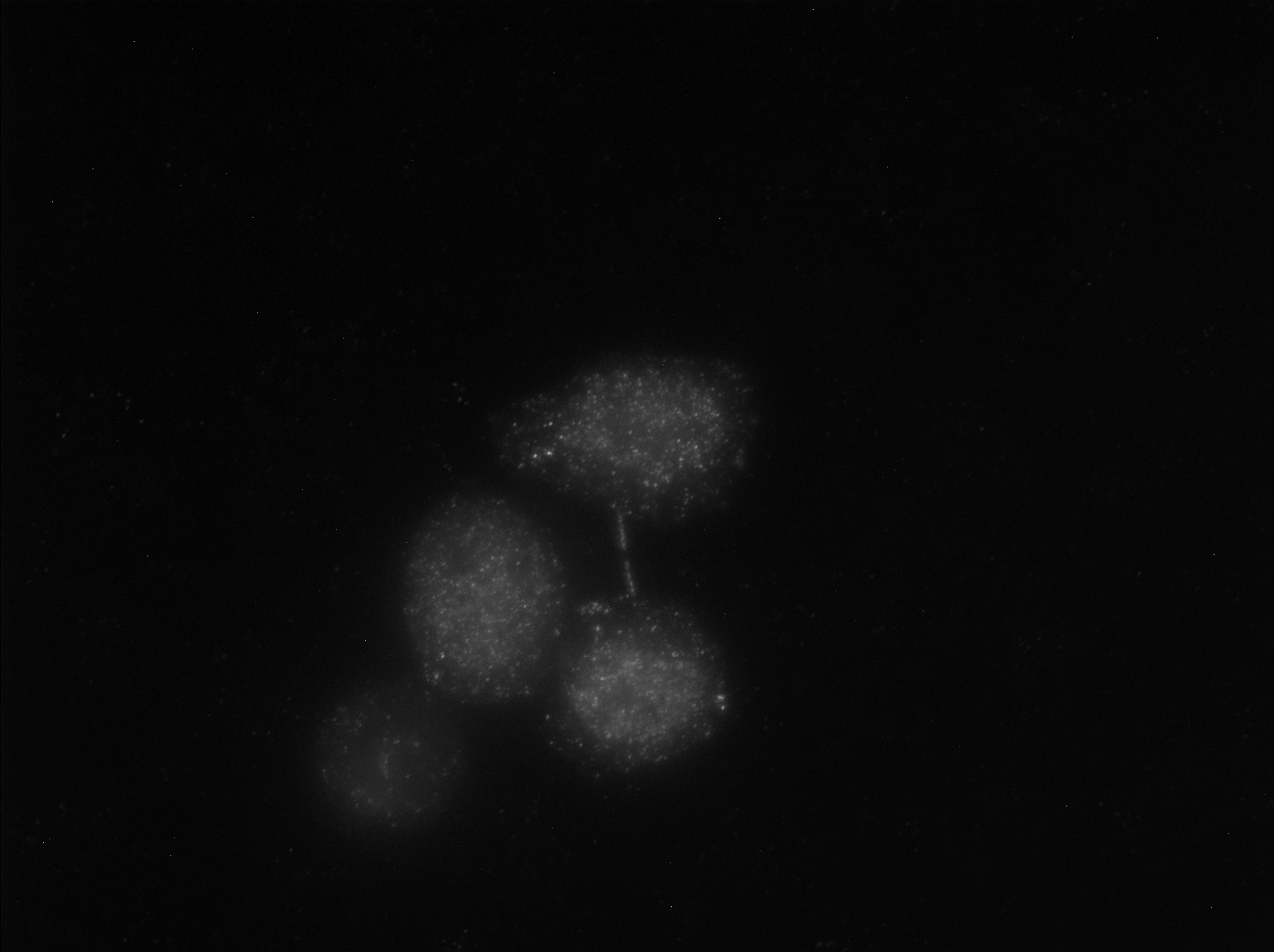

Supplement: Supplementary file 25 — Source data Fig. 7 part 1 [file 44319_2026_742_MOESM25_ESM.zip › Figure 7 Part 1/Fig 7acd Cas9 and TPGS1-ko rGT335 atubulin/Cas9 GT335recomb atub 3-24-25 R3 LT10.Project Maximum Z_XY1742851227_Z0_T0_C1.tif]

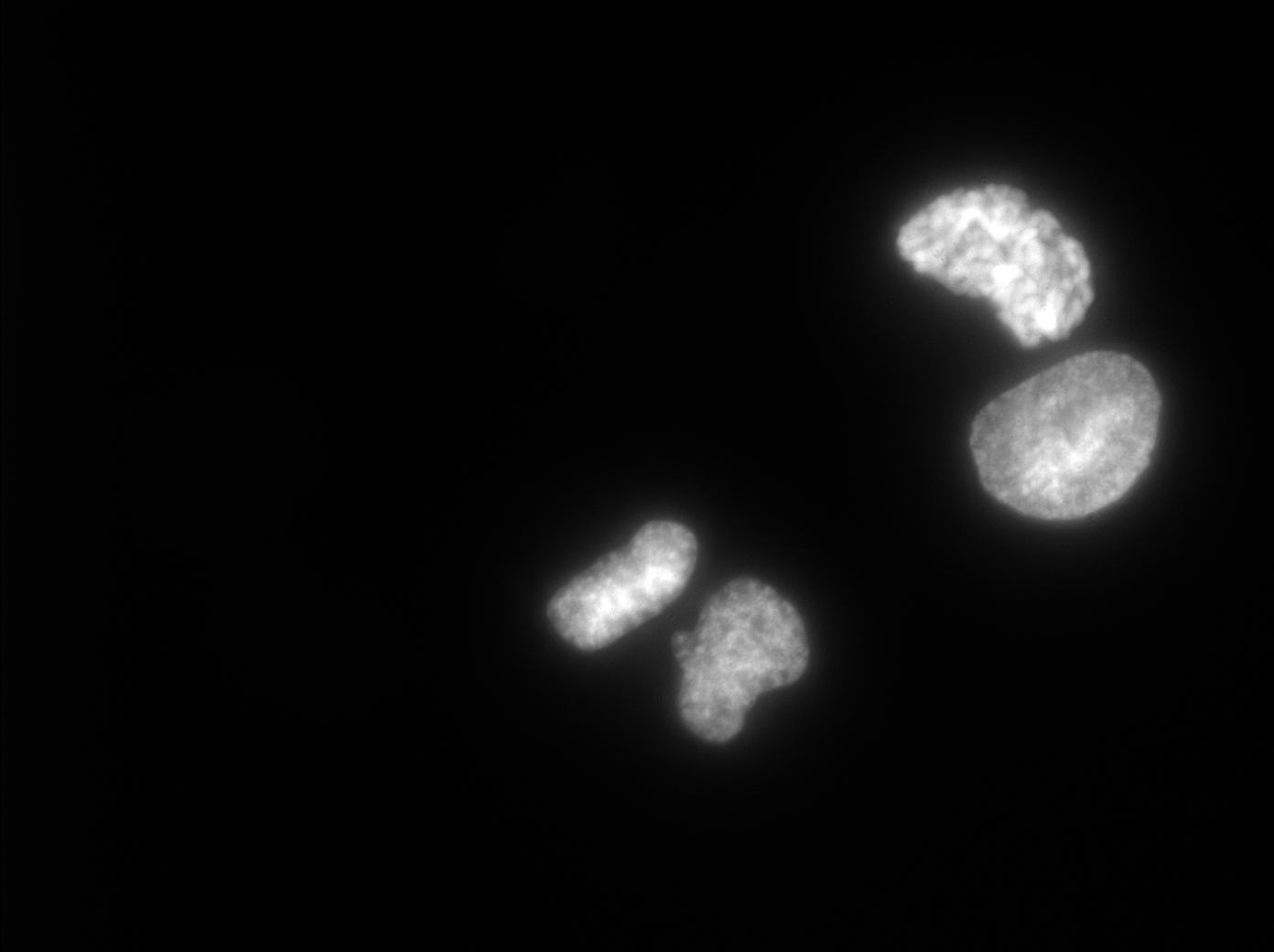

Supplement: Supplementary file 25 — Source data Fig. 7 part 1 [file 44319_2026_742_MOESM25_ESM.zip › Figure 7 Part 1/Fig 7acd Cas9 and TPGS1-ko rGT335 atubulin/Cas9 GT335recomb atub 3-24-25 R2 ET1.Project Maximum Z_XY1742845132_Z0_T0_C0.tif]

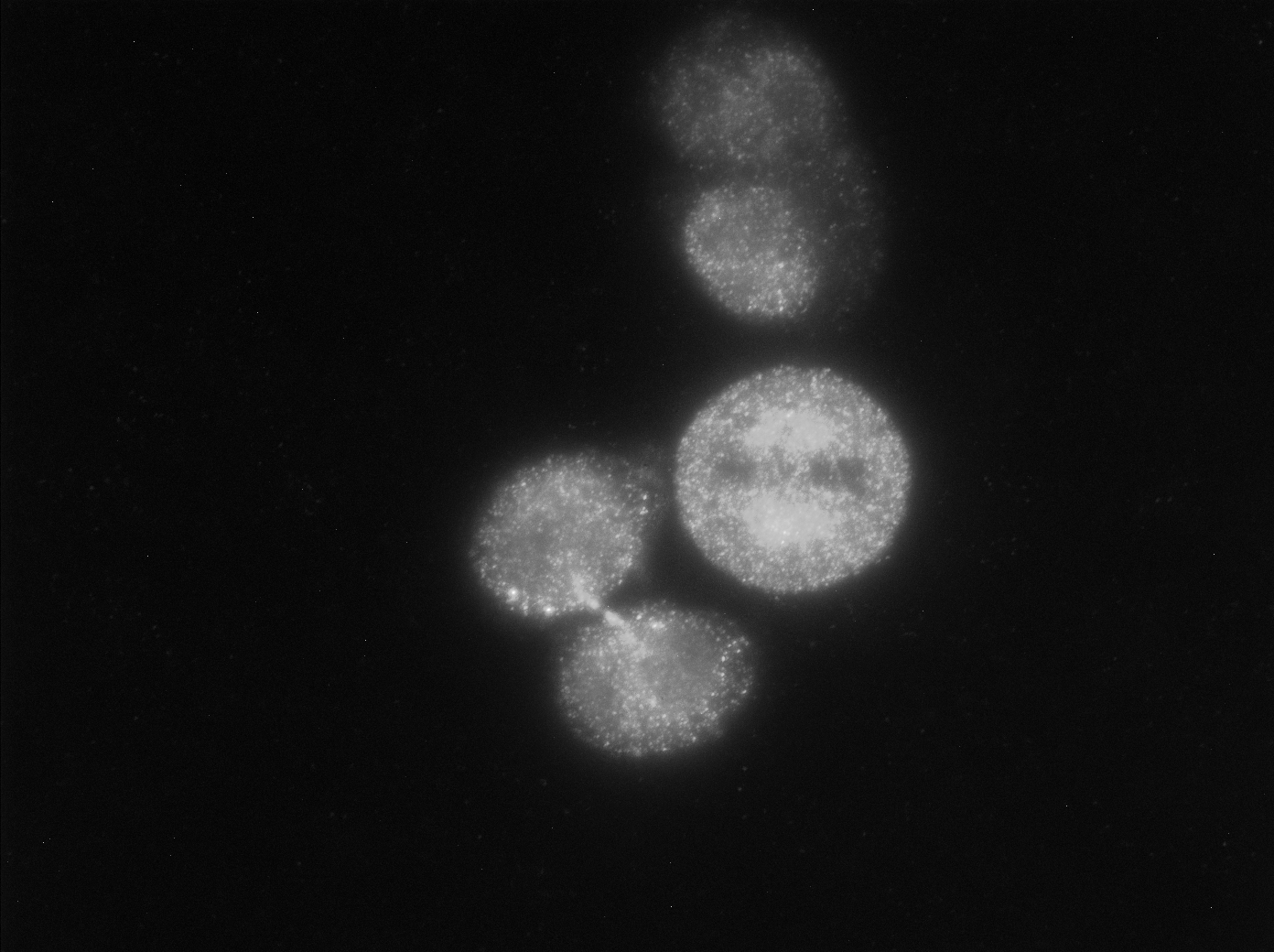

Supplement: Supplementary file 25 — Source data Fig. 7 part 1 [file 44319_2026_742_MOESM25_ESM.zip › Figure 7 Part 1/Fig 7acd Cas9 and TPGS1-ko rGT335 atubulin/Cas9 GT335recomb atub 3-24-25 R2 LT10 M1.Project Maximum Z_XY1742847842_Z0_T0_C1.tif]

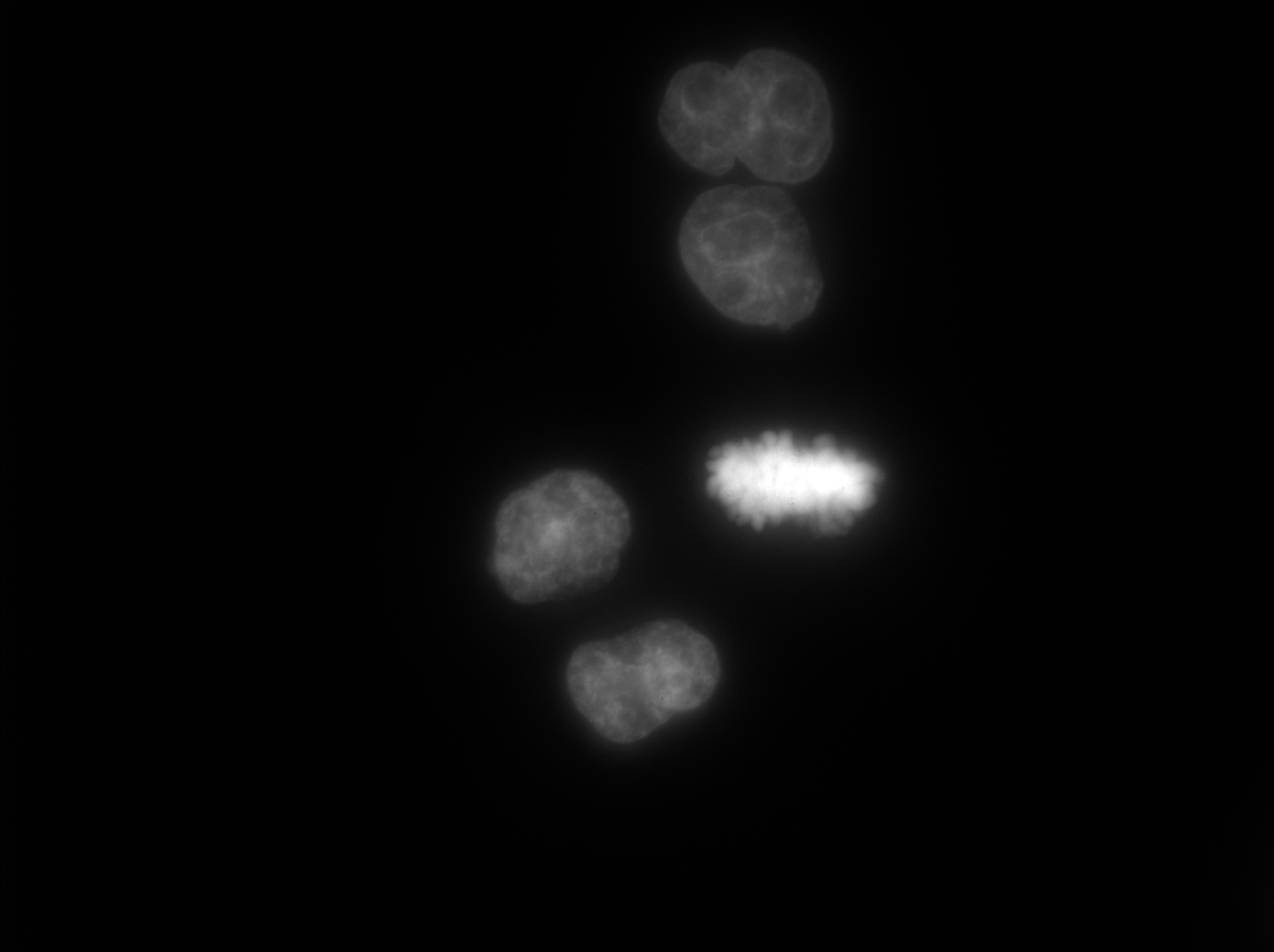

Supplement: Supplementary file 25 — Source data Fig. 7 part 1 [file 44319_2026_742_MOESM25_ESM.zip › Figure 7 Part 1/Fig 7acd Cas9 and TPGS1-ko rGT335 atubulin/Cas9 GT335recomb atub 3-24-25 R2 LT10 M1.Project Maximum Z_XY1742847842_Z0_T0_C0.tif]

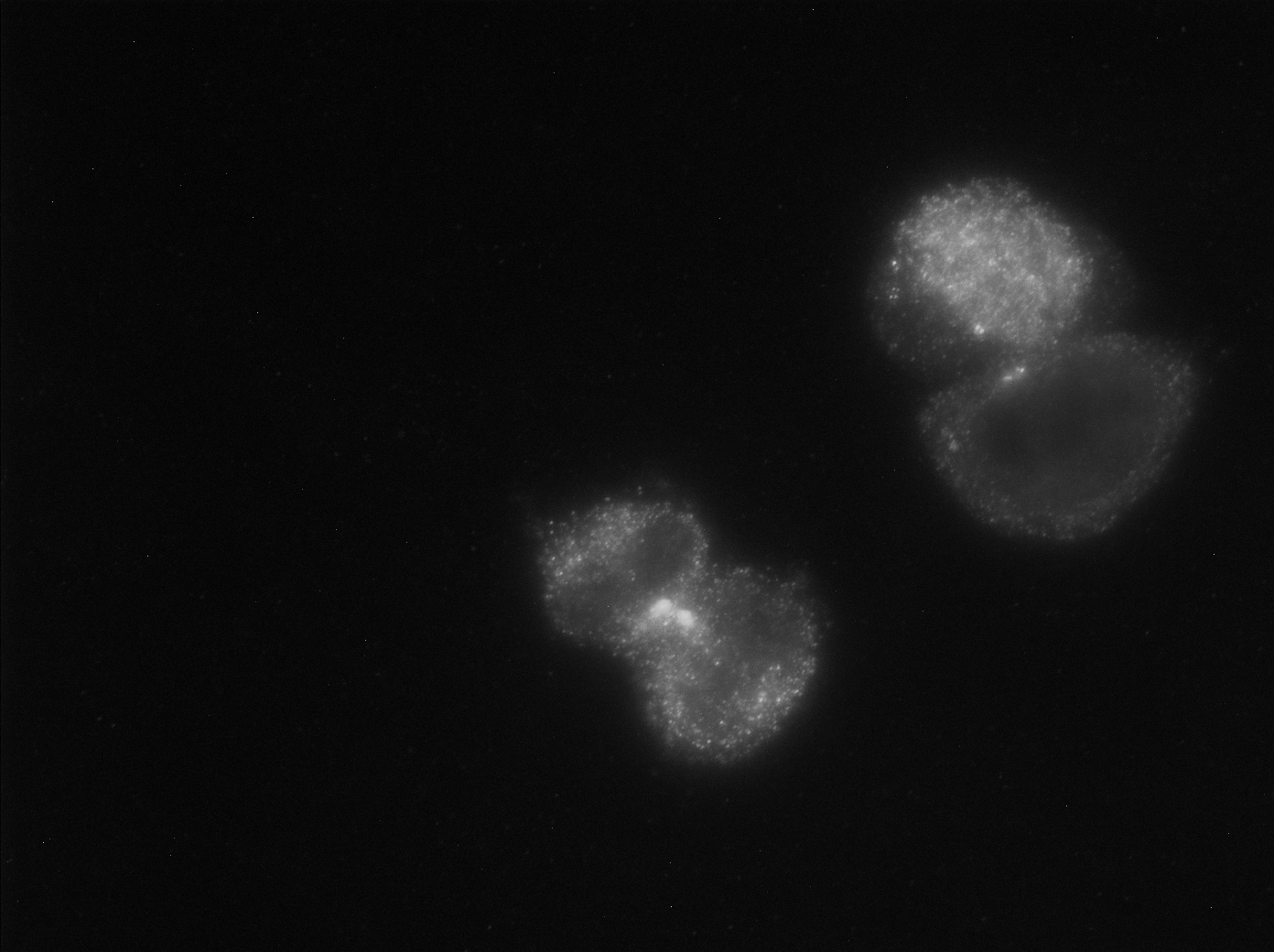

Supplement: Supplementary file 25 — Source data Fig. 7 part 1 [file 44319_2026_742_MOESM25_ESM.zip › Figure 7 Part 1/Fig 7acd Cas9 and TPGS1-ko rGT335 atubulin/Cas9 GT335recomb atub 3-24-25 R2 ET1.Project Maximum Z_XY1742845132_Z0_T0_C1.tif]

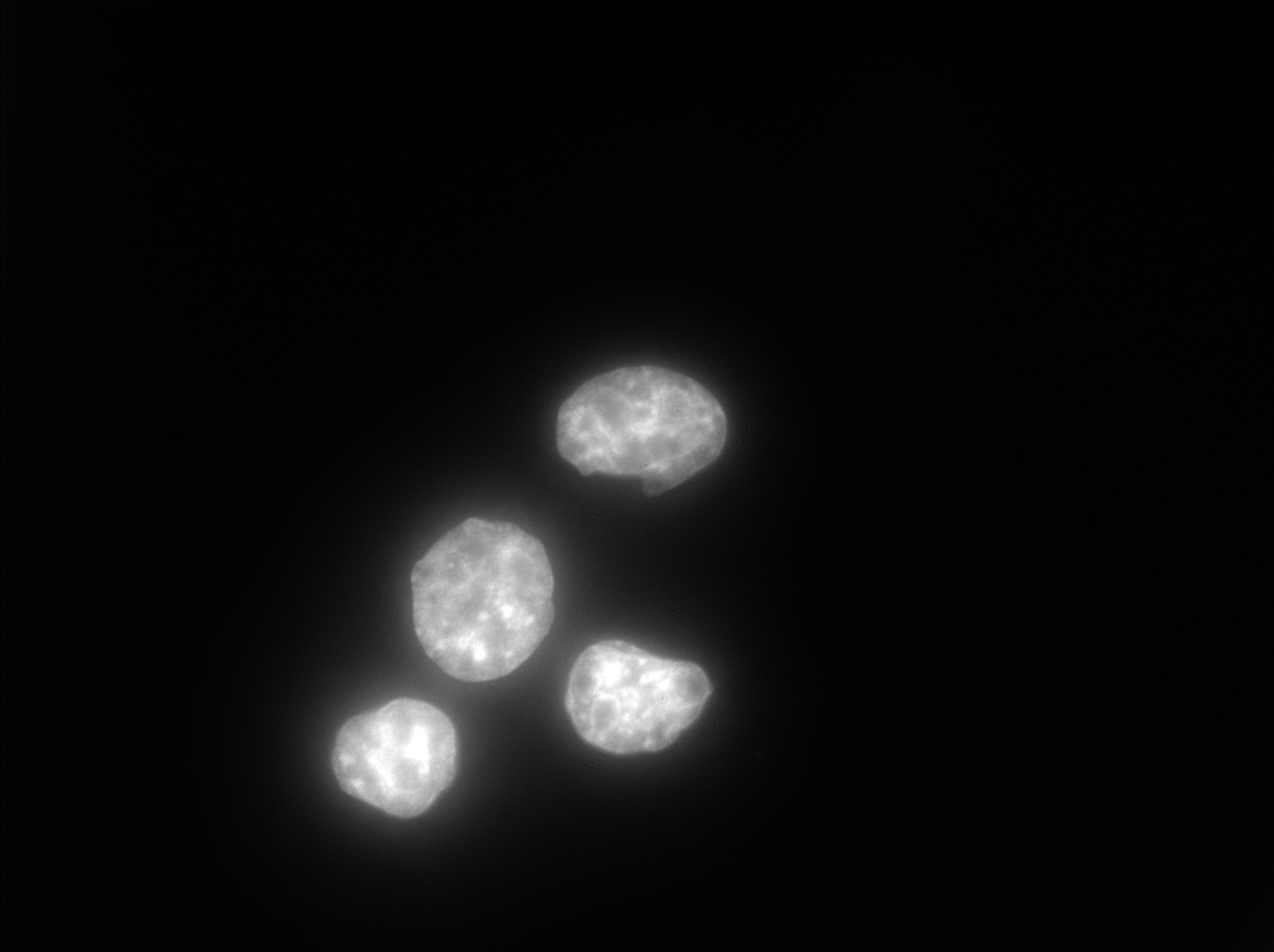

Supplement: Supplementary file 25 — Source data Fig. 7 part 1 [file 44319_2026_742_MOESM25_ESM.zip › Figure 7 Part 1/Fig 7acd Cas9 and TPGS1-ko rGT335 atubulin/Cas9 GT335recomb atub 3-24-25 R3 LT10.Project Maximum Z_XY1742851227_Z0_T0_C0.tif]

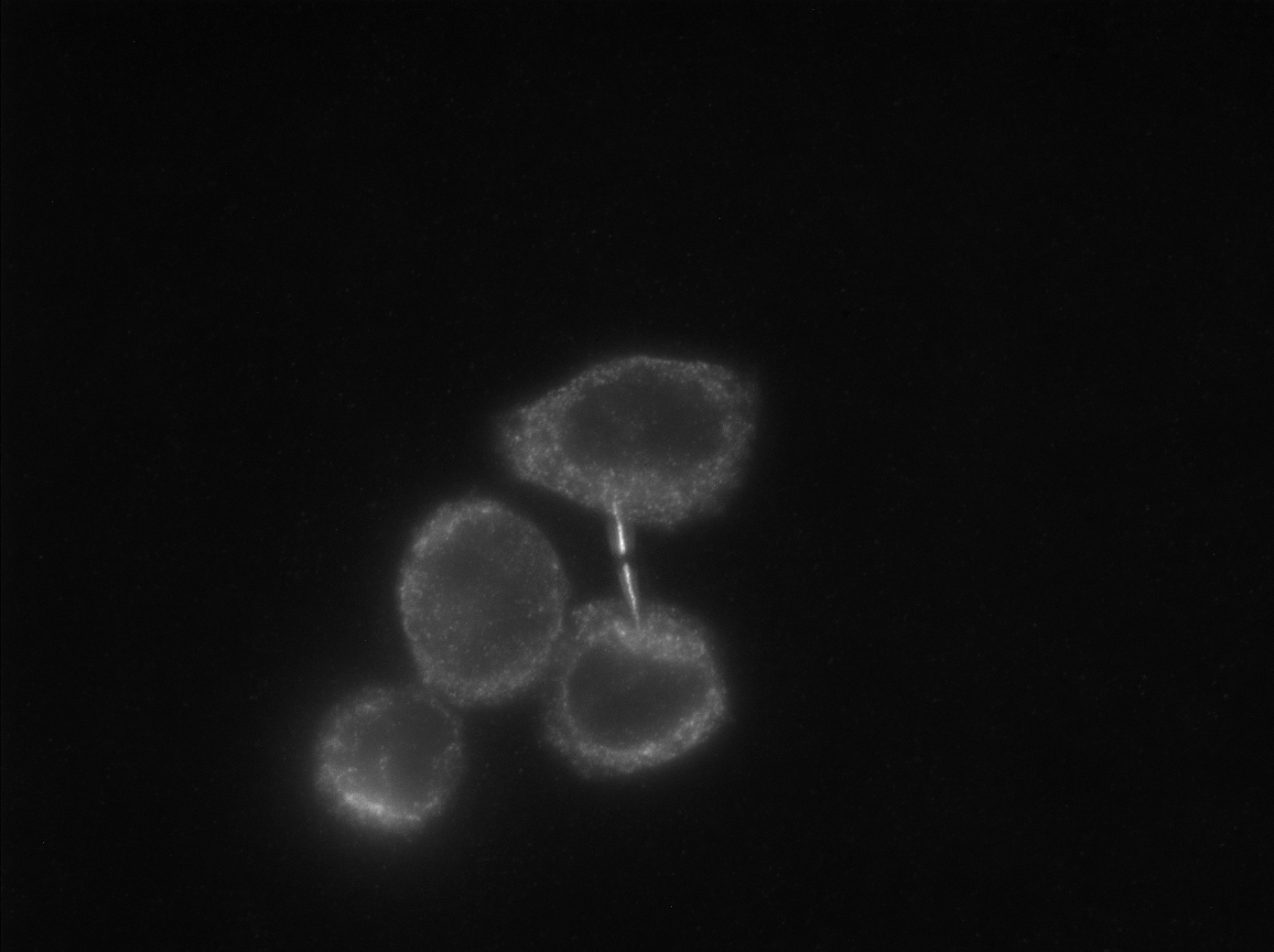

Supplement: Supplementary file 25 — Source data Fig. 7 part 1 [file 44319_2026_742_MOESM25_ESM.zip › Figure 7 Part 1/Fig 7acd Cas9 and TPGS1-ko rGT335 atubulin/Cas9 GT335recomb atub 3-24-25 R3 LT10.Project Maximum Z_XY1742851227_Z0_T0_C2.tif]

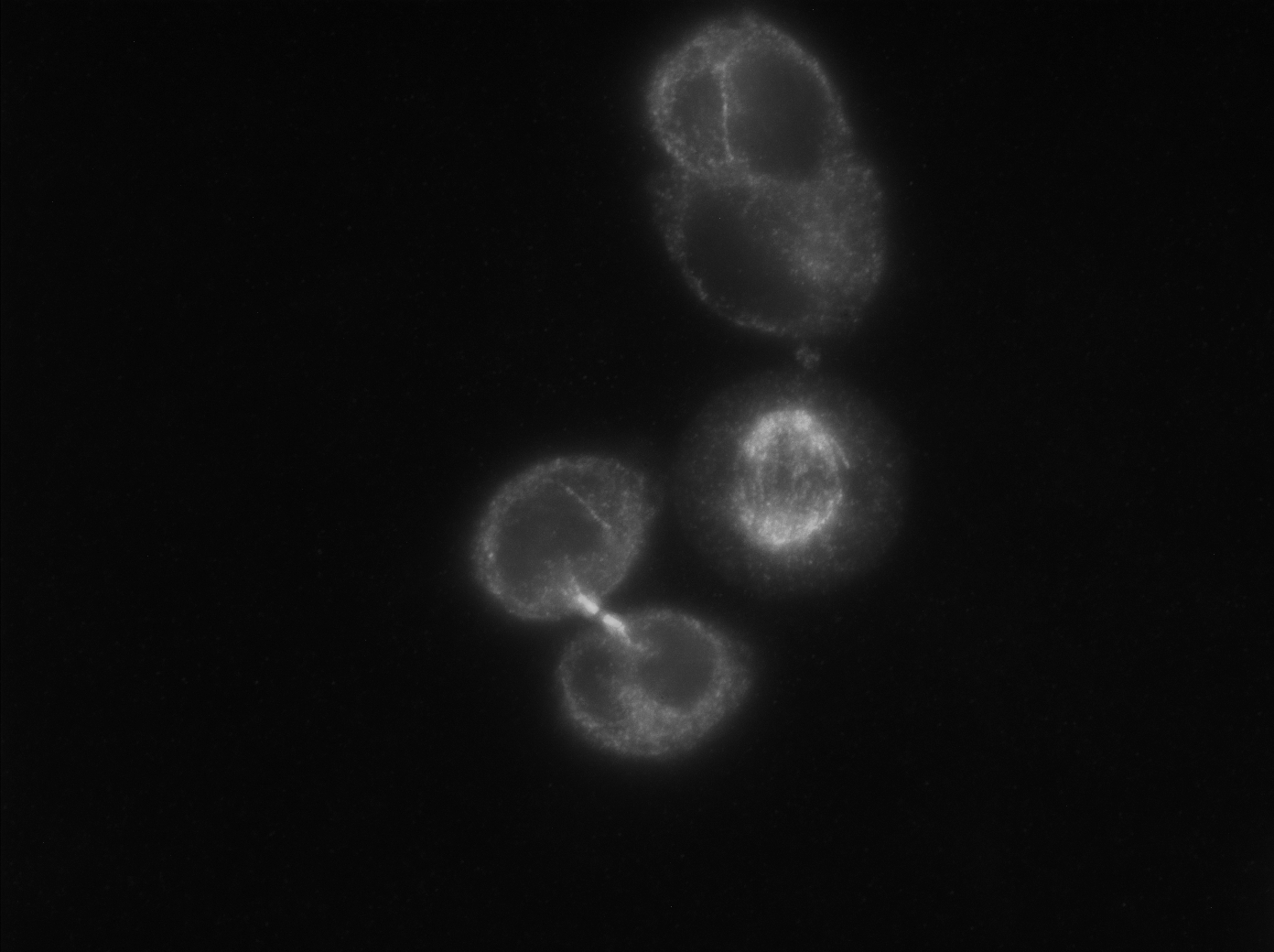

Supplement: Supplementary file 25 — Source data Fig. 7 part 1 [file 44319_2026_742_MOESM25_ESM.zip › Figure 7 Part 1/Fig 7acd Cas9 and TPGS1-ko rGT335 atubulin/Cas9 GT335recomb atub 3-24-25 R2 LT10 M1.Project Maximum Z_XY1742847842_Z0_T0_C2.tif]

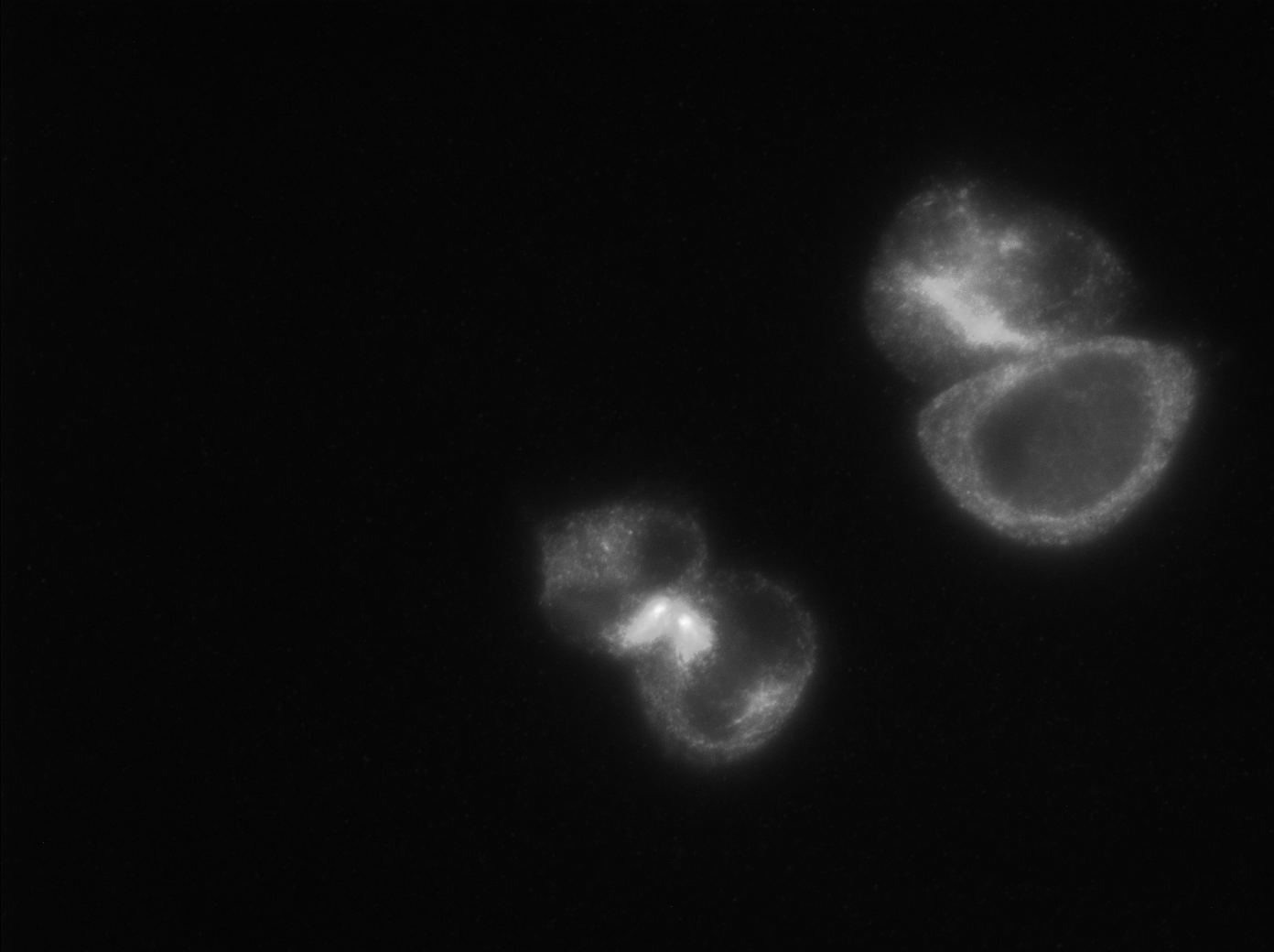

Supplement: Supplementary file 25 — Source data Fig. 7 part 1 [file 44319_2026_742_MOESM25_ESM.zip › Figure 7 Part 1/Fig 7acd Cas9 and TPGS1-ko rGT335 atubulin/Cas9 GT335recomb atub 3-24-25 R2 ET1.Project Maximum Z_XY1742845132_Z0_T0_C2.tif]

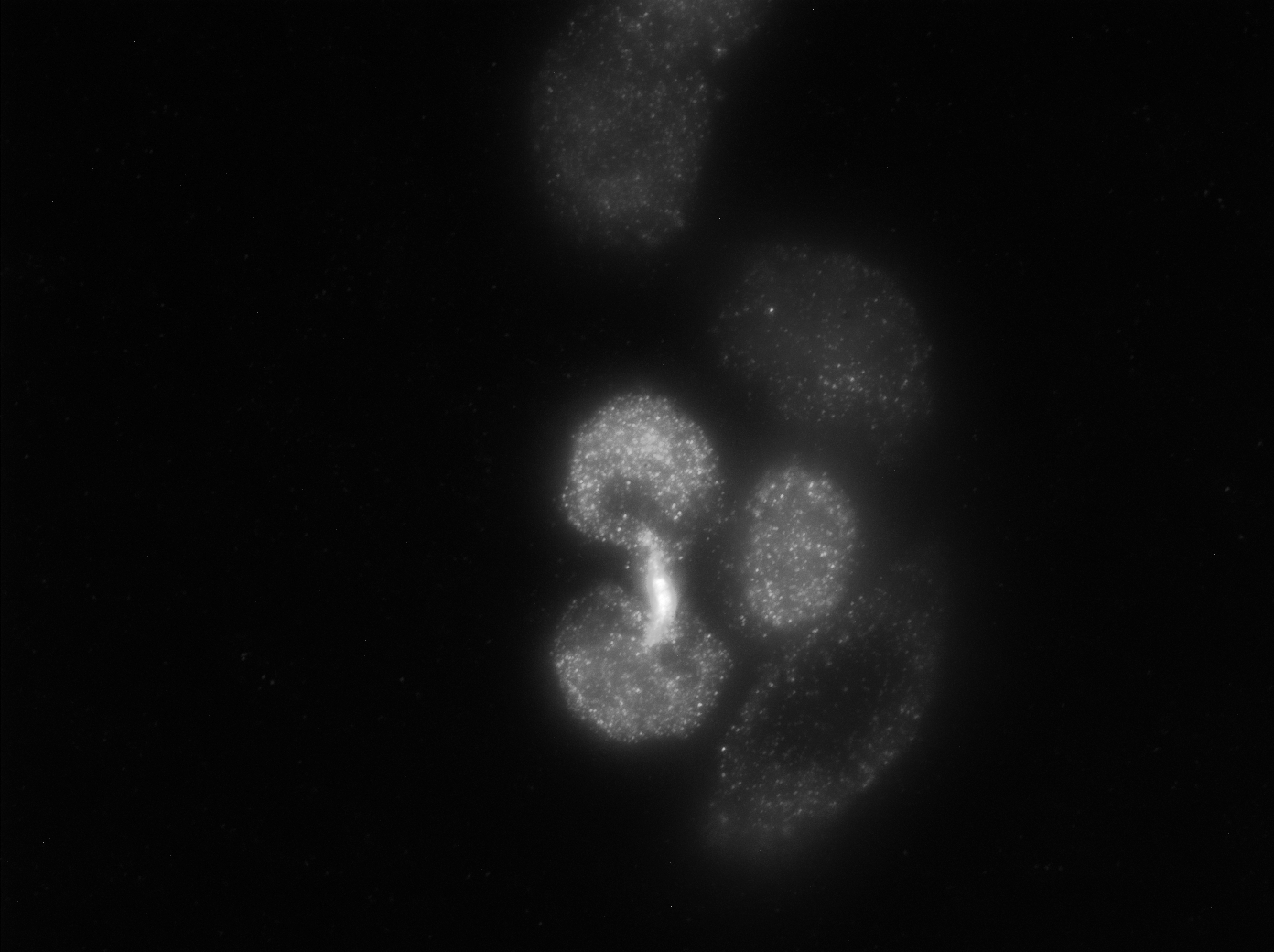

Supplement: Supplementary file 25 — Source data Fig. 7 part 1 [file 44319_2026_742_MOESM25_ESM.zip › Figure 7 Part 1/Fig 7acd Cas9 and TPGS1-ko rGT335 atubulin/Cas9 GT335recomb atub 3-24-25 R1 ET3.Project Maximum Z_XY1742835126_Z0_T0_C1.tif]

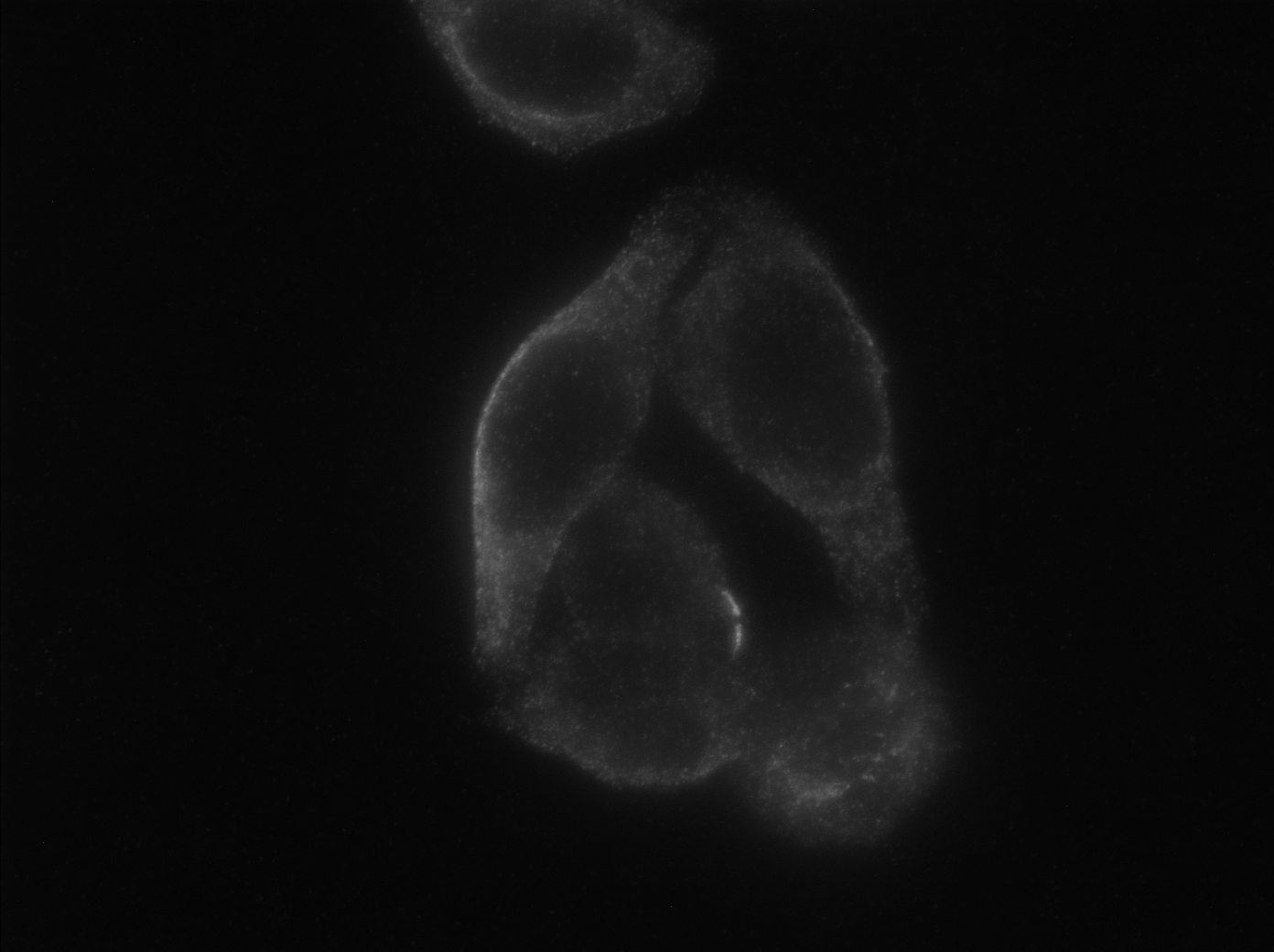

Supplement: Supplementary file 25 — Source data Fig. 7 part 1 [file 44319_2026_742_MOESM25_ESM.zip › Figure 7 Part 1/Fig 7acd Cas9 and TPGS1-ko rGT335 atubulin/Cas9 GT335recomb atub 3-24-25 R2 LT1.Project Maximum Z_XY1742845369_Z0_T0_C2.tif]

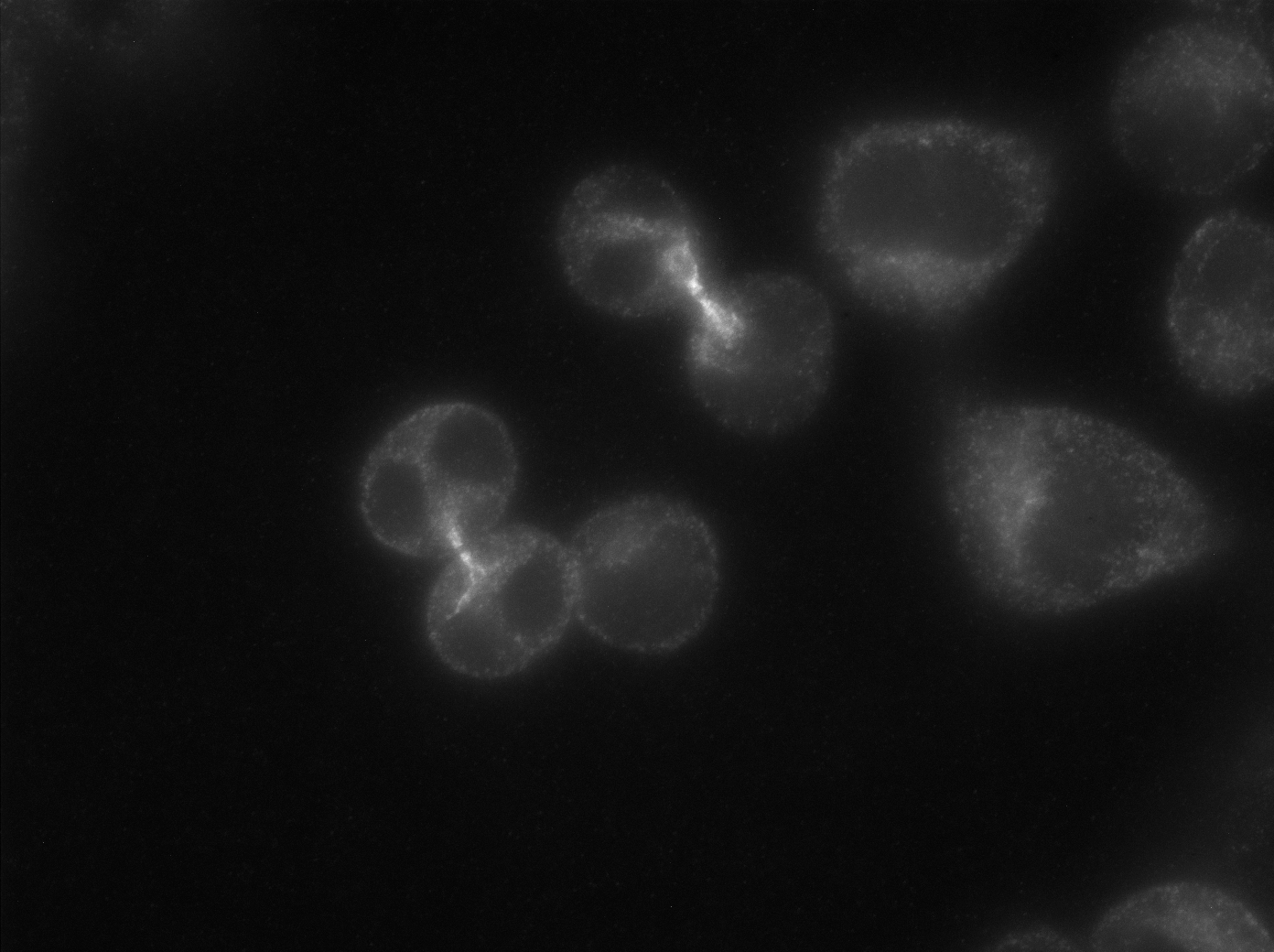

Supplement: Supplementary file 25 — Source data Fig. 7 part 1 [file 44319_2026_742_MOESM25_ESM.zip › Figure 7 Part 1/Fig 7acd Cas9 and TPGS1-ko rGT335 atubulin/Cas9 GT335recomb atub 3-24-25 R3 ET2ET3.Project Maximum Z_XY1742850085_Z0_T0_C2.tif]

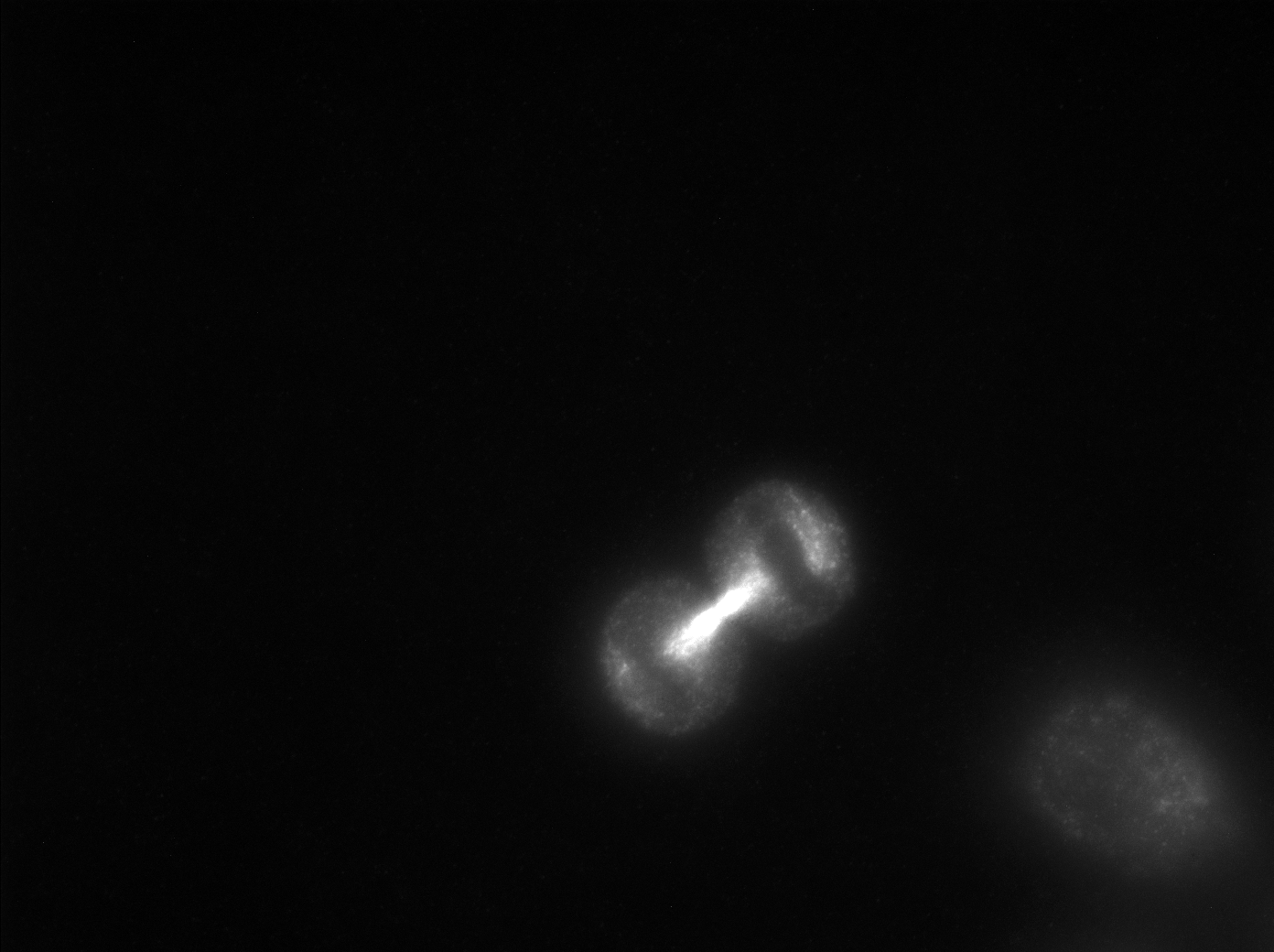

Supplement: Supplementary file 25 — Source data Fig. 7 part 1 [file 44319_2026_742_MOESM25_ESM.zip › Figure 7 Part 1/Fig 7acd Cas9 and TPGS1-ko rGT335 atubulin/Cas9 GT335recomb atub 3-24-25 R1 ET9.Project Maximum Z_XY1742836431_Z0_T0_C2.tif]

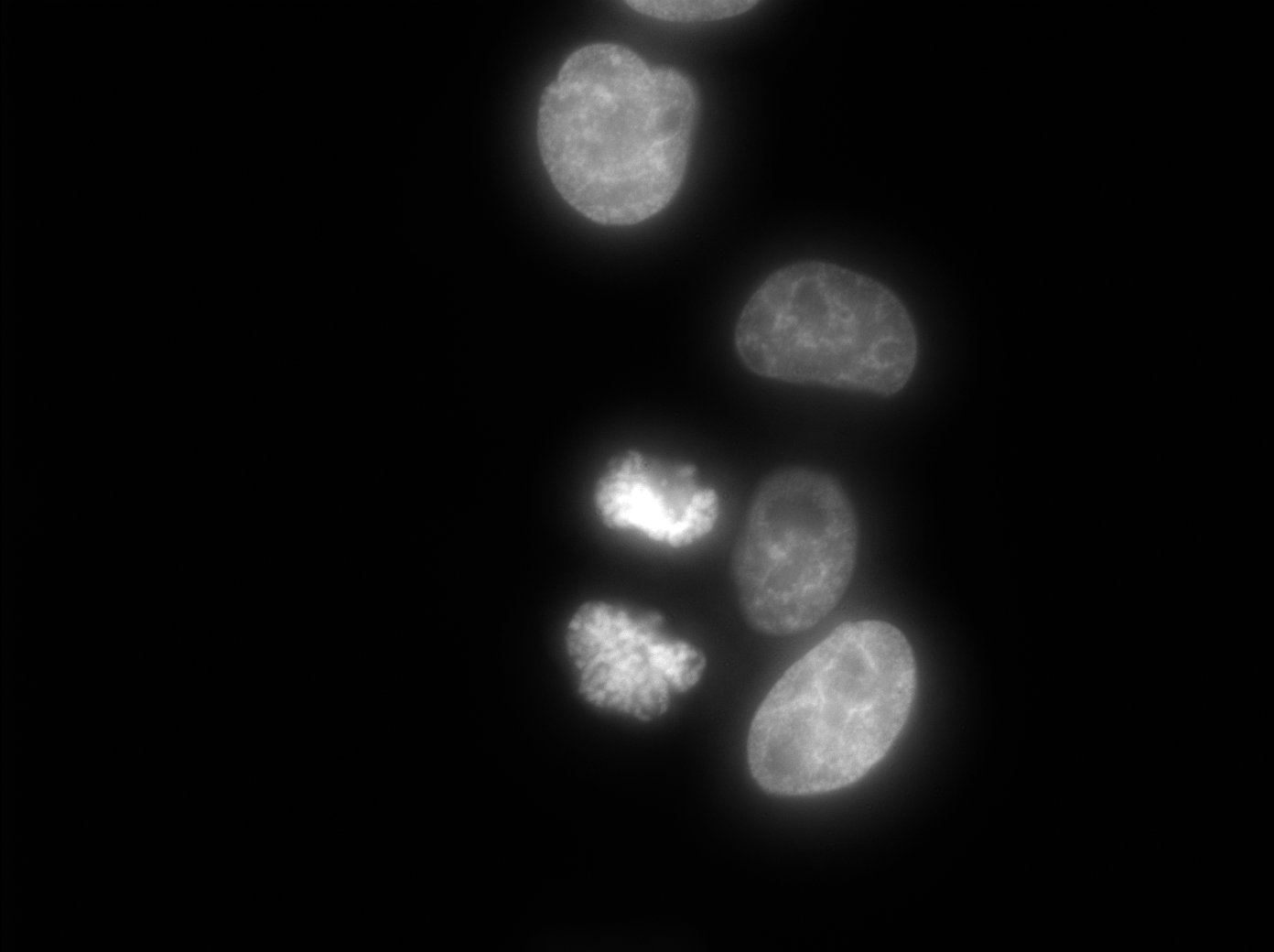

Supplement: Supplementary file 25 — Source data Fig. 7 part 1 [file 44319_2026_742_MOESM25_ESM.zip › Figure 7 Part 1/Fig 7acd Cas9 and TPGS1-ko rGT335 atubulin/Cas9 GT335recomb atub 3-24-25 R1 ET3.Project Maximum Z_XY1742835126_Z0_T0_C0.tif]

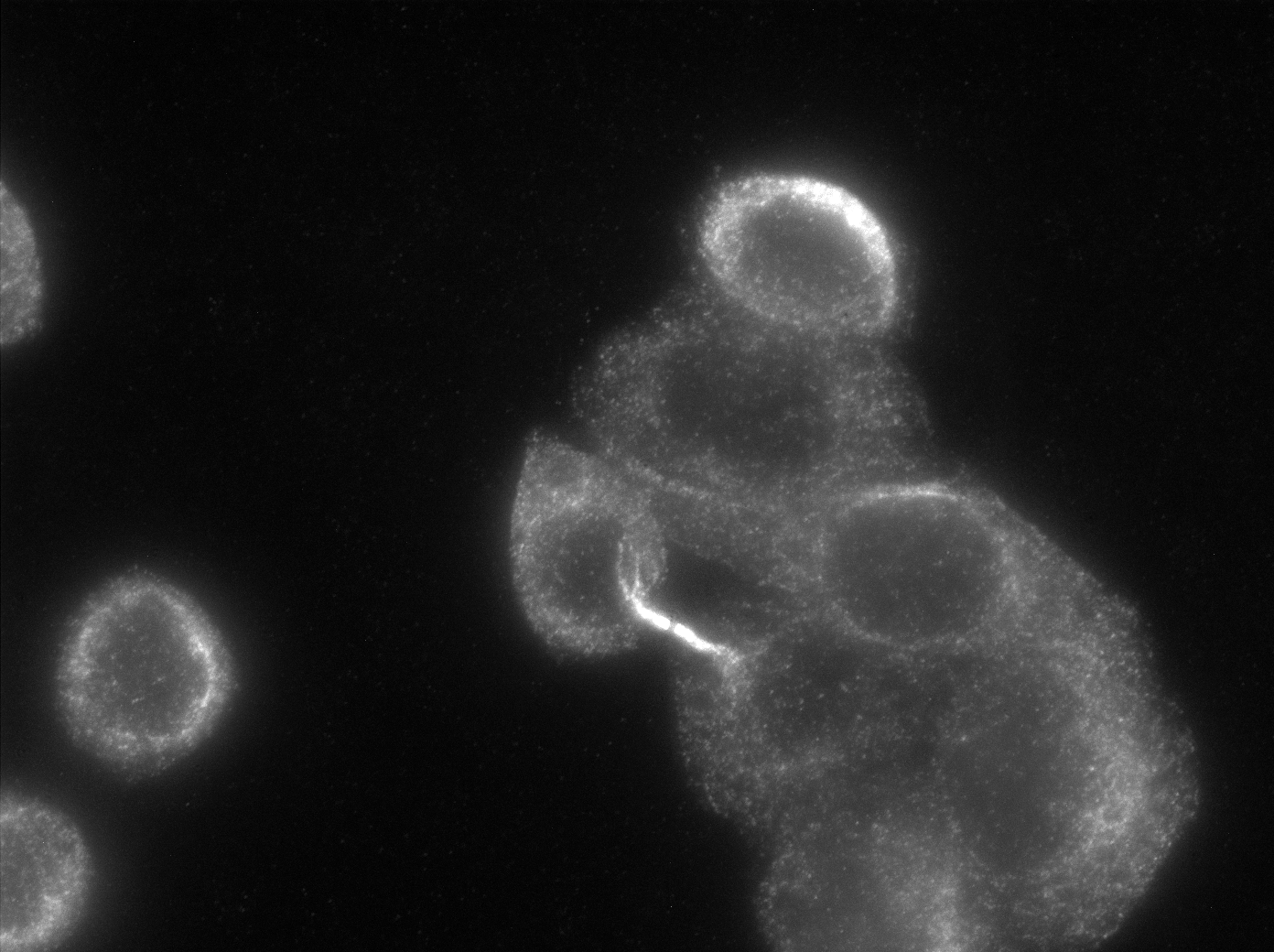

Supplement: Supplementary file 25 — Source data Fig. 7 part 1 [file 44319_2026_742_MOESM25_ESM.zip › Figure 7 Part 1/Fig 7acd Cas9 and TPGS1-ko rGT335 atubulin/Cas9 GT335recomb atub 3-24-25 R1 LT3.Project Maximum Z_XY1742835236_Z0_T0_C2.tif]

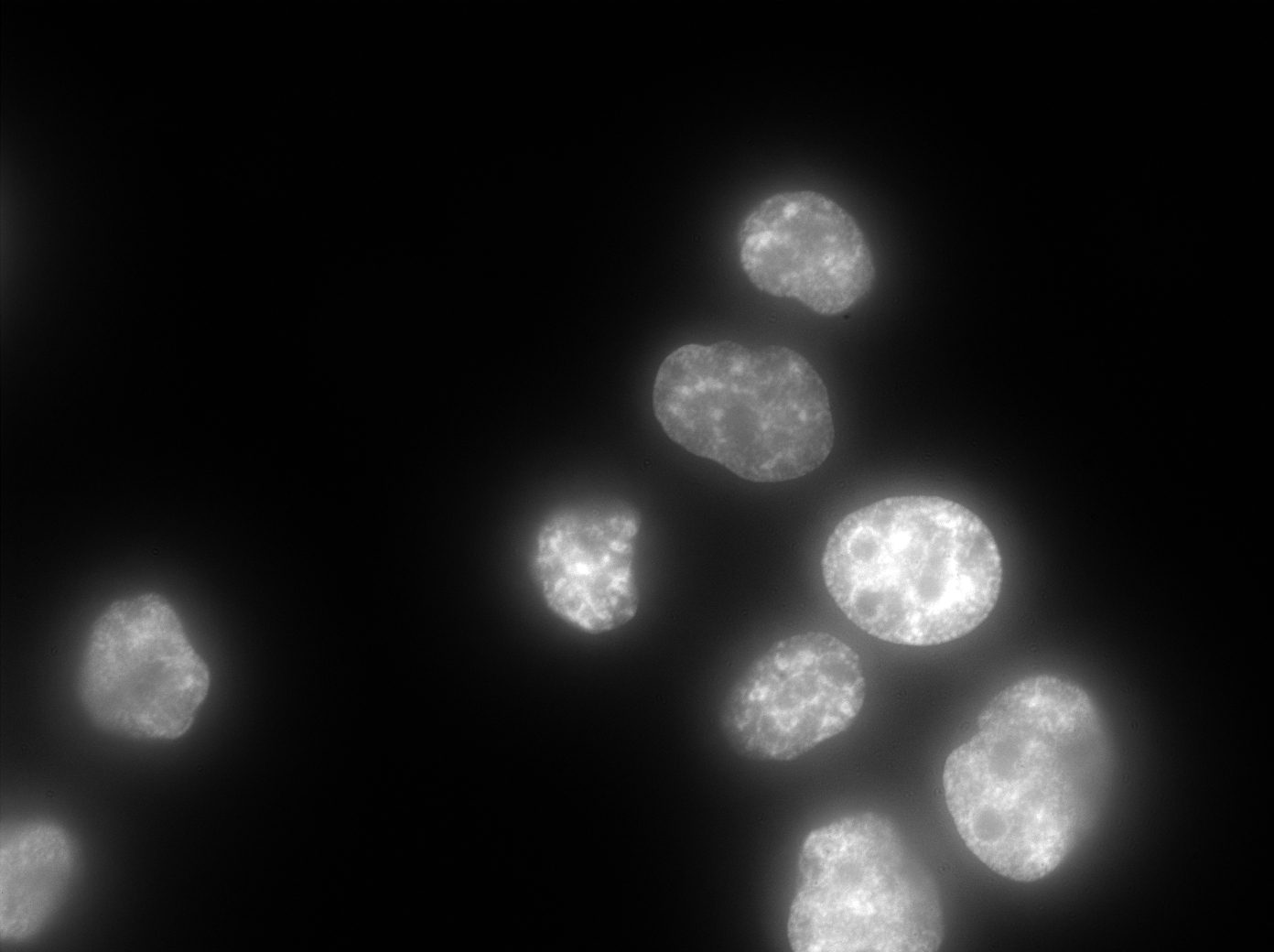

Supplement: Supplementary file 25 — Source data Fig. 7 part 1 [file 44319_2026_742_MOESM25_ESM.zip › Figure 7 Part 1/Fig 7acd Cas9 and TPGS1-ko rGT335 atubulin/Cas9 GT335recomb atub 3-24-25 R1 LT3.Project Maximum Z_XY1742835236_Z0_T0_C0.tif]

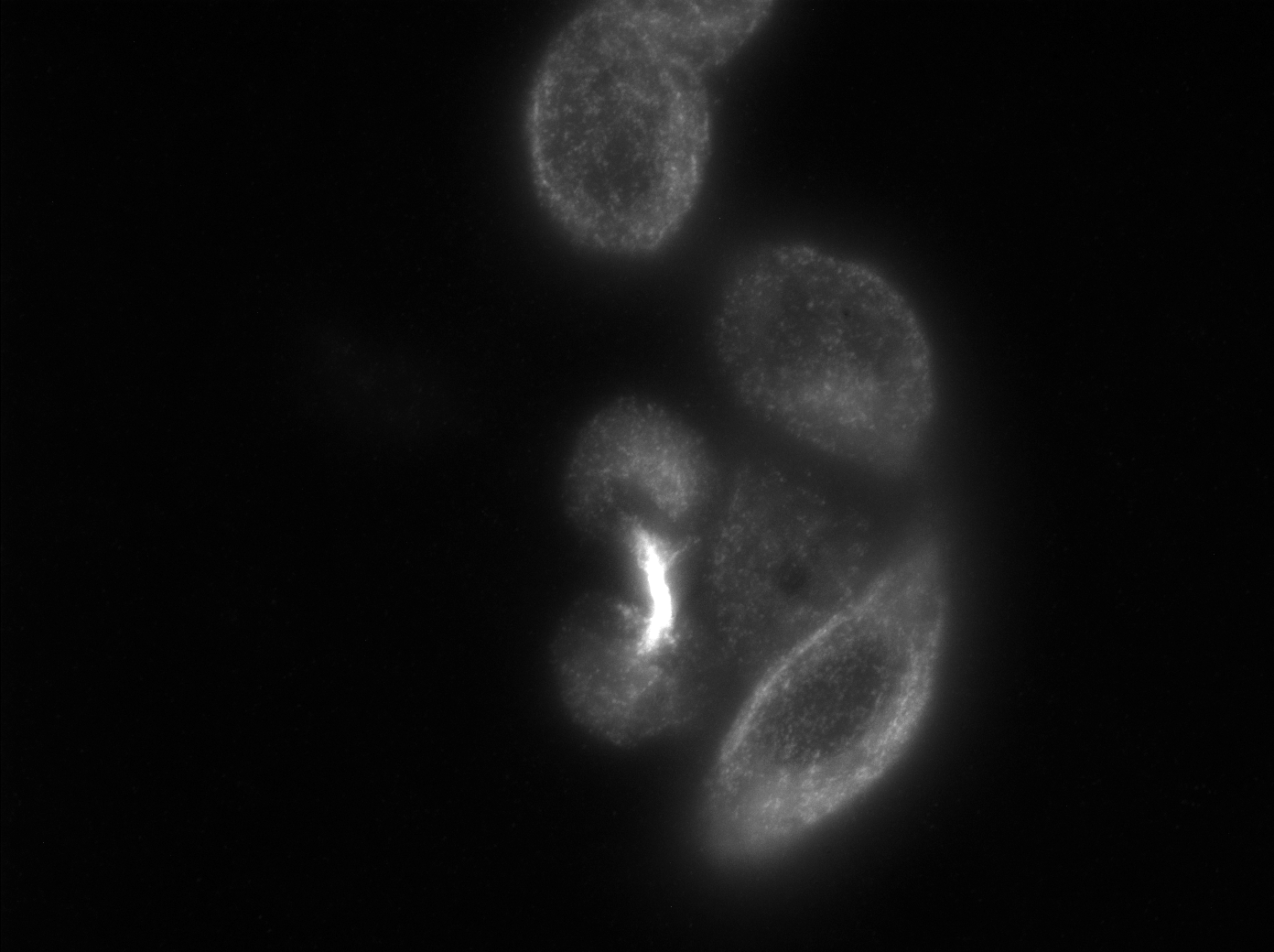

Supplement: Supplementary file 25 — Source data Fig. 7 part 1 [file 44319_2026_742_MOESM25_ESM.zip › Figure 7 Part 1/Fig 7acd Cas9 and TPGS1-ko rGT335 atubulin/Cas9 GT335recomb atub 3-24-25 R1 ET3.Project Maximum Z_XY1742835126_Z0_T0_C2.tif]

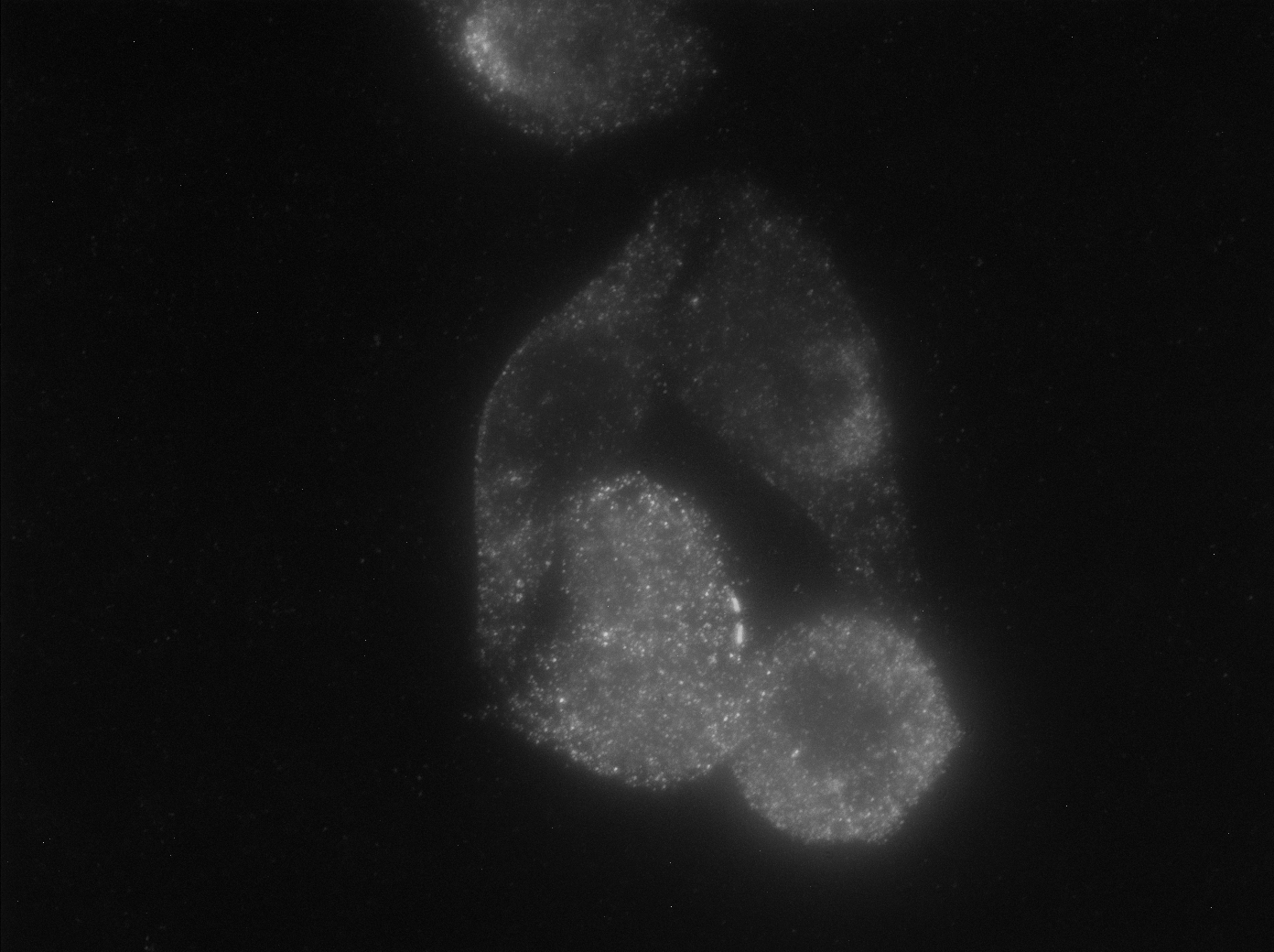

Supplement: Supplementary file 25 — Source data Fig. 7 part 1 [file 44319_2026_742_MOESM25_ESM.zip › Figure 7 Part 1/Fig 7acd Cas9 and TPGS1-ko rGT335 atubulin/Cas9 GT335recomb atub 3-24-25 R2 LT1.Project Maximum Z_XY1742845369_Z0_T0_C1.tif]

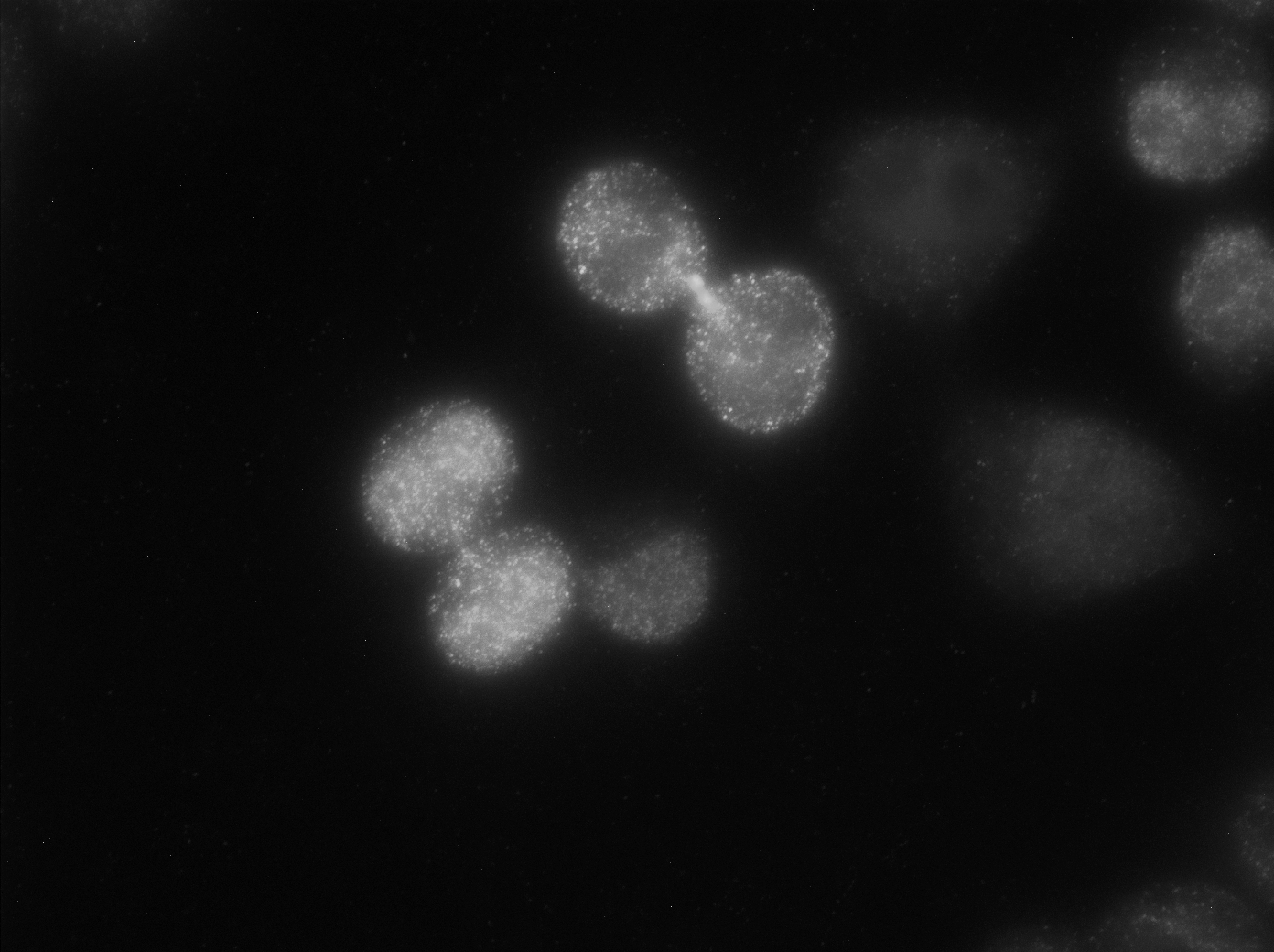

Supplement: Supplementary file 25 — Source data Fig. 7 part 1 [file 44319_2026_742_MOESM25_ESM.zip › Figure 7 Part 1/Fig 7acd Cas9 and TPGS1-ko rGT335 atubulin/Cas9 GT335recomb atub 3-24-25 R3 ET2ET3.Project Maximum Z_XY1742850085_Z0_T0_C1.tif]

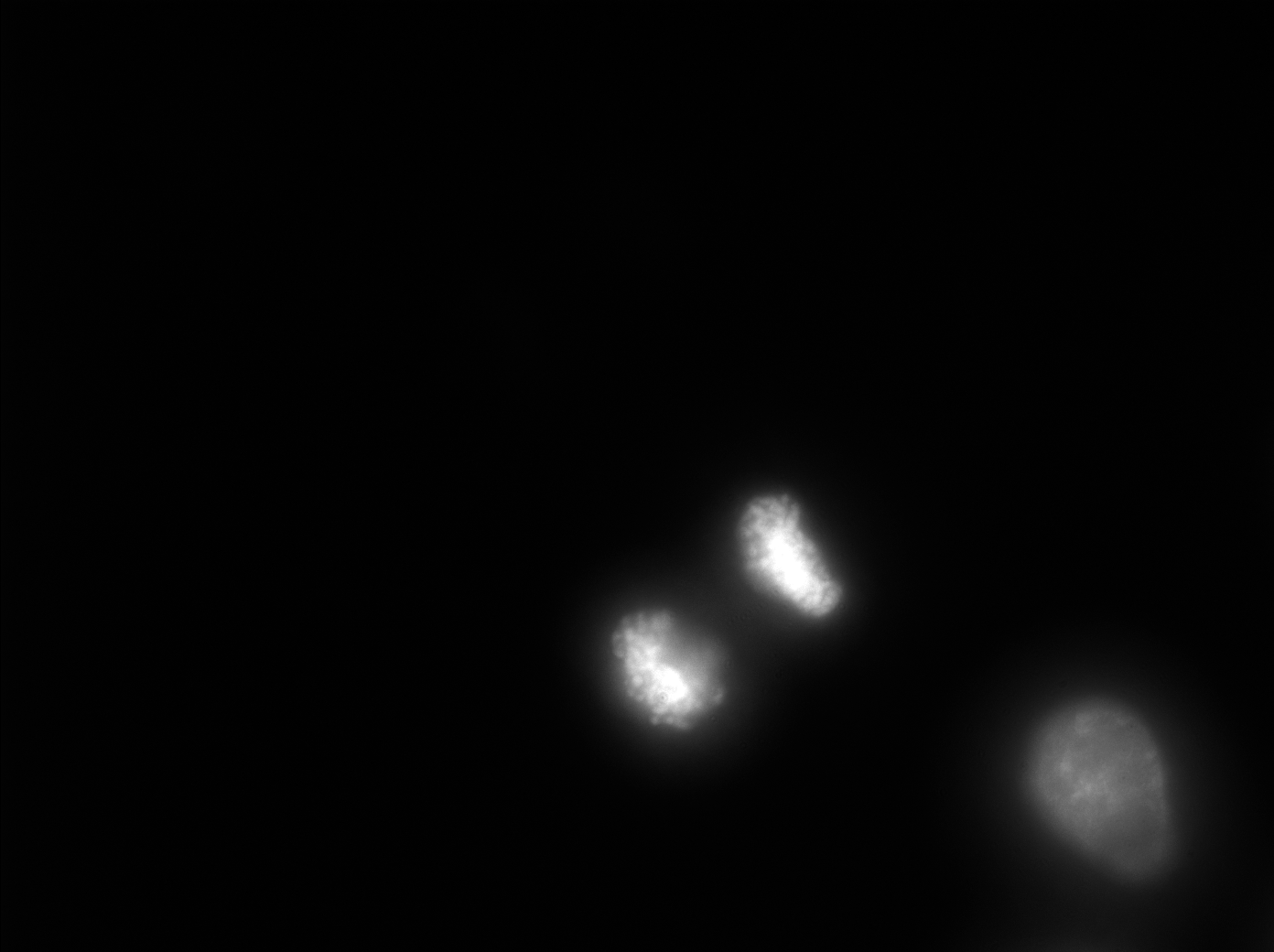

Supplement: Supplementary file 25 — Source data Fig. 7 part 1 [file 44319_2026_742_MOESM25_ESM.zip › Figure 7 Part 1/Fig 7acd Cas9 and TPGS1-ko rGT335 atubulin/Cas9 GT335recomb atub 3-24-25 R1 ET9.Project Maximum Z_XY1742836431_Z0_T0_C0.tif]

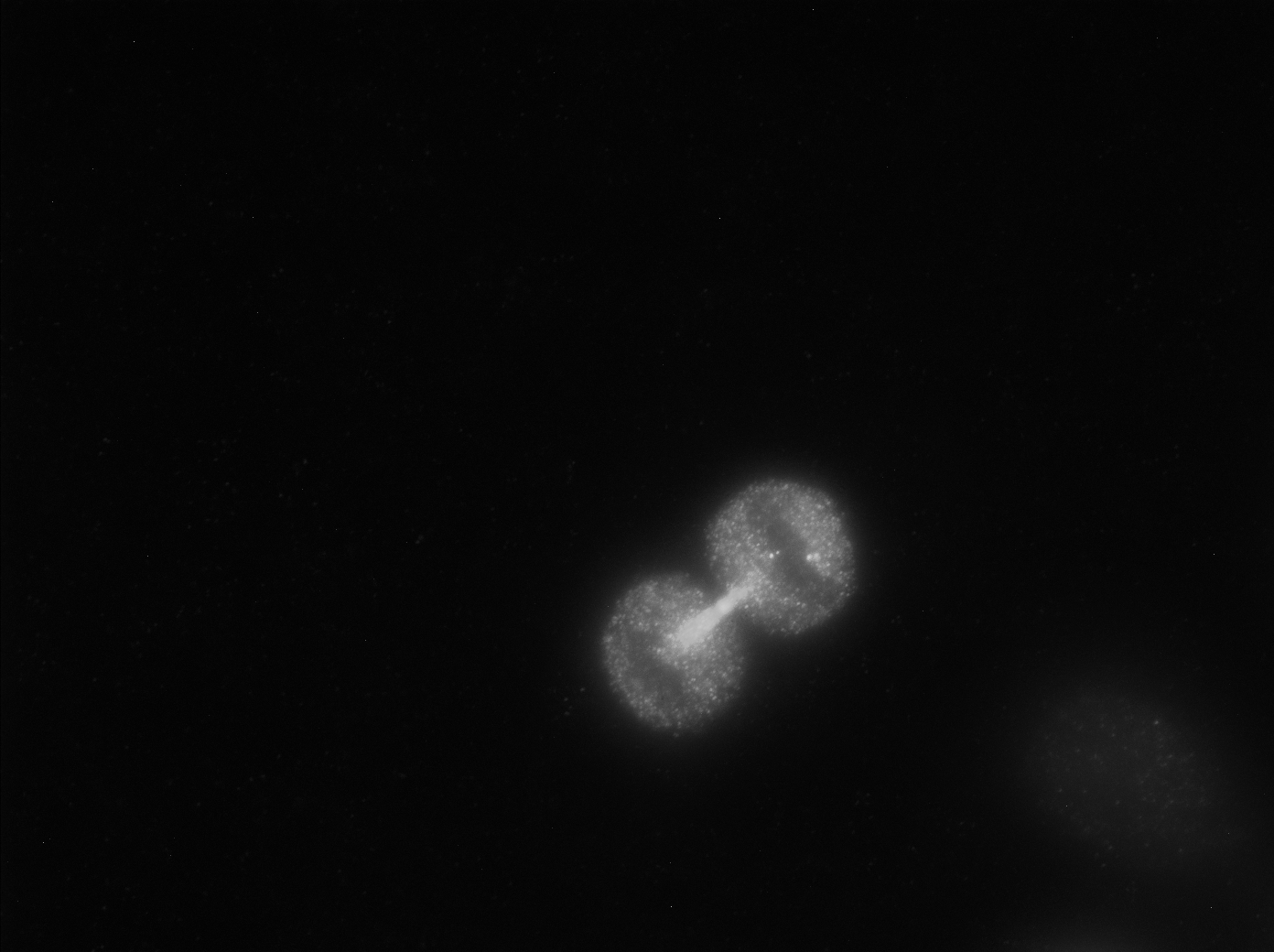

Supplement: Supplementary file 25 — Source data Fig. 7 part 1 [file 44319_2026_742_MOESM25_ESM.zip › Figure 7 Part 1/Fig 7acd Cas9 and TPGS1-ko rGT335 atubulin/Cas9 GT335recomb atub 3-24-25 R1 ET9.Project Maximum Z_XY1742836431_Z0_T0_C1.tif]

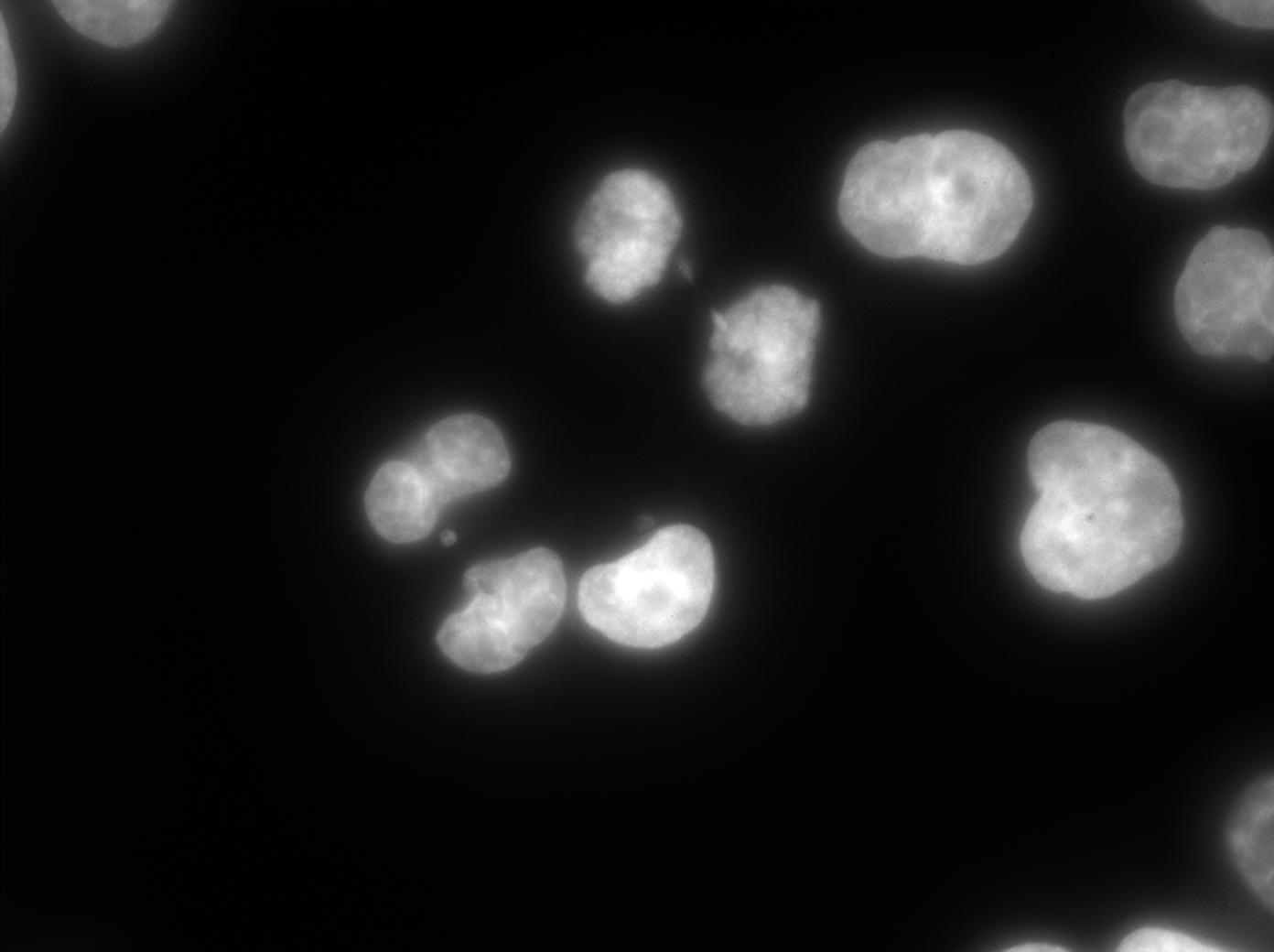

Supplement: Supplementary file 25 — Source data Fig. 7 part 1 [file 44319_2026_742_MOESM25_ESM.zip › Figure 7 Part 1/Fig 7acd Cas9 and TPGS1-ko rGT335 atubulin/Cas9 GT335recomb atub 3-24-25 R3 ET2ET3.Project Maximum Z_XY1742850085_Z0_T0_C0.tif]

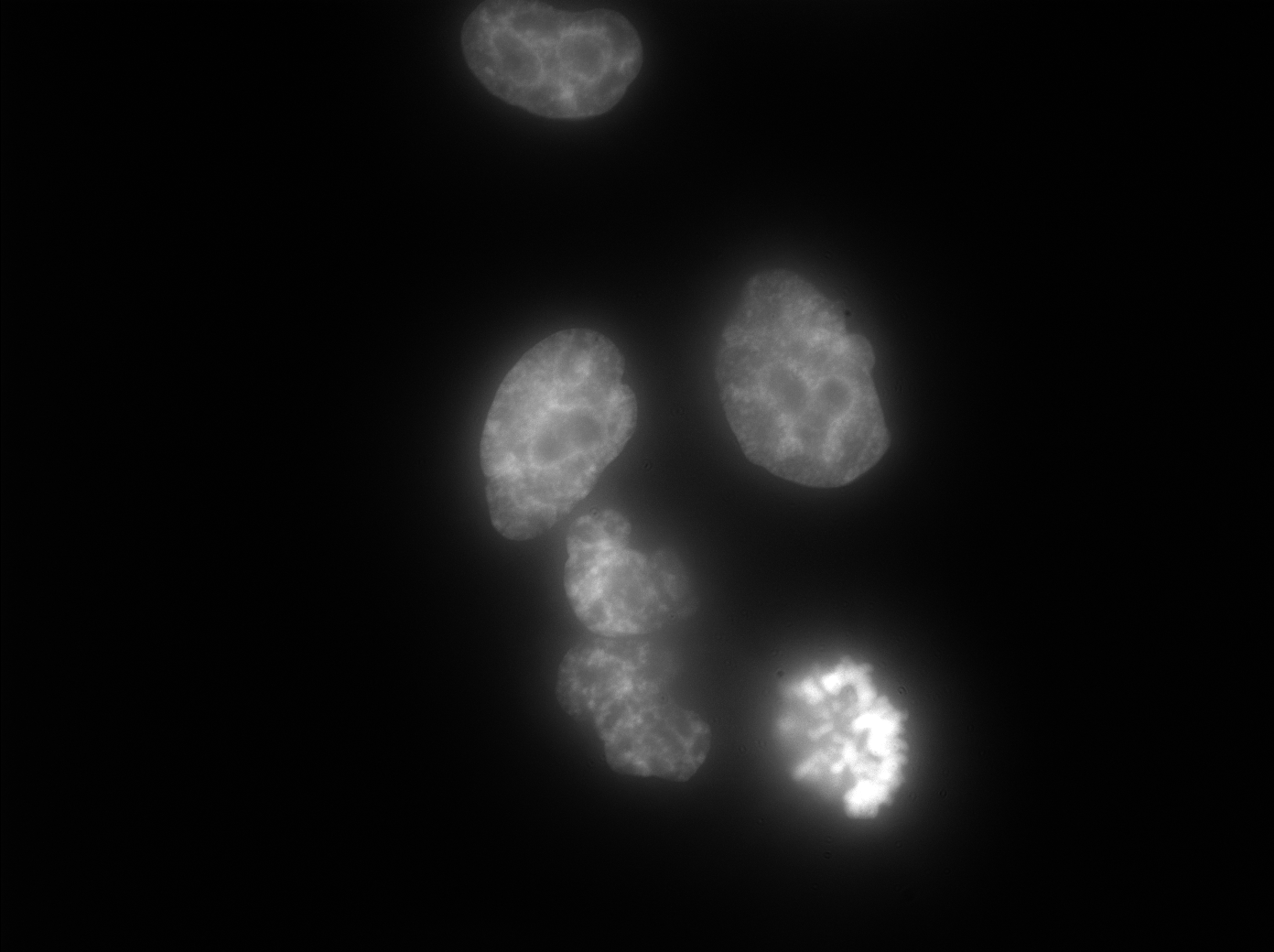

Supplement: Supplementary file 25 — Source data Fig. 7 part 1 [file 44319_2026_742_MOESM25_ESM.zip › Figure 7 Part 1/Fig 7acd Cas9 and TPGS1-ko rGT335 atubulin/Cas9 GT335recomb atub 3-24-25 R2 LT1.Project Maximum Z_XY1742845369_Z0_T0_C0.tif]

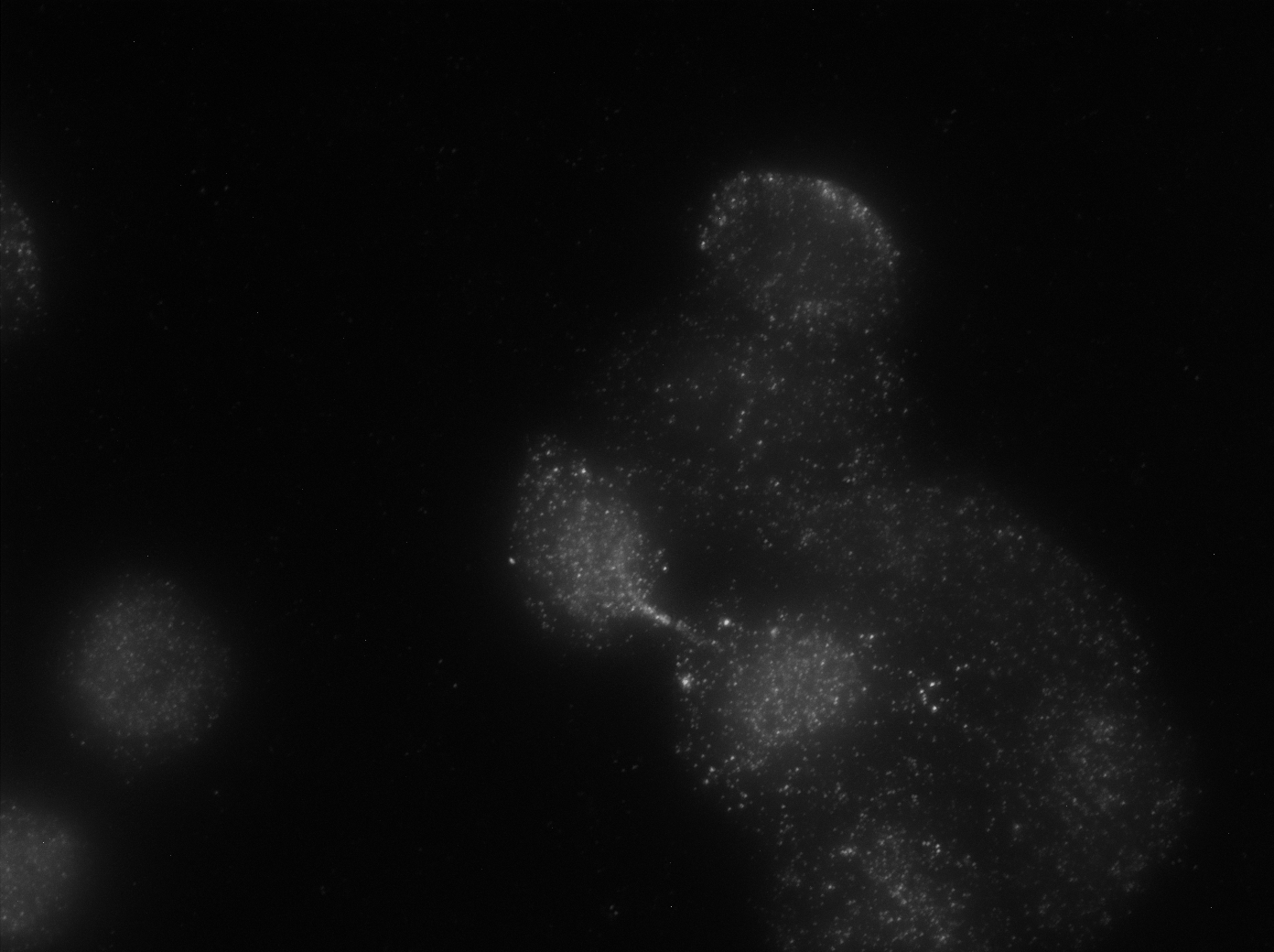

Supplement: Supplementary file 25 — Source data Fig. 7 part 1 [file 44319_2026_742_MOESM25_ESM.zip › Figure 7 Part 1/Fig 7acd Cas9 and TPGS1-ko rGT335 atubulin/Cas9 GT335recomb atub 3-24-25 R1 LT3.Project Maximum Z_XY1742835236_Z0_T0_C1.tif]

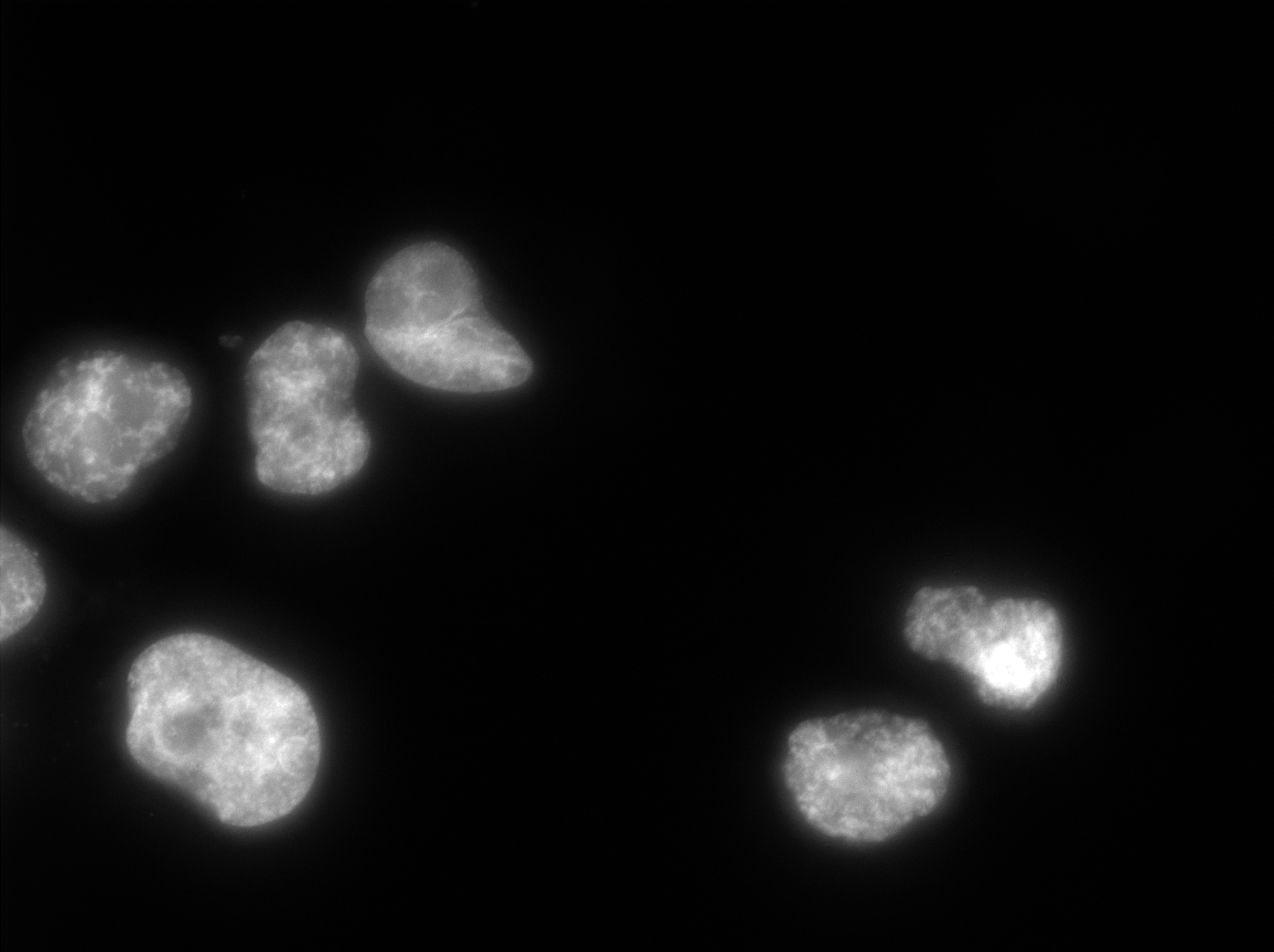

Supplement: Supplementary file 25 — Source data Fig. 7 part 1 [file 44319_2026_742_MOESM25_ESM.zip › Figure 7 Part 1/Fig 7acd Cas9 and TPGS1-ko rGT335 atubulin/Cas9 GT335recomb atub 3-24-25 R2 LT7 ET7.Project Maximum Z_XY1742846708_Z0_T0_C0.tif]

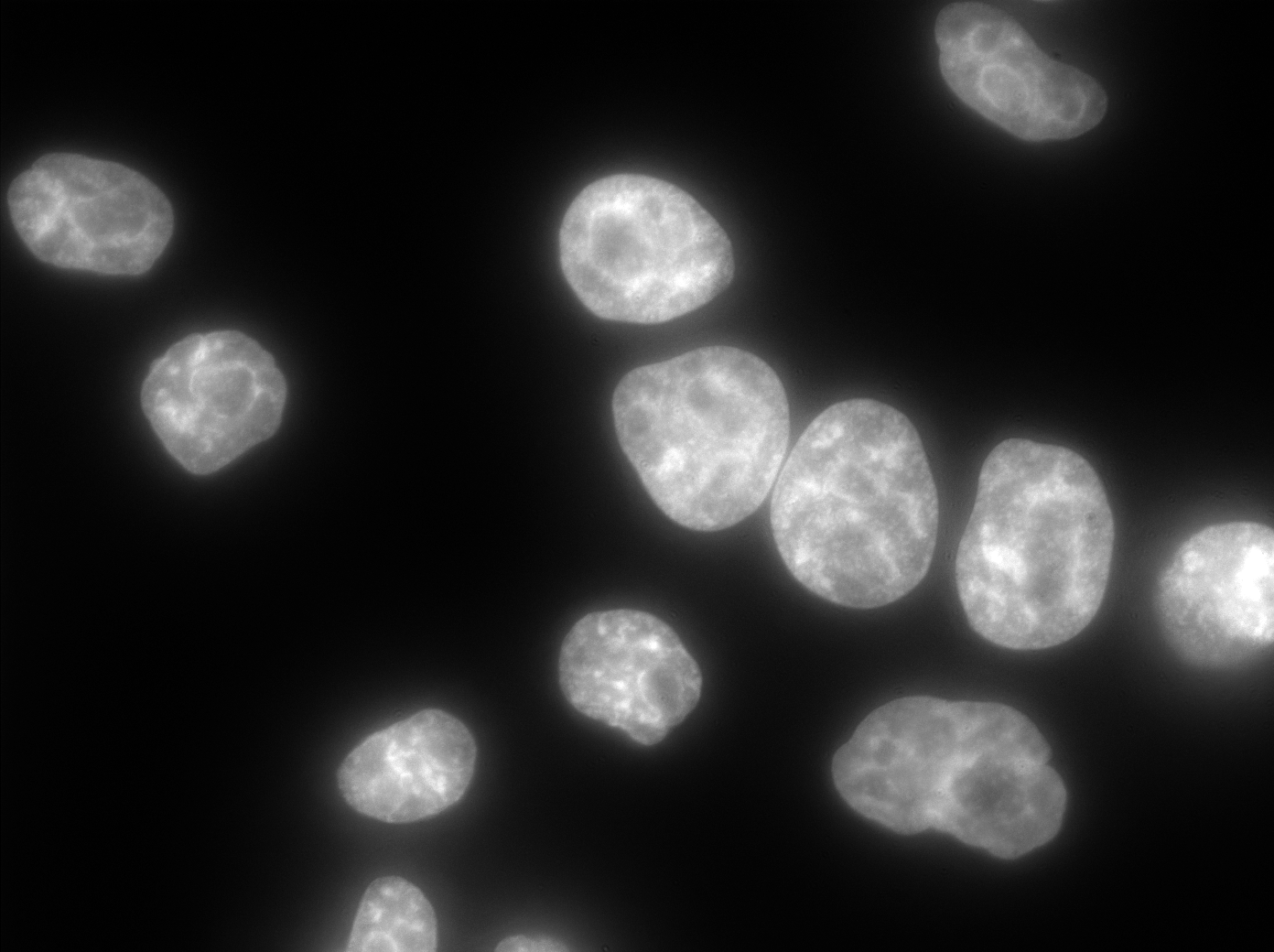

Supplement: Supplementary file 25 — Source data Fig. 7 part 1 [file 44319_2026_742_MOESM25_ESM.zip › Figure 7 Part 1/Fig 7acd Cas9 and TPGS1-ko rGT335 atubulin/Cas9 GT335recomb atub 3-24-25 R3 LT9.Project Maximum Z_XY1742851137_Z0_T0_C0.tif]

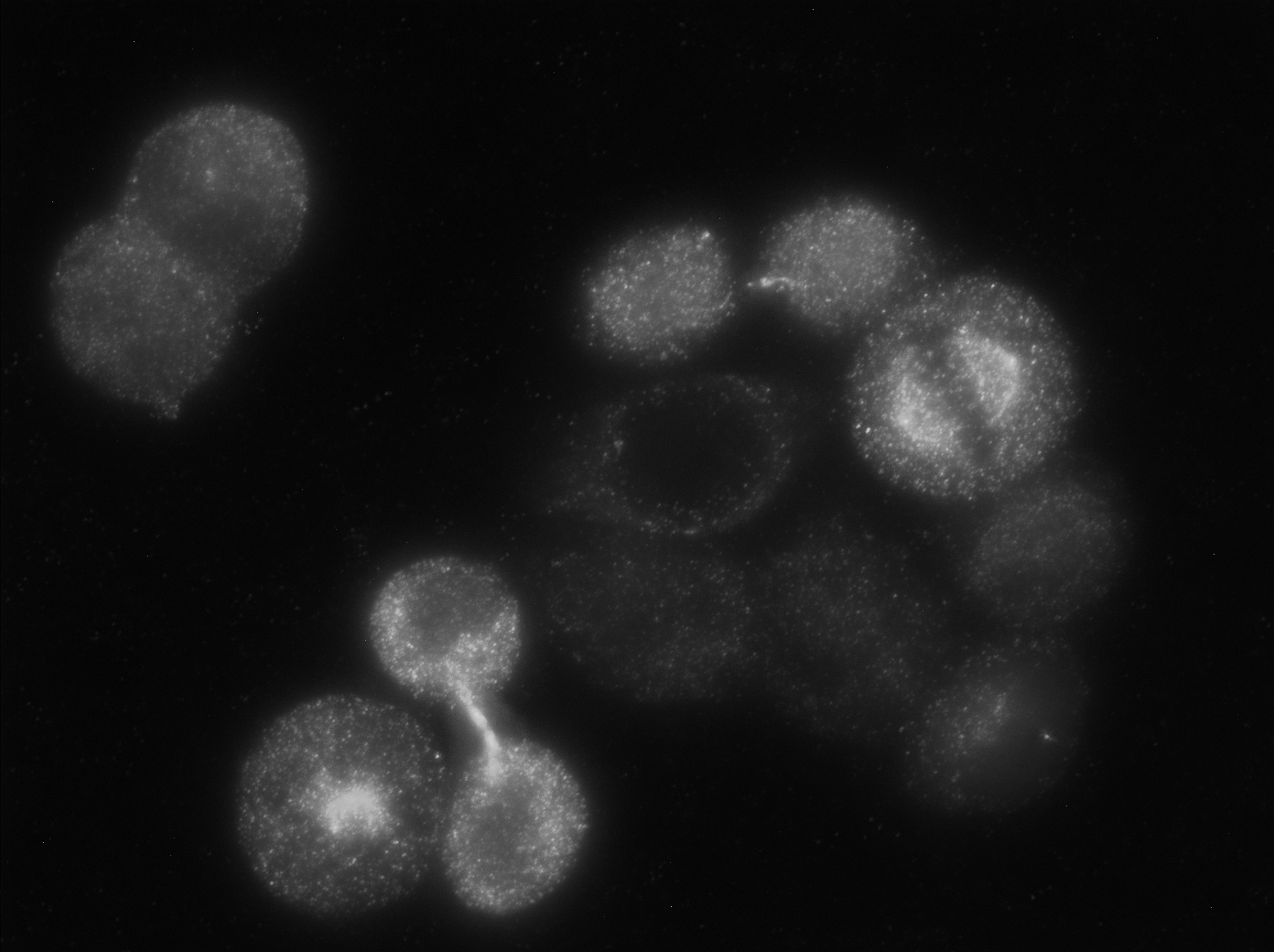

Supplement: Supplementary file 25 — Source data Fig. 7 part 1 [file 44319_2026_742_MOESM25_ESM.zip › Figure 7 Part 1/Fig 7acd Cas9 and TPGS1-ko rGT335 atubulin/Cas9 GT335recomb atub 3-24-25 R1 ET5 PA1 M1.Project Maximum Z_XY1742835552_Z0_T0_C1.tif]

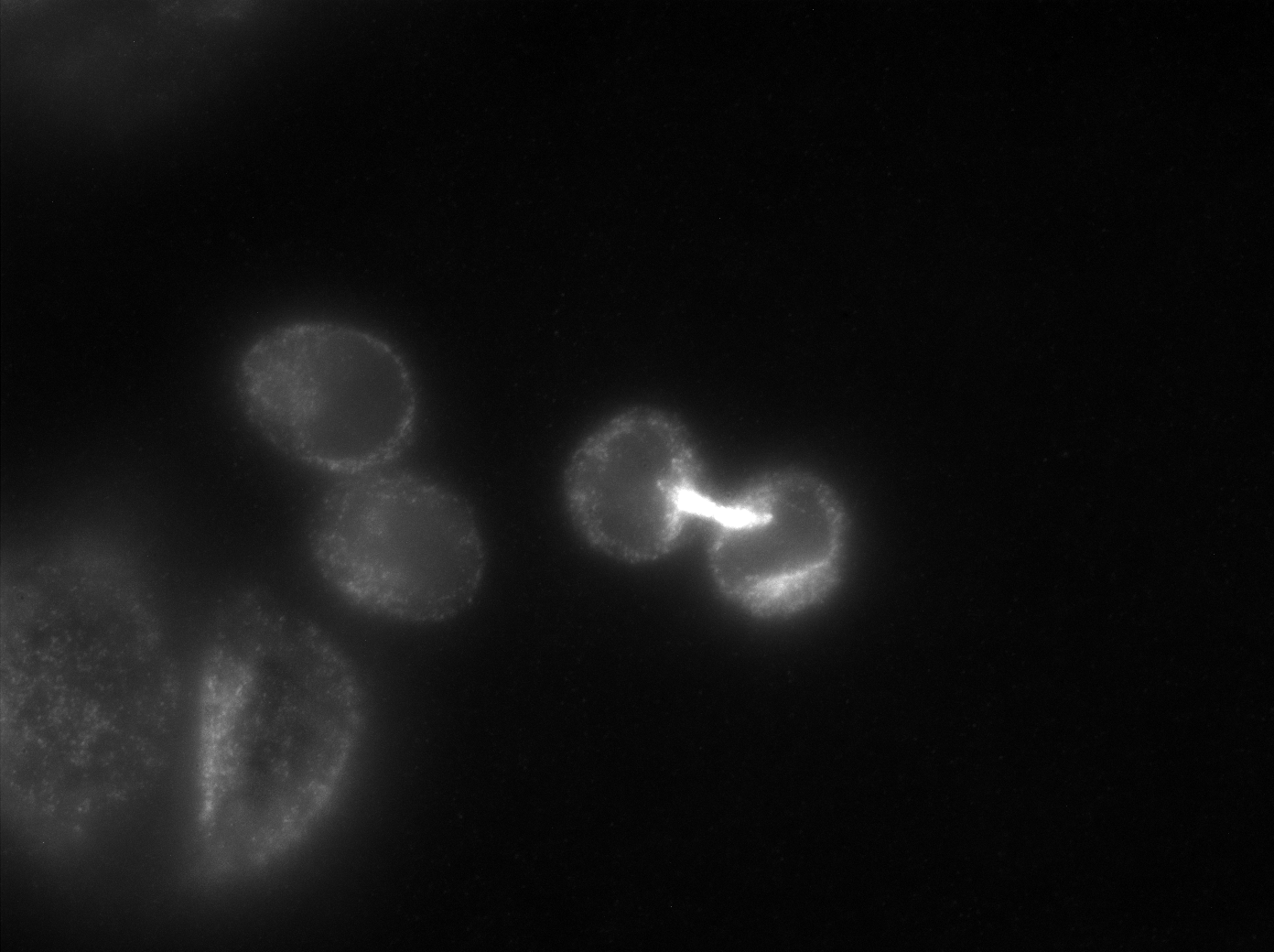

Supplement: Supplementary file 25 — Source data Fig. 7 part 1 [file 44319_2026_742_MOESM25_ESM.zip › Figure 7 Part 1/Fig 7acd Cas9 and TPGS1-ko rGT335 atubulin/Cas9 GT335recomb atub 3-24-25 R3 ET8.Project Maximum Z_XY1742850659_Z0_T0_C2.tif]

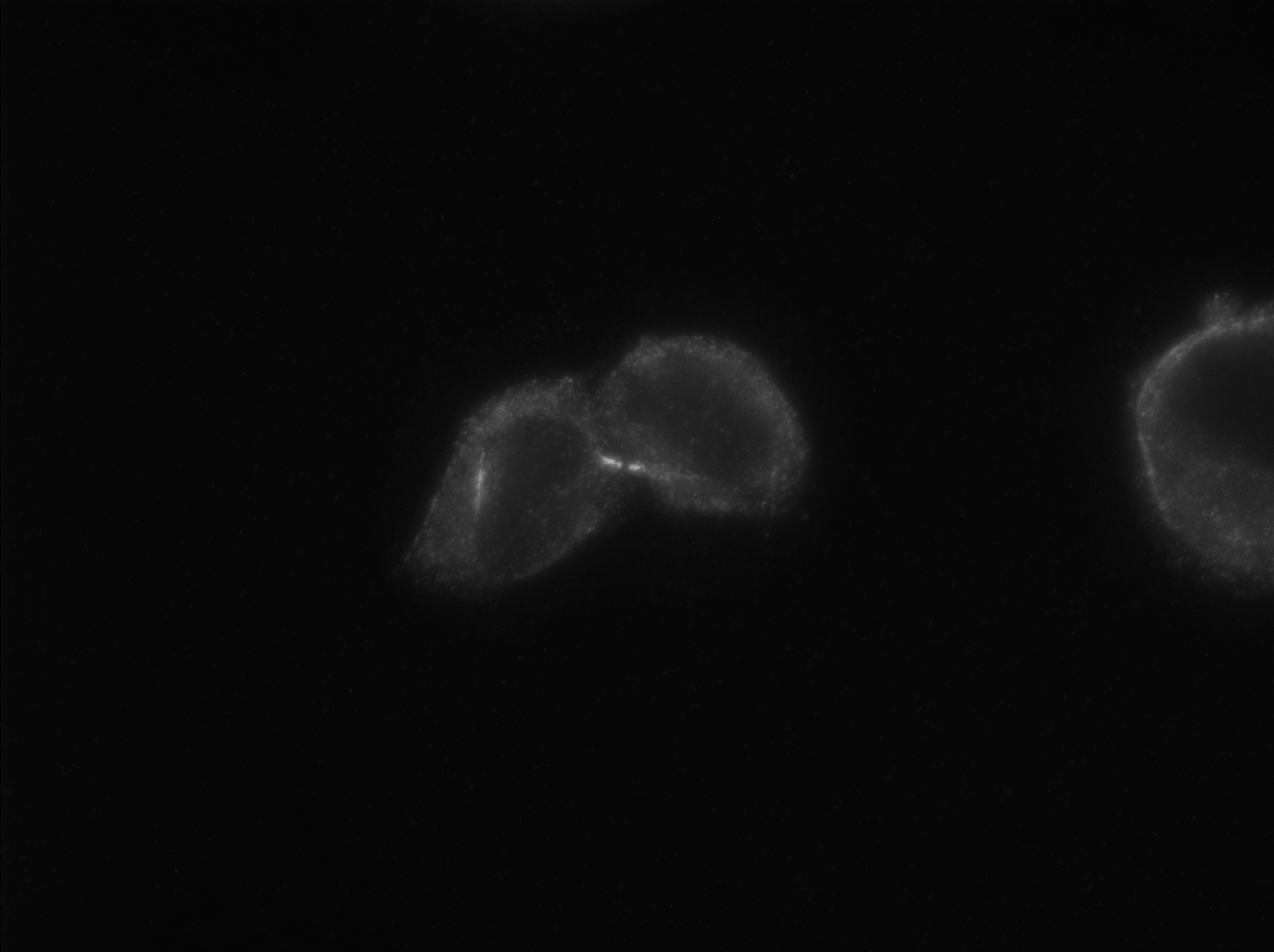

Supplement: Supplementary file 25 — Source data Fig. 7 part 1 [file 44319_2026_742_MOESM25_ESM.zip › Figure 7 Part 1/Fig 7acd Cas9 and TPGS1-ko rGT335 atubulin/Cas9 GT335recomb atub 3-24-25 R2 LT2.Project Maximum Z_XY1742845919_Z0_T0_C2.tif]

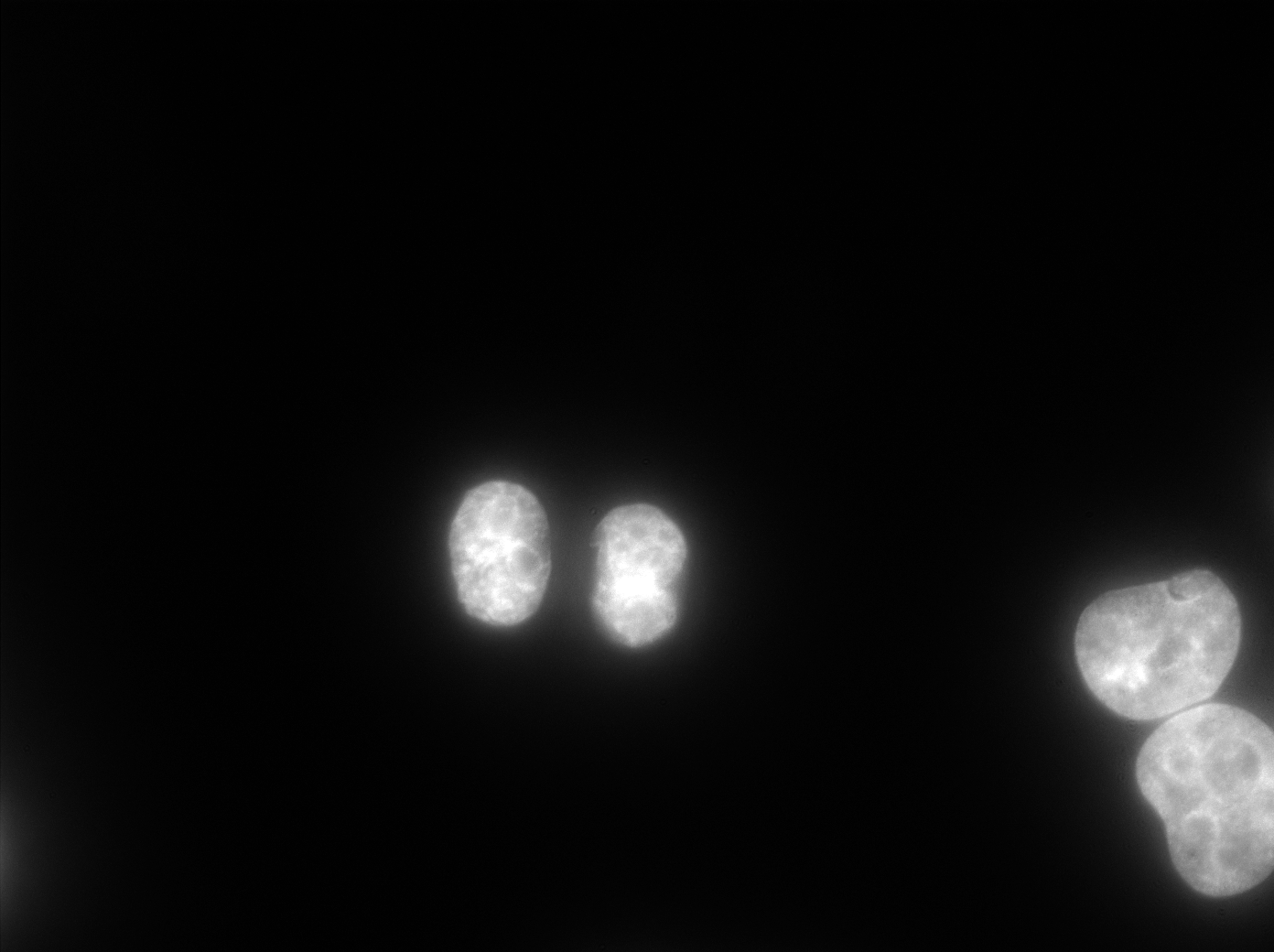

Supplement: Supplementary file 25 — Source data Fig. 7 part 1 [file 44319_2026_742_MOESM25_ESM.zip › Figure 7 Part 1/Fig 7acd Cas9 and TPGS1-ko rGT335 atubulin/Cas9 GT335recomb atub 3-24-25 R3 ET10.Project Maximum Z_XY1742850882_Z0_T0_C0.tif]

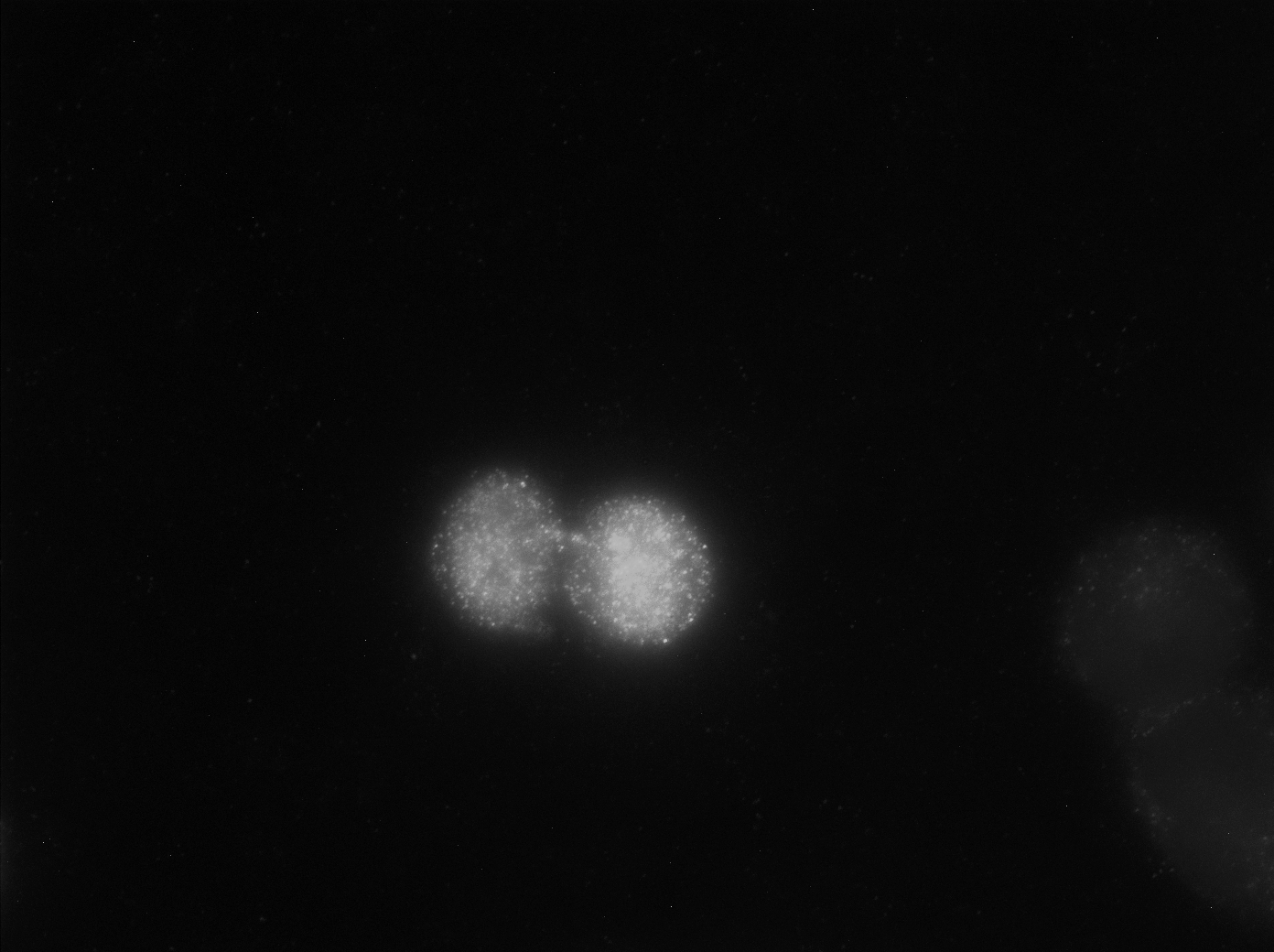

Supplement: Supplementary file 25 — Source data Fig. 7 part 1 [file 44319_2026_742_MOESM25_ESM.zip › Figure 7 Part 1/Fig 7acd Cas9 and TPGS1-ko rGT335 atubulin/Cas9 GT335recomb atub 3-24-25 R3 ET10.Project Maximum Z_XY1742850882_Z0_T0_C1.tif]

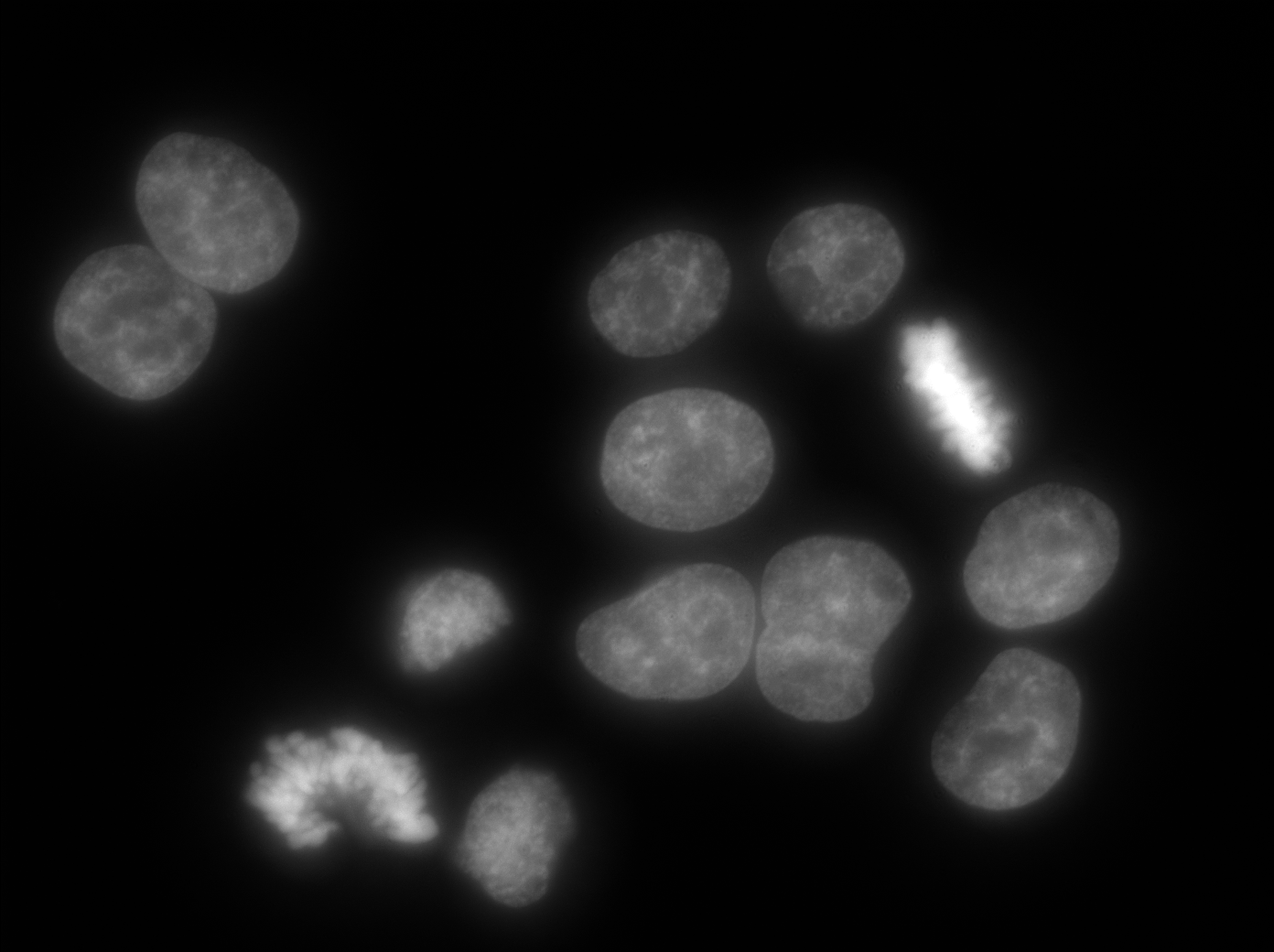

Supplement: Supplementary file 25 — Source data Fig. 7 part 1 [file 44319_2026_742_MOESM25_ESM.zip › Figure 7 Part 1/Fig 7acd Cas9 and TPGS1-ko rGT335 atubulin/Cas9 GT335recomb atub 3-24-25 R1 ET5 PA1 M1.Project Maximum Z_XY1742835552_Z0_T0_C0.tif]

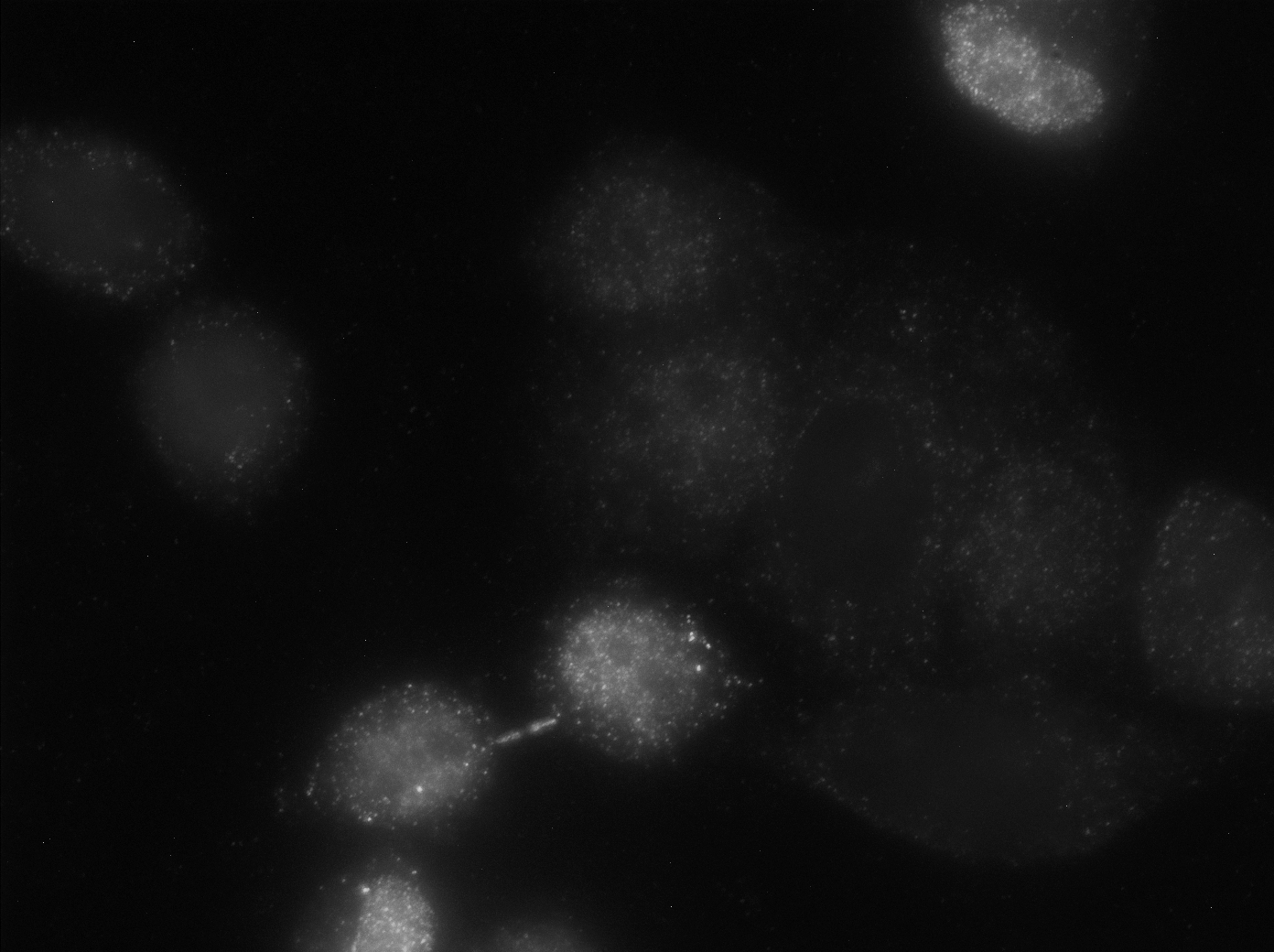

Supplement: Supplementary file 25 — Source data Fig. 7 part 1 [file 44319_2026_742_MOESM25_ESM.zip › Figure 7 Part 1/Fig 7acd Cas9 and TPGS1-ko rGT335 atubulin/Cas9 GT335recomb atub 3-24-25 R3 LT9.Project Maximum Z_XY1742851137_Z0_T0_C1.tif]

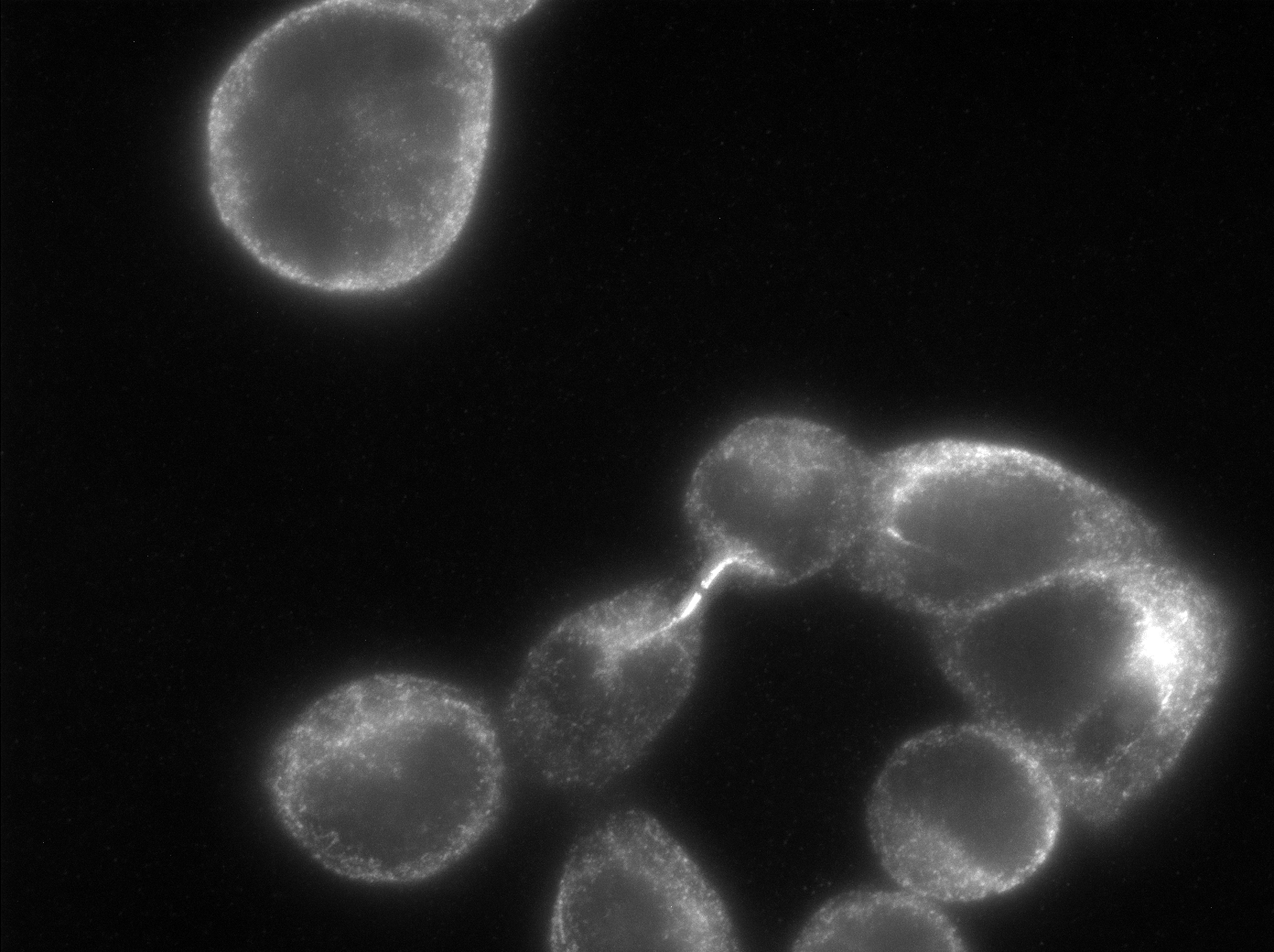

Supplement: Supplementary file 25 — Source data Fig. 7 part 1 [file 44319_2026_742_MOESM25_ESM.zip › Figure 7 Part 1/Fig 7acd Cas9 and TPGS1-ko rGT335 atubulin/Cas9 GT335recomb atub 3-24-25 R1 LT1.Project Maximum Z_XY1742834742_Z0_T0_C2.tif]

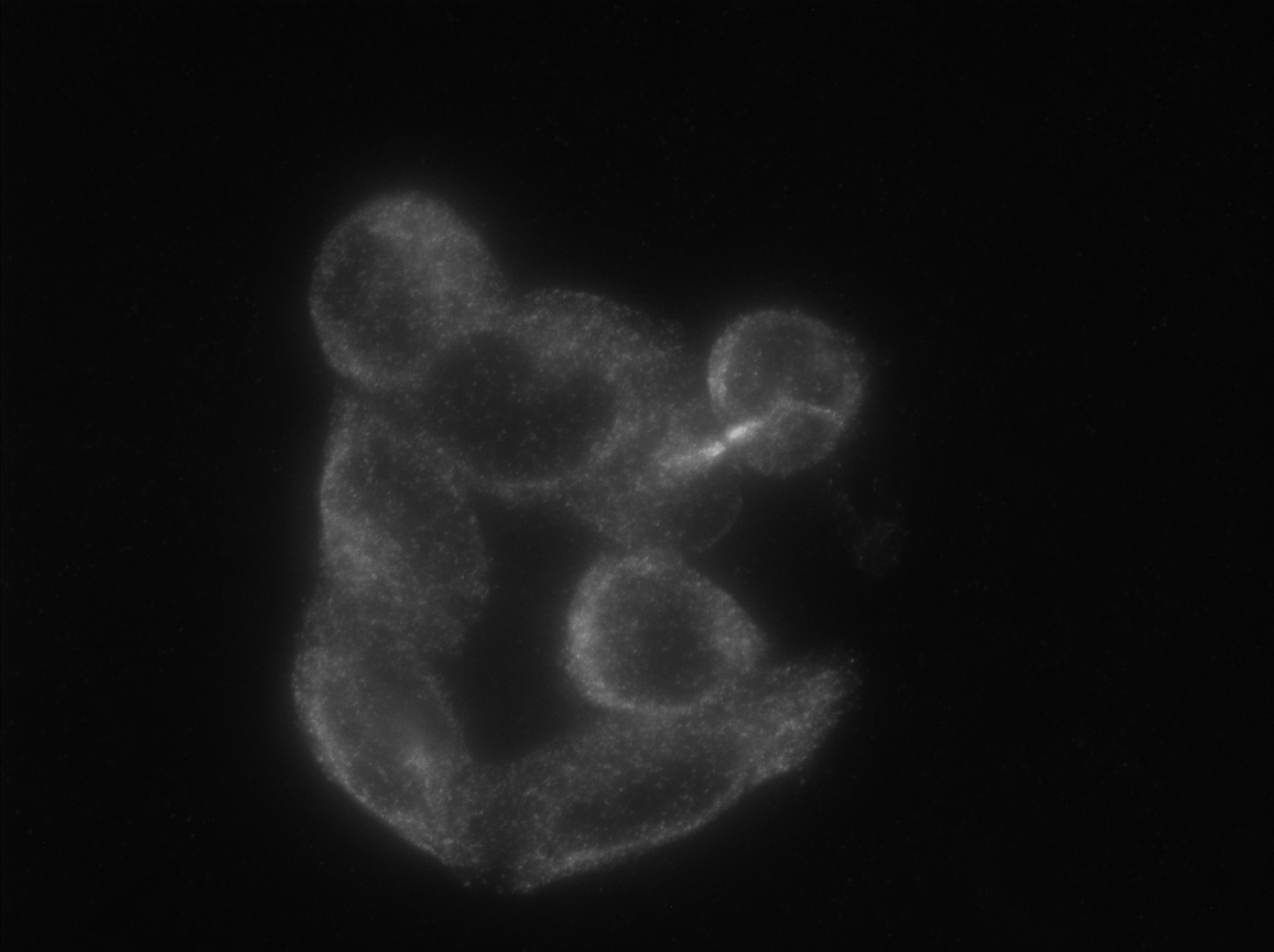

Supplement: Supplementary file 25 — Source data Fig. 7 part 1 [file 44319_2026_742_MOESM25_ESM.zip › Figure 7 Part 1/Fig 7acd Cas9 and TPGS1-ko rGT335 atubulin/Cas9 GT335recomb atub 3-24-25 R2 LT4.Project Maximum Z_XY1742846211_Z0_T0_C2.tif]

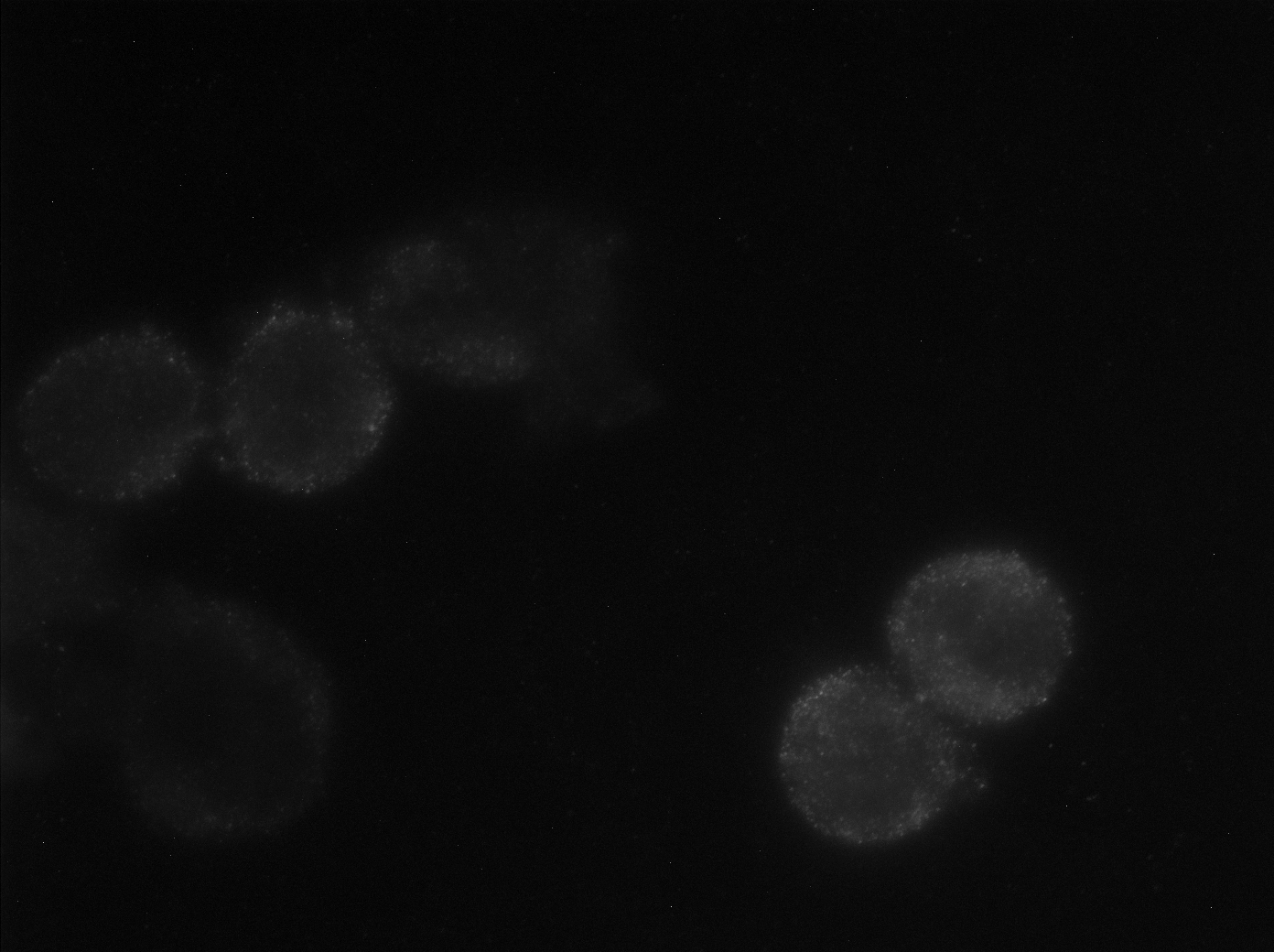

Supplement: Supplementary file 25 — Source data Fig. 7 part 1 [file 44319_2026_742_MOESM25_ESM.zip › Figure 7 Part 1/Fig 7acd Cas9 and TPGS1-ko rGT335 atubulin/Cas9 GT335recomb atub 3-24-25 R2 LT7 ET7.Project Maximum Z_XY1742846708_Z0_T0_C1.tif]

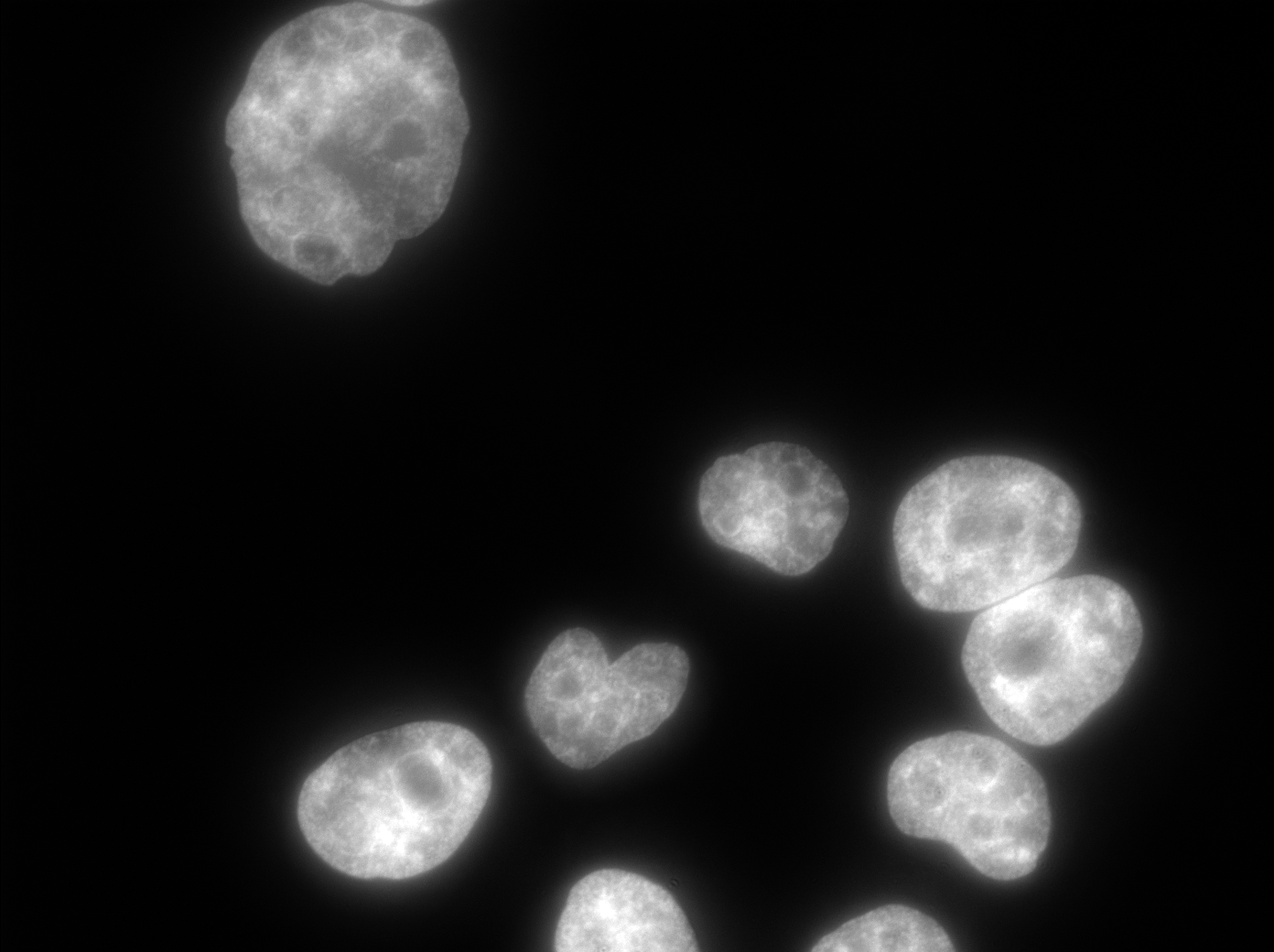

Supplement: Supplementary file 25 — Source data Fig. 7 part 1 [file 44319_2026_742_MOESM25_ESM.zip › Figure 7 Part 1/Fig 7acd Cas9 and TPGS1-ko rGT335 atubulin/Cas9 GT335recomb atub 3-24-25 R1 LT1.Project Maximum Z_XY1742834742_Z0_T0_C0.tif]

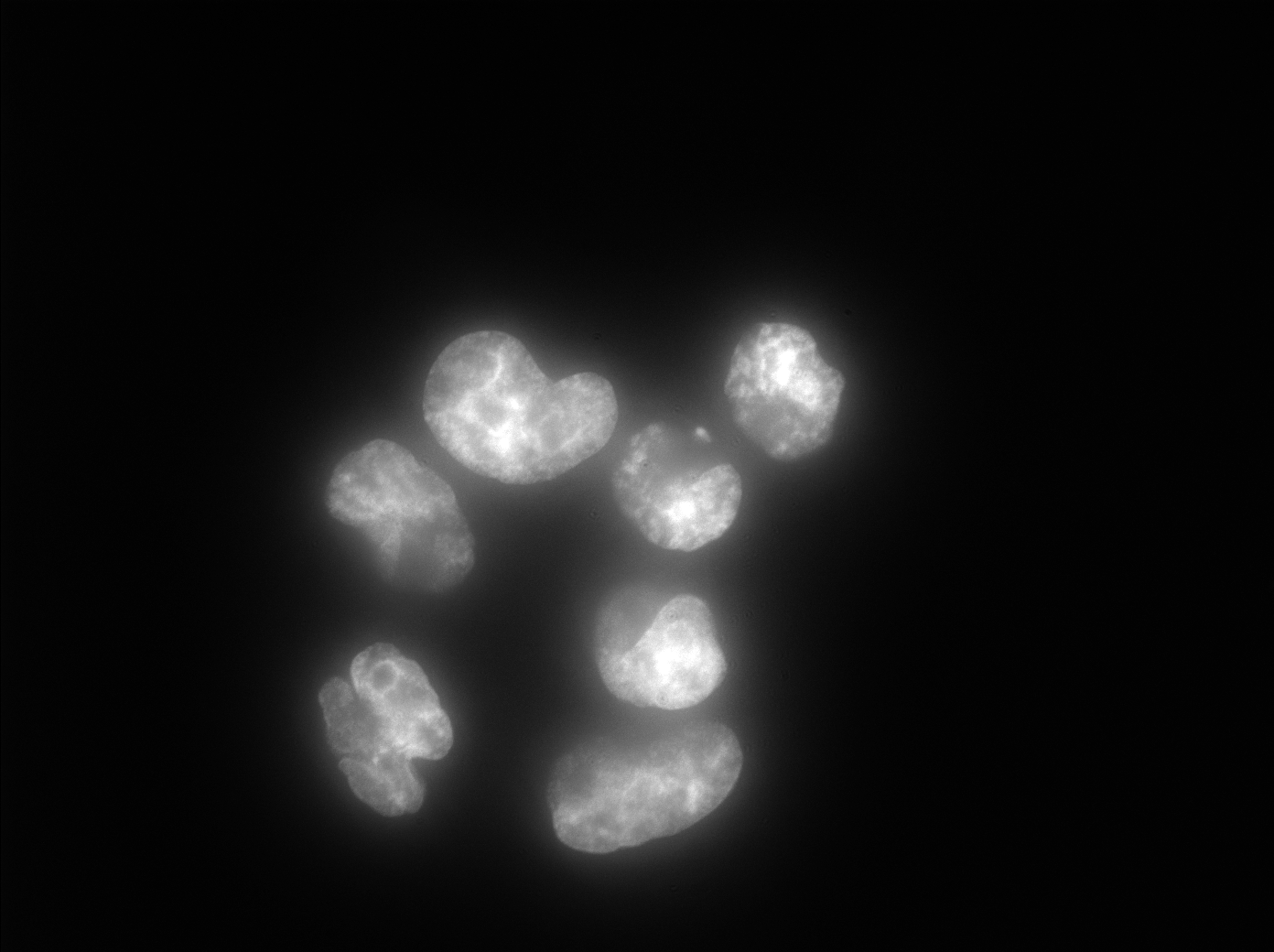

Supplement: Supplementary file 25 — Source data Fig. 7 part 1 [file 44319_2026_742_MOESM25_ESM.zip › Figure 7 Part 1/Fig 7acd Cas9 and TPGS1-ko rGT335 atubulin/Cas9 GT335recomb atub 3-24-25 R2 LT4.Project Maximum Z_XY1742846211_Z0_T0_C0.tif]

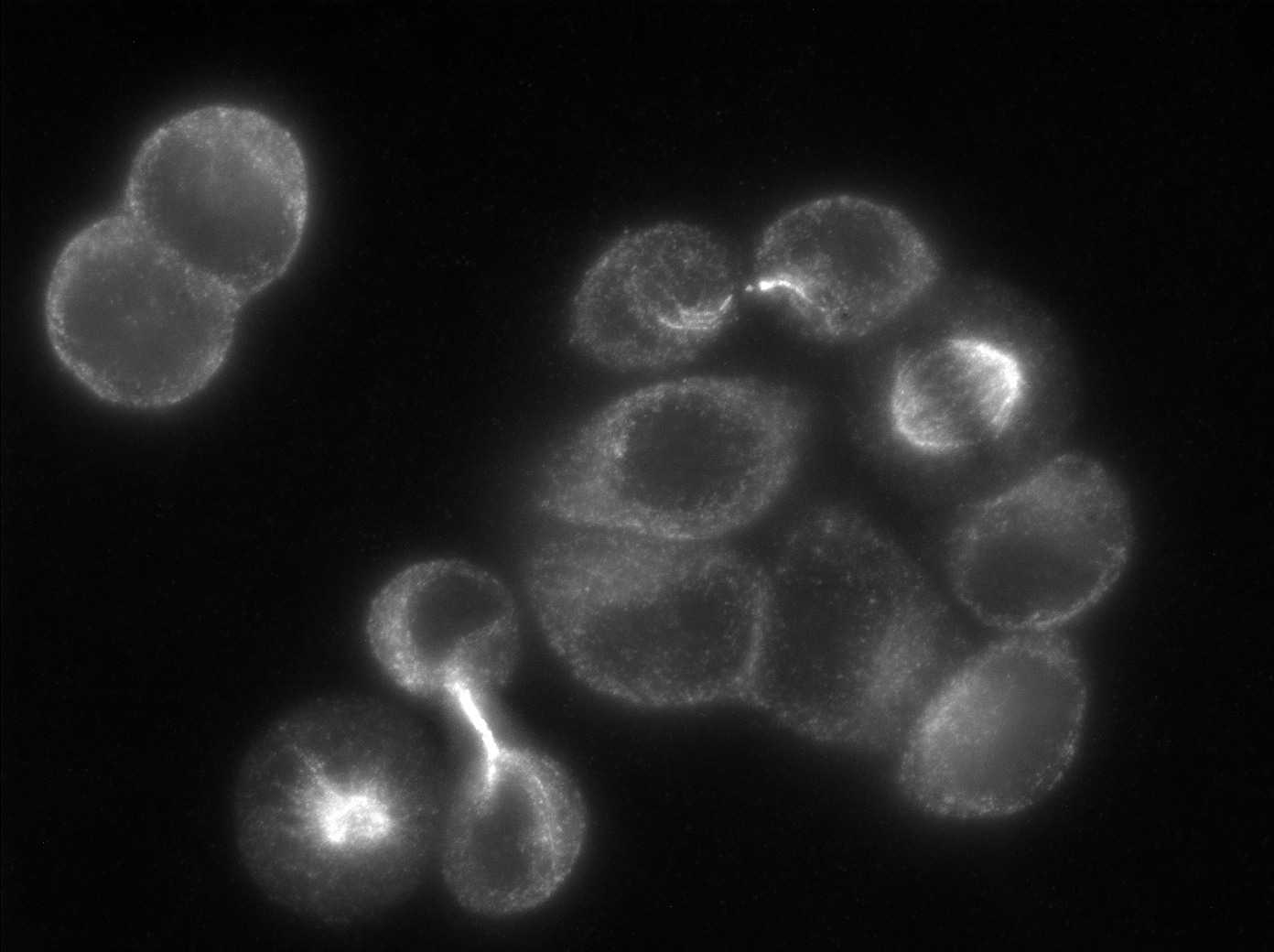

Supplement: Supplementary file 25 — Source data Fig. 7 part 1 [file 44319_2026_742_MOESM25_ESM.zip › Figure 7 Part 1/Fig 7acd Cas9 and TPGS1-ko rGT335 atubulin/Cas9 GT335recomb atub 3-24-25 R1 ET5 PA1 M1.Project Maximum Z_XY1742835552_Z0_T0_C2.tif]

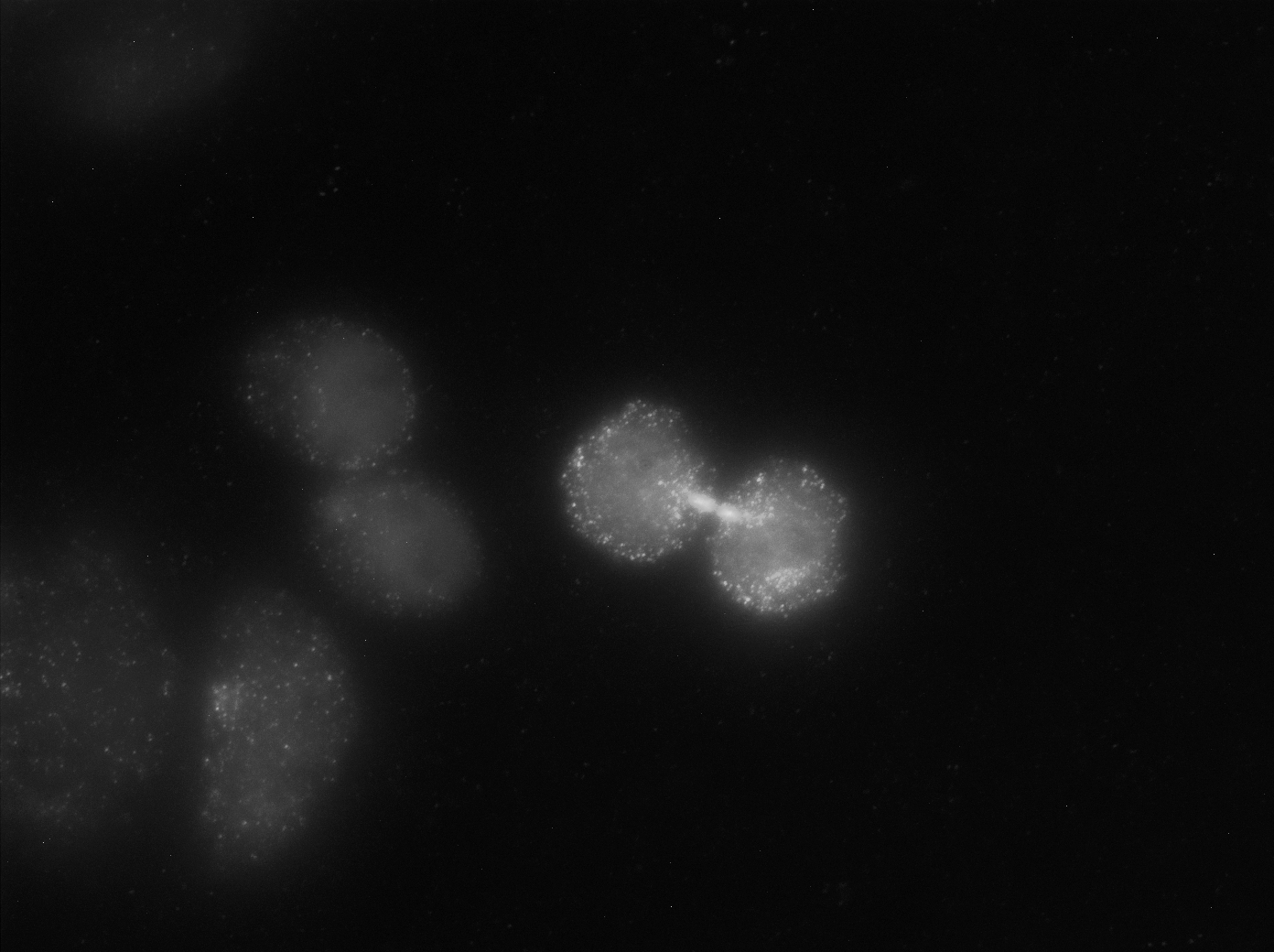

Supplement: Supplementary file 25 — Source data Fig. 7 part 1 [file 44319_2026_742_MOESM25_ESM.zip › Figure 7 Part 1/Fig 7acd Cas9 and TPGS1-ko rGT335 atubulin/Cas9 GT335recomb atub 3-24-25 R3 ET8.Project Maximum Z_XY1742850659_Z0_T0_C1.tif]

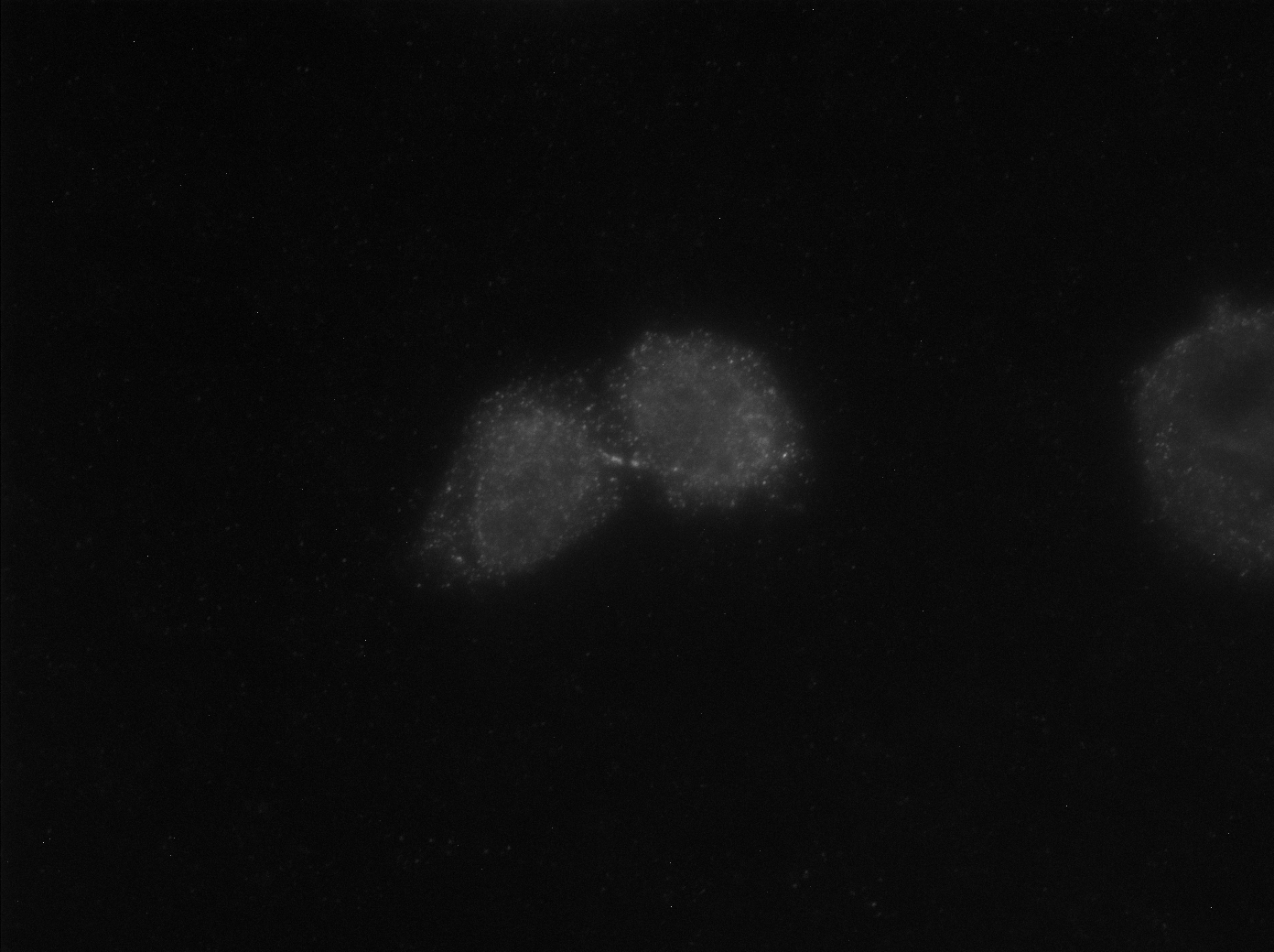

Supplement: Supplementary file 25 — Source data Fig. 7 part 1 [file 44319_2026_742_MOESM25_ESM.zip › Figure 7 Part 1/Fig 7acd Cas9 and TPGS1-ko rGT335 atubulin/Cas9 GT335recomb atub 3-24-25 R2 LT2.Project Maximum Z_XY1742845919_Z0_T0_C1.tif]

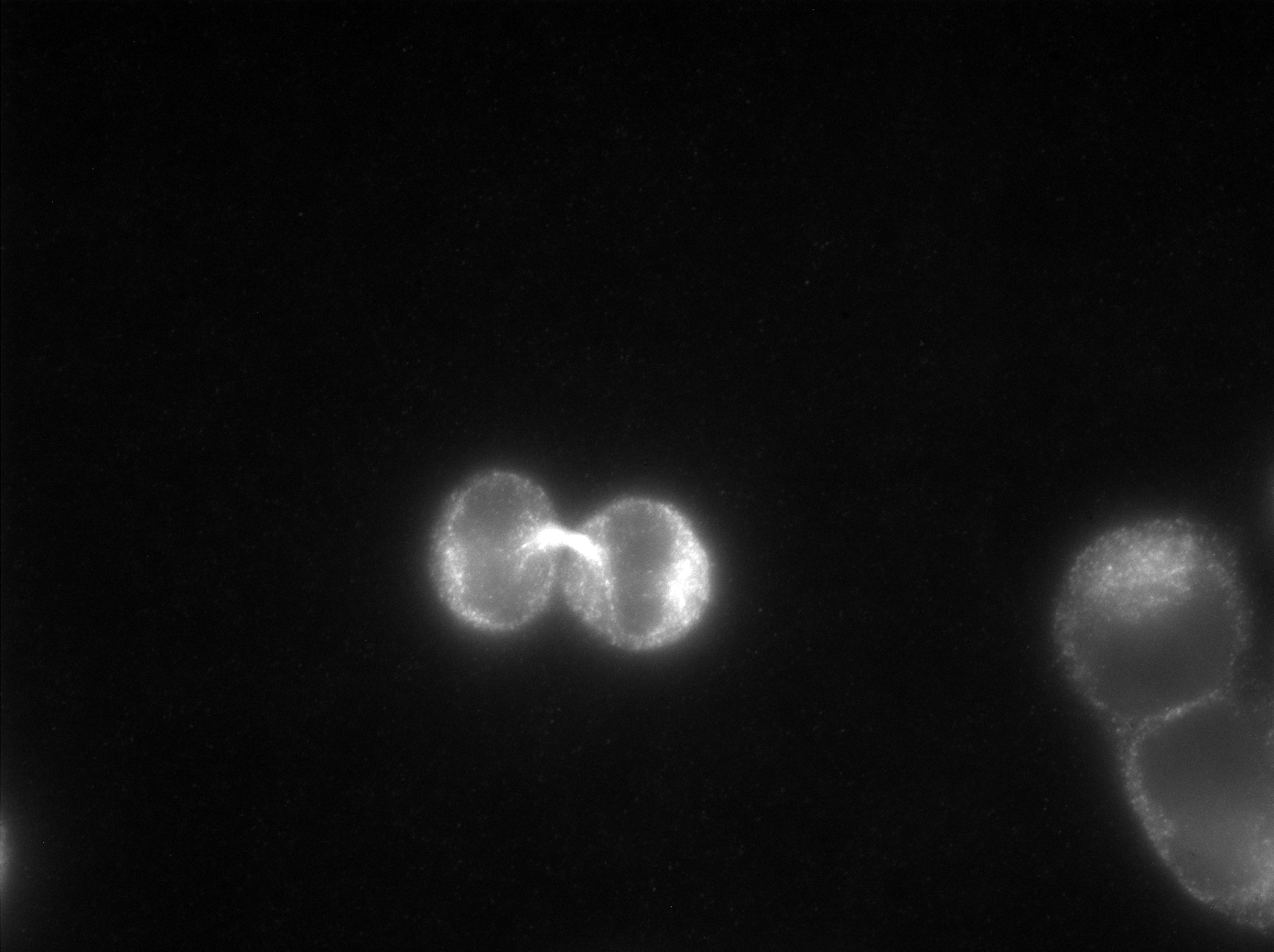

Supplement: Supplementary file 25 — Source data Fig. 7 part 1 [file 44319_2026_742_MOESM25_ESM.zip › Figure 7 Part 1/Fig 7acd Cas9 and TPGS1-ko rGT335 atubulin/Cas9 GT335recomb atub 3-24-25 R3 ET10.Project Maximum Z_XY1742850882_Z0_T0_C2.tif]

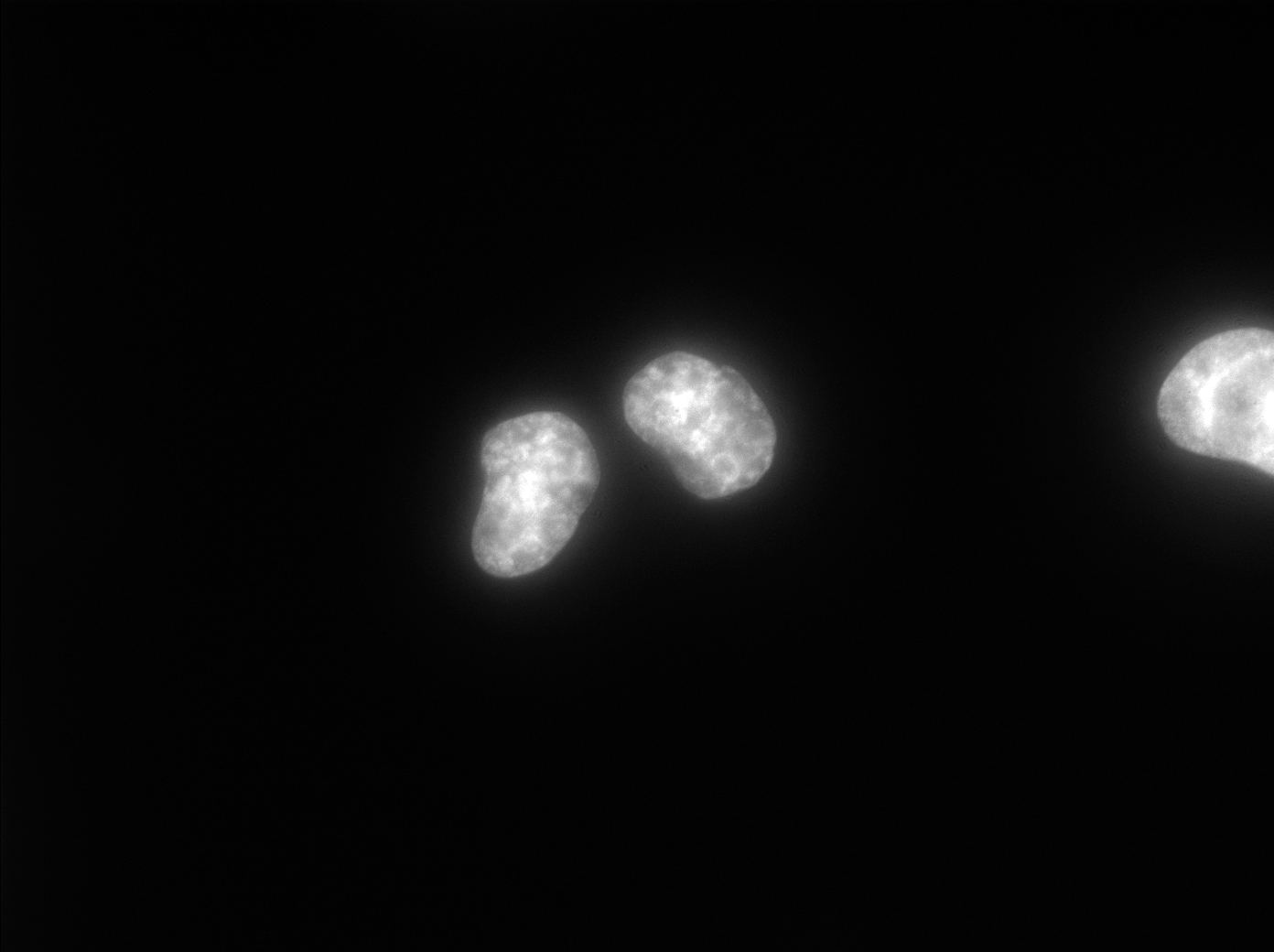

Supplement: Supplementary file 25 — Source data Fig. 7 part 1 [file 44319_2026_742_MOESM25_ESM.zip › Figure 7 Part 1/Fig 7acd Cas9 and TPGS1-ko rGT335 atubulin/Cas9 GT335recomb atub 3-24-25 R2 LT2.Project Maximum Z_XY1742845919_Z0_T0_C0.tif]

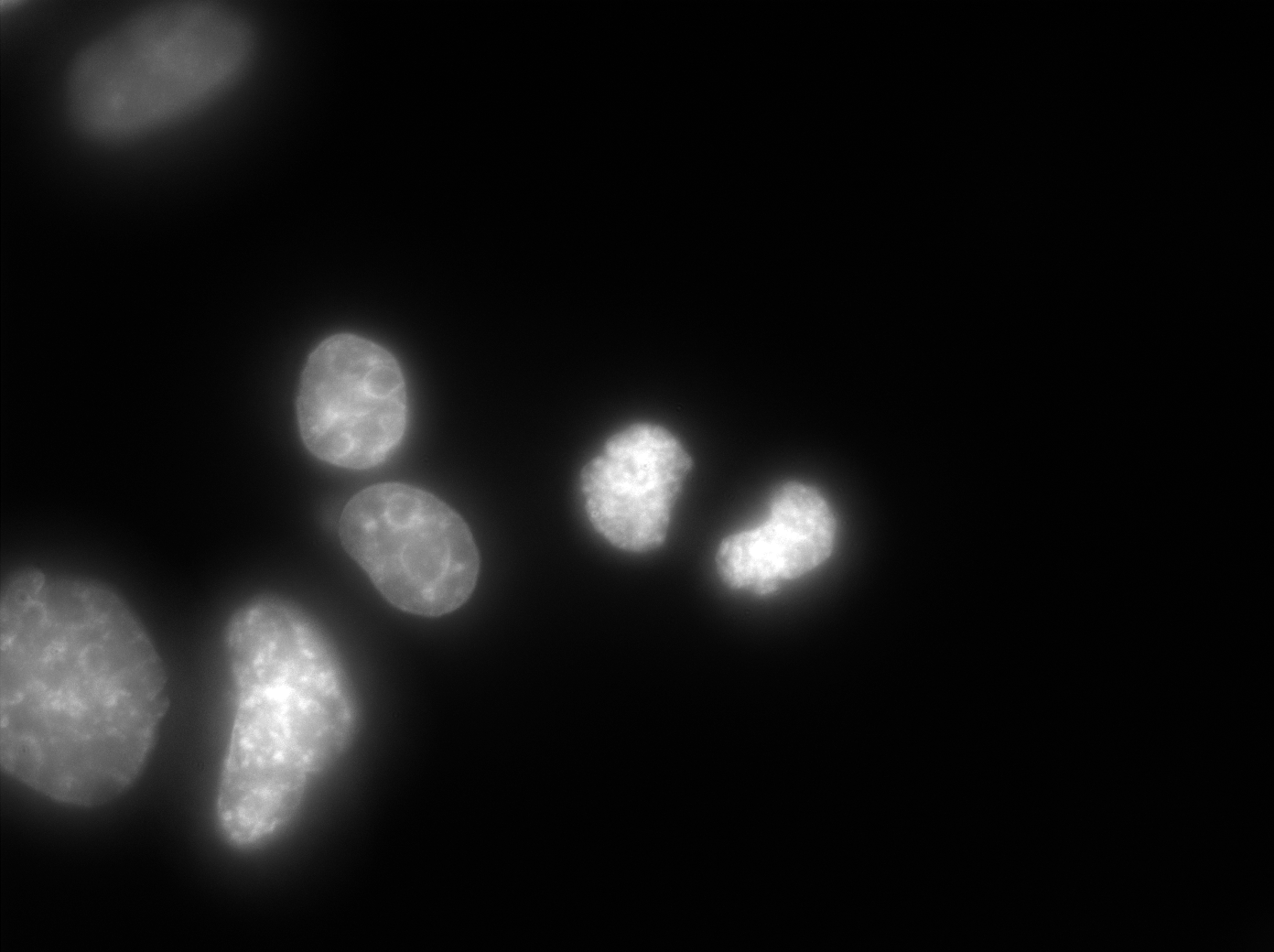

Supplement: Supplementary file 25 — Source data Fig. 7 part 1 [file 44319_2026_742_MOESM25_ESM.zip › Figure 7 Part 1/Fig 7acd Cas9 and TPGS1-ko rGT335 atubulin/Cas9 GT335recomb atub 3-24-25 R3 ET8.Project Maximum Z_XY1742850659_Z0_T0_C0.tif]

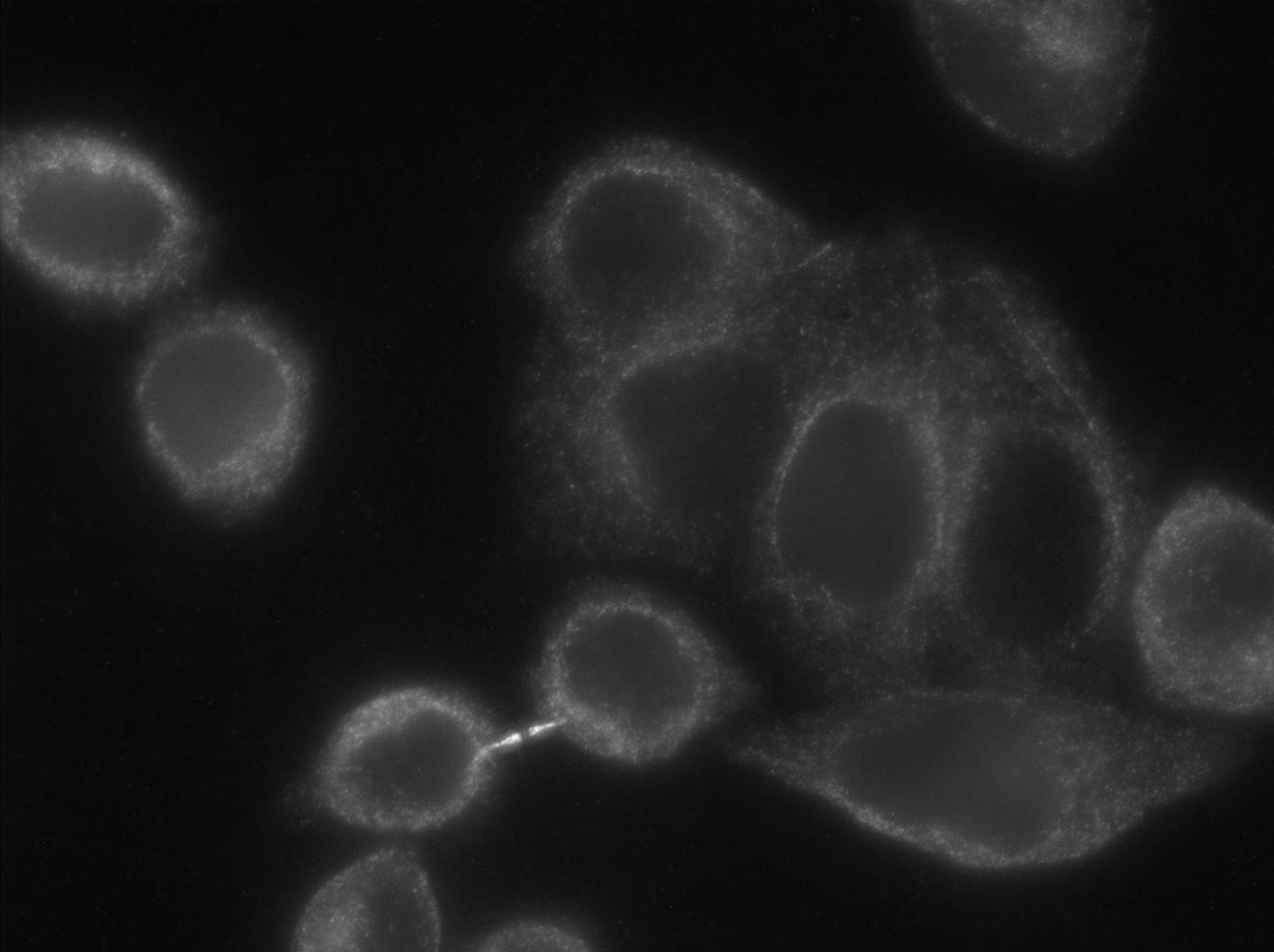

Supplement: Supplementary file 25 — Source data Fig. 7 part 1 [file 44319_2026_742_MOESM25_ESM.zip › Figure 7 Part 1/Fig 7acd Cas9 and TPGS1-ko rGT335 atubulin/Cas9 GT335recomb atub 3-24-25 R3 LT9.Project Maximum Z_XY1742851137_Z0_T0_C2.tif]

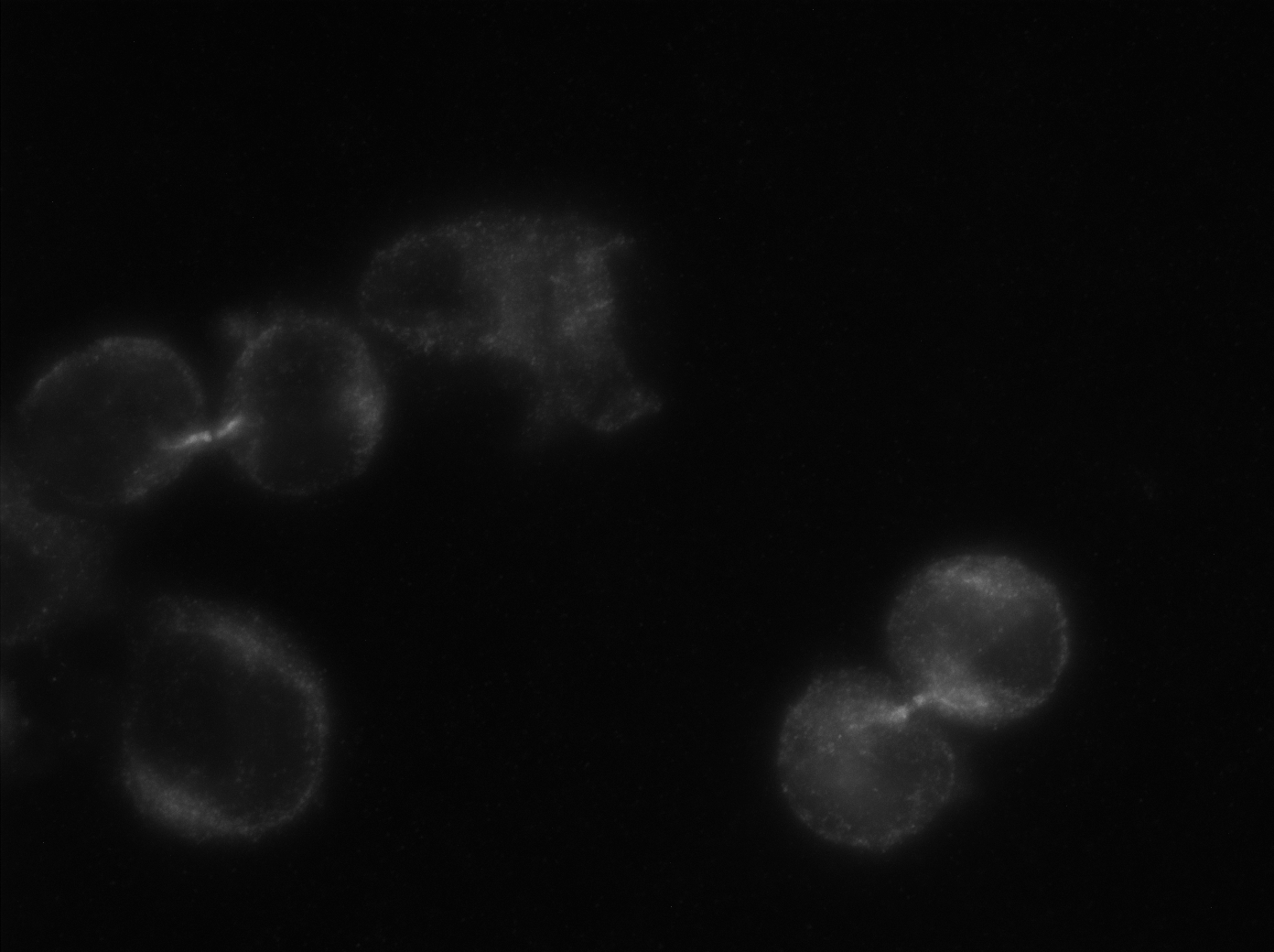

Supplement: Supplementary file 25 — Source data Fig. 7 part 1 [file 44319_2026_742_MOESM25_ESM.zip › Figure 7 Part 1/Fig 7acd Cas9 and TPGS1-ko rGT335 atubulin/Cas9 GT335recomb atub 3-24-25 R2 LT7 ET7.Project Maximum Z_XY1742846708_Z0_T0_C2.tif]

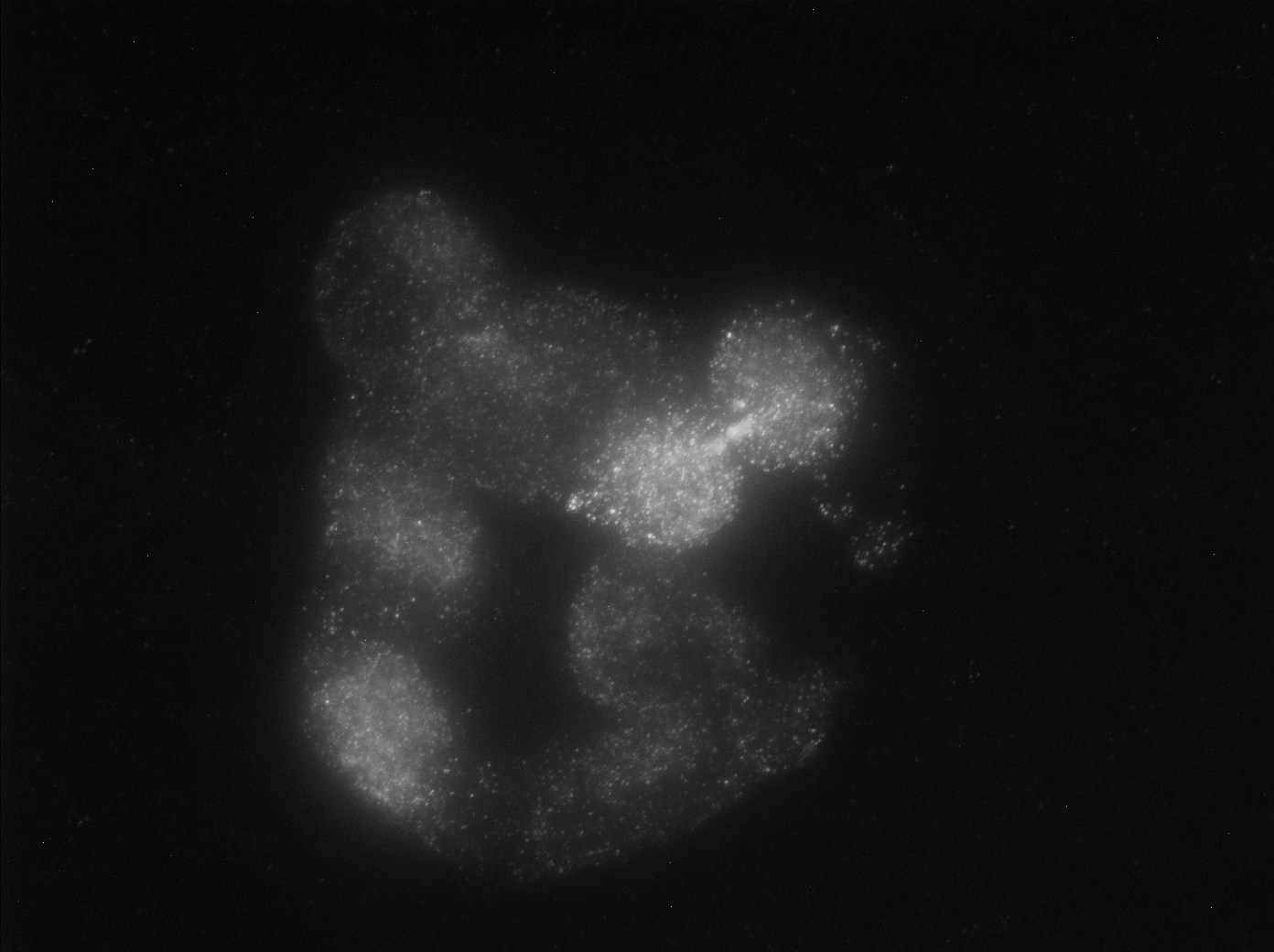

Supplement: Supplementary file 25 — Source data Fig. 7 part 1 [file 44319_2026_742_MOESM25_ESM.zip › Figure 7 Part 1/Fig 7acd Cas9 and TPGS1-ko rGT335 atubulin/Cas9 GT335recomb atub 3-24-25 R2 LT4.Project Maximum Z_XY1742846211_Z0_T0_C1.tif]

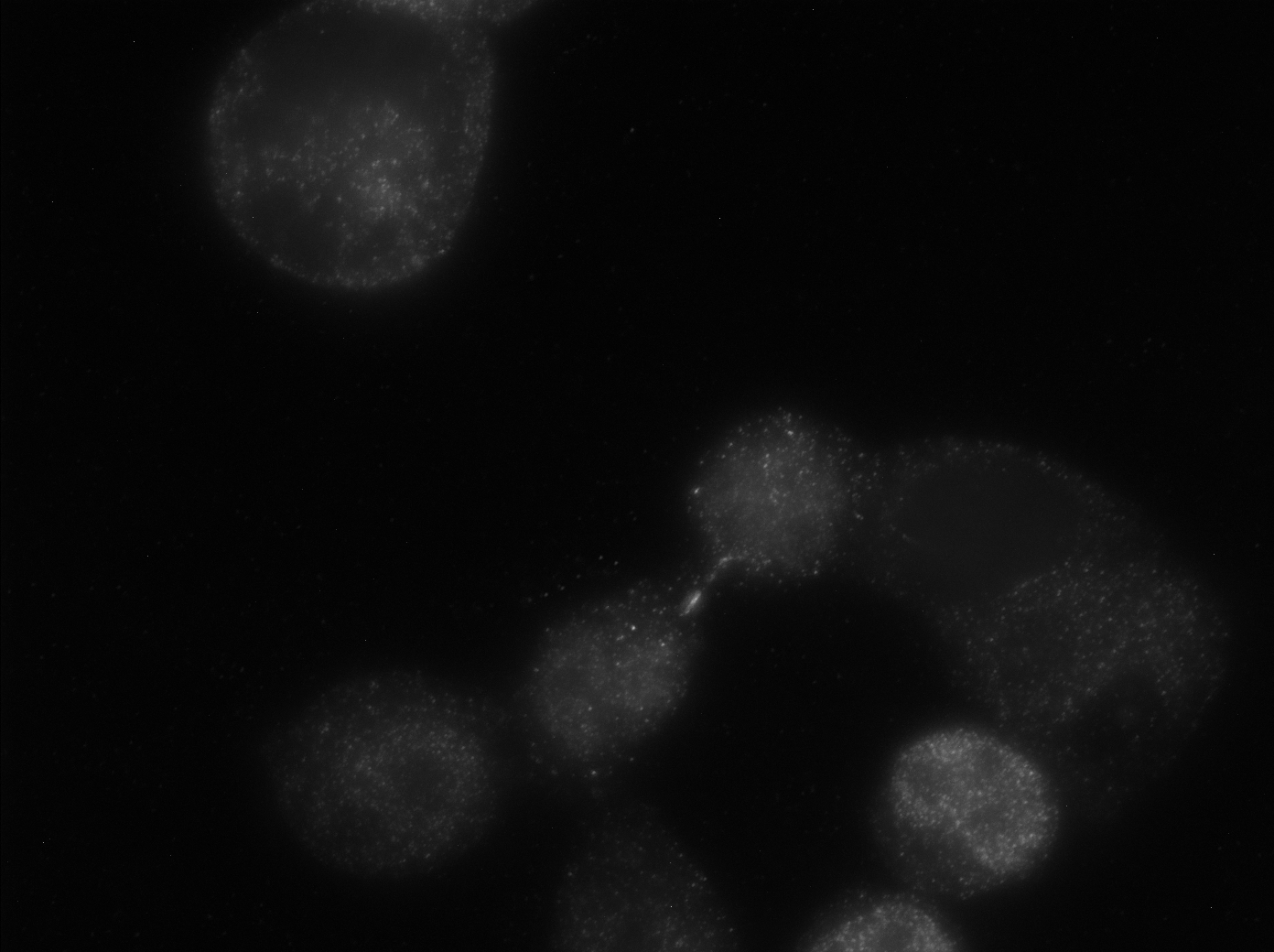

Supplement: Supplementary file 25 — Source data Fig. 7 part 1 [file 44319_2026_742_MOESM25_ESM.zip › Figure 7 Part 1/Fig 7acd Cas9 and TPGS1-ko rGT335 atubulin/Cas9 GT335recomb atub 3-24-25 R1 LT1.Project Maximum Z_XY1742834742_Z0_T0_C1.tif]

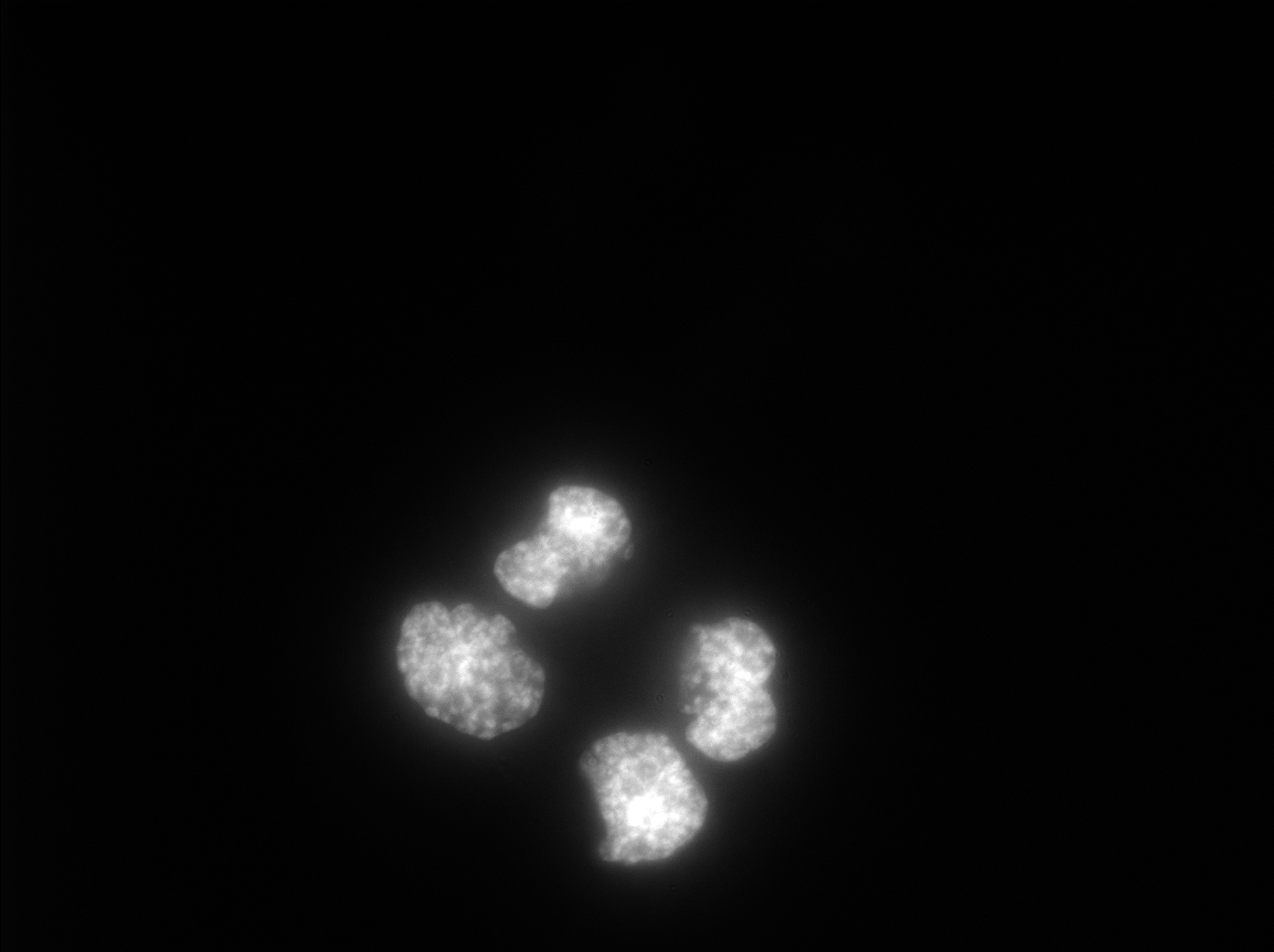

Supplement: Supplementary file 25 — Source data Fig. 7 part 1 [file 44319_2026_742_MOESM25_ESM.zip › Figure 7 Part 1/Fig 7acd Cas9 and TPGS1-ko rGT335 atubulin/Cas9 GT335recomb atub 3-24-25 R2 ET3ET4.Project Maximum Z_XY1742845467_Z0_T0_C0.tif]

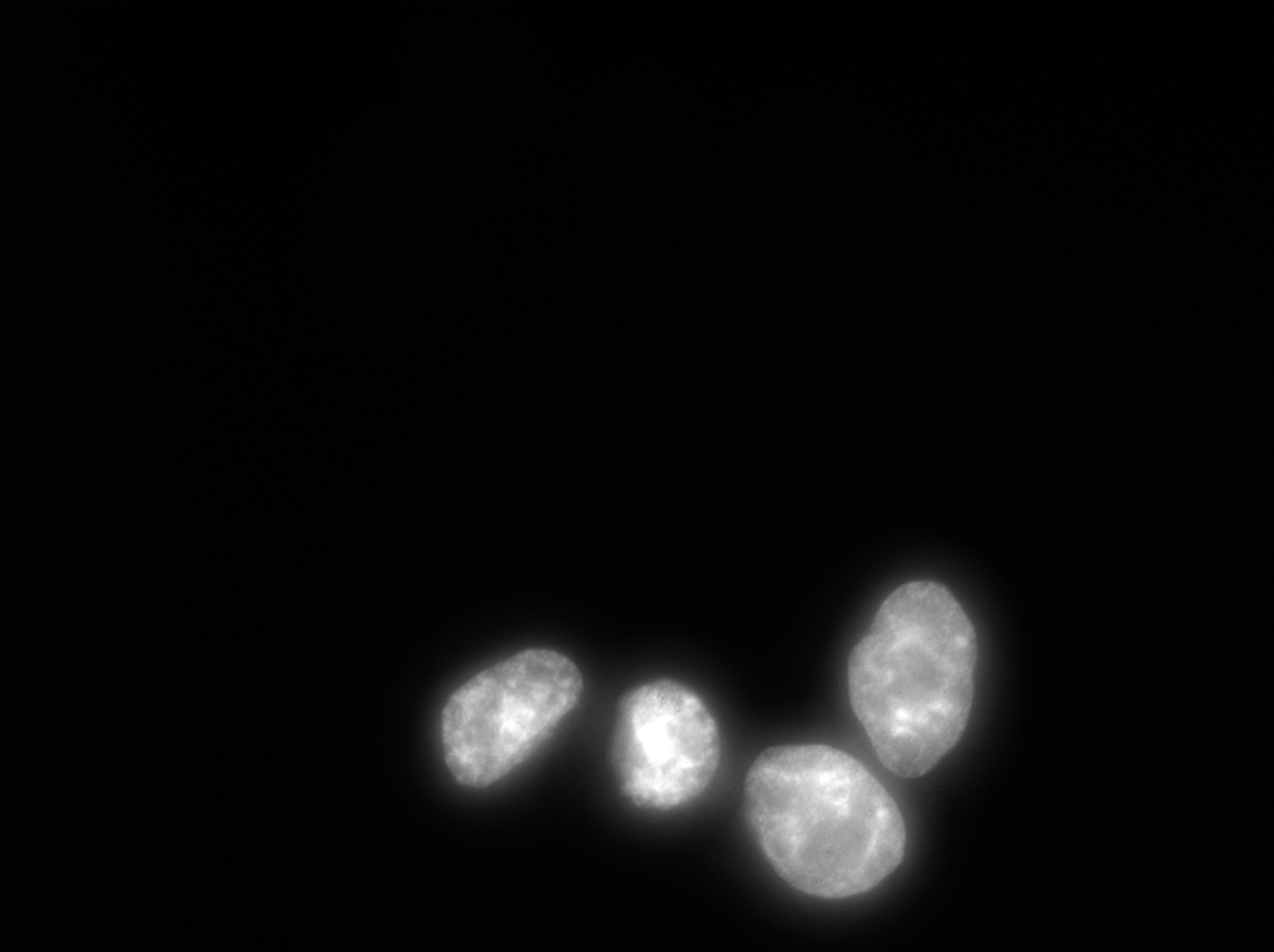

Supplement: Supplementary file 25 — Source data Fig. 7 part 1 [file 44319_2026_742_MOESM25_ESM.zip › Figure 7 Part 1/Fig 7acd Cas9 and TPGS1-ko rGT335 atubulin/Cas9 GT335recomb atub 3-24-25 R2 ET8.Project Maximum Z_XY1742847157_Z0_T0_C0.tif]

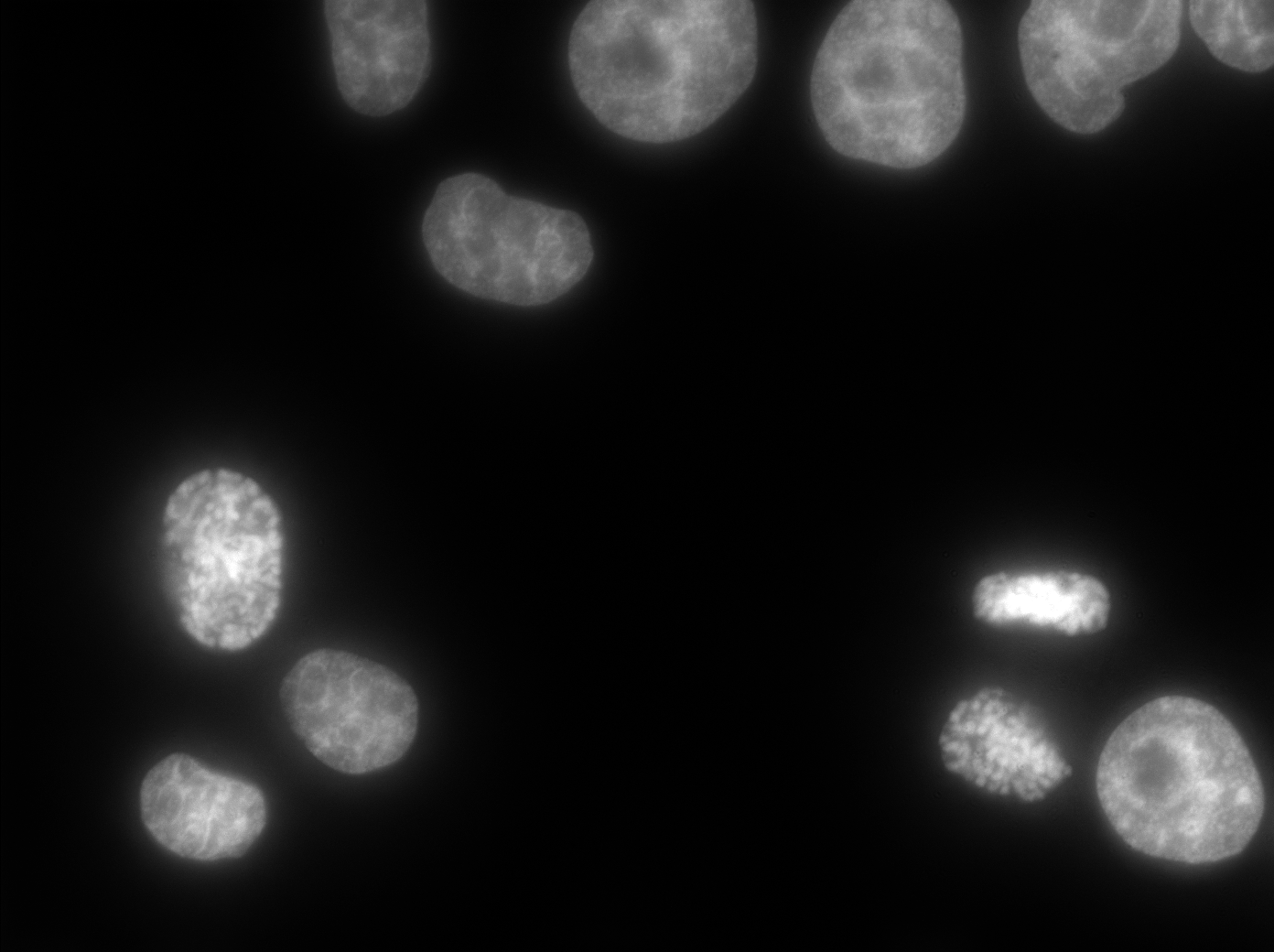

Supplement: Supplementary file 25 — Source data Fig. 7 part 1 [file 44319_2026_742_MOESM25_ESM.zip › Figure 7 Part 1/Fig 7acd Cas9 and TPGS1-ko rGT335 atubulin/Cas9 GT335recomb atub 3-24-25 R1 ET6 P1.Project Maximum Z_XY1742835965_Z0_T0_C0.tif]

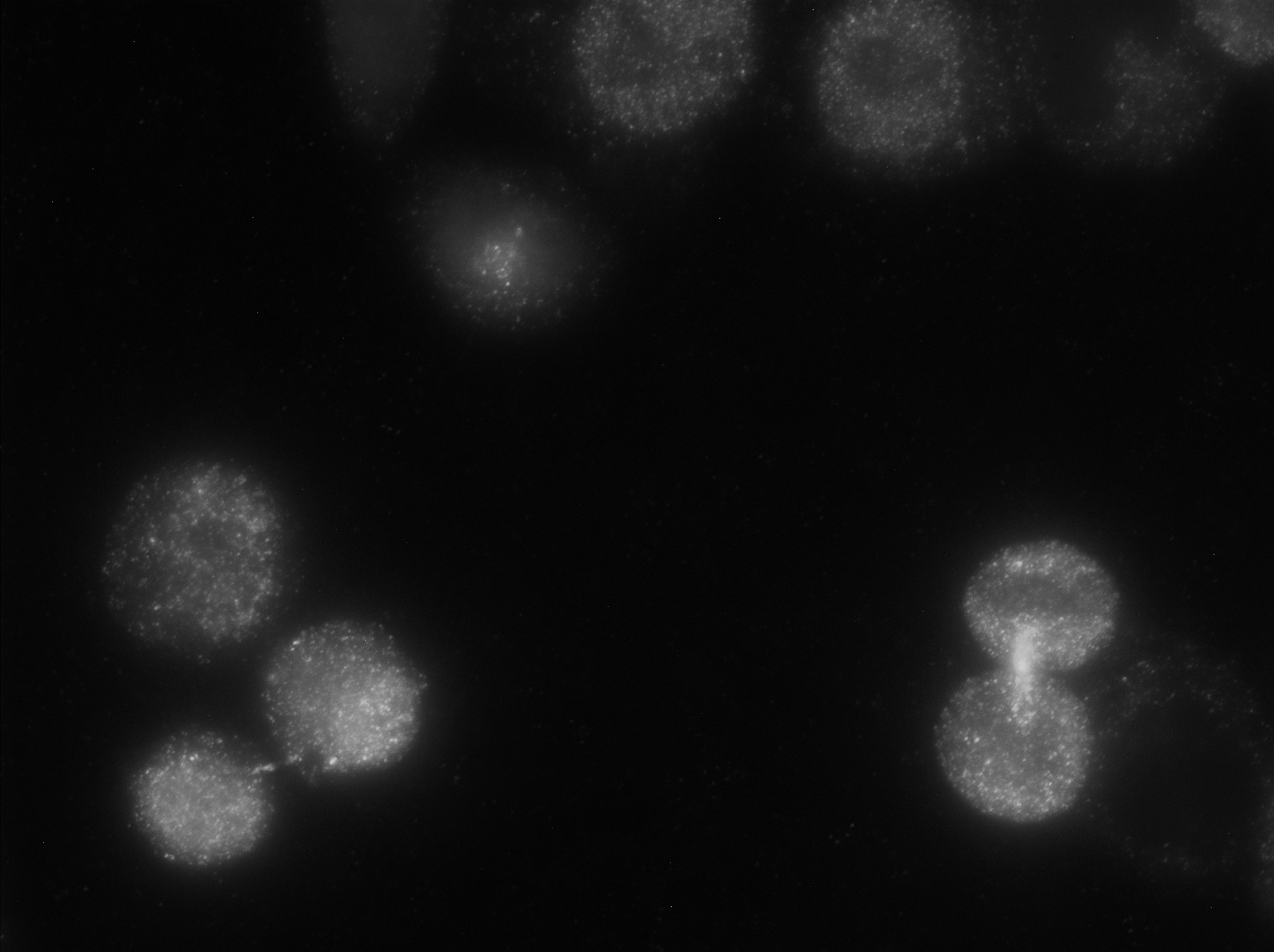

Supplement: Supplementary file 25 — Source data Fig. 7 part 1 [file 44319_2026_742_MOESM25_ESM.zip › Figure 7 Part 1/Fig 7acd Cas9 and TPGS1-ko rGT335 atubulin/Cas9 GT335recomb atub 3-24-25 R1 ET6 P1.Project Maximum Z_XY1742835965_Z0_T0_C1.tif]

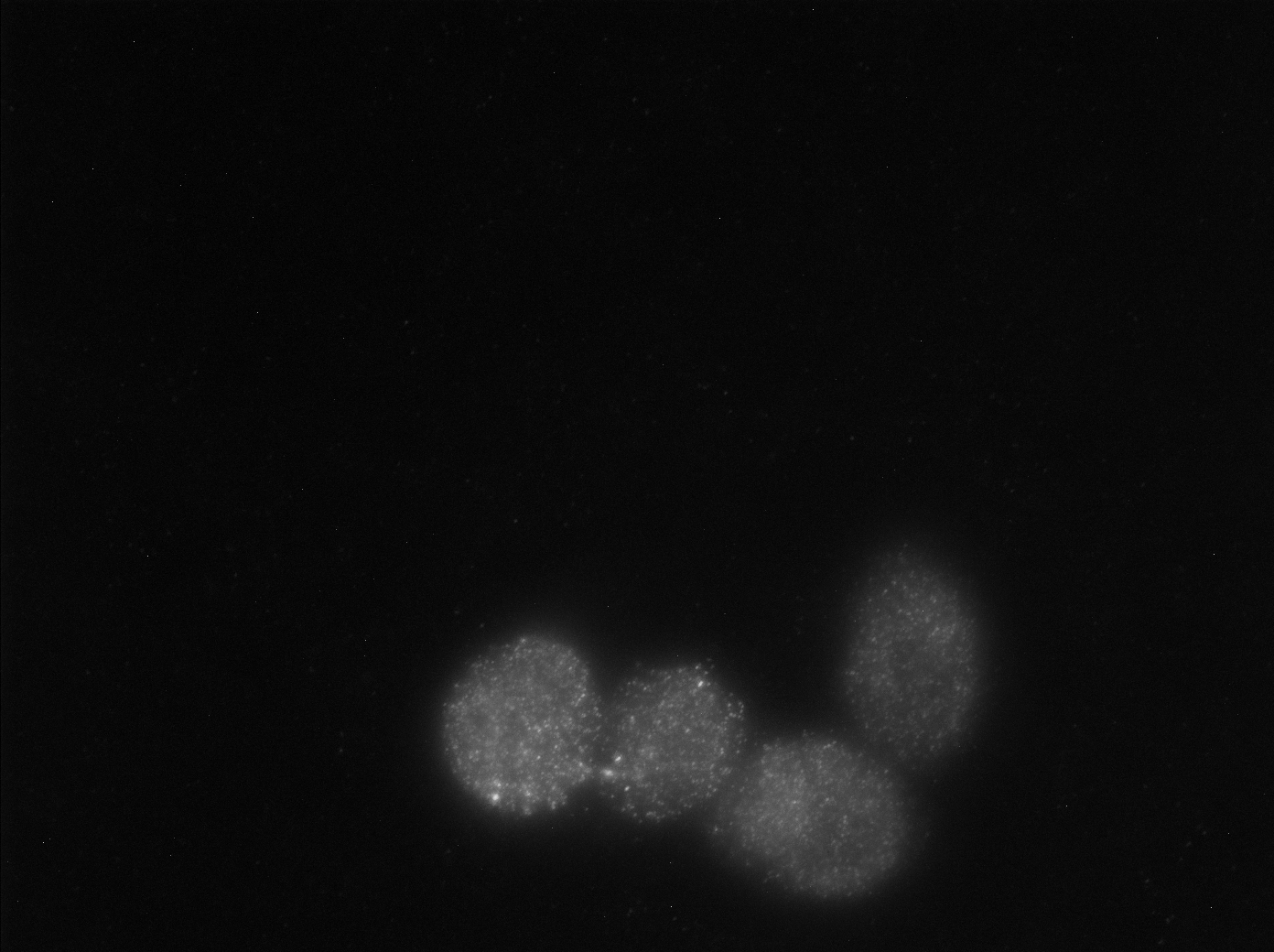

Supplement: Supplementary file 25 — Source data Fig. 7 part 1 [file 44319_2026_742_MOESM25_ESM.zip › Figure 7 Part 1/Fig 7acd Cas9 and TPGS1-ko rGT335 atubulin/Cas9 GT335recomb atub 3-24-25 R2 ET8.Project Maximum Z_XY1742847157_Z0_T0_C1.tif]

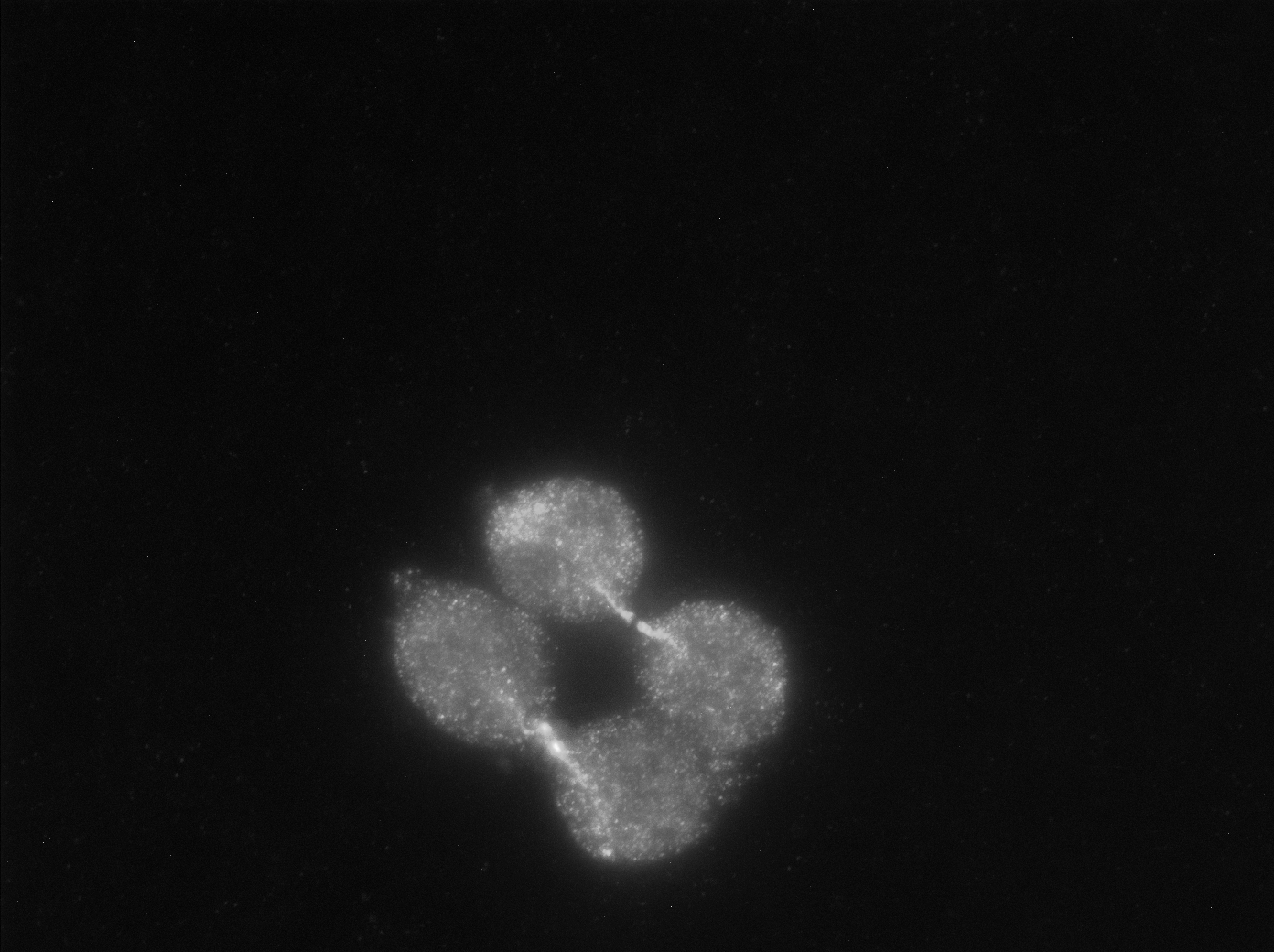

Supplement: Supplementary file 25 — Source data Fig. 7 part 1 [file 44319_2026_742_MOESM25_ESM.zip › Figure 7 Part 1/Fig 7acd Cas9 and TPGS1-ko rGT335 atubulin/Cas9 GT335recomb atub 3-24-25 R2 ET3ET4.Project Maximum Z_XY1742845467_Z0_T0_C1.tif]

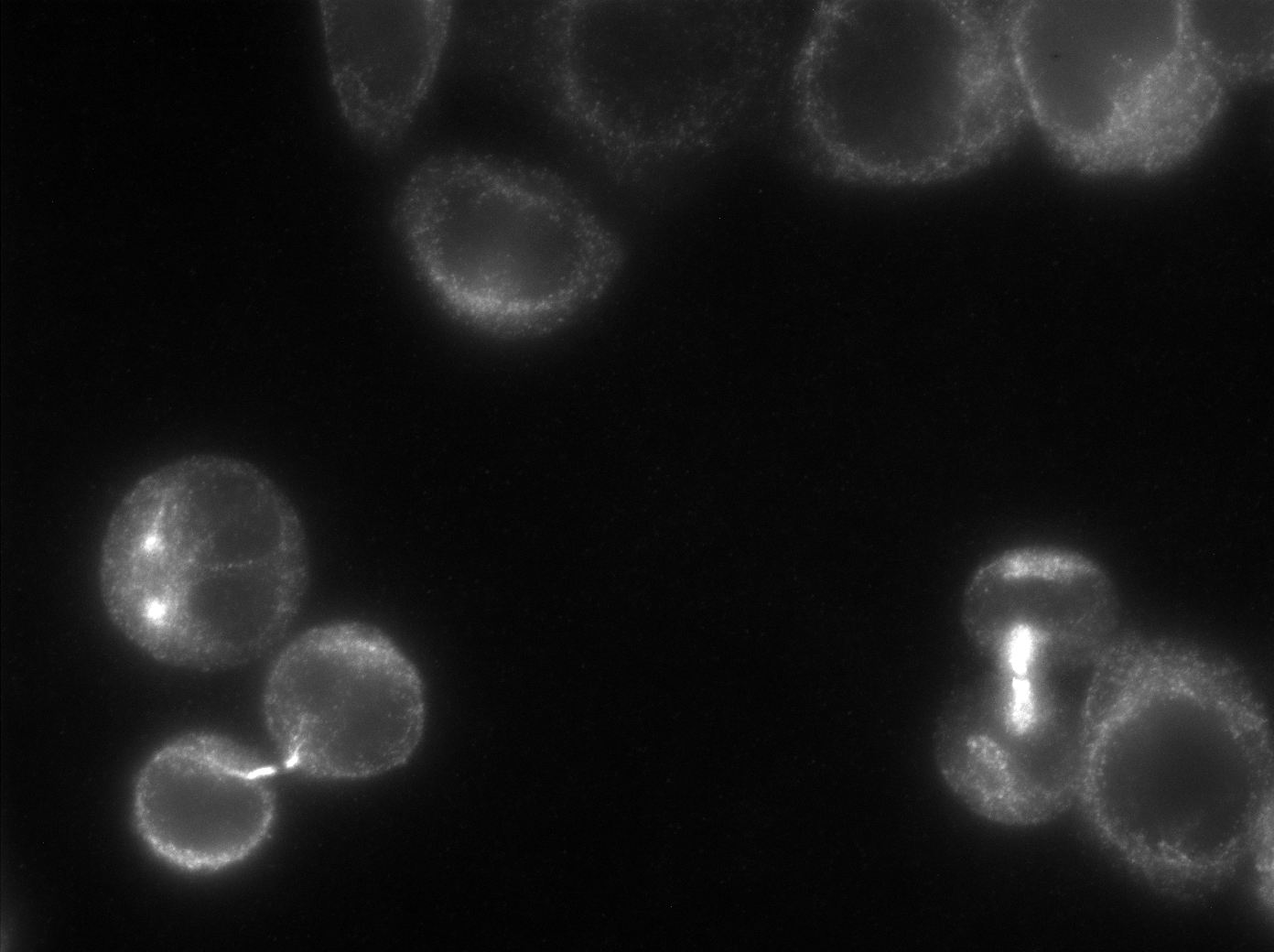

Supplement: Supplementary file 25 — Source data Fig. 7 part 1 [file 44319_2026_742_MOESM25_ESM.zip › Figure 7 Part 1/Fig 7acd Cas9 and TPGS1-ko rGT335 atubulin/Cas9 GT335recomb atub 3-24-25 R1 ET6 P1.Project Maximum Z_XY1742835965_Z0_T0_C2.tif]

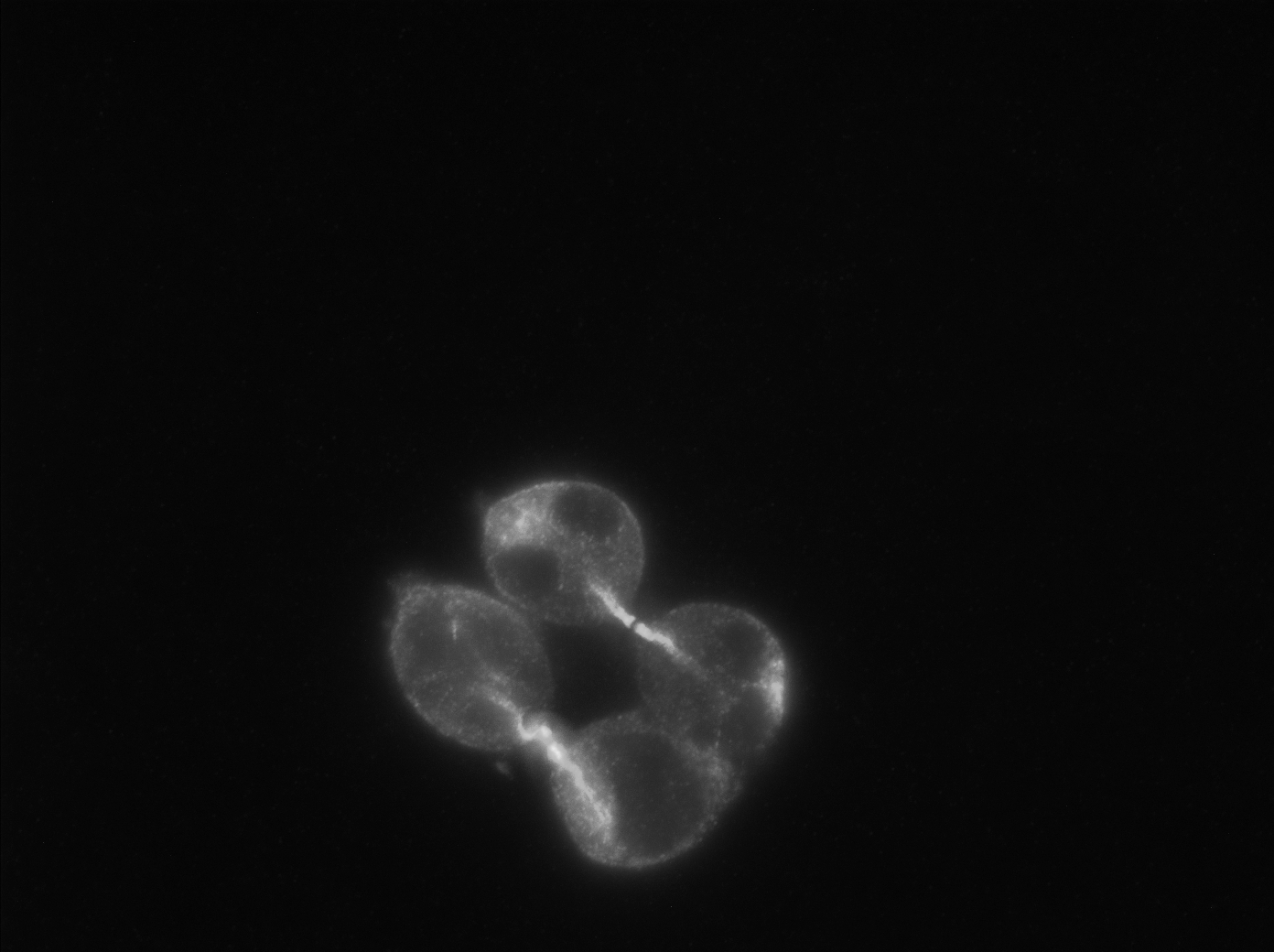

Supplement: Supplementary file 25 — Source data Fig. 7 part 1 [file 44319_2026_742_MOESM25_ESM.zip › Figure 7 Part 1/Fig 7acd Cas9 and TPGS1-ko rGT335 atubulin/Cas9 GT335recomb atub 3-24-25 R2 ET3ET4.Project Maximum Z_XY1742845467_Z0_T0_C2.tif]

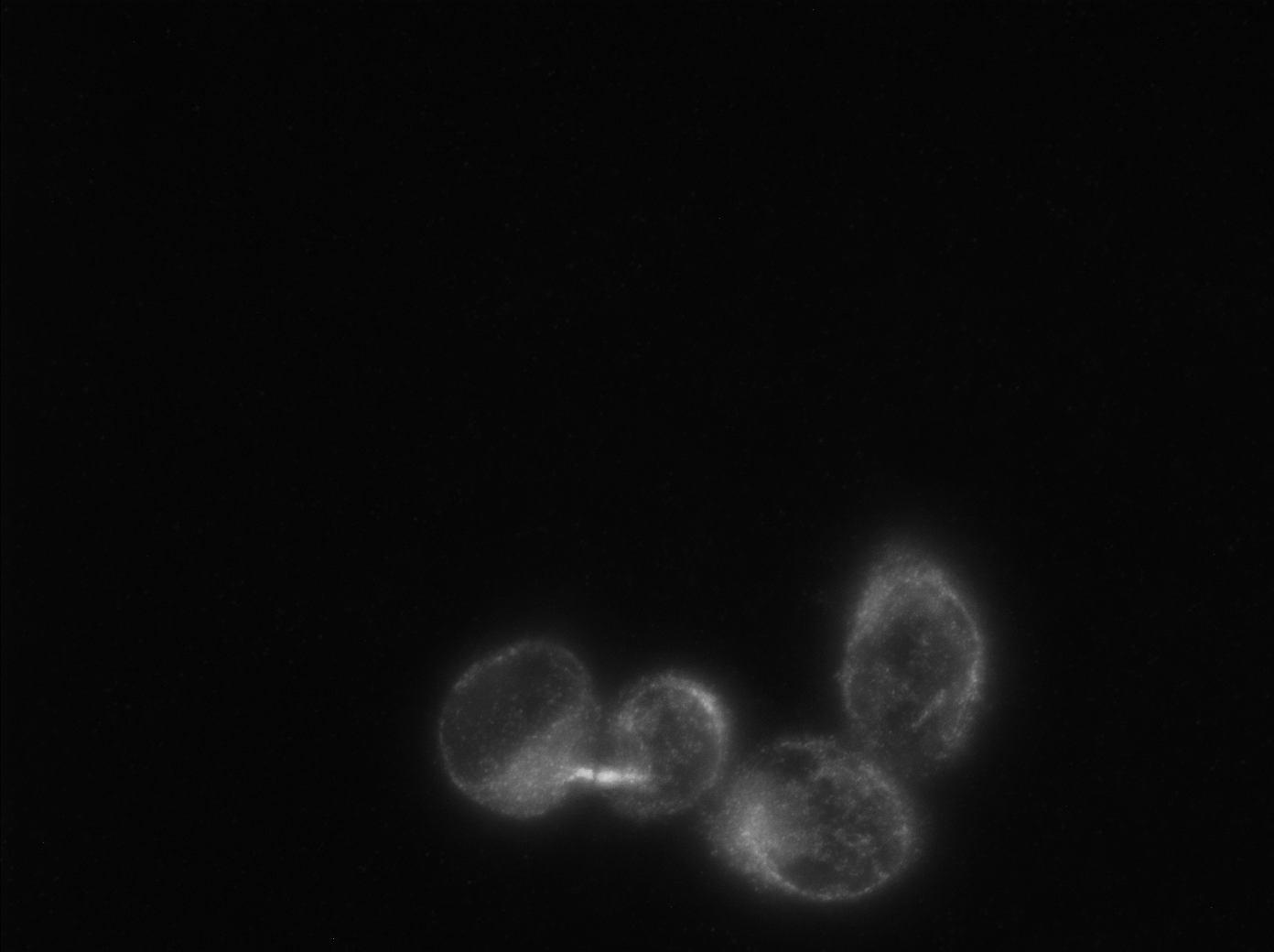

Supplement: Supplementary file 25 — Source data Fig. 7 part 1 [file 44319_2026_742_MOESM25_ESM.zip › Figure 7 Part 1/Fig 7acd Cas9 and TPGS1-ko rGT335 atubulin/Cas9 GT335recomb atub 3-24-25 R2 ET8.Project Maximum Z_XY1742847157_Z0_T0_C2.tif]

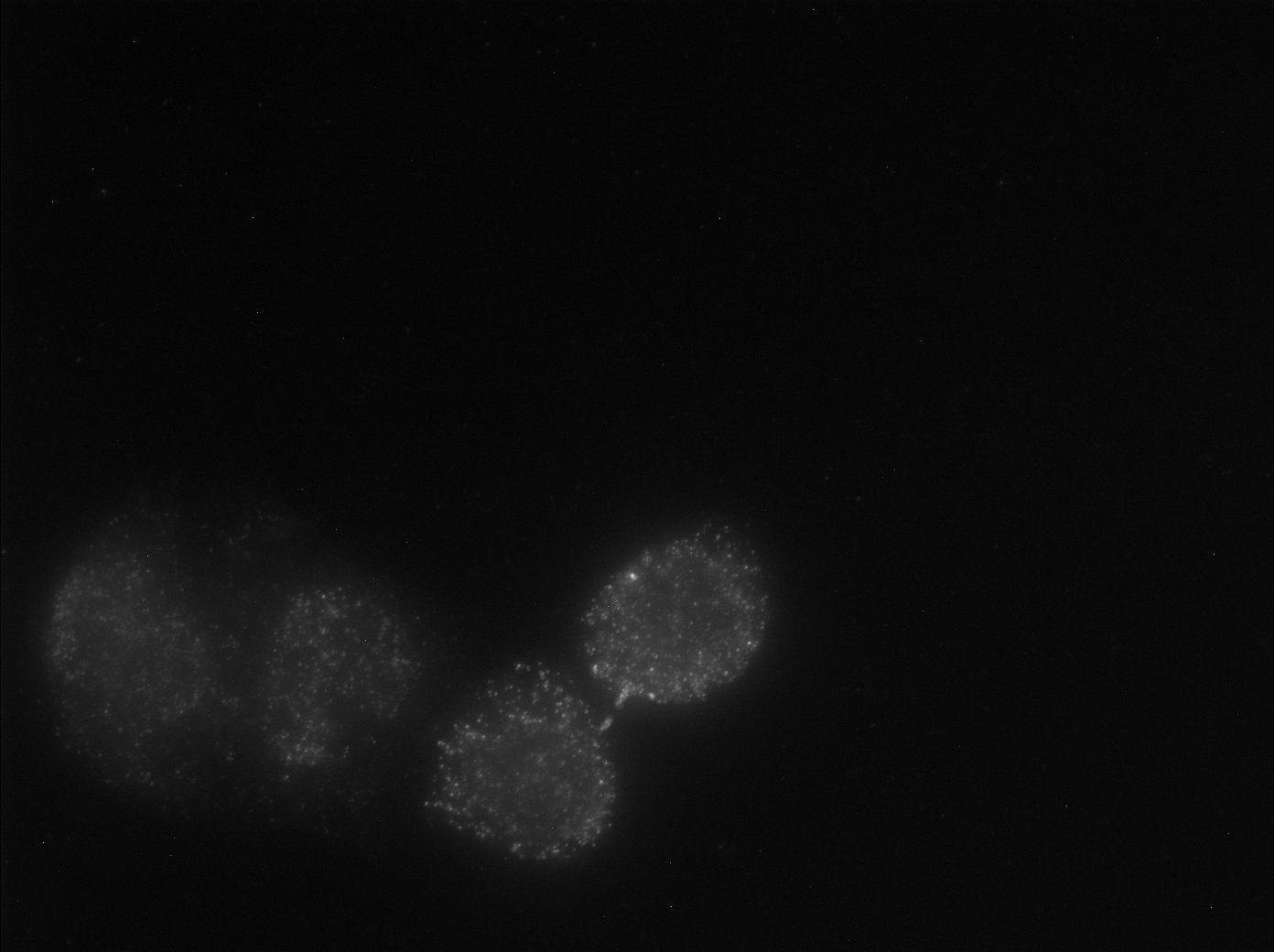

Supplement: Supplementary file 25 — Source data Fig. 7 part 1 [file 44319_2026_742_MOESM25_ESM.zip › Figure 7 Part 1/Fig 7acd Cas9 and TPGS1-ko rGT335 atubulin/Cas9 GT335recomb atub 3-24-25 R2 LT6.Project Maximum Z_XY1742846575_Z0_T0_C1.tif]

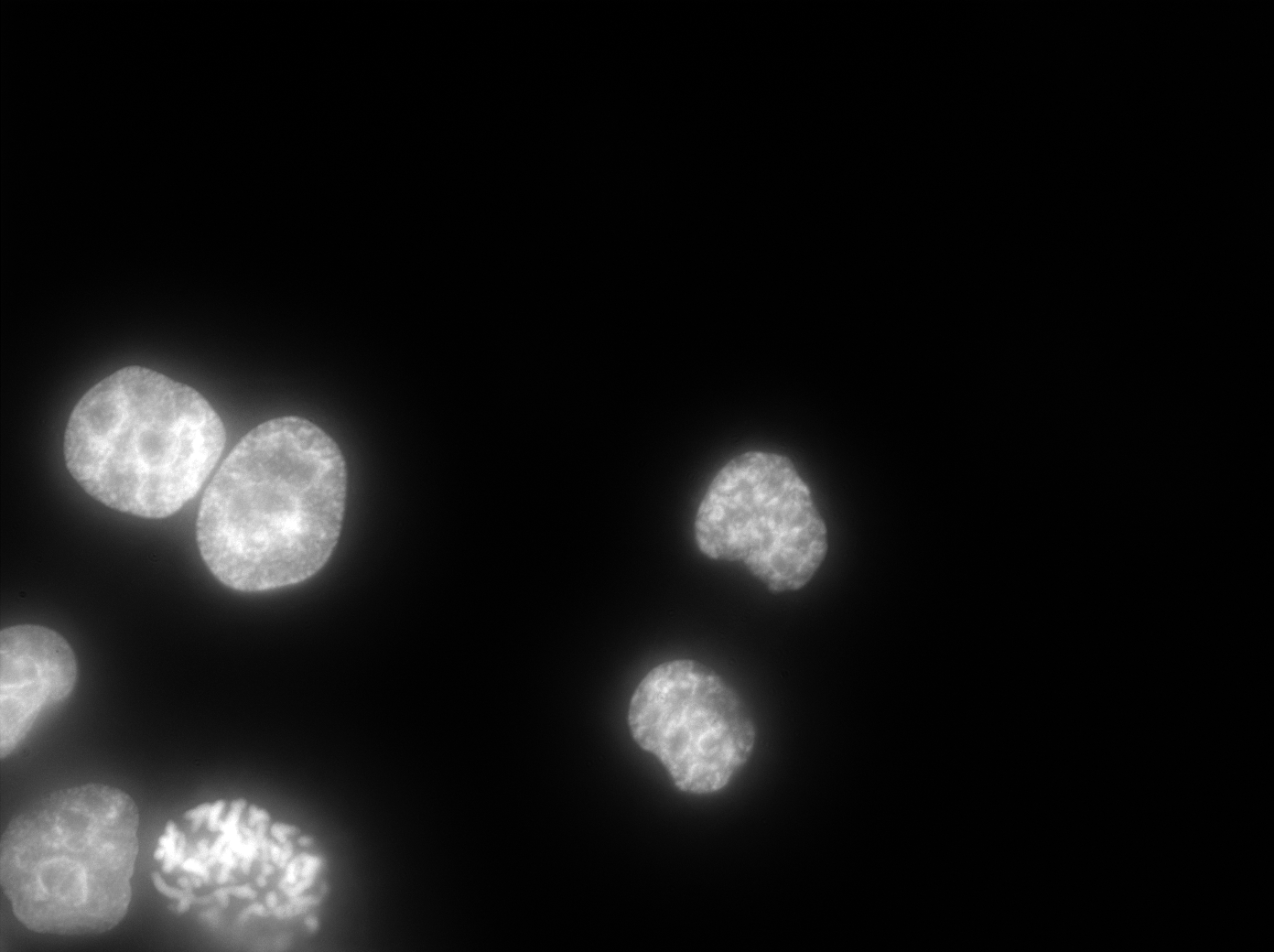

Supplement: Supplementary file 25 — Source data Fig. 7 part 1 [file 44319_2026_742_MOESM25_ESM.zip › Figure 7 Part 1/Fig 7acd Cas9 and TPGS1-ko rGT335 atubulin/Cas9 GT335recomb atub 3-24-25 R1 LT4.Project Maximum Z_XY1742835336_Z0_T0_C0.tif]

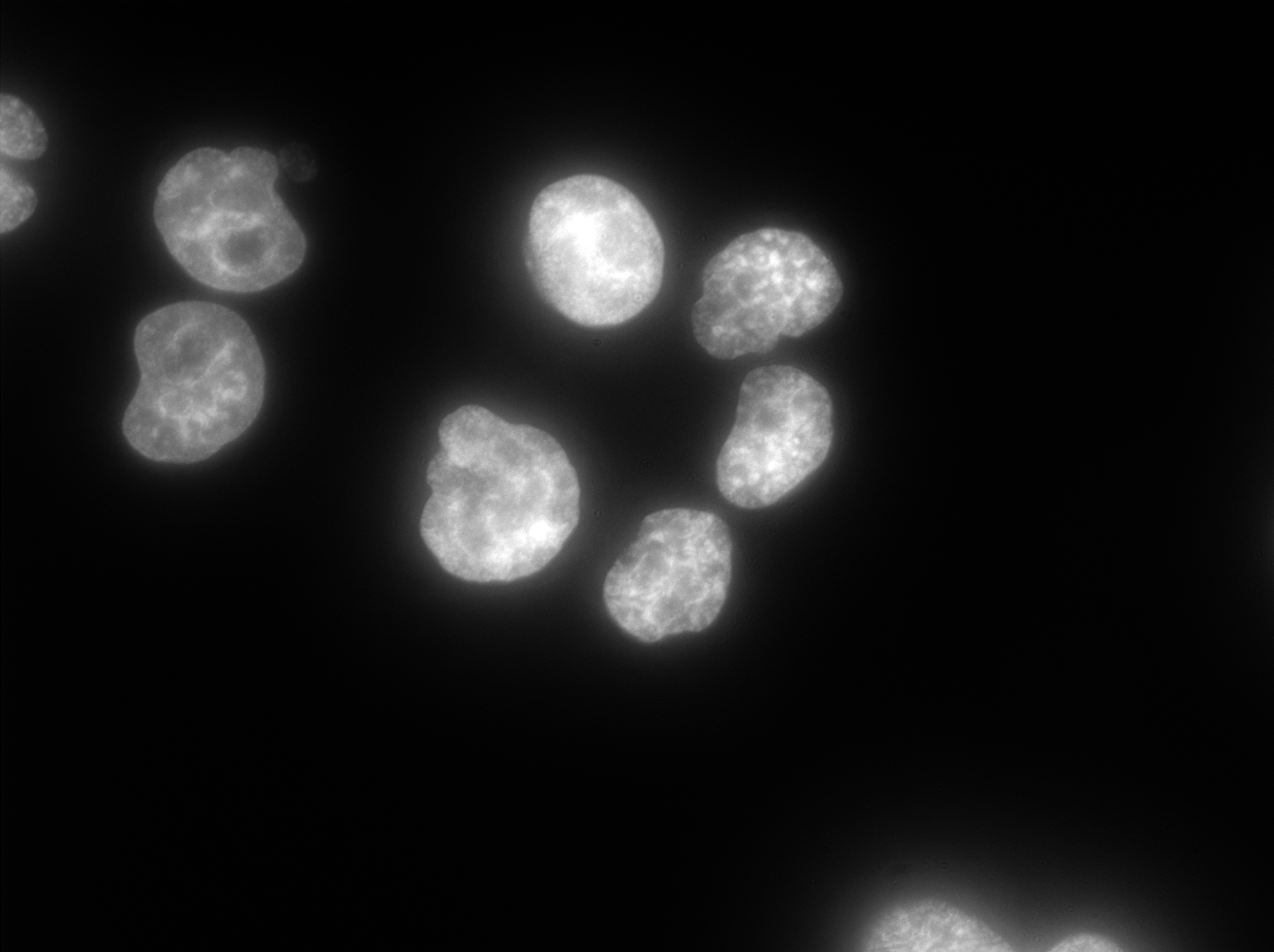

Supplement: Supplementary file 25 — Source data Fig. 7 part 1 [file 44319_2026_742_MOESM25_ESM.zip › Figure 7 Part 1/Fig 7acd Cas9 and TPGS1-ko rGT335 atubulin/Cas9 GT335recomb atub 3-24-25 R1 LT5 PA2.Project Maximum Z_XY1742835678_Z0_T0_C0.tif]

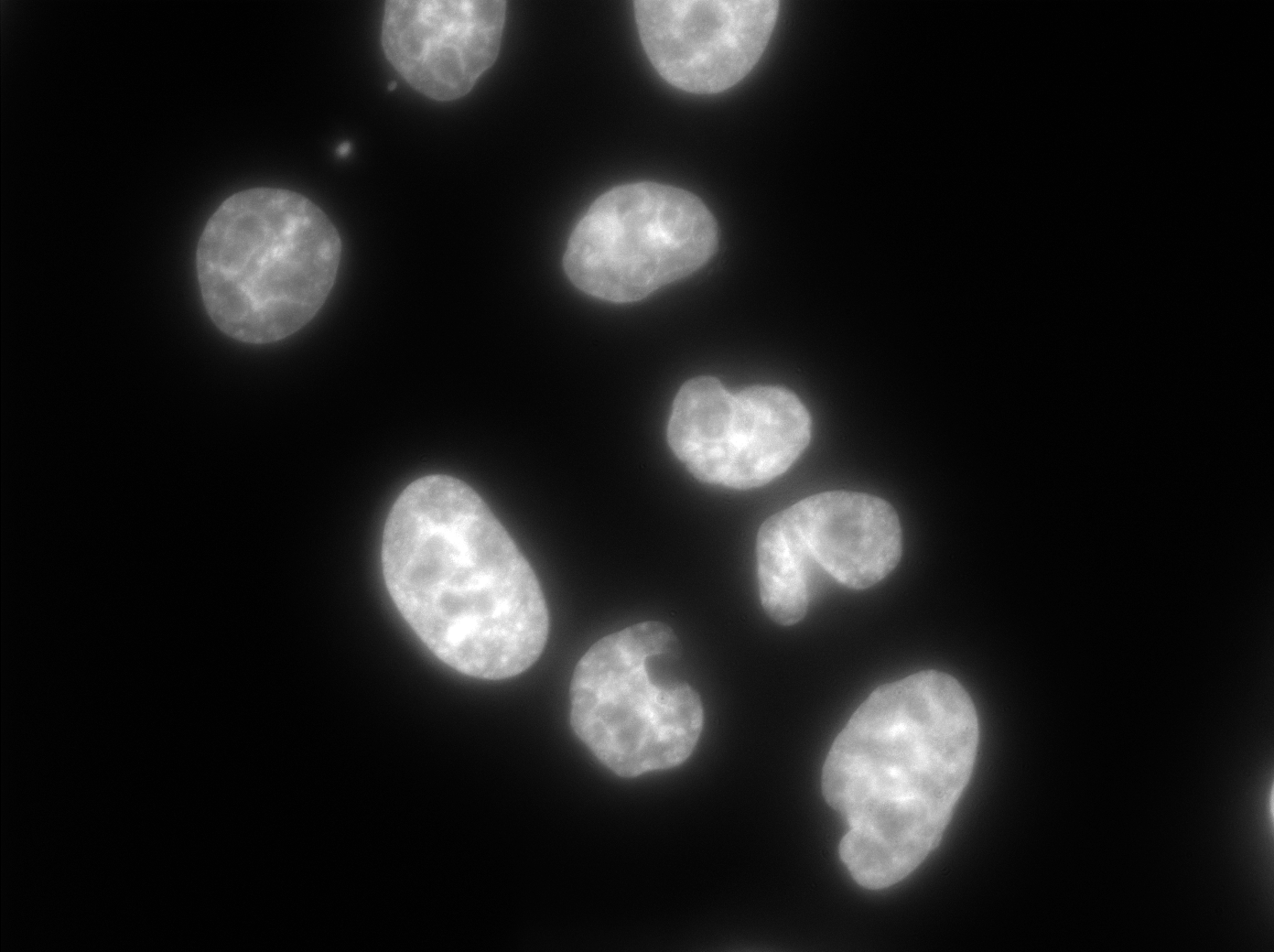

Supplement: Supplementary file 25 — Source data Fig. 7 part 1 [file 44319_2026_742_MOESM25_ESM.zip › Figure 7 Part 1/Fig 7acd Cas9 and TPGS1-ko rGT335 atubulin/Cas9 GT335recomb atub 3-24-25 R3 LT8.Project Maximum Z_XY1742849913_Z0_T0_C0.tif]

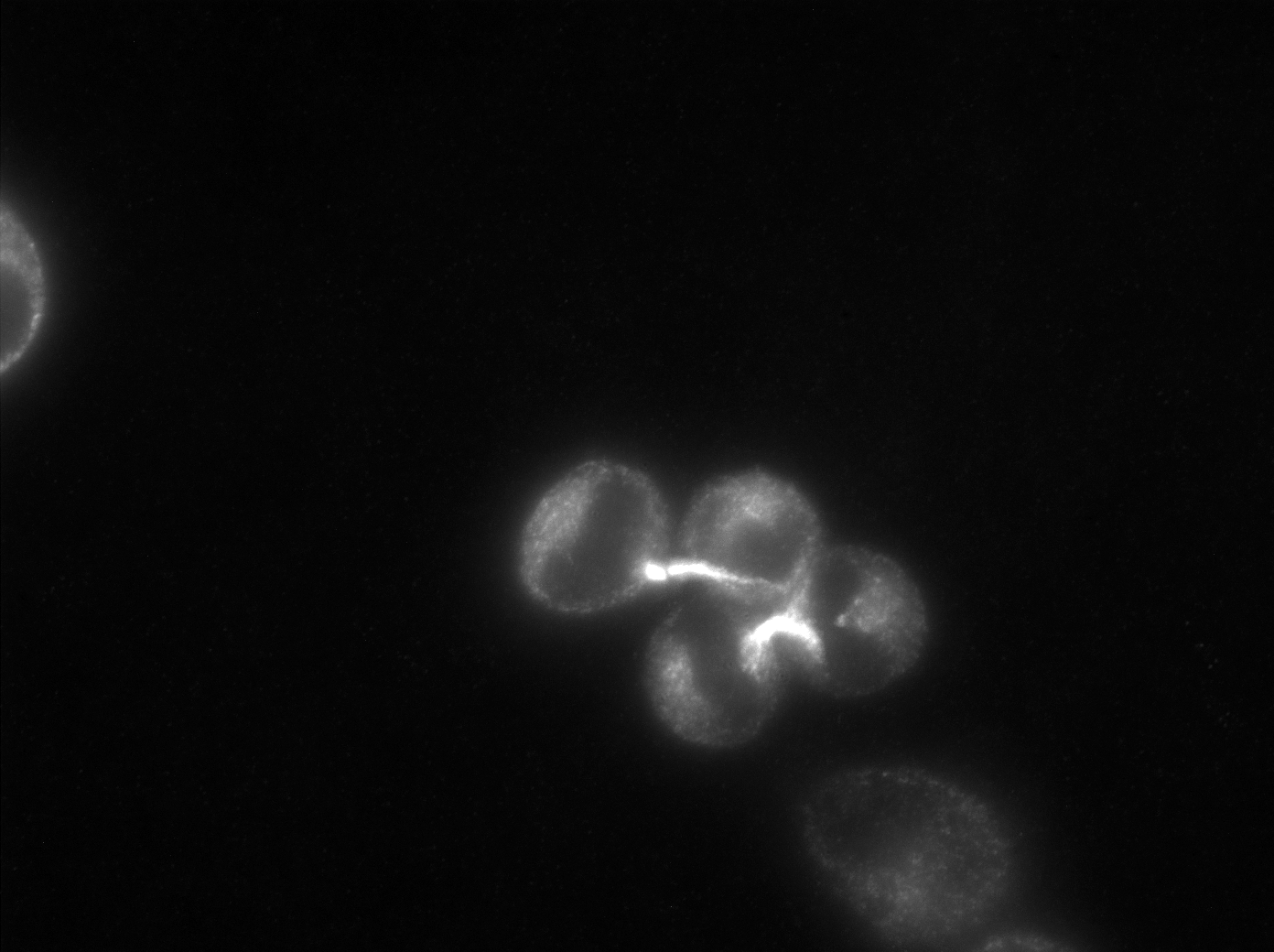

Supplement: Supplementary file 25 — Source data Fig. 7 part 1 [file 44319_2026_742_MOESM25_ESM.zip › Figure 7 Part 1/Fig 7acd Cas9 and TPGS1-ko rGT335 atubulin/Cas9 GT335recomb atub 3-24-25 R3 ET4ET5.Project Maximum Z_XY1742850252_Z0_T0_C2.tif]

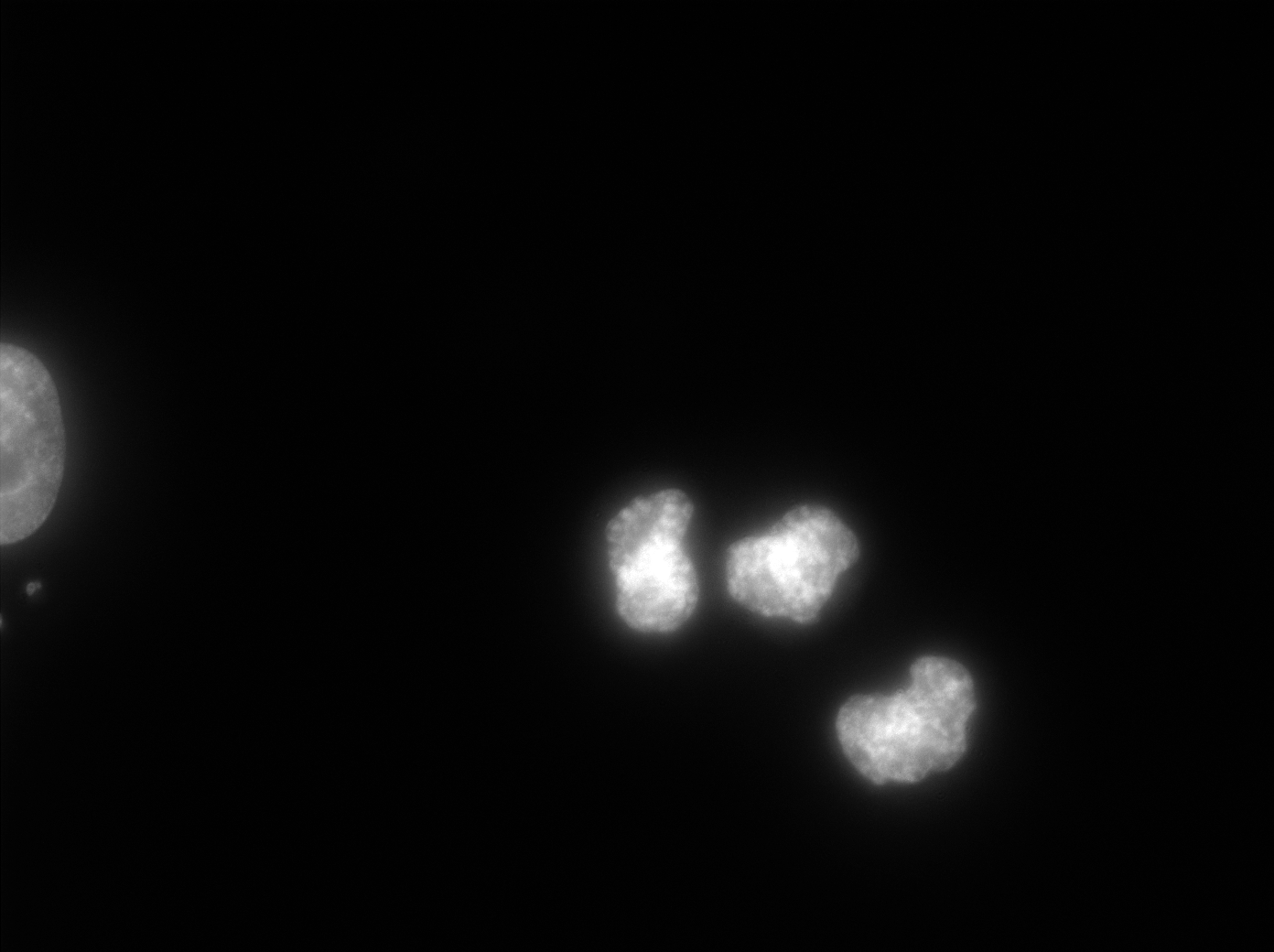

Supplement: Supplementary file 25 — Source data Fig. 7 part 1 [file 44319_2026_742_MOESM25_ESM.zip › Figure 7 Part 1/Fig 7acd Cas9 and TPGS1-ko rGT335 atubulin/Cas9 GT335recomb atub 3-24-25 R2 ET5.Project Maximum Z_XY1742845610_Z0_T0_C0.tif]

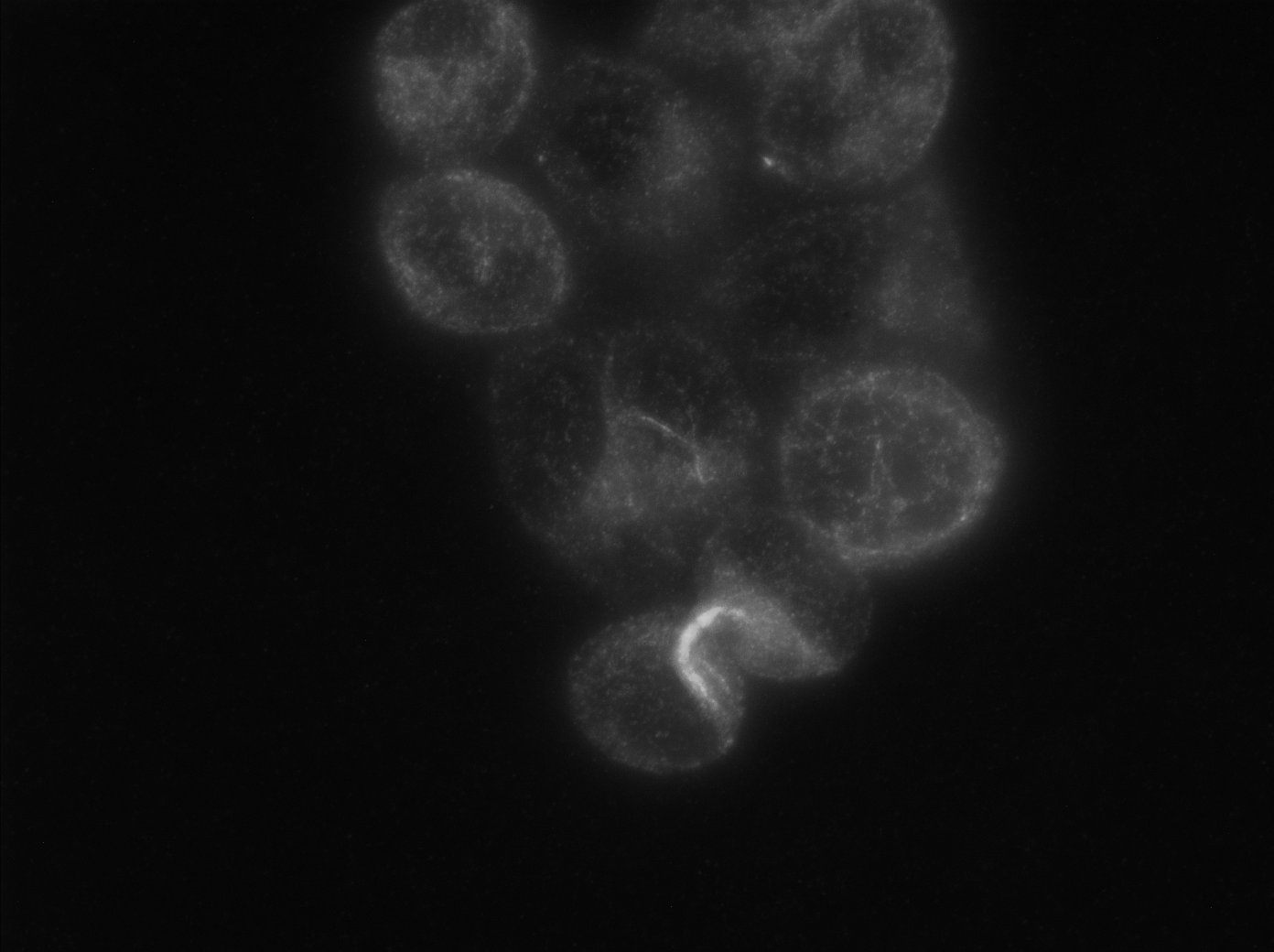

Supplement: Supplementary file 25 — Source data Fig. 7 part 1 [file 44319_2026_742_MOESM25_ESM.zip › Figure 7 Part 1/Fig 7acd Cas9 and TPGS1-ko rGT335 atubulin/Cas9 GT335recomb atub 3-24-25 R2 ET9.Project Maximum Z_XY1742847364_Z0_T0_C2.tif]

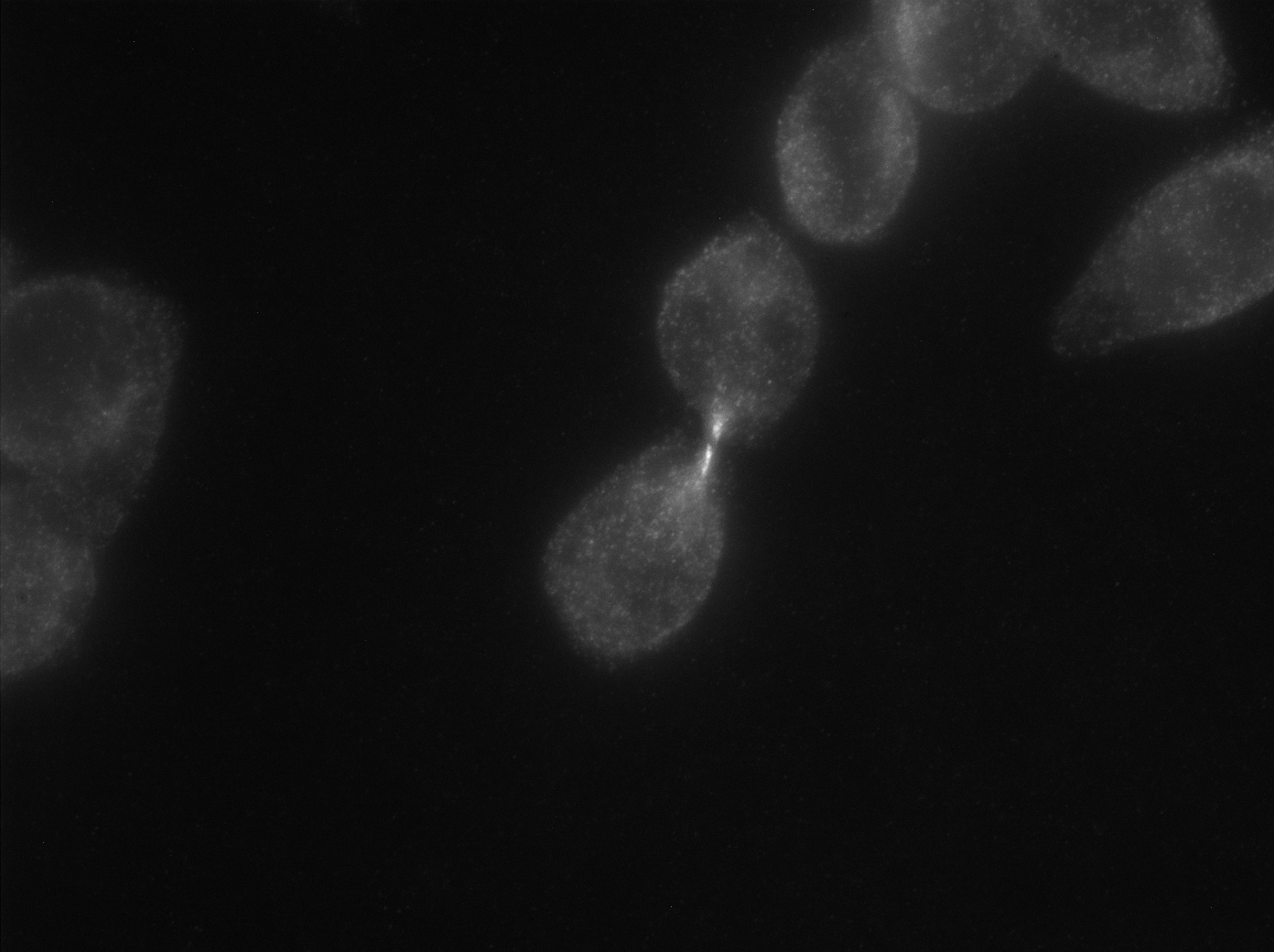

Supplement: Supplementary file 25 — Source data Fig. 7 part 1 [file 44319_2026_742_MOESM25_ESM.zip › Figure 7 Part 1/Fig 7acd Cas9 and TPGS1-ko rGT335 atubulin/Cas9 GT335recomb atub 3-24-25 R3 LT4.Project Maximum Z_XY1742849270_Z0_T0_C2.tif]

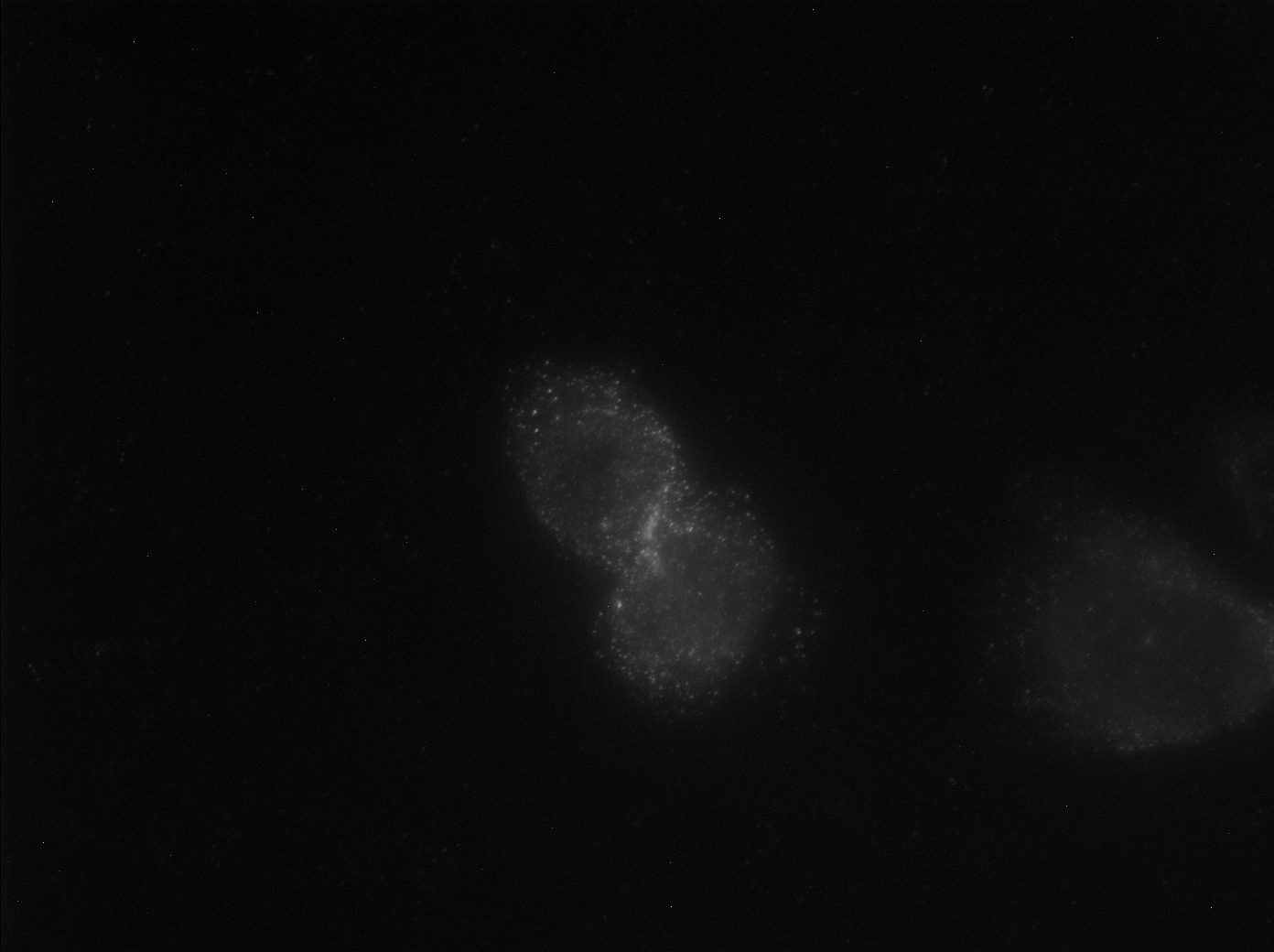

Supplement: Supplementary file 25 — Source data Fig. 7 part 1 [file 44319_2026_742_MOESM25_ESM.zip › Figure 7 Part 1/Fig 7acd Cas9 and TPGS1-ko rGT335 atubulin/Cas9 GT335recomb atub 3-24-25 R2 LT8.Project Maximum Z_XY1742846814_Z0_T0_C1.tif]

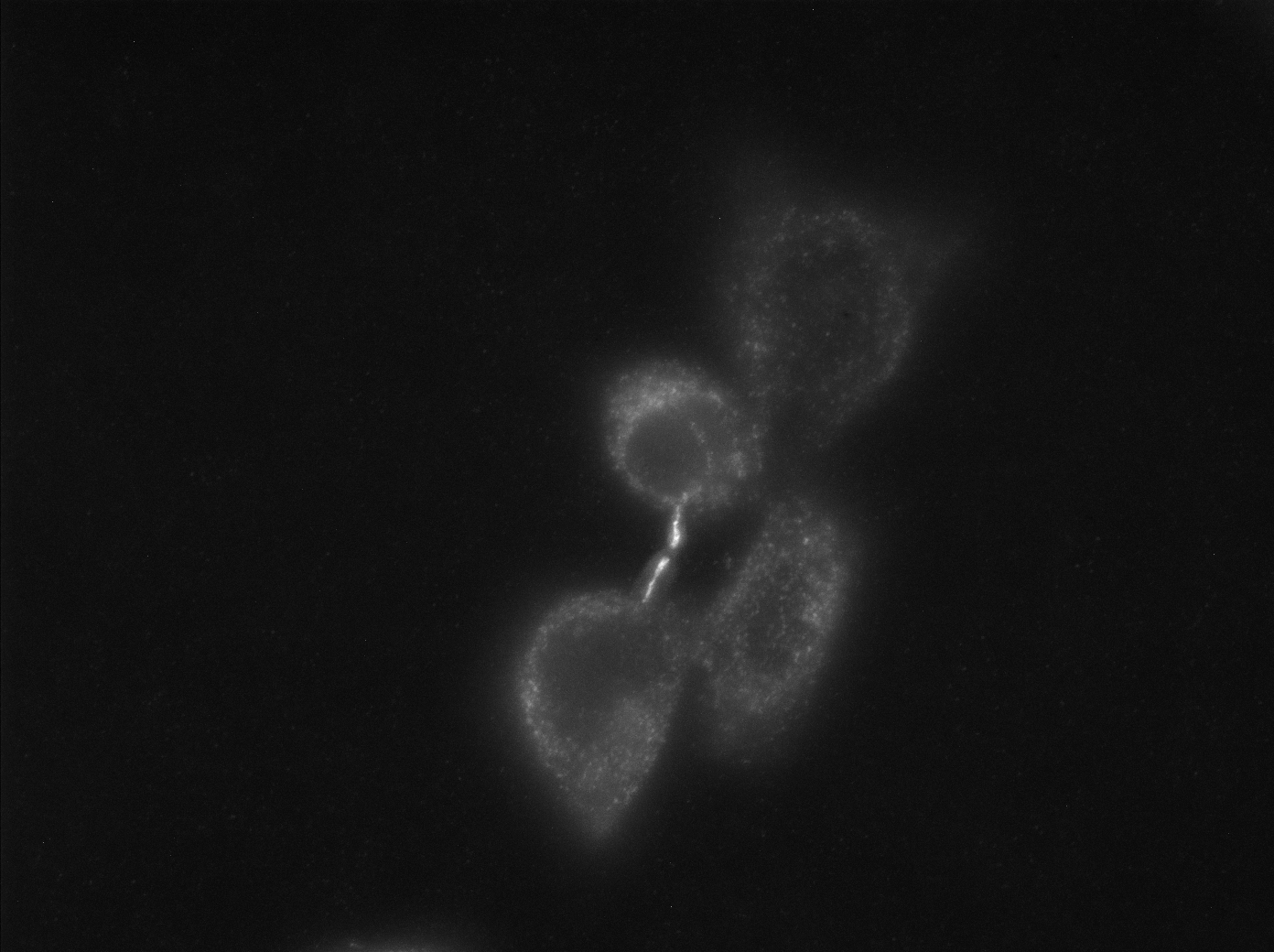

Supplement: Supplementary file 25 — Source data Fig. 7 part 1 [file 44319_2026_742_MOESM25_ESM.zip › Figure 7 Part 1/Fig 7acd Cas9 and TPGS1-ko rGT335 atubulin/Cas9 GT335recomb atub 3-24-25 R3 LT7.Project Maximum Z_XY1742849652_Z0_T0_C2.tif]

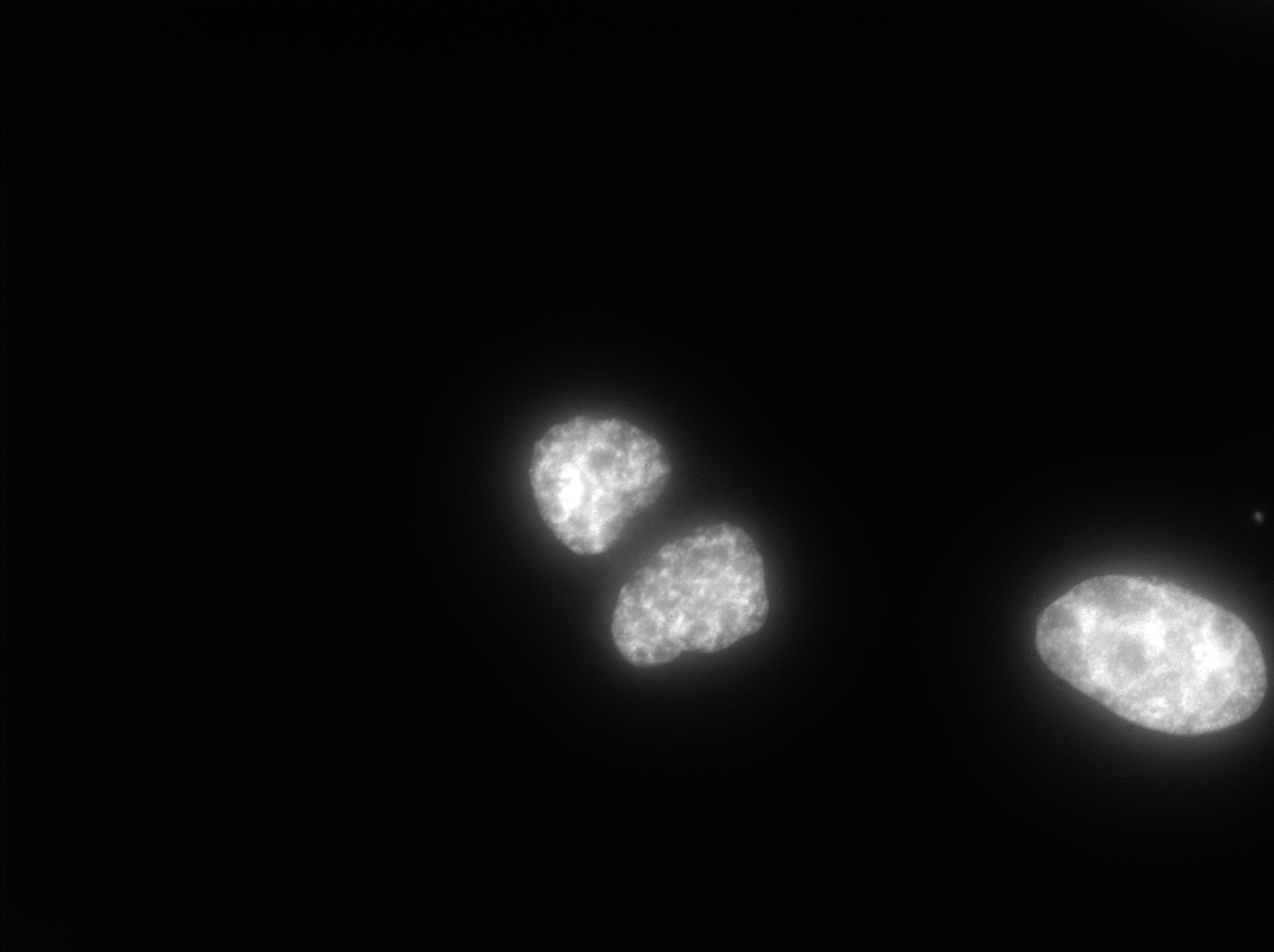

Supplement: Supplementary file 25 — Source data Fig. 7 part 1 [file 44319_2026_742_MOESM25_ESM.zip › Figure 7 Part 1/Fig 7acd Cas9 and TPGS1-ko rGT335 atubulin/Cas9 GT335recomb atub 3-24-25 R2 LT8.Project Maximum Z_XY1742846814_Z0_T0_C0.tif]

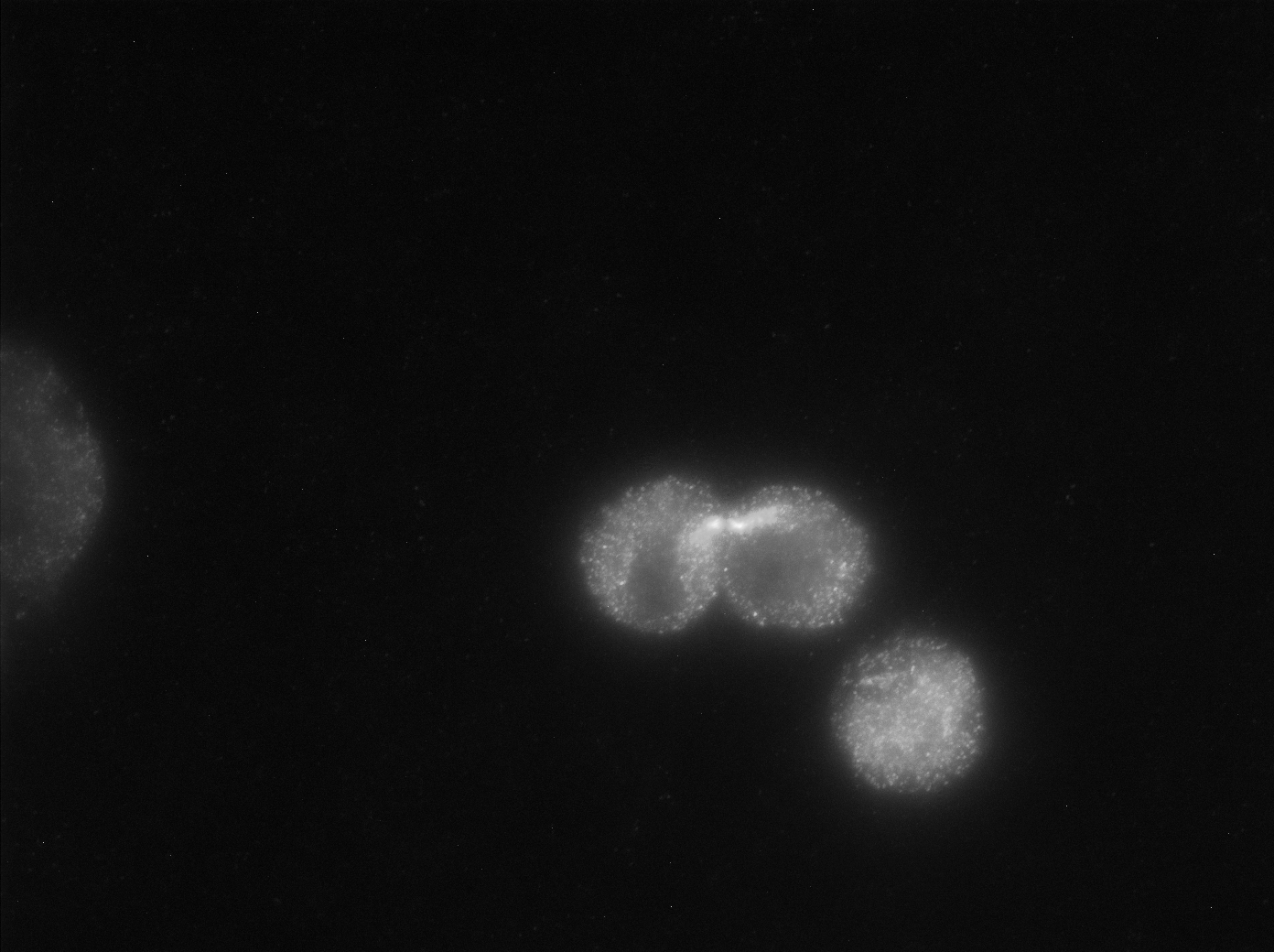

Supplement: Supplementary file 25 — Source data Fig. 7 part 1 [file 44319_2026_742_MOESM25_ESM.zip › Figure 7 Part 1/Fig 7acd Cas9 and TPGS1-ko rGT335 atubulin/Cas9 GT335recomb atub 3-24-25 R2 ET5.Project Maximum Z_XY1742845610_Z0_T0_C1.tif]

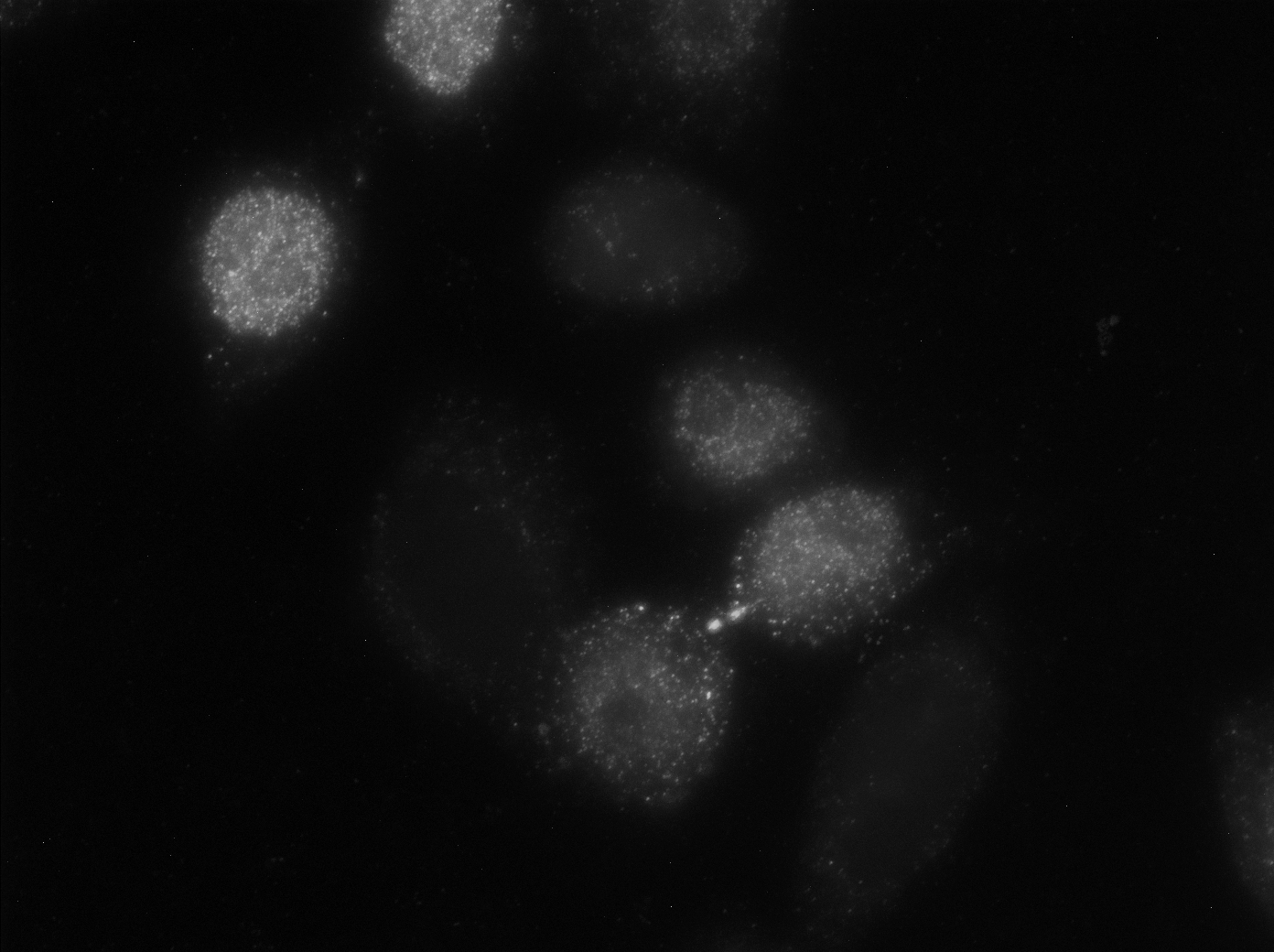

Supplement: Supplementary file 25 — Source data Fig. 7 part 1 [file 44319_2026_742_MOESM25_ESM.zip › Figure 7 Part 1/Fig 7acd Cas9 and TPGS1-ko rGT335 atubulin/Cas9 GT335recomb atub 3-24-25 R3 LT8.Project Maximum Z_XY1742849913_Z0_T0_C1.tif]

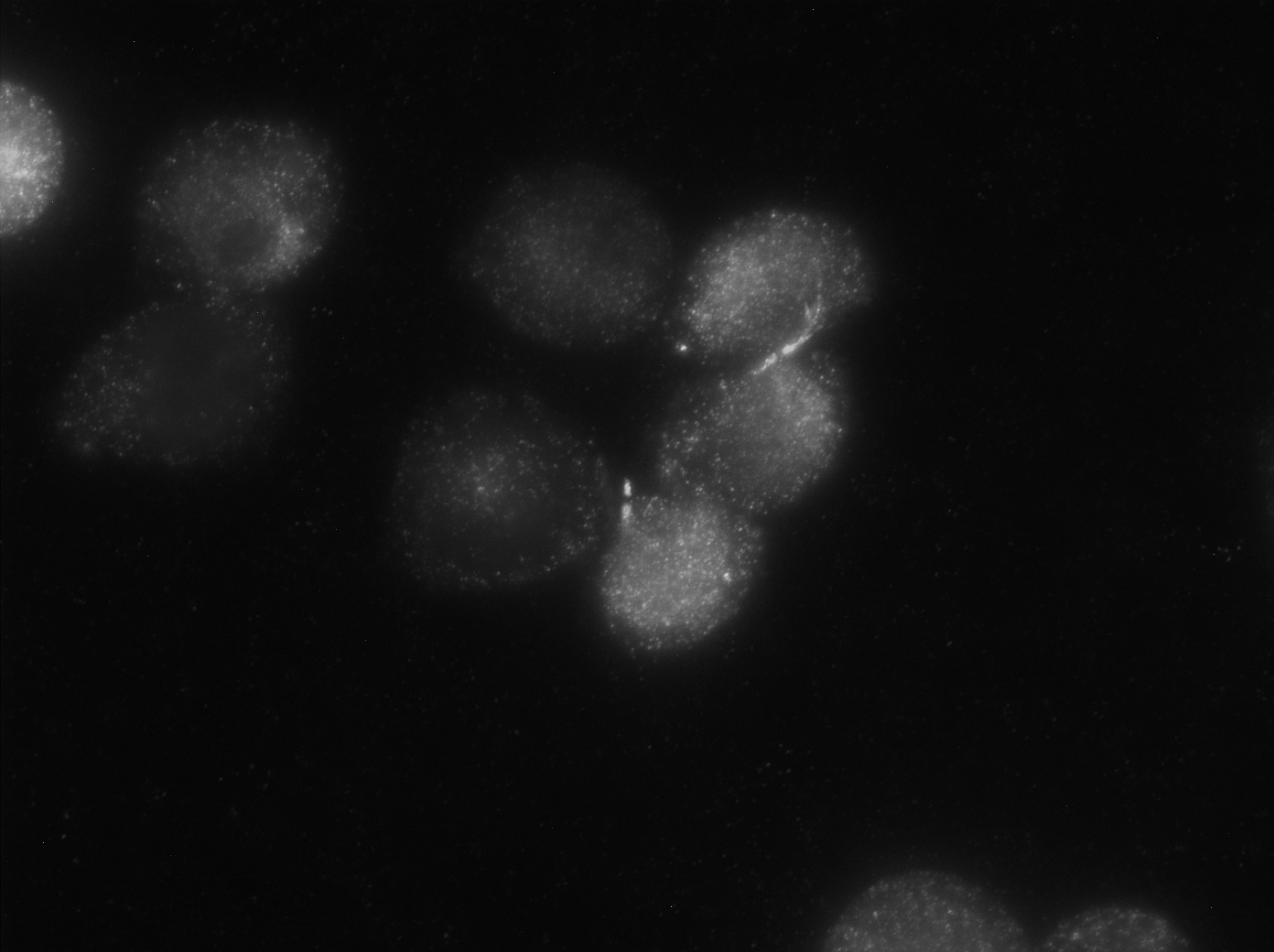

Supplement: Supplementary file 25 — Source data Fig. 7 part 1 [file 44319_2026_742_MOESM25_ESM.zip › Figure 7 Part 1/Fig 7acd Cas9 and TPGS1-ko rGT335 atubulin/Cas9 GT335recomb atub 3-24-25 R1 LT5 PA2.Project Maximum Z_XY1742835678_Z0_T0_C1.tif]

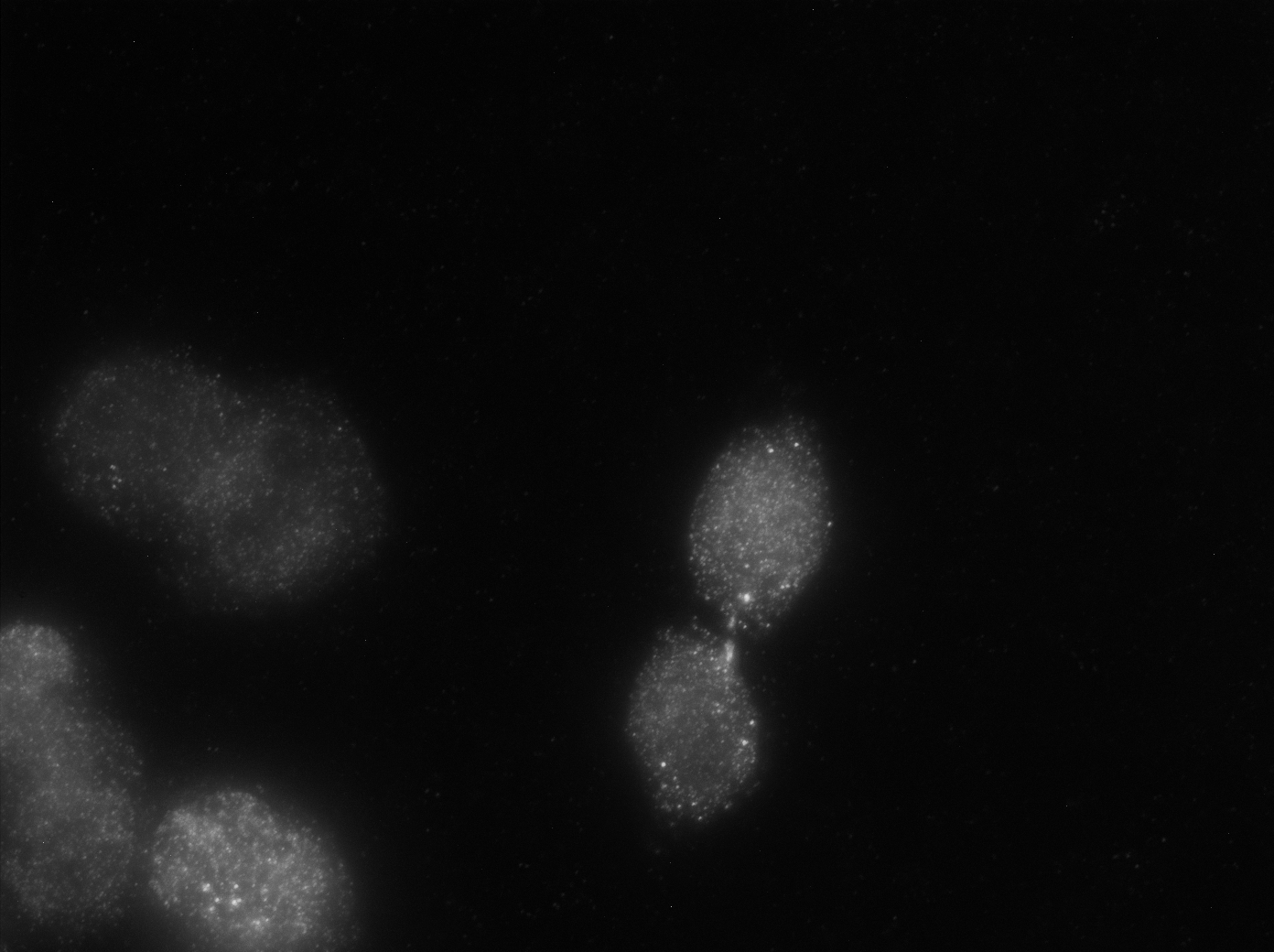

Supplement: Supplementary file 25 — Source data Fig. 7 part 1 [file 44319_2026_742_MOESM25_ESM.zip › Figure 7 Part 1/Fig 7acd Cas9 and TPGS1-ko rGT335 atubulin/Cas9 GT335recomb atub 3-24-25 R1 LT4.Project Maximum Z_XY1742835336_Z0_T0_C1.tif]

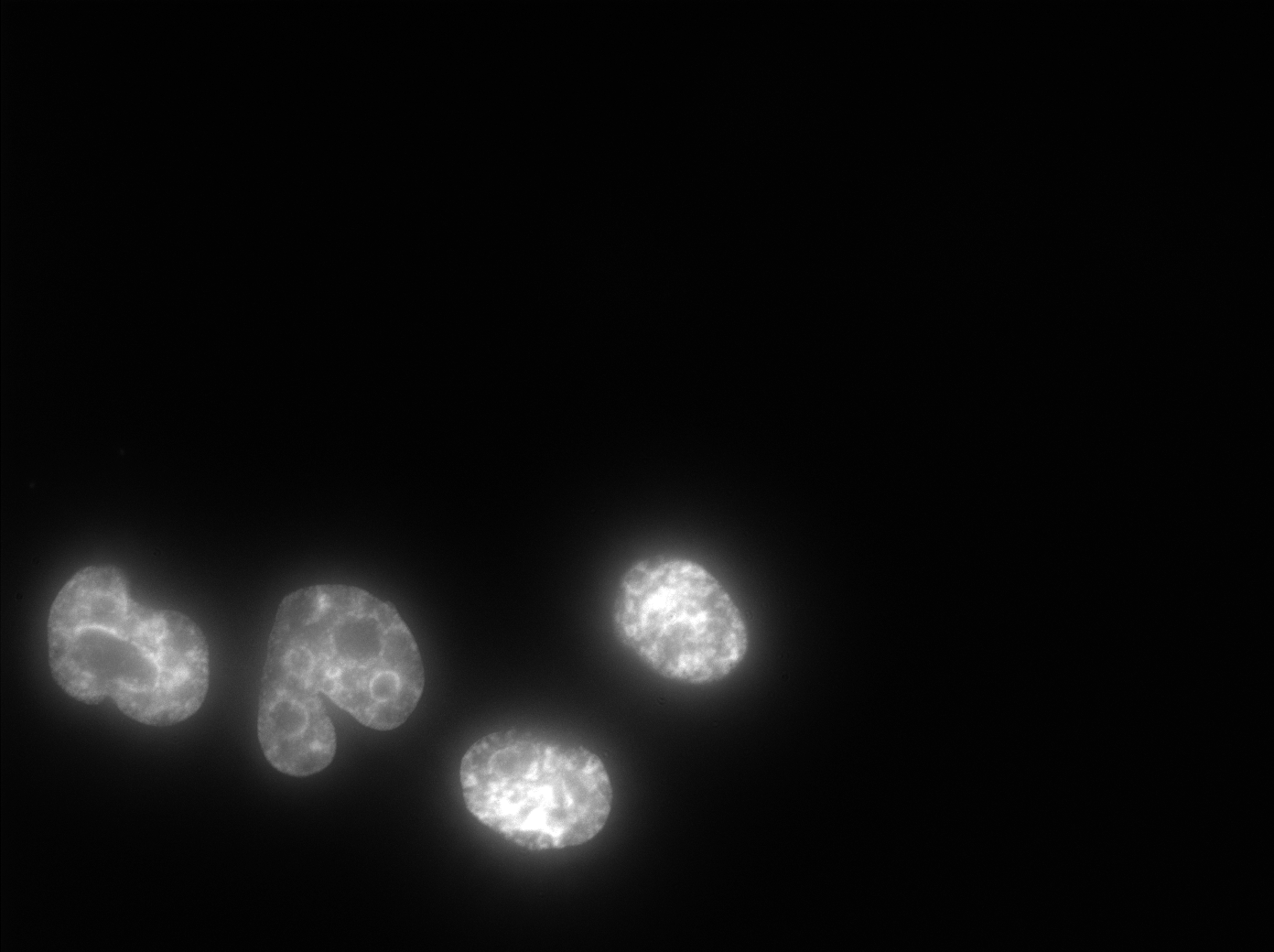

Supplement: Supplementary file 25 — Source data Fig. 7 part 1 [file 44319_2026_742_MOESM25_ESM.zip › Figure 7 Part 1/Fig 7acd Cas9 and TPGS1-ko rGT335 atubulin/Cas9 GT335recomb atub 3-24-25 R2 LT6.Project Maximum Z_XY1742846575_Z0_T0_C0.tif]

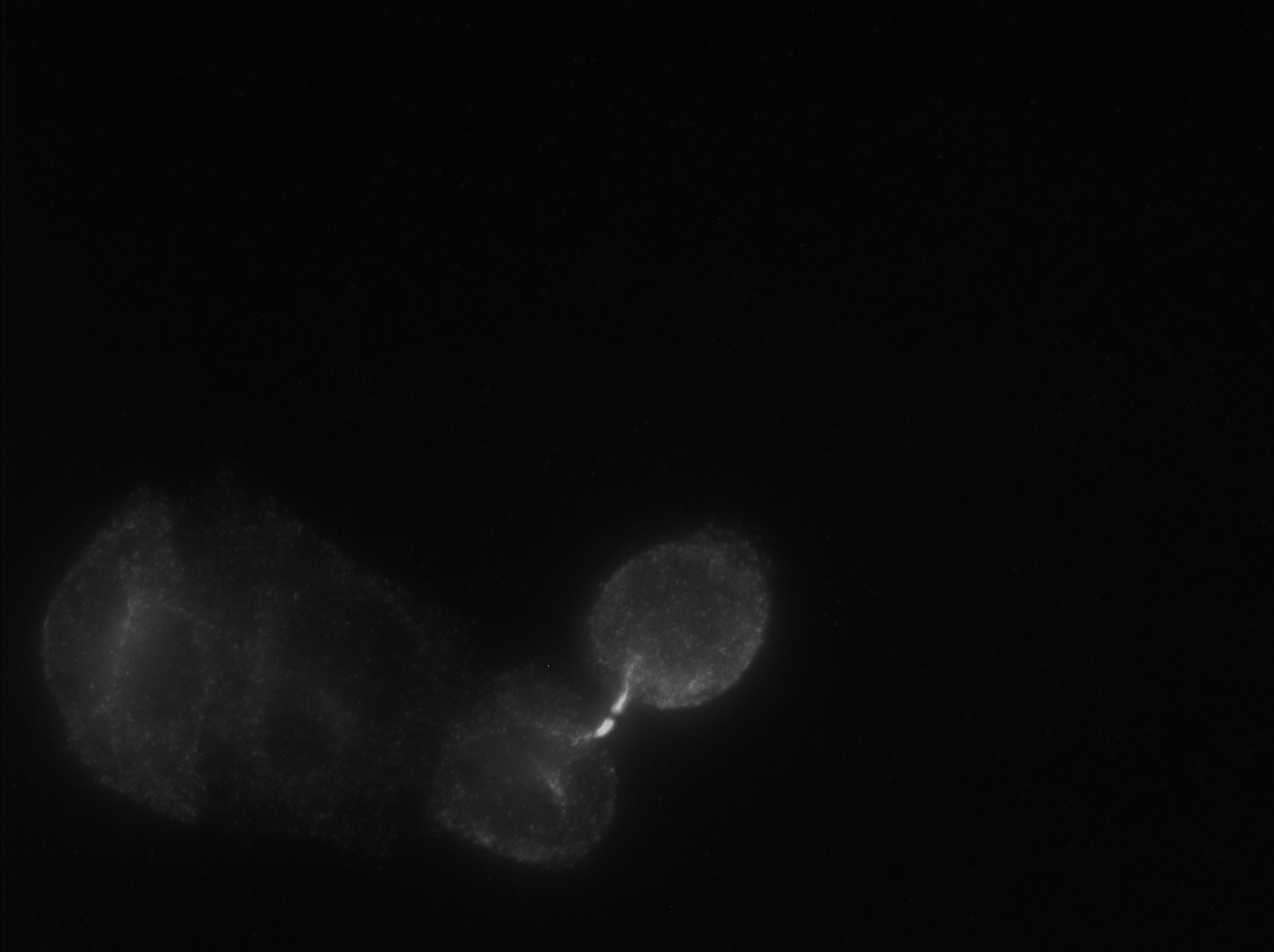

Supplement: Supplementary file 25 — Source data Fig. 7 part 1 [file 44319_2026_742_MOESM25_ESM.zip › Figure 7 Part 1/Fig 7acd Cas9 and TPGS1-ko rGT335 atubulin/Cas9 GT335recomb atub 3-24-25 R2 LT6.Project Maximum Z_XY1742846575_Z0_T0_C2.tif]

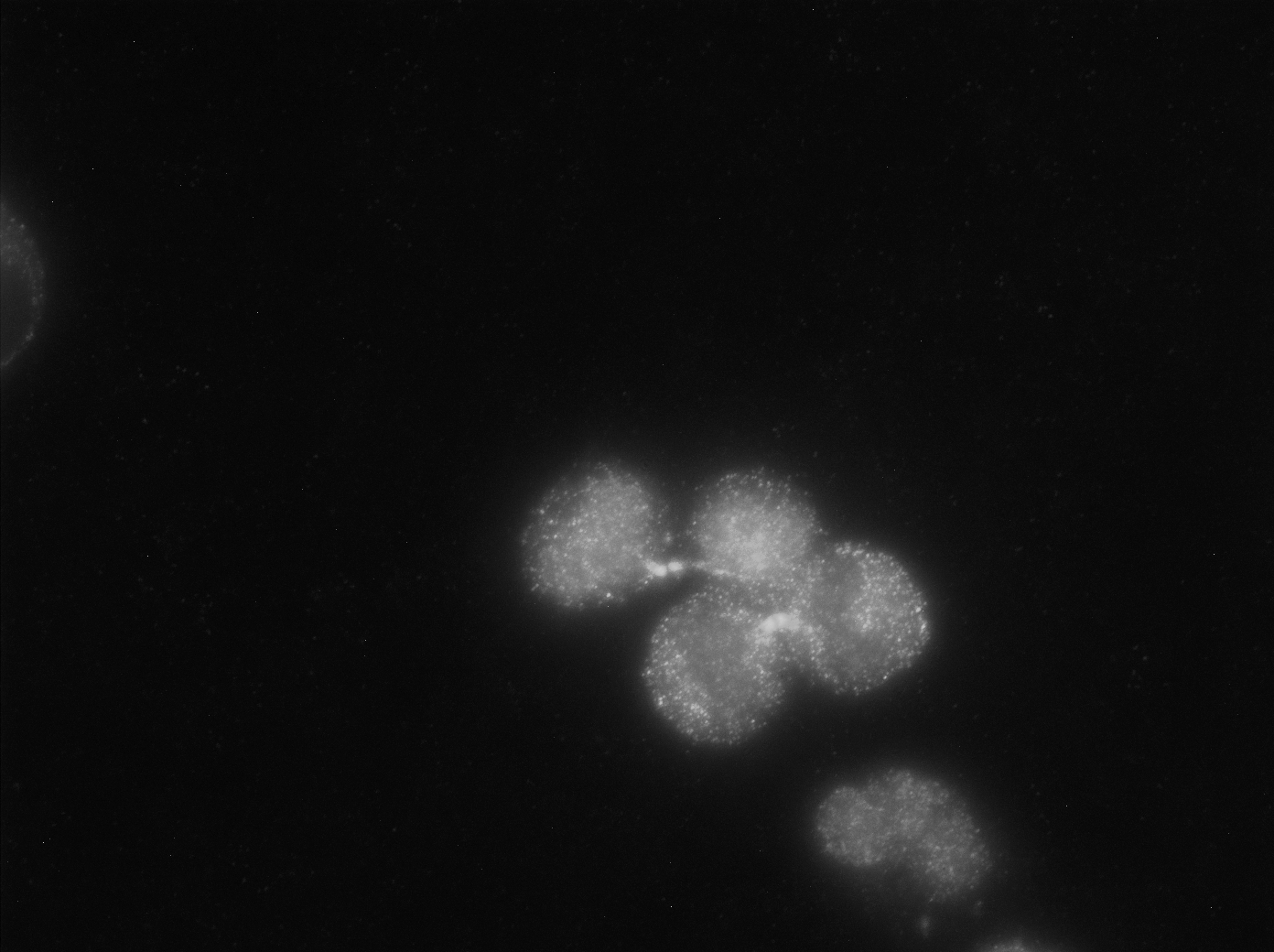

Supplement: Supplementary file 25 — Source data Fig. 7 part 1 [file 44319_2026_742_MOESM25_ESM.zip › Figure 7 Part 1/Fig 7acd Cas9 and TPGS1-ko rGT335 atubulin/Cas9 GT335recomb atub 3-24-25 R3 ET4ET5.Project Maximum Z_XY1742850252_Z0_T0_C1.tif]

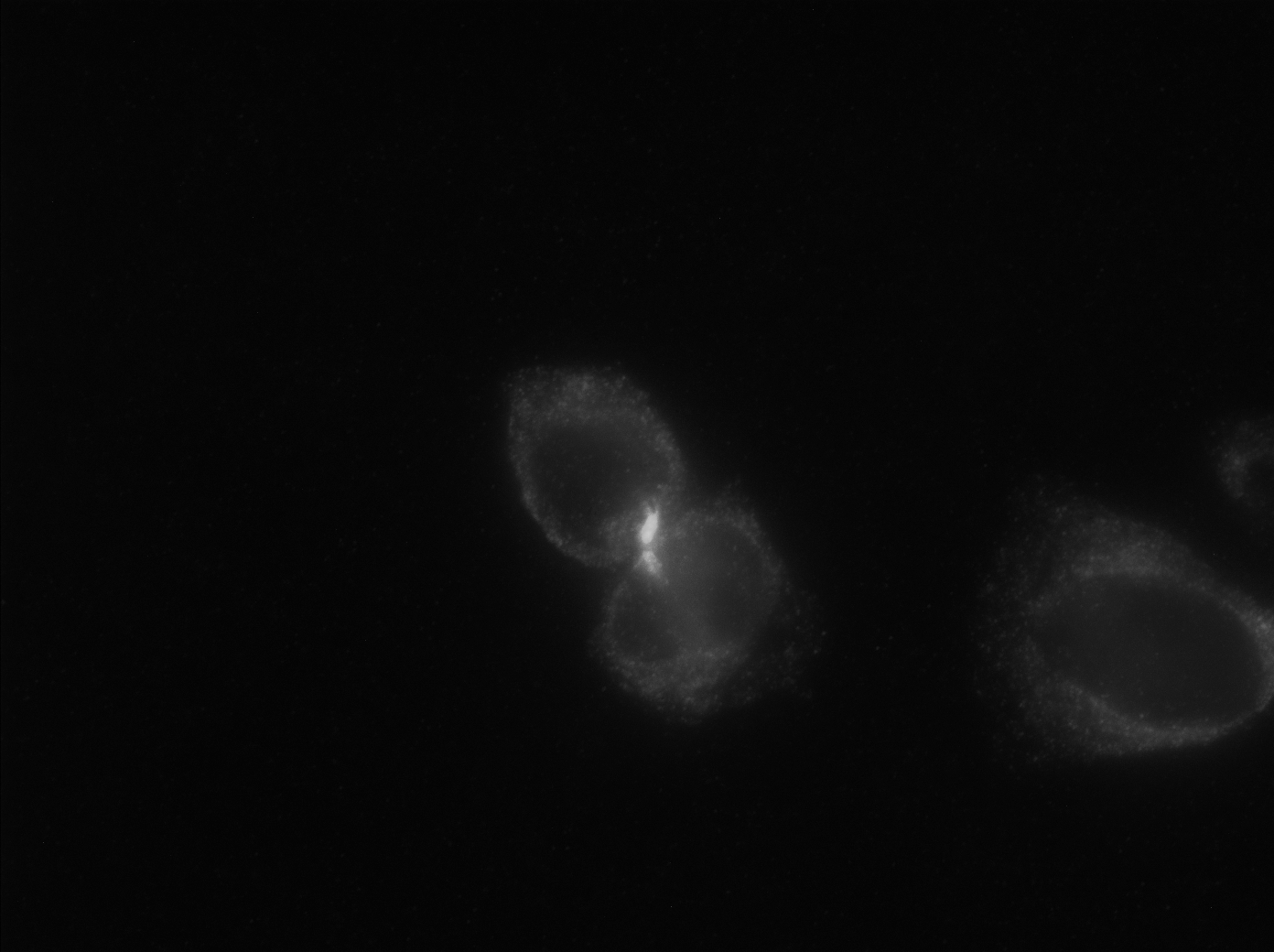

Supplement: Supplementary file 25 — Source data Fig. 7 part 1 [file 44319_2026_742_MOESM25_ESM.zip › Figure 7 Part 1/Fig 7acd Cas9 and TPGS1-ko rGT335 atubulin/Cas9 GT335recomb atub 3-24-25 R2 LT8.Project Maximum Z_XY1742846814_Z0_T0_C2.tif]

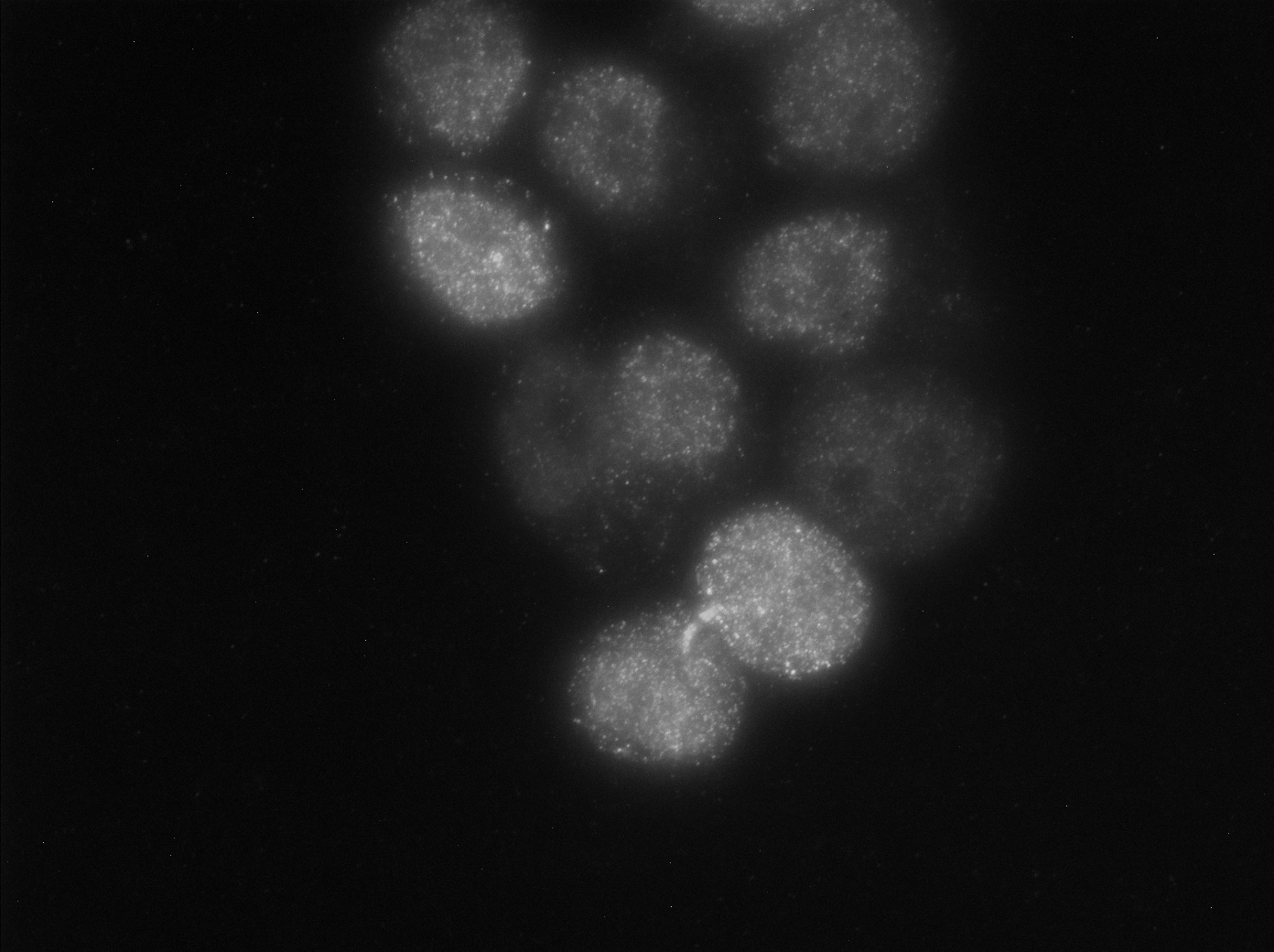

Supplement: Supplementary file 25 — Source data Fig. 7 part 1 [file 44319_2026_742_MOESM25_ESM.zip › Figure 7 Part 1/Fig 7acd Cas9 and TPGS1-ko rGT335 atubulin/Cas9 GT335recomb atub 3-24-25 R2 ET9.Project Maximum Z_XY1742847364_Z0_T0_C1.tif]

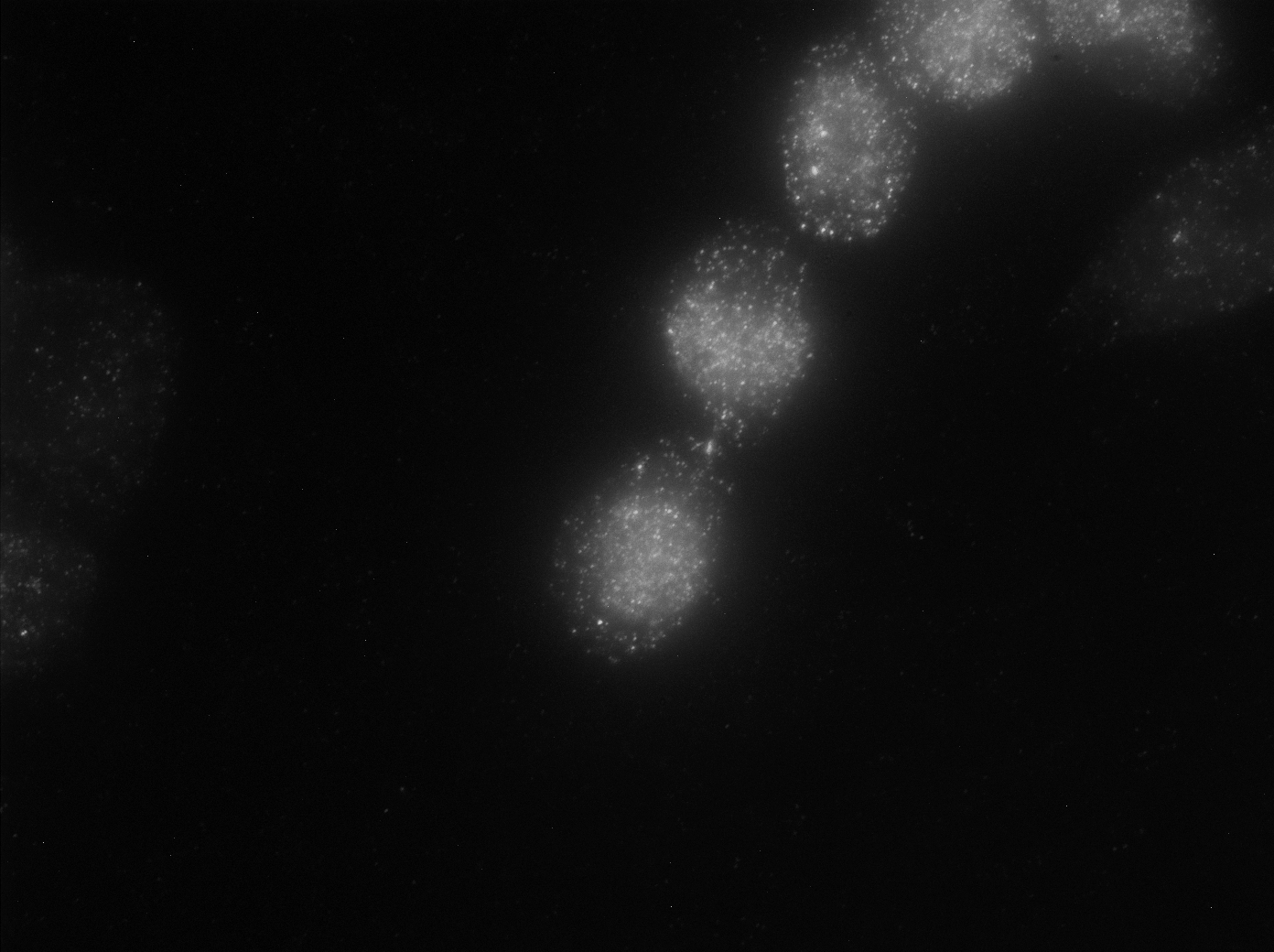

Supplement: Supplementary file 25 — Source data Fig. 7 part 1 [file 44319_2026_742_MOESM25_ESM.zip › Figure 7 Part 1/Fig 7acd Cas9 and TPGS1-ko rGT335 atubulin/Cas9 GT335recomb atub 3-24-25 R3 LT4.Project Maximum Z_XY1742849270_Z0_T0_C1.tif]

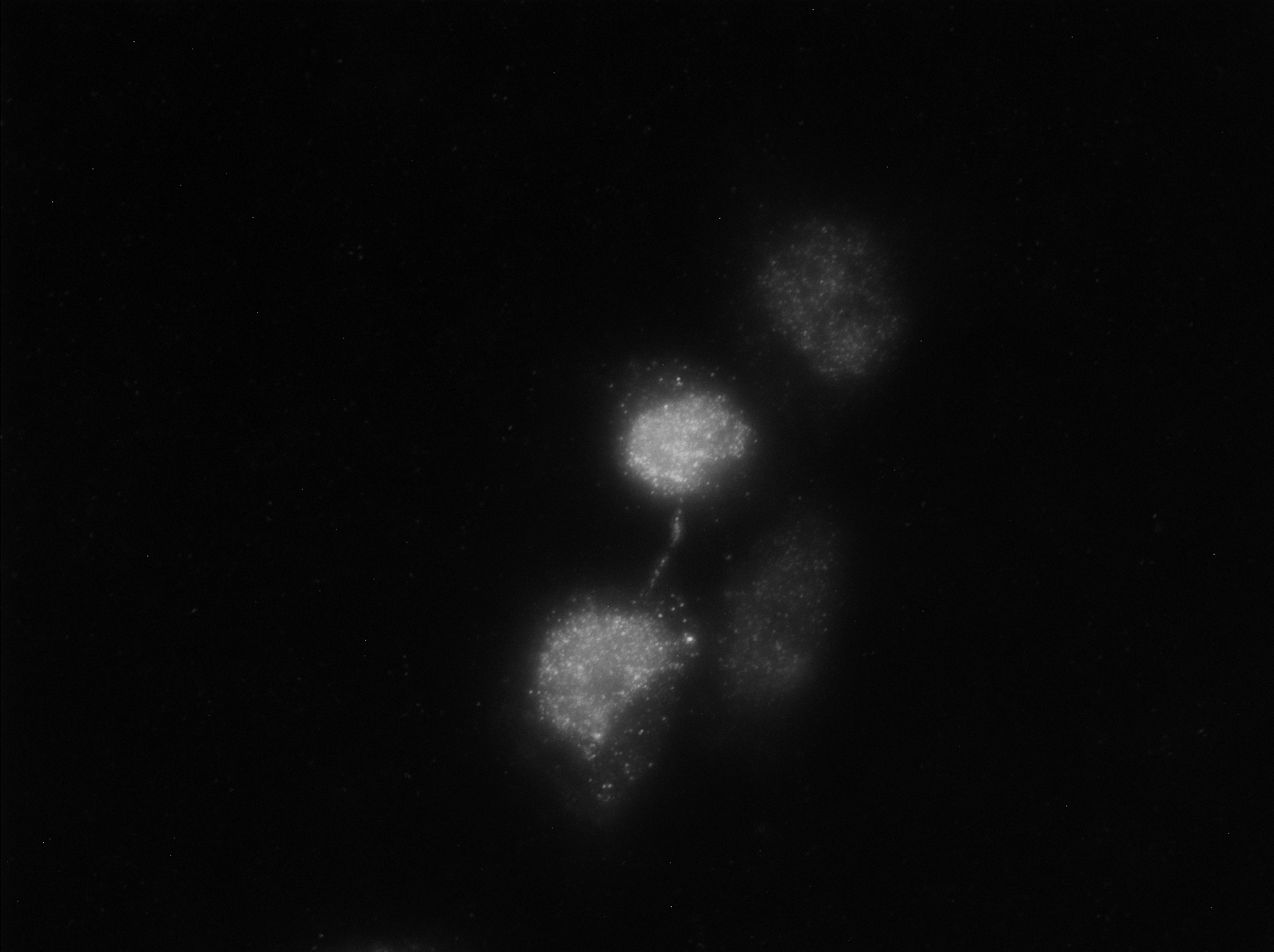

Supplement: Supplementary file 25 — Source data Fig. 7 part 1 [file 44319_2026_742_MOESM25_ESM.zip › Figure 7 Part 1/Fig 7acd Cas9 and TPGS1-ko rGT335 atubulin/Cas9 GT335recomb atub 3-24-25 R3 LT7.Project Maximum Z_XY1742849652_Z0_T0_C1.tif]

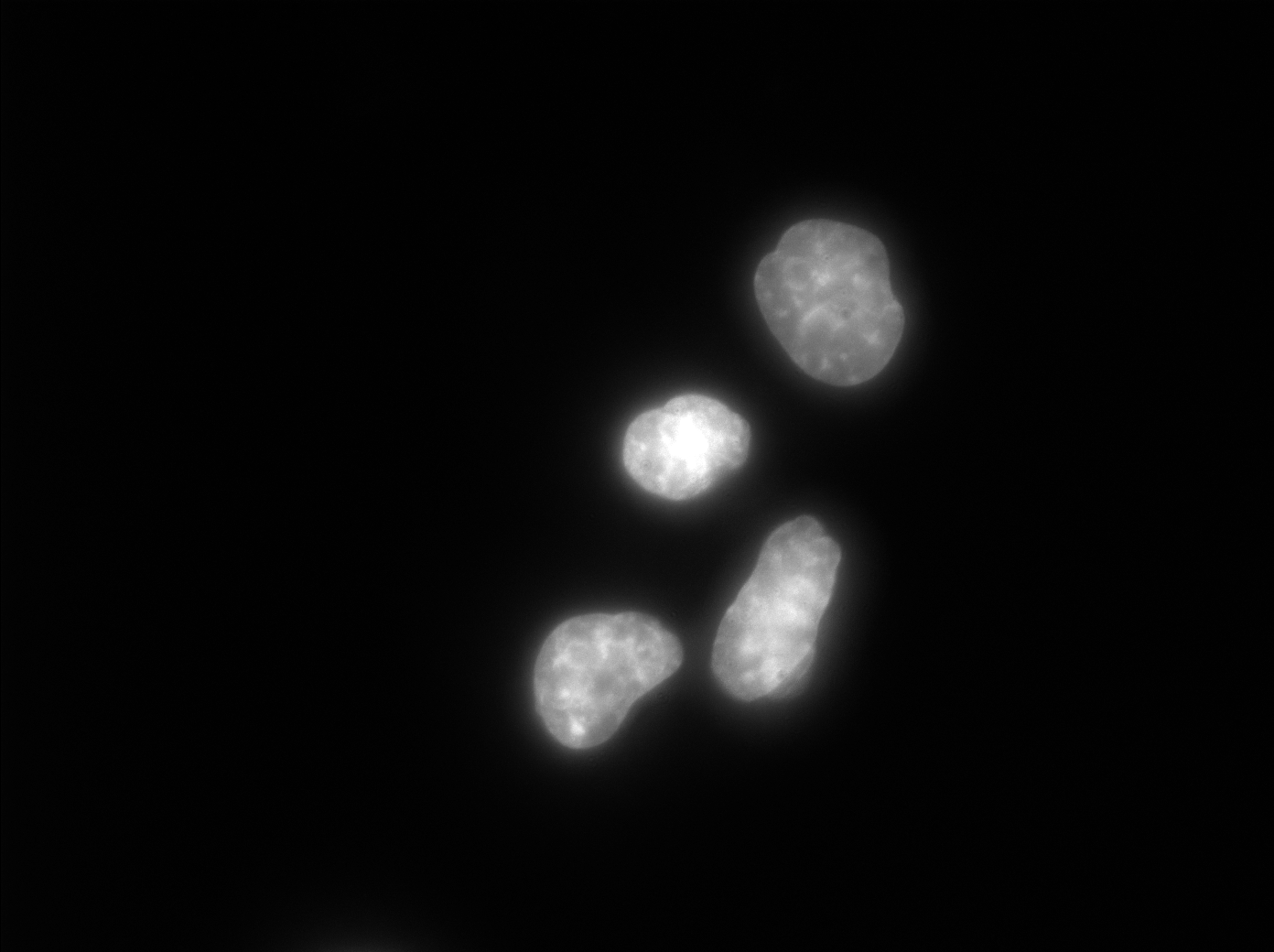

Supplement: Supplementary file 25 — Source data Fig. 7 part 1 [file 44319_2026_742_MOESM25_ESM.zip › Figure 7 Part 1/Fig 7acd Cas9 and TPGS1-ko rGT335 atubulin/Cas9 GT335recomb atub 3-24-25 R3 LT7.Project Maximum Z_XY1742849652_Z0_T0_C0.tif]

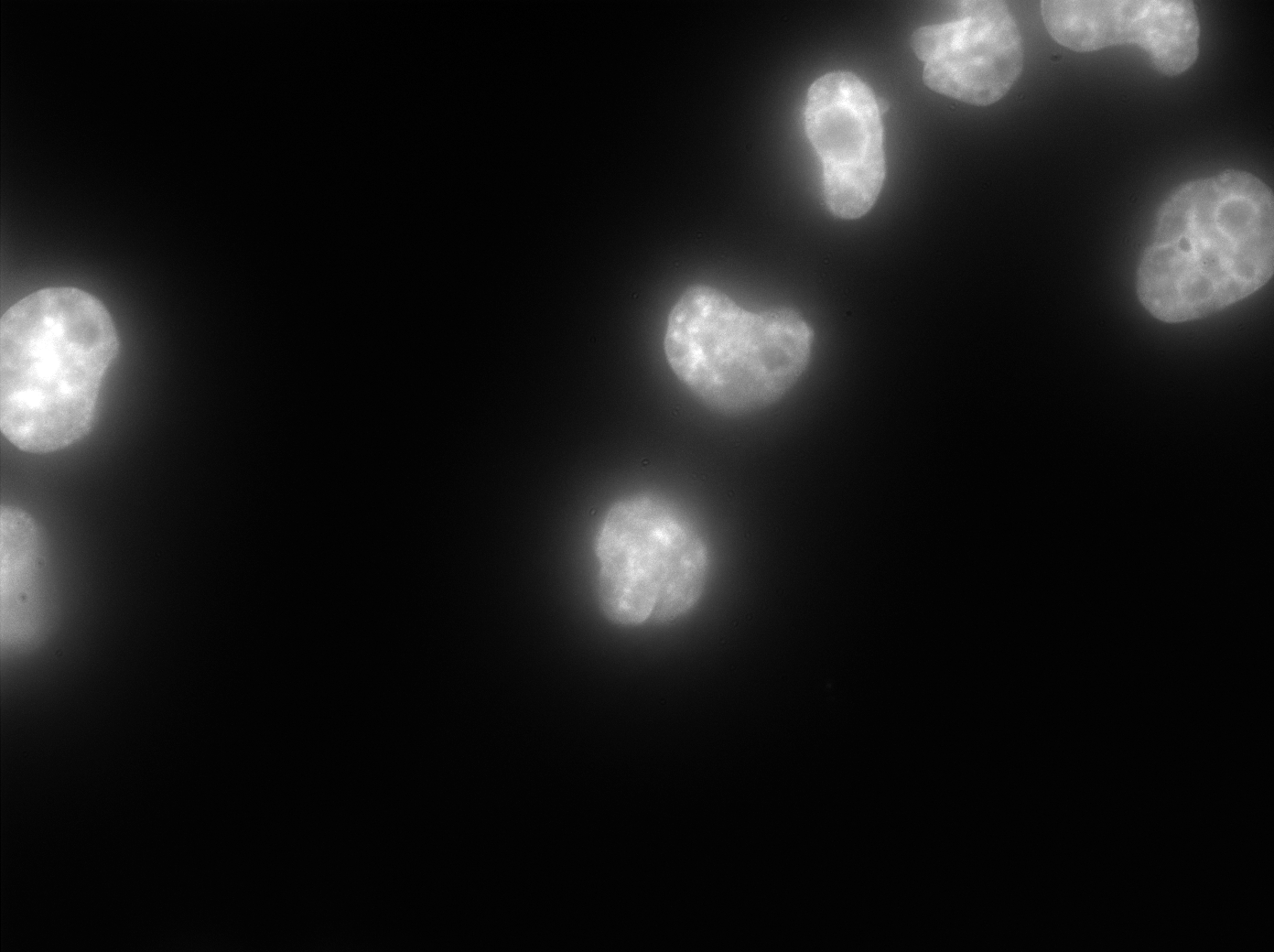

Supplement: Supplementary file 25 — Source data Fig. 7 part 1 [file 44319_2026_742_MOESM25_ESM.zip › Figure 7 Part 1/Fig 7acd Cas9 and TPGS1-ko rGT335 atubulin/Cas9 GT335recomb atub 3-24-25 R3 LT4.Project Maximum Z_XY1742849270_Z0_T0_C0.tif]

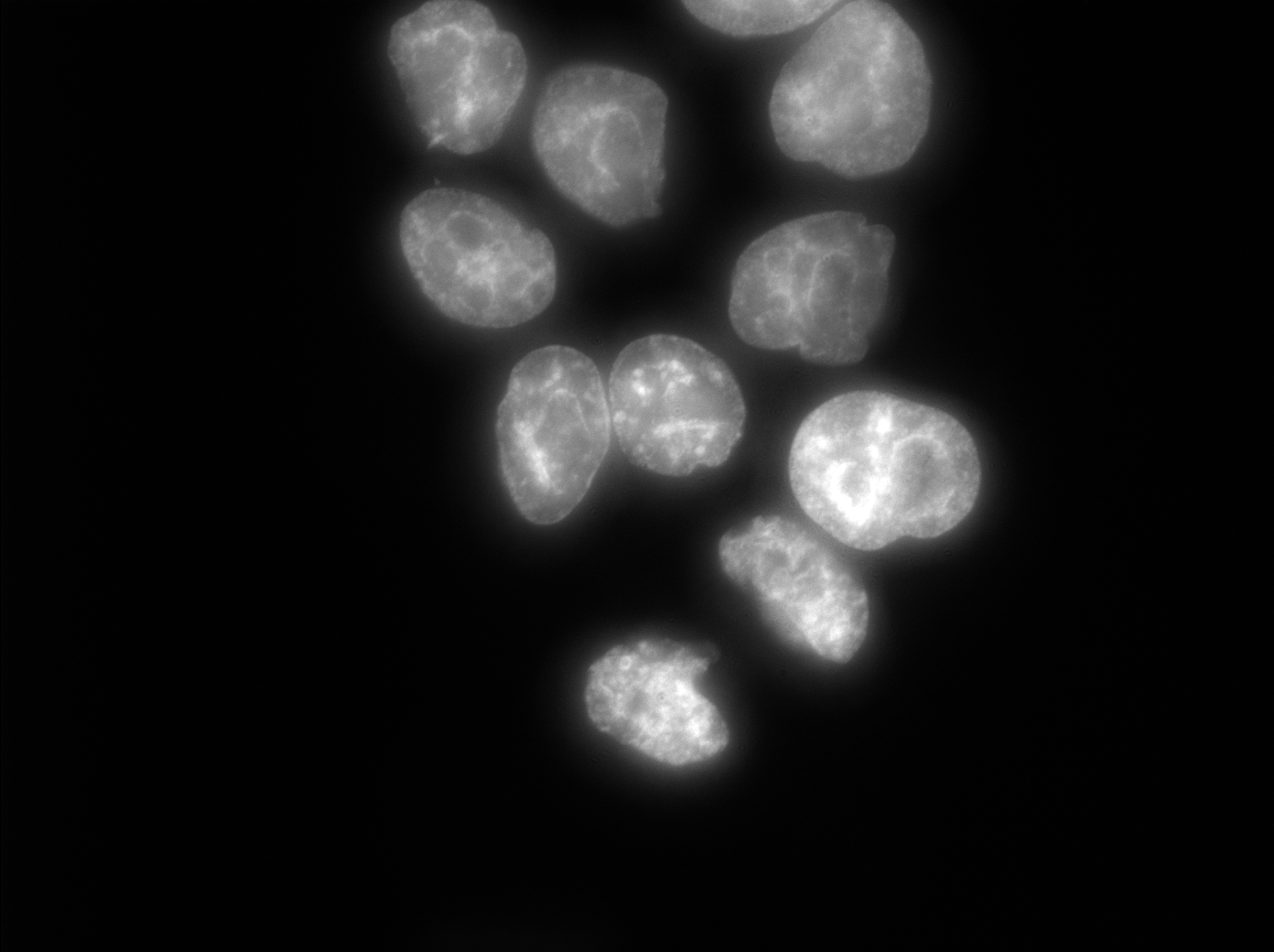

Supplement: Supplementary file 25 — Source data Fig. 7 part 1 [file 44319_2026_742_MOESM25_ESM.zip › Figure 7 Part 1/Fig 7acd Cas9 and TPGS1-ko rGT335 atubulin/Cas9 GT335recomb atub 3-24-25 R2 ET9.Project Maximum Z_XY1742847364_Z0_T0_C0.tif]

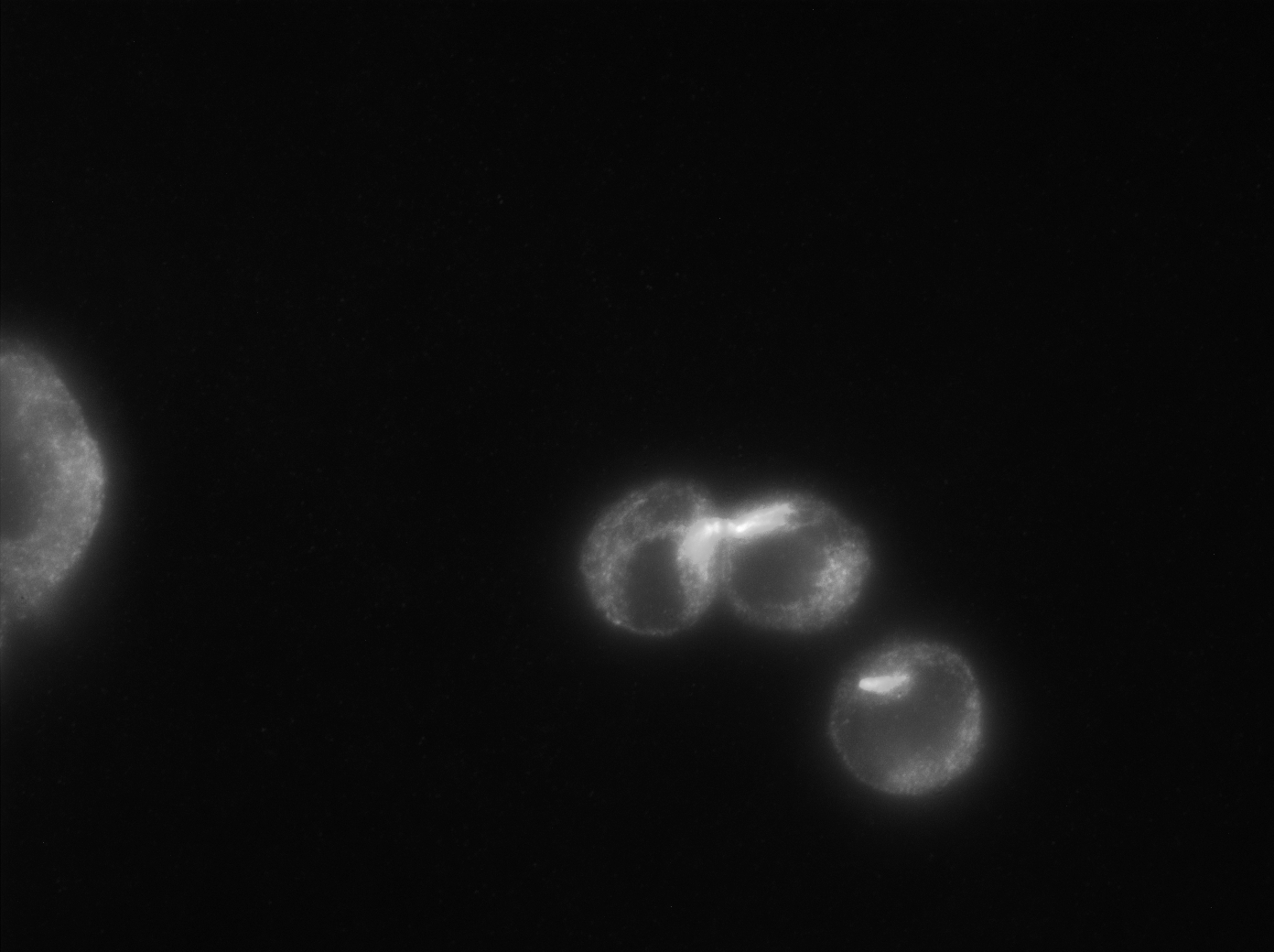

Supplement: Supplementary file 25 — Source data Fig. 7 part 1 [file 44319_2026_742_MOESM25_ESM.zip › Figure 7 Part 1/Fig 7acd Cas9 and TPGS1-ko rGT335 atubulin/Cas9 GT335recomb atub 3-24-25 R2 ET5.Project Maximum Z_XY1742845610_Z0_T0_C2.tif]

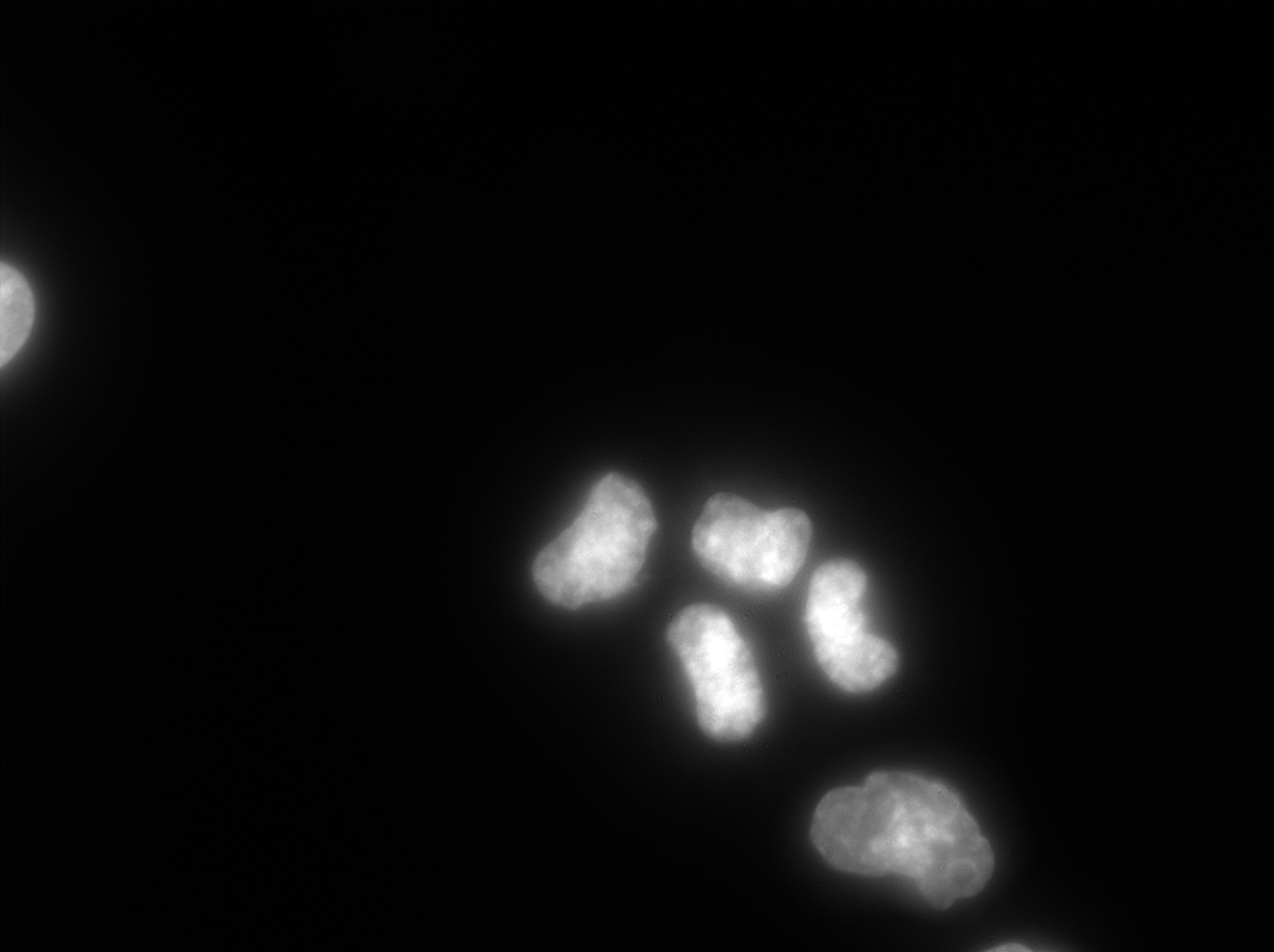

Supplement: Supplementary file 25 — Source data Fig. 7 part 1 [file 44319_2026_742_MOESM25_ESM.zip › Figure 7 Part 1/Fig 7acd Cas9 and TPGS1-ko rGT335 atubulin/Cas9 GT335recomb atub 3-24-25 R3 ET4ET5.Project Maximum Z_XY1742850252_Z0_T0_C0.tif]

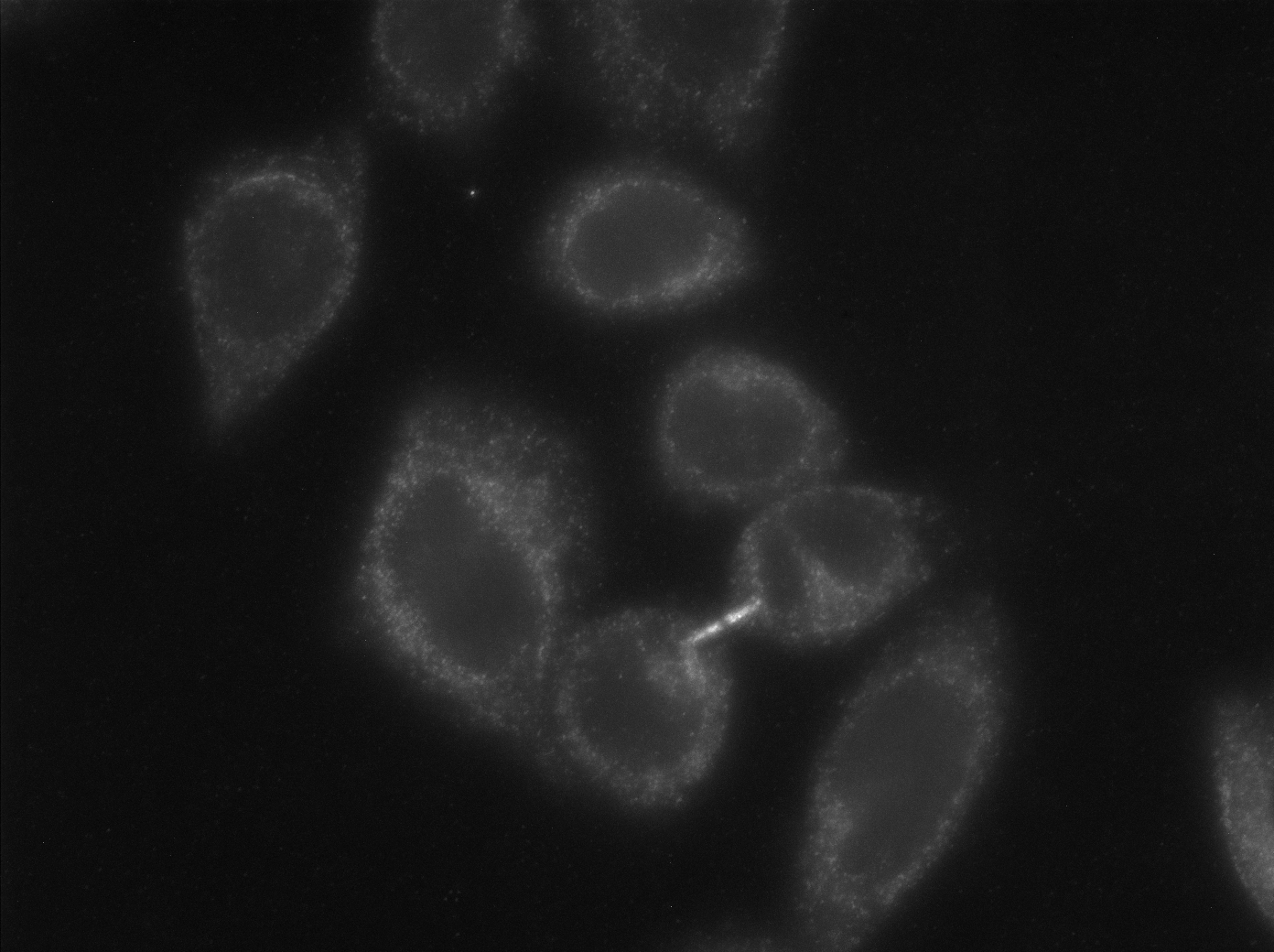

Supplement: Supplementary file 25 — Source data Fig. 7 part 1 [file 44319_2026_742_MOESM25_ESM.zip › Figure 7 Part 1/Fig 7acd Cas9 and TPGS1-ko rGT335 atubulin/Cas9 GT335recomb atub 3-24-25 R3 LT8.Project Maximum Z_XY1742849913_Z0_T0_C2.tif]

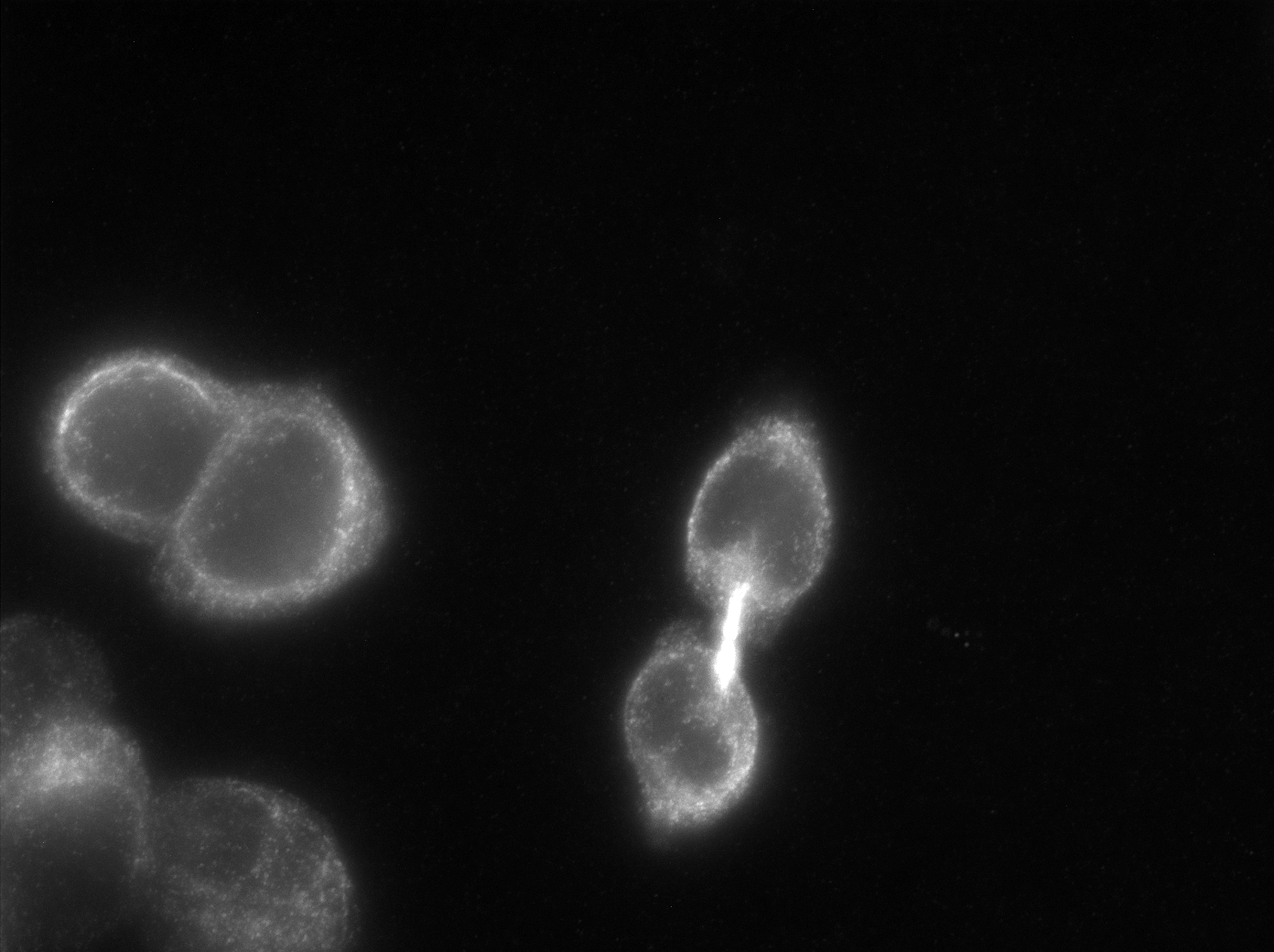

Supplement: Supplementary file 25 — Source data Fig. 7 part 1 [file 44319_2026_742_MOESM25_ESM.zip › Figure 7 Part 1/Fig 7acd Cas9 and TPGS1-ko rGT335 atubulin/Cas9 GT335recomb atub 3-24-25 R1 LT4.Project Maximum Z_XY1742835336_Z0_T0_C2.tif]

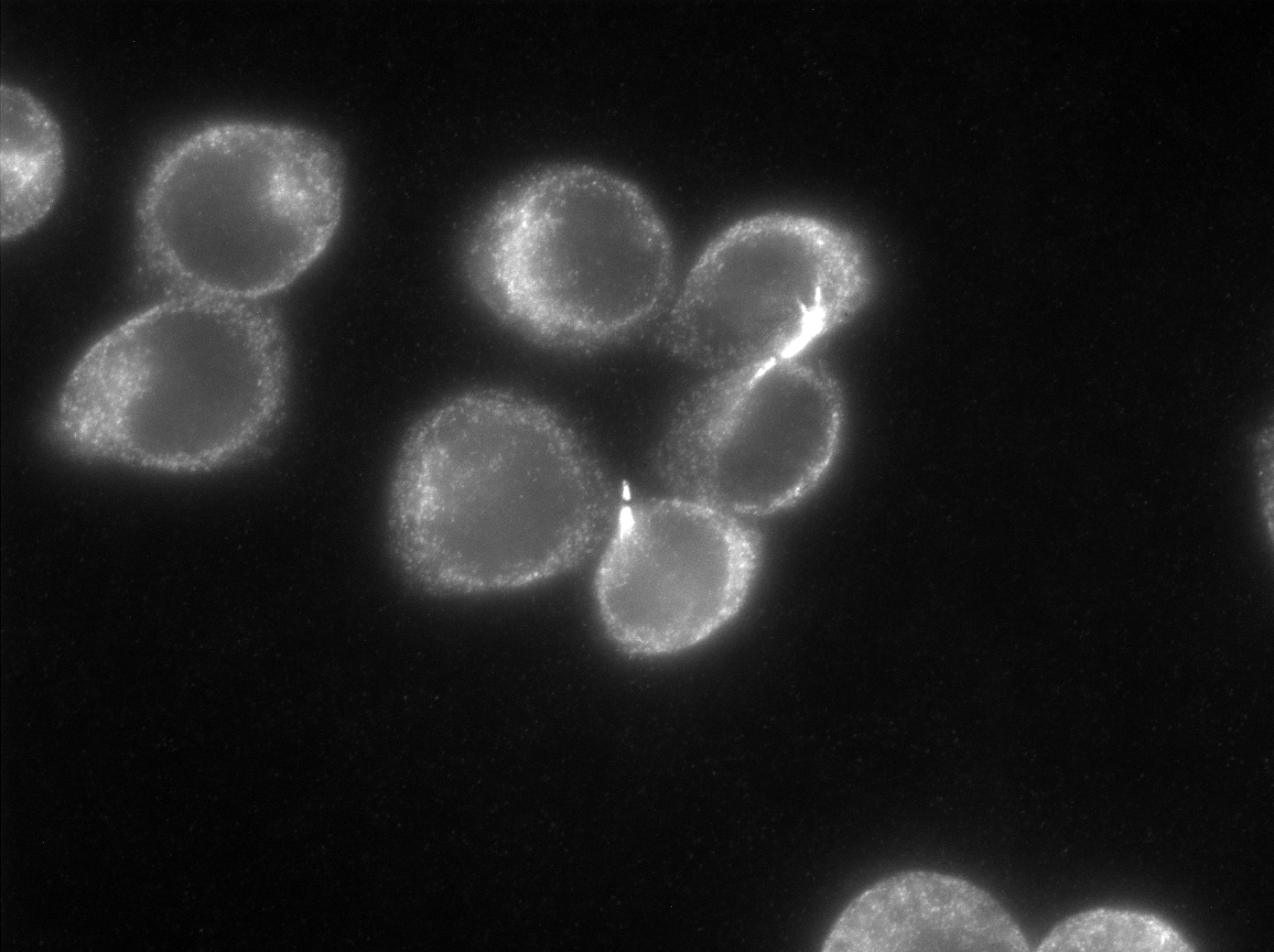

Supplement: Supplementary file 25 — Source data Fig. 7 part 1 [file 44319_2026_742_MOESM25_ESM.zip › Figure 7 Part 1/Fig 7acd Cas9 and TPGS1-ko rGT335 atubulin/Cas9 GT335recomb atub 3-24-25 R1 LT5 PA2.Project Maximum Z_XY1742835678_Z0_T0_C2.tif]

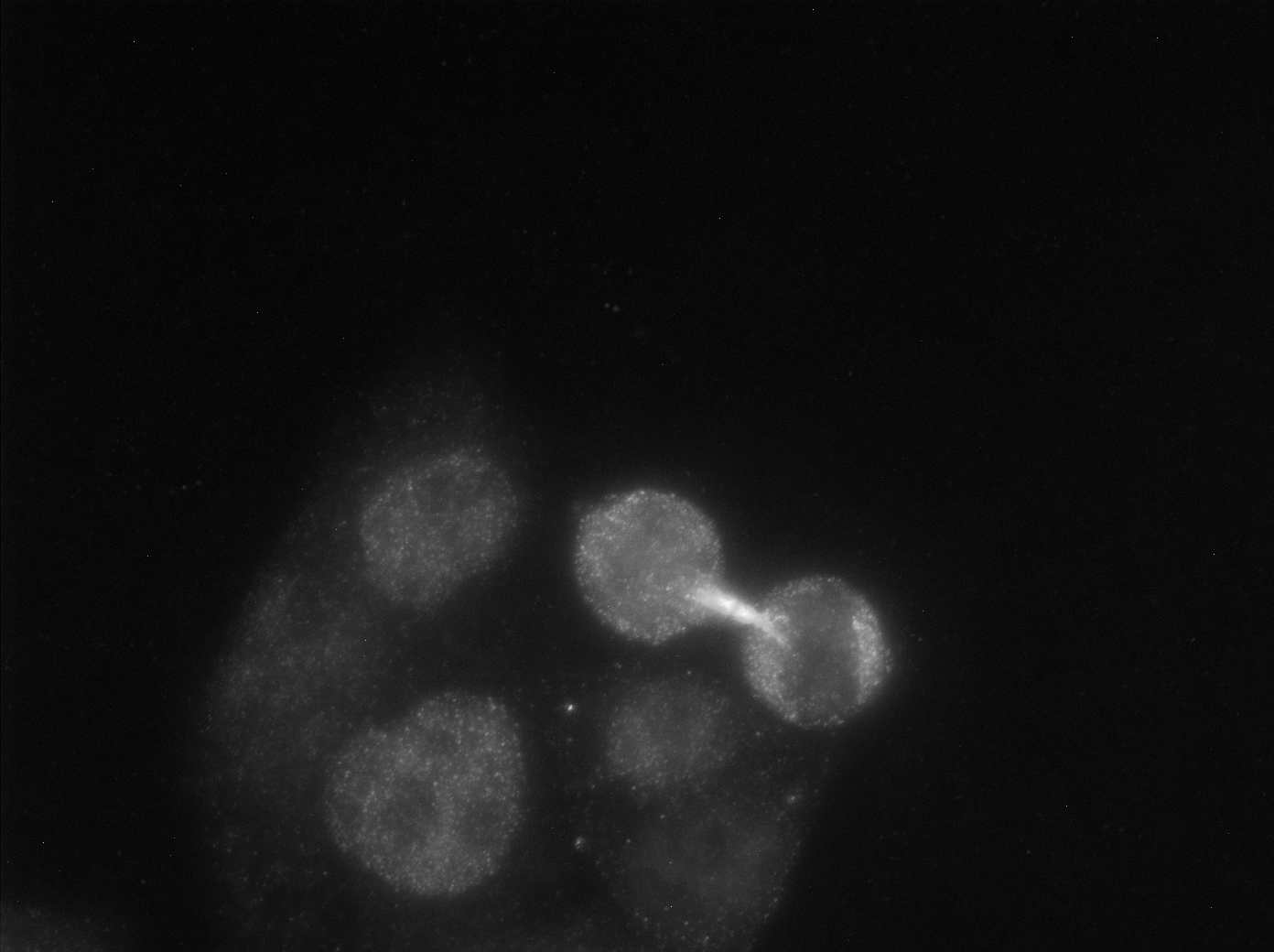

Supplement: Supplementary file 25 — Source data Fig. 7 part 1 [file 44319_2026_742_MOESM25_ESM.zip › Figure 7 Part 1/Fig 7acd Cas9 and TPGS1-ko rGT335 atubulin/Cas9 GT335recomb atub 3-24-25 R2 ET10.Project Maximum Z_XY1742847558_Z0_T0_C1.tif]

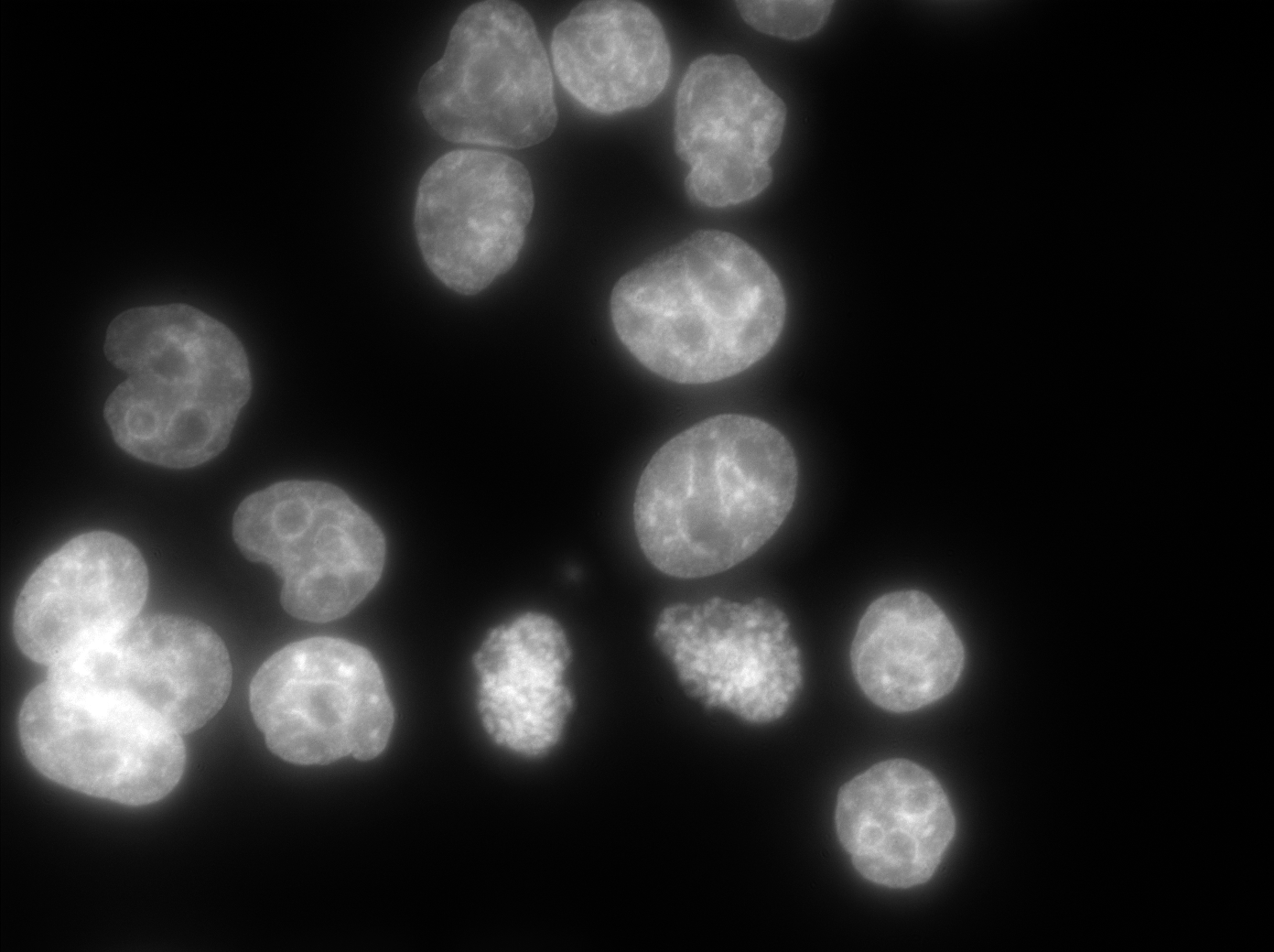

Supplement: Supplementary file 25 — Source data Fig. 7 part 1 [file 44319_2026_742_MOESM25_ESM.zip › Figure 7 Part 1/Fig 7acd Cas9 and TPGS1-ko rGT335 atubulin/Cas9 GT335recomb atub 3-24-25 R1 ET7.Project Maximum Z_XY1742836085_Z0_T0_C0.tif]

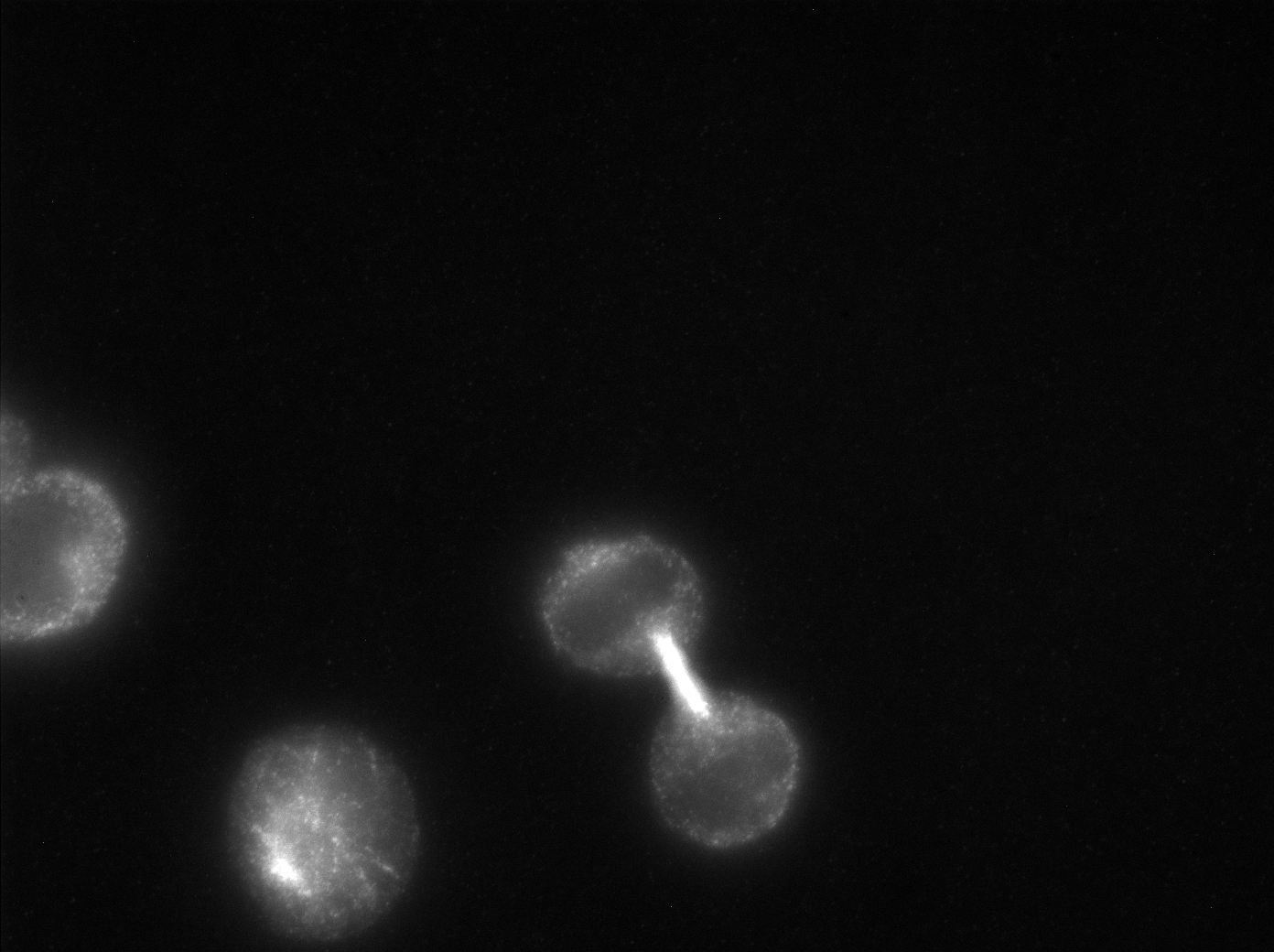

Supplement: Supplementary file 25 — Source data Fig. 7 part 1 [file 44319_2026_742_MOESM25_ESM.zip › Figure 7 Part 1/Fig 7acd Cas9 and TPGS1-ko rGT335 atubulin/Cas9 GT335recomb atub 3-24-25 R3 ET1.Project Maximum Z_XY1742848799_Z0_T0_C2.tif]

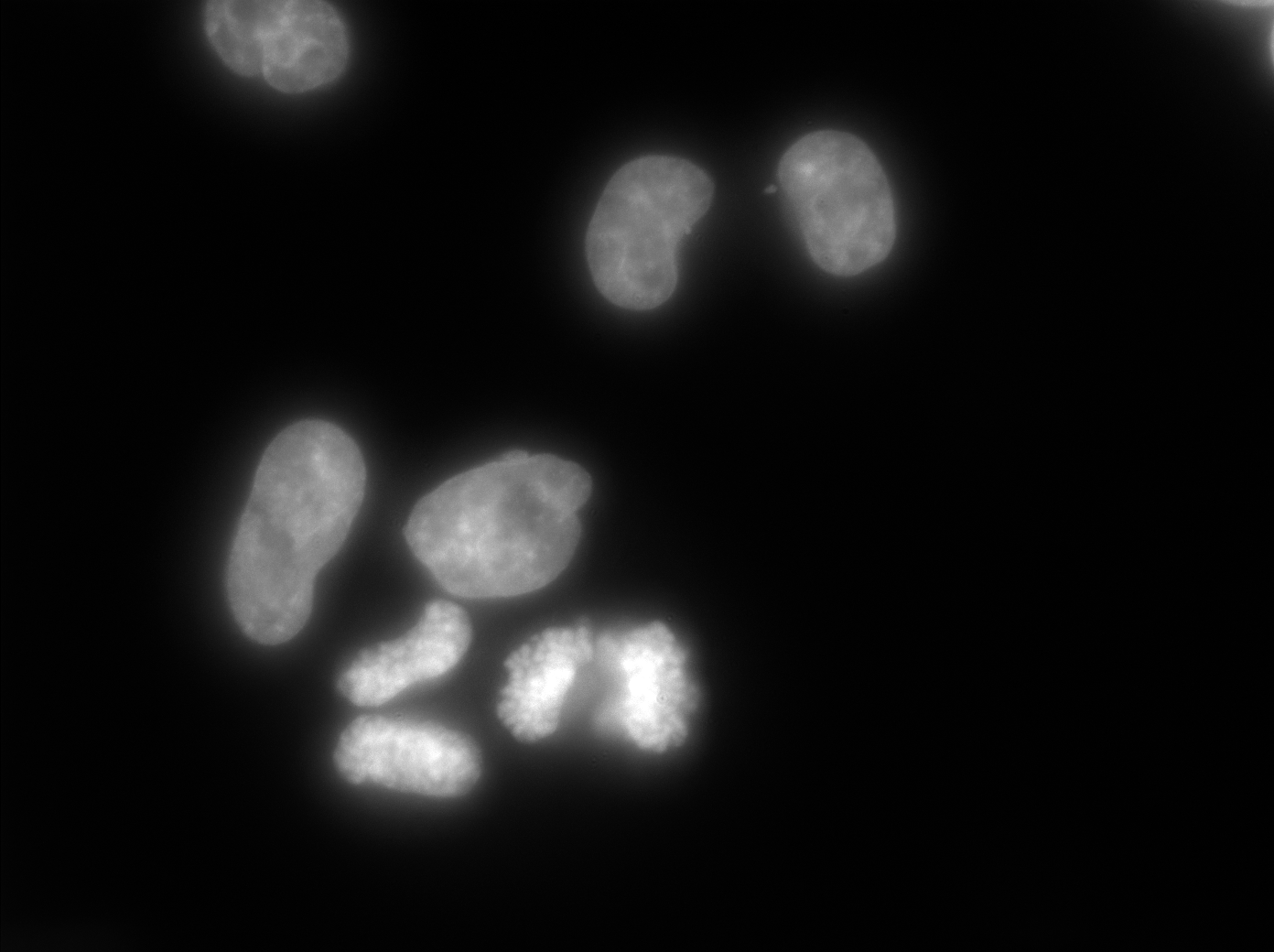

Supplement: Supplementary file 25 — Source data Fig. 7 part 1 [file 44319_2026_742_MOESM25_ESM.zip › Figure 7 Part 1/Fig 7acd Cas9 and TPGS1-ko rGT335 atubulin/Cas9 GT335recomb atub 3-24-25 R3 ET6ET7.Project Maximum Z_XY1742850373_Z0_T0_C0.tif]

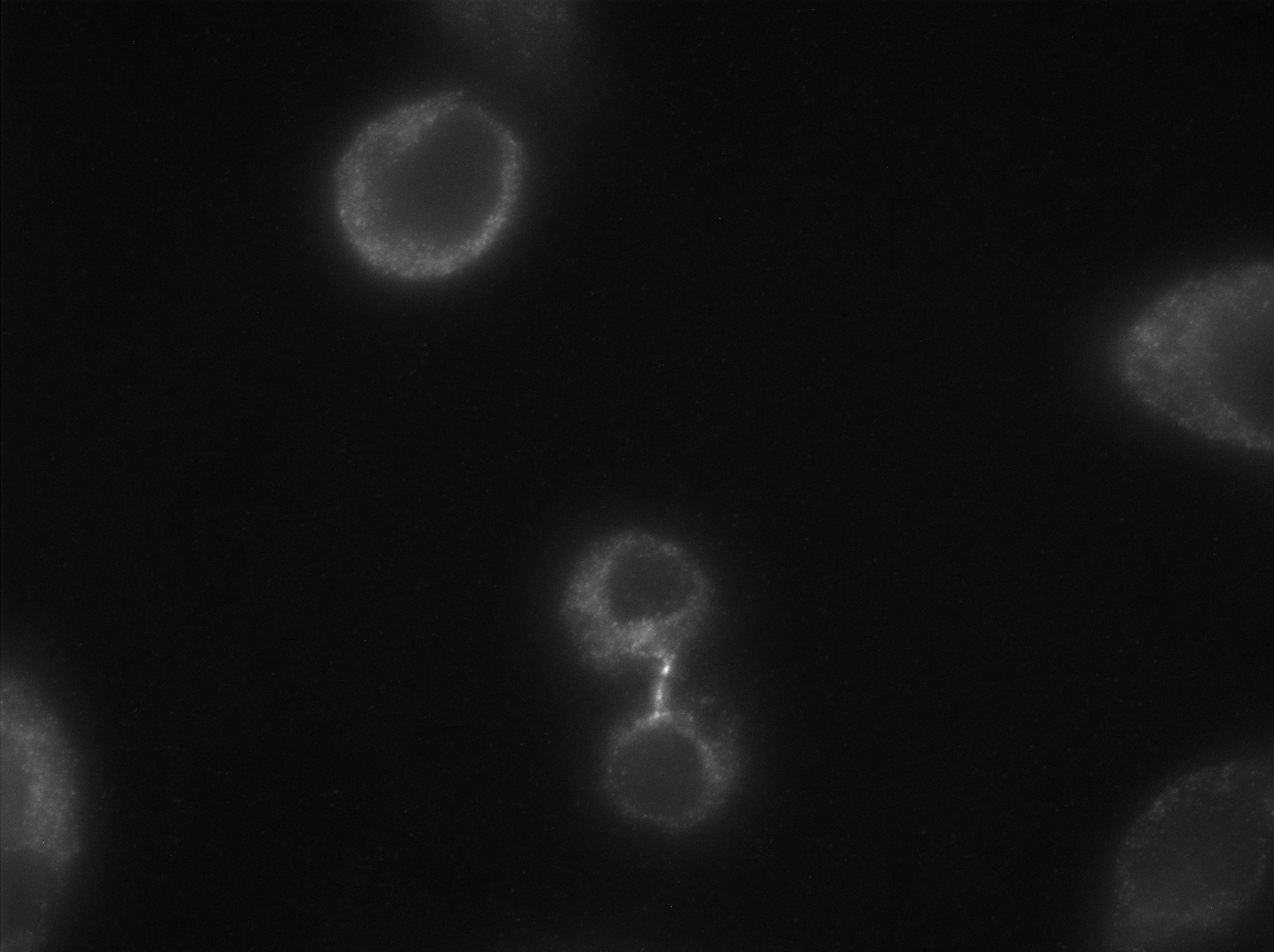

Supplement: Supplementary file 25 — Source data Fig. 7 part 1 [file 44319_2026_742_MOESM25_ESM.zip › Figure 7 Part 1/Fig 7acd Cas9 and TPGS1-ko rGT335 atubulin/Cas9 GT335recomb atub 3-24-25 R3 LT5.Project Maximum Z_XY1742849372_Z0_T0_C2.tif]

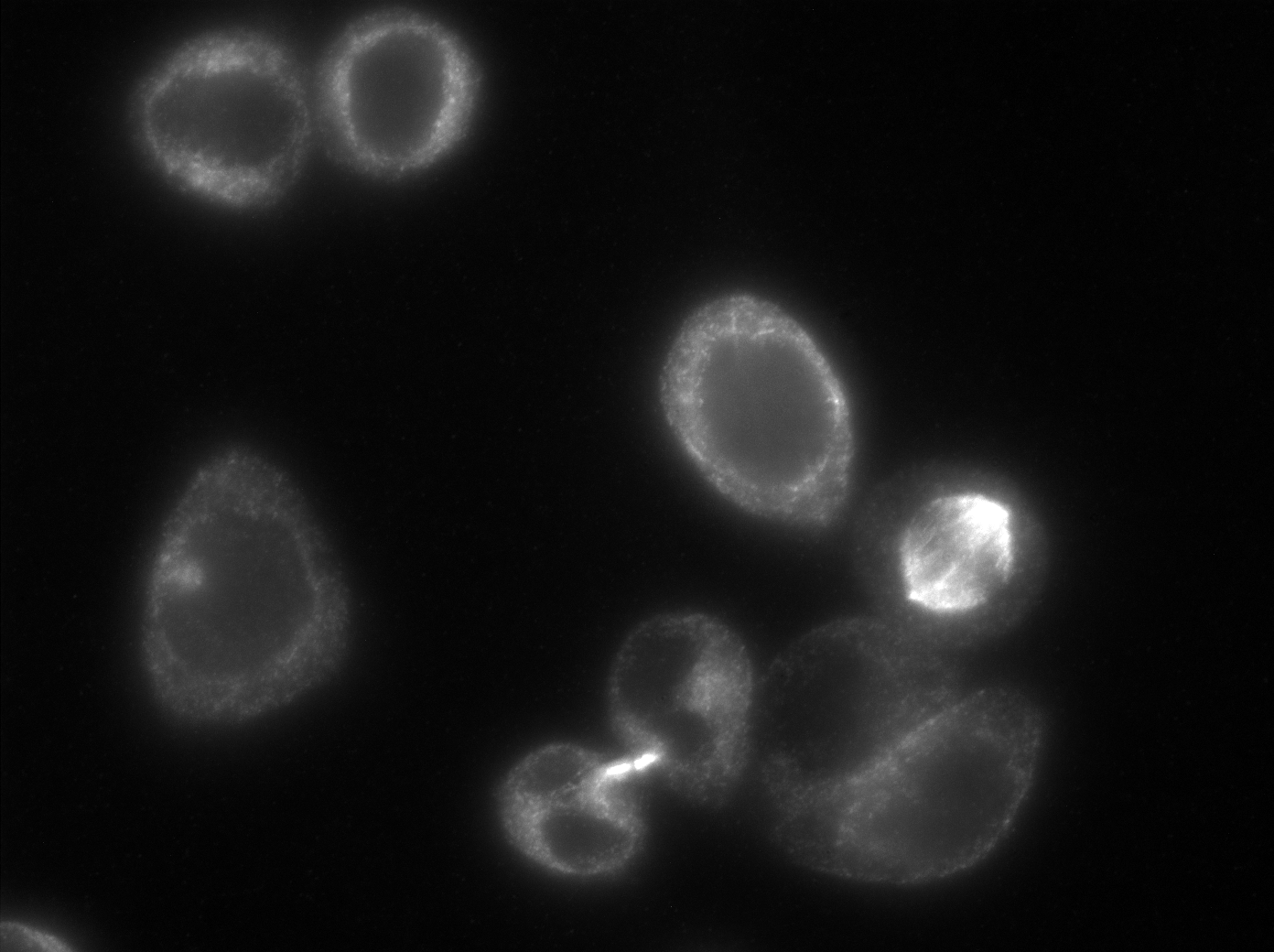

Supplement: Supplementary file 25 — Source data Fig. 7 part 1 [file 44319_2026_742_MOESM25_ESM.zip › Figure 7 Part 1/Fig 7acd Cas9 and TPGS1-ko rGT335 atubulin/Cas9 GT335recomb atub 3-24-25 R3 LT1 M1.Project Maximum Z_XY1742848535_Z0_T0_C2.tif]

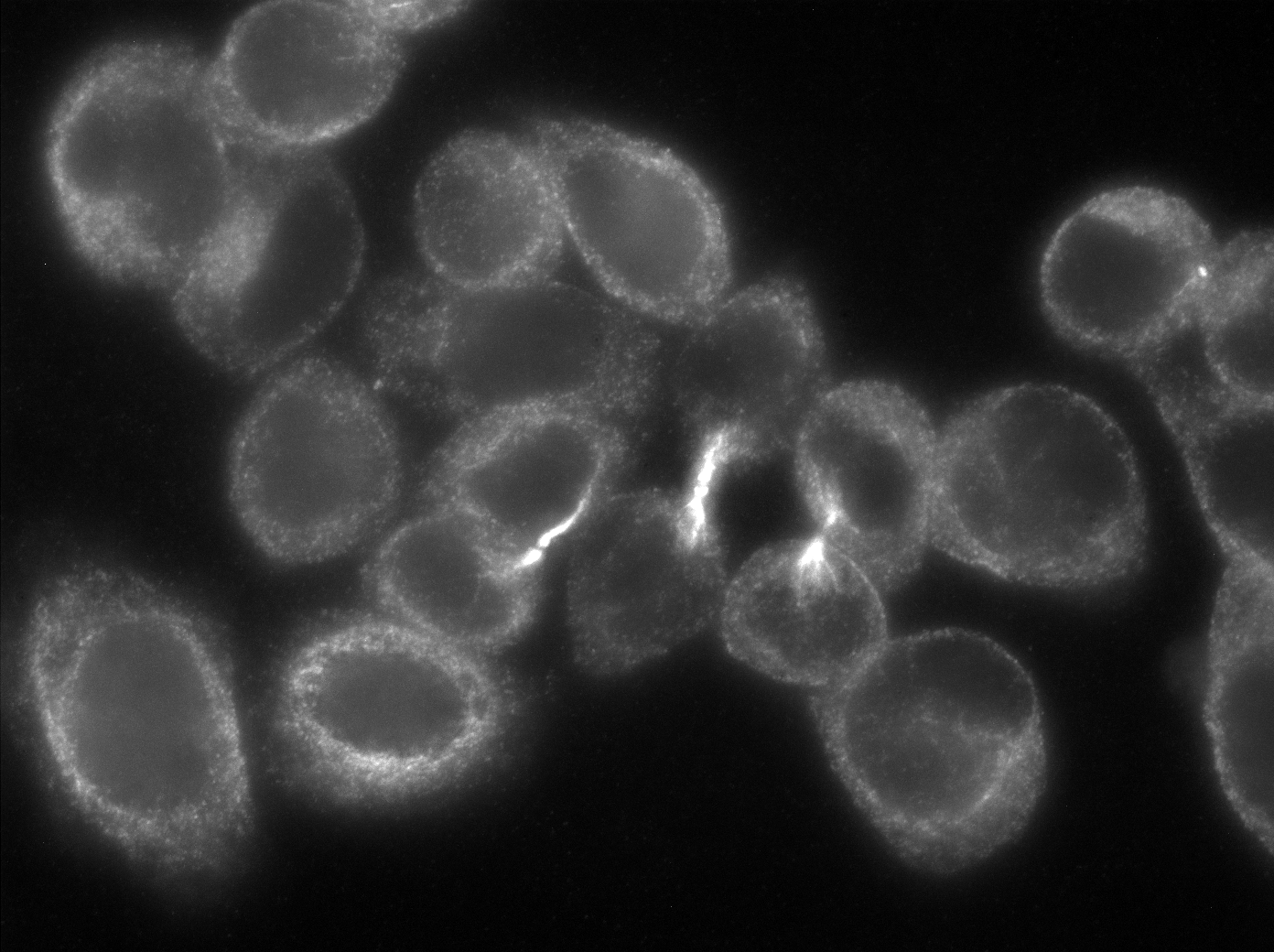

Supplement: Supplementary file 25 — Source data Fig. 7 part 1 [file 44319_2026_742_MOESM25_ESM.zip › Figure 7 Part 1/Fig 7acd Cas9 and TPGS1-ko rGT335 atubulin/Cas9 GT335recomb atub 3-24-25 R1 LT7LT8.Project Maximum Z_XY1742836175_Z0_T0_C2.tif]

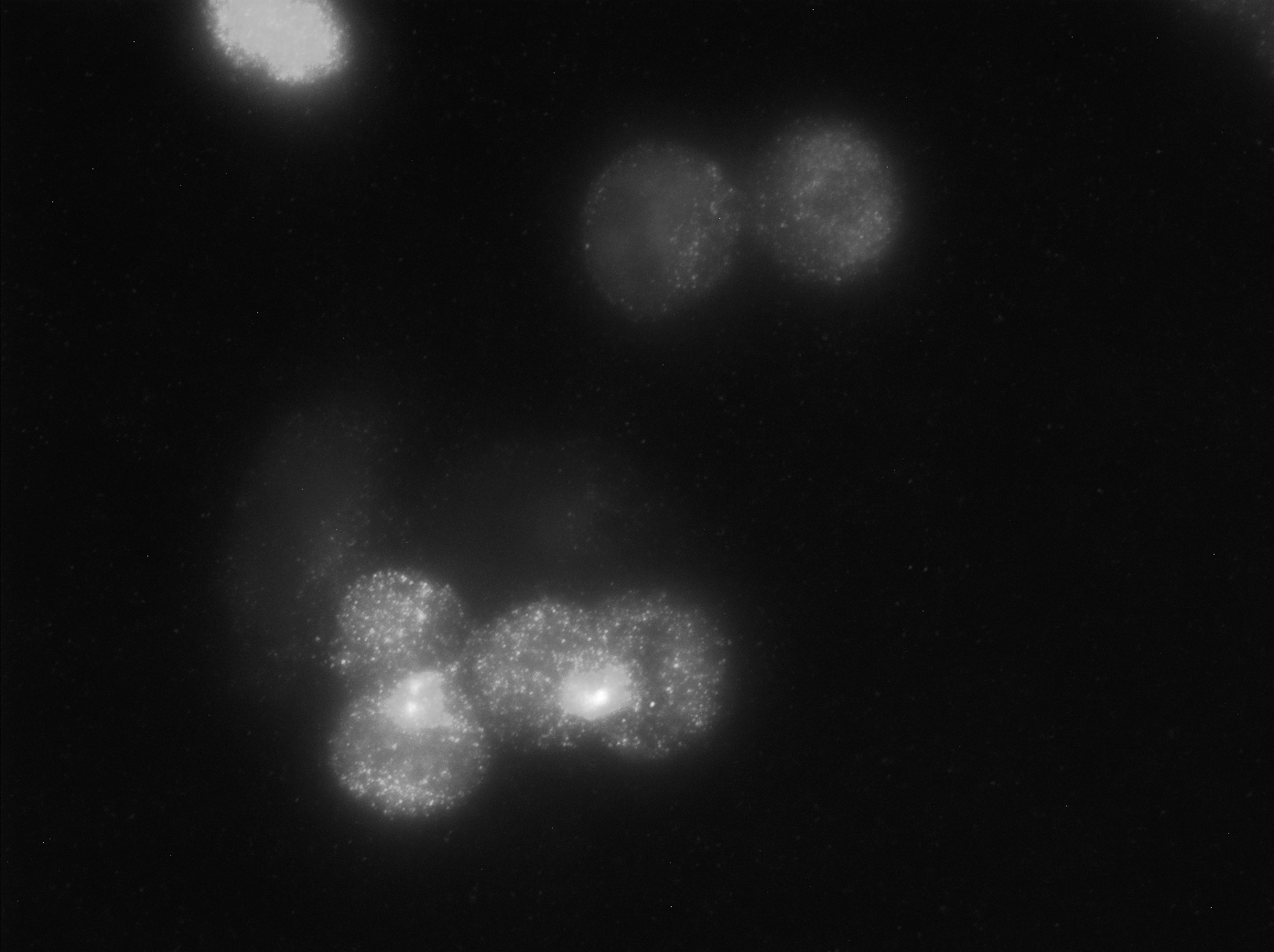

Supplement: Supplementary file 25 — Source data Fig. 7 part 1 [file 44319_2026_742_MOESM25_ESM.zip › Figure 7 Part 1/Fig 7acd Cas9 and TPGS1-ko rGT335 atubulin/Cas9 GT335recomb atub 3-24-25 R3 ET6ET7.Project Maximum Z_XY1742850373_Z0_T0_C1.tif]
