# Supplementary figures and images for: TPGS1 regulates central spindle microtubule glutamylation and remodeling during telophase and abscission (part 32 of 36)
Source: EMBO Rep. 2026 Mar 23;27(8):1944–63. doi: 10.1038/s44319-026-00742-3 (PMC13121839; doi:10.1038/s44319-026-00742-3)

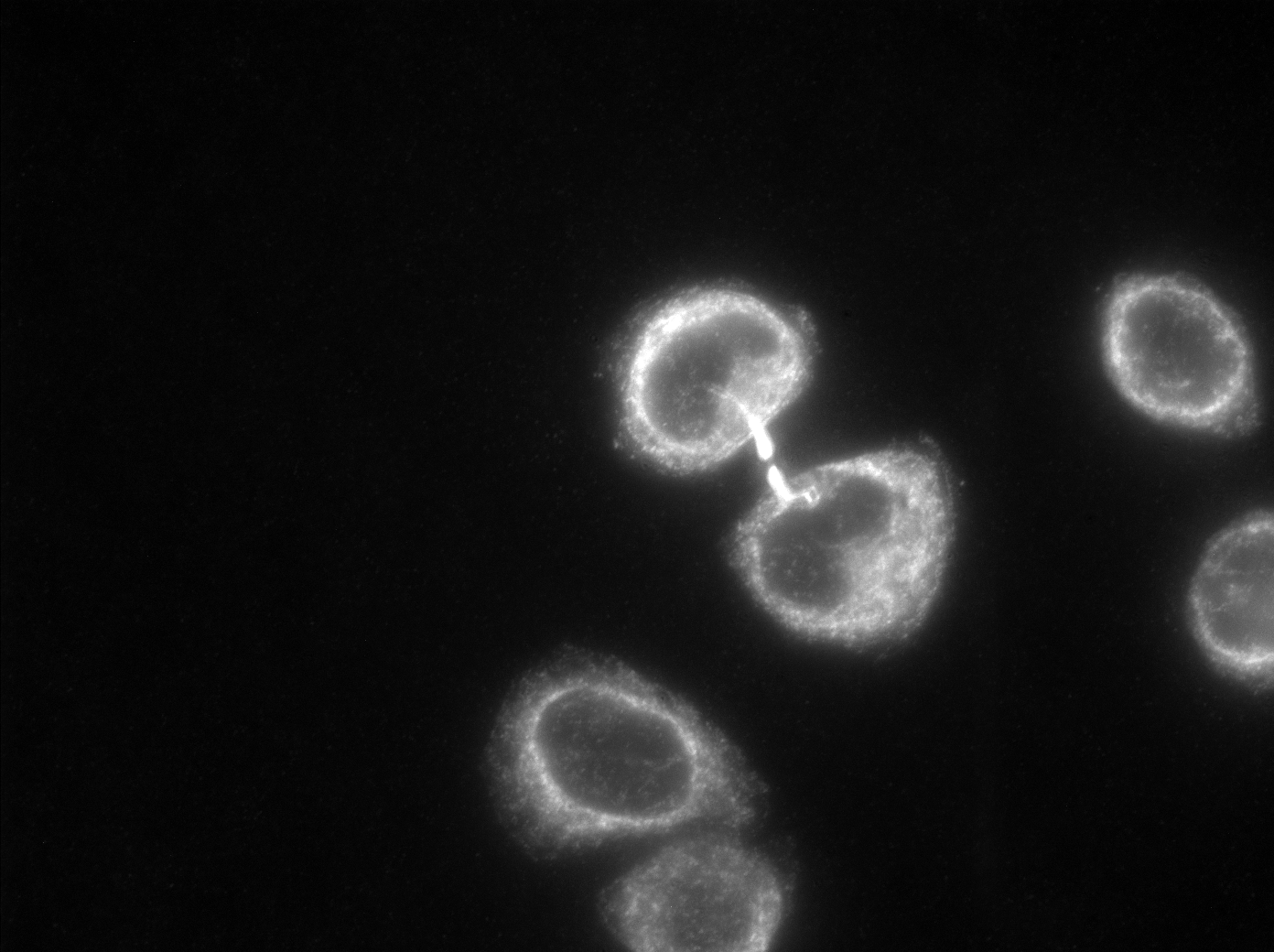

Supplement: Supplementary file 26 — Source data Fig. 7 part 2 [file 44319_2026_742_MOESM26_ESM.zip › Figure 7 Part 2/Fig 7acd Cas9 and TPGS1-ko rGT335 atubulin part 2/TPGS1-KO GT335recomb atub 3-24-25 R3 LT7.Project Maximum Z_XY1742853413_Z0_T0_C2.tif]

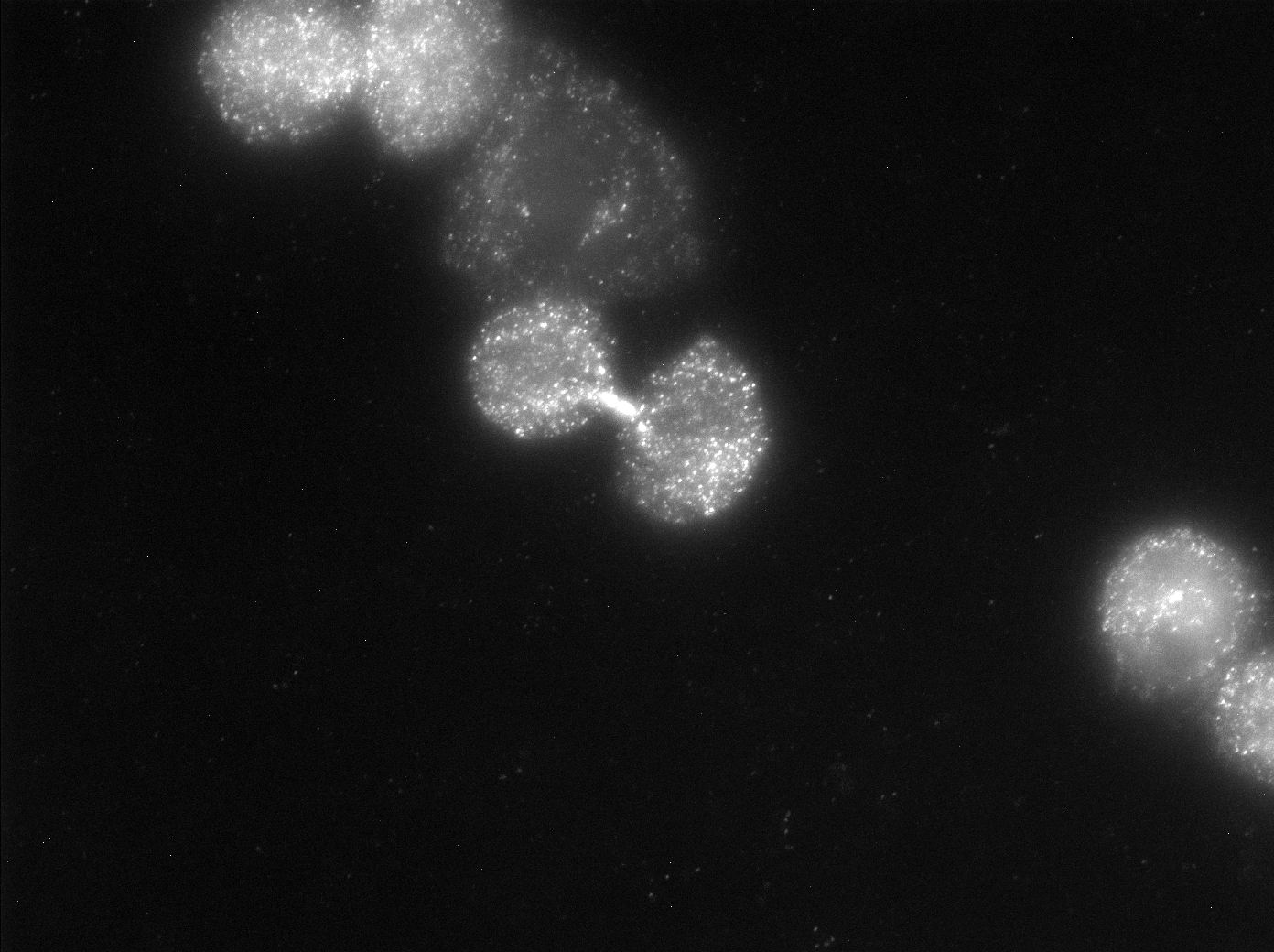

Supplement: Supplementary file 26 — Source data Fig. 7 part 2 [file 44319_2026_742_MOESM26_ESM.zip › Figure 7 Part 2/Fig 7acd Cas9 and TPGS1-ko rGT335 atubulin part 2/TPGS1-KO GT335recomb atub 3-24-25 R2 ET8.Project Maximum Z_XY1742842061_Z0_T0_C1.tif]

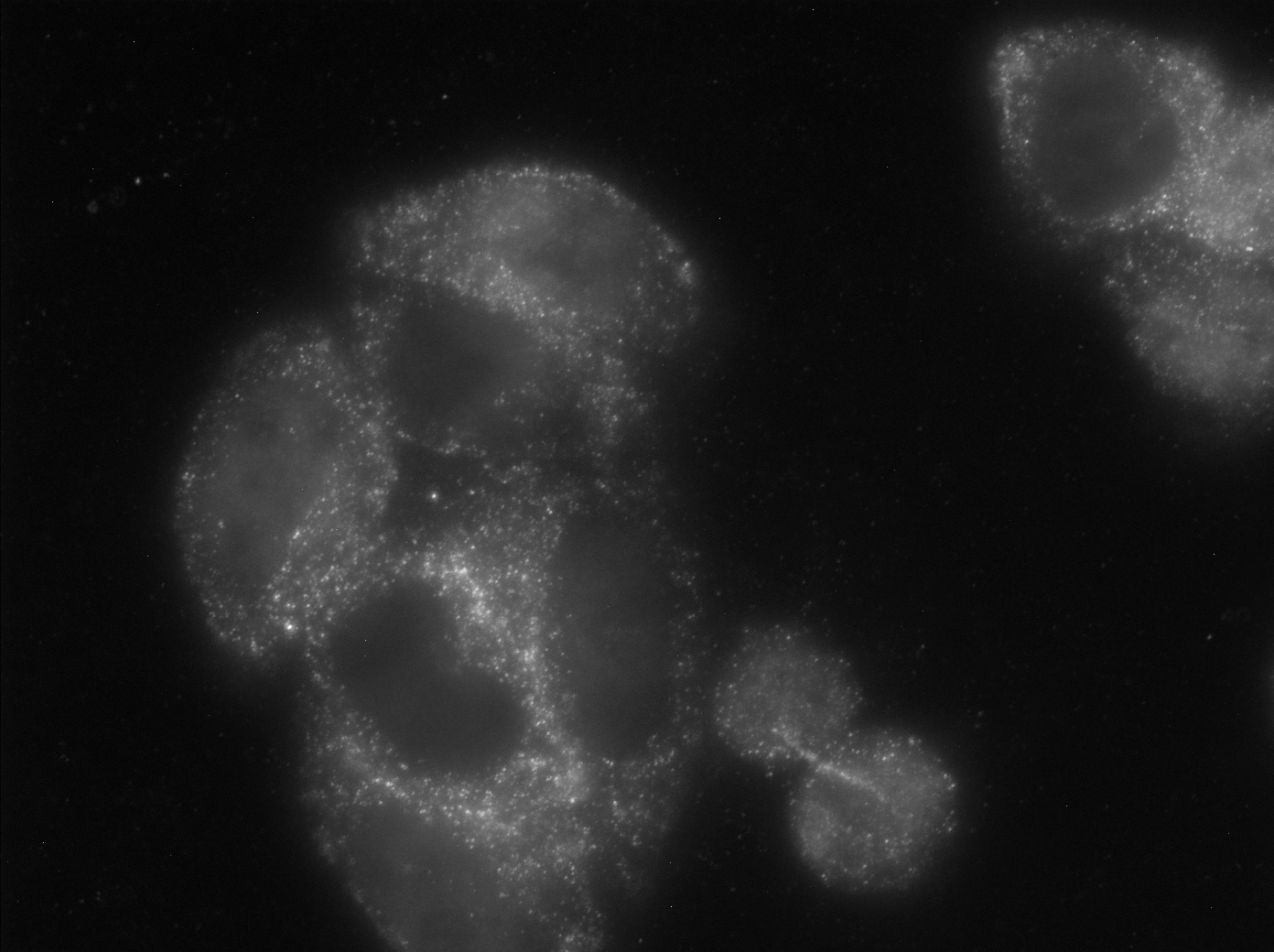

Supplement: Supplementary file 26 — Source data Fig. 7 part 2 [file 44319_2026_742_MOESM26_ESM.zip › Figure 7 Part 2/Fig 7acd Cas9 and TPGS1-ko rGT335 atubulin part 2/TPGS1-KO GT335recomb atub 3-24-25 R1 ET4.Project Maximum Z_XY1742840170_Z0_T0_C1.tif]

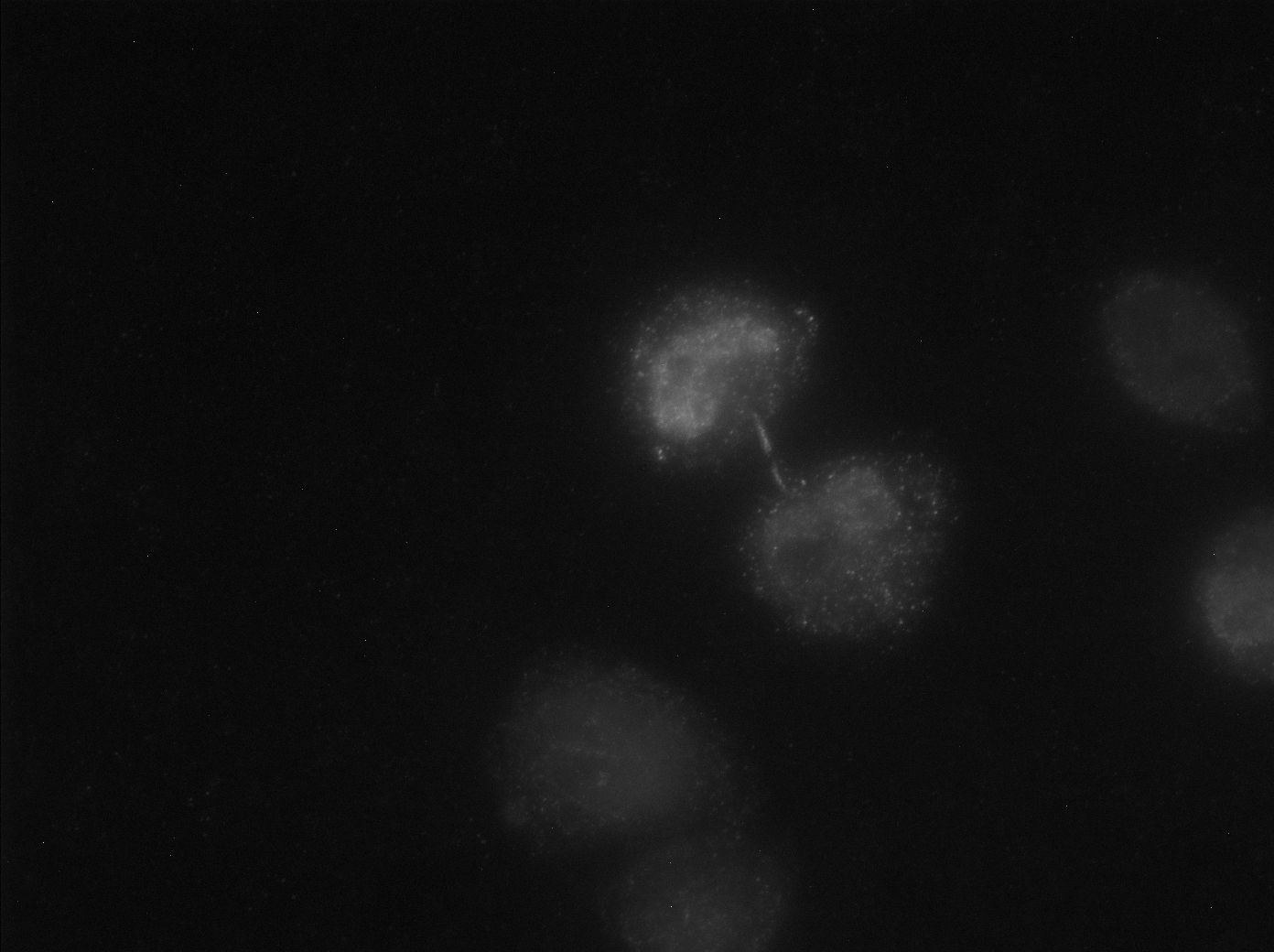

Supplement: Supplementary file 26 — Source data Fig. 7 part 2 [file 44319_2026_742_MOESM26_ESM.zip › Figure 7 Part 2/Fig 7acd Cas9 and TPGS1-ko rGT335 atubulin part 2/TPGS1-KO GT335recomb atub 3-24-25 R3 LT7.Project Maximum Z_XY1742853413_Z0_T0_C1.tif]

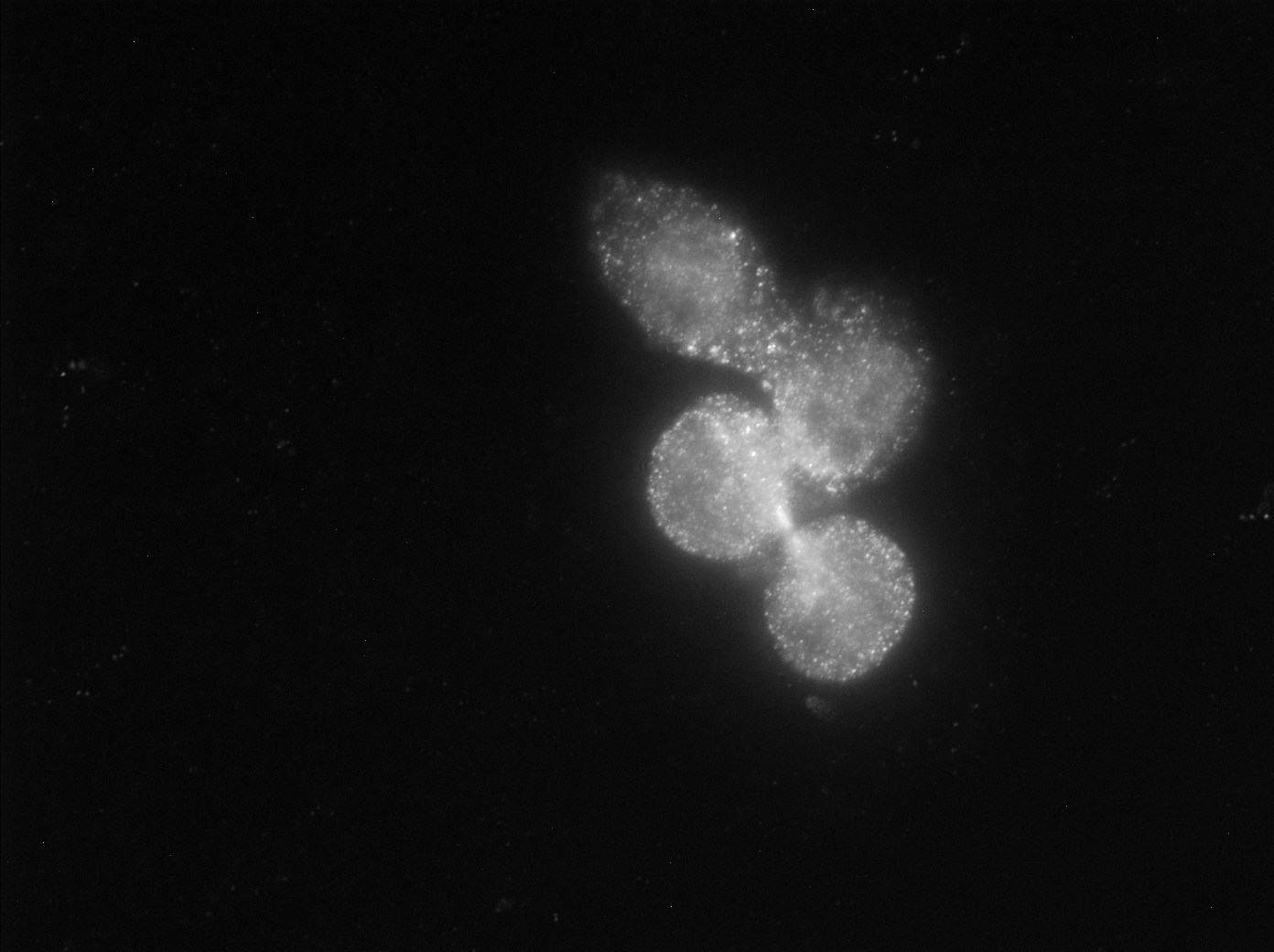

Supplement: Supplementary file 26 — Source data Fig. 7 part 2 [file 44319_2026_742_MOESM26_ESM.zip › Figure 7 Part 2/Fig 7acd Cas9 and TPGS1-ko rGT335 atubulin part 2/TPGS1-KO GT335recomb atub 3-24-25 R1 ET3 LT9.Project Maximum Z_XY1742840057_Z0_T0_C1.tif]

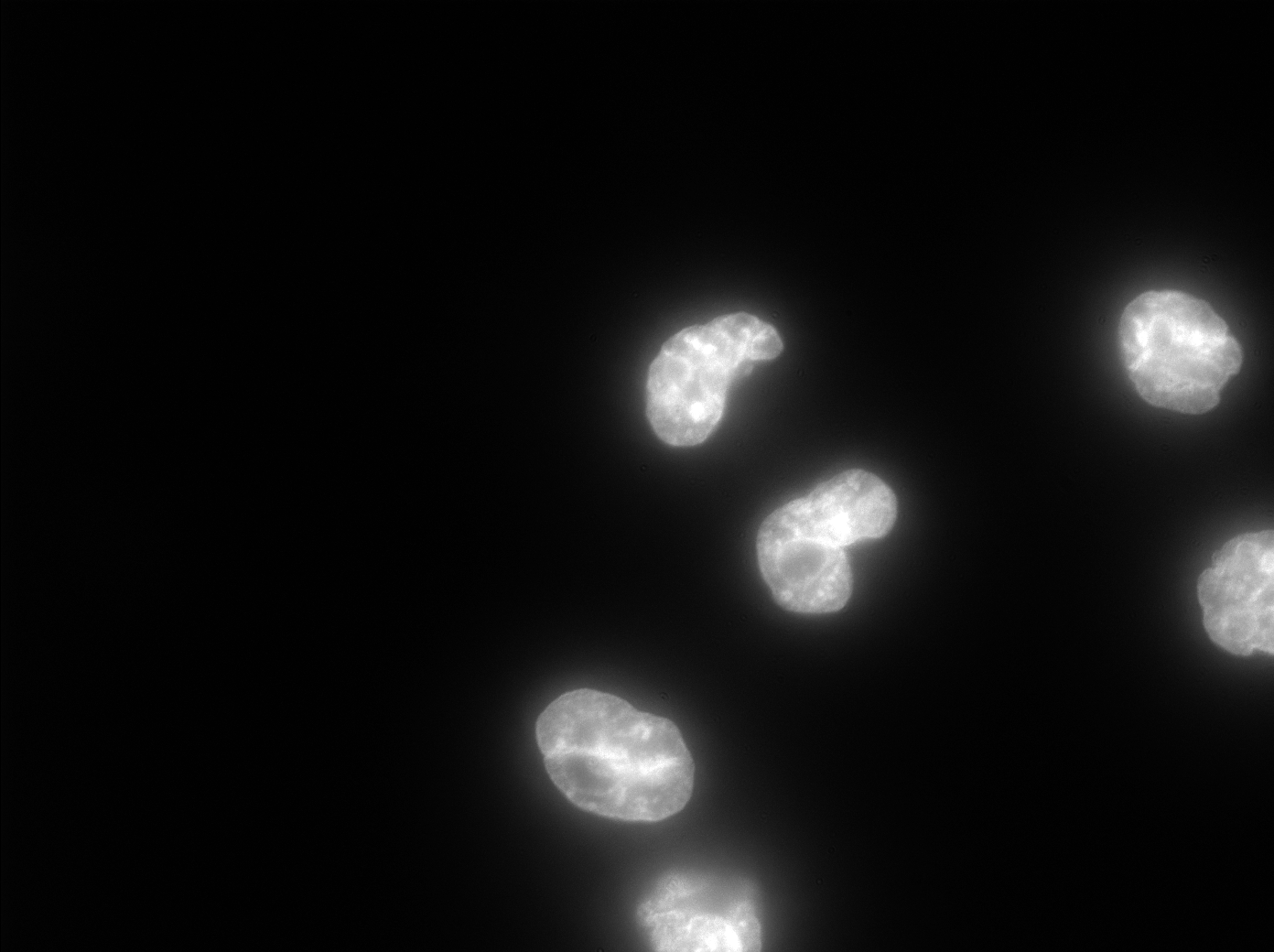

Supplement: Supplementary file 26 — Source data Fig. 7 part 2 [file 44319_2026_742_MOESM26_ESM.zip › Figure 7 Part 2/Fig 7acd Cas9 and TPGS1-ko rGT335 atubulin part 2/TPGS1-KO GT335recomb atub 3-24-25 R3 LT7.Project Maximum Z_XY1742853413_Z0_T0_C0.tif]

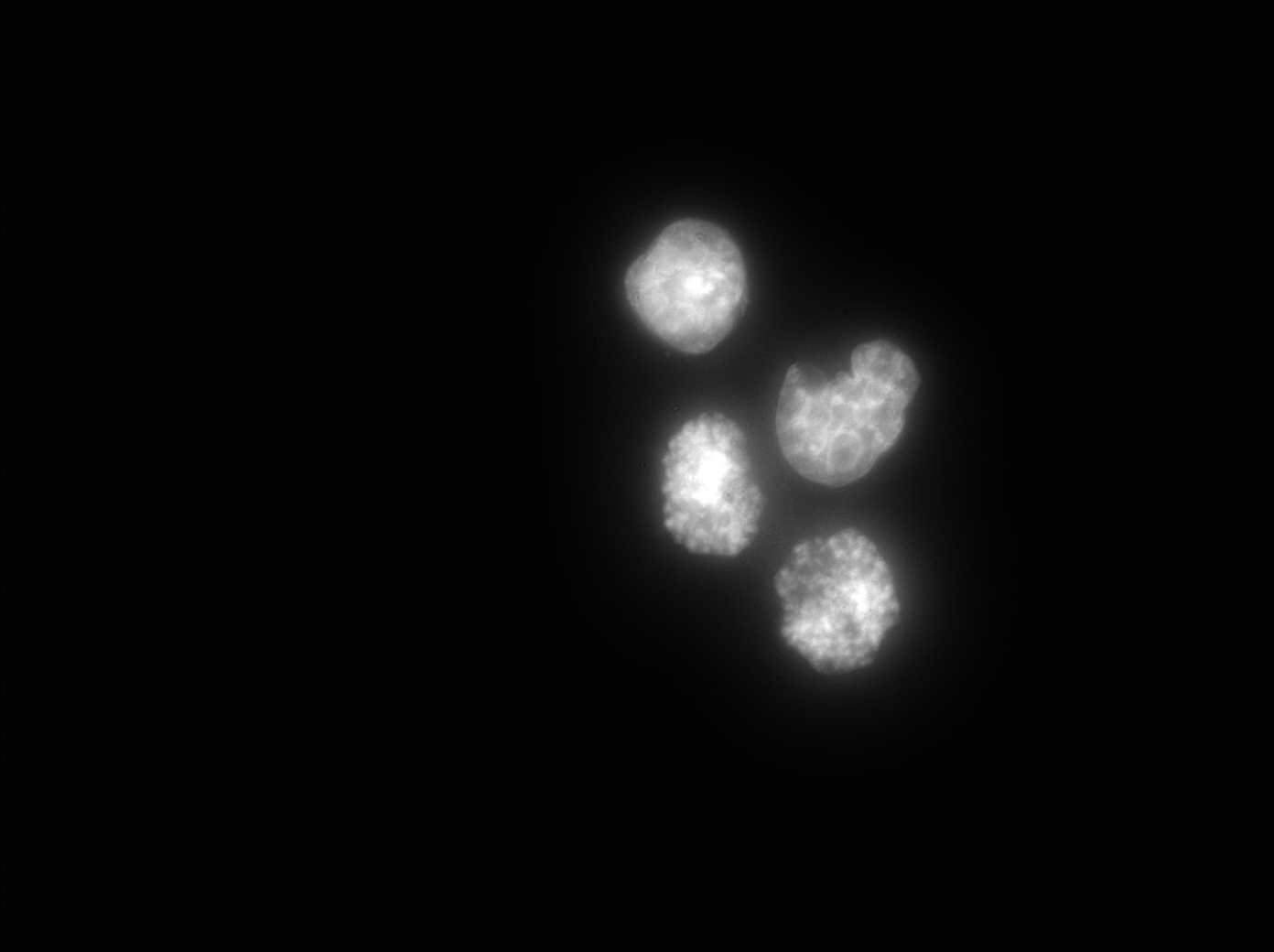

Supplement: Supplementary file 26 — Source data Fig. 7 part 2 [file 44319_2026_742_MOESM26_ESM.zip › Figure 7 Part 2/Fig 7acd Cas9 and TPGS1-ko rGT335 atubulin part 2/TPGS1-KO GT335recomb atub 3-24-25 R1 ET3 LT9.Project Maximum Z_XY1742840057_Z0_T0_C0.tif]

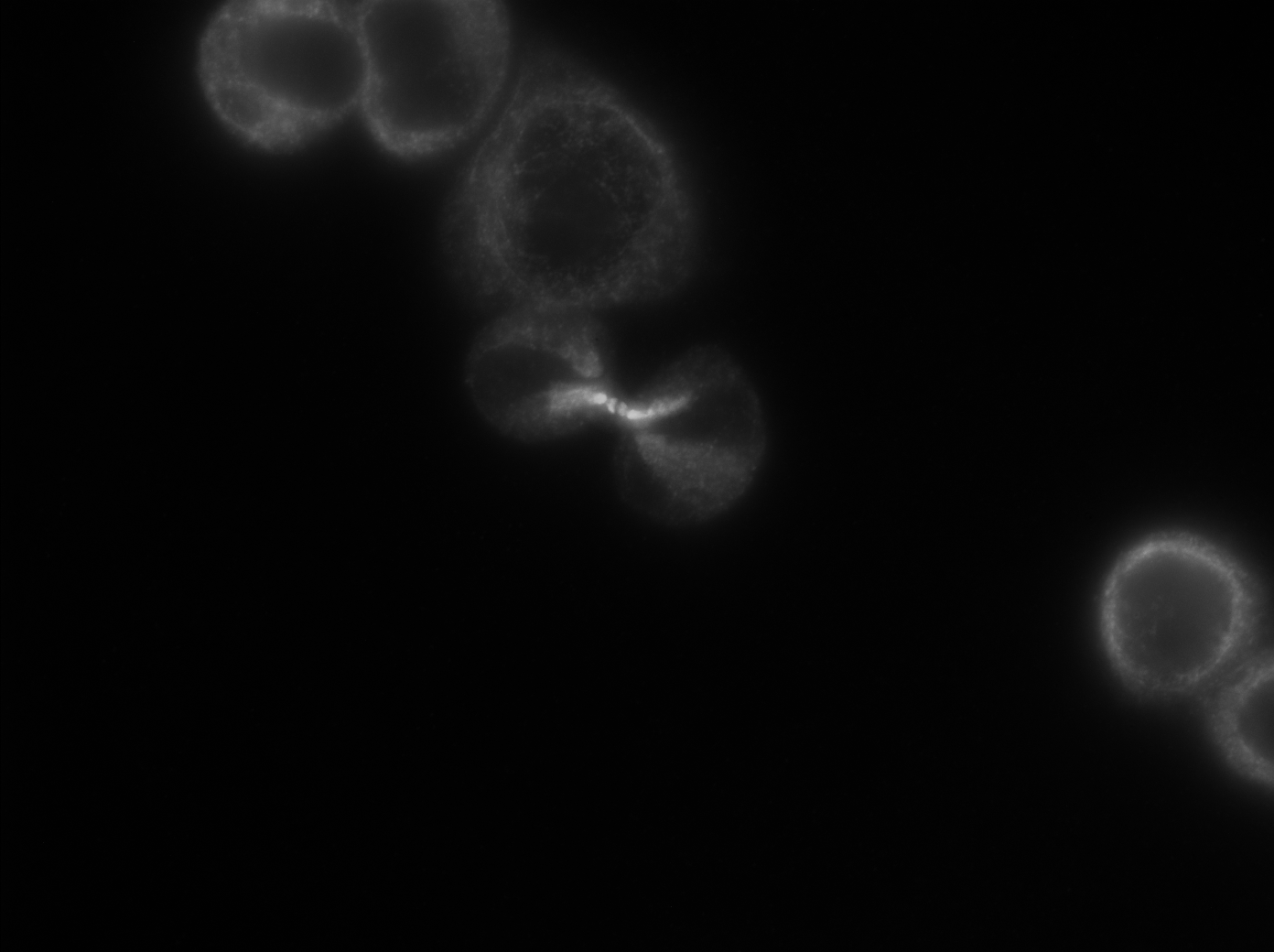

Supplement: Supplementary file 26 — Source data Fig. 7 part 2 [file 44319_2026_742_MOESM26_ESM.zip › Figure 7 Part 2/Fig 7acd Cas9 and TPGS1-ko rGT335 atubulin part 2/TPGS1-KO GT335recomb atub 3-24-25 R2 ET8.Project Maximum Z_XY1742842061_Z0_T0_C2.tif]

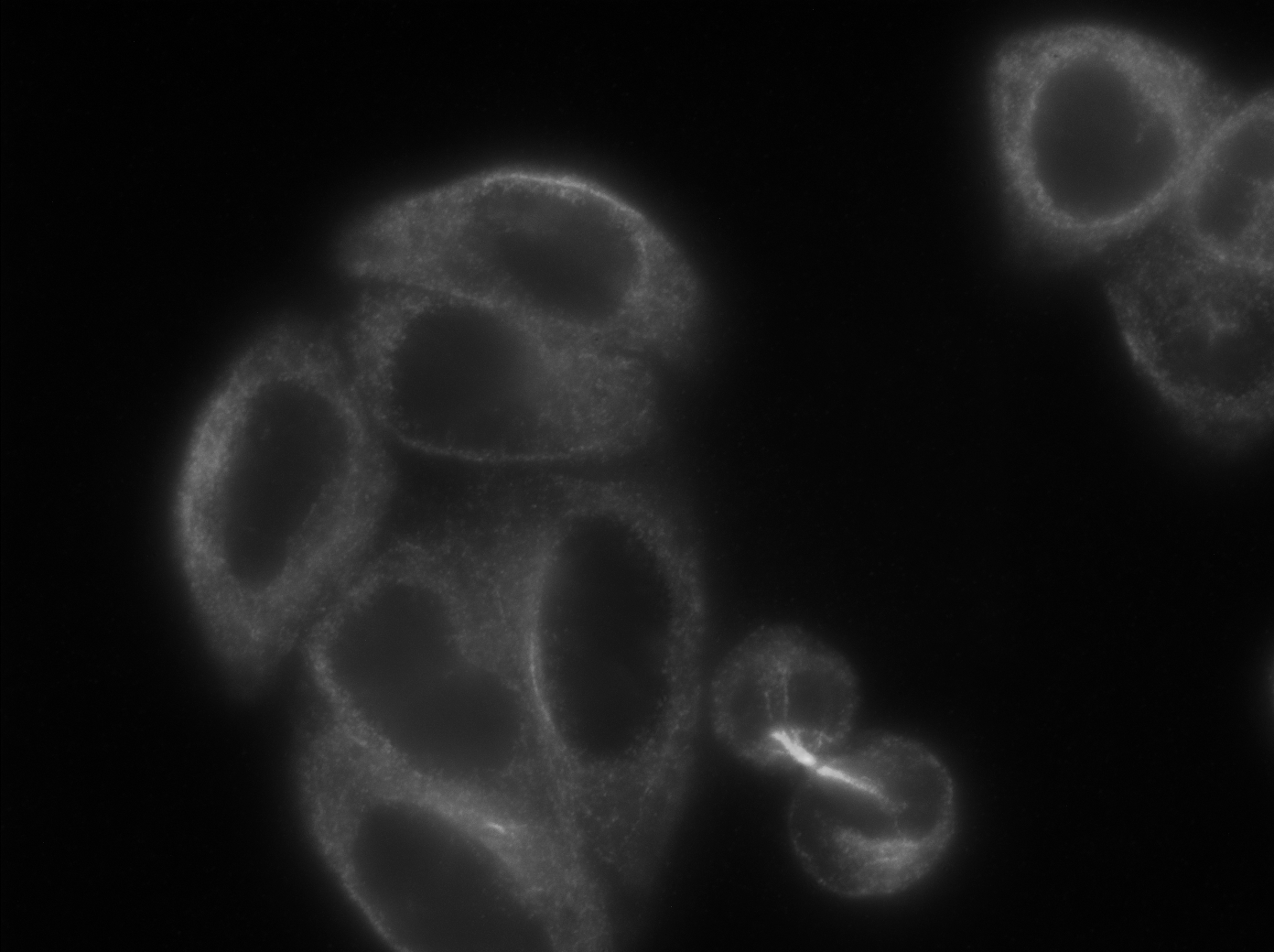

Supplement: Supplementary file 26 — Source data Fig. 7 part 2 [file 44319_2026_742_MOESM26_ESM.zip › Figure 7 Part 2/Fig 7acd Cas9 and TPGS1-ko rGT335 atubulin part 2/TPGS1-KO GT335recomb atub 3-24-25 R1 ET4.Project Maximum Z_XY1742840170_Z0_T0_C2.tif]

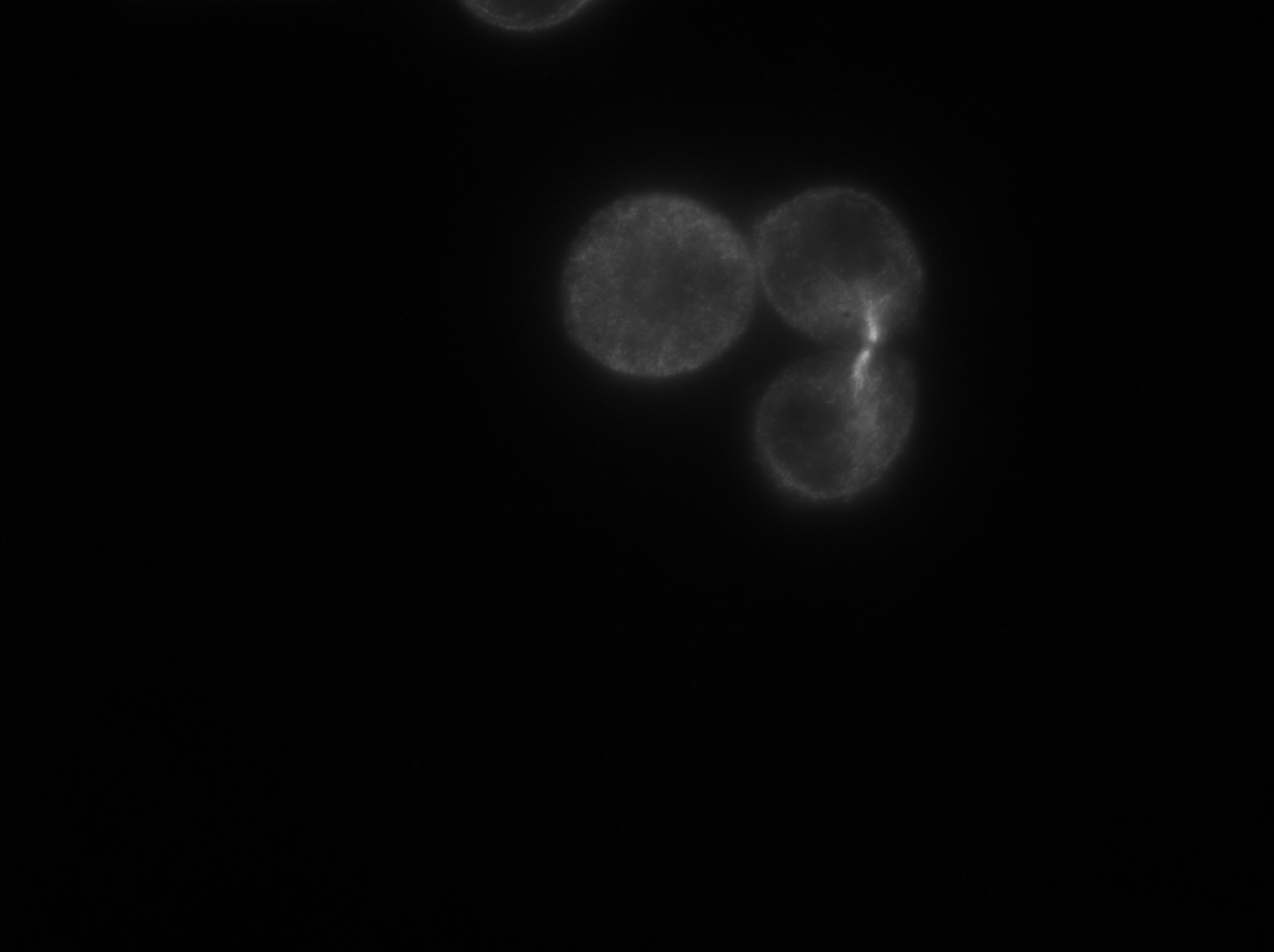

Supplement: Supplementary file 26 — Source data Fig. 7 part 2 [file 44319_2026_742_MOESM26_ESM.zip › Figure 7 Part 2/Fig 7acd Cas9 and TPGS1-ko rGT335 atubulin part 2/TPGS1-KO GT335recomb atub 3-24-25 R2 LT5.Project Maximum Z_XY1742841967_Z0_T0_C2.tif]

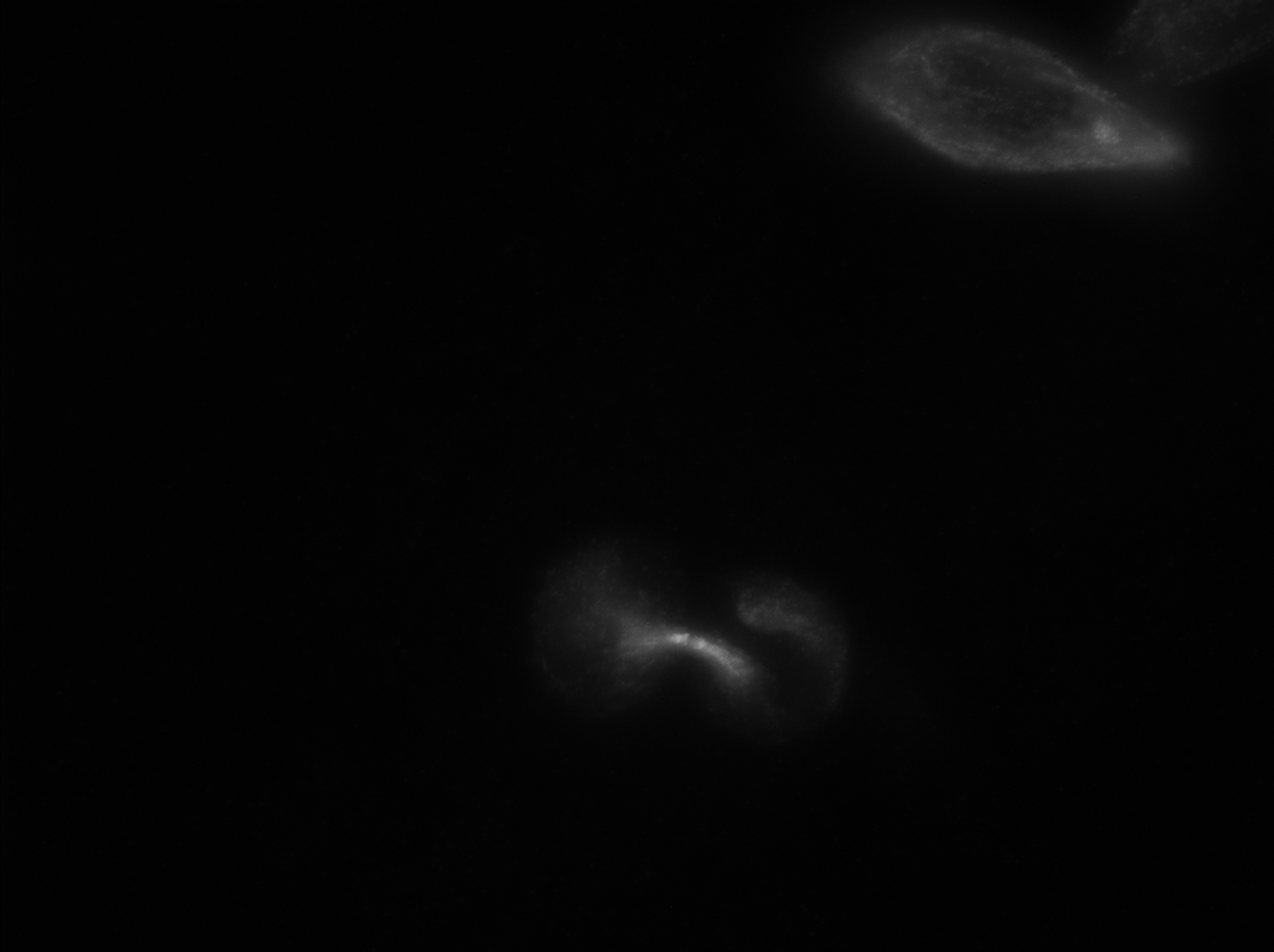

Supplement: Supplementary file 26 — Source data Fig. 7 part 2 [file 44319_2026_742_MOESM26_ESM.zip › Figure 7 Part 2/Fig 7acd Cas9 and TPGS1-ko rGT335 atubulin part 2/TPGS1-KO GT335recomb atub 3-24-25 R2 ET10.Project Maximum Z_XY1742842816_Z0_T0_C2.tif]

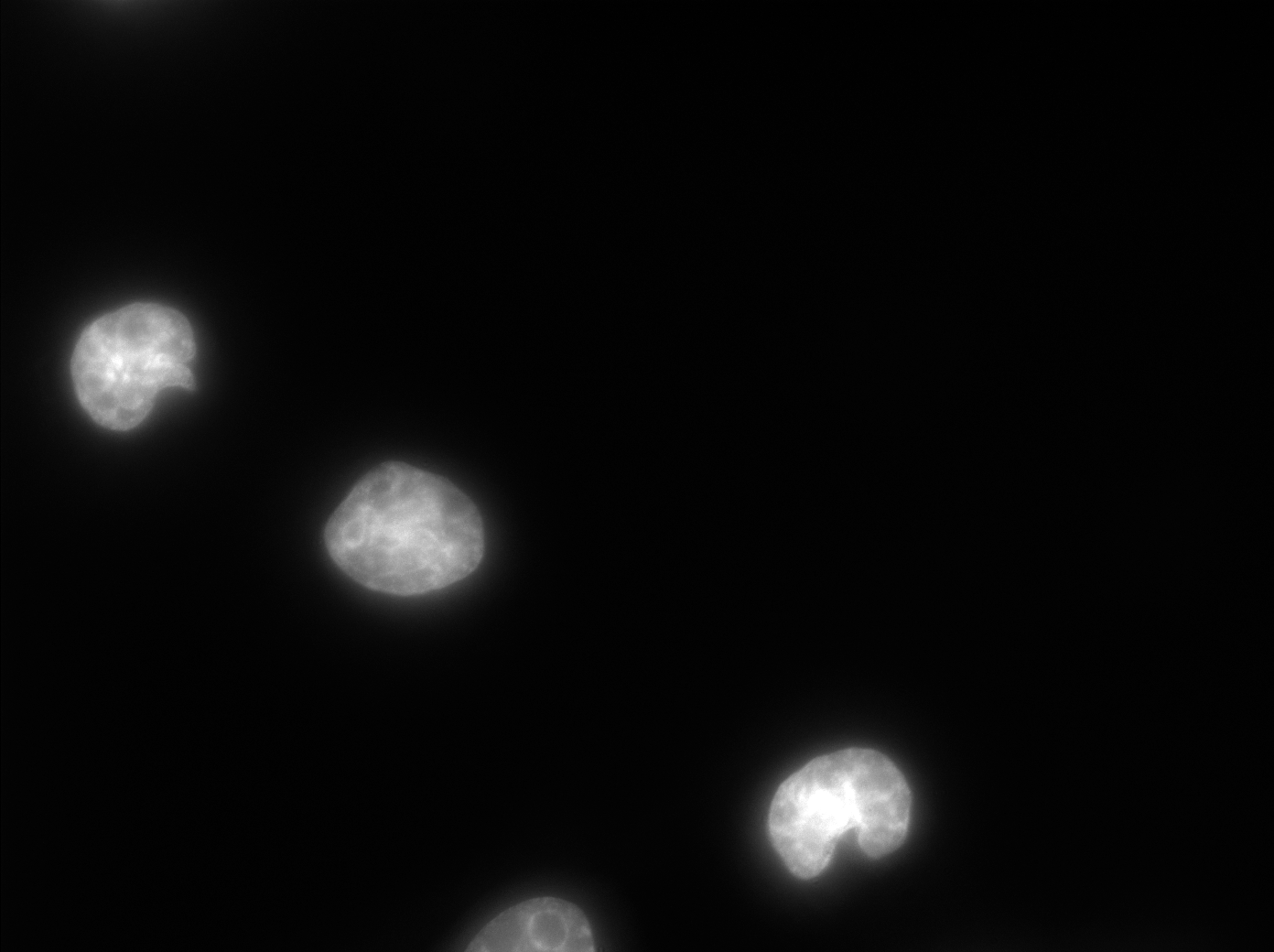

Supplement: Supplementary file 26 — Source data Fig. 7 part 2 [file 44319_2026_742_MOESM26_ESM.zip › Figure 7 Part 2/Fig 7acd Cas9 and TPGS1-ko rGT335 atubulin part 2/TPGS1-KO GT335recomb atub 3-24-25 R3 LT8.Project Maximum Z_XY1742853511_Z0_T0_C0.tif]

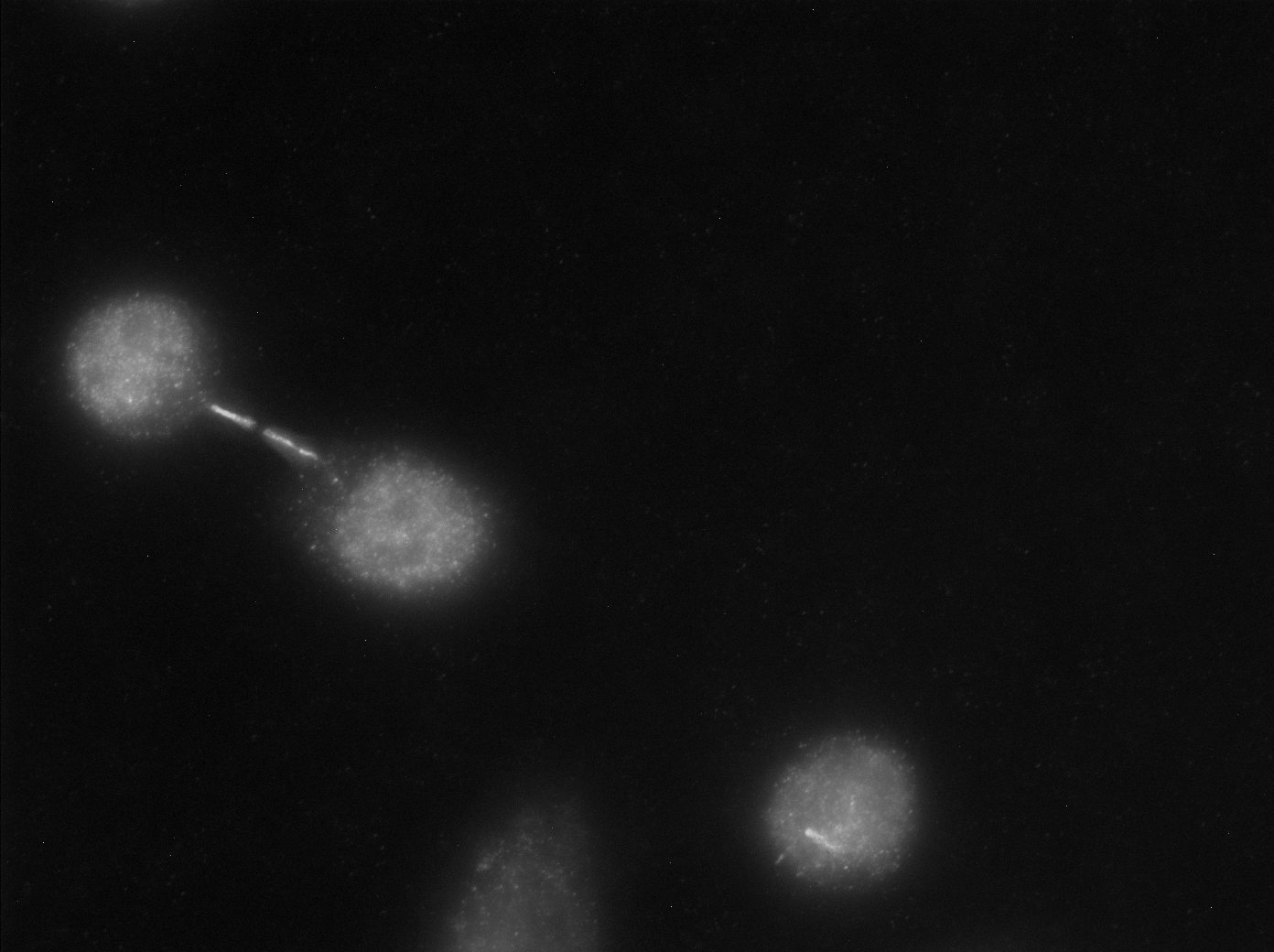

Supplement: Supplementary file 26 — Source data Fig. 7 part 2 [file 44319_2026_742_MOESM26_ESM.zip › Figure 7 Part 2/Fig 7acd Cas9 and TPGS1-ko rGT335 atubulin part 2/TPGS1-KO GT335recomb atub 3-24-25 R3 LT8.Project Maximum Z_XY1742853511_Z0_T0_C1.tif]

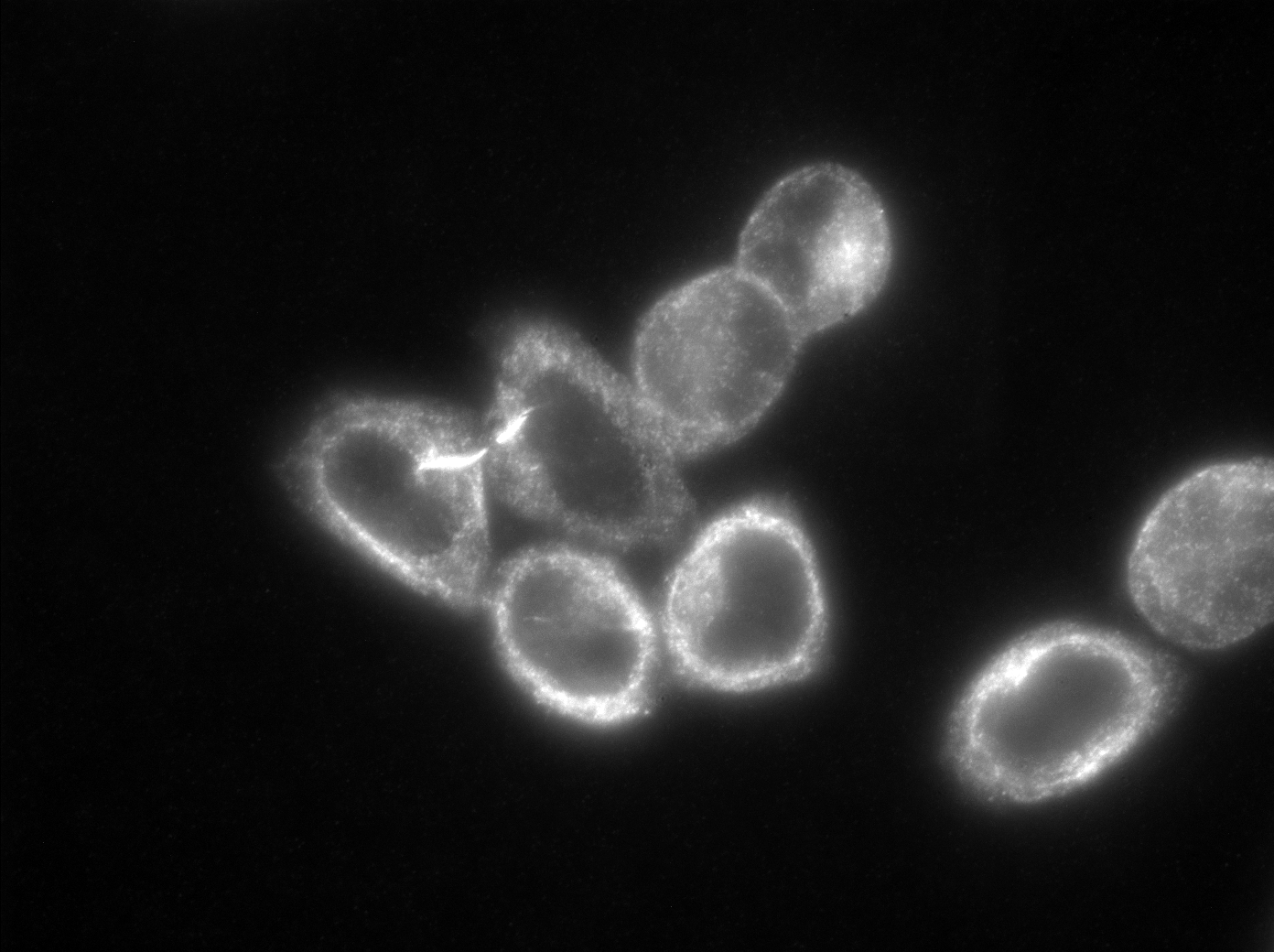

Supplement: Supplementary file 26 — Source data Fig. 7 part 2 [file 44319_2026_742_MOESM26_ESM.zip › Figure 7 Part 2/Fig 7acd Cas9 and TPGS1-ko rGT335 atubulin part 2/TPGS1-KO GT335recomb atub 3-24-25 R3 LT9.Project Maximum Z_XY1742853596_Z0_T0_C2.tif]

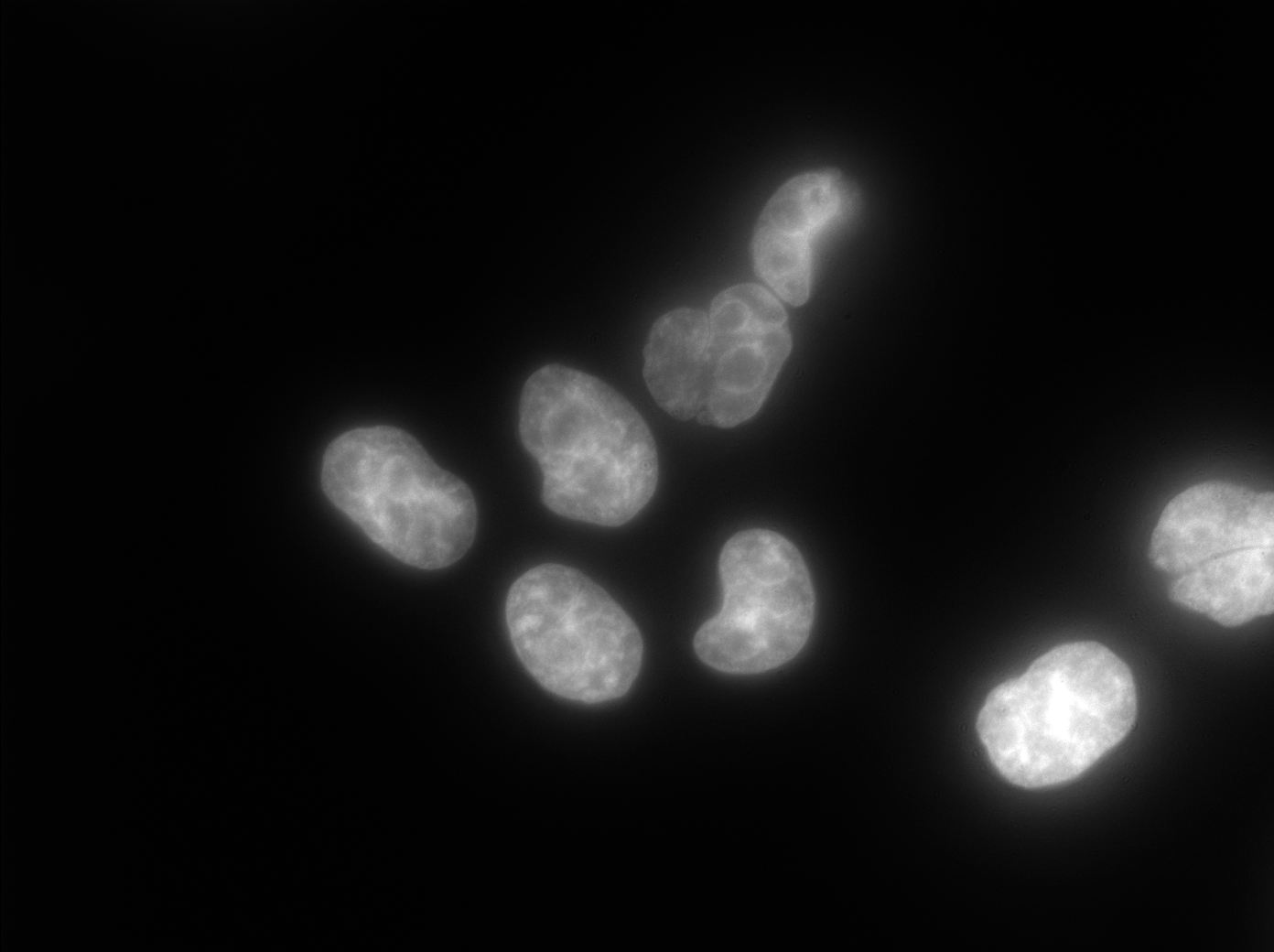

Supplement: Supplementary file 26 — Source data Fig. 7 part 2 [file 44319_2026_742_MOESM26_ESM.zip › Figure 7 Part 2/Fig 7acd Cas9 and TPGS1-ko rGT335 atubulin part 2/TPGS1-KO GT335recomb atub 3-24-25 R3 LT9.Project Maximum Z_XY1742853596_Z0_T0_C0.tif]

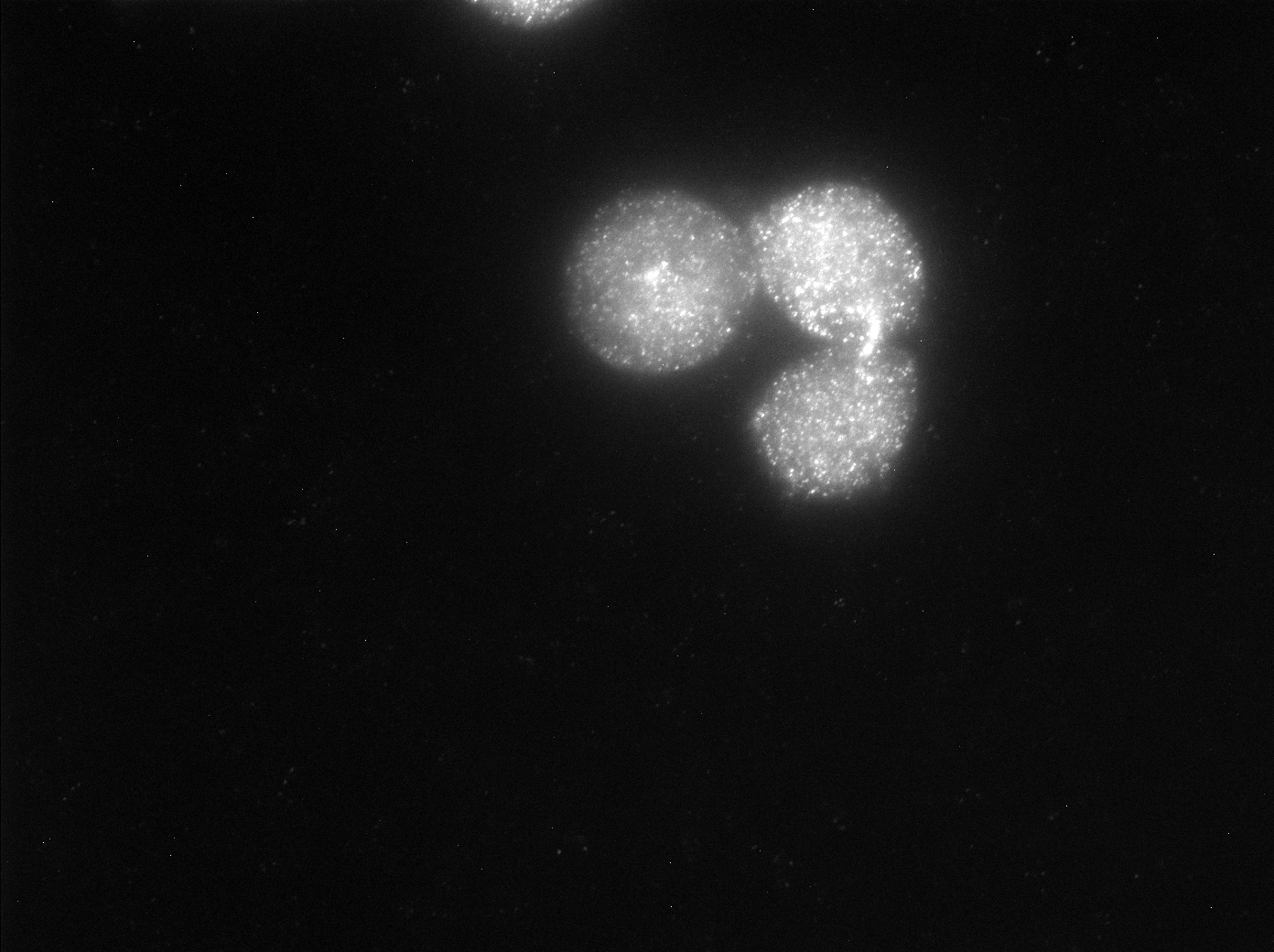

Supplement: Supplementary file 26 — Source data Fig. 7 part 2 [file 44319_2026_742_MOESM26_ESM.zip › Figure 7 Part 2/Fig 7acd Cas9 and TPGS1-ko rGT335 atubulin part 2/TPGS1-KO GT335recomb atub 3-24-25 R2 LT5.Project Maximum Z_XY1742841967_Z0_T0_C1.tif]

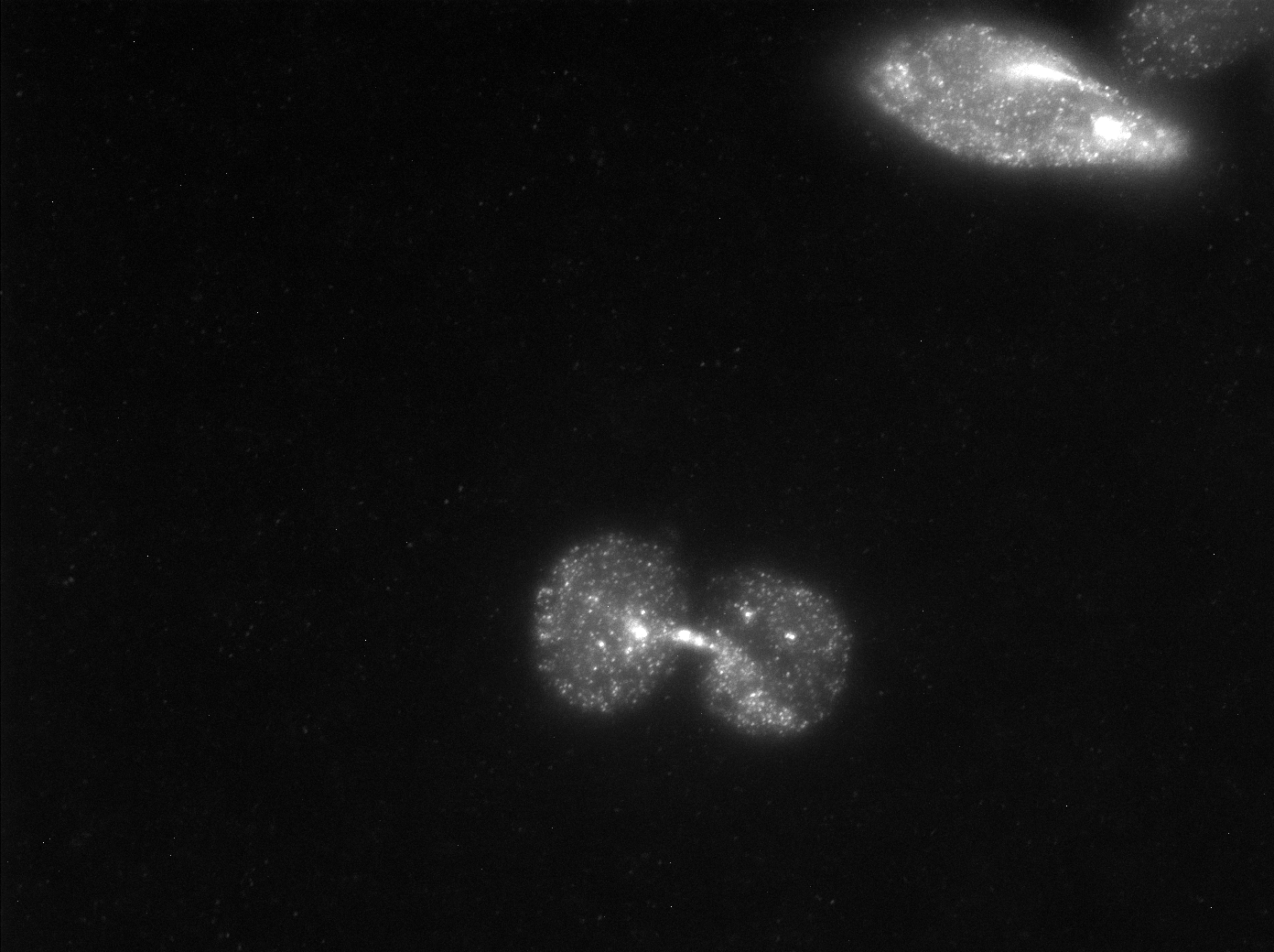

Supplement: Supplementary file 26 — Source data Fig. 7 part 2 [file 44319_2026_742_MOESM26_ESM.zip › Figure 7 Part 2/Fig 7acd Cas9 and TPGS1-ko rGT335 atubulin part 2/TPGS1-KO GT335recomb atub 3-24-25 R2 ET10.Project Maximum Z_XY1742842816_Z0_T0_C1.tif]

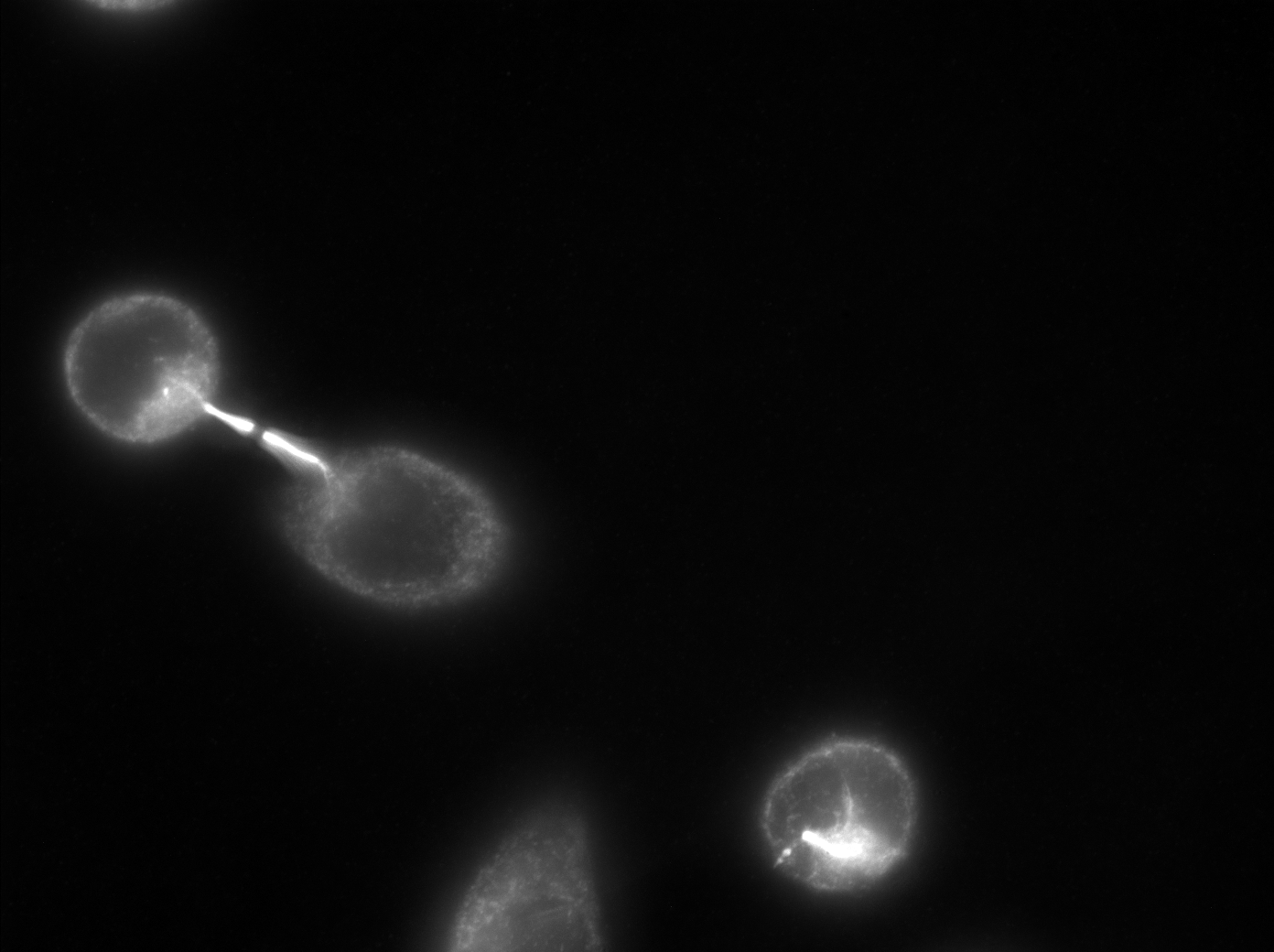

Supplement: Supplementary file 26 — Source data Fig. 7 part 2 [file 44319_2026_742_MOESM26_ESM.zip › Figure 7 Part 2/Fig 7acd Cas9 and TPGS1-ko rGT335 atubulin part 2/TPGS1-KO GT335recomb atub 3-24-25 R3 LT8.Project Maximum Z_XY1742853511_Z0_T0_C2.tif]

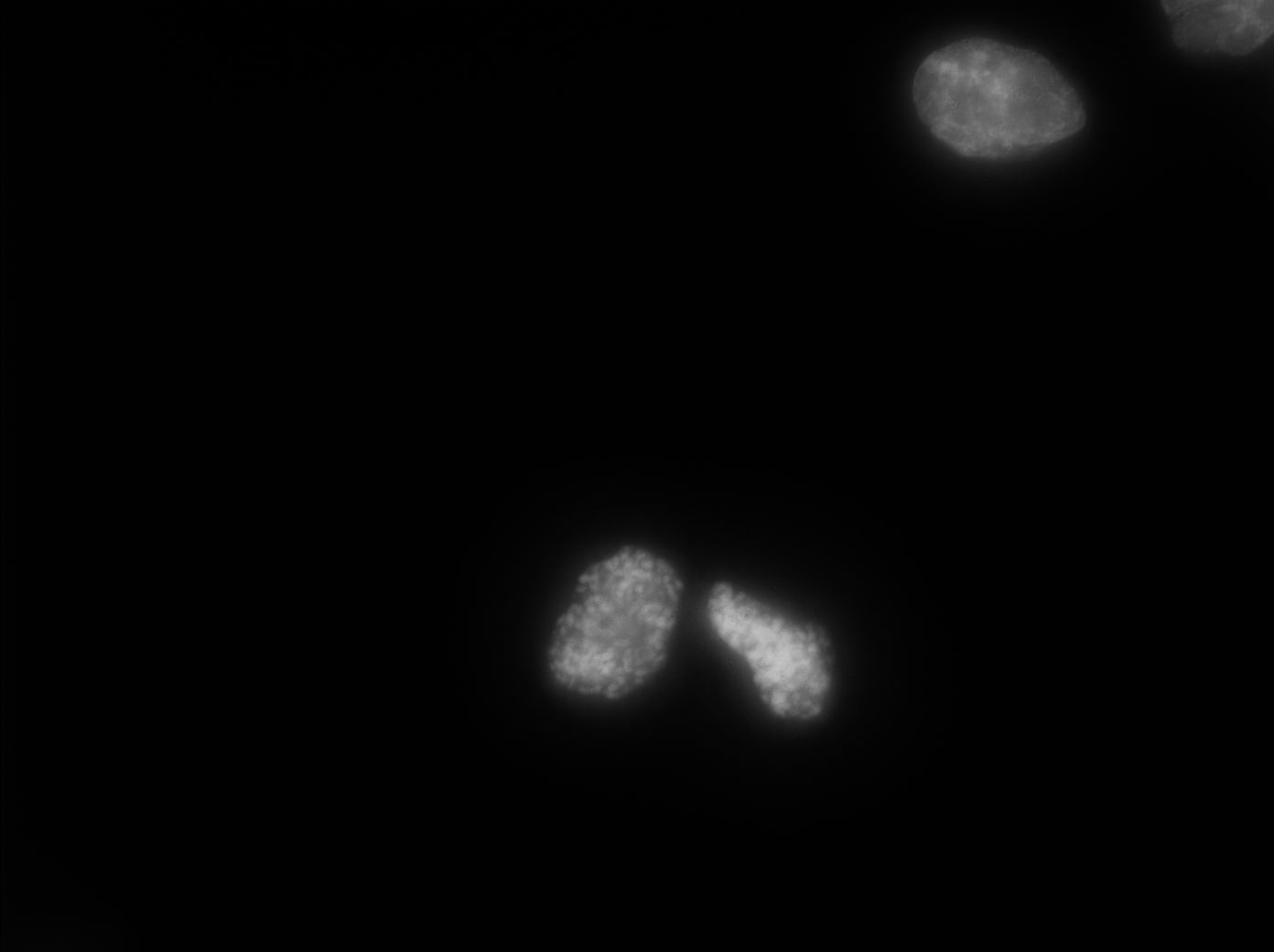

Supplement: Supplementary file 26 — Source data Fig. 7 part 2 [file 44319_2026_742_MOESM26_ESM.zip › Figure 7 Part 2/Fig 7acd Cas9 and TPGS1-ko rGT335 atubulin part 2/TPGS1-KO GT335recomb atub 3-24-25 R2 ET10.Project Maximum Z_XY1742842816_Z0_T0_C0.tif]

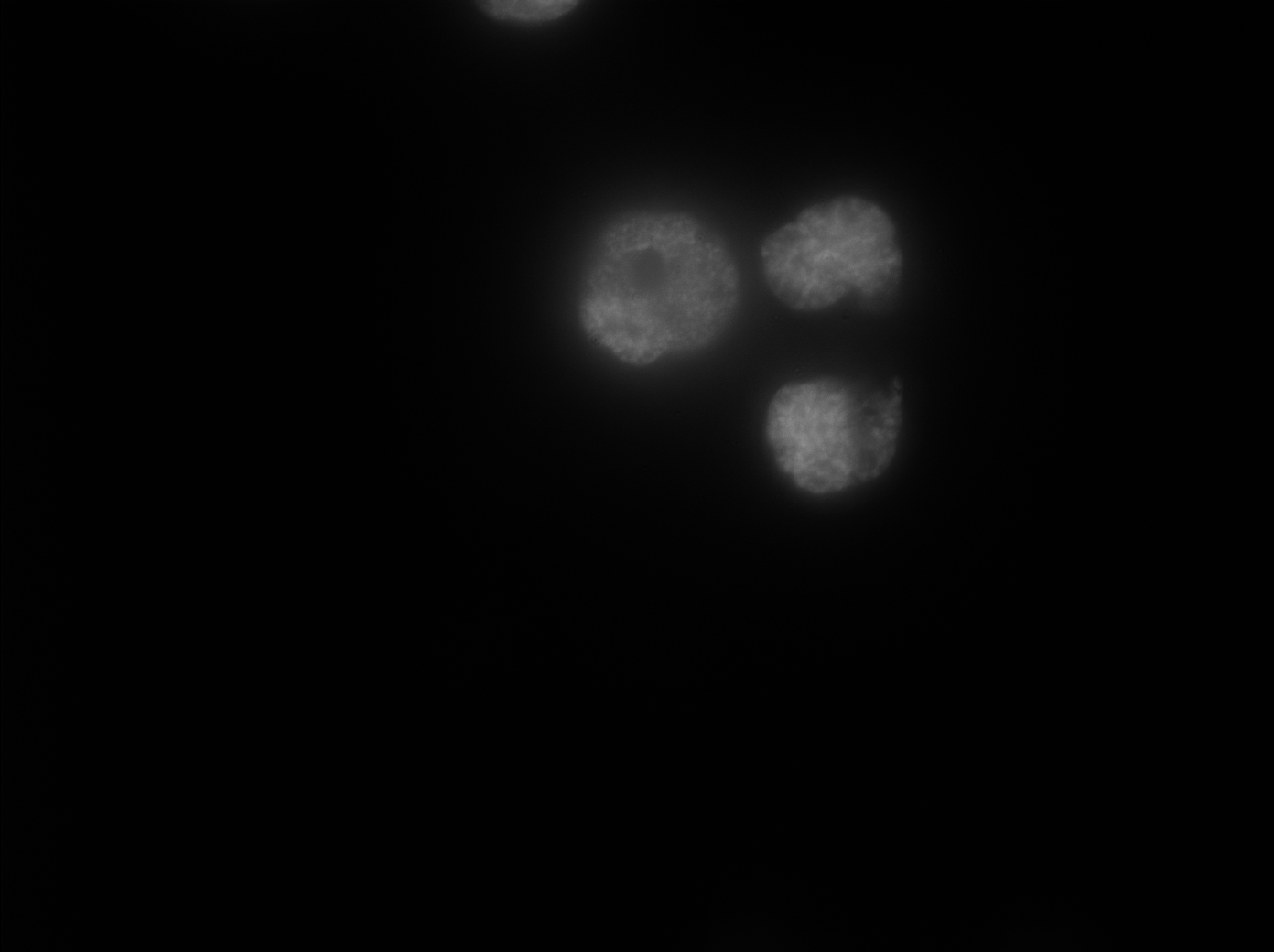

Supplement: Supplementary file 26 — Source data Fig. 7 part 2 [file 44319_2026_742_MOESM26_ESM.zip › Figure 7 Part 2/Fig 7acd Cas9 and TPGS1-ko rGT335 atubulin part 2/TPGS1-KO GT335recomb atub 3-24-25 R2 LT5.Project Maximum Z_XY1742841967_Z0_T0_C0.tif]

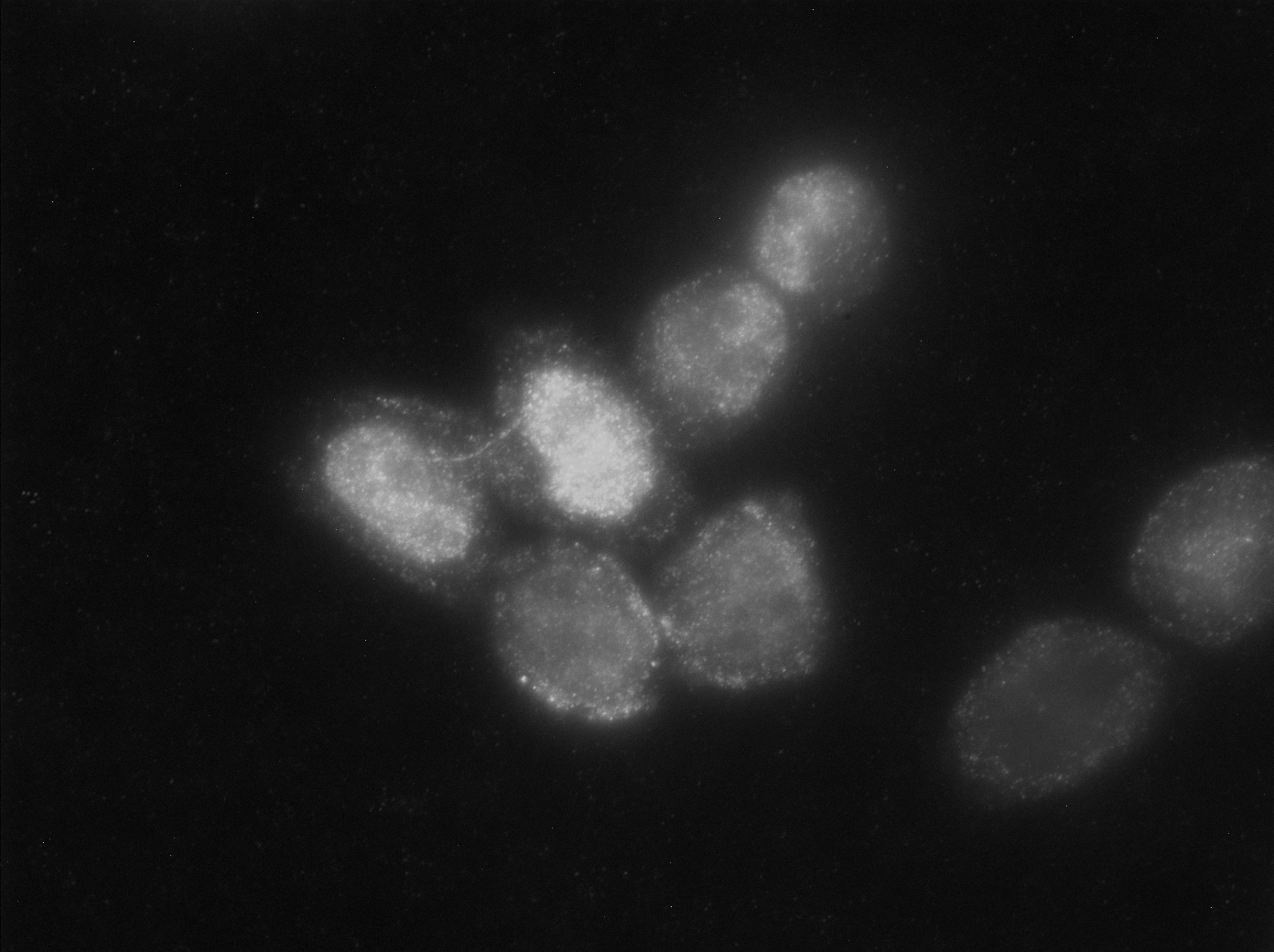

Supplement: Supplementary file 26 — Source data Fig. 7 part 2 [file 44319_2026_742_MOESM26_ESM.zip › Figure 7 Part 2/Fig 7acd Cas9 and TPGS1-ko rGT335 atubulin part 2/TPGS1-KO GT335recomb atub 3-24-25 R3 LT9.Project Maximum Z_XY1742853596_Z0_T0_C1.tif]

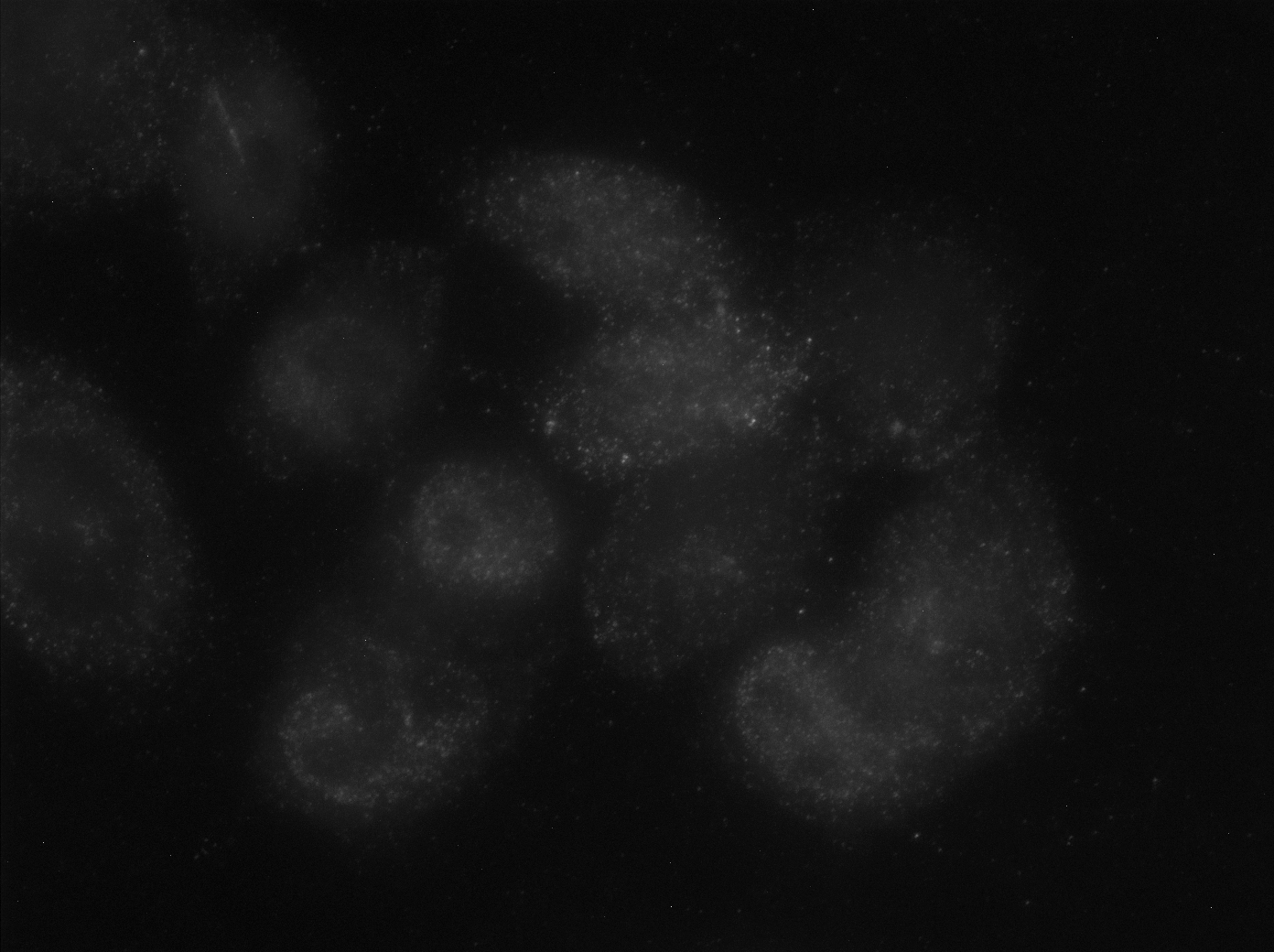

Supplement: Supplementary file 26 — Source data Fig. 7 part 2 [file 44319_2026_742_MOESM26_ESM.zip › Figure 7 Part 2/Fig 7acd Cas9 and TPGS1-ko rGT335 atubulin part 2/TPGS1-KO GT335recomb atub 3-24-25 R1 LT4.Project Maximum Z_XY1742839439_Z0_T0_C1.tif]

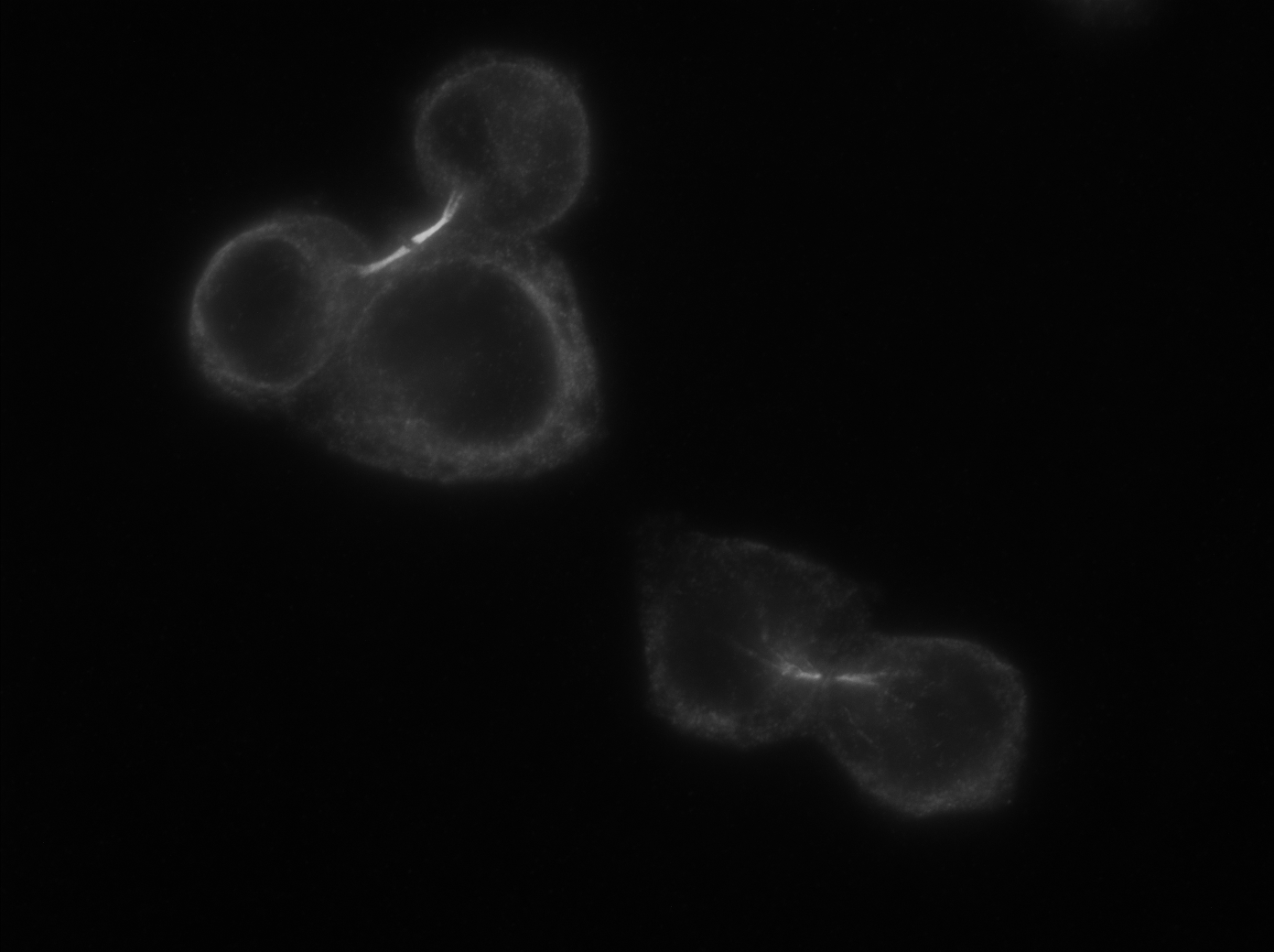

Supplement: Supplementary file 26 — Source data Fig. 7 part 2 [file 44319_2026_742_MOESM26_ESM.zip › Figure 7 Part 2/Fig 7acd Cas9 and TPGS1-ko rGT335 atubulin part 2/TPGS1-KO GT335recomb atub 3-24-25 R2 LT9LT10.Project Maximum Z_XY1742842932_Z0_T0_C2.tif]

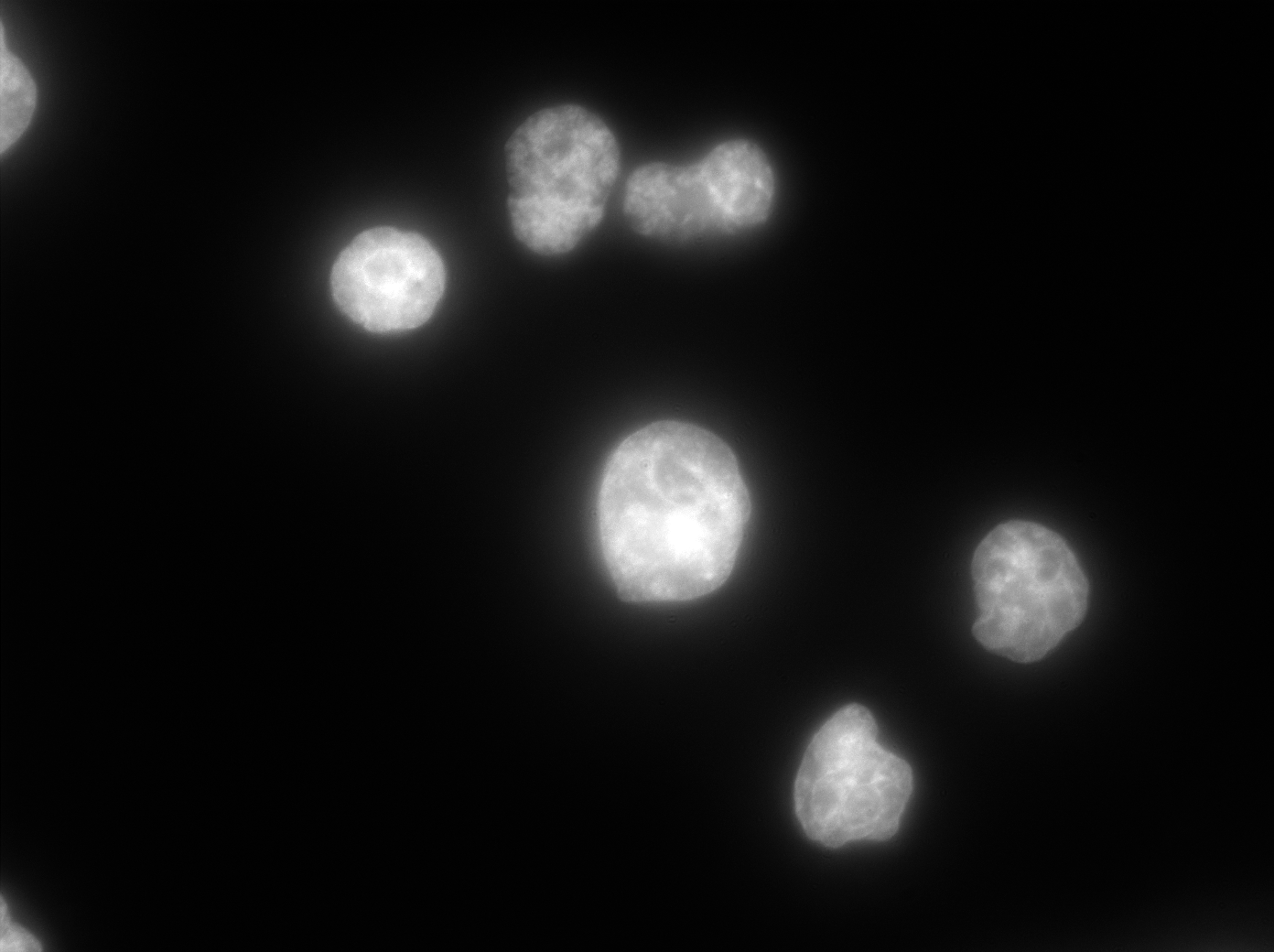

Supplement: Supplementary file 26 — Source data Fig. 7 part 2 [file 44319_2026_742_MOESM26_ESM.zip › Figure 7 Part 2/Fig 7acd Cas9 and TPGS1-ko rGT335 atubulin part 2/TPGS1-KO GT335recomb atub 3-24-25 R3 LT10 ET7.Project Maximum Z_XY1742853699_Z0_T0_C0.tif]

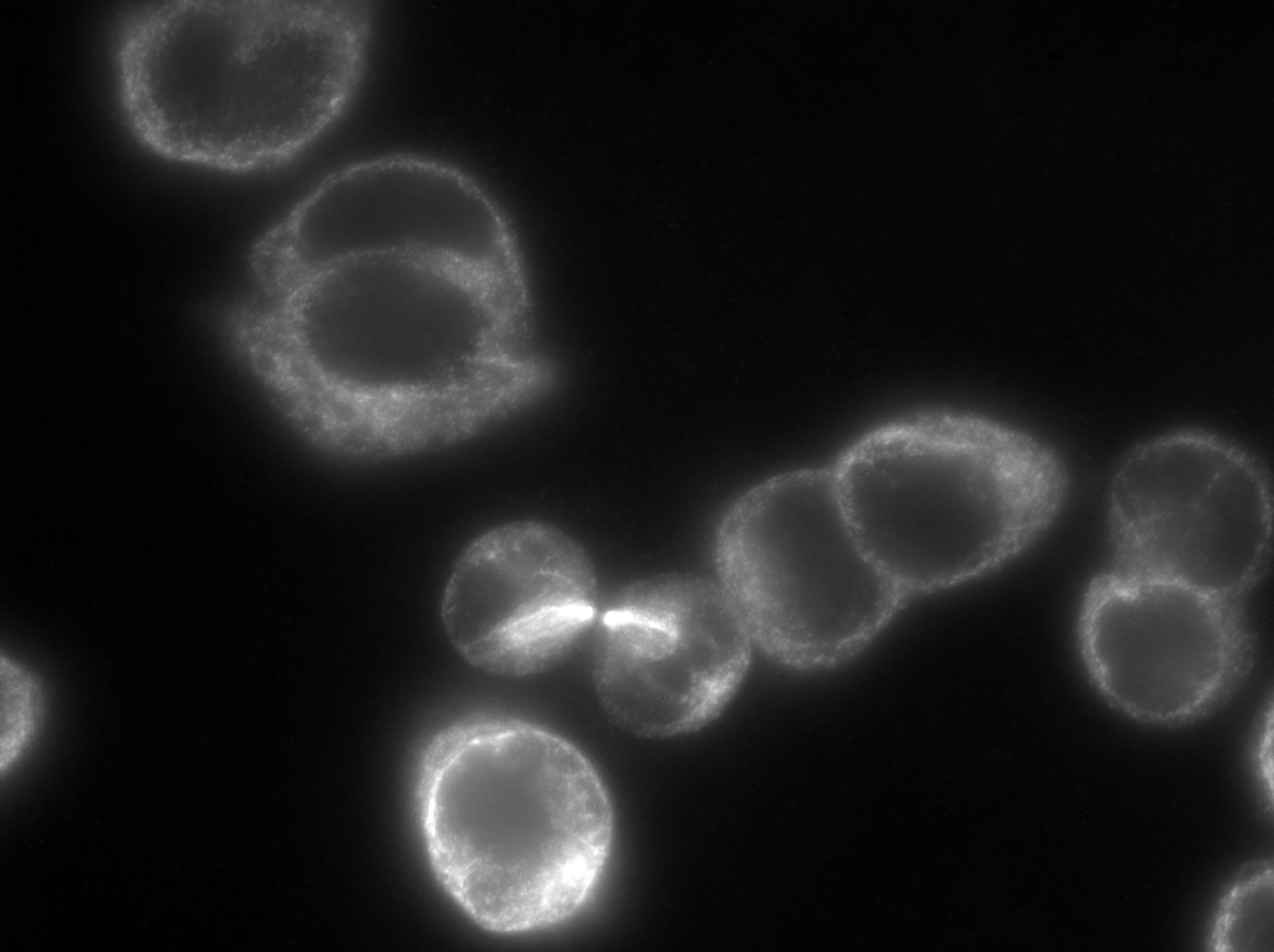

Supplement: Supplementary file 26 — Source data Fig. 7 part 2 [file 44319_2026_742_MOESM26_ESM.zip › Figure 7 Part 2/Fig 7acd Cas9 and TPGS1-ko rGT335 atubulin part 2/TPGS1-KO GT335recomb atub 3-24-25 R3 ET6.Project Maximum Z_XY1742853334_Z0_T0_C2.tif]

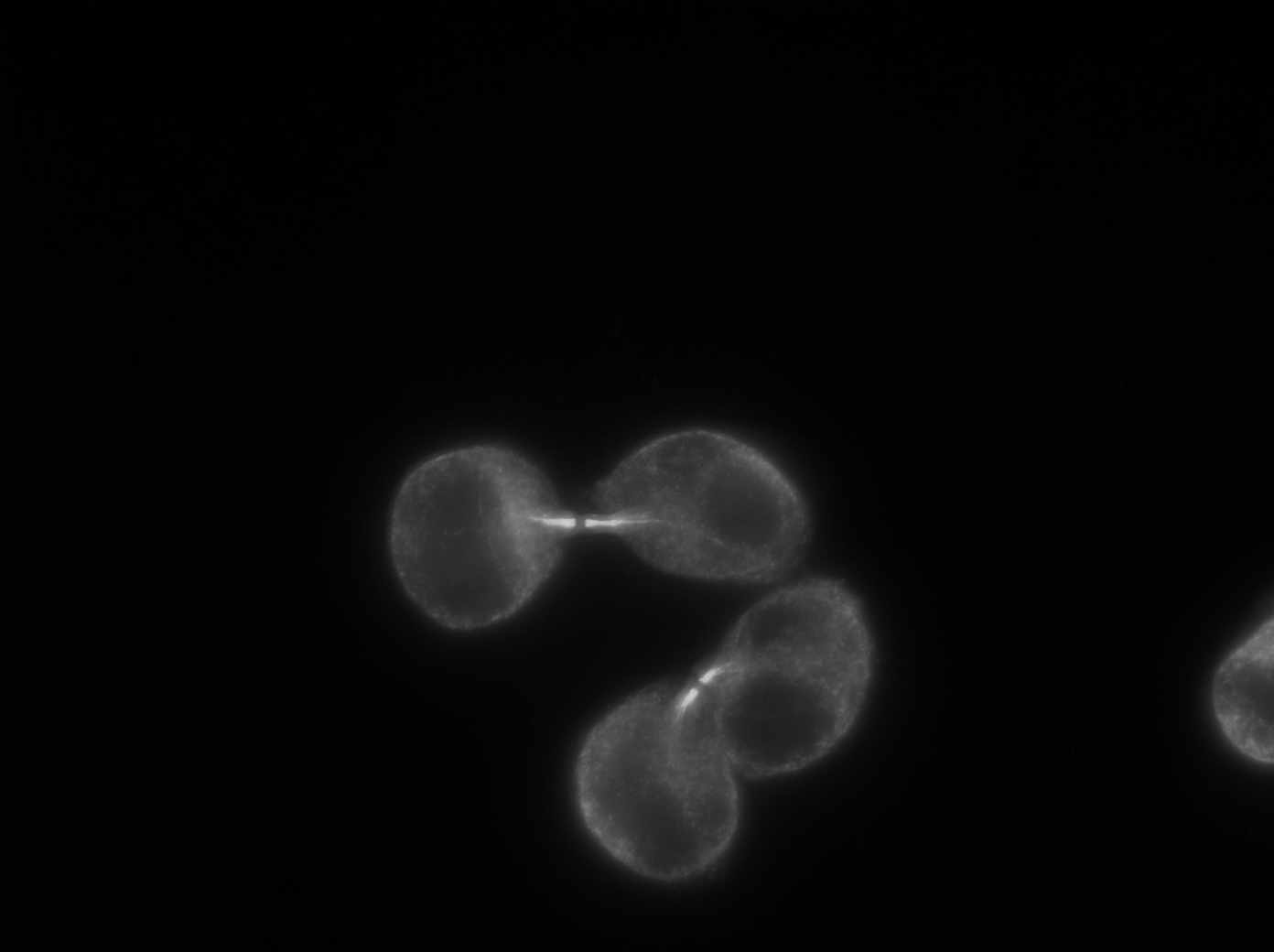

Supplement: Supplementary file 26 — Source data Fig. 7 part 2 [file 44319_2026_742_MOESM26_ESM.zip › Figure 7 Part 2/Fig 7acd Cas9 and TPGS1-ko rGT335 atubulin part 2/TPGS1-KO GT335recomb atub 3-24-25 R2 LT1LT2.Project Maximum Z_XY1742841626_Z0_T0_C2.tif]

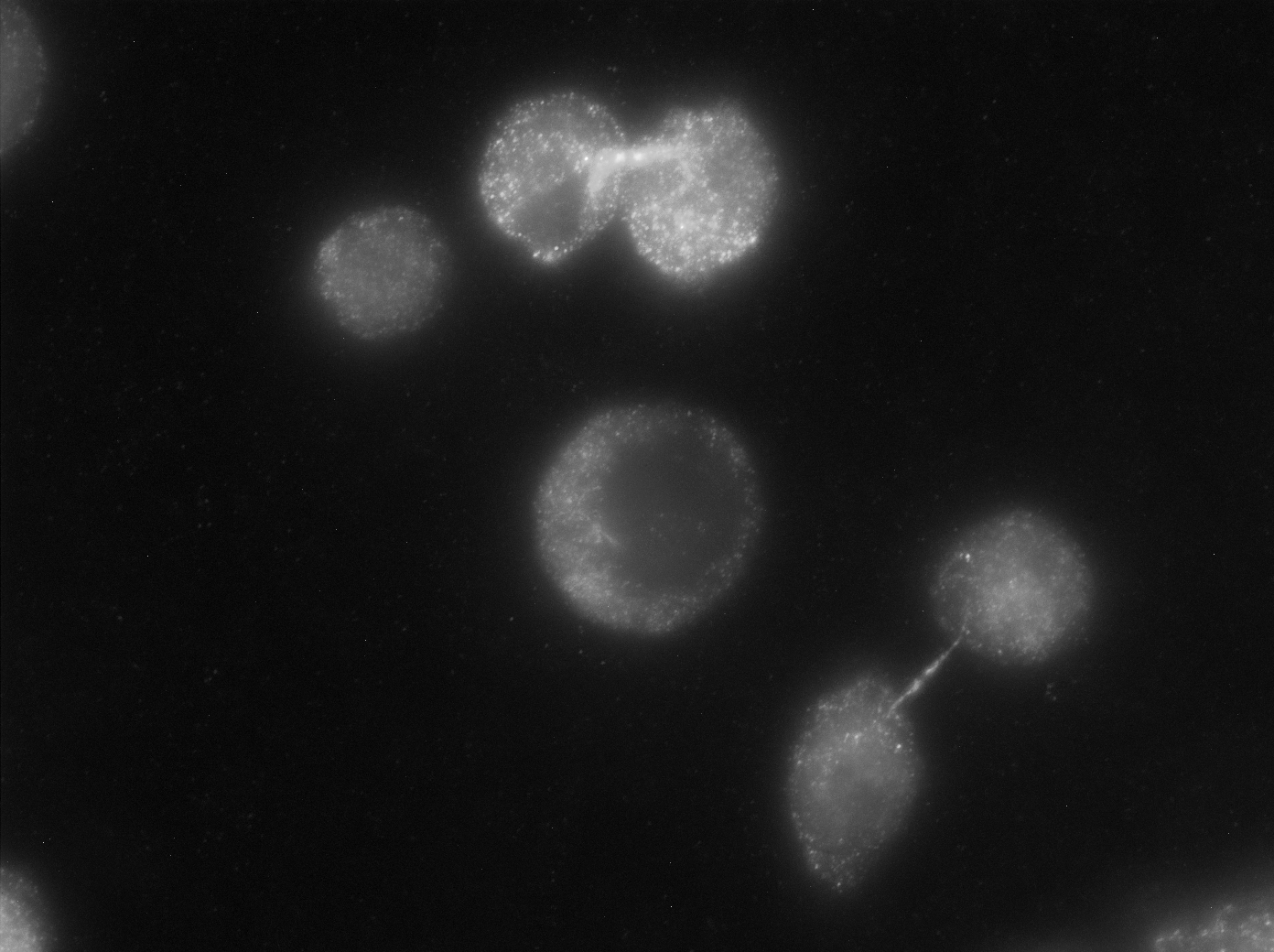

Supplement: Supplementary file 26 — Source data Fig. 7 part 2 [file 44319_2026_742_MOESM26_ESM.zip › Figure 7 Part 2/Fig 7acd Cas9 and TPGS1-ko rGT335 atubulin part 2/TPGS1-KO GT335recomb atub 3-24-25 R3 LT10 ET7.Project Maximum Z_XY1742853699_Z0_T0_C1.tif]

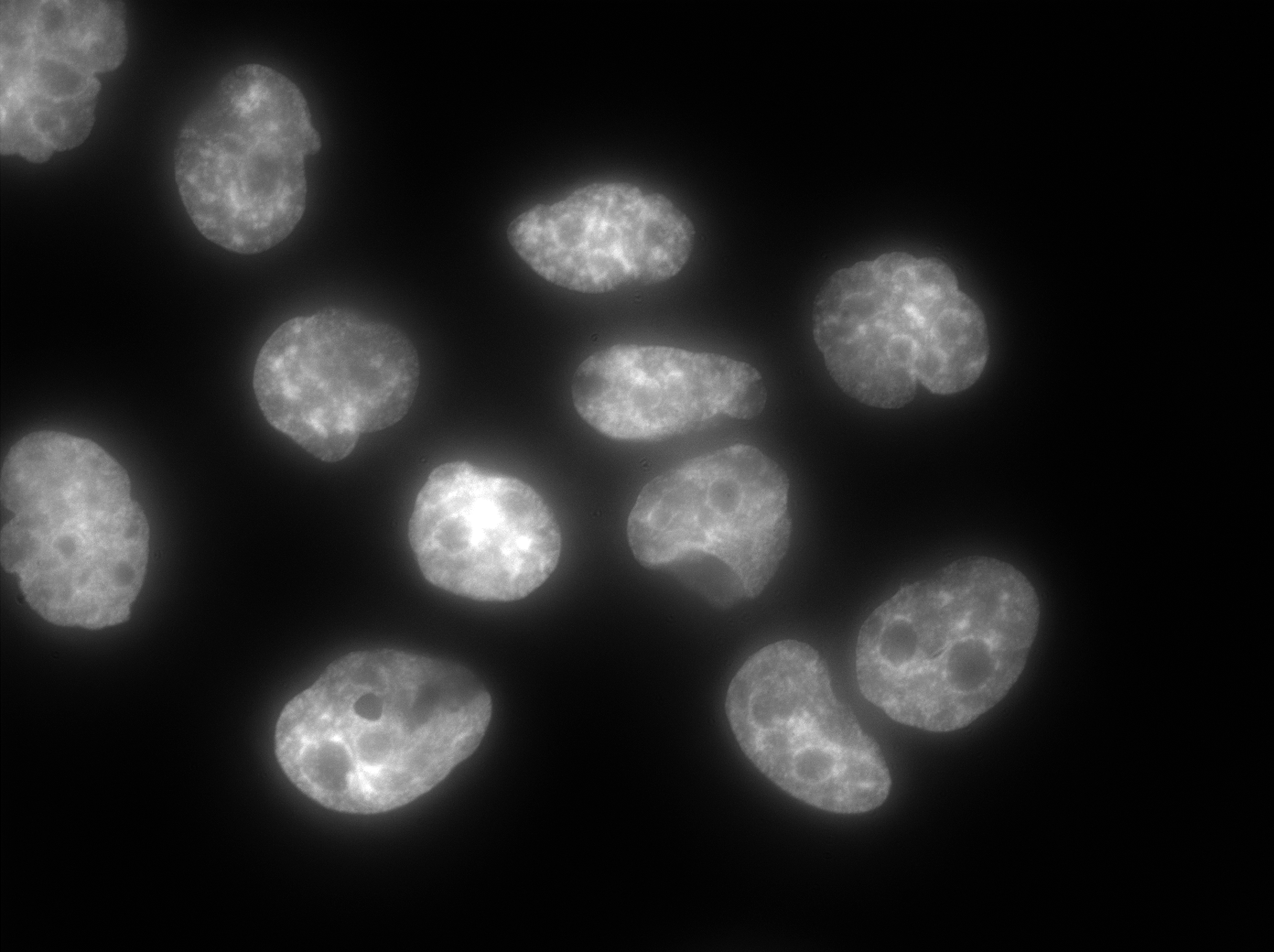

Supplement: Supplementary file 26 — Source data Fig. 7 part 2 [file 44319_2026_742_MOESM26_ESM.zip › Figure 7 Part 2/Fig 7acd Cas9 and TPGS1-ko rGT335 atubulin part 2/TPGS1-KO GT335recomb atub 3-24-25 R1 LT4.Project Maximum Z_XY1742839439_Z0_T0_C0.tif]

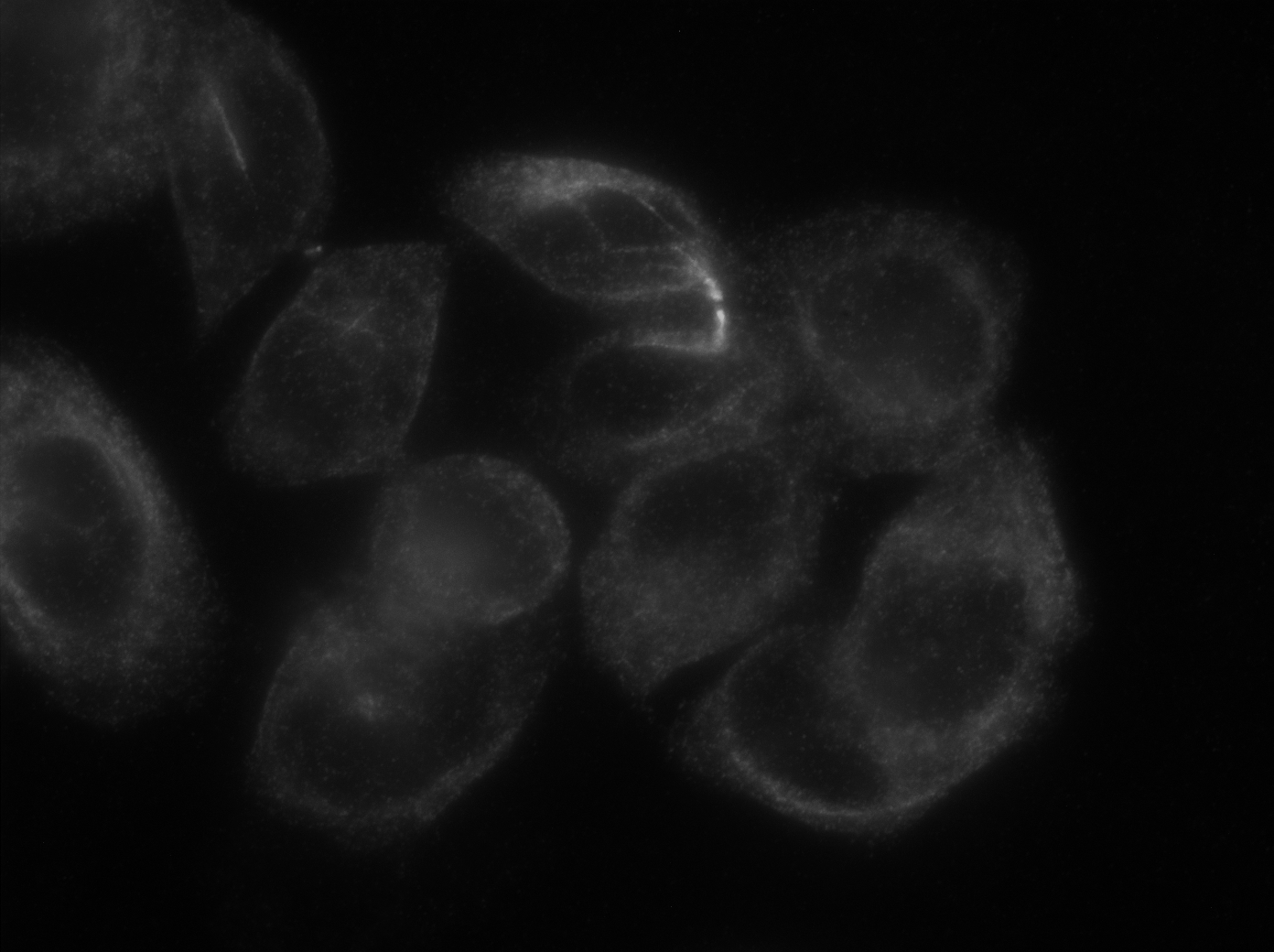

Supplement: Supplementary file 26 — Source data Fig. 7 part 2 [file 44319_2026_742_MOESM26_ESM.zip › Figure 7 Part 2/Fig 7acd Cas9 and TPGS1-ko rGT335 atubulin part 2/TPGS1-KO GT335recomb atub 3-24-25 R1 LT4.Project Maximum Z_XY1742839439_Z0_T0_C2.tif]

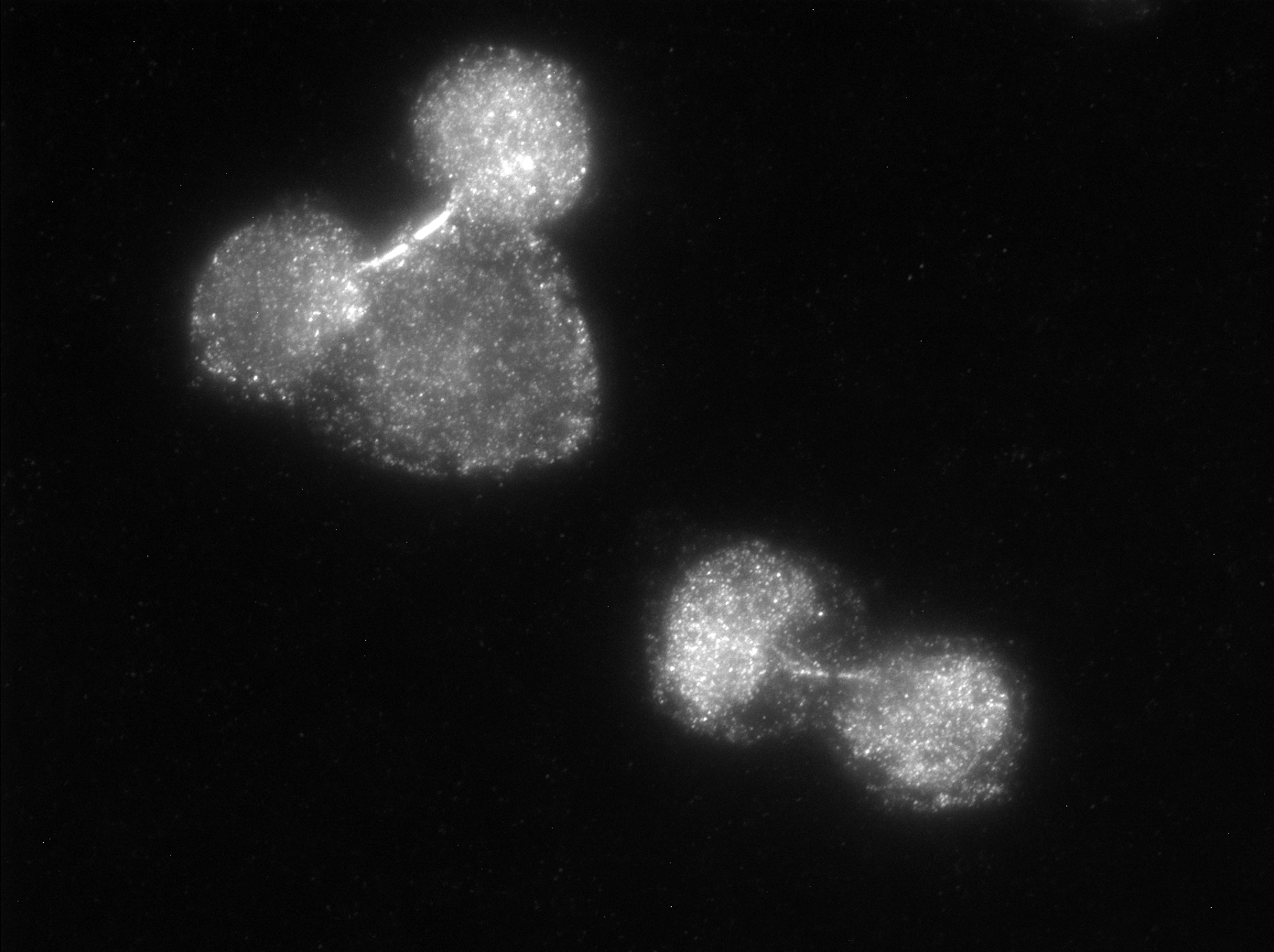

Supplement: Supplementary file 26 — Source data Fig. 7 part 2 [file 44319_2026_742_MOESM26_ESM.zip › Figure 7 Part 2/Fig 7acd Cas9 and TPGS1-ko rGT335 atubulin part 2/TPGS1-KO GT335recomb atub 3-24-25 R2 LT9LT10.Project Maximum Z_XY1742842932_Z0_T0_C1.tif]

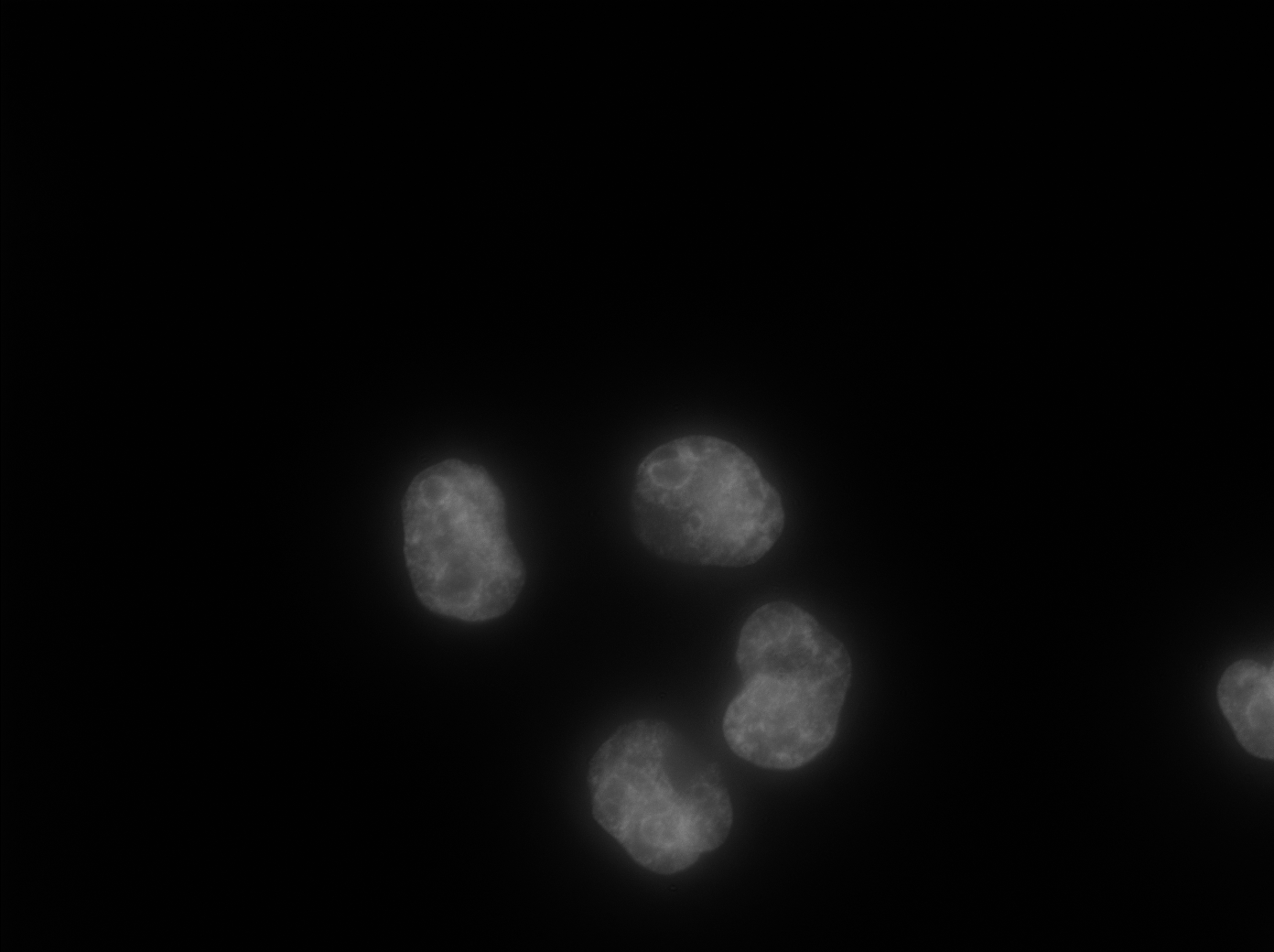

Supplement: Supplementary file 26 — Source data Fig. 7 part 2 [file 44319_2026_742_MOESM26_ESM.zip › Figure 7 Part 2/Fig 7acd Cas9 and TPGS1-ko rGT335 atubulin part 2/TPGS1-KO GT335recomb atub 3-24-25 R2 LT1LT2.Project Maximum Z_XY1742841626_Z0_T0_C0.tif]

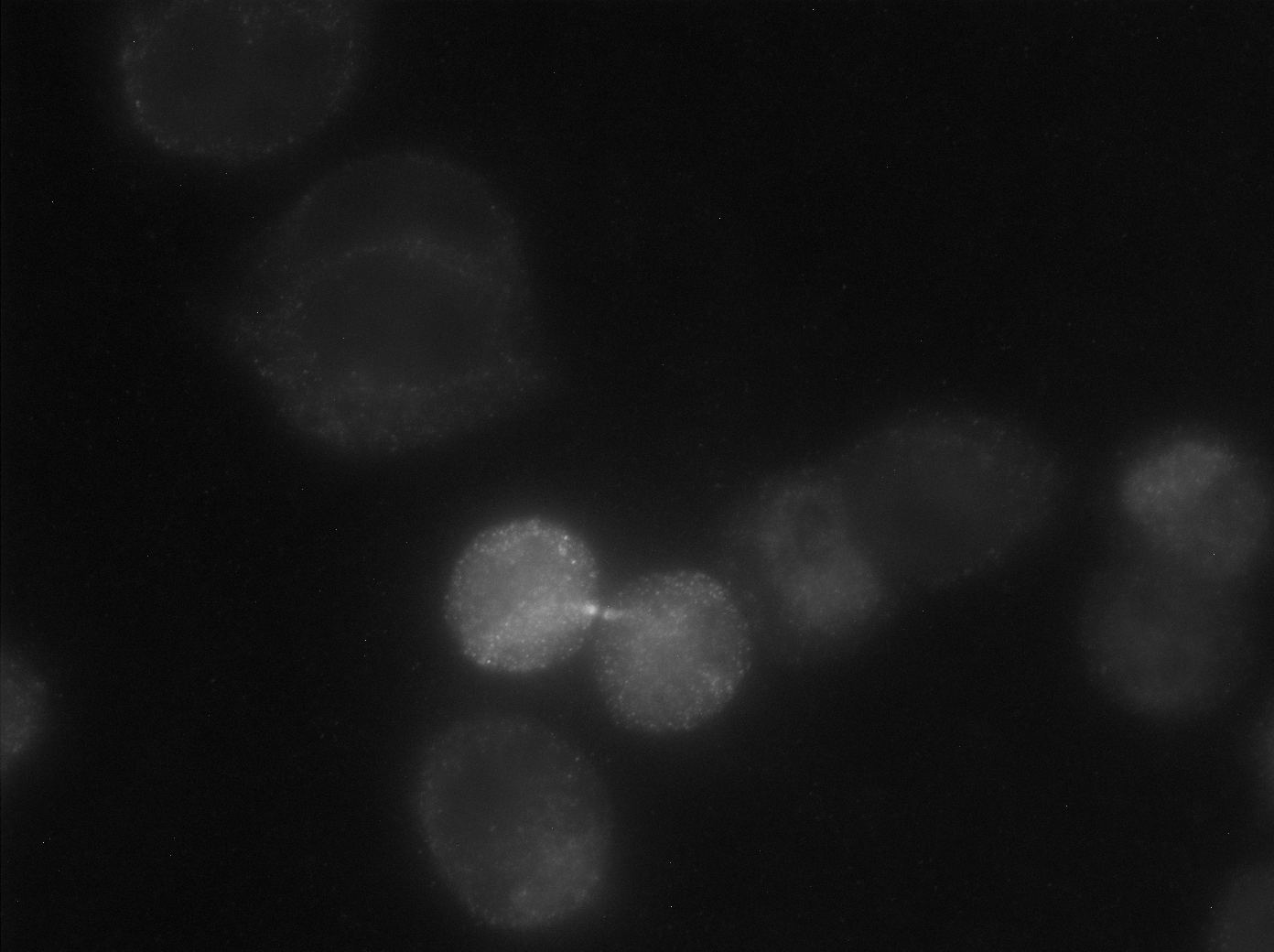

Supplement: Supplementary file 26 — Source data Fig. 7 part 2 [file 44319_2026_742_MOESM26_ESM.zip › Figure 7 Part 2/Fig 7acd Cas9 and TPGS1-ko rGT335 atubulin part 2/TPGS1-KO GT335recomb atub 3-24-25 R3 ET6.Project Maximum Z_XY1742853334_Z0_T0_C1.tif]

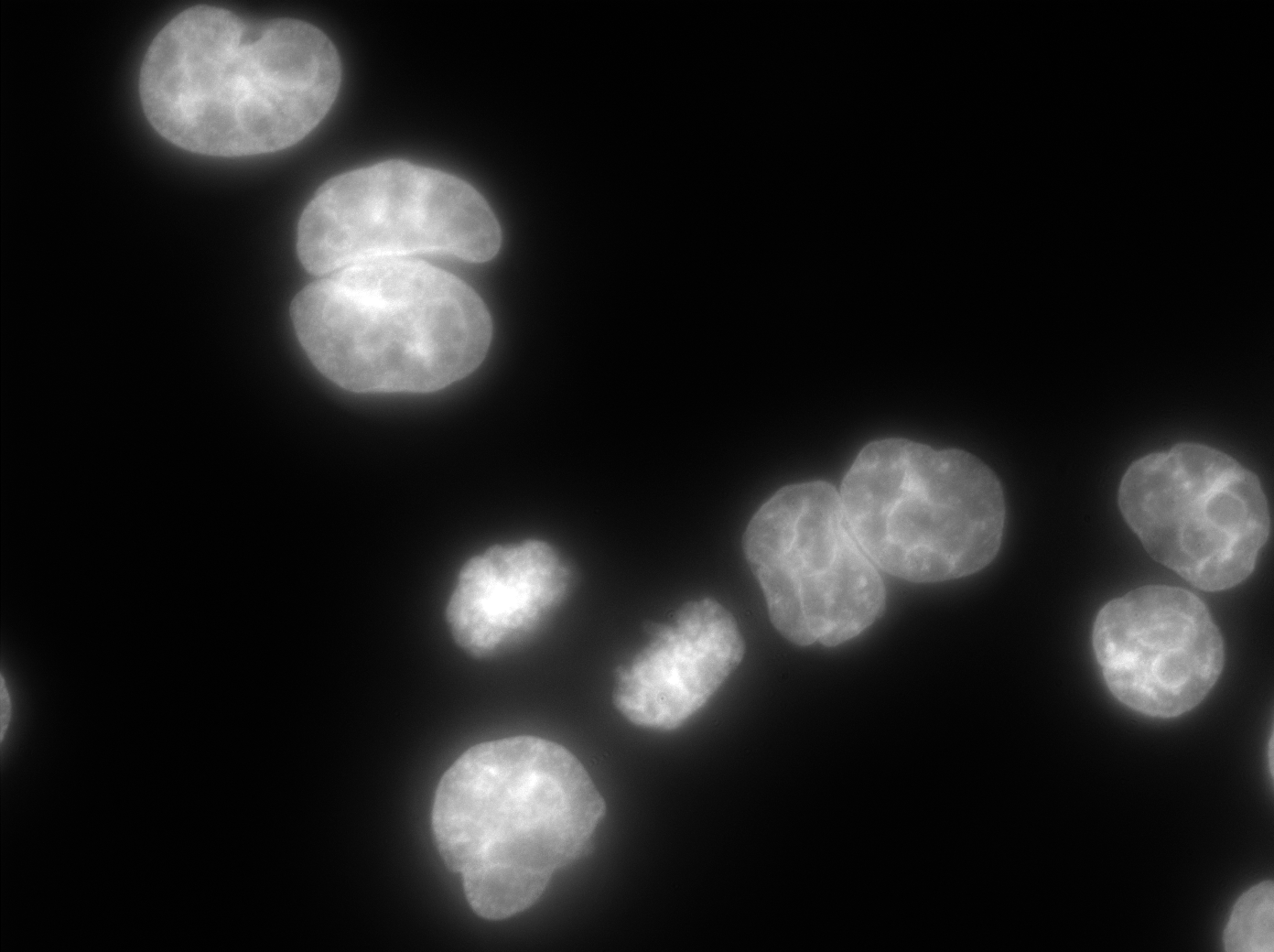

Supplement: Supplementary file 26 — Source data Fig. 7 part 2 [file 44319_2026_742_MOESM26_ESM.zip › Figure 7 Part 2/Fig 7acd Cas9 and TPGS1-ko rGT335 atubulin part 2/TPGS1-KO GT335recomb atub 3-24-25 R3 ET6.Project Maximum Z_XY1742853334_Z0_T0_C0.tif]

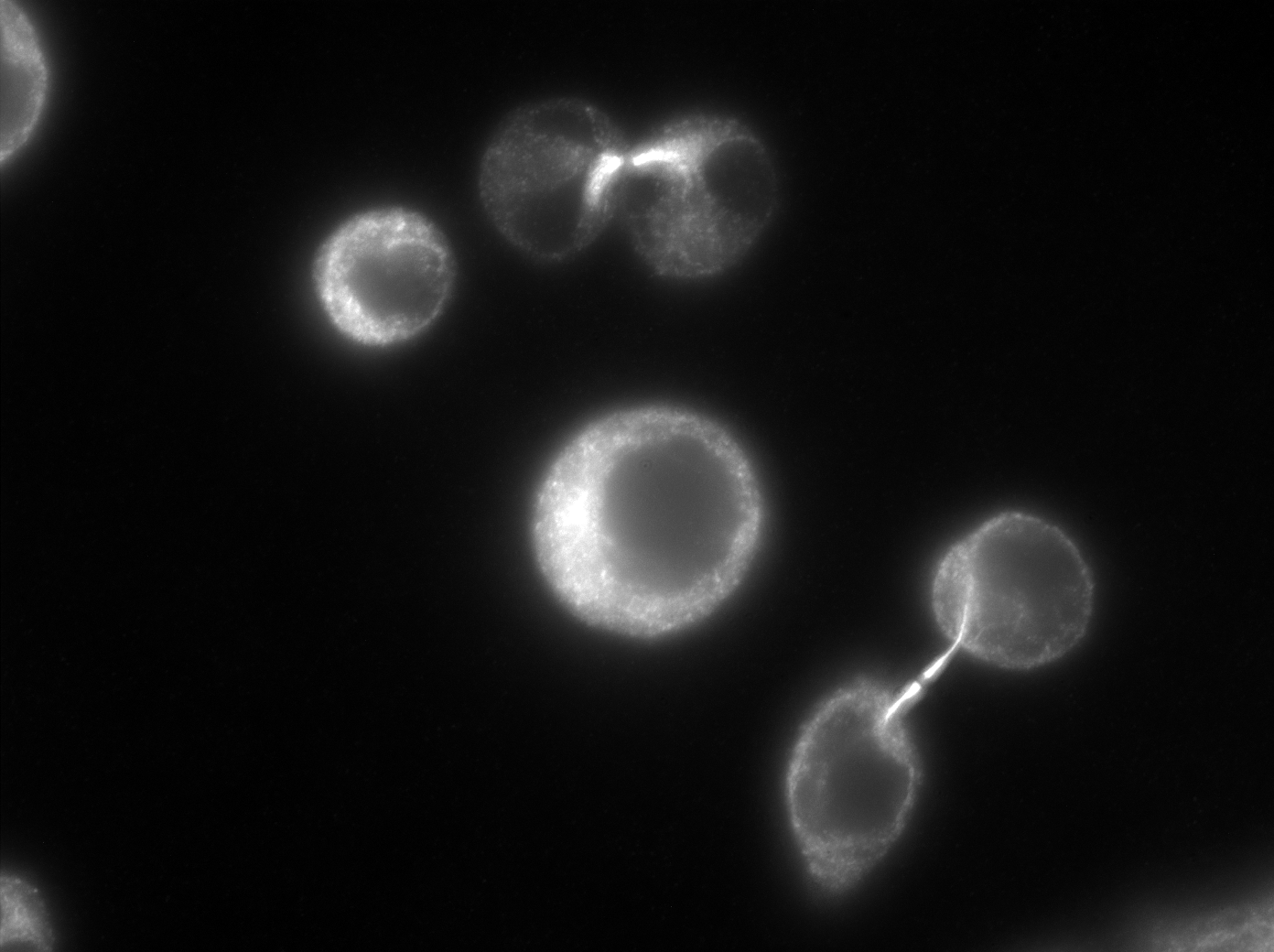

Supplement: Supplementary file 26 — Source data Fig. 7 part 2 [file 44319_2026_742_MOESM26_ESM.zip › Figure 7 Part 2/Fig 7acd Cas9 and TPGS1-ko rGT335 atubulin part 2/TPGS1-KO GT335recomb atub 3-24-25 R3 LT10 ET7.Project Maximum Z_XY1742853699_Z0_T0_C2.tif]

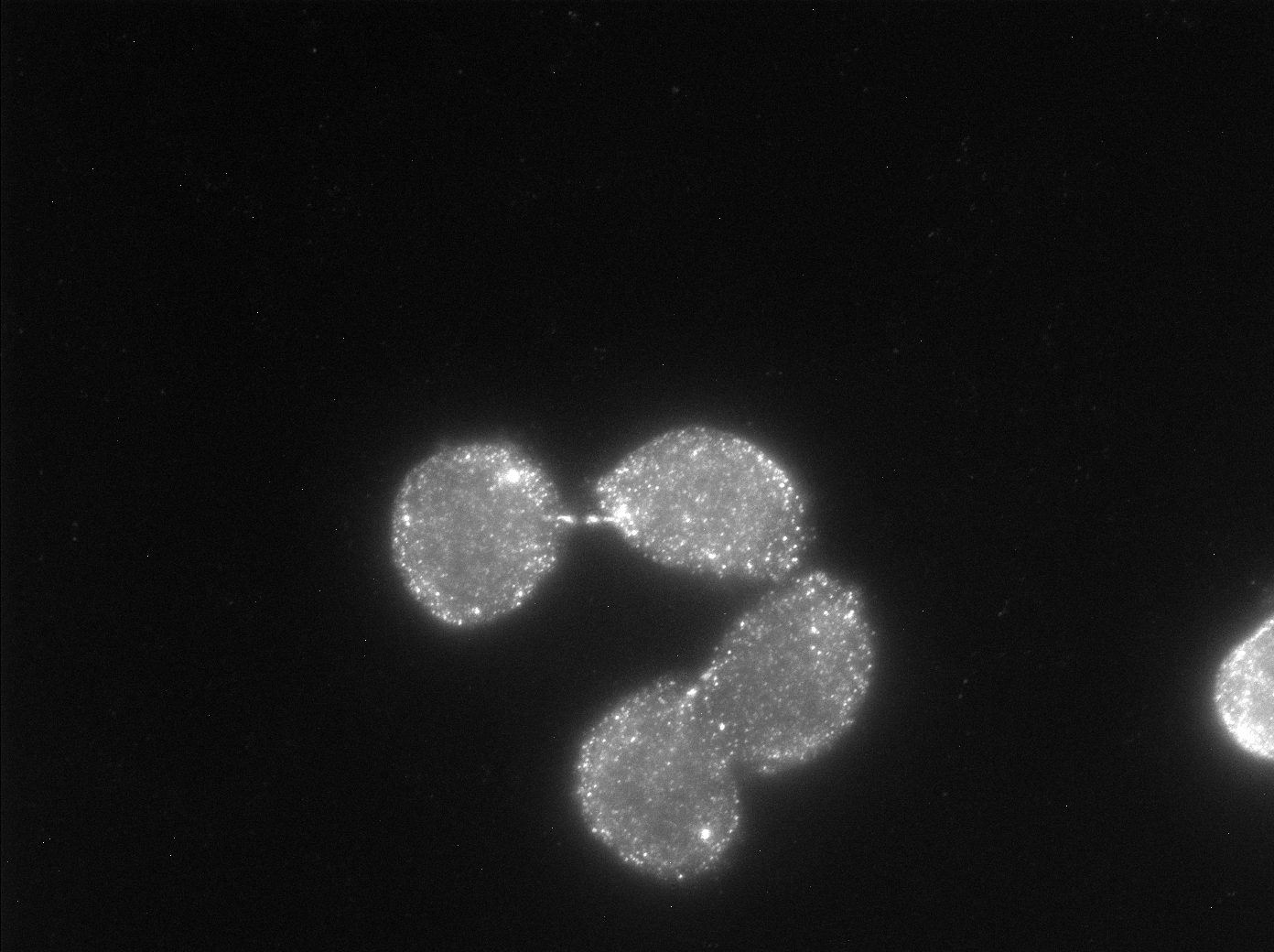

Supplement: Supplementary file 26 — Source data Fig. 7 part 2 [file 44319_2026_742_MOESM26_ESM.zip › Figure 7 Part 2/Fig 7acd Cas9 and TPGS1-ko rGT335 atubulin part 2/TPGS1-KO GT335recomb atub 3-24-25 R2 LT1LT2.Project Maximum Z_XY1742841626_Z0_T0_C1.tif]

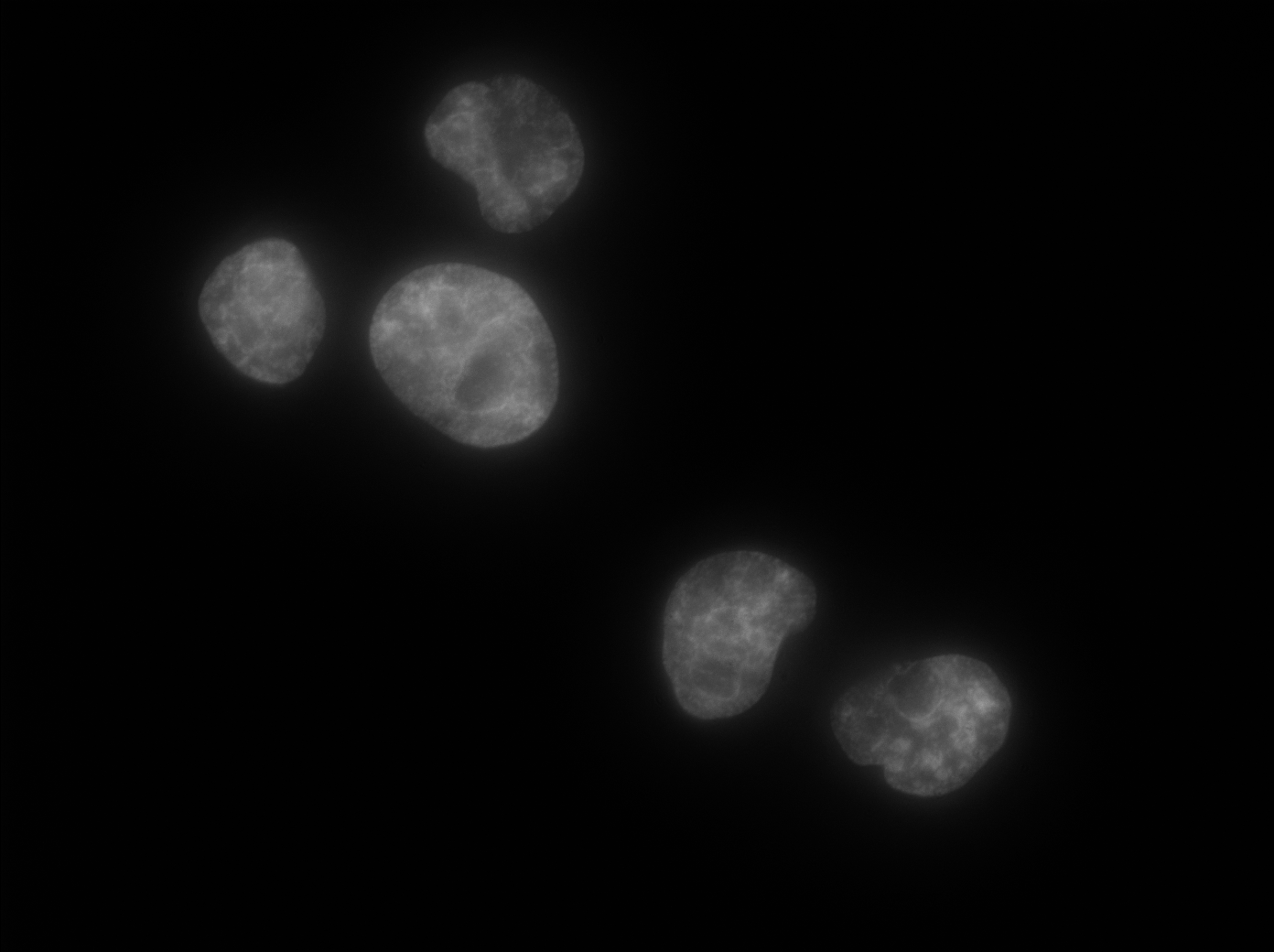

Supplement: Supplementary file 26 — Source data Fig. 7 part 2 [file 44319_2026_742_MOESM26_ESM.zip › Figure 7 Part 2/Fig 7acd Cas9 and TPGS1-ko rGT335 atubulin part 2/TPGS1-KO GT335recomb atub 3-24-25 R2 LT9LT10.Project Maximum Z_XY1742842932_Z0_T0_C0.tif]

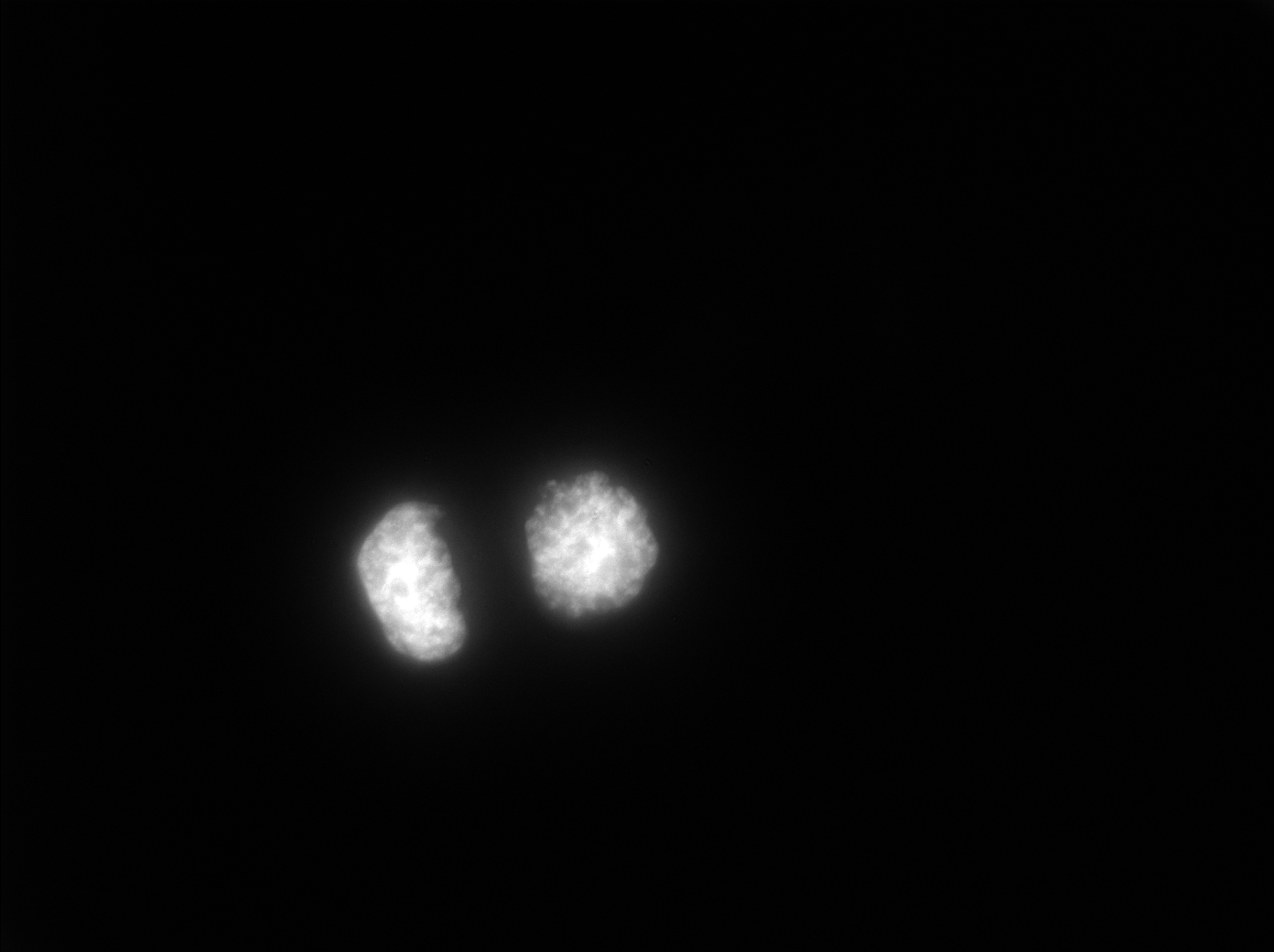

Supplement: Supplementary file 26 — Source data Fig. 7 part 2 [file 44319_2026_742_MOESM26_ESM.zip › Figure 7 Part 2/Fig 7acd Cas9 and TPGS1-ko rGT335 atubulin part 2/TPGS1-KO GT335recomb atub 3-24-25 R3 ET8.Project Maximum Z_XY1742853790_Z0_T0_C0.tif]

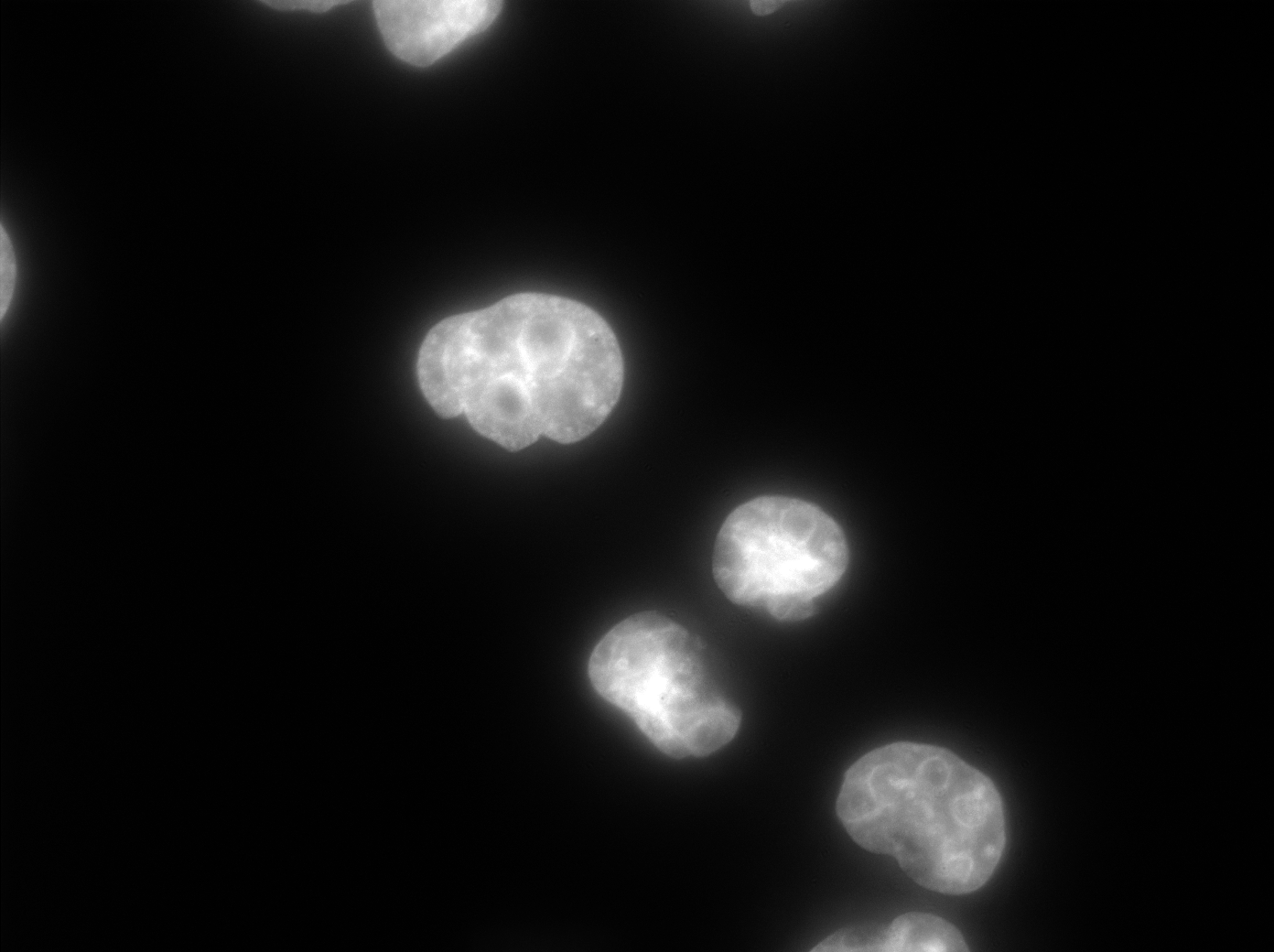

Supplement: Supplementary file 26 — Source data Fig. 7 part 2 [file 44319_2026_742_MOESM26_ESM.zip › Figure 7 Part 2/Fig 7acd Cas9 and TPGS1-ko rGT335 atubulin part 2/TPGS1-KO GT335recomb atub 3-24-25 R1 ET6.Project Maximum Z_XY1742840388_Z0_T0_C0.tif]

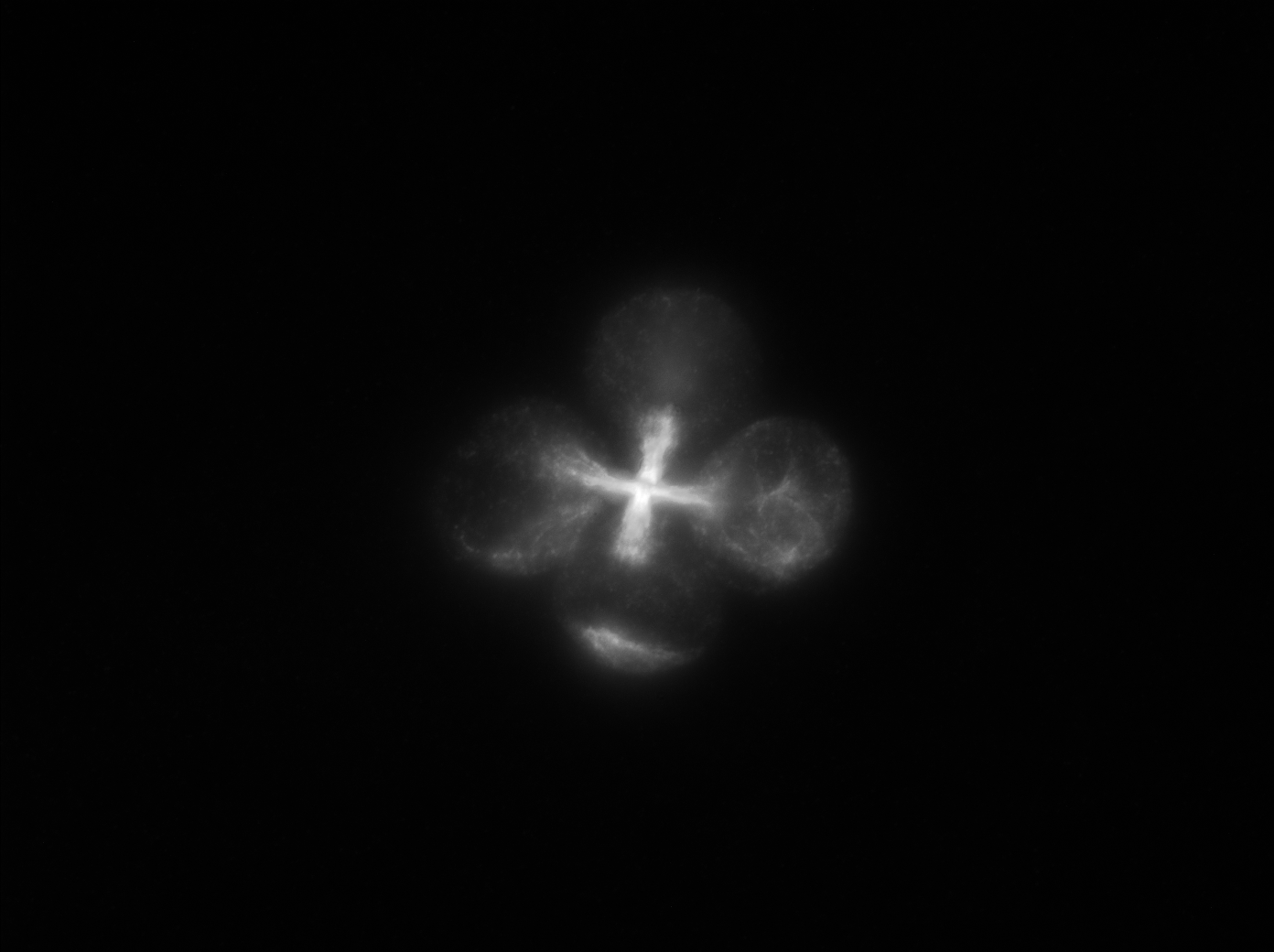

Supplement: Supplementary file 26 — Source data Fig. 7 part 2 [file 44319_2026_742_MOESM26_ESM.zip › Figure 7 Part 2/Fig 7acd Cas9 and TPGS1-ko rGT335 atubulin part 2/TPGS1-KO GT335recomb atub 3-24-25 R2 ETCROSS.Project Maximum Z_XY1742842646_Z0_T0_C2.tif]

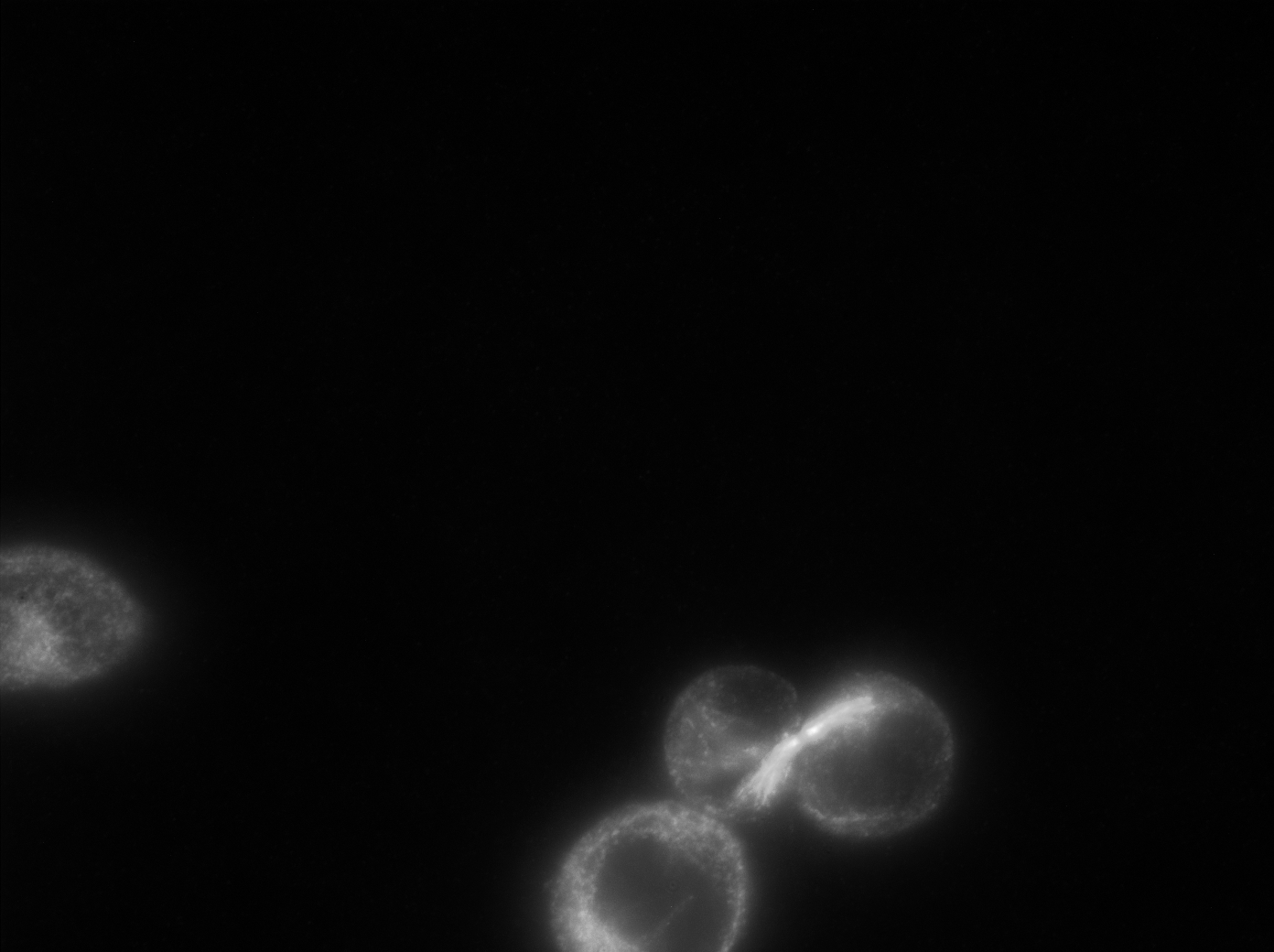

Supplement: Supplementary file 26 — Source data Fig. 7 part 2 [file 44319_2026_742_MOESM26_ESM.zip › Figure 7 Part 2/Fig 7acd Cas9 and TPGS1-ko rGT335 atubulin part 2/TPGS1-KO GT335recomb atub 3-24-25 R1 ET10.Project Maximum Z_XY1742840860_Z0_T0_C2.tif]

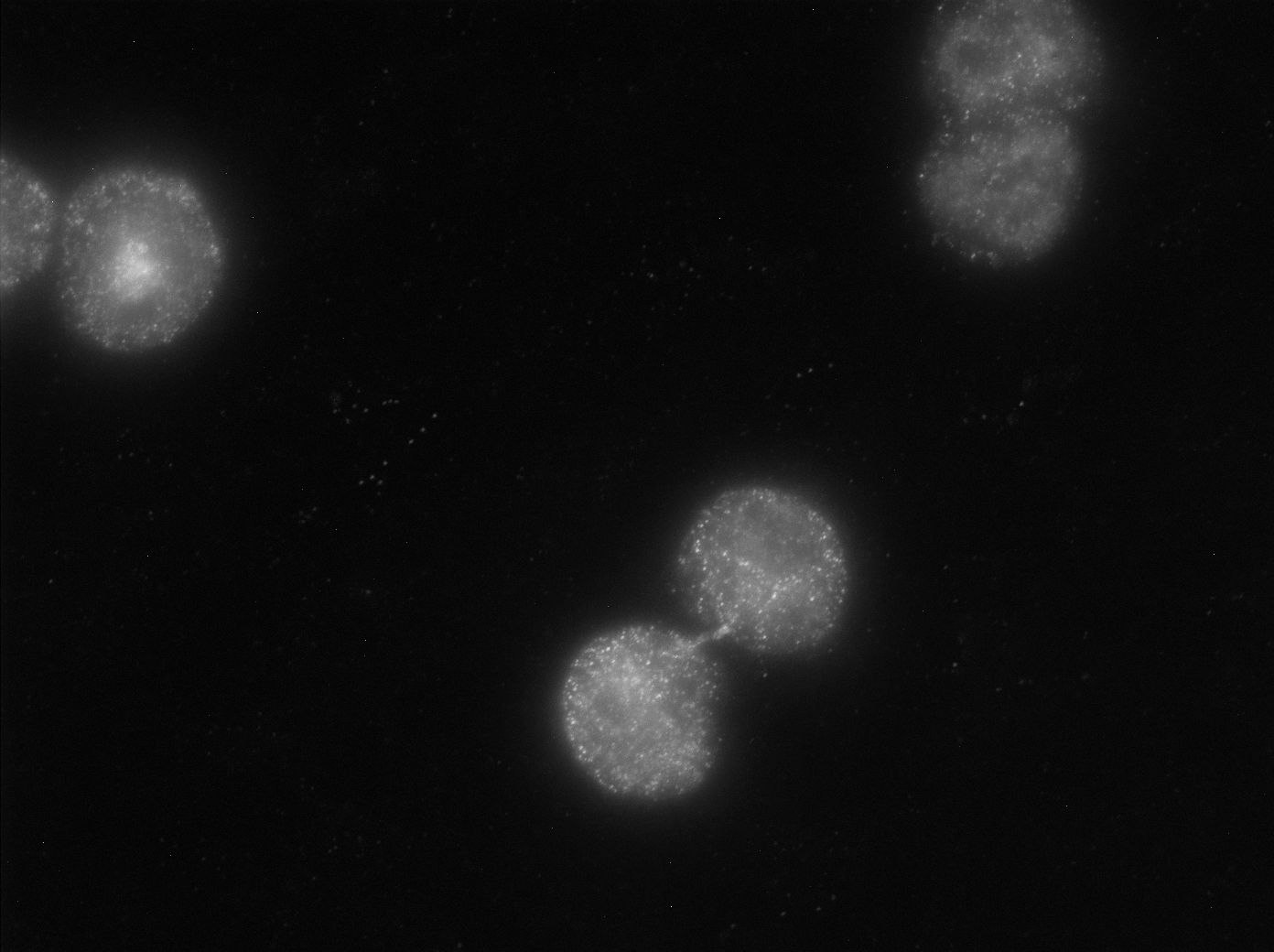

Supplement: Supplementary file 26 — Source data Fig. 7 part 2 [file 44319_2026_742_MOESM26_ESM.zip › Figure 7 Part 2/Fig 7acd Cas9 and TPGS1-ko rGT335 atubulin part 2/TPGS1-KO GT335recomb atub 3-24-25 R1 ET5.Project Maximum Z_XY1742840284_Z0_T0_C1.tif]

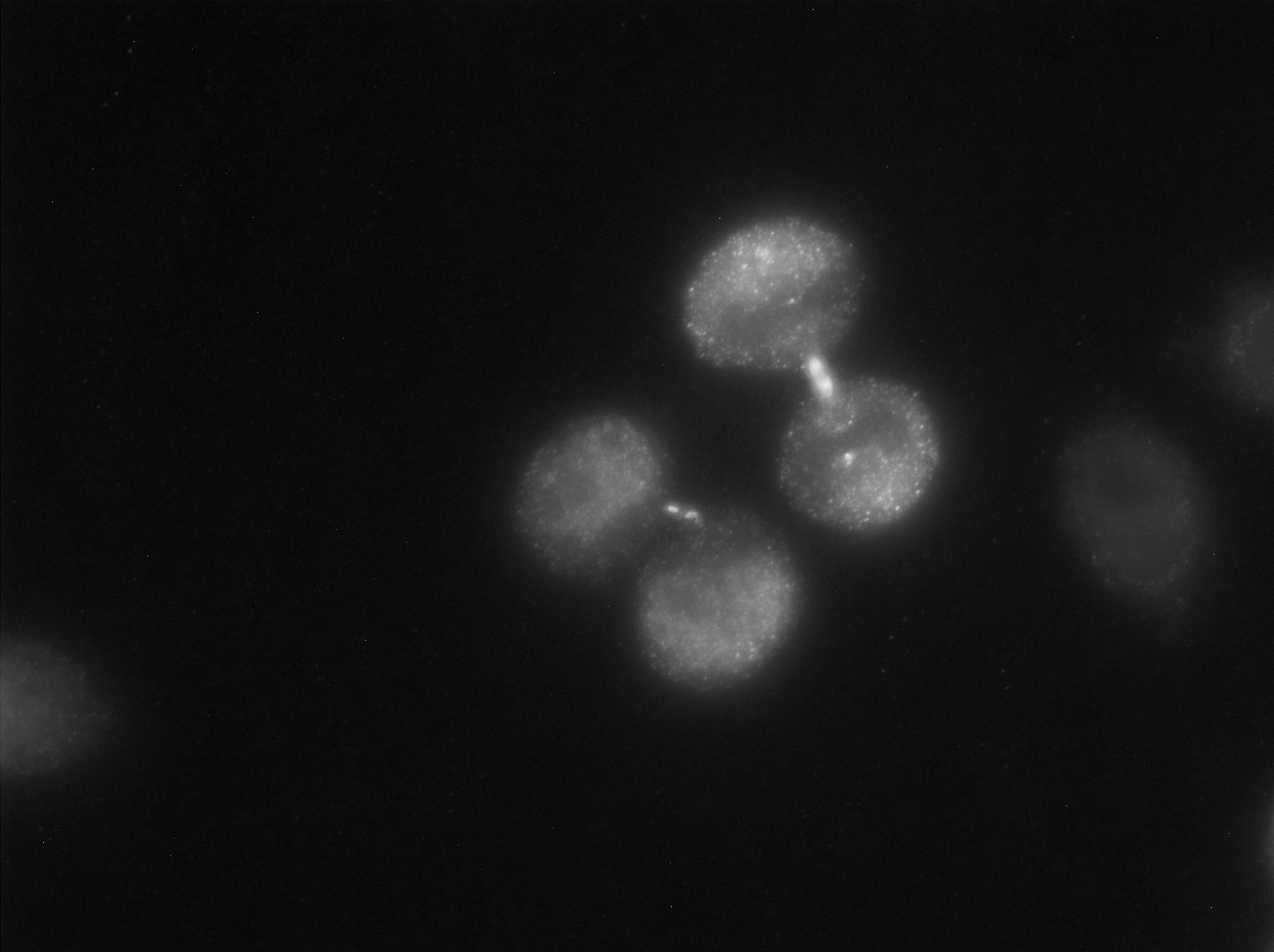

Supplement: Supplementary file 26 — Source data Fig. 7 part 2 [file 44319_2026_742_MOESM26_ESM.zip › Figure 7 Part 2/Fig 7acd Cas9 and TPGS1-ko rGT335 atubulin part 2/TPGS1-KO GT335recomb atub 3-24-25 R3 ET3 LT1.Project Maximum Z_XY1742851776_Z0_T0_C1.tif]

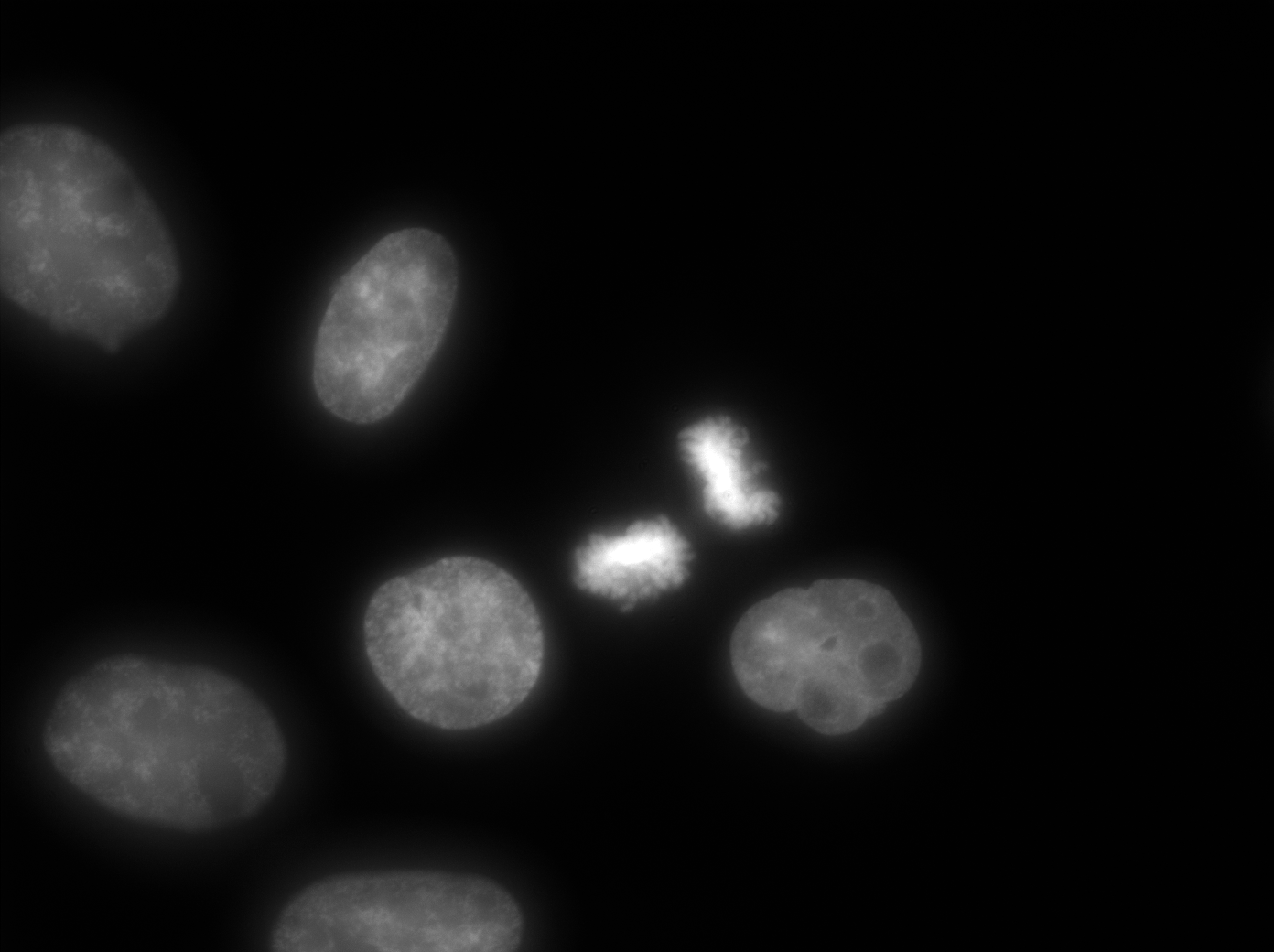

Supplement: Supplementary file 26 — Source data Fig. 7 part 2 [file 44319_2026_742_MOESM26_ESM.zip › Figure 7 Part 2/Fig 7acd Cas9 and TPGS1-ko rGT335 atubulin part 2/TPGS1-KO GT335recomb atub 3-24-25 R1 ET1.Project Maximum Z_XY1742839836_Z0_T0_C0.tif]

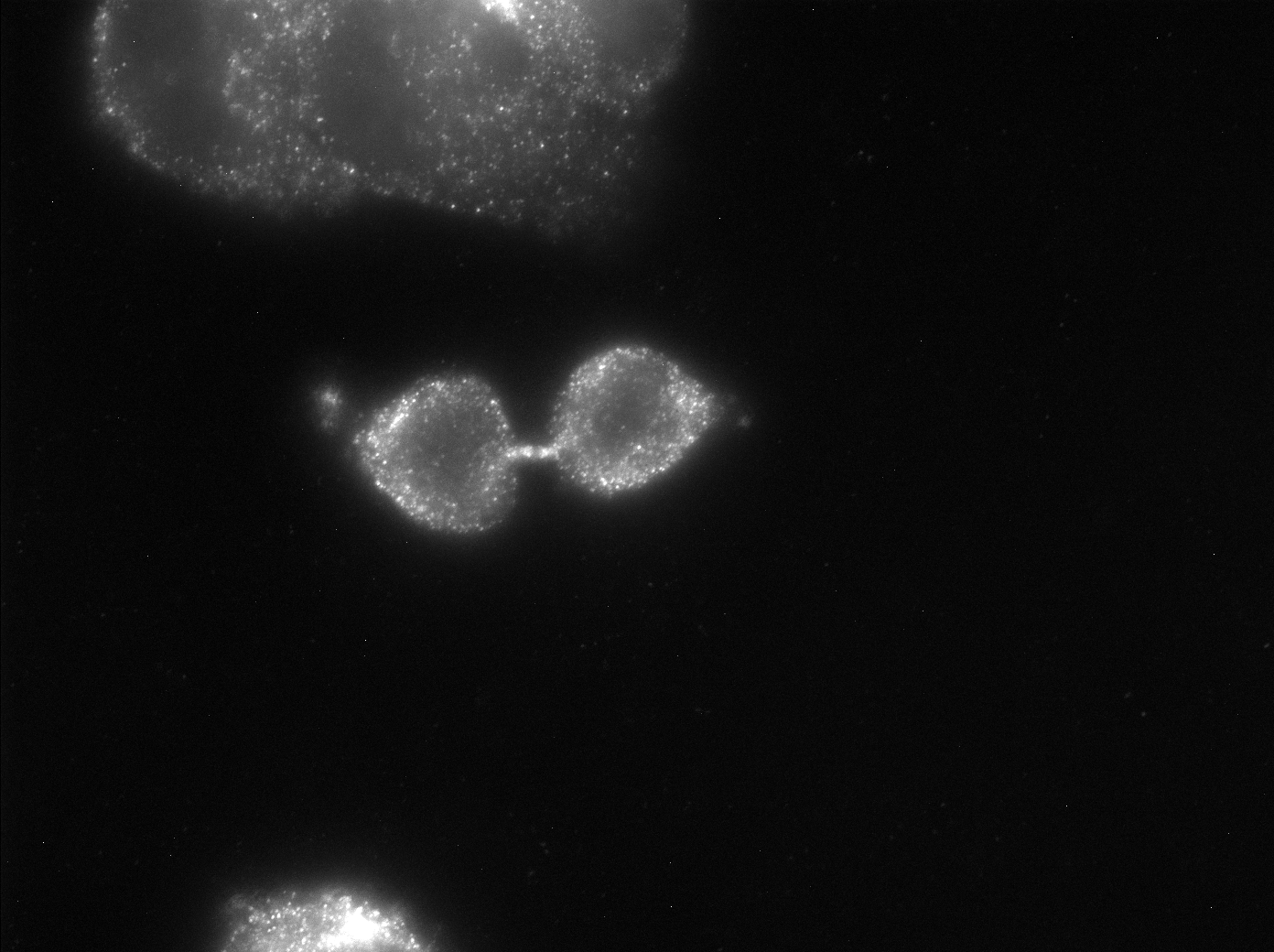

Supplement: Supplementary file 26 — Source data Fig. 7 part 2 [file 44319_2026_742_MOESM26_ESM.zip › Figure 7 Part 2/Fig 7acd Cas9 and TPGS1-ko rGT335 atubulin part 2/TPGS1-KO GT335recomb atub 3-24-25 R2 ET4.Project Maximum Z_XY1742841450_Z0_T0_C1.tif]

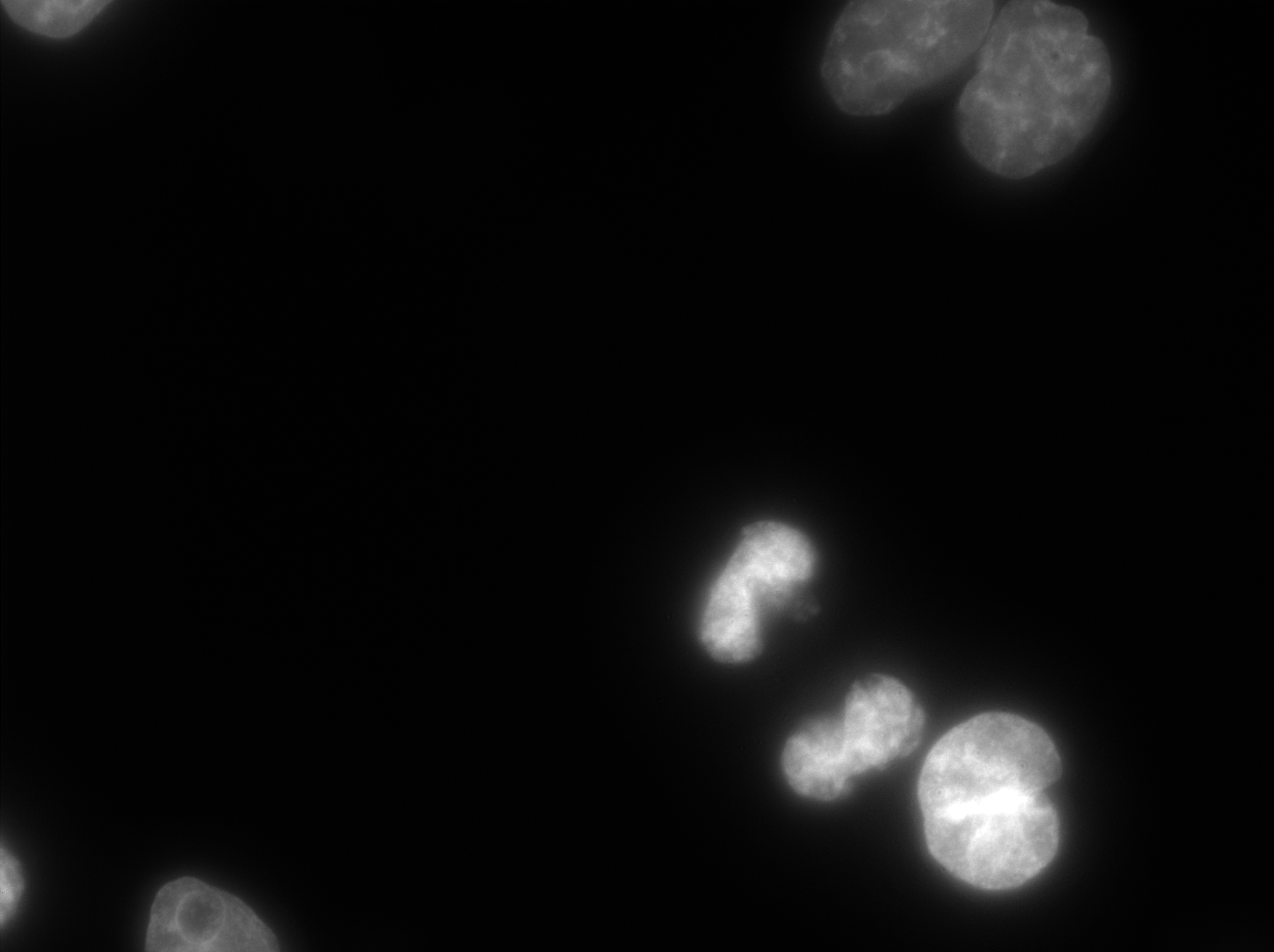

Supplement: Supplementary file 26 — Source data Fig. 7 part 2 [file 44319_2026_742_MOESM26_ESM.zip › Figure 7 Part 2/Fig 7acd Cas9 and TPGS1-ko rGT335 atubulin part 2/TPGS1-KO GT335recomb atub 3-24-25 R1 ET2.Project Maximum Z_XY1742839982_Z0_T0_C0.tif]

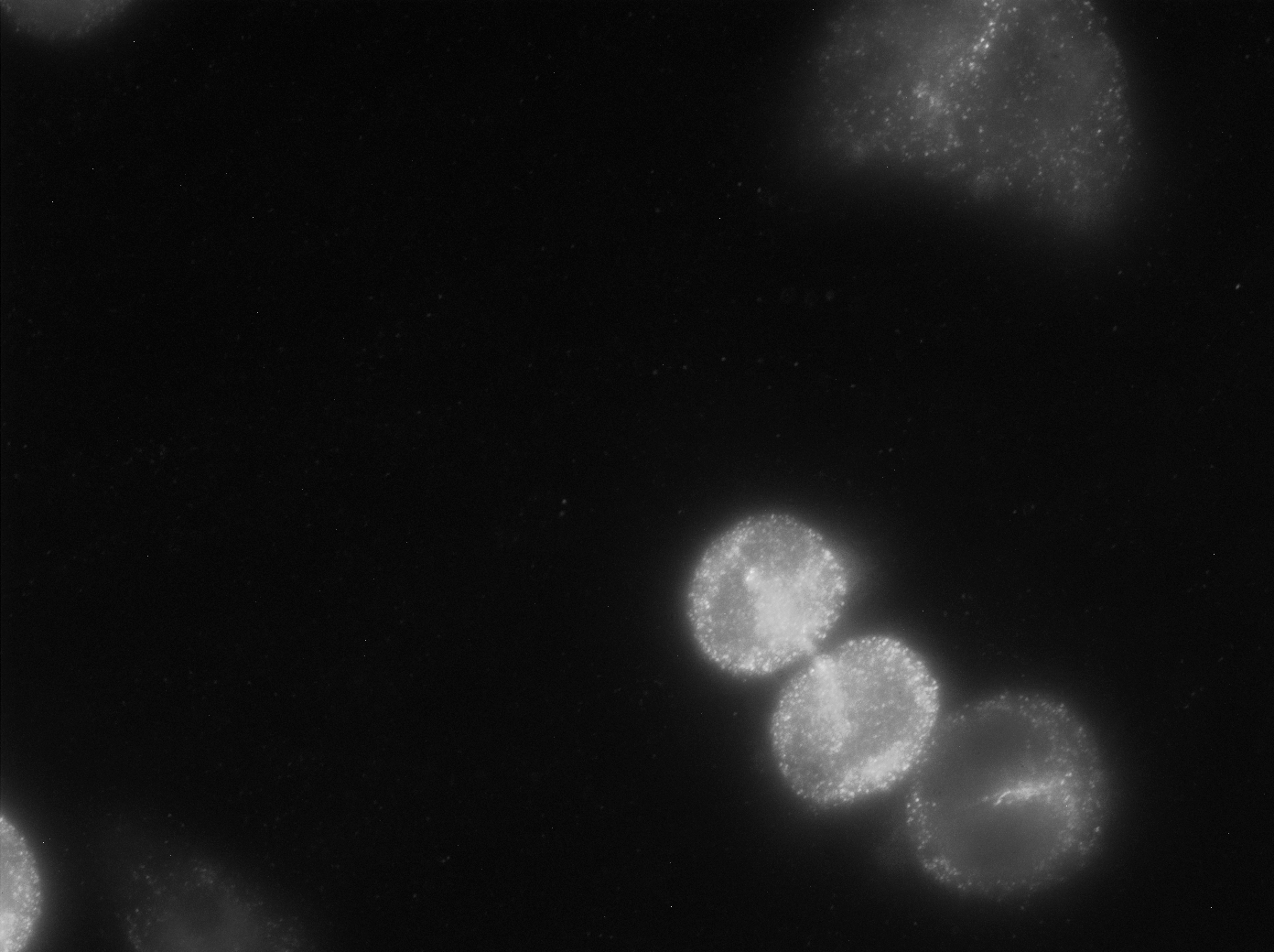

Supplement: Supplementary file 26 — Source data Fig. 7 part 2 [file 44319_2026_742_MOESM26_ESM.zip › Figure 7 Part 2/Fig 7acd Cas9 and TPGS1-ko rGT335 atubulin part 2/TPGS1-KO GT335recomb atub 3-24-25 R1 ET2.Project Maximum Z_XY1742839982_Z0_T0_C1.tif]

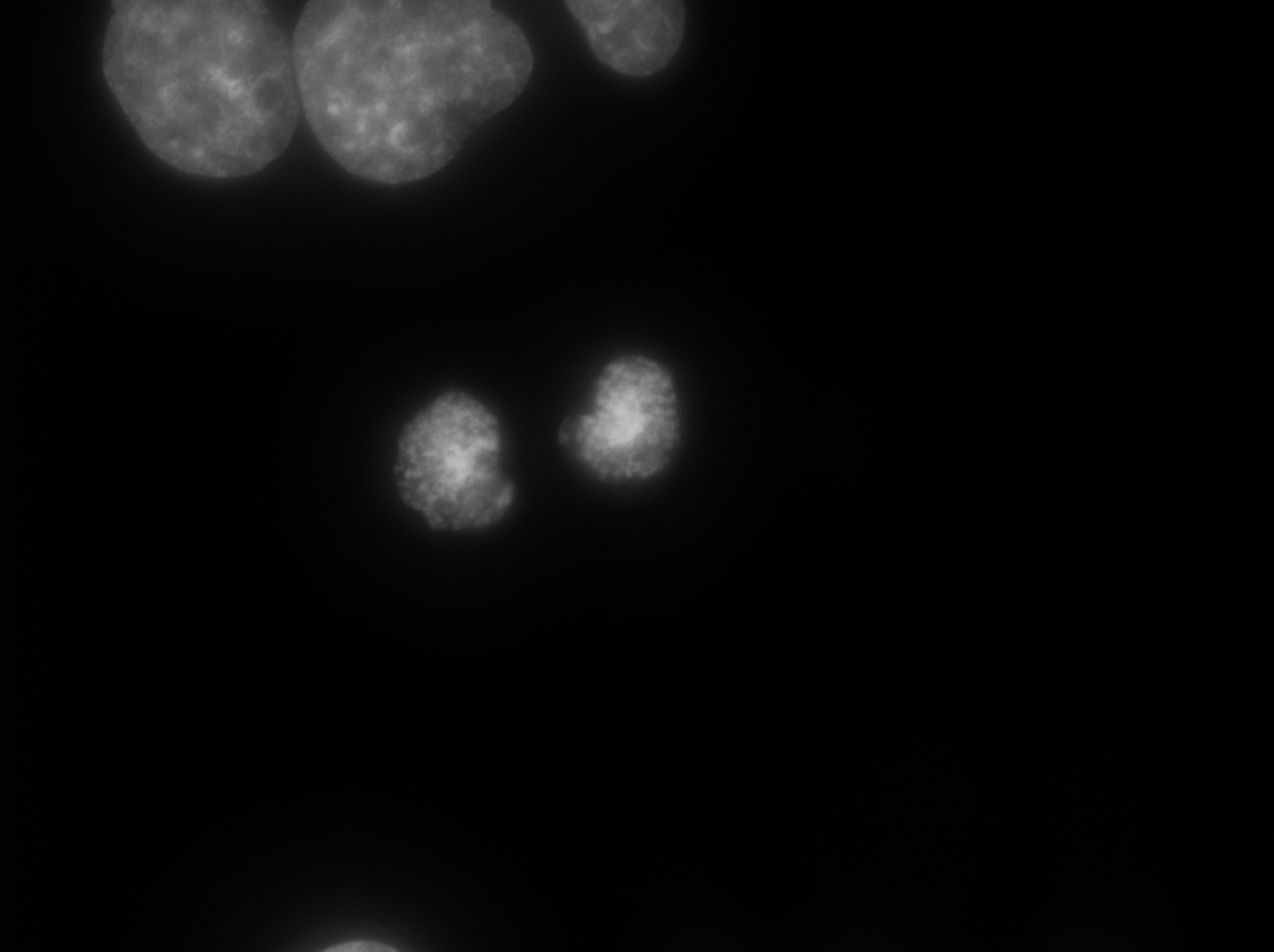

Supplement: Supplementary file 26 — Source data Fig. 7 part 2 [file 44319_2026_742_MOESM26_ESM.zip › Figure 7 Part 2/Fig 7acd Cas9 and TPGS1-ko rGT335 atubulin part 2/TPGS1-KO GT335recomb atub 3-24-25 R2 ET4.Project Maximum Z_XY1742841450_Z0_T0_C0.tif]

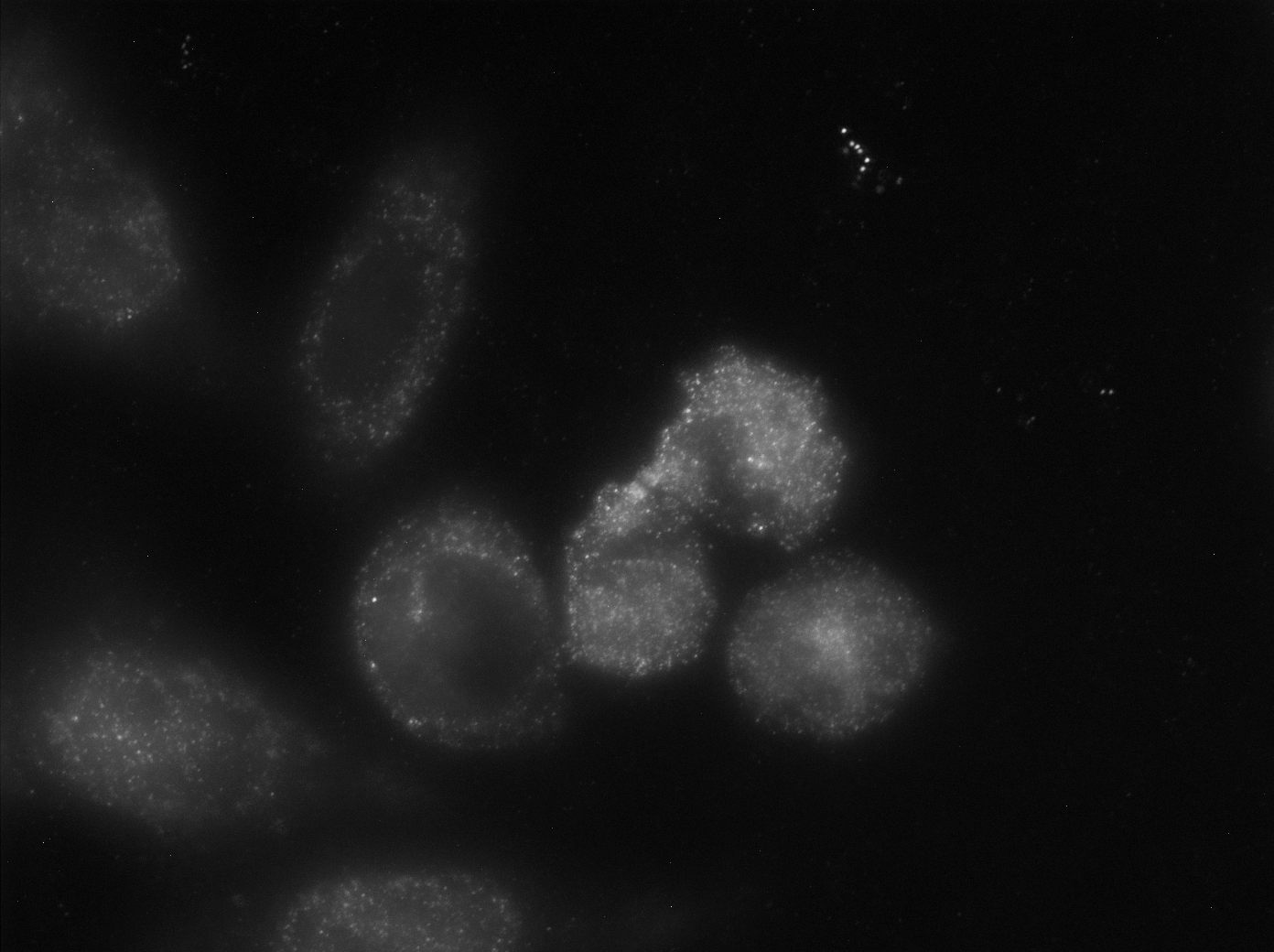

Supplement: Supplementary file 26 — Source data Fig. 7 part 2 [file 44319_2026_742_MOESM26_ESM.zip › Figure 7 Part 2/Fig 7acd Cas9 and TPGS1-ko rGT335 atubulin part 2/TPGS1-KO GT335recomb atub 3-24-25 R1 ET1.Project Maximum Z_XY1742839836_Z0_T0_C1.tif]

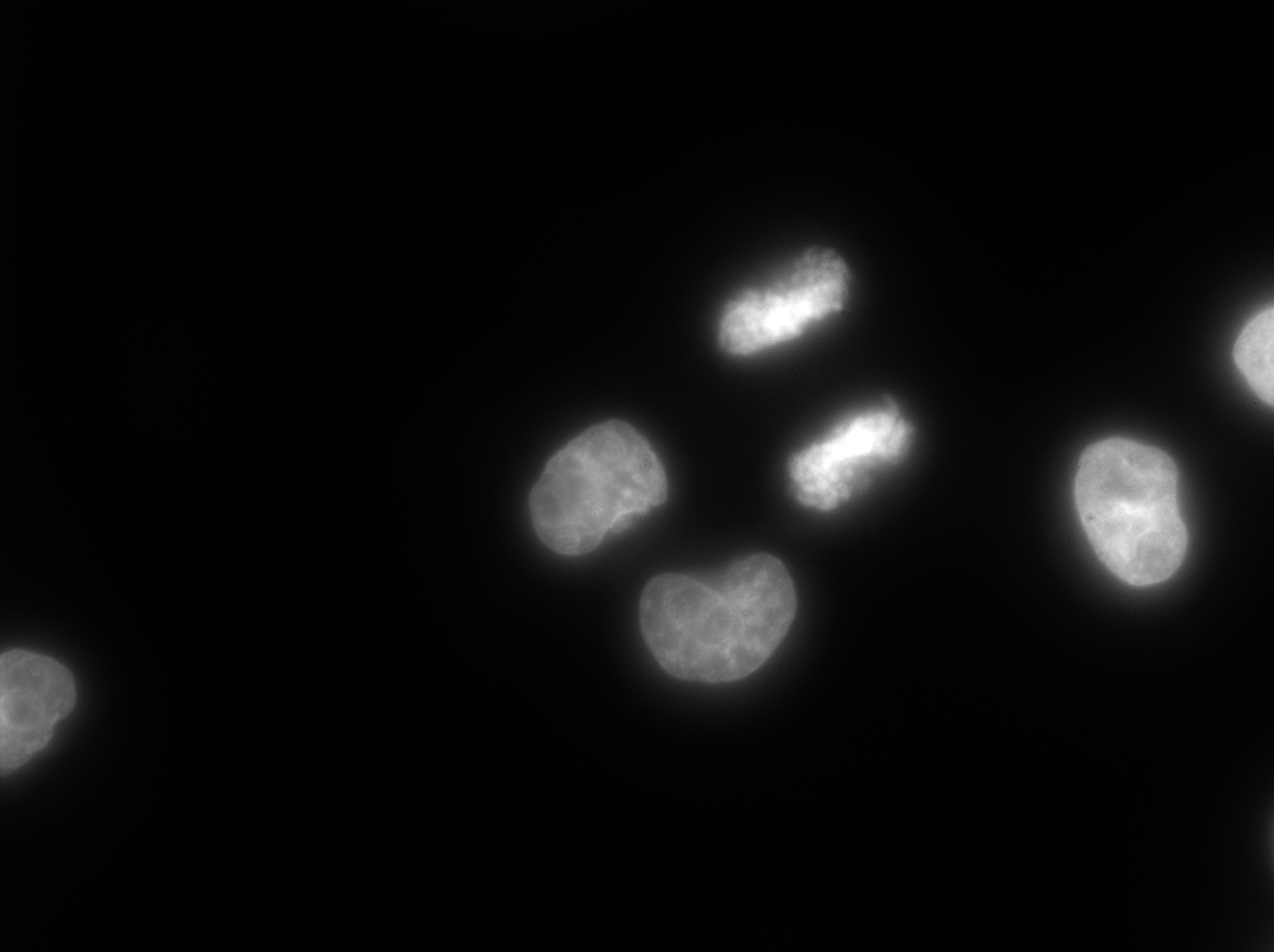

Supplement: Supplementary file 26 — Source data Fig. 7 part 2 [file 44319_2026_742_MOESM26_ESM.zip › Figure 7 Part 2/Fig 7acd Cas9 and TPGS1-ko rGT335 atubulin part 2/TPGS1-KO GT335recomb atub 3-24-25 R3 ET3 LT1.Project Maximum Z_XY1742851776_Z0_T0_C0.tif]

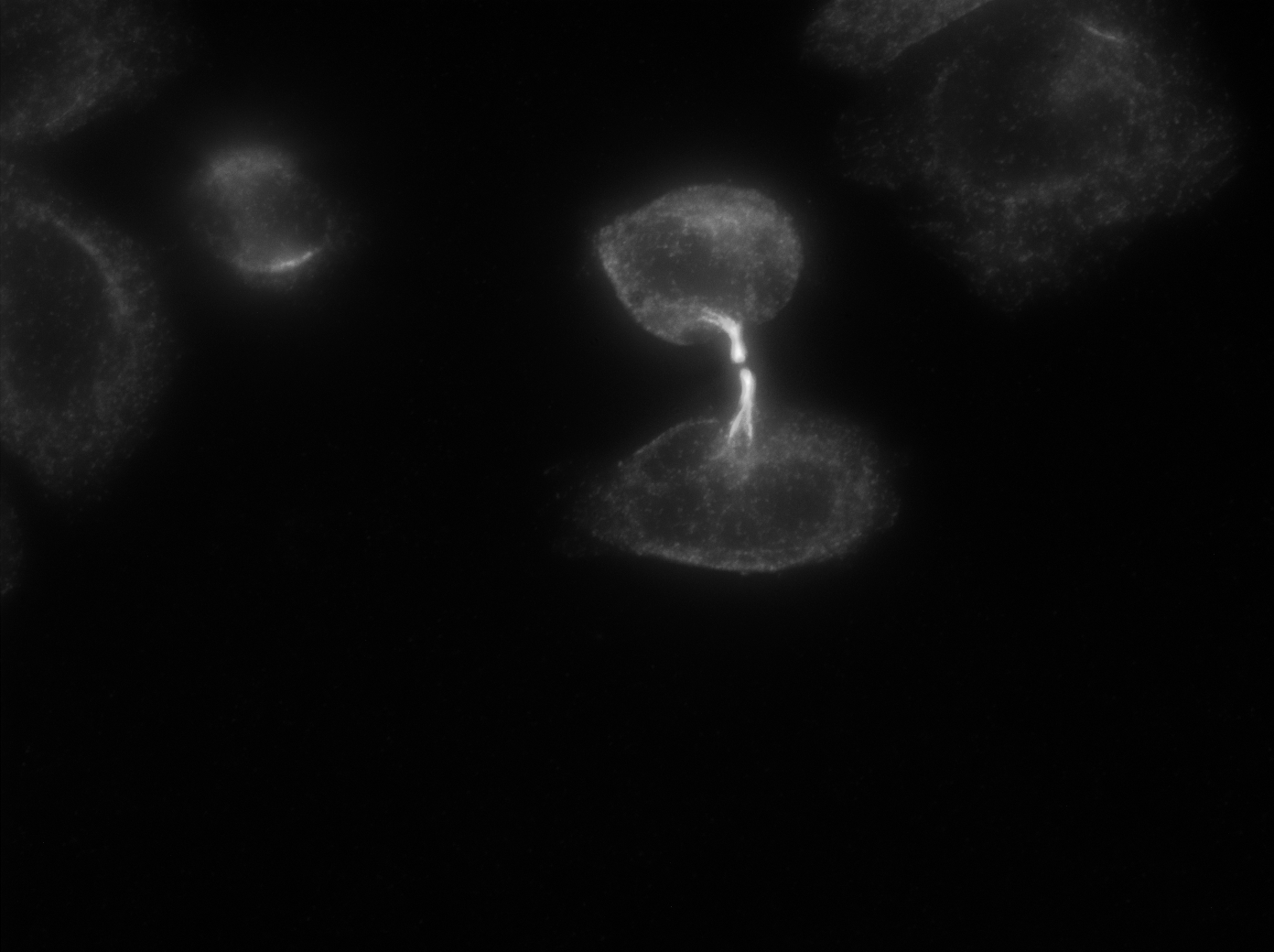

Supplement: Supplementary file 26 — Source data Fig. 7 part 2 [file 44319_2026_742_MOESM26_ESM.zip › Figure 7 Part 2/Fig 7acd Cas9 and TPGS1-ko rGT335 atubulin part 2/TPGS1-KO GT335recomb atub 3-24-25 R1 LT6.Project Maximum Z_XY1742839619_Z0_T0_C2.tif]

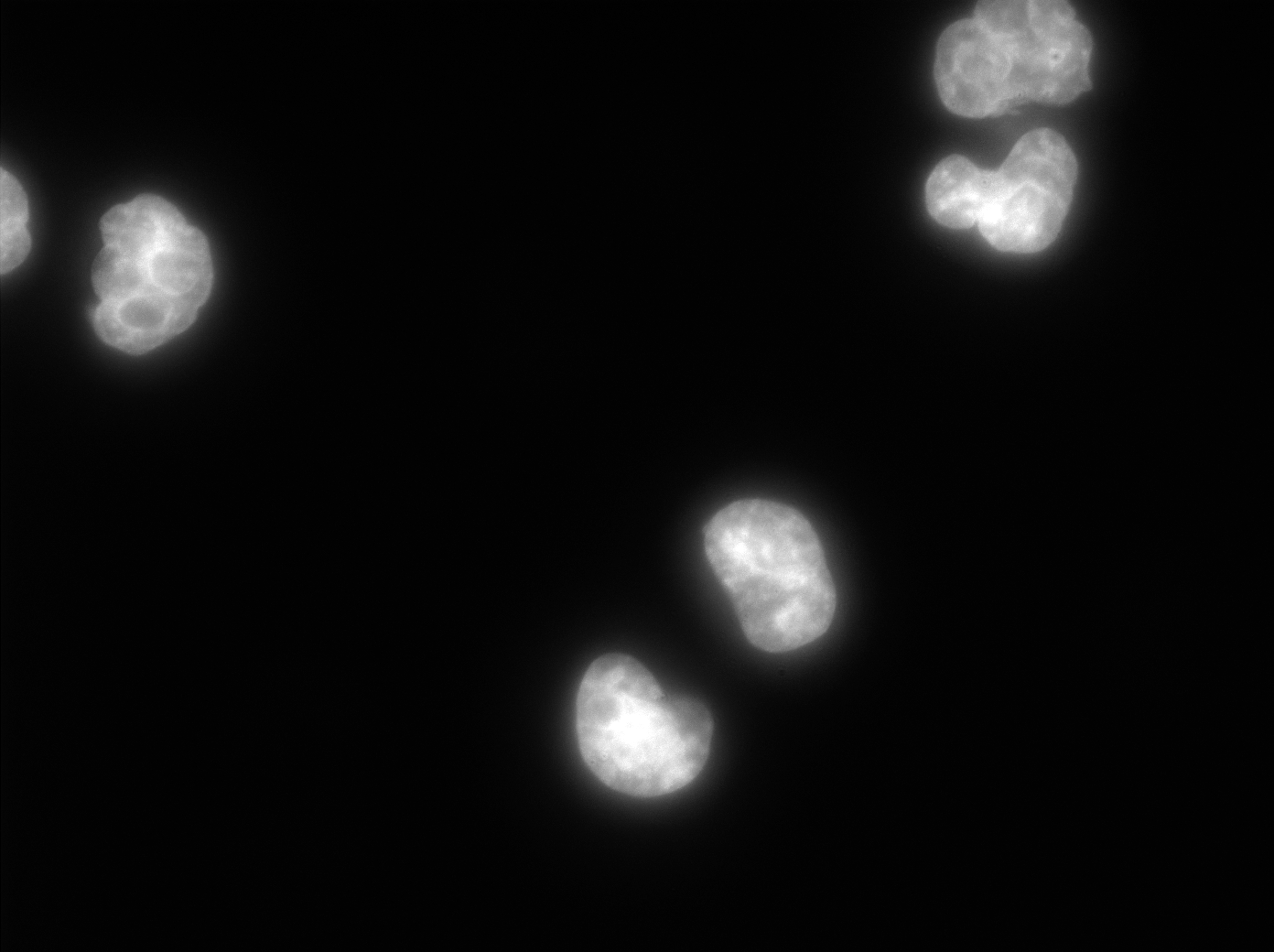

Supplement: Supplementary file 26 — Source data Fig. 7 part 2 [file 44319_2026_742_MOESM26_ESM.zip › Figure 7 Part 2/Fig 7acd Cas9 and TPGS1-ko rGT335 atubulin part 2/TPGS1-KO GT335recomb atub 3-24-25 R1 ET5.Project Maximum Z_XY1742840284_Z0_T0_C0.tif]

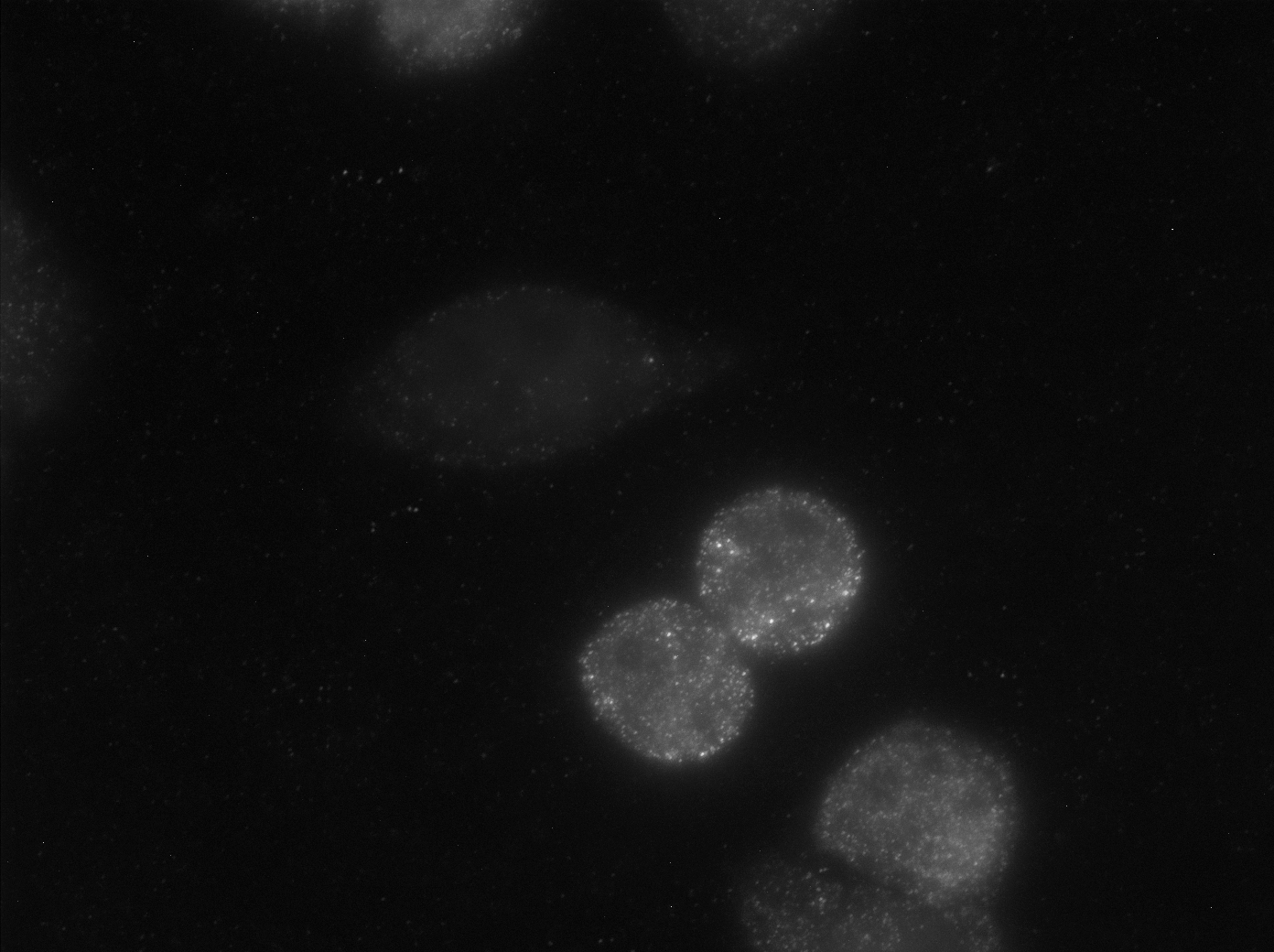

Supplement: Supplementary file 26 — Source data Fig. 7 part 2 [file 44319_2026_742_MOESM26_ESM.zip › Figure 7 Part 2/Fig 7acd Cas9 and TPGS1-ko rGT335 atubulin part 2/TPGS1-KO GT335recomb atub 3-24-25 R1 ET6.Project Maximum Z_XY1742840388_Z0_T0_C1.tif]

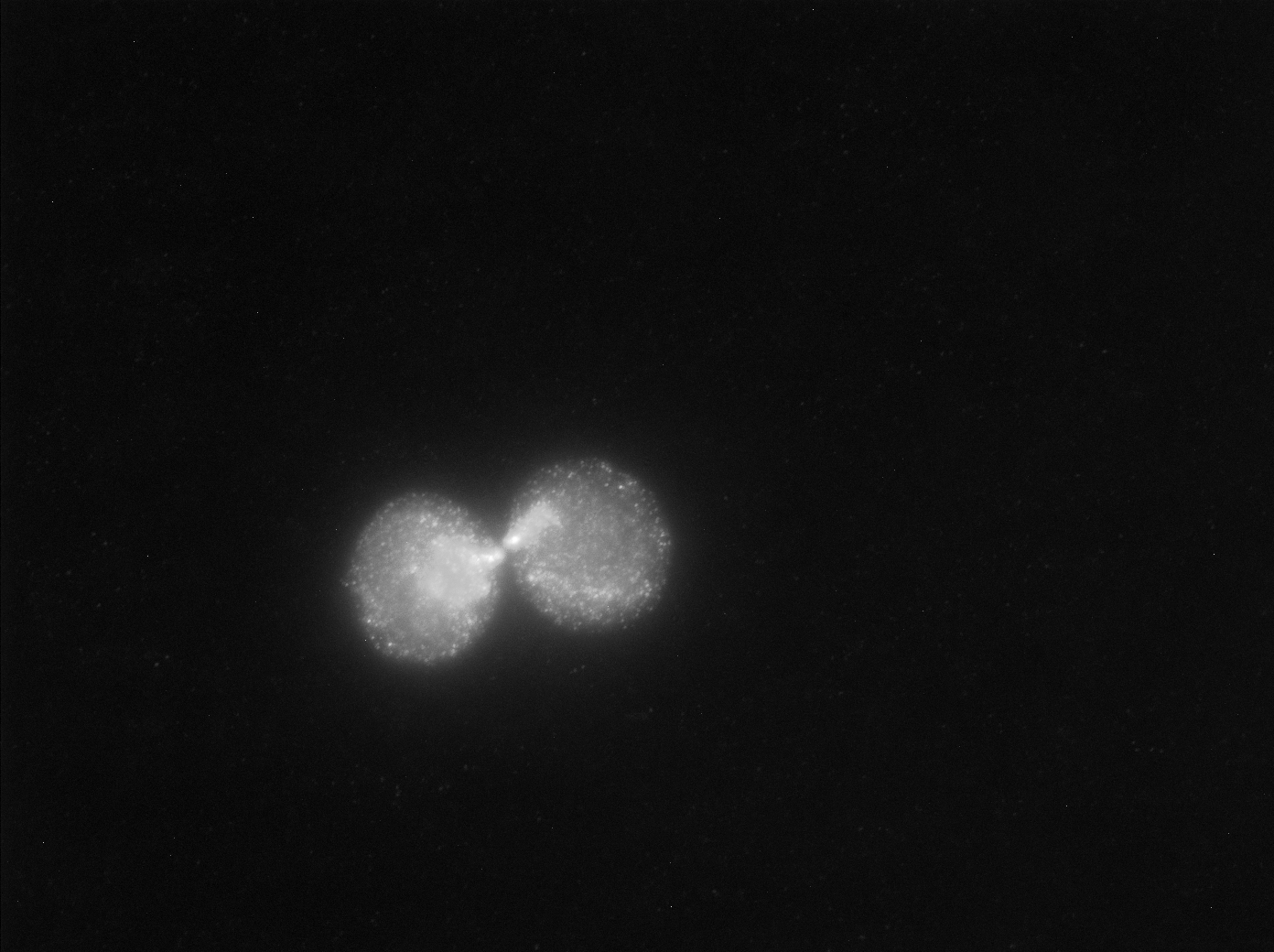

Supplement: Supplementary file 26 — Source data Fig. 7 part 2 [file 44319_2026_742_MOESM26_ESM.zip › Figure 7 Part 2/Fig 7acd Cas9 and TPGS1-ko rGT335 atubulin part 2/TPGS1-KO GT335recomb atub 3-24-25 R3 ET8.Project Maximum Z_XY1742853790_Z0_T0_C1.tif]

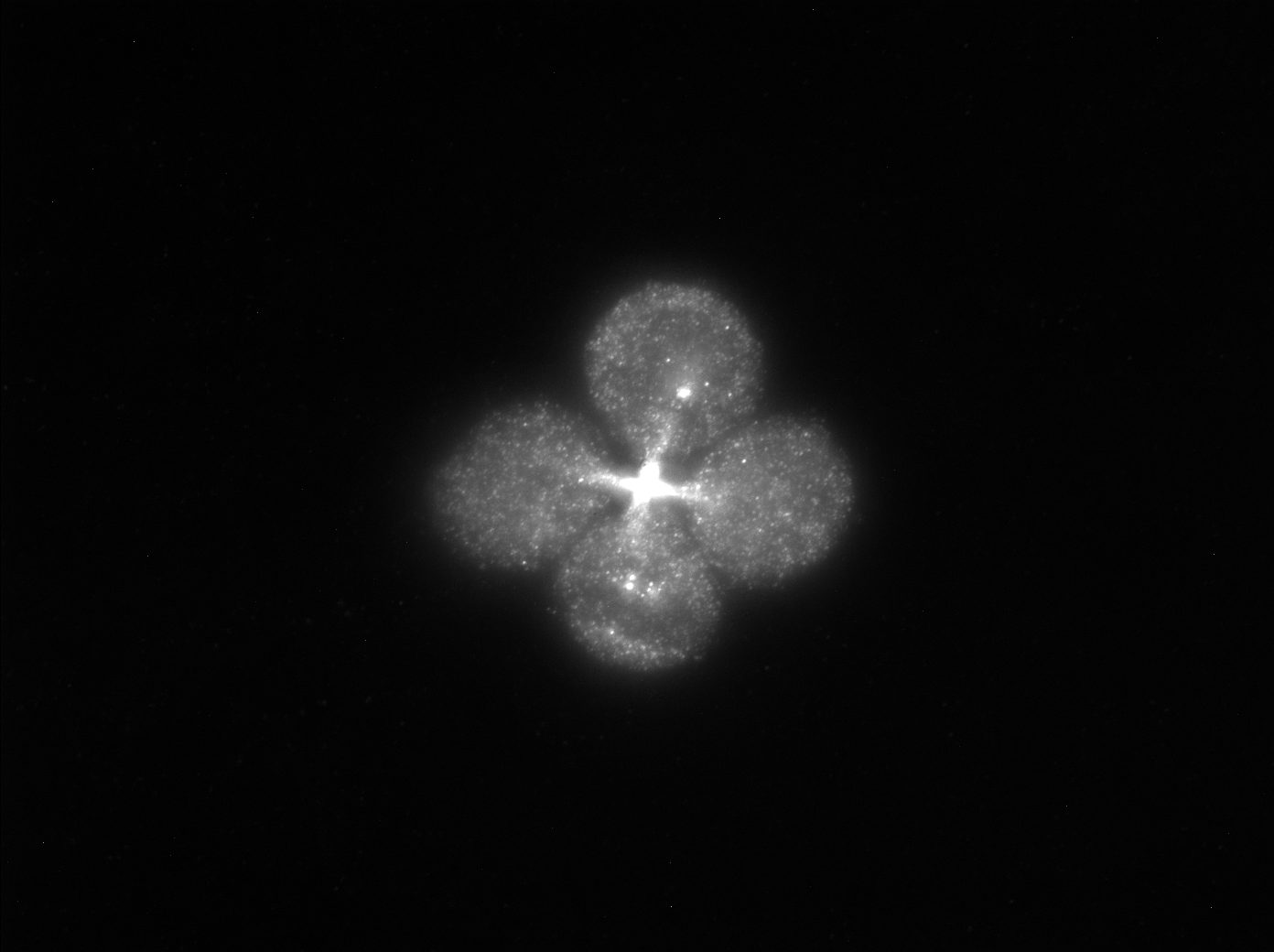

Supplement: Supplementary file 26 — Source data Fig. 7 part 2 [file 44319_2026_742_MOESM26_ESM.zip › Figure 7 Part 2/Fig 7acd Cas9 and TPGS1-ko rGT335 atubulin part 2/TPGS1-KO GT335recomb atub 3-24-25 R2 ETCROSS.Project Maximum Z_XY1742842646_Z0_T0_C1.tif]

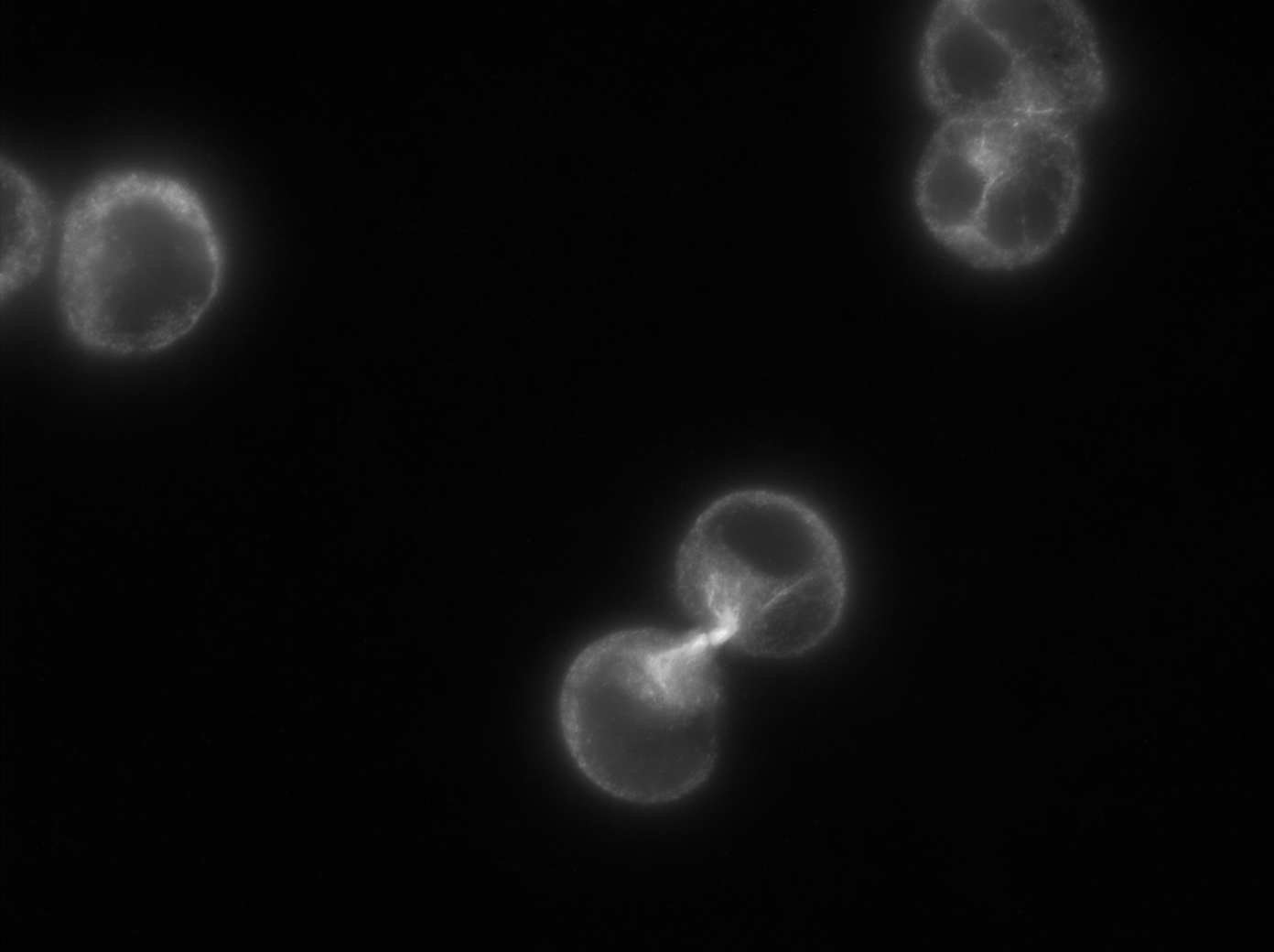

Supplement: Supplementary file 26 — Source data Fig. 7 part 2 [file 44319_2026_742_MOESM26_ESM.zip › Figure 7 Part 2/Fig 7acd Cas9 and TPGS1-ko rGT335 atubulin part 2/TPGS1-KO GT335recomb atub 3-24-25 R1 ET5.Project Maximum Z_XY1742840284_Z0_T0_C2.tif]

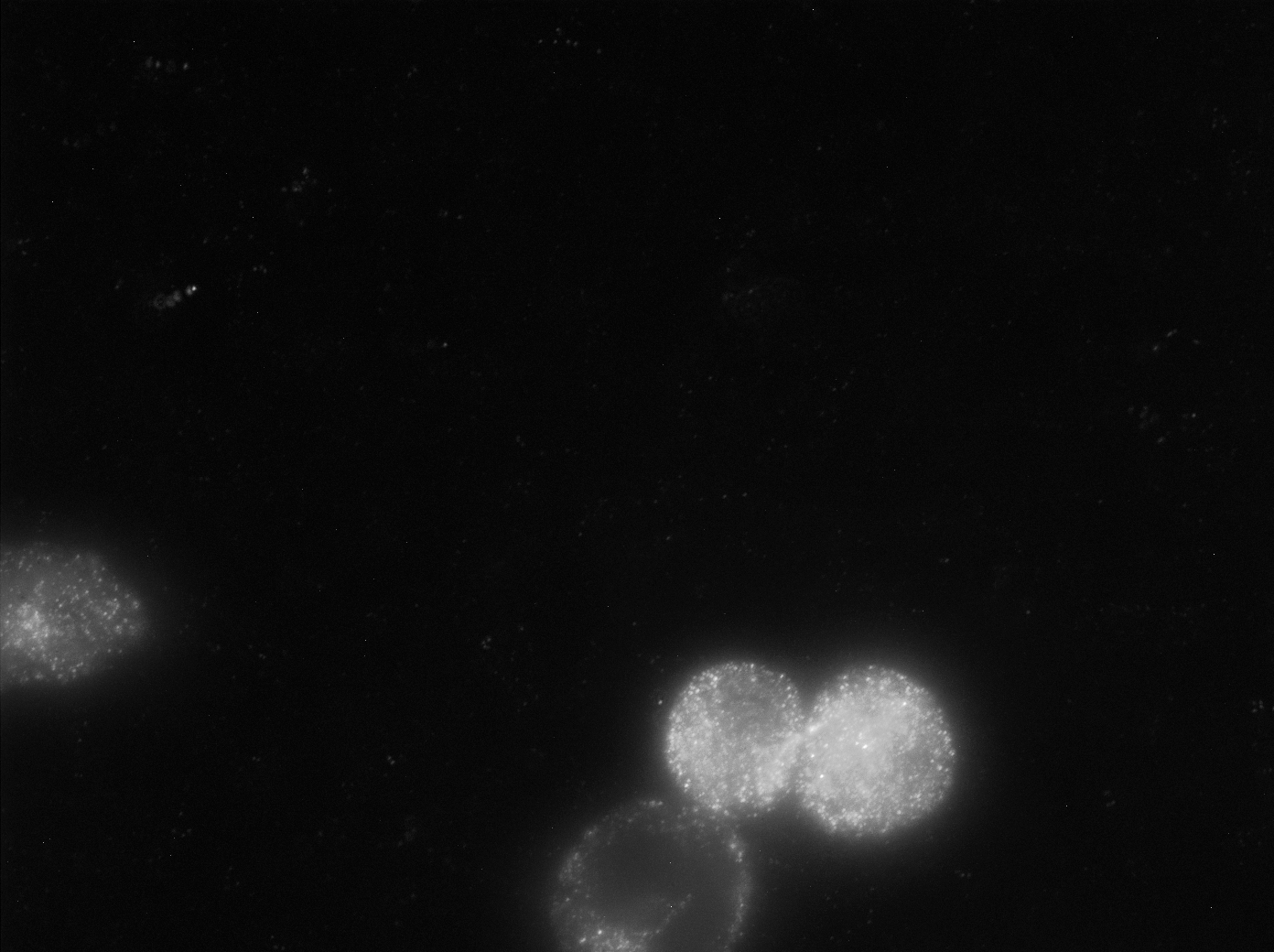

Supplement: Supplementary file 26 — Source data Fig. 7 part 2 [file 44319_2026_742_MOESM26_ESM.zip › Figure 7 Part 2/Fig 7acd Cas9 and TPGS1-ko rGT335 atubulin part 2/TPGS1-KO GT335recomb atub 3-24-25 R1 ET10.Project Maximum Z_XY1742840860_Z0_T0_C1.tif]

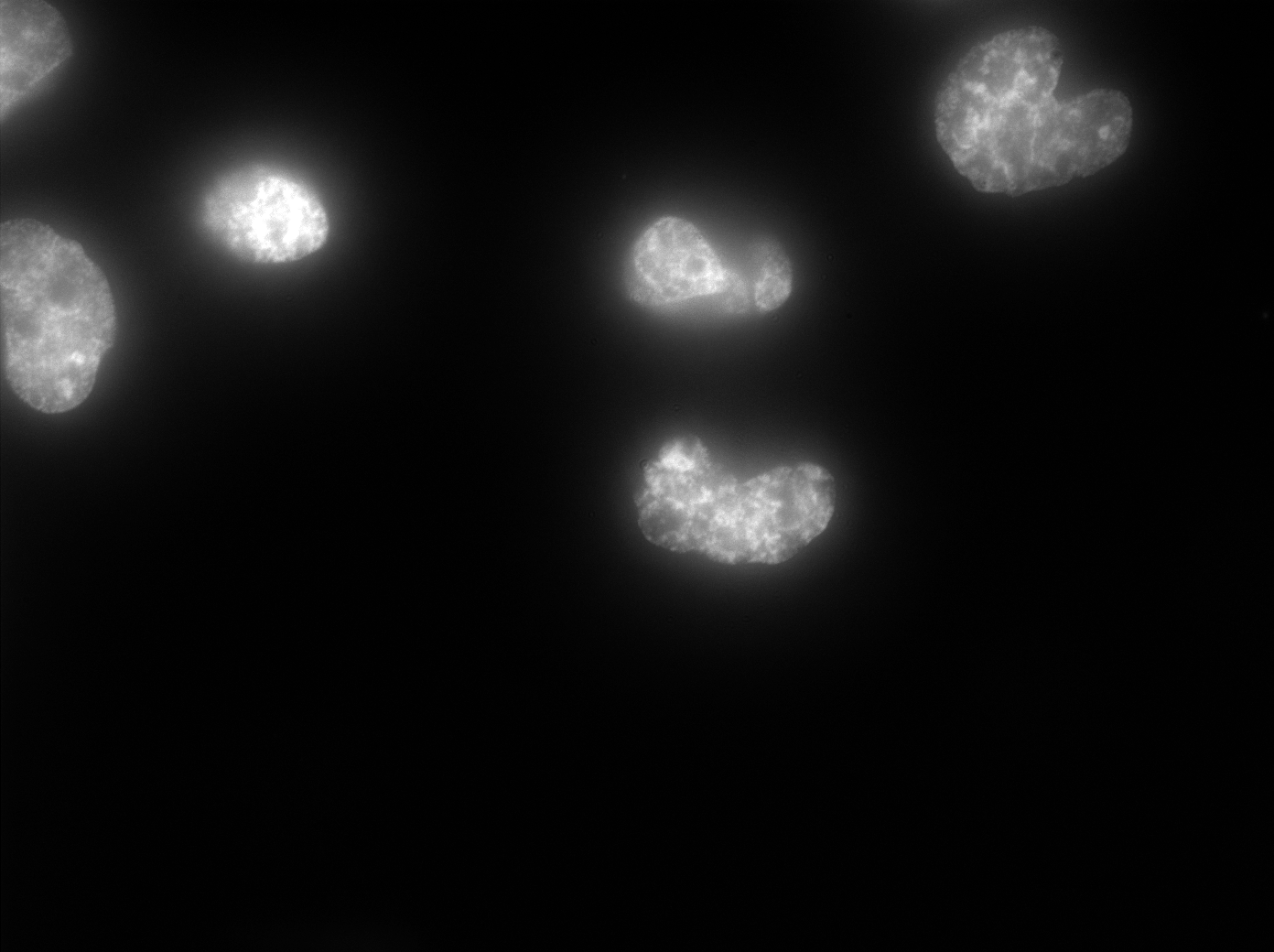

Supplement: Supplementary file 26 — Source data Fig. 7 part 2 [file 44319_2026_742_MOESM26_ESM.zip › Figure 7 Part 2/Fig 7acd Cas9 and TPGS1-ko rGT335 atubulin part 2/TPGS1-KO GT335recomb atub 3-24-25 R1 LT6.Project Maximum Z_XY1742839619_Z0_T0_C0.tif]

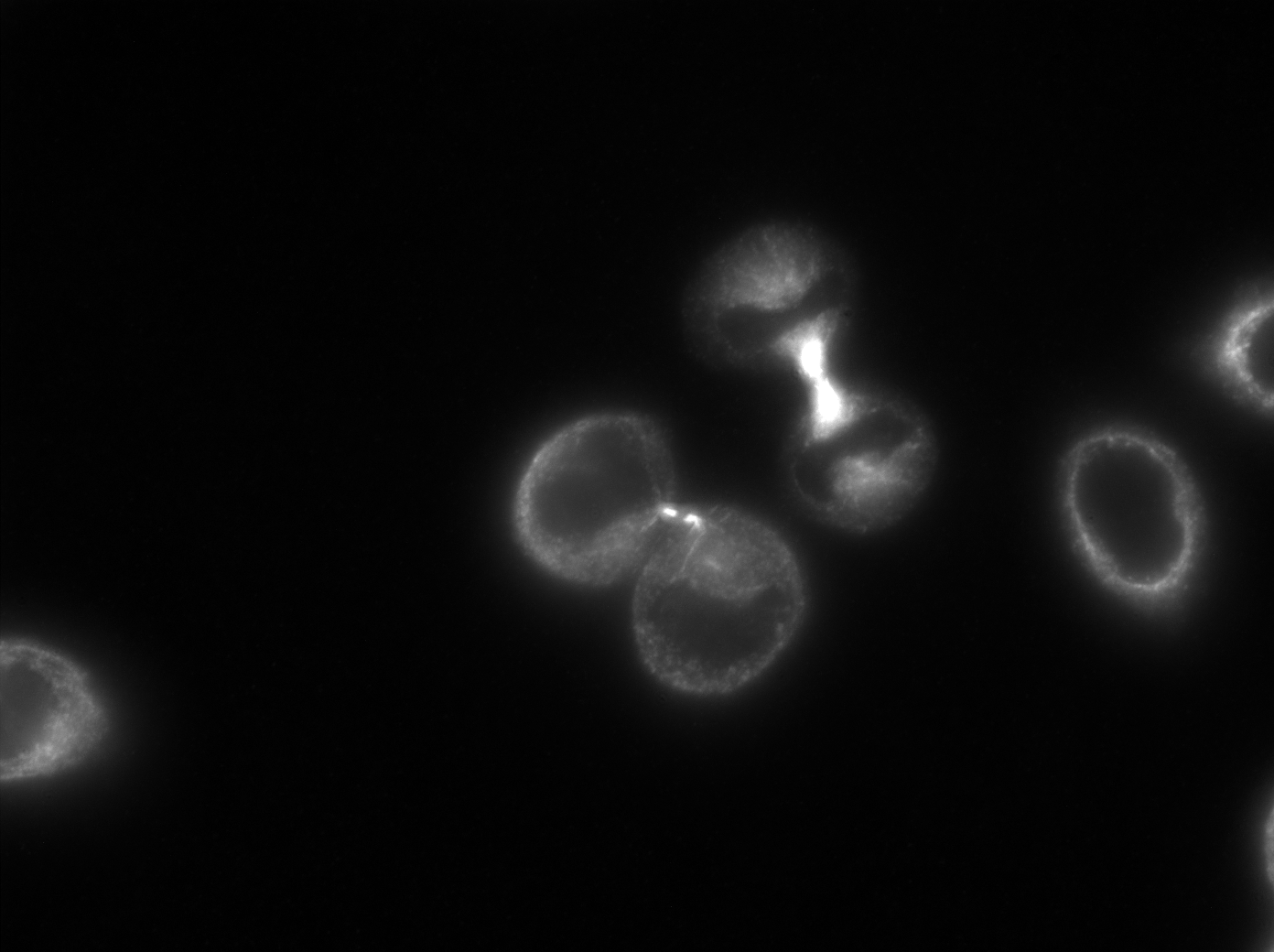

Supplement: Supplementary file 26 — Source data Fig. 7 part 2 [file 44319_2026_742_MOESM26_ESM.zip › Figure 7 Part 2/Fig 7acd Cas9 and TPGS1-ko rGT335 atubulin part 2/TPGS1-KO GT335recomb atub 3-24-25 R3 ET3 LT1.Project Maximum Z_XY1742851776_Z0_T0_C2.tif]

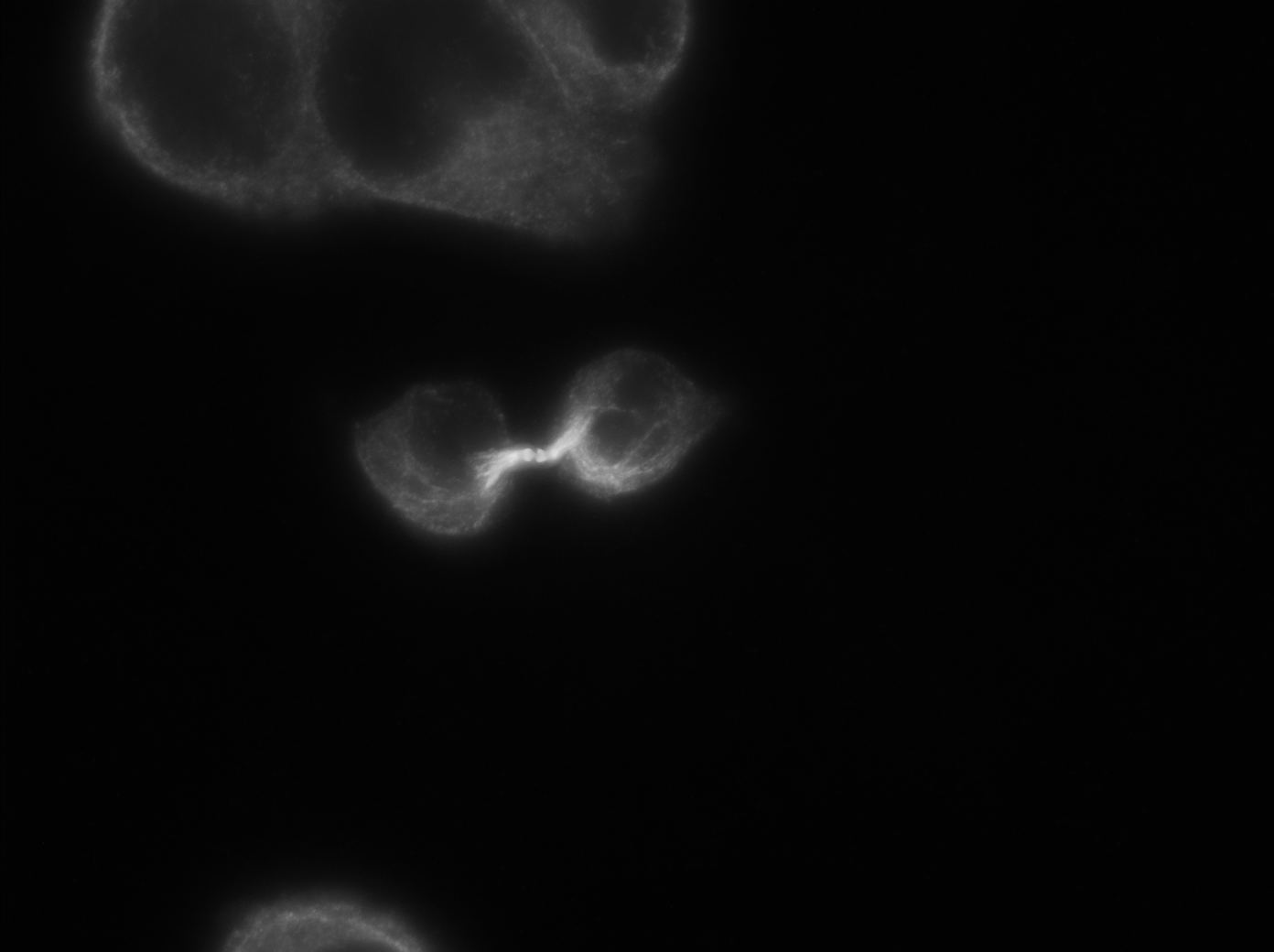

Supplement: Supplementary file 26 — Source data Fig. 7 part 2 [file 44319_2026_742_MOESM26_ESM.zip › Figure 7 Part 2/Fig 7acd Cas9 and TPGS1-ko rGT335 atubulin part 2/TPGS1-KO GT335recomb atub 3-24-25 R2 ET4.Project Maximum Z_XY1742841450_Z0_T0_C2.tif]

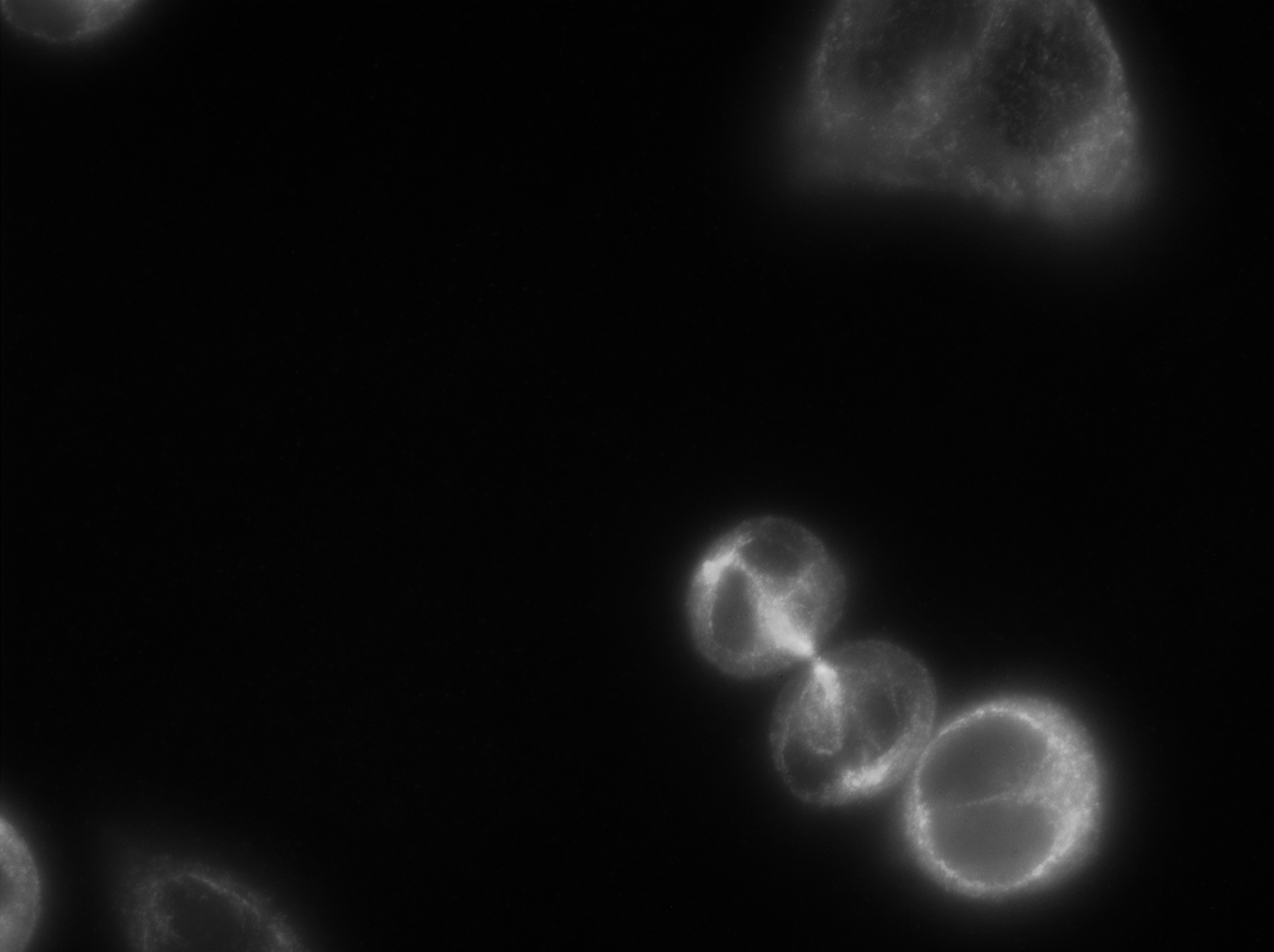

Supplement: Supplementary file 26 — Source data Fig. 7 part 2 [file 44319_2026_742_MOESM26_ESM.zip › Figure 7 Part 2/Fig 7acd Cas9 and TPGS1-ko rGT335 atubulin part 2/TPGS1-KO GT335recomb atub 3-24-25 R1 ET2.Project Maximum Z_XY1742839982_Z0_T0_C2.tif]

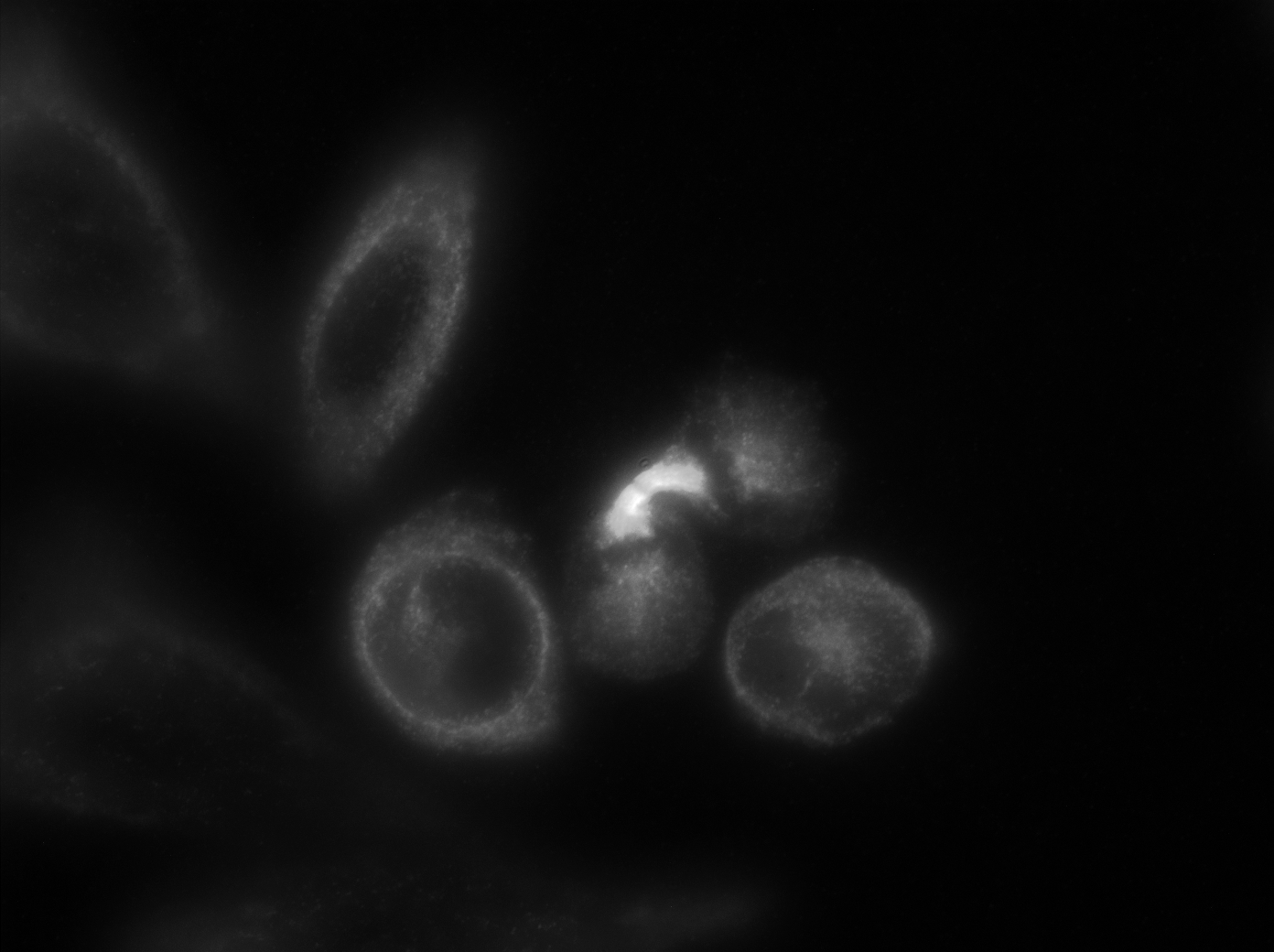

Supplement: Supplementary file 26 — Source data Fig. 7 part 2 [file 44319_2026_742_MOESM26_ESM.zip › Figure 7 Part 2/Fig 7acd Cas9 and TPGS1-ko rGT335 atubulin part 2/TPGS1-KO GT335recomb atub 3-24-25 R1 ET1.Project Maximum Z_XY1742839836_Z0_T0_C2.tif]

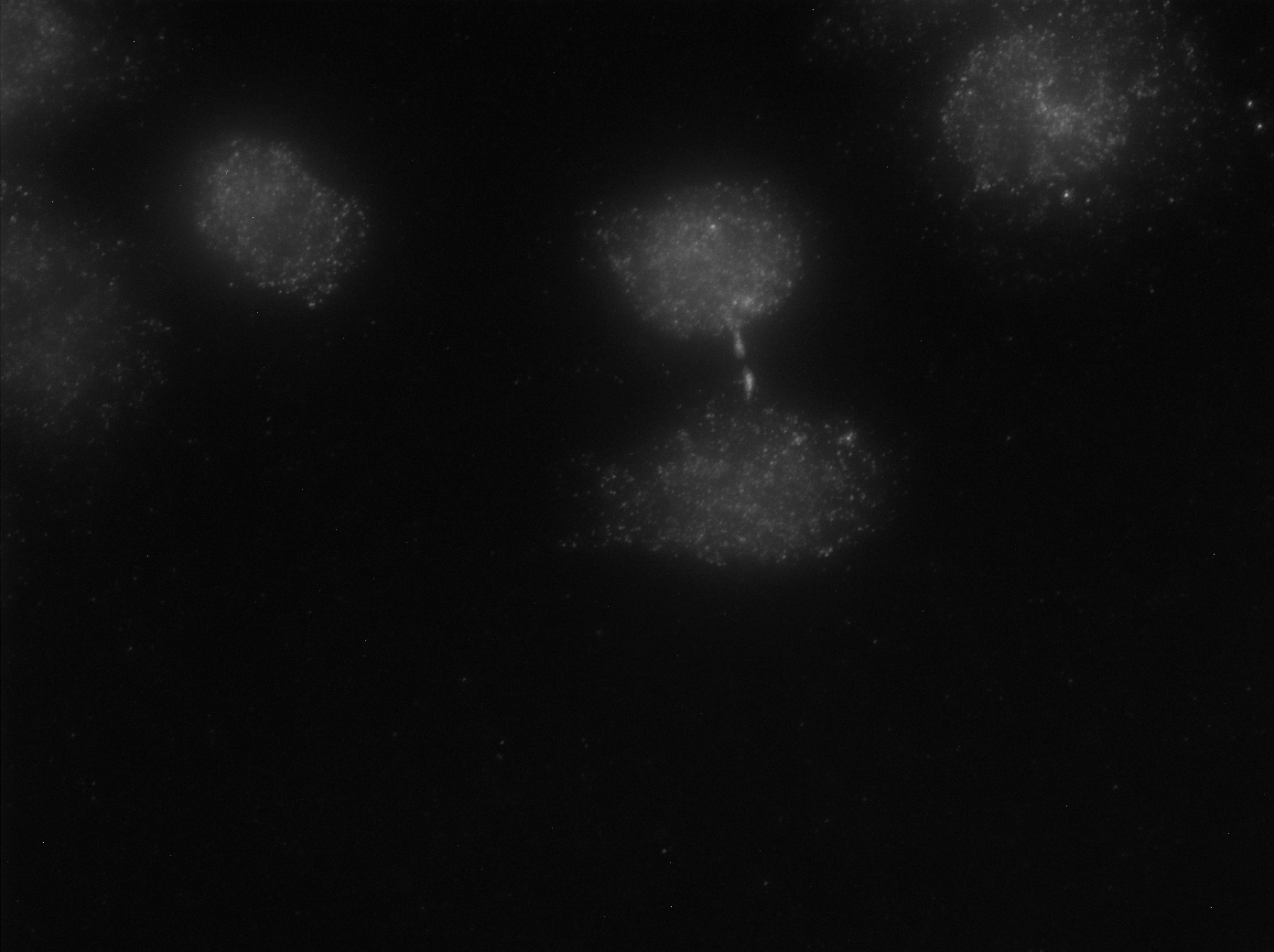

Supplement: Supplementary file 26 — Source data Fig. 7 part 2 [file 44319_2026_742_MOESM26_ESM.zip › Figure 7 Part 2/Fig 7acd Cas9 and TPGS1-ko rGT335 atubulin part 2/TPGS1-KO GT335recomb atub 3-24-25 R1 LT6.Project Maximum Z_XY1742839619_Z0_T0_C1.tif]

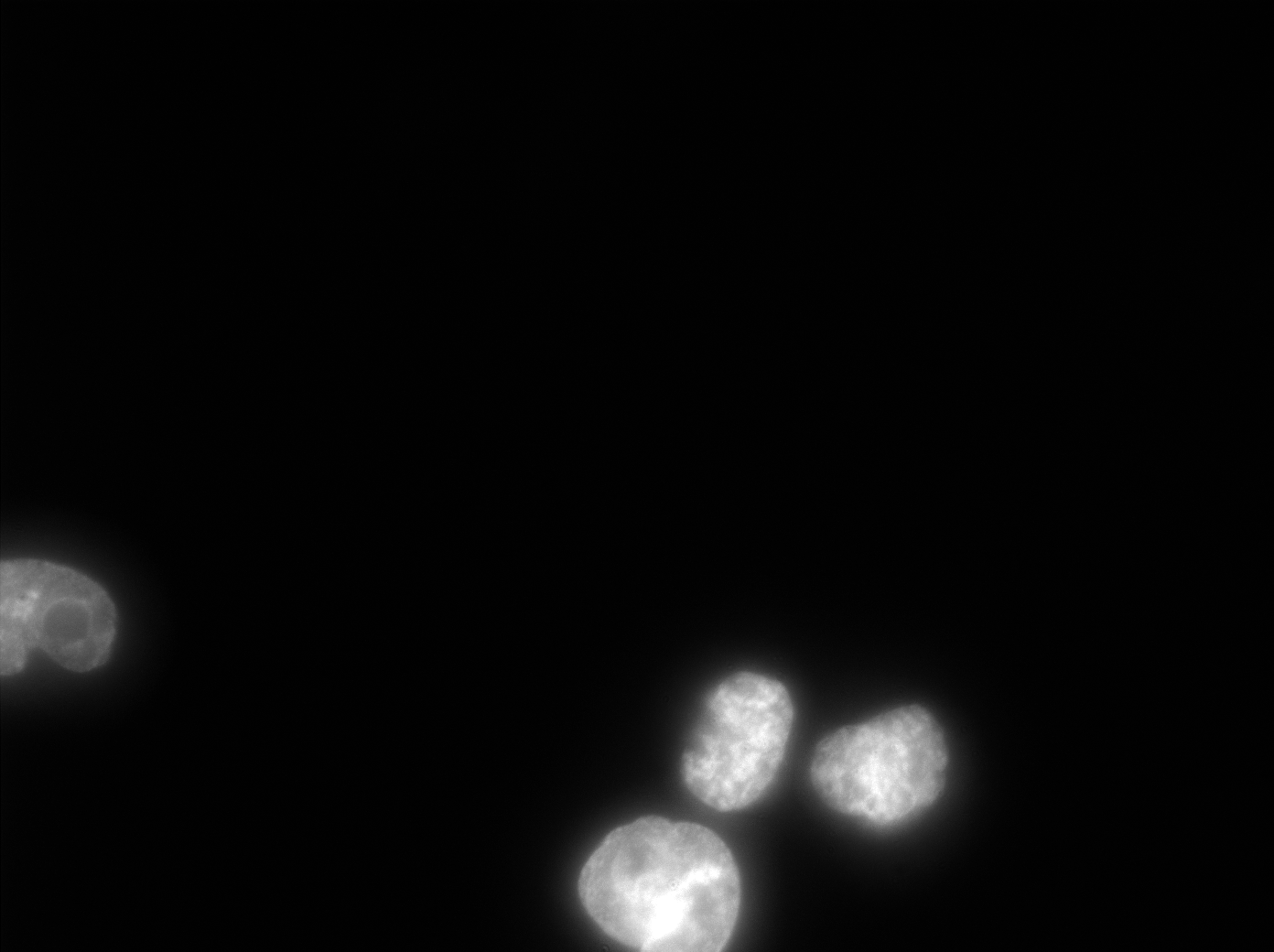

Supplement: Supplementary file 26 — Source data Fig. 7 part 2 [file 44319_2026_742_MOESM26_ESM.zip › Figure 7 Part 2/Fig 7acd Cas9 and TPGS1-ko rGT335 atubulin part 2/TPGS1-KO GT335recomb atub 3-24-25 R1 ET10.Project Maximum Z_XY1742840860_Z0_T0_C0.tif]

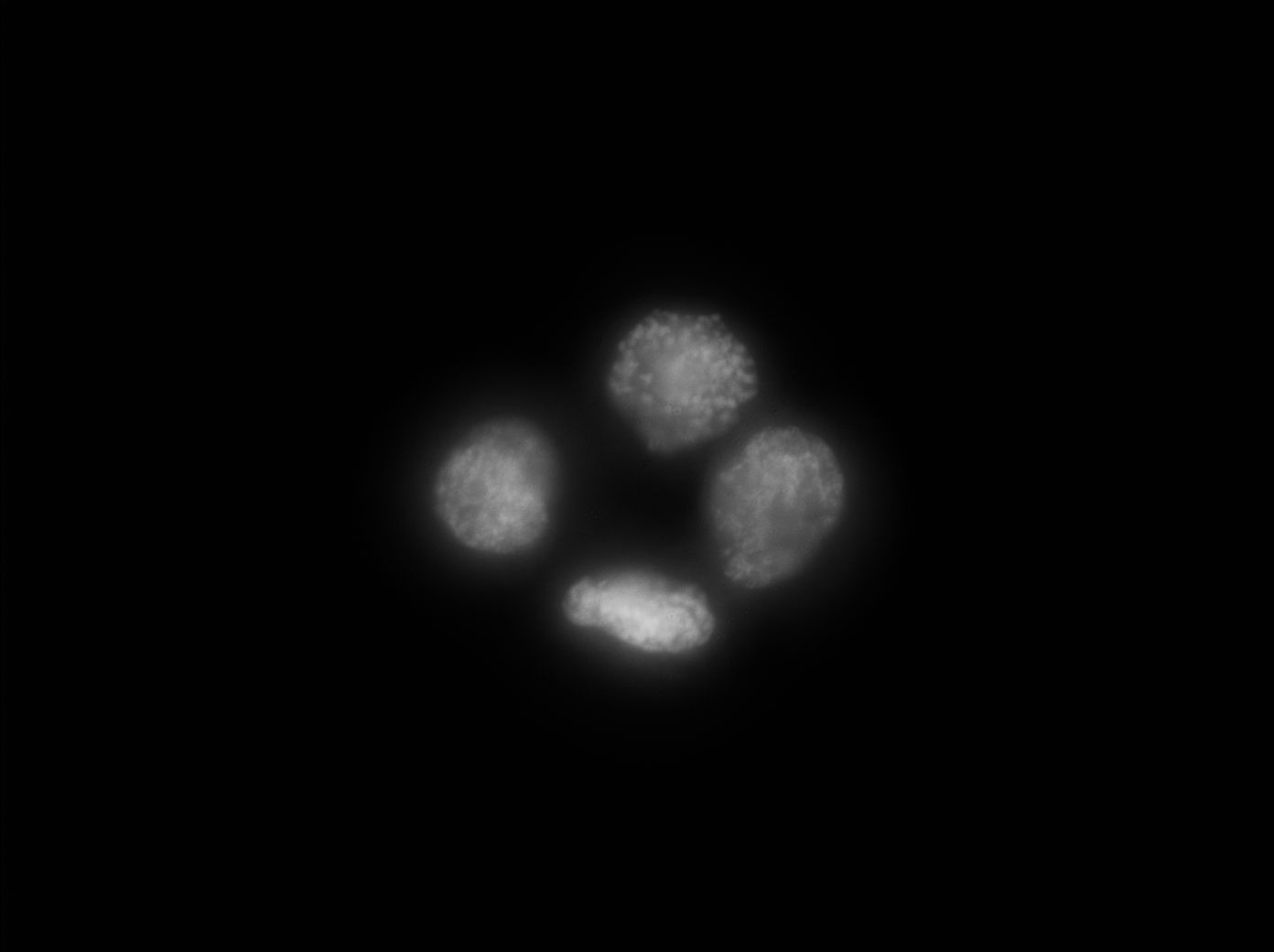

Supplement: Supplementary file 26 — Source data Fig. 7 part 2 [file 44319_2026_742_MOESM26_ESM.zip › Figure 7 Part 2/Fig 7acd Cas9 and TPGS1-ko rGT335 atubulin part 2/TPGS1-KO GT335recomb atub 3-24-25 R2 ETCROSS.Project Maximum Z_XY1742842646_Z0_T0_C0.tif]

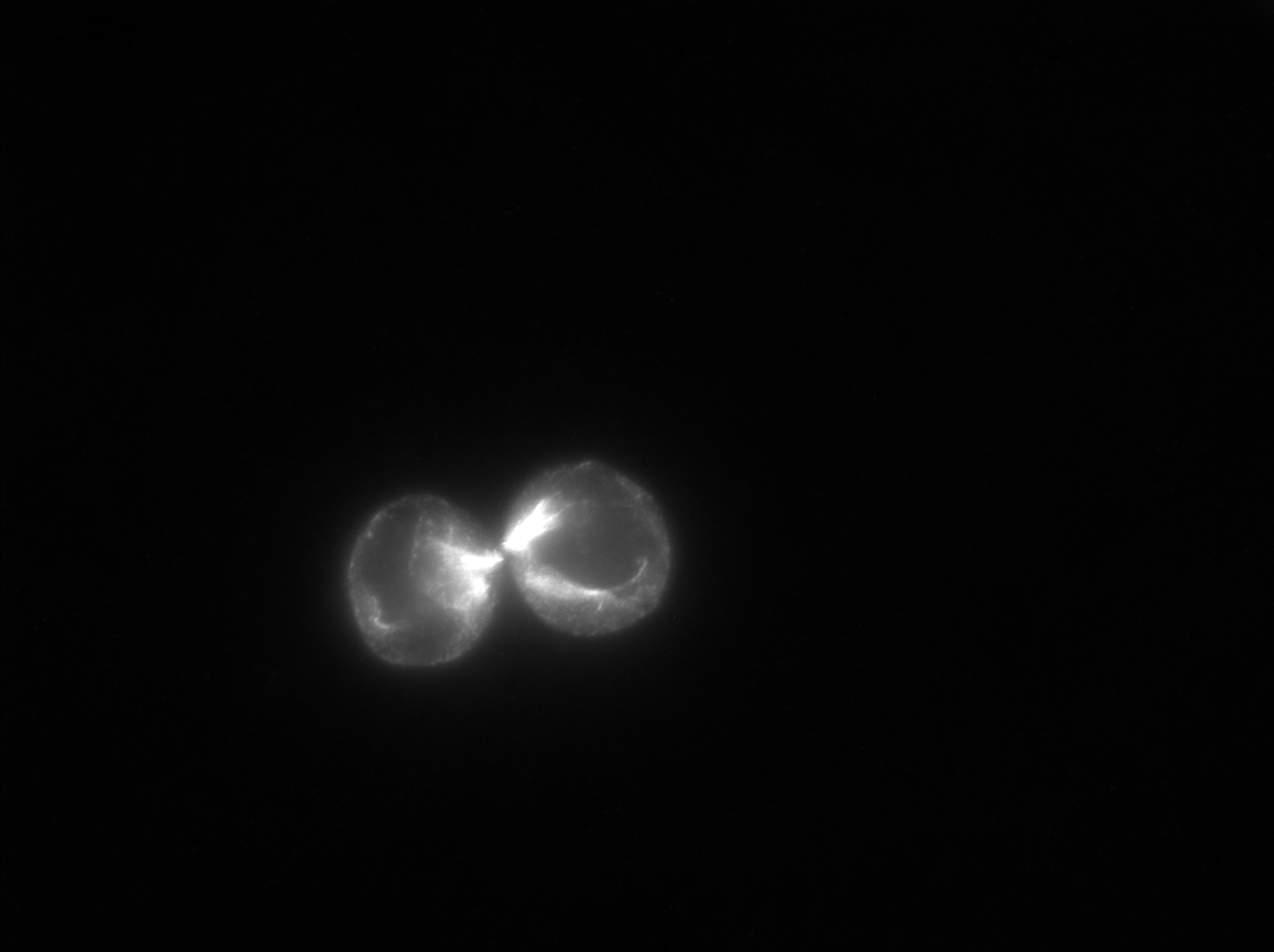

Supplement: Supplementary file 26 — Source data Fig. 7 part 2 [file 44319_2026_742_MOESM26_ESM.zip › Figure 7 Part 2/Fig 7acd Cas9 and TPGS1-ko rGT335 atubulin part 2/TPGS1-KO GT335recomb atub 3-24-25 R3 ET8.Project Maximum Z_XY1742853790_Z0_T0_C2.tif]

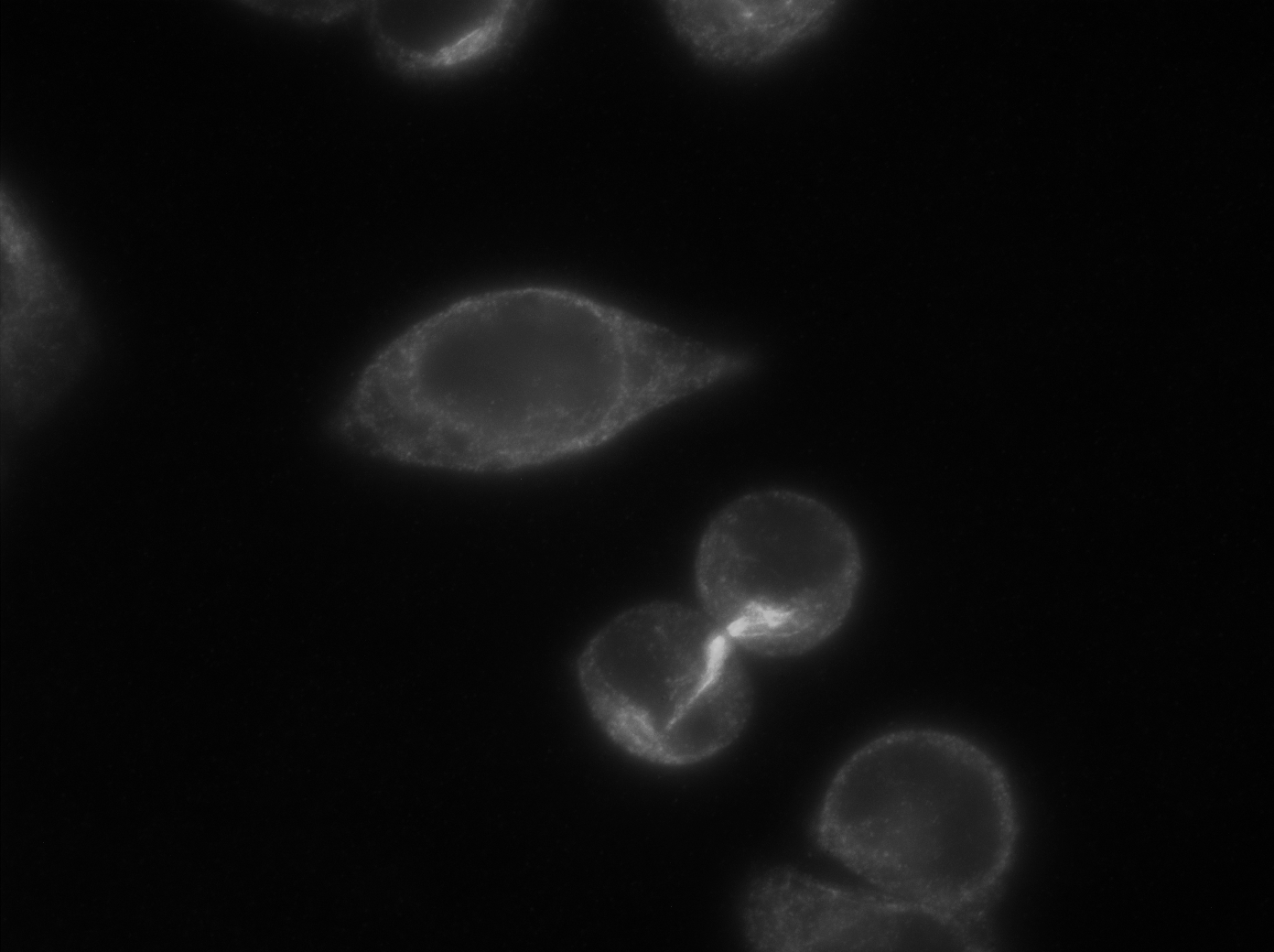

Supplement: Supplementary file 26 — Source data Fig. 7 part 2 [file 44319_2026_742_MOESM26_ESM.zip › Figure 7 Part 2/Fig 7acd Cas9 and TPGS1-ko rGT335 atubulin part 2/TPGS1-KO GT335recomb atub 3-24-25 R1 ET6.Project Maximum Z_XY1742840388_Z0_T0_C2.tif]

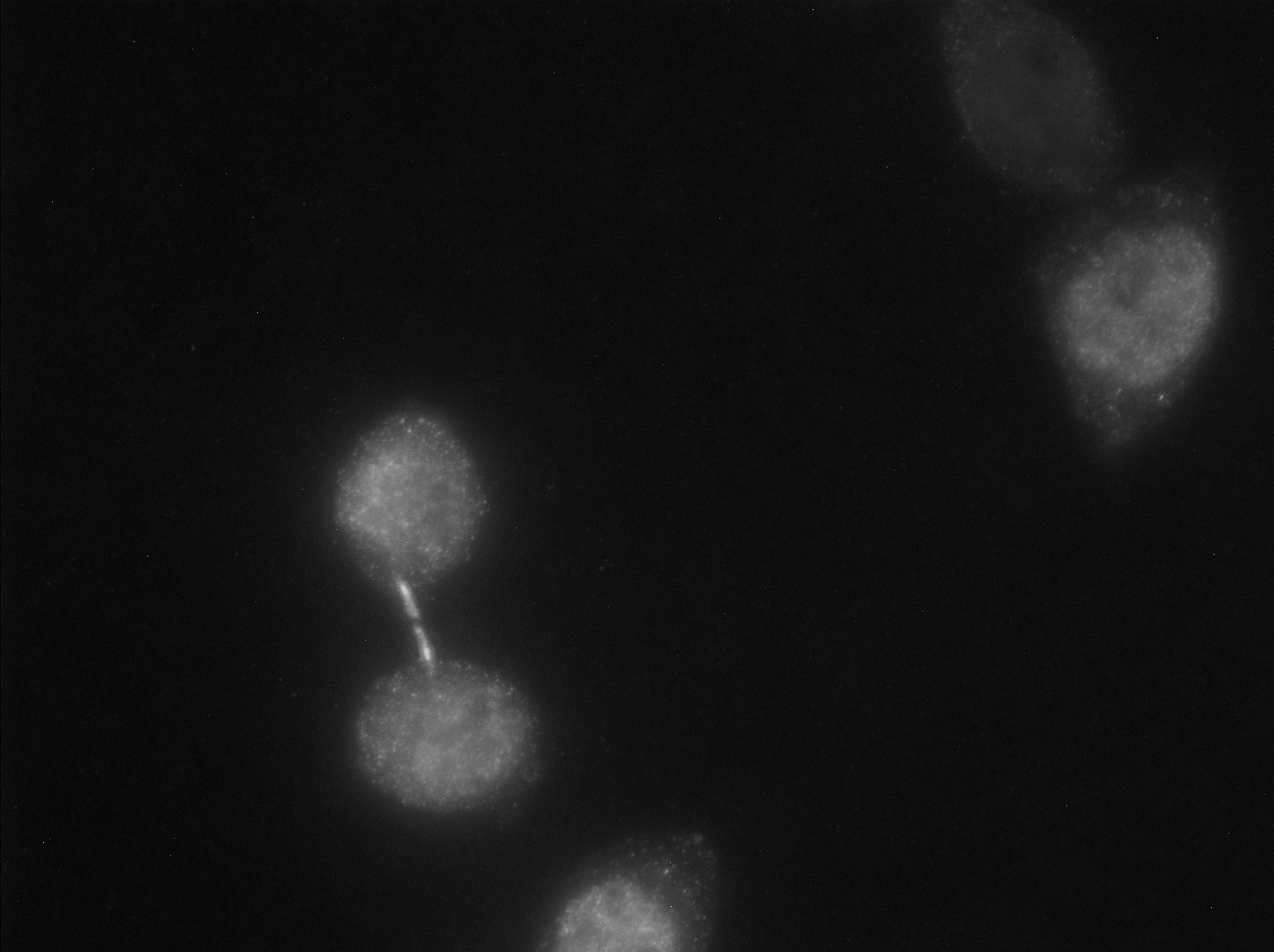

Supplement: Supplementary file 26 — Source data Fig. 7 part 2 [file 44319_2026_742_MOESM26_ESM.zip › Figure 7 Part 2/Fig 7acd Cas9 and TPGS1-ko rGT335 atubulin part 2/TPGS1-KO GT335recomb atub 3-24-25 R3 LT4.Project Maximum Z_XY1742852920_Z0_T0_C1.tif]

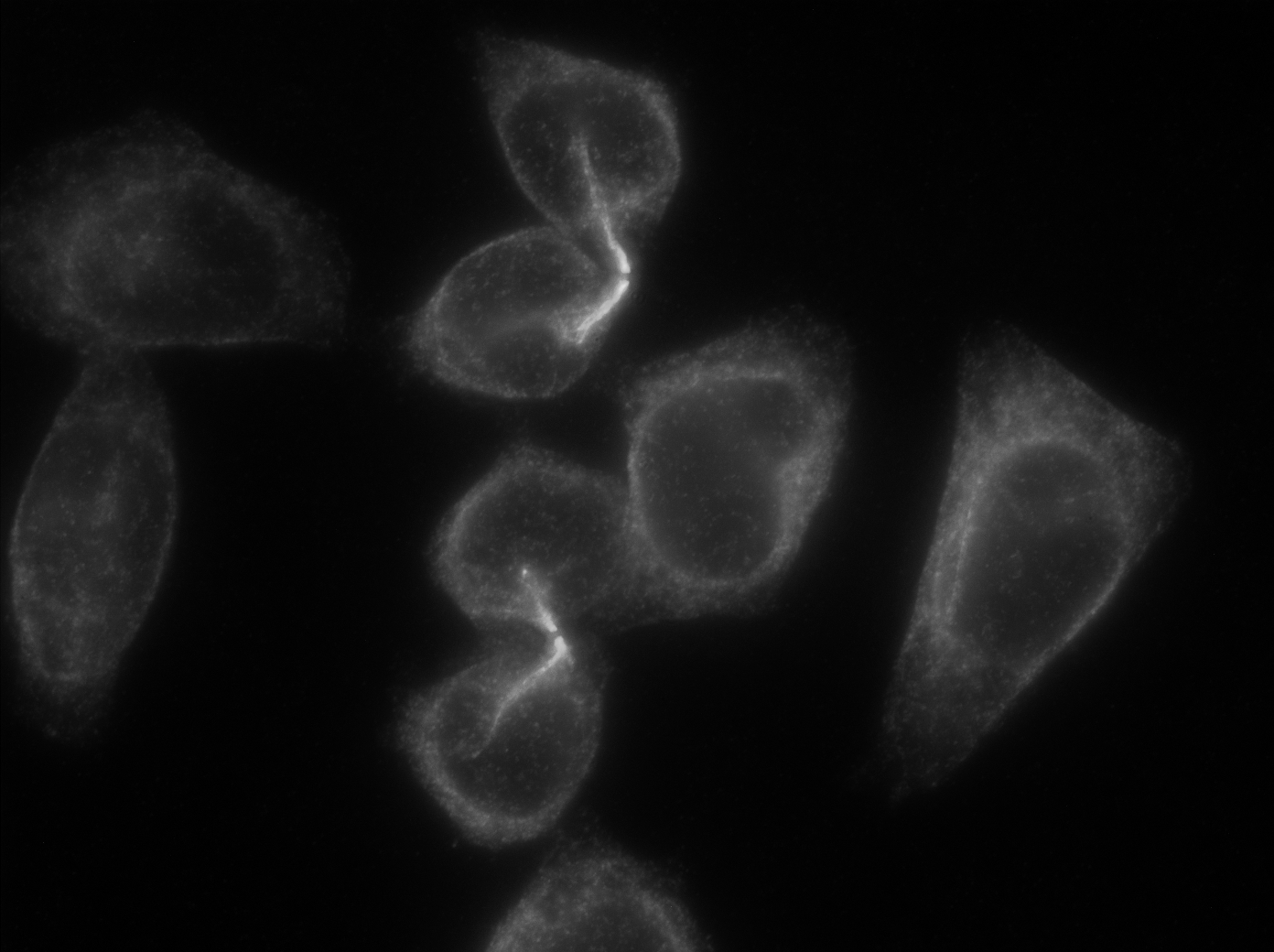

Supplement: Supplementary file 26 — Source data Fig. 7 part 2 [file 44319_2026_742_MOESM26_ESM.zip › Figure 7 Part 2/Fig 7acd Cas9 and TPGS1-ko rGT335 atubulin part 2/TPGS1-KO GT335recomb atub 3-24-25 R1 LT7LT8.Project Maximum Z_XY1742839726_Z0_T0_C2.tif]

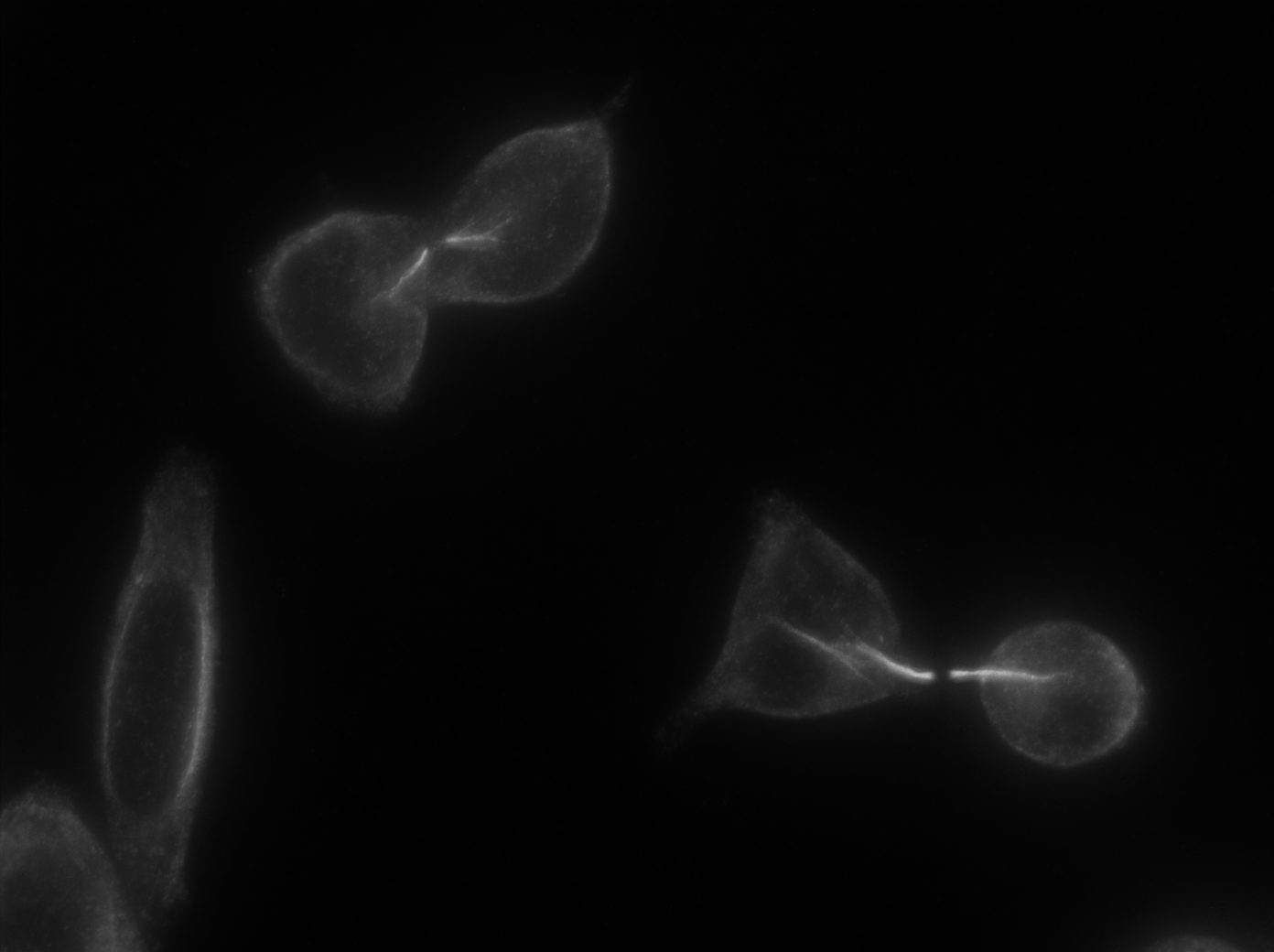

Supplement: Supplementary file 26 — Source data Fig. 7 part 2 [file 44319_2026_742_MOESM26_ESM.zip › Figure 7 Part 2/Fig 7acd Cas9 and TPGS1-ko rGT335 atubulin part 2/TPGS1-KO GT335recomb atub 3-24-25 R2 LT3LT4.Project Maximum Z_XY1742841894_Z0_T0_C2.tif]

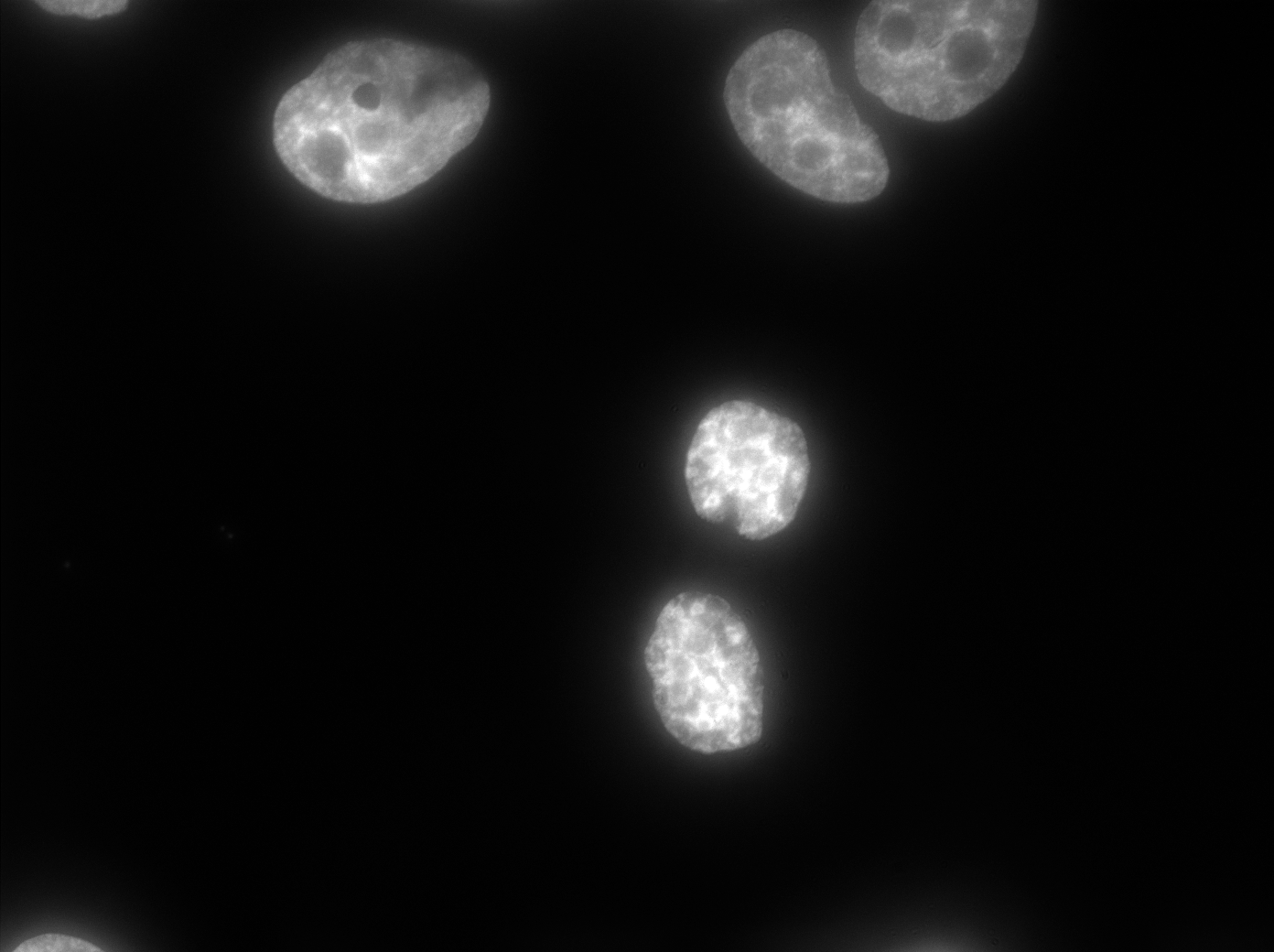

Supplement: Supplementary file 26 — Source data Fig. 7 part 2 [file 44319_2026_742_MOESM26_ESM.zip › Figure 7 Part 2/Fig 7acd Cas9 and TPGS1-ko rGT335 atubulin part 2/TPGS1-KO GT335recomb atub 3-24-25 R1 LT5.Project Maximum Z_XY1742839528_Z0_T0_C0.tif]

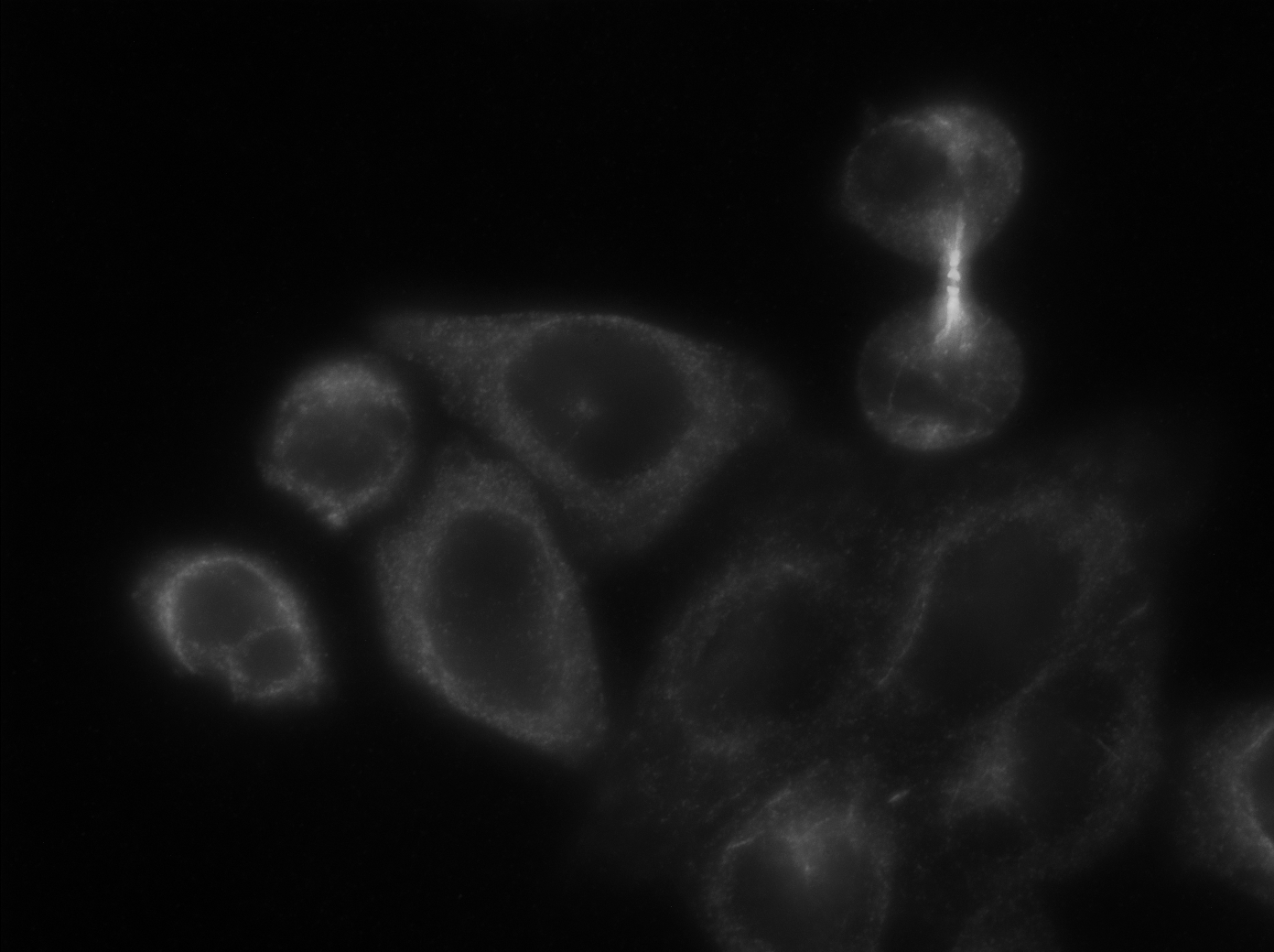

Supplement: Supplementary file 26 — Source data Fig. 7 part 2 [file 44319_2026_742_MOESM26_ESM.zip › Figure 7 Part 2/Fig 7acd Cas9 and TPGS1-ko rGT335 atubulin part 2/TPGS1-KO GT335recomb atub 3-24-25 R1 ET8.Project Maximum Z_XY1742840621_Z0_T0_C2.tif]

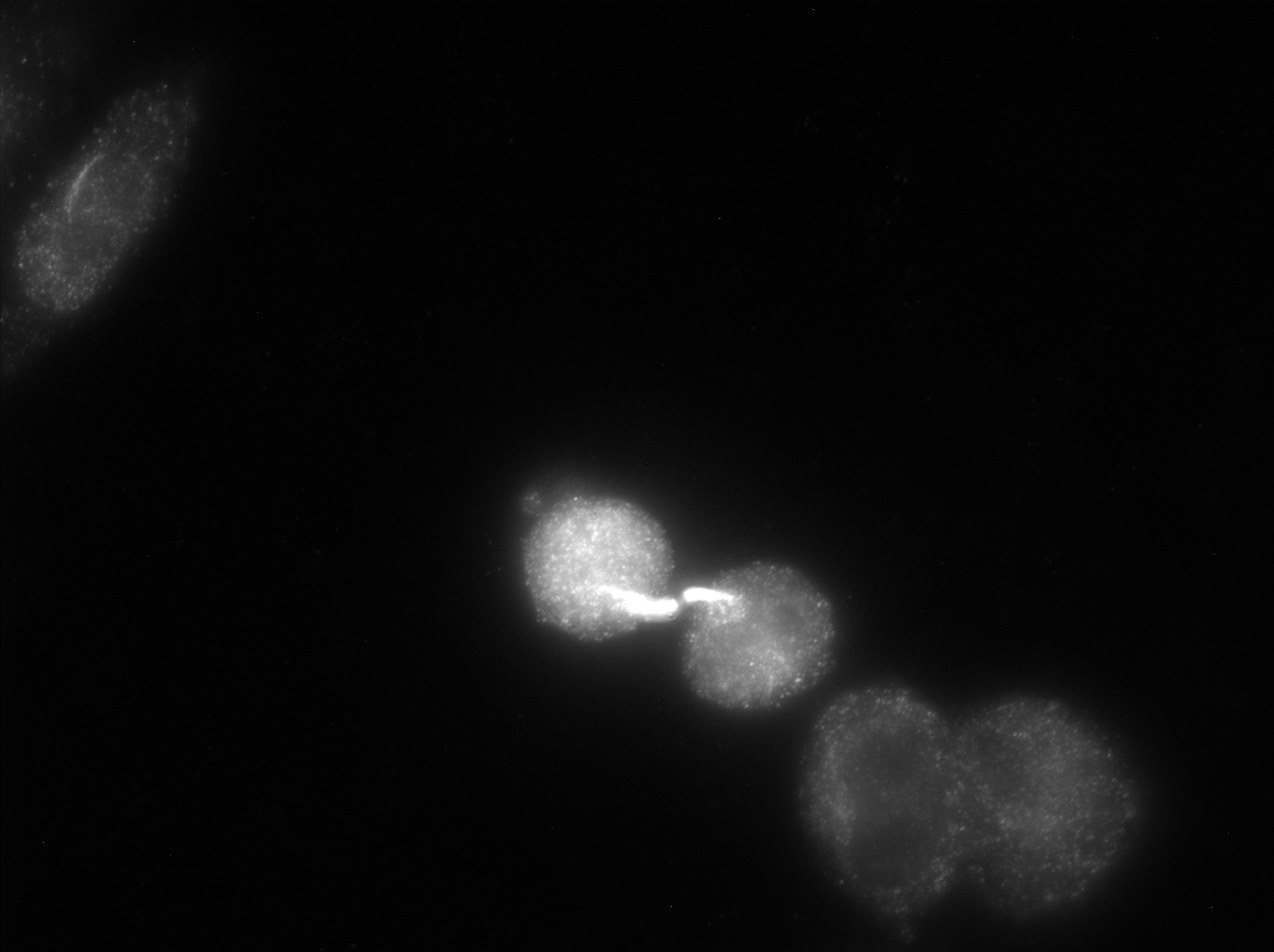

Supplement: Supplementary file 26 — Source data Fig. 7 part 2 [file 44319_2026_742_MOESM26_ESM.zip › Figure 7 Part 2/Fig 7acd Cas9 and TPGS1-ko rGT335 atubulin part 2/TPGS1-KO GT335recomb atub 3-24-25 R2 ET9.Project Maximum Z_XY1742842166_Z0_T0_C1.tif]

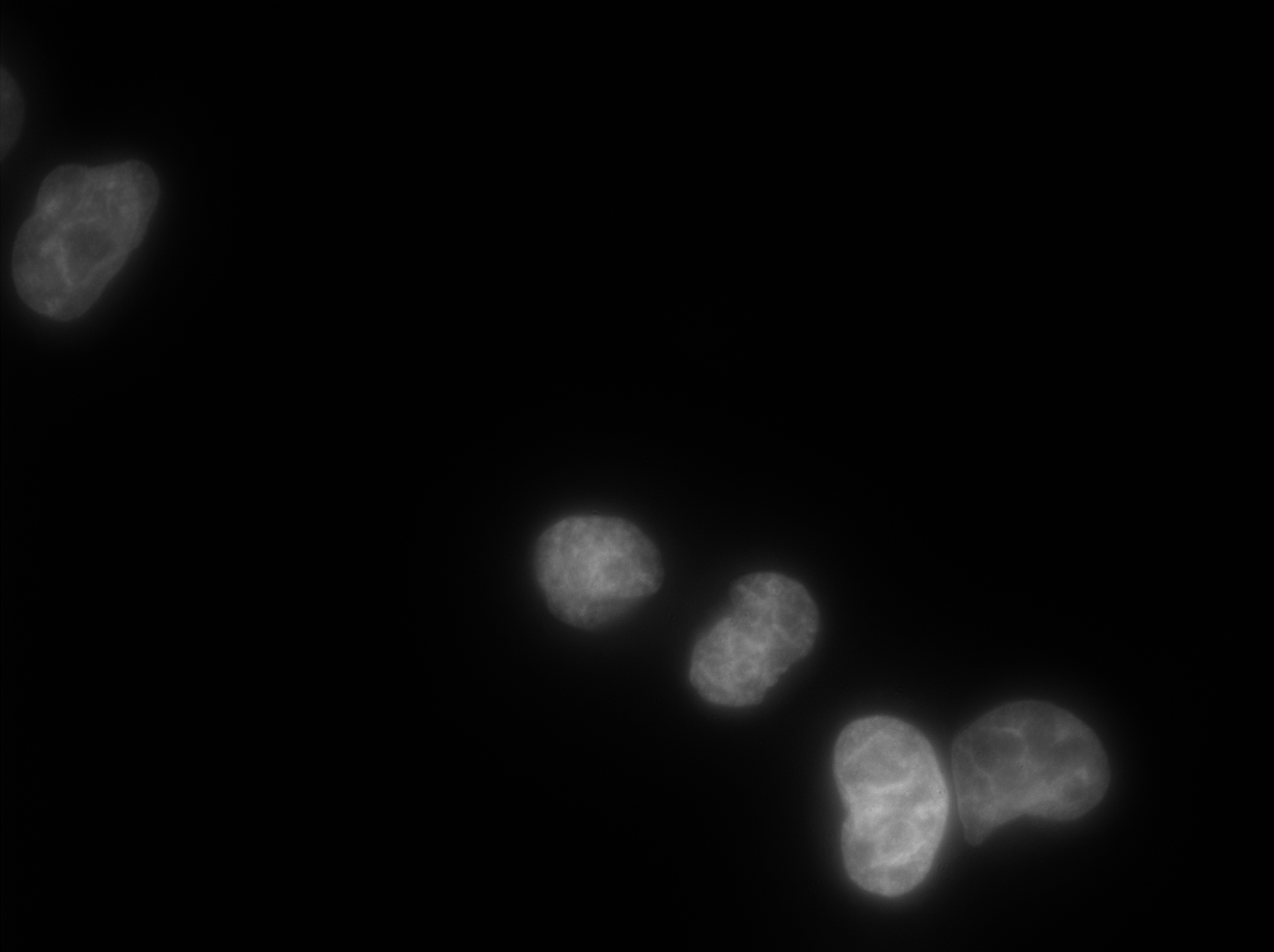

Supplement: Supplementary file 26 — Source data Fig. 7 part 2 [file 44319_2026_742_MOESM26_ESM.zip › Figure 7 Part 2/Fig 7acd Cas9 and TPGS1-ko rGT335 atubulin part 2/TPGS1-KO GT335recomb atub 3-24-25 R2 ET9.Project Maximum Z_XY1742842166_Z0_T0_C0.tif]

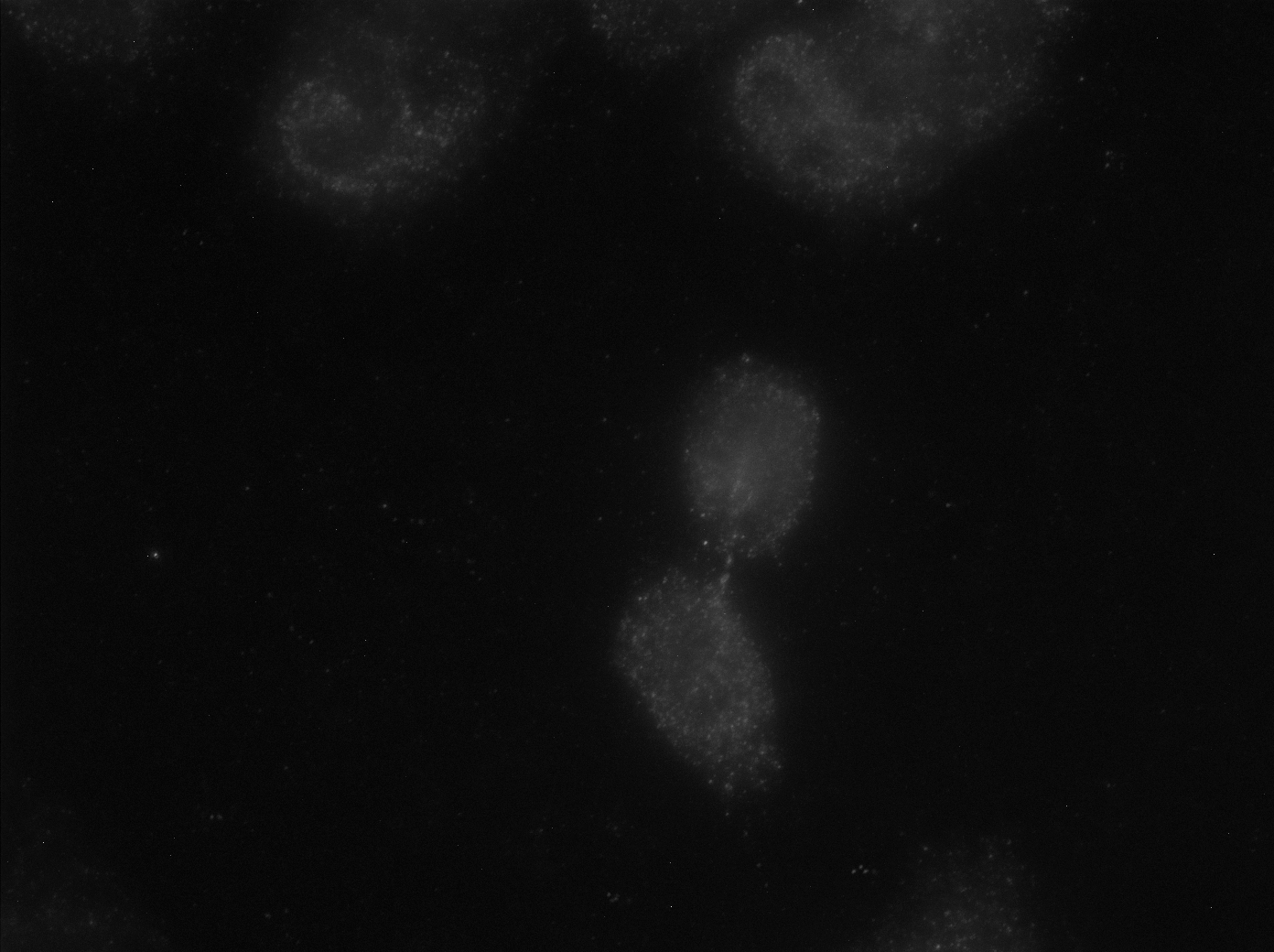

Supplement: Supplementary file 26 — Source data Fig. 7 part 2 [file 44319_2026_742_MOESM26_ESM.zip › Figure 7 Part 2/Fig 7acd Cas9 and TPGS1-ko rGT335 atubulin part 2/TPGS1-KO GT335recomb atub 3-24-25 R1 LT5.Project Maximum Z_XY1742839528_Z0_T0_C1.tif]

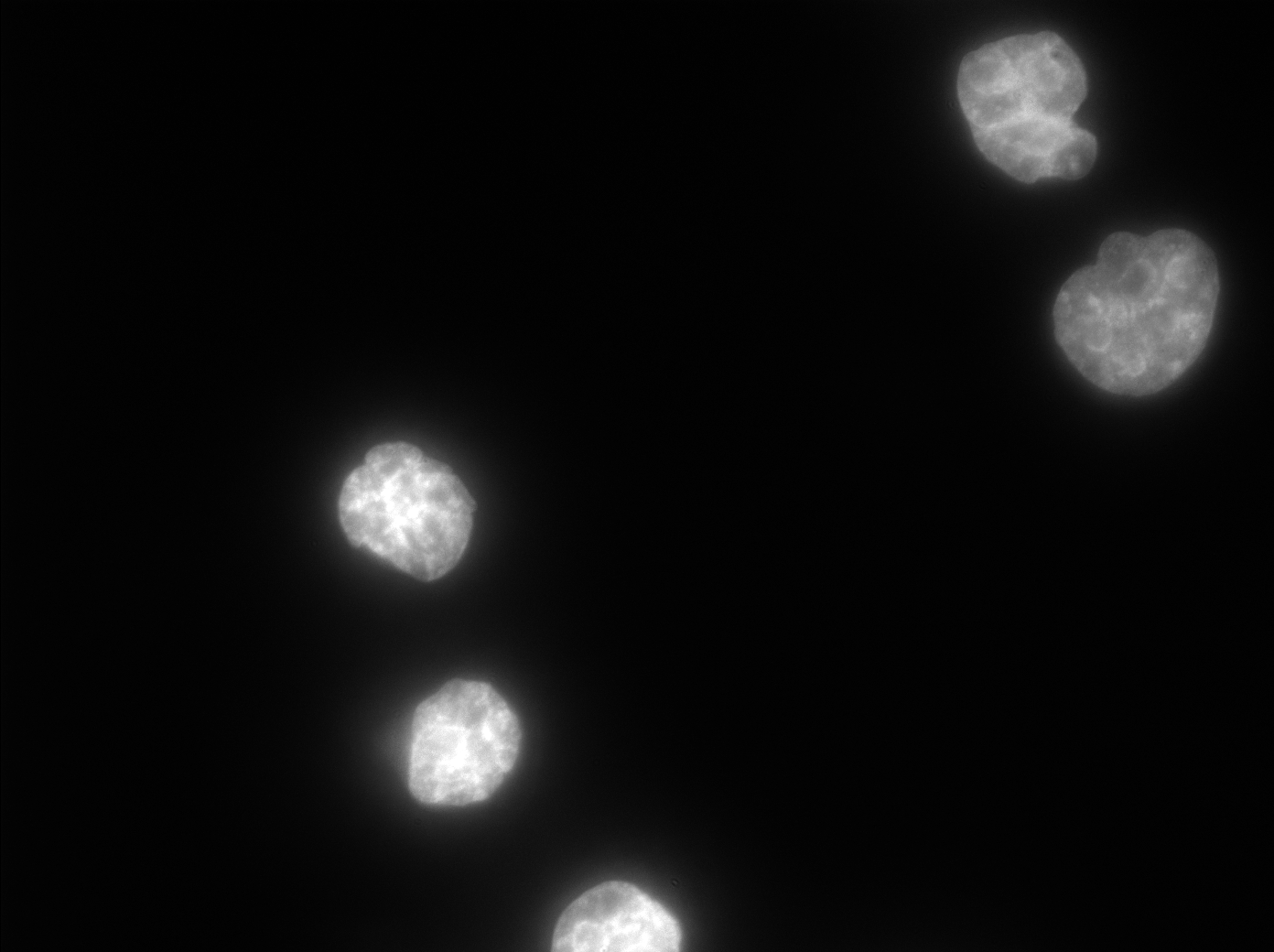

Supplement: Supplementary file 26 — Source data Fig. 7 part 2 [file 44319_2026_742_MOESM26_ESM.zip › Figure 7 Part 2/Fig 7acd Cas9 and TPGS1-ko rGT335 atubulin part 2/TPGS1-KO GT335recomb atub 3-24-25 R3 LT4.Project Maximum Z_XY1742852920_Z0_T0_C0.tif]

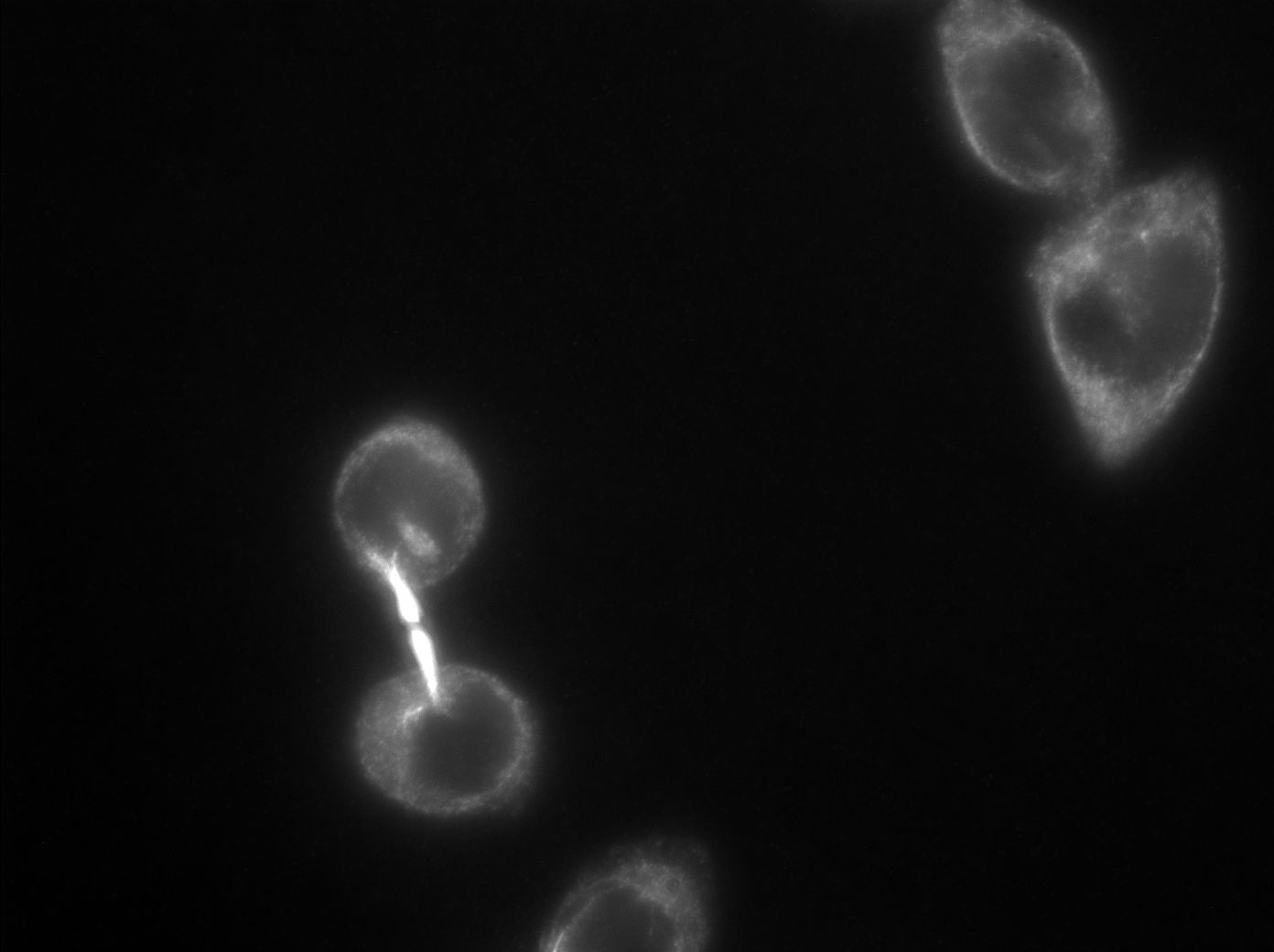

Supplement: Supplementary file 26 — Source data Fig. 7 part 2 [file 44319_2026_742_MOESM26_ESM.zip › Figure 7 Part 2/Fig 7acd Cas9 and TPGS1-ko rGT335 atubulin part 2/TPGS1-KO GT335recomb atub 3-24-25 R3 LT4.Project Maximum Z_XY1742852920_Z0_T0_C2.tif]

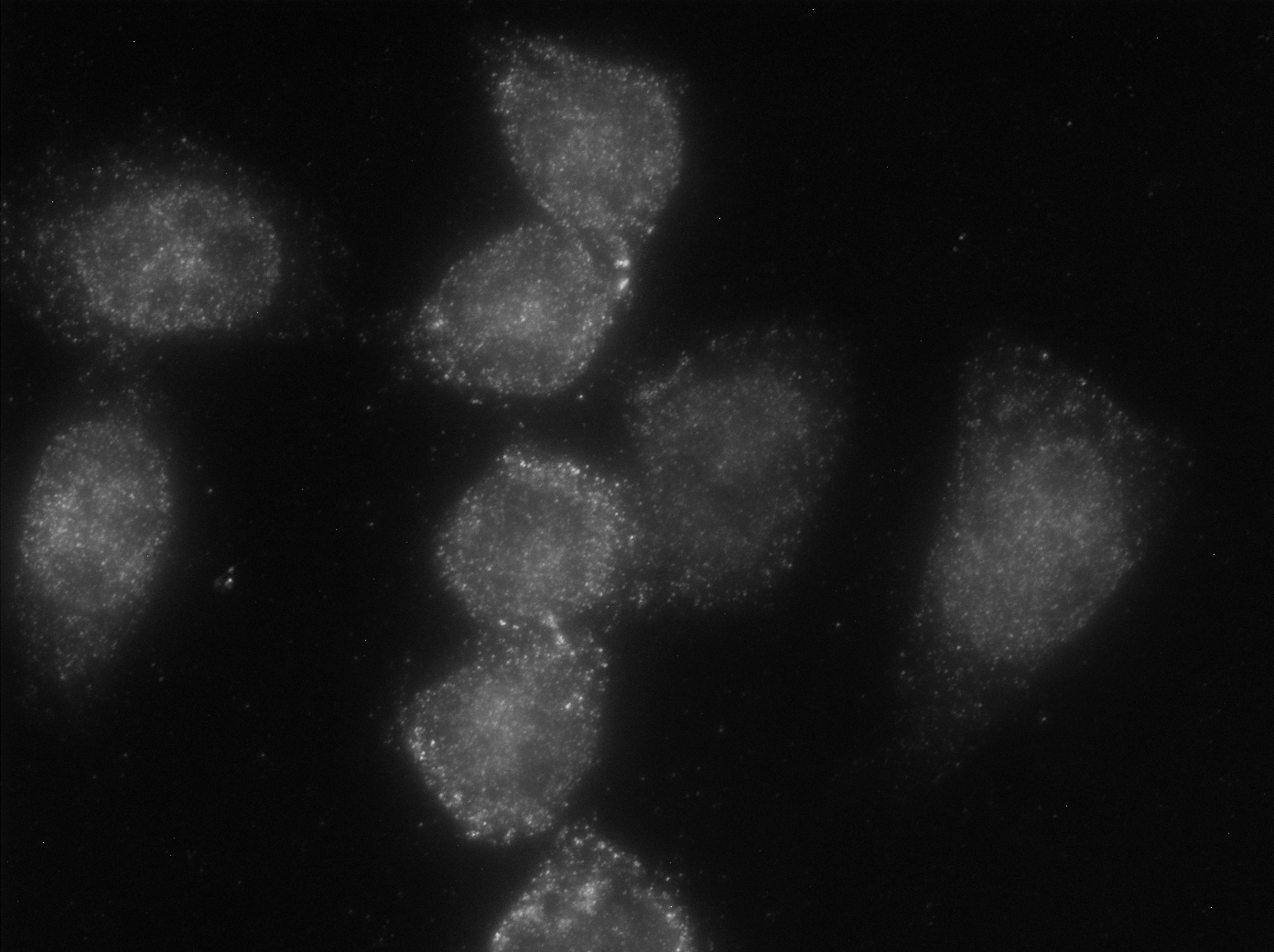

Supplement: Supplementary file 26 — Source data Fig. 7 part 2 [file 44319_2026_742_MOESM26_ESM.zip › Figure 7 Part 2/Fig 7acd Cas9 and TPGS1-ko rGT335 atubulin part 2/TPGS1-KO GT335recomb atub 3-24-25 R1 LT7LT8.Project Maximum Z_XY1742839726_Z0_T0_C1.tif]

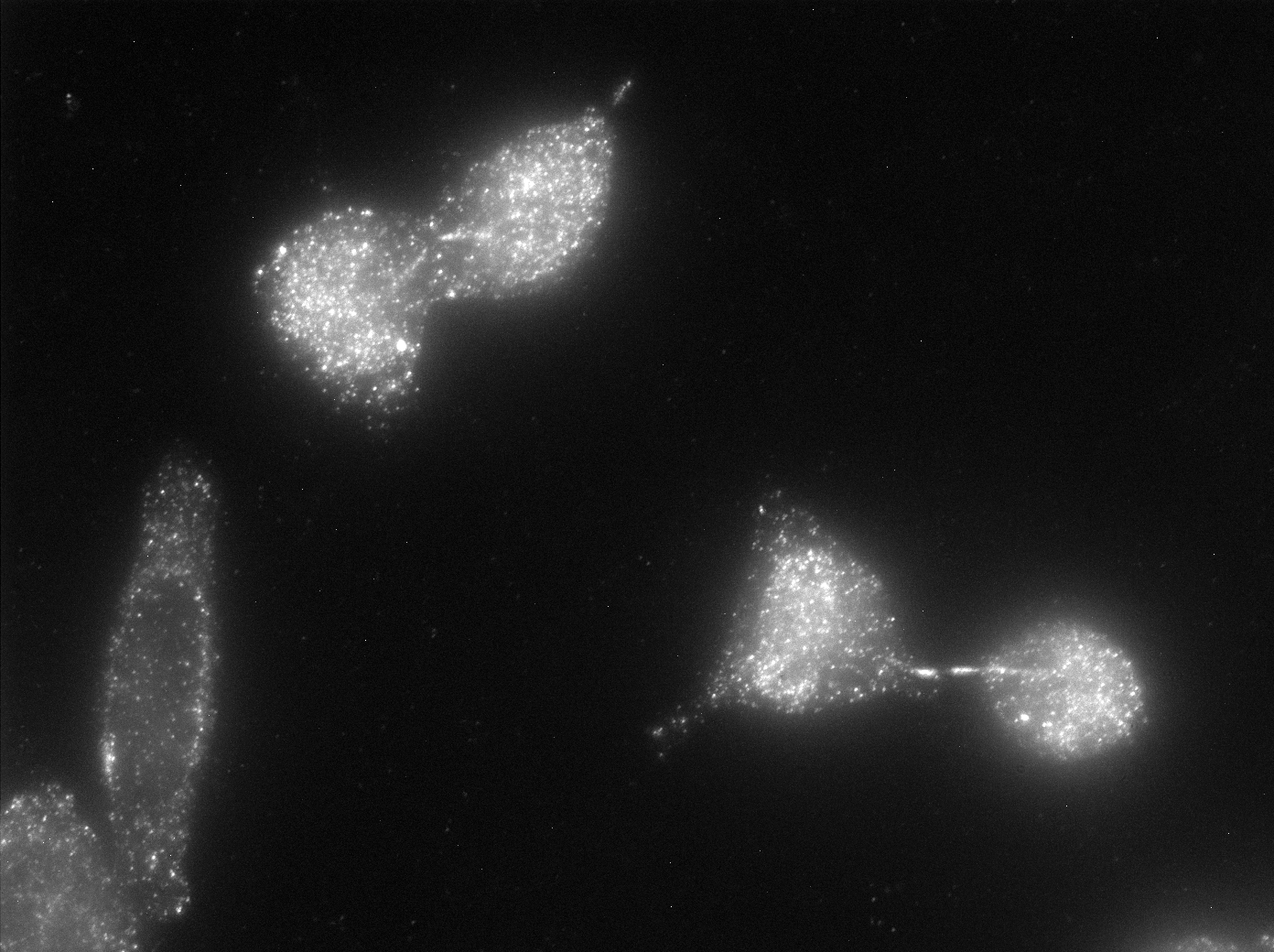

Supplement: Supplementary file 26 — Source data Fig. 7 part 2 [file 44319_2026_742_MOESM26_ESM.zip › Figure 7 Part 2/Fig 7acd Cas9 and TPGS1-ko rGT335 atubulin part 2/TPGS1-KO GT335recomb atub 3-24-25 R2 LT3LT4.Project Maximum Z_XY1742841894_Z0_T0_C1.tif]

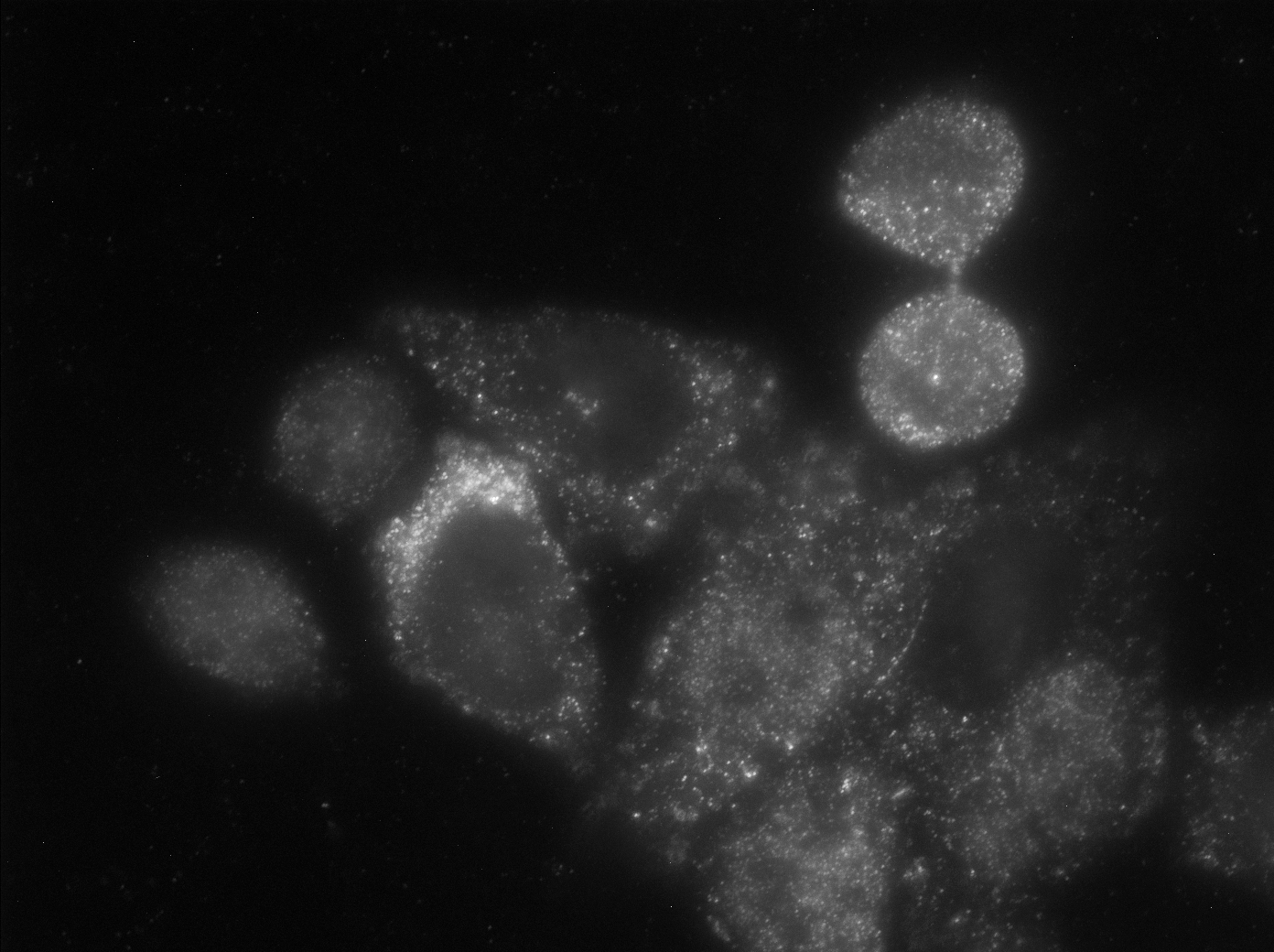

Supplement: Supplementary file 26 — Source data Fig. 7 part 2 [file 44319_2026_742_MOESM26_ESM.zip › Figure 7 Part 2/Fig 7acd Cas9 and TPGS1-ko rGT335 atubulin part 2/TPGS1-KO GT335recomb atub 3-24-25 R1 ET8.Project Maximum Z_XY1742840621_Z0_T0_C1.tif]

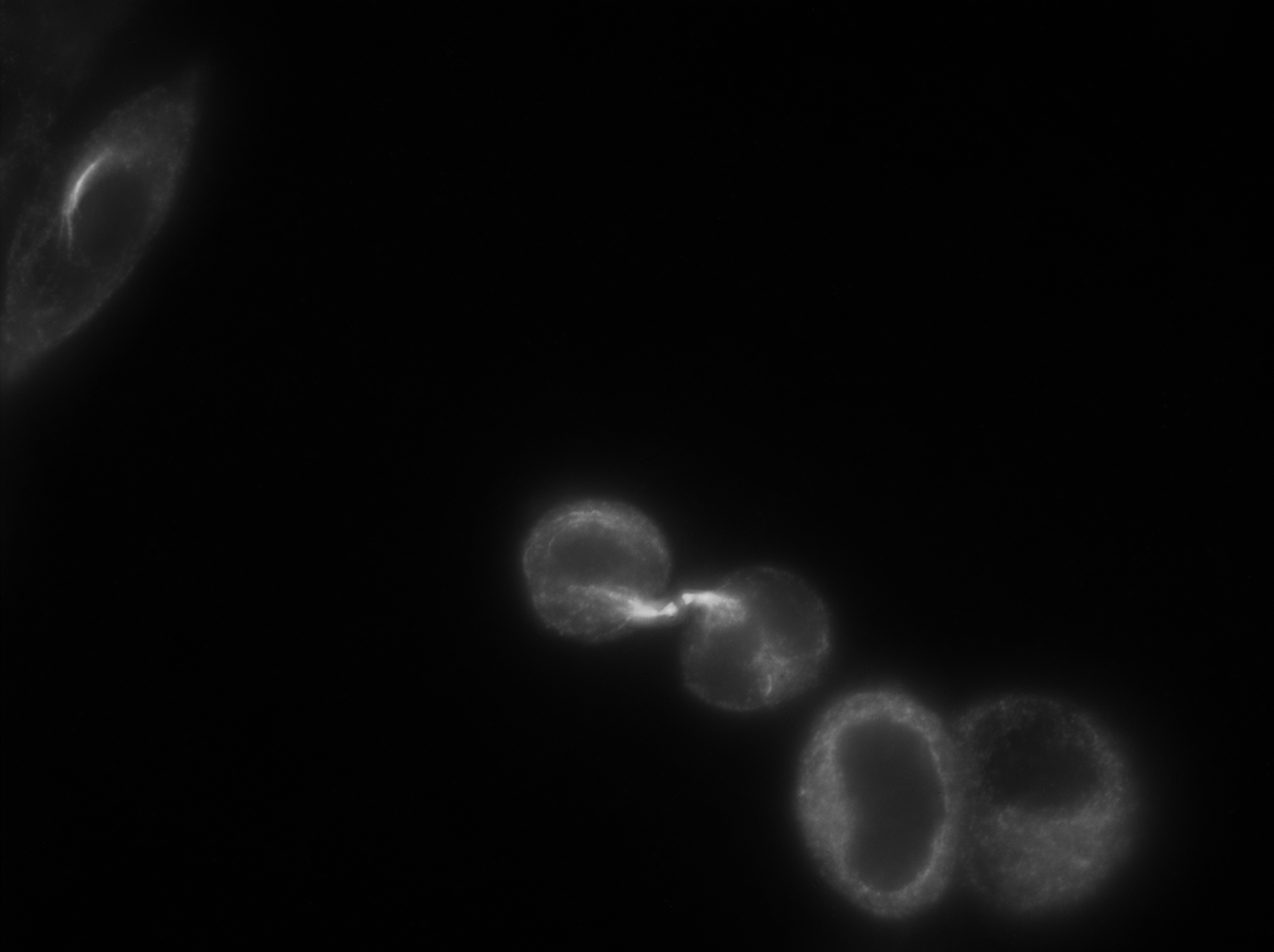

Supplement: Supplementary file 26 — Source data Fig. 7 part 2 [file 44319_2026_742_MOESM26_ESM.zip › Figure 7 Part 2/Fig 7acd Cas9 and TPGS1-ko rGT335 atubulin part 2/TPGS1-KO GT335recomb atub 3-24-25 R2 ET9.Project Maximum Z_XY1742842166_Z0_T0_C2.tif]

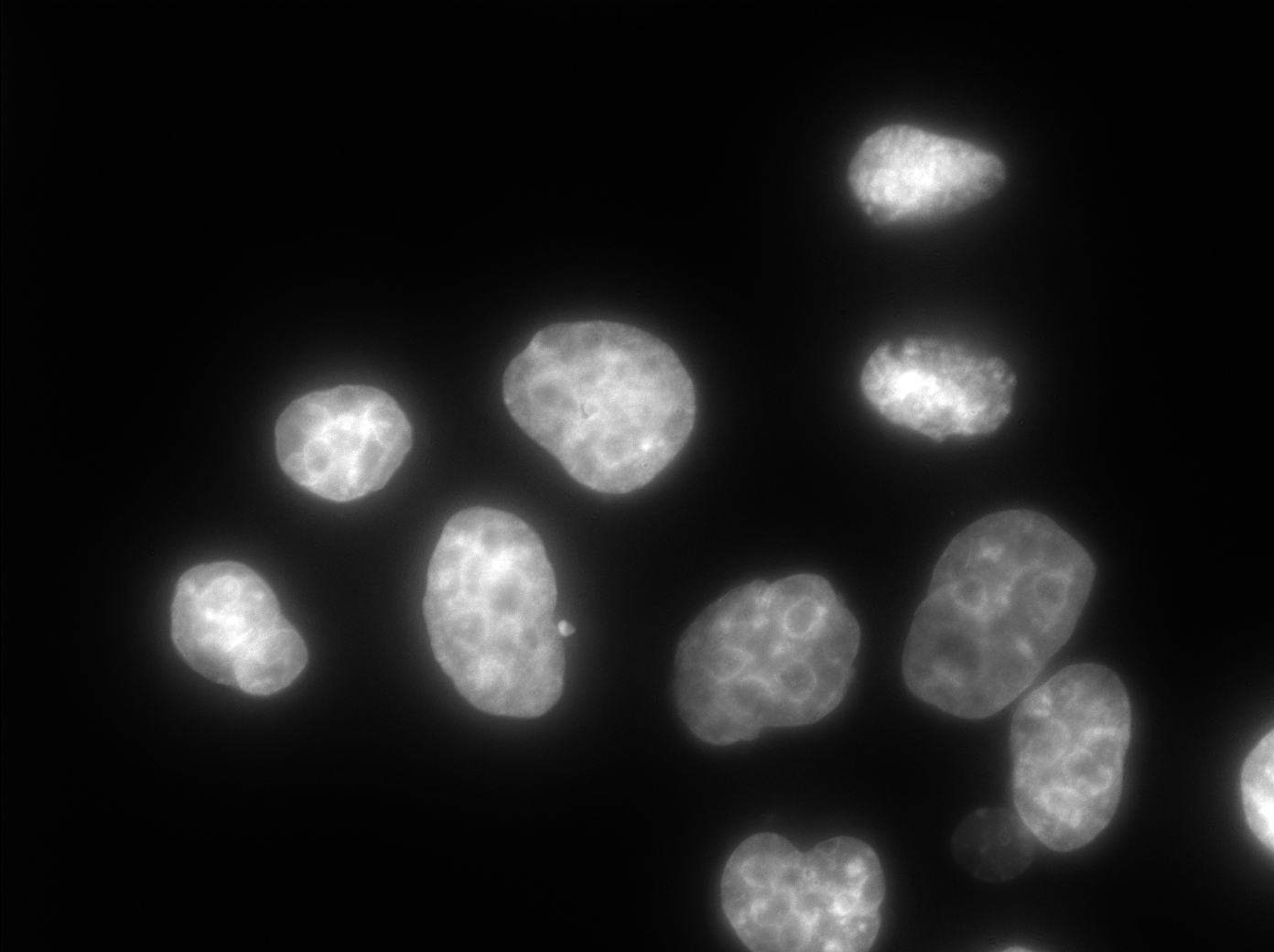

Supplement: Supplementary file 26 — Source data Fig. 7 part 2 [file 44319_2026_742_MOESM26_ESM.zip › Figure 7 Part 2/Fig 7acd Cas9 and TPGS1-ko rGT335 atubulin part 2/TPGS1-KO GT335recomb atub 3-24-25 R1 ET8.Project Maximum Z_XY1742840621_Z0_T0_C0.tif]

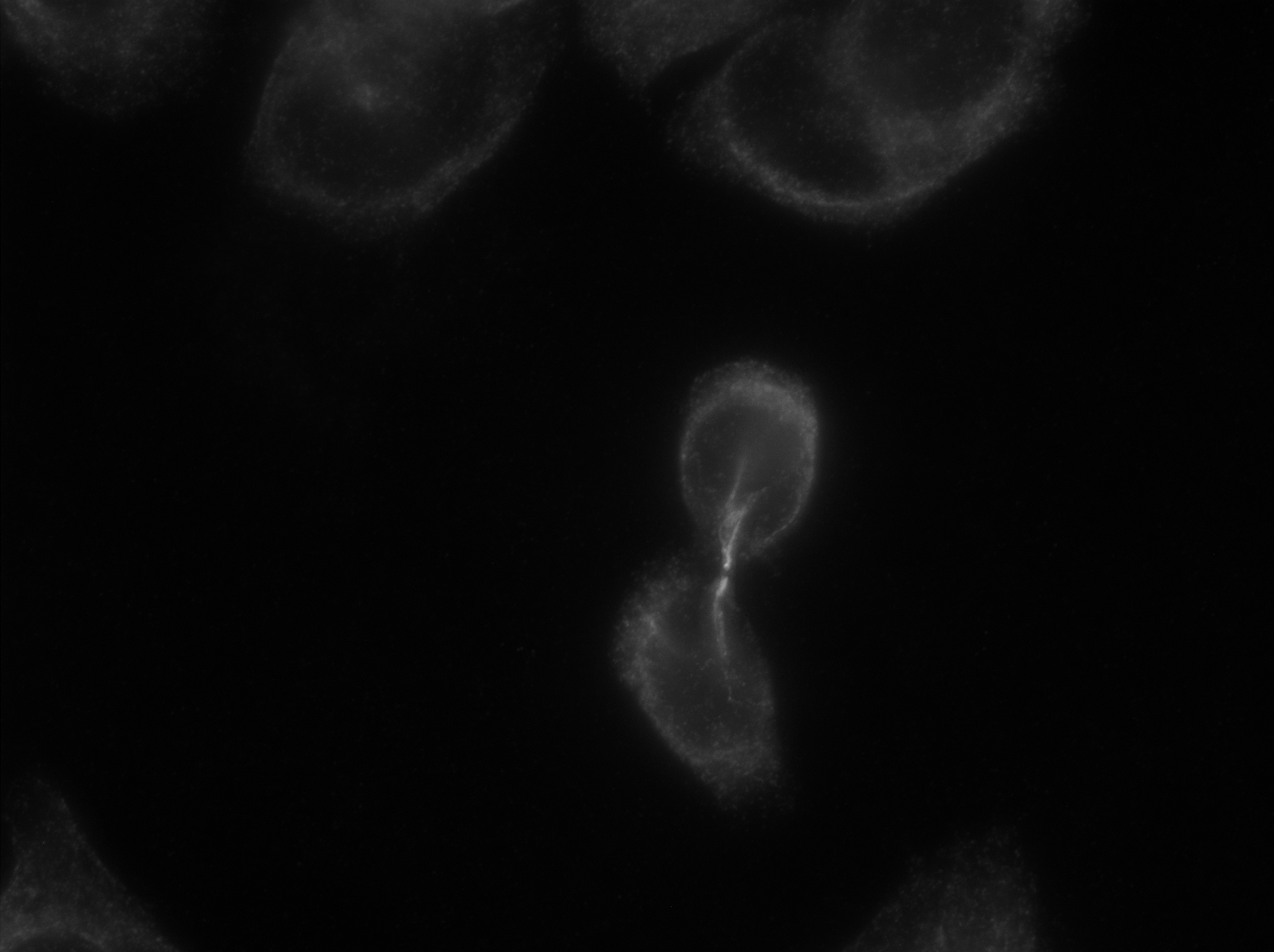

Supplement: Supplementary file 26 — Source data Fig. 7 part 2 [file 44319_2026_742_MOESM26_ESM.zip › Figure 7 Part 2/Fig 7acd Cas9 and TPGS1-ko rGT335 atubulin part 2/TPGS1-KO GT335recomb atub 3-24-25 R1 LT5.Project Maximum Z_XY1742839528_Z0_T0_C2.tif]

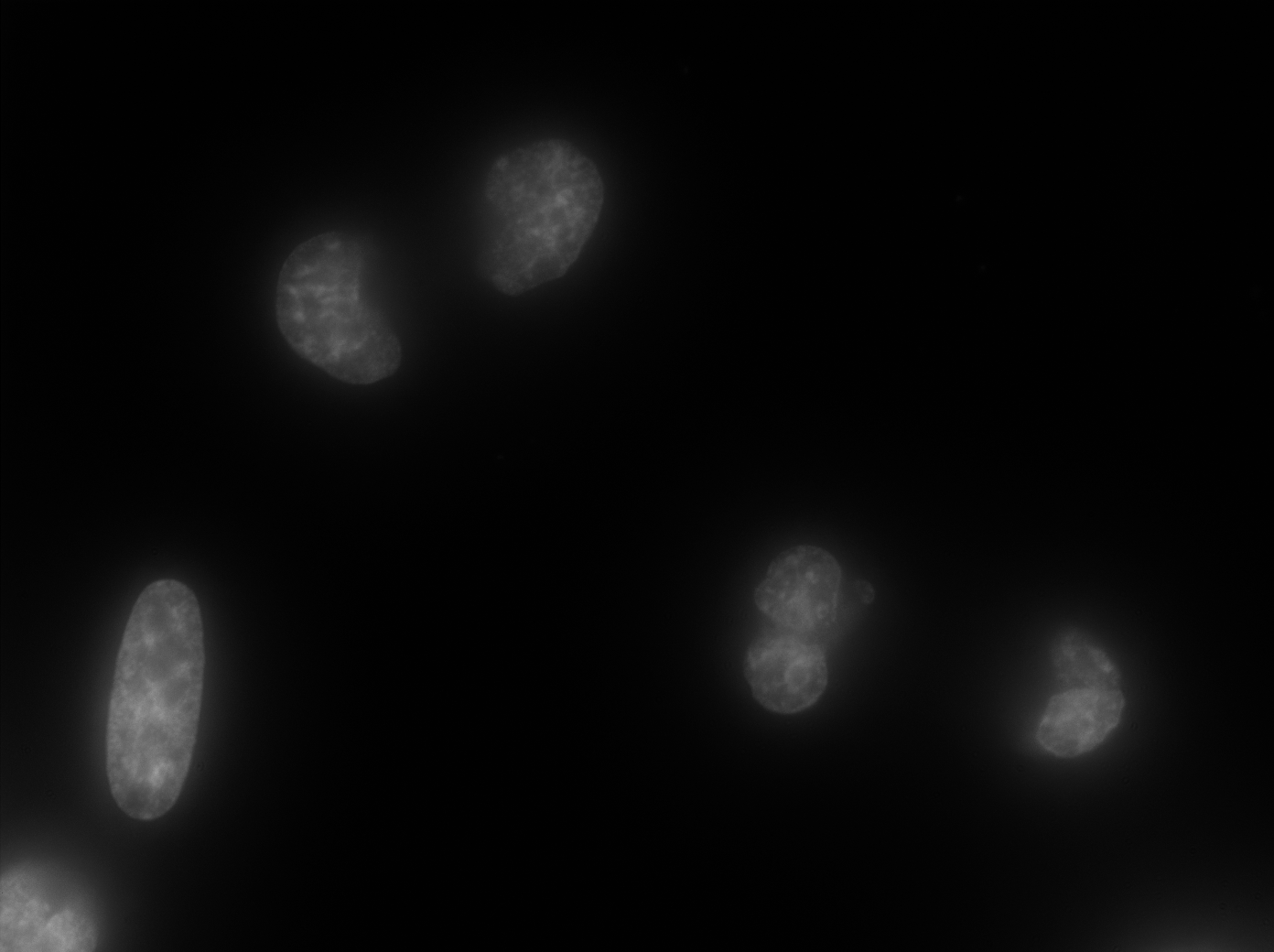

Supplement: Supplementary file 26 — Source data Fig. 7 part 2 [file 44319_2026_742_MOESM26_ESM.zip › Figure 7 Part 2/Fig 7acd Cas9 and TPGS1-ko rGT335 atubulin part 2/TPGS1-KO GT335recomb atub 3-24-25 R2 LT3LT4.Project Maximum Z_XY1742841894_Z0_T0_C0.tif]

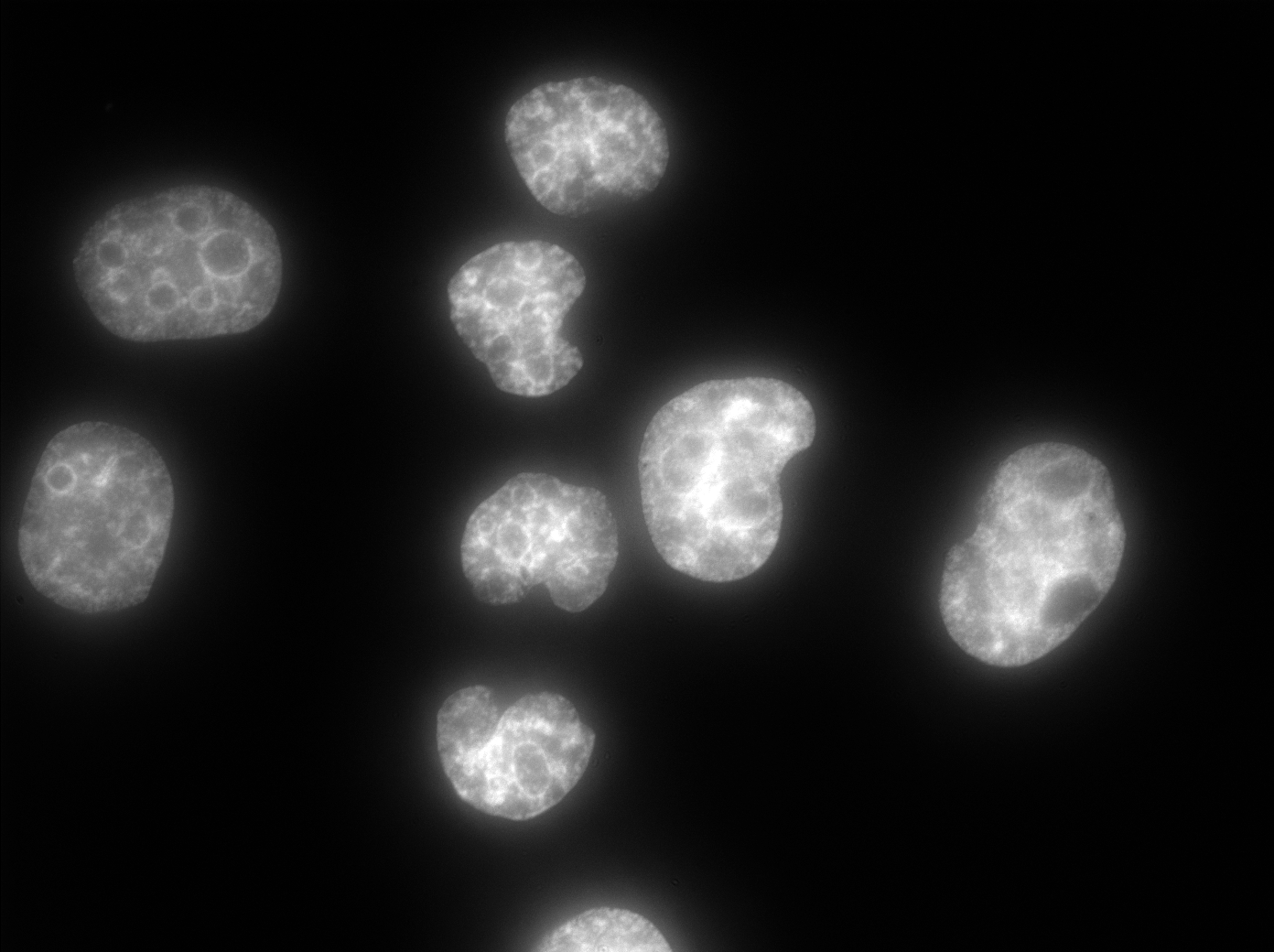

Supplement: Supplementary file 26 — Source data Fig. 7 part 2 [file 44319_2026_742_MOESM26_ESM.zip › Figure 7 Part 2/Fig 7acd Cas9 and TPGS1-ko rGT335 atubulin part 2/TPGS1-KO GT335recomb atub 3-24-25 R1 LT7LT8.Project Maximum Z_XY1742839726_Z0_T0_C0.tif]

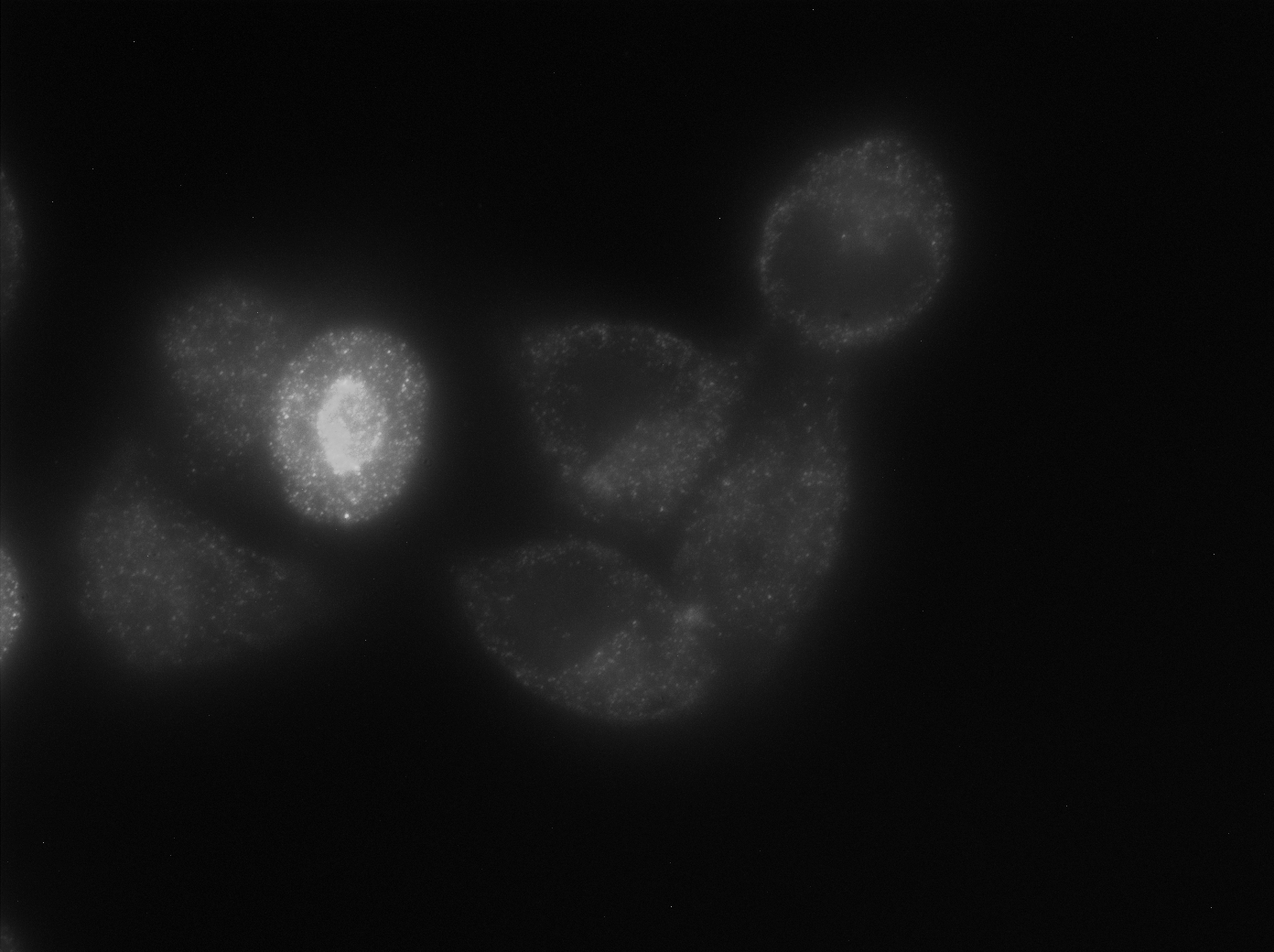

Supplement: Supplementary file 27 — Source data Fig. 7 part 3 [file 44319_2026_742_MOESM27_ESM.zip › Figure 7 Part 3/Fig 7be Cas9 and TPGS1-KO rGT335 atubulin/Cas9 5-2-25 rGT335 atub R3 M7.Project Maximum Z_XY1746217512_Z0_T0_C1.tif]

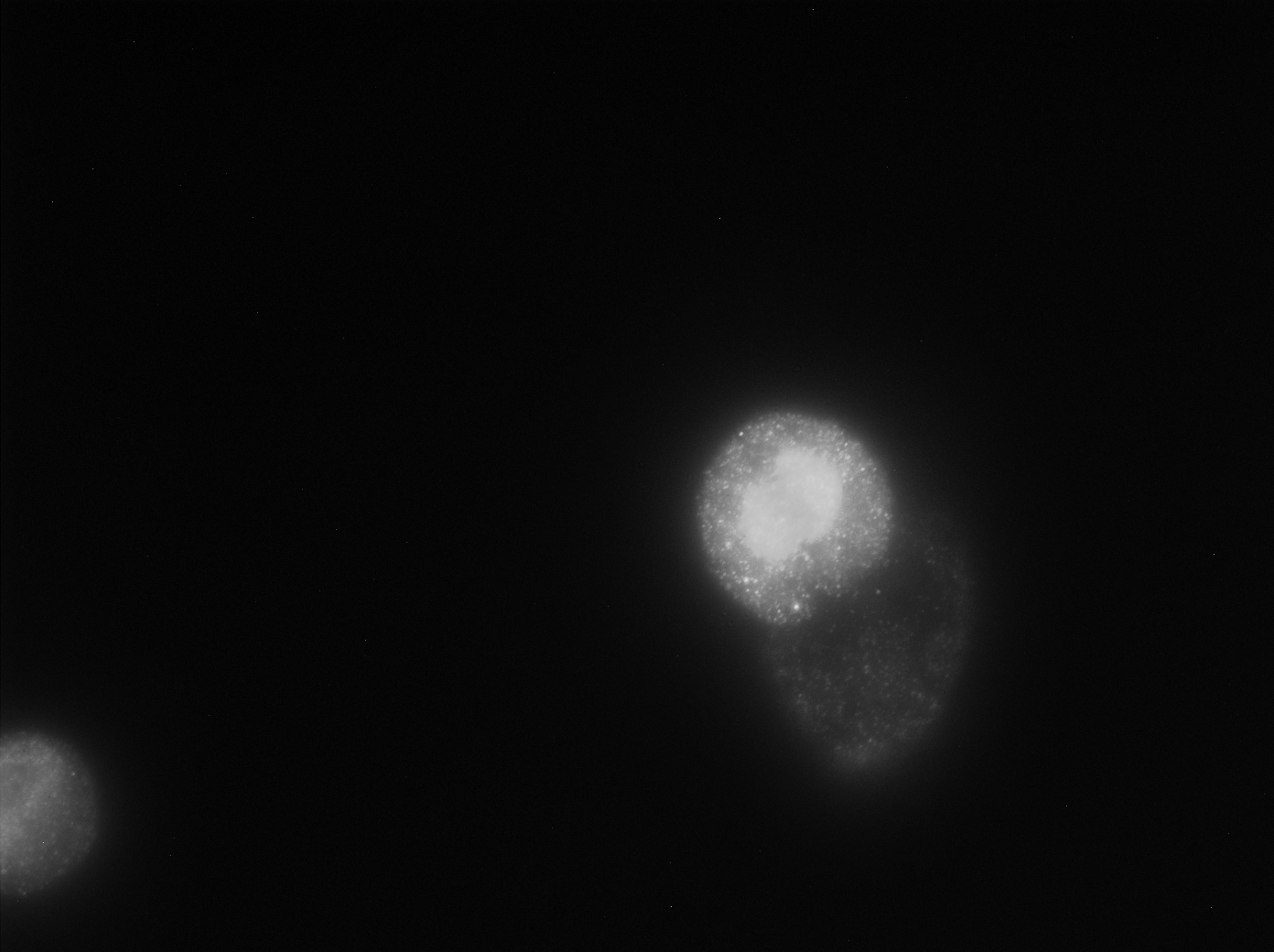

Supplement: Supplementary file 27 — Source data Fig. 7 part 3 [file 44319_2026_742_MOESM27_ESM.zip › Figure 7 Part 3/Fig 7be Cas9 and TPGS1-KO rGT335 atubulin/TPGS1-KO 5-2-25 rGT335 atub R3 M3.Project Maximum Z_XY1746219262_Z0_T0_C1.tif]

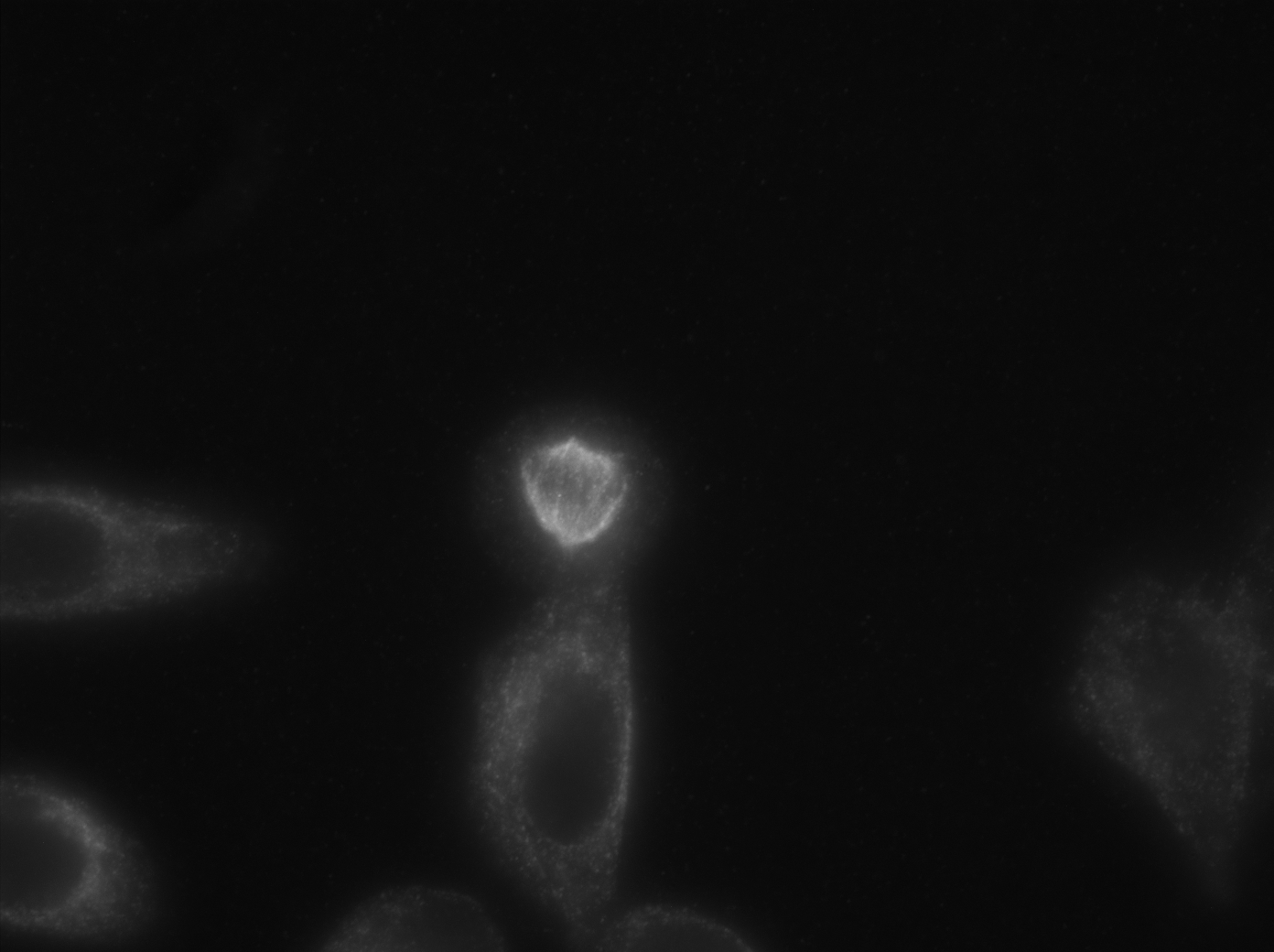

Supplement: Supplementary file 27 — Source data Fig. 7 part 3 [file 44319_2026_742_MOESM27_ESM.zip › Figure 7 Part 3/Fig 7be Cas9 and TPGS1-KO rGT335 atubulin/Cas9 5-2-25 rGT335 atub R3 M2.Project Maximum Z_XY1746214339_Z0_T0_C2.tif]

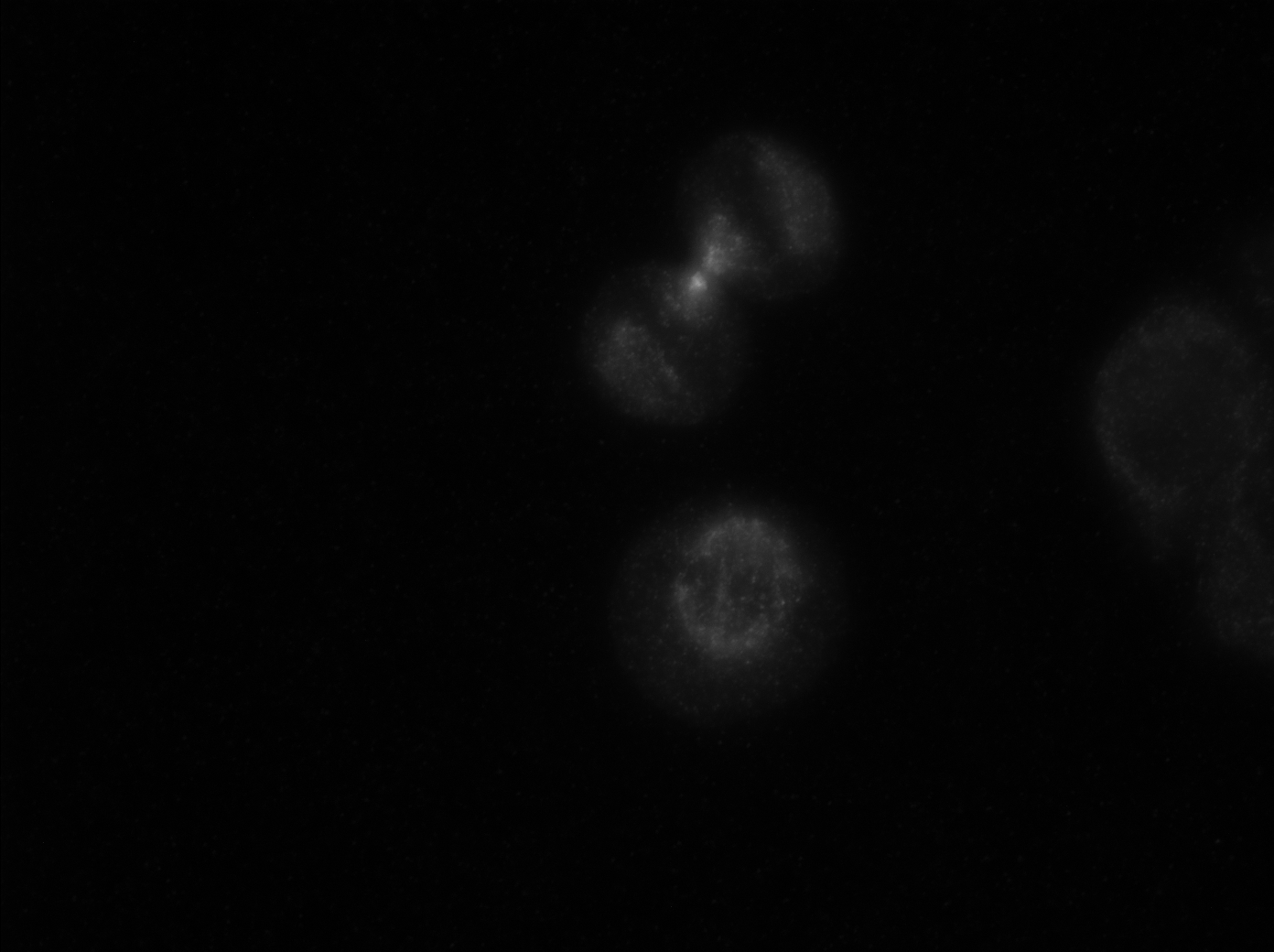

Supplement: Supplementary file 27 — Source data Fig. 7 part 3 [file 44319_2026_742_MOESM27_ESM.zip › Figure 7 Part 3/Fig 7be Cas9 and TPGS1-KO rGT335 atubulin/Cas9 5-2-25 rGT335 atub R2 M3.Project Maximum Z_XY1746559607_Z0_T0_C2.tif]

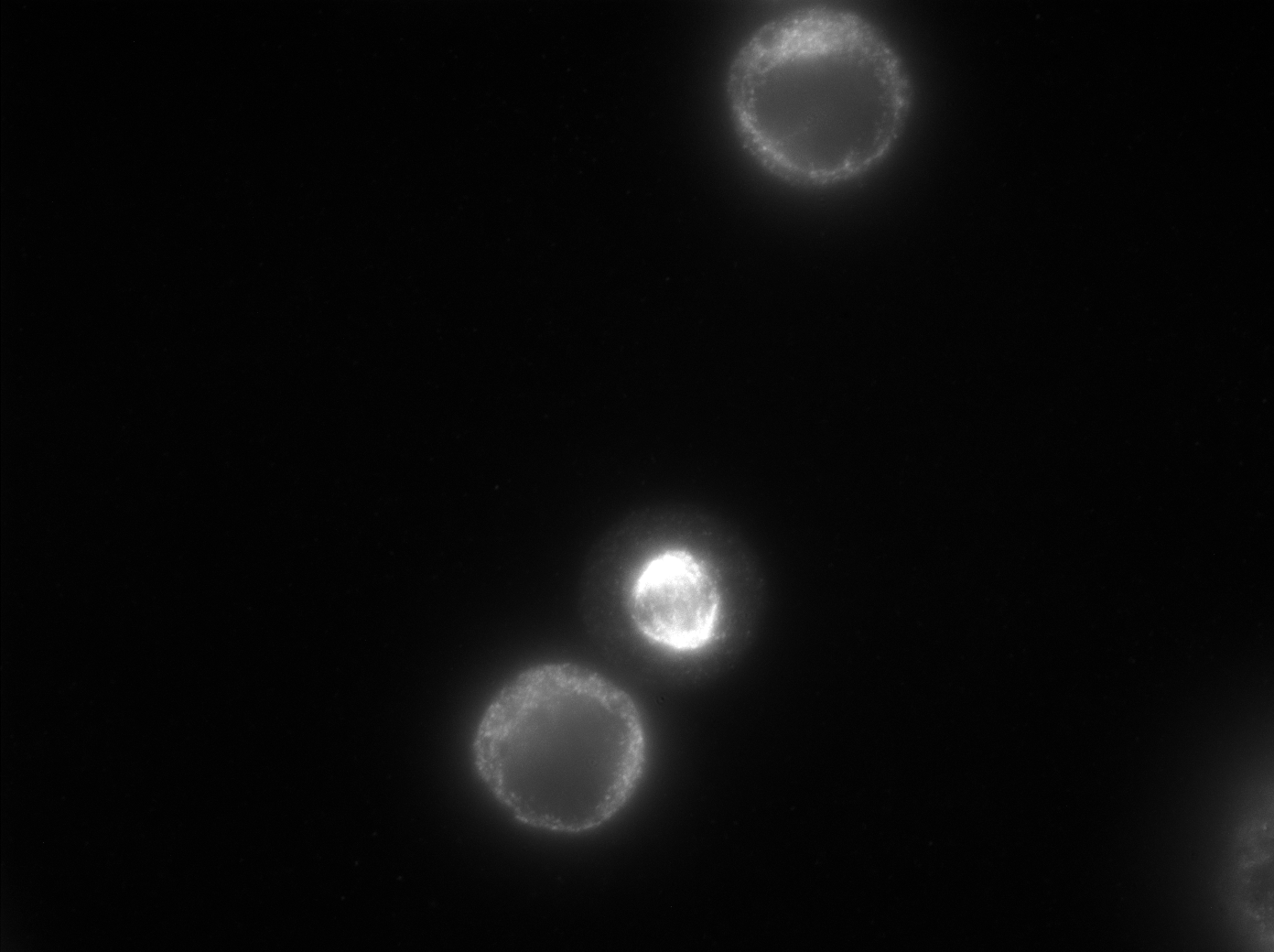

Supplement: Supplementary file 27 — Source data Fig. 7 part 3 [file 44319_2026_742_MOESM27_ESM.zip › Figure 7 Part 3/Fig 7be Cas9 and TPGS1-KO rGT335 atubulin/Cas9 5-2-25 rGT335 atub R3 M8.Project Maximum Z_XY1746217622_Z0_T0_C1 figutr (2).tif]

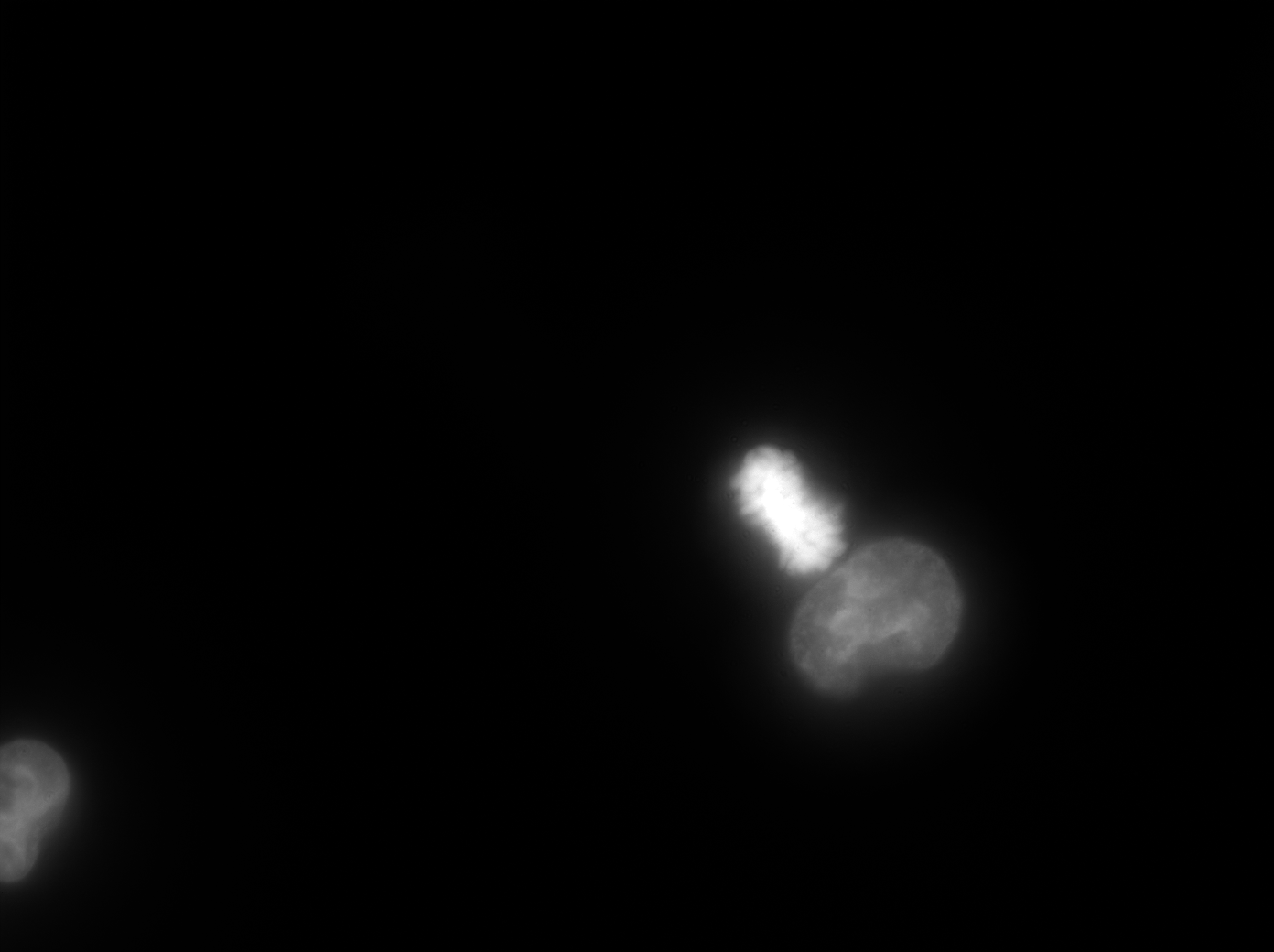

Supplement: Supplementary file 27 — Source data Fig. 7 part 3 [file 44319_2026_742_MOESM27_ESM.zip › Figure 7 Part 3/Fig 7be Cas9 and TPGS1-KO rGT335 atubulin/TPGS1-KO 5-2-25 rGT335 atub R3 M3.Project Maximum Z_XY1746219262_Z0_T0_C0.tif]

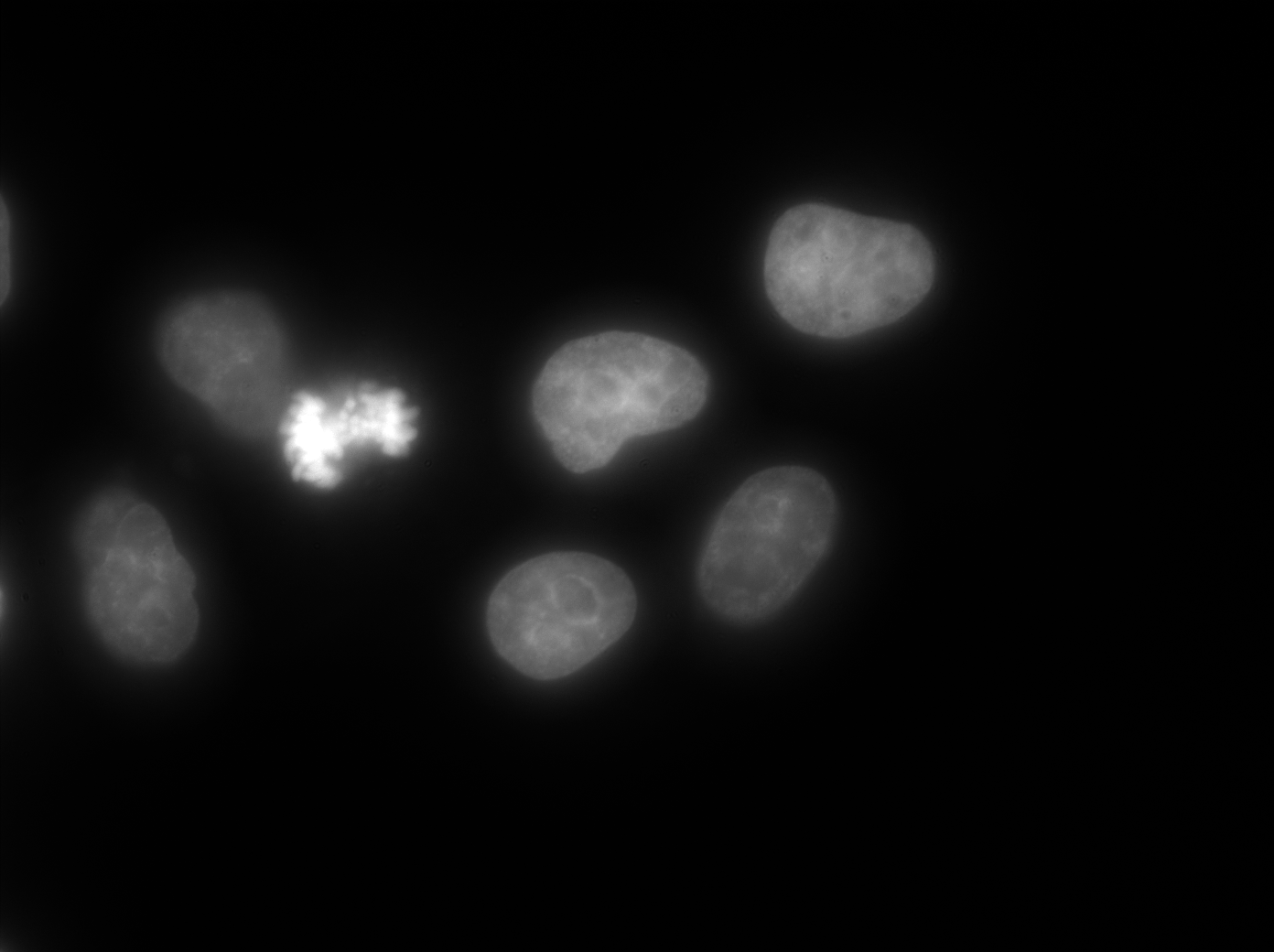

Supplement: Supplementary file 27 — Source data Fig. 7 part 3 [file 44319_2026_742_MOESM27_ESM.zip › Figure 7 Part 3/Fig 7be Cas9 and TPGS1-KO rGT335 atubulin/Cas9 5-2-25 rGT335 atub R3 M7.Project Maximum Z_XY1746217512_Z0_T0_C0.tif]

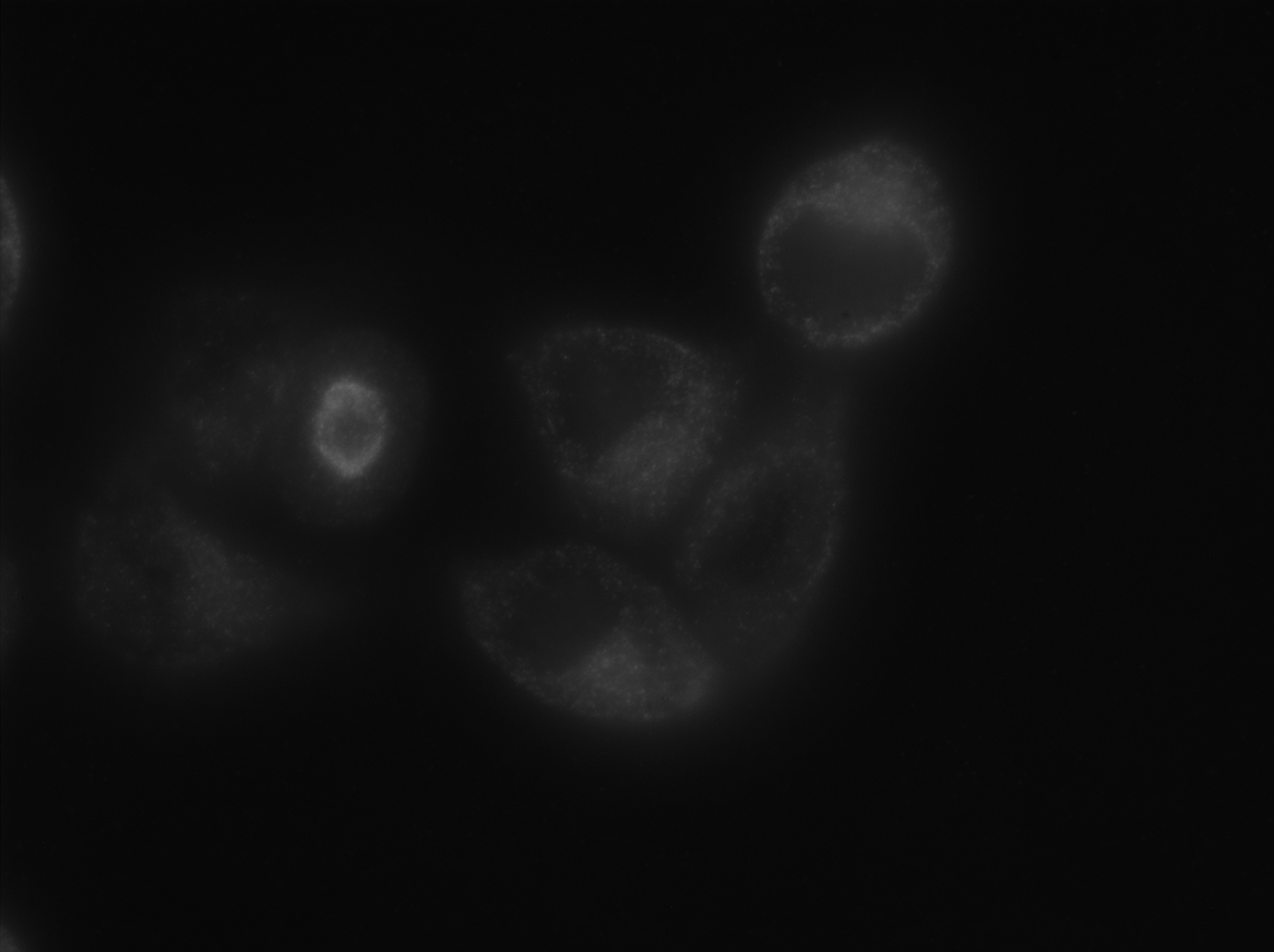

Supplement: Supplementary file 27 — Source data Fig. 7 part 3 [file 44319_2026_742_MOESM27_ESM.zip › Figure 7 Part 3/Fig 7be Cas9 and TPGS1-KO rGT335 atubulin/Cas9 5-2-25 rGT335 atub R3 M7.Project Maximum Z_XY1746217512_Z0_T0_C2.tif]

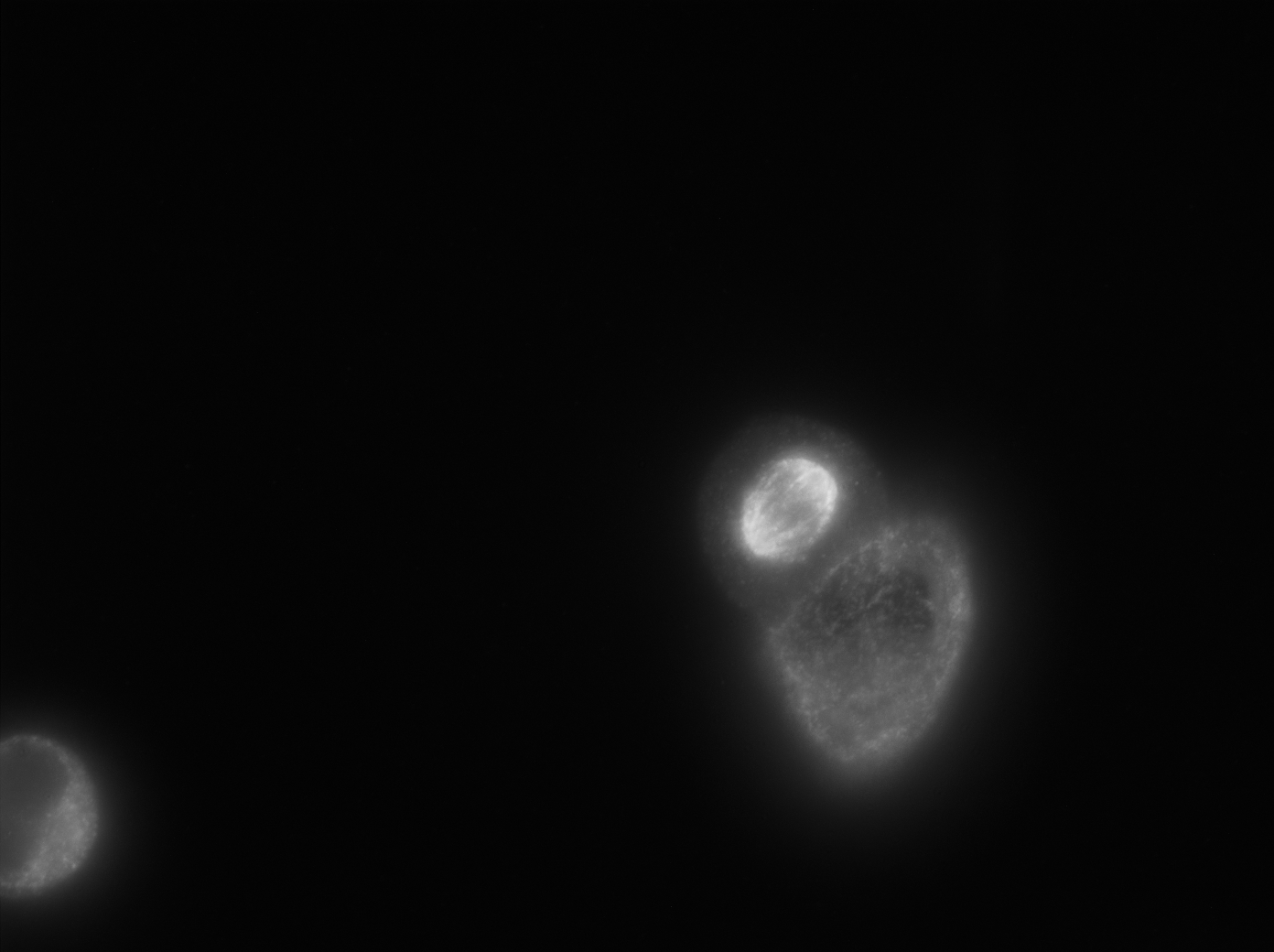

Supplement: Supplementary file 27 — Source data Fig. 7 part 3 [file 44319_2026_742_MOESM27_ESM.zip › Figure 7 Part 3/Fig 7be Cas9 and TPGS1-KO rGT335 atubulin/TPGS1-KO 5-2-25 rGT335 atub R3 M3.Project Maximum Z_XY1746219262_Z0_T0_C2.tif]

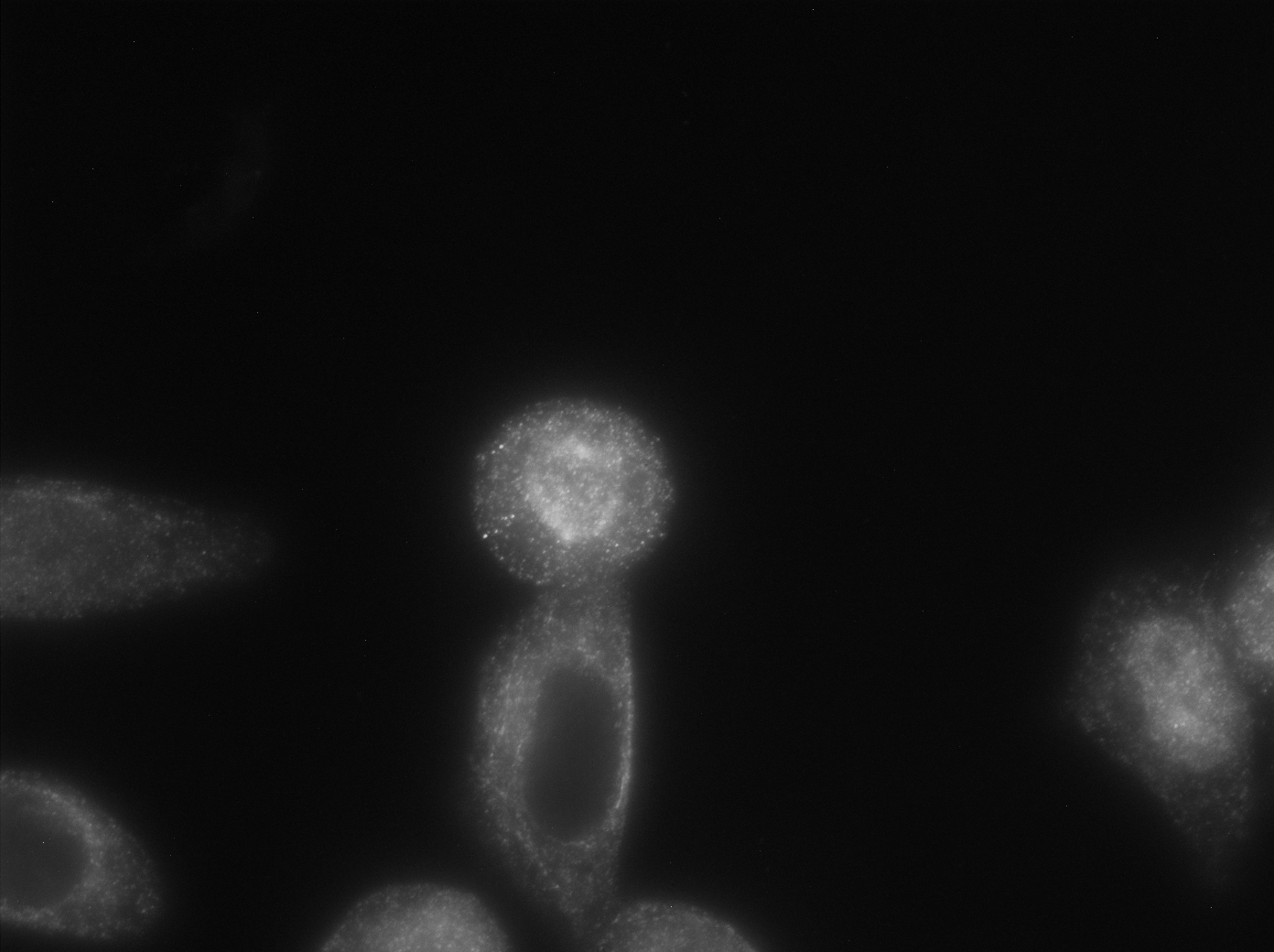

Supplement: Supplementary file 27 — Source data Fig. 7 part 3 [file 44319_2026_742_MOESM27_ESM.zip › Figure 7 Part 3/Fig 7be Cas9 and TPGS1-KO rGT335 atubulin/Cas9 5-2-25 rGT335 atub R3 M2.Project Maximum Z_XY1746214339_Z0_T0_C1.tif]

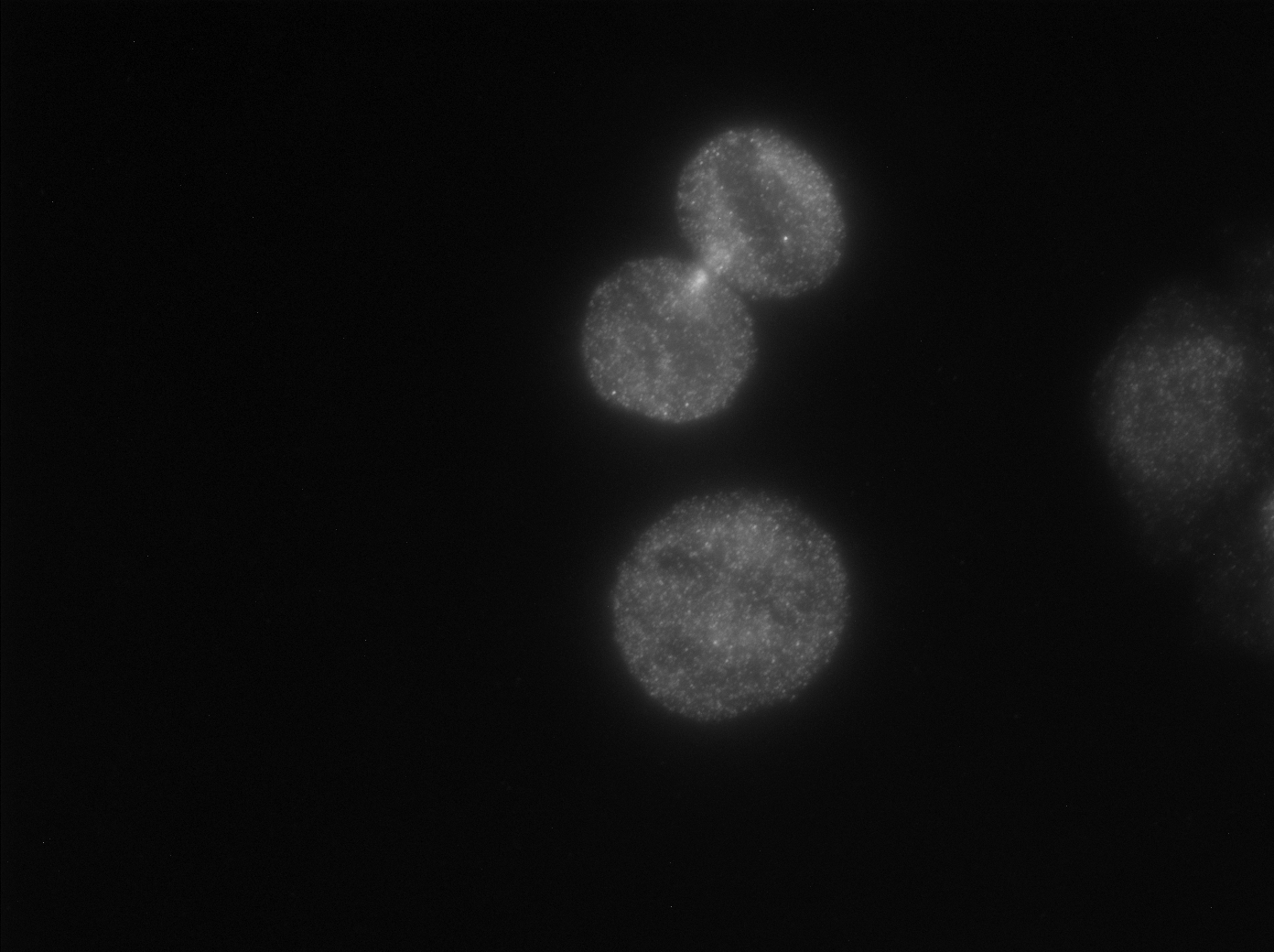

Supplement: Supplementary file 27 — Source data Fig. 7 part 3 [file 44319_2026_742_MOESM27_ESM.zip › Figure 7 Part 3/Fig 7be Cas9 and TPGS1-KO rGT335 atubulin/Cas9 5-2-25 rGT335 atub R2 M3.Project Maximum Z_XY1746559607_Z0_T0_C1.tif]

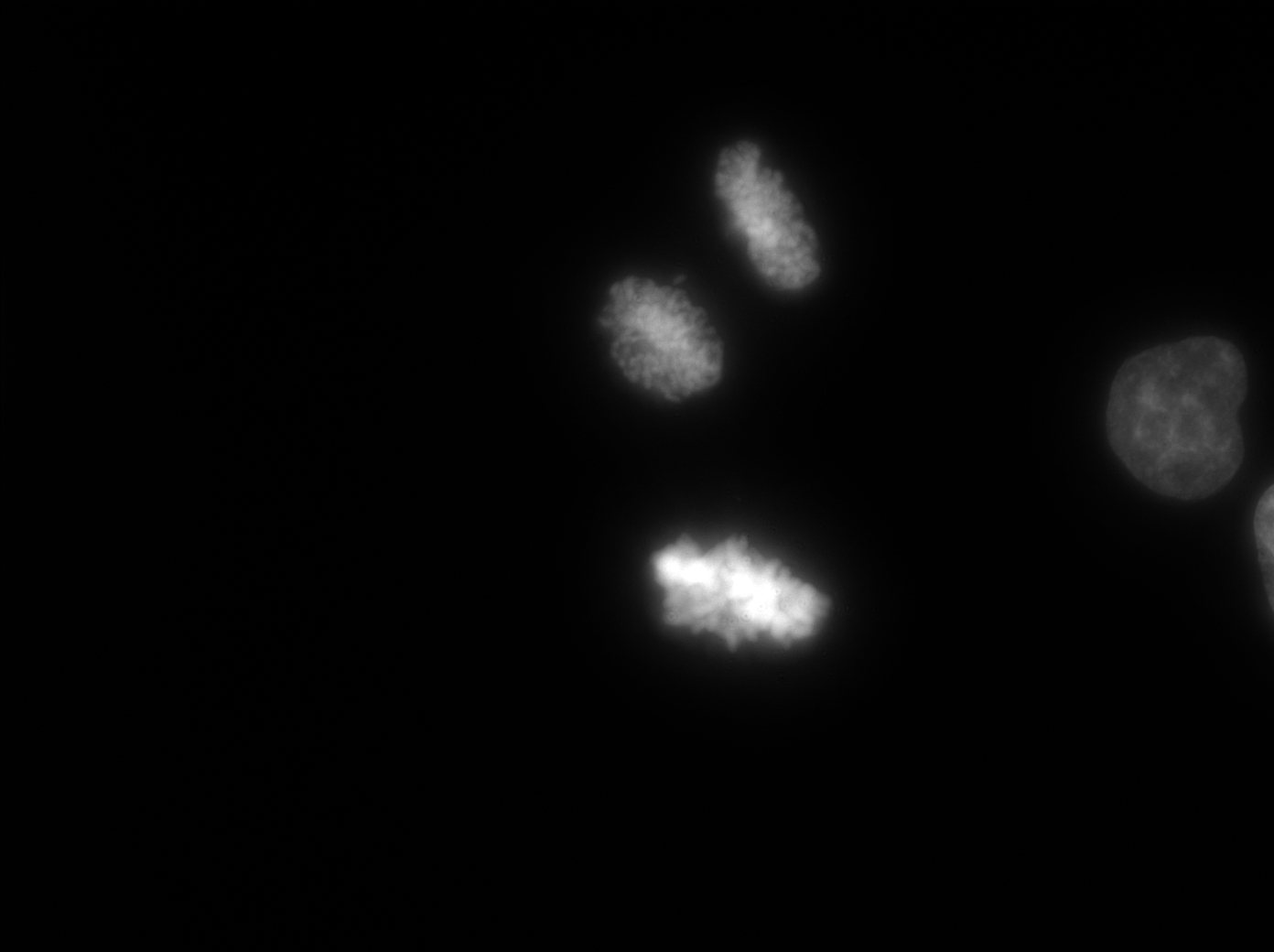

Supplement: Supplementary file 27 — Source data Fig. 7 part 3 [file 44319_2026_742_MOESM27_ESM.zip › Figure 7 Part 3/Fig 7be Cas9 and TPGS1-KO rGT335 atubulin/Cas9 5-2-25 rGT335 atub R2 M3.Project Maximum Z_XY1746559607_Z0_T0_C0.tif]

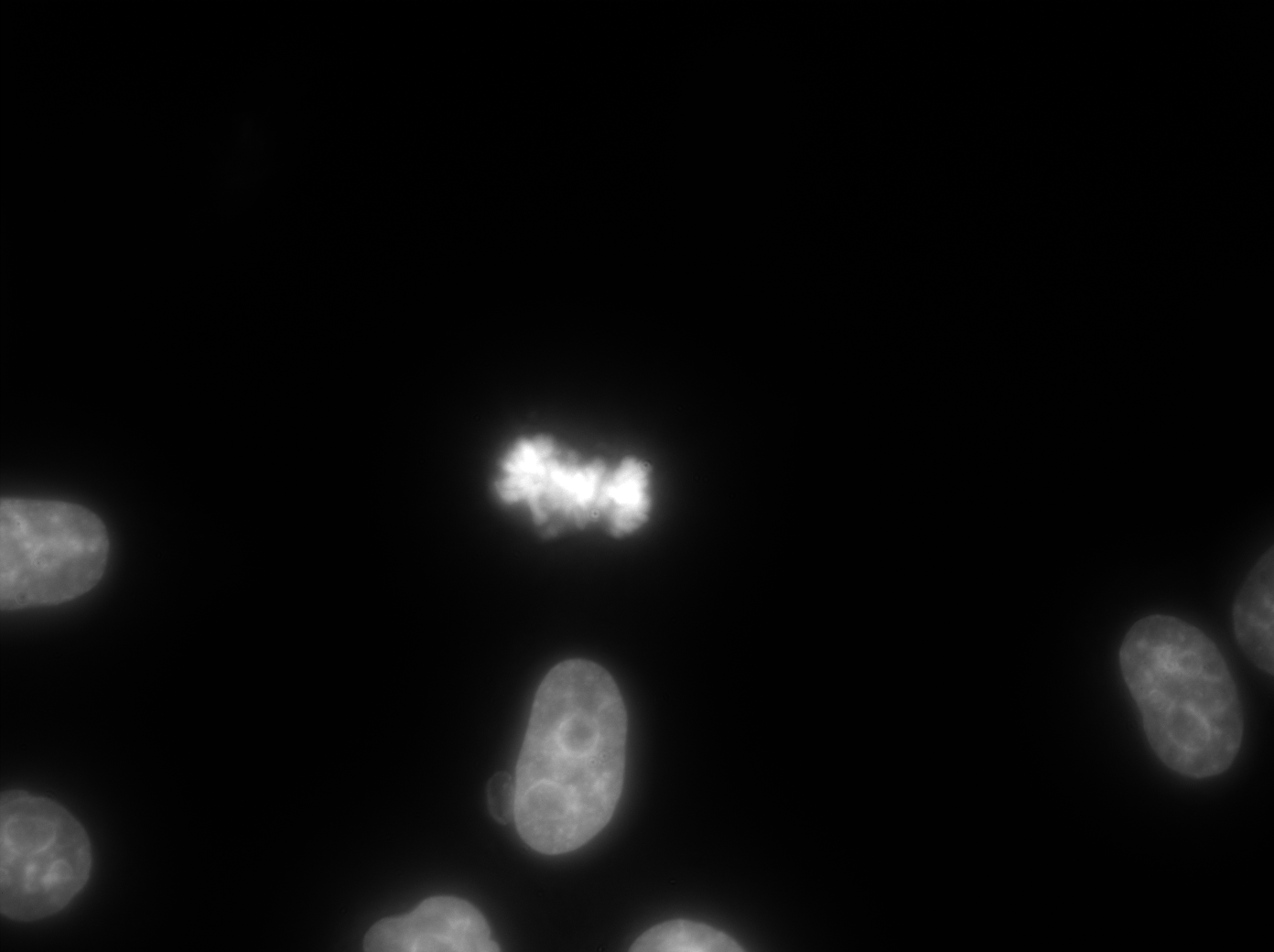

Supplement: Supplementary file 27 — Source data Fig. 7 part 3 [file 44319_2026_742_MOESM27_ESM.zip › Figure 7 Part 3/Fig 7be Cas9 and TPGS1-KO rGT335 atubulin/Cas9 5-2-25 rGT335 atub R3 M2.Project Maximum Z_XY1746214339_Z0_T0_C0.tif]

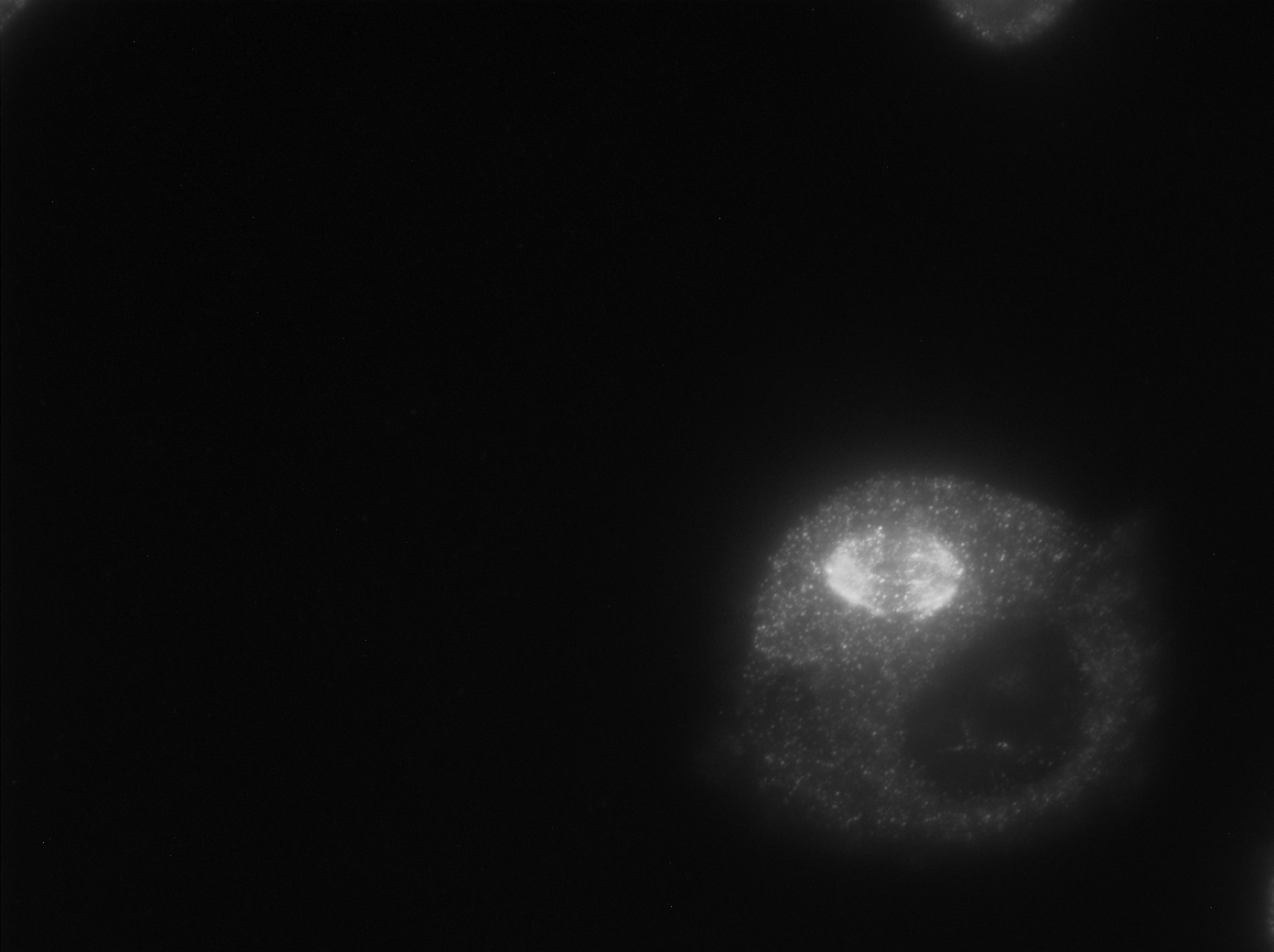

Supplement: Supplementary file 27 — Source data Fig. 7 part 3 [file 44319_2026_742_MOESM27_ESM.zip › Figure 7 Part 3/Fig 7be Cas9 and TPGS1-KO rGT335 atubulin/TPGS1-KO 5-2-25 rGT335 atub R2 M8.Project Maximum Z_XY1746564758_Z0_T0_C1.tif]

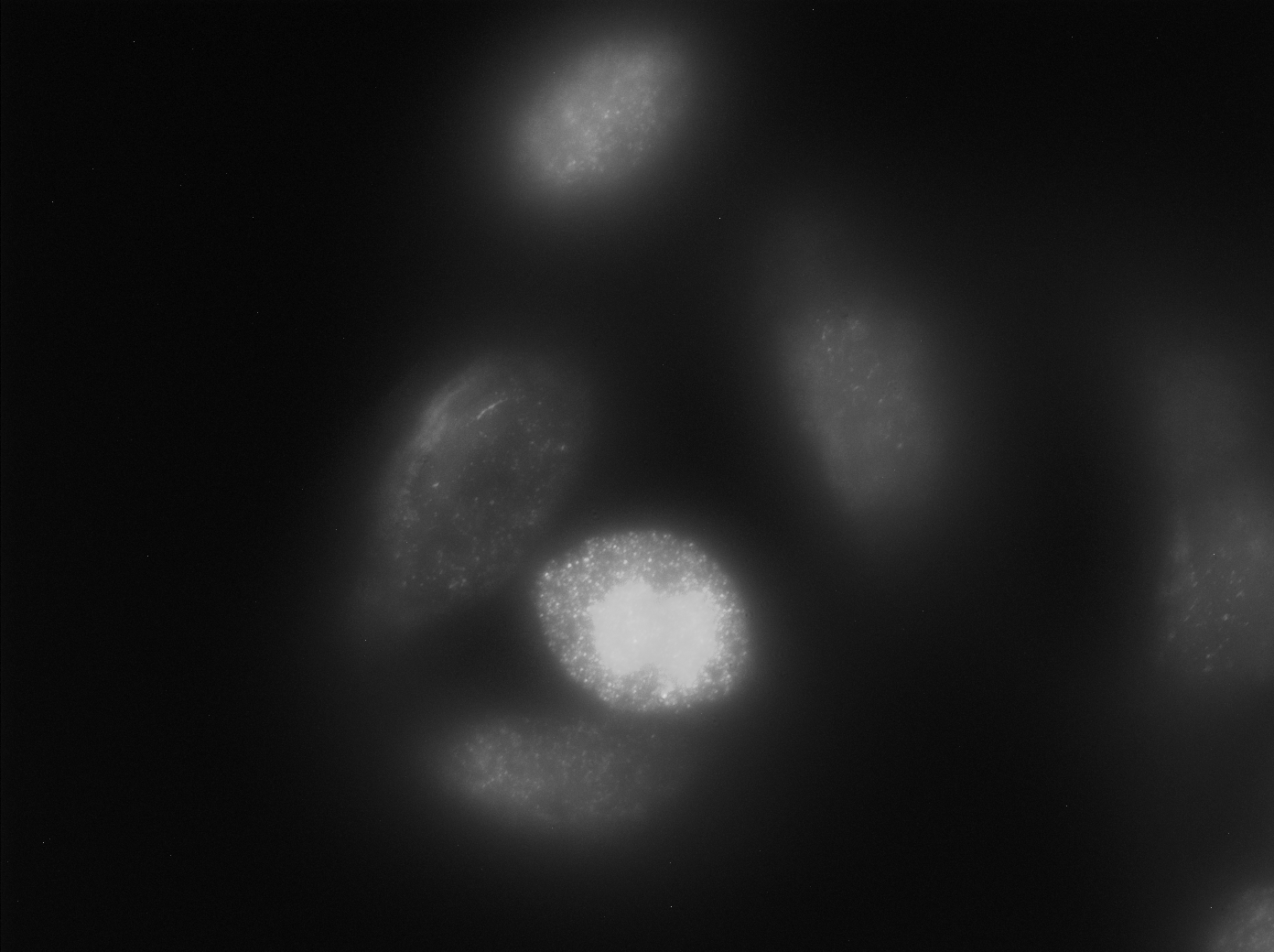

Supplement: Supplementary file 27 — Source data Fig. 7 part 3 [file 44319_2026_742_MOESM27_ESM.zip › Figure 7 Part 3/Fig 7be Cas9 and TPGS1-KO rGT335 atubulin/TPGS1-KO 5-2-25 rGT335 atub R1 M6.Project Maximum Z_XY1746221802_Z0_T0_C1.tif]

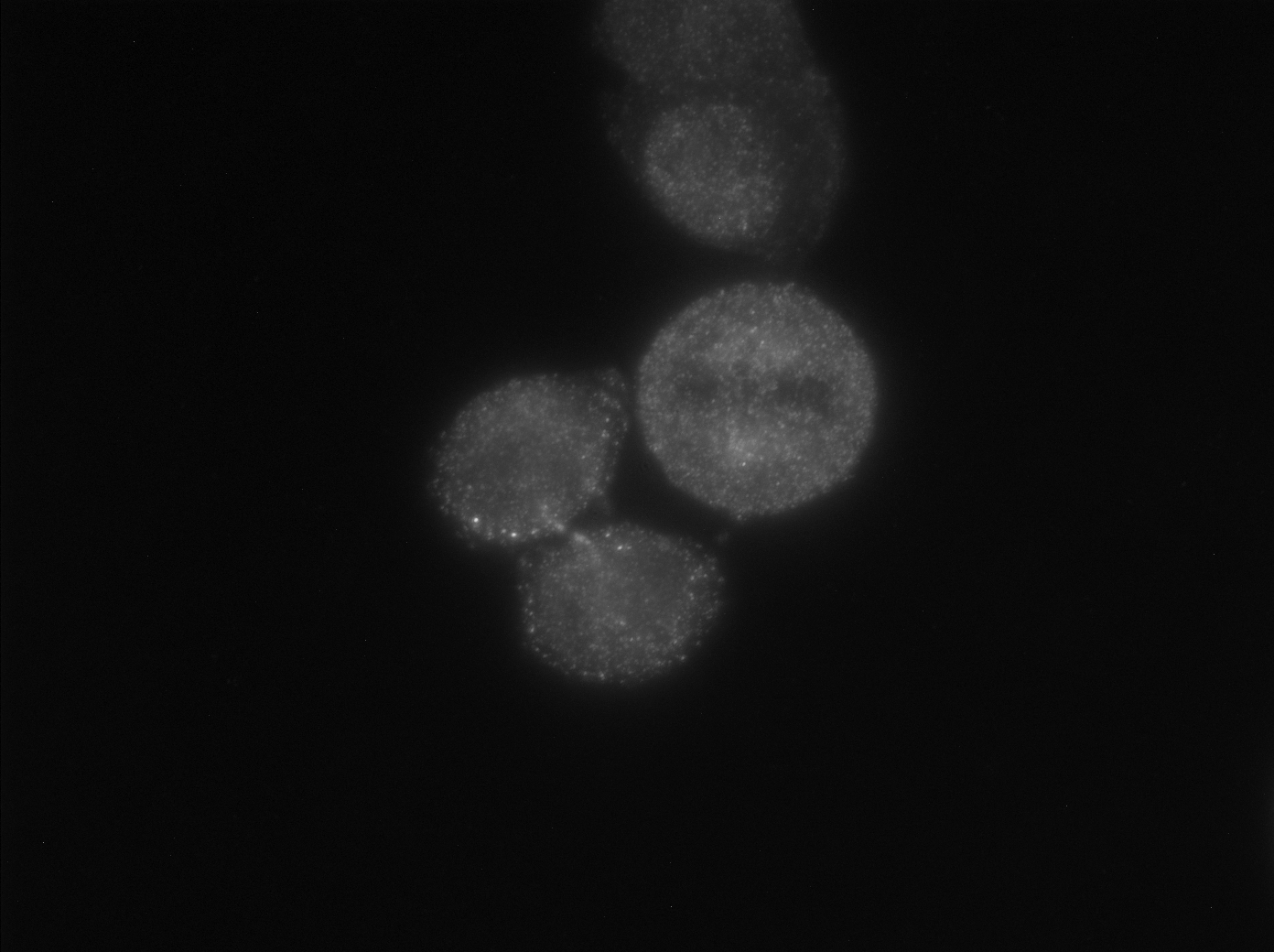

Supplement: Supplementary file 27 — Source data Fig. 7 part 3 [file 44319_2026_742_MOESM27_ESM.zip › Figure 7 Part 3/Fig 7be Cas9 and TPGS1-KO rGT335 atubulin/Cas9 5-2-25 rGT335 atub R2 M4.Project Maximum Z_XY1746561923_Z0_T0_C1.tif]
