# Supplementary figures and images for: TPGS1 regulates central spindle microtubule glutamylation and remodeling during telophase and abscission (part 34 of 36)
Source: EMBO Rep. 2026 Mar 23;27(8):1944–63. doi: 10.1038/s44319-026-00742-3 (PMC13121839; doi:10.1038/s44319-026-00742-3)

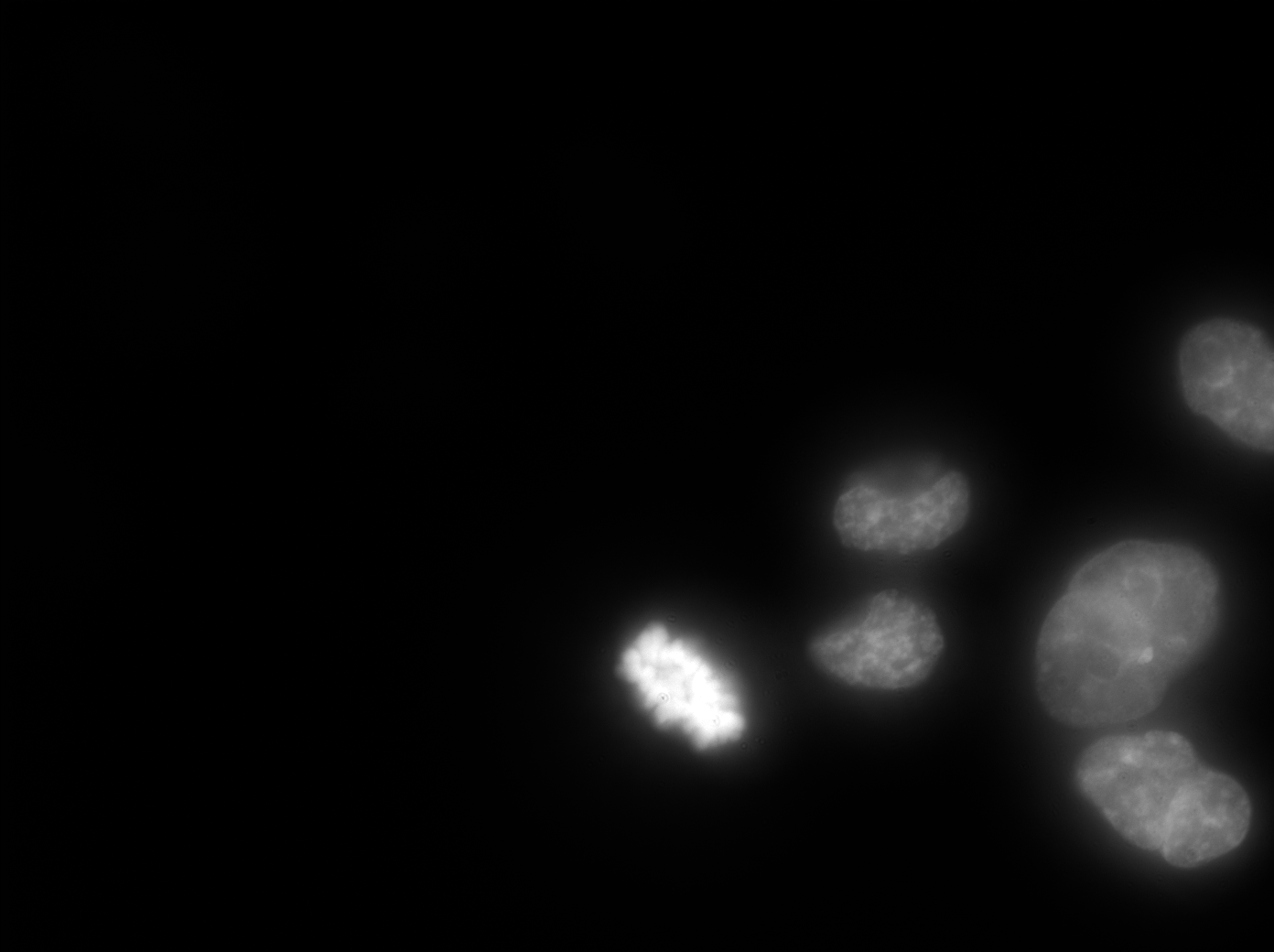

Supplement: Supplementary file 27 — Source data Fig. 7 part 3 [file 44319_2026_742_MOESM27_ESM.zip › Figure 7 Part 3/Fig 7be Cas9 and TPGS1-KO rGT335 atubulin/Cas9 5-2-25 rGT335 atub R1 M10.Project Maximum Z_XY1746558510_Z0_T0_C0.tif]

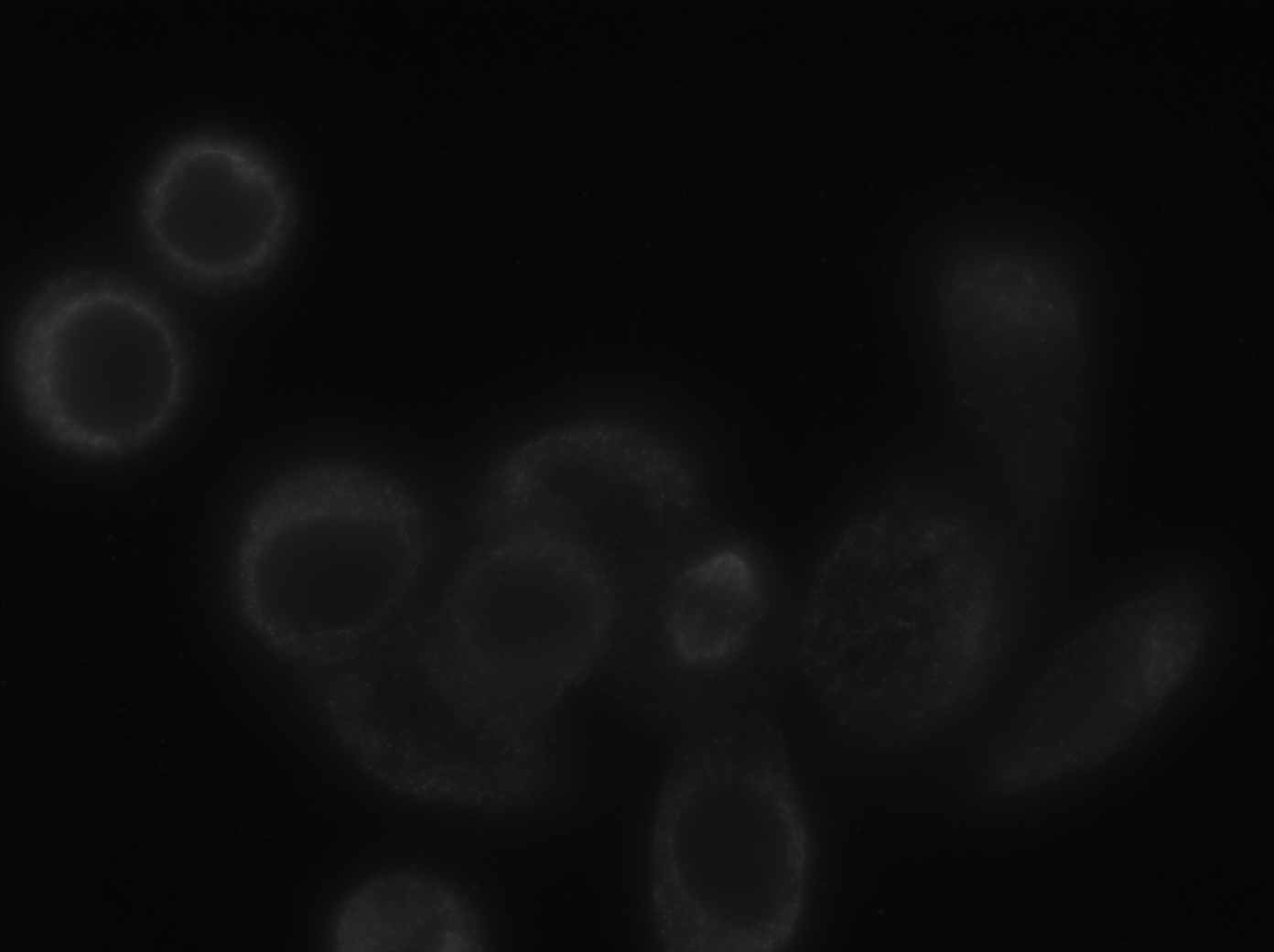

Supplement: Supplementary file 27 — Source data Fig. 7 part 3 [file 44319_2026_742_MOESM27_ESM.zip › Figure 7 Part 3/Fig 7be Cas9 and TPGS1-KO rGT335 atubulin/TPGS1-KO 5-2-25 rGT335 atub R3 M2.Project Maximum Z_XY1746218877_Z0_T0_C2.tif]

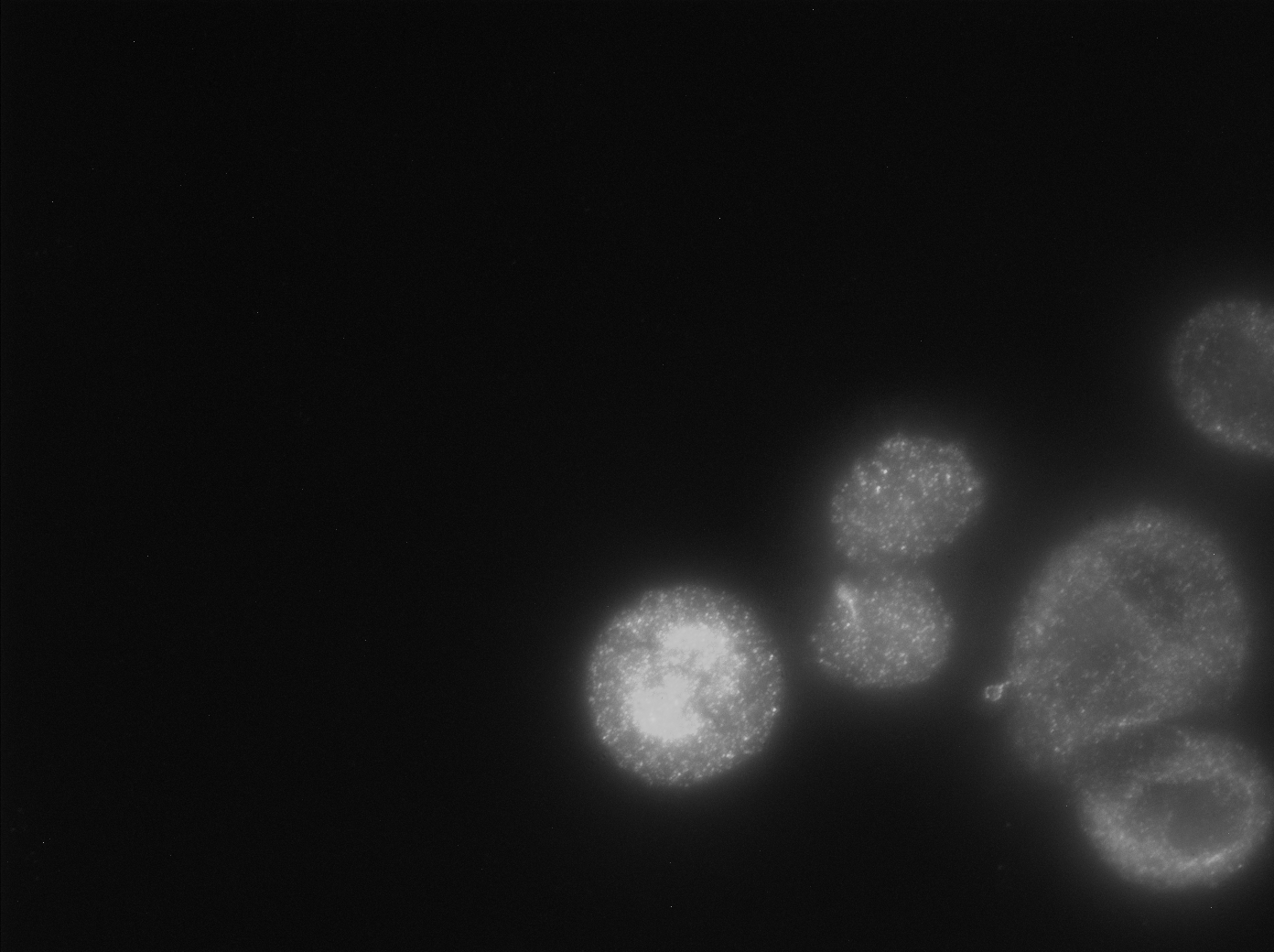

Supplement: Supplementary file 27 — Source data Fig. 7 part 3 [file 44319_2026_742_MOESM27_ESM.zip › Figure 7 Part 3/Fig 7be Cas9 and TPGS1-KO rGT335 atubulin/Cas9 5-2-25 rGT335 atub R1 M10.Project Maximum Z_XY1746558510_Z0_T0_C1.tif]

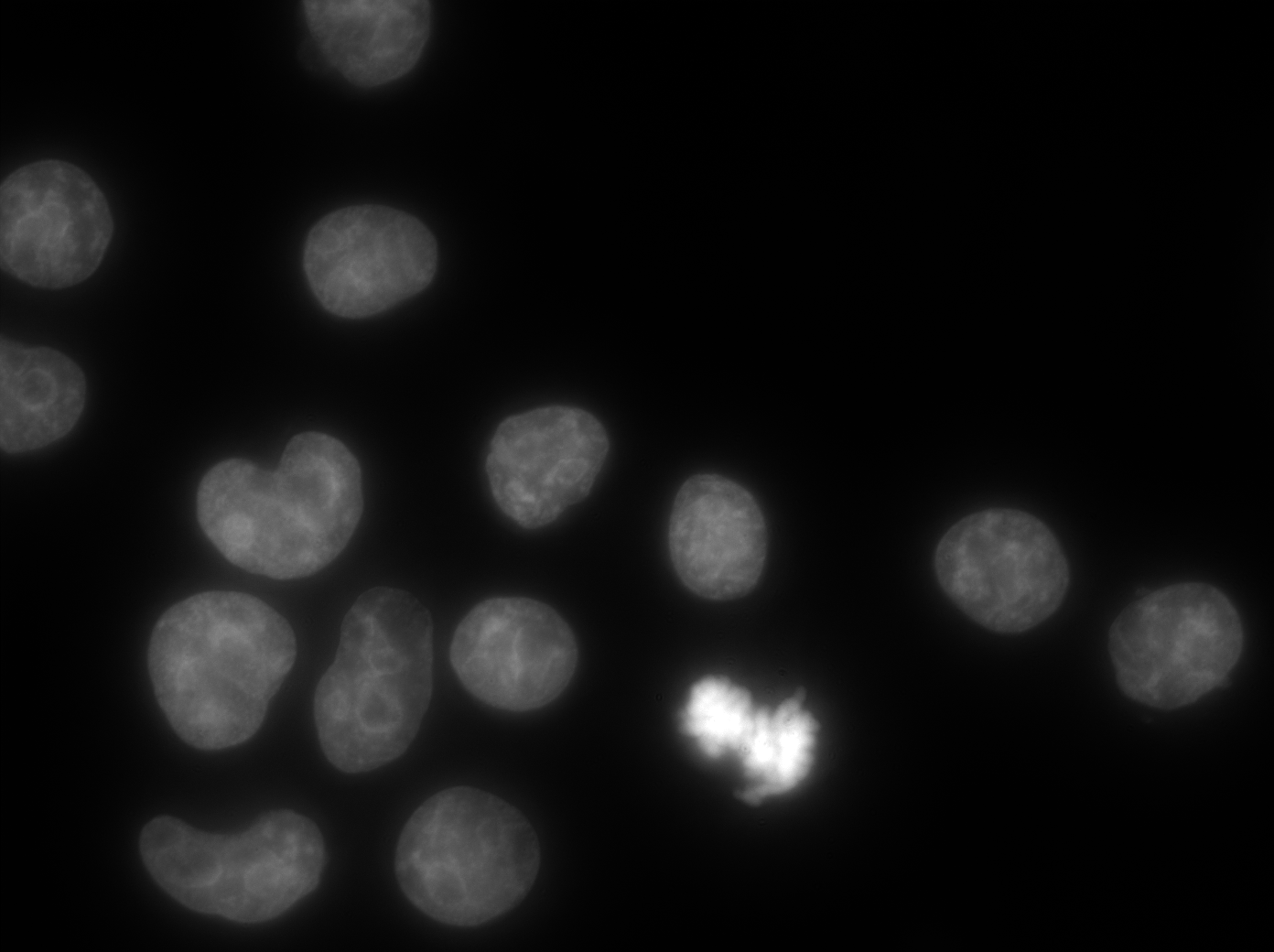

Supplement: Supplementary file 27 — Source data Fig. 7 part 3 [file 44319_2026_742_MOESM27_ESM.zip › Figure 7 Part 3/Fig 7be Cas9 and TPGS1-KO rGT335 atubulin/Cas9 5-2-25 rGT335 atub R2 M7.Project Maximum Z_XY1746562337_Z0_T0_C0.tif]

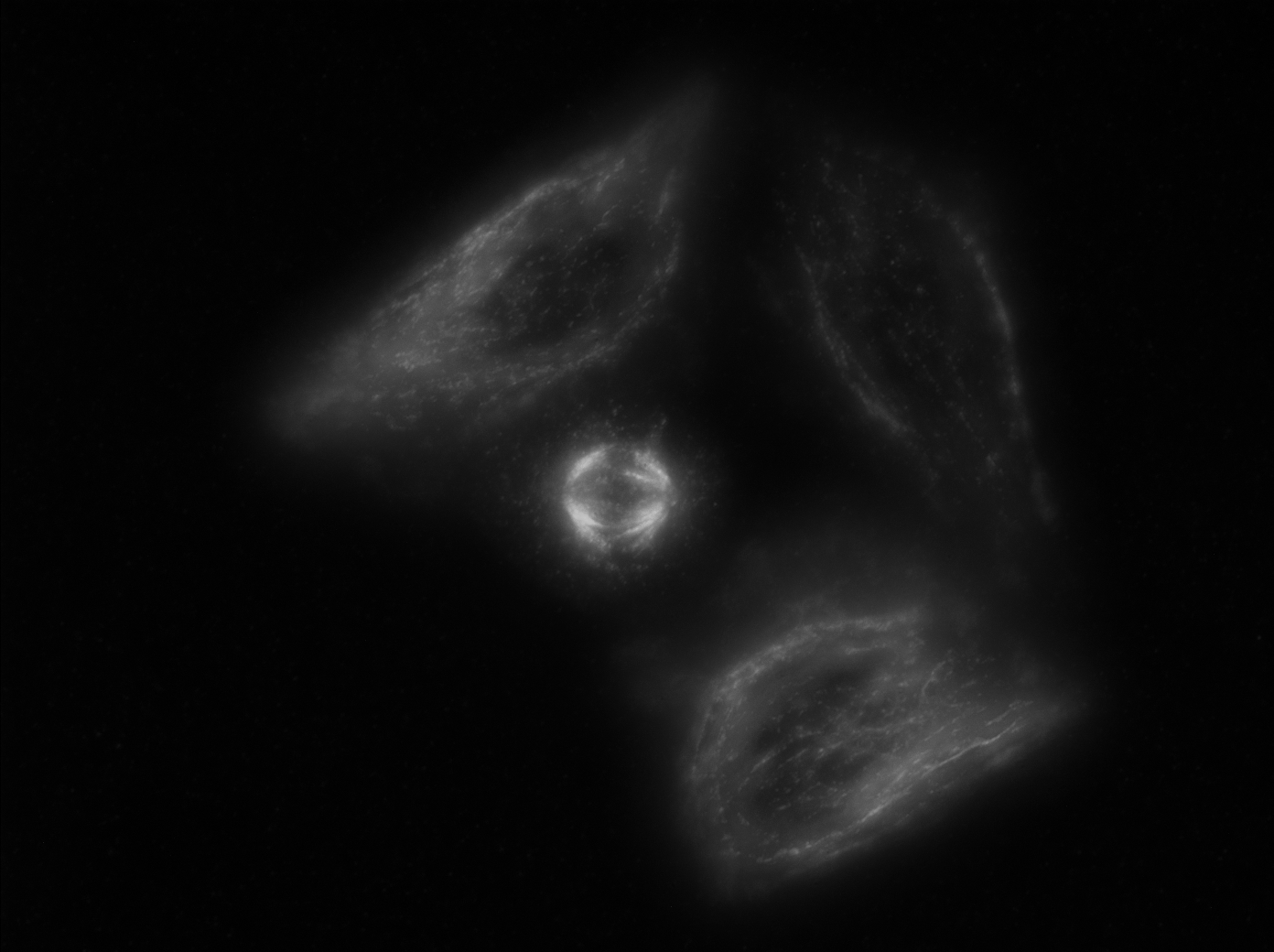

Supplement: Supplementary file 27 — Source data Fig. 7 part 3 [file 44319_2026_742_MOESM27_ESM.zip › Figure 7 Part 3/Fig 7be Cas9 and TPGS1-KO rGT335 atubulin/Cas9 5-2-25 rGT335 atub R2 M9.Project Maximum Z_XY1746562705_Z0_T0_C2.tif]

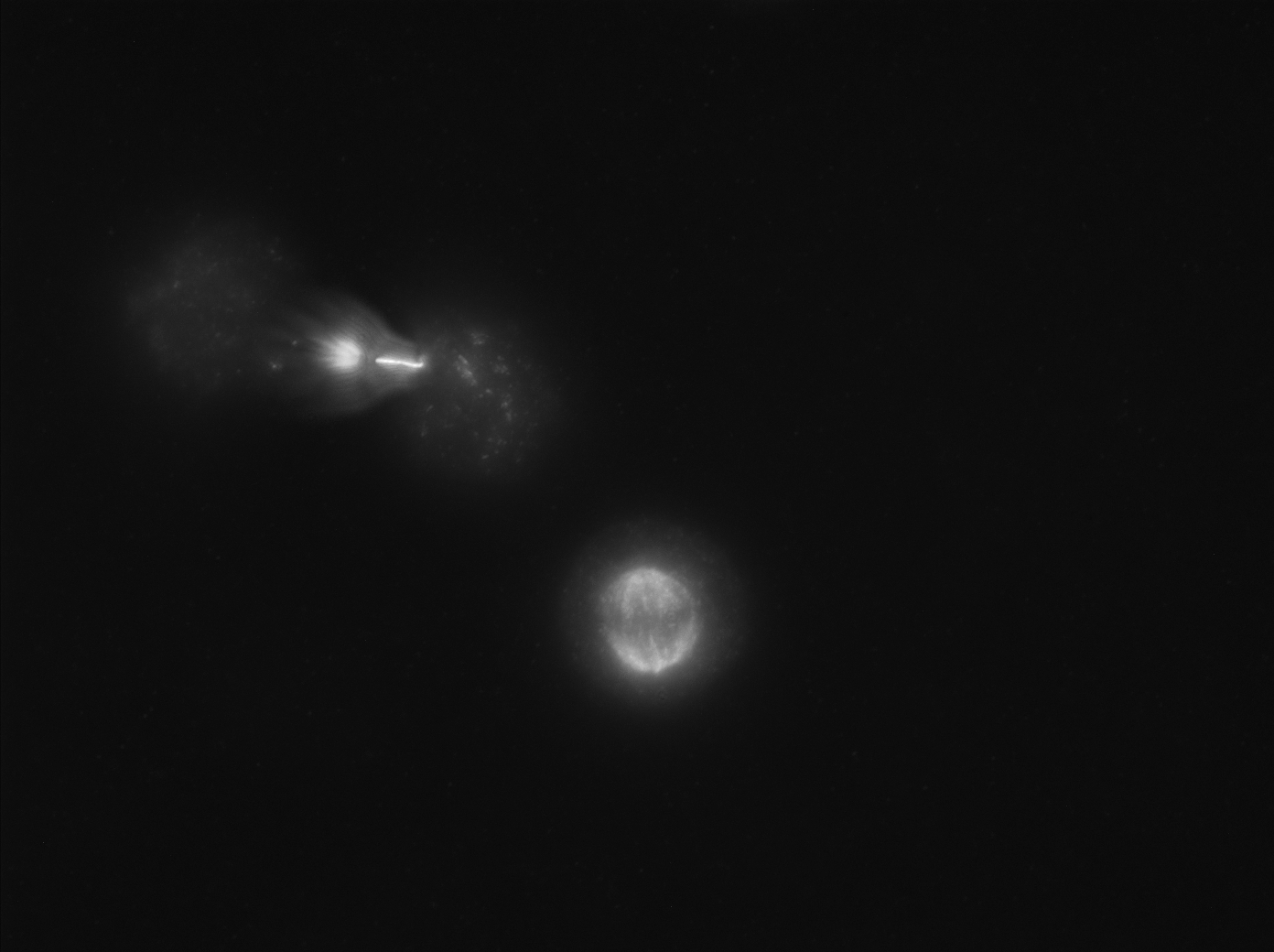

Supplement: Supplementary file 27 — Source data Fig. 7 part 3 [file 44319_2026_742_MOESM27_ESM.zip › Figure 7 Part 3/Fig 7be Cas9 and TPGS1-KO rGT335 atubulin/TPGS1-KO 5-2-25 rGT335 atub R1 M9.Project Maximum Z_XY1746222581_Z0_T0_C2.tif]

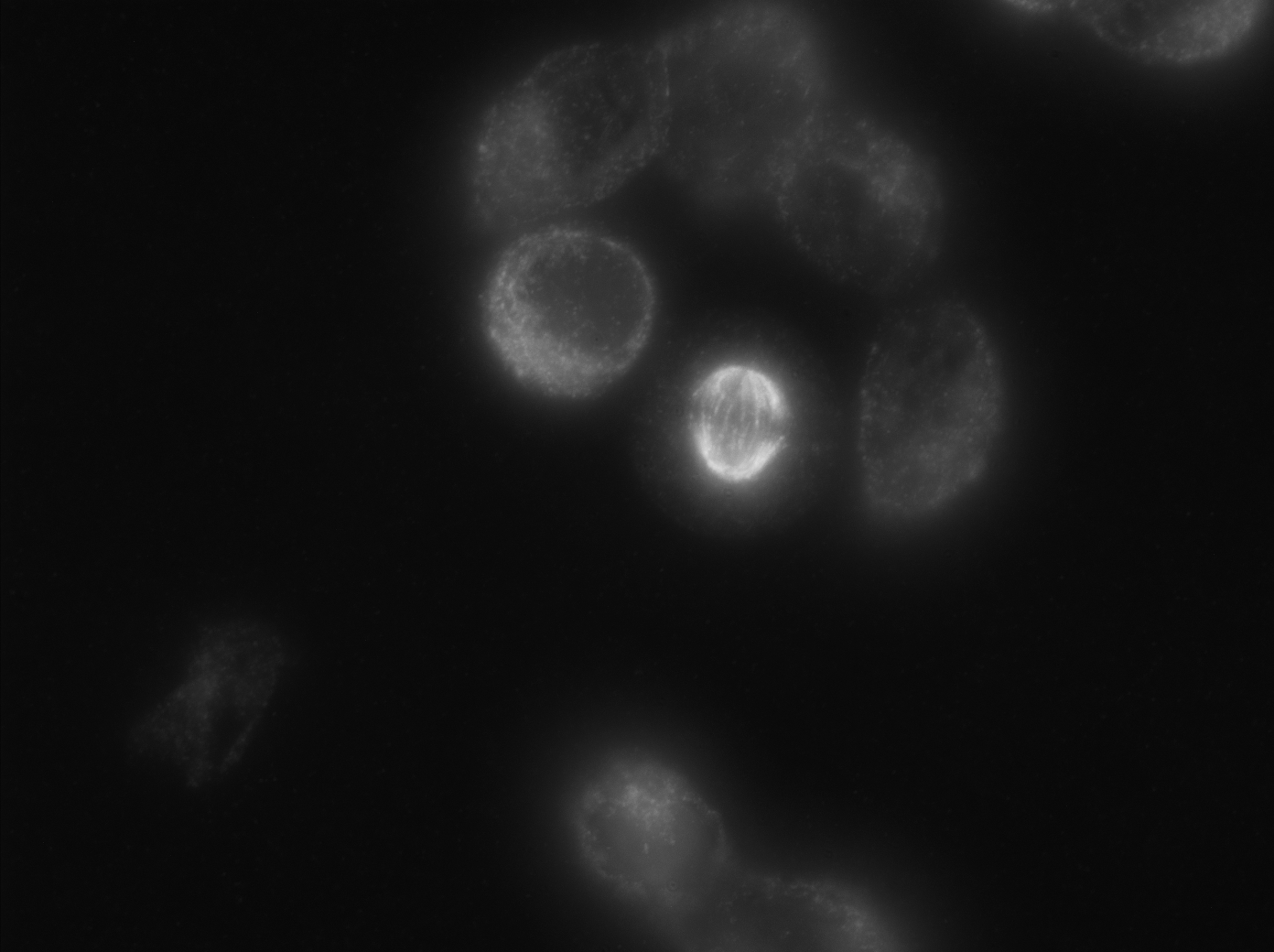

Supplement: Supplementary file 27 — Source data Fig. 7 part 3 [file 44319_2026_742_MOESM27_ESM.zip › Figure 7 Part 3/Fig 7be Cas9 and TPGS1-KO rGT335 atubulin/Cas9 5-2-25 rGT335 atub R3 M9.Project Maximum Z_XY1746218118_Z0_T0_C2.tif]

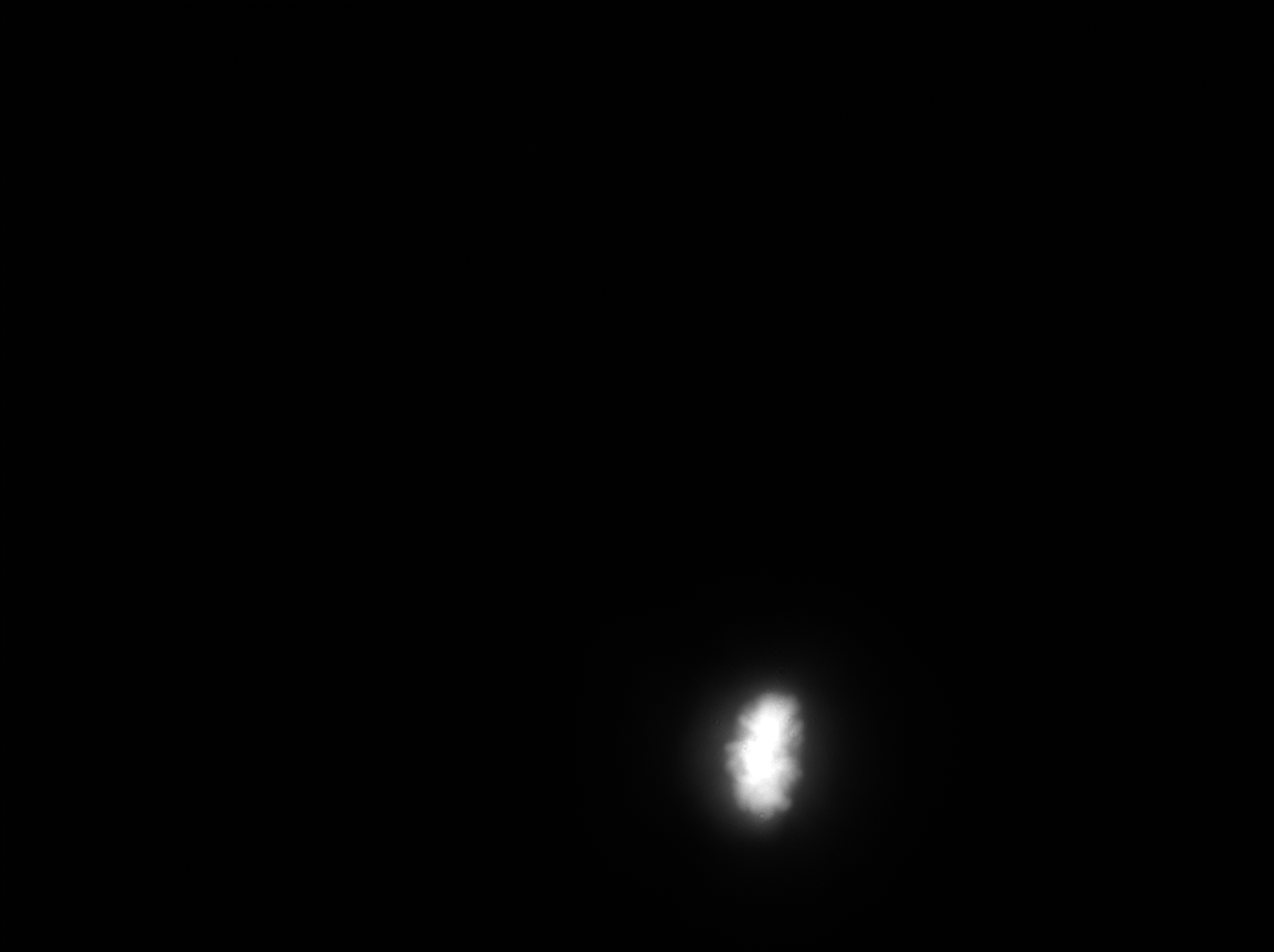

Supplement: Supplementary file 27 — Source data Fig. 7 part 3 [file 44319_2026_742_MOESM27_ESM.zip › Figure 7 Part 3/Fig 7be Cas9 and TPGS1-KO rGT335 atubulin/TPGS1-KO 5-2-25 rGT335 atub R1 M10.Project Maximum Z_XY1746222917_Z0_T0_C0.tif]

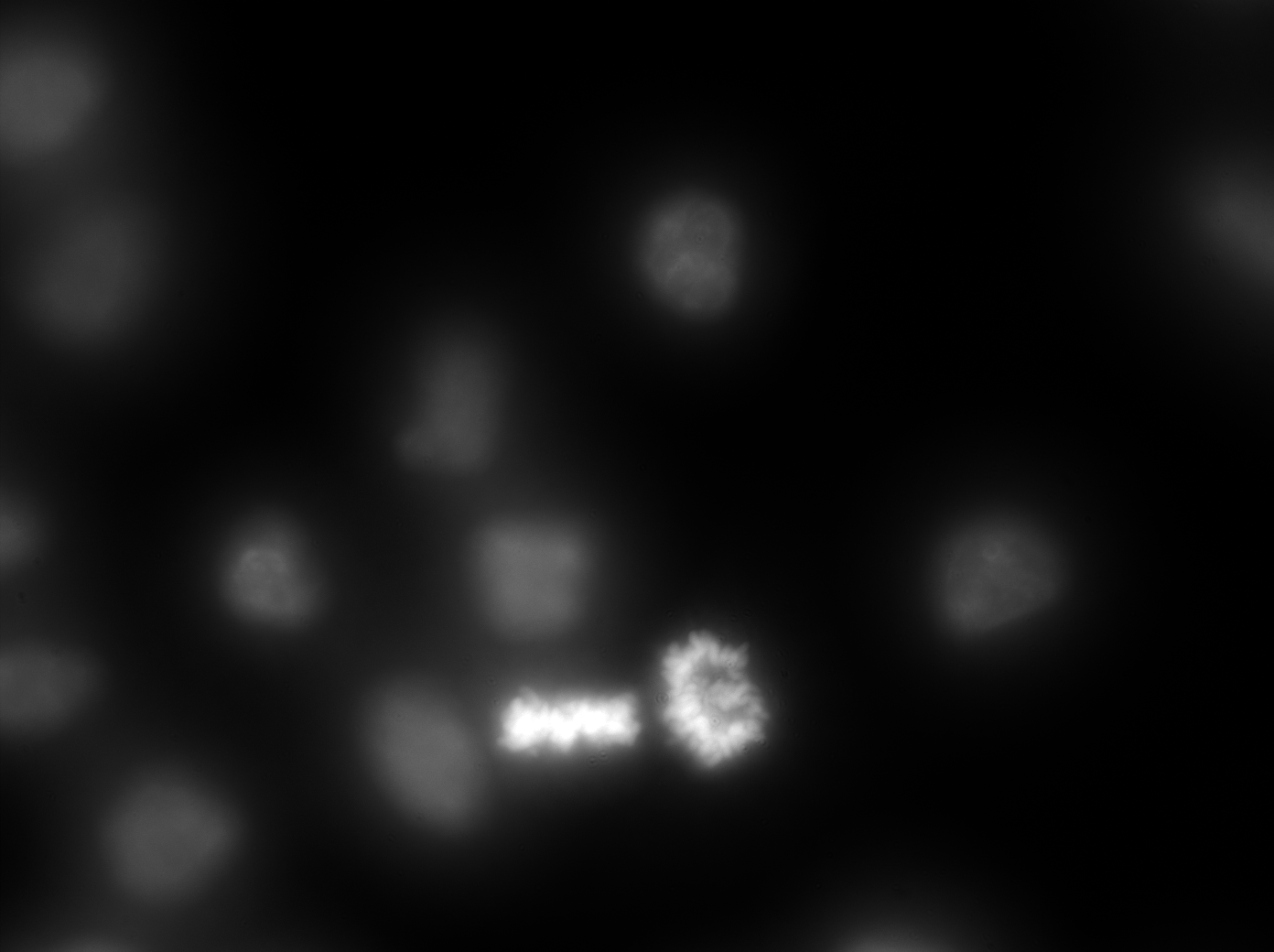

Supplement: Supplementary file 27 — Source data Fig. 7 part 3 [file 44319_2026_742_MOESM27_ESM.zip › Figure 7 Part 3/Fig 7be Cas9 and TPGS1-KO rGT335 atubulin/TPGS1-KO 5-2-25 rGT335 atub R1 M4M5.Project Maximum Z_XY1746221653_Z0_T0_C0.tif]

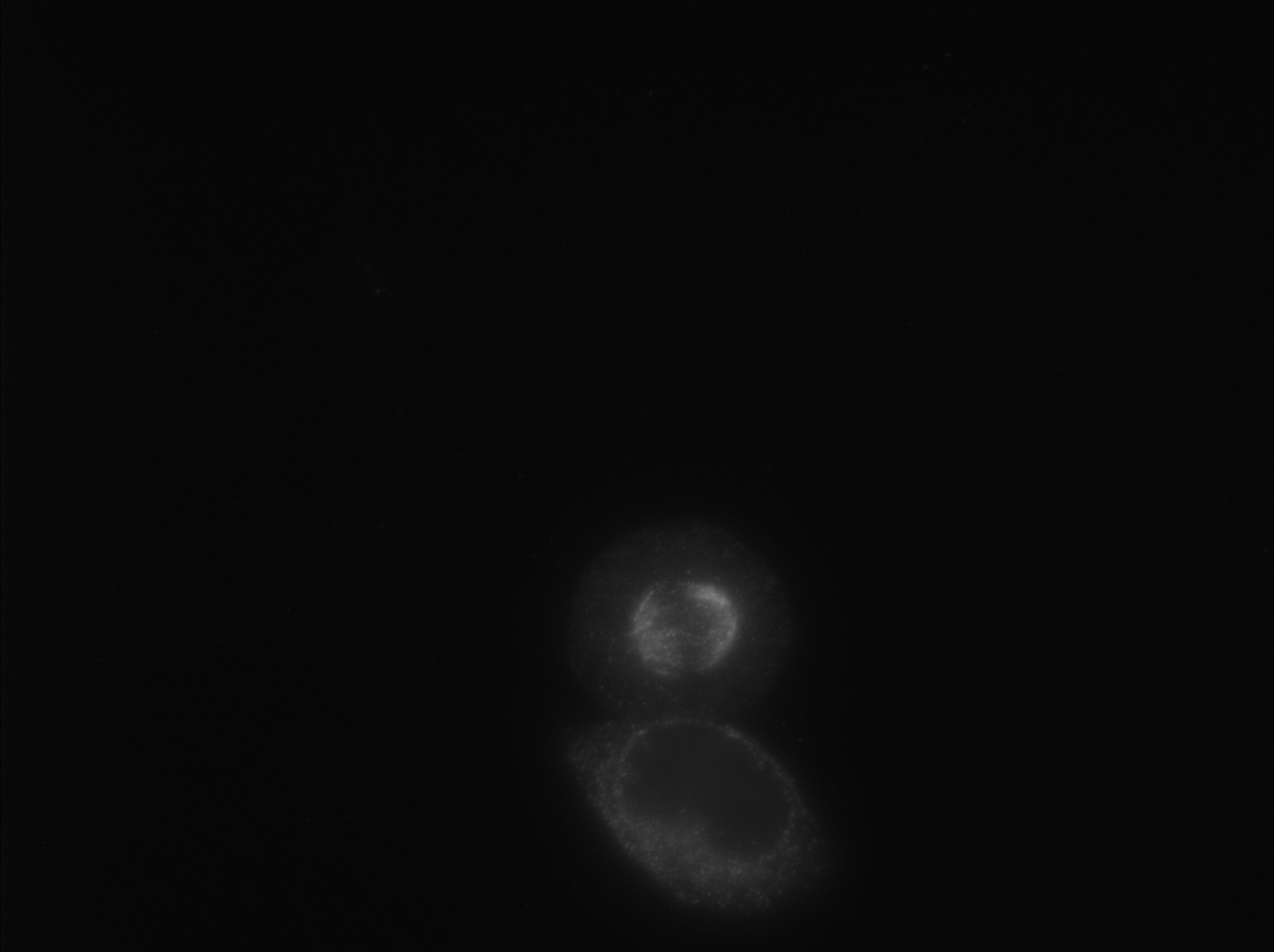

Supplement: Supplementary file 27 — Source data Fig. 7 part 3 [file 44319_2026_742_MOESM27_ESM.zip › Figure 7 Part 3/Fig 7be Cas9 and TPGS1-KO rGT335 atubulin/Cas9 5-2-25 rGT335 atub R1 M3.Project Maximum Z_XY1746557428_Z0_T0_C2.tif]

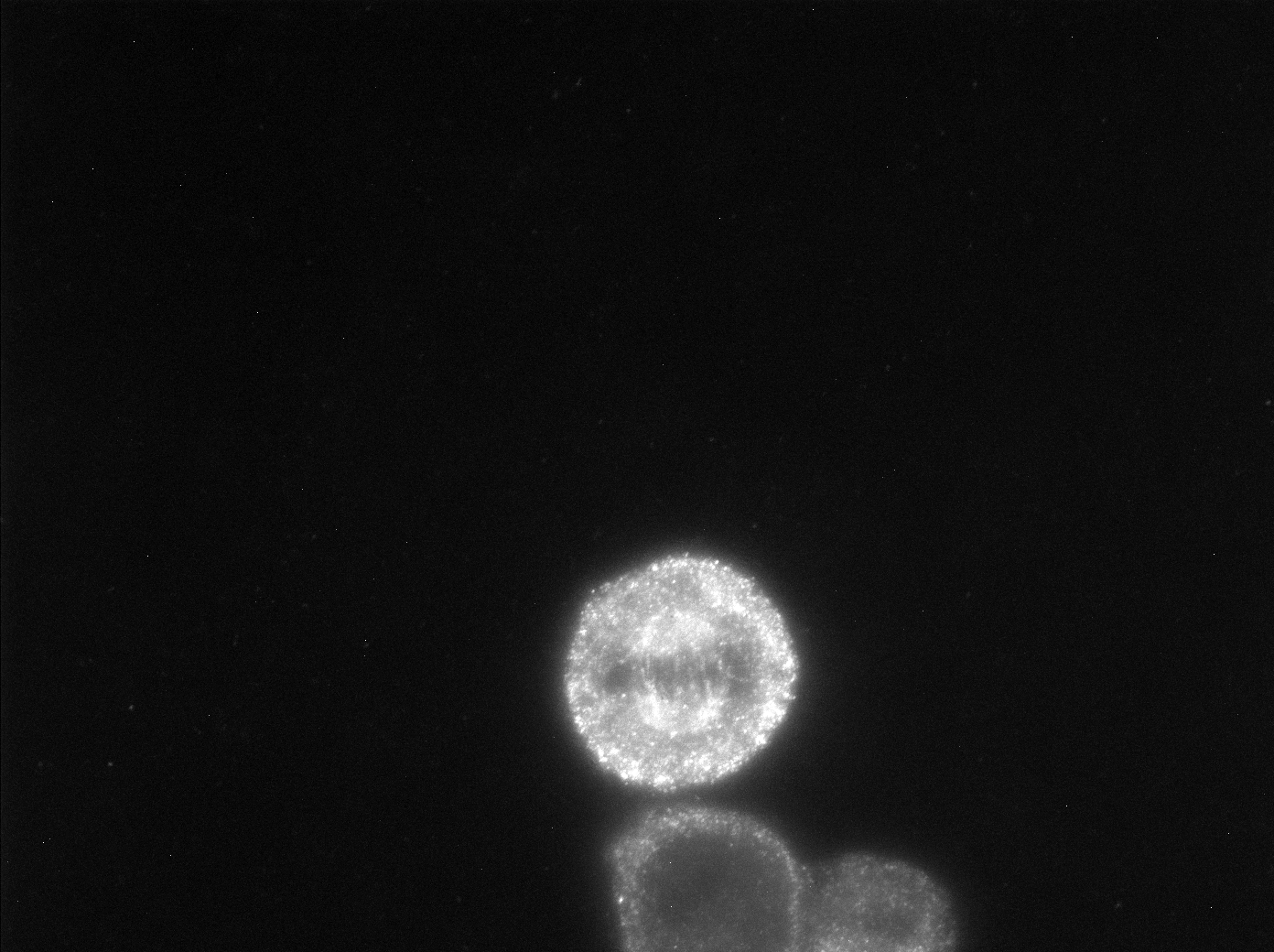

Supplement: Supplementary file 27 — Source data Fig. 7 part 3 [file 44319_2026_742_MOESM27_ESM.zip › Figure 7 Part 3/Fig 7be Cas9 and TPGS1-KO rGT335 atubulin/TPGS1-KO 5-2-25 rGT335 atub R2 M6.Project Maximum Z_XY1746564489_Z0_T0_C1 figure.tif]

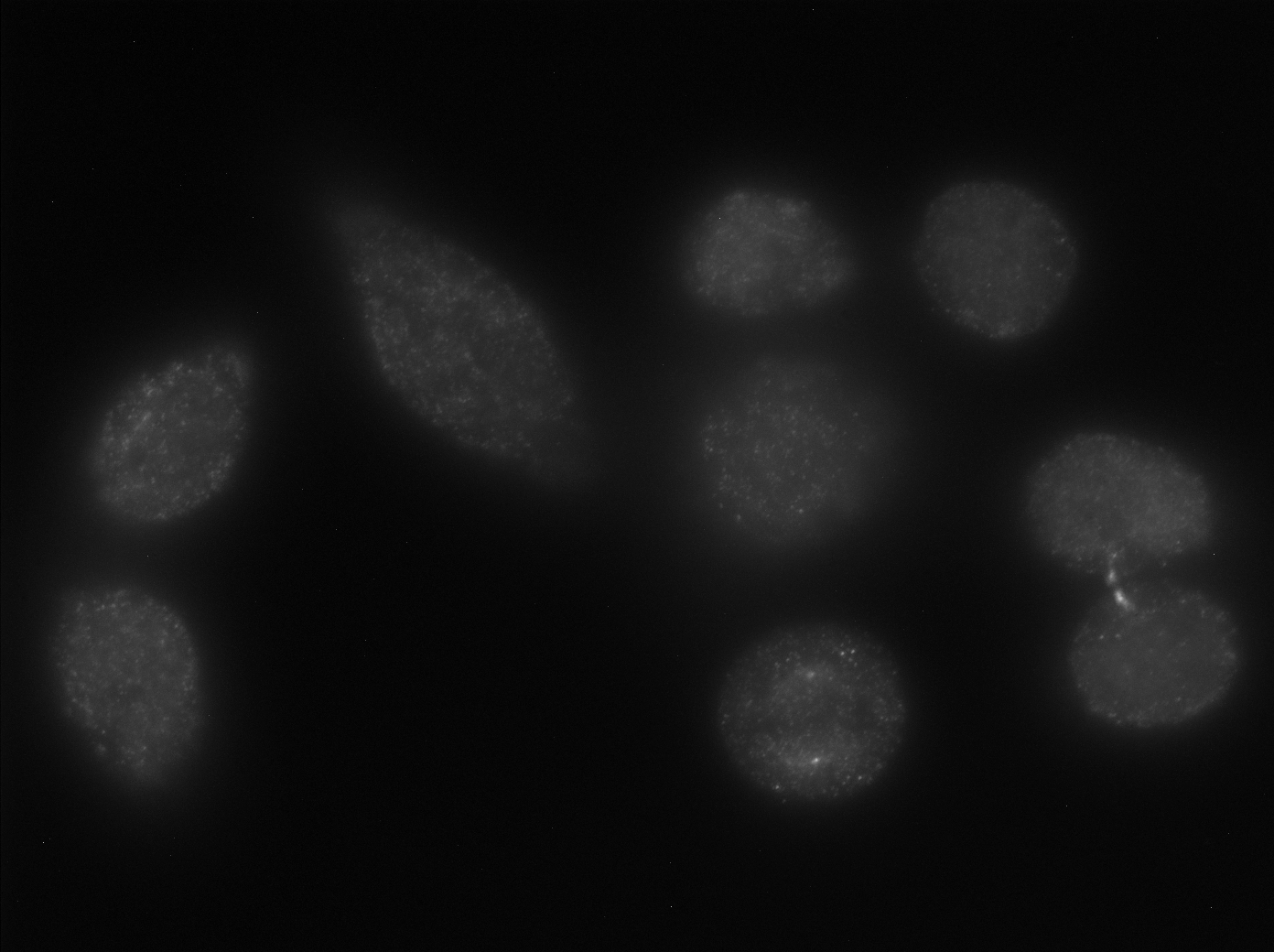

Supplement: Supplementary file 27 — Source data Fig. 7 part 3 [file 44319_2026_742_MOESM27_ESM.zip › Figure 7 Part 3/Fig 7be Cas9 and TPGS1-KO rGT335 atubulin/TPGS1-KO 5-2-25 rGT335 atub R3 M8.Project Maximum Z_XY1746220411_Z0_T0_C1.tif]

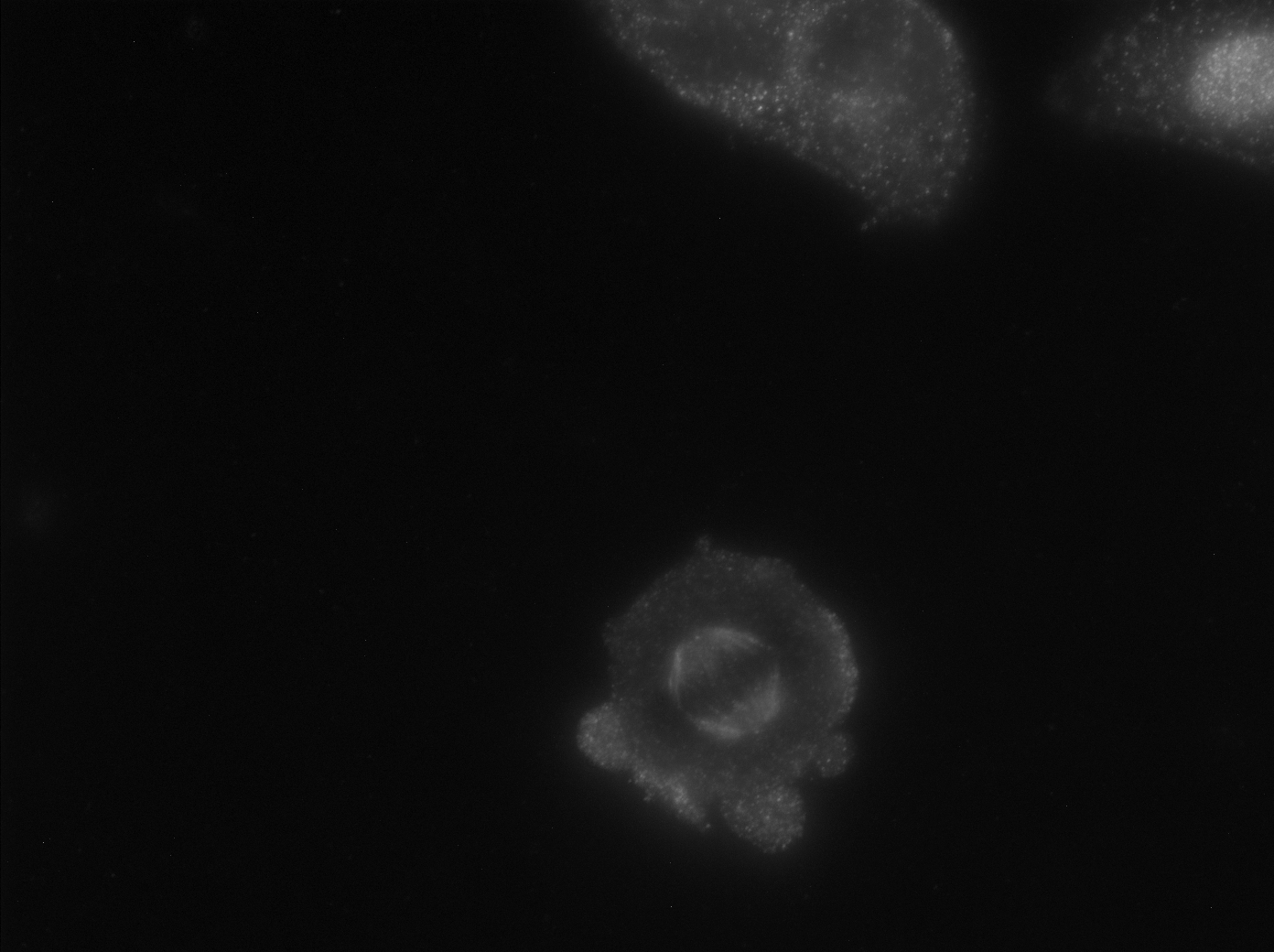

Supplement: Supplementary file 27 — Source data Fig. 7 part 3 [file 44319_2026_742_MOESM27_ESM.zip › Figure 7 Part 3/Fig 7be Cas9 and TPGS1-KO rGT335 atubulin/TPGS1-KO 5-2-25 rGT335 atub R2 M2.Project Maximum Z_XY1746563949_Z0_T0_C1.tif]

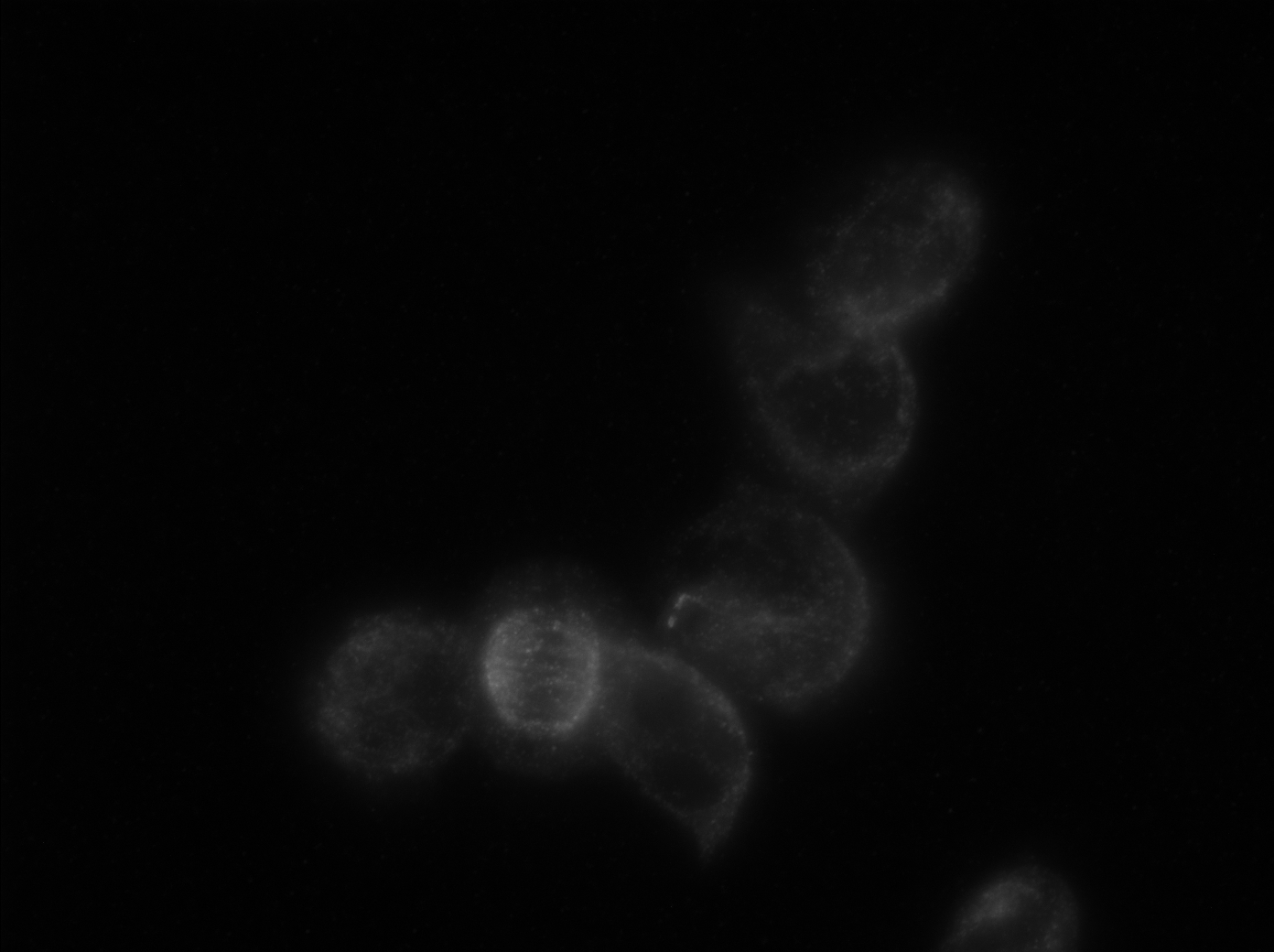

Supplement: Supplementary file 27 — Source data Fig. 7 part 3 [file 44319_2026_742_MOESM27_ESM.zip › Figure 7 Part 3/Fig 7be Cas9 and TPGS1-KO rGT335 atubulin/Cas9 5-2-25 rGT335 atub R2 M5.Project Maximum Z_XY1746562056_Z0_T0_C2.tif]

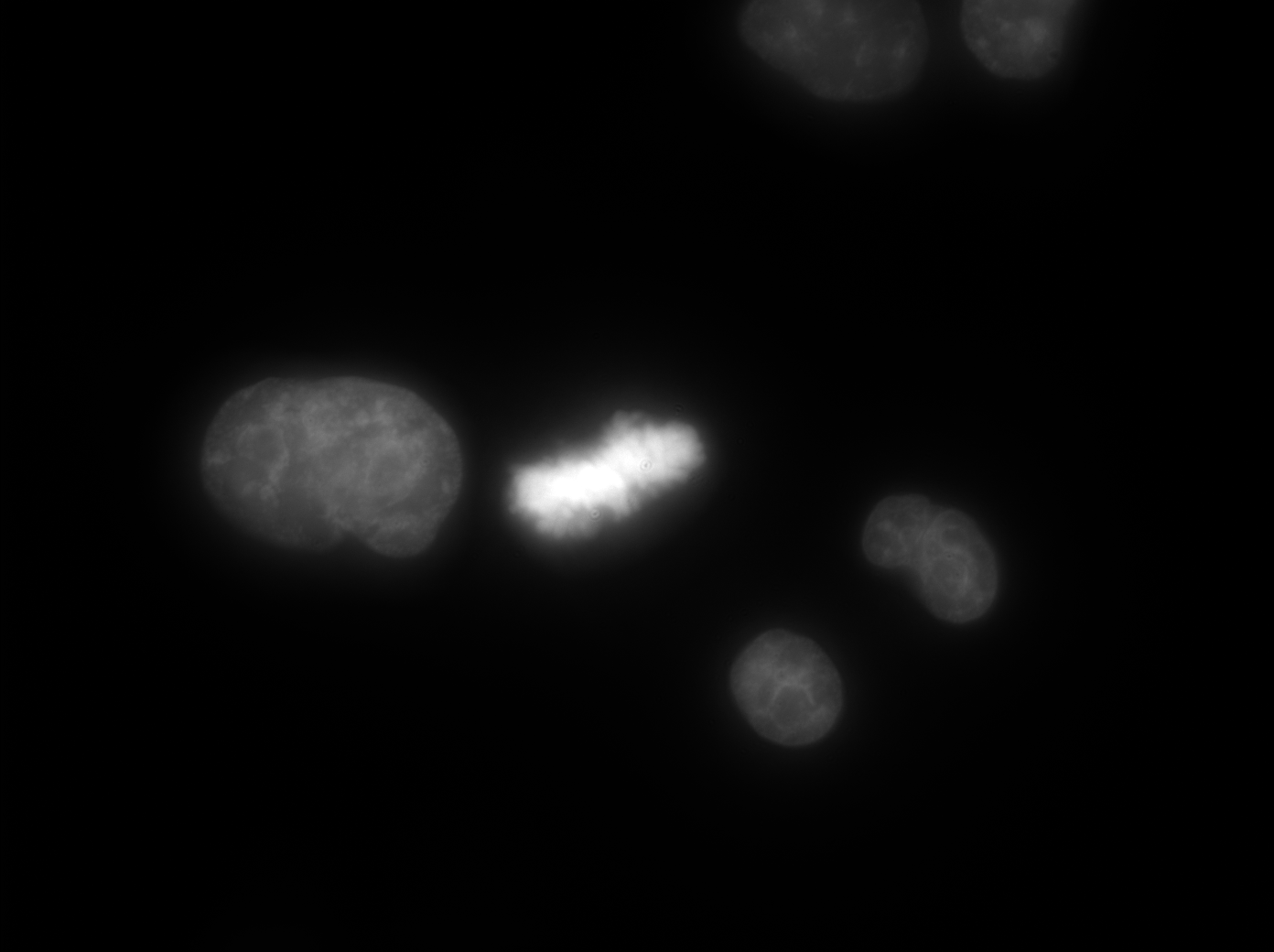

Supplement: Supplementary file 27 — Source data Fig. 7 part 3 [file 44319_2026_742_MOESM27_ESM.zip › Figure 7 Part 3/Fig 7be Cas9 and TPGS1-KO rGT335 atubulin/TPGS1-KO 5-2-25 rGT335 atub R2 M9.Project Maximum Z_XY1746564888_Z0_T0_C0.tif]

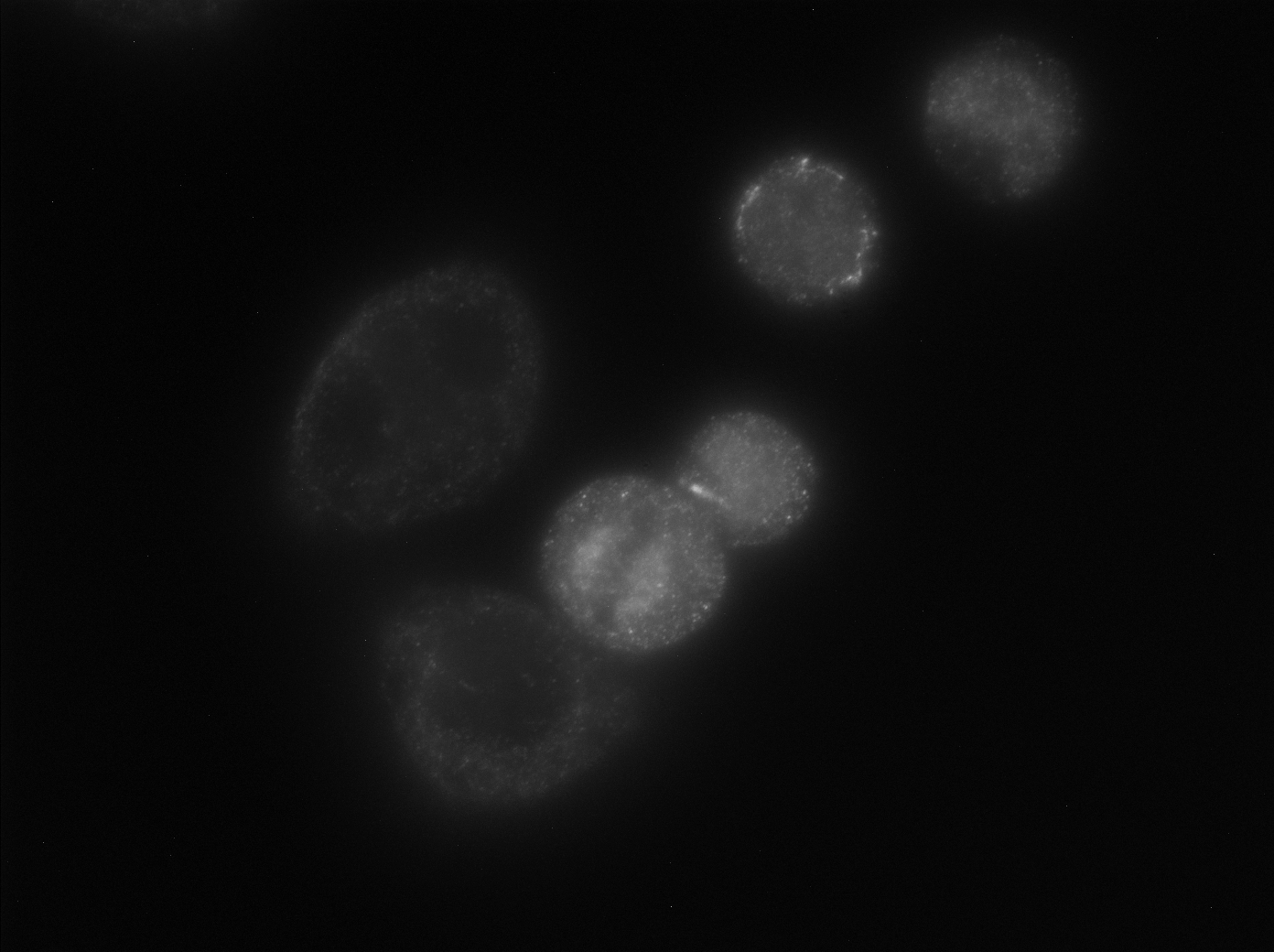

Supplement: Supplementary file 27 — Source data Fig. 7 part 3 [file 44319_2026_742_MOESM27_ESM.zip › Figure 7 Part 3/Fig 7be Cas9 and TPGS1-KO rGT335 atubulin/TPGS1-KO 5-2-25 rGT335 atub R3 M1.Project Maximum Z_XY1746218663_Z0_T0_C1.tif]

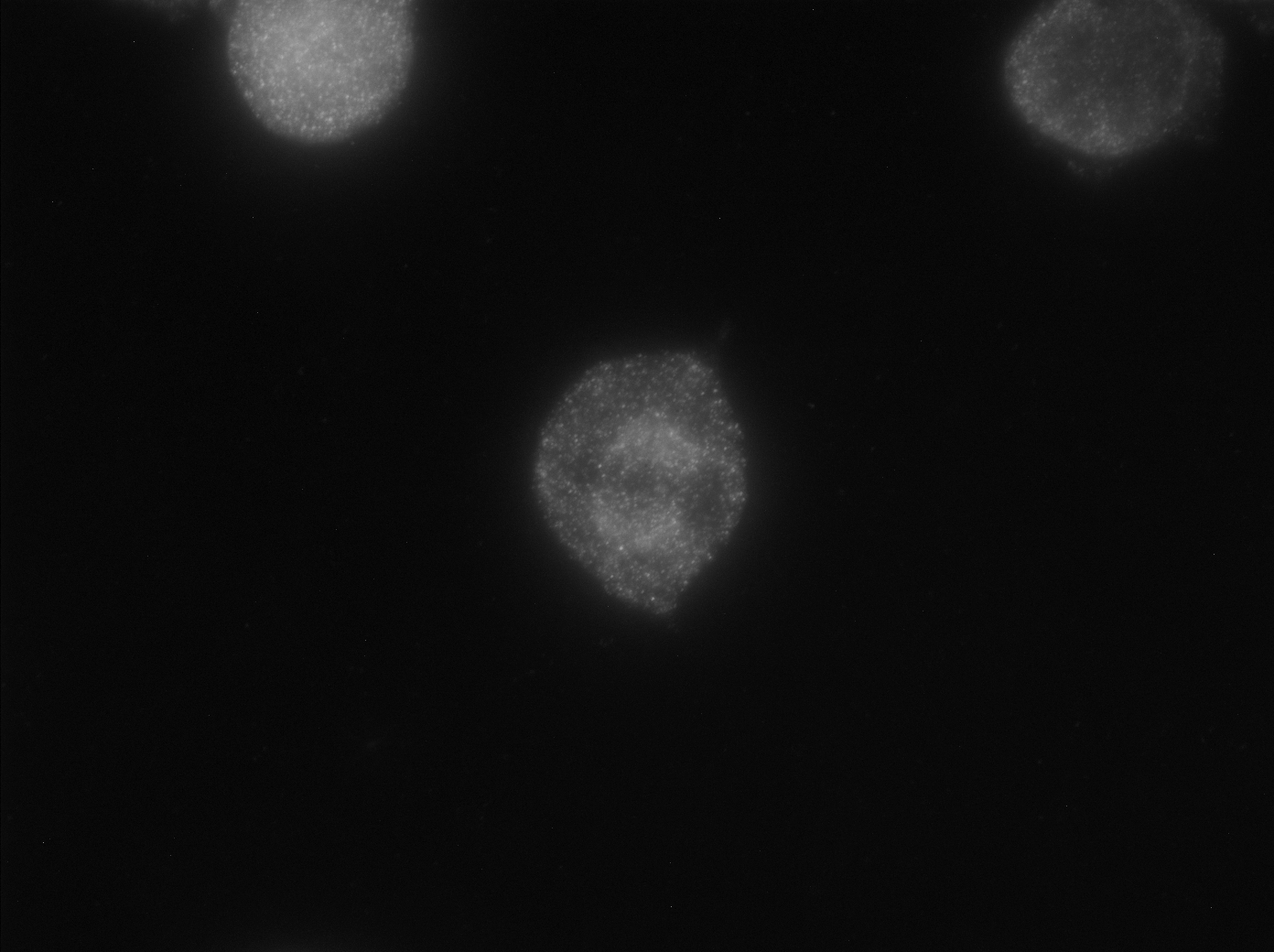

Supplement: Supplementary file 27 — Source data Fig. 7 part 3 [file 44319_2026_742_MOESM27_ESM.zip › Figure 7 Part 3/Fig 7be Cas9 and TPGS1-KO rGT335 atubulin/TPGS1-KO 5-2-25 rGT335 atub R2 M3.Project Maximum Z_XY1746564055_Z0_T0_C1.tif]

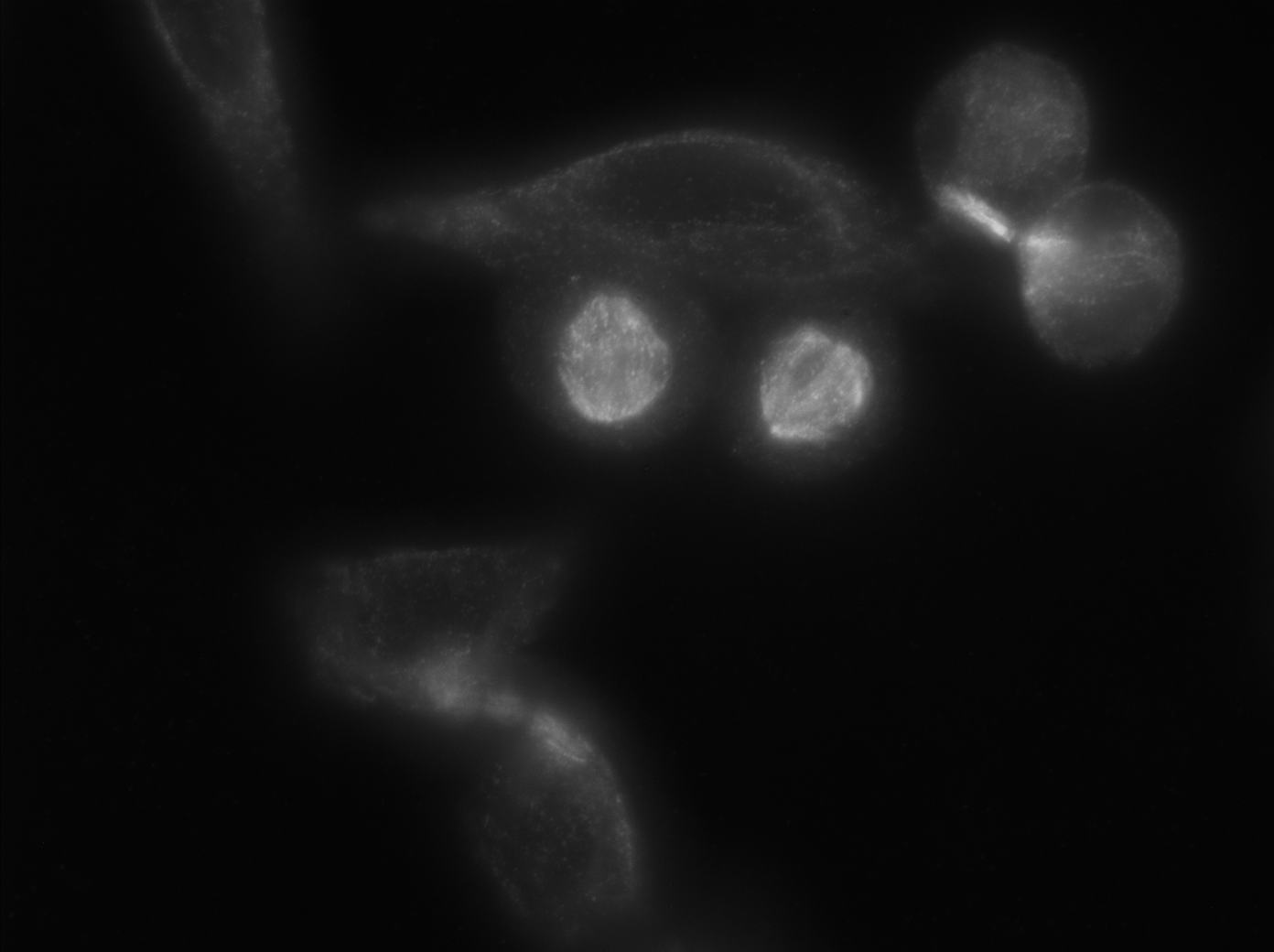

Supplement: Supplementary file 27 — Source data Fig. 7 part 3 [file 44319_2026_742_MOESM27_ESM.zip › Figure 7 Part 3/Fig 7be Cas9 and TPGS1-KO rGT335 atubulin/TPGS1-KO 5-2-25 rGT335 atub R3 M10M11.Project Maximum Z_XY1746220741_Z0_T0_C2.tif]

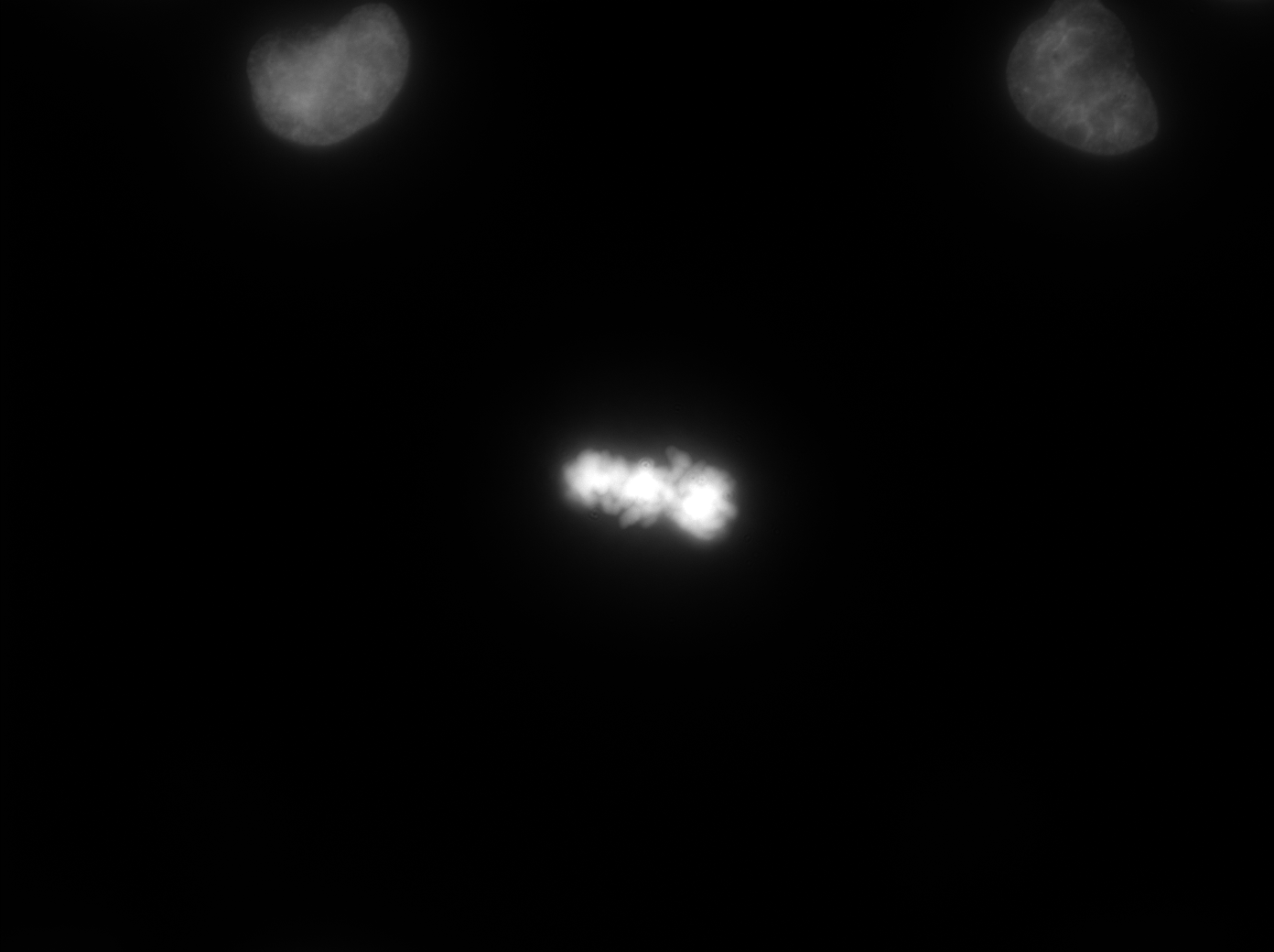

Supplement: Supplementary file 27 — Source data Fig. 7 part 3 [file 44319_2026_742_MOESM27_ESM.zip › Figure 7 Part 3/Fig 7be Cas9 and TPGS1-KO rGT335 atubulin/TPGS1-KO 5-2-25 rGT335 atub R2 M3.Project Maximum Z_XY1746564055_Z0_T0_C0.tif]

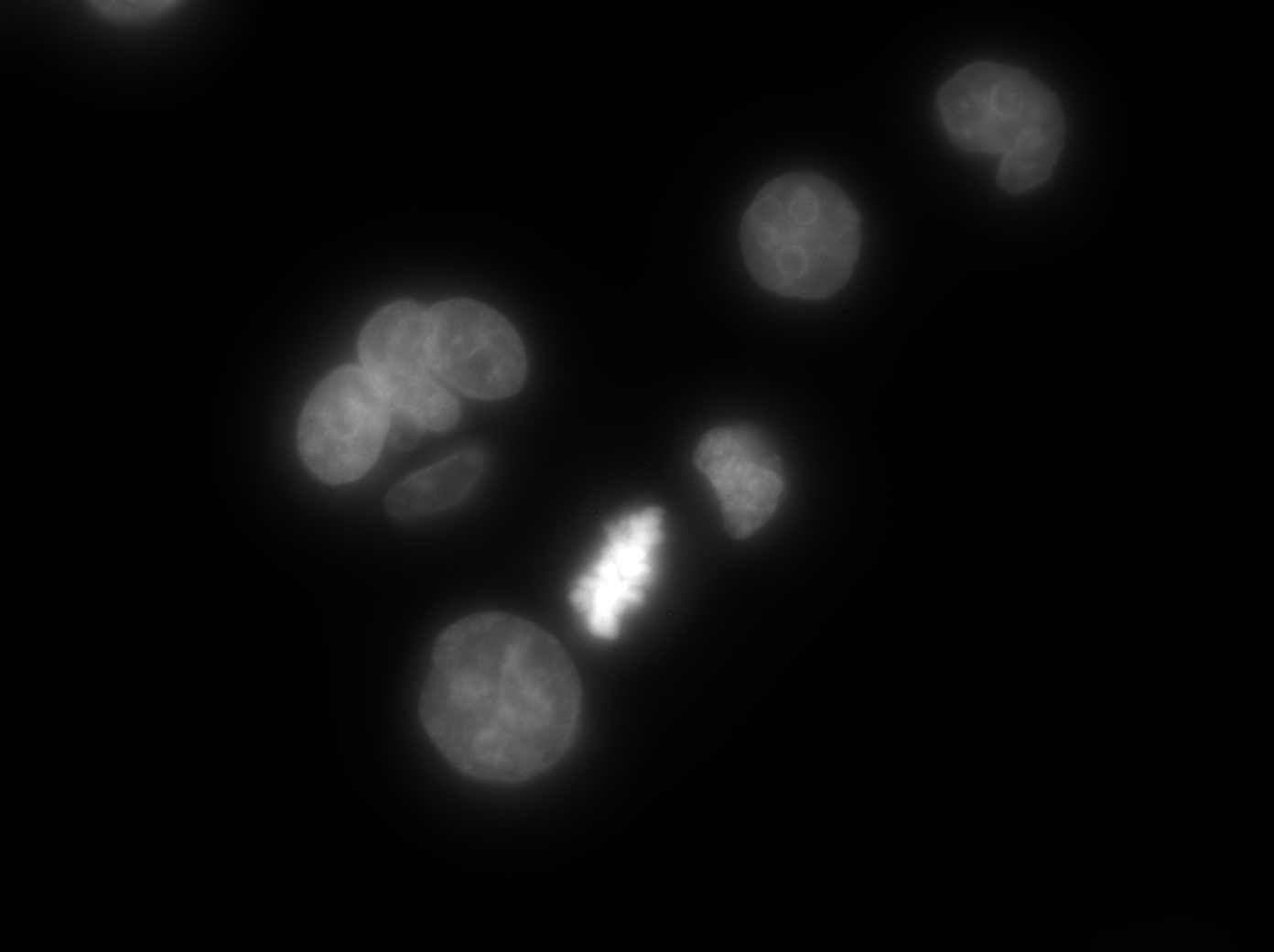

Supplement: Supplementary file 27 — Source data Fig. 7 part 3 [file 44319_2026_742_MOESM27_ESM.zip › Figure 7 Part 3/Fig 7be Cas9 and TPGS1-KO rGT335 atubulin/TPGS1-KO 5-2-25 rGT335 atub R3 M1.Project Maximum Z_XY1746218663_Z0_T0_C0.tif]

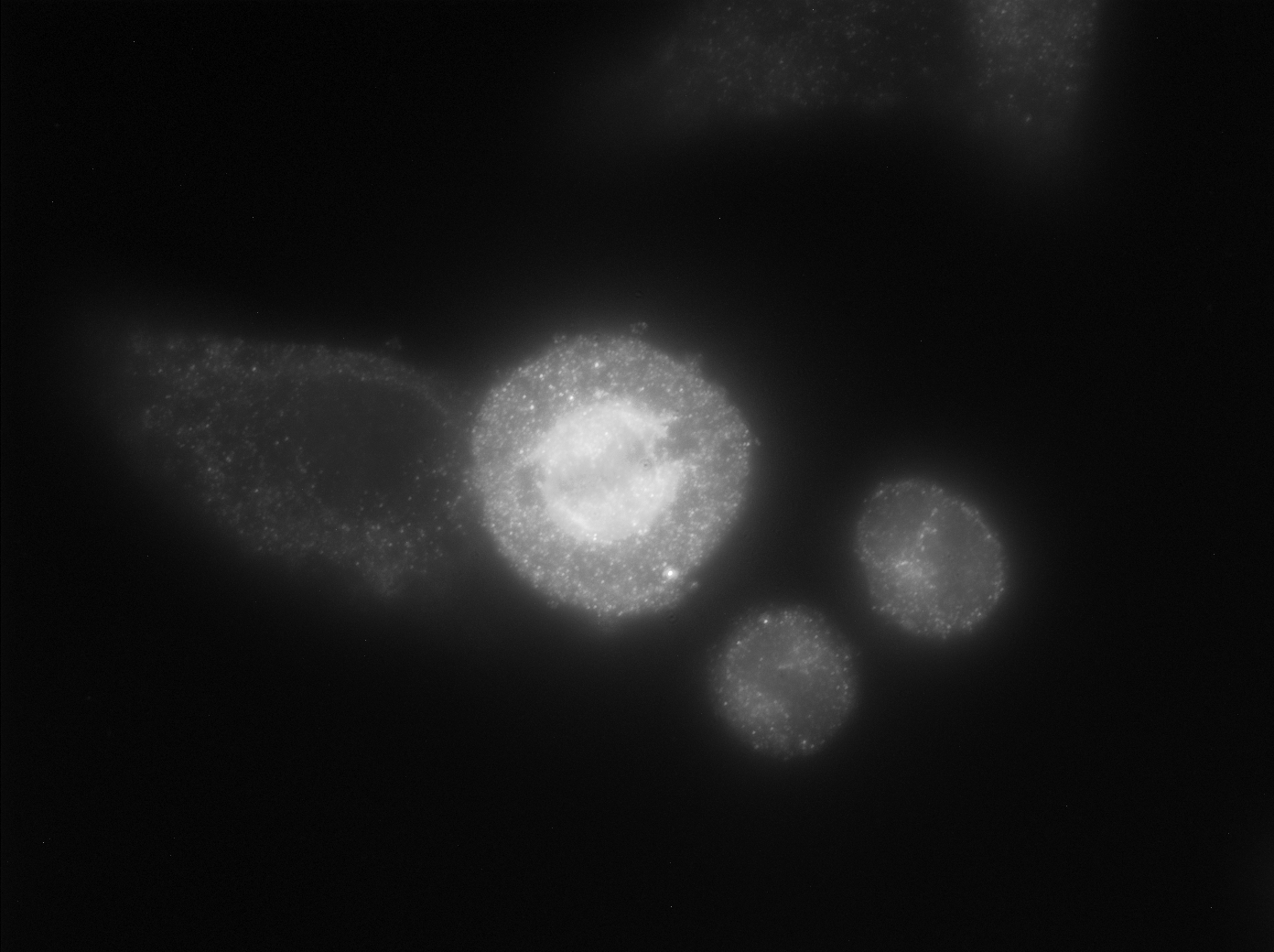

Supplement: Supplementary file 27 — Source data Fig. 7 part 3 [file 44319_2026_742_MOESM27_ESM.zip › Figure 7 Part 3/Fig 7be Cas9 and TPGS1-KO rGT335 atubulin/TPGS1-KO 5-2-25 rGT335 atub R2 M9.Project Maximum Z_XY1746564888_Z0_T0_C1.tif]

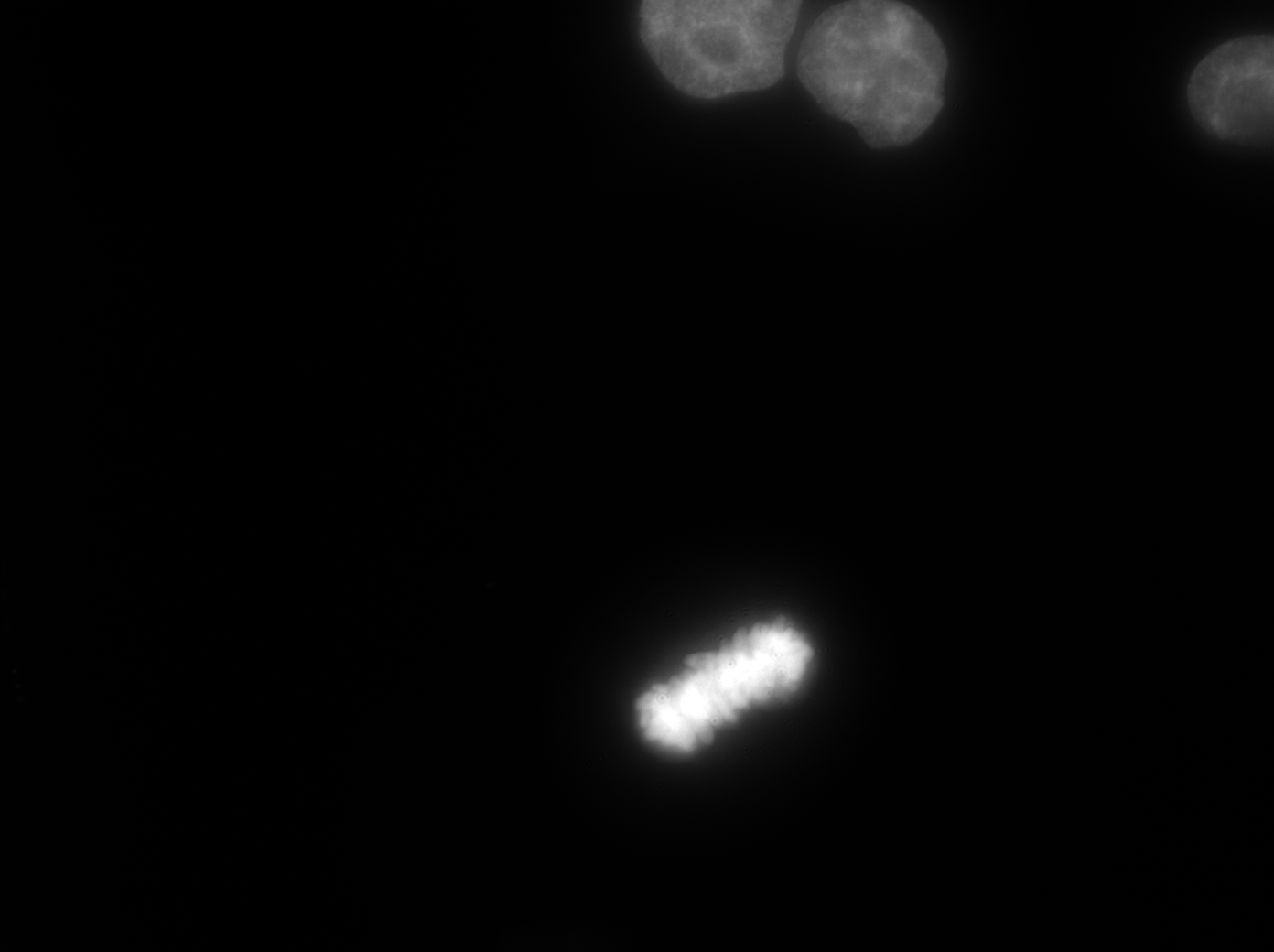

Supplement: Supplementary file 27 — Source data Fig. 7 part 3 [file 44319_2026_742_MOESM27_ESM.zip › Figure 7 Part 3/Fig 7be Cas9 and TPGS1-KO rGT335 atubulin/TPGS1-KO 5-2-25 rGT335 atub R2 M2.Project Maximum Z_XY1746563949_Z0_T0_C0.tif]

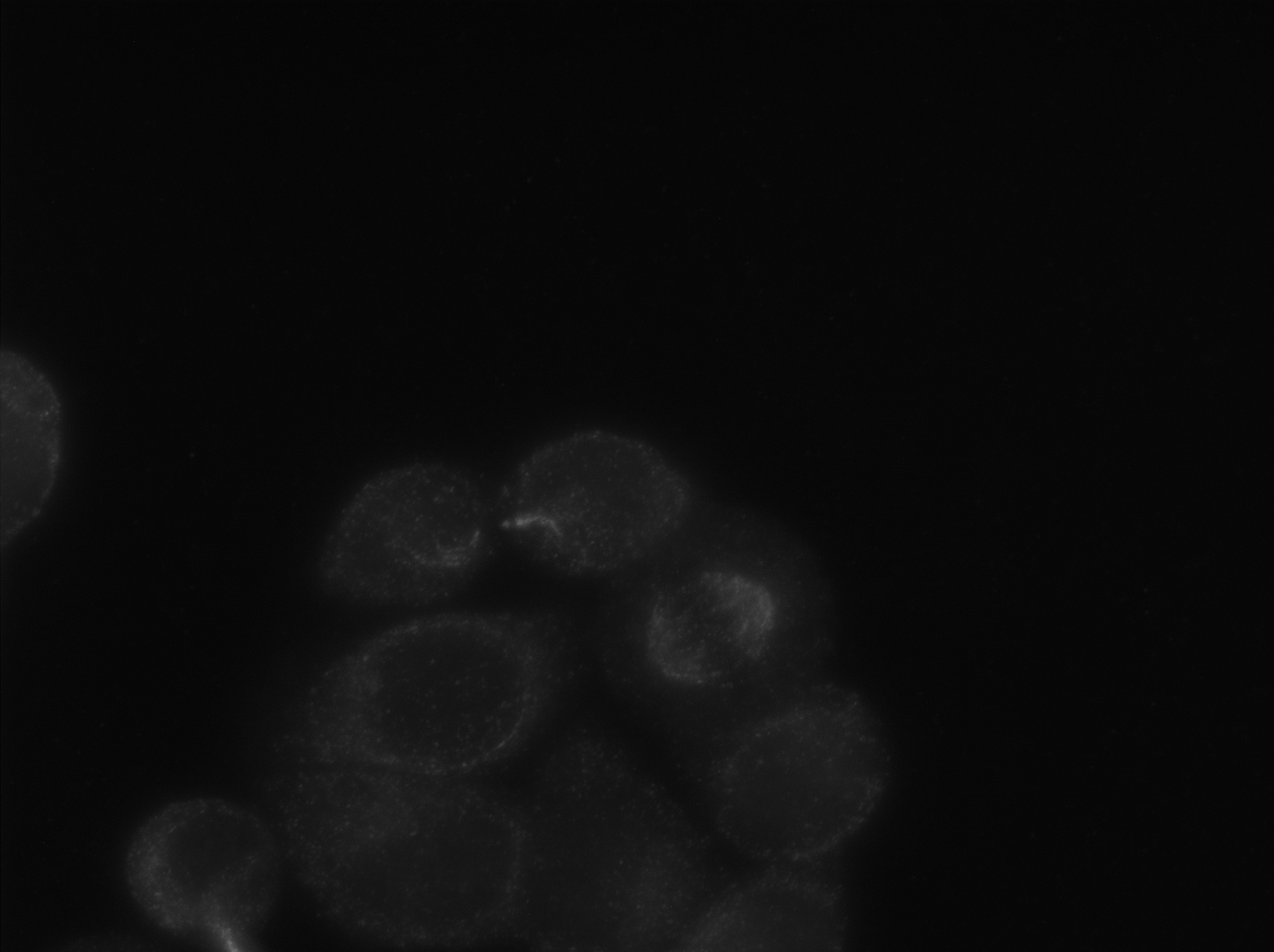

Supplement: Supplementary file 27 — Source data Fig. 7 part 3 [file 44319_2026_742_MOESM27_ESM.zip › Figure 7 Part 3/Fig 7be Cas9 and TPGS1-KO rGT335 atubulin/Cas9 5-2-25 rGT335 atub R1 M1.Project Maximum Z_XY1746557011_Z0_T0_C2.tif]

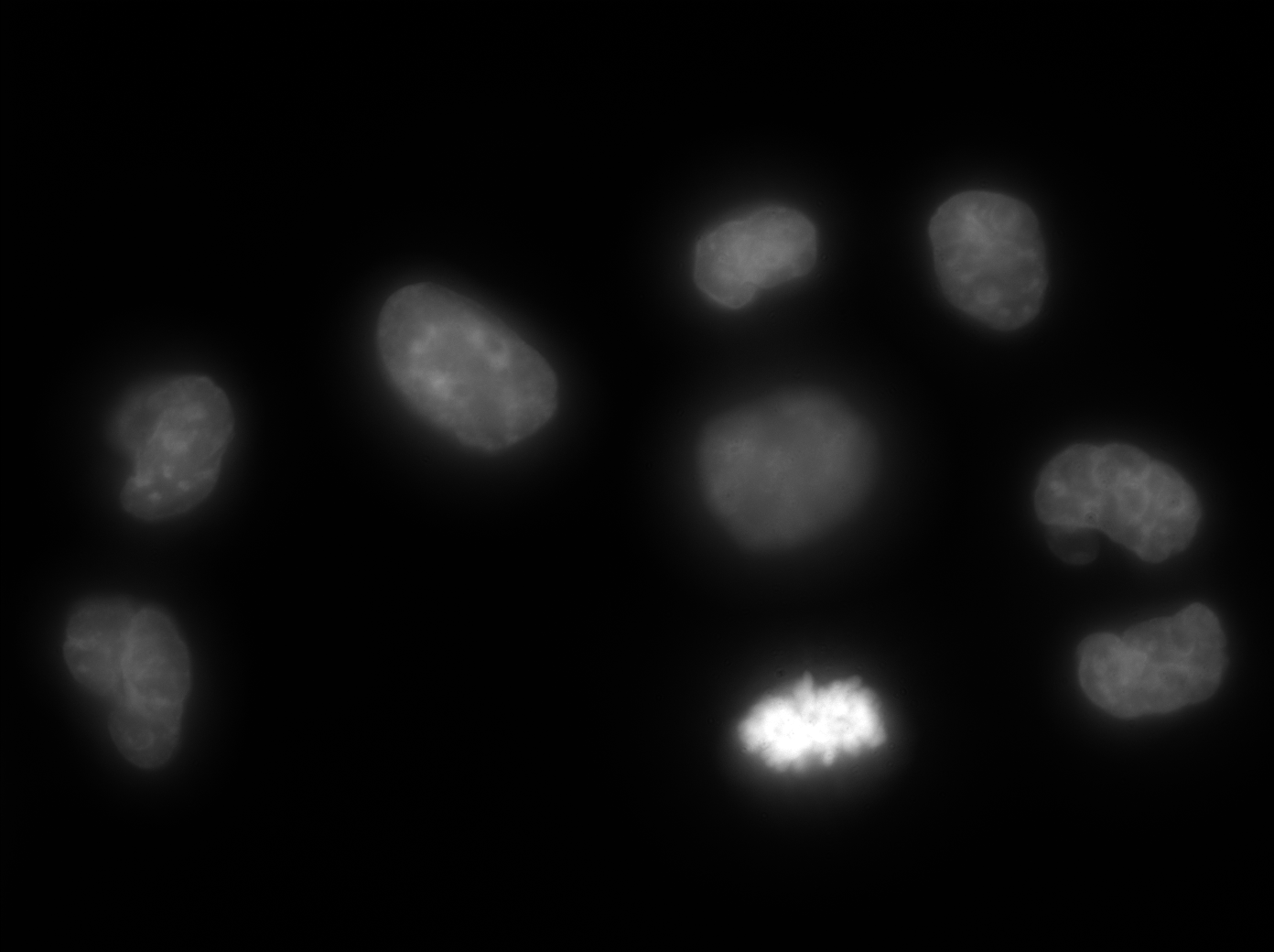

Supplement: Supplementary file 27 — Source data Fig. 7 part 3 [file 44319_2026_742_MOESM27_ESM.zip › Figure 7 Part 3/Fig 7be Cas9 and TPGS1-KO rGT335 atubulin/TPGS1-KO 5-2-25 rGT335 atub R3 M8.Project Maximum Z_XY1746220411_Z0_T0_C0.tif]

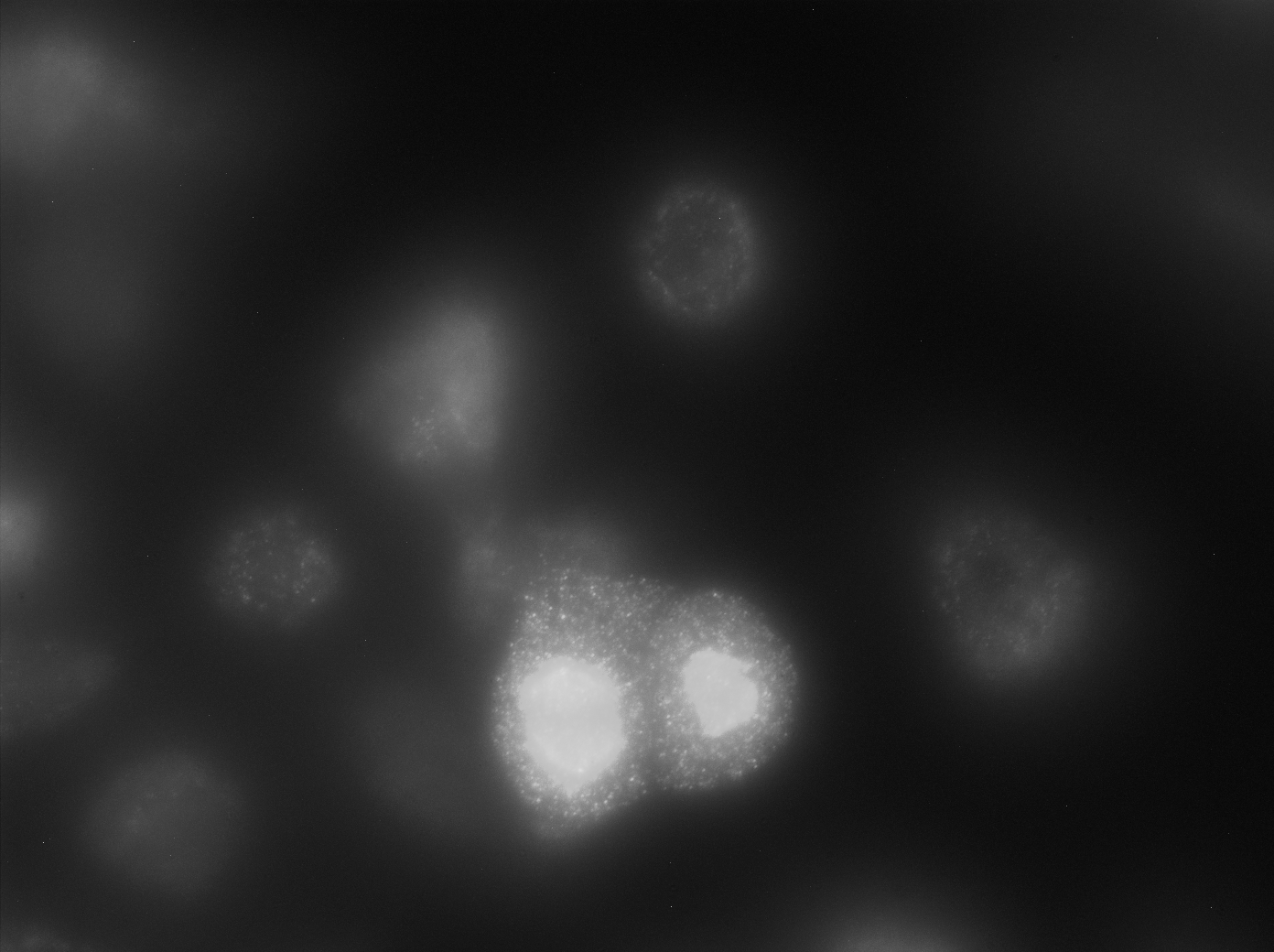

Supplement: Supplementary file 27 — Source data Fig. 7 part 3 [file 44319_2026_742_MOESM27_ESM.zip › Figure 7 Part 3/Fig 7be Cas9 and TPGS1-KO rGT335 atubulin/TPGS1-KO 5-2-25 rGT335 atub R1 M4M5.Project Maximum Z_XY1746221653_Z0_T0_C1.tif]

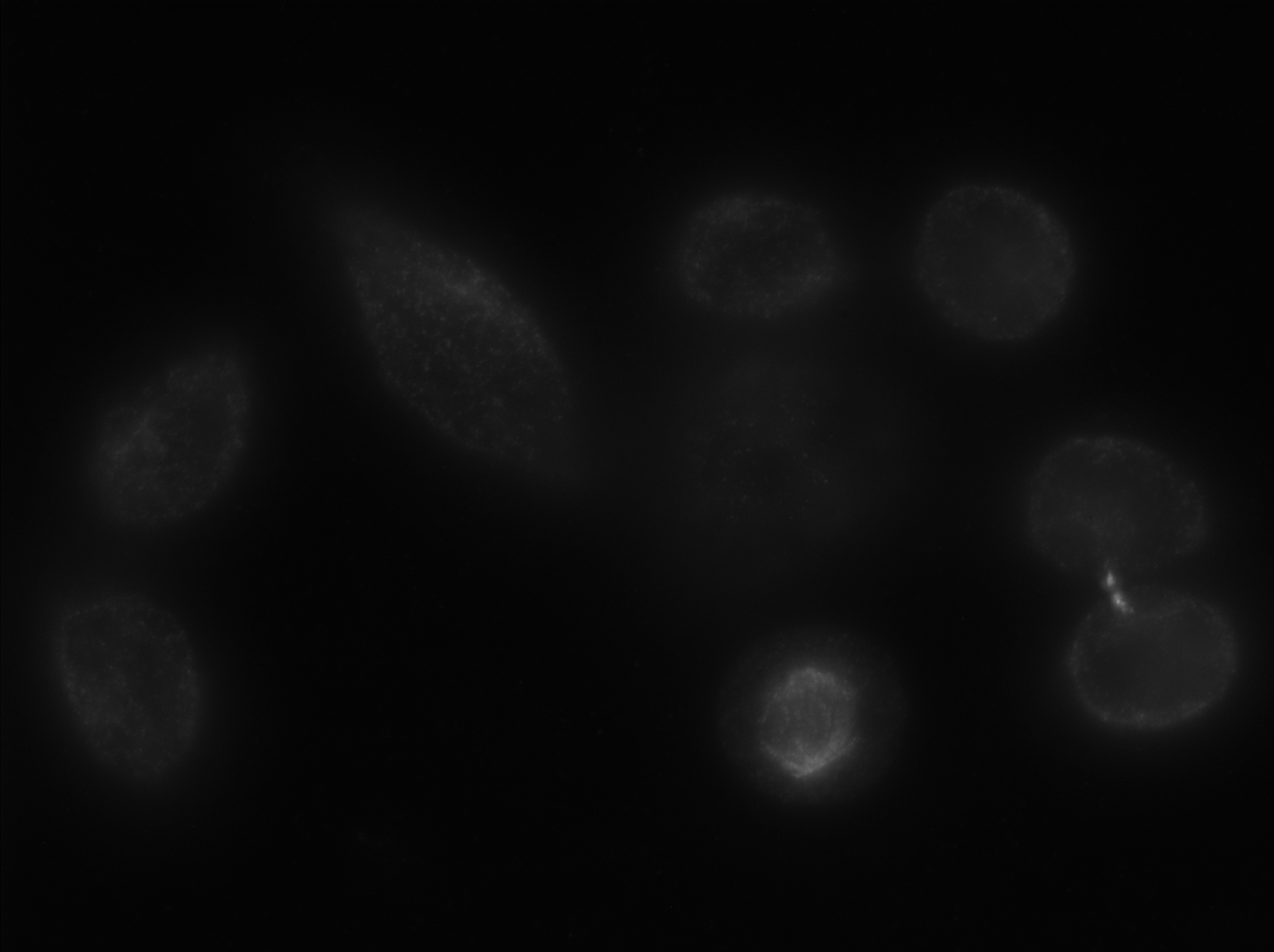

Supplement: Supplementary file 27 — Source data Fig. 7 part 3 [file 44319_2026_742_MOESM27_ESM.zip › Figure 7 Part 3/Fig 7be Cas9 and TPGS1-KO rGT335 atubulin/TPGS1-KO 5-2-25 rGT335 atub R3 M8.Project Maximum Z_XY1746220411_Z0_T0_C2.tif]

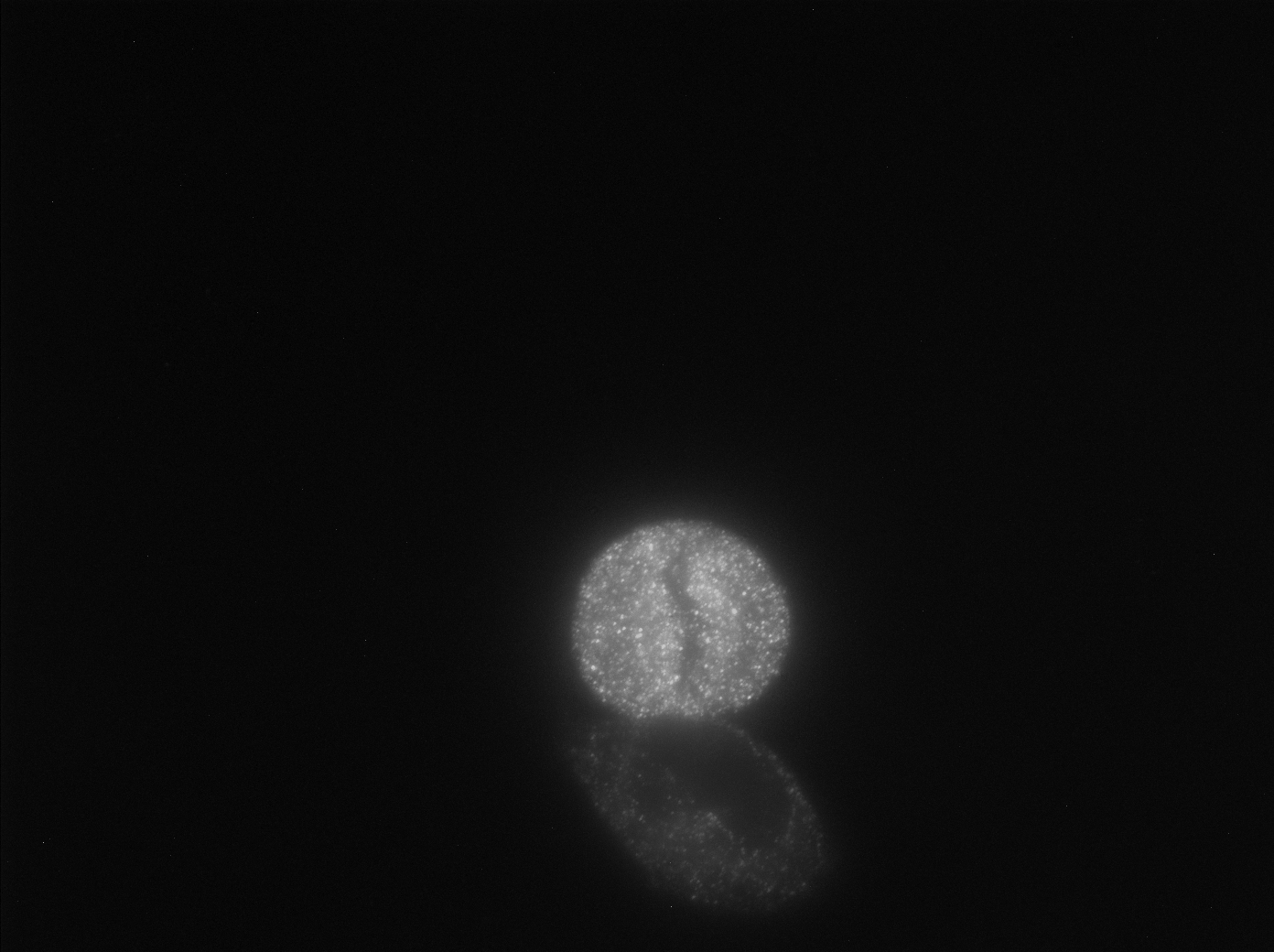

Supplement: Supplementary file 27 — Source data Fig. 7 part 3 [file 44319_2026_742_MOESM27_ESM.zip › Figure 7 Part 3/Fig 7be Cas9 and TPGS1-KO rGT335 atubulin/Cas9 5-2-25 rGT335 atub R1 M3.Project Maximum Z_XY1746557428_Z0_T0_C1.tif]

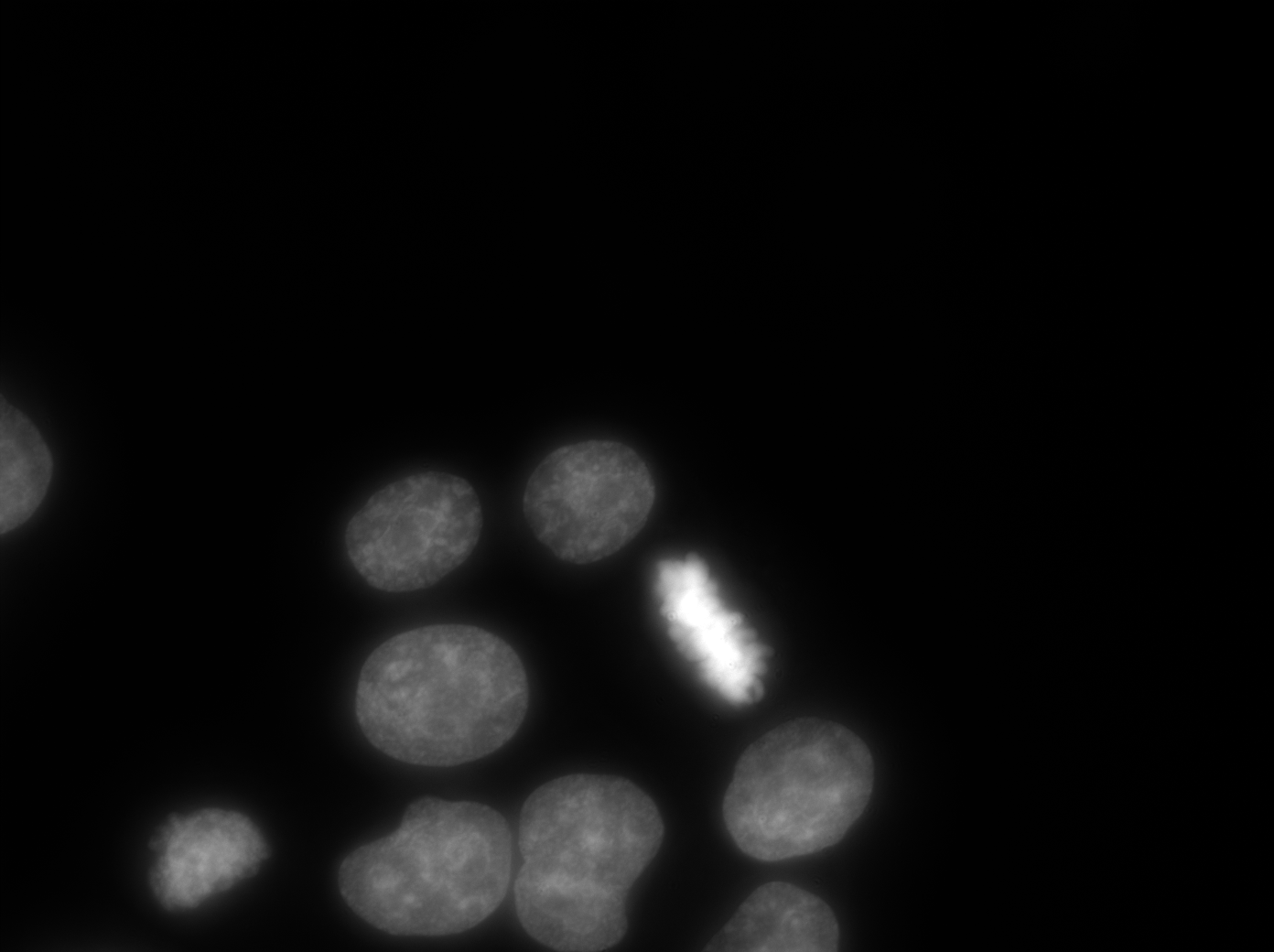

Supplement: Supplementary file 27 — Source data Fig. 7 part 3 [file 44319_2026_742_MOESM27_ESM.zip › Figure 7 Part 3/Fig 7be Cas9 and TPGS1-KO rGT335 atubulin/Cas9 5-2-25 rGT335 atub R1 M1.Project Maximum Z_XY1746557011_Z0_T0_C0.tif]

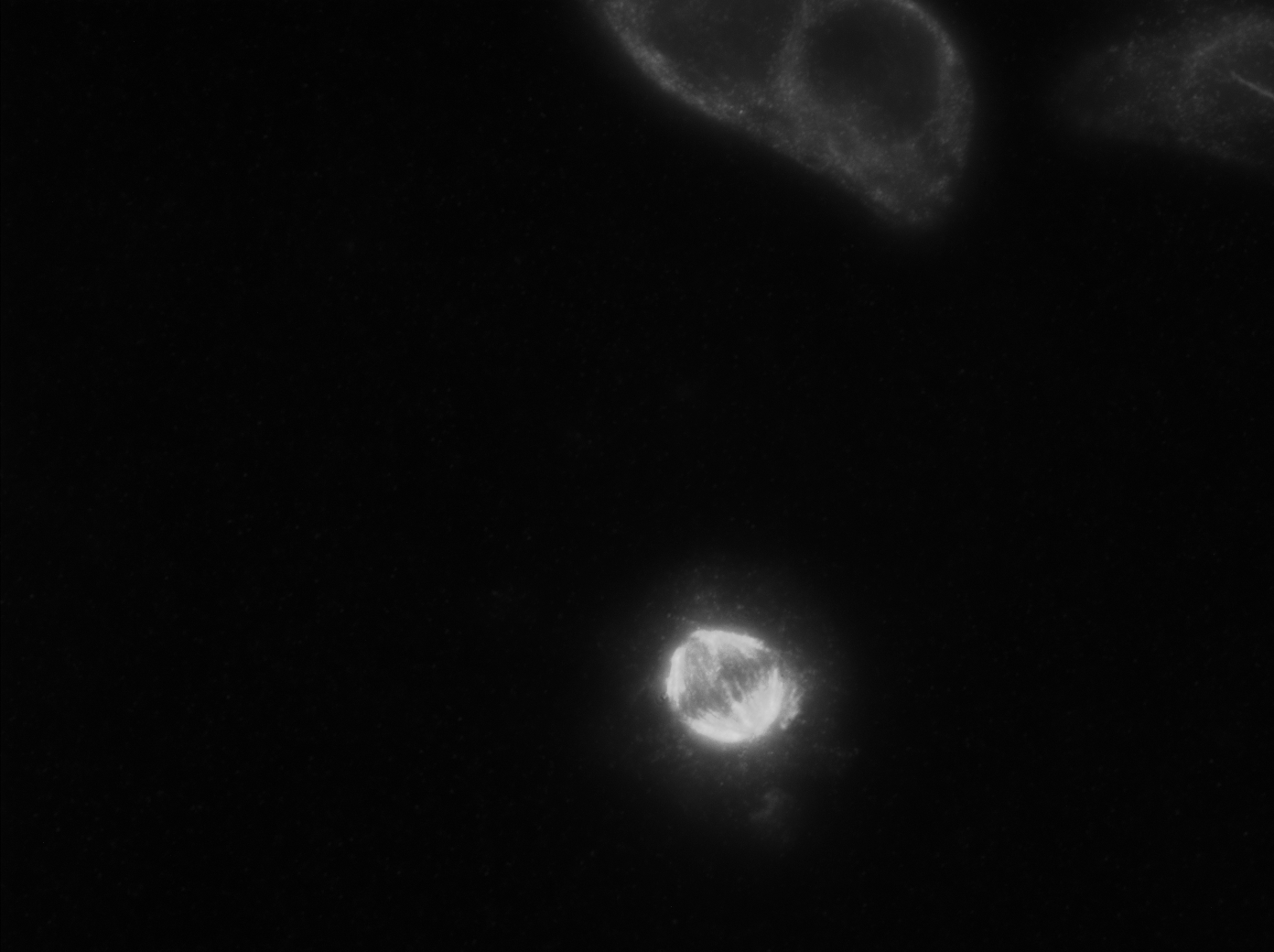

Supplement: Supplementary file 27 — Source data Fig. 7 part 3 [file 44319_2026_742_MOESM27_ESM.zip › Figure 7 Part 3/Fig 7be Cas9 and TPGS1-KO rGT335 atubulin/TPGS1-KO 5-2-25 rGT335 atub R2 M2.Project Maximum Z_XY1746563949_Z0_T0_C2.tif]

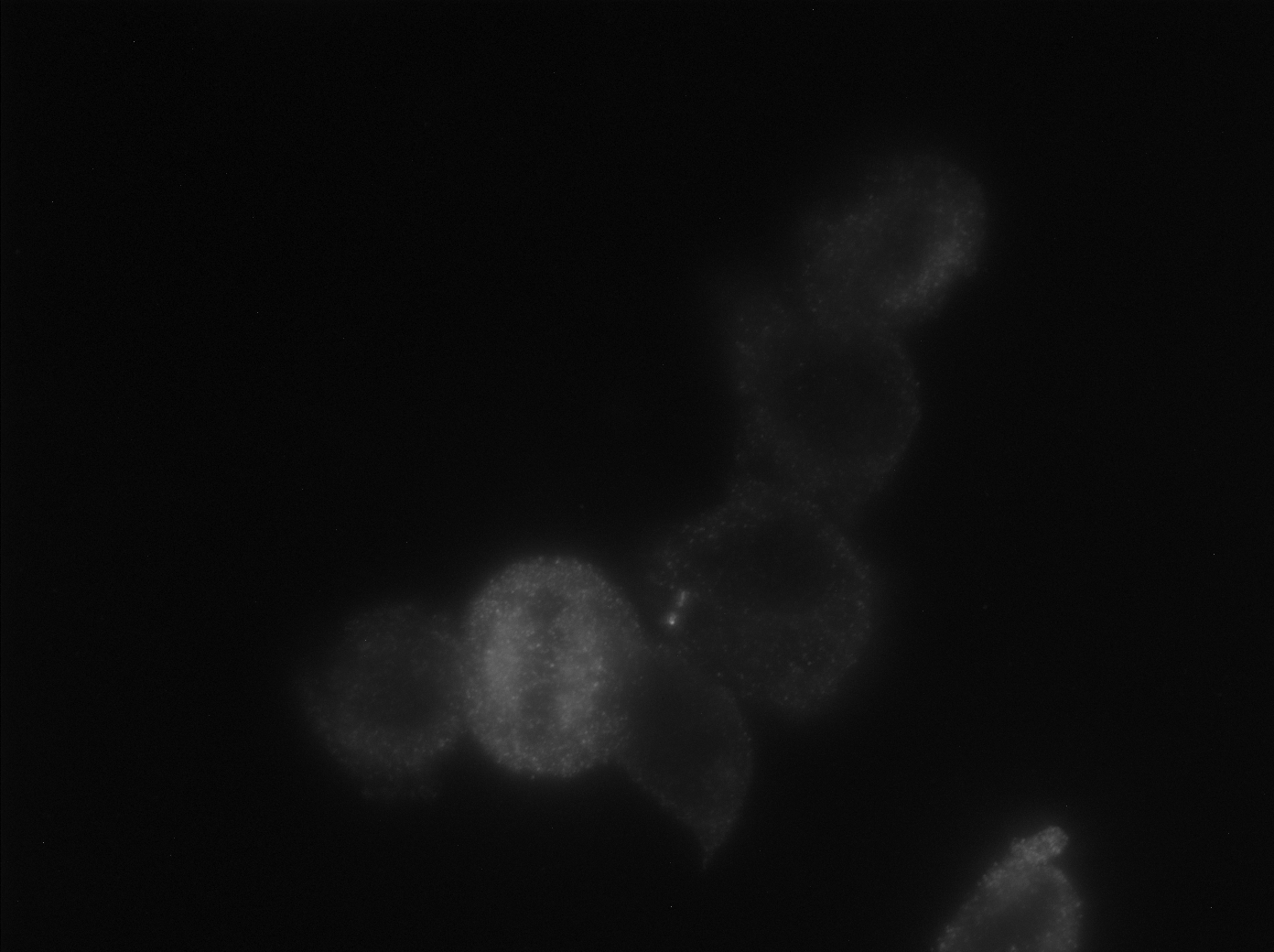

Supplement: Supplementary file 27 — Source data Fig. 7 part 3 [file 44319_2026_742_MOESM27_ESM.zip › Figure 7 Part 3/Fig 7be Cas9 and TPGS1-KO rGT335 atubulin/Cas9 5-2-25 rGT335 atub R2 M5.Project Maximum Z_XY1746562056_Z0_T0_C1.tif]

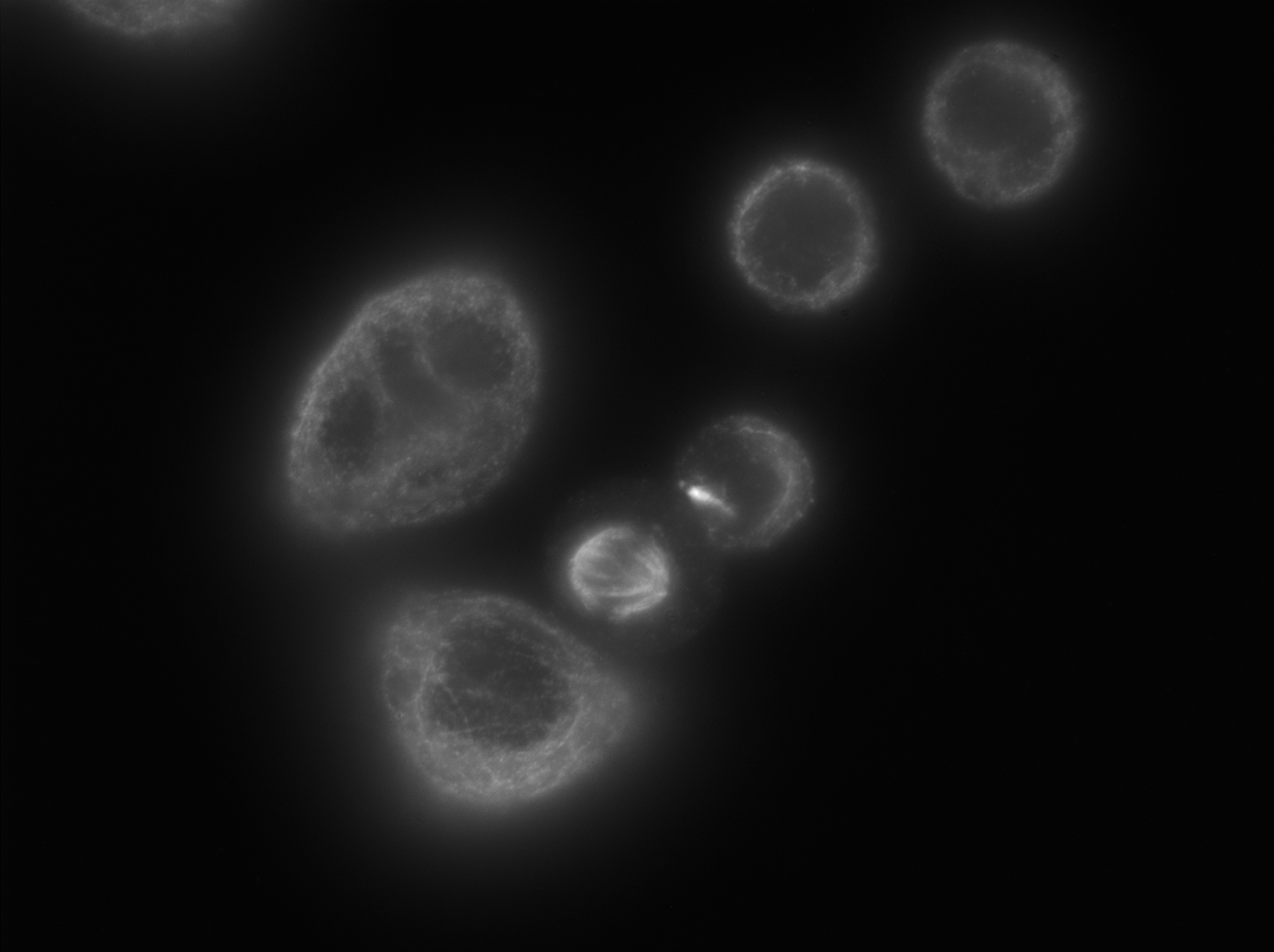

Supplement: Supplementary file 27 — Source data Fig. 7 part 3 [file 44319_2026_742_MOESM27_ESM.zip › Figure 7 Part 3/Fig 7be Cas9 and TPGS1-KO rGT335 atubulin/TPGS1-KO 5-2-25 rGT335 atub R3 M1.Project Maximum Z_XY1746218663_Z0_T0_C2.tif]

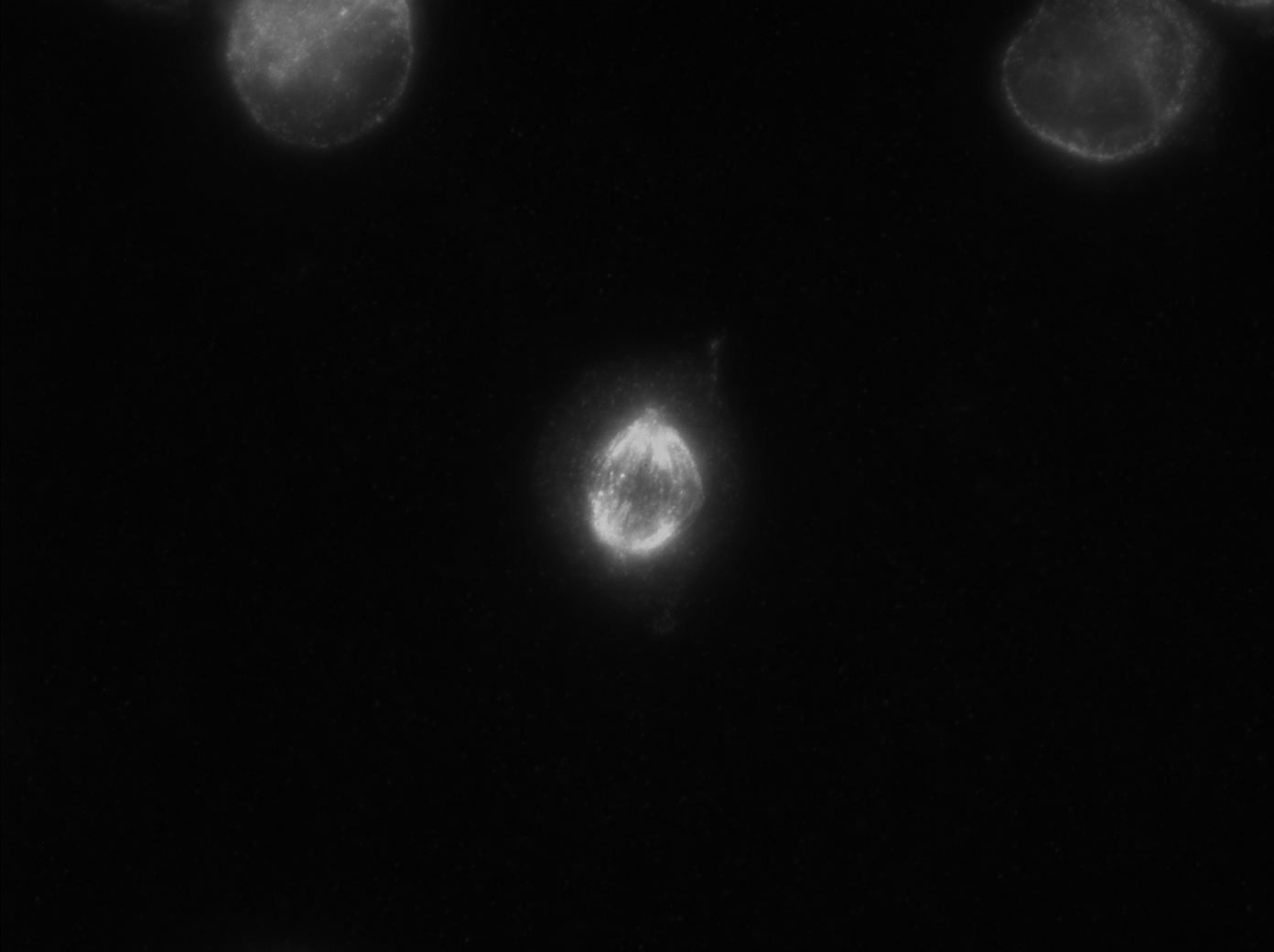

Supplement: Supplementary file 27 — Source data Fig. 7 part 3 [file 44319_2026_742_MOESM27_ESM.zip › Figure 7 Part 3/Fig 7be Cas9 and TPGS1-KO rGT335 atubulin/TPGS1-KO 5-2-25 rGT335 atub R2 M3.Project Maximum Z_XY1746564055_Z0_T0_C2.tif]

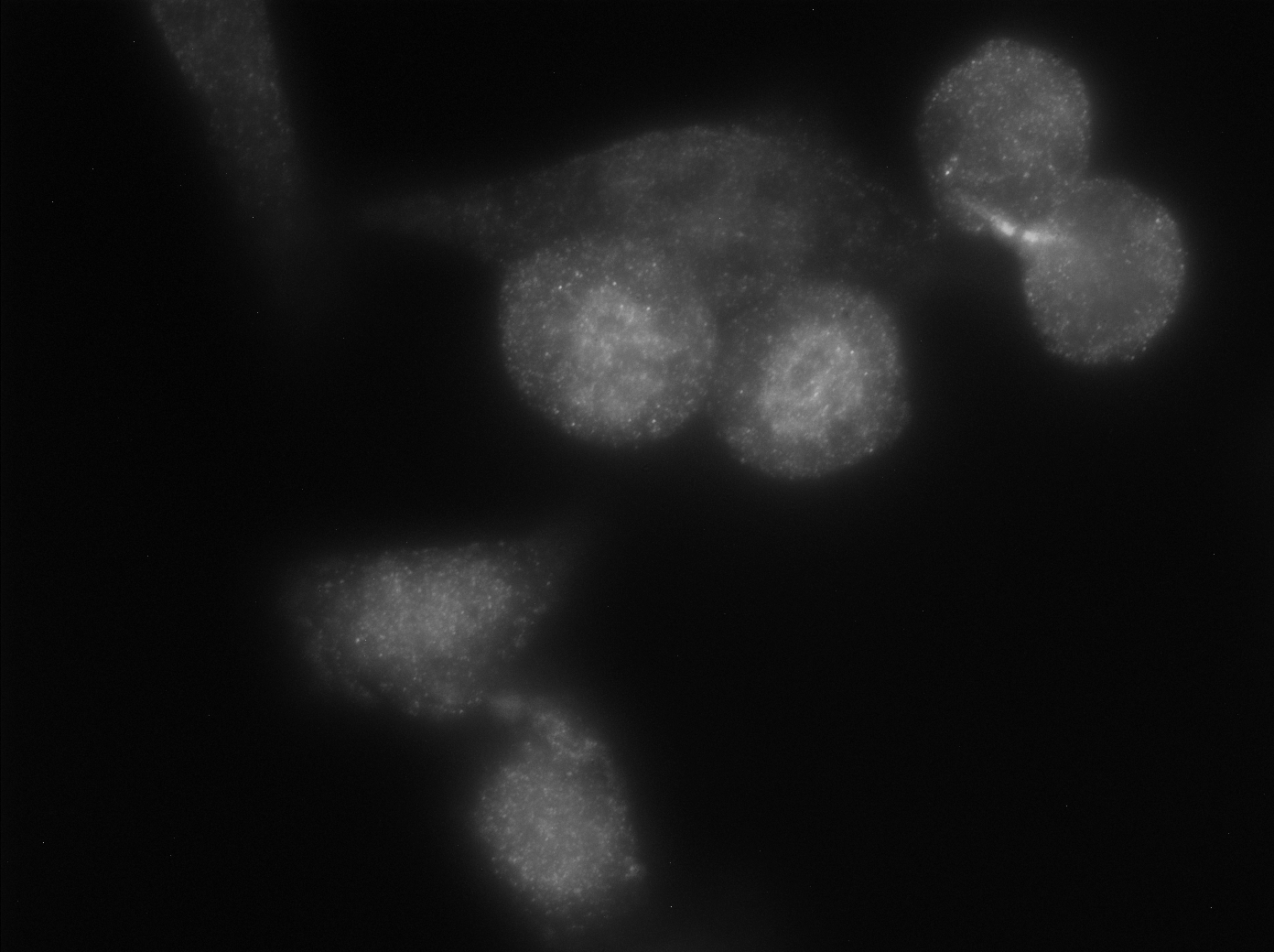

Supplement: Supplementary file 27 — Source data Fig. 7 part 3 [file 44319_2026_742_MOESM27_ESM.zip › Figure 7 Part 3/Fig 7be Cas9 and TPGS1-KO rGT335 atubulin/TPGS1-KO 5-2-25 rGT335 atub R3 M10M11.Project Maximum Z_XY1746220741_Z0_T0_C1.tif]

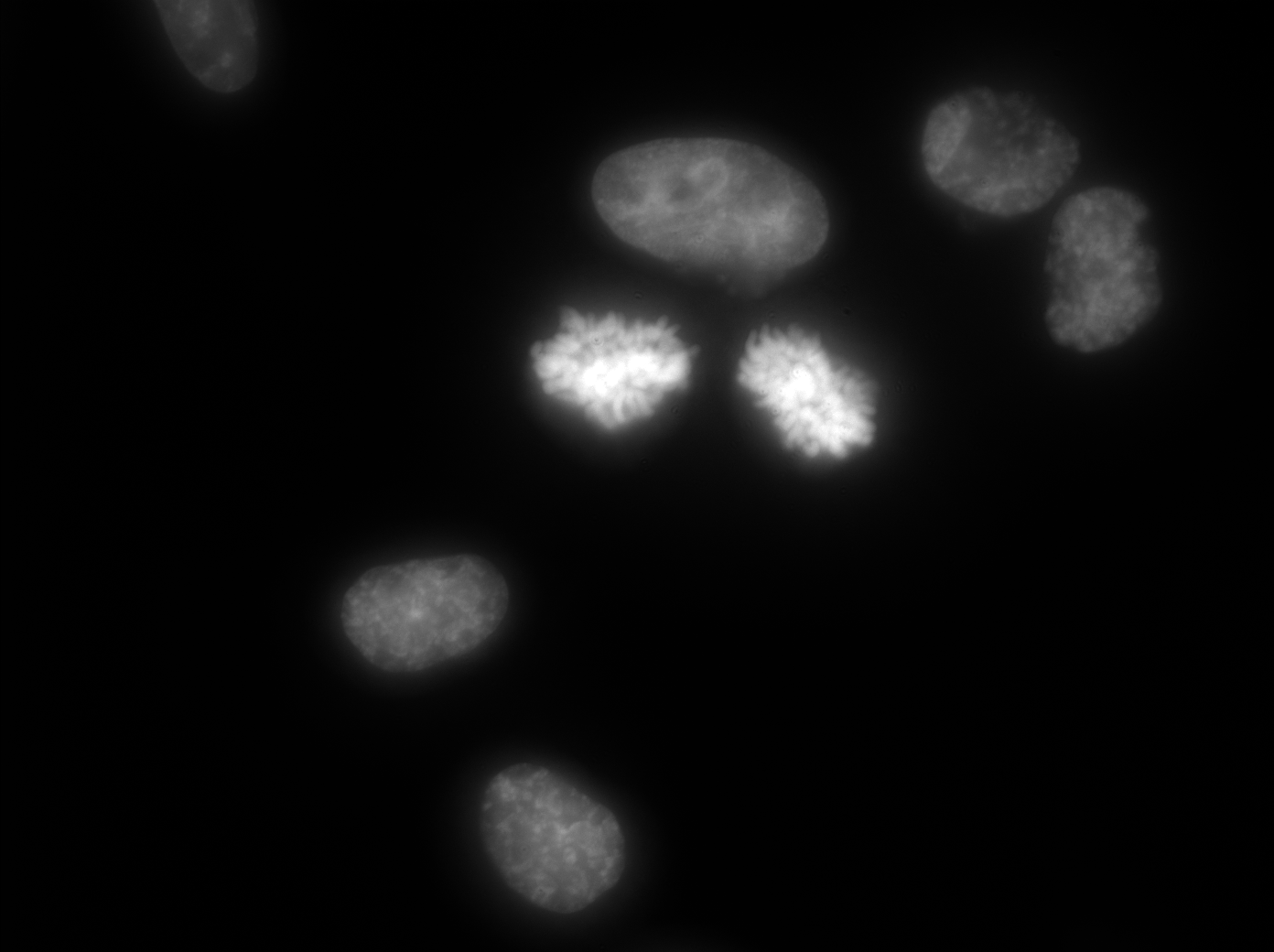

Supplement: Supplementary file 27 — Source data Fig. 7 part 3 [file 44319_2026_742_MOESM27_ESM.zip › Figure 7 Part 3/Fig 7be Cas9 and TPGS1-KO rGT335 atubulin/TPGS1-KO 5-2-25 rGT335 atub R3 M10M11.Project Maximum Z_XY1746220741_Z0_T0_C0.tif]

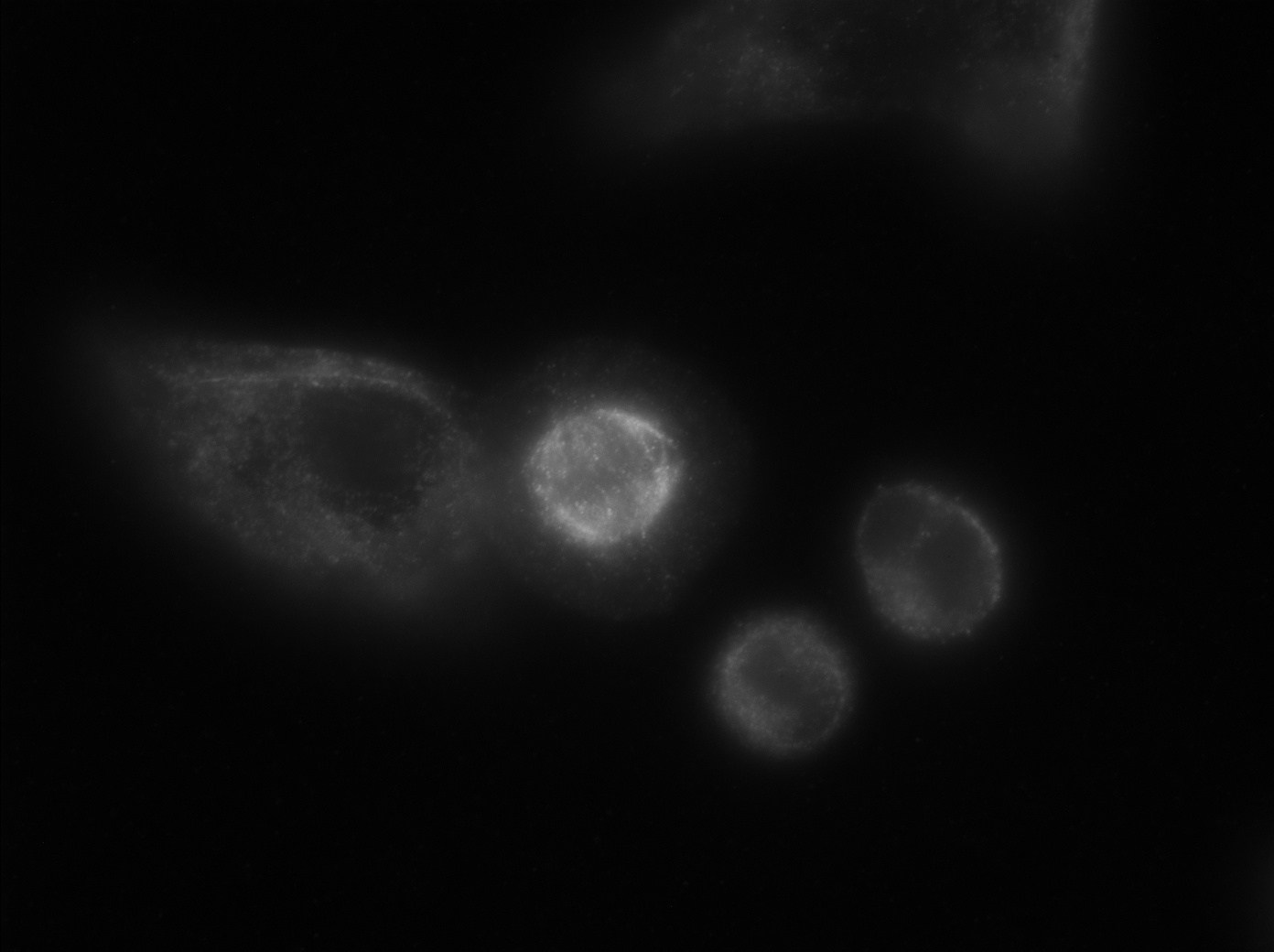

Supplement: Supplementary file 27 — Source data Fig. 7 part 3 [file 44319_2026_742_MOESM27_ESM.zip › Figure 7 Part 3/Fig 7be Cas9 and TPGS1-KO rGT335 atubulin/TPGS1-KO 5-2-25 rGT335 atub R2 M9.Project Maximum Z_XY1746564888_Z0_T0_C2.tif]

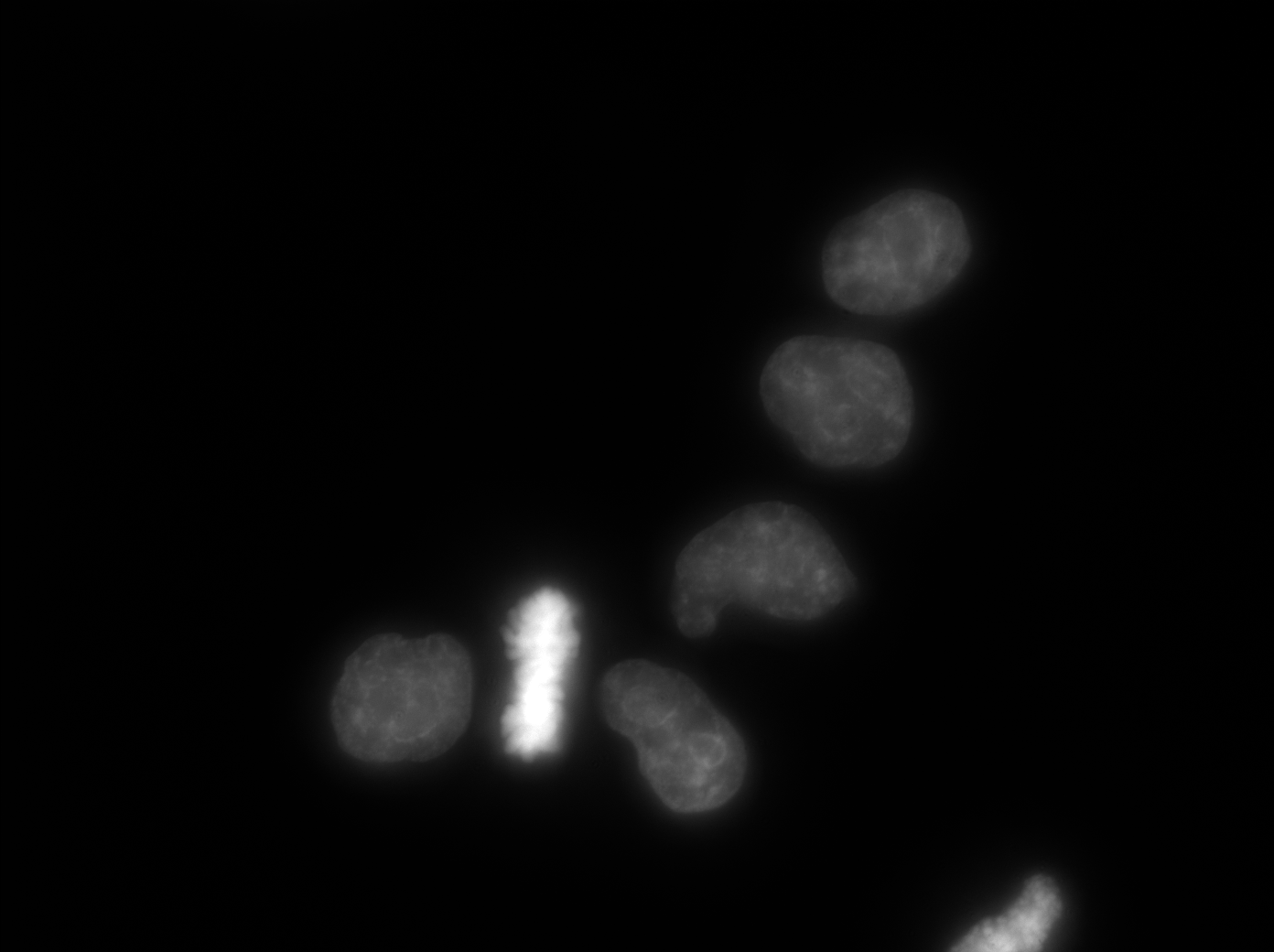

Supplement: Supplementary file 27 — Source data Fig. 7 part 3 [file 44319_2026_742_MOESM27_ESM.zip › Figure 7 Part 3/Fig 7be Cas9 and TPGS1-KO rGT335 atubulin/Cas9 5-2-25 rGT335 atub R2 M5.Project Maximum Z_XY1746562056_Z0_T0_C0.tif]

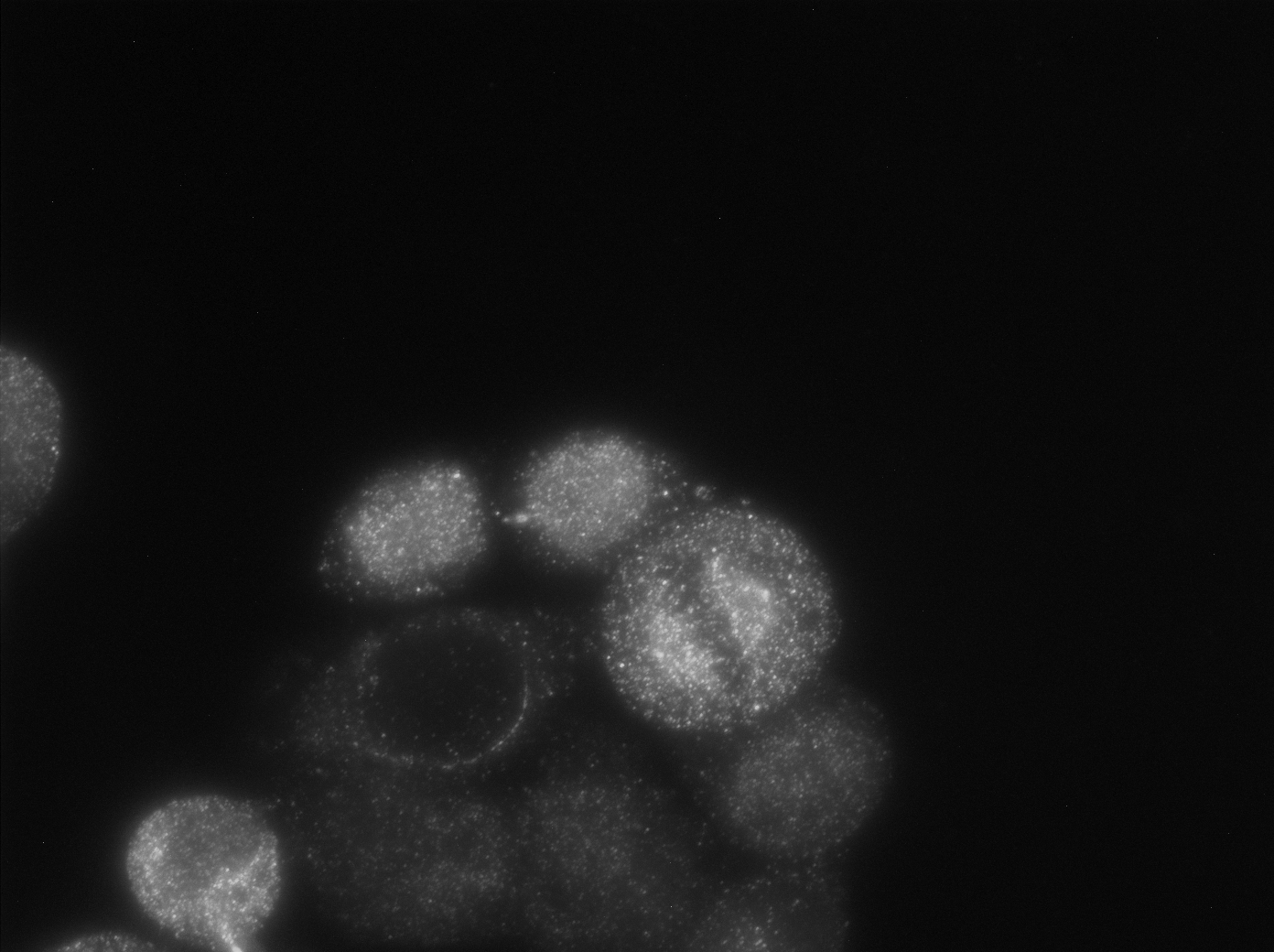

Supplement: Supplementary file 27 — Source data Fig. 7 part 3 [file 44319_2026_742_MOESM27_ESM.zip › Figure 7 Part 3/Fig 7be Cas9 and TPGS1-KO rGT335 atubulin/Cas9 5-2-25 rGT335 atub R1 M1.Project Maximum Z_XY1746557011_Z0_T0_C1.tif]

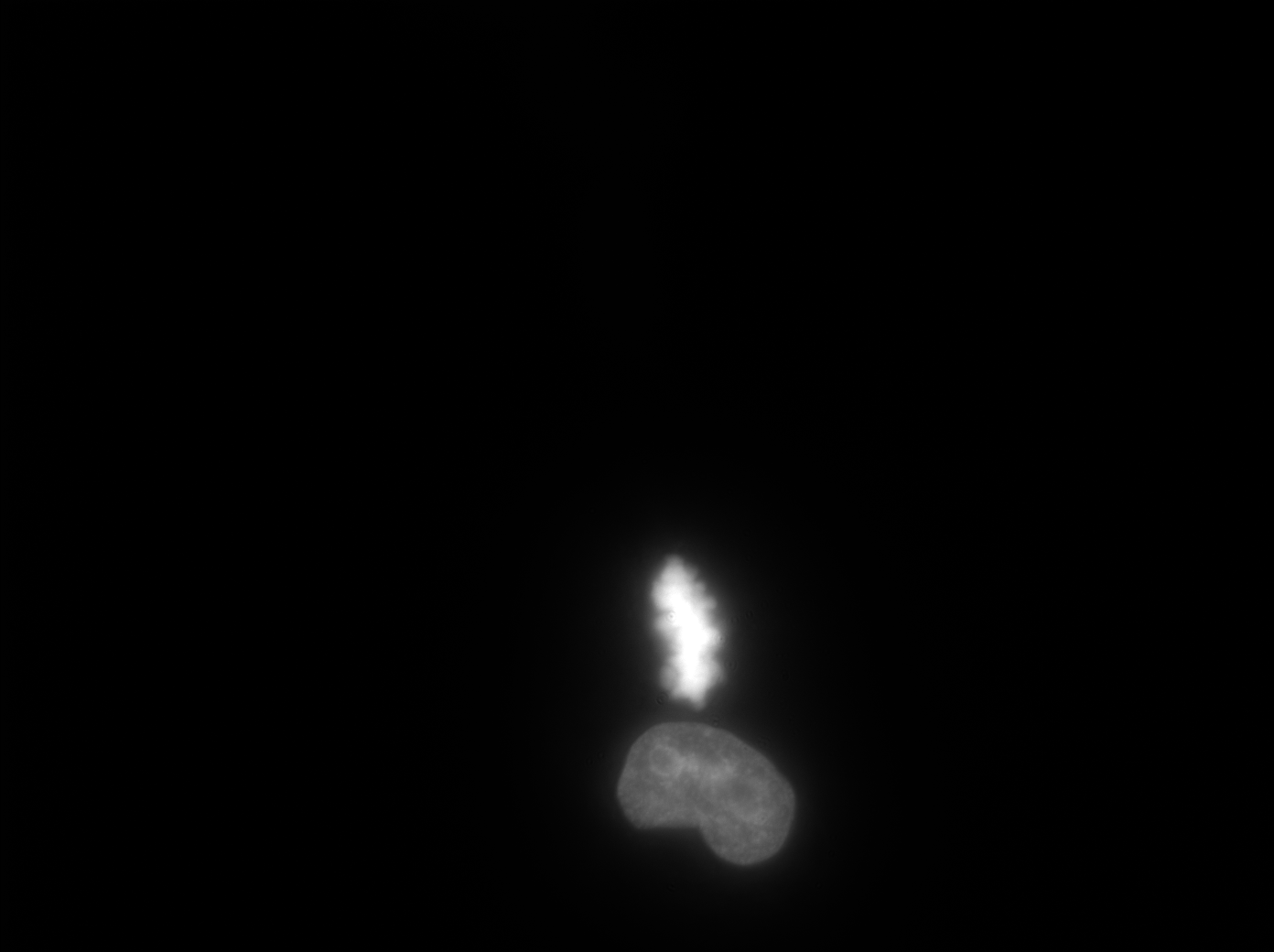

Supplement: Supplementary file 27 — Source data Fig. 7 part 3 [file 44319_2026_742_MOESM27_ESM.zip › Figure 7 Part 3/Fig 7be Cas9 and TPGS1-KO rGT335 atubulin/Cas9 5-2-25 rGT335 atub R1 M3.Project Maximum Z_XY1746557428_Z0_T0_C0.tif]

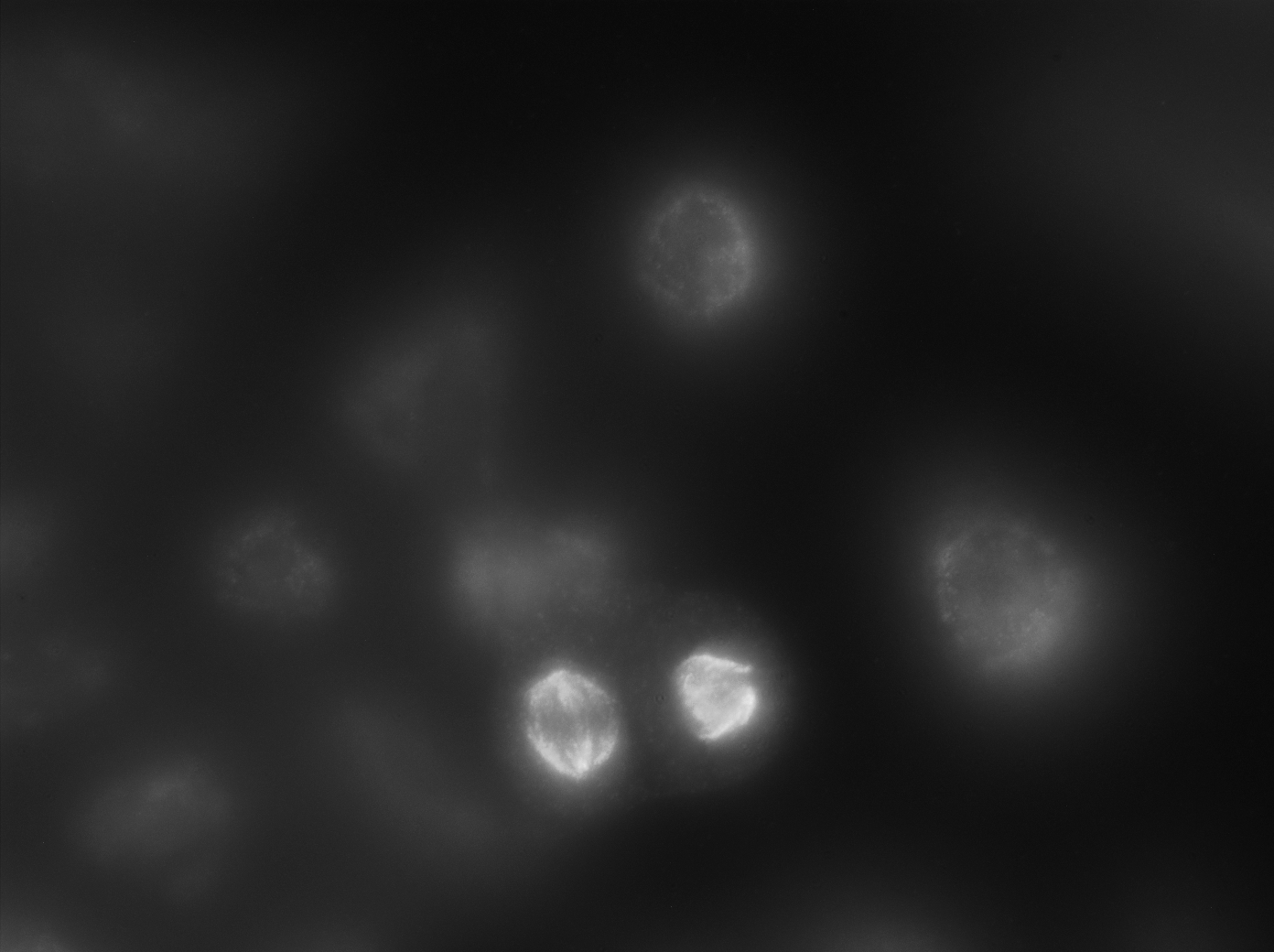

Supplement: Supplementary file 27 — Source data Fig. 7 part 3 [file 44319_2026_742_MOESM27_ESM.zip › Figure 7 Part 3/Fig 7be Cas9 and TPGS1-KO rGT335 atubulin/TPGS1-KO 5-2-25 rGT335 atub R1 M4M5.Project Maximum Z_XY1746221653_Z0_T0_C2.tif]

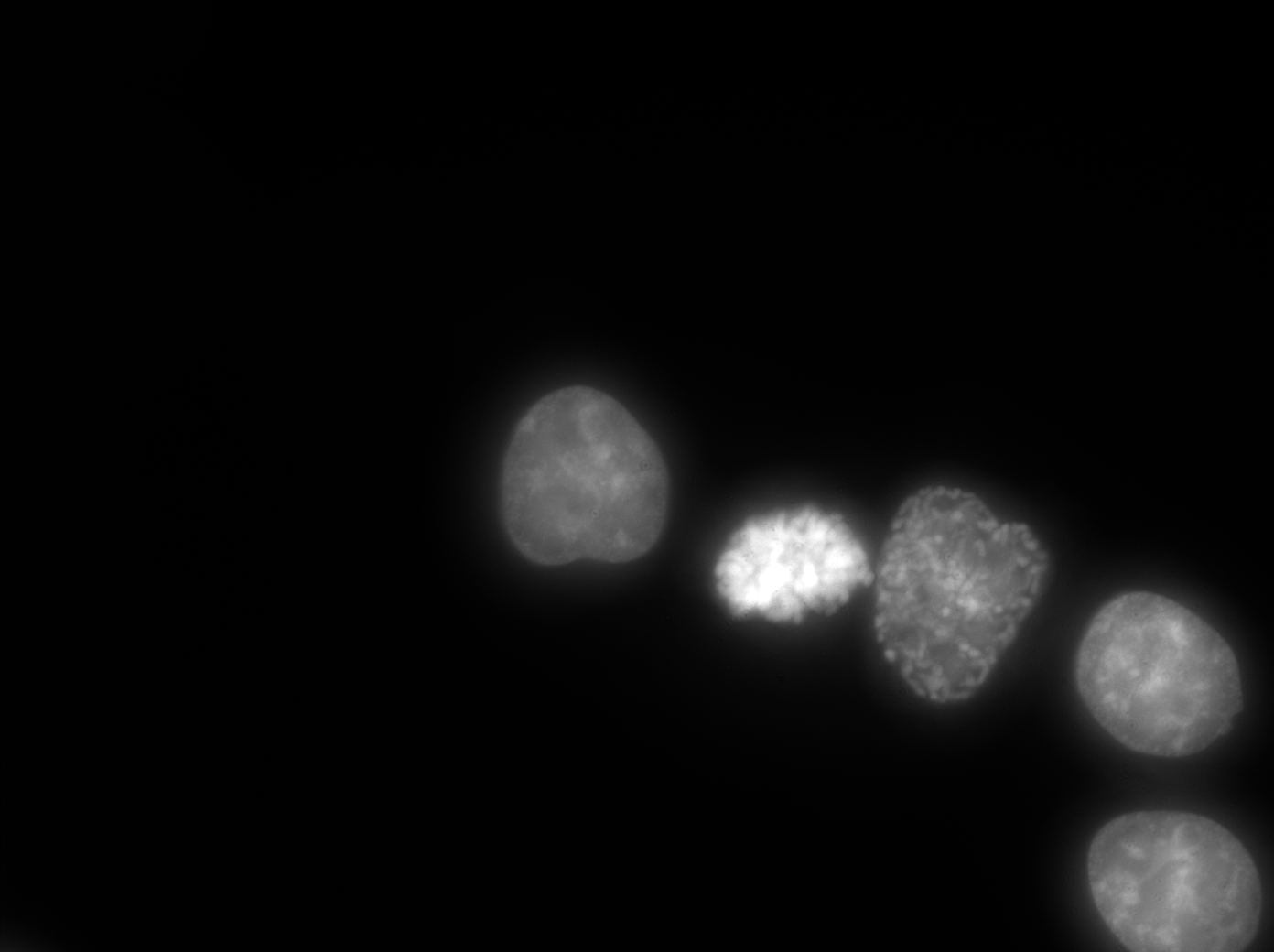

Supplement: Supplementary file 27 — Source data Fig. 7 part 3 [file 44319_2026_742_MOESM27_ESM.zip › Figure 7 Part 3/Fig 7be Cas9 and TPGS1-KO rGT335 atubulin/TPGS1-KO 5-2-25 rGT335 atub R3 M9.Project Maximum Z_XY1746220596_Z0_T0_C0.tif]

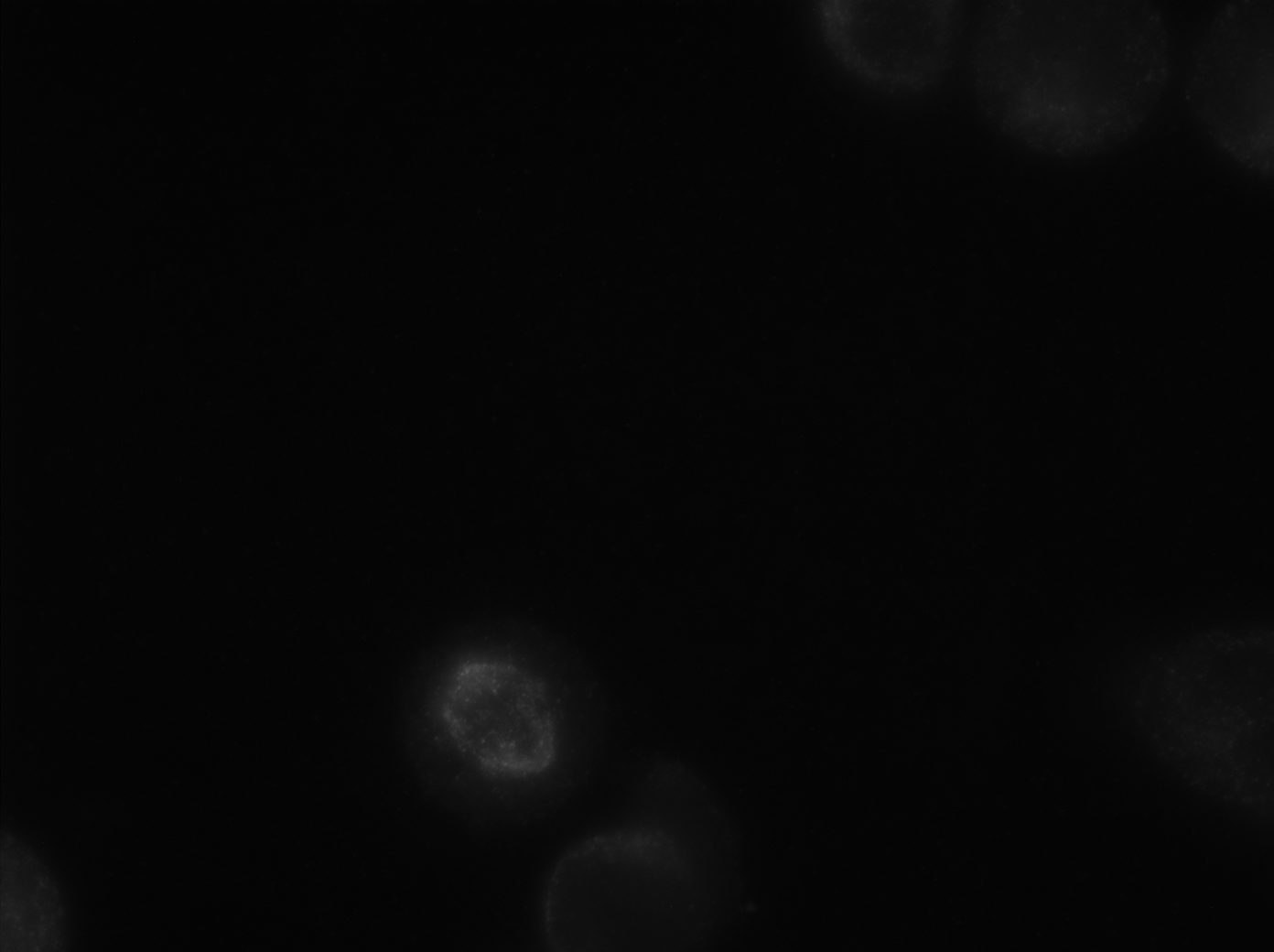

Supplement: Supplementary file 27 — Source data Fig. 7 part 3 [file 44319_2026_742_MOESM27_ESM.zip › Figure 7 Part 3/Fig 7be Cas9 and TPGS1-KO rGT335 atubulin/Cas9 5-2-25 rGT335 atub R3 M10.Project Maximum Z_XY1746218293_Z0_T0_C2.tif]

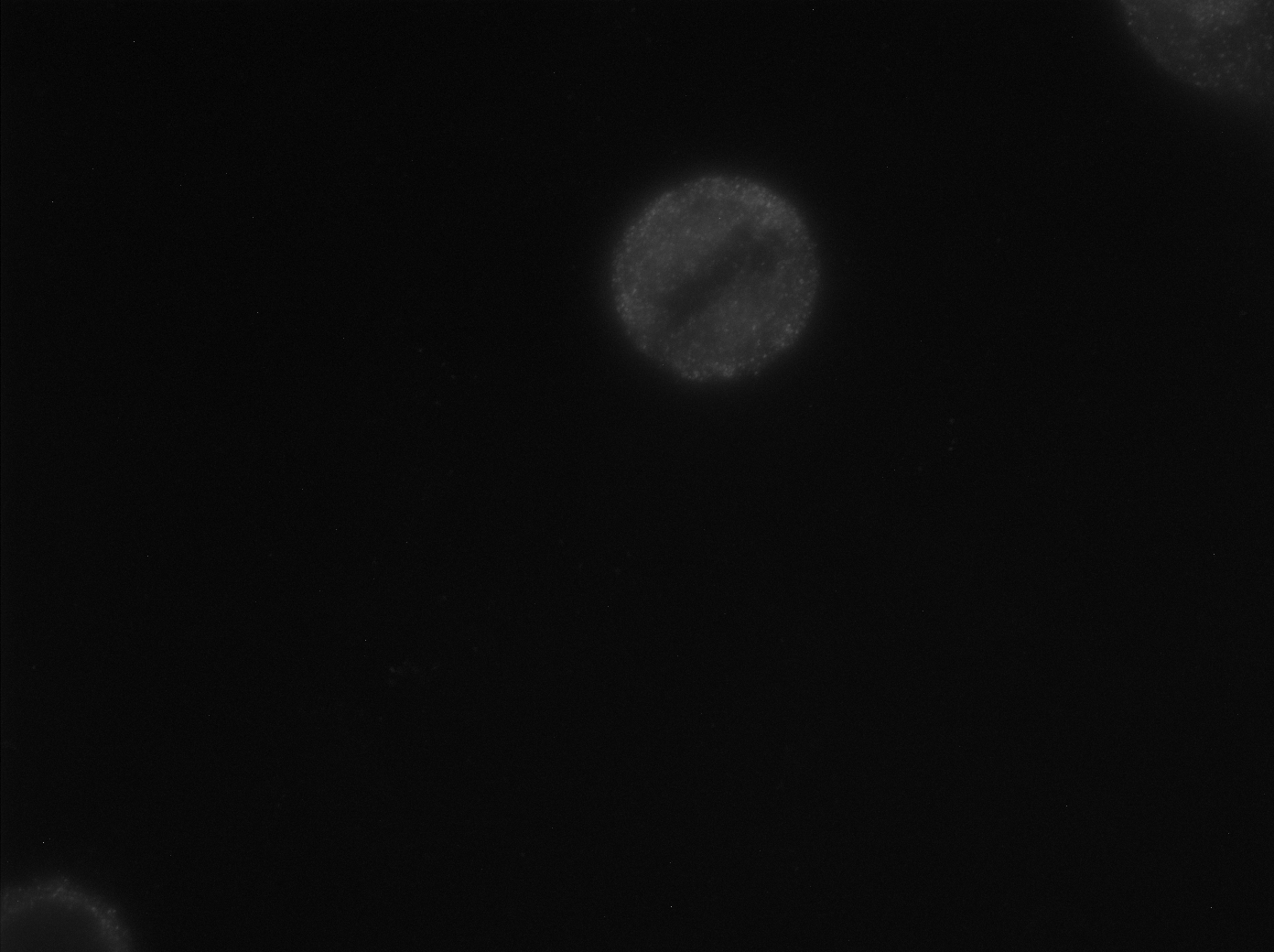

Supplement: Supplementary file 27 — Source data Fig. 7 part 3 [file 44319_2026_742_MOESM27_ESM.zip › Figure 7 Part 3/Fig 7be Cas9 and TPGS1-KO rGT335 atubulin/Cas9 5-2-25 rGT335 atub R1 M7.Project Maximum Z_XY1746558178_Z0_T0_C1.tif]

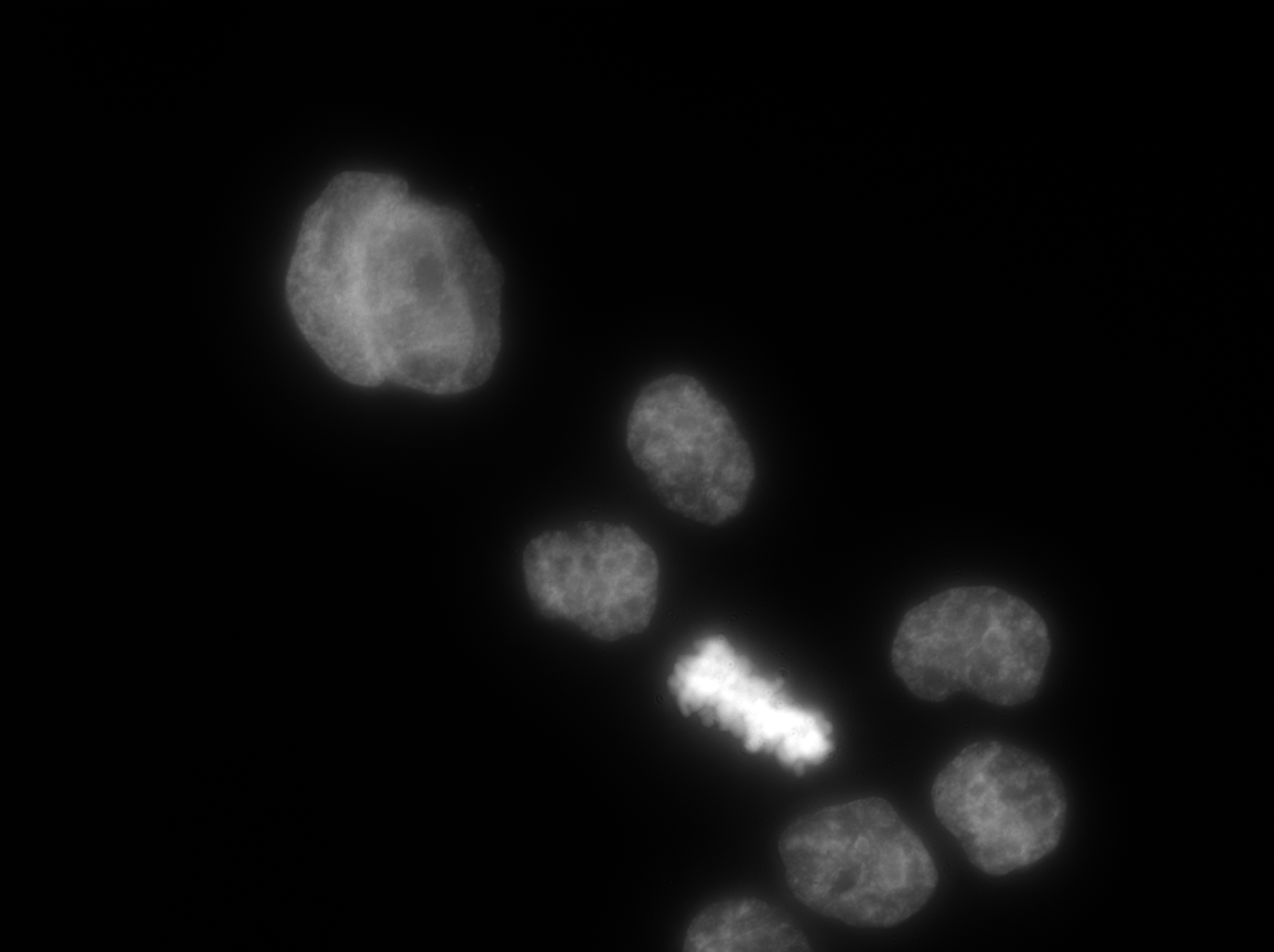

Supplement: Supplementary file 27 — Source data Fig. 7 part 3 [file 44319_2026_742_MOESM27_ESM.zip › Figure 7 Part 3/Fig 7be Cas9 and TPGS1-KO rGT335 atubulin/TPGS1-KO 5-2-25 rGT335 atub R3 M6.Project Maximum Z_XY1746220014_Z0_T0_C0.tif]

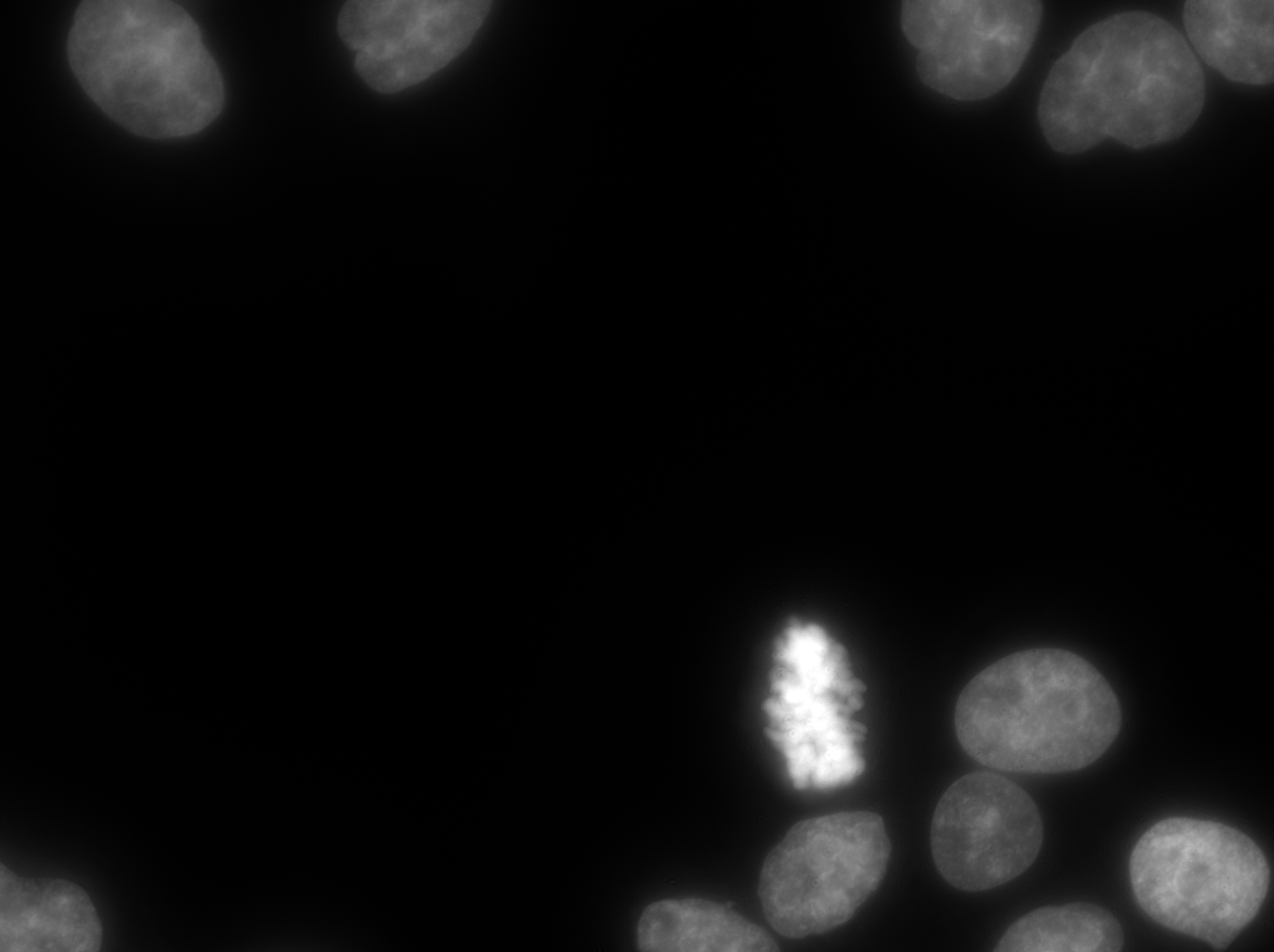

Supplement: Supplementary file 27 — Source data Fig. 7 part 3 [file 44319_2026_742_MOESM27_ESM.zip › Figure 7 Part 3/Fig 7be Cas9 and TPGS1-KO rGT335 atubulin/Cas9 5-2-25 rGT335 atub R1 M6.Project Maximum Z_XY1746558046_Z0_T0_C0.tif]

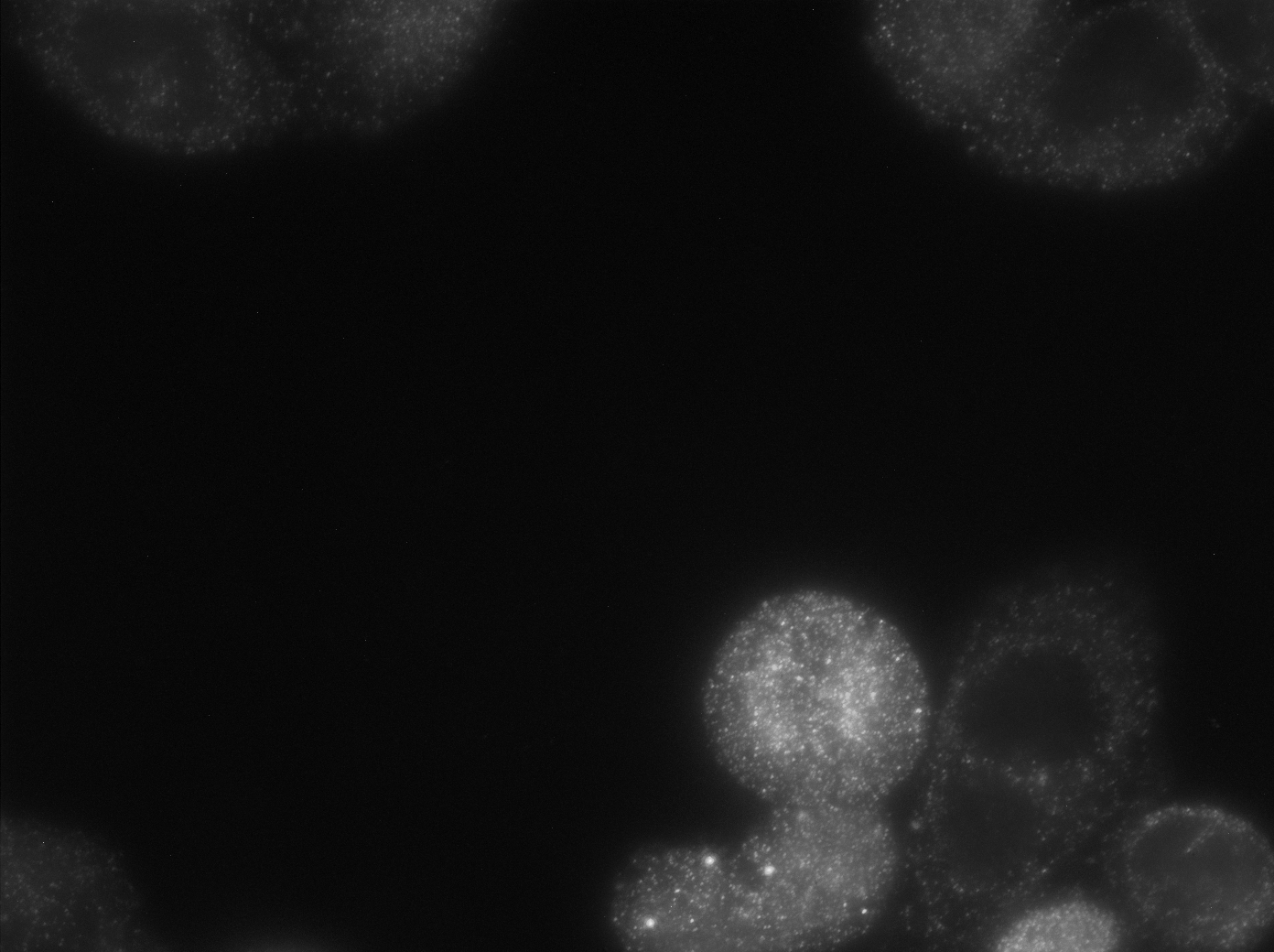

Supplement: Supplementary file 27 — Source data Fig. 7 part 3 [file 44319_2026_742_MOESM27_ESM.zip › Figure 7 Part 3/Fig 7be Cas9 and TPGS1-KO rGT335 atubulin/Cas9 5-2-25 rGT335 atub R1 M6.Project Maximum Z_XY1746558046_Z0_T0_C1.tif]

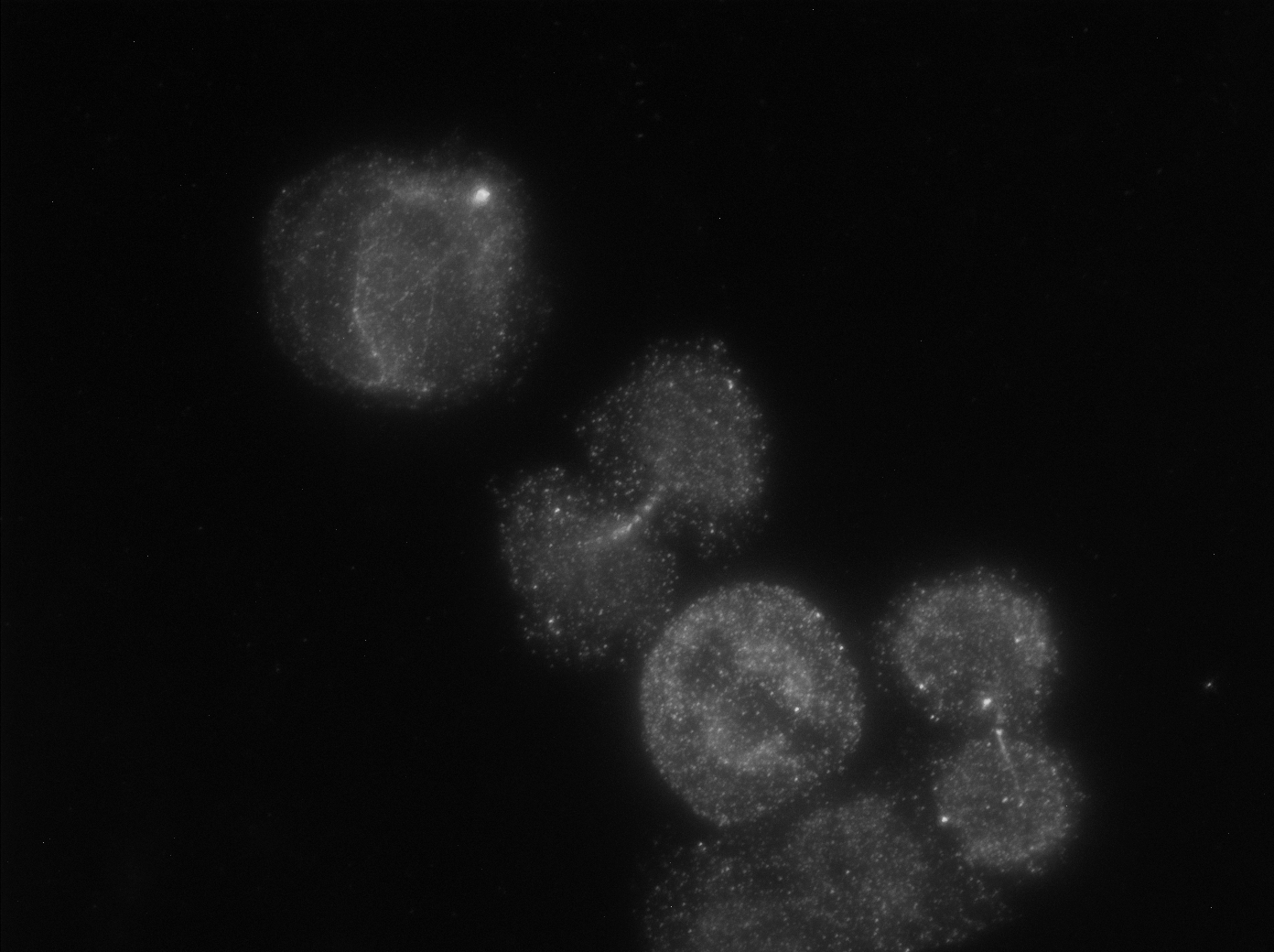

Supplement: Supplementary file 27 — Source data Fig. 7 part 3 [file 44319_2026_742_MOESM27_ESM.zip › Figure 7 Part 3/Fig 7be Cas9 and TPGS1-KO rGT335 atubulin/TPGS1-KO 5-2-25 rGT335 atub R3 M6.Project Maximum Z_XY1746220014_Z0_T0_C1.tif]

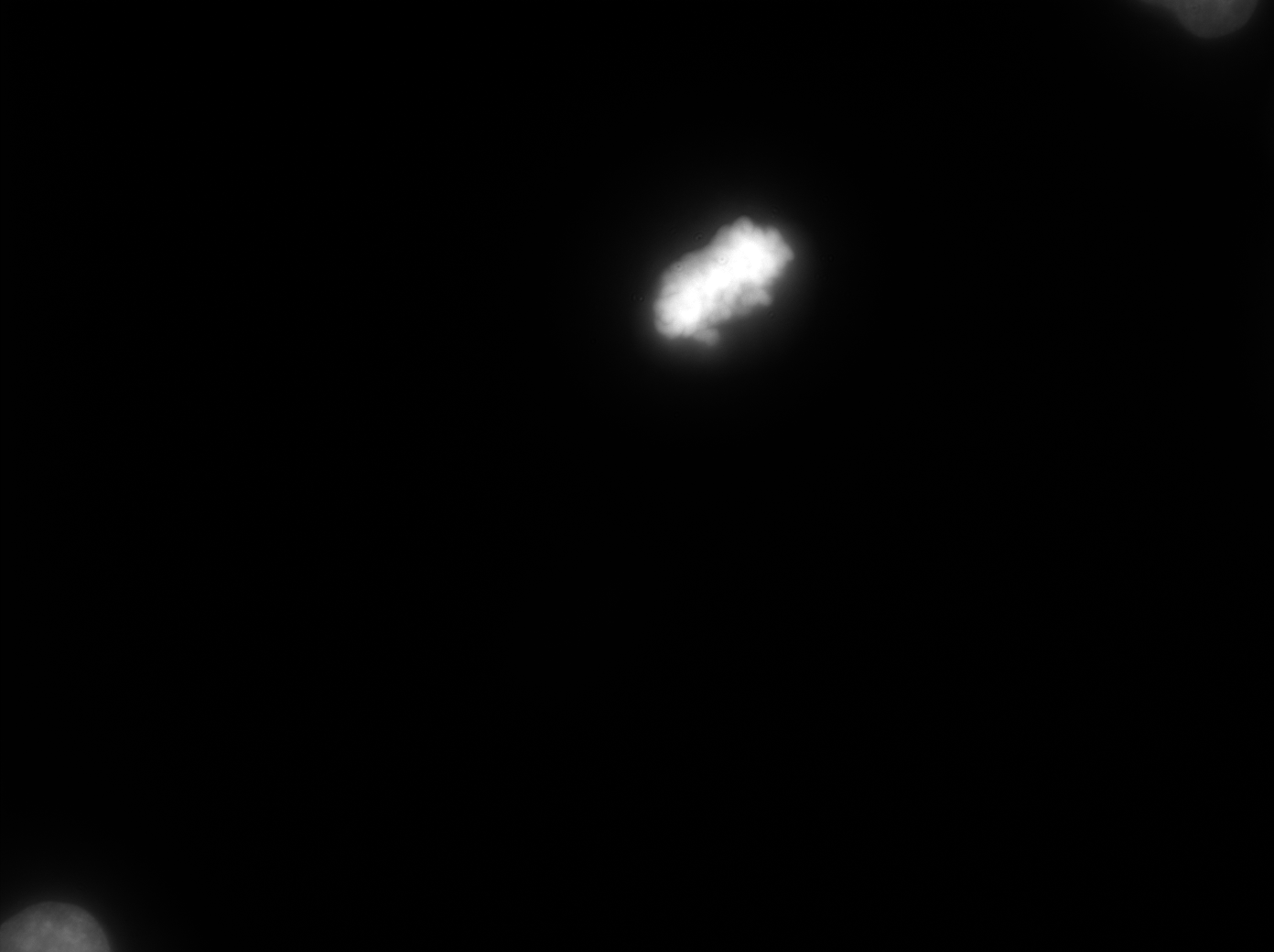

Supplement: Supplementary file 27 — Source data Fig. 7 part 3 [file 44319_2026_742_MOESM27_ESM.zip › Figure 7 Part 3/Fig 7be Cas9 and TPGS1-KO rGT335 atubulin/Cas9 5-2-25 rGT335 atub R1 M7.Project Maximum Z_XY1746558178_Z0_T0_C0.tif]

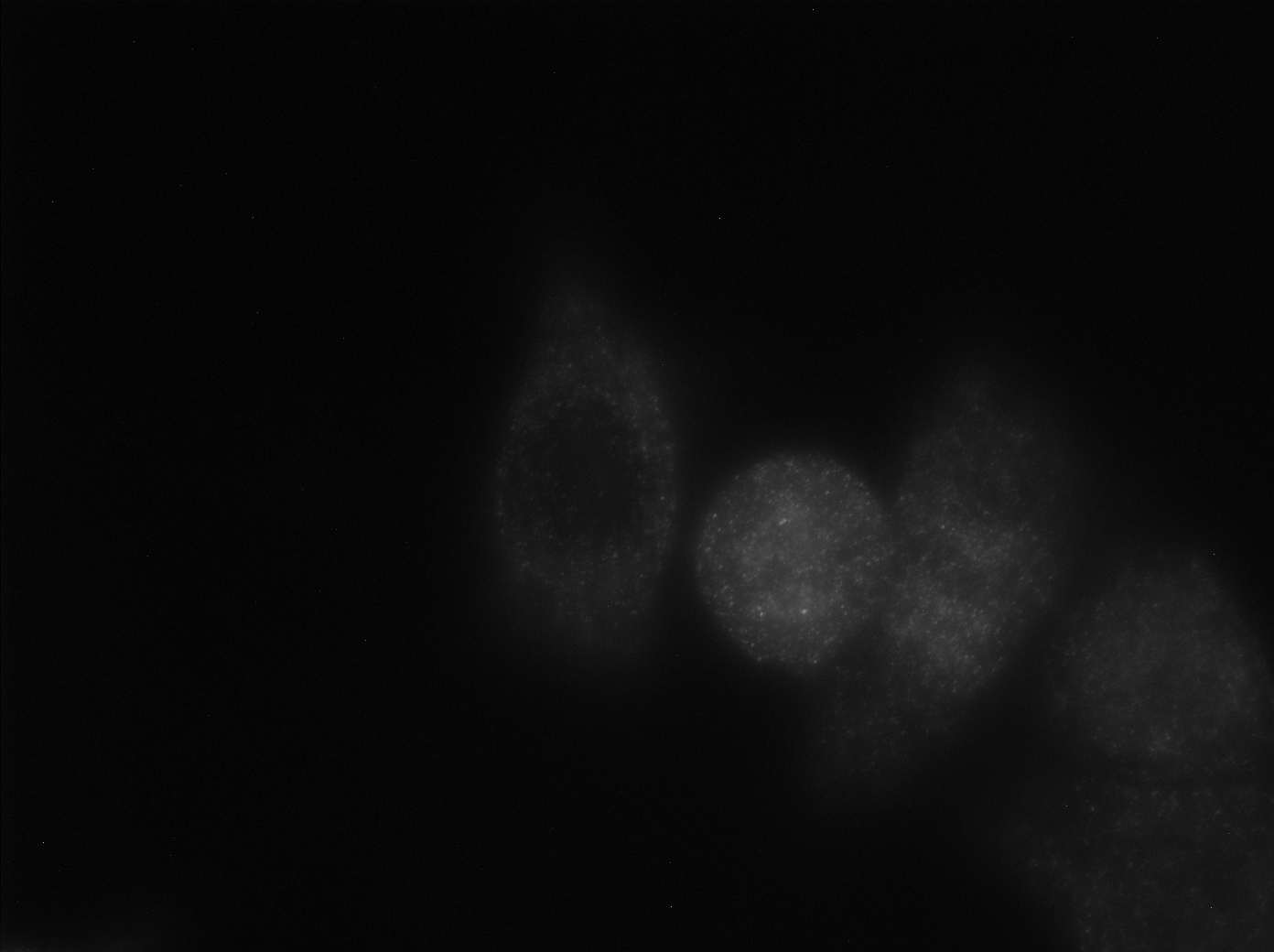

Supplement: Supplementary file 27 — Source data Fig. 7 part 3 [file 44319_2026_742_MOESM27_ESM.zip › Figure 7 Part 3/Fig 7be Cas9 and TPGS1-KO rGT335 atubulin/TPGS1-KO 5-2-25 rGT335 atub R3 M9.Project Maximum Z_XY1746220596_Z0_T0_C1.tif]

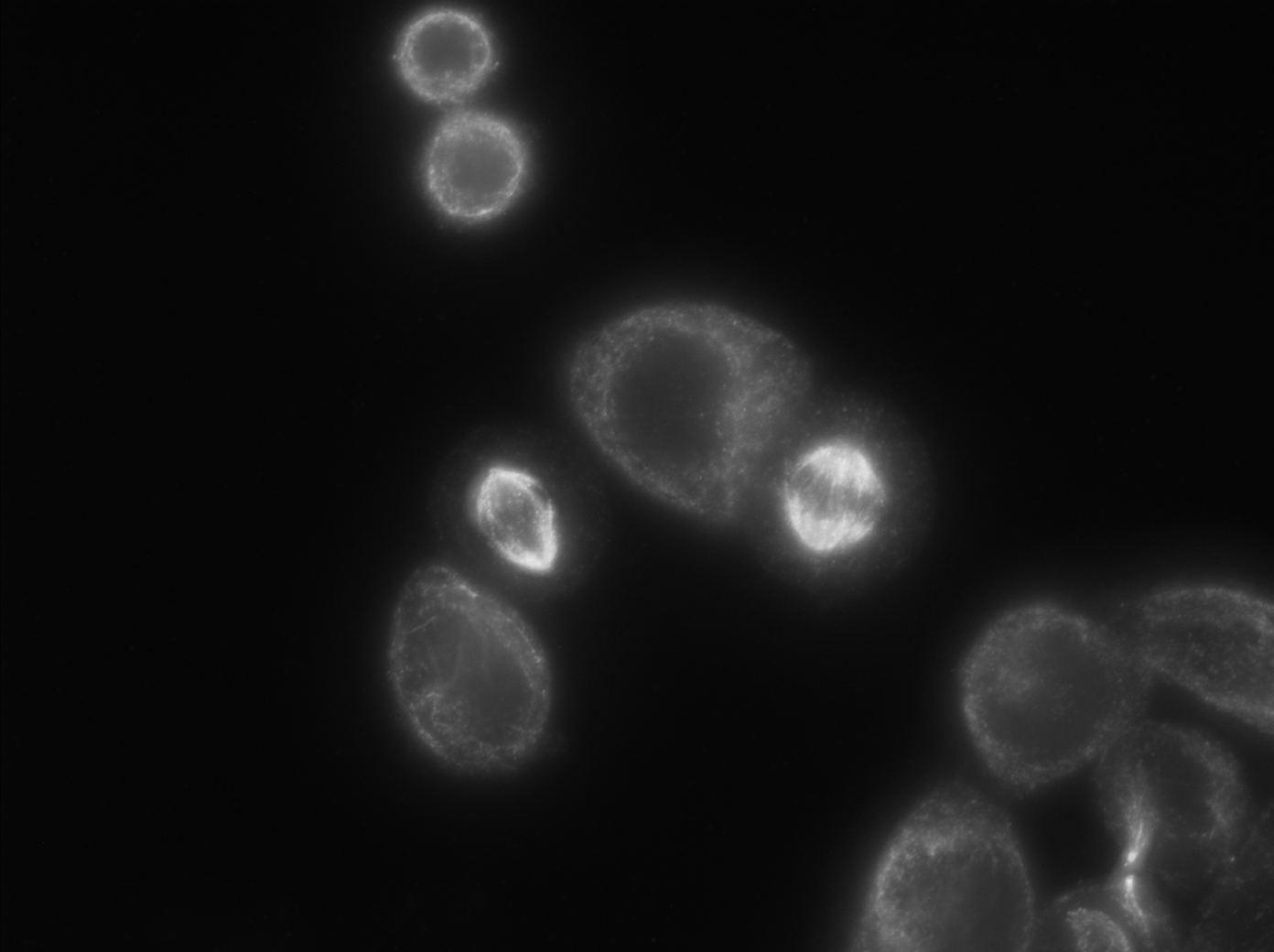

Supplement: Supplementary file 27 — Source data Fig. 7 part 3 [file 44319_2026_742_MOESM27_ESM.zip › Figure 7 Part 3/Fig 7be Cas9 and TPGS1-KO rGT335 atubulin/TPGS1-KO 5-2-25 rGT335 atub R3 M4M5.Project Maximum Z_XY1746219875_Z0_T0_C2.tif]

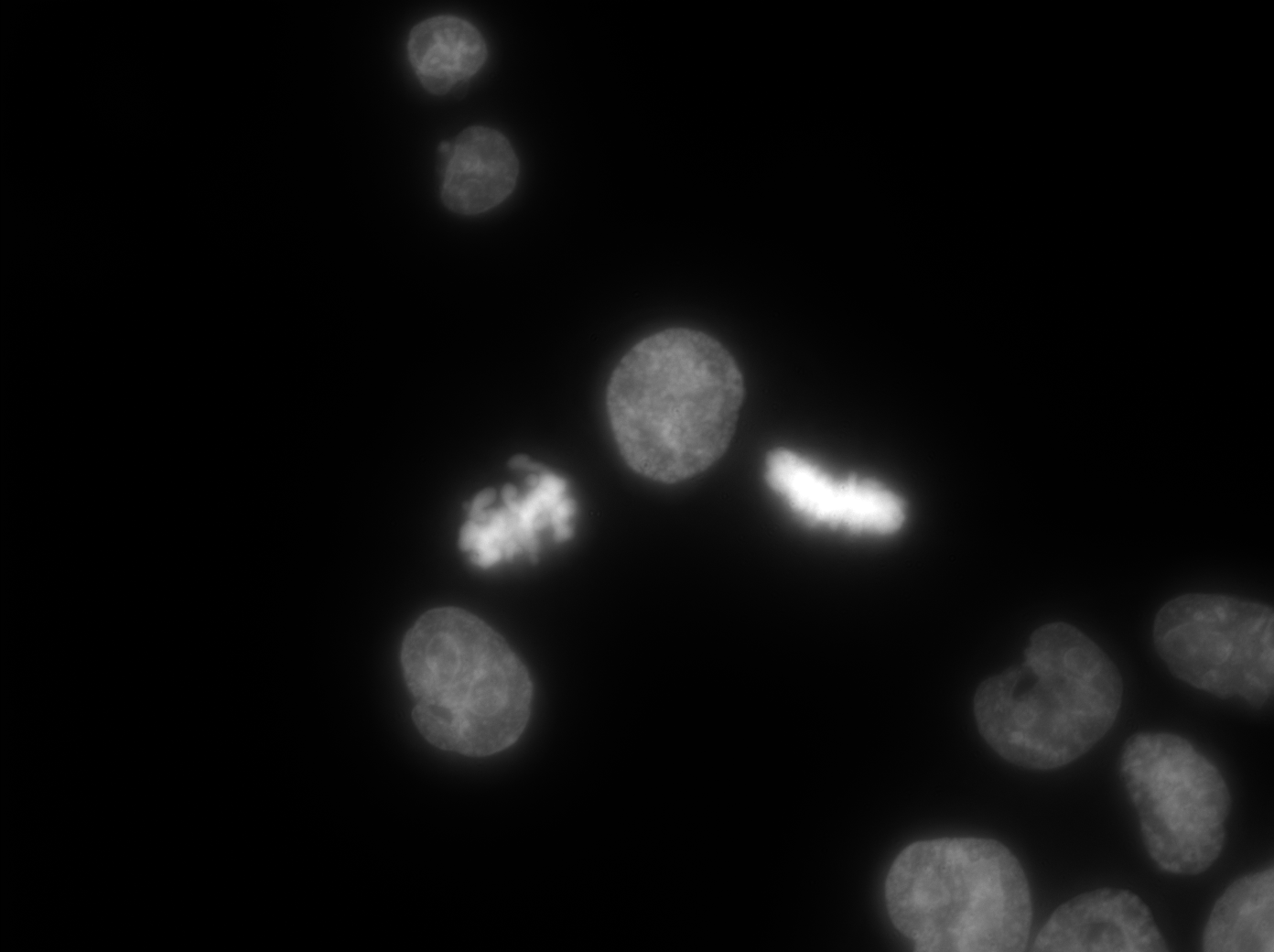

Supplement: Supplementary file 27 — Source data Fig. 7 part 3 [file 44319_2026_742_MOESM27_ESM.zip › Figure 7 Part 3/Fig 7be Cas9 and TPGS1-KO rGT335 atubulin/TPGS1-KO 5-2-25 rGT335 atub R3 M4M5.Project Maximum Z_XY1746219875_Z0_T0_C0.tif]

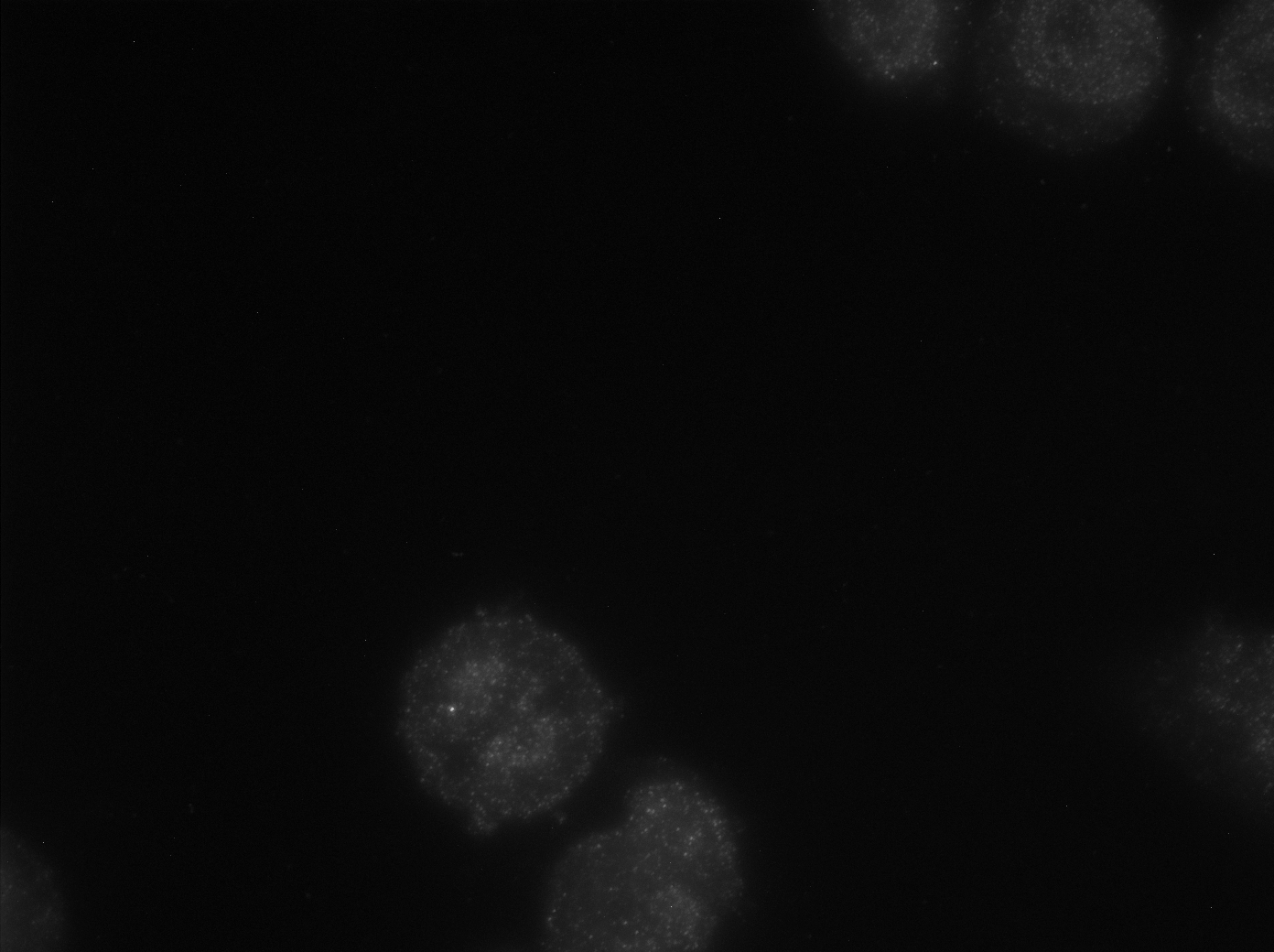

Supplement: Supplementary file 27 — Source data Fig. 7 part 3 [file 44319_2026_742_MOESM27_ESM.zip › Figure 7 Part 3/Fig 7be Cas9 and TPGS1-KO rGT335 atubulin/Cas9 5-2-25 rGT335 atub R3 M10.Project Maximum Z_XY1746218293_Z0_T0_C1.tif]

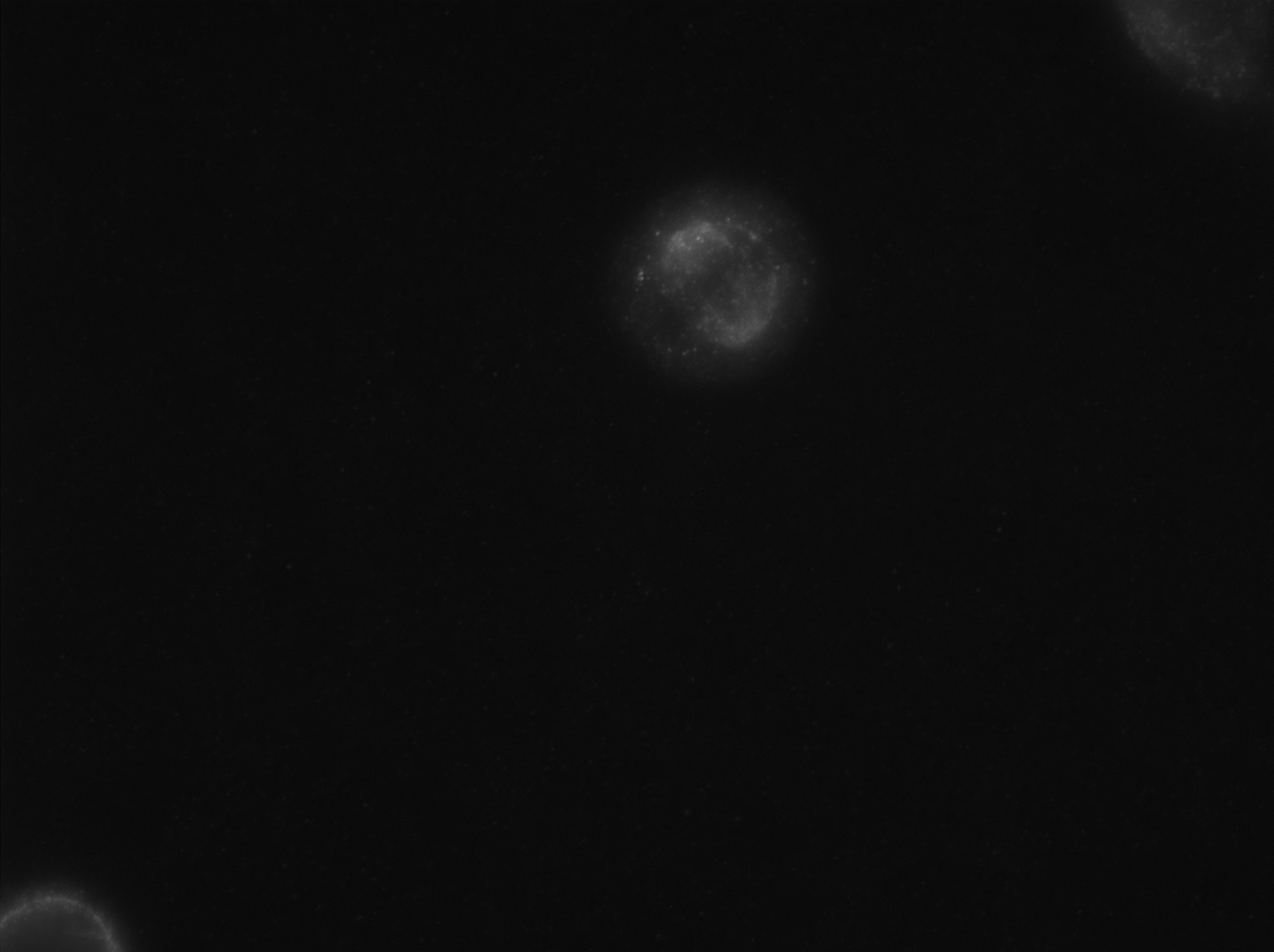

Supplement: Supplementary file 27 — Source data Fig. 7 part 3 [file 44319_2026_742_MOESM27_ESM.zip › Figure 7 Part 3/Fig 7be Cas9 and TPGS1-KO rGT335 atubulin/Cas9 5-2-25 rGT335 atub R1 M7.Project Maximum Z_XY1746558178_Z0_T0_C2.tif]

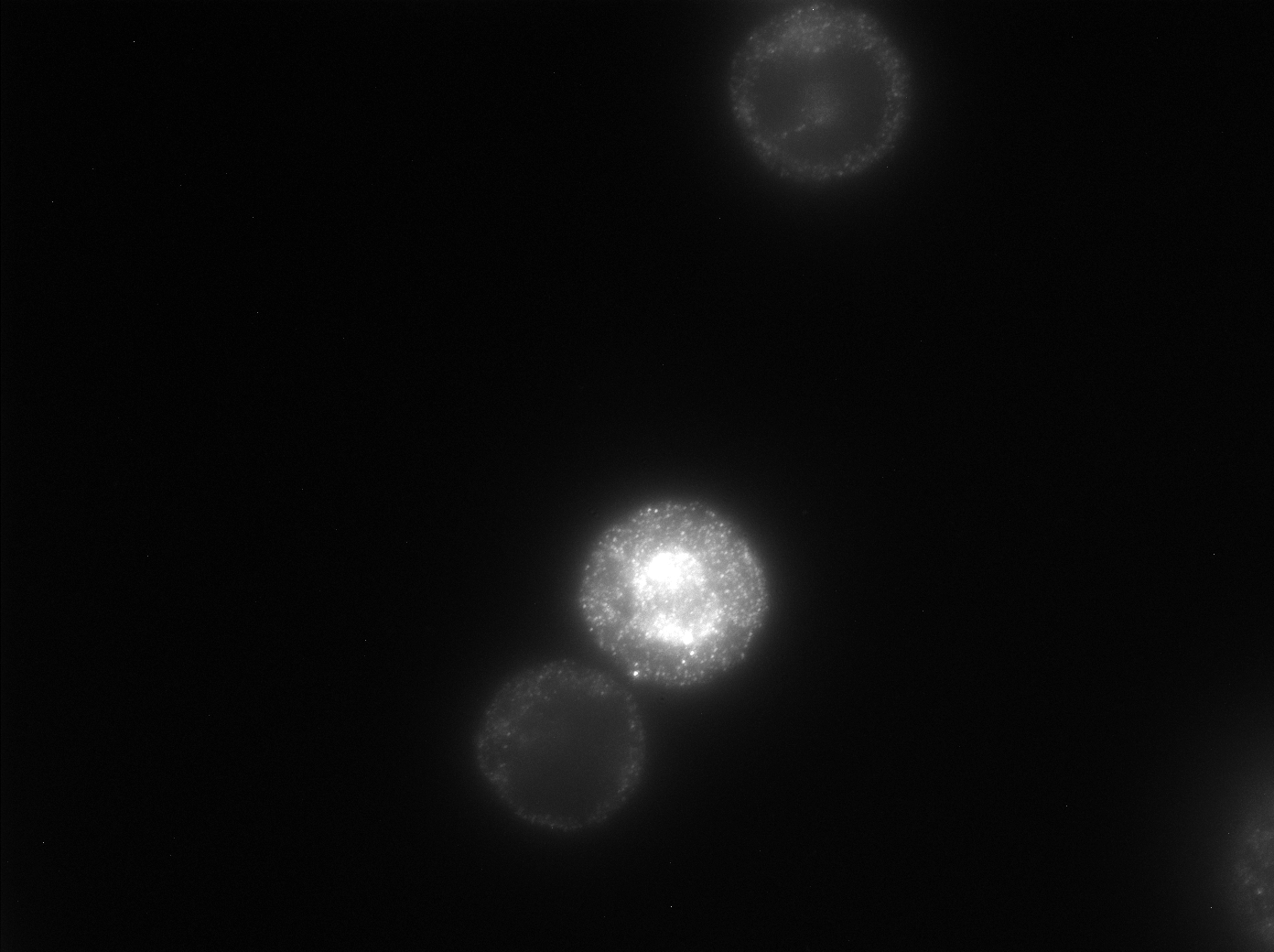

Supplement: Supplementary file 27 — Source data Fig. 7 part 3 [file 44319_2026_742_MOESM27_ESM.zip › Figure 7 Part 3/Fig 7be Cas9 and TPGS1-KO rGT335 atubulin/Cas9 5-2-25 rGT335 atub R3 M8.Project Maximum Z_XY1746217622_Z0_T0_C1 figutr (1).tif]

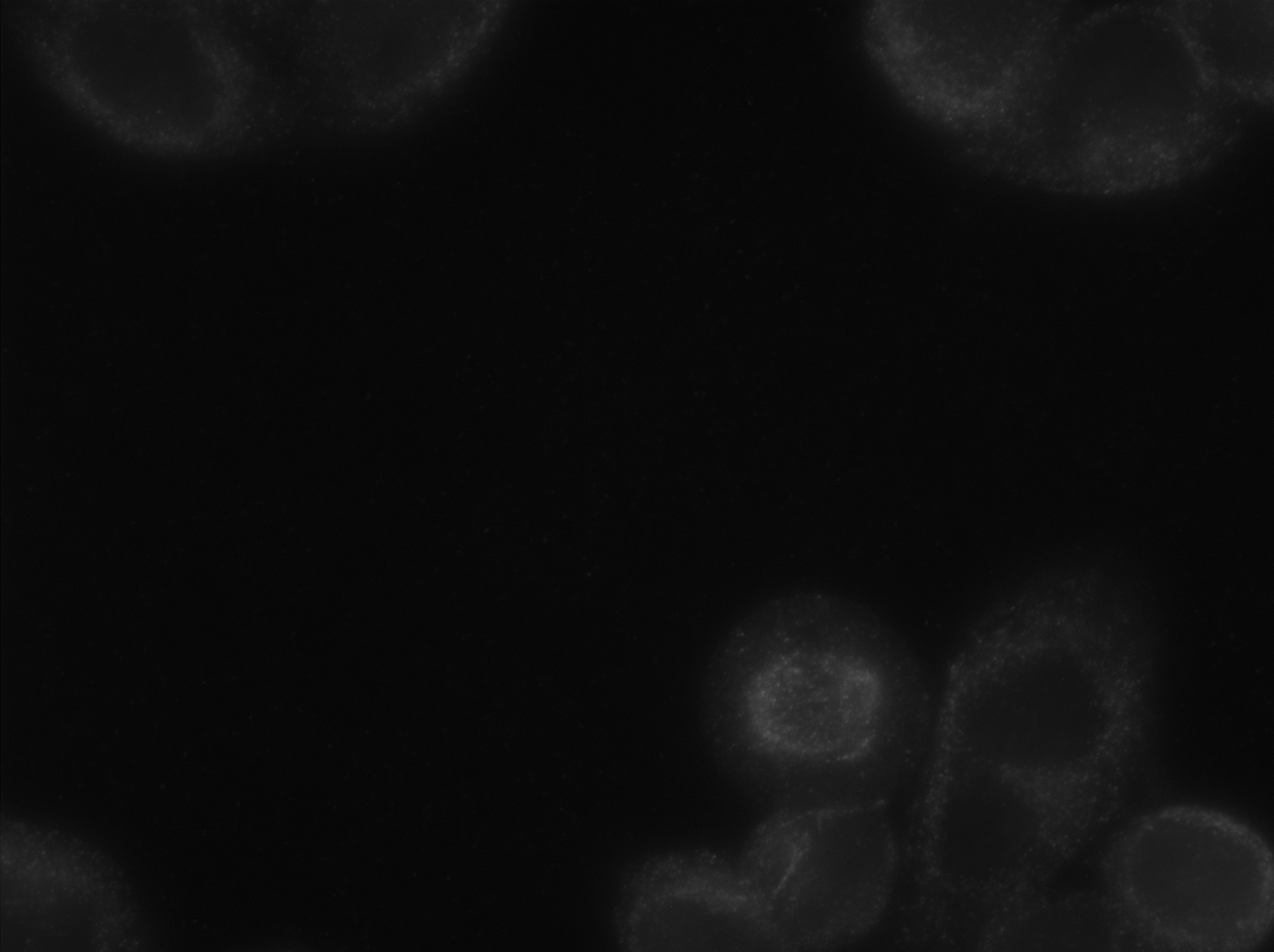

Supplement: Supplementary file 27 — Source data Fig. 7 part 3 [file 44319_2026_742_MOESM27_ESM.zip › Figure 7 Part 3/Fig 7be Cas9 and TPGS1-KO rGT335 atubulin/Cas9 5-2-25 rGT335 atub R1 M6.Project Maximum Z_XY1746558046_Z0_T0_C2.tif]

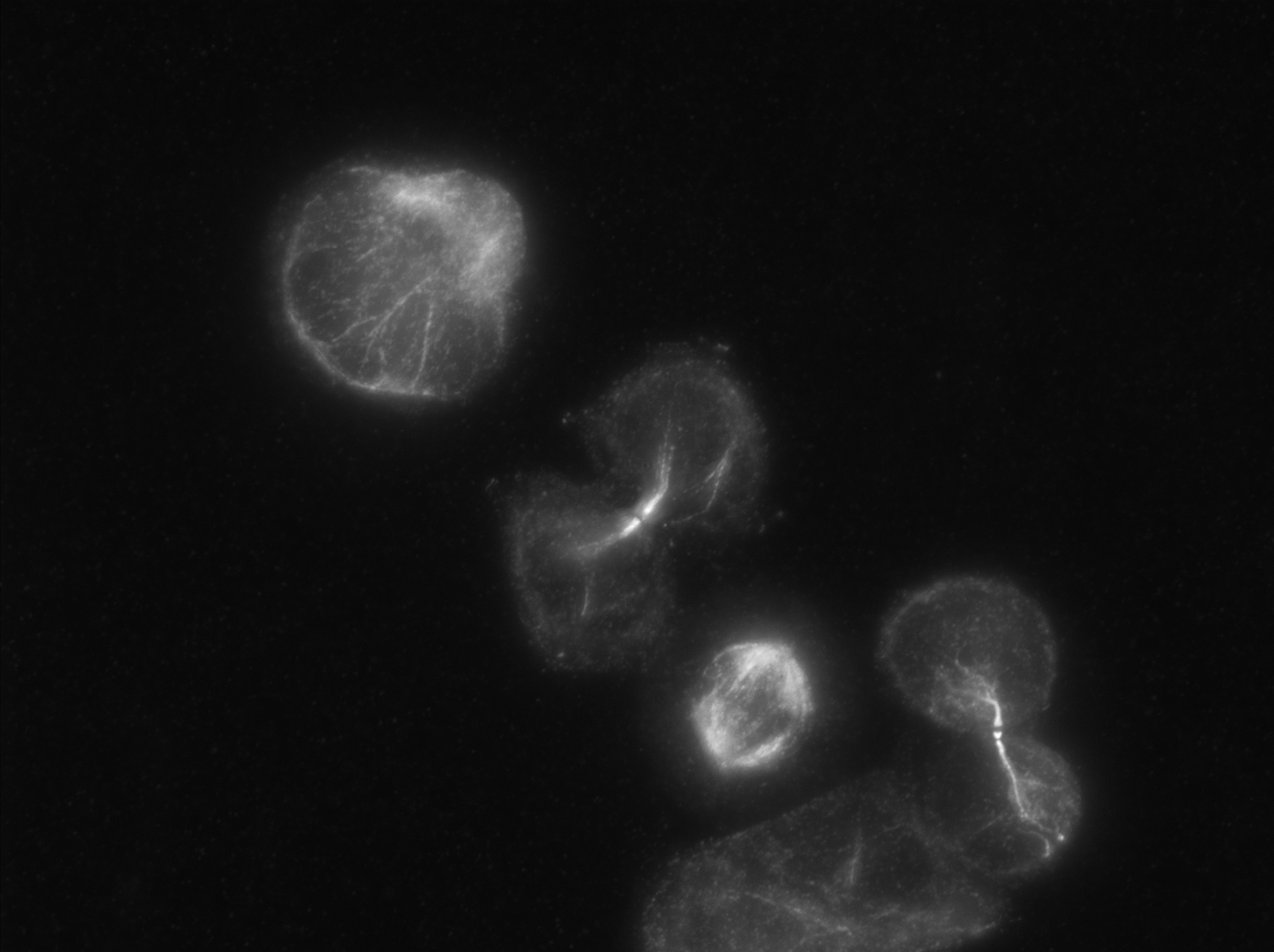

Supplement: Supplementary file 27 — Source data Fig. 7 part 3 [file 44319_2026_742_MOESM27_ESM.zip › Figure 7 Part 3/Fig 7be Cas9 and TPGS1-KO rGT335 atubulin/TPGS1-KO 5-2-25 rGT335 atub R3 M6.Project Maximum Z_XY1746220014_Z0_T0_C2.tif]

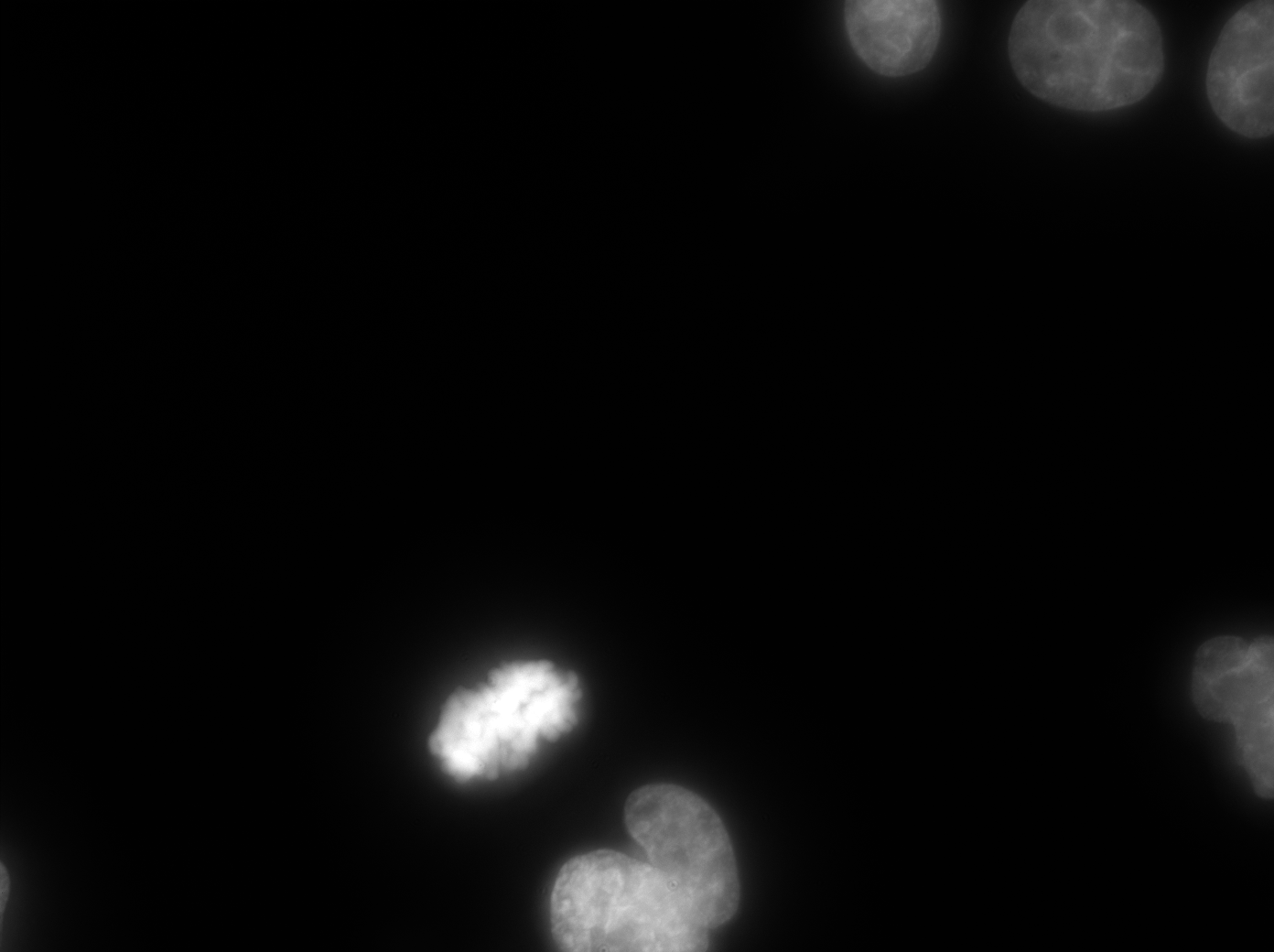

Supplement: Supplementary file 27 — Source data Fig. 7 part 3 [file 44319_2026_742_MOESM27_ESM.zip › Figure 7 Part 3/Fig 7be Cas9 and TPGS1-KO rGT335 atubulin/Cas9 5-2-25 rGT335 atub R3 M10.Project Maximum Z_XY1746218293_Z0_T0_C0.tif]

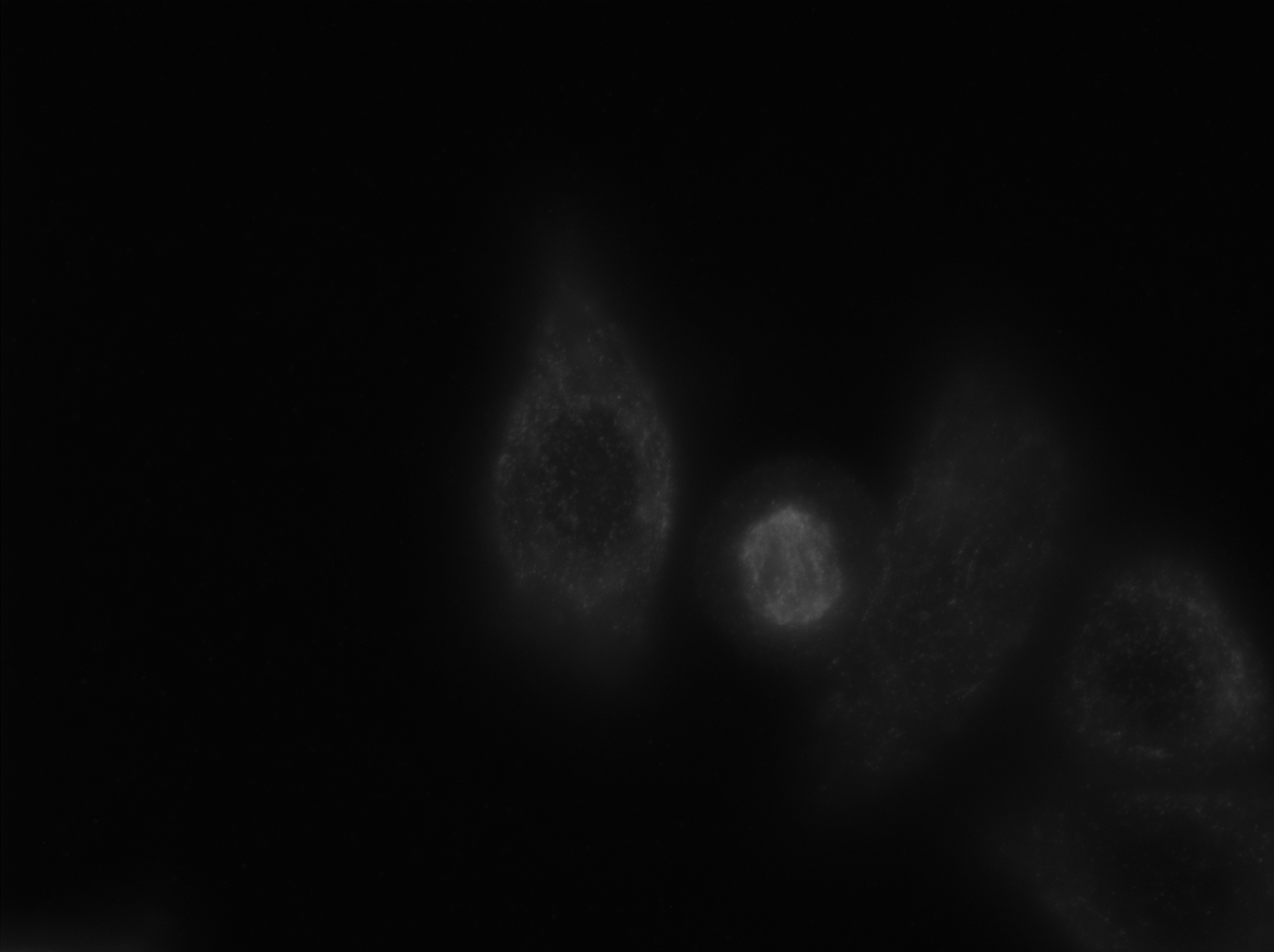

Supplement: Supplementary file 27 — Source data Fig. 7 part 3 [file 44319_2026_742_MOESM27_ESM.zip › Figure 7 Part 3/Fig 7be Cas9 and TPGS1-KO rGT335 atubulin/TPGS1-KO 5-2-25 rGT335 atub R3 M9.Project Maximum Z_XY1746220596_Z0_T0_C2.tif]

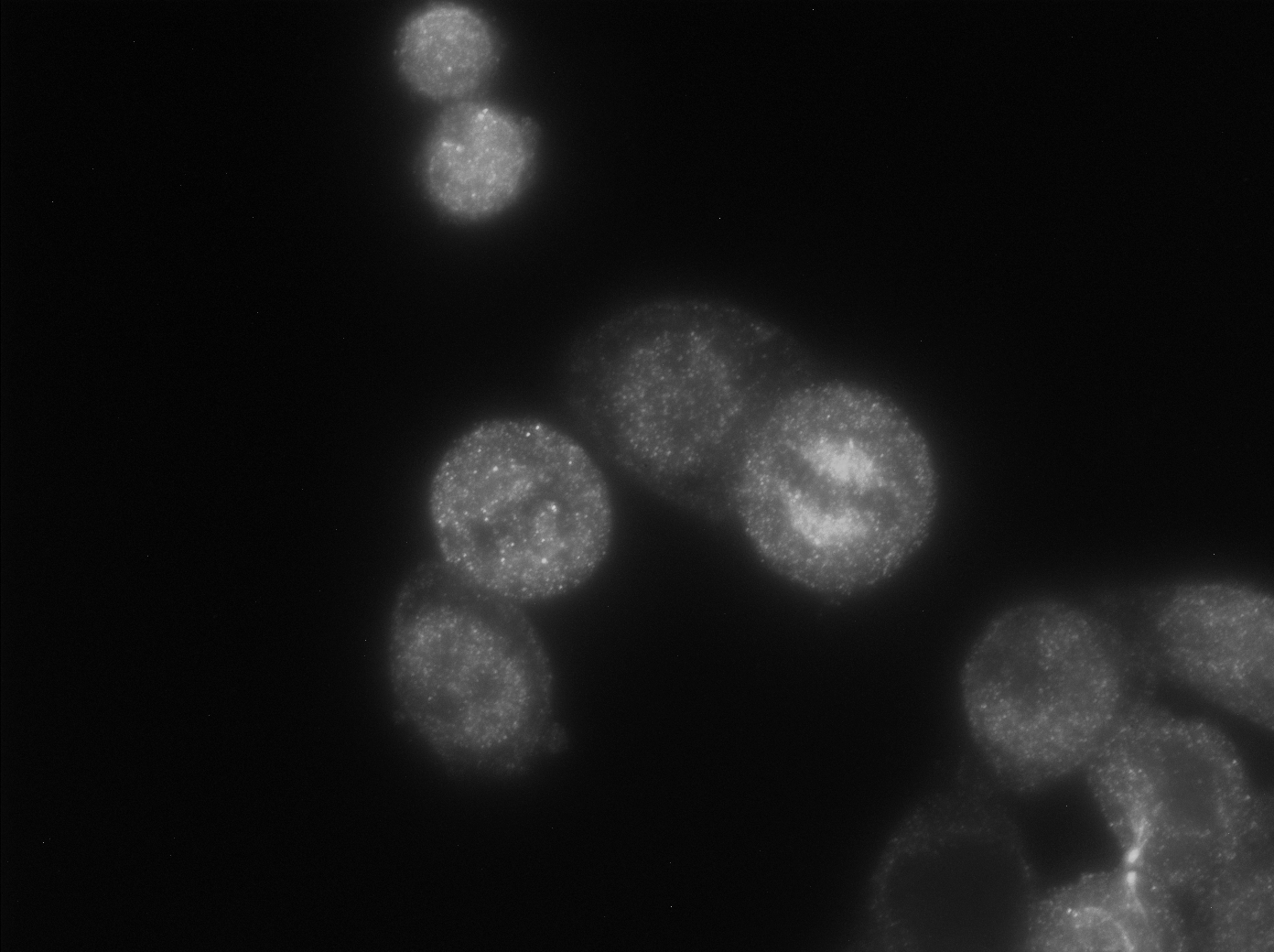

Supplement: Supplementary file 27 — Source data Fig. 7 part 3 [file 44319_2026_742_MOESM27_ESM.zip › Figure 7 Part 3/Fig 7be Cas9 and TPGS1-KO rGT335 atubulin/TPGS1-KO 5-2-25 rGT335 atub R3 M4M5.Project Maximum Z_XY1746219875_Z0_T0_C1.tif]

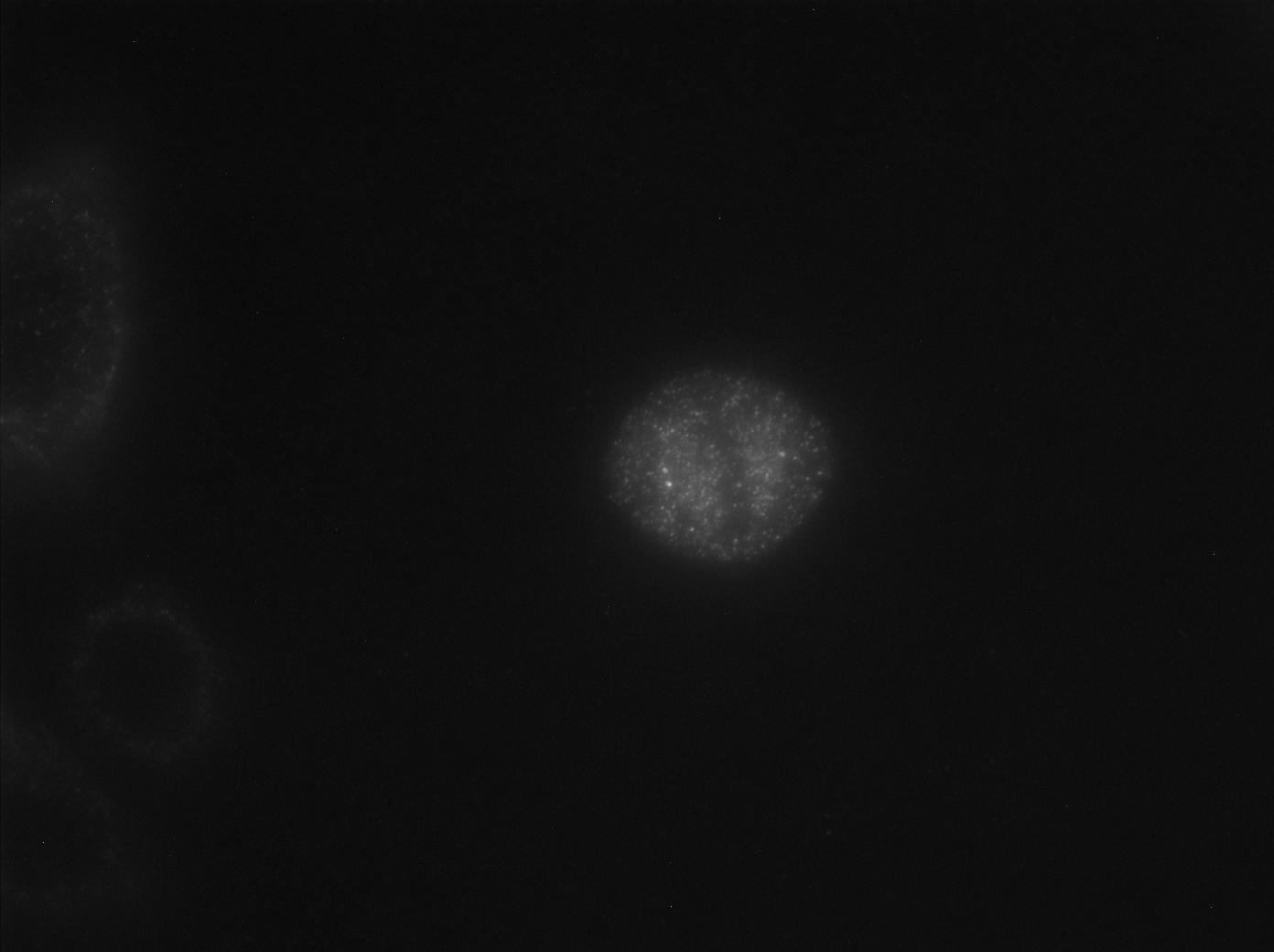

Supplement: Supplementary file 27 — Source data Fig. 7 part 3 [file 44319_2026_742_MOESM27_ESM.zip › Figure 7 Part 3/Fig 7be Cas9 and TPGS1-KO rGT335 atubulin/retakes/Cas9 5-2-25 rGT335 atub R1.2 M10.Project Maximum Z_XY1747769347_Z0_T0_C1.tif]

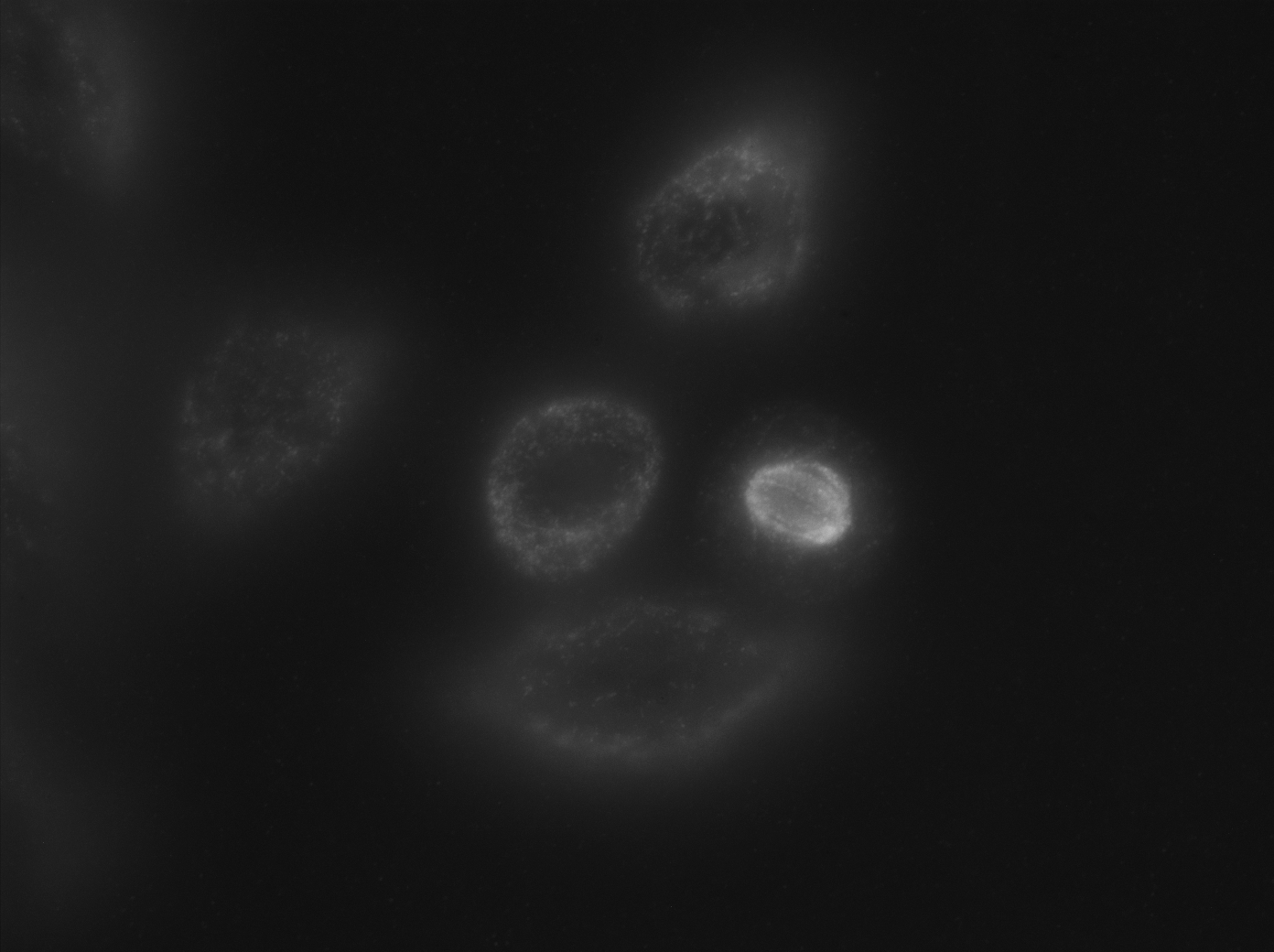

Supplement: Supplementary file 27 — Source data Fig. 7 part 3 [file 44319_2026_742_MOESM27_ESM.zip › Figure 7 Part 3/Fig 7be Cas9 and TPGS1-KO rGT335 atubulin/retakes/Cas9 5-2-25 rGT335 atub R1.1 M1.Project Maximum Z_XY1747762561_Z0_T0_C2.tif]

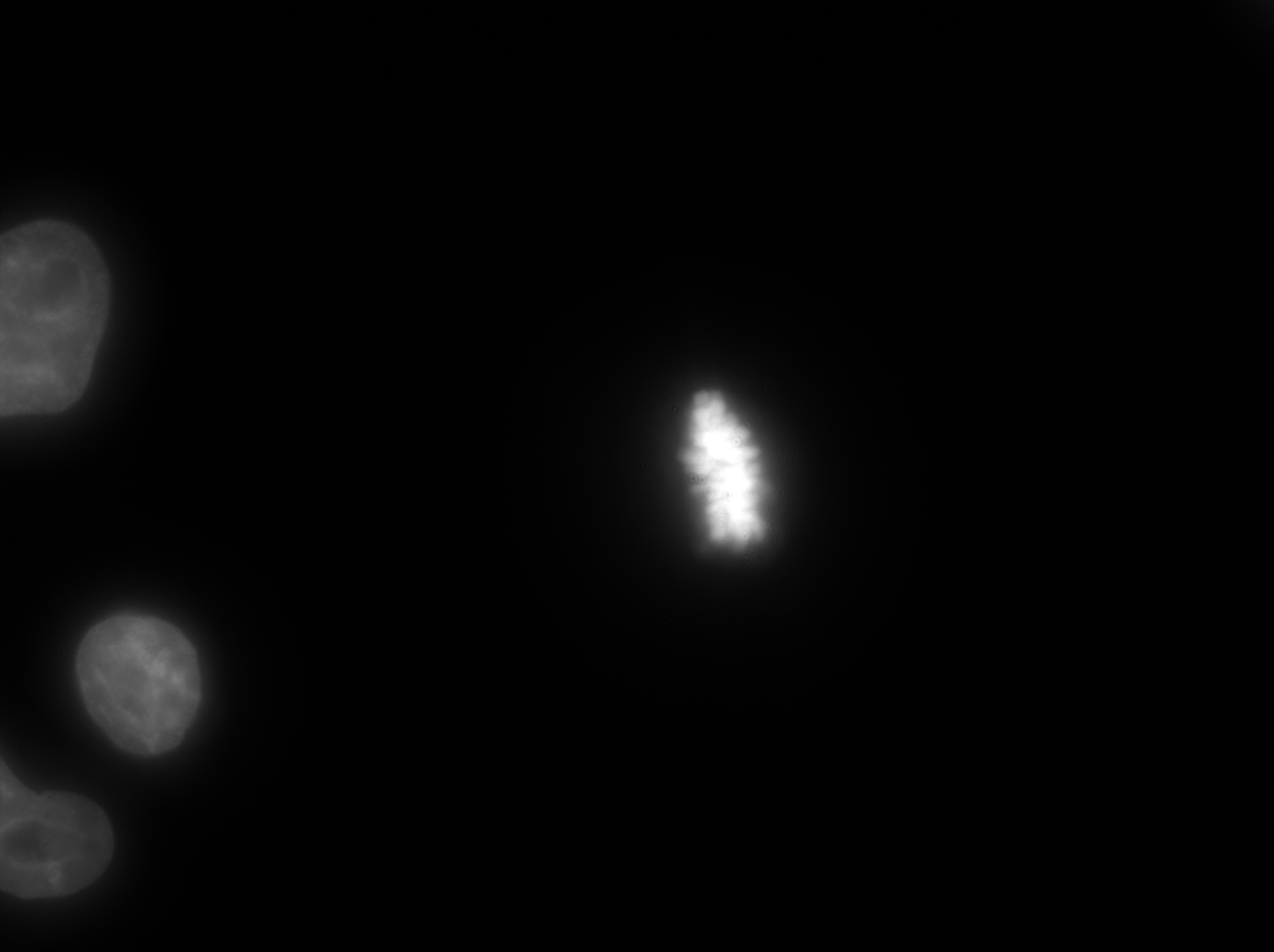

Supplement: Supplementary file 27 — Source data Fig. 7 part 3 [file 44319_2026_742_MOESM27_ESM.zip › Figure 7 Part 3/Fig 7be Cas9 and TPGS1-KO rGT335 atubulin/retakes/Cas9 5-2-25 rGT335 atub R1.2 M10.Project Maximum Z_XY1747769347_Z0_T0_C0.tif]

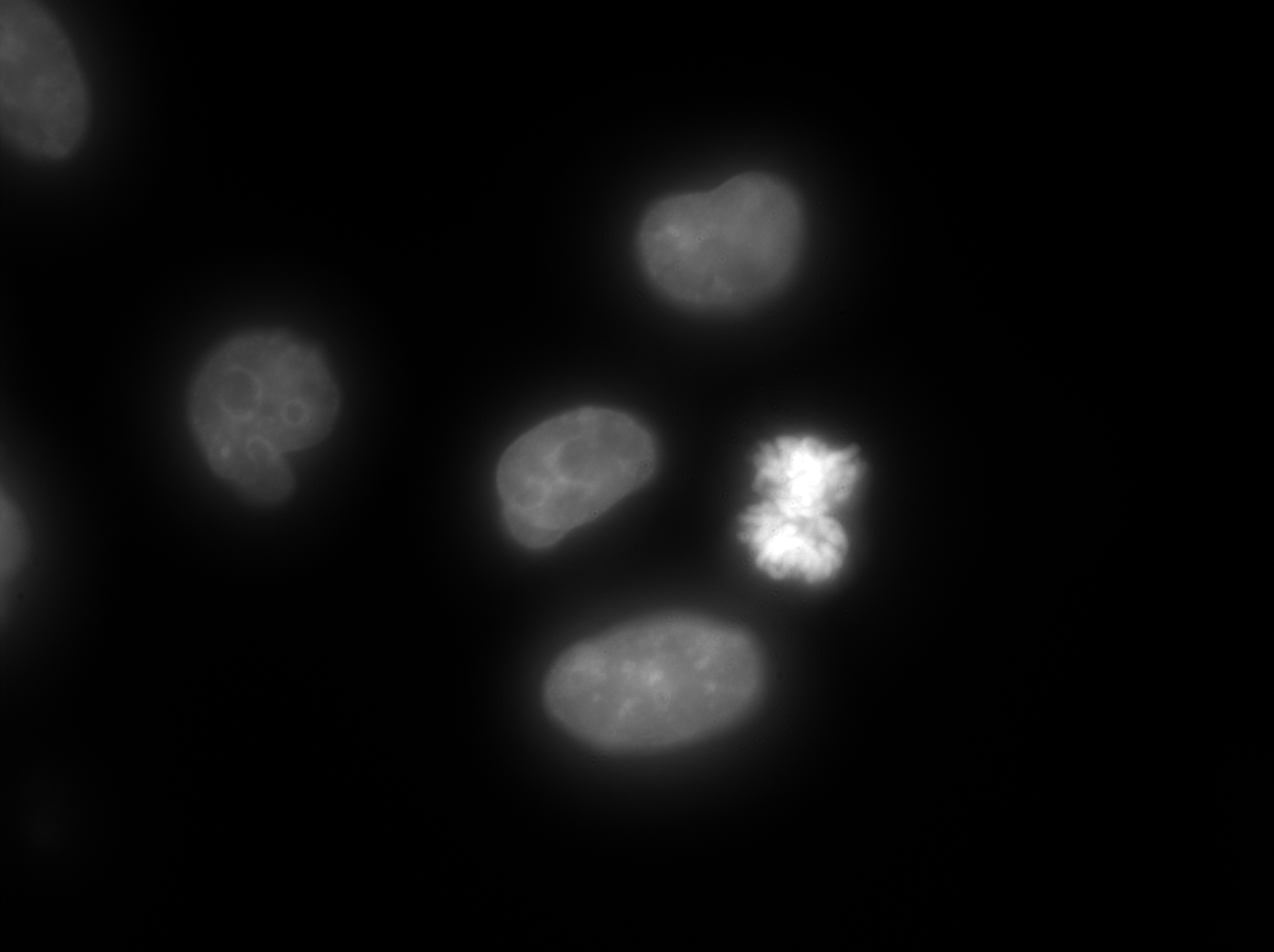

Supplement: Supplementary file 27 — Source data Fig. 7 part 3 [file 44319_2026_742_MOESM27_ESM.zip › Figure 7 Part 3/Fig 7be Cas9 and TPGS1-KO rGT335 atubulin/retakes/Cas9 5-2-25 rGT335 atub R1.1 M1.Project Maximum Z_XY1747762561_Z0_T0_C0.tif]

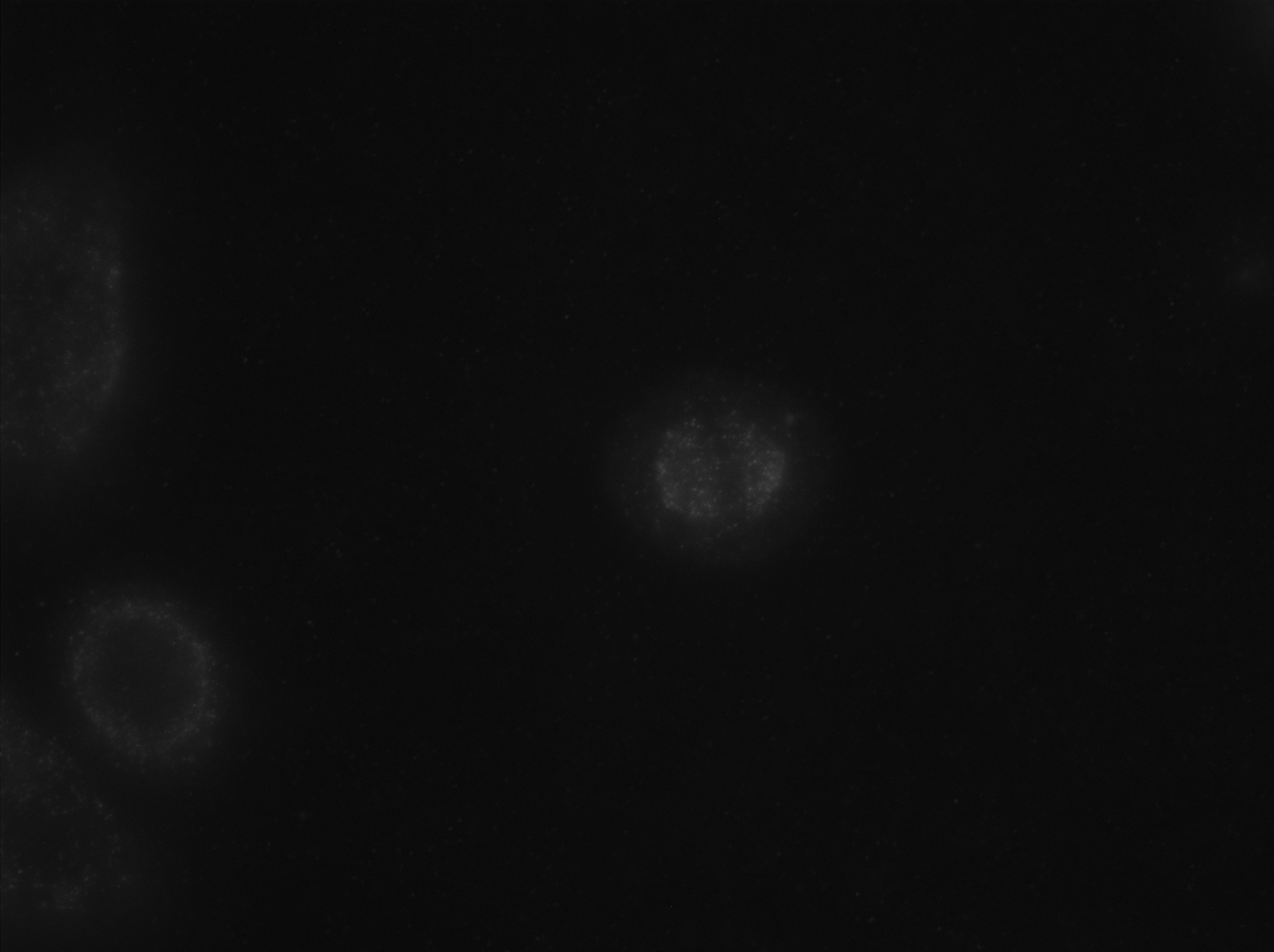

Supplement: Supplementary file 27 — Source data Fig. 7 part 3 [file 44319_2026_742_MOESM27_ESM.zip › Figure 7 Part 3/Fig 7be Cas9 and TPGS1-KO rGT335 atubulin/retakes/Cas9 5-2-25 rGT335 atub R1.2 M10.Project Maximum Z_XY1747769347_Z0_T0_C2.tif]

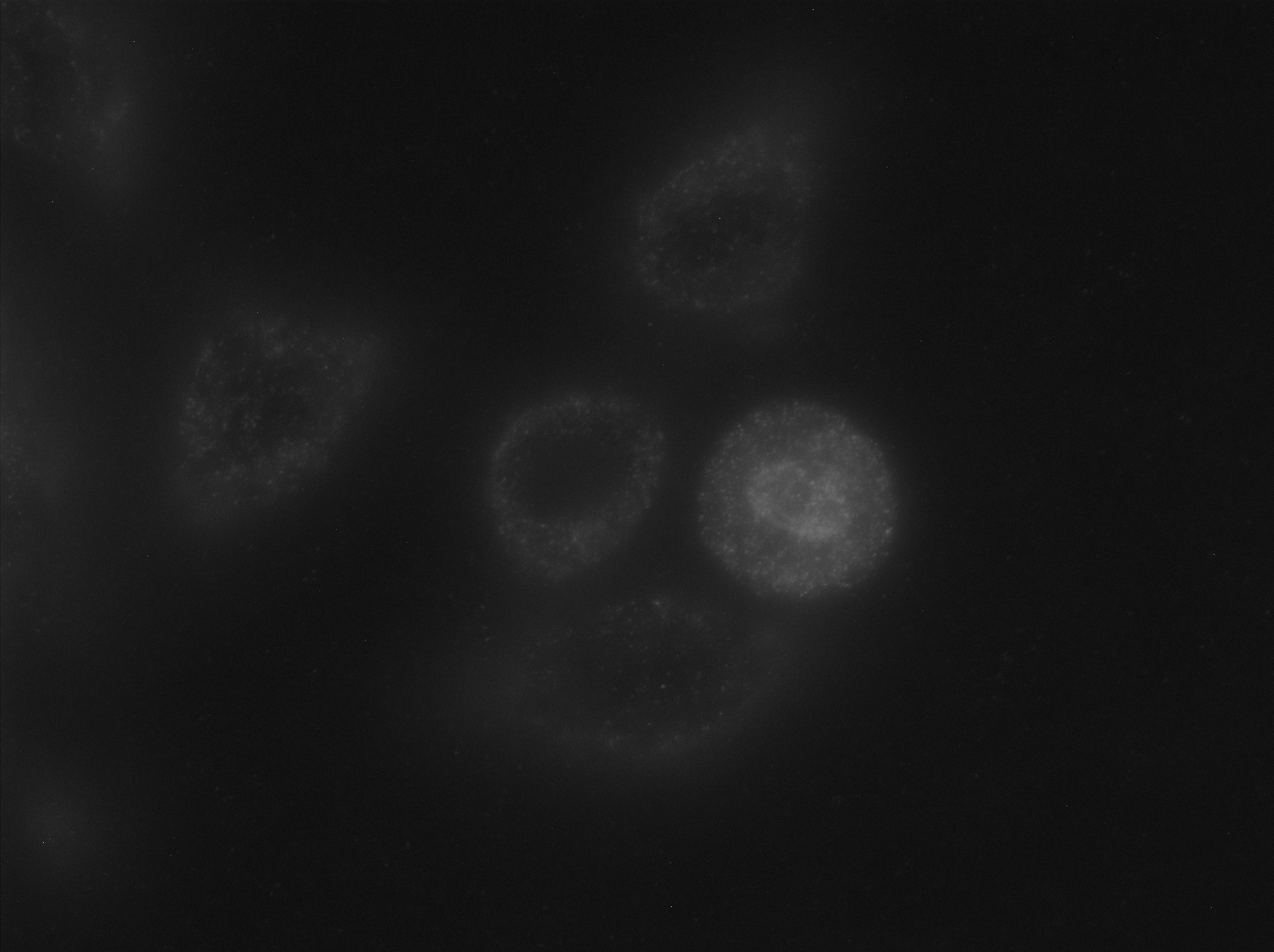

Supplement: Supplementary file 27 — Source data Fig. 7 part 3 [file 44319_2026_742_MOESM27_ESM.zip › Figure 7 Part 3/Fig 7be Cas9 and TPGS1-KO rGT335 atubulin/retakes/Cas9 5-2-25 rGT335 atub R1.1 M1.Project Maximum Z_XY1747762561_Z0_T0_C1.tif]

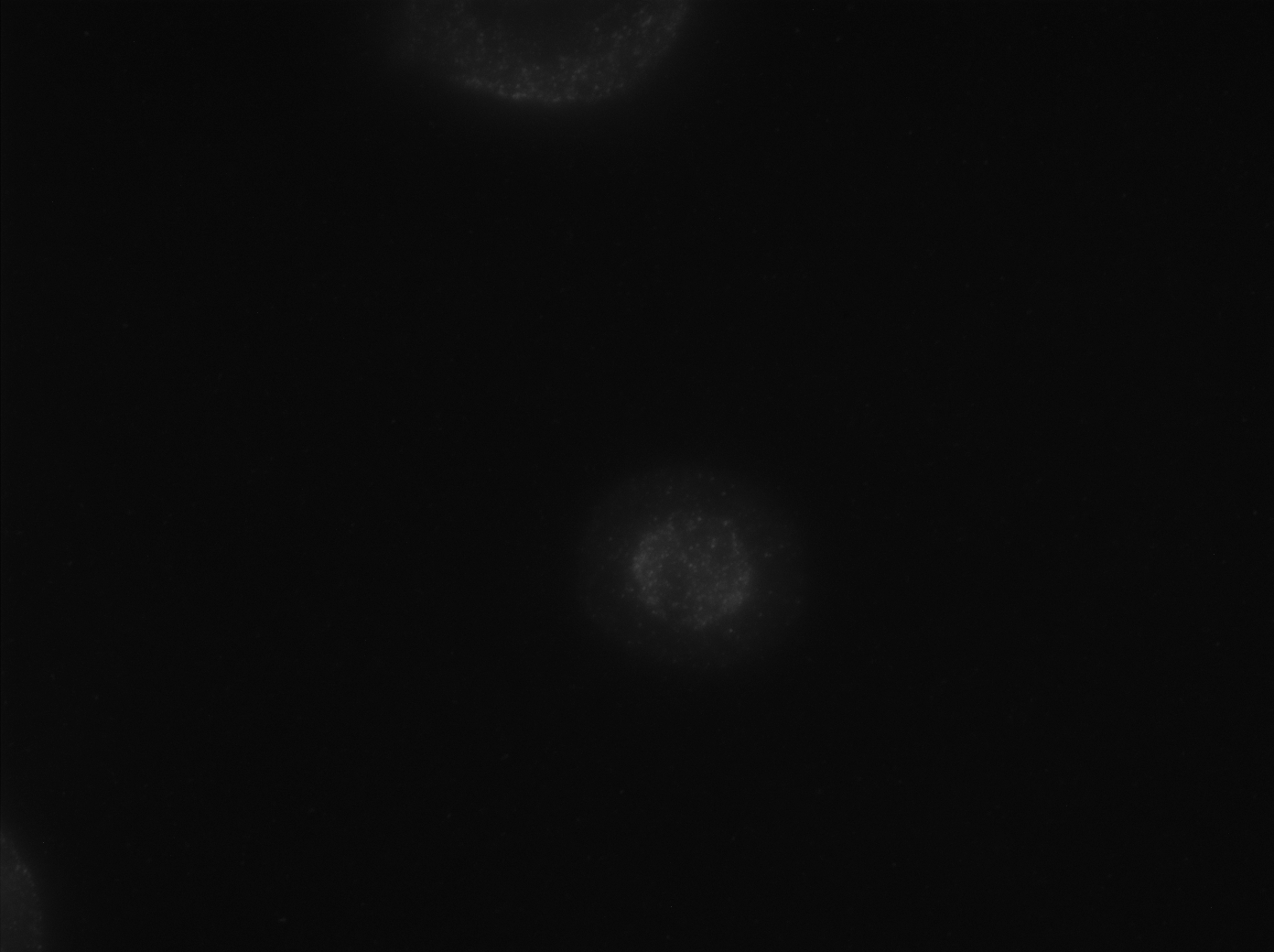

Supplement: Supplementary file 27 — Source data Fig. 7 part 3 [file 44319_2026_742_MOESM27_ESM.zip › Figure 7 Part 3/Fig 7be Cas9 and TPGS1-KO rGT335 atubulin/retakes/Cas9 5-2-25 rGT335 atub R1.2 M3.Project Maximum Z_XY1747767755_Z0_T0_C2.tif]

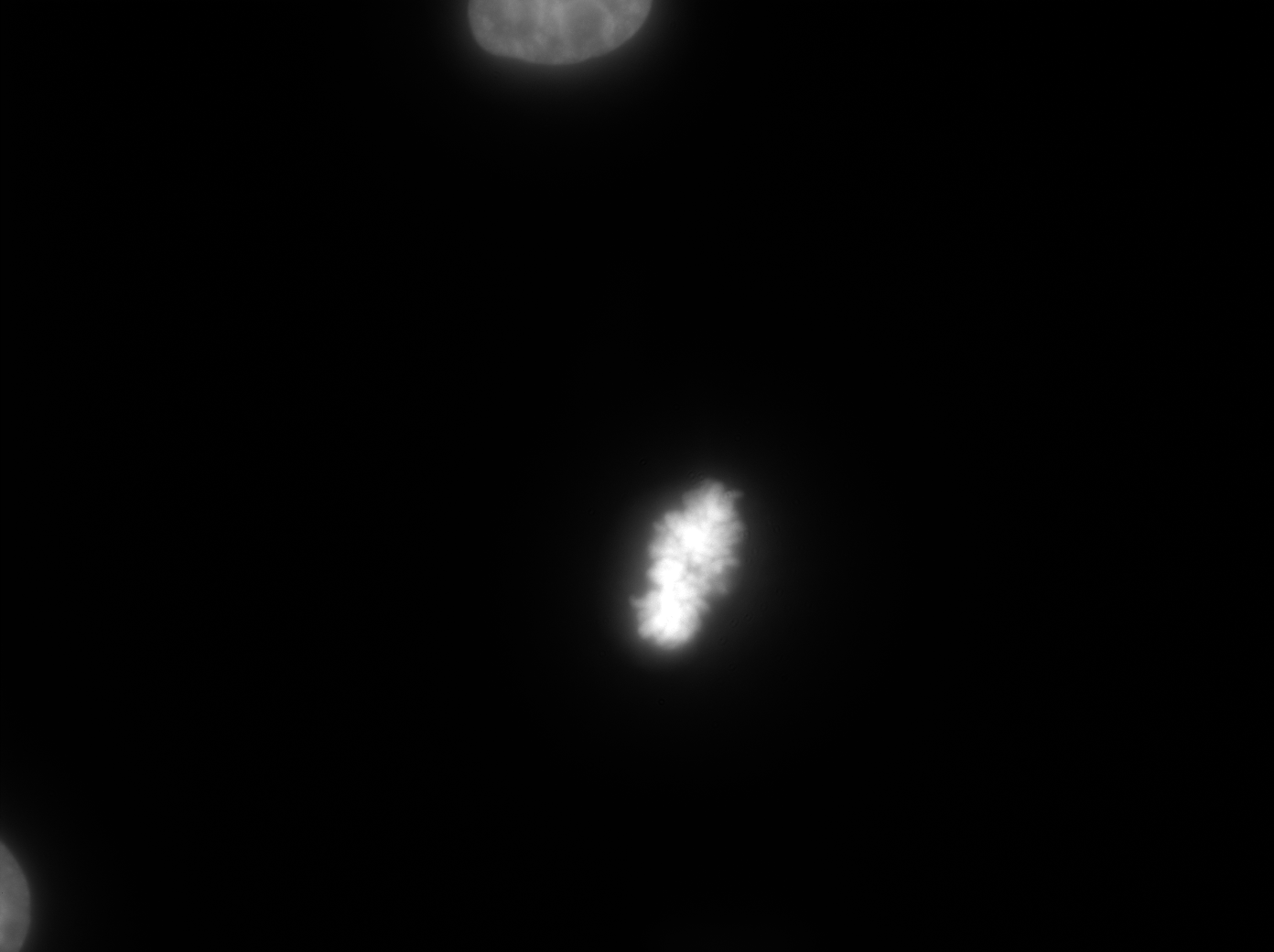

Supplement: Supplementary file 27 — Source data Fig. 7 part 3 [file 44319_2026_742_MOESM27_ESM.zip › Figure 7 Part 3/Fig 7be Cas9 and TPGS1-KO rGT335 atubulin/retakes/Cas9 5-2-25 rGT335 atub R1.2 M3.Project Maximum Z_XY1747767755_Z0_T0_C0.tif]

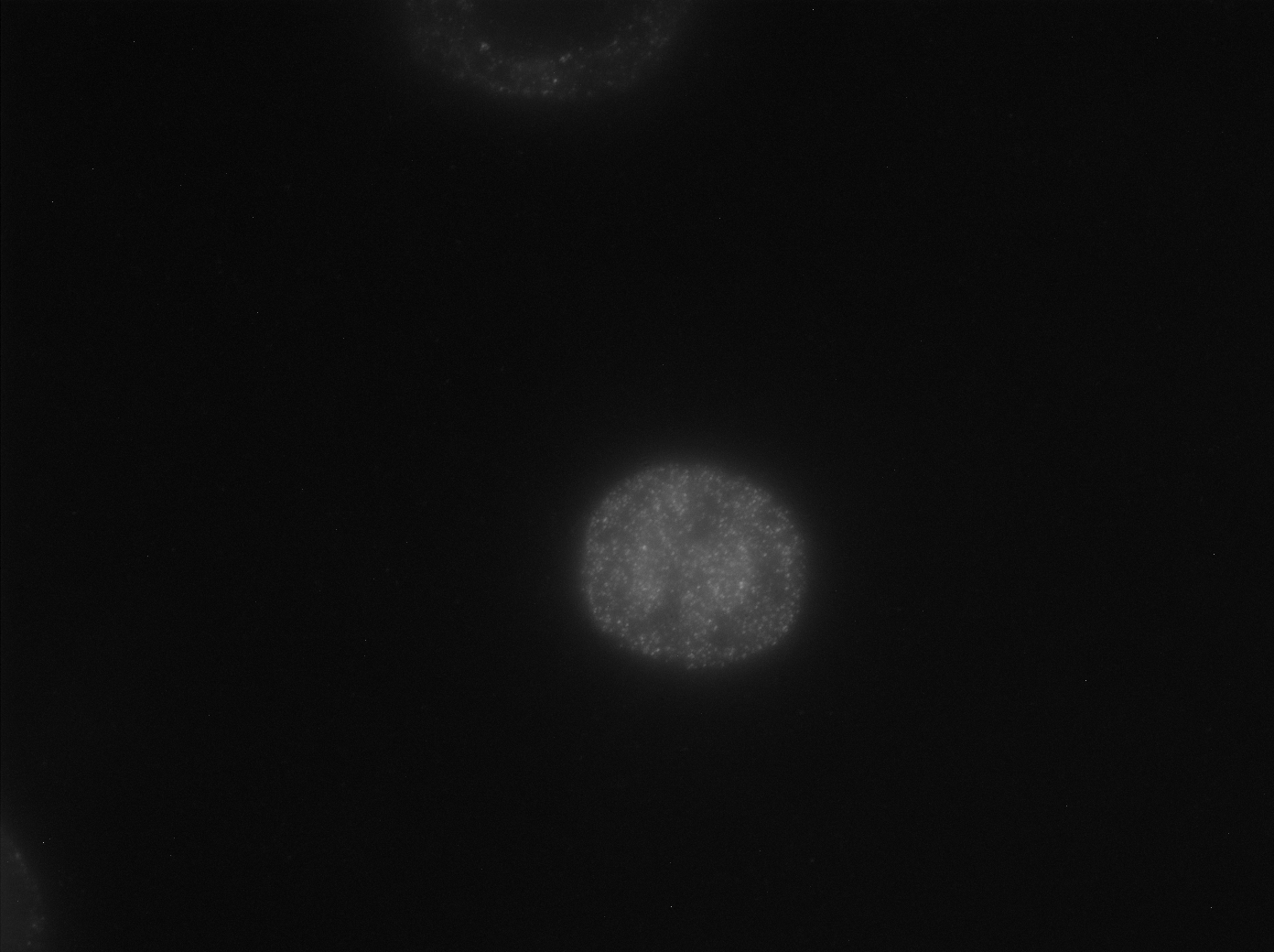

Supplement: Supplementary file 27 — Source data Fig. 7 part 3 [file 44319_2026_742_MOESM27_ESM.zip › Figure 7 Part 3/Fig 7be Cas9 and TPGS1-KO rGT335 atubulin/retakes/Cas9 5-2-25 rGT335 atub R1.2 M3.Project Maximum Z_XY1747767755_Z0_T0_C1.tif]

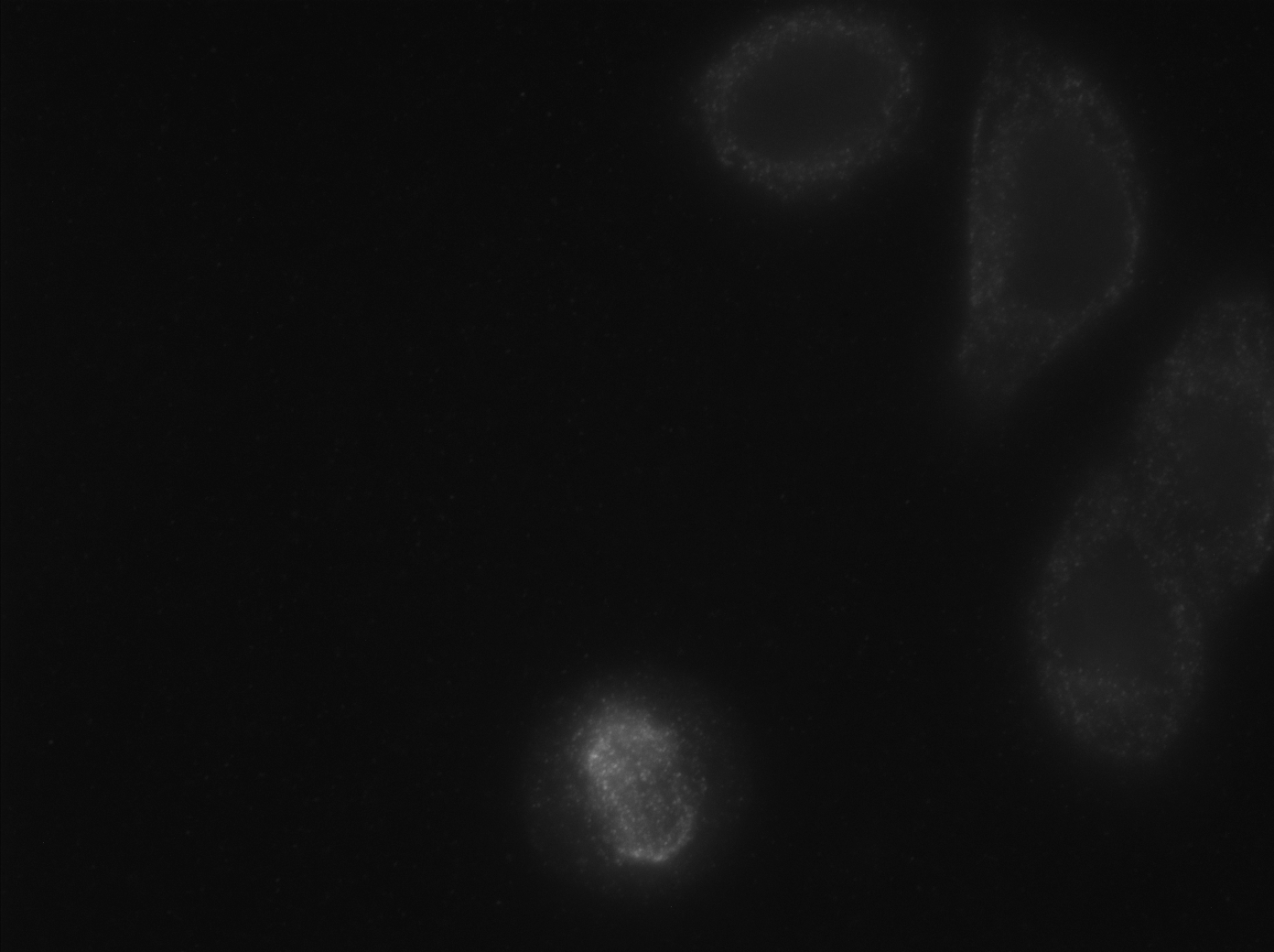

Supplement: Supplementary file 27 — Source data Fig. 7 part 3 [file 44319_2026_742_MOESM27_ESM.zip › Figure 7 Part 3/Fig 7be Cas9 and TPGS1-KO rGT335 atubulin/retakes/Cas9 5-2-25 rGT335 atub R1.2 M7.Project Maximum Z_XY1747768720_Z0_T0_C2.tif]

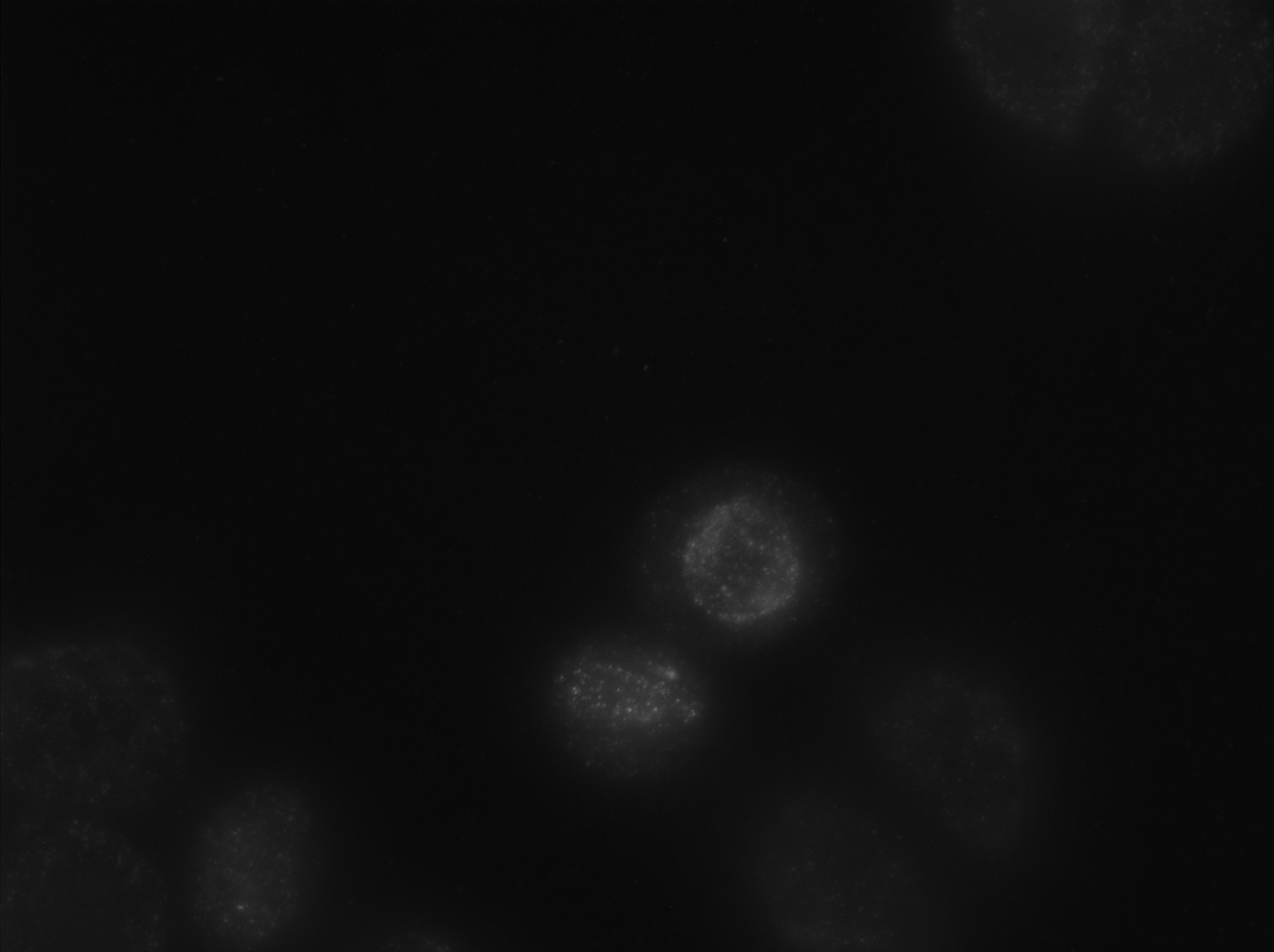

Supplement: Supplementary file 27 — Source data Fig. 7 part 3 [file 44319_2026_742_MOESM27_ESM.zip › Figure 7 Part 3/Fig 7be Cas9 and TPGS1-KO rGT335 atubulin/retakes/Cas9 5-2-25 rGT335 atub R1.2 M1.Project Maximum Z_XY1747767503_Z0_T0_C2.tif]

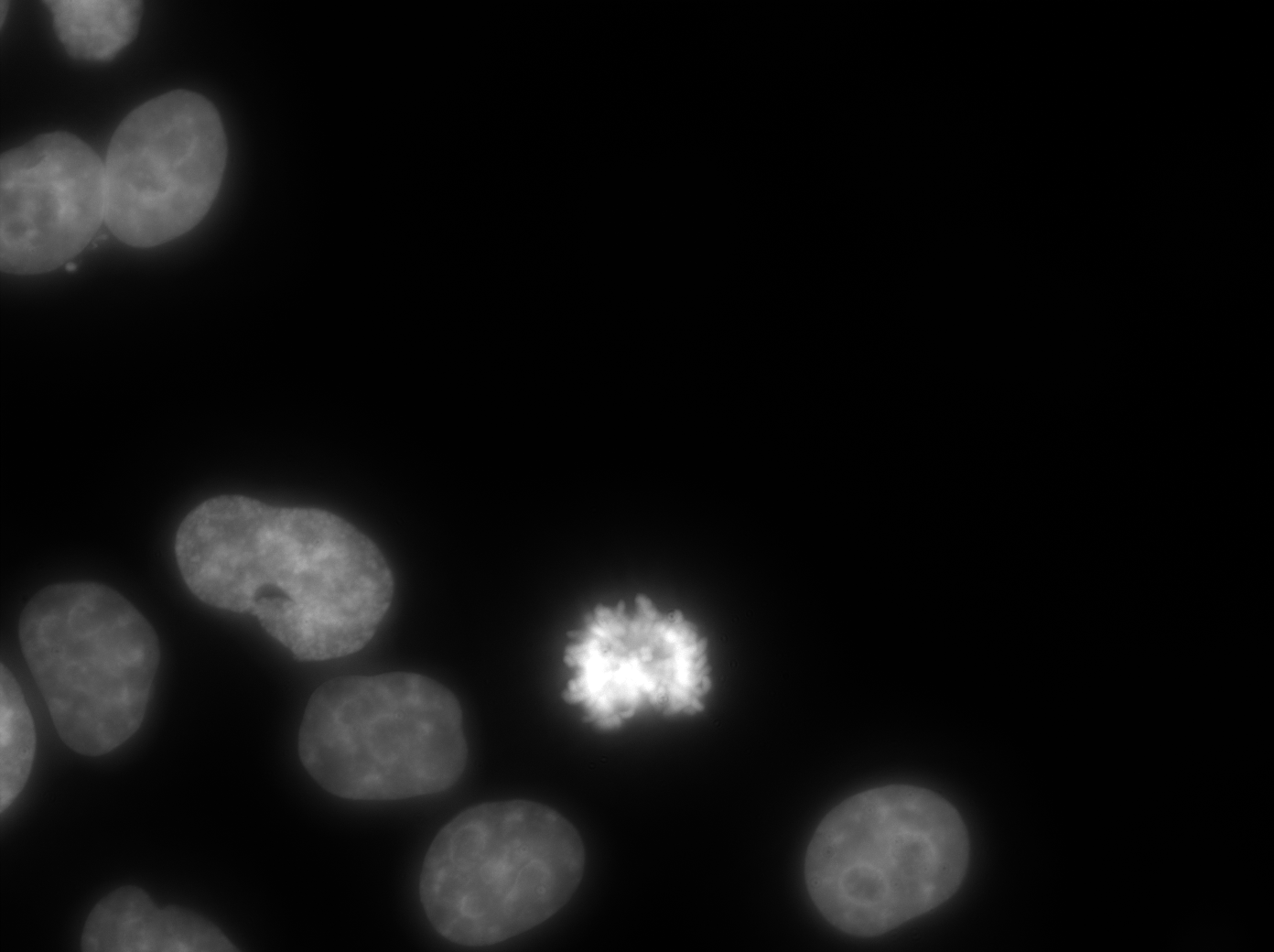

Supplement: Supplementary file 27 — Source data Fig. 7 part 3 [file 44319_2026_742_MOESM27_ESM.zip › Figure 7 Part 3/Fig 7be Cas9 and TPGS1-KO rGT335 atubulin/retakes/Cas9 5-2-25 rGT335 atub R1.2 M5.Project Maximum Z_XY1747768007_Z0_T0_C0.tif]

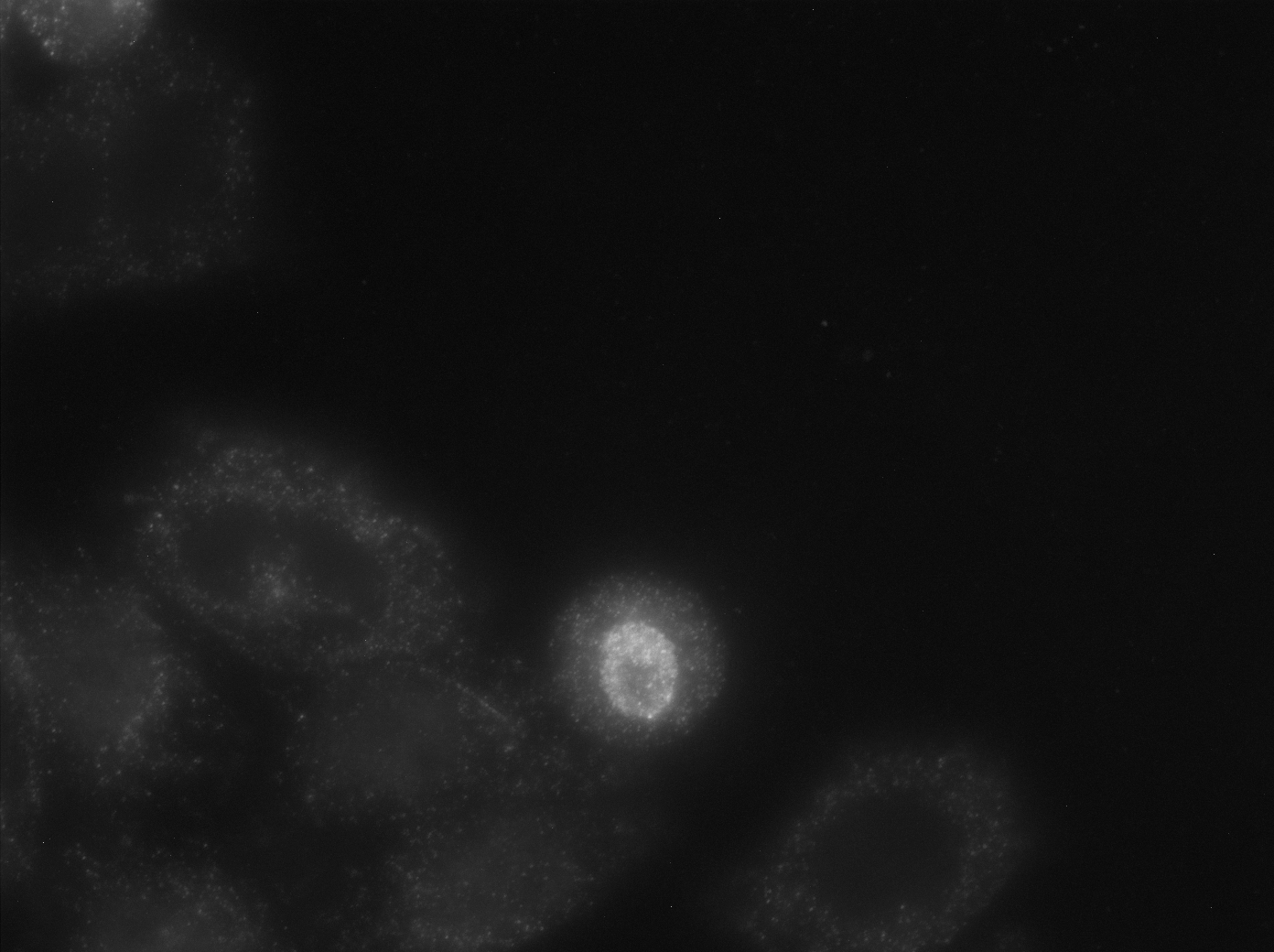

Supplement: Supplementary file 27 — Source data Fig. 7 part 3 [file 44319_2026_742_MOESM27_ESM.zip › Figure 7 Part 3/Fig 7be Cas9 and TPGS1-KO rGT335 atubulin/retakes/Cas9 5-2-25 rGT335 atub R1.2 M5.Project Maximum Z_XY1747768007_Z0_T0_C1.tif]

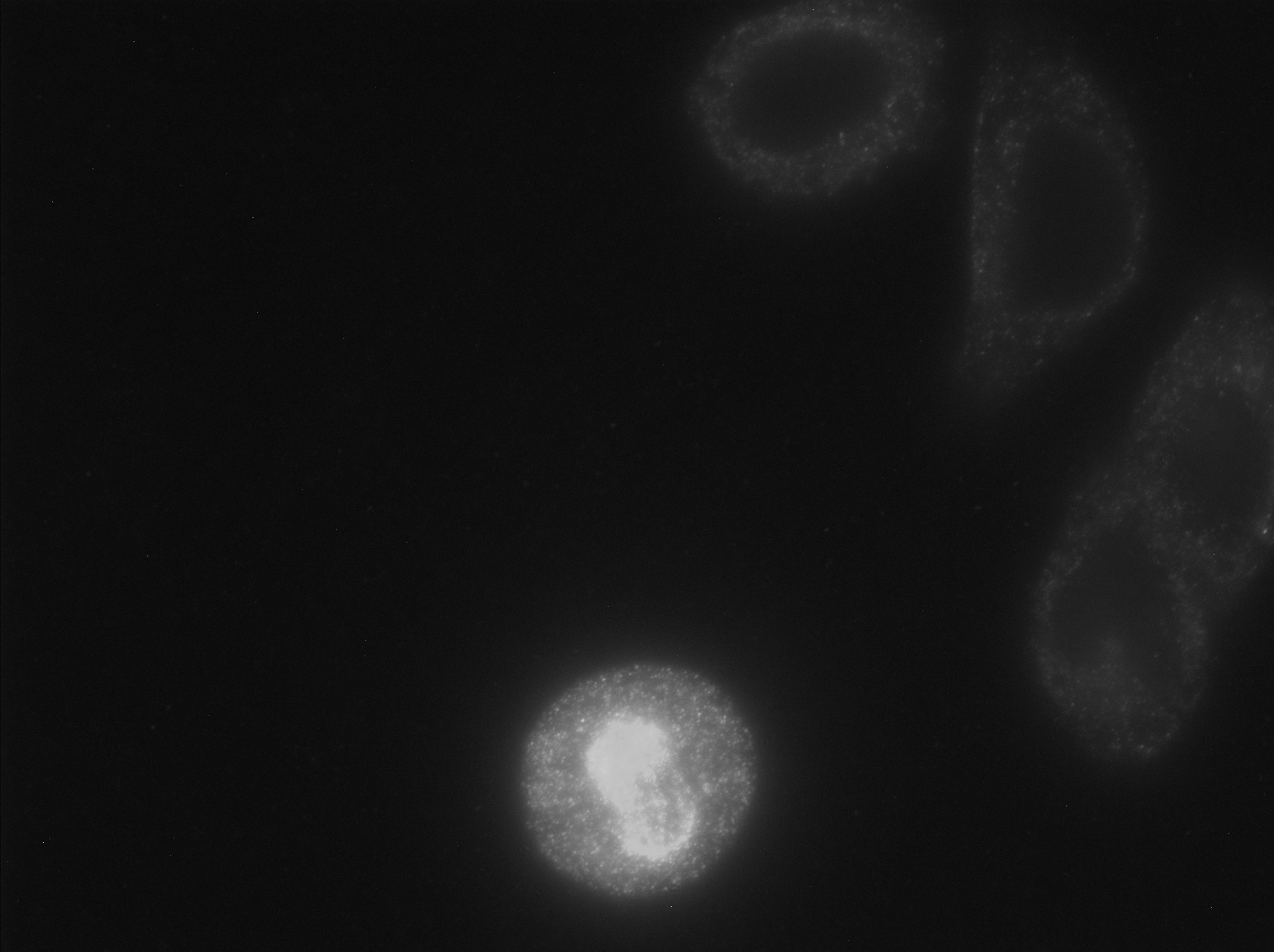

Supplement: Supplementary file 27 — Source data Fig. 7 part 3 [file 44319_2026_742_MOESM27_ESM.zip › Figure 7 Part 3/Fig 7be Cas9 and TPGS1-KO rGT335 atubulin/retakes/Cas9 5-2-25 rGT335 atub R1.2 M7.Project Maximum Z_XY1747768720_Z0_T0_C1.tif]

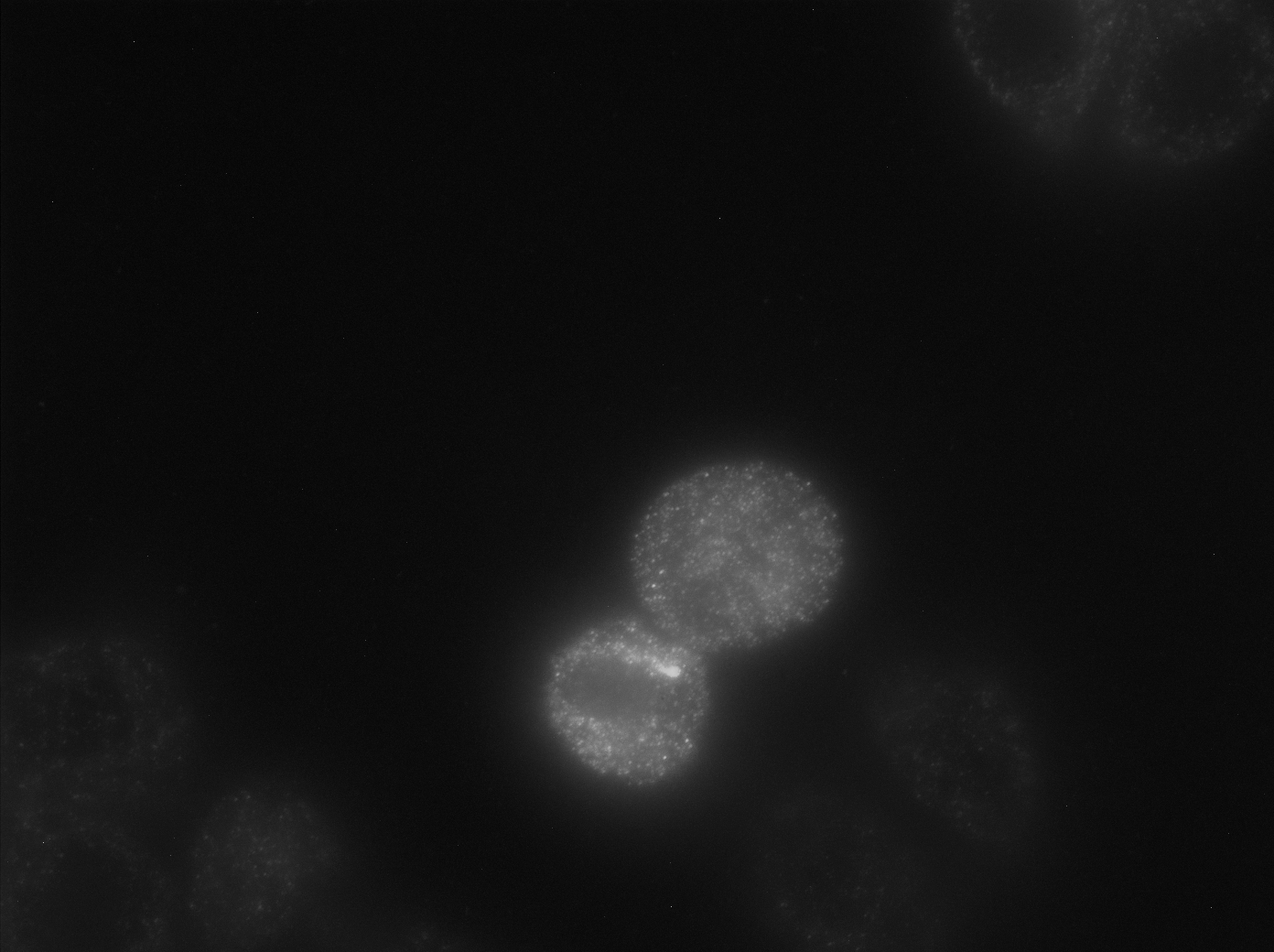

Supplement: Supplementary file 27 — Source data Fig. 7 part 3 [file 44319_2026_742_MOESM27_ESM.zip › Figure 7 Part 3/Fig 7be Cas9 and TPGS1-KO rGT335 atubulin/retakes/Cas9 5-2-25 rGT335 atub R1.2 M1.Project Maximum Z_XY1747767503_Z0_T0_C1.tif]

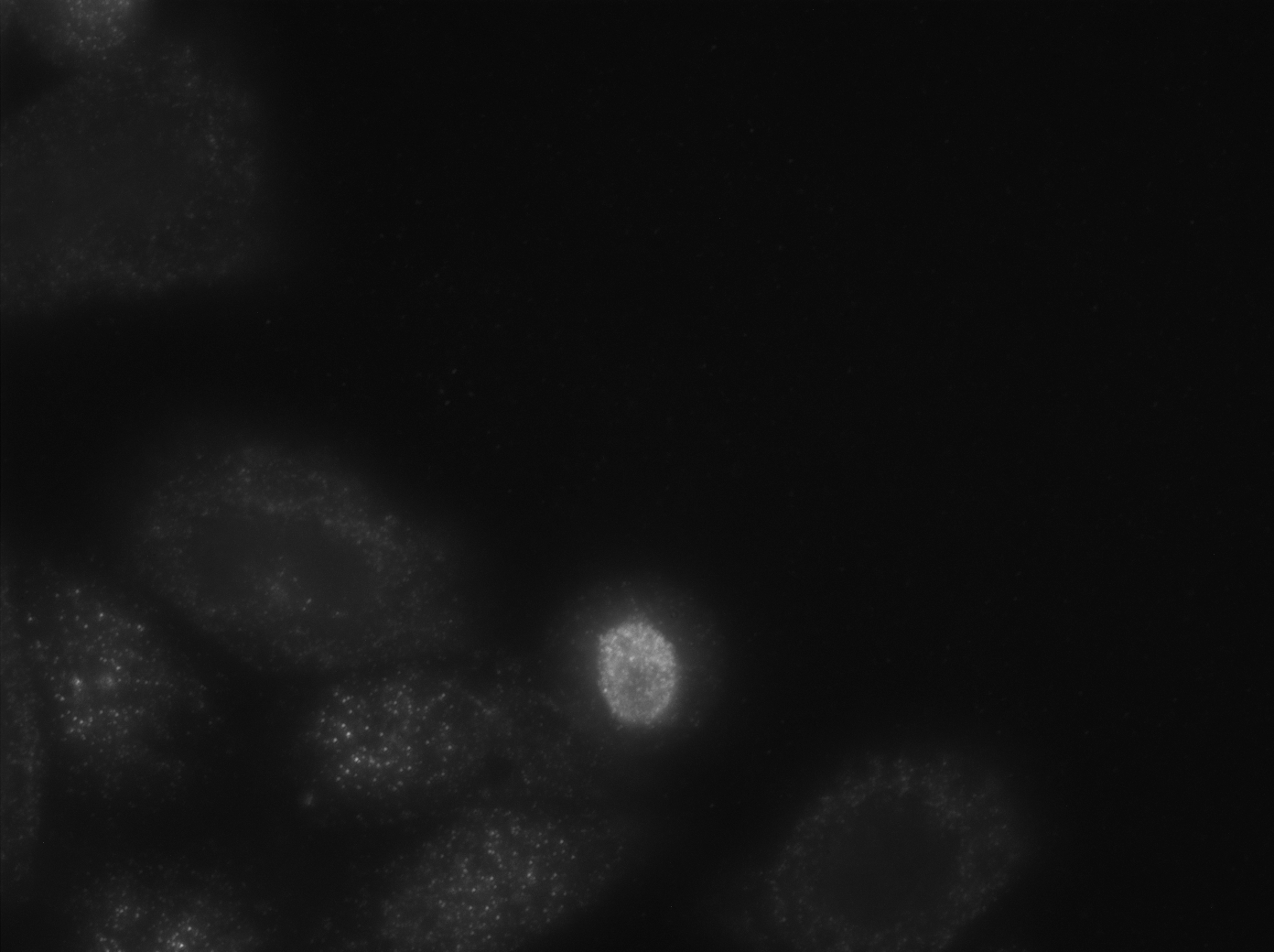

Supplement: Supplementary file 27 — Source data Fig. 7 part 3 [file 44319_2026_742_MOESM27_ESM.zip › Figure 7 Part 3/Fig 7be Cas9 and TPGS1-KO rGT335 atubulin/retakes/Cas9 5-2-25 rGT335 atub R1.2 M5.Project Maximum Z_XY1747768007_Z0_T0_C2.tif]

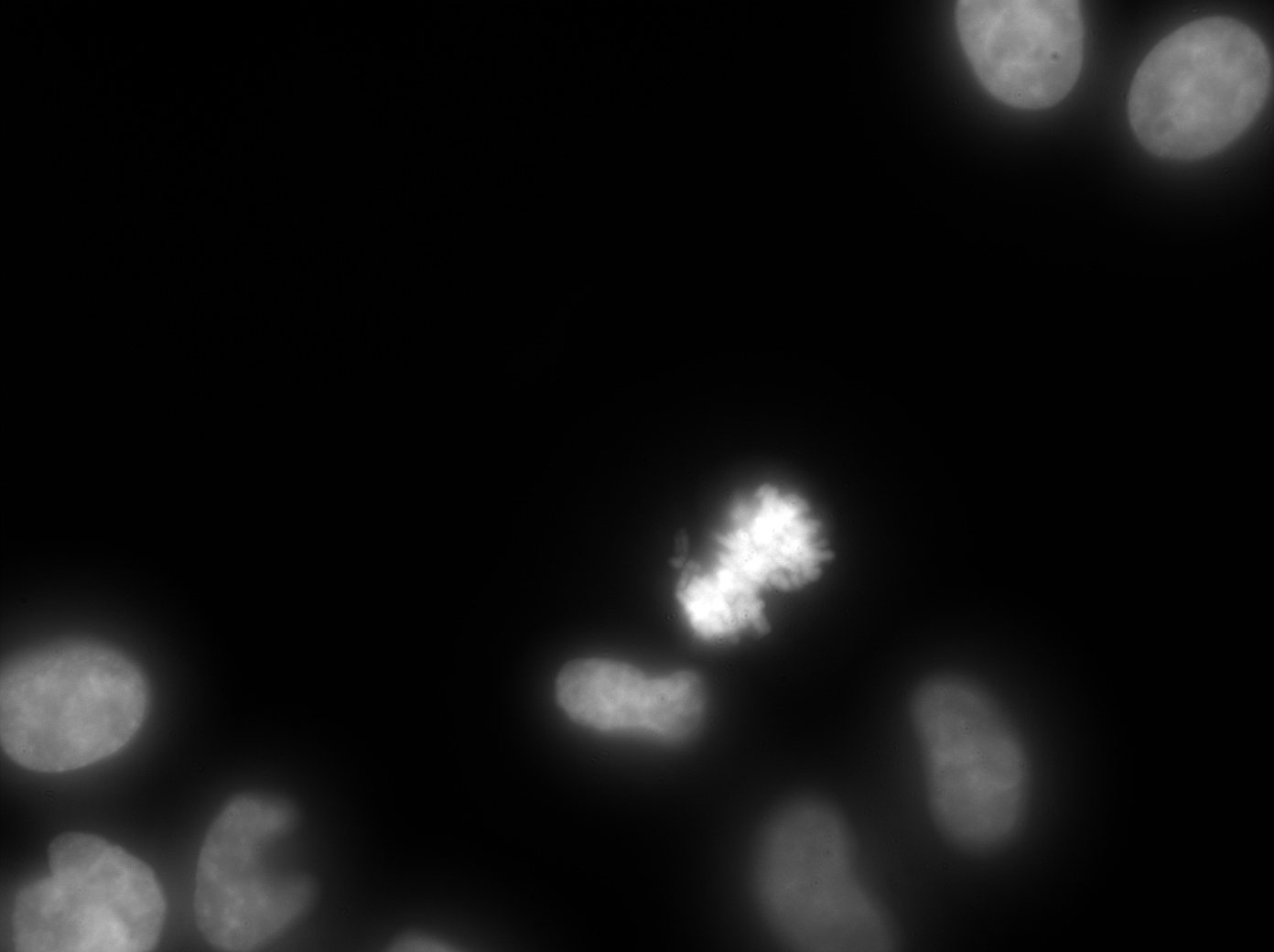

Supplement: Supplementary file 27 — Source data Fig. 7 part 3 [file 44319_2026_742_MOESM27_ESM.zip › Figure 7 Part 3/Fig 7be Cas9 and TPGS1-KO rGT335 atubulin/retakes/Cas9 5-2-25 rGT335 atub R1.2 M1.Project Maximum Z_XY1747767503_Z0_T0_C0.tif]

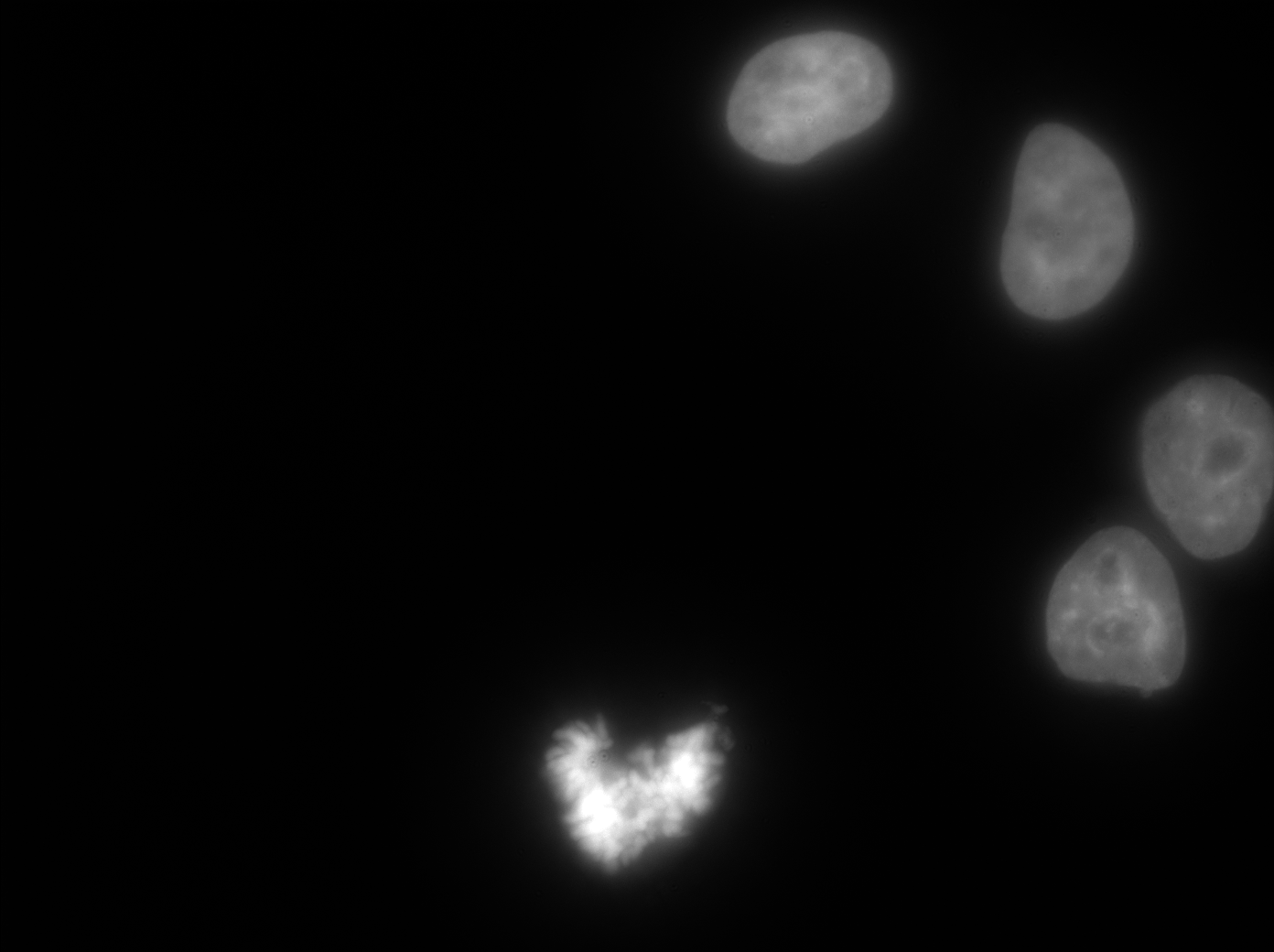

Supplement: Supplementary file 27 — Source data Fig. 7 part 3 [file 44319_2026_742_MOESM27_ESM.zip › Figure 7 Part 3/Fig 7be Cas9 and TPGS1-KO rGT335 atubulin/retakes/Cas9 5-2-25 rGT335 atub R1.2 M7.Project Maximum Z_XY1747768720_Z0_T0_C0.tif]

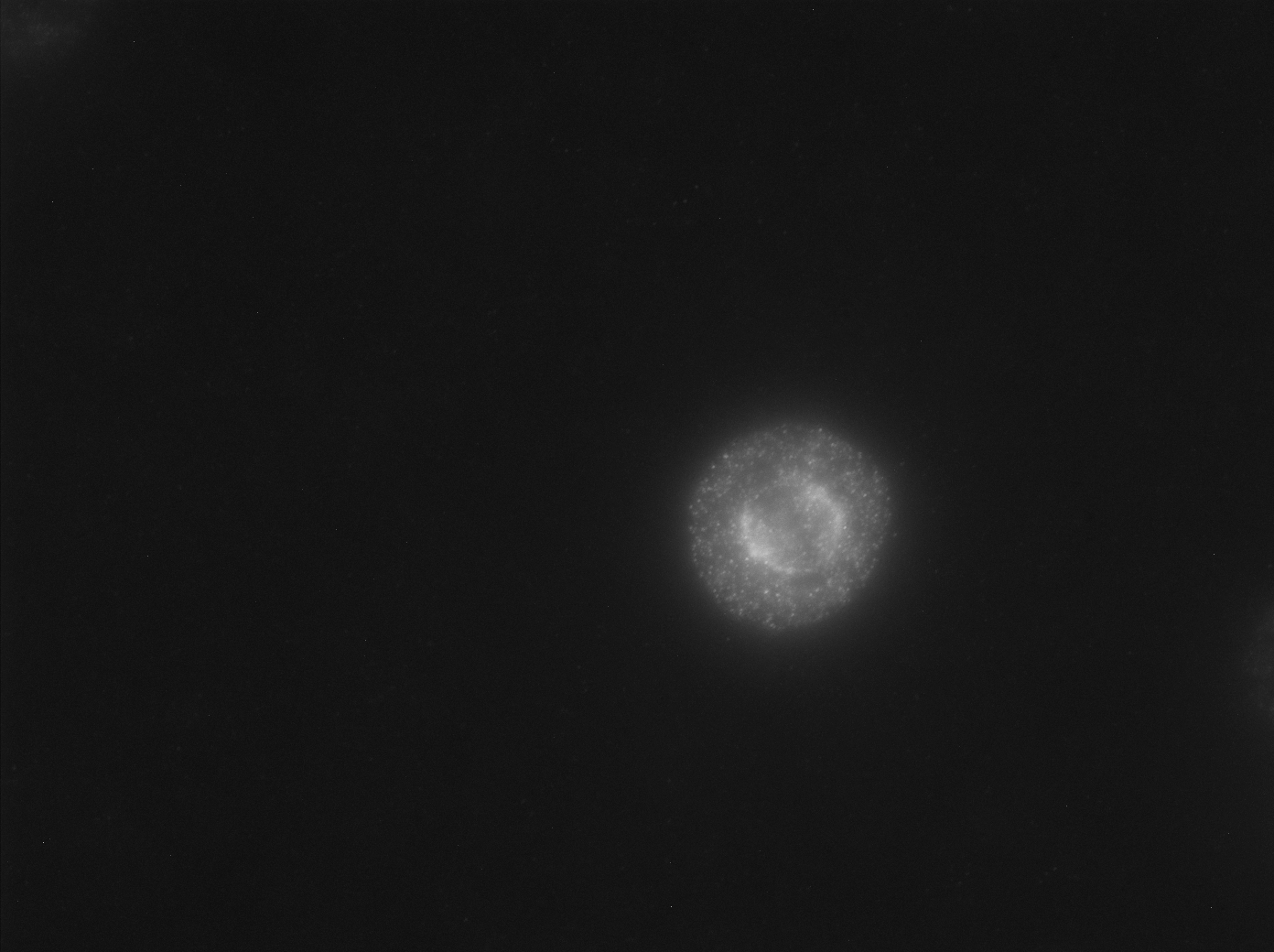

Supplement: Supplementary file 27 — Source data Fig. 7 part 3 [file 44319_2026_742_MOESM27_ESM.zip › Figure 7 Part 3/Fig 7be Cas9 and TPGS1-KO rGT335 atubulin/retakes/Cas9 5-2-25 rGT335 atub R1.1 M3.Project Maximum Z_XY1747763241_Z0_T0_C1.tif]

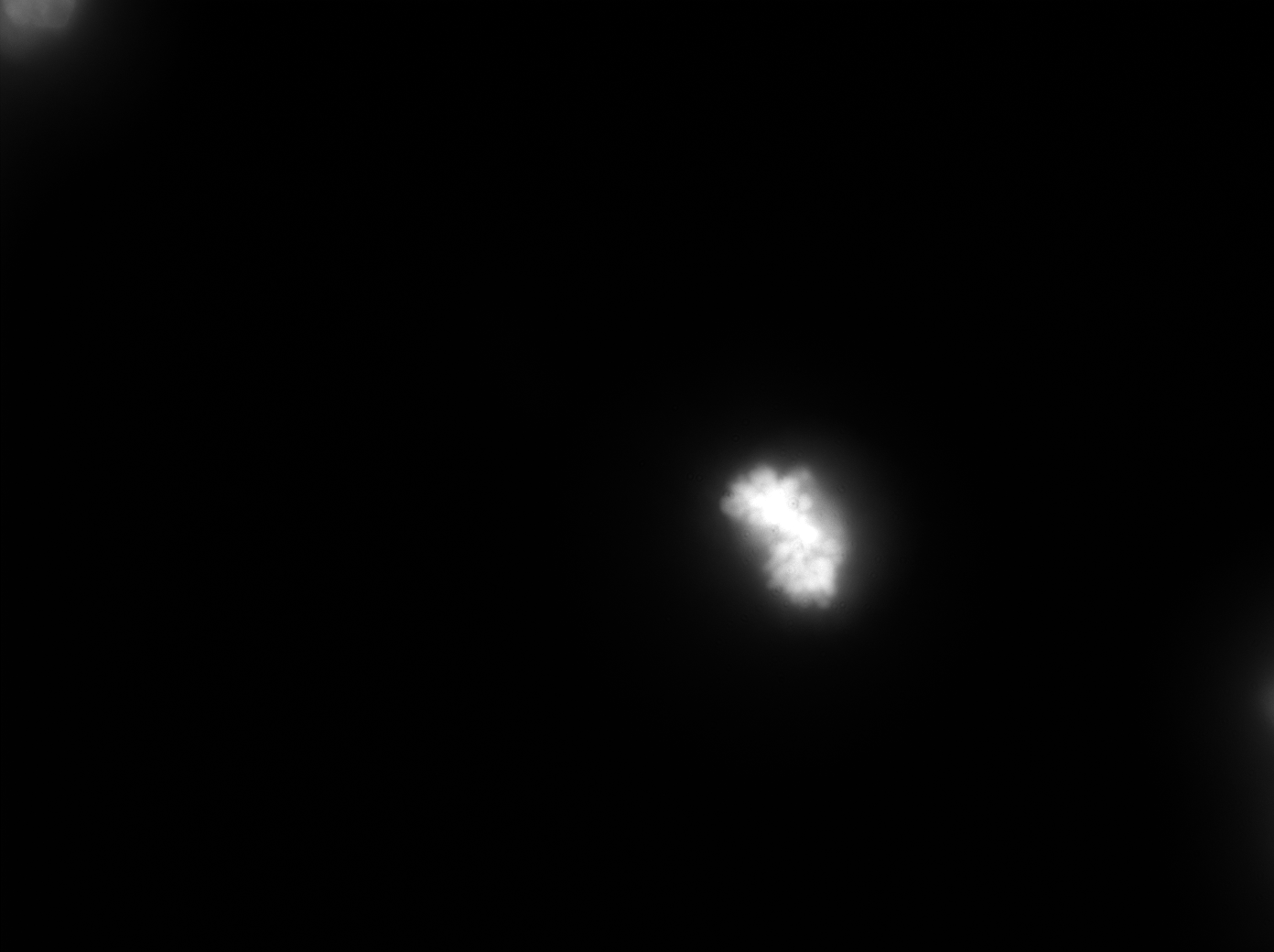

Supplement: Supplementary file 27 — Source data Fig. 7 part 3 [file 44319_2026_742_MOESM27_ESM.zip › Figure 7 Part 3/Fig 7be Cas9 and TPGS1-KO rGT335 atubulin/retakes/Cas9 5-2-25 rGT335 atub R1.1 M3.Project Maximum Z_XY1747763241_Z0_T0_C0.tif]

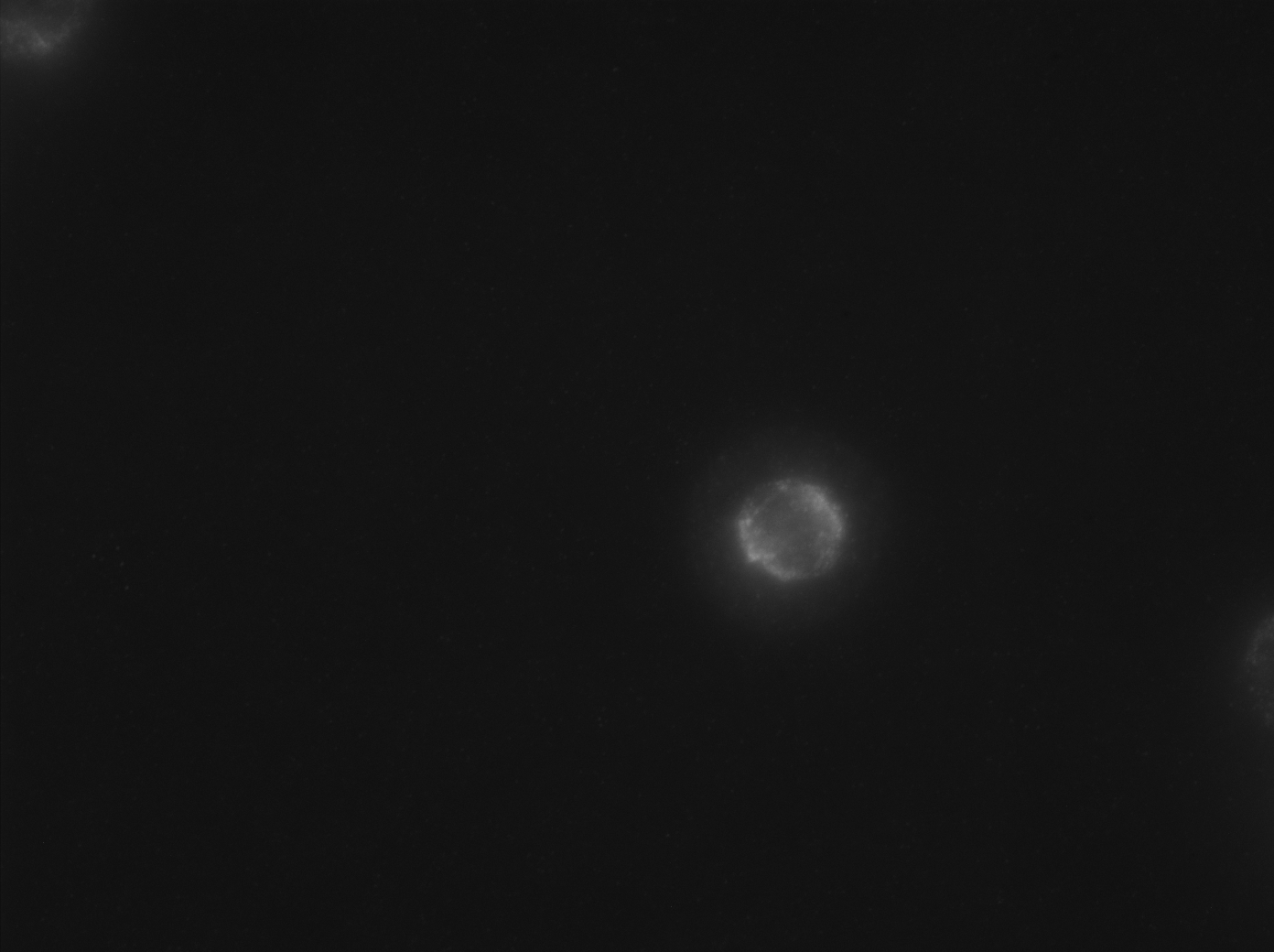

Supplement: Supplementary file 27 — Source data Fig. 7 part 3 [file 44319_2026_742_MOESM27_ESM.zip › Figure 7 Part 3/Fig 7be Cas9 and TPGS1-KO rGT335 atubulin/retakes/Cas9 5-2-25 rGT335 atub R1.1 M3.Project Maximum Z_XY1747763241_Z0_T0_C2.tif]

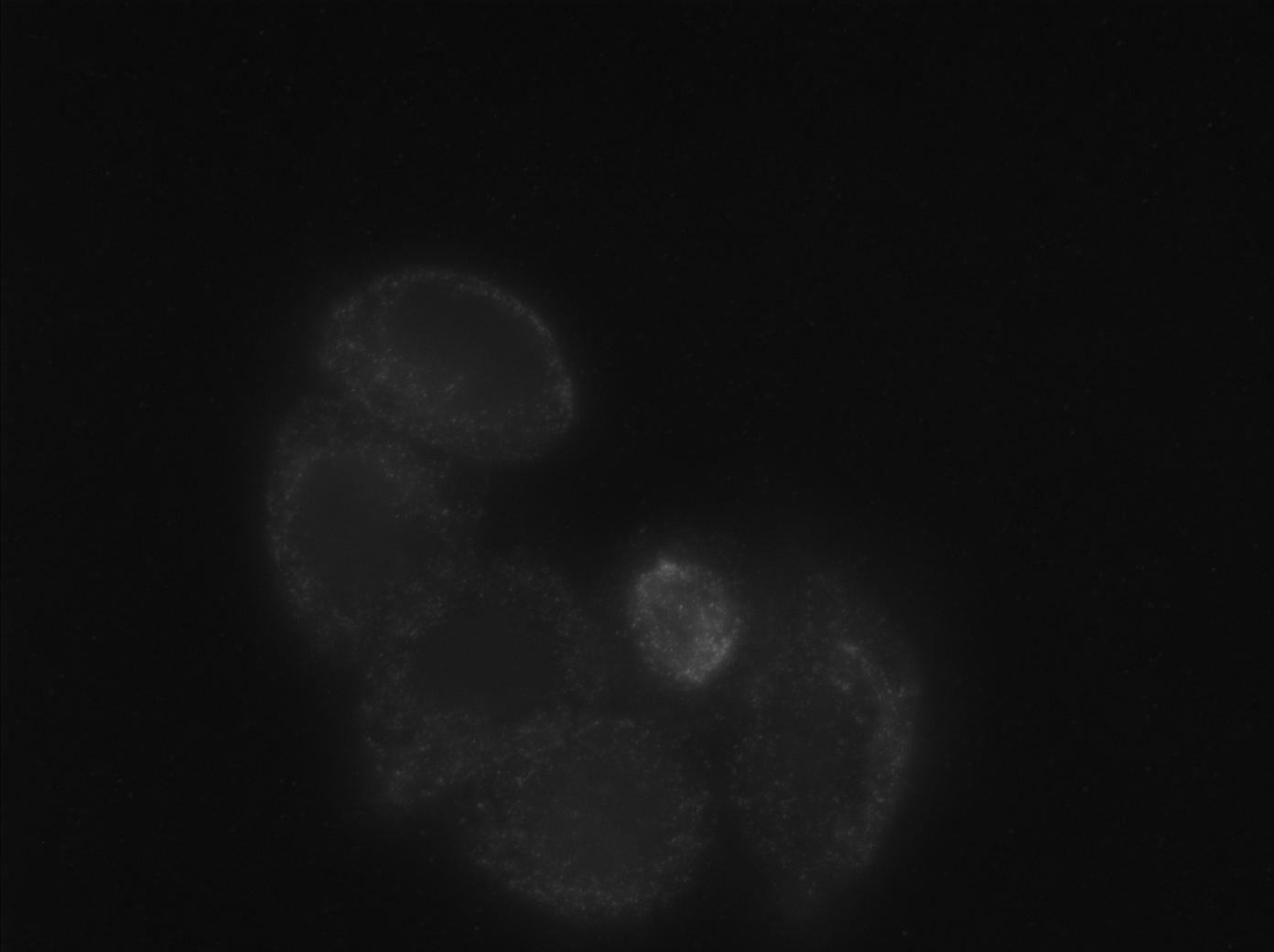

Supplement: Supplementary file 27 — Source data Fig. 7 part 3 [file 44319_2026_742_MOESM27_ESM.zip › Figure 7 Part 3/Fig 7be Cas9 and TPGS1-KO rGT335 atubulin/retakes/Cas9 5-2-25 rGT335 atub R1.2 M8.Project Maximum Z_XY1747768813_Z0_T0_C2.tif]

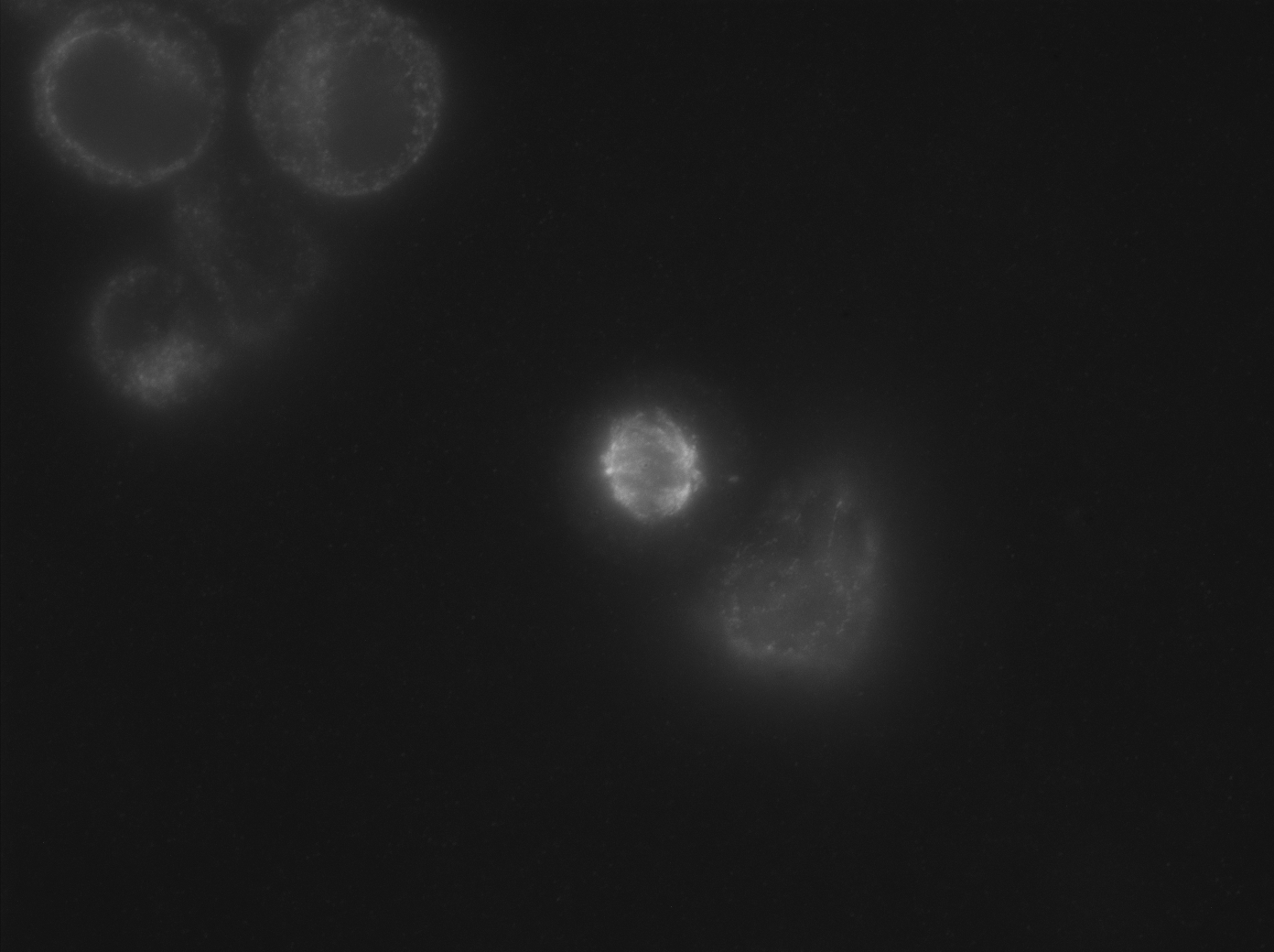

Supplement: Supplementary file 27 — Source data Fig. 7 part 3 [file 44319_2026_742_MOESM27_ESM.zip › Figure 7 Part 3/Fig 7be Cas9 and TPGS1-KO rGT335 atubulin/retakes/Cas9 5-2-25 rGT335 atub R1.1 M4.Project Maximum Z_XY1747763430_Z0_T0_C2.tif]

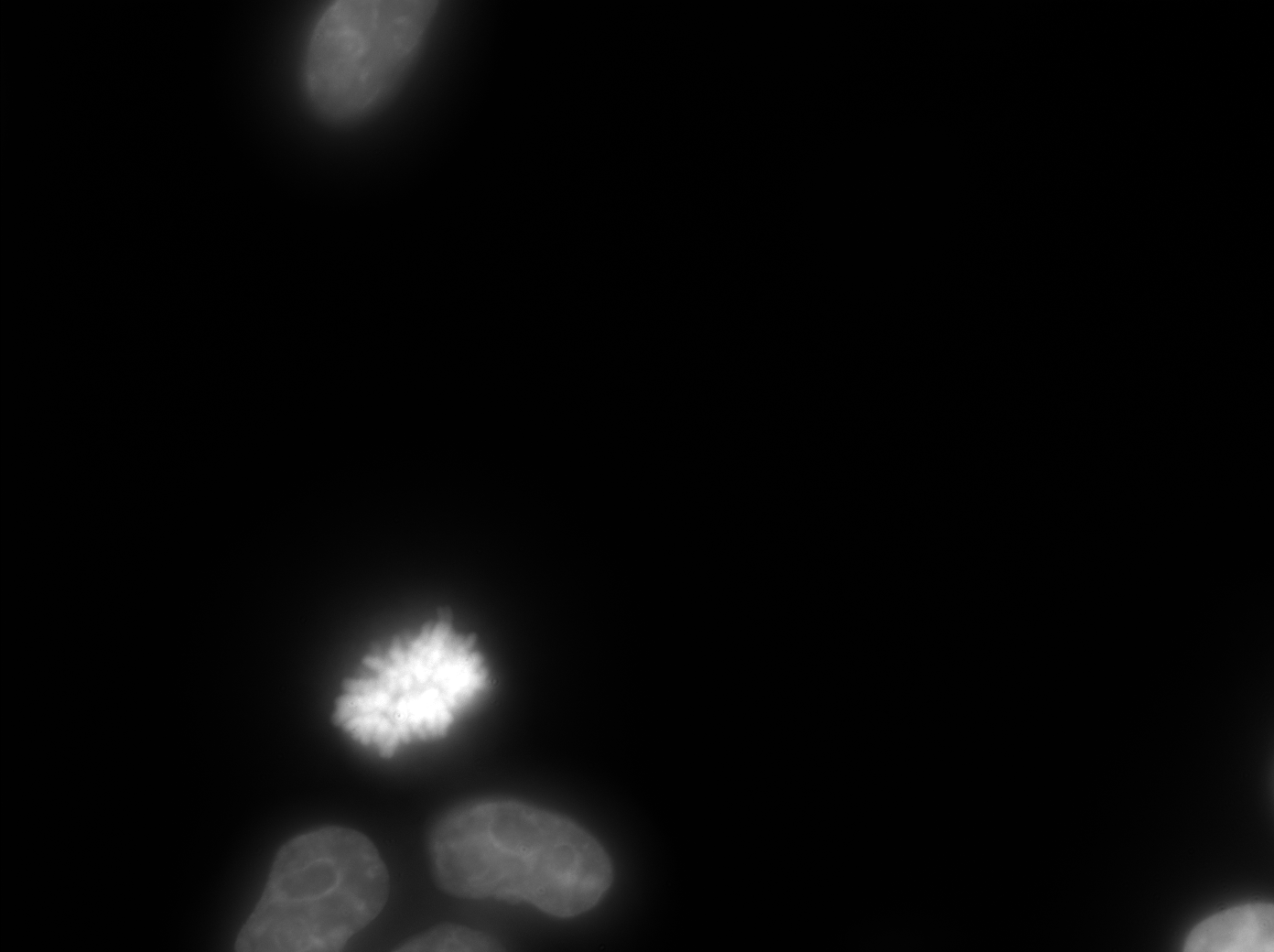

Supplement: Supplementary file 27 — Source data Fig. 7 part 3 [file 44319_2026_742_MOESM27_ESM.zip › Figure 7 Part 3/Fig 7be Cas9 and TPGS1-KO rGT335 atubulin/retakes/Cas9 5-2-25 rGT335 atub R1.2 M2.Project Maximum Z_XY1747767622_Z0_T0_C0.tif]

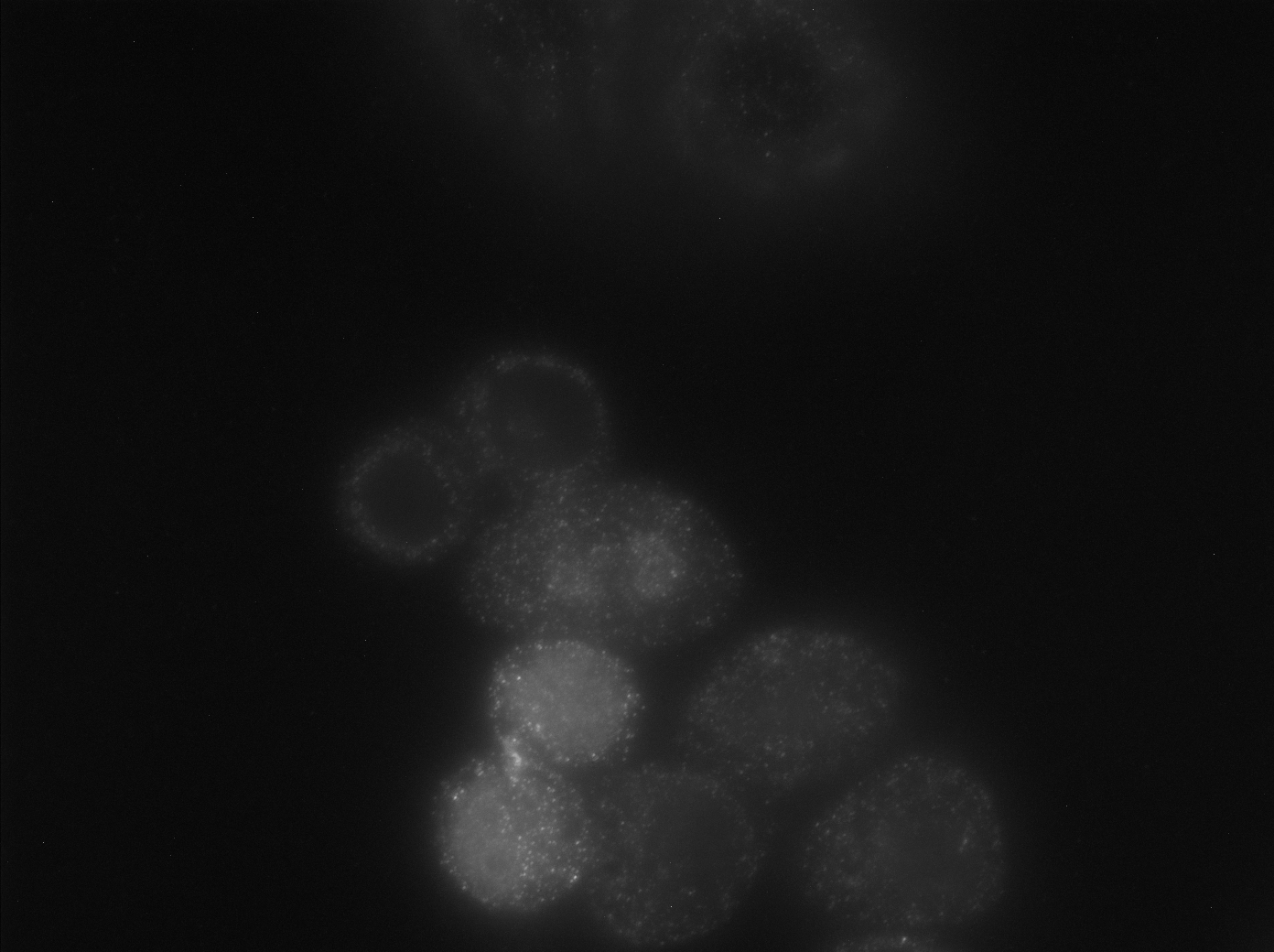

Supplement: Supplementary file 27 — Source data Fig. 7 part 3 [file 44319_2026_742_MOESM27_ESM.zip › Figure 7 Part 3/Fig 7be Cas9 and TPGS1-KO rGT335 atubulin/retakes/Cas9 5-2-25 rGT335 atub R1.2 M4.Project Maximum Z_XY1747767883_Z0_T0_C1.tif]

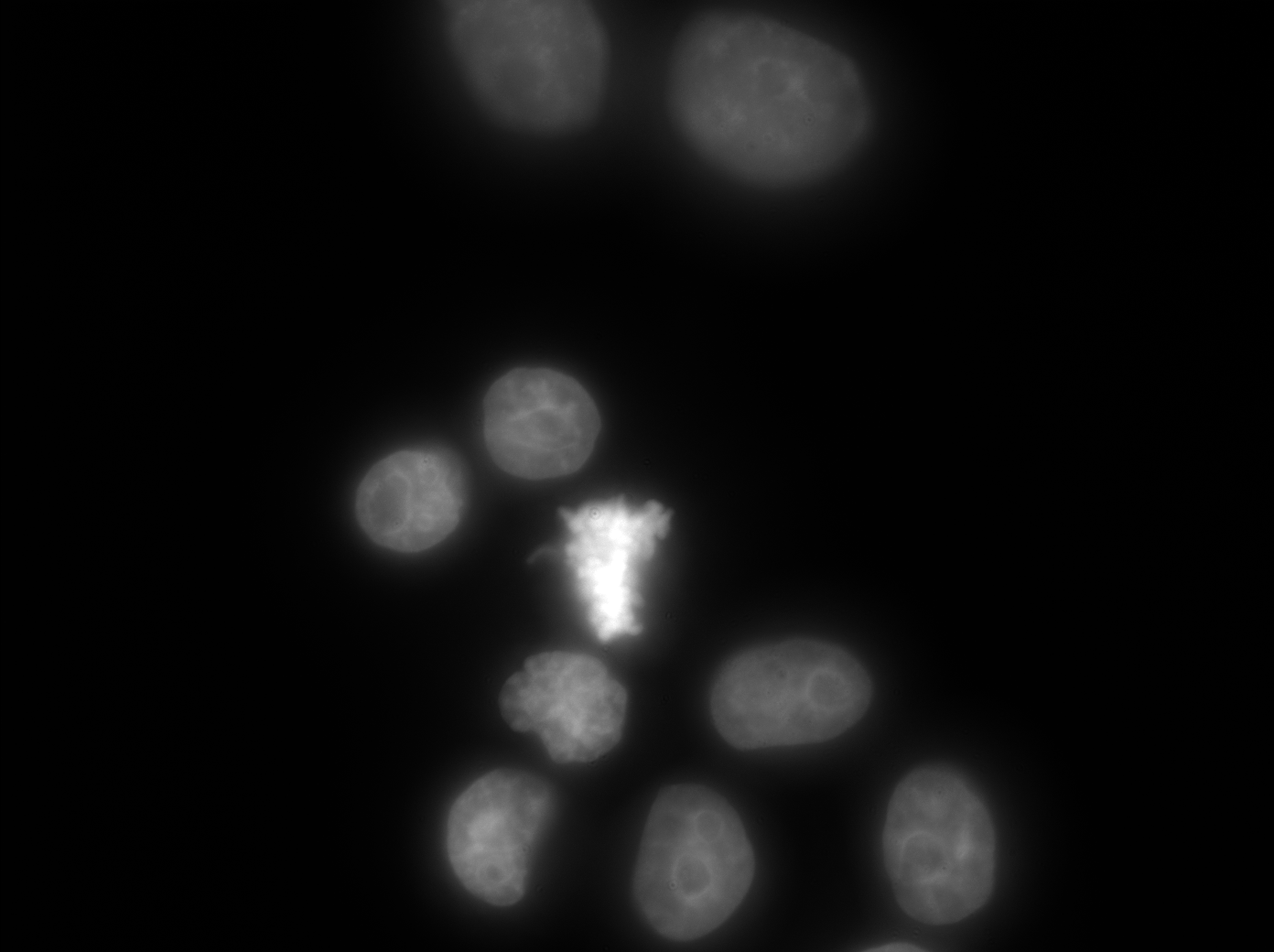

Supplement: Supplementary file 27 — Source data Fig. 7 part 3 [file 44319_2026_742_MOESM27_ESM.zip › Figure 7 Part 3/Fig 7be Cas9 and TPGS1-KO rGT335 atubulin/retakes/Cas9 5-2-25 rGT335 atub R1.2 M4.Project Maximum Z_XY1747767883_Z0_T0_C0.tif]

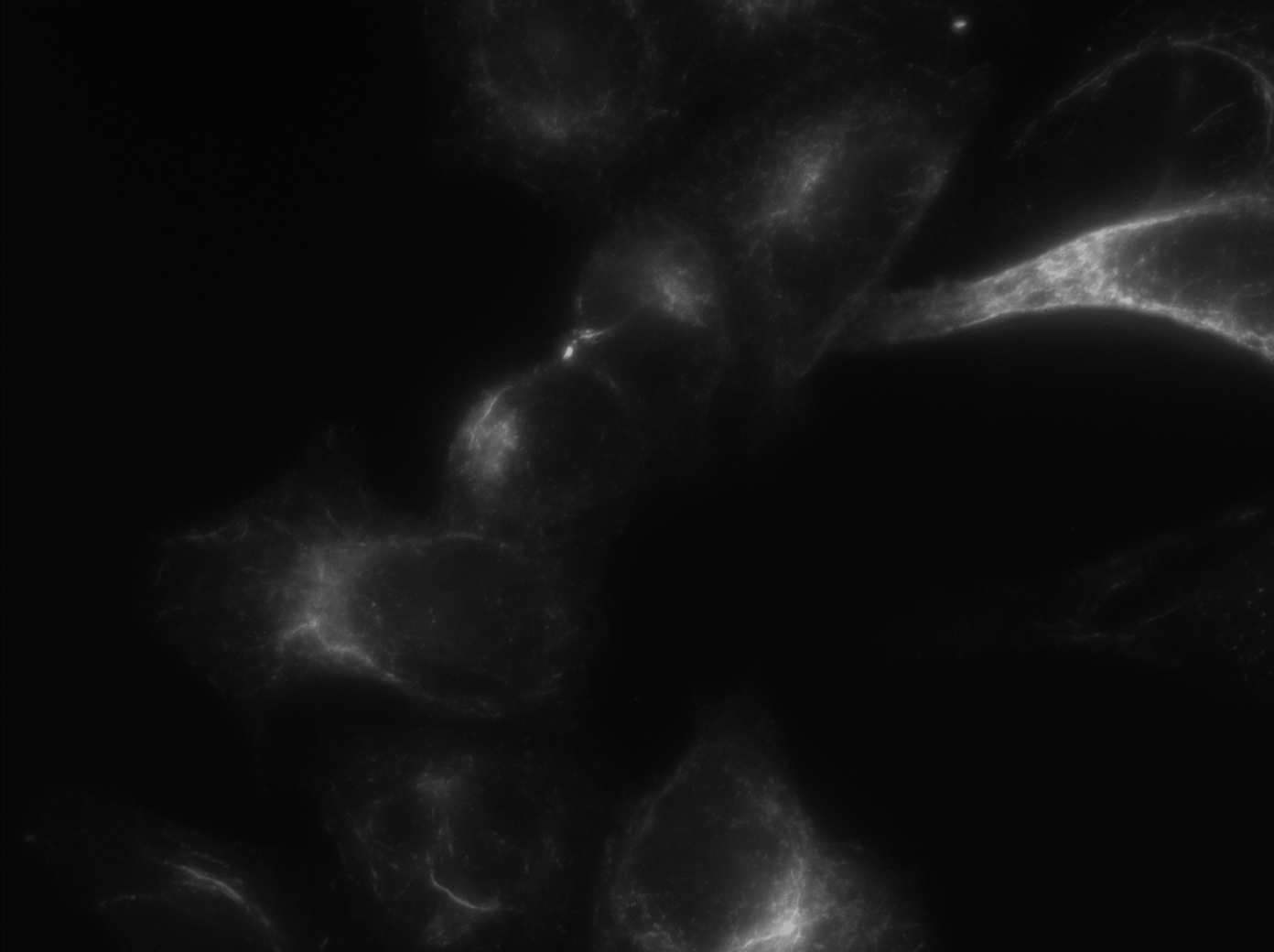

Supplement: Supplementary file 28 — Source data Fig. 7 part 4 [file 44319_2026_742_MOESM28_ESM.zip › Figure 7 Part 4/Fig 7fg Control and TPGS1-KO spastin acetylated tubulin/Cas9 spastin actub 4-1-25 R1 SI12.Project Maximum Z_XY1743531982_Z0_T0_C2.tif]

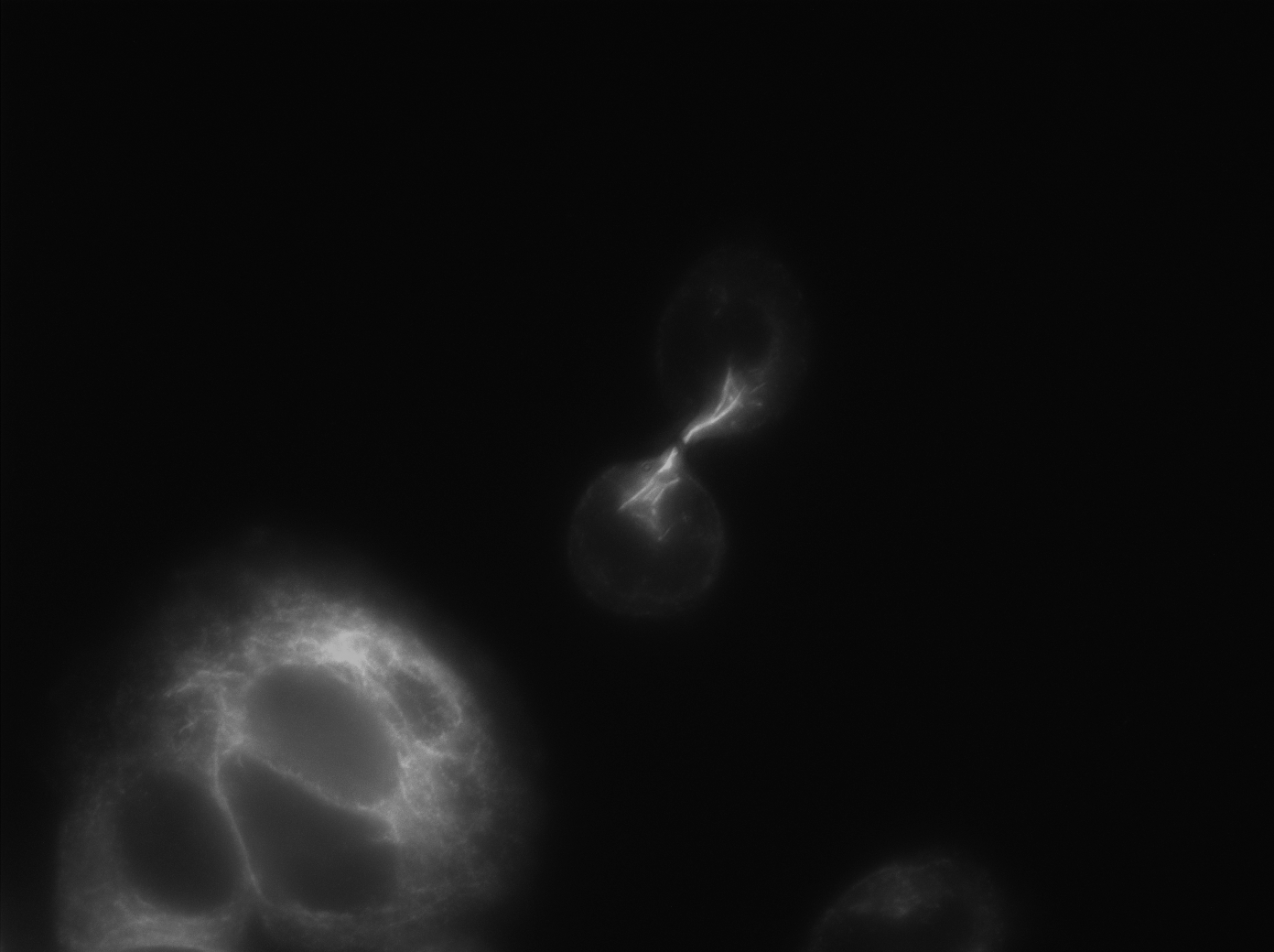

Supplement: Supplementary file 28 — Source data Fig. 7 part 4 [file 44319_2026_742_MOESM28_ESM.zip › Figure 7 Part 4/Fig 7fg Control and TPGS1-KO spastin acetylated tubulin/TPGS1-KO spastin actub 4-1-25 R1 SI16.Project Maximum Z_XY1743538181_Z0_T0_C2.tif]

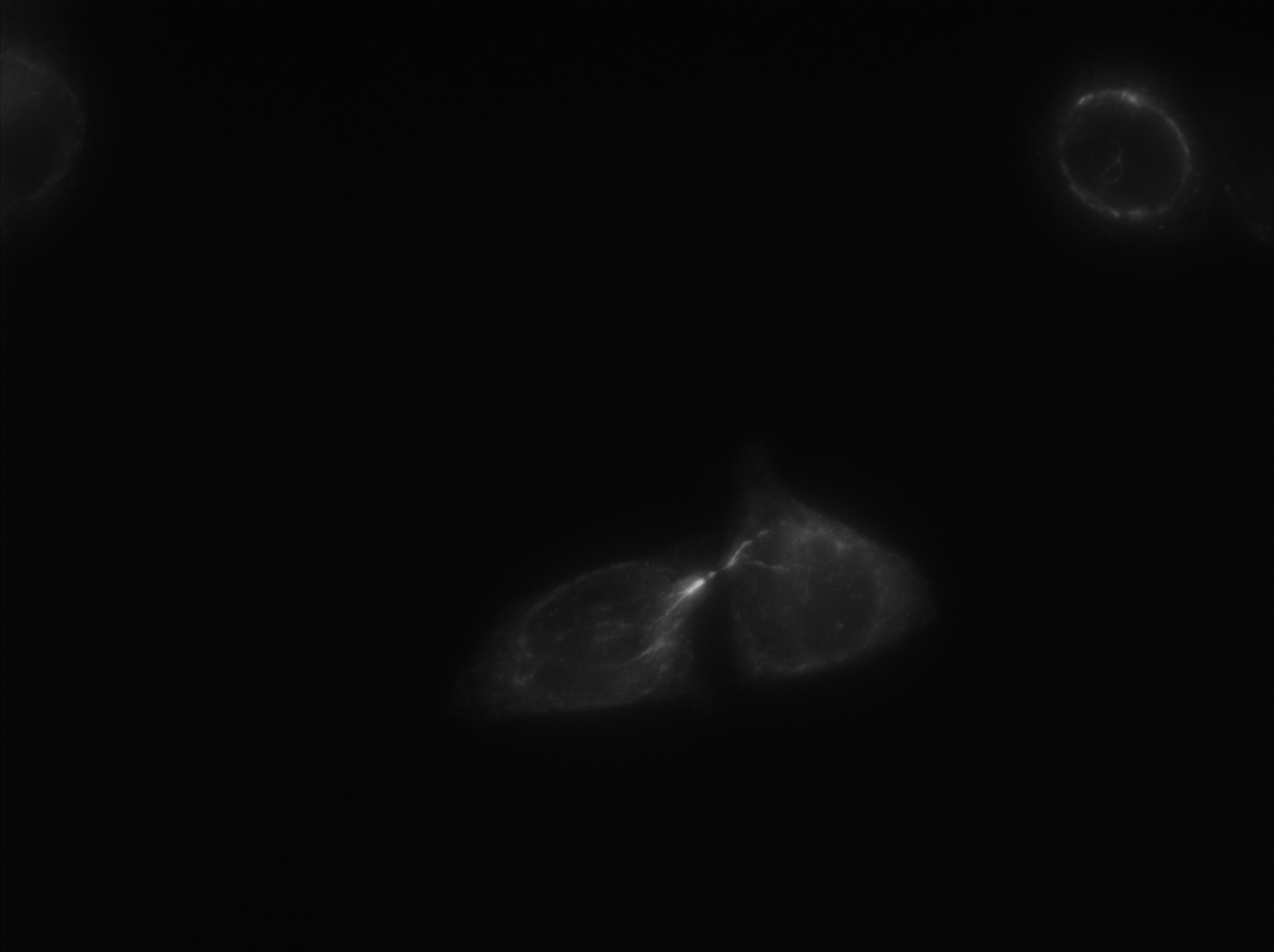

Supplement: Supplementary file 28 — Source data Fig. 7 part 4 [file 44319_2026_742_MOESM28_ESM.zip › Figure 7 Part 4/Fig 7fg Control and TPGS1-KO spastin acetylated tubulin/Cas9 spastin actub 4-1-25 R1 SI5.Project Maximum Z_XY1743530861_Z0_T0_C2.tif]

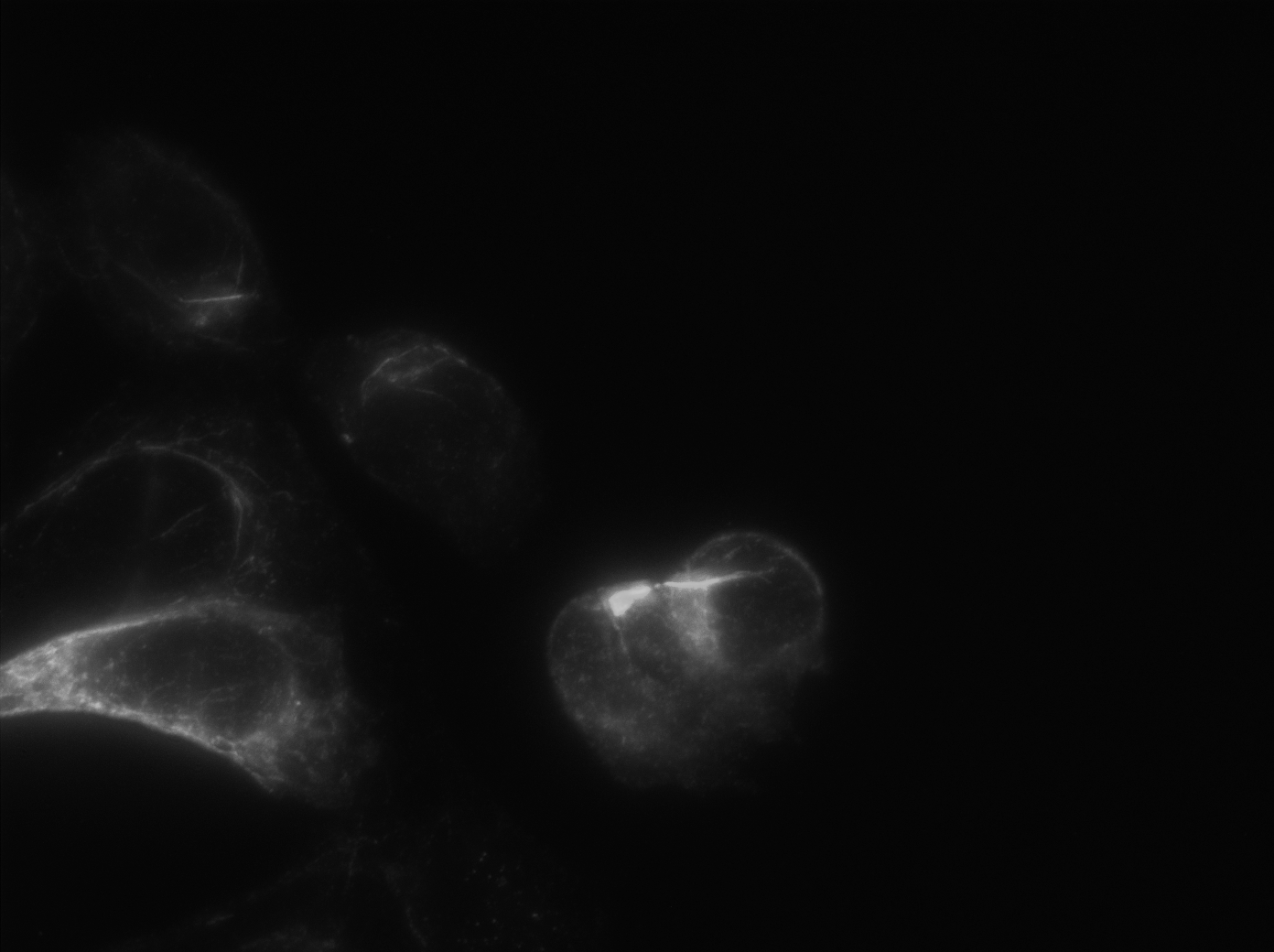

Supplement: Supplementary file 28 — Source data Fig. 7 part 4 [file 44319_2026_742_MOESM28_ESM.zip › Figure 7 Part 4/Fig 7fg Control and TPGS1-KO spastin acetylated tubulin/Cas9 spastin actub 4-1-25 R1 SI13.Project Maximum Z_XY1743532096_Z0_T0_C2.tif]

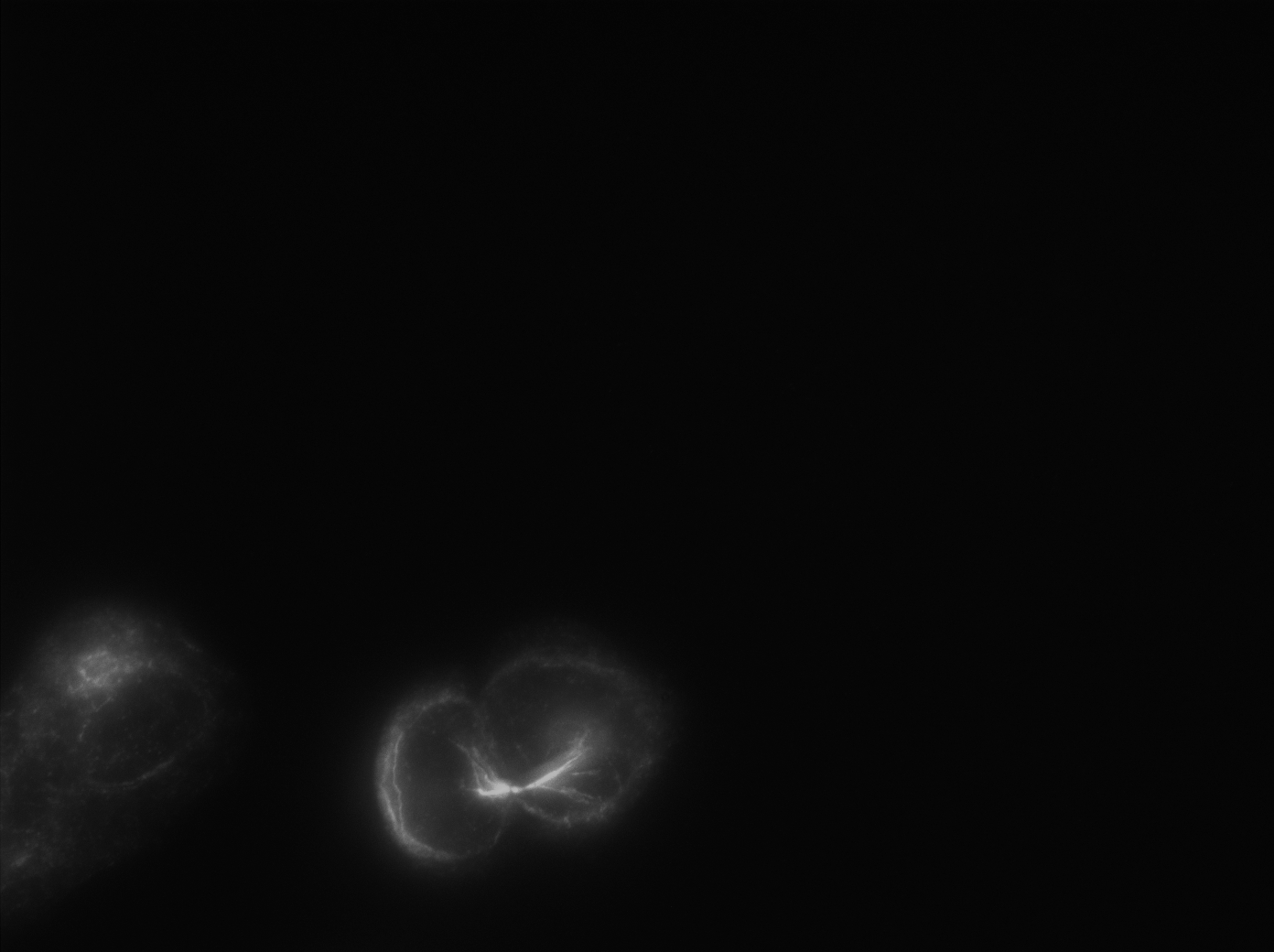

Supplement: Supplementary file 28 — Source data Fig. 7 part 4 [file 44319_2026_742_MOESM28_ESM.zip › Figure 7 Part 4/Fig 7fg Control and TPGS1-KO spastin acetylated tubulin/Cas9 spastin actub 4-1-25 R1 SI11.Project Maximum Z_XY1743531637_Z0_T0_C2.tif]

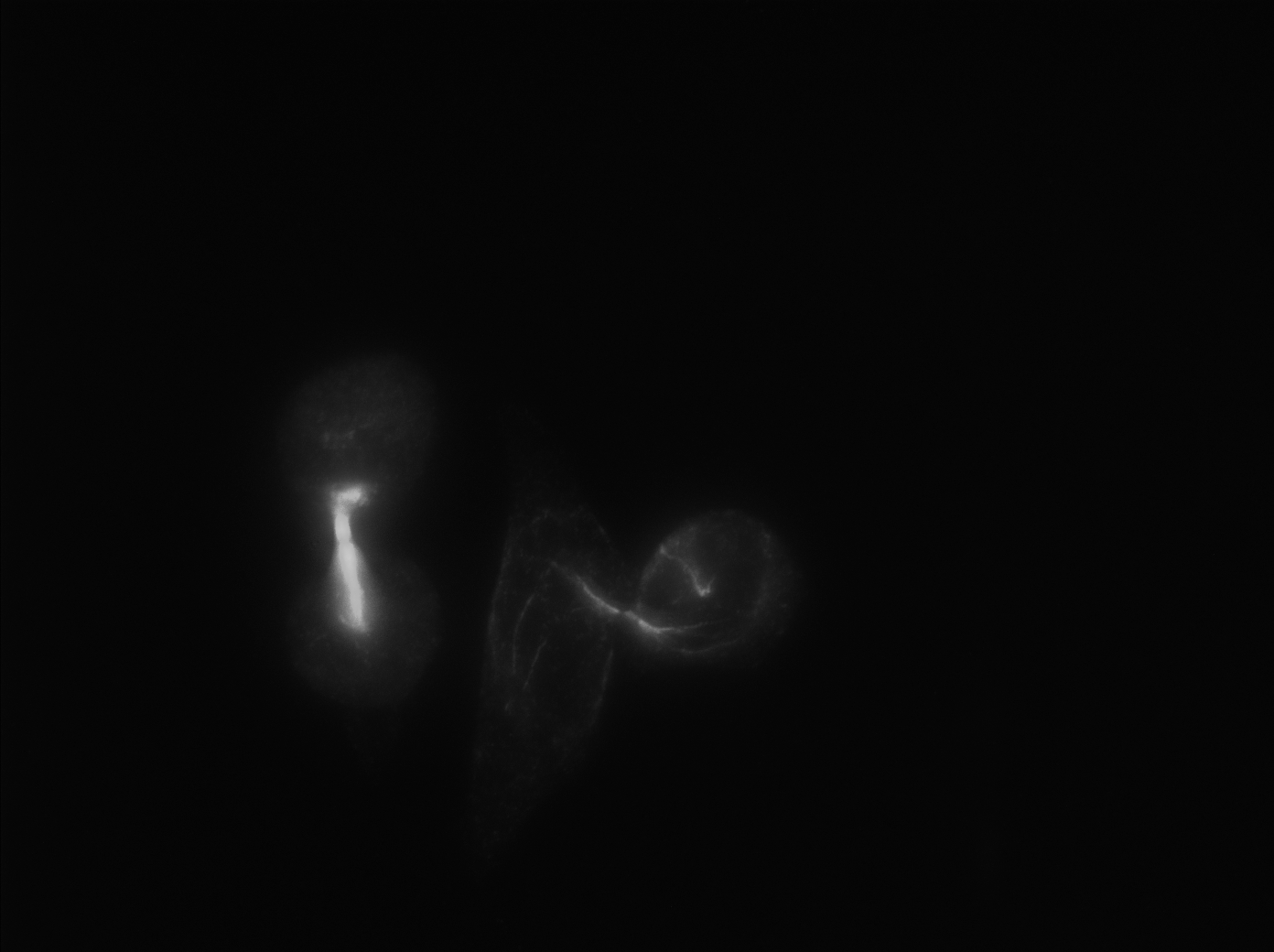

Supplement: Supplementary file 28 — Source data Fig. 7 part 4 [file 44319_2026_742_MOESM28_ESM.zip › Figure 7 Part 4/Fig 7fg Control and TPGS1-KO spastin acetylated tubulin/Cas9 spastin actub 4-1-25 R1 SI18.Project Maximum Z_XY1743534608_Z0_T0_C2.tif]

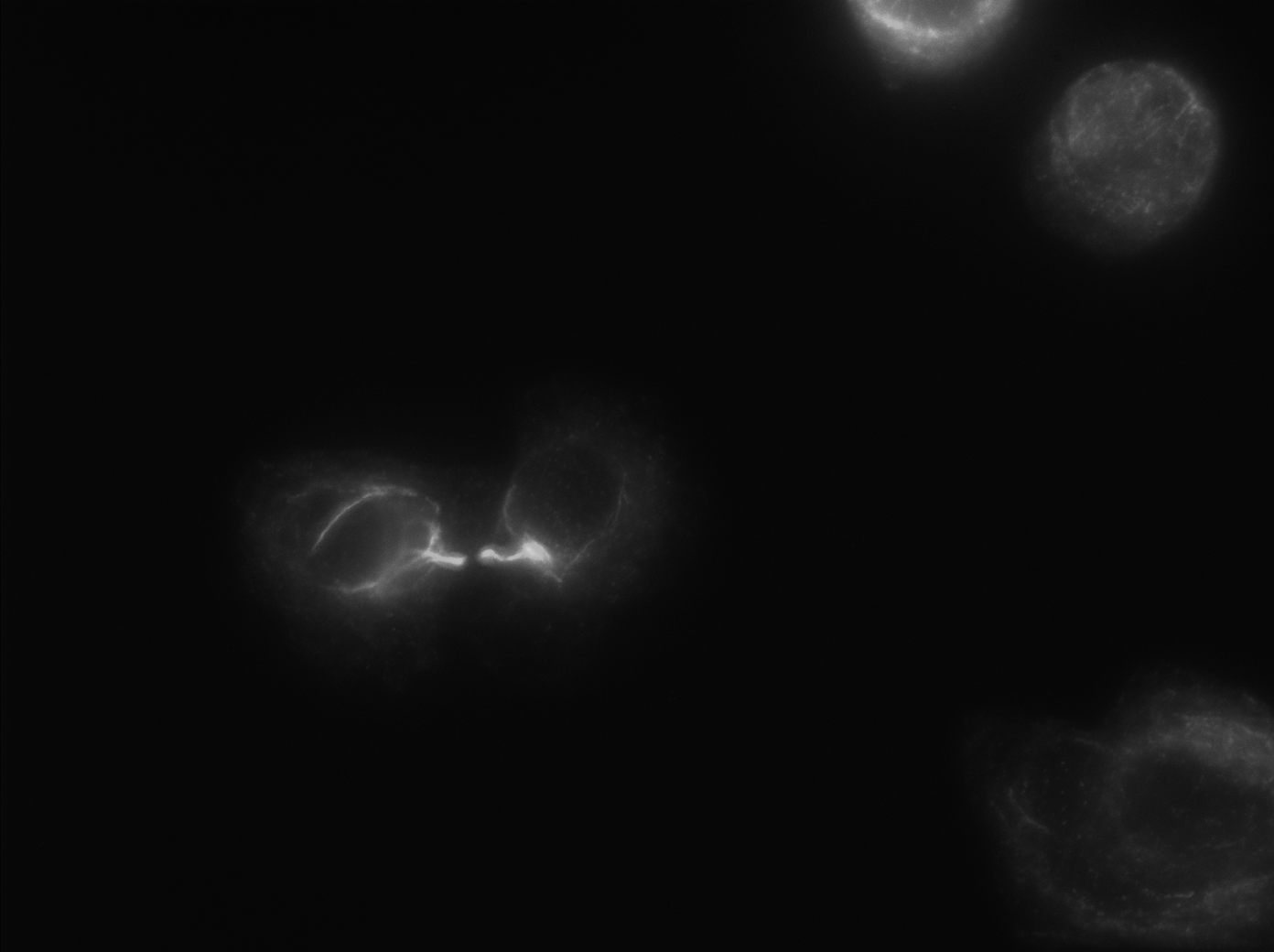

Supplement: Supplementary file 28 — Source data Fig. 7 part 4 [file 44319_2026_742_MOESM28_ESM.zip › Figure 7 Part 4/Fig 7fg Control and TPGS1-KO spastin acetylated tubulin/TPGS1-KO spastin actub 4-1-25 R1 SI9.Project Maximum Z_XY1743537099_Z0_T0_C2.tif]

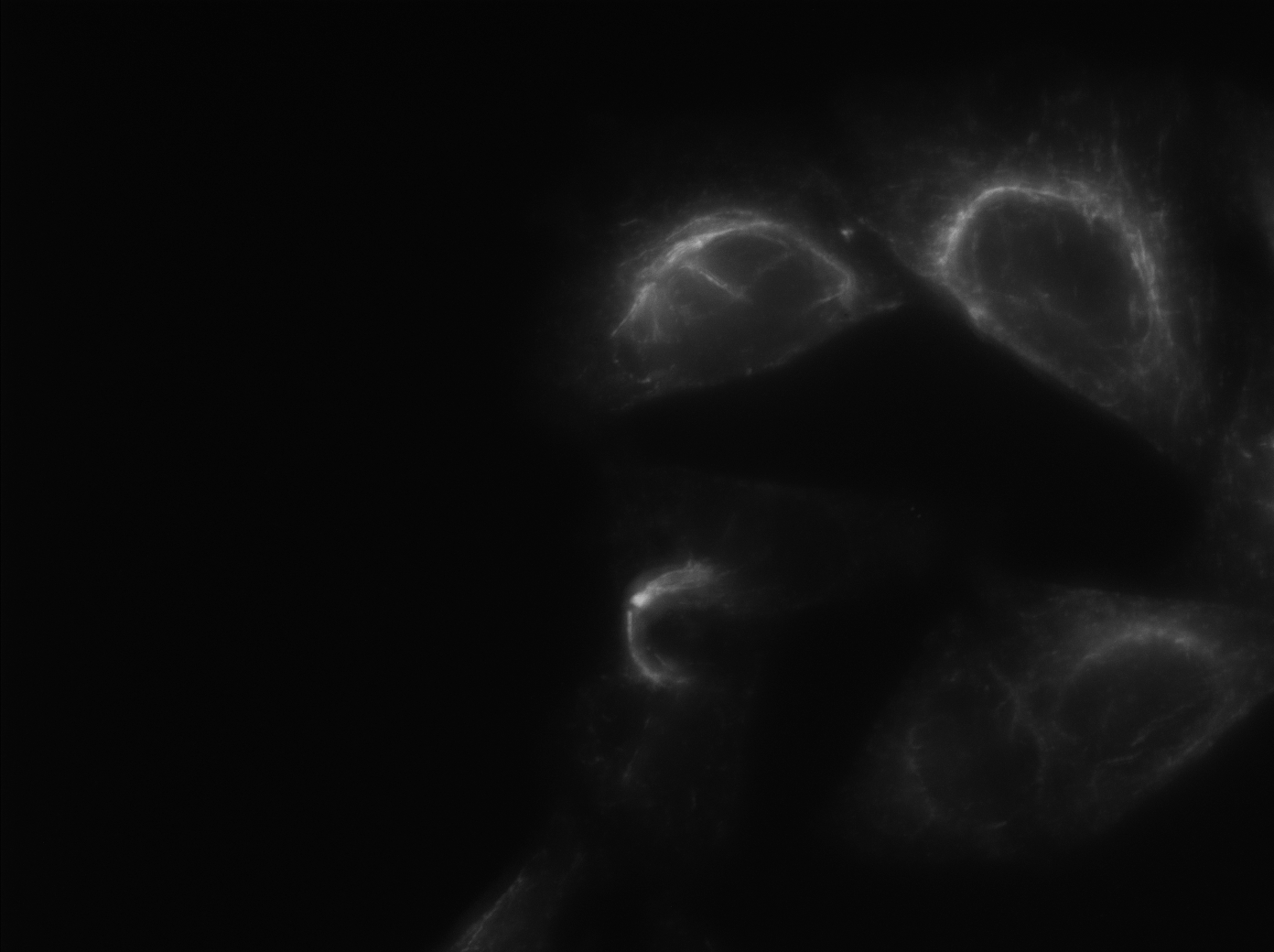

Supplement: Supplementary file 28 — Source data Fig. 7 part 4 [file 44319_2026_742_MOESM28_ESM.zip › Figure 7 Part 4/Fig 7fg Control and TPGS1-KO spastin acetylated tubulin/Cas9 spastin actub 4-1-25 R1 SI21.Project Maximum Z_XY1743535055_Z0_T0_C2.tif]

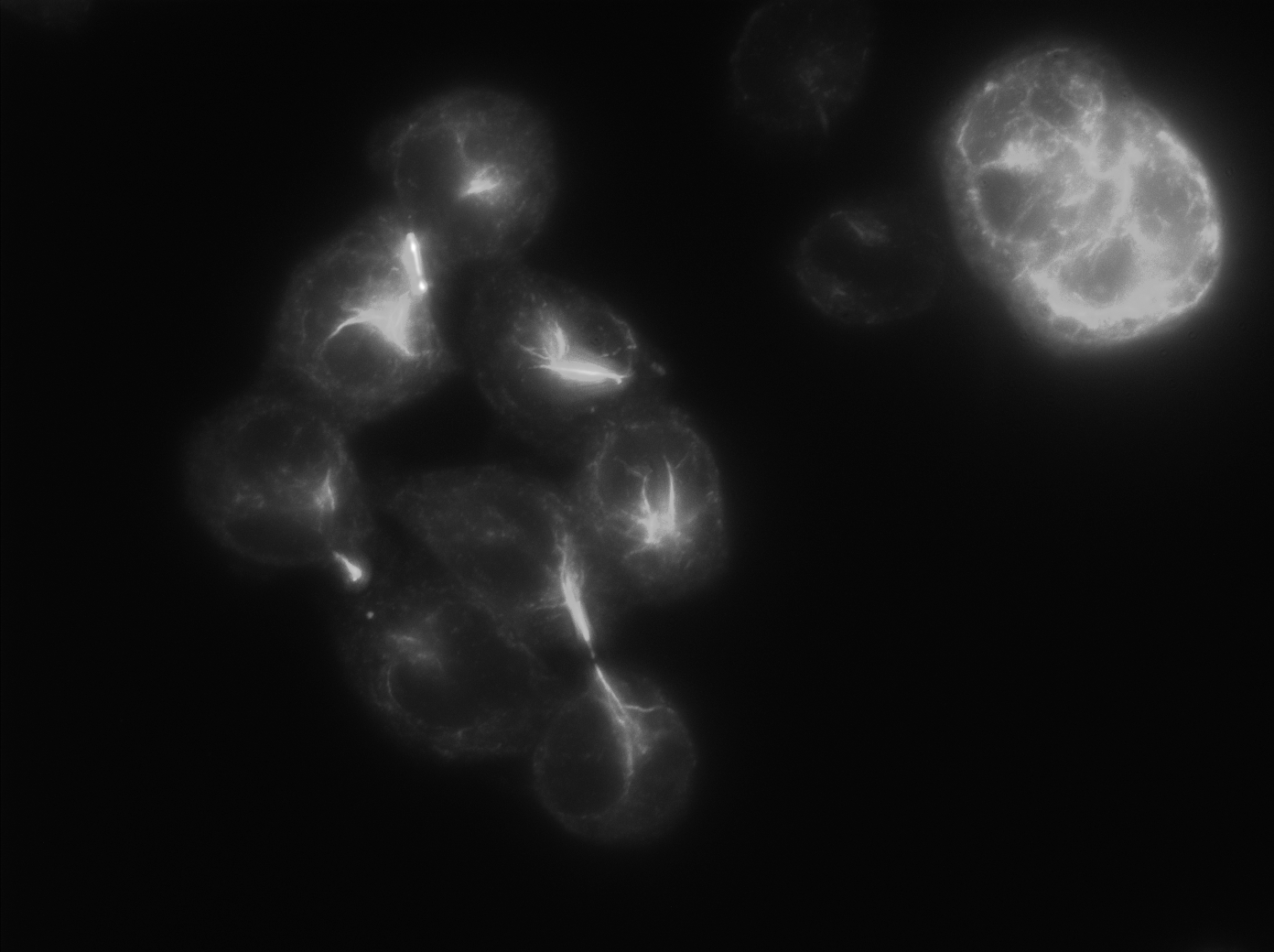

Supplement: Supplementary file 28 — Source data Fig. 7 part 4 [file 44319_2026_742_MOESM28_ESM.zip › Figure 7 Part 4/Fig 7fg Control and TPGS1-KO spastin acetylated tubulin/TPGS1-KO spastin actub 4-1-25 R1 SI11.Project Maximum Z_XY1743537437_Z0_T0_C2.tif]

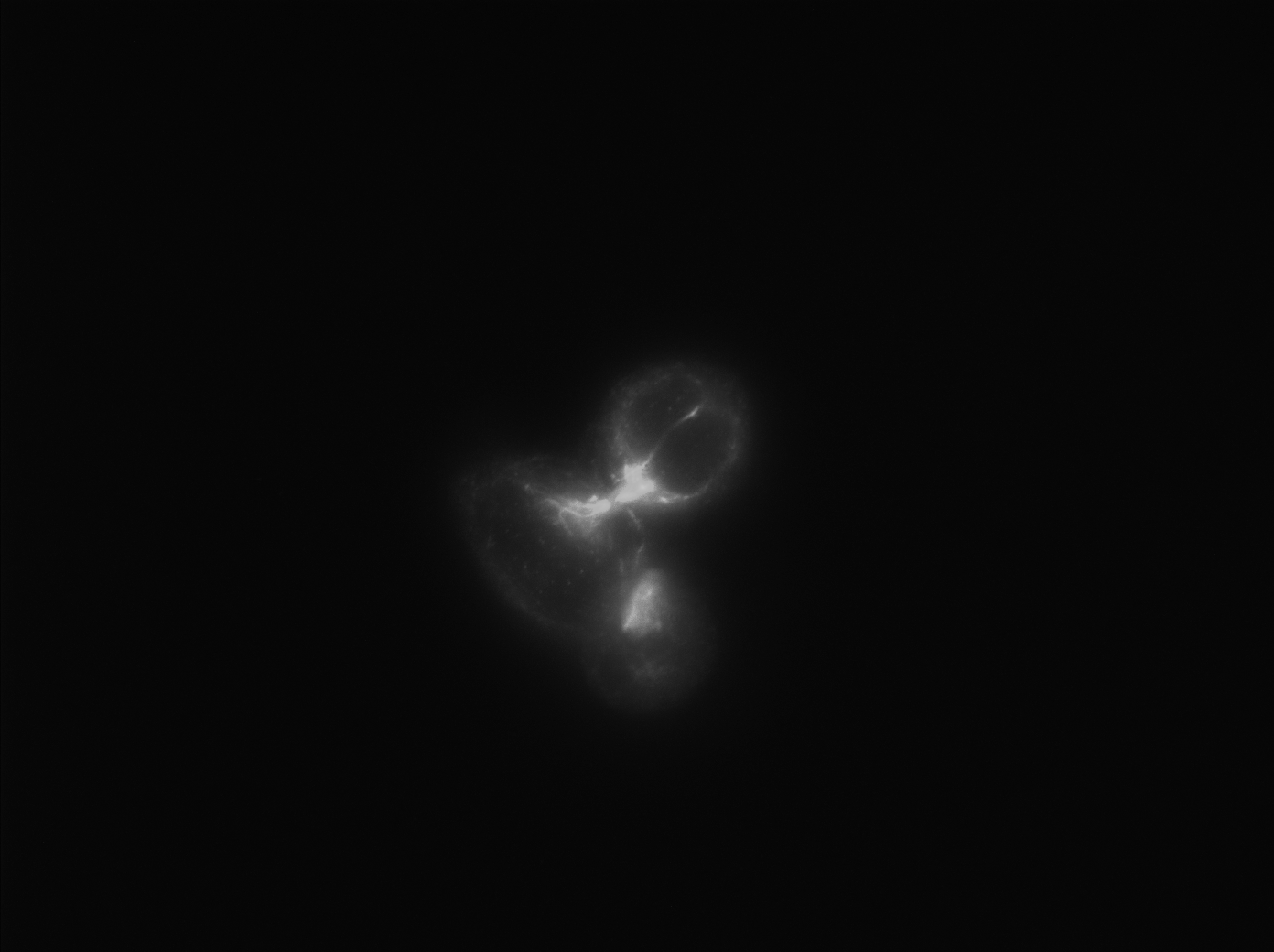

Supplement: Supplementary file 28 — Source data Fig. 7 part 4 [file 44319_2026_742_MOESM28_ESM.zip › Figure 7 Part 4/Fig 7fg Control and TPGS1-KO spastin acetylated tubulin/TPGS1-KO spastin actub 4-1-25 R1 SI12.Project Maximum Z_XY1743537613_Z0_T0_C2.tif]

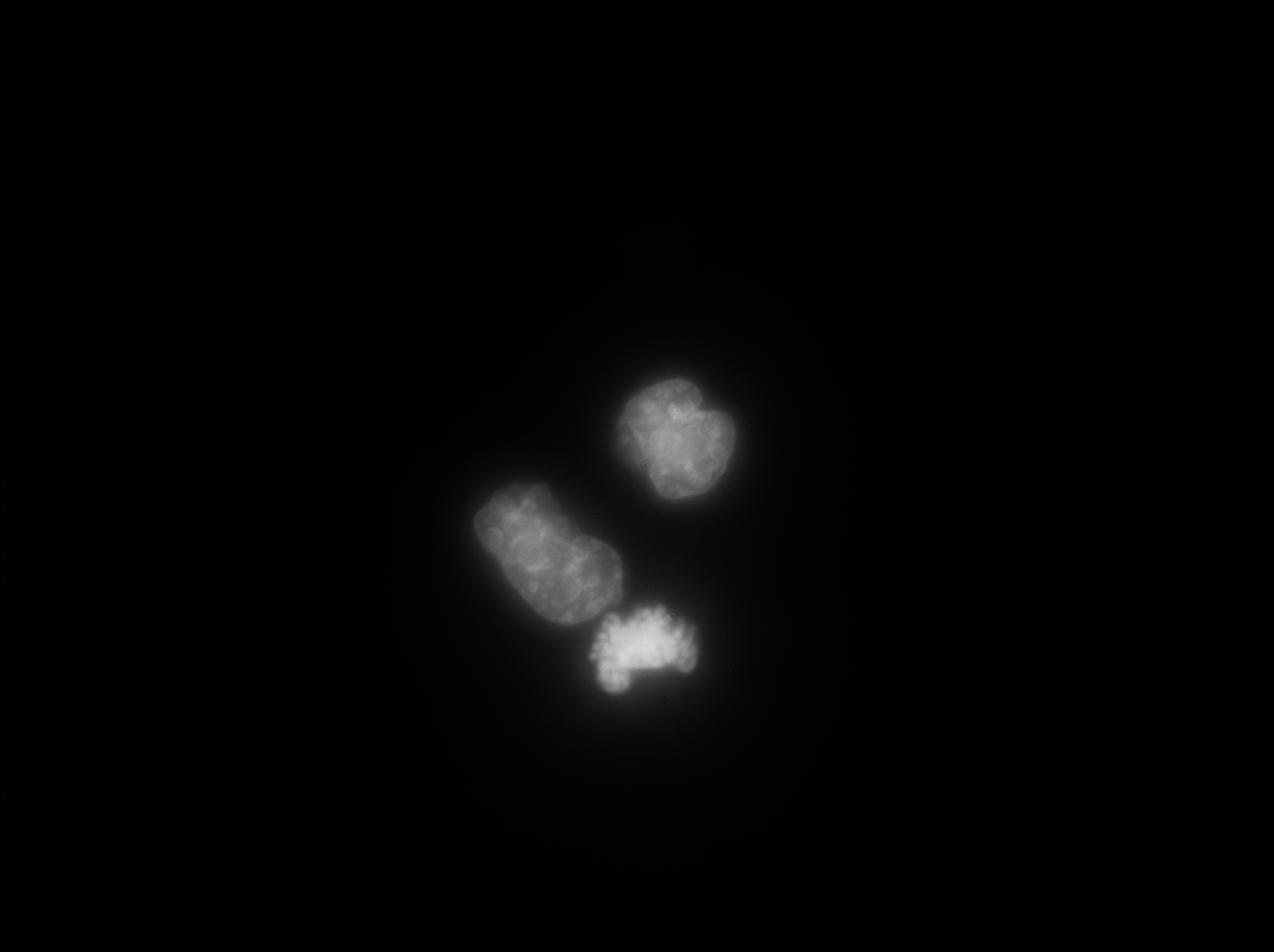

Supplement: Supplementary file 28 — Source data Fig. 7 part 4 [file 44319_2026_742_MOESM28_ESM.zip › Figure 7 Part 4/Fig 7fg Control and TPGS1-KO spastin acetylated tubulin/TPGS1-KO spastin actub 4-1-25 R1 SI12.Project Maximum Z_XY1743537613_Z0_T0_C0.tif]

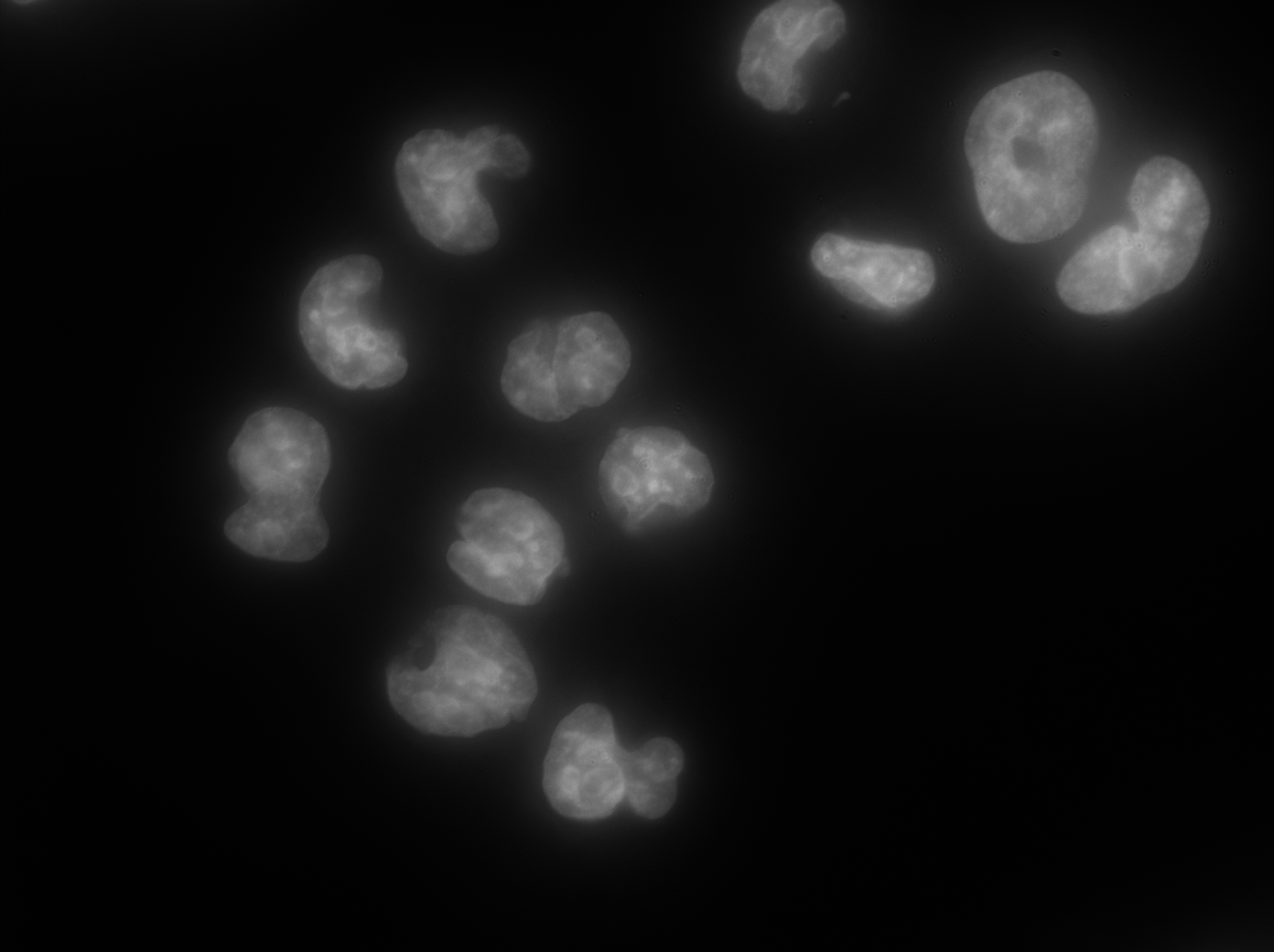

Supplement: Supplementary file 28 — Source data Fig. 7 part 4 [file 44319_2026_742_MOESM28_ESM.zip › Figure 7 Part 4/Fig 7fg Control and TPGS1-KO spastin acetylated tubulin/TPGS1-KO spastin actub 4-1-25 R1 SI11.Project Maximum Z_XY1743537437_Z0_T0_C0.tif]

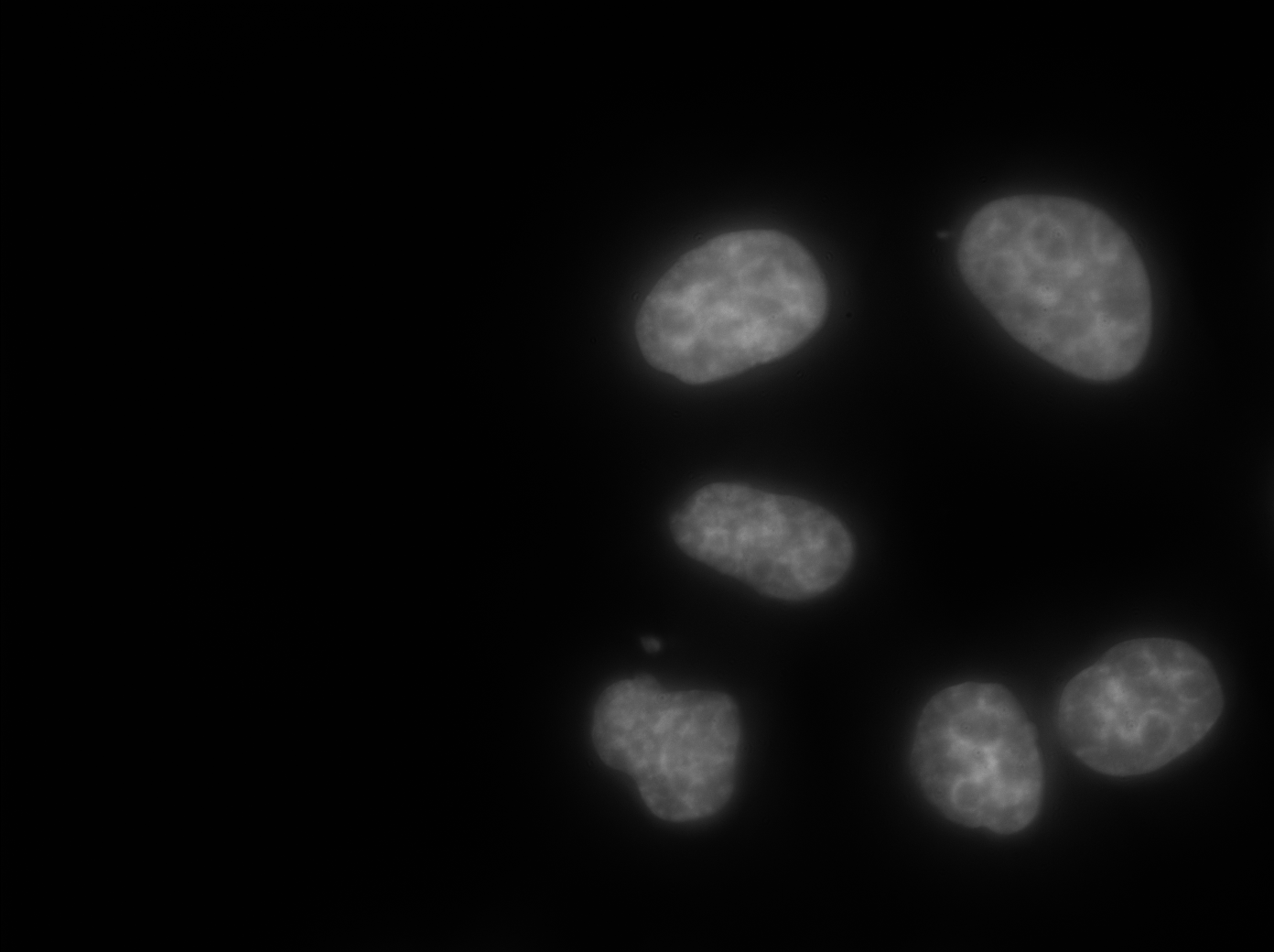

Supplement: Supplementary file 28 — Source data Fig. 7 part 4 [file 44319_2026_742_MOESM28_ESM.zip › Figure 7 Part 4/Fig 7fg Control and TPGS1-KO spastin acetylated tubulin/Cas9 spastin actub 4-1-25 R1 SI21.Project Maximum Z_XY1743535055_Z0_T0_C0.tif]

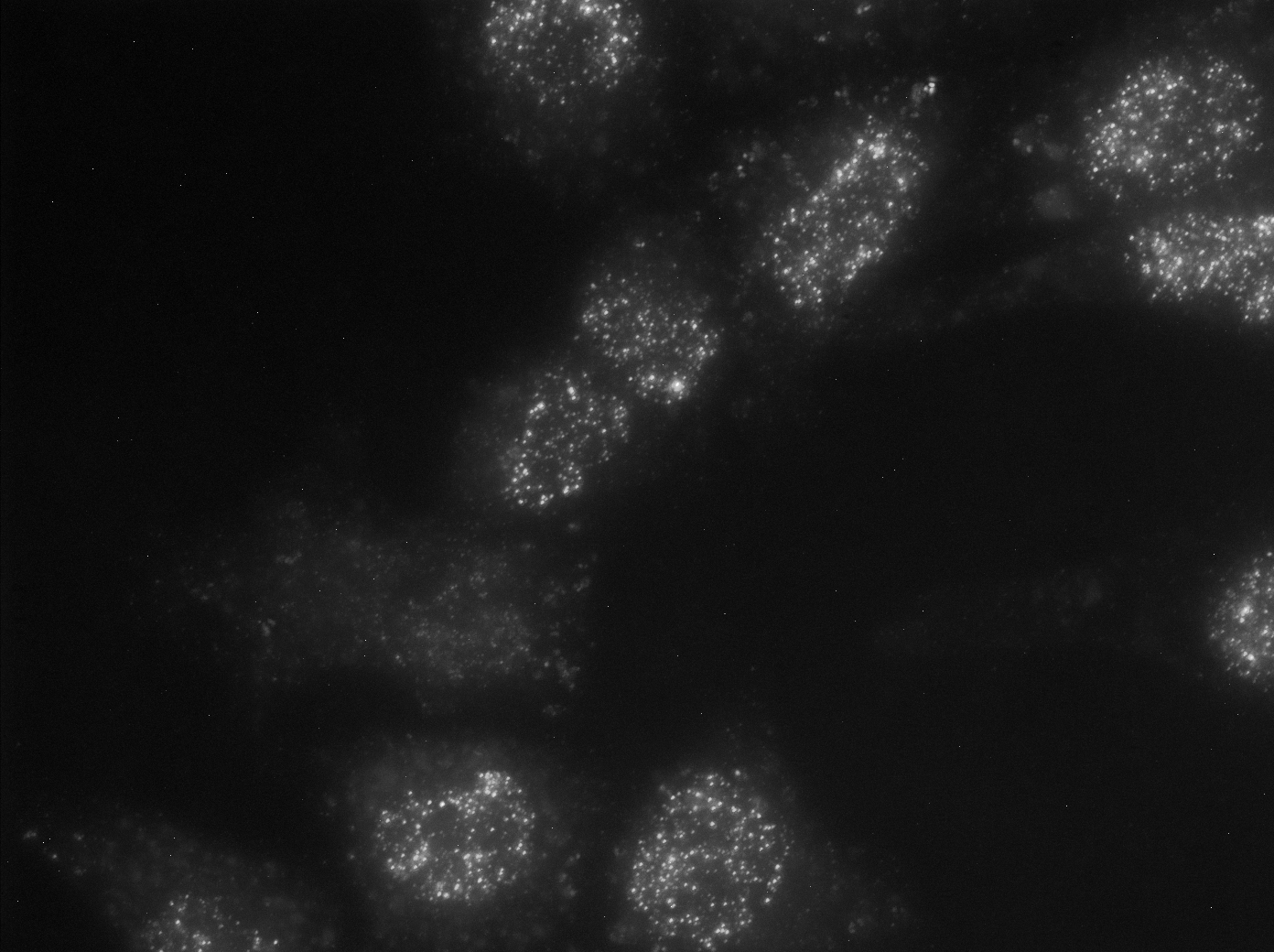

Supplement: Supplementary file 28 — Source data Fig. 7 part 4 [file 44319_2026_742_MOESM28_ESM.zip › Figure 7 Part 4/Fig 7fg Control and TPGS1-KO spastin acetylated tubulin/Cas9 spastin actub 4-1-25 R1 SI12.Project Maximum Z_XY1743531982_Z0_T0_C1.tif]

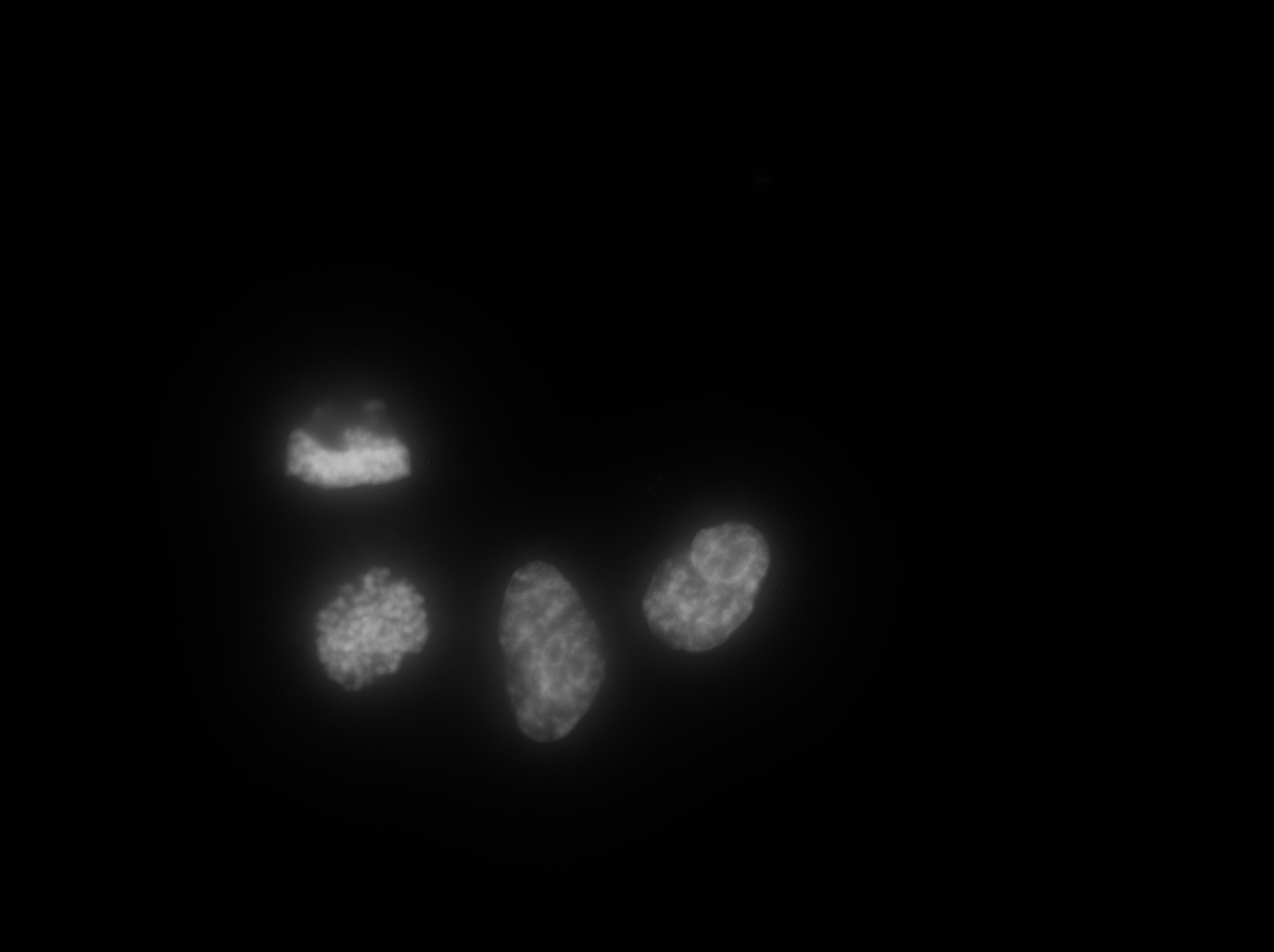

Supplement: Supplementary file 28 — Source data Fig. 7 part 4 [file 44319_2026_742_MOESM28_ESM.zip › Figure 7 Part 4/Fig 7fg Control and TPGS1-KO spastin acetylated tubulin/Cas9 spastin actub 4-1-25 R1 SI18.Project Maximum Z_XY1743534608_Z0_T0_C0.tif]

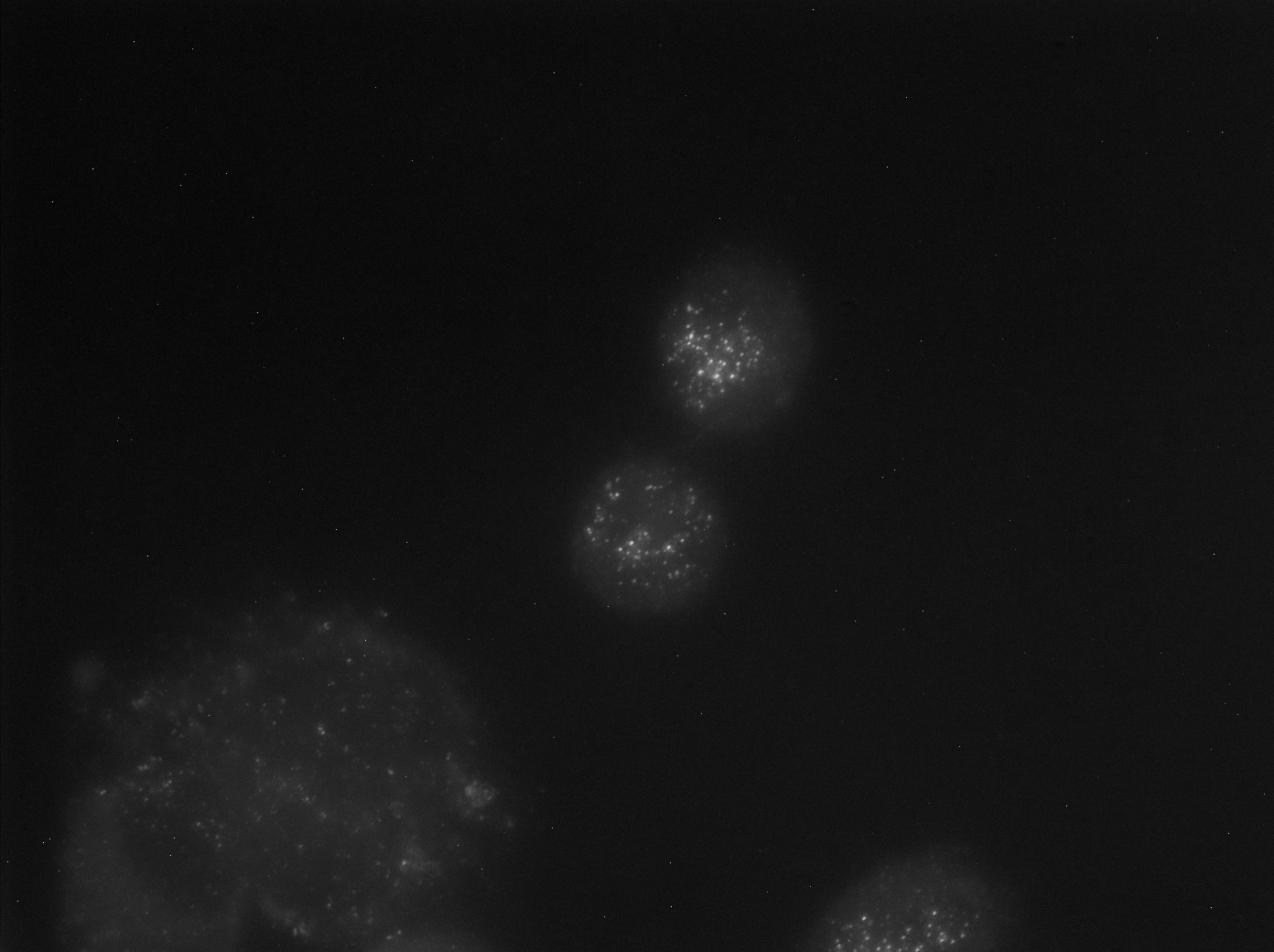

Supplement: Supplementary file 28 — Source data Fig. 7 part 4 [file 44319_2026_742_MOESM28_ESM.zip › Figure 7 Part 4/Fig 7fg Control and TPGS1-KO spastin acetylated tubulin/TPGS1-KO spastin actub 4-1-25 R1 SI16.Project Maximum Z_XY1743538181_Z0_T0_C1.tif]
