# Supplementary figures and images for: TPGS1 regulates central spindle microtubule glutamylation and remodeling during telophase and abscission (part 33 of 36)
Source: EMBO Rep. 2026 Mar 23;27(8):1944–63. doi: 10.1038/s44319-026-00742-3 (PMC13121839; doi:10.1038/s44319-026-00742-3)

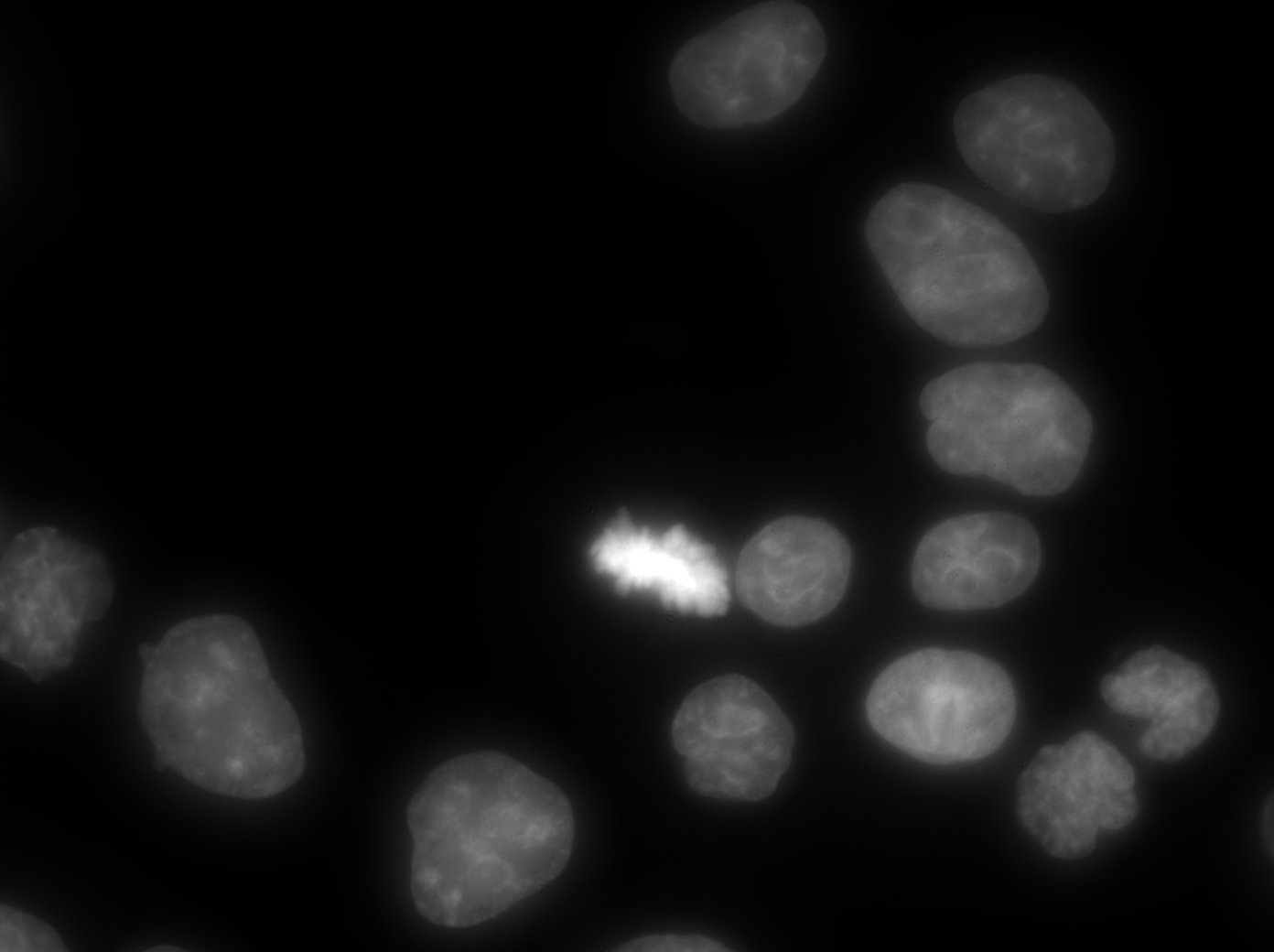

Supplement: Supplementary file 27 — Source data Fig. 7 part 3 [file 44319_2026_742_MOESM27_ESM.zip › Figure 7 Part 3/Fig 7be Cas9 and TPGS1-KO rGT335 atubulin/Cas9 5-2-25 rGT335 atub R3 M3.Project Maximum Z_XY1746214431_Z0_T0_C0.tif]

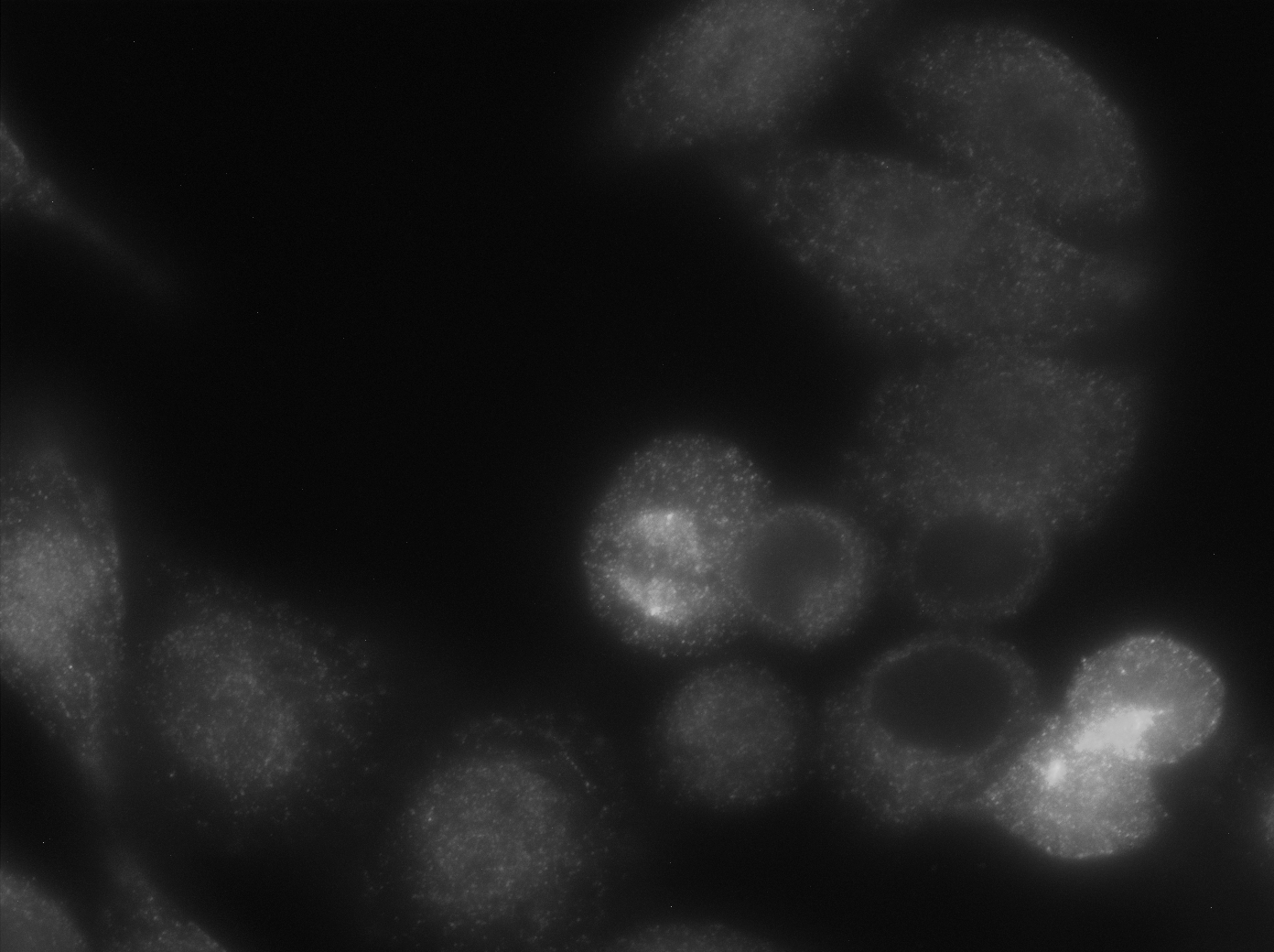

Supplement: Supplementary file 27 — Source data Fig. 7 part 3 [file 44319_2026_742_MOESM27_ESM.zip › Figure 7 Part 3/Fig 7be Cas9 and TPGS1-KO rGT335 atubulin/Cas9 5-2-25 rGT335 atub R3 M3.Project Maximum Z_XY1746214431_Z0_T0_C1.tif]

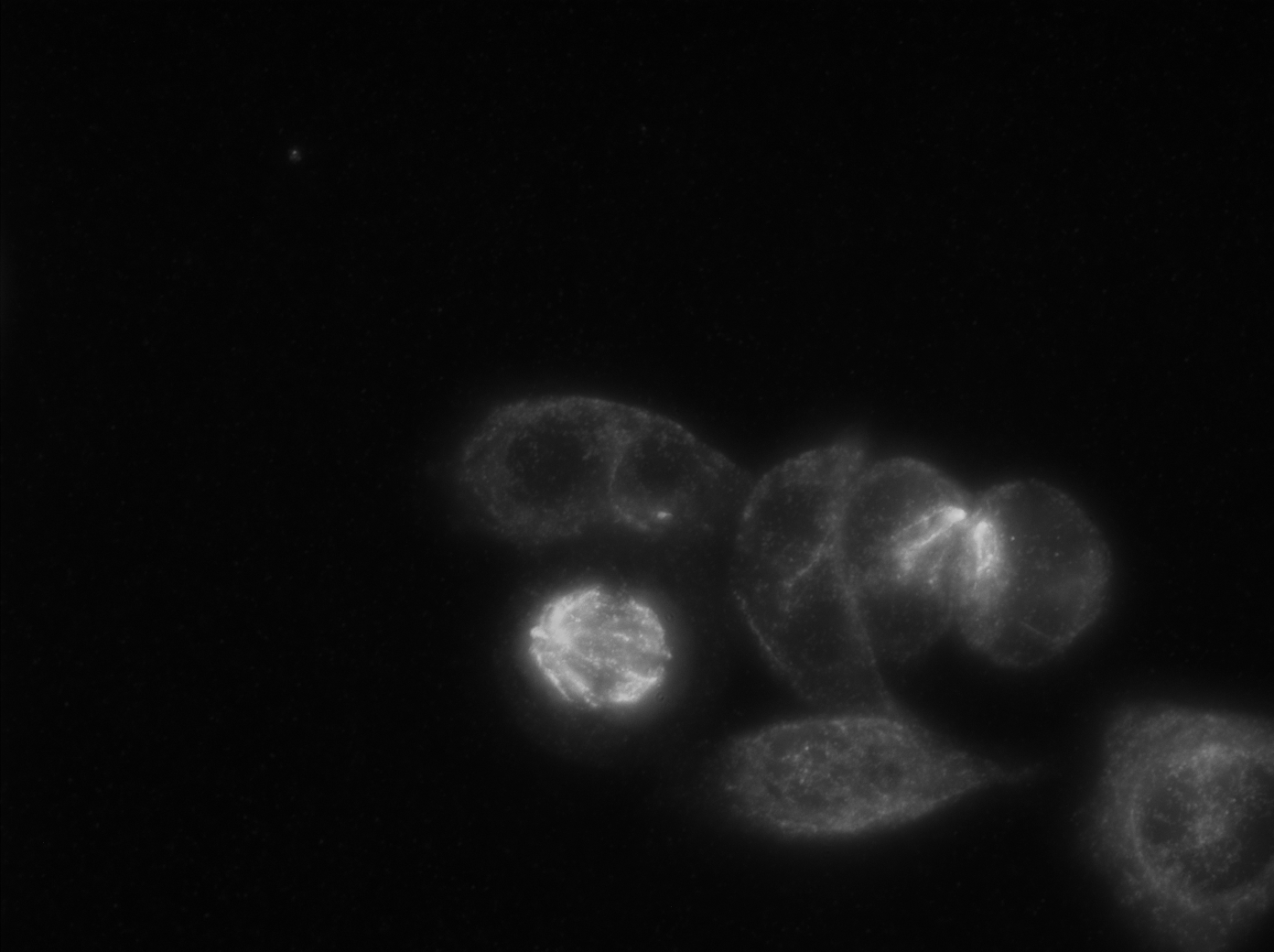

Supplement: Supplementary file 27 — Source data Fig. 7 part 3 [file 44319_2026_742_MOESM27_ESM.zip › Figure 7 Part 3/Fig 7be Cas9 and TPGS1-KO rGT335 atubulin/TPGS1-KO 5-2-25 rGT335 atub R2 M1.Project Maximum Z_XY1746563836_Z0_T0_C2.tif]

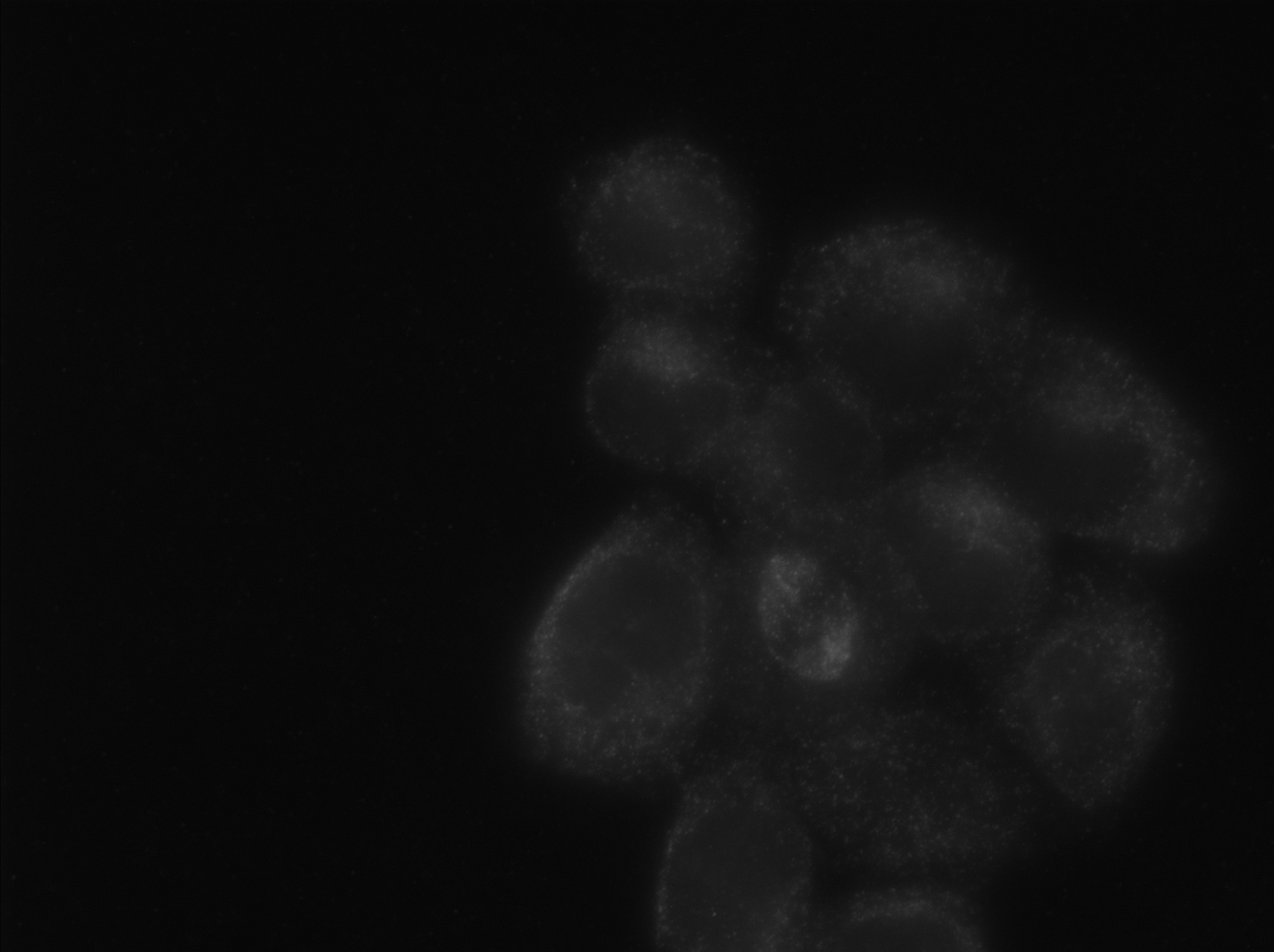

Supplement: Supplementary file 27 — Source data Fig. 7 part 3 [file 44319_2026_742_MOESM27_ESM.zip › Figure 7 Part 3/Fig 7be Cas9 and TPGS1-KO rGT335 atubulin/Cas9 5-2-25 rGT335 atub R1 M5.Project Maximum Z_XY1746557680_Z0_T0_C2.tif]

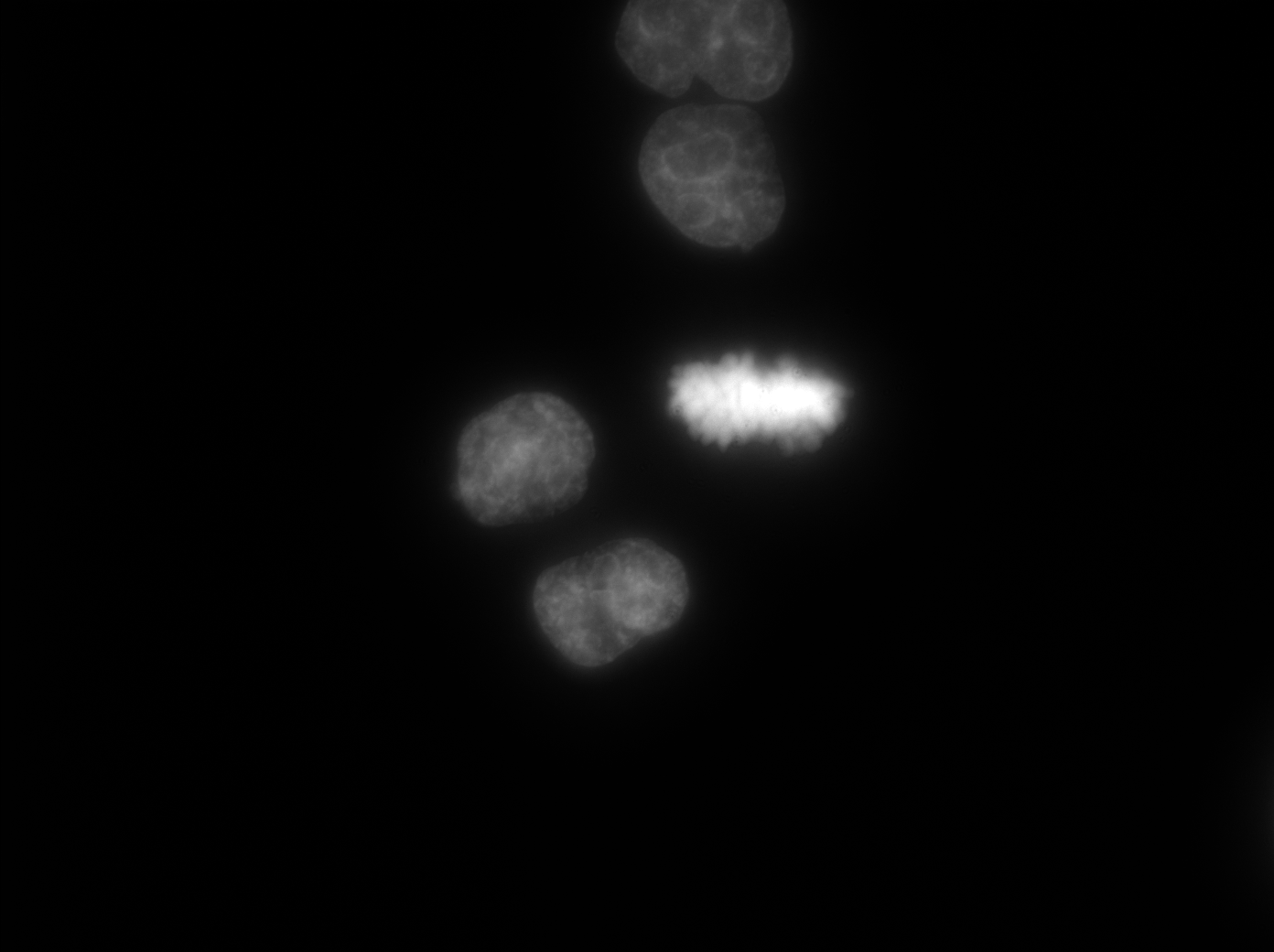

Supplement: Supplementary file 27 — Source data Fig. 7 part 3 [file 44319_2026_742_MOESM27_ESM.zip › Figure 7 Part 3/Fig 7be Cas9 and TPGS1-KO rGT335 atubulin/Cas9 5-2-25 rGT335 atub R2 M4.Project Maximum Z_XY1746561923_Z0_T0_C0.tif]

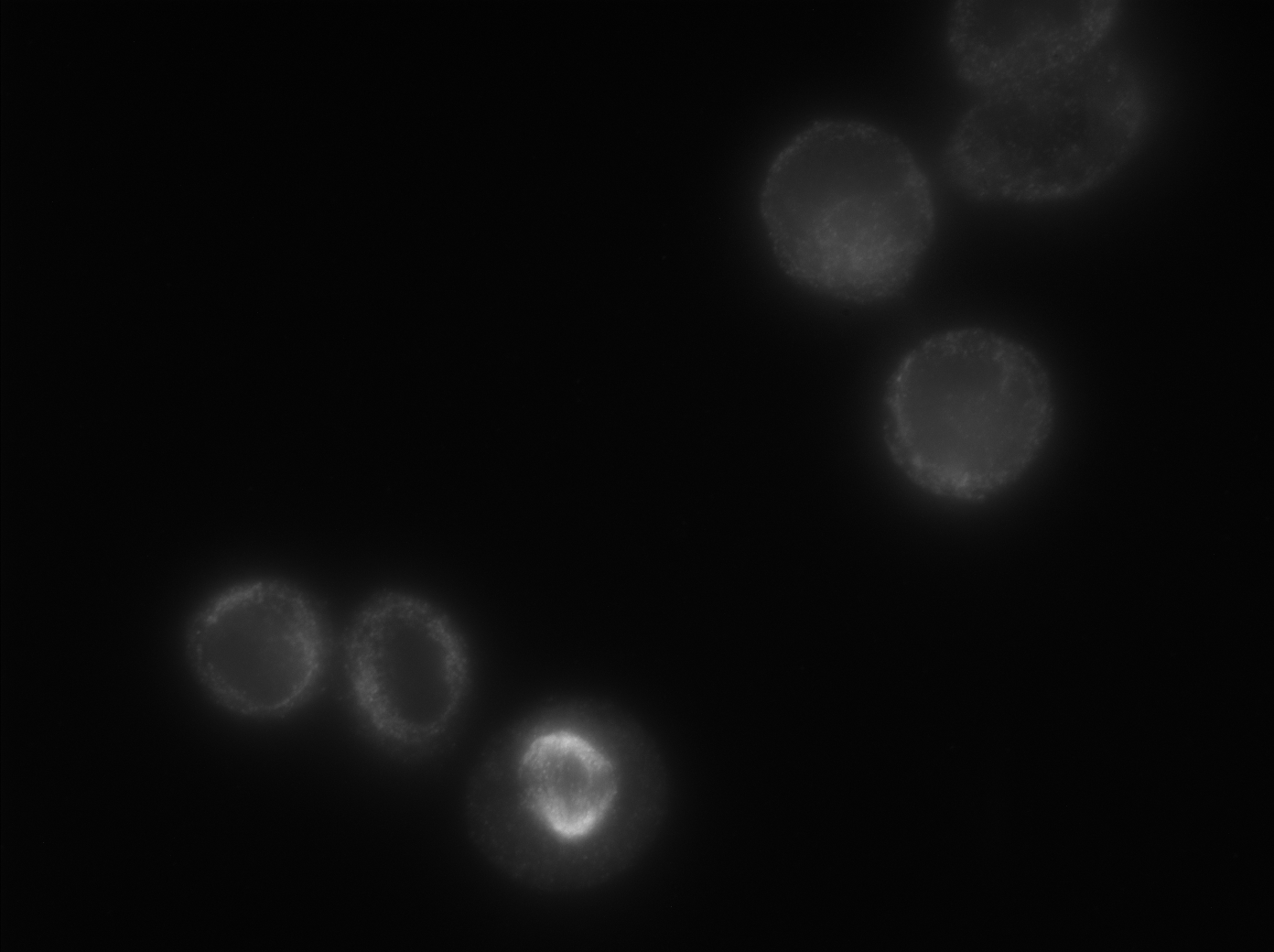

Supplement: Supplementary file 27 — Source data Fig. 7 part 3 [file 44319_2026_742_MOESM27_ESM.zip › Figure 7 Part 3/Fig 7be Cas9 and TPGS1-KO rGT335 atubulin/Cas9 5-2-25 rGT335 atub R3 M5.Project Maximum Z_XY1746217094_Z0_T0_C2.tif]

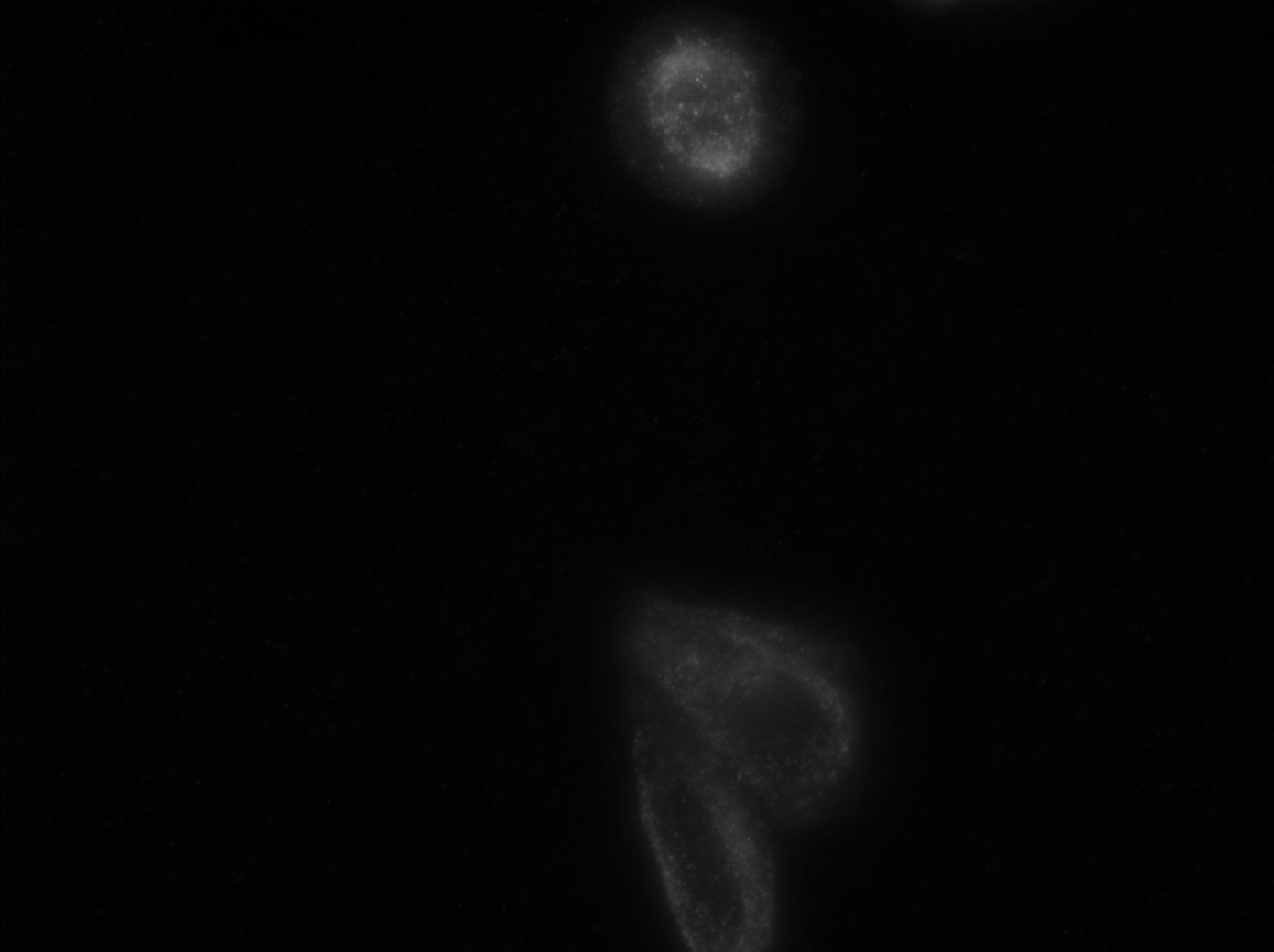

Supplement: Supplementary file 27 — Source data Fig. 7 part 3 [file 44319_2026_742_MOESM27_ESM.zip › Figure 7 Part 3/Fig 7be Cas9 and TPGS1-KO rGT335 atubulin/Cas9 5-2-25 rGT335 atub R2 M2.Project Maximum Z_XY1746559244_Z0_T0_C2.tif]

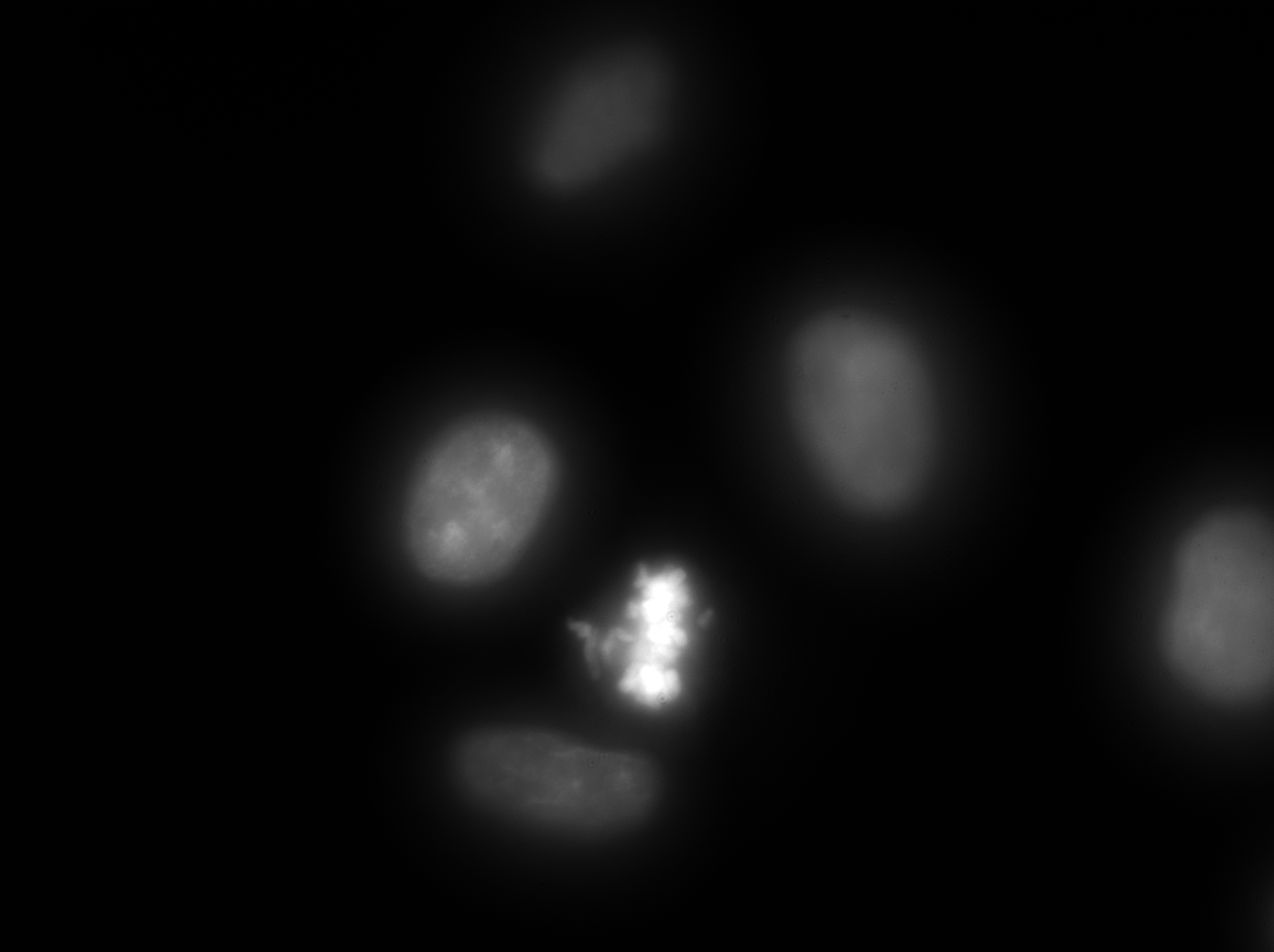

Supplement: Supplementary file 27 — Source data Fig. 7 part 3 [file 44319_2026_742_MOESM27_ESM.zip › Figure 7 Part 3/Fig 7be Cas9 and TPGS1-KO rGT335 atubulin/TPGS1-KO 5-2-25 rGT335 atub R1 M6.Project Maximum Z_XY1746221802_Z0_T0_C0.tif]

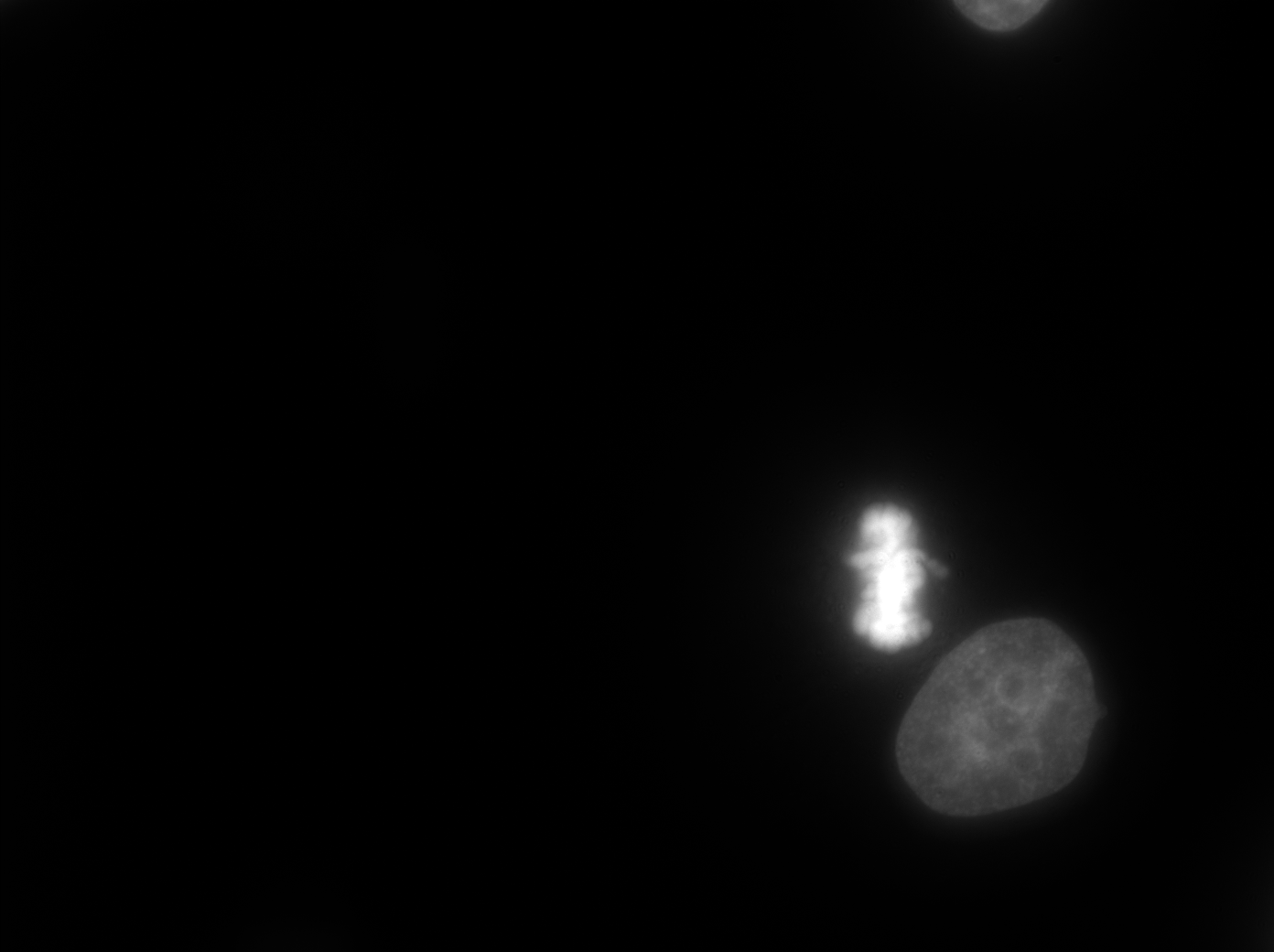

Supplement: Supplementary file 27 — Source data Fig. 7 part 3 [file 44319_2026_742_MOESM27_ESM.zip › Figure 7 Part 3/Fig 7be Cas9 and TPGS1-KO rGT335 atubulin/TPGS1-KO 5-2-25 rGT335 atub R2 M8.Project Maximum Z_XY1746564758_Z0_T0_C0.tif]

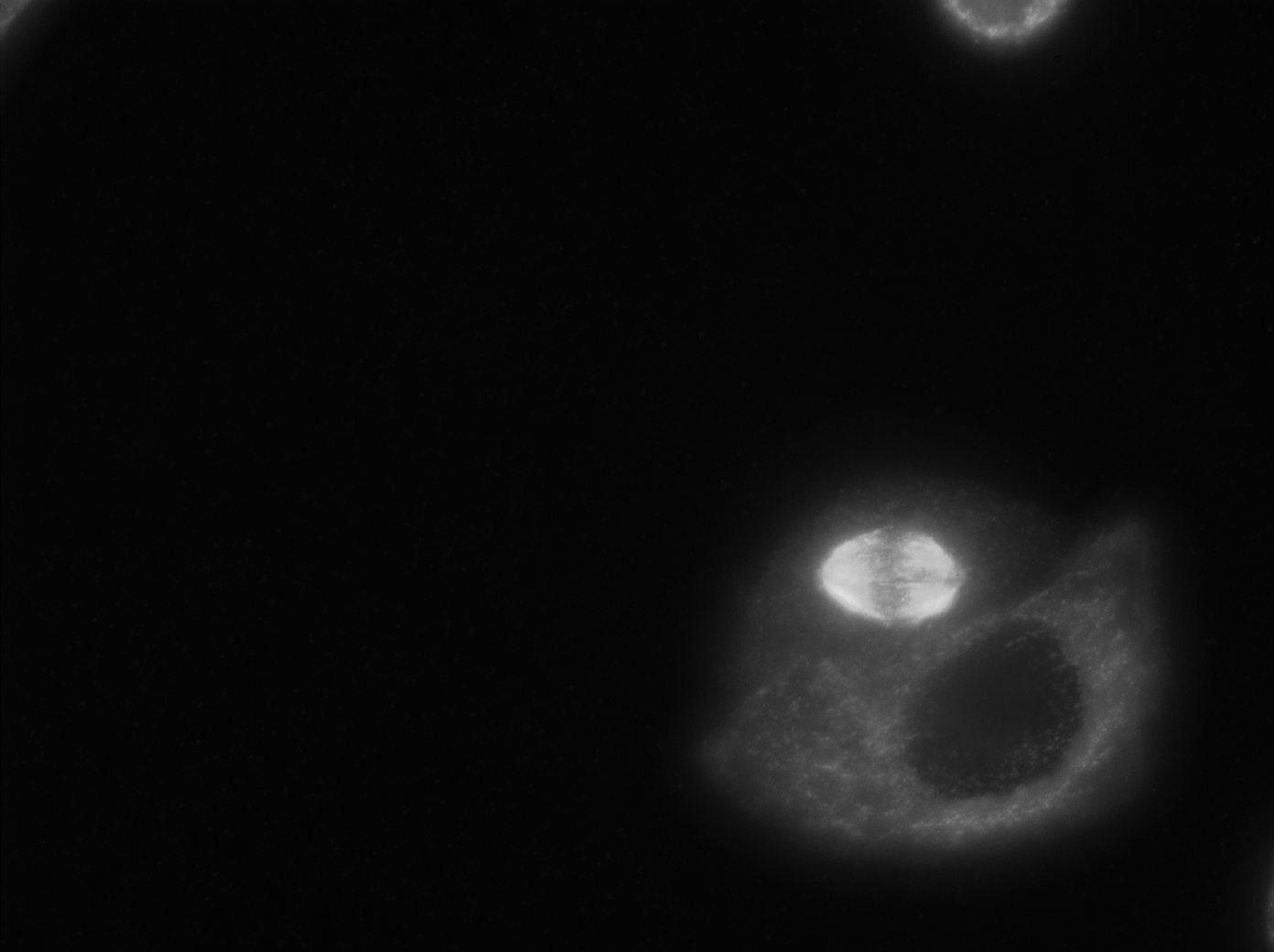

Supplement: Supplementary file 27 — Source data Fig. 7 part 3 [file 44319_2026_742_MOESM27_ESM.zip › Figure 7 Part 3/Fig 7be Cas9 and TPGS1-KO rGT335 atubulin/TPGS1-KO 5-2-25 rGT335 atub R2 M8.Project Maximum Z_XY1746564758_Z0_T0_C2.tif]

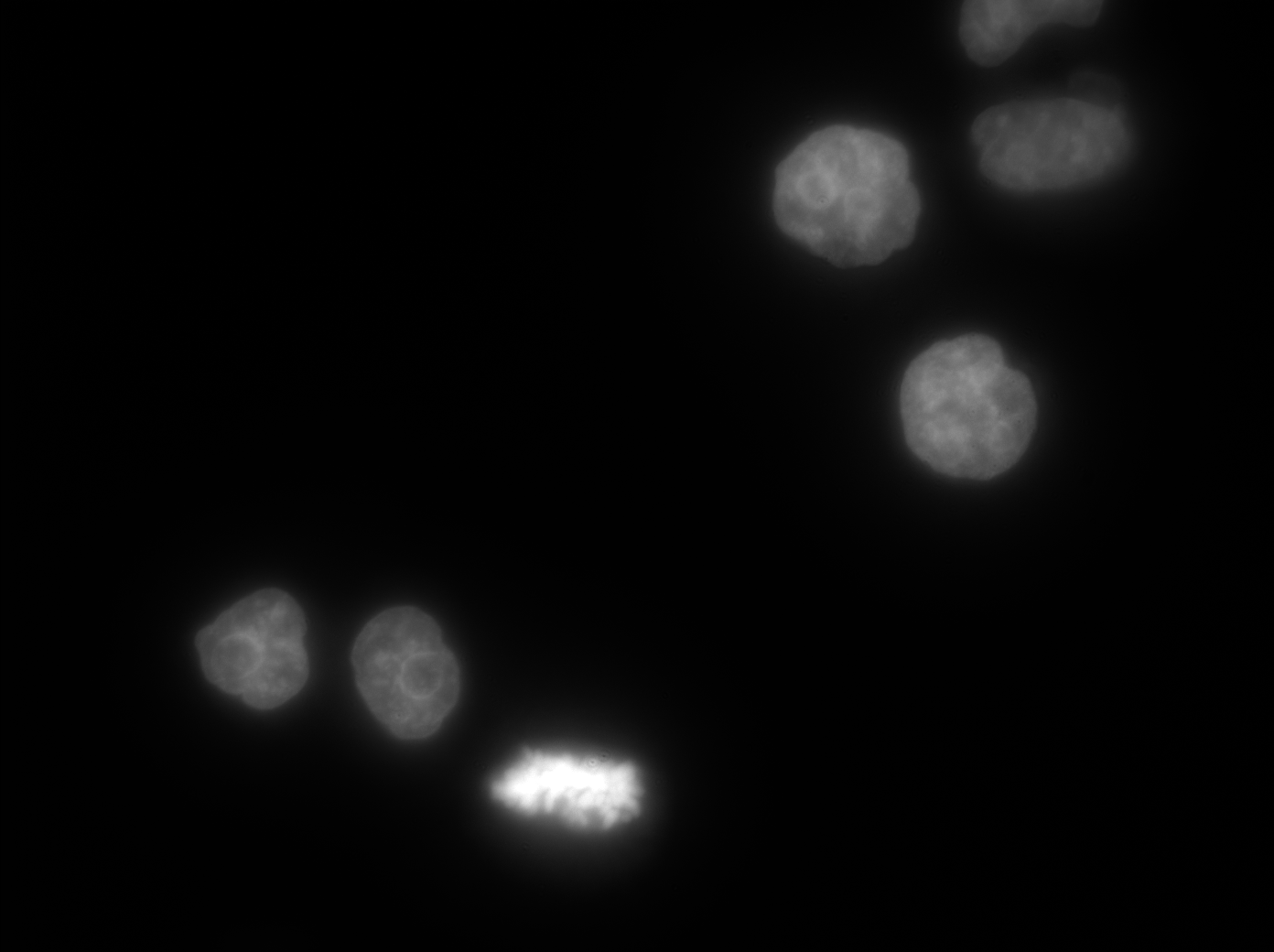

Supplement: Supplementary file 27 — Source data Fig. 7 part 3 [file 44319_2026_742_MOESM27_ESM.zip › Figure 7 Part 3/Fig 7be Cas9 and TPGS1-KO rGT335 atubulin/Cas9 5-2-25 rGT335 atub R3 M5.Project Maximum Z_XY1746217094_Z0_T0_C0.tif]

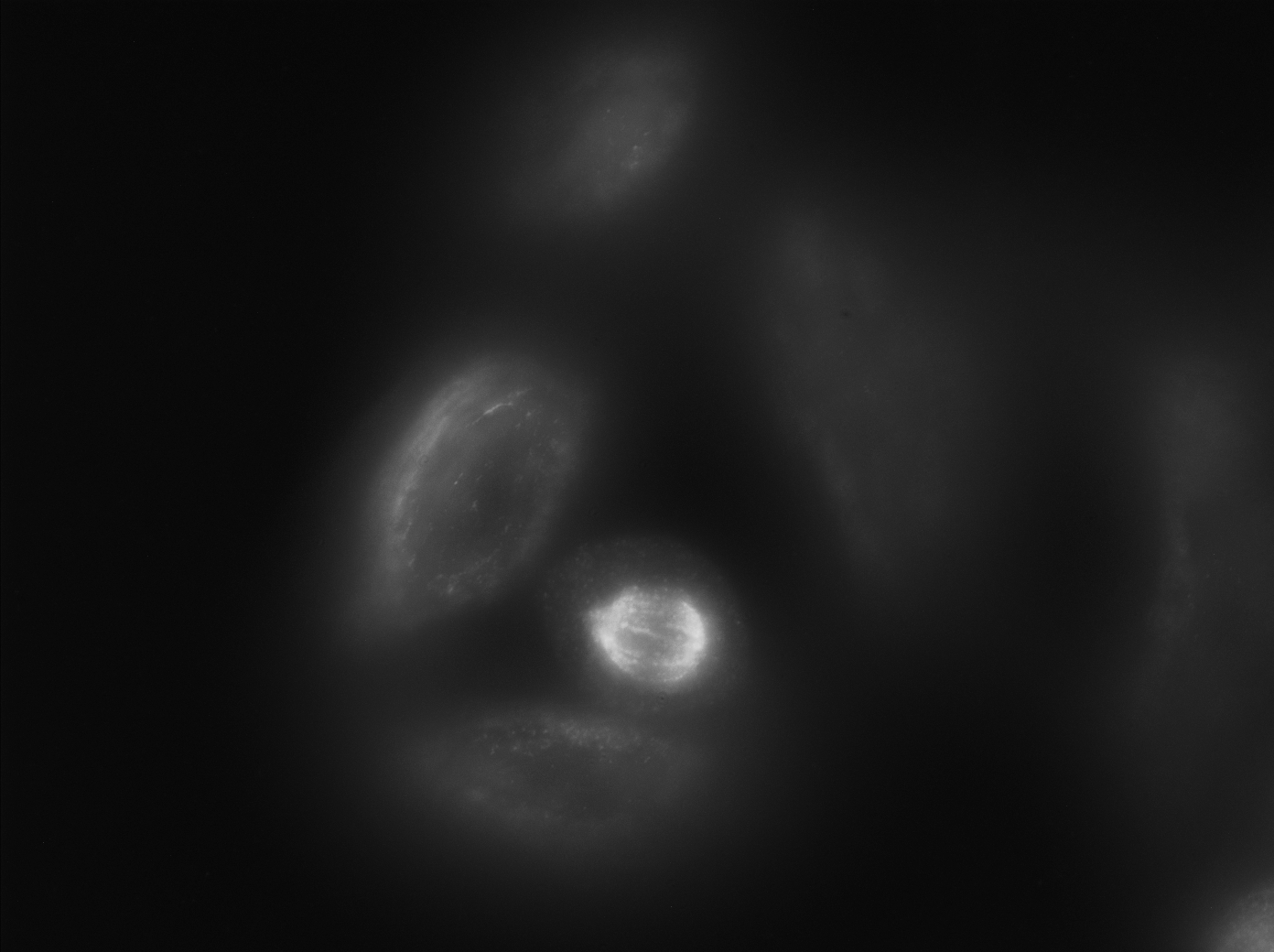

Supplement: Supplementary file 27 — Source data Fig. 7 part 3 [file 44319_2026_742_MOESM27_ESM.zip › Figure 7 Part 3/Fig 7be Cas9 and TPGS1-KO rGT335 atubulin/TPGS1-KO 5-2-25 rGT335 atub R1 M6.Project Maximum Z_XY1746221802_Z0_T0_C2.tif]

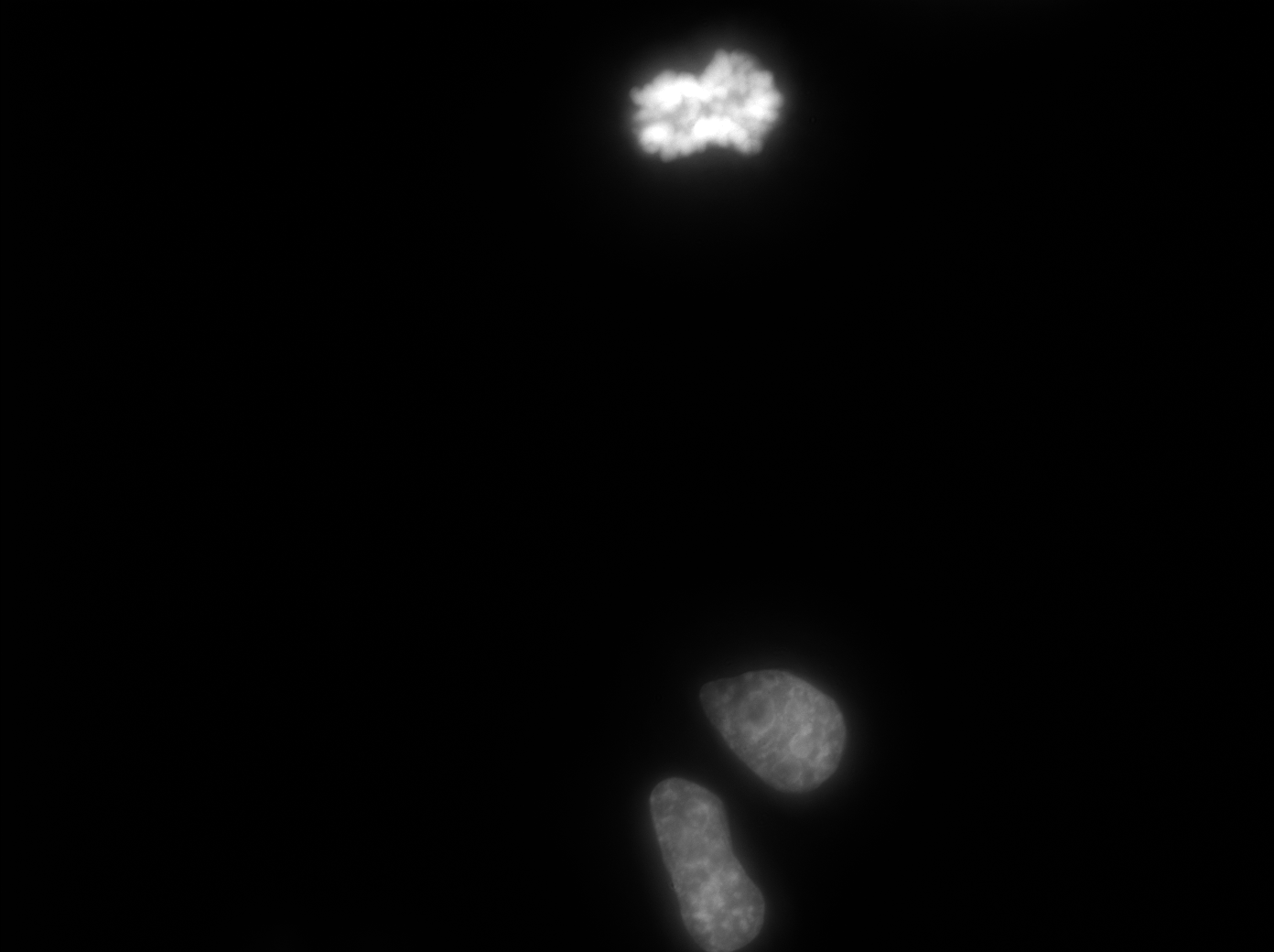

Supplement: Supplementary file 27 — Source data Fig. 7 part 3 [file 44319_2026_742_MOESM27_ESM.zip › Figure 7 Part 3/Fig 7be Cas9 and TPGS1-KO rGT335 atubulin/Cas9 5-2-25 rGT335 atub R2 M2.Project Maximum Z_XY1746559244_Z0_T0_C0.tif]

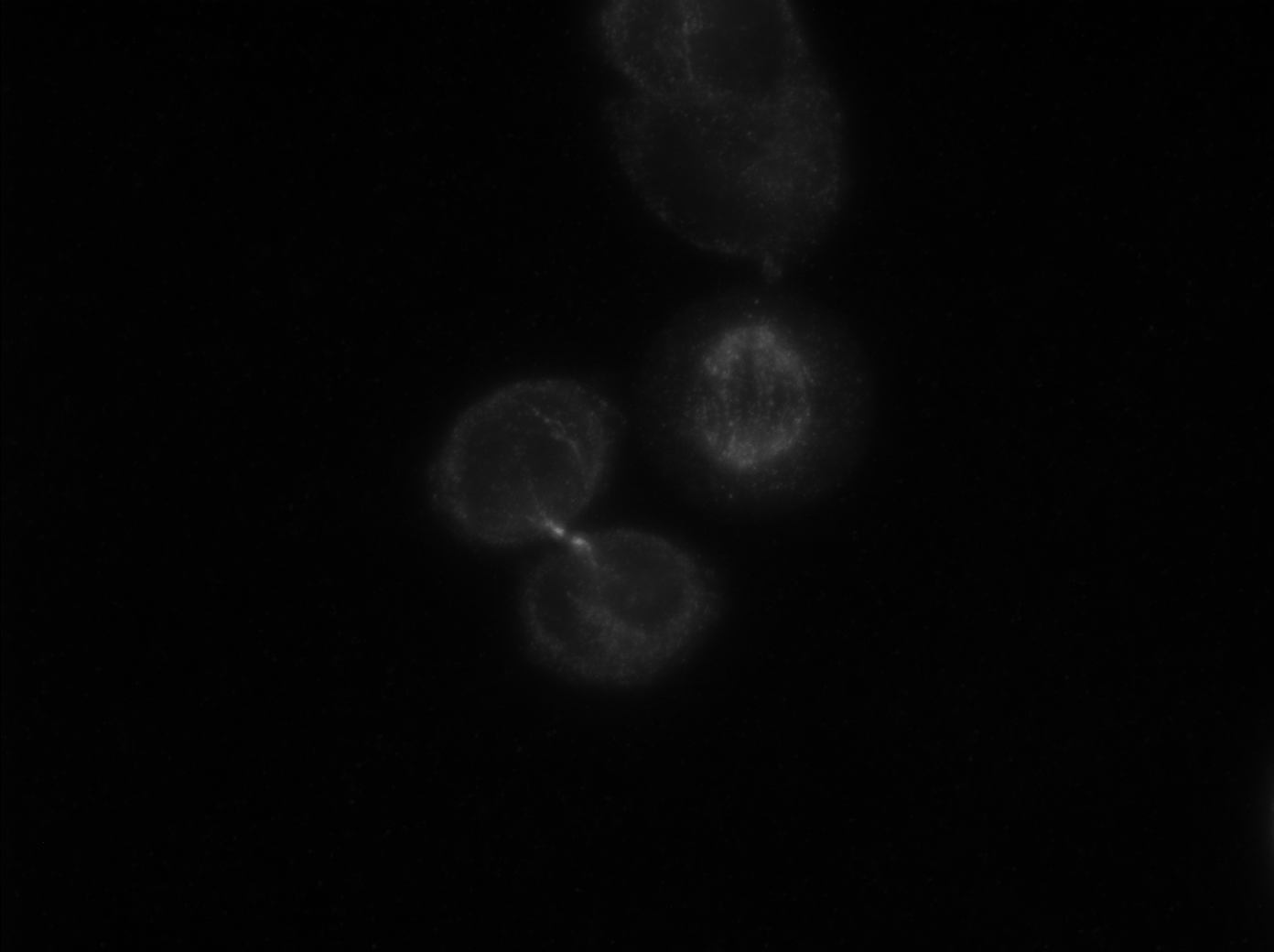

Supplement: Supplementary file 27 — Source data Fig. 7 part 3 [file 44319_2026_742_MOESM27_ESM.zip › Figure 7 Part 3/Fig 7be Cas9 and TPGS1-KO rGT335 atubulin/Cas9 5-2-25 rGT335 atub R2 M4.Project Maximum Z_XY1746561923_Z0_T0_C2.tif]

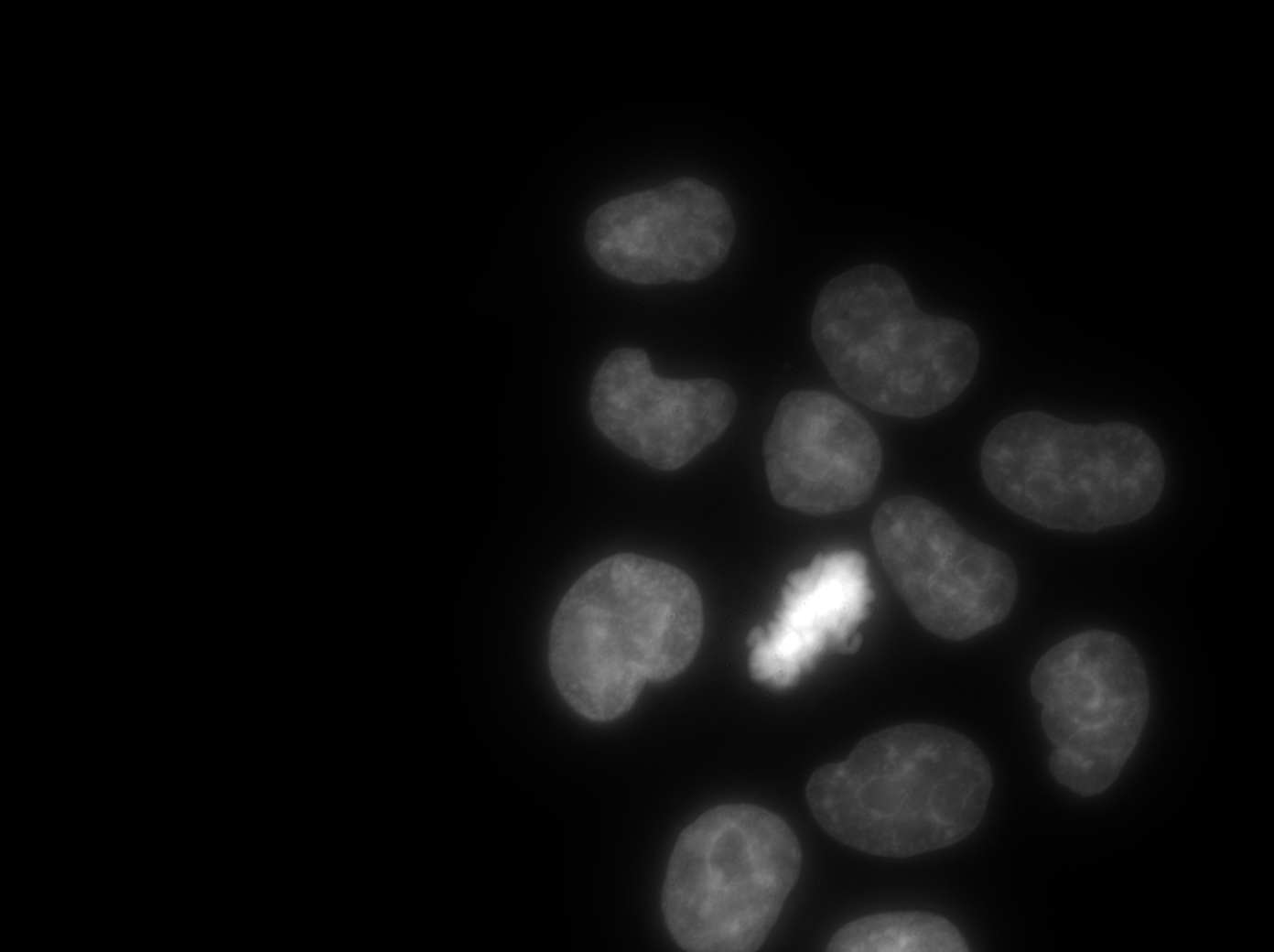

Supplement: Supplementary file 27 — Source data Fig. 7 part 3 [file 44319_2026_742_MOESM27_ESM.zip › Figure 7 Part 3/Fig 7be Cas9 and TPGS1-KO rGT335 atubulin/Cas9 5-2-25 rGT335 atub R1 M5.Project Maximum Z_XY1746557680_Z0_T0_C0.tif]

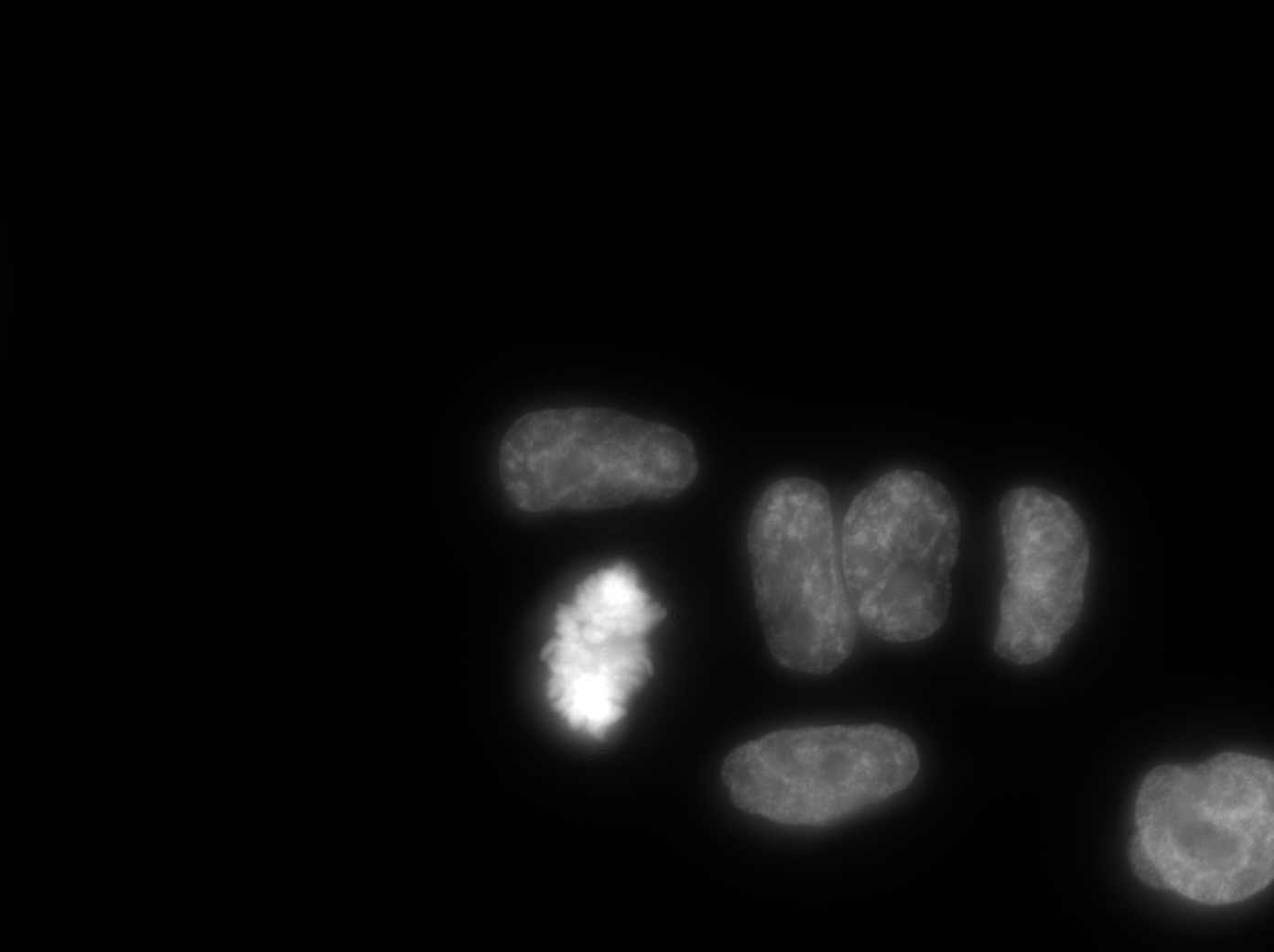

Supplement: Supplementary file 27 — Source data Fig. 7 part 3 [file 44319_2026_742_MOESM27_ESM.zip › Figure 7 Part 3/Fig 7be Cas9 and TPGS1-KO rGT335 atubulin/TPGS1-KO 5-2-25 rGT335 atub R2 M1.Project Maximum Z_XY1746563836_Z0_T0_C0.tif]

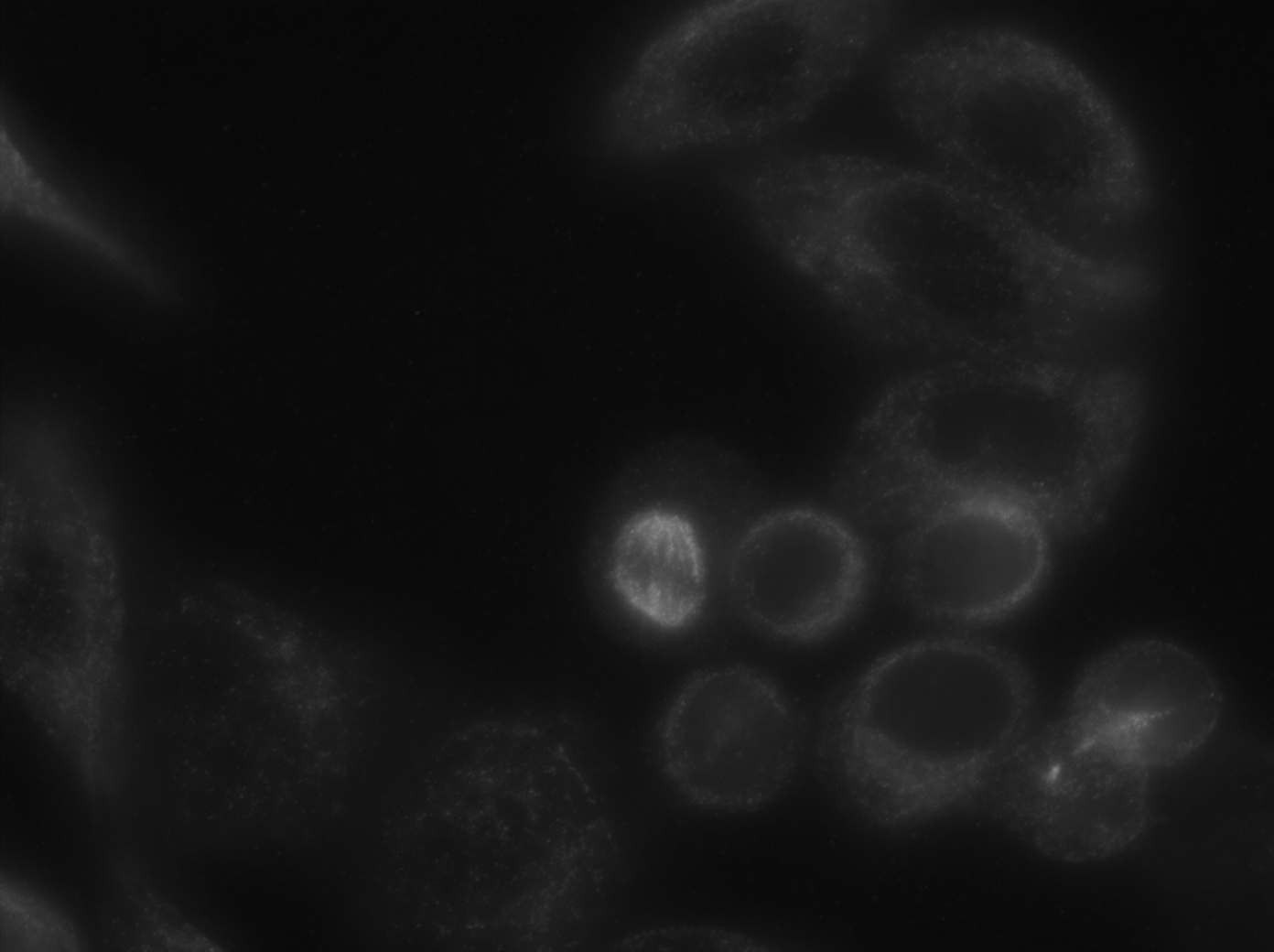

Supplement: Supplementary file 27 — Source data Fig. 7 part 3 [file 44319_2026_742_MOESM27_ESM.zip › Figure 7 Part 3/Fig 7be Cas9 and TPGS1-KO rGT335 atubulin/Cas9 5-2-25 rGT335 atub R3 M3.Project Maximum Z_XY1746214431_Z0_T0_C2.tif]

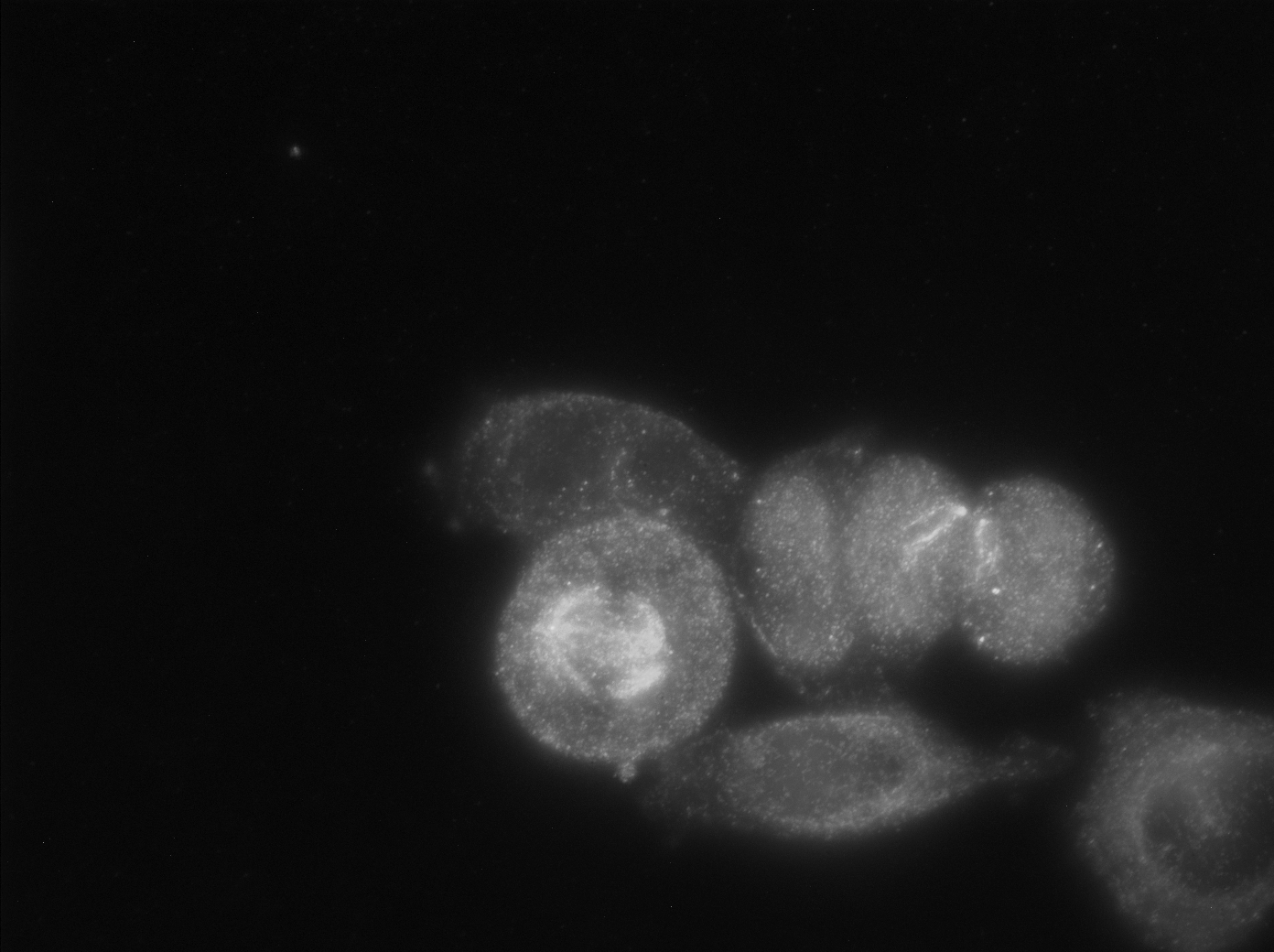

Supplement: Supplementary file 27 — Source data Fig. 7 part 3 [file 44319_2026_742_MOESM27_ESM.zip › Figure 7 Part 3/Fig 7be Cas9 and TPGS1-KO rGT335 atubulin/TPGS1-KO 5-2-25 rGT335 atub R2 M1.Project Maximum Z_XY1746563836_Z0_T0_C1.tif]

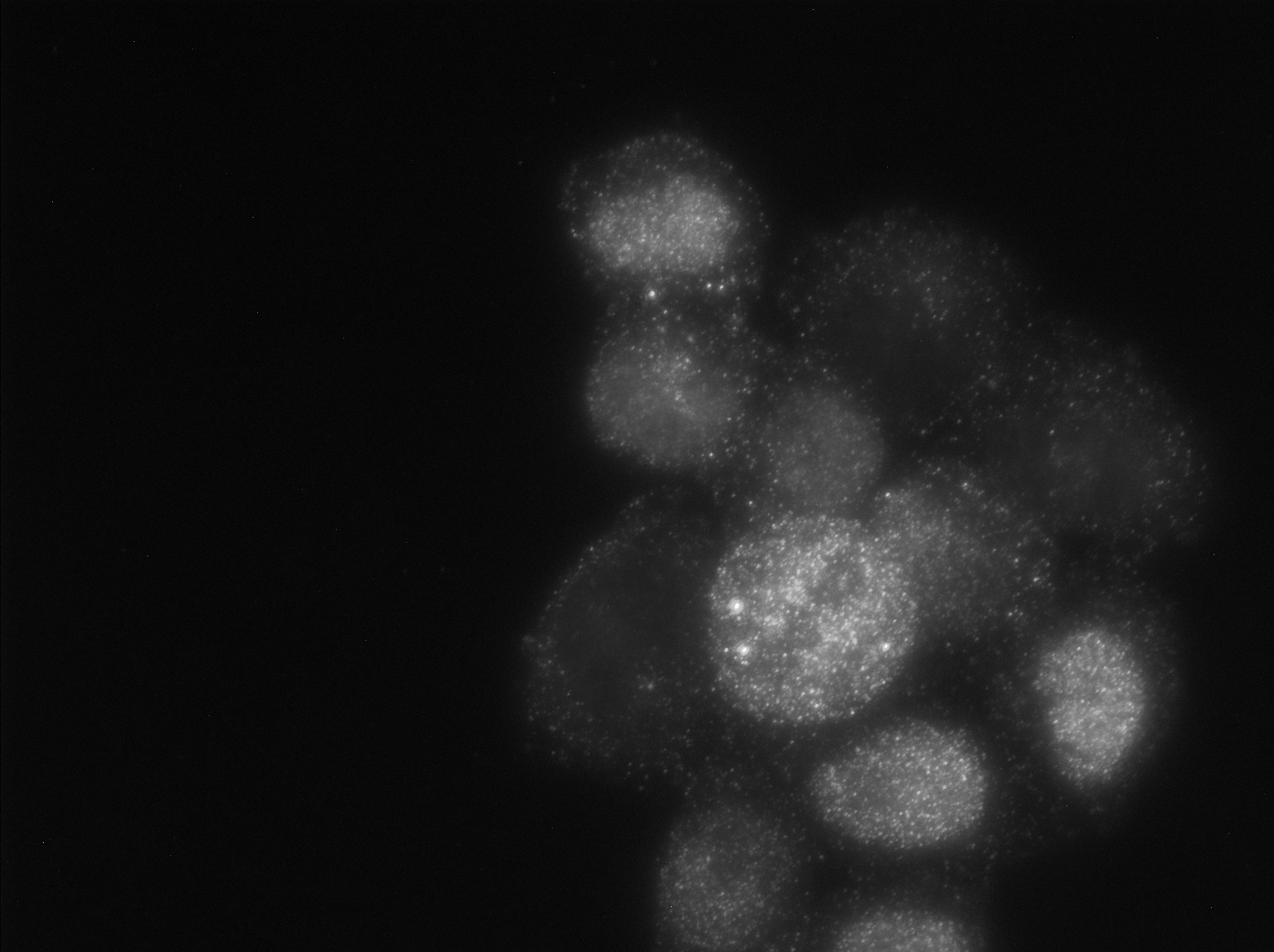

Supplement: Supplementary file 27 — Source data Fig. 7 part 3 [file 44319_2026_742_MOESM27_ESM.zip › Figure 7 Part 3/Fig 7be Cas9 and TPGS1-KO rGT335 atubulin/Cas9 5-2-25 rGT335 atub R1 M5.Project Maximum Z_XY1746557680_Z0_T0_C1.tif]

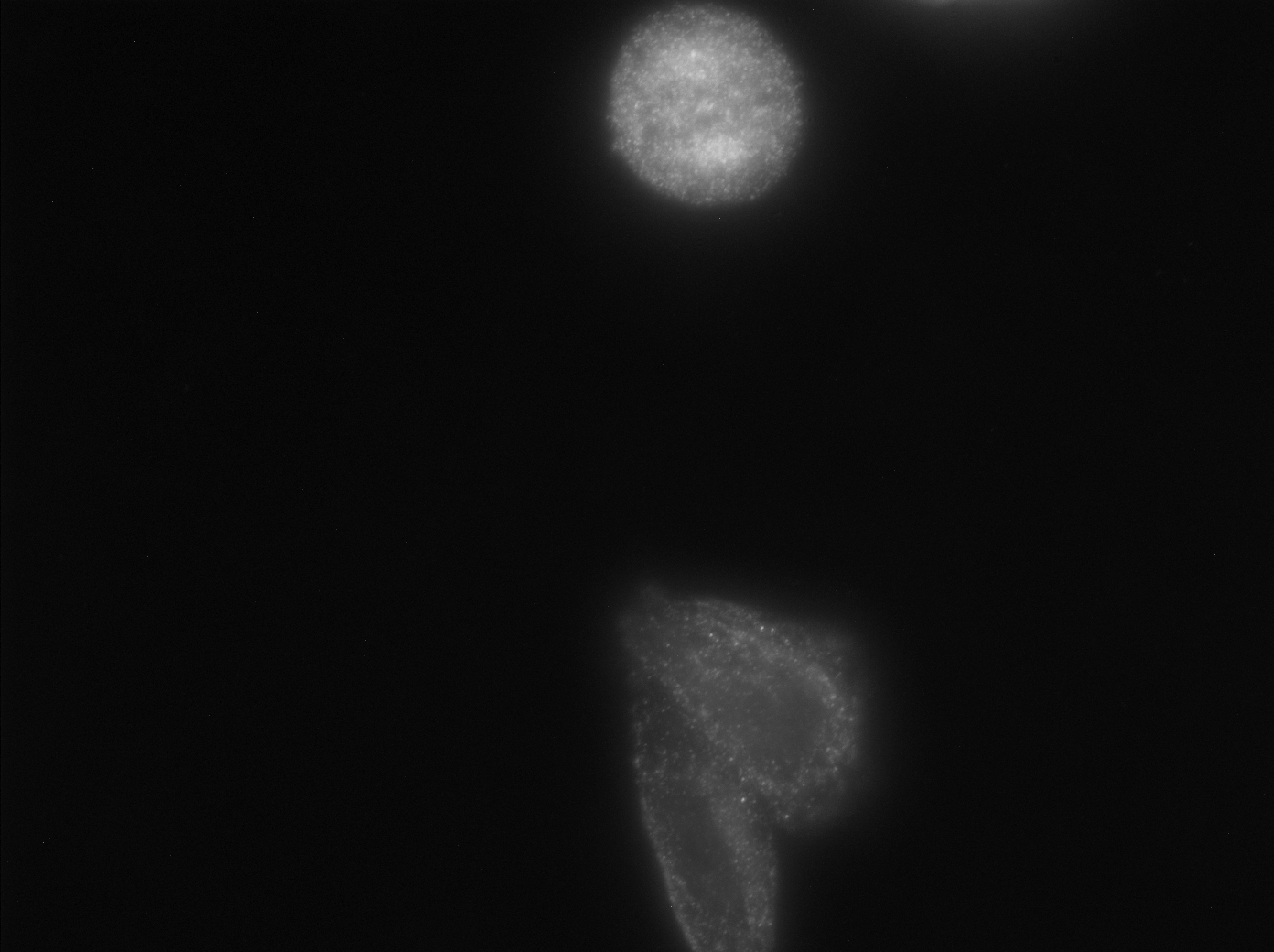

Supplement: Supplementary file 27 — Source data Fig. 7 part 3 [file 44319_2026_742_MOESM27_ESM.zip › Figure 7 Part 3/Fig 7be Cas9 and TPGS1-KO rGT335 atubulin/Cas9 5-2-25 rGT335 atub R2 M2.Project Maximum Z_XY1746559244_Z0_T0_C1.tif]

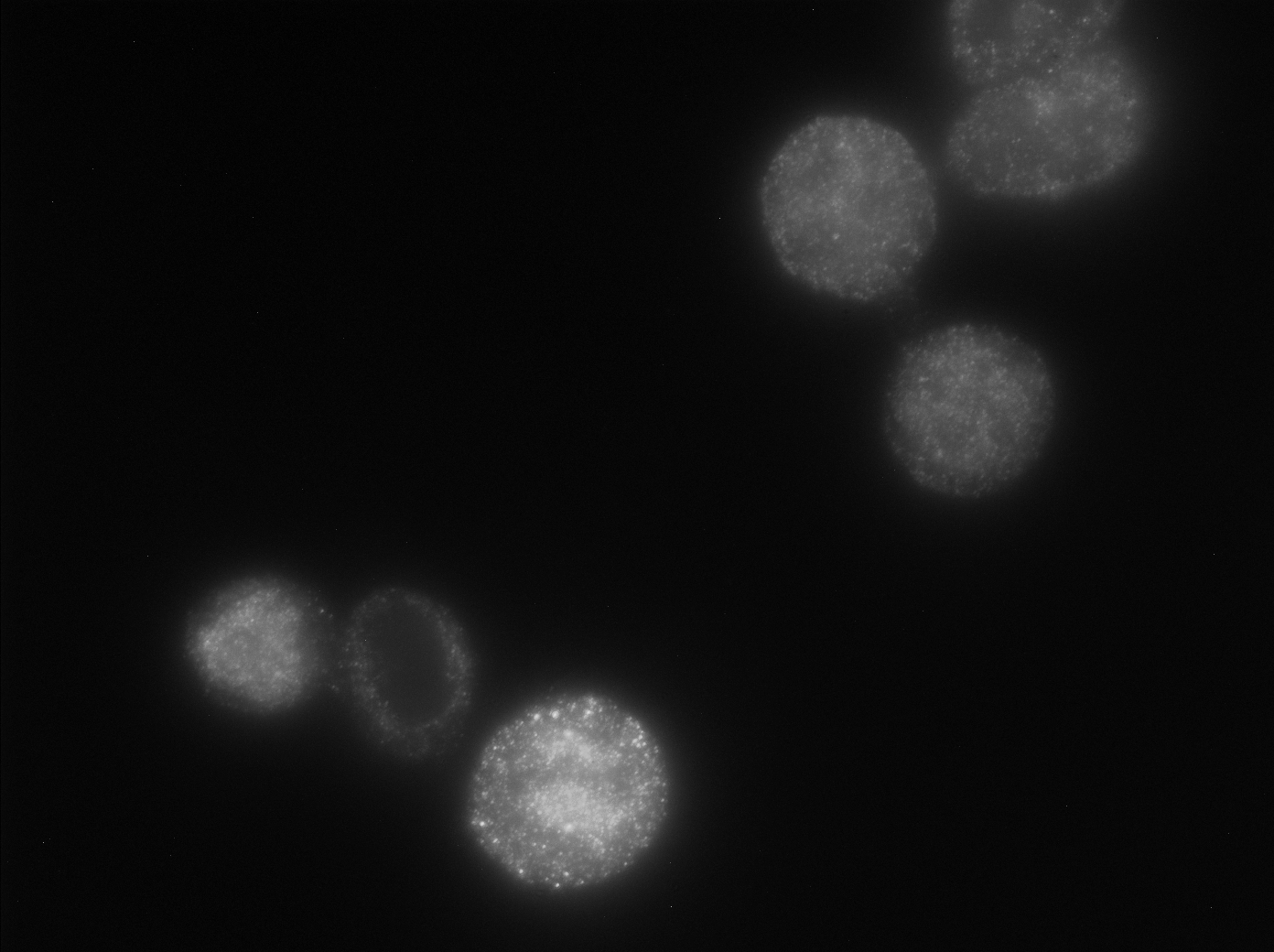

Supplement: Supplementary file 27 — Source data Fig. 7 part 3 [file 44319_2026_742_MOESM27_ESM.zip › Figure 7 Part 3/Fig 7be Cas9 and TPGS1-KO rGT335 atubulin/Cas9 5-2-25 rGT335 atub R3 M5.Project Maximum Z_XY1746217094_Z0_T0_C1.tif]

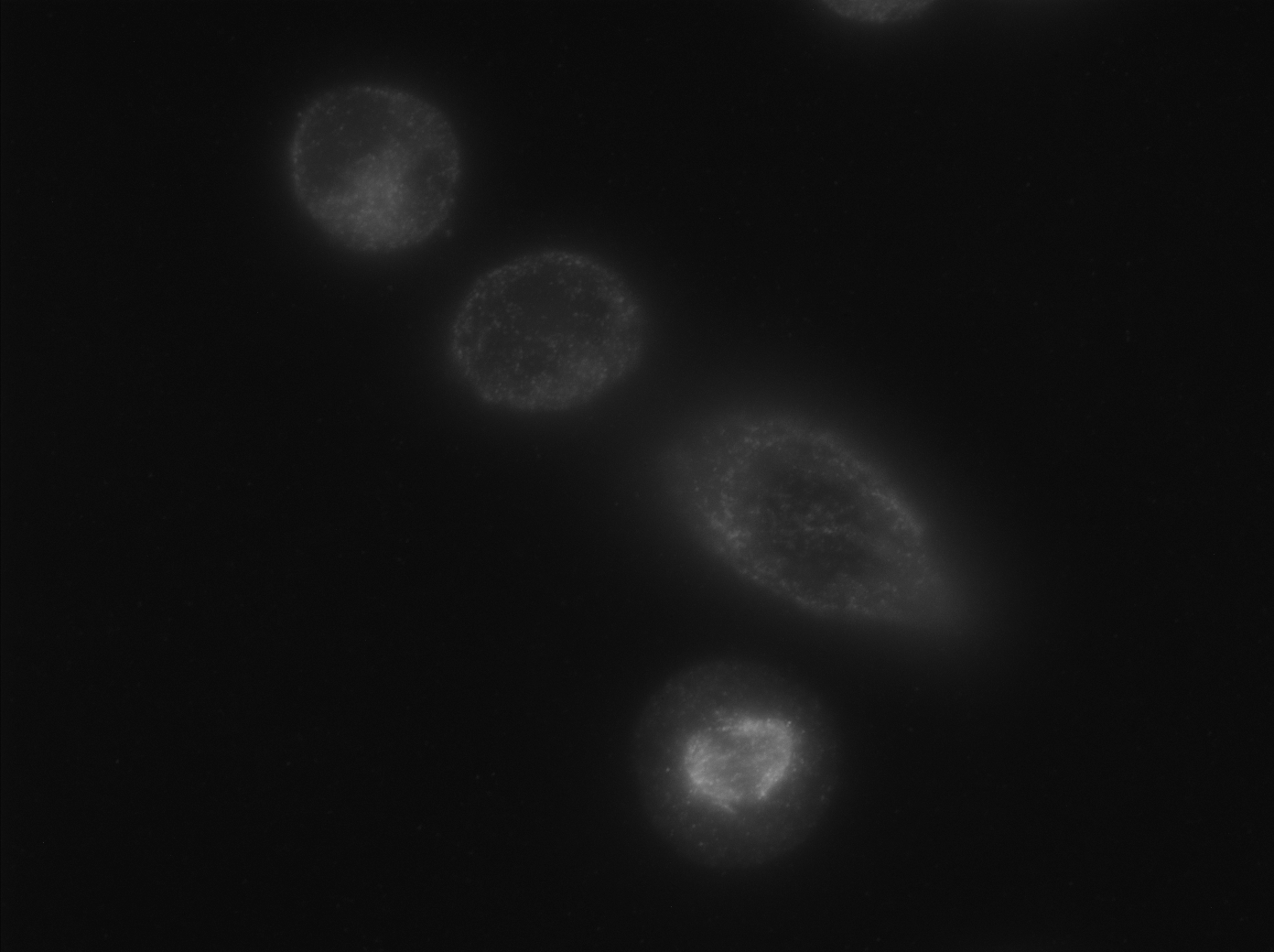

Supplement: Supplementary file 27 — Source data Fig. 7 part 3 [file 44319_2026_742_MOESM27_ESM.zip › Figure 7 Part 3/Fig 7be Cas9 and TPGS1-KO rGT335 atubulin/Cas9 5-2-25 rGT335 atub R1 M9.Project Maximum Z_XY1746558405_Z0_T0_C2.tif]

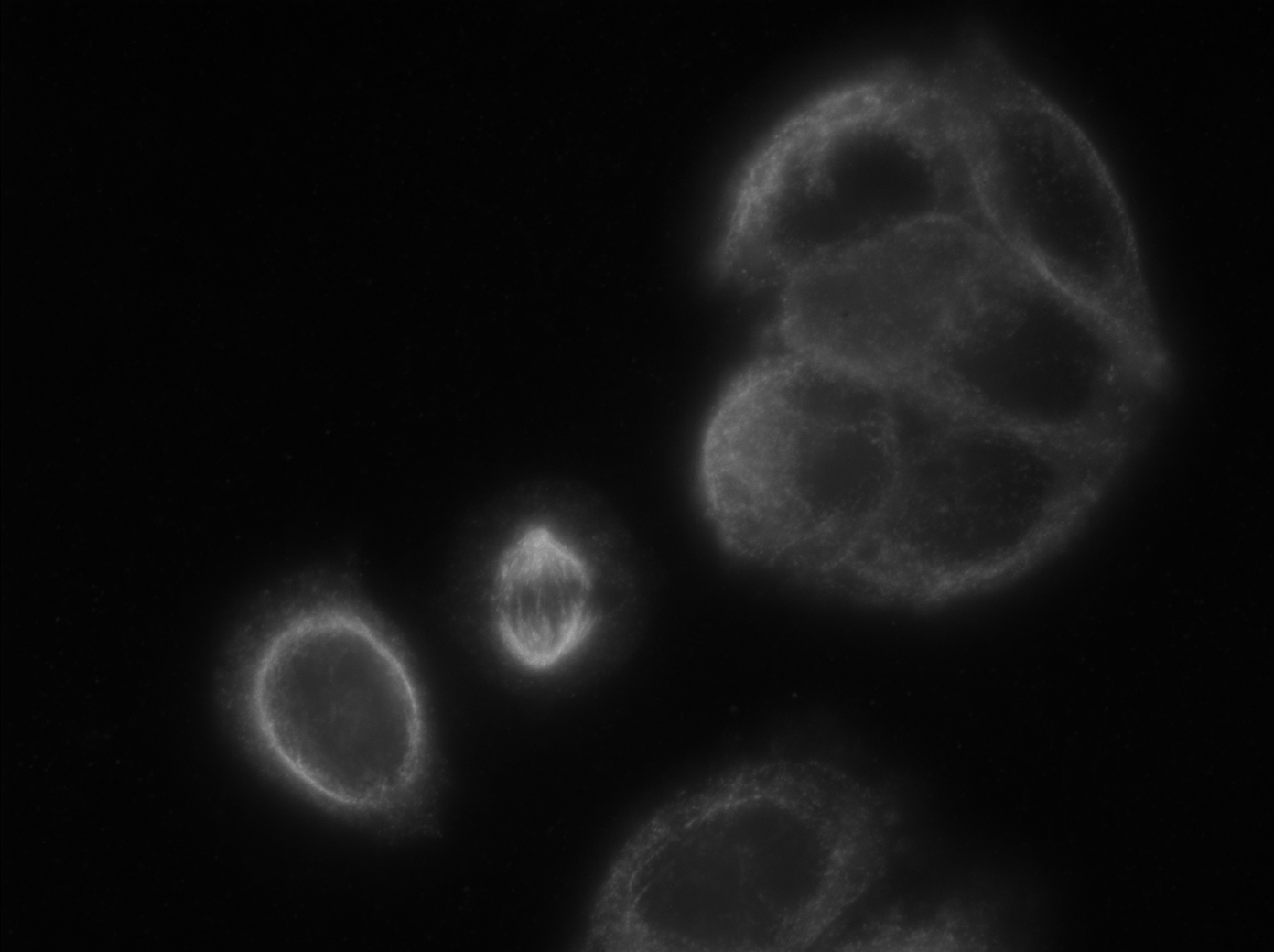

Supplement: Supplementary file 27 — Source data Fig. 7 part 3 [file 44319_2026_742_MOESM27_ESM.zip › Figure 7 Part 3/Fig 7be Cas9 and TPGS1-KO rGT335 atubulin/TPGS1-KO 5-2-25 rGT335 atub R2 M5.Project Maximum Z_XY1746564338_Z0_T0_C2.tif]

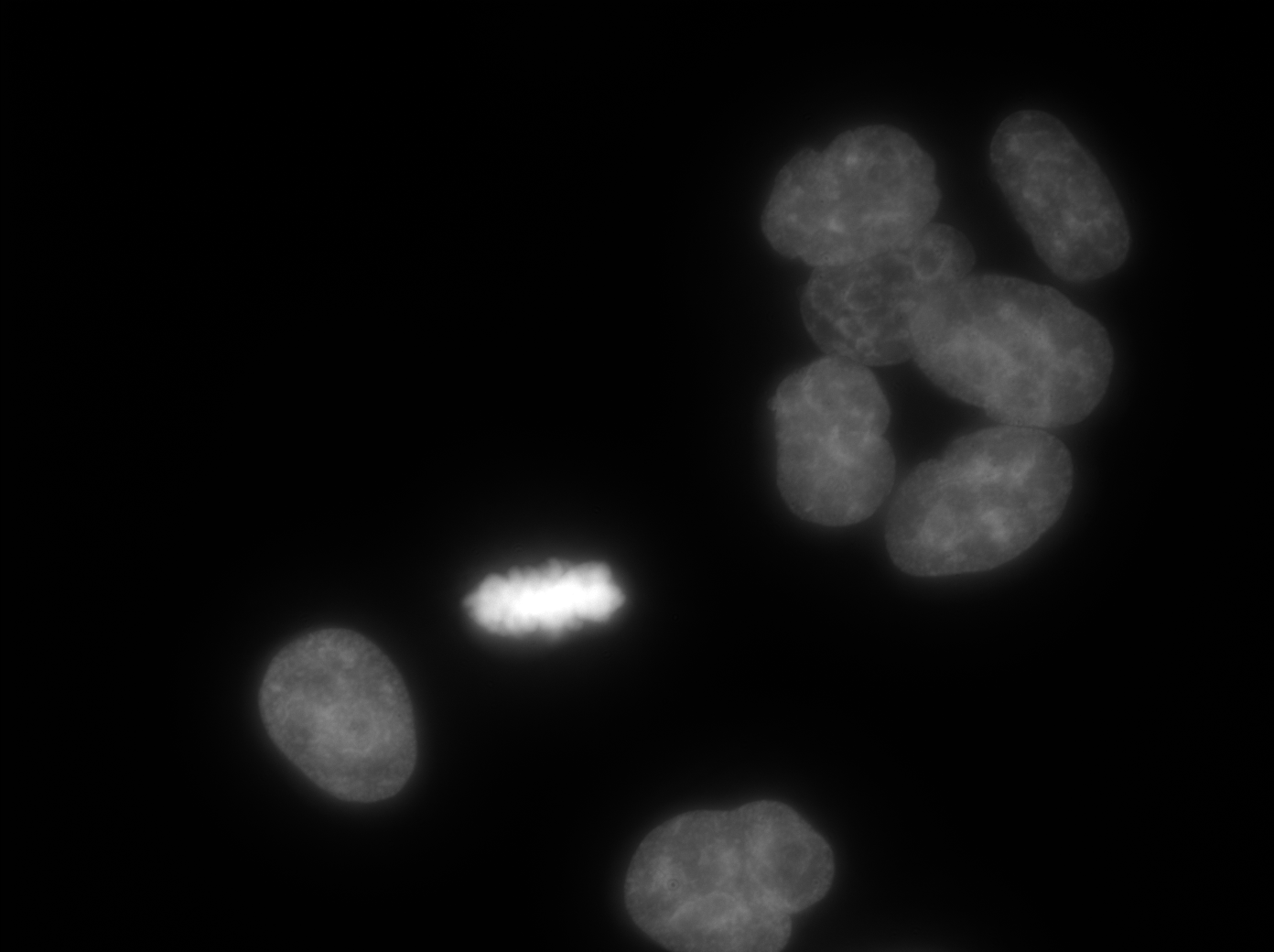

Supplement: Supplementary file 27 — Source data Fig. 7 part 3 [file 44319_2026_742_MOESM27_ESM.zip › Figure 7 Part 3/Fig 7be Cas9 and TPGS1-KO rGT335 atubulin/TPGS1-KO 5-2-25 rGT335 atub R2 M5.Project Maximum Z_XY1746564338_Z0_T0_C0.tif]

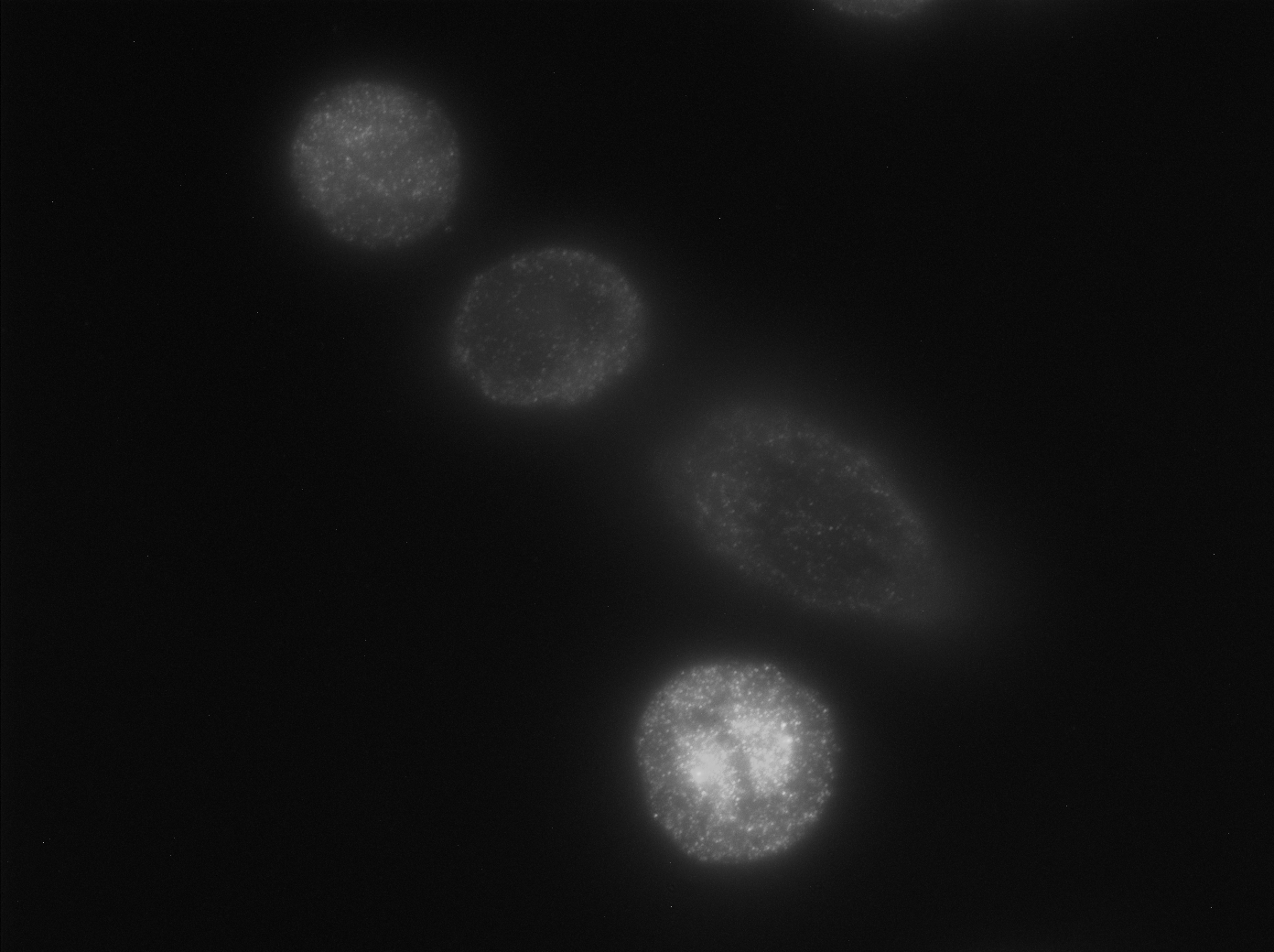

Supplement: Supplementary file 27 — Source data Fig. 7 part 3 [file 44319_2026_742_MOESM27_ESM.zip › Figure 7 Part 3/Fig 7be Cas9 and TPGS1-KO rGT335 atubulin/Cas9 5-2-25 rGT335 atub R1 M9.Project Maximum Z_XY1746558405_Z0_T0_C1.tif]

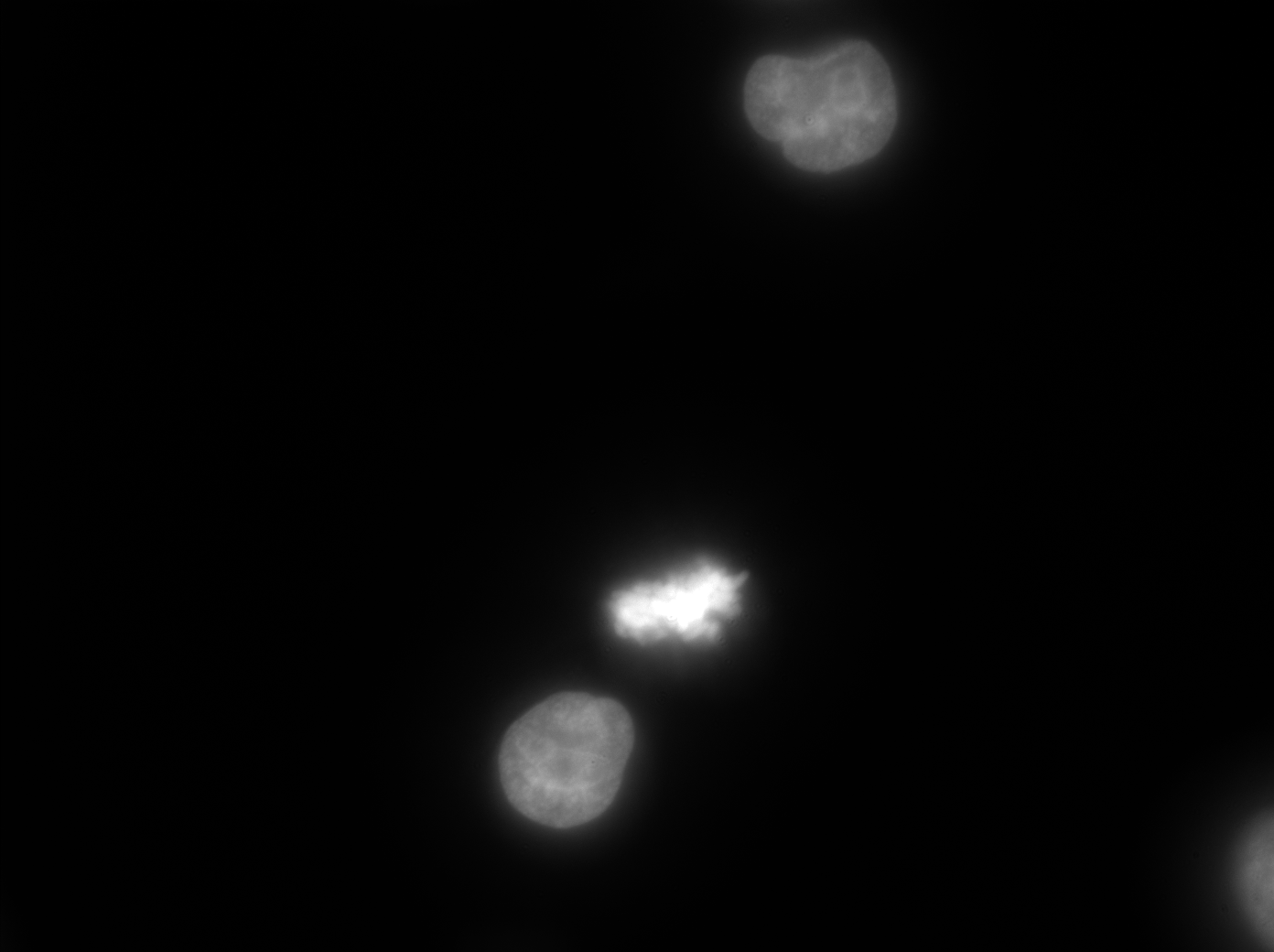

Supplement: Supplementary file 27 — Source data Fig. 7 part 3 [file 44319_2026_742_MOESM27_ESM.zip › Figure 7 Part 3/Fig 7be Cas9 and TPGS1-KO rGT335 atubulin/Cas9 5-2-25 rGT335 atub R3 M8.Project Maximum Z_XY1746217622_Z0_T0_C0.tif]

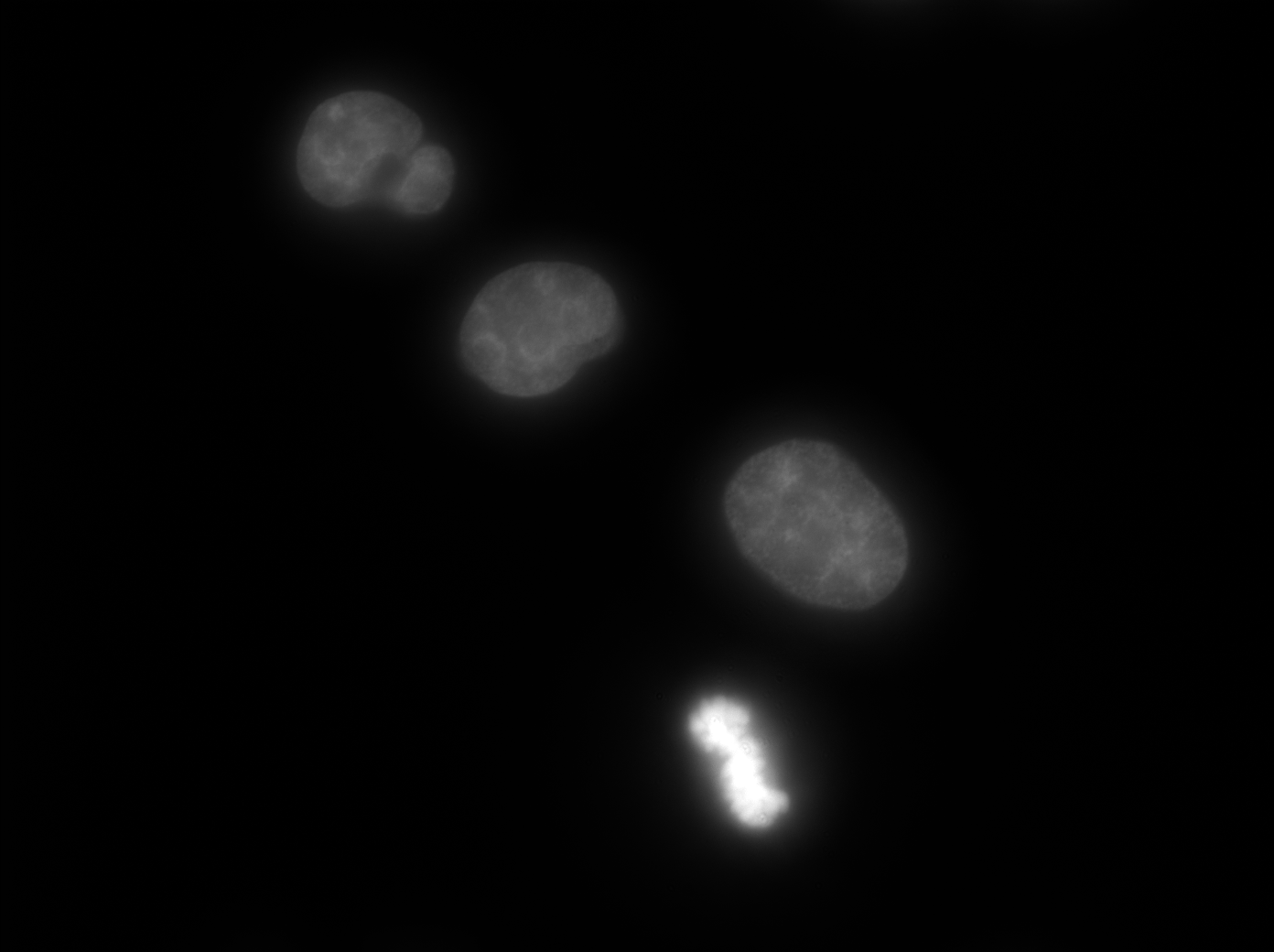

Supplement: Supplementary file 27 — Source data Fig. 7 part 3 [file 44319_2026_742_MOESM27_ESM.zip › Figure 7 Part 3/Fig 7be Cas9 and TPGS1-KO rGT335 atubulin/Cas9 5-2-25 rGT335 atub R1 M9.Project Maximum Z_XY1746558405_Z0_T0_C0.tif]

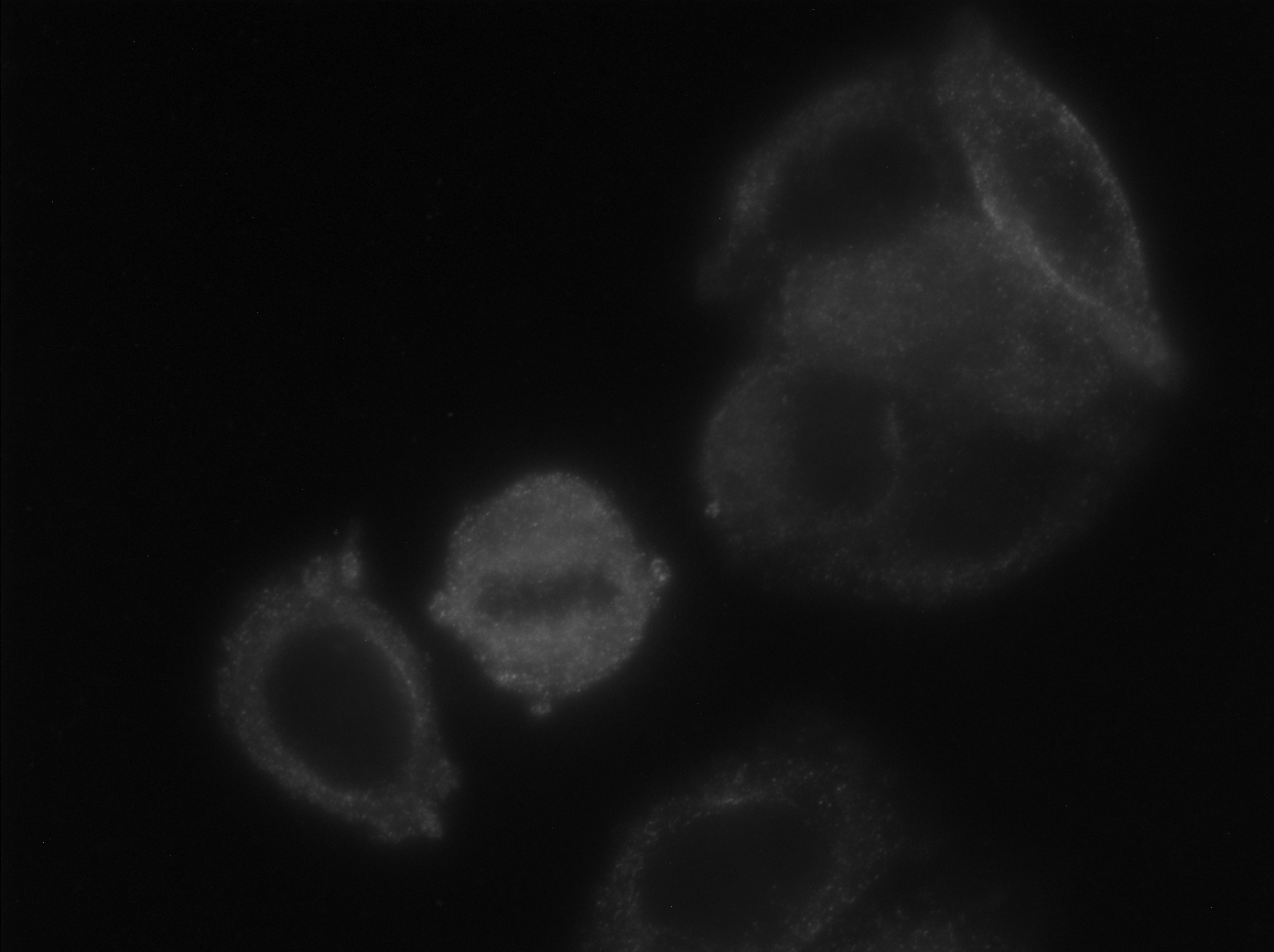

Supplement: Supplementary file 27 — Source data Fig. 7 part 3 [file 44319_2026_742_MOESM27_ESM.zip › Figure 7 Part 3/Fig 7be Cas9 and TPGS1-KO rGT335 atubulin/TPGS1-KO 5-2-25 rGT335 atub R2 M5.Project Maximum Z_XY1746564338_Z0_T0_C1.tif]

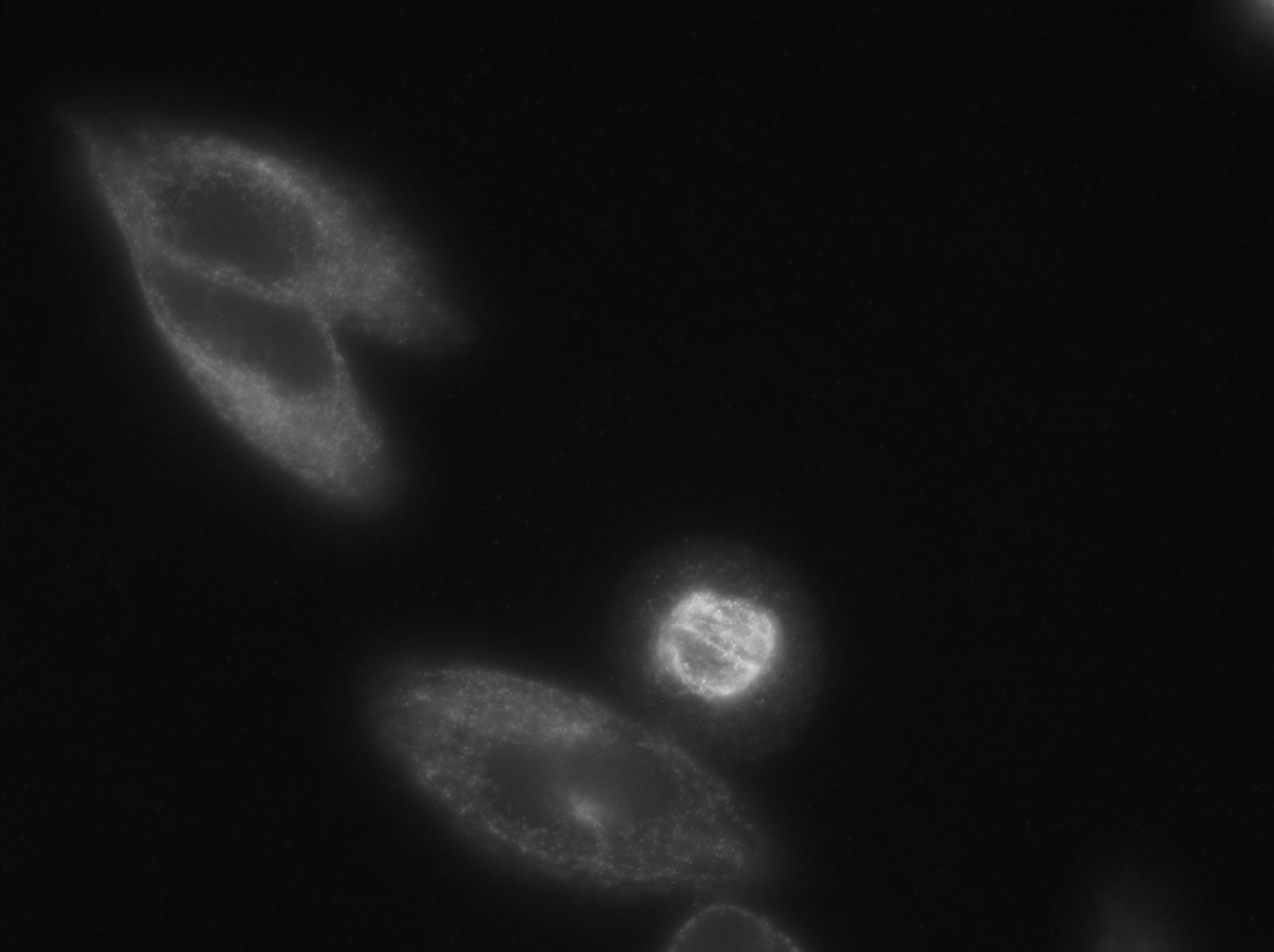

Supplement: Supplementary file 27 — Source data Fig. 7 part 3 [file 44319_2026_742_MOESM27_ESM.zip › Figure 7 Part 3/Fig 7be Cas9 and TPGS1-KO rGT335 atubulin/TPGS1-KO 5-2-25 rGT335 atub R3 M7.Project Maximum Z_XY1746220162_Z0_T0_C2.tif]

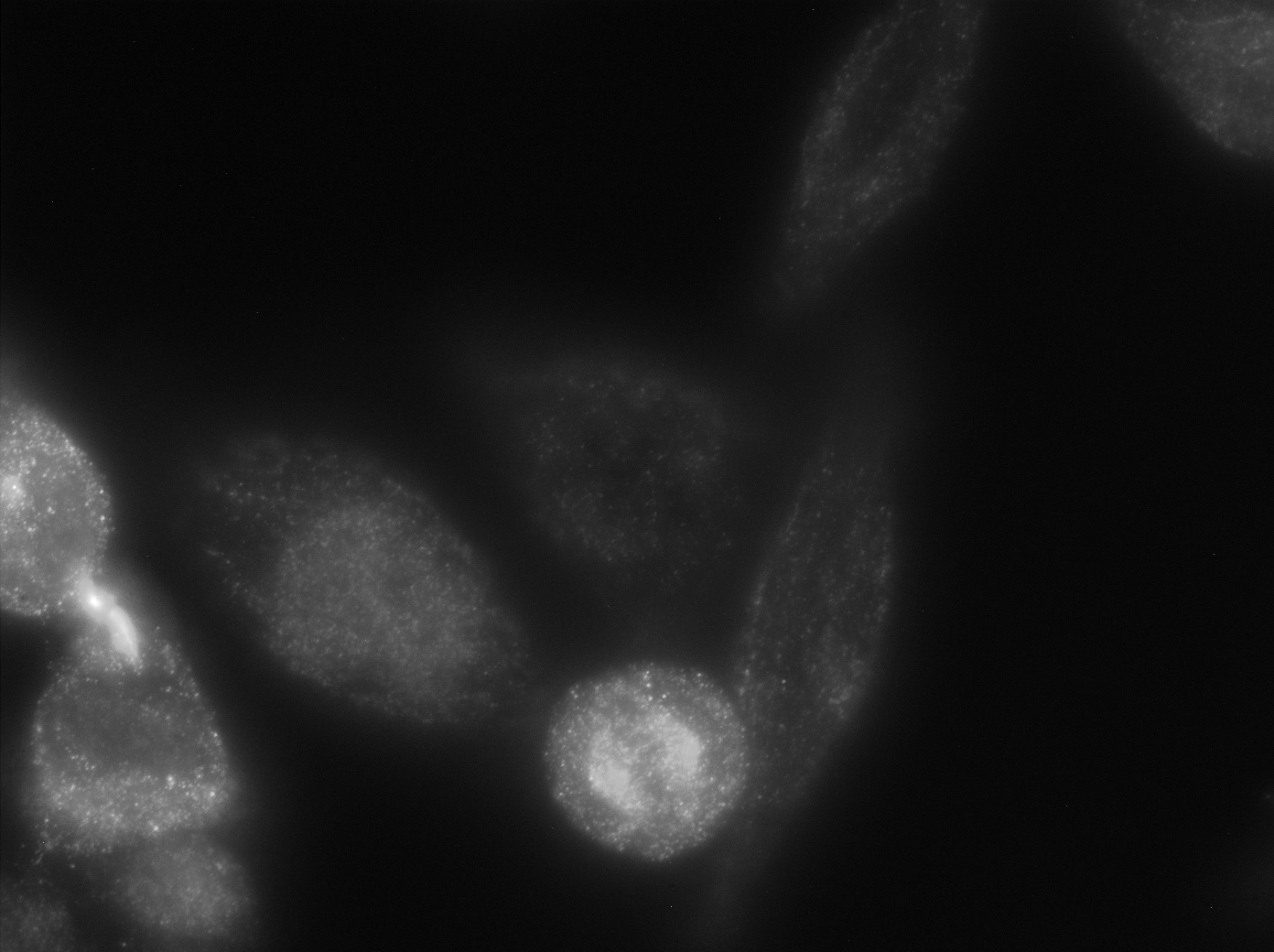

Supplement: Supplementary file 27 — Source data Fig. 7 part 3 [file 44319_2026_742_MOESM27_ESM.zip › Figure 7 Part 3/Fig 7be Cas9 and TPGS1-KO rGT335 atubulin/Cas9 5-2-25 rGT335 atub R3 M4.Project Maximum Z_XY1746216800_Z0_T0_C1.tif]

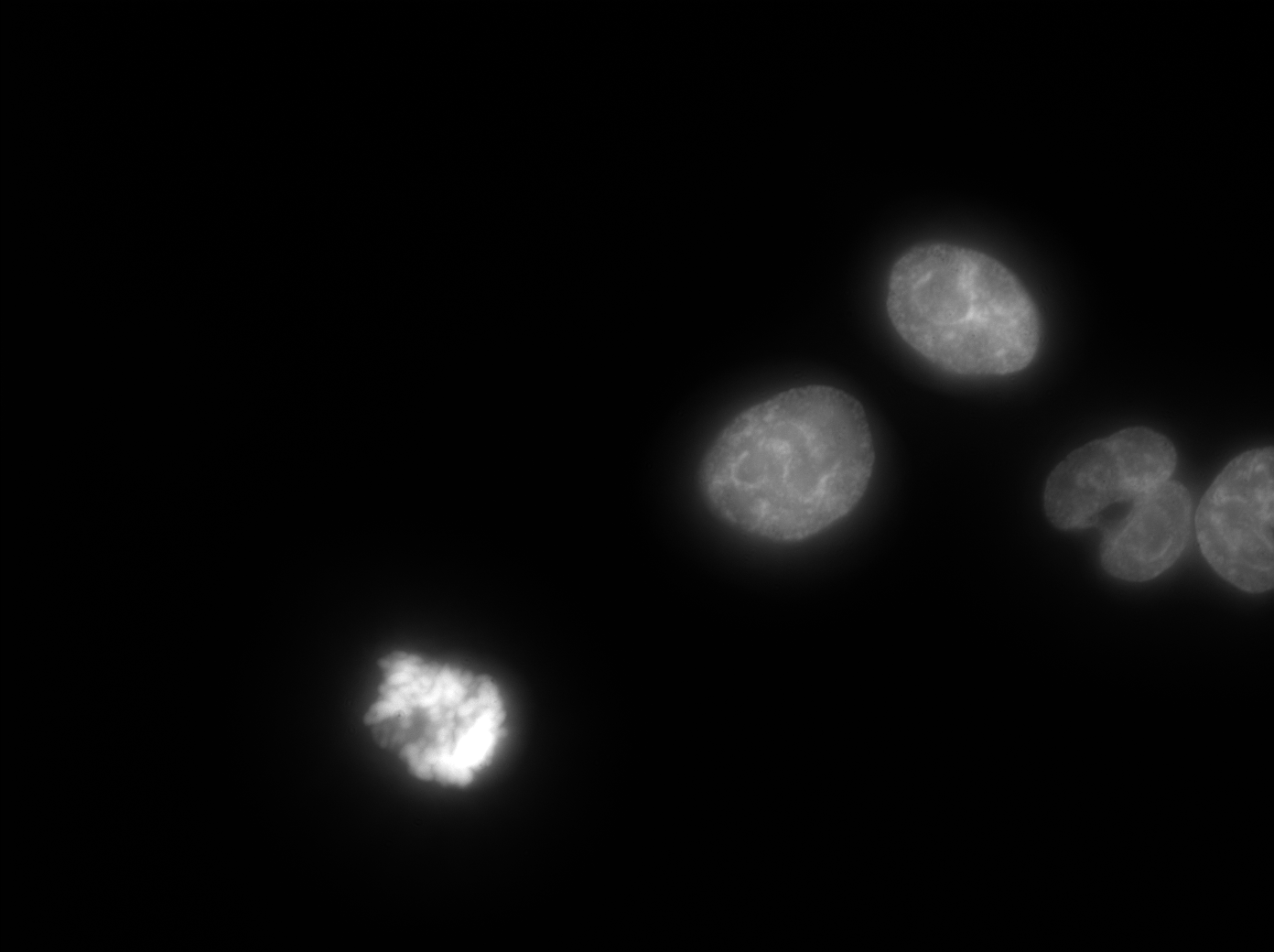

Supplement: Supplementary file 27 — Source data Fig. 7 part 3 [file 44319_2026_742_MOESM27_ESM.zip › Figure 7 Part 3/Fig 7be Cas9 and TPGS1-KO rGT335 atubulin/TPGS1-KO 5-2-25 rGT335 atub R1 M2.Project Maximum Z_XY1746221047_Z0_T0_C0.tif]

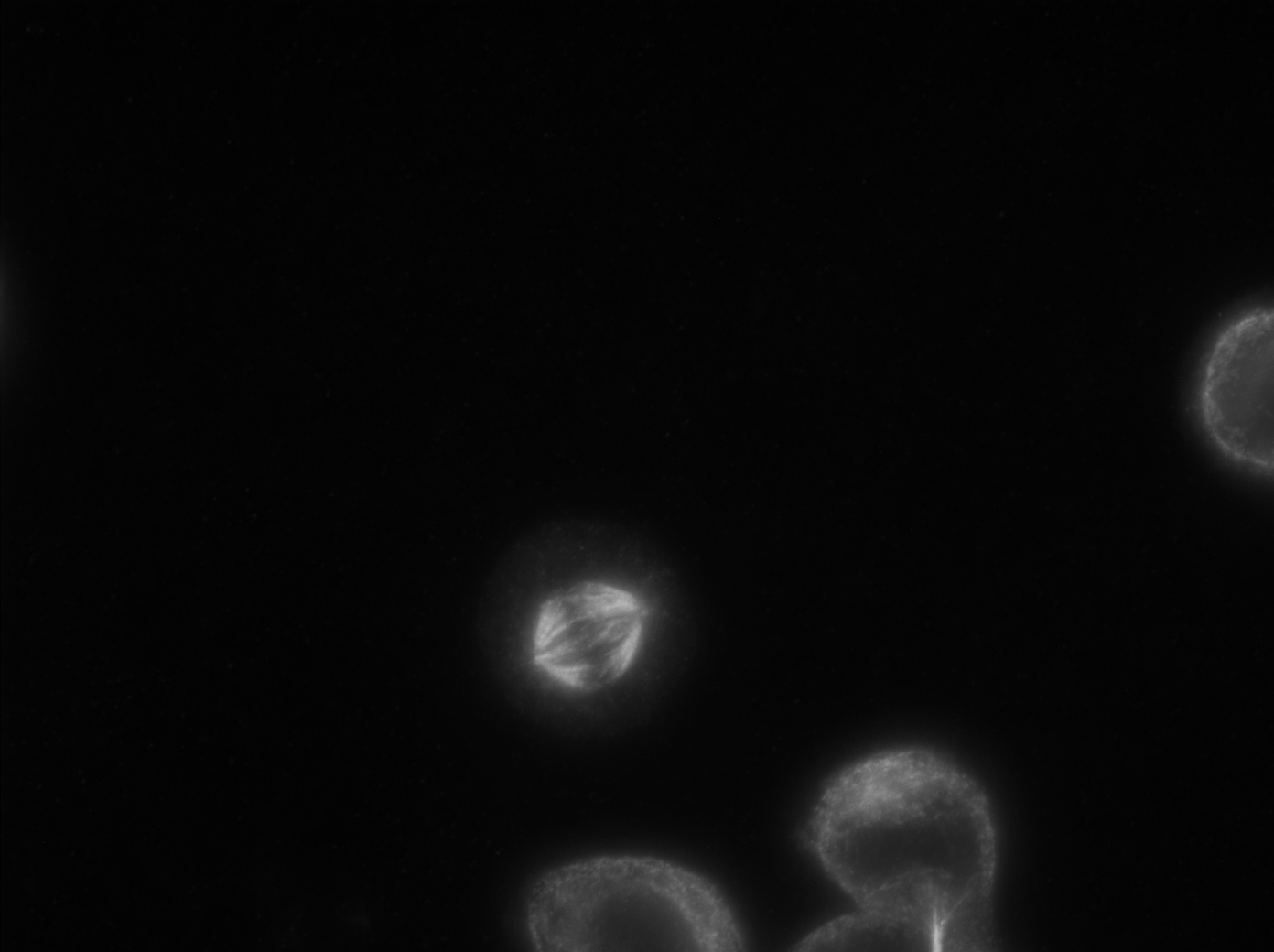

Supplement: Supplementary file 27 — Source data Fig. 7 part 3 [file 44319_2026_742_MOESM27_ESM.zip › Figure 7 Part 3/Fig 7be Cas9 and TPGS1-KO rGT335 atubulin/TPGS1-KO 5-2-25 rGT335 atub R2 M4.Project Maximum Z_XY1746564182_Z0_T0_C2.tif]

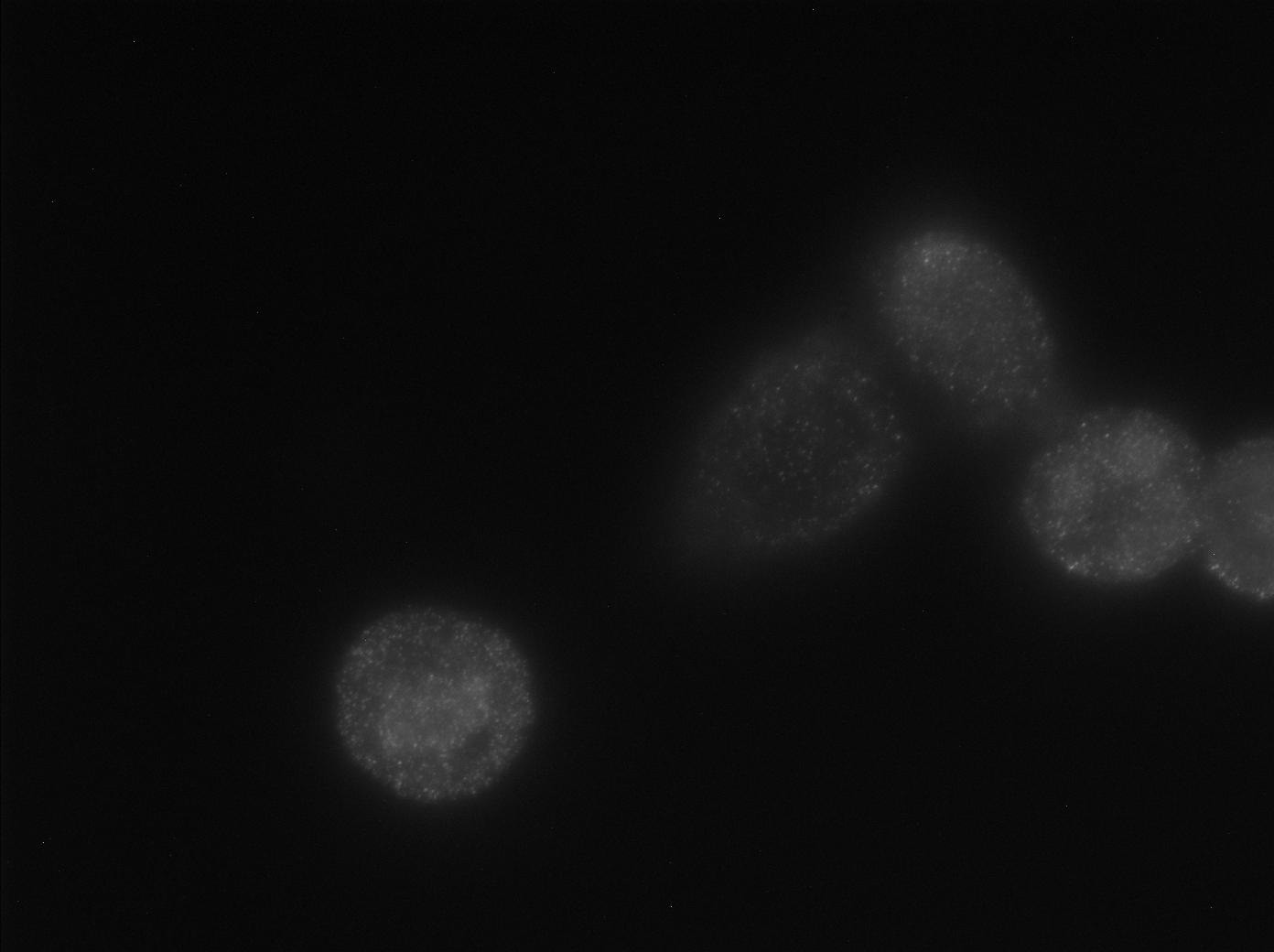

Supplement: Supplementary file 27 — Source data Fig. 7 part 3 [file 44319_2026_742_MOESM27_ESM.zip › Figure 7 Part 3/Fig 7be Cas9 and TPGS1-KO rGT335 atubulin/TPGS1-KO 5-2-25 rGT335 atub R1 M2.Project Maximum Z_XY1746221047_Z0_T0_C1.tif]

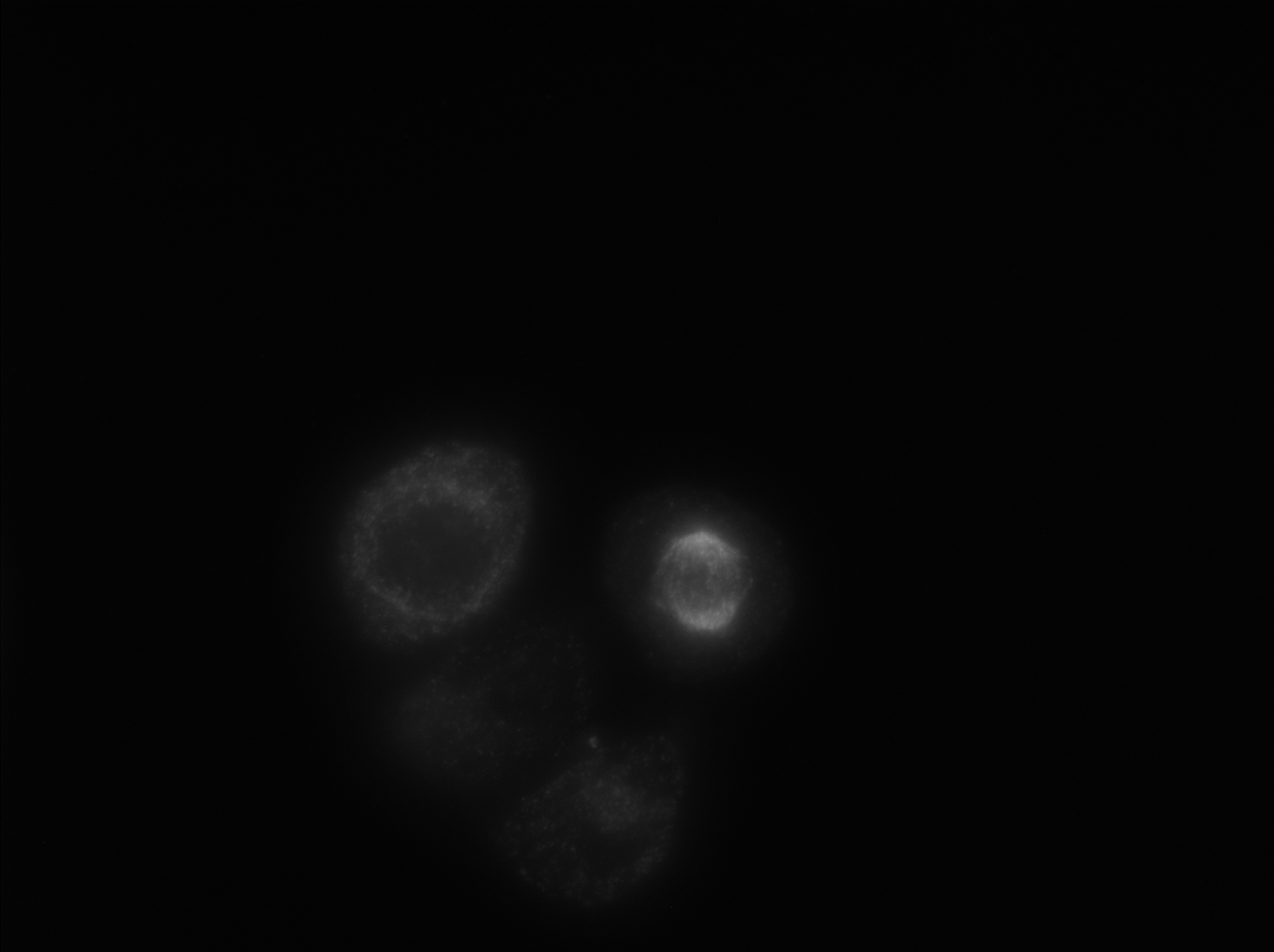

Supplement: Supplementary file 27 — Source data Fig. 7 part 3 [file 44319_2026_742_MOESM27_ESM.zip › Figure 7 Part 3/Fig 7be Cas9 and TPGS1-KO rGT335 atubulin/Cas9 5-2-25 rGT335 atub R3 M1.Project Maximum Z_XY1746214043_Z0_T0_C2.tif]

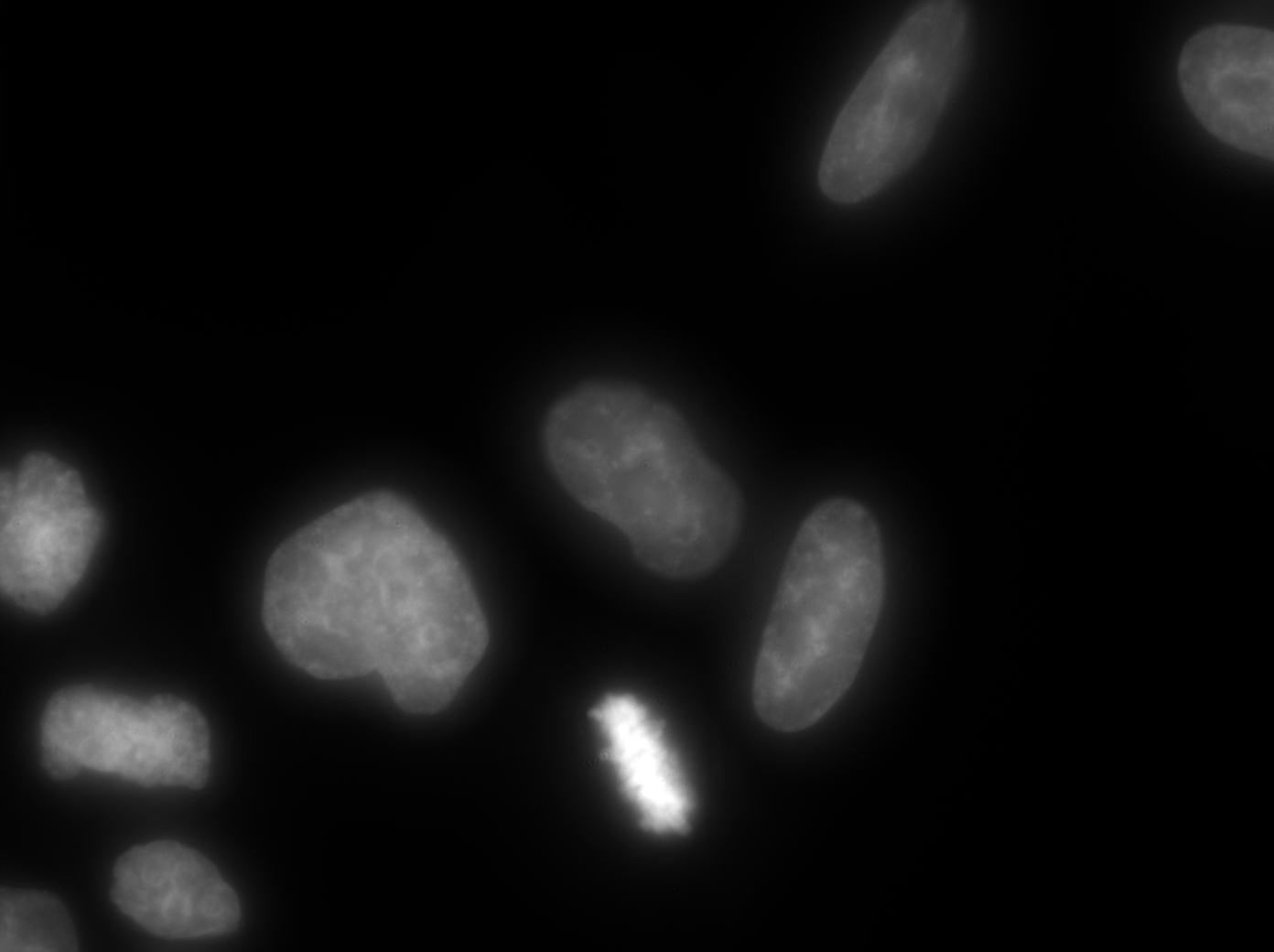

Supplement: Supplementary file 27 — Source data Fig. 7 part 3 [file 44319_2026_742_MOESM27_ESM.zip › Figure 7 Part 3/Fig 7be Cas9 and TPGS1-KO rGT335 atubulin/Cas9 5-2-25 rGT335 atub R3 M4.Project Maximum Z_XY1746216800_Z0_T0_C0.tif]

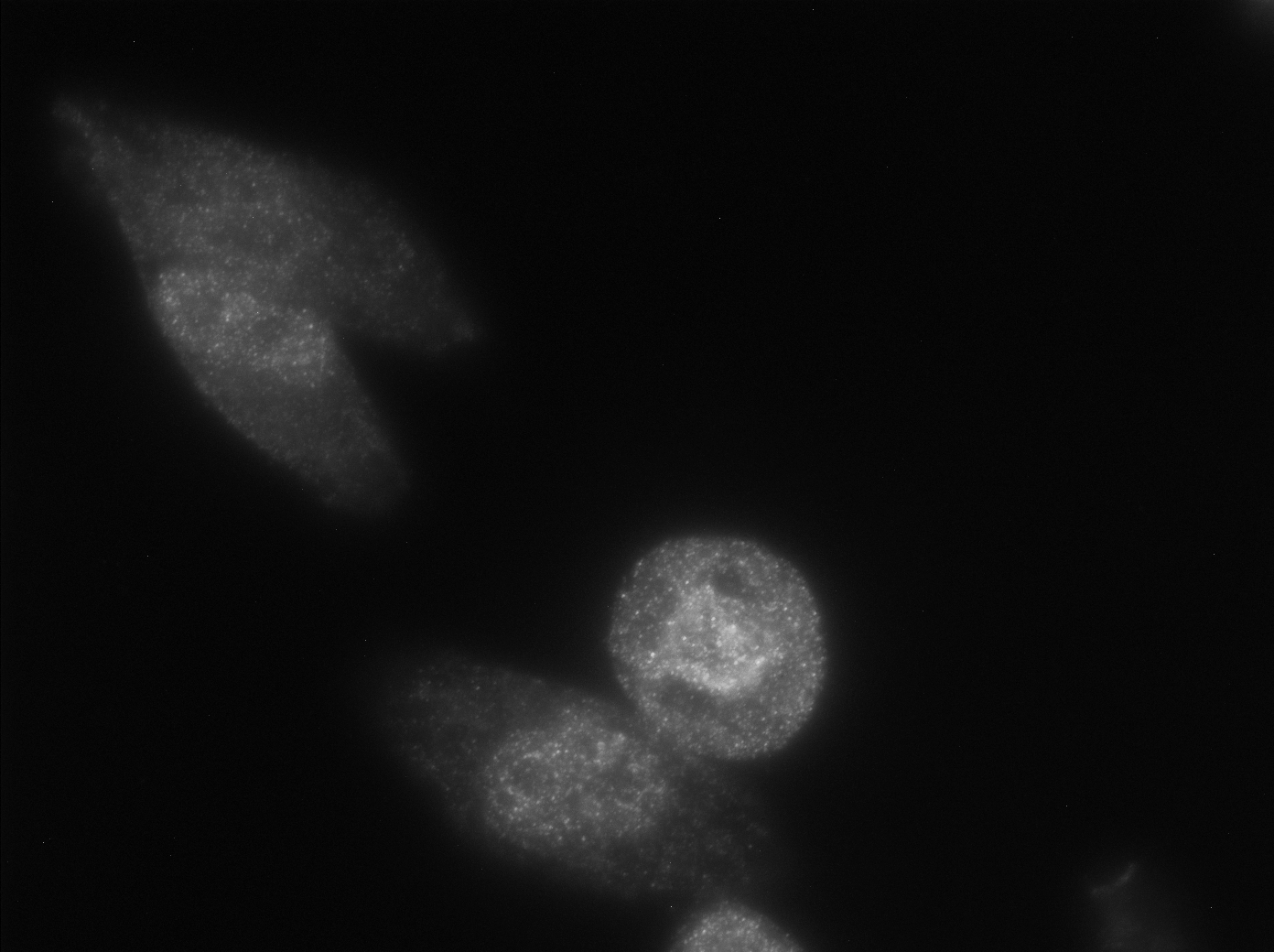

Supplement: Supplementary file 27 — Source data Fig. 7 part 3 [file 44319_2026_742_MOESM27_ESM.zip › Figure 7 Part 3/Fig 7be Cas9 and TPGS1-KO rGT335 atubulin/TPGS1-KO 5-2-25 rGT335 atub R3 M7.Project Maximum Z_XY1746220162_Z0_T0_C1.tif]

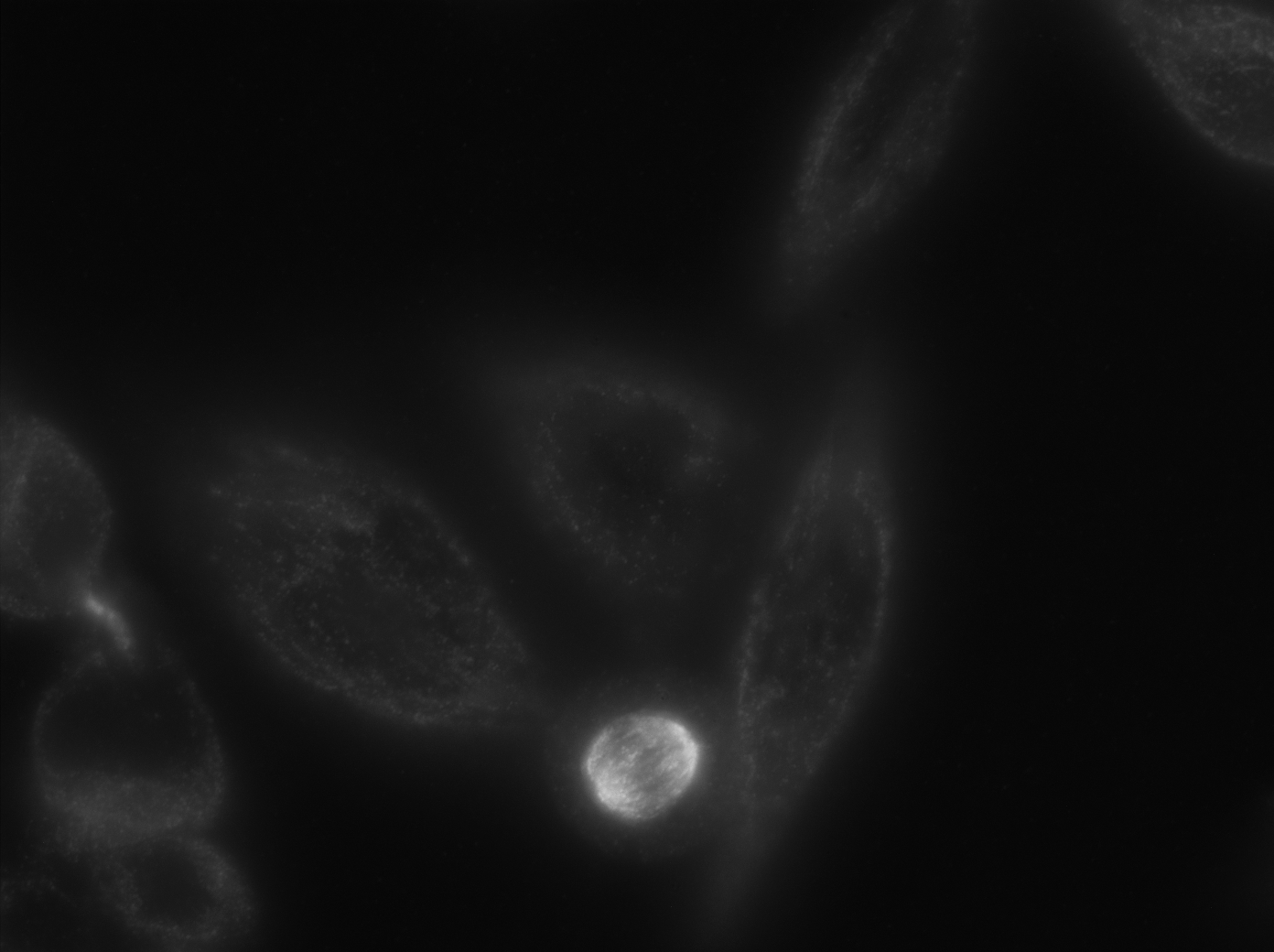

Supplement: Supplementary file 27 — Source data Fig. 7 part 3 [file 44319_2026_742_MOESM27_ESM.zip › Figure 7 Part 3/Fig 7be Cas9 and TPGS1-KO rGT335 atubulin/Cas9 5-2-25 rGT335 atub R3 M4.Project Maximum Z_XY1746216800_Z0_T0_C2.tif]

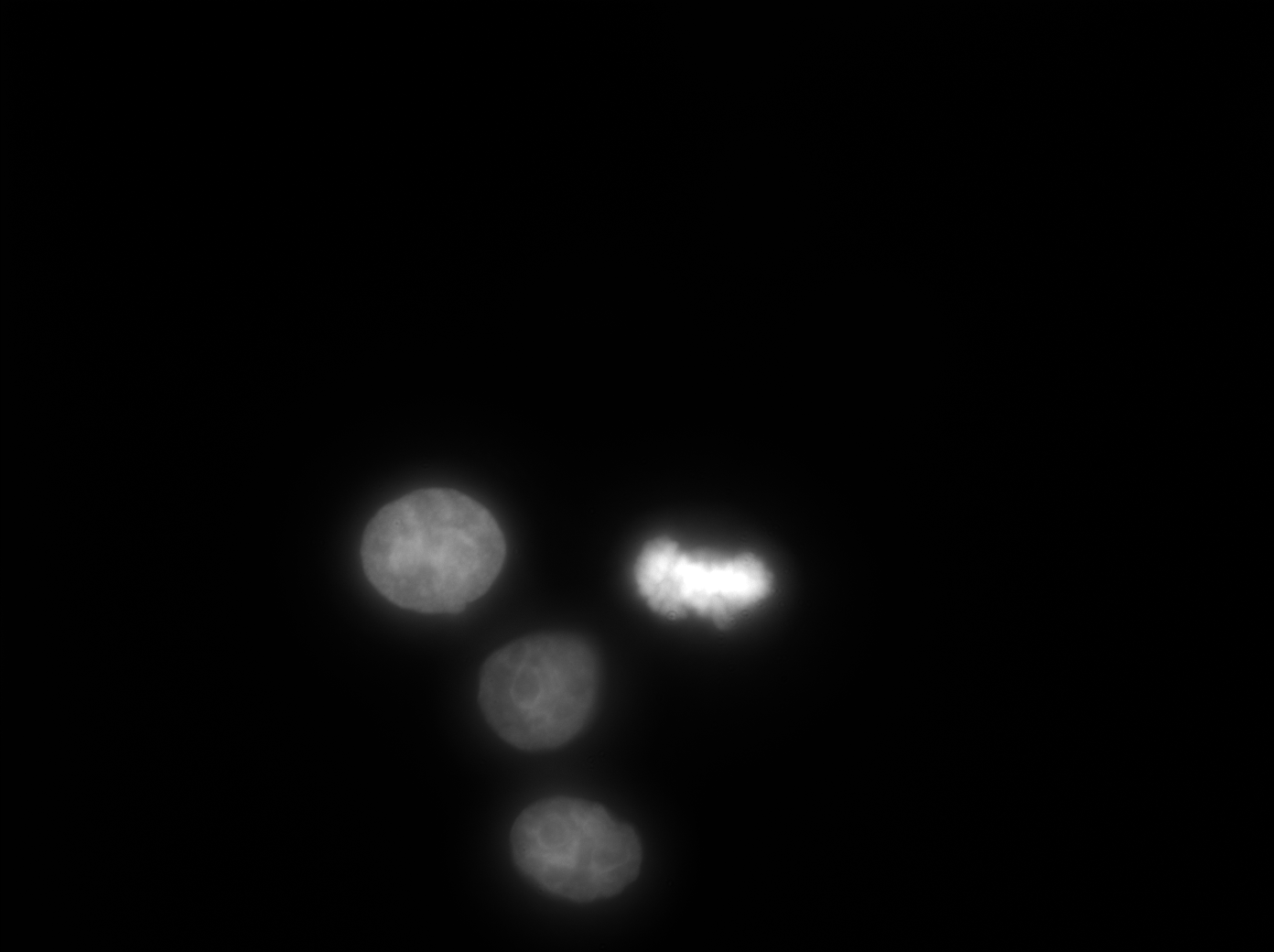

Supplement: Supplementary file 27 — Source data Fig. 7 part 3 [file 44319_2026_742_MOESM27_ESM.zip › Figure 7 Part 3/Fig 7be Cas9 and TPGS1-KO rGT335 atubulin/Cas9 5-2-25 rGT335 atub R3 M1.Project Maximum Z_XY1746214043_Z0_T0_C0.tif]

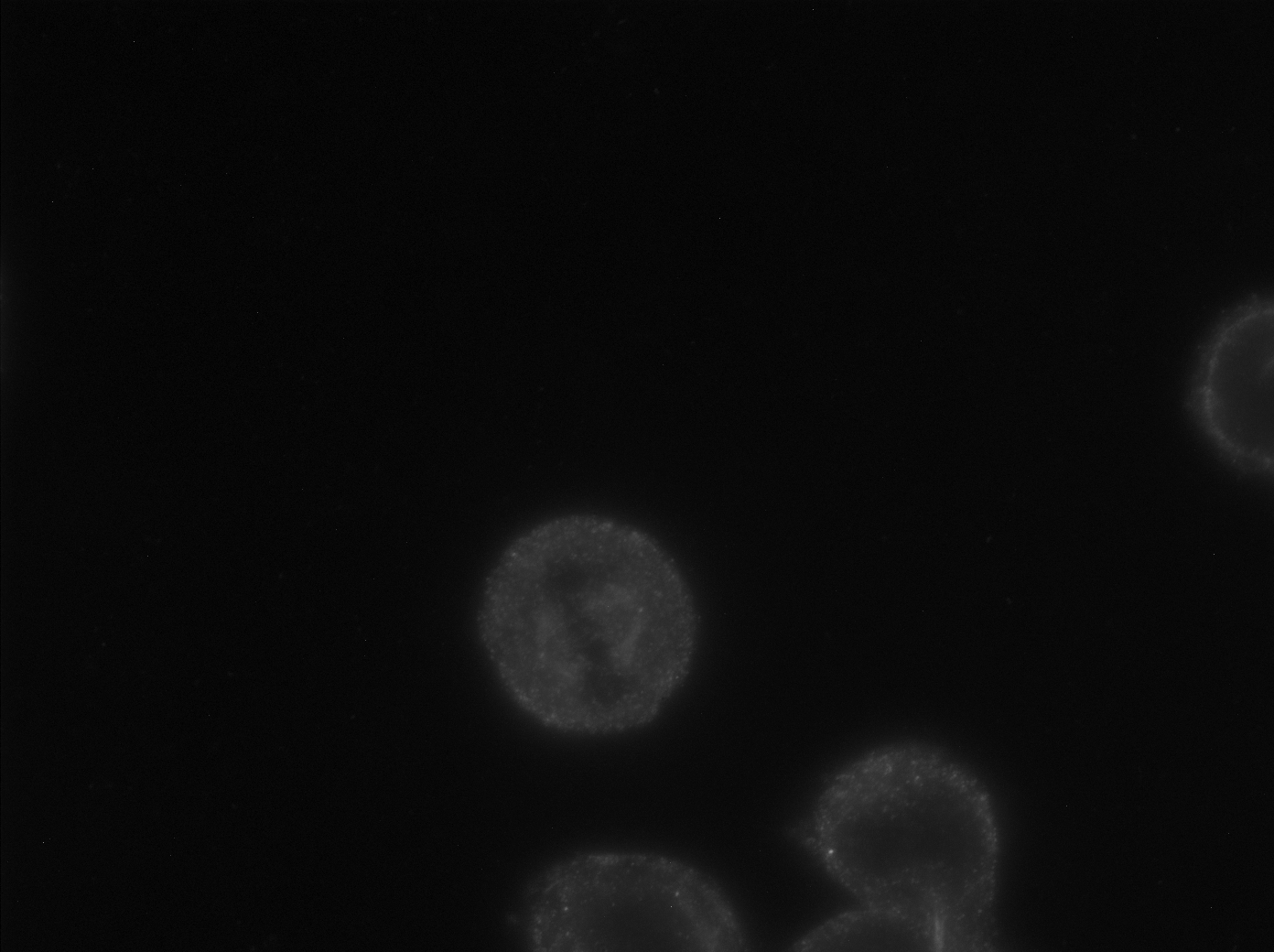

Supplement: Supplementary file 27 — Source data Fig. 7 part 3 [file 44319_2026_742_MOESM27_ESM.zip › Figure 7 Part 3/Fig 7be Cas9 and TPGS1-KO rGT335 atubulin/TPGS1-KO 5-2-25 rGT335 atub R2 M4.Project Maximum Z_XY1746564182_Z0_T0_C1.tif]

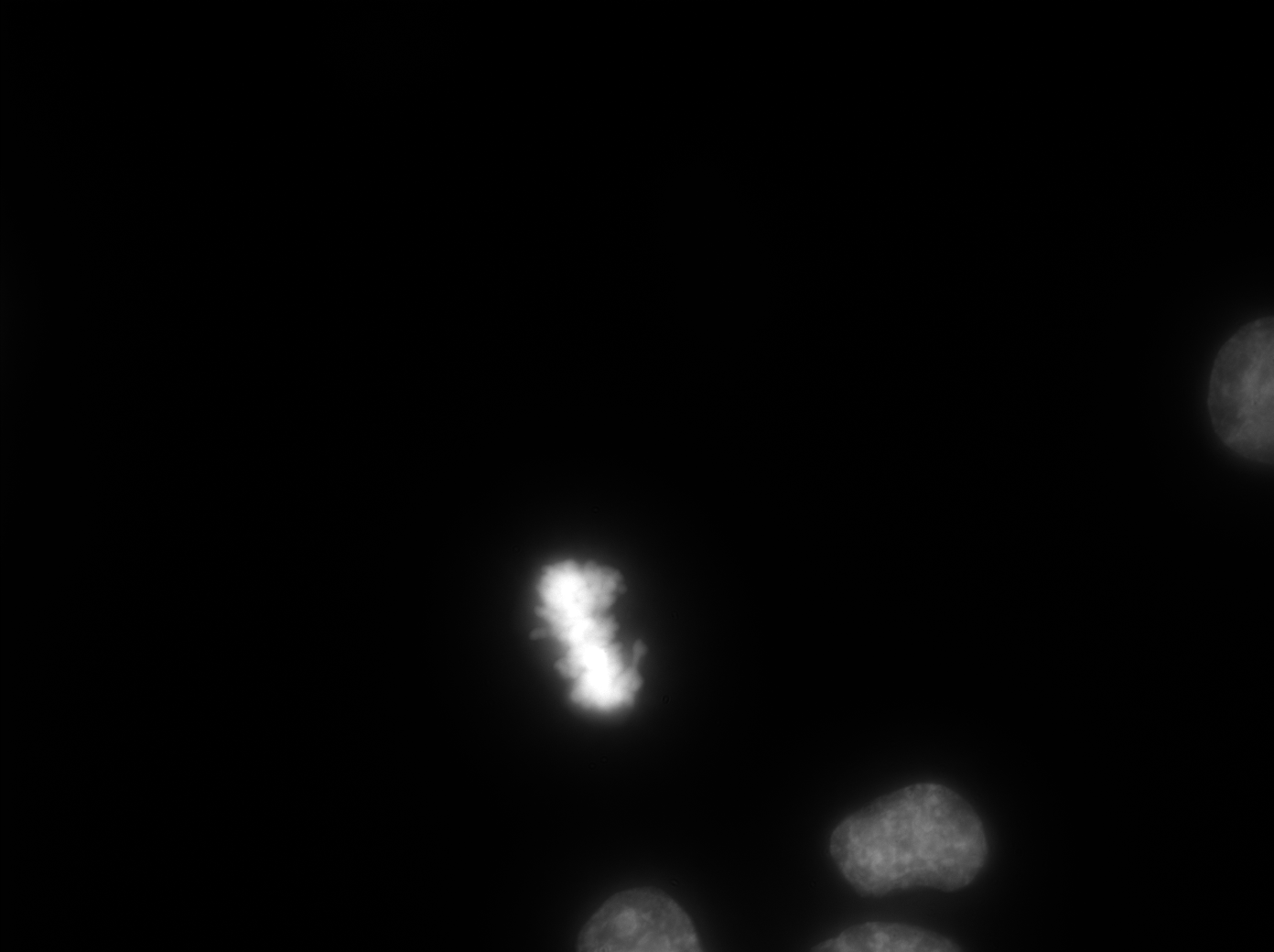

Supplement: Supplementary file 27 — Source data Fig. 7 part 3 [file 44319_2026_742_MOESM27_ESM.zip › Figure 7 Part 3/Fig 7be Cas9 and TPGS1-KO rGT335 atubulin/TPGS1-KO 5-2-25 rGT335 atub R2 M4.Project Maximum Z_XY1746564182_Z0_T0_C0.tif]

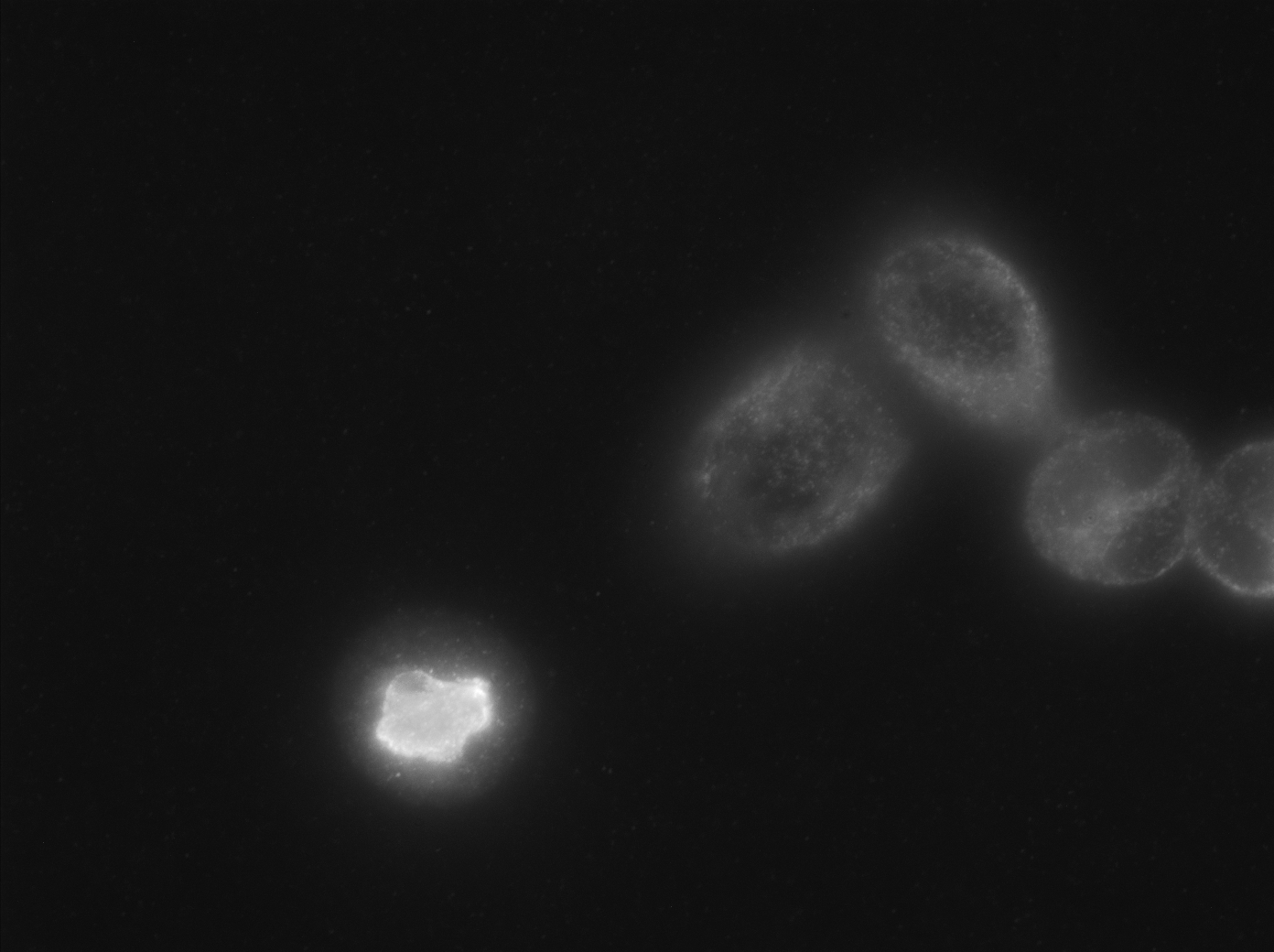

Supplement: Supplementary file 27 — Source data Fig. 7 part 3 [file 44319_2026_742_MOESM27_ESM.zip › Figure 7 Part 3/Fig 7be Cas9 and TPGS1-KO rGT335 atubulin/TPGS1-KO 5-2-25 rGT335 atub R1 M2.Project Maximum Z_XY1746221047_Z0_T0_C2.tif]

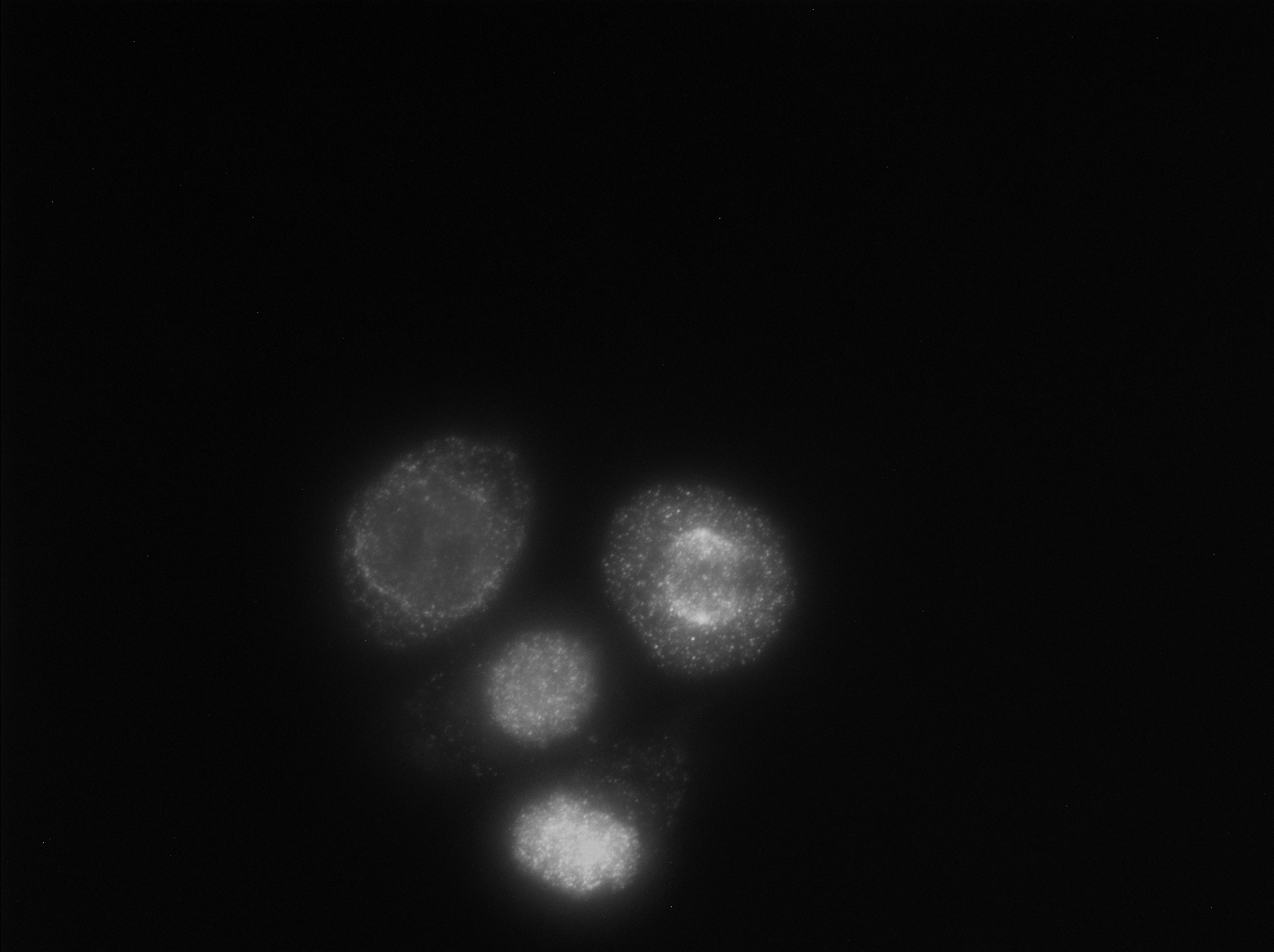

Supplement: Supplementary file 27 — Source data Fig. 7 part 3 [file 44319_2026_742_MOESM27_ESM.zip › Figure 7 Part 3/Fig 7be Cas9 and TPGS1-KO rGT335 atubulin/Cas9 5-2-25 rGT335 atub R3 M1.Project Maximum Z_XY1746214043_Z0_T0_C1.tif]

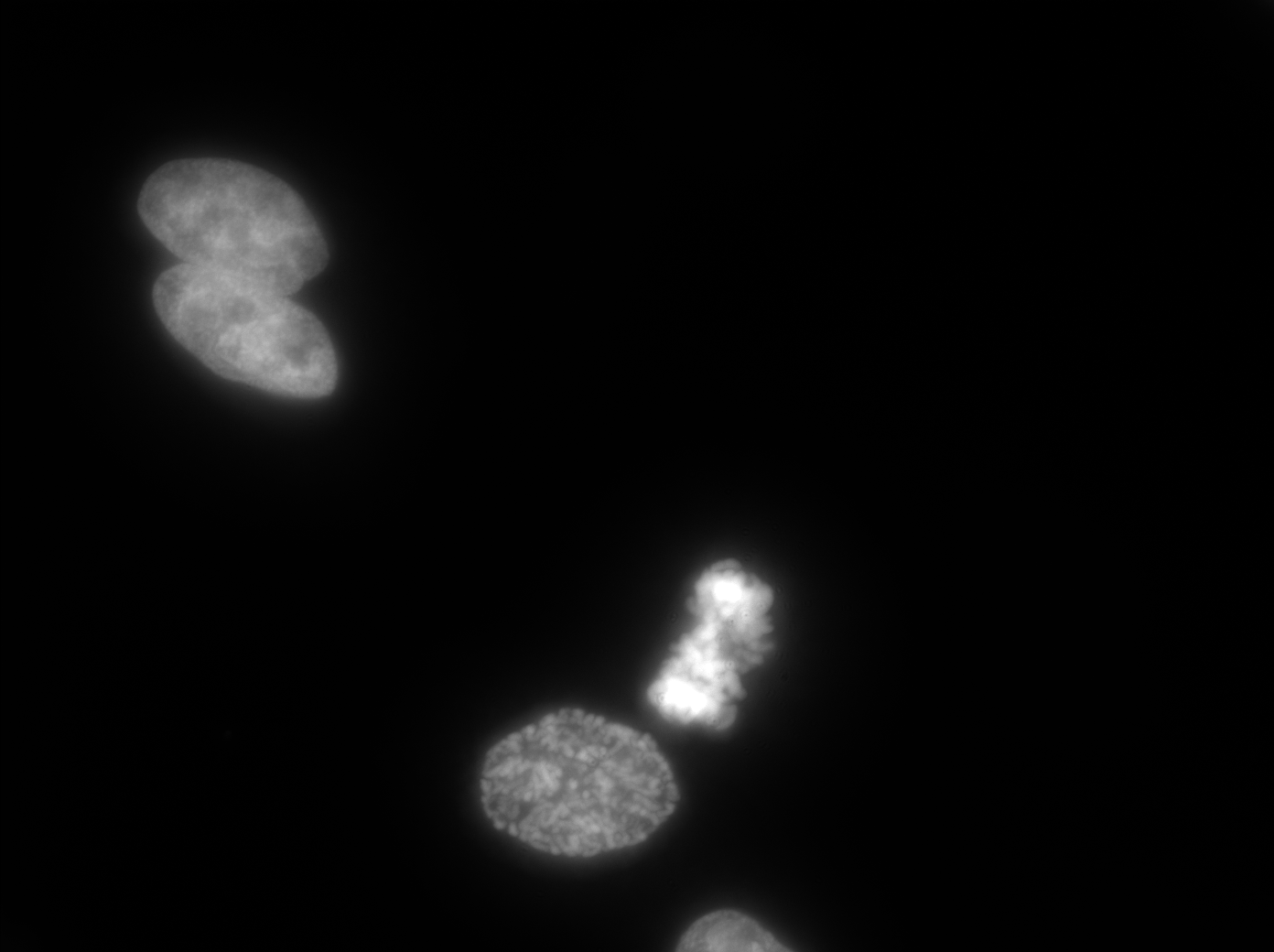

Supplement: Supplementary file 27 — Source data Fig. 7 part 3 [file 44319_2026_742_MOESM27_ESM.zip › Figure 7 Part 3/Fig 7be Cas9 and TPGS1-KO rGT335 atubulin/TPGS1-KO 5-2-25 rGT335 atub R3 M7.Project Maximum Z_XY1746220162_Z0_T0_C0.tif]

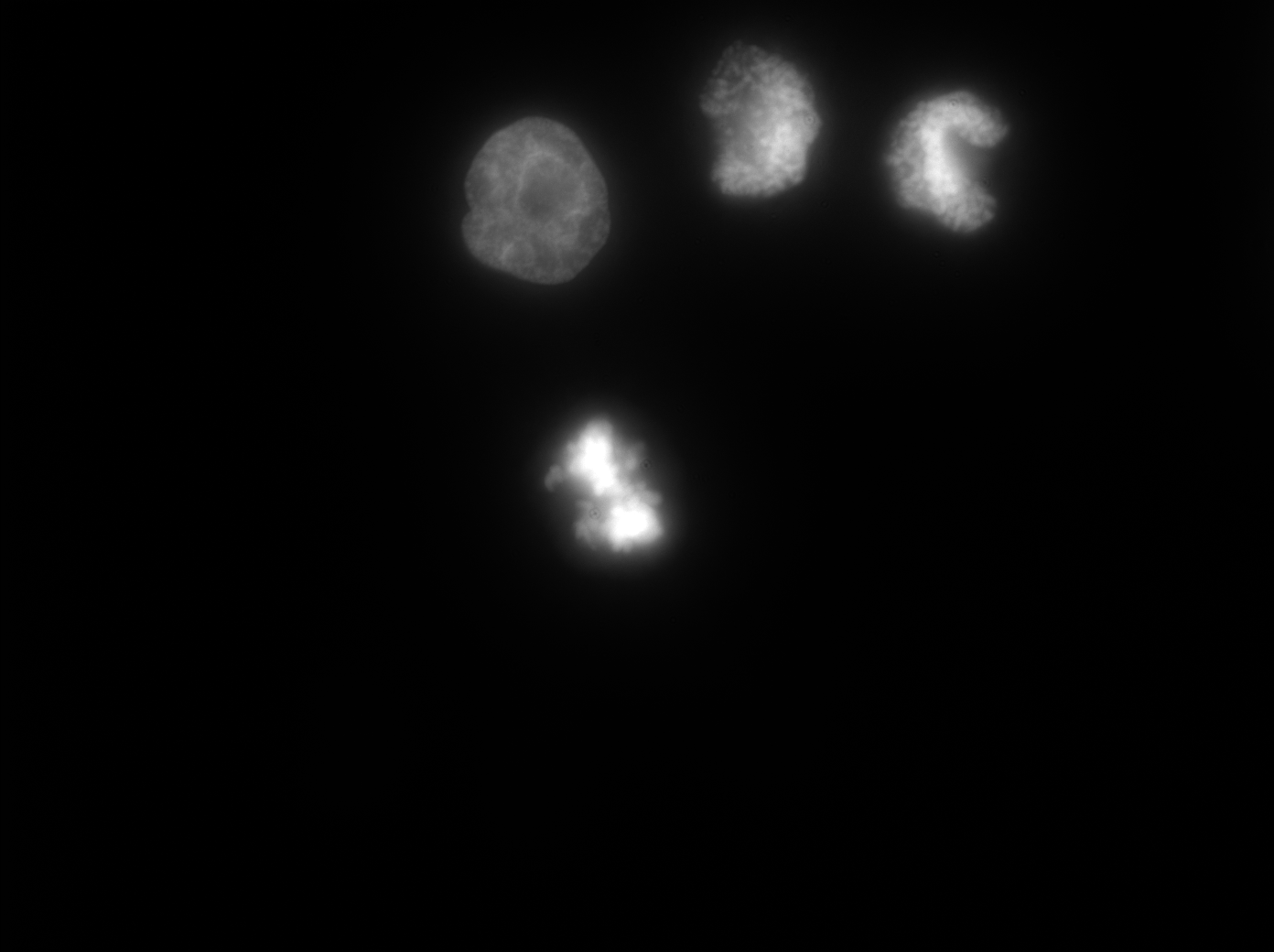

Supplement: Supplementary file 27 — Source data Fig. 7 part 3 [file 44319_2026_742_MOESM27_ESM.zip › Figure 7 Part 3/Fig 7be Cas9 and TPGS1-KO rGT335 atubulin/TPGS1-KO 5-2-25 rGT335 atub R1 M1.Project Maximum Z_XY1746220972_Z0_T0_C0.tif]

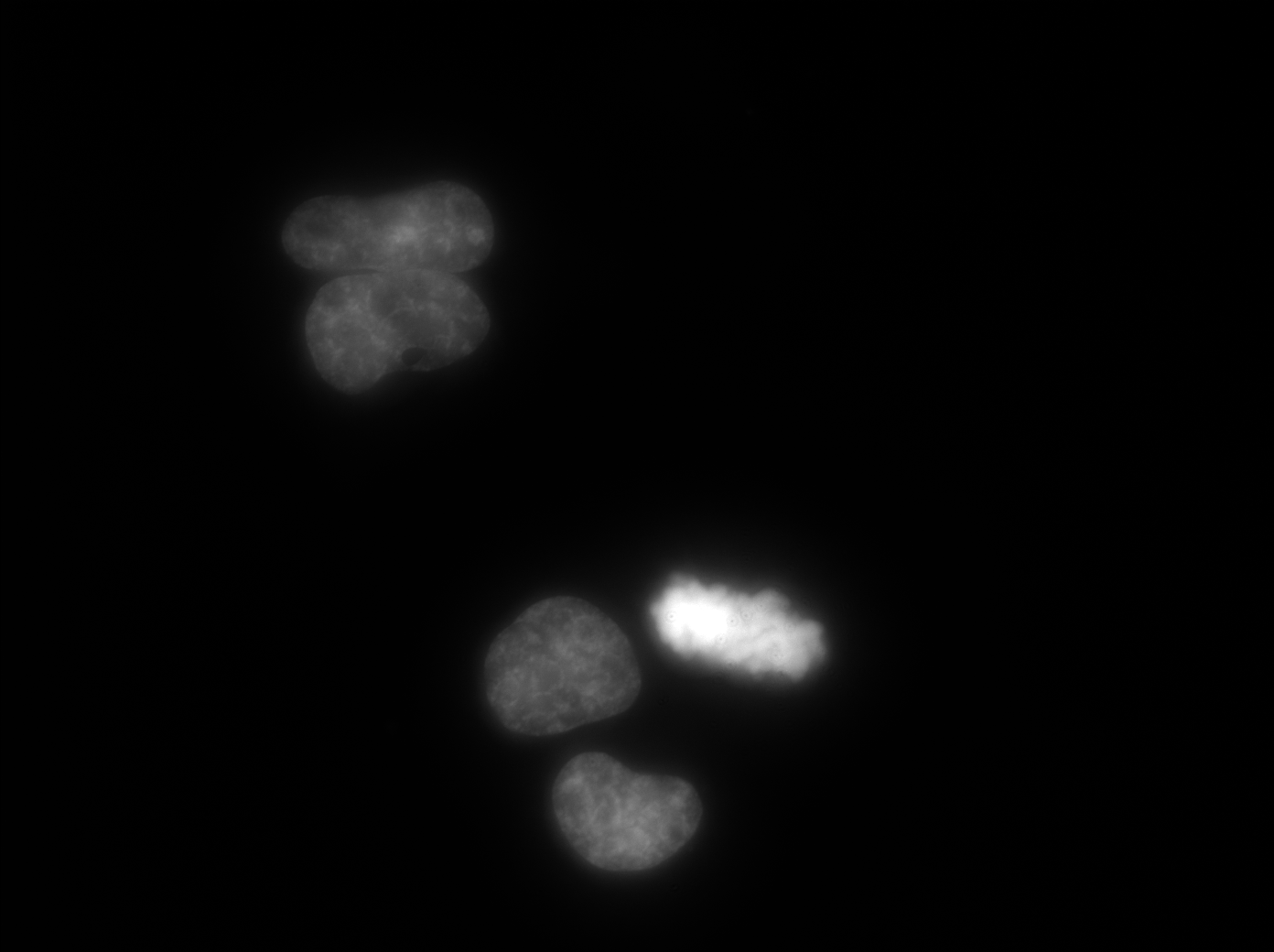

Supplement: Supplementary file 27 — Source data Fig. 7 part 3 [file 44319_2026_742_MOESM27_ESM.zip › Figure 7 Part 3/Fig 7be Cas9 and TPGS1-KO rGT335 atubulin/Cas9 5-2-25 rGT335 atub R2 M10.Project Maximum Z_XY1746562899_Z0_T0_C0.tif]

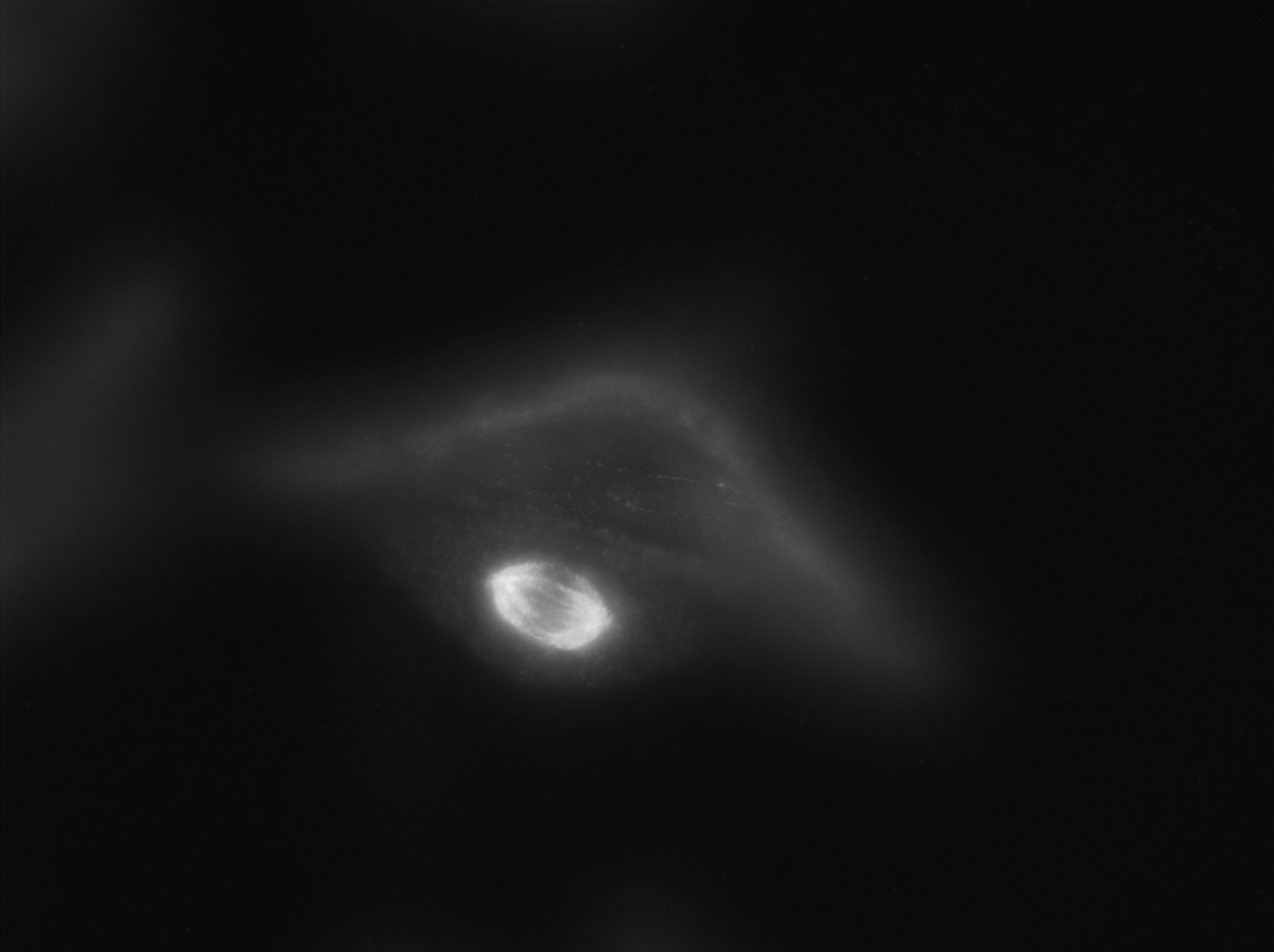

Supplement: Supplementary file 27 — Source data Fig. 7 part 3 [file 44319_2026_742_MOESM27_ESM.zip › Figure 7 Part 3/Fig 7be Cas9 and TPGS1-KO rGT335 atubulin/TPGS1-KO 5-2-25 rGT335 atub R1 M3.Project Maximum Z_XY1746221312_Z0_T0_C2.tif]

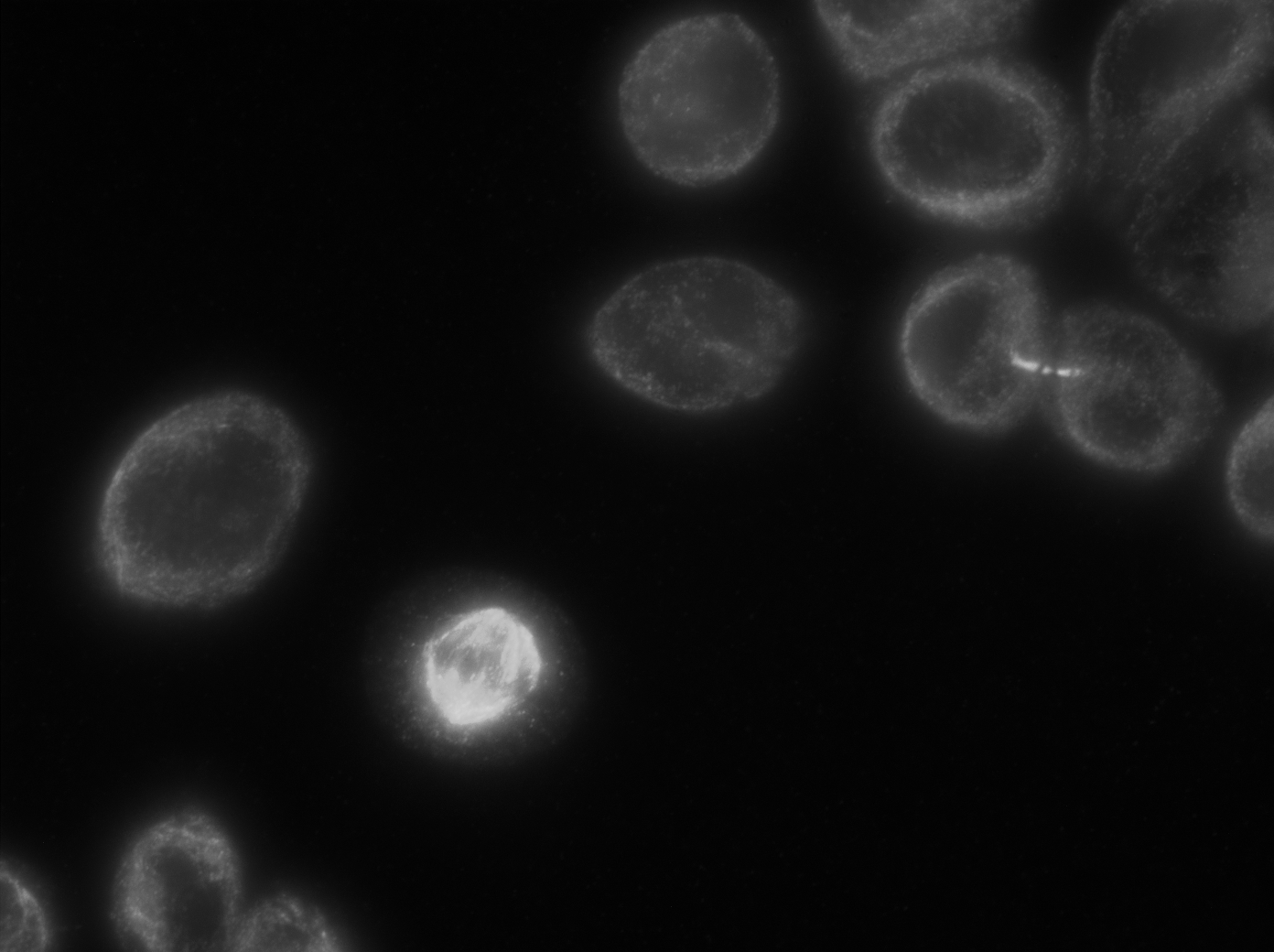

Supplement: Supplementary file 27 — Source data Fig. 7 part 3 [file 44319_2026_742_MOESM27_ESM.zip › Figure 7 Part 3/Fig 7be Cas9 and TPGS1-KO rGT335 atubulin/TPGS1-KO 5-2-25 rGT335 atub R2 M7.Project Maximum Z_XY1746564635_Z0_T0_C2.tif]

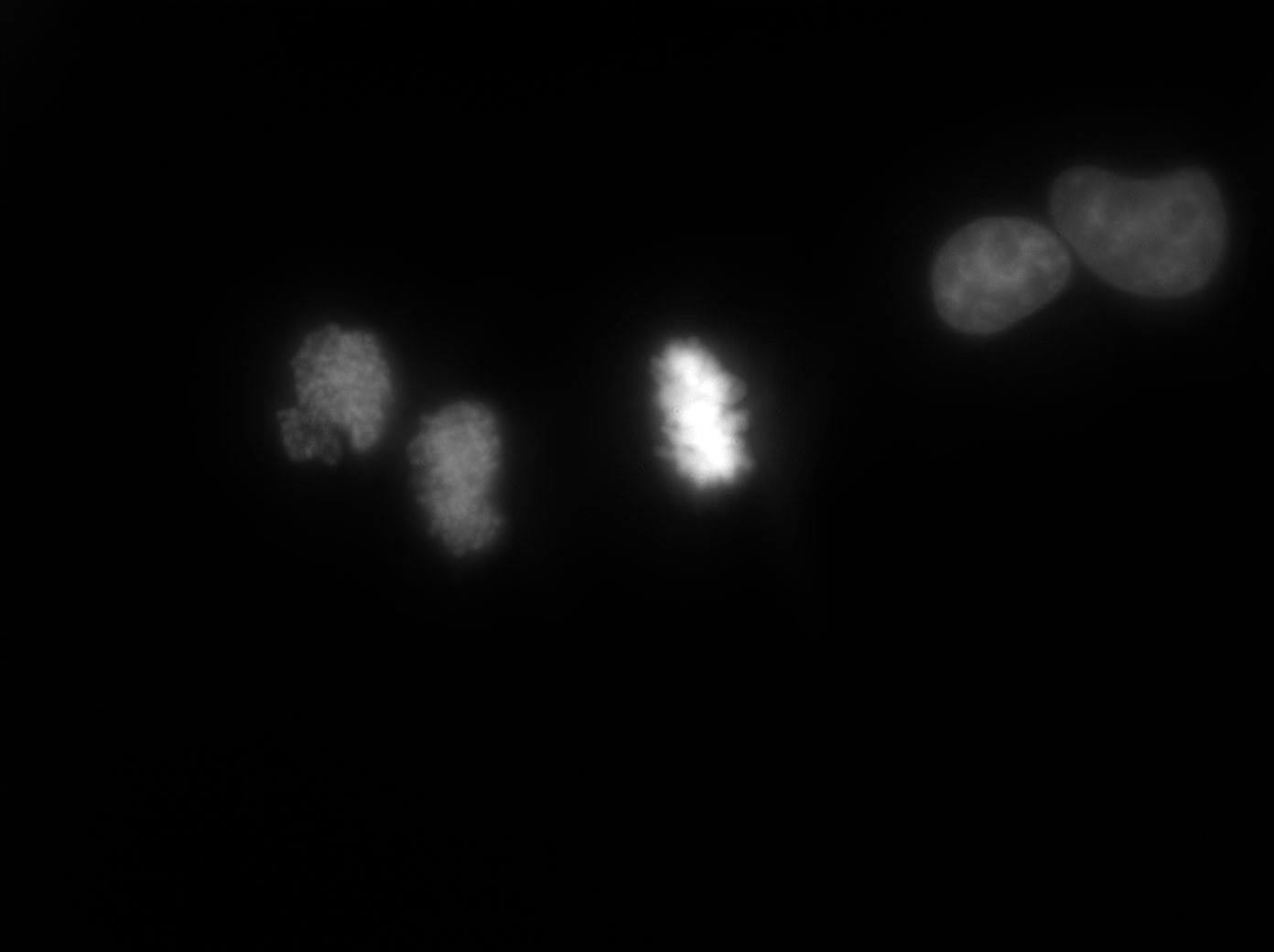

Supplement: Supplementary file 27 — Source data Fig. 7 part 3 [file 44319_2026_742_MOESM27_ESM.zip › Figure 7 Part 3/Fig 7be Cas9 and TPGS1-KO rGT335 atubulin/Cas9 5-2-25 rGT335 atub R2 M6.Project Maximum Z_XY1746562239_Z0_T0_C0.tif]

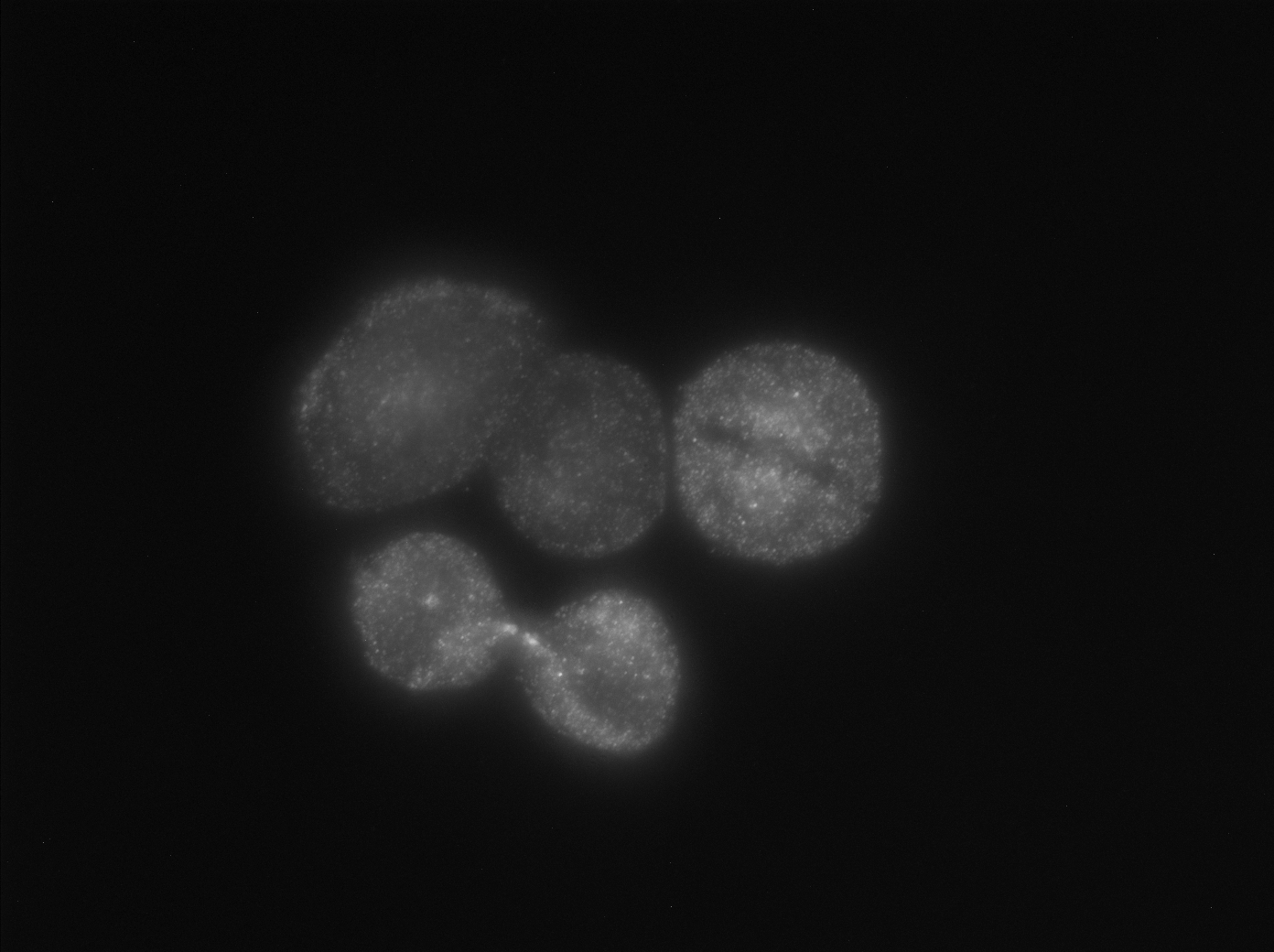

Supplement: Supplementary file 27 — Source data Fig. 7 part 3 [file 44319_2026_742_MOESM27_ESM.zip › Figure 7 Part 3/Fig 7be Cas9 and TPGS1-KO rGT335 atubulin/Cas9 5-2-25 rGT335 atub R2 M8.Project Maximum Z_XY1746562515_Z0_T0_C1.tif]

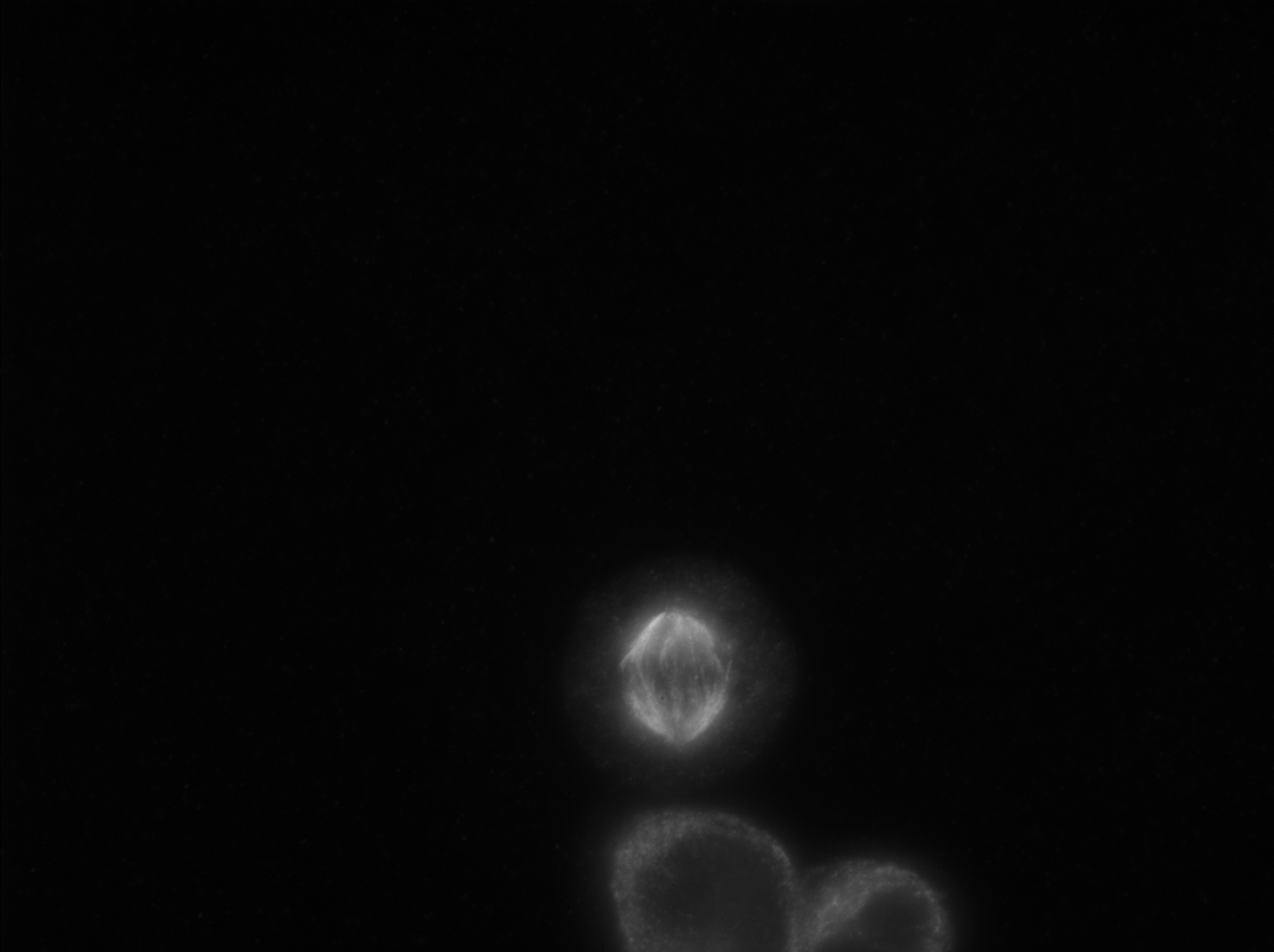

Supplement: Supplementary file 27 — Source data Fig. 7 part 3 [file 44319_2026_742_MOESM27_ESM.zip › Figure 7 Part 3/Fig 7be Cas9 and TPGS1-KO rGT335 atubulin/TPGS1-KO 5-2-25 rGT335 atub R2 M6.Project Maximum Z_XY1746564489_Z0_T0_C2.tif]

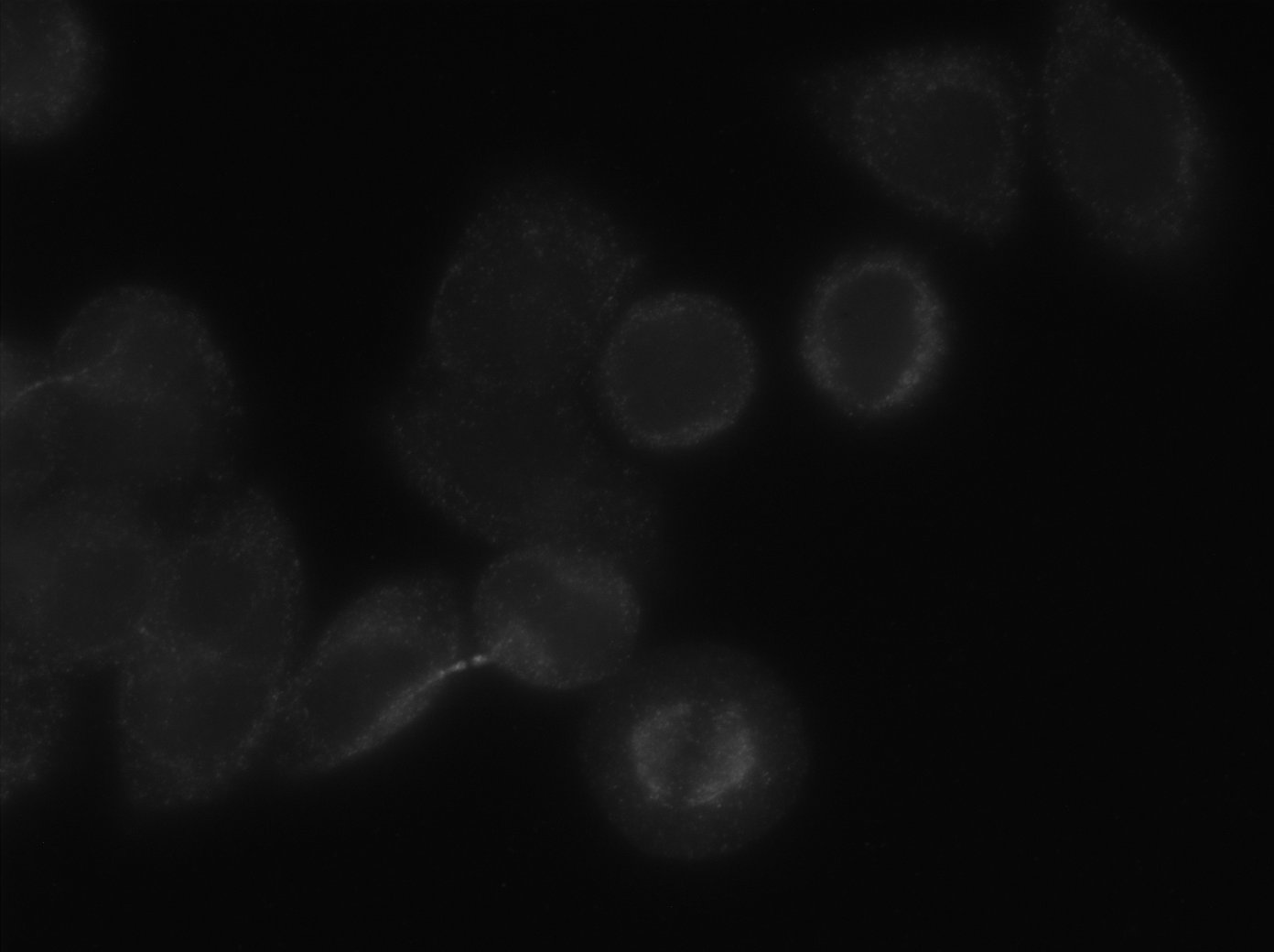

Supplement: Supplementary file 27 — Source data Fig. 7 part 3 [file 44319_2026_742_MOESM27_ESM.zip › Figure 7 Part 3/Fig 7be Cas9 and TPGS1-KO rGT335 atubulin/Cas9 5-2-25 rGT335 atub R1 M4.Project Maximum Z_XY1746557526_Z0_T0_C2.tif]

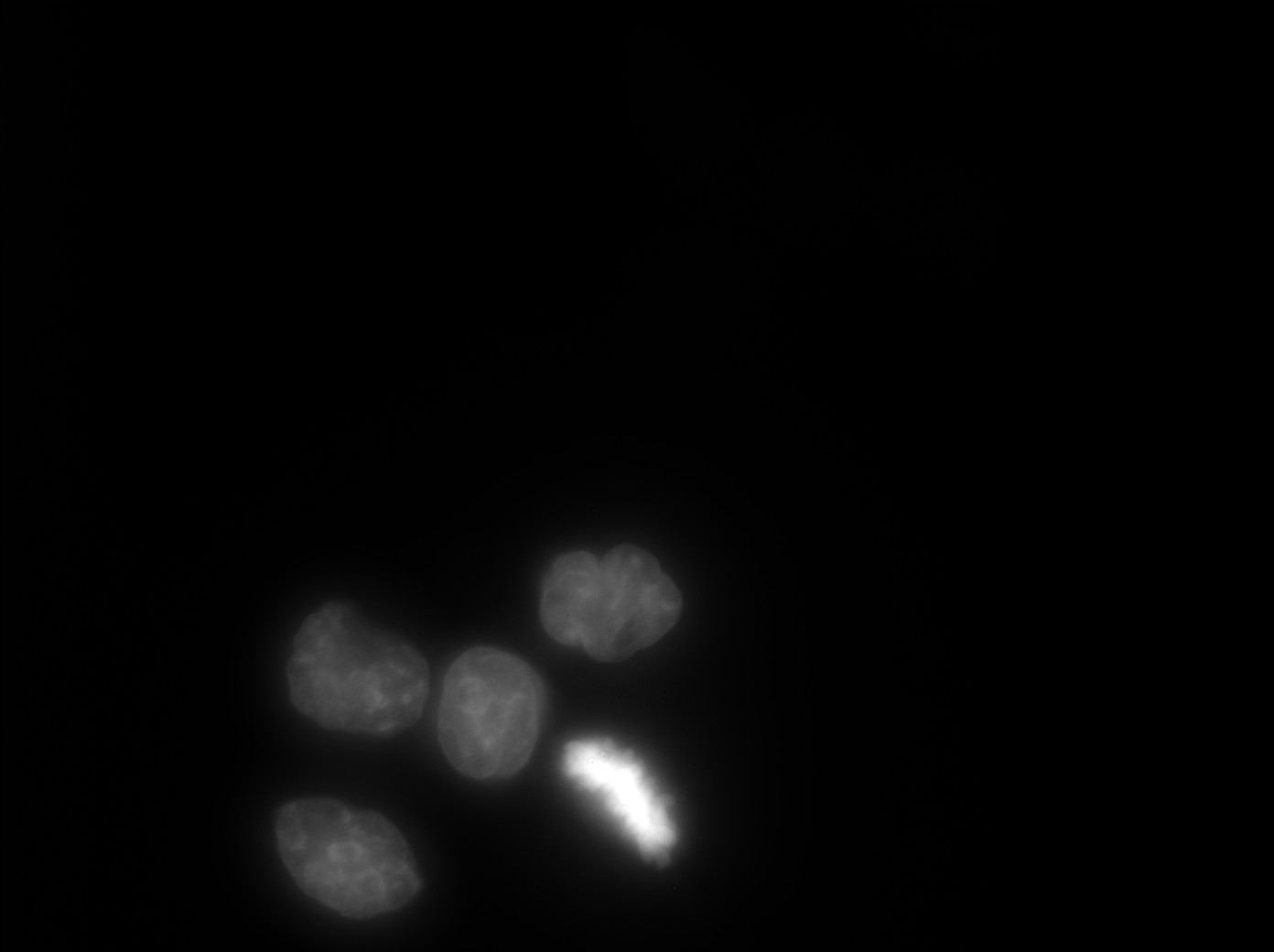

Supplement: Supplementary file 27 — Source data Fig. 7 part 3 [file 44319_2026_742_MOESM27_ESM.zip › Figure 7 Part 3/Fig 7be Cas9 and TPGS1-KO rGT335 atubulin/Cas9 5-2-25 rGT335 atub R3 M6.Project Maximum Z_XY1746217213_Z0_T0_C0.tif]

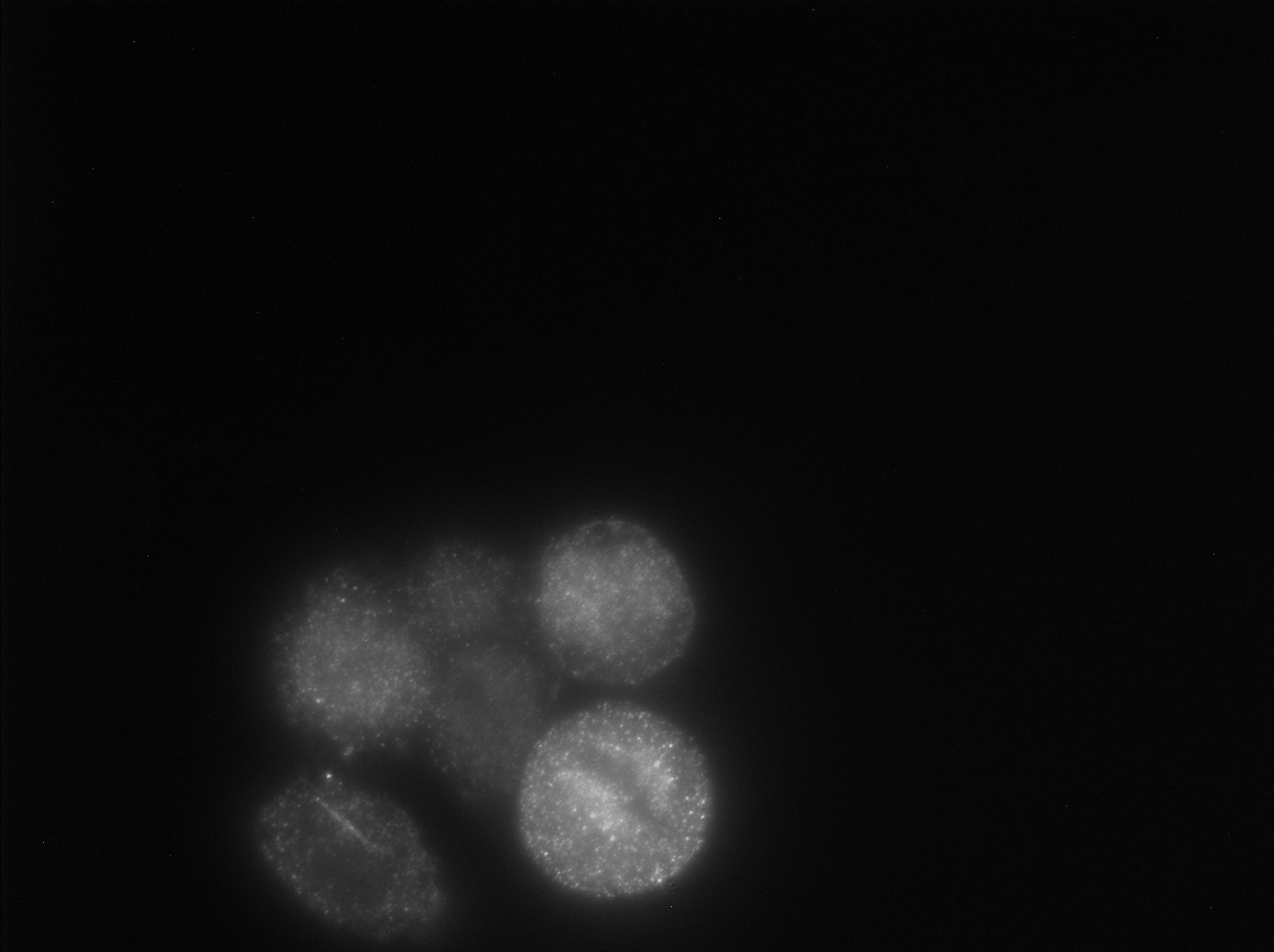

Supplement: Supplementary file 27 — Source data Fig. 7 part 3 [file 44319_2026_742_MOESM27_ESM.zip › Figure 7 Part 3/Fig 7be Cas9 and TPGS1-KO rGT335 atubulin/Cas9 5-2-25 rGT335 atub R3 M6.Project Maximum Z_XY1746217213_Z0_T0_C1.tif]

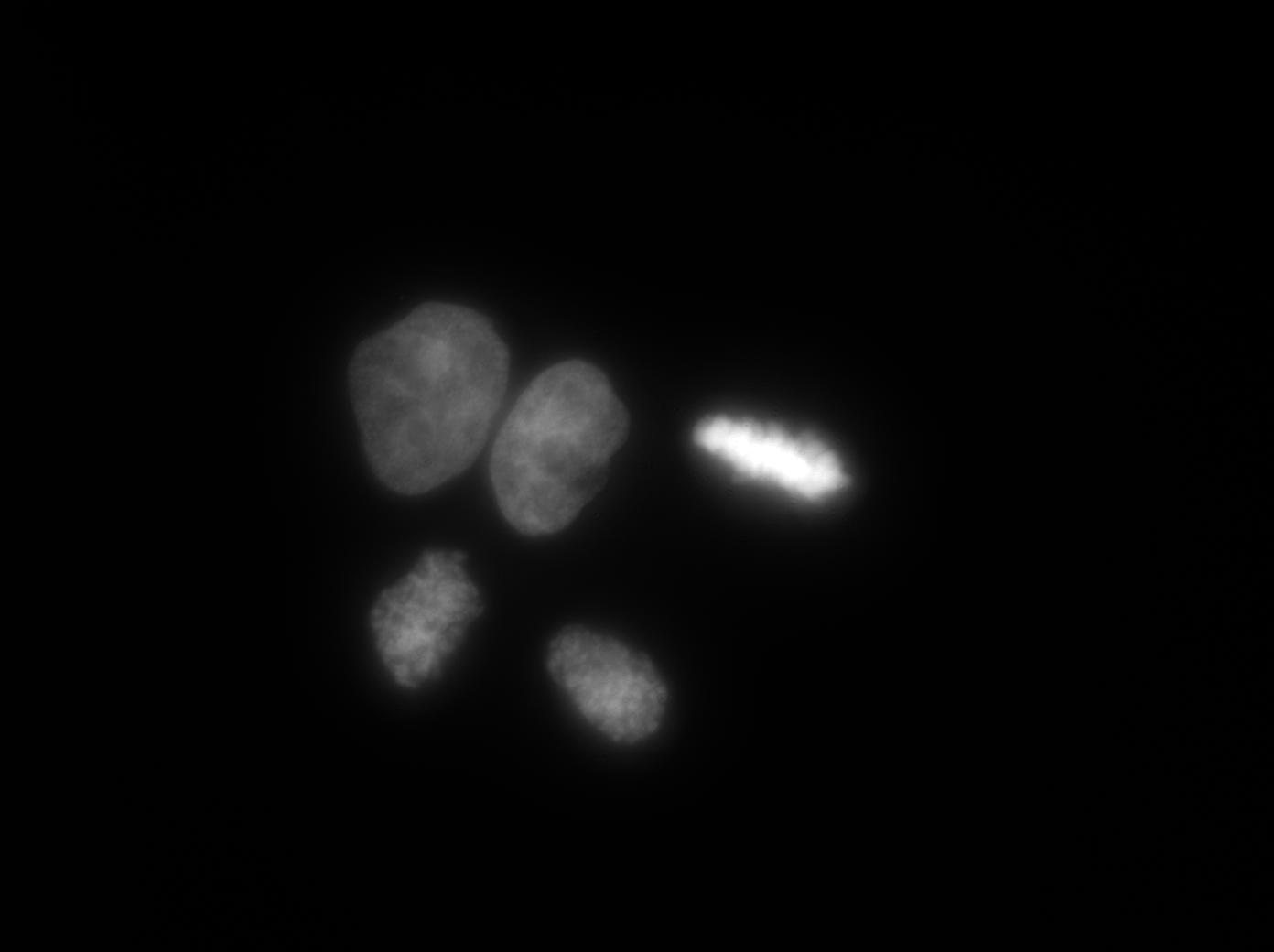

Supplement: Supplementary file 27 — Source data Fig. 7 part 3 [file 44319_2026_742_MOESM27_ESM.zip › Figure 7 Part 3/Fig 7be Cas9 and TPGS1-KO rGT335 atubulin/Cas9 5-2-25 rGT335 atub R2 M8.Project Maximum Z_XY1746562515_Z0_T0_C0.tif]

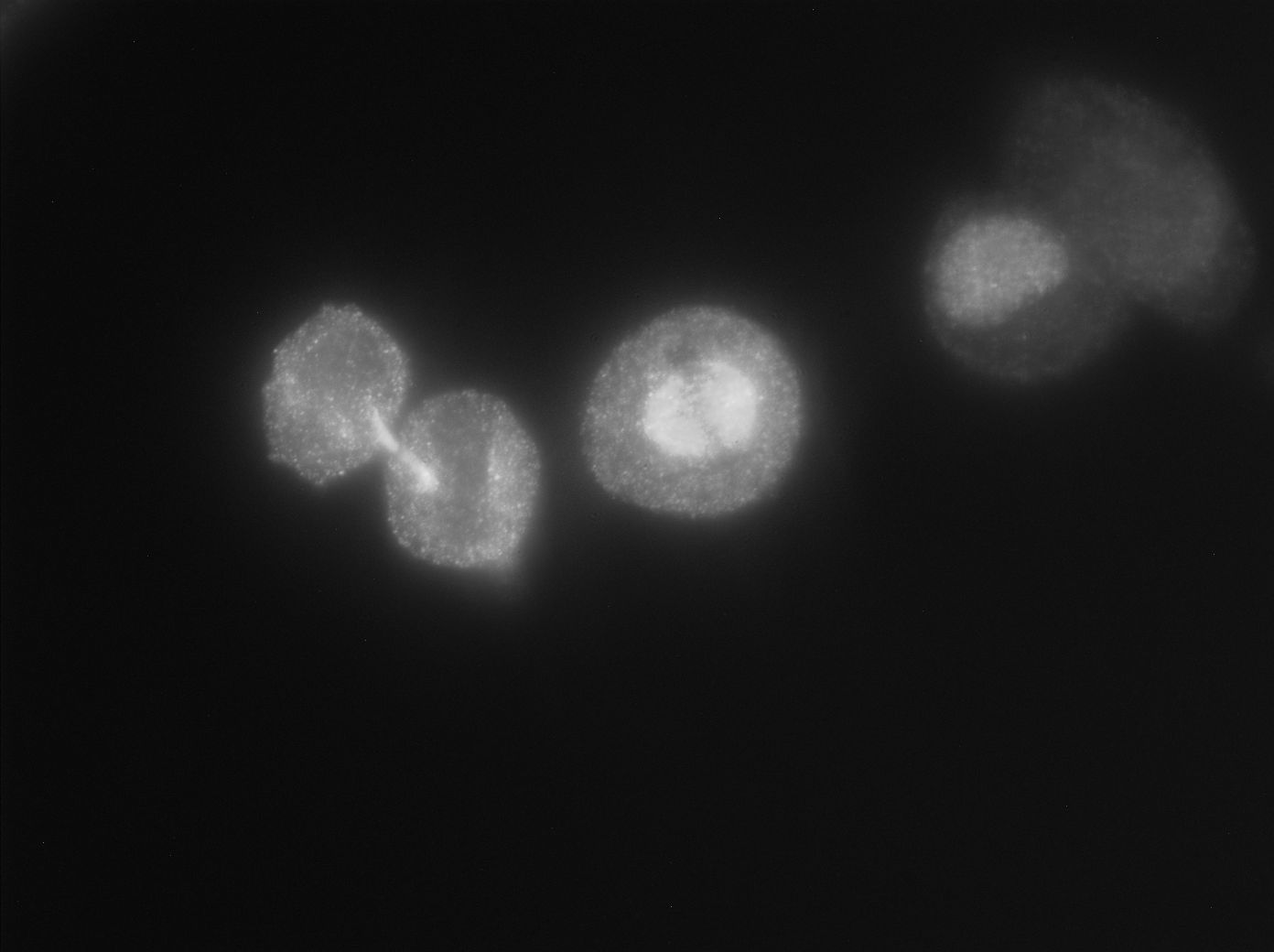

Supplement: Supplementary file 27 — Source data Fig. 7 part 3 [file 44319_2026_742_MOESM27_ESM.zip › Figure 7 Part 3/Fig 7be Cas9 and TPGS1-KO rGT335 atubulin/Cas9 5-2-25 rGT335 atub R2 M6.Project Maximum Z_XY1746562239_Z0_T0_C1.tif]

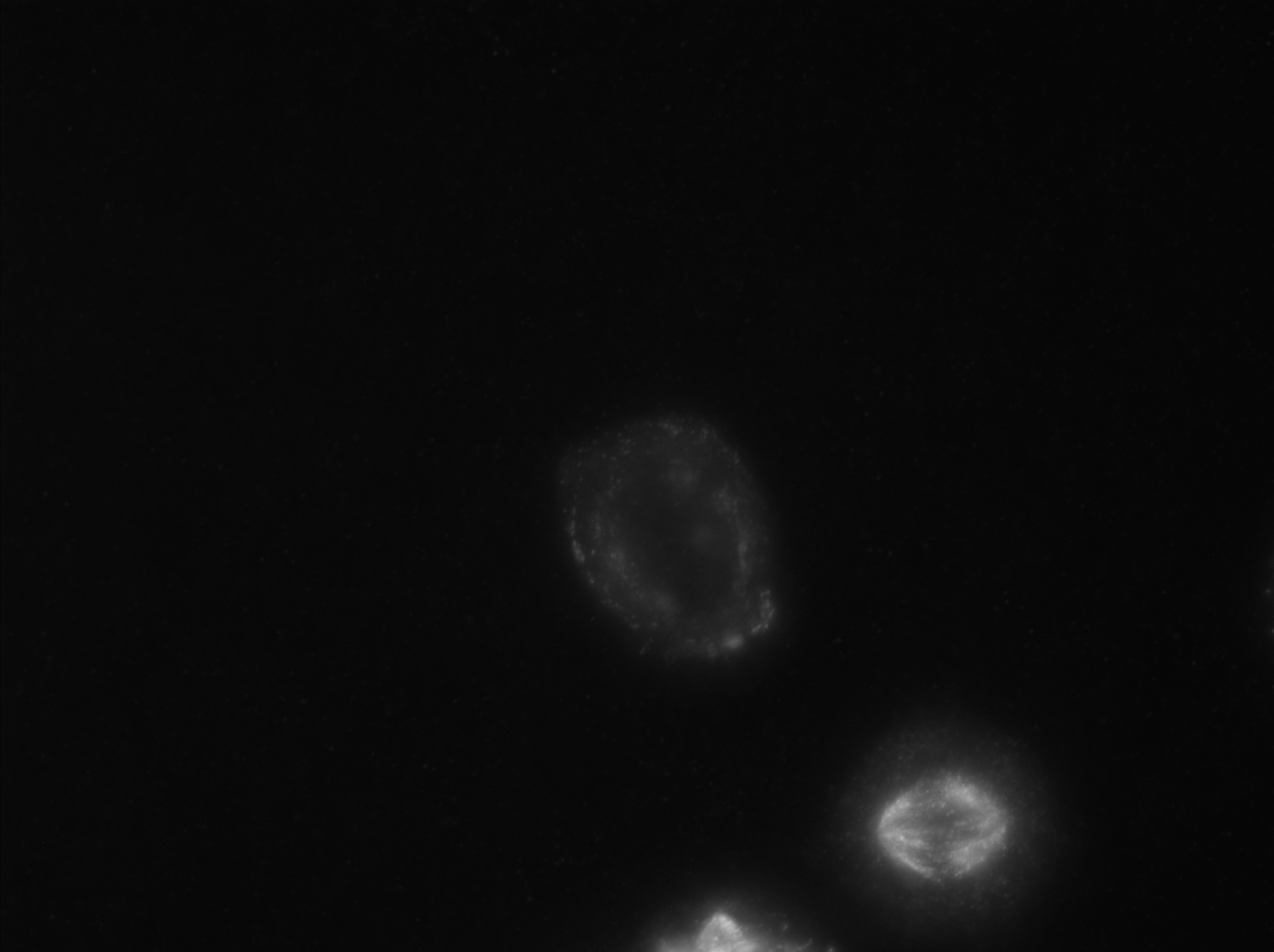

Supplement: Supplementary file 27 — Source data Fig. 7 part 3 [file 44319_2026_742_MOESM27_ESM.zip › Figure 7 Part 3/Fig 7be Cas9 and TPGS1-KO rGT335 atubulin/TPGS1-KO 5-2-25 rGT335 atub R2 M10.Project Maximum Z_XY1746565142_Z0_T0_C2.tif]

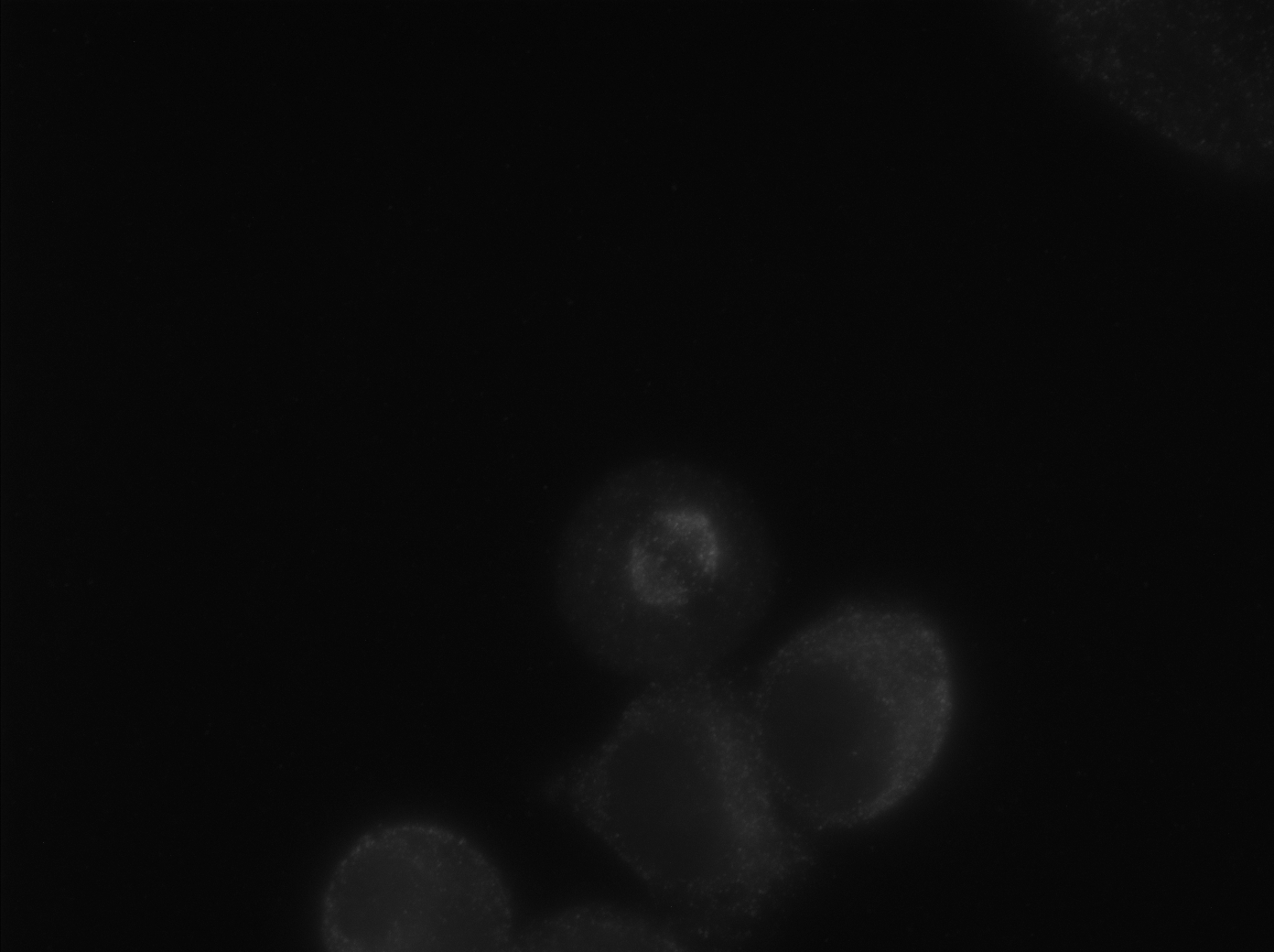

Supplement: Supplementary file 27 — Source data Fig. 7 part 3 [file 44319_2026_742_MOESM27_ESM.zip › Figure 7 Part 3/Fig 7be Cas9 and TPGS1-KO rGT335 atubulin/Cas9 5-2-25 rGT335 atub R1 M2.Project Maximum Z_XY1746557175_Z0_T0_C2.tif]

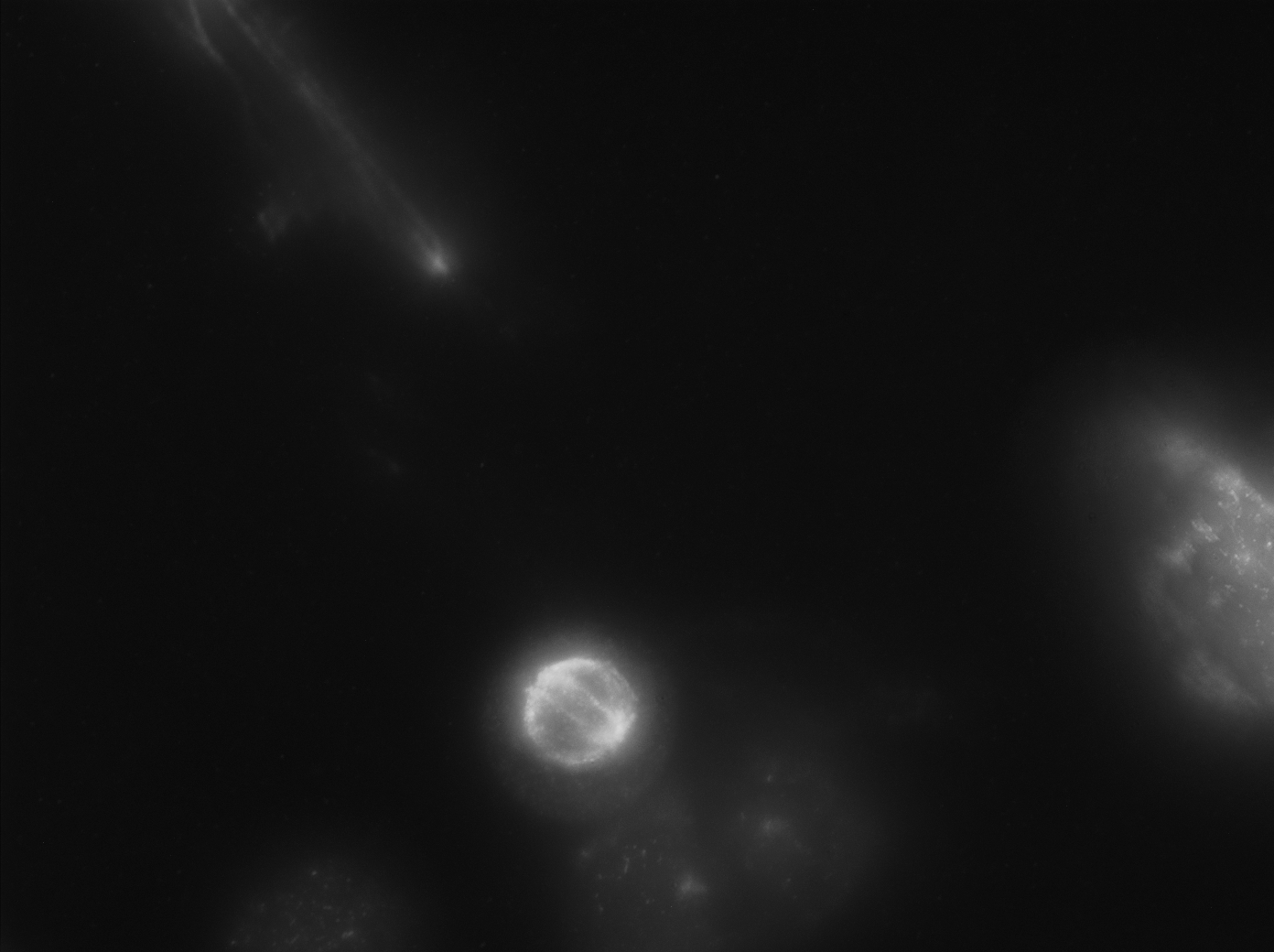

Supplement: Supplementary file 27 — Source data Fig. 7 part 3 [file 44319_2026_742_MOESM27_ESM.zip › Figure 7 Part 3/Fig 7be Cas9 and TPGS1-KO rGT335 atubulin/TPGS1-KO 5-2-25 rGT335 atub R1 M8.Project Maximum Z_XY1746222465_Z0_T0_C2.tif]

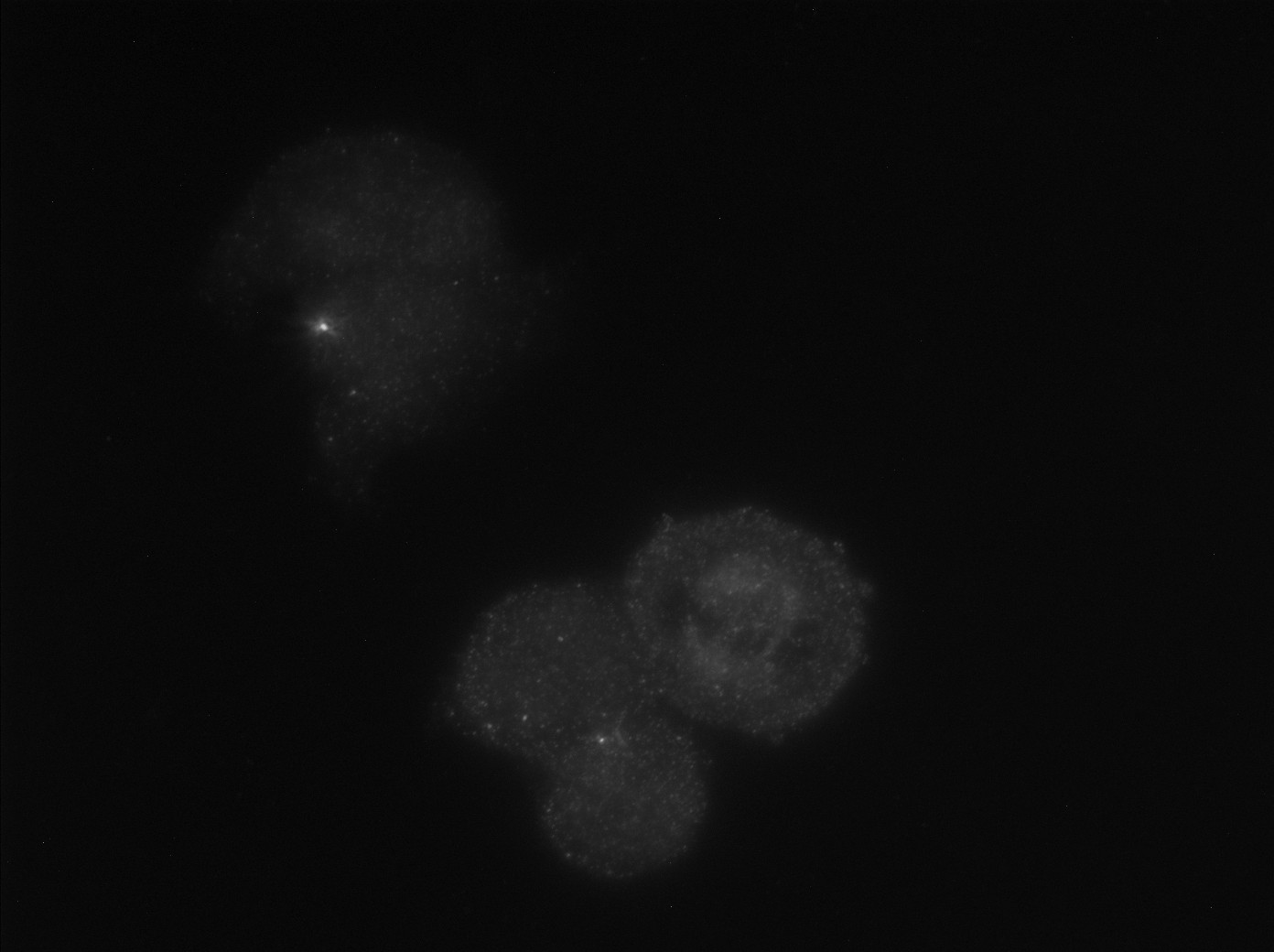

Supplement: Supplementary file 27 — Source data Fig. 7 part 3 [file 44319_2026_742_MOESM27_ESM.zip › Figure 7 Part 3/Fig 7be Cas9 and TPGS1-KO rGT335 atubulin/Cas9 5-2-25 rGT335 atub R2 M10.Project Maximum Z_XY1746562899_Z0_T0_C1.tif]

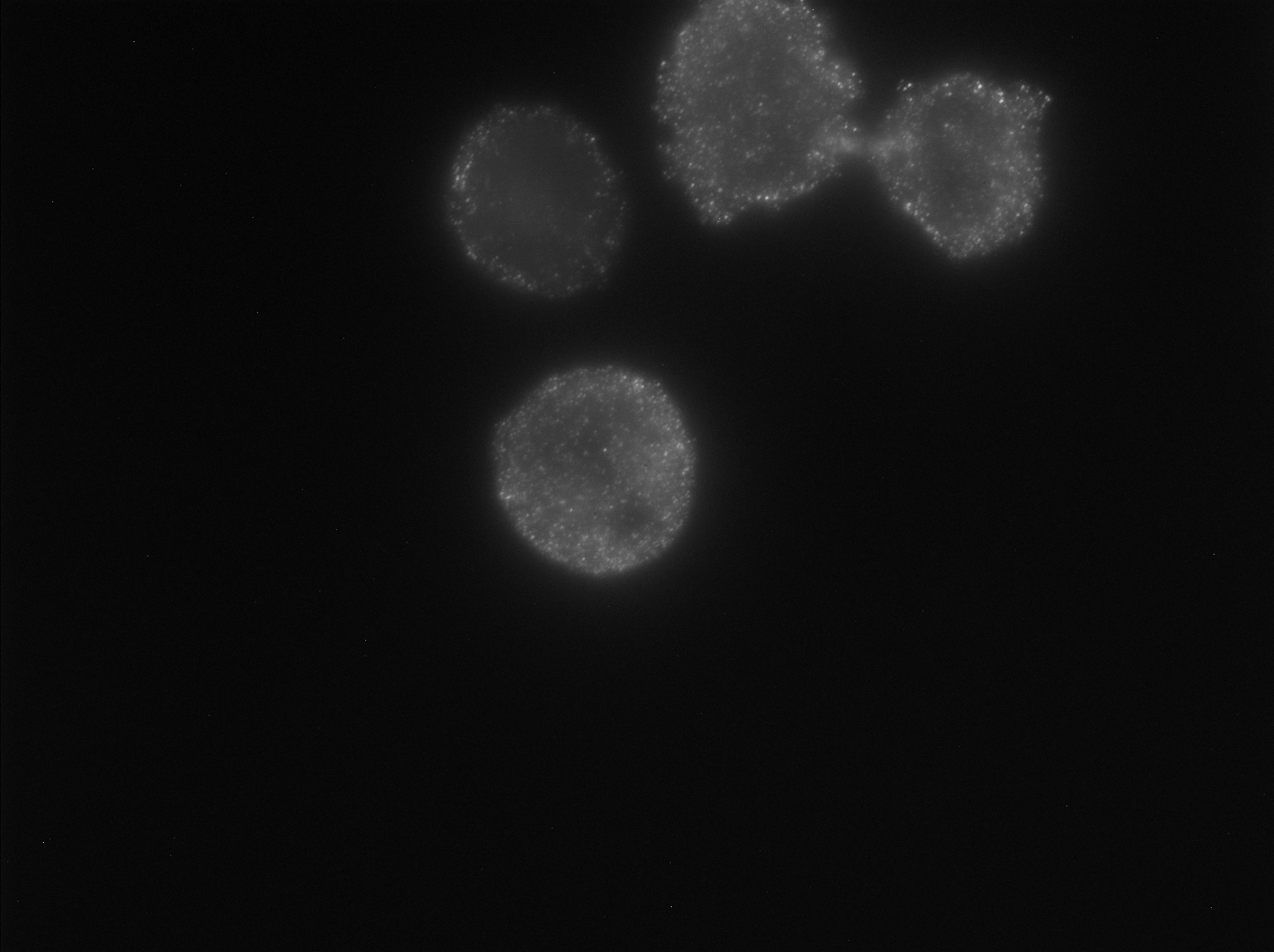

Supplement: Supplementary file 27 — Source data Fig. 7 part 3 [file 44319_2026_742_MOESM27_ESM.zip › Figure 7 Part 3/Fig 7be Cas9 and TPGS1-KO rGT335 atubulin/TPGS1-KO 5-2-25 rGT335 atub R1 M1.Project Maximum Z_XY1746220972_Z0_T0_C1.tif]

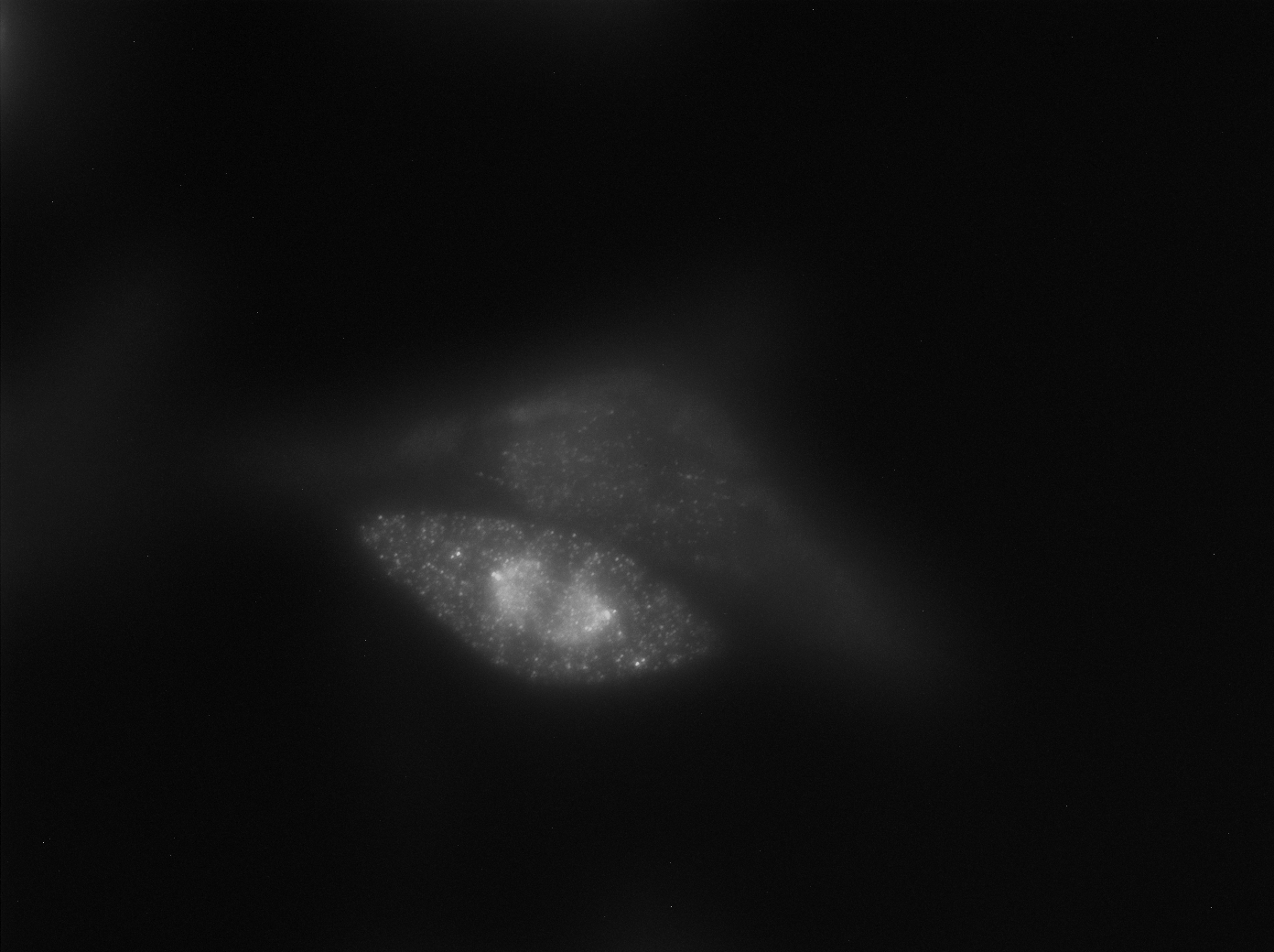

Supplement: Supplementary file 27 — Source data Fig. 7 part 3 [file 44319_2026_742_MOESM27_ESM.zip › Figure 7 Part 3/Fig 7be Cas9 and TPGS1-KO rGT335 atubulin/TPGS1-KO 5-2-25 rGT335 atub R1 M3.Project Maximum Z_XY1746221312_Z0_T0_C1.tif]

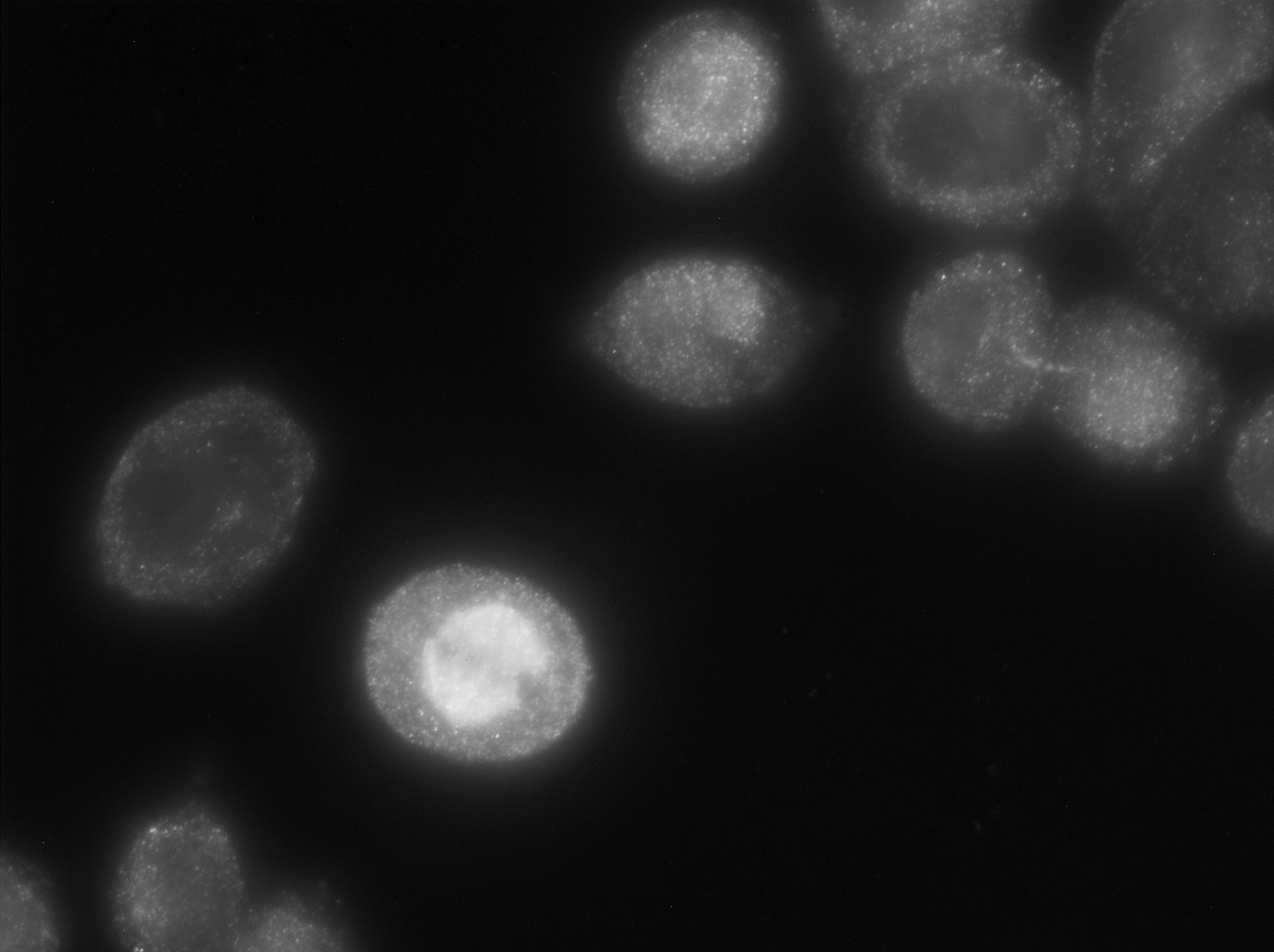

Supplement: Supplementary file 27 — Source data Fig. 7 part 3 [file 44319_2026_742_MOESM27_ESM.zip › Figure 7 Part 3/Fig 7be Cas9 and TPGS1-KO rGT335 atubulin/TPGS1-KO 5-2-25 rGT335 atub R2 M7.Project Maximum Z_XY1746564635_Z0_T0_C1.tif]

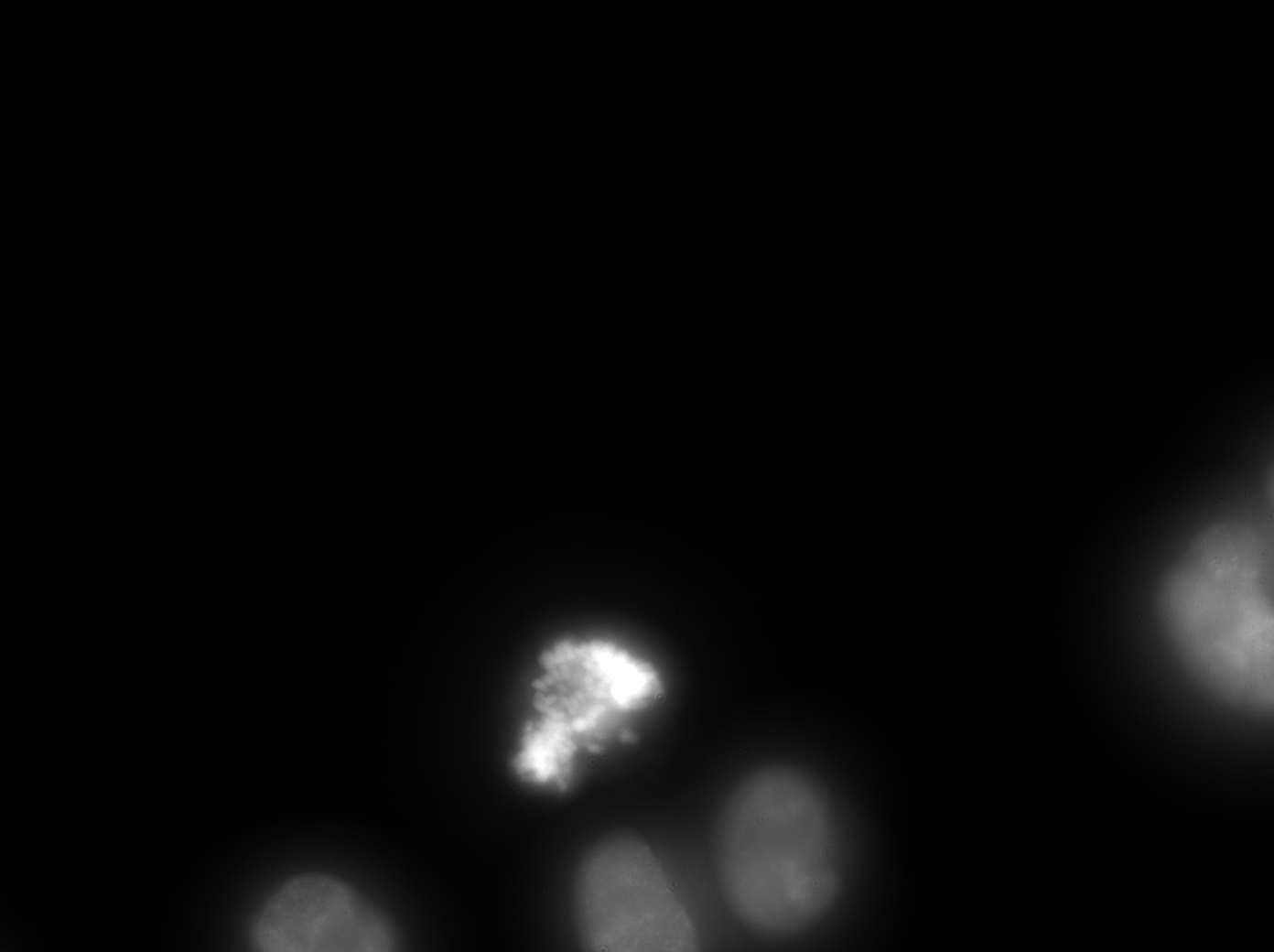

Supplement: Supplementary file 27 — Source data Fig. 7 part 3 [file 44319_2026_742_MOESM27_ESM.zip › Figure 7 Part 3/Fig 7be Cas9 and TPGS1-KO rGT335 atubulin/TPGS1-KO 5-2-25 rGT335 atub R1 M8.Project Maximum Z_XY1746222465_Z0_T0_C0.tif]

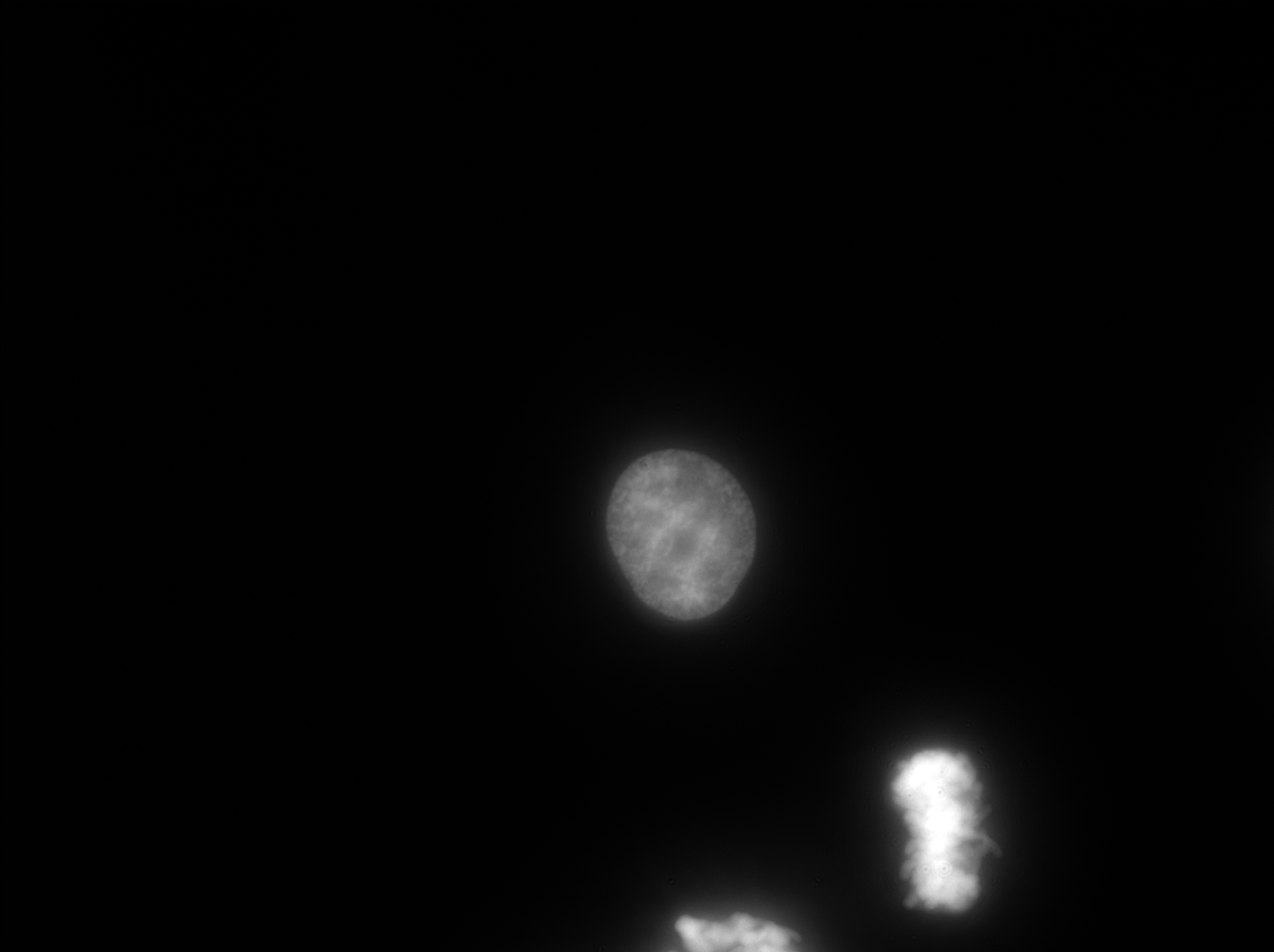

Supplement: Supplementary file 27 — Source data Fig. 7 part 3 [file 44319_2026_742_MOESM27_ESM.zip › Figure 7 Part 3/Fig 7be Cas9 and TPGS1-KO rGT335 atubulin/TPGS1-KO 5-2-25 rGT335 atub R2 M10.Project Maximum Z_XY1746565142_Z0_T0_C0.tif]

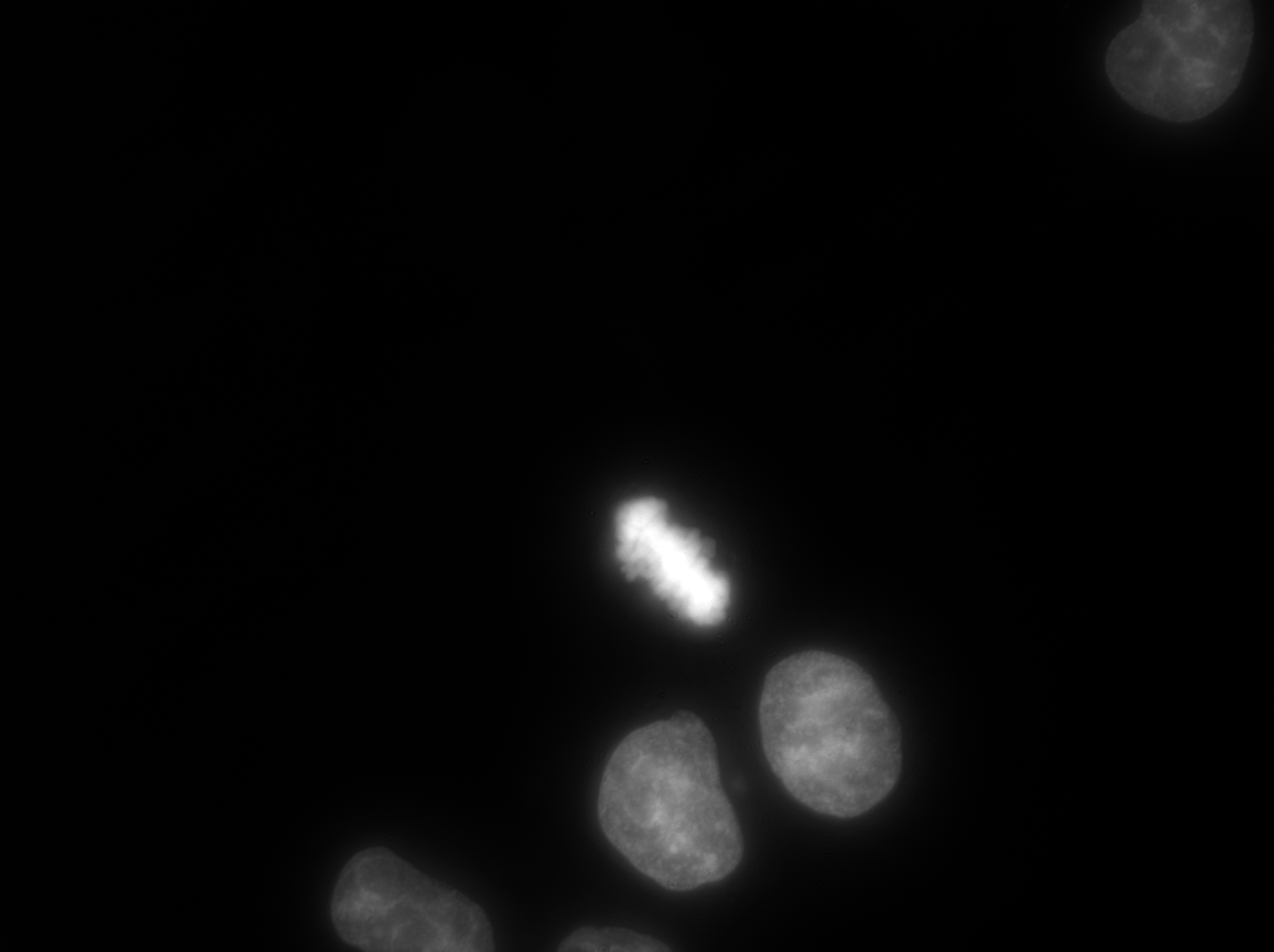

Supplement: Supplementary file 27 — Source data Fig. 7 part 3 [file 44319_2026_742_MOESM27_ESM.zip › Figure 7 Part 3/Fig 7be Cas9 and TPGS1-KO rGT335 atubulin/Cas9 5-2-25 rGT335 atub R1 M2.Project Maximum Z_XY1746557175_Z0_T0_C0.tif]

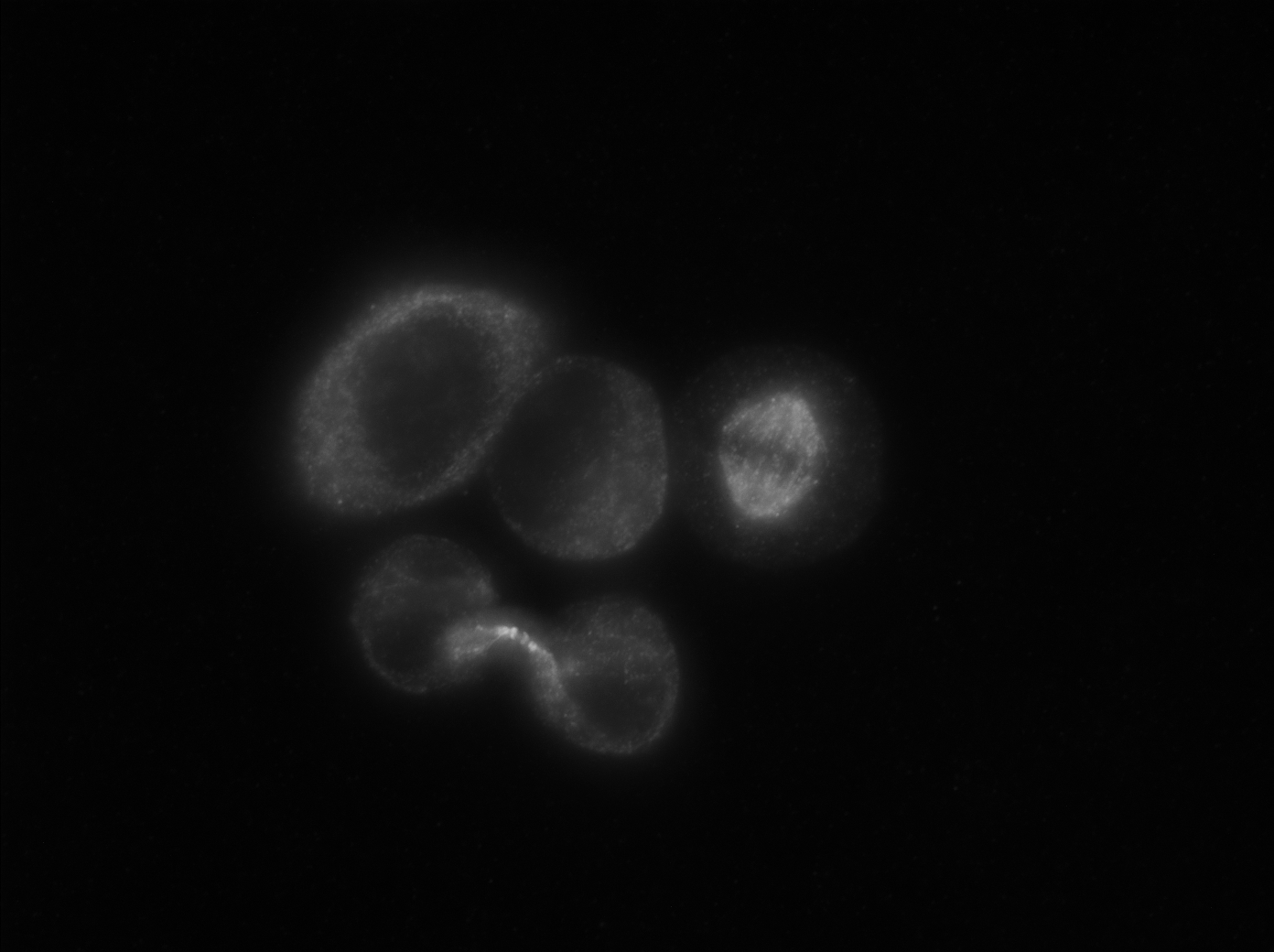

Supplement: Supplementary file 27 — Source data Fig. 7 part 3 [file 44319_2026_742_MOESM27_ESM.zip › Figure 7 Part 3/Fig 7be Cas9 and TPGS1-KO rGT335 atubulin/Cas9 5-2-25 rGT335 atub R2 M8.Project Maximum Z_XY1746562515_Z0_T0_C2.tif]

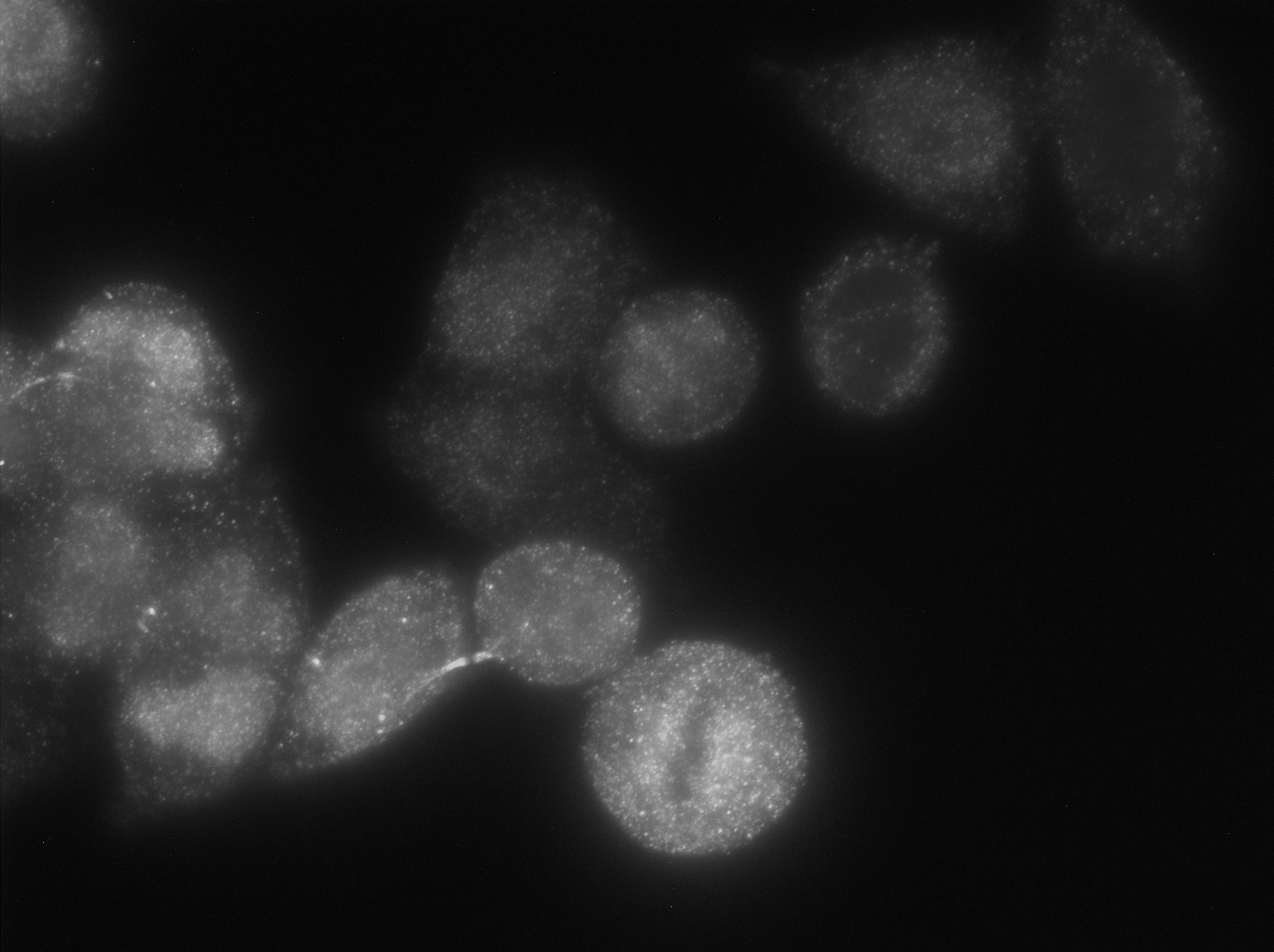

Supplement: Supplementary file 27 — Source data Fig. 7 part 3 [file 44319_2026_742_MOESM27_ESM.zip › Figure 7 Part 3/Fig 7be Cas9 and TPGS1-KO rGT335 atubulin/Cas9 5-2-25 rGT335 atub R1 M4.Project Maximum Z_XY1746557526_Z0_T0_C1.tif]

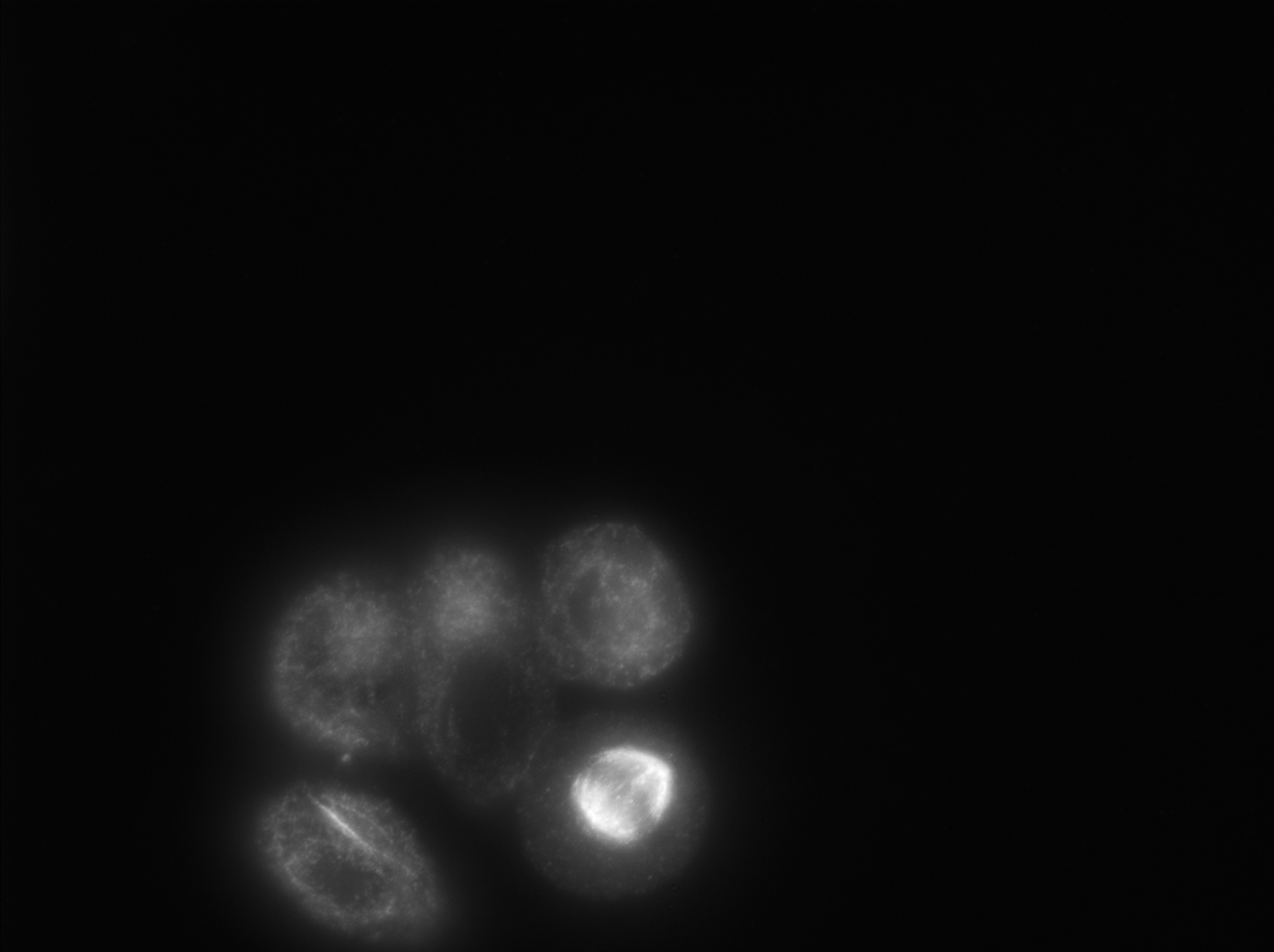

Supplement: Supplementary file 27 — Source data Fig. 7 part 3 [file 44319_2026_742_MOESM27_ESM.zip › Figure 7 Part 3/Fig 7be Cas9 and TPGS1-KO rGT335 atubulin/Cas9 5-2-25 rGT335 atub R3 M6.Project Maximum Z_XY1746217213_Z0_T0_C2.tif]

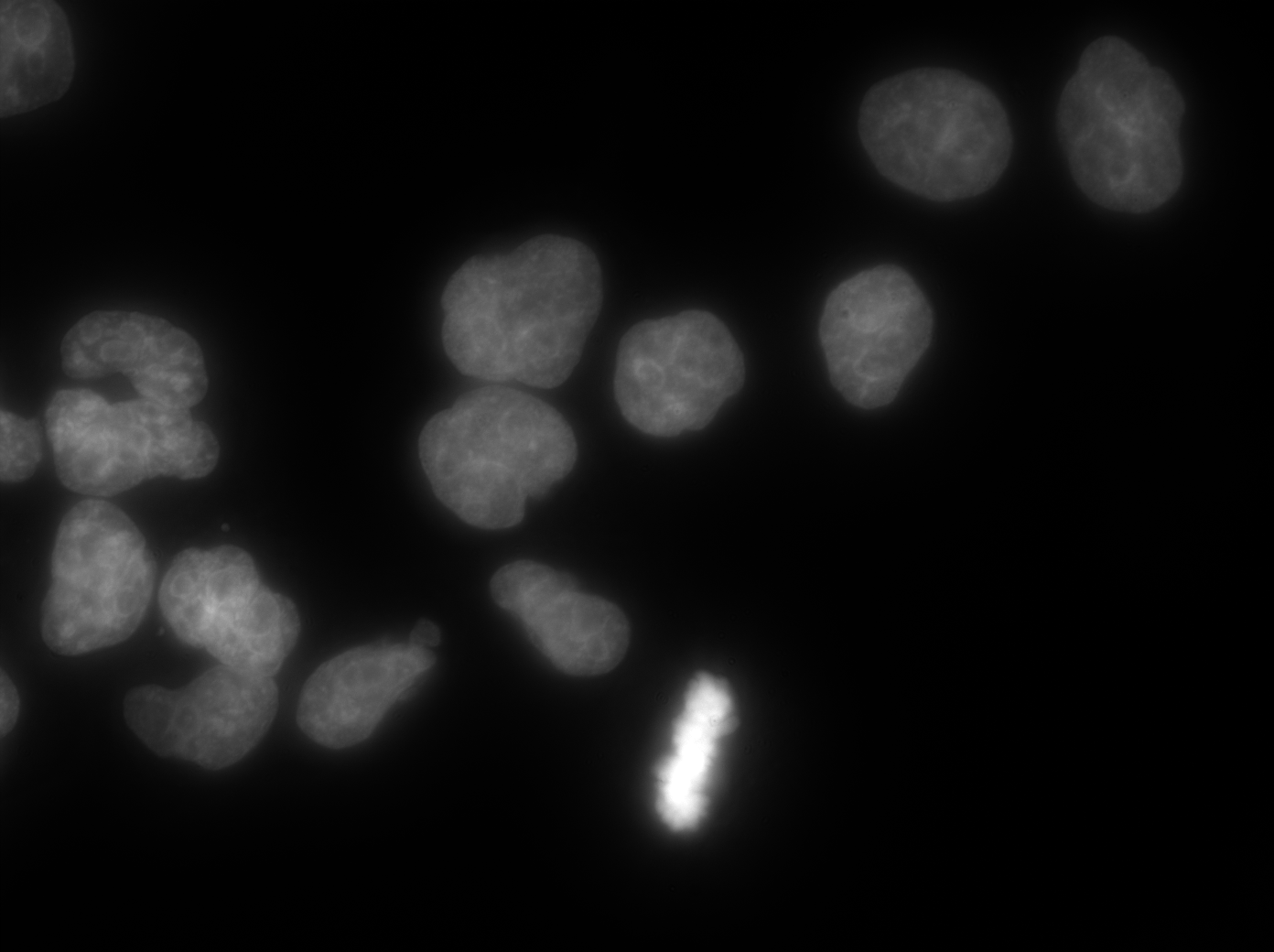

Supplement: Supplementary file 27 — Source data Fig. 7 part 3 [file 44319_2026_742_MOESM27_ESM.zip › Figure 7 Part 3/Fig 7be Cas9 and TPGS1-KO rGT335 atubulin/Cas9 5-2-25 rGT335 atub R1 M4.Project Maximum Z_XY1746557526_Z0_T0_C0.tif]

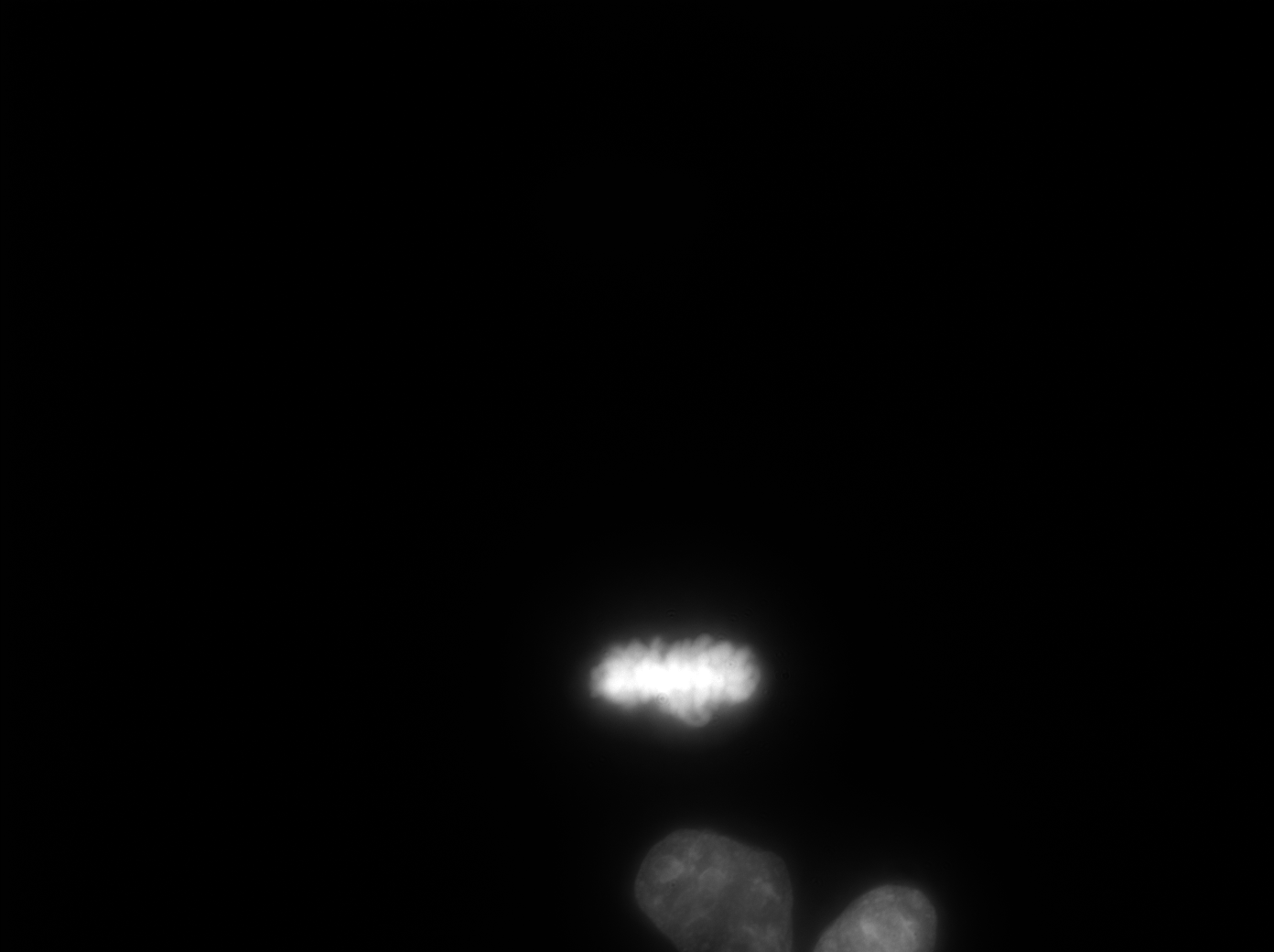

Supplement: Supplementary file 27 — Source data Fig. 7 part 3 [file 44319_2026_742_MOESM27_ESM.zip › Figure 7 Part 3/Fig 7be Cas9 and TPGS1-KO rGT335 atubulin/TPGS1-KO 5-2-25 rGT335 atub R2 M6.Project Maximum Z_XY1746564489_Z0_T0_C0.tif]

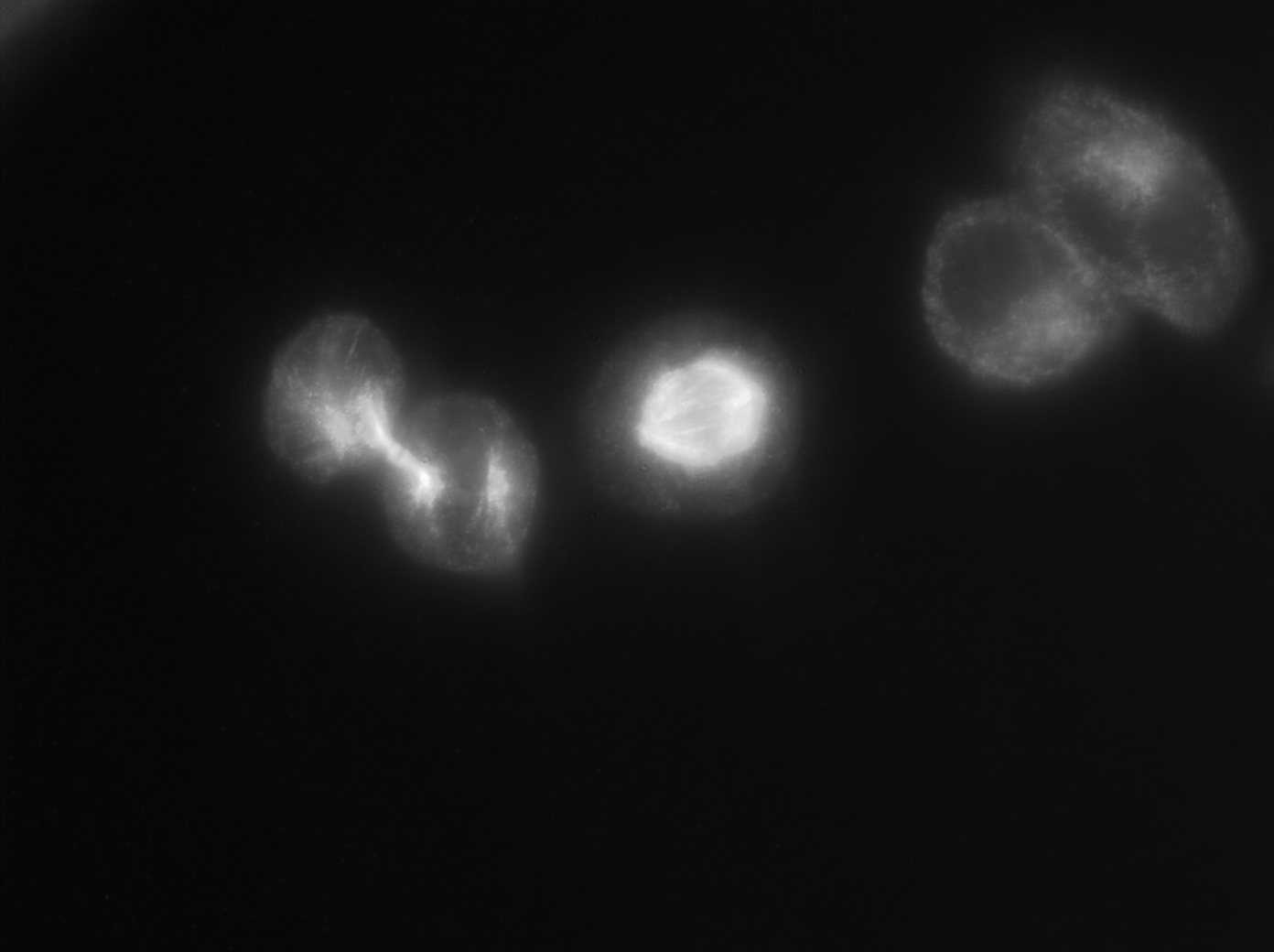

Supplement: Supplementary file 27 — Source data Fig. 7 part 3 [file 44319_2026_742_MOESM27_ESM.zip › Figure 7 Part 3/Fig 7be Cas9 and TPGS1-KO rGT335 atubulin/Cas9 5-2-25 rGT335 atub R2 M6.Project Maximum Z_XY1746562239_Z0_T0_C2.tif]

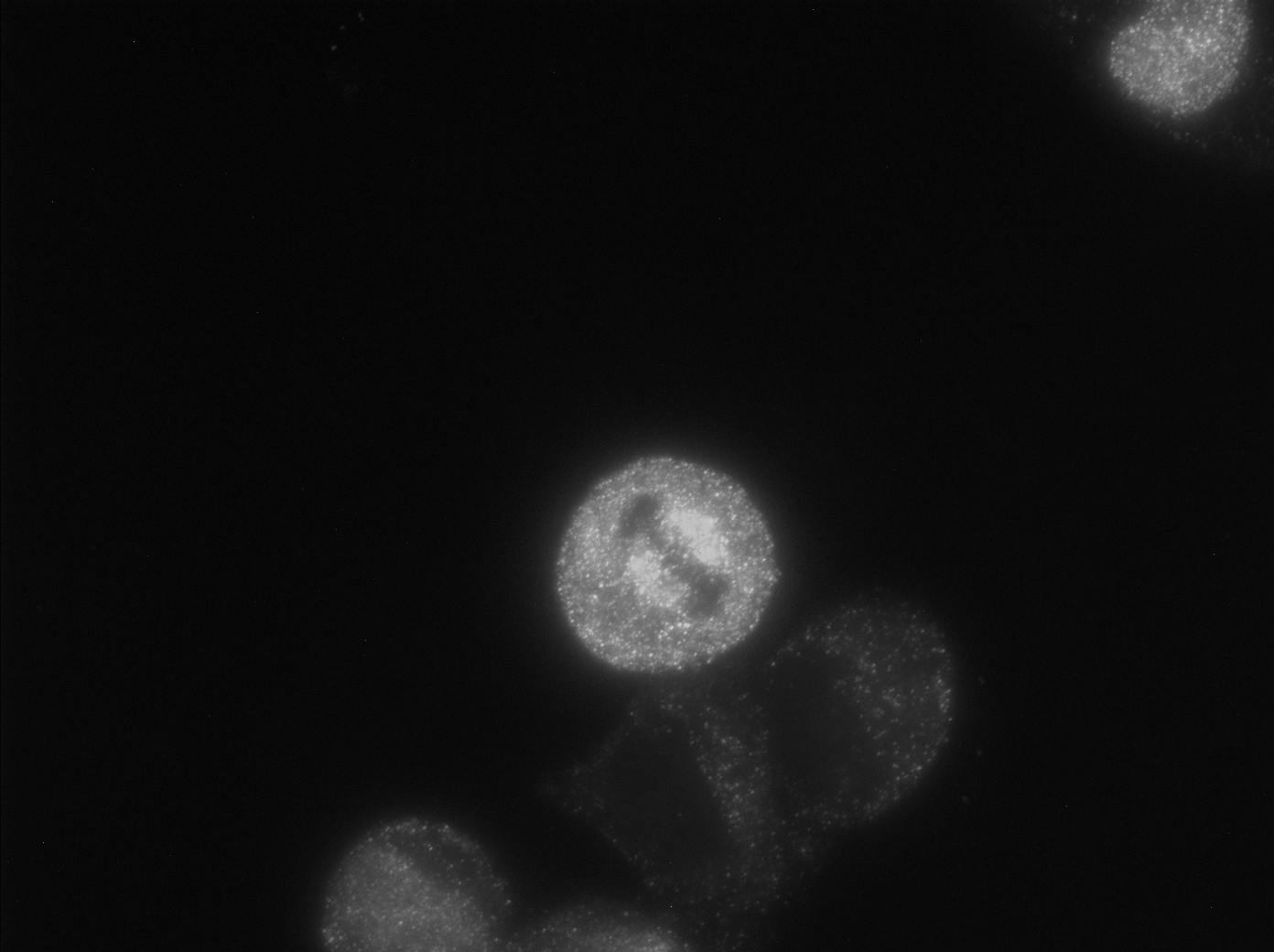

Supplement: Supplementary file 27 — Source data Fig. 7 part 3 [file 44319_2026_742_MOESM27_ESM.zip › Figure 7 Part 3/Fig 7be Cas9 and TPGS1-KO rGT335 atubulin/Cas9 5-2-25 rGT335 atub R1 M2.Project Maximum Z_XY1746557175_Z0_T0_C1.tif]

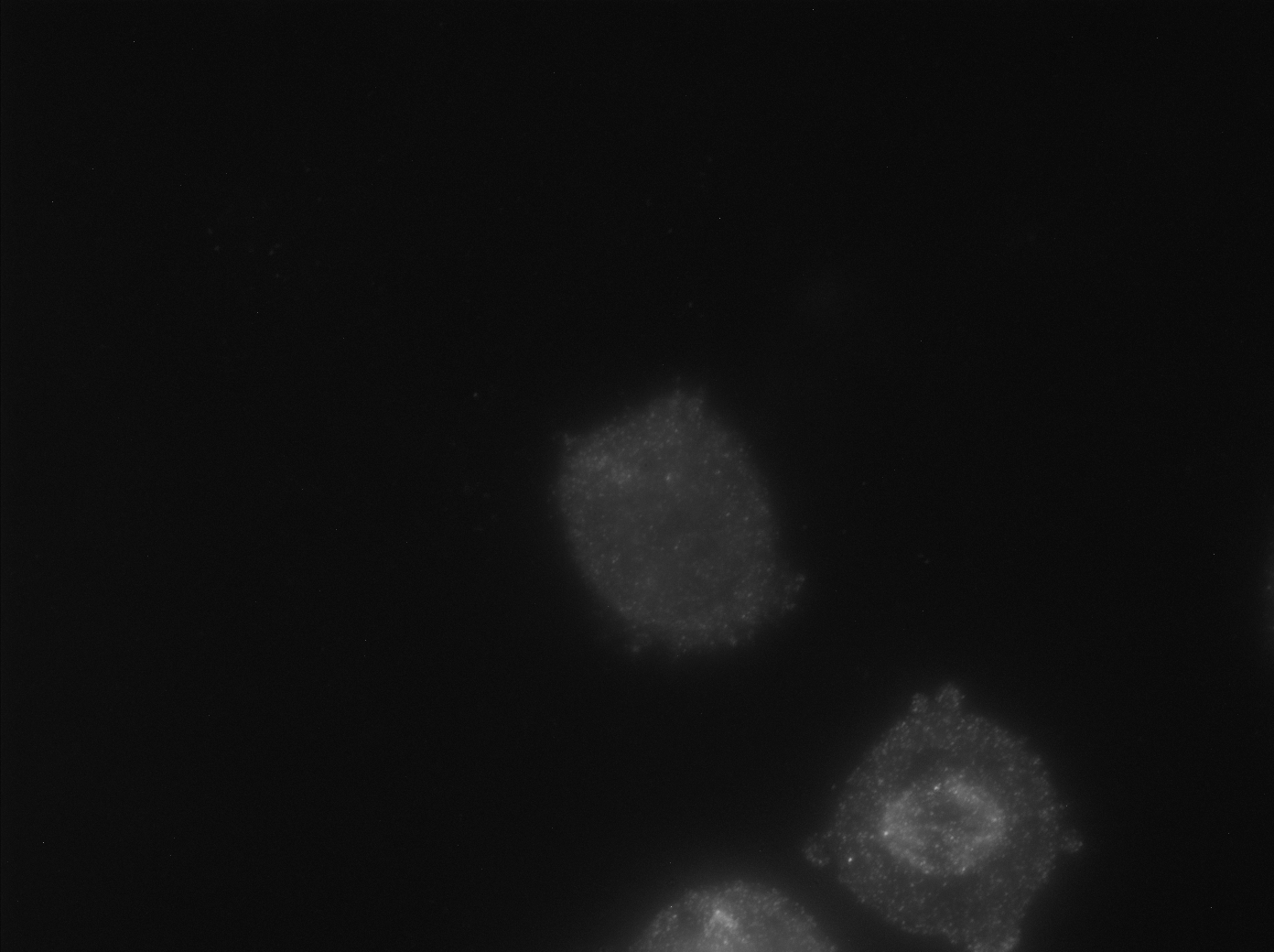

Supplement: Supplementary file 27 — Source data Fig. 7 part 3 [file 44319_2026_742_MOESM27_ESM.zip › Figure 7 Part 3/Fig 7be Cas9 and TPGS1-KO rGT335 atubulin/TPGS1-KO 5-2-25 rGT335 atub R2 M10.Project Maximum Z_XY1746565142_Z0_T0_C1.tif]

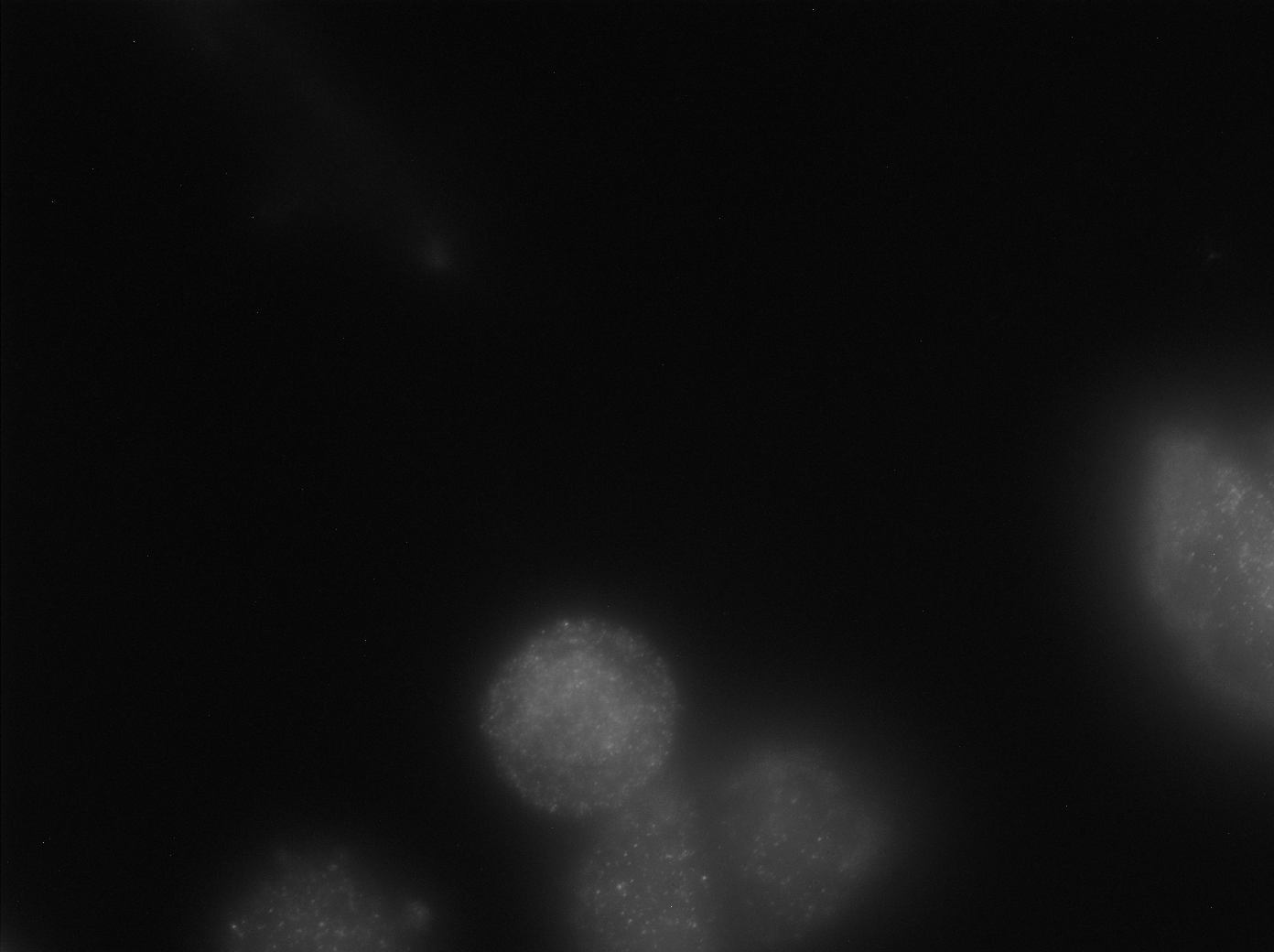

Supplement: Supplementary file 27 — Source data Fig. 7 part 3 [file 44319_2026_742_MOESM27_ESM.zip › Figure 7 Part 3/Fig 7be Cas9 and TPGS1-KO rGT335 atubulin/TPGS1-KO 5-2-25 rGT335 atub R1 M8.Project Maximum Z_XY1746222465_Z0_T0_C1.tif]

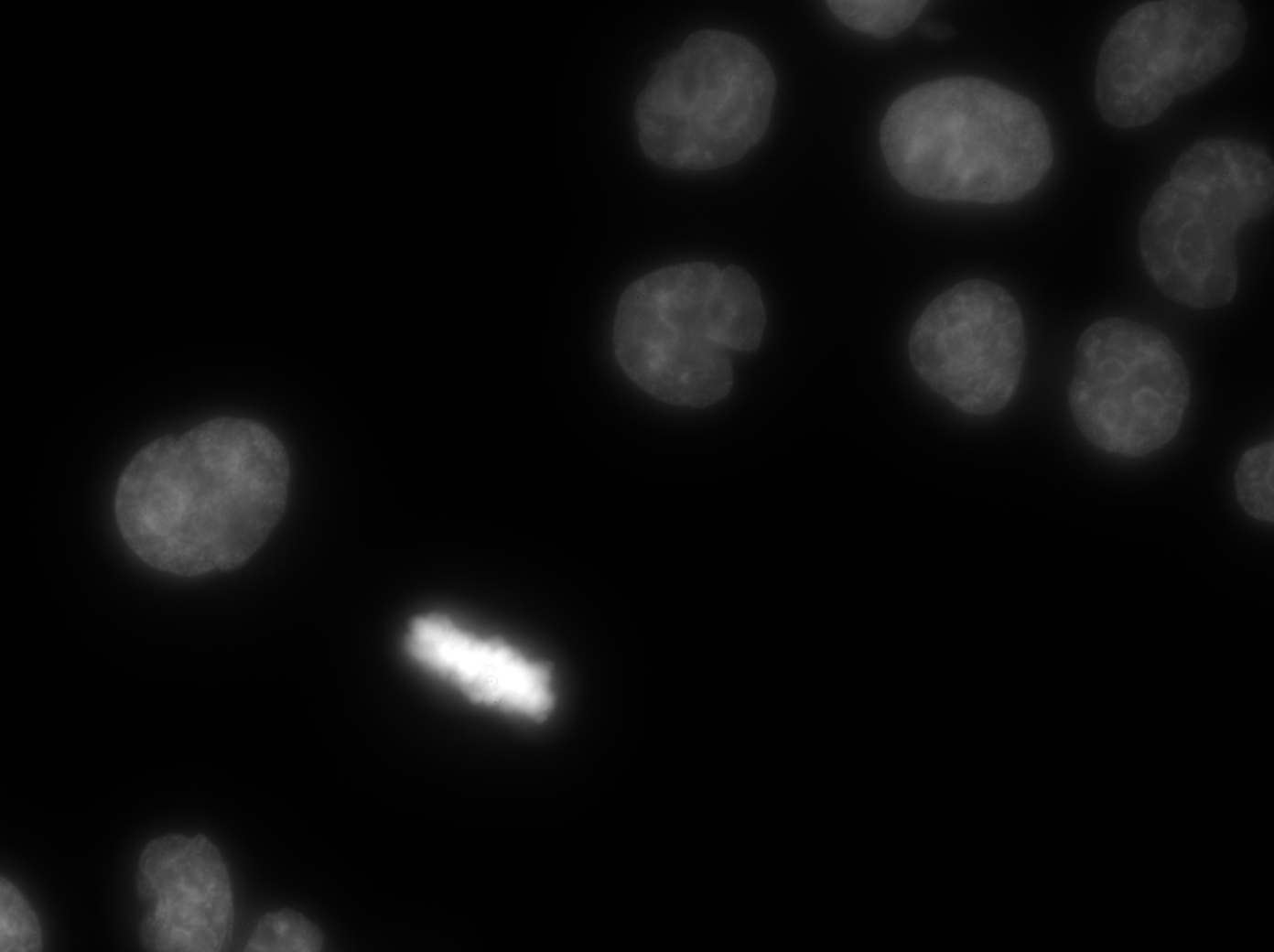

Supplement: Supplementary file 27 — Source data Fig. 7 part 3 [file 44319_2026_742_MOESM27_ESM.zip › Figure 7 Part 3/Fig 7be Cas9 and TPGS1-KO rGT335 atubulin/TPGS1-KO 5-2-25 rGT335 atub R2 M7.Project Maximum Z_XY1746564635_Z0_T0_C0.tif]

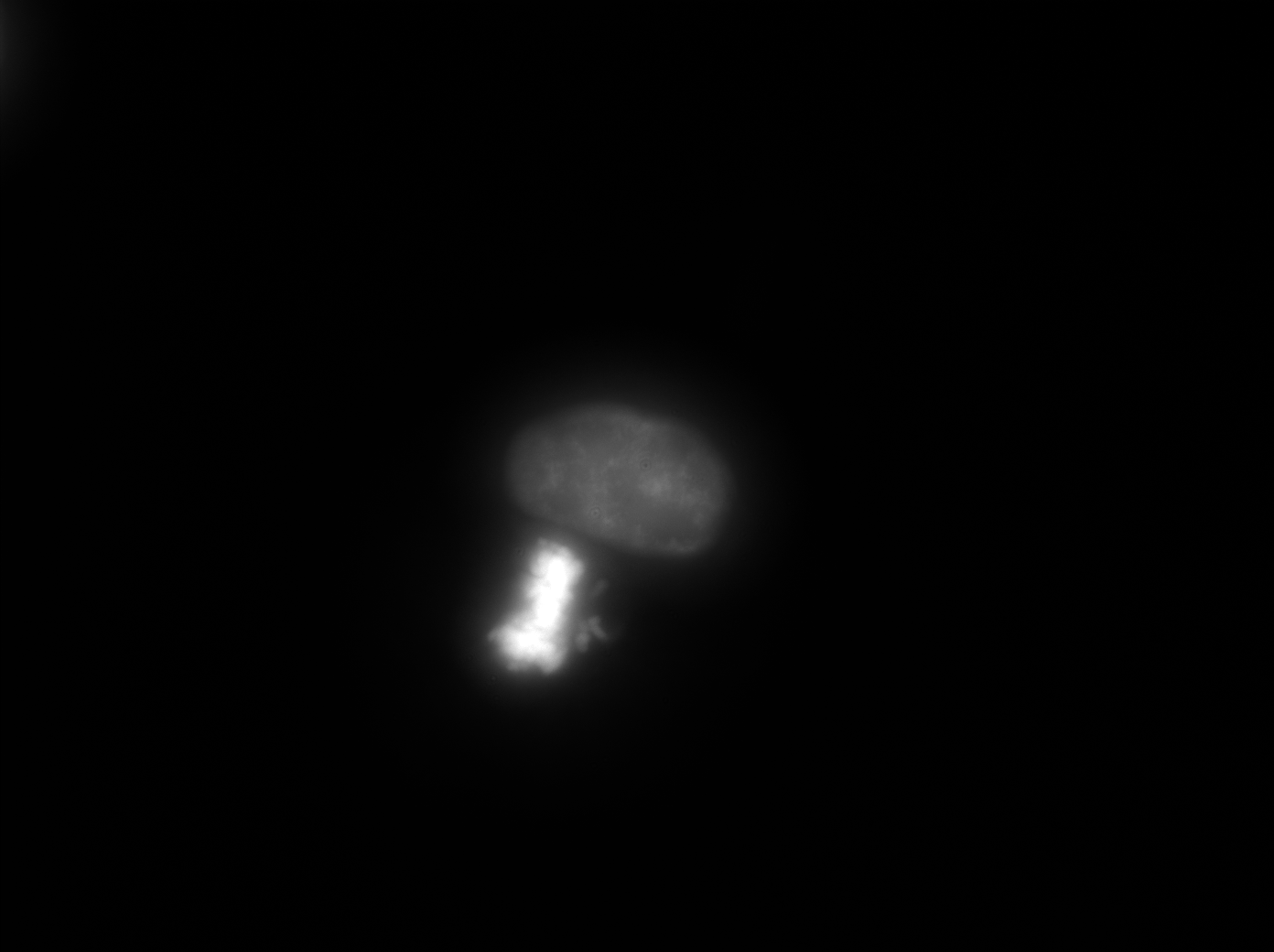

Supplement: Supplementary file 27 — Source data Fig. 7 part 3 [file 44319_2026_742_MOESM27_ESM.zip › Figure 7 Part 3/Fig 7be Cas9 and TPGS1-KO rGT335 atubulin/TPGS1-KO 5-2-25 rGT335 atub R1 M3.Project Maximum Z_XY1746221312_Z0_T0_C0.tif]

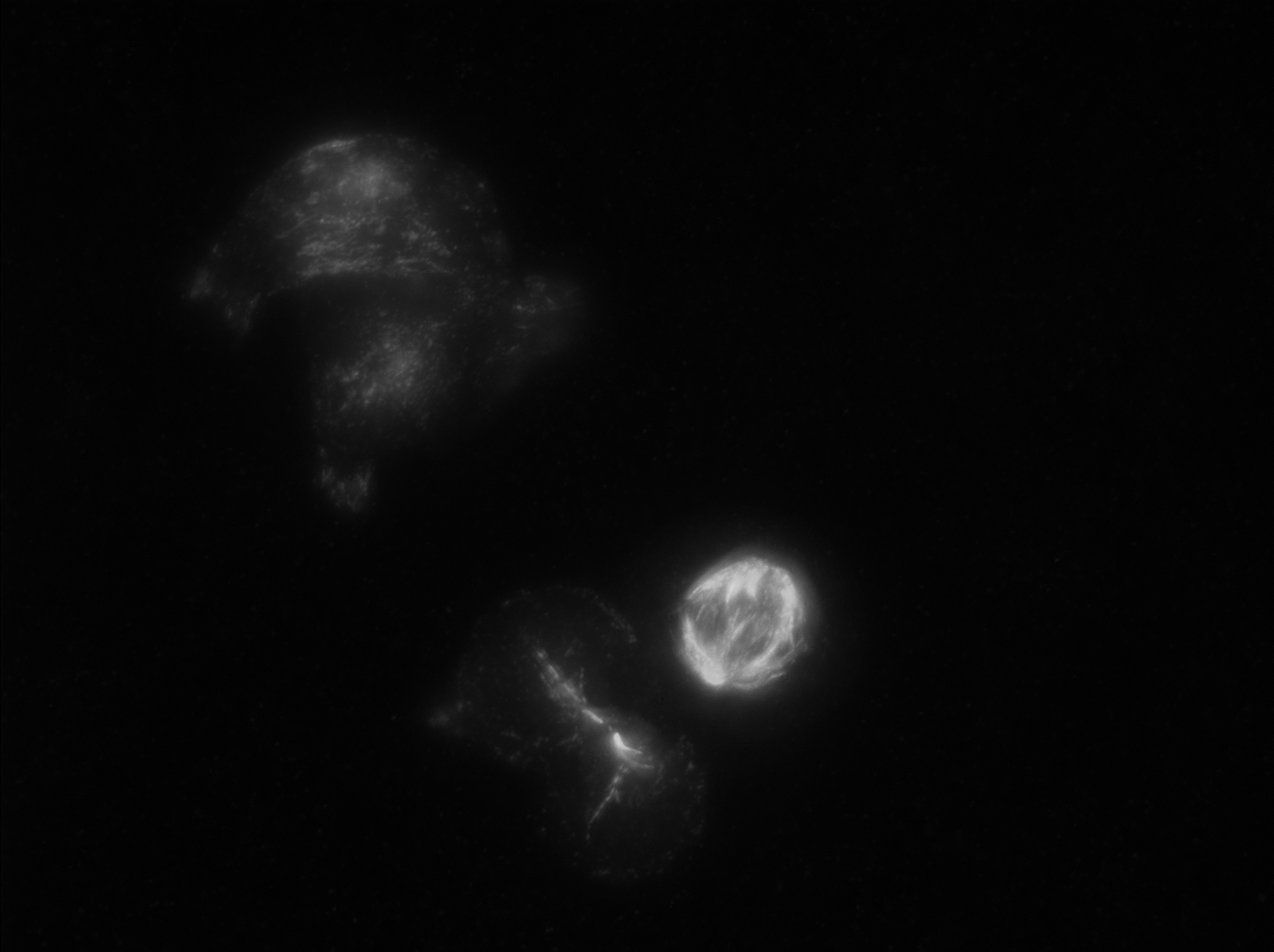

Supplement: Supplementary file 27 — Source data Fig. 7 part 3 [file 44319_2026_742_MOESM27_ESM.zip › Figure 7 Part 3/Fig 7be Cas9 and TPGS1-KO rGT335 atubulin/Cas9 5-2-25 rGT335 atub R2 M10.Project Maximum Z_XY1746562899_Z0_T0_C2.tif]

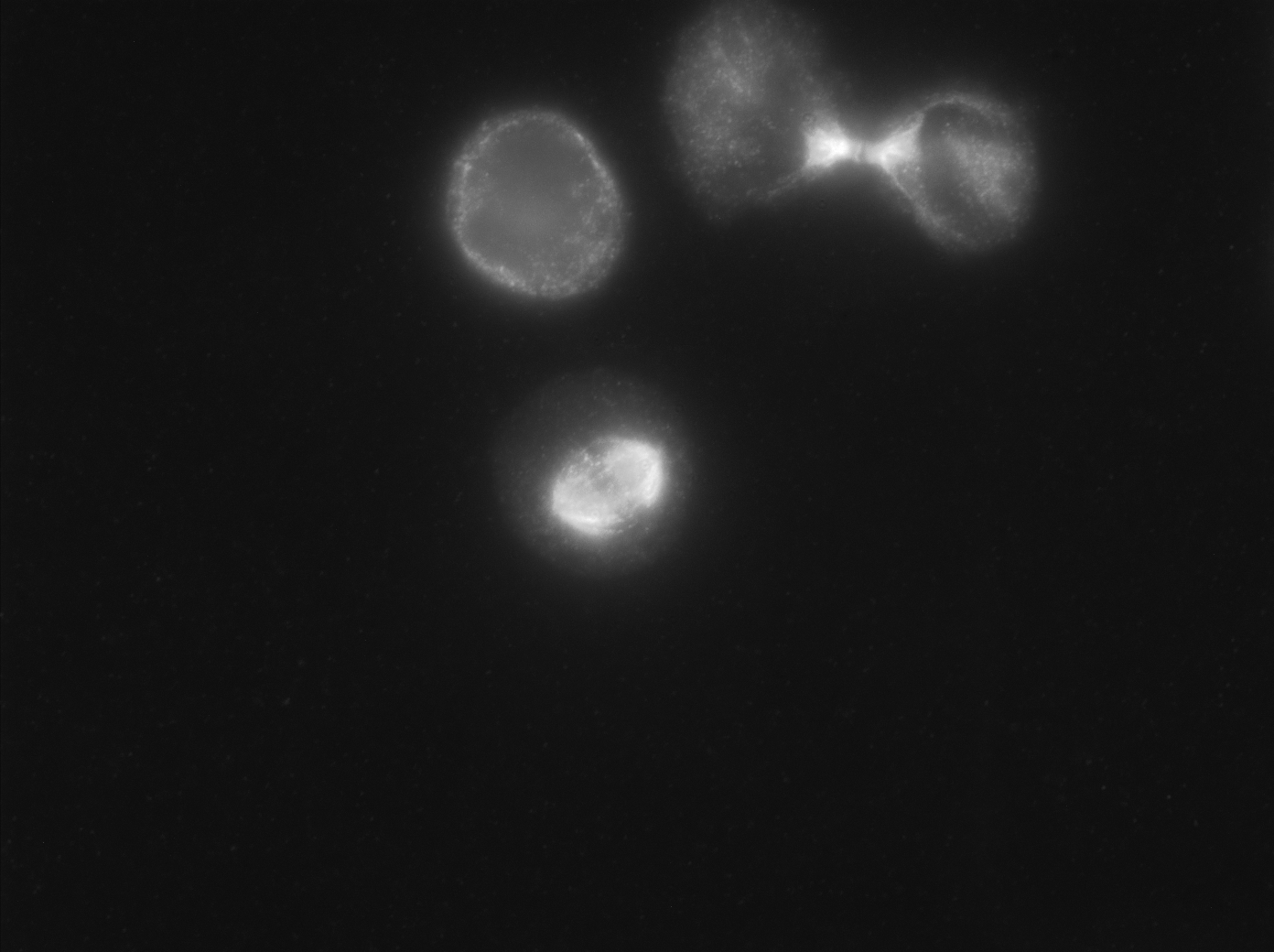

Supplement: Supplementary file 27 — Source data Fig. 7 part 3 [file 44319_2026_742_MOESM27_ESM.zip › Figure 7 Part 3/Fig 7be Cas9 and TPGS1-KO rGT335 atubulin/TPGS1-KO 5-2-25 rGT335 atub R1 M1.Project Maximum Z_XY1746220972_Z0_T0_C2.tif]

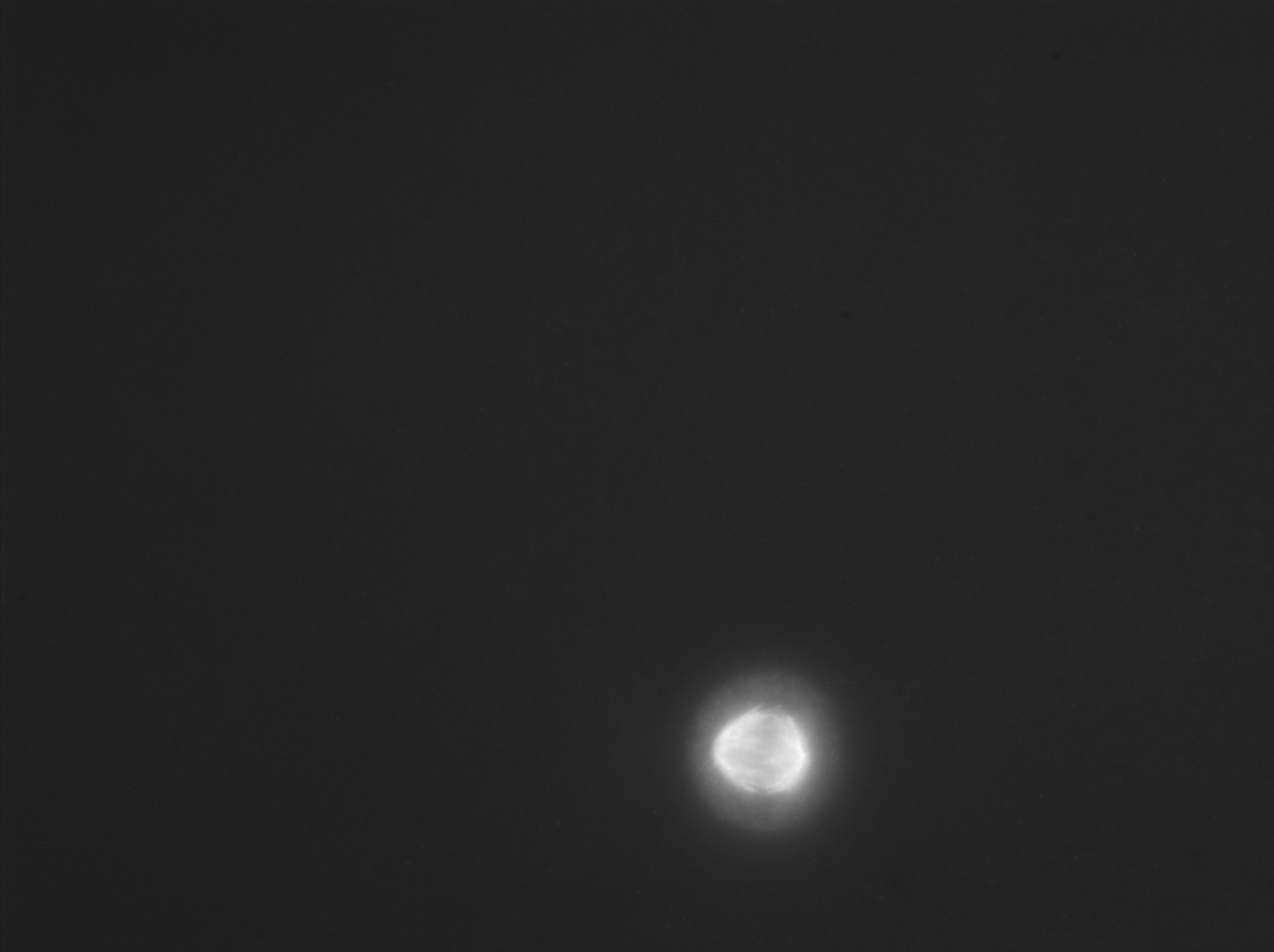

Supplement: Supplementary file 27 — Source data Fig. 7 part 3 [file 44319_2026_742_MOESM27_ESM.zip › Figure 7 Part 3/Fig 7be Cas9 and TPGS1-KO rGT335 atubulin/TPGS1-KO 5-2-25 rGT335 atub R1 M10.Project Maximum Z_XY1746222917_Z0_T0_C2.tif]

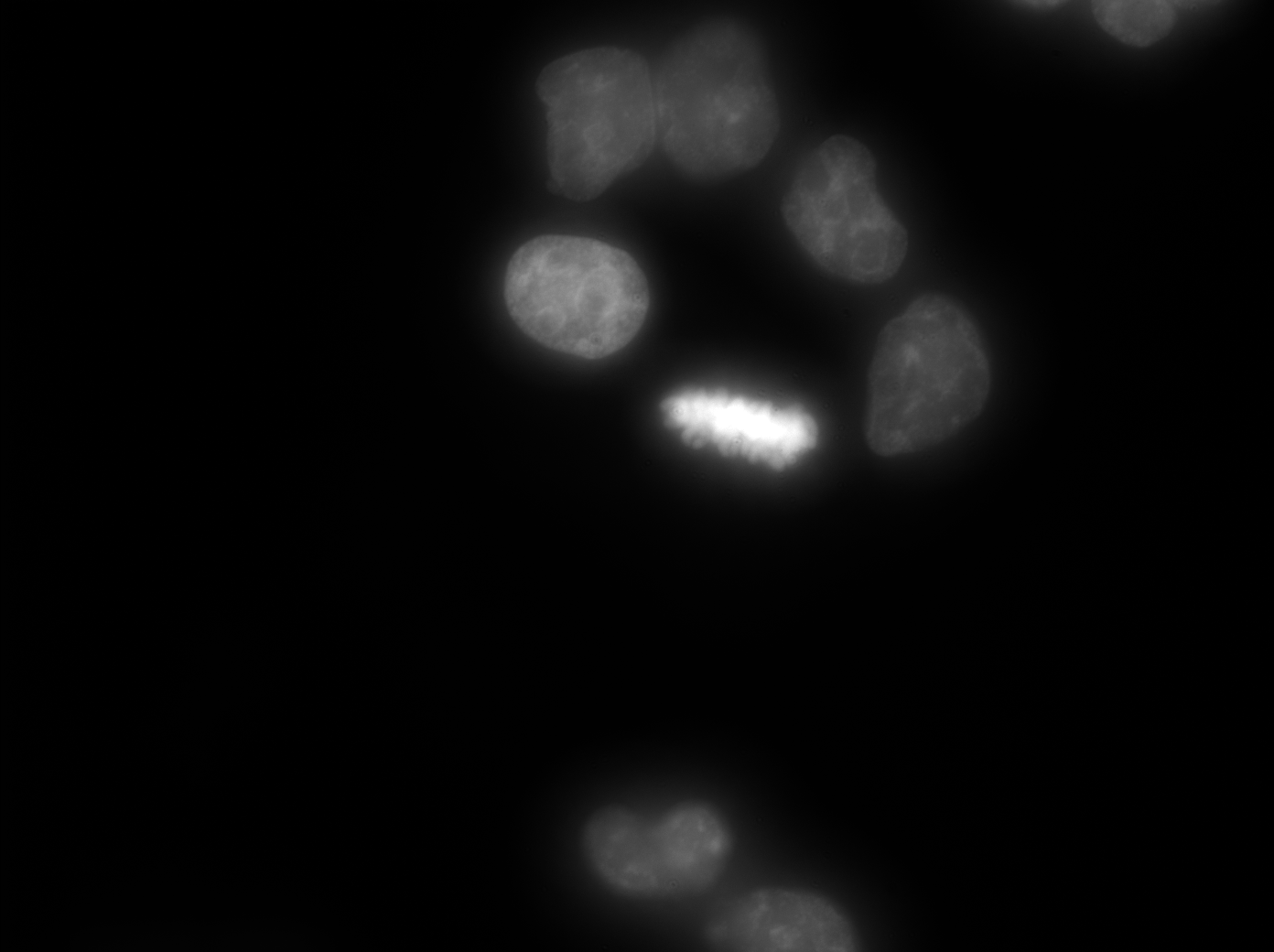

Supplement: Supplementary file 27 — Source data Fig. 7 part 3 [file 44319_2026_742_MOESM27_ESM.zip › Figure 7 Part 3/Fig 7be Cas9 and TPGS1-KO rGT335 atubulin/Cas9 5-2-25 rGT335 atub R3 M9.Project Maximum Z_XY1746218118_Z0_T0_C0.tif]

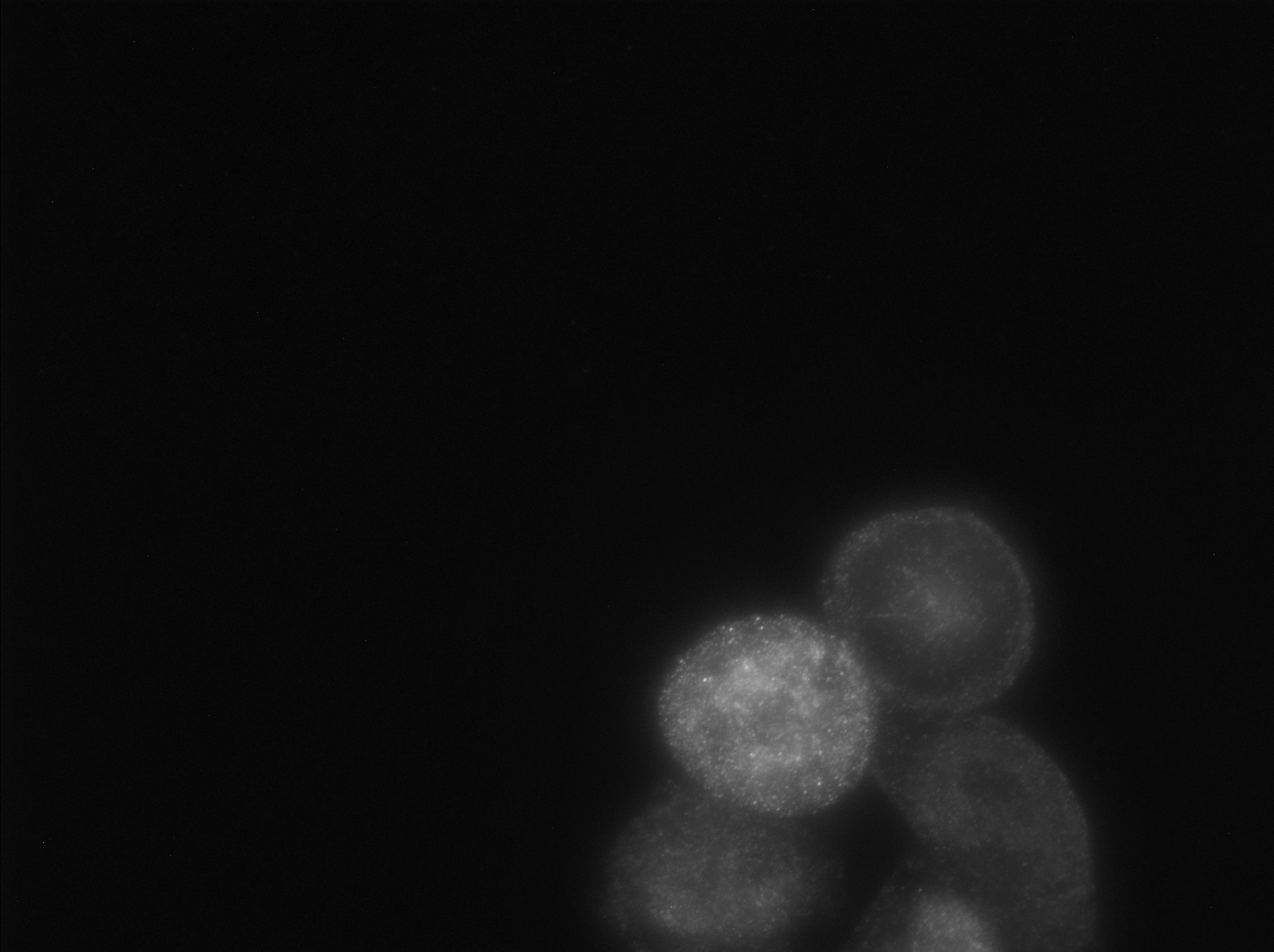

Supplement: Supplementary file 27 — Source data Fig. 7 part 3 [file 44319_2026_742_MOESM27_ESM.zip › Figure 7 Part 3/Fig 7be Cas9 and TPGS1-KO rGT335 atubulin/Cas9 5-2-25 rGT335 atub R2 M1.Project Maximum Z_XY1746558964_Z0_T0_C1.tif]

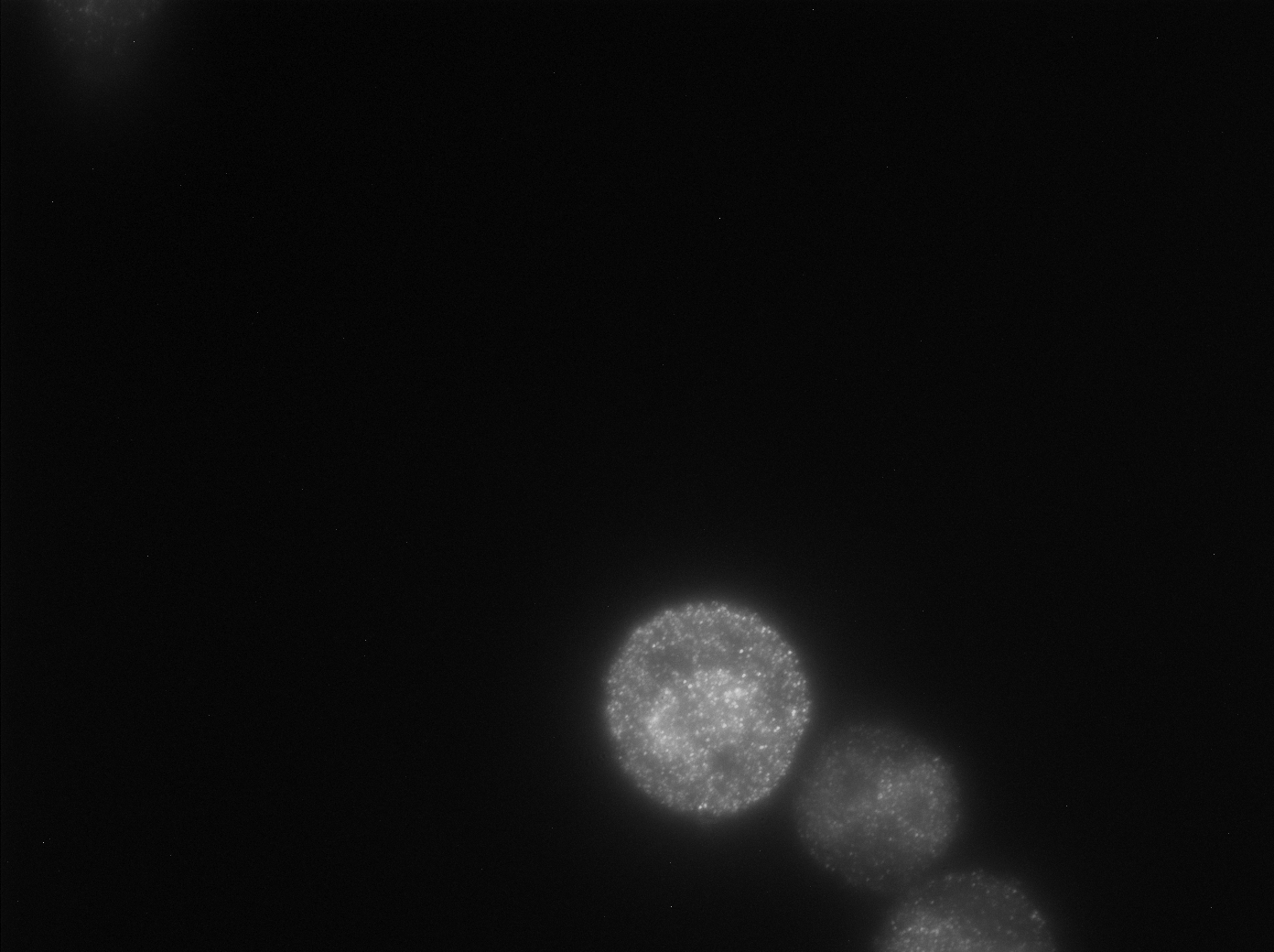

Supplement: Supplementary file 27 — Source data Fig. 7 part 3 [file 44319_2026_742_MOESM27_ESM.zip › Figure 7 Part 3/Fig 7be Cas9 and TPGS1-KO rGT335 atubulin/TPGS1-KO 5-2-25 rGT335 atub R1 M7.Project Maximum Z_XY1746222207_Z0_T0_C1.tif]

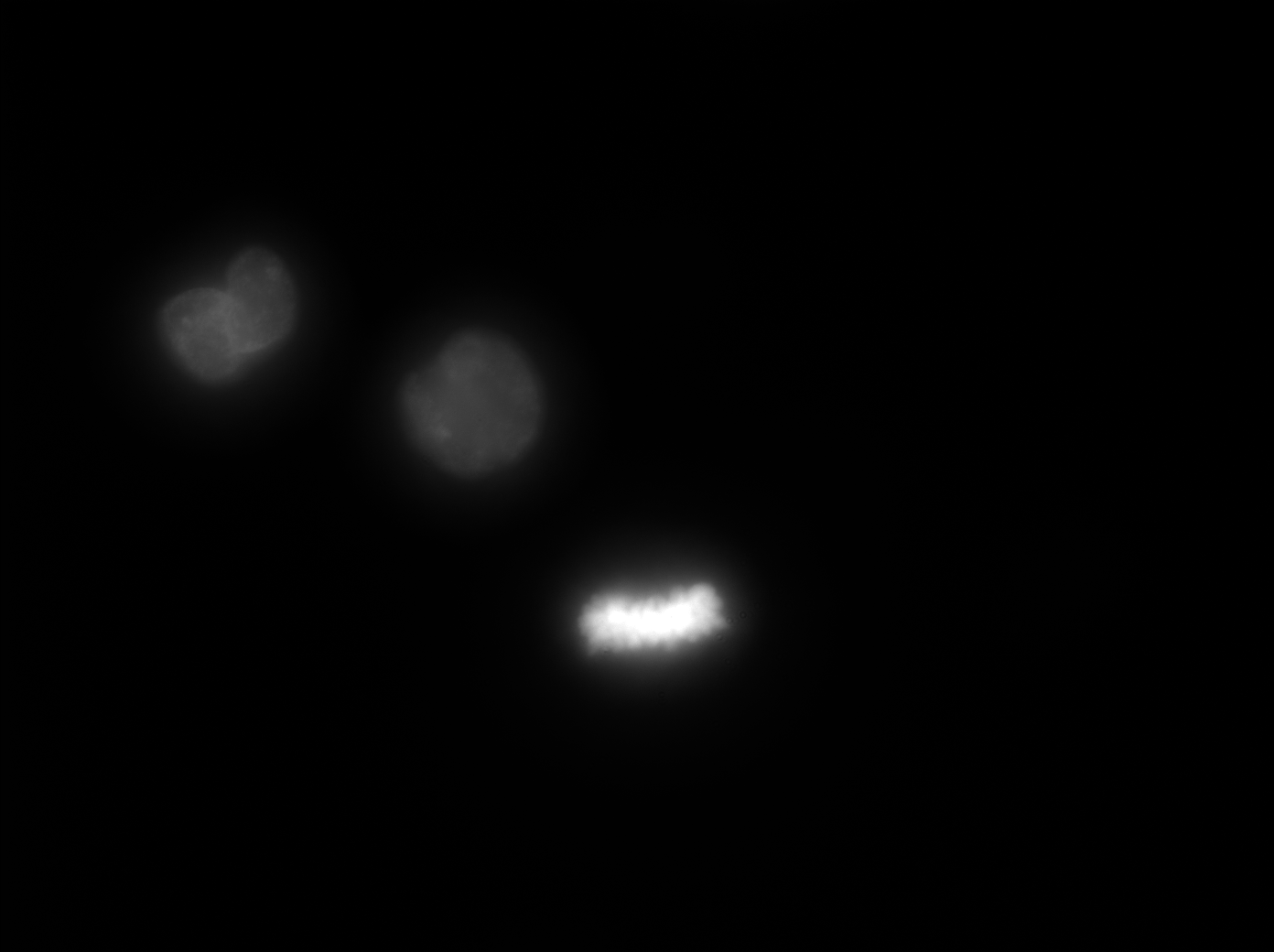

Supplement: Supplementary file 27 — Source data Fig. 7 part 3 [file 44319_2026_742_MOESM27_ESM.zip › Figure 7 Part 3/Fig 7be Cas9 and TPGS1-KO rGT335 atubulin/TPGS1-KO 5-2-25 rGT335 atub R1 M9.Project Maximum Z_XY1746222581_Z0_T0_C0.tif]

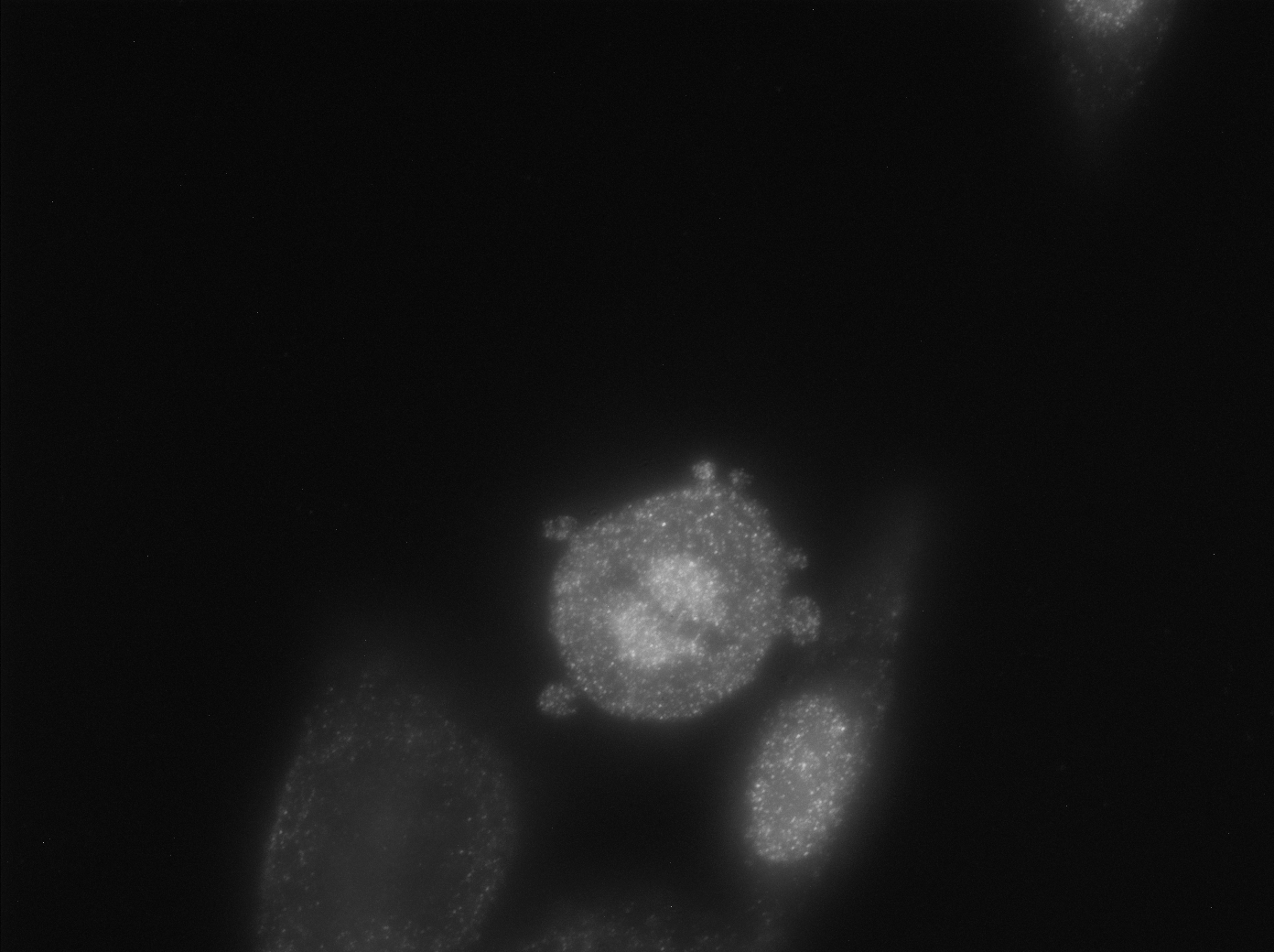

Supplement: Supplementary file 27 — Source data Fig. 7 part 3 [file 44319_2026_742_MOESM27_ESM.zip › Figure 7 Part 3/Fig 7be Cas9 and TPGS1-KO rGT335 atubulin/Cas9 5-2-25 rGT335 atub R1 M8.Project Maximum Z_XY1746558281_Z0_T0_C1.tif]

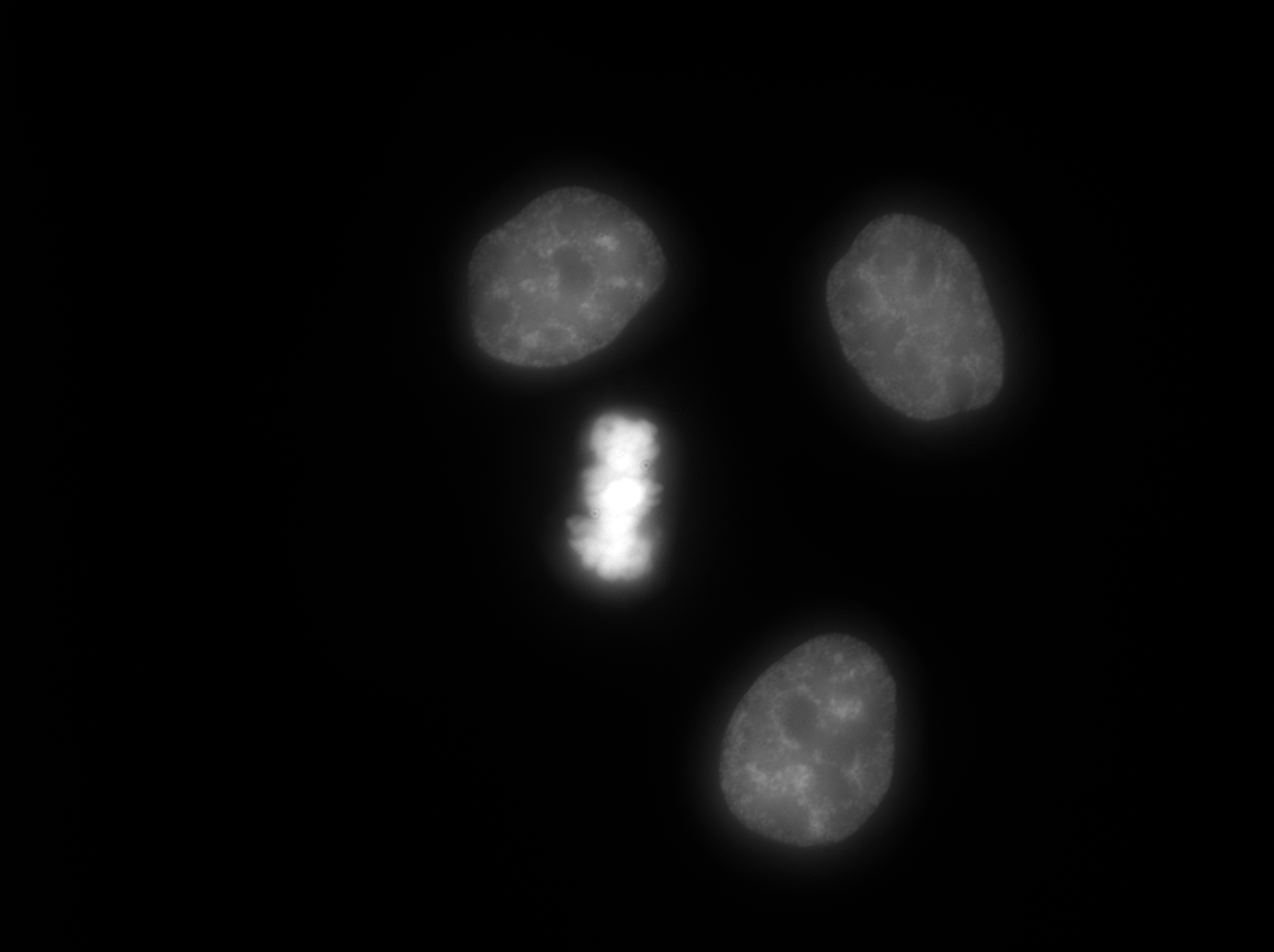

Supplement: Supplementary file 27 — Source data Fig. 7 part 3 [file 44319_2026_742_MOESM27_ESM.zip › Figure 7 Part 3/Fig 7be Cas9 and TPGS1-KO rGT335 atubulin/Cas9 5-2-25 rGT335 atub R2 M9.Project Maximum Z_XY1746562705_Z0_T0_C0.tif]

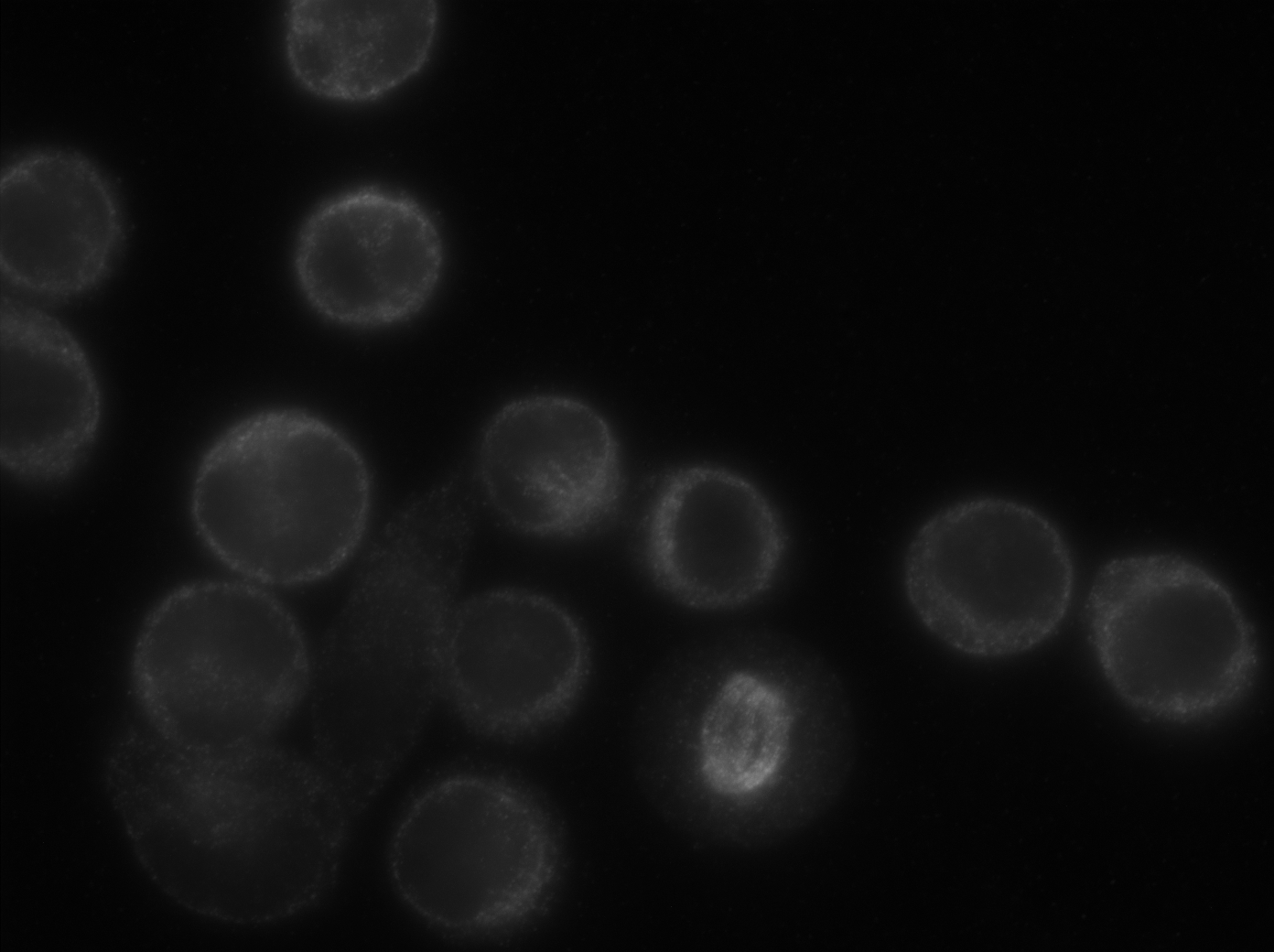

Supplement: Supplementary file 27 — Source data Fig. 7 part 3 [file 44319_2026_742_MOESM27_ESM.zip › Figure 7 Part 3/Fig 7be Cas9 and TPGS1-KO rGT335 atubulin/Cas9 5-2-25 rGT335 atub R2 M7.Project Maximum Z_XY1746562337_Z0_T0_C2.tif]

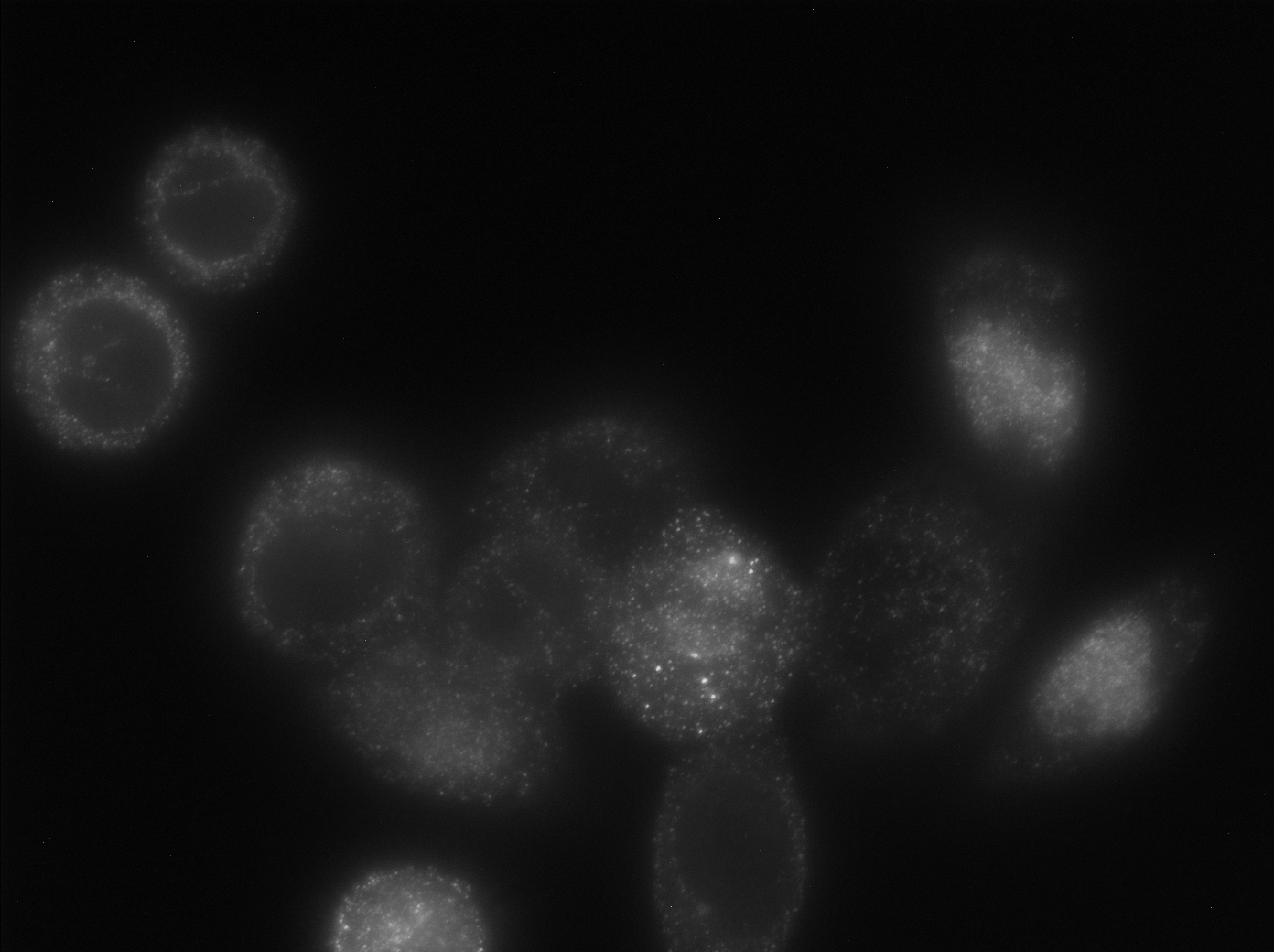

Supplement: Supplementary file 27 — Source data Fig. 7 part 3 [file 44319_2026_742_MOESM27_ESM.zip › Figure 7 Part 3/Fig 7be Cas9 and TPGS1-KO rGT335 atubulin/TPGS1-KO 5-2-25 rGT335 atub R3 M2.Project Maximum Z_XY1746218877_Z0_T0_C1.tif]

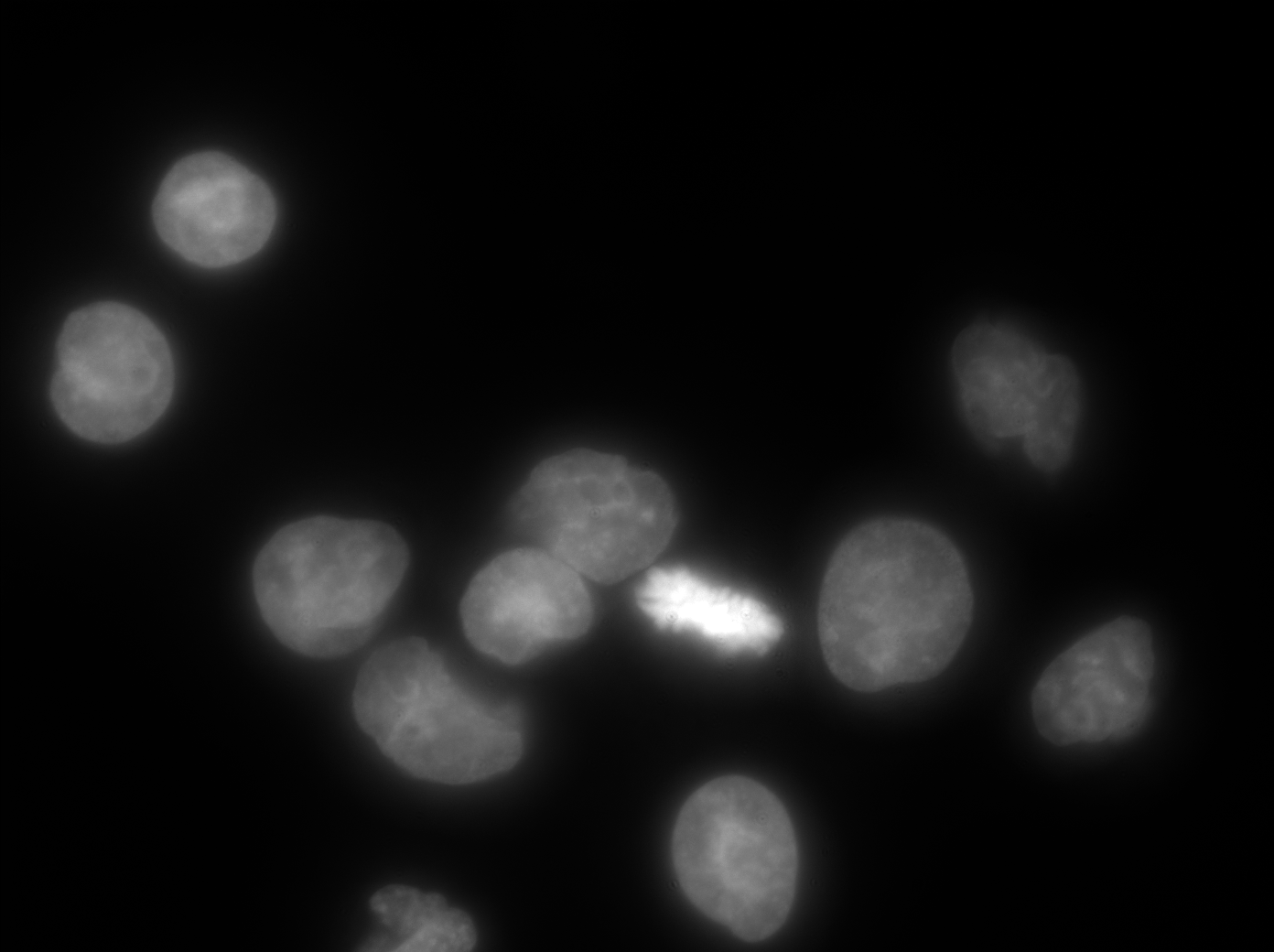

Supplement: Supplementary file 27 — Source data Fig. 7 part 3 [file 44319_2026_742_MOESM27_ESM.zip › Figure 7 Part 3/Fig 7be Cas9 and TPGS1-KO rGT335 atubulin/TPGS1-KO 5-2-25 rGT335 atub R3 M2.Project Maximum Z_XY1746218877_Z0_T0_C0.tif]

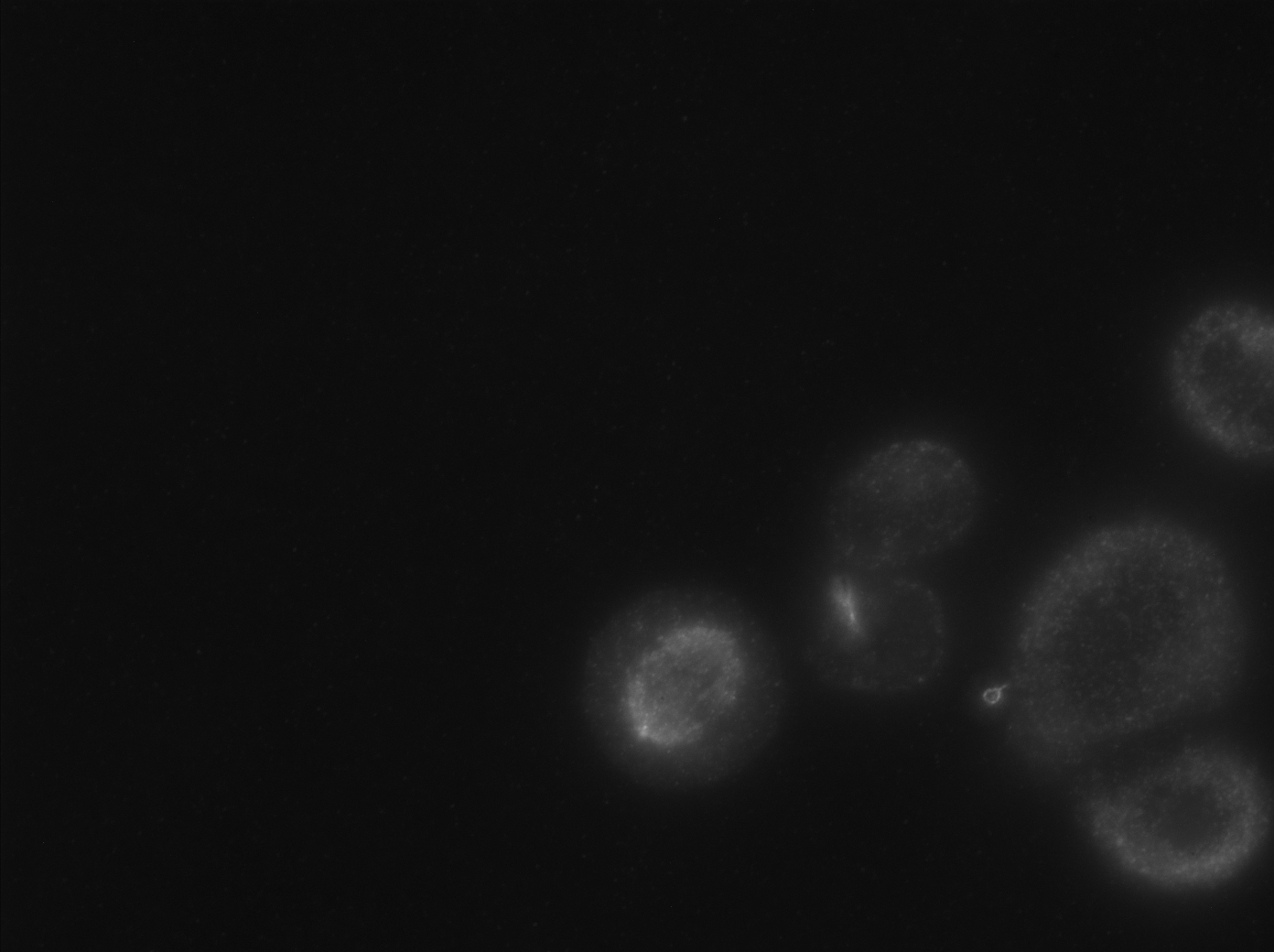

Supplement: Supplementary file 27 — Source data Fig. 7 part 3 [file 44319_2026_742_MOESM27_ESM.zip › Figure 7 Part 3/Fig 7be Cas9 and TPGS1-KO rGT335 atubulin/Cas9 5-2-25 rGT335 atub R1 M10.Project Maximum Z_XY1746558510_Z0_T0_C2.tif]

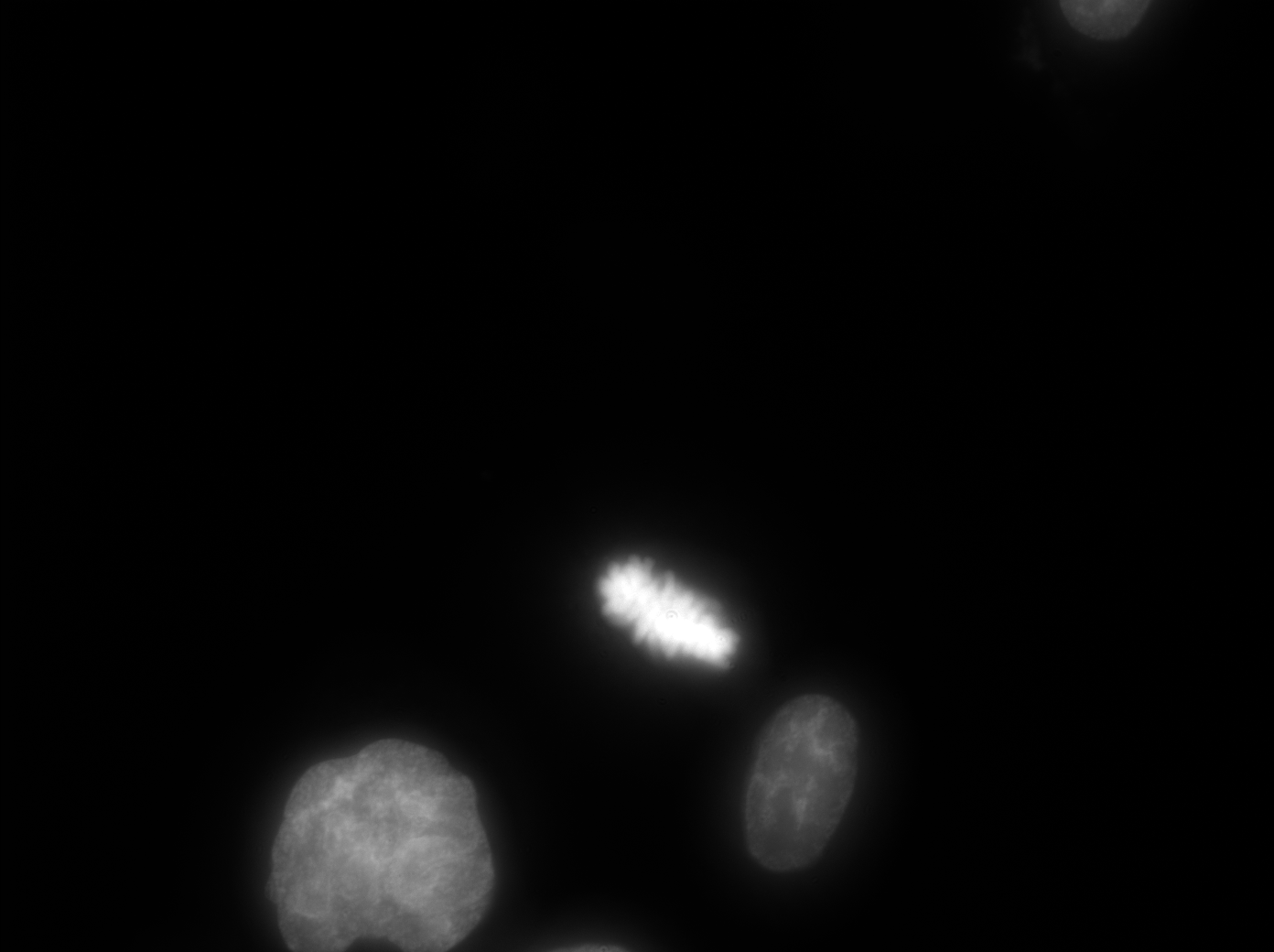

Supplement: Supplementary file 27 — Source data Fig. 7 part 3 [file 44319_2026_742_MOESM27_ESM.zip › Figure 7 Part 3/Fig 7be Cas9 and TPGS1-KO rGT335 atubulin/Cas9 5-2-25 rGT335 atub R1 M8.Project Maximum Z_XY1746558281_Z0_T0_C0.tif]

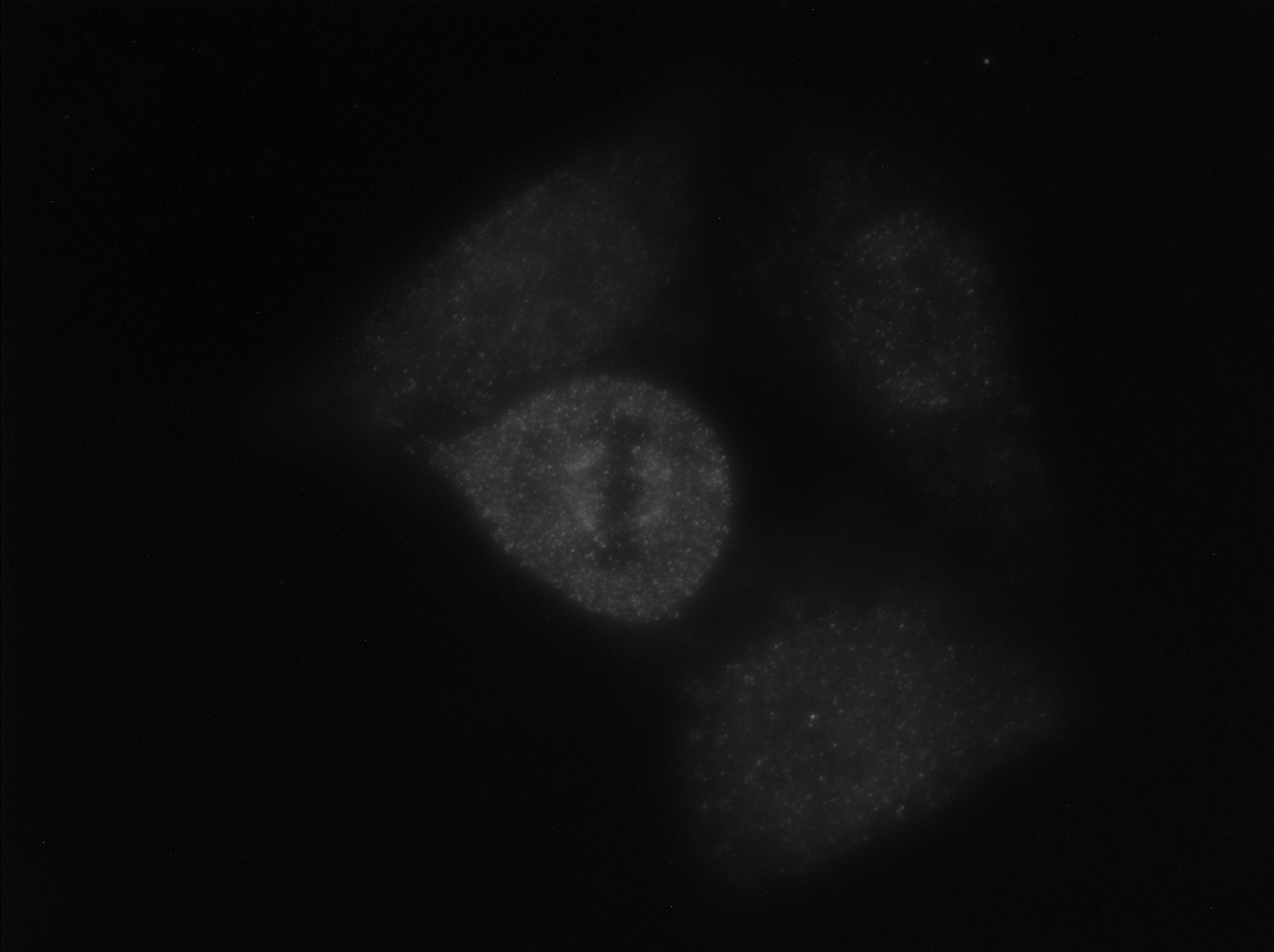

Supplement: Supplementary file 27 — Source data Fig. 7 part 3 [file 44319_2026_742_MOESM27_ESM.zip › Figure 7 Part 3/Fig 7be Cas9 and TPGS1-KO rGT335 atubulin/Cas9 5-2-25 rGT335 atub R2 M9.Project Maximum Z_XY1746562705_Z0_T0_C1.tif]

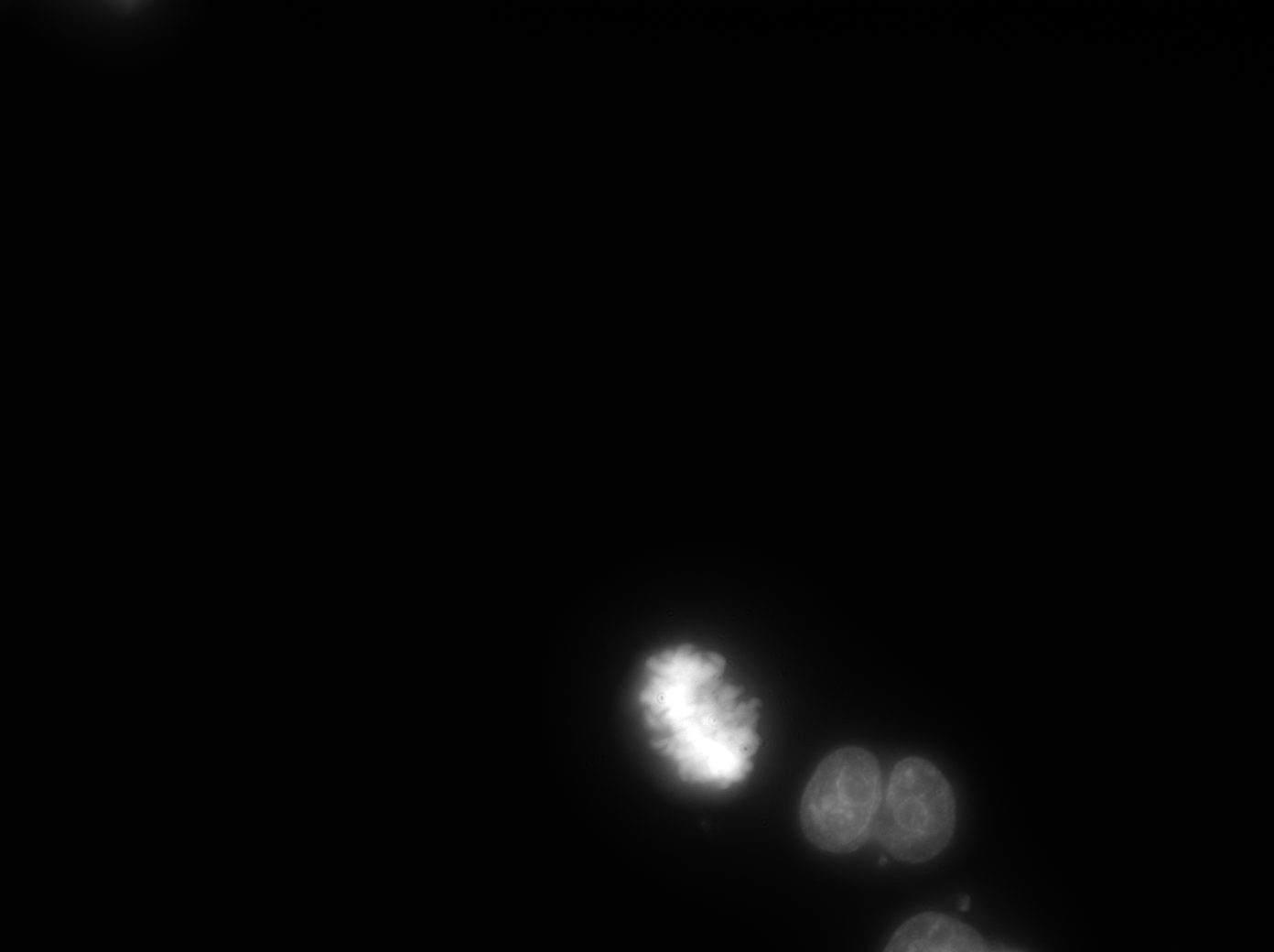

Supplement: Supplementary file 27 — Source data Fig. 7 part 3 [file 44319_2026_742_MOESM27_ESM.zip › Figure 7 Part 3/Fig 7be Cas9 and TPGS1-KO rGT335 atubulin/TPGS1-KO 5-2-25 rGT335 atub R1 M7.Project Maximum Z_XY1746222207_Z0_T0_C0.tif]

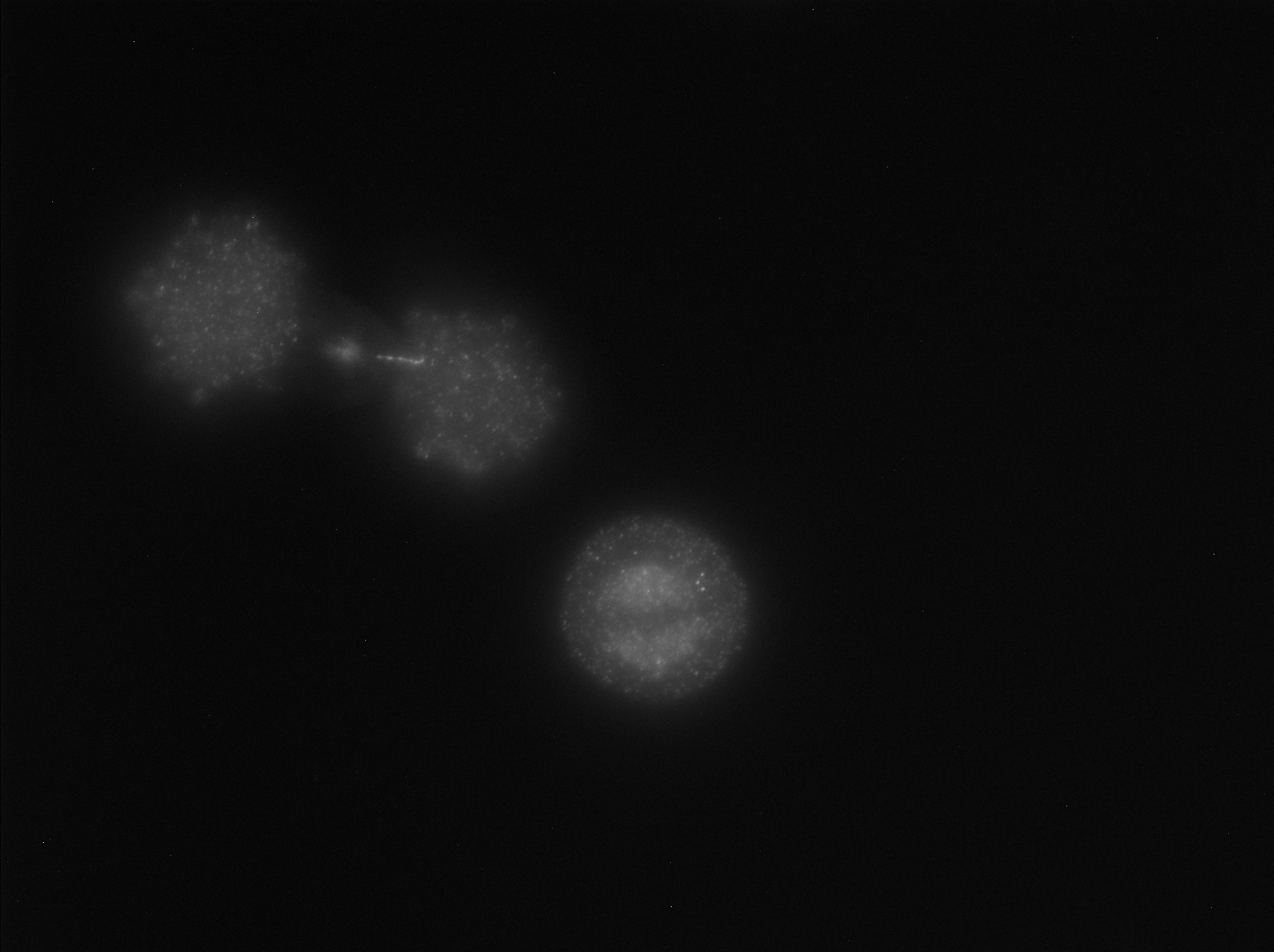

Supplement: Supplementary file 27 — Source data Fig. 7 part 3 [file 44319_2026_742_MOESM27_ESM.zip › Figure 7 Part 3/Fig 7be Cas9 and TPGS1-KO rGT335 atubulin/TPGS1-KO 5-2-25 rGT335 atub R1 M9.Project Maximum Z_XY1746222581_Z0_T0_C1.tif]

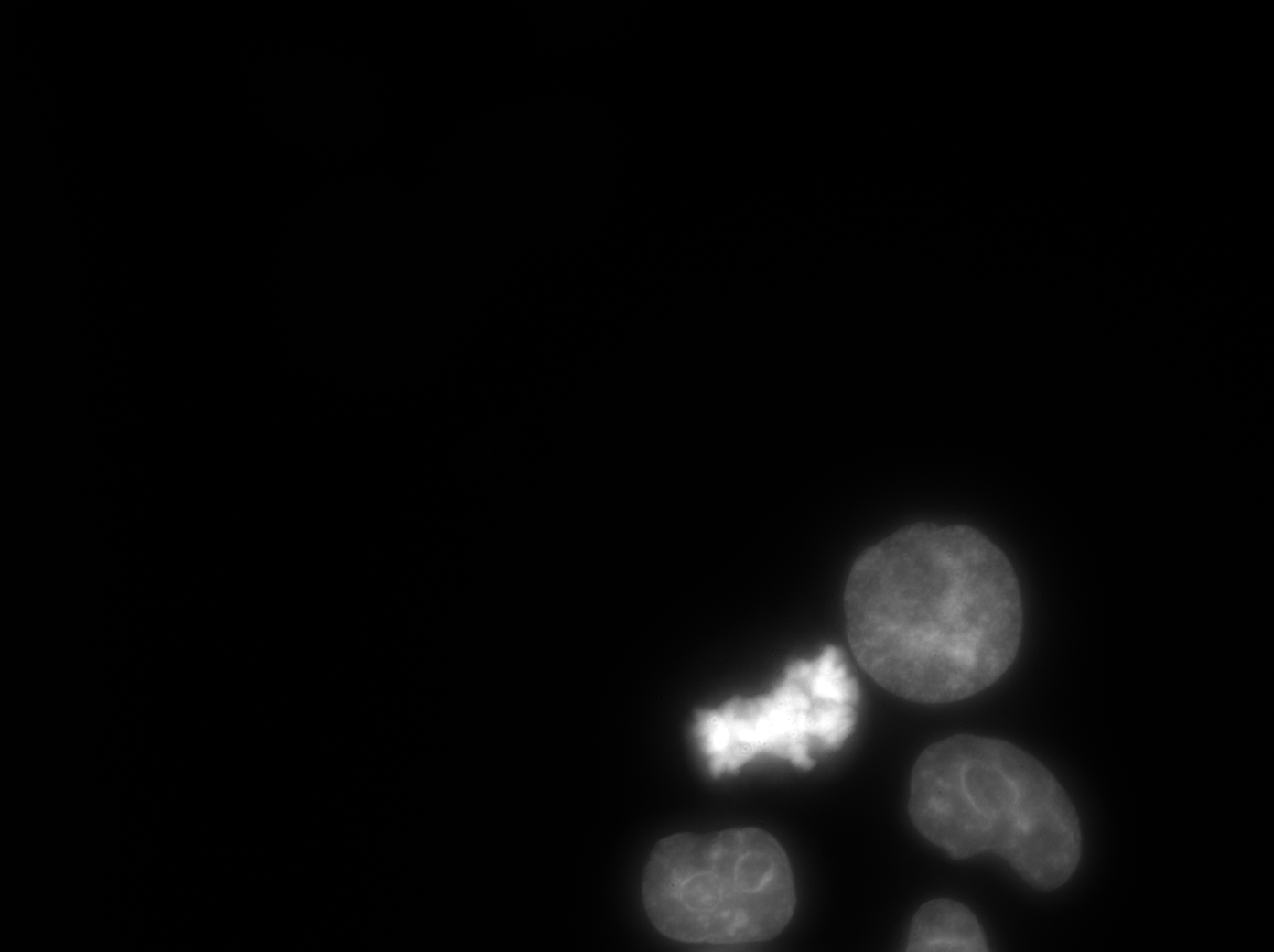

Supplement: Supplementary file 27 — Source data Fig. 7 part 3 [file 44319_2026_742_MOESM27_ESM.zip › Figure 7 Part 3/Fig 7be Cas9 and TPGS1-KO rGT335 atubulin/Cas9 5-2-25 rGT335 atub R2 M1.Project Maximum Z_XY1746558964_Z0_T0_C0.tif]

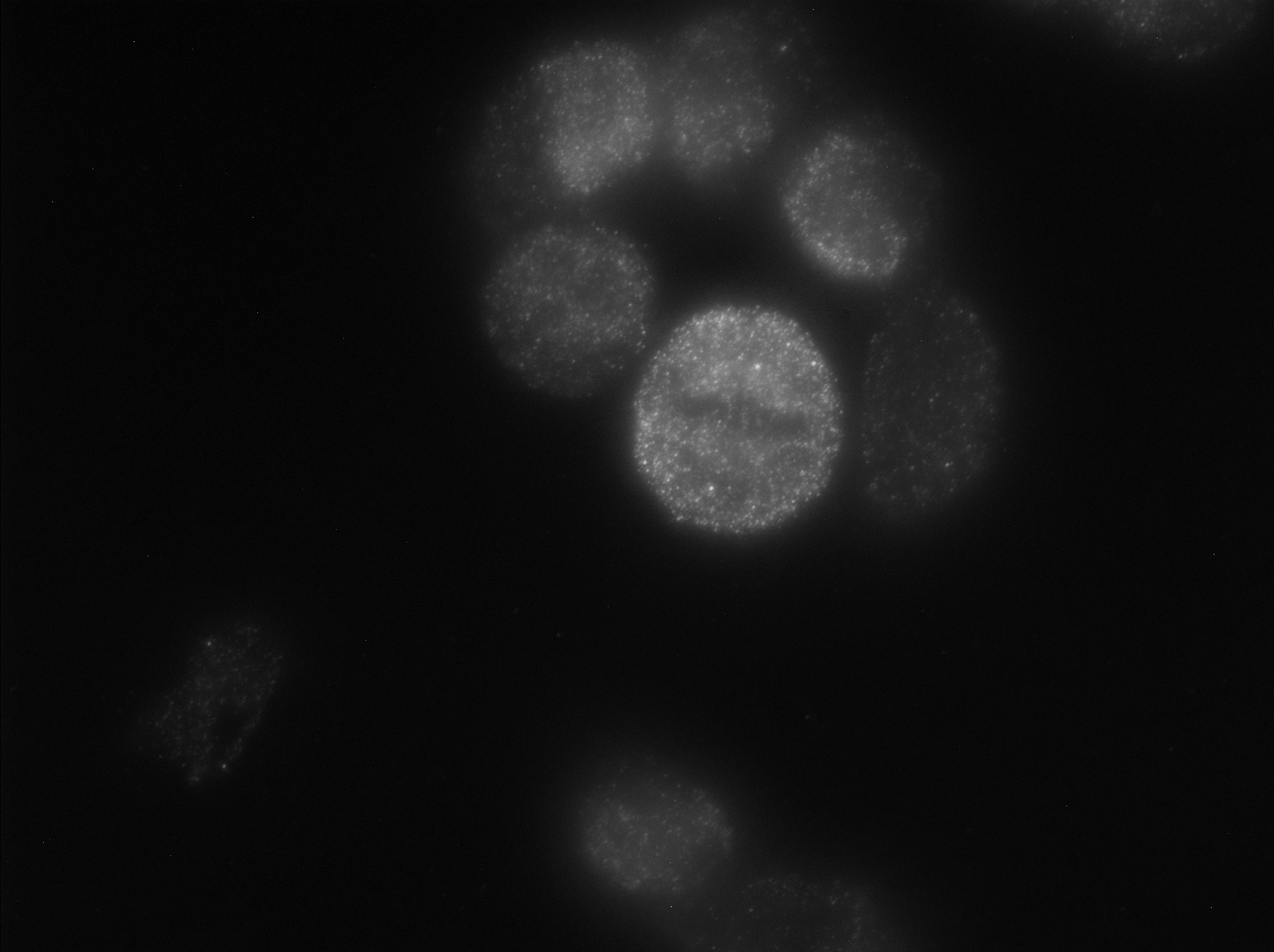

Supplement: Supplementary file 27 — Source data Fig. 7 part 3 [file 44319_2026_742_MOESM27_ESM.zip › Figure 7 Part 3/Fig 7be Cas9 and TPGS1-KO rGT335 atubulin/Cas9 5-2-25 rGT335 atub R3 M9.Project Maximum Z_XY1746218118_Z0_T0_C1.tif]

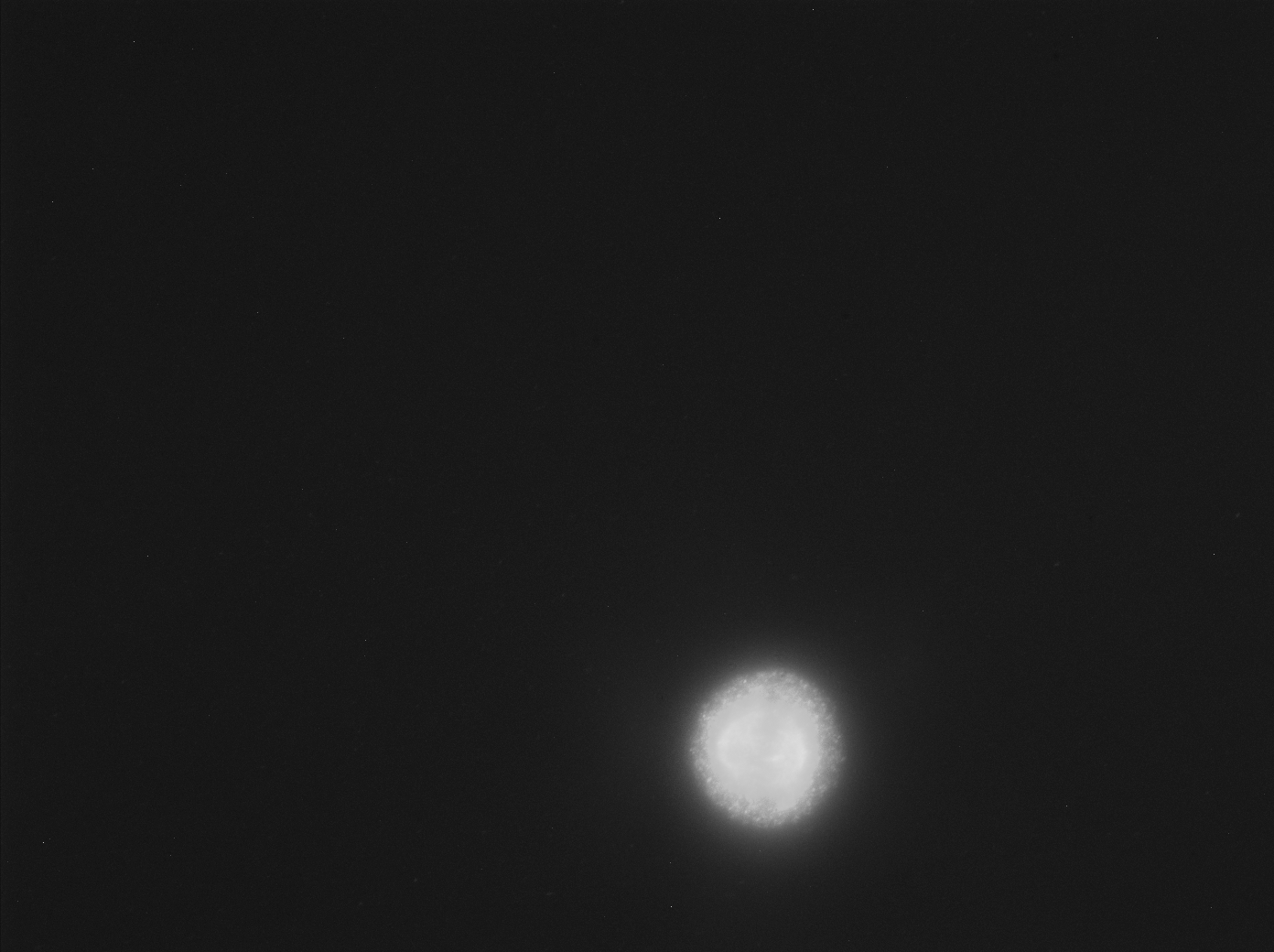

Supplement: Supplementary file 27 — Source data Fig. 7 part 3 [file 44319_2026_742_MOESM27_ESM.zip › Figure 7 Part 3/Fig 7be Cas9 and TPGS1-KO rGT335 atubulin/TPGS1-KO 5-2-25 rGT335 atub R1 M10.Project Maximum Z_XY1746222917_Z0_T0_C1.tif]

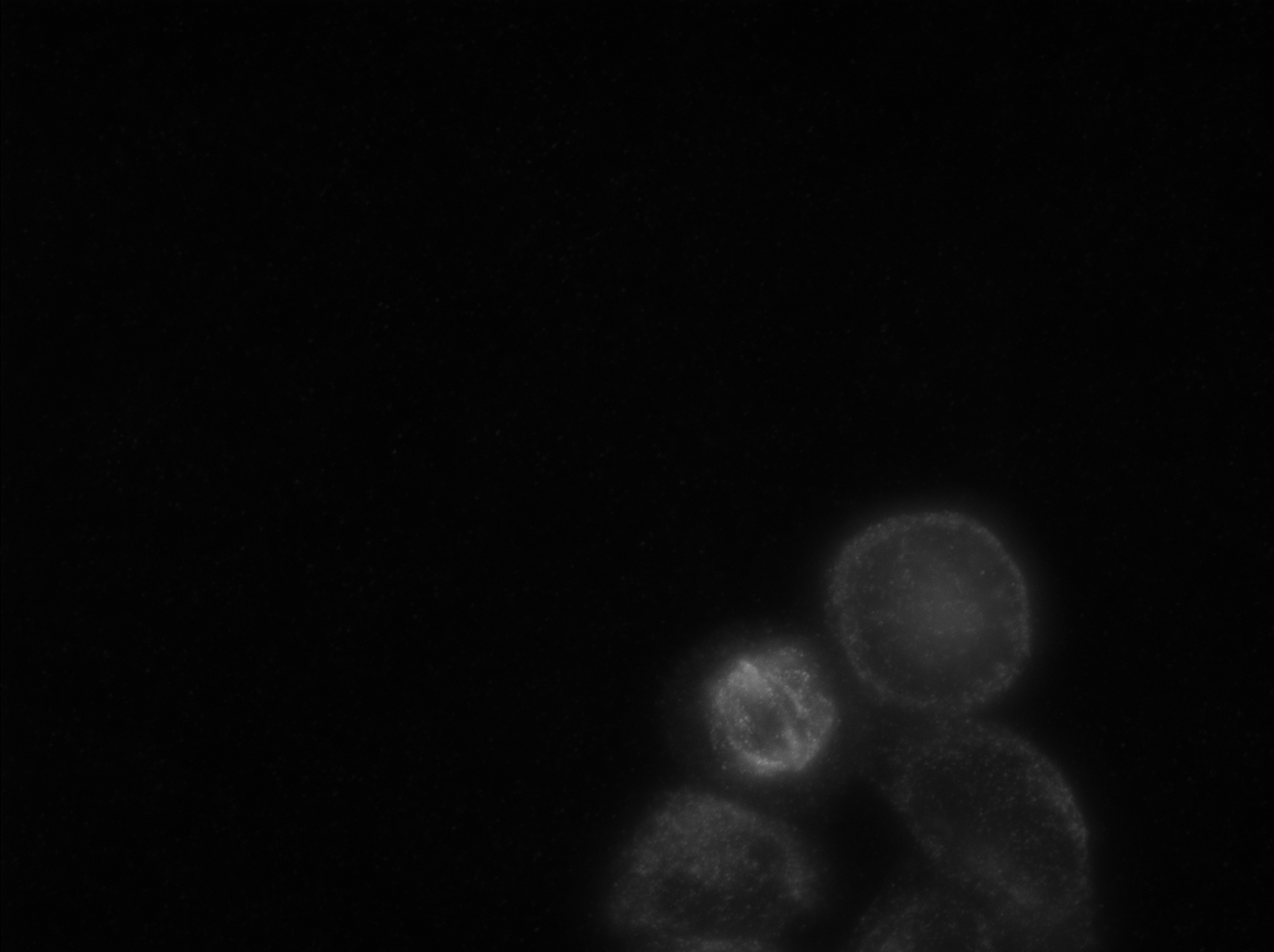

Supplement: Supplementary file 27 — Source data Fig. 7 part 3 [file 44319_2026_742_MOESM27_ESM.zip › Figure 7 Part 3/Fig 7be Cas9 and TPGS1-KO rGT335 atubulin/Cas9 5-2-25 rGT335 atub R2 M1.Project Maximum Z_XY1746558964_Z0_T0_C2.tif]

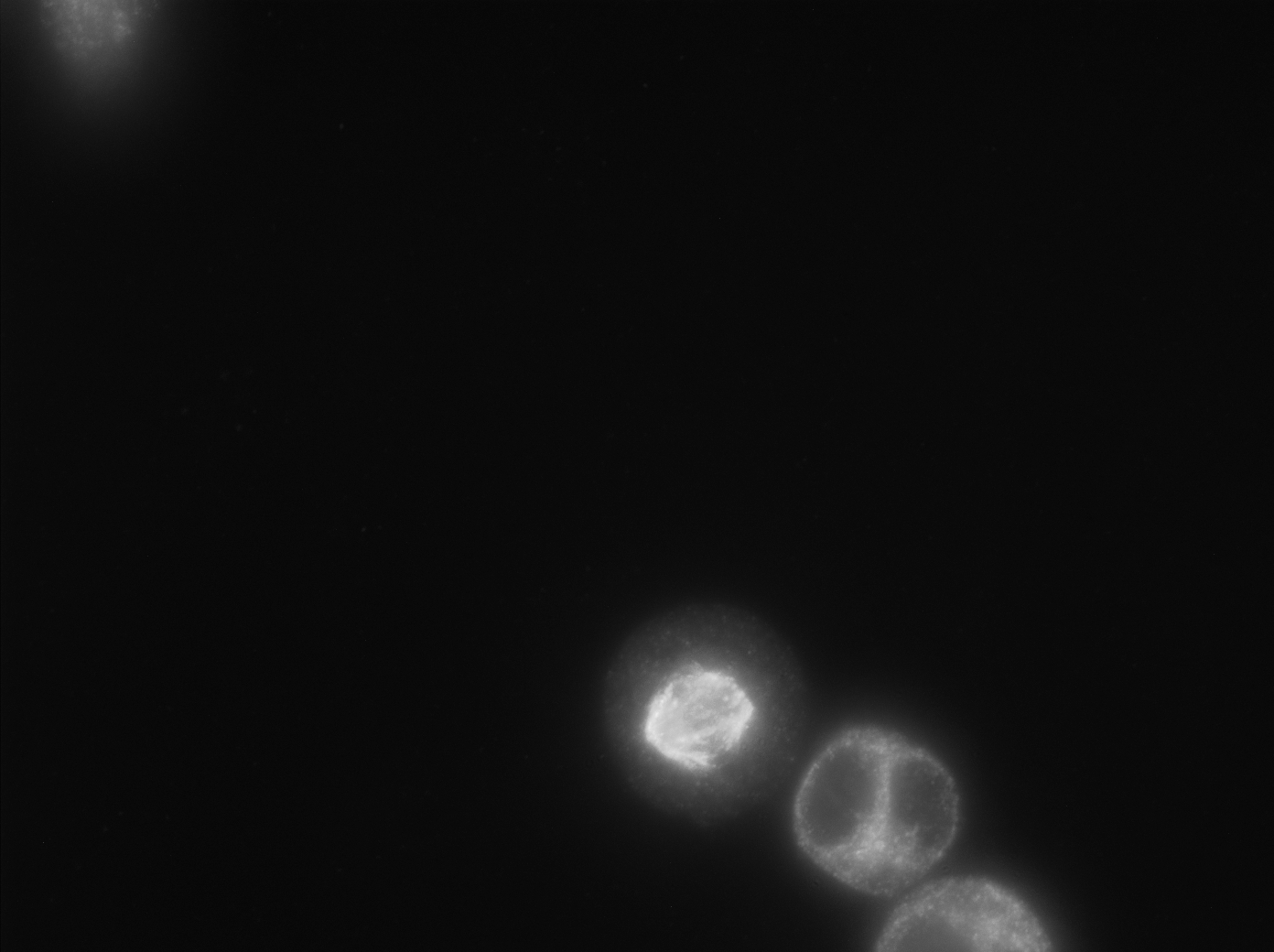

Supplement: Supplementary file 27 — Source data Fig. 7 part 3 [file 44319_2026_742_MOESM27_ESM.zip › Figure 7 Part 3/Fig 7be Cas9 and TPGS1-KO rGT335 atubulin/TPGS1-KO 5-2-25 rGT335 atub R1 M7.Project Maximum Z_XY1746222207_Z0_T0_C2.tif]

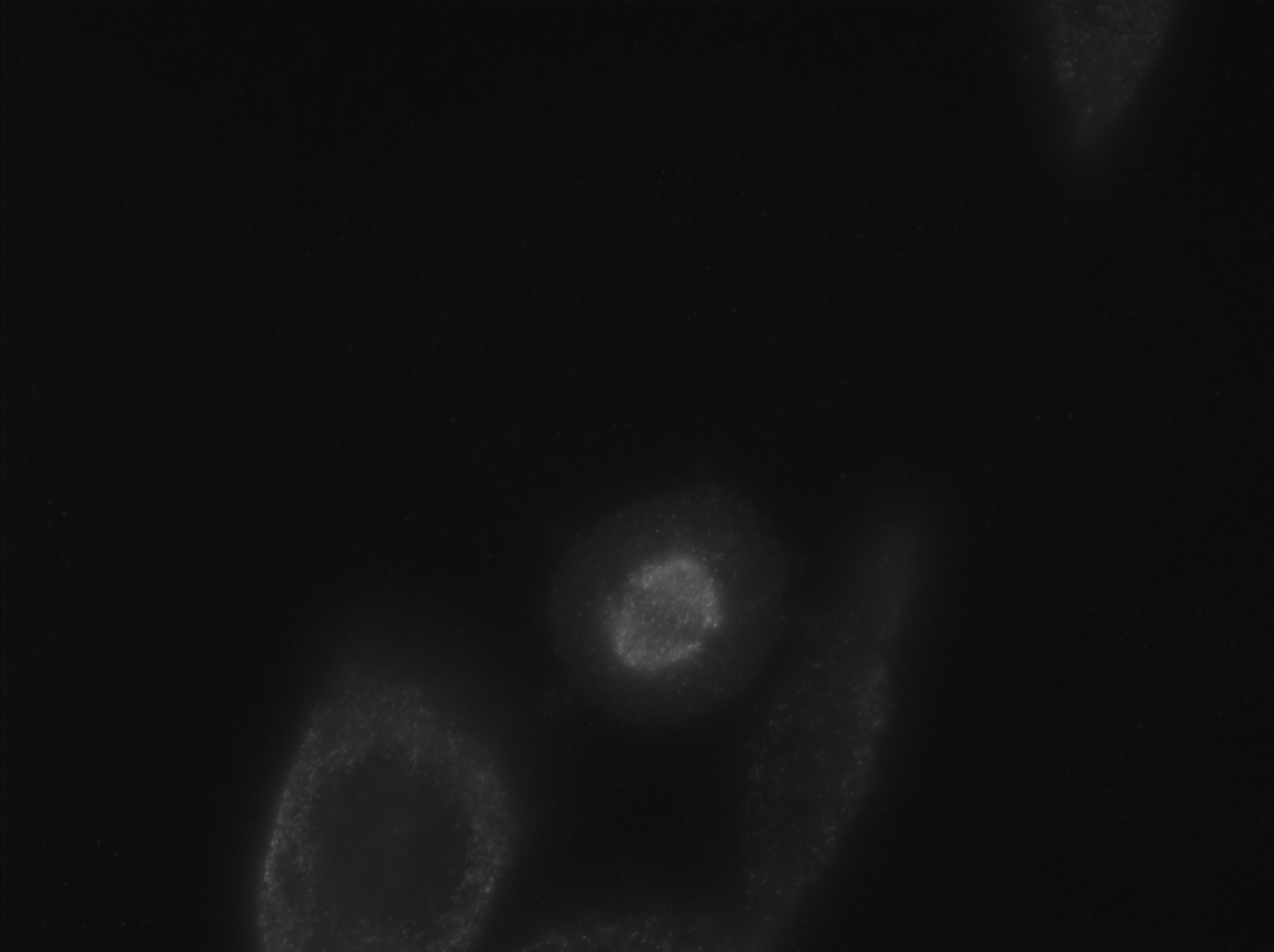

Supplement: Supplementary file 27 — Source data Fig. 7 part 3 [file 44319_2026_742_MOESM27_ESM.zip › Figure 7 Part 3/Fig 7be Cas9 and TPGS1-KO rGT335 atubulin/Cas9 5-2-25 rGT335 atub R1 M8.Project Maximum Z_XY1746558281_Z0_T0_C2.tif]

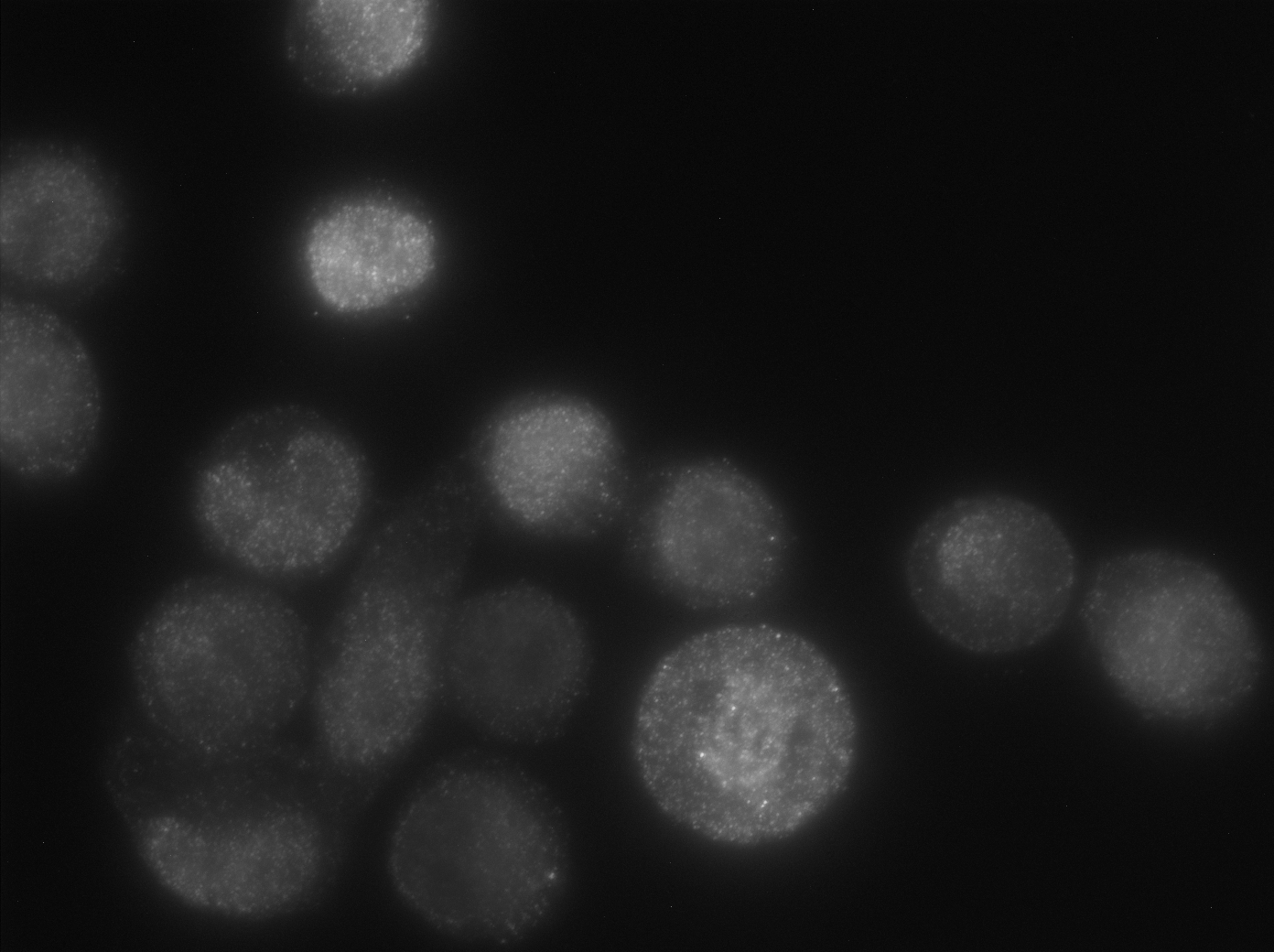

Supplement: Supplementary file 27 — Source data Fig. 7 part 3 [file 44319_2026_742_MOESM27_ESM.zip › Figure 7 Part 3/Fig 7be Cas9 and TPGS1-KO rGT335 atubulin/Cas9 5-2-25 rGT335 atub R2 M7.Project Maximum Z_XY1746562337_Z0_T0_C1.tif]
