# Supplementary figures and images for: TPGS1 regulates central spindle microtubule glutamylation and remodeling during telophase and abscission (part 36 of 36)
Source: EMBO Rep. 2026 Mar 23;27(8):1944–63. doi: 10.1038/s44319-026-00742-3 (PMC13121839; doi:10.1038/s44319-026-00742-3)

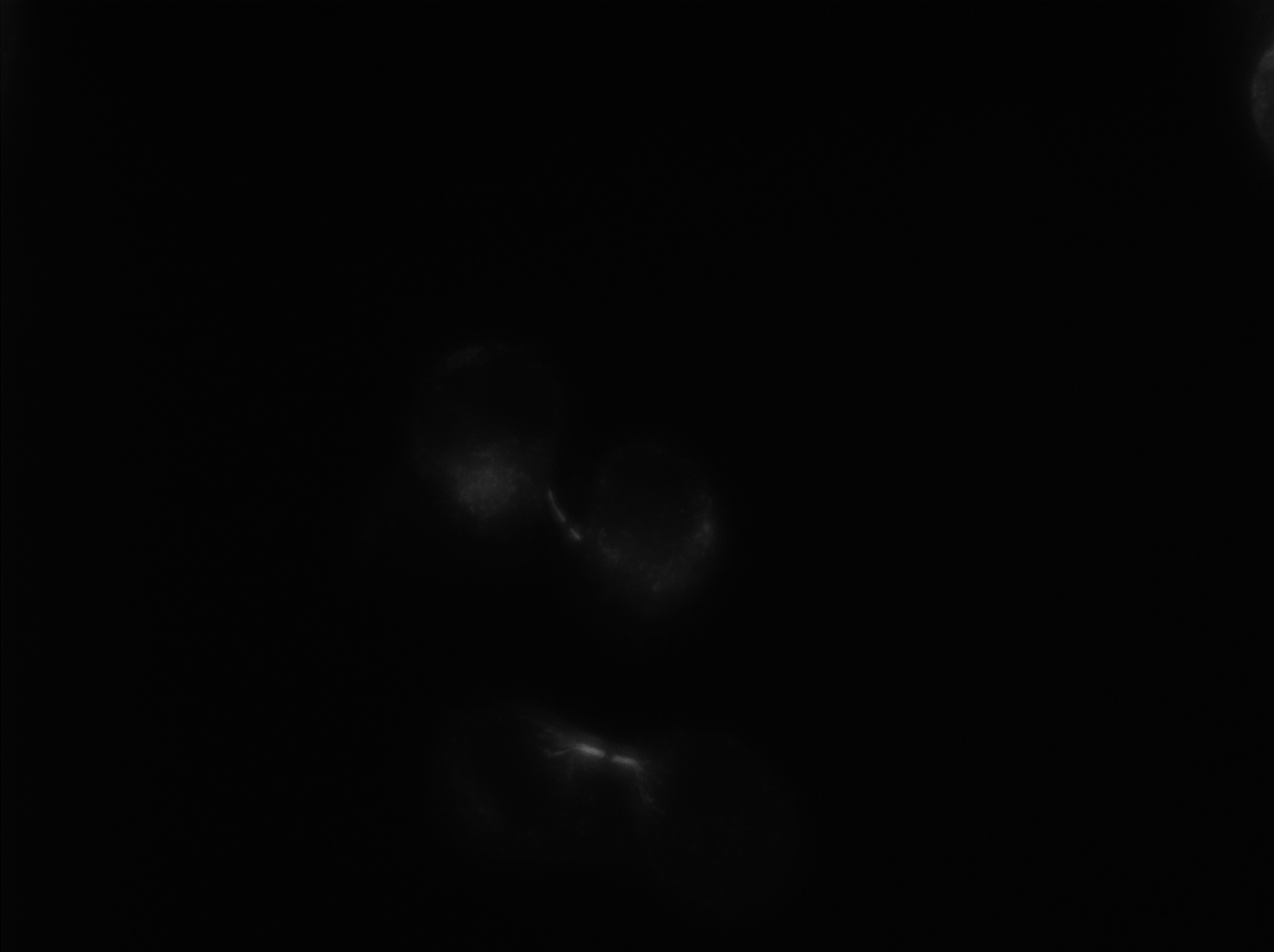

Supplement: Supplementary file 28 — Source data Fig. 7 part 4 [file 44319_2026_742_MOESM28_ESM.zip › Figure 7 Part 4/Fig 7fg Control and TPGS1-KO spastin acetylated tubulin/Cas9 spastin actub 4-1-25 R1 LT3LT4.Project Maximum Z_XY1743529831_Z0_T0_C2.tif]

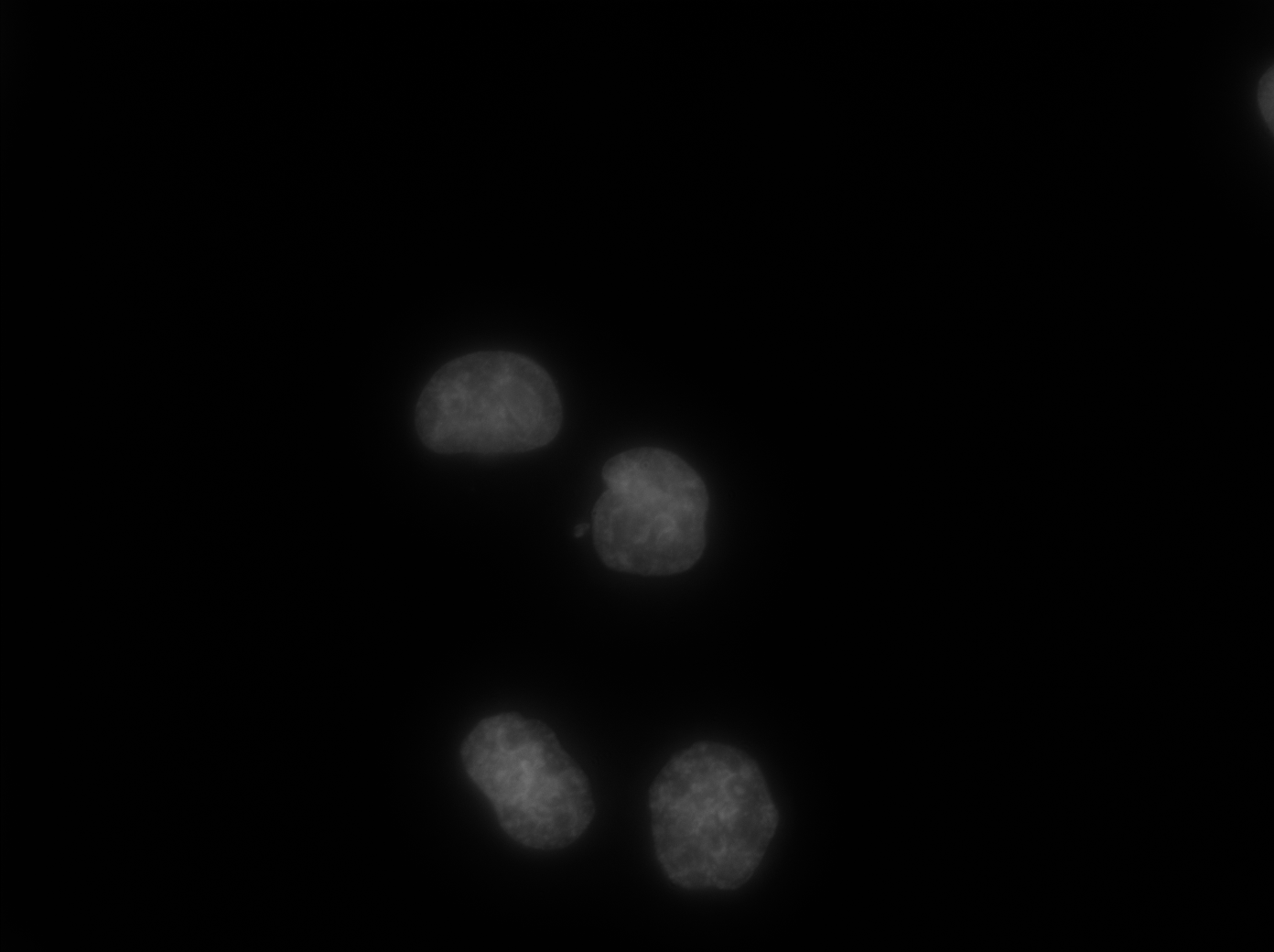

Supplement: Supplementary file 28 — Source data Fig. 7 part 4 [file 44319_2026_742_MOESM28_ESM.zip › Figure 7 Part 4/Fig 7fg Control and TPGS1-KO spastin acetylated tubulin/Cas9 spastin actub 4-1-25 R1 LT3LT4.Project Maximum Z_XY1743529831_Z0_T0_C0.tif]

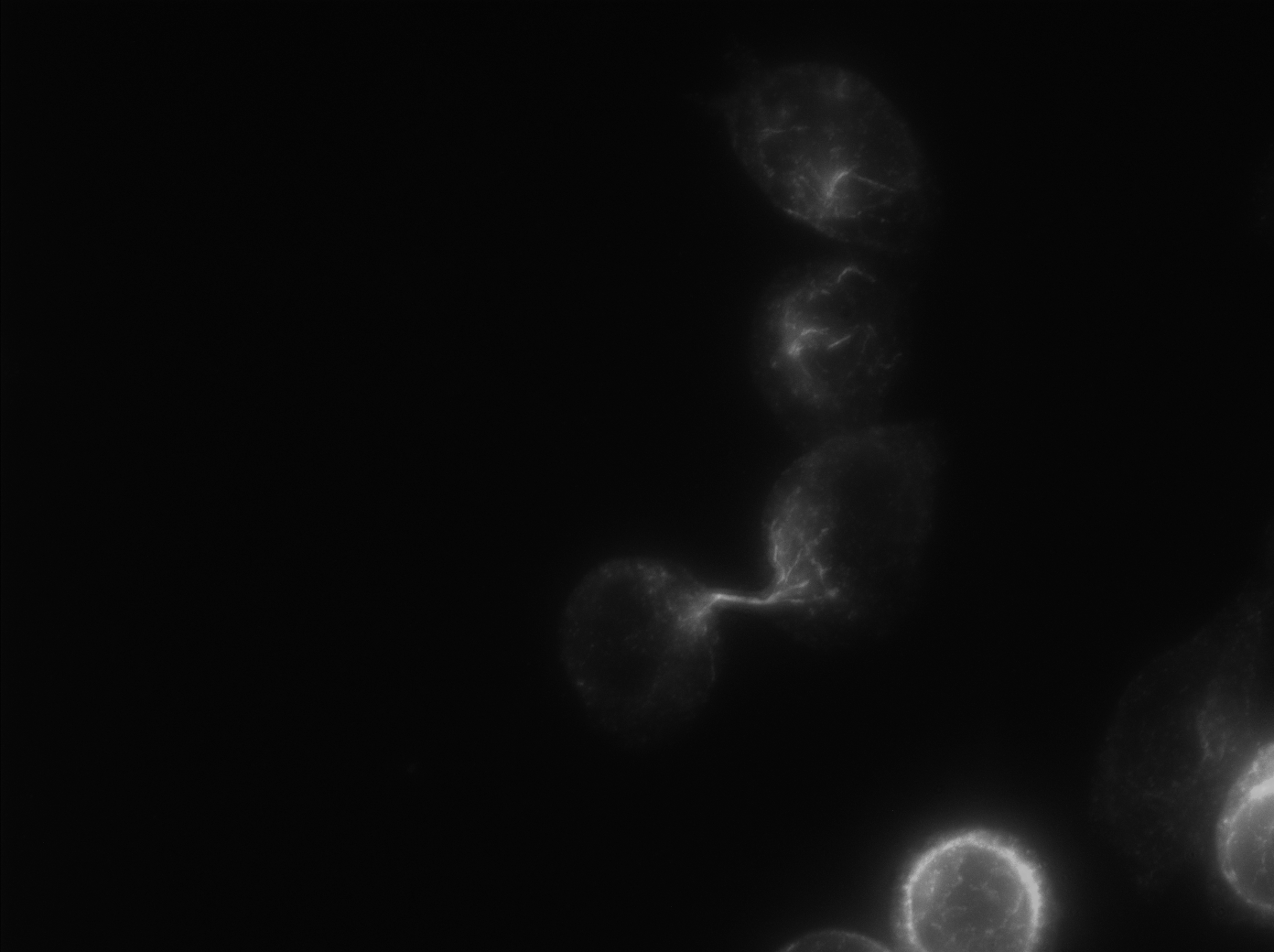

Supplement: Supplementary file 28 — Source data Fig. 7 part 4 [file 44319_2026_742_MOESM28_ESM.zip › Figure 7 Part 4/Fig 7fg Control and TPGS1-KO spastin acetylated tubulin/Cas9 spastin actub 4-1-25 R1 SI19.Project Maximum Z_XY1743534750_Z0_T0_C2.tif]

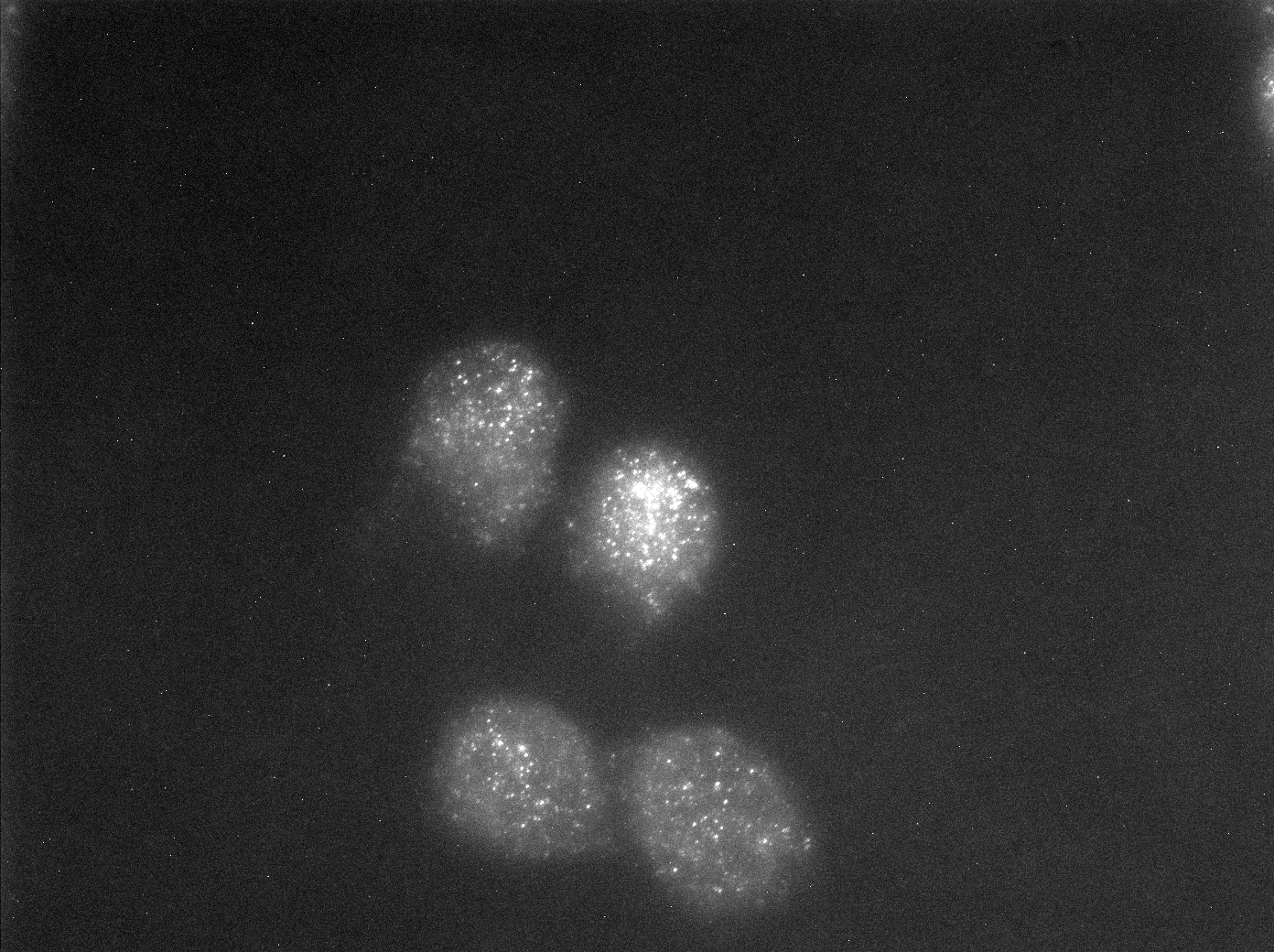

Supplement: Supplementary file 28 — Source data Fig. 7 part 4 [file 44319_2026_742_MOESM28_ESM.zip › Figure 7 Part 4/Fig 7fg Control and TPGS1-KO spastin acetylated tubulin/Cas9 spastin actub 4-1-25 R1 LT3LT4.Project Maximum Z_XY1743529831_Z0_T0_C1.tif]
