# Supplementary figures and images for: TPGS1 regulates central spindle microtubule glutamylation and remodeling during telophase and abscission (part 35 of 36)
Source: EMBO Rep. 2026 Mar 23;27(8):1944–63. doi: 10.1038/s44319-026-00742-3 (PMC13121839; doi:10.1038/s44319-026-00742-3)

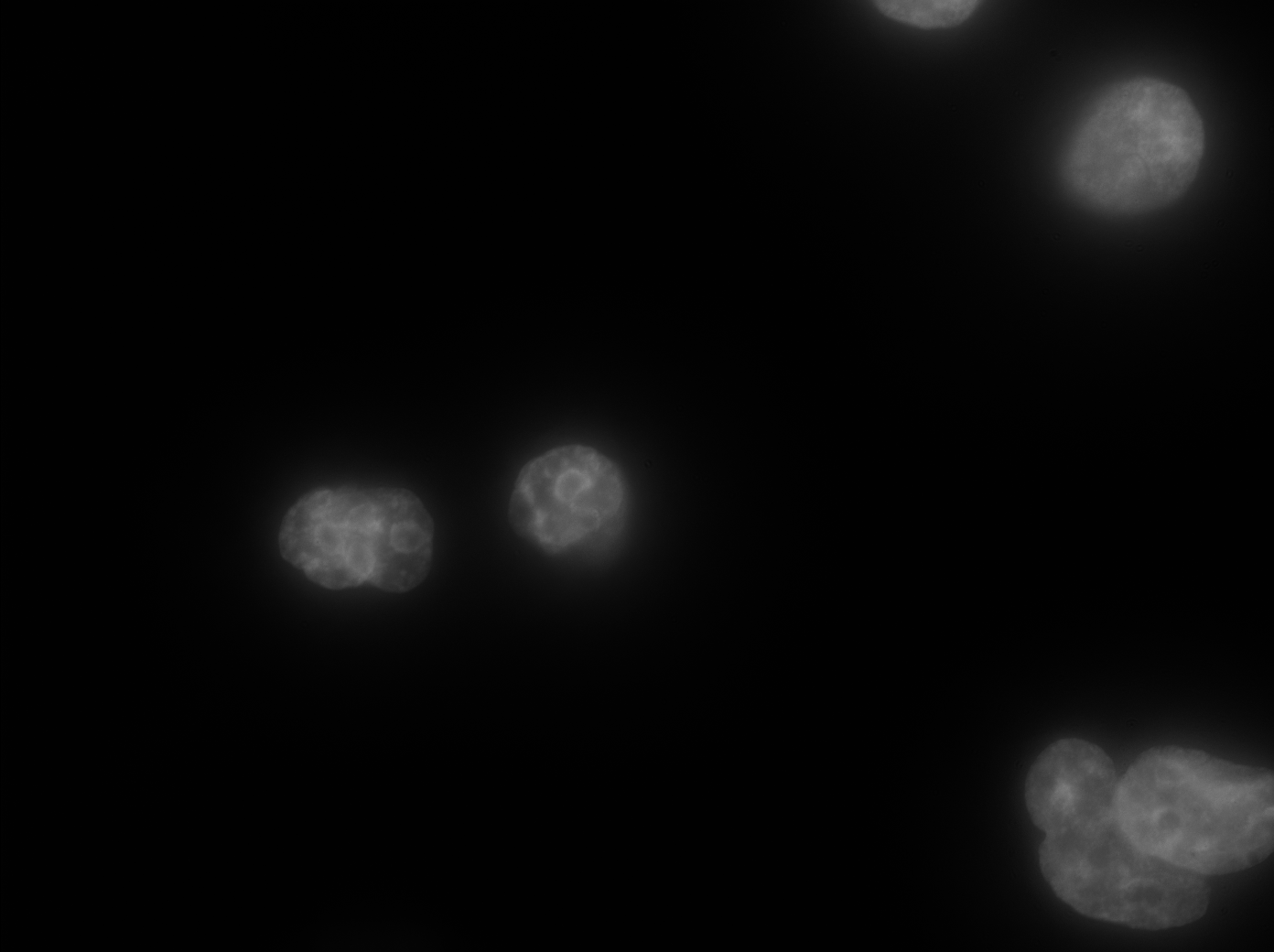

Supplement: Supplementary file 28 — Source data Fig. 7 part 4 [file 44319_2026_742_MOESM28_ESM.zip › Figure 7 Part 4/Fig 7fg Control and TPGS1-KO spastin acetylated tubulin/TPGS1-KO spastin actub 4-1-25 R1 SI9.Project Maximum Z_XY1743537099_Z0_T0_C0.tif]

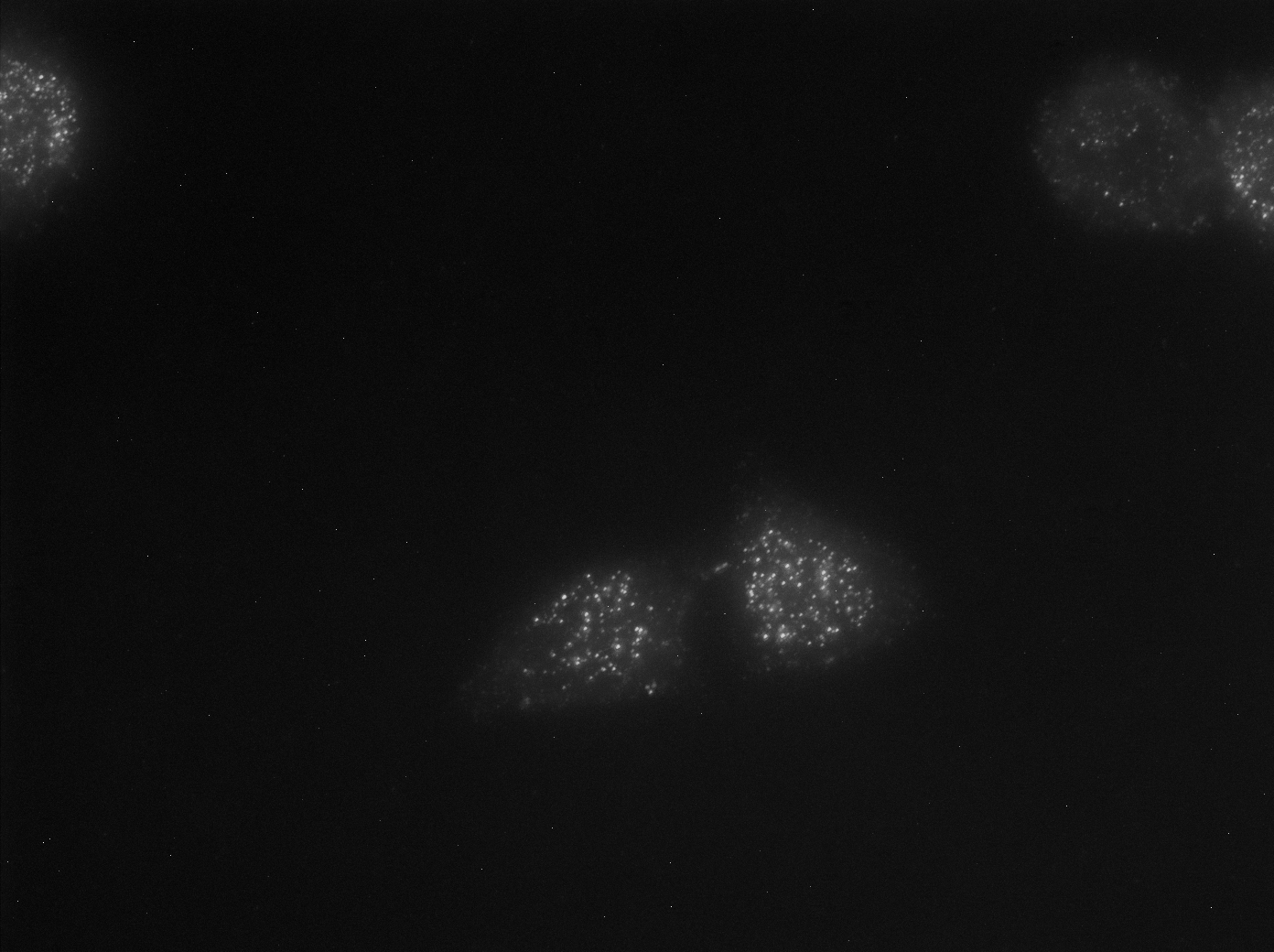

Supplement: Supplementary file 28 — Source data Fig. 7 part 4 [file 44319_2026_742_MOESM28_ESM.zip › Figure 7 Part 4/Fig 7fg Control and TPGS1-KO spastin acetylated tubulin/Cas9 spastin actub 4-1-25 R1 SI5.Project Maximum Z_XY1743530861_Z0_T0_C1.tif]

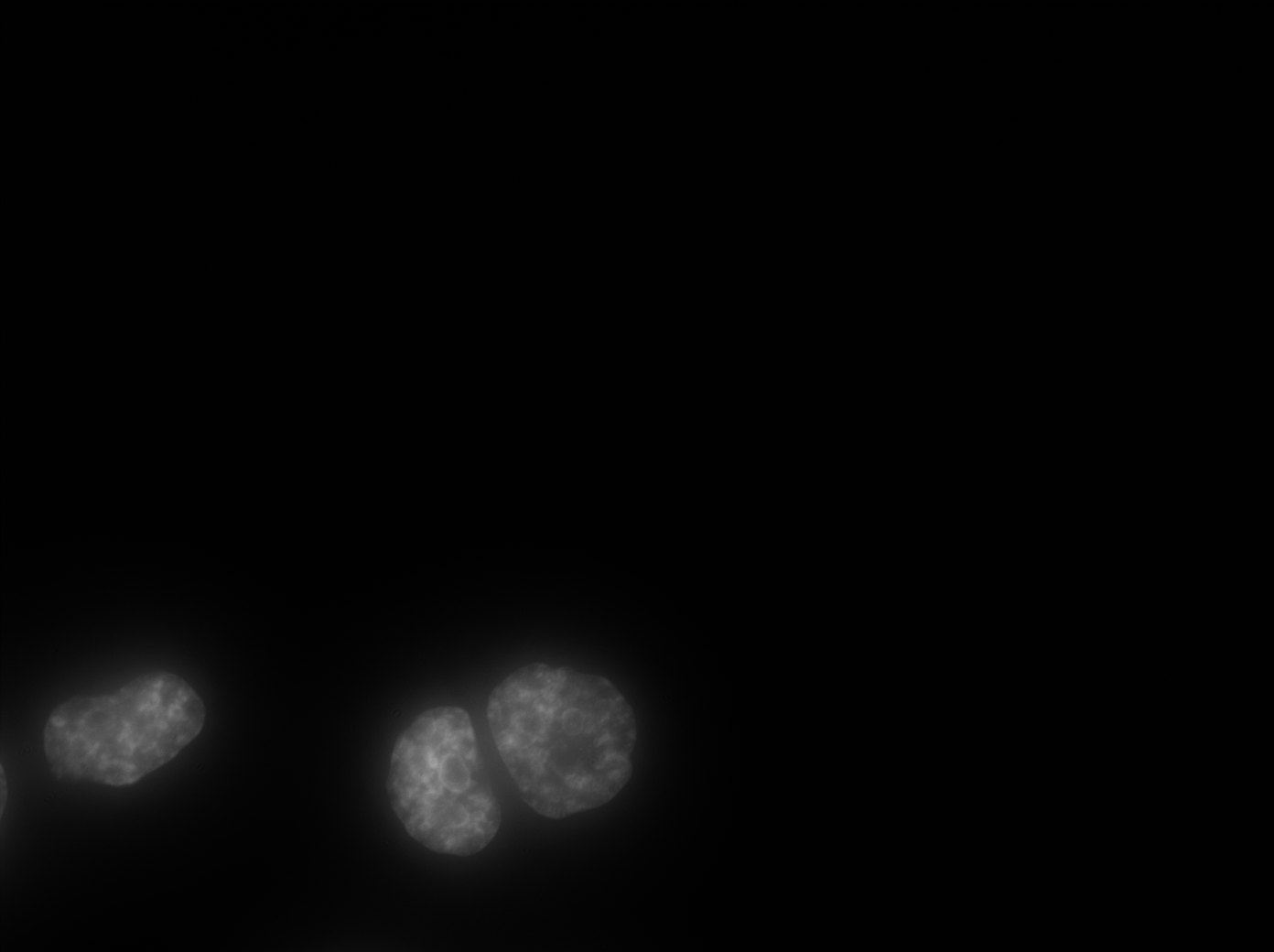

Supplement: Supplementary file 28 — Source data Fig. 7 part 4 [file 44319_2026_742_MOESM28_ESM.zip › Figure 7 Part 4/Fig 7fg Control and TPGS1-KO spastin acetylated tubulin/Cas9 spastin actub 4-1-25 R1 SI11.Project Maximum Z_XY1743531637_Z0_T0_C0.tif]

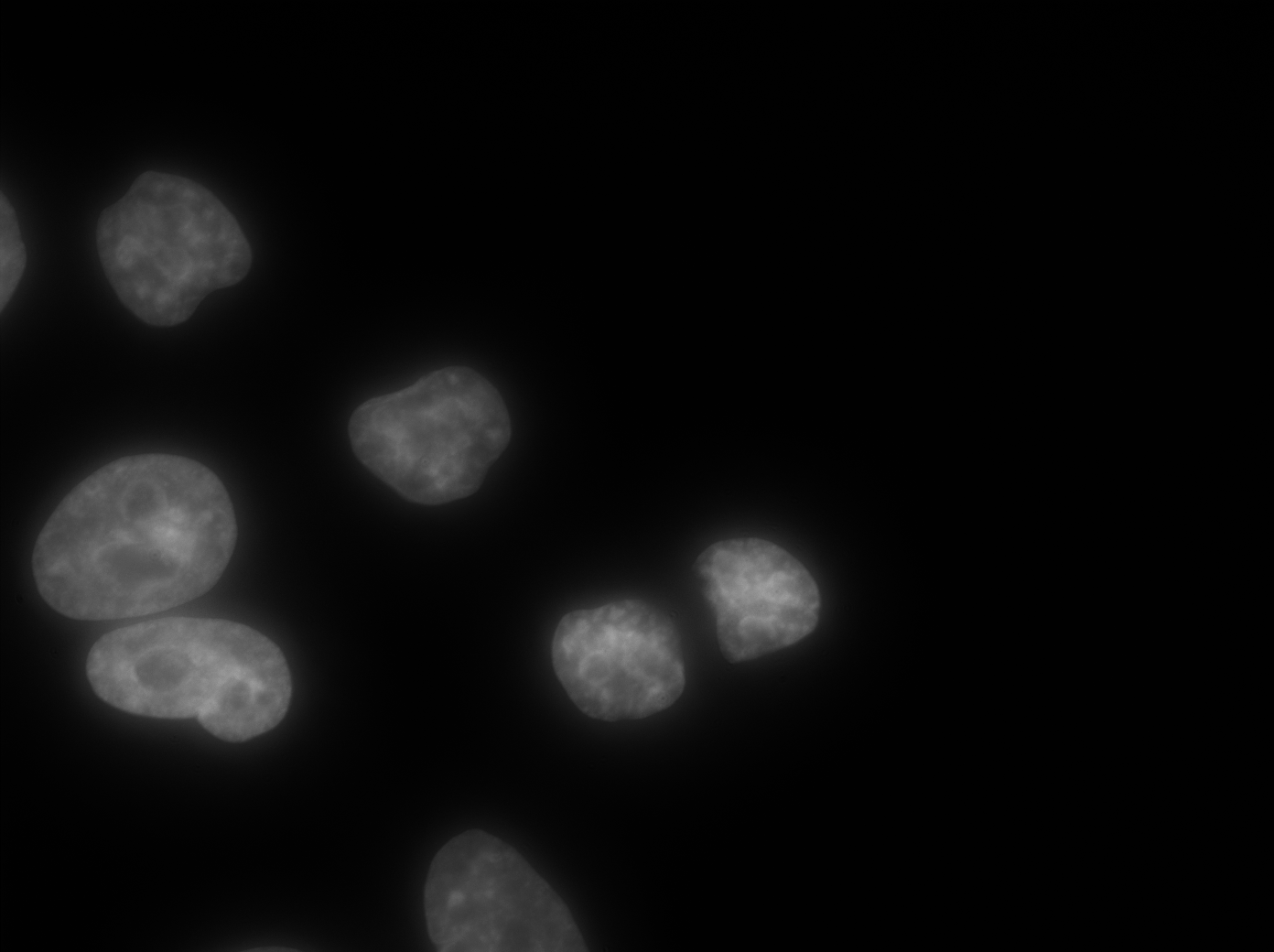

Supplement: Supplementary file 28 — Source data Fig. 7 part 4 [file 44319_2026_742_MOESM28_ESM.zip › Figure 7 Part 4/Fig 7fg Control and TPGS1-KO spastin acetylated tubulin/Cas9 spastin actub 4-1-25 R1 SI13.Project Maximum Z_XY1743532096_Z0_T0_C0.tif]

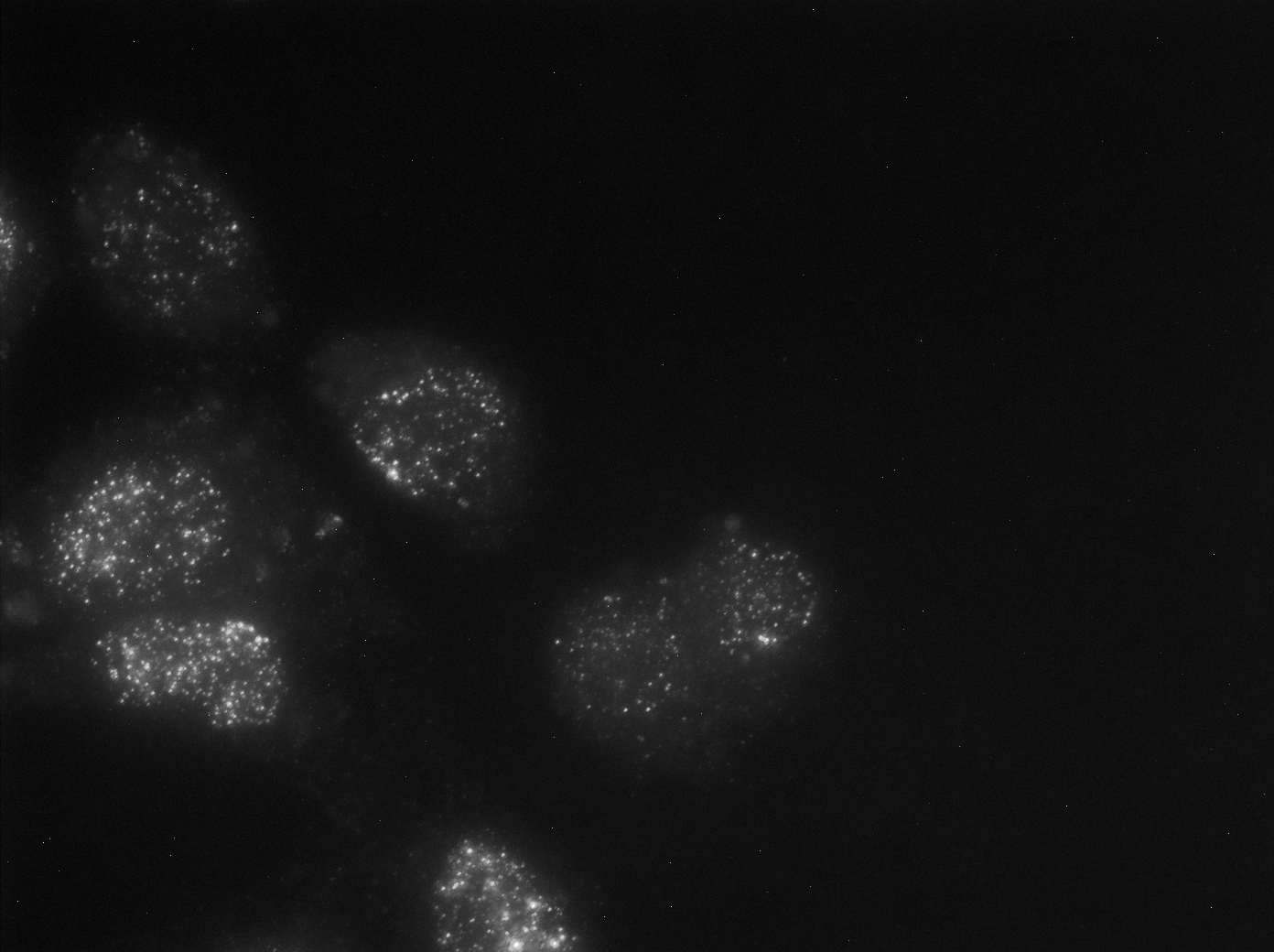

Supplement: Supplementary file 28 — Source data Fig. 7 part 4 [file 44319_2026_742_MOESM28_ESM.zip › Figure 7 Part 4/Fig 7fg Control and TPGS1-KO spastin acetylated tubulin/Cas9 spastin actub 4-1-25 R1 SI13.Project Maximum Z_XY1743532096_Z0_T0_C1.tif]

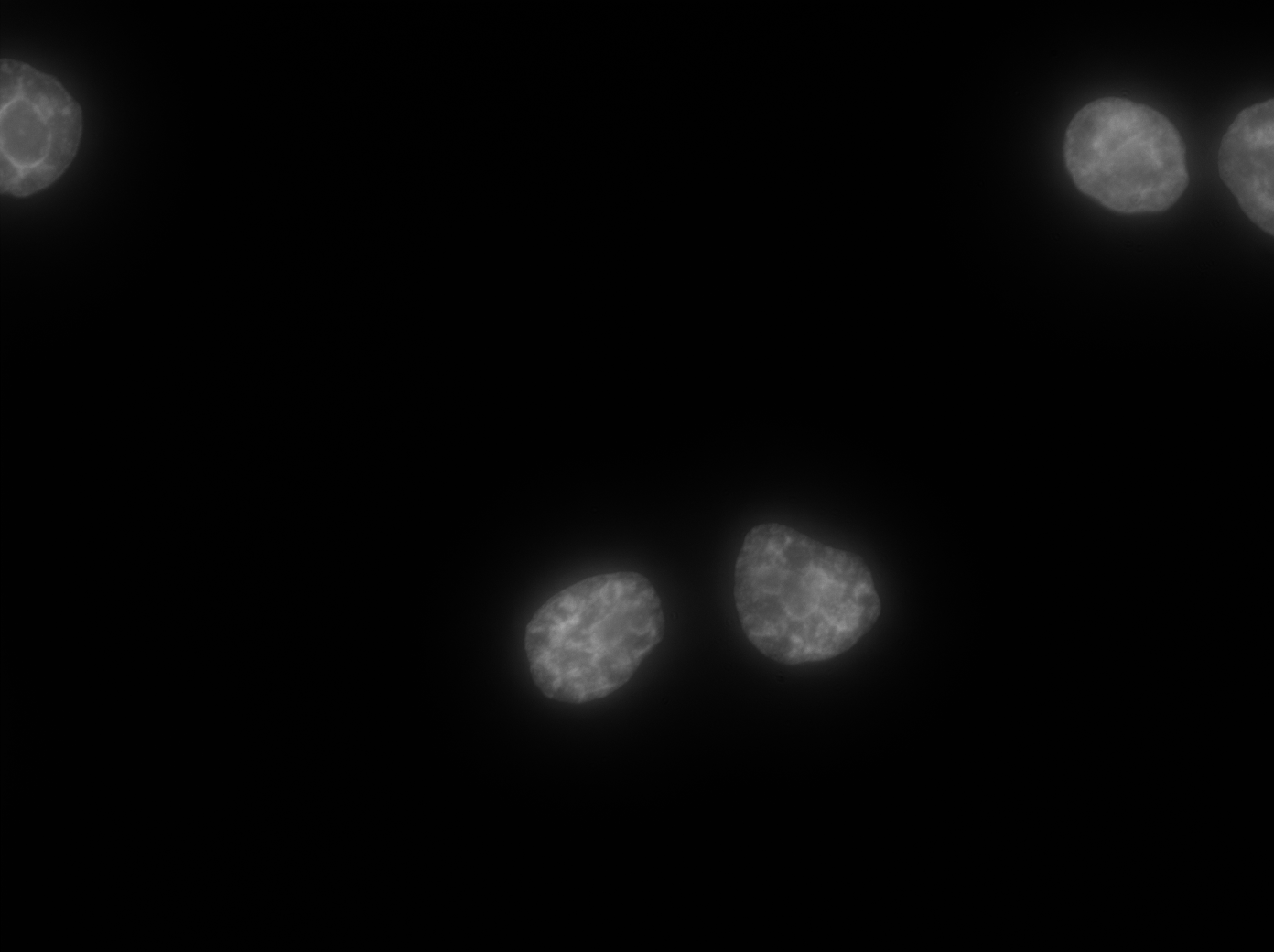

Supplement: Supplementary file 28 — Source data Fig. 7 part 4 [file 44319_2026_742_MOESM28_ESM.zip › Figure 7 Part 4/Fig 7fg Control and TPGS1-KO spastin acetylated tubulin/Cas9 spastin actub 4-1-25 R1 SI5.Project Maximum Z_XY1743530861_Z0_T0_C0.tif]

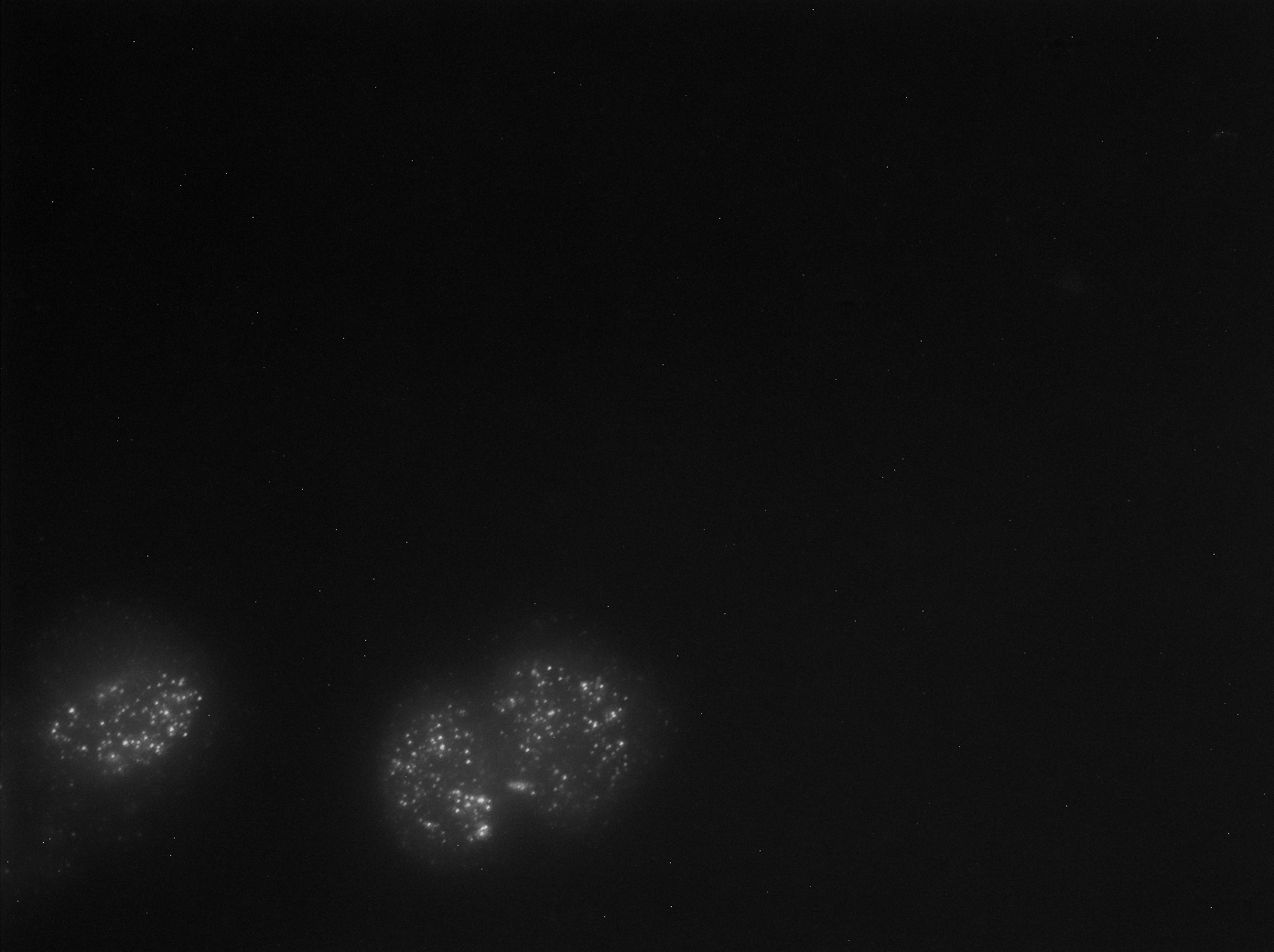

Supplement: Supplementary file 28 — Source data Fig. 7 part 4 [file 44319_2026_742_MOESM28_ESM.zip › Figure 7 Part 4/Fig 7fg Control and TPGS1-KO spastin acetylated tubulin/Cas9 spastin actub 4-1-25 R1 SI11.Project Maximum Z_XY1743531637_Z0_T0_C1.tif]

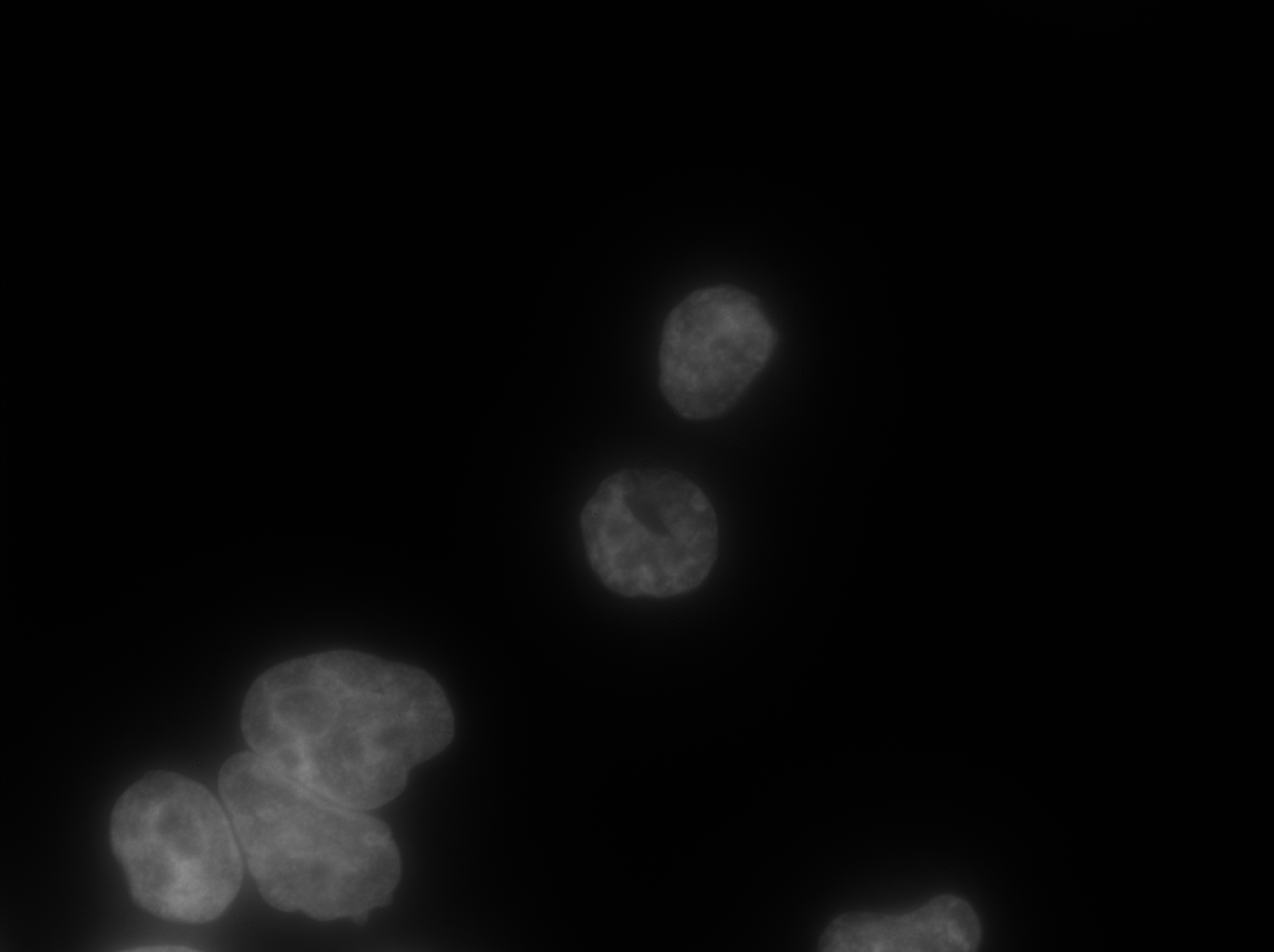

Supplement: Supplementary file 28 — Source data Fig. 7 part 4 [file 44319_2026_742_MOESM28_ESM.zip › Figure 7 Part 4/Fig 7fg Control and TPGS1-KO spastin acetylated tubulin/TPGS1-KO spastin actub 4-1-25 R1 SI16.Project Maximum Z_XY1743538181_Z0_T0_C0.tif]

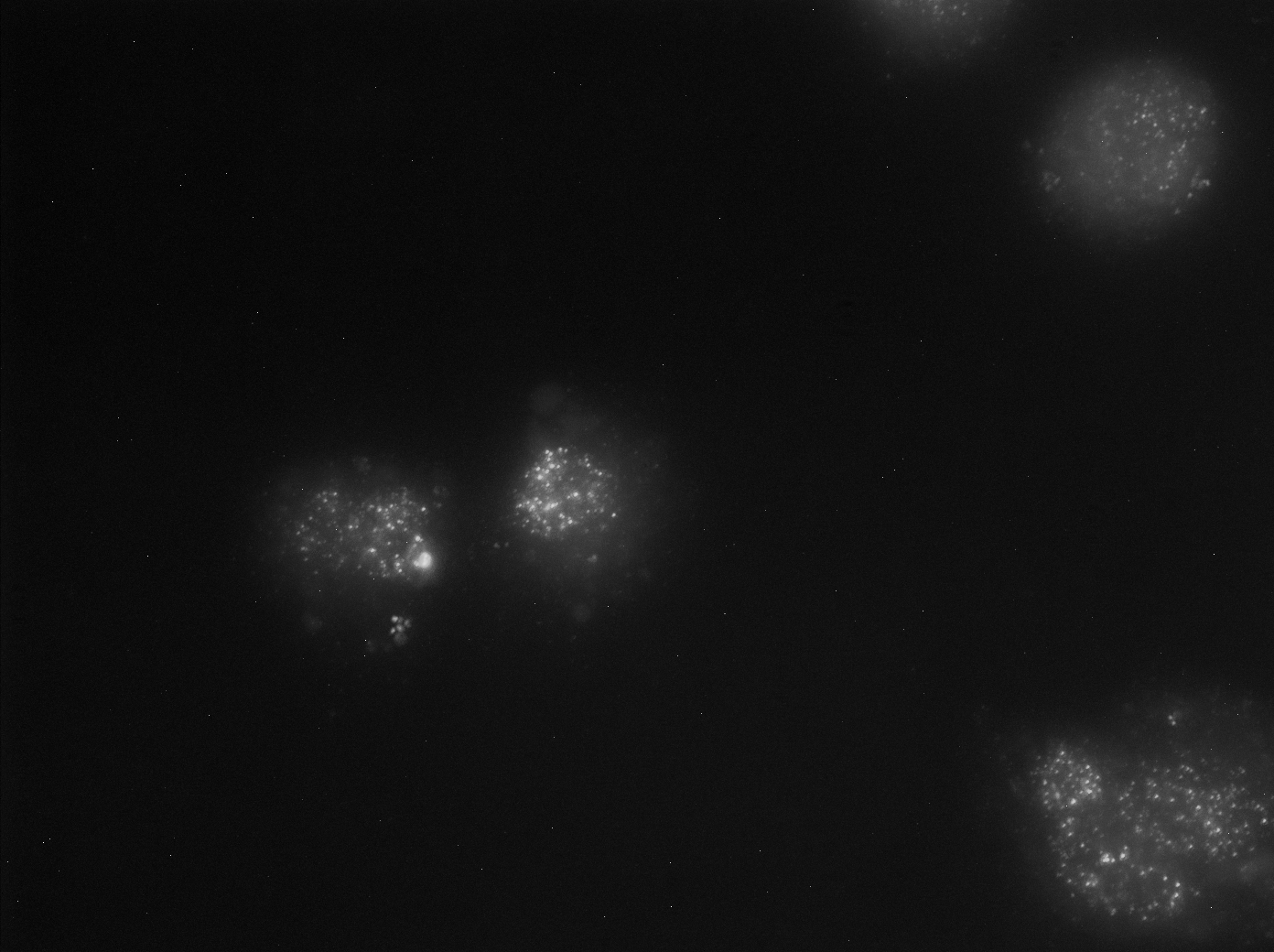

Supplement: Supplementary file 28 — Source data Fig. 7 part 4 [file 44319_2026_742_MOESM28_ESM.zip › Figure 7 Part 4/Fig 7fg Control and TPGS1-KO spastin acetylated tubulin/TPGS1-KO spastin actub 4-1-25 R1 SI9.Project Maximum Z_XY1743537099_Z0_T0_C1.tif]

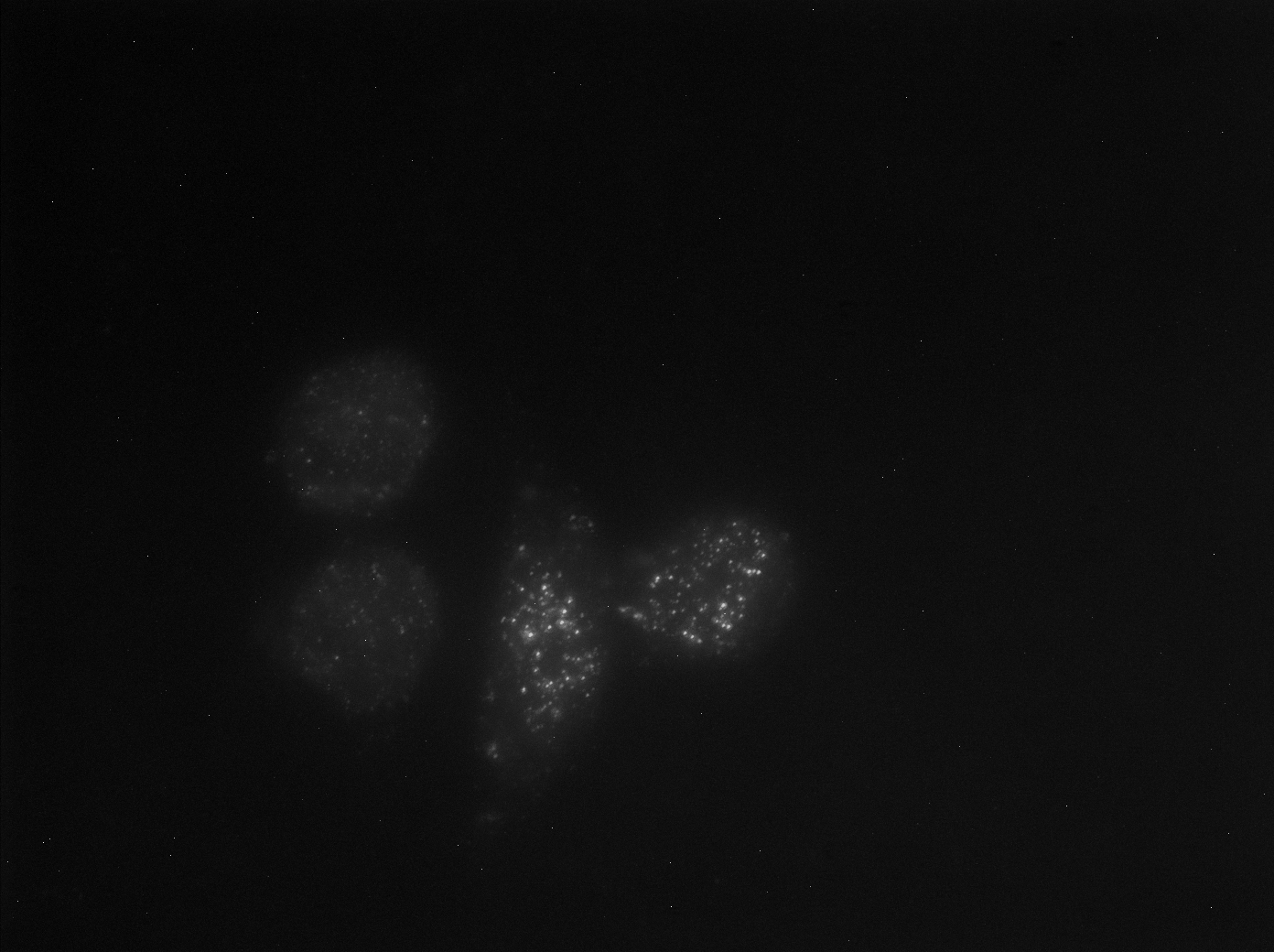

Supplement: Supplementary file 28 — Source data Fig. 7 part 4 [file 44319_2026_742_MOESM28_ESM.zip › Figure 7 Part 4/Fig 7fg Control and TPGS1-KO spastin acetylated tubulin/Cas9 spastin actub 4-1-25 R1 SI18.Project Maximum Z_XY1743534608_Z0_T0_C1.tif]

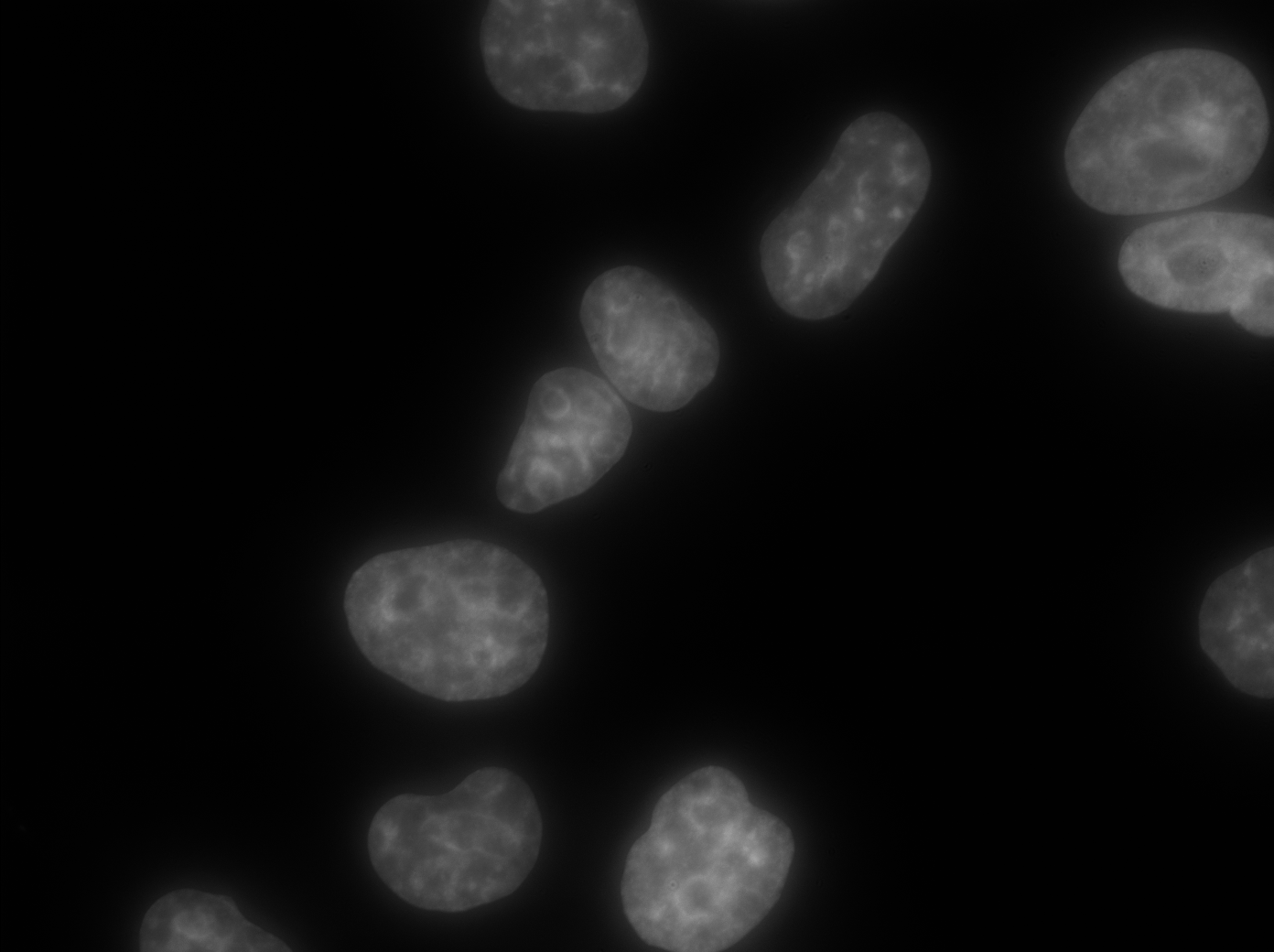

Supplement: Supplementary file 28 — Source data Fig. 7 part 4 [file 44319_2026_742_MOESM28_ESM.zip › Figure 7 Part 4/Fig 7fg Control and TPGS1-KO spastin acetylated tubulin/Cas9 spastin actub 4-1-25 R1 SI12.Project Maximum Z_XY1743531982_Z0_T0_C0.tif]

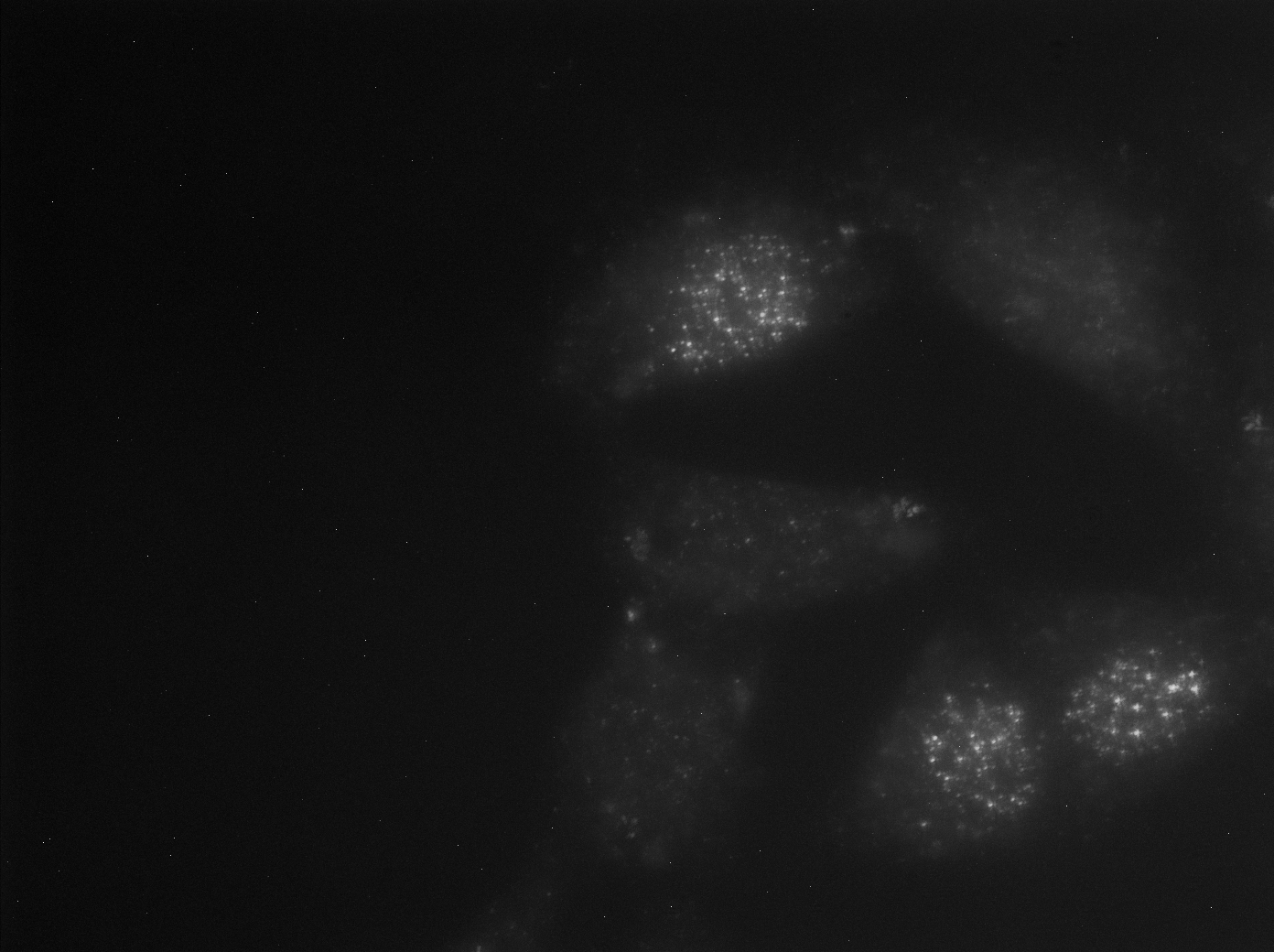

Supplement: Supplementary file 28 — Source data Fig. 7 part 4 [file 44319_2026_742_MOESM28_ESM.zip › Figure 7 Part 4/Fig 7fg Control and TPGS1-KO spastin acetylated tubulin/Cas9 spastin actub 4-1-25 R1 SI21.Project Maximum Z_XY1743535055_Z0_T0_C1.tif]

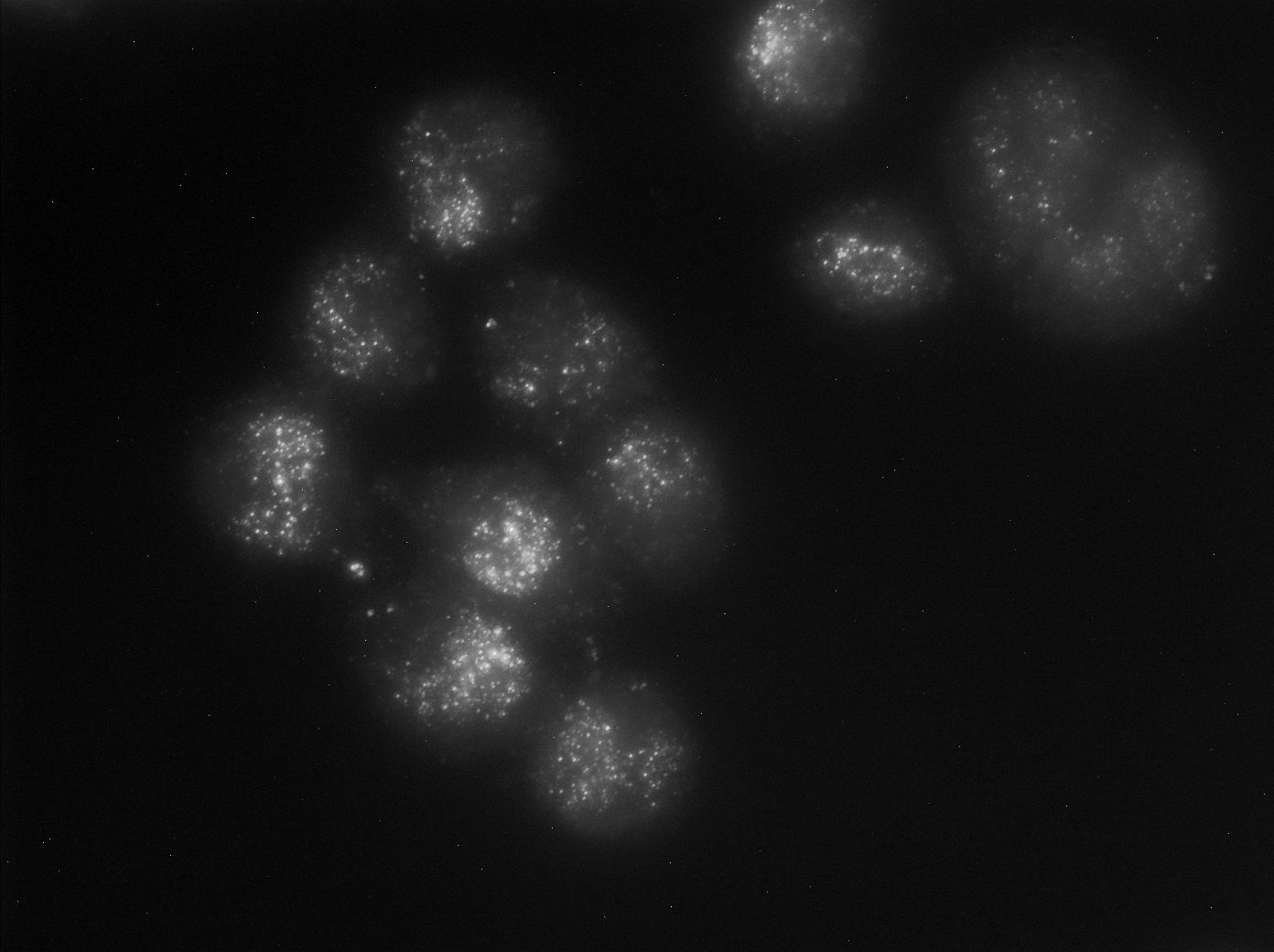

Supplement: Supplementary file 28 — Source data Fig. 7 part 4 [file 44319_2026_742_MOESM28_ESM.zip › Figure 7 Part 4/Fig 7fg Control and TPGS1-KO spastin acetylated tubulin/TPGS1-KO spastin actub 4-1-25 R1 SI11.Project Maximum Z_XY1743537437_Z0_T0_C1.tif]

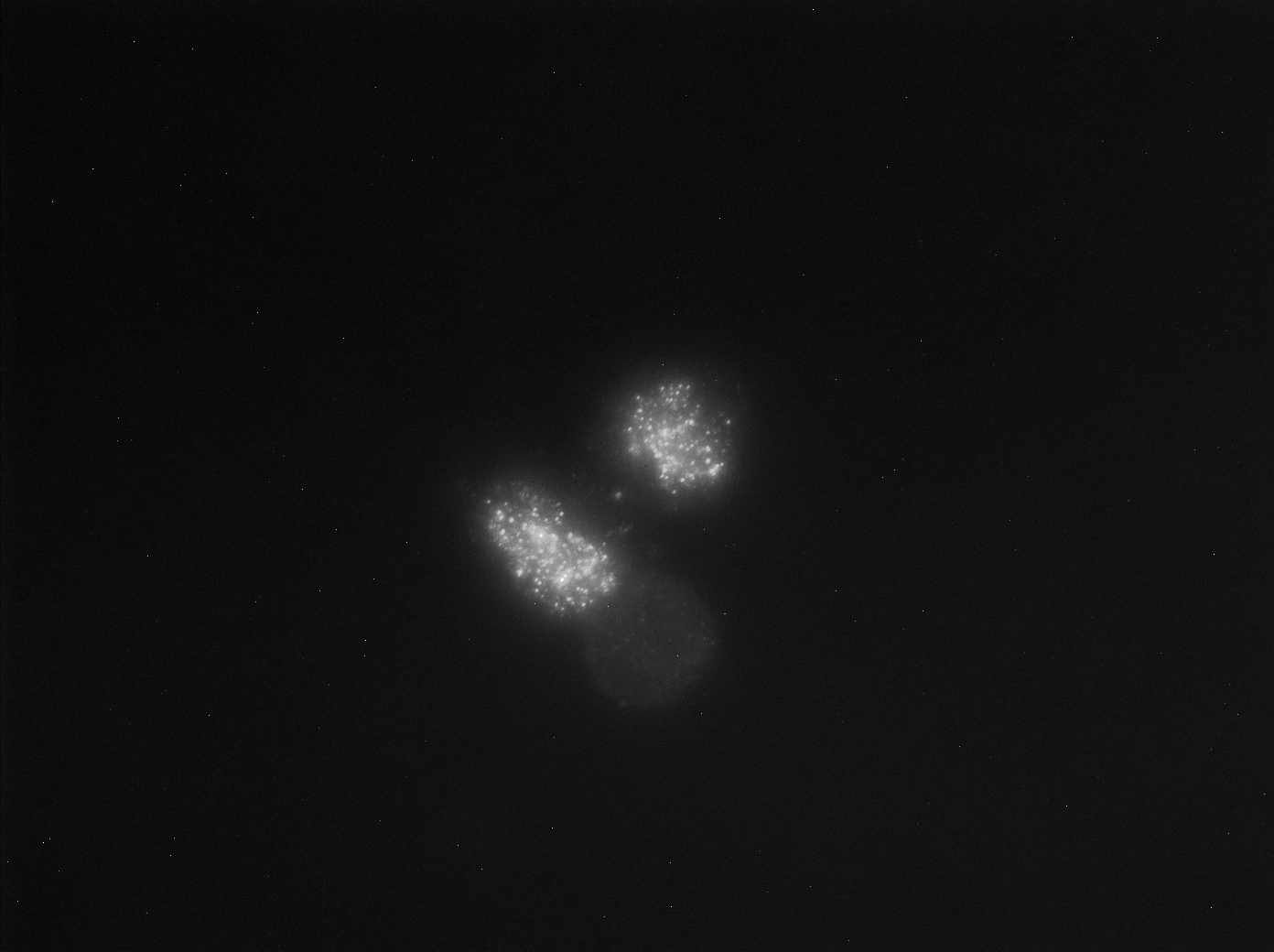

Supplement: Supplementary file 28 — Source data Fig. 7 part 4 [file 44319_2026_742_MOESM28_ESM.zip › Figure 7 Part 4/Fig 7fg Control and TPGS1-KO spastin acetylated tubulin/TPGS1-KO spastin actub 4-1-25 R1 SI12.Project Maximum Z_XY1743537613_Z0_T0_C1.tif]

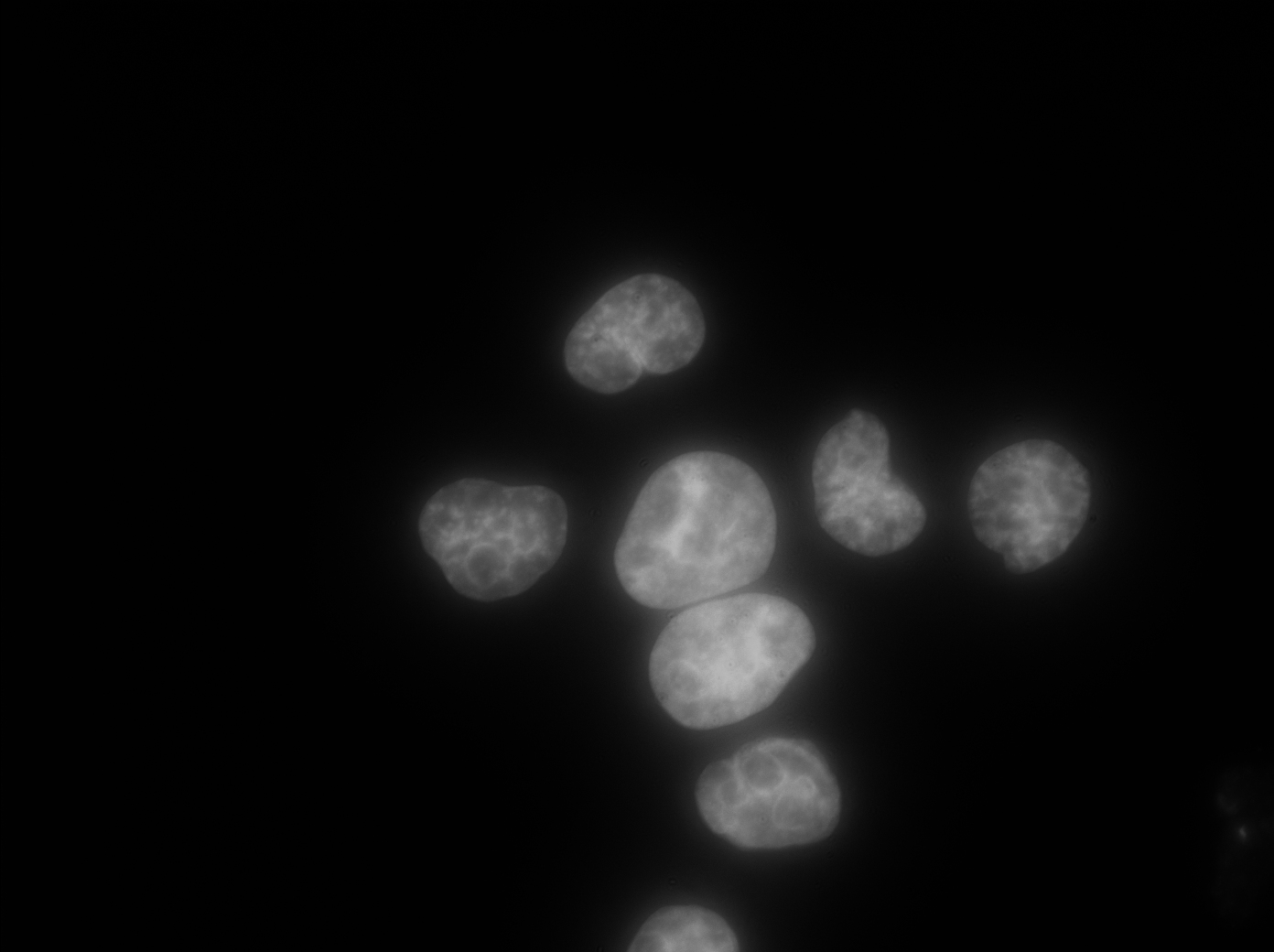

Supplement: Supplementary file 28 — Source data Fig. 7 part 4 [file 44319_2026_742_MOESM28_ESM.zip › Figure 7 Part 4/Fig 7fg Control and TPGS1-KO spastin acetylated tubulin/Cas9 spastin actub 4-1-25 R1 SI17.Project Maximum Z_XY1743534461_Z0_T0_C0.tif]

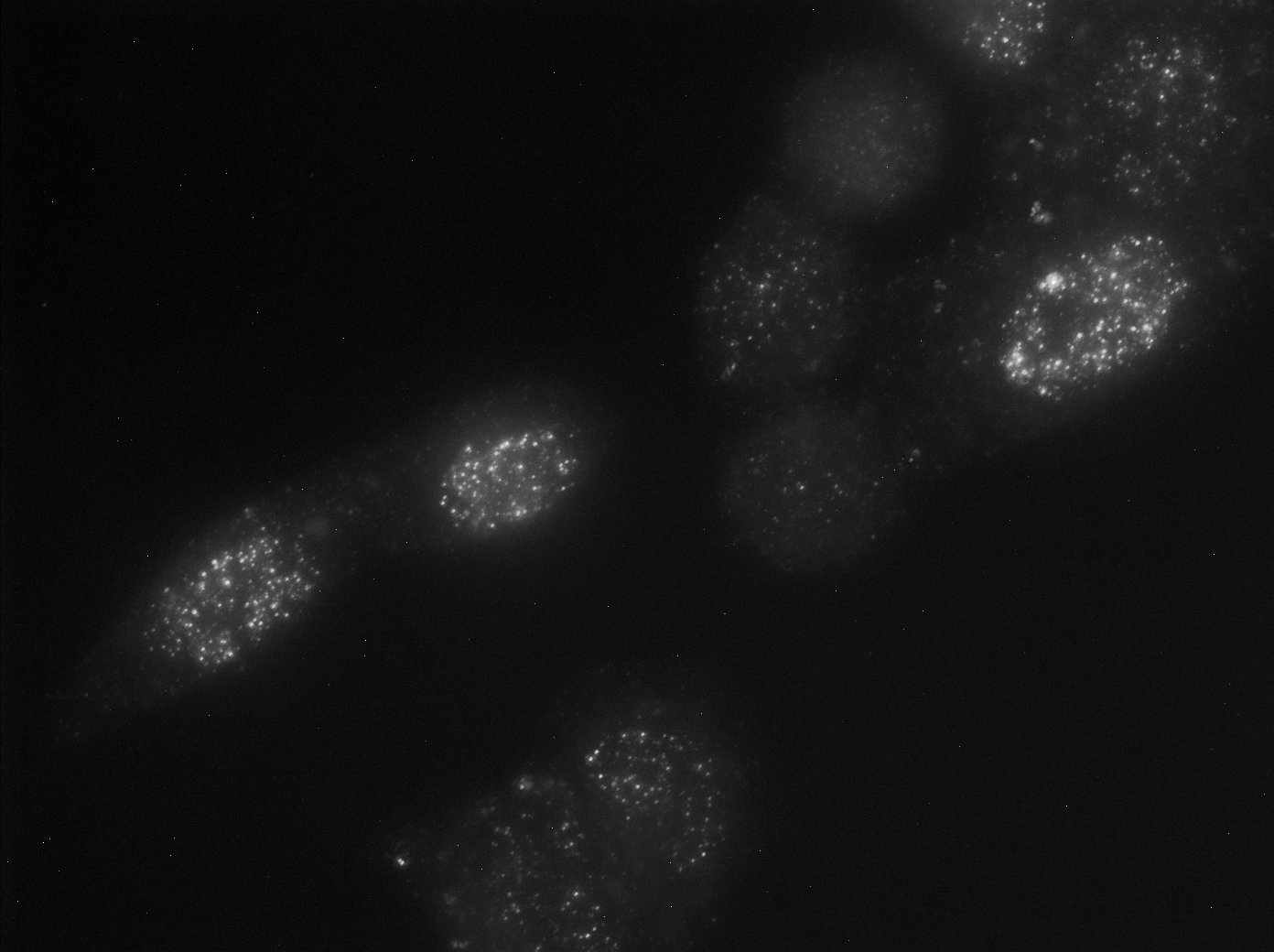

Supplement: Supplementary file 28 — Source data Fig. 7 part 4 [file 44319_2026_742_MOESM28_ESM.zip › Figure 7 Part 4/Fig 7fg Control and TPGS1-KO spastin acetylated tubulin/Cas9 spastin actub 4-1-25 R1 SI24.Project Maximum Z_XY1743535647_Z0_T0_C1.tif]

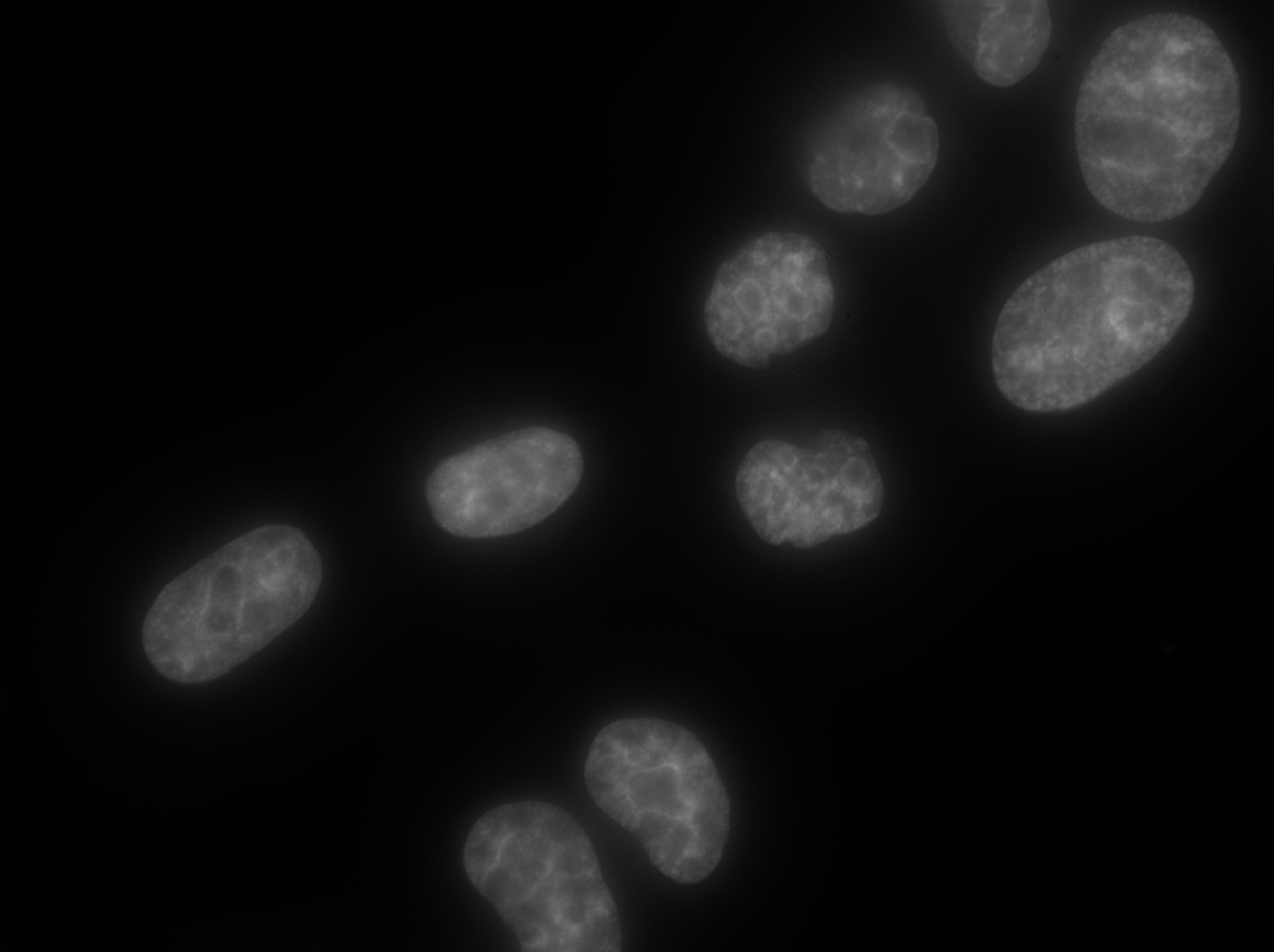

Supplement: Supplementary file 28 — Source data Fig. 7 part 4 [file 44319_2026_742_MOESM28_ESM.zip › Figure 7 Part 4/Fig 7fg Control and TPGS1-KO spastin acetylated tubulin/Cas9 spastin actub 4-1-25 R1 SI24.Project Maximum Z_XY1743535647_Z0_T0_C0.tif]

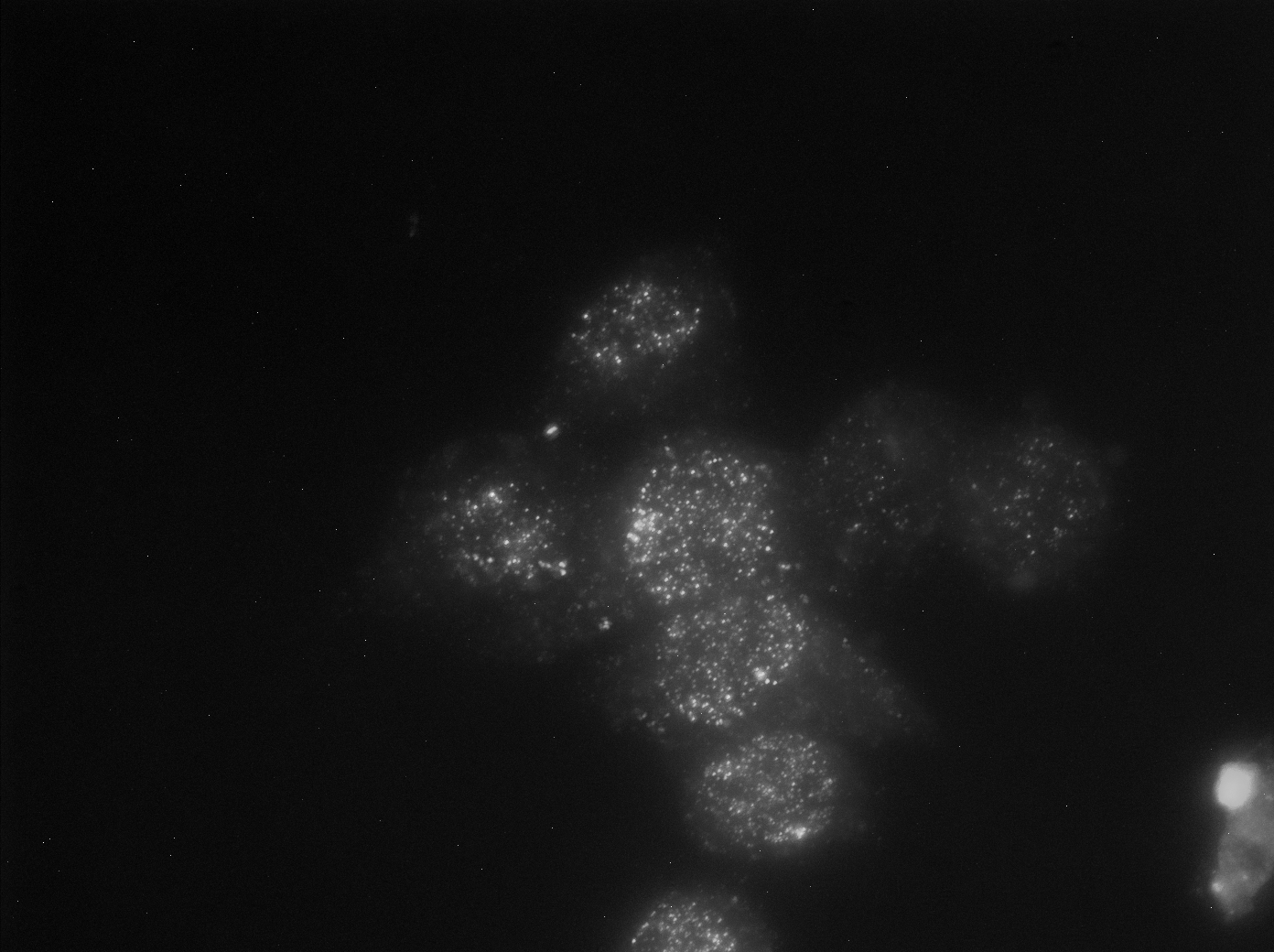

Supplement: Supplementary file 28 — Source data Fig. 7 part 4 [file 44319_2026_742_MOESM28_ESM.zip › Figure 7 Part 4/Fig 7fg Control and TPGS1-KO spastin acetylated tubulin/Cas9 spastin actub 4-1-25 R1 SI17.Project Maximum Z_XY1743534461_Z0_T0_C1.tif]

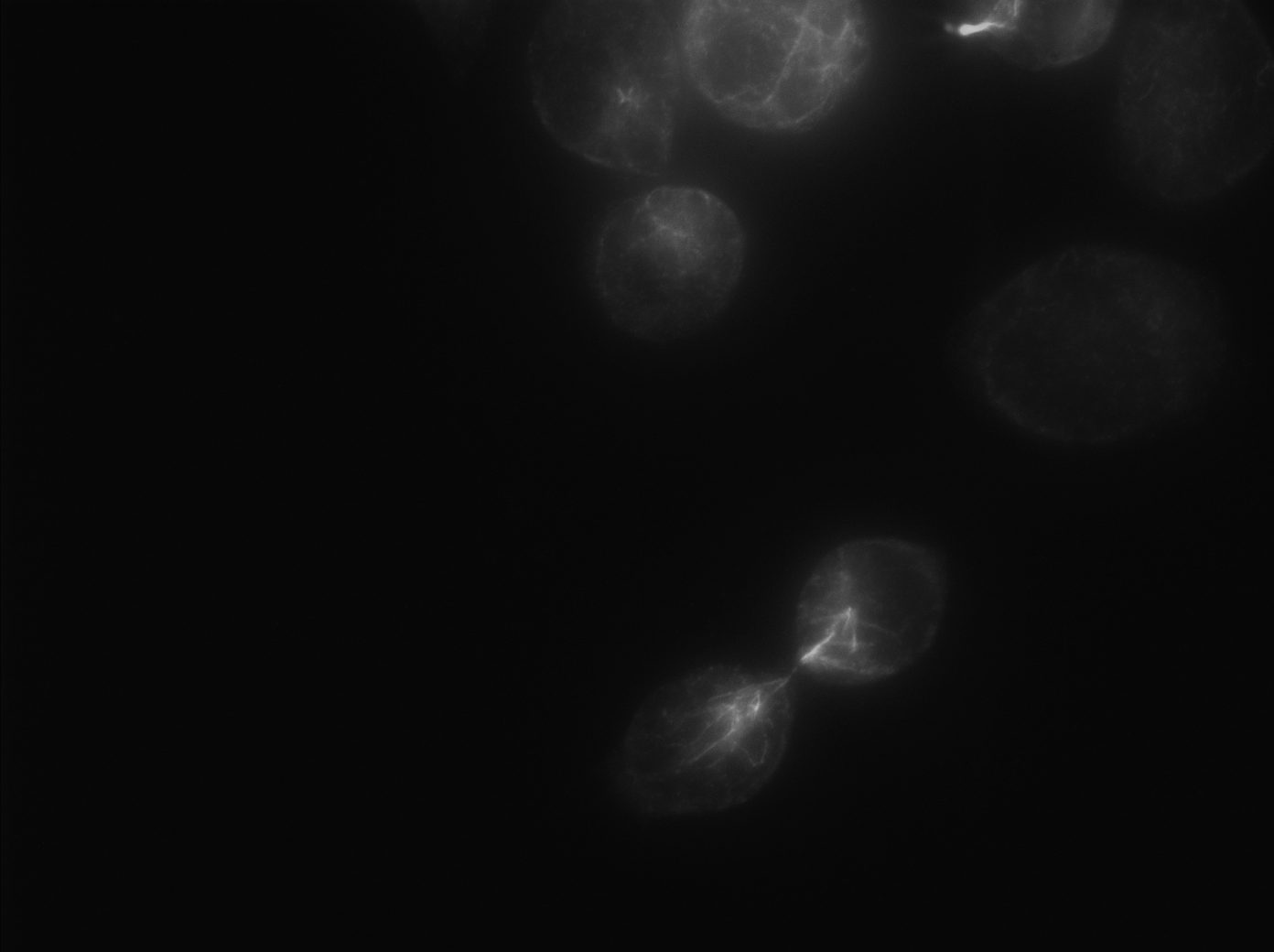

Supplement: Supplementary file 28 — Source data Fig. 7 part 4 [file 44319_2026_742_MOESM28_ESM.zip › Figure 7 Part 4/Fig 7fg Control and TPGS1-KO spastin acetylated tubulin/TPGS1-KO spastin actub 4-1-25 R1 SI22.Project Maximum Z_XY1743539238_Z0_T0_C2.tif]

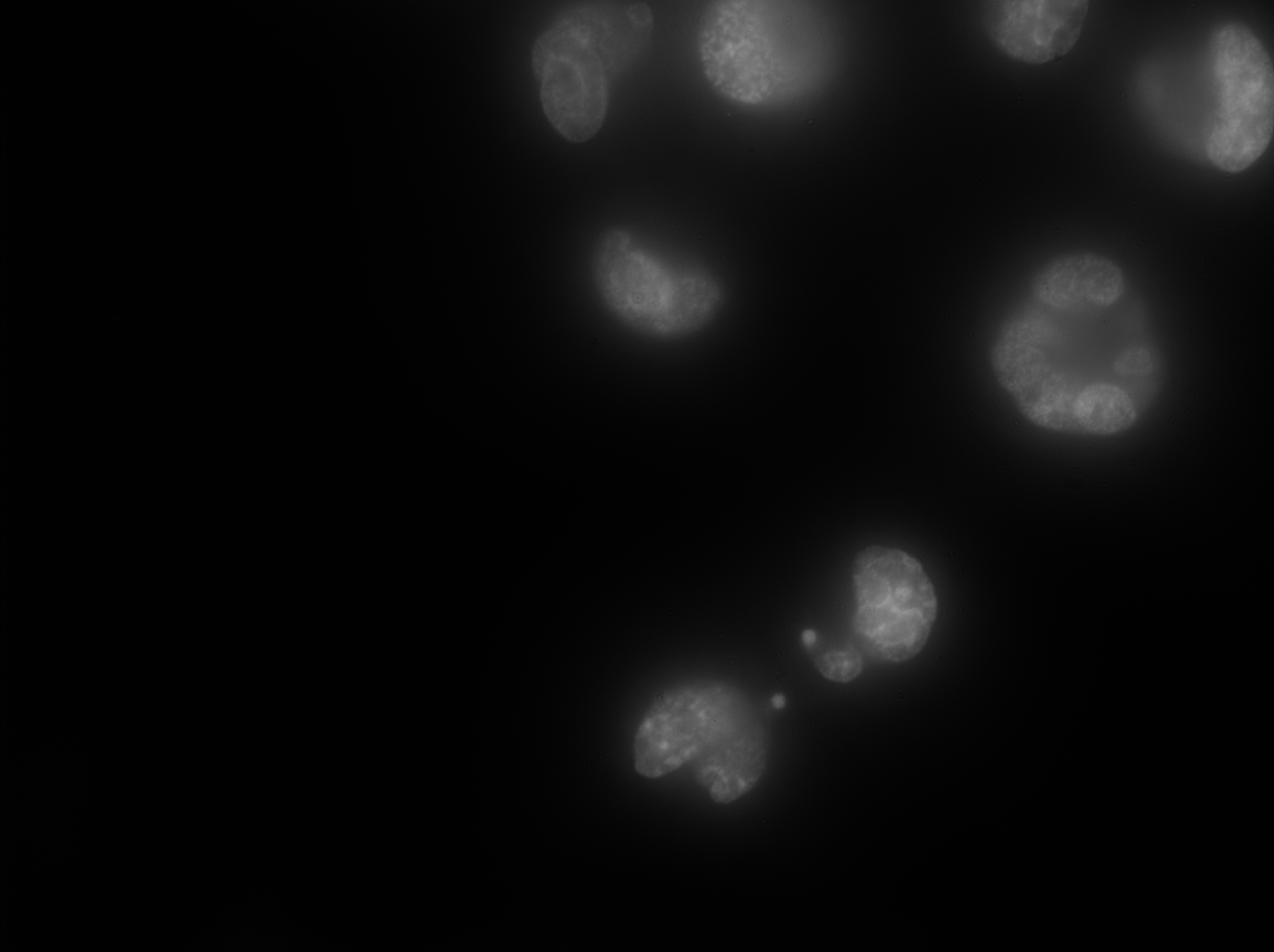

Supplement: Supplementary file 28 — Source data Fig. 7 part 4 [file 44319_2026_742_MOESM28_ESM.zip › Figure 7 Part 4/Fig 7fg Control and TPGS1-KO spastin acetylated tubulin/TPGS1-KO spastin actub 4-1-25 R1 SI22.Project Maximum Z_XY1743539238_Z0_T0_C0.tif]

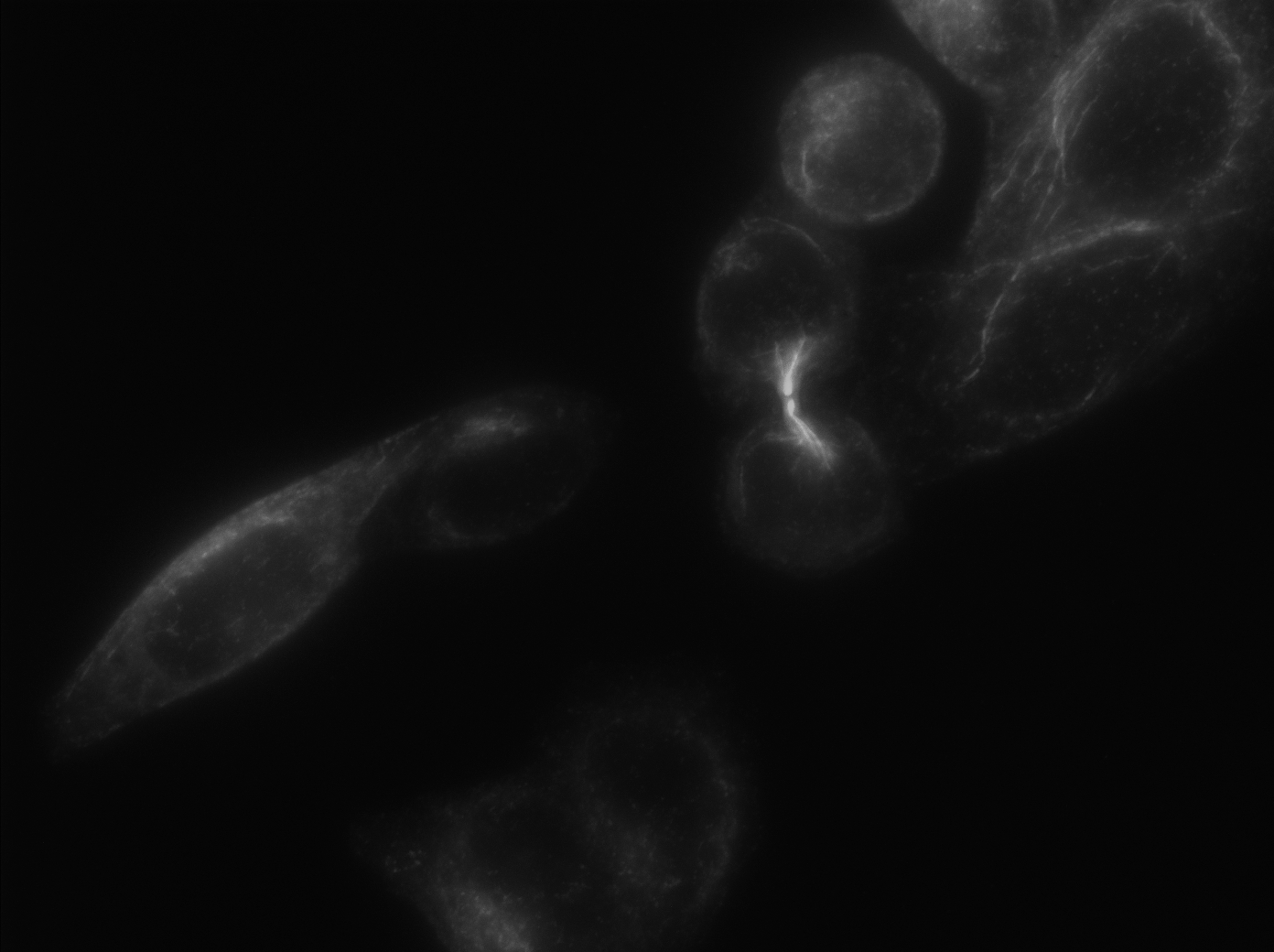

Supplement: Supplementary file 28 — Source data Fig. 7 part 4 [file 44319_2026_742_MOESM28_ESM.zip › Figure 7 Part 4/Fig 7fg Control and TPGS1-KO spastin acetylated tubulin/Cas9 spastin actub 4-1-25 R1 SI24.Project Maximum Z_XY1743535647_Z0_T0_C2.tif]

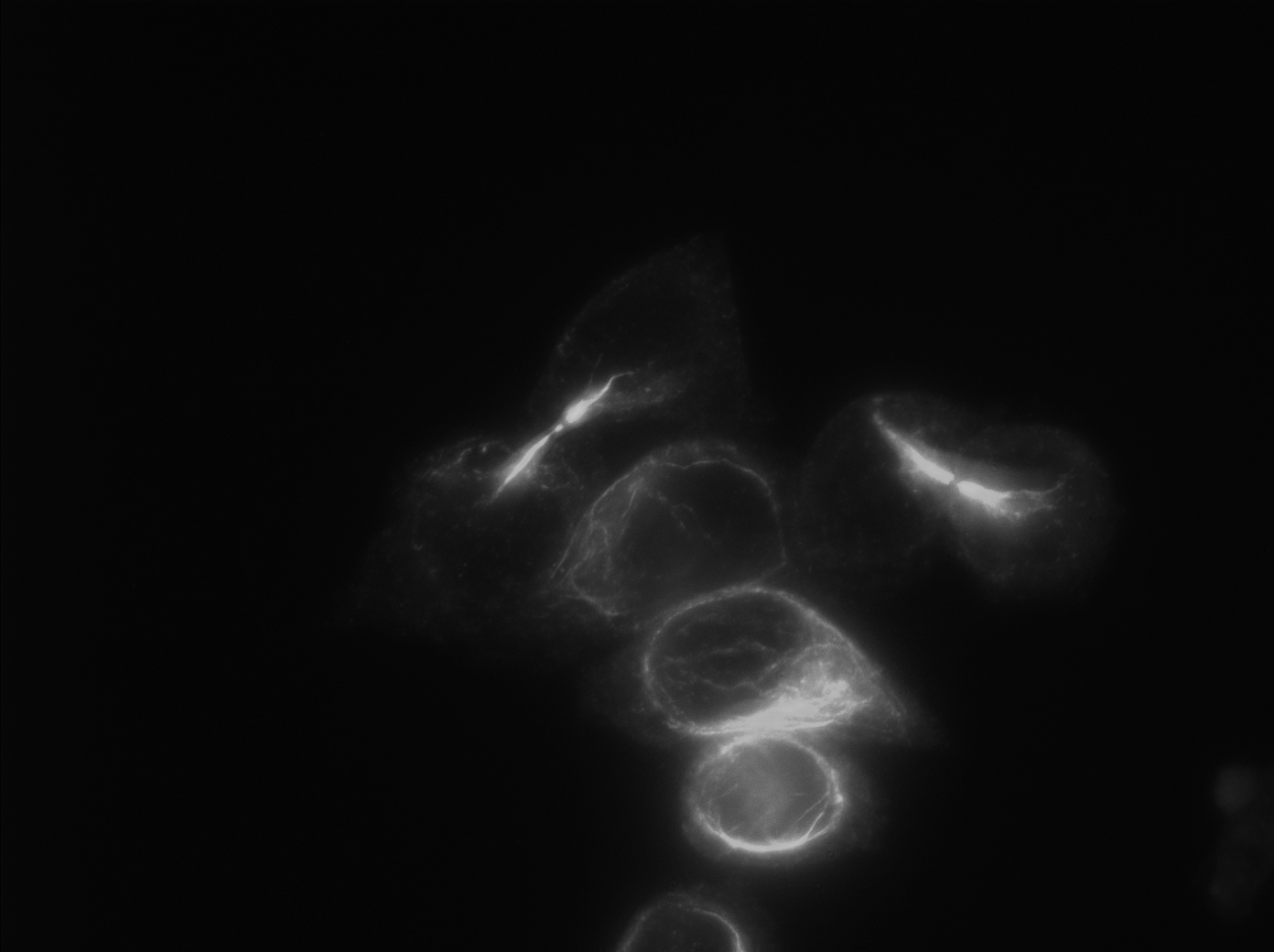

Supplement: Supplementary file 28 — Source data Fig. 7 part 4 [file 44319_2026_742_MOESM28_ESM.zip › Figure 7 Part 4/Fig 7fg Control and TPGS1-KO spastin acetylated tubulin/Cas9 spastin actub 4-1-25 R1 SI17.Project Maximum Z_XY1743534461_Z0_T0_C2.tif]

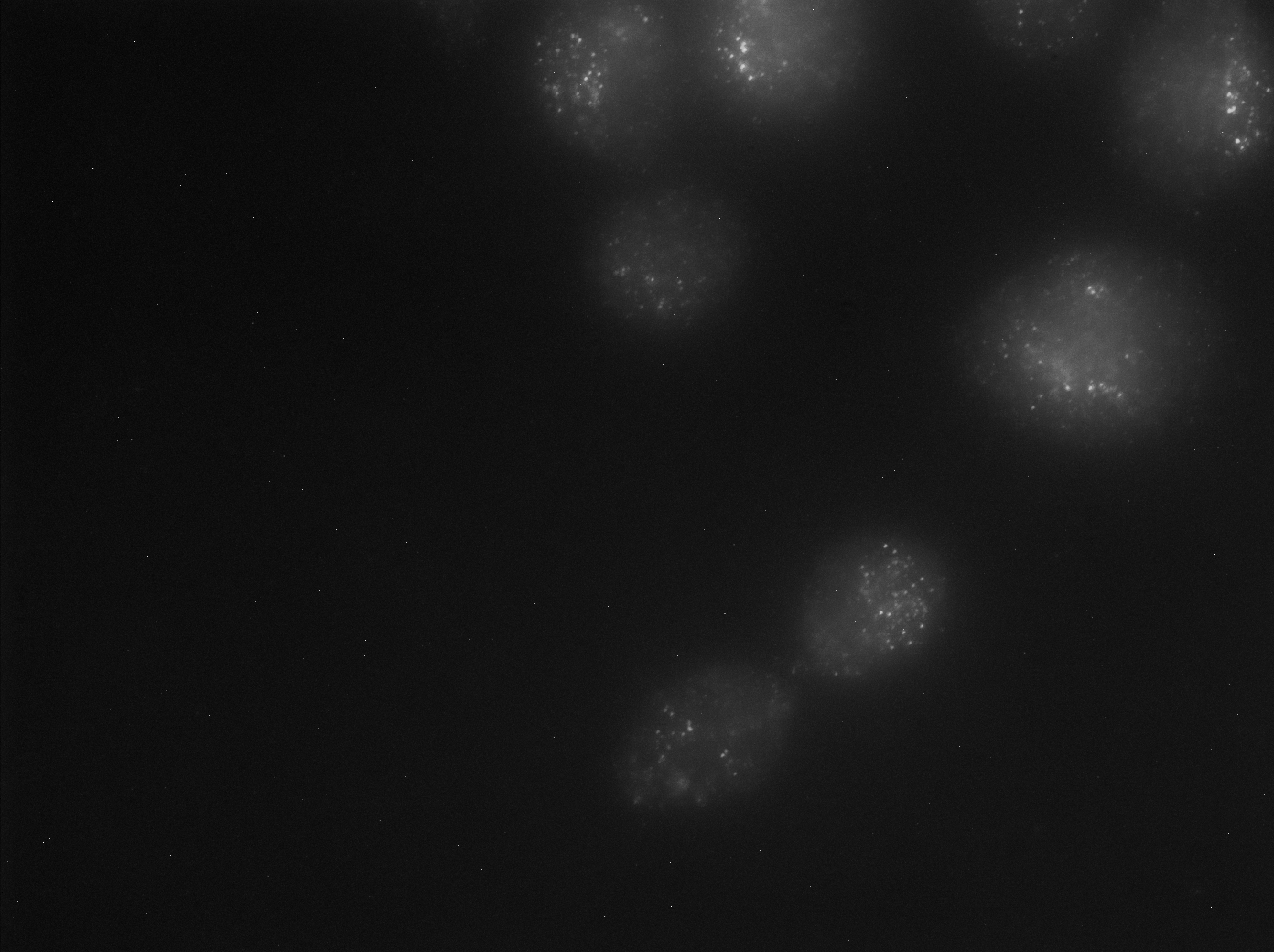

Supplement: Supplementary file 28 — Source data Fig. 7 part 4 [file 44319_2026_742_MOESM28_ESM.zip › Figure 7 Part 4/Fig 7fg Control and TPGS1-KO spastin acetylated tubulin/TPGS1-KO spastin actub 4-1-25 R1 SI22.Project Maximum Z_XY1743539238_Z0_T0_C1.tif]

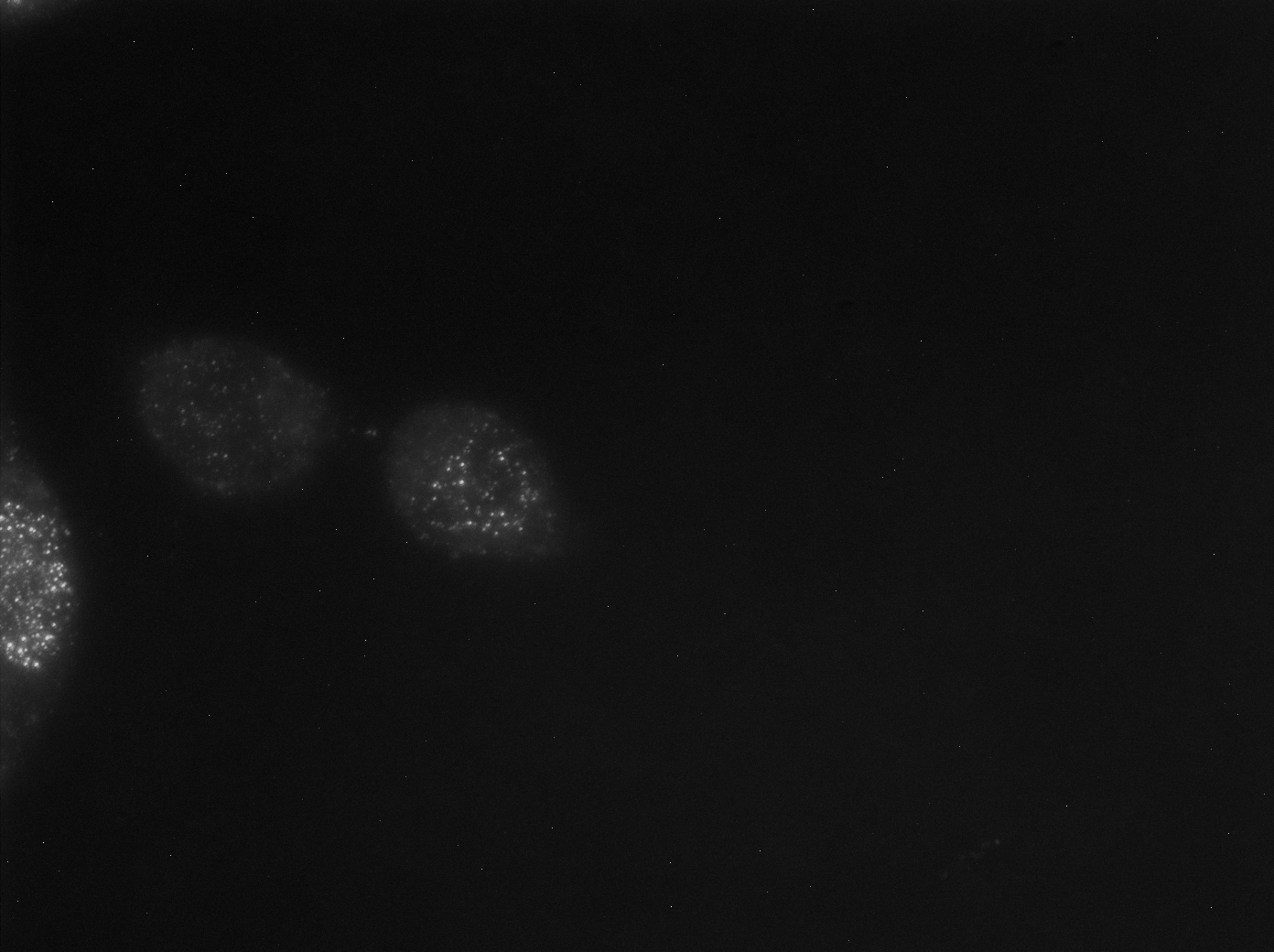

Supplement: Supplementary file 28 — Source data Fig. 7 part 4 [file 44319_2026_742_MOESM28_ESM.zip › Figure 7 Part 4/Fig 7fg Control and TPGS1-KO spastin acetylated tubulin/Cas9 spastin actub 4-1-25 R1 SI1.Project Maximum Z_XY1743530015_Z0_T0_C1.tif]

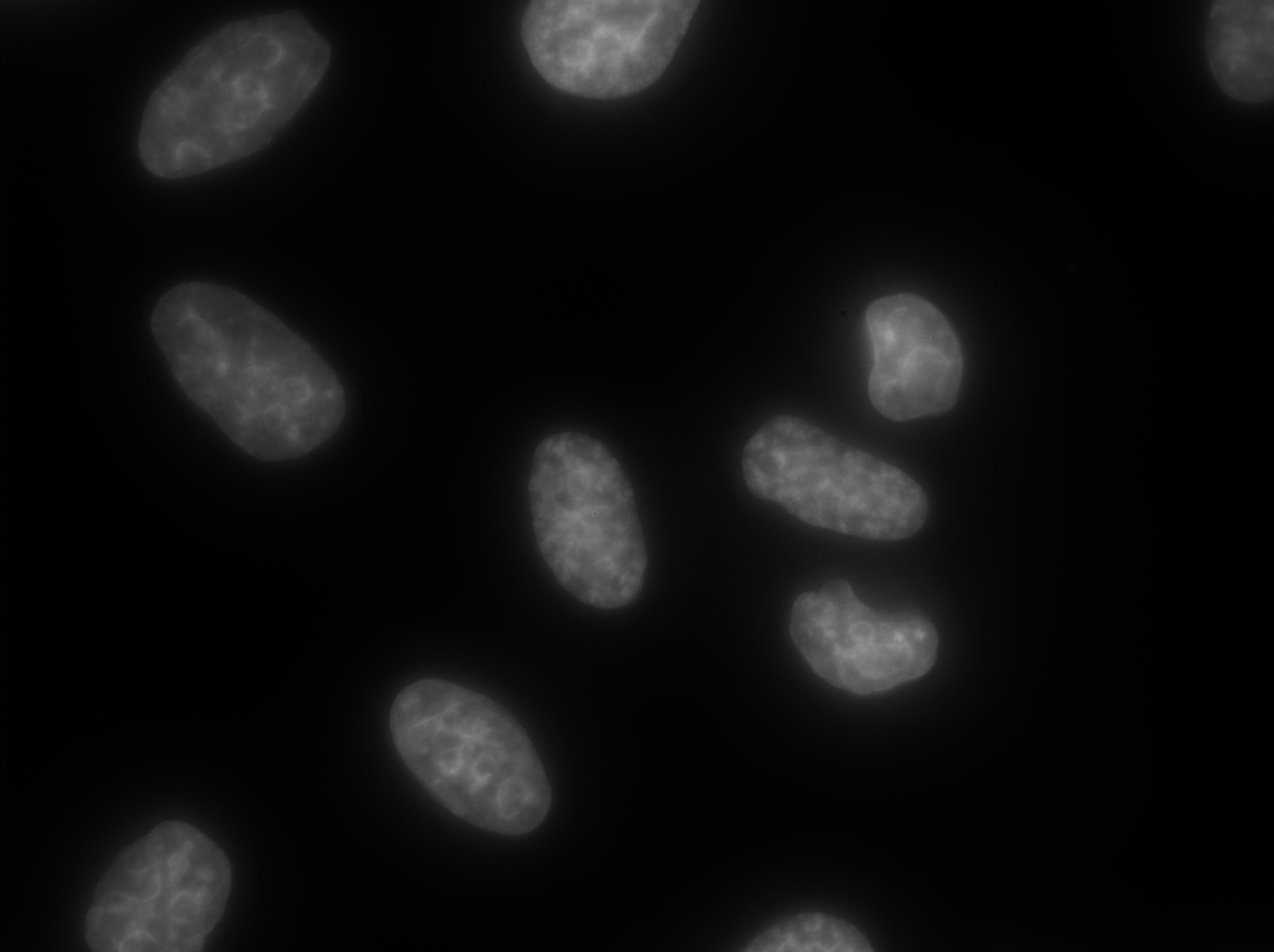

Supplement: Supplementary file 28 — Source data Fig. 7 part 4 [file 44319_2026_742_MOESM28_ESM.zip › Figure 7 Part 4/Fig 7fg Control and TPGS1-KO spastin acetylated tubulin/Cas9 spastin actub 4-1-25 R1 SI23.Project Maximum Z_XY1743535274_Z0_T0_C0.tif]

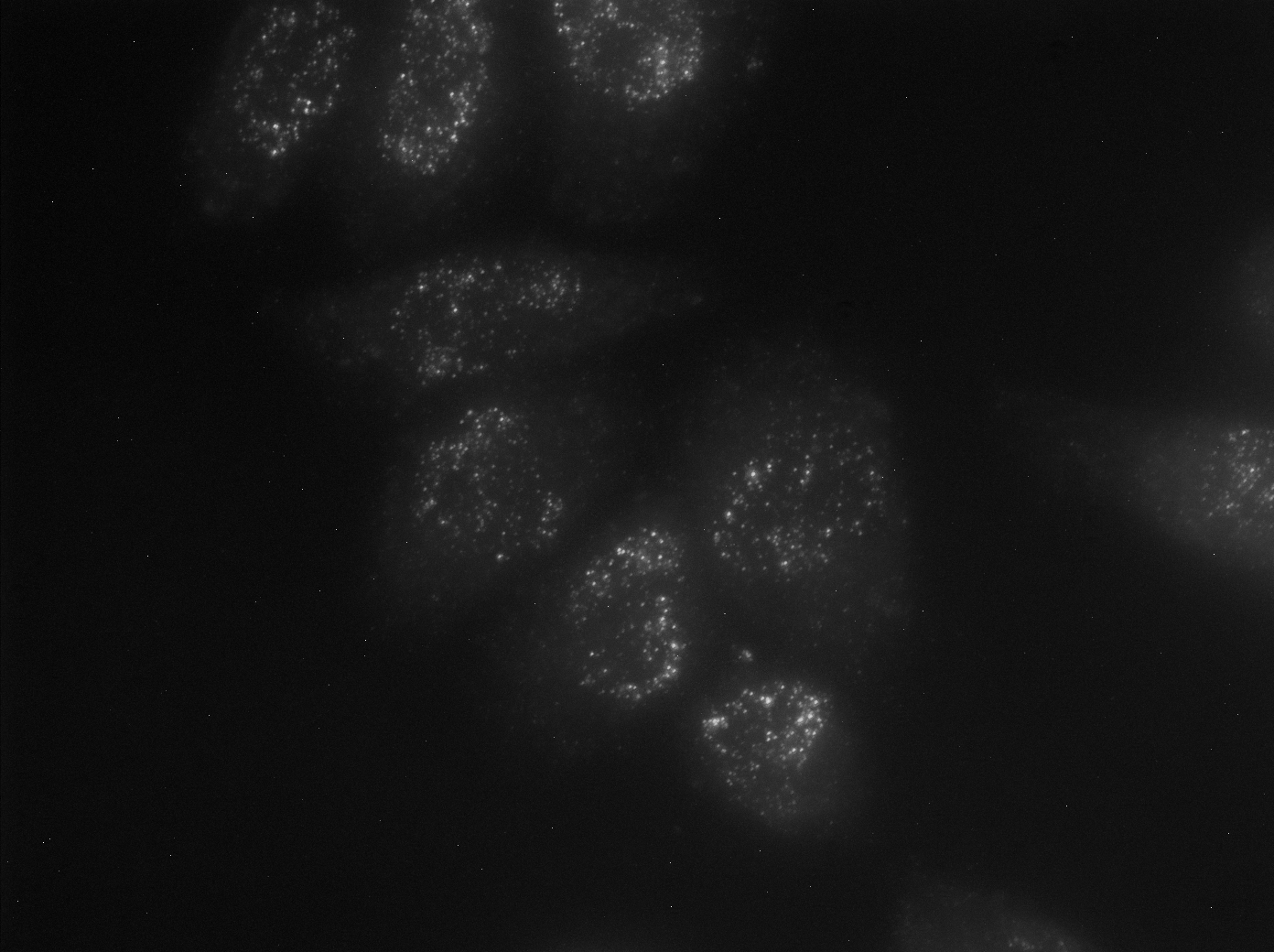

Supplement: Supplementary file 28 — Source data Fig. 7 part 4 [file 44319_2026_742_MOESM28_ESM.zip › Figure 7 Part 4/Fig 7fg Control and TPGS1-KO spastin acetylated tubulin/TPGS1-KO spastin actub 4-1-25 R1 SI7SI8.Project Maximum Z_XY1743536910_Z0_T0_C1.tif]

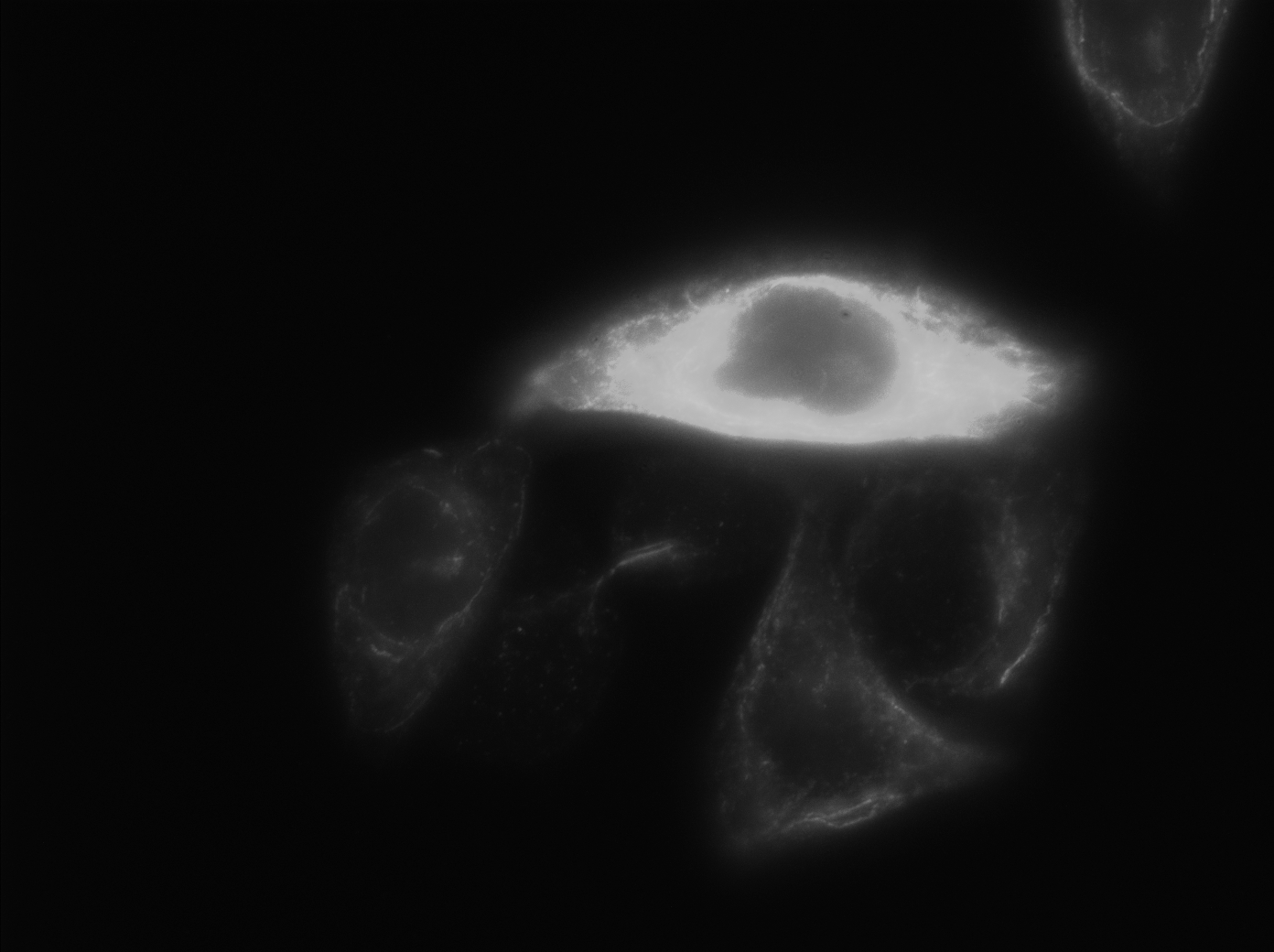

Supplement: Supplementary file 28 — Source data Fig. 7 part 4 [file 44319_2026_742_MOESM28_ESM.zip › Figure 7 Part 4/Fig 7fg Control and TPGS1-KO spastin acetylated tubulin/Cas9 spastin actub 4-1-25 R1 SI22.Project Maximum Z_XY1743535163_Z0_T0_C2.tif]

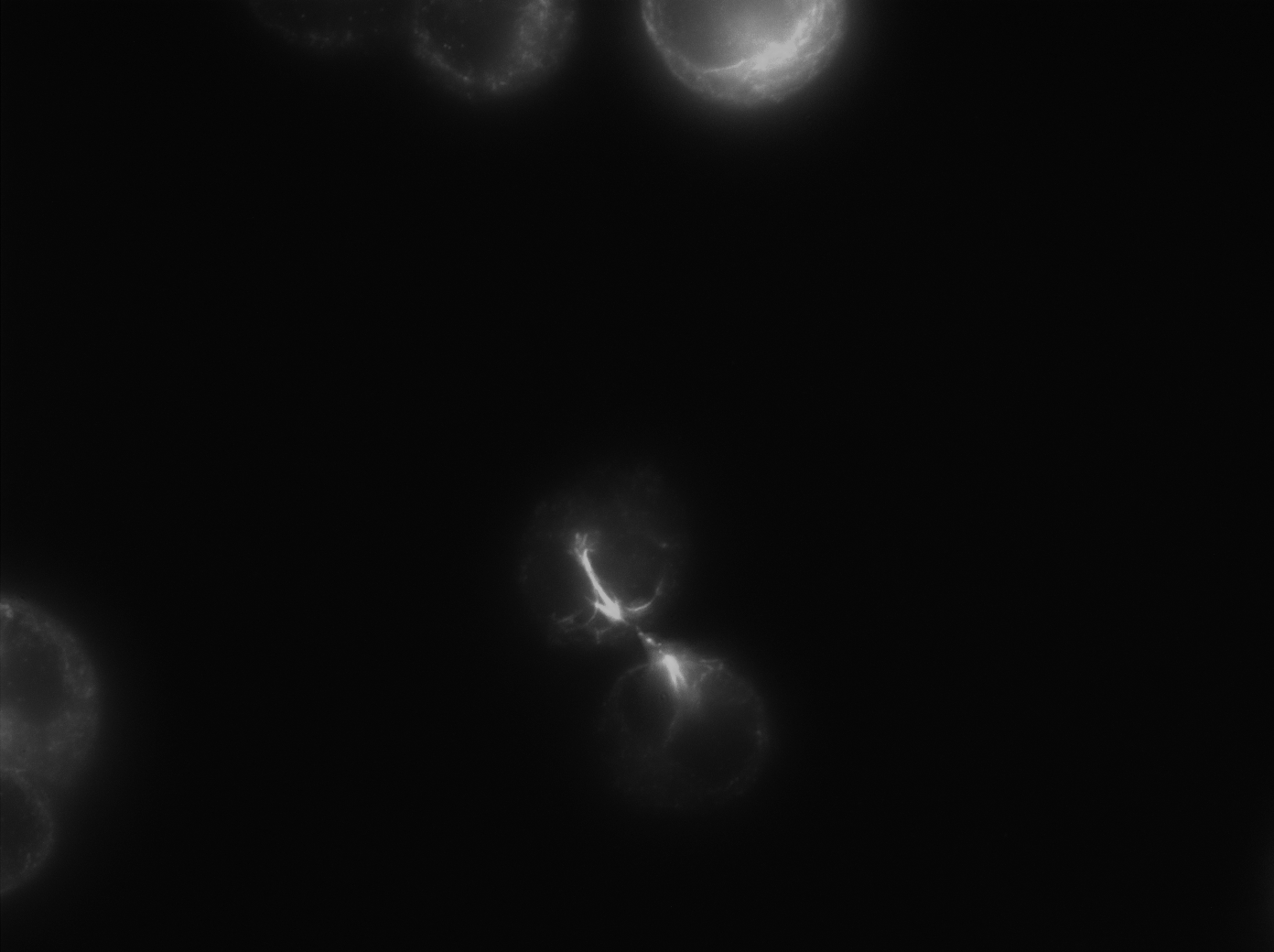

Supplement: Supplementary file 28 — Source data Fig. 7 part 4 [file 44319_2026_742_MOESM28_ESM.zip › Figure 7 Part 4/Fig 7fg Control and TPGS1-KO spastin acetylated tubulin/TPGS1-KO spastin actub 4-1-25 R1 SI10.Project Maximum Z_XY1743537217_Z0_T0_C2.tif]

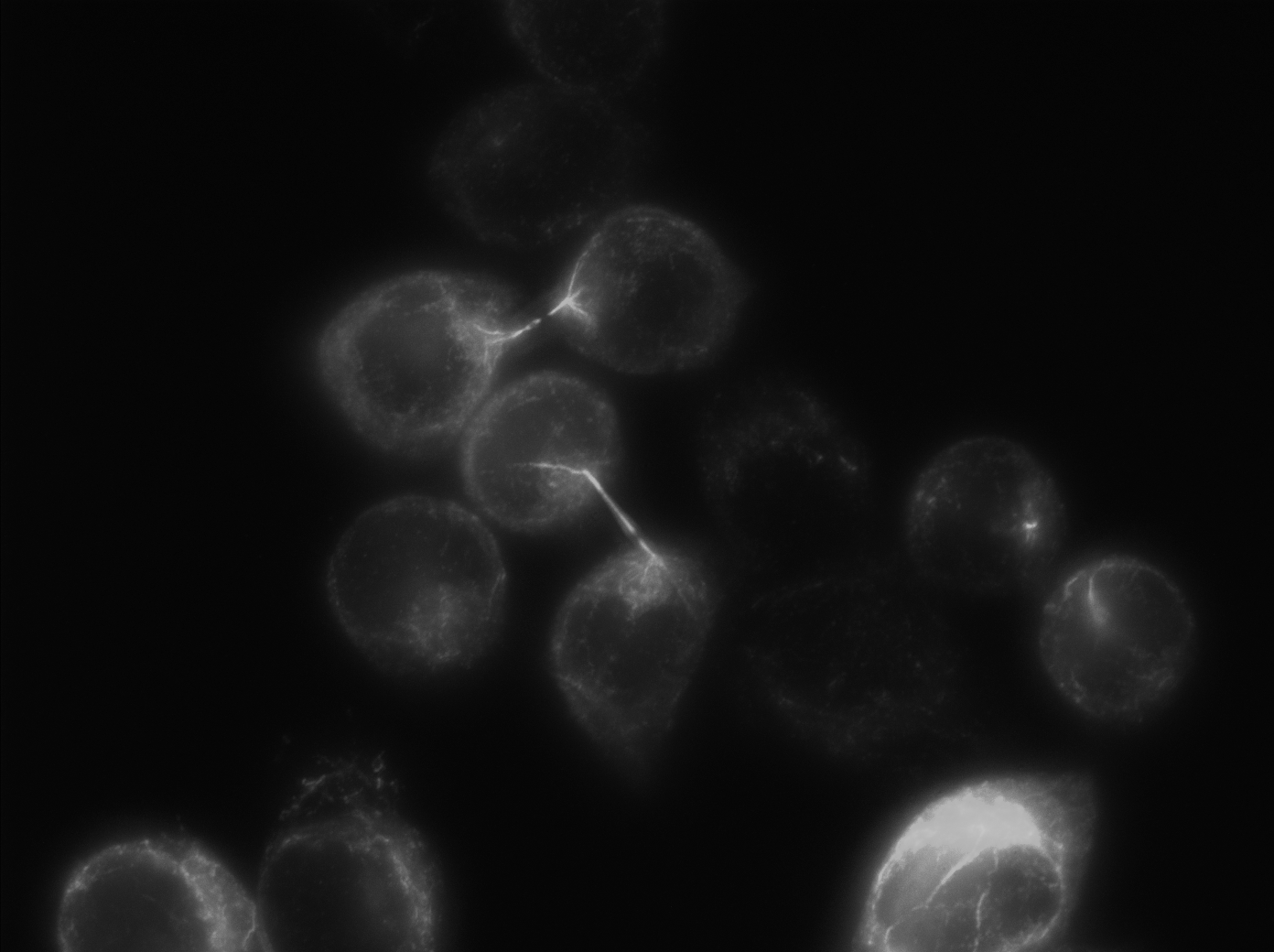

Supplement: Supplementary file 28 — Source data Fig. 7 part 4 [file 44319_2026_742_MOESM28_ESM.zip › Figure 7 Part 4/Fig 7fg Control and TPGS1-KO spastin acetylated tubulin/Cas9 spastin actub 4-1-25 R1 SI14SI15.Project Maximum Z_XY1743532298_Z0_T0_C2.tif]

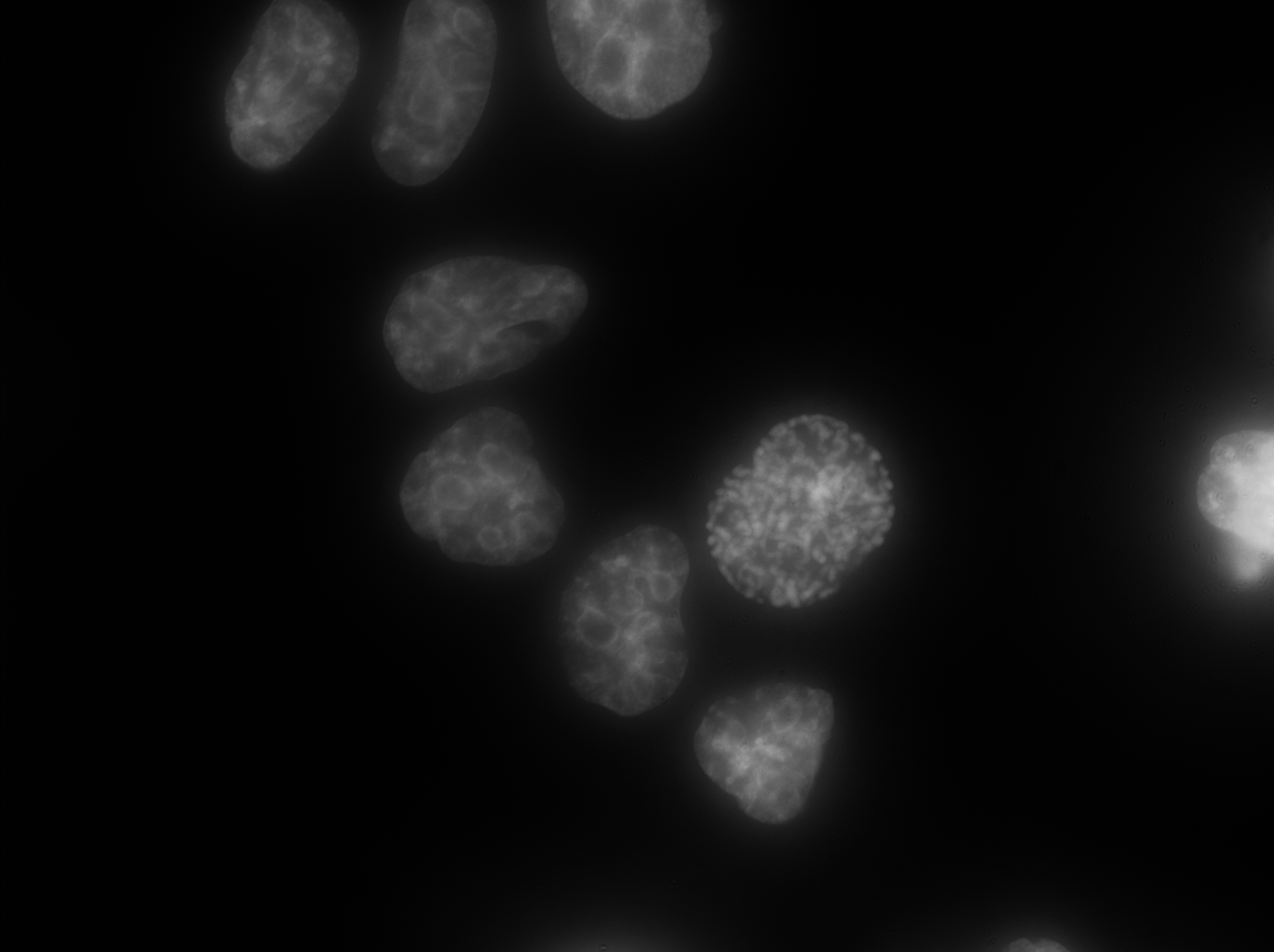

Supplement: Supplementary file 28 — Source data Fig. 7 part 4 [file 44319_2026_742_MOESM28_ESM.zip › Figure 7 Part 4/Fig 7fg Control and TPGS1-KO spastin acetylated tubulin/TPGS1-KO spastin actub 4-1-25 R1 SI7SI8.Project Maximum Z_XY1743536910_Z0_T0_C0.tif]

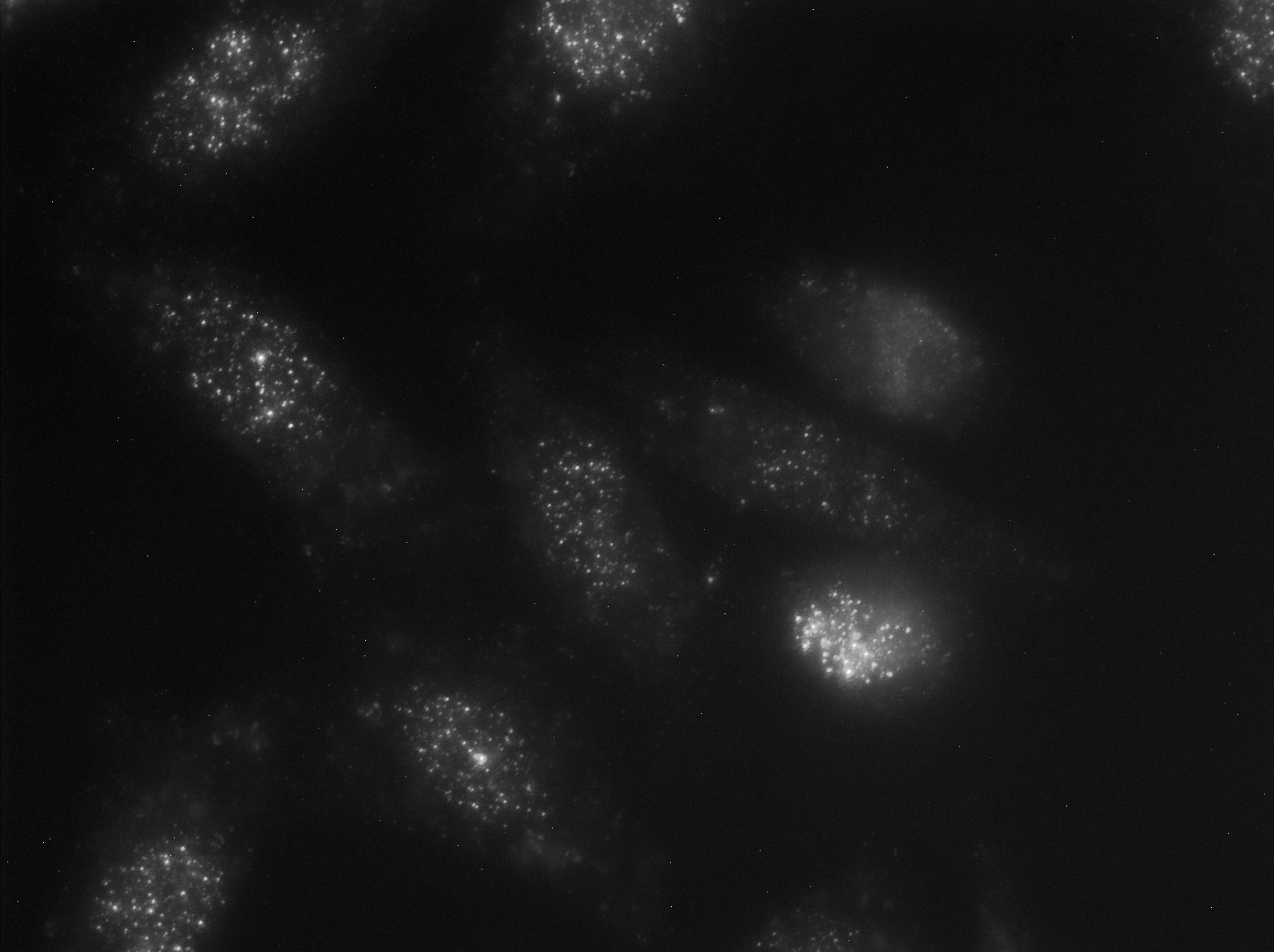

Supplement: Supplementary file 28 — Source data Fig. 7 part 4 [file 44319_2026_742_MOESM28_ESM.zip › Figure 7 Part 4/Fig 7fg Control and TPGS1-KO spastin acetylated tubulin/Cas9 spastin actub 4-1-25 R1 SI23.Project Maximum Z_XY1743535274_Z0_T0_C1.tif]

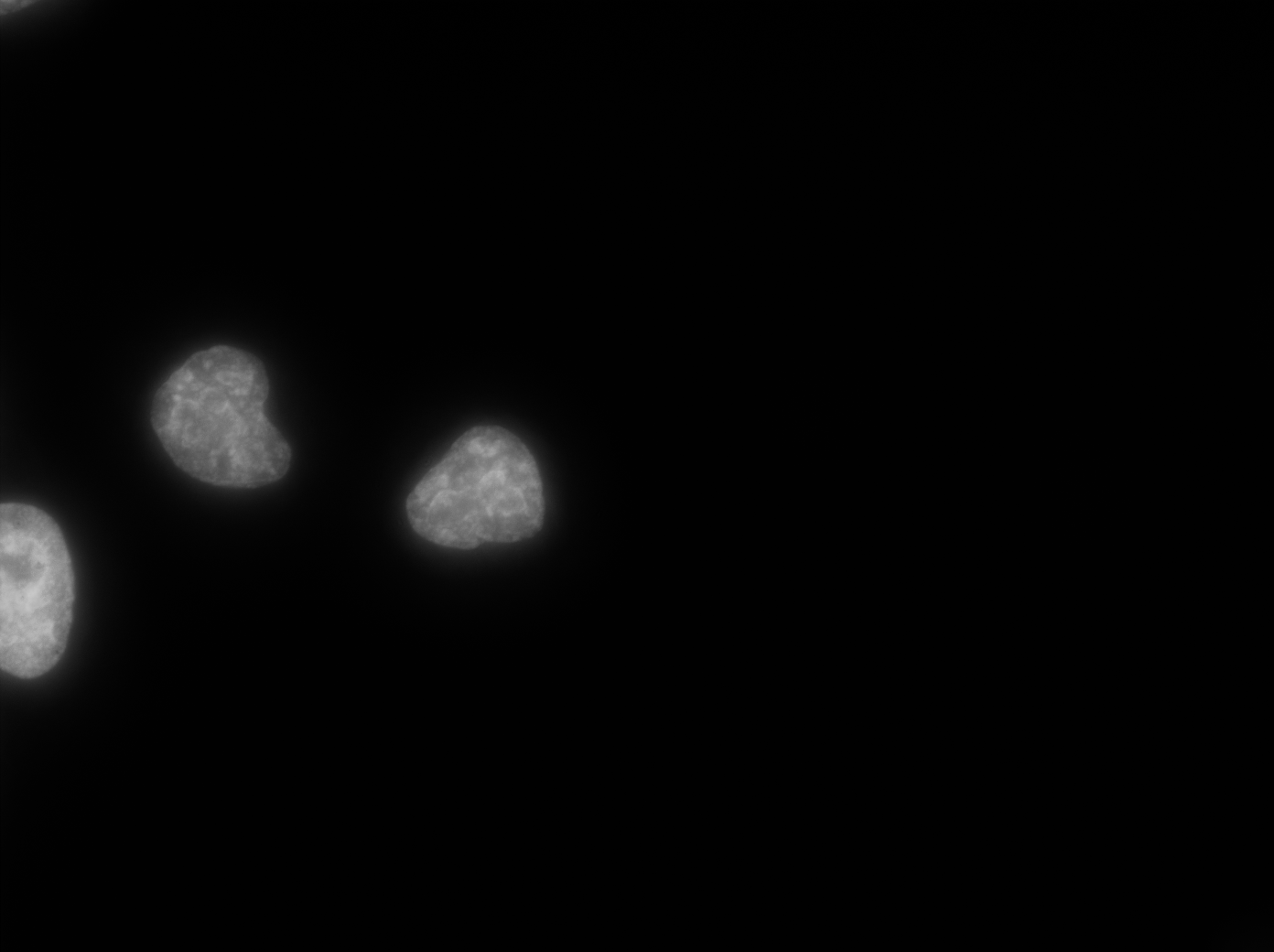

Supplement: Supplementary file 28 — Source data Fig. 7 part 4 [file 44319_2026_742_MOESM28_ESM.zip › Figure 7 Part 4/Fig 7fg Control and TPGS1-KO spastin acetylated tubulin/Cas9 spastin actub 4-1-25 R1 SI1.Project Maximum Z_XY1743530015_Z0_T0_C0.tif]

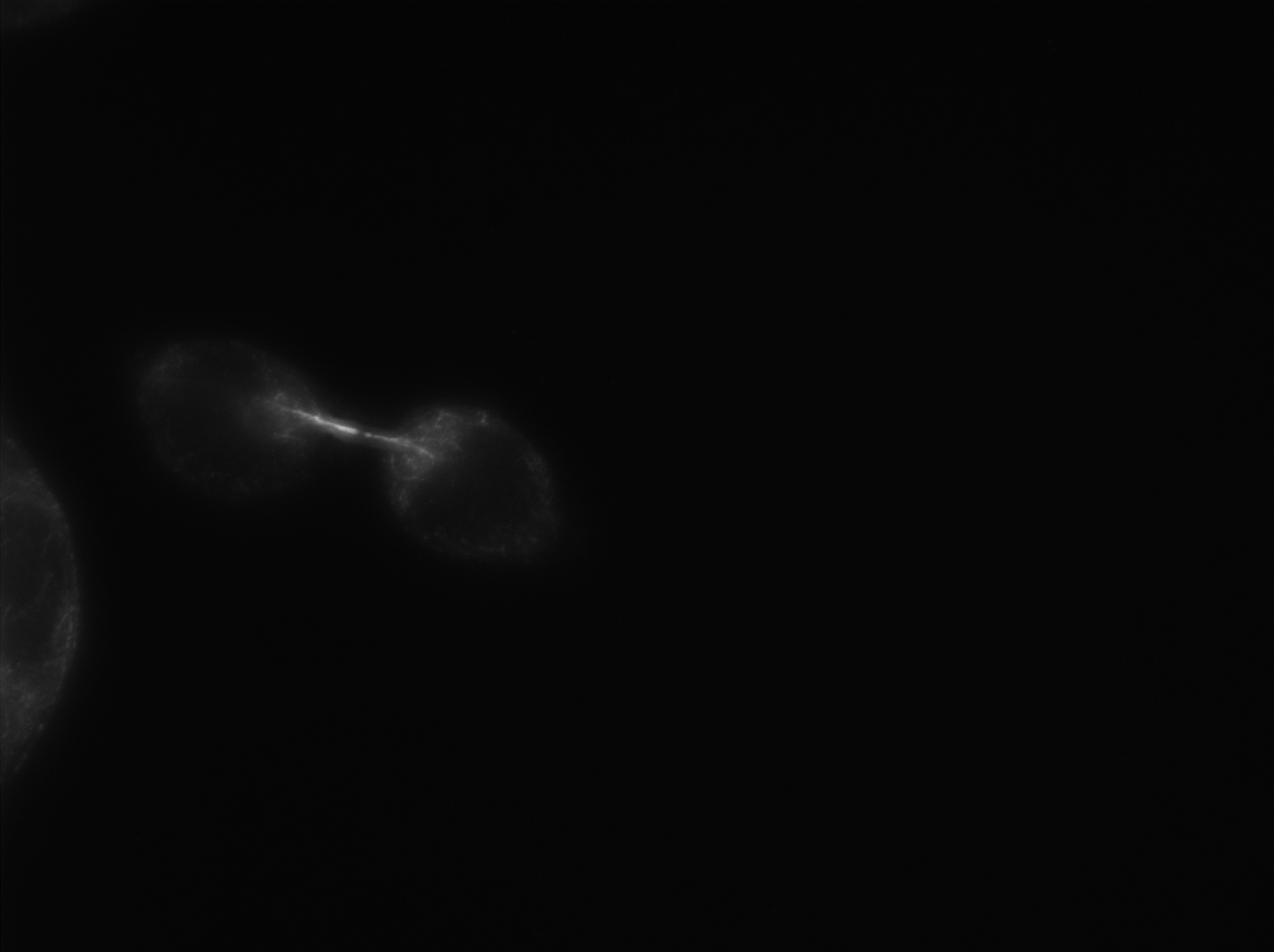

Supplement: Supplementary file 28 — Source data Fig. 7 part 4 [file 44319_2026_742_MOESM28_ESM.zip › Figure 7 Part 4/Fig 7fg Control and TPGS1-KO spastin acetylated tubulin/Cas9 spastin actub 4-1-25 R1 SI1.Project Maximum Z_XY1743530015_Z0_T0_C2.tif]

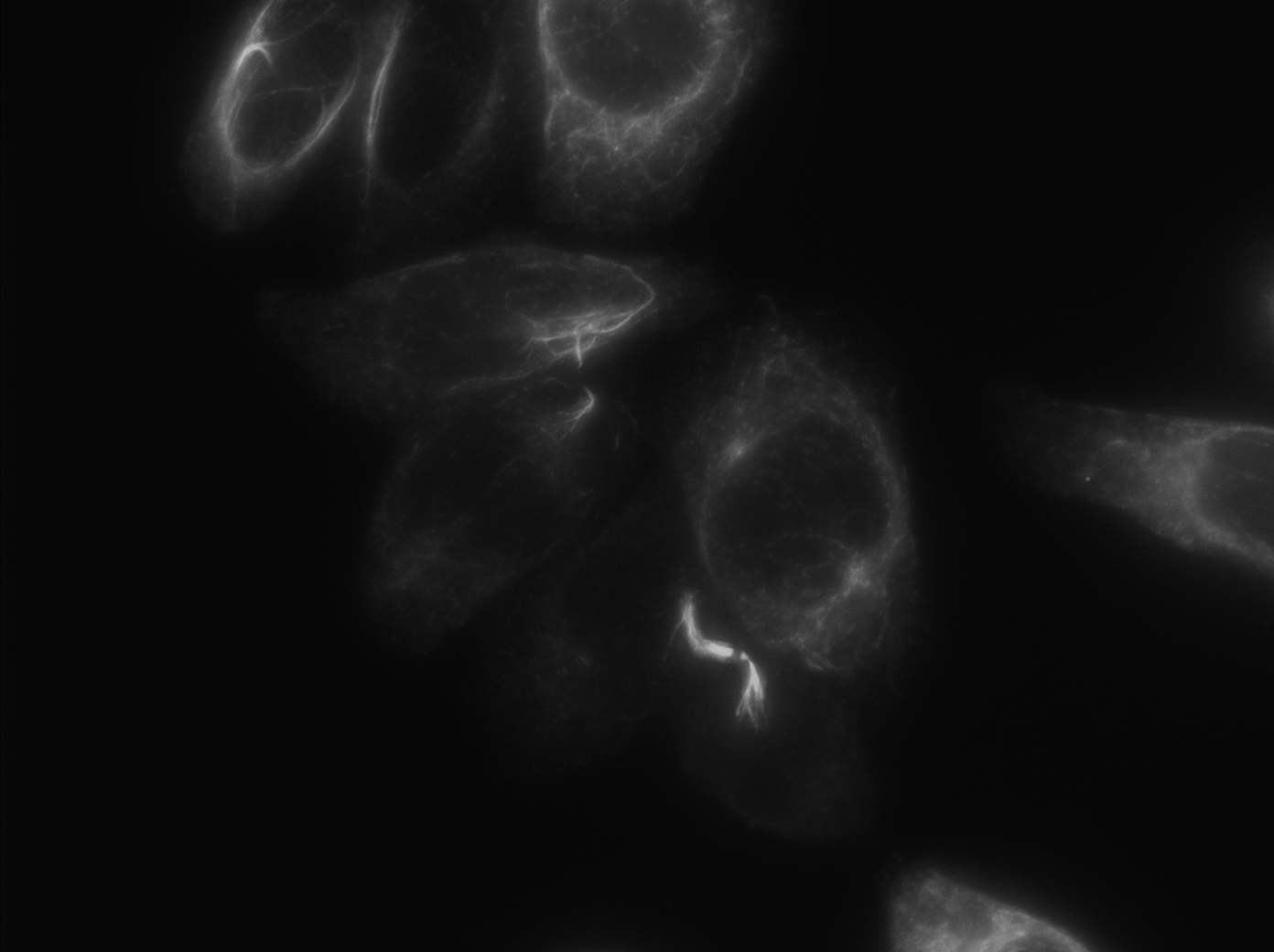

Supplement: Supplementary file 28 — Source data Fig. 7 part 4 [file 44319_2026_742_MOESM28_ESM.zip › Figure 7 Part 4/Fig 7fg Control and TPGS1-KO spastin acetylated tubulin/TPGS1-KO spastin actub 4-1-25 R1 SI7SI8.Project Maximum Z_XY1743536910_Z0_T0_C2.tif]

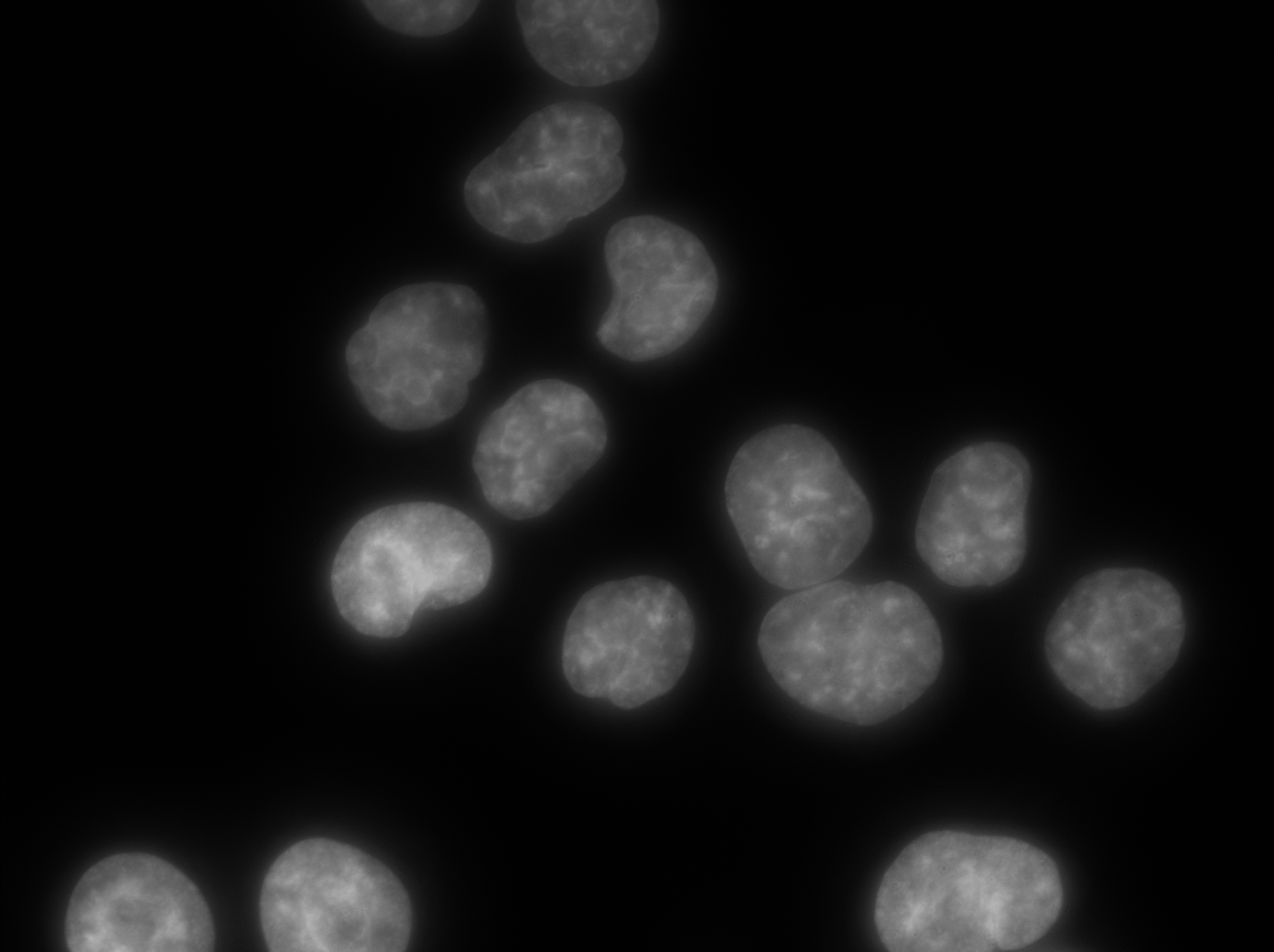

Supplement: Supplementary file 28 — Source data Fig. 7 part 4 [file 44319_2026_742_MOESM28_ESM.zip › Figure 7 Part 4/Fig 7fg Control and TPGS1-KO spastin acetylated tubulin/Cas9 spastin actub 4-1-25 R1 SI14SI15.Project Maximum Z_XY1743532298_Z0_T0_C0.tif]

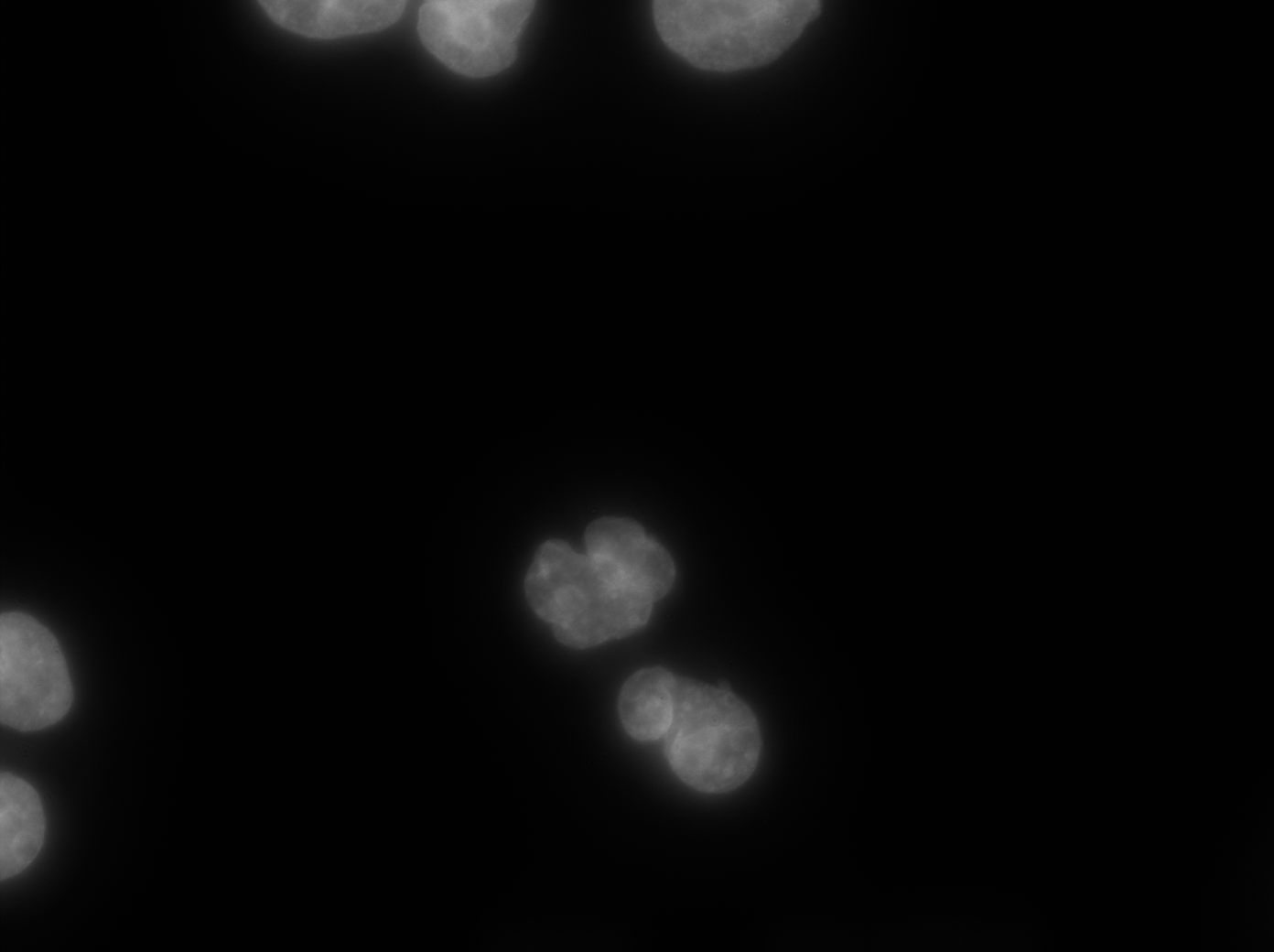

Supplement: Supplementary file 28 — Source data Fig. 7 part 4 [file 44319_2026_742_MOESM28_ESM.zip › Figure 7 Part 4/Fig 7fg Control and TPGS1-KO spastin acetylated tubulin/TPGS1-KO spastin actub 4-1-25 R1 SI10.Project Maximum Z_XY1743537217_Z0_T0_C0.tif]

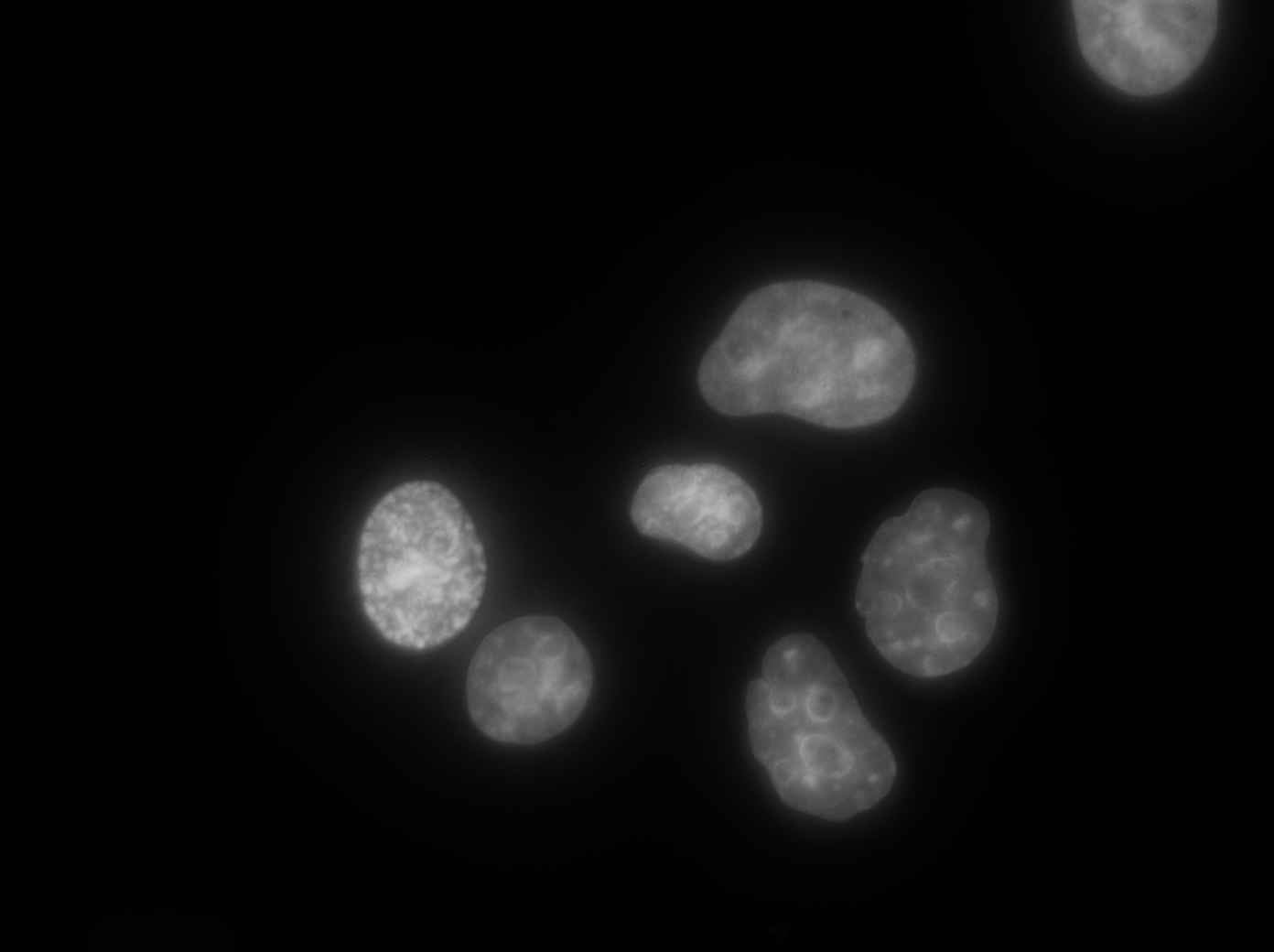

Supplement: Supplementary file 28 — Source data Fig. 7 part 4 [file 44319_2026_742_MOESM28_ESM.zip › Figure 7 Part 4/Fig 7fg Control and TPGS1-KO spastin acetylated tubulin/Cas9 spastin actub 4-1-25 R1 SI22.Project Maximum Z_XY1743535163_Z0_T0_C0.tif]

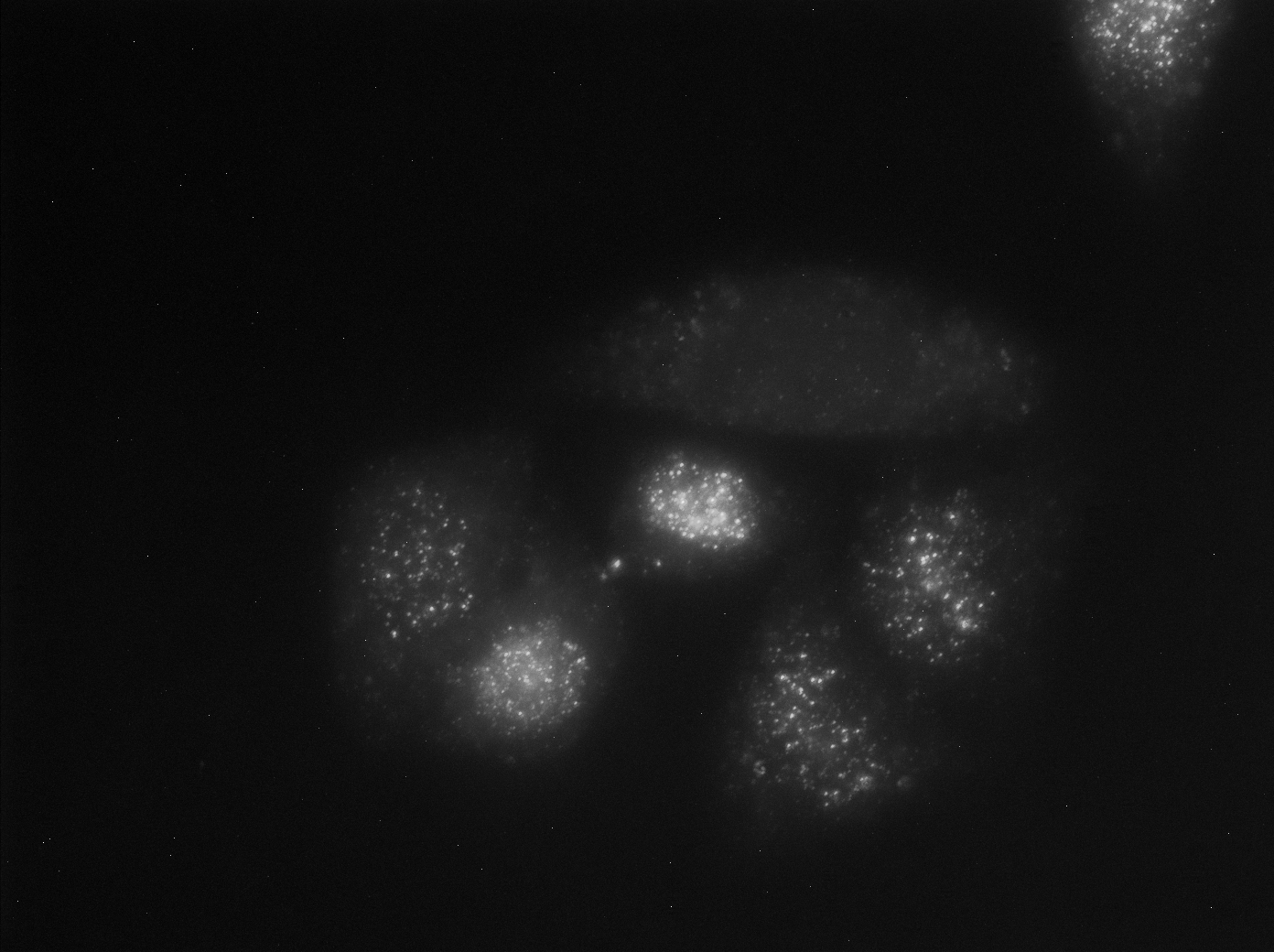

Supplement: Supplementary file 28 — Source data Fig. 7 part 4 [file 44319_2026_742_MOESM28_ESM.zip › Figure 7 Part 4/Fig 7fg Control and TPGS1-KO spastin acetylated tubulin/Cas9 spastin actub 4-1-25 R1 SI22.Project Maximum Z_XY1743535163_Z0_T0_C1.tif]

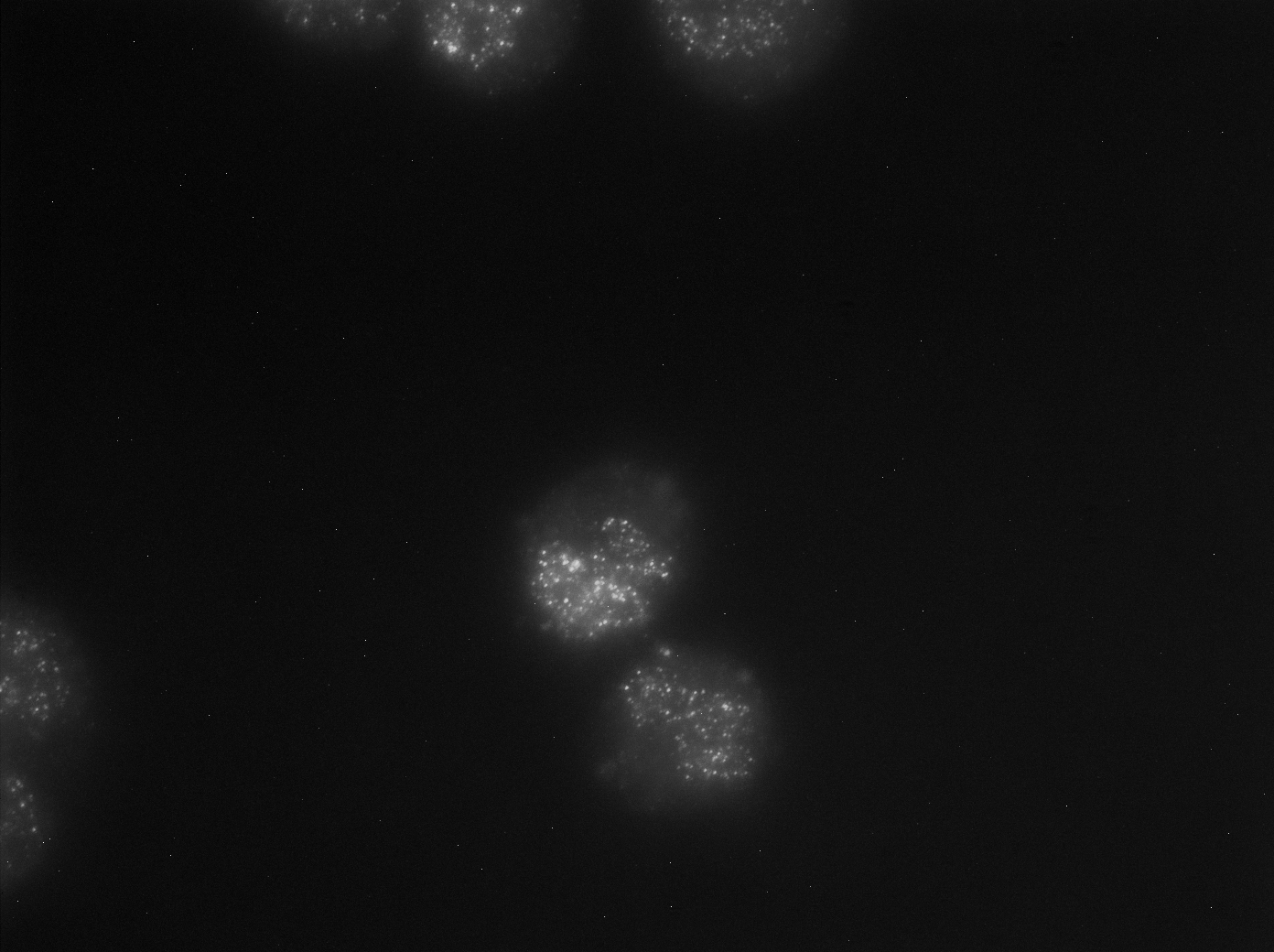

Supplement: Supplementary file 28 — Source data Fig. 7 part 4 [file 44319_2026_742_MOESM28_ESM.zip › Figure 7 Part 4/Fig 7fg Control and TPGS1-KO spastin acetylated tubulin/TPGS1-KO spastin actub 4-1-25 R1 SI10.Project Maximum Z_XY1743537217_Z0_T0_C1.tif]

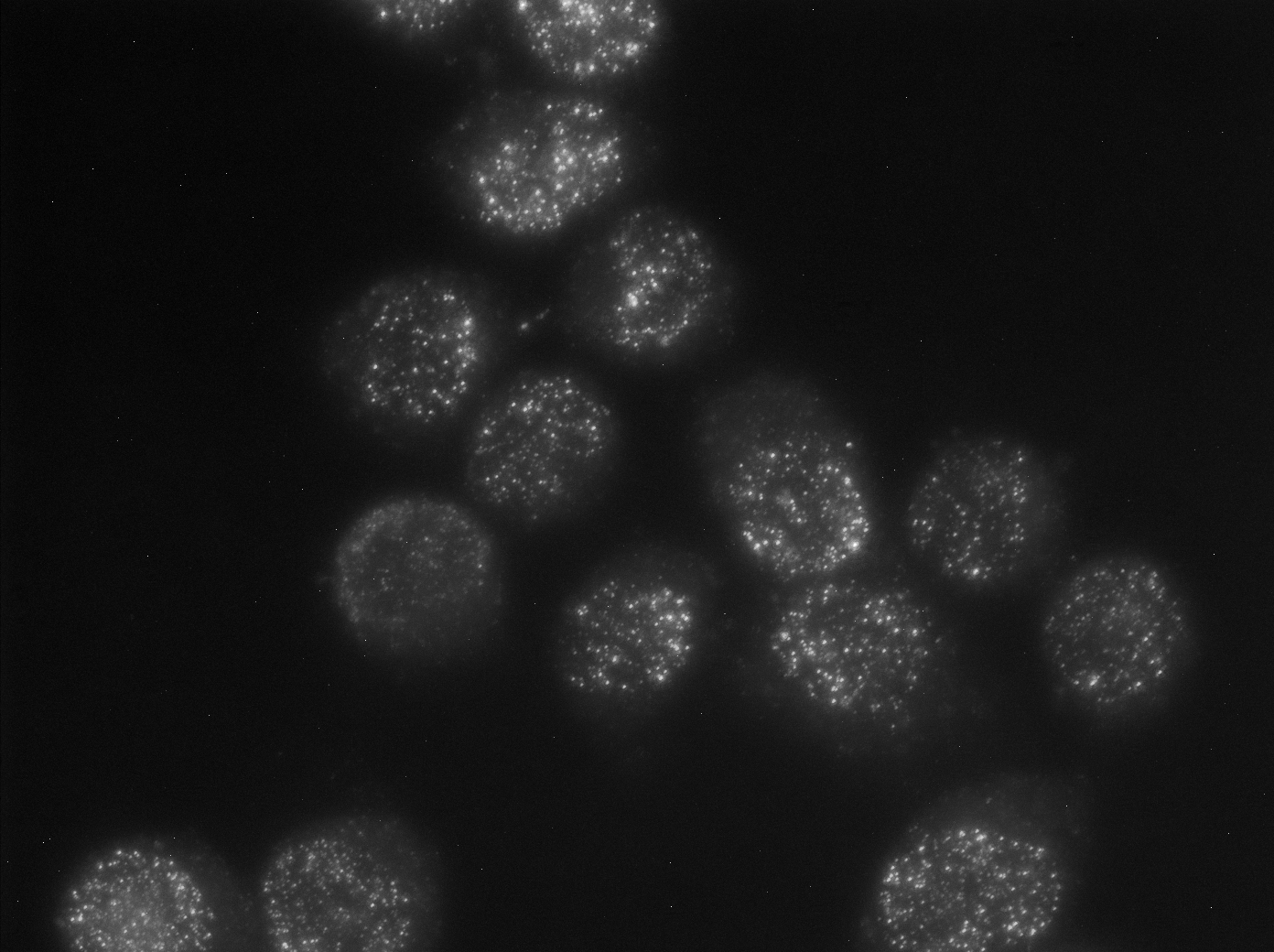

Supplement: Supplementary file 28 — Source data Fig. 7 part 4 [file 44319_2026_742_MOESM28_ESM.zip › Figure 7 Part 4/Fig 7fg Control and TPGS1-KO spastin acetylated tubulin/Cas9 spastin actub 4-1-25 R1 SI14SI15.Project Maximum Z_XY1743532298_Z0_T0_C1.tif]

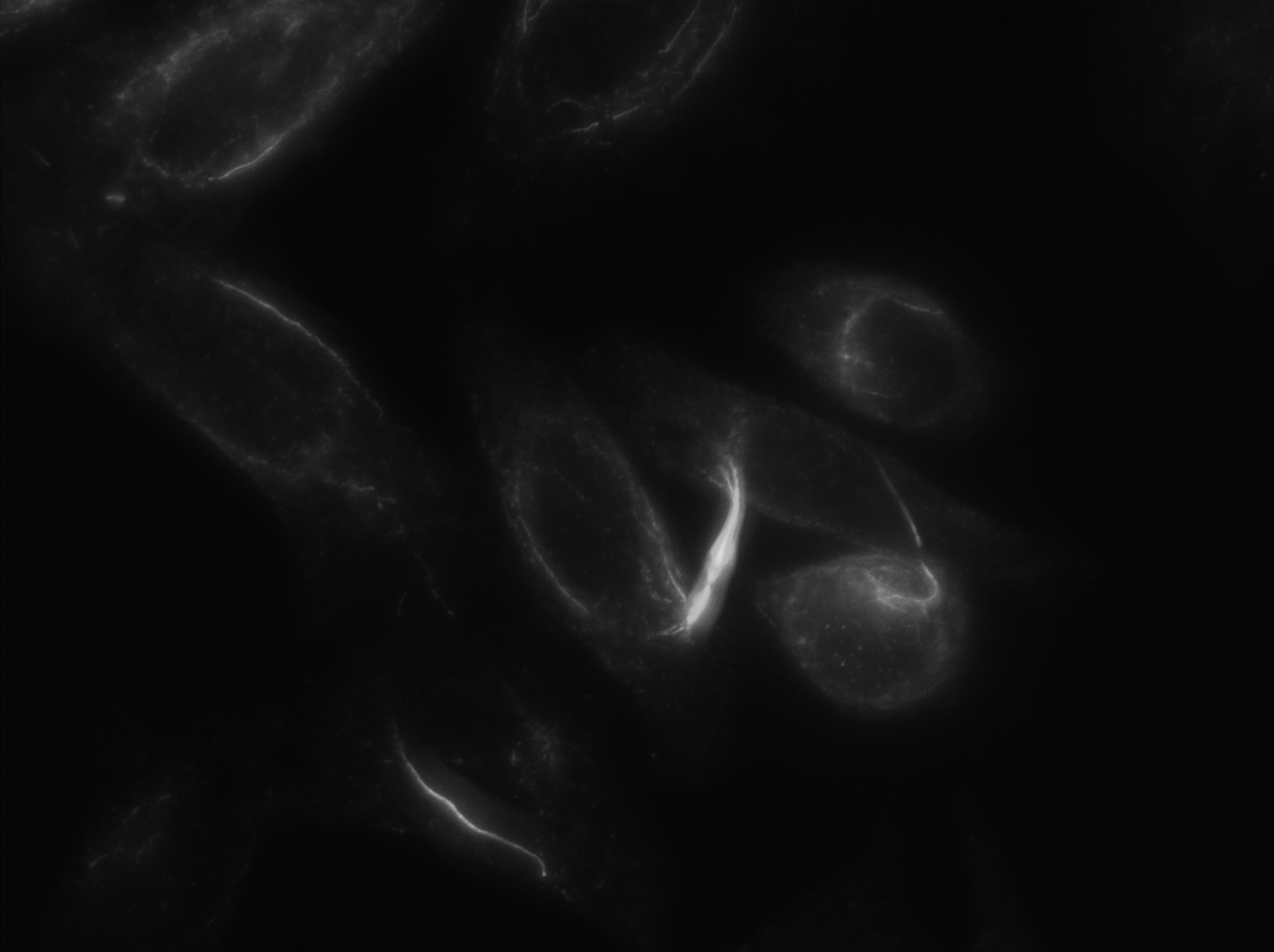

Supplement: Supplementary file 28 — Source data Fig. 7 part 4 [file 44319_2026_742_MOESM28_ESM.zip › Figure 7 Part 4/Fig 7fg Control and TPGS1-KO spastin acetylated tubulin/Cas9 spastin actub 4-1-25 R1 SI23.Project Maximum Z_XY1743535274_Z0_T0_C2.tif]

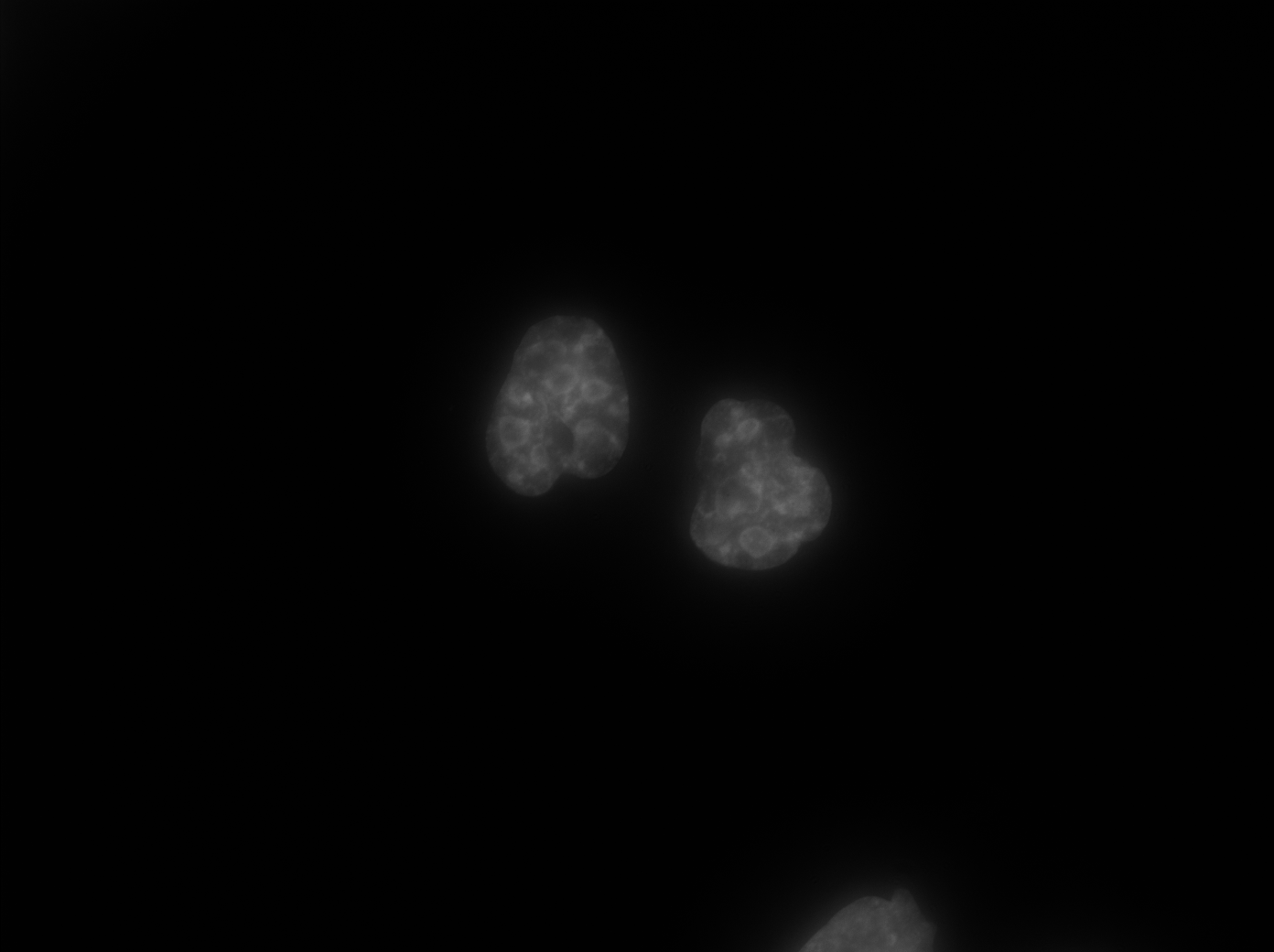

Supplement: Supplementary file 28 — Source data Fig. 7 part 4 [file 44319_2026_742_MOESM28_ESM.zip › Figure 7 Part 4/Fig 7fg Control and TPGS1-KO spastin acetylated tubulin/TPGS1-KO spastin actub 4-1-25 R1 SI6.Project Maximum Z_XY1743536775_Z0_T0_C0.tif]

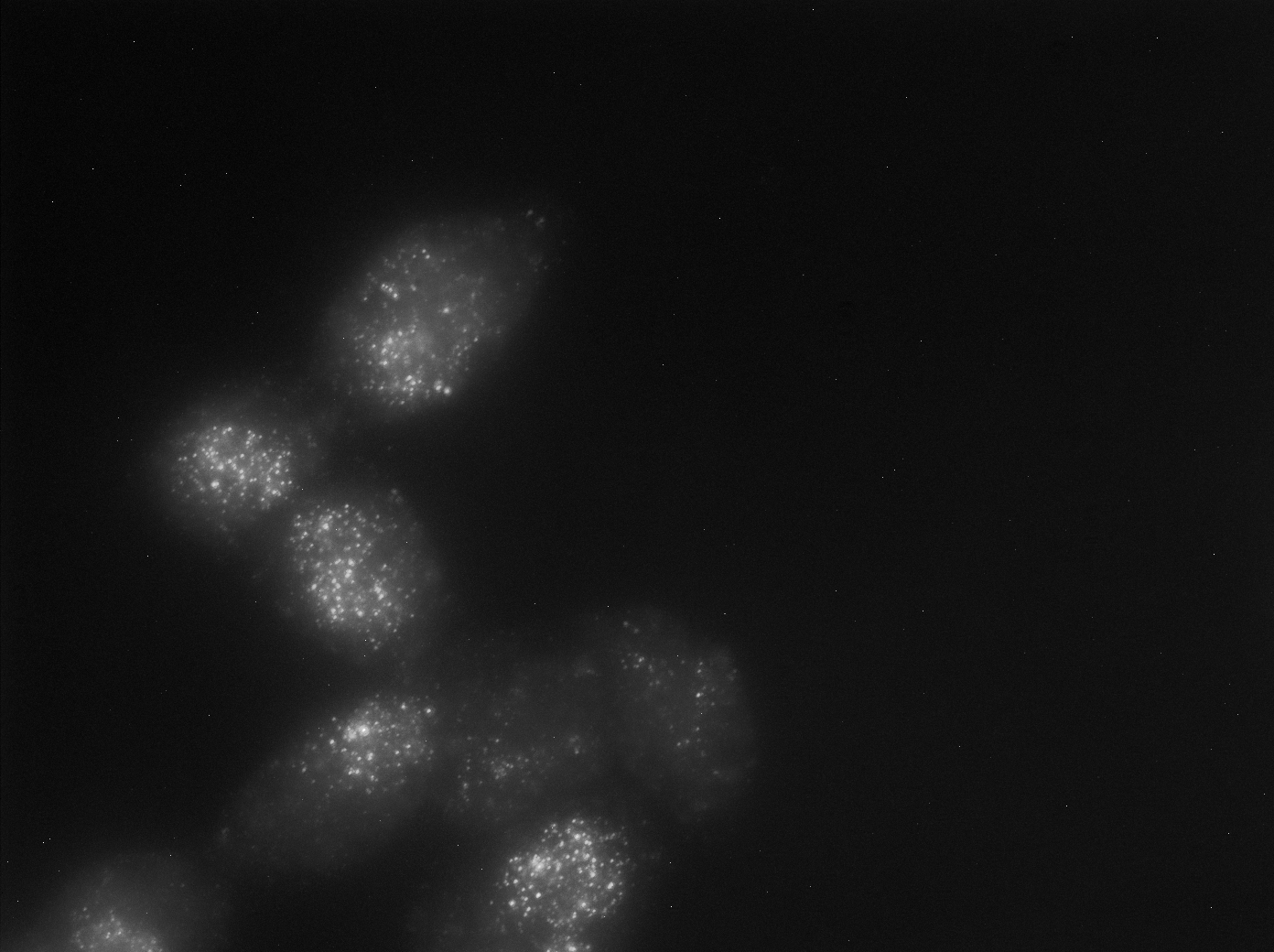

Supplement: Supplementary file 28 — Source data Fig. 7 part 4 [file 44319_2026_742_MOESM28_ESM.zip › Figure 7 Part 4/Fig 7fg Control and TPGS1-KO spastin acetylated tubulin/TPGS1-KO spastin actub 4-1-25 R1 SI13.Project Maximum Z_XY1743537735_Z0_T0_C1.tif]

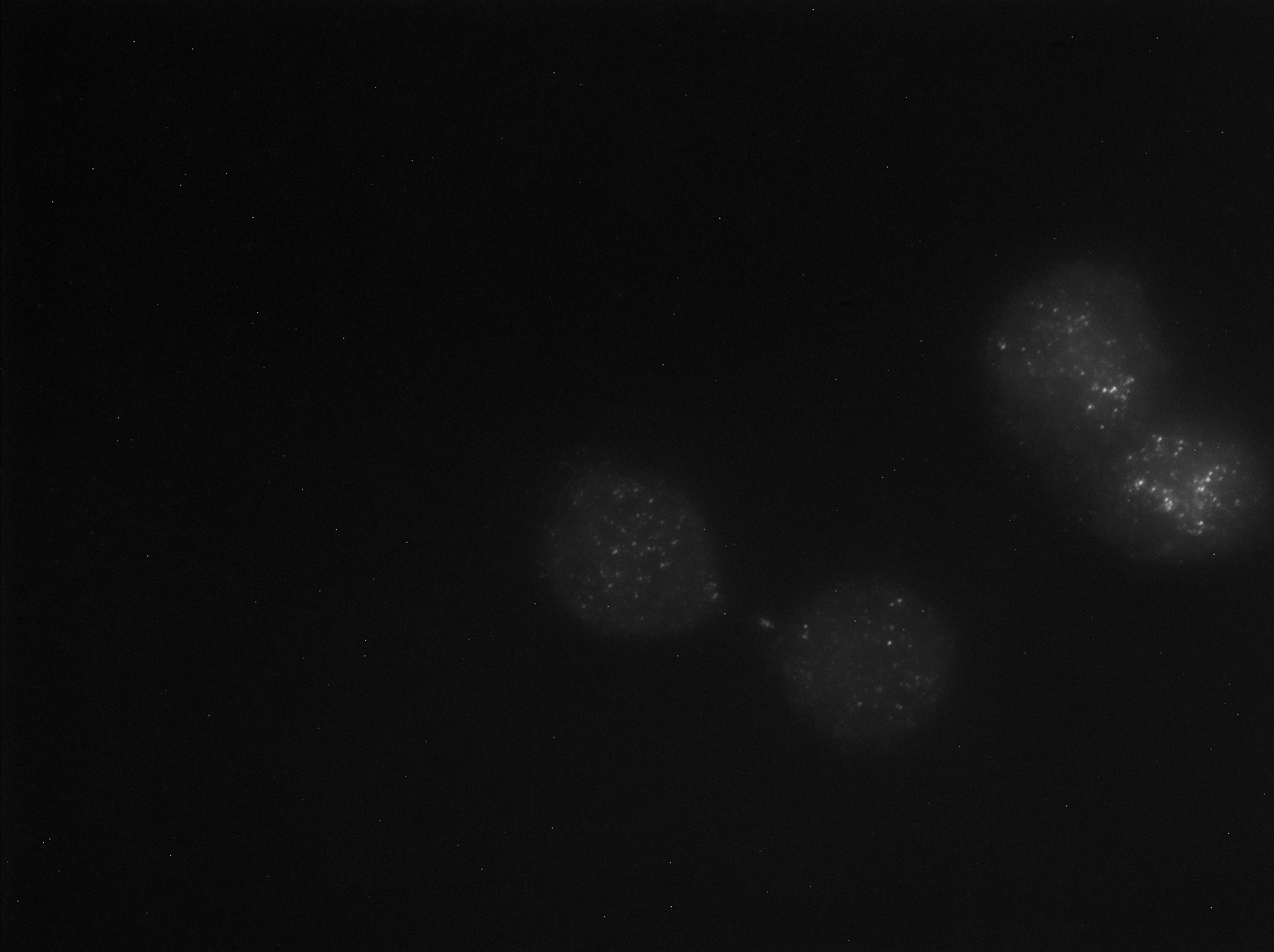

Supplement: Supplementary file 28 — Source data Fig. 7 part 4 [file 44319_2026_742_MOESM28_ESM.zip › Figure 7 Part 4/Fig 7fg Control and TPGS1-KO spastin acetylated tubulin/TPGS1-KO spastin actub 4-1-25 R1 SI21.Project Maximum Z_XY1743539120_Z0_T0_C1.tif]

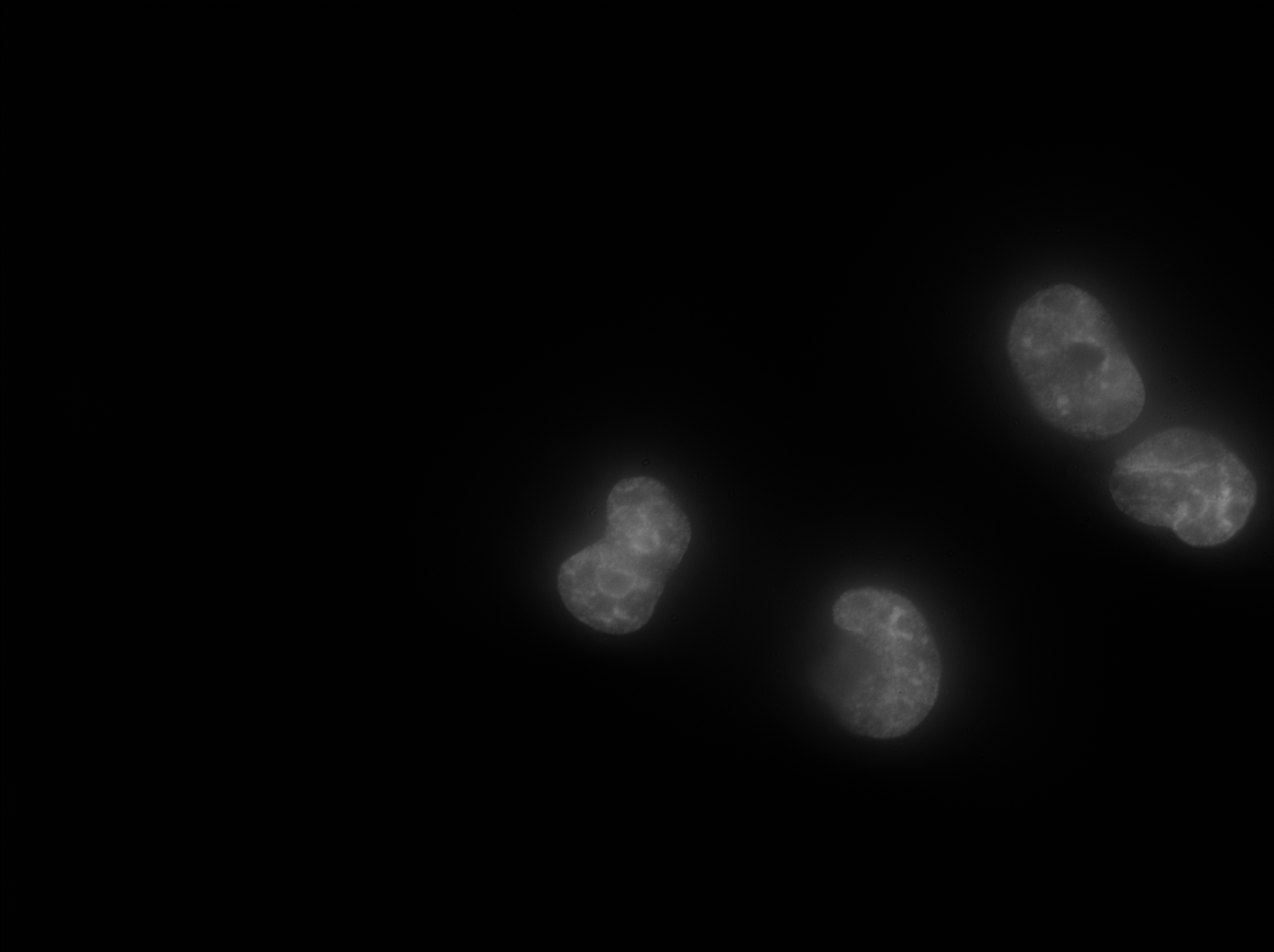

Supplement: Supplementary file 28 — Source data Fig. 7 part 4 [file 44319_2026_742_MOESM28_ESM.zip › Figure 7 Part 4/Fig 7fg Control and TPGS1-KO spastin acetylated tubulin/TPGS1-KO spastin actub 4-1-25 R1 SI21.Project Maximum Z_XY1743539120_Z0_T0_C0.tif]

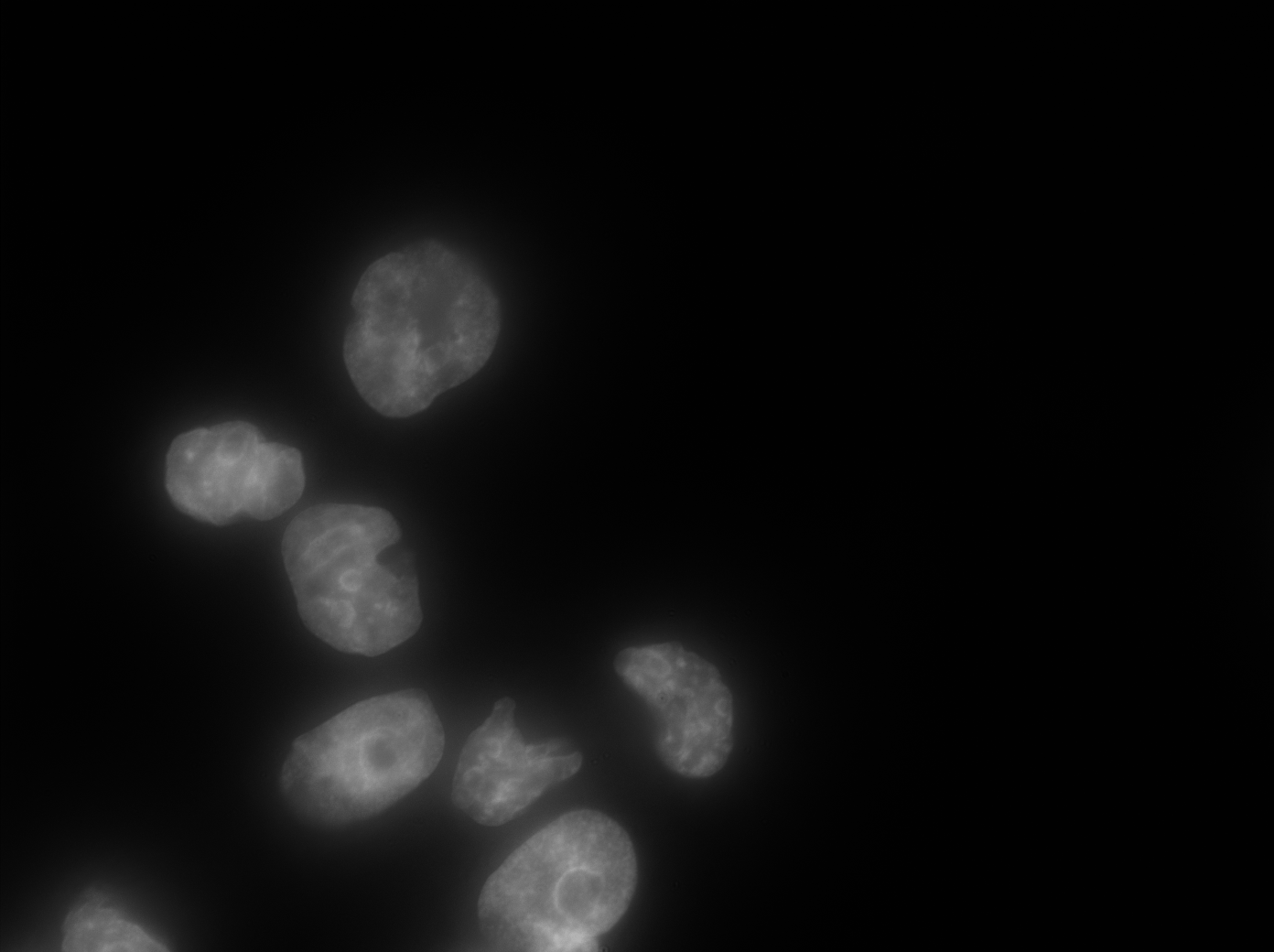

Supplement: Supplementary file 28 — Source data Fig. 7 part 4 [file 44319_2026_742_MOESM28_ESM.zip › Figure 7 Part 4/Fig 7fg Control and TPGS1-KO spastin acetylated tubulin/TPGS1-KO spastin actub 4-1-25 R1 SI13.Project Maximum Z_XY1743537735_Z0_T0_C0.tif]

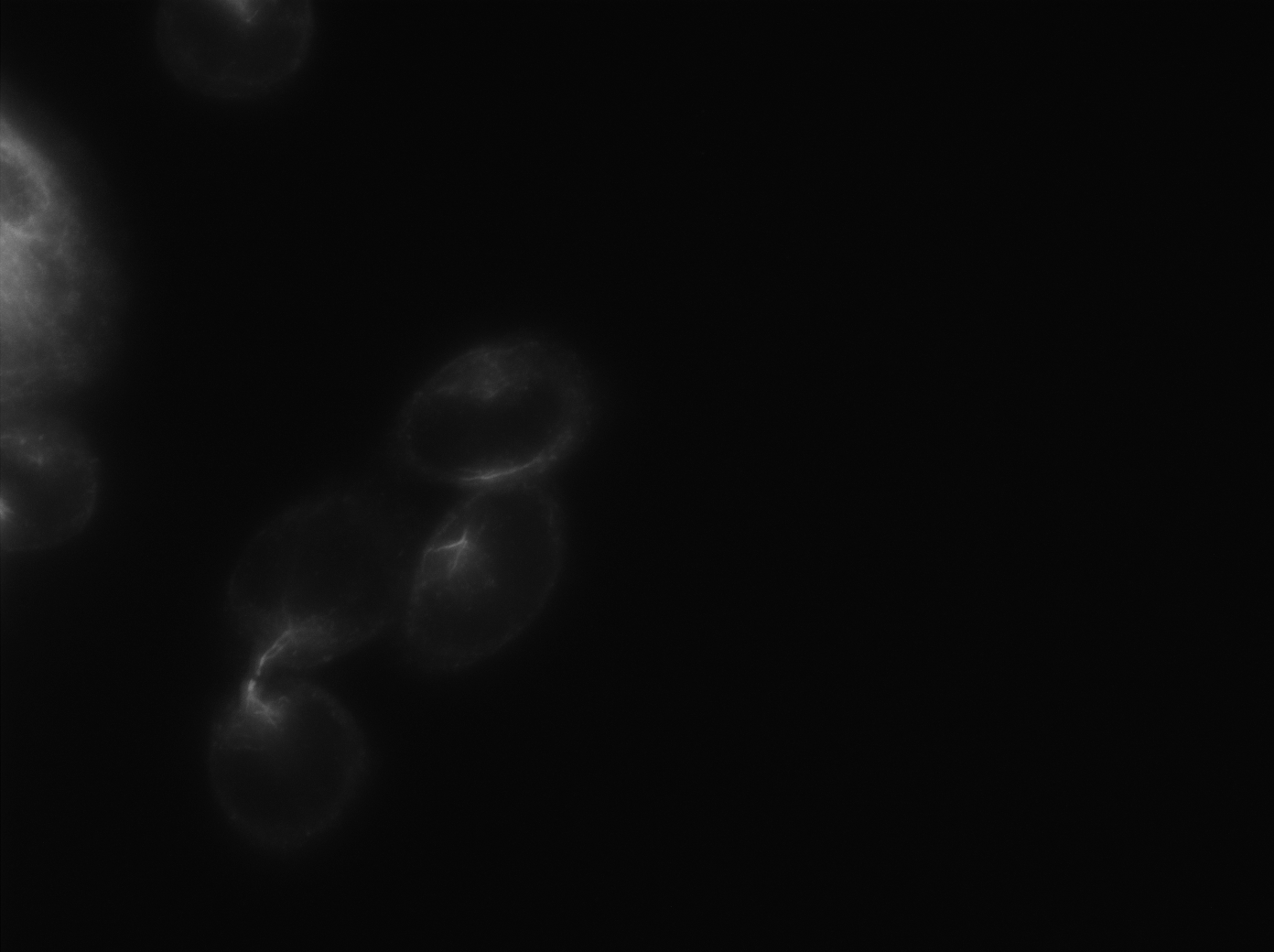

Supplement: Supplementary file 28 — Source data Fig. 7 part 4 [file 44319_2026_742_MOESM28_ESM.zip › Figure 7 Part 4/Fig 7fg Control and TPGS1-KO spastin acetylated tubulin/TPGS1-KO spastin actub 4-1-25 R1 SI17.Project Maximum Z_XY1743538264_Z0_T0_C2.tif]

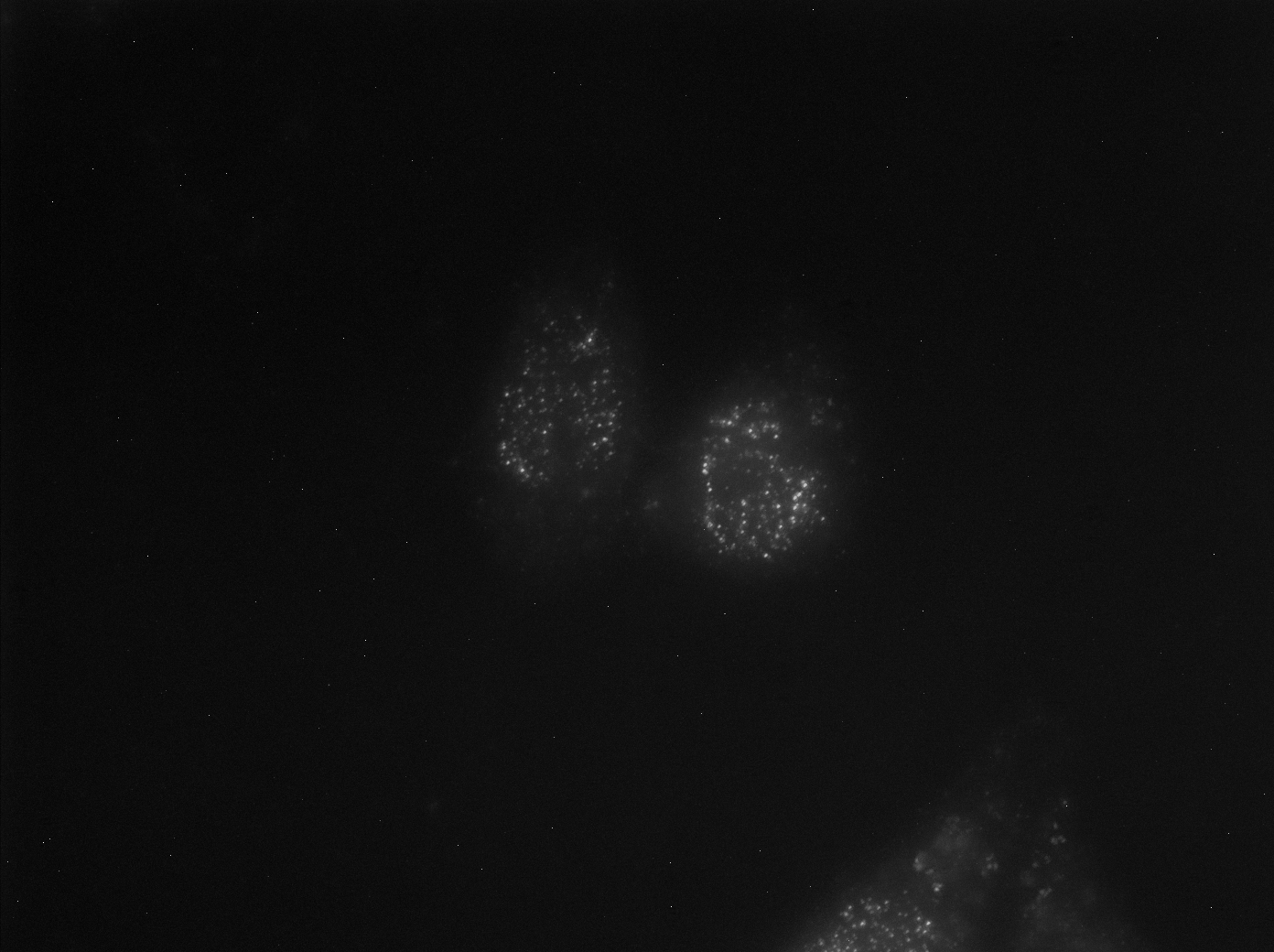

Supplement: Supplementary file 28 — Source data Fig. 7 part 4 [file 44319_2026_742_MOESM28_ESM.zip › Figure 7 Part 4/Fig 7fg Control and TPGS1-KO spastin acetylated tubulin/TPGS1-KO spastin actub 4-1-25 R1 SI6.Project Maximum Z_XY1743536775_Z0_T0_C1.tif]

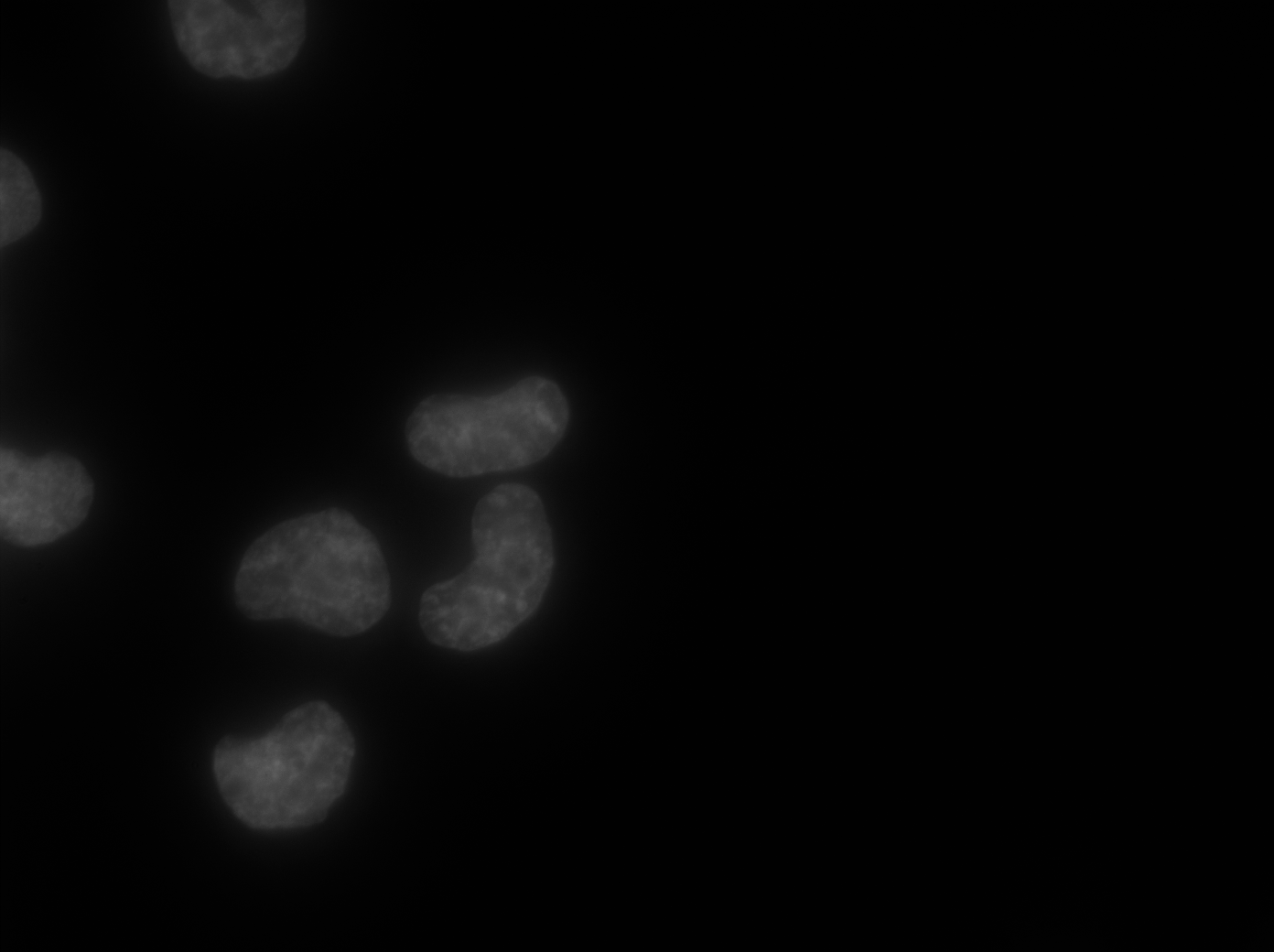

Supplement: Supplementary file 28 — Source data Fig. 7 part 4 [file 44319_2026_742_MOESM28_ESM.zip › Figure 7 Part 4/Fig 7fg Control and TPGS1-KO spastin acetylated tubulin/TPGS1-KO spastin actub 4-1-25 R1 SI17.Project Maximum Z_XY1743538264_Z0_T0_C0.tif]

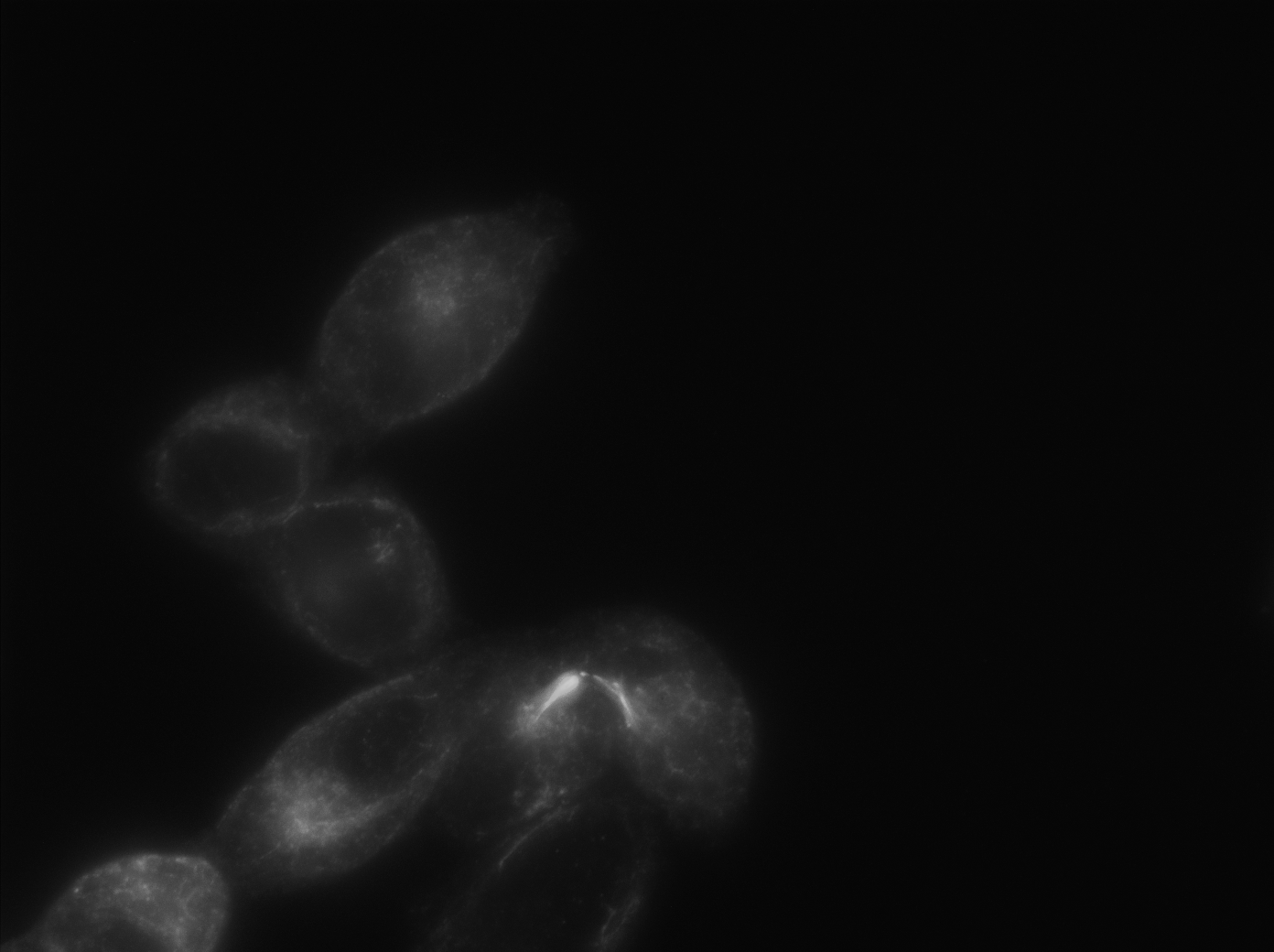

Supplement: Supplementary file 28 — Source data Fig. 7 part 4 [file 44319_2026_742_MOESM28_ESM.zip › Figure 7 Part 4/Fig 7fg Control and TPGS1-KO spastin acetylated tubulin/TPGS1-KO spastin actub 4-1-25 R1 SI13.Project Maximum Z_XY1743537735_Z0_T0_C2.tif]

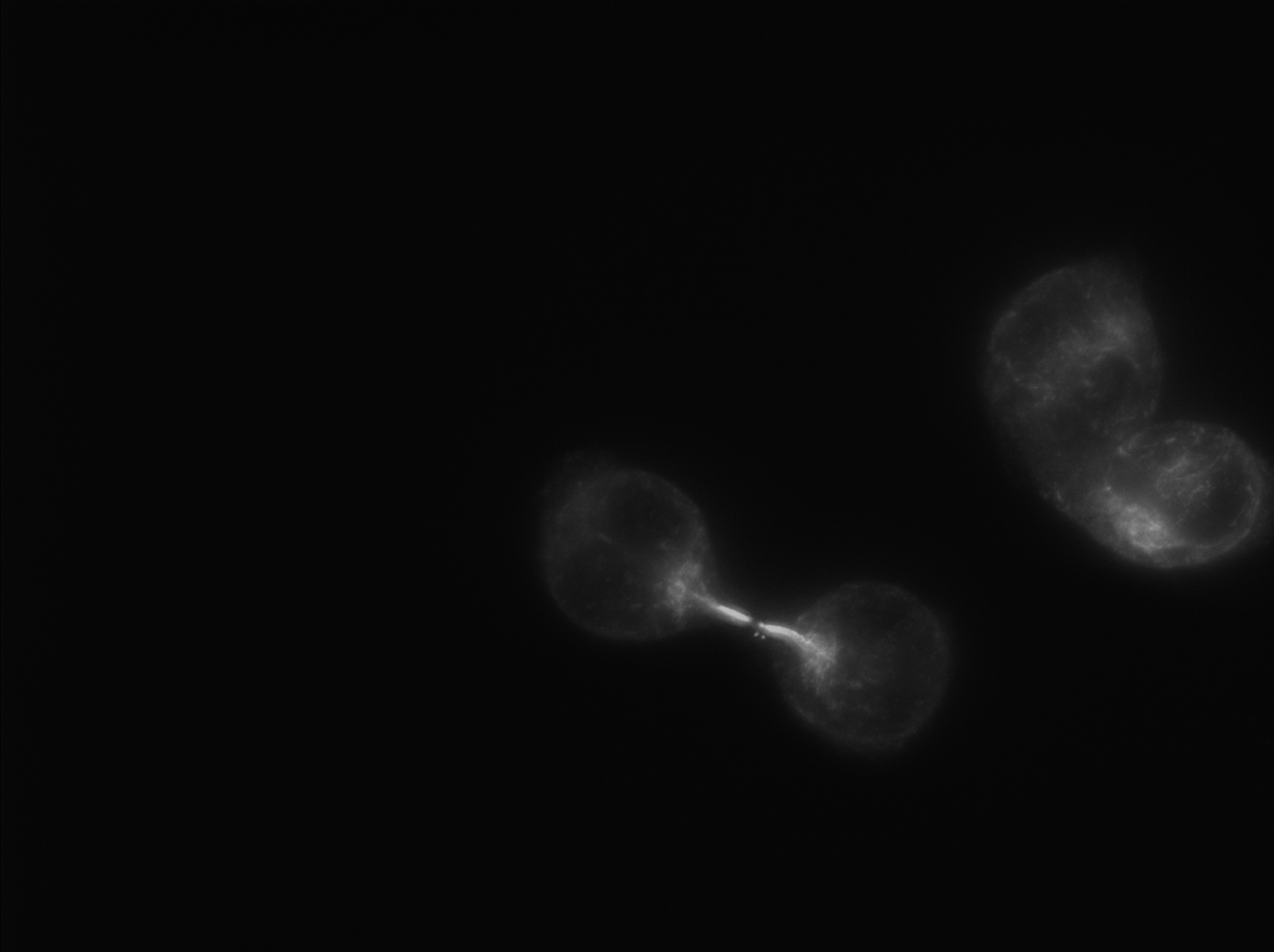

Supplement: Supplementary file 28 — Source data Fig. 7 part 4 [file 44319_2026_742_MOESM28_ESM.zip › Figure 7 Part 4/Fig 7fg Control and TPGS1-KO spastin acetylated tubulin/TPGS1-KO spastin actub 4-1-25 R1 SI21.Project Maximum Z_XY1743539120_Z0_T0_C2.tif]

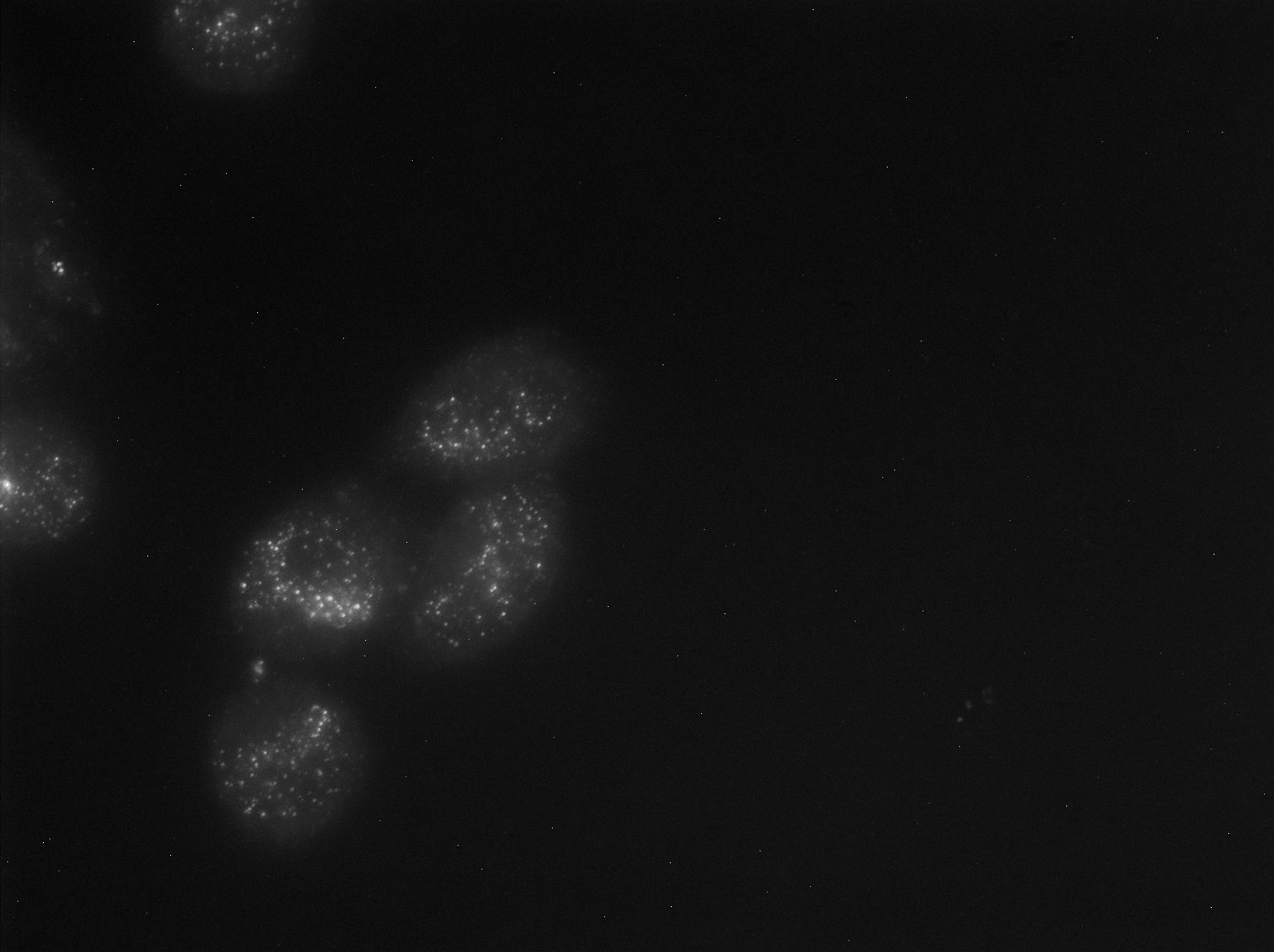

Supplement: Supplementary file 28 — Source data Fig. 7 part 4 [file 44319_2026_742_MOESM28_ESM.zip › Figure 7 Part 4/Fig 7fg Control and TPGS1-KO spastin acetylated tubulin/TPGS1-KO spastin actub 4-1-25 R1 SI17.Project Maximum Z_XY1743538264_Z0_T0_C1.tif]

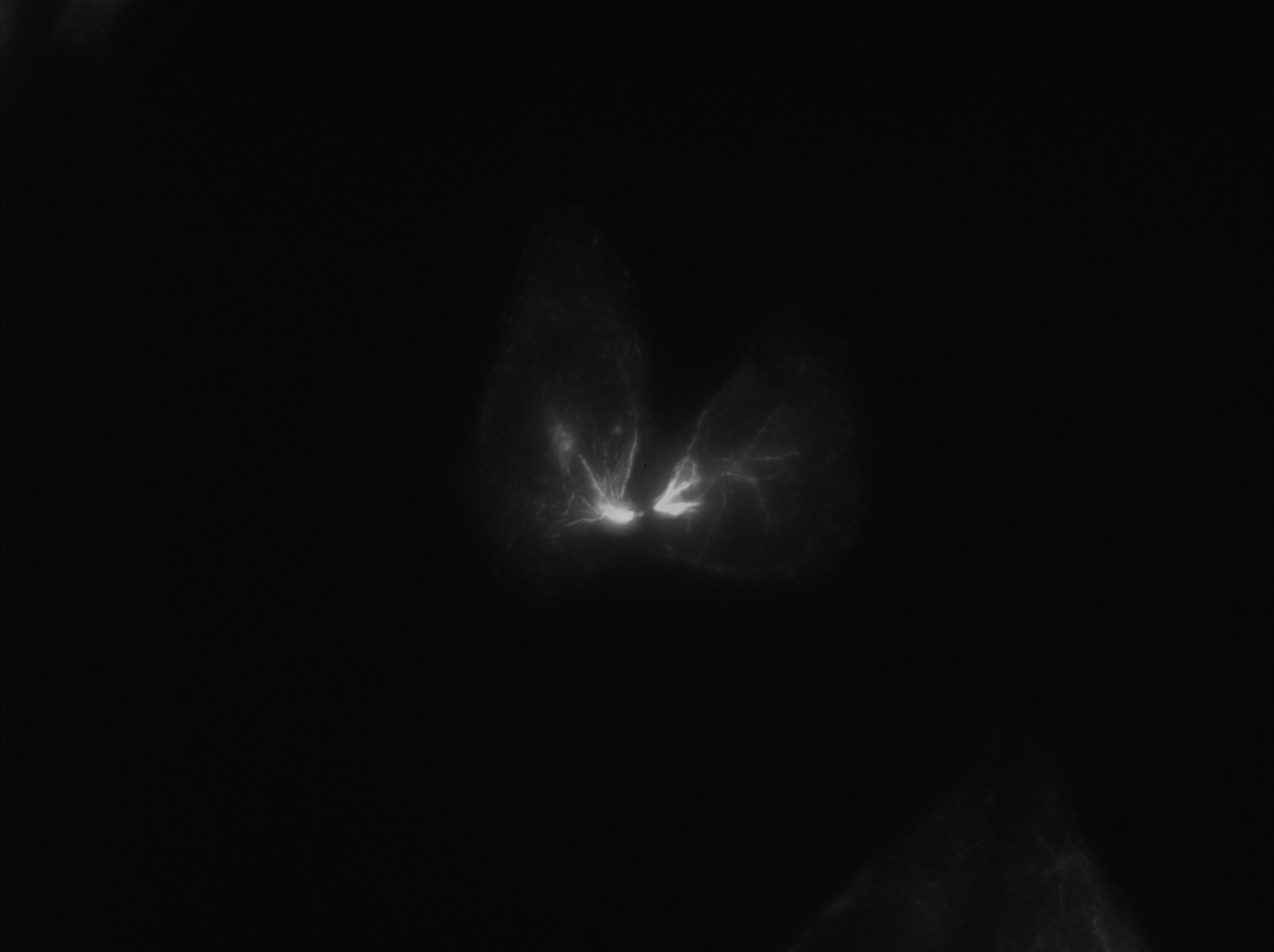

Supplement: Supplementary file 28 — Source data Fig. 7 part 4 [file 44319_2026_742_MOESM28_ESM.zip › Figure 7 Part 4/Fig 7fg Control and TPGS1-KO spastin acetylated tubulin/TPGS1-KO spastin actub 4-1-25 R1 SI6.Project Maximum Z_XY1743536775_Z0_T0_C2.tif]

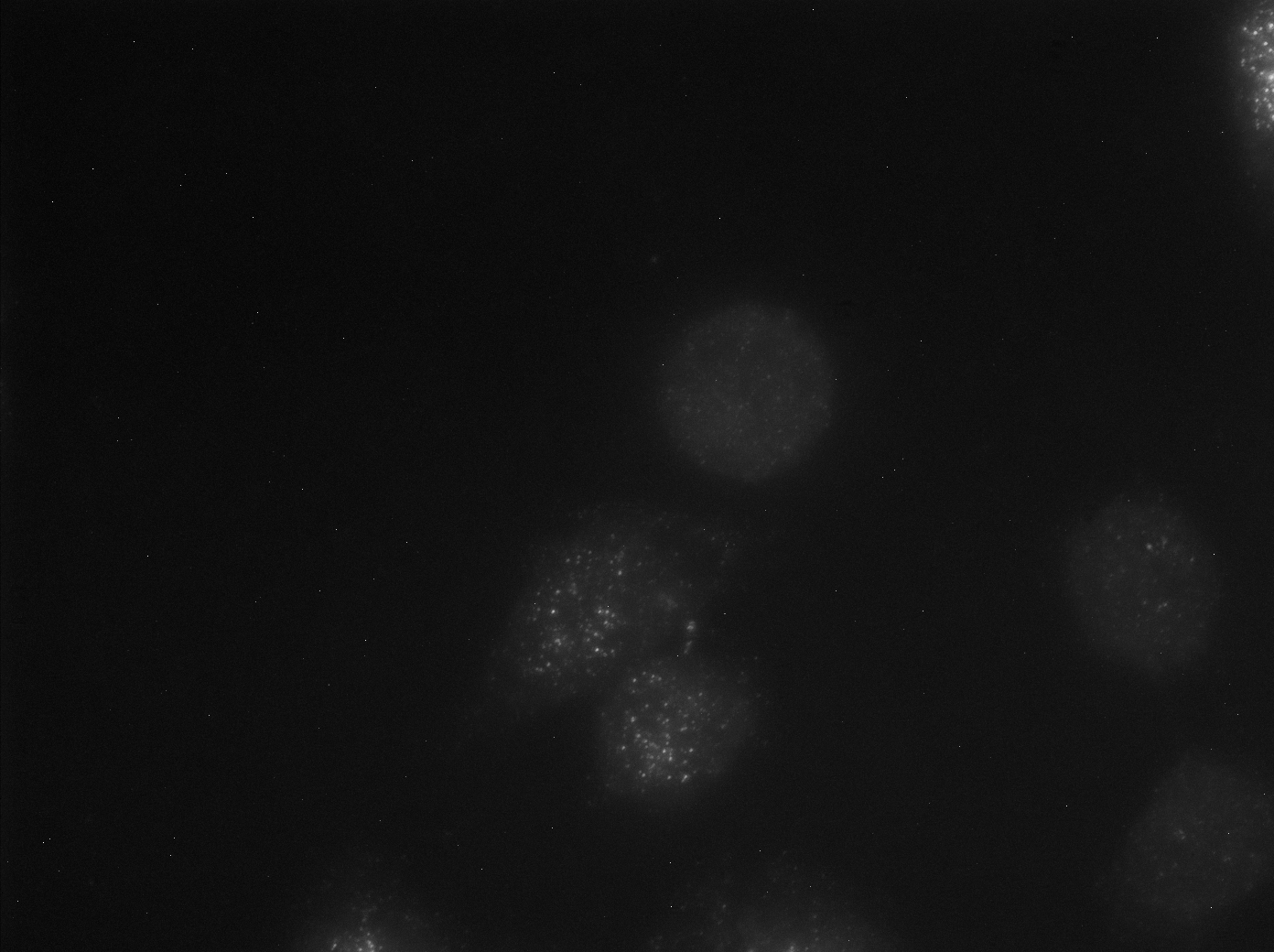

Supplement: Supplementary file 28 — Source data Fig. 7 part 4 [file 44319_2026_742_MOESM28_ESM.zip › Figure 7 Part 4/Fig 7fg Control and TPGS1-KO spastin acetylated tubulin/TPGS1-KO spastin actub 4-1-25 R1 SI20.Project Maximum Z_XY1743538950_Z0_T0_C1.tif]

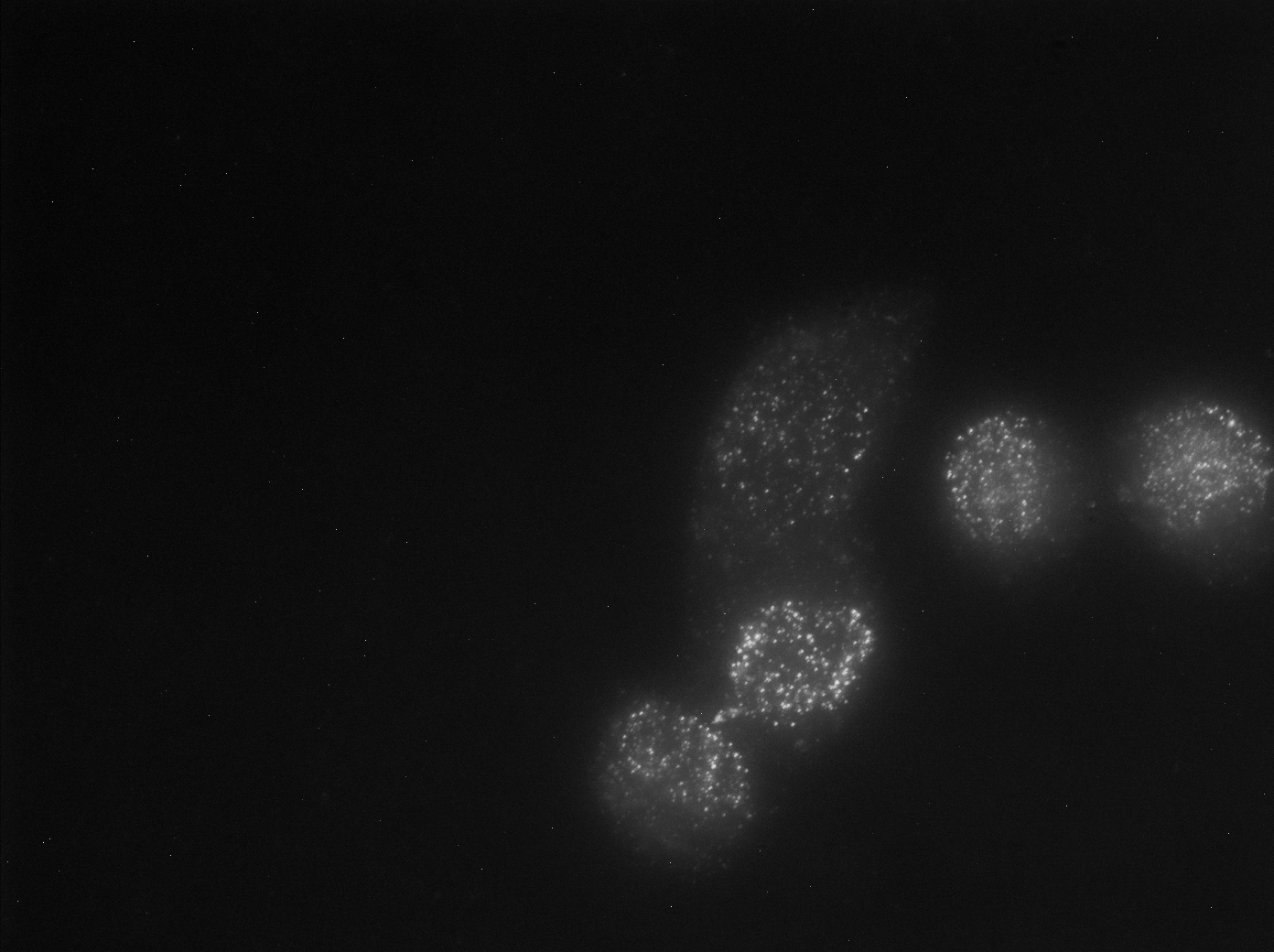

Supplement: Supplementary file 28 — Source data Fig. 7 part 4 [file 44319_2026_742_MOESM28_ESM.zip › Figure 7 Part 4/Fig 7fg Control and TPGS1-KO spastin acetylated tubulin/Cas9 spastin actub 4-1-25 R1 SI2.Project Maximum Z_XY1743530357_Z0_T0_C1.tif]

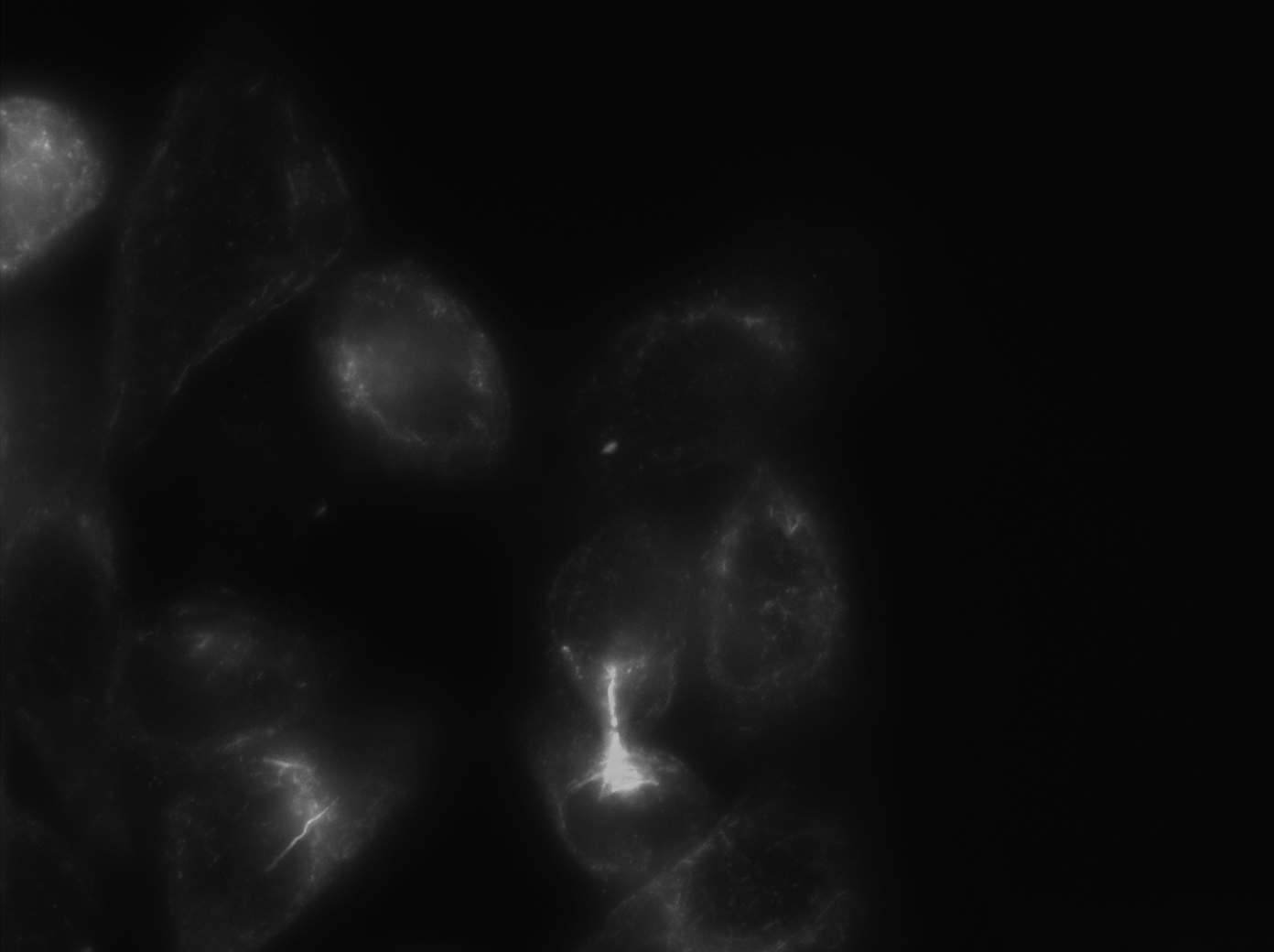

Supplement: Supplementary file 28 — Source data Fig. 7 part 4 [file 44319_2026_742_MOESM28_ESM.zip › Figure 7 Part 4/Fig 7fg Control and TPGS1-KO spastin acetylated tubulin/Cas9 spastin actub 4-1-25 R1 SI20.Project Maximum Z_XY1743534929_Z0_T0_C2.tif]

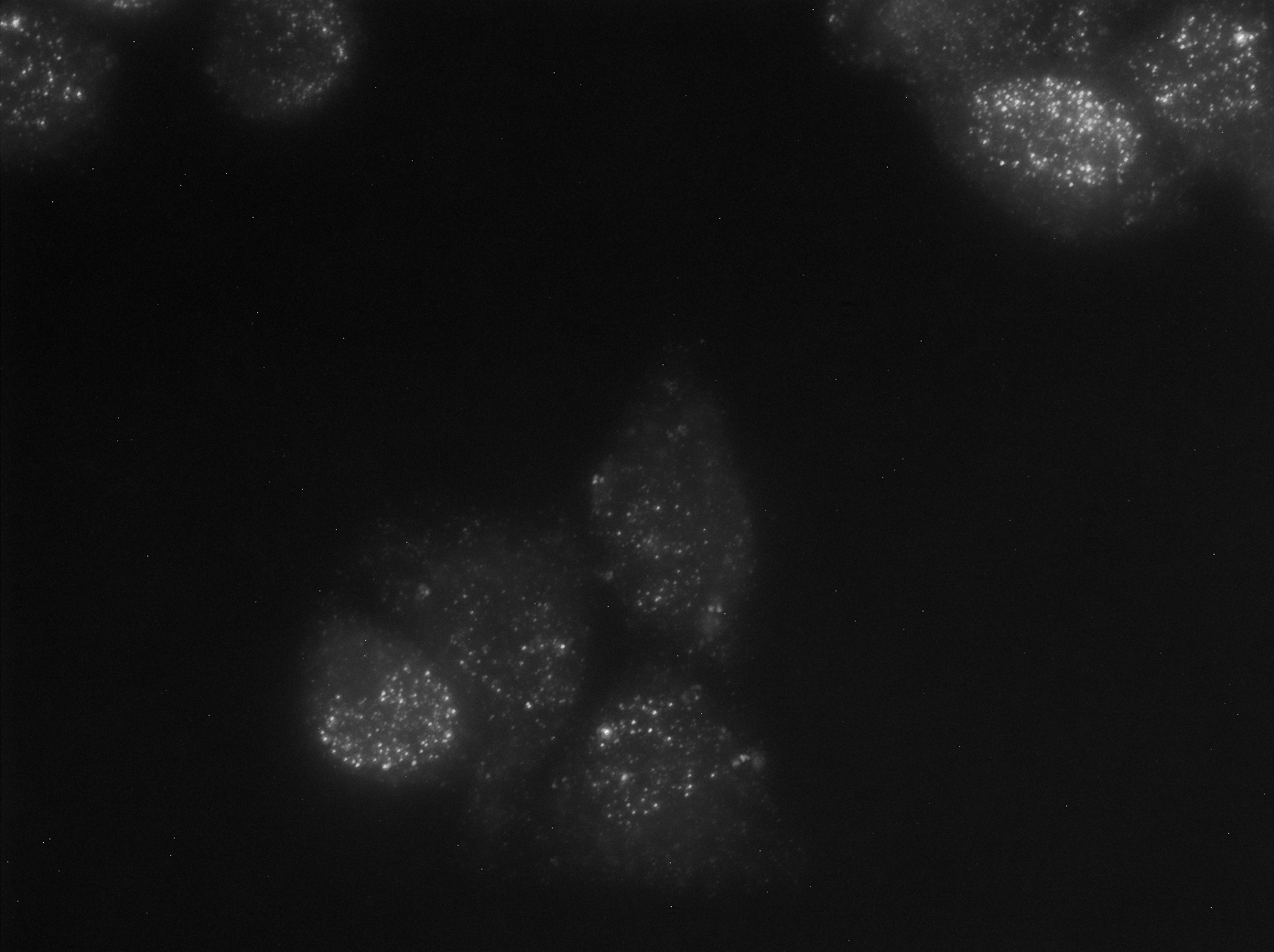

Supplement: Supplementary file 28 — Source data Fig. 7 part 4 [file 44319_2026_742_MOESM28_ESM.zip › Figure 7 Part 4/Fig 7fg Control and TPGS1-KO spastin acetylated tubulin/Cas9 spastin actub 4-1-25 R1 SI10.Project Maximum Z_XY1743531484_Z0_T0_C1.tif]

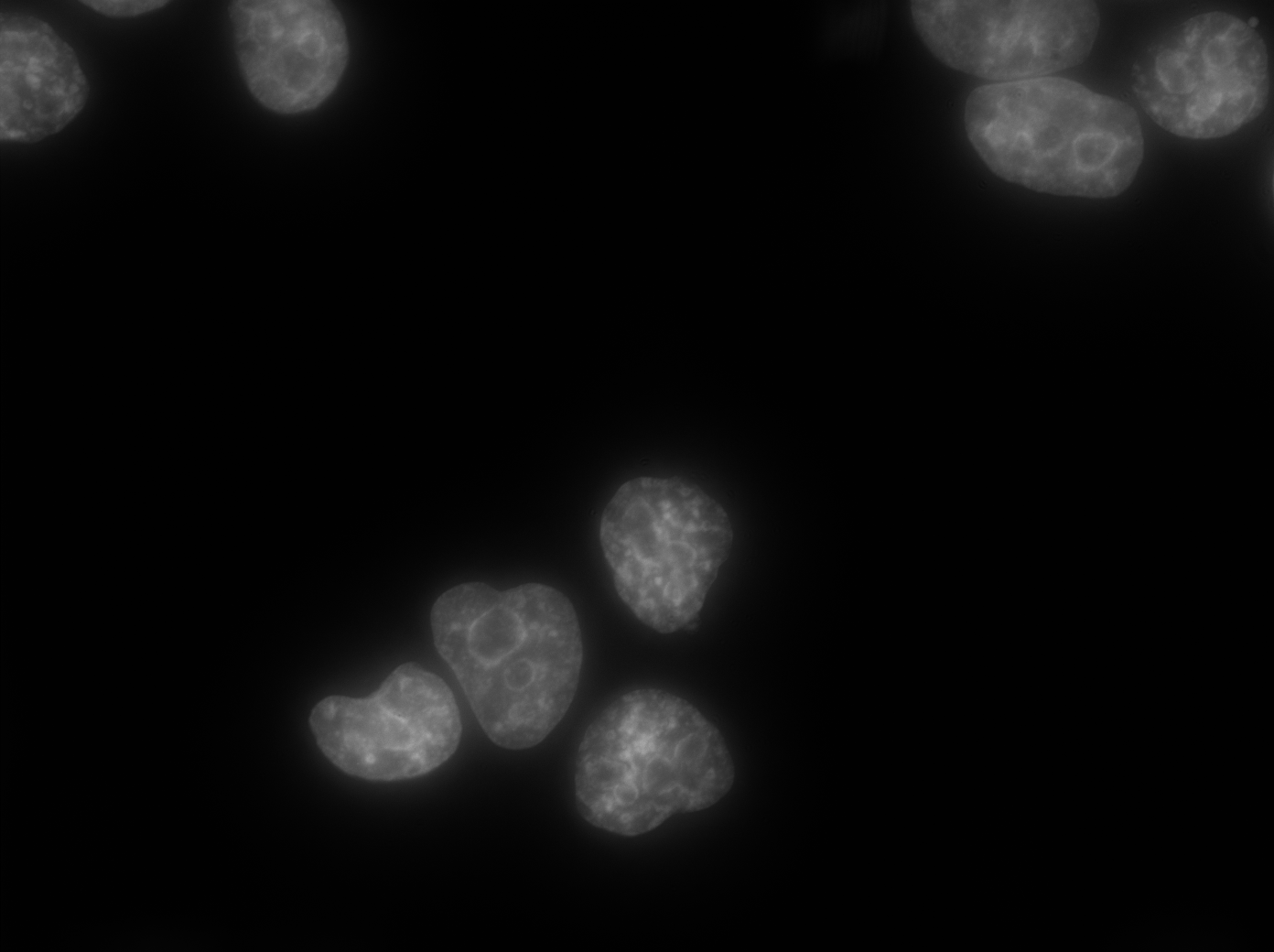

Supplement: Supplementary file 28 — Source data Fig. 7 part 4 [file 44319_2026_742_MOESM28_ESM.zip › Figure 7 Part 4/Fig 7fg Control and TPGS1-KO spastin acetylated tubulin/Cas9 spastin actub 4-1-25 R1 SI10.Project Maximum Z_XY1743531484_Z0_T0_C0.tif]

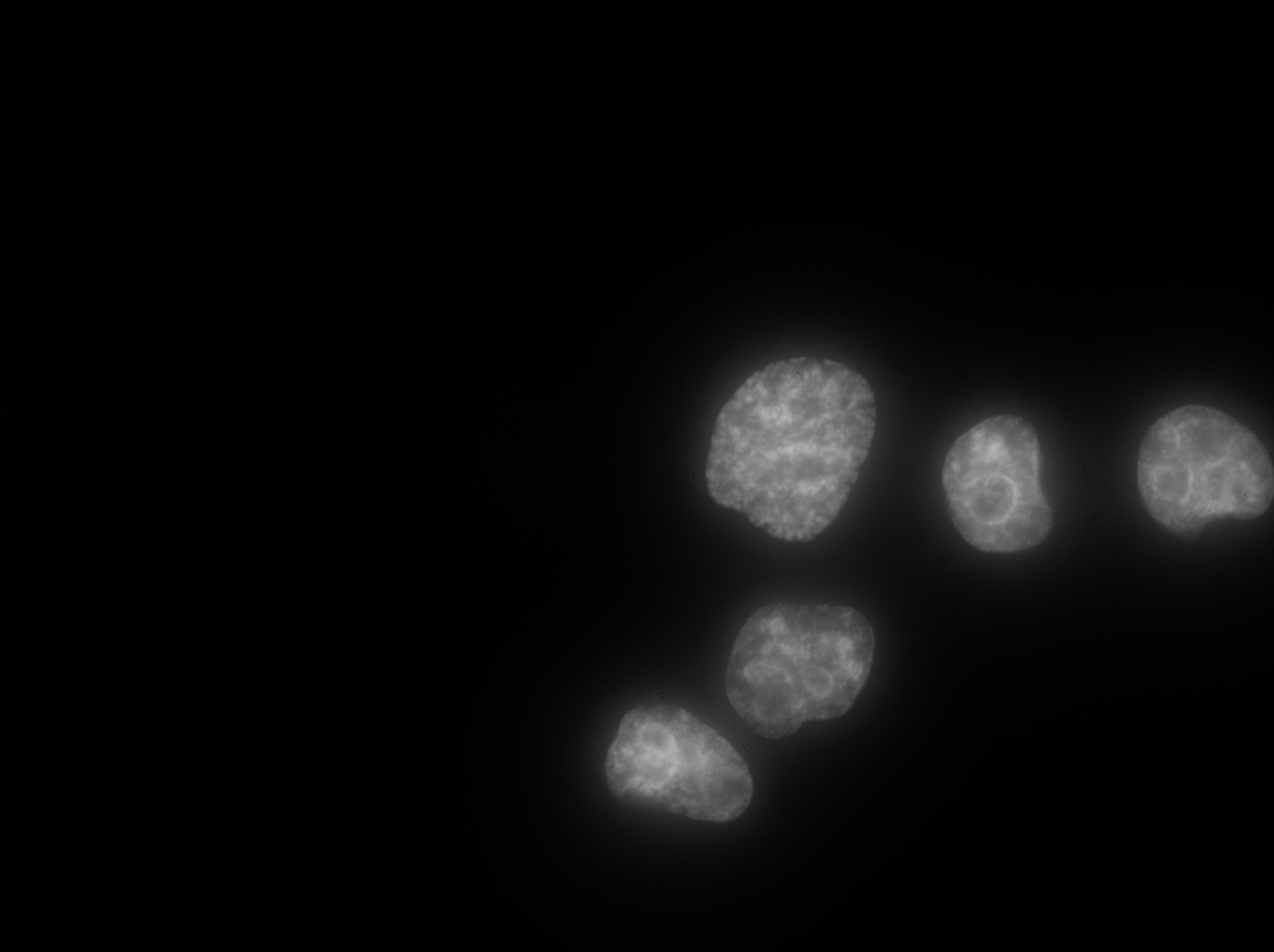

Supplement: Supplementary file 28 — Source data Fig. 7 part 4 [file 44319_2026_742_MOESM28_ESM.zip › Figure 7 Part 4/Fig 7fg Control and TPGS1-KO spastin acetylated tubulin/Cas9 spastin actub 4-1-25 R1 SI2.Project Maximum Z_XY1743530357_Z0_T0_C0.tif]

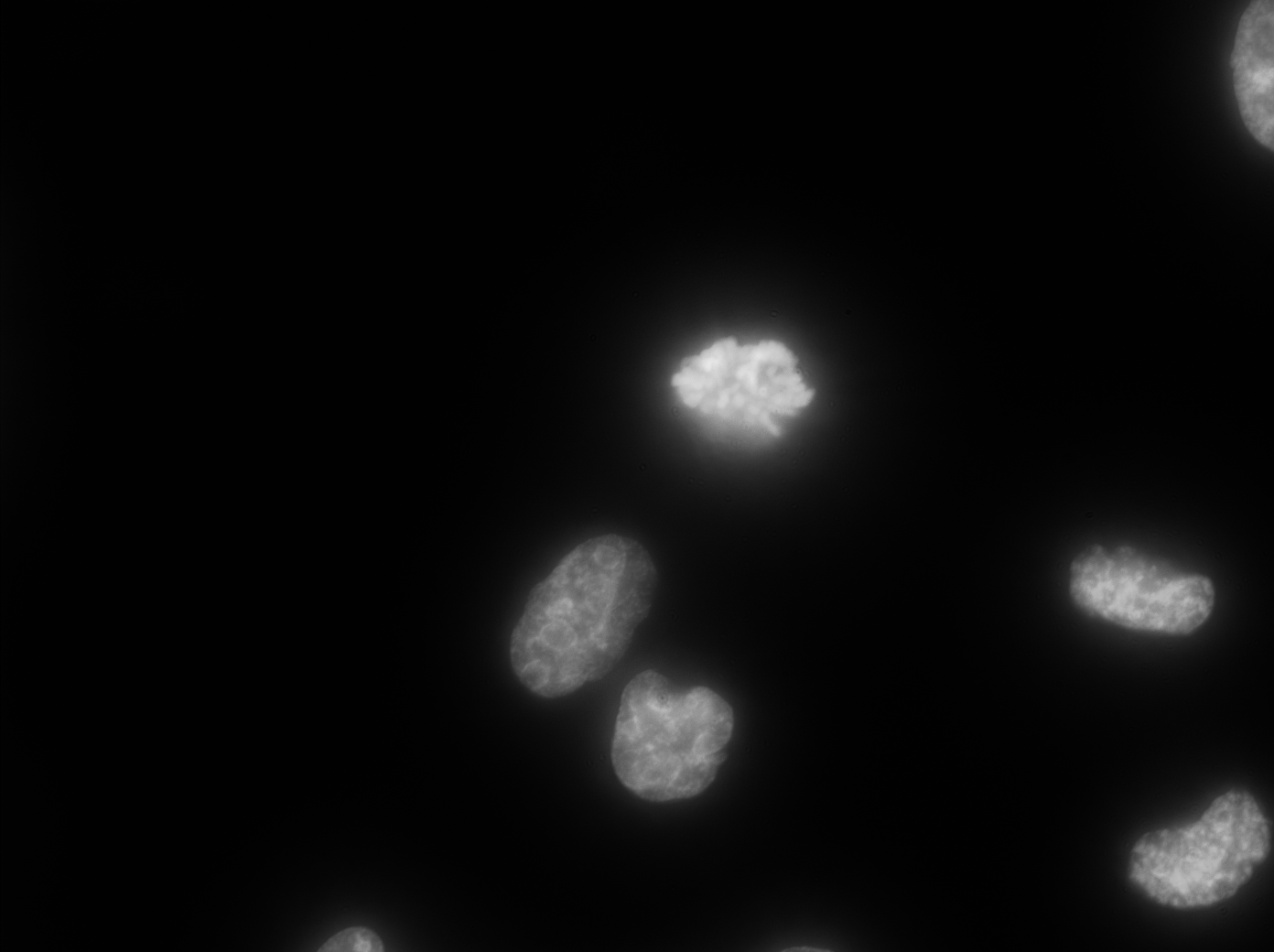

Supplement: Supplementary file 28 — Source data Fig. 7 part 4 [file 44319_2026_742_MOESM28_ESM.zip › Figure 7 Part 4/Fig 7fg Control and TPGS1-KO spastin acetylated tubulin/TPGS1-KO spastin actub 4-1-25 R1 SI20.Project Maximum Z_XY1743538950_Z0_T0_C0.tif]

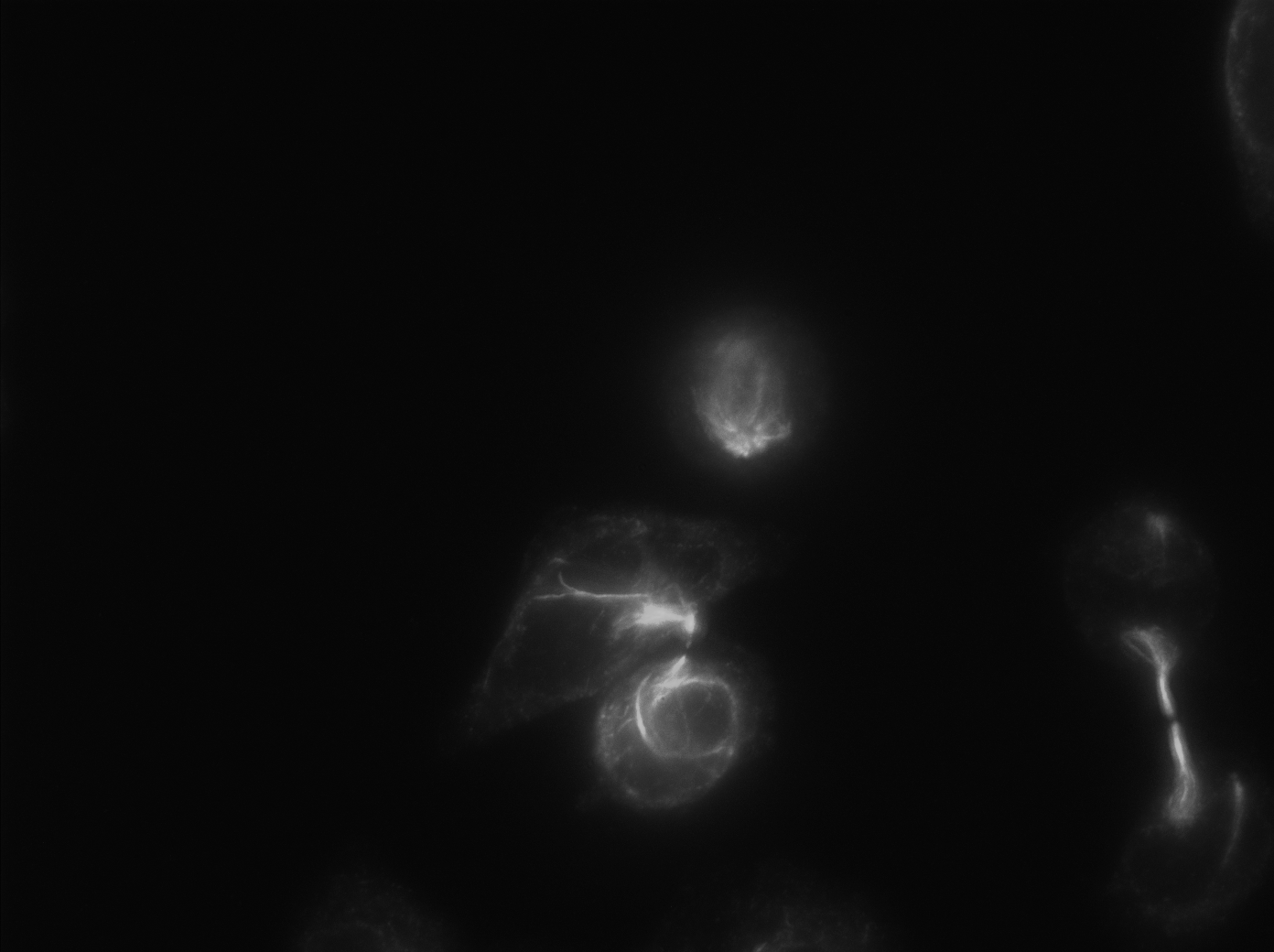

Supplement: Supplementary file 28 — Source data Fig. 7 part 4 [file 44319_2026_742_MOESM28_ESM.zip › Figure 7 Part 4/Fig 7fg Control and TPGS1-KO spastin acetylated tubulin/TPGS1-KO spastin actub 4-1-25 R1 SI20.Project Maximum Z_XY1743538950_Z0_T0_C2.tif]

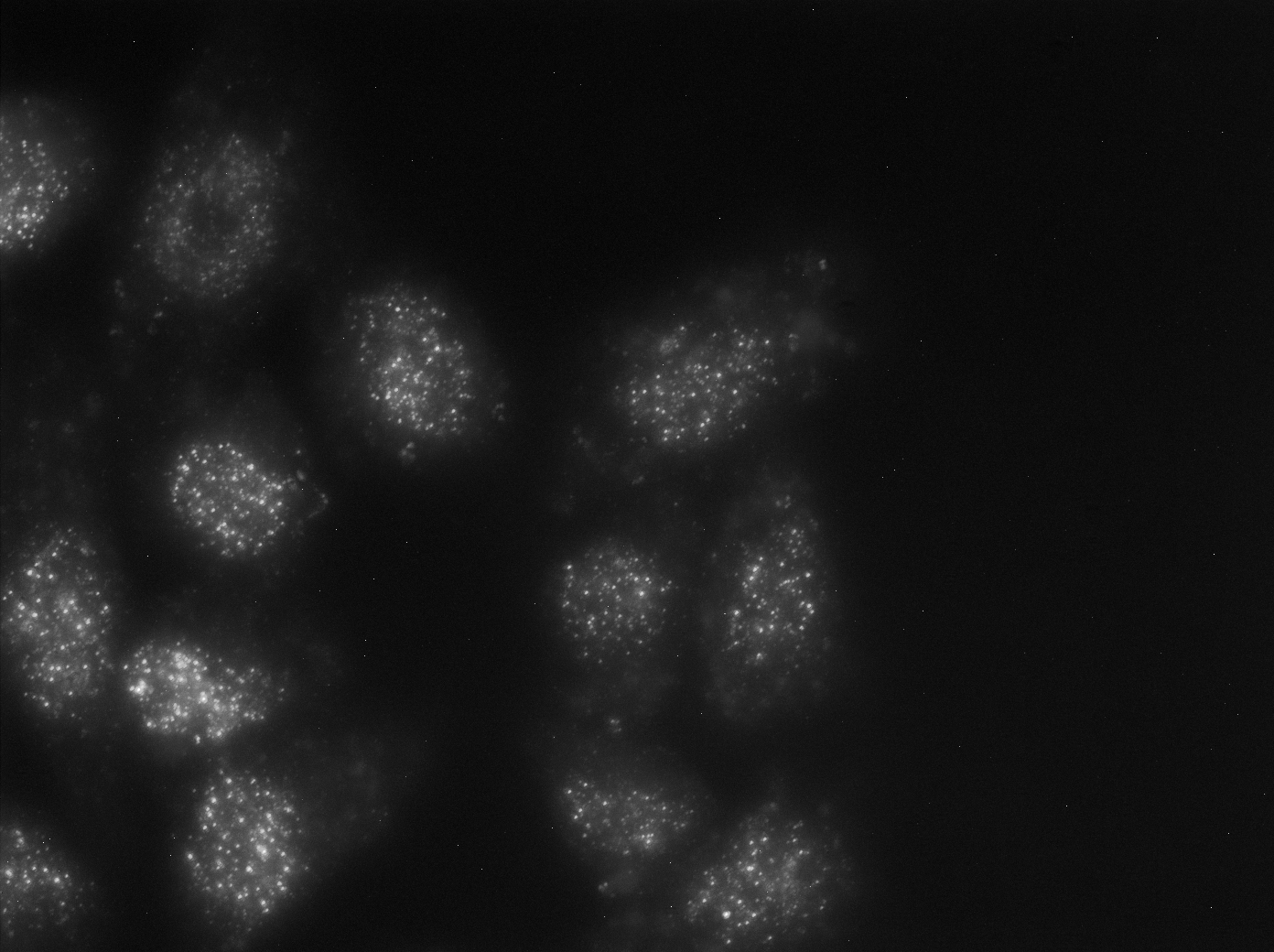

Supplement: Supplementary file 28 — Source data Fig. 7 part 4 [file 44319_2026_742_MOESM28_ESM.zip › Figure 7 Part 4/Fig 7fg Control and TPGS1-KO spastin acetylated tubulin/Cas9 spastin actub 4-1-25 R1 SI20.Project Maximum Z_XY1743534929_Z0_T0_C1.tif]

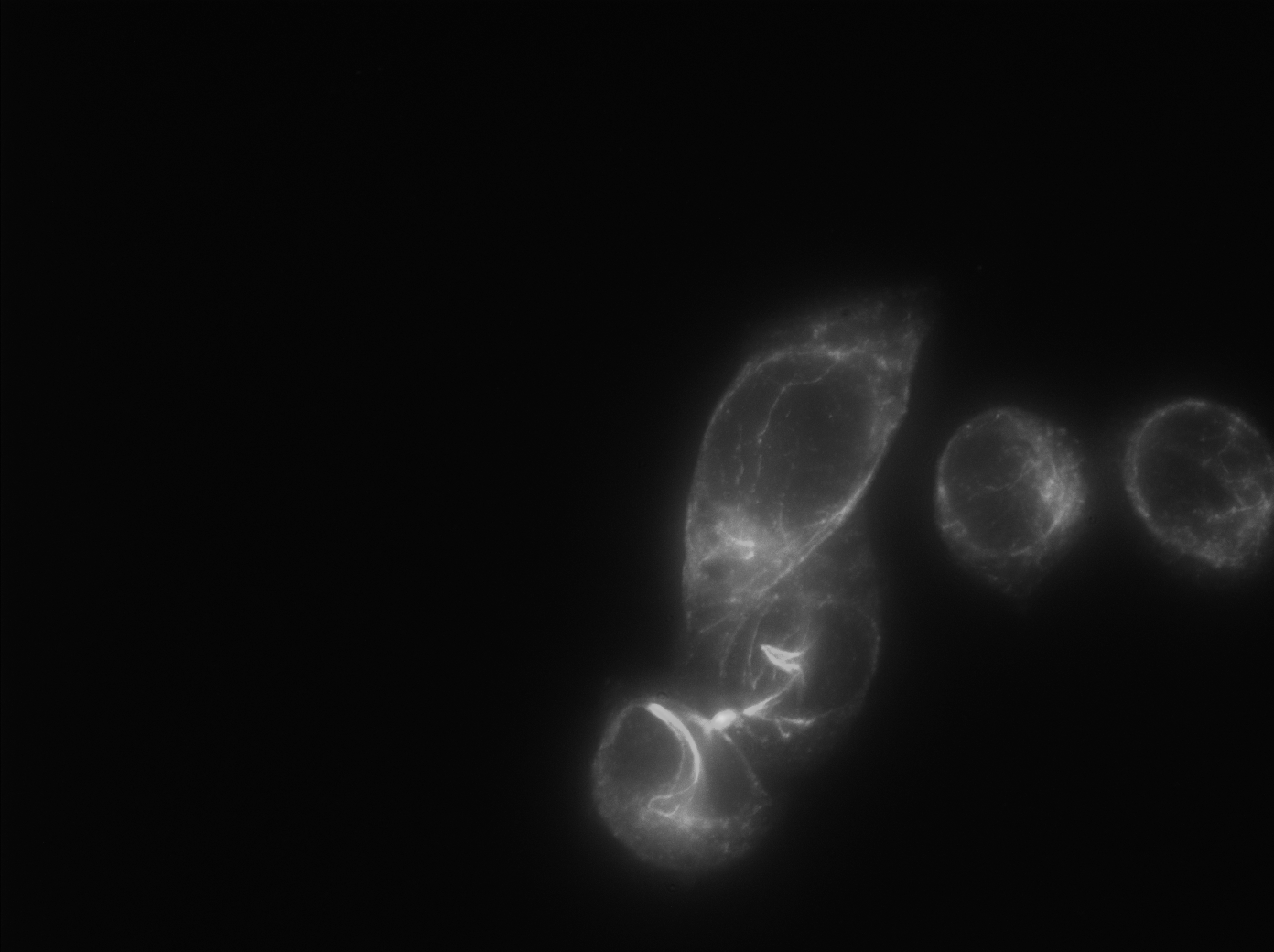

Supplement: Supplementary file 28 — Source data Fig. 7 part 4 [file 44319_2026_742_MOESM28_ESM.zip › Figure 7 Part 4/Fig 7fg Control and TPGS1-KO spastin acetylated tubulin/Cas9 spastin actub 4-1-25 R1 SI2.Project Maximum Z_XY1743530357_Z0_T0_C2.tif]

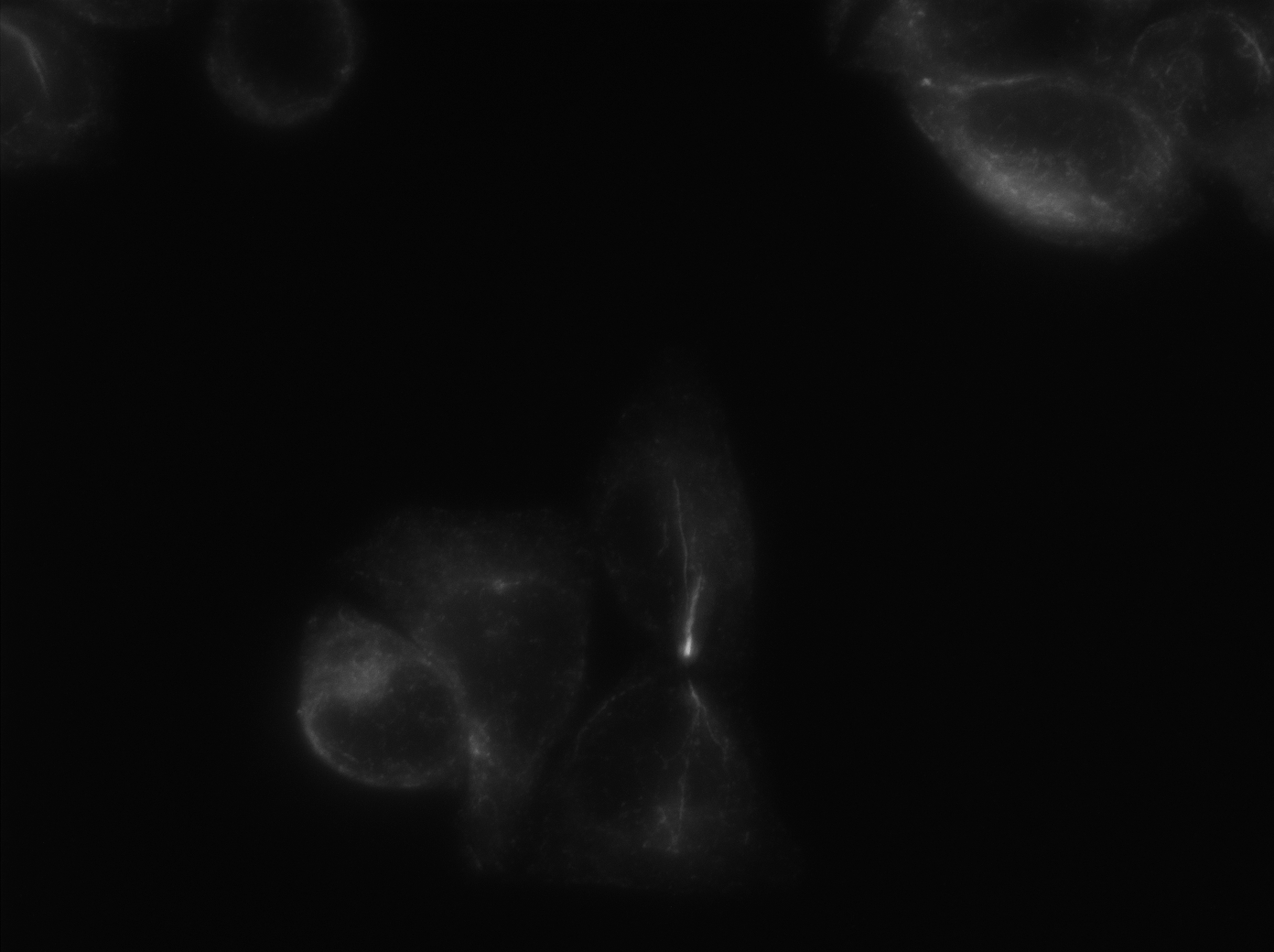

Supplement: Supplementary file 28 — Source data Fig. 7 part 4 [file 44319_2026_742_MOESM28_ESM.zip › Figure 7 Part 4/Fig 7fg Control and TPGS1-KO spastin acetylated tubulin/Cas9 spastin actub 4-1-25 R1 SI10.Project Maximum Z_XY1743531484_Z0_T0_C2.tif]

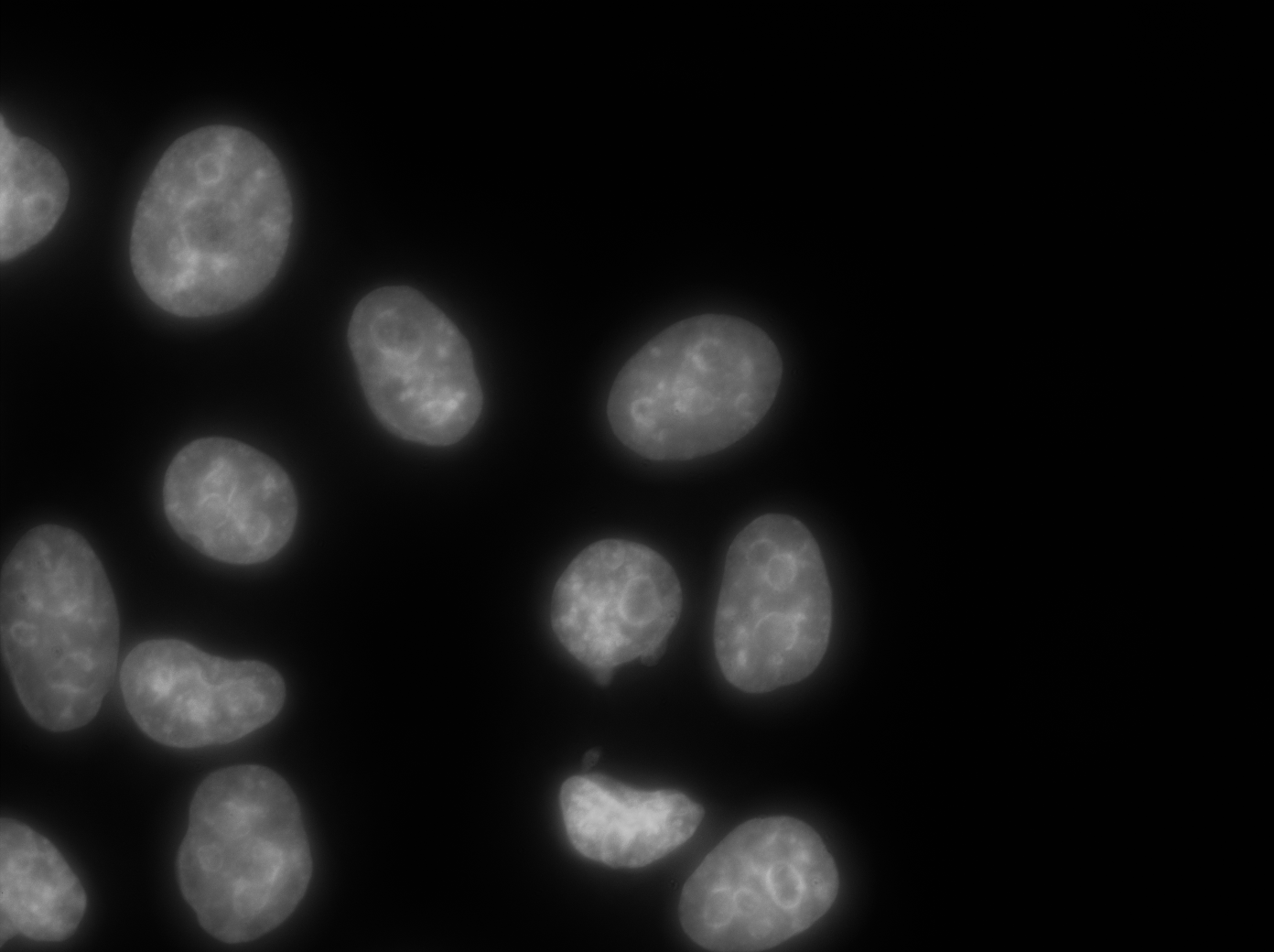

Supplement: Supplementary file 28 — Source data Fig. 7 part 4 [file 44319_2026_742_MOESM28_ESM.zip › Figure 7 Part 4/Fig 7fg Control and TPGS1-KO spastin acetylated tubulin/Cas9 spastin actub 4-1-25 R1 SI20.Project Maximum Z_XY1743534929_Z0_T0_C0.tif]

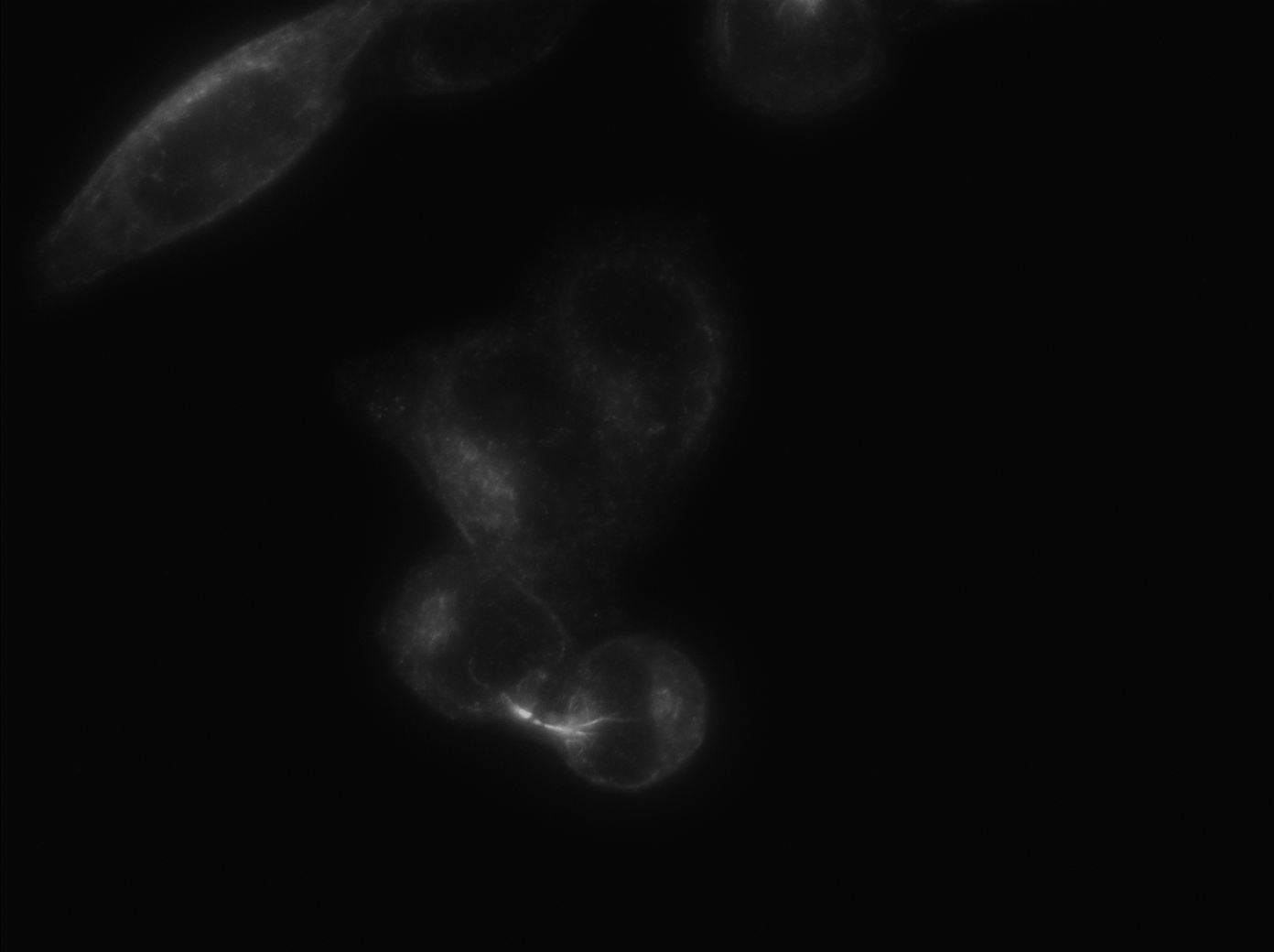

Supplement: Supplementary file 28 — Source data Fig. 7 part 4 [file 44319_2026_742_MOESM28_ESM.zip › Figure 7 Part 4/Fig 7fg Control and TPGS1-KO spastin acetylated tubulin/Cas9 spastin actub 4-1-25 R1 SI25.Project Maximum Z_XY1743535724_Z0_T0_C2.tif]

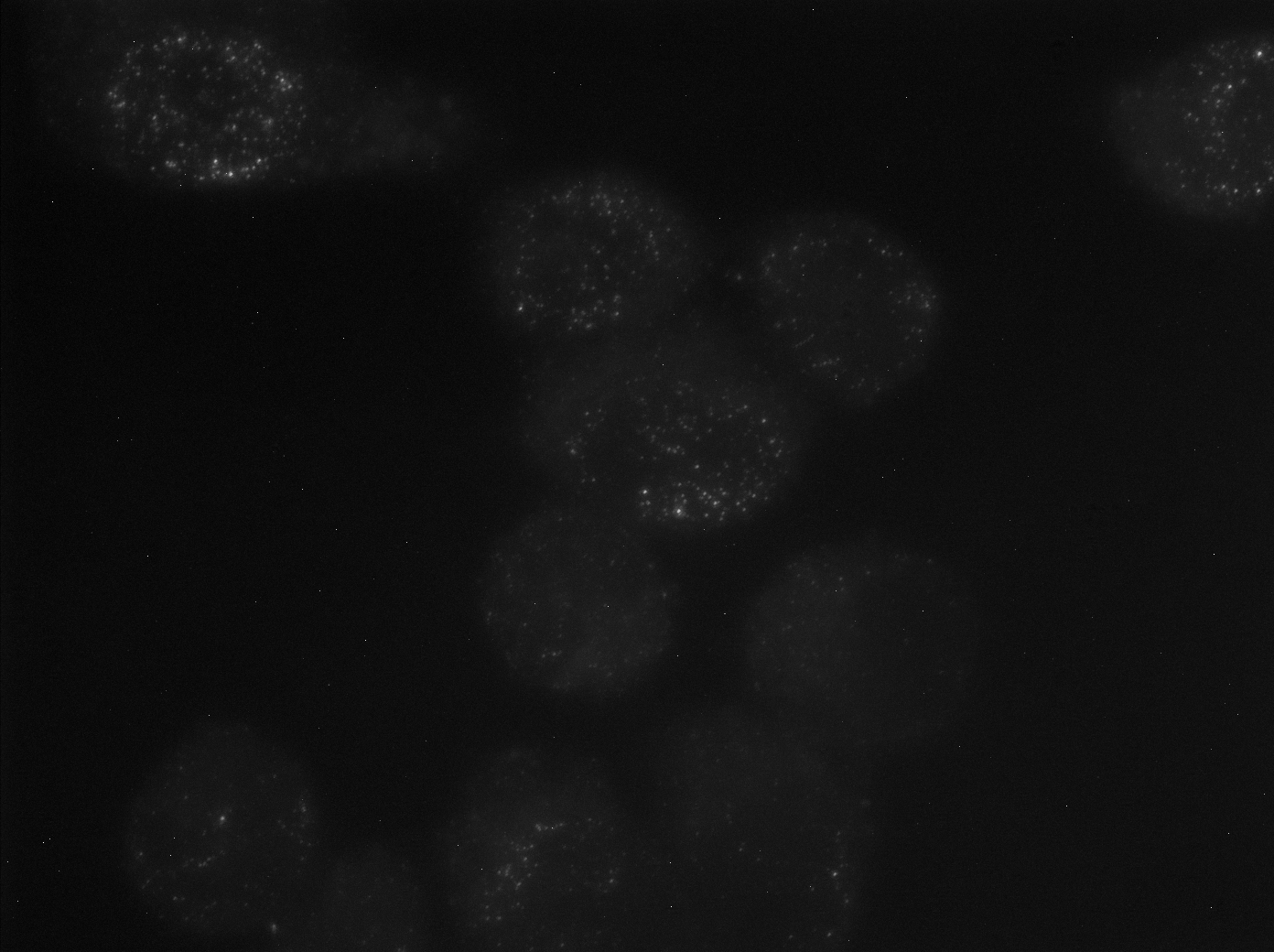

Supplement: Supplementary file 28 — Source data Fig. 7 part 4 [file 44319_2026_742_MOESM28_ESM.zip › Figure 7 Part 4/Fig 7fg Control and TPGS1-KO spastin acetylated tubulin/TPGS1-KO spastin actub 4-1-25 R1 SI14SI15.Project Maximum Z_XY1743537910_Z0_T0_C1.tif]

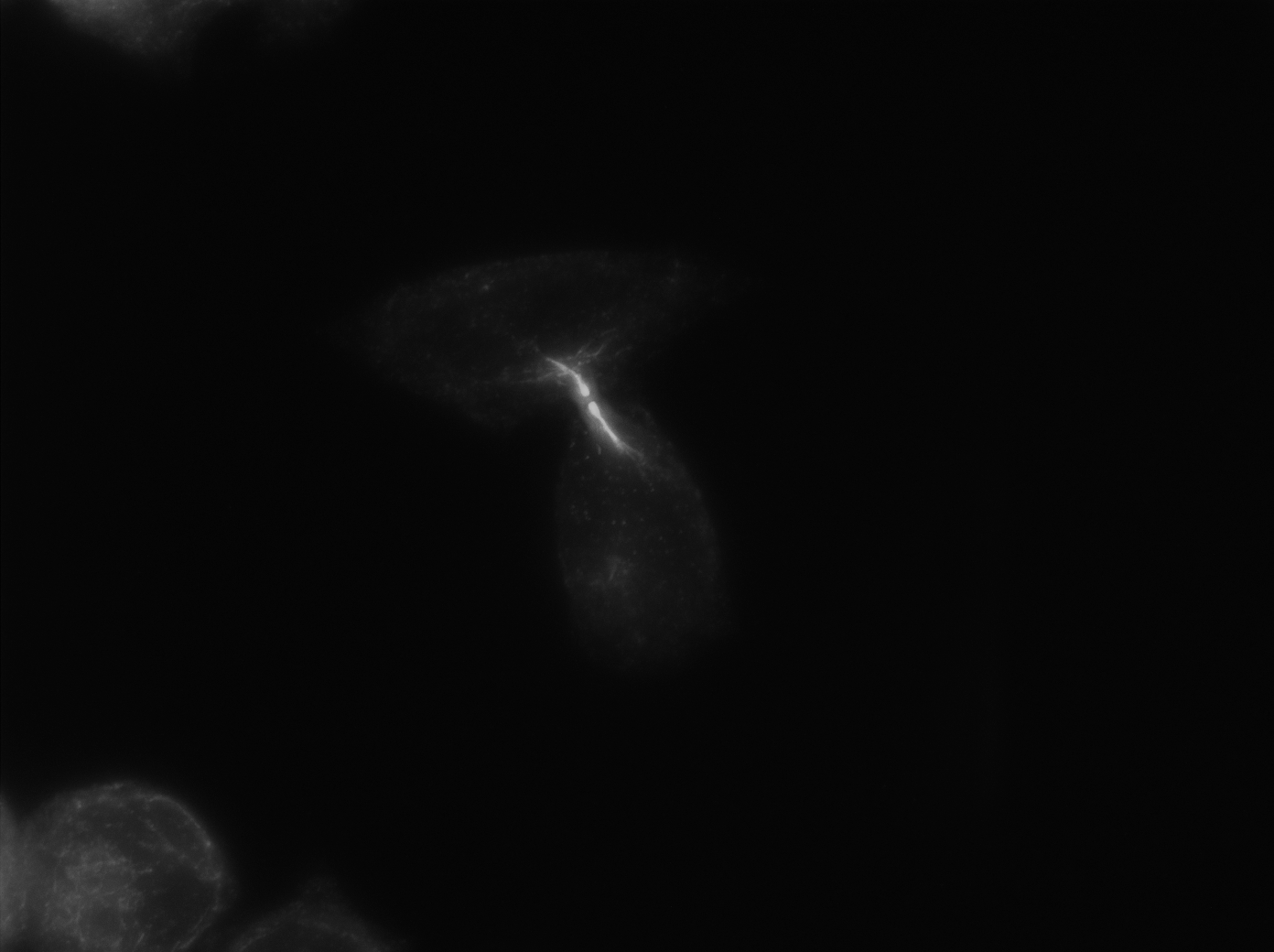

Supplement: Supplementary file 28 — Source data Fig. 7 part 4 [file 44319_2026_742_MOESM28_ESM.zip › Figure 7 Part 4/Fig 7fg Control and TPGS1-KO spastin acetylated tubulin/Cas9 spastin actub 4-1-25 R1 SI16.Project Maximum Z_XY1743534311_Z0_T0_C2.tif]

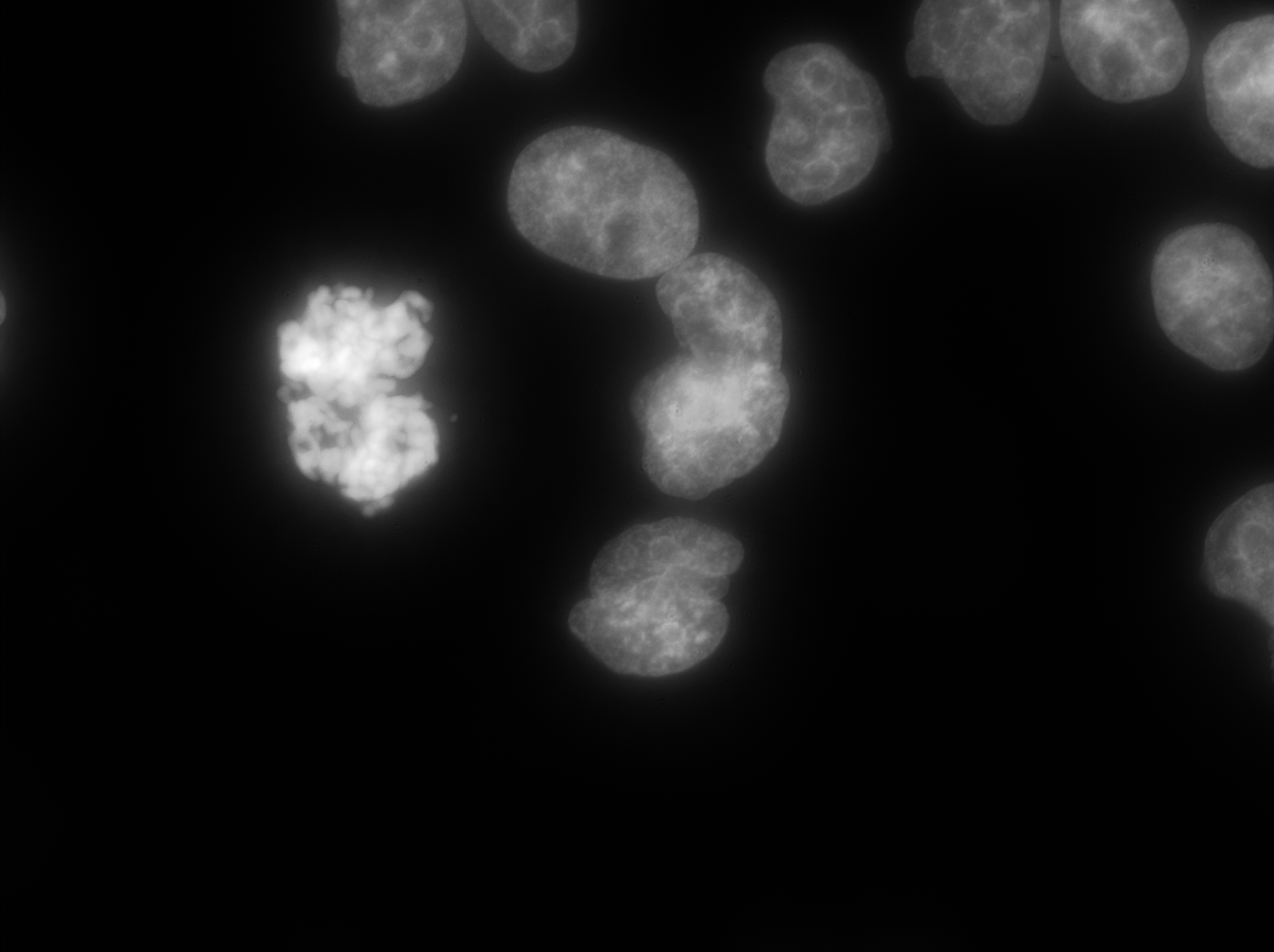

Supplement: Supplementary file 28 — Source data Fig. 7 part 4 [file 44319_2026_742_MOESM28_ESM.zip › Figure 7 Part 4/Fig 7fg Control and TPGS1-KO spastin acetylated tubulin/Cas9 spastin actub 4-1-25 R1 SI4.Project Maximum Z_XY1743530667_Z0_T0_C0.tif]

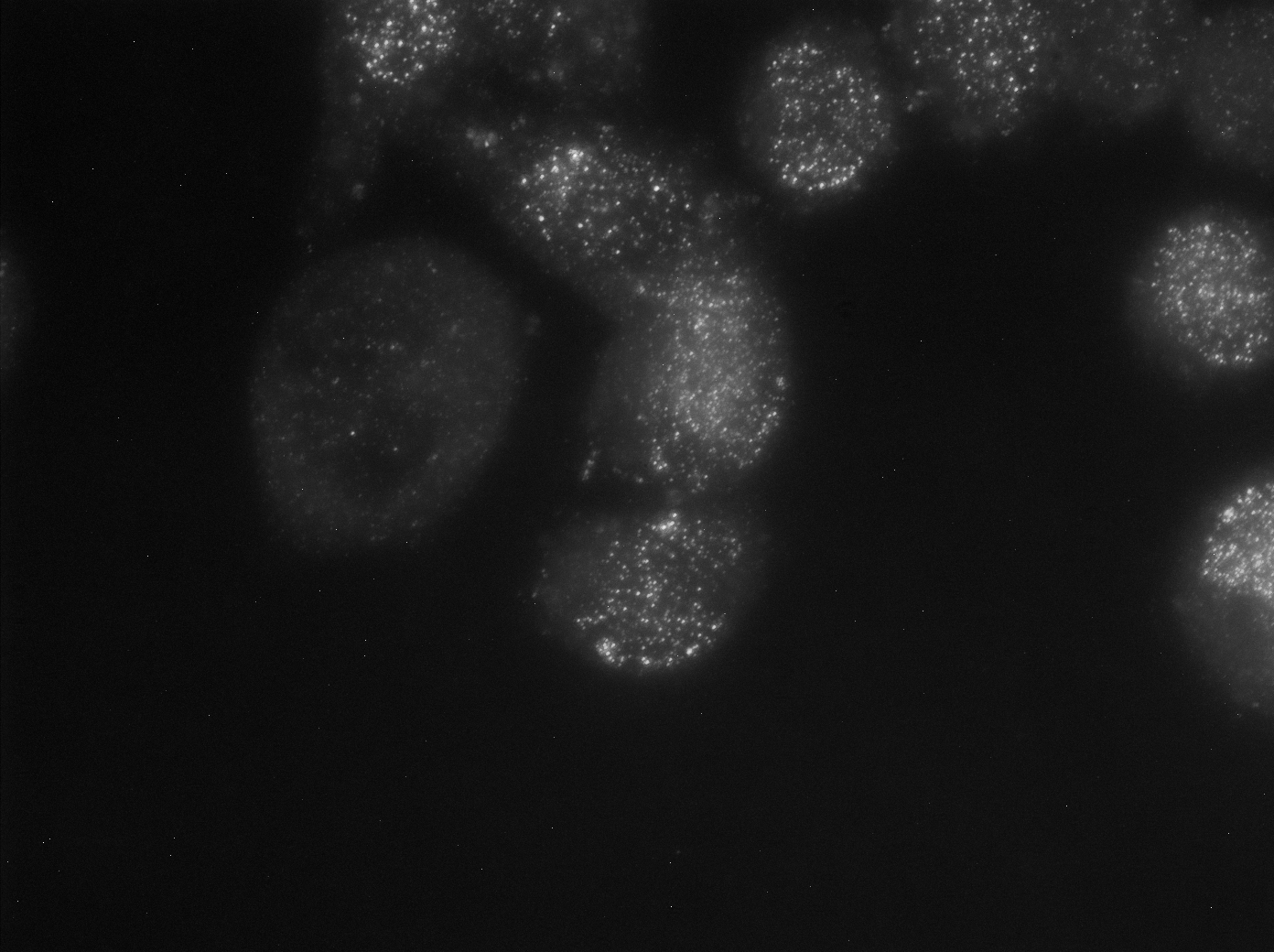

Supplement: Supplementary file 28 — Source data Fig. 7 part 4 [file 44319_2026_742_MOESM28_ESM.zip › Figure 7 Part 4/Fig 7fg Control and TPGS1-KO spastin acetylated tubulin/Cas9 spastin actub 4-1-25 R1 SI4.Project Maximum Z_XY1743530667_Z0_T0_C1.tif]

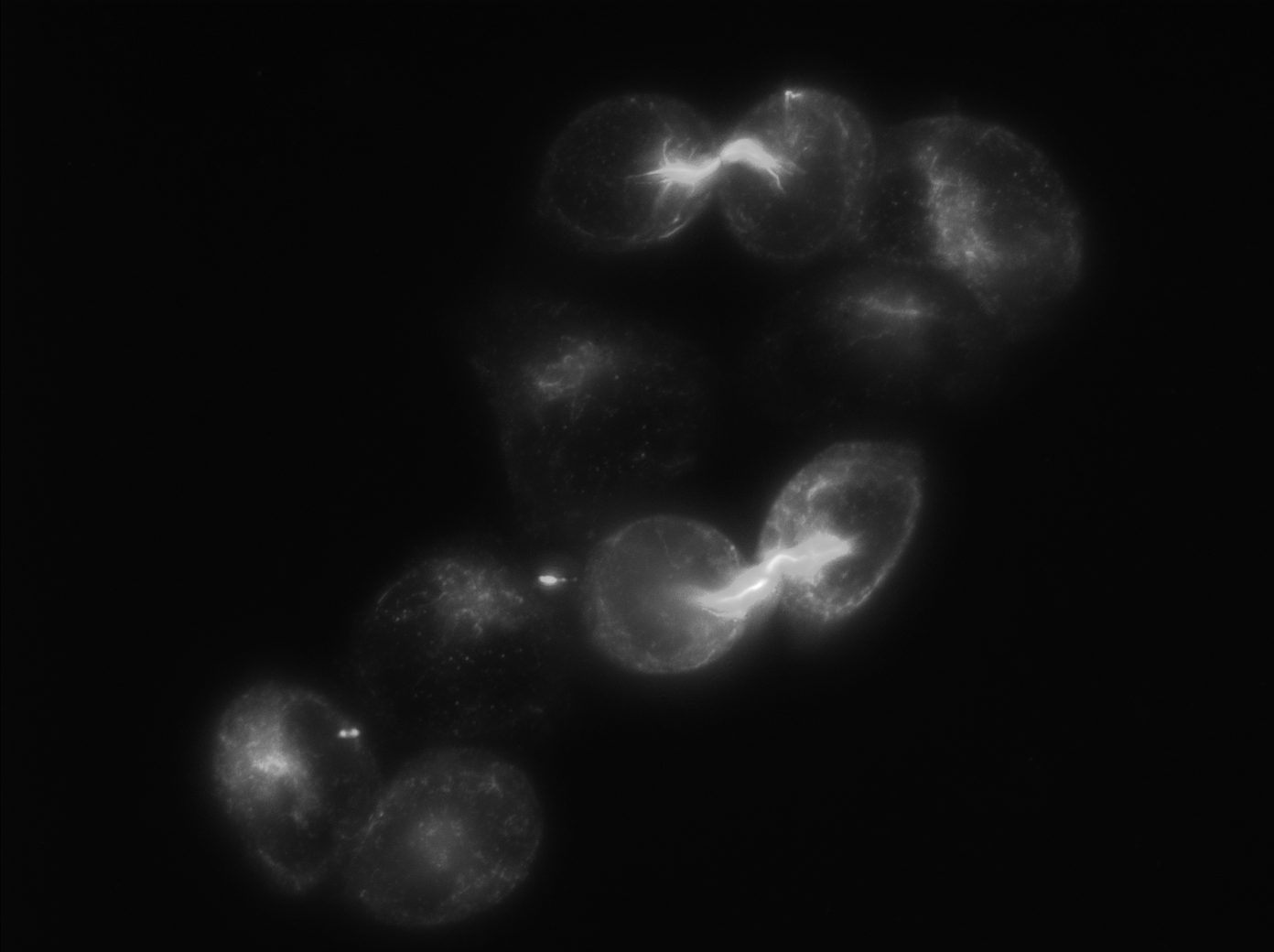

Supplement: Supplementary file 28 — Source data Fig. 7 part 4 [file 44319_2026_742_MOESM28_ESM.zip › Figure 7 Part 4/Fig 7fg Control and TPGS1-KO spastin acetylated tubulin/Cas9 spastin actub 4-1-25 R1 LT1LT2 PA1.Project Maximum Z_XY1743529647_Z0_T0_C2.tif]

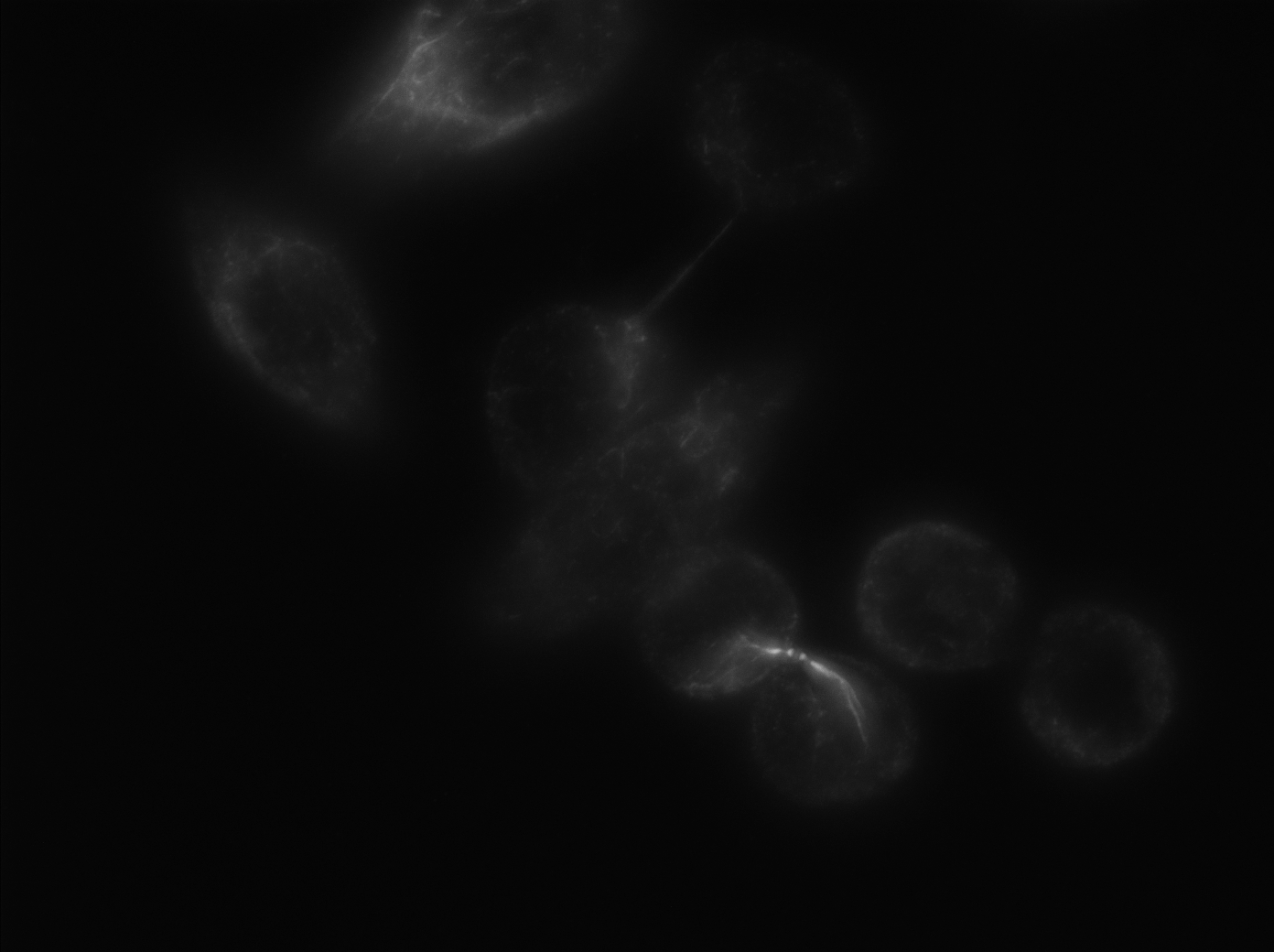

Supplement: Supplementary file 28 — Source data Fig. 7 part 4 [file 44319_2026_742_MOESM28_ESM.zip › Figure 7 Part 4/Fig 7fg Control and TPGS1-KO spastin acetylated tubulin/Cas9 spastin actub 4-1-25 R1 SI9.Project Maximum Z_XY1743531362_Z0_T0_C2.tif]

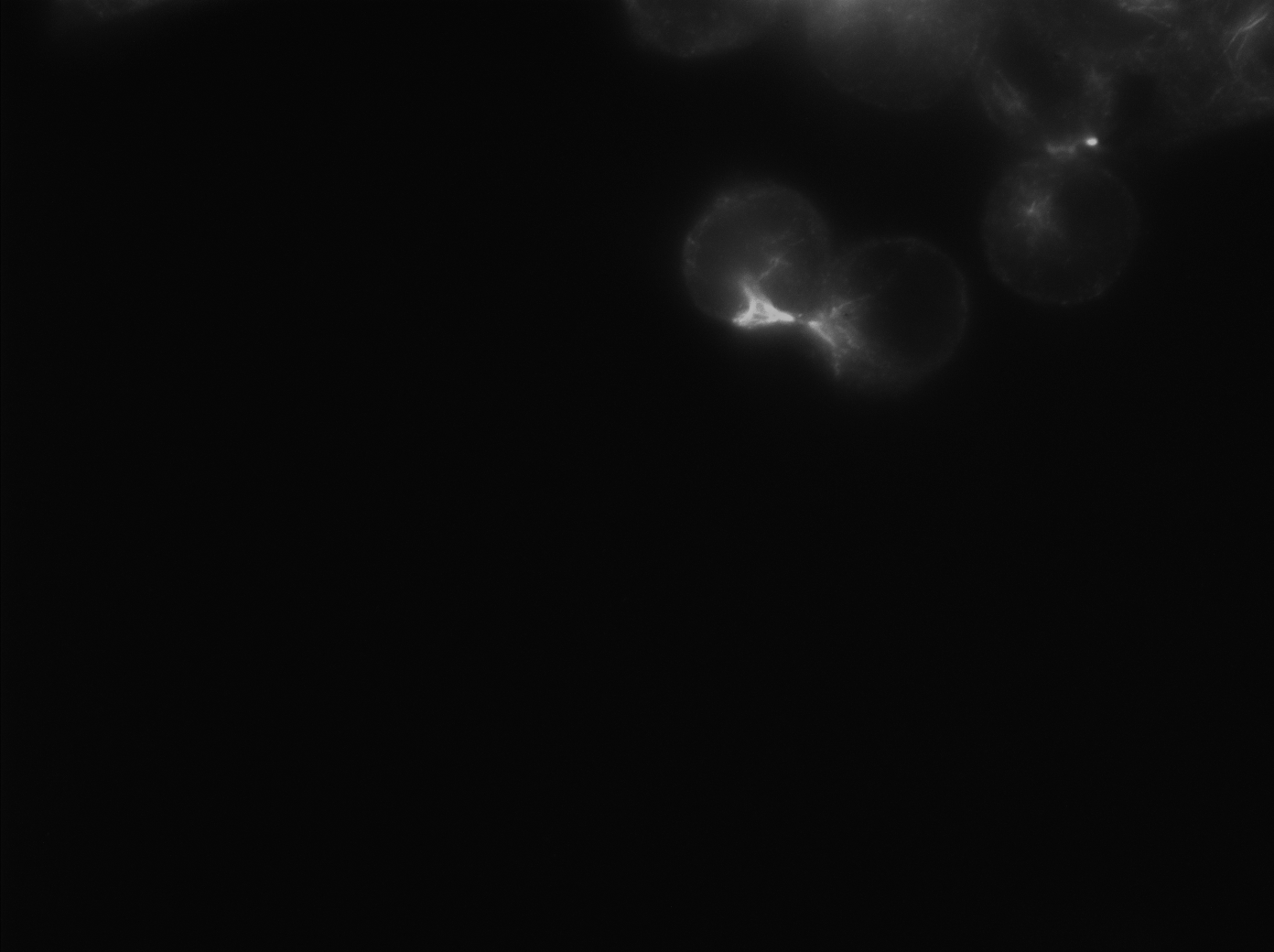

Supplement: Supplementary file 28 — Source data Fig. 7 part 4 [file 44319_2026_742_MOESM28_ESM.zip › Figure 7 Part 4/Fig 7fg Control and TPGS1-KO spastin acetylated tubulin/TPGS1-KO spastin actub 4-1-25 R1 SI18.Project Maximum Z_XY1743538687_Z0_T0_C2.tif]

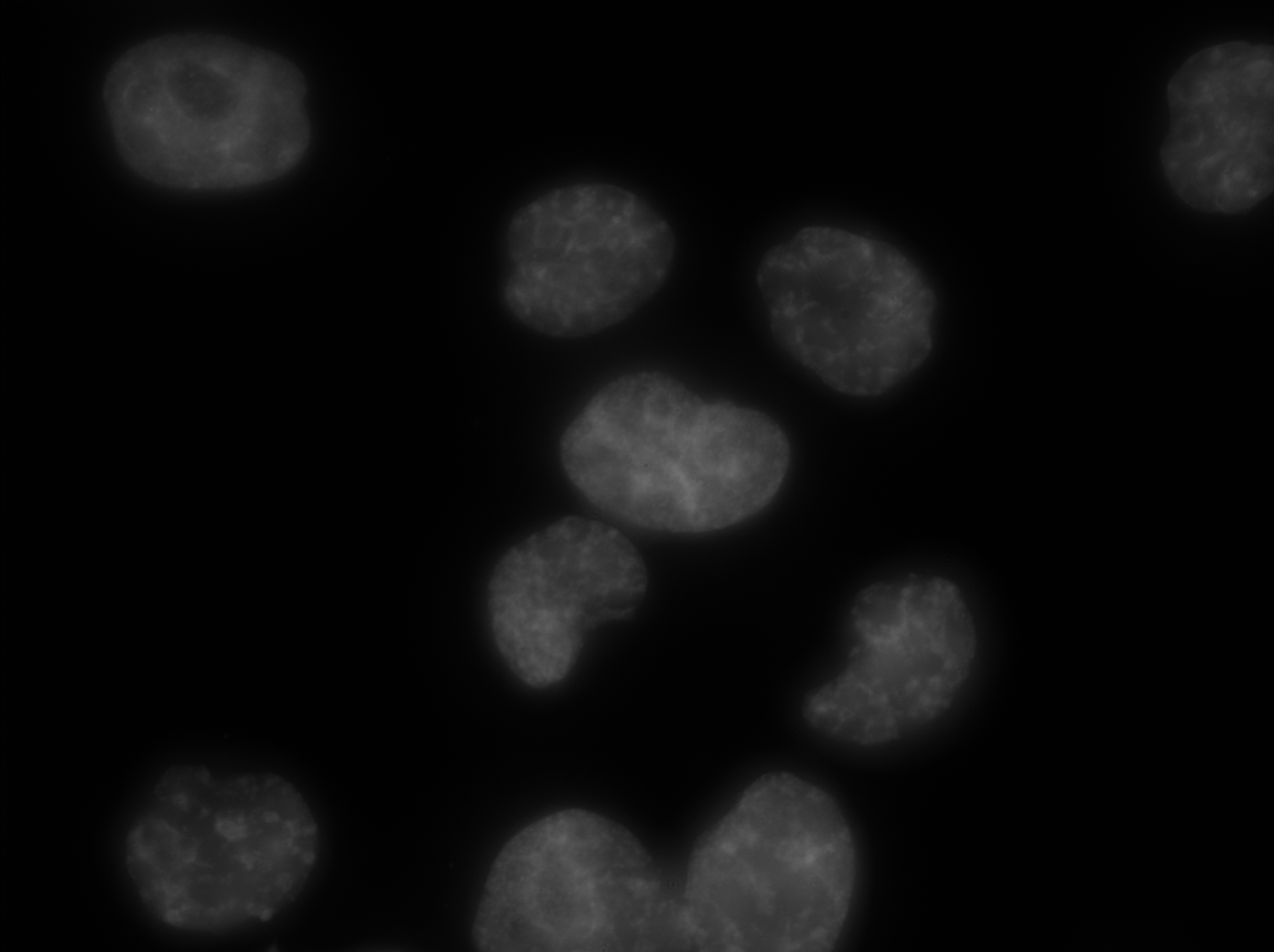

Supplement: Supplementary file 28 — Source data Fig. 7 part 4 [file 44319_2026_742_MOESM28_ESM.zip › Figure 7 Part 4/Fig 7fg Control and TPGS1-KO spastin acetylated tubulin/TPGS1-KO spastin actub 4-1-25 R1 SI14SI15.Project Maximum Z_XY1743537910_Z0_T0_C0.tif]

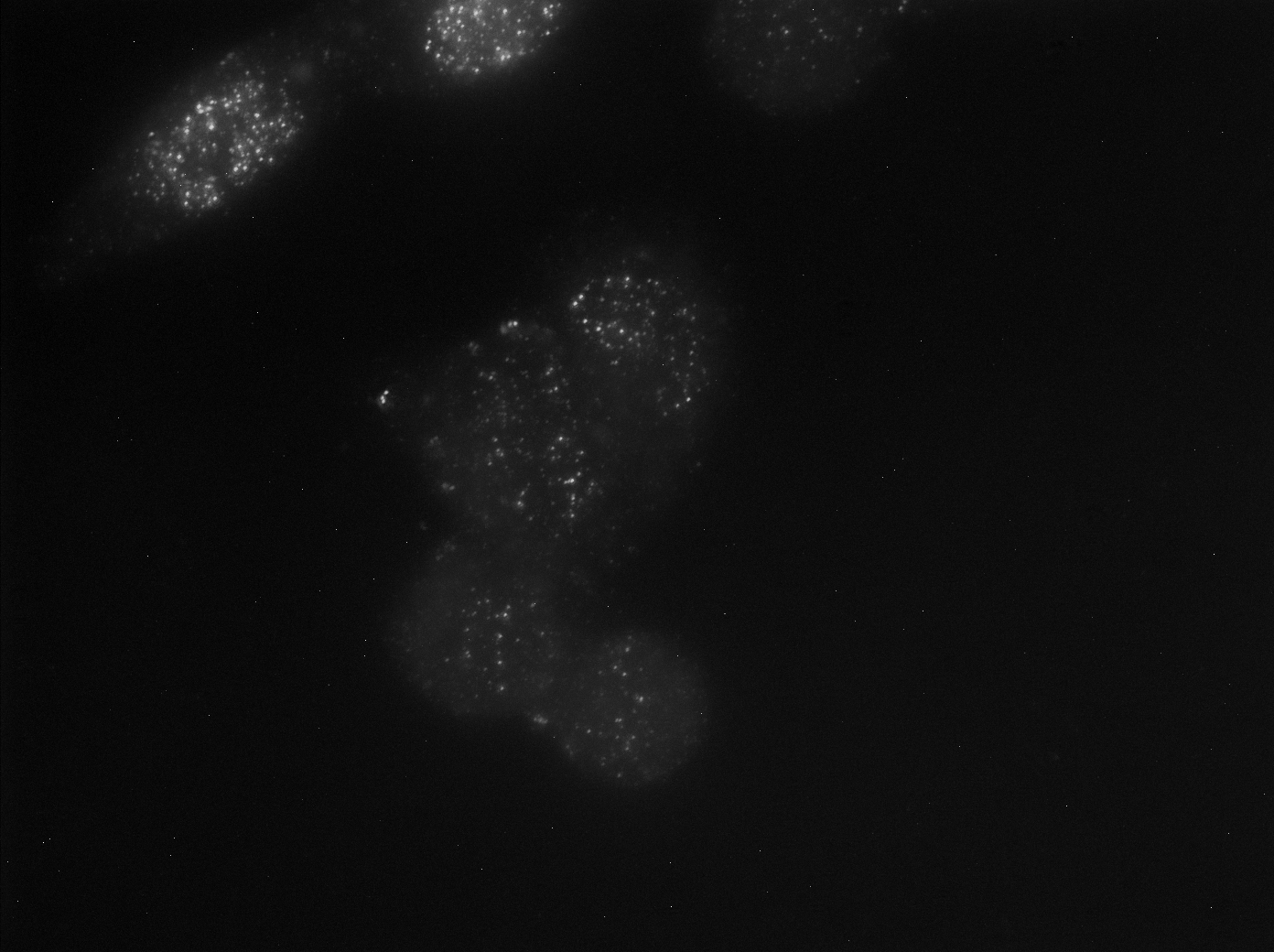

Supplement: Supplementary file 28 — Source data Fig. 7 part 4 [file 44319_2026_742_MOESM28_ESM.zip › Figure 7 Part 4/Fig 7fg Control and TPGS1-KO spastin acetylated tubulin/Cas9 spastin actub 4-1-25 R1 SI25.Project Maximum Z_XY1743535724_Z0_T0_C1.tif]

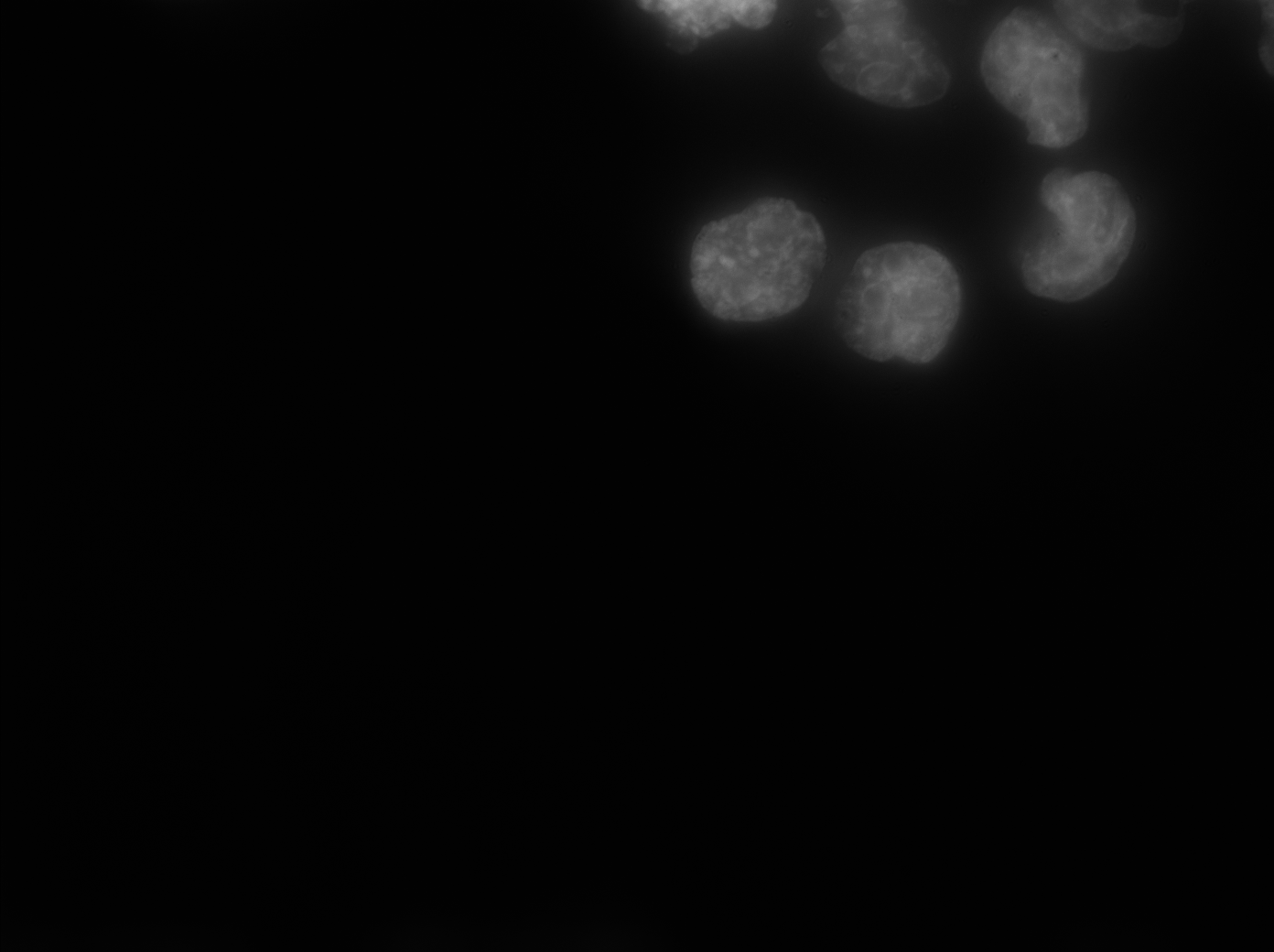

Supplement: Supplementary file 28 — Source data Fig. 7 part 4 [file 44319_2026_742_MOESM28_ESM.zip › Figure 7 Part 4/Fig 7fg Control and TPGS1-KO spastin acetylated tubulin/TPGS1-KO spastin actub 4-1-25 R1 SI18.Project Maximum Z_XY1743538687_Z0_T0_C0.tif]

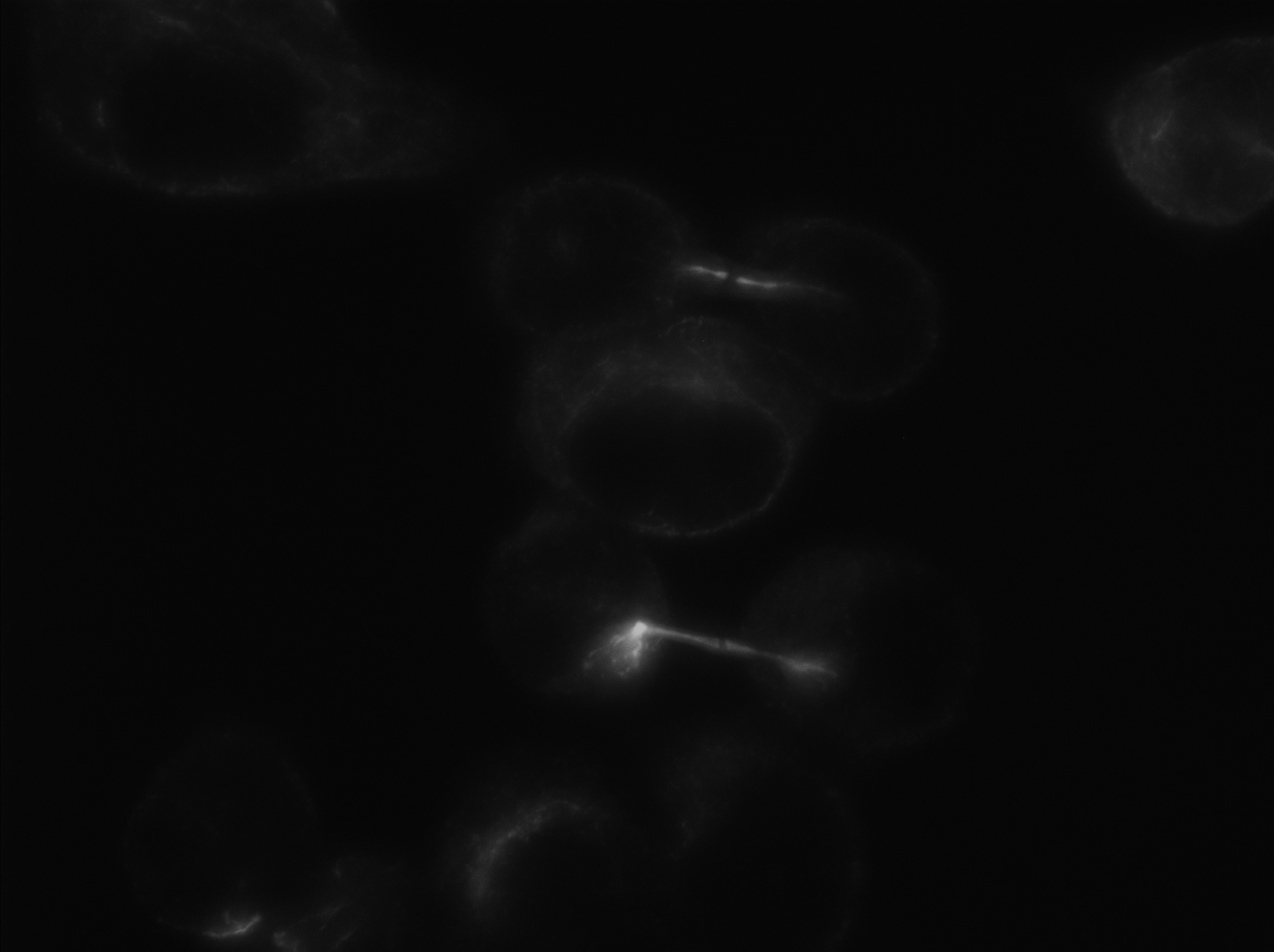

Supplement: Supplementary file 28 — Source data Fig. 7 part 4 [file 44319_2026_742_MOESM28_ESM.zip › Figure 7 Part 4/Fig 7fg Control and TPGS1-KO spastin acetylated tubulin/TPGS1-KO spastin actub 4-1-25 R1 SI14SI15.Project Maximum Z_XY1743537910_Z0_T0_C2.tif]

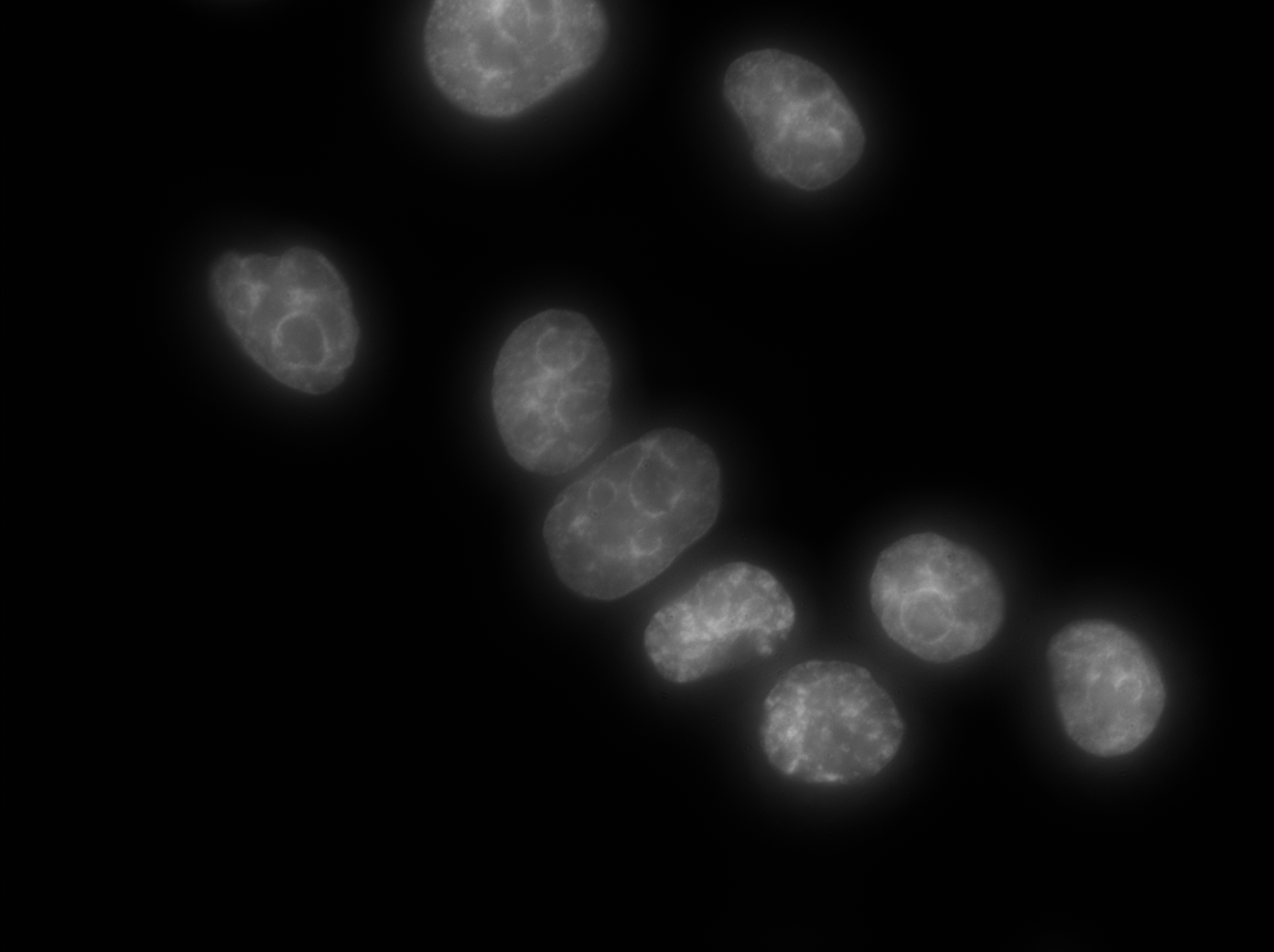

Supplement: Supplementary file 28 — Source data Fig. 7 part 4 [file 44319_2026_742_MOESM28_ESM.zip › Figure 7 Part 4/Fig 7fg Control and TPGS1-KO spastin acetylated tubulin/Cas9 spastin actub 4-1-25 R1 SI9.Project Maximum Z_XY1743531362_Z0_T0_C0.tif]

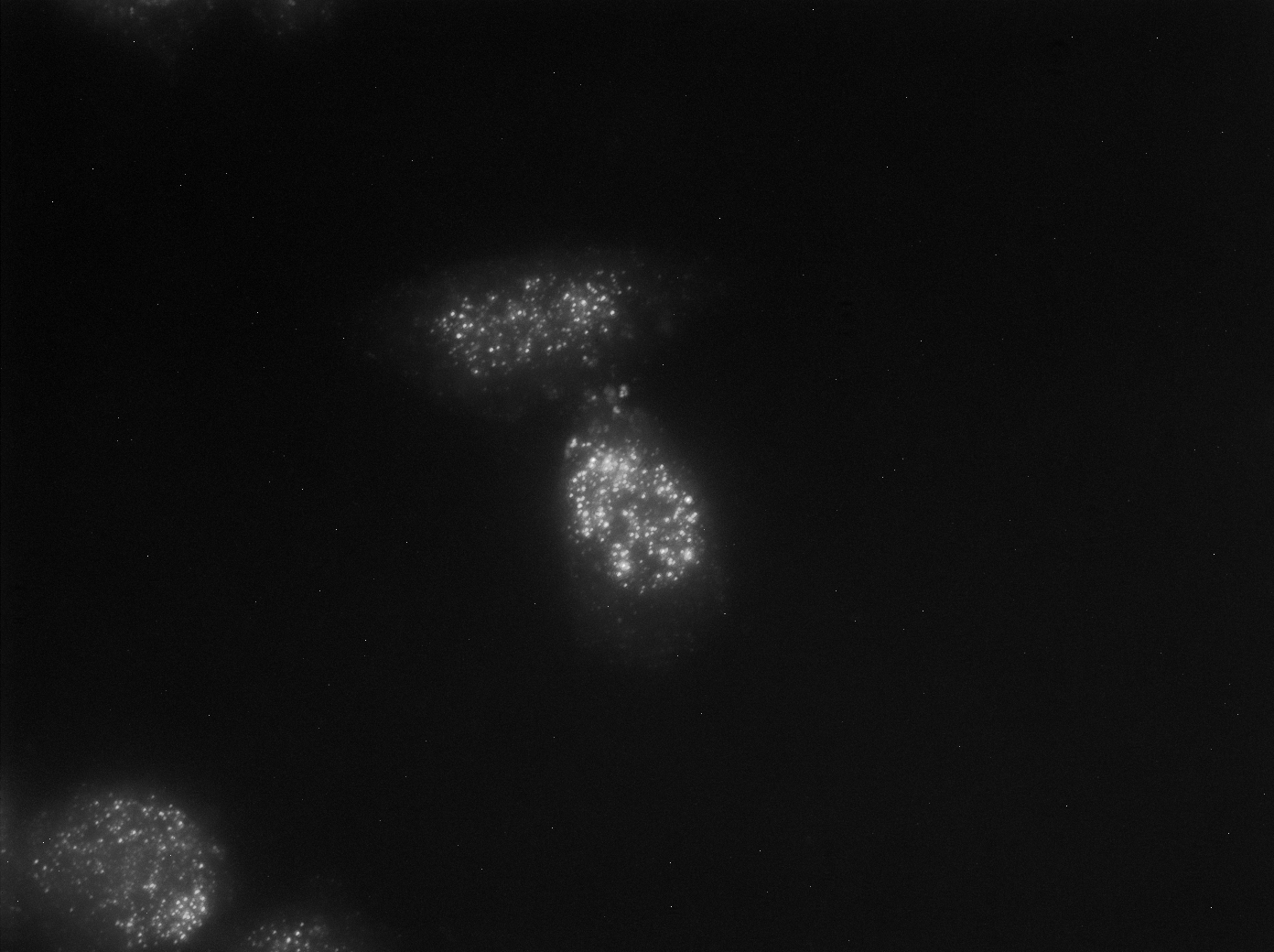

Supplement: Supplementary file 28 — Source data Fig. 7 part 4 [file 44319_2026_742_MOESM28_ESM.zip › Figure 7 Part 4/Fig 7fg Control and TPGS1-KO spastin acetylated tubulin/Cas9 spastin actub 4-1-25 R1 SI16.Project Maximum Z_XY1743534311_Z0_T0_C1.tif]

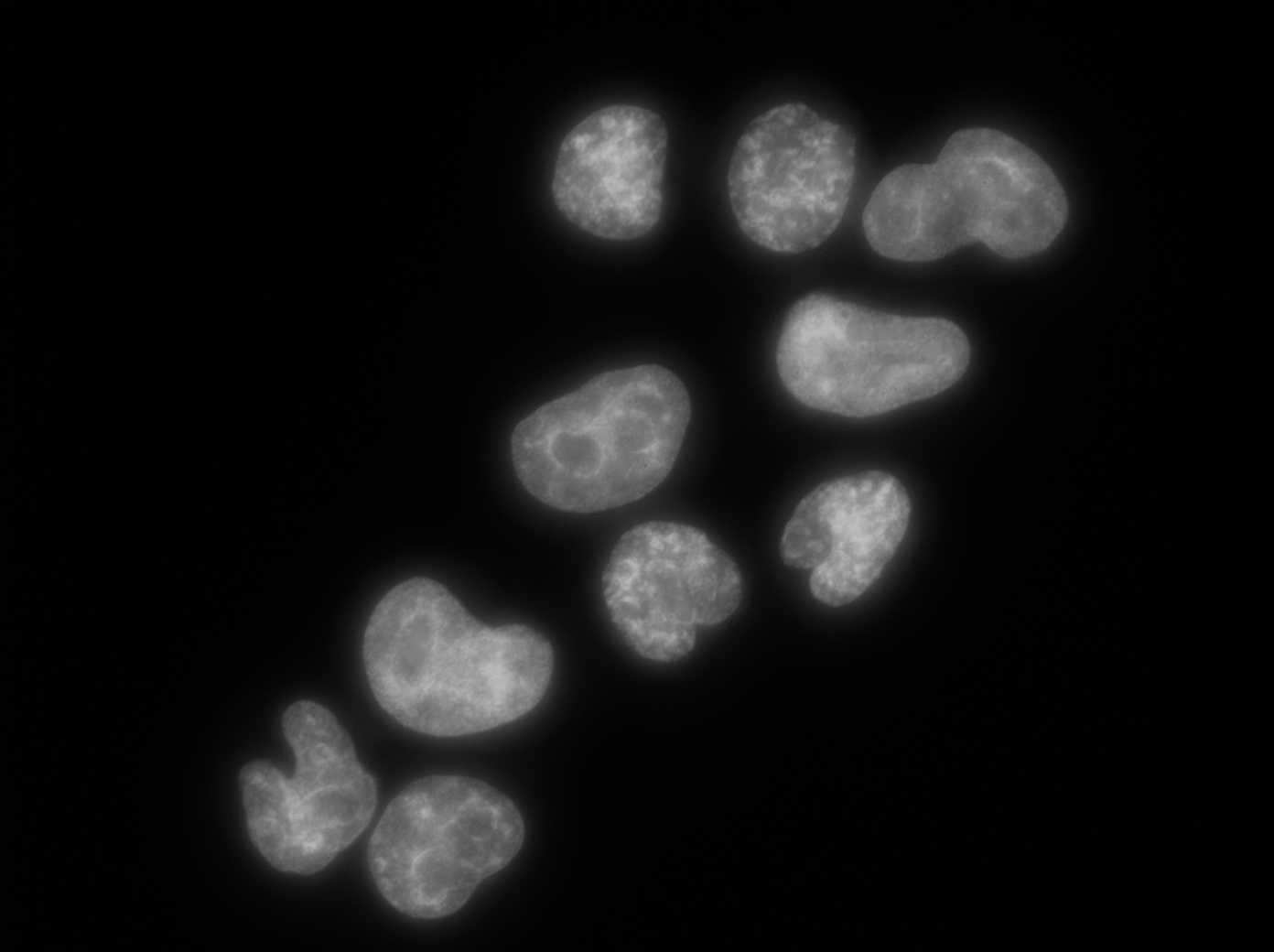

Supplement: Supplementary file 28 — Source data Fig. 7 part 4 [file 44319_2026_742_MOESM28_ESM.zip › Figure 7 Part 4/Fig 7fg Control and TPGS1-KO spastin acetylated tubulin/Cas9 spastin actub 4-1-25 R1 LT1LT2 PA1.Project Maximum Z_XY1743529647_Z0_T0_C0.tif]

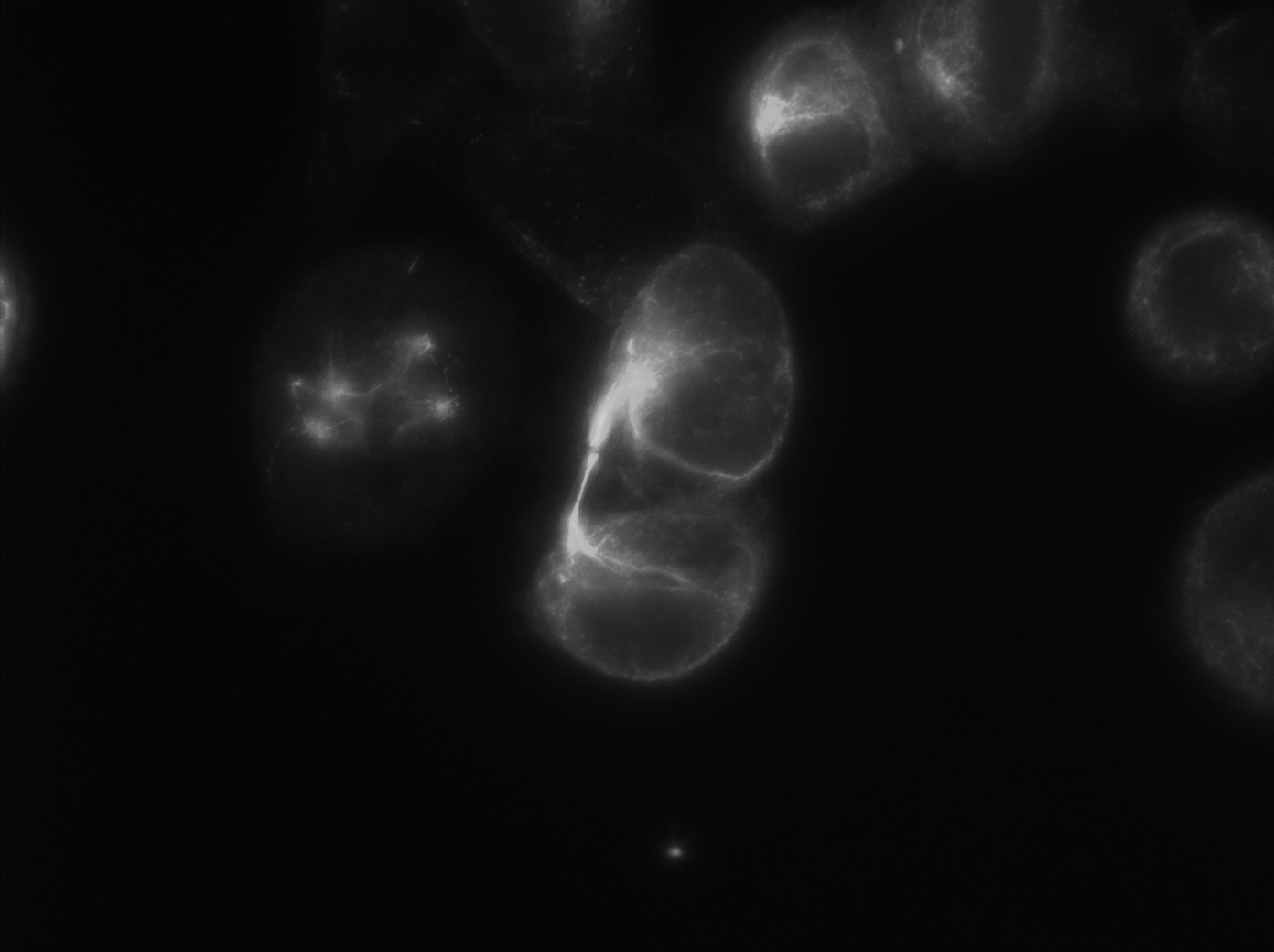

Supplement: Supplementary file 28 — Source data Fig. 7 part 4 [file 44319_2026_742_MOESM28_ESM.zip › Figure 7 Part 4/Fig 7fg Control and TPGS1-KO spastin acetylated tubulin/Cas9 spastin actub 4-1-25 R1 SI4.Project Maximum Z_XY1743530667_Z0_T0_C2.tif]

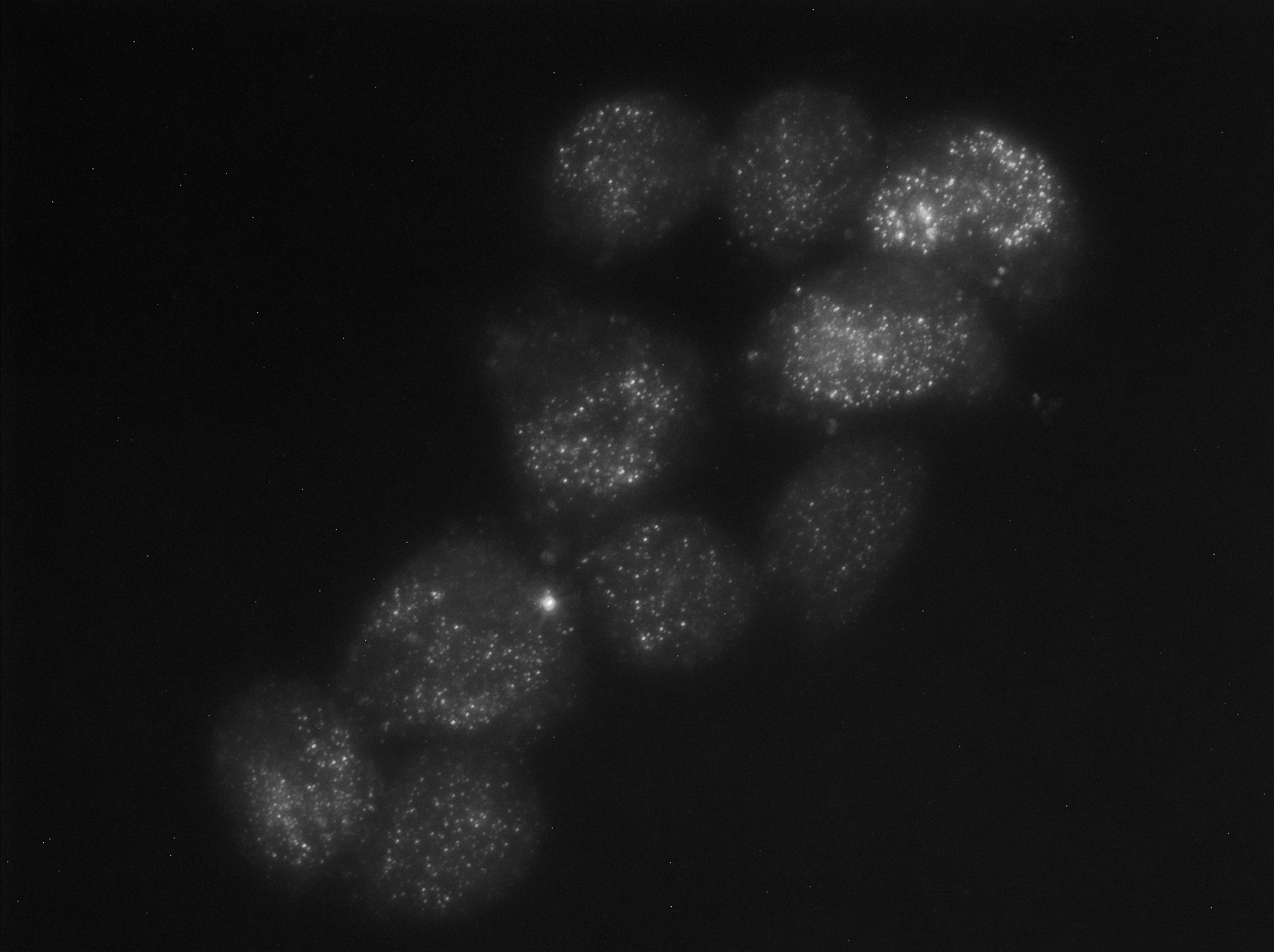

Supplement: Supplementary file 28 — Source data Fig. 7 part 4 [file 44319_2026_742_MOESM28_ESM.zip › Figure 7 Part 4/Fig 7fg Control and TPGS1-KO spastin acetylated tubulin/Cas9 spastin actub 4-1-25 R1 LT1LT2 PA1.Project Maximum Z_XY1743529647_Z0_T0_C1.tif]

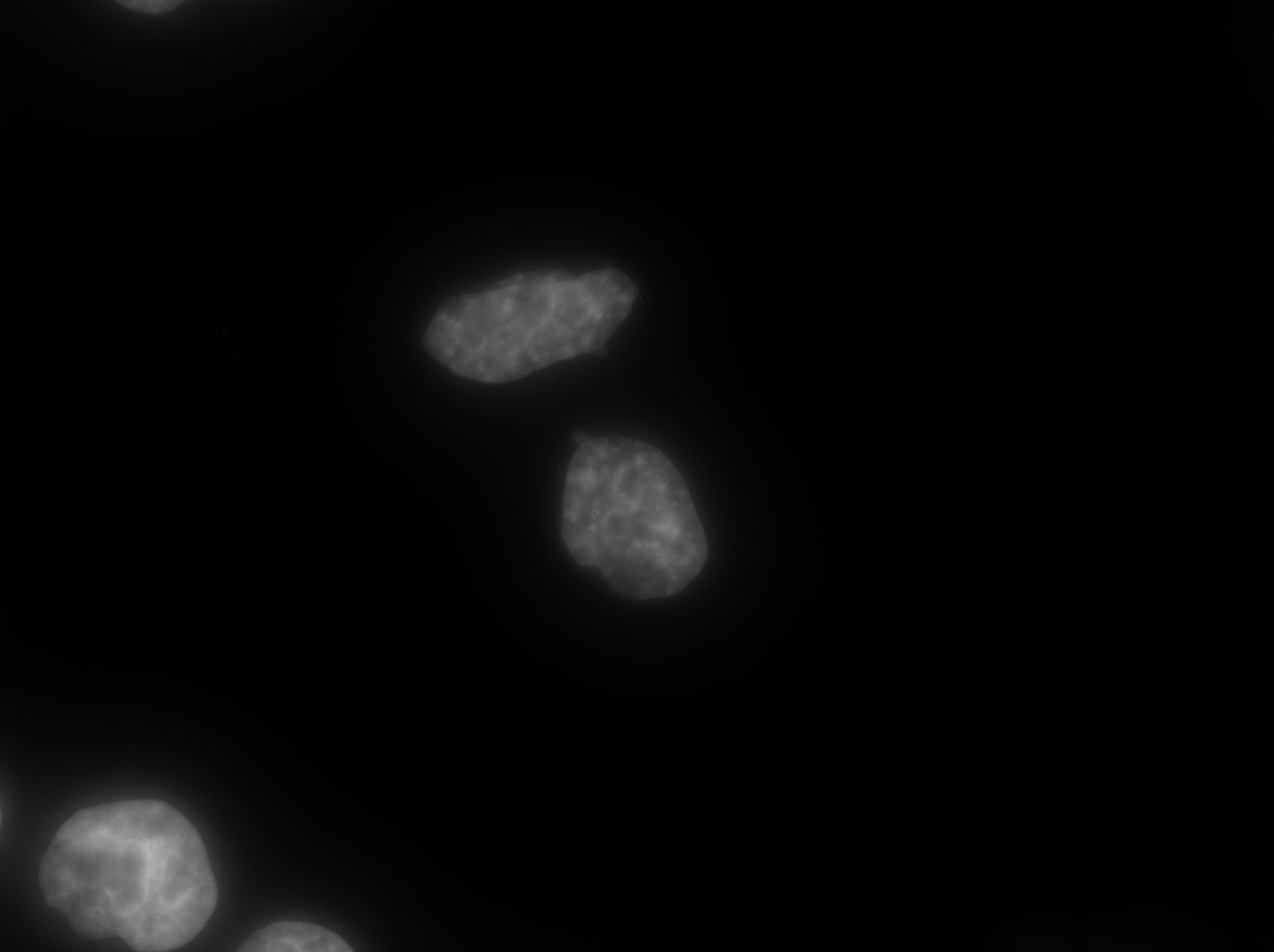

Supplement: Supplementary file 28 — Source data Fig. 7 part 4 [file 44319_2026_742_MOESM28_ESM.zip › Figure 7 Part 4/Fig 7fg Control and TPGS1-KO spastin acetylated tubulin/Cas9 spastin actub 4-1-25 R1 SI16.Project Maximum Z_XY1743534311_Z0_T0_C0.tif]

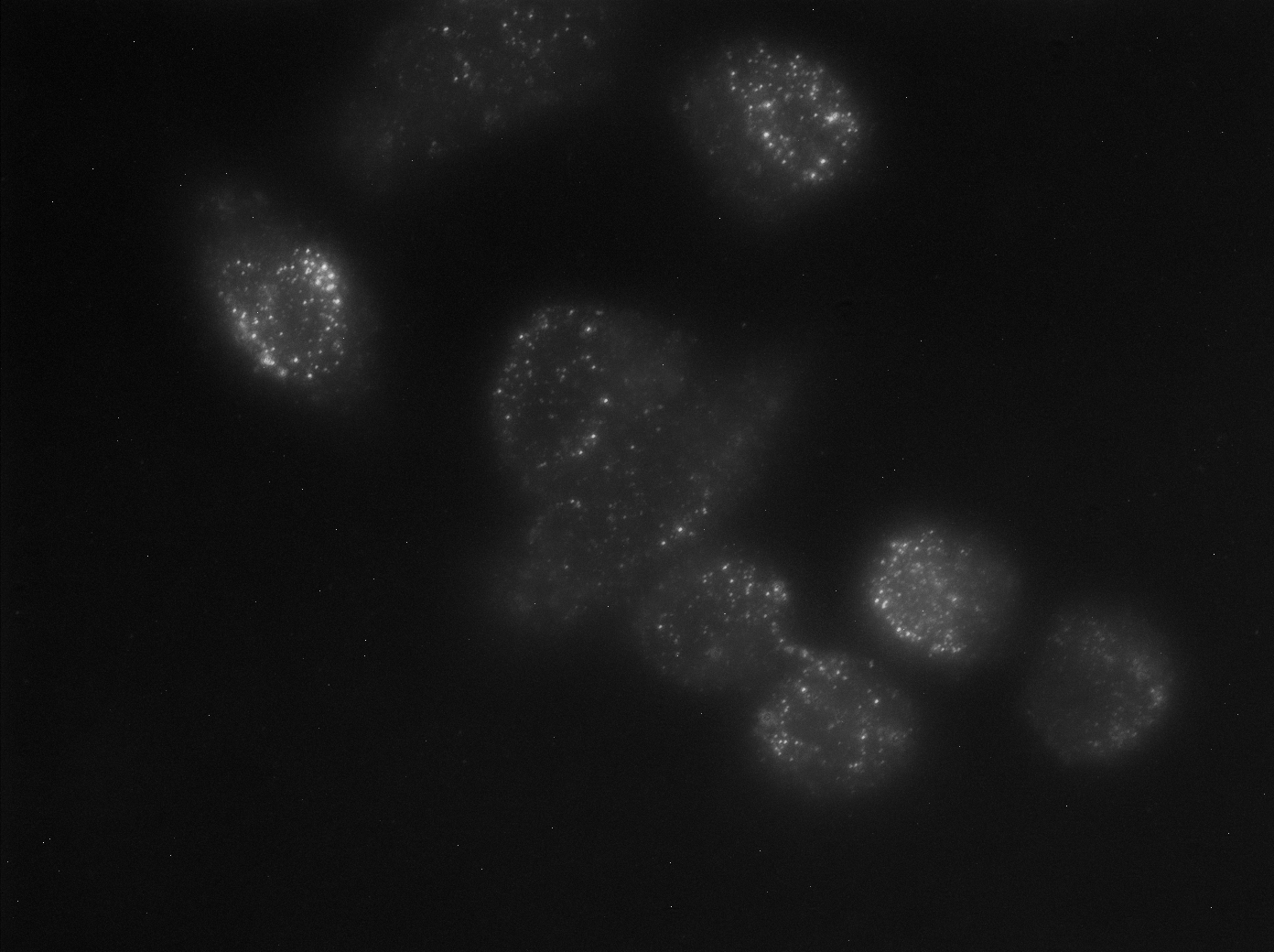

Supplement: Supplementary file 28 — Source data Fig. 7 part 4 [file 44319_2026_742_MOESM28_ESM.zip › Figure 7 Part 4/Fig 7fg Control and TPGS1-KO spastin acetylated tubulin/Cas9 spastin actub 4-1-25 R1 SI9.Project Maximum Z_XY1743531362_Z0_T0_C1.tif]

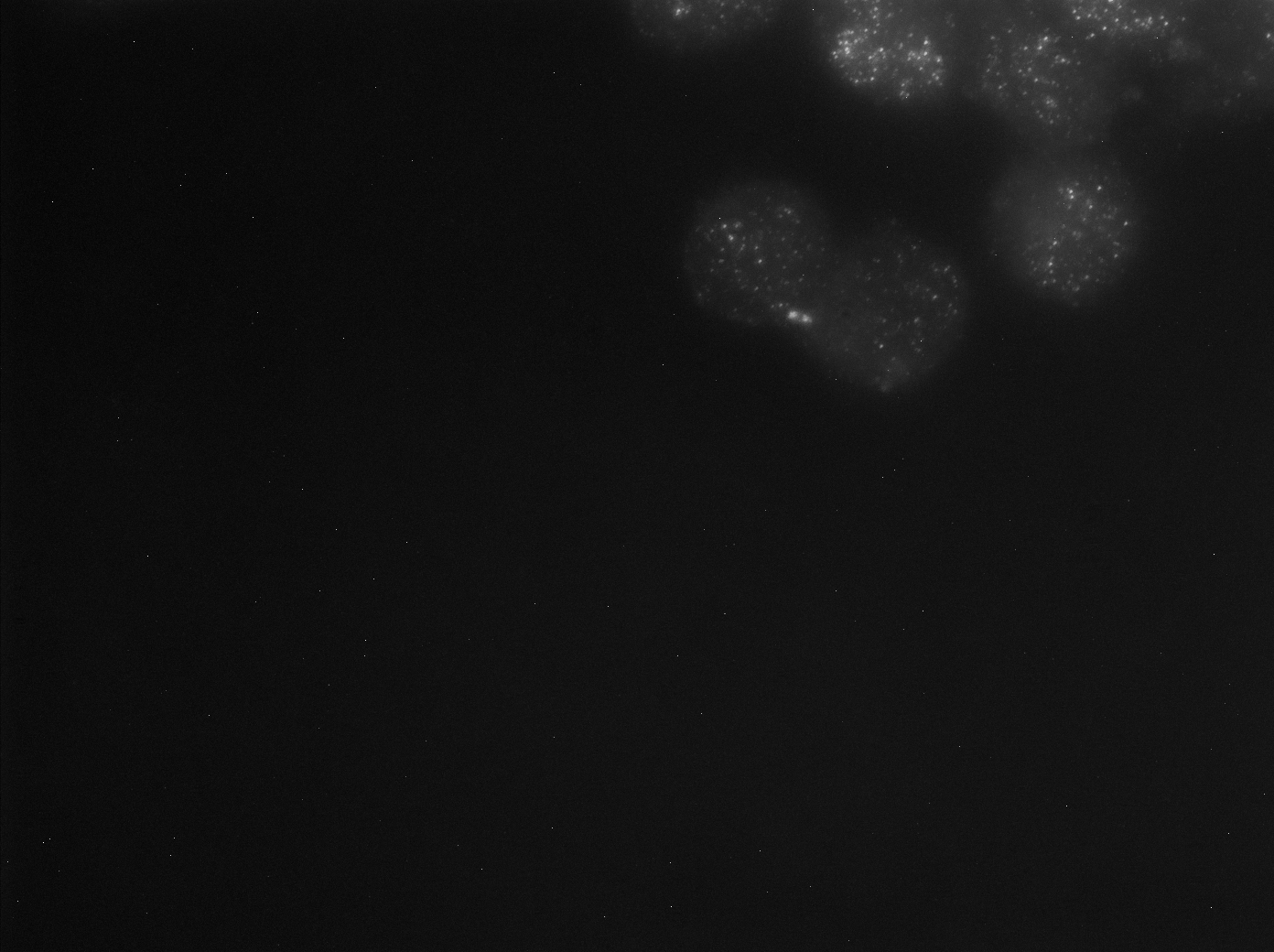

Supplement: Supplementary file 28 — Source data Fig. 7 part 4 [file 44319_2026_742_MOESM28_ESM.zip › Figure 7 Part 4/Fig 7fg Control and TPGS1-KO spastin acetylated tubulin/TPGS1-KO spastin actub 4-1-25 R1 SI18.Project Maximum Z_XY1743538687_Z0_T0_C1.tif]

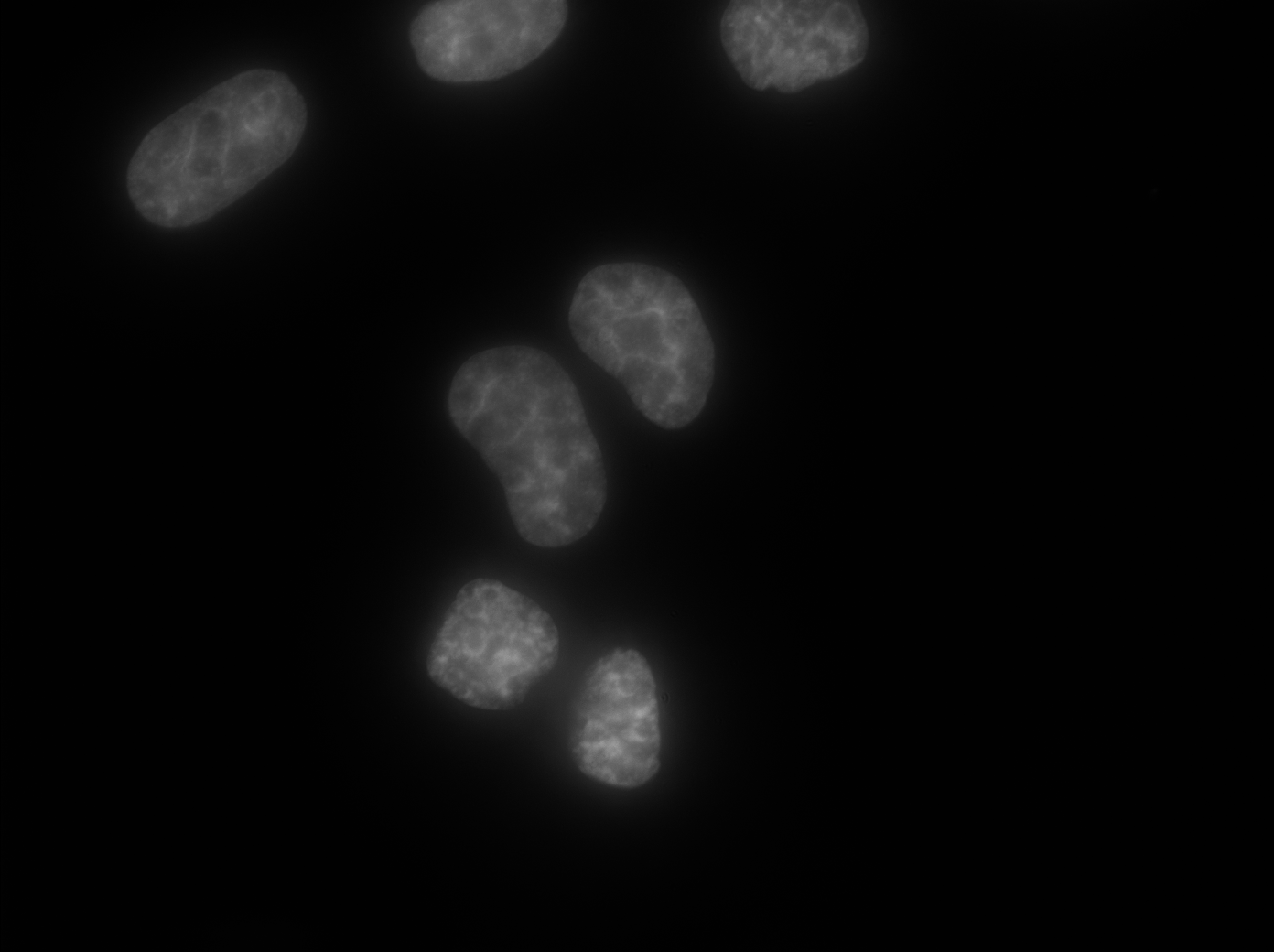

Supplement: Supplementary file 28 — Source data Fig. 7 part 4 [file 44319_2026_742_MOESM28_ESM.zip › Figure 7 Part 4/Fig 7fg Control and TPGS1-KO spastin acetylated tubulin/Cas9 spastin actub 4-1-25 R1 SI25.Project Maximum Z_XY1743535724_Z0_T0_C0.tif]

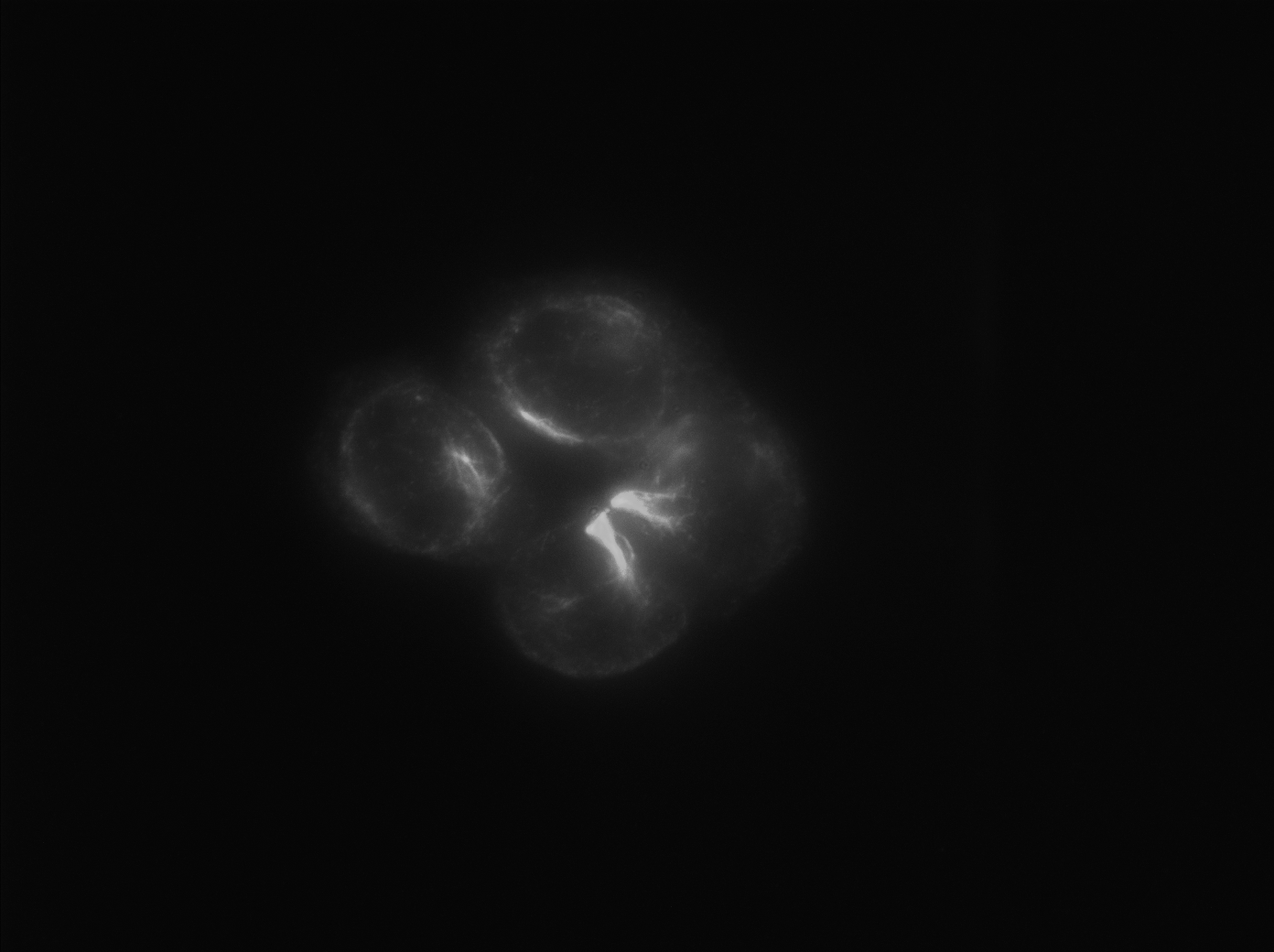

Supplement: Supplementary file 28 — Source data Fig. 7 part 4 [file 44319_2026_742_MOESM28_ESM.zip › Figure 7 Part 4/Fig 7fg Control and TPGS1-KO spastin acetylated tubulin/TPGS1-KO spastin actub 4-1-25 R1 SI19.Project Maximum Z_XY1743538782_Z0_T0_C2.tif]

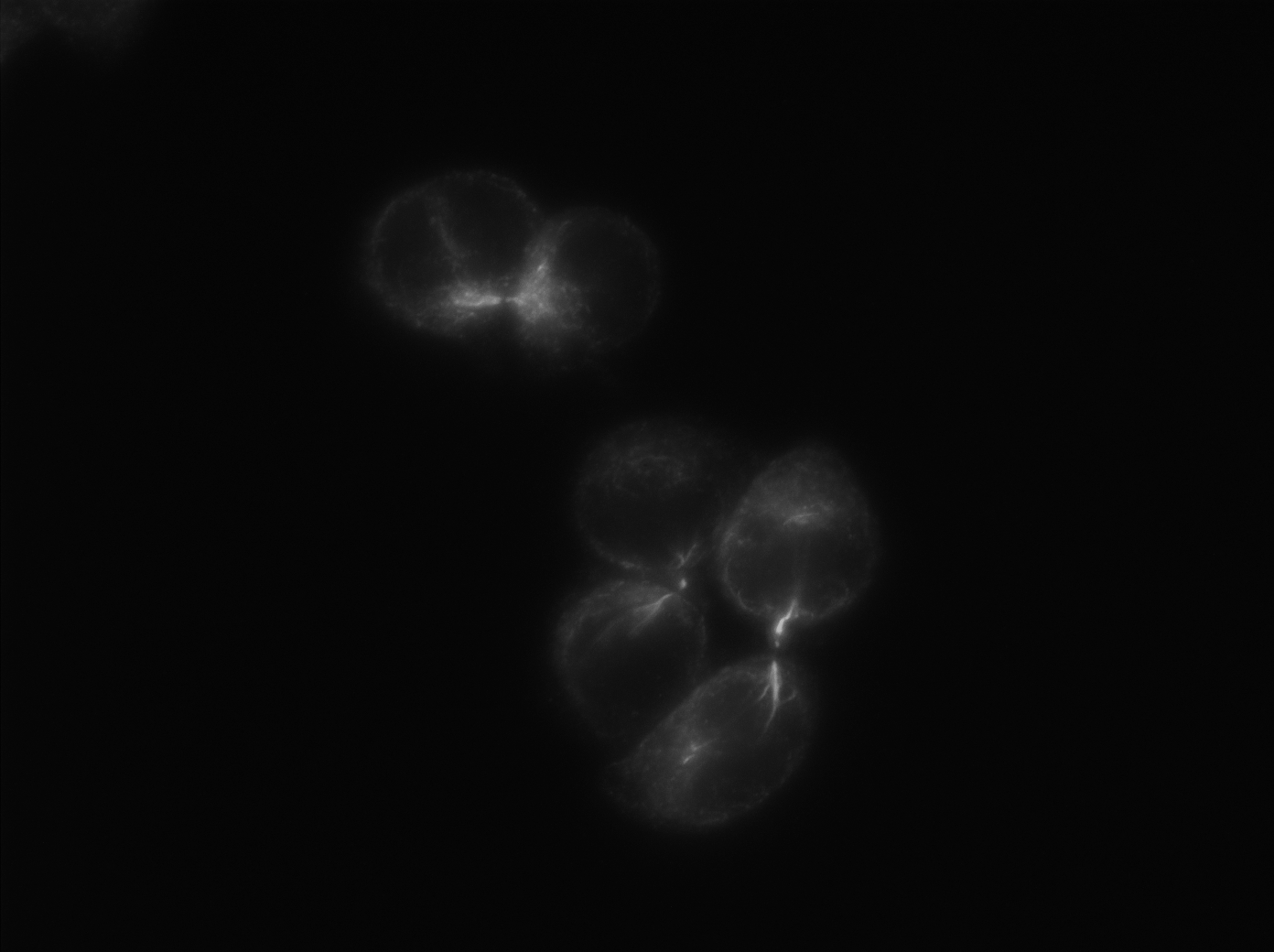

Supplement: Supplementary file 28 — Source data Fig. 7 part 4 [file 44319_2026_742_MOESM28_ESM.zip › Figure 7 Part 4/Fig 7fg Control and TPGS1-KO spastin acetylated tubulin/Cas9 spastin actub 4-1-25 R1 SI6SI7SI8.Project Maximum Z_XY1743531090_Z0_T0_C2.tif]

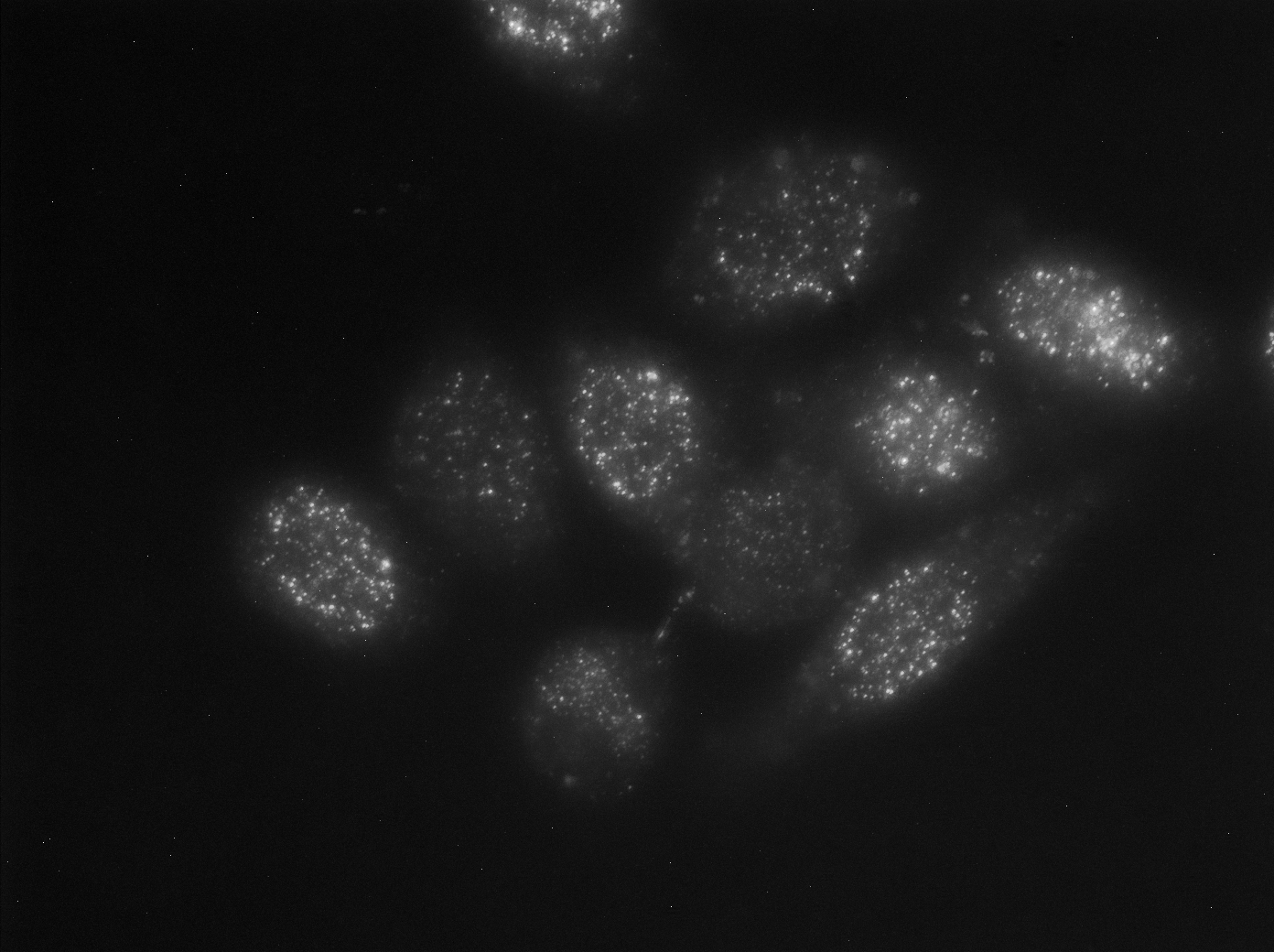

Supplement: Supplementary file 28 — Source data Fig. 7 part 4 [file 44319_2026_742_MOESM28_ESM.zip › Figure 7 Part 4/Fig 7fg Control and TPGS1-KO spastin acetylated tubulin/Cas9 spastin actub 4-1-25 R1 SI3.Project Maximum Z_XY1743530522_Z0_T0_C1.tif]

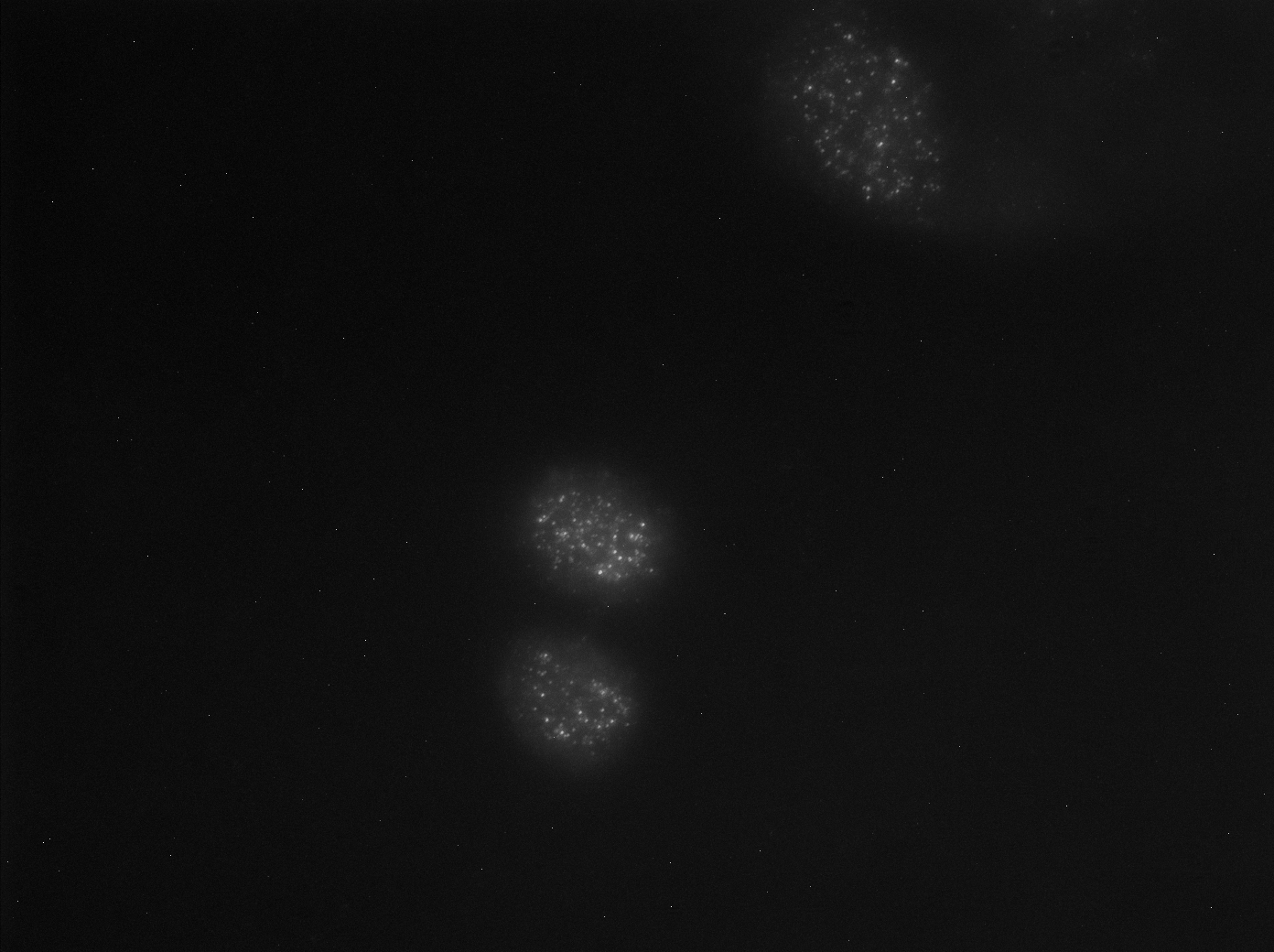

Supplement: Supplementary file 28 — Source data Fig. 7 part 4 [file 44319_2026_742_MOESM28_ESM.zip › Figure 7 Part 4/Fig 7fg Control and TPGS1-KO spastin acetylated tubulin/TPGS1-KO spastin actub 4-1-25 R1 SI5.Project Maximum Z_XY1743536653_Z0_T0_C1.tif]

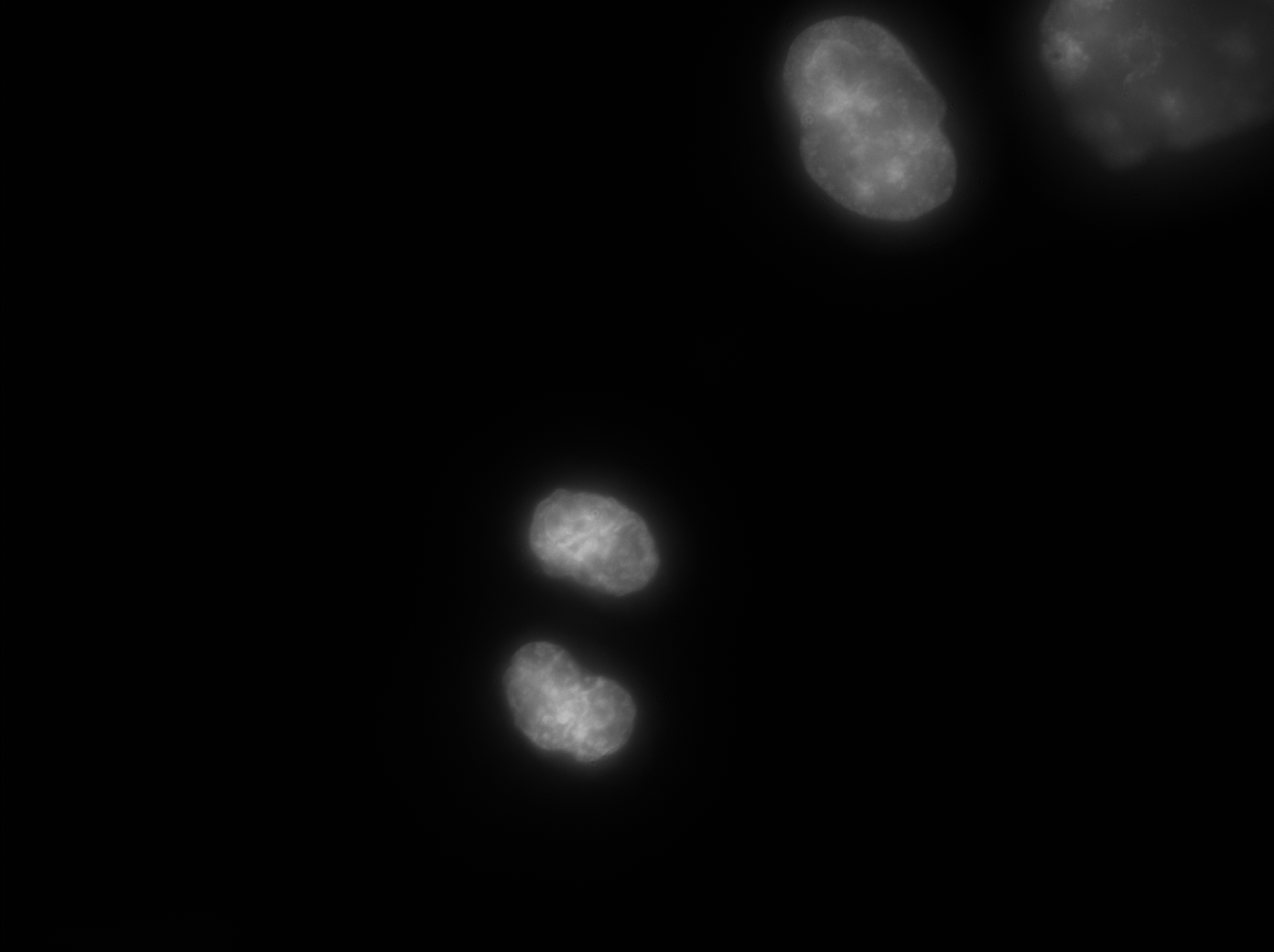

Supplement: Supplementary file 28 — Source data Fig. 7 part 4 [file 44319_2026_742_MOESM28_ESM.zip › Figure 7 Part 4/Fig 7fg Control and TPGS1-KO spastin acetylated tubulin/TPGS1-KO spastin actub 4-1-25 R1 SI5.Project Maximum Z_XY1743536653_Z0_T0_C0.tif]

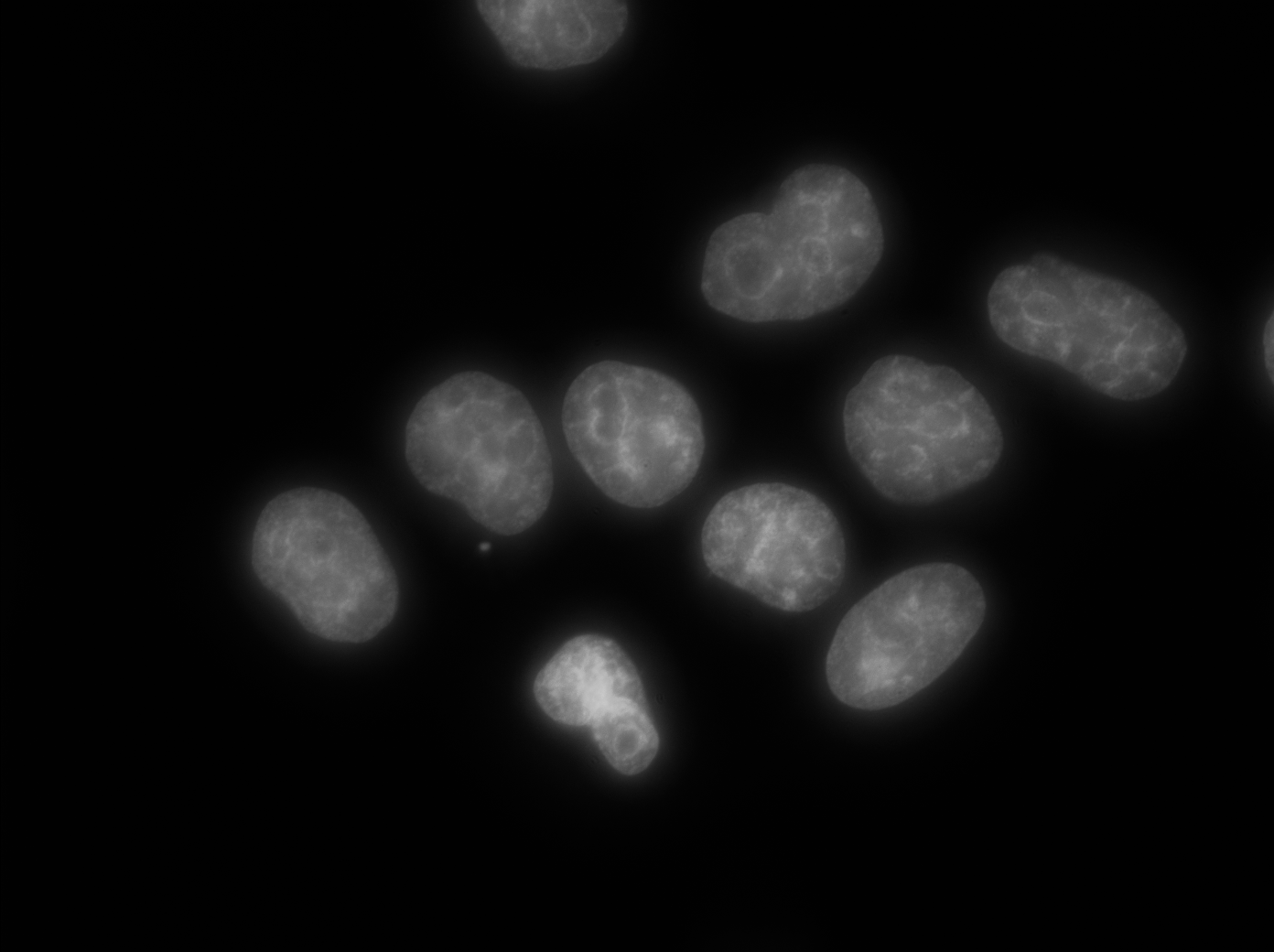

Supplement: Supplementary file 28 — Source data Fig. 7 part 4 [file 44319_2026_742_MOESM28_ESM.zip › Figure 7 Part 4/Fig 7fg Control and TPGS1-KO spastin acetylated tubulin/Cas9 spastin actub 4-1-25 R1 SI3.Project Maximum Z_XY1743530522_Z0_T0_C0.tif]

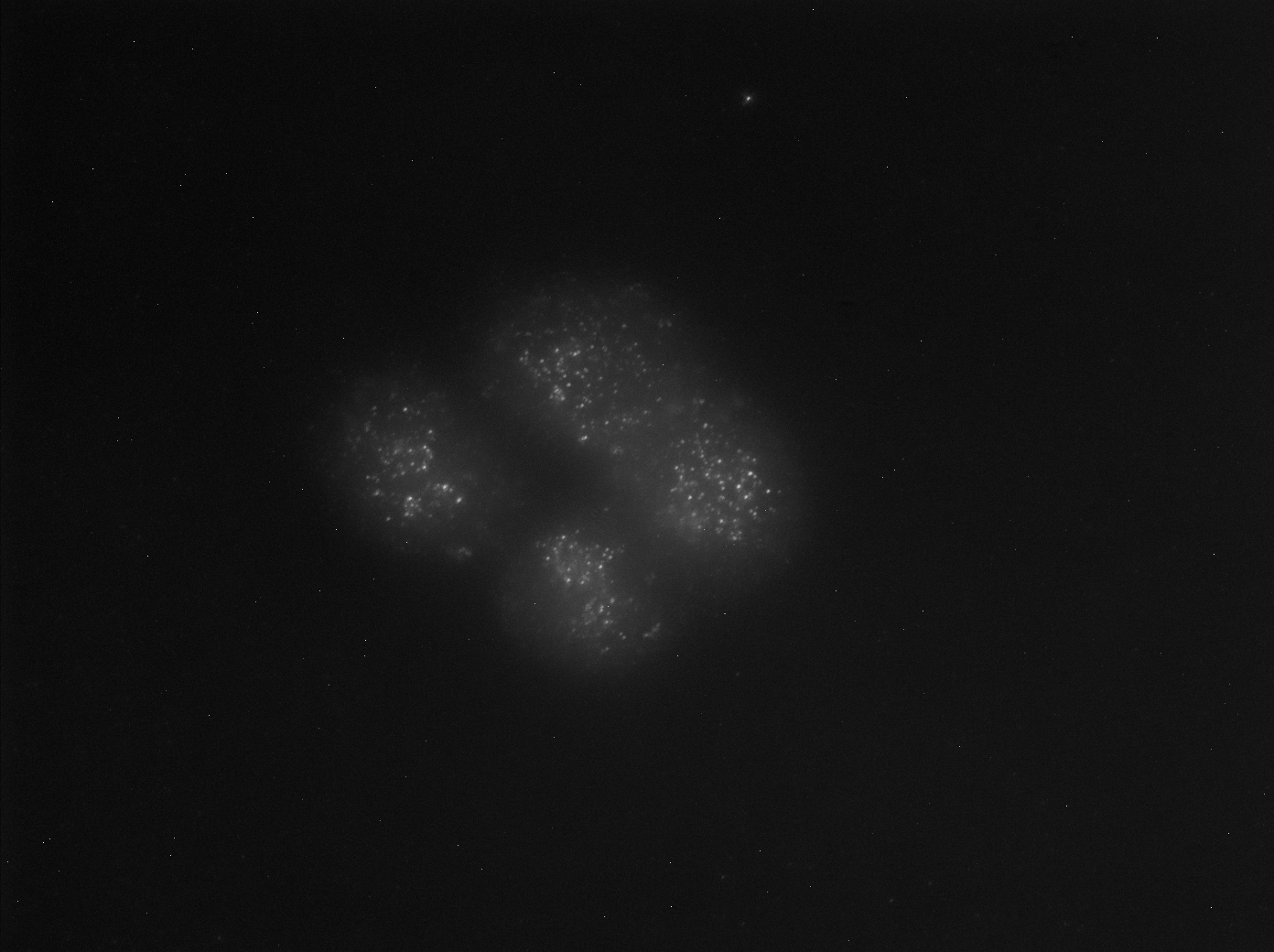

Supplement: Supplementary file 28 — Source data Fig. 7 part 4 [file 44319_2026_742_MOESM28_ESM.zip › Figure 7 Part 4/Fig 7fg Control and TPGS1-KO spastin acetylated tubulin/TPGS1-KO spastin actub 4-1-25 R1 SI19.Project Maximum Z_XY1743538782_Z0_T0_C1.tif]

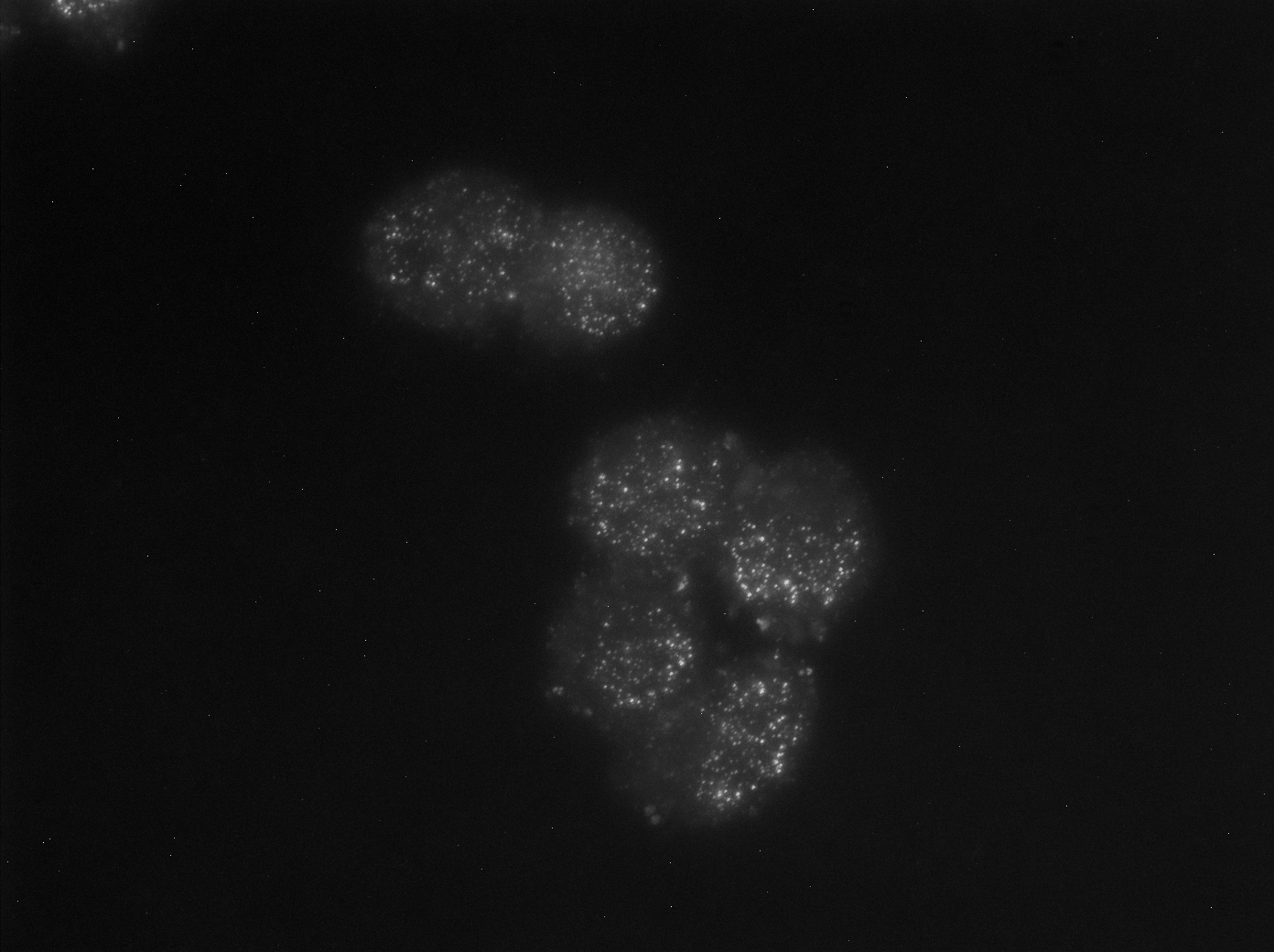

Supplement: Supplementary file 28 — Source data Fig. 7 part 4 [file 44319_2026_742_MOESM28_ESM.zip › Figure 7 Part 4/Fig 7fg Control and TPGS1-KO spastin acetylated tubulin/Cas9 spastin actub 4-1-25 R1 SI6SI7SI8.Project Maximum Z_XY1743531090_Z0_T0_C1.tif]

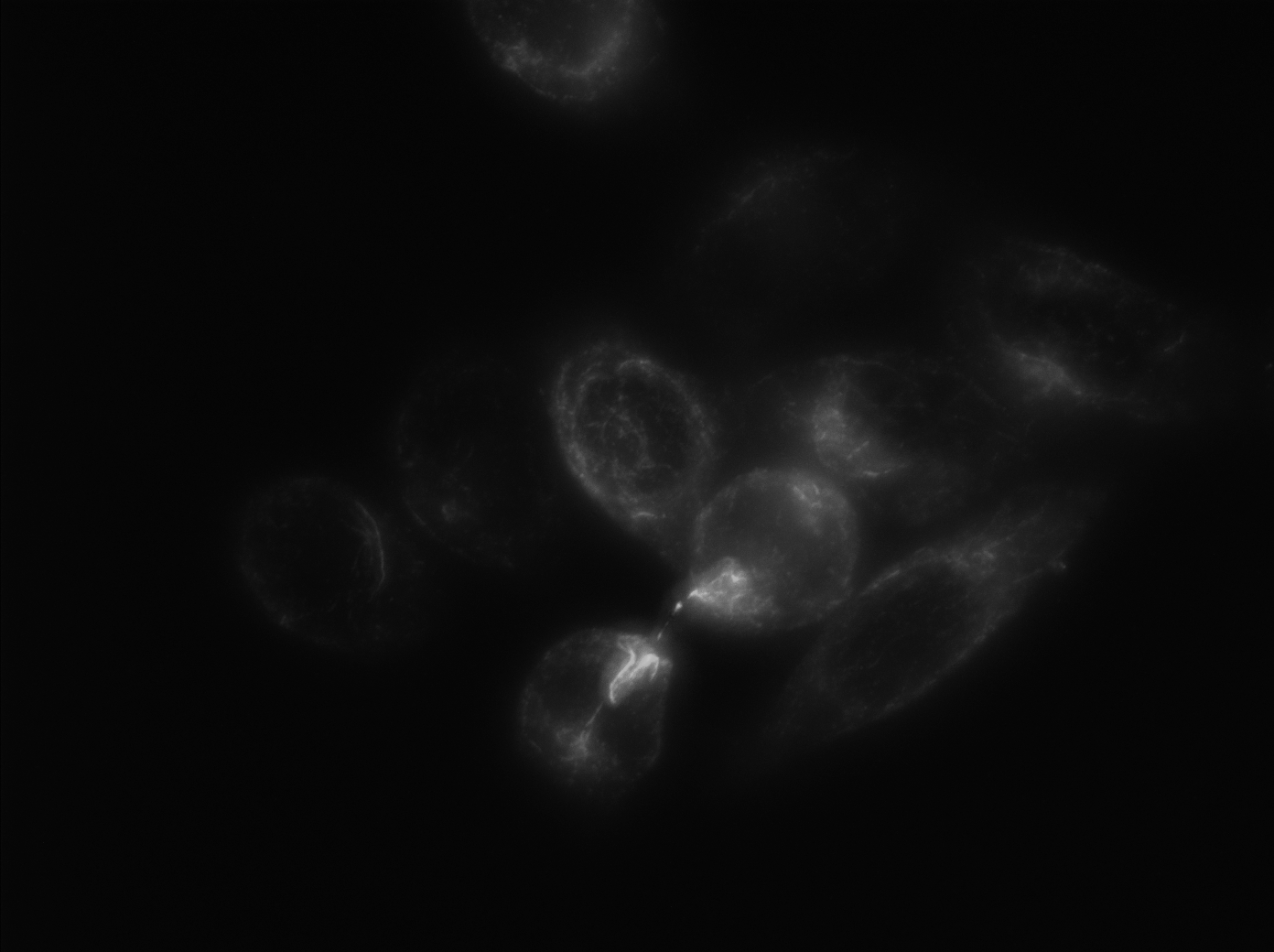

Supplement: Supplementary file 28 — Source data Fig. 7 part 4 [file 44319_2026_742_MOESM28_ESM.zip › Figure 7 Part 4/Fig 7fg Control and TPGS1-KO spastin acetylated tubulin/Cas9 spastin actub 4-1-25 R1 SI3.Project Maximum Z_XY1743530522_Z0_T0_C2.tif]

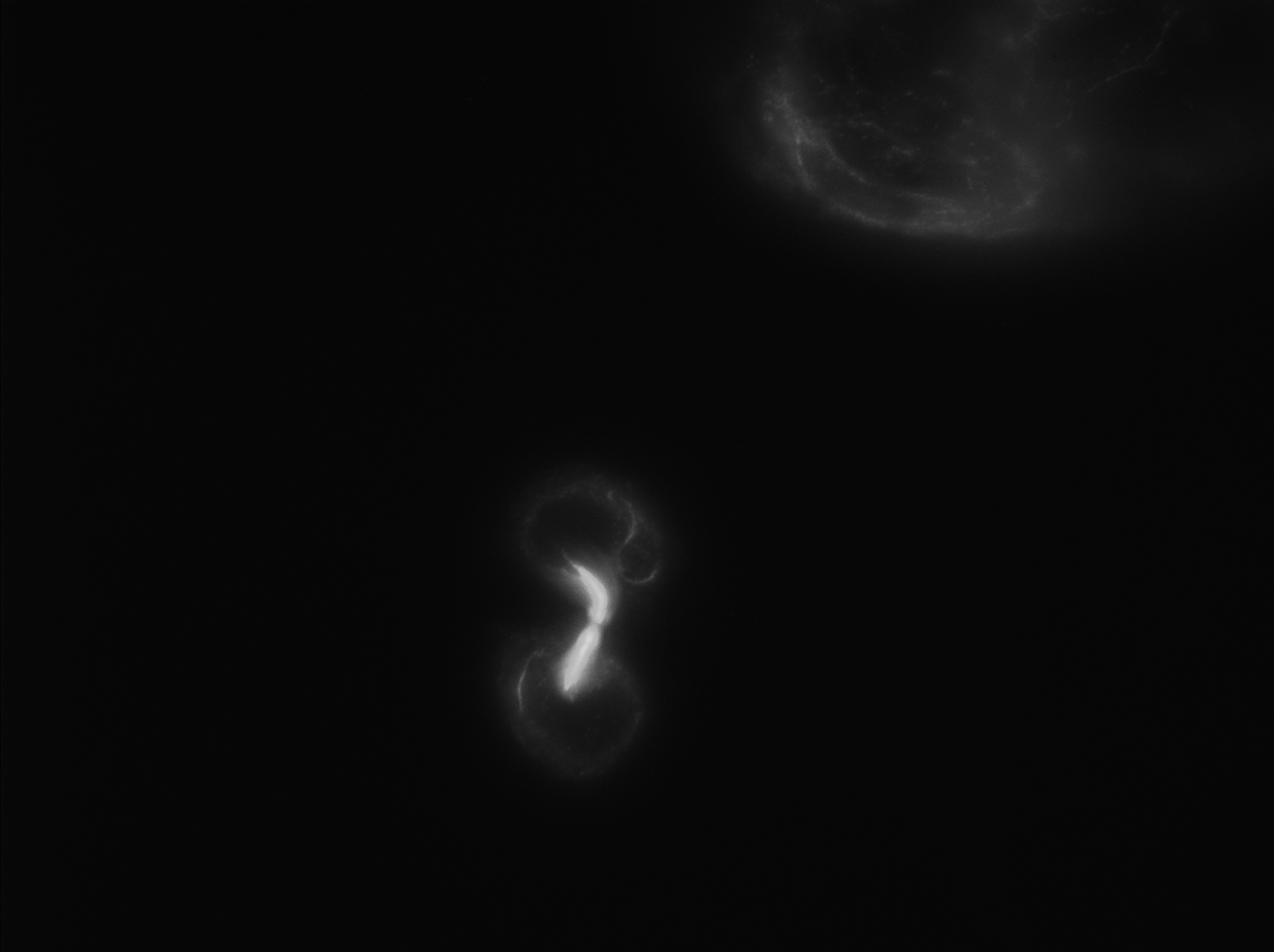

Supplement: Supplementary file 28 — Source data Fig. 7 part 4 [file 44319_2026_742_MOESM28_ESM.zip › Figure 7 Part 4/Fig 7fg Control and TPGS1-KO spastin acetylated tubulin/TPGS1-KO spastin actub 4-1-25 R1 SI5.Project Maximum Z_XY1743536653_Z0_T0_C2.tif]

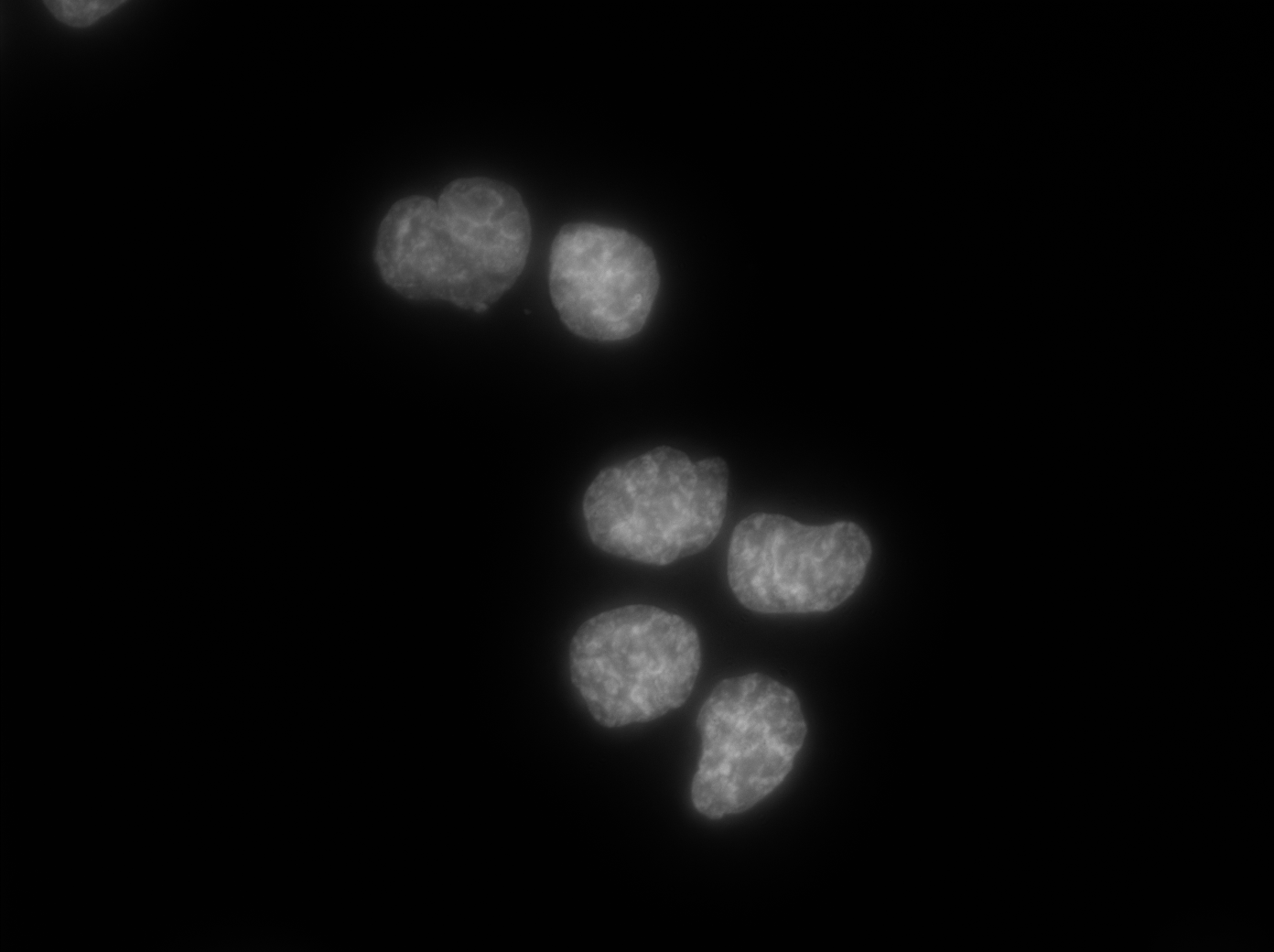

Supplement: Supplementary file 28 — Source data Fig. 7 part 4 [file 44319_2026_742_MOESM28_ESM.zip › Figure 7 Part 4/Fig 7fg Control and TPGS1-KO spastin acetylated tubulin/Cas9 spastin actub 4-1-25 R1 SI6SI7SI8.Project Maximum Z_XY1743531090_Z0_T0_C0.tif]

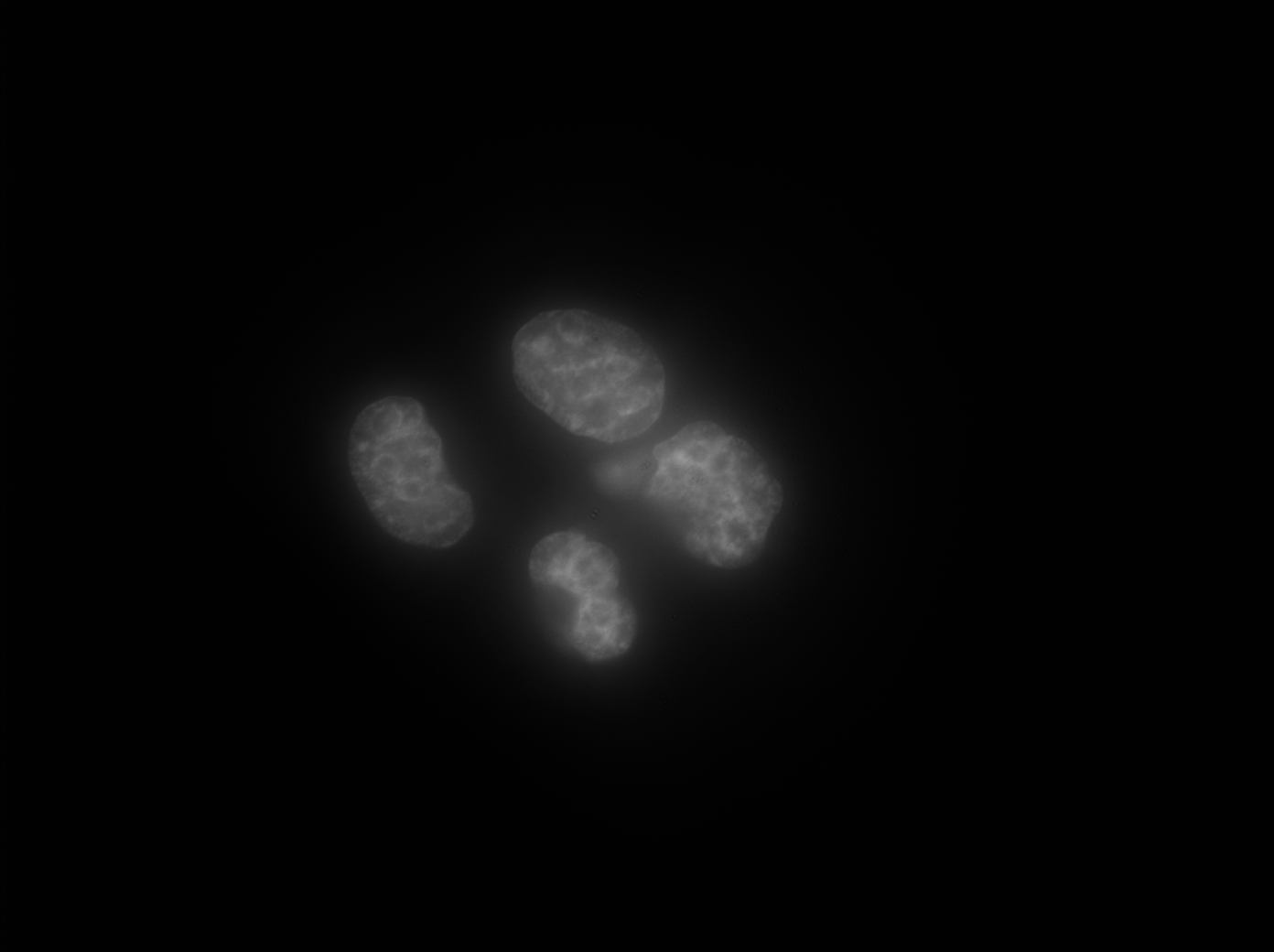

Supplement: Supplementary file 28 — Source data Fig. 7 part 4 [file 44319_2026_742_MOESM28_ESM.zip › Figure 7 Part 4/Fig 7fg Control and TPGS1-KO spastin acetylated tubulin/TPGS1-KO spastin actub 4-1-25 R1 SI19.Project Maximum Z_XY1743538782_Z0_T0_C0.tif]

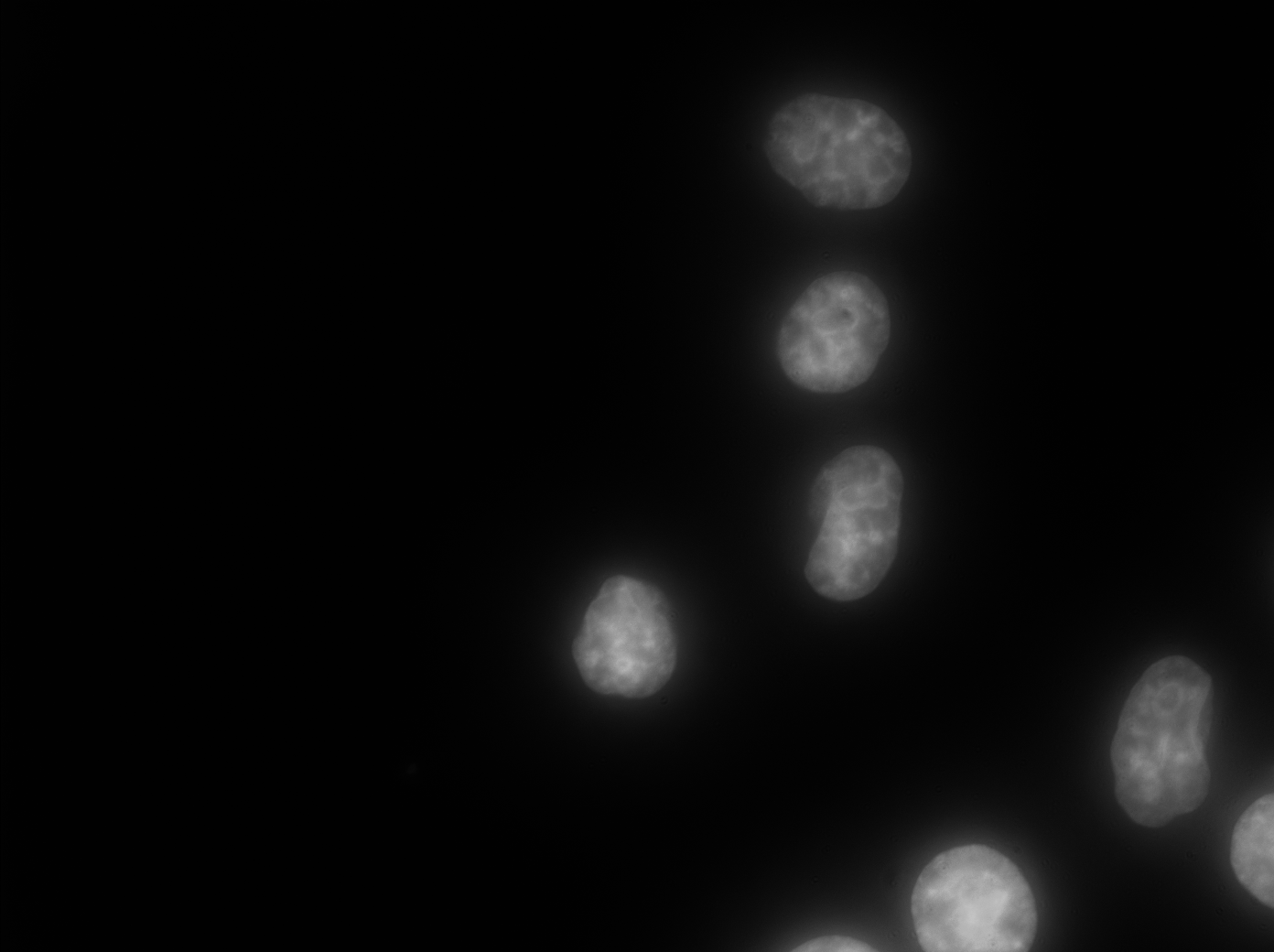

Supplement: Supplementary file 28 — Source data Fig. 7 part 4 [file 44319_2026_742_MOESM28_ESM.zip › Figure 7 Part 4/Fig 7fg Control and TPGS1-KO spastin acetylated tubulin/Cas9 spastin actub 4-1-25 R1 SI19.Project Maximum Z_XY1743534750_Z0_T0_C0.tif]

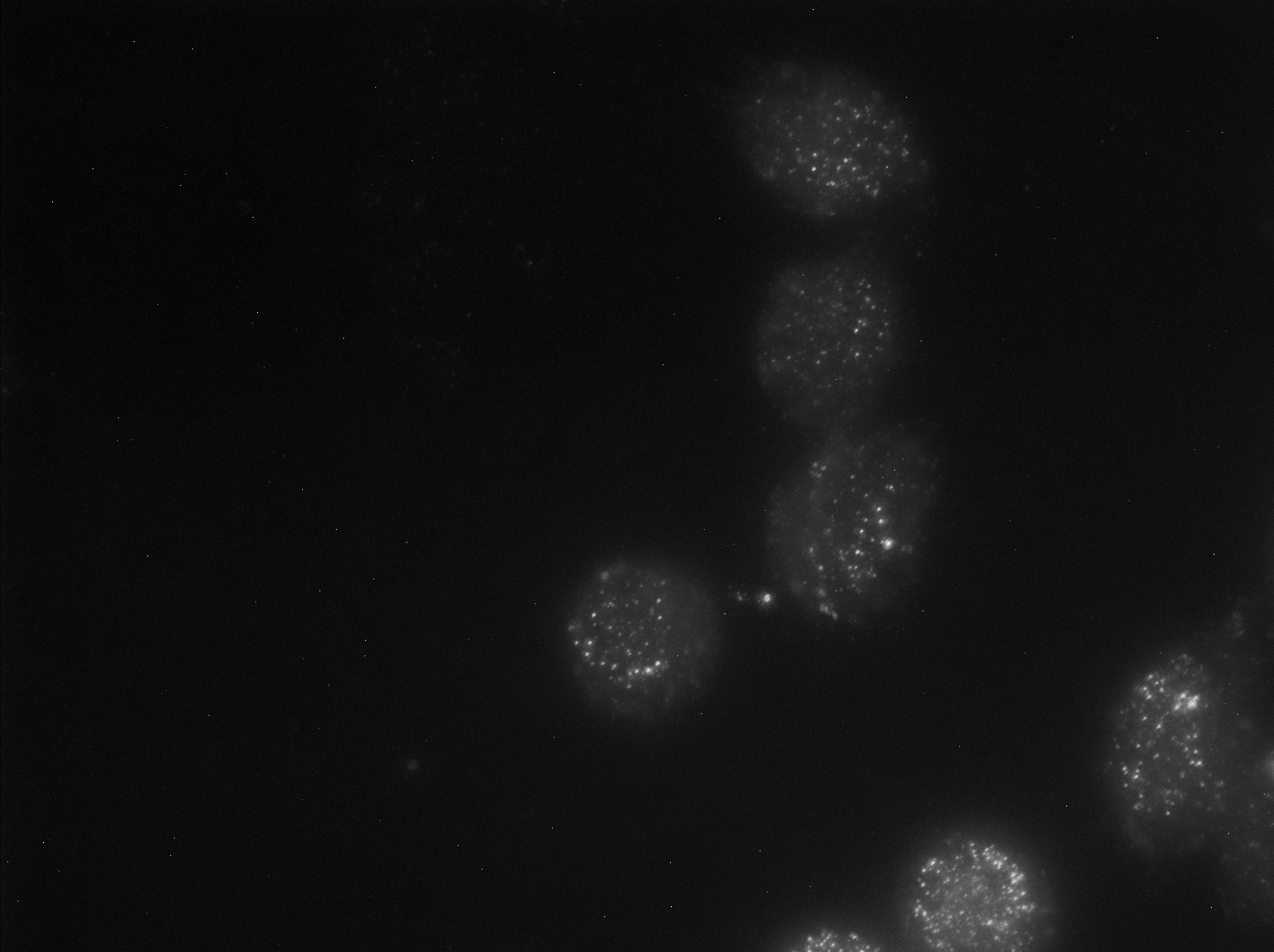

Supplement: Supplementary file 28 — Source data Fig. 7 part 4 [file 44319_2026_742_MOESM28_ESM.zip › Figure 7 Part 4/Fig 7fg Control and TPGS1-KO spastin acetylated tubulin/Cas9 spastin actub 4-1-25 R1 SI19.Project Maximum Z_XY1743534750_Z0_T0_C1.tif]
